# *In silico* prediction and characterization of secondary metabolite biosynthetic gene clusters in the wheat pathogen *Zymoseptoria tritici*

**DOI:** 10.1186/s12864-017-3969-y

**Published:** 2017-08-17

**Authors:** Timothy Cairns, Vera Meyer

**Affiliations:** Institute of Biotechnology, Department of Applied and Molecular Microbiology, Berlin University of Technology, Gustav-Meyer-Allee 25, 13355 Berlin, Germany

**Keywords:** *Zymoseptoria tritici*, *Mycosphaerella graminicola*, Secondary metabolism, Gene cluster, Toxin, Siderophore

## Abstract

**Background:**

Fungal pathogens of plants produce diverse repertoires of secondary metabolites, which have functions ranging from iron acquisition, defense against immune perturbation, to toxic assaults on the host. The wheat pathogen *Zymoseptoria tritici* causes Septoria tritici blotch, a foliar disease which is a significant threat to global food security. Currently, there is limited knowledge of the secondary metabolite arsenal produced by *Z. tritici*, which significantly restricts mechanistic understanding of infection. In this study, we analyzed the genome of *Z. tritici* isolate IP0323 to identify putative secondary metabolite biosynthetic gene clusters, and used comparative genomics to predict their encoded products.

**Results:**

We identified 32 putative secondary metabolite clusters. These were physically enriched at subtelomeric regions, which may facilitate diversification of cognate products by rapid gene rearrangement or mutations. Comparative genomics revealed a four gene cluster with significant similarity to the ferrichrome-A biosynthetic locus of the maize pathogen *Ustilago maydis*, suggesting this siderophore is deployed by *Z. tritici* to acquire iron. The *Z. tritici* genome also contains several isoprenoid biosynthetic gene clusters, including one with high similarity to a carotenoid/opsin producing locus in several fungi. Furthermore, we identify putative phytotoxin biosynthetic clusters, suggesting *Z. tritici* can produce an epipolythiodioxopiperazine, and a polyketide and non-ribosomal peptide with predicted structural similarities to fumonisin and the *Alternaria alternata* AM-toxin, respectively. Interrogation of an existing transcriptional dataset suggests stage specific deployment of numerous predicted loci during infection, indicating an important role of these secondary metabolites in *Z. tritici* disease.

**Conclusions:**

We were able to assign putative biosynthetic products to numerous clusters based on conservation amongst other fungi. However, analysis of the majority of secondary metabolite loci did not enable prediction of a cluster product, and consequently the capacity of these loci to play as yet undetermined roles in disease or other stages of the *Z. tritici* lifecycle is significant. These data will drive future experimentation for determining the role of these clusters and cognate secondary metabolite products in *Z. tritici* virulence, and may lead to discovery of novel bioactive molecules.

**Electronic supplementary material:**

The online version of this article (doi:10.1186/s12864-017-3969-y) contains supplementary material, which is available to authorized users.

## Background

The fungal pathogen *Zymoseptoria tritici* (previously *Mycosphaerella graminicola*) causes Septoria tritci blotch, an important foliar disease of wheat. Average yield losses attributed to this disease range from 5 to 10% annually, which can rise to 50% in some conditions [1]. Approximately 70% of European fungicides are deployed to control *Z. tritici* [2], which is likely to drive emergence of drug resistance in fungal pathogens of humans [3].

The infectious propagules for *Z. tritici* disease are multicellular, haline pycnidiospores (asexual) or ascospores (sexual), which are dispersed via rain splash or wind. After germination on the leaf surface, polarized hyphae enter the mesophyll through stomatal openings within 12–24 h [4], followed by a 9–16 day asymptomatic phase with very limited fungal growth. Subsequently, there is rapid onset of host cell death, a dramatic increase in fungal biomass, and finally production of spore-bearing reproductive structures (pycnidia) in necrotic plant tissue [5–7]. It was assumed that during the initial asymptomatic phase, a biotrophic interaction occurs, where the fungus acquires nutrients from the host tissue, and consequently, *Z. tritici* has been considered a hemibiotroph (i.e. that the lifecycle consists of both biotrophic and necrotrophic phases). Recent transcriptional profiling [6] has challenged the notion of fungal nutrient acquisition during the asymptomatic phase, suggesting nutrient acquisition predominantly occurs from dead host tissue, and consequently *Z. tritici* may more accurately be classified as a latent necrotroph [7, 8].

With regards to the molecular basis of *Z. tritici* disease, recent work has strongly suggested *Z. tritici* utilizes effector proteins to orchestrate infection. Fungal effectors are small molecular weight, cysteine rich, secreted proteins that manipulate or subvert host immunity. Sequencing of the *Z. tritici* genome revealed hundreds of genes predicted to encode fungal effectors [9], and transcriptional profiling has revealed phase-specific deployment of numerous putative effectors throughout infection, notably during the switch from asymptomatic to necrotrophic stages [6]. Functional characterization has revealed that secreted proteins protect *Z. tritici* pathogen associated molecular patterns from host recognition [10]. Additionally, the small secreted protein encoded by the *AvrStb6* gene is recognized by wheat cultivars carrying the *Stb6* resistance gene [7]. Such gene-for-gene interactions are a product of an evolutionary arms race between pathogen and host, resulting in either effector mediated disease susceptibility or effector triggered immunity. Taken together, these data indicate that small secreted effectors are likely a critical component of the *Z. tritici* virulence arsenal, and much work has been invested in functional analysis of these genes and their encoded products [7, 10–12].

In addition to secreted effectors, plant infecting fungi also utilize a diverse range of secondary metabolites during disease and other lifecycle stages. However, *Z. tritici* secondary metabolites remain largely unexplored, even though they may play crucial roles in the molecular basis of infection. Pathogens from the Dothideomycetes class are known to produce numerous secondary metabolite phytotoxins. For example, the HC-toxin produced by the maize pathogen *Cochliobolus carbonum* is a non-ribosomal peptide that functions as a histone deacetylase inhibitor, which enables pathogen modification of host chromatin structure and gene expression, and ultimately causes host cell death [13]. The AM-toxin produced by *Alternaria alternata* apple pathotype targets plasma membranes and chloroplast function, and is necessary for disease in susceptible cultivars [14]. In addition to toxins, secondary metabolites can play diverse roles during disease [15]. This includes structural functions, for example melanins in condial cell walls, as well as iron acquisition by siderophores, or modulation of host responses by production of plant hormones.

Most fungal secondary metabolites are produced by biosynthetic gene clusters, consisting of key enzymes, such as polyketide synthases (PKSs) or non-ribosomal peptide synthetases (NRPSs), and contiguous genes encoding tailoring enzymes and transporters [16]. Following a drastic increase in the number of available fungal genome sequences and improved bioinformatics tools, it is now possible to postulate the biosynthetic product of some clusters *in silico*. Previously, these *in silico* approaches have often relied solely on homology between key enzymes. For example, a *Z. tritici* gene predicted to encode a PKS (Mycgr3g96592) was hypothesized to catalyze the first step in the biosynthesis of the toxin dothistromin [17]. However, this gene has recently been demonstrated to be involved in melanin biosynthesis using quantitative trait locus mapping [18], highlighting the limitations of approaches based exclusively on key genes. A new approach for *in silico* predictions of secondary metabolite products is based on MultiGene Basic Local Alignment Search Tools (BLASTS), which identify significant conservation of gene cluster loci across the fungal kingdom based on conservation of gene architecture for both key biosynthetic and tailoring genes [19]. Where significant conservation is identified between a predicted cluster in a genome of interest, and a second locus for which the secondary metabolite has been experimentally defined in another species, it is possible to postulate a comparable biosynthetic product [20].

In this study we conducted *in silico* analysis of *Z. tritici* secondary metabolite loci. We firstly used existing bioinformatics pipelines to predict secondary metabolite loci in the sequenced isolate IP0323 [9], identifying 32 putative clusters. These loci are enriched at chromosome subtelomeres, and often contain genes encoding putative metabolite efflux proteins, strongly suggesting a diverse range of secreted metabolites. Subsequently, we used MultiGeneBLASTs to predict biosynthetic products of various clusters, and postulate roles of these metabolites during the *Z. tritici* lifecycle based on existing experimentation in other pathogenic fungi. Finally, we analyzed existing transcriptomic datasets for *Z. tritici* to study expression profiles of the predicted gene clusters during infection.

## Methods

The *Z. tritici* IP0323 genome [9] was retrieved from Genbank (accession number GCA_000219625.1). Secondary metabolite clusters were predicted using AntiSMASH [21] and SMURF [22] based domain searchers. 34 clusters predicted by identification of genes encoding putative biosynthetic enzymes (e.g. polyketide synthases, nonribosomal peptide synthases, or geranylgeranyl diphosphate synthases) and associated genes were identified, which were refined to a total of 32 following manual interrogation of gene content.

A MultiGeneBLAST algorithm [19] was used to detect homologous clusters across all Genbank genomes [23]. MultiGeneBLAST architecture searches were carried out as described previously [20], with the percent identity threshold set to 25%, the synteny weight set to 0 and the maximum intergenic distance set to 110% of the span of the corresponding *Z. tritici* cluster with a minimum bound of 25 kb. Other parameters were set to default. The input for each search was a multiFASTA file of the amino acid sequences of proteins predicted to be encoded by the genes in the region of the *Z. tritici* predicted cluster, erring on the side of AntiSMASH over-inclusion to detect cluster boundaries. Amino acid sequences were received from Ensembl database [24]. Both input and output files for MultiGeneBLAST are given in Additional file 1.

Subtelomeric regions were defined within 300 kb of the chromosome end, an approach used in other analyses of filamentous ascomycetes [25]. Any predicted clusters with one or more genes residing at these loci were considered subtelomeric (Additional file 2).

For assessment of gene cluster co-expression, we mined an existing RNA seq dataset [6] which previously profiled *Z. tritici* gene expression from two in vitro conditions (growth on potato dextrose or Czapek- Dox broth) and during key stages of disease (1, 4, 9, 14, 21 post infection). Only genes with detectable transcripts at all time points were considered. Average fragments per kilobase for each gene per million fragments (FPKM) at each timepoint ([6], Additional file 3) were analyzed using the FunGeneClusteRs programme using default parameters [26]. Only clusters with genes encoding 3 or more co-expressed transcripts were considered co-regulated. This analysis identified a total of 397 genes residing in 99 contiguous clusters, which were then mapped to the predicted secondary metabolite loci (Additional file 3).

Predicted paralogues for putative secondary metabolite genes were retrieved from the Ensemble database, which were limited to same-species paralogies [27]. Only paralogues that also resided in secondary metabolite loci were further analyzed.

## Results and discussion

### The *Z. tritici* genome contains 32 putative secondary metabolite clusters that are enriched at subtelomeric loci

Analysis of the *Z. tritici* genome identified numerous putative secondary metabolite clusters containing a predicted 682 genes (Table 1 and Additional file 4). When comparing both AntiSMASH and SMURF genome analysis pipelines (Table 1), AntiSMASH resulted in a greater number of clusters (33 vs 19), containing a greater number of total genes (669 vs 143) respectively, which is consistent with other studies [28]. SMURF identified only a single cluster that was not predicted by AntiSMASH (cluster 13, Table 1). While it is likely that these approaches overestimate the number of genes which are resident in secondary metabolite biosynthetic clusters [20], we did not manually curate cluster boundaries for two reasons. Firstly, we reasoned that for subsequent MultiGeneBLAST analysis, large cluster boundaries would maximize the chance of identifying homologous clusters from other species with a defined biosynthetic product. Secondly, fungal genomes are known to contain secondary metabolite super clusters composed of >50 genes [29].

**Table 1:** Predicted secondary metabolite loci in *Z. tritici*

Cluster number	Secondary metabolite class	Predicted key biosynthetic gene(s)	Predicted transporter	Resident transcription factor	Subtelomeric	Evidence of transcriptional co-expression?	AntiSMASH		SMURF	
							Cluster boundary	No. of genes	Cluster boundary	No. of genes
1	NRPS-Like	Mycgr3G107072	YES	NO	NO	NO	1:2,172,553:2,212,738	12	1:2,189,682:2,216,244	8
2	Transporter	Na	YES	NO	NO	NO	1:2,306,586:2,310,597	2	Na	Na
3	Terpene	Mycgr3G34236	NO	NO	NO	YES	1:2,366,583:2,438,578	28	Na	Na
4	Terpene	Mycgr3G33174	YES	NO	NO	NO	1:3,518,416:3,535,837	9	Na	Na
5	PKS	Mycgr3G83965	NO	NO	NO	NO	1:3,821,874:3,860,702	14	Na	Na
6	NRPS	Mycgr3G16590	YES	YES	NO	NO	1:5,128,643:5,192,491	23	Na	Na
7	PKS	Mycgr3G67477	YES	YES	NO	YES	1:5,513,274:5,566,181	17	1:5,524,934:5,578,932	20
8	PKS	Mycgr3G9788	YES	YES	YES	YES	2:31,258:149,728	37	2:123,001:157,676	11
9	NRPS	Mycgr3G19958	NO	NO	YES	NO	2:222,377:310,014	19	2:260,654:284,183	5
10	NRPS	Mycgr3G36951	YES	NO	NO	NO	2:420,337:471,819	14	2:433,742:453,243	2
11	NRPS	Mycgr3G90558	YES	NO	NO	NO	2:1,008,626:1,058,615	13	2:1,026,842:1,052,480	6
12	Terpene	Mycgr3G99148	NO	YES	NO	YES	2:1,934,751:2,046,004	31	Na	Na
13	PKS-Like	Mycgr3G39149	YES	NO	YES	YES	Na	Na	3:3,181,477:3,213,160	13
14	NRPS	Mycgr3G39931, Mycgr3G40534	YES	NO	YES	NO	4:12,877:43,949	7	Na	Na
15	PKS	Mycgr3G100089	YES	YES	YES	YES	5:85,463:149,465	22	5:94,206:129,292	12
16	NRPS-Like	Mycgr3G93235, Mycgr3G100227	NO	YES	NO	YES	5:1,291,545:1,380,182	31	5:1,309,417:1,324,422	4
17	PKS	Mycgr3G72709	YES	YES	YES	YES	6:72,339:170,251	36	6:106,974:121,853	4
18	NRPS	Mycgr3G72768	YES	NO	YES	YES	6:283,399:352,537	24	6:310,922:346,260	11
19	NRPS	Mycgr3G44313	NO	NO	NO	YES	6:1,808,394:1,845,239	16	6:1,821,703:1,841,259	9
20	NRPS	Mycgr3G109989	YES	YES	NO	NO	6:2,243,873:2,278,779	9	Na	Na
21	PKS	Mycgr3G45348	YES	NO	YES	NO	7:2,566,479:2,608,068	12	7:2,577,096:2,606,813	8
22	NRPS	Mycgr3G110642	YES	NO	YES	NO	8:2,346,378:2,374,211	8	8:2,352,913:2,362,665	2
23	NRPS-Like	Mycgr3G75370	YES	YES	NO	YES	9:483,924:714,190	65	Na	Na
24	PKS	Mycgr3G47832	YES	YES	NO	NO	9:1,474,679:1,516,611	12	9:1,494,054:1,506,383	3
25	Terpene	Mycgr3G76129	YES	YES	NO	NO	10:341,276:419,193	29	Na	Na
26	Hybrid PKS- NRPS	Mycgr3G62978	YES	NO	NO	YES	10:425,702:485,734	20	10:439,622:460,865	6
27	PKS	Mycgr3G101493	YES	NO	YES	YES	10:1,274,562:1,480,511	54	10:1,436,111:1,437,155	7
28	NRPS-Like	Mycgr3G49555	NO	NO	NO	YES	11:393,046:429,601	12	11:409,058:423,584	5
29	PKS	Mycgr3G96592	NO	YES	NO	NO	11:566,694:611,143	14	Na	Na
30	NRPS-Like	Mycgr3G50095	YES	YES	NO	NO	11:823,532:917,882	30	11:849,800:868,675	7
31	NRPS	Mycgr3G96900	YES	NO	YES	YES	12:157,368:195,467	15	Na	Na
32	NRPS-Like	Mycgr3G77312	NO	NO	NO	NO	12:434,096:471,899	9	Na	Na
33	PKS	Mycgr3G51018	NO	NO	NO	YES	13:656,107:712,077	17	Na	Na
34	Terpene	Mycgr3G101921, Mycgr3G12838	YES	NO	YES	NO	13:1,116,277:1,139,970	8	Na	Na

AntiSMASH and SMURF analysis pipelines were used to predict secondary metabolite clusters in *Z. tritici* isolate IP0323, identifying a total of 34 putative loci. Ensembl gene identifiers for predicted key biosynthetic genes identified by these analyses are given. Clusters were numbered in ascending numerical order based on their respective location in the *Z. tritici* genome. In order to identify genes encoding ABC or MFS transporters at each locus, genes were interrogated based on GO-terms GO:0055052 (ATP-binding cassette transporter complex) and GO:0055085 (transmembrane transporter). Transcription factors were identified by screening genes for GO-terms GO:0003677 (DNA binding) and GO:0003700 (transcription factor activity). Subtelomeric clusters were defined as any loci with predicted genes residing within 300 kb of the chromosome end. Interrogation of each individual cluster suggests the key gene of cluster 13 encodes a ketoacyl synthase domain-containing protein, and consequently is likely involved in fatty acid synthesis. Cluster 2 consists of two transport proteins, and lacks a gene encoding a putative key biosynthetic enzyme

Genes predicted to encode key biosynthetic enzymes resident in each cluster include 10 NRPSs, 6 NRPS-like enzymes, 10 PKSs and 1 hybrid PKS-NRPS (Table 1). Further interrogation of individual clusters suggested that the PKS of cluster 13 is actually a ketoacyl synthase domain-containing protein, and consequently is probably involved in fatty acid synthesis rather that production of a secondary metabolite. Our analysis also identified an additional 2 gene locus containing genes encoding ferric reductase like transmembrane transporters (cluster 2), which lacked any key biosynthetic genes. Consequently, clusters 2 and 13 are unlikely to be involved in secondary metabolite biosynthesis, giving a total of 32 predicted secondary metabolite biosynthetic clusters in the *Z. tritici* IP0323 genome. We did not identify any dimethylallyl tryptophan synthases (DMATs) which suggests *Z. tritici* does not produce any DMAT derived alkaloids (e.g. ergot alkaloid [30]). However, we identified 5 clusters with putative roles in isoprenoid biosynthesis based on the presence of genes predicted to encode geranylgeranyl diphosphate synthases and other key enzymes (Table 1).

The majority of the 32 putative clusters (*n* = 22) contain genes encoding a predicted ATP-binding cassette (ABC) transporter and/or major facilitator superfamily (MFS) transporter (Table 1 and Additional file 5). These are the main classes of transporters responsible for secondary metabolite efflux from fungal cells [31], and are often contiguously clustered with genes necessary for product biosynthesis [32, 33]. Our data therefore suggests that the products of these 22 gene clusters are extracellular, and consequently may biosynthesize molecules which mediate host-pathogen interactions during infection*.*

We identified 13 clusters that contain a predicted transcription factor (Table 1 and Additional file 6). Regulation of secondary metabolite gene expression in filamentous fungi is an multifaceted integrated system composed of epigenetic regulators, such as the velvet complex, which function at the level of chromatin remodeling, global transcription factors, including StuA and PacC, which link gene expression to development or environmental changes, and cluster-specific transcription factors which are physically located in respective clusters and control transcription of contiguous biosynthetic genes [34]. The velvet complex has been demonstrated to regulate secondary metabolism in *Z. tritici* [35], and our analyses has identified several genes that are likely important components for comprehensive understanding of cluster regulation in this pathogen.

**Fig. 1:**
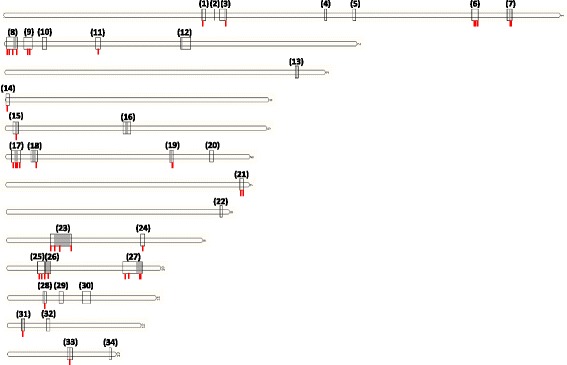
Physical distribution of predicted secondary metabolite clusters on the *Z. tritici* genome. Putative loci on the 13 *Z. tritici* core chromosomes are shown, and no clusters were predicted on the 8 dispensable chromosomes. Manual interrogation of clusters 2 and 13 demonstrated they are unlikely to biosynthesize a secondary metabolite (see main text), resulting in a total of 32 putative secondary metabolite clusters. Line boxes indicate boundaries of putative secondary metabolite cluster as predicted by AntiSMASH. Numbers in parentheses are the cluster number (Table 1). Shaded regions are loci that demonstrate evidence of gene co-regulation in transcriptional profiling. Red lines below secondary metabolite loci indicate regions containing genes that have one or more paralogues that also reside within a predicted biosynthetic gene cluster

With regards to physical distribution on the *Z. tritici* chromosomes (Fig. 1), all 32 clusters are located on core chromosomes [9], with 11 located at subtelomeric loci, representing 34% of the predicted clusters, and containing 36% of the putative secondary metabolite genes (Table 1). Given that the total number genes residing at subtelomeric loci in *Z. tritici* is 2501, or 22.8% of the genome (Additional file 2), our analysis suggests that subtelomeres and telomere proximal regions are enriched with secondary metabolite gene clusters, an observation consistent with the genomes of other ascomycetes [25, 36]. Subtelomeres of filamentous fungi are rich in repeat regions and transposable elements, and consequently undergo frequent chromosomal rearrangements. Additionally, repeat regions can lead to DNA polymerase ‘slippage’, resulting in elevated mutations in gene coding sequences when compared to telomere distal regions [37]. This had led to the duplication, diversification and differential gene loss (DDL) hypothesis, which suggests that subtelomeres are important for rapid evolution, gene expansion, and niche adaptation [38]. DDL events at subtelomeric secondary metabolite loci might result in novel biosynthetic products that could conceivably enhance *Z. tritici* virulence or expand pathogen host range. We therefore interrogated gene duplication amongst putative *Z. tritici* secondary metabolite loci in order to test if these events are more frequent at subtelomeres. We identified 72 genes residing in predicted biosynthetic gene clusters that had at least one or more paralogues at other secondary metabolite loci (Fig. 1 and Additional file 7). In support of the DDL hypothesis, 44% of these genes (*n* = 32) resided in subtelomeric loci, which represents enrichment of gene duplication relative to the total amount of secondary metabolite genes that are found at these loci (i.e. 36%). In several instances, local gene duplication events were found within specific subtelomeric clusters (Additional file 8). Other studies have also supported DDL in *Z. tritici*. For example, gene diversification is evidenced by the discovery of the avirulence gene *AvrStb6*, which resides in the subtelomere of chromosome 5, and encodes a secreted effector with numerous single nucleotide polymorphisms among tested isolates [7]. Additionally, PKS genes from clusters 7 and 8 (Table 1) are absent in certain field isolates, indicating gene loss also occurs at telomere proximal and subtelomeric loci [39]. Consequently, *Z. tritici* secondary metabolite clusters identified in this study are likely undergoing DDL processes, which may be more frequent at subtelomeric loci. Ultimately, this may result in modification or loss of cognate secondary metabolite products, which could result in enhanced virulence or expand pathogen cultivar or host range.

In order to postulate putative cluster products from the identified loci we conducted MultiGeneBLAST analyses of all clusters across the genomes present in the Genbank archive. This identified several clusters with high homology to loci in other fungi for which the biosynthetic product has been experimentally determined, allowing us to predict several metabolites that are produced by *Z. tritici*.

### Putative ferrichrome A biosynthetic gene cluster

**Fig. 2:**
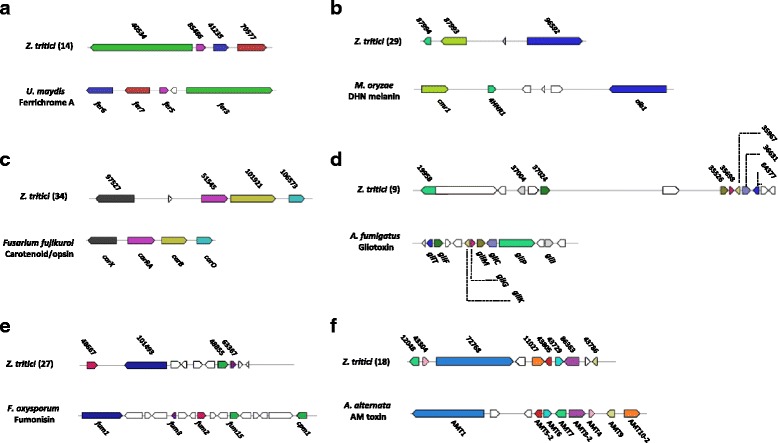
Schematic representation of conserved gene architecture between predicted *Z. tritici* loci and clusters from other fungi (**a**-**f**). Predicted gene boundaries are depicted by arrows and assigned Ensemble identifiers (*Z. tritici*) or gene names (other species). Identical colors between top and bottom loci are indicative of significant similarity at the level of encoded amino acid sequence (see Table 2). Non-colored arrows are predicted genes that lack an orthologue at the other respective locus. Numbers in parentheses are *Z. tritici* cluster number (see Table 1)

We identified a putative cluster (number 14, Table 1, Fig. 2a) which had significant similarity to the ferrichrome A biosynthetic locus (a total of 4 conserved genes) found in the basidiomycete pathogen of maize, *Ustilago maydis* [40]. Many fungi utilize small molecular weight, high-affinity iron-chelating NRPs termed siderophores for both internal iron storage and uptake from the external environment [41]. The most common fungal siderophore types are of the hydroxamate class, and include ferrichromes, coprogens or fusarinines.

In *U. maydis*, ferrichrome A production firstly requires synthesis of the precursor hydroxymethylglutaryl-CoA (HMG-CoA), by the HMG-CoA synthase Hcs1 [40]*. Z. tritici* also contains an orthologue of this gene (Mycgr3G54740, Table 2), and as in *U. maydis*, this gene is not part of the contiguous cluster. Comparable architecture between the *U. maydis* ferrichrome A biosynthetic gene cluster [40] and cluster 14 identified in this study included genes encoding the NRPS Fer3 and acylase Fer5, both of which are essential for production of this siderophore (Table 1 and Fig. 2a). Additionally, we found genes predicted to encode a multidrug resistance protein (*fer6*) and a siderophore transporter (*fer7*) which currently have uncharacterized functions but are co-regulated during iron perturbation in *U. maydis* [40, 42]. Interestingly, the enoyl-CoA reductase encoding gene *fer4*, which is essential for biosynthesis of this metabolite in *U. maydis*, is not present in the putative *Z. tritici* cluster, although there are orthologues of this gene at other genomic loci (e.g. Mycgr3G76805, Table 2), an observation that may reflect DDL events due to this clusters subtelomeric locus. These data suggest that despite this deviation in cluster architecture between these species, the *Z. tritici* genome still contains the necessary gene repertoire for biosynthesis of a ferrichrome A-like siderophore.

**Table 2:** BLAST analyses of predicted *Z. tritici* secondary metabolite loci across the Genbank sequence archive

Putative cluster in *Z. tritici*				Hit cluster with experimentally defined biosynthetic product				BLAST Results		
Putative product (cluster number)	Ensembl Gene ID	GenBank ID	Annotation in *Z. tritici*	Species Name	Ensembl Gene ID	GenBank ID	Annotation	% similarity	Sequence coverage	E value
Siderophore (14)	Mycgr3G40534	EGP88586	Non-ribosomal peptide synthetase	*Ustilago maydis*	UMAG_01433	23562457	Ferrichrome siderophore peptide synthetase *fer3*	48%	82%	8,00E-61
	Mycgr3G85486	EGP87766	putative siderophore biosynthesis protein		UMAG_01432	23562456	Putative lysine N-acyltransferase *fer5*	43%	89%	8,00E-103
	Mycgr3G41235	EGP87768	putative ABC transporter		UMAG_01431	23562455	Multidrug resistance-associated protein 1 *fer6*	42%	100%	0,00E + 00
	Mycgr3G70577	EGP87767	putative siderophore-dependent iron transporter		UMAG_01439	23562463	Siderophore iron transporter 3 *fer7*	39%	93%	3,00E-114
	(Mycgr3G76805)		Hypothetical protein		UMAG_01433		putative enoyl-CoA hydratase/isomerase *fer4*	39%	83%	2e-46
	(Mycgr3G5470)		HMG-CoA synthase		(UMAG_05362)		HMG-CoA synthase	54%	98%	3,00E-180
DHN melanin (29)	Mycgr3G87993	EGP83310	Hypothetical protein	*Magnaporthe oryzae*	MGG_07215	EHA55622	Transcription factor *cmr1*	40%	100%	0,00E + 00
	Mycgr3G87994	EGP83311	Hypothetical protein		MGG_07216	EHA55623	Versicolorin reductase 4HNR	48%	97%	2,00E-73
	Mycgr3G96592	EGP83620	Polyketide synthase		MGG_07219	EHA55627	Conidial pigment polyketide synthase *alb1*	45%	100%	0,00E + 00
Carotenoid (34)	Mycgr3G97527	EGP82655	Hypothetical protein	*Fusarium fujikuroi IMI 58289*	FFUJ_11801	CCT75764	related to lignostilbene alphabeta-dioxygenase I	57%	90%	0,00E + 00
	Mycgr3G51545	EGP82649	Hypothetical protein		FFUJ_11802	CCT76070	probable geranylgeranyl-diphosphate geranylgeranyltransferase	53%	99%	0,00E + 00
	Mycgr3G101921	EGP82650	Hypothetical protein		FFUJ_11803	CCT75765	probable phytoene dehydrogenase AL-1	58%	96%	0,00E + 00
	Mycgr3G106573	EGP82651	Hypothetical protein		FFUJ_11804	CCT75766	related to HSP30 heat shock protein Yro1p	68%	97%	0,00E + 00
	(Mycgr3G72713)		Hypothetical protein		(FFUJ_07962)	AM418467.1	Torulene oxygenase	41%	95%	2,00E-130
ETP (9)	Mycgr3G19958	EGP89696	Hypothetical protein	*Aspergillus fumigatus*	AFUA_6G09660	EAL88817	NRPS GliP	28%	100%	1,00E-146
	Mycgr3G37004	EGP89694	aminocyclopropane-1-carboxylate synthase-like protein		AFUA_6G09640	EAL88815	Aminotransferase gliI	34%	96%	4,00E-61
	Mycgr3G37024	EGP90779	putative P450 monooxygenase		AFUA_6G09730	EAL88824	cytochrome P450 oxidoreductase GliF	51%	97%	3,00E-168
	Mycgr3G35526	EGP90781	putative O-methyltransferase		AFUA_6G09680	EAL88819	O-methyltransferase GliM	42%	90%	2,00E-102
	Mycgr3G35698	EGP90782	putative glutathione S-transferase		AFUA_6G09690	EAL88820	glutathione S-transferase GliG	41%	87%	7,00E-55
	Mycgr3G35967	EGP89693	gliotoxin biosynthesis protein		AFUA_6G09700	EAL88821	gliotoxin biosynthesis protein GliK	35%	96%	1,00E-41
	Mycgr3G36631	EGP90783	putative P450 monooxygenase		AFUA_6G09670	EAL88818	Cytochrome P450 oxidoreductase gliC	34%	96%	7,00E-94
	Mycgr3G84377	EGP89692	putative pyridine nucleotide-disulfide oxidoreductase		AFUA_6G09740	EAL88825	Thioredoxin reductase gliT	44%	99%	3,00E-82
Fumonisin (27)	Mycgr3G101493	EGP83950	Polyketide synthase	*Fusarium oxysporum* FRC O-1890		ACB12550	Fum1 polyketide synthase	36%	87%	0,00E + 00
	Mycgr3G63387	EGP84006	Hypothetical protein			ACB12552	Fum3 cytochrome p450	52%	90%	8,00E-110
	Mycgr3G48687	EGP84002	putative P450 monooxygenase			ACB12551	Fum2 cytochrome p450	36%	97%	2,00E-79
	Mycgr3G48855	EGP84005	putative P450 monooxygenase			ACB12560	Fum15 cytochrome p450	28%	100%	4,00E-59
	Mycgr3G48855	EGP84005	putative P450 monooxygenase			ACB12565	Cpm1 cytochrome p450	38%	98%	3,00E-118
AM-toxin (18)	Mycgr3G72768	EGP86944	Hypothetical protein	*Alternatia alternata*		BAI44759	nonribosomal peptide synthetase AMT1	40%	84%	0,00E + 00
	Mycgr3G43805	EGP86331	Hypothetical protein			BAI44762	similar to branched-chain-amino-acid AMT5-2aminotransferase	55%	98%	5,00E-31
	Mycgr3G43729	EGP86946	Hypothetical protein			BAI44741	similar to 3-isopropylmalate dehydrogenase AMT6	60%	95%	3,00E-150
	Mycgr3G12048	EGP86333	Hypothetical protein			BAI44764	similar to 2-isopropylmalate synthase AMT7	58%	95%	0,00E + 00
	Mycgr3G86363	EGP86330	Hypothetical protein			BAI44765	aconitase family protein	58%	100%	0,00E + 00
	Mycgr3G43304	EGP86943	Hypothetical protein			BAI44766	thioesterase domain containing protein AMT4	38%	89%	2,00E-43
	Mycgr3G43786	EGP86329	Hypothetical protein			BAI44767	similar to methyltransferase AMT9	28%	100%	2,00E-13
	Mycgr3G11027	EGP86945	Hypothetical protein			BAI44768	nonribosomal peptide synthetase AMT10–2	44%	100%	0,00E + 00

MultiGeneBLAST analyses of predicted cluster loci (Table 1) were conducted across all available sequences in the Genbank archive (Clark et al. 2016). Loci with statistically significant similarity to *Z. tritici* query sequences were manually interrogated to identify biosynthetic gene clusters for which the secondary metabolite product has been experimentally confirmed. Results for statistically significant BLAST alignment between predicted amino acid sequences encoded by genes at each locus are reported. Gene identifiers in parentheses are not contiguously located at each respective cluster, but are necessary for biosynthesis of the experimentally confirmed product (see main text for details). These genes were identified by individual protein BLAST searches between respective genomes using the NCBI BLAST tool. Both Genbank and Ensembl gene identifiers are given where available. Gene annotations were retrieved from Ensembl, or where unavailable from Genbank

Residence of several transporters within *Z. tritici* cluster 14 (Mycgr3G99766, Mycgr3G41235) suggests that the putative siderophore product is also likely to be extracellular. In *U. maydis*, localization studies using fluorescently labeled siderophore analogs suggests ferrichrome A may function as both an extracellular and intracellular siderophore for iron scavenging and storage respectively [43]. Given this dual role in *U. maydis*, the putative ferrichrome A-like NRP produced by this locus in *Z. tritici* may also function as an extra and intracellular siderophore.

Our data suggests that *Z. tritici* has an unusual iron scavenging and/or storage strategy when compared to many pathogenic Dothideomycetes, which use the intracellular siderophore ferricrocin for iron storage and the extracellular siderophore triacetyl fusarine C (TAFC) for iron acquisition from the external environment [44, 45]. Indeed, previous comparative genomic analyses of 18 Dothideomycetes fungi revealed that *Z. tritici* is unique in lacking orthologues of the NRPS encoding genes required for ferricrocin and TAFC biosynthesis [46]. Our analysis supports these data, as we did not identify putative biosynthetic clusters for siderophores ferricrocin or TAFC. With regards to postulating a biological explanation for the lack of common Dothideomycetes siderophores in *Z. tritici*, it has recently been suggested that these structurally conserved, extracellular molecules may function as pathogen associated molecular patterns (PAMPs) which are recognized by host pattern recognition receptors (PRRs), resulting in subsequent activation of immune responses. Indeed, bacterial ferritin, and more recently fungal coprogen, have been demonstrated to activate host immunity in *Arabidopsis thaliana* and maize, respectively [47, 48]. Consequently, the absence of a TAFC biosynthetic gene cluster in *Z. tritici* may confer a selective advantage due to avoidance of host immune activation.

### Putative dihydroxynaphthalene melanin biosynthetic gene cluster

Our analyses identified a 14 gene cluster (cluster number 29, Table 1) containing a 3 gene sub-cluster which had significant similarity to putative or experimentally confirmed dihydroxynaphthalene (DHN) melanin biosynthetic gene clusters in numerous plant pathogens, including *Cochliobolus heterostrophus*, *Bipolaris maydis, Leptosphaeria maculans*, and *Magnaporthe oryzae* (Fig. 2b). This is the best studied secondary metabolite locus in *Z. tritici*, and our data is consistent with recent work by Lendenmann and colleagues, who used quantitative trait locus mapping to suggest that several genes required for DHN melanin biosynthesis reside at this locus [18]. Cluster architecture analysis identified a PKS encoding gene (Mycgr3G96592) with significant similarity to the *M. oryzae alb1* (Table 2, a total of 3 conserved genes). In the DHN melanin polyketide biosynthetic pathway this PKS synthesizes 1,3,6,8 tetrahydroxynaphthalene (1,3,6,8-THN) from acetyl-CoA and malonyl-CoA precursors [49]. Additionally, an orthologue for the *M. oryzae* tetrahydroxynaphthalene melanin reductase (4HNR, Table 2) required for reduction of 1,3,6,8-THN to form scytalone [50] is also present in this cluster (Fig. 2b and Table 2). Finally, the cluster also contains a gene encoding an orthologue of the transcription factor *cmr1* (Table 2, Fig. 2b), which regulates melanin production in several pathogenic fungi [51]. Interestingly, ∆*cmr1* strains in *M. oryzae* and *Colletotrichum lagenarium* were able to produce melanin in appressoria but not vegetative hyphae [51], and consequently we speculate this transcription factor may also regulate development and melanization in *Z. tritici.* Our analysis did not identify clusters responsible for the biosynthesis of other melanins in fungi, such as pyomelanin [52], suggesting that DHN-melanin might be the only melanin biosynthesized by this pathogen. This is consistent by work by Beltrán-García and colleagues, who demonstrated DHN-melanin was the only melanin in mycelium and culture filtrate of the closely related pathogen of banana *Mycosphaerella fijiensis* [53].

With regards to postulating a function of *Z. tritici* DHN melanin, in other fungi this molecule provides protection from various environmental stresses, such as antifungal agents, UV light, heavy metals, extreme temperatures and reactive oxygen species [54–56]. DHN melanin also plays diverse roles in fungal virulence. In *M. oryzae*, it is required for the high osmotic pressure in appressoria and consequently host penetration [57]. In the fungal pathogen of humans, *Aspergillus fumigatus*, DHN melanin inhibits acidification of phagolysosomes [58], and may shield pathogen associated molecular patterns from host pattern recognition receptors [59]. In addition to structural or defensive roles during fungal disease, recent work in *M. fijiensis* demonstrated that DHN-melanin generates highly reactive oxygen species that may facilitate host cell death [53]. We therefore predict that this cluster has important implications for *Z. tritici* infection.

### Putative carotenoid and opsin biosynthetic gene cluster

Our analysis also identified a putative carotenoid biosynthetic gene cluster in *Z. tritici* (cluster 34, Table 1), which is highly conserved in a variety of plant pathogens, including *Botryotinia fuckeliana*, *L. maculans*, *C. heterostrophus*, and *Fusarium fujikuroi*, amongst others. Carotenoid pigments may protect fungi from UV stress, and are also important intermediates for the biosynthesis of physiologically active apocarotenoids, such as retinal [60]. This cluster has been well characterized in *F. fujikuroi* (Fig. 2c, a total of 4 conserved genes), and contains genes encoding a bifunctional phytoene synthase/carotene cyclase (*carRA*) and a phytoene dehydrogenase (*carB*), which are required for biosynthesis of the red carotenoid torulene [61, 62]. Both these genes are conserved in cluster 34 (Fig. 2c), suggesting that this pigment may be part of the secondary metabolite content of *Z. tritici*, which may account for the light red/pink color of *Z. tritici* conidia when grown on rich agar. In subsequent secondary metabolic steps in both *F. graminearum* and *F. fujikuroi*, a carotenoid oxygenase that is not physically linked in the cluster, termed *carT*, converts the torulene precursor to neurosporaxanthin [63]. The hypothesis that *Z. tritici* may also produce a similar carotenoid is supported by the presence of a *carT* orthologue in the *Z. tritici* genome (Mycgr3G72713, Table 2). With regards to biosynthesis of physiologically active apocarotenoids, in *F. fujikuroi* this cluster is required for rential biosynthesis, containing genes encoding an opsin like protein (*carO*), and retinal synthesizing enzyme (*carX*) [64, 65]. Orthologues for both these genes are present in the corresponding *Z. tritici* cluster (Table 2 and Fig. 2c). Opsins are transmembrane proteins that bind retinal via a conserved lysine residue [65], and light mediated isomerization of retinal enables the opsins to act as light receptors at the fungal membrane. While *Z. tritici* light responses have not been comprehensively determined, the link between light and secondary metabolism has already been demonstrated, as the production of aerial mycelium in vitro is light dependent, and is regulated by the velvet complex, which also controls melanin production [35]. Taken together, we predict that cluster 34 (Table 1) may be multifunctional, biosynthesizing a torulene-like carotenoid pigment and light responsive opsin/chromophore, and ultimately this locus may co-ordinate light responses and pigment production in *Z. tritici*.

### Putative epipolythiodioxopiperazine biosynthetic gene cluster

Cluster 9 contains a total of 19 predicted genes, including a NRPS, and was highly comparable to epipolythiodioxopiperazine (ETP) biosynthetic clusters from numerous fungi. This included the gliotoxin and sirodesmin PL biosynthetic loci in *A. fumigatus* and *L. maculans*, with 8 and 6 conserved genes respectively (Fig. 2d and Additional file 1) [66, 67]. Both gliotoxin and sirodesmin PL are ETPs, which consist of a diketopiperazine core and contain a disulphide bridge [33]. This latter motif is important for ETP toxicity towards both plants and mammals, as it is required for protein-ETP conjugates [68]. For example, the presence of a *Z. tritici* orthologue for the thioredoxin reductase *gliT*, which is required for disulphide bridge formation during gliotoxin biosynthesis, suggests that this key moiety is also present on the putative *Z. tritici* ETP [69]. Interestingly, we did not find any gene encoding a putative toxin efflux pump or transporter in this cluster (Tables 1 and 2). Toxin efflux pumps are present in 15 out of 16 ETP clusters analyzed in filamentous ascomycetes [33]. In *A. fumigatus*, for example, the transporter is encoded by the *gliA* gene which is required for efflux of gliotoxin [32]. The absence of an orthologous gene in the *Z. tritici* cluster, and any putative transporters in the 18 predicted tailoring genes, suggests that the ETP may be intracellular. Consequently, the predicted ETP encoded by this locus in *Z. tritici* may not primarily function as a phytotoxin. Interestingly, ETPs have been shown to potently reduce H_2_0_2_ [70], one of the major reactive oxygen species encountered by *Z. tritici* in the host [71], and consequently, this putative ETP may act a defensive molecule during infection. Similar defensive functions related to detoxification have also been postulated for presumed toxins, including the carcinogenic polyketide aflatoxin [72].

With regards to regulation of this cluster, we did not identify a resident transcription factor (Table 1). Out of 16 ETP clusters surveyed amongst ascomycetes, resident transcription factors are only absent in *A. terreus* [33]. In *A. fumigatus*, for example, the resident transcription factor GliZ regulates genes expression of the ETP cluster and is essential for toxin biosynthesis [73]*.* These data suggest that in Z. *tritici* ETP gene expression does not rely on regulation by a resident transcription factor. Consequently global regulators of secondary metabolite biosynthesis, such as the transcription factor StuA or the velvet complex, may play important roles in regulation of this cluster in *Z. tritici* [34].

### Putative fumonisin biosynthetic gene cluster

The second largest cluster predicted by AntiSMASH analysis was number 27, with 54 putative genes surrounding a PKS (Mycgr3G101493, Table 1). SMURF predicted a considerably smaller cluster of 7 genes containing the same key enzyme. MultiGeneBLAST analysis demonstrated significant similarity of this locus to the fumonisin biosynthetic gene cluster in *Fusarium oxysporum* (a total of 4 conserved genes, Fig. 2e and Table 2) [74], in addition to predicted fumonisin clusters in various other species, including *Neosartorya fischeri*, *A. fumigatus* and *Aspergillus niger*. Fumonisins are a structurally diverse class of linear, 19–20-carbon backbone mycotoxins which cause significant crop contamination, and the genetics of their biosynthesis have been well characterized in various *Fusarium* species [75].

Interestingly, the *Z. tritici* cluster has some deviations from those of Fusarium spp., most notably the absence of genes encoding the oxoamine synthase Fum8 and the P450 monooxygenase Fum6, which are predicted to catalyze the second and third biosynthetic steps respectively [75], and both of which are essential for fumonisin production in *F. verticillioides* [76]. However we found significant homology between *Z. tritici* Mycgr3G101493 and PKS *fum1* (Table 2), which catalyzes the condensation of two methyl and nine acetate units to produce a linear polyketide in the first step in fumonisin biosynthesis in Fusarium spp. [75]. Additionally, we predict *Z. tritici* orthologues at this locus for *fum2* and *fum3*, which hydroxylate C-10 and C-5 in the fifth and final steps of fumonisin B1 biosynthesis [77]. Finally, a single *Z. tritici* gene had significant conservation with two cytochrome p450 encoding genes in the *Fusarium* cluster (*fum15* and *cpm1*) which presumably function to hydroxylate an as yet unknown carbon [75]. We therefore predict that *Z. tritici* produces a PKS which might be structurally similar to fungal fumonisins. This is of particular interest for necrotrophic pathogens, as fumonisins can induce plant cell death by depletion of extracellular ATP [78]. It is interesting to speculate that the product of this cluster may contribute to virulence by causing host cell death, a hypothesis supported by transcriptional upregulation of the PKS during necrotrophic phases of infection relative to laboratory culture [6].

### Putative AM-toxin biosynthetic gene cluster

Our analysis also identified a putative secondary metabolite locus in the subtelomeric region of chromosome six (cluster number 18, Table 1) with significant similarity to the AM-toxin biosynthetic gene cluster from the apple pathotype of *A. alternata* [14, 79]. Gene architecture between these two loci was highly conserved, with a total of 8 orthologous genes found at *Z. tritici* cluster and corresponding *A. alternata* locus (Fig. 2f and Table 2). This included the NRPS encoding gene *amt1*, which is essential for AM-toxin biosynthesis [79]. Although the functions of all genes within this cluster have not been elucidated in *A. alternata*, they are transcriptionally co-induced under AM-toxin producing conditions [80], and encode proteins associated with secondary metabolite biosynthesis, including thioesterases, methyltransferases, and dehydrogenases (Table 2). In *Z. tritici*, the cluster also includes two predicted transporters (Table 1) strongly suggesting that this metabolite is secreted. Interestingly, our analysis revealed poor conservation of this cluster in Dothideomycetes outside the *Mycosphaerella* genus (Additional file 1). In *A. alternata*, several toxin biosynthetic gene clusters, including the AM-toxin locus, reside on small (1.1–1.8 Mb) supernumerary chromosomes [80]. The biosynthetic products of these clusters are host specific toxins (HSTs) which are necessary for virulence of the various pathotypes, including apple, pear, strawberry or tangerine, but dispensable for normal development, growth, and cell viability [14]. *amt1* null mutants, for example, cannot produce the AM-toxin and are unable to cause disease symptoms on susceptible apple cultivars [79]. It has been suggested that supernumerary chromosomes are horizontally transferred across pathotypes and therefore facilitate host-range expansion [81]. Our data is indicative of horizontal gene transfer of this cluster between *Z. tritici* and *A. alternata*, and we postulate that *Z. tritici* produces a secondary metabolite similar to the *A. alternata* AM-toxin. With regards to predicting the structure and possible mechanism of action of this molecule, the AM-toxin is a cyclic depsipeptide with two sites of action, firstly, causing invagination and electrolyte loss across host plasma membranes and secondly membrane perturbation in choloroplasts, resulting in reduced chlorophyll content and photosynthesis [14]. It is interesting to speculate that this cluster in *Z. tritici* may biosynthesize an NRP with a similar structure and/or mechanism of action, which could conceivably be required for virulence and host or cultivar specificity.

### Gene expression analysis of predicted secondary metabolite loci

**Fig. 3:**
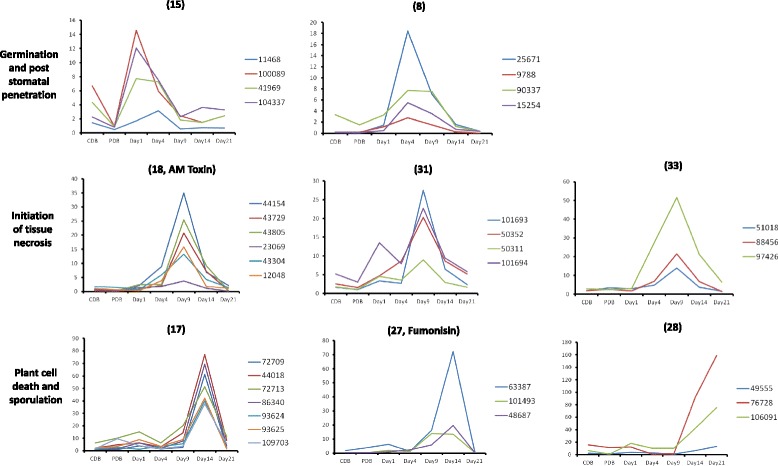
Transcriptional analysis reveals stage specific expression of numerous predicted secondary metabolite loci during infection. Numbers in parentheses indicate cluster number from this study (Table 1). Transcriptional values (y-axis) are average fragments per kilobase for each gene per million fragments (FPKM), with the Ensemble gene identifier numbers given. Data were taken from Rudd and colleagues Additional file 3: Table S3 ([6]). In this study, RNA samples were isolated from Czapek-Dox broth (CDB), potato dextrose broth (PDB), and from various days post inoculation in a virulence assay of IP0323 on ‘Riband’ wheat cultivar. We identified co-expressed contiguous loci using the FunGeneClusterS web interface [26]

We analyzed an existing RNA seq dataset [6] to determine if predicted secondary metabolite loci from our study demonstrated co-expression during in vitro growth and throughout a virulence model of *Z. tritici* infection (Table 1). This analysis suggested that 16 putative secondary metabolite loci demonstrate evidence of transcriptional co-regulation (Table 1 and Additional file 3). Several clusters demonstrated stage-specific transcriptional upregulation at key phases of infection (Fig. 3), for example during germination (day 1, cluster 15), asymptomatic growth (day 4, cluster 8), the switch to necrotrophic infection (day 9, clusters 18, 31, 33) and throughout rapid fungal growth and development of reproductive pycnidia (day 14 and 21, cluster 17, 27 and 28). These data indicate that the biosynthetic products of these clusters may play key roles at specific stages of disease, and it is possible use these expression patterns to suggest putative biological functions. For example, during the earliest stages of disease, transcriptionally upregulated metabolic clusters (e.g. cluster 15, Fig. 3) may biosynthesize germination inhibitors in order to spatially or temporally coordinate production of infectious hyphae [15]. Alternatively, initiating phases of infection characterized by slow, symptomless fungal growth may require extracellular metabolites for masking or counteracting host immune surveillance (cluster 15 and 8, Fig. 3). In contrast, the switch from symptomless disease to host tissue necrosis at 9 and 14 days post infection may require generalist or host-selective phytotoxins, an observation supported by the stage specific transcriptional upregulation of genes from clusters 18 and 27, which demonstrate cluster homology to known phytotoxins (Fig. 2). Consequently, it is possible to use transcriptional profiles of co-expressed secondary metabolite loci during disease to aid hypothesis construction regarding their biological function, which can then be validated using genetic and metabolomic approaches.

## Conclusions

This study has used *in silico* approaches to predict, and subsequently analyze, 32 secondary metabolite loci in the genome of the wheat pathogen *Z. tritici*. We were able to assign putative biosynthetic products to numerous clusters based on their conservation amongst other fungi. These analyses suggest a siderophore, melanin, carotenoid, opsin, ETP, fumonisin-like polyketide and AM-toxin-like NRP are part of the *Z. tritici* secondary metabolite repertoire. Interestingly, analysis of most secondary metabolite loci did not enable prediction of an experimentally confirmed product, yet several were transcriptionally deployed during infection, and consequently the capacity of these clusters to play as yet undetermined roles in disease or other stages of the *Z. tritici* lifecycle is significant.

While our analyses have been conducted using isolate IP0323, it is not currently clear if this is a representative or average genome for *Z. tritici*. Indeed, high rates of sexual reproduction [9] and gene flow [82] result in extreme genomic and phenotypic diversity in *Z. tritici* populations, which is likely to result in a range of secondary metabolite repertoires amongst field isolates. As noted earlier, genes predicted to encode key biosynthetic enzymes from the IP0323 genome are absent in certain field strains [39]. Future comparative genomic analyses of multiple *Z. tritici* isolates will enable identification of secondary metabolite loci that are essential for virulence or other phases of the pathogen lifecycle, and those that are either dispensable or required for cultivar specificity.

Clearly, to validate the products of the identified loci and comprehensively determine their biological function, gene functional characterization and metabolomics analyses will be essential, and numerous tools now exist for such experiments in *Z. tritici*. Finally, from a biotechnological perspective, these loci and cognate products may be promising for the discovery of new bioactive molecules.

## Additional files

Additional file 1: MultiGeneBLAST analysis of putative secondary metabolite clusters. All encoded amino acid sequences from genes residing in clusters predicted by AntiSMASH are given as FASTA file format. All output data from MultiGeneBLASTs are also provided. (ZIP 42911 kb)

Additional file 2: Subtelomeric co-ordinates and genes in the *Z. tritici* genome. Genome co-ordinates within 300 kb of each chromosome end are given. All genes located in these regions are provided. (XLSX 153 kb)

Additional file 3: FungalGeneClusteRs gene co-expression analysis. All co-regulated loci are given (Table 1), secondary metabolite co-regulated loci (Table 2), and FPKM expression values for each co-regulated secondary metabolite gene (Additional file 3: Table S3, [6]). (XLSX 143 kb)

Additional file 4: All putative secondary metabolite genes predicted in this study. (XLSX 59 kb)

Additional file 5: Genes encoding predicted ABC or MFS transporters located in putative secondary metabolite clusters. (XLSX 13 kb)

Additional file 6: Genes encoding predicted transcription factors located in putative secondary metabolite clusters. (XLSX 9 kb)

Additional file 7: Genes residing in secondary metabolite loci with at least one paralogue that also resides in a biosynthetic gene cluster. (XLSX 20 kb)

Additional file 8: Schematic representation of putative gene duplication events at exemplar secondary metabolite loci. Predicted gene boundaries are depicted by arrows and paralogous genes assigned Ensemble identifiers. Dashed lines indicate paralogous gene pairs. Ensemble identifiers for key biosynthetic genes (PKS, clusters 17 and 27, and an NRPS, cluster 31) are given in parentheses. (GIF 21 kb)

## Abbreviations

ABC: ATP-binding cassette
AntiSMASH: Antibiotics and secondary metabolite analysis shell
ATP: Adenosine triphosphate
BLAST: Basic local alignment search tool
CDB: Czapek-dox broth
CoA: Coenzyme A
DDL: Duplication, diversification and differential gene loss
DHN: Dihydroxynaphthalene
DNA: Deoxyribonucleic acid
ETP: Epipolythiodioxopiperazine
FPKM: Fragments per kilobase of transcript per million mapped reads
HMG-CoA: Hydroxymethylglutaryl-CoA
HST: Host specific toxins
MFS: Major facilitator superfamily
NRP: Non-ribosomal peptide
NRPS: Non-ribosomal peptide synthetase
PAMP: Pathogen associated molecular pattern
PDB: Potato dextrose broth
PKS: Polyketide synthase
PRR: Pattern recognition receptor
RNA: Ribonucleic acid
SMURF: Secondary metabolite unique regions finder
TAFC: Triacetyl fusarine C
THN: Tetrahydroxynapthalene
UV: Ultraviolet
