# Supplementary material for: In silico prediction and characterization of secondary metabolite biosynthetic gene clusters in the wheat pathogen Zymoseptoria tritici
Source: BMC Genomics. 2017 Aug 17;18:631. doi: 10.1186/s12864-017-3969-y (PMC5561558; doi:10.1186/s12864-017-3969-y)
Supplement: Supplementary file 1 — MultiGeneBLAST analysis of putative secondary metabolite clusters. All encoded amino acid sequences from genes residing in clusters predicted by AntiSMASH are given as FASTA file format. All output data from MultiGeneBLASTs are also provided. (ZIP 42911 kb) [file 12864_2017_3969_MOESM1_ESM.zip › Cluster MultiGene BLAST/out/Clusters_1_34/Cluster_1/displaypage1.xhtml]

xml version="1.0" encoding="UTF-8"?


Search Results
  
  
 Results pages: 1, 2, 3, 4, 5

**MultiGeneBlast hits**

Select gene cluster alignment
1. CM001196\_1 Mycosphaerella graminicola IPO323 chromosome 1, whole genome sh...
2. KB446537\_1 Dothistroma septosporum NZE10 unplaced genomic scaffold DOTSEsc...
3. KB445550\_1 Baudoinia compniacensis UAMH 10762 unplaced genomic scaffold BA...
4. KB446558\_1 Pseudocercospora fijiensis CIRAD86 unplaced genomic scaffold MY...
5. KB456262\_1 Mycosphaerella populorum SO2202 unplaced genomic scaffold SEPMU...
6. KB915926\_0 Neofusicoccum parvum UCRNP2 chromosome Unknown NP2\_03\_scaffold\_...
7. JH921454\_0 Marssonina brunnea f. sp. 'multigermtubi' MB\_m1 unplaced genomi...
8. KE145359\_0 Glarea lozoyensis ATCC 20868 chromosome Unknown GLAREA16, whole...
9. CH476635\_0 Sclerotinia sclerotiorum 1980 scaffold\_15 genomic scaffold, who...
10. AKHY01000097\_0 Aspergillus oryzae 3.042, whole genome shotgun sequencing ...
11. FQ790317\_0 Botryotinia fuckeliana T4 SuperContig\_168\_1 genomic supercontig.
12. KB708110\_0 Botryotinia fuckeliana BcDW1 unplaced genomic scaffold Scaffol...
13. DS027059\_1 Aspergillus clavatus NRRL 1 1099423829805 genomic scaffold, wh...
14. DS499594\_1 Aspergillus fumigatus A1163 scf\_000001 genomic scaffold, whole...
15. AAHF01000007\_0 Aspergillus fumigatus Af293, whole genome shotgun sequenci...
16. DS027688\_1 Neosartorya fischeri NRRL 181 1099437636249 genomic scaffold, ...
17. AKCU01000141\_0 Penicillium digitatum Pd1, whole genome shotgun sequencing...
18. AKCT01000207\_0 Penicillium digitatum PHI26, whole genome shotgun sequenci...
19. ACJE01000004\_0 Aspergillus niger ATCC 1015, whole genome shotgun sequenci...
20. AM920431\_3 Penicillium chrysogenum Wisconsin 54-1255 complete genome, con...
21. DS572753\_0 Paracoccidioides brasiliensis Pb18 supercont1.4 genomic scaffo...
22. GL573405\_0 Geomyces destructans 20631-21 unplaced genomic scaffold superc...
23. DS572812\_0 Paracoccidioides brasiliensis Pb01 supercont1.2 genomic scaffo...
24. AACD01000007\_0 Aspergillus nidulans FGSC A4, whole genome shotgun sequenc...
25. DF126464\_0 Aspergillus kawachii IFO 4308 DNA, contig: scaffold00018, whol...
26. GG700653\_2 Trichophyton rubrum CBS 118892 genomic scaffold supercont2.6, ...
27. GG749408\_0 Ajellomyces dermatitidis ATCC 18188 genomic scaffold supercont...
28. GG657458\_0 Ajellomyces dermatitidis SLH14081 genomic scaffold supercont1....
29. GG698513\_0 Trichophyton tonsurans CBS 112818 genomic scaffold supercont1....
30. EQ999975\_0 Ajellomyces dermatitidis ER-3 genomic scaffold supercont1.3, w...
31. DS995752\_0 Trichophyton equinum CBS 127.97 supercont1.35 genomic scaffold...
32. DS995903\_0 Penicillium marneffei ATCC 18224 scf\_1105668340984 genomic sca...
33. GG663363\_0 Ajellomyces capsulatus G186AR genomic scaffold supercont2.1, w...
34. GG692421\_1 Ajellomyces capsulatus H143 genomic scaffold supercont2.3, who...
35. DS990639\_1 Ajellomyces capsulatus H88 supercont1.4 genomic scaffold, whol...
36. DS989828\_1 Arthroderma gypseum CBS 118893 supercont1.7 genomic scaffold, ...
37. KB644410\_0 Penicillium oxalicum 114-2 unplaced genomic scaffold scaffold\_...
38. EQ962656\_1 Talaromyces stipitatus ATCC 10500 scf\_1105507295549 genomic sc...
39. DS995702\_2 Microsporum canis CBS 113480 supercont1.2 genomic scaffold, wh...
40. ACYE01000036\_0 Trichophyton verrucosum HKI 0517, whole genome shotgun seq...
41. AQGS01000844\_0 Dactylellina haptotyla CBS 200.50, whole genome shotgun se...
42. AP007155\_0 Aspergillus oryzae RIB40 DNA, SC003.
43. ADOT01000146\_0 Arthrobotrys oligospora ATCC 24927, whole genome shotgun s...
44. CH476657\_1 Ajellomyces capsulatus NAm1 scaffold\_3 genomic scaffold, whole...
45. DS544804\_0 Paracoccidioides brasiliensis Pb03 supercont1.2 genomic scaffo...
46. KB916820\_0 Neofusicoccum parvum UCRNP2 chromosome Unknown NP2\_03\_scaffold...
47. KB916240\_0 Neofusicoccum parvum UCRNP2 chromosome Unknown NP2\_03\_scaffold...
48. AHHD01000505\_0 Macrophomina phaseolina MS6, whole genome shotgun sequenci...
49. ABSU01000004\_2 Arthroderma benhamiae CBS 112371, whole genome shotgun seq...
50. JH767572\_0 Coniosporium apollinis CBS 100218 chromosome Unknown supercont...

Query: Architecture Search FASTA input

CM001196 : Mycosphaerella graminicola IPO323 chromosome 1    Total score: 12.0     Cumulative Blast bit score: 13049

Hit cluster cross-links:

Mycgr3G52686 Mycgr3T
  
Location: 0-861

Mycgr3G52686\_Mycgr3T

Mycgr3G102281 Mycgr3
  
Location: 961-1573

Mycgr3G102281\_Mycgr3

Mycgr3G89185 Mycgr3T
  
Location: 1673-2063

Mycgr3G89185\_Mycgr3T

Mycgr3G65725 Mycgr3T
  
Location: 2163-3612

Mycgr3G65725\_Mycgr3T

Mycgr3G102276 Mycgr3
  
Location: 3712-4801

Mycgr3G102276\_Mycgr3

Mycgr3G89189 Mycgr3T
  
Location: 4901-5564

Mycgr3G89189\_Mycgr3T

Mycgr3G52682 Mycgr3T
  
Location: 5664-9231

Mycgr3G52682\_Mycgr3T

Mycgr3G107072 Mycgr3
  
Location: 9331-13279

Mycgr3G107072\_Mycgr3

Mycgr3G34982 Mycgr3T
  
Location: 13379-15116

Mycgr3G34982\_Mycgr3T

Mycgr3G107069 Mycgr3
  
Location: 15216-17097

Mycgr3G107069\_Mycgr3

Mycgr3G32432 Mycgr3T
  
Location: 17197-19042

Mycgr3G32432\_Mycgr3T

Mycgr3G98385 Mycgr3T
  
Location: 19142-19898

Mycgr3G98385\_Mycgr3T

hypothetical protein
  
Accession: EGP91142
  
Location: 2170752-2171879
  
 NCBI BlastP on this gene

EGP91142

hypothetical protein
  
Accession: EGP91143
  
Location: 2172553-2173003
  
  
**BlastP hit with Mycgr3G89185\_Mycgr3T**
  
Percentage identity: 100 %
  
BlastP bit score: 270
  
Sequence coverage: 99 %
  
E-value: 7e-91
  
  
 NCBI BlastP on this gene

EGP91143

putative FRE ferric reductase-like transmembrane component
  
Accession: EGP92462
  
Location: 2173209-2175532
  
  
**BlastP hit with Mycgr3G107069\_Mycgr3**
  
Percentage identity: 100 %
  
BlastP bit score: 1291
  
Sequence coverage: 99 %
  
E-value: 0.0
  
  
 NCBI BlastP on this gene

EGP92462

hypothetical protein
  
Accession: EGP92461
  
Location: 2175948-2177095
  
  
**BlastP hit with Mycgr3G102276\_Mycgr3**
  
Percentage identity: 100 %
  
BlastP bit score: 747
  
Sequence coverage: 99 %
  
E-value: 0.0
  
  
 NCBI BlastP on this gene

EGP92461

glucose-methanol-choline oxidoreductase
  
Accession: EGP92460
  
Location: 2179138-2181288
  
  
**BlastP hit with Mycgr3G34982\_Mycgr3T**
  
Percentage identity: 100 %
  
BlastP bit score: 1204
  
Sequence coverage: 99 %
  
E-value: 0.0
  
  
 NCBI BlastP on this gene

EGP92460

hypothetical protein
  
Accession: EGP92459
  
Location: 2181964-2184459
  
 NCBI BlastP on this gene

EGP92459

hypothetical protein
  
Accession: EGP91144
  
Location: 2185647-2189560
  
  
**BlastP hit with Mycgr3G89189\_Mycgr3T**
  
Percentage identity: 100 %
  
BlastP bit score: 448
  
Sequence coverage: 99 %
  
E-value: 5e-158
  
  
 NCBI BlastP on this gene

EGP91144

hypothetical protein
  
Accession: EGP91145
  
Location: 2189682-2191185
  
  
**BlastP hit with Mycgr3G65725\_Mycgr3T**
  
Percentage identity: 100 %
  
BlastP bit score: 979
  
Sequence coverage: 99 %
  
E-value: 0.0
  
  
 NCBI BlastP on this gene

EGP91145

hypothetical protein
  
Accession: EGP92458
  
Location: 2191438-2195442
  
  
**BlastP hit with Mycgr3G107072\_Mycgr3**
  
Percentage identity: 100 %
  
BlastP bit score: 2672
  
Sequence coverage: 99 %
  
E-value: 0.0
  
  
 NCBI BlastP on this gene

EGP92458

hypothetical protein
  
Accession: EGP91146
  
Location: 2197464-2198749
  
  
**BlastP hit with Mycgr3G98385\_Mycgr3T**
  
Percentage identity: 100 %
  
BlastP bit score: 517
  
Sequence coverage: 99 %
  
E-value: 0.0
  
  
 NCBI BlastP on this gene

EGP91146

hypothetical protein
  
Accession: EGP91147
  
Location: 2201706-2202619
  
  
**BlastP hit with Mycgr3G102281\_Mycgr3**
  
Percentage identity: 100 %
  
BlastP bit score: 414
  
Sequence coverage: 99 %
  
E-value: 4e-145
  
  
 NCBI BlastP on this gene

EGP91147

hypothetical protein
  
Accession: EGP91148
  
Location: 2203268-2207035
  
  
**BlastP hit with Mycgr3G52682\_Mycgr3T**
  
Percentage identity: 100 %
  
BlastP bit score: 2444
  
Sequence coverage: 99 %
  
E-value: 0.0
  
  
 NCBI BlastP on this gene

EGP91148

hypothetical protein
  
Accession: EGP91149
  
Location: 2208268-2209189
  
  
**BlastP hit with Mycgr3G52686\_Mycgr3T**
  
Percentage identity: 100 %
  
BlastP bit score: 598
  
Sequence coverage: 99 %
  
E-value: 0.0
  
  
 NCBI BlastP on this gene

EGP91149

hypothetical protein
  
Accession: EGP91150
  
Location: 2210894-2212738
  
  
**BlastP hit with Mycgr3G32432\_Mycgr3T**
  
Percentage identity: 100 %
  
BlastP bit score: 1261
  
Sequence coverage: 99 %
  
E-value: 0.0
  
  
 NCBI BlastP on this gene

EGP91150

major facilitator superfamily MFS 1 protein
  
Accession: EGP91151
  
Location: 2214598-2216244
  
 NCBI BlastP on this gene

EGP91151

hypothetical protein
  
Accession: EGP92457
  
Location: 2216502-2218390
  
 NCBI BlastP on this gene

EGP92457

hypothetical protein
  
Accession: EGP91152
  
Location: 2218881-2220485
  
 NCBI BlastP on this gene

EGP91152

hypothetical protein
  
Accession: EGP92456
  
Location: 2220508-2220877
  
 NCBI BlastP on this gene

EGP92456

hypothetical protein
  
Accession: EGP92455
  
Location: 2222385-2224438
  
  
**BlastP hit with Mycgr3G32432\_Mycgr3T**
  
Percentage identity: 32 %
  
BlastP bit score: 204
  
Sequence coverage: 86 %
  
E-value: 7e-54
  
  
 NCBI BlastP on this gene

EGP92455

hypothetical protein
  
Accession: EGP92454
  
Location: 2226023-2226691
  
 NCBI BlastP on this gene

EGP92454

Query: Architecture Search FASTA input

KB446537 : Dothistroma septosporum NZE10 unplaced genomic scaffold DOTSEscaffold\_3    Total score: 5.0     Cumulative Blast bit score: 3674

Hit cluster cross-links:

Mycgr3G52686 Mycgr3T
  
Location: 0-861

Mycgr3G52686\_Mycgr3T

Mycgr3G102281 Mycgr3
  
Location: 961-1573

Mycgr3G102281\_Mycgr3

Mycgr3G89185 Mycgr3T
  
Location: 1673-2063

Mycgr3G89185\_Mycgr3T

Mycgr3G65725 Mycgr3T
  
Location: 2163-3612

Mycgr3G65725\_Mycgr3T

Mycgr3G102276 Mycgr3
  
Location: 3712-4801

Mycgr3G102276\_Mycgr3

Mycgr3G89189 Mycgr3T
  
Location: 4901-5564

Mycgr3G89189\_Mycgr3T

Mycgr3G52682 Mycgr3T
  
Location: 5664-9231

Mycgr3G52682\_Mycgr3T

Mycgr3G107072 Mycgr3
  
Location: 9331-13279

Mycgr3G107072\_Mycgr3

Mycgr3G34982 Mycgr3T
  
Location: 13379-15116

Mycgr3G34982\_Mycgr3T

Mycgr3G107069 Mycgr3
  
Location: 15216-17097

Mycgr3G107069\_Mycgr3

Mycgr3G32432 Mycgr3T
  
Location: 17197-19042

Mycgr3G32432\_Mycgr3T

Mycgr3G98385 Mycgr3T
  
Location: 19142-19898

Mycgr3G98385\_Mycgr3T

hypothetical protein
  
Accession: EME46357
  
Location: 1317727-1318303
  
 NCBI BlastP on this gene

EME46357

hypothetical protein
  
Accession: EME46358
  
Location: 1318868-1319824
  
 NCBI BlastP on this gene

EME46358

hypothetical protein
  
Accession: EME46359
  
Location: 1320439-1321649
  
  
**BlastP hit with Mycgr3G98385\_Mycgr3T**
  
Percentage identity: 87 %
  
BlastP bit score: 430
  
Sequence coverage: 91 %
  
E-value: 6e-150
  
  
 NCBI BlastP on this gene

EME46359

hypothetical protein
  
Accession: EME46360
  
Location: 1321862-1324100
  
 NCBI BlastP on this gene

EME46360

hypothetical protein
  
Accession: EME46361
  
Location: 1325002-1325769
  
  
**BlastP hit with Mycgr3G102281\_Mycgr3**
  
Percentage identity: 96 %
  
BlastP bit score: 384
  
Sequence coverage: 99 %
  
E-value: 3e-133
  
  
 NCBI BlastP on this gene

EME46361

hypothetical protein
  
Accession: EME46362
  
Location: 1326558-1330388
  
  
**BlastP hit with Mycgr3G52682\_Mycgr3T**
  
Percentage identity: 60 %
  
BlastP bit score: 1395
  
Sequence coverage: 108 %
  
E-value: 0.0
  
  
 NCBI BlastP on this gene

EME46362

hypothetical protein
  
Accession: EME46363
  
Location: 1332197-1333116
  
  
**BlastP hit with Mycgr3G52686\_Mycgr3T**
  
Percentage identity: 92 %
  
BlastP bit score: 555
  
Sequence coverage: 98 %
  
E-value: 0.0
  
  
 NCBI BlastP on this gene

EME46363

hypothetical protein
  
Accession: EME46364
  
Location: 1334461-1336650
  
  
**BlastP hit with Mycgr3G32432\_Mycgr3T**
  
Percentage identity: 57 %
  
BlastP bit score: 715
  
Sequence coverage: 99 %
  
E-value: 0.0
  
  
 NCBI BlastP on this gene

EME46364

hypothetical protein
  
Accession: EME46365
  
Location: 1338175-1340361
  
 NCBI BlastP on this gene

EME46365

hypothetical protein
  
Accession: EME46366
  
Location: 1341621-1343665
  
  
**BlastP hit with Mycgr3G32432\_Mycgr3T**
  
Percentage identity: 32 %
  
BlastP bit score: 195
  
Sequence coverage: 83 %
  
E-value: 2e-50
  
  
 NCBI BlastP on this gene

EME46366

hypothetical protein
  
Accession: EME46367
  
Location: 1346125-1348706
  
 NCBI BlastP on this gene

EME46367

Query: Architecture Search FASTA input

KB445550 : Baudoinia compniacensis UAMH 10762 unplaced genomic scaffold BAUCOscaffold\_1    Total score: 5.0     Cumulative Blast bit score: 3360

Hit cluster cross-links:

Mycgr3G52686 Mycgr3T
  
Location: 0-861

Mycgr3G52686\_Mycgr3T

Mycgr3G102281 Mycgr3
  
Location: 961-1573

Mycgr3G102281\_Mycgr3

Mycgr3G89185 Mycgr3T
  
Location: 1673-2063

Mycgr3G89185\_Mycgr3T

Mycgr3G65725 Mycgr3T
  
Location: 2163-3612

Mycgr3G65725\_Mycgr3T

Mycgr3G102276 Mycgr3
  
Location: 3712-4801

Mycgr3G102276\_Mycgr3

Mycgr3G89189 Mycgr3T
  
Location: 4901-5564

Mycgr3G89189\_Mycgr3T

Mycgr3G52682 Mycgr3T
  
Location: 5664-9231

Mycgr3G52682\_Mycgr3T

Mycgr3G107072 Mycgr3
  
Location: 9331-13279

Mycgr3G107072\_Mycgr3

Mycgr3G34982 Mycgr3T
  
Location: 13379-15116

Mycgr3G34982\_Mycgr3T

Mycgr3G107069 Mycgr3
  
Location: 15216-17097

Mycgr3G107069\_Mycgr3

Mycgr3G32432 Mycgr3T
  
Location: 17197-19042

Mycgr3G32432\_Mycgr3T

Mycgr3G98385 Mycgr3T
  
Location: 19142-19898

Mycgr3G98385\_Mycgr3T

hypothetical protein
  
Accession: EMD00554
  
Location: 450055-452751
  
 NCBI BlastP on this gene

EMD00554

hypothetical protein
  
Accession: EMD00555
  
Location: 453313-458175
  
  
**BlastP hit with Mycgr3G32432\_Mycgr3T**
  
Percentage identity: 53 %
  
BlastP bit score: 654
  
Sequence coverage: 99 %
  
E-value: 0.0
  
  
 NCBI BlastP on this gene

EMD00555

hypothetical protein
  
Accession: EMD00556
  
Location: 459219-460128
  
  
**BlastP hit with Mycgr3G52686\_Mycgr3T**
  
Percentage identity: 87 %
  
BlastP bit score: 522
  
Sequence coverage: 99 %
  
E-value: 0.0
  
  
 NCBI BlastP on this gene

EMD00556

hypothetical protein
  
Accession: EMD00557
  
Location: 461146-461416
  
 NCBI BlastP on this gene

EMD00557

hypothetical protein
  
Accession: EMD00558
  
Location: 461495-461716
  
 NCBI BlastP on this gene

EMD00558

hypothetical protein
  
Accession: EMD00559
  
Location: 462649-463545
  
  
**BlastP hit with Mycgr3G98385\_Mycgr3T**
  
Percentage identity: 78 %
  
BlastP bit score: 387
  
Sequence coverage: 92 %
  
E-value: 6e-133
  
  
 NCBI BlastP on this gene

EMD00559

hypothetical protein
  
Accession: EMD00560
  
Location: 463772-465522
  
 NCBI BlastP on this gene

EMD00560

hypothetical protein
  
Accession: EMD00561
  
Location: 466337-467127
  
  
**BlastP hit with Mycgr3G102281\_Mycgr3**
  
Percentage identity: 93 %
  
BlastP bit score: 373
  
Sequence coverage: 99 %
  
E-value: 4e-129
  
  
 NCBI BlastP on this gene

EMD00561

hypothetical protein
  
Accession: EMD00562
  
Location: 467807-471619
  
  
**BlastP hit with Mycgr3G52682\_Mycgr3T**
  
Percentage identity: 60 %
  
BlastP bit score: 1424
  
Sequence coverage: 104 %
  
E-value: 0.0
  
  
 NCBI BlastP on this gene

EMD00562

hypothetical protein
  
Accession: EMD00563
  
Location: 473144-473853
  
 NCBI BlastP on this gene

EMD00563

hypothetical protein
  
Accession: EMD00564
  
Location: 474183-476541
  
 NCBI BlastP on this gene

EMD00564

Query: Architecture Search FASTA input

KB446558 : Pseudocercospora fijiensis CIRAD86 unplaced genomic scaffold MYCFIscaffold\_4    Total score: 4.0     Cumulative Blast bit score: 3339

Hit cluster cross-links:

Mycgr3G52686 Mycgr3T
  
Location: 0-861

Mycgr3G52686\_Mycgr3T

Mycgr3G102281 Mycgr3
  
Location: 961-1573

Mycgr3G102281\_Mycgr3

Mycgr3G89185 Mycgr3T
  
Location: 1673-2063

Mycgr3G89185\_Mycgr3T

Mycgr3G65725 Mycgr3T
  
Location: 2163-3612

Mycgr3G65725\_Mycgr3T

Mycgr3G102276 Mycgr3
  
Location: 3712-4801

Mycgr3G102276\_Mycgr3

Mycgr3G89189 Mycgr3T
  
Location: 4901-5564

Mycgr3G89189\_Mycgr3T

Mycgr3G52682 Mycgr3T
  
Location: 5664-9231

Mycgr3G52682\_Mycgr3T

Mycgr3G107072 Mycgr3
  
Location: 9331-13279

Mycgr3G107072\_Mycgr3

Mycgr3G34982 Mycgr3T
  
Location: 13379-15116

Mycgr3G34982\_Mycgr3T

Mycgr3G107069 Mycgr3
  
Location: 15216-17097

Mycgr3G107069\_Mycgr3

Mycgr3G32432 Mycgr3T
  
Location: 17197-19042

Mycgr3G32432\_Mycgr3T

Mycgr3G98385 Mycgr3T
  
Location: 19142-19898

Mycgr3G98385\_Mycgr3T

hypothetical protein
  
Accession: EME83068
  
Location: 2482416-2483639
  
 NCBI BlastP on this gene

EME83068

hypothetical protein
  
Accession: EME83067
  
Location: 2480348-2481119
  
  
**BlastP hit with Mycgr3G102281\_Mycgr3**
  
Percentage identity: 97 %
  
BlastP bit score: 382
  
Sequence coverage: 99 %
  
E-value: 8e-133
  
  
 NCBI BlastP on this gene

EME83067

hypothetical protein
  
Accession: EME83066
  
Location: 2475708-2479547
  
  
**BlastP hit with Mycgr3G52682\_Mycgr3T**
  
Percentage identity: 63 %
  
BlastP bit score: 1492
  
Sequence coverage: 100 %
  
E-value: 0.0
  
  
 NCBI BlastP on this gene

EME83066

hypothetical protein
  
Accession: EME83065
  
Location: 2474397-2475663
  
 NCBI BlastP on this gene

EME83065

hypothetical protein
  
Accession: EME83064
  
Location: 2472201-2472761
  
 NCBI BlastP on this gene

EME83064

hypothetical protein
  
Accession: EME83063
  
Location: 2471438-2471731
  
 NCBI BlastP on this gene

EME83063

hypothetical protein
  
Accession: EME83062
  
Location: 2466546-2468667
  
 NCBI BlastP on this gene

EME83062

hypothetical protein
  
Accession: EME83061
  
Location: 2463367-2465063
  
  
**BlastP hit with Mycgr3G32432\_Mycgr3T**
  
Percentage identity: 33 %
  
BlastP bit score: 201
  
Sequence coverage: 79 %
  
E-value: 1e-53
  
  
 NCBI BlastP on this gene

EME83061

hypothetical protein
  
Accession: EME83060
  
Location: 2461958-2462672
  
 NCBI BlastP on this gene

EME83060

hypothetical protein
  
Accession: EME83059
  
Location: 2460308-2461927
  
 NCBI BlastP on this gene

EME83059

hypothetical protein
  
Accession: EME83058
  
Location: 2458178-2459996
  
 NCBI BlastP on this gene

EME83058

hypothetical protein
  
Accession: EME83057
  
Location: 2456508-2458077
  
 NCBI BlastP on this gene

EME83057

hypothetical protein
  
Accession: EME83056
  
Location: 2454953-2456042
  
 NCBI BlastP on this gene

EME83056

hypothetical protein
  
Accession: EME83055
  
Location: 2453199-2454124
  
  
**BlastP hit with Mycgr3G52686\_Mycgr3T**
  
Percentage identity: 92 %
  
BlastP bit score: 566
  
Sequence coverage: 99 %
  
E-value: 0.0
  
  
 NCBI BlastP on this gene

EME83055

hypothetical protein
  
Accession: EME83054
  
Location: 2430140-2435554
  
  
**BlastP hit with Mycgr3G32432\_Mycgr3T**
  
Percentage identity: 58 %
  
BlastP bit score: 698
  
Sequence coverage: 94 %
  
E-value: 0.0
  
  
 NCBI BlastP on this gene

EME83054

hypothetical protein
  
Accession: EME83053
  
Location: 2427769-2429696
  
 NCBI BlastP on this gene

EME83053

Query: Architecture Search FASTA input

KB456262 : Mycosphaerella populorum SO2202 unplaced genomic scaffold SEPMUscaffold\_3    Total score: 4.0     Cumulative Blast bit score: 3021

Hit cluster cross-links:

Mycgr3G52686 Mycgr3T
  
Location: 0-861

Mycgr3G52686\_Mycgr3T

Mycgr3G102281 Mycgr3
  
Location: 961-1573

Mycgr3G102281\_Mycgr3

Mycgr3G89185 Mycgr3T
  
Location: 1673-2063

Mycgr3G89185\_Mycgr3T

Mycgr3G65725 Mycgr3T
  
Location: 2163-3612

Mycgr3G65725\_Mycgr3T

Mycgr3G102276 Mycgr3
  
Location: 3712-4801

Mycgr3G102276\_Mycgr3

Mycgr3G89189 Mycgr3T
  
Location: 4901-5564

Mycgr3G89189\_Mycgr3T

Mycgr3G52682 Mycgr3T
  
Location: 5664-9231

Mycgr3G52682\_Mycgr3T

Mycgr3G107072 Mycgr3
  
Location: 9331-13279

Mycgr3G107072\_Mycgr3

Mycgr3G34982 Mycgr3T
  
Location: 13379-15116

Mycgr3G34982\_Mycgr3T

Mycgr3G107069 Mycgr3
  
Location: 15216-17097

Mycgr3G107069\_Mycgr3

Mycgr3G32432 Mycgr3T
  
Location: 17197-19042

Mycgr3G32432\_Mycgr3T

Mycgr3G98385 Mycgr3T
  
Location: 19142-19898

Mycgr3G98385\_Mycgr3T

hypothetical protein
  
Accession: EMF14506
  
Location: 1573351-1573593
  
 NCBI BlastP on this gene

EMF14506

hypothetical protein
  
Accession: EMF14507
  
Location: 1574940-1577108
  
 NCBI BlastP on this gene

EMF14507

ribosomal protein L15e
  
Accession: EMF14509
  
Location: 1578155-1578925
  
  
**BlastP hit with Mycgr3G102281\_Mycgr3**
  
Percentage identity: 95 %
  
BlastP bit score: 378
  
Sequence coverage: 99 %
  
E-value: 6e-131
  
  
 NCBI BlastP on this gene

EMF14509

HECT-domain-containing protein
  
Accession: EMF14510
  
Location: 1579846-1583691
  
  
**BlastP hit with Mycgr3G52682\_Mycgr3T**
  
Percentage identity: 59 %
  
BlastP bit score: 1416
  
Sequence coverage: 108 %
  
E-value: 0.0
  
  
 NCBI BlastP on this gene

EMF14510

hypothetical protein
  
Accession: EMF14511
  
Location: 1585300-1586225
  
  
**BlastP hit with Mycgr3G52686\_Mycgr3T**
  
Percentage identity: 89 %
  
BlastP bit score: 547
  
Sequence coverage: 99 %
  
E-value: 0.0
  
  
 NCBI BlastP on this gene

EMF14511

MFS general substrate transporter
  
Accession: EMF14512
  
Location: 1588321-1593561
  
  
**BlastP hit with Mycgr3G32432\_Mycgr3T**
  
Percentage identity: 57 %
  
BlastP bit score: 680
  
Sequence coverage: 93 %
  
E-value: 0.0
  
  
 NCBI BlastP on this gene

EMF14512

carbon-nitrogen hydrolase
  
Accession: EMF14513
  
Location: 1593988-1595061
  
 NCBI BlastP on this gene

EMF14513

D-hydantoinase
  
Accession: EMF14514
  
Location: 1595559-1597127
  
 NCBI BlastP on this gene

EMF14514

Query: Architecture Search FASTA input

KB915926 : Neofusicoccum parvum UCRNP2 chromosome Unknown NP2\_03\_scaffold\_288    Total score: 2.0     Cumulative Blast bit score: 1409

Hit cluster cross-links:

Mycgr3G52686 Mycgr3T
  
Location: 0-861

Mycgr3G52686\_Mycgr3T

Mycgr3G102281 Mycgr3
  
Location: 961-1573

Mycgr3G102281\_Mycgr3

Mycgr3G89185 Mycgr3T
  
Location: 1673-2063

Mycgr3G89185\_Mycgr3T

Mycgr3G65725 Mycgr3T
  
Location: 2163-3612

Mycgr3G65725\_Mycgr3T

Mycgr3G102276 Mycgr3
  
Location: 3712-4801

Mycgr3G102276\_Mycgr3

Mycgr3G89189 Mycgr3T
  
Location: 4901-5564

Mycgr3G89189\_Mycgr3T

Mycgr3G52682 Mycgr3T
  
Location: 5664-9231

Mycgr3G52682\_Mycgr3T

Mycgr3G107072 Mycgr3
  
Location: 9331-13279

Mycgr3G107072\_Mycgr3

Mycgr3G34982 Mycgr3T
  
Location: 13379-15116

Mycgr3G34982\_Mycgr3T

Mycgr3G107069 Mycgr3
  
Location: 15216-17097

Mycgr3G107069\_Mycgr3

Mycgr3G32432 Mycgr3T
  
Location: 17197-19042

Mycgr3G32432\_Mycgr3T

Mycgr3G98385 Mycgr3T
  
Location: 19142-19898

Mycgr3G98385\_Mycgr3T

putative sterigmatocystin 8-o-methyltransferase protein
  
Accession: EOD50854
  
Location: 48819-50255
  
 NCBI BlastP on this gene

EOD50854

putative Arylsulfotransferase protein
  
Accession: EOD50857
  
Location: 46555-48570
  
  
**BlastP hit with Mycgr3G32432\_Mycgr3T**
  
Percentage identity: 44 %
  
BlastP bit score: 432
  
Sequence coverage: 84 %
  
E-value: 2e-140
  
  
 NCBI BlastP on this gene

EOD50857

putative siderophore iron transporter mirb protein
  
Accession: EOD50856
  
Location: 45515-46046
  
 NCBI BlastP on this gene

EOD50856

putative abc multidrug transporter protein
  
Accession: EOD50849
  
Location: 39268-45487
  
 NCBI BlastP on this gene

EOD50849

putative d-alanine-poly ligase subunit 1 protein
  
Accession: EOD50859
  
Location: 32735-38229
  
 NCBI BlastP on this gene

EOD50859

putative siderophore iron transporter mirb protein
  
Accession: EOD50855
  
Location: 29575-31525
  
 NCBI BlastP on this gene

EOD50855

putative nad dependent epimerase protein
  
Accession: EOD50852
  
Location: 27540-28295
  
  
**BlastP hit with Mycgr3G52686\_Mycgr3T**
  
Percentage identity: 67 %
  
BlastP bit score: 313
  
Sequence coverage: 75 %
  
E-value: 5e-104
  
  
 NCBI BlastP on this gene

EOD50852

putative Arylsulfotransferase protein
  
Accession: EOD50858
  
Location: 25092-26898
  
  
**BlastP hit with Mycgr3G32432\_Mycgr3T**
  
Percentage identity: 47 %
  
BlastP bit score: 456
  
Sequence coverage: 82 %
  
E-value: 7e-150
  
  
 NCBI BlastP on this gene

EOD50858

hypothetical protein
  
Accession: EOD50850
  
Location: 21804-23345
  
  
**BlastP hit with Mycgr3G32432\_Mycgr3T**
  
Percentage identity: 30 %
  
BlastP bit score: 208
  
Sequence coverage: 73 %
  
E-value: 9e-57
  
  
 NCBI BlastP on this gene

EOD50850

Query: Architecture Search FASTA input

JH921454 : Marssonina brunnea f. sp. 'multigermtubi' MB\_m1 unplaced genomic scaffold M6\_S00027    Total score: 2.0     Cumulative Blast bit score: 1334

Hit cluster cross-links:

Mycgr3G52686 Mycgr3T
  
Location: 0-861

Mycgr3G52686\_Mycgr3T

Mycgr3G102281 Mycgr3
  
Location: 961-1573

Mycgr3G102281\_Mycgr3

Mycgr3G89185 Mycgr3T
  
Location: 1673-2063

Mycgr3G89185\_Mycgr3T

Mycgr3G65725 Mycgr3T
  
Location: 2163-3612

Mycgr3G65725\_Mycgr3T

Mycgr3G102276 Mycgr3
  
Location: 3712-4801

Mycgr3G102276\_Mycgr3

Mycgr3G89189 Mycgr3T
  
Location: 4901-5564

Mycgr3G89189\_Mycgr3T

Mycgr3G52682 Mycgr3T
  
Location: 5664-9231

Mycgr3G52682\_Mycgr3T

Mycgr3G107072 Mycgr3
  
Location: 9331-13279

Mycgr3G107072\_Mycgr3

Mycgr3G34982 Mycgr3T
  
Location: 13379-15116

Mycgr3G34982\_Mycgr3T

Mycgr3G107069 Mycgr3
  
Location: 15216-17097

Mycgr3G107069\_Mycgr3

Mycgr3G32432 Mycgr3T
  
Location: 17197-19042

Mycgr3G32432\_Mycgr3T

Mycgr3G98385 Mycgr3T
  
Location: 19142-19898

Mycgr3G98385\_Mycgr3T

zinc finger (HIT type) family protein
  
Accession: EKD12831
  
Location: 442357-443230
  
 NCBI BlastP on this gene

EKD12831

hypothetical protein
  
Accession: EKD12830
  
Location: 441159-442013
  
 NCBI BlastP on this gene

EKD12830

RasGEF domain-containing protein
  
Accession: EKD12829
  
Location: 436224-439468
  
 NCBI BlastP on this gene

EKD12829

hypothetical protein
  
Accession: EKD12828
  
Location: 434231-434962
  
 NCBI BlastP on this gene

EKD12828

60S ribosomal protein L15
  
Accession: EKD12827
  
Location: 432741-433595
  
  
**BlastP hit with Mycgr3G102281\_Mycgr3**
  
Percentage identity: 92 %
  
BlastP bit score: 356
  
Sequence coverage: 99 %
  
E-value: 3e-122
  
  
 NCBI BlastP on this gene

EKD12827

HECT-domain-containing protein
  
Accession: EKD12826
  
Location: 428047-431880
  
  
**BlastP hit with Mycgr3G52682\_Mycgr3T**
  
Percentage identity: 45 %
  
BlastP bit score: 978
  
Sequence coverage: 107 %
  
E-value: 0.0
  
  
 NCBI BlastP on this gene

EKD12826

50S ribosomal protein mrp49
  
Accession: EKD12825
  
Location: 426171-426975
  
 NCBI BlastP on this gene

EKD12825

hypothetical protein
  
Accession: EKD12824
  
Location: 423595-425996
  
 NCBI BlastP on this gene

EKD12824

homocitrate synthase
  
Accession: EKD12823
  
Location: 421133-422867
  
 NCBI BlastP on this gene

EKD12823

WD domain-containing protein
  
Accession: EKD12822
  
Location: 414825-418714
  
 NCBI BlastP on this gene

EKD12822

Query: Architecture Search FASTA input

KE145359 : Glarea lozoyensis ATCC 20868 chromosome Unknown GLAREA16    Total score: 2.0     Cumulative Blast bit score: 1330

Hit cluster cross-links:

Mycgr3G52686 Mycgr3T
  
Location: 0-861

Mycgr3G52686\_Mycgr3T

Mycgr3G102281 Mycgr3
  
Location: 961-1573

Mycgr3G102281\_Mycgr3

Mycgr3G89185 Mycgr3T
  
Location: 1673-2063

Mycgr3G89185\_Mycgr3T

Mycgr3G65725 Mycgr3T
  
Location: 2163-3612

Mycgr3G65725\_Mycgr3T

Mycgr3G102276 Mycgr3
  
Location: 3712-4801

Mycgr3G102276\_Mycgr3

Mycgr3G89189 Mycgr3T
  
Location: 4901-5564

Mycgr3G89189\_Mycgr3T

Mycgr3G52682 Mycgr3T
  
Location: 5664-9231

Mycgr3G52682\_Mycgr3T

Mycgr3G107072 Mycgr3
  
Location: 9331-13279

Mycgr3G107072\_Mycgr3

Mycgr3G34982 Mycgr3T
  
Location: 13379-15116

Mycgr3G34982\_Mycgr3T

Mycgr3G107069 Mycgr3
  
Location: 15216-17097

Mycgr3G107069\_Mycgr3

Mycgr3G32432 Mycgr3T
  
Location: 17197-19042

Mycgr3G32432\_Mycgr3T

Mycgr3G98385 Mycgr3T
  
Location: 19142-19898

Mycgr3G98385\_Mycgr3T

HIT/MYND zinc finger-like protein
  
Accession: EPE32615
  
Location: 1489540-1490336
  
 NCBI BlastP on this gene

EPE32615

hypothetical protein
  
Accession: EPE32616
  
Location: 1491137-1492113
  
 NCBI BlastP on this gene

EPE32616

Ras GEF
  
Accession: EPE32617
  
Location: 1495677-1499152
  
 NCBI BlastP on this gene

EPE32617

Ribosomal proteins S24e, L23 and L15e
  
Accession: EPE32618
  
Location: 1499849-1500688
  
  
**BlastP hit with Mycgr3G102281\_Mycgr3**
  
Percentage identity: 91 %
  
BlastP bit score: 358
  
Sequence coverage: 99 %
  
E-value: 2e-123
  
  
 NCBI BlastP on this gene

EPE32618

Hect, E3 ligase catalytic
  
Accession: EPE32619
  
Location: 1501342-1505141
  
  
**BlastP hit with Mycgr3G52682\_Mycgr3T**
  
Percentage identity: 45 %
  
BlastP bit score: 972
  
Sequence coverage: 100 %
  
E-value: 0.0
  
  
 NCBI BlastP on this gene

EPE32619

hypothetical protein
  
Accession: EPE32620
  
Location: 1505473-1506210
  
 NCBI BlastP on this gene

EPE32620

P-loop containing nucleoside triphosphate hydrolase
  
Accession: EPE32621
  
Location: 1506444-1508583
  
 NCBI BlastP on this gene

EPE32621

hypothetical protein
  
Accession: EPE32622
  
Location: 1508956-1509742
  
 NCBI BlastP on this gene

EPE32622

hypothetical protein
  
Accession: EPE32623
  
Location: 1510395-1511508
  
 NCBI BlastP on this gene

EPE32623

hypothetical protein
  
Accession: EPE32624
  
Location: 1512661-1514438
  
 NCBI BlastP on this gene

EPE32624

Query: Architecture Search FASTA input

CH476635 : Sclerotinia sclerotiorum 1980 scaffold\_15 genomic scaffold    Total score: 2.0     Cumulative Blast bit score: 1309

Hit cluster cross-links:

Mycgr3G52686 Mycgr3T
  
Location: 0-861

Mycgr3G52686\_Mycgr3T

Mycgr3G102281 Mycgr3
  
Location: 961-1573

Mycgr3G102281\_Mycgr3

Mycgr3G89185 Mycgr3T
  
Location: 1673-2063

Mycgr3G89185\_Mycgr3T

Mycgr3G65725 Mycgr3T
  
Location: 2163-3612

Mycgr3G65725\_Mycgr3T

Mycgr3G102276 Mycgr3
  
Location: 3712-4801

Mycgr3G102276\_Mycgr3

Mycgr3G89189 Mycgr3T
  
Location: 4901-5564

Mycgr3G89189\_Mycgr3T

Mycgr3G52682 Mycgr3T
  
Location: 5664-9231

Mycgr3G52682\_Mycgr3T

Mycgr3G107072 Mycgr3
  
Location: 9331-13279

Mycgr3G107072\_Mycgr3

Mycgr3G34982 Mycgr3T
  
Location: 13379-15116

Mycgr3G34982\_Mycgr3T

Mycgr3G107069 Mycgr3
  
Location: 15216-17097

Mycgr3G107069\_Mycgr3

Mycgr3G32432 Mycgr3T
  
Location: 17197-19042

Mycgr3G32432\_Mycgr3T

Mycgr3G98385 Mycgr3T
  
Location: 19142-19898

Mycgr3G98385\_Mycgr3T

predicted protein
  
Accession: EDN94634
  
Location: 1153251-1153468
  
 NCBI BlastP on this gene

EDN94634

predicted protein
  
Accession: EDN94635
  
Location: 1155526-1155883
  
 NCBI BlastP on this gene

EDN94635

hypothetical protein
  
Accession: EDN94636
  
Location: 1156302-1159106
  
 NCBI BlastP on this gene

EDN94636

predicted protein
  
Accession: EDN94637
  
Location: 1160592-1161856
  
 NCBI BlastP on this gene

EDN94637

hypothetical protein
  
Accession: EDN94638
  
Location: 1162787-1163627
  
  
**BlastP hit with Mycgr3G102281\_Mycgr3**
  
Percentage identity: 91 %
  
BlastP bit score: 355
  
Sequence coverage: 99 %
  
E-value: 6e-122
  
  
 NCBI BlastP on this gene

EDN94638

hypothetical protein
  
Accession: EDN94639
  
Location: 1164642-1168452
  
  
**BlastP hit with Mycgr3G52682\_Mycgr3T**
  
Percentage identity: 46 %
  
BlastP bit score: 954
  
Sequence coverage: 101 %
  
E-value: 0.0
  
  
 NCBI BlastP on this gene

EDN94639

predicted protein
  
Accession: EDN94640
  
Location: 1168580-1168897
  
 NCBI BlastP on this gene

EDN94640

hypothetical protein
  
Accession: EDN94641
  
Location: 1168982-1169815
  
 NCBI BlastP on this gene

EDN94641

hypothetical protein
  
Accession: EDN94642
  
Location: 1170428-1172215
  
 NCBI BlastP on this gene

EDN94642

predicted protein
  
Accession: EDN94643
  
Location: 1173080-1173178
  
 NCBI BlastP on this gene

EDN94643

predicted protein
  
Accession: EDN94644
  
Location: 1173525-1174727
  
 NCBI BlastP on this gene

EDN94644

hypothetical protein
  
Accession: EDN94645
  
Location: 1175079-1175859
  
 NCBI BlastP on this gene

EDN94645

hypothetical protein
  
Accession: EDN94646
  
Location: 1176452-1177160
  
 NCBI BlastP on this gene

EDN94646

hypothetical protein
  
Accession: EDN94647
  
Location: 1177426-1178317
  
 NCBI BlastP on this gene

EDN94647

Query: Architecture Search FASTA input

AKHY01000097 : Aspergillus oryzae 3.042    Total score: 2.0     Cumulative Blast bit score: 1309

Hit cluster cross-links:

Mycgr3G52686 Mycgr3T
  
Location: 0-861

Mycgr3G52686\_Mycgr3T

Mycgr3G102281 Mycgr3
  
Location: 961-1573

Mycgr3G102281\_Mycgr3

Mycgr3G89185 Mycgr3T
  
Location: 1673-2063

Mycgr3G89185\_Mycgr3T

Mycgr3G65725 Mycgr3T
  
Location: 2163-3612

Mycgr3G65725\_Mycgr3T

Mycgr3G102276 Mycgr3
  
Location: 3712-4801

Mycgr3G102276\_Mycgr3

Mycgr3G89189 Mycgr3T
  
Location: 4901-5564

Mycgr3G89189\_Mycgr3T

Mycgr3G52682 Mycgr3T
  
Location: 5664-9231

Mycgr3G52682\_Mycgr3T

Mycgr3G107072 Mycgr3
  
Location: 9331-13279

Mycgr3G107072\_Mycgr3

Mycgr3G34982 Mycgr3T
  
Location: 13379-15116

Mycgr3G34982\_Mycgr3T

Mycgr3G107069 Mycgr3
  
Location: 15216-17097

Mycgr3G107069\_Mycgr3

Mycgr3G32432 Mycgr3T
  
Location: 17197-19042

Mycgr3G32432\_Mycgr3T

Mycgr3G98385 Mycgr3T
  
Location: 19142-19898

Mycgr3G98385\_Mycgr3T

uncharacterized protein
  
Accession: EIT81800
  
Location: 118841-121552
  
 NCBI BlastP on this gene

EIT81800

60s ribosomal protein
  
Accession: EIT81821
  
Location: 122848-123792
  
  
**BlastP hit with Mycgr3G102281\_Mycgr3**
  
Percentage identity: 89 %
  
BlastP bit score: 380
  
Sequence coverage: 99 %
  
E-value: 6e-132
  
  
 NCBI BlastP on this gene

EIT81821

E3 ubiquitin protein ligase
  
Accession: EIT81787
  
Location: 125017-128694
  
  
**BlastP hit with Mycgr3G52682\_Mycgr3T**
  
Percentage identity: 44 %
  
BlastP bit score: 929
  
Sequence coverage: 105 %
  
E-value: 0.0
  
  
 NCBI BlastP on this gene

EIT81787

hypothetical protein
  
Accession: EIT81792
  
Location: 130363-130943
  
 NCBI BlastP on this gene

EIT81792

hypothetical protein
  
Accession: EIT81786
  
Location: 132688-134341
  
 NCBI BlastP on this gene

EIT81786

hypothetical protein
  
Accession: EIT81791
  
Location: 137925-139378
  
 NCBI BlastP on this gene

EIT81791

Query: Architecture Search FASTA input

FQ790317 : Botryotinia fuckeliana T4 SuperContig\_168\_1 genomic supercontig.    Total score: 2.0     Cumulative Blast bit score: 1288

Hit cluster cross-links:

Mycgr3G52686 Mycgr3T
  
Location: 0-861

Mycgr3G52686\_Mycgr3T

Mycgr3G102281 Mycgr3
  
Location: 961-1573

Mycgr3G102281\_Mycgr3

Mycgr3G89185 Mycgr3T
  
Location: 1673-2063

Mycgr3G89185\_Mycgr3T

Mycgr3G65725 Mycgr3T
  
Location: 2163-3612

Mycgr3G65725\_Mycgr3T

Mycgr3G102276 Mycgr3
  
Location: 3712-4801

Mycgr3G102276\_Mycgr3

Mycgr3G89189 Mycgr3T
  
Location: 4901-5564

Mycgr3G89189\_Mycgr3T

Mycgr3G52682 Mycgr3T
  
Location: 5664-9231

Mycgr3G52682\_Mycgr3T

Mycgr3G107072 Mycgr3
  
Location: 9331-13279

Mycgr3G107072\_Mycgr3

Mycgr3G34982 Mycgr3T
  
Location: 13379-15116

Mycgr3G34982\_Mycgr3T

Mycgr3G107069 Mycgr3
  
Location: 15216-17097

Mycgr3G107069\_Mycgr3

Mycgr3G32432 Mycgr3T
  
Location: 17197-19042

Mycgr3G32432\_Mycgr3T

Mycgr3G98385 Mycgr3T
  
Location: 19142-19898

Mycgr3G98385\_Mycgr3T

similar to PSP1 domain-containing protein
  
Accession: CCD34793
  
Location: 31254-34023
  
 NCBI BlastP on this gene

BofuT4\_P098520.1

hypothetical protein
  
Accession: CCD34792
  
Location: 28130-29233
  
 NCBI BlastP on this gene

BofuT4\_P098510.1

hypothetical protein
  
Accession: CCD34791
  
Location: 26208-27536
  
  
**BlastP hit with Mycgr3G102281\_Mycgr3**
  
Percentage identity: 91 %
  
BlastP bit score: 356
  
Sequence coverage: 99 %
  
E-value: 5e-121
  
  
 NCBI BlastP on this gene

BofuT4\_P098500.1

similar to ubiquitin-protein ligase E3
  
Accession: CCD34790
  
Location: 21386-25179
  
  
**BlastP hit with Mycgr3G52682\_Mycgr3T**
  
Percentage identity: 43 %
  
BlastP bit score: 932
  
Sequence coverage: 107 %
  
E-value: 0.0
  
  
 NCBI BlastP on this gene

BofuT4\_P098490.1

similar to 50S ribosomal protein Mrp49
  
Accession: CCD34789
  
Location: 19948-20791
  
 NCBI BlastP on this gene

BofuT4\_P098480.1

hypothetical protein
  
Accession: CCD34788
  
Location: 17580-19715
  
 NCBI BlastP on this gene

BofuT4\_P098470.1

hypothetical protein
  
Accession: CCD34787
  
Location: 16329-16781
  
 NCBI BlastP on this gene

BofuT4\_P098460.1

hypothetical protein
  
Accession: CCD34786
  
Location: 13592-15817
  
 NCBI BlastP on this gene

BofuT4\_P098450.1

hypothetical protein
  
Accession: CCD34785
  
Location: 12493-13260
  
 NCBI BlastP on this gene

BofuT4\_P098440.1

Query: Architecture Search FASTA input

KB708110 : Botryotinia fuckeliana BcDW1 unplaced genomic scaffold Scaffold\_438    Total score: 2.0     Cumulative Blast bit score: 1287

Hit cluster cross-links:

Mycgr3G52686 Mycgr3T
  
Location: 0-861

Mycgr3G52686\_Mycgr3T

Mycgr3G102281 Mycgr3
  
Location: 961-1573

Mycgr3G102281\_Mycgr3

Mycgr3G89185 Mycgr3T
  
Location: 1673-2063

Mycgr3G89185\_Mycgr3T

Mycgr3G65725 Mycgr3T
  
Location: 2163-3612

Mycgr3G65725\_Mycgr3T

Mycgr3G102276 Mycgr3
  
Location: 3712-4801

Mycgr3G102276\_Mycgr3

Mycgr3G89189 Mycgr3T
  
Location: 4901-5564

Mycgr3G89189\_Mycgr3T

Mycgr3G52682 Mycgr3T
  
Location: 5664-9231

Mycgr3G52682\_Mycgr3T

Mycgr3G107072 Mycgr3
  
Location: 9331-13279

Mycgr3G107072\_Mycgr3

Mycgr3G34982 Mycgr3T
  
Location: 13379-15116

Mycgr3G34982\_Mycgr3T

Mycgr3G107069 Mycgr3
  
Location: 15216-17097

Mycgr3G107069\_Mycgr3

Mycgr3G32432 Mycgr3T
  
Location: 17197-19042

Mycgr3G32432\_Mycgr3T

Mycgr3G98385 Mycgr3T
  
Location: 19142-19898

Mycgr3G98385\_Mycgr3T

putative psp1 domain-containing protein
  
Accession: EMR80688
  
Location: 38749-41518
  
 NCBI BlastP on this gene

EMR80688

hypothetical protein
  
Accession: EMR80687
  
Location: 35999-37102
  
 NCBI BlastP on this gene

EMR80687

putative 60s ribosomal protein l15 protein
  
Accession: EMR80686
  
Location: 34077-35405
  
  
**BlastP hit with Mycgr3G102281\_Mycgr3**
  
Percentage identity: 91 %
  
BlastP bit score: 356
  
Sequence coverage: 99 %
  
E-value: 5e-121
  
  
 NCBI BlastP on this gene

EMR80686

putative ubiquitin-protein ligase protein
  
Accession: EMR80685
  
Location: 29255-33048
  
  
**BlastP hit with Mycgr3G52682\_Mycgr3T**
  
Percentage identity: 43 %
  
BlastP bit score: 931
  
Sequence coverage: 107 %
  
E-value: 0.0
  
  
 NCBI BlastP on this gene

EMR80685

putative 50s ribosomal protein mrp49 protein
  
Accession: EMR80684
  
Location: 27839-28661
  
 NCBI BlastP on this gene

EMR80684

putative gtp-binding protein
  
Accession: EMR80683
  
Location: 25450-27585
  
 NCBI BlastP on this gene

EMR80683

hypothetical protein
  
Accession: EMR80682
  
Location: 21460-24651
  
 NCBI BlastP on this gene

EMR80682

putative ribosome biogenesis protein nhp2 protein
  
Accession: EMR80681
  
Location: 20361-21128
  
 NCBI BlastP on this gene

EMR80681

Query: Architecture Search FASTA input

DS027059 : Aspergillus clavatus NRRL 1 1099423829805 genomic scaffold    Total score: 2.0     Cumulative Blast bit score: 1278

Hit cluster cross-links:

Mycgr3G52686 Mycgr3T
  
Location: 0-861

Mycgr3G52686\_Mycgr3T

Mycgr3G102281 Mycgr3
  
Location: 961-1573

Mycgr3G102281\_Mycgr3

Mycgr3G89185 Mycgr3T
  
Location: 1673-2063

Mycgr3G89185\_Mycgr3T

Mycgr3G65725 Mycgr3T
  
Location: 2163-3612

Mycgr3G65725\_Mycgr3T

Mycgr3G102276 Mycgr3
  
Location: 3712-4801

Mycgr3G102276\_Mycgr3

Mycgr3G89189 Mycgr3T
  
Location: 4901-5564

Mycgr3G89189\_Mycgr3T

Mycgr3G52682 Mycgr3T
  
Location: 5664-9231

Mycgr3G52682\_Mycgr3T

Mycgr3G107072 Mycgr3
  
Location: 9331-13279

Mycgr3G107072\_Mycgr3

Mycgr3G34982 Mycgr3T
  
Location: 13379-15116

Mycgr3G34982\_Mycgr3T

Mycgr3G107069 Mycgr3
  
Location: 15216-17097

Mycgr3G107069\_Mycgr3

Mycgr3G32432 Mycgr3T
  
Location: 17197-19042

Mycgr3G32432\_Mycgr3T

Mycgr3G98385 Mycgr3T
  
Location: 19142-19898

Mycgr3G98385\_Mycgr3T

PSP1 domain protein
  
Accession: EAW08268
  
Location: 3603658-3606355
  
 NCBI BlastP on this gene

EAW08268

60S ribosomal protein L15
  
Accession: EAW08269
  
Location: 3607471-3608529
  
  
**BlastP hit with Mycgr3G102281\_Mycgr3**
  
Percentage identity: 89 %
  
BlastP bit score: 343
  
Sequence coverage: 99 %
  
E-value: 2e-117
  
  
 NCBI BlastP on this gene

EAW08269

ubiquitin-protein ligase (Hul4), putative
  
Accession: EAW08270
  
Location: 3609760-3613489
  
  
**BlastP hit with Mycgr3G52682\_Mycgr3T**
  
Percentage identity: 44 %
  
BlastP bit score: 935
  
Sequence coverage: 100 %
  
E-value: 0.0
  
  
 NCBI BlastP on this gene

EAW08270

alcohol dehydrogenase, zinc-containing, putative
  
Accession: EAW08271
  
Location: 3615663-3617156
  
 NCBI BlastP on this gene

EAW08271

conserved hypothetical protein
  
Accession: EAW08272
  
Location: 3617704-3618676
  
 NCBI BlastP on this gene

EAW08272

RING finger domain protein, putative
  
Accession: EAW08273
  
Location: 3619192-3620732
  
 NCBI BlastP on this gene

EAW08273

transcriptional regulator Ngg1, putative
  
Accession: EAW08274
  
Location: 3621997-3624159
  
 NCBI BlastP on this gene

EAW08274

Query: Architecture Search FASTA input

DS499594 : Aspergillus fumigatus A1163 scf\_000001 genomic scaffold    Total score: 2.0     Cumulative Blast bit score: 1275

Hit cluster cross-links:

Mycgr3G52686 Mycgr3T
  
Location: 0-861

Mycgr3G52686\_Mycgr3T

Mycgr3G102281 Mycgr3
  
Location: 961-1573

Mycgr3G102281\_Mycgr3

Mycgr3G89185 Mycgr3T
  
Location: 1673-2063

Mycgr3G89185\_Mycgr3T

Mycgr3G65725 Mycgr3T
  
Location: 2163-3612

Mycgr3G65725\_Mycgr3T

Mycgr3G102276 Mycgr3
  
Location: 3712-4801

Mycgr3G102276\_Mycgr3

Mycgr3G89189 Mycgr3T
  
Location: 4901-5564

Mycgr3G89189\_Mycgr3T

Mycgr3G52682 Mycgr3T
  
Location: 5664-9231

Mycgr3G52682\_Mycgr3T

Mycgr3G107072 Mycgr3
  
Location: 9331-13279

Mycgr3G107072\_Mycgr3

Mycgr3G34982 Mycgr3T
  
Location: 13379-15116

Mycgr3G34982\_Mycgr3T

Mycgr3G107069 Mycgr3
  
Location: 15216-17097

Mycgr3G107069\_Mycgr3

Mycgr3G32432 Mycgr3T
  
Location: 17197-19042

Mycgr3G32432\_Mycgr3T

Mycgr3G98385 Mycgr3T
  
Location: 19142-19898

Mycgr3G98385\_Mycgr3T

PSP1 domain protein
  
Accession: EDP55805
  
Location: 1399364-1402060
  
 NCBI BlastP on this gene

EDP55805

60S ribosomal protein L15
  
Accession: EDP55804
  
Location: 1397261-1398292
  
  
**BlastP hit with Mycgr3G102281\_Mycgr3**
  
Percentage identity: 79 %
  
BlastP bit score: 340
  
Sequence coverage: 113 %
  
E-value: 9e-116
  
  
 NCBI BlastP on this gene

EDP55804

ubiquitin-protein ligase (Hul4), putative
  
Accession: EDP55803
  
Location: 1392365-1396074
  
  
**BlastP hit with Mycgr3G52682\_Mycgr3T**
  
Percentage identity: 43 %
  
BlastP bit score: 935
  
Sequence coverage: 105 %
  
E-value: 0.0
  
  
 NCBI BlastP on this gene

EDP55803

hypothetical protein
  
Accession: EDP55802
  
Location: 1390745-1391132
  
 NCBI BlastP on this gene

EDP55802

alcohol dehydrogenase, zinc-containing, putative
  
Accession: EDP55801
  
Location: 1389233-1390568
  
 NCBI BlastP on this gene

EDP55801

conserved hypothetical protein
  
Accession: EDP55800
  
Location: 1387763-1388680
  
 NCBI BlastP on this gene

EDP55800

RING finger domain protein, putative
  
Accession: EDP55799
  
Location: 1385398-1387301
  
 NCBI BlastP on this gene

EDP55799

transcriptional regulator Ngg1, putative
  
Accession: EDP55798
  
Location: 1382242-1384493
  
 NCBI BlastP on this gene

EDP55798

Query: Architecture Search FASTA input

AAHF01000007 : Aspergillus fumigatus Af293    Total score: 2.0     Cumulative Blast bit score: 1275

Hit cluster cross-links:

Mycgr3G52686 Mycgr3T
  
Location: 0-861

Mycgr3G52686\_Mycgr3T

Mycgr3G102281 Mycgr3
  
Location: 961-1573

Mycgr3G102281\_Mycgr3

Mycgr3G89185 Mycgr3T
  
Location: 1673-2063

Mycgr3G89185\_Mycgr3T

Mycgr3G65725 Mycgr3T
  
Location: 2163-3612

Mycgr3G65725\_Mycgr3T

Mycgr3G102276 Mycgr3
  
Location: 3712-4801

Mycgr3G102276\_Mycgr3

Mycgr3G89189 Mycgr3T
  
Location: 4901-5564

Mycgr3G89189\_Mycgr3T

Mycgr3G52682 Mycgr3T
  
Location: 5664-9231

Mycgr3G52682\_Mycgr3T

Mycgr3G107072 Mycgr3
  
Location: 9331-13279

Mycgr3G107072\_Mycgr3

Mycgr3G34982 Mycgr3T
  
Location: 13379-15116

Mycgr3G34982\_Mycgr3T

Mycgr3G107069 Mycgr3
  
Location: 15216-17097

Mycgr3G107069\_Mycgr3

Mycgr3G32432 Mycgr3T
  
Location: 17197-19042

Mycgr3G32432\_Mycgr3T

Mycgr3G98385 Mycgr3T
  
Location: 19142-19898

Mycgr3G98385\_Mycgr3T

PSP1 domain protein
  
Accession: EAL88178
  
Location: 1319974-1322670
  
 NCBI BlastP on this gene

EAL88178

60S ribosomal protein L15
  
Accession: EAL88177
  
Location: 1317870-1318902
  
  
**BlastP hit with Mycgr3G102281\_Mycgr3**
  
Percentage identity: 79 %
  
BlastP bit score: 340
  
Sequence coverage: 113 %
  
E-value: 9e-116
  
  
 NCBI BlastP on this gene

EAL88177

ubiquitin-protein ligase (Hul4), putative
  
Accession: EAL88175
  
Location: 1311090-1314799
  
  
**BlastP hit with Mycgr3G52682\_Mycgr3T**
  
Percentage identity: 43 %
  
BlastP bit score: 935
  
Sequence coverage: 105 %
  
E-value: 0.0
  
  
 NCBI BlastP on this gene

EAL88175

hypothetical protein
  
Accession: EAL88174
  
Location: 1309470-1309857
  
 NCBI BlastP on this gene

EAL88174

alcohol dehydrogenase, zinc-containing, putative
  
Accession: EAL88173
  
Location: 1307958-1309293
  
 NCBI BlastP on this gene

EAL88173

conserved hypothetical protein
  
Accession: EAL88172
  
Location: 1306488-1307405
  
 NCBI BlastP on this gene

EAL88172

RING finger domain protein, putative
  
Accession: EAL88171
  
Location: 1304122-1306025
  
 NCBI BlastP on this gene

EAL88171

transcriptional regulator Ngg1, putative
  
Accession: EAL88170
  
Location: 1300966-1303217
  
 NCBI BlastP on this gene

EAL88170

Query: Architecture Search FASTA input

DS027688 : Neosartorya fischeri NRRL 181 1099437636249 genomic scaffold    Total score: 2.0     Cumulative Blast bit score: 1274

Hit cluster cross-links:

Mycgr3G52686 Mycgr3T
  
Location: 0-861

Mycgr3G52686\_Mycgr3T

Mycgr3G102281 Mycgr3
  
Location: 961-1573

Mycgr3G102281\_Mycgr3

Mycgr3G89185 Mycgr3T
  
Location: 1673-2063

Mycgr3G89185\_Mycgr3T

Mycgr3G65725 Mycgr3T
  
Location: 2163-3612

Mycgr3G65725\_Mycgr3T

Mycgr3G102276 Mycgr3
  
Location: 3712-4801

Mycgr3G102276\_Mycgr3

Mycgr3G89189 Mycgr3T
  
Location: 4901-5564

Mycgr3G89189\_Mycgr3T

Mycgr3G52682 Mycgr3T
  
Location: 5664-9231

Mycgr3G52682\_Mycgr3T

Mycgr3G107072 Mycgr3
  
Location: 9331-13279

Mycgr3G107072\_Mycgr3

Mycgr3G34982 Mycgr3T
  
Location: 13379-15116

Mycgr3G34982\_Mycgr3T

Mycgr3G107069 Mycgr3
  
Location: 15216-17097

Mycgr3G107069\_Mycgr3

Mycgr3G32432 Mycgr3T
  
Location: 17197-19042

Mycgr3G32432\_Mycgr3T

Mycgr3G98385 Mycgr3T
  
Location: 19142-19898

Mycgr3G98385\_Mycgr3T

PSP1 domain protein
  
Accession: EAW23301
  
Location: 3682103-3684800
  
 NCBI BlastP on this gene

EAW23301

60S ribosomal protein L15
  
Accession: EAW23302
  
Location: 3685931-3686967
  
  
**BlastP hit with Mycgr3G102281\_Mycgr3**
  
Percentage identity: 89 %
  
BlastP bit score: 345
  
Sequence coverage: 99 %
  
E-value: 5e-118
  
  
 NCBI BlastP on this gene

EAW23302

ubiquitin-protein ligase (Hul4), putative
  
Accession: EAW23303
  
Location: 3688177-3691889
  
  
**BlastP hit with Mycgr3G52682\_Mycgr3T**
  
Percentage identity: 45 %
  
BlastP bit score: 929
  
Sequence coverage: 96 %
  
E-value: 0.0
  
  
 NCBI BlastP on this gene

EAW23303

alcohol dehydrogenase, zinc-containing, putative
  
Accession: EAW23304
  
Location: 3693794-3695130
  
 NCBI BlastP on this gene

EAW23304

FRG1-like family protein
  
Accession: EAW23305
  
Location: 3695688-3696603
  
 NCBI BlastP on this gene

EAW23305

RING finger domain protein, putative
  
Accession: EAW23306
  
Location: 3697066-3698534
  
 NCBI BlastP on this gene

EAW23306

transcriptional regulator Ngg1, putative
  
Accession: EAW23307
  
Location: 3699904-3702154
  
 NCBI BlastP on this gene

EAW23307

Query: Architecture Search FASTA input

AKCU01000141 : Penicillium digitatum Pd1    Total score: 2.0     Cumulative Blast bit score: 1264

Hit cluster cross-links:

Mycgr3G52686 Mycgr3T
  
Location: 0-861

Mycgr3G52686\_Mycgr3T

Mycgr3G102281 Mycgr3
  
Location: 961-1573

Mycgr3G102281\_Mycgr3

Mycgr3G89185 Mycgr3T
  
Location: 1673-2063

Mycgr3G89185\_Mycgr3T

Mycgr3G65725 Mycgr3T
  
Location: 2163-3612

Mycgr3G65725\_Mycgr3T

Mycgr3G102276 Mycgr3
  
Location: 3712-4801

Mycgr3G102276\_Mycgr3

Mycgr3G89189 Mycgr3T
  
Location: 4901-5564

Mycgr3G89189\_Mycgr3T

Mycgr3G52682 Mycgr3T
  
Location: 5664-9231

Mycgr3G52682\_Mycgr3T

Mycgr3G107072 Mycgr3
  
Location: 9331-13279

Mycgr3G107072\_Mycgr3

Mycgr3G34982 Mycgr3T
  
Location: 13379-15116

Mycgr3G34982\_Mycgr3T

Mycgr3G107069 Mycgr3
  
Location: 15216-17097

Mycgr3G107069\_Mycgr3

Mycgr3G32432 Mycgr3T
  
Location: 17197-19042

Mycgr3G32432\_Mycgr3T

Mycgr3G98385 Mycgr3T
  
Location: 19142-19898

Mycgr3G98385\_Mycgr3T

hypothetical protein
  
Accession: EKV20036
  
Location: 55500-56393
  
 NCBI BlastP on this gene

EKV20036

Nitrogen permease regulator Npr2, putative
  
Accession: EKV20037
  
Location: 57754-59751
  
 NCBI BlastP on this gene

EKV20037

Transcriptional regulator Ngg1, putative
  
Accession: EKV20038
  
Location: 60078-62216
  
 NCBI BlastP on this gene

EKV20038

hypothetical protein
  
Accession: EKV20039
  
Location: 63571-66669
  
  
**BlastP hit with Mycgr3G102281\_Mycgr3**
  
Percentage identity: 91 %
  
BlastP bit score: 389
  
Sequence coverage: 99 %
  
E-value: 1e-130
  
  
 NCBI BlastP on this gene

EKV20039

Ubiquitin-protein ligase (Hul4), putative
  
Accession: EKV20040
  
Location: 68998-72682
  
  
**BlastP hit with Mycgr3G52682\_Mycgr3T**
  
Percentage identity: 42 %
  
BlastP bit score: 875
  
Sequence coverage: 105 %
  
E-value: 0.0
  
  
 NCBI BlastP on this gene

EKV20040

FAD dependent oxidoreductase, putative
  
Accession: EKV20041
  
Location: 73856-74984
  
 NCBI BlastP on this gene

EKV20041

hypothetical protein
  
Accession: EKV20042
  
Location: 75902-76045
  
 NCBI BlastP on this gene

EKV20042

hypothetical protein
  
Accession: EKV20043
  
Location: 76104-76217
  
 NCBI BlastP on this gene

EKV20043

hypothetical protein
  
Accession: EKV20044
  
Location: 76854-78100
  
 NCBI BlastP on this gene

EKV20044

Query: Architecture Search FASTA input

AKCT01000207 : Penicillium digitatum PHI26    Total score: 2.0     Cumulative Blast bit score: 1264

Hit cluster cross-links:

Mycgr3G52686 Mycgr3T
  
Location: 0-861

Mycgr3G52686\_Mycgr3T

Mycgr3G102281 Mycgr3
  
Location: 961-1573

Mycgr3G102281\_Mycgr3

Mycgr3G89185 Mycgr3T
  
Location: 1673-2063

Mycgr3G89185\_Mycgr3T

Mycgr3G65725 Mycgr3T
  
Location: 2163-3612

Mycgr3G65725\_Mycgr3T

Mycgr3G102276 Mycgr3
  
Location: 3712-4801

Mycgr3G102276\_Mycgr3

Mycgr3G89189 Mycgr3T
  
Location: 4901-5564

Mycgr3G89189\_Mycgr3T

Mycgr3G52682 Mycgr3T
  
Location: 5664-9231

Mycgr3G52682\_Mycgr3T

Mycgr3G107072 Mycgr3
  
Location: 9331-13279

Mycgr3G107072\_Mycgr3

Mycgr3G34982 Mycgr3T
  
Location: 13379-15116

Mycgr3G34982\_Mycgr3T

Mycgr3G107069 Mycgr3
  
Location: 15216-17097

Mycgr3G107069\_Mycgr3

Mycgr3G32432 Mycgr3T
  
Location: 17197-19042

Mycgr3G32432\_Mycgr3T

Mycgr3G98385 Mycgr3T
  
Location: 19142-19898

Mycgr3G98385\_Mycgr3T

hypothetical protein
  
Accession: EKV11305
  
Location: 126554-127447
  
 NCBI BlastP on this gene

EKV11305

Nitrogen permease regulator Npr2, putative
  
Accession: EKV11306
  
Location: 128808-130805
  
 NCBI BlastP on this gene

EKV11306

Transcriptional regulator Ngg1, putative
  
Accession: EKV11307
  
Location: 131134-133272
  
 NCBI BlastP on this gene

EKV11307

hypothetical protein
  
Accession: EKV11308
  
Location: 134627-137726
  
  
**BlastP hit with Mycgr3G102281\_Mycgr3**
  
Percentage identity: 91 %
  
BlastP bit score: 389
  
Sequence coverage: 99 %
  
E-value: 1e-130
  
  
 NCBI BlastP on this gene

EKV11308

Ubiquitin-protein ligase (Hul4), putative
  
Accession: EKV11309
  
Location: 140057-143741
  
  
**BlastP hit with Mycgr3G52682\_Mycgr3T**
  
Percentage identity: 42 %
  
BlastP bit score: 875
  
Sequence coverage: 105 %
  
E-value: 0.0
  
  
 NCBI BlastP on this gene

EKV11309

FAD dependent oxidoreductase, putative
  
Accession: EKV11310
  
Location: 144680-145868
  
 NCBI BlastP on this gene

EKV11310

hypothetical protein
  
Accession: EKV11311
  
Location: 146770-146931
  
 NCBI BlastP on this gene

EKV11311

hypothetical protein
  
Accession: EKV11312
  
Location: 146990-147103
  
 NCBI BlastP on this gene

EKV11312

hypothetical protein
  
Accession: EKV11313
  
Location: 147740-148986
  
 NCBI BlastP on this gene

EKV11313

Query: Architecture Search FASTA input

ACJE01000004 : Aspergillus niger ATCC 1015    Total score: 2.0     Cumulative Blast bit score: 1258

Hit cluster cross-links:

Mycgr3G52686 Mycgr3T
  
Location: 0-861

Mycgr3G52686\_Mycgr3T

Mycgr3G102281 Mycgr3
  
Location: 961-1573

Mycgr3G102281\_Mycgr3

Mycgr3G89185 Mycgr3T
  
Location: 1673-2063

Mycgr3G89185\_Mycgr3T

Mycgr3G65725 Mycgr3T
  
Location: 2163-3612

Mycgr3G65725\_Mycgr3T

Mycgr3G102276 Mycgr3
  
Location: 3712-4801

Mycgr3G102276\_Mycgr3

Mycgr3G89189 Mycgr3T
  
Location: 4901-5564

Mycgr3G89189\_Mycgr3T

Mycgr3G52682 Mycgr3T
  
Location: 5664-9231

Mycgr3G52682\_Mycgr3T

Mycgr3G107072 Mycgr3
  
Location: 9331-13279

Mycgr3G107072\_Mycgr3

Mycgr3G34982 Mycgr3T
  
Location: 13379-15116

Mycgr3G34982\_Mycgr3T

Mycgr3G107069 Mycgr3
  
Location: 15216-17097

Mycgr3G107069\_Mycgr3

Mycgr3G32432 Mycgr3T
  
Location: 17197-19042

Mycgr3G32432\_Mycgr3T

Mycgr3G98385 Mycgr3T
  
Location: 19142-19898

Mycgr3G98385\_Mycgr3T

hypothetical protein
  
Accession: EHA26331
  
Location: 961973-962589
  
 NCBI BlastP on this gene

EHA26331

hypothetical protein
  
Accession: EHA26332
  
Location: 965372-967910
  
 NCBI BlastP on this gene

EHA26332

hypothetical protein
  
Accession: EHA26333
  
Location: 969084-970054
  
  
**BlastP hit with Mycgr3G102281\_Mycgr3**
  
Percentage identity: 89 %
  
BlastP bit score: 381
  
Sequence coverage: 99 %
  
E-value: 4e-132
  
  
 NCBI BlastP on this gene

EHA26333

hypothetical protein
  
Accession: EHA26334
  
Location: 971349-975054
  
  
**BlastP hit with Mycgr3G52682\_Mycgr3T**
  
Percentage identity: 42 %
  
BlastP bit score: 877
  
Sequence coverage: 104 %
  
E-value: 0.0
  
  
 NCBI BlastP on this gene

EHA26334

hypothetical protein
  
Accession: EHA26335
  
Location: 976867-978222
  
 NCBI BlastP on this gene

EHA26335

hypothetical protein
  
Accession: EHA26336
  
Location: 978711-979725
  
 NCBI BlastP on this gene

EHA26336

hypothetical protein
  
Accession: EHA26337
  
Location: 979840-981055
  
 NCBI BlastP on this gene

EHA26337

hypothetical protein
  
Accession: EHA26338
  
Location: 982385-984530
  
 NCBI BlastP on this gene

EHA26338

Query: Architecture Search FASTA input

AM920431 : Penicillium chrysogenum Wisconsin 54-1255 complete genome, contig Pc00c16.    Total score: 2.0     Cumulative Blast bit score: 1255

Hit cluster cross-links:

Mycgr3G52686 Mycgr3T
  
Location: 0-861

Mycgr3G52686\_Mycgr3T

Mycgr3G102281 Mycgr3
  
Location: 961-1573

Mycgr3G102281\_Mycgr3

Mycgr3G89185 Mycgr3T
  
Location: 1673-2063

Mycgr3G89185\_Mycgr3T

Mycgr3G65725 Mycgr3T
  
Location: 2163-3612

Mycgr3G65725\_Mycgr3T

Mycgr3G102276 Mycgr3
  
Location: 3712-4801

Mycgr3G102276\_Mycgr3

Mycgr3G89189 Mycgr3T
  
Location: 4901-5564

Mycgr3G89189\_Mycgr3T

Mycgr3G52682 Mycgr3T
  
Location: 5664-9231

Mycgr3G52682\_Mycgr3T

Mycgr3G107072 Mycgr3
  
Location: 9331-13279

Mycgr3G107072\_Mycgr3

Mycgr3G34982 Mycgr3T
  
Location: 13379-15116

Mycgr3G34982\_Mycgr3T

Mycgr3G107069 Mycgr3
  
Location: 15216-17097

Mycgr3G107069\_Mycgr3

Mycgr3G32432 Mycgr3T
  
Location: 17197-19042

Mycgr3G32432\_Mycgr3T

Mycgr3G98385 Mycgr3T
  
Location: 19142-19898

Mycgr3G98385\_Mycgr3T

not annotated
  
Accession: CAP93590
  
Location: 2210127-2211017
  
 NCBI BlastP on this gene

Pc16g09200

not annotated
  
Accession: CAP93589
  
Location: 2207048-2208889
  
 NCBI BlastP on this gene

Pc16g09190

not annotated
  
Accession: CAP93588
  
Location: 2204414-2206548
  
 NCBI BlastP on this gene

Pc16g09180

not annotated
  
Accession: CAP93587
  
Location: 2201826-2203246
  
 NCBI BlastP on this gene

Pc16g09170

not annotated
  
Accession: CAP93586
  
Location: 2200142-2201117
  
  
**BlastP hit with Mycgr3G102281\_Mycgr3**
  
Percentage identity: 91 %
  
BlastP bit score: 381
  
Sequence coverage: 99 %
  
E-value: 3e-132
  
  
 NCBI BlastP on this gene

Pc16g09160

not annotated
  
Accession: CAP93585
  
Location: 2195577-2199256
  
  
**BlastP hit with Mycgr3G52682\_Mycgr3T**
  
Percentage identity: 42 %
  
BlastP bit score: 874
  
Sequence coverage: 104 %
  
E-value: 0.0
  
  
 NCBI BlastP on this gene

Pc16g09150

not annotated
  
Accession: CAP93584
  
Location: 2193251-2194882
  
 NCBI BlastP on this gene

Pc16g09140

not annotated
  
Accession: CAP93583
  
Location: 2189649-2192510
  
 NCBI BlastP on this gene

Pc16g09130

not annotated
  
Accession: CAP93582
  
Location: 2188182-2189453
  
 NCBI BlastP on this gene

Pc16g09120

not annotated
  
Accession: Pc16g09110
  
Location: 2186985-2187682
  
 NCBI BlastP on this gene

Pc16g09110

not annotated
  
Accession: CAP93580
  
Location: 2183682-2186583
  
 NCBI BlastP on this gene

Pc16g09100

Query: Architecture Search FASTA input

DS572753 : Paracoccidioides brasiliensis Pb18 supercont1.4 genomic scaffold    Total score: 2.0     Cumulative Blast bit score: 1254

Hit cluster cross-links:

Mycgr3G52686 Mycgr3T
  
Location: 0-861

Mycgr3G52686\_Mycgr3T

Mycgr3G102281 Mycgr3
  
Location: 961-1573

Mycgr3G102281\_Mycgr3

Mycgr3G89185 Mycgr3T
  
Location: 1673-2063

Mycgr3G89185\_Mycgr3T

Mycgr3G65725 Mycgr3T
  
Location: 2163-3612

Mycgr3G65725\_Mycgr3T

Mycgr3G102276 Mycgr3
  
Location: 3712-4801

Mycgr3G102276\_Mycgr3

Mycgr3G89189 Mycgr3T
  
Location: 4901-5564

Mycgr3G89189\_Mycgr3T

Mycgr3G52682 Mycgr3T
  
Location: 5664-9231

Mycgr3G52682\_Mycgr3T

Mycgr3G107072 Mycgr3
  
Location: 9331-13279

Mycgr3G107072\_Mycgr3

Mycgr3G34982 Mycgr3T
  
Location: 13379-15116

Mycgr3G34982\_Mycgr3T

Mycgr3G107069 Mycgr3
  
Location: 15216-17097

Mycgr3G107069\_Mycgr3

Mycgr3G32432 Mycgr3T
  
Location: 17197-19042

Mycgr3G32432\_Mycgr3T

Mycgr3G98385 Mycgr3T
  
Location: 19142-19898

Mycgr3G98385\_Mycgr3T

PSP1 domain-containing protein
  
Accession: EEH47771
  
Location: 1082443-1085228
  
 NCBI BlastP on this gene

EEH47771

60S ribosomal protein L15
  
Accession: EEH47772
  
Location: 1089359-1090343
  
  
**BlastP hit with Mycgr3G102281\_Mycgr3**
  
Percentage identity: 89 %
  
BlastP bit score: 375
  
Sequence coverage: 99 %
  
E-value: 1e-129
  
  
 NCBI BlastP on this gene

EEH47772

E3 ubiquitin-protein ligase HUWE1
  
Accession: EEH47773
  
Location: 1094074-1098658
  
  
**BlastP hit with Mycgr3G52682\_Mycgr3T**
  
Percentage identity: 42 %
  
BlastP bit score: 879
  
Sequence coverage: 103 %
  
E-value: 0.0
  
  
 NCBI BlastP on this gene

EEH47773

predicted protein
  
Accession: EEH47774
  
Location: 1098795-1100592
  
 NCBI BlastP on this gene

EEH47774

NADP-dependent mannitol dehydrogenase
  
Accession: EEH47775
  
Location: 1101321-1103167
  
 NCBI BlastP on this gene

EEH47775

conserved hypothetical protein
  
Accession: EEH47776
  
Location: 1105206-1106179
  
 NCBI BlastP on this gene

EEH47776

predicted protein
  
Accession: EEH47777
  
Location: 1106281-1106746
  
 NCBI BlastP on this gene

EEH47777

Query: Architecture Search FASTA input

GL573405 : Geomyces destructans 20631-21 unplaced genomic scaffold supercont1.237    Total score: 2.0     Cumulative Blast bit score: 1247

Hit cluster cross-links:

Mycgr3G52686 Mycgr3T
  
Location: 0-861

Mycgr3G52686\_Mycgr3T

Mycgr3G102281 Mycgr3
  
Location: 961-1573

Mycgr3G102281\_Mycgr3

Mycgr3G89185 Mycgr3T
  
Location: 1673-2063

Mycgr3G89185\_Mycgr3T

Mycgr3G65725 Mycgr3T
  
Location: 2163-3612

Mycgr3G65725\_Mycgr3T

Mycgr3G102276 Mycgr3
  
Location: 3712-4801

Mycgr3G102276\_Mycgr3

Mycgr3G89189 Mycgr3T
  
Location: 4901-5564

Mycgr3G89189\_Mycgr3T

Mycgr3G52682 Mycgr3T
  
Location: 5664-9231

Mycgr3G52682\_Mycgr3T

Mycgr3G107072 Mycgr3
  
Location: 9331-13279

Mycgr3G107072\_Mycgr3

Mycgr3G34982 Mycgr3T
  
Location: 13379-15116

Mycgr3G34982\_Mycgr3T

Mycgr3G107069 Mycgr3
  
Location: 15216-17097

Mycgr3G107069\_Mycgr3

Mycgr3G32432 Mycgr3T
  
Location: 17197-19042

Mycgr3G32432\_Mycgr3T

Mycgr3G98385 Mycgr3T
  
Location: 19142-19898

Mycgr3G98385\_Mycgr3T

hypothetical protein
  
Accession: ELR05721
  
Location: 7605-10437
  
 NCBI BlastP on this gene

ELR05721

60S ribosomal protein L15
  
Accession: ELR05722
  
Location: 11233-12050
  
  
**BlastP hit with Mycgr3G102281\_Mycgr3**
  
Percentage identity: 89 %
  
BlastP bit score: 350
  
Sequence coverage: 99 %
  
E-value: 5e-120
  
  
 NCBI BlastP on this gene

ELR05722

hypothetical protein
  
Accession: ELR05723
  
Location: 13646-14860
  
 NCBI BlastP on this gene

ELR05723

hypothetical protein
  
Accession: ELR05724
  
Location: 15501-16432
  
 NCBI BlastP on this gene

ELR05724

hypothetical protein
  
Accession: ELR05725
  
Location: 16491-17537
  
 NCBI BlastP on this gene

ELR05725

cytochrome c oxidase subunit VIb
  
Accession: ELR05726
  
Location: 18149-18668
  
 NCBI BlastP on this gene

ELR05726

hypothetical protein
  
Accession: ELR05727
  
Location: 19757-20286
  
 NCBI BlastP on this gene

ELR05727

hypothetical protein
  
Accession: ELR05728
  
Location: 20657-24512
  
 NCBI BlastP on this gene

ELR05728

hypothetical protein
  
Accession: ELR05729
  
Location: 25301-28987
  
  
**BlastP hit with Mycgr3G52682\_Mycgr3T**
  
Percentage identity: 45 %
  
BlastP bit score: 897
  
Sequence coverage: 94 %
  
E-value: 0.0
  
  
 NCBI BlastP on this gene

ELR05729

hypothetical protein
  
Accession: ELR05730
  
Location: 29219-29958
  
 NCBI BlastP on this gene

ELR05730

hypothetical protein
  
Accession: ELR05731
  
Location: 30219-32216
  
 NCBI BlastP on this gene

ELR05731

hypothetical protein
  
Accession: ELR05732
  
Location: 32336-33228
  
 NCBI BlastP on this gene

ELR05732

Query: Architecture Search FASTA input

DS572812 : Paracoccidioides brasiliensis Pb01 supercont1.2 genomic scaffold    Total score: 2.0     Cumulative Blast bit score: 1242

Hit cluster cross-links:

Mycgr3G52686 Mycgr3T
  
Location: 0-861

Mycgr3G52686\_Mycgr3T

Mycgr3G102281 Mycgr3
  
Location: 961-1573

Mycgr3G102281\_Mycgr3

Mycgr3G89185 Mycgr3T
  
Location: 1673-2063

Mycgr3G89185\_Mycgr3T

Mycgr3G65725 Mycgr3T
  
Location: 2163-3612

Mycgr3G65725\_Mycgr3T

Mycgr3G102276 Mycgr3
  
Location: 3712-4801

Mycgr3G102276\_Mycgr3

Mycgr3G89189 Mycgr3T
  
Location: 4901-5564

Mycgr3G89189\_Mycgr3T

Mycgr3G52682 Mycgr3T
  
Location: 5664-9231

Mycgr3G52682\_Mycgr3T

Mycgr3G107072 Mycgr3
  
Location: 9331-13279

Mycgr3G107072\_Mycgr3

Mycgr3G34982 Mycgr3T
  
Location: 13379-15116

Mycgr3G34982\_Mycgr3T

Mycgr3G107069 Mycgr3
  
Location: 15216-17097

Mycgr3G107069\_Mycgr3

Mycgr3G32432 Mycgr3T
  
Location: 17197-19042

Mycgr3G32432\_Mycgr3T

Mycgr3G98385 Mycgr3T
  
Location: 19142-19898

Mycgr3G98385\_Mycgr3T

PSP1 domain-containing protein
  
Accession: EEH38050
  
Location: 1033082-1035864
  
 NCBI BlastP on this gene

EEH38050

predicted protein
  
Accession: EEH38049
  
Location: 1029468-1031278
  
 NCBI BlastP on this gene

EEH38049

60S ribosomal protein L15
  
Accession: EEH38048
  
Location: 1027955-1028936
  
  
**BlastP hit with Mycgr3G102281\_Mycgr3**
  
Percentage identity: 89 %
  
BlastP bit score: 375
  
Sequence coverage: 99 %
  
E-value: 1e-129
  
  
 NCBI BlastP on this gene

EEH38048

E3 ubiquitin-protein ligase HUWE1
  
Accession: EEH38047
  
Location: 1019175-1023835
  
  
**BlastP hit with Mycgr3G52682\_Mycgr3T**
  
Percentage identity: 43 %
  
BlastP bit score: 867
  
Sequence coverage: 100 %
  
E-value: 0.0
  
  
 NCBI BlastP on this gene

EEH38047

predicted protein
  
Accession: EEH38046
  
Location: 1016713-1018802
  
 NCBI BlastP on this gene

EEH38046

L-threonine 3-dehydrogenase
  
Accession: EEH38045
  
Location: 1014399-1016140
  
 NCBI BlastP on this gene

EEH38045

predicted protein
  
Accession: EEH38044
  
Location: 1012325-1013503
  
 NCBI BlastP on this gene

EEH38044

conserved hypothetical protein
  
Accession: EEH38043
  
Location: 1011297-1012272
  
 NCBI BlastP on this gene

EEH38043

Query: Architecture Search FASTA input

AACD01000007 : Aspergillus nidulans FGSC A4    Total score: 2.0     Cumulative Blast bit score: 1238

Hit cluster cross-links:

Mycgr3G52686 Mycgr3T
  
Location: 0-861

Mycgr3G52686\_Mycgr3T

Mycgr3G102281 Mycgr3
  
Location: 961-1573

Mycgr3G102281\_Mycgr3

Mycgr3G89185 Mycgr3T
  
Location: 1673-2063

Mycgr3G89185\_Mycgr3T

Mycgr3G65725 Mycgr3T
  
Location: 2163-3612

Mycgr3G65725\_Mycgr3T

Mycgr3G102276 Mycgr3
  
Location: 3712-4801

Mycgr3G102276\_Mycgr3

Mycgr3G89189 Mycgr3T
  
Location: 4901-5564

Mycgr3G89189\_Mycgr3T

Mycgr3G52682 Mycgr3T
  
Location: 5664-9231

Mycgr3G52682\_Mycgr3T

Mycgr3G107072 Mycgr3
  
Location: 9331-13279

Mycgr3G107072\_Mycgr3

Mycgr3G34982 Mycgr3T
  
Location: 13379-15116

Mycgr3G34982\_Mycgr3T

Mycgr3G107069 Mycgr3
  
Location: 15216-17097

Mycgr3G107069\_Mycgr3

Mycgr3G32432 Mycgr3T
  
Location: 17197-19042

Mycgr3G32432\_Mycgr3T

Mycgr3G98385 Mycgr3T
  
Location: 19142-19898

Mycgr3G98385\_Mycgr3T

hypothetical protein
  
Accession: EAA66546
  
Location: 211697-212810
  
 NCBI BlastP on this gene

EAA66546

hypothetical protein
  
Accession: EAA66545
  
Location: 204111-206763
  
 NCBI BlastP on this gene

EAA66545

RL15 ASPNG 60S RIBOSOMAL PROTEIN L15
  
Accession: EAA66544
  
Location: 202260-203211
  
  
**BlastP hit with Mycgr3G102281\_Mycgr3**
  
Percentage identity: 90 %
  
BlastP bit score: 356
  
Sequence coverage: 99 %
  
E-value: 3e-122
  
  
 NCBI BlastP on this gene

EAA66544

hypothetical protein
  
Accession: EAA66543
  
Location: 197446-201058
  
  
**BlastP hit with Mycgr3G52682\_Mycgr3T**
  
Percentage identity: 44 %
  
BlastP bit score: 882
  
Sequence coverage: 91 %
  
E-value: 0.0
  
  
 NCBI BlastP on this gene

EAA66543

hypothetical protein
  
Accession: EAA66542
  
Location: 193897-195332
  
 NCBI BlastP on this gene

EAA66542

hypothetical protein
  
Accession: EAA66541
  
Location: 192532-193540
  
 NCBI BlastP on this gene

EAA66541

hypothetical protein
  
Accession: EAA66540
  
Location: 190754-192487
  
 NCBI BlastP on this gene

EAA66540

hypothetical protein
  
Accession: EAA66539
  
Location: 187586-189673
  
 NCBI BlastP on this gene

EAA66539

Query: Architecture Search FASTA input

DF126464 : Aspergillus kawachii IFO 4308 DNA, contig: scaffold00018    Total score: 2.0     Cumulative Blast bit score: 1236

Hit cluster cross-links:

Mycgr3G52686 Mycgr3T
  
Location: 0-861

Mycgr3G52686\_Mycgr3T

Mycgr3G102281 Mycgr3
  
Location: 961-1573

Mycgr3G102281\_Mycgr3

Mycgr3G89185 Mycgr3T
  
Location: 1673-2063

Mycgr3G89185\_Mycgr3T

Mycgr3G65725 Mycgr3T
  
Location: 2163-3612

Mycgr3G65725\_Mycgr3T

Mycgr3G102276 Mycgr3
  
Location: 3712-4801

Mycgr3G102276\_Mycgr3

Mycgr3G89189 Mycgr3T
  
Location: 4901-5564

Mycgr3G89189\_Mycgr3T

Mycgr3G52682 Mycgr3T
  
Location: 5664-9231

Mycgr3G52682\_Mycgr3T

Mycgr3G107072 Mycgr3
  
Location: 9331-13279

Mycgr3G107072\_Mycgr3

Mycgr3G34982 Mycgr3T
  
Location: 13379-15116

Mycgr3G34982\_Mycgr3T

Mycgr3G107069 Mycgr3
  
Location: 15216-17097

Mycgr3G107069\_Mycgr3

Mycgr3G32432 Mycgr3T
  
Location: 17197-19042

Mycgr3G32432\_Mycgr3T

Mycgr3G98385 Mycgr3T
  
Location: 19142-19898

Mycgr3G98385\_Mycgr3T

PSP1 domain protein
  
Accession: GAA88708
  
Location: 303428-305980
  
 NCBI BlastP on this gene

GAA88708

cytoplasmic ribosomal protein of the large subunit L15 (Rpl15)
  
Accession: GAA88707
  
Location: 301143-302114
  
  
**BlastP hit with Mycgr3G102281\_Mycgr3**
  
Percentage identity: 89 %
  
BlastP bit score: 381
  
Sequence coverage: 99 %
  
E-value: 4e-132
  
  
 NCBI BlastP on this gene

GAA88707

ubiquitin-protein ligase
  
Accession: GAA88706
  
Location: 296119-299594
  
  
**BlastP hit with Mycgr3G52682\_Mycgr3T**
  
Percentage identity: 50 %
  
BlastP bit score: 855
  
Sequence coverage: 75 %
  
E-value: 0.0
  
  
 NCBI BlastP on this gene

GAA88706

alcohol dehydrogenase, zinc-containing
  
Accession: GAA88705
  
Location: 292488-294170
  
 NCBI BlastP on this gene

GAA88705

FRG1-like family protein
  
Accession: GAA88704
  
Location: 290936-291991
  
 NCBI BlastP on this gene

GAA88704

RING finger domain protein
  
Accession: GAA88703
  
Location: 289330-290830
  
 NCBI BlastP on this gene

GAA88703

transcriptional regulator Ngg1
  
Accession: GAA88702
  
Location: 286129-288273
  
 NCBI BlastP on this gene

GAA88702

Query: Architecture Search FASTA input

GG700653 : Trichophyton rubrum CBS 118892 genomic scaffold supercont2.6    Total score: 2.0     Cumulative Blast bit score: 1222

Hit cluster cross-links:

Mycgr3G52686 Mycgr3T
  
Location: 0-861

Mycgr3G52686\_Mycgr3T

Mycgr3G102281 Mycgr3
  
Location: 961-1573

Mycgr3G102281\_Mycgr3

Mycgr3G89185 Mycgr3T
  
Location: 1673-2063

Mycgr3G89185\_Mycgr3T

Mycgr3G65725 Mycgr3T
  
Location: 2163-3612

Mycgr3G65725\_Mycgr3T

Mycgr3G102276 Mycgr3
  
Location: 3712-4801

Mycgr3G102276\_Mycgr3

Mycgr3G89189 Mycgr3T
  
Location: 4901-5564

Mycgr3G89189\_Mycgr3T

Mycgr3G52682 Mycgr3T
  
Location: 5664-9231

Mycgr3G52682\_Mycgr3T

Mycgr3G107072 Mycgr3
  
Location: 9331-13279

Mycgr3G107072\_Mycgr3

Mycgr3G34982 Mycgr3T
  
Location: 13379-15116

Mycgr3G34982\_Mycgr3T

Mycgr3G107069 Mycgr3
  
Location: 15216-17097

Mycgr3G107069\_Mycgr3

Mycgr3G32432 Mycgr3T
  
Location: 17197-19042

Mycgr3G32432\_Mycgr3T

Mycgr3G98385 Mycgr3T
  
Location: 19142-19898

Mycgr3G98385\_Mycgr3T

MFS multidrug transporter
  
Accession: EGD89567
  
Location: 1052542-1054563
  
 NCBI BlastP on this gene

EGD89567

EF hand domain-containing protein
  
Accession: EGD89566
  
Location: 1048002-1051373
  
 NCBI BlastP on this gene

EGD89566

mRNA splicing factor
  
Accession: EGD89565
  
Location: 1045869-1047665
  
 NCBI BlastP on this gene

EGD89565

hypothetical protein
  
Accession: EGD89564
  
Location: 1044292-1045359
  
 NCBI BlastP on this gene

EGD89564

60S ribosomal protein L15
  
Accession: EGD89563
  
Location: 1042901-1043866
  
  
**BlastP hit with Mycgr3G102281\_Mycgr3**
  
Percentage identity: 89 %
  
BlastP bit score: 372
  
Sequence coverage: 99 %
  
E-value: 2e-128
  
  
 NCBI BlastP on this gene

EGD89563

hypothetical protein
  
Accession: EGD89562
  
Location: 1038248-1041910
  
  
**BlastP hit with Mycgr3G52682\_Mycgr3T**
  
Percentage identity: 41 %
  
BlastP bit score: 850
  
Sequence coverage: 103 %
  
E-value: 0.0
  
  
 NCBI BlastP on this gene

EGD89562

alcohol dehydrogenase
  
Accession: EGD89561
  
Location: 1034385-1035935
  
 NCBI BlastP on this gene

EGD89561

hypothetical protein
  
Accession: EGD89560
  
Location: 1032823-1033726
  
 NCBI BlastP on this gene

EGD89560

hypothetical protein
  
Accession: EGD89559
  
Location: 1030480-1032252
  
 NCBI BlastP on this gene

EGD89559

transcriptional regulator Ngg1
  
Accession: EGD89558
  
Location: 1026530-1028706
  
 NCBI BlastP on this gene

EGD89558

Query: Architecture Search FASTA input

GG749408 : Ajellomyces dermatitidis ATCC 18188 genomic scaffold supercont1.2    Total score: 2.0     Cumulative Blast bit score: 1206

Hit cluster cross-links:

Mycgr3G52686 Mycgr3T
  
Location: 0-861

Mycgr3G52686\_Mycgr3T

Mycgr3G102281 Mycgr3
  
Location: 961-1573

Mycgr3G102281\_Mycgr3

Mycgr3G89185 Mycgr3T
  
Location: 1673-2063

Mycgr3G89185\_Mycgr3T

Mycgr3G65725 Mycgr3T
  
Location: 2163-3612

Mycgr3G65725\_Mycgr3T

Mycgr3G102276 Mycgr3
  
Location: 3712-4801

Mycgr3G102276\_Mycgr3

Mycgr3G89189 Mycgr3T
  
Location: 4901-5564

Mycgr3G89189\_Mycgr3T

Mycgr3G52682 Mycgr3T
  
Location: 5664-9231

Mycgr3G52682\_Mycgr3T

Mycgr3G107072 Mycgr3
  
Location: 9331-13279

Mycgr3G107072\_Mycgr3

Mycgr3G34982 Mycgr3T
  
Location: 13379-15116

Mycgr3G34982\_Mycgr3T

Mycgr3G107069 Mycgr3
  
Location: 15216-17097

Mycgr3G107069\_Mycgr3

Mycgr3G32432 Mycgr3T
  
Location: 17197-19042

Mycgr3G32432\_Mycgr3T

Mycgr3G98385 Mycgr3T
  
Location: 19142-19898

Mycgr3G98385\_Mycgr3T

PSP1 domain-containing protein
  
Accession: EGE77653
  
Location: 120451-123282
  
 NCBI BlastP on this gene

EGE77653

hypothetical protein
  
Accession: EGE77652
  
Location: 115656-117062
  
 NCBI BlastP on this gene

EGE77652

60S ribosomal protein L15
  
Accession: EGE77651
  
Location: 114156-115153
  
  
**BlastP hit with Mycgr3G102281\_Mycgr3**
  
Percentage identity: 87 %
  
BlastP bit score: 339
  
Sequence coverage: 99 %
  
E-value: 1e-115
  
  
 NCBI BlastP on this gene

EGE77651

secreted protein
  
Accession: EGE77650
  
Location: 112406-113160
  
 NCBI BlastP on this gene

EGE77650

hypothetical protein
  
Accession: EGE77649
  
Location: 106904-108989
  
 NCBI BlastP on this gene

EGE77649

ubiquitin-protein ligase
  
Accession: EGE77648
  
Location: 101411-105180
  
  
**BlastP hit with Mycgr3G52682\_Mycgr3T**
  
Percentage identity: 41 %
  
BlastP bit score: 867
  
Sequence coverage: 105 %
  
E-value: 0.0
  
  
 NCBI BlastP on this gene

EGE77648

NADP-dependent mannitol dehydrogenase
  
Accession: EGE77647
  
Location: 97218-98885
  
 NCBI BlastP on this gene

EGE77647

hypothetical protein
  
Accession: EGE77646
  
Location: 94955-95892
  
 NCBI BlastP on this gene

EGE77646

Query: Architecture Search FASTA input

GG657458 : Ajellomyces dermatitidis SLH14081 genomic scaffold supercont1.11    Total score: 2.0     Cumulative Blast bit score: 1206

Hit cluster cross-links:

Mycgr3G52686 Mycgr3T
  
Location: 0-861

Mycgr3G52686\_Mycgr3T

Mycgr3G102281 Mycgr3
  
Location: 961-1573

Mycgr3G102281\_Mycgr3

Mycgr3G89185 Mycgr3T
  
Location: 1673-2063

Mycgr3G89185\_Mycgr3T

Mycgr3G65725 Mycgr3T
  
Location: 2163-3612

Mycgr3G65725\_Mycgr3T

Mycgr3G102276 Mycgr3
  
Location: 3712-4801

Mycgr3G102276\_Mycgr3

Mycgr3G89189 Mycgr3T
  
Location: 4901-5564

Mycgr3G89189\_Mycgr3T

Mycgr3G52682 Mycgr3T
  
Location: 5664-9231

Mycgr3G52682\_Mycgr3T

Mycgr3G107072 Mycgr3
  
Location: 9331-13279

Mycgr3G107072\_Mycgr3

Mycgr3G34982 Mycgr3T
  
Location: 13379-15116

Mycgr3G34982\_Mycgr3T

Mycgr3G107069 Mycgr3
  
Location: 15216-17097

Mycgr3G107069\_Mycgr3

Mycgr3G32432 Mycgr3T
  
Location: 17197-19042

Mycgr3G32432\_Mycgr3T

Mycgr3G98385 Mycgr3T
  
Location: 19142-19898

Mycgr3G98385\_Mycgr3T

PSP1 domain-containing protein
  
Accession: EEQ70387
  
Location: 539294-542125
  
 NCBI BlastP on this gene

EEQ70387

conserved hypothetical protein
  
Accession: EEQ70386
  
Location: 535305-536691
  
 NCBI BlastP on this gene

EEQ70386

60S ribosomal protein L15
  
Accession: EEQ70385
  
Location: 533818-534815
  
  
**BlastP hit with Mycgr3G102281\_Mycgr3**
  
Percentage identity: 87 %
  
BlastP bit score: 339
  
Sequence coverage: 99 %
  
E-value: 1e-115
  
  
 NCBI BlastP on this gene

EEQ70385

secreted protein
  
Accession: EEQ70384
  
Location: 532068-532822
  
 NCBI BlastP on this gene

EEQ70384

ubiquitin-protein ligase
  
Accession: EEQ70383
  
Location: 521089-524858
  
  
**BlastP hit with Mycgr3G52682\_Mycgr3T**
  
Percentage identity: 41 %
  
BlastP bit score: 867
  
Sequence coverage: 105 %
  
E-value: 0.0
  
  
 NCBI BlastP on this gene

EEQ70383

NADP-dependent mannitol dehydrogenase
  
Accession: EEQ70382
  
Location: 516896-518563
  
 NCBI BlastP on this gene

EEQ70382

conserved hypothetical protein
  
Accession: EEQ70381
  
Location: 514634-515571
  
 NCBI BlastP on this gene

EEQ70381

Query: Architecture Search FASTA input

GG698513 : Trichophyton tonsurans CBS 112818 genomic scaffold supercont1.37    Total score: 2.0     Cumulative Blast bit score: 1204

Hit cluster cross-links:

Mycgr3G52686 Mycgr3T
  
Location: 0-861

Mycgr3G52686\_Mycgr3T

Mycgr3G102281 Mycgr3
  
Location: 961-1573

Mycgr3G102281\_Mycgr3

Mycgr3G89185 Mycgr3T
  
Location: 1673-2063

Mycgr3G89185\_Mycgr3T

Mycgr3G65725 Mycgr3T
  
Location: 2163-3612

Mycgr3G65725\_Mycgr3T

Mycgr3G102276 Mycgr3
  
Location: 3712-4801

Mycgr3G102276\_Mycgr3

Mycgr3G89189 Mycgr3T
  
Location: 4901-5564

Mycgr3G89189\_Mycgr3T

Mycgr3G52682 Mycgr3T
  
Location: 5664-9231

Mycgr3G52682\_Mycgr3T

Mycgr3G107072 Mycgr3
  
Location: 9331-13279

Mycgr3G107072\_Mycgr3

Mycgr3G34982 Mycgr3T
  
Location: 13379-15116

Mycgr3G34982\_Mycgr3T

Mycgr3G107069 Mycgr3
  
Location: 15216-17097

Mycgr3G107069\_Mycgr3

Mycgr3G32432 Mycgr3T
  
Location: 17197-19042

Mycgr3G32432\_Mycgr3T

Mycgr3G98385 Mycgr3T
  
Location: 19142-19898

Mycgr3G98385\_Mycgr3T

MFS multidrug transporter
  
Accession: EGD98638
  
Location: 92142-94166
  
 NCBI BlastP on this gene

EGD98638

EF hand domain-containing protein
  
Accession: EGD98637
  
Location: 87605-90973
  
 NCBI BlastP on this gene

EGD98637

mRNA splicing factor
  
Accession: EGD98636
  
Location: 85461-87268
  
 NCBI BlastP on this gene

EGD98636

hypothetical protein
  
Accession: EGD98635
  
Location: 83859-84929
  
 NCBI BlastP on this gene

EGD98635

60S ribosomal protein L15
  
Accession: EGD98634
  
Location: 82475-83438
  
  
**BlastP hit with Mycgr3G102281\_Mycgr3**
  
Percentage identity: 89 %
  
BlastP bit score: 372
  
Sequence coverage: 99 %
  
E-value: 2e-128
  
  
 NCBI BlastP on this gene

EGD98634

hypothetical protein
  
Accession: EGD98633
  
Location: 77800-81463
  
  
**BlastP hit with Mycgr3G52682\_Mycgr3T**
  
Percentage identity: 40 %
  
BlastP bit score: 832
  
Sequence coverage: 103 %
  
E-value: 0.0
  
  
 NCBI BlastP on this gene

EGD98633

alcohol dehydrogenase
  
Accession: EGD98632
  
Location: 74018-75451
  
 NCBI BlastP on this gene

EGD98632

hypothetical protein
  
Accession: EGD98631
  
Location: 72447-73349
  
 NCBI BlastP on this gene

EGD98631

hypothetical protein
  
Accession: EGD98630
  
Location: 70113-71927
  
 NCBI BlastP on this gene

EGD98630

transcriptional regulator Ngg1
  
Accession: EGD98629
  
Location: 66174-68347
  
 NCBI BlastP on this gene

EGD98629

Query: Architecture Search FASTA input

EQ999975 : Ajellomyces dermatitidis ER-3 genomic scaffold supercont1.3    Total score: 2.0     Cumulative Blast bit score: 1204

Hit cluster cross-links:

Mycgr3G52686 Mycgr3T
  
Location: 0-861

Mycgr3G52686\_Mycgr3T

Mycgr3G102281 Mycgr3
  
Location: 961-1573

Mycgr3G102281\_Mycgr3

Mycgr3G89185 Mycgr3T
  
Location: 1673-2063

Mycgr3G89185\_Mycgr3T

Mycgr3G65725 Mycgr3T
  
Location: 2163-3612

Mycgr3G65725\_Mycgr3T

Mycgr3G102276 Mycgr3
  
Location: 3712-4801

Mycgr3G102276\_Mycgr3

Mycgr3G89189 Mycgr3T
  
Location: 4901-5564

Mycgr3G89189\_Mycgr3T

Mycgr3G52682 Mycgr3T
  
Location: 5664-9231

Mycgr3G52682\_Mycgr3T

Mycgr3G107072 Mycgr3
  
Location: 9331-13279

Mycgr3G107072\_Mycgr3

Mycgr3G34982 Mycgr3T
  
Location: 13379-15116

Mycgr3G34982\_Mycgr3T

Mycgr3G107069 Mycgr3
  
Location: 15216-17097

Mycgr3G107069\_Mycgr3

Mycgr3G32432 Mycgr3T
  
Location: 17197-19042

Mycgr3G32432\_Mycgr3T

Mycgr3G98385 Mycgr3T
  
Location: 19142-19898

Mycgr3G98385\_Mycgr3T

PSP1 domain-containing protein
  
Accession: EEQ87748
  
Location: 1835613-1838444
  
 NCBI BlastP on this gene

EEQ87748

conserved hypothetical protein
  
Accession: EEQ87749
  
Location: 1841046-1842452
  
 NCBI BlastP on this gene

EEQ87749

60S ribosomal protein L15
  
Accession: EEQ87750
  
Location: 1842952-1843949
  
  
**BlastP hit with Mycgr3G102281\_Mycgr3**
  
Percentage identity: 87 %
  
BlastP bit score: 337
  
Sequence coverage: 99 %
  
E-value: 5e-115
  
  
 NCBI BlastP on this gene

EEQ87750

secreted protein
  
Accession: EEQ87751
  
Location: 1844945-1845699
  
 NCBI BlastP on this gene

EEQ87751

ubiquitin-protein ligase
  
Accession: EEQ87752
  
Location: 1852926-1856695
  
  
**BlastP hit with Mycgr3G52682\_Mycgr3T**
  
Percentage identity: 41 %
  
BlastP bit score: 867
  
Sequence coverage: 105 %
  
E-value: 0.0
  
  
 NCBI BlastP on this gene

EEQ87752

alcohol dehydrogenase
  
Accession: EEQ87753
  
Location: 1859221-1860888
  
 NCBI BlastP on this gene

EEQ87753

conserved hypothetical protein
  
Accession: EEQ87754
  
Location: 1862217-1863154
  
 NCBI BlastP on this gene

EEQ87754

Query: Architecture Search FASTA input

DS995752 : Trichophyton equinum CBS 127.97 supercont1.35 genomic scaffold    Total score: 2.0     Cumulative Blast bit score: 1204

Hit cluster cross-links:

Mycgr3G52686 Mycgr3T
  
Location: 0-861

Mycgr3G52686\_Mycgr3T

Mycgr3G102281 Mycgr3
  
Location: 961-1573

Mycgr3G102281\_Mycgr3

Mycgr3G89185 Mycgr3T
  
Location: 1673-2063

Mycgr3G89185\_Mycgr3T

Mycgr3G65725 Mycgr3T
  
Location: 2163-3612

Mycgr3G65725\_Mycgr3T

Mycgr3G102276 Mycgr3
  
Location: 3712-4801

Mycgr3G102276\_Mycgr3

Mycgr3G89189 Mycgr3T
  
Location: 4901-5564

Mycgr3G89189\_Mycgr3T

Mycgr3G52682 Mycgr3T
  
Location: 5664-9231

Mycgr3G52682\_Mycgr3T

Mycgr3G107072 Mycgr3
  
Location: 9331-13279

Mycgr3G107072\_Mycgr3

Mycgr3G34982 Mycgr3T
  
Location: 13379-15116

Mycgr3G34982\_Mycgr3T

Mycgr3G107069 Mycgr3
  
Location: 15216-17097

Mycgr3G107069\_Mycgr3

Mycgr3G32432 Mycgr3T
  
Location: 17197-19042

Mycgr3G32432\_Mycgr3T

Mycgr3G98385 Mycgr3T
  
Location: 19142-19898

Mycgr3G98385\_Mycgr3T

multidrug resistance protein fnx1
  
Accession: EGE06835
  
Location: 55556-57572
  
 NCBI BlastP on this gene

EGE06835

EF hand domain-containing protein
  
Accession: EGE06834
  
Location: 51019-54387
  
 NCBI BlastP on this gene

EGE06834

pre-mRNA-processing factor 17
  
Accession: EGE06833
  
Location: 48875-50682
  
 NCBI BlastP on this gene

EGE06833

hypothetical protein
  
Accession: EGE06832
  
Location: 47187-48343
  
 NCBI BlastP on this gene

EGE06832

ribosomal protein L15
  
Accession: EGE06831
  
Location: 45889-46852
  
  
**BlastP hit with Mycgr3G102281\_Mycgr3**
  
Percentage identity: 89 %
  
BlastP bit score: 372
  
Sequence coverage: 99 %
  
E-value: 2e-128
  
  
 NCBI BlastP on this gene

EGE06831

ubiquitin-protein ligase E3A
  
Accession: EGE06830
  
Location: 41214-44877
  
  
**BlastP hit with Mycgr3G52682\_Mycgr3T**
  
Percentage identity: 40 %
  
BlastP bit score: 832
  
Sequence coverage: 103 %
  
E-value: 0.0
  
  
 NCBI BlastP on this gene

EGE06830

alcohol dehydrogenase
  
Accession: EGE06829
  
Location: 37395-38887
  
 NCBI BlastP on this gene

EGE06829

hypothetical protein
  
Accession: EGE06828
  
Location: 35824-36726
  
 NCBI BlastP on this gene

EGE06828

RING finger domain-containing protein
  
Accession: EGE06827
  
Location: 33490-35304
  
 NCBI BlastP on this gene

EGE06827

transcriptional regulator Ngg1
  
Accession: EGE06826
  
Location: 29547-31720
  
 NCBI BlastP on this gene

EGE06826

Query: Architecture Search FASTA input

DS995903 : Penicillium marneffei ATCC 18224 scf\_1105668340984 genomic scaffold    Total score: 2.0     Cumulative Blast bit score: 1200

Hit cluster cross-links:

Mycgr3G52686 Mycgr3T
  
Location: 0-861

Mycgr3G52686\_Mycgr3T

Mycgr3G102281 Mycgr3
  
Location: 961-1573

Mycgr3G102281\_Mycgr3

Mycgr3G89185 Mycgr3T
  
Location: 1673-2063

Mycgr3G89185\_Mycgr3T

Mycgr3G65725 Mycgr3T
  
Location: 2163-3612

Mycgr3G65725\_Mycgr3T

Mycgr3G102276 Mycgr3
  
Location: 3712-4801

Mycgr3G102276\_Mycgr3

Mycgr3G89189 Mycgr3T
  
Location: 4901-5564

Mycgr3G89189\_Mycgr3T

Mycgr3G52682 Mycgr3T
  
Location: 5664-9231

Mycgr3G52682\_Mycgr3T

Mycgr3G107072 Mycgr3
  
Location: 9331-13279

Mycgr3G107072\_Mycgr3

Mycgr3G34982 Mycgr3T
  
Location: 13379-15116

Mycgr3G34982\_Mycgr3T

Mycgr3G107069 Mycgr3
  
Location: 15216-17097

Mycgr3G107069\_Mycgr3

Mycgr3G32432 Mycgr3T
  
Location: 17197-19042

Mycgr3G32432\_Mycgr3T

Mycgr3G98385 Mycgr3T
  
Location: 19142-19898

Mycgr3G98385\_Mycgr3T

HIT finger domain protein, putative
  
Accession: EEA21645
  
Location: 1177172-1177765
  
 NCBI BlastP on this gene

EEA21645

NifU-related protein
  
Accession: EEA21644
  
Location: 1175763-1176699
  
 NCBI BlastP on this gene

EEA21644

PSP1 domain protein
  
Accession: EEA21642
  
Location: 1169159-1171839
  
 NCBI BlastP on this gene

EEA21642

60S ribosomal protein L15
  
Accession: EEA21640
  
Location: 1166975-1168031
  
  
**BlastP hit with Mycgr3G102281\_Mycgr3**
  
Percentage identity: 88 %
  
BlastP bit score: 344
  
Sequence coverage: 99 %
  
E-value: 2e-117
  
  
 NCBI BlastP on this gene

EEA21640

ubiquitin-protein ligase (Hul4), putative
  
Accession: EEA21639
  
Location: 1162059-1165905
  
  
**BlastP hit with Mycgr3G52682\_Mycgr3T**
  
Percentage identity: 43 %
  
BlastP bit score: 856
  
Sequence coverage: 101 %
  
E-value: 0.0
  
  
 NCBI BlastP on this gene

EEA21639

conserved hypothetical protein
  
Accession: EEA21638
  
Location: 1160385-1161717
  
 NCBI BlastP on this gene

EEA21638

alcohol dehydrogenase, zinc-containing, putative
  
Accession: EEA21637
  
Location: 1156422-1157869
  
 NCBI BlastP on this gene

EEA21637

conserved hypothetical protein
  
Accession: EEA21636
  
Location: 1155003-1155997
  
 NCBI BlastP on this gene

EEA21636

DNA repair protein, putative
  
Accession: EEA21635
  
Location: 1152512-1154899
  
 NCBI BlastP on this gene

EEA21635

Query: Architecture Search FASTA input

GG663363 : Ajellomyces capsulatus G186AR genomic scaffold supercont2.1    Total score: 2.0     Cumulative Blast bit score: 1199

Hit cluster cross-links:

Mycgr3G52686 Mycgr3T
  
Location: 0-861

Mycgr3G52686\_Mycgr3T

Mycgr3G102281 Mycgr3
  
Location: 961-1573

Mycgr3G102281\_Mycgr3

Mycgr3G89185 Mycgr3T
  
Location: 1673-2063

Mycgr3G89185\_Mycgr3T

Mycgr3G65725 Mycgr3T
  
Location: 2163-3612

Mycgr3G65725\_Mycgr3T

Mycgr3G102276 Mycgr3
  
Location: 3712-4801

Mycgr3G102276\_Mycgr3

Mycgr3G89189 Mycgr3T
  
Location: 4901-5564

Mycgr3G89189\_Mycgr3T

Mycgr3G52682 Mycgr3T
  
Location: 5664-9231

Mycgr3G52682\_Mycgr3T

Mycgr3G107072 Mycgr3
  
Location: 9331-13279

Mycgr3G107072\_Mycgr3

Mycgr3G34982 Mycgr3T
  
Location: 13379-15116

Mycgr3G34982\_Mycgr3T

Mycgr3G107069 Mycgr3
  
Location: 15216-17097

Mycgr3G107069\_Mycgr3

Mycgr3G32432 Mycgr3T
  
Location: 17197-19042

Mycgr3G32432\_Mycgr3T

Mycgr3G98385 Mycgr3T
  
Location: 19142-19898

Mycgr3G98385\_Mycgr3T

conserved hypothetical protein
  
Accession: EEH11353
  
Location: 2516533-2517920
  
 NCBI BlastP on this gene

EEH11353

60S ribosomal protein L15
  
Accession: EEH11352
  
Location: 2515063-2516066
  
  
**BlastP hit with Mycgr3G102281\_Mycgr3**
  
Percentage identity: 88 %
  
BlastP bit score: 342
  
Sequence coverage: 99 %
  
E-value: 7e-117
  
  
 NCBI BlastP on this gene

EEH11352

secreted protein
  
Accession: EEH11351
  
Location: 2513250-2514005
  
 NCBI BlastP on this gene

EEH11351

conserved hypothetical protein
  
Accession: EEH11350
  
Location: 2504655-2507102
  
 NCBI BlastP on this gene

EEH11350

WD domain-containing protein
  
Accession: EEH11349
  
Location: 2501212-2501940
  
 NCBI BlastP on this gene

EEH11349

ubiquitin-protein ligase E3
  
Accession: EEH11348
  
Location: 2494617-2498367
  
  
**BlastP hit with Mycgr3G52682\_Mycgr3T**
  
Percentage identity: 42 %
  
BlastP bit score: 857
  
Sequence coverage: 102 %
  
E-value: 0.0
  
  
 NCBI BlastP on this gene

EEH11348

Query: Architecture Search FASTA input

GG692421 : Ajellomyces capsulatus H143 genomic scaffold supercont2.3    Total score: 2.0     Cumulative Blast bit score: 1194

Hit cluster cross-links:

Mycgr3G52686 Mycgr3T
  
Location: 0-861

Mycgr3G52686\_Mycgr3T

Mycgr3G102281 Mycgr3
  
Location: 961-1573

Mycgr3G102281\_Mycgr3

Mycgr3G89185 Mycgr3T
  
Location: 1673-2063

Mycgr3G89185\_Mycgr3T

Mycgr3G65725 Mycgr3T
  
Location: 2163-3612

Mycgr3G65725\_Mycgr3T

Mycgr3G102276 Mycgr3
  
Location: 3712-4801

Mycgr3G102276\_Mycgr3

Mycgr3G89189 Mycgr3T
  
Location: 4901-5564

Mycgr3G89189\_Mycgr3T

Mycgr3G52682 Mycgr3T
  
Location: 5664-9231

Mycgr3G52682\_Mycgr3T

Mycgr3G107072 Mycgr3
  
Location: 9331-13279

Mycgr3G107072\_Mycgr3

Mycgr3G34982 Mycgr3T
  
Location: 13379-15116

Mycgr3G34982\_Mycgr3T

Mycgr3G107069 Mycgr3
  
Location: 15216-17097

Mycgr3G107069\_Mycgr3

Mycgr3G32432 Mycgr3T
  
Location: 17197-19042

Mycgr3G32432\_Mycgr3T

Mycgr3G98385 Mycgr3T
  
Location: 19142-19898

Mycgr3G98385\_Mycgr3T

conserved hypothetical protein
  
Accession: EER43403
  
Location: 3138034-3139402
  
 NCBI BlastP on this gene

EER43403

60S ribosomal protein L15
  
Accession: EER43402
  
Location: 3136567-3137571
  
  
**BlastP hit with Mycgr3G102281\_Mycgr3**
  
Percentage identity: 88 %
  
BlastP bit score: 342
  
Sequence coverage: 99 %
  
E-value: 7e-117
  
  
 NCBI BlastP on this gene

EER43402

secreted protein
  
Accession: EER43401
  
Location: 3134737-3135492
  
 NCBI BlastP on this gene

EER43401

predicted protein
  
Accession: EER43400
  
Location: 3133059-3133794
  
 NCBI BlastP on this gene

EER43400

NACHT and WD40 domain-containing protein
  
Accession: EER43399
  
Location: 3128277-3129223
  
 NCBI BlastP on this gene

EER43399

predicted protein
  
Accession: EER43398
  
Location: 3127603-3127982
  
 NCBI BlastP on this gene

EER43398

ubiquitin-protein ligase E3
  
Accession: EER43397
  
Location: 3112354-3116104
  
  
**BlastP hit with Mycgr3G52682\_Mycgr3T**
  
Percentage identity: 43 %
  
BlastP bit score: 852
  
Sequence coverage: 100 %
  
E-value: 0.0
  
  
 NCBI BlastP on this gene

EER43397

hypothetical protein
  
Accession: EER43396
  
Location: 3110263-3110895
  
 NCBI BlastP on this gene

EER43396

Query: Architecture Search FASTA input

DS990639 : Ajellomyces capsulatus H88 supercont1.4 genomic scaffold    Total score: 2.0     Cumulative Blast bit score: 1194

Hit cluster cross-links:

Mycgr3G52686 Mycgr3T
  
Location: 0-861

Mycgr3G52686\_Mycgr3T

Mycgr3G102281 Mycgr3
  
Location: 961-1573

Mycgr3G102281\_Mycgr3

Mycgr3G89185 Mycgr3T
  
Location: 1673-2063

Mycgr3G89185\_Mycgr3T

Mycgr3G65725 Mycgr3T
  
Location: 2163-3612

Mycgr3G65725\_Mycgr3T

Mycgr3G102276 Mycgr3
  
Location: 3712-4801

Mycgr3G102276\_Mycgr3

Mycgr3G89189 Mycgr3T
  
Location: 4901-5564

Mycgr3G89189\_Mycgr3T

Mycgr3G52682 Mycgr3T
  
Location: 5664-9231

Mycgr3G52682\_Mycgr3T

Mycgr3G107072 Mycgr3
  
Location: 9331-13279

Mycgr3G107072\_Mycgr3

Mycgr3G34982 Mycgr3T
  
Location: 13379-15116

Mycgr3G34982\_Mycgr3T

Mycgr3G107069 Mycgr3
  
Location: 15216-17097

Mycgr3G107069\_Mycgr3

Mycgr3G32432 Mycgr3T
  
Location: 17197-19042

Mycgr3G32432\_Mycgr3T

Mycgr3G98385 Mycgr3T
  
Location: 19142-19898

Mycgr3G98385\_Mycgr3T

conserved hypothetical protein
  
Accession: EGC46337
  
Location: 3596461-3597844
  
 NCBI BlastP on this gene

EGC46337

60S ribosomal protein
  
Accession: EGC46336
  
Location: 3594990-3595994
  
  
**BlastP hit with Mycgr3G102281\_Mycgr3**
  
Percentage identity: 88 %
  
BlastP bit score: 342
  
Sequence coverage: 99 %
  
E-value: 7e-117
  
  
 NCBI BlastP on this gene

EGC46336

secreted protein
  
Accession: EGC46335
  
Location: 3593172-3593927
  
 NCBI BlastP on this gene

EGC46335

predicted protein
  
Accession: EGC46334
  
Location: 3591494-3592229
  
 NCBI BlastP on this gene

EGC46334

NACHT and WD40 domain-containing protein
  
Accession: EGC46333
  
Location: 3586379-3587325
  
 NCBI BlastP on this gene

EGC46333

predicted protein
  
Accession: EGC46332
  
Location: 3585705-3586084
  
 NCBI BlastP on this gene

EGC46332

ubiquitin-protein ligase E3
  
Accession: EGC46331
  
Location: 3579760-3583510
  
  
**BlastP hit with Mycgr3G52682\_Mycgr3T**
  
Percentage identity: 43 %
  
BlastP bit score: 852
  
Sequence coverage: 100 %
  
E-value: 0.0
  
  
 NCBI BlastP on this gene

EGC46331

hypothetical protein
  
Accession: EGC46330
  
Location: 3577669-3578301
  
 NCBI BlastP on this gene

EGC46330

NADP-dependent mannitol dehydrogenase
  
Accession: EGC46329
  
Location: 3575418-3577118
  
 NCBI BlastP on this gene

EGC46329

Query: Architecture Search FASTA input

DS989828 : Arthroderma gypseum CBS 118893 supercont1.7 genomic scaffold    Total score: 2.0     Cumulative Blast bit score: 1178

Hit cluster cross-links:

Mycgr3G52686 Mycgr3T
  
Location: 0-861

Mycgr3G52686\_Mycgr3T

Mycgr3G102281 Mycgr3
  
Location: 961-1573

Mycgr3G102281\_Mycgr3

Mycgr3G89185 Mycgr3T
  
Location: 1673-2063

Mycgr3G89185\_Mycgr3T

Mycgr3G65725 Mycgr3T
  
Location: 2163-3612

Mycgr3G65725\_Mycgr3T

Mycgr3G102276 Mycgr3
  
Location: 3712-4801

Mycgr3G102276\_Mycgr3

Mycgr3G89189 Mycgr3T
  
Location: 4901-5564

Mycgr3G89189\_Mycgr3T

Mycgr3G52682 Mycgr3T
  
Location: 5664-9231

Mycgr3G52682\_Mycgr3T

Mycgr3G107072 Mycgr3
  
Location: 9331-13279

Mycgr3G107072\_Mycgr3

Mycgr3G34982 Mycgr3T
  
Location: 13379-15116

Mycgr3G34982\_Mycgr3T

Mycgr3G107069 Mycgr3
  
Location: 15216-17097

Mycgr3G107069\_Mycgr3

Mycgr3G32432 Mycgr3T
  
Location: 17197-19042

Mycgr3G32432\_Mycgr3T

Mycgr3G98385 Mycgr3T
  
Location: 19142-19898

Mycgr3G98385\_Mycgr3T

multidrug resistance protein fnx1
  
Accession: EFR04443
  
Location: 417768-419780
  
 NCBI BlastP on this gene

EFR04443

EF hand domain-containing protein
  
Accession: EFR04442
  
Location: 413775-417134
  
 NCBI BlastP on this gene

EFR04442

pre-mRNA-processing factor 17
  
Accession: EFR04441
  
Location: 411647-413452
  
 NCBI BlastP on this gene

EFR04441

hypothetical protein
  
Accession: EFR04440
  
Location: 410034-411132
  
 NCBI BlastP on this gene

EFR04440

30S ribosomal protein S23
  
Accession: EFR04439
  
Location: 408650-409615
  
  
**BlastP hit with Mycgr3G102281\_Mycgr3**
  
Percentage identity: 80 %
  
BlastP bit score: 362
  
Sequence coverage: 110 %
  
E-value: 4e-124
  
  
 NCBI BlastP on this gene

EFR04439

ubiquitin-protein ligase E3A
  
Accession: EFR04438
  
Location: 404018-407671
  
  
**BlastP hit with Mycgr3G52682\_Mycgr3T**
  
Percentage identity: 40 %
  
BlastP bit score: 816
  
Sequence coverage: 103 %
  
E-value: 0.0
  
  
 NCBI BlastP on this gene

EFR04438

L-threonine 3-dehydrogenase
  
Accession: EFR04437
  
Location: 400269-401728
  
 NCBI BlastP on this gene

EFR04437

hypothetical protein
  
Accession: EFR04436
  
Location: 398730-399801
  
 NCBI BlastP on this gene

EFR04436

hypothetical protein
  
Accession: EFR04435
  
Location: 396376-398189
  
 NCBI BlastP on this gene

EFR04435

histone acetyltransferase transcription factor
  
Accession: EFR04434
  
Location: 392475-394647
  
 NCBI BlastP on this gene

EFR04434

Query: Architecture Search FASTA input

KB644410 : Penicillium oxalicum 114-2 unplaced genomic scaffold scaffold\_3    Total score: 2.0     Cumulative Blast bit score: 1168

Hit cluster cross-links:

Mycgr3G52686 Mycgr3T
  
Location: 0-861

Mycgr3G52686\_Mycgr3T

Mycgr3G102281 Mycgr3
  
Location: 961-1573

Mycgr3G102281\_Mycgr3

Mycgr3G89185 Mycgr3T
  
Location: 1673-2063

Mycgr3G89185\_Mycgr3T

Mycgr3G65725 Mycgr3T
  
Location: 2163-3612

Mycgr3G65725\_Mycgr3T

Mycgr3G102276 Mycgr3
  
Location: 3712-4801

Mycgr3G102276\_Mycgr3

Mycgr3G89189 Mycgr3T
  
Location: 4901-5564

Mycgr3G89189\_Mycgr3T

Mycgr3G52682 Mycgr3T
  
Location: 5664-9231

Mycgr3G52682\_Mycgr3T

Mycgr3G107072 Mycgr3
  
Location: 9331-13279

Mycgr3G107072\_Mycgr3

Mycgr3G34982 Mycgr3T
  
Location: 13379-15116

Mycgr3G34982\_Mycgr3T

Mycgr3G107069 Mycgr3
  
Location: 15216-17097

Mycgr3G107069\_Mycgr3

Mycgr3G32432 Mycgr3T
  
Location: 17197-19042

Mycgr3G32432\_Mycgr3T

Mycgr3G98385 Mycgr3T
  
Location: 19142-19898

Mycgr3G98385\_Mycgr3T

hypothetical protein
  
Accession: EPS27965
  
Location: 2448255-2450122
  
 NCBI BlastP on this gene

EPS27965

hypothetical protein
  
Accession: EPS27964
  
Location: 2445511-2447662
  
 NCBI BlastP on this gene

EPS27964

hypothetical protein
  
Accession: EPS27963
  
Location: 2442506-2443929
  
 NCBI BlastP on this gene

EPS27963

hypothetical protein
  
Accession: EPS27962
  
Location: 2440167-2441078
  
  
**BlastP hit with Mycgr3G102281\_Mycgr3**
  
Percentage identity: 89 %
  
BlastP bit score: 381
  
Sequence coverage: 99 %
  
E-value: 3e-132
  
  
 NCBI BlastP on this gene

EPS27962

hypothetical protein
  
Accession: EPS27961
  
Location: 2435482-2439235
  
  
**BlastP hit with Mycgr3G52682\_Mycgr3T**
  
Percentage identity: 47 %
  
BlastP bit score: 787
  
Sequence coverage: 74 %
  
E-value: 0.0
  
  
 NCBI BlastP on this gene

EPS27961

putative beta-glucosidase
  
Accession: EPS27960
  
Location: 2430467-2433245
  
 NCBI BlastP on this gene

EPS27960

hypothetical protein
  
Accession: EPS27959
  
Location: 2429192-2430064
  
 NCBI BlastP on this gene

EPS27959

hypothetical protein
  
Accession: EPS27958
  
Location: 2426642-2428921
  
 NCBI BlastP on this gene

EPS27958

putative d-4,5 unsaturated beta-glucuronyl hydrolase
  
Accession: EPS27957
  
Location: 2424725-2426259
  
 NCBI BlastP on this gene

EPS27957

Query: Architecture Search FASTA input

EQ962656 : Talaromyces stipitatus ATCC 10500 scf\_1105507295549 genomic scaffold    Total score: 2.0     Cumulative Blast bit score: 1164

Hit cluster cross-links:

Mycgr3G52686 Mycgr3T
  
Location: 0-861

Mycgr3G52686\_Mycgr3T

Mycgr3G102281 Mycgr3
  
Location: 961-1573

Mycgr3G102281\_Mycgr3

Mycgr3G89185 Mycgr3T
  
Location: 1673-2063

Mycgr3G89185\_Mycgr3T

Mycgr3G65725 Mycgr3T
  
Location: 2163-3612

Mycgr3G65725\_Mycgr3T

Mycgr3G102276 Mycgr3
  
Location: 3712-4801

Mycgr3G102276\_Mycgr3

Mycgr3G89189 Mycgr3T
  
Location: 4901-5564

Mycgr3G89189\_Mycgr3T

Mycgr3G52682 Mycgr3T
  
Location: 5664-9231

Mycgr3G52682\_Mycgr3T

Mycgr3G107072 Mycgr3
  
Location: 9331-13279

Mycgr3G107072\_Mycgr3

Mycgr3G34982 Mycgr3T
  
Location: 13379-15116

Mycgr3G34982\_Mycgr3T

Mycgr3G107069 Mycgr3
  
Location: 15216-17097

Mycgr3G107069\_Mycgr3

Mycgr3G32432 Mycgr3T
  
Location: 17197-19042

Mycgr3G32432\_Mycgr3T

Mycgr3G98385 Mycgr3T
  
Location: 19142-19898

Mycgr3G98385\_Mycgr3T

HIT finger domain protein, putative
  
Accession: EED16775
  
Location: 2701938-2702553
  
 NCBI BlastP on this gene

EED16775

NifU-related protein
  
Accession: EED16777
  
Location: 2703013-2703963
  
 NCBI BlastP on this gene

EED16777

PSP1 domain protein
  
Accession: EED16778
  
Location: 2707488-2710210
  
 NCBI BlastP on this gene

EED16778

60S ribosomal protein L15
  
Accession: EED16779
  
Location: 2711117-2712210
  
  
**BlastP hit with Mycgr3G102281\_Mycgr3**
  
Percentage identity: 89 %
  
BlastP bit score: 346
  
Sequence coverage: 99 %
  
E-value: 2e-118
  
  
 NCBI BlastP on this gene

EED16779

ubiquitin-protein ligase (Hul4), putative
  
Accession: EED16780
  
Location: 2713225-2717065
  
  
**BlastP hit with Mycgr3G52682\_Mycgr3T**
  
Percentage identity: 43 %
  
BlastP bit score: 818
  
Sequence coverage: 96 %
  
E-value: 0.0
  
  
 NCBI BlastP on this gene

EED16780

muramidase, putative
  
Accession: EED16781
  
Location: 2718431-2719732
  
 NCBI BlastP on this gene

EED16781

mucin-desulfating sulfatase, putative
  
Accession: EED16782
  
Location: 2721296-2722814
  
 NCBI BlastP on this gene

EED16782

conserved hypothetical protein
  
Accession: EED16783
  
Location: 2722991-2723565
  
 NCBI BlastP on this gene

EED16783

NAD(P)H-dependent FMN reductase LOT6, putative
  
Accession: EED16784
  
Location: 2723759-2724569
  
 NCBI BlastP on this gene

EED16784

conserved hypothetical protein
  
Accession: EED16785
  
Location: 2724814-2726290
  
 NCBI BlastP on this gene

EED16785

conserved hypothetical protein
  
Accession: EED16787
  
Location: 2725050-2726290
  
 NCBI BlastP on this gene

EED16787

Query: Architecture Search FASTA input

DS995702 : Microsporum canis CBS 113480 supercont1.2 genomic scaffold    Total score: 2.0     Cumulative Blast bit score: 1152

Hit cluster cross-links:

Mycgr3G52686 Mycgr3T
  
Location: 0-861

Mycgr3G52686\_Mycgr3T

Mycgr3G102281 Mycgr3
  
Location: 961-1573

Mycgr3G102281\_Mycgr3

Mycgr3G89185 Mycgr3T
  
Location: 1673-2063

Mycgr3G89185\_Mycgr3T

Mycgr3G65725 Mycgr3T
  
Location: 2163-3612

Mycgr3G65725\_Mycgr3T

Mycgr3G102276 Mycgr3
  
Location: 3712-4801

Mycgr3G102276\_Mycgr3

Mycgr3G89189 Mycgr3T
  
Location: 4901-5564

Mycgr3G89189\_Mycgr3T

Mycgr3G52682 Mycgr3T
  
Location: 5664-9231

Mycgr3G52682\_Mycgr3T

Mycgr3G107072 Mycgr3
  
Location: 9331-13279

Mycgr3G107072\_Mycgr3

Mycgr3G34982 Mycgr3T
  
Location: 13379-15116

Mycgr3G34982\_Mycgr3T

Mycgr3G107069 Mycgr3
  
Location: 15216-17097

Mycgr3G107069\_Mycgr3

Mycgr3G32432 Mycgr3T
  
Location: 17197-19042

Mycgr3G32432\_Mycgr3T

Mycgr3G98385 Mycgr3T
  
Location: 19142-19898

Mycgr3G98385\_Mycgr3T

conserved hypothetical protein
  
Accession: EEQ29627
  
Location: 2635519-2637069
  
 NCBI BlastP on this gene

EEQ29627

conserved hypothetical protein
  
Accession: EEQ29626
  
Location: 2634674-2635415
  
 NCBI BlastP on this gene

EEQ29626

EF hand domain-containing protein
  
Accession: EEQ29625
  
Location: 2630655-2634026
  
 NCBI BlastP on this gene

EEQ29625

pre-mRNA-processing factor 17
  
Accession: EEQ29624
  
Location: 2628417-2630213
  
 NCBI BlastP on this gene

EEQ29624

conserved hypothetical protein
  
Accession: EEQ29623
  
Location: 2626903-2628022
  
 NCBI BlastP on this gene

EEQ29623

60S ribosomal protein L15
  
Accession: EEQ29622
  
Location: 2625514-2626506
  
  
**BlastP hit with Mycgr3G102281\_Mycgr3**
  
Percentage identity: 73 %
  
BlastP bit score: 351
  
Sequence coverage: 120 %
  
E-value: 1e-119
  
  
 NCBI BlastP on this gene

EEQ29622

ubiquitin-protein ligase E3A
  
Accession: EEQ29621
  
Location: 2620922-2624570
  
  
**BlastP hit with Mycgr3G52682\_Mycgr3T**
  
Percentage identity: 40 %
  
BlastP bit score: 801
  
Sequence coverage: 104 %
  
E-value: 0.0
  
  
 NCBI BlastP on this gene

EEQ29621

L-threonine 3-dehydrogenase
  
Accession: EEQ29620
  
Location: 2617428-2618872
  
 NCBI BlastP on this gene

EEQ29620

conserved hypothetical protein
  
Accession: EEQ29619
  
Location: 2615604-2616537
  
 NCBI BlastP on this gene

EEQ29619

conserved hypothetical protein
  
Accession: EEQ29618
  
Location: 2613277-2614971
  
 NCBI BlastP on this gene

EEQ29618

predicted protein
  
Accession: EEQ29617
  
Location: 2612380-2612918
  
 NCBI BlastP on this gene

EEQ29617

transcriptional adapter 3
  
Accession: EEQ29616
  
Location: 2609357-2611540
  
 NCBI BlastP on this gene

EEQ29616

Query: Architecture Search FASTA input

ACYE01000036 : Trichophyton verrucosum HKI 0517    Total score: 2.0     Cumulative Blast bit score: 1152

Hit cluster cross-links:

Mycgr3G52686 Mycgr3T
  
Location: 0-861

Mycgr3G52686\_Mycgr3T

Mycgr3G102281 Mycgr3
  
Location: 961-1573

Mycgr3G102281\_Mycgr3

Mycgr3G89185 Mycgr3T
  
Location: 1673-2063

Mycgr3G89185\_Mycgr3T

Mycgr3G65725 Mycgr3T
  
Location: 2163-3612

Mycgr3G65725\_Mycgr3T

Mycgr3G102276 Mycgr3
  
Location: 3712-4801

Mycgr3G102276\_Mycgr3

Mycgr3G89189 Mycgr3T
  
Location: 4901-5564

Mycgr3G89189\_Mycgr3T

Mycgr3G52682 Mycgr3T
  
Location: 5664-9231

Mycgr3G52682\_Mycgr3T

Mycgr3G107072 Mycgr3
  
Location: 9331-13279

Mycgr3G107072\_Mycgr3

Mycgr3G34982 Mycgr3T
  
Location: 13379-15116

Mycgr3G34982\_Mycgr3T

Mycgr3G107069 Mycgr3
  
Location: 15216-17097

Mycgr3G107069\_Mycgr3

Mycgr3G32432 Mycgr3T
  
Location: 17197-19042

Mycgr3G32432\_Mycgr3T

Mycgr3G98385 Mycgr3T
  
Location: 19142-19898

Mycgr3G98385\_Mycgr3T

hypothetical protein
  
Accession: EFE44606
  
Location: 27524-28510
  
 NCBI BlastP on this gene

EFE44606

hypothetical protein
  
Accession: EFE44605
  
Location: 26120-27085
  
  
**BlastP hit with Mycgr3G102281\_Mycgr3**
  
Percentage identity: 83 %
  
BlastP bit score: 364
  
Sequence coverage: 105 %
  
E-value: 3e-125
  
  
 NCBI BlastP on this gene

EFE44605

ubiquitin-protein ligase (Hul4), putative
  
Accession: EFE44604
  
Location: 21474-25109
  
  
**BlastP hit with Mycgr3G52682\_Mycgr3T**
  
Percentage identity: 39 %
  
BlastP bit score: 788
  
Sequence coverage: 103 %
  
E-value: 0.0
  
  
 NCBI BlastP on this gene

EFE44604

zinc-containing alcohol dehydrogenase, putative
  
Accession: EFE44603
  
Location: 17661-19119
  
 NCBI BlastP on this gene

EFE44603

hypothetical protein
  
Accession: EFE44602
  
Location: 16047-16949
  
 NCBI BlastP on this gene

EFE44602

RING finger domain protein, putative
  
Accession: EFE44601
  
Location: 13834-15481
  
 NCBI BlastP on this gene

EFE44601

hypothetical protein
  
Accession: EFE44600
  
Location: 9751-11923
  
 NCBI BlastP on this gene

EFE44600

Query: Architecture Search FASTA input

AQGS01000844 : Dactylellina haptotyla CBS 200.50    Total score: 2.0     Cumulative Blast bit score: 1135

Hit cluster cross-links:

Mycgr3G52686 Mycgr3T
  
Location: 0-861

Mycgr3G52686\_Mycgr3T

Mycgr3G102281 Mycgr3
  
Location: 961-1573

Mycgr3G102281\_Mycgr3

Mycgr3G89185 Mycgr3T
  
Location: 1673-2063

Mycgr3G89185\_Mycgr3T

Mycgr3G65725 Mycgr3T
  
Location: 2163-3612

Mycgr3G65725\_Mycgr3T

Mycgr3G102276 Mycgr3
  
Location: 3712-4801

Mycgr3G102276\_Mycgr3

Mycgr3G89189 Mycgr3T
  
Location: 4901-5564

Mycgr3G89189\_Mycgr3T

Mycgr3G52682 Mycgr3T
  
Location: 5664-9231

Mycgr3G52682\_Mycgr3T

Mycgr3G107072 Mycgr3
  
Location: 9331-13279

Mycgr3G107072\_Mycgr3

Mycgr3G34982 Mycgr3T
  
Location: 13379-15116

Mycgr3G34982\_Mycgr3T

Mycgr3G107069 Mycgr3
  
Location: 15216-17097

Mycgr3G107069\_Mycgr3

Mycgr3G32432 Mycgr3T
  
Location: 17197-19042

Mycgr3G32432\_Mycgr3T

Mycgr3G98385 Mycgr3T
  
Location: 19142-19898

Mycgr3G98385\_Mycgr3T

hypothetical protein
  
Accession: EPS36652
  
Location: 4146-5394
  
 NCBI BlastP on this gene

EPS36652

hypothetical protein
  
Accession: EPS36657
  
Location: 9148-9929
  
 NCBI BlastP on this gene

EPS36657

hypothetical protein
  
Accession: EPS36660
  
Location: 12377-13270
  
 NCBI BlastP on this gene

EPS36660

hypothetical protein
  
Accession: EPS36651
  
Location: 13781-14694
  
  
**BlastP hit with Mycgr3G102281\_Mycgr3**
  
Percentage identity: 89 %
  
BlastP bit score: 372
  
Sequence coverage: 99 %
  
E-value: 8e-129
  
  
 NCBI BlastP on this gene

EPS36651

hypothetical protein
  
Accession: EPS36653
  
Location: 17055-20546
  
  
**BlastP hit with Mycgr3G52682\_Mycgr3T**
  
Percentage identity: 38 %
  
BlastP bit score: 763
  
Sequence coverage: 101 %
  
E-value: 0.0
  
  
 NCBI BlastP on this gene

EPS36653

hypothetical protein
  
Accession: EPS36648
  
Location: 21242-22254
  
 NCBI BlastP on this gene

EPS36648

hypothetical protein
  
Accession: EPS36658
  
Location: 23503-24360
  
 NCBI BlastP on this gene

EPS36658

hypothetical protein
  
Accession: EPS36659
  
Location: 24929-28056
  
 NCBI BlastP on this gene

EPS36659

hypothetical protein
  
Accession: EPS36654
  
Location: 29291-30379
  
 NCBI BlastP on this gene

EPS36654

Query: Architecture Search FASTA input

AP007155 : Aspergillus oryzae RIB40 DNA, SC003.    Total score: 2.0     Cumulative Blast bit score: 1124

Hit cluster cross-links:

Mycgr3G52686 Mycgr3T
  
Location: 0-861

Mycgr3G52686\_Mycgr3T

Mycgr3G102281 Mycgr3
  
Location: 961-1573

Mycgr3G102281\_Mycgr3

Mycgr3G89185 Mycgr3T
  
Location: 1673-2063

Mycgr3G89185\_Mycgr3T

Mycgr3G65725 Mycgr3T
  
Location: 2163-3612

Mycgr3G65725\_Mycgr3T

Mycgr3G102276 Mycgr3
  
Location: 3712-4801

Mycgr3G102276\_Mycgr3

Mycgr3G89189 Mycgr3T
  
Location: 4901-5564

Mycgr3G89189\_Mycgr3T

Mycgr3G52682 Mycgr3T
  
Location: 5664-9231

Mycgr3G52682\_Mycgr3T

Mycgr3G107072 Mycgr3
  
Location: 9331-13279

Mycgr3G107072\_Mycgr3

Mycgr3G34982 Mycgr3T
  
Location: 13379-15116

Mycgr3G34982\_Mycgr3T

Mycgr3G107069 Mycgr3
  
Location: 15216-17097

Mycgr3G107069\_Mycgr3

Mycgr3G32432 Mycgr3T
  
Location: 17197-19042

Mycgr3G32432\_Mycgr3T

Mycgr3G98385 Mycgr3T
  
Location: 19142-19898

Mycgr3G98385\_Mycgr3T

not annotated
  
Accession: BAE58014
  
Location: 2425217-2427928
  
 NCBI BlastP on this gene

AO090003000896

not annotated
  
Accession: BAE58013
  
Location: 2422976-2423920
  
  
**BlastP hit with Mycgr3G102281\_Mycgr3**
  
Percentage identity: 89 %
  
BlastP bit score: 380
  
Sequence coverage: 99 %
  
E-value: 6e-132
  
  
 NCBI BlastP on this gene

AO090003000895

not annotated
  
Accession: BAE58012
  
Location: 2418074-2421607
  
  
**BlastP hit with Mycgr3G52682\_Mycgr3T**
  
Percentage identity: 54 %
  
BlastP bit score: 744
  
Sequence coverage: 57 %
  
E-value: 0.0
  
  
 NCBI BlastP on this gene

AO090003000894

not annotated
  
Accession: BAE58011
  
Location: 2416603-2417863
  
 NCBI BlastP on this gene

AO090003000893

not annotated
  
Accession: BAE58010
  
Location: 2415168-2416059
  
 NCBI BlastP on this gene

AO090003000892

not annotated
  
Accession: BAE58009
  
Location: 2410256-2411200
  
 NCBI BlastP on this gene

AO090003000890

Query: Architecture Search FASTA input

ADOT01000146 : Arthrobotrys oligospora ATCC 24927    Total score: 2.0     Cumulative Blast bit score: 1098

Hit cluster cross-links:

Mycgr3G52686 Mycgr3T
  
Location: 0-861

Mycgr3G52686\_Mycgr3T

Mycgr3G102281 Mycgr3
  
Location: 961-1573

Mycgr3G102281\_Mycgr3

Mycgr3G89185 Mycgr3T
  
Location: 1673-2063

Mycgr3G89185\_Mycgr3T

Mycgr3G65725 Mycgr3T
  
Location: 2163-3612

Mycgr3G65725\_Mycgr3T

Mycgr3G102276 Mycgr3
  
Location: 3712-4801

Mycgr3G102276\_Mycgr3

Mycgr3G89189 Mycgr3T
  
Location: 4901-5564

Mycgr3G89189\_Mycgr3T

Mycgr3G52682 Mycgr3T
  
Location: 5664-9231

Mycgr3G52682\_Mycgr3T

Mycgr3G107072 Mycgr3
  
Location: 9331-13279

Mycgr3G107072\_Mycgr3

Mycgr3G34982 Mycgr3T
  
Location: 13379-15116

Mycgr3G34982\_Mycgr3T

Mycgr3G107069 Mycgr3
  
Location: 15216-17097

Mycgr3G107069\_Mycgr3

Mycgr3G32432 Mycgr3T
  
Location: 17197-19042

Mycgr3G32432\_Mycgr3T

Mycgr3G98385 Mycgr3T
  
Location: 19142-19898

Mycgr3G98385\_Mycgr3T

hypothetical protein
  
Accession: EGX48066
  
Location: 80605-81411
  
 NCBI BlastP on this gene

EGX48066

hypothetical protein
  
Accession: EGX48065
  
Location: 76572-77534
  
 NCBI BlastP on this gene

EGX48065

hypothetical protein
  
Accession: EGX48064
  
Location: 75108-76037
  
  
**BlastP hit with Mycgr3G102281\_Mycgr3**
  
Percentage identity: 91 %
  
BlastP bit score: 384
  
Sequence coverage: 99 %
  
E-value: 3e-133
  
  
 NCBI BlastP on this gene

EGX48064

hypothetical protein
  
Accession: EGX48063
  
Location: 69053-72651
  
  
**BlastP hit with Mycgr3G52682\_Mycgr3T**
  
Percentage identity: 46 %
  
BlastP bit score: 714
  
Sequence coverage: 66 %
  
E-value: 0.0
  
  
 NCBI BlastP on this gene

EGX48063

hypothetical protein
  
Accession: EGX48062
  
Location: 67503-68497
  
 NCBI BlastP on this gene

EGX48062

hypothetical protein
  
Accession: EGX48061
  
Location: 65117-66217
  
 NCBI BlastP on this gene

EGX48061

hypothetical protein
  
Accession: EGX48060
  
Location: 63456-64079
  
 NCBI BlastP on this gene

EGX48060

hypothetical protein
  
Accession: EGX48059
  
Location: 62033-62916
  
 NCBI BlastP on this gene

EGX48059

hypothetical protein
  
Accession: EGX48058
  
Location: 60193-60816
  
 NCBI BlastP on this gene

EGX48058

Query: Architecture Search FASTA input

CH476657 : Ajellomyces capsulatus NAm1 scaffold\_3 genomic scaffold    Total score: 2.0     Cumulative Blast bit score: 1081

Hit cluster cross-links:

Mycgr3G52686 Mycgr3T
  
Location: 0-861

Mycgr3G52686\_Mycgr3T

Mycgr3G102281 Mycgr3
  
Location: 961-1573

Mycgr3G102281\_Mycgr3

Mycgr3G89185 Mycgr3T
  
Location: 1673-2063

Mycgr3G89185\_Mycgr3T

Mycgr3G65725 Mycgr3T
  
Location: 2163-3612

Mycgr3G65725\_Mycgr3T

Mycgr3G102276 Mycgr3
  
Location: 3712-4801

Mycgr3G102276\_Mycgr3

Mycgr3G89189 Mycgr3T
  
Location: 4901-5564

Mycgr3G89189\_Mycgr3T

Mycgr3G52682 Mycgr3T
  
Location: 5664-9231

Mycgr3G52682\_Mycgr3T

Mycgr3G107072 Mycgr3
  
Location: 9331-13279

Mycgr3G107072\_Mycgr3

Mycgr3G34982 Mycgr3T
  
Location: 13379-15116

Mycgr3G34982\_Mycgr3T

Mycgr3G107069 Mycgr3
  
Location: 15216-17097

Mycgr3G107069\_Mycgr3

Mycgr3G32432 Mycgr3T
  
Location: 17197-19042

Mycgr3G32432\_Mycgr3T

Mycgr3G98385 Mycgr3T
  
Location: 19142-19898

Mycgr3G98385\_Mycgr3T

predicted protein
  
Accession: EDN07081
  
Location: 1599444-1600830
  
 NCBI BlastP on this gene

EDN07081

60S ribosomal protein L15
  
Accession: EDN07080
  
Location: 1597982-1598988
  
  
**BlastP hit with Mycgr3G102281\_Mycgr3**
  
Percentage identity: 88 %
  
BlastP bit score: 342
  
Sequence coverage: 99 %
  
E-value: 7e-117
  
  
 NCBI BlastP on this gene

EDN07080

predicted protein
  
Accession: EDN07079
  
Location: 1596158-1596913
  
 NCBI BlastP on this gene

EDN07079

predicted protein
  
Accession: EDN07078
  
Location: 1591765-1592762
  
 NCBI BlastP on this gene

EDN07078

predicted protein
  
Accession: EDN07077
  
Location: 1590804-1591154
  
 NCBI BlastP on this gene

EDN07077

predicted protein
  
Accession: EDN07076
  
Location: 1589009-1589949
  
 NCBI BlastP on this gene

EDN07076

conserved hypothetical protein
  
Accession: EDN07075
  
Location: 1582818-1585479
  
  
**BlastP hit with Mycgr3G52682\_Mycgr3T**
  
Percentage identity: 47 %
  
BlastP bit score: 739
  
Sequence coverage: 74 %
  
E-value: 0.0
  
  
 NCBI BlastP on this gene

EDN07075

hypothetical protein
  
Accession: EDN07074
  
Location: 1578350-1580048
  
 NCBI BlastP on this gene

EDN07074

Query: Architecture Search FASTA input

DS544804 : Paracoccidioides brasiliensis Pb03 supercont1.2 genomic scaffold    Total score: 2.0     Cumulative Blast bit score: 1070

Hit cluster cross-links:

Mycgr3G52686 Mycgr3T
  
Location: 0-861

Mycgr3G52686\_Mycgr3T

Mycgr3G102281 Mycgr3
  
Location: 961-1573

Mycgr3G102281\_Mycgr3

Mycgr3G89185 Mycgr3T
  
Location: 1673-2063

Mycgr3G89185\_Mycgr3T

Mycgr3G65725 Mycgr3T
  
Location: 2163-3612

Mycgr3G65725\_Mycgr3T

Mycgr3G102276 Mycgr3
  
Location: 3712-4801

Mycgr3G102276\_Mycgr3

Mycgr3G89189 Mycgr3T
  
Location: 4901-5564

Mycgr3G89189\_Mycgr3T

Mycgr3G52682 Mycgr3T
  
Location: 5664-9231

Mycgr3G52682\_Mycgr3T

Mycgr3G107072 Mycgr3
  
Location: 9331-13279

Mycgr3G107072\_Mycgr3

Mycgr3G34982 Mycgr3T
  
Location: 13379-15116

Mycgr3G34982\_Mycgr3T

Mycgr3G107069 Mycgr3
  
Location: 15216-17097

Mycgr3G107069\_Mycgr3

Mycgr3G32432 Mycgr3T
  
Location: 17197-19042

Mycgr3G32432\_Mycgr3T

Mycgr3G98385 Mycgr3T
  
Location: 19142-19898

Mycgr3G98385\_Mycgr3T

conserved hypothetical protein
  
Accession: EEH18958
  
Location: 943075-945859
  
 NCBI BlastP on this gene

EEH18958

60S ribosomal protein L15
  
Accession: EEH18959
  
Location: 949969-950953
  
  
**BlastP hit with Mycgr3G102281\_Mycgr3**
  
Percentage identity: 89 %
  
BlastP bit score: 375
  
Sequence coverage: 99 %
  
E-value: 1e-129
  
  
 NCBI BlastP on this gene

EEH18959

E3 ubiquitin-protein ligase HUWE1
  
Accession: EEH18960
  
Location: 955442-959279
  
  
**BlastP hit with Mycgr3G52682\_Mycgr3T**
  
Percentage identity: 55 %
  
BlastP bit score: 695
  
Sequence coverage: 52 %
  
E-value: 0.0
  
  
 NCBI BlastP on this gene

EEH18960

L-threonine 3-dehydrogenase
  
Accession: EEH18961
  
Location: 962712-963721
  
 NCBI BlastP on this gene

EEH18961

conserved hypothetical protein
  
Accession: EEH18962
  
Location: 965763-966739
  
 NCBI BlastP on this gene

EEH18962

Query: Architecture Search FASTA input

KB916820 : Neofusicoccum parvum UCRNP2 chromosome Unknown NP2\_03\_scaffold\_1182    Total score: 2.0     Cumulative Blast bit score: 1049

Hit cluster cross-links:

Mycgr3G52686 Mycgr3T
  
Location: 0-861

Mycgr3G52686\_Mycgr3T

Mycgr3G102281 Mycgr3
  
Location: 961-1573

Mycgr3G102281\_Mycgr3

Mycgr3G89185 Mycgr3T
  
Location: 1673-2063

Mycgr3G89185\_Mycgr3T

Mycgr3G65725 Mycgr3T
  
Location: 2163-3612

Mycgr3G65725\_Mycgr3T

Mycgr3G102276 Mycgr3
  
Location: 3712-4801

Mycgr3G102276\_Mycgr3

Mycgr3G89189 Mycgr3T
  
Location: 4901-5564

Mycgr3G89189\_Mycgr3T

Mycgr3G52682 Mycgr3T
  
Location: 5664-9231

Mycgr3G52682\_Mycgr3T

Mycgr3G107072 Mycgr3
  
Location: 9331-13279

Mycgr3G107072\_Mycgr3

Mycgr3G34982 Mycgr3T
  
Location: 13379-15116

Mycgr3G34982\_Mycgr3T

Mycgr3G107069 Mycgr3
  
Location: 15216-17097

Mycgr3G107069\_Mycgr3

Mycgr3G32432 Mycgr3T
  
Location: 17197-19042

Mycgr3G32432\_Mycgr3T

Mycgr3G98385 Mycgr3T
  
Location: 19142-19898

Mycgr3G98385\_Mycgr3T

putative u-box domain-containing protein
  
Accession: EOD43493
  
Location: 240687-241855
  
 NCBI BlastP on this gene

EOD43493

putative 60s ribosomal protein l15 protein
  
Accession: EOD43521
  
Location: 242596-243460
  
  
**BlastP hit with Mycgr3G102281\_Mycgr3**
  
Percentage identity: 92 %
  
BlastP bit score: 368
  
Sequence coverage: 99 %
  
E-value: 6e-127
  
  
 NCBI BlastP on this gene

EOD43521

putative swi-snf complex subunit protein
  
Accession: EOD43504
  
Location: 248340-249873
  
 NCBI BlastP on this gene

EOD43504

putative cytosine deaminase protein
  
Accession: EOD43508
  
Location: 250678-251991
  
 NCBI BlastP on this gene

EOD43508

putative ubiquitin-protein ligase protein
  
Accession: EOD43522
  
Location: 256411-257990
  
  
**BlastP hit with Mycgr3G52682\_Mycgr3T**
  
Percentage identity: 64 %
  
BlastP bit score: 681
  
Sequence coverage: 43 %
  
E-value: 0.0
  
  
 NCBI BlastP on this gene

EOD43522

putative mfs monocarboxylate transporter protein
  
Accession: EOD43515
  
Location: 261386-263419
  
 NCBI BlastP on this gene

EOD43515

Query: Architecture Search FASTA input

KB916240 : Neofusicoccum parvum UCRNP2 chromosome Unknown NP2\_03\_scaffold\_602    Total score: 2.0     Cumulative Blast bit score: 1049

Hit cluster cross-links:

Mycgr3G52686 Mycgr3T
  
Location: 0-861

Mycgr3G52686\_Mycgr3T

Mycgr3G102281 Mycgr3
  
Location: 961-1573

Mycgr3G102281\_Mycgr3

Mycgr3G89185 Mycgr3T
  
Location: 1673-2063

Mycgr3G89185\_Mycgr3T

Mycgr3G65725 Mycgr3T
  
Location: 2163-3612

Mycgr3G65725\_Mycgr3T

Mycgr3G102276 Mycgr3
  
Location: 3712-4801

Mycgr3G102276\_Mycgr3

Mycgr3G89189 Mycgr3T
  
Location: 4901-5564

Mycgr3G89189\_Mycgr3T

Mycgr3G52682 Mycgr3T
  
Location: 5664-9231

Mycgr3G52682\_Mycgr3T

Mycgr3G107072 Mycgr3
  
Location: 9331-13279

Mycgr3G107072\_Mycgr3

Mycgr3G34982 Mycgr3T
  
Location: 13379-15116

Mycgr3G34982\_Mycgr3T

Mycgr3G107069 Mycgr3
  
Location: 15216-17097

Mycgr3G107069\_Mycgr3

Mycgr3G32432 Mycgr3T
  
Location: 17197-19042

Mycgr3G32432\_Mycgr3T

Mycgr3G98385 Mycgr3T
  
Location: 19142-19898

Mycgr3G98385\_Mycgr3T

putative ureidoglycolate hydrolase protein
  
Accession: EOD48161
  
Location: 222965-223837
  
 NCBI BlastP on this gene

EOD48161

putative hmg box-containing protein
  
Accession: EOD48132
  
Location: 218394-218929
  
 NCBI BlastP on this gene

EOD48132

putative nad dependent epimerase protein
  
Accession: EOD48100
  
Location: 213865-214900
  
  
**BlastP hit with Mycgr3G52686\_Mycgr3T**
  
Percentage identity: 76 %
  
BlastP bit score: 461
  
Sequence coverage: 97 %
  
E-value: 4e-161
  
  
 NCBI BlastP on this gene

EOD48100

putative Arylsulfotransferase protein
  
Accession: EOD48168
  
Location: 210935-213387
  
  
**BlastP hit with Mycgr3G32432\_Mycgr3T**
  
Percentage identity: 57 %
  
BlastP bit score: 588
  
Sequence coverage: 82 %
  
E-value: 0.0
  
  
 NCBI BlastP on this gene

EOD48168

putative mfs transporter protein
  
Accession: EOD48146
  
Location: 208158-210090
  
 NCBI BlastP on this gene

EOD48146

putative r3h and g-patch domain-containing protein
  
Accession: EOD48141
  
Location: 205266-206462
  
 NCBI BlastP on this gene

EOD48141

putative cysteine protease atg4 protein
  
Accession: EOD48149
  
Location: 202528-203567
  
 NCBI BlastP on this gene

EOD48149

putative histone h2b protein
  
Accession: EOD48114
  
Location: 200887-201497
  
 NCBI BlastP on this gene

EOD48114

Query: Architecture Search FASTA input

AHHD01000505 : Macrophomina phaseolina MS6    Total score: 2.0     Cumulative Blast bit score: 1040

Hit cluster cross-links:

Mycgr3G52686 Mycgr3T
  
Location: 0-861

Mycgr3G52686\_Mycgr3T

Mycgr3G102281 Mycgr3
  
Location: 961-1573

Mycgr3G102281\_Mycgr3

Mycgr3G89185 Mycgr3T
  
Location: 1673-2063

Mycgr3G89185\_Mycgr3T

Mycgr3G65725 Mycgr3T
  
Location: 2163-3612

Mycgr3G65725\_Mycgr3T

Mycgr3G102276 Mycgr3
  
Location: 3712-4801

Mycgr3G102276\_Mycgr3

Mycgr3G89189 Mycgr3T
  
Location: 4901-5564

Mycgr3G89189\_Mycgr3T

Mycgr3G52682 Mycgr3T
  
Location: 5664-9231

Mycgr3G52682\_Mycgr3T

Mycgr3G107072 Mycgr3
  
Location: 9331-13279

Mycgr3G107072\_Mycgr3

Mycgr3G34982 Mycgr3T
  
Location: 13379-15116

Mycgr3G34982\_Mycgr3T

Mycgr3G107069 Mycgr3
  
Location: 15216-17097

Mycgr3G107069\_Mycgr3

Mycgr3G32432 Mycgr3T
  
Location: 17197-19042

Mycgr3G32432\_Mycgr3T

Mycgr3G98385 Mycgr3T
  
Location: 19142-19898

Mycgr3G98385\_Mycgr3T

hypothetical protein
  
Accession: EKG10726
  
Location: 148090-148557
  
 NCBI BlastP on this gene

EKG10726

Ureidoglycolate hydrolase
  
Accession: EKG10727
  
Location: 150063-150929
  
 NCBI BlastP on this gene

EKG10727

protein of unknown function SprT-like protein
  
Accession: EKG10728
  
Location: 153533-155936
  
 NCBI BlastP on this gene

EKG10728

hypothetical protein
  
Accession: EKG10729
  
Location: 157429-158463
  
  
**BlastP hit with Mycgr3G52686\_Mycgr3T**
  
Percentage identity: 75 %
  
BlastP bit score: 453
  
Sequence coverage: 97 %
  
E-value: 6e-158
  
  
 NCBI BlastP on this gene

EKG10729

Arylsulfotransferase
  
Accession: EKG10730
  
Location: 159426-161469
  
  
**BlastP hit with Mycgr3G32432\_Mycgr3T**
  
Percentage identity: 56 %
  
BlastP bit score: 587
  
Sequence coverage: 82 %
  
E-value: 0.0
  
  
 NCBI BlastP on this gene

EKG10730

Major facilitator superfamily
  
Accession: EKG10731
  
Location: 162352-164129
  
 NCBI BlastP on this gene

EKG10731

hypothetical protein
  
Accession: EKG10732
  
Location: 165532-167691
  
 NCBI BlastP on this gene

EKG10732

Peptidase C54
  
Accession: EKG10733
  
Location: 168427-169890
  
 NCBI BlastP on this gene

EKG10733

Histone H2B
  
Accession: EKG10734
  
Location: 170436-170988
  
 NCBI BlastP on this gene

EKG10734

Histone H2A
  
Accession: EKG10735
  
Location: 171487-172117
  
 NCBI BlastP on this gene

EKG10735

Query: Architecture Search FASTA input

ABSU01000004 : Arthroderma benhamiae CBS 112371    Total score: 2.0     Cumulative Blast bit score: 1000

Hit cluster cross-links:

Mycgr3G52686 Mycgr3T
  
Location: 0-861

Mycgr3G52686\_Mycgr3T

Mycgr3G102281 Mycgr3
  
Location: 961-1573

Mycgr3G102281\_Mycgr3

Mycgr3G89185 Mycgr3T
  
Location: 1673-2063

Mycgr3G89185\_Mycgr3T

Mycgr3G65725 Mycgr3T
  
Location: 2163-3612

Mycgr3G65725\_Mycgr3T

Mycgr3G102276 Mycgr3
  
Location: 3712-4801

Mycgr3G102276\_Mycgr3

Mycgr3G89189 Mycgr3T
  
Location: 4901-5564

Mycgr3G89189\_Mycgr3T

Mycgr3G52682 Mycgr3T
  
Location: 5664-9231

Mycgr3G52682\_Mycgr3T

Mycgr3G107072 Mycgr3
  
Location: 9331-13279

Mycgr3G107072\_Mycgr3

Mycgr3G34982 Mycgr3T
  
Location: 13379-15116

Mycgr3G34982\_Mycgr3T

Mycgr3G107069 Mycgr3
  
Location: 15216-17097

Mycgr3G107069\_Mycgr3

Mycgr3G32432 Mycgr3T
  
Location: 17197-19042

Mycgr3G32432\_Mycgr3T

Mycgr3G98385 Mycgr3T
  
Location: 19142-19898

Mycgr3G98385\_Mycgr3T

hypothetical protein
  
Accession: EFE35223
  
Location: 993283-994290
  
 NCBI BlastP on this gene

EFE35223

EF hand domain protein
  
Accession: EFE35222
  
Location: 989296-992667
  
 NCBI BlastP on this gene

EFE35222

hypothetical protein
  
Accession: EFE35221
  
Location: 987373-988956
  
 NCBI BlastP on this gene

EFE35221

hypothetical protein
  
Accession: EFE35220
  
Location: 985558-986543
  
 NCBI BlastP on this gene

EFE35220

hypothetical protein
  
Accession: EFE35219
  
Location: 984262-985138
  
  
**BlastP hit with Mycgr3G102281\_Mycgr3**
  
Percentage identity: 74 %
  
BlastP bit score: 312
  
Sequence coverage: 105 %
  
E-value: 4e-105
  
  
 NCBI BlastP on this gene

EFE35219

ubiquitin-protein ligase (Hul4), putative
  
Accession: EFE35218
  
Location: 979498-983173
  
  
**BlastP hit with Mycgr3G52682\_Mycgr3T**
  
Percentage identity: 44 %
  
BlastP bit score: 688
  
Sequence coverage: 69 %
  
E-value: 0.0
  
  
 NCBI BlastP on this gene

EFE35218

hypothetical protein
  
Accession: EFE35217
  
Location: 977759-978674
  
 NCBI BlastP on this gene

EFE35217

zinc-containing alcohol dehydrogenase, putative
  
Accession: EFE35216
  
Location: 975770-977227
  
 NCBI BlastP on this gene

EFE35216

hypothetical protein
  
Accession: EFE35215
  
Location: 974169-975071
  
 NCBI BlastP on this gene

EFE35215

RING finger domain protein, putative
  
Accession: EFE35214
  
Location: 972212-973598
  
 NCBI BlastP on this gene

EFE35214

hypothetical protein
  
Accession: EFE35213
  
Location: 967873-970046
  
 NCBI BlastP on this gene

EFE35213

Query: Architecture Search FASTA input

JH767572 : Coniosporium apollinis CBS 100218 chromosome Unknown supercont1.19    Total score: 2.0     Cumulative Blast bit score: 988

Hit cluster cross-links:

Mycgr3G52686 Mycgr3T
  
Location: 0-861

Mycgr3G52686\_Mycgr3T

Mycgr3G102281 Mycgr3
  
Location: 961-1573

Mycgr3G102281\_Mycgr3

Mycgr3G89185 Mycgr3T
  
Location: 1673-2063

Mycgr3G89185\_Mycgr3T

Mycgr3G65725 Mycgr3T
  
Location: 2163-3612

Mycgr3G65725\_Mycgr3T

Mycgr3G102276 Mycgr3
  
Location: 3712-4801

Mycgr3G102276\_Mycgr3

Mycgr3G89189 Mycgr3T
  
Location: 4901-5564

Mycgr3G89189\_Mycgr3T

Mycgr3G52682 Mycgr3T
  
Location: 5664-9231

Mycgr3G52682\_Mycgr3T

Mycgr3G107072 Mycgr3
  
Location: 9331-13279

Mycgr3G107072\_Mycgr3

Mycgr3G34982 Mycgr3T
  
Location: 13379-15116

Mycgr3G34982\_Mycgr3T

Mycgr3G107069 Mycgr3
  
Location: 15216-17097

Mycgr3G107069\_Mycgr3

Mycgr3G32432 Mycgr3T
  
Location: 17197-19042

Mycgr3G32432\_Mycgr3T

Mycgr3G98385 Mycgr3T
  
Location: 19142-19898

Mycgr3G98385\_Mycgr3T

hypothetical protein
  
Accession: EON65152
  
Location: 226885-228285
  
 NCBI BlastP on this gene

EON65152

hypothetical protein
  
Accession: EON65151
  
Location: 223875-226298
  
 NCBI BlastP on this gene

EON65151

hypothetical protein
  
Accession: EON65150
  
Location: 220014-221900
  
 NCBI BlastP on this gene

EON65150

hypothetical protein
  
Accession: EON65149
  
Location: 217137-218206
  
  
**BlastP hit with Mycgr3G52686\_Mycgr3T**
  
Percentage identity: 72 %
  
BlastP bit score: 427
  
Sequence coverage: 99 %
  
E-value: 1e-147
  
  
 NCBI BlastP on this gene

EON65149

hypothetical protein
  
Accession: EON65148
  
Location: 214431-216468
  
  
**BlastP hit with Mycgr3G32432\_Mycgr3T**
  
Percentage identity: 52 %
  
BlastP bit score: 561
  
Sequence coverage: 86 %
  
E-value: 0.0
  
  
 NCBI BlastP on this gene

EON65148

Query: Architecture Search FASTA input

1. :  CM001196 Mycosphaerella graminicola IPO323 chromosome 1     Total score: 12.0     Cumulative Blast bit score: 13049

Mycgr3G52686 Mycgr3T
  
Location: 0-861
  
 NCBI BlastP on this gene

Mycgr3G52686\_Mycgr3T

Mycgr3G102281 Mycgr3
  
Location: 961-1573
  
 NCBI BlastP on this gene

Mycgr3G102281\_Mycgr3

Mycgr3G89185 Mycgr3T
  
Location: 1673-2063
  
 NCBI BlastP on this gene

Mycgr3G89185\_Mycgr3T

Mycgr3G65725 Mycgr3T
  
Location: 2163-3612
  
 NCBI BlastP on this gene

Mycgr3G65725\_Mycgr3T

Mycgr3G102276 Mycgr3
  
Location: 3712-4801
  
 NCBI BlastP on this gene

Mycgr3G102276\_Mycgr3

Mycgr3G89189 Mycgr3T
  
Location: 4901-5564
  
 NCBI BlastP on this gene

Mycgr3G89189\_Mycgr3T

Mycgr3G52682 Mycgr3T
  
Location: 5664-9231
  
 NCBI BlastP on this gene

Mycgr3G52682\_Mycgr3T

Mycgr3G107072 Mycgr3
  
Location: 9331-13279
  
 NCBI BlastP on this gene

Mycgr3G107072\_Mycgr3

Mycgr3G34982 Mycgr3T
  
Location: 13379-15116
  
 NCBI BlastP on this gene

Mycgr3G34982\_Mycgr3T

Mycgr3G107069 Mycgr3
  
Location: 15216-17097
  
 NCBI BlastP on this gene

Mycgr3G107069\_Mycgr3

Mycgr3G32432 Mycgr3T
  
Location: 17197-19042
  
 NCBI BlastP on this gene

Mycgr3G32432\_Mycgr3T

Mycgr3G98385 Mycgr3T
  
Location: 19142-19898
  
 NCBI BlastP on this gene

Mycgr3G98385\_Mycgr3T

hypothetical protein
  
Accession: EGP91142
  
Location: 2170752-2171879
  
 NCBI BlastP on this gene

EGP91142

hypothetical protein
  
Accession: EGP91143
  
Location: 2172553-2173003
  
  
**BlastP hit with Mycgr3G89185\_Mycgr3T**
  
Percentage identity: 100 %
  
BlastP bit score: 270
  
Sequence coverage: 99 %
  
E-value: 7e-91
  
  
 NCBI BlastP on this gene

EGP91143

putative FRE ferric reductase-like transmembrane component
  
Accession: EGP92462
  
Location: 2173209-2175532
  
  
**BlastP hit with Mycgr3G107069\_Mycgr3**
  
Percentage identity: 100 %
  
BlastP bit score: 1291
  
Sequence coverage: 99 %
  
E-value: 0.0
  
  
 NCBI BlastP on this gene

EGP92462

hypothetical protein
  
Accession: EGP92461
  
Location: 2175948-2177095
  
  
**BlastP hit with Mycgr3G102276\_Mycgr3**
  
Percentage identity: 100 %
  
BlastP bit score: 747
  
Sequence coverage: 99 %
  
E-value: 0.0
  
  
 NCBI BlastP on this gene

EGP92461

glucose-methanol-choline oxidoreductase
  
Accession: EGP92460
  
Location: 2179138-2181288
  
  
**BlastP hit with Mycgr3G34982\_Mycgr3T**
  
Percentage identity: 100 %
  
BlastP bit score: 1204
  
Sequence coverage: 99 %
  
E-value: 0.0
  
  
 NCBI BlastP on this gene

EGP92460

hypothetical protein
  
Accession: EGP92459
  
Location: 2181964-2184459
  
 NCBI BlastP on this gene

EGP92459

hypothetical protein
  
Accession: EGP91144
  
Location: 2185647-2189560
  
  
**BlastP hit with Mycgr3G89189\_Mycgr3T**
  
Percentage identity: 100 %
  
BlastP bit score: 448
  
Sequence coverage: 99 %
  
E-value: 5e-158
  
  
 NCBI BlastP on this gene

EGP91144

hypothetical protein
  
Accession: EGP91145
  
Location: 2189682-2191185
  
  
**BlastP hit with Mycgr3G65725\_Mycgr3T**
  
Percentage identity: 100 %
  
BlastP bit score: 979
  
Sequence coverage: 99 %
  
E-value: 0.0
  
  
 NCBI BlastP on this gene

EGP91145

hypothetical protein
  
Accession: EGP92458
  
Location: 2191438-2195442
  
  
**BlastP hit with Mycgr3G107072\_Mycgr3**
  
Percentage identity: 100 %
  
BlastP bit score: 2672
  
Sequence coverage: 99 %
  
E-value: 0.0
  
  
 NCBI BlastP on this gene

EGP92458

hypothetical protein
  
Accession: EGP91146
  
Location: 2197464-2198749
  
  
**BlastP hit with Mycgr3G98385\_Mycgr3T**
  
Percentage identity: 100 %
  
BlastP bit score: 517
  
Sequence coverage: 99 %
  
E-value: 0.0
  
  
 NCBI BlastP on this gene

EGP91146

hypothetical protein
  
Accession: EGP91147
  
Location: 2201706-2202619
  
  
**BlastP hit with Mycgr3G102281\_Mycgr3**
  
Percentage identity: 100 %
  
BlastP bit score: 414
  
Sequence coverage: 99 %
  
E-value: 4e-145
  
  
 NCBI BlastP on this gene

EGP91147

hypothetical protein
  
Accession: EGP91148
  
Location: 2203268-2207035
  
  
**BlastP hit with Mycgr3G52682\_Mycgr3T**
  
Percentage identity: 100 %
  
BlastP bit score: 2444
  
Sequence coverage: 99 %
  
E-value: 0.0
  
  
 NCBI BlastP on this gene

EGP91148

hypothetical protein
  
Accession: EGP91149
  
Location: 2208268-2209189
  
  
**BlastP hit with Mycgr3G52686\_Mycgr3T**
  
Percentage identity: 100 %
  
BlastP bit score: 598
  
Sequence coverage: 99 %
  
E-value: 0.0
  
  
 NCBI BlastP on this gene

EGP91149

hypothetical protein
  
Accession: EGP91150
  
Location: 2210894-2212738
  
  
**BlastP hit with Mycgr3G32432\_Mycgr3T**
  
Percentage identity: 100 %
  
BlastP bit score: 1261
  
Sequence coverage: 99 %
  
E-value: 0.0
  
  
 NCBI BlastP on this gene

EGP91150

major facilitator superfamily MFS 1 protein
  
Accession: EGP91151
  
Location: 2214598-2216244
  
 NCBI BlastP on this gene

EGP91151

hypothetical protein
  
Accession: EGP92457
  
Location: 2216502-2218390
  
 NCBI BlastP on this gene

EGP92457

hypothetical protein
  
Accession: EGP91152
  
Location: 2218881-2220485
  
 NCBI BlastP on this gene

EGP91152

hypothetical protein
  
Accession: EGP92456
  
Location: 2220508-2220877
  
 NCBI BlastP on this gene

EGP92456

hypothetical protein
  
Accession: EGP92455
  
Location: 2222385-2224438
  
  
**BlastP hit with Mycgr3G32432\_Mycgr3T**
  
Percentage identity: 32 %
  
BlastP bit score: 204
  
Sequence coverage: 86 %
  
E-value: 7e-54
  
  
 NCBI BlastP on this gene

EGP92455

hypothetical protein
  
Accession: EGP92454
  
Location: 2226023-2226691
  
 NCBI BlastP on this gene

EGP92454

2. :  KB446537 Dothistroma septosporum NZE10 unplaced genomic scaffold DOTSEscaffold\_3     Total score: 5.0     Cumulative Blast bit score: 3674

hypothetical protein
  
Accession: EME46350
  
Location: 1303006-1304185
  
 NCBI BlastP on this gene

EME46350

hypothetical protein
  
Accession: EME46351
  
Location: 1304475-1304894
  
 NCBI BlastP on this gene

EME46351

hypothetical protein
  
Accession: EME46352
  
Location: 1307111-1308454
  
 NCBI BlastP on this gene

EME46352

hypothetical protein
  
Accession: EME46354
  
Location: 1310373-1311053
  
 NCBI BlastP on this gene

EME46354

hypothetical protein
  
Accession: EME46355
  
Location: 1313053-1315275
  
 NCBI BlastP on this gene

EME46355

hypothetical protein
  
Accession: EME46356
  
Location: 1316087-1317274
  
 NCBI BlastP on this gene

EME46356

hypothetical protein
  
Accession: EME46357
  
Location: 1317727-1318303
  
 NCBI BlastP on this gene

EME46357

hypothetical protein
  
Accession: EME46358
  
Location: 1318868-1319824
  
 NCBI BlastP on this gene

EME46358

hypothetical protein
  
Accession: EME46359
  
Location: 1320439-1321649
  
  
**BlastP hit with Mycgr3G98385\_Mycgr3T**
  
Percentage identity: 87 %
  
BlastP bit score: 430
  
Sequence coverage: 91 %
  
E-value: 6e-150
  
  
 NCBI BlastP on this gene

EME46359

hypothetical protein
  
Accession: EME46360
  
Location: 1321862-1324100
  
 NCBI BlastP on this gene

EME46360

hypothetical protein
  
Accession: EME46361
  
Location: 1325002-1325769
  
  
**BlastP hit with Mycgr3G102281\_Mycgr3**
  
Percentage identity: 96 %
  
BlastP bit score: 384
  
Sequence coverage: 99 %
  
E-value: 3e-133
  
  
 NCBI BlastP on this gene

EME46361

hypothetical protein
  
Accession: EME46362
  
Location: 1326558-1330388
  
  
**BlastP hit with Mycgr3G52682\_Mycgr3T**
  
Percentage identity: 60 %
  
BlastP bit score: 1395
  
Sequence coverage: 108 %
  
E-value: 0.0
  
  
 NCBI BlastP on this gene

EME46362

hypothetical protein
  
Accession: EME46363
  
Location: 1332197-1333116
  
  
**BlastP hit with Mycgr3G52686\_Mycgr3T**
  
Percentage identity: 92 %
  
BlastP bit score: 555
  
Sequence coverage: 98 %
  
E-value: 0.0
  
  
 NCBI BlastP on this gene

EME46363

hypothetical protein
  
Accession: EME46364
  
Location: 1334461-1336650
  
  
**BlastP hit with Mycgr3G32432\_Mycgr3T**
  
Percentage identity: 57 %
  
BlastP bit score: 715
  
Sequence coverage: 99 %
  
E-value: 0.0
  
  
 NCBI BlastP on this gene

EME46364

hypothetical protein
  
Accession: EME46365
  
Location: 1338175-1340361
  
 NCBI BlastP on this gene

EME46365

hypothetical protein
  
Accession: EME46366
  
Location: 1341621-1343665
  
  
**BlastP hit with Mycgr3G32432\_Mycgr3T**
  
Percentage identity: 32 %
  
BlastP bit score: 195
  
Sequence coverage: 83 %
  
E-value: 2e-50
  
  
 NCBI BlastP on this gene

EME46366

hypothetical protein
  
Accession: EME46367
  
Location: 1346125-1348706
  
 NCBI BlastP on this gene

EME46367

hypothetical protein
  
Accession: EME46368
  
Location: 1349547-1350757
  
 NCBI BlastP on this gene

EME46368

hypothetical protein
  
Accession: EME46369
  
Location: 1350976-1352022
  
 NCBI BlastP on this gene

EME46369

hypothetical protein
  
Accession: EME46370
  
Location: 1352633-1357498
  
 NCBI BlastP on this gene

EME46370

glycoside hydrolase family 20 protein
  
Accession: EME46371
  
Location: 1357883-1360057
  
 NCBI BlastP on this gene

EME46371

hypothetical protein
  
Accession: EME46372
  
Location: 1360348-1366949
  
 NCBI BlastP on this gene

EME46372

3. :  KB445550 Baudoinia compniacensis UAMH 10762 unplaced genomic scaffold BAUCOscaffold\_1     Total score: 5.0     Cumulative Blast bit score: 3360

hypothetical protein
  
Accession: EMD00546
  
Location: 433737-434102
  
 NCBI BlastP on this gene

EMD00546

hypothetical protein
  
Accession: EMD00547
  
Location: 434307-436220
  
 NCBI BlastP on this gene

EMD00547

hypothetical protein
  
Accession: EMD00548
  
Location: 436393-436947
  
 NCBI BlastP on this gene

EMD00548

hypothetical protein
  
Accession: EMD00549
  
Location: 437387-438424
  
 NCBI BlastP on this gene

EMD00549

hypothetical protein
  
Accession: EMD00550
  
Location: 440099-443235
  
 NCBI BlastP on this gene

EMD00550

hypothetical protein
  
Accession: EMD00551
  
Location: 443938-445549
  
 NCBI BlastP on this gene

EMD00551

hypothetical protein
  
Accession: EMD00552
  
Location: 445900-446595
  
 NCBI BlastP on this gene

EMD00552

glycoside hydrolase family 72 protein
  
Accession: EMD00553
  
Location: 447761-449236
  
 NCBI BlastP on this gene

EMD00553

hypothetical protein
  
Accession: EMD00554
  
Location: 450055-452751
  
 NCBI BlastP on this gene

EMD00554

hypothetical protein
  
Accession: EMD00555
  
Location: 453313-458175
  
  
**BlastP hit with Mycgr3G32432\_Mycgr3T**
  
Percentage identity: 53 %
  
BlastP bit score: 654
  
Sequence coverage: 99 %
  
E-value: 0.0
  
  
 NCBI BlastP on this gene

EMD00555

hypothetical protein
  
Accession: EMD00556
  
Location: 459219-460128
  
  
**BlastP hit with Mycgr3G52686\_Mycgr3T**
  
Percentage identity: 87 %
  
BlastP bit score: 522
  
Sequence coverage: 99 %
  
E-value: 0.0
  
  
 NCBI BlastP on this gene

EMD00556

hypothetical protein
  
Accession: EMD00557
  
Location: 461146-461416
  
 NCBI BlastP on this gene

EMD00557

hypothetical protein
  
Accession: EMD00558
  
Location: 461495-461716
  
 NCBI BlastP on this gene

EMD00558

hypothetical protein
  
Accession: EMD00559
  
Location: 462649-463545
  
  
**BlastP hit with Mycgr3G98385\_Mycgr3T**
  
Percentage identity: 78 %
  
BlastP bit score: 387
  
Sequence coverage: 92 %
  
E-value: 6e-133
  
  
 NCBI BlastP on this gene

EMD00559

hypothetical protein
  
Accession: EMD00560
  
Location: 463772-465522
  
 NCBI BlastP on this gene

EMD00560

hypothetical protein
  
Accession: EMD00561
  
Location: 466337-467127
  
  
**BlastP hit with Mycgr3G102281\_Mycgr3**
  
Percentage identity: 93 %
  
BlastP bit score: 373
  
Sequence coverage: 99 %
  
E-value: 4e-129
  
  
 NCBI BlastP on this gene

EMD00561

hypothetical protein
  
Accession: EMD00562
  
Location: 467807-471619
  
  
**BlastP hit with Mycgr3G52682\_Mycgr3T**
  
Percentage identity: 60 %
  
BlastP bit score: 1424
  
Sequence coverage: 104 %
  
E-value: 0.0
  
  
 NCBI BlastP on this gene

EMD00562

hypothetical protein
  
Accession: EMD00563
  
Location: 473144-473853
  
 NCBI BlastP on this gene

EMD00563

hypothetical protein
  
Accession: EMD00564
  
Location: 474183-476541
  
 NCBI BlastP on this gene

EMD00564

hypothetical protein
  
Accession: EMD00565
  
Location: 476730-477821
  
 NCBI BlastP on this gene

EMD00565

hypothetical protein
  
Accession: EMD00566
  
Location: 478800-480767
  
 NCBI BlastP on this gene

EMD00566

glycosyltransferase family 8 protein
  
Accession: EMD00567
  
Location: 481419-483351
  
 NCBI BlastP on this gene

EMD00567

hypothetical protein
  
Accession: EMD00568
  
Location: 483556-484380
  
 NCBI BlastP on this gene

EMD00568

hypothetical protein
  
Accession: EMD00569
  
Location: 484951-485499
  
 NCBI BlastP on this gene

EMD00569

hypothetical protein
  
Accession: EMD00570
  
Location: 485770-486060
  
 NCBI BlastP on this gene

EMD00570

hypothetical protein
  
Accession: EMD00571
  
Location: 486459-487701
  
 NCBI BlastP on this gene

EMD00571

hypothetical protein
  
Accession: EMD00572
  
Location: 488070-490673
  
 NCBI BlastP on this gene

EMD00572

hypothetical protein
  
Accession: EMD00573
  
Location: 490897-491640
  
 NCBI BlastP on this gene

EMD00573

4. :  KB446558 Pseudocercospora fijiensis CIRAD86 unplaced genomic scaffold MYCFIscaffold\_4     Total score: 4.0     Cumulative Blast bit score: 3339

hypothetical protein
  
Accession: EME83068
  
Location: 2482416-2483639
  
 NCBI BlastP on this gene

EME83068

hypothetical protein
  
Accession: EME83067
  
Location: 2480348-2481119
  
  
**BlastP hit with Mycgr3G102281\_Mycgr3**
  
Percentage identity: 97 %
  
BlastP bit score: 382
  
Sequence coverage: 99 %
  
E-value: 8e-133
  
  
 NCBI BlastP on this gene

EME83067

hypothetical protein
  
Accession: EME83066
  
Location: 2475708-2479547
  
  
**BlastP hit with Mycgr3G52682\_Mycgr3T**
  
Percentage identity: 63 %
  
BlastP bit score: 1492
  
Sequence coverage: 100 %
  
E-value: 0.0
  
  
 NCBI BlastP on this gene

EME83066

hypothetical protein
  
Accession: EME83065
  
Location: 2474397-2475663
  
 NCBI BlastP on this gene

EME83065

hypothetical protein
  
Accession: EME83064
  
Location: 2472201-2472761
  
 NCBI BlastP on this gene

EME83064

hypothetical protein
  
Accession: EME83063
  
Location: 2471438-2471731
  
 NCBI BlastP on this gene

EME83063

hypothetical protein
  
Accession: EME83062
  
Location: 2466546-2468667
  
 NCBI BlastP on this gene

EME83062

hypothetical protein
  
Accession: EME83061
  
Location: 2463367-2465063
  
  
**BlastP hit with Mycgr3G32432\_Mycgr3T**
  
Percentage identity: 33 %
  
BlastP bit score: 201
  
Sequence coverage: 79 %
  
E-value: 1e-53
  
  
 NCBI BlastP on this gene

EME83061

hypothetical protein
  
Accession: EME83060
  
Location: 2461958-2462672
  
 NCBI BlastP on this gene

EME83060

hypothetical protein
  
Accession: EME83059
  
Location: 2460308-2461927
  
 NCBI BlastP on this gene

EME83059

hypothetical protein
  
Accession: EME83058
  
Location: 2458178-2459996
  
 NCBI BlastP on this gene

EME83058

hypothetical protein
  
Accession: EME83057
  
Location: 2456508-2458077
  
 NCBI BlastP on this gene

EME83057

hypothetical protein
  
Accession: EME83056
  
Location: 2454953-2456042
  
 NCBI BlastP on this gene

EME83056

hypothetical protein
  
Accession: EME83055
  
Location: 2453199-2454124
  
  
**BlastP hit with Mycgr3G52686\_Mycgr3T**
  
Percentage identity: 92 %
  
BlastP bit score: 566
  
Sequence coverage: 99 %
  
E-value: 0.0
  
  
 NCBI BlastP on this gene

EME83055

hypothetical protein
  
Accession: EME83054
  
Location: 2430140-2435554
  
  
**BlastP hit with Mycgr3G32432\_Mycgr3T**
  
Percentage identity: 58 %
  
BlastP bit score: 698
  
Sequence coverage: 94 %
  
E-value: 0.0
  
  
 NCBI BlastP on this gene

EME83054

hypothetical protein
  
Accession: EME83053
  
Location: 2427769-2429696
  
 NCBI BlastP on this gene

EME83053

hypothetical protein
  
Accession: EME83052
  
Location: 2424876-2427420
  
 NCBI BlastP on this gene

EME83052

5. :  KB456262 Mycosphaerella populorum SO2202 unplaced genomic scaffold SEPMUscaffold\_3     Total score: 4.0     Cumulative Blast bit score: 3021

hypothetical protein
  
Accession: EMF14497
  
Location: 1559999-1560703
  
 NCBI BlastP on this gene

EMF14497

hypothetical protein
  
Accession: EMF14498
  
Location: 1561125-1562378
  
 NCBI BlastP on this gene

EMF14498

vacuolar protein sorting-associated protein Vps28
  
Accession: EMF14499
  
Location: 1563023-1563837
  
 NCBI BlastP on this gene

EMF14499

Noc2-domain-containing protein
  
Accession: EMF14500
  
Location: 1564102-1566348
  
 NCBI BlastP on this gene

EMF14500

hypothetical protein
  
Accession: EMF14502
  
Location: 1567961-1568254
  
 NCBI BlastP on this gene

EMF14502

hypothetical protein
  
Accession: EMF14503
  
Location: 1568838-1569353
  
 NCBI BlastP on this gene

EMF14503

hypothetical protein
  
Accession: EMF14504
  
Location: 1569944-1570448
  
 NCBI BlastP on this gene

EMF14504

carbohydrate esterase family 1 protein
  
Accession: EMF14505
  
Location: 1571722-1572746
  
 NCBI BlastP on this gene

EMF14505

hypothetical protein
  
Accession: EMF14506
  
Location: 1573351-1573593
  
 NCBI BlastP on this gene

EMF14506

hypothetical protein
  
Accession: EMF14507
  
Location: 1574940-1577108
  
 NCBI BlastP on this gene

EMF14507

ribosomal protein L15e
  
Accession: EMF14509
  
Location: 1578155-1578925
  
  
**BlastP hit with Mycgr3G102281\_Mycgr3**
  
Percentage identity: 95 %
  
BlastP bit score: 378
  
Sequence coverage: 99 %
  
E-value: 6e-131
  
  
 NCBI BlastP on this gene

EMF14509

HECT-domain-containing protein
  
Accession: EMF14510
  
Location: 1579846-1583691
  
  
**BlastP hit with Mycgr3G52682\_Mycgr3T**
  
Percentage identity: 59 %
  
BlastP bit score: 1416
  
Sequence coverage: 108 %
  
E-value: 0.0
  
  
 NCBI BlastP on this gene

EMF14510

hypothetical protein
  
Accession: EMF14511
  
Location: 1585300-1586225
  
  
**BlastP hit with Mycgr3G52686\_Mycgr3T**
  
Percentage identity: 89 %
  
BlastP bit score: 547
  
Sequence coverage: 99 %
  
E-value: 0.0
  
  
 NCBI BlastP on this gene

EMF14511

MFS general substrate transporter
  
Accession: EMF14512
  
Location: 1588321-1593561
  
  
**BlastP hit with Mycgr3G32432\_Mycgr3T**
  
Percentage identity: 57 %
  
BlastP bit score: 680
  
Sequence coverage: 93 %
  
E-value: 0.0
  
  
 NCBI BlastP on this gene

EMF14512

carbon-nitrogen hydrolase
  
Accession: EMF14513
  
Location: 1593988-1595061
  
 NCBI BlastP on this gene

EMF14513

D-hydantoinase
  
Accession: EMF14514
  
Location: 1595559-1597127
  
 NCBI BlastP on this gene

EMF14514

hypothetical protein
  
Accession: EMF14516
  
Location: 1599249-1600005
  
 NCBI BlastP on this gene

EMF14516

ubiquitin-conjugating enzyme E2 2
  
Accession: EMF14517
  
Location: 1601184-1601859
  
 NCBI BlastP on this gene

EMF14517

CTP transf 1-domain-containing protein
  
Accession: EMF14518
  
Location: 1602270-1603520
  
 NCBI BlastP on this gene

EMF14518

histidinol-phosphate aminotransferase
  
Accession: EMF14519
  
Location: 1604173-1605574
  
 NCBI BlastP on this gene

EMF14519

hypothetical protein
  
Accession: EMF14520
  
Location: 1605809-1606837
  
 NCBI BlastP on this gene

EMF14520

glycoside hydrolase family 47 protein
  
Accession: EMF14521
  
Location: 1607080-1609045
  
 NCBI BlastP on this gene

EMF14521

hypothetical protein
  
Accession: EMF14522
  
Location: 1609901-1610113
  
 NCBI BlastP on this gene

EMF14522

glycoside hydrolase family 64 protein
  
Accession: EMF14523
  
Location: 1611101-1612039
  
 NCBI BlastP on this gene

EMF14523

6. :  KB915926 Neofusicoccum parvum UCRNP2 chromosome Unknown NP2\_03\_scaffold\_288     Total score: 2.0     Cumulative Blast bit score: 1409

putative sterigmatocystin 8-o-methyltransferase protein
  
Accession: EOD50854
  
Location: 48819-50255
  
 NCBI BlastP on this gene

EOD50854

putative Arylsulfotransferase protein
  
Accession: EOD50857
  
Location: 46555-48570
  
  
**BlastP hit with Mycgr3G32432\_Mycgr3T**
  
Percentage identity: 44 %
  
BlastP bit score: 432
  
Sequence coverage: 84 %
  
E-value: 2e-140
  
  
 NCBI BlastP on this gene

EOD50857

putative siderophore iron transporter mirb protein
  
Accession: EOD50856
  
Location: 45515-46046
  
 NCBI BlastP on this gene

EOD50856

putative abc multidrug transporter protein
  
Accession: EOD50849
  
Location: 39268-45487
  
 NCBI BlastP on this gene

EOD50849

putative d-alanine-poly ligase subunit 1 protein
  
Accession: EOD50859
  
Location: 32735-38229
  
 NCBI BlastP on this gene

EOD50859

putative siderophore iron transporter mirb protein
  
Accession: EOD50855
  
Location: 29575-31525
  
 NCBI BlastP on this gene

EOD50855

putative nad dependent epimerase protein
  
Accession: EOD50852
  
Location: 27540-28295
  
  
**BlastP hit with Mycgr3G52686\_Mycgr3T**
  
Percentage identity: 67 %
  
BlastP bit score: 313
  
Sequence coverage: 75 %
  
E-value: 5e-104
  
  
 NCBI BlastP on this gene

EOD50852

putative Arylsulfotransferase protein
  
Accession: EOD50858
  
Location: 25092-26898
  
  
**BlastP hit with Mycgr3G32432\_Mycgr3T**
  
Percentage identity: 47 %
  
BlastP bit score: 456
  
Sequence coverage: 82 %
  
E-value: 7e-150
  
  
 NCBI BlastP on this gene

EOD50858

hypothetical protein
  
Accession: EOD50850
  
Location: 21804-23345
  
  
**BlastP hit with Mycgr3G32432\_Mycgr3T**
  
Percentage identity: 30 %
  
BlastP bit score: 208
  
Sequence coverage: 73 %
  
E-value: 9e-57
  
  
 NCBI BlastP on this gene

EOD50850

putative siderophore biosynthesis acetylase protein
  
Accession: EOD50851
  
Location: 16297-17390
  
 NCBI BlastP on this gene

EOD50851

7. :  JH921454 Marssonina brunnea f. sp. 'multigermtubi' MB\_m1 unplaced genomic scaffold M6\_S00027     Total score: 2.0     Cumulative Blast bit score: 1334

hypothetical protein
  
Accession: EKD12835
  
Location: 453462-454716
  
 NCBI BlastP on this gene

EKD12835

PSP1 domain-containing protein
  
Accession: EKD12834
  
Location: 448515-452333
  
 NCBI BlastP on this gene

EKD12834

hypothetical protein
  
Accession: EKD12833
  
Location: 445035-446741
  
 NCBI BlastP on this gene

EKD12833

NifU-like protein
  
Accession: EKD12832
  
Location: 443532-444521
  
 NCBI BlastP on this gene

EKD12832

zinc finger (HIT type) family protein
  
Accession: EKD12831
  
Location: 442357-443230
  
 NCBI BlastP on this gene

EKD12831

hypothetical protein
  
Accession: EKD12830
  
Location: 441159-442013
  
 NCBI BlastP on this gene

EKD12830

RasGEF domain-containing protein
  
Accession: EKD12829
  
Location: 436224-439468
  
 NCBI BlastP on this gene

EKD12829

hypothetical protein
  
Accession: EKD12828
  
Location: 434231-434962
  
 NCBI BlastP on this gene

EKD12828

60S ribosomal protein L15
  
Accession: EKD12827
  
Location: 432741-433595
  
  
**BlastP hit with Mycgr3G102281\_Mycgr3**
  
Percentage identity: 92 %
  
BlastP bit score: 356
  
Sequence coverage: 99 %
  
E-value: 3e-122
  
  
 NCBI BlastP on this gene

EKD12827

HECT-domain-containing protein
  
Accession: EKD12826
  
Location: 428047-431880
  
  
**BlastP hit with Mycgr3G52682\_Mycgr3T**
  
Percentage identity: 45 %
  
BlastP bit score: 978
  
Sequence coverage: 107 %
  
E-value: 0.0
  
  
 NCBI BlastP on this gene

EKD12826

50S ribosomal protein mrp49
  
Accession: EKD12825
  
Location: 426171-426975
  
 NCBI BlastP on this gene

EKD12825

hypothetical protein
  
Accession: EKD12824
  
Location: 423595-425996
  
 NCBI BlastP on this gene

EKD12824

homocitrate synthase
  
Accession: EKD12823
  
Location: 421133-422867
  
 NCBI BlastP on this gene

EKD12823

WD domain-containing protein
  
Accession: EKD12822
  
Location: 414825-418714
  
 NCBI BlastP on this gene

EKD12822

nuclear cap-binding protein
  
Accession: EKD12821
  
Location: 413909-414492
  
 NCBI BlastP on this gene

EKD12821

hypothetical protein
  
Accession: EKD12820
  
Location: 412982-413460
  
 NCBI BlastP on this gene

EKD12820

cytochrome c oxidase polypeptide VIb
  
Accession: EKD12819
  
Location: 412217-412722
  
 NCBI BlastP on this gene

EKD12819

N-carbamoyl-L-amino acid hydrolase
  
Accession: EKD12818
  
Location: 410006-412058
  
 NCBI BlastP on this gene

EKD12818

hypothetical protein
  
Accession: EKD12817
  
Location: 408354-409213
  
 NCBI BlastP on this gene

EKD12817

8. :  KE145359 Glarea lozoyensis ATCC 20868 chromosome Unknown GLAREA16     Total score: 2.0     Cumulative Blast bit score: 1330

hypothetical protein
  
Accession: EPE32613
  
Location: 1481132-1483897
  
 NCBI BlastP on this gene

EPE32613

Fe-S cluster assembly (FSCA)
  
Accession: EPE32614
  
Location: 1488324-1489289
  
 NCBI BlastP on this gene

EPE32614

HIT/MYND zinc finger-like protein
  
Accession: EPE32615
  
Location: 1489540-1490336
  
 NCBI BlastP on this gene

EPE32615

hypothetical protein
  
Accession: EPE32616
  
Location: 1491137-1492113
  
 NCBI BlastP on this gene

EPE32616

Ras GEF
  
Accession: EPE32617
  
Location: 1495677-1499152
  
 NCBI BlastP on this gene

EPE32617

Ribosomal proteins S24e, L23 and L15e
  
Accession: EPE32618
  
Location: 1499849-1500688
  
  
**BlastP hit with Mycgr3G102281\_Mycgr3**
  
Percentage identity: 91 %
  
BlastP bit score: 358
  
Sequence coverage: 99 %
  
E-value: 2e-123
  
  
 NCBI BlastP on this gene

EPE32618

Hect, E3 ligase catalytic
  
Accession: EPE32619
  
Location: 1501342-1505141
  
  
**BlastP hit with Mycgr3G52682\_Mycgr3T**
  
Percentage identity: 45 %
  
BlastP bit score: 972
  
Sequence coverage: 100 %
  
E-value: 0.0
  
  
 NCBI BlastP on this gene

EPE32619

hypothetical protein
  
Accession: EPE32620
  
Location: 1505473-1506210
  
 NCBI BlastP on this gene

EPE32620

P-loop containing nucleoside triphosphate hydrolase
  
Accession: EPE32621
  
Location: 1506444-1508583
  
 NCBI BlastP on this gene

EPE32621

hypothetical protein
  
Accession: EPE32622
  
Location: 1508956-1509742
  
 NCBI BlastP on this gene

EPE32622

hypothetical protein
  
Accession: EPE32623
  
Location: 1510395-1511508
  
 NCBI BlastP on this gene

EPE32623

hypothetical protein
  
Accession: EPE32624
  
Location: 1512661-1514438
  
 NCBI BlastP on this gene

EPE32624

CSL zinc finger
  
Accession: EPE32625
  
Location: 1515585-1516157
  
 NCBI BlastP on this gene

EPE32625

Aldolase
  
Accession: EPE32626
  
Location: 1517134-1518874
  
 NCBI BlastP on this gene

EPE32626

S-adenosyl-L-methionine-dependent methyltransferase
  
Accession: EPE32627
  
Location: 1521215-1522713
  
 NCBI BlastP on this gene

EPE32627

hypothetical protein
  
Accession: EPE32628
  
Location: 1524542-1525487
  
 NCBI BlastP on this gene

EPE32628

9. :  CH476635 Sclerotinia sclerotiorum 1980 scaffold\_15 genomic scaffold     Total score: 2.0     Cumulative Blast bit score: 1309

predicted protein
  
Accession: EDN94629
  
Location: 1144445-1145150
  
 NCBI BlastP on this gene

EDN94629

predicted protein
  
Accession: EDN94630
  
Location: 1145569-1146071
  
 NCBI BlastP on this gene

EDN94630

predicted protein
  
Accession: EDN94631
  
Location: 1147023-1147994
  
 NCBI BlastP on this gene

EDN94631

hypothetical protein
  
Accession: EDN94632
  
Location: 1148355-1149146
  
 NCBI BlastP on this gene

EDN94632

hypothetical protein
  
Accession: EDN94633
  
Location: 1149755-1151261
  
 NCBI BlastP on this gene

EDN94633

predicted protein
  
Accession: EDN94634
  
Location: 1153251-1153468
  
 NCBI BlastP on this gene

EDN94634

predicted protein
  
Accession: EDN94635
  
Location: 1155526-1155883
  
 NCBI BlastP on this gene

EDN94635

hypothetical protein
  
Accession: EDN94636
  
Location: 1156302-1159106
  
 NCBI BlastP on this gene

EDN94636

predicted protein
  
Accession: EDN94637
  
Location: 1160592-1161856
  
 NCBI BlastP on this gene

EDN94637

hypothetical protein
  
Accession: EDN94638
  
Location: 1162787-1163627
  
  
**BlastP hit with Mycgr3G102281\_Mycgr3**
  
Percentage identity: 91 %
  
BlastP bit score: 355
  
Sequence coverage: 99 %
  
E-value: 6e-122
  
  
 NCBI BlastP on this gene

EDN94638

hypothetical protein
  
Accession: EDN94639
  
Location: 1164642-1168452
  
  
**BlastP hit with Mycgr3G52682\_Mycgr3T**
  
Percentage identity: 46 %
  
BlastP bit score: 954
  
Sequence coverage: 101 %
  
E-value: 0.0
  
  
 NCBI BlastP on this gene

EDN94639

predicted protein
  
Accession: EDN94640
  
Location: 1168580-1168897
  
 NCBI BlastP on this gene

EDN94640

hypothetical protein
  
Accession: EDN94641
  
Location: 1168982-1169815
  
 NCBI BlastP on this gene

EDN94641

hypothetical protein
  
Accession: EDN94642
  
Location: 1170428-1172215
  
 NCBI BlastP on this gene

EDN94642

predicted protein
  
Accession: EDN94643
  
Location: 1173080-1173178
  
 NCBI BlastP on this gene

EDN94643

predicted protein
  
Accession: EDN94644
  
Location: 1173525-1174727
  
 NCBI BlastP on this gene

EDN94644

hypothetical protein
  
Accession: EDN94645
  
Location: 1175079-1175859
  
 NCBI BlastP on this gene

EDN94645

hypothetical protein
  
Accession: EDN94646
  
Location: 1176452-1177160
  
 NCBI BlastP on this gene

EDN94646

hypothetical protein
  
Accession: EDN94647
  
Location: 1177426-1178317
  
 NCBI BlastP on this gene

EDN94647

hypothetical protein
  
Accession: EDN94648
  
Location: 1180392-1180719
  
 NCBI BlastP on this gene

EDN94648

predicted protein
  
Accession: EDN94649
  
Location: 1181846-1182043
  
 NCBI BlastP on this gene

EDN94649

10. :  AKHY01000097 Aspergillus oryzae 3.042     Total score: 2.0     Cumulative Blast bit score: 1309

hypothetical protein
  
Accession: EIT81776
  
Location: 107553-108269
  
 NCBI BlastP on this gene

EIT81776

hypothetical protein
  
Accession: EIT81813
  
Location: 109670-110665
  
 NCBI BlastP on this gene

EIT81813

hypothetical protein
  
Accession: EIT81803
  
Location: 112400-113122
  
 NCBI BlastP on this gene

EIT81803

uncharacterized protein
  
Accession: EIT81800
  
Location: 118841-121552
  
 NCBI BlastP on this gene

EIT81800

60s ribosomal protein
  
Accession: EIT81821
  
Location: 122848-123792
  
  
**BlastP hit with Mycgr3G102281\_Mycgr3**
  
Percentage identity: 89 %
  
BlastP bit score: 380
  
Sequence coverage: 99 %
  
E-value: 6e-132
  
  
 NCBI BlastP on this gene

EIT81821

E3 ubiquitin protein ligase
  
Accession: EIT81787
  
Location: 125017-128694
  
  
**BlastP hit with Mycgr3G52682\_Mycgr3T**
  
Percentage identity: 44 %
  
BlastP bit score: 929
  
Sequence coverage: 105 %
  
E-value: 0.0
  
  
 NCBI BlastP on this gene

EIT81787

hypothetical protein
  
Accession: EIT81792
  
Location: 130363-130943
  
 NCBI BlastP on this gene

EIT81792

hypothetical protein
  
Accession: EIT81786
  
Location: 132688-134341
  
 NCBI BlastP on this gene

EIT81786

hypothetical protein
  
Accession: EIT81791
  
Location: 137925-139378
  
 NCBI BlastP on this gene

EIT81791

hypothetical protein
  
Accession: EIT81785
  
Location: 139833-140855
  
 NCBI BlastP on this gene

EIT81785

RING finger domain protein, putative
  
Accession: EIT81795
  
Location: 141485-142222
  
 NCBI BlastP on this gene

EIT81795

histone acetyltransferases PCAF/SAGA/ADA, subunit TADA3L/NGG1
  
Accession: EIT81820
  
Location: 144154-146292
  
 NCBI BlastP on this gene

EIT81820

nitrogen permease regulator NLRG/NPR2
  
Accession: EIT81804
  
Location: 146640-148441
  
 NCBI BlastP on this gene

EIT81804

11. :  FQ790317 Botryotinia fuckeliana T4 SuperContig\_168\_1 genomic supercontig.     Total score: 2.0     Cumulative Blast bit score: 1288

predicted protein
  
Accession: CCD34800
  
Location: 44944-45097
  
 NCBI BlastP on this gene

BofuT4\_uP098590.1

hypothetical protein
  
Accession: CCD34799
  
Location: 42828-43822
  
 NCBI BlastP on this gene

BofuT4\_P098580.1

hypothetical protein
  
Accession: CCD34798
  
Location: 41676-42465
  
 NCBI BlastP on this gene

BofuT4\_P098570.1

similar to HIRA-interacting protein 5
  
Accession: CCD34797
  
Location: 40112-41131
  
 NCBI BlastP on this gene

BofuT4\_P098560.1

hypothetical protein
  
Accession: CCD34796
  
Location: 39468-39682
  
 NCBI BlastP on this gene

BofuT4\_uP098550.1

hypothetical protein
  
Accession: CCD34795
  
Location: 38377-38833
  
 NCBI BlastP on this gene

BofuT4\_uP098540.1

hypothetical protein
  
Accession: CCD34794
  
Location: 37493-37810
  
 NCBI BlastP on this gene

BofuT4\_uP098530.1

similar to PSP1 domain-containing protein
  
Accession: CCD34793
  
Location: 31254-34023
  
 NCBI BlastP on this gene

BofuT4\_P098520.1

hypothetical protein
  
Accession: CCD34792
  
Location: 28130-29233
  
 NCBI BlastP on this gene

BofuT4\_P098510.1

hypothetical protein
  
Accession: CCD34791
  
Location: 26208-27536
  
  
**BlastP hit with Mycgr3G102281\_Mycgr3**
  
Percentage identity: 91 %
  
BlastP bit score: 356
  
Sequence coverage: 99 %
  
E-value: 5e-121
  
  
 NCBI BlastP on this gene

BofuT4\_P098500.1

similar to ubiquitin-protein ligase E3
  
Accession: CCD34790
  
Location: 21386-25179
  
  
**BlastP hit with Mycgr3G52682\_Mycgr3T**
  
Percentage identity: 43 %
  
BlastP bit score: 932
  
Sequence coverage: 107 %
  
E-value: 0.0
  
  
 NCBI BlastP on this gene

BofuT4\_P098490.1

similar to 50S ribosomal protein Mrp49
  
Accession: CCD34789
  
Location: 19948-20791
  
 NCBI BlastP on this gene

BofuT4\_P098480.1

hypothetical protein
  
Accession: CCD34788
  
Location: 17580-19715
  
 NCBI BlastP on this gene

BofuT4\_P098470.1

hypothetical protein
  
Accession: CCD34787
  
Location: 16329-16781
  
 NCBI BlastP on this gene

BofuT4\_P098460.1

hypothetical protein
  
Accession: CCD34786
  
Location: 13592-15817
  
 NCBI BlastP on this gene

BofuT4\_P098450.1

hypothetical protein
  
Accession: CCD34785
  
Location: 12493-13260
  
 NCBI BlastP on this gene

BofuT4\_P098440.1

similar to ubiquinol-cytochrome c reductase complex ubiquinone-binding protein
  
Accession: CCD34784
  
Location: 11186-11913
  
 NCBI BlastP on this gene

BofuT4\_P098430.1

similar to prohibitin
  
Accession: CCD34783
  
Location: 10020-10906
  
 NCBI BlastP on this gene

BofuT4\_P098420.1

hypothetical protein
  
Accession: CCD34782
  
Location: 8494-8643
  
 NCBI BlastP on this gene

BofuT4\_uP098410.1

hypothetical protein
  
Accession: CCD34781
  
Location: 7291-8152
  
 NCBI BlastP on this gene

BofuT4\_P098400.1

hypothetical protein
  
Accession: CCD34780
  
Location: 6252-6780
  
 NCBI BlastP on this gene

BofuT4\_P098390.1

similar to nucleoside-diphosphate-sugar epimerase
  
Accession: CCD34779
  
Location: 3877-4566
  
 NCBI BlastP on this gene

BofuT4\_P098380.1

hypothetical protein
  
Accession: CCD34778
  
Location: 2850-3380
  
 NCBI BlastP on this gene

BofuT4\_P098370.1

hypothetical protein
  
Accession: CCD34777
  
Location: 1582-2018
  
 NCBI BlastP on this gene

BofuT4\_uP098360.1

12. :  KB708110 Botryotinia fuckeliana BcDW1 unplaced genomic scaffold Scaffold\_438     Total score: 2.0     Cumulative Blast bit score: 1287

putative zinc finger (hit type) family protein
  
Accession: EMR80690
  
Location: 49176-49965
  
 NCBI BlastP on this gene

EMR80690

putative hira-interacting protein 5 protein
  
Accession: EMR80689
  
Location: 47612-48631
  
 NCBI BlastP on this gene

EMR80689

putative psp1 domain-containing protein
  
Accession: EMR80688
  
Location: 38749-41518
  
 NCBI BlastP on this gene

EMR80688

hypothetical protein
  
Accession: EMR80687
  
Location: 35999-37102
  
 NCBI BlastP on this gene

EMR80687

putative 60s ribosomal protein l15 protein
  
Accession: EMR80686
  
Location: 34077-35405
  
  
**BlastP hit with Mycgr3G102281\_Mycgr3**
  
Percentage identity: 91 %
  
BlastP bit score: 356
  
Sequence coverage: 99 %
  
E-value: 5e-121
  
  
 NCBI BlastP on this gene

EMR80686

putative ubiquitin-protein ligase protein
  
Accession: EMR80685
  
Location: 29255-33048
  
  
**BlastP hit with Mycgr3G52682\_Mycgr3T**
  
Percentage identity: 43 %
  
BlastP bit score: 931
  
Sequence coverage: 107 %
  
E-value: 0.0
  
  
 NCBI BlastP on this gene

EMR80685

putative 50s ribosomal protein mrp49 protein
  
Accession: EMR80684
  
Location: 27839-28661
  
 NCBI BlastP on this gene

EMR80684

putative gtp-binding protein
  
Accession: EMR80683
  
Location: 25450-27585
  
 NCBI BlastP on this gene

EMR80683

hypothetical protein
  
Accession: EMR80682
  
Location: 21460-24651
  
 NCBI BlastP on this gene

EMR80682

putative ribosome biogenesis protein nhp2 protein
  
Accession: EMR80681
  
Location: 20361-21128
  
 NCBI BlastP on this gene

EMR80681

putative ubiquinol-cytochrome c reductase complex subunit protein
  
Accession: EMR80680
  
Location: 19054-19781
  
 NCBI BlastP on this gene

EMR80680

putative prohibitin protein
  
Accession: EMR80679
  
Location: 17888-18774
  
 NCBI BlastP on this gene

EMR80679

putative cytochrome p450 alkane hydroxylase protein
  
Accession: EMR80678
  
Location: 14659-16196
  
 NCBI BlastP on this gene

EMR80678

putative nucleoside-diphosphate-sugar epimerase protein
  
Accession: EMR80677
  
Location: 12693-13382
  
 NCBI BlastP on this gene

EMR80677

putative dimethylallyl tryptophan synthase protein
  
Accession: EMR80676
  
Location: 10404-12196
  
 NCBI BlastP on this gene

EMR80676

13. :  DS027059 Aspergillus clavatus NRRL 1 1099423829805 genomic scaffold     Total score: 2.0     Cumulative Blast bit score: 1278

HIT finger domain protein, putative
  
Accession: EAW08266
  
Location: 3592958-3593608
  
 NCBI BlastP on this gene

EAW08266

NifU-related protein
  
Accession: EAW08267
  
Location: 3596151-3597249
  
 NCBI BlastP on this gene

EAW08267

PSP1 domain protein
  
Accession: EAW08268
  
Location: 3603658-3606355
  
 NCBI BlastP on this gene

EAW08268

60S ribosomal protein L15
  
Accession: EAW08269
  
Location: 3607471-3608529
  
  
**BlastP hit with Mycgr3G102281\_Mycgr3**
  
Percentage identity: 89 %
  
BlastP bit score: 343
  
Sequence coverage: 99 %
  
E-value: 2e-117
  
  
 NCBI BlastP on this gene

EAW08269

ubiquitin-protein ligase (Hul4), putative
  
Accession: EAW08270
  
Location: 3609760-3613489
  
  
**BlastP hit with Mycgr3G52682\_Mycgr3T**
  
Percentage identity: 44 %
  
BlastP bit score: 935
  
Sequence coverage: 100 %
  
E-value: 0.0
  
  
 NCBI BlastP on this gene

EAW08270

alcohol dehydrogenase, zinc-containing, putative
  
Accession: EAW08271
  
Location: 3615663-3617156
  
 NCBI BlastP on this gene

EAW08271

conserved hypothetical protein
  
Accession: EAW08272
  
Location: 3617704-3618676
  
 NCBI BlastP on this gene

EAW08272

RING finger domain protein, putative
  
Accession: EAW08273
  
Location: 3619192-3620732
  
 NCBI BlastP on this gene

EAW08273

transcriptional regulator Ngg1, putative
  
Accession: EAW08274
  
Location: 3621997-3624159
  
 NCBI BlastP on this gene

EAW08274

nitrogen permease regulator Npr2, putative
  
Accession: EAW08275
  
Location: 3624604-3626503
  
 NCBI BlastP on this gene

EAW08275

conserved hypothetical protein
  
Accession: EAW08276
  
Location: 3627215-3628126
  
 NCBI BlastP on this gene

EAW08276

hypothetical protein
  
Accession: EAW08277
  
Location: 3630222-3632878
  
 NCBI BlastP on this gene

EAW08277

Urease alpha-subunit, N-terminal domain protein
  
Accession: EAW08278
  
Location: 3633178-3636092
  
 NCBI BlastP on this gene

EAW08278

14. :  DS499594 Aspergillus fumigatus A1163 scf\_000001 genomic scaffold     Total score: 2.0     Cumulative Blast bit score: 1275

PP-loop ATPase superfamily protein, putative
  
Accession: EDP55809
  
Location: 1417676-1418938
  
 NCBI BlastP on this gene

EDP55809

Ras guanyl-nucleotide exchange factor RasGEF, putative
  
Accession: EDP55808
  
Location: 1413142-1416410
  
 NCBI BlastP on this gene

EDP55808

HIT finger domain protein, putative
  
Accession: EDP55807
  
Location: 1409970-1410598
  
 NCBI BlastP on this gene

EDP55807

NifU-related protein
  
Accession: EDP55806
  
Location: 1408003-1409110
  
 NCBI BlastP on this gene

EDP55806

PSP1 domain protein
  
Accession: EDP55805
  
Location: 1399364-1402060
  
 NCBI BlastP on this gene

EDP55805

60S ribosomal protein L15
  
Accession: EDP55804
  
Location: 1397261-1398292
  
  
**BlastP hit with Mycgr3G102281\_Mycgr3**
  
Percentage identity: 79 %
  
BlastP bit score: 340
  
Sequence coverage: 113 %
  
E-value: 9e-116
  
  
 NCBI BlastP on this gene

EDP55804

ubiquitin-protein ligase (Hul4), putative
  
Accession: EDP55803
  
Location: 1392365-1396074
  
  
**BlastP hit with Mycgr3G52682\_Mycgr3T**
  
Percentage identity: 43 %
  
BlastP bit score: 935
  
Sequence coverage: 105 %
  
E-value: 0.0
  
  
 NCBI BlastP on this gene

EDP55803

hypothetical protein
  
Accession: EDP55802
  
Location: 1390745-1391132
  
 NCBI BlastP on this gene

EDP55802

alcohol dehydrogenase, zinc-containing, putative
  
Accession: EDP55801
  
Location: 1389233-1390568
  
 NCBI BlastP on this gene

EDP55801

conserved hypothetical protein
  
Accession: EDP55800
  
Location: 1387763-1388680
  
 NCBI BlastP on this gene

EDP55800

RING finger domain protein, putative
  
Accession: EDP55799
  
Location: 1385398-1387301
  
 NCBI BlastP on this gene

EDP55799

transcriptional regulator Ngg1, putative
  
Accession: EDP55798
  
Location: 1382242-1384493
  
 NCBI BlastP on this gene

EDP55798

nitrogen permease regulator Npr2, putative
  
Accession: EDP55797
  
Location: 1379835-1381761
  
 NCBI BlastP on this gene

EDP55797

conserved hypothetical protein
  
Accession: EDP55796
  
Location: 1378211-1379122
  
 NCBI BlastP on this gene

EDP55796

DNA repair protein, putative
  
Accession: EDP55795
  
Location: 1374369-1376216
  
 NCBI BlastP on this gene

EDP55795

15. :  AAHF01000007 Aspergillus fumigatus Af293     Total score: 2.0     Cumulative Blast bit score: 1275

PP-loop ATPase superfamily protein, putative
  
Accession: EAL88182
  
Location: 1338318-1339580
  
 NCBI BlastP on this gene

EAL88182

Ras guanyl-nucleotide exchange factor RasGEF, putative
  
Accession: EAL88181
  
Location: 1333784-1337052
  
 NCBI BlastP on this gene

EAL88181

HIT finger domain protein, putative
  
Accession: EAL88180
  
Location: 1330615-1331243
  
 NCBI BlastP on this gene

EAL88180

NifU-related protein
  
Accession: EAL88179
  
Location: 1328649-1329756
  
 NCBI BlastP on this gene

EAL88179

PSP1 domain protein
  
Accession: EAL88178
  
Location: 1319974-1322670
  
 NCBI BlastP on this gene

EAL88178

60S ribosomal protein L15
  
Accession: EAL88177
  
Location: 1317870-1318902
  
  
**BlastP hit with Mycgr3G102281\_Mycgr3**
  
Percentage identity: 79 %
  
BlastP bit score: 340
  
Sequence coverage: 113 %
  
E-value: 9e-116
  
  
 NCBI BlastP on this gene

EAL88177

ubiquitin-protein ligase (Hul4), putative
  
Accession: EAL88175
  
Location: 1311090-1314799
  
  
**BlastP hit with Mycgr3G52682\_Mycgr3T**
  
Percentage identity: 43 %
  
BlastP bit score: 935
  
Sequence coverage: 105 %
  
E-value: 0.0
  
  
 NCBI BlastP on this gene

EAL88175

hypothetical protein
  
Accession: EAL88174
  
Location: 1309470-1309857
  
 NCBI BlastP on this gene

EAL88174

alcohol dehydrogenase, zinc-containing, putative
  
Accession: EAL88173
  
Location: 1307958-1309293
  
 NCBI BlastP on this gene

EAL88173

conserved hypothetical protein
  
Accession: EAL88172
  
Location: 1306488-1307405
  
 NCBI BlastP on this gene

EAL88172

RING finger domain protein, putative
  
Accession: EAL88171
  
Location: 1304122-1306025
  
 NCBI BlastP on this gene

EAL88171

transcriptional regulator Ngg1, putative
  
Accession: EAL88170
  
Location: 1300966-1303217
  
 NCBI BlastP on this gene

EAL88170

nitrogen permease regulator Npr2, putative
  
Accession: EAL88169
  
Location: 1298568-1300485
  
 NCBI BlastP on this gene

EAL88169

conserved hypothetical protein
  
Accession: EAL88168
  
Location: 1296944-1297855
  
 NCBI BlastP on this gene

EAL88168

DNA repair protein, putative
  
Accession: EAL88167
  
Location: 1292511-1294943
  
 NCBI BlastP on this gene

EAL88167

16. :  DS027688 Neosartorya fischeri NRRL 181 1099437636249 genomic scaffold     Total score: 2.0     Cumulative Blast bit score: 1274

hypothetical protein
  
Accession: EAW23297
  
Location: 3666894-3667223
  
 NCBI BlastP on this gene

EAW23297

Ras guanyl-nucleotide exchange factor RasGEF, putative
  
Accession: EAW23298
  
Location: 3667711-3671115
  
 NCBI BlastP on this gene

EAW23298

HIT finger domain protein, putative
  
Accession: EAW23299
  
Location: 3673557-3674186
  
 NCBI BlastP on this gene

EAW23299

NifU-related protein
  
Accession: EAW23300
  
Location: 3675004-3676111
  
 NCBI BlastP on this gene

EAW23300

PSP1 domain protein
  
Accession: EAW23301
  
Location: 3682103-3684800
  
 NCBI BlastP on this gene

EAW23301

60S ribosomal protein L15
  
Accession: EAW23302
  
Location: 3685931-3686967
  
  
**BlastP hit with Mycgr3G102281\_Mycgr3**
  
Percentage identity: 89 %
  
BlastP bit score: 345
  
Sequence coverage: 99 %
  
E-value: 5e-118
  
  
 NCBI BlastP on this gene

EAW23302

ubiquitin-protein ligase (Hul4), putative
  
Accession: EAW23303
  
Location: 3688177-3691889
  
  
**BlastP hit with Mycgr3G52682\_Mycgr3T**
  
Percentage identity: 45 %
  
BlastP bit score: 929
  
Sequence coverage: 96 %
  
E-value: 0.0
  
  
 NCBI BlastP on this gene

EAW23303

alcohol dehydrogenase, zinc-containing, putative
  
Accession: EAW23304
  
Location: 3693794-3695130
  
 NCBI BlastP on this gene

EAW23304

FRG1-like family protein
  
Accession: EAW23305
  
Location: 3695688-3696603
  
 NCBI BlastP on this gene

EAW23305

RING finger domain protein, putative
  
Accession: EAW23306
  
Location: 3697066-3698534
  
 NCBI BlastP on this gene

EAW23306

transcriptional regulator Ngg1, putative
  
Accession: EAW23307
  
Location: 3699904-3702154
  
 NCBI BlastP on this gene

EAW23307

nitrogen permease regulator Npr2, putative
  
Accession: EAW23308
  
Location: 3702618-3704529
  
 NCBI BlastP on this gene

EAW23308

conserved hypothetical protein
  
Accession: EAW23309
  
Location: 3705227-3706138
  
 NCBI BlastP on this gene

EAW23309

conserved hypothetical protein
  
Accession: EAW23310
  
Location: 3708317-3710585
  
 NCBI BlastP on this gene

EAW23310

urease, putative
  
Accession: EAW23311
  
Location: 3710766-3713634
  
 NCBI BlastP on this gene

EAW23311

17. :  AKCU01000141 Penicillium digitatum Pd1     Total score: 2.0     Cumulative Blast bit score: 1264

HMG box protein, putative
  
Accession: EKV20031
  
Location: 44751-46094
  
 NCBI BlastP on this gene

EKV20031

Urease
  
Accession: EKV20032
  
Location: 46740-49550
  
 NCBI BlastP on this gene

EKV20032

hypothetical protein
  
Accession: EKV20033
  
Location: 50235-50609
  
 NCBI BlastP on this gene

EKV20033

hypothetical protein
  
Accession: EKV20034
  
Location: 50831-51250
  
 NCBI BlastP on this gene

EKV20034

DNA repair protein, putative
  
Accession: EKV20035
  
Location: 52197-54397
  
 NCBI BlastP on this gene

EKV20035

hypothetical protein
  
Accession: EKV20036
  
Location: 55500-56393
  
 NCBI BlastP on this gene

EKV20036

Nitrogen permease regulator Npr2, putative
  
Accession: EKV20037
  
Location: 57754-59751
  
 NCBI BlastP on this gene

EKV20037

Transcriptional regulator Ngg1, putative
  
Accession: EKV20038
  
Location: 60078-62216
  
 NCBI BlastP on this gene

EKV20038

hypothetical protein
  
Accession: EKV20039
  
Location: 63571-66669
  
  
**BlastP hit with Mycgr3G102281\_Mycgr3**
  
Percentage identity: 91 %
  
BlastP bit score: 389
  
Sequence coverage: 99 %
  
E-value: 1e-130
  
  
 NCBI BlastP on this gene

EKV20039

Ubiquitin-protein ligase (Hul4), putative
  
Accession: EKV20040
  
Location: 68998-72682
  
  
**BlastP hit with Mycgr3G52682\_Mycgr3T**
  
Percentage identity: 42 %
  
BlastP bit score: 875
  
Sequence coverage: 105 %
  
E-value: 0.0
  
  
 NCBI BlastP on this gene

EKV20040

FAD dependent oxidoreductase, putative
  
Accession: EKV20041
  
Location: 73856-74984
  
 NCBI BlastP on this gene

EKV20041

hypothetical protein
  
Accession: EKV20042
  
Location: 75902-76045
  
 NCBI BlastP on this gene

EKV20042

hypothetical protein
  
Accession: EKV20043
  
Location: 76104-76217
  
 NCBI BlastP on this gene

EKV20043

hypothetical protein
  
Accession: EKV20044
  
Location: 76854-78100
  
 NCBI BlastP on this gene

EKV20044

18. :  AKCT01000207 Penicillium digitatum PHI26     Total score: 2.0     Cumulative Blast bit score: 1264

HMG box protein, putative
  
Accession: EKV11300
  
Location: 115518-116861
  
 NCBI BlastP on this gene

EKV11300

Urease
  
Accession: EKV11301
  
Location: 117790-120601
  
 NCBI BlastP on this gene

EKV11301

hypothetical protein
  
Accession: EKV11302
  
Location: 121287-121661
  
 NCBI BlastP on this gene

EKV11302

hypothetical protein
  
Accession: EKV11303
  
Location: 121884-122303
  
 NCBI BlastP on this gene

EKV11303

DNA repair protein, putative
  
Accession: EKV11304
  
Location: 123250-125450
  
 NCBI BlastP on this gene

EKV11304

hypothetical protein
  
Accession: EKV11305
  
Location: 126554-127447
  
 NCBI BlastP on this gene

EKV11305

Nitrogen permease regulator Npr2, putative
  
Accession: EKV11306
  
Location: 128808-130805
  
 NCBI BlastP on this gene

EKV11306

Transcriptional regulator Ngg1, putative
  
Accession: EKV11307
  
Location: 131134-133272
  
 NCBI BlastP on this gene

EKV11307

hypothetical protein
  
Accession: EKV11308
  
Location: 134627-137726
  
  
**BlastP hit with Mycgr3G102281\_Mycgr3**
  
Percentage identity: 91 %
  
BlastP bit score: 389
  
Sequence coverage: 99 %
  
E-value: 1e-130
  
  
 NCBI BlastP on this gene

EKV11308

Ubiquitin-protein ligase (Hul4), putative
  
Accession: EKV11309
  
Location: 140057-143741
  
  
**BlastP hit with Mycgr3G52682\_Mycgr3T**
  
Percentage identity: 42 %
  
BlastP bit score: 875
  
Sequence coverage: 105 %
  
E-value: 0.0
  
  
 NCBI BlastP on this gene

EKV11309

FAD dependent oxidoreductase, putative
  
Accession: EKV11310
  
Location: 144680-145868
  
 NCBI BlastP on this gene

EKV11310

hypothetical protein
  
Accession: EKV11311
  
Location: 146770-146931
  
 NCBI BlastP on this gene

EKV11311

hypothetical protein
  
Accession: EKV11312
  
Location: 146990-147103
  
 NCBI BlastP on this gene

EKV11312

hypothetical protein
  
Accession: EKV11313
  
Location: 147740-148986
  
 NCBI BlastP on this gene

EKV11313

hypothetical protein
  
Accession: EKV11314
  
Location: 154912-155076
  
 NCBI BlastP on this gene

EKV11314

hypothetical protein
  
Accession: EKV11315
  
Location: 155199-155303
  
 NCBI BlastP on this gene

EKV11315

hypothetical protein
  
Accession: EKV11316
  
Location: 156256-156588
  
 NCBI BlastP on this gene

EKV11316

hypothetical protein
  
Accession: EKV11317
  
Location: 157353-157748
  
 NCBI BlastP on this gene

EKV11317

hypothetical protein
  
Accession: EKV11318
  
Location: 158694-158819
  
 NCBI BlastP on this gene

EKV11318

hypothetical protein
  
Accession: EKV11319
  
Location: 158960-159151
  
 NCBI BlastP on this gene

EKV11319

Polyadenylate-binding protein
  
Accession: EKV11320
  
Location: 159910-162232
  
 NCBI BlastP on this gene

EKV11320

hypothetical protein
  
Accession: EKV11321
  
Location: 163074-166880
  
 NCBI BlastP on this gene

EKV11321

19. :  ACJE01000004 Aspergillus niger ATCC 1015     Total score: 2.0     Cumulative Blast bit score: 1258

hypothetical protein
  
Accession: EHA26329
  
Location: 952717-953324
  
 NCBI BlastP on this gene

EHA26329

hypothetical protein
  
Accession: EHA26330
  
Location: 956445-957586
  
 NCBI BlastP on this gene

EHA26330

hypothetical protein
  
Accession: EHA26331
  
Location: 961973-962589
  
 NCBI BlastP on this gene

EHA26331

hypothetical protein
  
Accession: EHA26332
  
Location: 965372-967910
  
 NCBI BlastP on this gene

EHA26332

hypothetical protein
  
Accession: EHA26333
  
Location: 969084-970054
  
  
**BlastP hit with Mycgr3G102281\_Mycgr3**
  
Percentage identity: 89 %
  
BlastP bit score: 381
  
Sequence coverage: 99 %
  
E-value: 4e-132
  
  
 NCBI BlastP on this gene

EHA26333

hypothetical protein
  
Accession: EHA26334
  
Location: 971349-975054
  
  
**BlastP hit with Mycgr3G52682\_Mycgr3T**
  
Percentage identity: 42 %
  
BlastP bit score: 877
  
Sequence coverage: 104 %
  
E-value: 0.0
  
  
 NCBI BlastP on this gene

EHA26334

hypothetical protein
  
Accession: EHA26335
  
Location: 976867-978222
  
 NCBI BlastP on this gene

EHA26335

hypothetical protein
  
Accession: EHA26336
  
Location: 978711-979725
  
 NCBI BlastP on this gene

EHA26336

hypothetical protein
  
Accession: EHA26337
  
Location: 979840-981055
  
 NCBI BlastP on this gene

EHA26337

hypothetical protein
  
Accession: EHA26338
  
Location: 982385-984530
  
 NCBI BlastP on this gene

EHA26338

hypothetical protein
  
Accession: EHA26339
  
Location: 984968-986838
  
 NCBI BlastP on this gene

EHA26339

hypothetical protein
  
Accession: EHA26340
  
Location: 987358-988268
  
 NCBI BlastP on this gene

EHA26340

hypothetical protein
  
Accession: EHA26341
  
Location: 990044-992802
  
 NCBI BlastP on this gene

EHA26341

20. :  AM920431 Penicillium chrysogenum Wisconsin 54-1255 complete genome, contig Pc00c16.     Total score: 2.0     Cumulative Blast bit score: 1255

not annotated
  
Accession: CAP93594
  
Location: 2220411-2221497
  
 NCBI BlastP on this gene

Pc16g09240

not annotated
  
Accession: CAP93593
  
Location: 2216931-2219734
  
 NCBI BlastP on this gene

Pc16g09230

hypothetical protein
  
Accession: CAP93592
  
Location: 2215213-2216685
  
 NCBI BlastP on this gene

Pc16g09220

not annotated
  
Accession: CAP93591
  
Location: 2211811-2214339
  
 NCBI BlastP on this gene

Pc16g09210

not annotated
  
Accession: CAP93590
  
Location: 2210127-2211017
  
 NCBI BlastP on this gene

Pc16g09200

not annotated
  
Accession: CAP93589
  
Location: 2207048-2208889
  
 NCBI BlastP on this gene

Pc16g09190

not annotated
  
Accession: CAP93588
  
Location: 2204414-2206548
  
 NCBI BlastP on this gene

Pc16g09180

not annotated
  
Accession: CAP93587
  
Location: 2201826-2203246
  
 NCBI BlastP on this gene

Pc16g09170

not annotated
  
Accession: CAP93586
  
Location: 2200142-2201117
  
  
**BlastP hit with Mycgr3G102281\_Mycgr3**
  
Percentage identity: 91 %
  
BlastP bit score: 381
  
Sequence coverage: 99 %
  
E-value: 3e-132
  
  
 NCBI BlastP on this gene

Pc16g09160

not annotated
  
Accession: CAP93585
  
Location: 2195577-2199256
  
  
**BlastP hit with Mycgr3G52682\_Mycgr3T**
  
Percentage identity: 42 %
  
BlastP bit score: 874
  
Sequence coverage: 104 %
  
E-value: 0.0
  
  
 NCBI BlastP on this gene

Pc16g09150

not annotated
  
Accession: CAP93584
  
Location: 2193251-2194882
  
 NCBI BlastP on this gene

Pc16g09140

not annotated
  
Accession: CAP93583
  
Location: 2189649-2192510
  
 NCBI BlastP on this gene

Pc16g09130

not annotated
  
Accession: CAP93582
  
Location: 2188182-2189453
  
 NCBI BlastP on this gene

Pc16g09120

not annotated
  
Accession: Pc16g09110
  
Location: 2186985-2187682
  
 NCBI BlastP on this gene

Pc16g09110

not annotated
  
Accession: CAP93580
  
Location: 2183682-2186583
  
 NCBI BlastP on this gene

Pc16g09100

unnamed
  
Accession: CAP93579
  
Location: 2182648-2183128
  
 NCBI BlastP on this gene

Pc16g09090

hypothetical protein
  
Accession: CAP93578
  
Location: 2179106-2181392
  
 NCBI BlastP on this gene

Pc16g09080

not annotated
  
Accession: CAP93577
  
Location: 2177146-2179035
  
 NCBI BlastP on this gene

Pc16g09070

not annotated
  
Accession: CAP93576
  
Location: 2175745-2176889
  
 NCBI BlastP on this gene

Pc16g09060

21. :  DS572753 Paracoccidioides brasiliensis Pb18 supercont1.4 genomic scaffold     Total score: 2.0     Cumulative Blast bit score: 1254

HIRA interacting protein
  
Accession: EEH47768
  
Location: 1072496-1073785
  
 NCBI BlastP on this gene

EEH47768

predicted protein
  
Accession: EEH47769
  
Location: 1078975-1079689
  
 NCBI BlastP on this gene

EEH47769

predicted protein
  
Accession: EEH47770
  
Location: 1079861-1081532
  
 NCBI BlastP on this gene

EEH47770

PSP1 domain-containing protein
  
Accession: EEH47771
  
Location: 1082443-1085228
  
 NCBI BlastP on this gene

EEH47771

60S ribosomal protein L15
  
Accession: EEH47772
  
Location: 1089359-1090343
  
  
**BlastP hit with Mycgr3G102281\_Mycgr3**
  
Percentage identity: 89 %
  
BlastP bit score: 375
  
Sequence coverage: 99 %
  
E-value: 1e-129
  
  
 NCBI BlastP on this gene

EEH47772

E3 ubiquitin-protein ligase HUWE1
  
Accession: EEH47773
  
Location: 1094074-1098658
  
  
**BlastP hit with Mycgr3G52682\_Mycgr3T**
  
Percentage identity: 42 %
  
BlastP bit score: 879
  
Sequence coverage: 103 %
  
E-value: 0.0
  
  
 NCBI BlastP on this gene

EEH47773

predicted protein
  
Accession: EEH47774
  
Location: 1098795-1100592
  
 NCBI BlastP on this gene

EEH47774

NADP-dependent mannitol dehydrogenase
  
Accession: EEH47775
  
Location: 1101321-1103167
  
 NCBI BlastP on this gene

EEH47775

conserved hypothetical protein
  
Accession: EEH47776
  
Location: 1105206-1106179
  
 NCBI BlastP on this gene

EEH47776

predicted protein
  
Accession: EEH47777
  
Location: 1106281-1106746
  
 NCBI BlastP on this gene

EEH47777

conserved hypothetical protein
  
Accession: EEH47778
  
Location: 1107519-1108965
  
 NCBI BlastP on this gene

EEH47778

conserved hypothetical protein
  
Accession: EEH47779
  
Location: 1111981-1114262
  
 NCBI BlastP on this gene

EEH47779

conserved hypothetical protein
  
Accession: EEH47780
  
Location: 1114891-1116794
  
 NCBI BlastP on this gene

EEH47780

conserved hypothetical protein
  
Accession: EEH47781
  
Location: 1117824-1118765
  
 NCBI BlastP on this gene

EEH47781

22. :  GL573405 Geomyces destructans 20631-21 unplaced genomic scaffold supercont1.237     Total score: 2.0     Cumulative Blast bit score: 1247

hypothetical protein
  
Accession: ELR05718
  
Location: 756-2653
  
 NCBI BlastP on this gene

ELR05718

hypothetical protein
  
Accession: ELR05719
  
Location: 3219-4107
  
 NCBI BlastP on this gene

ELR05719

hypothetical protein
  
Accession: ELR05720
  
Location: 4335-5348
  
 NCBI BlastP on this gene

ELR05720

hypothetical protein
  
Accession: ELR05721
  
Location: 7605-10437
  
 NCBI BlastP on this gene

ELR05721

60S ribosomal protein L15
  
Accession: ELR05722
  
Location: 11233-12050
  
  
**BlastP hit with Mycgr3G102281\_Mycgr3**
  
Percentage identity: 89 %
  
BlastP bit score: 350
  
Sequence coverage: 99 %
  
E-value: 5e-120
  
  
 NCBI BlastP on this gene

ELR05722

hypothetical protein
  
Accession: ELR05723
  
Location: 13646-14860
  
 NCBI BlastP on this gene

ELR05723

hypothetical protein
  
Accession: ELR05724
  
Location: 15501-16432
  
 NCBI BlastP on this gene

ELR05724

hypothetical protein
  
Accession: ELR05725
  
Location: 16491-17537
  
 NCBI BlastP on this gene

ELR05725

cytochrome c oxidase subunit VIb
  
Accession: ELR05726
  
Location: 18149-18668
  
 NCBI BlastP on this gene

ELR05726

hypothetical protein
  
Accession: ELR05727
  
Location: 19757-20286
  
 NCBI BlastP on this gene

ELR05727

hypothetical protein
  
Accession: ELR05728
  
Location: 20657-24512
  
 NCBI BlastP on this gene

ELR05728

hypothetical protein
  
Accession: ELR05729
  
Location: 25301-28987
  
  
**BlastP hit with Mycgr3G52682\_Mycgr3T**
  
Percentage identity: 45 %
  
BlastP bit score: 897
  
Sequence coverage: 94 %
  
E-value: 0.0
  
  
 NCBI BlastP on this gene

ELR05729

hypothetical protein
  
Accession: ELR05730
  
Location: 29219-29958
  
 NCBI BlastP on this gene

ELR05730

hypothetical protein
  
Accession: ELR05731
  
Location: 30219-32216
  
 NCBI BlastP on this gene

ELR05731

hypothetical protein
  
Accession: ELR05732
  
Location: 32336-33228
  
 NCBI BlastP on this gene

ELR05732

hypothetical protein
  
Accession: ELR05733
  
Location: 33596-36946
  
 NCBI BlastP on this gene

ELR05733

hypothetical protein
  
Accession: ELR05734
  
Location: 37507-39402
  
 NCBI BlastP on this gene

ELR05734

23. :  DS572812 Paracoccidioides brasiliensis Pb01 supercont1.2 genomic scaffold     Total score: 2.0     Cumulative Blast bit score: 1242

conserved hypothetical protein
  
Accession: EEH38056
  
Location: 1047322-1051093
  
 NCBI BlastP on this gene

EEH38056

LiPid Depleted family member
  
Accession: EEH38055
  
Location: 1044687-1045990
  
 NCBI BlastP on this gene

EEH38055

predicted protein
  
Accession: EEH38054
  
Location: 1043805-1044258
  
 NCBI BlastP on this gene

EEH38054

predicted protein
  
Accession: EEH38053
  
Location: 1041034-1042117
  
 NCBI BlastP on this gene

EEH38053

predicted protein
  
Accession: EEH38052
  
Location: 1040637-1040999
  
 NCBI BlastP on this gene

EEH38052

predicted protein
  
Accession: EEH38051
  
Location: 1036731-1039569
  
 NCBI BlastP on this gene

EEH38051

PSP1 domain-containing protein
  
Accession: EEH38050
  
Location: 1033082-1035864
  
 NCBI BlastP on this gene

EEH38050

predicted protein
  
Accession: EEH38049
  
Location: 1029468-1031278
  
 NCBI BlastP on this gene

EEH38049

60S ribosomal protein L15
  
Accession: EEH38048
  
Location: 1027955-1028936
  
  
**BlastP hit with Mycgr3G102281\_Mycgr3**
  
Percentage identity: 89 %
  
BlastP bit score: 375
  
Sequence coverage: 99 %
  
E-value: 1e-129
  
  
 NCBI BlastP on this gene

EEH38048

E3 ubiquitin-protein ligase HUWE1
  
Accession: EEH38047
  
Location: 1019175-1023835
  
  
**BlastP hit with Mycgr3G52682\_Mycgr3T**
  
Percentage identity: 43 %
  
BlastP bit score: 867
  
Sequence coverage: 100 %
  
E-value: 0.0
  
  
 NCBI BlastP on this gene

EEH38047

predicted protein
  
Accession: EEH38046
  
Location: 1016713-1018802
  
 NCBI BlastP on this gene

EEH38046

L-threonine 3-dehydrogenase
  
Accession: EEH38045
  
Location: 1014399-1016140
  
 NCBI BlastP on this gene

EEH38045

predicted protein
  
Accession: EEH38044
  
Location: 1012325-1013503
  
 NCBI BlastP on this gene

EEH38044

conserved hypothetical protein
  
Accession: EEH38043
  
Location: 1011297-1012272
  
 NCBI BlastP on this gene

EEH38043

conserved hypothetical protein
  
Accession: EEH38042
  
Location: 1007667-1009641
  
 NCBI BlastP on this gene

EEH38042

conserved hypothetical protein
  
Accession: EEH38041
  
Location: 1003078-1005358
  
 NCBI BlastP on this gene

EEH38041

conserved hypothetical protein
  
Accession: EEH38040
  
Location: 1000584-1002486
  
 NCBI BlastP on this gene

EEH38040

24. :  AACD01000007 Aspergillus nidulans FGSC A4     Total score: 2.0     Cumulative Blast bit score: 1238

hypothetical protein
  
Accession: EAA66550
  
Location: 222912-223819
  
 NCBI BlastP on this gene

EAA66550

hypothetical protein
  
Accession: EAA66549
  
Location: 221502-222770
  
 NCBI BlastP on this gene

EAA66549

hypothetical protein
  
Accession: EAA66548
  
Location: 216852-221132
  
 NCBI BlastP on this gene

EAA66548

hypothetical protein
  
Accession: EAA66547
  
Location: 214579-215187
  
 NCBI BlastP on this gene

EAA66547

hypothetical protein
  
Accession: EAA66546
  
Location: 211697-212810
  
 NCBI BlastP on this gene

EAA66546

hypothetical protein
  
Accession: EAA66545
  
Location: 204111-206763
  
 NCBI BlastP on this gene

EAA66545

RL15 ASPNG 60S RIBOSOMAL PROTEIN L15
  
Accession: EAA66544
  
Location: 202260-203211
  
  
**BlastP hit with Mycgr3G102281\_Mycgr3**
  
Percentage identity: 90 %
  
BlastP bit score: 356
  
Sequence coverage: 99 %
  
E-value: 3e-122
  
  
 NCBI BlastP on this gene

EAA66544

hypothetical protein
  
Accession: EAA66543
  
Location: 197446-201058
  
  
**BlastP hit with Mycgr3G52682\_Mycgr3T**
  
Percentage identity: 44 %
  
BlastP bit score: 882
  
Sequence coverage: 91 %
  
E-value: 0.0
  
  
 NCBI BlastP on this gene

EAA66543

hypothetical protein
  
Accession: EAA66542
  
Location: 193897-195332
  
 NCBI BlastP on this gene

EAA66542

hypothetical protein
  
Accession: EAA66541
  
Location: 192532-193540
  
 NCBI BlastP on this gene

EAA66541

hypothetical protein
  
Accession: EAA66540
  
Location: 190754-192487
  
 NCBI BlastP on this gene

EAA66540

hypothetical protein
  
Accession: EAA66539
  
Location: 187586-189673
  
 NCBI BlastP on this gene

EAA66539

hypothetical protein
  
Accession: EAA66538
  
Location: 185431-187294
  
 NCBI BlastP on this gene

EAA66538

hypothetical protein
  
Accession: EAA66537
  
Location: 183861-184763
  
 NCBI BlastP on this gene

EAA66537

hypothetical protein
  
Accession: EAA66536
  
Location: 179260-182788
  
 NCBI BlastP on this gene

EAA66536

25. :  DF126464 Aspergillus kawachii IFO 4308 DNA, contig: scaffold00018     Total score: 2.0     Cumulative Blast bit score: 1236

Ras guanyl-nucleotide exchange factor RasGEF
  
Accession: GAA88712
  
Location: 321473-324878
  
 NCBI BlastP on this gene

GAA88712

HIT finger domain protein
  
Accession: GAA88711
  
Location: 318044-318686
  
 NCBI BlastP on this gene

GAA88711

hypothetical protein
  
Accession: GAA88710
  
Location: 315696-317509
  
 NCBI BlastP on this gene

GAA88710

NifU-related protein
  
Accession: GAA88709
  
Location: 313912-315058
  
 NCBI BlastP on this gene

GAA88709

PSP1 domain protein
  
Accession: GAA88708
  
Location: 303428-305980
  
 NCBI BlastP on this gene

GAA88708

cytoplasmic ribosomal protein of the large subunit L15 (Rpl15)
  
Accession: GAA88707
  
Location: 301143-302114
  
  
**BlastP hit with Mycgr3G102281\_Mycgr3**
  
Percentage identity: 89 %
  
BlastP bit score: 381
  
Sequence coverage: 99 %
  
E-value: 4e-132
  
  
 NCBI BlastP on this gene

GAA88707

ubiquitin-protein ligase
  
Accession: GAA88706
  
Location: 296119-299594
  
  
**BlastP hit with Mycgr3G52682\_Mycgr3T**
  
Percentage identity: 50 %
  
BlastP bit score: 855
  
Sequence coverage: 75 %
  
E-value: 0.0
  
  
 NCBI BlastP on this gene

GAA88706

alcohol dehydrogenase, zinc-containing
  
Accession: GAA88705
  
Location: 292488-294170
  
 NCBI BlastP on this gene

GAA88705

FRG1-like family protein
  
Accession: GAA88704
  
Location: 290936-291991
  
 NCBI BlastP on this gene

GAA88704

RING finger domain protein
  
Accession: GAA88703
  
Location: 289330-290830
  
 NCBI BlastP on this gene

GAA88703

transcriptional regulator Ngg1
  
Accession: GAA88702
  
Location: 286129-288273
  
 NCBI BlastP on this gene

GAA88702

nitrogen permease regulator Npr2
  
Accession: GAA88701
  
Location: 283798-285667
  
 NCBI BlastP on this gene

GAA88701

similar to An01g03530
  
Accession: GAA88700
  
Location: 282351-283262
  
 NCBI BlastP on this gene

GAA88700

DNA repair protein
  
Accession: GAA88699
  
Location: 277771-280187
  
 NCBI BlastP on this gene

GAA88699

26. :  GG700653 Trichophyton rubrum CBS 118892 genomic scaffold supercont2.6     Total score: 2.0     Cumulative Blast bit score: 1222

hypothetical protein
  
Accession: EGD89571
  
Location: 1063163-1063781
  
 NCBI BlastP on this gene

EGD89571

hypothetical protein
  
Accession: EGD89570
  
Location: 1060369-1061406
  
 NCBI BlastP on this gene

EGD89570

kynurenine 3-monooxygenase
  
Accession: EGD89569
  
Location: 1057653-1059467
  
 NCBI BlastP on this gene

EGD89569

methionine aminopeptidase
  
Accession: EGD89568
  
Location: 1055225-1056555
  
 NCBI BlastP on this gene

EGD89568

MFS multidrug transporter
  
Accession: EGD89567
  
Location: 1052542-1054563
  
 NCBI BlastP on this gene

EGD89567

EF hand domain-containing protein
  
Accession: EGD89566
  
Location: 1048002-1051373
  
 NCBI BlastP on this gene

EGD89566

mRNA splicing factor
  
Accession: EGD89565
  
Location: 1045869-1047665
  
 NCBI BlastP on this gene

EGD89565

hypothetical protein
  
Accession: EGD89564
  
Location: 1044292-1045359
  
 NCBI BlastP on this gene

EGD89564

60S ribosomal protein L15
  
Accession: EGD89563
  
Location: 1042901-1043866
  
  
**BlastP hit with Mycgr3G102281\_Mycgr3**
  
Percentage identity: 89 %
  
BlastP bit score: 372
  
Sequence coverage: 99 %
  
E-value: 2e-128
  
  
 NCBI BlastP on this gene

EGD89563

hypothetical protein
  
Accession: EGD89562
  
Location: 1038248-1041910
  
  
**BlastP hit with Mycgr3G52682\_Mycgr3T**
  
Percentage identity: 41 %
  
BlastP bit score: 850
  
Sequence coverage: 103 %
  
E-value: 0.0
  
  
 NCBI BlastP on this gene

EGD89562

alcohol dehydrogenase
  
Accession: EGD89561
  
Location: 1034385-1035935
  
 NCBI BlastP on this gene

EGD89561

hypothetical protein
  
Accession: EGD89560
  
Location: 1032823-1033726
  
 NCBI BlastP on this gene

EGD89560

hypothetical protein
  
Accession: EGD89559
  
Location: 1030480-1032252
  
 NCBI BlastP on this gene

EGD89559

transcriptional regulator Ngg1
  
Accession: EGD89558
  
Location: 1026530-1028706
  
 NCBI BlastP on this gene

EGD89558

nitrogen permease regulator Npr2
  
Accession: EGD89557
  
Location: 1024375-1026298
  
 NCBI BlastP on this gene

EGD89557

hypothetical protein
  
Accession: EGD89556
  
Location: 1021772-1023242
  
 NCBI BlastP on this gene

EGD89556

hypothetical protein
  
Accession: EGD89555
  
Location: 1019343-1020374
  
 NCBI BlastP on this gene

EGD89555

27. :  GG749408 Ajellomyces dermatitidis ATCC 18188 genomic scaffold supercont1.2     Total score: 2.0     Cumulative Blast bit score: 1206

rap guanine nucleotide exchange factor 5
  
Accession: EGE77657
  
Location: 134626-138654
  
 NCBI BlastP on this gene

EGE77657

HIRA-interacting protein 5
  
Accession: EGE77656
  
Location: 131640-132894
  
 NCBI BlastP on this gene

EGE77656

hypothetical protein
  
Accession: EGE77655
  
Location: 127816-128726
  
 NCBI BlastP on this gene

EGE77655

hypothetical protein
  
Accession: EGE77654
  
Location: 124779-126632
  
 NCBI BlastP on this gene

EGE77654

PSP1 domain-containing protein
  
Accession: EGE77653
  
Location: 120451-123282
  
 NCBI BlastP on this gene

EGE77653

hypothetical protein
  
Accession: EGE77652
  
Location: 115656-117062
  
 NCBI BlastP on this gene

EGE77652

60S ribosomal protein L15
  
Accession: EGE77651
  
Location: 114156-115153
  
  
**BlastP hit with Mycgr3G102281\_Mycgr3**
  
Percentage identity: 87 %
  
BlastP bit score: 339
  
Sequence coverage: 99 %
  
E-value: 1e-115
  
  
 NCBI BlastP on this gene

EGE77651

secreted protein
  
Accession: EGE77650
  
Location: 112406-113160
  
 NCBI BlastP on this gene

EGE77650

hypothetical protein
  
Accession: EGE77649
  
Location: 106904-108989
  
 NCBI BlastP on this gene

EGE77649

ubiquitin-protein ligase
  
Accession: EGE77648
  
Location: 101411-105180
  
  
**BlastP hit with Mycgr3G52682\_Mycgr3T**
  
Percentage identity: 41 %
  
BlastP bit score: 867
  
Sequence coverage: 105 %
  
E-value: 0.0
  
  
 NCBI BlastP on this gene

EGE77648

NADP-dependent mannitol dehydrogenase
  
Accession: EGE77647
  
Location: 97218-98885
  
 NCBI BlastP on this gene

EGE77647

hypothetical protein
  
Accession: EGE77646
  
Location: 94955-95892
  
 NCBI BlastP on this gene

EGE77646

hypothetical protein
  
Accession: EGE77645
  
Location: 91523-93818
  
 NCBI BlastP on this gene

EGE77645

transcriptional regulator Ngg1
  
Accession: EGE77644
  
Location: 86224-88495
  
 NCBI BlastP on this gene

EGE77644

nitrogen permease regulator Npr2
  
Accession: EGE77643
  
Location: 83709-85638
  
 NCBI BlastP on this gene

EGE77643

hypothetical protein
  
Accession: EGE77642
  
Location: 81731-82621
  
 NCBI BlastP on this gene

EGE77642

28. :  GG657458 Ajellomyces dermatitidis SLH14081 genomic scaffold supercont1.11     Total score: 2.0     Cumulative Blast bit score: 1206

conserved hypothetical protein
  
Accession: EEQ70391
  
Location: 553451-557473
  
 NCBI BlastP on this gene

EEQ70391

HIRA-interacting protein 5
  
Accession: EEQ70390
  
Location: 550479-551733
  
 NCBI BlastP on this gene

EEQ70390

predicted protein
  
Accession: EEQ70389
  
Location: 546044-547356
  
 NCBI BlastP on this gene

EEQ70389

predicted protein
  
Accession: EEQ70388
  
Location: 543621-545471
  
 NCBI BlastP on this gene

EEQ70388

PSP1 domain-containing protein
  
Accession: EEQ70387
  
Location: 539294-542125
  
 NCBI BlastP on this gene

EEQ70387

conserved hypothetical protein
  
Accession: EEQ70386
  
Location: 535305-536691
  
 NCBI BlastP on this gene

EEQ70386

60S ribosomal protein L15
  
Accession: EEQ70385
  
Location: 533818-534815
  
  
**BlastP hit with Mycgr3G102281\_Mycgr3**
  
Percentage identity: 87 %
  
BlastP bit score: 339
  
Sequence coverage: 99 %
  
E-value: 1e-115
  
  
 NCBI BlastP on this gene

EEQ70385

secreted protein
  
Accession: EEQ70384
  
Location: 532068-532822
  
 NCBI BlastP on this gene

EEQ70384

ubiquitin-protein ligase
  
Accession: EEQ70383
  
Location: 521089-524858
  
  
**BlastP hit with Mycgr3G52682\_Mycgr3T**
  
Percentage identity: 41 %
  
BlastP bit score: 867
  
Sequence coverage: 105 %
  
E-value: 0.0
  
  
 NCBI BlastP on this gene

EEQ70383

NADP-dependent mannitol dehydrogenase
  
Accession: EEQ70382
  
Location: 516896-518563
  
 NCBI BlastP on this gene

EEQ70382

conserved hypothetical protein
  
Accession: EEQ70381
  
Location: 514634-515571
  
 NCBI BlastP on this gene

EEQ70381

RING finger domain-containing protein
  
Accession: EEQ70380
  
Location: 511201-513497
  
 NCBI BlastP on this gene

EEQ70380

transcriptional regulator Ngg1
  
Accession: EEQ70379
  
Location: 504379-506650
  
 NCBI BlastP on this gene

EEQ70379

nitrogen permease regulator Npr2
  
Accession: EEQ70378
  
Location: 501864-503793
  
 NCBI BlastP on this gene

EEQ70378

29. :  GG698513 Trichophyton tonsurans CBS 112818 genomic scaffold supercont1.37     Total score: 2.0     Cumulative Blast bit score: 1204

hypothetical protein
  
Accession: EGD98641
  
Location: 99981-101018
  
 NCBI BlastP on this gene

EGD98641

kynurenine 3-monooxygenase
  
Accession: EGD98640
  
Location: 97281-99103
  
 NCBI BlastP on this gene

EGD98640

methionine aminopeptidase
  
Accession: EGD98639
  
Location: 94855-96188
  
 NCBI BlastP on this gene

EGD98639

MFS multidrug transporter
  
Accession: EGD98638
  
Location: 92142-94166
  
 NCBI BlastP on this gene

EGD98638

EF hand domain-containing protein
  
Accession: EGD98637
  
Location: 87605-90973
  
 NCBI BlastP on this gene

EGD98637

mRNA splicing factor
  
Accession: EGD98636
  
Location: 85461-87268
  
 NCBI BlastP on this gene

EGD98636

hypothetical protein
  
Accession: EGD98635
  
Location: 83859-84929
  
 NCBI BlastP on this gene

EGD98635

60S ribosomal protein L15
  
Accession: EGD98634
  
Location: 82475-83438
  
  
**BlastP hit with Mycgr3G102281\_Mycgr3**
  
Percentage identity: 89 %
  
BlastP bit score: 372
  
Sequence coverage: 99 %
  
E-value: 2e-128
  
  
 NCBI BlastP on this gene

EGD98634

hypothetical protein
  
Accession: EGD98633
  
Location: 77800-81463
  
  
**BlastP hit with Mycgr3G52682\_Mycgr3T**
  
Percentage identity: 40 %
  
BlastP bit score: 832
  
Sequence coverage: 103 %
  
E-value: 0.0
  
  
 NCBI BlastP on this gene

EGD98633

alcohol dehydrogenase
  
Accession: EGD98632
  
Location: 74018-75451
  
 NCBI BlastP on this gene

EGD98632

hypothetical protein
  
Accession: EGD98631
  
Location: 72447-73349
  
 NCBI BlastP on this gene

EGD98631

hypothetical protein
  
Accession: EGD98630
  
Location: 70113-71927
  
 NCBI BlastP on this gene

EGD98630

transcriptional regulator Ngg1
  
Accession: EGD98629
  
Location: 66174-68347
  
 NCBI BlastP on this gene

EGD98629

nitrogen permease regulator Npr2
  
Accession: EGD98628
  
Location: 64014-65960
  
 NCBI BlastP on this gene

EGD98628

hypothetical protein
  
Accession: EGD98627
  
Location: 61985-63043
  
 NCBI BlastP on this gene

EGD98627

30. :  EQ999975 Ajellomyces dermatitidis ER-3 genomic scaffold supercont1.3     Total score: 2.0     Cumulative Blast bit score: 1204

HIRA-interacting protein 5
  
Accession: EEQ87746
  
Location: 1825982-1827236
  
 NCBI BlastP on this gene

EEQ87746

predicted protein
  
Accession: EEQ87747
  
Location: 1832261-1834115
  
 NCBI BlastP on this gene

EEQ87747

PSP1 domain-containing protein
  
Accession: EEQ87748
  
Location: 1835613-1838444
  
 NCBI BlastP on this gene

EEQ87748

conserved hypothetical protein
  
Accession: EEQ87749
  
Location: 1841046-1842452
  
 NCBI BlastP on this gene

EEQ87749

60S ribosomal protein L15
  
Accession: EEQ87750
  
Location: 1842952-1843949
  
  
**BlastP hit with Mycgr3G102281\_Mycgr3**
  
Percentage identity: 87 %
  
BlastP bit score: 337
  
Sequence coverage: 99 %
  
E-value: 5e-115
  
  
 NCBI BlastP on this gene

EEQ87750

secreted protein
  
Accession: EEQ87751
  
Location: 1844945-1845699
  
 NCBI BlastP on this gene

EEQ87751

ubiquitin-protein ligase
  
Accession: EEQ87752
  
Location: 1852926-1856695
  
  
**BlastP hit with Mycgr3G52682\_Mycgr3T**
  
Percentage identity: 41 %
  
BlastP bit score: 867
  
Sequence coverage: 105 %
  
E-value: 0.0
  
  
 NCBI BlastP on this gene

EEQ87752

alcohol dehydrogenase
  
Accession: EEQ87753
  
Location: 1859221-1860888
  
 NCBI BlastP on this gene

EEQ87753

conserved hypothetical protein
  
Accession: EEQ87754
  
Location: 1862217-1863154
  
 NCBI BlastP on this gene

EEQ87754

RING finger domain-containing protein
  
Accession: EEQ87755
  
Location: 1864291-1866587
  
 NCBI BlastP on this gene

EEQ87755

transcriptional regulator Ngg1
  
Accession: EEQ87756
  
Location: 1868841-1871108
  
 NCBI BlastP on this gene

EEQ87756

nitrogen permease regulator Npr2
  
Accession: EEQ87757
  
Location: 1871695-1873624
  
 NCBI BlastP on this gene

EEQ87757

conserved hypothetical protein
  
Accession: EEQ87758
  
Location: 1874712-1875659
  
 NCBI BlastP on this gene

EEQ87758

31. :  DS995752 Trichophyton equinum CBS 127.97 supercont1.35 genomic scaffold     Total score: 2.0     Cumulative Blast bit score: 1204

hypothetical protein
  
Accession: EGE06838
  
Location: 63379-64416
  
 NCBI BlastP on this gene

EGE06838

kynurenine 3-monooxygenase
  
Accession: EGE06837
  
Location: 60678-62501
  
 NCBI BlastP on this gene

EGE06837

methionine aminopeptidase
  
Accession: EGE06836
  
Location: 58255-59588
  
 NCBI BlastP on this gene

EGE06836

multidrug resistance protein fnx1
  
Accession: EGE06835
  
Location: 55556-57572
  
 NCBI BlastP on this gene

EGE06835

EF hand domain-containing protein
  
Accession: EGE06834
  
Location: 51019-54387
  
 NCBI BlastP on this gene

EGE06834

pre-mRNA-processing factor 17
  
Accession: EGE06833
  
Location: 48875-50682
  
 NCBI BlastP on this gene

EGE06833

hypothetical protein
  
Accession: EGE06832
  
Location: 47187-48343
  
 NCBI BlastP on this gene

EGE06832

ribosomal protein L15
  
Accession: EGE06831
  
Location: 45889-46852
  
  
**BlastP hit with Mycgr3G102281\_Mycgr3**
  
Percentage identity: 89 %
  
BlastP bit score: 372
  
Sequence coverage: 99 %
  
E-value: 2e-128
  
  
 NCBI BlastP on this gene

EGE06831

ubiquitin-protein ligase E3A
  
Accession: EGE06830
  
Location: 41214-44877
  
  
**BlastP hit with Mycgr3G52682\_Mycgr3T**
  
Percentage identity: 40 %
  
BlastP bit score: 832
  
Sequence coverage: 103 %
  
E-value: 0.0
  
  
 NCBI BlastP on this gene

EGE06830

alcohol dehydrogenase
  
Accession: EGE06829
  
Location: 37395-38887
  
 NCBI BlastP on this gene

EGE06829

hypothetical protein
  
Accession: EGE06828
  
Location: 35824-36726
  
 NCBI BlastP on this gene

EGE06828

RING finger domain-containing protein
  
Accession: EGE06827
  
Location: 33490-35304
  
 NCBI BlastP on this gene

EGE06827

transcriptional regulator Ngg1
  
Accession: EGE06826
  
Location: 29547-31720
  
 NCBI BlastP on this gene

EGE06826

nitrogen permease regulator Npr2
  
Accession: EGE06825
  
Location: 27387-29333
  
 NCBI BlastP on this gene

EGE06825

hypothetical protein
  
Accession: EGE06824
  
Location: 25407-26471
  
 NCBI BlastP on this gene

EGE06824

32. :  DS995903 Penicillium marneffei ATCC 18224 scf\_1105668340984 genomic scaffold     Total score: 2.0     Cumulative Blast bit score: 1200

C-8 sterol isomerase (Erg-1), putative
  
Accession: EEA21649
  
Location: 1185210-1186007
  
 NCBI BlastP on this gene

EEA21649

PP-loop ATPase superfamily protein, putative
  
Accession: EEA21648
  
Location: 1182405-1183664
  
 NCBI BlastP on this gene

EEA21648

conserved hypothetical protein
  
Accession: EEA21647
  
Location: 1181581-1182122
  
 NCBI BlastP on this gene

EEA21647

Ras guanyl-nucleotide exchange factor RasGEF, putative
  
Accession: EEA21646
  
Location: 1179199-1180969
  
 NCBI BlastP on this gene

EEA21646

HIT finger domain protein, putative
  
Accession: EEA21645
  
Location: 1177172-1177765
  
 NCBI BlastP on this gene

EEA21645

NifU-related protein
  
Accession: EEA21644
  
Location: 1175763-1176699
  
 NCBI BlastP on this gene

EEA21644

PSP1 domain protein
  
Accession: EEA21642
  
Location: 1169159-1171839
  
 NCBI BlastP on this gene

EEA21642

60S ribosomal protein L15
  
Accession: EEA21640
  
Location: 1166975-1168031
  
  
**BlastP hit with Mycgr3G102281\_Mycgr3**
  
Percentage identity: 88 %
  
BlastP bit score: 344
  
Sequence coverage: 99 %
  
E-value: 2e-117
  
  
 NCBI BlastP on this gene

EEA21640

ubiquitin-protein ligase (Hul4), putative
  
Accession: EEA21639
  
Location: 1162059-1165905
  
  
**BlastP hit with Mycgr3G52682\_Mycgr3T**
  
Percentage identity: 43 %
  
BlastP bit score: 856
  
Sequence coverage: 101 %
  
E-value: 0.0
  
  
 NCBI BlastP on this gene

EEA21639

conserved hypothetical protein
  
Accession: EEA21638
  
Location: 1160385-1161717
  
 NCBI BlastP on this gene

EEA21638

alcohol dehydrogenase, zinc-containing, putative
  
Accession: EEA21637
  
Location: 1156422-1157869
  
 NCBI BlastP on this gene

EEA21637

conserved hypothetical protein
  
Accession: EEA21636
  
Location: 1155003-1155997
  
 NCBI BlastP on this gene

EEA21636

DNA repair protein, putative
  
Accession: EEA21635
  
Location: 1152512-1154899
  
 NCBI BlastP on this gene

EEA21635

conserved hypothetical protein
  
Accession: EEA21634
  
Location: 1148908-1149789
  
 NCBI BlastP on this gene

EEA21634

nitrogen permease regulator Npr2, putative
  
Accession: EEA21632
  
Location: 1146184-1148263
  
 NCBI BlastP on this gene

EEA21632

transcriptional regulator Ngg1, putative
  
Accession: EEA21631
  
Location: 1143884-1145980
  
 NCBI BlastP on this gene

EEA21631

33. :  GG663363 Ajellomyces capsulatus G186AR genomic scaffold supercont2.1     Total score: 2.0     Cumulative Blast bit score: 1199

HIRA-interacting protein
  
Accession: EEH11358
  
Location: 2531592-2532832
  
 NCBI BlastP on this gene

EEH11358

hypothetical protein
  
Accession: EEH11357
  
Location: 2530565-2530684
  
 NCBI BlastP on this gene

EEH11357

predicted protein
  
Accession: EEH11356
  
Location: 2526155-2526845
  
 NCBI BlastP on this gene

EEH11356

predicted protein
  
Accession: EEH11355
  
Location: 2524933-2525700
  
 NCBI BlastP on this gene

EEH11355

PSP1 domain-containing protein
  
Accession: EEH11354
  
Location: 2520531-2523368
  
 NCBI BlastP on this gene

EEH11354

conserved hypothetical protein
  
Accession: EEH11353
  
Location: 2516533-2517920
  
 NCBI BlastP on this gene

EEH11353

60S ribosomal protein L15
  
Accession: EEH11352
  
Location: 2515063-2516066
  
  
**BlastP hit with Mycgr3G102281\_Mycgr3**
  
Percentage identity: 88 %
  
BlastP bit score: 342
  
Sequence coverage: 99 %
  
E-value: 7e-117
  
  
 NCBI BlastP on this gene

EEH11352

secreted protein
  
Accession: EEH11351
  
Location: 2513250-2514005
  
 NCBI BlastP on this gene

EEH11351

conserved hypothetical protein
  
Accession: EEH11350
  
Location: 2504655-2507102
  
 NCBI BlastP on this gene

EEH11350

WD domain-containing protein
  
Accession: EEH11349
  
Location: 2501212-2501940
  
 NCBI BlastP on this gene

EEH11349

ubiquitin-protein ligase E3
  
Accession: EEH11348
  
Location: 2494617-2498367
  
  
**BlastP hit with Mycgr3G52682\_Mycgr3T**
  
Percentage identity: 42 %
  
BlastP bit score: 857
  
Sequence coverage: 102 %
  
E-value: 0.0
  
  
 NCBI BlastP on this gene

EEH11348

NADP-dependent mannitol dehydrogenase
  
Accession: EEH11347
  
Location: 2490145-2491842
  
 NCBI BlastP on this gene

EEH11347

conserved hypothetical protein
  
Accession: EEH11346
  
Location: 2487619-2488583
  
 NCBI BlastP on this gene

EEH11346

conserved hypothetical protein
  
Accession: EEH11345
  
Location: 2484412-2486364
  
 NCBI BlastP on this gene

EEH11345

ada histone acetyltransferase complex component
  
Accession: EEH11344
  
Location: 2479779-2482049
  
 NCBI BlastP on this gene

EEH11344

nitrogen permease regulator family
  
Accession: EEH11343
  
Location: 2477343-2479176
  
 NCBI BlastP on this gene

EEH11343

34. :  GG692421 Ajellomyces capsulatus H143 genomic scaffold supercont2.3     Total score: 2.0     Cumulative Blast bit score: 1194

rap guanine nucleotide exchange factor 5
  
Accession: EER43408
  
Location: 3152701-3154353
  
 NCBI BlastP on this gene

EER43408

predicted protein
  
Accession: EER43407
  
Location: 3148564-3149395
  
 NCBI BlastP on this gene

EER43407

predicted protein
  
Accession: EER43406
  
Location: 3147522-3148041
  
 NCBI BlastP on this gene

EER43406

predicted protein
  
Accession: EER43405
  
Location: 3146794-3147257
  
 NCBI BlastP on this gene

EER43405

PSP1 domain-containing protein
  
Accession: EER43404
  
Location: 3142047-3144885
  
 NCBI BlastP on this gene

EER43404

conserved hypothetical protein
  
Accession: EER43403
  
Location: 3138034-3139402
  
 NCBI BlastP on this gene

EER43403

60S ribosomal protein L15
  
Accession: EER43402
  
Location: 3136567-3137571
  
  
**BlastP hit with Mycgr3G102281\_Mycgr3**
  
Percentage identity: 88 %
  
BlastP bit score: 342
  
Sequence coverage: 99 %
  
E-value: 7e-117
  
  
 NCBI BlastP on this gene

EER43402

secreted protein
  
Accession: EER43401
  
Location: 3134737-3135492
  
 NCBI BlastP on this gene

EER43401

predicted protein
  
Accession: EER43400
  
Location: 3133059-3133794
  
 NCBI BlastP on this gene

EER43400

NACHT and WD40 domain-containing protein
  
Accession: EER43399
  
Location: 3128277-3129223
  
 NCBI BlastP on this gene

EER43399

predicted protein
  
Accession: EER43398
  
Location: 3127603-3127982
  
 NCBI BlastP on this gene

EER43398

ubiquitin-protein ligase E3
  
Accession: EER43397
  
Location: 3112354-3116104
  
  
**BlastP hit with Mycgr3G52682\_Mycgr3T**
  
Percentage identity: 43 %
  
BlastP bit score: 852
  
Sequence coverage: 100 %
  
E-value: 0.0
  
  
 NCBI BlastP on this gene

EER43397

hypothetical protein
  
Accession: EER43396
  
Location: 3110263-3110895
  
 NCBI BlastP on this gene

EER43396

NADP-dependent mannitol dehydrogenase
  
Accession: EER43395
  
Location: 3108012-3109712
  
 NCBI BlastP on this gene

EER43395

conserved hypothetical protein
  
Accession: EER43394
  
Location: 3105556-3106520
  
 NCBI BlastP on this gene

EER43394

RING finger domain-containing protein
  
Accession: EER43393
  
Location: 3102312-3104385
  
 NCBI BlastP on this gene

EER43393

predicted protein
  
Accession: EER43392
  
Location: 3100469-3102049
  
 NCBI BlastP on this gene

EER43392

transcriptional regulator Ngg1
  
Accession: EER43391
  
Location: 3097685-3099976
  
 NCBI BlastP on this gene

EER43391

nitrogen permease regulator family
  
Accession: EER43390
  
Location: 3095249-3097082
  
 NCBI BlastP on this gene

EER43390

35. :  DS990639 Ajellomyces capsulatus H88 supercont1.4 genomic scaffold     Total score: 2.0     Cumulative Blast bit score: 1194

rap guanine nucleotide exchange factor 5
  
Accession: EGC46343
  
Location: 3614157-3618163
  
 NCBI BlastP on this gene

EGC46343

HIRA-interacting protein
  
Accession: EGC46342
  
Location: 3611475-3612716
  
 NCBI BlastP on this gene

EGC46342

predicted protein
  
Accession: EGC46341
  
Location: 3610626-3610958
  
 NCBI BlastP on this gene

EGC46341

predicted protein
  
Accession: EGC46340
  
Location: 3607118-3607841
  
 NCBI BlastP on this gene

EGC46340

predicted protein
  
Accession: EGC46339
  
Location: 3605215-3605682
  
 NCBI BlastP on this gene

EGC46339

PSP1 domain-containing protein
  
Accession: EGC46338
  
Location: 3600478-3603316
  
 NCBI BlastP on this gene

EGC46338

conserved hypothetical protein
  
Accession: EGC46337
  
Location: 3596461-3597844
  
 NCBI BlastP on this gene

EGC46337

60S ribosomal protein
  
Accession: EGC46336
  
Location: 3594990-3595994
  
  
**BlastP hit with Mycgr3G102281\_Mycgr3**
  
Percentage identity: 88 %
  
BlastP bit score: 342
  
Sequence coverage: 99 %
  
E-value: 7e-117
  
  
 NCBI BlastP on this gene

EGC46336

secreted protein
  
Accession: EGC46335
  
Location: 3593172-3593927
  
 NCBI BlastP on this gene

EGC46335

predicted protein
  
Accession: EGC46334
  
Location: 3591494-3592229
  
 NCBI BlastP on this gene

EGC46334

NACHT and WD40 domain-containing protein
  
Accession: EGC46333
  
Location: 3586379-3587325
  
 NCBI BlastP on this gene

EGC46333

predicted protein
  
Accession: EGC46332
  
Location: 3585705-3586084
  
 NCBI BlastP on this gene

EGC46332

ubiquitin-protein ligase E3
  
Accession: EGC46331
  
Location: 3579760-3583510
  
  
**BlastP hit with Mycgr3G52682\_Mycgr3T**
  
Percentage identity: 43 %
  
BlastP bit score: 852
  
Sequence coverage: 100 %
  
E-value: 0.0
  
  
 NCBI BlastP on this gene

EGC46331

hypothetical protein
  
Accession: EGC46330
  
Location: 3577669-3578301
  
 NCBI BlastP on this gene

EGC46330

NADP-dependent mannitol dehydrogenase
  
Accession: EGC46329
  
Location: 3575418-3577118
  
 NCBI BlastP on this gene

EGC46329

conserved hypothetical protein
  
Accession: EGC46328
  
Location: 3572962-3573926
  
 NCBI BlastP on this gene

EGC46328

RING finger domain-containing protein
  
Accession: EGC46327
  
Location: 3569716-3571789
  
 NCBI BlastP on this gene

EGC46327

predicted protein
  
Accession: EGC46326
  
Location: 3568564-3569453
  
 NCBI BlastP on this gene

EGC46326

ada histone acetyltransferase complex component
  
Accession: EGC46325
  
Location: 3565110-3567380
  
 NCBI BlastP on this gene

EGC46325

nitrogen permease regulator family
  
Accession: EGC46324
  
Location: 3562674-3564507
  
 NCBI BlastP on this gene

EGC46324

conserved hypothetical protein
  
Accession: EGC46323
  
Location: 3560797-3561735
  
 NCBI BlastP on this gene

EGC46323

36. :  DS989828 Arthroderma gypseum CBS 118893 supercont1.7 genomic scaffold     Total score: 2.0     Cumulative Blast bit score: 1178

hypothetical protein
  
Accession: EFR04446
  
Location: 425479-426516
  
 NCBI BlastP on this gene

EFR04446

kynurenine 3-monooxygenase
  
Accession: EFR04445
  
Location: 422766-424589
  
 NCBI BlastP on this gene

EFR04445

methionine aminopeptidase 1
  
Accession: EFR04444
  
Location: 420409-421739
  
 NCBI BlastP on this gene

EFR04444

multidrug resistance protein fnx1
  
Accession: EFR04443
  
Location: 417768-419780
  
 NCBI BlastP on this gene

EFR04443

EF hand domain-containing protein
  
Accession: EFR04442
  
Location: 413775-417134
  
 NCBI BlastP on this gene

EFR04442

pre-mRNA-processing factor 17
  
Accession: EFR04441
  
Location: 411647-413452
  
 NCBI BlastP on this gene

EFR04441

hypothetical protein
  
Accession: EFR04440
  
Location: 410034-411132
  
 NCBI BlastP on this gene

EFR04440

30S ribosomal protein S23
  
Accession: EFR04439
  
Location: 408650-409615
  
  
**BlastP hit with Mycgr3G102281\_Mycgr3**
  
Percentage identity: 80 %
  
BlastP bit score: 362
  
Sequence coverage: 110 %
  
E-value: 4e-124
  
  
 NCBI BlastP on this gene

EFR04439

ubiquitin-protein ligase E3A
  
Accession: EFR04438
  
Location: 404018-407671
  
  
**BlastP hit with Mycgr3G52682\_Mycgr3T**
  
Percentage identity: 40 %
  
BlastP bit score: 816
  
Sequence coverage: 103 %
  
E-value: 0.0
  
  
 NCBI BlastP on this gene

EFR04438

L-threonine 3-dehydrogenase
  
Accession: EFR04437
  
Location: 400269-401728
  
 NCBI BlastP on this gene

EFR04437

hypothetical protein
  
Accession: EFR04436
  
Location: 398730-399801
  
 NCBI BlastP on this gene

EFR04436

hypothetical protein
  
Accession: EFR04435
  
Location: 396376-398189
  
 NCBI BlastP on this gene

EFR04435

histone acetyltransferase transcription factor
  
Accession: EFR04434
  
Location: 392475-394647
  
 NCBI BlastP on this gene

EFR04434

hypothetical protein
  
Accession: EFR04433
  
Location: 390272-392214
  
 NCBI BlastP on this gene

EFR04433

hypothetical protein
  
Accession: EFR04432
  
Location: 388374-389381
  
 NCBI BlastP on this gene

EFR04432

37. :  KB644410 Penicillium oxalicum 114-2 unplaced genomic scaffold scaffold\_3     Total score: 2.0     Cumulative Blast bit score: 1168

hypothetical protein
  
Accession: EPS27968
  
Location: 2457379-2460682
  
 NCBI BlastP on this gene

EPS27968

hypothetical protein
  
Accession: EPS27967
  
Location: 2453118-2456030
  
 NCBI BlastP on this gene

EPS27967

hypothetical protein
  
Accession: EPS27966
  
Location: 2450916-2451986
  
 NCBI BlastP on this gene

EPS27966

hypothetical protein
  
Accession: EPS27965
  
Location: 2448255-2450122
  
 NCBI BlastP on this gene

EPS27965

hypothetical protein
  
Accession: EPS27964
  
Location: 2445511-2447662
  
 NCBI BlastP on this gene

EPS27964

hypothetical protein
  
Accession: EPS27963
  
Location: 2442506-2443929
  
 NCBI BlastP on this gene

EPS27963

hypothetical protein
  
Accession: EPS27962
  
Location: 2440167-2441078
  
  
**BlastP hit with Mycgr3G102281\_Mycgr3**
  
Percentage identity: 89 %
  
BlastP bit score: 381
  
Sequence coverage: 99 %
  
E-value: 3e-132
  
  
 NCBI BlastP on this gene

EPS27962

hypothetical protein
  
Accession: EPS27961
  
Location: 2435482-2439235
  
  
**BlastP hit with Mycgr3G52682\_Mycgr3T**
  
Percentage identity: 47 %
  
BlastP bit score: 787
  
Sequence coverage: 74 %
  
E-value: 0.0
  
  
 NCBI BlastP on this gene

EPS27961

putative beta-glucosidase
  
Accession: EPS27960
  
Location: 2430467-2433245
  
 NCBI BlastP on this gene

EPS27960

hypothetical protein
  
Accession: EPS27959
  
Location: 2429192-2430064
  
 NCBI BlastP on this gene

EPS27959

hypothetical protein
  
Accession: EPS27958
  
Location: 2426642-2428921
  
 NCBI BlastP on this gene

EPS27958

putative d-4,5 unsaturated beta-glucuronyl hydrolase
  
Accession: EPS27957
  
Location: 2424725-2426259
  
 NCBI BlastP on this gene

EPS27957

hypothetical protein
  
Accession: EPS27956
  
Location: 2421195-2424218
  
 NCBI BlastP on this gene

EPS27956

hypothetical protein
  
Accession: EPS27955
  
Location: 2418812-2420416
  
 NCBI BlastP on this gene

EPS27955

38. :  EQ962656 Talaromyces stipitatus ATCC 10500 scf\_1105507295549 genomic scaffold     Total score: 2.0     Cumulative Blast bit score: 1164

C-8 sterol isomerase (Erg-1), putative
  
Accession: EED16771
  
Location: 2691594-2692335
  
 NCBI BlastP on this gene

EED16771

conserved hypothetical protein
  
Accession: EED16772
  
Location: 2694330-2695693
  
 NCBI BlastP on this gene

EED16772

PP-loop ATPase superfamily protein, putative
  
Accession: EED16773
  
Location: 2696157-2697650
  
 NCBI BlastP on this gene

EED16773

Ras guanyl-nucleotide exchange factor RasGEF, putative
  
Accession: EED16774
  
Location: 2697809-2700497
  
 NCBI BlastP on this gene

EED16774

HIT finger domain protein, putative
  
Accession: EED16775
  
Location: 2701938-2702553
  
 NCBI BlastP on this gene

EED16775

NifU-related protein
  
Accession: EED16777
  
Location: 2703013-2703963
  
 NCBI BlastP on this gene

EED16777

PSP1 domain protein
  
Accession: EED16778
  
Location: 2707488-2710210
  
 NCBI BlastP on this gene

EED16778

60S ribosomal protein L15
  
Accession: EED16779
  
Location: 2711117-2712210
  
  
**BlastP hit with Mycgr3G102281\_Mycgr3**
  
Percentage identity: 89 %
  
BlastP bit score: 346
  
Sequence coverage: 99 %
  
E-value: 2e-118
  
  
 NCBI BlastP on this gene

EED16779

ubiquitin-protein ligase (Hul4), putative
  
Accession: EED16780
  
Location: 2713225-2717065
  
  
**BlastP hit with Mycgr3G52682\_Mycgr3T**
  
Percentage identity: 43 %
  
BlastP bit score: 818
  
Sequence coverage: 96 %
  
E-value: 0.0
  
  
 NCBI BlastP on this gene

EED16780

muramidase, putative
  
Accession: EED16781
  
Location: 2718431-2719732
  
 NCBI BlastP on this gene

EED16781

mucin-desulfating sulfatase, putative
  
Accession: EED16782
  
Location: 2721296-2722814
  
 NCBI BlastP on this gene

EED16782

conserved hypothetical protein
  
Accession: EED16783
  
Location: 2722991-2723565
  
 NCBI BlastP on this gene

EED16783

NAD(P)H-dependent FMN reductase LOT6, putative
  
Accession: EED16784
  
Location: 2723759-2724569
  
 NCBI BlastP on this gene

EED16784

conserved hypothetical protein
  
Accession: EED16785
  
Location: 2724814-2726290
  
 NCBI BlastP on this gene

EED16785

conserved hypothetical protein
  
Accession: EED16787
  
Location: 2725050-2726290
  
 NCBI BlastP on this gene

EED16787

alcohol dehydrogenase, zinc-containing, putative
  
Accession: EED16788
  
Location: 2727381-2728815
  
 NCBI BlastP on this gene

EED16788

conserved hypothetical protein
  
Accession: EED16790
  
Location: 2729138-2730084
  
 NCBI BlastP on this gene

EED16790

DNA repair protein, putative
  
Accession: EED16791
  
Location: 2730193-2732408
  
 NCBI BlastP on this gene

EED16791

conserved hypothetical protein
  
Accession: EED16792
  
Location: 2732718-2734653
  
 NCBI BlastP on this gene

EED16792

conserved hypothetical protein
  
Accession: EED16793
  
Location: 2735875-2736734
  
 NCBI BlastP on this gene

EED16793

39. :  DS995702 Microsporum canis CBS 113480 supercont1.2 genomic scaffold     Total score: 2.0     Cumulative Blast bit score: 1152

1-phosphatidylinositol-3-phosphate 5-kinase FAB1
  
Accession: EEQ29630
  
Location: 2644698-2652262
  
 NCBI BlastP on this gene

EEQ29630

predicted protein
  
Accession: EEQ29629
  
Location: 2640632-2644088
  
 NCBI BlastP on this gene

EEQ29629

conserved hypothetical protein
  
Accession: EEQ29628
  
Location: 2637328-2639718
  
 NCBI BlastP on this gene

EEQ29628

conserved hypothetical protein
  
Accession: EEQ29627
  
Location: 2635519-2637069
  
 NCBI BlastP on this gene

EEQ29627

conserved hypothetical protein
  
Accession: EEQ29626
  
Location: 2634674-2635415
  
 NCBI BlastP on this gene

EEQ29626

EF hand domain-containing protein
  
Accession: EEQ29625
  
Location: 2630655-2634026
  
 NCBI BlastP on this gene

EEQ29625

pre-mRNA-processing factor 17
  
Accession: EEQ29624
  
Location: 2628417-2630213
  
 NCBI BlastP on this gene

EEQ29624

conserved hypothetical protein
  
Accession: EEQ29623
  
Location: 2626903-2628022
  
 NCBI BlastP on this gene

EEQ29623

60S ribosomal protein L15
  
Accession: EEQ29622
  
Location: 2625514-2626506
  
  
**BlastP hit with Mycgr3G102281\_Mycgr3**
  
Percentage identity: 73 %
  
BlastP bit score: 351
  
Sequence coverage: 120 %
  
E-value: 1e-119
  
  
 NCBI BlastP on this gene

EEQ29622

ubiquitin-protein ligase E3A
  
Accession: EEQ29621
  
Location: 2620922-2624570
  
  
**BlastP hit with Mycgr3G52682\_Mycgr3T**
  
Percentage identity: 40 %
  
BlastP bit score: 801
  
Sequence coverage: 104 %
  
E-value: 0.0
  
  
 NCBI BlastP on this gene

EEQ29621

L-threonine 3-dehydrogenase
  
Accession: EEQ29620
  
Location: 2617428-2618872
  
 NCBI BlastP on this gene

EEQ29620

conserved hypothetical protein
  
Accession: EEQ29619
  
Location: 2615604-2616537
  
 NCBI BlastP on this gene

EEQ29619

conserved hypothetical protein
  
Accession: EEQ29618
  
Location: 2613277-2614971
  
 NCBI BlastP on this gene

EEQ29618

predicted protein
  
Accession: EEQ29617
  
Location: 2612380-2612918
  
 NCBI BlastP on this gene

EEQ29617

transcriptional adapter 3
  
Accession: EEQ29616
  
Location: 2609357-2611540
  
 NCBI BlastP on this gene

EEQ29616

nitrogen permease regulator 2
  
Accession: EEQ29615
  
Location: 2607215-2609126
  
 NCBI BlastP on this gene

EEQ29615

conserved hypothetical protein
  
Accession: EEQ29614
  
Location: 2605415-2606368
  
 NCBI BlastP on this gene

EEQ29614

40. :  ACYE01000036 Trichophyton verrucosum HKI 0517     Total score: 2.0     Cumulative Blast bit score: 1152

hypothetical protein
  
Accession: EFE44606
  
Location: 27524-28510
  
 NCBI BlastP on this gene

EFE44606

hypothetical protein
  
Accession: EFE44605
  
Location: 26120-27085
  
  
**BlastP hit with Mycgr3G102281\_Mycgr3**
  
Percentage identity: 83 %
  
BlastP bit score: 364
  
Sequence coverage: 105 %
  
E-value: 3e-125
  
  
 NCBI BlastP on this gene

EFE44605

ubiquitin-protein ligase (Hul4), putative
  
Accession: EFE44604
  
Location: 21474-25109
  
  
**BlastP hit with Mycgr3G52682\_Mycgr3T**
  
Percentage identity: 39 %
  
BlastP bit score: 788
  
Sequence coverage: 103 %
  
E-value: 0.0
  
  
 NCBI BlastP on this gene

EFE44604

zinc-containing alcohol dehydrogenase, putative
  
Accession: EFE44603
  
Location: 17661-19119
  
 NCBI BlastP on this gene

EFE44603

hypothetical protein
  
Accession: EFE44602
  
Location: 16047-16949
  
 NCBI BlastP on this gene

EFE44602

RING finger domain protein, putative
  
Accession: EFE44601
  
Location: 13834-15481
  
 NCBI BlastP on this gene

EFE44601

hypothetical protein
  
Accession: EFE44600
  
Location: 9751-11923
  
 NCBI BlastP on this gene

EFE44600

hypothetical protein
  
Accession: EFE44599
  
Location: 7918-9528
  
 NCBI BlastP on this gene

EFE44599

hypothetical protein
  
Accession: EFE44598
  
Location: 4613-6522
  
 NCBI BlastP on this gene

EFE44598

hypothetical protein
  
Accession: EFE44597
  
Location: 2608-4114
  
 NCBI BlastP on this gene

EFE44597

41. :  AQGS01000844 Dactylellina haptotyla CBS 200.50     Total score: 2.0     Cumulative Blast bit score: 1135

hypothetical protein
  
Accession: EPS36649
  
Location: 172-1414
  
 NCBI BlastP on this gene

EPS36649

hypothetical protein
  
Accession: EPS36652
  
Location: 4146-5394
  
 NCBI BlastP on this gene

EPS36652

hypothetical protein
  
Accession: EPS36657
  
Location: 9148-9929
  
 NCBI BlastP on this gene

EPS36657

hypothetical protein
  
Accession: EPS36660
  
Location: 12377-13270
  
 NCBI BlastP on this gene

EPS36660

hypothetical protein
  
Accession: EPS36651
  
Location: 13781-14694
  
  
**BlastP hit with Mycgr3G102281\_Mycgr3**
  
Percentage identity: 89 %
  
BlastP bit score: 372
  
Sequence coverage: 99 %
  
E-value: 8e-129
  
  
 NCBI BlastP on this gene

EPS36651

hypothetical protein
  
Accession: EPS36653
  
Location: 17055-20546
  
  
**BlastP hit with Mycgr3G52682\_Mycgr3T**
  
Percentage identity: 38 %
  
BlastP bit score: 763
  
Sequence coverage: 101 %
  
E-value: 0.0
  
  
 NCBI BlastP on this gene

EPS36653

hypothetical protein
  
Accession: EPS36648
  
Location: 21242-22254
  
 NCBI BlastP on this gene

EPS36648

hypothetical protein
  
Accession: EPS36658
  
Location: 23503-24360
  
 NCBI BlastP on this gene

EPS36658

hypothetical protein
  
Accession: EPS36659
  
Location: 24929-28056
  
 NCBI BlastP on this gene

EPS36659

hypothetical protein
  
Accession: EPS36654
  
Location: 29291-30379
  
 NCBI BlastP on this gene

EPS36654

hypothetical protein
  
Accession: EPS36645
  
Location: 31016-33303
  
 NCBI BlastP on this gene

EPS36645

hypothetical protein
  
Accession: EPS36650
  
Location: 34034-35312
  
 NCBI BlastP on this gene

EPS36650

hypothetical protein
  
Accession: EPS36647
  
Location: 36170-36996
  
 NCBI BlastP on this gene

EPS36647

hypothetical protein
  
Accession: EPS36646
  
Location: 37945-40245
  
 NCBI BlastP on this gene

EPS36646

42. :  AP007155 Aspergillus oryzae RIB40 DNA, SC003.     Total score: 2.0     Cumulative Blast bit score: 1124

not annotated
  
Accession: BAE58018
  
Location: 2443615-2445170
  
 NCBI BlastP on this gene

AO090003000901

not annotated
  
Accession: BAE58017
  
Location: 2438558-2439218
  
 NCBI BlastP on this gene

AO090003000900

not annotated
  
Accession: BAE58016
  
Location: 2436107-2437102
  
 NCBI BlastP on this gene

AO090003000898

not annotated
  
Accession: BAE58015
  
Location: 2433671-2434393
  
 NCBI BlastP on this gene

AO090003000897

not annotated
  
Accession: BAE58014
  
Location: 2425217-2427928
  
 NCBI BlastP on this gene

AO090003000896

not annotated
  
Accession: BAE58013
  
Location: 2422976-2423920
  
  
**BlastP hit with Mycgr3G102281\_Mycgr3**
  
Percentage identity: 89 %
  
BlastP bit score: 380
  
Sequence coverage: 99 %
  
E-value: 6e-132
  
  
 NCBI BlastP on this gene

AO090003000895

not annotated
  
Accession: BAE58012
  
Location: 2418074-2421607
  
  
**BlastP hit with Mycgr3G52682\_Mycgr3T**
  
Percentage identity: 54 %
  
BlastP bit score: 744
  
Sequence coverage: 57 %
  
E-value: 0.0
  
  
 NCBI BlastP on this gene

AO090003000894

not annotated
  
Accession: BAE58011
  
Location: 2416603-2417863
  
 NCBI BlastP on this gene

AO090003000893

not annotated
  
Accession: BAE58010
  
Location: 2415168-2416059
  
 NCBI BlastP on this gene

AO090003000892

not annotated
  
Accession: BAE58009
  
Location: 2410256-2411200
  
 NCBI BlastP on this gene

AO090003000890

not annotated
  
Accession: BAE58008
  
Location: 2405518-2406365
  
 NCBI BlastP on this gene

AO090003000888

not annotated
  
Accession: BAE58007
  
Location: 2404042-2405063
  
 NCBI BlastP on this gene

AO090003000887

not annotated
  
Accession: BAE58006
  
Location: 2402675-2403412
  
 NCBI BlastP on this gene

AO090003000886

not annotated
  
Accession: BAE58005
  
Location: 2398605-2400743
  
 NCBI BlastP on this gene

AO090003000885

43. :  ADOT01000146 Arthrobotrys oligospora ATCC 24927     Total score: 2.0     Cumulative Blast bit score: 1098

hypothetical protein
  
Accession: EGX48068
  
Location: 89170-91940
  
 NCBI BlastP on this gene

EGX48068

hypothetical protein
  
Accession: EGX48067
  
Location: 85120-86335
  
 NCBI BlastP on this gene

EGX48067

hypothetical protein
  
Accession: EGX48066
  
Location: 80605-81411
  
 NCBI BlastP on this gene

EGX48066

hypothetical protein
  
Accession: EGX48065
  
Location: 76572-77534
  
 NCBI BlastP on this gene

EGX48065

hypothetical protein
  
Accession: EGX48064
  
Location: 75108-76037
  
  
**BlastP hit with Mycgr3G102281\_Mycgr3**
  
Percentage identity: 91 %
  
BlastP bit score: 384
  
Sequence coverage: 99 %
  
E-value: 3e-133
  
  
 NCBI BlastP on this gene

EGX48064

hypothetical protein
  
Accession: EGX48063
  
Location: 69053-72651
  
  
**BlastP hit with Mycgr3G52682\_Mycgr3T**
  
Percentage identity: 46 %
  
BlastP bit score: 714
  
Sequence coverage: 66 %
  
E-value: 0.0
  
  
 NCBI BlastP on this gene

EGX48063

hypothetical protein
  
Accession: EGX48062
  
Location: 67503-68497
  
 NCBI BlastP on this gene

EGX48062

hypothetical protein
  
Accession: EGX48061
  
Location: 65117-66217
  
 NCBI BlastP on this gene

EGX48061

hypothetical protein
  
Accession: EGX48060
  
Location: 63456-64079
  
 NCBI BlastP on this gene

EGX48060

hypothetical protein
  
Accession: EGX48059
  
Location: 62033-62916
  
 NCBI BlastP on this gene

EGX48059

hypothetical protein
  
Accession: EGX48058
  
Location: 60193-60816
  
 NCBI BlastP on this gene

EGX48058

hypothetical protein
  
Accession: EGX48057
  
Location: 55661-56512
  
 NCBI BlastP on this gene

EGX48057

hypothetical protein
  
Accession: EGX48056
  
Location: 54774-55217
  
 NCBI BlastP on this gene

EGX48056

hypothetical protein
  
Accession: EGX48055
  
Location: 50075-54380
  
 NCBI BlastP on this gene

EGX48055

44. :  CH476657 Ajellomyces capsulatus NAm1 scaffold\_3 genomic scaffold     Total score: 2.0     Cumulative Blast bit score: 1081

conserved hypothetical protein
  
Accession: EDN07086
  
Location: 1617275-1621291
  
 NCBI BlastP on this gene

EDN07086

conserved hypothetical protein
  
Accession: EDN07085
  
Location: 1614601-1615738
  
 NCBI BlastP on this gene

EDN07085

predicted protein
  
Accession: EDN07084
  
Location: 1610447-1610752
  
 NCBI BlastP on this gene

EDN07084

predicted protein
  
Accession: EDN07083
  
Location: 1606427-1606976
  
 NCBI BlastP on this gene

EDN07083

conserved hypothetical protein
  
Accession: EDN07082
  
Location: 1603498-1605564
  
 NCBI BlastP on this gene

EDN07082

predicted protein
  
Accession: EDN07081
  
Location: 1599444-1600830
  
 NCBI BlastP on this gene

EDN07081

60S ribosomal protein L15
  
Accession: EDN07080
  
Location: 1597982-1598988
  
  
**BlastP hit with Mycgr3G102281\_Mycgr3**
  
Percentage identity: 88 %
  
BlastP bit score: 342
  
Sequence coverage: 99 %
  
E-value: 7e-117
  
  
 NCBI BlastP on this gene

EDN07080

predicted protein
  
Accession: EDN07079
  
Location: 1596158-1596913
  
 NCBI BlastP on this gene

EDN07079

predicted protein
  
Accession: EDN07078
  
Location: 1591765-1592762
  
 NCBI BlastP on this gene

EDN07078

predicted protein
  
Accession: EDN07077
  
Location: 1590804-1591154
  
 NCBI BlastP on this gene

EDN07077

predicted protein
  
Accession: EDN07076
  
Location: 1589009-1589949
  
 NCBI BlastP on this gene

EDN07076

conserved hypothetical protein
  
Accession: EDN07075
  
Location: 1582818-1585479
  
  
**BlastP hit with Mycgr3G52682\_Mycgr3T**
  
Percentage identity: 47 %
  
BlastP bit score: 739
  
Sequence coverage: 74 %
  
E-value: 0.0
  
  
 NCBI BlastP on this gene

EDN07075

hypothetical protein
  
Accession: EDN07074
  
Location: 1578350-1580048
  
 NCBI BlastP on this gene

EDN07074

conserved hypothetical protein
  
Accession: EDN07073
  
Location: 1575806-1576770
  
 NCBI BlastP on this gene

EDN07073

predicted protein
  
Accession: EDN07072
  
Location: 1572644-1574714
  
 NCBI BlastP on this gene

EDN07072

conserved hypothetical protein
  
Accession: EDN07071
  
Location: 1568043-1570312
  
 NCBI BlastP on this gene

EDN07071

conserved hypothetical protein
  
Accession: EDN07070
  
Location: 1565609-1567442
  
 NCBI BlastP on this gene

EDN07070

predicted protein
  
Accession: EDN07069
  
Location: 1563728-1564608
  
 NCBI BlastP on this gene

EDN07069

45. :  DS544804 Paracoccidioides brasiliensis Pb03 supercont1.2 genomic scaffold     Total score: 2.0     Cumulative Blast bit score: 1070

HIRA-interacting protein
  
Accession: EEH18956
  
Location: 933149-934438
  
 NCBI BlastP on this gene

EEH18956

predicted protein
  
Accession: EEH18957
  
Location: 940496-942172
  
 NCBI BlastP on this gene

EEH18957

conserved hypothetical protein
  
Accession: EEH18958
  
Location: 943075-945859
  
 NCBI BlastP on this gene

EEH18958

60S ribosomal protein L15
  
Accession: EEH18959
  
Location: 949969-950953
  
  
**BlastP hit with Mycgr3G102281\_Mycgr3**
  
Percentage identity: 89 %
  
BlastP bit score: 375
  
Sequence coverage: 99 %
  
E-value: 1e-129
  
  
 NCBI BlastP on this gene

EEH18959

E3 ubiquitin-protein ligase HUWE1
  
Accession: EEH18960
  
Location: 955442-959279
  
  
**BlastP hit with Mycgr3G52682\_Mycgr3T**
  
Percentage identity: 55 %
  
BlastP bit score: 695
  
Sequence coverage: 52 %
  
E-value: 0.0
  
  
 NCBI BlastP on this gene

EEH18960

L-threonine 3-dehydrogenase
  
Accession: EEH18961
  
Location: 962712-963721
  
 NCBI BlastP on this gene

EEH18961

conserved hypothetical protein
  
Accession: EEH18962
  
Location: 965763-966739
  
 NCBI BlastP on this gene

EEH18962

conserved hypothetical protein
  
Accession: EEH18963
  
Location: 968092-969536
  
 NCBI BlastP on this gene

EEH18963

alteration deficiency activation 3 protein
  
Accession: EEH18964
  
Location: 972549-974812
  
 NCBI BlastP on this gene

EEH18964

candidate tumor suppressor gene 21 protein
  
Accession: EEH18965
  
Location: 975433-977336
  
 NCBI BlastP on this gene

EEH18965

conserved hypothetical protein
  
Accession: EEH18966
  
Location: 978366-979307
  
 NCBI BlastP on this gene

EEH18966

46. :  KB916820 Neofusicoccum parvum UCRNP2 chromosome Unknown NP2\_03\_scaffold\_1182     Total score: 2.0     Cumulative Blast bit score: 1049

hypothetical protein
  
Accession: EOD43457
  
Location: 226834-228684
  
 NCBI BlastP on this gene

EOD43457

putative zinc knuckle protein
  
Accession: EOD43511
  
Location: 231119-231893
  
 NCBI BlastP on this gene

EOD43511

putative serine threonine protein kinase protein
  
Accession: EOD43494
  
Location: 233980-236509
  
 NCBI BlastP on this gene

EOD43494

putative u-box domain-containing protein
  
Accession: EOD43493
  
Location: 240687-241855
  
 NCBI BlastP on this gene

EOD43493

putative 60s ribosomal protein l15 protein
  
Accession: EOD43521
  
Location: 242596-243460
  
  
**BlastP hit with Mycgr3G102281\_Mycgr3**
  
Percentage identity: 92 %
  
BlastP bit score: 368
  
Sequence coverage: 99 %
  
E-value: 6e-127
  
  
 NCBI BlastP on this gene

EOD43521

putative swi-snf complex subunit protein
  
Accession: EOD43504
  
Location: 248340-249873
  
 NCBI BlastP on this gene

EOD43504

putative cytosine deaminase protein
  
Accession: EOD43508
  
Location: 250678-251991
  
 NCBI BlastP on this gene

EOD43508

putative ubiquitin-protein ligase protein
  
Accession: EOD43522
  
Location: 256411-257990
  
  
**BlastP hit with Mycgr3G52682\_Mycgr3T**
  
Percentage identity: 64 %
  
BlastP bit score: 681
  
Sequence coverage: 43 %
  
E-value: 0.0
  
  
 NCBI BlastP on this gene

EOD43522

putative mfs monocarboxylate transporter protein
  
Accession: EOD43515
  
Location: 261386-263419
  
 NCBI BlastP on this gene

EOD43515

putative paraben-hydrolyzing esterase precursor protein
  
Accession: EOD43519
  
Location: 264576-266231
  
 NCBI BlastP on this gene

EOD43519

putative candidate carboxylesterase from carbohydrate esterase family ce10 protein
  
Accession: EOD43465
  
Location: 266474-269215
  
 NCBI BlastP on this gene

EOD43465

putative phd finger and set domain protein
  
Accession: EOD43439
  
Location: 270989-272874
  
 NCBI BlastP on this gene

EOD43439

putative nadh-ubiquinone oxidoreductase 299 kda protein
  
Accession: EOD43458
  
Location: 275272-276103
  
 NCBI BlastP on this gene

EOD43458

putative disulfide isomerase protein
  
Accession: EOD43453
  
Location: 276462-278697
  
 NCBI BlastP on this gene

EOD43453

47. :  KB916240 Neofusicoccum parvum UCRNP2 chromosome Unknown NP2\_03\_scaffold\_602     Total score: 2.0     Cumulative Blast bit score: 1049

putative cp2 transcription factor protein
  
Accession: EOD48106
  
Location: 227417-230120
  
 NCBI BlastP on this gene

EOD48106

putative ureidoglycolate hydrolase protein
  
Accession: EOD48161
  
Location: 222965-223837
  
 NCBI BlastP on this gene

EOD48161

putative hmg box-containing protein
  
Accession: EOD48132
  
Location: 218394-218929
  
 NCBI BlastP on this gene

EOD48132

putative nad dependent epimerase protein
  
Accession: EOD48100
  
Location: 213865-214900
  
  
**BlastP hit with Mycgr3G52686\_Mycgr3T**
  
Percentage identity: 76 %
  
BlastP bit score: 461
  
Sequence coverage: 97 %
  
E-value: 4e-161
  
  
 NCBI BlastP on this gene

EOD48100

putative Arylsulfotransferase protein
  
Accession: EOD48168
  
Location: 210935-213387
  
  
**BlastP hit with Mycgr3G32432\_Mycgr3T**
  
Percentage identity: 57 %
  
BlastP bit score: 588
  
Sequence coverage: 82 %
  
E-value: 0.0
  
  
 NCBI BlastP on this gene

EOD48168

putative mfs transporter protein
  
Accession: EOD48146
  
Location: 208158-210090
  
 NCBI BlastP on this gene

EOD48146

putative r3h and g-patch domain-containing protein
  
Accession: EOD48141
  
Location: 205266-206462
  
 NCBI BlastP on this gene

EOD48141

putative cysteine protease atg4 protein
  
Accession: EOD48149
  
Location: 202528-203567
  
 NCBI BlastP on this gene

EOD48149

putative histone h2b protein
  
Accession: EOD48114
  
Location: 200887-201497
  
 NCBI BlastP on this gene

EOD48114

putative histone h2a protein
  
Accession: EOD48140
  
Location: 199729-200386
  
 NCBI BlastP on this gene

EOD48140

putative nadh dehydrogenase 1 beta subcomplex subunit 9 protein
  
Accession: EOD48179
  
Location: 198340-198855
  
 NCBI BlastP on this gene

EOD48179

putative dihydrolipoamide succinyltransferase protein
  
Accession: EOD48121
  
Location: 196392-197892
  
 NCBI BlastP on this gene

EOD48121

putative glycoside hydrolase family 2 protein
  
Accession: EOD48097
  
Location: 191542-193718
  
 NCBI BlastP on this gene

EOD48097

48. :  AHHD01000505 Macrophomina phaseolina MS6     Total score: 2.0     Cumulative Blast bit score: 1040

Ribosomal protein L29e
  
Accession: EKG10723
  
Location: 137494-137889
  
 NCBI BlastP on this gene

EKG10723

hypothetical protein
  
Accession: EKG10724
  
Location: 139451-141289
  
 NCBI BlastP on this gene

EKG10724

CP2 transcription factor
  
Accession: EKG10725
  
Location: 144023-146718
  
 NCBI BlastP on this gene

EKG10725

hypothetical protein
  
Accession: EKG10726
  
Location: 148090-148557
  
 NCBI BlastP on this gene

EKG10726

Ureidoglycolate hydrolase
  
Accession: EKG10727
  
Location: 150063-150929
  
 NCBI BlastP on this gene

EKG10727

protein of unknown function SprT-like protein
  
Accession: EKG10728
  
Location: 153533-155936
  
 NCBI BlastP on this gene

EKG10728

hypothetical protein
  
Accession: EKG10729
  
Location: 157429-158463
  
  
**BlastP hit with Mycgr3G52686\_Mycgr3T**
  
Percentage identity: 75 %
  
BlastP bit score: 453
  
Sequence coverage: 97 %
  
E-value: 6e-158
  
  
 NCBI BlastP on this gene

EKG10729

Arylsulfotransferase
  
Accession: EKG10730
  
Location: 159426-161469
  
  
**BlastP hit with Mycgr3G32432\_Mycgr3T**
  
Percentage identity: 56 %
  
BlastP bit score: 587
  
Sequence coverage: 82 %
  
E-value: 0.0
  
  
 NCBI BlastP on this gene

EKG10730

Major facilitator superfamily
  
Accession: EKG10731
  
Location: 162352-164129
  
 NCBI BlastP on this gene

EKG10731

hypothetical protein
  
Accession: EKG10732
  
Location: 165532-167691
  
 NCBI BlastP on this gene

EKG10732

Peptidase C54
  
Accession: EKG10733
  
Location: 168427-169890
  
 NCBI BlastP on this gene

EKG10733

Histone H2B
  
Accession: EKG10734
  
Location: 170436-170988
  
 NCBI BlastP on this gene

EKG10734

Histone H2A
  
Accession: EKG10735
  
Location: 171487-172117
  
 NCBI BlastP on this gene

EKG10735

Complex 1 LYR protein
  
Accession: EKG10736
  
Location: 172946-173459
  
 NCBI BlastP on this gene

EKG10736

Biotin/lipoyl attachment
  
Accession: EKG10737
  
Location: 173894-175373
  
 NCBI BlastP on this gene

EKG10737

Glycoside hydrolase family 2 immunoglobulin-like beta-sandwich
  
Accession: EKG10738
  
Location: 176200-178372
  
 NCBI BlastP on this gene

EKG10738

49. :  ABSU01000004 Arthroderma benhamiae CBS 112371     Total score: 2.0     Cumulative Blast bit score: 1000

hypothetical protein
  
Accession: EFE35227
  
Location: 1001160-1002197
  
 NCBI BlastP on this gene

EFE35227

hypothetical protein
  
Accession: EFE35226
  
Location: 998835-1000252
  
 NCBI BlastP on this gene

EFE35226

methionine aminopeptidase, type I, putative
  
Accession: EFE35225
  
Location: 995979-996804
  
 NCBI BlastP on this gene

EFE35225

hypothetical protein
  
Accession: EFE35224
  
Location: 994979-995302
  
 NCBI BlastP on this gene

EFE35224

hypothetical protein
  
Accession: EFE35223
  
Location: 993283-994290
  
 NCBI BlastP on this gene

EFE35223

EF hand domain protein
  
Accession: EFE35222
  
Location: 989296-992667
  
 NCBI BlastP on this gene

EFE35222

hypothetical protein
  
Accession: EFE35221
  
Location: 987373-988956
  
 NCBI BlastP on this gene

EFE35221

hypothetical protein
  
Accession: EFE35220
  
Location: 985558-986543
  
 NCBI BlastP on this gene

EFE35220

hypothetical protein
  
Accession: EFE35219
  
Location: 984262-985138
  
  
**BlastP hit with Mycgr3G102281\_Mycgr3**
  
Percentage identity: 74 %
  
BlastP bit score: 312
  
Sequence coverage: 105 %
  
E-value: 4e-105
  
  
 NCBI BlastP on this gene

EFE35219

ubiquitin-protein ligase (Hul4), putative
  
Accession: EFE35218
  
Location: 979498-983173
  
  
**BlastP hit with Mycgr3G52682\_Mycgr3T**
  
Percentage identity: 44 %
  
BlastP bit score: 688
  
Sequence coverage: 69 %
  
E-value: 0.0
  
  
 NCBI BlastP on this gene

EFE35218

hypothetical protein
  
Accession: EFE35217
  
Location: 977759-978674
  
 NCBI BlastP on this gene

EFE35217

zinc-containing alcohol dehydrogenase, putative
  
Accession: EFE35216
  
Location: 975770-977227
  
 NCBI BlastP on this gene

EFE35216

hypothetical protein
  
Accession: EFE35215
  
Location: 974169-975071
  
 NCBI BlastP on this gene

EFE35215

RING finger domain protein, putative
  
Accession: EFE35214
  
Location: 972212-973598
  
 NCBI BlastP on this gene

EFE35214

hypothetical protein
  
Accession: EFE35213
  
Location: 967873-970046
  
 NCBI BlastP on this gene

EFE35213

hypothetical protein
  
Accession: EFE35212
  
Location: 965707-967630
  
 NCBI BlastP on this gene

EFE35212

hypothetical protein
  
Accession: EFE35211
  
Location: 962714-964623
  
 NCBI BlastP on this gene

EFE35211

hypothetical protein
  
Accession: EFE35210
  
Location: 960734-962219
  
 NCBI BlastP on this gene

EFE35210

50. :  JH767572 Coniosporium apollinis CBS 100218 chromosome Unknown supercont1.19     Total score: 2.0     Cumulative Blast bit score: 988

STE/STE11/BCK1 protein kinase
  
Accession: EON65158
  
Location: 237975-243674
  
 NCBI BlastP on this gene

EON65158

pyruvate decarboxylase
  
Accession: EON65157
  
Location: 234612-236768
  
 NCBI BlastP on this gene

EON65157

dihydrolipoyllysine-residue succinyltransferase, E2 component
  
Accession: EON65156
  
Location: 232722-234305
  
 NCBI BlastP on this gene

EON65156

hypothetical protein
  
Accession: EON65155
  
Location: 231615-232102
  
 NCBI BlastP on this gene

EON65155

histone H2A
  
Accession: EON65154
  
Location: 230190-230800
  
 NCBI BlastP on this gene

EON65154

histone H2B
  
Accession: EON65153
  
Location: 229035-229675
  
 NCBI BlastP on this gene

EON65153

hypothetical protein
  
Accession: EON65152
  
Location: 226885-228285
  
 NCBI BlastP on this gene

EON65152

hypothetical protein
  
Accession: EON65151
  
Location: 223875-226298
  
 NCBI BlastP on this gene

EON65151

hypothetical protein
  
Accession: EON65150
  
Location: 220014-221900
  
 NCBI BlastP on this gene

EON65150

hypothetical protein
  
Accession: EON65149
  
Location: 217137-218206
  
  
**BlastP hit with Mycgr3G52686\_Mycgr3T**
  
Percentage identity: 72 %
  
BlastP bit score: 427
  
Sequence coverage: 99 %
  
E-value: 1e-147
  
  
 NCBI BlastP on this gene

EON65149

hypothetical protein
  
Accession: EON65148
  
Location: 214431-216468
  
  
**BlastP hit with Mycgr3G32432\_Mycgr3T**
  
Percentage identity: 52 %
  
BlastP bit score: 561
  
Sequence coverage: 86 %
  
E-value: 0.0
  
  
 NCBI BlastP on this gene

EON65148

Detecting sequence homology at the gene cluster level with MultiGeneBlast.
  
Marnix H. Medema, Rainer Breitling & Eriko Takano (2013)
  
*Molecular Biology and Evolution* , 30: 1218-1223.
