# Supplementary material for: In silico prediction and characterization of secondary metabolite biosynthetic gene clusters in the wheat pathogen Zymoseptoria tritici
Source: BMC Genomics. 2017 Aug 17;18:631. doi: 10.1186/s12864-017-3969-y (PMC5561558; doi:10.1186/s12864-017-3969-y)
Supplement: Supplementary file 1 — MultiGeneBLAST analysis of putative secondary metabolite clusters. All encoded amino acid sequences from genes residing in clusters predicted by AntiSMASH are given as FASTA file format. All output data from MultiGeneBLASTs are also provided. (ZIP 42911 kb) [file 12864_2017_3969_MOESM1_ESM.zip › Cluster MultiGene BLAST/out/Clusters_1_34/Cluster_1/displaypage2.xhtml]

xml version="1.0" encoding="UTF-8"?


Search Results
  
  
 Results pages: 1, 2, 3, 4, 5

**MultiGeneBlast hits**

Select gene cluster alignment
51. DS499594\_2 Aspergillus fumigatus A1163 scf\_000001 genomic scaffold, whole...
52. DF126459\_0 Aspergillus kawachii IFO 4308 DNA, contig: scaffold00013, whol...
53. AAHF01000004\_0 Aspergillus fumigatus Af293, whole genome shotgun sequenci...
54. DS027688\_0 Neosartorya fischeri NRRL 181 1099437636249 genomic scaffold, ...
55. DS027059\_0 Aspergillus clavatus NRRL 1 1099423829805 genomic scaffold, wh...
56. JH226131\_1 Exophiala dermatitidis NIH/UT8656 unplaced genomic scaffold su...
57. EQ963474\_1 Aspergillus flavus NRRL3357 scf\_1106286417496 genomic scaffold...
58. BX649607\_0 Aspergillus fumigatus BAC pilot project supercontig; segment 3/3.
59. KB644408\_0 Penicillium oxalicum 114-2 unplaced genomic scaffold scaffold\_...
60. ACJE01000021\_2 Aspergillus niger ATCC 1015, whole genome shotgun sequenci...
61. AM270171\_0 Aspergillus niger contig An08c0160, genomic contig.
62. AM920431\_5 Penicillium chrysogenum Wisconsin 54-1255 complete genome, con...
63. AACD01000011\_0 Aspergillus nidulans FGSC A4, whole genome shotgun sequenc...
64. CH476594\_0 Aspergillus terreus NIH2624 scaffold\_1 genomic scaffold, whole...
65. AHHD01000271\_0 Macrophomina phaseolina MS6, whole genome shotgun sequenci...
66. EQ963485\_3 Aspergillus flavus NRRL3357 scf\_1106286419448 genomic scaffold...
67. GG697417\_0 Glomerella graminicola M1.001 genomic scaffold supercont1.87, ...
68. KB726028\_0 Colletotrichum orbiculare MAFF 240422 unplaced genomic scaffol...
69. CH476597\_0 Aspergillus terreus NIH2624 scaffold\_4 genomic scaffold, whole...
70. AP007174\_0 Aspergillus oryzae RIB40 DNA, SC103.
71. AKHY01000199\_0 Aspergillus oryzae 3.042, whole genome shotgun sequencing ...
72. DS027060\_1 Aspergillus clavatus NRRL 1 1099423829806 genomic scaffold, wh...
73. DS499594\_0 Aspergillus fumigatus A1163 scf\_000001 genomic scaffold, whole...
74. AAHF01000006\_1 Aspergillus fumigatus Af293, whole genome shotgun sequenci...
75. DS027698\_1 Neosartorya fischeri NRRL 181 1099437636266 genomic scaffold, ...
76. EQ963486\_0 Aspergillus flavus NRRL3357 scf\_1106286417242 genomic scaffold...
77. KE124052\_0 Mucor circinelloides f. circinelloides 1006PhL unplaced genomi...
78. KE148163\_0 Ophiostoma piceae UAMH 11346 chromosome Unknown scf18, whole g...
79. KB706899\_0 Eutypa lata UCREL1 unplaced genomic scaffold EL1\_03\_scaffold\_1...
80. GL698480\_0 Metarhizium acridum CQMa 102 unplaced genomic scaffold Scf\_011...
81. KB707843\_0 Botryotinia fuckeliana BcDW1 unplaced genomic scaffold Scaffol...
82. FQ790301\_0 Botryotinia fuckeliana T4 SuperContig\_226\_1 genomic supercontig.
83. CM001199\_0 Mycosphaerella graminicola IPO323 chromosome 4, whole genome s...
84. AMYD01000021\_0 Colletotrichum gloeosporioides Cg-14, whole genome shotgun...
85. DS985218\_0 Verticillium albo-atrum VaMs.102 supercont1.5 genomic scaffold...
86. KB916913\_0 Neofusicoccum parvum UCRNP2 chromosome Unknown NP2\_03\_scaffold...
87. DS544807\_0 Paracoccidioides brasiliensis Pb03 supercont1.5 genomic scaffo...
88. GG663377\_0 Ajellomyces capsulatus G186AR genomic scaffold supercont2.15, ...
89. EQ999987\_0 Ajellomyces dermatitidis ER-3 genomic scaffold supercont1.15, ...
90. GG749478\_0 Ajellomyces dermatitidis ATCC 18188 genomic scaffold supercont...
91. GG657474\_0 Ajellomyces dermatitidis SLH14081 genomic scaffold supercont1....
92. DS572750\_0 Paracoccidioides brasiliensis Pb18 supercont1.1 genomic scaffo...
93. DS572813\_0 Paracoccidioides brasiliensis Pb01 supercont1.3 genomic scaffo...
94. AB530986\_0 Streptomyces sp. SANK 60405 DNA, A-90289 biosynthetic gene clu...
95. KB456266\_0 Mycosphaerella populorum SO2202 unplaced genomic scaffold SEPM...
96. KB446546\_1 Dothistroma septosporum NZE10 unplaced genomic scaffold DOTSEs...
97. KB446556\_1 Pseudocercospora fijiensis CIRAD86 unplaced genomic scaffold M...
98. JH226130\_2 Exophiala dermatitidis NIH/UT8656 unplaced genomic scaffold su...
99. DS499597\_1 Aspergillus fumigatus A1163 scf\_000004 genomic scaffold, whole...
100. DS027696\_1 Neosartorya fischeri NRRL 181 1099437636264 genomic scaffold,...

Query: Architecture Search FASTA input

DS499594 : Aspergillus fumigatus A1163 scf\_000001 genomic scaffold    Total score: 2.0     Cumulative Blast bit score: 882

Hit cluster cross-links:

Mycgr3G52686 Mycgr3T
  
Location: 0-861

Mycgr3G52686\_Mycgr3T

Mycgr3G102281 Mycgr3
  
Location: 961-1573

Mycgr3G102281\_Mycgr3

Mycgr3G89185 Mycgr3T
  
Location: 1673-2063

Mycgr3G89185\_Mycgr3T

Mycgr3G65725 Mycgr3T
  
Location: 2163-3612

Mycgr3G65725\_Mycgr3T

Mycgr3G102276 Mycgr3
  
Location: 3712-4801

Mycgr3G102276\_Mycgr3

Mycgr3G89189 Mycgr3T
  
Location: 4901-5564

Mycgr3G89189\_Mycgr3T

Mycgr3G52682 Mycgr3T
  
Location: 5664-9231

Mycgr3G52682\_Mycgr3T

Mycgr3G107072 Mycgr3
  
Location: 9331-13279

Mycgr3G107072\_Mycgr3

Mycgr3G34982 Mycgr3T
  
Location: 13379-15116

Mycgr3G34982\_Mycgr3T

Mycgr3G107069 Mycgr3
  
Location: 15216-17097

Mycgr3G107069\_Mycgr3

Mycgr3G32432 Mycgr3T
  
Location: 17197-19042

Mycgr3G32432\_Mycgr3T

Mycgr3G98385 Mycgr3T
  
Location: 19142-19898

Mycgr3G98385\_Mycgr3T

blue light-inducible protein Bli-3
  
Accession: EDP56641
  
Location: 3820018-3820699
  
 NCBI BlastP on this gene

EDP56641

SAGA-like transcriptional regulatory complex subunit Spt3, putative
  
Accession: EDP56640
  
Location: 3818601-3819741
  
 NCBI BlastP on this gene

EDP56640

diphthine synthase, putative
  
Accession: EDP56639
  
Location: 3816704-3817677
  
 NCBI BlastP on this gene

EDP56639

AP-2 adaptor complex subunit sigma, putative
  
Accession: EDP56638
  
Location: 3814335-3815066
  
 NCBI BlastP on this gene

EDP56638

hypothetical protein
  
Accession: EDP56637
  
Location: 3813053-3813361
  
 NCBI BlastP on this gene

EDP56637

conserved hypothetical protein
  
Accession: EDP56636
  
Location: 3810215-3811242
  
  
**BlastP hit with Mycgr3G52686\_Mycgr3T**
  
Percentage identity: 62 %
  
BlastP bit score: 370
  
Sequence coverage: 95 %
  
E-value: 2e-125
  
  
 NCBI BlastP on this gene

EDP56636

conserved hypothetical protein
  
Accession: EDP56635
  
Location: 3806264-3808238
  
  
**BlastP hit with Mycgr3G32432\_Mycgr3T**
  
Percentage identity: 50 %
  
BlastP bit score: 513
  
Sequence coverage: 82 %
  
E-value: 1e-171
  
  
 NCBI BlastP on this gene

EDP56635

MFS transporter, putative
  
Accession: EDP56634
  
Location: 3802346-3804571
  
 NCBI BlastP on this gene

EDP56634

DUF292 domian protein
  
Accession: EDP56633
  
Location: 3800264-3801835
  
 NCBI BlastP on this gene

EDP56633

short-chain oxidoreductase, putative
  
Accession: EDP56632
  
Location: 3798446-3799362
  
 NCBI BlastP on this gene

EDP56632

SUN domain protein (Adg3), putative
  
Accession: EDP56631
  
Location: 3796167-3797696
  
 NCBI BlastP on this gene

EDP56631

Query: Architecture Search FASTA input

DF126459 : Aspergillus kawachii IFO 4308 DNA, contig: scaffold00013    Total score: 2.0     Cumulative Blast bit score: 882

Hit cluster cross-links:

Mycgr3G52686 Mycgr3T
  
Location: 0-861

Mycgr3G52686\_Mycgr3T

Mycgr3G102281 Mycgr3
  
Location: 961-1573

Mycgr3G102281\_Mycgr3

Mycgr3G89185 Mycgr3T
  
Location: 1673-2063

Mycgr3G89185\_Mycgr3T

Mycgr3G65725 Mycgr3T
  
Location: 2163-3612

Mycgr3G65725\_Mycgr3T

Mycgr3G102276 Mycgr3
  
Location: 3712-4801

Mycgr3G102276\_Mycgr3

Mycgr3G89189 Mycgr3T
  
Location: 4901-5564

Mycgr3G89189\_Mycgr3T

Mycgr3G52682 Mycgr3T
  
Location: 5664-9231

Mycgr3G52682\_Mycgr3T

Mycgr3G107072 Mycgr3
  
Location: 9331-13279

Mycgr3G107072\_Mycgr3

Mycgr3G34982 Mycgr3T
  
Location: 13379-15116

Mycgr3G34982\_Mycgr3T

Mycgr3G107069 Mycgr3
  
Location: 15216-17097

Mycgr3G107069\_Mycgr3

Mycgr3G32432 Mycgr3T
  
Location: 17197-19042

Mycgr3G32432\_Mycgr3T

Mycgr3G98385 Mycgr3T
  
Location: 19142-19898

Mycgr3G98385\_Mycgr3T

diphthine synthase
  
Accession: GAA87446
  
Location: 511314-512313
  
 NCBI BlastP on this gene

GAA87446

AorFlbE
  
Accession: GAA87447
  
Location: 512508-513199
  
 NCBI BlastP on this gene

GAA87447

AP-2 complex subunit sigma
  
Accession: GAA87448
  
Location: 514403-515158
  
 NCBI BlastP on this gene

GAA87448

NAD dependent epimerase/dehydratase
  
Accession: GAA87449
  
Location: 520295-521322
  
  
**BlastP hit with Mycgr3G52686\_Mycgr3T**
  
Percentage identity: 64 %
  
BlastP bit score: 370
  
Sequence coverage: 95 %
  
E-value: 4e-125
  
  
 NCBI BlastP on this gene

GAA87449

similar to An08g07120
  
Accession: GAA87450
  
Location: 525042-527293
  
  
**BlastP hit with Mycgr3G32432\_Mycgr3T**
  
Percentage identity: 51 %
  
BlastP bit score: 513
  
Sequence coverage: 80 %
  
E-value: 2e-170
  
  
 NCBI BlastP on this gene

GAA87450

MFS transporter
  
Accession: GAA87451
  
Location: 528679-530887
  
 NCBI BlastP on this gene

GAA87451

SUN domain protein
  
Accession: GAA87452
  
Location: 533064-534637
  
 NCBI BlastP on this gene

GAA87452

leucine carboxyl methyltransferase superfamily
  
Accession: GAA87453
  
Location: 536058-537210
  
 NCBI BlastP on this gene

GAA87453

Query: Architecture Search FASTA input

AAHF01000004 : Aspergillus fumigatus Af293    Total score: 2.0     Cumulative Blast bit score: 882

Hit cluster cross-links:

Mycgr3G52686 Mycgr3T
  
Location: 0-861

Mycgr3G52686\_Mycgr3T

Mycgr3G102281 Mycgr3
  
Location: 961-1573

Mycgr3G102281\_Mycgr3

Mycgr3G89185 Mycgr3T
  
Location: 1673-2063

Mycgr3G89185\_Mycgr3T

Mycgr3G65725 Mycgr3T
  
Location: 2163-3612

Mycgr3G65725\_Mycgr3T

Mycgr3G102276 Mycgr3
  
Location: 3712-4801

Mycgr3G102276\_Mycgr3

Mycgr3G89189 Mycgr3T
  
Location: 4901-5564

Mycgr3G89189\_Mycgr3T

Mycgr3G52682 Mycgr3T
  
Location: 5664-9231

Mycgr3G52682\_Mycgr3T

Mycgr3G107072 Mycgr3
  
Location: 9331-13279

Mycgr3G107072\_Mycgr3

Mycgr3G34982 Mycgr3T
  
Location: 13379-15116

Mycgr3G34982\_Mycgr3T

Mycgr3G107069 Mycgr3
  
Location: 15216-17097

Mycgr3G107069\_Mycgr3

Mycgr3G32432 Mycgr3T
  
Location: 17197-19042

Mycgr3G32432\_Mycgr3T

Mycgr3G98385 Mycgr3T
  
Location: 19142-19898

Mycgr3G98385\_Mycgr3T

blue light-inducible protein Bli-3
  
Accession: EAL90736
  
Location: 1504992-1505673
  
 NCBI BlastP on this gene

EAL90736

SAGA-like transcriptional regulatory complex subunit Spt3, putative
  
Accession: EAL90735
  
Location: 1503575-1504715
  
 NCBI BlastP on this gene

EAL90735

diphthine synthase, putative
  
Accession: EAL90734
  
Location: 1501678-1502651
  
 NCBI BlastP on this gene

EAL90734

AP-2 adaptor complex subunit sigma, putative
  
Accession: EAL90733
  
Location: 1499309-1500040
  
 NCBI BlastP on this gene

EAL90733

hypothetical protein
  
Accession: EAL90732
  
Location: 1498027-1498335
  
 NCBI BlastP on this gene

EAL90732

conserved hypothetical protein
  
Accession: EAL90731
  
Location: 1495189-1496216
  
  
**BlastP hit with Mycgr3G52686\_Mycgr3T**
  
Percentage identity: 62 %
  
BlastP bit score: 370
  
Sequence coverage: 95 %
  
E-value: 2e-125
  
  
 NCBI BlastP on this gene

EAL90731

conserved hypothetical protein
  
Accession: EAL90730
  
Location: 1491238-1493212
  
  
**BlastP hit with Mycgr3G32432\_Mycgr3T**
  
Percentage identity: 50 %
  
BlastP bit score: 513
  
Sequence coverage: 82 %
  
E-value: 1e-171
  
  
 NCBI BlastP on this gene

EAL90730

MFS transporter, putative
  
Accession: EAL90729
  
Location: 1487320-1489545
  
 NCBI BlastP on this gene

EAL90729

DUF292 domian protein
  
Accession: EAL90728
  
Location: 1485238-1486809
  
 NCBI BlastP on this gene

EAL90728

short-chain oxidoreductase, putative
  
Accession: EAL90727
  
Location: 1483420-1484336
  
 NCBI BlastP on this gene

EAL90727

SUN domain protein (Adg3), putative
  
Accession: EAL90726
  
Location: 1481141-1482670
  
 NCBI BlastP on this gene

EAL90726

Query: Architecture Search FASTA input

DS027688 : Neosartorya fischeri NRRL 181 1099437636249 genomic scaffold    Total score: 2.0     Cumulative Blast bit score: 882

Hit cluster cross-links:

Mycgr3G52686 Mycgr3T
  
Location: 0-861

Mycgr3G52686\_Mycgr3T

Mycgr3G102281 Mycgr3
  
Location: 961-1573

Mycgr3G102281\_Mycgr3

Mycgr3G89185 Mycgr3T
  
Location: 1673-2063

Mycgr3G89185\_Mycgr3T

Mycgr3G65725 Mycgr3T
  
Location: 2163-3612

Mycgr3G65725\_Mycgr3T

Mycgr3G102276 Mycgr3
  
Location: 3712-4801

Mycgr3G102276\_Mycgr3

Mycgr3G89189 Mycgr3T
  
Location: 4901-5564

Mycgr3G89189\_Mycgr3T

Mycgr3G52682 Mycgr3T
  
Location: 5664-9231

Mycgr3G52682\_Mycgr3T

Mycgr3G107072 Mycgr3
  
Location: 9331-13279

Mycgr3G107072\_Mycgr3

Mycgr3G34982 Mycgr3T
  
Location: 13379-15116

Mycgr3G34982\_Mycgr3T

Mycgr3G107069 Mycgr3
  
Location: 15216-17097

Mycgr3G107069\_Mycgr3

Mycgr3G32432 Mycgr3T
  
Location: 17197-19042

Mycgr3G32432\_Mycgr3T

Mycgr3G98385 Mycgr3T
  
Location: 19142-19898

Mycgr3G98385\_Mycgr3T

blue light-inducible protein Bli-3
  
Accession: EAW22455
  
Location: 1192849-1193528
  
 NCBI BlastP on this gene

EAW22455

SAGA-like transcriptional regulatory complex subunit Spt3, putative
  
Accession: EAW22456
  
Location: 1193910-1195555
  
 NCBI BlastP on this gene

EAW22456

diphthine synthase, putative
  
Accession: EAW22457
  
Location: 1195872-1196845
  
 NCBI BlastP on this gene

EAW22457

conserved hypothetical protein
  
Accession: EAW22458
  
Location: 1197378-1198046
  
 NCBI BlastP on this gene

EAW22458

clathrin coat assembly protein ap17
  
Accession: EAW22459
  
Location: 1198490-1199221
  
 NCBI BlastP on this gene

EAW22459

conserved hypothetical protein
  
Accession: EAW22460
  
Location: 1202338-1203369
  
  
**BlastP hit with Mycgr3G52686\_Mycgr3T**
  
Percentage identity: 63 %
  
BlastP bit score: 371
  
Sequence coverage: 95 %
  
E-value: 1e-125
  
  
 NCBI BlastP on this gene

EAW22460

conserved hypothetical protein
  
Accession: EAW22461
  
Location: 1205380-1207619
  
  
**BlastP hit with Mycgr3G32432\_Mycgr3T**
  
Percentage identity: 49 %
  
BlastP bit score: 511
  
Sequence coverage: 82 %
  
E-value: 2e-169
  
  
 NCBI BlastP on this gene

EAW22461

MFS transporter, putative
  
Accession: EAW22462
  
Location: 1209061-1211293
  
 NCBI BlastP on this gene

EAW22462

conserved hypothetical protein
  
Accession: EAW22463
  
Location: 1211795-1213192
  
 NCBI BlastP on this gene

EAW22463

short-chain oxidoreductase, putative
  
Accession: EAW22464
  
Location: 1214267-1215182
  
 NCBI BlastP on this gene

EAW22464

SUN domain protein (Adg3), putative
  
Accession: EAW22465
  
Location: 1215902-1217441
  
 NCBI BlastP on this gene

EAW22465

Query: Architecture Search FASTA input

DS027059 : Aspergillus clavatus NRRL 1 1099423829805 genomic scaffold    Total score: 2.0     Cumulative Blast bit score: 880

Hit cluster cross-links:

Mycgr3G52686 Mycgr3T
  
Location: 0-861

Mycgr3G52686\_Mycgr3T

Mycgr3G102281 Mycgr3
  
Location: 961-1573

Mycgr3G102281\_Mycgr3

Mycgr3G89185 Mycgr3T
  
Location: 1673-2063

Mycgr3G89185\_Mycgr3T

Mycgr3G65725 Mycgr3T
  
Location: 2163-3612

Mycgr3G65725\_Mycgr3T

Mycgr3G102276 Mycgr3
  
Location: 3712-4801

Mycgr3G102276\_Mycgr3

Mycgr3G89189 Mycgr3T
  
Location: 4901-5564

Mycgr3G89189\_Mycgr3T

Mycgr3G52682 Mycgr3T
  
Location: 5664-9231

Mycgr3G52682\_Mycgr3T

Mycgr3G107072 Mycgr3
  
Location: 9331-13279

Mycgr3G107072\_Mycgr3

Mycgr3G34982 Mycgr3T
  
Location: 13379-15116

Mycgr3G34982\_Mycgr3T

Mycgr3G107069 Mycgr3
  
Location: 15216-17097

Mycgr3G107069\_Mycgr3

Mycgr3G32432 Mycgr3T
  
Location: 17197-19042

Mycgr3G32432\_Mycgr3T

Mycgr3G98385 Mycgr3T
  
Location: 19142-19898

Mycgr3G98385\_Mycgr3T

SAGA-like transcriptional regulatory complex subunit Spt3, putative
  
Accession: EAW07430
  
Location: 994139-995886
  
 NCBI BlastP on this gene

EAW07430

diphthine synthase, putative
  
Accession: EAW07431
  
Location: 996215-997192
  
 NCBI BlastP on this gene

EAW07431

conserved hypothetical protein
  
Accession: EAW07432
  
Location: 997755-998503
  
 NCBI BlastP on this gene

EAW07432

AP-2 adaptor complex subunit sigma, putative
  
Accession: EAW07433
  
Location: 998910-999642
  
 NCBI BlastP on this gene

EAW07433

conserved hypothetical protein
  
Accession: EAW07434
  
Location: 1003408-1004436
  
  
**BlastP hit with Mycgr3G52686\_Mycgr3T**
  
Percentage identity: 63 %
  
BlastP bit score: 374
  
Sequence coverage: 95 %
  
E-value: 6e-127
  
  
 NCBI BlastP on this gene

EAW07434

conserved hypothetical protein
  
Accession: EAW07435
  
Location: 1007098-1009288
  
  
**BlastP hit with Mycgr3G32432\_Mycgr3T**
  
Percentage identity: 50 %
  
BlastP bit score: 506
  
Sequence coverage: 82 %
  
E-value: 4e-168
  
  
 NCBI BlastP on this gene

EAW07435

MFS transporter, putative
  
Accession: EAW07436
  
Location: 1011181-1013070
  
 NCBI BlastP on this gene

EAW07436

DUF292 domian protein
  
Accession: EAW07437
  
Location: 1013654-1015034
  
 NCBI BlastP on this gene

EAW07437

SUN domain protein (Adg3), putative
  
Accession: EAW07438
  
Location: 1015712-1017315
  
 NCBI BlastP on this gene

EAW07438

Leucine carboxyl methyltransferase superfamily
  
Accession: EAW07439
  
Location: 1018602-1020021
  
 NCBI BlastP on this gene

EAW07439

Query: Architecture Search FASTA input

JH226131 : Exophiala dermatitidis NIH/UT8656 unplaced genomic scaffold supercont1.2    Total score: 2.0     Cumulative Blast bit score: 878

Hit cluster cross-links:

Mycgr3G52686 Mycgr3T
  
Location: 0-861

Mycgr3G52686\_Mycgr3T

Mycgr3G102281 Mycgr3
  
Location: 961-1573

Mycgr3G102281\_Mycgr3

Mycgr3G89185 Mycgr3T
  
Location: 1673-2063

Mycgr3G89185\_Mycgr3T

Mycgr3G65725 Mycgr3T
  
Location: 2163-3612

Mycgr3G65725\_Mycgr3T

Mycgr3G102276 Mycgr3
  
Location: 3712-4801

Mycgr3G102276\_Mycgr3

Mycgr3G89189 Mycgr3T
  
Location: 4901-5564

Mycgr3G89189\_Mycgr3T

Mycgr3G52682 Mycgr3T
  
Location: 5664-9231

Mycgr3G52682\_Mycgr3T

Mycgr3G107072 Mycgr3
  
Location: 9331-13279

Mycgr3G107072\_Mycgr3

Mycgr3G34982 Mycgr3T
  
Location: 13379-15116

Mycgr3G34982\_Mycgr3T

Mycgr3G107069 Mycgr3
  
Location: 15216-17097

Mycgr3G107069\_Mycgr3

Mycgr3G32432 Mycgr3T
  
Location: 17197-19042

Mycgr3G32432\_Mycgr3T

Mycgr3G98385 Mycgr3T
  
Location: 19142-19898

Mycgr3G98385\_Mycgr3T

hypothetical protein
  
Accession: EHY53904
  
Location: 1590077-1591486
  
 NCBI BlastP on this gene

EHY53904

N-acetyltransferase (Nat5)
  
Accession: EHY53905
  
Location: 1592037-1592680
  
 NCBI BlastP on this gene

EHY53905

cytidine deaminase
  
Accession: EHY53906
  
Location: 1593373-1594098
  
 NCBI BlastP on this gene

EHY53906

hypothetical protein
  
Accession: EHY53907
  
Location: 1595248-1596165
  
 NCBI BlastP on this gene

EHY53907

valyl-tRNA synthetase
  
Accession: EHY53908
  
Location: 1597264-1598483
  
 NCBI BlastP on this gene

EHY53908

hypothetical protein
  
Accession: EHY53909
  
Location: 1600926-1601816
  
  
**BlastP hit with Mycgr3G52686\_Mycgr3T**
  
Percentage identity: 68 %
  
BlastP bit score: 413
  
Sequence coverage: 98 %
  
E-value: 7e-142
  
  
 NCBI BlastP on this gene

EHY53909

hypothetical protein
  
Accession: EHY53910
  
Location: 1603049-1605163
  
  
**BlastP hit with Mycgr3G32432\_Mycgr3T**
  
Percentage identity: 46 %
  
BlastP bit score: 466
  
Sequence coverage: 88 %
  
E-value: 1e-152
  
  
 NCBI BlastP on this gene

EHY53910

hypothetical protein
  
Accession: EHY53911
  
Location: 1605668-1606129
  
 NCBI BlastP on this gene

EHY53911

hypothetical protein
  
Accession: EHY53912
  
Location: 1607129-1608790
  
 NCBI BlastP on this gene

EHY53912

hypothetical protein
  
Accession: EHY53913
  
Location: 1609643-1610249
  
 NCBI BlastP on this gene

EHY53913

hypothetical protein
  
Accession: EHY53914
  
Location: 1610978-1612513
  
 NCBI BlastP on this gene

EHY53914

general transcription factor IIIA
  
Accession: EHY53915
  
Location: 1613899-1615626
  
 NCBI BlastP on this gene

EHY53915

Query: Architecture Search FASTA input

EQ963474 : Aspergillus flavus NRRL3357 scf\_1106286417496 genomic scaffold    Total score: 2.0     Cumulative Blast bit score: 878

Hit cluster cross-links:

Mycgr3G52686 Mycgr3T
  
Location: 0-861

Mycgr3G52686\_Mycgr3T

Mycgr3G102281 Mycgr3
  
Location: 961-1573

Mycgr3G102281\_Mycgr3

Mycgr3G89185 Mycgr3T
  
Location: 1673-2063

Mycgr3G89185\_Mycgr3T

Mycgr3G65725 Mycgr3T
  
Location: 2163-3612

Mycgr3G65725\_Mycgr3T

Mycgr3G102276 Mycgr3
  
Location: 3712-4801

Mycgr3G102276\_Mycgr3

Mycgr3G89189 Mycgr3T
  
Location: 4901-5564

Mycgr3G89189\_Mycgr3T

Mycgr3G52682 Mycgr3T
  
Location: 5664-9231

Mycgr3G52682\_Mycgr3T

Mycgr3G107072 Mycgr3
  
Location: 9331-13279

Mycgr3G107072\_Mycgr3

Mycgr3G34982 Mycgr3T
  
Location: 13379-15116

Mycgr3G34982\_Mycgr3T

Mycgr3G107069 Mycgr3
  
Location: 15216-17097

Mycgr3G107069\_Mycgr3

Mycgr3G32432 Mycgr3T
  
Location: 17197-19042

Mycgr3G32432\_Mycgr3T

Mycgr3G98385 Mycgr3T
  
Location: 19142-19898

Mycgr3G98385\_Mycgr3T

blue light-inducible protein Bli-3
  
Accession: EED54489
  
Location: 1225760-1226524
  
 NCBI BlastP on this gene

EED54489

SAGA-like transcriptional regulatory complex subunit Spt3, putative
  
Accession: EED54488
  
Location: 1223861-1225386
  
 NCBI BlastP on this gene

EED54488

diphthine synthase, putative
  
Accession: EED54487
  
Location: 1222569-1223557
  
 NCBI BlastP on this gene

EED54487

conserved hypothetical protein
  
Accession: EED54486
  
Location: 1221758-1222420
  
 NCBI BlastP on this gene

EED54486

AP-2 adaptor complex subunit sigma, putative
  
Accession: EED54485
  
Location: 1220458-1221185
  
 NCBI BlastP on this gene

EED54485

NAD dependent epimerase/dehydratase, putative
  
Accession: EED54484
  
Location: 1216447-1217421
  
  
**BlastP hit with Mycgr3G52686\_Mycgr3T**
  
Percentage identity: 64 %
  
BlastP bit score: 372
  
Sequence coverage: 95 %
  
E-value: 5e-126
  
  
 NCBI BlastP on this gene

EED54484

conserved hypothetical protein
  
Accession: EED54483
  
Location: 1211726-1213997
  
  
**BlastP hit with Mycgr3G32432\_Mycgr3T**
  
Percentage identity: 51 %
  
BlastP bit score: 506
  
Sequence coverage: 82 %
  
E-value: 2e-167
  
  
 NCBI BlastP on this gene

EED54483

MFS transporter, putative
  
Accession: EED54482
  
Location: 1208403-1210609
  
 NCBI BlastP on this gene

EED54482

DUF292 domain protein
  
Accession: EED54481
  
Location: 1206766-1208010
  
 NCBI BlastP on this gene

EED54481

conserved hypothetical protein
  
Accession: EED54480
  
Location: 1204256-1204715
  
 NCBI BlastP on this gene

EED54480

hypothetical protein
  
Accession: EED54479
  
Location: 1203361-1203556
  
 NCBI BlastP on this gene

EED54479

cytochrome P450 oxidoreductase OrdA-like, putative
  
Accession: EED54478
  
Location: 1200588-1202512
  
 NCBI BlastP on this gene

EED54478

Query: Architecture Search FASTA input

BX649607 : Aspergillus fumigatus BAC pilot project supercontig; segment 3/3.    Total score: 2.0     Cumulative Blast bit score: 871

Hit cluster cross-links:

Mycgr3G52686 Mycgr3T
  
Location: 0-861

Mycgr3G52686\_Mycgr3T

Mycgr3G102281 Mycgr3
  
Location: 961-1573

Mycgr3G102281\_Mycgr3

Mycgr3G89185 Mycgr3T
  
Location: 1673-2063

Mycgr3G89185\_Mycgr3T

Mycgr3G65725 Mycgr3T
  
Location: 2163-3612

Mycgr3G65725\_Mycgr3T

Mycgr3G102276 Mycgr3
  
Location: 3712-4801

Mycgr3G102276\_Mycgr3

Mycgr3G89189 Mycgr3T
  
Location: 4901-5564

Mycgr3G89189\_Mycgr3T

Mycgr3G52682 Mycgr3T
  
Location: 5664-9231

Mycgr3G52682\_Mycgr3T

Mycgr3G107072 Mycgr3
  
Location: 9331-13279

Mycgr3G107072\_Mycgr3

Mycgr3G34982 Mycgr3T
  
Location: 13379-15116

Mycgr3G34982\_Mycgr3T

Mycgr3G107069 Mycgr3
  
Location: 15216-17097

Mycgr3G107069\_Mycgr3

Mycgr3G32432 Mycgr3T
  
Location: 17197-19042

Mycgr3G32432\_Mycgr3T

Mycgr3G98385 Mycgr3T
  
Location: 19142-19898

Mycgr3G98385\_Mycgr3T

Bli-3 protein, putative
  
Accession: CAF32114
  
Location: 101527-102208
  
 NCBI BlastP on this gene

AfA31E11.020c

transcription factor spt3, putative
  
Accession: CAF32113
  
Location: 100110-101250
  
 NCBI BlastP on this gene

AfA31E11.015

diphthine synthase, putative
  
Accession: CAF32112
  
Location: 98213-99186
  
 NCBI BlastP on this gene

AfA31E11.010c

hypothetical protein
  
Accession: CAF32111
  
Location: 97012-97680
  
 NCBI BlastP on this gene

AfA31E11.005c

clathrin coat assembly protein, putative
  
Accession: CAF32110
  
Location: 95929-96492
  
 NCBI BlastP on this gene

AfA31E11.001c

hypothetical protein
  
Accession: CAF32109
  
Location: 94016-94870
  
 NCBI BlastP on this gene

AfA19D12.105

hypothetical protein, conserved
  
Accession: CAF32108
  
Location: 91724-92751
  
  
**BlastP hit with Mycgr3G52686\_Mycgr3T**
  
Percentage identity: 61 %
  
BlastP bit score: 358
  
Sequence coverage: 95 %
  
E-value: 9e-121
  
  
 NCBI BlastP on this gene

AfA19D12.100c

hypothetical protein
  
Accession: CAF32107
  
Location: 87773-89747
  
  
**BlastP hit with Mycgr3G32432\_Mycgr3T**
  
Percentage identity: 50 %
  
BlastP bit score: 513
  
Sequence coverage: 82 %
  
E-value: 1e-171
  
  
 NCBI BlastP on this gene

AfA19D12.095c

possible transporter-like protein
  
Accession: CAF32106
  
Location: 83855-86080
  
 NCBI BlastP on this gene

AfA19D12.090c

hypothetical protein with DUF292 domain, putative
  
Accession: CAF32105
  
Location: 81536-83344
  
 NCBI BlastP on this gene

AfA19D12.085

short-chain oxidoreductase, putative
  
Accession: CAF32104
  
Location: 79955-80871
  
 NCBI BlastP on this gene

AfA19D12.080

SUN family protein, putative
  
Accession: CAF32103
  
Location: 77676-79205
  
 NCBI BlastP on this gene

AfA19D12.075

Query: Architecture Search FASTA input

KB644408 : Penicillium oxalicum 114-2 unplaced genomic scaffold scaffold\_1    Total score: 2.0     Cumulative Blast bit score: 869

Hit cluster cross-links:

Mycgr3G52686 Mycgr3T
  
Location: 0-861

Mycgr3G52686\_Mycgr3T

Mycgr3G102281 Mycgr3
  
Location: 961-1573

Mycgr3G102281\_Mycgr3

Mycgr3G89185 Mycgr3T
  
Location: 1673-2063

Mycgr3G89185\_Mycgr3T

Mycgr3G65725 Mycgr3T
  
Location: 2163-3612

Mycgr3G65725\_Mycgr3T

Mycgr3G102276 Mycgr3
  
Location: 3712-4801

Mycgr3G102276\_Mycgr3

Mycgr3G89189 Mycgr3T
  
Location: 4901-5564

Mycgr3G89189\_Mycgr3T

Mycgr3G52682 Mycgr3T
  
Location: 5664-9231

Mycgr3G52682\_Mycgr3T

Mycgr3G107072 Mycgr3
  
Location: 9331-13279

Mycgr3G107072\_Mycgr3

Mycgr3G34982 Mycgr3T
  
Location: 13379-15116

Mycgr3G34982\_Mycgr3T

Mycgr3G107069 Mycgr3
  
Location: 15216-17097

Mycgr3G107069\_Mycgr3

Mycgr3G32432 Mycgr3T
  
Location: 17197-19042

Mycgr3G32432\_Mycgr3T

Mycgr3G98385 Mycgr3T
  
Location: 19142-19898

Mycgr3G98385\_Mycgr3T

hypothetical protein
  
Accession: EPS25319
  
Location: 677025-678409
  
 NCBI BlastP on this gene

EPS25319

hypothetical protein
  
Accession: EPS25320
  
Location: 679678-680383
  
 NCBI BlastP on this gene

EPS25320

hypothetical protein
  
Accession: EPS25321
  
Location: 681023-681767
  
 NCBI BlastP on this gene

EPS25321

hypothetical protein
  
Accession: EPS25322
  
Location: 686425-687370
  
  
**BlastP hit with Mycgr3G52686\_Mycgr3T**
  
Percentage identity: 63 %
  
BlastP bit score: 372
  
Sequence coverage: 95 %
  
E-value: 5e-126
  
  
 NCBI BlastP on this gene

EPS25322

hypothetical protein
  
Accession: EPS25323
  
Location: 692132-694516
  
  
**BlastP hit with Mycgr3G32432\_Mycgr3T**
  
Percentage identity: 50 %
  
BlastP bit score: 497
  
Sequence coverage: 79 %
  
E-value: 3e-164
  
  
 NCBI BlastP on this gene

EPS25323

hypothetical protein
  
Accession: EPS25324
  
Location: 696590-697364
  
 NCBI BlastP on this gene

EPS25324

hypothetical protein
  
Accession: EPS25325
  
Location: 698294-700585
  
 NCBI BlastP on this gene

EPS25325

hypothetical protein
  
Accession: EPS25326
  
Location: 701087-702353
  
 NCBI BlastP on this gene

EPS25326

hypothetical protein
  
Accession: EPS25327
  
Location: 702575-703620
  
 NCBI BlastP on this gene

EPS25327

Query: Architecture Search FASTA input

ACJE01000021 : Aspergillus niger ATCC 1015    Total score: 2.0     Cumulative Blast bit score: 840

Hit cluster cross-links:

Mycgr3G52686 Mycgr3T
  
Location: 0-861

Mycgr3G52686\_Mycgr3T

Mycgr3G102281 Mycgr3
  
Location: 961-1573

Mycgr3G102281\_Mycgr3

Mycgr3G89185 Mycgr3T
  
Location: 1673-2063

Mycgr3G89185\_Mycgr3T

Mycgr3G65725 Mycgr3T
  
Location: 2163-3612

Mycgr3G65725\_Mycgr3T

Mycgr3G102276 Mycgr3
  
Location: 3712-4801

Mycgr3G102276\_Mycgr3

Mycgr3G89189 Mycgr3T
  
Location: 4901-5564

Mycgr3G89189\_Mycgr3T

Mycgr3G52682 Mycgr3T
  
Location: 5664-9231

Mycgr3G52682\_Mycgr3T

Mycgr3G107072 Mycgr3
  
Location: 9331-13279

Mycgr3G107072\_Mycgr3

Mycgr3G34982 Mycgr3T
  
Location: 13379-15116

Mycgr3G34982\_Mycgr3T

Mycgr3G107069 Mycgr3
  
Location: 15216-17097

Mycgr3G107069\_Mycgr3

Mycgr3G32432 Mycgr3T
  
Location: 17197-19042

Mycgr3G32432\_Mycgr3T

Mycgr3G98385 Mycgr3T
  
Location: 19142-19898

Mycgr3G98385\_Mycgr3T

hypothetical protein
  
Accession: EHA18248
  
Location: 1722236-1722932
  
 NCBI BlastP on this gene

EHA18248

hypothetical protein
  
Accession: EHA18247
  
Location: 1720724-1721467
  
 NCBI BlastP on this gene

EHA18247

hypothetical protein
  
Accession: EHA18246
  
Location: 1714610-1715635
  
  
**BlastP hit with Mycgr3G52686\_Mycgr3T**
  
Percentage identity: 64 %
  
BlastP bit score: 370
  
Sequence coverage: 95 %
  
E-value: 4e-125
  
  
 NCBI BlastP on this gene

EHA18246

hypothetical protein
  
Accession: EHA18245
  
Location: 1705321-1711158
  
  
**BlastP hit with Mycgr3G32432\_Mycgr3T**
  
Percentage identity: 43 %
  
BlastP bit score: 470
  
Sequence coverage: 95 %
  
E-value: 8e-147
  
  
 NCBI BlastP on this gene

EHA18245

hypothetical protein
  
Accession: EHA18244
  
Location: 1703861-1705003
  
 NCBI BlastP on this gene

EHA18244

hypothetical protein
  
Accession: EHA18243
  
Location: 1701660-1702591
  
 NCBI BlastP on this gene

EHA18243

hypothetical protein
  
Accession: EHA18242
  
Location: 1699014-1700188
  
 NCBI BlastP on this gene

EHA18242

hypothetical protein
  
Accession: EHA18241
  
Location: 1693378-1698619
  
 NCBI BlastP on this gene

EHA18241

Query: Architecture Search FASTA input

AM270171 : Aspergillus niger contig An08c0160, genomic contig.    Total score: 2.0     Cumulative Blast bit score: 831

Hit cluster cross-links:

Mycgr3G52686 Mycgr3T
  
Location: 0-861

Mycgr3G52686\_Mycgr3T

Mycgr3G102281 Mycgr3
  
Location: 961-1573

Mycgr3G102281\_Mycgr3

Mycgr3G89185 Mycgr3T
  
Location: 1673-2063

Mycgr3G89185\_Mycgr3T

Mycgr3G65725 Mycgr3T
  
Location: 2163-3612

Mycgr3G65725\_Mycgr3T

Mycgr3G102276 Mycgr3
  
Location: 3712-4801

Mycgr3G102276\_Mycgr3

Mycgr3G89189 Mycgr3T
  
Location: 4901-5564

Mycgr3G89189\_Mycgr3T

Mycgr3G52682 Mycgr3T
  
Location: 5664-9231

Mycgr3G52682\_Mycgr3T

Mycgr3G107072 Mycgr3
  
Location: 9331-13279

Mycgr3G107072\_Mycgr3

Mycgr3G34982 Mycgr3T
  
Location: 13379-15116

Mycgr3G34982\_Mycgr3T

Mycgr3G107069 Mycgr3
  
Location: 15216-17097

Mycgr3G107069\_Mycgr3

Mycgr3G32432 Mycgr3T
  
Location: 17197-19042

Mycgr3G32432\_Mycgr3T

Mycgr3G98385 Mycgr3T
  
Location: 19142-19898

Mycgr3G98385\_Mycgr3T

not annotated
  
Accession: CAK39969
  
Location: 47433-48432
  
 NCBI BlastP on this gene

An08g07220

not annotated
  
Accession: CAK39968
  
Location: 46540-47236
  
 NCBI BlastP on this gene

An08g07210

not annotated
  
Accession: CAK39967
  
Location: 45028-45771
  
 NCBI BlastP on this gene

An08g07200

hypothetical protein
  
Accession: CAK39966
  
Location: 43840-44423
  
 NCBI BlastP on this gene

An08g07190

hypothetical protein
  
Accession: CAK39965
  
Location: 43023-43773
  
 NCBI BlastP on this gene

An08g07180

hypothetical protein
  
Accession: CAK39964
  
Location: 41517-42768
  
 NCBI BlastP on this gene

An08g07170

hypothetical protein
  
Accession: CAK39963
  
Location: 40404-41318
  
 NCBI BlastP on this gene

An08g07160

not annotated
  
Accession: CAK39962
  
Location: 38914-39939
  
  
**BlastP hit with Mycgr3G52686\_Mycgr3T**
  
Percentage identity: 64 %
  
BlastP bit score: 370
  
Sequence coverage: 95 %
  
E-value: 4e-125
  
  
 NCBI BlastP on this gene

An08g07150

hypothetical protein
  
Accession: CAK39961
  
Location: 38216-38566
  
 NCBI BlastP on this gene

An08g07140

hypothetical protein
  
Accession: CAK39960
  
Location: 35580-36294
  
 NCBI BlastP on this gene

An08g07130

unnamed
  
Accession: CAK39959
  
Location: 33245-35496
  
  
**BlastP hit with Mycgr3G32432\_Mycgr3T**
  
Percentage identity: 50 %
  
BlastP bit score: 462
  
Sequence coverage: 75 %
  
E-value: 3e-150
  
  
 NCBI BlastP on this gene

An08g07120

not annotated
  
Accession: CAK39958
  
Location: 29616-32985
  
 NCBI BlastP on this gene

An08g07110

not annotated
  
Accession: CAK39957
  
Location: 28342-29301
  
 NCBI BlastP on this gene

An08g07100

unnamed
  
Accession: CAK39956
  
Location: 25832-27453
  
 NCBI BlastP on this gene

An08g07090

not annotated
  
Accession: CAK39955
  
Location: 23306-24480
  
 NCBI BlastP on this gene

An08g07080

Query: Architecture Search FASTA input

AM920431 : Penicillium chrysogenum Wisconsin 54-1255 complete genome, contig Pc00c16.    Total score: 2.0     Cumulative Blast bit score: 831

Hit cluster cross-links:

Mycgr3G52686 Mycgr3T
  
Location: 0-861

Mycgr3G52686\_Mycgr3T

Mycgr3G102281 Mycgr3
  
Location: 961-1573

Mycgr3G102281\_Mycgr3

Mycgr3G89185 Mycgr3T
  
Location: 1673-2063

Mycgr3G89185\_Mycgr3T

Mycgr3G65725 Mycgr3T
  
Location: 2163-3612

Mycgr3G65725\_Mycgr3T

Mycgr3G102276 Mycgr3
  
Location: 3712-4801

Mycgr3G102276\_Mycgr3

Mycgr3G89189 Mycgr3T
  
Location: 4901-5564

Mycgr3G89189\_Mycgr3T

Mycgr3G52682 Mycgr3T
  
Location: 5664-9231

Mycgr3G52682\_Mycgr3T

Mycgr3G107072 Mycgr3
  
Location: 9331-13279

Mycgr3G107072\_Mycgr3

Mycgr3G34982 Mycgr3T
  
Location: 13379-15116

Mycgr3G34982\_Mycgr3T

Mycgr3G107069 Mycgr3
  
Location: 15216-17097

Mycgr3G107069\_Mycgr3

Mycgr3G32432 Mycgr3T
  
Location: 17197-19042

Mycgr3G32432\_Mycgr3T

Mycgr3G98385 Mycgr3T
  
Location: 19142-19898

Mycgr3G98385\_Mycgr3T

not annotated
  
Accession: CAP93913
  
Location: 3001787-3003441
  
 NCBI BlastP on this gene

Pc16g12430

not annotated
  
Accession: CAP93914
  
Location: 3003693-3004926
  
 NCBI BlastP on this gene

Pc16g12440

not annotated
  
Accession: CAP93915
  
Location: 3006066-3007271
  
 NCBI BlastP on this gene

Pc16g12450

not annotated
  
Accession: CAP93916
  
Location: 3008120-3008962
  
 NCBI BlastP on this gene

Pc16g12460

not annotated
  
Accession: CAP93917
  
Location: 3009295-3010046
  
 NCBI BlastP on this gene

Pc16g12470

not annotated
  
Accession: CAP93918
  
Location: 3012331-3013349
  
  
**BlastP hit with Mycgr3G52686\_Mycgr3T**
  
Percentage identity: 61 %
  
BlastP bit score: 370
  
Sequence coverage: 96 %
  
E-value: 2e-125
  
  
 NCBI BlastP on this gene

Pc16g12480

hypothetical protein
  
Accession: CAP93919
  
Location: 3013767-3014206
  
 NCBI BlastP on this gene

Pc16g12490

hypothetical protein
  
Accession: CAP93920
  
Location: 3014487-3015207
  
 NCBI BlastP on this gene

Pc16g12500

not annotated
  
Accession: CAP93921
  
Location: 3016159-3018178
  
  
**BlastP hit with Mycgr3G32432\_Mycgr3T**
  
Percentage identity: 51 %
  
BlastP bit score: 461
  
Sequence coverage: 75 %
  
E-value: 7e-151
  
  
 NCBI BlastP on this gene

Pc16g12510

not annotated
  
Accession: CAP93922
  
Location: 3019061-3020960
  
 NCBI BlastP on this gene

Pc16g12520

not annotated
  
Accession: CAP93923
  
Location: 3021262-3021954
  
 NCBI BlastP on this gene

Pc16g12530

not annotated
  
Accession: CAP93924
  
Location: 3023555-3025592
  
 NCBI BlastP on this gene

Pc16g12540

not annotated
  
Accession: CAP93925
  
Location: 3026127-3027138
  
 NCBI BlastP on this gene

Pc16g12550

Query: Architecture Search FASTA input

AACD01000011 : Aspergillus nidulans FGSC A4    Total score: 2.0     Cumulative Blast bit score: 797

Hit cluster cross-links:

Mycgr3G52686 Mycgr3T
  
Location: 0-861

Mycgr3G52686\_Mycgr3T

Mycgr3G102281 Mycgr3
  
Location: 961-1573

Mycgr3G102281\_Mycgr3

Mycgr3G89185 Mycgr3T
  
Location: 1673-2063

Mycgr3G89185\_Mycgr3T

Mycgr3G65725 Mycgr3T
  
Location: 2163-3612

Mycgr3G65725\_Mycgr3T

Mycgr3G102276 Mycgr3
  
Location: 3712-4801

Mycgr3G102276\_Mycgr3

Mycgr3G89189 Mycgr3T
  
Location: 4901-5564

Mycgr3G89189\_Mycgr3T

Mycgr3G52682 Mycgr3T
  
Location: 5664-9231

Mycgr3G52682\_Mycgr3T

Mycgr3G107072 Mycgr3
  
Location: 9331-13279

Mycgr3G107072\_Mycgr3

Mycgr3G34982 Mycgr3T
  
Location: 13379-15116

Mycgr3G34982\_Mycgr3T

Mycgr3G107069 Mycgr3
  
Location: 15216-17097

Mycgr3G107069\_Mycgr3

Mycgr3G32432 Mycgr3T
  
Location: 17197-19042

Mycgr3G32432\_Mycgr3T

Mycgr3G98385 Mycgr3T
  
Location: 19142-19898

Mycgr3G98385\_Mycgr3T

predicted protein
  
Accession: EAA65195
  
Location: 37621-39864
  
 NCBI BlastP on this gene

EAA65195

hypothetical protein
  
Accession: EAA65196
  
Location: 40229-41916
  
 NCBI BlastP on this gene

EAA65196

hypothetical protein
  
Accession: EAA65197
  
Location: 42190-43190
  
 NCBI BlastP on this gene

EAA65197

hypothetical protein
  
Accession: EAA65198
  
Location: 43370-44051
  
 NCBI BlastP on this gene

EAA65198

conserved hypothetical protein
  
Accession: EAA65199
  
Location: 44685-45382
  
 NCBI BlastP on this gene

EAA65199

hypothetical protein
  
Accession: EAA65200
  
Location: 48130-49104
  
  
**BlastP hit with Mycgr3G52686\_Mycgr3T**
  
Percentage identity: 60 %
  
BlastP bit score: 334
  
Sequence coverage: 95 %
  
E-value: 3e-111
  
  
 NCBI BlastP on this gene

EAA65200

hypothetical protein
  
Accession: EAA65201
  
Location: 51163-56292
  
  
**BlastP hit with Mycgr3G32432\_Mycgr3T**
  
Percentage identity: 47 %
  
BlastP bit score: 464
  
Sequence coverage: 84 %
  
E-value: 1e-144
  
  
 NCBI BlastP on this gene

EAA65201

hypothetical protein
  
Accession: EAA65202
  
Location: 56626-58016
  
 NCBI BlastP on this gene

EAA65202

hypothetical protein
  
Accession: EAA65203
  
Location: 58580-60037
  
 NCBI BlastP on this gene

EAA65203

Query: Architecture Search FASTA input

CH476594 : Aspergillus terreus NIH2624 scaffold\_1 genomic scaffold    Total score: 2.0     Cumulative Blast bit score: 766

Hit cluster cross-links:

Mycgr3G52686 Mycgr3T
  
Location: 0-861

Mycgr3G52686\_Mycgr3T

Mycgr3G102281 Mycgr3
  
Location: 961-1573

Mycgr3G102281\_Mycgr3

Mycgr3G89185 Mycgr3T
  
Location: 1673-2063

Mycgr3G89185\_Mycgr3T

Mycgr3G65725 Mycgr3T
  
Location: 2163-3612

Mycgr3G65725\_Mycgr3T

Mycgr3G102276 Mycgr3
  
Location: 3712-4801

Mycgr3G102276\_Mycgr3

Mycgr3G89189 Mycgr3T
  
Location: 4901-5564

Mycgr3G89189\_Mycgr3T

Mycgr3G52682 Mycgr3T
  
Location: 5664-9231

Mycgr3G52682\_Mycgr3T

Mycgr3G107072 Mycgr3
  
Location: 9331-13279

Mycgr3G107072\_Mycgr3

Mycgr3G34982 Mycgr3T
  
Location: 13379-15116

Mycgr3G34982\_Mycgr3T

Mycgr3G107069 Mycgr3
  
Location: 15216-17097

Mycgr3G107069\_Mycgr3

Mycgr3G32432 Mycgr3T
  
Location: 17197-19042

Mycgr3G32432\_Mycgr3T

Mycgr3G98385 Mycgr3T
  
Location: 19142-19898

Mycgr3G98385\_Mycgr3T

protein spt3
  
Accession: EAU39249
  
Location: 1703089-1704288
  
 NCBI BlastP on this gene

EAU39249

diphthine synthase
  
Accession: EAU39248
  
Location: 1701698-1702576
  
 NCBI BlastP on this gene

EAU39248

conserved hypothetical protein
  
Accession: EAU39247
  
Location: 1700730-1701396
  
 NCBI BlastP on this gene

EAU39247

AP-2 complex subunit sigma
  
Accession: EAU39246
  
Location: 1699460-1700178
  
 NCBI BlastP on this gene

EAU39246

conserved hypothetical protein
  
Accession: EAU39245
  
Location: 1695023-1696063
  
  
**BlastP hit with Mycgr3G52686\_Mycgr3T**
  
Percentage identity: 64 %
  
BlastP bit score: 376
  
Sequence coverage: 95 %
  
E-value: 2e-127
  
  
 NCBI BlastP on this gene

EAU39245

conserved hypothetical protein
  
Accession: EAU39244
  
Location: 1687460-1692182
  
  
**BlastP hit with Mycgr3G32432\_Mycgr3T**
  
Percentage identity: 48 %
  
BlastP bit score: 390
  
Sequence coverage: 67 %
  
E-value: 3e-118
  
  
 NCBI BlastP on this gene

EAU39244

conserved hypothetical protein
  
Accession: EAU39243
  
Location: 1686159-1686870
  
 NCBI BlastP on this gene

EAU39243

conserved hypothetical protein
  
Accession: EAU39242
  
Location: 1684136-1685596
  
 NCBI BlastP on this gene

EAU39242

conserved hypothetical protein
  
Accession: EAU39241
  
Location: 1676743-1683104
  
 NCBI BlastP on this gene

EAU39241

Query: Architecture Search FASTA input

AHHD01000271 : Macrophomina phaseolina MS6    Total score: 2.0     Cumulative Blast bit score: 734

Hit cluster cross-links:

Mycgr3G52686 Mycgr3T
  
Location: 0-861

Mycgr3G52686\_Mycgr3T

Mycgr3G102281 Mycgr3
  
Location: 961-1573

Mycgr3G102281\_Mycgr3

Mycgr3G89185 Mycgr3T
  
Location: 1673-2063

Mycgr3G89185\_Mycgr3T

Mycgr3G65725 Mycgr3T
  
Location: 2163-3612

Mycgr3G65725\_Mycgr3T

Mycgr3G102276 Mycgr3
  
Location: 3712-4801

Mycgr3G102276\_Mycgr3

Mycgr3G89189 Mycgr3T
  
Location: 4901-5564

Mycgr3G89189\_Mycgr3T

Mycgr3G52682 Mycgr3T
  
Location: 5664-9231

Mycgr3G52682\_Mycgr3T

Mycgr3G107072 Mycgr3
  
Location: 9331-13279

Mycgr3G107072\_Mycgr3

Mycgr3G34982 Mycgr3T
  
Location: 13379-15116

Mycgr3G34982\_Mycgr3T

Mycgr3G107069 Mycgr3
  
Location: 15216-17097

Mycgr3G107069\_Mycgr3

Mycgr3G32432 Mycgr3T
  
Location: 17197-19042

Mycgr3G32432\_Mycgr3T

Mycgr3G98385 Mycgr3T
  
Location: 19142-19898

Mycgr3G98385\_Mycgr3T

hypothetical protein
  
Accession: EKG16380
  
Location: 24272-25749
  
 NCBI BlastP on this gene

EKG16380

NmrA-like protein
  
Accession: EKG16379
  
Location: 21930-22625
  
 NCBI BlastP on this gene

EKG16379

Alcohol dehydrogenase superfamily zinc-containing
  
Accession: EKG16378
  
Location: 16979-18055
  
  
**BlastP hit with Mycgr3G102276\_Mycgr3**
  
Percentage identity: 63 %
  
BlastP bit score: 442
  
Sequence coverage: 99 %
  
E-value: 2e-151
  
  
 NCBI BlastP on this gene

EKG16378

hypothetical protein
  
Accession: EKG16377
  
Location: 13637-14186
  
 NCBI BlastP on this gene

EKG16377

Pyoverdine biosynthesis
  
Accession: EKG16376
  
Location: 11193-11875
  
 NCBI BlastP on this gene

EKG16376

Pyoverdine biosynthesis
  
Accession: EKG16375
  
Location: 9867-11067
  
 NCBI BlastP on this gene

EKG16375

Major facilitator superfamily
  
Accession: EKG16374
  
Location: 7404-8776
  
  
**BlastP hit with Mycgr3G65725\_Mycgr3T**
  
Percentage identity: 37 %
  
BlastP bit score: 292
  
Sequence coverage: 84 %
  
E-value: 5e-90
  
  
 NCBI BlastP on this gene

EKG16374

hypothetical protein
  
Accession: EKG16373
  
Location: 3703-4524
  
 NCBI BlastP on this gene

EKG16373

Query: Architecture Search FASTA input

EQ963485 : Aspergillus flavus NRRL3357 scf\_1106286419448 genomic scaffold    Total score: 2.0     Cumulative Blast bit score: 700

Hit cluster cross-links:

Mycgr3G52686 Mycgr3T
  
Location: 0-861

Mycgr3G52686\_Mycgr3T

Mycgr3G102281 Mycgr3
  
Location: 961-1573

Mycgr3G102281\_Mycgr3

Mycgr3G89185 Mycgr3T
  
Location: 1673-2063

Mycgr3G89185\_Mycgr3T

Mycgr3G65725 Mycgr3T
  
Location: 2163-3612

Mycgr3G65725\_Mycgr3T

Mycgr3G102276 Mycgr3
  
Location: 3712-4801

Mycgr3G102276\_Mycgr3

Mycgr3G89189 Mycgr3T
  
Location: 4901-5564

Mycgr3G89189\_Mycgr3T

Mycgr3G52682 Mycgr3T
  
Location: 5664-9231

Mycgr3G52682\_Mycgr3T

Mycgr3G107072 Mycgr3
  
Location: 9331-13279

Mycgr3G107072\_Mycgr3

Mycgr3G34982 Mycgr3T
  
Location: 13379-15116

Mycgr3G34982\_Mycgr3T

Mycgr3G107069 Mycgr3
  
Location: 15216-17097

Mycgr3G107069\_Mycgr3

Mycgr3G32432 Mycgr3T
  
Location: 17197-19042

Mycgr3G32432\_Mycgr3T

Mycgr3G98385 Mycgr3T
  
Location: 19142-19898

Mycgr3G98385\_Mycgr3T

conserved hypothetical protein
  
Accession: EED45922
  
Location: 1476852-1477769
  
 NCBI BlastP on this gene

EED45922

NRPS-like enzyme, putative
  
Accession: EED45923
  
Location: 1479298-1482390
  
  
**BlastP hit with Mycgr3G107072\_Mycgr3**
  
Percentage identity: 27 %
  
BlastP bit score: 301
  
Sequence coverage: 77 %
  
E-value: 4e-82
  
  
 NCBI BlastP on this gene

EED45923

NADH-dependent flavin oxidoreductase, putative
  
Accession: EED45924
  
Location: 1483117-1484250
  
 NCBI BlastP on this gene

EED45924

MFS multidrug transporter, putative
  
Accession: EED45925
  
Location: 1485551-1487055
  
 NCBI BlastP on this gene

EED45925

conserved hypothetical protein
  
Accession: EED45926
  
Location: 1491278-1492114
  
 NCBI BlastP on this gene

EED45926

conserved hypothetical protein
  
Accession: EED45927
  
Location: 1492324-1493409
  
 NCBI BlastP on this gene

EED45927

conserved hypothetical protein
  
Accession: EED45928
  
Location: 1495959-1496569
  
 NCBI BlastP on this gene

EED45928

C-4 methyl sterol oxidase, putative
  
Accession: EED45929
  
Location: 1496865-1497827
  
 NCBI BlastP on this gene

EED45929

zinc-binding alcohol dehydrogenase, putative
  
Accession: EED45930
  
Location: 1500353-1501492
  
  
**BlastP hit with Mycgr3G102276\_Mycgr3**
  
Percentage identity: 57 %
  
BlastP bit score: 399
  
Sequence coverage: 99 %
  
E-value: 3e-134
  
  
 NCBI BlastP on this gene

EED45930

conserved hypothetical protein
  
Accession: EED45931
  
Location: 1501551-1502374
  
 NCBI BlastP on this gene

EED45931

cytochrome P450, putative
  
Accession: EED45932
  
Location: 1502766-1504429
  
 NCBI BlastP on this gene

EED45932

Query: Architecture Search FASTA input

GG697417 : Glomerella graminicola M1.001 genomic scaffold supercont1.87    Total score: 2.0     Cumulative Blast bit score: 648

Hit cluster cross-links:

Mycgr3G52686 Mycgr3T
  
Location: 0-861

Mycgr3G52686\_Mycgr3T

Mycgr3G102281 Mycgr3
  
Location: 961-1573

Mycgr3G102281\_Mycgr3

Mycgr3G89185 Mycgr3T
  
Location: 1673-2063

Mycgr3G89185\_Mycgr3T

Mycgr3G65725 Mycgr3T
  
Location: 2163-3612

Mycgr3G65725\_Mycgr3T

Mycgr3G102276 Mycgr3
  
Location: 3712-4801

Mycgr3G102276\_Mycgr3

Mycgr3G89189 Mycgr3T
  
Location: 4901-5564

Mycgr3G89189\_Mycgr3T

Mycgr3G52682 Mycgr3T
  
Location: 5664-9231

Mycgr3G52682\_Mycgr3T

Mycgr3G107072 Mycgr3
  
Location: 9331-13279

Mycgr3G107072\_Mycgr3

Mycgr3G34982 Mycgr3T
  
Location: 13379-15116

Mycgr3G34982\_Mycgr3T

Mycgr3G107069 Mycgr3
  
Location: 15216-17097

Mycgr3G107069\_Mycgr3

Mycgr3G32432 Mycgr3T
  
Location: 17197-19042

Mycgr3G32432\_Mycgr3T

Mycgr3G98385 Mycgr3T
  
Location: 19142-19898

Mycgr3G98385\_Mycgr3T

hypothetical protein
  
Accession: EFQ36299
  
Location: 83651-85458
  
  
**BlastP hit with Mycgr3G32432\_Mycgr3T**
  
Percentage identity: 38 %
  
BlastP bit score: 326
  
Sequence coverage: 80 %
  
E-value: 3e-100
  
  
 NCBI BlastP on this gene

EFQ36299

hypothetical protein
  
Accession: EFQ36298
  
Location: 82673-83130
  
 NCBI BlastP on this gene

EFQ36298

hypothetical protein
  
Accession: EFQ36297
  
Location: 74594-75450
  
  
**BlastP hit with Mycgr3G52686\_Mycgr3T**
  
Percentage identity: 61 %
  
BlastP bit score: 322
  
Sequence coverage: 85 %
  
E-value: 8e-107
  
  
 NCBI BlastP on this gene

EFQ36297

hypothetical protein
  
Accession: EFQ36296
  
Location: 73093-73794
  
 NCBI BlastP on this gene

EFQ36296

hypothetical protein
  
Accession: EFQ36295
  
Location: 68758-69467
  
 NCBI BlastP on this gene

EFQ36295

Query: Architecture Search FASTA input

KB726028 : Colletotrichum orbiculare MAFF 240422 unplaced genomic scaffold Scaffold\_454    Total score: 2.0     Cumulative Blast bit score: 615

Hit cluster cross-links:

Mycgr3G52686 Mycgr3T
  
Location: 0-861

Mycgr3G52686\_Mycgr3T

Mycgr3G102281 Mycgr3
  
Location: 961-1573

Mycgr3G102281\_Mycgr3

Mycgr3G89185 Mycgr3T
  
Location: 1673-2063

Mycgr3G89185\_Mycgr3T

Mycgr3G65725 Mycgr3T
  
Location: 2163-3612

Mycgr3G65725\_Mycgr3T

Mycgr3G102276 Mycgr3
  
Location: 3712-4801

Mycgr3G102276\_Mycgr3

Mycgr3G89189 Mycgr3T
  
Location: 4901-5564

Mycgr3G89189\_Mycgr3T

Mycgr3G52682 Mycgr3T
  
Location: 5664-9231

Mycgr3G52682\_Mycgr3T

Mycgr3G107072 Mycgr3
  
Location: 9331-13279

Mycgr3G107072\_Mycgr3

Mycgr3G34982 Mycgr3T
  
Location: 13379-15116

Mycgr3G34982\_Mycgr3T

Mycgr3G107069 Mycgr3
  
Location: 15216-17097

Mycgr3G107069\_Mycgr3

Mycgr3G32432 Mycgr3T
  
Location: 17197-19042

Mycgr3G32432\_Mycgr3T

Mycgr3G98385 Mycgr3T
  
Location: 19142-19898

Mycgr3G98385\_Mycgr3T

ankyrin repeat protein
  
Accession: ENH79132
  
Location: 350291-351484
  
 NCBI BlastP on this gene

ENH79132

hypothetical protein
  
Accession: ENH79133
  
Location: 352068-353891
  
  
**BlastP hit with Mycgr3G32432\_Mycgr3T**
  
Percentage identity: 43 %
  
BlastP bit score: 366
  
Sequence coverage: 80 %
  
E-value: 3e-115
  
  
 NCBI BlastP on this gene

ENH79133

gram-positive signal ysirk family
  
Accession: ENH79134
  
Location: 354256-354718
  
 NCBI BlastP on this gene

ENH79134

nad dependent epimerase
  
Accession: ENH79135
  
Location: 355525-358086
  
  
**BlastP hit with Mycgr3G52686\_Mycgr3T**
  
Percentage identity: 57 %
  
BlastP bit score: 249
  
Sequence coverage: 70 %
  
E-value: 8e-74
  
  
 NCBI BlastP on this gene

ENH79135

Query: Architecture Search FASTA input

CH476597 : Aspergillus terreus NIH2624 scaffold\_4 genomic scaffold    Total score: 2.0     Cumulative Blast bit score: 615

Hit cluster cross-links:

Mycgr3G52686 Mycgr3T
  
Location: 0-861

Mycgr3G52686\_Mycgr3T

Mycgr3G102281 Mycgr3
  
Location: 961-1573

Mycgr3G102281\_Mycgr3

Mycgr3G89185 Mycgr3T
  
Location: 1673-2063

Mycgr3G89185\_Mycgr3T

Mycgr3G65725 Mycgr3T
  
Location: 2163-3612

Mycgr3G65725\_Mycgr3T

Mycgr3G102276 Mycgr3
  
Location: 3712-4801

Mycgr3G102276\_Mycgr3

Mycgr3G89189 Mycgr3T
  
Location: 4901-5564

Mycgr3G89189\_Mycgr3T

Mycgr3G52682 Mycgr3T
  
Location: 5664-9231

Mycgr3G52682\_Mycgr3T

Mycgr3G107072 Mycgr3
  
Location: 9331-13279

Mycgr3G107072\_Mycgr3

Mycgr3G34982 Mycgr3T
  
Location: 13379-15116

Mycgr3G34982\_Mycgr3T

Mycgr3G107069 Mycgr3
  
Location: 15216-17097

Mycgr3G107069\_Mycgr3

Mycgr3G32432 Mycgr3T
  
Location: 17197-19042

Mycgr3G32432\_Mycgr3T

Mycgr3G98385 Mycgr3T
  
Location: 19142-19898

Mycgr3G98385\_Mycgr3T

hypothetical protein
  
Accession: EAU36720
  
Location: 1835763-1843945
  
 NCBI BlastP on this gene

EAU36720

predicted protein
  
Accession: EAU36721
  
Location: 1844712-1845911
  
 NCBI BlastP on this gene

EAU36721

conserved hypothetical protein
  
Accession: EAU36722
  
Location: 1847139-1847581
  
 NCBI BlastP on this gene

EAU36722

conserved hypothetical protein
  
Accession: EAU36723
  
Location: 1848099-1849330
  
 NCBI BlastP on this gene

EAU36723

predicted protein
  
Accession: EAU36724
  
Location: 1849784-1851077
  
 NCBI BlastP on this gene

EAU36724

conserved hypothetical protein
  
Accession: EAU36725
  
Location: 1851718-1852477
  
 NCBI BlastP on this gene

EAU36725

conserved hypothetical protein
  
Accession: EAU36726
  
Location: 1852607-1853534
  
  
**BlastP hit with Mycgr3G52686\_Mycgr3T**
  
Percentage identity: 33 %
  
BlastP bit score: 123
  
Sequence coverage: 93 %
  
E-value: 1e-29
  
  
 NCBI BlastP on this gene

EAU36726

predicted protein
  
Accession: EAU36727
  
Location: 1854395-1855967
  
 NCBI BlastP on this gene

EAU36727

conserved hypothetical protein
  
Accession: EAU36728
  
Location: 1856614-1858599
  
  
**BlastP hit with Mycgr3G107069\_Mycgr3**
  
Percentage identity: 43 %
  
BlastP bit score: 492
  
Sequence coverage: 100 %
  
E-value: 3e-162
  
  
 NCBI BlastP on this gene

EAU36728

conserved hypothetical protein
  
Accession: EAU36729
  
Location: 1859547-1860491
  
 NCBI BlastP on this gene

EAU36729

predicted protein
  
Accession: EAU36730
  
Location: 1860895-1863542
  
 NCBI BlastP on this gene

EAU36730

predicted protein
  
Accession: EAU36731
  
Location: 1863869-1865716
  
 NCBI BlastP on this gene

EAU36731

Query: Architecture Search FASTA input

AP007174 : Aspergillus oryzae RIB40 DNA, SC103.    Total score: 2.0     Cumulative Blast bit score: 613

Hit cluster cross-links:

Mycgr3G52686 Mycgr3T
  
Location: 0-861

Mycgr3G52686\_Mycgr3T

Mycgr3G102281 Mycgr3
  
Location: 961-1573

Mycgr3G102281\_Mycgr3

Mycgr3G89185 Mycgr3T
  
Location: 1673-2063

Mycgr3G89185\_Mycgr3T

Mycgr3G65725 Mycgr3T
  
Location: 2163-3612

Mycgr3G65725\_Mycgr3T

Mycgr3G102276 Mycgr3
  
Location: 3712-4801

Mycgr3G102276\_Mycgr3

Mycgr3G89189 Mycgr3T
  
Location: 4901-5564

Mycgr3G89189\_Mycgr3T

Mycgr3G52682 Mycgr3T
  
Location: 5664-9231

Mycgr3G52682\_Mycgr3T

Mycgr3G107072 Mycgr3
  
Location: 9331-13279

Mycgr3G107072\_Mycgr3

Mycgr3G34982 Mycgr3T
  
Location: 13379-15116

Mycgr3G34982\_Mycgr3T

Mycgr3G107069 Mycgr3
  
Location: 15216-17097

Mycgr3G107069\_Mycgr3

Mycgr3G32432 Mycgr3T
  
Location: 17197-19042

Mycgr3G32432\_Mycgr3T

Mycgr3G98385 Mycgr3T
  
Location: 19142-19898

Mycgr3G98385\_Mycgr3T

not annotated
  
Accession: BAE65798
  
Location: 952615-953968
  
 NCBI BlastP on this gene

AO090103000363

not annotated
  
Accession: BAE65799
  
Location: 954644-955834
  
 NCBI BlastP on this gene

AO090103000364

not annotated
  
Accession: BAE65800
  
Location: 957630-958541
  
 NCBI BlastP on this gene

AO090103000365

not annotated
  
Accession: BAE65801
  
Location: 958936-961522
  
 NCBI BlastP on this gene

AO090103000366

not annotated
  
Accession: BAE65802
  
Location: 962403-964382
  
  
**BlastP hit with Mycgr3G107069\_Mycgr3**
  
Percentage identity: 41 %
  
BlastP bit score: 494
  
Sequence coverage: 104 %
  
E-value: 4e-163
  
  
 NCBI BlastP on this gene

AO090103000367

not annotated
  
Accession: BAE65803
  
Location: 965455-966704
  
 NCBI BlastP on this gene

AO090103000369

not annotated
  
Accession: BAE65804
  
Location: 966912-967949
  
 NCBI BlastP on this gene

AO090103000370

not annotated
  
Accession: BAE65805
  
Location: 968818-969783
  
  
**BlastP hit with Mycgr3G52686\_Mycgr3T**
  
Percentage identity: 31 %
  
BlastP bit score: 119
  
Sequence coverage: 93 %
  
E-value: 4e-28
  
  
 NCBI BlastP on this gene

AO090103000371

not annotated
  
Accession: BAE65806
  
Location: 970247-971113
  
 NCBI BlastP on this gene

AO090103000372

not annotated
  
Accession: BAE65807
  
Location: 975581-976603
  
 NCBI BlastP on this gene

AO090103000374

not annotated
  
Accession: BAE65808
  
Location: 977041-977674
  
 NCBI BlastP on this gene

AO090103000375

Query: Architecture Search FASTA input

AKHY01000199 : Aspergillus oryzae 3.042    Total score: 2.0     Cumulative Blast bit score: 609

Hit cluster cross-links:

Mycgr3G52686 Mycgr3T
  
Location: 0-861

Mycgr3G52686\_Mycgr3T

Mycgr3G102281 Mycgr3
  
Location: 961-1573

Mycgr3G102281\_Mycgr3

Mycgr3G89185 Mycgr3T
  
Location: 1673-2063

Mycgr3G89185\_Mycgr3T

Mycgr3G65725 Mycgr3T
  
Location: 2163-3612

Mycgr3G65725\_Mycgr3T

Mycgr3G102276 Mycgr3
  
Location: 3712-4801

Mycgr3G102276\_Mycgr3

Mycgr3G89189 Mycgr3T
  
Location: 4901-5564

Mycgr3G89189\_Mycgr3T

Mycgr3G52682 Mycgr3T
  
Location: 5664-9231

Mycgr3G52682\_Mycgr3T

Mycgr3G107072 Mycgr3
  
Location: 9331-13279

Mycgr3G107072\_Mycgr3

Mycgr3G34982 Mycgr3T
  
Location: 13379-15116

Mycgr3G34982\_Mycgr3T

Mycgr3G107069 Mycgr3
  
Location: 15216-17097

Mycgr3G107069\_Mycgr3

Mycgr3G32432 Mycgr3T
  
Location: 17197-19042

Mycgr3G32432\_Mycgr3T

Mycgr3G98385 Mycgr3T
  
Location: 19142-19898

Mycgr3G98385\_Mycgr3T

hypothetical protein
  
Accession: EIT73701
  
Location: 24760-26113
  
 NCBI BlastP on this gene

EIT73701

hypothetical protein
  
Accession: EIT73608
  
Location: 22895-24084
  
 NCBI BlastP on this gene

EIT73608

hypothetical protein
  
Accession: EIT73545
  
Location: 20188-21099
  
 NCBI BlastP on this gene

EIT73545

dehydrogenase with different specificitie
  
Accession: EIT73825
  
Location: 17219-19793
  
 NCBI BlastP on this gene

EIT73825

ferric reductase, NADH/NADPH oxidase
  
Accession: EIT73566
  
Location: 14359-16338
  
  
**BlastP hit with Mycgr3G107069\_Mycgr3**
  
Percentage identity: 41 %
  
BlastP bit score: 488
  
Sequence coverage: 104 %
  
E-value: 1e-160
  
  
 NCBI BlastP on this gene

EIT73566

hypothetical protein
  
Accession: EIT73711
  
Location: 12042-13291
  
 NCBI BlastP on this gene

EIT73711

proline racemase
  
Accession: EIT73709
  
Location: 10797-11834
  
 NCBI BlastP on this gene

EIT73709

hypothetical protein
  
Accession: EIT73723
  
Location: 8963-9928
  
  
**BlastP hit with Mycgr3G52686\_Mycgr3T**
  
Percentage identity: 31 %
  
BlastP bit score: 121
  
Sequence coverage: 93 %
  
E-value: 1e-28
  
  
 NCBI BlastP on this gene

EIT73723

hypothetical protein
  
Accession: EIT73531
  
Location: 7633-8247
  
 NCBI BlastP on this gene

EIT73531

Query: Architecture Search FASTA input

DS027060 : Aspergillus clavatus NRRL 1 1099423829806 genomic scaffold    Total score: 2.0     Cumulative Blast bit score: 605

Hit cluster cross-links:

Mycgr3G52686 Mycgr3T
  
Location: 0-861

Mycgr3G52686\_Mycgr3T

Mycgr3G102281 Mycgr3
  
Location: 961-1573

Mycgr3G102281\_Mycgr3

Mycgr3G89185 Mycgr3T
  
Location: 1673-2063

Mycgr3G89185\_Mycgr3T

Mycgr3G65725 Mycgr3T
  
Location: 2163-3612

Mycgr3G65725\_Mycgr3T

Mycgr3G102276 Mycgr3
  
Location: 3712-4801

Mycgr3G102276\_Mycgr3

Mycgr3G89189 Mycgr3T
  
Location: 4901-5564

Mycgr3G89189\_Mycgr3T

Mycgr3G52682 Mycgr3T
  
Location: 5664-9231

Mycgr3G52682\_Mycgr3T

Mycgr3G107072 Mycgr3
  
Location: 9331-13279

Mycgr3G107072\_Mycgr3

Mycgr3G34982 Mycgr3T
  
Location: 13379-15116

Mycgr3G34982\_Mycgr3T

Mycgr3G107069 Mycgr3
  
Location: 15216-17097

Mycgr3G107069\_Mycgr3

Mycgr3G32432 Mycgr3T
  
Location: 17197-19042

Mycgr3G32432\_Mycgr3T

Mycgr3G98385 Mycgr3T
  
Location: 19142-19898

Mycgr3G98385\_Mycgr3T

conserved hypothetical protein
  
Accession: EAW07011
  
Location: 2302787-2303782
  
 NCBI BlastP on this gene

EAW07011

conserved hypothetical protein
  
Accession: EAW07010
  
Location: 2301086-2301589
  
 NCBI BlastP on this gene

EAW07010

conserved hypothetical protein
  
Accession: EAW07009
  
Location: 2298771-2299792
  
  
**BlastP hit with Mycgr3G52686\_Mycgr3T**
  
Percentage identity: 34 %
  
BlastP bit score: 120
  
Sequence coverage: 92 %
  
E-value: 3e-28
  
  
 NCBI BlastP on this gene

EAW07009

ferric-chelate reductase, putative
  
Accession: EAW07008
  
Location: 2296265-2298169
  
  
**BlastP hit with Mycgr3G107069\_Mycgr3**
  
Percentage identity: 41 %
  
BlastP bit score: 485
  
Sequence coverage: 98 %
  
E-value: 5e-160
  
  
 NCBI BlastP on this gene

EAW07008

alpha-1,2-mannosidase, putative subfamily
  
Accession: EAW07007
  
Location: 2293312-2295749
  
 NCBI BlastP on this gene

EAW07007

PHD finger domain protein
  
Accession: EAW07006
  
Location: 2292223-2293232
  
 NCBI BlastP on this gene

EAW07006

C6 finger domain protein, putative
  
Accession: EAW07005
  
Location: 2289332-2291734
  
 NCBI BlastP on this gene

EAW07005

MFS multidrug transporter, putative
  
Accession: EAW07004
  
Location: 2286351-2288214
  
 NCBI BlastP on this gene

EAW07004

Query: Architecture Search FASTA input

DS499594 : Aspergillus fumigatus A1163 scf\_000001 genomic scaffold    Total score: 2.0     Cumulative Blast bit score: 594

Hit cluster cross-links:

Mycgr3G52686 Mycgr3T
  
Location: 0-861

Mycgr3G52686\_Mycgr3T

Mycgr3G102281 Mycgr3
  
Location: 961-1573

Mycgr3G102281\_Mycgr3

Mycgr3G89185 Mycgr3T
  
Location: 1673-2063

Mycgr3G89185\_Mycgr3T

Mycgr3G65725 Mycgr3T
  
Location: 2163-3612

Mycgr3G65725\_Mycgr3T

Mycgr3G102276 Mycgr3
  
Location: 3712-4801

Mycgr3G102276\_Mycgr3

Mycgr3G89189 Mycgr3T
  
Location: 4901-5564

Mycgr3G89189\_Mycgr3T

Mycgr3G52682 Mycgr3T
  
Location: 5664-9231

Mycgr3G52682\_Mycgr3T

Mycgr3G107072 Mycgr3
  
Location: 9331-13279

Mycgr3G107072\_Mycgr3

Mycgr3G34982 Mycgr3T
  
Location: 13379-15116

Mycgr3G34982\_Mycgr3T

Mycgr3G107069 Mycgr3
  
Location: 15216-17097

Mycgr3G107069\_Mycgr3

Mycgr3G32432 Mycgr3T
  
Location: 17197-19042

Mycgr3G32432\_Mycgr3T

Mycgr3G98385 Mycgr3T
  
Location: 19142-19898

Mycgr3G98385\_Mycgr3T

hypothetical protein
  
Accession: EDP55412
  
Location: 267538-269914
  
 NCBI BlastP on this gene

EDP55412

class V chitinase, putative
  
Accession: EDP55411
  
Location: 262383-266843
  
 NCBI BlastP on this gene

EDP55411

hypothetical protein
  
Accession: EDP55410
  
Location: 260465-260632
  
 NCBI BlastP on this gene

EDP55410

conserved hypothetical protein
  
Accession: EDP55409
  
Location: 257484-258438
  
  
**BlastP hit with Mycgr3G52686\_Mycgr3T**
  
Percentage identity: 36 %
  
BlastP bit score: 128
  
Sequence coverage: 94 %
  
E-value: 4e-31
  
  
 NCBI BlastP on this gene

EDP55409

ferric-chelate reductase, putative
  
Accession: EDP55408
  
Location: 255084-257042
  
  
**BlastP hit with Mycgr3G107069\_Mycgr3**
  
Percentage identity: 39 %
  
BlastP bit score: 466
  
Sequence coverage: 100 %
  
E-value: 2e-152
  
  
 NCBI BlastP on this gene

EDP55408

alpha-1,2-mannosidase, putative subfamily
  
Accession: EDP55407
  
Location: 252516-254960
  
 NCBI BlastP on this gene

EDP55407

PHD finger protein
  
Accession: EDP55406
  
Location: 250552-251515
  
 NCBI BlastP on this gene

EDP55406

C6 finger domain protein, putative
  
Accession: EDP55405
  
Location: 247785-250137
  
 NCBI BlastP on this gene

EDP55405

MFS multidrug transporter, putative
  
Accession: EDP55404
  
Location: 245078-246960
  
 NCBI BlastP on this gene

EDP55404

Query: Architecture Search FASTA input

AAHF01000006 : Aspergillus fumigatus Af293    Total score: 2.0     Cumulative Blast bit score: 590

Hit cluster cross-links:

Mycgr3G52686 Mycgr3T
  
Location: 0-861

Mycgr3G52686\_Mycgr3T

Mycgr3G102281 Mycgr3
  
Location: 961-1573

Mycgr3G102281\_Mycgr3

Mycgr3G89185 Mycgr3T
  
Location: 1673-2063

Mycgr3G89185\_Mycgr3T

Mycgr3G65725 Mycgr3T
  
Location: 2163-3612

Mycgr3G65725\_Mycgr3T

Mycgr3G102276 Mycgr3
  
Location: 3712-4801

Mycgr3G102276\_Mycgr3

Mycgr3G89189 Mycgr3T
  
Location: 4901-5564

Mycgr3G89189\_Mycgr3T

Mycgr3G52682 Mycgr3T
  
Location: 5664-9231

Mycgr3G52682\_Mycgr3T

Mycgr3G107072 Mycgr3
  
Location: 9331-13279

Mycgr3G107072\_Mycgr3

Mycgr3G34982 Mycgr3T
  
Location: 13379-15116

Mycgr3G34982\_Mycgr3T

Mycgr3G107069 Mycgr3
  
Location: 15216-17097

Mycgr3G107069\_Mycgr3

Mycgr3G32432 Mycgr3T
  
Location: 17197-19042

Mycgr3G32432\_Mycgr3T

Mycgr3G98385 Mycgr3T
  
Location: 19142-19898

Mycgr3G98385\_Mycgr3T

hypothetical protein
  
Accession: EAL89209
  
Location: 2180080-2182456
  
 NCBI BlastP on this gene

EAL89209

class V chitinase, putative
  
Accession: EAL89210
  
Location: 2183151-2187611
  
 NCBI BlastP on this gene

EAL89210

hypothetical protein
  
Accession: EAL89211
  
Location: 2189362-2189529
  
 NCBI BlastP on this gene

EAL89211

conserved hypothetical protein
  
Accession: EAL89212
  
Location: 2191556-2192510
  
  
**BlastP hit with Mycgr3G52686\_Mycgr3T**
  
Percentage identity: 36 %
  
BlastP bit score: 128
  
Sequence coverage: 94 %
  
E-value: 4e-31
  
  
 NCBI BlastP on this gene

EAL89212

ferric-chelate reductase, putative
  
Accession: EAL89213
  
Location: 2192952-2194910
  
  
**BlastP hit with Mycgr3G107069\_Mycgr3**
  
Percentage identity: 39 %
  
BlastP bit score: 462
  
Sequence coverage: 100 %
  
E-value: 9e-151
  
  
 NCBI BlastP on this gene

EAL89213

alpha-1,2-mannosidase, putative subfamily
  
Accession: EAL89214
  
Location: 2195034-2197478
  
 NCBI BlastP on this gene

EAL89214

PHD finger protein
  
Accession: EBA27370
  
Location: 2198479-2199442
  
 NCBI BlastP on this gene

EBA27370

C6 finger domain protein, putative
  
Accession: EAL89215
  
Location: 2199857-2202209
  
 NCBI BlastP on this gene

EAL89215

MFS multidrug transporter, putative
  
Accession: EAL89216
  
Location: 2203034-2204916
  
 NCBI BlastP on this gene

EAL89216

Query: Architecture Search FASTA input

DS027698 : Neosartorya fischeri NRRL 181 1099437636266 genomic scaffold    Total score: 2.0     Cumulative Blast bit score: 589

Hit cluster cross-links:

Mycgr3G52686 Mycgr3T
  
Location: 0-861

Mycgr3G52686\_Mycgr3T

Mycgr3G102281 Mycgr3
  
Location: 961-1573

Mycgr3G102281\_Mycgr3

Mycgr3G89185 Mycgr3T
  
Location: 1673-2063

Mycgr3G89185\_Mycgr3T

Mycgr3G65725 Mycgr3T
  
Location: 2163-3612

Mycgr3G65725\_Mycgr3T

Mycgr3G102276 Mycgr3
  
Location: 3712-4801

Mycgr3G102276\_Mycgr3

Mycgr3G89189 Mycgr3T
  
Location: 4901-5564

Mycgr3G89189\_Mycgr3T

Mycgr3G52682 Mycgr3T
  
Location: 5664-9231

Mycgr3G52682\_Mycgr3T

Mycgr3G107072 Mycgr3
  
Location: 9331-13279

Mycgr3G107072\_Mycgr3

Mycgr3G34982 Mycgr3T
  
Location: 13379-15116

Mycgr3G34982\_Mycgr3T

Mycgr3G107069 Mycgr3
  
Location: 15216-17097

Mycgr3G107069\_Mycgr3

Mycgr3G32432 Mycgr3T
  
Location: 17197-19042

Mycgr3G32432\_Mycgr3T

Mycgr3G98385 Mycgr3T
  
Location: 19142-19898

Mycgr3G98385\_Mycgr3T

MFS transporter, putative
  
Accession: EAW16630
  
Location: 3763835-3765635
  
 NCBI BlastP on this gene

EAW16630

conserved hypothetical protein
  
Accession: EAW16629
  
Location: 3761984-3762463
  
 NCBI BlastP on this gene

EAW16629

hypothetical protein
  
Accession: EAW16628
  
Location: 3759595-3760197
  
 NCBI BlastP on this gene

EAW16628

hypothetical protein
  
Accession: EAW16627
  
Location: 3757699-3758088
  
 NCBI BlastP on this gene

EAW16627

hypothetical protein
  
Accession: EAW16626
  
Location: 3755622-3756096
  
 NCBI BlastP on this gene

EAW16626

conserved hypothetical protein
  
Accession: EAW16625
  
Location: 3753886-3754840
  
  
**BlastP hit with Mycgr3G52686\_Mycgr3T**
  
Percentage identity: 34 %
  
BlastP bit score: 125
  
Sequence coverage: 95 %
  
E-value: 3e-30
  
  
 NCBI BlastP on this gene

EAW16625

ferric-chelate reductase, putative
  
Accession: EAW16624
  
Location: 3751461-3753422
  
  
**BlastP hit with Mycgr3G107069\_Mycgr3**
  
Percentage identity: 38 %
  
BlastP bit score: 464
  
Sequence coverage: 101 %
  
E-value: 2e-151
  
  
 NCBI BlastP on this gene

EAW16624

alpha-1,2-mannosidase, putative subfamily
  
Accession: EAW16623
  
Location: 3748905-3751346
  
 NCBI BlastP on this gene

EAW16623

C6 finger domain protein, putative
  
Accession: EAW16622
  
Location: 3744220-3746595
  
 NCBI BlastP on this gene

EAW16622

MFS multidrug transporter, putative
  
Accession: EAW16621
  
Location: 3741443-3743324
  
 NCBI BlastP on this gene

EAW16621

Query: Architecture Search FASTA input

EQ963486 : Aspergillus flavus NRRL3357 scf\_1106286417242 genomic scaffold    Total score: 2.0     Cumulative Blast bit score: 583

Hit cluster cross-links:

Mycgr3G52686 Mycgr3T
  
Location: 0-861

Mycgr3G52686\_Mycgr3T

Mycgr3G102281 Mycgr3
  
Location: 961-1573

Mycgr3G102281\_Mycgr3

Mycgr3G89185 Mycgr3T
  
Location: 1673-2063

Mycgr3G89185\_Mycgr3T

Mycgr3G65725 Mycgr3T
  
Location: 2163-3612

Mycgr3G65725\_Mycgr3T

Mycgr3G102276 Mycgr3
  
Location: 3712-4801

Mycgr3G102276\_Mycgr3

Mycgr3G89189 Mycgr3T
  
Location: 4901-5564

Mycgr3G89189\_Mycgr3T

Mycgr3G52682 Mycgr3T
  
Location: 5664-9231

Mycgr3G52682\_Mycgr3T

Mycgr3G107072 Mycgr3
  
Location: 9331-13279

Mycgr3G107072\_Mycgr3

Mycgr3G34982 Mycgr3T
  
Location: 13379-15116

Mycgr3G34982\_Mycgr3T

Mycgr3G107069 Mycgr3
  
Location: 15216-17097

Mycgr3G107069\_Mycgr3

Mycgr3G32432 Mycgr3T
  
Location: 17197-19042

Mycgr3G32432\_Mycgr3T

Mycgr3G98385 Mycgr3T
  
Location: 19142-19898

Mycgr3G98385\_Mycgr3T

conserved hypothetical protein
  
Accession: EED44984
  
Location: 315538-316891
  
 NCBI BlastP on this gene

EED44984

conserved hypothetical protein
  
Accession: EED44983
  
Location: 313672-314862
  
 NCBI BlastP on this gene

EED44983

hypothetical protein
  
Accession: EED44982
  
Location: 310965-312334
  
 NCBI BlastP on this gene

EED44982

short-chain dehydrogenases/reductase, putative
  
Accession: EED44981
  
Location: 309992-310570
  
 NCBI BlastP on this gene

EED44981

ferric-chelate reductase, putative
  
Accession: EED44980
  
Location: 305145-306821
  
  
**BlastP hit with Mycgr3G107069\_Mycgr3**
  
Percentage identity: 44 %
  
BlastP bit score: 462
  
Sequence coverage: 85 %
  
E-value: 4e-152
  
  
 NCBI BlastP on this gene

EED44980

hypothetical protein
  
Accession: EED44979
  
Location: 304610-304918
  
 NCBI BlastP on this gene

EED44979

ankyrin repeat-containing protein, putative
  
Accession: EED44978
  
Location: 301826-304077
  
 NCBI BlastP on this gene

EED44978

hypothetical protein
  
Accession: EED44977
  
Location: 301584-301823
  
 NCBI BlastP on this gene

EED44977

conserved hypothetical protein
  
Accession: EED44976
  
Location: 299748-300713
  
  
**BlastP hit with Mycgr3G52686\_Mycgr3T**
  
Percentage identity: 31 %
  
BlastP bit score: 121
  
Sequence coverage: 93 %
  
E-value: 9e-29
  
  
 NCBI BlastP on this gene

EED44976

conserved hypothetical protein
  
Accession: EED44975
  
Location: 298018-299284
  
 NCBI BlastP on this gene

EED44975

hypothetical protein
  
Accession: EED44974
  
Location: 297631-298016
  
 NCBI BlastP on this gene

EED44974

conserved hypothetical protein
  
Accession: EED44973
  
Location: 292561-294479
  
 NCBI BlastP on this gene

EED44973

Query: Architecture Search FASTA input

KE124052 : Mucor circinelloides f. circinelloides 1006PhL unplaced genomic scaffold supercont1.157    Total score: 2.0     Cumulative Blast bit score: 581

Hit cluster cross-links:

Mycgr3G52686 Mycgr3T
  
Location: 0-861

Mycgr3G52686\_Mycgr3T

Mycgr3G102281 Mycgr3
  
Location: 961-1573

Mycgr3G102281\_Mycgr3

Mycgr3G89185 Mycgr3T
  
Location: 1673-2063

Mycgr3G89185\_Mycgr3T

Mycgr3G65725 Mycgr3T
  
Location: 2163-3612

Mycgr3G65725\_Mycgr3T

Mycgr3G102276 Mycgr3
  
Location: 3712-4801

Mycgr3G102276\_Mycgr3

Mycgr3G89189 Mycgr3T
  
Location: 4901-5564

Mycgr3G89189\_Mycgr3T

Mycgr3G52682 Mycgr3T
  
Location: 5664-9231

Mycgr3G52682\_Mycgr3T

Mycgr3G107072 Mycgr3
  
Location: 9331-13279

Mycgr3G107072\_Mycgr3

Mycgr3G34982 Mycgr3T
  
Location: 13379-15116

Mycgr3G34982\_Mycgr3T

Mycgr3G107069 Mycgr3
  
Location: 15216-17097

Mycgr3G107069\_Mycgr3

Mycgr3G32432 Mycgr3T
  
Location: 17197-19042

Mycgr3G32432\_Mycgr3T

Mycgr3G98385 Mycgr3T
  
Location: 19142-19898

Mycgr3G98385\_Mycgr3T

hypothetical protein
  
Accession: EPB83985
  
Location: 36880-38296
  
 NCBI BlastP on this gene

EPB83985

hypothetical protein
  
Accession: EPB83986
  
Location: 40726-42138
  
  
**BlastP hit with Mycgr3G102276\_Mycgr3**
  
Percentage identity: 41 %
  
BlastP bit score: 289
  
Sequence coverage: 96 %
  
E-value: 2e-91
  
  
 NCBI BlastP on this gene

EPB83986

hypothetical protein
  
Accession: EPB83987
  
Location: 42584-44059
  
 NCBI BlastP on this gene

EPB83987

hypothetical protein
  
Accession: EPB83988
  
Location: 44764-46381
  
 NCBI BlastP on this gene

EPB83988

ribose 5-phosphate isomerase A
  
Accession: EPB83989
  
Location: 47474-48318
  
 NCBI BlastP on this gene

EPB83989

histone H1/5
  
Accession: EPB83990
  
Location: 48821-49762
  
 NCBI BlastP on this gene

EPB83990

hypothetical protein
  
Accession: EPB83991
  
Location: 53647-54143
  
 NCBI BlastP on this gene

EPB83991

hypothetical protein
  
Accession: EPB83992
  
Location: 54395-55291
  
 NCBI BlastP on this gene

EPB83992

hypothetical protein
  
Accession: EPB83993
  
Location: 55633-57195
  
 NCBI BlastP on this gene

EPB83993

hypothetical protein
  
Accession: EPB83994
  
Location: 57604-58729
  
 NCBI BlastP on this gene

EPB83994

hypothetical protein
  
Accession: EPB83995
  
Location: 60643-62548
  
 NCBI BlastP on this gene

EPB83995

hypothetical protein
  
Accession: EPB83996
  
Location: 62606-62806
  
 NCBI BlastP on this gene

EPB83996

hypothetical protein
  
Accession: EPB83997
  
Location: 63209-64067
  
 NCBI BlastP on this gene

EPB83997

hypothetical protein
  
Accession: EPB83998
  
Location: 66052-66246
  
 NCBI BlastP on this gene

EPB83998

peroxidase
  
Accession: EPB83999
  
Location: 66747-67515
  
  
**BlastP hit with Mycgr3G98385\_Mycgr3T**
  
Percentage identity: 66 %
  
BlastP bit score: 292
  
Sequence coverage: 83 %
  
E-value: 4e-96
  
  
 NCBI BlastP on this gene

EPB83999

FK506-binding protein 14
  
Accession: EPB84000
  
Location: 68192-68890
  
 NCBI BlastP on this gene

EPB84000

cyclin domain-containing protein
  
Accession: EPB84001
  
Location: 69117-69726
  
 NCBI BlastP on this gene

EPB84001

Query: Architecture Search FASTA input

KE148163 : Ophiostoma piceae UAMH 11346 chromosome Unknown scf18    Total score: 2.0     Cumulative Blast bit score: 555

Hit cluster cross-links:

Mycgr3G52686 Mycgr3T
  
Location: 0-861

Mycgr3G52686\_Mycgr3T

Mycgr3G102281 Mycgr3
  
Location: 961-1573

Mycgr3G102281\_Mycgr3

Mycgr3G89185 Mycgr3T
  
Location: 1673-2063

Mycgr3G89185\_Mycgr3T

Mycgr3G65725 Mycgr3T
  
Location: 2163-3612

Mycgr3G65725\_Mycgr3T

Mycgr3G102276 Mycgr3
  
Location: 3712-4801

Mycgr3G102276\_Mycgr3

Mycgr3G89189 Mycgr3T
  
Location: 4901-5564

Mycgr3G89189\_Mycgr3T

Mycgr3G52682 Mycgr3T
  
Location: 5664-9231

Mycgr3G52682\_Mycgr3T

Mycgr3G107072 Mycgr3
  
Location: 9331-13279

Mycgr3G107072\_Mycgr3

Mycgr3G34982 Mycgr3T
  
Location: 13379-15116

Mycgr3G34982\_Mycgr3T

Mycgr3G107069 Mycgr3
  
Location: 15216-17097

Mycgr3G107069\_Mycgr3

Mycgr3G32432 Mycgr3T
  
Location: 17197-19042

Mycgr3G32432\_Mycgr3T

Mycgr3G98385 Mycgr3T
  
Location: 19142-19898

Mycgr3G98385\_Mycgr3T

hypothetical protein
  
Accession: EPE04025
  
Location: 321457-323058
  
 NCBI BlastP on this gene

EPE04025

hypothetical protein
  
Accession: EPE04024
  
Location: 319029-320252
  
 NCBI BlastP on this gene

EPE04024

hypothetical protein
  
Accession: EPE04023
  
Location: 317812-318660
  
 NCBI BlastP on this gene

EPE04023

hypothetical protein
  
Accession: EPE04022
  
Location: 316845-317165
  
 NCBI BlastP on this gene

EPE04022

hypothetical protein
  
Accession: EPE04021
  
Location: 315258-316265
  
 NCBI BlastP on this gene

EPE04021

nad dependent epimerase
  
Accession: EPE04020
  
Location: 313171-314254
  
  
**BlastP hit with Mycgr3G52686\_Mycgr3T**
  
Percentage identity: 60 %
  
BlastP bit score: 332
  
Sequence coverage: 93 %
  
E-value: 2e-109
  
  
 NCBI BlastP on this gene

EPE04020

mfs transporter
  
Accession: EPE04019
  
Location: 310162-312107
  
 NCBI BlastP on this gene

EPE04019

hypothetical protein
  
Accession: EPE04018
  
Location: 306586-308460
  
  
**BlastP hit with Mycgr3G32432\_Mycgr3T**
  
Percentage identity: 34 %
  
BlastP bit score: 223
  
Sequence coverage: 79 %
  
E-value: 2e-60
  
  
 NCBI BlastP on this gene

EPE04018

hypothetical protein
  
Accession: EPE04017
  
Location: 303564-305957
  
 NCBI BlastP on this gene

EPE04017

hypothetical protein
  
Accession: EPE04016
  
Location: 302946-303221
  
 NCBI BlastP on this gene

EPE04016

hypothetical protein
  
Accession: EPE04015
  
Location: 301074-301805
  
 NCBI BlastP on this gene

EPE04015

n-acetylglucosaminyl-phosphatidylinositol de-n-acetylase
  
Accession: EPE04014
  
Location: 299304-300280
  
 NCBI BlastP on this gene

EPE04014

u6 snrnp-associated protein lsm3
  
Accession: EPE04013
  
Location: 297518-298039
  
 NCBI BlastP on this gene

EPE04013

Query: Architecture Search FASTA input

KB706899 : Eutypa lata UCREL1 unplaced genomic scaffold EL1\_03\_scaffold\_1561    Total score: 2.0     Cumulative Blast bit score: 550

Hit cluster cross-links:

Mycgr3G52686 Mycgr3T
  
Location: 0-861

Mycgr3G52686\_Mycgr3T

Mycgr3G102281 Mycgr3
  
Location: 961-1573

Mycgr3G102281\_Mycgr3

Mycgr3G89185 Mycgr3T
  
Location: 1673-2063

Mycgr3G89185\_Mycgr3T

Mycgr3G65725 Mycgr3T
  
Location: 2163-3612

Mycgr3G65725\_Mycgr3T

Mycgr3G102276 Mycgr3
  
Location: 3712-4801

Mycgr3G102276\_Mycgr3

Mycgr3G89189 Mycgr3T
  
Location: 4901-5564

Mycgr3G89189\_Mycgr3T

Mycgr3G52682 Mycgr3T
  
Location: 5664-9231

Mycgr3G52682\_Mycgr3T

Mycgr3G107072 Mycgr3
  
Location: 9331-13279

Mycgr3G107072\_Mycgr3

Mycgr3G34982 Mycgr3T
  
Location: 13379-15116

Mycgr3G34982\_Mycgr3T

Mycgr3G107069 Mycgr3
  
Location: 15216-17097

Mycgr3G107069\_Mycgr3

Mycgr3G32432 Mycgr3T
  
Location: 17197-19042

Mycgr3G32432\_Mycgr3T

Mycgr3G98385 Mycgr3T
  
Location: 19142-19898

Mycgr3G98385\_Mycgr3T

hypothetical protein
  
Accession: EMR65278
  
Location: 31200-32175
  
 NCBI BlastP on this gene

EMR65278

putative proline rich protein 5 protein
  
Accession: EMR65250
  
Location: 33885-34956
  
 NCBI BlastP on this gene

EMR65250

putative nad dependent epimerase dehydratase protein
  
Accession: EMR65281
  
Location: 41834-42794
  
  
**BlastP hit with Mycgr3G52686\_Mycgr3T**
  
Percentage identity: 64 %
  
BlastP bit score: 354
  
Sequence coverage: 94 %
  
E-value: 8e-119
  
  
 NCBI BlastP on this gene

EMR65281

hypothetical protein
  
Accession: EMR65261
  
Location: 44493-46326
  
  
**BlastP hit with Mycgr3G32432\_Mycgr3T**
  
Percentage identity: 30 %
  
BlastP bit score: 196
  
Sequence coverage: 79 %
  
E-value: 1e-51
  
  
 NCBI BlastP on this gene

EMR65261

putative mfs transporter protein
  
Accession: EMR65252
  
Location: 48696-50573
  
 NCBI BlastP on this gene

EMR65252

putative mfs sugar protein
  
Accession: EMR65266
  
Location: 51754-53655
  
 NCBI BlastP on this gene

EMR65266

Query: Architecture Search FASTA input

GL698480 : Metarhizium acridum CQMa 102 unplaced genomic scaffold Scf\_011    Total score: 2.0     Cumulative Blast bit score: 550

Hit cluster cross-links:

Mycgr3G52686 Mycgr3T
  
Location: 0-861

Mycgr3G52686\_Mycgr3T

Mycgr3G102281 Mycgr3
  
Location: 961-1573

Mycgr3G102281\_Mycgr3

Mycgr3G89185 Mycgr3T
  
Location: 1673-2063

Mycgr3G89185\_Mycgr3T

Mycgr3G65725 Mycgr3T
  
Location: 2163-3612

Mycgr3G65725\_Mycgr3T

Mycgr3G102276 Mycgr3
  
Location: 3712-4801

Mycgr3G102276\_Mycgr3

Mycgr3G89189 Mycgr3T
  
Location: 4901-5564

Mycgr3G89189\_Mycgr3T

Mycgr3G52682 Mycgr3T
  
Location: 5664-9231

Mycgr3G52682\_Mycgr3T

Mycgr3G107072 Mycgr3
  
Location: 9331-13279

Mycgr3G107072\_Mycgr3

Mycgr3G34982 Mycgr3T
  
Location: 13379-15116

Mycgr3G34982\_Mycgr3T

Mycgr3G107069 Mycgr3
  
Location: 15216-17097

Mycgr3G107069\_Mycgr3

Mycgr3G32432 Mycgr3T
  
Location: 17197-19042

Mycgr3G32432\_Mycgr3T

Mycgr3G98385 Mycgr3T
  
Location: 19142-19898

Mycgr3G98385\_Mycgr3T

AAA family ATPase, putative
  
Accession: EFY91698
  
Location: 333791-335389
  
 NCBI BlastP on this gene

EFY91698

indoleamine 2,3-dioxygenase family protein
  
Accession: EFY91697
  
Location: 331415-332676
  
 NCBI BlastP on this gene

EFY91697

hypothetical protein
  
Accession: EFY91696
  
Location: 329724-330950
  
 NCBI BlastP on this gene

EFY91696

MFS transporter, putative
  
Accession: EFY91695
  
Location: 327537-328004
  
 NCBI BlastP on this gene

EFY91695

hypothetical protein
  
Accession: EFY91694
  
Location: 323506-325175
  
  
**BlastP hit with Mycgr3G32432\_Mycgr3T**
  
Percentage identity: 33 %
  
BlastP bit score: 194
  
Sequence coverage: 72 %
  
E-value: 5e-51
  
  
 NCBI BlastP on this gene

EFY91694

NAD dependent epimerase/dehydratase, putative
  
Accession: EFY91693
  
Location: 318822-321100
  
  
**BlastP hit with Mycgr3G52686\_Mycgr3T**
  
Percentage identity: 62 %
  
BlastP bit score: 356
  
Sequence coverage: 98 %
  
E-value: 5e-119
  
  
 NCBI BlastP on this gene

EFY91693

Query: Architecture Search FASTA input

KB707843 : Botryotinia fuckeliana BcDW1 unplaced genomic scaffold Scaffold\_171    Total score: 2.0     Cumulative Blast bit score: 545

Hit cluster cross-links:

Mycgr3G52686 Mycgr3T
  
Location: 0-861

Mycgr3G52686\_Mycgr3T

Mycgr3G102281 Mycgr3
  
Location: 961-1573

Mycgr3G102281\_Mycgr3

Mycgr3G89185 Mycgr3T
  
Location: 1673-2063

Mycgr3G89185\_Mycgr3T

Mycgr3G65725 Mycgr3T
  
Location: 2163-3612

Mycgr3G65725\_Mycgr3T

Mycgr3G102276 Mycgr3
  
Location: 3712-4801

Mycgr3G102276\_Mycgr3

Mycgr3G89189 Mycgr3T
  
Location: 4901-5564

Mycgr3G89189\_Mycgr3T

Mycgr3G52682 Mycgr3T
  
Location: 5664-9231

Mycgr3G52682\_Mycgr3T

Mycgr3G107072 Mycgr3
  
Location: 9331-13279

Mycgr3G107072\_Mycgr3

Mycgr3G34982 Mycgr3T
  
Location: 13379-15116

Mycgr3G34982\_Mycgr3T

Mycgr3G107069 Mycgr3
  
Location: 15216-17097

Mycgr3G107069\_Mycgr3

Mycgr3G32432 Mycgr3T
  
Location: 17197-19042

Mycgr3G32432\_Mycgr3T

Mycgr3G98385 Mycgr3T
  
Location: 19142-19898

Mycgr3G98385\_Mycgr3T

putative nonribosomal peptide synthetase 12 protein
  
Accession: EMR87076
  
Location: 75670-77148
  
 NCBI BlastP on this gene

EMR87076

putative nad dependent epimerase dehydratase protein
  
Accession: EMR87077
  
Location: 84585-85505
  
  
**BlastP hit with Mycgr3G52686\_Mycgr3T**
  
Percentage identity: 60 %
  
BlastP bit score: 345
  
Sequence coverage: 95 %
  
E-value: 1e-115
  
  
 NCBI BlastP on this gene

EMR87077

hypothetical protein
  
Accession: EMR87078
  
Location: 86938-88801
  
  
**BlastP hit with Mycgr3G32432\_Mycgr3T**
  
Percentage identity: 34 %
  
BlastP bit score: 200
  
Sequence coverage: 70 %
  
E-value: 1e-52
  
  
 NCBI BlastP on this gene

EMR87078

putative mfs transporter protein
  
Accession: EMR87079
  
Location: 90801-92942
  
 NCBI BlastP on this gene

EMR87079

hypothetical protein
  
Accession: EMR87080
  
Location: 93928-95816
  
 NCBI BlastP on this gene

EMR87080

putative flavin-binding monooxygenase-like protein
  
Accession: EMR87081
  
Location: 98126-99957
  
 NCBI BlastP on this gene

EMR87081

Query: Architecture Search FASTA input

FQ790301 : Botryotinia fuckeliana T4 SuperContig\_226\_1 genomic supercontig.    Total score: 2.0     Cumulative Blast bit score: 545

Hit cluster cross-links:

Mycgr3G52686 Mycgr3T
  
Location: 0-861

Mycgr3G52686\_Mycgr3T

Mycgr3G102281 Mycgr3
  
Location: 961-1573

Mycgr3G102281\_Mycgr3

Mycgr3G89185 Mycgr3T
  
Location: 1673-2063

Mycgr3G89185\_Mycgr3T

Mycgr3G65725 Mycgr3T
  
Location: 2163-3612

Mycgr3G65725\_Mycgr3T

Mycgr3G102276 Mycgr3
  
Location: 3712-4801

Mycgr3G102276\_Mycgr3

Mycgr3G89189 Mycgr3T
  
Location: 4901-5564

Mycgr3G89189\_Mycgr3T

Mycgr3G52682 Mycgr3T
  
Location: 5664-9231

Mycgr3G52682\_Mycgr3T

Mycgr3G107072 Mycgr3
  
Location: 9331-13279

Mycgr3G107072\_Mycgr3

Mycgr3G34982 Mycgr3T
  
Location: 13379-15116

Mycgr3G34982\_Mycgr3T

Mycgr3G107069 Mycgr3
  
Location: 15216-17097

Mycgr3G107069\_Mycgr3

Mycgr3G32432 Mycgr3T
  
Location: 17197-19042

Mycgr3G32432\_Mycgr3T

Mycgr3G98385 Mycgr3T
  
Location: 19142-19898

Mycgr3G98385\_Mycgr3T

similar to flavin-binding monooxygenase-like protein
  
Accession: CCD34107
  
Location: 38609-40440
  
 NCBI BlastP on this gene

BofuT4\_P105340.1

predicted protein
  
Accession: CCD34108
  
Location: 41291-41488
  
 NCBI BlastP on this gene

BofuT4\_uP105350.1

hypothetical protein
  
Accession: CCD34109
  
Location: 42750-44638
  
 NCBI BlastP on this gene

BofuT4\_P105360.1

similar to MFS transporter
  
Accession: CCD34110
  
Location: 45624-47765
  
 NCBI BlastP on this gene

BofuT4\_P105370.1

hypothetical protein
  
Accession: CCD34111
  
Location: 48311-48685
  
 NCBI BlastP on this gene

BofuT4\_P105380.1

hypothetical protein
  
Accession: CCD34112
  
Location: 49765-51628
  
  
**BlastP hit with Mycgr3G32432\_Mycgr3T**
  
Percentage identity: 34 %
  
BlastP bit score: 200
  
Sequence coverage: 70 %
  
E-value: 1e-52
  
  
 NCBI BlastP on this gene

BofuT4\_P105390.1

hypothetical protein
  
Accession: CCD34113
  
Location: 53061-53981
  
  
**BlastP hit with Mycgr3G52686\_Mycgr3T**
  
Percentage identity: 60 %
  
BlastP bit score: 345
  
Sequence coverage: 95 %
  
E-value: 1e-115
  
  
 NCBI BlastP on this gene

BofuT4\_P105400.1

predicted protein
  
Accession: CCD34114
  
Location: 56067-56273
  
 NCBI BlastP on this gene

BofuT4\_uP105410.1

similar to integral membrane protein TmpA
  
Accession: CCD34115
  
Location: 61419-62897
  
 NCBI BlastP on this gene

BofuT4\_P105420.1

hypothetical protein
  
Accession: CCD34116
  
Location: 63603-63948
  
 NCBI BlastP on this gene

BofuT4\_uP105430.1

Query: Architecture Search FASTA input

CM001199 : Mycosphaerella graminicola IPO323 chromosome 4    Total score: 2.0     Cumulative Blast bit score: 500

Hit cluster cross-links:

Mycgr3G52686 Mycgr3T
  
Location: 0-861

Mycgr3G52686\_Mycgr3T

Mycgr3G102281 Mycgr3
  
Location: 961-1573

Mycgr3G102281\_Mycgr3

Mycgr3G89185 Mycgr3T
  
Location: 1673-2063

Mycgr3G89185\_Mycgr3T

Mycgr3G65725 Mycgr3T
  
Location: 2163-3612

Mycgr3G65725\_Mycgr3T

Mycgr3G102276 Mycgr3
  
Location: 3712-4801

Mycgr3G102276\_Mycgr3

Mycgr3G89189 Mycgr3T
  
Location: 4901-5564

Mycgr3G89189\_Mycgr3T

Mycgr3G52682 Mycgr3T
  
Location: 5664-9231

Mycgr3G52682\_Mycgr3T

Mycgr3G107072 Mycgr3
  
Location: 9331-13279

Mycgr3G107072\_Mycgr3

Mycgr3G34982 Mycgr3T
  
Location: 13379-15116

Mycgr3G34982\_Mycgr3T

Mycgr3G107069 Mycgr3
  
Location: 15216-17097

Mycgr3G107069\_Mycgr3

Mycgr3G32432 Mycgr3T
  
Location: 17197-19042

Mycgr3G32432\_Mycgr3T

Mycgr3G98385 Mycgr3T
  
Location: 19142-19898

Mycgr3G98385\_Mycgr3T

hypothetical protein
  
Accession: EGP88057
  
Location: 2051228-2053476
  
 NCBI BlastP on this gene

EGP88057

glucose-methanol-choline oxidoreductase
  
Accession: EGP88058
  
Location: 2054269-2056342
  
  
**BlastP hit with Mycgr3G34982\_Mycgr3T**
  
Percentage identity: 29 %
  
BlastP bit score: 203
  
Sequence coverage: 102 %
  
E-value: 1e-53
  
  
 NCBI BlastP on this gene

EGP88058

hypothetical protein
  
Accession: EGP88059
  
Location: 2056681-2058114
  
 NCBI BlastP on this gene

EGP88059

hypothetical protein
  
Accession: EGP88305
  
Location: 2058152-2058694
  
 NCBI BlastP on this gene

EGP88305

hypothetical protein
  
Accession: EGP88060
  
Location: 2059620-2060863
  
 NCBI BlastP on this gene

EGP88060

hypothetical protein
  
Accession: EGP88061
  
Location: 2061289-2062505
  
 NCBI BlastP on this gene

EGP88061

hypothetical protein
  
Accession: EGP88304
  
Location: 2062819-2063436
  
 NCBI BlastP on this gene

EGP88304

hypothetical protein
  
Accession: EGP88062
  
Location: 2064048-2066336
  
 NCBI BlastP on this gene

EGP88062

GTA glutaminase A
  
Accession: EGP88303
  
Location: 2066537-2068990
  
 NCBI BlastP on this gene

EGP88303

hypothetical protein
  
Accession: EGP88063
  
Location: 2070297-2072538
  
 NCBI BlastP on this gene

EGP88063

hypothetical protein
  
Accession: EGP88302
  
Location: 2072808-2074588
  
 NCBI BlastP on this gene

EGP88302

hypothetical protein
  
Accession: EGP88064
  
Location: 2077946-2078819
  
  
**BlastP hit with Mycgr3G98385\_Mycgr3T**
  
Percentage identity: 66 %
  
BlastP bit score: 298
  
Sequence coverage: 84 %
  
E-value: 2e-98
  
  
 NCBI BlastP on this gene

EGP88064

Query: Architecture Search FASTA input

AMYD01000021 : Colletotrichum gloeosporioides Cg-14    Total score: 2.0     Cumulative Blast bit score: 496

Hit cluster cross-links:

Mycgr3G52686 Mycgr3T
  
Location: 0-861

Mycgr3G52686\_Mycgr3T

Mycgr3G102281 Mycgr3
  
Location: 961-1573

Mycgr3G102281\_Mycgr3

Mycgr3G89185 Mycgr3T
  
Location: 1673-2063

Mycgr3G89185\_Mycgr3T

Mycgr3G65725 Mycgr3T
  
Location: 2163-3612

Mycgr3G65725\_Mycgr3T

Mycgr3G102276 Mycgr3
  
Location: 3712-4801

Mycgr3G102276\_Mycgr3

Mycgr3G89189 Mycgr3T
  
Location: 4901-5564

Mycgr3G89189\_Mycgr3T

Mycgr3G52682 Mycgr3T
  
Location: 5664-9231

Mycgr3G52682\_Mycgr3T

Mycgr3G107072 Mycgr3
  
Location: 9331-13279

Mycgr3G107072\_Mycgr3

Mycgr3G34982 Mycgr3T
  
Location: 13379-15116

Mycgr3G34982\_Mycgr3T

Mycgr3G107069 Mycgr3
  
Location: 15216-17097

Mycgr3G107069\_Mycgr3

Mycgr3G32432 Mycgr3T
  
Location: 17197-19042

Mycgr3G32432\_Mycgr3T

Mycgr3G98385 Mycgr3T
  
Location: 19142-19898

Mycgr3G98385\_Mycgr3T

hypothetical protein
  
Accession: EQB59521
  
Location: 7793-9915
  
 NCBI BlastP on this gene

EQB59521

UDP-glucoronosyl and UDP-glucosyl transferase
  
Accession: EQB59522
  
Location: 13138-13586
  
 NCBI BlastP on this gene

EQB59522

hypothetical protein
  
Accession: EQB59523
  
Location: 14032-14667
  
 NCBI BlastP on this gene

EQB59523

hypothetical protein
  
Accession: EQB59524
  
Location: 15399-16242
  
  
**BlastP hit with Mycgr3G52686\_Mycgr3T**
  
Percentage identity: 57 %
  
BlastP bit score: 246
  
Sequence coverage: 71 %
  
E-value: 2e-77
  
  
 NCBI BlastP on this gene

EQB59524

hypothetical protein
  
Accession: EQB59525
  
Location: 16946-17177
  
 NCBI BlastP on this gene

EQB59525

integral membrane protein DUF6
  
Accession: EQB59526
  
Location: 18279-19657
  
 NCBI BlastP on this gene

EQB59526

hypothetical protein
  
Accession: EQB59527
  
Location: 19820-21202
  
  
**BlastP hit with Mycgr3G32432\_Mycgr3T**
  
Percentage identity: 38 %
  
BlastP bit score: 251
  
Sequence coverage: 64 %
  
E-value: 1e-72
  
  
 NCBI BlastP on this gene

EQB59527

Query: Architecture Search FASTA input

DS985218 : Verticillium albo-atrum VaMs.102 supercont1.5 genomic scaffold    Total score: 2.0     Cumulative Blast bit score: 491

Hit cluster cross-links:

Mycgr3G52686 Mycgr3T
  
Location: 0-861

Mycgr3G52686\_Mycgr3T

Mycgr3G102281 Mycgr3
  
Location: 961-1573

Mycgr3G102281\_Mycgr3

Mycgr3G89185 Mycgr3T
  
Location: 1673-2063

Mycgr3G89185\_Mycgr3T

Mycgr3G65725 Mycgr3T
  
Location: 2163-3612

Mycgr3G65725\_Mycgr3T

Mycgr3G102276 Mycgr3
  
Location: 3712-4801

Mycgr3G102276\_Mycgr3

Mycgr3G89189 Mycgr3T
  
Location: 4901-5564

Mycgr3G89189\_Mycgr3T

Mycgr3G52682 Mycgr3T
  
Location: 5664-9231

Mycgr3G52682\_Mycgr3T

Mycgr3G107072 Mycgr3
  
Location: 9331-13279

Mycgr3G107072\_Mycgr3

Mycgr3G34982 Mycgr3T
  
Location: 13379-15116

Mycgr3G34982\_Mycgr3T

Mycgr3G107069 Mycgr3
  
Location: 15216-17097

Mycgr3G107069\_Mycgr3

Mycgr3G32432 Mycgr3T
  
Location: 17197-19042

Mycgr3G32432\_Mycgr3T

Mycgr3G98385 Mycgr3T
  
Location: 19142-19898

Mycgr3G98385\_Mycgr3T

conserved hypothetical protein
  
Accession: EEY18397
  
Location: 598679-599221
  
 NCBI BlastP on this gene

EEY18397

predicted protein
  
Accession: EEY18398
  
Location: 599706-600026
  
 NCBI BlastP on this gene

EEY18398

cytosolic Cu/Zn superoxide dismutase
  
Accession: EEY18399
  
Location: 600863-601758
  
 NCBI BlastP on this gene

EEY18399

hypothetical protein
  
Accession: EEY18400
  
Location: 602936-603367
  
 NCBI BlastP on this gene

EEY18400

conserved hypothetical protein
  
Accession: EEY18401
  
Location: 605114-607472
  
 NCBI BlastP on this gene

EEY18401

conserved hypothetical protein
  
Accession: EEY18402
  
Location: 608507-610343
  
  
**BlastP hit with Mycgr3G32432\_Mycgr3T**
  
Percentage identity: 35 %
  
BlastP bit score: 248
  
Sequence coverage: 71 %
  
E-value: 6e-70
  
  
 NCBI BlastP on this gene

EEY18402

conserved hypothetical protein
  
Accession: EEY18403
  
Location: 610537-612335
  
 NCBI BlastP on this gene

EEY18403

conserved hypothetical protein
  
Accession: EEY18404
  
Location: 612982-614171
  
  
**BlastP hit with Mycgr3G52686\_Mycgr3T**
  
Percentage identity: 60 %
  
BlastP bit score: 243
  
Sequence coverage: 65 %
  
E-value: 3e-74
  
  
 NCBI BlastP on this gene

EEY18404

WSC domain-containing protein
  
Accession: EEY18405
  
Location: 614778-618208
  
 NCBI BlastP on this gene

EEY18405

predicted protein
  
Accession: EEY18406
  
Location: 618982-619529
  
 NCBI BlastP on this gene

EEY18406

Query: Architecture Search FASTA input

KB916913 : Neofusicoccum parvum UCRNP2 chromosome Unknown NP2\_03\_scaffold\_1275    Total score: 2.0     Cumulative Blast bit score: 464

Hit cluster cross-links:

Mycgr3G52686 Mycgr3T
  
Location: 0-861

Mycgr3G52686\_Mycgr3T

Mycgr3G102281 Mycgr3
  
Location: 961-1573

Mycgr3G102281\_Mycgr3

Mycgr3G89185 Mycgr3T
  
Location: 1673-2063

Mycgr3G89185\_Mycgr3T

Mycgr3G65725 Mycgr3T
  
Location: 2163-3612

Mycgr3G65725\_Mycgr3T

Mycgr3G102276 Mycgr3
  
Location: 3712-4801

Mycgr3G102276\_Mycgr3

Mycgr3G89189 Mycgr3T
  
Location: 4901-5564

Mycgr3G89189\_Mycgr3T

Mycgr3G52682 Mycgr3T
  
Location: 5664-9231

Mycgr3G52682\_Mycgr3T

Mycgr3G107072 Mycgr3
  
Location: 9331-13279

Mycgr3G107072\_Mycgr3

Mycgr3G34982 Mycgr3T
  
Location: 13379-15116

Mycgr3G34982\_Mycgr3T

Mycgr3G107069 Mycgr3
  
Location: 15216-17097

Mycgr3G107069\_Mycgr3

Mycgr3G32432 Mycgr3T
  
Location: 17197-19042

Mycgr3G32432\_Mycgr3T

Mycgr3G98385 Mycgr3T
  
Location: 19142-19898

Mycgr3G98385\_Mycgr3T

putative mfs drug efflux transporter protein
  
Accession: EOD43059
  
Location: 21401-23459
  
 NCBI BlastP on this gene

EOD43059

putative zinc-binding oxidoreductase protein
  
Accession: EOD43062
  
Location: 24203-25243
  
 NCBI BlastP on this gene

EOD43062

putative lactonohydrolase protein
  
Accession: EOD43063
  
Location: 25713-27059
  
 NCBI BlastP on this gene

EOD43063

putative mfs monocarboxylate transporter protein
  
Accession: EOD43064
  
Location: 27901-29269
  
  
**BlastP hit with Mycgr3G65725\_Mycgr3T**
  
Percentage identity: 38 %
  
BlastP bit score: 276
  
Sequence coverage: 82 %
  
E-value: 7e-84
  
  
 NCBI BlastP on this gene

EOD43064

putative pyoverdine dityrosine biosynthesis protein
  
Accession: EOD43066
  
Location: 30797-32480
  
 NCBI BlastP on this gene

EOD43066

putative laccase-1 precursor protein
  
Accession: EOD43061
  
Location: 33609-35508
  
 NCBI BlastP on this gene

EOD43061

putative gmc oxidoreductase protein
  
Accession: EOD43060
  
Location: 37016-38988
  
  
**BlastP hit with Mycgr3G34982\_Mycgr3T**
  
Percentage identity: 27 %
  
BlastP bit score: 188
  
Sequence coverage: 102 %
  
E-value: 1e-48
  
  
 NCBI BlastP on this gene

EOD43060

Query: Architecture Search FASTA input

DS544807 : Paracoccidioides brasiliensis Pb03 supercont1.5 genomic scaffold    Total score: 2.0     Cumulative Blast bit score: 456

Hit cluster cross-links:

Mycgr3G52686 Mycgr3T
  
Location: 0-861

Mycgr3G52686\_Mycgr3T

Mycgr3G102281 Mycgr3
  
Location: 961-1573

Mycgr3G102281\_Mycgr3

Mycgr3G89185 Mycgr3T
  
Location: 1673-2063

Mycgr3G89185\_Mycgr3T

Mycgr3G65725 Mycgr3T
  
Location: 2163-3612

Mycgr3G65725\_Mycgr3T

Mycgr3G102276 Mycgr3
  
Location: 3712-4801

Mycgr3G102276\_Mycgr3

Mycgr3G89189 Mycgr3T
  
Location: 4901-5564

Mycgr3G89189\_Mycgr3T

Mycgr3G52682 Mycgr3T
  
Location: 5664-9231

Mycgr3G52682\_Mycgr3T

Mycgr3G107072 Mycgr3
  
Location: 9331-13279

Mycgr3G107072\_Mycgr3

Mycgr3G34982 Mycgr3T
  
Location: 13379-15116

Mycgr3G34982\_Mycgr3T

Mycgr3G107069 Mycgr3
  
Location: 15216-17097

Mycgr3G107069\_Mycgr3

Mycgr3G32432 Mycgr3T
  
Location: 17197-19042

Mycgr3G32432\_Mycgr3T

Mycgr3G98385 Mycgr3T
  
Location: 19142-19898

Mycgr3G98385\_Mycgr3T

conserved hypothetical protein
  
Accession: EEH21382
  
Location: 122426-124300
  
 NCBI BlastP on this gene

EEH21382

glutamyl-tRNA(Gln) amidotransferase subunit A
  
Accession: EEH21383
  
Location: 124702-126471
  
 NCBI BlastP on this gene

EEH21383

predicted protein
  
Accession: EEH21384
  
Location: 126839-129005
  
 NCBI BlastP on this gene

EEH21384

conserved hypothetical protein
  
Accession: EEH21385
  
Location: 129531-130724
  
 NCBI BlastP on this gene

EEH21385

gramicidin S synthetase 1
  
Accession: EEH21386
  
Location: 131609-134758
  
  
**BlastP hit with Mycgr3G107072\_Mycgr3**
  
Percentage identity: 27 %
  
BlastP bit score: 306
  
Sequence coverage: 81 %
  
E-value: 8e-84
  
  
 NCBI BlastP on this gene

EEH21386

conserved hypothetical protein
  
Accession: EEH21387
  
Location: 135246-136513
  
 NCBI BlastP on this gene

EEH21387

conserved hypothetical protein
  
Accession: EEH21388
  
Location: 138050-138958
  
  
**BlastP hit with Mycgr3G52686\_Mycgr3T**
  
Percentage identity: 37 %
  
BlastP bit score: 150
  
Sequence coverage: 79 %
  
E-value: 7e-40
  
  
 NCBI BlastP on this gene

EEH21388

predicted protein
  
Accession: EEH21389
  
Location: 139127-139651
  
 NCBI BlastP on this gene

EEH21389

phenol hydroxylase
  
Accession: EEH21390
  
Location: 139999-142075
  
 NCBI BlastP on this gene

EEH21390

predicted protein
  
Accession: EEH21391
  
Location: 142221-142700
  
 NCBI BlastP on this gene

EEH21391

multidrug resistance protein fnx1
  
Accession: EEH21392
  
Location: 142866-144703
  
 NCBI BlastP on this gene

EEH21392

conserved hypothetical protein
  
Accession: EEH21393
  
Location: 145527-146456
  
 NCBI BlastP on this gene

EEH21393

Query: Architecture Search FASTA input

GG663377 : Ajellomyces capsulatus G186AR genomic scaffold supercont2.15    Total score: 2.0     Cumulative Blast bit score: 455

Hit cluster cross-links:

Mycgr3G52686 Mycgr3T
  
Location: 0-861

Mycgr3G52686\_Mycgr3T

Mycgr3G102281 Mycgr3
  
Location: 961-1573

Mycgr3G102281\_Mycgr3

Mycgr3G89185 Mycgr3T
  
Location: 1673-2063

Mycgr3G89185\_Mycgr3T

Mycgr3G65725 Mycgr3T
  
Location: 2163-3612

Mycgr3G65725\_Mycgr3T

Mycgr3G102276 Mycgr3
  
Location: 3712-4801

Mycgr3G102276\_Mycgr3

Mycgr3G89189 Mycgr3T
  
Location: 4901-5564

Mycgr3G89189\_Mycgr3T

Mycgr3G52682 Mycgr3T
  
Location: 5664-9231

Mycgr3G52682\_Mycgr3T

Mycgr3G107072 Mycgr3
  
Location: 9331-13279

Mycgr3G107072\_Mycgr3

Mycgr3G34982 Mycgr3T
  
Location: 13379-15116

Mycgr3G34982\_Mycgr3T

Mycgr3G107069 Mycgr3
  
Location: 15216-17097

Mycgr3G107069\_Mycgr3

Mycgr3G32432 Mycgr3T
  
Location: 17197-19042

Mycgr3G32432\_Mycgr3T

Mycgr3G98385 Mycgr3T
  
Location: 19142-19898

Mycgr3G98385\_Mycgr3T

hypothetical protein
  
Accession: EEH03444
  
Location: 453180-455429
  
 NCBI BlastP on this gene

EEH03444

predicted protein
  
Accession: EEH03445
  
Location: 456970-457572
  
 NCBI BlastP on this gene

EEH03445

antibiotic synthetase
  
Accession: EEH03446
  
Location: 460656-463817
  
  
**BlastP hit with Mycgr3G107072\_Mycgr3**
  
Percentage identity: 27 %
  
BlastP bit score: 301
  
Sequence coverage: 81 %
  
E-value: 4e-82
  
  
 NCBI BlastP on this gene

EEH03446

NmrA family protein
  
Accession: EEH03447
  
Location: 464306-465544
  
 NCBI BlastP on this gene

EEH03447

conserved hypothetical protein
  
Accession: EEH03448
  
Location: 466332-467221
  
  
**BlastP hit with Mycgr3G52686\_Mycgr3T**
  
Percentage identity: 37 %
  
BlastP bit score: 154
  
Sequence coverage: 78 %
  
E-value: 1e-41
  
  
 NCBI BlastP on this gene

EEH03448

FAD binding domain-containing protein
  
Accession: EEH03449
  
Location: 468182-470249
  
 NCBI BlastP on this gene

EEH03449

multidrug resistance protein fnx1
  
Accession: EEH03450
  
Location: 470911-473399
  
 NCBI BlastP on this gene

EEH03450

conserved hypothetical protein
  
Accession: EEH03451
  
Location: 474062-474991
  
 NCBI BlastP on this gene

EEH03451

Query: Architecture Search FASTA input

EQ999987 : Ajellomyces dermatitidis ER-3 genomic scaffold supercont1.15    Total score: 2.0     Cumulative Blast bit score: 455

Hit cluster cross-links:

Mycgr3G52686 Mycgr3T
  
Location: 0-861

Mycgr3G52686\_Mycgr3T

Mycgr3G102281 Mycgr3
  
Location: 961-1573

Mycgr3G102281\_Mycgr3

Mycgr3G89185 Mycgr3T
  
Location: 1673-2063

Mycgr3G89185\_Mycgr3T

Mycgr3G65725 Mycgr3T
  
Location: 2163-3612

Mycgr3G65725\_Mycgr3T

Mycgr3G102276 Mycgr3
  
Location: 3712-4801

Mycgr3G102276\_Mycgr3

Mycgr3G89189 Mycgr3T
  
Location: 4901-5564

Mycgr3G89189\_Mycgr3T

Mycgr3G52682 Mycgr3T
  
Location: 5664-9231

Mycgr3G52682\_Mycgr3T

Mycgr3G107072 Mycgr3
  
Location: 9331-13279

Mycgr3G107072\_Mycgr3

Mycgr3G34982 Mycgr3T
  
Location: 13379-15116

Mycgr3G34982\_Mycgr3T

Mycgr3G107069 Mycgr3
  
Location: 15216-17097

Mycgr3G107069\_Mycgr3

Mycgr3G32432 Mycgr3T
  
Location: 17197-19042

Mycgr3G32432\_Mycgr3T

Mycgr3G98385 Mycgr3T
  
Location: 19142-19898

Mycgr3G98385\_Mycgr3T

predicted protein
  
Accession: EEQ86129
  
Location: 696888-698114
  
 NCBI BlastP on this gene

EEQ86129

predicted protein
  
Accession: EEQ86130
  
Location: 699757-700698
  
 NCBI BlastP on this gene

EEQ86130

capsule polysaccharide biosynthesis protein
  
Accession: EEQ86131
  
Location: 701135-702328
  
 NCBI BlastP on this gene

EEQ86131

antibiotic synthetase
  
Accession: EEQ86132
  
Location: 703326-706487
  
  
**BlastP hit with Mycgr3G107072\_Mycgr3**
  
Percentage identity: 27 %
  
BlastP bit score: 302
  
Sequence coverage: 81 %
  
E-value: 3e-82
  
  
 NCBI BlastP on this gene

EEQ86132

conserved hypothetical protein
  
Accession: EEQ86133
  
Location: 707037-708263
  
 NCBI BlastP on this gene

EEQ86133

conserved hypothetical protein
  
Accession: EEQ86134
  
Location: 709046-709896
  
  
**BlastP hit with Mycgr3G52686\_Mycgr3T**
  
Percentage identity: 37 %
  
BlastP bit score: 153
  
Sequence coverage: 79 %
  
E-value: 6e-41
  
  
 NCBI BlastP on this gene

EEQ86134

phenol monooxygenase
  
Accession: EEQ86135
  
Location: 710855-712930
  
 NCBI BlastP on this gene

EEQ86135

MFS multidrug transporter
  
Accession: EEQ86136
  
Location: 713738-715498
  
 NCBI BlastP on this gene

EEQ86136

conserved hypothetical protein
  
Accession: EEQ86137
  
Location: 717016-717945
  
 NCBI BlastP on this gene

EEQ86137

Query: Architecture Search FASTA input

GG749478 : Ajellomyces dermatitidis ATCC 18188 genomic scaffold supercont1.72    Total score: 2.0     Cumulative Blast bit score: 454

Hit cluster cross-links:

Mycgr3G52686 Mycgr3T
  
Location: 0-861

Mycgr3G52686\_Mycgr3T

Mycgr3G102281 Mycgr3
  
Location: 961-1573

Mycgr3G102281\_Mycgr3

Mycgr3G89185 Mycgr3T
  
Location: 1673-2063

Mycgr3G89185\_Mycgr3T

Mycgr3G65725 Mycgr3T
  
Location: 2163-3612

Mycgr3G65725\_Mycgr3T

Mycgr3G102276 Mycgr3
  
Location: 3712-4801

Mycgr3G102276\_Mycgr3

Mycgr3G89189 Mycgr3T
  
Location: 4901-5564

Mycgr3G89189\_Mycgr3T

Mycgr3G52682 Mycgr3T
  
Location: 5664-9231

Mycgr3G52682\_Mycgr3T

Mycgr3G107072 Mycgr3
  
Location: 9331-13279

Mycgr3G107072\_Mycgr3

Mycgr3G34982 Mycgr3T
  
Location: 13379-15116

Mycgr3G34982\_Mycgr3T

Mycgr3G107069 Mycgr3
  
Location: 15216-17097

Mycgr3G107069\_Mycgr3

Mycgr3G32432 Mycgr3T
  
Location: 17197-19042

Mycgr3G32432\_Mycgr3T

Mycgr3G98385 Mycgr3T
  
Location: 19142-19898

Mycgr3G98385\_Mycgr3T

capsule polysaccharide biosynthesis protein
  
Accession: EGE84872
  
Location: 210018-211211
  
 NCBI BlastP on this gene

EGE84872

antibiotic synthetase
  
Accession: EGE84873
  
Location: 212204-215368
  
  
**BlastP hit with Mycgr3G107072\_Mycgr3**
  
Percentage identity: 27 %
  
BlastP bit score: 301
  
Sequence coverage: 81 %
  
E-value: 4e-82
  
  
 NCBI BlastP on this gene

EGE84873

NmrA family protein
  
Accession: EGE84874
  
Location: 215915-217141
  
 NCBI BlastP on this gene

EGE84874

hypothetical protein
  
Accession: EGE84875
  
Location: 217921-218771
  
  
**BlastP hit with Mycgr3G52686\_Mycgr3T**
  
Percentage identity: 37 %
  
BlastP bit score: 153
  
Sequence coverage: 79 %
  
E-value: 6e-41
  
  
 NCBI BlastP on this gene

EGE84875

phenol monooxygenase
  
Accession: EGE84876
  
Location: 219730-221805
  
 NCBI BlastP on this gene

EGE84876

MFS multidrug transporter
  
Accession: EGE84877
  
Location: 222615-224375
  
 NCBI BlastP on this gene

EGE84877

hypothetical protein
  
Accession: EGE84878
  
Location: 225893-226822
  
 NCBI BlastP on this gene

EGE84878

Query: Architecture Search FASTA input

GG657474 : Ajellomyces dermatitidis SLH14081 genomic scaffold supercont1.27    Total score: 2.0     Cumulative Blast bit score: 451

Hit cluster cross-links:

Mycgr3G52686 Mycgr3T
  
Location: 0-861

Mycgr3G52686\_Mycgr3T

Mycgr3G102281 Mycgr3
  
Location: 961-1573

Mycgr3G102281\_Mycgr3

Mycgr3G89185 Mycgr3T
  
Location: 1673-2063

Mycgr3G89185\_Mycgr3T

Mycgr3G65725 Mycgr3T
  
Location: 2163-3612

Mycgr3G65725\_Mycgr3T

Mycgr3G102276 Mycgr3
  
Location: 3712-4801

Mycgr3G102276\_Mycgr3

Mycgr3G89189 Mycgr3T
  
Location: 4901-5564

Mycgr3G89189\_Mycgr3T

Mycgr3G52682 Mycgr3T
  
Location: 5664-9231

Mycgr3G52682\_Mycgr3T

Mycgr3G107072 Mycgr3
  
Location: 9331-13279

Mycgr3G107072\_Mycgr3

Mycgr3G34982 Mycgr3T
  
Location: 13379-15116

Mycgr3G34982\_Mycgr3T

Mycgr3G107069 Mycgr3
  
Location: 15216-17097

Mycgr3G107069\_Mycgr3

Mycgr3G32432 Mycgr3T
  
Location: 17197-19042

Mycgr3G32432\_Mycgr3T

Mycgr3G98385 Mycgr3T
  
Location: 19142-19898

Mycgr3G98385\_Mycgr3T

predicted protein
  
Accession: EEQ74448
  
Location: 40276-41166
  
 NCBI BlastP on this gene

EEQ74448

predicted protein
  
Accession: EEQ74449
  
Location: 42811-43752
  
 NCBI BlastP on this gene

EEQ74449

capsule polysaccharide biosynthesis protein
  
Accession: EEQ74450
  
Location: 44196-45389
  
 NCBI BlastP on this gene

EEQ74450

antibiotic synthetase
  
Accession: EEQ74451
  
Location: 46385-49546
  
  
**BlastP hit with Mycgr3G107072\_Mycgr3**
  
Percentage identity: 27 %
  
BlastP bit score: 298
  
Sequence coverage: 81 %
  
E-value: 4e-81
  
  
 NCBI BlastP on this gene

EEQ74451

conserved hypothetical protein
  
Accession: EEQ74452
  
Location: 50097-51323
  
 NCBI BlastP on this gene

EEQ74452

conserved hypothetical protein
  
Accession: EEQ74453
  
Location: 52106-52956
  
  
**BlastP hit with Mycgr3G52686\_Mycgr3T**
  
Percentage identity: 37 %
  
BlastP bit score: 153
  
Sequence coverage: 79 %
  
E-value: 6e-41
  
  
 NCBI BlastP on this gene

EEQ74453

phenol monooxygenase
  
Accession: EEQ74454
  
Location: 53913-55988
  
 NCBI BlastP on this gene

EEQ74454

MFS multidrug transporter
  
Accession: EEQ74455
  
Location: 56759-58519
  
 NCBI BlastP on this gene

EEQ74455

conserved hypothetical protein
  
Accession: EEQ74456
  
Location: 60037-60966
  
 NCBI BlastP on this gene

EEQ74456

Query: Architecture Search FASTA input

DS572750 : Paracoccidioides brasiliensis Pb18 supercont1.1 genomic scaffold    Total score: 2.0     Cumulative Blast bit score: 449

Hit cluster cross-links:

Mycgr3G52686 Mycgr3T
  
Location: 0-861

Mycgr3G52686\_Mycgr3T

Mycgr3G102281 Mycgr3
  
Location: 961-1573

Mycgr3G102281\_Mycgr3

Mycgr3G89185 Mycgr3T
  
Location: 1673-2063

Mycgr3G89185\_Mycgr3T

Mycgr3G65725 Mycgr3T
  
Location: 2163-3612

Mycgr3G65725\_Mycgr3T

Mycgr3G102276 Mycgr3
  
Location: 3712-4801

Mycgr3G102276\_Mycgr3

Mycgr3G89189 Mycgr3T
  
Location: 4901-5564

Mycgr3G89189\_Mycgr3T

Mycgr3G52682 Mycgr3T
  
Location: 5664-9231

Mycgr3G52682\_Mycgr3T

Mycgr3G107072 Mycgr3
  
Location: 9331-13279

Mycgr3G107072\_Mycgr3

Mycgr3G34982 Mycgr3T
  
Location: 13379-15116

Mycgr3G34982\_Mycgr3T

Mycgr3G107069 Mycgr3
  
Location: 15216-17097

Mycgr3G107069\_Mycgr3

Mycgr3G32432 Mycgr3T
  
Location: 17197-19042

Mycgr3G32432\_Mycgr3T

Mycgr3G98385 Mycgr3T
  
Location: 19142-19898

Mycgr3G98385\_Mycgr3T

conserved hypothetical protein
  
Accession: EEH43938
  
Location: 780149-781278
  
 NCBI BlastP on this gene

EEH43938

multidrug resistance protein fnx1
  
Accession: EEH43939
  
Location: 782099-783884
  
 NCBI BlastP on this gene

EEH43939

conserved hypothetical protein
  
Accession: EEH43940
  
Location: 784672-786748
  
 NCBI BlastP on this gene

EEH43940

predicted protein
  
Accession: EEH43941
  
Location: 787257-787620
  
 NCBI BlastP on this gene

EEH43941

conserved hypothetical protein
  
Accession: EEH43942
  
Location: 787764-788672
  
  
**BlastP hit with Mycgr3G52686\_Mycgr3T**
  
Percentage identity: 37 %
  
BlastP bit score: 150
  
Sequence coverage: 79 %
  
E-value: 8e-40
  
  
 NCBI BlastP on this gene

EEH43942

conserved hypothetical protein
  
Accession: EEH43943
  
Location: 790221-791488
  
 NCBI BlastP on this gene

EEH43943

D-alanine-poly(phosphoribitol) ligase subunit 1
  
Accession: EEH43944
  
Location: 791976-795125
  
  
**BlastP hit with Mycgr3G107072\_Mycgr3**
  
Percentage identity: 27 %
  
BlastP bit score: 300
  
Sequence coverage: 76 %
  
E-value: 1e-81
  
  
 NCBI BlastP on this gene

EEH43944

conserved hypothetical protein
  
Accession: EEH43945
  
Location: 796299-797198
  
 NCBI BlastP on this gene

EEH43945

predicted protein
  
Accession: EEH43946
  
Location: 798191-799561
  
 NCBI BlastP on this gene

EEH43946

glutamyl-tRNA(Gln) amidotransferase subunit A
  
Accession: EEH43947
  
Location: 800260-802029
  
 NCBI BlastP on this gene

EEH43947

conserved hypothetical protein
  
Accession: EEH43948
  
Location: 802432-804307
  
 NCBI BlastP on this gene

EEH43948

Query: Architecture Search FASTA input

DS572813 : Paracoccidioides brasiliensis Pb01 supercont1.3 genomic scaffold    Total score: 2.0     Cumulative Blast bit score: 445

Hit cluster cross-links:

Mycgr3G52686 Mycgr3T
  
Location: 0-861

Mycgr3G52686\_Mycgr3T

Mycgr3G102281 Mycgr3
  
Location: 961-1573

Mycgr3G102281\_Mycgr3

Mycgr3G89185 Mycgr3T
  
Location: 1673-2063

Mycgr3G89185\_Mycgr3T

Mycgr3G65725 Mycgr3T
  
Location: 2163-3612

Mycgr3G65725\_Mycgr3T

Mycgr3G102276 Mycgr3
  
Location: 3712-4801

Mycgr3G102276\_Mycgr3

Mycgr3G89189 Mycgr3T
  
Location: 4901-5564

Mycgr3G89189\_Mycgr3T

Mycgr3G52682 Mycgr3T
  
Location: 5664-9231

Mycgr3G52682\_Mycgr3T

Mycgr3G107072 Mycgr3
  
Location: 9331-13279

Mycgr3G107072\_Mycgr3

Mycgr3G34982 Mycgr3T
  
Location: 13379-15116

Mycgr3G34982\_Mycgr3T

Mycgr3G107069 Mycgr3
  
Location: 15216-17097

Mycgr3G107069\_Mycgr3

Mycgr3G32432 Mycgr3T
  
Location: 17197-19042

Mycgr3G32432\_Mycgr3T

Mycgr3G98385 Mycgr3T
  
Location: 19142-19898

Mycgr3G98385\_Mycgr3T

conserved hypothetical protein
  
Accession: EEH39079
  
Location: 727410-729197
  
 NCBI BlastP on this gene

EEH39079

glutamyl-tRNA(Gln) amidotransferase subunit A
  
Accession: EEH39080
  
Location: 729679-731450
  
 NCBI BlastP on this gene

EEH39080

conserved hypothetical protein
  
Accession: EEH39081
  
Location: 732046-734771
  
 NCBI BlastP on this gene

EEH39081

conserved hypothetical protein
  
Accession: EEH39082
  
Location: 735456-736533
  
 NCBI BlastP on this gene

EEH39082

D-alanine-poly(phosphoribitol) ligase subunit 1
  
Accession: EEH39083
  
Location: 737495-740644
  
  
**BlastP hit with Mycgr3G107072\_Mycgr3**
  
Percentage identity: 27 %
  
BlastP bit score: 297
  
Sequence coverage: 73 %
  
E-value: 6e-81
  
  
 NCBI BlastP on this gene

EEH39083

conserved hypothetical protein
  
Accession: EEH39084
  
Location: 741131-742398
  
 NCBI BlastP on this gene

EEH39084

conserved hypothetical protein
  
Accession: EEH39085
  
Location: 743913-744821
  
  
**BlastP hit with Mycgr3G52686\_Mycgr3T**
  
Percentage identity: 36 %
  
BlastP bit score: 148
  
Sequence coverage: 78 %
  
E-value: 4e-39
  
  
 NCBI BlastP on this gene

EEH39085

phenol 2-monooxygenase
  
Accession: EEH39086
  
Location: 745822-747898
  
 NCBI BlastP on this gene

EEH39086

multidrug resistance protein fnx1
  
Accession: EEH39087
  
Location: 748704-750487
  
 NCBI BlastP on this gene

EEH39087

conserved hypothetical protein
  
Accession: EEH39088
  
Location: 751280-752340
  
 NCBI BlastP on this gene

EEH39088

predicted protein
  
Accession: EEH39089
  
Location: 752578-753394
  
 NCBI BlastP on this gene

EEH39089

predicted protein
  
Accession: EEH39090
  
Location: 753434-756958
  
 NCBI BlastP on this gene

EEH39090

Query: Architecture Search FASTA input

AB530986 : Streptomyces sp. SANK 60405 DNA, A-90289 biosynthetic gene cluster region    Total score: 2.0     Cumulative Blast bit score: 341

Hit cluster cross-links:

Mycgr3G52686 Mycgr3T
  
Location: 0-861

Mycgr3G52686\_Mycgr3T

Mycgr3G102281 Mycgr3
  
Location: 961-1573

Mycgr3G102281\_Mycgr3

Mycgr3G89185 Mycgr3T
  
Location: 1673-2063

Mycgr3G89185\_Mycgr3T

Mycgr3G65725 Mycgr3T
  
Location: 2163-3612

Mycgr3G65725\_Mycgr3T

Mycgr3G102276 Mycgr3
  
Location: 3712-4801

Mycgr3G102276\_Mycgr3

Mycgr3G89189 Mycgr3T
  
Location: 4901-5564

Mycgr3G89189\_Mycgr3T

Mycgr3G52682 Mycgr3T
  
Location: 5664-9231

Mycgr3G52682\_Mycgr3T

Mycgr3G107072 Mycgr3
  
Location: 9331-13279

Mycgr3G107072\_Mycgr3

Mycgr3G34982 Mycgr3T
  
Location: 13379-15116

Mycgr3G34982\_Mycgr3T

Mycgr3G107069 Mycgr3
  
Location: 15216-17097

Mycgr3G107069\_Mycgr3

Mycgr3G32432 Mycgr3T
  
Location: 17197-19042

Mycgr3G32432\_Mycgr3T

Mycgr3G98385 Mycgr3T
  
Location: 19142-19898

Mycgr3G98385\_Mycgr3T

hypothetical protein
  
Accession: BAJ05876
  
Location: 1-546
  
 NCBI BlastP on this gene

lipA

putative pirin-like protein
  
Accession: BAJ05877
  
Location: 855-1820
  
 NCBI BlastP on this gene

BAJ05877

aryl sulfotransferase
  
Accession: BAJ05878
  
Location: 1894-3432
  
  
**BlastP hit with Mycgr3G32432\_Mycgr3T**
  
Percentage identity: 33 %
  
BlastP bit score: 222
  
Sequence coverage: 80 %
  
E-value: 4e-61
  
  
 NCBI BlastP on this gene

lipB

putative 3-hydroxy-3-methylglutaryl-CoA synthase
  
Accession: BAJ05879
  
Location: 3452-4714
  
 NCBI BlastP on this gene

lipC

putative type III polyketide synthase
  
Accession: BAJ05880
  
Location: 5013-6062
  
 NCBI BlastP on this gene

lipD

hypothetical protein
  
Accession: BAJ05881
  
Location: 6619-7257
  
  
**BlastP hit with Mycgr3G52686\_Mycgr3T**
  
Percentage identity: 33 %
  
BlastP bit score: 119
  
Sequence coverage: 77 %
  
E-value: 1e-28
  
  
 NCBI BlastP on this gene

lipE

putative AraC family transcriptional regulator
  
Accession: BAJ05882
  
Location: 7475-8464
  
 NCBI BlastP on this gene

lipF

putative beta-hydroxylase
  
Accession: BAJ05883
  
Location: 8518-9165
  
 NCBI BlastP on this gene

lipG

putative SAM-dependent methyltransferase
  
Accession: BAJ05884
  
Location: 9198-9818
  
 NCBI BlastP on this gene

lipH

putative TmrB-like protein
  
Accession: BAJ05885
  
Location: 9815-10384
  
 NCBI BlastP on this gene

lipI

putative aminotransferase
  
Accession: BAJ05886
  
Location: 10449-11771
  
 NCBI BlastP on this gene

lipJ

putative serine hydroxymethyltransferase
  
Accession: BAJ05887
  
Location: 11776-13050
  
 NCBI BlastP on this gene

lipK

putative dioxygenase
  
Accession: BAJ05888
  
Location: 13132-13956
  
 NCBI BlastP on this gene

lipL

putative nucleotidyltransferase
  
Accession: BAJ05889
  
Location: 13953-14654
  
 NCBI BlastP on this gene

lipM

putative glycosyltransferase
  
Accession: BAJ05890
  
Location: 14654-15787
  
 NCBI BlastP on this gene

lipN

putative aminotransferase
  
Accession: BAJ05891
  
Location: 15784-17058
  
 NCBI BlastP on this gene

lipO

Query: Architecture Search FASTA input

KB456266 : Mycosphaerella populorum SO2202 unplaced genomic scaffold SEPMUscaffold\_7    Total score: 1.0     Cumulative Blast bit score: 1893

Hit cluster cross-links:

Mycgr3G52686 Mycgr3T
  
Location: 0-861

Mycgr3G52686\_Mycgr3T

Mycgr3G102281 Mycgr3
  
Location: 961-1573

Mycgr3G102281\_Mycgr3

Mycgr3G89185 Mycgr3T
  
Location: 1673-2063

Mycgr3G89185\_Mycgr3T

Mycgr3G65725 Mycgr3T
  
Location: 2163-3612

Mycgr3G65725\_Mycgr3T

Mycgr3G102276 Mycgr3
  
Location: 3712-4801

Mycgr3G102276\_Mycgr3

Mycgr3G89189 Mycgr3T
  
Location: 4901-5564

Mycgr3G89189\_Mycgr3T

Mycgr3G52682 Mycgr3T
  
Location: 5664-9231

Mycgr3G52682\_Mycgr3T

Mycgr3G107072 Mycgr3
  
Location: 9331-13279

Mycgr3G107072\_Mycgr3

Mycgr3G34982 Mycgr3T
  
Location: 13379-15116

Mycgr3G34982\_Mycgr3T

Mycgr3G107069 Mycgr3
  
Location: 15216-17097

Mycgr3G107069\_Mycgr3

Mycgr3G32432 Mycgr3T
  
Location: 17197-19042

Mycgr3G32432\_Mycgr3T

Mycgr3G98385 Mycgr3T
  
Location: 19142-19898

Mycgr3G98385\_Mycgr3T

hexose carrier protein
  
Accession: EMF11162
  
Location: 543277-545044
  
 NCBI BlastP on this gene

EMF11162

hypothetical protein
  
Accession: EMF11163
  
Location: 545167-546613
  
 NCBI BlastP on this gene

EMF11163

hypothetical protein
  
Accession: EMF11164
  
Location: 548135-549061
  
 NCBI BlastP on this gene

EMF11164

mitochondrial chaperone BCS1
  
Accession: EMF11165
  
Location: 550358-551898
  
 NCBI BlastP on this gene

EMF11165

NRPS-like enzyme
  
Accession: EMF11166
  
Location: 553284-557222
  
  
**BlastP hit with Mycgr3G107072\_Mycgr3**
  
Percentage identity: 71 %
  
BlastP bit score: 1893
  
Sequence coverage: 100 %
  
E-value: 0.0
  
  
 NCBI BlastP on this gene

EMF11166

exonuclease family protein
  
Accession: EMF11167
  
Location: 558053-558637
  
 NCBI BlastP on this gene

EMF11167

NAD(P)-binding protein
  
Accession: EMF11168
  
Location: 559130-560716
  
 NCBI BlastP on this gene

EMF11168

NAD(P)-binding protein
  
Accession: EMF11169
  
Location: 561138-562259
  
 NCBI BlastP on this gene

EMF11169

WSC-domain-containing protein
  
Accession: EMF11170
  
Location: 564079-564736
  
 NCBI BlastP on this gene

EMF11170

autophagy protein
  
Accession: EMF11171
  
Location: 565731-568041
  
 NCBI BlastP on this gene

EMF11171

Query: Architecture Search FASTA input

KB446546 : Dothistroma septosporum NZE10 unplaced genomic scaffold DOTSEscaffold\_12    Total score: 1.0     Cumulative Blast bit score: 1883

Hit cluster cross-links:

Mycgr3G52686 Mycgr3T
  
Location: 0-861

Mycgr3G52686\_Mycgr3T

Mycgr3G102281 Mycgr3
  
Location: 961-1573

Mycgr3G102281\_Mycgr3

Mycgr3G89185 Mycgr3T
  
Location: 1673-2063

Mycgr3G89185\_Mycgr3T

Mycgr3G65725 Mycgr3T
  
Location: 2163-3612

Mycgr3G65725\_Mycgr3T

Mycgr3G102276 Mycgr3
  
Location: 3712-4801

Mycgr3G102276\_Mycgr3

Mycgr3G89189 Mycgr3T
  
Location: 4901-5564

Mycgr3G89189\_Mycgr3T

Mycgr3G52682 Mycgr3T
  
Location: 5664-9231

Mycgr3G52682\_Mycgr3T

Mycgr3G107072 Mycgr3
  
Location: 9331-13279

Mycgr3G107072\_Mycgr3

Mycgr3G34982 Mycgr3T
  
Location: 13379-15116

Mycgr3G34982\_Mycgr3T

Mycgr3G107069 Mycgr3
  
Location: 15216-17097

Mycgr3G107069\_Mycgr3

Mycgr3G32432 Mycgr3T
  
Location: 17197-19042

Mycgr3G32432\_Mycgr3T

Mycgr3G98385 Mycgr3T
  
Location: 19142-19898

Mycgr3G98385\_Mycgr3T

hypothetical protein
  
Accession: EME38801
  
Location: 445611-447007
  
 NCBI BlastP on this gene

EME38801

hypothetical protein
  
Accession: EME38800
  
Location: 444148-445035
  
 NCBI BlastP on this gene

EME38800

hypothetical protein
  
Accession: EME38799
  
Location: 442016-443428
  
 NCBI BlastP on this gene

EME38799

hypothetical protein
  
Accession: EME38798
  
Location: 439471-441636
  
 NCBI BlastP on this gene

EME38798

hypothetical protein
  
Accession: EME38797
  
Location: 438328-438775
  
 NCBI BlastP on this gene

EME38797

hypothetical protein
  
Accession: EME38796
  
Location: 430986-436638
  
  
**BlastP hit with Mycgr3G107072\_Mycgr3**
  
Percentage identity: 69 %
  
BlastP bit score: 1883
  
Sequence coverage: 102 %
  
E-value: 0.0
  
  
 NCBI BlastP on this gene

EME38796

hypothetical protein
  
Accession: EME38795
  
Location: 429631-430779
  
 NCBI BlastP on this gene

EME38795

hypothetical protein
  
Accession: EME38794
  
Location: 428509-429447
  
 NCBI BlastP on this gene

EME38794

hypothetical protein
  
Accession: EME38793
  
Location: 426133-426426
  
 NCBI BlastP on this gene

EME38793

hypothetical protein
  
Accession: EME38792
  
Location: 424669-425902
  
 NCBI BlastP on this gene

EME38792

Query: Architecture Search FASTA input

KB446556 : Pseudocercospora fijiensis CIRAD86 unplaced genomic scaffold MYCFIscaffold\_2    Total score: 1.0     Cumulative Blast bit score: 1880

Hit cluster cross-links:

Mycgr3G52686 Mycgr3T
  
Location: 0-861

Mycgr3G52686\_Mycgr3T

Mycgr3G102281 Mycgr3
  
Location: 961-1573

Mycgr3G102281\_Mycgr3

Mycgr3G89185 Mycgr3T
  
Location: 1673-2063

Mycgr3G89185\_Mycgr3T

Mycgr3G65725 Mycgr3T
  
Location: 2163-3612

Mycgr3G65725\_Mycgr3T

Mycgr3G102276 Mycgr3
  
Location: 3712-4801

Mycgr3G102276\_Mycgr3

Mycgr3G89189 Mycgr3T
  
Location: 4901-5564

Mycgr3G89189\_Mycgr3T

Mycgr3G52682 Mycgr3T
  
Location: 5664-9231

Mycgr3G52682\_Mycgr3T

Mycgr3G107072 Mycgr3
  
Location: 9331-13279

Mycgr3G107072\_Mycgr3

Mycgr3G34982 Mycgr3T
  
Location: 13379-15116

Mycgr3G34982\_Mycgr3T

Mycgr3G107069 Mycgr3
  
Location: 15216-17097

Mycgr3G107069\_Mycgr3

Mycgr3G32432 Mycgr3T
  
Location: 17197-19042

Mycgr3G32432\_Mycgr3T

Mycgr3G98385 Mycgr3T
  
Location: 19142-19898

Mycgr3G98385\_Mycgr3T

hypothetical protein
  
Accession: EME85369
  
Location: 706543-706979
  
 NCBI BlastP on this gene

EME85369

hypothetical protein
  
Accession: EME85368
  
Location: 704858-706466
  
 NCBI BlastP on this gene

EME85368

hypothetical protein
  
Accession: EME85367
  
Location: 702645-703791
  
 NCBI BlastP on this gene

EME85367

hypothetical protein
  
Accession: EME85366
  
Location: 697078-700965
  
  
**BlastP hit with Mycgr3G107072\_Mycgr3**
  
Percentage identity: 70 %
  
BlastP bit score: 1880
  
Sequence coverage: 100 %
  
E-value: 0.0
  
  
 NCBI BlastP on this gene

EME85366

Query: Architecture Search FASTA input

JH226130 : Exophiala dermatitidis NIH/UT8656 unplaced genomic scaffold supercont1.1    Total score: 1.0     Cumulative Blast bit score: 1796

Hit cluster cross-links:

Mycgr3G52686 Mycgr3T
  
Location: 0-861

Mycgr3G52686\_Mycgr3T

Mycgr3G102281 Mycgr3
  
Location: 961-1573

Mycgr3G102281\_Mycgr3

Mycgr3G89185 Mycgr3T
  
Location: 1673-2063

Mycgr3G89185\_Mycgr3T

Mycgr3G65725 Mycgr3T
  
Location: 2163-3612

Mycgr3G65725\_Mycgr3T

Mycgr3G102276 Mycgr3
  
Location: 3712-4801

Mycgr3G102276\_Mycgr3

Mycgr3G89189 Mycgr3T
  
Location: 4901-5564

Mycgr3G89189\_Mycgr3T

Mycgr3G52682 Mycgr3T
  
Location: 5664-9231

Mycgr3G52682\_Mycgr3T

Mycgr3G107072 Mycgr3
  
Location: 9331-13279

Mycgr3G107072\_Mycgr3

Mycgr3G34982 Mycgr3T
  
Location: 13379-15116

Mycgr3G34982\_Mycgr3T

Mycgr3G107069 Mycgr3
  
Location: 15216-17097

Mycgr3G107069\_Mycgr3

Mycgr3G32432 Mycgr3T
  
Location: 17197-19042

Mycgr3G32432\_Mycgr3T

Mycgr3G98385 Mycgr3T
  
Location: 19142-19898

Mycgr3G98385\_Mycgr3T

glutaminyl-peptide cyclotransferase
  
Accession: EHY52380
  
Location: 1642717-1643874
  
 NCBI BlastP on this gene

EHY52380

hypothetical protein
  
Accession: EHY52381
  
Location: 1644292-1645410
  
 NCBI BlastP on this gene

EHY52381

hypothetical protein
  
Accession: EHY52382
  
Location: 1645701-1646695
  
 NCBI BlastP on this gene

EHY52382

myosin-crossreactive antigen
  
Accession: EHY52383
  
Location: 1650043-1651824
  
 NCBI BlastP on this gene

EHY52383

linear gramicidin synthetase subunit C
  
Accession: EHY52384
  
Location: 1652960-1656802
  
  
**BlastP hit with Mycgr3G107072\_Mycgr3**
  
Percentage identity: 67 %
  
BlastP bit score: 1796
  
Sequence coverage: 100 %
  
E-value: 0.0
  
  
 NCBI BlastP on this gene

EHY52384

hypothetical protein
  
Accession: EHY52385
  
Location: 1657750-1658226
  
 NCBI BlastP on this gene

EHY52385

histone-lysine N-methyltransferase SETD1
  
Accession: EHY52386
  
Location: 1659708-1663602
  
 NCBI BlastP on this gene

EHY52386

hypothetical protein
  
Accession: EHY52387
  
Location: 1664676-1665563
  
 NCBI BlastP on this gene

EHY52387

Query: Architecture Search FASTA input

DS499597 : Aspergillus fumigatus A1163 scf\_000004 genomic scaffold    Total score: 1.0     Cumulative Blast bit score: 1739

Hit cluster cross-links:

Mycgr3G52686 Mycgr3T
  
Location: 0-861

Mycgr3G52686\_Mycgr3T

Mycgr3G102281 Mycgr3
  
Location: 961-1573

Mycgr3G102281\_Mycgr3

Mycgr3G89185 Mycgr3T
  
Location: 1673-2063

Mycgr3G89185\_Mycgr3T

Mycgr3G65725 Mycgr3T
  
Location: 2163-3612

Mycgr3G65725\_Mycgr3T

Mycgr3G102276 Mycgr3
  
Location: 3712-4801

Mycgr3G102276\_Mycgr3

Mycgr3G89189 Mycgr3T
  
Location: 4901-5564

Mycgr3G89189\_Mycgr3T

Mycgr3G52682 Mycgr3T
  
Location: 5664-9231

Mycgr3G52682\_Mycgr3T

Mycgr3G107072 Mycgr3
  
Location: 9331-13279

Mycgr3G107072\_Mycgr3

Mycgr3G34982 Mycgr3T
  
Location: 13379-15116

Mycgr3G34982\_Mycgr3T

Mycgr3G107069 Mycgr3
  
Location: 15216-17097

Mycgr3G107069\_Mycgr3

Mycgr3G32432 Mycgr3T
  
Location: 17197-19042

Mycgr3G32432\_Mycgr3T

Mycgr3G98385 Mycgr3T
  
Location: 19142-19898

Mycgr3G98385\_Mycgr3T

cytochrome b5 reductase, putative
  
Accession: EDP51747
  
Location: 2488541-2490003
  
 NCBI BlastP on this gene

EDP51747

3-hydroxyacyl-CoA dehydrogenase, putative
  
Accession: EDP51748
  
Location: 2490846-2491666
  
 NCBI BlastP on this gene

EDP51748

hypothetical protein
  
Accession: EDP51749
  
Location: 2492006-2492813
  
 NCBI BlastP on this gene

EDP51749

conserved hypothetical protein
  
Accession: EDP51750
  
Location: 2492955-2494828
  
 NCBI BlastP on this gene

EDP51750

3-demethylubiquinone-9 3-methyltransferase, putative
  
Accession: EDP51751
  
Location: 2495764-2496264
  
 NCBI BlastP on this gene

EDP51751

conserved hypothetical protein
  
Accession: EDP51752
  
Location: 2497790-2498145
  
 NCBI BlastP on this gene

EDP51752

conserved hypothetical protein
  
Accession: EDP51753
  
Location: 2498322-2498873
  
 NCBI BlastP on this gene

EDP51753

hybrid NRPS/PKS enzyme, putative
  
Accession: EDP51754
  
Location: 2500293-2504117
  
  
**BlastP hit with Mycgr3G107072\_Mycgr3**
  
Percentage identity: 66 %
  
BlastP bit score: 1739
  
Sequence coverage: 100 %
  
E-value: 0.0
  
  
 NCBI BlastP on this gene

EDP51754

Query: Architecture Search FASTA input

DS027696 : Neosartorya fischeri NRRL 181 1099437636264 genomic scaffold    Total score: 1.0     Cumulative Blast bit score: 1738

Hit cluster cross-links:

Mycgr3G52686 Mycgr3T
  
Location: 0-861

Mycgr3G52686\_Mycgr3T

Mycgr3G102281 Mycgr3
  
Location: 961-1573

Mycgr3G102281\_Mycgr3

Mycgr3G89185 Mycgr3T
  
Location: 1673-2063

Mycgr3G89185\_Mycgr3T

Mycgr3G65725 Mycgr3T
  
Location: 2163-3612

Mycgr3G65725\_Mycgr3T

Mycgr3G102276 Mycgr3
  
Location: 3712-4801

Mycgr3G102276\_Mycgr3

Mycgr3G89189 Mycgr3T
  
Location: 4901-5564

Mycgr3G89189\_Mycgr3T

Mycgr3G52682 Mycgr3T
  
Location: 5664-9231

Mycgr3G52682\_Mycgr3T

Mycgr3G107072 Mycgr3
  
Location: 9331-13279

Mycgr3G107072\_Mycgr3

Mycgr3G34982 Mycgr3T
  
Location: 13379-15116

Mycgr3G34982\_Mycgr3T

Mycgr3G107069 Mycgr3
  
Location: 15216-17097

Mycgr3G107069\_Mycgr3

Mycgr3G32432 Mycgr3T
  
Location: 17197-19042

Mycgr3G32432\_Mycgr3T

Mycgr3G98385 Mycgr3T
  
Location: 19142-19898

Mycgr3G98385\_Mycgr3T

hybrid NRPS/PKS enzyme, putative
  
Accession: EAW17784
  
Location: 1341334-1345155
  
  
**BlastP hit with Mycgr3G107072\_Mycgr3**
  
Percentage identity: 66 %
  
BlastP bit score: 1738
  
Sequence coverage: 100 %
  
E-value: 0.0
  
  
 NCBI BlastP on this gene

EAW17784

bZIP transcription factor, putative
  
Accession: EAW17783
  
Location: 1339691-1340479
  
 NCBI BlastP on this gene

EAW17783

conserved hypothetical protein
  
Accession: EAW17782
  
Location: 1337402-1339289
  
 NCBI BlastP on this gene

EAW17782

hypothetical protein
  
Accession: EAW17781
  
Location: 1335195-1336685
  
 NCBI BlastP on this gene

EAW17781

short-chain dehydrogenase/reductase, putative
  
Accession: EAW17780
  
Location: 1333863-1334953
  
 NCBI BlastP on this gene

EAW17780

conserved hypothetical protein
  
Accession: EAW17779
  
Location: 1332402-1333394
  
 NCBI BlastP on this gene

EAW17779

NmrA-like family protein
  
Accession: EAW17778
  
Location: 1330667-1331636
  
 NCBI BlastP on this gene

EAW17778

Query: Architecture Search FASTA input

51. :  DS499594 Aspergillus fumigatus A1163 scf\_000001 genomic scaffold     Total score: 2.0     Cumulative Blast bit score: 882

Mycgr3G52686 Mycgr3T
  
Location: 0-861
  
 NCBI BlastP on this gene

Mycgr3G52686\_Mycgr3T

Mycgr3G102281 Mycgr3
  
Location: 961-1573
  
 NCBI BlastP on this gene

Mycgr3G102281\_Mycgr3

Mycgr3G89185 Mycgr3T
  
Location: 1673-2063
  
 NCBI BlastP on this gene

Mycgr3G89185\_Mycgr3T

Mycgr3G65725 Mycgr3T
  
Location: 2163-3612
  
 NCBI BlastP on this gene

Mycgr3G65725\_Mycgr3T

Mycgr3G102276 Mycgr3
  
Location: 3712-4801
  
 NCBI BlastP on this gene

Mycgr3G102276\_Mycgr3

Mycgr3G89189 Mycgr3T
  
Location: 4901-5564
  
 NCBI BlastP on this gene

Mycgr3G89189\_Mycgr3T

Mycgr3G52682 Mycgr3T
  
Location: 5664-9231
  
 NCBI BlastP on this gene

Mycgr3G52682\_Mycgr3T

Mycgr3G107072 Mycgr3
  
Location: 9331-13279
  
 NCBI BlastP on this gene

Mycgr3G107072\_Mycgr3

Mycgr3G34982 Mycgr3T
  
Location: 13379-15116
  
 NCBI BlastP on this gene

Mycgr3G34982\_Mycgr3T

Mycgr3G107069 Mycgr3
  
Location: 15216-17097
  
 NCBI BlastP on this gene

Mycgr3G107069\_Mycgr3

Mycgr3G32432 Mycgr3T
  
Location: 17197-19042
  
 NCBI BlastP on this gene

Mycgr3G32432\_Mycgr3T

Mycgr3G98385 Mycgr3T
  
Location: 19142-19898
  
 NCBI BlastP on this gene

Mycgr3G98385\_Mycgr3T

chromosome segregation protein BIR1, putative
  
Accession: EDP56644
  
Location: 3824181-3827000
  
 NCBI BlastP on this gene

EDP56644

C2H2 finger domain protein, putative
  
Accession: EDP56643
  
Location: 3823245-3823901
  
 NCBI BlastP on this gene

EDP56643

F-box domain protein
  
Accession: EDP56642
  
Location: 3822112-3822588
  
 NCBI BlastP on this gene

EDP56642

blue light-inducible protein Bli-3
  
Accession: EDP56641
  
Location: 3820018-3820699
  
 NCBI BlastP on this gene

EDP56641

SAGA-like transcriptional regulatory complex subunit Spt3, putative
  
Accession: EDP56640
  
Location: 3818601-3819741
  
 NCBI BlastP on this gene

EDP56640

diphthine synthase, putative
  
Accession: EDP56639
  
Location: 3816704-3817677
  
 NCBI BlastP on this gene

EDP56639

AP-2 adaptor complex subunit sigma, putative
  
Accession: EDP56638
  
Location: 3814335-3815066
  
 NCBI BlastP on this gene

EDP56638

hypothetical protein
  
Accession: EDP56637
  
Location: 3813053-3813361
  
 NCBI BlastP on this gene

EDP56637

conserved hypothetical protein
  
Accession: EDP56636
  
Location: 3810215-3811242
  
  
**BlastP hit with Mycgr3G52686\_Mycgr3T**
  
Percentage identity: 62 %
  
BlastP bit score: 370
  
Sequence coverage: 95 %
  
E-value: 2e-125
  
  
 NCBI BlastP on this gene

EDP56636

conserved hypothetical protein
  
Accession: EDP56635
  
Location: 3806264-3808238
  
  
**BlastP hit with Mycgr3G32432\_Mycgr3T**
  
Percentage identity: 50 %
  
BlastP bit score: 513
  
Sequence coverage: 82 %
  
E-value: 1e-171
  
  
 NCBI BlastP on this gene

EDP56635

MFS transporter, putative
  
Accession: EDP56634
  
Location: 3802346-3804571
  
 NCBI BlastP on this gene

EDP56634

DUF292 domian protein
  
Accession: EDP56633
  
Location: 3800264-3801835
  
 NCBI BlastP on this gene

EDP56633

short-chain oxidoreductase, putative
  
Accession: EDP56632
  
Location: 3798446-3799362
  
 NCBI BlastP on this gene

EDP56632

SUN domain protein (Adg3), putative
  
Accession: EDP56631
  
Location: 3796167-3797696
  
 NCBI BlastP on this gene

EDP56631

Leucine carboxyl methyltransferase superfamily
  
Accession: EDP56630
  
Location: 3793605-3794865
  
 NCBI BlastP on this gene

EDP56630

RING zinc finger protein, putative
  
Accession: EDP56629
  
Location: 3788048-3793310
  
 NCBI BlastP on this gene

EDP56629

52. :  DF126459 Aspergillus kawachii IFO 4308 DNA, contig: scaffold00013     Total score: 2.0     Cumulative Blast bit score: 882

blue light-inducible protein Bli-3
  
Accession: GAA87444
  
Location: 507993-508791
  
 NCBI BlastP on this gene

GAA87444

SAGA-like transcriptional regulatory complex subunit Spt3
  
Accession: GAA87445
  
Location: 509284-510538
  
 NCBI BlastP on this gene

GAA87445

diphthine synthase
  
Accession: GAA87446
  
Location: 511314-512313
  
 NCBI BlastP on this gene

GAA87446

AorFlbE
  
Accession: GAA87447
  
Location: 512508-513199
  
 NCBI BlastP on this gene

GAA87447

AP-2 complex subunit sigma
  
Accession: GAA87448
  
Location: 514403-515158
  
 NCBI BlastP on this gene

GAA87448

NAD dependent epimerase/dehydratase
  
Accession: GAA87449
  
Location: 520295-521322
  
  
**BlastP hit with Mycgr3G52686\_Mycgr3T**
  
Percentage identity: 64 %
  
BlastP bit score: 370
  
Sequence coverage: 95 %
  
E-value: 4e-125
  
  
 NCBI BlastP on this gene

GAA87449

similar to An08g07120
  
Accession: GAA87450
  
Location: 525042-527293
  
  
**BlastP hit with Mycgr3G32432\_Mycgr3T**
  
Percentage identity: 51 %
  
BlastP bit score: 513
  
Sequence coverage: 80 %
  
E-value: 2e-170
  
  
 NCBI BlastP on this gene

GAA87450

MFS transporter
  
Accession: GAA87451
  
Location: 528679-530887
  
 NCBI BlastP on this gene

GAA87451

SUN domain protein
  
Accession: GAA87452
  
Location: 533064-534637
  
 NCBI BlastP on this gene

GAA87452

leucine carboxyl methyltransferase superfamily
  
Accession: GAA87453
  
Location: 536058-537210
  
 NCBI BlastP on this gene

GAA87453

hypothetical protein
  
Accession: GAA87454
  
Location: 537784-538473
  
 NCBI BlastP on this gene

GAA87454

RING zinc finger protein
  
Accession: GAA87455
  
Location: 538605-543844
  
 NCBI BlastP on this gene

GAA87455

53. :  AAHF01000004 Aspergillus fumigatus Af293     Total score: 2.0     Cumulative Blast bit score: 882

chromosome segregation protein BIR1, putative
  
Accession: EAL90739
  
Location: 1509155-1511974
  
 NCBI BlastP on this gene

EAL90739

C2H2 finger domain protein, putative
  
Accession: EAL90738
  
Location: 1508219-1508875
  
 NCBI BlastP on this gene

EAL90738

F-box domain protein
  
Accession: EAL90737
  
Location: 1507086-1507562
  
 NCBI BlastP on this gene

EAL90737

blue light-inducible protein Bli-3
  
Accession: EAL90736
  
Location: 1504992-1505673
  
 NCBI BlastP on this gene

EAL90736

SAGA-like transcriptional regulatory complex subunit Spt3, putative
  
Accession: EAL90735
  
Location: 1503575-1504715
  
 NCBI BlastP on this gene

EAL90735

diphthine synthase, putative
  
Accession: EAL90734
  
Location: 1501678-1502651
  
 NCBI BlastP on this gene

EAL90734

AP-2 adaptor complex subunit sigma, putative
  
Accession: EAL90733
  
Location: 1499309-1500040
  
 NCBI BlastP on this gene

EAL90733

hypothetical protein
  
Accession: EAL90732
  
Location: 1498027-1498335
  
 NCBI BlastP on this gene

EAL90732

conserved hypothetical protein
  
Accession: EAL90731
  
Location: 1495189-1496216
  
  
**BlastP hit with Mycgr3G52686\_Mycgr3T**
  
Percentage identity: 62 %
  
BlastP bit score: 370
  
Sequence coverage: 95 %
  
E-value: 2e-125
  
  
 NCBI BlastP on this gene

EAL90731

conserved hypothetical protein
  
Accession: EAL90730
  
Location: 1491238-1493212
  
  
**BlastP hit with Mycgr3G32432\_Mycgr3T**
  
Percentage identity: 50 %
  
BlastP bit score: 513
  
Sequence coverage: 82 %
  
E-value: 1e-171
  
  
 NCBI BlastP on this gene

EAL90730

MFS transporter, putative
  
Accession: EAL90729
  
Location: 1487320-1489545
  
 NCBI BlastP on this gene

EAL90729

DUF292 domian protein
  
Accession: EAL90728
  
Location: 1485238-1486809
  
 NCBI BlastP on this gene

EAL90728

short-chain oxidoreductase, putative
  
Accession: EAL90727
  
Location: 1483420-1484336
  
 NCBI BlastP on this gene

EAL90727

SUN domain protein (Adg3), putative
  
Accession: EAL90726
  
Location: 1481141-1482670
  
 NCBI BlastP on this gene

EAL90726

Leucine carboxyl methyltransferase superfamily
  
Accession: EAL90725
  
Location: 1478579-1479839
  
 NCBI BlastP on this gene

EAL90725

RING zinc finger protein, putative
  
Accession: EAL90724
  
Location: 1473022-1478284
  
 NCBI BlastP on this gene

EAL90724

54. :  DS027688 Neosartorya fischeri NRRL 181 1099437636249 genomic scaffold     Total score: 2.0     Cumulative Blast bit score: 882

chromosome segregation protein BIR1, putative
  
Accession: EAW22452
  
Location: 1186497-1189319
  
 NCBI BlastP on this gene

EAW22452

C2H2 finger domain protein, putative
  
Accession: EAW22453
  
Location: 1189599-1190255
  
 NCBI BlastP on this gene

EAW22453

F-box domain protein
  
Accession: EAW22454
  
Location: 1190938-1191414
  
 NCBI BlastP on this gene

EAW22454

blue light-inducible protein Bli-3
  
Accession: EAW22455
  
Location: 1192849-1193528
  
 NCBI BlastP on this gene

EAW22455

SAGA-like transcriptional regulatory complex subunit Spt3, putative
  
Accession: EAW22456
  
Location: 1193910-1195555
  
 NCBI BlastP on this gene

EAW22456

diphthine synthase, putative
  
Accession: EAW22457
  
Location: 1195872-1196845
  
 NCBI BlastP on this gene

EAW22457

conserved hypothetical protein
  
Accession: EAW22458
  
Location: 1197378-1198046
  
 NCBI BlastP on this gene

EAW22458

clathrin coat assembly protein ap17
  
Accession: EAW22459
  
Location: 1198490-1199221
  
 NCBI BlastP on this gene

EAW22459

conserved hypothetical protein
  
Accession: EAW22460
  
Location: 1202338-1203369
  
  
**BlastP hit with Mycgr3G52686\_Mycgr3T**
  
Percentage identity: 63 %
  
BlastP bit score: 371
  
Sequence coverage: 95 %
  
E-value: 1e-125
  
  
 NCBI BlastP on this gene

EAW22460

conserved hypothetical protein
  
Accession: EAW22461
  
Location: 1205380-1207619
  
  
**BlastP hit with Mycgr3G32432\_Mycgr3T**
  
Percentage identity: 49 %
  
BlastP bit score: 511
  
Sequence coverage: 82 %
  
E-value: 2e-169
  
  
 NCBI BlastP on this gene

EAW22461

MFS transporter, putative
  
Accession: EAW22462
  
Location: 1209061-1211293
  
 NCBI BlastP on this gene

EAW22462

conserved hypothetical protein
  
Accession: EAW22463
  
Location: 1211795-1213192
  
 NCBI BlastP on this gene

EAW22463

short-chain oxidoreductase, putative
  
Accession: EAW22464
  
Location: 1214267-1215182
  
 NCBI BlastP on this gene

EAW22464

SUN domain protein (Adg3), putative
  
Accession: EAW22465
  
Location: 1215902-1217441
  
 NCBI BlastP on this gene

EAW22465

Leucine carboxyl methyltransferase superfamily
  
Accession: EAW22466
  
Location: 1218697-1219957
  
 NCBI BlastP on this gene

EAW22466

RING zinc finger protein, putative
  
Accession: EAW22467
  
Location: 1220253-1225518
  
 NCBI BlastP on this gene

EAW22467

55. :  DS027059 Aspergillus clavatus NRRL 1 1099423829805 genomic scaffold     Total score: 2.0     Cumulative Blast bit score: 880

F-box domain protein
  
Accession: EAW07428
  
Location: 991065-991760
  
 NCBI BlastP on this gene

EAW07428

blue light-inducible protein Bli-3
  
Accession: EAW07429
  
Location: 993015-993817
  
 NCBI BlastP on this gene

EAW07429

SAGA-like transcriptional regulatory complex subunit Spt3, putative
  
Accession: EAW07430
  
Location: 994139-995886
  
 NCBI BlastP on this gene

EAW07430

diphthine synthase, putative
  
Accession: EAW07431
  
Location: 996215-997192
  
 NCBI BlastP on this gene

EAW07431

conserved hypothetical protein
  
Accession: EAW07432
  
Location: 997755-998503
  
 NCBI BlastP on this gene

EAW07432

AP-2 adaptor complex subunit sigma, putative
  
Accession: EAW07433
  
Location: 998910-999642
  
 NCBI BlastP on this gene

EAW07433

conserved hypothetical protein
  
Accession: EAW07434
  
Location: 1003408-1004436
  
  
**BlastP hit with Mycgr3G52686\_Mycgr3T**
  
Percentage identity: 63 %
  
BlastP bit score: 374
  
Sequence coverage: 95 %
  
E-value: 6e-127
  
  
 NCBI BlastP on this gene

EAW07434

conserved hypothetical protein
  
Accession: EAW07435
  
Location: 1007098-1009288
  
  
**BlastP hit with Mycgr3G32432\_Mycgr3T**
  
Percentage identity: 50 %
  
BlastP bit score: 506
  
Sequence coverage: 82 %
  
E-value: 4e-168
  
  
 NCBI BlastP on this gene

EAW07435

MFS transporter, putative
  
Accession: EAW07436
  
Location: 1011181-1013070
  
 NCBI BlastP on this gene

EAW07436

DUF292 domian protein
  
Accession: EAW07437
  
Location: 1013654-1015034
  
 NCBI BlastP on this gene

EAW07437

SUN domain protein (Adg3), putative
  
Accession: EAW07438
  
Location: 1015712-1017315
  
 NCBI BlastP on this gene

EAW07438

Leucine carboxyl methyltransferase superfamily
  
Accession: EAW07439
  
Location: 1018602-1020021
  
 NCBI BlastP on this gene

EAW07439

RING zinc finger protein, putative
  
Accession: EAW07440
  
Location: 1020197-1025458
  
 NCBI BlastP on this gene

EAW07440

56. :  JH226131 Exophiala dermatitidis NIH/UT8656 unplaced genomic scaffold supercont1.2     Total score: 2.0     Cumulative Blast bit score: 878

hypothetical protein
  
Accession: EHY53903
  
Location: 1587601-1588527
  
 NCBI BlastP on this gene

EHY53903

hypothetical protein
  
Accession: EHY53904
  
Location: 1590077-1591486
  
 NCBI BlastP on this gene

EHY53904

N-acetyltransferase (Nat5)
  
Accession: EHY53905
  
Location: 1592037-1592680
  
 NCBI BlastP on this gene

EHY53905

cytidine deaminase
  
Accession: EHY53906
  
Location: 1593373-1594098
  
 NCBI BlastP on this gene

EHY53906

hypothetical protein
  
Accession: EHY53907
  
Location: 1595248-1596165
  
 NCBI BlastP on this gene

EHY53907

valyl-tRNA synthetase
  
Accession: EHY53908
  
Location: 1597264-1598483
  
 NCBI BlastP on this gene

EHY53908

hypothetical protein
  
Accession: EHY53909
  
Location: 1600926-1601816
  
  
**BlastP hit with Mycgr3G52686\_Mycgr3T**
  
Percentage identity: 68 %
  
BlastP bit score: 413
  
Sequence coverage: 98 %
  
E-value: 7e-142
  
  
 NCBI BlastP on this gene

EHY53909

hypothetical protein
  
Accession: EHY53910
  
Location: 1603049-1605163
  
  
**BlastP hit with Mycgr3G32432\_Mycgr3T**
  
Percentage identity: 46 %
  
BlastP bit score: 466
  
Sequence coverage: 88 %
  
E-value: 1e-152
  
  
 NCBI BlastP on this gene

EHY53910

hypothetical protein
  
Accession: EHY53911
  
Location: 1605668-1606129
  
 NCBI BlastP on this gene

EHY53911

hypothetical protein
  
Accession: EHY53912
  
Location: 1607129-1608790
  
 NCBI BlastP on this gene

EHY53912

hypothetical protein
  
Accession: EHY53913
  
Location: 1609643-1610249
  
 NCBI BlastP on this gene

EHY53913

hypothetical protein
  
Accession: EHY53914
  
Location: 1610978-1612513
  
 NCBI BlastP on this gene

EHY53914

general transcription factor IIIA
  
Accession: EHY53915
  
Location: 1613899-1615626
  
 NCBI BlastP on this gene

EHY53915

DNA-directed RNA Polymerase II subunit F
  
Accession: EHY53916
  
Location: 1616067-1616700
  
 NCBI BlastP on this gene

EHY53916

hypothetical protein
  
Accession: EHY53917
  
Location: 1618524-1619282
  
 NCBI BlastP on this gene

EHY53917

57. :  EQ963474 Aspergillus flavus NRRL3357 scf\_1106286417496 genomic scaffold     Total score: 2.0     Cumulative Blast bit score: 878

conserved hypothetical protein
  
Accession: EED54491
  
Location: 1229288-1229926
  
 NCBI BlastP on this gene

EED54491

conserved hypothetical protein
  
Accession: EED54490
  
Location: 1227372-1228214
  
 NCBI BlastP on this gene

EED54490

blue light-inducible protein Bli-3
  
Accession: EED54489
  
Location: 1225760-1226524
  
 NCBI BlastP on this gene

EED54489

SAGA-like transcriptional regulatory complex subunit Spt3, putative
  
Accession: EED54488
  
Location: 1223861-1225386
  
 NCBI BlastP on this gene

EED54488

diphthine synthase, putative
  
Accession: EED54487
  
Location: 1222569-1223557
  
 NCBI BlastP on this gene

EED54487

conserved hypothetical protein
  
Accession: EED54486
  
Location: 1221758-1222420
  
 NCBI BlastP on this gene

EED54486

AP-2 adaptor complex subunit sigma, putative
  
Accession: EED54485
  
Location: 1220458-1221185
  
 NCBI BlastP on this gene

EED54485

NAD dependent epimerase/dehydratase, putative
  
Accession: EED54484
  
Location: 1216447-1217421
  
  
**BlastP hit with Mycgr3G52686\_Mycgr3T**
  
Percentage identity: 64 %
  
BlastP bit score: 372
  
Sequence coverage: 95 %
  
E-value: 5e-126
  
  
 NCBI BlastP on this gene

EED54484

conserved hypothetical protein
  
Accession: EED54483
  
Location: 1211726-1213997
  
  
**BlastP hit with Mycgr3G32432\_Mycgr3T**
  
Percentage identity: 51 %
  
BlastP bit score: 506
  
Sequence coverage: 82 %
  
E-value: 2e-167
  
  
 NCBI BlastP on this gene

EED54483

MFS transporter, putative
  
Accession: EED54482
  
Location: 1208403-1210609
  
 NCBI BlastP on this gene

EED54482

DUF292 domain protein
  
Accession: EED54481
  
Location: 1206766-1208010
  
 NCBI BlastP on this gene

EED54481

conserved hypothetical protein
  
Accession: EED54480
  
Location: 1204256-1204715
  
 NCBI BlastP on this gene

EED54480

hypothetical protein
  
Accession: EED54479
  
Location: 1203361-1203556
  
 NCBI BlastP on this gene

EED54479

cytochrome P450 oxidoreductase OrdA-like, putative
  
Accession: EED54478
  
Location: 1200588-1202512
  
 NCBI BlastP on this gene

EED54478

conserved hypothetical protein
  
Accession: EED54477
  
Location: 1196727-1199144
  
 NCBI BlastP on this gene

EED54477

58. :  BX649607 Aspergillus fumigatus BAC pilot project supercontig; segment 3/3.     Total score: 2.0     Cumulative Blast bit score: 871

hypothetical protein
  
Accession: CAF32117
  
Location: 105690-107846
  
 NCBI BlastP on this gene

AfA33H4.010c

possible zinc finger protein
  
Accession: CAF32116
  
Location: 104516-105410
  
 NCBI BlastP on this gene

AfA33H4.005

hypothetical protein
  
Accession: CAF32115
  
Location: 103621-104429
  
 NCBI BlastP on this gene

AfA33H4.001c

Bli-3 protein, putative
  
Accession: CAF32114
  
Location: 101527-102208
  
 NCBI BlastP on this gene

AfA31E11.020c

transcription factor spt3, putative
  
Accession: CAF32113
  
Location: 100110-101250
  
 NCBI BlastP on this gene

AfA31E11.015

diphthine synthase, putative
  
Accession: CAF32112
  
Location: 98213-99186
  
 NCBI BlastP on this gene

AfA31E11.010c

hypothetical protein
  
Accession: CAF32111
  
Location: 97012-97680
  
 NCBI BlastP on this gene

AfA31E11.005c

clathrin coat assembly protein, putative
  
Accession: CAF32110
  
Location: 95929-96492
  
 NCBI BlastP on this gene

AfA31E11.001c

hypothetical protein
  
Accession: CAF32109
  
Location: 94016-94870
  
 NCBI BlastP on this gene

AfA19D12.105

hypothetical protein, conserved
  
Accession: CAF32108
  
Location: 91724-92751
  
  
**BlastP hit with Mycgr3G52686\_Mycgr3T**
  
Percentage identity: 61 %
  
BlastP bit score: 358
  
Sequence coverage: 95 %
  
E-value: 9e-121
  
  
 NCBI BlastP on this gene

AfA19D12.100c

hypothetical protein
  
Accession: CAF32107
  
Location: 87773-89747
  
  
**BlastP hit with Mycgr3G32432\_Mycgr3T**
  
Percentage identity: 50 %
  
BlastP bit score: 513
  
Sequence coverage: 82 %
  
E-value: 1e-171
  
  
 NCBI BlastP on this gene

AfA19D12.095c

possible transporter-like protein
  
Accession: CAF32106
  
Location: 83855-86080
  
 NCBI BlastP on this gene

AfA19D12.090c

hypothetical protein with DUF292 domain, putative
  
Accession: CAF32105
  
Location: 81536-83344
  
 NCBI BlastP on this gene

AfA19D12.085

short-chain oxidoreductase, putative
  
Accession: CAF32104
  
Location: 79955-80871
  
 NCBI BlastP on this gene

AfA19D12.080

SUN family protein, putative
  
Accession: CAF32103
  
Location: 77676-79205
  
 NCBI BlastP on this gene

AfA19D12.075

hypothetical protein, conserved
  
Accession: CAF32102
  
Location: 74889-76374
  
 NCBI BlastP on this gene

AfA19D12.070

zinc finger protein, putative
  
Accession: CAF32101
  
Location: 69557-74819
  
 NCBI BlastP on this gene

AfA19D12.065

59. :  KB644408 Penicillium oxalicum 114-2 unplaced genomic scaffold scaffold\_1     Total score: 2.0     Cumulative Blast bit score: 869

hypothetical protein
  
Accession: EPS25317
  
Location: 674257-674825
  
 NCBI BlastP on this gene

EPS25317

hypothetical protein
  
Accession: EPS25318
  
Location: 675732-676502
  
 NCBI BlastP on this gene

EPS25318

hypothetical protein
  
Accession: EPS25319
  
Location: 677025-678409
  
 NCBI BlastP on this gene

EPS25319

hypothetical protein
  
Accession: EPS25320
  
Location: 679678-680383
  
 NCBI BlastP on this gene

EPS25320

hypothetical protein
  
Accession: EPS25321
  
Location: 681023-681767
  
 NCBI BlastP on this gene

EPS25321

hypothetical protein
  
Accession: EPS25322
  
Location: 686425-687370
  
  
**BlastP hit with Mycgr3G52686\_Mycgr3T**
  
Percentage identity: 63 %
  
BlastP bit score: 372
  
Sequence coverage: 95 %
  
E-value: 5e-126
  
  
 NCBI BlastP on this gene

EPS25322

hypothetical protein
  
Accession: EPS25323
  
Location: 692132-694516
  
  
**BlastP hit with Mycgr3G32432\_Mycgr3T**
  
Percentage identity: 50 %
  
BlastP bit score: 497
  
Sequence coverage: 79 %
  
E-value: 3e-164
  
  
 NCBI BlastP on this gene

EPS25323

hypothetical protein
  
Accession: EPS25324
  
Location: 696590-697364
  
 NCBI BlastP on this gene

EPS25324

hypothetical protein
  
Accession: EPS25325
  
Location: 698294-700585
  
 NCBI BlastP on this gene

EPS25325

hypothetical protein
  
Accession: EPS25326
  
Location: 701087-702353
  
 NCBI BlastP on this gene

EPS25326

hypothetical protein
  
Accession: EPS25327
  
Location: 702575-703620
  
 NCBI BlastP on this gene

EPS25327

hypothetical protein
  
Accession: EPS25328
  
Location: 705205-706268
  
 NCBI BlastP on this gene

EPS25328

60. :  ACJE01000021 Aspergillus niger ATCC 1015     Total score: 2.0     Cumulative Blast bit score: 840

hypothetical protein
  
Accession: EHA18250
  
Location: 1724480-1726119
  
 NCBI BlastP on this gene

EHA18250

hypothetical protein
  
Accession: EHA18249
  
Location: 1723129-1724128
  
 NCBI BlastP on this gene

EHA18249

hypothetical protein
  
Accession: EHA18248
  
Location: 1722236-1722932
  
 NCBI BlastP on this gene

EHA18248

hypothetical protein
  
Accession: EHA18247
  
Location: 1720724-1721467
  
 NCBI BlastP on this gene

EHA18247

hypothetical protein
  
Accession: EHA18246
  
Location: 1714610-1715635
  
  
**BlastP hit with Mycgr3G52686\_Mycgr3T**
  
Percentage identity: 64 %
  
BlastP bit score: 370
  
Sequence coverage: 95 %
  
E-value: 4e-125
  
  
 NCBI BlastP on this gene

EHA18246

hypothetical protein
  
Accession: EHA18245
  
Location: 1705321-1711158
  
  
**BlastP hit with Mycgr3G32432\_Mycgr3T**
  
Percentage identity: 43 %
  
BlastP bit score: 470
  
Sequence coverage: 95 %
  
E-value: 8e-147
  
  
 NCBI BlastP on this gene

EHA18245

hypothetical protein
  
Accession: EHA18244
  
Location: 1703861-1705003
  
 NCBI BlastP on this gene

EHA18244

hypothetical protein
  
Accession: EHA18243
  
Location: 1701660-1702591
  
 NCBI BlastP on this gene

EHA18243

hypothetical protein
  
Accession: EHA18242
  
Location: 1699014-1700188
  
 NCBI BlastP on this gene

EHA18242

hypothetical protein
  
Accession: EHA18241
  
Location: 1693378-1698619
  
 NCBI BlastP on this gene

EHA18241

61. :  AM270171 Aspergillus niger contig An08c0160, genomic contig.     Total score: 2.0     Cumulative Blast bit score: 831

not annotated
  
Accession: CAK39972
  
Location: 52122-55076
  
 NCBI BlastP on this gene

An08g07250

not annotated
  
Accession: CAK39971
  
Location: 50952-51754
  
 NCBI BlastP on this gene

An08g07240

not annotated
  
Accession: CAK39970
  
Location: 49356-50438
  
 NCBI BlastP on this gene

An08g07230

not annotated
  
Accession: CAK39969
  
Location: 47433-48432
  
 NCBI BlastP on this gene

An08g07220

not annotated
  
Accession: CAK39968
  
Location: 46540-47236
  
 NCBI BlastP on this gene

An08g07210

not annotated
  
Accession: CAK39967
  
Location: 45028-45771
  
 NCBI BlastP on this gene

An08g07200

hypothetical protein
  
Accession: CAK39966
  
Location: 43840-44423
  
 NCBI BlastP on this gene

An08g07190

hypothetical protein
  
Accession: CAK39965
  
Location: 43023-43773
  
 NCBI BlastP on this gene

An08g07180

hypothetical protein
  
Accession: CAK39964
  
Location: 41517-42768
  
 NCBI BlastP on this gene

An08g07170

hypothetical protein
  
Accession: CAK39963
  
Location: 40404-41318
  
 NCBI BlastP on this gene

An08g07160

not annotated
  
Accession: CAK39962
  
Location: 38914-39939
  
  
**BlastP hit with Mycgr3G52686\_Mycgr3T**
  
Percentage identity: 64 %
  
BlastP bit score: 370
  
Sequence coverage: 95 %
  
E-value: 4e-125
  
  
 NCBI BlastP on this gene

An08g07150

hypothetical protein
  
Accession: CAK39961
  
Location: 38216-38566
  
 NCBI BlastP on this gene

An08g07140

hypothetical protein
  
Accession: CAK39960
  
Location: 35580-36294
  
 NCBI BlastP on this gene

An08g07130

unnamed
  
Accession: CAK39959
  
Location: 33245-35496
  
  
**BlastP hit with Mycgr3G32432\_Mycgr3T**
  
Percentage identity: 50 %
  
BlastP bit score: 462
  
Sequence coverage: 75 %
  
E-value: 3e-150
  
  
 NCBI BlastP on this gene

An08g07120

not annotated
  
Accession: CAK39958
  
Location: 29616-32985
  
 NCBI BlastP on this gene

An08g07110

not annotated
  
Accession: CAK39957
  
Location: 28342-29301
  
 NCBI BlastP on this gene

An08g07100

unnamed
  
Accession: CAK39956
  
Location: 25832-27453
  
 NCBI BlastP on this gene

An08g07090

not annotated
  
Accession: CAK39955
  
Location: 23306-24480
  
 NCBI BlastP on this gene

An08g07080

unnamed
  
Accession: CAK39954
  
Location: 17670-22911
  
 NCBI BlastP on this gene

An08g07070

62. :  AM920431 Penicillium chrysogenum Wisconsin 54-1255 complete genome, contig Pc00c16.     Total score: 2.0     Cumulative Blast bit score: 831

not annotated
  
Accession: CAP93912
  
Location: 2999719-3000278
  
 NCBI BlastP on this gene

Pc16g12420

not annotated
  
Accession: CAP93913
  
Location: 3001787-3003441
  
 NCBI BlastP on this gene

Pc16g12430

not annotated
  
Accession: CAP93914
  
Location: 3003693-3004926
  
 NCBI BlastP on this gene

Pc16g12440

not annotated
  
Accession: CAP93915
  
Location: 3006066-3007271
  
 NCBI BlastP on this gene

Pc16g12450

not annotated
  
Accession: CAP93916
  
Location: 3008120-3008962
  
 NCBI BlastP on this gene

Pc16g12460

not annotated
  
Accession: CAP93917
  
Location: 3009295-3010046
  
 NCBI BlastP on this gene

Pc16g12470

not annotated
  
Accession: CAP93918
  
Location: 3012331-3013349
  
  
**BlastP hit with Mycgr3G52686\_Mycgr3T**
  
Percentage identity: 61 %
  
BlastP bit score: 370
  
Sequence coverage: 96 %
  
E-value: 2e-125
  
  
 NCBI BlastP on this gene

Pc16g12480

hypothetical protein
  
Accession: CAP93919
  
Location: 3013767-3014206
  
 NCBI BlastP on this gene

Pc16g12490

hypothetical protein
  
Accession: CAP93920
  
Location: 3014487-3015207
  
 NCBI BlastP on this gene

Pc16g12500

not annotated
  
Accession: CAP93921
  
Location: 3016159-3018178
  
  
**BlastP hit with Mycgr3G32432\_Mycgr3T**
  
Percentage identity: 51 %
  
BlastP bit score: 461
  
Sequence coverage: 75 %
  
E-value: 7e-151
  
  
 NCBI BlastP on this gene

Pc16g12510

not annotated
  
Accession: CAP93922
  
Location: 3019061-3020960
  
 NCBI BlastP on this gene

Pc16g12520

not annotated
  
Accession: CAP93923
  
Location: 3021262-3021954
  
 NCBI BlastP on this gene

Pc16g12530

not annotated
  
Accession: CAP93924
  
Location: 3023555-3025592
  
 NCBI BlastP on this gene

Pc16g12540

not annotated
  
Accession: CAP93925
  
Location: 3026127-3027138
  
 NCBI BlastP on this gene

Pc16g12550

not annotated
  
Accession: CAP93926
  
Location: 3028446-3030024
  
 NCBI BlastP on this gene

Pc16g12560

not annotated
  
Accession: CAP93927
  
Location: 3030460-3030812
  
 NCBI BlastP on this gene

Pc16g12570

not annotated
  
Accession: CAP93928
  
Location: 3031007-3031765
  
 NCBI BlastP on this gene

Pc16g12580

63. :  AACD01000011 Aspergillus nidulans FGSC A4     Total score: 2.0     Cumulative Blast bit score: 797

hypothetical protein
  
Accession: EAA65194
  
Location: 35933-37340
  
 NCBI BlastP on this gene

EAA65194

predicted protein
  
Accession: EAA65195
  
Location: 37621-39864
  
 NCBI BlastP on this gene

EAA65195

hypothetical protein
  
Accession: EAA65196
  
Location: 40229-41916
  
 NCBI BlastP on this gene

EAA65196

hypothetical protein
  
Accession: EAA65197
  
Location: 42190-43190
  
 NCBI BlastP on this gene

EAA65197

hypothetical protein
  
Accession: EAA65198
  
Location: 43370-44051
  
 NCBI BlastP on this gene

EAA65198

conserved hypothetical protein
  
Accession: EAA65199
  
Location: 44685-45382
  
 NCBI BlastP on this gene

EAA65199

hypothetical protein
  
Accession: EAA65200
  
Location: 48130-49104
  
  
**BlastP hit with Mycgr3G52686\_Mycgr3T**
  
Percentage identity: 60 %
  
BlastP bit score: 334
  
Sequence coverage: 95 %
  
E-value: 3e-111
  
  
 NCBI BlastP on this gene

EAA65200

hypothetical protein
  
Accession: EAA65201
  
Location: 51163-56292
  
  
**BlastP hit with Mycgr3G32432\_Mycgr3T**
  
Percentage identity: 47 %
  
BlastP bit score: 464
  
Sequence coverage: 84 %
  
E-value: 1e-144
  
  
 NCBI BlastP on this gene

EAA65201

hypothetical protein
  
Accession: EAA65202
  
Location: 56626-58016
  
 NCBI BlastP on this gene

EAA65202

hypothetical protein
  
Accession: EAA65203
  
Location: 58580-60037
  
 NCBI BlastP on this gene

EAA65203

64. :  CH476594 Aspergillus terreus NIH2624 scaffold\_1 genomic scaffold     Total score: 2.0     Cumulative Blast bit score: 766

predicted protein
  
Accession: EAU39251
  
Location: 1706227-1707220
  
 NCBI BlastP on this gene

EAU39251

conserved hypothetical protein
  
Accession: EAU39250
  
Location: 1704543-1705277
  
 NCBI BlastP on this gene

EAU39250

protein spt3
  
Accession: EAU39249
  
Location: 1703089-1704288
  
 NCBI BlastP on this gene

EAU39249

diphthine synthase
  
Accession: EAU39248
  
Location: 1701698-1702576
  
 NCBI BlastP on this gene

EAU39248

conserved hypothetical protein
  
Accession: EAU39247
  
Location: 1700730-1701396
  
 NCBI BlastP on this gene

EAU39247

AP-2 complex subunit sigma
  
Accession: EAU39246
  
Location: 1699460-1700178
  
 NCBI BlastP on this gene

EAU39246

conserved hypothetical protein
  
Accession: EAU39245
  
Location: 1695023-1696063
  
  
**BlastP hit with Mycgr3G52686\_Mycgr3T**
  
Percentage identity: 64 %
  
BlastP bit score: 376
  
Sequence coverage: 95 %
  
E-value: 2e-127
  
  
 NCBI BlastP on this gene

EAU39245

conserved hypothetical protein
  
Accession: EAU39244
  
Location: 1687460-1692182
  
  
**BlastP hit with Mycgr3G32432\_Mycgr3T**
  
Percentage identity: 48 %
  
BlastP bit score: 390
  
Sequence coverage: 67 %
  
E-value: 3e-118
  
  
 NCBI BlastP on this gene

EAU39244

conserved hypothetical protein
  
Accession: EAU39243
  
Location: 1686159-1686870
  
 NCBI BlastP on this gene

EAU39243

conserved hypothetical protein
  
Accession: EAU39242
  
Location: 1684136-1685596
  
 NCBI BlastP on this gene

EAU39242

conserved hypothetical protein
  
Accession: EAU39241
  
Location: 1676743-1683104
  
 NCBI BlastP on this gene

EAU39241

DNA repair helicase RAD3
  
Accession: EAU39240
  
Location: 1673612-1676101
  
 NCBI BlastP on this gene

EAU39240

65. :  AHHD01000271 Macrophomina phaseolina MS6     Total score: 2.0     Cumulative Blast bit score: 734

hypothetical protein
  
Accession: EKG16381
  
Location: 27795-28720
  
 NCBI BlastP on this gene

EKG16381

hypothetical protein
  
Accession: EKG16380
  
Location: 24272-25749
  
 NCBI BlastP on this gene

EKG16380

NmrA-like protein
  
Accession: EKG16379
  
Location: 21930-22625
  
 NCBI BlastP on this gene

EKG16379

Alcohol dehydrogenase superfamily zinc-containing
  
Accession: EKG16378
  
Location: 16979-18055
  
  
**BlastP hit with Mycgr3G102276\_Mycgr3**
  
Percentage identity: 63 %
  
BlastP bit score: 442
  
Sequence coverage: 99 %
  
E-value: 2e-151
  
  
 NCBI BlastP on this gene

EKG16378

hypothetical protein
  
Accession: EKG16377
  
Location: 13637-14186
  
 NCBI BlastP on this gene

EKG16377

Pyoverdine biosynthesis
  
Accession: EKG16376
  
Location: 11193-11875
  
 NCBI BlastP on this gene

EKG16376

Pyoverdine biosynthesis
  
Accession: EKG16375
  
Location: 9867-11067
  
 NCBI BlastP on this gene

EKG16375

Major facilitator superfamily
  
Accession: EKG16374
  
Location: 7404-8776
  
  
**BlastP hit with Mycgr3G65725\_Mycgr3T**
  
Percentage identity: 37 %
  
BlastP bit score: 292
  
Sequence coverage: 84 %
  
E-value: 5e-90
  
  
 NCBI BlastP on this gene

EKG16374

hypothetical protein
  
Accession: EKG16373
  
Location: 3703-4524
  
 NCBI BlastP on this gene

EKG16373

66. :  EQ963485 Aspergillus flavus NRRL3357 scf\_1106286419448 genomic scaffold     Total score: 2.0     Cumulative Blast bit score: 700

cytochrome P450, putative
  
Accession: EED45921
  
Location: 1474631-1476355
  
 NCBI BlastP on this gene

EED45921

conserved hypothetical protein
  
Accession: EED45922
  
Location: 1476852-1477769
  
 NCBI BlastP on this gene

EED45922

NRPS-like enzyme, putative
  
Accession: EED45923
  
Location: 1479298-1482390
  
  
**BlastP hit with Mycgr3G107072\_Mycgr3**
  
Percentage identity: 27 %
  
BlastP bit score: 301
  
Sequence coverage: 77 %
  
E-value: 4e-82
  
  
 NCBI BlastP on this gene

EED45923

NADH-dependent flavin oxidoreductase, putative
  
Accession: EED45924
  
Location: 1483117-1484250
  
 NCBI BlastP on this gene

EED45924

MFS multidrug transporter, putative
  
Accession: EED45925
  
Location: 1485551-1487055
  
 NCBI BlastP on this gene

EED45925

conserved hypothetical protein
  
Accession: EED45926
  
Location: 1491278-1492114
  
 NCBI BlastP on this gene

EED45926

conserved hypothetical protein
  
Accession: EED45927
  
Location: 1492324-1493409
  
 NCBI BlastP on this gene

EED45927

conserved hypothetical protein
  
Accession: EED45928
  
Location: 1495959-1496569
  
 NCBI BlastP on this gene

EED45928

C-4 methyl sterol oxidase, putative
  
Accession: EED45929
  
Location: 1496865-1497827
  
 NCBI BlastP on this gene

EED45929

zinc-binding alcohol dehydrogenase, putative
  
Accession: EED45930
  
Location: 1500353-1501492
  
  
**BlastP hit with Mycgr3G102276\_Mycgr3**
  
Percentage identity: 57 %
  
BlastP bit score: 399
  
Sequence coverage: 99 %
  
E-value: 3e-134
  
  
 NCBI BlastP on this gene

EED45930

conserved hypothetical protein
  
Accession: EED45931
  
Location: 1501551-1502374
  
 NCBI BlastP on this gene

EED45931

cytochrome P450, putative
  
Accession: EED45932
  
Location: 1502766-1504429
  
 NCBI BlastP on this gene

EED45932

conserved hypothetical protein
  
Accession: EED45933
  
Location: 1505326-1506363
  
 NCBI BlastP on this gene

EED45933

67. :  GG697417 Glomerella graminicola M1.001 genomic scaffold supercont1.87     Total score: 2.0     Cumulative Blast bit score: 648

hypothetical protein
  
Accession: EFQ36299
  
Location: 83651-85458
  
  
**BlastP hit with Mycgr3G32432\_Mycgr3T**
  
Percentage identity: 38 %
  
BlastP bit score: 326
  
Sequence coverage: 80 %
  
E-value: 3e-100
  
  
 NCBI BlastP on this gene

EFQ36299

hypothetical protein
  
Accession: EFQ36298
  
Location: 82673-83130
  
 NCBI BlastP on this gene

EFQ36298

hypothetical protein
  
Accession: EFQ36297
  
Location: 74594-75450
  
  
**BlastP hit with Mycgr3G52686\_Mycgr3T**
  
Percentage identity: 61 %
  
BlastP bit score: 322
  
Sequence coverage: 85 %
  
E-value: 8e-107
  
  
 NCBI BlastP on this gene

EFQ36297

hypothetical protein
  
Accession: EFQ36296
  
Location: 73093-73794
  
 NCBI BlastP on this gene

EFQ36296

hypothetical protein
  
Accession: EFQ36295
  
Location: 68758-69467
  
 NCBI BlastP on this gene

EFQ36295

short chain dehydrogenase
  
Accession: EFQ36294
  
Location: 66252-67464
  
 NCBI BlastP on this gene

EFQ36294

capsule polysaccharide biosynthesis protein
  
Accession: EFQ36293
  
Location: 64203-65486
  
 NCBI BlastP on this gene

EFQ36293

68. :  KB726028 Colletotrichum orbiculare MAFF 240422 unplaced genomic scaffold Scaffold\_454     Total score: 2.0     Cumulative Blast bit score: 615

ankyrin repeat protein
  
Accession: ENH79132
  
Location: 350291-351484
  
 NCBI BlastP on this gene

ENH79132

hypothetical protein
  
Accession: ENH79133
  
Location: 352068-353891
  
  
**BlastP hit with Mycgr3G32432\_Mycgr3T**
  
Percentage identity: 43 %
  
BlastP bit score: 366
  
Sequence coverage: 80 %
  
E-value: 3e-115
  
  
 NCBI BlastP on this gene

ENH79133

gram-positive signal ysirk family
  
Accession: ENH79134
  
Location: 354256-354718
  
 NCBI BlastP on this gene

ENH79134

nad dependent epimerase
  
Accession: ENH79135
  
Location: 355525-358086
  
  
**BlastP hit with Mycgr3G52686\_Mycgr3T**
  
Percentage identity: 57 %
  
BlastP bit score: 249
  
Sequence coverage: 70 %
  
E-value: 8e-74
  
  
 NCBI BlastP on this gene

ENH79135

C6 transcription factor
  
Accession: ENH79136
  
Location: 368157-370414
  
 NCBI BlastP on this gene

ENH79136

69. :  CH476597 Aspergillus terreus NIH2624 scaffold\_4 genomic scaffold     Total score: 2.0     Cumulative Blast bit score: 615

hypothetical protein
  
Accession: EAU36720
  
Location: 1835763-1843945
  
 NCBI BlastP on this gene

EAU36720

predicted protein
  
Accession: EAU36721
  
Location: 1844712-1845911
  
 NCBI BlastP on this gene

EAU36721

conserved hypothetical protein
  
Accession: EAU36722
  
Location: 1847139-1847581
  
 NCBI BlastP on this gene

EAU36722

conserved hypothetical protein
  
Accession: EAU36723
  
Location: 1848099-1849330
  
 NCBI BlastP on this gene

EAU36723

predicted protein
  
Accession: EAU36724
  
Location: 1849784-1851077
  
 NCBI BlastP on this gene

EAU36724

conserved hypothetical protein
  
Accession: EAU36725
  
Location: 1851718-1852477
  
 NCBI BlastP on this gene

EAU36725

conserved hypothetical protein
  
Accession: EAU36726
  
Location: 1852607-1853534
  
  
**BlastP hit with Mycgr3G52686\_Mycgr3T**
  
Percentage identity: 33 %
  
BlastP bit score: 123
  
Sequence coverage: 93 %
  
E-value: 1e-29
  
  
 NCBI BlastP on this gene

EAU36726

predicted protein
  
Accession: EAU36727
  
Location: 1854395-1855967
  
 NCBI BlastP on this gene

EAU36727

conserved hypothetical protein
  
Accession: EAU36728
  
Location: 1856614-1858599
  
  
**BlastP hit with Mycgr3G107069\_Mycgr3**
  
Percentage identity: 43 %
  
BlastP bit score: 492
  
Sequence coverage: 100 %
  
E-value: 3e-162
  
  
 NCBI BlastP on this gene

EAU36728

conserved hypothetical protein
  
Accession: EAU36729
  
Location: 1859547-1860491
  
 NCBI BlastP on this gene

EAU36729

predicted protein
  
Accession: EAU36730
  
Location: 1860895-1863542
  
 NCBI BlastP on this gene

EAU36730

predicted protein
  
Accession: EAU36731
  
Location: 1863869-1865716
  
 NCBI BlastP on this gene

EAU36731

conserved hypothetical protein
  
Accession: EAU36732
  
Location: 1868322-1873754
  
 NCBI BlastP on this gene

EAU36732

70. :  AP007174 Aspergillus oryzae RIB40 DNA, SC103.     Total score: 2.0     Cumulative Blast bit score: 613

not annotated
  
Accession: BAE65796
  
Location: 948513-950495
  
 NCBI BlastP on this gene

AO090103000361

not annotated
  
Accession: BAE65797
  
Location: 951716-952170
  
 NCBI BlastP on this gene

AO090103000362

not annotated
  
Accession: BAE65798
  
Location: 952615-953968
  
 NCBI BlastP on this gene

AO090103000363

not annotated
  
Accession: BAE65799
  
Location: 954644-955834
  
 NCBI BlastP on this gene

AO090103000364

not annotated
  
Accession: BAE65800
  
Location: 957630-958541
  
 NCBI BlastP on this gene

AO090103000365

not annotated
  
Accession: BAE65801
  
Location: 958936-961522
  
 NCBI BlastP on this gene

AO090103000366

not annotated
  
Accession: BAE65802
  
Location: 962403-964382
  
  
**BlastP hit with Mycgr3G107069\_Mycgr3**
  
Percentage identity: 41 %
  
BlastP bit score: 494
  
Sequence coverage: 104 %
  
E-value: 4e-163
  
  
 NCBI BlastP on this gene

AO090103000367

not annotated
  
Accession: BAE65803
  
Location: 965455-966704
  
 NCBI BlastP on this gene

AO090103000369

not annotated
  
Accession: BAE65804
  
Location: 966912-967949
  
 NCBI BlastP on this gene

AO090103000370

not annotated
  
Accession: BAE65805
  
Location: 968818-969783
  
  
**BlastP hit with Mycgr3G52686\_Mycgr3T**
  
Percentage identity: 31 %
  
BlastP bit score: 119
  
Sequence coverage: 93 %
  
E-value: 4e-28
  
  
 NCBI BlastP on this gene

AO090103000371

not annotated
  
Accession: BAE65806
  
Location: 970247-971113
  
 NCBI BlastP on this gene

AO090103000372

not annotated
  
Accession: BAE65807
  
Location: 975581-976603
  
 NCBI BlastP on this gene

AO090103000374

not annotated
  
Accession: BAE65808
  
Location: 977041-977674
  
 NCBI BlastP on this gene

AO090103000375

not annotated
  
Accession: BAE65809
  
Location: 979245-982605
  
 NCBI BlastP on this gene

AO090103000376

71. :  AKHY01000199 Aspergillus oryzae 3.042     Total score: 2.0     Cumulative Blast bit score: 609

putative flavoprotein involved in K+ transport
  
Accession: EIT73798
  
Location: 28234-30216
  
 NCBI BlastP on this gene

EIT73798

hypothetical protein
  
Accession: EIT73790
  
Location: 26659-27012
  
 NCBI BlastP on this gene

EIT73790

hypothetical protein
  
Accession: EIT73701
  
Location: 24760-26113
  
 NCBI BlastP on this gene

EIT73701

hypothetical protein
  
Accession: EIT73608
  
Location: 22895-24084
  
 NCBI BlastP on this gene

EIT73608

hypothetical protein
  
Accession: EIT73545
  
Location: 20188-21099
  
 NCBI BlastP on this gene

EIT73545

dehydrogenase with different specificitie
  
Accession: EIT73825
  
Location: 17219-19793
  
 NCBI BlastP on this gene

EIT73825

ferric reductase, NADH/NADPH oxidase
  
Accession: EIT73566
  
Location: 14359-16338
  
  
**BlastP hit with Mycgr3G107069\_Mycgr3**
  
Percentage identity: 41 %
  
BlastP bit score: 488
  
Sequence coverage: 104 %
  
E-value: 1e-160
  
  
 NCBI BlastP on this gene

EIT73566

hypothetical protein
  
Accession: EIT73711
  
Location: 12042-13291
  
 NCBI BlastP on this gene

EIT73711

proline racemase
  
Accession: EIT73709
  
Location: 10797-11834
  
 NCBI BlastP on this gene

EIT73709

hypothetical protein
  
Accession: EIT73723
  
Location: 8963-9928
  
  
**BlastP hit with Mycgr3G52686\_Mycgr3T**
  
Percentage identity: 31 %
  
BlastP bit score: 121
  
Sequence coverage: 93 %
  
E-value: 1e-28
  
  
 NCBI BlastP on this gene

EIT73723

hypothetical protein
  
Accession: EIT73531
  
Location: 7633-8247
  
 NCBI BlastP on this gene

EIT73531

72. :  DS027060 Aspergillus clavatus NRRL 1 1099423829806 genomic scaffold     Total score: 2.0     Cumulative Blast bit score: 605

conserved hypothetical protein
  
Accession: EAW07011
  
Location: 2302787-2303782
  
 NCBI BlastP on this gene

EAW07011

conserved hypothetical protein
  
Accession: EAW07010
  
Location: 2301086-2301589
  
 NCBI BlastP on this gene

EAW07010

conserved hypothetical protein
  
Accession: EAW07009
  
Location: 2298771-2299792
  
  
**BlastP hit with Mycgr3G52686\_Mycgr3T**
  
Percentage identity: 34 %
  
BlastP bit score: 120
  
Sequence coverage: 92 %
  
E-value: 3e-28
  
  
 NCBI BlastP on this gene

EAW07009

ferric-chelate reductase, putative
  
Accession: EAW07008
  
Location: 2296265-2298169
  
  
**BlastP hit with Mycgr3G107069\_Mycgr3**
  
Percentage identity: 41 %
  
BlastP bit score: 485
  
Sequence coverage: 98 %
  
E-value: 5e-160
  
  
 NCBI BlastP on this gene

EAW07008

alpha-1,2-mannosidase, putative subfamily
  
Accession: EAW07007
  
Location: 2293312-2295749
  
 NCBI BlastP on this gene

EAW07007

PHD finger domain protein
  
Accession: EAW07006
  
Location: 2292223-2293232
  
 NCBI BlastP on this gene

EAW07006

C6 finger domain protein, putative
  
Accession: EAW07005
  
Location: 2289332-2291734
  
 NCBI BlastP on this gene

EAW07005

MFS multidrug transporter, putative
  
Accession: EAW07004
  
Location: 2286351-2288214
  
 NCBI BlastP on this gene

EAW07004

flavin-binding monooxygenase, putative
  
Accession: EAW07003
  
Location: 2283231-2285275
  
 NCBI BlastP on this gene

EAW07003

conserved hypothetical protein
  
Accession: EAW07002
  
Location: 2280987-2282342
  
 NCBI BlastP on this gene

EAW07002

73. :  DS499594 Aspergillus fumigatus A1163 scf\_000001 genomic scaffold     Total score: 2.0     Cumulative Blast bit score: 594

DNA topoisomerase II
  
Accession: EDP55414
  
Location: 272389-277511
  
 NCBI BlastP on this gene

EDP55414

hypothetical protein
  
Accession: EDP55413
  
Location: 270109-271038
  
 NCBI BlastP on this gene

EDP55413

hypothetical protein
  
Accession: EDP55412
  
Location: 267538-269914
  
 NCBI BlastP on this gene

EDP55412

class V chitinase, putative
  
Accession: EDP55411
  
Location: 262383-266843
  
 NCBI BlastP on this gene

EDP55411

hypothetical protein
  
Accession: EDP55410
  
Location: 260465-260632
  
 NCBI BlastP on this gene

EDP55410

conserved hypothetical protein
  
Accession: EDP55409
  
Location: 257484-258438
  
  
**BlastP hit with Mycgr3G52686\_Mycgr3T**
  
Percentage identity: 36 %
  
BlastP bit score: 128
  
Sequence coverage: 94 %
  
E-value: 4e-31
  
  
 NCBI BlastP on this gene

EDP55409

ferric-chelate reductase, putative
  
Accession: EDP55408
  
Location: 255084-257042
  
  
**BlastP hit with Mycgr3G107069\_Mycgr3**
  
Percentage identity: 39 %
  
BlastP bit score: 466
  
Sequence coverage: 100 %
  
E-value: 2e-152
  
  
 NCBI BlastP on this gene

EDP55408

alpha-1,2-mannosidase, putative subfamily
  
Accession: EDP55407
  
Location: 252516-254960
  
 NCBI BlastP on this gene

EDP55407

PHD finger protein
  
Accession: EDP55406
  
Location: 250552-251515
  
 NCBI BlastP on this gene

EDP55406

C6 finger domain protein, putative
  
Accession: EDP55405
  
Location: 247785-250137
  
 NCBI BlastP on this gene

EDP55405

MFS multidrug transporter, putative
  
Accession: EDP55404
  
Location: 245078-246960
  
 NCBI BlastP on this gene

EDP55404

flavin-binding monooxygenase, putative
  
Accession: EDP55403
  
Location: 242088-244128
  
 NCBI BlastP on this gene

EDP55403

integral membrane protein Pth11-like, putative
  
Accession: EDP55402
  
Location: 239789-241182
  
 NCBI BlastP on this gene

EDP55402

74. :  AAHF01000006 Aspergillus fumigatus Af293     Total score: 2.0     Cumulative Blast bit score: 590

DNA topoisomerase II
  
Accession: EAL89207
  
Location: 2172483-2177605
  
 NCBI BlastP on this gene

EAL89207

hypothetical protein
  
Accession: EAL89208
  
Location: 2178956-2179885
  
 NCBI BlastP on this gene

EAL89208

hypothetical protein
  
Accession: EAL89209
  
Location: 2180080-2182456
  
 NCBI BlastP on this gene

EAL89209

class V chitinase, putative
  
Accession: EAL89210
  
Location: 2183151-2187611
  
 NCBI BlastP on this gene

EAL89210

hypothetical protein
  
Accession: EAL89211
  
Location: 2189362-2189529
  
 NCBI BlastP on this gene

EAL89211

conserved hypothetical protein
  
Accession: EAL89212
  
Location: 2191556-2192510
  
  
**BlastP hit with Mycgr3G52686\_Mycgr3T**
  
Percentage identity: 36 %
  
BlastP bit score: 128
  
Sequence coverage: 94 %
  
E-value: 4e-31
  
  
 NCBI BlastP on this gene

EAL89212

ferric-chelate reductase, putative
  
Accession: EAL89213
  
Location: 2192952-2194910
  
  
**BlastP hit with Mycgr3G107069\_Mycgr3**
  
Percentage identity: 39 %
  
BlastP bit score: 462
  
Sequence coverage: 100 %
  
E-value: 9e-151
  
  
 NCBI BlastP on this gene

EAL89213

alpha-1,2-mannosidase, putative subfamily
  
Accession: EAL89214
  
Location: 2195034-2197478
  
 NCBI BlastP on this gene

EAL89214

PHD finger protein
  
Accession: EBA27370
  
Location: 2198479-2199442
  
 NCBI BlastP on this gene

EBA27370

C6 finger domain protein, putative
  
Accession: EAL89215
  
Location: 2199857-2202209
  
 NCBI BlastP on this gene

EAL89215

MFS multidrug transporter, putative
  
Accession: EAL89216
  
Location: 2203034-2204916
  
 NCBI BlastP on this gene

EAL89216

flavin-binding monooxygenase, putative
  
Accession: EAL89217
  
Location: 2205866-2207906
  
 NCBI BlastP on this gene

EAL89217

integral membrane protein Pth11-like, putative
  
Accession: EAL89218
  
Location: 2208812-2210205
  
 NCBI BlastP on this gene

EAL89218

75. :  DS027698 Neosartorya fischeri NRRL 181 1099437636266 genomic scaffold     Total score: 2.0     Cumulative Blast bit score: 589

conserved hypothetical protein
  
Accession: EAW16632
  
Location: 3767498-3768872
  
 NCBI BlastP on this gene

EAW16632

conserved hypothetical protein
  
Accession: EAW16631
  
Location: 3765792-3766778
  
 NCBI BlastP on this gene

EAW16631

MFS transporter, putative
  
Accession: EAW16630
  
Location: 3763835-3765635
  
 NCBI BlastP on this gene

EAW16630

conserved hypothetical protein
  
Accession: EAW16629
  
Location: 3761984-3762463
  
 NCBI BlastP on this gene

EAW16629

hypothetical protein
  
Accession: EAW16628
  
Location: 3759595-3760197
  
 NCBI BlastP on this gene

EAW16628

hypothetical protein
  
Accession: EAW16627
  
Location: 3757699-3758088
  
 NCBI BlastP on this gene

EAW16627

hypothetical protein
  
Accession: EAW16626
  
Location: 3755622-3756096
  
 NCBI BlastP on this gene

EAW16626

conserved hypothetical protein
  
Accession: EAW16625
  
Location: 3753886-3754840
  
  
**BlastP hit with Mycgr3G52686\_Mycgr3T**
  
Percentage identity: 34 %
  
BlastP bit score: 125
  
Sequence coverage: 95 %
  
E-value: 3e-30
  
  
 NCBI BlastP on this gene

EAW16625

ferric-chelate reductase, putative
  
Accession: EAW16624
  
Location: 3751461-3753422
  
  
**BlastP hit with Mycgr3G107069\_Mycgr3**
  
Percentage identity: 38 %
  
BlastP bit score: 464
  
Sequence coverage: 101 %
  
E-value: 2e-151
  
  
 NCBI BlastP on this gene

EAW16624

alpha-1,2-mannosidase, putative subfamily
  
Accession: EAW16623
  
Location: 3748905-3751346
  
 NCBI BlastP on this gene

EAW16623

C6 finger domain protein, putative
  
Accession: EAW16622
  
Location: 3744220-3746595
  
 NCBI BlastP on this gene

EAW16622

MFS multidrug transporter, putative
  
Accession: EAW16621
  
Location: 3741443-3743324
  
 NCBI BlastP on this gene

EAW16621

flavin-binding monooxygenase, putative
  
Accession: EAW16620
  
Location: 3738355-3740374
  
 NCBI BlastP on this gene

EAW16620

conserved hypothetical protein
  
Accession: EAW16619
  
Location: 3736051-3737444
  
 NCBI BlastP on this gene

EAW16619

76. :  EQ963486 Aspergillus flavus NRRL3357 scf\_1106286417242 genomic scaffold     Total score: 2.0     Cumulative Blast bit score: 583

monooxygenase, putative
  
Accession: EED44986
  
Location: 318905-319923
  
 NCBI BlastP on this gene

EED44986

conserved hypothetical protein
  
Accession: EED44985
  
Location: 317335-317789
  
 NCBI BlastP on this gene

EED44985

conserved hypothetical protein
  
Accession: EED44984
  
Location: 315538-316891
  
 NCBI BlastP on this gene

EED44984

conserved hypothetical protein
  
Accession: EED44983
  
Location: 313672-314862
  
 NCBI BlastP on this gene

EED44983

hypothetical protein
  
Accession: EED44982
  
Location: 310965-312334
  
 NCBI BlastP on this gene

EED44982

short-chain dehydrogenases/reductase, putative
  
Accession: EED44981
  
Location: 309992-310570
  
 NCBI BlastP on this gene

EED44981

ferric-chelate reductase, putative
  
Accession: EED44980
  
Location: 305145-306821
  
  
**BlastP hit with Mycgr3G107069\_Mycgr3**
  
Percentage identity: 44 %
  
BlastP bit score: 462
  
Sequence coverage: 85 %
  
E-value: 4e-152
  
  
 NCBI BlastP on this gene

EED44980

hypothetical protein
  
Accession: EED44979
  
Location: 304610-304918
  
 NCBI BlastP on this gene

EED44979

ankyrin repeat-containing protein, putative
  
Accession: EED44978
  
Location: 301826-304077
  
 NCBI BlastP on this gene

EED44978

hypothetical protein
  
Accession: EED44977
  
Location: 301584-301823
  
 NCBI BlastP on this gene

EED44977

conserved hypothetical protein
  
Accession: EED44976
  
Location: 299748-300713
  
  
**BlastP hit with Mycgr3G52686\_Mycgr3T**
  
Percentage identity: 31 %
  
BlastP bit score: 121
  
Sequence coverage: 93 %
  
E-value: 9e-29
  
  
 NCBI BlastP on this gene

EED44976

conserved hypothetical protein
  
Accession: EED44975
  
Location: 298018-299284
  
 NCBI BlastP on this gene

EED44975

hypothetical protein
  
Accession: EED44974
  
Location: 297631-298016
  
 NCBI BlastP on this gene

EED44974

conserved hypothetical protein
  
Accession: EED44973
  
Location: 292561-294479
  
 NCBI BlastP on this gene

EED44973

alpha-1,2-mannosidase, putative subfamily
  
Accession: EED44972
  
Location: 286729-288908
  
 NCBI BlastP on this gene

EED44972

77. :  KE124052 Mucor circinelloides f. circinelloides 1006PhL unplaced genomic scaffold supercont1.157     Total score: 2.0     Cumulative Blast bit score: 581

hypothetical protein
  
Accession: EPB83985
  
Location: 36880-38296
  
 NCBI BlastP on this gene

EPB83985

hypothetical protein
  
Accession: EPB83986
  
Location: 40726-42138
  
  
**BlastP hit with Mycgr3G102276\_Mycgr3**
  
Percentage identity: 41 %
  
BlastP bit score: 289
  
Sequence coverage: 96 %
  
E-value: 2e-91
  
  
 NCBI BlastP on this gene

EPB83986

hypothetical protein
  
Accession: EPB83987
  
Location: 42584-44059
  
 NCBI BlastP on this gene

EPB83987

hypothetical protein
  
Accession: EPB83988
  
Location: 44764-46381
  
 NCBI BlastP on this gene

EPB83988

ribose 5-phosphate isomerase A
  
Accession: EPB83989
  
Location: 47474-48318
  
 NCBI BlastP on this gene

EPB83989

histone H1/5
  
Accession: EPB83990
  
Location: 48821-49762
  
 NCBI BlastP on this gene

EPB83990

hypothetical protein
  
Accession: EPB83991
  
Location: 53647-54143
  
 NCBI BlastP on this gene

EPB83991

hypothetical protein
  
Accession: EPB83992
  
Location: 54395-55291
  
 NCBI BlastP on this gene

EPB83992

hypothetical protein
  
Accession: EPB83993
  
Location: 55633-57195
  
 NCBI BlastP on this gene

EPB83993

hypothetical protein
  
Accession: EPB83994
  
Location: 57604-58729
  
 NCBI BlastP on this gene

EPB83994

hypothetical protein
  
Accession: EPB83995
  
Location: 60643-62548
  
 NCBI BlastP on this gene

EPB83995

hypothetical protein
  
Accession: EPB83996
  
Location: 62606-62806
  
 NCBI BlastP on this gene

EPB83996

hypothetical protein
  
Accession: EPB83997
  
Location: 63209-64067
  
 NCBI BlastP on this gene

EPB83997

hypothetical protein
  
Accession: EPB83998
  
Location: 66052-66246
  
 NCBI BlastP on this gene

EPB83998

peroxidase
  
Accession: EPB83999
  
Location: 66747-67515
  
  
**BlastP hit with Mycgr3G98385\_Mycgr3T**
  
Percentage identity: 66 %
  
BlastP bit score: 292
  
Sequence coverage: 83 %
  
E-value: 4e-96
  
  
 NCBI BlastP on this gene

EPB83999

FK506-binding protein 14
  
Accession: EPB84000
  
Location: 68192-68890
  
 NCBI BlastP on this gene

EPB84000

cyclin domain-containing protein
  
Accession: EPB84001
  
Location: 69117-69726
  
 NCBI BlastP on this gene

EPB84001

78. :  KE148163 Ophiostoma piceae UAMH 11346 chromosome Unknown scf18     Total score: 2.0     Cumulative Blast bit score: 555

aspartic-type endopeptidase
  
Accession: EPE04026
  
Location: 324603-326335
  
 NCBI BlastP on this gene

EPE04026

hypothetical protein
  
Accession: EPE04025
  
Location: 321457-323058
  
 NCBI BlastP on this gene

EPE04025

hypothetical protein
  
Accession: EPE04024
  
Location: 319029-320252
  
 NCBI BlastP on this gene

EPE04024

hypothetical protein
  
Accession: EPE04023
  
Location: 317812-318660
  
 NCBI BlastP on this gene

EPE04023

hypothetical protein
  
Accession: EPE04022
  
Location: 316845-317165
  
 NCBI BlastP on this gene

EPE04022

hypothetical protein
  
Accession: EPE04021
  
Location: 315258-316265
  
 NCBI BlastP on this gene

EPE04021

nad dependent epimerase
  
Accession: EPE04020
  
Location: 313171-314254
  
  
**BlastP hit with Mycgr3G52686\_Mycgr3T**
  
Percentage identity: 60 %
  
BlastP bit score: 332
  
Sequence coverage: 93 %
  
E-value: 2e-109
  
  
 NCBI BlastP on this gene

EPE04020

mfs transporter
  
Accession: EPE04019
  
Location: 310162-312107
  
 NCBI BlastP on this gene

EPE04019

hypothetical protein
  
Accession: EPE04018
  
Location: 306586-308460
  
  
**BlastP hit with Mycgr3G32432\_Mycgr3T**
  
Percentage identity: 34 %
  
BlastP bit score: 223
  
Sequence coverage: 79 %
  
E-value: 2e-60
  
  
 NCBI BlastP on this gene

EPE04018

hypothetical protein
  
Accession: EPE04017
  
Location: 303564-305957
  
 NCBI BlastP on this gene

EPE04017

hypothetical protein
  
Accession: EPE04016
  
Location: 302946-303221
  
 NCBI BlastP on this gene

EPE04016

hypothetical protein
  
Accession: EPE04015
  
Location: 301074-301805
  
 NCBI BlastP on this gene

EPE04015

n-acetylglucosaminyl-phosphatidylinositol de-n-acetylase
  
Accession: EPE04014
  
Location: 299304-300280
  
 NCBI BlastP on this gene

EPE04014

u6 snrnp-associated protein lsm3
  
Accession: EPE04013
  
Location: 297518-298039
  
 NCBI BlastP on this gene

EPE04013

hypothetical protein
  
Accession: EPE04012
  
Location: 296199-296660
  
 NCBI BlastP on this gene

EPE04012

79. :  KB706899 Eutypa lata UCREL1 unplaced genomic scaffold EL1\_03\_scaffold\_1561     Total score: 2.0     Cumulative Blast bit score: 550

hypothetical protein
  
Accession: EMR65278
  
Location: 31200-32175
  
 NCBI BlastP on this gene

EMR65278

putative proline rich protein 5 protein
  
Accession: EMR65250
  
Location: 33885-34956
  
 NCBI BlastP on this gene

EMR65250

putative nad dependent epimerase dehydratase protein
  
Accession: EMR65281
  
Location: 41834-42794
  
  
**BlastP hit with Mycgr3G52686\_Mycgr3T**
  
Percentage identity: 64 %
  
BlastP bit score: 354
  
Sequence coverage: 94 %
  
E-value: 8e-119
  
  
 NCBI BlastP on this gene

EMR65281

hypothetical protein
  
Accession: EMR65261
  
Location: 44493-46326
  
  
**BlastP hit with Mycgr3G32432\_Mycgr3T**
  
Percentage identity: 30 %
  
BlastP bit score: 196
  
Sequence coverage: 79 %
  
E-value: 1e-51
  
  
 NCBI BlastP on this gene

EMR65261

putative mfs transporter protein
  
Accession: EMR65252
  
Location: 48696-50573
  
 NCBI BlastP on this gene

EMR65252

putative mfs sugar protein
  
Accession: EMR65266
  
Location: 51754-53655
  
 NCBI BlastP on this gene

EMR65266

putative indoleamine -dioxygenase protein
  
Accession: EMR65254
  
Location: 58519-60222
  
 NCBI BlastP on this gene

EMR65254

80. :  GL698480 Metarhizium acridum CQMa 102 unplaced genomic scaffold Scf\_011     Total score: 2.0     Cumulative Blast bit score: 550

AAA family ATPase, putative
  
Accession: EFY91698
  
Location: 333791-335389
  
 NCBI BlastP on this gene

EFY91698

indoleamine 2,3-dioxygenase family protein
  
Accession: EFY91697
  
Location: 331415-332676
  
 NCBI BlastP on this gene

EFY91697

hypothetical protein
  
Accession: EFY91696
  
Location: 329724-330950
  
 NCBI BlastP on this gene

EFY91696

MFS transporter, putative
  
Accession: EFY91695
  
Location: 327537-328004
  
 NCBI BlastP on this gene

EFY91695

hypothetical protein
  
Accession: EFY91694
  
Location: 323506-325175
  
  
**BlastP hit with Mycgr3G32432\_Mycgr3T**
  
Percentage identity: 33 %
  
BlastP bit score: 194
  
Sequence coverage: 72 %
  
E-value: 5e-51
  
  
 NCBI BlastP on this gene

EFY91694

NAD dependent epimerase/dehydratase, putative
  
Accession: EFY91693
  
Location: 318822-321100
  
  
**BlastP hit with Mycgr3G52686\_Mycgr3T**
  
Percentage identity: 62 %
  
BlastP bit score: 356
  
Sequence coverage: 98 %
  
E-value: 5e-119
  
  
 NCBI BlastP on this gene

EFY91693

81. :  KB707843 Botryotinia fuckeliana BcDW1 unplaced genomic scaffold Scaffold\_171     Total score: 2.0     Cumulative Blast bit score: 545

putative methionine type protein
  
Accession: EMR87075
  
Location: 70792-72531
  
 NCBI BlastP on this gene

EMR87075

putative nonribosomal peptide synthetase 12 protein
  
Accession: EMR87076
  
Location: 75670-77148
  
 NCBI BlastP on this gene

EMR87076

putative nad dependent epimerase dehydratase protein
  
Accession: EMR87077
  
Location: 84585-85505
  
  
**BlastP hit with Mycgr3G52686\_Mycgr3T**
  
Percentage identity: 60 %
  
BlastP bit score: 345
  
Sequence coverage: 95 %
  
E-value: 1e-115
  
  
 NCBI BlastP on this gene

EMR87077

hypothetical protein
  
Accession: EMR87078
  
Location: 86938-88801
  
  
**BlastP hit with Mycgr3G32432\_Mycgr3T**
  
Percentage identity: 34 %
  
BlastP bit score: 200
  
Sequence coverage: 70 %
  
E-value: 1e-52
  
  
 NCBI BlastP on this gene

EMR87078

putative mfs transporter protein
  
Accession: EMR87079
  
Location: 90801-92942
  
 NCBI BlastP on this gene

EMR87079

hypothetical protein
  
Accession: EMR87080
  
Location: 93928-95816
  
 NCBI BlastP on this gene

EMR87080

putative flavin-binding monooxygenase-like protein
  
Accession: EMR87081
  
Location: 98126-99957
  
 NCBI BlastP on this gene

EMR87081

putative 3-ketodihydrosphingosine reductase tsc10 protein
  
Accession: EMR87082
  
Location: 101027-102325
  
 NCBI BlastP on this gene

EMR87082

82. :  FQ790301 Botryotinia fuckeliana T4 SuperContig\_226\_1 genomic supercontig.     Total score: 2.0     Cumulative Blast bit score: 545

similar to 3-ketodihydrosphingosine reductase tsc10
  
Accession: CCD34106
  
Location: 36241-37539
  
 NCBI BlastP on this gene

BofuT4\_P105330.1

similar to flavin-binding monooxygenase-like protein
  
Accession: CCD34107
  
Location: 38609-40440
  
 NCBI BlastP on this gene

BofuT4\_P105340.1

predicted protein
  
Accession: CCD34108
  
Location: 41291-41488
  
 NCBI BlastP on this gene

BofuT4\_uP105350.1

hypothetical protein
  
Accession: CCD34109
  
Location: 42750-44638
  
 NCBI BlastP on this gene

BofuT4\_P105360.1

similar to MFS transporter
  
Accession: CCD34110
  
Location: 45624-47765
  
 NCBI BlastP on this gene

BofuT4\_P105370.1

hypothetical protein
  
Accession: CCD34111
  
Location: 48311-48685
  
 NCBI BlastP on this gene

BofuT4\_P105380.1

hypothetical protein
  
Accession: CCD34112
  
Location: 49765-51628
  
  
**BlastP hit with Mycgr3G32432\_Mycgr3T**
  
Percentage identity: 34 %
  
BlastP bit score: 200
  
Sequence coverage: 70 %
  
E-value: 1e-52
  
  
 NCBI BlastP on this gene

BofuT4\_P105390.1

hypothetical protein
  
Accession: CCD34113
  
Location: 53061-53981
  
  
**BlastP hit with Mycgr3G52686\_Mycgr3T**
  
Percentage identity: 60 %
  
BlastP bit score: 345
  
Sequence coverage: 95 %
  
E-value: 1e-115
  
  
 NCBI BlastP on this gene

BofuT4\_P105400.1

predicted protein
  
Accession: CCD34114
  
Location: 56067-56273
  
 NCBI BlastP on this gene

BofuT4\_uP105410.1

similar to integral membrane protein TmpA
  
Accession: CCD34115
  
Location: 61419-62897
  
 NCBI BlastP on this gene

BofuT4\_P105420.1

hypothetical protein
  
Accession: CCD34116
  
Location: 63603-63948
  
 NCBI BlastP on this gene

BofuT4\_uP105430.1

predicted protein
  
Accession: CCD34117
  
Location: 64992-65252
  
 NCBI BlastP on this gene

BofuT4\_uP105440.1

similar to aminopeptidase P
  
Accession: CCD34118
  
Location: 66275-67776
  
 NCBI BlastP on this gene

BofuT4\_P105450.1

83. :  CM001199 Mycosphaerella graminicola IPO323 chromosome 4     Total score: 2.0     Cumulative Blast bit score: 500

hypothetical protein
  
Accession: EGP88057
  
Location: 2051228-2053476
  
 NCBI BlastP on this gene

EGP88057

glucose-methanol-choline oxidoreductase
  
Accession: EGP88058
  
Location: 2054269-2056342
  
  
**BlastP hit with Mycgr3G34982\_Mycgr3T**
  
Percentage identity: 29 %
  
BlastP bit score: 203
  
Sequence coverage: 102 %
  
E-value: 1e-53
  
  
 NCBI BlastP on this gene

EGP88058

hypothetical protein
  
Accession: EGP88059
  
Location: 2056681-2058114
  
 NCBI BlastP on this gene

EGP88059

hypothetical protein
  
Accession: EGP88305
  
Location: 2058152-2058694
  
 NCBI BlastP on this gene

EGP88305

hypothetical protein
  
Accession: EGP88060
  
Location: 2059620-2060863
  
 NCBI BlastP on this gene

EGP88060

hypothetical protein
  
Accession: EGP88061
  
Location: 2061289-2062505
  
 NCBI BlastP on this gene

EGP88061

hypothetical protein
  
Accession: EGP88304
  
Location: 2062819-2063436
  
 NCBI BlastP on this gene

EGP88304

hypothetical protein
  
Accession: EGP88062
  
Location: 2064048-2066336
  
 NCBI BlastP on this gene

EGP88062

GTA glutaminase A
  
Accession: EGP88303
  
Location: 2066537-2068990
  
 NCBI BlastP on this gene

EGP88303

hypothetical protein
  
Accession: EGP88063
  
Location: 2070297-2072538
  
 NCBI BlastP on this gene

EGP88063

hypothetical protein
  
Accession: EGP88302
  
Location: 2072808-2074588
  
 NCBI BlastP on this gene

EGP88302

hypothetical protein
  
Accession: EGP88064
  
Location: 2077946-2078819
  
  
**BlastP hit with Mycgr3G98385\_Mycgr3T**
  
Percentage identity: 66 %
  
BlastP bit score: 298
  
Sequence coverage: 84 %
  
E-value: 2e-98
  
  
 NCBI BlastP on this gene

EGP88064

hypothetical protein
  
Accession: EGP88065
  
Location: 2082208-2084625
  
 NCBI BlastP on this gene

EGP88065

84. :  AMYD01000021 Colletotrichum gloeosporioides Cg-14     Total score: 2.0     Cumulative Blast bit score: 496

hypothetical protein
  
Accession: EQB59521
  
Location: 7793-9915
  
 NCBI BlastP on this gene

EQB59521

UDP-glucoronosyl and UDP-glucosyl transferase
  
Accession: EQB59522
  
Location: 13138-13586
  
 NCBI BlastP on this gene

EQB59522

hypothetical protein
  
Accession: EQB59523
  
Location: 14032-14667
  
 NCBI BlastP on this gene

EQB59523

hypothetical protein
  
Accession: EQB59524
  
Location: 15399-16242
  
  
**BlastP hit with Mycgr3G52686\_Mycgr3T**
  
Percentage identity: 57 %
  
BlastP bit score: 246
  
Sequence coverage: 71 %
  
E-value: 2e-77
  
  
 NCBI BlastP on this gene

EQB59524

hypothetical protein
  
Accession: EQB59525
  
Location: 16946-17177
  
 NCBI BlastP on this gene

EQB59525

integral membrane protein DUF6
  
Accession: EQB59526
  
Location: 18279-19657
  
 NCBI BlastP on this gene

EQB59526

hypothetical protein
  
Accession: EQB59527
  
Location: 19820-21202
  
  
**BlastP hit with Mycgr3G32432\_Mycgr3T**
  
Percentage identity: 38 %
  
BlastP bit score: 251
  
Sequence coverage: 64 %
  
E-value: 1e-72
  
  
 NCBI BlastP on this gene

EQB59527

85. :  DS985218 Verticillium albo-atrum VaMs.102 supercont1.5 genomic scaffold     Total score: 2.0     Cumulative Blast bit score: 491

predicted protein
  
Accession: EEY18396
  
Location: 596724-597635
  
 NCBI BlastP on this gene

EEY18396

conserved hypothetical protein
  
Accession: EEY18397
  
Location: 598679-599221
  
 NCBI BlastP on this gene

EEY18397

predicted protein
  
Accession: EEY18398
  
Location: 599706-600026
  
 NCBI BlastP on this gene

EEY18398

cytosolic Cu/Zn superoxide dismutase
  
Accession: EEY18399
  
Location: 600863-601758
  
 NCBI BlastP on this gene

EEY18399

hypothetical protein
  
Accession: EEY18400
  
Location: 602936-603367
  
 NCBI BlastP on this gene

EEY18400

conserved hypothetical protein
  
Accession: EEY18401
  
Location: 605114-607472
  
 NCBI BlastP on this gene

EEY18401

conserved hypothetical protein
  
Accession: EEY18402
  
Location: 608507-610343
  
  
**BlastP hit with Mycgr3G32432\_Mycgr3T**
  
Percentage identity: 35 %
  
BlastP bit score: 248
  
Sequence coverage: 71 %
  
E-value: 6e-70
  
  
 NCBI BlastP on this gene

EEY18402

conserved hypothetical protein
  
Accession: EEY18403
  
Location: 610537-612335
  
 NCBI BlastP on this gene

EEY18403

conserved hypothetical protein
  
Accession: EEY18404
  
Location: 612982-614171
  
  
**BlastP hit with Mycgr3G52686\_Mycgr3T**
  
Percentage identity: 60 %
  
BlastP bit score: 243
  
Sequence coverage: 65 %
  
E-value: 3e-74
  
  
 NCBI BlastP on this gene

EEY18404

WSC domain-containing protein
  
Accession: EEY18405
  
Location: 614778-618208
  
 NCBI BlastP on this gene

EEY18405

predicted protein
  
Accession: EEY18406
  
Location: 618982-619529
  
 NCBI BlastP on this gene

EEY18406

shuttle craft
  
Accession: EEY18407
  
Location: 623851-627366
  
 NCBI BlastP on this gene

EEY18407

86. :  KB916913 Neofusicoccum parvum UCRNP2 chromosome Unknown NP2\_03\_scaffold\_1275     Total score: 2.0     Cumulative Blast bit score: 464

putative major royal jelly protein
  
Accession: EOD43065
  
Location: 19123-20658
  
 NCBI BlastP on this gene

EOD43065

putative mfs drug efflux transporter protein
  
Accession: EOD43059
  
Location: 21401-23459
  
 NCBI BlastP on this gene

EOD43059

putative zinc-binding oxidoreductase protein
  
Accession: EOD43062
  
Location: 24203-25243
  
 NCBI BlastP on this gene

EOD43062

putative lactonohydrolase protein
  
Accession: EOD43063
  
Location: 25713-27059
  
 NCBI BlastP on this gene

EOD43063

putative mfs monocarboxylate transporter protein
  
Accession: EOD43064
  
Location: 27901-29269
  
  
**BlastP hit with Mycgr3G65725\_Mycgr3T**
  
Percentage identity: 38 %
  
BlastP bit score: 276
  
Sequence coverage: 82 %
  
E-value: 7e-84
  
  
 NCBI BlastP on this gene

EOD43064

putative pyoverdine dityrosine biosynthesis protein
  
Accession: EOD43066
  
Location: 30797-32480
  
 NCBI BlastP on this gene

EOD43066

putative laccase-1 precursor protein
  
Accession: EOD43061
  
Location: 33609-35508
  
 NCBI BlastP on this gene

EOD43061

putative gmc oxidoreductase protein
  
Accession: EOD43060
  
Location: 37016-38988
  
  
**BlastP hit with Mycgr3G34982\_Mycgr3T**
  
Percentage identity: 27 %
  
BlastP bit score: 188
  
Sequence coverage: 102 %
  
E-value: 1e-48
  
  
 NCBI BlastP on this gene

EOD43060

87. :  DS544807 Paracoccidioides brasiliensis Pb03 supercont1.5 genomic scaffold     Total score: 2.0     Cumulative Blast bit score: 456

bystin-family protein
  
Accession: EEH21381
  
Location: 118598-120289
  
 NCBI BlastP on this gene

EEH21381

conserved hypothetical protein
  
Accession: EEH21382
  
Location: 122426-124300
  
 NCBI BlastP on this gene

EEH21382

glutamyl-tRNA(Gln) amidotransferase subunit A
  
Accession: EEH21383
  
Location: 124702-126471
  
 NCBI BlastP on this gene

EEH21383

predicted protein
  
Accession: EEH21384
  
Location: 126839-129005
  
 NCBI BlastP on this gene

EEH21384

conserved hypothetical protein
  
Accession: EEH21385
  
Location: 129531-130724
  
 NCBI BlastP on this gene

EEH21385

gramicidin S synthetase 1
  
Accession: EEH21386
  
Location: 131609-134758
  
  
**BlastP hit with Mycgr3G107072\_Mycgr3**
  
Percentage identity: 27 %
  
BlastP bit score: 306
  
Sequence coverage: 81 %
  
E-value: 8e-84
  
  
 NCBI BlastP on this gene

EEH21386

conserved hypothetical protein
  
Accession: EEH21387
  
Location: 135246-136513
  
 NCBI BlastP on this gene

EEH21387

conserved hypothetical protein
  
Accession: EEH21388
  
Location: 138050-138958
  
  
**BlastP hit with Mycgr3G52686\_Mycgr3T**
  
Percentage identity: 37 %
  
BlastP bit score: 150
  
Sequence coverage: 79 %
  
E-value: 7e-40
  
  
 NCBI BlastP on this gene

EEH21388

predicted protein
  
Accession: EEH21389
  
Location: 139127-139651
  
 NCBI BlastP on this gene

EEH21389

phenol hydroxylase
  
Accession: EEH21390
  
Location: 139999-142075
  
 NCBI BlastP on this gene

EEH21390

predicted protein
  
Accession: EEH21391
  
Location: 142221-142700
  
 NCBI BlastP on this gene

EEH21391

multidrug resistance protein fnx1
  
Accession: EEH21392
  
Location: 142866-144703
  
 NCBI BlastP on this gene

EEH21392

conserved hypothetical protein
  
Accession: EEH21393
  
Location: 145527-146456
  
 NCBI BlastP on this gene

EEH21393

predicted protein
  
Accession: EEH21394
  
Location: 147996-148923
  
 NCBI BlastP on this gene

EEH21394

88. :  GG663377 Ajellomyces capsulatus G186AR genomic scaffold supercont2.15     Total score: 2.0     Cumulative Blast bit score: 455

hypothetical protein
  
Accession: EEH03444
  
Location: 453180-455429
  
 NCBI BlastP on this gene

EEH03444

predicted protein
  
Accession: EEH03445
  
Location: 456970-457572
  
 NCBI BlastP on this gene

EEH03445

antibiotic synthetase
  
Accession: EEH03446
  
Location: 460656-463817
  
  
**BlastP hit with Mycgr3G107072\_Mycgr3**
  
Percentage identity: 27 %
  
BlastP bit score: 301
  
Sequence coverage: 81 %
  
E-value: 4e-82
  
  
 NCBI BlastP on this gene

EEH03446

NmrA family protein
  
Accession: EEH03447
  
Location: 464306-465544
  
 NCBI BlastP on this gene

EEH03447

conserved hypothetical protein
  
Accession: EEH03448
  
Location: 466332-467221
  
  
**BlastP hit with Mycgr3G52686\_Mycgr3T**
  
Percentage identity: 37 %
  
BlastP bit score: 154
  
Sequence coverage: 78 %
  
E-value: 1e-41
  
  
 NCBI BlastP on this gene

EEH03448

FAD binding domain-containing protein
  
Accession: EEH03449
  
Location: 468182-470249
  
 NCBI BlastP on this gene

EEH03449

multidrug resistance protein fnx1
  
Accession: EEH03450
  
Location: 470911-473399
  
 NCBI BlastP on this gene

EEH03450

conserved hypothetical protein
  
Accession: EEH03451
  
Location: 474062-474991
  
 NCBI BlastP on this gene

EEH03451

predicted protein
  
Accession: EEH03452
  
Location: 476459-477586
  
 NCBI BlastP on this gene

EEH03452

conserved hypothetical protein
  
Accession: EEH03453
  
Location: 479333-480319
  
 NCBI BlastP on this gene

EEH03453

89. :  EQ999987 Ajellomyces dermatitidis ER-3 genomic scaffold supercont1.15     Total score: 2.0     Cumulative Blast bit score: 455

SNF7 family protein Fti1/Did2
  
Accession: EEQ86127
  
Location: 690091-690921
  
 NCBI BlastP on this gene

EEQ86127

bZIP transcription factor HacA
  
Accession: EEQ86128
  
Location: 691870-693581
  
 NCBI BlastP on this gene

EEQ86128

predicted protein
  
Accession: EEQ86129
  
Location: 696888-698114
  
 NCBI BlastP on this gene

EEQ86129

predicted protein
  
Accession: EEQ86130
  
Location: 699757-700698
  
 NCBI BlastP on this gene

EEQ86130

capsule polysaccharide biosynthesis protein
  
Accession: EEQ86131
  
Location: 701135-702328
  
 NCBI BlastP on this gene

EEQ86131

antibiotic synthetase
  
Accession: EEQ86132
  
Location: 703326-706487
  
  
**BlastP hit with Mycgr3G107072\_Mycgr3**
  
Percentage identity: 27 %
  
BlastP bit score: 302
  
Sequence coverage: 81 %
  
E-value: 3e-82
  
  
 NCBI BlastP on this gene

EEQ86132

conserved hypothetical protein
  
Accession: EEQ86133
  
Location: 707037-708263
  
 NCBI BlastP on this gene

EEQ86133

conserved hypothetical protein
  
Accession: EEQ86134
  
Location: 709046-709896
  
  
**BlastP hit with Mycgr3G52686\_Mycgr3T**
  
Percentage identity: 37 %
  
BlastP bit score: 153
  
Sequence coverage: 79 %
  
E-value: 6e-41
  
  
 NCBI BlastP on this gene

EEQ86134

phenol monooxygenase
  
Accession: EEQ86135
  
Location: 710855-712930
  
 NCBI BlastP on this gene

EEQ86135

MFS multidrug transporter
  
Accession: EEQ86136
  
Location: 713738-715498
  
 NCBI BlastP on this gene

EEQ86136

conserved hypothetical protein
  
Accession: EEQ86137
  
Location: 717016-717945
  
 NCBI BlastP on this gene

EEQ86137

90. :  GG749478 Ajellomyces dermatitidis ATCC 18188 genomic scaffold supercont1.72     Total score: 2.0     Cumulative Blast bit score: 454

capsule polysaccharide biosynthesis protein
  
Accession: EGE84872
  
Location: 210018-211211
  
 NCBI BlastP on this gene

EGE84872

antibiotic synthetase
  
Accession: EGE84873
  
Location: 212204-215368
  
  
**BlastP hit with Mycgr3G107072\_Mycgr3**
  
Percentage identity: 27 %
  
BlastP bit score: 301
  
Sequence coverage: 81 %
  
E-value: 4e-82
  
  
 NCBI BlastP on this gene

EGE84873

NmrA family protein
  
Accession: EGE84874
  
Location: 215915-217141
  
 NCBI BlastP on this gene

EGE84874

hypothetical protein
  
Accession: EGE84875
  
Location: 217921-218771
  
  
**BlastP hit with Mycgr3G52686\_Mycgr3T**
  
Percentage identity: 37 %
  
BlastP bit score: 153
  
Sequence coverage: 79 %
  
E-value: 6e-41
  
  
 NCBI BlastP on this gene

EGE84875

phenol monooxygenase
  
Accession: EGE84876
  
Location: 219730-221805
  
 NCBI BlastP on this gene

EGE84876

MFS multidrug transporter
  
Accession: EGE84877
  
Location: 222615-224375
  
 NCBI BlastP on this gene

EGE84877

hypothetical protein
  
Accession: EGE84878
  
Location: 225893-226822
  
 NCBI BlastP on this gene

EGE84878

91. :  GG657474 Ajellomyces dermatitidis SLH14081 genomic scaffold supercont1.27     Total score: 2.0     Cumulative Blast bit score: 451

SNF7 family protein
  
Accession: EEQ74446
  
Location: 33473-34304
  
 NCBI BlastP on this gene

EEQ74446

bZIP transcription factor HacA
  
Accession: EEQ74447
  
Location: 35251-36968
  
 NCBI BlastP on this gene

EEQ74447

predicted protein
  
Accession: EEQ74448
  
Location: 40276-41166
  
 NCBI BlastP on this gene

EEQ74448

predicted protein
  
Accession: EEQ74449
  
Location: 42811-43752
  
 NCBI BlastP on this gene

EEQ74449

capsule polysaccharide biosynthesis protein
  
Accession: EEQ74450
  
Location: 44196-45389
  
 NCBI BlastP on this gene

EEQ74450

antibiotic synthetase
  
Accession: EEQ74451
  
Location: 46385-49546
  
  
**BlastP hit with Mycgr3G107072\_Mycgr3**
  
Percentage identity: 27 %
  
BlastP bit score: 298
  
Sequence coverage: 81 %
  
E-value: 4e-81
  
  
 NCBI BlastP on this gene

EEQ74451

conserved hypothetical protein
  
Accession: EEQ74452
  
Location: 50097-51323
  
 NCBI BlastP on this gene

EEQ74452

conserved hypothetical protein
  
Accession: EEQ74453
  
Location: 52106-52956
  
  
**BlastP hit with Mycgr3G52686\_Mycgr3T**
  
Percentage identity: 37 %
  
BlastP bit score: 153
  
Sequence coverage: 79 %
  
E-value: 6e-41
  
  
 NCBI BlastP on this gene

EEQ74453

phenol monooxygenase
  
Accession: EEQ74454
  
Location: 53913-55988
  
 NCBI BlastP on this gene

EEQ74454

MFS multidrug transporter
  
Accession: EEQ74455
  
Location: 56759-58519
  
 NCBI BlastP on this gene

EEQ74455

conserved hypothetical protein
  
Accession: EEQ74456
  
Location: 60037-60966
  
 NCBI BlastP on this gene

EEQ74456

conserved hypothetical protein
  
Accession: EEQ74457
  
Location: 64471-65487
  
 NCBI BlastP on this gene

EEQ74457

92. :  DS572750 Paracoccidioides brasiliensis Pb18 supercont1.1 genomic scaffold     Total score: 2.0     Cumulative Blast bit score: 449

predicted protein
  
Accession: EEH43937
  
Location: 775881-777102
  
 NCBI BlastP on this gene

EEH43937

conserved hypothetical protein
  
Accession: EEH43938
  
Location: 780149-781278
  
 NCBI BlastP on this gene

EEH43938

multidrug resistance protein fnx1
  
Accession: EEH43939
  
Location: 782099-783884
  
 NCBI BlastP on this gene

EEH43939

conserved hypothetical protein
  
Accession: EEH43940
  
Location: 784672-786748
  
 NCBI BlastP on this gene

EEH43940

predicted protein
  
Accession: EEH43941
  
Location: 787257-787620
  
 NCBI BlastP on this gene

EEH43941

conserved hypothetical protein
  
Accession: EEH43942
  
Location: 787764-788672
  
  
**BlastP hit with Mycgr3G52686\_Mycgr3T**
  
Percentage identity: 37 %
  
BlastP bit score: 150
  
Sequence coverage: 79 %
  
E-value: 8e-40
  
  
 NCBI BlastP on this gene

EEH43942

conserved hypothetical protein
  
Accession: EEH43943
  
Location: 790221-791488
  
 NCBI BlastP on this gene

EEH43943

D-alanine-poly(phosphoribitol) ligase subunit 1
  
Accession: EEH43944
  
Location: 791976-795125
  
  
**BlastP hit with Mycgr3G107072\_Mycgr3**
  
Percentage identity: 27 %
  
BlastP bit score: 300
  
Sequence coverage: 76 %
  
E-value: 1e-81
  
  
 NCBI BlastP on this gene

EEH43944

conserved hypothetical protein
  
Accession: EEH43945
  
Location: 796299-797198
  
 NCBI BlastP on this gene

EEH43945

predicted protein
  
Accession: EEH43946
  
Location: 798191-799561
  
 NCBI BlastP on this gene

EEH43946

glutamyl-tRNA(Gln) amidotransferase subunit A
  
Accession: EEH43947
  
Location: 800260-802029
  
 NCBI BlastP on this gene

EEH43947

conserved hypothetical protein
  
Accession: EEH43948
  
Location: 802432-804307
  
 NCBI BlastP on this gene

EEH43948

predicted protein
  
Accession: EEH43949
  
Location: 804616-805389
  
 NCBI BlastP on this gene

EEH43949

bystin
  
Accession: EEH43950
  
Location: 806438-808594
  
 NCBI BlastP on this gene

EEH43950

93. :  DS572813 Paracoccidioides brasiliensis Pb01 supercont1.3 genomic scaffold     Total score: 2.0     Cumulative Blast bit score: 445

bystin
  
Accession: EEH39078
  
Location: 723571-726243
  
 NCBI BlastP on this gene

EEH39078

conserved hypothetical protein
  
Accession: EEH39079
  
Location: 727410-729197
  
 NCBI BlastP on this gene

EEH39079

glutamyl-tRNA(Gln) amidotransferase subunit A
  
Accession: EEH39080
  
Location: 729679-731450
  
 NCBI BlastP on this gene

EEH39080

conserved hypothetical protein
  
Accession: EEH39081
  
Location: 732046-734771
  
 NCBI BlastP on this gene

EEH39081

conserved hypothetical protein
  
Accession: EEH39082
  
Location: 735456-736533
  
 NCBI BlastP on this gene

EEH39082

D-alanine-poly(phosphoribitol) ligase subunit 1
  
Accession: EEH39083
  
Location: 737495-740644
  
  
**BlastP hit with Mycgr3G107072\_Mycgr3**
  
Percentage identity: 27 %
  
BlastP bit score: 297
  
Sequence coverage: 73 %
  
E-value: 6e-81
  
  
 NCBI BlastP on this gene

EEH39083

conserved hypothetical protein
  
Accession: EEH39084
  
Location: 741131-742398
  
 NCBI BlastP on this gene

EEH39084

conserved hypothetical protein
  
Accession: EEH39085
  
Location: 743913-744821
  
  
**BlastP hit with Mycgr3G52686\_Mycgr3T**
  
Percentage identity: 36 %
  
BlastP bit score: 148
  
Sequence coverage: 78 %
  
E-value: 4e-39
  
  
 NCBI BlastP on this gene

EEH39085

phenol 2-monooxygenase
  
Accession: EEH39086
  
Location: 745822-747898
  
 NCBI BlastP on this gene

EEH39086

multidrug resistance protein fnx1
  
Accession: EEH39087
  
Location: 748704-750487
  
 NCBI BlastP on this gene

EEH39087

conserved hypothetical protein
  
Accession: EEH39088
  
Location: 751280-752340
  
 NCBI BlastP on this gene

EEH39088

predicted protein
  
Accession: EEH39089
  
Location: 752578-753394
  
 NCBI BlastP on this gene

EEH39089

predicted protein
  
Accession: EEH39090
  
Location: 753434-756958
  
 NCBI BlastP on this gene

EEH39090

catalase B
  
Accession: EEH39091
  
Location: 756982-759653
  
 NCBI BlastP on this gene

EEH39091

94. :  AB530986 Streptomyces sp. SANK 60405 DNA, A-90289 biosynthetic gene cluster region     Total score: 2.0     Cumulative Blast bit score: 341

hypothetical protein
  
Accession: BAJ05876
  
Location: 1-546
  
 NCBI BlastP on this gene

lipA

putative pirin-like protein
  
Accession: BAJ05877
  
Location: 855-1820
  
 NCBI BlastP on this gene

BAJ05877

aryl sulfotransferase
  
Accession: BAJ05878
  
Location: 1894-3432
  
  
**BlastP hit with Mycgr3G32432\_Mycgr3T**
  
Percentage identity: 33 %
  
BlastP bit score: 222
  
Sequence coverage: 80 %
  
E-value: 4e-61
  
  
 NCBI BlastP on this gene

lipB

putative 3-hydroxy-3-methylglutaryl-CoA synthase
  
Accession: BAJ05879
  
Location: 3452-4714
  
 NCBI BlastP on this gene

lipC

putative type III polyketide synthase
  
Accession: BAJ05880
  
Location: 5013-6062
  
 NCBI BlastP on this gene

lipD

hypothetical protein
  
Accession: BAJ05881
  
Location: 6619-7257
  
  
**BlastP hit with Mycgr3G52686\_Mycgr3T**
  
Percentage identity: 33 %
  
BlastP bit score: 119
  
Sequence coverage: 77 %
  
E-value: 1e-28
  
  
 NCBI BlastP on this gene

lipE

putative AraC family transcriptional regulator
  
Accession: BAJ05882
  
Location: 7475-8464
  
 NCBI BlastP on this gene

lipF

putative beta-hydroxylase
  
Accession: BAJ05883
  
Location: 8518-9165
  
 NCBI BlastP on this gene

lipG

putative SAM-dependent methyltransferase
  
Accession: BAJ05884
  
Location: 9198-9818
  
 NCBI BlastP on this gene

lipH

putative TmrB-like protein
  
Accession: BAJ05885
  
Location: 9815-10384
  
 NCBI BlastP on this gene

lipI

putative aminotransferase
  
Accession: BAJ05886
  
Location: 10449-11771
  
 NCBI BlastP on this gene

lipJ

putative serine hydroxymethyltransferase
  
Accession: BAJ05887
  
Location: 11776-13050
  
 NCBI BlastP on this gene

lipK

putative dioxygenase
  
Accession: BAJ05888
  
Location: 13132-13956
  
 NCBI BlastP on this gene

lipL

putative nucleotidyltransferase
  
Accession: BAJ05889
  
Location: 13953-14654
  
 NCBI BlastP on this gene

lipM

putative glycosyltransferase
  
Accession: BAJ05890
  
Location: 14654-15787
  
 NCBI BlastP on this gene

lipN

putative aminotransferase
  
Accession: BAJ05891
  
Location: 15784-17058
  
 NCBI BlastP on this gene

lipO

putative pyrimidine-nucleoside phosphorylase
  
Accession: BAJ05892
  
Location: 17069-18436
  
 NCBI BlastP on this gene

lipP

putative acyl-CoA synthase
  
Accession: BAJ05893
  
Location: 18429-19493
  
 NCBI BlastP on this gene

lipQ

putative acyltransferase
  
Accession: BAJ05894
  
Location: 19493-20971
  
 NCBI BlastP on this gene

lipR

95. :  KB456266 Mycosphaerella populorum SO2202 unplaced genomic scaffold SEPMUscaffold\_7     Total score: 1.0     Cumulative Blast bit score: 1893

DUF185-domain-containing protein
  
Accession: EMF11160
  
Location: 538466-540171
  
 NCBI BlastP on this gene

EMF11160

kinase-like protein
  
Accession: EMF11161
  
Location: 540331-541795
  
 NCBI BlastP on this gene

EMF11161

hexose carrier protein
  
Accession: EMF11162
  
Location: 543277-545044
  
 NCBI BlastP on this gene

EMF11162

hypothetical protein
  
Accession: EMF11163
  
Location: 545167-546613
  
 NCBI BlastP on this gene

EMF11163

hypothetical protein
  
Accession: EMF11164
  
Location: 548135-549061
  
 NCBI BlastP on this gene

EMF11164

mitochondrial chaperone BCS1
  
Accession: EMF11165
  
Location: 550358-551898
  
 NCBI BlastP on this gene

EMF11165

NRPS-like enzyme
  
Accession: EMF11166
  
Location: 553284-557222
  
  
**BlastP hit with Mycgr3G107072\_Mycgr3**
  
Percentage identity: 71 %
  
BlastP bit score: 1893
  
Sequence coverage: 100 %
  
E-value: 0.0
  
  
 NCBI BlastP on this gene

EMF11166

exonuclease family protein
  
Accession: EMF11167
  
Location: 558053-558637
  
 NCBI BlastP on this gene

EMF11167

NAD(P)-binding protein
  
Accession: EMF11168
  
Location: 559130-560716
  
 NCBI BlastP on this gene

EMF11168

NAD(P)-binding protein
  
Accession: EMF11169
  
Location: 561138-562259
  
 NCBI BlastP on this gene

EMF11169

WSC-domain-containing protein
  
Accession: EMF11170
  
Location: 564079-564736
  
 NCBI BlastP on this gene

EMF11170

autophagy protein
  
Accession: EMF11171
  
Location: 565731-568041
  
 NCBI BlastP on this gene

EMF11171

Glucosamine iso-domain-containing protein
  
Accession: EMF11172
  
Location: 568891-570187
  
 NCBI BlastP on this gene

EMF11172

96. :  KB446546 Dothistroma septosporum NZE10 unplaced genomic scaffold DOTSEscaffold\_12     Total score: 1.0     Cumulative Blast bit score: 1883

hypothetical protein
  
Accession: EME38802
  
Location: 447856-449747
  
 NCBI BlastP on this gene

EME38802

hypothetical protein
  
Accession: EME38801
  
Location: 445611-447007
  
 NCBI BlastP on this gene

EME38801

hypothetical protein
  
Accession: EME38800
  
Location: 444148-445035
  
 NCBI BlastP on this gene

EME38800

hypothetical protein
  
Accession: EME38799
  
Location: 442016-443428
  
 NCBI BlastP on this gene

EME38799

hypothetical protein
  
Accession: EME38798
  
Location: 439471-441636
  
 NCBI BlastP on this gene

EME38798

hypothetical protein
  
Accession: EME38797
  
Location: 438328-438775
  
 NCBI BlastP on this gene

EME38797

hypothetical protein
  
Accession: EME38796
  
Location: 430986-436638
  
  
**BlastP hit with Mycgr3G107072\_Mycgr3**
  
Percentage identity: 69 %
  
BlastP bit score: 1883
  
Sequence coverage: 102 %
  
E-value: 0.0
  
  
 NCBI BlastP on this gene

EME38796

hypothetical protein
  
Accession: EME38795
  
Location: 429631-430779
  
 NCBI BlastP on this gene

EME38795

hypothetical protein
  
Accession: EME38794
  
Location: 428509-429447
  
 NCBI BlastP on this gene

EME38794

hypothetical protein
  
Accession: EME38793
  
Location: 426133-426426
  
 NCBI BlastP on this gene

EME38793

hypothetical protein
  
Accession: EME38792
  
Location: 424669-425902
  
 NCBI BlastP on this gene

EME38792

hypothetical protein
  
Accession: EME38791
  
Location: 417506-418550
  
 NCBI BlastP on this gene

EME38791

97. :  KB446556 Pseudocercospora fijiensis CIRAD86 unplaced genomic scaffold MYCFIscaffold\_2     Total score: 1.0     Cumulative Blast bit score: 1880

hypothetical protein
  
Accession: EME85369
  
Location: 706543-706979
  
 NCBI BlastP on this gene

EME85369

hypothetical protein
  
Accession: EME85368
  
Location: 704858-706466
  
 NCBI BlastP on this gene

EME85368

hypothetical protein
  
Accession: EME85367
  
Location: 702645-703791
  
 NCBI BlastP on this gene

EME85367

hypothetical protein
  
Accession: EME85366
  
Location: 697078-700965
  
  
**BlastP hit with Mycgr3G107072\_Mycgr3**
  
Percentage identity: 70 %
  
BlastP bit score: 1880
  
Sequence coverage: 100 %
  
E-value: 0.0
  
  
 NCBI BlastP on this gene

EME85366

98. :  JH226130 Exophiala dermatitidis NIH/UT8656 unplaced genomic scaffold supercont1.1     Total score: 1.0     Cumulative Blast bit score: 1796

ABC bile acid transporter
  
Accession: EHY52379
  
Location: 1636972-1641897
  
 NCBI BlastP on this gene

EHY52379

glutaminyl-peptide cyclotransferase
  
Accession: EHY52380
  
Location: 1642717-1643874
  
 NCBI BlastP on this gene

EHY52380

hypothetical protein
  
Accession: EHY52381
  
Location: 1644292-1645410
  
 NCBI BlastP on this gene

EHY52381

hypothetical protein
  
Accession: EHY52382
  
Location: 1645701-1646695
  
 NCBI BlastP on this gene

EHY52382

myosin-crossreactive antigen
  
Accession: EHY52383
  
Location: 1650043-1651824
  
 NCBI BlastP on this gene

EHY52383

linear gramicidin synthetase subunit C
  
Accession: EHY52384
  
Location: 1652960-1656802
  
  
**BlastP hit with Mycgr3G107072\_Mycgr3**
  
Percentage identity: 67 %
  
BlastP bit score: 1796
  
Sequence coverage: 100 %
  
E-value: 0.0
  
  
 NCBI BlastP on this gene

EHY52384

hypothetical protein
  
Accession: EHY52385
  
Location: 1657750-1658226
  
 NCBI BlastP on this gene

EHY52385

histone-lysine N-methyltransferase SETD1
  
Accession: EHY52386
  
Location: 1659708-1663602
  
 NCBI BlastP on this gene

EHY52386

hypothetical protein
  
Accession: EHY52387
  
Location: 1664676-1665563
  
 NCBI BlastP on this gene

EHY52387

glucosamine-fructose-6-phosphate aminotransferase
  
Accession: EHY52388
  
Location: 1667884-1670201
  
 NCBI BlastP on this gene

EHY52388

phosphatidylinositol glycan, class B
  
Accession: EHY52389
  
Location: 1670754-1673329
  
 NCBI BlastP on this gene

EHY52389

99. :  DS499597 Aspergillus fumigatus A1163 scf\_000004 genomic scaffold     Total score: 1.0     Cumulative Blast bit score: 1739

cytochrome P450 monooxygenase, putative
  
Accession: EDP51746
  
Location: 2486277-2488057
  
 NCBI BlastP on this gene

EDP51746

cytochrome b5 reductase, putative
  
Accession: EDP51747
  
Location: 2488541-2490003
  
 NCBI BlastP on this gene

EDP51747

3-hydroxyacyl-CoA dehydrogenase, putative
  
Accession: EDP51748
  
Location: 2490846-2491666
  
 NCBI BlastP on this gene

EDP51748

hypothetical protein
  
Accession: EDP51749
  
Location: 2492006-2492813
  
 NCBI BlastP on this gene

EDP51749

conserved hypothetical protein
  
Accession: EDP51750
  
Location: 2492955-2494828
  
 NCBI BlastP on this gene

EDP51750

3-demethylubiquinone-9 3-methyltransferase, putative
  
Accession: EDP51751
  
Location: 2495764-2496264
  
 NCBI BlastP on this gene

EDP51751

conserved hypothetical protein
  
Accession: EDP51752
  
Location: 2497790-2498145
  
 NCBI BlastP on this gene

EDP51752

conserved hypothetical protein
  
Accession: EDP51753
  
Location: 2498322-2498873
  
 NCBI BlastP on this gene

EDP51753

hybrid NRPS/PKS enzyme, putative
  
Accession: EDP51754
  
Location: 2500293-2504117
  
  
**BlastP hit with Mycgr3G107072\_Mycgr3**
  
Percentage identity: 66 %
  
BlastP bit score: 1739
  
Sequence coverage: 100 %
  
E-value: 0.0
  
  
 NCBI BlastP on this gene

EDP51754

100. :  DS027696 Neosartorya fischeri NRRL 181 1099437636264 genomic scaffold     Total score: 1.0     Cumulative Blast bit score: 1738

hybrid NRPS/PKS enzyme, putative
  
Accession: EAW17784
  
Location: 1341334-1345155
  
  
**BlastP hit with Mycgr3G107072\_Mycgr3**
  
Percentage identity: 66 %
  
BlastP bit score: 1738
  
Sequence coverage: 100 %
  
E-value: 0.0
  
  
 NCBI BlastP on this gene

EAW17784

bZIP transcription factor, putative
  
Accession: EAW17783
  
Location: 1339691-1340479
  
 NCBI BlastP on this gene

EAW17783

conserved hypothetical protein
  
Accession: EAW17782
  
Location: 1337402-1339289
  
 NCBI BlastP on this gene

EAW17782

hypothetical protein
  
Accession: EAW17781
  
Location: 1335195-1336685
  
 NCBI BlastP on this gene

EAW17781

short-chain dehydrogenase/reductase, putative
  
Accession: EAW17780
  
Location: 1333863-1334953
  
 NCBI BlastP on this gene

EAW17780

conserved hypothetical protein
  
Accession: EAW17779
  
Location: 1332402-1333394
  
 NCBI BlastP on this gene

EAW17779

NmrA-like family protein
  
Accession: EAW17778
  
Location: 1330667-1331636
  
 NCBI BlastP on this gene

EAW17778

pectate lyase, putative
  
Accession: EAW17777
  
Location: 1329153-1330464
  
 NCBI BlastP on this gene

EAW17777

salicylate hydroxylase, putative
  
Accession: EAW17776
  
Location: 1326885-1328387
  
 NCBI BlastP on this gene

EAW17776

Detecting sequence homology at the gene cluster level with MultiGeneBlast.
  
Marnix H. Medema, Rainer Breitling & Eriko Takano (2013)
  
*Molecular Biology and Evolution* , 30: 1218-1223.
