# Supplementary material for: In silico prediction and characterization of secondary metabolite biosynthetic gene clusters in the wheat pathogen Zymoseptoria tritici
Source: BMC Genomics. 2017 Aug 17;18:631. doi: 10.1186/s12864-017-3969-y (PMC5561558; doi:10.1186/s12864-017-3969-y)
Supplement: Supplementary file 1 — MultiGeneBLAST analysis of putative secondary metabolite clusters. All encoded amino acid sequences from genes residing in clusters predicted by AntiSMASH are given as FASTA file format. All output data from MultiGeneBLASTs are also provided. (ZIP 42911 kb) [file 12864_2017_3969_MOESM1_ESM.zip › Cluster MultiGene BLAST/out/Clusters_1_34/Cluster_1/displaypage3.xhtml]

xml version="1.0" encoding="UTF-8"?


Search Results
  
  
 Results pages: 1, 2, 3, 4, 5

**MultiGeneBlast hits**

Select gene cluster alignment
101. DF126495\_1 Aspergillus kawachii IFO 4308 DNA, contig: scaffold00049, who...
102. AACD01000093\_0 Aspergillus nidulans FGSC A4, whole genome shotgun sequen...
103. EQ963472\_2 Aspergillus flavus NRRL3357 scf\_1106286418772 genomic scaffol...
104. AP007151\_2 Aspergillus oryzae RIB40 DNA, SC005.
105. DS990638\_0 Ajellomyces capsulatus H88 supercont1.3 genomic scaffold, who...
106. DS231615\_1 Pyrenophora tritici-repentis Pt-1C-BFP supercont1.1 genomic s...
107. KB644414\_0 Penicillium oxalicum 114-2 unplaced genomic scaffold scaffold...
108. EQ962653\_0 Talaromyces stipitatus ATCC 10500 scf\_1105507295527 genomic s...
109. DS995900\_0 Penicillium marneffei ATCC 18224 scf\_1105668340758 genomic sc...
110. KB733447\_1 Bipolaris maydis ATCC 48331 unplaced genomic scaffold COCC4sc...
111. KB445576\_0 Cochliobolus heterostrophus C5 unplaced genomic scaffold COCH...
112. GG663364\_0 Ajellomyces capsulatus G186AR genomic scaffold supercont2.2, ...
113. AM920433\_0 Penicillium chrysogenum Wisconsin 54-1255 complete genome, co...
114. CM001234\_2 Magnaporthe oryzae 70-15 chromosome 4, whole genome shotgun s...
115. KB725930\_1 Colletotrichum orbiculare MAFF 240422 unplaced genomic scaffo...
116. DS985216\_0 Verticillium albo-atrum VaMs.102 supercont1.3 genomic scaffol...
117. DS572721\_1 Verticillium dahliae VdLs.17 supercont1.27 genomic scaffold, ...
118. HF679025\_1 Fusarium fujikuroi IMI 58289 draft genome, chromosome FFUJ\_ch...
119. ABDF02000092\_0 Trichoderma virens Gv29-8, whole genome shotgun sequencin...
120. ABDG02000027\_1 Trichoderma atroviride IMI 206040, whole genome shotgun s...
121. DF196785\_0 Pseudozyma antarctica T-34 DNA, contig: scaffold00019, whole ...
122. FQ311472\_1 Sporisorium reilianum SRZ2 chromosome 7 complete DNA sequence.
123. KE148153\_1 Ophiostoma piceae UAMH 11346 chromosome Unknown scf08, whole ...
124. KB310677\_0 Capitella teleta unplaced genomic scaffold CAPTEscaffold\_748,...
125. DS572752\_0 Paracoccidioides brasiliensis Pb18 supercont1.3 genomic scaff...
126. JH767588\_1 Coniosporium apollinis CBS 100218 chromosome Unknown supercon...
127. GL985056\_0 Trichoderma reesei QM6a unplaced genomic scaffold TRIREscaffo...
128. KB446555\_2 Pseudocercospora fijiensis CIRAD86 unplaced genomic scaffold ...
129. GG698898\_0 Nectria haematococca mpVI 77-13-4 chromosome 4 genomic scaffo...
130. KB730248\_0 Fusarium oxysporum f. sp. cubense race 1 unplaced genomic sca...
131. KB726554\_1 Fusarium oxysporum f. sp. cubense race 4 unplaced genomic sca...
132. HF679026\_1 Fusarium fujikuroi IMI 58289 draft genome, chromosome FFUJ\_ch...
133. ABDF02000004\_1 Trichoderma virens Gv29-8, whole genome shotgun sequencin...
134. JH226131\_0 Exophiala dermatitidis NIH/UT8656 unplaced genomic scaffold s...
135. GG704914\_0 Coccidioides immitis RS genomic scaffold supercont3.4, whole ...
136. DS572697\_0 Verticillium dahliae VdLs.17 supercont1.3 genomic scaffold, w...
137. GL636503\_0 Coccidioides posadasii str. Silveira unplaced genomic scaffol...
138. ACFW01000009\_1 Coccidioides posadasii C735 delta SOWgp, whole genome sho...
139. ABDG02000017\_1 Trichoderma atroviride IMI 206040, whole genome shotgun s...
140. GL891305\_2 Neurospora tetrasperma FGSC 2508 unplaced genomic scaffold NE...
141. GL891247\_0 Neurospora tetrasperma FGSC 2509 unplaced genomic scaffold NE...
142. KB456260\_0 Mycosphaerella populorum SO2202 unplaced genomic scaffold SEP...
143. CU638743\_0 Podospora anserina S mat+ genomic DNA chromosome 3, supercont...
144. CM001235\_0 Magnaporthe oryzae 70-15 chromosome 5, whole genome shotgun s...
145. EQ963473\_1 Aspergillus flavus NRRL3357 scf\_1106286417600 genomic scaffol...
146. GL629729\_0 Grosmannia clavigera kw1407 unplaced genomic scaffold GCSC\_10...
147. KE123956\_0 Mucor circinelloides f. circinelloides 1006PhL unplaced genom...
148. CP003008\_0 Myceliophthora thermophila ATCC 42464 chromosome 7, complete ...
149. CP004025\_0 Myxococcus stipitatus DSM 14675, complete genome.
150. DS985245\_0 Trichoplax adhaerens TRIADscaffold\_5 genomic scaffold, whole ...

Query: Architecture Search FASTA input

DF126495 : Aspergillus kawachii IFO 4308 DNA, contig: scaffold00049    Total score: 1.0     Cumulative Blast bit score: 1712

Hit cluster cross-links:

Mycgr3G52686 Mycgr3T
  
Location: 0-861

Mycgr3G52686\_Mycgr3T

Mycgr3G102281 Mycgr3
  
Location: 961-1573

Mycgr3G102281\_Mycgr3

Mycgr3G89185 Mycgr3T
  
Location: 1673-2063

Mycgr3G89185\_Mycgr3T

Mycgr3G65725 Mycgr3T
  
Location: 2163-3612

Mycgr3G65725\_Mycgr3T

Mycgr3G102276 Mycgr3
  
Location: 3712-4801

Mycgr3G102276\_Mycgr3

Mycgr3G89189 Mycgr3T
  
Location: 4901-5564

Mycgr3G89189\_Mycgr3T

Mycgr3G52682 Mycgr3T
  
Location: 5664-9231

Mycgr3G52682\_Mycgr3T

Mycgr3G107072 Mycgr3
  
Location: 9331-13279

Mycgr3G107072\_Mycgr3

Mycgr3G34982 Mycgr3T
  
Location: 13379-15116

Mycgr3G34982\_Mycgr3T

Mycgr3G107069 Mycgr3
  
Location: 15216-17097

Mycgr3G107069\_Mycgr3

Mycgr3G32432 Mycgr3T
  
Location: 17197-19042

Mycgr3G32432\_Mycgr3T

Mycgr3G98385 Mycgr3T
  
Location: 19142-19898

Mycgr3G98385\_Mycgr3T

sodium/phosphate symporter
  
Accession: GAA92507
  
Location: 89202-91156
  
 NCBI BlastP on this gene

GAA92507

hypothetical protein
  
Accession: GAA92508
  
Location: 91369-91694
  
 NCBI BlastP on this gene

GAA92508

amidase family protein
  
Accession: GAA92509
  
Location: 92266-94131
  
 NCBI BlastP on this gene

GAA92509

hypothetical protein
  
Accession: GAA92510
  
Location: 95540-96938
  
 NCBI BlastP on this gene

GAA92510

hybrid NRPS/PKS enzyme
  
Accession: GAA92511
  
Location: 99856-103686
  
  
**BlastP hit with Mycgr3G107072\_Mycgr3**
  
Percentage identity: 64 %
  
BlastP bit score: 1712
  
Sequence coverage: 100 %
  
E-value: 0.0
  
  
 NCBI BlastP on this gene

GAA92511

Query: Architecture Search FASTA input

AACD01000093 : Aspergillus nidulans FGSC A4    Total score: 1.0     Cumulative Blast bit score: 1705

Hit cluster cross-links:

Mycgr3G52686 Mycgr3T
  
Location: 0-861

Mycgr3G52686\_Mycgr3T

Mycgr3G102281 Mycgr3
  
Location: 961-1573

Mycgr3G102281\_Mycgr3

Mycgr3G89185 Mycgr3T
  
Location: 1673-2063

Mycgr3G89185\_Mycgr3T

Mycgr3G65725 Mycgr3T
  
Location: 2163-3612

Mycgr3G65725\_Mycgr3T

Mycgr3G102276 Mycgr3
  
Location: 3712-4801

Mycgr3G102276\_Mycgr3

Mycgr3G89189 Mycgr3T
  
Location: 4901-5564

Mycgr3G89189\_Mycgr3T

Mycgr3G52682 Mycgr3T
  
Location: 5664-9231

Mycgr3G52682\_Mycgr3T

Mycgr3G107072 Mycgr3
  
Location: 9331-13279

Mycgr3G107072\_Mycgr3

Mycgr3G34982 Mycgr3T
  
Location: 13379-15116

Mycgr3G34982\_Mycgr3T

Mycgr3G107069 Mycgr3
  
Location: 15216-17097

Mycgr3G107069\_Mycgr3

Mycgr3G32432 Mycgr3T
  
Location: 17197-19042

Mycgr3G32432\_Mycgr3T

Mycgr3G98385 Mycgr3T
  
Location: 19142-19898

Mycgr3G98385\_Mycgr3T

hypothetical protein
  
Accession: EAA62474
  
Location: 153912-155547
  
 NCBI BlastP on this gene

EAA62474

predicted protein
  
Accession: EAA62475
  
Location: 156458-159074
  
 NCBI BlastP on this gene

EAA62475

predicted protein
  
Accession: EAA62476
  
Location: 159441-160143
  
 NCBI BlastP on this gene

EAA62476

hypothetical protein
  
Accession: EAA62477
  
Location: 160727-161650
  
 NCBI BlastP on this gene

EAA62477

hypothetical protein
  
Accession: EAA62478
  
Location: 164592-168404
  
  
**BlastP hit with Mycgr3G107072\_Mycgr3**
  
Percentage identity: 64 %
  
BlastP bit score: 1705
  
Sequence coverage: 100 %
  
E-value: 0.0
  
  
 NCBI BlastP on this gene

EAA62478

predicted protein
  
Accession: EAA62479
  
Location: 171837-172913
  
 NCBI BlastP on this gene

EAA62479

hypothetical protein
  
Accession: EAA62480
  
Location: 173388-175076
  
 NCBI BlastP on this gene

EAA62480

hypothetical protein
  
Accession: EAA62481
  
Location: 175181-177062
  
 NCBI BlastP on this gene

EAA62481

predicted protein
  
Accession: EAA62482
  
Location: 177262-177640
  
 NCBI BlastP on this gene

EAA62482

hypothetical protein
  
Accession: EAA62483
  
Location: 178783-180921
  
 NCBI BlastP on this gene

EAA62483

Query: Architecture Search FASTA input

EQ963472 : Aspergillus flavus NRRL3357 scf\_1106286418772 genomic scaffold    Total score: 1.0     Cumulative Blast bit score: 1704

Hit cluster cross-links:

Mycgr3G52686 Mycgr3T
  
Location: 0-861

Mycgr3G52686\_Mycgr3T

Mycgr3G102281 Mycgr3
  
Location: 961-1573

Mycgr3G102281\_Mycgr3

Mycgr3G89185 Mycgr3T
  
Location: 1673-2063

Mycgr3G89185\_Mycgr3T

Mycgr3G65725 Mycgr3T
  
Location: 2163-3612

Mycgr3G65725\_Mycgr3T

Mycgr3G102276 Mycgr3
  
Location: 3712-4801

Mycgr3G102276\_Mycgr3

Mycgr3G89189 Mycgr3T
  
Location: 4901-5564

Mycgr3G89189\_Mycgr3T

Mycgr3G52682 Mycgr3T
  
Location: 5664-9231

Mycgr3G52682\_Mycgr3T

Mycgr3G107072 Mycgr3
  
Location: 9331-13279

Mycgr3G107072\_Mycgr3

Mycgr3G34982 Mycgr3T
  
Location: 13379-15116

Mycgr3G34982\_Mycgr3T

Mycgr3G107069 Mycgr3
  
Location: 15216-17097

Mycgr3G107069\_Mycgr3

Mycgr3G32432 Mycgr3T
  
Location: 17197-19042

Mycgr3G32432\_Mycgr3T

Mycgr3G98385 Mycgr3T
  
Location: 19142-19898

Mycgr3G98385\_Mycgr3T

conserved hypothetical protein
  
Accession: EED57240
  
Location: 1890073-1890807
  
 NCBI BlastP on this gene

EED57240

PKS-like enzyme, putative
  
Accession: EED57241
  
Location: 1892237-1892994
  
 NCBI BlastP on this gene

EED57241

hypothetical protein
  
Accession: EED57242
  
Location: 1894296-1894484
  
 NCBI BlastP on this gene

EED57242

NRPS-like enzyme, putative
  
Accession: EED57243
  
Location: 1894656-1897651
  
 NCBI BlastP on this gene

EED57243

hypothetical protein
  
Accession: EED57244
  
Location: 1898598-1899094
  
 NCBI BlastP on this gene

EED57244

NRPS-like enzyme, putative
  
Accession: EED57245
  
Location: 1901221-1905057
  
  
**BlastP hit with Mycgr3G107072\_Mycgr3**
  
Percentage identity: 65 %
  
BlastP bit score: 1704
  
Sequence coverage: 100 %
  
E-value: 0.0
  
  
 NCBI BlastP on this gene

EED57245

MFS transporter, putative
  
Accession: EED57246
  
Location: 1905565-1907524
  
 NCBI BlastP on this gene

EED57246

conserved hypothetical protein
  
Accession: EED57247
  
Location: 1907742-1908365
  
 NCBI BlastP on this gene

EED57247

amidase, putative
  
Accession: EED57248
  
Location: 1910291-1911075
  
 NCBI BlastP on this gene

EED57248

allantoate permease, putative
  
Accession: EED57249
  
Location: 1912113-1913531
  
 NCBI BlastP on this gene

EED57249

ureidoglycolate hydrolase, putative
  
Accession: EED57250
  
Location: 1913795-1914532
  
 NCBI BlastP on this gene

EED57250

DNA mismatch repair protein Msh1, putative
  
Accession: EED57251
  
Location: 1914711-1917746
  
 NCBI BlastP on this gene

EED57251

Query: Architecture Search FASTA input

AP007151 : Aspergillus oryzae RIB40 DNA, SC005.    Total score: 1.0     Cumulative Blast bit score: 1703

Hit cluster cross-links:

Mycgr3G52686 Mycgr3T
  
Location: 0-861

Mycgr3G52686\_Mycgr3T

Mycgr3G102281 Mycgr3
  
Location: 961-1573

Mycgr3G102281\_Mycgr3

Mycgr3G89185 Mycgr3T
  
Location: 1673-2063

Mycgr3G89185\_Mycgr3T

Mycgr3G65725 Mycgr3T
  
Location: 2163-3612

Mycgr3G65725\_Mycgr3T

Mycgr3G102276 Mycgr3
  
Location: 3712-4801

Mycgr3G102276\_Mycgr3

Mycgr3G89189 Mycgr3T
  
Location: 4901-5564

Mycgr3G89189\_Mycgr3T

Mycgr3G52682 Mycgr3T
  
Location: 5664-9231

Mycgr3G52682\_Mycgr3T

Mycgr3G107072 Mycgr3
  
Location: 9331-13279

Mycgr3G107072\_Mycgr3

Mycgr3G34982 Mycgr3T
  
Location: 13379-15116

Mycgr3G34982\_Mycgr3T

Mycgr3G107069 Mycgr3
  
Location: 15216-17097

Mycgr3G107069\_Mycgr3

Mycgr3G32432 Mycgr3T
  
Location: 17197-19042

Mycgr3G32432\_Mycgr3T

Mycgr3G98385 Mycgr3T
  
Location: 19142-19898

Mycgr3G98385\_Mycgr3T

not annotated
  
Accession: BAE55721
  
Location: 1812269-1813031
  
 NCBI BlastP on this gene

AO090005000687

not annotated
  
Accession: BAE55722
  
Location: 1814333-1817688
  
 NCBI BlastP on this gene

AO090005000688

not annotated
  
Accession: BAE55723
  
Location: 1818636-1819288
  
 NCBI BlastP on this gene

AO090005000689

not annotated
  
Accession: BAE55724
  
Location: 1821261-1825097
  
  
**BlastP hit with Mycgr3G107072\_Mycgr3**
  
Percentage identity: 65 %
  
BlastP bit score: 1703
  
Sequence coverage: 100 %
  
E-value: 0.0
  
  
 NCBI BlastP on this gene

AO090005000690

not annotated
  
Accession: BAE55725
  
Location: 1827787-1828410
  
 NCBI BlastP on this gene

AO090005000691

not annotated
  
Accession: BAE55726
  
Location: 1829266-1831255
  
 NCBI BlastP on this gene

AO090005000692

not annotated
  
Accession: BAE55727
  
Location: 1832167-1833585
  
 NCBI BlastP on this gene

AO090005000693

not annotated
  
Accession: BAE55728
  
Location: 1833850-1834587
  
 NCBI BlastP on this gene

AO090005000694

not annotated
  
Accession: BAE55729
  
Location: 1834766-1837801
  
 NCBI BlastP on this gene

AO090005000695

Query: Architecture Search FASTA input

DS990638 : Ajellomyces capsulatus H88 supercont1.3 genomic scaffold    Total score: 1.0     Cumulative Blast bit score: 1688

Hit cluster cross-links:

Mycgr3G52686 Mycgr3T
  
Location: 0-861

Mycgr3G52686\_Mycgr3T

Mycgr3G102281 Mycgr3
  
Location: 961-1573

Mycgr3G102281\_Mycgr3

Mycgr3G89185 Mycgr3T
  
Location: 1673-2063

Mycgr3G89185\_Mycgr3T

Mycgr3G65725 Mycgr3T
  
Location: 2163-3612

Mycgr3G65725\_Mycgr3T

Mycgr3G102276 Mycgr3
  
Location: 3712-4801

Mycgr3G102276\_Mycgr3

Mycgr3G89189 Mycgr3T
  
Location: 4901-5564

Mycgr3G89189\_Mycgr3T

Mycgr3G52682 Mycgr3T
  
Location: 5664-9231

Mycgr3G52682\_Mycgr3T

Mycgr3G107072 Mycgr3
  
Location: 9331-13279

Mycgr3G107072\_Mycgr3

Mycgr3G34982 Mycgr3T
  
Location: 13379-15116

Mycgr3G34982\_Mycgr3T

Mycgr3G107069 Mycgr3
  
Location: 15216-17097

Mycgr3G107069\_Mycgr3

Mycgr3G32432 Mycgr3T
  
Location: 17197-19042

Mycgr3G32432\_Mycgr3T

Mycgr3G98385 Mycgr3T
  
Location: 19142-19898

Mycgr3G98385\_Mycgr3T

conserved hypothetical protein
  
Accession: EGC44737
  
Location: 2320159-2321674
  
 NCBI BlastP on this gene

EGC44737

conserved hypothetical protein
  
Accession: EGC44736
  
Location: 2318370-2319284
  
 NCBI BlastP on this gene

EGC44736

ATP synthase subunit 4
  
Accession: EGC44735
  
Location: 2317153-2318132
  
 NCBI BlastP on this gene

EGC44735

CBF/Mak21 family
  
Accession: EGC44734
  
Location: 2315100-2316893
  
 NCBI BlastP on this gene

EGC44734

conserved hypothetical protein
  
Accession: EGC44733
  
Location: 2313619-2314611
  
 NCBI BlastP on this gene

EGC44733

nonribosomal peptide synthetase
  
Accession: EGC44732
  
Location: 2307588-2311418
  
  
**BlastP hit with Mycgr3G107072\_Mycgr3**
  
Percentage identity: 64 %
  
BlastP bit score: 1688
  
Sequence coverage: 100 %
  
E-value: 0.0
  
  
 NCBI BlastP on this gene

EGC44732

predicted protein
  
Accession: EGC44731
  
Location: 2305770-2306498
  
 NCBI BlastP on this gene

EGC44731

oxidoreductase
  
Accession: EGC44730
  
Location: 2301960-2303005
  
 NCBI BlastP on this gene

EGC44730

conserved hypothetical protein
  
Accession: EGC44729
  
Location: 2298579-2299423
  
 NCBI BlastP on this gene

EGC44729

conserved hypothetical protein
  
Accession: EGC44728
  
Location: 2297108-2297531
  
 NCBI BlastP on this gene

EGC44728

Query: Architecture Search FASTA input

DS231615 : Pyrenophora tritici-repentis Pt-1C-BFP supercont1.1 genomic scaffold    Total score: 1.0     Cumulative Blast bit score: 1675

Hit cluster cross-links:

Mycgr3G52686 Mycgr3T
  
Location: 0-861

Mycgr3G52686\_Mycgr3T

Mycgr3G102281 Mycgr3
  
Location: 961-1573

Mycgr3G102281\_Mycgr3

Mycgr3G89185 Mycgr3T
  
Location: 1673-2063

Mycgr3G89185\_Mycgr3T

Mycgr3G65725 Mycgr3T
  
Location: 2163-3612

Mycgr3G65725\_Mycgr3T

Mycgr3G102276 Mycgr3
  
Location: 3712-4801

Mycgr3G102276\_Mycgr3

Mycgr3G89189 Mycgr3T
  
Location: 4901-5564

Mycgr3G89189\_Mycgr3T

Mycgr3G52682 Mycgr3T
  
Location: 5664-9231

Mycgr3G52682\_Mycgr3T

Mycgr3G107072 Mycgr3
  
Location: 9331-13279

Mycgr3G107072\_Mycgr3

Mycgr3G34982 Mycgr3T
  
Location: 13379-15116

Mycgr3G34982\_Mycgr3T

Mycgr3G107069 Mycgr3
  
Location: 15216-17097

Mycgr3G107069\_Mycgr3

Mycgr3G32432 Mycgr3T
  
Location: 17197-19042

Mycgr3G32432\_Mycgr3T

Mycgr3G98385 Mycgr3T
  
Location: 19142-19898

Mycgr3G98385\_Mycgr3T

splicing factor 3a subunit 2
  
Accession: EDU39879
  
Location: 1173289-1174047
  
 NCBI BlastP on this gene

EDU39879

tubulin-specific chaperone E
  
Accession: EDU39880
  
Location: 1174612-1176403
  
 NCBI BlastP on this gene

EDU39880

hypothetical protein
  
Accession: EDU39881
  
Location: 1176447-1177473
  
 NCBI BlastP on this gene

EDU39881

c-myc binding protein
  
Accession: EDU39882
  
Location: 1177648-1178202
  
 NCBI BlastP on this gene

EDU39882

predicted protein
  
Accession: EDU39883
  
Location: 1178506-1180523
  
 NCBI BlastP on this gene

EDU39883

mitochondrial ribosomal protein subunit L23
  
Accession: EDU39884
  
Location: 1181378-1182091
  
 NCBI BlastP on this gene

EDU39884

tyrocidine synthetase 1
  
Accession: EDU39885
  
Location: 1184080-1187928
  
  
**BlastP hit with Mycgr3G107072\_Mycgr3**
  
Percentage identity: 63 %
  
BlastP bit score: 1675
  
Sequence coverage: 100 %
  
E-value: 0.0
  
  
 NCBI BlastP on this gene

EDU39885

Query: Architecture Search FASTA input

KB644414 : Penicillium oxalicum 114-2 unplaced genomic scaffold scaffold\_7    Total score: 1.0     Cumulative Blast bit score: 1665

Hit cluster cross-links:

Mycgr3G52686 Mycgr3T
  
Location: 0-861

Mycgr3G52686\_Mycgr3T

Mycgr3G102281 Mycgr3
  
Location: 961-1573

Mycgr3G102281\_Mycgr3

Mycgr3G89185 Mycgr3T
  
Location: 1673-2063

Mycgr3G89185\_Mycgr3T

Mycgr3G65725 Mycgr3T
  
Location: 2163-3612

Mycgr3G65725\_Mycgr3T

Mycgr3G102276 Mycgr3
  
Location: 3712-4801

Mycgr3G102276\_Mycgr3

Mycgr3G89189 Mycgr3T
  
Location: 4901-5564

Mycgr3G89189\_Mycgr3T

Mycgr3G52682 Mycgr3T
  
Location: 5664-9231

Mycgr3G52682\_Mycgr3T

Mycgr3G107072 Mycgr3
  
Location: 9331-13279

Mycgr3G107072\_Mycgr3

Mycgr3G34982 Mycgr3T
  
Location: 13379-15116

Mycgr3G34982\_Mycgr3T

Mycgr3G107069 Mycgr3
  
Location: 15216-17097

Mycgr3G107069\_Mycgr3

Mycgr3G32432 Mycgr3T
  
Location: 17197-19042

Mycgr3G32432\_Mycgr3T

Mycgr3G98385 Mycgr3T
  
Location: 19142-19898

Mycgr3G98385\_Mycgr3T

hypothetical protein
  
Accession: EPS32408
  
Location: 2332945-2335027
  
 NCBI BlastP on this gene

EPS32408

hypothetical protein
  
Accession: EPS32409
  
Location: 2335899-2336219
  
 NCBI BlastP on this gene

EPS32409

hypothetical protein
  
Accession: EPS32410
  
Location: 2337112-2337831
  
 NCBI BlastP on this gene

EPS32410

hypothetical protein
  
Accession: EPS32411
  
Location: 2338462-2340785
  
 NCBI BlastP on this gene

EPS32411

hypothetical protein
  
Accession: EPS32412
  
Location: 2341213-2342406
  
 NCBI BlastP on this gene

EPS32412

hypothetical protein
  
Accession: EPS32413
  
Location: 2344043-2347876
  
  
**BlastP hit with Mycgr3G107072\_Mycgr3**
  
Percentage identity: 64 %
  
BlastP bit score: 1665
  
Sequence coverage: 100 %
  
E-value: 0.0
  
  
 NCBI BlastP on this gene

EPS32413

hypothetical protein
  
Accession: EPS32414
  
Location: 2350568-2350867
  
 NCBI BlastP on this gene

EPS32414

hypothetical protein
  
Accession: EPS32415
  
Location: 2351379-2352656
  
 NCBI BlastP on this gene

EPS32415

hypothetical protein
  
Accession: EPS32416
  
Location: 2354988-2355850
  
 NCBI BlastP on this gene

EPS32416

Query: Architecture Search FASTA input

EQ962653 : Talaromyces stipitatus ATCC 10500 scf\_1105507295527 genomic scaffold    Total score: 1.0     Cumulative Blast bit score: 1663

Hit cluster cross-links:

Mycgr3G52686 Mycgr3T
  
Location: 0-861

Mycgr3G52686\_Mycgr3T

Mycgr3G102281 Mycgr3
  
Location: 961-1573

Mycgr3G102281\_Mycgr3

Mycgr3G89185 Mycgr3T
  
Location: 1673-2063

Mycgr3G89185\_Mycgr3T

Mycgr3G65725 Mycgr3T
  
Location: 2163-3612

Mycgr3G65725\_Mycgr3T

Mycgr3G102276 Mycgr3
  
Location: 3712-4801

Mycgr3G102276\_Mycgr3

Mycgr3G89189 Mycgr3T
  
Location: 4901-5564

Mycgr3G89189\_Mycgr3T

Mycgr3G52682 Mycgr3T
  
Location: 5664-9231

Mycgr3G52682\_Mycgr3T

Mycgr3G107072 Mycgr3
  
Location: 9331-13279

Mycgr3G107072\_Mycgr3

Mycgr3G34982 Mycgr3T
  
Location: 13379-15116

Mycgr3G34982\_Mycgr3T

Mycgr3G107069 Mycgr3
  
Location: 15216-17097

Mycgr3G107069\_Mycgr3

Mycgr3G32432 Mycgr3T
  
Location: 17197-19042

Mycgr3G32432\_Mycgr3T

Mycgr3G98385 Mycgr3T
  
Location: 19142-19898

Mycgr3G98385\_Mycgr3T

hypothetical protein
  
Accession: EED20985
  
Location: 473981-474277
  
 NCBI BlastP on this gene

EED20985

short-chain dehydrogenase, putative
  
Accession: EED20984
  
Location: 472336-473226
  
 NCBI BlastP on this gene

EED20984

hypothetical protein
  
Accession: EED20983
  
Location: 470334-471027
  
 NCBI BlastP on this gene

EED20983

amino acid transporter, putative
  
Accession: EED20982
  
Location: 467450-468920
  
 NCBI BlastP on this gene

EED20982

NRPS-like enzyme, putative
  
Accession: EED20981
  
Location: 460829-464674
  
  
**BlastP hit with Mycgr3G107072\_Mycgr3**
  
Percentage identity: 62 %
  
BlastP bit score: 1664
  
Sequence coverage: 100 %
  
E-value: 0.0
  
  
 NCBI BlastP on this gene

EED20981

conserved hypothetical protein
  
Accession: EED20980
  
Location: 459043-460450
  
 NCBI BlastP on this gene

EED20980

beta-xylosidase XylA
  
Accession: EED20979
  
Location: 456506-458899
  
 NCBI BlastP on this gene

EED20979

mucin-1 precursor, putative
  
Accession: EED20978
  
Location: 453058-455335
  
 NCBI BlastP on this gene

EED20978

hypothetical protein
  
Accession: EED20977
  
Location: 450993-452204
  
 NCBI BlastP on this gene

EED20977

Query: Architecture Search FASTA input

DS995900 : Penicillium marneffei ATCC 18224 scf\_1105668340758 genomic scaffold    Total score: 1.0     Cumulative Blast bit score: 1663

Hit cluster cross-links:

Mycgr3G52686 Mycgr3T
  
Location: 0-861

Mycgr3G52686\_Mycgr3T

Mycgr3G102281 Mycgr3
  
Location: 961-1573

Mycgr3G102281\_Mycgr3

Mycgr3G89185 Mycgr3T
  
Location: 1673-2063

Mycgr3G89185\_Mycgr3T

Mycgr3G65725 Mycgr3T
  
Location: 2163-3612

Mycgr3G65725\_Mycgr3T

Mycgr3G102276 Mycgr3
  
Location: 3712-4801

Mycgr3G102276\_Mycgr3

Mycgr3G89189 Mycgr3T
  
Location: 4901-5564

Mycgr3G89189\_Mycgr3T

Mycgr3G52682 Mycgr3T
  
Location: 5664-9231

Mycgr3G52682\_Mycgr3T

Mycgr3G107072 Mycgr3
  
Location: 9331-13279

Mycgr3G107072\_Mycgr3

Mycgr3G34982 Mycgr3T
  
Location: 13379-15116

Mycgr3G34982\_Mycgr3T

Mycgr3G107069 Mycgr3
  
Location: 15216-17097

Mycgr3G107069\_Mycgr3

Mycgr3G32432 Mycgr3T
  
Location: 17197-19042

Mycgr3G32432\_Mycgr3T

Mycgr3G98385 Mycgr3T
  
Location: 19142-19898

Mycgr3G98385\_Mycgr3T

conserved hypothetical protein
  
Accession: EEA25140
  
Location: 238306-238827
  
 NCBI BlastP on this gene

EEA25140

short-chain dehydrogenase, putative
  
Accession: EEA25139
  
Location: 235856-236831
  
 NCBI BlastP on this gene

EEA25139

amino acid transporter, putative
  
Accession: EEA25138
  
Location: 232134-233605
  
 NCBI BlastP on this gene

EEA25138

NRPS-like enzyme, putative
  
Accession: EEA25137
  
Location: 227129-230968
  
  
**BlastP hit with Mycgr3G107072\_Mycgr3**
  
Percentage identity: 62 %
  
BlastP bit score: 1663
  
Sequence coverage: 100 %
  
E-value: 0.0
  
  
 NCBI BlastP on this gene

EEA25137

conserved hypothetical protein
  
Accession: EEA25136
  
Location: 225283-226690
  
 NCBI BlastP on this gene

EEA25136

beta-xylosidase XylA
  
Accession: EEA25135
  
Location: 222711-225110
  
 NCBI BlastP on this gene

EEA25135

dienelactone hydrolase family protein
  
Accession: EEA25134
  
Location: 220334-221185
  
 NCBI BlastP on this gene

EEA25134

extracellular exo-polygalacturonase, putative
  
Accession: EEA25131
  
Location: 218226-219610
  
 NCBI BlastP on this gene

EEA25131

conserved hypothetical protein
  
Accession: EEA25130
  
Location: 217199-217975
  
 NCBI BlastP on this gene

EEA25130

Query: Architecture Search FASTA input

KB733447 : Bipolaris maydis ATCC 48331 unplaced genomic scaffold COCC4scaffold\_4    Total score: 1.0     Cumulative Blast bit score: 1661

Hit cluster cross-links:

Mycgr3G52686 Mycgr3T
  
Location: 0-861

Mycgr3G52686\_Mycgr3T

Mycgr3G102281 Mycgr3
  
Location: 961-1573

Mycgr3G102281\_Mycgr3

Mycgr3G89185 Mycgr3T
  
Location: 1673-2063

Mycgr3G89185\_Mycgr3T

Mycgr3G65725 Mycgr3T
  
Location: 2163-3612

Mycgr3G65725\_Mycgr3T

Mycgr3G102276 Mycgr3
  
Location: 3712-4801

Mycgr3G102276\_Mycgr3

Mycgr3G89189 Mycgr3T
  
Location: 4901-5564

Mycgr3G89189\_Mycgr3T

Mycgr3G52682 Mycgr3T
  
Location: 5664-9231

Mycgr3G52682\_Mycgr3T

Mycgr3G107072 Mycgr3
  
Location: 9331-13279

Mycgr3G107072\_Mycgr3

Mycgr3G34982 Mycgr3T
  
Location: 13379-15116

Mycgr3G34982\_Mycgr3T

Mycgr3G107069 Mycgr3
  
Location: 15216-17097

Mycgr3G107069\_Mycgr3

Mycgr3G32432 Mycgr3T
  
Location: 17197-19042

Mycgr3G32432\_Mycgr3T

Mycgr3G98385 Mycgr3T
  
Location: 19142-19898

Mycgr3G98385\_Mycgr3T

hypothetical protein
  
Accession: ENI08913
  
Location: 1369227-1370809
  
 NCBI BlastP on this gene

ENI08913

hypothetical protein
  
Accession: ENI08914
  
Location: 1371908-1373208
  
 NCBI BlastP on this gene

ENI08914

hypothetical protein
  
Accession: ENI08915
  
Location: 1374944-1378034
  
 NCBI BlastP on this gene

ENI08915

hypothetical protein
  
Accession: ENI08916
  
Location: 1379319-1383167
  
  
**BlastP hit with Mycgr3G107072\_Mycgr3**
  
Percentage identity: 63 %
  
BlastP bit score: 1661
  
Sequence coverage: 100 %
  
E-value: 0.0
  
  
 NCBI BlastP on this gene

ENI08916

Query: Architecture Search FASTA input

KB445576 : Cochliobolus heterostrophus C5 unplaced genomic scaffold COCHEscaffold\_8    Total score: 1.0     Cumulative Blast bit score: 1661

Hit cluster cross-links:

Mycgr3G52686 Mycgr3T
  
Location: 0-861

Mycgr3G52686\_Mycgr3T

Mycgr3G102281 Mycgr3
  
Location: 961-1573

Mycgr3G102281\_Mycgr3

Mycgr3G89185 Mycgr3T
  
Location: 1673-2063

Mycgr3G89185\_Mycgr3T

Mycgr3G65725 Mycgr3T
  
Location: 2163-3612

Mycgr3G65725\_Mycgr3T

Mycgr3G102276 Mycgr3
  
Location: 3712-4801

Mycgr3G102276\_Mycgr3

Mycgr3G89189 Mycgr3T
  
Location: 4901-5564

Mycgr3G89189\_Mycgr3T

Mycgr3G52682 Mycgr3T
  
Location: 5664-9231

Mycgr3G52682\_Mycgr3T

Mycgr3G107072 Mycgr3
  
Location: 9331-13279

Mycgr3G107072\_Mycgr3

Mycgr3G34982 Mycgr3T
  
Location: 13379-15116

Mycgr3G34982\_Mycgr3T

Mycgr3G107069 Mycgr3
  
Location: 15216-17097

Mycgr3G107069\_Mycgr3

Mycgr3G32432 Mycgr3T
  
Location: 17197-19042

Mycgr3G32432\_Mycgr3T

Mycgr3G98385 Mycgr3T
  
Location: 19142-19898

Mycgr3G98385\_Mycgr3T

hypothetical protein
  
Accession: EMD91330
  
Location: 261837-263419
  
 NCBI BlastP on this gene

EMD91330

hypothetical protein
  
Accession: EMD91329
  
Location: 259438-260738
  
 NCBI BlastP on this gene

EMD91329

hypothetical protein
  
Accession: EMD91328
  
Location: 254612-257702
  
 NCBI BlastP on this gene

EMD91328

hypothetical protein
  
Accession: EMD91327
  
Location: 249479-253327
  
  
**BlastP hit with Mycgr3G107072\_Mycgr3**
  
Percentage identity: 63 %
  
BlastP bit score: 1661
  
Sequence coverage: 100 %
  
E-value: 0.0
  
  
 NCBI BlastP on this gene

EMD91327

hypothetical protein
  
Accession: EMD91326
  
Location: 247978-248883
  
 NCBI BlastP on this gene

EMD91326

hypothetical protein
  
Accession: EMD91325
  
Location: 246711-247712
  
 NCBI BlastP on this gene

EMD91325

hypothetical protein
  
Accession: EMD91324
  
Location: 245190-246351
  
 NCBI BlastP on this gene

EMD91324

hypothetical protein
  
Accession: EMD91323
  
Location: 243382-244599
  
 NCBI BlastP on this gene

EMD91323

hypothetical protein
  
Accession: EMD91322
  
Location: 240529-242217
  
 NCBI BlastP on this gene

EMD91322

hypothetical protein
  
Accession: EMD91321
  
Location: 239356-240256
  
 NCBI BlastP on this gene

EMD91321

hypothetical protein
  
Accession: EMD91320
  
Location: 237537-238963
  
 NCBI BlastP on this gene

EMD91320

Query: Architecture Search FASTA input

GG663364 : Ajellomyces capsulatus G186AR genomic scaffold supercont2.2    Total score: 1.0     Cumulative Blast bit score: 1661

Hit cluster cross-links:

Mycgr3G52686 Mycgr3T
  
Location: 0-861

Mycgr3G52686\_Mycgr3T

Mycgr3G102281 Mycgr3
  
Location: 961-1573

Mycgr3G102281\_Mycgr3

Mycgr3G89185 Mycgr3T
  
Location: 1673-2063

Mycgr3G89185\_Mycgr3T

Mycgr3G65725 Mycgr3T
  
Location: 2163-3612

Mycgr3G65725\_Mycgr3T

Mycgr3G102276 Mycgr3
  
Location: 3712-4801

Mycgr3G102276\_Mycgr3

Mycgr3G89189 Mycgr3T
  
Location: 4901-5564

Mycgr3G89189\_Mycgr3T

Mycgr3G52682 Mycgr3T
  
Location: 5664-9231

Mycgr3G52682\_Mycgr3T

Mycgr3G107072 Mycgr3
  
Location: 9331-13279

Mycgr3G107072\_Mycgr3

Mycgr3G34982 Mycgr3T
  
Location: 13379-15116

Mycgr3G34982\_Mycgr3T

Mycgr3G107069 Mycgr3
  
Location: 15216-17097

Mycgr3G107069\_Mycgr3

Mycgr3G32432 Mycgr3T
  
Location: 17197-19042

Mycgr3G32432\_Mycgr3T

Mycgr3G98385 Mycgr3T
  
Location: 19142-19898

Mycgr3G98385\_Mycgr3T

conserved hypothetical protein
  
Accession: EEH10265
  
Location: 2040059-2041565
  
 NCBI BlastP on this gene

EEH10265

conserved hypothetical protein
  
Accession: EEH10264
  
Location: 2038120-2039034
  
 NCBI BlastP on this gene

EEH10264

ATP synthase subunit 4
  
Accession: EEH10263
  
Location: 2036901-2037880
  
 NCBI BlastP on this gene

EEH10263

CBF/Mak21 family
  
Accession: EEH10262
  
Location: 2034825-2036639
  
 NCBI BlastP on this gene

EEH10262

conserved hypothetical protein
  
Accession: EEH10261
  
Location: 2033345-2034337
  
 NCBI BlastP on this gene

EEH10261

nonribosomal peptide synthetase
  
Accession: EEH10260
  
Location: 2027391-2031221
  
  
**BlastP hit with Mycgr3G107072\_Mycgr3**
  
Percentage identity: 64 %
  
BlastP bit score: 1661
  
Sequence coverage: 100 %
  
E-value: 0.0
  
  
 NCBI BlastP on this gene

EEH10260

predicted protein
  
Accession: EEH10259
  
Location: 2025626-2026756
  
 NCBI BlastP on this gene

EEH10259

oxidoreductase
  
Accession: EEH10258
  
Location: 2021808-2022853
  
 NCBI BlastP on this gene

EEH10258

predicted protein
  
Accession: EEH10257
  
Location: 2018388-2019402
  
 NCBI BlastP on this gene

EEH10257

predicted protein
  
Accession: EEH10256
  
Location: 2016663-2017109
  
 NCBI BlastP on this gene

EEH10256

Query: Architecture Search FASTA input

AM920433 : Penicillium chrysogenum Wisconsin 54-1255 complete genome, contig Pc00c18.    Total score: 1.0     Cumulative Blast bit score: 1635

Hit cluster cross-links:

Mycgr3G52686 Mycgr3T
  
Location: 0-861

Mycgr3G52686\_Mycgr3T

Mycgr3G102281 Mycgr3
  
Location: 961-1573

Mycgr3G102281\_Mycgr3

Mycgr3G89185 Mycgr3T
  
Location: 1673-2063

Mycgr3G89185\_Mycgr3T

Mycgr3G65725 Mycgr3T
  
Location: 2163-3612

Mycgr3G65725\_Mycgr3T

Mycgr3G102276 Mycgr3
  
Location: 3712-4801

Mycgr3G102276\_Mycgr3

Mycgr3G89189 Mycgr3T
  
Location: 4901-5564

Mycgr3G89189\_Mycgr3T

Mycgr3G52682 Mycgr3T
  
Location: 5664-9231

Mycgr3G52682\_Mycgr3T

Mycgr3G107072 Mycgr3
  
Location: 9331-13279

Mycgr3G107072\_Mycgr3

Mycgr3G34982 Mycgr3T
  
Location: 13379-15116

Mycgr3G34982\_Mycgr3T

Mycgr3G107069 Mycgr3
  
Location: 15216-17097

Mycgr3G107069\_Mycgr3

Mycgr3G32432 Mycgr3T
  
Location: 17197-19042

Mycgr3G32432\_Mycgr3T

Mycgr3G98385 Mycgr3T
  
Location: 19142-19898

Mycgr3G98385\_Mycgr3T

not annotated
  
Accession: CAP94258
  
Location: 78530-81058
  
 NCBI BlastP on this gene

Pc18g00340

hypothetical protein
  
Accession: CAP94259
  
Location: 81990-82980
  
 NCBI BlastP on this gene

Pc18g00350

not annotated
  
Accession: CAP94260
  
Location: 86036-88019
  
 NCBI BlastP on this gene

Pc18g00360

hypothetical protein
  
Accession: CAP94261
  
Location: 88198-88757
  
 NCBI BlastP on this gene

Pc18g00370

not annotated
  
Accession: CAP94262
  
Location: 89193-93023
  
  
**BlastP hit with Mycgr3G107072\_Mycgr3**
  
Percentage identity: 63 %
  
BlastP bit score: 1635
  
Sequence coverage: 100 %
  
E-value: 0.0
  
  
 NCBI BlastP on this gene

Pc18g00380

unnamed
  
Accession: CAP94263
  
Location: 94040-95770
  
 NCBI BlastP on this gene

Pc18g00390

not annotated
  
Accession: CAP94264
  
Location: 97407-100685
  
 NCBI BlastP on this gene

Pc18g00400

not annotated
  
Accession: CAP94265
  
Location: 101599-102624
  
 NCBI BlastP on this gene

Pc18g00410

Query: Architecture Search FASTA input

CM001234 : Magnaporthe oryzae 70-15 chromosome 4    Total score: 1.0     Cumulative Blast bit score: 1549

Hit cluster cross-links:

Mycgr3G52686 Mycgr3T
  
Location: 0-861

Mycgr3G52686\_Mycgr3T

Mycgr3G102281 Mycgr3
  
Location: 961-1573

Mycgr3G102281\_Mycgr3

Mycgr3G89185 Mycgr3T
  
Location: 1673-2063

Mycgr3G89185\_Mycgr3T

Mycgr3G65725 Mycgr3T
  
Location: 2163-3612

Mycgr3G65725\_Mycgr3T

Mycgr3G102276 Mycgr3
  
Location: 3712-4801

Mycgr3G102276\_Mycgr3

Mycgr3G89189 Mycgr3T
  
Location: 4901-5564

Mycgr3G89189\_Mycgr3T

Mycgr3G52682 Mycgr3T
  
Location: 5664-9231

Mycgr3G52682\_Mycgr3T

Mycgr3G107072 Mycgr3
  
Location: 9331-13279

Mycgr3G107072\_Mycgr3

Mycgr3G34982 Mycgr3T
  
Location: 13379-15116

Mycgr3G34982\_Mycgr3T

Mycgr3G107069 Mycgr3
  
Location: 15216-17097

Mycgr3G107069\_Mycgr3

Mycgr3G32432 Mycgr3T
  
Location: 17197-19042

Mycgr3G32432\_Mycgr3T

Mycgr3G98385 Mycgr3T
  
Location: 19142-19898

Mycgr3G98385\_Mycgr3T

hypothetical protein
  
Accession: EHA50349
  
Location: 2365176-2366261
  
 NCBI BlastP on this gene

EHA50349

hypothetical protein
  
Accession: EHA50350
  
Location: 2366847-2369294
  
 NCBI BlastP on this gene

EHA50350

SDA1 domain-containing protein
  
Accession: EHA50351
  
Location: 2370973-2373347
  
 NCBI BlastP on this gene

EHA50351

hypothetical protein
  
Accession: EHA50352
  
Location: 2374972-2375246
  
 NCBI BlastP on this gene

EHA50352

N-(5-amino-5-carboxypentanoyl)-L-cysteinyl-D- valine synthase
  
Accession: EHA50353
  
Location: 2376126-2379968
  
  
**BlastP hit with Mycgr3G107072\_Mycgr3**
  
Percentage identity: 59 %
  
BlastP bit score: 1549
  
Sequence coverage: 100 %
  
E-value: 0.0
  
  
 NCBI BlastP on this gene

EHA50353

Query: Architecture Search FASTA input

KB725930 : Colletotrichum orbiculare MAFF 240422 unplaced genomic scaffold Scaffold\_366    Total score: 1.0     Cumulative Blast bit score: 1518

Hit cluster cross-links:

Mycgr3G52686 Mycgr3T
  
Location: 0-861

Mycgr3G52686\_Mycgr3T

Mycgr3G102281 Mycgr3
  
Location: 961-1573

Mycgr3G102281\_Mycgr3

Mycgr3G89185 Mycgr3T
  
Location: 1673-2063

Mycgr3G89185\_Mycgr3T

Mycgr3G65725 Mycgr3T
  
Location: 2163-3612

Mycgr3G65725\_Mycgr3T

Mycgr3G102276 Mycgr3
  
Location: 3712-4801

Mycgr3G102276\_Mycgr3

Mycgr3G89189 Mycgr3T
  
Location: 4901-5564

Mycgr3G89189\_Mycgr3T

Mycgr3G52682 Mycgr3T
  
Location: 5664-9231

Mycgr3G52682\_Mycgr3T

Mycgr3G107072 Mycgr3
  
Location: 9331-13279

Mycgr3G107072\_Mycgr3

Mycgr3G34982 Mycgr3T
  
Location: 13379-15116

Mycgr3G34982\_Mycgr3T

Mycgr3G107069 Mycgr3
  
Location: 15216-17097

Mycgr3G107069\_Mycgr3

Mycgr3G32432 Mycgr3T
  
Location: 17197-19042

Mycgr3G32432\_Mycgr3T

Mycgr3G98385 Mycgr3T
  
Location: 19142-19898

Mycgr3G98385\_Mycgr3T

stress protein ddr48-like protein
  
Accession: ENH82698
  
Location: 1494991-1497313
  
 NCBI BlastP on this gene

ENH82698

phospholipase carboxylesterase superfamily
  
Accession: ENH82699
  
Location: 1497712-1498494
  
 NCBI BlastP on this gene

ENH82699

small secreted protein
  
Accession: ENH82700
  
Location: 1499989-1500908
  
 NCBI BlastP on this gene

ENH82700

hypothetical protein
  
Accession: ENH82701
  
Location: 1503050-1503490
  
 NCBI BlastP on this gene

ENH82701

lccl domain containing protein
  
Accession: ENH82702
  
Location: 1503707-1505692
  
 NCBI BlastP on this gene

ENH82702

nonribosomal peptide synthetase 10
  
Accession: ENH82703
  
Location: 1507531-1511370
  
  
**BlastP hit with Mycgr3G107072\_Mycgr3**
  
Percentage identity: 58 %
  
BlastP bit score: 1518
  
Sequence coverage: 100 %
  
E-value: 0.0
  
  
 NCBI BlastP on this gene

ENH82703

Query: Architecture Search FASTA input

DS985216 : Verticillium albo-atrum VaMs.102 supercont1.3 genomic scaffold    Total score: 1.0     Cumulative Blast bit score: 1503

Hit cluster cross-links:

Mycgr3G52686 Mycgr3T
  
Location: 0-861

Mycgr3G52686\_Mycgr3T

Mycgr3G102281 Mycgr3
  
Location: 961-1573

Mycgr3G102281\_Mycgr3

Mycgr3G89185 Mycgr3T
  
Location: 1673-2063

Mycgr3G89185\_Mycgr3T

Mycgr3G65725 Mycgr3T
  
Location: 2163-3612

Mycgr3G65725\_Mycgr3T

Mycgr3G102276 Mycgr3
  
Location: 3712-4801

Mycgr3G102276\_Mycgr3

Mycgr3G89189 Mycgr3T
  
Location: 4901-5564

Mycgr3G89189\_Mycgr3T

Mycgr3G52682 Mycgr3T
  
Location: 5664-9231

Mycgr3G52682\_Mycgr3T

Mycgr3G107072 Mycgr3
  
Location: 9331-13279

Mycgr3G107072\_Mycgr3

Mycgr3G34982 Mycgr3T
  
Location: 13379-15116

Mycgr3G34982\_Mycgr3T

Mycgr3G107069 Mycgr3
  
Location: 15216-17097

Mycgr3G107069\_Mycgr3

Mycgr3G32432 Mycgr3T
  
Location: 17197-19042

Mycgr3G32432\_Mycgr3T

Mycgr3G98385 Mycgr3T
  
Location: 19142-19898

Mycgr3G98385\_Mycgr3T

cryptochrome-1
  
Accession: EEY16458
  
Location: 158346-160390
  
 NCBI BlastP on this gene

EEY16458

veA protein
  
Accession: EEY16459
  
Location: 162732-163651
  
 NCBI BlastP on this gene

EEY16459

enterobactin synthetase component F
  
Accession: EEY16460
  
Location: 170075-173929
  
  
**BlastP hit with Mycgr3G107072\_Mycgr3**
  
Percentage identity: 58 %
  
BlastP bit score: 1503
  
Sequence coverage: 100 %
  
E-value: 0.0
  
  
 NCBI BlastP on this gene

EEY16460

conserved hypothetical protein
  
Accession: EEY16461
  
Location: 177680-178561
  
 NCBI BlastP on this gene

EEY16461

monooxygenase
  
Accession: EEY16462
  
Location: 183082-184226
  
 NCBI BlastP on this gene

EEY16462

Query: Architecture Search FASTA input

DS572721 : Verticillium dahliae VdLs.17 supercont1.27 genomic scaffold    Total score: 1.0     Cumulative Blast bit score: 1501

Hit cluster cross-links:

Mycgr3G52686 Mycgr3T
  
Location: 0-861

Mycgr3G52686\_Mycgr3T

Mycgr3G102281 Mycgr3
  
Location: 961-1573

Mycgr3G102281\_Mycgr3

Mycgr3G89185 Mycgr3T
  
Location: 1673-2063

Mycgr3G89185\_Mycgr3T

Mycgr3G65725 Mycgr3T
  
Location: 2163-3612

Mycgr3G65725\_Mycgr3T

Mycgr3G102276 Mycgr3
  
Location: 3712-4801

Mycgr3G102276\_Mycgr3

Mycgr3G89189 Mycgr3T
  
Location: 4901-5564

Mycgr3G89189\_Mycgr3T

Mycgr3G52682 Mycgr3T
  
Location: 5664-9231

Mycgr3G52682\_Mycgr3T

Mycgr3G107072 Mycgr3
  
Location: 9331-13279

Mycgr3G107072\_Mycgr3

Mycgr3G34982 Mycgr3T
  
Location: 13379-15116

Mycgr3G34982\_Mycgr3T

Mycgr3G107069 Mycgr3
  
Location: 15216-17097

Mycgr3G107069\_Mycgr3

Mycgr3G32432 Mycgr3T
  
Location: 17197-19042

Mycgr3G32432\_Mycgr3T

Mycgr3G98385 Mycgr3T
  
Location: 19142-19898

Mycgr3G98385\_Mycgr3T

cryptochrome-1
  
Accession: EGY19300
  
Location: 159383-161430
  
 NCBI BlastP on this gene

EGY19300

VosA
  
Accession: EGY19301
  
Location: 163241-164084
  
 NCBI BlastP on this gene

EGY19301

hypothetical protein
  
Accession: EGY19302
  
Location: 169160-169387
  
 NCBI BlastP on this gene

EGY19302

enterobactin synthetase component F
  
Accession: EGY19303
  
Location: 170836-174690
  
  
**BlastP hit with Mycgr3G107072\_Mycgr3**
  
Percentage identity: 58 %
  
BlastP bit score: 1501
  
Sequence coverage: 100 %
  
E-value: 0.0
  
  
 NCBI BlastP on this gene

EGY19303

Query: Architecture Search FASTA input

HF679025 : Fusarium fujikuroi IMI 58289 draft genome, chromosome FFUJ\_chr03.    Total score: 1.0     Cumulative Blast bit score: 1500

Hit cluster cross-links:

Mycgr3G52686 Mycgr3T
  
Location: 0-861

Mycgr3G52686\_Mycgr3T

Mycgr3G102281 Mycgr3
  
Location: 961-1573

Mycgr3G102281\_Mycgr3

Mycgr3G89185 Mycgr3T
  
Location: 1673-2063

Mycgr3G89185\_Mycgr3T

Mycgr3G65725 Mycgr3T
  
Location: 2163-3612

Mycgr3G65725\_Mycgr3T

Mycgr3G102276 Mycgr3
  
Location: 3712-4801

Mycgr3G102276\_Mycgr3

Mycgr3G89189 Mycgr3T
  
Location: 4901-5564

Mycgr3G89189\_Mycgr3T

Mycgr3G52682 Mycgr3T
  
Location: 5664-9231

Mycgr3G52682\_Mycgr3T

Mycgr3G107072 Mycgr3
  
Location: 9331-13279

Mycgr3G107072\_Mycgr3

Mycgr3G34982 Mycgr3T
  
Location: 13379-15116

Mycgr3G34982\_Mycgr3T

Mycgr3G107069 Mycgr3
  
Location: 15216-17097

Mycgr3G107069\_Mycgr3

Mycgr3G32432 Mycgr3T
  
Location: 17197-19042

Mycgr3G32432\_Mycgr3T

Mycgr3G98385 Mycgr3T
  
Location: 19142-19898

Mycgr3G98385\_Mycgr3T

related to alpha-aminoadipate reductase large subunit
  
Accession: CCT66472
  
Location: 4537841-4541695
  
  
**BlastP hit with Mycgr3G107072\_Mycgr3**
  
Percentage identity: 57 %
  
BlastP bit score: 1500
  
Sequence coverage: 100 %
  
E-value: 0.0
  
  
 NCBI BlastP on this gene

FFUJ\_03506

uncharacterized protein
  
Accession: CCT66471
  
Location: 4534907-4536163
  
 NCBI BlastP on this gene

FFUJ\_03505

related to methyltransferase
  
Accession: CCT66470
  
Location: 4532643-4533731
  
 NCBI BlastP on this gene

FFUJ\_03504

uncharacterized protein
  
Accession: CCT66469
  
Location: 4530500-4531313
  
 NCBI BlastP on this gene

FFUJ\_03503

related to protein involved in authophagy (APG17)
  
Accession: CCT66468
  
Location: 4528232-4529829
  
 NCBI BlastP on this gene

FFUJ\_03502

related to calpain-like protein
  
Accession: CCT66467
  
Location: 4524509-4527462
  
 NCBI BlastP on this gene

FFUJ\_03501

Query: Architecture Search FASTA input

ABDF02000092 : Trichoderma virens Gv29-8    Total score: 1.0     Cumulative Blast bit score: 1474

Hit cluster cross-links:

Mycgr3G52686 Mycgr3T
  
Location: 0-861

Mycgr3G52686\_Mycgr3T

Mycgr3G102281 Mycgr3
  
Location: 961-1573

Mycgr3G102281\_Mycgr3

Mycgr3G89185 Mycgr3T
  
Location: 1673-2063

Mycgr3G89185\_Mycgr3T

Mycgr3G65725 Mycgr3T
  
Location: 2163-3612

Mycgr3G65725\_Mycgr3T

Mycgr3G102276 Mycgr3
  
Location: 3712-4801

Mycgr3G102276\_Mycgr3

Mycgr3G89189 Mycgr3T
  
Location: 4901-5564

Mycgr3G89189\_Mycgr3T

Mycgr3G52682 Mycgr3T
  
Location: 5664-9231

Mycgr3G52682\_Mycgr3T

Mycgr3G107072 Mycgr3
  
Location: 9331-13279

Mycgr3G107072\_Mycgr3

Mycgr3G34982 Mycgr3T
  
Location: 13379-15116

Mycgr3G34982\_Mycgr3T

Mycgr3G107069 Mycgr3
  
Location: 15216-17097

Mycgr3G107069\_Mycgr3

Mycgr3G32432 Mycgr3T
  
Location: 17197-19042

Mycgr3G32432\_Mycgr3T

Mycgr3G98385 Mycgr3T
  
Location: 19142-19898

Mycgr3G98385\_Mycgr3T

hypothetical protein
  
Accession: EHK15304
  
Location: 258006-259229
  
 NCBI BlastP on this gene

EHK15304

hypothetical protein
  
Accession: EHK15305
  
Location: 259548-260884
  
 NCBI BlastP on this gene

EHK15305

hypothetical protein
  
Accession: EHK15306
  
Location: 265690-267276
  
 NCBI BlastP on this gene

EHK15306

non-ribosomal peptide synthetase
  
Accession: EHK15307
  
Location: 268628-272479
  
  
**BlastP hit with Mycgr3G107072\_Mycgr3**
  
Percentage identity: 58 %
  
BlastP bit score: 1474
  
Sequence coverage: 100 %
  
E-value: 0.0
  
  
 NCBI BlastP on this gene

EHK15307

hypothetical protein
  
Accession: EHK15308
  
Location: 273906-275859
  
 NCBI BlastP on this gene

EHK15308

hypothetical protein
  
Accession: EHK15309
  
Location: 276048-277775
  
 NCBI BlastP on this gene

EHK15309

hypothetical protein
  
Accession: EHK15310
  
Location: 278437-282408
  
 NCBI BlastP on this gene

EHK15310

hypothetical protein
  
Accession: EHK15311
  
Location: 282901-285655
  
 NCBI BlastP on this gene

EHK15311

Query: Architecture Search FASTA input

ABDG02000027 : Trichoderma atroviride IMI 206040    Total score: 1.0     Cumulative Blast bit score: 1456

Hit cluster cross-links:

Mycgr3G52686 Mycgr3T
  
Location: 0-861

Mycgr3G52686\_Mycgr3T

Mycgr3G102281 Mycgr3
  
Location: 961-1573

Mycgr3G102281\_Mycgr3

Mycgr3G89185 Mycgr3T
  
Location: 1673-2063

Mycgr3G89185\_Mycgr3T

Mycgr3G65725 Mycgr3T
  
Location: 2163-3612

Mycgr3G65725\_Mycgr3T

Mycgr3G102276 Mycgr3
  
Location: 3712-4801

Mycgr3G102276\_Mycgr3

Mycgr3G89189 Mycgr3T
  
Location: 4901-5564

Mycgr3G89189\_Mycgr3T

Mycgr3G52682 Mycgr3T
  
Location: 5664-9231

Mycgr3G52682\_Mycgr3T

Mycgr3G107072 Mycgr3
  
Location: 9331-13279

Mycgr3G107072\_Mycgr3

Mycgr3G34982 Mycgr3T
  
Location: 13379-15116

Mycgr3G34982\_Mycgr3T

Mycgr3G107069 Mycgr3
  
Location: 15216-17097

Mycgr3G107069\_Mycgr3

Mycgr3G32432 Mycgr3T
  
Location: 17197-19042

Mycgr3G32432\_Mycgr3T

Mycgr3G98385 Mycgr3T
  
Location: 19142-19898

Mycgr3G98385\_Mycgr3T

non-ribosomal peptide synthetase
  
Accession: EHK41519
  
Location: 3289356-3293213
  
  
**BlastP hit with Mycgr3G107072\_Mycgr3**
  
Percentage identity: 57 %
  
BlastP bit score: 1456
  
Sequence coverage: 100 %
  
E-value: 0.0
  
  
 NCBI BlastP on this gene

EHK41519

hypothetical protein
  
Accession: EHK41518
  
Location: 3286231-3287918
  
 NCBI BlastP on this gene

EHK41518

hypothetical protein
  
Accession: EHK41517
  
Location: 3281497-3285565
  
 NCBI BlastP on this gene

EHK41517

hypothetical protein
  
Accession: EHK41516
  
Location: 3278335-3281071
  
 NCBI BlastP on this gene

EHK41516

Query: Architecture Search FASTA input

DF196785 : Pseudozyma antarctica T-34 DNA, contig: scaffold00019    Total score: 1.0     Cumulative Blast bit score: 1434

Hit cluster cross-links:

Mycgr3G52686 Mycgr3T
  
Location: 0-861

Mycgr3G52686\_Mycgr3T

Mycgr3G102281 Mycgr3
  
Location: 961-1573

Mycgr3G102281\_Mycgr3

Mycgr3G89185 Mycgr3T
  
Location: 1673-2063

Mycgr3G89185\_Mycgr3T

Mycgr3G65725 Mycgr3T
  
Location: 2163-3612

Mycgr3G65725\_Mycgr3T

Mycgr3G102276 Mycgr3
  
Location: 3712-4801

Mycgr3G102276\_Mycgr3

Mycgr3G89189 Mycgr3T
  
Location: 4901-5564

Mycgr3G89189\_Mycgr3T

Mycgr3G52682 Mycgr3T
  
Location: 5664-9231

Mycgr3G52682\_Mycgr3T

Mycgr3G107072 Mycgr3
  
Location: 9331-13279

Mycgr3G107072\_Mycgr3

Mycgr3G34982 Mycgr3T
  
Location: 13379-15116

Mycgr3G34982\_Mycgr3T

Mycgr3G107069 Mycgr3
  
Location: 15216-17097

Mycgr3G107069\_Mycgr3

Mycgr3G32432 Mycgr3T
  
Location: 17197-19042

Mycgr3G32432\_Mycgr3T

Mycgr3G98385 Mycgr3T
  
Location: 19142-19898

Mycgr3G98385\_Mycgr3T

triosephosphate isomerase
  
Accession: GAC75902
  
Location: 46282-47115
  
 NCBI BlastP on this gene

GAC75902

hypothetical protein
  
Accession: GAC75901
  
Location: 45336-46100
  
 NCBI BlastP on this gene

GAC75901

uncharacterized conserved protein
  
Accession: GAC75900
  
Location: 44122-45133
  
 NCBI BlastP on this gene

GAC75900

hypothetical protein
  
Accession: GAC75899
  
Location: 39715-44038
  
 NCBI BlastP on this gene

GAC75899

RNA polymerase subunit K
  
Accession: GAC75898
  
Location: 37059-39446
  
 NCBI BlastP on this gene

GAC75898

non-ribosomal peptide synthetase
  
Accession: GAC75897
  
Location: 31970-35968
  
  
**BlastP hit with Mycgr3G107072\_Mycgr3**
  
Percentage identity: 55 %
  
BlastP bit score: 1434
  
Sequence coverage: 102 %
  
E-value: 0.0
  
  
 NCBI BlastP on this gene

GAC75897

transport protein Sec61, alpha subunit
  
Accession: GAC75896
  
Location: 30911-31864
  
 NCBI BlastP on this gene

GAC75896

Fe2+/Zn2+ regulated transporter
  
Accession: GAC75895
  
Location: 28690-30153
  
 NCBI BlastP on this gene

GAC75895

hypothetical protein
  
Accession: GAC75894
  
Location: 26594-28612
  
 NCBI BlastP on this gene

GAC75894

hypothetical protein
  
Accession: GAC75893
  
Location: 24266-25498
  
 NCBI BlastP on this gene

GAC75893

hypothetical protein
  
Accession: GAC75892
  
Location: 17921-23870
  
 NCBI BlastP on this gene

GAC75892

Query: Architecture Search FASTA input

FQ311472 : Sporisorium reilianum SRZ2 chromosome 7 complete DNA sequence.    Total score: 1.0     Cumulative Blast bit score: 1394

Hit cluster cross-links:

Mycgr3G52686 Mycgr3T
  
Location: 0-861

Mycgr3G52686\_Mycgr3T

Mycgr3G102281 Mycgr3
  
Location: 961-1573

Mycgr3G102281\_Mycgr3

Mycgr3G89185 Mycgr3T
  
Location: 1673-2063

Mycgr3G89185\_Mycgr3T

Mycgr3G65725 Mycgr3T
  
Location: 2163-3612

Mycgr3G65725\_Mycgr3T

Mycgr3G102276 Mycgr3
  
Location: 3712-4801

Mycgr3G102276\_Mycgr3

Mycgr3G89189 Mycgr3T
  
Location: 4901-5564

Mycgr3G89189\_Mycgr3T

Mycgr3G52682 Mycgr3T
  
Location: 5664-9231

Mycgr3G52682\_Mycgr3T

Mycgr3G107072 Mycgr3
  
Location: 9331-13279

Mycgr3G107072\_Mycgr3

Mycgr3G34982 Mycgr3T
  
Location: 13379-15116

Mycgr3G34982\_Mycgr3T

Mycgr3G107069 Mycgr3
  
Location: 15216-17097

Mycgr3G107069\_Mycgr3

Mycgr3G32432 Mycgr3T
  
Location: 17197-19042

Mycgr3G32432\_Mycgr3T

Mycgr3G98385 Mycgr3T
  
Location: 19142-19898

Mycgr3G98385\_Mycgr3T

conserved hypothetical protein
  
Accession: CBQ73509
  
Location: 842577-843187
  
 NCBI BlastP on this gene

sr14166

conserved hypothetical protein
  
Accession: CBQ73510
  
Location: 843638-844691
  
 NCBI BlastP on this gene

sr14167

conserved hypothetical protein
  
Accession: CBQ73511
  
Location: 845597-849456
  
 NCBI BlastP on this gene

sr14168

conserved hypothetical protein
  
Accession: CBQ73512
  
Location: 849740-852085
  
 NCBI BlastP on this gene

sr14169

related to Aminoadipate-semialdehyde dehydrogenase
  
Accession: CBQ73513
  
Location: 852961-856890
  
  
**BlastP hit with Mycgr3G107072\_Mycgr3**
  
Percentage identity: 53 %
  
BlastP bit score: 1394
  
Sequence coverage: 102 %
  
E-value: 0.0
  
  
 NCBI BlastP on this gene

sr14170

Query: Architecture Search FASTA input

KE148153 : Ophiostoma piceae UAMH 11346 chromosome Unknown scf08    Total score: 1.0     Cumulative Blast bit score: 1215

Hit cluster cross-links:

Mycgr3G52686 Mycgr3T
  
Location: 0-861

Mycgr3G52686\_Mycgr3T

Mycgr3G102281 Mycgr3
  
Location: 961-1573

Mycgr3G102281\_Mycgr3

Mycgr3G89185 Mycgr3T
  
Location: 1673-2063

Mycgr3G89185\_Mycgr3T

Mycgr3G65725 Mycgr3T
  
Location: 2163-3612

Mycgr3G65725\_Mycgr3T

Mycgr3G102276 Mycgr3
  
Location: 3712-4801

Mycgr3G102276\_Mycgr3

Mycgr3G89189 Mycgr3T
  
Location: 4901-5564

Mycgr3G89189\_Mycgr3T

Mycgr3G52682 Mycgr3T
  
Location: 5664-9231

Mycgr3G52682\_Mycgr3T

Mycgr3G107072 Mycgr3
  
Location: 9331-13279

Mycgr3G107072\_Mycgr3

Mycgr3G34982 Mycgr3T
  
Location: 13379-15116

Mycgr3G34982\_Mycgr3T

Mycgr3G107069 Mycgr3
  
Location: 15216-17097

Mycgr3G107069\_Mycgr3

Mycgr3G32432 Mycgr3T
  
Location: 17197-19042

Mycgr3G32432\_Mycgr3T

Mycgr3G98385 Mycgr3T
  
Location: 19142-19898

Mycgr3G98385\_Mycgr3T

non-imprinted in prader-willi angelman syndrome region protein 2
  
Accession: EPE06500
  
Location: 980949-983336
  
 NCBI BlastP on this gene

EPE06500

nonribosomal peptide synthetase 10
  
Accession: EPE06501
  
Location: 991797-995894
  
  
**BlastP hit with Mycgr3G107072\_Mycgr3**
  
Percentage identity: 50 %
  
BlastP bit score: 1215
  
Sequence coverage: 106 %
  
E-value: 0.0
  
  
 NCBI BlastP on this gene

EPE06501

Query: Architecture Search FASTA input

KB310677 : Capitella teleta unplaced genomic scaffold CAPTEscaffold\_748    Total score: 1.0     Cumulative Blast bit score: 1171

Hit cluster cross-links:

Mycgr3G52686 Mycgr3T
  
Location: 0-861

Mycgr3G52686\_Mycgr3T

Mycgr3G102281 Mycgr3
  
Location: 961-1573

Mycgr3G102281\_Mycgr3

Mycgr3G89185 Mycgr3T
  
Location: 1673-2063

Mycgr3G89185\_Mycgr3T

Mycgr3G65725 Mycgr3T
  
Location: 2163-3612

Mycgr3G65725\_Mycgr3T

Mycgr3G102276 Mycgr3
  
Location: 3712-4801

Mycgr3G102276\_Mycgr3

Mycgr3G89189 Mycgr3T
  
Location: 4901-5564

Mycgr3G89189\_Mycgr3T

Mycgr3G52682 Mycgr3T
  
Location: 5664-9231

Mycgr3G52682\_Mycgr3T

Mycgr3G107072 Mycgr3
  
Location: 9331-13279

Mycgr3G107072\_Mycgr3

Mycgr3G34982 Mycgr3T
  
Location: 13379-15116

Mycgr3G34982\_Mycgr3T

Mycgr3G107069 Mycgr3
  
Location: 15216-17097

Mycgr3G107069\_Mycgr3

Mycgr3G32432 Mycgr3T
  
Location: 17197-19042

Mycgr3G32432\_Mycgr3T

Mycgr3G98385 Mycgr3T
  
Location: 19142-19898

Mycgr3G98385\_Mycgr3T

hypothetical protein
  
Accession: ELT91031
  
Location: 24875-31914
  
  
**BlastP hit with Mycgr3G107072\_Mycgr3**
  
Percentage identity: 36 %
  
BlastP bit score: 724
  
Sequence coverage: 101 %
  
E-value: 0.0
  
  
 NCBI BlastP on this gene

ELT91031

hypothetical protein
  
Accession: ELT91030
  
Location: 19105-22341
  
 NCBI BlastP on this gene

ELT91030

hypothetical protein
  
Accession: ELT91029
  
Location: 13911-15737
  
 NCBI BlastP on this gene

ELT91029

hypothetical protein
  
Accession: ELT91028
  
Location: 12442-13587
  
 NCBI BlastP on this gene

ELT91028

hypothetical protein
  
Accession: ELT91027
  
Location: 4631-9906
  
  
**BlastP hit with Mycgr3G107072\_Mycgr3**
  
Percentage identity: 32 %
  
BlastP bit score: 447
  
Sequence coverage: 74 %
  
E-value: 9e-134
  
  
 NCBI BlastP on this gene

ELT91027

Query: Architecture Search FASTA input

DS572752 : Paracoccidioides brasiliensis Pb18 supercont1.3 genomic scaffold    Total score: 1.0     Cumulative Blast bit score: 1068

Hit cluster cross-links:

Mycgr3G52686 Mycgr3T
  
Location: 0-861

Mycgr3G52686\_Mycgr3T

Mycgr3G102281 Mycgr3
  
Location: 961-1573

Mycgr3G102281\_Mycgr3

Mycgr3G89185 Mycgr3T
  
Location: 1673-2063

Mycgr3G89185\_Mycgr3T

Mycgr3G65725 Mycgr3T
  
Location: 2163-3612

Mycgr3G65725\_Mycgr3T

Mycgr3G102276 Mycgr3
  
Location: 3712-4801

Mycgr3G102276\_Mycgr3

Mycgr3G89189 Mycgr3T
  
Location: 4901-5564

Mycgr3G89189\_Mycgr3T

Mycgr3G52682 Mycgr3T
  
Location: 5664-9231

Mycgr3G52682\_Mycgr3T

Mycgr3G107072 Mycgr3
  
Location: 9331-13279

Mycgr3G107072\_Mycgr3

Mycgr3G34982 Mycgr3T
  
Location: 13379-15116

Mycgr3G34982\_Mycgr3T

Mycgr3G107069 Mycgr3
  
Location: 15216-17097

Mycgr3G107069\_Mycgr3

Mycgr3G32432 Mycgr3T
  
Location: 17197-19042

Mycgr3G32432\_Mycgr3T

Mycgr3G98385 Mycgr3T
  
Location: 19142-19898

Mycgr3G98385\_Mycgr3T

conserved hypothetical protein
  
Accession: EEH46483
  
Location: 570339-571860
  
 NCBI BlastP on this gene

EEH46483

predicted protein
  
Accession: EEH46482
  
Location: 569318-569806
  
 NCBI BlastP on this gene

EEH46482

conserved hypothetical protein
  
Accession: EEH46481
  
Location: 568068-568997
  
 NCBI BlastP on this gene

EEH46481

ATP synthase subunit 4
  
Accession: EEH46480
  
Location: 566899-567868
  
 NCBI BlastP on this gene

EEH46480

CBF/Mak21 family protein
  
Accession: EEH46479
  
Location: 564840-566656
  
 NCBI BlastP on this gene

EEH46479

conserved hypothetical protein
  
Accession: EEH46478
  
Location: 563292-564305
  
 NCBI BlastP on this gene

EEH46478

polyketide synthase hetM
  
Accession: EEH46477
  
Location: 557234-561063
  
  
**BlastP hit with Mycgr3G107072\_Mycgr3**
  
Percentage identity: 61 %
  
BlastP bit score: 1068
  
Sequence coverage: 67 %
  
E-value: 0.0
  
  
 NCBI BlastP on this gene

EEH46477

predicted protein
  
Accession: EEH46476
  
Location: 555411-556562
  
 NCBI BlastP on this gene

EEH46476

conserved hypothetical protein
  
Accession: EEH46475
  
Location: 554020-555161
  
 NCBI BlastP on this gene

EEH46475

predicted protein
  
Accession: EEH46474
  
Location: 552571-553781
  
 NCBI BlastP on this gene

EEH46474

predicted protein
  
Accession: EEH46473
  
Location: 549897-552421
  
 NCBI BlastP on this gene

EEH46473

predicted protein
  
Accession: EEH46472
  
Location: 548094-549339
  
 NCBI BlastP on this gene

EEH46472

hypothetical protein
  
Accession: EEH46471
  
Location: 546913-547687
  
 NCBI BlastP on this gene

EEH46471

Query: Architecture Search FASTA input

JH767588 : Coniosporium apollinis CBS 100218 chromosome Unknown supercont1.35    Total score: 1.0     Cumulative Blast bit score: 1009

Hit cluster cross-links:

Mycgr3G52686 Mycgr3T
  
Location: 0-861

Mycgr3G52686\_Mycgr3T

Mycgr3G102281 Mycgr3
  
Location: 961-1573

Mycgr3G102281\_Mycgr3

Mycgr3G89185 Mycgr3T
  
Location: 1673-2063

Mycgr3G89185\_Mycgr3T

Mycgr3G65725 Mycgr3T
  
Location: 2163-3612

Mycgr3G65725\_Mycgr3T

Mycgr3G102276 Mycgr3
  
Location: 3712-4801

Mycgr3G102276\_Mycgr3

Mycgr3G89189 Mycgr3T
  
Location: 4901-5564

Mycgr3G89189\_Mycgr3T

Mycgr3G52682 Mycgr3T
  
Location: 5664-9231

Mycgr3G52682\_Mycgr3T

Mycgr3G107072 Mycgr3
  
Location: 9331-13279

Mycgr3G107072\_Mycgr3

Mycgr3G34982 Mycgr3T
  
Location: 13379-15116

Mycgr3G34982\_Mycgr3T

Mycgr3G107069 Mycgr3
  
Location: 15216-17097

Mycgr3G107069\_Mycgr3

Mycgr3G32432 Mycgr3T
  
Location: 17197-19042

Mycgr3G32432\_Mycgr3T

Mycgr3G98385 Mycgr3T
  
Location: 19142-19898

Mycgr3G98385\_Mycgr3T

hypothetical protein
  
Accession: EON67609
  
Location: 311565-312140
  
 NCBI BlastP on this gene

EON67609

hypothetical protein
  
Accession: EON67610
  
Location: 314154-317231
  
 NCBI BlastP on this gene

EON67610

hypothetical protein
  
Accession: EON67611
  
Location: 319536-320305
  
 NCBI BlastP on this gene

EON67611

hypothetical protein
  
Accession: EON67612
  
Location: 321636-325454
  
  
**BlastP hit with Mycgr3G52682\_Mycgr3T**
  
Percentage identity: 56 %
  
BlastP bit score: 1009
  
Sequence coverage: 78 %
  
E-value: 0.0
  
  
 NCBI BlastP on this gene

EON67612

Query: Architecture Search FASTA input

GL985056 : Trichoderma reesei QM6a unplaced genomic scaffold TRIREscaffold\_1    Total score: 1.0     Cumulative Blast bit score: 910

Hit cluster cross-links:

Mycgr3G52686 Mycgr3T
  
Location: 0-861

Mycgr3G52686\_Mycgr3T

Mycgr3G102281 Mycgr3
  
Location: 961-1573

Mycgr3G102281\_Mycgr3

Mycgr3G89185 Mycgr3T
  
Location: 1673-2063

Mycgr3G89185\_Mycgr3T

Mycgr3G65725 Mycgr3T
  
Location: 2163-3612

Mycgr3G65725\_Mycgr3T

Mycgr3G102276 Mycgr3
  
Location: 3712-4801

Mycgr3G102276\_Mycgr3

Mycgr3G89189 Mycgr3T
  
Location: 4901-5564

Mycgr3G89189\_Mycgr3T

Mycgr3G52682 Mycgr3T
  
Location: 5664-9231

Mycgr3G52682\_Mycgr3T

Mycgr3G107072 Mycgr3
  
Location: 9331-13279

Mycgr3G107072\_Mycgr3

Mycgr3G34982 Mycgr3T
  
Location: 13379-15116

Mycgr3G34982\_Mycgr3T

Mycgr3G107069 Mycgr3
  
Location: 15216-17097

Mycgr3G107069\_Mycgr3

Mycgr3G32432 Mycgr3T
  
Location: 17197-19042

Mycgr3G32432\_Mycgr3T

Mycgr3G98385 Mycgr3T
  
Location: 19142-19898

Mycgr3G98385\_Mycgr3T

predicted protein
  
Accession: EGR52828
  
Location: 1514592-1516554
  
 NCBI BlastP on this gene

EGR52828

predicted protein
  
Accession: EGR52827
  
Location: 1511324-1513991
  
 NCBI BlastP on this gene

EGR52827

predicted protein
  
Accession: EGR52305
  
Location: 1509679-1510809
  
 NCBI BlastP on this gene

EGR52305

dihydrolipoyllysine-residue acetyltransferase-like protein
  
Accession: EGR52826
  
Location: 1506237-1507614
  
 NCBI BlastP on this gene

EGR52826

predicted protein
  
Accession: EGR52825
  
Location: 1501361-1504994
  
  
**BlastP hit with Mycgr3G52682\_Mycgr3T**
  
Percentage identity: 44 %
  
BlastP bit score: 910
  
Sequence coverage: 98 %
  
E-value: 0.0
  
  
 NCBI BlastP on this gene

EGR52825

predicted protein
  
Accession: EGR52304
  
Location: 1500478-1500978
  
 NCBI BlastP on this gene

EGR52304

predicted protein
  
Accession: EGR52824
  
Location: 1497445-1499682
  
 NCBI BlastP on this gene

EGR52824

predicted protein
  
Accession: EGR52823
  
Location: 1496157-1496768
  
 NCBI BlastP on this gene

EGR52823

predicted protein
  
Accession: EGR52303
  
Location: 1495331-1495789
  
 NCBI BlastP on this gene

EGR52303

vesicle fusion protein
  
Accession: EGR52822
  
Location: 1493399-1494656
  
 NCBI BlastP on this gene

EGR52822

predicted protein
  
Accession: EGR52302
  
Location: 1490666-1492814
  
 NCBI BlastP on this gene

EGR52302

Query: Architecture Search FASTA input

KB446555 : Pseudocercospora fijiensis CIRAD86 unplaced genomic scaffold MYCFIscaffold\_1    Total score: 1.0     Cumulative Blast bit score: 886

Hit cluster cross-links:

Mycgr3G52686 Mycgr3T
  
Location: 0-861

Mycgr3G52686\_Mycgr3T

Mycgr3G102281 Mycgr3
  
Location: 961-1573

Mycgr3G102281\_Mycgr3

Mycgr3G89185 Mycgr3T
  
Location: 1673-2063

Mycgr3G89185\_Mycgr3T

Mycgr3G65725 Mycgr3T
  
Location: 2163-3612

Mycgr3G65725\_Mycgr3T

Mycgr3G102276 Mycgr3
  
Location: 3712-4801

Mycgr3G102276\_Mycgr3

Mycgr3G89189 Mycgr3T
  
Location: 4901-5564

Mycgr3G89189\_Mycgr3T

Mycgr3G52682 Mycgr3T
  
Location: 5664-9231

Mycgr3G52682\_Mycgr3T

Mycgr3G107072 Mycgr3
  
Location: 9331-13279

Mycgr3G107072\_Mycgr3

Mycgr3G34982 Mycgr3T
  
Location: 13379-15116

Mycgr3G34982\_Mycgr3T

Mycgr3G107069 Mycgr3
  
Location: 15216-17097

Mycgr3G107069\_Mycgr3

Mycgr3G32432 Mycgr3T
  
Location: 17197-19042

Mycgr3G32432\_Mycgr3T

Mycgr3G98385 Mycgr3T
  
Location: 19142-19898

Mycgr3G98385\_Mycgr3T

hypothetical protein
  
Accession: EME89630
  
Location: 11163995-11165942
  
  
**BlastP hit with Mycgr3G107069\_Mycgr3**
  
Percentage identity: 68 %
  
BlastP bit score: 886
  
Sequence coverage: 99 %
  
E-value: 0.0
  
  
 NCBI BlastP on this gene

EME89630

hypothetical protein
  
Accession: EME89629
  
Location: 11160690-11163097
  
 NCBI BlastP on this gene

EME89629

hypothetical protein
  
Accession: EME89628
  
Location: 11158814-11160364
  
 NCBI BlastP on this gene

EME89628

hypothetical protein
  
Accession: EME89627
  
Location: 11156449-11158166
  
 NCBI BlastP on this gene

EME89627

hypothetical protein
  
Accession: EME89626
  
Location: 11154512-11155099
  
 NCBI BlastP on this gene

EME89626

hypothetical protein
  
Accession: EME89625
  
Location: 11153146-11154000
  
 NCBI BlastP on this gene

EME89625

Query: Architecture Search FASTA input

GG698898 : Nectria haematococca mpVI 77-13-4 chromosome 4 genomic scaffold NECHAsca\_3\_chr4\_2\_0    Total score: 1.0     Cumulative Blast bit score: 878

Hit cluster cross-links:

Mycgr3G52686 Mycgr3T
  
Location: 0-861

Mycgr3G52686\_Mycgr3T

Mycgr3G102281 Mycgr3
  
Location: 961-1573

Mycgr3G102281\_Mycgr3

Mycgr3G89185 Mycgr3T
  
Location: 1673-2063

Mycgr3G89185\_Mycgr3T

Mycgr3G65725 Mycgr3T
  
Location: 2163-3612

Mycgr3G65725\_Mycgr3T

Mycgr3G102276 Mycgr3
  
Location: 3712-4801

Mycgr3G102276\_Mycgr3

Mycgr3G89189 Mycgr3T
  
Location: 4901-5564

Mycgr3G89189\_Mycgr3T

Mycgr3G52682 Mycgr3T
  
Location: 5664-9231

Mycgr3G52682\_Mycgr3T

Mycgr3G107072 Mycgr3
  
Location: 9331-13279

Mycgr3G107072\_Mycgr3

Mycgr3G34982 Mycgr3T
  
Location: 13379-15116

Mycgr3G34982\_Mycgr3T

Mycgr3G107069 Mycgr3
  
Location: 15216-17097

Mycgr3G107069\_Mycgr3

Mycgr3G32432 Mycgr3T
  
Location: 17197-19042

Mycgr3G32432\_Mycgr3T

Mycgr3G98385 Mycgr3T
  
Location: 19142-19898

Mycgr3G98385\_Mycgr3T

predicted protein
  
Accession: EEU45839
  
Location: 516576-517421
  
 NCBI BlastP on this gene

EEU45839

hypothetical protein
  
Accession: EEU46296
  
Location: 515677-516241
  
 NCBI BlastP on this gene

EEU46296

hypothetical protein
  
Accession: EEU46295
  
Location: 511101-512913
  
 NCBI BlastP on this gene

EEU46295

hypothetical protein
  
Accession: EEU46294
  
Location: 509032-509959
  
 NCBI BlastP on this gene

EEU46294

hypothetical protein
  
Accession: EEU46293
  
Location: 503226-506977
  
  
**BlastP hit with Mycgr3G52682\_Mycgr3T**
  
Percentage identity: 43 %
  
BlastP bit score: 879
  
Sequence coverage: 98 %
  
E-value: 0.0
  
  
 NCBI BlastP on this gene

EEU46293

hypothetical protein
  
Accession: EEU45838
  
Location: 502051-502827
  
 NCBI BlastP on this gene

EEU45838

predicted protein
  
Accession: EEU46292
  
Location: 499500-501753
  
 NCBI BlastP on this gene

EEU46292

expressed protein
  
Accession: EEU46291
  
Location: 498666-499102
  
 NCBI BlastP on this gene

EEU46291

hypothetical protein
  
Accession: EEU45837
  
Location: 497769-498353
  
 NCBI BlastP on this gene

EEU45837

predicted protein
  
Accession: EEU46290
  
Location: 496292-497137
  
 NCBI BlastP on this gene

EEU46290

hypothetical protein
  
Accession: EEU45836
  
Location: 493425-495591
  
 NCBI BlastP on this gene

EEU45836

Query: Architecture Search FASTA input

KB730248 : Fusarium oxysporum f. sp. cubense race 1 unplaced genomic scaffold scaffold101    Total score: 1.0     Cumulative Blast bit score: 877

Hit cluster cross-links:

Mycgr3G52686 Mycgr3T
  
Location: 0-861

Mycgr3G52686\_Mycgr3T

Mycgr3G102281 Mycgr3
  
Location: 961-1573

Mycgr3G102281\_Mycgr3

Mycgr3G89185 Mycgr3T
  
Location: 1673-2063

Mycgr3G89185\_Mycgr3T

Mycgr3G65725 Mycgr3T
  
Location: 2163-3612

Mycgr3G65725\_Mycgr3T

Mycgr3G102276 Mycgr3
  
Location: 3712-4801

Mycgr3G102276\_Mycgr3

Mycgr3G89189 Mycgr3T
  
Location: 4901-5564

Mycgr3G89189\_Mycgr3T

Mycgr3G52682 Mycgr3T
  
Location: 5664-9231

Mycgr3G52682\_Mycgr3T

Mycgr3G107072 Mycgr3
  
Location: 9331-13279

Mycgr3G107072\_Mycgr3

Mycgr3G34982 Mycgr3T
  
Location: 13379-15116

Mycgr3G34982\_Mycgr3T

Mycgr3G107069 Mycgr3
  
Location: 15216-17097

Mycgr3G107069\_Mycgr3

Mycgr3G32432 Mycgr3T
  
Location: 17197-19042

Mycgr3G32432\_Mycgr3T

Mycgr3G98385 Mycgr3T
  
Location: 19142-19898

Mycgr3G98385\_Mycgr3T

Protein transport protein SEC31
  
Accession: ENH68404
  
Location: 503704-507644
  
 NCBI BlastP on this gene

ENH68404

Peptidyl-prolyl cis-trans isomerase E
  
Accession: ENH68403
  
Location: 502072-502554
  
 NCBI BlastP on this gene

ENH68403

hypothetical protein
  
Accession: ENH68402
  
Location: 501281-501823
  
 NCBI BlastP on this gene

ENH68402

Cytochrome c oxidase subunit 6B
  
Accession: ENH68401
  
Location: 500267-500886
  
 NCBI BlastP on this gene

ENH68401

UPF0667 family protein C31G5.18c
  
Accession: ENH68400
  
Location: 499059-499907
  
 NCBI BlastP on this gene

ENH68400

40S ribosomal protein S10-A
  
Accession: ENH68399
  
Location: 498029-498695
  
 NCBI BlastP on this gene

ENH68399

hypothetical protein
  
Accession: ENH68398
  
Location: 495177-496090
  
 NCBI BlastP on this gene

ENH68398

Putative E3 ubiquitin-protein ligase mug30
  
Accession: ENH68397
  
Location: 489831-493572
  
  
**BlastP hit with Mycgr3G52682\_Mycgr3T**
  
Percentage identity: 42 %
  
BlastP bit score: 877
  
Sequence coverage: 100 %
  
E-value: 0.0
  
  
 NCBI BlastP on this gene

ENH68397

Putative 60S ribosomal protein MRP49, mitochondrial
  
Accession: ENH68396
  
Location: 488697-489474
  
 NCBI BlastP on this gene

ENH68396

hypothetical protein
  
Accession: ENH68395
  
Location: 486157-488363
  
 NCBI BlastP on this gene

ENH68395

Type 1 phosphatases regulator ypi-1
  
Accession: ENH68394
  
Location: 485000-485527
  
 NCBI BlastP on this gene

ENH68394

hypothetical protein
  
Accession: ENH68393
  
Location: 484162-484734
  
 NCBI BlastP on this gene

ENH68393

Putative vesicular-fusion protein sec17 like protein
  
Accession: ENH68392
  
Location: 482705-483772
  
 NCBI BlastP on this gene

ENH68392

Glucoamylase
  
Accession: ENH68391
  
Location: 479866-482053
  
 NCBI BlastP on this gene

ENH68391

Query: Architecture Search FASTA input

KB726554 : Fusarium oxysporum f. sp. cubense race 4 unplaced genomic scaffold scaffold44    Total score: 1.0     Cumulative Blast bit score: 872

Hit cluster cross-links:

Mycgr3G52686 Mycgr3T
  
Location: 0-861

Mycgr3G52686\_Mycgr3T

Mycgr3G102281 Mycgr3
  
Location: 961-1573

Mycgr3G102281\_Mycgr3

Mycgr3G89185 Mycgr3T
  
Location: 1673-2063

Mycgr3G89185\_Mycgr3T

Mycgr3G65725 Mycgr3T
  
Location: 2163-3612

Mycgr3G65725\_Mycgr3T

Mycgr3G102276 Mycgr3
  
Location: 3712-4801

Mycgr3G102276\_Mycgr3

Mycgr3G89189 Mycgr3T
  
Location: 4901-5564

Mycgr3G89189\_Mycgr3T

Mycgr3G52682 Mycgr3T
  
Location: 5664-9231

Mycgr3G52682\_Mycgr3T

Mycgr3G107072 Mycgr3
  
Location: 9331-13279

Mycgr3G107072\_Mycgr3

Mycgr3G34982 Mycgr3T
  
Location: 13379-15116

Mycgr3G34982\_Mycgr3T

Mycgr3G107069 Mycgr3
  
Location: 15216-17097

Mycgr3G107069\_Mycgr3

Mycgr3G32432 Mycgr3T
  
Location: 17197-19042

Mycgr3G32432\_Mycgr3T

Mycgr3G98385 Mycgr3T
  
Location: 19142-19898

Mycgr3G98385\_Mycgr3T

hypothetical protein
  
Accession: EMT68538
  
Location: 1974565-1975119
  
 NCBI BlastP on this gene

EMT68538

Cytochrome c oxidase subunit 6B
  
Accession: EMT68539
  
Location: 1975514-1976133
  
 NCBI BlastP on this gene

EMT68539

UPF0667 family protein C31G5.18c
  
Accession: EMT68540
  
Location: 1976493-1977341
  
 NCBI BlastP on this gene

EMT68540

40S ribosomal protein S10-A
  
Accession: EMT68541
  
Location: 1977705-1978371
  
 NCBI BlastP on this gene

EMT68541

hypothetical protein
  
Accession: EMT68542
  
Location: 1980309-1981222
  
 NCBI BlastP on this gene

EMT68542

hypothetical protein
  
Accession: EMT68543
  
Location: 1982404-1983317
  
 NCBI BlastP on this gene

EMT68543

Putative E3 ubiquitin-protein ligase mug30
  
Accession: EMT68544
  
Location: 1984908-1988641
  
  
**BlastP hit with Mycgr3G52682\_Mycgr3T**
  
Percentage identity: 42 %
  
BlastP bit score: 872
  
Sequence coverage: 100 %
  
E-value: 0.0
  
  
 NCBI BlastP on this gene

EMT68544

Query: Architecture Search FASTA input

HF679026 : Fusarium fujikuroi IMI 58289 draft genome, chromosome FFUJ\_chr04.    Total score: 1.0     Cumulative Blast bit score: 867

Hit cluster cross-links:

Mycgr3G52686 Mycgr3T
  
Location: 0-861

Mycgr3G52686\_Mycgr3T

Mycgr3G102281 Mycgr3
  
Location: 961-1573

Mycgr3G102281\_Mycgr3

Mycgr3G89185 Mycgr3T
  
Location: 1673-2063

Mycgr3G89185\_Mycgr3T

Mycgr3G65725 Mycgr3T
  
Location: 2163-3612

Mycgr3G65725\_Mycgr3T

Mycgr3G102276 Mycgr3
  
Location: 3712-4801

Mycgr3G102276\_Mycgr3

Mycgr3G89189 Mycgr3T
  
Location: 4901-5564

Mycgr3G89189\_Mycgr3T

Mycgr3G52682 Mycgr3T
  
Location: 5664-9231

Mycgr3G52682\_Mycgr3T

Mycgr3G107072 Mycgr3
  
Location: 9331-13279

Mycgr3G107072\_Mycgr3

Mycgr3G34982 Mycgr3T
  
Location: 13379-15116

Mycgr3G34982\_Mycgr3T

Mycgr3G107069 Mycgr3
  
Location: 15216-17097

Mycgr3G107069\_Mycgr3

Mycgr3G32432 Mycgr3T
  
Location: 17197-19042

Mycgr3G32432\_Mycgr3T

Mycgr3G98385 Mycgr3T
  
Location: 19142-19898

Mycgr3G98385\_Mycgr3T

related to SEC31 protein
  
Accession: CCT67297
  
Location: 1602093-1606027
  
 NCBI BlastP on this gene

FFUJ\_13498

related to cyclophilin
  
Accession: CCT67298
  
Location: 1606914-1607396
  
 NCBI BlastP on this gene

FFUJ\_13499

uncharacterized protein
  
Accession: CCT67299
  
Location: 1607645-1608199
  
 NCBI BlastP on this gene

FFUJ\_13500

probable COX12-cytochrome-c oxidase, subunit VIB
  
Accession: CCT67300
  
Location: 1608603-1609219
  
 NCBI BlastP on this gene

FFUJ\_13501

uncharacterized protein
  
Accession: CCT67301
  
Location: 1609574-1610422
  
 NCBI BlastP on this gene

FFUJ\_13502

probable 40s ribosomal protein s10-b
  
Accession: CCT67302
  
Location: 1610799-1611467
  
 NCBI BlastP on this gene

FFUJ\_13503

uncharacterized protein
  
Accession: CCT67303
  
Location: 1613541-1614460
  
 NCBI BlastP on this gene

FFUJ\_13504

related to ubiquitin-protein ligase HUL4
  
Accession: CCT67304
  
Location: 1616188-1619914
  
  
**BlastP hit with Mycgr3G52682\_Mycgr3T**
  
Percentage identity: 42 %
  
BlastP bit score: 867
  
Sequence coverage: 99 %
  
E-value: 0.0
  
  
 NCBI BlastP on this gene

FFUJ\_13505

related to ribosomal protein MRP49
  
Accession: CCT67535
  
Location: 1620279-1621055
  
 NCBI BlastP on this gene

FFUJ\_13506

uncharacterized protein
  
Accession: CCT67305
  
Location: 1621380-1623586
  
 NCBI BlastP on this gene

FFUJ\_13507

related to Type 1 phosphatases regulator ypi-1
  
Accession: CCT67306
  
Location: 1624242-1624769
  
 NCBI BlastP on this gene

FFUJ\_13508

related to a-agglutinin core protein AGA1
  
Accession: CCT67307
  
Location: 1625036-1625608
  
 NCBI BlastP on this gene

FFUJ\_13509

probable transport vesicle fusion protein SEC17
  
Accession: CCT67308
  
Location: 1626025-1627092
  
 NCBI BlastP on this gene

FFUJ\_13510

probable glucan 1,4-alpha-glucosidase
  
Accession: CCT67309
  
Location: 1627724-1629992
  
 NCBI BlastP on this gene

FFUJ\_13511

Query: Architecture Search FASTA input

ABDF02000004 : Trichoderma virens Gv29-8    Total score: 1.0     Cumulative Blast bit score: 865

Hit cluster cross-links:

Mycgr3G52686 Mycgr3T
  
Location: 0-861

Mycgr3G52686\_Mycgr3T

Mycgr3G102281 Mycgr3
  
Location: 961-1573

Mycgr3G102281\_Mycgr3

Mycgr3G89185 Mycgr3T
  
Location: 1673-2063

Mycgr3G89185\_Mycgr3T

Mycgr3G65725 Mycgr3T
  
Location: 2163-3612

Mycgr3G65725\_Mycgr3T

Mycgr3G102276 Mycgr3
  
Location: 3712-4801

Mycgr3G102276\_Mycgr3

Mycgr3G89189 Mycgr3T
  
Location: 4901-5564

Mycgr3G89189\_Mycgr3T

Mycgr3G52682 Mycgr3T
  
Location: 5664-9231

Mycgr3G52682\_Mycgr3T

Mycgr3G107072 Mycgr3
  
Location: 9331-13279

Mycgr3G107072\_Mycgr3

Mycgr3G34982 Mycgr3T
  
Location: 13379-15116

Mycgr3G34982\_Mycgr3T

Mycgr3G107069 Mycgr3
  
Location: 15216-17097

Mycgr3G107069\_Mycgr3

Mycgr3G32432 Mycgr3T
  
Location: 17197-19042

Mycgr3G32432\_Mycgr3T

Mycgr3G98385 Mycgr3T
  
Location: 19142-19898

Mycgr3G98385\_Mycgr3T

hypothetical protein
  
Accession: EHK24451
  
Location: 796394-800034
  
  
**BlastP hit with Mycgr3G52682\_Mycgr3T**
  
Percentage identity: 43 %
  
BlastP bit score: 866
  
Sequence coverage: 99 %
  
E-value: 0.0
  
  
 NCBI BlastP on this gene

EHK24451

hypothetical protein
  
Accession: EHK24450
  
Location: 795572-796051
  
 NCBI BlastP on this gene

EHK24450

hypothetical protein
  
Accession: EHK24449
  
Location: 792677-794896
  
 NCBI BlastP on this gene

EHK24449

hypothetical protein
  
Accession: EHK24448
  
Location: 791542-792142
  
 NCBI BlastP on this gene

EHK24448

hypothetical protein
  
Accession: EHK24447
  
Location: 790653-791231
  
 NCBI BlastP on this gene

EHK24447

hypothetical protein
  
Accession: EHK24446
  
Location: 789064-790209
  
 NCBI BlastP on this gene

EHK24446

glycoside hydrolase family 15 protein
  
Accession: EHK25059
  
Location: 786393-788541
  
 NCBI BlastP on this gene

EHK25059

Query: Architecture Search FASTA input

JH226131 : Exophiala dermatitidis NIH/UT8656 unplaced genomic scaffold supercont1.2    Total score: 1.0     Cumulative Blast bit score: 863

Hit cluster cross-links:

Mycgr3G52686 Mycgr3T
  
Location: 0-861

Mycgr3G52686\_Mycgr3T

Mycgr3G102281 Mycgr3
  
Location: 961-1573

Mycgr3G102281\_Mycgr3

Mycgr3G89185 Mycgr3T
  
Location: 1673-2063

Mycgr3G89185\_Mycgr3T

Mycgr3G65725 Mycgr3T
  
Location: 2163-3612

Mycgr3G65725\_Mycgr3T

Mycgr3G102276 Mycgr3
  
Location: 3712-4801

Mycgr3G102276\_Mycgr3

Mycgr3G89189 Mycgr3T
  
Location: 4901-5564

Mycgr3G89189\_Mycgr3T

Mycgr3G52682 Mycgr3T
  
Location: 5664-9231

Mycgr3G52682\_Mycgr3T

Mycgr3G107072 Mycgr3
  
Location: 9331-13279

Mycgr3G107072\_Mycgr3

Mycgr3G34982 Mycgr3T
  
Location: 13379-15116

Mycgr3G34982\_Mycgr3T

Mycgr3G107069 Mycgr3
  
Location: 15216-17097

Mycgr3G107069\_Mycgr3

Mycgr3G32432 Mycgr3T
  
Location: 17197-19042

Mycgr3G32432\_Mycgr3T

Mycgr3G98385 Mycgr3T
  
Location: 19142-19898

Mycgr3G98385\_Mycgr3T

other hect domain ubiquitin protein ligase E3
  
Accession: EHY53349
  
Location: 99189-102809
  
  
**BlastP hit with Mycgr3G52682\_Mycgr3T**
  
Percentage identity: 43 %
  
BlastP bit score: 863
  
Sequence coverage: 102 %
  
E-value: 0.0
  
  
 NCBI BlastP on this gene

EHY53349

alcohol dehydrogenase, zinc-containing
  
Accession: EHY53350
  
Location: 104525-105819
  
 NCBI BlastP on this gene

EHY53350

hypothetical protein
  
Accession: EHY53351
  
Location: 107144-108004
  
 NCBI BlastP on this gene

EHY53351

40S ribosomal protein S8-B
  
Accession: EHY53352
  
Location: 108947-109673
  
 NCBI BlastP on this gene

EHY53352

30S ribosomal protein S10e
  
Accession: EHY53353
  
Location: 110257-110894
  
 NCBI BlastP on this gene

EHY53353

hypothetical protein
  
Accession: EHY53354
  
Location: 111916-112264
  
 NCBI BlastP on this gene

EHY53354

sulfite oxidase
  
Accession: EHY53355
  
Location: 113158-114350
  
 NCBI BlastP on this gene

EHY53355

Query: Architecture Search FASTA input

GG704914 : Coccidioides immitis RS genomic scaffold supercont3.4    Total score: 1.0     Cumulative Blast bit score: 847

Hit cluster cross-links:

Mycgr3G52686 Mycgr3T
  
Location: 0-861

Mycgr3G52686\_Mycgr3T

Mycgr3G102281 Mycgr3
  
Location: 961-1573

Mycgr3G102281\_Mycgr3

Mycgr3G89185 Mycgr3T
  
Location: 1673-2063

Mycgr3G89185\_Mycgr3T

Mycgr3G65725 Mycgr3T
  
Location: 2163-3612

Mycgr3G65725\_Mycgr3T

Mycgr3G102276 Mycgr3
  
Location: 3712-4801

Mycgr3G102276\_Mycgr3

Mycgr3G89189 Mycgr3T
  
Location: 4901-5564

Mycgr3G89189\_Mycgr3T

Mycgr3G52682 Mycgr3T
  
Location: 5664-9231

Mycgr3G52682\_Mycgr3T

Mycgr3G107072 Mycgr3
  
Location: 9331-13279

Mycgr3G107072\_Mycgr3

Mycgr3G34982 Mycgr3T
  
Location: 13379-15116

Mycgr3G34982\_Mycgr3T

Mycgr3G107069 Mycgr3
  
Location: 15216-17097

Mycgr3G107069\_Mycgr3

Mycgr3G32432 Mycgr3T
  
Location: 17197-19042

Mycgr3G32432\_Mycgr3T

Mycgr3G98385 Mycgr3T
  
Location: 19142-19898

Mycgr3G98385\_Mycgr3T

hypothetical protein
  
Accession: EAS34190
  
Location: 1913600-1914927
  
 NCBI BlastP on this gene

EAS34190

hypothetical protein
  
Accession: EAS34189
  
Location: 1911449-1912423
  
 NCBI BlastP on this gene

EAS34189

hypothetical protein
  
Accession: EAS34188
  
Location: 1909269-1910759
  
 NCBI BlastP on this gene

EAS34188

arsenical-resistance protein
  
Accession: EAS34187
  
Location: 1907074-1908425
  
 NCBI BlastP on this gene

EAS34187

cytoplasmic tRNA 2-thiolation protein 1
  
Accession: EAS34186
  
Location: 1905434-1906729
  
 NCBI BlastP on this gene

EAS34186

hypothetical protein
  
Accession: EJB11488
  
Location: 1904707-1905027
  
 NCBI BlastP on this gene

EJB11488

ubiquitin-protein ligase
  
Accession: EAS34185
  
Location: 1900449-1904362
  
  
**BlastP hit with Mycgr3G52682\_Mycgr3T**
  
Percentage identity: 43 %
  
BlastP bit score: 847
  
Sequence coverage: 100 %
  
E-value: 0.0
  
  
 NCBI BlastP on this gene

EAS34185

chlorophyll synthesis pathway protein BchC
  
Accession: EAS34184
  
Location: 1896047-1897613
  
 NCBI BlastP on this gene

EAS34184

hypothetical protein
  
Accession: EAS34183
  
Location: 1894547-1895476
  
 NCBI BlastP on this gene

EAS34183

hypothetical protein
  
Accession: EAS34182
  
Location: 1891785-1893635
  
 NCBI BlastP on this gene

EAS34182

hypothetical protein
  
Accession: EAS34181
  
Location: 1890788-1891254
  
 NCBI BlastP on this gene

EAS34181

Query: Architecture Search FASTA input

DS572697 : Verticillium dahliae VdLs.17 supercont1.3 genomic scaffold    Total score: 1.0     Cumulative Blast bit score: 841

Hit cluster cross-links:

Mycgr3G52686 Mycgr3T
  
Location: 0-861

Mycgr3G52686\_Mycgr3T

Mycgr3G102281 Mycgr3
  
Location: 961-1573

Mycgr3G102281\_Mycgr3

Mycgr3G89185 Mycgr3T
  
Location: 1673-2063

Mycgr3G89185\_Mycgr3T

Mycgr3G65725 Mycgr3T
  
Location: 2163-3612

Mycgr3G65725\_Mycgr3T

Mycgr3G102276 Mycgr3
  
Location: 3712-4801

Mycgr3G102276\_Mycgr3

Mycgr3G89189 Mycgr3T
  
Location: 4901-5564

Mycgr3G89189\_Mycgr3T

Mycgr3G52682 Mycgr3T
  
Location: 5664-9231

Mycgr3G52682\_Mycgr3T

Mycgr3G107072 Mycgr3
  
Location: 9331-13279

Mycgr3G107072\_Mycgr3

Mycgr3G34982 Mycgr3T
  
Location: 13379-15116

Mycgr3G34982\_Mycgr3T

Mycgr3G107069 Mycgr3
  
Location: 15216-17097

Mycgr3G107069\_Mycgr3

Mycgr3G32432 Mycgr3T
  
Location: 17197-19042

Mycgr3G32432\_Mycgr3T

Mycgr3G98385 Mycgr3T
  
Location: 19142-19898

Mycgr3G98385\_Mycgr3T

suppressor of Mek1
  
Accession: EGY19923
  
Location: 1020123-1023035
  
 NCBI BlastP on this gene

EGY19923

AFG2 protein
  
Accession: EGY19924
  
Location: 1023775-1026045
  
 NCBI BlastP on this gene

EGY19924

hypothetical protein
  
Accession: EGY19925
  
Location: 1027278-1028498
  
 NCBI BlastP on this gene

EGY19925

GTP-binding protein SAS1
  
Accession: EGY19926
  
Location: 1029249-1030098
  
 NCBI BlastP on this gene

EGY19926

ubiquitin-protein ligase E3A
  
Accession: EGY19927
  
Location: 1032174-1035898
  
  
**BlastP hit with Mycgr3G52682\_Mycgr3T**
  
Percentage identity: 42 %
  
BlastP bit score: 841
  
Sequence coverage: 99 %
  
E-value: 0.0
  
  
 NCBI BlastP on this gene

EGY19927

50S ribosomal protein Mrp49
  
Accession: EGY19928
  
Location: 1036239-1037076
  
 NCBI BlastP on this gene

EGY19928

hypothetical protein
  
Accession: EGY19929
  
Location: 1037518-1039652
  
 NCBI BlastP on this gene

EGY19929

hypothetical protein
  
Accession: EGY19930
  
Location: 1040077-1041018
  
 NCBI BlastP on this gene

EGY19930

hypothetical protein
  
Accession: EGY19931
  
Location: 1042089-1042678
  
 NCBI BlastP on this gene

EGY19931

urease accessory protein ureG
  
Accession: EGY19932
  
Location: 1043412-1044332
  
 NCBI BlastP on this gene

EGY19932

ribosome biogenesis protein TSR1
  
Accession: EGY19933
  
Location: 1044574-1047143
  
 NCBI BlastP on this gene

EGY19933

Query: Architecture Search FASTA input

GL636503 : Coccidioides posadasii str. Silveira unplaced genomic scaffold supercont2.18    Total score: 1.0     Cumulative Blast bit score: 838

Hit cluster cross-links:

Mycgr3G52686 Mycgr3T
  
Location: 0-861

Mycgr3G52686\_Mycgr3T

Mycgr3G102281 Mycgr3
  
Location: 961-1573

Mycgr3G102281\_Mycgr3

Mycgr3G89185 Mycgr3T
  
Location: 1673-2063

Mycgr3G89185\_Mycgr3T

Mycgr3G65725 Mycgr3T
  
Location: 2163-3612

Mycgr3G65725\_Mycgr3T

Mycgr3G102276 Mycgr3
  
Location: 3712-4801

Mycgr3G102276\_Mycgr3

Mycgr3G89189 Mycgr3T
  
Location: 4901-5564

Mycgr3G89189\_Mycgr3T

Mycgr3G52682 Mycgr3T
  
Location: 5664-9231

Mycgr3G52682\_Mycgr3T

Mycgr3G107072 Mycgr3
  
Location: 9331-13279

Mycgr3G107072\_Mycgr3

Mycgr3G34982 Mycgr3T
  
Location: 13379-15116

Mycgr3G34982\_Mycgr3T

Mycgr3G107069 Mycgr3
  
Location: 15216-17097

Mycgr3G107069\_Mycgr3

Mycgr3G32432 Mycgr3T
  
Location: 17197-19042

Mycgr3G32432\_Mycgr3T

Mycgr3G98385 Mycgr3T
  
Location: 19142-19898

Mycgr3G98385\_Mycgr3T

conserved hypothetical protein
  
Accession: EFW14799
  
Location: 149729-150580
  
 NCBI BlastP on this gene

EFW14799

conserved hypothetical protein
  
Accession: EFW14798
  
Location: 146980-148386
  
 NCBI BlastP on this gene

EFW14798

conserved hypothetical protein
  
Accession: EFW14797
  
Location: 144961-146451
  
 NCBI BlastP on this gene

EFW14797

arsenical-resistance protein
  
Accession: EFW14796
  
Location: 142769-144120
  
 NCBI BlastP on this gene

EFW14796

PP-loop ATPase superfamily protein
  
Accession: EFW14795
  
Location: 141144-142439
  
 NCBI BlastP on this gene

EFW14795

E3 ubiquitin-protein ligase HUWE1
  
Accession: EFW14794
  
Location: 135995-139645
  
  
**BlastP hit with Mycgr3G52682\_Mycgr3T**
  
Percentage identity: 42 %
  
BlastP bit score: 838
  
Sequence coverage: 99 %
  
E-value: 0.0
  
  
 NCBI BlastP on this gene

EFW14794

alcohol dehydrogenase
  
Accession: EFW14793
  
Location: 131568-133133
  
 NCBI BlastP on this gene

EFW14793

conserved hypothetical protein
  
Accession: EFW14792
  
Location: 130075-131001
  
 NCBI BlastP on this gene

EFW14792

conserved hypothetical protein
  
Accession: EFW14791
  
Location: 127313-129164
  
 NCBI BlastP on this gene

EFW14791

predicted protein
  
Accession: EFW14790
  
Location: 126325-126791
  
 NCBI BlastP on this gene

EFW14790

transcriptional regulator Ngg1
  
Accession: EFW14789
  
Location: 123184-125505
  
 NCBI BlastP on this gene

EFW14789

Query: Architecture Search FASTA input

ACFW01000009 : Coccidioides posadasii C735 delta SOWgp    Total score: 1.0     Cumulative Blast bit score: 835

Hit cluster cross-links:

Mycgr3G52686 Mycgr3T
  
Location: 0-861

Mycgr3G52686\_Mycgr3T

Mycgr3G102281 Mycgr3
  
Location: 961-1573

Mycgr3G102281\_Mycgr3

Mycgr3G89185 Mycgr3T
  
Location: 1673-2063

Mycgr3G89185\_Mycgr3T

Mycgr3G65725 Mycgr3T
  
Location: 2163-3612

Mycgr3G65725\_Mycgr3T

Mycgr3G102276 Mycgr3
  
Location: 3712-4801

Mycgr3G102276\_Mycgr3

Mycgr3G89189 Mycgr3T
  
Location: 4901-5564

Mycgr3G89189\_Mycgr3T

Mycgr3G52682 Mycgr3T
  
Location: 5664-9231

Mycgr3G52682\_Mycgr3T

Mycgr3G107072 Mycgr3
  
Location: 9331-13279

Mycgr3G107072\_Mycgr3

Mycgr3G34982 Mycgr3T
  
Location: 13379-15116

Mycgr3G34982\_Mycgr3T

Mycgr3G107069 Mycgr3
  
Location: 15216-17097

Mycgr3G107069\_Mycgr3

Mycgr3G32432 Mycgr3T
  
Location: 17197-19042

Mycgr3G32432\_Mycgr3T

Mycgr3G98385 Mycgr3T
  
Location: 19142-19898

Mycgr3G98385\_Mycgr3T

kinase domain containing protein
  
Accession: EER29247
  
Location: 485768-486619
  
 NCBI BlastP on this gene

EER29247

hypothetical protein
  
Accession: EER29248
  
Location: 488022-489065
  
 NCBI BlastP on this gene

EER29248

Major Facilitator Superfamily protein
  
Accession: EER29249
  
Location: 489901-491391
  
 NCBI BlastP on this gene

EER29249

Sodium Bile acid symporter family protein
  
Accession: EER29250
  
Location: 492232-493583
  
 NCBI BlastP on this gene

EER29250

PP-loop family protein
  
Accession: EER29251
  
Location: 493913-495208
  
 NCBI BlastP on this gene

EER29251

HECT-domain containing protein
  
Accession: EER29252
  
Location: 496722-500372
  
  
**BlastP hit with Mycgr3G52682\_Mycgr3T**
  
Percentage identity: 42 %
  
BlastP bit score: 836
  
Sequence coverage: 99 %
  
E-value: 0.0
  
  
 NCBI BlastP on this gene

EER29252

Query: Architecture Search FASTA input

ABDG02000017 : Trichoderma atroviride IMI 206040    Total score: 1.0     Cumulative Blast bit score: 835

Hit cluster cross-links:

Mycgr3G52686 Mycgr3T
  
Location: 0-861

Mycgr3G52686\_Mycgr3T

Mycgr3G102281 Mycgr3
  
Location: 961-1573

Mycgr3G102281\_Mycgr3

Mycgr3G89185 Mycgr3T
  
Location: 1673-2063

Mycgr3G89185\_Mycgr3T

Mycgr3G65725 Mycgr3T
  
Location: 2163-3612

Mycgr3G65725\_Mycgr3T

Mycgr3G102276 Mycgr3
  
Location: 3712-4801

Mycgr3G102276\_Mycgr3

Mycgr3G89189 Mycgr3T
  
Location: 4901-5564

Mycgr3G89189\_Mycgr3T

Mycgr3G52682 Mycgr3T
  
Location: 5664-9231

Mycgr3G52682\_Mycgr3T

Mycgr3G107072 Mycgr3
  
Location: 9331-13279

Mycgr3G107072\_Mycgr3

Mycgr3G34982 Mycgr3T
  
Location: 13379-15116

Mycgr3G34982\_Mycgr3T

Mycgr3G107069 Mycgr3
  
Location: 15216-17097

Mycgr3G107069\_Mycgr3

Mycgr3G32432 Mycgr3T
  
Location: 17197-19042

Mycgr3G32432\_Mycgr3T

Mycgr3G98385 Mycgr3T
  
Location: 19142-19898

Mycgr3G98385\_Mycgr3T

hypothetical protein
  
Accession: EHK49020
  
Location: 474289-474843
  
 NCBI BlastP on this gene

EHK49020

hypothetical protein
  
Accession: EHK49021
  
Location: 475159-475664
  
 NCBI BlastP on this gene

EHK49021

hypothetical protein
  
Accession: EHK49022
  
Location: 476042-476705
  
 NCBI BlastP on this gene

EHK49022

hypothetical protein
  
Accession: EHK49023
  
Location: 477045-477896
  
 NCBI BlastP on this gene

EHK49023

hypothetical protein
  
Accession: EHK49024
  
Location: 478394-479286
  
 NCBI BlastP on this gene

EHK49024

hypothetical protein
  
Accession: EHK49025
  
Location: 479642-479896
  
 NCBI BlastP on this gene

EHK49025

hypothetical protein
  
Accession: EHK49026
  
Location: 480459-481373
  
 NCBI BlastP on this gene

EHK49026

hypothetical protein
  
Accession: EHK49027
  
Location: 482143-482913
  
 NCBI BlastP on this gene

EHK49027

hypothetical protein
  
Accession: EHK49028
  
Location: 484273-487756
  
  
**BlastP hit with Mycgr3G52682\_Mycgr3T**
  
Percentage identity: 42 %
  
BlastP bit score: 836
  
Sequence coverage: 95 %
  
E-value: 0.0
  
  
 NCBI BlastP on this gene

EHK49028

hypothetical protein
  
Accession: EHK49029
  
Location: 488221-489050
  
 NCBI BlastP on this gene

EHK49029

hypothetical protein
  
Accession: EHK49030
  
Location: 489386-491616
  
 NCBI BlastP on this gene

EHK49030

hypothetical protein
  
Accession: EHK49031
  
Location: 492153-492777
  
 NCBI BlastP on this gene

EHK49031

hypothetical protein
  
Accession: EHK49032
  
Location: 493117-493599
  
 NCBI BlastP on this gene

EHK49032

hypothetical protein
  
Accession: EHK49033
  
Location: 494052-495245
  
 NCBI BlastP on this gene

EHK49033

glycoside hydrolase family 15 protein
  
Accession: EHK49034
  
Location: 495781-497869
  
 NCBI BlastP on this gene

EHK49034

Query: Architecture Search FASTA input

GL891305 : Neurospora tetrasperma FGSC 2508 unplaced genomic scaffold NEUTE1scaffold\_4    Total score: 1.0     Cumulative Blast bit score: 830

Hit cluster cross-links:

Mycgr3G52686 Mycgr3T
  
Location: 0-861

Mycgr3G52686\_Mycgr3T

Mycgr3G102281 Mycgr3
  
Location: 961-1573

Mycgr3G102281\_Mycgr3

Mycgr3G89185 Mycgr3T
  
Location: 1673-2063

Mycgr3G89185\_Mycgr3T

Mycgr3G65725 Mycgr3T
  
Location: 2163-3612

Mycgr3G65725\_Mycgr3T

Mycgr3G102276 Mycgr3
  
Location: 3712-4801

Mycgr3G102276\_Mycgr3

Mycgr3G89189 Mycgr3T
  
Location: 4901-5564

Mycgr3G89189\_Mycgr3T

Mycgr3G52682 Mycgr3T
  
Location: 5664-9231

Mycgr3G52682\_Mycgr3T

Mycgr3G107072 Mycgr3
  
Location: 9331-13279

Mycgr3G107072\_Mycgr3

Mycgr3G34982 Mycgr3T
  
Location: 13379-15116

Mycgr3G34982\_Mycgr3T

Mycgr3G107069 Mycgr3
  
Location: 15216-17097

Mycgr3G107069\_Mycgr3

Mycgr3G32432 Mycgr3T
  
Location: 17197-19042

Mycgr3G32432\_Mycgr3T

Mycgr3G98385 Mycgr3T
  
Location: 19142-19898

Mycgr3G98385\_Mycgr3T

hypothetical protein
  
Accession: EGO56667
  
Location: 2183624-2184468
  
 NCBI BlastP on this gene

EGO56667

hypothetical protein
  
Accession: EGO56668
  
Location: 2185111-2186275
  
 NCBI BlastP on this gene

EGO56668

hypothetical protein
  
Accession: EGO56669
  
Location: 2187805-2190542
  
 NCBI BlastP on this gene

EGO56669

hypothetical protein
  
Accession: EGO56670
  
Location: 2191799-2192636
  
 NCBI BlastP on this gene

EGO56670

hypothetical protein
  
Accession: EGO56671
  
Location: 2194848-2198959
  
  
**BlastP hit with Mycgr3G52682\_Mycgr3T**
  
Percentage identity: 42 %
  
BlastP bit score: 830
  
Sequence coverage: 100 %
  
E-value: 0.0
  
  
 NCBI BlastP on this gene

EGO56671

Query: Architecture Search FASTA input

GL891247 : Neurospora tetrasperma FGSC 2509 unplaced genomic scaffold NEUTE2scaffold\_5    Total score: 1.0     Cumulative Blast bit score: 830

Hit cluster cross-links:

Mycgr3G52686 Mycgr3T
  
Location: 0-861

Mycgr3G52686\_Mycgr3T

Mycgr3G102281 Mycgr3
  
Location: 961-1573

Mycgr3G102281\_Mycgr3

Mycgr3G89185 Mycgr3T
  
Location: 1673-2063

Mycgr3G89185\_Mycgr3T

Mycgr3G65725 Mycgr3T
  
Location: 2163-3612

Mycgr3G65725\_Mycgr3T

Mycgr3G102276 Mycgr3
  
Location: 3712-4801

Mycgr3G102276\_Mycgr3

Mycgr3G89189 Mycgr3T
  
Location: 4901-5564

Mycgr3G89189\_Mycgr3T

Mycgr3G52682 Mycgr3T
  
Location: 5664-9231

Mycgr3G52682\_Mycgr3T

Mycgr3G107072 Mycgr3
  
Location: 9331-13279

Mycgr3G107072\_Mycgr3

Mycgr3G34982 Mycgr3T
  
Location: 13379-15116

Mycgr3G34982\_Mycgr3T

Mycgr3G107069 Mycgr3
  
Location: 15216-17097

Mycgr3G107069\_Mycgr3

Mycgr3G32432 Mycgr3T
  
Location: 17197-19042

Mycgr3G32432\_Mycgr3T

Mycgr3G98385 Mycgr3T
  
Location: 19142-19898

Mycgr3G98385\_Mycgr3T

hypothetical protein
  
Accession: EGZ70458
  
Location: 2028880-2029724
  
 NCBI BlastP on this gene

EGZ70458

hypothetical protein
  
Accession: EGZ70457
  
Location: 2027073-2028237
  
 NCBI BlastP on this gene

EGZ70457

hypothetical protein
  
Accession: EGZ70456
  
Location: 2022806-2025543
  
 NCBI BlastP on this gene

EGZ70456

hypothetical protein
  
Accession: EGZ70455
  
Location: 2020712-2022405
  
 NCBI BlastP on this gene

EGZ70455

HECT-domain-containing protein
  
Accession: EGZ70454
  
Location: 2014391-2018502
  
  
**BlastP hit with Mycgr3G52682\_Mycgr3T**
  
Percentage identity: 42 %
  
BlastP bit score: 830
  
Sequence coverage: 100 %
  
E-value: 0.0
  
  
 NCBI BlastP on this gene

EGZ70454

DNA/RNA polymerase
  
Accession: EGZ70453
  
Location: 2010983-2013736
  
 NCBI BlastP on this gene

EGZ70453

WD40 repeat-like protein
  
Accession: EGZ70452
  
Location: 2005817-2009323
  
 NCBI BlastP on this gene

EGZ70452

hypothetical protein
  
Accession: EGZ70451
  
Location: 2002652-2004917
  
 NCBI BlastP on this gene

EGZ70451

Query: Architecture Search FASTA input

KB456260 : Mycosphaerella populorum SO2202 unplaced genomic scaffold SEPMUscaffold\_1    Total score: 1.0     Cumulative Blast bit score: 825

Hit cluster cross-links:

Mycgr3G52686 Mycgr3T
  
Location: 0-861

Mycgr3G52686\_Mycgr3T

Mycgr3G102281 Mycgr3
  
Location: 961-1573

Mycgr3G102281\_Mycgr3

Mycgr3G89185 Mycgr3T
  
Location: 1673-2063

Mycgr3G89185\_Mycgr3T

Mycgr3G65725 Mycgr3T
  
Location: 2163-3612

Mycgr3G65725\_Mycgr3T

Mycgr3G102276 Mycgr3
  
Location: 3712-4801

Mycgr3G102276\_Mycgr3

Mycgr3G89189 Mycgr3T
  
Location: 4901-5564

Mycgr3G89189\_Mycgr3T

Mycgr3G52682 Mycgr3T
  
Location: 5664-9231

Mycgr3G52682\_Mycgr3T

Mycgr3G107072 Mycgr3
  
Location: 9331-13279

Mycgr3G107072\_Mycgr3

Mycgr3G34982 Mycgr3T
  
Location: 13379-15116

Mycgr3G34982\_Mycgr3T

Mycgr3G107069 Mycgr3
  
Location: 15216-17097

Mycgr3G107069\_Mycgr3

Mycgr3G32432 Mycgr3T
  
Location: 17197-19042

Mycgr3G32432\_Mycgr3T

Mycgr3G98385 Mycgr3T
  
Location: 19142-19898

Mycgr3G98385\_Mycgr3T

hypothetical protein
  
Accession: EMF16384
  
Location: 632327-633968
  
 NCBI BlastP on this gene

EMF16384

glycoside hydrolase family 5 protein
  
Accession: EMF16385
  
Location: 638298-639653
  
 NCBI BlastP on this gene

EMF16385

phosphoglycerate mutase-like protein
  
Accession: EMF16386
  
Location: 641807-643390
  
 NCBI BlastP on this gene

EMF16386

Ferric reduct-domain-containing protein
  
Accession: EMF16387
  
Location: 643957-645981
  
  
**BlastP hit with Mycgr3G107069\_Mycgr3**
  
Percentage identity: 61 %
  
BlastP bit score: 825
  
Sequence coverage: 103 %
  
E-value: 0.0
  
  
 NCBI BlastP on this gene

EMF16387

hexose transport-related protein
  
Accession: EMF16388
  
Location: 646746-648564
  
 NCBI BlastP on this gene

EMF16388

hypothetical protein
  
Accession: EMF16389
  
Location: 653310-655146
  
 NCBI BlastP on this gene

EMF16389

hypothetical protein
  
Accession: EMF16391
  
Location: 655880-656431
  
 NCBI BlastP on this gene

EMF16391

Query: Architecture Search FASTA input

CU638743 : Podospora anserina S mat+ genomic DNA chromosome 3, supercontig 2.    Total score: 1.0     Cumulative Blast bit score: 810

Hit cluster cross-links:

Mycgr3G52686 Mycgr3T
  
Location: 0-861

Mycgr3G52686\_Mycgr3T

Mycgr3G102281 Mycgr3
  
Location: 961-1573

Mycgr3G102281\_Mycgr3

Mycgr3G89185 Mycgr3T
  
Location: 1673-2063

Mycgr3G89185\_Mycgr3T

Mycgr3G65725 Mycgr3T
  
Location: 2163-3612

Mycgr3G65725\_Mycgr3T

Mycgr3G102276 Mycgr3
  
Location: 3712-4801

Mycgr3G102276\_Mycgr3

Mycgr3G89189 Mycgr3T
  
Location: 4901-5564

Mycgr3G89189\_Mycgr3T

Mycgr3G52682 Mycgr3T
  
Location: 5664-9231

Mycgr3G52682\_Mycgr3T

Mycgr3G107072 Mycgr3
  
Location: 9331-13279

Mycgr3G107072\_Mycgr3

Mycgr3G34982 Mycgr3T
  
Location: 13379-15116

Mycgr3G34982\_Mycgr3T

Mycgr3G107069 Mycgr3
  
Location: 15216-17097

Mycgr3G107069\_Mycgr3

Mycgr3G32432 Mycgr3T
  
Location: 17197-19042

Mycgr3G32432\_Mycgr3T

Mycgr3G98385 Mycgr3T
  
Location: 19142-19898

Mycgr3G98385\_Mycgr3T

not annotated
  
Accession: CAP70770
  
Location: 2280484-2282511
  
 NCBI BlastP on this gene

CAP70770

not annotated
  
Accession: CAP70769
  
Location: 2276681-2279472
  
 NCBI BlastP on this gene

CAP70769

not annotated
  
Accession: CAP70768
  
Location: 2267853-2271588
  
  
**BlastP hit with Mycgr3G52682\_Mycgr3T**
  
Percentage identity: 41 %
  
BlastP bit score: 811
  
Sequence coverage: 101 %
  
E-value: 0.0
  
  
 NCBI BlastP on this gene

CAP70768

not annotated
  
Accession: CAP70767
  
Location: 2264201-2265355
  
 NCBI BlastP on this gene

CAP70767

not annotated
  
Accession: CAP70766
  
Location: 2261265-2263268
  
 NCBI BlastP on this gene

CAP70766

not annotated
  
Accession: CAP70765
  
Location: 2260494-2261101
  
 NCBI BlastP on this gene

CAP70765

not annotated
  
Accession: CAP70764
  
Location: 2256504-2258593
  
 NCBI BlastP on this gene

CAP70764

Query: Architecture Search FASTA input

CM001235 : Magnaporthe oryzae 70-15 chromosome 5    Total score: 1.0     Cumulative Blast bit score: 810

Hit cluster cross-links:

Mycgr3G52686 Mycgr3T
  
Location: 0-861

Mycgr3G52686\_Mycgr3T

Mycgr3G102281 Mycgr3
  
Location: 961-1573

Mycgr3G102281\_Mycgr3

Mycgr3G89185 Mycgr3T
  
Location: 1673-2063

Mycgr3G89185\_Mycgr3T

Mycgr3G65725 Mycgr3T
  
Location: 2163-3612

Mycgr3G65725\_Mycgr3T

Mycgr3G102276 Mycgr3
  
Location: 3712-4801

Mycgr3G102276\_Mycgr3

Mycgr3G89189 Mycgr3T
  
Location: 4901-5564

Mycgr3G89189\_Mycgr3T

Mycgr3G52682 Mycgr3T
  
Location: 5664-9231

Mycgr3G52682\_Mycgr3T

Mycgr3G107072 Mycgr3
  
Location: 9331-13279

Mycgr3G107072\_Mycgr3

Mycgr3G34982 Mycgr3T
  
Location: 13379-15116

Mycgr3G34982\_Mycgr3T

Mycgr3G107069 Mycgr3
  
Location: 15216-17097

Mycgr3G107069\_Mycgr3

Mycgr3G32432 Mycgr3T
  
Location: 17197-19042

Mycgr3G32432\_Mycgr3T

Mycgr3G98385 Mycgr3T
  
Location: 19142-19898

Mycgr3G98385\_Mycgr3T

nuclear cap-binding protein
  
Accession: EHA48241
  
Location: 485496-486030
  
 NCBI BlastP on this gene

EHA48241

hypothetical protein
  
Accession: EHA48240
  
Location: 484589-485186
  
 NCBI BlastP on this gene

EHA48240

cytochrome c oxidase subunit 6B
  
Accession: EHA48239
  
Location: 483533-484138
  
 NCBI BlastP on this gene

EHA48239

hypothetical protein
  
Accession: EHA48238
  
Location: 482251-483147
  
 NCBI BlastP on this gene

EHA48238

40S ribosomal protein S10-A
  
Accession: EHA48237
  
Location: 480900-481785
  
 NCBI BlastP on this gene

EHA48237

NADP-dependent mannitol dehydrogenase
  
Accession: EHA48236
  
Location: 477746-479121
  
 NCBI BlastP on this gene

EHA48236

ubiquitin-protein ligase E3A
  
Accession: EHA48235
  
Location: 471321-475547
  
  
**BlastP hit with Mycgr3G52682\_Mycgr3T**
  
Percentage identity: 42 %
  
BlastP bit score: 810
  
Sequence coverage: 100 %
  
E-value: 0.0
  
  
 NCBI BlastP on this gene

EHA48235

hypothetical protein
  
Accession: EHA48234
  
Location: 470066-470907
  
 NCBI BlastP on this gene

EHA48234

hypothetical protein
  
Accession: EHA48233
  
Location: 467346-469568
  
 NCBI BlastP on this gene

EHA48233

hypothetical protein
  
Accession: EHA48232
  
Location: 465302-466834
  
 NCBI BlastP on this gene

EHA48232

transmembrane and coiled-coil domain-containing protein 4
  
Accession: EHA48231
  
Location: 461603-464179
  
 NCBI BlastP on this gene

EHA48231

hypothetical protein
  
Accession: EHA48230
  
Location: 460502-461161
  
 NCBI BlastP on this gene

EHA48230

Query: Architecture Search FASTA input

EQ963473 : Aspergillus flavus NRRL3357 scf\_1106286417600 genomic scaffold    Total score: 1.0     Cumulative Blast bit score: 803

Hit cluster cross-links:

Mycgr3G52686 Mycgr3T
  
Location: 0-861

Mycgr3G52686\_Mycgr3T

Mycgr3G102281 Mycgr3
  
Location: 961-1573

Mycgr3G102281\_Mycgr3

Mycgr3G89185 Mycgr3T
  
Location: 1673-2063

Mycgr3G89185\_Mycgr3T

Mycgr3G65725 Mycgr3T
  
Location: 2163-3612

Mycgr3G65725\_Mycgr3T

Mycgr3G102276 Mycgr3
  
Location: 3712-4801

Mycgr3G102276\_Mycgr3

Mycgr3G89189 Mycgr3T
  
Location: 4901-5564

Mycgr3G89189\_Mycgr3T

Mycgr3G52682 Mycgr3T
  
Location: 5664-9231

Mycgr3G52682\_Mycgr3T

Mycgr3G107072 Mycgr3
  
Location: 9331-13279

Mycgr3G107072\_Mycgr3

Mycgr3G34982 Mycgr3T
  
Location: 13379-15116

Mycgr3G34982\_Mycgr3T

Mycgr3G107069 Mycgr3
  
Location: 15216-17097

Mycgr3G107069\_Mycgr3

Mycgr3G32432 Mycgr3T
  
Location: 17197-19042

Mycgr3G32432\_Mycgr3T

Mycgr3G98385 Mycgr3T
  
Location: 19142-19898

Mycgr3G98385\_Mycgr3T

PSP1 domain protein
  
Accession: EED55653
  
Location: 1726176-1728887
  
 NCBI BlastP on this gene

EED55653

60S ribosomal protein L15, putative
  
Accession: EED55654
  
Location: 1730183-1731275
  
 NCBI BlastP on this gene

EED55654

hypothetical protein
  
Accession: EED55655
  
Location: 1732496-1732921
  
 NCBI BlastP on this gene

EED55655

ubiquitin-protein ligase (Hul4), putative
  
Accession: EED55656
  
Location: 1733752-1736029
  
  
**BlastP hit with Mycgr3G52682\_Mycgr3T**
  
Percentage identity: 53 %
  
BlastP bit score: 803
  
Sequence coverage: 64 %
  
E-value: 0.0
  
  
 NCBI BlastP on this gene

EED55656

Query: Architecture Search FASTA input

GL629729 : Grosmannia clavigera kw1407 unplaced genomic scaffold GCSC\_108    Total score: 1.0     Cumulative Blast bit score: 797

Hit cluster cross-links:

Mycgr3G52686 Mycgr3T
  
Location: 0-861

Mycgr3G52686\_Mycgr3T

Mycgr3G102281 Mycgr3
  
Location: 961-1573

Mycgr3G102281\_Mycgr3

Mycgr3G89185 Mycgr3T
  
Location: 1673-2063

Mycgr3G89185\_Mycgr3T

Mycgr3G65725 Mycgr3T
  
Location: 2163-3612

Mycgr3G65725\_Mycgr3T

Mycgr3G102276 Mycgr3
  
Location: 3712-4801

Mycgr3G102276\_Mycgr3

Mycgr3G89189 Mycgr3T
  
Location: 4901-5564

Mycgr3G89189\_Mycgr3T

Mycgr3G52682 Mycgr3T
  
Location: 5664-9231

Mycgr3G52682\_Mycgr3T

Mycgr3G107072 Mycgr3
  
Location: 9331-13279

Mycgr3G107072\_Mycgr3

Mycgr3G34982 Mycgr3T
  
Location: 13379-15116

Mycgr3G34982\_Mycgr3T

Mycgr3G107069 Mycgr3
  
Location: 15216-17097

Mycgr3G107069\_Mycgr3

Mycgr3G32432 Mycgr3T
  
Location: 17197-19042

Mycgr3G32432\_Mycgr3T

Mycgr3G98385 Mycgr3T
  
Location: 19142-19898

Mycgr3G98385\_Mycgr3T

protein transport protein
  
Accession: EFX06673
  
Location: 64938-68981
  
 NCBI BlastP on this gene

EFX06673

peptidyl prolyl cis-trans isomerase
  
Accession: EFX06598
  
Location: 63847-64416
  
 NCBI BlastP on this gene

EFX06598

hypothetical protein
  
Accession: EFX06604
  
Location: 62979-63534
  
 NCBI BlastP on this gene

EFX06604

cytochrome c oxidase polypeptide vib
  
Accession: EFX06706
  
Location: 61757-62448
  
 NCBI BlastP on this gene

EFX06706

hypothetical protein
  
Accession: EFX06287
  
Location: 60228-61157
  
 NCBI BlastP on this gene

EFX06287

40S ribosomal protein s10b
  
Accession: EFX06236
  
Location: 58984-59839
  
 NCBI BlastP on this gene

EFX06236

vesicular-fusion protein sec17
  
Accession: EFX06269
  
Location: 57165-58247
  
 NCBI BlastP on this gene

EFX06269

duf726 domain containing protein
  
Accession: EFX06273
  
Location: 56233-56823
  
 NCBI BlastP on this gene

EFX06273

ubiquitin-protein ligase
  
Accession: EFX06337
  
Location: 51336-55121
  
  
**BlastP hit with Mycgr3G52682\_Mycgr3T**
  
Percentage identity: 39 %
  
BlastP bit score: 798
  
Sequence coverage: 105 %
  
E-value: 0.0
  
  
 NCBI BlastP on this gene

EFX06337

50S ribosomal protein mrp49
  
Accession: EFX06292
  
Location: 50376-51217
  
 NCBI BlastP on this gene

EFX06292

duf974 domain containing protein
  
Accession: EFX06401
  
Location: 46647-50081
  
 NCBI BlastP on this gene

EFX06401

ddenn domain containing protein
  
Accession: EFX06468
  
Location: 41005-44757
  
 NCBI BlastP on this gene

EFX06468

Query: Architecture Search FASTA input

KE123956 : Mucor circinelloides f. circinelloides 1006PhL unplaced genomic scaffold supercont1.61    Total score: 1.0     Cumulative Blast bit score: 784

Hit cluster cross-links:

Mycgr3G52686 Mycgr3T
  
Location: 0-861

Mycgr3G52686\_Mycgr3T

Mycgr3G102281 Mycgr3
  
Location: 961-1573

Mycgr3G102281\_Mycgr3

Mycgr3G89185 Mycgr3T
  
Location: 1673-2063

Mycgr3G89185\_Mycgr3T

Mycgr3G65725 Mycgr3T
  
Location: 2163-3612

Mycgr3G65725\_Mycgr3T

Mycgr3G102276 Mycgr3
  
Location: 3712-4801

Mycgr3G102276\_Mycgr3

Mycgr3G89189 Mycgr3T
  
Location: 4901-5564

Mycgr3G89189\_Mycgr3T

Mycgr3G52682 Mycgr3T
  
Location: 5664-9231

Mycgr3G52682\_Mycgr3T

Mycgr3G107072 Mycgr3
  
Location: 9331-13279

Mycgr3G107072\_Mycgr3

Mycgr3G34982 Mycgr3T
  
Location: 13379-15116

Mycgr3G34982\_Mycgr3T

Mycgr3G107069 Mycgr3
  
Location: 15216-17097

Mycgr3G107069\_Mycgr3

Mycgr3G32432 Mycgr3T
  
Location: 17197-19042

Mycgr3G32432\_Mycgr3T

Mycgr3G98385 Mycgr3T
  
Location: 19142-19898

Mycgr3G98385\_Mycgr3T

AGC/RSK/RSK-UNCLASSIFIED protein kinase
  
Accession: EPB88109
  
Location: 38814-40628
  
 NCBI BlastP on this gene

EPB88109

hypothetical protein
  
Accession: EPB88110
  
Location: 40889-42065
  
 NCBI BlastP on this gene

EPB88110

hypothetical protein
  
Accession: EPB88111
  
Location: 42268-45000
  
  
**BlastP hit with Mycgr3G52682\_Mycgr3T**
  
Percentage identity: 40 %
  
BlastP bit score: 525
  
Sequence coverage: 59 %
  
E-value: 7e-167
  
  
 NCBI BlastP on this gene

EPB88111

hypothetical protein
  
Accession: EPB88112
  
Location: 46318-47424
  
 NCBI BlastP on this gene

EPB88112

hypothetical protein
  
Accession: EPB88113
  
Location: 47493-49301
  
 NCBI BlastP on this gene

EPB88113

hypothetical protein
  
Accession: EPB88114
  
Location: 49825-51432
  
 NCBI BlastP on this gene

EPB88114

hypothetical protein
  
Accession: EPB88115
  
Location: 53897-55786
  
 NCBI BlastP on this gene

EPB88115

6-phosphofructokinase
  
Accession: EPB88116
  
Location: 55983-59037
  
 NCBI BlastP on this gene

EPB88116

other hect domain ubiquitin protein ligase E3
  
Accession: EPB88117
  
Location: 62129-63045
  
  
**BlastP hit with Mycgr3G52682\_Mycgr3T**
  
Percentage identity: 49 %
  
BlastP bit score: 260
  
Sequence coverage: 20 %
  
E-value: 9e-76
  
  
 NCBI BlastP on this gene

EPB88117

STE/STE20/PAKA protein kinase
  
Accession: EPB88118
  
Location: 64184-65886
  
 NCBI BlastP on this gene

EPB88118

Query: Architecture Search FASTA input

CP003008 : Myceliophthora thermophila ATCC 42464 chromosome 7    Total score: 1.0     Cumulative Blast bit score: 784

Hit cluster cross-links:

Mycgr3G52686 Mycgr3T
  
Location: 0-861

Mycgr3G52686\_Mycgr3T

Mycgr3G102281 Mycgr3
  
Location: 961-1573

Mycgr3G102281\_Mycgr3

Mycgr3G89185 Mycgr3T
  
Location: 1673-2063

Mycgr3G89185\_Mycgr3T

Mycgr3G65725 Mycgr3T
  
Location: 2163-3612

Mycgr3G65725\_Mycgr3T

Mycgr3G102276 Mycgr3
  
Location: 3712-4801

Mycgr3G102276\_Mycgr3

Mycgr3G89189 Mycgr3T
  
Location: 4901-5564

Mycgr3G89189\_Mycgr3T

Mycgr3G52682 Mycgr3T
  
Location: 5664-9231

Mycgr3G52682\_Mycgr3T

Mycgr3G107072 Mycgr3
  
Location: 9331-13279

Mycgr3G107072\_Mycgr3

Mycgr3G34982 Mycgr3T
  
Location: 13379-15116

Mycgr3G34982\_Mycgr3T

Mycgr3G107069 Mycgr3
  
Location: 15216-17097

Mycgr3G107069\_Mycgr3

Mycgr3G32432 Mycgr3T
  
Location: 17197-19042

Mycgr3G32432\_Mycgr3T

Mycgr3G98385 Mycgr3T
  
Location: 19142-19898

Mycgr3G98385\_Mycgr3T

hypothetical protein
  
Accession: AEO61597
  
Location: 1618110-1618982
  
 NCBI BlastP on this gene

MYCTH\_2311907

hypothetical protein
  
Accession: AEO61598
  
Location: 1620342-1621907
  
 NCBI BlastP on this gene

MYCTH\_2311910

hypothetical protein
  
Accession: AEO61599
  
Location: 1622486-1626363
  
 NCBI BlastP on this gene

MYCTH\_2311912

hypothetical protein
  
Accession: AEO61600
  
Location: 1628505-1632331
  
  
**BlastP hit with Mycgr3G52682\_Mycgr3T**
  
Percentage identity: 41 %
  
BlastP bit score: 784
  
Sequence coverage: 101 %
  
E-value: 0.0
  
  
 NCBI BlastP on this gene

MYCTH\_2311917

hypothetical protein
  
Accession: AEO61601
  
Location: 1634929-1636131
  
 NCBI BlastP on this gene

MYCTH\_2311919

hypothetical protein
  
Accession: AEO61602
  
Location: 1637270-1639367
  
 NCBI BlastP on this gene

MYCTH\_2311921

hypothetical protein
  
Accession: AEO61603
  
Location: 1639624-1640358
  
 NCBI BlastP on this gene

MYCTH\_104267

hypothetical protein
  
Accession: AEO61604
  
Location: 1642046-1644003
  
 NCBI BlastP on this gene

MYCTH\_56385

Query: Architecture Search FASTA input

CP004025 : Myxococcus stipitatus DSM 14675    Total score: 1.0     Cumulative Blast bit score: 774

Hit cluster cross-links:

Mycgr3G52686 Mycgr3T
  
Location: 0-861

Mycgr3G52686\_Mycgr3T

Mycgr3G102281 Mycgr3
  
Location: 961-1573

Mycgr3G102281\_Mycgr3

Mycgr3G89185 Mycgr3T
  
Location: 1673-2063

Mycgr3G89185\_Mycgr3T

Mycgr3G65725 Mycgr3T
  
Location: 2163-3612

Mycgr3G65725\_Mycgr3T

Mycgr3G102276 Mycgr3
  
Location: 3712-4801

Mycgr3G102276\_Mycgr3

Mycgr3G89189 Mycgr3T
  
Location: 4901-5564

Mycgr3G89189\_Mycgr3T

Mycgr3G52682 Mycgr3T
  
Location: 5664-9231

Mycgr3G52682\_Mycgr3T

Mycgr3G107072 Mycgr3
  
Location: 9331-13279

Mycgr3G107072\_Mycgr3

Mycgr3G34982 Mycgr3T
  
Location: 13379-15116

Mycgr3G34982\_Mycgr3T

Mycgr3G107069 Mycgr3
  
Location: 15216-17097

Mycgr3G107069\_Mycgr3

Mycgr3G32432 Mycgr3T
  
Location: 17197-19042

Mycgr3G32432\_Mycgr3T

Mycgr3G98385 Mycgr3T
  
Location: 19142-19898

Mycgr3G98385\_Mycgr3T

2,3-dihydroxybenzoate-AMP ligase
  
Accession: AGC45605
  
Location: 5495561-5497186
  
 NCBI BlastP on this gene

MYSTI\_04307

isochorismatase
  
Accession: AGC45606
  
Location: 5497223-5498158
  
 NCBI BlastP on this gene

MYSTI\_04308

non-ribosomal peptide synthetase
  
Accession: AGC45607
  
Location: 5498193-5502659
  
  
**BlastP hit with Mycgr3G107072\_Mycgr3**
  
Percentage identity: 30 %
  
BlastP bit score: 330
  
Sequence coverage: 82 %
  
E-value: 4e-90
  
  
 NCBI BlastP on this gene

MYSTI\_04309

3-deoxy-7-phosphoheptulonate synthase
  
Accession: AGC45608
  
Location: 5502659-5504032
  
 NCBI BlastP on this gene

MYSTI\_04310

TonB family protein
  
Accession: AGC45609
  
Location: 5504087-5506708
  
 NCBI BlastP on this gene

MYSTI\_04311

hypothetical protein
  
Accession: AGC45610
  
Location: 5506765-5507988
  
 NCBI BlastP on this gene

MYSTI\_04312

MotA/TolQ/ExbB proton channel family protein
  
Accession: AGC45611
  
Location: 5508116-5508796
  
 NCBI BlastP on this gene

MYSTI\_04313

ExbD/TolR family transport energizing protein
  
Accession: AGC45612
  
Location: 5508799-5509233
  
 NCBI BlastP on this gene

MYSTI\_04314

hypothetical protein
  
Accession: AGC45613
  
Location: 5509220-5510044
  
 NCBI BlastP on this gene

MYSTI\_04315

major facilitator family transporter
  
Accession: AGC45614
  
Location: 5510076-5511299
  
 NCBI BlastP on this gene

MYSTI\_04316

siderophore biosynthesis aminotransferase
  
Accession: AGC45615
  
Location: 5511334-5512602
  
 NCBI BlastP on this gene

MYSTI\_04317

iron-chelator utilization protein
  
Accession: AGC45616
  
Location: 5512656-5513477
  
 NCBI BlastP on this gene

MYSTI\_04318

hypothetical protein
  
Accession: AGC45617
  
Location: 5513696-5515117
  
 NCBI BlastP on this gene

MYSTI\_04319

non-ribosomal peptide synthetase
  
Accession: AGC45618
  
Location: 5515262-5519788
  
  
**BlastP hit with Mycgr3G107072\_Mycgr3**
  
Percentage identity: 34 %
  
BlastP bit score: 444
  
Sequence coverage: 74 %
  
E-value: 7e-129
  
  
 NCBI BlastP on this gene

MYSTI\_04320

polyketide synthase
  
Accession: AGC45619
  
Location: 5519785-5526216
  
 NCBI BlastP on this gene

MYSTI\_04321

Query: Architecture Search FASTA input

DS985245 : Trichoplax adhaerens TRIADscaffold\_5 genomic scaffold    Total score: 1.0     Cumulative Blast bit score: 772

Hit cluster cross-links:

Mycgr3G52686 Mycgr3T
  
Location: 0-861

Mycgr3G52686\_Mycgr3T

Mycgr3G102281 Mycgr3
  
Location: 961-1573

Mycgr3G102281\_Mycgr3

Mycgr3G89185 Mycgr3T
  
Location: 1673-2063

Mycgr3G89185\_Mycgr3T

Mycgr3G65725 Mycgr3T
  
Location: 2163-3612

Mycgr3G65725\_Mycgr3T

Mycgr3G102276 Mycgr3
  
Location: 3712-4801

Mycgr3G102276\_Mycgr3

Mycgr3G89189 Mycgr3T
  
Location: 4901-5564

Mycgr3G89189\_Mycgr3T

Mycgr3G52682 Mycgr3T
  
Location: 5664-9231

Mycgr3G52682\_Mycgr3T

Mycgr3G107072 Mycgr3
  
Location: 9331-13279

Mycgr3G107072\_Mycgr3

Mycgr3G34982 Mycgr3T
  
Location: 13379-15116

Mycgr3G34982\_Mycgr3T

Mycgr3G107069 Mycgr3
  
Location: 15216-17097

Mycgr3G107069\_Mycgr3

Mycgr3G32432 Mycgr3T
  
Location: 17197-19042

Mycgr3G32432\_Mycgr3T

Mycgr3G98385 Mycgr3T
  
Location: 19142-19898

Mycgr3G98385\_Mycgr3T

hypothetical protein
  
Accession: EDV24532
  
Location: 2823722-2831955
  
  
**BlastP hit with Mycgr3G107072\_Mycgr3**
  
Percentage identity: 37 %
  
BlastP bit score: 772
  
Sequence coverage: 100 %
  
E-value: 0.0
  
  
 NCBI BlastP on this gene

EDV24532

hypothetical protein
  
Accession: EDV24971
  
Location: 2833494-2836864
  
 NCBI BlastP on this gene

EDV24971

Query: Architecture Search FASTA input

101. :  DF126495 Aspergillus kawachii IFO 4308 DNA, contig: scaffold00049     Total score: 1.0     Cumulative Blast bit score: 1712

Mycgr3G52686 Mycgr3T
  
Location: 0-861
  
 NCBI BlastP on this gene

Mycgr3G52686\_Mycgr3T

Mycgr3G102281 Mycgr3
  
Location: 961-1573
  
 NCBI BlastP on this gene

Mycgr3G102281\_Mycgr3

Mycgr3G89185 Mycgr3T
  
Location: 1673-2063
  
 NCBI BlastP on this gene

Mycgr3G89185\_Mycgr3T

Mycgr3G65725 Mycgr3T
  
Location: 2163-3612
  
 NCBI BlastP on this gene

Mycgr3G65725\_Mycgr3T

Mycgr3G102276 Mycgr3
  
Location: 3712-4801
  
 NCBI BlastP on this gene

Mycgr3G102276\_Mycgr3

Mycgr3G89189 Mycgr3T
  
Location: 4901-5564
  
 NCBI BlastP on this gene

Mycgr3G89189\_Mycgr3T

Mycgr3G52682 Mycgr3T
  
Location: 5664-9231
  
 NCBI BlastP on this gene

Mycgr3G52682\_Mycgr3T

Mycgr3G107072 Mycgr3
  
Location: 9331-13279
  
 NCBI BlastP on this gene

Mycgr3G107072\_Mycgr3

Mycgr3G34982 Mycgr3T
  
Location: 13379-15116
  
 NCBI BlastP on this gene

Mycgr3G34982\_Mycgr3T

Mycgr3G107069 Mycgr3
  
Location: 15216-17097
  
 NCBI BlastP on this gene

Mycgr3G107069\_Mycgr3

Mycgr3G32432 Mycgr3T
  
Location: 17197-19042
  
 NCBI BlastP on this gene

Mycgr3G32432\_Mycgr3T

Mycgr3G98385 Mycgr3T
  
Location: 19142-19898
  
 NCBI BlastP on this gene

Mycgr3G98385\_Mycgr3T

hydantoinase/oxoprolinase
  
Accession: GAA92506
  
Location: 85752-88810
  
 NCBI BlastP on this gene

GAA92506

sodium/phosphate symporter
  
Accession: GAA92507
  
Location: 89202-91156
  
 NCBI BlastP on this gene

GAA92507

hypothetical protein
  
Accession: GAA92508
  
Location: 91369-91694
  
 NCBI BlastP on this gene

GAA92508

amidase family protein
  
Accession: GAA92509
  
Location: 92266-94131
  
 NCBI BlastP on this gene

GAA92509

hypothetical protein
  
Accession: GAA92510
  
Location: 95540-96938
  
 NCBI BlastP on this gene

GAA92510

hybrid NRPS/PKS enzyme
  
Accession: GAA92511
  
Location: 99856-103686
  
  
**BlastP hit with Mycgr3G107072\_Mycgr3**
  
Percentage identity: 64 %
  
BlastP bit score: 1712
  
Sequence coverage: 100 %
  
E-value: 0.0
  
  
 NCBI BlastP on this gene

GAA92511

102. :  AACD01000093 Aspergillus nidulans FGSC A4     Total score: 1.0     Cumulative Blast bit score: 1705

predicted protein
  
Accession: EAA62473
  
Location: 150190-150685
  
 NCBI BlastP on this gene

EAA62473

hypothetical protein
  
Accession: EAA62474
  
Location: 153912-155547
  
 NCBI BlastP on this gene

EAA62474

predicted protein
  
Accession: EAA62475
  
Location: 156458-159074
  
 NCBI BlastP on this gene

EAA62475

predicted protein
  
Accession: EAA62476
  
Location: 159441-160143
  
 NCBI BlastP on this gene

EAA62476

hypothetical protein
  
Accession: EAA62477
  
Location: 160727-161650
  
 NCBI BlastP on this gene

EAA62477

hypothetical protein
  
Accession: EAA62478
  
Location: 164592-168404
  
  
**BlastP hit with Mycgr3G107072\_Mycgr3**
  
Percentage identity: 64 %
  
BlastP bit score: 1705
  
Sequence coverage: 100 %
  
E-value: 0.0
  
  
 NCBI BlastP on this gene

EAA62478

predicted protein
  
Accession: EAA62479
  
Location: 171837-172913
  
 NCBI BlastP on this gene

EAA62479

hypothetical protein
  
Accession: EAA62480
  
Location: 173388-175076
  
 NCBI BlastP on this gene

EAA62480

hypothetical protein
  
Accession: EAA62481
  
Location: 175181-177062
  
 NCBI BlastP on this gene

EAA62481

predicted protein
  
Accession: EAA62482
  
Location: 177262-177640
  
 NCBI BlastP on this gene

EAA62482

hypothetical protein
  
Accession: EAA62483
  
Location: 178783-180921
  
 NCBI BlastP on this gene

EAA62483

103. :  EQ963472 Aspergillus flavus NRRL3357 scf\_1106286418772 genomic scaffold     Total score: 1.0     Cumulative Blast bit score: 1704

dienelactone hydrolase, putative
  
Accession: EED57239
  
Location: 1886382-1887372
  
 NCBI BlastP on this gene

EED57239

conserved hypothetical protein
  
Accession: EED57240
  
Location: 1890073-1890807
  
 NCBI BlastP on this gene

EED57240

PKS-like enzyme, putative
  
Accession: EED57241
  
Location: 1892237-1892994
  
 NCBI BlastP on this gene

EED57241

hypothetical protein
  
Accession: EED57242
  
Location: 1894296-1894484
  
 NCBI BlastP on this gene

EED57242

NRPS-like enzyme, putative
  
Accession: EED57243
  
Location: 1894656-1897651
  
 NCBI BlastP on this gene

EED57243

hypothetical protein
  
Accession: EED57244
  
Location: 1898598-1899094
  
 NCBI BlastP on this gene

EED57244

NRPS-like enzyme, putative
  
Accession: EED57245
  
Location: 1901221-1905057
  
  
**BlastP hit with Mycgr3G107072\_Mycgr3**
  
Percentage identity: 65 %
  
BlastP bit score: 1704
  
Sequence coverage: 100 %
  
E-value: 0.0
  
  
 NCBI BlastP on this gene

EED57245

MFS transporter, putative
  
Accession: EED57246
  
Location: 1905565-1907524
  
 NCBI BlastP on this gene

EED57246

conserved hypothetical protein
  
Accession: EED57247
  
Location: 1907742-1908365
  
 NCBI BlastP on this gene

EED57247

amidase, putative
  
Accession: EED57248
  
Location: 1910291-1911075
  
 NCBI BlastP on this gene

EED57248

allantoate permease, putative
  
Accession: EED57249
  
Location: 1912113-1913531
  
 NCBI BlastP on this gene

EED57249

ureidoglycolate hydrolase, putative
  
Accession: EED57250
  
Location: 1913795-1914532
  
 NCBI BlastP on this gene

EED57250

DNA mismatch repair protein Msh1, putative
  
Accession: EED57251
  
Location: 1914711-1917746
  
 NCBI BlastP on this gene

EED57251

pre-mRNA splicing helicase, putative
  
Accession: EED57252
  
Location: 1918357-1924989
  
 NCBI BlastP on this gene

EED57252

104. :  AP007151 Aspergillus oryzae RIB40 DNA, SC005.     Total score: 1.0     Cumulative Blast bit score: 1703

not annotated
  
Accession: BAE55721
  
Location: 1812269-1813031
  
 NCBI BlastP on this gene

AO090005000687

not annotated
  
Accession: BAE55722
  
Location: 1814333-1817688
  
 NCBI BlastP on this gene

AO090005000688

not annotated
  
Accession: BAE55723
  
Location: 1818636-1819288
  
 NCBI BlastP on this gene

AO090005000689

not annotated
  
Accession: BAE55724
  
Location: 1821261-1825097
  
  
**BlastP hit with Mycgr3G107072\_Mycgr3**
  
Percentage identity: 65 %
  
BlastP bit score: 1703
  
Sequence coverage: 100 %
  
E-value: 0.0
  
  
 NCBI BlastP on this gene

AO090005000690

not annotated
  
Accession: BAE55725
  
Location: 1827787-1828410
  
 NCBI BlastP on this gene

AO090005000691

not annotated
  
Accession: BAE55726
  
Location: 1829266-1831255
  
 NCBI BlastP on this gene

AO090005000692

not annotated
  
Accession: BAE55727
  
Location: 1832167-1833585
  
 NCBI BlastP on this gene

AO090005000693

not annotated
  
Accession: BAE55728
  
Location: 1833850-1834587
  
 NCBI BlastP on this gene

AO090005000694

not annotated
  
Accession: BAE55729
  
Location: 1834766-1837801
  
 NCBI BlastP on this gene

AO090005000695

not annotated
  
Accession: BAE55730
  
Location: 1838412-1845044
  
 NCBI BlastP on this gene

AO090005000696

105. :  DS990638 Ajellomyces capsulatus H88 supercont1.3 genomic scaffold     Total score: 1.0     Cumulative Blast bit score: 1688

bZIP transcription factor
  
Accession: EGC44738
  
Location: 2323807-2326827
  
 NCBI BlastP on this gene

EGC44738

conserved hypothetical protein
  
Accession: EGC44737
  
Location: 2320159-2321674
  
 NCBI BlastP on this gene

EGC44737

conserved hypothetical protein
  
Accession: EGC44736
  
Location: 2318370-2319284
  
 NCBI BlastP on this gene

EGC44736

ATP synthase subunit 4
  
Accession: EGC44735
  
Location: 2317153-2318132
  
 NCBI BlastP on this gene

EGC44735

CBF/Mak21 family
  
Accession: EGC44734
  
Location: 2315100-2316893
  
 NCBI BlastP on this gene

EGC44734

conserved hypothetical protein
  
Accession: EGC44733
  
Location: 2313619-2314611
  
 NCBI BlastP on this gene

EGC44733

nonribosomal peptide synthetase
  
Accession: EGC44732
  
Location: 2307588-2311418
  
  
**BlastP hit with Mycgr3G107072\_Mycgr3**
  
Percentage identity: 64 %
  
BlastP bit score: 1688
  
Sequence coverage: 100 %
  
E-value: 0.0
  
  
 NCBI BlastP on this gene

EGC44732

predicted protein
  
Accession: EGC44731
  
Location: 2305770-2306498
  
 NCBI BlastP on this gene

EGC44731

oxidoreductase
  
Accession: EGC44730
  
Location: 2301960-2303005
  
 NCBI BlastP on this gene

EGC44730

conserved hypothetical protein
  
Accession: EGC44729
  
Location: 2298579-2299423
  
 NCBI BlastP on this gene

EGC44729

conserved hypothetical protein
  
Accession: EGC44728
  
Location: 2297108-2297531
  
 NCBI BlastP on this gene

EGC44728

extracellular matrix protein
  
Accession: EGC44727
  
Location: 2294502-2295299
  
 NCBI BlastP on this gene

EGC44727

106. :  DS231615 Pyrenophora tritici-repentis Pt-1C-BFP supercont1.1 genomic scaffold     Total score: 1.0     Cumulative Blast bit score: 1675

predicted protein
  
Accession: EDU39878
  
Location: 1171380-1171544
  
 NCBI BlastP on this gene

EDU39878

splicing factor 3a subunit 2
  
Accession: EDU39879
  
Location: 1173289-1174047
  
 NCBI BlastP on this gene

EDU39879

tubulin-specific chaperone E
  
Accession: EDU39880
  
Location: 1174612-1176403
  
 NCBI BlastP on this gene

EDU39880

hypothetical protein
  
Accession: EDU39881
  
Location: 1176447-1177473
  
 NCBI BlastP on this gene

EDU39881

c-myc binding protein
  
Accession: EDU39882
  
Location: 1177648-1178202
  
 NCBI BlastP on this gene

EDU39882

predicted protein
  
Accession: EDU39883
  
Location: 1178506-1180523
  
 NCBI BlastP on this gene

EDU39883

mitochondrial ribosomal protein subunit L23
  
Accession: EDU39884
  
Location: 1181378-1182091
  
 NCBI BlastP on this gene

EDU39884

tyrocidine synthetase 1
  
Accession: EDU39885
  
Location: 1184080-1187928
  
  
**BlastP hit with Mycgr3G107072\_Mycgr3**
  
Percentage identity: 63 %
  
BlastP bit score: 1675
  
Sequence coverage: 100 %
  
E-value: 0.0
  
  
 NCBI BlastP on this gene

EDU39885

107. :  KB644414 Penicillium oxalicum 114-2 unplaced genomic scaffold scaffold\_7     Total score: 1.0     Cumulative Blast bit score: 1665

hypothetical protein
  
Accession: EPS32406
  
Location: 2329530-2330172
  
 NCBI BlastP on this gene

EPS32406

hypothetical protein
  
Accession: EPS32407
  
Location: 2330664-2332388
  
 NCBI BlastP on this gene

EPS32407

hypothetical protein
  
Accession: EPS32408
  
Location: 2332945-2335027
  
 NCBI BlastP on this gene

EPS32408

hypothetical protein
  
Accession: EPS32409
  
Location: 2335899-2336219
  
 NCBI BlastP on this gene

EPS32409

hypothetical protein
  
Accession: EPS32410
  
Location: 2337112-2337831
  
 NCBI BlastP on this gene

EPS32410

hypothetical protein
  
Accession: EPS32411
  
Location: 2338462-2340785
  
 NCBI BlastP on this gene

EPS32411

hypothetical protein
  
Accession: EPS32412
  
Location: 2341213-2342406
  
 NCBI BlastP on this gene

EPS32412

hypothetical protein
  
Accession: EPS32413
  
Location: 2344043-2347876
  
  
**BlastP hit with Mycgr3G107072\_Mycgr3**
  
Percentage identity: 64 %
  
BlastP bit score: 1665
  
Sequence coverage: 100 %
  
E-value: 0.0
  
  
 NCBI BlastP on this gene

EPS32413

hypothetical protein
  
Accession: EPS32414
  
Location: 2350568-2350867
  
 NCBI BlastP on this gene

EPS32414

hypothetical protein
  
Accession: EPS32415
  
Location: 2351379-2352656
  
 NCBI BlastP on this gene

EPS32415

hypothetical protein
  
Accession: EPS32416
  
Location: 2354988-2355850
  
 NCBI BlastP on this gene

EPS32416

hypothetical protein
  
Accession: EPS32417
  
Location: 2358451-2358875
  
 NCBI BlastP on this gene

EPS32417

hypothetical protein
  
Accession: EPS32418
  
Location: 2360848-2363328
  
 NCBI BlastP on this gene

EPS32418

108. :  EQ962653 Talaromyces stipitatus ATCC 10500 scf\_1105507295527 genomic scaffold     Total score: 1.0     Cumulative Blast bit score: 1663

NRPS-like enzyme, putative
  
Accession: EED20986
  
Location: 475973-479177
  
 NCBI BlastP on this gene

EED20986

hypothetical protein
  
Accession: EED20985
  
Location: 473981-474277
  
 NCBI BlastP on this gene

EED20985

short-chain dehydrogenase, putative
  
Accession: EED20984
  
Location: 472336-473226
  
 NCBI BlastP on this gene

EED20984

hypothetical protein
  
Accession: EED20983
  
Location: 470334-471027
  
 NCBI BlastP on this gene

EED20983

amino acid transporter, putative
  
Accession: EED20982
  
Location: 467450-468920
  
 NCBI BlastP on this gene

EED20982

NRPS-like enzyme, putative
  
Accession: EED20981
  
Location: 460829-464674
  
  
**BlastP hit with Mycgr3G107072\_Mycgr3**
  
Percentage identity: 62 %
  
BlastP bit score: 1664
  
Sequence coverage: 100 %
  
E-value: 0.0
  
  
 NCBI BlastP on this gene

EED20981

conserved hypothetical protein
  
Accession: EED20980
  
Location: 459043-460450
  
 NCBI BlastP on this gene

EED20980

beta-xylosidase XylA
  
Accession: EED20979
  
Location: 456506-458899
  
 NCBI BlastP on this gene

EED20979

mucin-1 precursor, putative
  
Accession: EED20978
  
Location: 453058-455335
  
 NCBI BlastP on this gene

EED20978

hypothetical protein
  
Accession: EED20977
  
Location: 450993-452204
  
 NCBI BlastP on this gene

EED20977

hypothetical protein
  
Accession: EED20976
  
Location: 449291-450201
  
 NCBI BlastP on this gene

EED20976

109. :  DS995900 Penicillium marneffei ATCC 18224 scf\_1105668340758 genomic scaffold     Total score: 1.0     Cumulative Blast bit score: 1663

12-oxophytodienoate reductase, putative
  
Accession: EEA25142
  
Location: 241868-242909
  
 NCBI BlastP on this gene

EEA25142

12-oxophytodienoate reductase, putative
  
Accession: EEA25141
  
Location: 241504-242909
  
 NCBI BlastP on this gene

EEA25141

conserved hypothetical protein
  
Accession: EEA25140
  
Location: 238306-238827
  
 NCBI BlastP on this gene

EEA25140

short-chain dehydrogenase, putative
  
Accession: EEA25139
  
Location: 235856-236831
  
 NCBI BlastP on this gene

EEA25139

amino acid transporter, putative
  
Accession: EEA25138
  
Location: 232134-233605
  
 NCBI BlastP on this gene

EEA25138

NRPS-like enzyme, putative
  
Accession: EEA25137
  
Location: 227129-230968
  
  
**BlastP hit with Mycgr3G107072\_Mycgr3**
  
Percentage identity: 62 %
  
BlastP bit score: 1663
  
Sequence coverage: 100 %
  
E-value: 0.0
  
  
 NCBI BlastP on this gene

EEA25137

conserved hypothetical protein
  
Accession: EEA25136
  
Location: 225283-226690
  
 NCBI BlastP on this gene

EEA25136

beta-xylosidase XylA
  
Accession: EEA25135
  
Location: 222711-225110
  
 NCBI BlastP on this gene

EEA25135

dienelactone hydrolase family protein
  
Accession: EEA25134
  
Location: 220334-221185
  
 NCBI BlastP on this gene

EEA25134

extracellular exo-polygalacturonase, putative
  
Accession: EEA25131
  
Location: 218226-219610
  
 NCBI BlastP on this gene

EEA25131

conserved hypothetical protein
  
Accession: EEA25130
  
Location: 217199-217975
  
 NCBI BlastP on this gene

EEA25130

hypothetical protein
  
Accession: EEA25129
  
Location: 214782-215486
  
 NCBI BlastP on this gene

EEA25129

ammonium transporter, putative
  
Accession: EEA25127
  
Location: 212731-214581
  
 NCBI BlastP on this gene

EEA25127

110. :  KB733447 Bipolaris maydis ATCC 48331 unplaced genomic scaffold COCC4scaffold\_4     Total score: 1.0     Cumulative Blast bit score: 1661

hypothetical protein
  
Accession: ENI08913
  
Location: 1369227-1370809
  
 NCBI BlastP on this gene

ENI08913

hypothetical protein
  
Accession: ENI08914
  
Location: 1371908-1373208
  
 NCBI BlastP on this gene

ENI08914

hypothetical protein
  
Accession: ENI08915
  
Location: 1374944-1378034
  
 NCBI BlastP on this gene

ENI08915

hypothetical protein
  
Accession: ENI08916
  
Location: 1379319-1383167
  
  
**BlastP hit with Mycgr3G107072\_Mycgr3**
  
Percentage identity: 63 %
  
BlastP bit score: 1661
  
Sequence coverage: 100 %
  
E-value: 0.0
  
  
 NCBI BlastP on this gene

ENI08916

111. :  KB445576 Cochliobolus heterostrophus C5 unplaced genomic scaffold COCHEscaffold\_8     Total score: 1.0     Cumulative Blast bit score: 1661

hypothetical protein
  
Accession: EMD91330
  
Location: 261837-263419
  
 NCBI BlastP on this gene

EMD91330

hypothetical protein
  
Accession: EMD91329
  
Location: 259438-260738
  
 NCBI BlastP on this gene

EMD91329

hypothetical protein
  
Accession: EMD91328
  
Location: 254612-257702
  
 NCBI BlastP on this gene

EMD91328

hypothetical protein
  
Accession: EMD91327
  
Location: 249479-253327
  
  
**BlastP hit with Mycgr3G107072\_Mycgr3**
  
Percentage identity: 63 %
  
BlastP bit score: 1661
  
Sequence coverage: 100 %
  
E-value: 0.0
  
  
 NCBI BlastP on this gene

EMD91327

hypothetical protein
  
Accession: EMD91326
  
Location: 247978-248883
  
 NCBI BlastP on this gene

EMD91326

hypothetical protein
  
Accession: EMD91325
  
Location: 246711-247712
  
 NCBI BlastP on this gene

EMD91325

hypothetical protein
  
Accession: EMD91324
  
Location: 245190-246351
  
 NCBI BlastP on this gene

EMD91324

hypothetical protein
  
Accession: EMD91323
  
Location: 243382-244599
  
 NCBI BlastP on this gene

EMD91323

hypothetical protein
  
Accession: EMD91322
  
Location: 240529-242217
  
 NCBI BlastP on this gene

EMD91322

hypothetical protein
  
Accession: EMD91321
  
Location: 239356-240256
  
 NCBI BlastP on this gene

EMD91321

hypothetical protein
  
Accession: EMD91320
  
Location: 237537-238963
  
 NCBI BlastP on this gene

EMD91320

hypothetical protein
  
Accession: EMD91319
  
Location: 235040-236960
  
 NCBI BlastP on this gene

EMD91319

112. :  GG663364 Ajellomyces capsulatus G186AR genomic scaffold supercont2.2     Total score: 1.0     Cumulative Blast bit score: 1661

conserved hypothetical protein
  
Accession: EEH10266
  
Location: 2044401-2046728
  
 NCBI BlastP on this gene

EEH10266

conserved hypothetical protein
  
Accession: EEH10265
  
Location: 2040059-2041565
  
 NCBI BlastP on this gene

EEH10265

conserved hypothetical protein
  
Accession: EEH10264
  
Location: 2038120-2039034
  
 NCBI BlastP on this gene

EEH10264

ATP synthase subunit 4
  
Accession: EEH10263
  
Location: 2036901-2037880
  
 NCBI BlastP on this gene

EEH10263

CBF/Mak21 family
  
Accession: EEH10262
  
Location: 2034825-2036639
  
 NCBI BlastP on this gene

EEH10262

conserved hypothetical protein
  
Accession: EEH10261
  
Location: 2033345-2034337
  
 NCBI BlastP on this gene

EEH10261

nonribosomal peptide synthetase
  
Accession: EEH10260
  
Location: 2027391-2031221
  
  
**BlastP hit with Mycgr3G107072\_Mycgr3**
  
Percentage identity: 64 %
  
BlastP bit score: 1661
  
Sequence coverage: 100 %
  
E-value: 0.0
  
  
 NCBI BlastP on this gene

EEH10260

predicted protein
  
Accession: EEH10259
  
Location: 2025626-2026756
  
 NCBI BlastP on this gene

EEH10259

oxidoreductase
  
Accession: EEH10258
  
Location: 2021808-2022853
  
 NCBI BlastP on this gene

EEH10258

predicted protein
  
Accession: EEH10257
  
Location: 2018388-2019402
  
 NCBI BlastP on this gene

EEH10257

predicted protein
  
Accession: EEH10256
  
Location: 2016663-2017109
  
 NCBI BlastP on this gene

EEH10256

extracellular matrix protein
  
Accession: EEH10255
  
Location: 2014300-2015092
  
 NCBI BlastP on this gene

EEH10255

predicted protein
  
Accession: EEH10254
  
Location: 2013289-2014042
  
 NCBI BlastP on this gene

EEH10254

113. :  AM920433 Penicillium chrysogenum Wisconsin 54-1255 complete genome, contig Pc00c18.     Total score: 1.0     Cumulative Blast bit score: 1635

not annotated
  
Accession: CAP94257
  
Location: 74166-76767
  
 NCBI BlastP on this gene

Pc18g00330

not annotated
  
Accession: CAP94258
  
Location: 78530-81058
  
 NCBI BlastP on this gene

Pc18g00340

hypothetical protein
  
Accession: CAP94259
  
Location: 81990-82980
  
 NCBI BlastP on this gene

Pc18g00350

not annotated
  
Accession: CAP94260
  
Location: 86036-88019
  
 NCBI BlastP on this gene

Pc18g00360

hypothetical protein
  
Accession: CAP94261
  
Location: 88198-88757
  
 NCBI BlastP on this gene

Pc18g00370

not annotated
  
Accession: CAP94262
  
Location: 89193-93023
  
  
**BlastP hit with Mycgr3G107072\_Mycgr3**
  
Percentage identity: 63 %
  
BlastP bit score: 1635
  
Sequence coverage: 100 %
  
E-value: 0.0
  
  
 NCBI BlastP on this gene

Pc18g00380

unnamed
  
Accession: CAP94263
  
Location: 94040-95770
  
 NCBI BlastP on this gene

Pc18g00390

not annotated
  
Accession: CAP94264
  
Location: 97407-100685
  
 NCBI BlastP on this gene

Pc18g00400

not annotated
  
Accession: CAP94265
  
Location: 101599-102624
  
 NCBI BlastP on this gene

Pc18g00410

transcription factor pacC-Penicillium chrysogenum
  
Accession: CAP94266
  
Location: 103869-105856
  
 NCBI BlastP on this gene

pacC

114. :  CM001234 Magnaporthe oryzae 70-15 chromosome 4     Total score: 1.0     Cumulative Blast bit score: 1549

IBR domain-containing protein
  
Accession: EHA50348
  
Location: 2362178-2364688
  
 NCBI BlastP on this gene

EHA50348

hypothetical protein
  
Accession: EHA50349
  
Location: 2365176-2366261
  
 NCBI BlastP on this gene

EHA50349

hypothetical protein
  
Accession: EHA50350
  
Location: 2366847-2369294
  
 NCBI BlastP on this gene

EHA50350

SDA1 domain-containing protein
  
Accession: EHA50351
  
Location: 2370973-2373347
  
 NCBI BlastP on this gene

EHA50351

hypothetical protein
  
Accession: EHA50352
  
Location: 2374972-2375246
  
 NCBI BlastP on this gene

EHA50352

N-(5-amino-5-carboxypentanoyl)-L-cysteinyl-D- valine synthase
  
Accession: EHA50353
  
Location: 2376126-2379968
  
  
**BlastP hit with Mycgr3G107072\_Mycgr3**
  
Percentage identity: 59 %
  
BlastP bit score: 1549
  
Sequence coverage: 100 %
  
E-value: 0.0
  
  
 NCBI BlastP on this gene

EHA50353

115. :  KB725930 Colletotrichum orbiculare MAFF 240422 unplaced genomic scaffold Scaffold\_366     Total score: 1.0     Cumulative Blast bit score: 1518

WD domain-containing protein
  
Accession: ENH82697
  
Location: 1492394-1494400
  
 NCBI BlastP on this gene

ENH82697

stress protein ddr48-like protein
  
Accession: ENH82698
  
Location: 1494991-1497313
  
 NCBI BlastP on this gene

ENH82698

phospholipase carboxylesterase superfamily
  
Accession: ENH82699
  
Location: 1497712-1498494
  
 NCBI BlastP on this gene

ENH82699

small secreted protein
  
Accession: ENH82700
  
Location: 1499989-1500908
  
 NCBI BlastP on this gene

ENH82700

hypothetical protein
  
Accession: ENH82701
  
Location: 1503050-1503490
  
 NCBI BlastP on this gene

ENH82701

lccl domain containing protein
  
Accession: ENH82702
  
Location: 1503707-1505692
  
 NCBI BlastP on this gene

ENH82702

nonribosomal peptide synthetase 10
  
Accession: ENH82703
  
Location: 1507531-1511370
  
  
**BlastP hit with Mycgr3G107072\_Mycgr3**
  
Percentage identity: 58 %
  
BlastP bit score: 1518
  
Sequence coverage: 100 %
  
E-value: 0.0
  
  
 NCBI BlastP on this gene

ENH82703

116. :  DS985216 Verticillium albo-atrum VaMs.102 supercont1.3 genomic scaffold     Total score: 1.0     Cumulative Blast bit score: 1503

integral membrane protein
  
Accession: EEY16457
  
Location: 155408-157295
  
 NCBI BlastP on this gene

EEY16457

cryptochrome-1
  
Accession: EEY16458
  
Location: 158346-160390
  
 NCBI BlastP on this gene

EEY16458

veA protein
  
Accession: EEY16459
  
Location: 162732-163651
  
 NCBI BlastP on this gene

EEY16459

enterobactin synthetase component F
  
Accession: EEY16460
  
Location: 170075-173929
  
  
**BlastP hit with Mycgr3G107072\_Mycgr3**
  
Percentage identity: 58 %
  
BlastP bit score: 1503
  
Sequence coverage: 100 %
  
E-value: 0.0
  
  
 NCBI BlastP on this gene

EEY16460

conserved hypothetical protein
  
Accession: EEY16461
  
Location: 177680-178561
  
 NCBI BlastP on this gene

EEY16461

monooxygenase
  
Accession: EEY16462
  
Location: 183082-184226
  
 NCBI BlastP on this gene

EEY16462

multidrug resistance protein
  
Accession: EEY16463
  
Location: 186246-190312
  
 NCBI BlastP on this gene

EEY16463

117. :  DS572721 Verticillium dahliae VdLs.17 supercont1.27 genomic scaffold     Total score: 1.0     Cumulative Blast bit score: 1501

integral membrane protein
  
Accession: EGY19299
  
Location: 156448-158313
  
 NCBI BlastP on this gene

EGY19299

cryptochrome-1
  
Accession: EGY19300
  
Location: 159383-161430
  
 NCBI BlastP on this gene

EGY19300

VosA
  
Accession: EGY19301
  
Location: 163241-164084
  
 NCBI BlastP on this gene

EGY19301

hypothetical protein
  
Accession: EGY19302
  
Location: 169160-169387
  
 NCBI BlastP on this gene

EGY19302

enterobactin synthetase component F
  
Accession: EGY19303
  
Location: 170836-174690
  
  
**BlastP hit with Mycgr3G107072\_Mycgr3**
  
Percentage identity: 58 %
  
BlastP bit score: 1501
  
Sequence coverage: 100 %
  
E-value: 0.0
  
  
 NCBI BlastP on this gene

EGY19303

118. :  HF679025 Fusarium fujikuroi IMI 58289 draft genome, chromosome FFUJ\_chr03.     Total score: 1.0     Cumulative Blast bit score: 1500

related to alpha-aminoadipate reductase large subunit
  
Accession: CCT66472
  
Location: 4537841-4541695
  
  
**BlastP hit with Mycgr3G107072\_Mycgr3**
  
Percentage identity: 57 %
  
BlastP bit score: 1500
  
Sequence coverage: 100 %
  
E-value: 0.0
  
  
 NCBI BlastP on this gene

FFUJ\_03506

uncharacterized protein
  
Accession: CCT66471
  
Location: 4534907-4536163
  
 NCBI BlastP on this gene

FFUJ\_03505

related to methyltransferase
  
Accession: CCT66470
  
Location: 4532643-4533731
  
 NCBI BlastP on this gene

FFUJ\_03504

uncharacterized protein
  
Accession: CCT66469
  
Location: 4530500-4531313
  
 NCBI BlastP on this gene

FFUJ\_03503

related to protein involved in authophagy (APG17)
  
Accession: CCT66468
  
Location: 4528232-4529829
  
 NCBI BlastP on this gene

FFUJ\_03502

related to calpain-like protein
  
Accession: CCT66467
  
Location: 4524509-4527462
  
 NCBI BlastP on this gene

FFUJ\_03501

119. :  ABDF02000092 Trichoderma virens Gv29-8     Total score: 1.0     Cumulative Blast bit score: 1474

hypothetical protein
  
Accession: EHK15303
  
Location: 254901-256454
  
 NCBI BlastP on this gene

EHK15303

hypothetical protein
  
Accession: EHK15304
  
Location: 258006-259229
  
 NCBI BlastP on this gene

EHK15304

hypothetical protein
  
Accession: EHK15305
  
Location: 259548-260884
  
 NCBI BlastP on this gene

EHK15305

hypothetical protein
  
Accession: EHK15306
  
Location: 265690-267276
  
 NCBI BlastP on this gene

EHK15306

non-ribosomal peptide synthetase
  
Accession: EHK15307
  
Location: 268628-272479
  
  
**BlastP hit with Mycgr3G107072\_Mycgr3**
  
Percentage identity: 58 %
  
BlastP bit score: 1474
  
Sequence coverage: 100 %
  
E-value: 0.0
  
  
 NCBI BlastP on this gene

EHK15307

hypothetical protein
  
Accession: EHK15308
  
Location: 273906-275859
  
 NCBI BlastP on this gene

EHK15308

hypothetical protein
  
Accession: EHK15309
  
Location: 276048-277775
  
 NCBI BlastP on this gene

EHK15309

hypothetical protein
  
Accession: EHK15310
  
Location: 278437-282408
  
 NCBI BlastP on this gene

EHK15310

hypothetical protein
  
Accession: EHK15311
  
Location: 282901-285655
  
 NCBI BlastP on this gene

EHK15311

hypothetical protein
  
Accession: EHK15312
  
Location: 286257-288004
  
 NCBI BlastP on this gene

EHK15312

120. :  ABDG02000027 Trichoderma atroviride IMI 206040     Total score: 1.0     Cumulative Blast bit score: 1456

non-ribosomal peptide synthetase
  
Accession: EHK41519
  
Location: 3289356-3293213
  
  
**BlastP hit with Mycgr3G107072\_Mycgr3**
  
Percentage identity: 57 %
  
BlastP bit score: 1456
  
Sequence coverage: 100 %
  
E-value: 0.0
  
  
 NCBI BlastP on this gene

EHK41519

hypothetical protein
  
Accession: EHK41518
  
Location: 3286231-3287918
  
 NCBI BlastP on this gene

EHK41518

hypothetical protein
  
Accession: EHK41517
  
Location: 3281497-3285565
  
 NCBI BlastP on this gene

EHK41517

hypothetical protein
  
Accession: EHK41516
  
Location: 3278335-3281071
  
 NCBI BlastP on this gene

EHK41516

hypothetical protein
  
Accession: EHK41515
  
Location: 3275927-3277662
  
 NCBI BlastP on this gene

EHK41515

121. :  DF196785 Pseudozyma antarctica T-34 DNA, contig: scaffold00019     Total score: 1.0     Cumulative Blast bit score: 1434

hypothetical protein
  
Accession: GAC75904
  
Location: 48895-52149
  
 NCBI BlastP on this gene

GAC75904

hypothetical protein
  
Accession: GAC75903
  
Location: 47888-48370
  
 NCBI BlastP on this gene

GAC75903

triosephosphate isomerase
  
Accession: GAC75902
  
Location: 46282-47115
  
 NCBI BlastP on this gene

GAC75902

hypothetical protein
  
Accession: GAC75901
  
Location: 45336-46100
  
 NCBI BlastP on this gene

GAC75901

uncharacterized conserved protein
  
Accession: GAC75900
  
Location: 44122-45133
  
 NCBI BlastP on this gene

GAC75900

hypothetical protein
  
Accession: GAC75899
  
Location: 39715-44038
  
 NCBI BlastP on this gene

GAC75899

RNA polymerase subunit K
  
Accession: GAC75898
  
Location: 37059-39446
  
 NCBI BlastP on this gene

GAC75898

non-ribosomal peptide synthetase
  
Accession: GAC75897
  
Location: 31970-35968
  
  
**BlastP hit with Mycgr3G107072\_Mycgr3**
  
Percentage identity: 55 %
  
BlastP bit score: 1434
  
Sequence coverage: 102 %
  
E-value: 0.0
  
  
 NCBI BlastP on this gene

GAC75897

transport protein Sec61, alpha subunit
  
Accession: GAC75896
  
Location: 30911-31864
  
 NCBI BlastP on this gene

GAC75896

Fe2+/Zn2+ regulated transporter
  
Accession: GAC75895
  
Location: 28690-30153
  
 NCBI BlastP on this gene

GAC75895

hypothetical protein
  
Accession: GAC75894
  
Location: 26594-28612
  
 NCBI BlastP on this gene

GAC75894

hypothetical protein
  
Accession: GAC75893
  
Location: 24266-25498
  
 NCBI BlastP on this gene

GAC75893

hypothetical protein
  
Accession: GAC75892
  
Location: 17921-23870
  
 NCBI BlastP on this gene

GAC75892

122. :  FQ311472 Sporisorium reilianum SRZ2 chromosome 7 complete DNA sequence.     Total score: 1.0     Cumulative Blast bit score: 1394

conserved hypothetical protein
  
Accession: CBQ73506
  
Location: 836225-839515
  
 NCBI BlastP on this gene

sr14163

probable ribose-5-phosphate isomerase
  
Accession: CBQ73507
  
Location: 840093-840578
  
 NCBI BlastP on this gene

sr14164

related to triose-phosphate isomerase
  
Accession: CBQ73508
  
Location: 841576-842415
  
 NCBI BlastP on this gene

sr14165

conserved hypothetical protein
  
Accession: CBQ73509
  
Location: 842577-843187
  
 NCBI BlastP on this gene

sr14166

conserved hypothetical protein
  
Accession: CBQ73510
  
Location: 843638-844691
  
 NCBI BlastP on this gene

sr14167

conserved hypothetical protein
  
Accession: CBQ73511
  
Location: 845597-849456
  
 NCBI BlastP on this gene

sr14168

conserved hypothetical protein
  
Accession: CBQ73512
  
Location: 849740-852085
  
 NCBI BlastP on this gene

sr14169

related to Aminoadipate-semialdehyde dehydrogenase
  
Accession: CBQ73513
  
Location: 852961-856890
  
  
**BlastP hit with Mycgr3G107072\_Mycgr3**
  
Percentage identity: 53 %
  
BlastP bit score: 1394
  
Sequence coverage: 102 %
  
E-value: 0.0
  
  
 NCBI BlastP on this gene

sr14170

123. :  KE148153 Ophiostoma piceae UAMH 11346 chromosome Unknown scf08     Total score: 1.0     Cumulative Blast bit score: 1215

u-box domain-containing protein
  
Accession: EPE06498
  
Location: 977002-977893
  
 NCBI BlastP on this gene

EPE06498

hypothetical protein
  
Accession: EPE06499
  
Location: 978183-979921
  
 NCBI BlastP on this gene

EPE06499

non-imprinted in prader-willi angelman syndrome region protein 2
  
Accession: EPE06500
  
Location: 980949-983336
  
 NCBI BlastP on this gene

EPE06500

nonribosomal peptide synthetase 10
  
Accession: EPE06501
  
Location: 991797-995894
  
  
**BlastP hit with Mycgr3G107072\_Mycgr3**
  
Percentage identity: 50 %
  
BlastP bit score: 1215
  
Sequence coverage: 106 %
  
E-value: 0.0
  
  
 NCBI BlastP on this gene

EPE06501

124. :  KB310677 Capitella teleta unplaced genomic scaffold CAPTEscaffold\_748     Total score: 1.0     Cumulative Blast bit score: 1171

hypothetical protein
  
Accession: ELT91031
  
Location: 24875-31914
  
  
**BlastP hit with Mycgr3G107072\_Mycgr3**
  
Percentage identity: 36 %
  
BlastP bit score: 724
  
Sequence coverage: 101 %
  
E-value: 0.0
  
  
 NCBI BlastP on this gene

ELT91031

hypothetical protein
  
Accession: ELT91030
  
Location: 19105-22341
  
 NCBI BlastP on this gene

ELT91030

hypothetical protein
  
Accession: ELT91029
  
Location: 13911-15737
  
 NCBI BlastP on this gene

ELT91029

hypothetical protein
  
Accession: ELT91028
  
Location: 12442-13587
  
 NCBI BlastP on this gene

ELT91028

hypothetical protein
  
Accession: ELT91027
  
Location: 4631-9906
  
  
**BlastP hit with Mycgr3G107072\_Mycgr3**
  
Percentage identity: 32 %
  
BlastP bit score: 447
  
Sequence coverage: 74 %
  
E-value: 9e-134
  
  
 NCBI BlastP on this gene

ELT91027

125. :  DS572752 Paracoccidioides brasiliensis Pb18 supercont1.3 genomic scaffold     Total score: 1.0     Cumulative Blast bit score: 1068

predicted protein
  
Accession: EEH46484
  
Location: 572342-572633
  
 NCBI BlastP on this gene

EEH46484

conserved hypothetical protein
  
Accession: EEH46483
  
Location: 570339-571860
  
 NCBI BlastP on this gene

EEH46483

predicted protein
  
Accession: EEH46482
  
Location: 569318-569806
  
 NCBI BlastP on this gene

EEH46482

conserved hypothetical protein
  
Accession: EEH46481
  
Location: 568068-568997
  
 NCBI BlastP on this gene

EEH46481

ATP synthase subunit 4
  
Accession: EEH46480
  
Location: 566899-567868
  
 NCBI BlastP on this gene

EEH46480

CBF/Mak21 family protein
  
Accession: EEH46479
  
Location: 564840-566656
  
 NCBI BlastP on this gene

EEH46479

conserved hypothetical protein
  
Accession: EEH46478
  
Location: 563292-564305
  
 NCBI BlastP on this gene

EEH46478

polyketide synthase hetM
  
Accession: EEH46477
  
Location: 557234-561063
  
  
**BlastP hit with Mycgr3G107072\_Mycgr3**
  
Percentage identity: 61 %
  
BlastP bit score: 1068
  
Sequence coverage: 67 %
  
E-value: 0.0
  
  
 NCBI BlastP on this gene

EEH46477

predicted protein
  
Accession: EEH46476
  
Location: 555411-556562
  
 NCBI BlastP on this gene

EEH46476

conserved hypothetical protein
  
Accession: EEH46475
  
Location: 554020-555161
  
 NCBI BlastP on this gene

EEH46475

predicted protein
  
Accession: EEH46474
  
Location: 552571-553781
  
 NCBI BlastP on this gene

EEH46474

predicted protein
  
Accession: EEH46473
  
Location: 549897-552421
  
 NCBI BlastP on this gene

EEH46473

predicted protein
  
Accession: EEH46472
  
Location: 548094-549339
  
 NCBI BlastP on this gene

EEH46472

hypothetical protein
  
Accession: EEH46471
  
Location: 546913-547687
  
 NCBI BlastP on this gene

EEH46471

conserved hypothetical protein
  
Accession: EEH46470
  
Location: 543221-544424
  
 NCBI BlastP on this gene

EEH46470

126. :  JH767588 Coniosporium apollinis CBS 100218 chromosome Unknown supercont1.35     Total score: 1.0     Cumulative Blast bit score: 1009

hypothetical protein
  
Accession: EON67607
  
Location: 306691-307722
  
 NCBI BlastP on this gene

EON67607

hypothetical protein
  
Accession: EON67608
  
Location: 308950-309663
  
 NCBI BlastP on this gene

EON67608

hypothetical protein
  
Accession: EON67609
  
Location: 311565-312140
  
 NCBI BlastP on this gene

EON67609

hypothetical protein
  
Accession: EON67610
  
Location: 314154-317231
  
 NCBI BlastP on this gene

EON67610

hypothetical protein
  
Accession: EON67611
  
Location: 319536-320305
  
 NCBI BlastP on this gene

EON67611

hypothetical protein
  
Accession: EON67612
  
Location: 321636-325454
  
  
**BlastP hit with Mycgr3G52682\_Mycgr3T**
  
Percentage identity: 56 %
  
BlastP bit score: 1009
  
Sequence coverage: 78 %
  
E-value: 0.0
  
  
 NCBI BlastP on this gene

EON67612

127. :  GL985056 Trichoderma reesei QM6a unplaced genomic scaffold TRIREscaffold\_1     Total score: 1.0     Cumulative Blast bit score: 910

hypothetical protein
  
Accession: EGR52829
  
Location: 1519162-1520393
  
 NCBI BlastP on this gene

EGR52829

predicted protein
  
Accession: EGR52306
  
Location: 1517768-1518787
  
 NCBI BlastP on this gene

EGR52306

predicted protein
  
Accession: EGR52828
  
Location: 1514592-1516554
  
 NCBI BlastP on this gene

EGR52828

predicted protein
  
Accession: EGR52827
  
Location: 1511324-1513991
  
 NCBI BlastP on this gene

EGR52827

predicted protein
  
Accession: EGR52305
  
Location: 1509679-1510809
  
 NCBI BlastP on this gene

EGR52305

dihydrolipoyllysine-residue acetyltransferase-like protein
  
Accession: EGR52826
  
Location: 1506237-1507614
  
 NCBI BlastP on this gene

EGR52826

predicted protein
  
Accession: EGR52825
  
Location: 1501361-1504994
  
  
**BlastP hit with Mycgr3G52682\_Mycgr3T**
  
Percentage identity: 44 %
  
BlastP bit score: 910
  
Sequence coverage: 98 %
  
E-value: 0.0
  
  
 NCBI BlastP on this gene

EGR52825

predicted protein
  
Accession: EGR52304
  
Location: 1500478-1500978
  
 NCBI BlastP on this gene

EGR52304

predicted protein
  
Accession: EGR52824
  
Location: 1497445-1499682
  
 NCBI BlastP on this gene

EGR52824

predicted protein
  
Accession: EGR52823
  
Location: 1496157-1496768
  
 NCBI BlastP on this gene

EGR52823

predicted protein
  
Accession: EGR52303
  
Location: 1495331-1495789
  
 NCBI BlastP on this gene

EGR52303

vesicle fusion protein
  
Accession: EGR52822
  
Location: 1493399-1494656
  
 NCBI BlastP on this gene

EGR52822

predicted protein
  
Accession: EGR52302
  
Location: 1490666-1492814
  
 NCBI BlastP on this gene

EGR52302

predicted protein
  
Accession: EGR52821
  
Location: 1485729-1487768
  
 NCBI BlastP on this gene

EGR52821

128. :  KB446555 Pseudocercospora fijiensis CIRAD86 unplaced genomic scaffold MYCFIscaffold\_1     Total score: 1.0     Cumulative Blast bit score: 886

hypothetical protein
  
Accession: EME89630
  
Location: 11163995-11165942
  
  
**BlastP hit with Mycgr3G107069\_Mycgr3**
  
Percentage identity: 68 %
  
BlastP bit score: 886
  
Sequence coverage: 99 %
  
E-value: 0.0
  
  
 NCBI BlastP on this gene

EME89630

hypothetical protein
  
Accession: EME89629
  
Location: 11160690-11163097
  
 NCBI BlastP on this gene

EME89629

hypothetical protein
  
Accession: EME89628
  
Location: 11158814-11160364
  
 NCBI BlastP on this gene

EME89628

hypothetical protein
  
Accession: EME89627
  
Location: 11156449-11158166
  
 NCBI BlastP on this gene

EME89627

hypothetical protein
  
Accession: EME89626
  
Location: 11154512-11155099
  
 NCBI BlastP on this gene

EME89626

hypothetical protein
  
Accession: EME89625
  
Location: 11153146-11154000
  
 NCBI BlastP on this gene

EME89625

129. :  GG698898 Nectria haematococca mpVI 77-13-4 chromosome 4 genomic scaffold NECHAsca\_3\_chr4\_2\_0     Total score: 1.0     Cumulative Blast bit score: 878

hypothetical protein
  
Accession: EEU45840
  
Location: 521034-524980
  
 NCBI BlastP on this gene

EEU45840

hypothetical protein
  
Accession: EEU46298
  
Location: 519518-520023
  
 NCBI BlastP on this gene

EEU46298

predicted protein
  
Accession: EEU46297
  
Location: 517784-518385
  
 NCBI BlastP on this gene

EEU46297

predicted protein
  
Accession: EEU45839
  
Location: 516576-517421
  
 NCBI BlastP on this gene

EEU45839

hypothetical protein
  
Accession: EEU46296
  
Location: 515677-516241
  
 NCBI BlastP on this gene

EEU46296

hypothetical protein
  
Accession: EEU46295
  
Location: 511101-512913
  
 NCBI BlastP on this gene

EEU46295

hypothetical protein
  
Accession: EEU46294
  
Location: 509032-509959
  
 NCBI BlastP on this gene

EEU46294

hypothetical protein
  
Accession: EEU46293
  
Location: 503226-506977
  
  
**BlastP hit with Mycgr3G52682\_Mycgr3T**
  
Percentage identity: 43 %
  
BlastP bit score: 879
  
Sequence coverage: 98 %
  
E-value: 0.0
  
  
 NCBI BlastP on this gene

EEU46293

hypothetical protein
  
Accession: EEU45838
  
Location: 502051-502827
  
 NCBI BlastP on this gene

EEU45838

predicted protein
  
Accession: EEU46292
  
Location: 499500-501753
  
 NCBI BlastP on this gene

EEU46292

expressed protein
  
Accession: EEU46291
  
Location: 498666-499102
  
 NCBI BlastP on this gene

EEU46291

hypothetical protein
  
Accession: EEU45837
  
Location: 497769-498353
  
 NCBI BlastP on this gene

EEU45837

predicted protein
  
Accession: EEU46290
  
Location: 496292-497137
  
 NCBI BlastP on this gene

EEU46290

hypothetical protein
  
Accession: EEU45836
  
Location: 493425-495591
  
 NCBI BlastP on this gene

EEU45836

hypothetical protein
  
Accession: EEU46289
  
Location: 489480-491489
  
 NCBI BlastP on this gene

EEU46289

130. :  KB730248 Fusarium oxysporum f. sp. cubense race 1 unplaced genomic scaffold scaffold101     Total score: 1.0     Cumulative Blast bit score: 877

Protein transport protein SEC31
  
Accession: ENH68404
  
Location: 503704-507644
  
 NCBI BlastP on this gene

ENH68404

Peptidyl-prolyl cis-trans isomerase E
  
Accession: ENH68403
  
Location: 502072-502554
  
 NCBI BlastP on this gene

ENH68403

hypothetical protein
  
Accession: ENH68402
  
Location: 501281-501823
  
 NCBI BlastP on this gene

ENH68402

Cytochrome c oxidase subunit 6B
  
Accession: ENH68401
  
Location: 500267-500886
  
 NCBI BlastP on this gene

ENH68401

UPF0667 family protein C31G5.18c
  
Accession: ENH68400
  
Location: 499059-499907
  
 NCBI BlastP on this gene

ENH68400

40S ribosomal protein S10-A
  
Accession: ENH68399
  
Location: 498029-498695
  
 NCBI BlastP on this gene

ENH68399

hypothetical protein
  
Accession: ENH68398
  
Location: 495177-496090
  
 NCBI BlastP on this gene

ENH68398

Putative E3 ubiquitin-protein ligase mug30
  
Accession: ENH68397
  
Location: 489831-493572
  
  
**BlastP hit with Mycgr3G52682\_Mycgr3T**
  
Percentage identity: 42 %
  
BlastP bit score: 877
  
Sequence coverage: 100 %
  
E-value: 0.0
  
  
 NCBI BlastP on this gene

ENH68397

Putative 60S ribosomal protein MRP49, mitochondrial
  
Accession: ENH68396
  
Location: 488697-489474
  
 NCBI BlastP on this gene

ENH68396

hypothetical protein
  
Accession: ENH68395
  
Location: 486157-488363
  
 NCBI BlastP on this gene

ENH68395

Type 1 phosphatases regulator ypi-1
  
Accession: ENH68394
  
Location: 485000-485527
  
 NCBI BlastP on this gene

ENH68394

hypothetical protein
  
Accession: ENH68393
  
Location: 484162-484734
  
 NCBI BlastP on this gene

ENH68393

Putative vesicular-fusion protein sec17 like protein
  
Accession: ENH68392
  
Location: 482705-483772
  
 NCBI BlastP on this gene

ENH68392

Glucoamylase
  
Accession: ENH68391
  
Location: 479866-482053
  
 NCBI BlastP on this gene

ENH68391

U4/U6.U5 tri-snRNP-associated protein snu66
  
Accession: ENH68390
  
Location: 476009-478003
  
 NCBI BlastP on this gene

ENH68390

131. :  KB726554 Fusarium oxysporum f. sp. cubense race 4 unplaced genomic scaffold scaffold44     Total score: 1.0     Cumulative Blast bit score: 872

Protein transport protein SEC31
  
Accession: EMT68536
  
Location: 1968745-1972685
  
 NCBI BlastP on this gene

EMT68536

Peptidyl-prolyl cis-trans isomerase E
  
Accession: EMT68537
  
Location: 1973834-1974316
  
 NCBI BlastP on this gene

EMT68537

hypothetical protein
  
Accession: EMT68538
  
Location: 1974565-1975119
  
 NCBI BlastP on this gene

EMT68538

Cytochrome c oxidase subunit 6B
  
Accession: EMT68539
  
Location: 1975514-1976133
  
 NCBI BlastP on this gene

EMT68539

UPF0667 family protein C31G5.18c
  
Accession: EMT68540
  
Location: 1976493-1977341
  
 NCBI BlastP on this gene

EMT68540

40S ribosomal protein S10-A
  
Accession: EMT68541
  
Location: 1977705-1978371
  
 NCBI BlastP on this gene

EMT68541

hypothetical protein
  
Accession: EMT68542
  
Location: 1980309-1981222
  
 NCBI BlastP on this gene

EMT68542

hypothetical protein
  
Accession: EMT68543
  
Location: 1982404-1983317
  
 NCBI BlastP on this gene

EMT68543

Putative E3 ubiquitin-protein ligase mug30
  
Accession: EMT68544
  
Location: 1984908-1988641
  
  
**BlastP hit with Mycgr3G52682\_Mycgr3T**
  
Percentage identity: 42 %
  
BlastP bit score: 872
  
Sequence coverage: 100 %
  
E-value: 0.0
  
  
 NCBI BlastP on this gene

EMT68544

132. :  HF679026 Fusarium fujikuroi IMI 58289 draft genome, chromosome FFUJ\_chr04.     Total score: 1.0     Cumulative Blast bit score: 867

related to SEC31 protein
  
Accession: CCT67297
  
Location: 1602093-1606027
  
 NCBI BlastP on this gene

FFUJ\_13498

related to cyclophilin
  
Accession: CCT67298
  
Location: 1606914-1607396
  
 NCBI BlastP on this gene

FFUJ\_13499

uncharacterized protein
  
Accession: CCT67299
  
Location: 1607645-1608199
  
 NCBI BlastP on this gene

FFUJ\_13500

probable COX12-cytochrome-c oxidase, subunit VIB
  
Accession: CCT67300
  
Location: 1608603-1609219
  
 NCBI BlastP on this gene

FFUJ\_13501

uncharacterized protein
  
Accession: CCT67301
  
Location: 1609574-1610422
  
 NCBI BlastP on this gene

FFUJ\_13502

probable 40s ribosomal protein s10-b
  
Accession: CCT67302
  
Location: 1610799-1611467
  
 NCBI BlastP on this gene

FFUJ\_13503

uncharacterized protein
  
Accession: CCT67303
  
Location: 1613541-1614460
  
 NCBI BlastP on this gene

FFUJ\_13504

related to ubiquitin-protein ligase HUL4
  
Accession: CCT67304
  
Location: 1616188-1619914
  
  
**BlastP hit with Mycgr3G52682\_Mycgr3T**
  
Percentage identity: 42 %
  
BlastP bit score: 867
  
Sequence coverage: 99 %
  
E-value: 0.0
  
  
 NCBI BlastP on this gene

FFUJ\_13505

related to ribosomal protein MRP49
  
Accession: CCT67535
  
Location: 1620279-1621055
  
 NCBI BlastP on this gene

FFUJ\_13506

uncharacterized protein
  
Accession: CCT67305
  
Location: 1621380-1623586
  
 NCBI BlastP on this gene

FFUJ\_13507

related to Type 1 phosphatases regulator ypi-1
  
Accession: CCT67306
  
Location: 1624242-1624769
  
 NCBI BlastP on this gene

FFUJ\_13508

related to a-agglutinin core protein AGA1
  
Accession: CCT67307
  
Location: 1625036-1625608
  
 NCBI BlastP on this gene

FFUJ\_13509

probable transport vesicle fusion protein SEC17
  
Accession: CCT67308
  
Location: 1626025-1627092
  
 NCBI BlastP on this gene

FFUJ\_13510

probable glucan 1,4-alpha-glucosidase
  
Accession: CCT67309
  
Location: 1627724-1629992
  
 NCBI BlastP on this gene

FFUJ\_13511

related to DNA binding protein SART-1
  
Accession: CCT67310
  
Location: 1631877-1633871
  
 NCBI BlastP on this gene

FFUJ\_13512

133. :  ABDF02000004 Trichoderma virens Gv29-8     Total score: 1.0     Cumulative Blast bit score: 865

hypothetical protein
  
Accession: EHK24451
  
Location: 796394-800034
  
  
**BlastP hit with Mycgr3G52682\_Mycgr3T**
  
Percentage identity: 43 %
  
BlastP bit score: 866
  
Sequence coverage: 99 %
  
E-value: 0.0
  
  
 NCBI BlastP on this gene

EHK24451

hypothetical protein
  
Accession: EHK24450
  
Location: 795572-796051
  
 NCBI BlastP on this gene

EHK24450

hypothetical protein
  
Accession: EHK24449
  
Location: 792677-794896
  
 NCBI BlastP on this gene

EHK24449

hypothetical protein
  
Accession: EHK24448
  
Location: 791542-792142
  
 NCBI BlastP on this gene

EHK24448

hypothetical protein
  
Accession: EHK24447
  
Location: 790653-791231
  
 NCBI BlastP on this gene

EHK24447

hypothetical protein
  
Accession: EHK24446
  
Location: 789064-790209
  
 NCBI BlastP on this gene

EHK24446

glycoside hydrolase family 15 protein
  
Accession: EHK25059
  
Location: 786393-788541
  
 NCBI BlastP on this gene

EHK25059

hypothetical protein
  
Accession: EHK24445
  
Location: 781828-783884
  
 NCBI BlastP on this gene

EHK24445

134. :  JH226131 Exophiala dermatitidis NIH/UT8656 unplaced genomic scaffold supercont1.2     Total score: 1.0     Cumulative Blast bit score: 863

other hect domain ubiquitin protein ligase E3
  
Accession: EHY53349
  
Location: 99189-102809
  
  
**BlastP hit with Mycgr3G52682\_Mycgr3T**
  
Percentage identity: 43 %
  
BlastP bit score: 863
  
Sequence coverage: 102 %
  
E-value: 0.0
  
  
 NCBI BlastP on this gene

EHY53349

alcohol dehydrogenase, zinc-containing
  
Accession: EHY53350
  
Location: 104525-105819
  
 NCBI BlastP on this gene

EHY53350

hypothetical protein
  
Accession: EHY53351
  
Location: 107144-108004
  
 NCBI BlastP on this gene

EHY53351

40S ribosomal protein S8-B
  
Accession: EHY53352
  
Location: 108947-109673
  
 NCBI BlastP on this gene

EHY53352

30S ribosomal protein S10e
  
Accession: EHY53353
  
Location: 110257-110894
  
 NCBI BlastP on this gene

EHY53353

hypothetical protein
  
Accession: EHY53354
  
Location: 111916-112264
  
 NCBI BlastP on this gene

EHY53354

sulfite oxidase
  
Accession: EHY53355
  
Location: 113158-114350
  
 NCBI BlastP on this gene

EHY53355

hypothetical protein
  
Accession: EHY53356
  
Location: 115744-116175
  
 NCBI BlastP on this gene

EHY53356

hypothetical protein
  
Accession: EHY53357
  
Location: 116748-118852
  
 NCBI BlastP on this gene

EHY53357

135. :  GG704914 Coccidioides immitis RS genomic scaffold supercont3.4     Total score: 1.0     Cumulative Blast bit score: 847

hypothetical protein
  
Accession: EAS34193
  
Location: 1918050-1918566
  
 NCBI BlastP on this gene

EAS34193

hypothetical protein, variant
  
Accession: EJB11490
  
Location: 1916477-1917349
  
 NCBI BlastP on this gene

EJB11490

hypothetical protein
  
Accession: EAS34191
  
Location: 1915296-1915744
  
 NCBI BlastP on this gene

EAS34191

hypothetical protein
  
Accession: EAS34190
  
Location: 1913600-1914927
  
 NCBI BlastP on this gene

EAS34190

hypothetical protein
  
Accession: EAS34189
  
Location: 1911449-1912423
  
 NCBI BlastP on this gene

EAS34189

hypothetical protein
  
Accession: EAS34188
  
Location: 1909269-1910759
  
 NCBI BlastP on this gene

EAS34188

arsenical-resistance protein
  
Accession: EAS34187
  
Location: 1907074-1908425
  
 NCBI BlastP on this gene

EAS34187

cytoplasmic tRNA 2-thiolation protein 1
  
Accession: EAS34186
  
Location: 1905434-1906729
  
 NCBI BlastP on this gene

EAS34186

hypothetical protein
  
Accession: EJB11488
  
Location: 1904707-1905027
  
 NCBI BlastP on this gene

EJB11488

ubiquitin-protein ligase
  
Accession: EAS34185
  
Location: 1900449-1904362
  
  
**BlastP hit with Mycgr3G52682\_Mycgr3T**
  
Percentage identity: 43 %
  
BlastP bit score: 847
  
Sequence coverage: 100 %
  
E-value: 0.0
  
  
 NCBI BlastP on this gene

EAS34185

chlorophyll synthesis pathway protein BchC
  
Accession: EAS34184
  
Location: 1896047-1897613
  
 NCBI BlastP on this gene

EAS34184

hypothetical protein
  
Accession: EAS34183
  
Location: 1894547-1895476
  
 NCBI BlastP on this gene

EAS34183

hypothetical protein
  
Accession: EAS34182
  
Location: 1891785-1893635
  
 NCBI BlastP on this gene

EAS34182

hypothetical protein
  
Accession: EAS34181
  
Location: 1890788-1891254
  
 NCBI BlastP on this gene

EAS34181

transcriptional regulator Ngg1
  
Accession: EAS34180
  
Location: 1887630-1889950
  
 NCBI BlastP on this gene

EAS34180

nitrogen permease regulator Npr2
  
Accession: EAS34179
  
Location: 1885388-1887207
  
 NCBI BlastP on this gene

EAS34179

136. :  DS572697 Verticillium dahliae VdLs.17 supercont1.3 genomic scaffold     Total score: 1.0     Cumulative Blast bit score: 841

suppressor of Mek1
  
Accession: EGY19923
  
Location: 1020123-1023035
  
 NCBI BlastP on this gene

EGY19923

AFG2 protein
  
Accession: EGY19924
  
Location: 1023775-1026045
  
 NCBI BlastP on this gene

EGY19924

hypothetical protein
  
Accession: EGY19925
  
Location: 1027278-1028498
  
 NCBI BlastP on this gene

EGY19925

GTP-binding protein SAS1
  
Accession: EGY19926
  
Location: 1029249-1030098
  
 NCBI BlastP on this gene

EGY19926

ubiquitin-protein ligase E3A
  
Accession: EGY19927
  
Location: 1032174-1035898
  
  
**BlastP hit with Mycgr3G52682\_Mycgr3T**
  
Percentage identity: 42 %
  
BlastP bit score: 841
  
Sequence coverage: 99 %
  
E-value: 0.0
  
  
 NCBI BlastP on this gene

EGY19927

50S ribosomal protein Mrp49
  
Accession: EGY19928
  
Location: 1036239-1037076
  
 NCBI BlastP on this gene

EGY19928

hypothetical protein
  
Accession: EGY19929
  
Location: 1037518-1039652
  
 NCBI BlastP on this gene

EGY19929

hypothetical protein
  
Accession: EGY19930
  
Location: 1040077-1041018
  
 NCBI BlastP on this gene

EGY19930

hypothetical protein
  
Accession: EGY19931
  
Location: 1042089-1042678
  
 NCBI BlastP on this gene

EGY19931

urease accessory protein ureG
  
Accession: EGY19932
  
Location: 1043412-1044332
  
 NCBI BlastP on this gene

EGY19932

ribosome biogenesis protein TSR1
  
Accession: EGY19933
  
Location: 1044574-1047143
  
 NCBI BlastP on this gene

EGY19933

U3 small nucleolar RNA-associated protein
  
Accession: EGY19934
  
Location: 1047752-1049473
  
 NCBI BlastP on this gene

EGY19934

peptidyl-tRNA hydrolase
  
Accession: EGY19935
  
Location: 1049958-1050719
  
 NCBI BlastP on this gene

EGY19935

137. :  GL636503 Coccidioides posadasii str. Silveira unplaced genomic scaffold supercont2.18     Total score: 1.0     Cumulative Blast bit score: 838

predicted protein
  
Accession: EFW14801
  
Location: 152271-153049
  
 NCBI BlastP on this gene

EFW14801

predicted protein
  
Accession: EFW14800
  
Location: 150950-151413
  
 NCBI BlastP on this gene

EFW14800

conserved hypothetical protein
  
Accession: EFW14799
  
Location: 149729-150580
  
 NCBI BlastP on this gene

EFW14799

conserved hypothetical protein
  
Accession: EFW14798
  
Location: 146980-148386
  
 NCBI BlastP on this gene

EFW14798

conserved hypothetical protein
  
Accession: EFW14797
  
Location: 144961-146451
  
 NCBI BlastP on this gene

EFW14797

arsenical-resistance protein
  
Accession: EFW14796
  
Location: 142769-144120
  
 NCBI BlastP on this gene

EFW14796

PP-loop ATPase superfamily protein
  
Accession: EFW14795
  
Location: 141144-142439
  
 NCBI BlastP on this gene

EFW14795

E3 ubiquitin-protein ligase HUWE1
  
Accession: EFW14794
  
Location: 135995-139645
  
  
**BlastP hit with Mycgr3G52682\_Mycgr3T**
  
Percentage identity: 42 %
  
BlastP bit score: 838
  
Sequence coverage: 99 %
  
E-value: 0.0
  
  
 NCBI BlastP on this gene

EFW14794

alcohol dehydrogenase
  
Accession: EFW14793
  
Location: 131568-133133
  
 NCBI BlastP on this gene

EFW14793

conserved hypothetical protein
  
Accession: EFW14792
  
Location: 130075-131001
  
 NCBI BlastP on this gene

EFW14792

conserved hypothetical protein
  
Accession: EFW14791
  
Location: 127313-129164
  
 NCBI BlastP on this gene

EFW14791

predicted protein
  
Accession: EFW14790
  
Location: 126325-126791
  
 NCBI BlastP on this gene

EFW14790

transcriptional regulator Ngg1
  
Accession: EFW14789
  
Location: 123184-125505
  
 NCBI BlastP on this gene

EFW14789

nitrogen permease regulator Npr2
  
Accession: EFW14788
  
Location: 120944-122763
  
 NCBI BlastP on this gene

EFW14788

138. :  ACFW01000009 Coccidioides posadasii C735 delta SOWgp     Total score: 1.0     Cumulative Blast bit score: 835

kinase domain containing protein
  
Accession: EER29247
  
Location: 485768-486619
  
 NCBI BlastP on this gene

EER29247

hypothetical protein
  
Accession: EER29248
  
Location: 488022-489065
  
 NCBI BlastP on this gene

EER29248

Major Facilitator Superfamily protein
  
Accession: EER29249
  
Location: 489901-491391
  
 NCBI BlastP on this gene

EER29249

Sodium Bile acid symporter family protein
  
Accession: EER29250
  
Location: 492232-493583
  
 NCBI BlastP on this gene

EER29250

PP-loop family protein
  
Accession: EER29251
  
Location: 493913-495208
  
 NCBI BlastP on this gene

EER29251

HECT-domain containing protein
  
Accession: EER29252
  
Location: 496722-500372
  
  
**BlastP hit with Mycgr3G52682\_Mycgr3T**
  
Percentage identity: 42 %
  
BlastP bit score: 836
  
Sequence coverage: 99 %
  
E-value: 0.0
  
  
 NCBI BlastP on this gene

EER29252

139. :  ABDG02000017 Trichoderma atroviride IMI 206040     Total score: 1.0     Cumulative Blast bit score: 835

hypothetical protein
  
Accession: EHK49019
  
Location: 469464-473345
  
 NCBI BlastP on this gene

EHK49019

hypothetical protein
  
Accession: EHK49020
  
Location: 474289-474843
  
 NCBI BlastP on this gene

EHK49020

hypothetical protein
  
Accession: EHK49021
  
Location: 475159-475664
  
 NCBI BlastP on this gene

EHK49021

hypothetical protein
  
Accession: EHK49022
  
Location: 476042-476705
  
 NCBI BlastP on this gene

EHK49022

hypothetical protein
  
Accession: EHK49023
  
Location: 477045-477896
  
 NCBI BlastP on this gene

EHK49023

hypothetical protein
  
Accession: EHK49024
  
Location: 478394-479286
  
 NCBI BlastP on this gene

EHK49024

hypothetical protein
  
Accession: EHK49025
  
Location: 479642-479896
  
 NCBI BlastP on this gene

EHK49025

hypothetical protein
  
Accession: EHK49026
  
Location: 480459-481373
  
 NCBI BlastP on this gene

EHK49026

hypothetical protein
  
Accession: EHK49027
  
Location: 482143-482913
  
 NCBI BlastP on this gene

EHK49027

hypothetical protein
  
Accession: EHK49028
  
Location: 484273-487756
  
  
**BlastP hit with Mycgr3G52682\_Mycgr3T**
  
Percentage identity: 42 %
  
BlastP bit score: 836
  
Sequence coverage: 95 %
  
E-value: 0.0
  
  
 NCBI BlastP on this gene

EHK49028

hypothetical protein
  
Accession: EHK49029
  
Location: 488221-489050
  
 NCBI BlastP on this gene

EHK49029

hypothetical protein
  
Accession: EHK49030
  
Location: 489386-491616
  
 NCBI BlastP on this gene

EHK49030

hypothetical protein
  
Accession: EHK49031
  
Location: 492153-492777
  
 NCBI BlastP on this gene

EHK49031

hypothetical protein
  
Accession: EHK49032
  
Location: 493117-493599
  
 NCBI BlastP on this gene

EHK49032

hypothetical protein
  
Accession: EHK49033
  
Location: 494052-495245
  
 NCBI BlastP on this gene

EHK49033

glycoside hydrolase family 15 protein
  
Accession: EHK49034
  
Location: 495781-497869
  
 NCBI BlastP on this gene

EHK49034

hypothetical protein
  
Accession: EHK49035
  
Location: 500506-502566
  
 NCBI BlastP on this gene

EHK49035

140. :  GL891305 Neurospora tetrasperma FGSC 2508 unplaced genomic scaffold NEUTE1scaffold\_4     Total score: 1.0     Cumulative Blast bit score: 830

hypothetical protein
  
Accession: EGO56666
  
Location: 2179709-2182989
  
 NCBI BlastP on this gene

EGO56666

hypothetical protein
  
Accession: EGO56667
  
Location: 2183624-2184468
  
 NCBI BlastP on this gene

EGO56667

hypothetical protein
  
Accession: EGO56668
  
Location: 2185111-2186275
  
 NCBI BlastP on this gene

EGO56668

hypothetical protein
  
Accession: EGO56669
  
Location: 2187805-2190542
  
 NCBI BlastP on this gene

EGO56669

hypothetical protein
  
Accession: EGO56670
  
Location: 2191799-2192636
  
 NCBI BlastP on this gene

EGO56670

hypothetical protein
  
Accession: EGO56671
  
Location: 2194848-2198959
  
  
**BlastP hit with Mycgr3G52682\_Mycgr3T**
  
Percentage identity: 42 %
  
BlastP bit score: 830
  
Sequence coverage: 100 %
  
E-value: 0.0
  
  
 NCBI BlastP on this gene

EGO56671

141. :  GL891247 Neurospora tetrasperma FGSC 2509 unplaced genomic scaffold NEUTE2scaffold\_5     Total score: 1.0     Cumulative Blast bit score: 830

hypothetical protein
  
Accession: EGZ70459
  
Location: 2030359-2033639
  
 NCBI BlastP on this gene

EGZ70459

hypothetical protein
  
Accession: EGZ70458
  
Location: 2028880-2029724
  
 NCBI BlastP on this gene

EGZ70458

hypothetical protein
  
Accession: EGZ70457
  
Location: 2027073-2028237
  
 NCBI BlastP on this gene

EGZ70457

hypothetical protein
  
Accession: EGZ70456
  
Location: 2022806-2025543
  
 NCBI BlastP on this gene

EGZ70456

hypothetical protein
  
Accession: EGZ70455
  
Location: 2020712-2022405
  
 NCBI BlastP on this gene

EGZ70455

HECT-domain-containing protein
  
Accession: EGZ70454
  
Location: 2014391-2018502
  
  
**BlastP hit with Mycgr3G52682\_Mycgr3T**
  
Percentage identity: 42 %
  
BlastP bit score: 830
  
Sequence coverage: 100 %
  
E-value: 0.0
  
  
 NCBI BlastP on this gene

EGZ70454

DNA/RNA polymerase
  
Accession: EGZ70453
  
Location: 2010983-2013736
  
 NCBI BlastP on this gene

EGZ70453

WD40 repeat-like protein
  
Accession: EGZ70452
  
Location: 2005817-2009323
  
 NCBI BlastP on this gene

EGZ70452

hypothetical protein
  
Accession: EGZ70451
  
Location: 2002652-2004917
  
 NCBI BlastP on this gene

EGZ70451

142. :  KB456260 Mycosphaerella populorum SO2202 unplaced genomic scaffold SEPMUscaffold\_1     Total score: 1.0     Cumulative Blast bit score: 825

hypothetical protein
  
Accession: EMF16383
  
Location: 629638-630448
  
 NCBI BlastP on this gene

EMF16383

hypothetical protein
  
Accession: EMF16384
  
Location: 632327-633968
  
 NCBI BlastP on this gene

EMF16384

glycoside hydrolase family 5 protein
  
Accession: EMF16385
  
Location: 638298-639653
  
 NCBI BlastP on this gene

EMF16385

phosphoglycerate mutase-like protein
  
Accession: EMF16386
  
Location: 641807-643390
  
 NCBI BlastP on this gene

EMF16386

Ferric reduct-domain-containing protein
  
Accession: EMF16387
  
Location: 643957-645981
  
  
**BlastP hit with Mycgr3G107069\_Mycgr3**
  
Percentage identity: 61 %
  
BlastP bit score: 825
  
Sequence coverage: 103 %
  
E-value: 0.0
  
  
 NCBI BlastP on this gene

EMF16387

hexose transport-related protein
  
Accession: EMF16388
  
Location: 646746-648564
  
 NCBI BlastP on this gene

EMF16388

hypothetical protein
  
Accession: EMF16389
  
Location: 653310-655146
  
 NCBI BlastP on this gene

EMF16389

hypothetical protein
  
Accession: EMF16391
  
Location: 655880-656431
  
 NCBI BlastP on this gene

EMF16391

pyruvate kinase
  
Accession: EMF16392
  
Location: 658503-660235
  
 NCBI BlastP on this gene

EMF16392

143. :  CU638743 Podospora anserina S mat+ genomic DNA chromosome 3, supercontig 2.     Total score: 1.0     Cumulative Blast bit score: 810

not annotated
  
Accession: CAP70771
  
Location: 2284707-2287168
  
 NCBI BlastP on this gene

CAP70771

not annotated
  
Accession: CAP70770
  
Location: 2280484-2282511
  
 NCBI BlastP on this gene

CAP70770

not annotated
  
Accession: CAP70769
  
Location: 2276681-2279472
  
 NCBI BlastP on this gene

CAP70769

not annotated
  
Accession: CAP70768
  
Location: 2267853-2271588
  
  
**BlastP hit with Mycgr3G52682\_Mycgr3T**
  
Percentage identity: 41 %
  
BlastP bit score: 811
  
Sequence coverage: 101 %
  
E-value: 0.0
  
  
 NCBI BlastP on this gene

CAP70768

not annotated
  
Accession: CAP70767
  
Location: 2264201-2265355
  
 NCBI BlastP on this gene

CAP70767

not annotated
  
Accession: CAP70766
  
Location: 2261265-2263268
  
 NCBI BlastP on this gene

CAP70766

not annotated
  
Accession: CAP70765
  
Location: 2260494-2261101
  
 NCBI BlastP on this gene

CAP70765

not annotated
  
Accession: CAP70764
  
Location: 2256504-2258593
  
 NCBI BlastP on this gene

CAP70764

not annotated
  
Accession: CAP70763
  
Location: 2254885-2255613
  
 NCBI BlastP on this gene

CAP70763

144. :  CM001235 Magnaporthe oryzae 70-15 chromosome 5     Total score: 1.0     Cumulative Blast bit score: 810

protein transporter SEC31
  
Accession: EHA48242
  
Location: 486645-490641
  
 NCBI BlastP on this gene

EHA48242

nuclear cap-binding protein
  
Accession: EHA48241
  
Location: 485496-486030
  
 NCBI BlastP on this gene

EHA48241

hypothetical protein
  
Accession: EHA48240
  
Location: 484589-485186
  
 NCBI BlastP on this gene

EHA48240

cytochrome c oxidase subunit 6B
  
Accession: EHA48239
  
Location: 483533-484138
  
 NCBI BlastP on this gene

EHA48239

hypothetical protein
  
Accession: EHA48238
  
Location: 482251-483147
  
 NCBI BlastP on this gene

EHA48238

40S ribosomal protein S10-A
  
Accession: EHA48237
  
Location: 480900-481785
  
 NCBI BlastP on this gene

EHA48237

NADP-dependent mannitol dehydrogenase
  
Accession: EHA48236
  
Location: 477746-479121
  
 NCBI BlastP on this gene

EHA48236

ubiquitin-protein ligase E3A
  
Accession: EHA48235
  
Location: 471321-475547
  
  
**BlastP hit with Mycgr3G52682\_Mycgr3T**
  
Percentage identity: 42 %
  
BlastP bit score: 810
  
Sequence coverage: 100 %
  
E-value: 0.0
  
  
 NCBI BlastP on this gene

EHA48235

hypothetical protein
  
Accession: EHA48234
  
Location: 470066-470907
  
 NCBI BlastP on this gene

EHA48234

hypothetical protein
  
Accession: EHA48233
  
Location: 467346-469568
  
 NCBI BlastP on this gene

EHA48233

hypothetical protein
  
Accession: EHA48232
  
Location: 465302-466834
  
 NCBI BlastP on this gene

EHA48232

transmembrane and coiled-coil domain-containing protein 4
  
Accession: EHA48231
  
Location: 461603-464179
  
 NCBI BlastP on this gene

EHA48231

hypothetical protein
  
Accession: EHA48230
  
Location: 460502-461161
  
 NCBI BlastP on this gene

EHA48230

vesicular-fusion protein SEC17
  
Accession: EHA48229
  
Location: 458862-460062
  
 NCBI BlastP on this gene

EHA48229

hypothetical protein
  
Accession: EHA48228
  
Location: 457757-458440
  
 NCBI BlastP on this gene

EHA48228

145. :  EQ963473 Aspergillus flavus NRRL3357 scf\_1106286417600 genomic scaffold     Total score: 1.0     Cumulative Blast bit score: 803

NifU-related protein
  
Accession: EED55652
  
Location: 1719311-1720439
  
 NCBI BlastP on this gene

EED55652

PSP1 domain protein
  
Accession: EED55653
  
Location: 1726176-1728887
  
 NCBI BlastP on this gene

EED55653

60S ribosomal protein L15, putative
  
Accession: EED55654
  
Location: 1730183-1731275
  
 NCBI BlastP on this gene

EED55654

hypothetical protein
  
Accession: EED55655
  
Location: 1732496-1732921
  
 NCBI BlastP on this gene

EED55655

ubiquitin-protein ligase (Hul4), putative
  
Accession: EED55656
  
Location: 1733752-1736029
  
  
**BlastP hit with Mycgr3G52682\_Mycgr3T**
  
Percentage identity: 53 %
  
BlastP bit score: 803
  
Sequence coverage: 64 %
  
E-value: 0.0
  
  
 NCBI BlastP on this gene

EED55656

146. :  GL629729 Grosmannia clavigera kw1407 unplaced genomic scaffold GCSC\_108     Total score: 1.0     Cumulative Blast bit score: 797

hypothetical protein
  
Accession: EFX06488
  
Location: 69254-70298
  
 NCBI BlastP on this gene

EFX06488

protein transport protein
  
Accession: EFX06673
  
Location: 64938-68981
  
 NCBI BlastP on this gene

EFX06673

peptidyl prolyl cis-trans isomerase
  
Accession: EFX06598
  
Location: 63847-64416
  
 NCBI BlastP on this gene

EFX06598

hypothetical protein
  
Accession: EFX06604
  
Location: 62979-63534
  
 NCBI BlastP on this gene

EFX06604

cytochrome c oxidase polypeptide vib
  
Accession: EFX06706
  
Location: 61757-62448
  
 NCBI BlastP on this gene

EFX06706

hypothetical protein
  
Accession: EFX06287
  
Location: 60228-61157
  
 NCBI BlastP on this gene

EFX06287

40S ribosomal protein s10b
  
Accession: EFX06236
  
Location: 58984-59839
  
 NCBI BlastP on this gene

EFX06236

vesicular-fusion protein sec17
  
Accession: EFX06269
  
Location: 57165-58247
  
 NCBI BlastP on this gene

EFX06269

duf726 domain containing protein
  
Accession: EFX06273
  
Location: 56233-56823
  
 NCBI BlastP on this gene

EFX06273

ubiquitin-protein ligase
  
Accession: EFX06337
  
Location: 51336-55121
  
  
**BlastP hit with Mycgr3G52682\_Mycgr3T**
  
Percentage identity: 39 %
  
BlastP bit score: 798
  
Sequence coverage: 105 %
  
E-value: 0.0
  
  
 NCBI BlastP on this gene

EFX06337

50S ribosomal protein mrp49
  
Accession: EFX06292
  
Location: 50376-51217
  
 NCBI BlastP on this gene

EFX06292

duf974 domain containing protein
  
Accession: EFX06401
  
Location: 46647-50081
  
 NCBI BlastP on this gene

EFX06401

ddenn domain containing protein
  
Accession: EFX06468
  
Location: 41005-44757
  
 NCBI BlastP on this gene

EFX06468

kh domain containing protein
  
Accession: EFX06349
  
Location: 37440-40592
  
 NCBI BlastP on this gene

EFX06349

147. :  KE123956 Mucor circinelloides f. circinelloides 1006PhL unplaced genomic scaffold supercont1.61     Total score: 1.0     Cumulative Blast bit score: 784

hypothetical protein
  
Accession: EPB88108
  
Location: 32848-38290
  
 NCBI BlastP on this gene

EPB88108

AGC/RSK/RSK-UNCLASSIFIED protein kinase
  
Accession: EPB88109
  
Location: 38814-40628
  
 NCBI BlastP on this gene

EPB88109

hypothetical protein
  
Accession: EPB88110
  
Location: 40889-42065
  
 NCBI BlastP on this gene

EPB88110

hypothetical protein
  
Accession: EPB88111
  
Location: 42268-45000
  
  
**BlastP hit with Mycgr3G52682\_Mycgr3T**
  
Percentage identity: 40 %
  
BlastP bit score: 525
  
Sequence coverage: 59 %
  
E-value: 7e-167
  
  
 NCBI BlastP on this gene

EPB88111

hypothetical protein
  
Accession: EPB88112
  
Location: 46318-47424
  
 NCBI BlastP on this gene

EPB88112

hypothetical protein
  
Accession: EPB88113
  
Location: 47493-49301
  
 NCBI BlastP on this gene

EPB88113

hypothetical protein
  
Accession: EPB88114
  
Location: 49825-51432
  
 NCBI BlastP on this gene

EPB88114

hypothetical protein
  
Accession: EPB88115
  
Location: 53897-55786
  
 NCBI BlastP on this gene

EPB88115

6-phosphofructokinase
  
Accession: EPB88116
  
Location: 55983-59037
  
 NCBI BlastP on this gene

EPB88116

other hect domain ubiquitin protein ligase E3
  
Accession: EPB88117
  
Location: 62129-63045
  
  
**BlastP hit with Mycgr3G52682\_Mycgr3T**
  
Percentage identity: 49 %
  
BlastP bit score: 260
  
Sequence coverage: 20 %
  
E-value: 9e-76
  
  
 NCBI BlastP on this gene

EPB88117

STE/STE20/PAKA protein kinase
  
Accession: EPB88118
  
Location: 64184-65886
  
 NCBI BlastP on this gene

EPB88118

hypothetical protein
  
Accession: EPB88119
  
Location: 67786-68055
  
 NCBI BlastP on this gene

EPB88119

148. :  CP003008 Myceliophthora thermophila ATCC 42464 chromosome 7     Total score: 1.0     Cumulative Blast bit score: 784

hypothetical protein
  
Accession: AEO61595
  
Location: 1613901-1614404
  
 NCBI BlastP on this gene

MYCTH\_17562

hypothetical protein
  
Accession: AEO61596
  
Location: 1614855-1617304
  
 NCBI BlastP on this gene

MYCTH\_2311906

hypothetical protein
  
Accession: AEO61597
  
Location: 1618110-1618982
  
 NCBI BlastP on this gene

MYCTH\_2311907

hypothetical protein
  
Accession: AEO61598
  
Location: 1620342-1621907
  
 NCBI BlastP on this gene

MYCTH\_2311910

hypothetical protein
  
Accession: AEO61599
  
Location: 1622486-1626363
  
 NCBI BlastP on this gene

MYCTH\_2311912

hypothetical protein
  
Accession: AEO61600
  
Location: 1628505-1632331
  
  
**BlastP hit with Mycgr3G52682\_Mycgr3T**
  
Percentage identity: 41 %
  
BlastP bit score: 784
  
Sequence coverage: 101 %
  
E-value: 0.0
  
  
 NCBI BlastP on this gene

MYCTH\_2311917

hypothetical protein
  
Accession: AEO61601
  
Location: 1634929-1636131
  
 NCBI BlastP on this gene

MYCTH\_2311919

hypothetical protein
  
Accession: AEO61602
  
Location: 1637270-1639367
  
 NCBI BlastP on this gene

MYCTH\_2311921

hypothetical protein
  
Accession: AEO61603
  
Location: 1639624-1640358
  
 NCBI BlastP on this gene

MYCTH\_104267

hypothetical protein
  
Accession: AEO61604
  
Location: 1642046-1644003
  
 NCBI BlastP on this gene

MYCTH\_56385

149. :  CP004025 Myxococcus stipitatus DSM 14675     Total score: 1.0     Cumulative Blast bit score: 774

Crp/Fnr family transcriptional regulator
  
Accession: AGC45602
  
Location: 5492527-5493210
  
 NCBI BlastP on this gene

MYSTI\_04304

2,3-dihydroxybenzoate-2,3-dehydrogenase
  
Accession: AGC45603
  
Location: 5493451-5494224
  
 NCBI BlastP on this gene

MYSTI\_04305

isochorismate synthase DhbC
  
Accession: AGC45604
  
Location: 5494332-5495564
  
 NCBI BlastP on this gene

MYSTI\_04306

2,3-dihydroxybenzoate-AMP ligase
  
Accession: AGC45605
  
Location: 5495561-5497186
  
 NCBI BlastP on this gene

MYSTI\_04307

isochorismatase
  
Accession: AGC45606
  
Location: 5497223-5498158
  
 NCBI BlastP on this gene

MYSTI\_04308

non-ribosomal peptide synthetase
  
Accession: AGC45607
  
Location: 5498193-5502659
  
  
**BlastP hit with Mycgr3G107072\_Mycgr3**
  
Percentage identity: 30 %
  
BlastP bit score: 330
  
Sequence coverage: 82 %
  
E-value: 4e-90
  
  
 NCBI BlastP on this gene

MYSTI\_04309

3-deoxy-7-phosphoheptulonate synthase
  
Accession: AGC45608
  
Location: 5502659-5504032
  
 NCBI BlastP on this gene

MYSTI\_04310

TonB family protein
  
Accession: AGC45609
  
Location: 5504087-5506708
  
 NCBI BlastP on this gene

MYSTI\_04311

hypothetical protein
  
Accession: AGC45610
  
Location: 5506765-5507988
  
 NCBI BlastP on this gene

MYSTI\_04312

MotA/TolQ/ExbB proton channel family protein
  
Accession: AGC45611
  
Location: 5508116-5508796
  
 NCBI BlastP on this gene

MYSTI\_04313

ExbD/TolR family transport energizing protein
  
Accession: AGC45612
  
Location: 5508799-5509233
  
 NCBI BlastP on this gene

MYSTI\_04314

hypothetical protein
  
Accession: AGC45613
  
Location: 5509220-5510044
  
 NCBI BlastP on this gene

MYSTI\_04315

major facilitator family transporter
  
Accession: AGC45614
  
Location: 5510076-5511299
  
 NCBI BlastP on this gene

MYSTI\_04316

siderophore biosynthesis aminotransferase
  
Accession: AGC45615
  
Location: 5511334-5512602
  
 NCBI BlastP on this gene

MYSTI\_04317

iron-chelator utilization protein
  
Accession: AGC45616
  
Location: 5512656-5513477
  
 NCBI BlastP on this gene

MYSTI\_04318

hypothetical protein
  
Accession: AGC45617
  
Location: 5513696-5515117
  
 NCBI BlastP on this gene

MYSTI\_04319

non-ribosomal peptide synthetase
  
Accession: AGC45618
  
Location: 5515262-5519788
  
  
**BlastP hit with Mycgr3G107072\_Mycgr3**
  
Percentage identity: 34 %
  
BlastP bit score: 444
  
Sequence coverage: 74 %
  
E-value: 7e-129
  
  
 NCBI BlastP on this gene

MYSTI\_04320

polyketide synthase
  
Accession: AGC45619
  
Location: 5519785-5526216
  
 NCBI BlastP on this gene

MYSTI\_04321

150. :  DS985245 Trichoplax adhaerens TRIADscaffold\_5 genomic scaffold     Total score: 1.0     Cumulative Blast bit score: 772

hypothetical protein
  
Accession: EDV24532
  
Location: 2823722-2831955
  
  
**BlastP hit with Mycgr3G107072\_Mycgr3**
  
Percentage identity: 37 %
  
BlastP bit score: 772
  
Sequence coverage: 100 %
  
E-value: 0.0
  
  
 NCBI BlastP on this gene

EDV24532

hypothetical protein
  
Accession: EDV24971
  
Location: 2833494-2836864
  
 NCBI BlastP on this gene

EDV24971

hypothetical protein
  
Accession: EDV24533
  
Location: 2841156-2844522
  
 NCBI BlastP on this gene

EDV24533

Detecting sequence homology at the gene cluster level with MultiGeneBlast.
  
Marnix H. Medema, Rainer Breitling & Eriko Takano (2013)
  
*Molecular Biology and Evolution* , 30: 1218-1223.
