# Supplementary material for: In silico prediction and characterization of secondary metabolite biosynthetic gene clusters in the wheat pathogen Zymoseptoria tritici
Source: BMC Genomics. 2017 Aug 17;18:631. doi: 10.1186/s12864-017-3969-y (PMC5561558; doi:10.1186/s12864-017-3969-y)
Supplement: Supplementary file 1 — MultiGeneBLAST analysis of putative secondary metabolite clusters. All encoded amino acid sequences from genes residing in clusters predicted by AntiSMASH are given as FASTA file format. All output data from MultiGeneBLASTs are also provided. (ZIP 42911 kb) [file 12864_2017_3969_MOESM1_ESM.zip › Cluster MultiGene BLAST/out/Clusters_1_34/Cluster_1/displaypage4.xhtml]

xml version="1.0" encoding="UTF-8"?


Search Results
  
  
 Results pages: 1, 2, 3, 4, 5

**MultiGeneBlast hits**

Select gene cluster alignment
151. CABT02000003\_1 Sordaria macrospora k-hell, whole genome shotgun sequenci...
152. JH126401\_0 Cordyceps militaris CM01 unplaced genomic scaffold CCM\_S00003...
153. KB722656\_0 Rhodosporidium toruloides NP11 unplaced genomic scaffold Scaf...
154. CH476618\_1 Uncinocarpus reesii 1704 scaffold\_4 genomic scaffold, whole g...
155. JH930469\_2 Phanerochaete carnosa HHB-10118-sp unplaced genomic scaffold ...
156. EQ966375\_0 Postia placenta Mad-698-R POSPLscaffold\_143 genomic scaffold,...
157. KB726077\_0 Colletotrichum orbiculare MAFF 240422 unplaced genomic scaffo...
158. KB369422\_0 Tupaia chinensis unplaced genomic scaffold Scaffold148626\_1, ...
159. JH711784\_0 Trametes versicolor FP-101664 SS1 unplaced genomic scaffold T...
160. KB446538\_2 Dothistroma septosporum NZE10 unplaced genomic scaffold DOTSE...
161. GL988032\_2 Chaetomium thermophilum var. thermophilum DSM 1495 unplaced g...
162. KB446537\_2 Dothistroma septosporum NZE10 unplaced genomic scaffold DOTSE...
163. KB469296\_0 Gloeophyllum trabeum ATCC 11539 unplaced genomic scaffold GLO...
164. KB456262\_2 Mycosphaerella populorum SO2202 unplaced genomic scaffold SEP...
165. KB446537\_0 Dothistroma septosporum NZE10 unplaced genomic scaffold DOTSE...
166. KB446560\_2 Pseudocercospora fijiensis CIRAD86 unplaced genomic scaffold ...
167. KB733456\_0 Bipolaris maydis ATCC 48331 unplaced genomic scaffold COCC4sc...
168. KB445574\_1 Cochliobolus heterostrophus C5 unplaced genomic scaffold COCH...
169. CH471151\_0 Homo sapiens 211000035831495 genomic scaffold, whole genome s...
170. KB445644\_1 Cochliobolus sativus ND90Pr unplaced genomic scaffold COCSAsc...
171. GG698901\_0 Nectria haematococca mpVI 77-13-4 chromosome 1 genomic scaffo...
172. AAAB01008807\_0 Anopheles gambiae str. PEST whole genome shotgun sequenci...
173. KB446558\_0 Pseudocercospora fijiensis CIRAD86 unplaced genomic scaffold ...
174. KB733480\_0 Bipolaris maydis ATCC 48331 unplaced genomic scaffold COCC4sc...
175. KB445573\_1 Cochliobolus heterostrophus C5 unplaced genomic scaffold COCH...
176. KE145357\_1 Glarea lozoyensis ATCC 20868 chromosome Unknown GLAREA14, who...
177. KB725793\_0 Colletotrichum orbiculare MAFF 240422 unplaced genomic scaffo...
178. CH476636\_1 Sclerotinia sclerotiorum 1980 scaffold\_16 genomic scaffold, w...
179. KB445646\_0 Cochliobolus sativus ND90Pr unplaced genomic scaffold COCSAsc...
180. KB706647\_1 Eutypa lata UCREL1 unplaced genomic scaffold EL1\_03\_scaffold\_...
181. JH226130\_0 Exophiala dermatitidis NIH/UT8656 unplaced genomic scaffold s...
182. DS985223\_2 Verticillium albo-atrum VaMs.102 supercont1.10 genomic scaffo...
183. DS572705\_0 Verticillium dahliae VdLs.17 supercont1.11 genomic scaffold, ...
184. KB445550\_0 Baudoinia compniacensis UAMH 10762 unplaced genomic scaffold ...
185. CM001233\_4 Magnaporthe oryzae 70-15 chromosome 3, whole genome shotgun s...
186. CH408159\_0 Pichia guilliermondii ATCC 6260 scaffold\_5 genomic scaffold, ...
187. AFNW01000605\_1 Fusarium pseudograminearum CS3096, whole genome shotgun s...
188. KB644415\_1 Penicillium oxalicum 114-2 unplaced genomic scaffold scaffold...
189. CM001235\_1 Magnaporthe oryzae 70-15 chromosome 5, whole genome shotgun s...
190. KB726554\_0 Fusarium oxysporum f. sp. cubense race 4 unplaced genomic sca...
191. HF679023\_0 Fusarium fujikuroi IMI 58289 draft genome, chromosome FFUJ\_ch...
192. KB730248\_1 Fusarium oxysporum f. sp. cubense race 1 unplaced genomic sca...
193. ACJE01000009\_1 Aspergillus niger ATCC 1015, whole genome shotgun sequenc...
194. HF679026\_0 Fusarium fujikuroi IMI 58289 draft genome, chromosome FFUJ\_ch...
195. DS231620\_1 Pyrenophora tritici-repentis Pt-1C-BFP supercont1.6 genomic s...
196. CP003014\_0 Thielavia terrestris NRRL 8126 chromosome 6, complete sequence.
197. GG698898\_1 Nectria haematococca mpVI 77-13-4 chromosome 4 genomic scaffo...
198. KB456262\_3 Mycosphaerella populorum SO2202 unplaced genomic scaffold SEP...
199. GL629729\_1 Grosmannia clavigera kw1407 unplaced genomic scaffold GCSC\_10...
200. DS572697\_1 Verticillium dahliae VdLs.17 supercont1.3 genomic scaffold, w...

Query: Architecture Search FASTA input

CABT02000003 : Sordaria macrospora k-hell    Total score: 1.0     Cumulative Blast bit score: 769

Hit cluster cross-links:

Mycgr3G52686 Mycgr3T
  
Location: 0-861

Mycgr3G52686\_Mycgr3T

Mycgr3G102281 Mycgr3
  
Location: 961-1573

Mycgr3G102281\_Mycgr3

Mycgr3G89185 Mycgr3T
  
Location: 1673-2063

Mycgr3G89185\_Mycgr3T

Mycgr3G65725 Mycgr3T
  
Location: 2163-3612

Mycgr3G65725\_Mycgr3T

Mycgr3G102276 Mycgr3
  
Location: 3712-4801

Mycgr3G102276\_Mycgr3

Mycgr3G89189 Mycgr3T
  
Location: 4901-5564

Mycgr3G89189\_Mycgr3T

Mycgr3G52682 Mycgr3T
  
Location: 5664-9231

Mycgr3G52682\_Mycgr3T

Mycgr3G107072 Mycgr3
  
Location: 9331-13279

Mycgr3G107072\_Mycgr3

Mycgr3G34982 Mycgr3T
  
Location: 13379-15116

Mycgr3G34982\_Mycgr3T

Mycgr3G107069 Mycgr3
  
Location: 15216-17097

Mycgr3G107069\_Mycgr3

Mycgr3G32432 Mycgr3T
  
Location: 17197-19042

Mycgr3G32432\_Mycgr3T

Mycgr3G98385 Mycgr3T
  
Location: 19142-19898

Mycgr3G98385\_Mycgr3T

not annotated
  
Accession: CCC07543
  
Location: 1365922-1368687
  
 NCBI BlastP on this gene

CCC07543

not annotated
  
Accession: CCC07544
  
Location: 1373014-1374096
  
 NCBI BlastP on this gene

CCC07544

not annotated
  
Accession: CCC07545
  
Location: 1374719-1377143
  
  
**BlastP hit with Mycgr3G52682\_Mycgr3T**
  
Percentage identity: 47 %
  
BlastP bit score: 769
  
Sequence coverage: 70 %
  
E-value: 0.0
  
  
 NCBI BlastP on this gene

CCC07545

not annotated
  
Accession: CCC07546
  
Location: 1377509-1380006
  
 NCBI BlastP on this gene

CCC07546

not annotated
  
Accession: CCC07547
  
Location: 1382161-1385733
  
 NCBI BlastP on this gene

CCC07547

not annotated
  
Accession: CCC07548
  
Location: 1386763-1389137
  
 NCBI BlastP on this gene

CCC07548

Query: Architecture Search FASTA input

JH126401 : Cordyceps militaris CM01 unplaced genomic scaffold CCM\_S00003    Total score: 1.0     Cumulative Blast bit score: 758

Hit cluster cross-links:

Mycgr3G52686 Mycgr3T
  
Location: 0-861

Mycgr3G52686\_Mycgr3T

Mycgr3G102281 Mycgr3
  
Location: 961-1573

Mycgr3G102281\_Mycgr3

Mycgr3G89185 Mycgr3T
  
Location: 1673-2063

Mycgr3G89185\_Mycgr3T

Mycgr3G65725 Mycgr3T
  
Location: 2163-3612

Mycgr3G65725\_Mycgr3T

Mycgr3G102276 Mycgr3
  
Location: 3712-4801

Mycgr3G102276\_Mycgr3

Mycgr3G89189 Mycgr3T
  
Location: 4901-5564

Mycgr3G89189\_Mycgr3T

Mycgr3G52682 Mycgr3T
  
Location: 5664-9231

Mycgr3G52682\_Mycgr3T

Mycgr3G107072 Mycgr3
  
Location: 9331-13279

Mycgr3G107072\_Mycgr3

Mycgr3G34982 Mycgr3T
  
Location: 13379-15116

Mycgr3G34982\_Mycgr3T

Mycgr3G107069 Mycgr3
  
Location: 15216-17097

Mycgr3G107069\_Mycgr3

Mycgr3G32432 Mycgr3T
  
Location: 17197-19042

Mycgr3G32432\_Mycgr3T

Mycgr3G98385 Mycgr3T
  
Location: 19142-19898

Mycgr3G98385\_Mycgr3T

protein transport protein (SEC31), putative
  
Accession: EGX93172
  
Location: 2922175-2925688
  
 NCBI BlastP on this gene

EGX93172

RNA recognition protein, RNP-1
  
Accession: EGX93171
  
Location: 2920935-2921425
  
 NCBI BlastP on this gene

EGX93171

hypothetical protein
  
Accession: EGX93170
  
Location: 2920234-2920695
  
 NCBI BlastP on this gene

EGX93170

cytochrome c oxidase polypeptide VIb
  
Accession: EGX93169
  
Location: 2919354-2919908
  
 NCBI BlastP on this gene

EGX93169

hypothetical protein
  
Accession: EGX93168
  
Location: 2918187-2919029
  
 NCBI BlastP on this gene

EGX93168

40S ribosomal protein S10
  
Accession: EGX93167
  
Location: 2917247-2917877
  
 NCBI BlastP on this gene

EGX93167

ubiquitin-protein ligase (Hul4), putative
  
Accession: EGX93166
  
Location: 2911801-2915416
  
  
**BlastP hit with Mycgr3G52682\_Mycgr3T**
  
Percentage identity: 39 %
  
BlastP bit score: 758
  
Sequence coverage: 100 %
  
E-value: 0.0
  
  
 NCBI BlastP on this gene

EGX93166

50S ribosomal protein Mrp49
  
Accession: EGX93165
  
Location: 2910934-2911604
  
 NCBI BlastP on this gene

EGX93165

Protein phosphatase inhibitor
  
Accession: EGX93164
  
Location: 2907279-2910704
  
 NCBI BlastP on this gene

EGX93164

DUF726 domain protein
  
Accession: EGX93163
  
Location: 2906052-2906621
  
 NCBI BlastP on this gene

EGX93163

vesicular-fusion protein sec17
  
Accession: EGX93162
  
Location: 2904660-2905761
  
 NCBI BlastP on this gene

EGX93162

Armadillo-type fold domain containing protein
  
Accession: EGX93161
  
Location: 2900846-2903994
  
 NCBI BlastP on this gene

EGX93161

Query: Architecture Search FASTA input

KB722656 : Rhodosporidium toruloides NP11 unplaced genomic scaffold Scaffold15    Total score: 1.0     Cumulative Blast bit score: 694

Hit cluster cross-links:

Mycgr3G52686 Mycgr3T
  
Location: 0-861

Mycgr3G52686\_Mycgr3T

Mycgr3G102281 Mycgr3
  
Location: 961-1573

Mycgr3G102281\_Mycgr3

Mycgr3G89185 Mycgr3T
  
Location: 1673-2063

Mycgr3G89185\_Mycgr3T

Mycgr3G65725 Mycgr3T
  
Location: 2163-3612

Mycgr3G65725\_Mycgr3T

Mycgr3G102276 Mycgr3
  
Location: 3712-4801

Mycgr3G102276\_Mycgr3

Mycgr3G89189 Mycgr3T
  
Location: 4901-5564

Mycgr3G89189\_Mycgr3T

Mycgr3G52682 Mycgr3T
  
Location: 5664-9231

Mycgr3G52682\_Mycgr3T

Mycgr3G107072 Mycgr3
  
Location: 9331-13279

Mycgr3G107072\_Mycgr3

Mycgr3G34982 Mycgr3T
  
Location: 13379-15116

Mycgr3G34982\_Mycgr3T

Mycgr3G107069 Mycgr3
  
Location: 15216-17097

Mycgr3G107069\_Mycgr3

Mycgr3G32432 Mycgr3T
  
Location: 17197-19042

Mycgr3G32432\_Mycgr3T

Mycgr3G98385 Mycgr3T
  
Location: 19142-19898

Mycgr3G98385\_Mycgr3T

sulfide:quinone oxidoreductase, mitochondrial precursor
  
Accession: EMS21454
  
Location: 452989-455066
  
 NCBI BlastP on this gene

EMS21454

metallo-beta-lactamase domain containing protein
  
Accession: EMS21455
  
Location: 455336-456605
  
 NCBI BlastP on this gene

EMS21455

alcohol dehydrogenase (NADP+)
  
Accession: EMS21456
  
Location: 457177-459036
  
  
**BlastP hit with Mycgr3G102276\_Mycgr3**
  
Percentage identity: 53 %
  
BlastP bit score: 353
  
Sequence coverage: 99 %
  
E-value: 3e-116
  
  
 NCBI BlastP on this gene

EMS21456

MFS transporter, sugar transporter
  
Accession: EMS21457
  
Location: 459922-462605
  
 NCBI BlastP on this gene

EMS21457

hypothetical protein
  
Accession: EMS21458
  
Location: 462980-463903
  
 NCBI BlastP on this gene

EMS21458

protein of Emopamil-binding protein family
  
Accession: EMS21459
  
Location: 464025-465036
  
 NCBI BlastP on this gene

EMS21459

WSC domain protein
  
Accession: EMS21460
  
Location: 465561-467475
  
 NCBI BlastP on this gene

EMS21460

exportin-1
  
Accession: EMS21461
  
Location: 467866-472563
  
 NCBI BlastP on this gene

EMS21461

hypothetical protein
  
Accession: EMS21462
  
Location: 473166-473961
  
 NCBI BlastP on this gene

EMS21462

MFS nicotinic acid transporter
  
Accession: EMS21463
  
Location: 474746-477830
  
 NCBI BlastP on this gene

EMS21463

alcohol dehydrogenase (NADP+)
  
Accession: EMS21464
  
Location: 478333-480152
  
  
**BlastP hit with Mycgr3G102276\_Mycgr3**
  
Percentage identity: 52 %
  
BlastP bit score: 341
  
Sequence coverage: 103 %
  
E-value: 2e-111
  
  
 NCBI BlastP on this gene

EMS21464

glycoside hydrolase family 61 protein
  
Accession: EMS21465
  
Location: 481201-484560
  
 NCBI BlastP on this gene

EMS21465

Query: Architecture Search FASTA input

CH476618 : Uncinocarpus reesii 1704 scaffold\_4 genomic scaffold    Total score: 1.0     Cumulative Blast bit score: 677

Hit cluster cross-links:

Mycgr3G52686 Mycgr3T
  
Location: 0-861

Mycgr3G52686\_Mycgr3T

Mycgr3G102281 Mycgr3
  
Location: 961-1573

Mycgr3G102281\_Mycgr3

Mycgr3G89185 Mycgr3T
  
Location: 1673-2063

Mycgr3G89185\_Mycgr3T

Mycgr3G65725 Mycgr3T
  
Location: 2163-3612

Mycgr3G65725\_Mycgr3T

Mycgr3G102276 Mycgr3
  
Location: 3712-4801

Mycgr3G102276\_Mycgr3

Mycgr3G89189 Mycgr3T
  
Location: 4901-5564

Mycgr3G89189\_Mycgr3T

Mycgr3G52682 Mycgr3T
  
Location: 5664-9231

Mycgr3G52682\_Mycgr3T

Mycgr3G107072 Mycgr3
  
Location: 9331-13279

Mycgr3G107072\_Mycgr3

Mycgr3G34982 Mycgr3T
  
Location: 13379-15116

Mycgr3G34982\_Mycgr3T

Mycgr3G107069 Mycgr3
  
Location: 15216-17097

Mycgr3G107069\_Mycgr3

Mycgr3G32432 Mycgr3T
  
Location: 17197-19042

Mycgr3G32432\_Mycgr3T

Mycgr3G98385 Mycgr3T
  
Location: 19142-19898

Mycgr3G98385\_Mycgr3T

predicted protein
  
Accession: EEP81735
  
Location: 1420413-1421338
  
 NCBI BlastP on this gene

EEP81735

predicted protein
  
Accession: EEP81736
  
Location: 1422412-1423696
  
 NCBI BlastP on this gene

EEP81736

arsenical-resistance protein
  
Accession: EEP81737
  
Location: 1425296-1426587
  
 NCBI BlastP on this gene

EEP81737

VEG136 protein
  
Accession: EEP81738
  
Location: 1427714-1428361
  
 NCBI BlastP on this gene

EEP81738

conserved hypothetical protein
  
Accession: EEP81739
  
Location: 1429464-1433013
  
  
**BlastP hit with Mycgr3G52682\_Mycgr3T**
  
Percentage identity: 54 %
  
BlastP bit score: 677
  
Sequence coverage: 53 %
  
E-value: 0.0
  
  
 NCBI BlastP on this gene

EEP81739

predicted protein
  
Accession: EEP81740
  
Location: 1433658-1435001
  
 NCBI BlastP on this gene

EEP81740

hypothetical protein
  
Accession: EEP81741
  
Location: 1435514-1436963
  
 NCBI BlastP on this gene

EEP81741

conserved hypothetical protein
  
Accession: EEP81742
  
Location: 1437571-1438287
  
 NCBI BlastP on this gene

EEP81742

predicted protein
  
Accession: EEP81743
  
Location: 1439267-1440539
  
 NCBI BlastP on this gene

EEP81743

predicted protein
  
Accession: EEP81744
  
Location: 1441738-1441935
  
 NCBI BlastP on this gene

EEP81744

conserved hypothetical protein
  
Accession: EEP81745
  
Location: 1442527-1444839
  
 NCBI BlastP on this gene

EEP81745

Query: Architecture Search FASTA input

JH930469 : Phanerochaete carnosa HHB-10118-sp unplaced genomic scaffold PHACAscaffold\_2    Total score: 1.0     Cumulative Blast bit score: 650

Hit cluster cross-links:

Mycgr3G52686 Mycgr3T
  
Location: 0-861

Mycgr3G52686\_Mycgr3T

Mycgr3G102281 Mycgr3
  
Location: 961-1573

Mycgr3G102281\_Mycgr3

Mycgr3G89185 Mycgr3T
  
Location: 1673-2063

Mycgr3G89185\_Mycgr3T

Mycgr3G65725 Mycgr3T
  
Location: 2163-3612

Mycgr3G65725\_Mycgr3T

Mycgr3G102276 Mycgr3
  
Location: 3712-4801

Mycgr3G102276\_Mycgr3

Mycgr3G89189 Mycgr3T
  
Location: 4901-5564

Mycgr3G89189\_Mycgr3T

Mycgr3G52682 Mycgr3T
  
Location: 5664-9231

Mycgr3G52682\_Mycgr3T

Mycgr3G107072 Mycgr3
  
Location: 9331-13279

Mycgr3G107072\_Mycgr3

Mycgr3G34982 Mycgr3T
  
Location: 13379-15116

Mycgr3G34982\_Mycgr3T

Mycgr3G107069 Mycgr3
  
Location: 15216-17097

Mycgr3G107069\_Mycgr3

Mycgr3G32432 Mycgr3T
  
Location: 17197-19042

Mycgr3G32432\_Mycgr3T

Mycgr3G98385 Mycgr3T
  
Location: 19142-19898

Mycgr3G98385\_Mycgr3T

hypothetical protein
  
Accession: EKM59947
  
Location: 3780636-3782113
  
 NCBI BlastP on this gene

EKM59947

hypothetical protein
  
Accession: EKM59946
  
Location: 3780083-3780284
  
 NCBI BlastP on this gene

EKM59946

hypothetical protein
  
Accession: EKM59945
  
Location: 3772177-3774961
  
  
**BlastP hit with Mycgr3G34982\_Mycgr3T**
  
Percentage identity: 30 %
  
BlastP bit score: 232
  
Sequence coverage: 103 %
  
E-value: 3e-64
  
  
 NCBI BlastP on this gene

EKM59945

hypothetical protein
  
Accession: EKM59944
  
Location: 3767756-3770742
  
  
**BlastP hit with Mycgr3G34982\_Mycgr3T**
  
Percentage identity: 28 %
  
BlastP bit score: 195
  
Sequence coverage: 101 %
  
E-value: 6e-51
  
  
 NCBI BlastP on this gene

EKM59944

hypothetical protein
  
Accession: EKM59943
  
Location: 3763276-3766056
  
  
**BlastP hit with Mycgr3G34982\_Mycgr3T**
  
Percentage identity: 28 %
  
BlastP bit score: 223
  
Sequence coverage: 106 %
  
E-value: 6e-61
  
  
 NCBI BlastP on this gene

EKM59943

hypothetical protein
  
Accession: EKM59942
  
Location: 3757671-3761810
  
 NCBI BlastP on this gene

EKM59942

Query: Architecture Search FASTA input

EQ966375 : Postia placenta Mad-698-R POSPLscaffold\_143 genomic scaffold    Total score: 1.0     Cumulative Blast bit score: 638

Hit cluster cross-links:

Mycgr3G52686 Mycgr3T
  
Location: 0-861

Mycgr3G52686\_Mycgr3T

Mycgr3G102281 Mycgr3
  
Location: 961-1573

Mycgr3G102281\_Mycgr3

Mycgr3G89185 Mycgr3T
  
Location: 1673-2063

Mycgr3G89185\_Mycgr3T

Mycgr3G65725 Mycgr3T
  
Location: 2163-3612

Mycgr3G65725\_Mycgr3T

Mycgr3G102276 Mycgr3
  
Location: 3712-4801

Mycgr3G102276\_Mycgr3

Mycgr3G89189 Mycgr3T
  
Location: 4901-5564

Mycgr3G89189\_Mycgr3T

Mycgr3G52682 Mycgr3T
  
Location: 5664-9231

Mycgr3G52682\_Mycgr3T

Mycgr3G107072 Mycgr3
  
Location: 9331-13279

Mycgr3G107072\_Mycgr3

Mycgr3G34982 Mycgr3T
  
Location: 13379-15116

Mycgr3G34982\_Mycgr3T

Mycgr3G107069 Mycgr3
  
Location: 15216-17097

Mycgr3G107069\_Mycgr3

Mycgr3G32432 Mycgr3T
  
Location: 17197-19042

Mycgr3G32432\_Mycgr3T

Mycgr3G98385 Mycgr3T
  
Location: 19142-19898

Mycgr3G98385\_Mycgr3T

hypothetical triacylglycerol lipase
  
Accession: EED79575
  
Location: 72278-74743
  
 NCBI BlastP on this gene

EED79575

predicted protein
  
Accession: EED79587
  
Location: 75340-75838
  
 NCBI BlastP on this gene

EED79587

60S ribosomal protein L15
  
Accession: EED79576
  
Location: 76397-77216
  
  
**BlastP hit with Mycgr3G102281\_Mycgr3**
  
Percentage identity: 78 %
  
BlastP bit score: 319
  
Sequence coverage: 99 %
  
E-value: 7e-108
  
  
 NCBI BlastP on this gene

EED79576

predicted protein
  
Accession: EED79588
  
Location: 80222-80705
  
 NCBI BlastP on this gene

EED79588

60S ribosomal protein L15
  
Accession: EED79577
  
Location: 81218-82037
  
  
**BlastP hit with Mycgr3G102281\_Mycgr3**
  
Percentage identity: 78 %
  
BlastP bit score: 319
  
Sequence coverage: 99 %
  
E-value: 7e-108
  
  
 NCBI BlastP on this gene

EED79577

predicted protein
  
Accession: EED79578
  
Location: 83360-83961
  
 NCBI BlastP on this gene

EED79578

predicted protein
  
Accession: EED79579
  
Location: 89339-94114
  
 NCBI BlastP on this gene

EED79579

Query: Architecture Search FASTA input

KB726077 : Colletotrichum orbiculare MAFF 240422 unplaced genomic scaffold Scaffold\_499    Total score: 1.0     Cumulative Blast bit score: 630

Hit cluster cross-links:

Mycgr3G52686 Mycgr3T
  
Location: 0-861

Mycgr3G52686\_Mycgr3T

Mycgr3G102281 Mycgr3
  
Location: 961-1573

Mycgr3G102281\_Mycgr3

Mycgr3G89185 Mycgr3T
  
Location: 1673-2063

Mycgr3G89185\_Mycgr3T

Mycgr3G65725 Mycgr3T
  
Location: 2163-3612

Mycgr3G65725\_Mycgr3T

Mycgr3G102276 Mycgr3
  
Location: 3712-4801

Mycgr3G102276\_Mycgr3

Mycgr3G89189 Mycgr3T
  
Location: 4901-5564

Mycgr3G89189\_Mycgr3T

Mycgr3G52682 Mycgr3T
  
Location: 5664-9231

Mycgr3G52682\_Mycgr3T

Mycgr3G107072 Mycgr3
  
Location: 9331-13279

Mycgr3G107072\_Mycgr3

Mycgr3G34982 Mycgr3T
  
Location: 13379-15116

Mycgr3G34982\_Mycgr3T

Mycgr3G107069 Mycgr3
  
Location: 15216-17097

Mycgr3G107069\_Mycgr3

Mycgr3G32432 Mycgr3T
  
Location: 17197-19042

Mycgr3G32432\_Mycgr3T

Mycgr3G98385 Mycgr3T
  
Location: 19142-19898

Mycgr3G98385\_Mycgr3T

secreted protein
  
Accession: ENH77497
  
Location: 79361-81238
  
 NCBI BlastP on this gene

ENH77497

MFS monocarboxylate transporter
  
Accession: ENH77498
  
Location: 83778-85270
  
  
**BlastP hit with Mycgr3G65725\_Mycgr3T**
  
Percentage identity: 38 %
  
BlastP bit score: 308
  
Sequence coverage: 92 %
  
E-value: 4e-96
  
  
 NCBI BlastP on this gene

ENH77498

alkaline serine protease alp1
  
Accession: ENH77499
  
Location: 85427-86737
  
 NCBI BlastP on this gene

ENH77499

naphthalene -dioxygenase subunit alpha
  
Accession: ENH77500
  
Location: 88732-90113
  
 NCBI BlastP on this gene

ENH77500

hypothetical protein
  
Accession: ENH77501
  
Location: 91113-91517
  
 NCBI BlastP on this gene

ENH77501

sarcosine oxidase
  
Accession: ENH77502
  
Location: 92283-93740
  
 NCBI BlastP on this gene

ENH77502

C6 transcription factor
  
Accession: ENH77503
  
Location: 95122-97373
  
 NCBI BlastP on this gene

ENH77503

nad dehydrogenase
  
Accession: ENH77504
  
Location: 99210-101699
  
 NCBI BlastP on this gene

ENH77504

MFS monocarboxylate transporter
  
Accession: ENH77505
  
Location: 102026-103572
  
  
**BlastP hit with Mycgr3G65725\_Mycgr3T**
  
Percentage identity: 41 %
  
BlastP bit score: 322
  
Sequence coverage: 91 %
  
E-value: 3e-101
  
  
 NCBI BlastP on this gene

ENH77505

hypothetical protein
  
Accession: ENH77506
  
Location: 104221-104433
  
 NCBI BlastP on this gene

ENH77506

hypothetical protein
  
Accession: ENH77507
  
Location: 105912-106254
  
 NCBI BlastP on this gene

ENH77507

Query: Architecture Search FASTA input

KB369422 : Tupaia chinensis unplaced genomic scaffold Scaffold148626\_1    Total score: 1.0     Cumulative Blast bit score: 626

Hit cluster cross-links:

Mycgr3G52686 Mycgr3T
  
Location: 0-861

Mycgr3G52686\_Mycgr3T

Mycgr3G102281 Mycgr3
  
Location: 961-1573

Mycgr3G102281\_Mycgr3

Mycgr3G89185 Mycgr3T
  
Location: 1673-2063

Mycgr3G89185\_Mycgr3T

Mycgr3G65725 Mycgr3T
  
Location: 2163-3612

Mycgr3G65725\_Mycgr3T

Mycgr3G102276 Mycgr3
  
Location: 3712-4801

Mycgr3G102276\_Mycgr3

Mycgr3G89189 Mycgr3T
  
Location: 4901-5564

Mycgr3G89189\_Mycgr3T

Mycgr3G52682 Mycgr3T
  
Location: 5664-9231

Mycgr3G52682\_Mycgr3T

Mycgr3G107072 Mycgr3
  
Location: 9331-13279

Mycgr3G107072\_Mycgr3

Mycgr3G34982 Mycgr3T
  
Location: 13379-15116

Mycgr3G34982\_Mycgr3T

Mycgr3G107069 Mycgr3
  
Location: 15216-17097

Mycgr3G107069\_Mycgr3

Mycgr3G32432 Mycgr3T
  
Location: 17197-19042

Mycgr3G32432\_Mycgr3T

Mycgr3G98385 Mycgr3T
  
Location: 19142-19898

Mycgr3G98385\_Mycgr3T

NF-kappa-B inhibitor-interacting Ras-like protein 1
  
Accession: ELV09863
  
Location: 849780-854737
  
 NCBI BlastP on this gene

ELV09863

60S ribosomal protein L15
  
Accession: ELV09862
  
Location: 842822-844705
  
  
**BlastP hit with Mycgr3G102281\_Mycgr3**
  
Percentage identity: 72 %
  
BlastP bit score: 313
  
Sequence coverage: 100 %
  
E-value: 2e-105
  
  
 NCBI BlastP on this gene

ELV09862

60S ribosomal protein L15
  
Accession: ELV09861
  
Location: 835857-837740
  
  
**BlastP hit with Mycgr3G102281\_Mycgr3**
  
Percentage identity: 72 %
  
BlastP bit score: 313
  
Sequence coverage: 100 %
  
E-value: 2e-105
  
  
 NCBI BlastP on this gene

ELV09861

Query: Architecture Search FASTA input

JH711784 : Trametes versicolor FP-101664 SS1 unplaced genomic scaffold TRAVEscaffold\_2    Total score: 1.0     Cumulative Blast bit score: 608

Hit cluster cross-links:

Mycgr3G52686 Mycgr3T
  
Location: 0-861

Mycgr3G52686\_Mycgr3T

Mycgr3G102281 Mycgr3
  
Location: 961-1573

Mycgr3G102281\_Mycgr3

Mycgr3G89185 Mycgr3T
  
Location: 1673-2063

Mycgr3G89185\_Mycgr3T

Mycgr3G65725 Mycgr3T
  
Location: 2163-3612

Mycgr3G65725\_Mycgr3T

Mycgr3G102276 Mycgr3
  
Location: 3712-4801

Mycgr3G102276\_Mycgr3

Mycgr3G89189 Mycgr3T
  
Location: 4901-5564

Mycgr3G89189\_Mycgr3T

Mycgr3G52682 Mycgr3T
  
Location: 5664-9231

Mycgr3G52682\_Mycgr3T

Mycgr3G107072 Mycgr3
  
Location: 9331-13279

Mycgr3G107072\_Mycgr3

Mycgr3G34982 Mycgr3T
  
Location: 13379-15116

Mycgr3G34982\_Mycgr3T

Mycgr3G107069 Mycgr3
  
Location: 15216-17097

Mycgr3G107069\_Mycgr3

Mycgr3G32432 Mycgr3T
  
Location: 17197-19042

Mycgr3G32432\_Mycgr3T

Mycgr3G98385 Mycgr3T
  
Location: 19142-19898

Mycgr3G98385\_Mycgr3T

cytochrome P450
  
Accession: EIW62528
  
Location: 458490-460938
  
 NCBI BlastP on this gene

EIW62528

TFIIH basal transcription factor complex subunit SSL1
  
Accession: EIW62529
  
Location: 462928-464633
  
 NCBI BlastP on this gene

EIW62529

GroES-like protein
  
Accession: EIW62530
  
Location: 465439-466669
  
  
**BlastP hit with Mycgr3G102276\_Mycgr3**
  
Percentage identity: 45 %
  
BlastP bit score: 307
  
Sequence coverage: 93 %
  
E-value: 3e-98
  
  
 NCBI BlastP on this gene

EIW62530

nmrA-family protein
  
Accession: EIW62531
  
Location: 467827-469063
  
 NCBI BlastP on this gene

EIW62531

hypothetical protein
  
Accession: EIW62532
  
Location: 469720-470182
  
 NCBI BlastP on this gene

EIW62532

hypothetical protein
  
Accession: EIW62533
  
Location: 472701-473037
  
 NCBI BlastP on this gene

EIW62533

GroES-like protein
  
Accession: EIW62534
  
Location: 476151-477408
  
  
**BlastP hit with Mycgr3G102276\_Mycgr3**
  
Percentage identity: 47 %
  
BlastP bit score: 301
  
Sequence coverage: 98 %
  
E-value: 6e-96
  
  
 NCBI BlastP on this gene

EIW62534

hypothetical protein
  
Accession: EIW62535
  
Location: 480180-480584
  
 NCBI BlastP on this gene

EIW62535

acyl-CoA dehydrogenase NM domain-like protein
  
Accession: EIW62536
  
Location: 482884-484939
  
 NCBI BlastP on this gene

EIW62536

Query: Architecture Search FASTA input

KB446538 : Dothistroma septosporum NZE10 unplaced genomic scaffold DOTSEscaffold\_4    Total score: 1.0     Cumulative Blast bit score: 591

Hit cluster cross-links:

Mycgr3G52686 Mycgr3T
  
Location: 0-861

Mycgr3G52686\_Mycgr3T

Mycgr3G102281 Mycgr3
  
Location: 961-1573

Mycgr3G102281\_Mycgr3

Mycgr3G89185 Mycgr3T
  
Location: 1673-2063

Mycgr3G89185\_Mycgr3T

Mycgr3G65725 Mycgr3T
  
Location: 2163-3612

Mycgr3G65725\_Mycgr3T

Mycgr3G102276 Mycgr3
  
Location: 3712-4801

Mycgr3G102276\_Mycgr3

Mycgr3G89189 Mycgr3T
  
Location: 4901-5564

Mycgr3G89189\_Mycgr3T

Mycgr3G52682 Mycgr3T
  
Location: 5664-9231

Mycgr3G52682\_Mycgr3T

Mycgr3G107072 Mycgr3
  
Location: 9331-13279

Mycgr3G107072\_Mycgr3

Mycgr3G34982 Mycgr3T
  
Location: 13379-15116

Mycgr3G34982\_Mycgr3T

Mycgr3G107069 Mycgr3
  
Location: 15216-17097

Mycgr3G107069\_Mycgr3

Mycgr3G32432 Mycgr3T
  
Location: 17197-19042

Mycgr3G32432\_Mycgr3T

Mycgr3G98385 Mycgr3T
  
Location: 19142-19898

Mycgr3G98385\_Mycgr3T

hypothetical protein
  
Accession: EME45384
  
Location: 1344560-1344772
  
 NCBI BlastP on this gene

EME45384

hypothetical protein
  
Accession: EME45383
  
Location: 1339753-1342092
  
 NCBI BlastP on this gene

EME45383

hypothetical protein
  
Accession: EME45382
  
Location: 1337265-1339022
  
 NCBI BlastP on this gene

EME45382

alcohol dehydrogenase-like protein
  
Accession: EME45381
  
Location: 1335391-1336531
  
  
**BlastP hit with Mycgr3G102276\_Mycgr3**
  
Percentage identity: 81 %
  
BlastP bit score: 591
  
Sequence coverage: 99 %
  
E-value: 0.0
  
  
 NCBI BlastP on this gene

EME45381

Non-ribosomal peptide synthetase-like protein
  
Accession: EME45380
  
Location: 1327192-1335024
  
 NCBI BlastP on this gene

EME45380

hypothetical protein
  
Accession: EME45379
  
Location: 1325632-1326337
  
 NCBI BlastP on this gene

EME45379

Query: Architecture Search FASTA input

GL988032 : Chaetomium thermophilum var. thermophilum DSM 1495 unplaced genomic scaffold scf7180000...    Total score: 1.0     Cumulative Blast bit score: 590

Hit cluster cross-links:

Mycgr3G52686 Mycgr3T
  
Location: 0-861

Mycgr3G52686\_Mycgr3T

Mycgr3G102281 Mycgr3
  
Location: 961-1573

Mycgr3G102281\_Mycgr3

Mycgr3G89185 Mycgr3T
  
Location: 1673-2063

Mycgr3G89185\_Mycgr3T

Mycgr3G65725 Mycgr3T
  
Location: 2163-3612

Mycgr3G65725\_Mycgr3T

Mycgr3G102276 Mycgr3
  
Location: 3712-4801

Mycgr3G102276\_Mycgr3

Mycgr3G89189 Mycgr3T
  
Location: 4901-5564

Mycgr3G89189\_Mycgr3T

Mycgr3G52682 Mycgr3T
  
Location: 5664-9231

Mycgr3G52682\_Mycgr3T

Mycgr3G107072 Mycgr3
  
Location: 9331-13279

Mycgr3G107072\_Mycgr3

Mycgr3G34982 Mycgr3T
  
Location: 13379-15116

Mycgr3G34982\_Mycgr3T

Mycgr3G107069 Mycgr3
  
Location: 15216-17097

Mycgr3G107069\_Mycgr3

Mycgr3G32432 Mycgr3T
  
Location: 17197-19042

Mycgr3G32432\_Mycgr3T

Mycgr3G98385 Mycgr3T
  
Location: 19142-19898

Mycgr3G98385\_Mycgr3T

putative pyridoxal phosphate binding protein
  
Accession: EGS23544
  
Location: 689202-690843
  
 NCBI BlastP on this gene

EGS23544

hypothetical protein
  
Accession: EGS23545
  
Location: 692818-696603
  
 NCBI BlastP on this gene

EGS23545

ubiquitin-protein ligase-like protein
  
Accession: EGS23546
  
Location: 699282-703480
  
  
**BlastP hit with Mycgr3G52682\_Mycgr3T**
  
Percentage identity: 36 %
  
BlastP bit score: 590
  
Sequence coverage: 102 %
  
E-value: 0.0
  
  
 NCBI BlastP on this gene

EGS23546

hypothetical protein
  
Accession: EGS23547
  
Location: 707149-708360
  
 NCBI BlastP on this gene

EGS23547

hypothetical protein
  
Accession: EGS23548
  
Location: 709375-711357
  
 NCBI BlastP on this gene

EGS23548

hypothetical protein
  
Accession: EGS23549
  
Location: 711597-712278
  
 NCBI BlastP on this gene

EGS23549

Query: Architecture Search FASTA input

KB446537 : Dothistroma septosporum NZE10 unplaced genomic scaffold DOTSEscaffold\_3    Total score: 1.0     Cumulative Blast bit score: 585

Hit cluster cross-links:

Mycgr3G52686 Mycgr3T
  
Location: 0-861

Mycgr3G52686\_Mycgr3T

Mycgr3G102281 Mycgr3
  
Location: 961-1573

Mycgr3G102281\_Mycgr3

Mycgr3G89185 Mycgr3T
  
Location: 1673-2063

Mycgr3G89185\_Mycgr3T

Mycgr3G65725 Mycgr3T
  
Location: 2163-3612

Mycgr3G65725\_Mycgr3T

Mycgr3G102276 Mycgr3
  
Location: 3712-4801

Mycgr3G102276\_Mycgr3

Mycgr3G89189 Mycgr3T
  
Location: 4901-5564

Mycgr3G89189\_Mycgr3T

Mycgr3G52682 Mycgr3T
  
Location: 5664-9231

Mycgr3G52682\_Mycgr3T

Mycgr3G107072 Mycgr3
  
Location: 9331-13279

Mycgr3G107072\_Mycgr3

Mycgr3G34982 Mycgr3T
  
Location: 13379-15116

Mycgr3G34982\_Mycgr3T

Mycgr3G107069 Mycgr3
  
Location: 15216-17097

Mycgr3G107069\_Mycgr3

Mycgr3G32432 Mycgr3T
  
Location: 17197-19042

Mycgr3G32432\_Mycgr3T

Mycgr3G98385 Mycgr3T
  
Location: 19142-19898

Mycgr3G98385\_Mycgr3T

glycoside hydrolase family 31 protein
  
Accession: EME46579
  
Location: 1850121-1853350
  
 NCBI BlastP on this gene

EME46579

hypothetical protein
  
Accession: EME46580
  
Location: 1854224-1856905
  
 NCBI BlastP on this gene

EME46580

hypothetical protein
  
Accession: EME46581
  
Location: 1857568-1858655
  
 NCBI BlastP on this gene

EME46581

hypothetical protein
  
Accession: EME46582
  
Location: 1859294-1860091
  
 NCBI BlastP on this gene

EME46582

hypothetical protein
  
Accession: EME46583
  
Location: 1860438-1861013
  
 NCBI BlastP on this gene

EME46583

hypothetical protein
  
Accession: EME46584
  
Location: 1863045-1864610
  
  
**BlastP hit with Mycgr3G65725\_Mycgr3T**
  
Percentage identity: 59 %
  
BlastP bit score: 585
  
Sequence coverage: 98 %
  
E-value: 0.0
  
  
 NCBI BlastP on this gene

EME46584

hypothetical protein
  
Accession: EME46585
  
Location: 1865508-1867113
  
 NCBI BlastP on this gene

EME46585

hypothetical protein
  
Accession: EME46586
  
Location: 1868028-1868414
  
 NCBI BlastP on this gene

EME46586

hypothetical protein
  
Accession: EME46587
  
Location: 1868724-1868960
  
 NCBI BlastP on this gene

EME46587

hypothetical protein
  
Accession: EME46588
  
Location: 1869809-1871643
  
 NCBI BlastP on this gene

EME46588

hypothetical protein
  
Accession: EME46589
  
Location: 1875008-1875946
  
 NCBI BlastP on this gene

EME46589

Query: Architecture Search FASTA input

KB469296 : Gloeophyllum trabeum ATCC 11539 unplaced genomic scaffold GLOTRscaffold\_00001    Total score: 1.0     Cumulative Blast bit score: 582

Hit cluster cross-links:

Mycgr3G52686 Mycgr3T
  
Location: 0-861

Mycgr3G52686\_Mycgr3T

Mycgr3G102281 Mycgr3
  
Location: 961-1573

Mycgr3G102281\_Mycgr3

Mycgr3G89185 Mycgr3T
  
Location: 1673-2063

Mycgr3G89185\_Mycgr3T

Mycgr3G65725 Mycgr3T
  
Location: 2163-3612

Mycgr3G65725\_Mycgr3T

Mycgr3G102276 Mycgr3
  
Location: 3712-4801

Mycgr3G102276\_Mycgr3

Mycgr3G89189 Mycgr3T
  
Location: 4901-5564

Mycgr3G89189\_Mycgr3T

Mycgr3G52682 Mycgr3T
  
Location: 5664-9231

Mycgr3G52682\_Mycgr3T

Mycgr3G107072 Mycgr3
  
Location: 9331-13279

Mycgr3G107072\_Mycgr3

Mycgr3G34982 Mycgr3T
  
Location: 13379-15116

Mycgr3G34982\_Mycgr3T

Mycgr3G107069 Mycgr3
  
Location: 15216-17097

Mycgr3G107069\_Mycgr3

Mycgr3G32432 Mycgr3T
  
Location: 17197-19042

Mycgr3G32432\_Mycgr3T

Mycgr3G98385 Mycgr3T
  
Location: 19142-19898

Mycgr3G98385\_Mycgr3T

hypothetical protein
  
Accession: EPQ60827
  
Location: 2585534-2586492
  
 NCBI BlastP on this gene

EPQ60827

hypothetical protein
  
Accession: EPQ60828
  
Location: 2586666-2587477
  
 NCBI BlastP on this gene

EPQ60828

P-loop containing nucleoside triphosphate hydrolase protein
  
Accession: EPQ60829
  
Location: 2589456-2590132
  
 NCBI BlastP on this gene

EPQ60829

hypothetical protein
  
Accession: EPQ60830
  
Location: 2590224-2591050
  
 NCBI BlastP on this gene

EPQ60830

hypothetical protein
  
Accession: EPQ60831
  
Location: 2591435-2593833
  
 NCBI BlastP on this gene

EPQ60831

GroES-like protein
  
Accession: EPQ60832
  
Location: 2595514-2597063
  
  
**BlastP hit with Mycgr3G102276\_Mycgr3**
  
Percentage identity: 46 %
  
BlastP bit score: 290
  
Sequence coverage: 97 %
  
E-value: 1e-91
  
  
 NCBI BlastP on this gene

EPQ60832

GroES-like protein
  
Accession: EPQ60833
  
Location: 2597653-2599221
  
  
**BlastP hit with Mycgr3G102276\_Mycgr3**
  
Percentage identity: 46 %
  
BlastP bit score: 292
  
Sequence coverage: 100 %
  
E-value: 1e-92
  
  
 NCBI BlastP on this gene

EPQ60833

GroES-like protein
  
Accession: EPQ60834
  
Location: 2600234-2601568
  
 NCBI BlastP on this gene

EPQ60834

hypothetical protein
  
Accession: EPQ60835
  
Location: 2607896-2608246
  
 NCBI BlastP on this gene

EPQ60835

Query: Architecture Search FASTA input

KB456262 : Mycosphaerella populorum SO2202 unplaced genomic scaffold SEPMUscaffold\_3    Total score: 1.0     Cumulative Blast bit score: 578

Hit cluster cross-links:

Mycgr3G52686 Mycgr3T
  
Location: 0-861

Mycgr3G52686\_Mycgr3T

Mycgr3G102281 Mycgr3
  
Location: 961-1573

Mycgr3G102281\_Mycgr3

Mycgr3G89185 Mycgr3T
  
Location: 1673-2063

Mycgr3G89185\_Mycgr3T

Mycgr3G65725 Mycgr3T
  
Location: 2163-3612

Mycgr3G65725\_Mycgr3T

Mycgr3G102276 Mycgr3
  
Location: 3712-4801

Mycgr3G102276\_Mycgr3

Mycgr3G89189 Mycgr3T
  
Location: 4901-5564

Mycgr3G89189\_Mycgr3T

Mycgr3G52682 Mycgr3T
  
Location: 5664-9231

Mycgr3G52682\_Mycgr3T

Mycgr3G107072 Mycgr3
  
Location: 9331-13279

Mycgr3G107072\_Mycgr3

Mycgr3G34982 Mycgr3T
  
Location: 13379-15116

Mycgr3G34982\_Mycgr3T

Mycgr3G107069 Mycgr3
  
Location: 15216-17097

Mycgr3G107069\_Mycgr3

Mycgr3G32432 Mycgr3T
  
Location: 17197-19042

Mycgr3G32432\_Mycgr3T

Mycgr3G98385 Mycgr3T
  
Location: 19142-19898

Mycgr3G98385\_Mycgr3T

40S ribosomal protein SA
  
Accession: EMF14620
  
Location: 1899575-1900652
  
 NCBI BlastP on this gene

EMF14620

Pentafunctional AroM protein
  
Accession: EMF14621
  
Location: 1901417-1906261
  
 NCBI BlastP on this gene

EMF14621

glycoside hydrolase family 20 protein
  
Accession: EMF14622
  
Location: 1907051-1909497
  
 NCBI BlastP on this gene

EMF14622

cyanamide hydratase
  
Accession: EMF14623
  
Location: 1909650-1910466
  
 NCBI BlastP on this gene

EMF14623

MFS general substrate transporter
  
Accession: EMF14624
  
Location: 1911693-1913404
  
  
**BlastP hit with Mycgr3G65725\_Mycgr3T**
  
Percentage identity: 57 %
  
BlastP bit score: 578
  
Sequence coverage: 102 %
  
E-value: 0.0
  
  
 NCBI BlastP on this gene

EMF14624

hypothetical protein
  
Accession: EMF14625
  
Location: 1914196-1915452
  
 NCBI BlastP on this gene

EMF14625

hypothetical protein
  
Accession: EMF14626
  
Location: 1916268-1918433
  
 NCBI BlastP on this gene

EMF14626

bleomycin hydrolase
  
Accession: EMF14627
  
Location: 1919438-1921188
  
 NCBI BlastP on this gene

EMF14627

carboxyvinyl-carboxyphosphonate phosphorylmutase
  
Accession: EMF14628
  
Location: 1924575-1925525
  
 NCBI BlastP on this gene

EMF14628

Query: Architecture Search FASTA input

KB446537 : Dothistroma septosporum NZE10 unplaced genomic scaffold DOTSEscaffold\_3    Total score: 1.0     Cumulative Blast bit score: 574

Hit cluster cross-links:

Mycgr3G52686 Mycgr3T
  
Location: 0-861

Mycgr3G52686\_Mycgr3T

Mycgr3G102281 Mycgr3
  
Location: 961-1573

Mycgr3G102281\_Mycgr3

Mycgr3G89185 Mycgr3T
  
Location: 1673-2063

Mycgr3G89185\_Mycgr3T

Mycgr3G65725 Mycgr3T
  
Location: 2163-3612

Mycgr3G65725\_Mycgr3T

Mycgr3G102276 Mycgr3
  
Location: 3712-4801

Mycgr3G102276\_Mycgr3

Mycgr3G89189 Mycgr3T
  
Location: 4901-5564

Mycgr3G89189\_Mycgr3T

Mycgr3G52682 Mycgr3T
  
Location: 5664-9231

Mycgr3G52682\_Mycgr3T

Mycgr3G107072 Mycgr3
  
Location: 9331-13279

Mycgr3G107072\_Mycgr3

Mycgr3G34982 Mycgr3T
  
Location: 13379-15116

Mycgr3G34982\_Mycgr3T

Mycgr3G107069 Mycgr3
  
Location: 15216-17097

Mycgr3G107069\_Mycgr3

Mycgr3G32432 Mycgr3T
  
Location: 17197-19042

Mycgr3G32432\_Mycgr3T

Mycgr3G98385 Mycgr3T
  
Location: 19142-19898

Mycgr3G98385\_Mycgr3T

glycoside hydrolase family 79 protein
  
Accession: EME46224
  
Location: 980417-982141
  
 NCBI BlastP on this gene

EME46224

hypothetical protein
  
Accession: EME46223
  
Location: 978877-979431
  
 NCBI BlastP on this gene

EME46223

hypothetical protein
  
Accession: EME46222
  
Location: 974667-975911
  
 NCBI BlastP on this gene

EME46222

hypothetical protein
  
Accession: EME46221
  
Location: 973130-974326
  
 NCBI BlastP on this gene

EME46221

hypothetical protein
  
Accession: EME46220
  
Location: 968169-969949
  
  
**BlastP hit with Mycgr3G34982\_Mycgr3T**
  
Percentage identity: 55 %
  
BlastP bit score: 574
  
Sequence coverage: 88 %
  
E-value: 0.0
  
  
 NCBI BlastP on this gene

EME46220

hypothetical protein
  
Accession: EME46219
  
Location: 965489-967363
  
 NCBI BlastP on this gene

EME46219

hypothetical protein
  
Accession: EME46218
  
Location: 960958-962170
  
 NCBI BlastP on this gene

EME46218

hypothetical protein
  
Accession: EME46217
  
Location: 959502-960049
  
 NCBI BlastP on this gene

EME46217

hypothetical protein
  
Accession: EME46216
  
Location: 957799-958567
  
 NCBI BlastP on this gene

EME46216

Query: Architecture Search FASTA input

KB446560 : Pseudocercospora fijiensis CIRAD86 unplaced genomic scaffold MYCFIscaffold\_6    Total score: 1.0     Cumulative Blast bit score: 570

Hit cluster cross-links:

Mycgr3G52686 Mycgr3T
  
Location: 0-861

Mycgr3G52686\_Mycgr3T

Mycgr3G102281 Mycgr3
  
Location: 961-1573

Mycgr3G102281\_Mycgr3

Mycgr3G89185 Mycgr3T
  
Location: 1673-2063

Mycgr3G89185\_Mycgr3T

Mycgr3G65725 Mycgr3T
  
Location: 2163-3612

Mycgr3G65725\_Mycgr3T

Mycgr3G102276 Mycgr3
  
Location: 3712-4801

Mycgr3G102276\_Mycgr3

Mycgr3G89189 Mycgr3T
  
Location: 4901-5564

Mycgr3G89189\_Mycgr3T

Mycgr3G52682 Mycgr3T
  
Location: 5664-9231

Mycgr3G52682\_Mycgr3T

Mycgr3G107072 Mycgr3
  
Location: 9331-13279

Mycgr3G107072\_Mycgr3

Mycgr3G34982 Mycgr3T
  
Location: 13379-15116

Mycgr3G34982\_Mycgr3T

Mycgr3G107069 Mycgr3
  
Location: 15216-17097

Mycgr3G107069\_Mycgr3

Mycgr3G32432 Mycgr3T
  
Location: 17197-19042

Mycgr3G32432\_Mycgr3T

Mycgr3G98385 Mycgr3T
  
Location: 19142-19898

Mycgr3G98385\_Mycgr3T

hypothetical protein
  
Accession: EME81246
  
Location: 3017539-3018200
  
 NCBI BlastP on this gene

EME81246

hypothetical protein
  
Accession: EME81247
  
Location: 3018579-3019836
  
  
**BlastP hit with Mycgr3G102276\_Mycgr3**
  
Percentage identity: 78 %
  
BlastP bit score: 570
  
Sequence coverage: 99 %
  
E-value: 0.0
  
  
 NCBI BlastP on this gene

EME81247

Query: Architecture Search FASTA input

KB733456 : Bipolaris maydis ATCC 48331 unplaced genomic scaffold COCC4scaffold\_13    Total score: 1.0     Cumulative Blast bit score: 549

Hit cluster cross-links:

Mycgr3G52686 Mycgr3T
  
Location: 0-861

Mycgr3G52686\_Mycgr3T

Mycgr3G102281 Mycgr3
  
Location: 961-1573

Mycgr3G102281\_Mycgr3

Mycgr3G89185 Mycgr3T
  
Location: 1673-2063

Mycgr3G89185\_Mycgr3T

Mycgr3G65725 Mycgr3T
  
Location: 2163-3612

Mycgr3G65725\_Mycgr3T

Mycgr3G102276 Mycgr3
  
Location: 3712-4801

Mycgr3G102276\_Mycgr3

Mycgr3G89189 Mycgr3T
  
Location: 4901-5564

Mycgr3G89189\_Mycgr3T

Mycgr3G52682 Mycgr3T
  
Location: 5664-9231

Mycgr3G52682\_Mycgr3T

Mycgr3G107072 Mycgr3
  
Location: 9331-13279

Mycgr3G107072\_Mycgr3

Mycgr3G34982 Mycgr3T
  
Location: 13379-15116

Mycgr3G34982\_Mycgr3T

Mycgr3G107069 Mycgr3
  
Location: 15216-17097

Mycgr3G107069\_Mycgr3

Mycgr3G32432 Mycgr3T
  
Location: 17197-19042

Mycgr3G32432\_Mycgr3T

Mycgr3G98385 Mycgr3T
  
Location: 19142-19898

Mycgr3G98385\_Mycgr3T

hypothetical protein
  
Accession: ENI04832
  
Location: 777525-778834
  
 NCBI BlastP on this gene

ENI04832

hypothetical protein
  
Accession: ENI04831
  
Location: 774643-775837
  
 NCBI BlastP on this gene

ENI04831

hypothetical protein
  
Accession: ENI04830
  
Location: 773905-774239
  
 NCBI BlastP on this gene

ENI04830

hypothetical protein
  
Accession: ENI04829
  
Location: 772650-773666
  
 NCBI BlastP on this gene

ENI04829

hypothetical protein
  
Accession: ENI04828
  
Location: 770593-772243
  
 NCBI BlastP on this gene

ENI04828

hypothetical protein
  
Accession: ENI04827
  
Location: 768617-769800
  
  
**BlastP hit with Mycgr3G102276\_Mycgr3**
  
Percentage identity: 76 %
  
BlastP bit score: 549
  
Sequence coverage: 96 %
  
E-value: 0.0
  
  
 NCBI BlastP on this gene

ENI04827

hypothetical protein
  
Accession: ENI04826
  
Location: 764419-765669
  
 NCBI BlastP on this gene

ENI04826

hypothetical protein
  
Accession: ENI04825
  
Location: 756765-760101
  
 NCBI BlastP on this gene

ENI04825

Query: Architecture Search FASTA input

KB445574 : Cochliobolus heterostrophus C5 unplaced genomic scaffold COCHEscaffold\_6    Total score: 1.0     Cumulative Blast bit score: 549

Hit cluster cross-links:

Mycgr3G52686 Mycgr3T
  
Location: 0-861

Mycgr3G52686\_Mycgr3T

Mycgr3G102281 Mycgr3
  
Location: 961-1573

Mycgr3G102281\_Mycgr3

Mycgr3G89185 Mycgr3T
  
Location: 1673-2063

Mycgr3G89185\_Mycgr3T

Mycgr3G65725 Mycgr3T
  
Location: 2163-3612

Mycgr3G65725\_Mycgr3T

Mycgr3G102276 Mycgr3
  
Location: 3712-4801

Mycgr3G102276\_Mycgr3

Mycgr3G89189 Mycgr3T
  
Location: 4901-5564

Mycgr3G89189\_Mycgr3T

Mycgr3G52682 Mycgr3T
  
Location: 5664-9231

Mycgr3G52682\_Mycgr3T

Mycgr3G107072 Mycgr3
  
Location: 9331-13279

Mycgr3G107072\_Mycgr3

Mycgr3G34982 Mycgr3T
  
Location: 13379-15116

Mycgr3G34982\_Mycgr3T

Mycgr3G107069 Mycgr3
  
Location: 15216-17097

Mycgr3G107069\_Mycgr3

Mycgr3G32432 Mycgr3T
  
Location: 17197-19042

Mycgr3G32432\_Mycgr3T

Mycgr3G98385 Mycgr3T
  
Location: 19142-19898

Mycgr3G98385\_Mycgr3T

hypothetical protein
  
Accession: EMD92780
  
Location: 201125-202434
  
 NCBI BlastP on this gene

EMD92780

hypothetical protein
  
Accession: EMD92781
  
Location: 204122-205316
  
 NCBI BlastP on this gene

EMD92781

hypothetical protein
  
Accession: EMD92782
  
Location: 205720-206054
  
 NCBI BlastP on this gene

EMD92782

hypothetical protein
  
Accession: EMD92783
  
Location: 206293-207309
  
 NCBI BlastP on this gene

EMD92783

hypothetical protein
  
Accession: EMD92784
  
Location: 207716-209366
  
 NCBI BlastP on this gene

EMD92784

hypothetical protein
  
Accession: EMD92785
  
Location: 210159-211342
  
  
**BlastP hit with Mycgr3G102276\_Mycgr3**
  
Percentage identity: 76 %
  
BlastP bit score: 549
  
Sequence coverage: 96 %
  
E-value: 0.0
  
  
 NCBI BlastP on this gene

EMD92785

Query: Architecture Search FASTA input

CH471151 : Homo sapiens 211000035831495 genomic scaffold    Total score: 1.0     Cumulative Blast bit score: 548

Hit cluster cross-links:

Mycgr3G52686 Mycgr3T
  
Location: 0-861

Mycgr3G52686\_Mycgr3T

Mycgr3G102281 Mycgr3
  
Location: 961-1573

Mycgr3G102281\_Mycgr3

Mycgr3G89185 Mycgr3T
  
Location: 1673-2063

Mycgr3G89185\_Mycgr3T

Mycgr3G65725 Mycgr3T
  
Location: 2163-3612

Mycgr3G65725\_Mycgr3T

Mycgr3G102276 Mycgr3
  
Location: 3712-4801

Mycgr3G102276\_Mycgr3

Mycgr3G89189 Mycgr3T
  
Location: 4901-5564

Mycgr3G89189\_Mycgr3T

Mycgr3G52682 Mycgr3T
  
Location: 5664-9231

Mycgr3G52682\_Mycgr3T

Mycgr3G107072 Mycgr3
  
Location: 9331-13279

Mycgr3G107072\_Mycgr3

Mycgr3G34982 Mycgr3T
  
Location: 13379-15116

Mycgr3G34982\_Mycgr3T

Mycgr3G107069 Mycgr3
  
Location: 15216-17097

Mycgr3G107069\_Mycgr3

Mycgr3G32432 Mycgr3T
  
Location: 17197-19042

Mycgr3G32432\_Mycgr3T

Mycgr3G98385 Mycgr3T
  
Location: 19142-19898

Mycgr3G98385\_Mycgr3T

ubiquitin protein ligase E3A (human papilloma
  
Accession: CP1918456
  
Location: 1967266-2033573
  
  
**BlastP hit with Mycgr3G52682\_Mycgr3T**
  
Percentage identity: 35 %
  
BlastP bit score: 274
  
Sequence coverage: 39 %
  
E-value: 2e-74
  
  
 NCBI BlastP on this gene

CP1918456

ubiquitin protein ligase E3A (human papilloma
  
Accession: CP1752652
  
Location: 1967264-2033573
  
  
**BlastP hit with Mycgr3G52682\_Mycgr3T**
  
Percentage identity: 35 %
  
BlastP bit score: 274
  
Sequence coverage: 39 %
  
E-value: 2e-74
  
  
 NCBI BlastP on this gene

CP1752652

Query: Architecture Search FASTA input

KB445644 : Cochliobolus sativus ND90Pr unplaced genomic scaffold COCSAscaffold\_8    Total score: 1.0     Cumulative Blast bit score: 540

Hit cluster cross-links:

Mycgr3G52686 Mycgr3T
  
Location: 0-861

Mycgr3G52686\_Mycgr3T

Mycgr3G102281 Mycgr3
  
Location: 961-1573

Mycgr3G102281\_Mycgr3

Mycgr3G89185 Mycgr3T
  
Location: 1673-2063

Mycgr3G89185\_Mycgr3T

Mycgr3G65725 Mycgr3T
  
Location: 2163-3612

Mycgr3G65725\_Mycgr3T

Mycgr3G102276 Mycgr3
  
Location: 3712-4801

Mycgr3G102276\_Mycgr3

Mycgr3G89189 Mycgr3T
  
Location: 4901-5564

Mycgr3G89189\_Mycgr3T

Mycgr3G52682 Mycgr3T
  
Location: 5664-9231

Mycgr3G52682\_Mycgr3T

Mycgr3G107072 Mycgr3
  
Location: 9331-13279

Mycgr3G107072\_Mycgr3

Mycgr3G34982 Mycgr3T
  
Location: 13379-15116

Mycgr3G34982\_Mycgr3T

Mycgr3G107069 Mycgr3
  
Location: 15216-17097

Mycgr3G107069\_Mycgr3

Mycgr3G32432 Mycgr3T
  
Location: 17197-19042

Mycgr3G32432\_Mycgr3T

Mycgr3G98385 Mycgr3T
  
Location: 19142-19898

Mycgr3G98385\_Mycgr3T

hypothetical protein
  
Accession: EMD63595
  
Location: 617839-619148
  
 NCBI BlastP on this gene

EMD63595

hypothetical protein
  
Accession: EMD63596
  
Location: 620804-621998
  
 NCBI BlastP on this gene

EMD63596

hypothetical protein
  
Accession: EMD63597
  
Location: 622400-622736
  
 NCBI BlastP on this gene

EMD63597

hypothetical protein
  
Accession: EMD63598
  
Location: 622975-623991
  
 NCBI BlastP on this gene

EMD63598

hypothetical protein
  
Accession: EMD63599
  
Location: 624399-626049
  
 NCBI BlastP on this gene

EMD63599

hypothetical protein
  
Accession: EMD63600
  
Location: 626828-628011
  
  
**BlastP hit with Mycgr3G102276\_Mycgr3**
  
Percentage identity: 74 %
  
BlastP bit score: 540
  
Sequence coverage: 96 %
  
E-value: 0.0
  
  
 NCBI BlastP on this gene

EMD63600

Query: Architecture Search FASTA input

GG698901 : Nectria haematococca mpVI 77-13-4 chromosome 1 genomic scaffold NECHAsca\_8\_chr1\_1\_0    Total score: 1.0     Cumulative Blast bit score: 530

Hit cluster cross-links:

Mycgr3G52686 Mycgr3T
  
Location: 0-861

Mycgr3G52686\_Mycgr3T

Mycgr3G102281 Mycgr3
  
Location: 961-1573

Mycgr3G102281\_Mycgr3

Mycgr3G89185 Mycgr3T
  
Location: 1673-2063

Mycgr3G89185\_Mycgr3T

Mycgr3G65725 Mycgr3T
  
Location: 2163-3612

Mycgr3G65725\_Mycgr3T

Mycgr3G102276 Mycgr3
  
Location: 3712-4801

Mycgr3G102276\_Mycgr3

Mycgr3G89189 Mycgr3T
  
Location: 4901-5564

Mycgr3G89189\_Mycgr3T

Mycgr3G52682 Mycgr3T
  
Location: 5664-9231

Mycgr3G52682\_Mycgr3T

Mycgr3G107072 Mycgr3
  
Location: 9331-13279

Mycgr3G107072\_Mycgr3

Mycgr3G34982 Mycgr3T
  
Location: 13379-15116

Mycgr3G34982\_Mycgr3T

Mycgr3G107069 Mycgr3
  
Location: 15216-17097

Mycgr3G107069\_Mycgr3

Mycgr3G32432 Mycgr3T
  
Location: 17197-19042

Mycgr3G32432\_Mycgr3T

Mycgr3G98385 Mycgr3T
  
Location: 19142-19898

Mycgr3G98385\_Mycgr3T

predicted protein
  
Accession: EEU44046
  
Location: 240806-243590
  
 NCBI BlastP on this gene

EEU44046

hypothetical protein
  
Accession: EEU44047
  
Location: 244064-245893
  
 NCBI BlastP on this gene

EEU44047

hypothetical protein
  
Accession: EEU43842
  
Location: 246403-247352
  
 NCBI BlastP on this gene

EEU43842

hypothetical protein
  
Accession: EEU44048
  
Location: 247807-249605
  
 NCBI BlastP on this gene

EEU44048

hypothetical protein
  
Accession: EEU43843
  
Location: 251504-252123
  
 NCBI BlastP on this gene

EEU43843

hypothetical protein
  
Accession: EEU44049
  
Location: 252477-253343
  
 NCBI BlastP on this gene

EEU44049

hypothetical protein
  
Accession: EEU43844
  
Location: 254151-255298
  
  
**BlastP hit with Mycgr3G102276\_Mycgr3**
  
Percentage identity: 72 %
  
BlastP bit score: 530
  
Sequence coverage: 99 %
  
E-value: 0.0
  
  
 NCBI BlastP on this gene

EEU43844

hypothetical protein
  
Accession: EEU43845
  
Location: 255580-256081
  
 NCBI BlastP on this gene

EEU43845

hypothetical protein
  
Accession: EEU44050
  
Location: 256668-261897
  
 NCBI BlastP on this gene

EEU44050

Query: Architecture Search FASTA input

AAAB01008807 : Anopheles gambiae str. PEST whole genome shotgun sequencing project.    Total score: 1.0     Cumulative Blast bit score: 523

Hit cluster cross-links:

Mycgr3G52686 Mycgr3T
  
Location: 0-861

Mycgr3G52686\_Mycgr3T

Mycgr3G102281 Mycgr3
  
Location: 961-1573

Mycgr3G102281\_Mycgr3

Mycgr3G89185 Mycgr3T
  
Location: 1673-2063

Mycgr3G89185\_Mycgr3T

Mycgr3G65725 Mycgr3T
  
Location: 2163-3612

Mycgr3G65725\_Mycgr3T

Mycgr3G102276 Mycgr3
  
Location: 3712-4801

Mycgr3G102276\_Mycgr3

Mycgr3G89189 Mycgr3T
  
Location: 4901-5564

Mycgr3G89189\_Mycgr3T

Mycgr3G52682 Mycgr3T
  
Location: 5664-9231

Mycgr3G52682\_Mycgr3T

Mycgr3G107072 Mycgr3
  
Location: 9331-13279

Mycgr3G107072\_Mycgr3

Mycgr3G34982 Mycgr3T
  
Location: 13379-15116

Mycgr3G34982\_Mycgr3T

Mycgr3G107069 Mycgr3
  
Location: 15216-17097

Mycgr3G107069\_Mycgr3

Mycgr3G32432 Mycgr3T
  
Location: 17197-19042

Mycgr3G32432\_Mycgr3T

Mycgr3G98385 Mycgr3T
  
Location: 19142-19898

Mycgr3G98385\_Mycgr3T

AGAP006995-PA
  
Accession: EAA04298
  
Location: 8832531-8842084
  
 NCBI BlastP on this gene

EAA04298

AGAP006994-PA
  
Accession: EAA04764
  
Location: 8845926-8850697
  
  
**BlastP hit with Mycgr3G52682\_Mycgr3T**
  
Percentage identity: 32 %
  
BlastP bit score: 262
  
Sequence coverage: 40 %
  
E-value: 2e-69
  
  
 NCBI BlastP on this gene

EAA04764

AGAP006994-PB
  
Accession: EDO64729
  
Location: 8845926-8850697
  
  
**BlastP hit with Mycgr3G52682\_Mycgr3T**
  
Percentage identity: 32 %
  
BlastP bit score: 261
  
Sequence coverage: 40 %
  
E-value: 3e-69
  
  
 NCBI BlastP on this gene

EDO64729

AGAP006993-PA
  
Accession: EAA04662
  
Location: 8851431-8854563
  
 NCBI BlastP on this gene

EAA04662

AGAP006992-PA
  
Accession: EAA04338
  
Location: 8858030-8863842
  
 NCBI BlastP on this gene

EAA04338

Query: Architecture Search FASTA input

KB446558 : Pseudocercospora fijiensis CIRAD86 unplaced genomic scaffold MYCFIscaffold\_4    Total score: 1.0     Cumulative Blast bit score: 522

Hit cluster cross-links:

Mycgr3G52686 Mycgr3T
  
Location: 0-861

Mycgr3G52686\_Mycgr3T

Mycgr3G102281 Mycgr3
  
Location: 961-1573

Mycgr3G102281\_Mycgr3

Mycgr3G89185 Mycgr3T
  
Location: 1673-2063

Mycgr3G89185\_Mycgr3T

Mycgr3G65725 Mycgr3T
  
Location: 2163-3612

Mycgr3G65725\_Mycgr3T

Mycgr3G102276 Mycgr3
  
Location: 3712-4801

Mycgr3G102276\_Mycgr3

Mycgr3G89189 Mycgr3T
  
Location: 4901-5564

Mycgr3G89189\_Mycgr3T

Mycgr3G52682 Mycgr3T
  
Location: 5664-9231

Mycgr3G52682\_Mycgr3T

Mycgr3G107072 Mycgr3
  
Location: 9331-13279

Mycgr3G107072\_Mycgr3

Mycgr3G34982 Mycgr3T
  
Location: 13379-15116

Mycgr3G34982\_Mycgr3T

Mycgr3G107069 Mycgr3
  
Location: 15216-17097

Mycgr3G107069\_Mycgr3

Mycgr3G32432 Mycgr3T
  
Location: 17197-19042

Mycgr3G32432\_Mycgr3T

Mycgr3G98385 Mycgr3T
  
Location: 19142-19898

Mycgr3G98385\_Mycgr3T

hypothetical protein
  
Accession: EME82816
  
Location: 1433967-1435085
  
 NCBI BlastP on this gene

EME82816

hypothetical protein
  
Accession: EME82815
  
Location: 1433195-1433554
  
 NCBI BlastP on this gene

EME82815

hypothetical protein
  
Accession: EME82814
  
Location: 1431548-1432747
  
 NCBI BlastP on this gene

EME82814

hypothetical protein
  
Accession: EME82813
  
Location: 1425634-1428400
  
 NCBI BlastP on this gene

EME82813

hypothetical protein
  
Accession: EME82812
  
Location: 1424770-1425075
  
 NCBI BlastP on this gene

EME82812

hypothetical protein
  
Accession: EME82811
  
Location: 1421940-1424008
  
  
**BlastP hit with Mycgr3G65725\_Mycgr3T**
  
Percentage identity: 67 %
  
BlastP bit score: 522
  
Sequence coverage: 87 %
  
E-value: 1e-179
  
  
 NCBI BlastP on this gene

EME82811

hypothetical protein
  
Accession: EME82810
  
Location: 1418937-1421807
  
 NCBI BlastP on this gene

EME82810

hypothetical protein
  
Accession: EME82809
  
Location: 1417673-1418901
  
 NCBI BlastP on this gene

EME82809

hypothetical protein
  
Accession: EME82808
  
Location: 1415615-1416584
  
 NCBI BlastP on this gene

EME82808

hypothetical protein
  
Accession: EME82807
  
Location: 1410914-1413538
  
 NCBI BlastP on this gene

EME82807

Query: Architecture Search FASTA input

KB733480 : Bipolaris maydis ATCC 48331 unplaced genomic scaffold COCC4scaffold\_37    Total score: 1.0     Cumulative Blast bit score: 520

Hit cluster cross-links:

Mycgr3G52686 Mycgr3T
  
Location: 0-861

Mycgr3G52686\_Mycgr3T

Mycgr3G102281 Mycgr3
  
Location: 961-1573

Mycgr3G102281\_Mycgr3

Mycgr3G89185 Mycgr3T
  
Location: 1673-2063

Mycgr3G89185\_Mycgr3T

Mycgr3G65725 Mycgr3T
  
Location: 2163-3612

Mycgr3G65725\_Mycgr3T

Mycgr3G102276 Mycgr3
  
Location: 3712-4801

Mycgr3G102276\_Mycgr3

Mycgr3G89189 Mycgr3T
  
Location: 4901-5564

Mycgr3G89189\_Mycgr3T

Mycgr3G52682 Mycgr3T
  
Location: 5664-9231

Mycgr3G52682\_Mycgr3T

Mycgr3G107072 Mycgr3
  
Location: 9331-13279

Mycgr3G107072\_Mycgr3

Mycgr3G34982 Mycgr3T
  
Location: 13379-15116

Mycgr3G34982\_Mycgr3T

Mycgr3G107069 Mycgr3
  
Location: 15216-17097

Mycgr3G107069\_Mycgr3

Mycgr3G32432 Mycgr3T
  
Location: 17197-19042

Mycgr3G32432\_Mycgr3T

Mycgr3G98385 Mycgr3T
  
Location: 19142-19898

Mycgr3G98385\_Mycgr3T

hypothetical protein
  
Accession: ENH99911
  
Location: 112109-112593
  
 NCBI BlastP on this gene

ENH99911

hypothetical protein
  
Accession: ENH99910
  
Location: 108541-110567
  
 NCBI BlastP on this gene

ENH99910

hypothetical protein
  
Accession: ENH99909
  
Location: 107049-108451
  
 NCBI BlastP on this gene

ENH99909

hypothetical protein
  
Accession: ENH99908
  
Location: 102225-103300
  
 NCBI BlastP on this gene

ENH99908

hypothetical protein
  
Accession: ENH99907
  
Location: 99060-101486
  
  
**BlastP hit with Mycgr3G107069\_Mycgr3**
  
Percentage identity: 42 %
  
BlastP bit score: 520
  
Sequence coverage: 106 %
  
E-value: 1e-172
  
  
 NCBI BlastP on this gene

ENH99907

hypothetical protein
  
Accession: ENH99906
  
Location: 96300-96770
  
 NCBI BlastP on this gene

ENH99906

hypothetical protein
  
Accession: ENH99905
  
Location: 94457-94790
  
 NCBI BlastP on this gene

ENH99905

hypothetical protein
  
Accession: ENH99904
  
Location: 93917-94075
  
 NCBI BlastP on this gene

ENH99904

hypothetical protein
  
Accession: ENH99903
  
Location: 93431-93911
  
 NCBI BlastP on this gene

ENH99903

hypothetical protein
  
Accession: ENH99902
  
Location: 87533-90674
  
 NCBI BlastP on this gene

ENH99902

Query: Architecture Search FASTA input

KB445573 : Cochliobolus heterostrophus C5 unplaced genomic scaffold COCHEscaffold\_5    Total score: 1.0     Cumulative Blast bit score: 520

Hit cluster cross-links:

Mycgr3G52686 Mycgr3T
  
Location: 0-861

Mycgr3G52686\_Mycgr3T

Mycgr3G102281 Mycgr3
  
Location: 961-1573

Mycgr3G102281\_Mycgr3

Mycgr3G89185 Mycgr3T
  
Location: 1673-2063

Mycgr3G89185\_Mycgr3T

Mycgr3G65725 Mycgr3T
  
Location: 2163-3612

Mycgr3G65725\_Mycgr3T

Mycgr3G102276 Mycgr3
  
Location: 3712-4801

Mycgr3G102276\_Mycgr3

Mycgr3G89189 Mycgr3T
  
Location: 4901-5564

Mycgr3G89189\_Mycgr3T

Mycgr3G52682 Mycgr3T
  
Location: 5664-9231

Mycgr3G52682\_Mycgr3T

Mycgr3G107072 Mycgr3
  
Location: 9331-13279

Mycgr3G107072\_Mycgr3

Mycgr3G34982 Mycgr3T
  
Location: 13379-15116

Mycgr3G34982\_Mycgr3T

Mycgr3G107069 Mycgr3
  
Location: 15216-17097

Mycgr3G107069\_Mycgr3

Mycgr3G32432 Mycgr3T
  
Location: 17197-19042

Mycgr3G32432\_Mycgr3T

Mycgr3G98385 Mycgr3T
  
Location: 19142-19898

Mycgr3G98385\_Mycgr3T

hypothetical protein
  
Accession: EMD93795
  
Location: 1741862-1742346
  
 NCBI BlastP on this gene

EMD93795

hypothetical protein
  
Accession: EMD93796
  
Location: 1743888-1745914
  
 NCBI BlastP on this gene

EMD93796

hypothetical protein
  
Accession: EMD93797
  
Location: 1746004-1747406
  
 NCBI BlastP on this gene

EMD93797

hypothetical protein
  
Accession: EMD93798
  
Location: 1751155-1752230
  
 NCBI BlastP on this gene

EMD93798

hypothetical protein
  
Accession: EMD93799
  
Location: 1752969-1755395
  
  
**BlastP hit with Mycgr3G107069\_Mycgr3**
  
Percentage identity: 42 %
  
BlastP bit score: 520
  
Sequence coverage: 106 %
  
E-value: 1e-172
  
  
 NCBI BlastP on this gene

EMD93799

Query: Architecture Search FASTA input

KE145357 : Glarea lozoyensis ATCC 20868 chromosome Unknown GLAREA14    Total score: 1.0     Cumulative Blast bit score: 519

Hit cluster cross-links:

Mycgr3G52686 Mycgr3T
  
Location: 0-861

Mycgr3G52686\_Mycgr3T

Mycgr3G102281 Mycgr3
  
Location: 961-1573

Mycgr3G102281\_Mycgr3

Mycgr3G89185 Mycgr3T
  
Location: 1673-2063

Mycgr3G89185\_Mycgr3T

Mycgr3G65725 Mycgr3T
  
Location: 2163-3612

Mycgr3G65725\_Mycgr3T

Mycgr3G102276 Mycgr3
  
Location: 3712-4801

Mycgr3G102276\_Mycgr3

Mycgr3G89189 Mycgr3T
  
Location: 4901-5564

Mycgr3G89189\_Mycgr3T

Mycgr3G52682 Mycgr3T
  
Location: 5664-9231

Mycgr3G52682\_Mycgr3T

Mycgr3G107072 Mycgr3
  
Location: 9331-13279

Mycgr3G107072\_Mycgr3

Mycgr3G34982 Mycgr3T
  
Location: 13379-15116

Mycgr3G34982\_Mycgr3T

Mycgr3G107069 Mycgr3
  
Location: 15216-17097

Mycgr3G107069\_Mycgr3

Mycgr3G32432 Mycgr3T
  
Location: 17197-19042

Mycgr3G32432\_Mycgr3T

Mycgr3G98385 Mycgr3T
  
Location: 19142-19898

Mycgr3G98385\_Mycgr3T

Ferredoxin reductase-like, C-terminal NADP-linked
  
Accession: EPE34197
  
Location: 1873318-1875462
  
  
**BlastP hit with Mycgr3G107069\_Mycgr3**
  
Percentage identity: 43 %
  
BlastP bit score: 519
  
Sequence coverage: 106 %
  
E-value: 7e-173
  
  
 NCBI BlastP on this gene

EPE34197

P-loop containing nucleoside triphosphate hydrolase
  
Accession: EPE34196
  
Location: 1864809-1871532
  
 NCBI BlastP on this gene

EPE34196

hypothetical protein
  
Accession: EPE34195
  
Location: 1861226-1862928
  
 NCBI BlastP on this gene

EPE34195

Query: Architecture Search FASTA input

KB725793 : Colletotrichum orbiculare MAFF 240422 unplaced genomic scaffold Scaffold\_242    Total score: 1.0     Cumulative Blast bit score: 517

Hit cluster cross-links:

Mycgr3G52686 Mycgr3T
  
Location: 0-861

Mycgr3G52686\_Mycgr3T

Mycgr3G102281 Mycgr3
  
Location: 961-1573

Mycgr3G102281\_Mycgr3

Mycgr3G89185 Mycgr3T
  
Location: 1673-2063

Mycgr3G89185\_Mycgr3T

Mycgr3G65725 Mycgr3T
  
Location: 2163-3612

Mycgr3G65725\_Mycgr3T

Mycgr3G102276 Mycgr3
  
Location: 3712-4801

Mycgr3G102276\_Mycgr3

Mycgr3G89189 Mycgr3T
  
Location: 4901-5564

Mycgr3G89189\_Mycgr3T

Mycgr3G52682 Mycgr3T
  
Location: 5664-9231

Mycgr3G52682\_Mycgr3T

Mycgr3G107072 Mycgr3
  
Location: 9331-13279

Mycgr3G107072\_Mycgr3

Mycgr3G34982 Mycgr3T
  
Location: 13379-15116

Mycgr3G34982\_Mycgr3T

Mycgr3G107069 Mycgr3
  
Location: 15216-17097

Mycgr3G107069\_Mycgr3

Mycgr3G32432 Mycgr3T
  
Location: 17197-19042

Mycgr3G32432\_Mycgr3T

Mycgr3G98385 Mycgr3T
  
Location: 19142-19898

Mycgr3G98385\_Mycgr3T

hypothetical protein
  
Accession: ENH85055
  
Location: 74063-74892
  
 NCBI BlastP on this gene

ENH85055

hypothetical protein
  
Accession: ENH85054
  
Location: 72488-72770
  
 NCBI BlastP on this gene

ENH85054

hypothetical protein
  
Accession: ENH85053
  
Location: 70099-70420
  
 NCBI BlastP on this gene

ENH85053

hypothetical protein
  
Accession: ENH85052
  
Location: 68748-69981
  
 NCBI BlastP on this gene

ENH85052

integral membrane protein
  
Accession: ENH85051
  
Location: 65741-67137
  
 NCBI BlastP on this gene

ENH85051

nadp-dependent alcohol dehydrogenase
  
Accession: ENH85050
  
Location: 62126-63315
  
  
**BlastP hit with Mycgr3G102276\_Mycgr3**
  
Percentage identity: 71 %
  
BlastP bit score: 517
  
Sequence coverage: 98 %
  
E-value: 1e-180
  
  
 NCBI BlastP on this gene

ENH85050

hypothetical protein
  
Accession: ENH85049
  
Location: 60672-61654
  
 NCBI BlastP on this gene

ENH85049

hypothetical protein
  
Accession: ENH85048
  
Location: 56309-58216
  
 NCBI BlastP on this gene

ENH85048

hypothetical protein
  
Accession: ENH85047
  
Location: 54337-54971
  
 NCBI BlastP on this gene

ENH85047

hypothetical protein
  
Accession: ENH85046
  
Location: 53439-53790
  
 NCBI BlastP on this gene

ENH85046

extracellular exo-polygalacturonase, putative
  
Accession: ENH85045
  
Location: 51601-52947
  
 NCBI BlastP on this gene

ENH85045

exopolygalacturonase
  
Accession: ENH85044
  
Location: 49629-51131
  
 NCBI BlastP on this gene

ENH85044

Query: Architecture Search FASTA input

CH476636 : Sclerotinia sclerotiorum 1980 scaffold\_16 genomic scaffold    Total score: 1.0     Cumulative Blast bit score: 515

Hit cluster cross-links:

Mycgr3G52686 Mycgr3T
  
Location: 0-861

Mycgr3G52686\_Mycgr3T

Mycgr3G102281 Mycgr3
  
Location: 961-1573

Mycgr3G102281\_Mycgr3

Mycgr3G89185 Mycgr3T
  
Location: 1673-2063

Mycgr3G89185\_Mycgr3T

Mycgr3G65725 Mycgr3T
  
Location: 2163-3612

Mycgr3G65725\_Mycgr3T

Mycgr3G102276 Mycgr3
  
Location: 3712-4801

Mycgr3G102276\_Mycgr3

Mycgr3G89189 Mycgr3T
  
Location: 4901-5564

Mycgr3G89189\_Mycgr3T

Mycgr3G52682 Mycgr3T
  
Location: 5664-9231

Mycgr3G52682\_Mycgr3T

Mycgr3G107072 Mycgr3
  
Location: 9331-13279

Mycgr3G107072\_Mycgr3

Mycgr3G34982 Mycgr3T
  
Location: 13379-15116

Mycgr3G34982\_Mycgr3T

Mycgr3G107069 Mycgr3
  
Location: 15216-17097

Mycgr3G107069\_Mycgr3

Mycgr3G32432 Mycgr3T
  
Location: 17197-19042

Mycgr3G32432\_Mycgr3T

Mycgr3G98385 Mycgr3T
  
Location: 19142-19898

Mycgr3G98385\_Mycgr3T

hypothetical protein
  
Accession: EDN94816
  
Location: 408757-410900
  
  
**BlastP hit with Mycgr3G107069\_Mycgr3**
  
Percentage identity: 44 %
  
BlastP bit score: 515
  
Sequence coverage: 100 %
  
E-value: 2e-171
  
  
 NCBI BlastP on this gene

EDN94816

predicted protein
  
Accession: EDN94815
  
Location: 406156-407226
  
 NCBI BlastP on this gene

EDN94815

hypothetical protein
  
Accession: EDN94814
  
Location: 403607-404080
  
 NCBI BlastP on this gene

EDN94814

hypothetical protein
  
Accession: EDN94813
  
Location: 402641-402982
  
 NCBI BlastP on this gene

EDN94813

predicted protein
  
Accession: EDN94812
  
Location: 400767-401837
  
 NCBI BlastP on this gene

EDN94812

predicted protein
  
Accession: EDN94811
  
Location: 397524-398246
  
 NCBI BlastP on this gene

EDN94811

Query: Architecture Search FASTA input

KB445646 : Cochliobolus sativus ND90Pr unplaced genomic scaffold COCSAscaffold\_10    Total score: 1.0     Cumulative Blast bit score: 511

Hit cluster cross-links:

Mycgr3G52686 Mycgr3T
  
Location: 0-861

Mycgr3G52686\_Mycgr3T

Mycgr3G102281 Mycgr3
  
Location: 961-1573

Mycgr3G102281\_Mycgr3

Mycgr3G89185 Mycgr3T
  
Location: 1673-2063

Mycgr3G89185\_Mycgr3T

Mycgr3G65725 Mycgr3T
  
Location: 2163-3612

Mycgr3G65725\_Mycgr3T

Mycgr3G102276 Mycgr3
  
Location: 3712-4801

Mycgr3G102276\_Mycgr3

Mycgr3G89189 Mycgr3T
  
Location: 4901-5564

Mycgr3G89189\_Mycgr3T

Mycgr3G52682 Mycgr3T
  
Location: 5664-9231

Mycgr3G52682\_Mycgr3T

Mycgr3G107072 Mycgr3
  
Location: 9331-13279

Mycgr3G107072\_Mycgr3

Mycgr3G34982 Mycgr3T
  
Location: 13379-15116

Mycgr3G34982\_Mycgr3T

Mycgr3G107069 Mycgr3
  
Location: 15216-17097

Mycgr3G107069\_Mycgr3

Mycgr3G32432 Mycgr3T
  
Location: 17197-19042

Mycgr3G32432\_Mycgr3T

Mycgr3G98385 Mycgr3T
  
Location: 19142-19898

Mycgr3G98385\_Mycgr3T

hypothetical protein
  
Accession: EMD62511
  
Location: 776458-776942
  
 NCBI BlastP on this gene

EMD62511

hypothetical protein
  
Accession: EMD62510
  
Location: 772982-774998
  
 NCBI BlastP on this gene

EMD62510

hypothetical protein
  
Accession: EMD62509
  
Location: 771488-772893
  
 NCBI BlastP on this gene

EMD62509

hypothetical protein
  
Accession: EMD62508
  
Location: 766575-767650
  
 NCBI BlastP on this gene

EMD62508

hypothetical protein
  
Accession: EMD62507
  
Location: 763400-765826
  
  
**BlastP hit with Mycgr3G107069\_Mycgr3**
  
Percentage identity: 40 %
  
BlastP bit score: 511
  
Sequence coverage: 105 %
  
E-value: 3e-169
  
  
 NCBI BlastP on this gene

EMD62507

hypothetical protein
  
Accession: EMD62506
  
Location: 760640-761101
  
 NCBI BlastP on this gene

EMD62506

hypothetical protein
  
Accession: EMD62505
  
Location: 756158-758524
  
 NCBI BlastP on this gene

EMD62505

hypothetical protein
  
Accession: EMD62504
  
Location: 755121-755993
  
 NCBI BlastP on this gene

EMD62504

hypothetical protein
  
Accession: EMD62503
  
Location: 753268-754473
  
 NCBI BlastP on this gene

EMD62503

hypothetical protein
  
Accession: EMD62502
  
Location: 752379-753099
  
 NCBI BlastP on this gene

EMD62502

Query: Architecture Search FASTA input

KB706647 : Eutypa lata UCREL1 unplaced genomic scaffold EL1\_03\_scaffold\_1309    Total score: 1.0     Cumulative Blast bit score: 509

Hit cluster cross-links:

Mycgr3G52686 Mycgr3T
  
Location: 0-861

Mycgr3G52686\_Mycgr3T

Mycgr3G102281 Mycgr3
  
Location: 961-1573

Mycgr3G102281\_Mycgr3

Mycgr3G89185 Mycgr3T
  
Location: 1673-2063

Mycgr3G89185\_Mycgr3T

Mycgr3G65725 Mycgr3T
  
Location: 2163-3612

Mycgr3G65725\_Mycgr3T

Mycgr3G102276 Mycgr3
  
Location: 3712-4801

Mycgr3G102276\_Mycgr3

Mycgr3G89189 Mycgr3T
  
Location: 4901-5564

Mycgr3G89189\_Mycgr3T

Mycgr3G52682 Mycgr3T
  
Location: 5664-9231

Mycgr3G52682\_Mycgr3T

Mycgr3G107072 Mycgr3
  
Location: 9331-13279

Mycgr3G107072\_Mycgr3

Mycgr3G34982 Mycgr3T
  
Location: 13379-15116

Mycgr3G34982\_Mycgr3T

Mycgr3G107069 Mycgr3
  
Location: 15216-17097

Mycgr3G107069\_Mycgr3

Mycgr3G32432 Mycgr3T
  
Location: 17197-19042

Mycgr3G32432\_Mycgr3T

Mycgr3G98385 Mycgr3T
  
Location: 19142-19898

Mycgr3G98385\_Mycgr3T

putative ubiquitin-protein ligase e3a protein
  
Accession: EMR66472
  
Location: 165832-167291
  
  
**BlastP hit with Mycgr3G52682\_Mycgr3T**
  
Percentage identity: 53 %
  
BlastP bit score: 509
  
Sequence coverage: 40 %
  
E-value: 4e-165
  
  
 NCBI BlastP on this gene

EMR66472

hypothetical protein
  
Accession: EMR66453
  
Location: 162027-163157
  
 NCBI BlastP on this gene

EMR66453

hypothetical protein
  
Accession: EMR66443
  
Location: 154756-155691
  
 NCBI BlastP on this gene

EMR66443

Query: Architecture Search FASTA input

JH226130 : Exophiala dermatitidis NIH/UT8656 unplaced genomic scaffold supercont1.1    Total score: 1.0     Cumulative Blast bit score: 508

Hit cluster cross-links:

Mycgr3G52686 Mycgr3T
  
Location: 0-861

Mycgr3G52686\_Mycgr3T

Mycgr3G102281 Mycgr3
  
Location: 961-1573

Mycgr3G102281\_Mycgr3

Mycgr3G89185 Mycgr3T
  
Location: 1673-2063

Mycgr3G89185\_Mycgr3T

Mycgr3G65725 Mycgr3T
  
Location: 2163-3612

Mycgr3G65725\_Mycgr3T

Mycgr3G102276 Mycgr3
  
Location: 3712-4801

Mycgr3G102276\_Mycgr3

Mycgr3G89189 Mycgr3T
  
Location: 4901-5564

Mycgr3G89189\_Mycgr3T

Mycgr3G52682 Mycgr3T
  
Location: 5664-9231

Mycgr3G52682\_Mycgr3T

Mycgr3G107072 Mycgr3
  
Location: 9331-13279

Mycgr3G107072\_Mycgr3

Mycgr3G34982 Mycgr3T
  
Location: 13379-15116

Mycgr3G34982\_Mycgr3T

Mycgr3G107069 Mycgr3
  
Location: 15216-17097

Mycgr3G107069\_Mycgr3

Mycgr3G32432 Mycgr3T
  
Location: 17197-19042

Mycgr3G32432\_Mycgr3T

Mycgr3G98385 Mycgr3T
  
Location: 19142-19898

Mycgr3G98385\_Mycgr3T

MFS transporter, DHA1 family, multidrug resistance protein
  
Accession: EHY51797
  
Location: 65999-67652
  
 NCBI BlastP on this gene

EHY51797

gamma-glutamyltransferase
  
Accession: EHY51799
  
Location: 68902-70801
  
 NCBI BlastP on this gene

EHY51799

two-component system, NarL family, sensor histidine kinase BarA
  
Accession: EHY51800
  
Location: 73542-76235
  
 NCBI BlastP on this gene

EHY51800

hypothetical protein
  
Accession: EHY51801
  
Location: 76668-77427
  
 NCBI BlastP on this gene

EHY51801

ferric-chelate reductase
  
Accession: EHY51802
  
Location: 78169-80178
  
  
**BlastP hit with Mycgr3G107069\_Mycgr3**
  
Percentage identity: 40 %
  
BlastP bit score: 508
  
Sequence coverage: 104 %
  
E-value: 2e-168
  
  
 NCBI BlastP on this gene

EHY51802

methionyl-tRNA synthetase
  
Accession: EHY51803
  
Location: 81895-83781
  
 NCBI BlastP on this gene

EHY51803

hypothetical protein
  
Accession: EHY51804
  
Location: 84231-85354
  
 NCBI BlastP on this gene

EHY51804

hypothetical protein
  
Accession: EHY51805
  
Location: 86653-88947
  
 NCBI BlastP on this gene

EHY51805

solute carrier family 12 member 2
  
Accession: EHY51806
  
Location: 90369-94420
  
 NCBI BlastP on this gene

EHY51806

Query: Architecture Search FASTA input

DS985223 : Verticillium albo-atrum VaMs.102 supercont1.10 genomic scaffold    Total score: 1.0     Cumulative Blast bit score: 502

Hit cluster cross-links:

Mycgr3G52686 Mycgr3T
  
Location: 0-861

Mycgr3G52686\_Mycgr3T

Mycgr3G102281 Mycgr3
  
Location: 961-1573

Mycgr3G102281\_Mycgr3

Mycgr3G89185 Mycgr3T
  
Location: 1673-2063

Mycgr3G89185\_Mycgr3T

Mycgr3G65725 Mycgr3T
  
Location: 2163-3612

Mycgr3G65725\_Mycgr3T

Mycgr3G102276 Mycgr3
  
Location: 3712-4801

Mycgr3G102276\_Mycgr3

Mycgr3G89189 Mycgr3T
  
Location: 4901-5564

Mycgr3G89189\_Mycgr3T

Mycgr3G52682 Mycgr3T
  
Location: 5664-9231

Mycgr3G52682\_Mycgr3T

Mycgr3G107072 Mycgr3
  
Location: 9331-13279

Mycgr3G107072\_Mycgr3

Mycgr3G34982 Mycgr3T
  
Location: 13379-15116

Mycgr3G34982\_Mycgr3T

Mycgr3G107069 Mycgr3
  
Location: 15216-17097

Mycgr3G107069\_Mycgr3

Mycgr3G32432 Mycgr3T
  
Location: 17197-19042

Mycgr3G32432\_Mycgr3T

Mycgr3G98385 Mycgr3T
  
Location: 19142-19898

Mycgr3G98385\_Mycgr3T

NADP-dependent alcohol dehydrogenase
  
Accession: EEY21586
  
Location: 790600-791741
  
  
**BlastP hit with Mycgr3G102276\_Mycgr3**
  
Percentage identity: 68 %
  
BlastP bit score: 502
  
Sequence coverage: 98 %
  
E-value: 8e-175
  
  
 NCBI BlastP on this gene

EEY21586

predicted protein
  
Accession: EEY21585
  
Location: 786666-787184
  
 NCBI BlastP on this gene

EEY21585

BNR/Asp-box repeat domain-containing protein
  
Accession: EEY21584
  
Location: 783700-784943
  
 NCBI BlastP on this gene

EEY21584

Query: Architecture Search FASTA input

DS572705 : Verticillium dahliae VdLs.17 supercont1.11 genomic scaffold    Total score: 1.0     Cumulative Blast bit score: 500

Hit cluster cross-links:

Mycgr3G52686 Mycgr3T
  
Location: 0-861

Mycgr3G52686\_Mycgr3T

Mycgr3G102281 Mycgr3
  
Location: 961-1573

Mycgr3G102281\_Mycgr3

Mycgr3G89185 Mycgr3T
  
Location: 1673-2063

Mycgr3G89185\_Mycgr3T

Mycgr3G65725 Mycgr3T
  
Location: 2163-3612

Mycgr3G65725\_Mycgr3T

Mycgr3G102276 Mycgr3
  
Location: 3712-4801

Mycgr3G102276\_Mycgr3

Mycgr3G89189 Mycgr3T
  
Location: 4901-5564

Mycgr3G89189\_Mycgr3T

Mycgr3G52682 Mycgr3T
  
Location: 5664-9231

Mycgr3G52682\_Mycgr3T

Mycgr3G107072 Mycgr3
  
Location: 9331-13279

Mycgr3G107072\_Mycgr3

Mycgr3G34982 Mycgr3T
  
Location: 13379-15116

Mycgr3G34982\_Mycgr3T

Mycgr3G107069 Mycgr3
  
Location: 15216-17097

Mycgr3G107069\_Mycgr3

Mycgr3G32432 Mycgr3T
  
Location: 17197-19042

Mycgr3G32432\_Mycgr3T

Mycgr3G98385 Mycgr3T
  
Location: 19142-19898

Mycgr3G98385\_Mycgr3T

hypothetical protein
  
Accession: EGY14479
  
Location: 36863-41450
  
 NCBI BlastP on this gene

EGY14479

hypothetical protein
  
Accession: EGY14480
  
Location: 42108-46021
  
 NCBI BlastP on this gene

EGY14480

hypothetical protein
  
Accession: EGY14481
  
Location: 47858-49517
  
 NCBI BlastP on this gene

EGY14481

NADP-dependent alcohol dehydrogenase
  
Accession: EGY14482
  
Location: 51297-52438
  
  
**BlastP hit with Mycgr3G102276\_Mycgr3**
  
Percentage identity: 67 %
  
BlastP bit score: 501
  
Sequence coverage: 98 %
  
E-value: 2e-174
  
  
 NCBI BlastP on this gene

EGY14482

hypothetical protein
  
Accession: EGY14483
  
Location: 53692-54132
  
 NCBI BlastP on this gene

EGY14483

hypothetical protein
  
Accession: EGY14484
  
Location: 55209-55721
  
 NCBI BlastP on this gene

EGY14484

BNR/Asp-box repeat domain-containing protein
  
Accession: EGY14485
  
Location: 57630-58880
  
 NCBI BlastP on this gene

EGY14485

trypsin
  
Accession: EGY14486
  
Location: 62617-63509
  
 NCBI BlastP on this gene

EGY14486

Query: Architecture Search FASTA input

KB445550 : Baudoinia compniacensis UAMH 10762 unplaced genomic scaffold BAUCOscaffold\_1    Total score: 1.0     Cumulative Blast bit score: 498

Hit cluster cross-links:

Mycgr3G52686 Mycgr3T
  
Location: 0-861

Mycgr3G52686\_Mycgr3T

Mycgr3G102281 Mycgr3
  
Location: 961-1573

Mycgr3G102281\_Mycgr3

Mycgr3G89185 Mycgr3T
  
Location: 1673-2063

Mycgr3G89185\_Mycgr3T

Mycgr3G65725 Mycgr3T
  
Location: 2163-3612

Mycgr3G65725\_Mycgr3T

Mycgr3G102276 Mycgr3
  
Location: 3712-4801

Mycgr3G102276\_Mycgr3

Mycgr3G89189 Mycgr3T
  
Location: 4901-5564

Mycgr3G89189\_Mycgr3T

Mycgr3G52682 Mycgr3T
  
Location: 5664-9231

Mycgr3G52682\_Mycgr3T

Mycgr3G107072 Mycgr3
  
Location: 9331-13279

Mycgr3G107072\_Mycgr3

Mycgr3G34982 Mycgr3T
  
Location: 13379-15116

Mycgr3G34982\_Mycgr3T

Mycgr3G107069 Mycgr3
  
Location: 15216-17097

Mycgr3G107069\_Mycgr3

Mycgr3G32432 Mycgr3T
  
Location: 17197-19042

Mycgr3G32432\_Mycgr3T

Mycgr3G98385 Mycgr3T
  
Location: 19142-19898

Mycgr3G98385\_Mycgr3T

hypothetical protein
  
Accession: EMD00532
  
Location: 406961-409192
  
 NCBI BlastP on this gene

EMD00532

hypothetical protein
  
Accession: EMD00531
  
Location: 405830-406508
  
 NCBI BlastP on this gene

EMD00531

hypothetical protein
  
Accession: EMD00530
  
Location: 402847-405169
  
 NCBI BlastP on this gene

EMD00530

hypothetical protein
  
Accession: EMD00529
  
Location: 400834-402102
  
 NCBI BlastP on this gene

EMD00529

hypothetical protein
  
Accession: EMD00528
  
Location: 397387-400266
  
 NCBI BlastP on this gene

EMD00528

hypothetical protein
  
Accession: EMD00527
  
Location: 394976-396532
  
  
**BlastP hit with Mycgr3G65725\_Mycgr3T**
  
Percentage identity: 54 %
  
BlastP bit score: 499
  
Sequence coverage: 96 %
  
E-value: 3e-170
  
  
 NCBI BlastP on this gene

EMD00527

hypothetical protein
  
Accession: EMD00526
  
Location: 393529-394442
  
 NCBI BlastP on this gene

EMD00526

hypothetical protein
  
Accession: EMD00525
  
Location: 391193-392953
  
 NCBI BlastP on this gene

EMD00525

hypothetical protein
  
Accession: EMD00524
  
Location: 389876-390133
  
 NCBI BlastP on this gene

EMD00524

hypothetical protein
  
Accession: EMD00523
  
Location: 389175-389522
  
 NCBI BlastP on this gene

EMD00523

hypothetical protein
  
Accession: EMD00522
  
Location: 386293-388842
  
 NCBI BlastP on this gene

EMD00522

hypothetical protein
  
Accession: EMD00521
  
Location: 384537-385891
  
 NCBI BlastP on this gene

EMD00521

hypothetical protein
  
Accession: EMD00520
  
Location: 382376-383995
  
 NCBI BlastP on this gene

EMD00520

Query: Architecture Search FASTA input

CM001233 : Magnaporthe oryzae 70-15 chromosome 3    Total score: 1.0     Cumulative Blast bit score: 498

Hit cluster cross-links:

Mycgr3G52686 Mycgr3T
  
Location: 0-861

Mycgr3G52686\_Mycgr3T

Mycgr3G102281 Mycgr3
  
Location: 961-1573

Mycgr3G102281\_Mycgr3

Mycgr3G89185 Mycgr3T
  
Location: 1673-2063

Mycgr3G89185\_Mycgr3T

Mycgr3G65725 Mycgr3T
  
Location: 2163-3612

Mycgr3G65725\_Mycgr3T

Mycgr3G102276 Mycgr3
  
Location: 3712-4801

Mycgr3G102276\_Mycgr3

Mycgr3G89189 Mycgr3T
  
Location: 4901-5564

Mycgr3G89189\_Mycgr3T

Mycgr3G52682 Mycgr3T
  
Location: 5664-9231

Mycgr3G52682\_Mycgr3T

Mycgr3G107072 Mycgr3
  
Location: 9331-13279

Mycgr3G107072\_Mycgr3

Mycgr3G34982 Mycgr3T
  
Location: 13379-15116

Mycgr3G34982\_Mycgr3T

Mycgr3G107069 Mycgr3
  
Location: 15216-17097

Mycgr3G107069\_Mycgr3

Mycgr3G32432 Mycgr3T
  
Location: 17197-19042

Mycgr3G32432\_Mycgr3T

Mycgr3G98385 Mycgr3T
  
Location: 19142-19898

Mycgr3G98385\_Mycgr3T

thiamine transporter
  
Accession: EHA53309
  
Location: 6129201-6131130
  
 NCBI BlastP on this gene

EHA53309

hypothetical protein
  
Accession: EHA53310
  
Location: 6131392-6132327
  
 NCBI BlastP on this gene

EHA53310

hypothetical protein
  
Accession: EHA53311
  
Location: 6132495-6133792
  
 NCBI BlastP on this gene

EHA53311

hypothetical protein
  
Accession: EHA53312
  
Location: 6134160-6135504
  
 NCBI BlastP on this gene

EHA53312

hypothetical protein
  
Accession: EHA53313
  
Location: 6136901-6137664
  
 NCBI BlastP on this gene

EHA53313

hypothetical protein
  
Accession: EHA53314
  
Location: 6138147-6139085
  
 NCBI BlastP on this gene

EHA53314

hypothetical protein
  
Accession: EHA53315
  
Location: 6139181-6140297
  
 NCBI BlastP on this gene

EHA53315

hypothetical protein
  
Accession: EHA53316
  
Location: 6141449-6142950
  
  
**BlastP hit with Mycgr3G65725\_Mycgr3T**
  
Percentage identity: 54 %
  
BlastP bit score: 498
  
Sequence coverage: 93 %
  
E-value: 2e-169
  
  
 NCBI BlastP on this gene

EHA53316

Query: Architecture Search FASTA input

CH408159 : Pichia guilliermondii ATCC 6260 scaffold\_5 genomic scaffold    Total score: 1.0     Cumulative Blast bit score: 492

Hit cluster cross-links:

Mycgr3G52686 Mycgr3T
  
Location: 0-861

Mycgr3G52686\_Mycgr3T

Mycgr3G102281 Mycgr3
  
Location: 961-1573

Mycgr3G102281\_Mycgr3

Mycgr3G89185 Mycgr3T
  
Location: 1673-2063

Mycgr3G89185\_Mycgr3T

Mycgr3G65725 Mycgr3T
  
Location: 2163-3612

Mycgr3G65725\_Mycgr3T

Mycgr3G102276 Mycgr3
  
Location: 3712-4801

Mycgr3G102276\_Mycgr3

Mycgr3G89189 Mycgr3T
  
Location: 4901-5564

Mycgr3G89189\_Mycgr3T

Mycgr3G52682 Mycgr3T
  
Location: 5664-9231

Mycgr3G52682\_Mycgr3T

Mycgr3G107072 Mycgr3
  
Location: 9331-13279

Mycgr3G107072\_Mycgr3

Mycgr3G34982 Mycgr3T
  
Location: 13379-15116

Mycgr3G34982\_Mycgr3T

Mycgr3G107069 Mycgr3
  
Location: 15216-17097

Mycgr3G107069\_Mycgr3

Mycgr3G32432 Mycgr3T
  
Location: 17197-19042

Mycgr3G32432\_Mycgr3T

Mycgr3G98385 Mycgr3T
  
Location: 19142-19898

Mycgr3G98385\_Mycgr3T

hypothetical protein
  
Accession: EDK40396
  
Location: 1000268-1001707
  
 NCBI BlastP on this gene

EDK40396

hypothetical protein
  
Accession: EDK40395
  
Location: 998405-999988
  
 NCBI BlastP on this gene

EDK40395

hypothetical protein
  
Accession: EDK40394
  
Location: 996542-998167
  
 NCBI BlastP on this gene

EDK40394

hypothetical protein
  
Accession: EDK40393
  
Location: 994801-996441
  
  
**BlastP hit with Mycgr3G32432\_Mycgr3T**
  
Percentage identity: 35 %
  
BlastP bit score: 251
  
Sequence coverage: 82 %
  
E-value: 1e-71
  
  
 NCBI BlastP on this gene

EDK40393

hypothetical protein
  
Accession: EDK40392
  
Location: 992705-994345
  
  
**BlastP hit with Mycgr3G32432\_Mycgr3T**
  
Percentage identity: 34 %
  
BlastP bit score: 241
  
Sequence coverage: 82 %
  
E-value: 5e-68
  
  
 NCBI BlastP on this gene

EDK40392

hypothetical protein
  
Accession: EDK40391
  
Location: 991384-992694
  
 NCBI BlastP on this gene

EDK40391

hypothetical protein
  
Accession: EDK40390
  
Location: 990627-991382
  
 NCBI BlastP on this gene

EDK40390

hypothetical protein
  
Accession: EDK40389
  
Location: 989059-990261
  
 NCBI BlastP on this gene

EDK40389

hypothetical protein
  
Accession: EDK40388
  
Location: 988068-988718
  
 NCBI BlastP on this gene

EDK40388

hypothetical protein
  
Accession: EDK40387
  
Location: 986552-987733
  
 NCBI BlastP on this gene

EDK40387

hypothetical protein
  
Accession: EDK40386
  
Location: 985257-986336
  
 NCBI BlastP on this gene

EDK40386

hypothetical protein
  
Accession: EDK40385
  
Location: 984219-985118
  
 NCBI BlastP on this gene

EDK40385

hypothetical protein
  
Accession: EDK40384
  
Location: 982308-984023
  
 NCBI BlastP on this gene

EDK40384

conserved hypothetical protein
  
Accession: EDK40383
  
Location: 980863-982254
  
 NCBI BlastP on this gene

EDK40383

Query: Architecture Search FASTA input

AFNW01000605 : Fusarium pseudograminearum CS3096    Total score: 1.0     Cumulative Blast bit score: 489

Hit cluster cross-links:

Mycgr3G52686 Mycgr3T
  
Location: 0-861

Mycgr3G52686\_Mycgr3T

Mycgr3G102281 Mycgr3
  
Location: 961-1573

Mycgr3G102281\_Mycgr3

Mycgr3G89185 Mycgr3T
  
Location: 1673-2063

Mycgr3G89185\_Mycgr3T

Mycgr3G65725 Mycgr3T
  
Location: 2163-3612

Mycgr3G65725\_Mycgr3T

Mycgr3G102276 Mycgr3
  
Location: 3712-4801

Mycgr3G102276\_Mycgr3

Mycgr3G89189 Mycgr3T
  
Location: 4901-5564

Mycgr3G89189\_Mycgr3T

Mycgr3G52682 Mycgr3T
  
Location: 5664-9231

Mycgr3G52682\_Mycgr3T

Mycgr3G107072 Mycgr3
  
Location: 9331-13279

Mycgr3G107072\_Mycgr3

Mycgr3G34982 Mycgr3T
  
Location: 13379-15116

Mycgr3G34982\_Mycgr3T

Mycgr3G107069 Mycgr3
  
Location: 15216-17097

Mycgr3G107069\_Mycgr3

Mycgr3G32432 Mycgr3T
  
Location: 17197-19042

Mycgr3G32432\_Mycgr3T

Mycgr3G98385 Mycgr3T
  
Location: 19142-19898

Mycgr3G98385\_Mycgr3T

hypothetical protein
  
Accession: EKJ68466
  
Location: 390314-391450
  
  
**BlastP hit with Mycgr3G102276\_Mycgr3**
  
Percentage identity: 66 %
  
BlastP bit score: 489
  
Sequence coverage: 98 %
  
E-value: 1e-169
  
  
 NCBI BlastP on this gene

EKJ68466

hypothetical protein
  
Accession: EKJ68465
  
Location: 388453-389153
  
 NCBI BlastP on this gene

EKJ68465

hypothetical protein
  
Accession: EKJ68464
  
Location: 386389-388254
  
 NCBI BlastP on this gene

EKJ68464

hypothetical protein
  
Accession: EKJ68463
  
Location: 385658-386146
  
 NCBI BlastP on this gene

EKJ68463

hypothetical protein
  
Accession: EKJ68462
  
Location: 379803-385130
  
 NCBI BlastP on this gene

EKJ68462

Query: Architecture Search FASTA input

KB644415 : Penicillium oxalicum 114-2 unplaced genomic scaffold scaffold\_8    Total score: 1.0     Cumulative Blast bit score: 488

Hit cluster cross-links:

Mycgr3G52686 Mycgr3T
  
Location: 0-861

Mycgr3G52686\_Mycgr3T

Mycgr3G102281 Mycgr3
  
Location: 961-1573

Mycgr3G102281\_Mycgr3

Mycgr3G89185 Mycgr3T
  
Location: 1673-2063

Mycgr3G89185\_Mycgr3T

Mycgr3G65725 Mycgr3T
  
Location: 2163-3612

Mycgr3G65725\_Mycgr3T

Mycgr3G102276 Mycgr3
  
Location: 3712-4801

Mycgr3G102276\_Mycgr3

Mycgr3G89189 Mycgr3T
  
Location: 4901-5564

Mycgr3G89189\_Mycgr3T

Mycgr3G52682 Mycgr3T
  
Location: 5664-9231

Mycgr3G52682\_Mycgr3T

Mycgr3G107072 Mycgr3
  
Location: 9331-13279

Mycgr3G107072\_Mycgr3

Mycgr3G34982 Mycgr3T
  
Location: 13379-15116

Mycgr3G34982\_Mycgr3T

Mycgr3G107069 Mycgr3
  
Location: 15216-17097

Mycgr3G107069\_Mycgr3

Mycgr3G32432 Mycgr3T
  
Location: 17197-19042

Mycgr3G32432\_Mycgr3T

Mycgr3G98385 Mycgr3T
  
Location: 19142-19898

Mycgr3G98385\_Mycgr3T

hypothetical protein
  
Accession: EPS33799
  
Location: 2190597-2192417
  
 NCBI BlastP on this gene

EPS33799

hypothetical protein
  
Accession: EPS33798
  
Location: 2189106-2189899
  
 NCBI BlastP on this gene

EPS33798

hypothetical protein
  
Accession: EPS33797
  
Location: 2187057-2188459
  
 NCBI BlastP on this gene

EPS33797

hypothetical protein
  
Accession: EPS33796
  
Location: 2184893-2185951
  
 NCBI BlastP on this gene

EPS33796

hypothetical protein
  
Accession: EPS33795
  
Location: 2183628-2184560
  
 NCBI BlastP on this gene

EPS33795

hypothetical protein
  
Accession: EPS33794
  
Location: 2182007-2182607
  
 NCBI BlastP on this gene

EPS33794

hypothetical protein
  
Accession: EPS33793
  
Location: 2179468-2181405
  
  
**BlastP hit with Mycgr3G107069\_Mycgr3**
  
Percentage identity: 41 %
  
BlastP bit score: 488
  
Sequence coverage: 99 %
  
E-value: 4e-161
  
  
 NCBI BlastP on this gene

EPS33793

hypothetical protein
  
Accession: EPS33792
  
Location: 2177016-2178518
  
 NCBI BlastP on this gene

EPS33792

hypothetical protein
  
Accession: EPS33791
  
Location: 2174118-2176009
  
 NCBI BlastP on this gene

EPS33791

hypothetical protein
  
Accession: EPS33790
  
Location: 2172785-2173895
  
 NCBI BlastP on this gene

EPS33790

hypothetical protein
  
Accession: EPS33789
  
Location: 2171554-2172279
  
 NCBI BlastP on this gene

EPS33789

hypothetical protein
  
Accession: EPS33788
  
Location: 2169164-2170296
  
 NCBI BlastP on this gene

EPS33788

Query: Architecture Search FASTA input

CM001235 : Magnaporthe oryzae 70-15 chromosome 5    Total score: 1.0     Cumulative Blast bit score: 488

Hit cluster cross-links:

Mycgr3G52686 Mycgr3T
  
Location: 0-861

Mycgr3G52686\_Mycgr3T

Mycgr3G102281 Mycgr3
  
Location: 961-1573

Mycgr3G102281\_Mycgr3

Mycgr3G89185 Mycgr3T
  
Location: 1673-2063

Mycgr3G89185\_Mycgr3T

Mycgr3G65725 Mycgr3T
  
Location: 2163-3612

Mycgr3G65725\_Mycgr3T

Mycgr3G102276 Mycgr3
  
Location: 3712-4801

Mycgr3G102276\_Mycgr3

Mycgr3G89189 Mycgr3T
  
Location: 4901-5564

Mycgr3G89189\_Mycgr3T

Mycgr3G52682 Mycgr3T
  
Location: 5664-9231

Mycgr3G52682\_Mycgr3T

Mycgr3G107072 Mycgr3
  
Location: 9331-13279

Mycgr3G107072\_Mycgr3

Mycgr3G34982 Mycgr3T
  
Location: 13379-15116

Mycgr3G34982\_Mycgr3T

Mycgr3G107069 Mycgr3
  
Location: 15216-17097

Mycgr3G107069\_Mycgr3

Mycgr3G32432 Mycgr3T
  
Location: 17197-19042

Mycgr3G32432\_Mycgr3T

Mycgr3G98385 Mycgr3T
  
Location: 19142-19898

Mycgr3G98385\_Mycgr3T

hypothetical protein
  
Accession: EHA49272
  
Location: 3522578-3524296
  
 NCBI BlastP on this gene

EHA49272

hypothetical protein
  
Accession: EHA49273
  
Location: 3526037-3529629
  
 NCBI BlastP on this gene

EHA49273

hypothetical protein
  
Accession: EHA49274
  
Location: 3532241-3533576
  
 NCBI BlastP on this gene

EHA49274

NADP-dependent alcohol dehydrogenase 6
  
Accession: EHA49275
  
Location: 3534096-3535270
  
  
**BlastP hit with Mycgr3G102276\_Mycgr3**
  
Percentage identity: 66 %
  
BlastP bit score: 488
  
Sequence coverage: 98 %
  
E-value: 2e-169
  
  
 NCBI BlastP on this gene

EHA49275

hypothetical protein
  
Accession: EHA49276
  
Location: 3535607-3535914
  
 NCBI BlastP on this gene

EHA49276

hypothetical protein
  
Accession: EHA49277
  
Location: 3536972-3537602
  
 NCBI BlastP on this gene

EHA49277

15-hydroxyprostaglandin dehydrogenase
  
Accession: EHA49278
  
Location: 3540107-3541354
  
 NCBI BlastP on this gene

EHA49278

hypothetical protein
  
Accession: EHA49279
  
Location: 3541990-3544311
  
 NCBI BlastP on this gene

EHA49279

hypothetical protein
  
Accession: EHA49280
  
Location: 3547028-3548056
  
 NCBI BlastP on this gene

EHA49280

Query: Architecture Search FASTA input

KB726554 : Fusarium oxysporum f. sp. cubense race 4 unplaced genomic scaffold scaffold44    Total score: 1.0     Cumulative Blast bit score: 481

Hit cluster cross-links:

Mycgr3G52686 Mycgr3T
  
Location: 0-861

Mycgr3G52686\_Mycgr3T

Mycgr3G102281 Mycgr3
  
Location: 961-1573

Mycgr3G102281\_Mycgr3

Mycgr3G89185 Mycgr3T
  
Location: 1673-2063

Mycgr3G89185\_Mycgr3T

Mycgr3G65725 Mycgr3T
  
Location: 2163-3612

Mycgr3G65725\_Mycgr3T

Mycgr3G102276 Mycgr3
  
Location: 3712-4801

Mycgr3G102276\_Mycgr3

Mycgr3G89189 Mycgr3T
  
Location: 4901-5564

Mycgr3G89189\_Mycgr3T

Mycgr3G52682 Mycgr3T
  
Location: 5664-9231

Mycgr3G52682\_Mycgr3T

Mycgr3G107072 Mycgr3
  
Location: 9331-13279

Mycgr3G107072\_Mycgr3

Mycgr3G34982 Mycgr3T
  
Location: 13379-15116

Mycgr3G34982\_Mycgr3T

Mycgr3G107069 Mycgr3
  
Location: 15216-17097

Mycgr3G107069\_Mycgr3

Mycgr3G32432 Mycgr3T
  
Location: 17197-19042

Mycgr3G32432\_Mycgr3T

Mycgr3G98385 Mycgr3T
  
Location: 19142-19898

Mycgr3G98385\_Mycgr3T

DNA polymerase epsilon catalytic subunit A
  
Accession: EMT68272
  
Location: 1091587-1098454
  
 NCBI BlastP on this gene

EMT68272

Cytochrome c oxidase protein 20, mitochondrial
  
Accession: EMT68271
  
Location: 1088484-1089048
  
 NCBI BlastP on this gene

EMT68271

hypothetical protein
  
Accession: EMT68270
  
Location: 1087139-1088053
  
 NCBI BlastP on this gene

EMT68270

Protein VTS1
  
Accession: EMT68269
  
Location: 1085610-1086158
  
 NCBI BlastP on this gene

EMT68269

Copper transport protein ctr4
  
Accession: EMT68268
  
Location: 1083516-1084115
  
 NCBI BlastP on this gene

EMT68268

Copper transport protein ctr4
  
Accession: EMT68267
  
Location: 1082356-1082955
  
 NCBI BlastP on this gene

EMT68267

Ferric reductase transmembrane component 5
  
Accession: EMT68266
  
Location: 1079347-1081302
  
  
**BlastP hit with Mycgr3G107069\_Mycgr3**
  
Percentage identity: 39 %
  
BlastP bit score: 482
  
Sequence coverage: 102 %
  
E-value: 9e-159
  
  
 NCBI BlastP on this gene

EMT68266

Bifunctional protein GAL10
  
Accession: EMT68265
  
Location: 1077456-1078409
  
 NCBI BlastP on this gene

EMT68265

Bifunctional protein GAL10
  
Accession: EMT68264
  
Location: 1075221-1076243
  
 NCBI BlastP on this gene

EMT68264

hypothetical protein
  
Accession: EMT68263
  
Location: 1073664-1075007
  
 NCBI BlastP on this gene

EMT68263

Mitochondrial distribution and morphology protein 35
  
Accession: EMT68262
  
Location: 1072683-1073133
  
 NCBI BlastP on this gene

EMT68262

N-acetylglucosaminyl-phosphatidylinositol biosynthetic protein gpi1
  
Accession: EMT68261
  
Location: 1067307-1070023
  
 NCBI BlastP on this gene

EMT68261

Query: Architecture Search FASTA input

HF679023 : Fusarium fujikuroi IMI 58289 draft genome, chromosome FFUJ\_chr01.    Total score: 1.0     Cumulative Blast bit score: 481

Hit cluster cross-links:

Mycgr3G52686 Mycgr3T
  
Location: 0-861

Mycgr3G52686\_Mycgr3T

Mycgr3G102281 Mycgr3
  
Location: 961-1573

Mycgr3G102281\_Mycgr3

Mycgr3G89185 Mycgr3T
  
Location: 1673-2063

Mycgr3G89185\_Mycgr3T

Mycgr3G65725 Mycgr3T
  
Location: 2163-3612

Mycgr3G65725\_Mycgr3T

Mycgr3G102276 Mycgr3
  
Location: 3712-4801

Mycgr3G102276\_Mycgr3

Mycgr3G89189 Mycgr3T
  
Location: 4901-5564

Mycgr3G89189\_Mycgr3T

Mycgr3G52682 Mycgr3T
  
Location: 5664-9231

Mycgr3G52682\_Mycgr3T

Mycgr3G107072 Mycgr3
  
Location: 9331-13279

Mycgr3G107072\_Mycgr3

Mycgr3G34982 Mycgr3T
  
Location: 13379-15116

Mycgr3G34982\_Mycgr3T

Mycgr3G107069 Mycgr3
  
Location: 15216-17097

Mycgr3G107069\_Mycgr3

Mycgr3G32432 Mycgr3T
  
Location: 17197-19042

Mycgr3G32432\_Mycgr3T

Mycgr3G98385 Mycgr3T
  
Location: 19142-19898

Mycgr3G98385\_Mycgr3T

uncharacterized protein
  
Accession: CCT61593
  
Location: 457396-461111
  
 NCBI BlastP on this gene

FFUJ\_01920

uncharacterized protein
  
Accession: CCT61594
  
Location: 461569-463296
  
 NCBI BlastP on this gene

FFUJ\_01919

related to alpha-mannosidase
  
Accession: CCT61595
  
Location: 463792-465538
  
 NCBI BlastP on this gene

FFUJ\_01918

uncharacterized protein
  
Accession: CCT61596
  
Location: 466393-467066
  
 NCBI BlastP on this gene

FFUJ\_01917

uncharacterized protein
  
Accession: CCT61597
  
Location: 467226-468127
  
 NCBI BlastP on this gene

FFUJ\_01916

related to alcohol dehydrogenase, class C
  
Accession: CCT61598
  
Location: 469026-470164
  
  
**BlastP hit with Mycgr3G102276\_Mycgr3**
  
Percentage identity: 65 %
  
BlastP bit score: 482
  
Sequence coverage: 98 %
  
E-value: 7e-167
  
  
 NCBI BlastP on this gene

FFUJ\_01915

related to MGMT family protein
  
Accession: CCT61599
  
Location: 470427-470925
  
 NCBI BlastP on this gene

FFUJ\_01914

related to kinesin-like protein
  
Accession: CCT61600
  
Location: 471525-476841
  
 NCBI BlastP on this gene

FFUJ\_01913

uncharacterized protein
  
Accession: CCT61601
  
Location: 481068-483098
  
 NCBI BlastP on this gene

FFUJ\_01912

Query: Architecture Search FASTA input

KB730248 : Fusarium oxysporum f. sp. cubense race 1 unplaced genomic scaffold scaffold101    Total score: 1.0     Cumulative Blast bit score: 479

Hit cluster cross-links:

Mycgr3G52686 Mycgr3T
  
Location: 0-861

Mycgr3G52686\_Mycgr3T

Mycgr3G102281 Mycgr3
  
Location: 961-1573

Mycgr3G102281\_Mycgr3

Mycgr3G89185 Mycgr3T
  
Location: 1673-2063

Mycgr3G89185\_Mycgr3T

Mycgr3G65725 Mycgr3T
  
Location: 2163-3612

Mycgr3G65725\_Mycgr3T

Mycgr3G102276 Mycgr3
  
Location: 3712-4801

Mycgr3G102276\_Mycgr3

Mycgr3G89189 Mycgr3T
  
Location: 4901-5564

Mycgr3G89189\_Mycgr3T

Mycgr3G52682 Mycgr3T
  
Location: 5664-9231

Mycgr3G52682\_Mycgr3T

Mycgr3G107072 Mycgr3
  
Location: 9331-13279

Mycgr3G107072\_Mycgr3

Mycgr3G34982 Mycgr3T
  
Location: 13379-15116

Mycgr3G34982\_Mycgr3T

Mycgr3G107069 Mycgr3
  
Location: 15216-17097

Mycgr3G107069\_Mycgr3

Mycgr3G32432 Mycgr3T
  
Location: 17197-19042

Mycgr3G32432\_Mycgr3T

Mycgr3G98385 Mycgr3T
  
Location: 19142-19898

Mycgr3G98385\_Mycgr3T

DNA polymerase epsilon catalytic subunit A
  
Accession: ENH68646
  
Location: 1231996-1238863
  
 NCBI BlastP on this gene

ENH68646

Cytochrome c oxidase protein 20, mitochondrial
  
Accession: ENH68647
  
Location: 1239331-1239895
  
 NCBI BlastP on this gene

ENH68647

hypothetical protein
  
Accession: ENH68648
  
Location: 1240336-1241250
  
 NCBI BlastP on this gene

ENH68648

Protein VTS1
  
Accession: ENH68649
  
Location: 1242228-1242776
  
 NCBI BlastP on this gene

ENH68649

Copper transport protein ctr4
  
Accession: ENH68650
  
Location: 1244275-1244874
  
 NCBI BlastP on this gene

ENH68650

Ferric reductase transmembrane component 5
  
Accession: ENH68651
  
Location: 1245821-1247776
  
  
**BlastP hit with Mycgr3G107069\_Mycgr3**
  
Percentage identity: 39 %
  
BlastP bit score: 480
  
Sequence coverage: 101 %
  
E-value: 7e-158
  
  
 NCBI BlastP on this gene

ENH68651

Query: Architecture Search FASTA input

ACJE01000009 : Aspergillus niger ATCC 1015    Total score: 1.0     Cumulative Blast bit score: 476

Hit cluster cross-links:

Mycgr3G52686 Mycgr3T
  
Location: 0-861

Mycgr3G52686\_Mycgr3T

Mycgr3G102281 Mycgr3
  
Location: 961-1573

Mycgr3G102281\_Mycgr3

Mycgr3G89185 Mycgr3T
  
Location: 1673-2063

Mycgr3G89185\_Mycgr3T

Mycgr3G65725 Mycgr3T
  
Location: 2163-3612

Mycgr3G65725\_Mycgr3T

Mycgr3G102276 Mycgr3
  
Location: 3712-4801

Mycgr3G102276\_Mycgr3

Mycgr3G89189 Mycgr3T
  
Location: 4901-5564

Mycgr3G89189\_Mycgr3T

Mycgr3G52682 Mycgr3T
  
Location: 5664-9231

Mycgr3G52682\_Mycgr3T

Mycgr3G107072 Mycgr3
  
Location: 9331-13279

Mycgr3G107072\_Mycgr3

Mycgr3G34982 Mycgr3T
  
Location: 13379-15116

Mycgr3G34982\_Mycgr3T

Mycgr3G107069 Mycgr3
  
Location: 15216-17097

Mycgr3G107069\_Mycgr3

Mycgr3G32432 Mycgr3T
  
Location: 17197-19042

Mycgr3G32432\_Mycgr3T

Mycgr3G98385 Mycgr3T
  
Location: 19142-19898

Mycgr3G98385\_Mycgr3T

hypothetical protein
  
Accession: EHA24029
  
Location: 1232878-1233938
  
 NCBI BlastP on this gene

EHA24029

hypothetical protein
  
Accession: EHA24030
  
Location: 1234603-1235331
  
 NCBI BlastP on this gene

EHA24030

hypothetical protein
  
Accession: EHA24031
  
Location: 1236231-1236740
  
 NCBI BlastP on this gene

EHA24031

hypothetical protein
  
Accession: EHA24032
  
Location: 1237326-1238318
  
 NCBI BlastP on this gene

EHA24032

hypothetical protein
  
Accession: EHA24033
  
Location: 1239648-1240826
  
 NCBI BlastP on this gene

EHA24033

hypothetical protein
  
Accession: EHA24034
  
Location: 1241093-1243192
  
 NCBI BlastP on this gene

EHA24034

hypothetical protein
  
Accession: EHA24035
  
Location: 1243959-1244501
  
 NCBI BlastP on this gene

EHA24035

hypothetical protein
  
Accession: EHA24036
  
Location: 1244759-1246747
  
  
**BlastP hit with Mycgr3G107069\_Mycgr3**
  
Percentage identity: 41 %
  
BlastP bit score: 476
  
Sequence coverage: 100 %
  
E-value: 3e-156
  
  
 NCBI BlastP on this gene

EHA24036

Query: Architecture Search FASTA input

HF679026 : Fusarium fujikuroi IMI 58289 draft genome, chromosome FFUJ\_chr04.    Total score: 1.0     Cumulative Blast bit score: 475

Hit cluster cross-links:

Mycgr3G52686 Mycgr3T
  
Location: 0-861

Mycgr3G52686\_Mycgr3T

Mycgr3G102281 Mycgr3
  
Location: 961-1573

Mycgr3G102281\_Mycgr3

Mycgr3G89185 Mycgr3T
  
Location: 1673-2063

Mycgr3G89185\_Mycgr3T

Mycgr3G65725 Mycgr3T
  
Location: 2163-3612

Mycgr3G65725\_Mycgr3T

Mycgr3G102276 Mycgr3
  
Location: 3712-4801

Mycgr3G102276\_Mycgr3

Mycgr3G89189 Mycgr3T
  
Location: 4901-5564

Mycgr3G89189\_Mycgr3T

Mycgr3G52682 Mycgr3T
  
Location: 5664-9231

Mycgr3G52682\_Mycgr3T

Mycgr3G107072 Mycgr3
  
Location: 9331-13279

Mycgr3G107072\_Mycgr3

Mycgr3G34982 Mycgr3T
  
Location: 13379-15116

Mycgr3G34982\_Mycgr3T

Mycgr3G107069 Mycgr3
  
Location: 15216-17097

Mycgr3G107069\_Mycgr3

Mycgr3G32432 Mycgr3T
  
Location: 17197-19042

Mycgr3G32432\_Mycgr3T

Mycgr3G98385 Mycgr3T
  
Location: 19142-19898

Mycgr3G98385\_Mycgr3T

probable DNA polymerase epsilon, calytic chain POL2
  
Accession: CCT67076
  
Location: 856451-863318
  
 NCBI BlastP on this gene

FFUJ\_13258

uncharacterized protein
  
Accession: CCT67075
  
Location: 855424-855987
  
 NCBI BlastP on this gene

FFUJ\_13257

uncharacterized protein
  
Accession: CCT67074
  
Location: 854103-855017
  
 NCBI BlastP on this gene

FFUJ\_13256

uncharacterized protein
  
Accession: CCT67073
  
Location: 852566-853142
  
 NCBI BlastP on this gene

FFUJ\_13255

related to copper transport protein
  
Accession: CCT67072
  
Location: 850476-851075
  
 NCBI BlastP on this gene

FFUJ\_13254

related to ferric reductase FRE2 precursor
  
Accession: CCT67071
  
Location: 847545-849500
  
  
**BlastP hit with Mycgr3G107069\_Mycgr3**
  
Percentage identity: 39 %
  
BlastP bit score: 475
  
Sequence coverage: 102 %
  
E-value: 7e-156
  
  
 NCBI BlastP on this gene

FFUJ\_13253

related to GAL10-UDP-glucose 4-epimerase
  
Accession: CCT67070
  
Location: 845579-846601
  
 NCBI BlastP on this gene

FFUJ\_13252

uncharacterized protein
  
Accession: CCT67069
  
Location: 844048-845374
  
 NCBI BlastP on this gene

FFUJ\_13251

related to MDM35 Mitochondrial Distribution and Morphology protein
  
Accession: CCT67554
  
Location: 843070-843500
  
 NCBI BlastP on this gene

FFUJ\_14434

related to
  
Accession: CCT67504
  
Location: 839767-842482
  
 NCBI BlastP on this gene

FFUJ\_13250

uncharacterized protein
  
Accession: CCT67068
  
Location: 838109-838660
  
 NCBI BlastP on this gene

FFUJ\_13249

probable tubulin folding cofactor C
  
Accession: CCT67067
  
Location: 836616-837872
  
 NCBI BlastP on this gene

FFUJ\_13248

uncharacterized protein
  
Accession: CCT67066
  
Location: 835950-836390
  
 NCBI BlastP on this gene

FFUJ\_13247

Query: Architecture Search FASTA input

DS231620 : Pyrenophora tritici-repentis Pt-1C-BFP supercont1.6 genomic scaffold    Total score: 1.0     Cumulative Blast bit score: 469

Hit cluster cross-links:

Mycgr3G52686 Mycgr3T
  
Location: 0-861

Mycgr3G52686\_Mycgr3T

Mycgr3G102281 Mycgr3
  
Location: 961-1573

Mycgr3G102281\_Mycgr3

Mycgr3G89185 Mycgr3T
  
Location: 1673-2063

Mycgr3G89185\_Mycgr3T

Mycgr3G65725 Mycgr3T
  
Location: 2163-3612

Mycgr3G65725\_Mycgr3T

Mycgr3G102276 Mycgr3
  
Location: 3712-4801

Mycgr3G102276\_Mycgr3

Mycgr3G89189 Mycgr3T
  
Location: 4901-5564

Mycgr3G89189\_Mycgr3T

Mycgr3G52682 Mycgr3T
  
Location: 5664-9231

Mycgr3G52682\_Mycgr3T

Mycgr3G107072 Mycgr3
  
Location: 9331-13279

Mycgr3G107072\_Mycgr3

Mycgr3G34982 Mycgr3T
  
Location: 13379-15116

Mycgr3G34982\_Mycgr3T

Mycgr3G107069 Mycgr3
  
Location: 15216-17097

Mycgr3G107069\_Mycgr3

Mycgr3G32432 Mycgr3T
  
Location: 17197-19042

Mycgr3G32432\_Mycgr3T

Mycgr3G98385 Mycgr3T
  
Location: 19142-19898

Mycgr3G98385\_Mycgr3T

conserved hypothetical protein
  
Accession: EDU49698
  
Location: 1666091-1667896
  
 NCBI BlastP on this gene

EDU49698

siderophore iron transporter mirB
  
Accession: EDU49699
  
Location: 1669879-1671777
  
 NCBI BlastP on this gene

EDU49699

conserved hypothetical protein
  
Accession: EDU49700
  
Location: 1672065-1673187
  
 NCBI BlastP on this gene

EDU49700

predicted protein
  
Accession: EDU49701
  
Location: 1675575-1677260
  
 NCBI BlastP on this gene

EDU49701

high affinity copper transporter
  
Accession: EDU49702
  
Location: 1678126-1678752
  
 NCBI BlastP on this gene

EDU49702

ferric reductase
  
Accession: EDU49703
  
Location: 1679262-1681283
  
  
**BlastP hit with Mycgr3G107069\_Mycgr3**
  
Percentage identity: 40 %
  
BlastP bit score: 469
  
Sequence coverage: 105 %
  
E-value: 4e-153
  
  
 NCBI BlastP on this gene

EDU49703

Query: Architecture Search FASTA input

CP003014 : Thielavia terrestris NRRL 8126 chromosome 6    Total score: 1.0     Cumulative Blast bit score: 468

Hit cluster cross-links:

Mycgr3G52686 Mycgr3T
  
Location: 0-861

Mycgr3G52686\_Mycgr3T

Mycgr3G102281 Mycgr3
  
Location: 961-1573

Mycgr3G102281\_Mycgr3

Mycgr3G89185 Mycgr3T
  
Location: 1673-2063

Mycgr3G89185\_Mycgr3T

Mycgr3G65725 Mycgr3T
  
Location: 2163-3612

Mycgr3G65725\_Mycgr3T

Mycgr3G102276 Mycgr3
  
Location: 3712-4801

Mycgr3G102276\_Mycgr3

Mycgr3G89189 Mycgr3T
  
Location: 4901-5564

Mycgr3G89189\_Mycgr3T

Mycgr3G52682 Mycgr3T
  
Location: 5664-9231

Mycgr3G52682\_Mycgr3T

Mycgr3G107072 Mycgr3
  
Location: 9331-13279

Mycgr3G107072\_Mycgr3

Mycgr3G34982 Mycgr3T
  
Location: 13379-15116

Mycgr3G34982\_Mycgr3T

Mycgr3G107069 Mycgr3
  
Location: 15216-17097

Mycgr3G107069\_Mycgr3

Mycgr3G32432 Mycgr3T
  
Location: 17197-19042

Mycgr3G32432\_Mycgr3T

Mycgr3G98385 Mycgr3T
  
Location: 19142-19898

Mycgr3G98385\_Mycgr3T

hypothetical protein
  
Accession: AEO71360
  
Location: 1426212-1427384
  
 NCBI BlastP on this gene

THITE\_2123618

hypothetical protein
  
Accession: AEO71359
  
Location: 1418007-1421857
  
 NCBI BlastP on this gene

THITE\_124350

hypothetical protein
  
Accession: AEO71358
  
Location: 1413240-1416021
  
  
**BlastP hit with Mycgr3G52682\_Mycgr3T**
  
Percentage identity: 38 %
  
BlastP bit score: 468
  
Sequence coverage: 70 %
  
E-value: 2e-143
  
  
 NCBI BlastP on this gene

THITE\_2123607

hypothetical protein
  
Accession: AEO71357
  
Location: 1409562-1411101
  
 NCBI BlastP on this gene

THITE\_2123604

hypothetical protein
  
Accession: AEO71356
  
Location: 1407378-1408108
  
 NCBI BlastP on this gene

THITE\_2123602

hypothetical protein
  
Accession: AEO71355
  
Location: 1404021-1406434
  
 NCBI BlastP on this gene

THITE\_2123600

hypothetical protein
  
Accession: AEO71354
  
Location: 1402962-1403508
  
 NCBI BlastP on this gene

THITE\_2123598

Query: Architecture Search FASTA input

GG698898 : Nectria haematococca mpVI 77-13-4 chromosome 4 genomic scaffold NECHAsca\_3\_chr4\_2\_0    Total score: 1.0     Cumulative Blast bit score: 466

Hit cluster cross-links:

Mycgr3G52686 Mycgr3T
  
Location: 0-861

Mycgr3G52686\_Mycgr3T

Mycgr3G102281 Mycgr3
  
Location: 961-1573

Mycgr3G102281\_Mycgr3

Mycgr3G89185 Mycgr3T
  
Location: 1673-2063

Mycgr3G89185\_Mycgr3T

Mycgr3G65725 Mycgr3T
  
Location: 2163-3612

Mycgr3G65725\_Mycgr3T

Mycgr3G102276 Mycgr3
  
Location: 3712-4801

Mycgr3G102276\_Mycgr3

Mycgr3G89189 Mycgr3T
  
Location: 4901-5564

Mycgr3G89189\_Mycgr3T

Mycgr3G52682 Mycgr3T
  
Location: 5664-9231

Mycgr3G52682\_Mycgr3T

Mycgr3G107072 Mycgr3
  
Location: 9331-13279

Mycgr3G107072\_Mycgr3

Mycgr3G34982 Mycgr3T
  
Location: 13379-15116

Mycgr3G34982\_Mycgr3T

Mycgr3G107069 Mycgr3
  
Location: 15216-17097

Mycgr3G107069\_Mycgr3

Mycgr3G32432 Mycgr3T
  
Location: 17197-19042

Mycgr3G32432\_Mycgr3T

Mycgr3G98385 Mycgr3T
  
Location: 19142-19898

Mycgr3G98385\_Mycgr3T

hypothetical protein
  
Accession: EEU46364
  
Location: 1029943-1031895
  
  
**BlastP hit with Mycgr3G107069\_Mycgr3**
  
Percentage identity: 38 %
  
BlastP bit score: 466
  
Sequence coverage: 102 %
  
E-value: 2e-152
  
  
 NCBI BlastP on this gene

EEU46364

hypothetical protein
  
Accession: EEU46363
  
Location: 1027977-1028999
  
 NCBI BlastP on this gene

EEU46363

hypothetical protein
  
Accession: EEU45920
  
Location: 1026402-1027715
  
 NCBI BlastP on this gene

EEU45920

hypothetical protein
  
Accession: EEU46362
  
Location: 1022117-1025891
  
 NCBI BlastP on this gene

EEU46362

Query: Architecture Search FASTA input

KB456262 : Mycosphaerella populorum SO2202 unplaced genomic scaffold SEPMUscaffold\_3    Total score: 1.0     Cumulative Blast bit score: 464

Hit cluster cross-links:

Mycgr3G52686 Mycgr3T
  
Location: 0-861

Mycgr3G52686\_Mycgr3T

Mycgr3G102281 Mycgr3
  
Location: 961-1573

Mycgr3G102281\_Mycgr3

Mycgr3G89185 Mycgr3T
  
Location: 1673-2063

Mycgr3G89185\_Mycgr3T

Mycgr3G65725 Mycgr3T
  
Location: 2163-3612

Mycgr3G65725\_Mycgr3T

Mycgr3G102276 Mycgr3
  
Location: 3712-4801

Mycgr3G102276\_Mycgr3

Mycgr3G89189 Mycgr3T
  
Location: 4901-5564

Mycgr3G89189\_Mycgr3T

Mycgr3G52682 Mycgr3T
  
Location: 5664-9231

Mycgr3G52682\_Mycgr3T

Mycgr3G107072 Mycgr3
  
Location: 9331-13279

Mycgr3G107072\_Mycgr3

Mycgr3G34982 Mycgr3T
  
Location: 13379-15116

Mycgr3G34982\_Mycgr3T

Mycgr3G107069 Mycgr3
  
Location: 15216-17097

Mycgr3G107069\_Mycgr3

Mycgr3G32432 Mycgr3T
  
Location: 17197-19042

Mycgr3G32432\_Mycgr3T

Mycgr3G98385 Mycgr3T
  
Location: 19142-19898

Mycgr3G98385\_Mycgr3T

ARM repeat-containing protein
  
Accession: EMF14807
  
Location: 2480643-2483825
  
 NCBI BlastP on this gene

EMF14807

hypothetical protein
  
Accession: EMF14808
  
Location: 2485131-2486066
  
 NCBI BlastP on this gene

EMF14808

zinc-binding alcohol dehydrogenase
  
Accession: EMF14809
  
Location: 2487157-2488401
  
  
**BlastP hit with Mycgr3G102276\_Mycgr3**
  
Percentage identity: 66 %
  
BlastP bit score: 465
  
Sequence coverage: 100 %
  
E-value: 3e-160
  
  
 NCBI BlastP on this gene

EMF14809

acyl-CoA dehydrogenase
  
Accession: EMF14810
  
Location: 2488722-2490501
  
 NCBI BlastP on this gene

EMF14810

alpha/beta-hydrolase
  
Accession: EMF14811
  
Location: 2491113-2492668
  
 NCBI BlastP on this gene

EMF14811

hypothetical protein
  
Accession: EMF14812
  
Location: 2493069-2493818
  
 NCBI BlastP on this gene

EMF14812

Metallo-hydrolase/oxidoreductase
  
Accession: EMF14813
  
Location: 2494600-2495607
  
 NCBI BlastP on this gene

EMF14813

hypothetical protein
  
Accession: EMF14814
  
Location: 2496241-2497651
  
 NCBI BlastP on this gene

EMF14814

Query: Architecture Search FASTA input

GL629729 : Grosmannia clavigera kw1407 unplaced genomic scaffold GCSC\_108    Total score: 1.0     Cumulative Blast bit score: 462

Hit cluster cross-links:

Mycgr3G52686 Mycgr3T
  
Location: 0-861

Mycgr3G52686\_Mycgr3T

Mycgr3G102281 Mycgr3
  
Location: 961-1573

Mycgr3G102281\_Mycgr3

Mycgr3G89185 Mycgr3T
  
Location: 1673-2063

Mycgr3G89185\_Mycgr3T

Mycgr3G65725 Mycgr3T
  
Location: 2163-3612

Mycgr3G65725\_Mycgr3T

Mycgr3G102276 Mycgr3
  
Location: 3712-4801

Mycgr3G102276\_Mycgr3

Mycgr3G89189 Mycgr3T
  
Location: 4901-5564

Mycgr3G89189\_Mycgr3T

Mycgr3G52682 Mycgr3T
  
Location: 5664-9231

Mycgr3G52682\_Mycgr3T

Mycgr3G107072 Mycgr3
  
Location: 9331-13279

Mycgr3G107072\_Mycgr3

Mycgr3G34982 Mycgr3T
  
Location: 13379-15116

Mycgr3G34982\_Mycgr3T

Mycgr3G107069 Mycgr3
  
Location: 15216-17097

Mycgr3G107069\_Mycgr3

Mycgr3G32432 Mycgr3T
  
Location: 17197-19042

Mycgr3G32432\_Mycgr3T

Mycgr3G98385 Mycgr3T
  
Location: 19142-19898

Mycgr3G98385\_Mycgr3T

tbc domain protein
  
Accession: EFX06333
  
Location: 253396-257247
  
 NCBI BlastP on this gene

EFX06333

ankyrin unc44
  
Accession: EFX06213
  
Location: 258978-260324
  
 NCBI BlastP on this gene

EFX06213

mitochondrial ATPase, aaa-type
  
Accession: EFX06609
  
Location: 261444-262908
  
 NCBI BlastP on this gene

EFX06609

high affinity copper transporter
  
Accession: EFX06270
  
Location: 263930-264388
  
 NCBI BlastP on this gene

EFX06270

ferric-chelate reductase
  
Accession: EFX06529
  
Location: 264730-266796
  
  
**BlastP hit with Mycgr3G107069\_Mycgr3**
  
Percentage identity: 39 %
  
BlastP bit score: 462
  
Sequence coverage: 105 %
  
E-value: 3e-150
  
  
 NCBI BlastP on this gene

EFX06529

Query: Architecture Search FASTA input

DS572697 : Verticillium dahliae VdLs.17 supercont1.3 genomic scaffold    Total score: 1.0     Cumulative Blast bit score: 462

Hit cluster cross-links:

Mycgr3G52686 Mycgr3T
  
Location: 0-861

Mycgr3G52686\_Mycgr3T

Mycgr3G102281 Mycgr3
  
Location: 961-1573

Mycgr3G102281\_Mycgr3

Mycgr3G89185 Mycgr3T
  
Location: 1673-2063

Mycgr3G89185\_Mycgr3T

Mycgr3G65725 Mycgr3T
  
Location: 2163-3612

Mycgr3G65725\_Mycgr3T

Mycgr3G102276 Mycgr3
  
Location: 3712-4801

Mycgr3G102276\_Mycgr3

Mycgr3G89189 Mycgr3T
  
Location: 4901-5564

Mycgr3G89189\_Mycgr3T

Mycgr3G52682 Mycgr3T
  
Location: 5664-9231

Mycgr3G52682\_Mycgr3T

Mycgr3G107072 Mycgr3
  
Location: 9331-13279

Mycgr3G107072\_Mycgr3

Mycgr3G34982 Mycgr3T
  
Location: 13379-15116

Mycgr3G34982\_Mycgr3T

Mycgr3G107069 Mycgr3
  
Location: 15216-17097

Mycgr3G107069\_Mycgr3

Mycgr3G32432 Mycgr3T
  
Location: 17197-19042

Mycgr3G32432\_Mycgr3T

Mycgr3G98385 Mycgr3T
  
Location: 19142-19898

Mycgr3G98385\_Mycgr3T

hypothetical protein
  
Accession: EGY20026
  
Location: 1312899-1313567
  
 NCBI BlastP on this gene

EGY20026

chitosanase
  
Accession: EGY20027
  
Location: 1314130-1316053
  
 NCBI BlastP on this gene

EGY20027

hypothetical protein
  
Accession: EGY20028
  
Location: 1317811-1318140
  
 NCBI BlastP on this gene

EGY20028

high affinity copper transporter
  
Accession: EGY20029
  
Location: 1319730-1320631
  
 NCBI BlastP on this gene

EGY20029

ferric reductase transmembrane component 5
  
Accession: EGY20030
  
Location: 1321680-1323702
  
  
**BlastP hit with Mycgr3G107069\_Mycgr3**
  
Percentage identity: 40 %
  
BlastP bit score: 462
  
Sequence coverage: 102 %
  
E-value: 5e-151
  
  
 NCBI BlastP on this gene

EGY20030

hypothetical protein
  
Accession: EGY20031
  
Location: 1324271-1328173
  
 NCBI BlastP on this gene

EGY20031

MFS transporter
  
Accession: EGY20032
  
Location: 1328374-1330048
  
 NCBI BlastP on this gene

EGY20032

hypothetical protein
  
Accession: EGY20033
  
Location: 1331774-1332237
  
 NCBI BlastP on this gene

EGY20033

hypothetical protein
  
Accession: EGY20034
  
Location: 1334315-1336389
  
 NCBI BlastP on this gene

EGY20034

Query: Architecture Search FASTA input

151. :  CABT02000003 Sordaria macrospora k-hell     Total score: 1.0     Cumulative Blast bit score: 769

Mycgr3G52686 Mycgr3T
  
Location: 0-861
  
 NCBI BlastP on this gene

Mycgr3G52686\_Mycgr3T

Mycgr3G102281 Mycgr3
  
Location: 961-1573
  
 NCBI BlastP on this gene

Mycgr3G102281\_Mycgr3

Mycgr3G89185 Mycgr3T
  
Location: 1673-2063
  
 NCBI BlastP on this gene

Mycgr3G89185\_Mycgr3T

Mycgr3G65725 Mycgr3T
  
Location: 2163-3612
  
 NCBI BlastP on this gene

Mycgr3G65725\_Mycgr3T

Mycgr3G102276 Mycgr3
  
Location: 3712-4801
  
 NCBI BlastP on this gene

Mycgr3G102276\_Mycgr3

Mycgr3G89189 Mycgr3T
  
Location: 4901-5564
  
 NCBI BlastP on this gene

Mycgr3G89189\_Mycgr3T

Mycgr3G52682 Mycgr3T
  
Location: 5664-9231
  
 NCBI BlastP on this gene

Mycgr3G52682\_Mycgr3T

Mycgr3G107072 Mycgr3
  
Location: 9331-13279
  
 NCBI BlastP on this gene

Mycgr3G107072\_Mycgr3

Mycgr3G34982 Mycgr3T
  
Location: 13379-15116
  
 NCBI BlastP on this gene

Mycgr3G34982\_Mycgr3T

Mycgr3G107069 Mycgr3
  
Location: 15216-17097
  
 NCBI BlastP on this gene

Mycgr3G107069\_Mycgr3

Mycgr3G32432 Mycgr3T
  
Location: 17197-19042
  
 NCBI BlastP on this gene

Mycgr3G32432\_Mycgr3T

Mycgr3G98385 Mycgr3T
  
Location: 19142-19898
  
 NCBI BlastP on this gene

Mycgr3G98385\_Mycgr3T

not annotated
  
Accession: CCC07539
  
Location: 1355850-1356825
  
 NCBI BlastP on this gene

CCC07539

not annotated
  
Accession: CCC07540
  
Location: 1357036-1357871
  
 NCBI BlastP on this gene

CCC07540

not annotated
  
Accession: CCC07541
  
Location: 1358329-1361612
  
 NCBI BlastP on this gene

CCC07541

not annotated
  
Accession: CCC07542
  
Location: 1362201-1363053
  
 NCBI BlastP on this gene

CCC07542

not annotated
  
Accession: CCC07543
  
Location: 1365922-1368687
  
 NCBI BlastP on this gene

CCC07543

not annotated
  
Accession: CCC07544
  
Location: 1373014-1374096
  
 NCBI BlastP on this gene

CCC07544

not annotated
  
Accession: CCC07545
  
Location: 1374719-1377143
  
  
**BlastP hit with Mycgr3G52682\_Mycgr3T**
  
Percentage identity: 47 %
  
BlastP bit score: 769
  
Sequence coverage: 70 %
  
E-value: 0.0
  
  
 NCBI BlastP on this gene

CCC07545

not annotated
  
Accession: CCC07546
  
Location: 1377509-1380006
  
 NCBI BlastP on this gene

CCC07546

not annotated
  
Accession: CCC07547
  
Location: 1382161-1385733
  
 NCBI BlastP on this gene

CCC07547

not annotated
  
Accession: CCC07548
  
Location: 1386763-1389137
  
 NCBI BlastP on this gene

CCC07548

not annotated
  
Accession: CCC07549
  
Location: 1392097-1393850
  
 NCBI BlastP on this gene

CCC07549

152. :  JH126401 Cordyceps militaris CM01 unplaced genomic scaffold CCM\_S00003     Total score: 1.0     Cumulative Blast bit score: 758

Helix-loop-helix DNA-binding protein
  
Accession: EGX93174
  
Location: 2934859-2936380
  
 NCBI BlastP on this gene

EGX93174

integral membrane protein
  
Accession: EGX93173
  
Location: 2927512-2929221
  
 NCBI BlastP on this gene

EGX93173

protein transport protein (SEC31), putative
  
Accession: EGX93172
  
Location: 2922175-2925688
  
 NCBI BlastP on this gene

EGX93172

RNA recognition protein, RNP-1
  
Accession: EGX93171
  
Location: 2920935-2921425
  
 NCBI BlastP on this gene

EGX93171

hypothetical protein
  
Accession: EGX93170
  
Location: 2920234-2920695
  
 NCBI BlastP on this gene

EGX93170

cytochrome c oxidase polypeptide VIb
  
Accession: EGX93169
  
Location: 2919354-2919908
  
 NCBI BlastP on this gene

EGX93169

hypothetical protein
  
Accession: EGX93168
  
Location: 2918187-2919029
  
 NCBI BlastP on this gene

EGX93168

40S ribosomal protein S10
  
Accession: EGX93167
  
Location: 2917247-2917877
  
 NCBI BlastP on this gene

EGX93167

ubiquitin-protein ligase (Hul4), putative
  
Accession: EGX93166
  
Location: 2911801-2915416
  
  
**BlastP hit with Mycgr3G52682\_Mycgr3T**
  
Percentage identity: 39 %
  
BlastP bit score: 758
  
Sequence coverage: 100 %
  
E-value: 0.0
  
  
 NCBI BlastP on this gene

EGX93166

50S ribosomal protein Mrp49
  
Accession: EGX93165
  
Location: 2910934-2911604
  
 NCBI BlastP on this gene

EGX93165

Protein phosphatase inhibitor
  
Accession: EGX93164
  
Location: 2907279-2910704
  
 NCBI BlastP on this gene

EGX93164

DUF726 domain protein
  
Accession: EGX93163
  
Location: 2906052-2906621
  
 NCBI BlastP on this gene

EGX93163

vesicular-fusion protein sec17
  
Accession: EGX93162
  
Location: 2904660-2905761
  
 NCBI BlastP on this gene

EGX93162

Armadillo-type fold domain containing protein
  
Accession: EGX93161
  
Location: 2900846-2903994
  
 NCBI BlastP on this gene

EGX93161

hypothetical protein
  
Accession: EGX93160
  
Location: 2893304-2894565
  
 NCBI BlastP on this gene

EGX93160

153. :  KB722656 Rhodosporidium toruloides NP11 unplaced genomic scaffold Scaffold15     Total score: 1.0     Cumulative Blast bit score: 694

large subunit ribosomal protein L14
  
Accession: EMS21448
  
Location: 441859-442591
  
 NCBI BlastP on this gene

EMS21448

precorrin-2 dehydrogenase / sirohydrochlorin ferrochelatase
  
Accession: EMS21449
  
Location: 442899-443882
  
 NCBI BlastP on this gene

EMS21449

hypothetical protein
  
Accession: EMS21450
  
Location: 444643-445191
  
 NCBI BlastP on this gene

EMS21450

glycoside hydrolase family 5 protein
  
Accession: EMS21451
  
Location: 445777-449181
  
 NCBI BlastP on this gene

EMS21451

monooxygenase
  
Accession: EMS21452
  
Location: 449303-451375
  
 NCBI BlastP on this gene

EMS21452

hypothetical protein
  
Accession: EMS21453
  
Location: 451729-452760
  
 NCBI BlastP on this gene

EMS21453

sulfide:quinone oxidoreductase, mitochondrial precursor
  
Accession: EMS21454
  
Location: 452989-455066
  
 NCBI BlastP on this gene

EMS21454

metallo-beta-lactamase domain containing protein
  
Accession: EMS21455
  
Location: 455336-456605
  
 NCBI BlastP on this gene

EMS21455

alcohol dehydrogenase (NADP+)
  
Accession: EMS21456
  
Location: 457177-459036
  
  
**BlastP hit with Mycgr3G102276\_Mycgr3**
  
Percentage identity: 53 %
  
BlastP bit score: 353
  
Sequence coverage: 99 %
  
E-value: 3e-116
  
  
 NCBI BlastP on this gene

EMS21456

MFS transporter, sugar transporter
  
Accession: EMS21457
  
Location: 459922-462605
  
 NCBI BlastP on this gene

EMS21457

hypothetical protein
  
Accession: EMS21458
  
Location: 462980-463903
  
 NCBI BlastP on this gene

EMS21458

protein of Emopamil-binding protein family
  
Accession: EMS21459
  
Location: 464025-465036
  
 NCBI BlastP on this gene

EMS21459

WSC domain protein
  
Accession: EMS21460
  
Location: 465561-467475
  
 NCBI BlastP on this gene

EMS21460

exportin-1
  
Accession: EMS21461
  
Location: 467866-472563
  
 NCBI BlastP on this gene

EMS21461

hypothetical protein
  
Accession: EMS21462
  
Location: 473166-473961
  
 NCBI BlastP on this gene

EMS21462

MFS nicotinic acid transporter
  
Accession: EMS21463
  
Location: 474746-477830
  
 NCBI BlastP on this gene

EMS21463

alcohol dehydrogenase (NADP+)
  
Accession: EMS21464
  
Location: 478333-480152
  
  
**BlastP hit with Mycgr3G102276\_Mycgr3**
  
Percentage identity: 52 %
  
BlastP bit score: 341
  
Sequence coverage: 103 %
  
E-value: 2e-111
  
  
 NCBI BlastP on this gene

EMS21464

glycoside hydrolase family 61 protein
  
Accession: EMS21465
  
Location: 481201-484560
  
 NCBI BlastP on this gene

EMS21465

DNA-directed RNA polymerase II subunit RPB9
  
Accession: EMS21466
  
Location: 485565-486541
  
 NCBI BlastP on this gene

EMS21466

60S ribosomal protein l39
  
Accession: EMS21467
  
Location: 487564-488183
  
 NCBI BlastP on this gene

EMS21467

154. :  CH476618 Uncinocarpus reesii 1704 scaffold\_4 genomic scaffold     Total score: 1.0     Cumulative Blast bit score: 677

predicted protein
  
Accession: EEP81732
  
Location: 1412087-1412848
  
 NCBI BlastP on this gene

EEP81732

predicted protein
  
Accession: EEP81733
  
Location: 1413939-1415838
  
 NCBI BlastP on this gene

EEP81733

predicted protein
  
Accession: EEP81734
  
Location: 1417329-1418672
  
 NCBI BlastP on this gene

EEP81734

predicted protein
  
Accession: EEP81735
  
Location: 1420413-1421338
  
 NCBI BlastP on this gene

EEP81735

predicted protein
  
Accession: EEP81736
  
Location: 1422412-1423696
  
 NCBI BlastP on this gene

EEP81736

arsenical-resistance protein
  
Accession: EEP81737
  
Location: 1425296-1426587
  
 NCBI BlastP on this gene

EEP81737

VEG136 protein
  
Accession: EEP81738
  
Location: 1427714-1428361
  
 NCBI BlastP on this gene

EEP81738

conserved hypothetical protein
  
Accession: EEP81739
  
Location: 1429464-1433013
  
  
**BlastP hit with Mycgr3G52682\_Mycgr3T**
  
Percentage identity: 54 %
  
BlastP bit score: 677
  
Sequence coverage: 53 %
  
E-value: 0.0
  
  
 NCBI BlastP on this gene

EEP81739

predicted protein
  
Accession: EEP81740
  
Location: 1433658-1435001
  
 NCBI BlastP on this gene

EEP81740

hypothetical protein
  
Accession: EEP81741
  
Location: 1435514-1436963
  
 NCBI BlastP on this gene

EEP81741

conserved hypothetical protein
  
Accession: EEP81742
  
Location: 1437571-1438287
  
 NCBI BlastP on this gene

EEP81742

predicted protein
  
Accession: EEP81743
  
Location: 1439267-1440539
  
 NCBI BlastP on this gene

EEP81743

predicted protein
  
Accession: EEP81744
  
Location: 1441738-1441935
  
 NCBI BlastP on this gene

EEP81744

conserved hypothetical protein
  
Accession: EEP81745
  
Location: 1442527-1444839
  
 NCBI BlastP on this gene

EEP81745

conserved hypothetical protein
  
Accession: EEP81746
  
Location: 1445240-1447046
  
 NCBI BlastP on this gene

EEP81746

conserved hypothetical protein
  
Accession: EEP81747
  
Location: 1447919-1450347
  
 NCBI BlastP on this gene

EEP81747

conserved hypothetical protein
  
Accession: EEP81748
  
Location: 1450818-1452408
  
 NCBI BlastP on this gene

EEP81748

155. :  JH930469 Phanerochaete carnosa HHB-10118-sp unplaced genomic scaffold PHACAscaffold\_2     Total score: 1.0     Cumulative Blast bit score: 650

hypothetical protein
  
Accession: EKM59949
  
Location: 3788856-3789176
  
 NCBI BlastP on this gene

EKM59949

hypothetical protein
  
Accession: EKM59948
  
Location: 3782225-3783426
  
 NCBI BlastP on this gene

EKM59948

hypothetical protein
  
Accession: EKM59947
  
Location: 3780636-3782113
  
 NCBI BlastP on this gene

EKM59947

hypothetical protein
  
Accession: EKM59946
  
Location: 3780083-3780284
  
 NCBI BlastP on this gene

EKM59946

hypothetical protein
  
Accession: EKM59945
  
Location: 3772177-3774961
  
  
**BlastP hit with Mycgr3G34982\_Mycgr3T**
  
Percentage identity: 30 %
  
BlastP bit score: 232
  
Sequence coverage: 103 %
  
E-value: 3e-64
  
  
 NCBI BlastP on this gene

EKM59945

hypothetical protein
  
Accession: EKM59944
  
Location: 3767756-3770742
  
  
**BlastP hit with Mycgr3G34982\_Mycgr3T**
  
Percentage identity: 28 %
  
BlastP bit score: 195
  
Sequence coverage: 101 %
  
E-value: 6e-51
  
  
 NCBI BlastP on this gene

EKM59944

hypothetical protein
  
Accession: EKM59943
  
Location: 3763276-3766056
  
  
**BlastP hit with Mycgr3G34982\_Mycgr3T**
  
Percentage identity: 28 %
  
BlastP bit score: 223
  
Sequence coverage: 106 %
  
E-value: 6e-61
  
  
 NCBI BlastP on this gene

EKM59943

hypothetical protein
  
Accession: EKM59942
  
Location: 3757671-3761810
  
 NCBI BlastP on this gene

EKM59942

hypothetical protein
  
Accession: EKM59941
  
Location: 3755860-3756516
  
 NCBI BlastP on this gene

EKM59941

hypothetical protein
  
Accession: EKM59940
  
Location: 3754072-3755568
  
 NCBI BlastP on this gene

EKM59940

hypothetical protein
  
Accession: EKM59939
  
Location: 3750028-3753346
  
 NCBI BlastP on this gene

EKM59939

156. :  EQ966375 Postia placenta Mad-698-R POSPLscaffold\_143 genomic scaffold     Total score: 1.0     Cumulative Blast bit score: 638

hypothetical triacylglycerol lipase
  
Accession: EED79575
  
Location: 72278-74743
  
 NCBI BlastP on this gene

EED79575

predicted protein
  
Accession: EED79587
  
Location: 75340-75838
  
 NCBI BlastP on this gene

EED79587

60S ribosomal protein L15
  
Accession: EED79576
  
Location: 76397-77216
  
  
**BlastP hit with Mycgr3G102281\_Mycgr3**
  
Percentage identity: 78 %
  
BlastP bit score: 319
  
Sequence coverage: 99 %
  
E-value: 7e-108
  
  
 NCBI BlastP on this gene

EED79576

predicted protein
  
Accession: EED79588
  
Location: 80222-80705
  
 NCBI BlastP on this gene

EED79588

60S ribosomal protein L15
  
Accession: EED79577
  
Location: 81218-82037
  
  
**BlastP hit with Mycgr3G102281\_Mycgr3**
  
Percentage identity: 78 %
  
BlastP bit score: 319
  
Sequence coverage: 99 %
  
E-value: 7e-108
  
  
 NCBI BlastP on this gene

EED79577

predicted protein
  
Accession: EED79578
  
Location: 83360-83961
  
 NCBI BlastP on this gene

EED79578

predicted protein
  
Accession: EED79579
  
Location: 89339-94114
  
 NCBI BlastP on this gene

EED79579

40S ribosomal protein S3
  
Accession: EED79589
  
Location: 101476-102423
  
 NCBI BlastP on this gene

EED79589

157. :  KB726077 Colletotrichum orbiculare MAFF 240422 unplaced genomic scaffold Scaffold\_499     Total score: 1.0     Cumulative Blast bit score: 630

2-amino-3-carboxymuconate-6-semialdehyde decarboxylase
  
Accession: ENH77490
  
Location: 65440-66429
  
 NCBI BlastP on this gene

ENH77490

polysaccharide deacetylase
  
Accession: ENH77491
  
Location: 67098-67922
  
 NCBI BlastP on this gene

ENH77491

2-keto-4-pentenoate hydratase 2-oxohepta-3-ene-dioic acid hydratase
  
Accession: ENH77492
  
Location: 68320-69243
  
 NCBI BlastP on this gene

ENH77492

HypA-like protein, putative
  
Accession: ENH77493
  
Location: 69834-71371
  
 NCBI BlastP on this gene

ENH77493

carbohydrate binding family 6
  
Accession: ENH77494
  
Location: 72576-73525
  
 NCBI BlastP on this gene

ENH77494

fmn binding protein
  
Accession: ENH77495
  
Location: 76209-77622
  
 NCBI BlastP on this gene

ENH77495

beta-lactamase domain protein
  
Accession: ENH77496
  
Location: 78281-79267
  
 NCBI BlastP on this gene

ENH77496

secreted protein
  
Accession: ENH77497
  
Location: 79361-81238
  
 NCBI BlastP on this gene

ENH77497

MFS monocarboxylate transporter
  
Accession: ENH77498
  
Location: 83778-85270
  
  
**BlastP hit with Mycgr3G65725\_Mycgr3T**
  
Percentage identity: 38 %
  
BlastP bit score: 308
  
Sequence coverage: 92 %
  
E-value: 4e-96
  
  
 NCBI BlastP on this gene

ENH77498

alkaline serine protease alp1
  
Accession: ENH77499
  
Location: 85427-86737
  
 NCBI BlastP on this gene

ENH77499

naphthalene -dioxygenase subunit alpha
  
Accession: ENH77500
  
Location: 88732-90113
  
 NCBI BlastP on this gene

ENH77500

hypothetical protein
  
Accession: ENH77501
  
Location: 91113-91517
  
 NCBI BlastP on this gene

ENH77501

sarcosine oxidase
  
Accession: ENH77502
  
Location: 92283-93740
  
 NCBI BlastP on this gene

ENH77502

C6 transcription factor
  
Accession: ENH77503
  
Location: 95122-97373
  
 NCBI BlastP on this gene

ENH77503

nad dehydrogenase
  
Accession: ENH77504
  
Location: 99210-101699
  
 NCBI BlastP on this gene

ENH77504

MFS monocarboxylate transporter
  
Accession: ENH77505
  
Location: 102026-103572
  
  
**BlastP hit with Mycgr3G65725\_Mycgr3T**
  
Percentage identity: 41 %
  
BlastP bit score: 322
  
Sequence coverage: 91 %
  
E-value: 3e-101
  
  
 NCBI BlastP on this gene

ENH77505

hypothetical protein
  
Accession: ENH77506
  
Location: 104221-104433
  
 NCBI BlastP on this gene

ENH77506

hypothetical protein
  
Accession: ENH77507
  
Location: 105912-106254
  
 NCBI BlastP on this gene

ENH77507

zinc-binding alcohol dehydrogenase
  
Accession: ENH77508
  
Location: 106460-107586
  
 NCBI BlastP on this gene

ENH77508

alpha-mannosyltransferase och1
  
Accession: ENH77509
  
Location: 108632-109858
  
 NCBI BlastP on this gene

ENH77509

endo-beta-glucanase
  
Accession: ENH77510
  
Location: 112636-116641
  
 NCBI BlastP on this gene

ENH77510

hypothetical protein
  
Accession: ENH77511
  
Location: 116972-117450
  
 NCBI BlastP on this gene

ENH77511

hypothetical protein
  
Accession: ENH77512
  
Location: 120084-120365
  
 NCBI BlastP on this gene

ENH77512

hypothetical protein
  
Accession: ENH77513
  
Location: 123558-126465
  
 NCBI BlastP on this gene

ENH77513

158. :  KB369422 Tupaia chinensis unplaced genomic scaffold Scaffold148626\_1     Total score: 1.0     Cumulative Blast bit score: 626

Ubiquitin-conjugating enzyme E2 E1
  
Accession: ELV09864
  
Location: 856969-932526
  
 NCBI BlastP on this gene

ELV09864

NF-kappa-B inhibitor-interacting Ras-like protein 1
  
Accession: ELV09863
  
Location: 849780-854737
  
 NCBI BlastP on this gene

ELV09863

60S ribosomal protein L15
  
Accession: ELV09862
  
Location: 842822-844705
  
  
**BlastP hit with Mycgr3G102281\_Mycgr3**
  
Percentage identity: 72 %
  
BlastP bit score: 313
  
Sequence coverage: 100 %
  
E-value: 2e-105
  
  
 NCBI BlastP on this gene

ELV09862

60S ribosomal protein L15
  
Accession: ELV09861
  
Location: 835857-837740
  
  
**BlastP hit with Mycgr3G102281\_Mycgr3**
  
Percentage identity: 72 %
  
BlastP bit score: 313
  
Sequence coverage: 100 %
  
E-value: 2e-105
  
  
 NCBI BlastP on this gene

ELV09861

159. :  JH711784 Trametes versicolor FP-101664 SS1 unplaced genomic scaffold TRAVEscaffold\_2     Total score: 1.0     Cumulative Blast bit score: 608

FAD/NAD-P-binding domain-containing protein
  
Accession: EIW62525
  
Location: 445523-447087
  
 NCBI BlastP on this gene

EIW62525

hypothetical protein
  
Accession: EIW62526
  
Location: 448193-451984
  
 NCBI BlastP on this gene

EIW62526

ARM repeat-containing protein
  
Accession: EIW62527
  
Location: 452503-456838
  
 NCBI BlastP on this gene

EIW62527

cytochrome P450
  
Accession: EIW62528
  
Location: 458490-460938
  
 NCBI BlastP on this gene

EIW62528

TFIIH basal transcription factor complex subunit SSL1
  
Accession: EIW62529
  
Location: 462928-464633
  
 NCBI BlastP on this gene

EIW62529

GroES-like protein
  
Accession: EIW62530
  
Location: 465439-466669
  
  
**BlastP hit with Mycgr3G102276\_Mycgr3**
  
Percentage identity: 45 %
  
BlastP bit score: 307
  
Sequence coverage: 93 %
  
E-value: 3e-98
  
  
 NCBI BlastP on this gene

EIW62530

nmrA-family protein
  
Accession: EIW62531
  
Location: 467827-469063
  
 NCBI BlastP on this gene

EIW62531

hypothetical protein
  
Accession: EIW62532
  
Location: 469720-470182
  
 NCBI BlastP on this gene

EIW62532

hypothetical protein
  
Accession: EIW62533
  
Location: 472701-473037
  
 NCBI BlastP on this gene

EIW62533

GroES-like protein
  
Accession: EIW62534
  
Location: 476151-477408
  
  
**BlastP hit with Mycgr3G102276\_Mycgr3**
  
Percentage identity: 47 %
  
BlastP bit score: 301
  
Sequence coverage: 98 %
  
E-value: 6e-96
  
  
 NCBI BlastP on this gene

EIW62534

hypothetical protein
  
Accession: EIW62535
  
Location: 480180-480584
  
 NCBI BlastP on this gene

EIW62535

acyl-CoA dehydrogenase NM domain-like protein
  
Accession: EIW62536
  
Location: 482884-484939
  
 NCBI BlastP on this gene

EIW62536

acyl-CoA dehydrogenase NM domain-like protein
  
Accession: EIW62537
  
Location: 488548-490528
  
 NCBI BlastP on this gene

EIW62537

hypothetical protein
  
Accession: EIW62538
  
Location: 491153-493773
  
 NCBI BlastP on this gene

EIW62538

UPF0041-domain-containing protein
  
Accession: EIW62539
  
Location: 495178-495702
  
 NCBI BlastP on this gene

EIW62539

160. :  KB446538 Dothistroma septosporum NZE10 unplaced genomic scaffold DOTSEscaffold\_4     Total score: 1.0     Cumulative Blast bit score: 591

hypothetical protein
  
Accession: EME45386
  
Location: 1356362-1357705
  
 NCBI BlastP on this gene

EME45386

hypothetical protein
  
Accession: EME45385
  
Location: 1348823-1350958
  
 NCBI BlastP on this gene

EME45385

hypothetical protein
  
Accession: EME45384
  
Location: 1344560-1344772
  
 NCBI BlastP on this gene

EME45384

hypothetical protein
  
Accession: EME45383
  
Location: 1339753-1342092
  
 NCBI BlastP on this gene

EME45383

hypothetical protein
  
Accession: EME45382
  
Location: 1337265-1339022
  
 NCBI BlastP on this gene

EME45382

alcohol dehydrogenase-like protein
  
Accession: EME45381
  
Location: 1335391-1336531
  
  
**BlastP hit with Mycgr3G102276\_Mycgr3**
  
Percentage identity: 81 %
  
BlastP bit score: 591
  
Sequence coverage: 99 %
  
E-value: 0.0
  
  
 NCBI BlastP on this gene

EME45381

Non-ribosomal peptide synthetase-like protein
  
Accession: EME45380
  
Location: 1327192-1335024
  
 NCBI BlastP on this gene

EME45380

hypothetical protein
  
Accession: EME45379
  
Location: 1325632-1326337
  
 NCBI BlastP on this gene

EME45379

hypothetical protein
  
Accession: EME45378
  
Location: 1321126-1322834
  
 NCBI BlastP on this gene

EME45378

hypothetical protein
  
Accession: EME45377
  
Location: 1318933-1320510
  
 NCBI BlastP on this gene

EME45377

hypothetical protein
  
Accession: EME45376
  
Location: 1315838-1316122
  
 NCBI BlastP on this gene

EME45376

161. :  GL988032 Chaetomium thermophilum var. thermophilum DSM 1495 unplaced genomic scaffold scf7180000...     Total score: 1.0     Cumulative Blast bit score: 590

hypothetical protein
  
Accession: EGS23540
  
Location: 679365-681436
  
 NCBI BlastP on this gene

EGS23540

hypothetical protein
  
Accession: EGS23541
  
Location: 682412-682938
  
 NCBI BlastP on this gene

EGS23541

hypothetical protein
  
Accession: EGS23542
  
Location: 683346-685777
  
 NCBI BlastP on this gene

EGS23542

putative vacuolar protein sorting-associated protein
  
Accession: EGS23543
  
Location: 686559-687354
  
 NCBI BlastP on this gene

EGS23543

putative pyridoxal phosphate binding protein
  
Accession: EGS23544
  
Location: 689202-690843
  
 NCBI BlastP on this gene

EGS23544

hypothetical protein
  
Accession: EGS23545
  
Location: 692818-696603
  
 NCBI BlastP on this gene

EGS23545

ubiquitin-protein ligase-like protein
  
Accession: EGS23546
  
Location: 699282-703480
  
  
**BlastP hit with Mycgr3G52682\_Mycgr3T**
  
Percentage identity: 36 %
  
BlastP bit score: 590
  
Sequence coverage: 102 %
  
E-value: 0.0
  
  
 NCBI BlastP on this gene

EGS23546

hypothetical protein
  
Accession: EGS23547
  
Location: 707149-708360
  
 NCBI BlastP on this gene

EGS23547

hypothetical protein
  
Accession: EGS23548
  
Location: 709375-711357
  
 NCBI BlastP on this gene

EGS23548

hypothetical protein
  
Accession: EGS23549
  
Location: 711597-712278
  
 NCBI BlastP on this gene

EGS23549

hypothetical protein
  
Accession: EGS23550
  
Location: 715027-717156
  
 NCBI BlastP on this gene

EGS23550

162. :  KB446537 Dothistroma septosporum NZE10 unplaced genomic scaffold DOTSEscaffold\_3     Total score: 1.0     Cumulative Blast bit score: 585

hypothetical protein
  
Accession: EME46577
  
Location: 1843047-1844339
  
 NCBI BlastP on this gene

EME46577

hypothetical protein
  
Accession: EME46578
  
Location: 1844751-1846669
  
 NCBI BlastP on this gene

EME46578

glycoside hydrolase family 31 protein
  
Accession: EME46579
  
Location: 1850121-1853350
  
 NCBI BlastP on this gene

EME46579

hypothetical protein
  
Accession: EME46580
  
Location: 1854224-1856905
  
 NCBI BlastP on this gene

EME46580

hypothetical protein
  
Accession: EME46581
  
Location: 1857568-1858655
  
 NCBI BlastP on this gene

EME46581

hypothetical protein
  
Accession: EME46582
  
Location: 1859294-1860091
  
 NCBI BlastP on this gene

EME46582

hypothetical protein
  
Accession: EME46583
  
Location: 1860438-1861013
  
 NCBI BlastP on this gene

EME46583

hypothetical protein
  
Accession: EME46584
  
Location: 1863045-1864610
  
  
**BlastP hit with Mycgr3G65725\_Mycgr3T**
  
Percentage identity: 59 %
  
BlastP bit score: 585
  
Sequence coverage: 98 %
  
E-value: 0.0
  
  
 NCBI BlastP on this gene

EME46584

hypothetical protein
  
Accession: EME46585
  
Location: 1865508-1867113
  
 NCBI BlastP on this gene

EME46585

hypothetical protein
  
Accession: EME46586
  
Location: 1868028-1868414
  
 NCBI BlastP on this gene

EME46586

hypothetical protein
  
Accession: EME46587
  
Location: 1868724-1868960
  
 NCBI BlastP on this gene

EME46587

hypothetical protein
  
Accession: EME46588
  
Location: 1869809-1871643
  
 NCBI BlastP on this gene

EME46588

hypothetical protein
  
Accession: EME46589
  
Location: 1875008-1875946
  
 NCBI BlastP on this gene

EME46589

hypothetical protein
  
Accession: EME46590
  
Location: 1877646-1877939
  
 NCBI BlastP on this gene

EME46590

hypothetical protein
  
Accession: EME46591
  
Location: 1878442-1879103
  
 NCBI BlastP on this gene

EME46591

hypothetical protein
  
Accession: EME46592
  
Location: 1881517-1883325
  
 NCBI BlastP on this gene

EME46592

hypothetical protein
  
Accession: EME46593
  
Location: 1883815-1884949
  
 NCBI BlastP on this gene

EME46593

163. :  KB469296 Gloeophyllum trabeum ATCC 11539 unplaced genomic scaffold GLOTRscaffold\_00001     Total score: 1.0     Cumulative Blast bit score: 582

cyclin-like protein
  
Accession: EPQ60824
  
Location: 2579289-2580520
  
 NCBI BlastP on this gene

EPQ60824

hypothetical protein
  
Accession: EPQ60825
  
Location: 2580736-2581894
  
 NCBI BlastP on this gene

EPQ60825

hypothetical protein
  
Accession: EPQ60826
  
Location: 2582035-2583898
  
 NCBI BlastP on this gene

EPQ60826

hypothetical protein
  
Accession: EPQ60827
  
Location: 2585534-2586492
  
 NCBI BlastP on this gene

EPQ60827

hypothetical protein
  
Accession: EPQ60828
  
Location: 2586666-2587477
  
 NCBI BlastP on this gene

EPQ60828

P-loop containing nucleoside triphosphate hydrolase protein
  
Accession: EPQ60829
  
Location: 2589456-2590132
  
 NCBI BlastP on this gene

EPQ60829

hypothetical protein
  
Accession: EPQ60830
  
Location: 2590224-2591050
  
 NCBI BlastP on this gene

EPQ60830

hypothetical protein
  
Accession: EPQ60831
  
Location: 2591435-2593833
  
 NCBI BlastP on this gene

EPQ60831

GroES-like protein
  
Accession: EPQ60832
  
Location: 2595514-2597063
  
  
**BlastP hit with Mycgr3G102276\_Mycgr3**
  
Percentage identity: 46 %
  
BlastP bit score: 290
  
Sequence coverage: 97 %
  
E-value: 1e-91
  
  
 NCBI BlastP on this gene

EPQ60832

GroES-like protein
  
Accession: EPQ60833
  
Location: 2597653-2599221
  
  
**BlastP hit with Mycgr3G102276\_Mycgr3**
  
Percentage identity: 46 %
  
BlastP bit score: 292
  
Sequence coverage: 100 %
  
E-value: 1e-92
  
  
 NCBI BlastP on this gene

EPQ60833

GroES-like protein
  
Accession: EPQ60834
  
Location: 2600234-2601568
  
 NCBI BlastP on this gene

EPQ60834

hypothetical protein
  
Accession: EPQ60835
  
Location: 2607896-2608246
  
 NCBI BlastP on this gene

EPQ60835

hypothetical protein
  
Accession: EPQ60836
  
Location: 2610443-2611773
  
 NCBI BlastP on this gene

EPQ60836

hypothetical protein
  
Accession: EPQ60837
  
Location: 2611780-2612052
  
 NCBI BlastP on this gene

EPQ60837

hypothetical protein
  
Accession: EPQ60838
  
Location: 2612352-2613536
  
 NCBI BlastP on this gene

EPQ60838

hypothetical protein
  
Accession: EPQ60839
  
Location: 2614151-2615494
  
 NCBI BlastP on this gene

EPQ60839

TTL-domain-containing protein
  
Accession: EPQ60840
  
Location: 2616817-2618433
  
 NCBI BlastP on this gene

EPQ60840

hypothetical protein
  
Accession: EPQ60841
  
Location: 2618714-2622266
  
 NCBI BlastP on this gene

EPQ60841

164. :  KB456262 Mycosphaerella populorum SO2202 unplaced genomic scaffold SEPMUscaffold\_3     Total score: 1.0     Cumulative Blast bit score: 578

hypothetical protein
  
Accession: EMF14618
  
Location: 1895413-1896090
  
 NCBI BlastP on this gene

EMF14618

hypothetical protein
  
Accession: EMF14619
  
Location: 1898181-1899338
  
 NCBI BlastP on this gene

EMF14619

40S ribosomal protein SA
  
Accession: EMF14620
  
Location: 1899575-1900652
  
 NCBI BlastP on this gene

EMF14620

Pentafunctional AroM protein
  
Accession: EMF14621
  
Location: 1901417-1906261
  
 NCBI BlastP on this gene

EMF14621

glycoside hydrolase family 20 protein
  
Accession: EMF14622
  
Location: 1907051-1909497
  
 NCBI BlastP on this gene

EMF14622

cyanamide hydratase
  
Accession: EMF14623
  
Location: 1909650-1910466
  
 NCBI BlastP on this gene

EMF14623

MFS general substrate transporter
  
Accession: EMF14624
  
Location: 1911693-1913404
  
  
**BlastP hit with Mycgr3G65725\_Mycgr3T**
  
Percentage identity: 57 %
  
BlastP bit score: 578
  
Sequence coverage: 102 %
  
E-value: 0.0
  
  
 NCBI BlastP on this gene

EMF14624

hypothetical protein
  
Accession: EMF14625
  
Location: 1914196-1915452
  
 NCBI BlastP on this gene

EMF14625

hypothetical protein
  
Accession: EMF14626
  
Location: 1916268-1918433
  
 NCBI BlastP on this gene

EMF14626

bleomycin hydrolase
  
Accession: EMF14627
  
Location: 1919438-1921188
  
 NCBI BlastP on this gene

EMF14627

carboxyvinyl-carboxyphosphonate phosphorylmutase
  
Accession: EMF14628
  
Location: 1924575-1925525
  
 NCBI BlastP on this gene

EMF14628

sterol-binding-like protein
  
Accession: EMF14630
  
Location: 1926172-1926699
  
 NCBI BlastP on this gene

EMF14630

165. :  KB446537 Dothistroma septosporum NZE10 unplaced genomic scaffold DOTSEscaffold\_3     Total score: 1.0     Cumulative Blast bit score: 574

hypothetical protein
  
Accession: EME46227
  
Location: 987630-991871
  
 NCBI BlastP on this gene

EME46227

hypothetical protein
  
Accession: EME46226
  
Location: 985323-986140
  
 NCBI BlastP on this gene

EME46226

hypothetical protein
  
Accession: EME46225
  
Location: 983140-983778
  
 NCBI BlastP on this gene

EME46225

glycoside hydrolase family 79 protein
  
Accession: EME46224
  
Location: 980417-982141
  
 NCBI BlastP on this gene

EME46224

hypothetical protein
  
Accession: EME46223
  
Location: 978877-979431
  
 NCBI BlastP on this gene

EME46223

hypothetical protein
  
Accession: EME46222
  
Location: 974667-975911
  
 NCBI BlastP on this gene

EME46222

hypothetical protein
  
Accession: EME46221
  
Location: 973130-974326
  
 NCBI BlastP on this gene

EME46221

hypothetical protein
  
Accession: EME46220
  
Location: 968169-969949
  
  
**BlastP hit with Mycgr3G34982\_Mycgr3T**
  
Percentage identity: 55 %
  
BlastP bit score: 574
  
Sequence coverage: 88 %
  
E-value: 0.0
  
  
 NCBI BlastP on this gene

EME46220

hypothetical protein
  
Accession: EME46219
  
Location: 965489-967363
  
 NCBI BlastP on this gene

EME46219

hypothetical protein
  
Accession: EME46218
  
Location: 960958-962170
  
 NCBI BlastP on this gene

EME46218

hypothetical protein
  
Accession: EME46217
  
Location: 959502-960049
  
 NCBI BlastP on this gene

EME46217

hypothetical protein
  
Accession: EME46216
  
Location: 957799-958567
  
 NCBI BlastP on this gene

EME46216

hypothetical protein
  
Accession: EME46215
  
Location: 954080-955585
  
 NCBI BlastP on this gene

EME46215

hypothetical protein
  
Accession: EME46214
  
Location: 951120-953138
  
 NCBI BlastP on this gene

EME46214

hypothetical protein
  
Accession: EME46213
  
Location: 948418-949359
  
 NCBI BlastP on this gene

EME46213

166. :  KB446560 Pseudocercospora fijiensis CIRAD86 unplaced genomic scaffold MYCFIscaffold\_6     Total score: 1.0     Cumulative Blast bit score: 570

hypothetical protein
  
Accession: EME81244
  
Location: 3002088-3003013
  
 NCBI BlastP on this gene

EME81244

hypothetical protein
  
Accession: EME81245
  
Location: 3003831-3005058
  
 NCBI BlastP on this gene

EME81245

hypothetical protein
  
Accession: EME81246
  
Location: 3017539-3018200
  
 NCBI BlastP on this gene

EME81246

hypothetical protein
  
Accession: EME81247
  
Location: 3018579-3019836
  
  
**BlastP hit with Mycgr3G102276\_Mycgr3**
  
Percentage identity: 78 %
  
BlastP bit score: 570
  
Sequence coverage: 99 %
  
E-value: 0.0
  
  
 NCBI BlastP on this gene

EME81247

167. :  KB733456 Bipolaris maydis ATCC 48331 unplaced genomic scaffold COCC4scaffold\_13     Total score: 1.0     Cumulative Blast bit score: 549

hypothetical protein
  
Accession: ENI04836
  
Location: 787761-793480
  
 NCBI BlastP on this gene

ENI04836

hypothetical protein
  
Accession: ENI04835
  
Location: 785956-786711
  
 NCBI BlastP on this gene

ENI04835

hypothetical protein
  
Accession: ENI04834
  
Location: 784544-785248
  
 NCBI BlastP on this gene

ENI04834

hypothetical protein
  
Accession: ENI04833
  
Location: 782211-784262
  
 NCBI BlastP on this gene

ENI04833

hypothetical protein
  
Accession: ENI04832
  
Location: 777525-778834
  
 NCBI BlastP on this gene

ENI04832

hypothetical protein
  
Accession: ENI04831
  
Location: 774643-775837
  
 NCBI BlastP on this gene

ENI04831

hypothetical protein
  
Accession: ENI04830
  
Location: 773905-774239
  
 NCBI BlastP on this gene

ENI04830

hypothetical protein
  
Accession: ENI04829
  
Location: 772650-773666
  
 NCBI BlastP on this gene

ENI04829

hypothetical protein
  
Accession: ENI04828
  
Location: 770593-772243
  
 NCBI BlastP on this gene

ENI04828

hypothetical protein
  
Accession: ENI04827
  
Location: 768617-769800
  
  
**BlastP hit with Mycgr3G102276\_Mycgr3**
  
Percentage identity: 76 %
  
BlastP bit score: 549
  
Sequence coverage: 96 %
  
E-value: 0.0
  
  
 NCBI BlastP on this gene

ENI04827

hypothetical protein
  
Accession: ENI04826
  
Location: 764419-765669
  
 NCBI BlastP on this gene

ENI04826

hypothetical protein
  
Accession: ENI04825
  
Location: 756765-760101
  
 NCBI BlastP on this gene

ENI04825

hypothetical protein
  
Accession: ENI04824
  
Location: 754195-754795
  
 NCBI BlastP on this gene

ENI04824

168. :  KB445574 Cochliobolus heterostrophus C5 unplaced genomic scaffold COCHEscaffold\_6     Total score: 1.0     Cumulative Blast bit score: 549

hypothetical protein
  
Accession: EMD92777
  
Location: 193225-193980
  
 NCBI BlastP on this gene

EMD92777

hypothetical protein
  
Accession: EMD92778
  
Location: 194688-195392
  
 NCBI BlastP on this gene

EMD92778

hypothetical protein
  
Accession: EMD92779
  
Location: 195674-197725
  
 NCBI BlastP on this gene

EMD92779

hypothetical protein
  
Accession: EMD92780
  
Location: 201125-202434
  
 NCBI BlastP on this gene

EMD92780

hypothetical protein
  
Accession: EMD92781
  
Location: 204122-205316
  
 NCBI BlastP on this gene

EMD92781

hypothetical protein
  
Accession: EMD92782
  
Location: 205720-206054
  
 NCBI BlastP on this gene

EMD92782

hypothetical protein
  
Accession: EMD92783
  
Location: 206293-207309
  
 NCBI BlastP on this gene

EMD92783

hypothetical protein
  
Accession: EMD92784
  
Location: 207716-209366
  
 NCBI BlastP on this gene

EMD92784

hypothetical protein
  
Accession: EMD92785
  
Location: 210159-211342
  
  
**BlastP hit with Mycgr3G102276\_Mycgr3**
  
Percentage identity: 76 %
  
BlastP bit score: 549
  
Sequence coverage: 96 %
  
E-value: 0.0
  
  
 NCBI BlastP on this gene

EMD92785

169. :  CH471151 Homo sapiens 211000035831495 genomic scaffold     Total score: 1.0     Cumulative Blast bit score: 548

ubiquitin protein ligase E3A (human papilloma
  
Accession: CP1918456
  
Location: 1967266-2033573
  
  
**BlastP hit with Mycgr3G52682\_Mycgr3T**
  
Percentage identity: 35 %
  
BlastP bit score: 274
  
Sequence coverage: 39 %
  
E-value: 2e-74
  
  
 NCBI BlastP on this gene

CP1918456

ubiquitin protein ligase E3A (human papilloma
  
Accession: CP1752652
  
Location: 1967264-2033573
  
  
**BlastP hit with Mycgr3G52682\_Mycgr3T**
  
Percentage identity: 35 %
  
BlastP bit score: 274
  
Sequence coverage: 39 %
  
E-value: 2e-74
  
  
 NCBI BlastP on this gene

CP1752652

170. :  KB445644 Cochliobolus sativus ND90Pr unplaced genomic scaffold COCSAscaffold\_8     Total score: 1.0     Cumulative Blast bit score: 540

hypothetical protein
  
Accession: EMD63592
  
Location: 610197-610952
  
 NCBI BlastP on this gene

EMD63592

hypothetical protein
  
Accession: EMD63593
  
Location: 611625-612336
  
 NCBI BlastP on this gene

EMD63593

hypothetical protein
  
Accession: EMD63594
  
Location: 612617-614674
  
 NCBI BlastP on this gene

EMD63594

hypothetical protein
  
Accession: EMD63595
  
Location: 617839-619148
  
 NCBI BlastP on this gene

EMD63595

hypothetical protein
  
Accession: EMD63596
  
Location: 620804-621998
  
 NCBI BlastP on this gene

EMD63596

hypothetical protein
  
Accession: EMD63597
  
Location: 622400-622736
  
 NCBI BlastP on this gene

EMD63597

hypothetical protein
  
Accession: EMD63598
  
Location: 622975-623991
  
 NCBI BlastP on this gene

EMD63598

hypothetical protein
  
Accession: EMD63599
  
Location: 624399-626049
  
 NCBI BlastP on this gene

EMD63599

hypothetical protein
  
Accession: EMD63600
  
Location: 626828-628011
  
  
**BlastP hit with Mycgr3G102276\_Mycgr3**
  
Percentage identity: 74 %
  
BlastP bit score: 540
  
Sequence coverage: 96 %
  
E-value: 0.0
  
  
 NCBI BlastP on this gene

EMD63600

171. :  GG698901 Nectria haematococca mpVI 77-13-4 chromosome 1 genomic scaffold NECHAsca\_8\_chr1\_1\_0     Total score: 1.0     Cumulative Blast bit score: 530

hypothetical protein
  
Accession: EEU43841
  
Location: 236224-240570
  
 NCBI BlastP on this gene

EEU43841

predicted protein
  
Accession: EEU44046
  
Location: 240806-243590
  
 NCBI BlastP on this gene

EEU44046

hypothetical protein
  
Accession: EEU44047
  
Location: 244064-245893
  
 NCBI BlastP on this gene

EEU44047

hypothetical protein
  
Accession: EEU43842
  
Location: 246403-247352
  
 NCBI BlastP on this gene

EEU43842

hypothetical protein
  
Accession: EEU44048
  
Location: 247807-249605
  
 NCBI BlastP on this gene

EEU44048

hypothetical protein
  
Accession: EEU43843
  
Location: 251504-252123
  
 NCBI BlastP on this gene

EEU43843

hypothetical protein
  
Accession: EEU44049
  
Location: 252477-253343
  
 NCBI BlastP on this gene

EEU44049

hypothetical protein
  
Accession: EEU43844
  
Location: 254151-255298
  
  
**BlastP hit with Mycgr3G102276\_Mycgr3**
  
Percentage identity: 72 %
  
BlastP bit score: 530
  
Sequence coverage: 99 %
  
E-value: 0.0
  
  
 NCBI BlastP on this gene

EEU43844

hypothetical protein
  
Accession: EEU43845
  
Location: 255580-256081
  
 NCBI BlastP on this gene

EEU43845

hypothetical protein
  
Accession: EEU44050
  
Location: 256668-261897
  
 NCBI BlastP on this gene

EEU44050

hypothetical protein
  
Accession: EEU43846
  
Location: 267179-268339
  
 NCBI BlastP on this gene

EEU43846

hypothetical protein
  
Accession: EEU44051
  
Location: 268605-269519
  
 NCBI BlastP on this gene

EEU44051

hypothetical protein
  
Accession: EEU44052
  
Location: 269678-274005
  
 NCBI BlastP on this gene

EEU44052

hypothetical protein
  
Accession: EEU44053
  
Location: 274932-275564
  
 NCBI BlastP on this gene

EEU44053

172. :  AAAB01008807 Anopheles gambiae str. PEST whole genome shotgun sequencing project.     Total score: 1.0     Cumulative Blast bit score: 523

AGAP006996-PA
  
Accession: EAA04167
  
Location: 8826191-8827821
  
 NCBI BlastP on this gene

EAA04167

AGAP006995-PA
  
Accession: EAA04298
  
Location: 8832531-8842084
  
 NCBI BlastP on this gene

EAA04298

AGAP006994-PA
  
Accession: EAA04764
  
Location: 8845926-8850697
  
  
**BlastP hit with Mycgr3G52682\_Mycgr3T**
  
Percentage identity: 32 %
  
BlastP bit score: 262
  
Sequence coverage: 40 %
  
E-value: 2e-69
  
  
 NCBI BlastP on this gene

EAA04764

AGAP006994-PB
  
Accession: EDO64729
  
Location: 8845926-8850697
  
  
**BlastP hit with Mycgr3G52682\_Mycgr3T**
  
Percentage identity: 32 %
  
BlastP bit score: 261
  
Sequence coverage: 40 %
  
E-value: 3e-69
  
  
 NCBI BlastP on this gene

EDO64729

AGAP006993-PA
  
Accession: EAA04662
  
Location: 8851431-8854563
  
 NCBI BlastP on this gene

EAA04662

AGAP006992-PA
  
Accession: EAA04338
  
Location: 8858030-8863842
  
 NCBI BlastP on this gene

EAA04338

AGAP006991-PA
  
Accession: EAA04414
  
Location: 8866894-8867260
  
 NCBI BlastP on this gene

EAA04414

AGAP006990-PB
  
Accession: EAA04740
  
Location: 8868459-8878334
  
 NCBI BlastP on this gene

EAA04740

AGAP006990-PA
  
Accession: EDO64730
  
Location: 8868459-8878334
  
 NCBI BlastP on this gene

EDO64730

173. :  KB446558 Pseudocercospora fijiensis CIRAD86 unplaced genomic scaffold MYCFIscaffold\_4     Total score: 1.0     Cumulative Blast bit score: 522

glycoside hydrolase family 31 protein
  
Accession: EME82820
  
Location: 1442756-1445056
  
 NCBI BlastP on this gene

EME82820

hypothetical protein
  
Accession: EME82819
  
Location: 1440901-1442707
  
 NCBI BlastP on this gene

EME82819

hypothetical protein
  
Accession: EME82818
  
Location: 1439207-1440017
  
 NCBI BlastP on this gene

EME82818

glycoside hydrolase family 37 protein
  
Accession: EME82817
  
Location: 1435606-1438138
  
 NCBI BlastP on this gene

EME82817

hypothetical protein
  
Accession: EME82816
  
Location: 1433967-1435085
  
 NCBI BlastP on this gene

EME82816

hypothetical protein
  
Accession: EME82815
  
Location: 1433195-1433554
  
 NCBI BlastP on this gene

EME82815

hypothetical protein
  
Accession: EME82814
  
Location: 1431548-1432747
  
 NCBI BlastP on this gene

EME82814

hypothetical protein
  
Accession: EME82813
  
Location: 1425634-1428400
  
 NCBI BlastP on this gene

EME82813

hypothetical protein
  
Accession: EME82812
  
Location: 1424770-1425075
  
 NCBI BlastP on this gene

EME82812

hypothetical protein
  
Accession: EME82811
  
Location: 1421940-1424008
  
  
**BlastP hit with Mycgr3G65725\_Mycgr3T**
  
Percentage identity: 67 %
  
BlastP bit score: 522
  
Sequence coverage: 87 %
  
E-value: 1e-179
  
  
 NCBI BlastP on this gene

EME82811

hypothetical protein
  
Accession: EME82810
  
Location: 1418937-1421807
  
 NCBI BlastP on this gene

EME82810

hypothetical protein
  
Accession: EME82809
  
Location: 1417673-1418901
  
 NCBI BlastP on this gene

EME82809

hypothetical protein
  
Accession: EME82808
  
Location: 1415615-1416584
  
 NCBI BlastP on this gene

EME82808

hypothetical protein
  
Accession: EME82807
  
Location: 1410914-1413538
  
 NCBI BlastP on this gene

EME82807

hypothetical protein
  
Accession: EME82806
  
Location: 1408884-1410408
  
 NCBI BlastP on this gene

EME82806

hypothetical protein
  
Accession: EME82805
  
Location: 1407603-1408330
  
 NCBI BlastP on this gene

EME82805

hypothetical protein
  
Accession: EME82804
  
Location: 1405022-1406076
  
 NCBI BlastP on this gene

EME82804

174. :  KB733480 Bipolaris maydis ATCC 48331 unplaced genomic scaffold COCC4scaffold\_37     Total score: 1.0     Cumulative Blast bit score: 520

hypothetical protein
  
Accession: ENH99916
  
Location: 121196-124534
  
 NCBI BlastP on this gene

ENH99916

hypothetical protein
  
Accession: ENH99915
  
Location: 118840-119524
  
 NCBI BlastP on this gene

ENH99915

hypothetical protein
  
Accession: ENH99914
  
Location: 116692-118410
  
 NCBI BlastP on this gene

ENH99914

glycoside hydrolase family 31 protein
  
Accession: ENH99913
  
Location: 113660-116183
  
 NCBI BlastP on this gene

ENH99913

hypothetical protein
  
Accession: ENH99912
  
Location: 112961-113194
  
 NCBI BlastP on this gene

ENH99912

hypothetical protein
  
Accession: ENH99911
  
Location: 112109-112593
  
 NCBI BlastP on this gene

ENH99911

hypothetical protein
  
Accession: ENH99910
  
Location: 108541-110567
  
 NCBI BlastP on this gene

ENH99910

hypothetical protein
  
Accession: ENH99909
  
Location: 107049-108451
  
 NCBI BlastP on this gene

ENH99909

hypothetical protein
  
Accession: ENH99908
  
Location: 102225-103300
  
 NCBI BlastP on this gene

ENH99908

hypothetical protein
  
Accession: ENH99907
  
Location: 99060-101486
  
  
**BlastP hit with Mycgr3G107069\_Mycgr3**
  
Percentage identity: 42 %
  
BlastP bit score: 520
  
Sequence coverage: 106 %
  
E-value: 1e-172
  
  
 NCBI BlastP on this gene

ENH99907

hypothetical protein
  
Accession: ENH99906
  
Location: 96300-96770
  
 NCBI BlastP on this gene

ENH99906

hypothetical protein
  
Accession: ENH99905
  
Location: 94457-94790
  
 NCBI BlastP on this gene

ENH99905

hypothetical protein
  
Accession: ENH99904
  
Location: 93917-94075
  
 NCBI BlastP on this gene

ENH99904

hypothetical protein
  
Accession: ENH99903
  
Location: 93431-93911
  
 NCBI BlastP on this gene

ENH99903

hypothetical protein
  
Accession: ENH99902
  
Location: 87533-90674
  
 NCBI BlastP on this gene

ENH99902

hypothetical protein
  
Accession: ENH99901
  
Location: 86310-87187
  
 NCBI BlastP on this gene

ENH99901

hypothetical protein
  
Accession: ENH99900
  
Location: 84466-85668
  
 NCBI BlastP on this gene

ENH99900

hypothetical protein
  
Accession: ENH99899
  
Location: 83592-84312
  
 NCBI BlastP on this gene

ENH99899

hypothetical protein
  
Accession: ENH99898
  
Location: 82024-83124
  
 NCBI BlastP on this gene

ENH99898

hypothetical protein
  
Accession: ENH99897
  
Location: 81308-81943
  
 NCBI BlastP on this gene

ENH99897

hypothetical protein
  
Accession: ENH99896
  
Location: 79343-80720
  
 NCBI BlastP on this gene

ENH99896

175. :  KB445573 Cochliobolus heterostrophus C5 unplaced genomic scaffold COCHEscaffold\_5     Total score: 1.0     Cumulative Blast bit score: 520

hypothetical protein
  
Accession: EMD93791
  
Location: 1734931-1735615
  
 NCBI BlastP on this gene

EMD93791

hypothetical protein
  
Accession: EMD93792
  
Location: 1736045-1737763
  
 NCBI BlastP on this gene

EMD93792

glycoside hydrolase family 31 protein
  
Accession: EMD93793
  
Location: 1738272-1740795
  
 NCBI BlastP on this gene

EMD93793

hypothetical protein
  
Accession: EMD93794
  
Location: 1741153-1741494
  
 NCBI BlastP on this gene

EMD93794

hypothetical protein
  
Accession: EMD93795
  
Location: 1741862-1742346
  
 NCBI BlastP on this gene

EMD93795

hypothetical protein
  
Accession: EMD93796
  
Location: 1743888-1745914
  
 NCBI BlastP on this gene

EMD93796

hypothetical protein
  
Accession: EMD93797
  
Location: 1746004-1747406
  
 NCBI BlastP on this gene

EMD93797

hypothetical protein
  
Accession: EMD93798
  
Location: 1751155-1752230
  
 NCBI BlastP on this gene

EMD93798

hypothetical protein
  
Accession: EMD93799
  
Location: 1752969-1755395
  
  
**BlastP hit with Mycgr3G107069\_Mycgr3**
  
Percentage identity: 42 %
  
BlastP bit score: 520
  
Sequence coverage: 106 %
  
E-value: 1e-172
  
  
 NCBI BlastP on this gene

EMD93799

176. :  KE145357 Glarea lozoyensis ATCC 20868 chromosome Unknown GLAREA14     Total score: 1.0     Cumulative Blast bit score: 519

Ferredoxin reductase-like, C-terminal NADP-linked
  
Accession: EPE34197
  
Location: 1873318-1875462
  
  
**BlastP hit with Mycgr3G107069\_Mycgr3**
  
Percentage identity: 43 %
  
BlastP bit score: 519
  
Sequence coverage: 106 %
  
E-value: 7e-173
  
  
 NCBI BlastP on this gene

EPE34197

P-loop containing nucleoside triphosphate hydrolase
  
Accession: EPE34196
  
Location: 1864809-1871532
  
 NCBI BlastP on this gene

EPE34196

hypothetical protein
  
Accession: EPE34195
  
Location: 1861226-1862928
  
 NCBI BlastP on this gene

EPE34195

177. :  KB725793 Colletotrichum orbiculare MAFF 240422 unplaced genomic scaffold Scaffold\_242     Total score: 1.0     Cumulative Blast bit score: 517

hypothetical protein
  
Accession: ENH85058
  
Location: 80492-80929
  
 NCBI BlastP on this gene

ENH85058

hypothetical protein
  
Accession: ENH85057
  
Location: 80122-80464
  
 NCBI BlastP on this gene

ENH85057

hypothetical protein
  
Accession: ENH85056
  
Location: 78043-79548
  
 NCBI BlastP on this gene

ENH85056

hypothetical protein
  
Accession: ENH85055
  
Location: 74063-74892
  
 NCBI BlastP on this gene

ENH85055

hypothetical protein
  
Accession: ENH85054
  
Location: 72488-72770
  
 NCBI BlastP on this gene

ENH85054

hypothetical protein
  
Accession: ENH85053
  
Location: 70099-70420
  
 NCBI BlastP on this gene

ENH85053

hypothetical protein
  
Accession: ENH85052
  
Location: 68748-69981
  
 NCBI BlastP on this gene

ENH85052

integral membrane protein
  
Accession: ENH85051
  
Location: 65741-67137
  
 NCBI BlastP on this gene

ENH85051

nadp-dependent alcohol dehydrogenase
  
Accession: ENH85050
  
Location: 62126-63315
  
  
**BlastP hit with Mycgr3G102276\_Mycgr3**
  
Percentage identity: 71 %
  
BlastP bit score: 517
  
Sequence coverage: 98 %
  
E-value: 1e-180
  
  
 NCBI BlastP on this gene

ENH85050

hypothetical protein
  
Accession: ENH85049
  
Location: 60672-61654
  
 NCBI BlastP on this gene

ENH85049

hypothetical protein
  
Accession: ENH85048
  
Location: 56309-58216
  
 NCBI BlastP on this gene

ENH85048

hypothetical protein
  
Accession: ENH85047
  
Location: 54337-54971
  
 NCBI BlastP on this gene

ENH85047

hypothetical protein
  
Accession: ENH85046
  
Location: 53439-53790
  
 NCBI BlastP on this gene

ENH85046

extracellular exo-polygalacturonase, putative
  
Accession: ENH85045
  
Location: 51601-52947
  
 NCBI BlastP on this gene

ENH85045

exopolygalacturonase
  
Accession: ENH85044
  
Location: 49629-51131
  
 NCBI BlastP on this gene

ENH85044

hypothetical protein
  
Accession: ENH85043
  
Location: 47009-47746
  
 NCBI BlastP on this gene

ENH85043

lactose permease
  
Accession: ENH85042
  
Location: 44908-46657
  
 NCBI BlastP on this gene

ENH85042

178. :  CH476636 Sclerotinia sclerotiorum 1980 scaffold\_16 genomic scaffold     Total score: 1.0     Cumulative Blast bit score: 515

hypothetical protein
  
Accession: EDN94816
  
Location: 408757-410900
  
  
**BlastP hit with Mycgr3G107069\_Mycgr3**
  
Percentage identity: 44 %
  
BlastP bit score: 515
  
Sequence coverage: 100 %
  
E-value: 2e-171
  
  
 NCBI BlastP on this gene

EDN94816

predicted protein
  
Accession: EDN94815
  
Location: 406156-407226
  
 NCBI BlastP on this gene

EDN94815

hypothetical protein
  
Accession: EDN94814
  
Location: 403607-404080
  
 NCBI BlastP on this gene

EDN94814

hypothetical protein
  
Accession: EDN94813
  
Location: 402641-402982
  
 NCBI BlastP on this gene

EDN94813

predicted protein
  
Accession: EDN94812
  
Location: 400767-401837
  
 NCBI BlastP on this gene

EDN94812

predicted protein
  
Accession: EDN94811
  
Location: 397524-398246
  
 NCBI BlastP on this gene

EDN94811

predicted protein
  
Accession: EDN94810
  
Location: 396199-396306
  
 NCBI BlastP on this gene

EDN94810

hypothetical protein
  
Accession: EDN94809
  
Location: 392675-395181
  
 NCBI BlastP on this gene

EDN94809

hypothetical protein
  
Accession: EDN94808
  
Location: 388791-390194
  
 NCBI BlastP on this gene

EDN94808

179. :  KB445646 Cochliobolus sativus ND90Pr unplaced genomic scaffold COCSAscaffold\_10     Total score: 1.0     Cumulative Blast bit score: 511

hypothetical protein
  
Accession: EMD62515
  
Location: 785535-788870
  
 NCBI BlastP on this gene

EMD62515

hypothetical protein
  
Accession: EMD62514
  
Location: 781039-782757
  
 NCBI BlastP on this gene

EMD62514

glycoside hydrolase family 31 protein
  
Accession: EMD62513
  
Location: 778119-780530
  
 NCBI BlastP on this gene

EMD62513

hypothetical protein
  
Accession: EMD62512
  
Location: 777310-777543
  
 NCBI BlastP on this gene

EMD62512

hypothetical protein
  
Accession: EMD62511
  
Location: 776458-776942
  
 NCBI BlastP on this gene

EMD62511

hypothetical protein
  
Accession: EMD62510
  
Location: 772982-774998
  
 NCBI BlastP on this gene

EMD62510

hypothetical protein
  
Accession: EMD62509
  
Location: 771488-772893
  
 NCBI BlastP on this gene

EMD62509

hypothetical protein
  
Accession: EMD62508
  
Location: 766575-767650
  
 NCBI BlastP on this gene

EMD62508

hypothetical protein
  
Accession: EMD62507
  
Location: 763400-765826
  
  
**BlastP hit with Mycgr3G107069\_Mycgr3**
  
Percentage identity: 40 %
  
BlastP bit score: 511
  
Sequence coverage: 105 %
  
E-value: 3e-169
  
  
 NCBI BlastP on this gene

EMD62507

hypothetical protein
  
Accession: EMD62506
  
Location: 760640-761101
  
 NCBI BlastP on this gene

EMD62506

hypothetical protein
  
Accession: EMD62505
  
Location: 756158-758524
  
 NCBI BlastP on this gene

EMD62505

hypothetical protein
  
Accession: EMD62504
  
Location: 755121-755993
  
 NCBI BlastP on this gene

EMD62504

hypothetical protein
  
Accession: EMD62503
  
Location: 753268-754473
  
 NCBI BlastP on this gene

EMD62503

hypothetical protein
  
Accession: EMD62502
  
Location: 752379-753099
  
 NCBI BlastP on this gene

EMD62502

hypothetical protein
  
Accession: EMD62501
  
Location: 750777-751877
  
 NCBI BlastP on this gene

EMD62501

hypothetical protein
  
Accession: EMD62500
  
Location: 749774-750673
  
 NCBI BlastP on this gene

EMD62500

hypothetical protein
  
Accession: EMD62499
  
Location: 748101-749473
  
 NCBI BlastP on this gene

EMD62499

hypothetical protein
  
Accession: EMD62498
  
Location: 747694-747930
  
 NCBI BlastP on this gene

EMD62498

180. :  KB706647 Eutypa lata UCREL1 unplaced genomic scaffold EL1\_03\_scaffold\_1309     Total score: 1.0     Cumulative Blast bit score: 509

putative ubiquitin-protein ligase e3a protein
  
Accession: EMR66472
  
Location: 165832-167291
  
  
**BlastP hit with Mycgr3G52682\_Mycgr3T**
  
Percentage identity: 53 %
  
BlastP bit score: 509
  
Sequence coverage: 40 %
  
E-value: 4e-165
  
  
 NCBI BlastP on this gene

EMR66472

hypothetical protein
  
Accession: EMR66453
  
Location: 162027-163157
  
 NCBI BlastP on this gene

EMR66453

hypothetical protein
  
Accession: EMR66443
  
Location: 154756-155691
  
 NCBI BlastP on this gene

EMR66443

hypothetical protein
  
Accession: EMR66463
  
Location: 146881-147750
  
 NCBI BlastP on this gene

EMR66463

181. :  JH226130 Exophiala dermatitidis NIH/UT8656 unplaced genomic scaffold supercont1.1     Total score: 1.0     Cumulative Blast bit score: 508

tRNA (guanine-N7-)-methyltransferase
  
Accession: EHY51795
  
Location: 59200-60201
  
 NCBI BlastP on this gene

EHY51795

hypothetical protein
  
Accession: EHY51796
  
Location: 60708-63778
  
 NCBI BlastP on this gene

EHY51796

MFS transporter, DHA1 family, multidrug resistance protein
  
Accession: EHY51797
  
Location: 65999-67652
  
 NCBI BlastP on this gene

EHY51797

gamma-glutamyltransferase
  
Accession: EHY51799
  
Location: 68902-70801
  
 NCBI BlastP on this gene

EHY51799

two-component system, NarL family, sensor histidine kinase BarA
  
Accession: EHY51800
  
Location: 73542-76235
  
 NCBI BlastP on this gene

EHY51800

hypothetical protein
  
Accession: EHY51801
  
Location: 76668-77427
  
 NCBI BlastP on this gene

EHY51801

ferric-chelate reductase
  
Accession: EHY51802
  
Location: 78169-80178
  
  
**BlastP hit with Mycgr3G107069\_Mycgr3**
  
Percentage identity: 40 %
  
BlastP bit score: 508
  
Sequence coverage: 104 %
  
E-value: 2e-168
  
  
 NCBI BlastP on this gene

EHY51802

methionyl-tRNA synthetase
  
Accession: EHY51803
  
Location: 81895-83781
  
 NCBI BlastP on this gene

EHY51803

hypothetical protein
  
Accession: EHY51804
  
Location: 84231-85354
  
 NCBI BlastP on this gene

EHY51804

hypothetical protein
  
Accession: EHY51805
  
Location: 86653-88947
  
 NCBI BlastP on this gene

EHY51805

solute carrier family 12 member 2
  
Accession: EHY51806
  
Location: 90369-94420
  
 NCBI BlastP on this gene

EHY51806

30S ribosomal protein S13
  
Accession: EHY51807
  
Location: 94757-95242
  
 NCBI BlastP on this gene

EHY51807

peptidyl-prolyl cis-trans isomerase B
  
Accession: EHY51808
  
Location: 95606-96337
  
 NCBI BlastP on this gene

EHY51808

hypothetical protein
  
Accession: EHY51809
  
Location: 96378-96647
  
 NCBI BlastP on this gene

EHY51809

ATP-dependent RNA helicase ded1
  
Accession: EHY51810
  
Location: 97617-99724
  
 NCBI BlastP on this gene

EHY51810

182. :  DS985223 Verticillium albo-atrum VaMs.102 supercont1.10 genomic scaffold     Total score: 1.0     Cumulative Blast bit score: 502

NADP-dependent alcohol dehydrogenase
  
Accession: EEY21586
  
Location: 790600-791741
  
  
**BlastP hit with Mycgr3G102276\_Mycgr3**
  
Percentage identity: 68 %
  
BlastP bit score: 502
  
Sequence coverage: 98 %
  
E-value: 8e-175
  
  
 NCBI BlastP on this gene

EEY21586

predicted protein
  
Accession: EEY21585
  
Location: 786666-787184
  
 NCBI BlastP on this gene

EEY21585

BNR/Asp-box repeat domain-containing protein
  
Accession: EEY21584
  
Location: 783700-784943
  
 NCBI BlastP on this gene

EEY21584

predicted protein
  
Accession: EEY21583
  
Location: 776364-778217
  
 NCBI BlastP on this gene

EEY21583

trypsin
  
Accession: EEY21582
  
Location: 772647-773539
  
 NCBI BlastP on this gene

EEY21582

conserved hypothetical protein
  
Accession: EEY21581
  
Location: 770837-771426
  
 NCBI BlastP on this gene

EEY21581

183. :  DS572705 Verticillium dahliae VdLs.17 supercont1.11 genomic scaffold     Total score: 1.0     Cumulative Blast bit score: 500

hypothetical protein
  
Accession: EGY14477
  
Location: 31715-32635
  
 NCBI BlastP on this gene

EGY14477

hypothetical protein
  
Accession: EGY14478
  
Location: 33389-36476
  
 NCBI BlastP on this gene

EGY14478

hypothetical protein
  
Accession: EGY14479
  
Location: 36863-41450
  
 NCBI BlastP on this gene

EGY14479

hypothetical protein
  
Accession: EGY14480
  
Location: 42108-46021
  
 NCBI BlastP on this gene

EGY14480

hypothetical protein
  
Accession: EGY14481
  
Location: 47858-49517
  
 NCBI BlastP on this gene

EGY14481

NADP-dependent alcohol dehydrogenase
  
Accession: EGY14482
  
Location: 51297-52438
  
  
**BlastP hit with Mycgr3G102276\_Mycgr3**
  
Percentage identity: 67 %
  
BlastP bit score: 501
  
Sequence coverage: 98 %
  
E-value: 2e-174
  
  
 NCBI BlastP on this gene

EGY14482

hypothetical protein
  
Accession: EGY14483
  
Location: 53692-54132
  
 NCBI BlastP on this gene

EGY14483

hypothetical protein
  
Accession: EGY14484
  
Location: 55209-55721
  
 NCBI BlastP on this gene

EGY14484

BNR/Asp-box repeat domain-containing protein
  
Accession: EGY14485
  
Location: 57630-58880
  
 NCBI BlastP on this gene

EGY14485

trypsin
  
Accession: EGY14486
  
Location: 62617-63509
  
 NCBI BlastP on this gene

EGY14486

hypothetical protein
  
Accession: EGY14487
  
Location: 64730-65319
  
 NCBI BlastP on this gene

EGY14487

hypothetical protein
  
Accession: EGY14488
  
Location: 67209-69421
  
 NCBI BlastP on this gene

EGY14488

thioesterase family protein
  
Accession: EGY14489
  
Location: 70080-70574
  
 NCBI BlastP on this gene

EGY14489

hypothetical protein
  
Accession: EGY14490
  
Location: 71070-72896
  
 NCBI BlastP on this gene

EGY14490

184. :  KB445550 Baudoinia compniacensis UAMH 10762 unplaced genomic scaffold BAUCOscaffold\_1     Total score: 1.0     Cumulative Blast bit score: 498

hypothetical protein
  
Accession: EMD00539
  
Location: 416523-418712
  
 NCBI BlastP on this gene

EMD00539

hypothetical protein
  
Accession: EMD00538
  
Location: 415095-416378
  
 NCBI BlastP on this gene

EMD00538

hypothetical protein
  
Accession: EMD00537
  
Location: 413932-414865
  
 NCBI BlastP on this gene

EMD00537

hypothetical protein
  
Accession: EMD00536
  
Location: 413042-413569
  
 NCBI BlastP on this gene

EMD00536

hypothetical protein
  
Accession: EMD00535
  
Location: 411567-411761
  
 NCBI BlastP on this gene

EMD00535

hypothetical protein
  
Accession: EMD00534
  
Location: 410428-410931
  
 NCBI BlastP on this gene

EMD00534

hypothetical protein
  
Accession: EMD00533
  
Location: 409539-409763
  
 NCBI BlastP on this gene

EMD00533

hypothetical protein
  
Accession: EMD00532
  
Location: 406961-409192
  
 NCBI BlastP on this gene

EMD00532

hypothetical protein
  
Accession: EMD00531
  
Location: 405830-406508
  
 NCBI BlastP on this gene

EMD00531

hypothetical protein
  
Accession: EMD00530
  
Location: 402847-405169
  
 NCBI BlastP on this gene

EMD00530

hypothetical protein
  
Accession: EMD00529
  
Location: 400834-402102
  
 NCBI BlastP on this gene

EMD00529

hypothetical protein
  
Accession: EMD00528
  
Location: 397387-400266
  
 NCBI BlastP on this gene

EMD00528

hypothetical protein
  
Accession: EMD00527
  
Location: 394976-396532
  
  
**BlastP hit with Mycgr3G65725\_Mycgr3T**
  
Percentage identity: 54 %
  
BlastP bit score: 499
  
Sequence coverage: 96 %
  
E-value: 3e-170
  
  
 NCBI BlastP on this gene

EMD00527

hypothetical protein
  
Accession: EMD00526
  
Location: 393529-394442
  
 NCBI BlastP on this gene

EMD00526

hypothetical protein
  
Accession: EMD00525
  
Location: 391193-392953
  
 NCBI BlastP on this gene

EMD00525

hypothetical protein
  
Accession: EMD00524
  
Location: 389876-390133
  
 NCBI BlastP on this gene

EMD00524

hypothetical protein
  
Accession: EMD00523
  
Location: 389175-389522
  
 NCBI BlastP on this gene

EMD00523

hypothetical protein
  
Accession: EMD00522
  
Location: 386293-388842
  
 NCBI BlastP on this gene

EMD00522

hypothetical protein
  
Accession: EMD00521
  
Location: 384537-385891
  
 NCBI BlastP on this gene

EMD00521

hypothetical protein
  
Accession: EMD00520
  
Location: 382376-383995
  
 NCBI BlastP on this gene

EMD00520

hypothetical protein
  
Accession: EMD00519
  
Location: 380170-380554
  
 NCBI BlastP on this gene

EMD00519

hypothetical protein
  
Accession: EMD00518
  
Location: 379339-379545
  
 NCBI BlastP on this gene

EMD00518

hypothetical protein
  
Accession: EMD00517
  
Location: 376652-378012
  
 NCBI BlastP on this gene

EMD00517

185. :  CM001233 Magnaporthe oryzae 70-15 chromosome 3     Total score: 1.0     Cumulative Blast bit score: 498

hypothetical protein
  
Accession: EHA53306
  
Location: 6122187-6122497
  
 NCBI BlastP on this gene

EHA53306

hypothetical protein
  
Accession: EHA53307
  
Location: 6123472-6124600
  
 NCBI BlastP on this gene

EHA53307

dipeptidyl-peptidase V
  
Accession: EHA53308
  
Location: 6125331-6127751
  
 NCBI BlastP on this gene

EHA53308

thiamine transporter
  
Accession: EHA53309
  
Location: 6129201-6131130
  
 NCBI BlastP on this gene

EHA53309

hypothetical protein
  
Accession: EHA53310
  
Location: 6131392-6132327
  
 NCBI BlastP on this gene

EHA53310

hypothetical protein
  
Accession: EHA53311
  
Location: 6132495-6133792
  
 NCBI BlastP on this gene

EHA53311

hypothetical protein
  
Accession: EHA53312
  
Location: 6134160-6135504
  
 NCBI BlastP on this gene

EHA53312

hypothetical protein
  
Accession: EHA53313
  
Location: 6136901-6137664
  
 NCBI BlastP on this gene

EHA53313

hypothetical protein
  
Accession: EHA53314
  
Location: 6138147-6139085
  
 NCBI BlastP on this gene

EHA53314

hypothetical protein
  
Accession: EHA53315
  
Location: 6139181-6140297
  
 NCBI BlastP on this gene

EHA53315

hypothetical protein
  
Accession: EHA53316
  
Location: 6141449-6142950
  
  
**BlastP hit with Mycgr3G65725\_Mycgr3T**
  
Percentage identity: 54 %
  
BlastP bit score: 498
  
Sequence coverage: 93 %
  
E-value: 2e-169
  
  
 NCBI BlastP on this gene

EHA53316

186. :  CH408159 Pichia guilliermondii ATCC 6260 scaffold\_5 genomic scaffold     Total score: 1.0     Cumulative Blast bit score: 492

predicted protein
  
Accession: EDK40397
  
Location: 1011000-1011737
  
 NCBI BlastP on this gene

EDK40397

hypothetical protein
  
Accession: EDK40396
  
Location: 1000268-1001707
  
 NCBI BlastP on this gene

EDK40396

hypothetical protein
  
Accession: EDK40395
  
Location: 998405-999988
  
 NCBI BlastP on this gene

EDK40395

hypothetical protein
  
Accession: EDK40394
  
Location: 996542-998167
  
 NCBI BlastP on this gene

EDK40394

hypothetical protein
  
Accession: EDK40393
  
Location: 994801-996441
  
  
**BlastP hit with Mycgr3G32432\_Mycgr3T**
  
Percentage identity: 35 %
  
BlastP bit score: 251
  
Sequence coverage: 82 %
  
E-value: 1e-71
  
  
 NCBI BlastP on this gene

EDK40393

hypothetical protein
  
Accession: EDK40392
  
Location: 992705-994345
  
  
**BlastP hit with Mycgr3G32432\_Mycgr3T**
  
Percentage identity: 34 %
  
BlastP bit score: 241
  
Sequence coverage: 82 %
  
E-value: 5e-68
  
  
 NCBI BlastP on this gene

EDK40392

hypothetical protein
  
Accession: EDK40391
  
Location: 991384-992694
  
 NCBI BlastP on this gene

EDK40391

hypothetical protein
  
Accession: EDK40390
  
Location: 990627-991382
  
 NCBI BlastP on this gene

EDK40390

hypothetical protein
  
Accession: EDK40389
  
Location: 989059-990261
  
 NCBI BlastP on this gene

EDK40389

hypothetical protein
  
Accession: EDK40388
  
Location: 988068-988718
  
 NCBI BlastP on this gene

EDK40388

hypothetical protein
  
Accession: EDK40387
  
Location: 986552-987733
  
 NCBI BlastP on this gene

EDK40387

hypothetical protein
  
Accession: EDK40386
  
Location: 985257-986336
  
 NCBI BlastP on this gene

EDK40386

hypothetical protein
  
Accession: EDK40385
  
Location: 984219-985118
  
 NCBI BlastP on this gene

EDK40385

hypothetical protein
  
Accession: EDK40384
  
Location: 982308-984023
  
 NCBI BlastP on this gene

EDK40384

conserved hypothetical protein
  
Accession: EDK40383
  
Location: 980863-982254
  
 NCBI BlastP on this gene

EDK40383

hypothetical protein
  
Accession: EDK40382
  
Location: 979531-980733
  
 NCBI BlastP on this gene

EDK40382

hypothetical protein
  
Accession: EDK40381
  
Location: 978367-979503
  
 NCBI BlastP on this gene

EDK40381

hypothetical protein
  
Accession: EDK40380
  
Location: 975955-978165
  
 NCBI BlastP on this gene

EDK40380

hypothetical protein
  
Accession: EDK40379
  
Location: 975178-975831
  
 NCBI BlastP on this gene

EDK40379

ubiquitin
  
Accession: EDK40378
  
Location: 973177-973632
  
 NCBI BlastP on this gene

EDK40378

187. :  AFNW01000605 Fusarium pseudograminearum CS3096     Total score: 1.0     Cumulative Blast bit score: 489

hypothetical protein
  
Accession: EKJ68466
  
Location: 390314-391450
  
  
**BlastP hit with Mycgr3G102276\_Mycgr3**
  
Percentage identity: 66 %
  
BlastP bit score: 489
  
Sequence coverage: 98 %
  
E-value: 1e-169
  
  
 NCBI BlastP on this gene

EKJ68466

hypothetical protein
  
Accession: EKJ68465
  
Location: 388453-389153
  
 NCBI BlastP on this gene

EKJ68465

hypothetical protein
  
Accession: EKJ68464
  
Location: 386389-388254
  
 NCBI BlastP on this gene

EKJ68464

hypothetical protein
  
Accession: EKJ68463
  
Location: 385658-386146
  
 NCBI BlastP on this gene

EKJ68463

hypothetical protein
  
Accession: EKJ68462
  
Location: 379803-385130
  
 NCBI BlastP on this gene

EKJ68462

hypothetical protein
  
Accession: EKJ68461
  
Location: 373320-375347
  
 NCBI BlastP on this gene

EKJ68461

hypothetical protein
  
Accession: EKJ68460
  
Location: 372279-373043
  
 NCBI BlastP on this gene

EKJ68460

188. :  KB644415 Penicillium oxalicum 114-2 unplaced genomic scaffold scaffold\_8     Total score: 1.0     Cumulative Blast bit score: 488

hypothetical protein
  
Accession: EPS33803
  
Location: 2200504-2201421
  
 NCBI BlastP on this gene

EPS33803

hypothetical protein
  
Accession: EPS33802
  
Location: 2198299-2200123
  
 NCBI BlastP on this gene

EPS33802

hypothetical protein
  
Accession: EPS33801
  
Location: 2196123-2197829
  
 NCBI BlastP on this gene

EPS33801

hypothetical protein
  
Accession: EPS33800
  
Location: 2193570-2195266
  
 NCBI BlastP on this gene

EPS33800

hypothetical protein
  
Accession: EPS33799
  
Location: 2190597-2192417
  
 NCBI BlastP on this gene

EPS33799

hypothetical protein
  
Accession: EPS33798
  
Location: 2189106-2189899
  
 NCBI BlastP on this gene

EPS33798

hypothetical protein
  
Accession: EPS33797
  
Location: 2187057-2188459
  
 NCBI BlastP on this gene

EPS33797

hypothetical protein
  
Accession: EPS33796
  
Location: 2184893-2185951
  
 NCBI BlastP on this gene

EPS33796

hypothetical protein
  
Accession: EPS33795
  
Location: 2183628-2184560
  
 NCBI BlastP on this gene

EPS33795

hypothetical protein
  
Accession: EPS33794
  
Location: 2182007-2182607
  
 NCBI BlastP on this gene

EPS33794

hypothetical protein
  
Accession: EPS33793
  
Location: 2179468-2181405
  
  
**BlastP hit with Mycgr3G107069\_Mycgr3**
  
Percentage identity: 41 %
  
BlastP bit score: 488
  
Sequence coverage: 99 %
  
E-value: 4e-161
  
  
 NCBI BlastP on this gene

EPS33793

hypothetical protein
  
Accession: EPS33792
  
Location: 2177016-2178518
  
 NCBI BlastP on this gene

EPS33792

hypothetical protein
  
Accession: EPS33791
  
Location: 2174118-2176009
  
 NCBI BlastP on this gene

EPS33791

hypothetical protein
  
Accession: EPS33790
  
Location: 2172785-2173895
  
 NCBI BlastP on this gene

EPS33790

hypothetical protein
  
Accession: EPS33789
  
Location: 2171554-2172279
  
 NCBI BlastP on this gene

EPS33789

hypothetical protein
  
Accession: EPS33788
  
Location: 2169164-2170296
  
 NCBI BlastP on this gene

EPS33788

hypothetical protein
  
Accession: EPS33787
  
Location: 2167127-2167851
  
 NCBI BlastP on this gene

EPS33787

hypothetical protein
  
Accession: EPS33786
  
Location: 2166079-2167016
  
 NCBI BlastP on this gene

EPS33786

hypothetical protein
  
Accession: EPS33785
  
Location: 2162785-2164446
  
 NCBI BlastP on this gene

EPS33785

189. :  CM001235 Magnaporthe oryzae 70-15 chromosome 5     Total score: 1.0     Cumulative Blast bit score: 488

hypothetical protein
  
Accession: EHA49267
  
Location: 3514201-3514419
  
 NCBI BlastP on this gene

EHA49267

ornithine decarboxylase antizyme
  
Accession: EHA49268
  
Location: 3514867-3515946
  
 NCBI BlastP on this gene

EHA49268

imidazole glycerol phosphate synthase hisHF
  
Accession: EHA49269
  
Location: 3516536-3518280
  
 NCBI BlastP on this gene

EHA49269

hypothetical protein
  
Accession: EHA49270
  
Location: 3518908-3520416
  
 NCBI BlastP on this gene

EHA49270

dimethyladenosine transferase
  
Accession: EHA49271
  
Location: 3520805-3522101
  
 NCBI BlastP on this gene

EHA49271

hypothetical protein
  
Accession: EHA49272
  
Location: 3522578-3524296
  
 NCBI BlastP on this gene

EHA49272

hypothetical protein
  
Accession: EHA49273
  
Location: 3526037-3529629
  
 NCBI BlastP on this gene

EHA49273

hypothetical protein
  
Accession: EHA49274
  
Location: 3532241-3533576
  
 NCBI BlastP on this gene

EHA49274

NADP-dependent alcohol dehydrogenase 6
  
Accession: EHA49275
  
Location: 3534096-3535270
  
  
**BlastP hit with Mycgr3G102276\_Mycgr3**
  
Percentage identity: 66 %
  
BlastP bit score: 488
  
Sequence coverage: 98 %
  
E-value: 2e-169
  
  
 NCBI BlastP on this gene

EHA49275

hypothetical protein
  
Accession: EHA49276
  
Location: 3535607-3535914
  
 NCBI BlastP on this gene

EHA49276

hypothetical protein
  
Accession: EHA49277
  
Location: 3536972-3537602
  
 NCBI BlastP on this gene

EHA49277

15-hydroxyprostaglandin dehydrogenase
  
Accession: EHA49278
  
Location: 3540107-3541354
  
 NCBI BlastP on this gene

EHA49278

hypothetical protein
  
Accession: EHA49279
  
Location: 3541990-3544311
  
 NCBI BlastP on this gene

EHA49279

hypothetical protein
  
Accession: EHA49280
  
Location: 3547028-3548056
  
 NCBI BlastP on this gene

EHA49280

polyketide synthase
  
Accession: EHA49281
  
Location: 3548461-3555648
  
 NCBI BlastP on this gene

EHA49281

190. :  KB726554 Fusarium oxysporum f. sp. cubense race 4 unplaced genomic scaffold scaffold44     Total score: 1.0     Cumulative Blast bit score: 481

hypothetical protein
  
Accession: EMT68273
  
Location: 1099537-1099800
  
 NCBI BlastP on this gene

EMT68273

DNA polymerase epsilon catalytic subunit A
  
Accession: EMT68272
  
Location: 1091587-1098454
  
 NCBI BlastP on this gene

EMT68272

Cytochrome c oxidase protein 20, mitochondrial
  
Accession: EMT68271
  
Location: 1088484-1089048
  
 NCBI BlastP on this gene

EMT68271

hypothetical protein
  
Accession: EMT68270
  
Location: 1087139-1088053
  
 NCBI BlastP on this gene

EMT68270

Protein VTS1
  
Accession: EMT68269
  
Location: 1085610-1086158
  
 NCBI BlastP on this gene

EMT68269

Copper transport protein ctr4
  
Accession: EMT68268
  
Location: 1083516-1084115
  
 NCBI BlastP on this gene

EMT68268

Copper transport protein ctr4
  
Accession: EMT68267
  
Location: 1082356-1082955
  
 NCBI BlastP on this gene

EMT68267

Ferric reductase transmembrane component 5
  
Accession: EMT68266
  
Location: 1079347-1081302
  
  
**BlastP hit with Mycgr3G107069\_Mycgr3**
  
Percentage identity: 39 %
  
BlastP bit score: 482
  
Sequence coverage: 102 %
  
E-value: 9e-159
  
  
 NCBI BlastP on this gene

EMT68266

Bifunctional protein GAL10
  
Accession: EMT68265
  
Location: 1077456-1078409
  
 NCBI BlastP on this gene

EMT68265

Bifunctional protein GAL10
  
Accession: EMT68264
  
Location: 1075221-1076243
  
 NCBI BlastP on this gene

EMT68264

hypothetical protein
  
Accession: EMT68263
  
Location: 1073664-1075007
  
 NCBI BlastP on this gene

EMT68263

Mitochondrial distribution and morphology protein 35
  
Accession: EMT68262
  
Location: 1072683-1073133
  
 NCBI BlastP on this gene

EMT68262

N-acetylglucosaminyl-phosphatidylinositol biosynthetic protein gpi1
  
Accession: EMT68261
  
Location: 1067307-1070023
  
 NCBI BlastP on this gene

EMT68261

Tubulin-specific chaperone C
  
Accession: EMT68260
  
Location: 1064097-1065354
  
 NCBI BlastP on this gene

EMT68260

hypothetical protein
  
Accession: EMT68259
  
Location: 1063435-1063881
  
 NCBI BlastP on this gene

EMT68259

2,3-bisphosphoglycerate-independent phosphoglycerate mutase
  
Accession: EMT68258
  
Location: 1060834-1062760
  
 NCBI BlastP on this gene

EMT68258

191. :  HF679023 Fusarium fujikuroi IMI 58289 draft genome, chromosome FFUJ\_chr01.     Total score: 1.0     Cumulative Blast bit score: 481

uncharacterized protein
  
Accession: CCT61590
  
Location: 450795-452457
  
 NCBI BlastP on this gene

FFUJ\_01923

uncharacterized protein
  
Accession: CCT61591
  
Location: 454578-455051
  
 NCBI BlastP on this gene

FFUJ\_01922

uncharacterized protein
  
Accession: CCT61592
  
Location: 455799-457027
  
 NCBI BlastP on this gene

FFUJ\_01921

uncharacterized protein
  
Accession: CCT61593
  
Location: 457396-461111
  
 NCBI BlastP on this gene

FFUJ\_01920

uncharacterized protein
  
Accession: CCT61594
  
Location: 461569-463296
  
 NCBI BlastP on this gene

FFUJ\_01919

related to alpha-mannosidase
  
Accession: CCT61595
  
Location: 463792-465538
  
 NCBI BlastP on this gene

FFUJ\_01918

uncharacterized protein
  
Accession: CCT61596
  
Location: 466393-467066
  
 NCBI BlastP on this gene

FFUJ\_01917

uncharacterized protein
  
Accession: CCT61597
  
Location: 467226-468127
  
 NCBI BlastP on this gene

FFUJ\_01916

related to alcohol dehydrogenase, class C
  
Accession: CCT61598
  
Location: 469026-470164
  
  
**BlastP hit with Mycgr3G102276\_Mycgr3**
  
Percentage identity: 65 %
  
BlastP bit score: 482
  
Sequence coverage: 98 %
  
E-value: 7e-167
  
  
 NCBI BlastP on this gene

FFUJ\_01915

related to MGMT family protein
  
Accession: CCT61599
  
Location: 470427-470925
  
 NCBI BlastP on this gene

FFUJ\_01914

related to kinesin-like protein
  
Accession: CCT61600
  
Location: 471525-476841
  
 NCBI BlastP on this gene

FFUJ\_01913

uncharacterized protein
  
Accession: CCT61601
  
Location: 481068-483098
  
 NCBI BlastP on this gene

FFUJ\_01912

uncharacterized protein
  
Accession: CCT61602
  
Location: 483363-488706
  
 NCBI BlastP on this gene

FFUJ\_01911

uncharacterized protein
  
Accession: CCT61603
  
Location: 489771-490550
  
 NCBI BlastP on this gene

FFUJ\_01910

192. :  KB730248 Fusarium oxysporum f. sp. cubense race 1 unplaced genomic scaffold scaffold101     Total score: 1.0     Cumulative Blast bit score: 479

Activating signal cointegrator 1 complex subunit 2
  
Accession: ENH68643
  
Location: 1226138-1228151
  
 NCBI BlastP on this gene

ENH68643

hypothetical protein
  
Accession: ENH68644
  
Location: 1228152-1229405
  
 NCBI BlastP on this gene

ENH68644

hypothetical protein
  
Accession: ENH68645
  
Location: 1229840-1230112
  
 NCBI BlastP on this gene

ENH68645

DNA polymerase epsilon catalytic subunit A
  
Accession: ENH68646
  
Location: 1231996-1238863
  
 NCBI BlastP on this gene

ENH68646

Cytochrome c oxidase protein 20, mitochondrial
  
Accession: ENH68647
  
Location: 1239331-1239895
  
 NCBI BlastP on this gene

ENH68647

hypothetical protein
  
Accession: ENH68648
  
Location: 1240336-1241250
  
 NCBI BlastP on this gene

ENH68648

Protein VTS1
  
Accession: ENH68649
  
Location: 1242228-1242776
  
 NCBI BlastP on this gene

ENH68649

Copper transport protein ctr4
  
Accession: ENH68650
  
Location: 1244275-1244874
  
 NCBI BlastP on this gene

ENH68650

Ferric reductase transmembrane component 5
  
Accession: ENH68651
  
Location: 1245821-1247776
  
  
**BlastP hit with Mycgr3G107069\_Mycgr3**
  
Percentage identity: 39 %
  
BlastP bit score: 480
  
Sequence coverage: 101 %
  
E-value: 7e-158
  
  
 NCBI BlastP on this gene

ENH68651

193. :  ACJE01000009 Aspergillus niger ATCC 1015     Total score: 1.0     Cumulative Blast bit score: 476

hypothetical protein
  
Accession: EHA24027
  
Location: 1227262-1230748
  
 NCBI BlastP on this gene

EHA24027

hypothetical protein
  
Accession: EHA24028
  
Location: 1231661-1231996
  
 NCBI BlastP on this gene

EHA24028

hypothetical protein
  
Accession: EHA24029
  
Location: 1232878-1233938
  
 NCBI BlastP on this gene

EHA24029

hypothetical protein
  
Accession: EHA24030
  
Location: 1234603-1235331
  
 NCBI BlastP on this gene

EHA24030

hypothetical protein
  
Accession: EHA24031
  
Location: 1236231-1236740
  
 NCBI BlastP on this gene

EHA24031

hypothetical protein
  
Accession: EHA24032
  
Location: 1237326-1238318
  
 NCBI BlastP on this gene

EHA24032

hypothetical protein
  
Accession: EHA24033
  
Location: 1239648-1240826
  
 NCBI BlastP on this gene

EHA24033

hypothetical protein
  
Accession: EHA24034
  
Location: 1241093-1243192
  
 NCBI BlastP on this gene

EHA24034

hypothetical protein
  
Accession: EHA24035
  
Location: 1243959-1244501
  
 NCBI BlastP on this gene

EHA24035

hypothetical protein
  
Accession: EHA24036
  
Location: 1244759-1246747
  
  
**BlastP hit with Mycgr3G107069\_Mycgr3**
  
Percentage identity: 41 %
  
BlastP bit score: 476
  
Sequence coverage: 100 %
  
E-value: 3e-156
  
  
 NCBI BlastP on this gene

EHA24036

194. :  HF679026 Fusarium fujikuroi IMI 58289 draft genome, chromosome FFUJ\_chr04.     Total score: 1.0     Cumulative Blast bit score: 475

related to CUE3 Meiotic induced gene, protein has a CUE domain that binds ubiquitin
  
Accession: CCT67077
  
Location: 867907-869923
  
 NCBI BlastP on this gene

FFUJ\_13260

probable DNA polymerase epsilon, calytic chain POL2
  
Accession: CCT67519
  
Location: 865217-867878
  
 NCBI BlastP on this gene

FFUJ\_13259

probable DNA polymerase epsilon, calytic chain POL2
  
Accession: CCT67076
  
Location: 856451-863318
  
 NCBI BlastP on this gene

FFUJ\_13258

uncharacterized protein
  
Accession: CCT67075
  
Location: 855424-855987
  
 NCBI BlastP on this gene

FFUJ\_13257

uncharacterized protein
  
Accession: CCT67074
  
Location: 854103-855017
  
 NCBI BlastP on this gene

FFUJ\_13256

uncharacterized protein
  
Accession: CCT67073
  
Location: 852566-853142
  
 NCBI BlastP on this gene

FFUJ\_13255

related to copper transport protein
  
Accession: CCT67072
  
Location: 850476-851075
  
 NCBI BlastP on this gene

FFUJ\_13254

related to ferric reductase FRE2 precursor
  
Accession: CCT67071
  
Location: 847545-849500
  
  
**BlastP hit with Mycgr3G107069\_Mycgr3**
  
Percentage identity: 39 %
  
BlastP bit score: 475
  
Sequence coverage: 102 %
  
E-value: 7e-156
  
  
 NCBI BlastP on this gene

FFUJ\_13253

related to GAL10-UDP-glucose 4-epimerase
  
Accession: CCT67070
  
Location: 845579-846601
  
 NCBI BlastP on this gene

FFUJ\_13252

uncharacterized protein
  
Accession: CCT67069
  
Location: 844048-845374
  
 NCBI BlastP on this gene

FFUJ\_13251

related to MDM35 Mitochondrial Distribution and Morphology protein
  
Accession: CCT67554
  
Location: 843070-843500
  
 NCBI BlastP on this gene

FFUJ\_14434

related to
  
Accession: CCT67504
  
Location: 839767-842482
  
 NCBI BlastP on this gene

FFUJ\_13250

uncharacterized protein
  
Accession: CCT67068
  
Location: 838109-838660
  
 NCBI BlastP on this gene

FFUJ\_13249

probable tubulin folding cofactor C
  
Accession: CCT67067
  
Location: 836616-837872
  
 NCBI BlastP on this gene

FFUJ\_13248

uncharacterized protein
  
Accession: CCT67066
  
Location: 835950-836390
  
 NCBI BlastP on this gene

FFUJ\_13247

probable phosphoglyceromutase
  
Accession: CCT67065
  
Location: 833353-835279
  
 NCBI BlastP on this gene

FFUJ\_13246

195. :  DS231620 Pyrenophora tritici-repentis Pt-1C-BFP supercont1.6 genomic scaffold     Total score: 1.0     Cumulative Blast bit score: 469

hypothetical protein
  
Accession: EDU49697
  
Location: 1659801-1659947
  
 NCBI BlastP on this gene

EDU49697

conserved hypothetical protein
  
Accession: EDU49698
  
Location: 1666091-1667896
  
 NCBI BlastP on this gene

EDU49698

siderophore iron transporter mirB
  
Accession: EDU49699
  
Location: 1669879-1671777
  
 NCBI BlastP on this gene

EDU49699

conserved hypothetical protein
  
Accession: EDU49700
  
Location: 1672065-1673187
  
 NCBI BlastP on this gene

EDU49700

predicted protein
  
Accession: EDU49701
  
Location: 1675575-1677260
  
 NCBI BlastP on this gene

EDU49701

high affinity copper transporter
  
Accession: EDU49702
  
Location: 1678126-1678752
  
 NCBI BlastP on this gene

EDU49702

ferric reductase
  
Accession: EDU49703
  
Location: 1679262-1681283
  
  
**BlastP hit with Mycgr3G107069\_Mycgr3**
  
Percentage identity: 40 %
  
BlastP bit score: 469
  
Sequence coverage: 105 %
  
E-value: 4e-153
  
  
 NCBI BlastP on this gene

EDU49703

196. :  CP003014 Thielavia terrestris NRRL 8126 chromosome 6     Total score: 1.0     Cumulative Blast bit score: 468

hypothetical protein
  
Accession: AEO71363
  
Location: 1433564-1435577
  
 NCBI BlastP on this gene

THITE\_50845

hypothetical protein
  
Accession: AEO71362
  
Location: 1431091-1431390
  
 NCBI BlastP on this gene

THITE\_2034030

hypothetical protein
  
Accession: AEO71361
  
Location: 1428451-1430486
  
 NCBI BlastP on this gene

THITE\_2071253

hypothetical protein
  
Accession: AEO71360
  
Location: 1426212-1427384
  
 NCBI BlastP on this gene

THITE\_2123618

hypothetical protein
  
Accession: AEO71359
  
Location: 1418007-1421857
  
 NCBI BlastP on this gene

THITE\_124350

hypothetical protein
  
Accession: AEO71358
  
Location: 1413240-1416021
  
  
**BlastP hit with Mycgr3G52682\_Mycgr3T**
  
Percentage identity: 38 %
  
BlastP bit score: 468
  
Sequence coverage: 70 %
  
E-value: 2e-143
  
  
 NCBI BlastP on this gene

THITE\_2123607

hypothetical protein
  
Accession: AEO71357
  
Location: 1409562-1411101
  
 NCBI BlastP on this gene

THITE\_2123604

hypothetical protein
  
Accession: AEO71356
  
Location: 1407378-1408108
  
 NCBI BlastP on this gene

THITE\_2123602

hypothetical protein
  
Accession: AEO71355
  
Location: 1404021-1406434
  
 NCBI BlastP on this gene

THITE\_2123600

hypothetical protein
  
Accession: AEO71354
  
Location: 1402962-1403508
  
 NCBI BlastP on this gene

THITE\_2123598

hypothetical protein
  
Accession: AEO71353
  
Location: 1399910-1402058
  
 NCBI BlastP on this gene

THITE\_2123596

hypothetical protein
  
Accession: AEO71352
  
Location: 1396407-1398503
  
 NCBI BlastP on this gene

THITE\_2123595

hypothetical protein
  
Accession: AEO71351
  
Location: 1395320-1395601
  
 NCBI BlastP on this gene

THITE\_2123594

hypothetical protein
  
Accession: AEO71350
  
Location: 1394526-1394867
  
 NCBI BlastP on this gene

THITE\_2123593

197. :  GG698898 Nectria haematococca mpVI 77-13-4 chromosome 4 genomic scaffold NECHAsca\_3\_chr4\_2\_0     Total score: 1.0     Cumulative Blast bit score: 466

hypothetical protein
  
Accession: EEU46364
  
Location: 1029943-1031895
  
  
**BlastP hit with Mycgr3G107069\_Mycgr3**
  
Percentage identity: 38 %
  
BlastP bit score: 466
  
Sequence coverage: 102 %
  
E-value: 2e-152
  
  
 NCBI BlastP on this gene

EEU46364

hypothetical protein
  
Accession: EEU46363
  
Location: 1027977-1028999
  
 NCBI BlastP on this gene

EEU46363

hypothetical protein
  
Accession: EEU45920
  
Location: 1026402-1027715
  
 NCBI BlastP on this gene

EEU45920

hypothetical protein
  
Accession: EEU46362
  
Location: 1022117-1025891
  
 NCBI BlastP on this gene

EEU46362

hypothetical protein
  
Accession: EEU46361
  
Location: 1015796-1017052
  
 NCBI BlastP on this gene

EEU46361

hypothetical protein
  
Accession: EEU45919
  
Location: 1015144-1015569
  
 NCBI BlastP on this gene

EEU45919

predicted protein
  
Accession: EEU46360
  
Location: 1012469-1014430
  
 NCBI BlastP on this gene

EEU46360

198. :  KB456262 Mycosphaerella populorum SO2202 unplaced genomic scaffold SEPMUscaffold\_3     Total score: 1.0     Cumulative Blast bit score: 464

hypothetical protein
  
Accession: EMF14805
  
Location: 2467457-2468379
  
 NCBI BlastP on this gene

EMF14805

hypothetical protein
  
Accession: EMF14806
  
Location: 2470685-2471068
  
 NCBI BlastP on this gene

EMF14806

ARM repeat-containing protein
  
Accession: EMF14807
  
Location: 2480643-2483825
  
 NCBI BlastP on this gene

EMF14807

hypothetical protein
  
Accession: EMF14808
  
Location: 2485131-2486066
  
 NCBI BlastP on this gene

EMF14808

zinc-binding alcohol dehydrogenase
  
Accession: EMF14809
  
Location: 2487157-2488401
  
  
**BlastP hit with Mycgr3G102276\_Mycgr3**
  
Percentage identity: 66 %
  
BlastP bit score: 465
  
Sequence coverage: 100 %
  
E-value: 3e-160
  
  
 NCBI BlastP on this gene

EMF14809

acyl-CoA dehydrogenase
  
Accession: EMF14810
  
Location: 2488722-2490501
  
 NCBI BlastP on this gene

EMF14810

alpha/beta-hydrolase
  
Accession: EMF14811
  
Location: 2491113-2492668
  
 NCBI BlastP on this gene

EMF14811

hypothetical protein
  
Accession: EMF14812
  
Location: 2493069-2493818
  
 NCBI BlastP on this gene

EMF14812

Metallo-hydrolase/oxidoreductase
  
Accession: EMF14813
  
Location: 2494600-2495607
  
 NCBI BlastP on this gene

EMF14813

hypothetical protein
  
Accession: EMF14814
  
Location: 2496241-2497651
  
 NCBI BlastP on this gene

EMF14814

hypothetical protein
  
Accession: EMF14815
  
Location: 2501296-2502357
  
 NCBI BlastP on this gene

EMF14815

Hydroxyisourate hydrolase
  
Accession: EMF14817
  
Location: 2502717-2503103
  
 NCBI BlastP on this gene

EMF14817

199. :  GL629729 Grosmannia clavigera kw1407 unplaced genomic scaffold GCSC\_108     Total score: 1.0     Cumulative Blast bit score: 462

glutathione reductase
  
Accession: EFX06457
  
Location: 245670-250446
  
 NCBI BlastP on this gene

EFX06457

exocyst complex component
  
Accession: EFX06522
  
Location: 250736-253069
  
 NCBI BlastP on this gene

EFX06522

tbc domain protein
  
Accession: EFX06333
  
Location: 253396-257247
  
 NCBI BlastP on this gene

EFX06333

ankyrin unc44
  
Accession: EFX06213
  
Location: 258978-260324
  
 NCBI BlastP on this gene

EFX06213

mitochondrial ATPase, aaa-type
  
Accession: EFX06609
  
Location: 261444-262908
  
 NCBI BlastP on this gene

EFX06609

high affinity copper transporter
  
Accession: EFX06270
  
Location: 263930-264388
  
 NCBI BlastP on this gene

EFX06270

ferric-chelate reductase
  
Accession: EFX06529
  
Location: 264730-266796
  
  
**BlastP hit with Mycgr3G107069\_Mycgr3**
  
Percentage identity: 39 %
  
BlastP bit score: 462
  
Sequence coverage: 105 %
  
E-value: 3e-150
  
  
 NCBI BlastP on this gene

EFX06529

200. :  DS572697 Verticillium dahliae VdLs.17 supercont1.3 genomic scaffold     Total score: 1.0     Cumulative Blast bit score: 462

hypothetical protein
  
Accession: EGY20023
  
Location: 1302675-1303842
  
 NCBI BlastP on this gene

EGY20023

carboxylesterase A
  
Accession: EGY20024
  
Location: 1303985-1305298
  
 NCBI BlastP on this gene

EGY20024

aspartyl-tRNA synthetase
  
Accession: EGY20025
  
Location: 1307008-1309659
  
 NCBI BlastP on this gene

EGY20025

hypothetical protein
  
Accession: EGY20026
  
Location: 1312899-1313567
  
 NCBI BlastP on this gene

EGY20026

chitosanase
  
Accession: EGY20027
  
Location: 1314130-1316053
  
 NCBI BlastP on this gene

EGY20027

hypothetical protein
  
Accession: EGY20028
  
Location: 1317811-1318140
  
 NCBI BlastP on this gene

EGY20028

high affinity copper transporter
  
Accession: EGY20029
  
Location: 1319730-1320631
  
 NCBI BlastP on this gene

EGY20029

ferric reductase transmembrane component 5
  
Accession: EGY20030
  
Location: 1321680-1323702
  
  
**BlastP hit with Mycgr3G107069\_Mycgr3**
  
Percentage identity: 40 %
  
BlastP bit score: 462
  
Sequence coverage: 102 %
  
E-value: 5e-151
  
  
 NCBI BlastP on this gene

EGY20030

hypothetical protein
  
Accession: EGY20031
  
Location: 1324271-1328173
  
 NCBI BlastP on this gene

EGY20031

MFS transporter
  
Accession: EGY20032
  
Location: 1328374-1330048
  
 NCBI BlastP on this gene

EGY20032

hypothetical protein
  
Accession: EGY20033
  
Location: 1331774-1332237
  
 NCBI BlastP on this gene

EGY20033

hypothetical protein
  
Accession: EGY20034
  
Location: 1334315-1336389
  
 NCBI BlastP on this gene

EGY20034

high-affinity glucose transporter ght2
  
Accession: EGY20035
  
Location: 1338883-1339749
  
 NCBI BlastP on this gene

EGY20035

hypothetical protein
  
Accession: EGY20036
  
Location: 1341959-1342687
  
 NCBI BlastP on this gene

EGY20036

hypothetical protein
  
Accession: EGY20037
  
Location: 1343181-1344185
  
 NCBI BlastP on this gene

EGY20037

Detecting sequence homology at the gene cluster level with MultiGeneBlast.
  
Marnix H. Medema, Rainer Breitling & Eriko Takano (2013)
  
*Molecular Biology and Evolution* , 30: 1218-1223.
