# Supplementary material for: In silico prediction and characterization of secondary metabolite biosynthetic gene clusters in the wheat pathogen Zymoseptoria tritici
Source: BMC Genomics. 2017 Aug 17;18:631. doi: 10.1186/s12864-017-3969-y (PMC5561558; doi:10.1186/s12864-017-3969-y)
Supplement: Supplementary file 1 — MultiGeneBLAST analysis of putative secondary metabolite clusters. All encoded amino acid sequences from genes residing in clusters predicted by AntiSMASH are given as FASTA file format. All output data from MultiGeneBLASTs are also provided. (ZIP 42911 kb) [file 12864_2017_3969_MOESM1_ESM.zip › Cluster MultiGene BLAST/out/Clusters_1_34/Cluster_1/displaypage5.xhtml]

xml version="1.0" encoding="UTF-8"?


Search Results
  
  
 Results pages: 1, 2, 3, 4, 5

**MultiGeneBlast hits**

Select gene cluster alignment
201. AM920431\_2 Penicillium chrysogenum Wisconsin 54-1255 complete genome, co...
202. GL385395\_0 Gaeumannomyces graminis var. tritici R3-111a-1 unplaced genom...
203. KB445643\_0 Cochliobolus sativus ND90Pr unplaced genomic scaffold COCSAsc...
204. AACD01000093\_1 Aspergillus nidulans FGSC A4, whole genome shotgun sequen...
205. CH408033\_3 Chaetomium globosum CBS 148.51 scaffold\_5 genomic scaffold, w...
206. KB733454\_0 Bipolaris maydis ATCC 48331 unplaced genomic scaffold COCC4sc...
207. KB445580\_1 Cochliobolus heterostrophus C5 unplaced genomic scaffold COCH...
208. DF126453\_1 Aspergillus kawachii IFO 4308 DNA, contig: scaffold00007, who...
209. CU633899\_1 Podospora anserina S mat+ genomic DNA chromosome 1, supercont...
210. KB908570\_1 Setosphaeria turcica Et28A unplaced genomic scaffold SETTUsca...
211. KB725930\_0 Colletotrichum orbiculare MAFF 240422 unplaced genomic scaffo...
212. GL385395\_2 Gaeumannomyces graminis var. tritici R3-111a-1 unplaced genom...
213. CM001233\_2 Magnaporthe oryzae 70-15 chromosome 3, whole genome shotgun s...
214. KB446540\_0 Dothistroma septosporum NZE10 unplaced genomic scaffold DOTSE...
215. DS178270\_0 Puccinia graminis f. sp. tritici CRL 75-36-700-3 supercont2.9...
216. CP000113\_1 Myxococcus xanthus DK 1622, complete genome.
217. CM001196\_0 Mycosphaerella graminicola IPO323 chromosome 1, whole genome ...
218. KB908548\_1 Setosphaeria turcica Et28A unplaced genomic scaffold SETTUsca...
219. GL385398\_0 Gaeumannomyces graminis var. tritici R3-111a-1 unplaced genom...
220. JH126405\_2 Cordyceps militaris CM01 unplaced genomic scaffold CCM\_S00007...
221. DS027694\_0 Neosartorya fischeri NRRL 181 1099437636262 genomic scaffold,...
222. CM001197\_0 Mycosphaerella graminicola IPO323 chromosome 2, whole genome ...
223. KE145356\_1 Glarea lozoyensis ATCC 20868 chromosome Unknown GLAREA13, who...
224. KB446558\_2 Pseudocercospora fijiensis CIRAD86 unplaced genomic scaffold ...
225. DS990637\_1 Ajellomyces capsulatus H88 unplaced genomic scaffold supercon...
226. KB644408\_2 Penicillium oxalicum 114-2 unplaced genomic scaffold scaffold...
227. DS499600\_1 Aspergillus fumigatus A1163 scf\_000007 genomic scaffold, whol...
228. GL377303\_1 Schizophyllum commune H4-8 unplaced genomic scaffold SCHCOsca...
229. AM920431\_0 Penicillium chrysogenum Wisconsin 54-1255 complete genome, co...
230. GL988032\_3 Chaetomium thermophilum var. thermophilum DSM 1495 unplaced g...
231. KB446537\_3 Dothistroma septosporum NZE10 unplaced genomic scaffold DOTSE...
232. AP012319\_0 Actinoplanes missouriensis 431 DNA, complete genome.
233. JH795880\_0 Dacryopinax sp. DJM-731 SS1 chromosome Unknown DACRYscaffold\_...
234. KB726020\_1 Colletotrichum orbiculare MAFF 240422 unplaced genomic scaffo...
235. CM001234\_0 Magnaporthe oryzae 70-15 chromosome 4, whole genome shotgun s...
236. CAIF01000017\_0 Wickerhamomyces ciferrii strain NRRL Y-1031 F-60-10, whol...
237. KB446546\_0 Dothistroma septosporum NZE10 unplaced genomic scaffold DOTSE...
238. CP003008\_2 Myceliophthora thermophila ATCC 42464 chromosome 7, complete ...
239. CM001200\_1 Mycosphaerella graminicola IPO323 chromosome 5, whole genome ...
240. GL988032\_0 Chaetomium thermophilum var. thermophilum DSM 1495 unplaced g...
241. EQ963472\_3 Aspergillus flavus NRRL3357 scf\_1106286418772 genomic scaffol...
242. DS985216\_1 Verticillium albo-atrum VaMs.102 supercont1.3 genomic scaffol...
243. AP007175\_3 Aspergillus oryzae RIB40 DNA, SC010.
244. AKHY01000175\_0 Aspergillus oryzae 3.042, whole genome shotgun sequencing...
245. JH668231\_0 Wallemia sebi CBS 633.66 unplaced genomic scaffold WALSEscaff...
246. GL629765\_0 Grosmannia clavigera kw1407 unplaced genomic scaffold GCSC\_14...
247. EQ963475\_1 Aspergillus flavus NRRL3357 scf\_1106286419142 genomic scaffol...
248. KB644409\_1 Penicillium oxalicum 114-2 unplaced genomic scaffold scaffold...
249. AP007157\_2 Aspergillus oryzae RIB40 DNA, SC023.
250. AP007151\_3 Aspergillus oryzae RIB40 DNA, SC005.

Query: Architecture Search FASTA input

AM920431 : Penicillium chrysogenum Wisconsin 54-1255 complete genome, contig Pc00c16.    Total score: 1.0     Cumulative Blast bit score: 462

Hit cluster cross-links:

Mycgr3G52686 Mycgr3T
  
Location: 0-861

Mycgr3G52686\_Mycgr3T

Mycgr3G102281 Mycgr3
  
Location: 961-1573

Mycgr3G102281\_Mycgr3

Mycgr3G89185 Mycgr3T
  
Location: 1673-2063

Mycgr3G89185\_Mycgr3T

Mycgr3G65725 Mycgr3T
  
Location: 2163-3612

Mycgr3G65725\_Mycgr3T

Mycgr3G102276 Mycgr3
  
Location: 3712-4801

Mycgr3G102276\_Mycgr3

Mycgr3G89189 Mycgr3T
  
Location: 4901-5564

Mycgr3G89189\_Mycgr3T

Mycgr3G52682 Mycgr3T
  
Location: 5664-9231

Mycgr3G52682\_Mycgr3T

Mycgr3G107072 Mycgr3
  
Location: 9331-13279

Mycgr3G107072\_Mycgr3

Mycgr3G34982 Mycgr3T
  
Location: 13379-15116

Mycgr3G34982\_Mycgr3T

Mycgr3G107069 Mycgr3
  
Location: 15216-17097

Mycgr3G107069\_Mycgr3

Mycgr3G32432 Mycgr3T
  
Location: 17197-19042

Mycgr3G32432\_Mycgr3T

Mycgr3G98385 Mycgr3T
  
Location: 19142-19898

Mycgr3G98385\_Mycgr3T

not annotated
  
Accession: CAP93315
  
Location: 1492540-1493617
  
 NCBI BlastP on this gene

Pc16g06450

hypothetical protein
  
Accession: CAP93314
  
Location: 1490079-1490520
  
 NCBI BlastP on this gene

Pc16g06440

hypothetical protein
  
Accession: CAP93313
  
Location: 1489135-1489584
  
 NCBI BlastP on this gene

Pc16g06430

unnamed
  
Accession: CAP93312
  
Location: 1486682-1488721
  
 NCBI BlastP on this gene

Pc16g06420

hypothetical protein
  
Accession: CAP93311
  
Location: 1484802-1485429
  
 NCBI BlastP on this gene

Pc16g06410

not annotated
  
Accession: CAP93310
  
Location: 1483942-1484514
  
 NCBI BlastP on this gene

Pc16g06400

not annotated
  
Accession: CAP93309
  
Location: 1481588-1483540
  
  
**BlastP hit with Mycgr3G107069\_Mycgr3**
  
Percentage identity: 40 %
  
BlastP bit score: 462
  
Sequence coverage: 100 %
  
E-value: 5e-151
  
  
 NCBI BlastP on this gene

Pc16g06390

not annotated
  
Accession: CAP93308
  
Location: 1479091-1480851
  
 NCBI BlastP on this gene

Pc16g06380

unnamed
  
Accession: CAP93307
  
Location: 1476259-1478399
  
 NCBI BlastP on this gene

Pc16g06370

not annotated
  
Accession: CAP93306
  
Location: 1473535-1474860
  
 NCBI BlastP on this gene

Pc16g06360

unnamed
  
Accession: CAP93305
  
Location: 1470706-1471610
  
 NCBI BlastP on this gene

Pc16g06350

not annotated
  
Accession: CAP93304
  
Location: 1469323-1470437
  
 NCBI BlastP on this gene

Pc16g06340

Query: Architecture Search FASTA input

GL385395 : Gaeumannomyces graminis var. tritici R3-111a-1 unplaced genomic scaffold supercont2.1    Total score: 1.0     Cumulative Blast bit score: 461

Hit cluster cross-links:

Mycgr3G52686 Mycgr3T
  
Location: 0-861

Mycgr3G52686\_Mycgr3T

Mycgr3G102281 Mycgr3
  
Location: 961-1573

Mycgr3G102281\_Mycgr3

Mycgr3G89185 Mycgr3T
  
Location: 1673-2063

Mycgr3G89185\_Mycgr3T

Mycgr3G65725 Mycgr3T
  
Location: 2163-3612

Mycgr3G65725\_Mycgr3T

Mycgr3G102276 Mycgr3
  
Location: 3712-4801

Mycgr3G102276\_Mycgr3

Mycgr3G89189 Mycgr3T
  
Location: 4901-5564

Mycgr3G89189\_Mycgr3T

Mycgr3G52682 Mycgr3T
  
Location: 5664-9231

Mycgr3G52682\_Mycgr3T

Mycgr3G107072 Mycgr3
  
Location: 9331-13279

Mycgr3G107072\_Mycgr3

Mycgr3G34982 Mycgr3T
  
Location: 13379-15116

Mycgr3G34982\_Mycgr3T

Mycgr3G107069 Mycgr3
  
Location: 15216-17097

Mycgr3G107069\_Mycgr3

Mycgr3G32432 Mycgr3T
  
Location: 17197-19042

Mycgr3G32432\_Mycgr3T

Mycgr3G98385 Mycgr3T
  
Location: 19142-19898

Mycgr3G98385\_Mycgr3T

hypothetical protein
  
Accession: EJT82169
  
Location: 6799243-6803077
  
 NCBI BlastP on this gene

EJT82169

hypothetical protein
  
Accession: EJT82170
  
Location: 6804421-6804789
  
 NCBI BlastP on this gene

EJT82170

oligopeptide transporter 2
  
Accession: EJT82171
  
Location: 6805724-6808756
  
 NCBI BlastP on this gene

EJT82171

NADP-dependent alcohol dehydrogenase 6
  
Accession: EJT82172
  
Location: 6810657-6811868
  
  
**BlastP hit with Mycgr3G102276\_Mycgr3**
  
Percentage identity: 61 %
  
BlastP bit score: 461
  
Sequence coverage: 99 %
  
E-value: 3e-158
  
  
 NCBI BlastP on this gene

EJT82172

hypothetical protein
  
Accession: EJT82173
  
Location: 6813295-6814057
  
 NCBI BlastP on this gene

EJT82173

hypothetical protein
  
Accession: EJT82174
  
Location: 6815075-6816808
  
 NCBI BlastP on this gene

EJT82174

hypothetical protein
  
Accession: EJT82175
  
Location: 6818556-6821952
  
 NCBI BlastP on this gene

EJT82175

hypothetical protein
  
Accession: EJT82176
  
Location: 6823240-6826141
  
 NCBI BlastP on this gene

EJT82176

Query: Architecture Search FASTA input

KB445643 : Cochliobolus sativus ND90Pr unplaced genomic scaffold COCSAscaffold\_7    Total score: 1.0     Cumulative Blast bit score: 459

Hit cluster cross-links:

Mycgr3G52686 Mycgr3T
  
Location: 0-861

Mycgr3G52686\_Mycgr3T

Mycgr3G102281 Mycgr3
  
Location: 961-1573

Mycgr3G102281\_Mycgr3

Mycgr3G89185 Mycgr3T
  
Location: 1673-2063

Mycgr3G89185\_Mycgr3T

Mycgr3G65725 Mycgr3T
  
Location: 2163-3612

Mycgr3G65725\_Mycgr3T

Mycgr3G102276 Mycgr3
  
Location: 3712-4801

Mycgr3G102276\_Mycgr3

Mycgr3G89189 Mycgr3T
  
Location: 4901-5564

Mycgr3G89189\_Mycgr3T

Mycgr3G52682 Mycgr3T
  
Location: 5664-9231

Mycgr3G52682\_Mycgr3T

Mycgr3G107072 Mycgr3
  
Location: 9331-13279

Mycgr3G107072\_Mycgr3

Mycgr3G34982 Mycgr3T
  
Location: 13379-15116

Mycgr3G34982\_Mycgr3T

Mycgr3G107069 Mycgr3
  
Location: 15216-17097

Mycgr3G107069\_Mycgr3

Mycgr3G32432 Mycgr3T
  
Location: 17197-19042

Mycgr3G32432\_Mycgr3T

Mycgr3G98385 Mycgr3T
  
Location: 19142-19898

Mycgr3G98385\_Mycgr3T

hypothetical protein
  
Accession: EMD64395
  
Location: 1242621-1244524
  
 NCBI BlastP on this gene

EMD64395

carbohydrate esterase family 5 protein
  
Accession: EMD64394
  
Location: 1241182-1242309
  
 NCBI BlastP on this gene

EMD64394

hypothetical protein
  
Accession: EMD64393
  
Location: 1237229-1238832
  
 NCBI BlastP on this gene

EMD64393

hypothetical protein
  
Accession: EMD64392
  
Location: 1233718-1235665
  
 NCBI BlastP on this gene

EMD64392

hypothetical protein
  
Accession: EMD64391
  
Location: 1231839-1232497
  
 NCBI BlastP on this gene

EMD64391

hypothetical protein
  
Accession: EMD64390
  
Location: 1229281-1231314
  
  
**BlastP hit with Mycgr3G107069\_Mycgr3**
  
Percentage identity: 38 %
  
BlastP bit score: 459
  
Sequence coverage: 104 %
  
E-value: 2e-149
  
  
 NCBI BlastP on this gene

EMD64390

hypothetical protein
  
Accession: EMD64389
  
Location: 1227049-1228671
  
 NCBI BlastP on this gene

EMD64389

carbohydrate-binding module family 35 protein
  
Accession: EMD64388
  
Location: 1221798-1226464
  
 NCBI BlastP on this gene

EMD64388

glycoside hydrolase family 3 protein
  
Accession: EMD64387
  
Location: 1218750-1221089
  
 NCBI BlastP on this gene

EMD64387

Query: Architecture Search FASTA input

AACD01000093 : Aspergillus nidulans FGSC A4    Total score: 1.0     Cumulative Blast bit score: 459

Hit cluster cross-links:

Mycgr3G52686 Mycgr3T
  
Location: 0-861

Mycgr3G52686\_Mycgr3T

Mycgr3G102281 Mycgr3
  
Location: 961-1573

Mycgr3G102281\_Mycgr3

Mycgr3G89185 Mycgr3T
  
Location: 1673-2063

Mycgr3G89185\_Mycgr3T

Mycgr3G65725 Mycgr3T
  
Location: 2163-3612

Mycgr3G65725\_Mycgr3T

Mycgr3G102276 Mycgr3
  
Location: 3712-4801

Mycgr3G102276\_Mycgr3

Mycgr3G89189 Mycgr3T
  
Location: 4901-5564

Mycgr3G89189\_Mycgr3T

Mycgr3G52682 Mycgr3T
  
Location: 5664-9231

Mycgr3G52682\_Mycgr3T

Mycgr3G107072 Mycgr3
  
Location: 9331-13279

Mycgr3G107072\_Mycgr3

Mycgr3G34982 Mycgr3T
  
Location: 13379-15116

Mycgr3G34982\_Mycgr3T

Mycgr3G107069 Mycgr3
  
Location: 15216-17097

Mycgr3G107069\_Mycgr3

Mycgr3G32432 Mycgr3T
  
Location: 17197-19042

Mycgr3G32432\_Mycgr3T

Mycgr3G98385 Mycgr3T
  
Location: 19142-19898

Mycgr3G98385\_Mycgr3T

hypothetical protein
  
Accession: EAA62511
  
Location: 255546-256217
  
 NCBI BlastP on this gene

EAA62511

hypothetical protein
  
Accession: EAA62512
  
Location: 256895-260575
  
 NCBI BlastP on this gene

EAA62512

hypothetical protein
  
Accession: EAA62513
  
Location: 261614-262385
  
 NCBI BlastP on this gene

EAA62513

hypothetical protein
  
Accession: EAA62514
  
Location: 265652-266799
  
 NCBI BlastP on this gene

EAA62514

hypothetical protein
  
Accession: EAA62515
  
Location: 267703-268853
  
  
**BlastP hit with Mycgr3G102276\_Mycgr3**
  
Percentage identity: 64 %
  
BlastP bit score: 459
  
Sequence coverage: 98 %
  
E-value: 6e-158
  
  
 NCBI BlastP on this gene

EAA62515

Query: Architecture Search FASTA input

CH408033 : Chaetomium globosum CBS 148.51 scaffold\_5 genomic scaffold    Total score: 1.0     Cumulative Blast bit score: 457

Hit cluster cross-links:

Mycgr3G52686 Mycgr3T
  
Location: 0-861

Mycgr3G52686\_Mycgr3T

Mycgr3G102281 Mycgr3
  
Location: 961-1573

Mycgr3G102281\_Mycgr3

Mycgr3G89185 Mycgr3T
  
Location: 1673-2063

Mycgr3G89185\_Mycgr3T

Mycgr3G65725 Mycgr3T
  
Location: 2163-3612

Mycgr3G65725\_Mycgr3T

Mycgr3G102276 Mycgr3
  
Location: 3712-4801

Mycgr3G102276\_Mycgr3

Mycgr3G89189 Mycgr3T
  
Location: 4901-5564

Mycgr3G89189\_Mycgr3T

Mycgr3G52682 Mycgr3T
  
Location: 5664-9231

Mycgr3G52682\_Mycgr3T

Mycgr3G107072 Mycgr3
  
Location: 9331-13279

Mycgr3G107072\_Mycgr3

Mycgr3G34982 Mycgr3T
  
Location: 13379-15116

Mycgr3G34982\_Mycgr3T

Mycgr3G107069 Mycgr3
  
Location: 15216-17097

Mycgr3G107069\_Mycgr3

Mycgr3G32432 Mycgr3T
  
Location: 17197-19042

Mycgr3G32432\_Mycgr3T

Mycgr3G98385 Mycgr3T
  
Location: 19142-19898

Mycgr3G98385\_Mycgr3T

hypothetical protein
  
Accession: EAQ86698
  
Location: 3404753-3408736
  
  
**BlastP hit with Mycgr3G52682\_Mycgr3T**
  
Percentage identity: 37 %
  
BlastP bit score: 457
  
Sequence coverage: 69 %
  
E-value: 1e-137
  
  
 NCBI BlastP on this gene

EAQ86698

hypothetical protein
  
Accession: EAQ86697
  
Location: 3401198-3402406
  
 NCBI BlastP on this gene

EAQ86697

T-complex protein 1
  
Accession: EAQ86696
  
Location: 3398198-3400130
  
 NCBI BlastP on this gene

EAQ86696

hypothetical protein
  
Accession: EAQ86695
  
Location: 3397401-3398075
  
 NCBI BlastP on this gene

EAQ86695

hypothetical protein
  
Accession: EAQ86694
  
Location: 3394164-3396102
  
 NCBI BlastP on this gene

EAQ86694

Query: Architecture Search FASTA input

KB733454 : Bipolaris maydis ATCC 48331 unplaced genomic scaffold COCC4scaffold\_11    Total score: 1.0     Cumulative Blast bit score: 456

Hit cluster cross-links:

Mycgr3G52686 Mycgr3T
  
Location: 0-861

Mycgr3G52686\_Mycgr3T

Mycgr3G102281 Mycgr3
  
Location: 961-1573

Mycgr3G102281\_Mycgr3

Mycgr3G89185 Mycgr3T
  
Location: 1673-2063

Mycgr3G89185\_Mycgr3T

Mycgr3G65725 Mycgr3T
  
Location: 2163-3612

Mycgr3G65725\_Mycgr3T

Mycgr3G102276 Mycgr3
  
Location: 3712-4801

Mycgr3G102276\_Mycgr3

Mycgr3G89189 Mycgr3T
  
Location: 4901-5564

Mycgr3G89189\_Mycgr3T

Mycgr3G52682 Mycgr3T
  
Location: 5664-9231

Mycgr3G52682\_Mycgr3T

Mycgr3G107072 Mycgr3
  
Location: 9331-13279

Mycgr3G107072\_Mycgr3

Mycgr3G34982 Mycgr3T
  
Location: 13379-15116

Mycgr3G34982\_Mycgr3T

Mycgr3G107069 Mycgr3
  
Location: 15216-17097

Mycgr3G107069\_Mycgr3

Mycgr3G32432 Mycgr3T
  
Location: 17197-19042

Mycgr3G32432\_Mycgr3T

Mycgr3G98385 Mycgr3T
  
Location: 19142-19898

Mycgr3G98385\_Mycgr3T

hypothetical protein
  
Accession: ENI05574
  
Location: 697758-699661
  
 NCBI BlastP on this gene

ENI05574

carbohydrate esterase family 5 protein
  
Accession: ENI05573
  
Location: 696265-697434
  
 NCBI BlastP on this gene

ENI05573

hypothetical protein
  
Accession: ENI05572
  
Location: 690765-692757
  
 NCBI BlastP on this gene

ENI05572

hypothetical protein
  
Accession: ENI05571
  
Location: 688888-689547
  
 NCBI BlastP on this gene

ENI05571

hypothetical protein
  
Accession: ENI05570
  
Location: 686330-688363
  
  
**BlastP hit with Mycgr3G107069\_Mycgr3**
  
Percentage identity: 38 %
  
BlastP bit score: 456
  
Sequence coverage: 104 %
  
E-value: 2e-148
  
  
 NCBI BlastP on this gene

ENI05570

hypothetical protein
  
Accession: ENI05569
  
Location: 684122-685714
  
 NCBI BlastP on this gene

ENI05569

carbohydrate-binding module family 35 protein
  
Accession: ENI05568
  
Location: 677735-682396
  
 NCBI BlastP on this gene

ENI05568

glycoside hydrolase family 3 protein
  
Accession: ENI05567
  
Location: 674693-677032
  
 NCBI BlastP on this gene

ENI05567

Query: Architecture Search FASTA input

KB445580 : Cochliobolus heterostrophus C5 unplaced genomic scaffold COCHEscaffold\_12    Total score: 1.0     Cumulative Blast bit score: 456

Hit cluster cross-links:

Mycgr3G52686 Mycgr3T
  
Location: 0-861

Mycgr3G52686\_Mycgr3T

Mycgr3G102281 Mycgr3
  
Location: 961-1573

Mycgr3G102281\_Mycgr3

Mycgr3G89185 Mycgr3T
  
Location: 1673-2063

Mycgr3G89185\_Mycgr3T

Mycgr3G65725 Mycgr3T
  
Location: 2163-3612

Mycgr3G65725\_Mycgr3T

Mycgr3G102276 Mycgr3
  
Location: 3712-4801

Mycgr3G102276\_Mycgr3

Mycgr3G89189 Mycgr3T
  
Location: 4901-5564

Mycgr3G89189\_Mycgr3T

Mycgr3G52682 Mycgr3T
  
Location: 5664-9231

Mycgr3G52682\_Mycgr3T

Mycgr3G107072 Mycgr3
  
Location: 9331-13279

Mycgr3G107072\_Mycgr3

Mycgr3G34982 Mycgr3T
  
Location: 13379-15116

Mycgr3G34982\_Mycgr3T

Mycgr3G107069 Mycgr3
  
Location: 15216-17097

Mycgr3G107069\_Mycgr3

Mycgr3G32432 Mycgr3T
  
Location: 17197-19042

Mycgr3G32432\_Mycgr3T

Mycgr3G98385 Mycgr3T
  
Location: 19142-19898

Mycgr3G98385\_Mycgr3T

hypothetical protein
  
Accession: EMD88709
  
Location: 1146030-1147933
  
 NCBI BlastP on this gene

EMD88709

carbohydrate esterase family 5 protein
  
Accession: EMD88710
  
Location: 1148257-1149427
  
 NCBI BlastP on this gene

EMD88710

hypothetical protein
  
Accession: EMD88711
  
Location: 1152935-1154927
  
 NCBI BlastP on this gene

EMD88711

hypothetical protein
  
Accession: EMD88712
  
Location: 1156145-1156804
  
 NCBI BlastP on this gene

EMD88712

hypothetical protein
  
Accession: EMD88713
  
Location: 1157329-1159362
  
  
**BlastP hit with Mycgr3G107069\_Mycgr3**
  
Percentage identity: 38 %
  
BlastP bit score: 456
  
Sequence coverage: 104 %
  
E-value: 2e-148
  
  
 NCBI BlastP on this gene

EMD88713

Query: Architecture Search FASTA input

DF126453 : Aspergillus kawachii IFO 4308 DNA, contig: scaffold00007    Total score: 1.0     Cumulative Blast bit score: 456

Hit cluster cross-links:

Mycgr3G52686 Mycgr3T
  
Location: 0-861

Mycgr3G52686\_Mycgr3T

Mycgr3G102281 Mycgr3
  
Location: 961-1573

Mycgr3G102281\_Mycgr3

Mycgr3G89185 Mycgr3T
  
Location: 1673-2063

Mycgr3G89185\_Mycgr3T

Mycgr3G65725 Mycgr3T
  
Location: 2163-3612

Mycgr3G65725\_Mycgr3T

Mycgr3G102276 Mycgr3
  
Location: 3712-4801

Mycgr3G102276\_Mycgr3

Mycgr3G89189 Mycgr3T
  
Location: 4901-5564

Mycgr3G89189\_Mycgr3T

Mycgr3G52682 Mycgr3T
  
Location: 5664-9231

Mycgr3G52682\_Mycgr3T

Mycgr3G107072 Mycgr3
  
Location: 9331-13279

Mycgr3G107072\_Mycgr3

Mycgr3G34982 Mycgr3T
  
Location: 13379-15116

Mycgr3G34982\_Mycgr3T

Mycgr3G107069 Mycgr3
  
Location: 15216-17097

Mycgr3G107069\_Mycgr3

Mycgr3G32432 Mycgr3T
  
Location: 17197-19042

Mycgr3G32432\_Mycgr3T

Mycgr3G98385 Mycgr3T
  
Location: 19142-19898

Mycgr3G98385\_Mycgr3T

conserved serine proline-rich protein
  
Accession: GAA85232
  
Location: 165718-168188
  
 NCBI BlastP on this gene

GAA85232

similar to An07g05420
  
Accession: GAA85233
  
Location: 169030-169365
  
 NCBI BlastP on this gene

GAA85233

hypothetical protein
  
Accession: GAA85234
  
Location: 170012-171069
  
 NCBI BlastP on this gene

GAA85234

short-chain dehydrogenase
  
Accession: GAA85235
  
Location: 172398-173174
  
 NCBI BlastP on this gene

GAA85235

similar to An07g05450
  
Accession: GAA85236
  
Location: 174135-174659
  
 NCBI BlastP on this gene

GAA85236

methyltransferase family protein
  
Accession: GAA85237
  
Location: 175211-176206
  
 NCBI BlastP on this gene

GAA85237

stage V sporulation protein k
  
Accession: GAA85238
  
Location: 177005-177824
  
 NCBI BlastP on this gene

GAA85238

hypothetical protein
  
Accession: GAA85239
  
Location: 178247-178941
  
 NCBI BlastP on this gene

GAA85239

ferric-chelate reductase
  
Accession: GAA85240
  
Location: 179188-181176
  
  
**BlastP hit with Mycgr3G107069\_Mycgr3**
  
Percentage identity: 41 %
  
BlastP bit score: 456
  
Sequence coverage: 106 %
  
E-value: 2e-148
  
  
 NCBI BlastP on this gene

GAA85240

Query: Architecture Search FASTA input

CU633899 : Podospora anserina S mat+ genomic DNA chromosome 1, supercontig 4.    Total score: 1.0     Cumulative Blast bit score: 456

Hit cluster cross-links:

Mycgr3G52686 Mycgr3T
  
Location: 0-861

Mycgr3G52686\_Mycgr3T

Mycgr3G102281 Mycgr3
  
Location: 961-1573

Mycgr3G102281\_Mycgr3

Mycgr3G89185 Mycgr3T
  
Location: 1673-2063

Mycgr3G89185\_Mycgr3T

Mycgr3G65725 Mycgr3T
  
Location: 2163-3612

Mycgr3G65725\_Mycgr3T

Mycgr3G102276 Mycgr3
  
Location: 3712-4801

Mycgr3G102276\_Mycgr3

Mycgr3G89189 Mycgr3T
  
Location: 4901-5564

Mycgr3G89189\_Mycgr3T

Mycgr3G52682 Mycgr3T
  
Location: 5664-9231

Mycgr3G52682\_Mycgr3T

Mycgr3G107072 Mycgr3
  
Location: 9331-13279

Mycgr3G107072\_Mycgr3

Mycgr3G34982 Mycgr3T
  
Location: 13379-15116

Mycgr3G34982\_Mycgr3T

Mycgr3G107069 Mycgr3
  
Location: 15216-17097

Mycgr3G107069\_Mycgr3

Mycgr3G32432 Mycgr3T
  
Location: 17197-19042

Mycgr3G32432\_Mycgr3T

Mycgr3G98385 Mycgr3T
  
Location: 19142-19898

Mycgr3G98385\_Mycgr3T

not annotated
  
Accession: CAP67747
  
Location: 697446-700892
  
 NCBI BlastP on this gene

CAP67747

not annotated
  
Accession: CAP67748
  
Location: 701001-702607
  
 NCBI BlastP on this gene

CAP67748

not annotated
  
Accession: CAP67749
  
Location: 704205-706226
  
 NCBI BlastP on this gene

CAP67749

not annotated
  
Accession: CAP67750
  
Location: 707784-709349
  
 NCBI BlastP on this gene

CAP67750

not annotated
  
Accession: CAP67751
  
Location: 709764-710465
  
 NCBI BlastP on this gene

CAP67751

not annotated
  
Accession: CAP67752
  
Location: 711799-713760
  
  
**BlastP hit with Mycgr3G107069\_Mycgr3**
  
Percentage identity: 38 %
  
BlastP bit score: 456
  
Sequence coverage: 103 %
  
E-value: 2e-148
  
  
 NCBI BlastP on this gene

CAP67752

Query: Architecture Search FASTA input

KB908570 : Setosphaeria turcica Et28A unplaced genomic scaffold SETTUscaffold\_18    Total score: 1.0     Cumulative Blast bit score: 455

Hit cluster cross-links:

Mycgr3G52686 Mycgr3T
  
Location: 0-861

Mycgr3G52686\_Mycgr3T

Mycgr3G102281 Mycgr3
  
Location: 961-1573

Mycgr3G102281\_Mycgr3

Mycgr3G89185 Mycgr3T
  
Location: 1673-2063

Mycgr3G89185\_Mycgr3T

Mycgr3G65725 Mycgr3T
  
Location: 2163-3612

Mycgr3G65725\_Mycgr3T

Mycgr3G102276 Mycgr3
  
Location: 3712-4801

Mycgr3G102276\_Mycgr3

Mycgr3G89189 Mycgr3T
  
Location: 4901-5564

Mycgr3G89189\_Mycgr3T

Mycgr3G52682 Mycgr3T
  
Location: 5664-9231

Mycgr3G52682\_Mycgr3T

Mycgr3G107072 Mycgr3
  
Location: 9331-13279

Mycgr3G107072\_Mycgr3

Mycgr3G34982 Mycgr3T
  
Location: 13379-15116

Mycgr3G34982\_Mycgr3T

Mycgr3G107069 Mycgr3
  
Location: 15216-17097

Mycgr3G107069\_Mycgr3

Mycgr3G32432 Mycgr3T
  
Location: 17197-19042

Mycgr3G32432\_Mycgr3T

Mycgr3G98385 Mycgr3T
  
Location: 19142-19898

Mycgr3G98385\_Mycgr3T

hypothetical protein
  
Accession: EOA87797
  
Location: 534968-536815
  
  
**BlastP hit with Mycgr3G107069\_Mycgr3**
  
Percentage identity: 41 %
  
BlastP bit score: 455
  
Sequence coverage: 95 %
  
E-value: 1e-148
  
  
 NCBI BlastP on this gene

EOA87797

hypothetical protein
  
Accession: EOA87796
  
Location: 534478-534888
  
 NCBI BlastP on this gene

EOA87796

hypothetical protein
  
Accession: EOA87795
  
Location: 533642-533821
  
 NCBI BlastP on this gene

EOA87795

hypothetical protein
  
Accession: EOA87794
  
Location: 532418-533065
  
 NCBI BlastP on this gene

EOA87794

hypothetical protein
  
Accession: EOA87793
  
Location: 529970-531338
  
 NCBI BlastP on this gene

EOA87793

hypothetical protein
  
Accession: EOA87792
  
Location: 524512-528896
  
 NCBI BlastP on this gene

EOA87792

hypothetical protein
  
Accession: EOA87791
  
Location: 521513-523816
  
 NCBI BlastP on this gene

EOA87791

Query: Architecture Search FASTA input

KB725930 : Colletotrichum orbiculare MAFF 240422 unplaced genomic scaffold Scaffold\_366    Total score: 1.0     Cumulative Blast bit score: 451

Hit cluster cross-links:

Mycgr3G52686 Mycgr3T
  
Location: 0-861

Mycgr3G52686\_Mycgr3T

Mycgr3G102281 Mycgr3
  
Location: 961-1573

Mycgr3G102281\_Mycgr3

Mycgr3G89185 Mycgr3T
  
Location: 1673-2063

Mycgr3G89185\_Mycgr3T

Mycgr3G65725 Mycgr3T
  
Location: 2163-3612

Mycgr3G65725\_Mycgr3T

Mycgr3G102276 Mycgr3
  
Location: 3712-4801

Mycgr3G102276\_Mycgr3

Mycgr3G89189 Mycgr3T
  
Location: 4901-5564

Mycgr3G89189\_Mycgr3T

Mycgr3G52682 Mycgr3T
  
Location: 5664-9231

Mycgr3G52682\_Mycgr3T

Mycgr3G107072 Mycgr3
  
Location: 9331-13279

Mycgr3G107072\_Mycgr3

Mycgr3G34982 Mycgr3T
  
Location: 13379-15116

Mycgr3G34982\_Mycgr3T

Mycgr3G107069 Mycgr3
  
Location: 15216-17097

Mycgr3G107069\_Mycgr3

Mycgr3G32432 Mycgr3T
  
Location: 17197-19042

Mycgr3G32432\_Mycgr3T

Mycgr3G98385 Mycgr3T
  
Location: 19142-19898

Mycgr3G98385\_Mycgr3T

wsc domain-containing protein
  
Accession: ENH82408
  
Location: 540858-546223
  
 NCBI BlastP on this gene

ENH82408

cytochrome p450
  
Accession: ENH82407
  
Location: 538278-540096
  
 NCBI BlastP on this gene

ENH82407

high affinity copper transporter
  
Accession: ENH82406
  
Location: 537176-537780
  
 NCBI BlastP on this gene

ENH82406

ferric reductase transmembrane component 2
  
Accession: ENH82405
  
Location: 533798-535957
  
  
**BlastP hit with Mycgr3G107069\_Mycgr3**
  
Percentage identity: 40 %
  
BlastP bit score: 452
  
Sequence coverage: 102 %
  
E-value: 1e-146
  
  
 NCBI BlastP on this gene

ENH82405

dynamin family protein
  
Accession: ENH82404
  
Location: 529946-532460
  
 NCBI BlastP on this gene

ENH82404

metalloprotease mep1
  
Accession: ENH82403
  
Location: 527744-528718
  
 NCBI BlastP on this gene

ENH82403

hypothetical protein
  
Accession: ENH82402
  
Location: 526667-527108
  
 NCBI BlastP on this gene

ENH82402

platelet-activating factor acetylhydrolase precursor
  
Accession: ENH82401
  
Location: 524331-526171
  
 NCBI BlastP on this gene

ENH82401

fatty acid oxygenase
  
Accession: ENH82400
  
Location: 518936-522797
  
 NCBI BlastP on this gene

ENH82400

Query: Architecture Search FASTA input

GL385395 : Gaeumannomyces graminis var. tritici R3-111a-1 unplaced genomic scaffold supercont2.1    Total score: 1.0     Cumulative Blast bit score: 451

Hit cluster cross-links:

Mycgr3G52686 Mycgr3T
  
Location: 0-861

Mycgr3G52686\_Mycgr3T

Mycgr3G102281 Mycgr3
  
Location: 961-1573

Mycgr3G102281\_Mycgr3

Mycgr3G89185 Mycgr3T
  
Location: 1673-2063

Mycgr3G89185\_Mycgr3T

Mycgr3G65725 Mycgr3T
  
Location: 2163-3612

Mycgr3G65725\_Mycgr3T

Mycgr3G102276 Mycgr3
  
Location: 3712-4801

Mycgr3G102276\_Mycgr3

Mycgr3G89189 Mycgr3T
  
Location: 4901-5564

Mycgr3G89189\_Mycgr3T

Mycgr3G52682 Mycgr3T
  
Location: 5664-9231

Mycgr3G52682\_Mycgr3T

Mycgr3G107072 Mycgr3
  
Location: 9331-13279

Mycgr3G107072\_Mycgr3

Mycgr3G34982 Mycgr3T
  
Location: 13379-15116

Mycgr3G34982\_Mycgr3T

Mycgr3G107069 Mycgr3
  
Location: 15216-17097

Mycgr3G107069\_Mycgr3

Mycgr3G32432 Mycgr3T
  
Location: 17197-19042

Mycgr3G32432\_Mycgr3T

Mycgr3G98385 Mycgr3T
  
Location: 19142-19898

Mycgr3G98385\_Mycgr3T

integral membrane protein
  
Accession: EJT82546
  
Location: 7876483-7877619
  
 NCBI BlastP on this gene

EJT82546

hypothetical protein
  
Accession: EJT82547
  
Location: 7880535-7882603
  
 NCBI BlastP on this gene

EJT82547

hypothetical protein
  
Accession: EJT82548
  
Location: 7883762-7885938
  
 NCBI BlastP on this gene

EJT82548

hypothetical protein
  
Accession: EJT82549
  
Location: 7888106-7889923
  
  
**BlastP hit with Mycgr3G65725\_Mycgr3T**
  
Percentage identity: 55 %
  
BlastP bit score: 452
  
Sequence coverage: 88 %
  
E-value: 7e-151
  
  
 NCBI BlastP on this gene

EJT82549

Query: Architecture Search FASTA input

CM001233 : Magnaporthe oryzae 70-15 chromosome 3    Total score: 1.0     Cumulative Blast bit score: 451

Hit cluster cross-links:

Mycgr3G52686 Mycgr3T
  
Location: 0-861

Mycgr3G52686\_Mycgr3T

Mycgr3G102281 Mycgr3
  
Location: 961-1573

Mycgr3G102281\_Mycgr3

Mycgr3G89185 Mycgr3T
  
Location: 1673-2063

Mycgr3G89185\_Mycgr3T

Mycgr3G65725 Mycgr3T
  
Location: 2163-3612

Mycgr3G65725\_Mycgr3T

Mycgr3G102276 Mycgr3
  
Location: 3712-4801

Mycgr3G102276\_Mycgr3

Mycgr3G89189 Mycgr3T
  
Location: 4901-5564

Mycgr3G89189\_Mycgr3T

Mycgr3G52682 Mycgr3T
  
Location: 5664-9231

Mycgr3G52682\_Mycgr3T

Mycgr3G107072 Mycgr3
  
Location: 9331-13279

Mycgr3G107072\_Mycgr3

Mycgr3G34982 Mycgr3T
  
Location: 13379-15116

Mycgr3G34982\_Mycgr3T

Mycgr3G107069 Mycgr3
  
Location: 15216-17097

Mycgr3G107069\_Mycgr3

Mycgr3G32432 Mycgr3T
  
Location: 17197-19042

Mycgr3G32432\_Mycgr3T

Mycgr3G98385 Mycgr3T
  
Location: 19142-19898

Mycgr3G98385\_Mycgr3T

glutamyl-tRNA(Gln) amidotransferase subunit A
  
Accession: EHA53257
  
Location: 5963961-5966063
  
 NCBI BlastP on this gene

EHA53257

nonselective cation channel
  
Accession: EHA53258
  
Location: 5967077-5969391
  
 NCBI BlastP on this gene

EHA53258

hypothetical protein
  
Accession: EHA53259
  
Location: 5970031-5971771
  
 NCBI BlastP on this gene

EHA53259

phytanoyl-CoA dioxygenase
  
Accession: EHA53260
  
Location: 5972051-5973168
  
 NCBI BlastP on this gene

EHA53260

high affinity copper transporter
  
Accession: EHA53261
  
Location: 5973738-5974452
  
 NCBI BlastP on this gene

EHA53261

ferric reductase
  
Accession: EHA53262
  
Location: 5976475-5978571
  
  
**BlastP hit with Mycgr3G107069\_Mycgr3**
  
Percentage identity: 39 %
  
BlastP bit score: 452
  
Sequence coverage: 107 %
  
E-value: 2e-146
  
  
 NCBI BlastP on this gene

EHA53262

hypothetical protein
  
Accession: EHA53263
  
Location: 5979581-5979943
  
 NCBI BlastP on this gene

EHA53263

hypothetical protein
  
Accession: EHA53264
  
Location: 5982855-5983352
  
 NCBI BlastP on this gene

EHA53264

hypothetical protein
  
Accession: EHA53265
  
Location: 5984940-5985395
  
 NCBI BlastP on this gene

EHA53265

beta-fructofuranosidase
  
Accession: EHA53266
  
Location: 5986465-5988793
  
 NCBI BlastP on this gene

EHA53266

Query: Architecture Search FASTA input

KB446540 : Dothistroma septosporum NZE10 unplaced genomic scaffold DOTSEscaffold\_6    Total score: 1.0     Cumulative Blast bit score: 449

Hit cluster cross-links:

Mycgr3G52686 Mycgr3T
  
Location: 0-861

Mycgr3G52686\_Mycgr3T

Mycgr3G102281 Mycgr3
  
Location: 961-1573

Mycgr3G102281\_Mycgr3

Mycgr3G89185 Mycgr3T
  
Location: 1673-2063

Mycgr3G89185\_Mycgr3T

Mycgr3G65725 Mycgr3T
  
Location: 2163-3612

Mycgr3G65725\_Mycgr3T

Mycgr3G102276 Mycgr3
  
Location: 3712-4801

Mycgr3G102276\_Mycgr3

Mycgr3G89189 Mycgr3T
  
Location: 4901-5564

Mycgr3G89189\_Mycgr3T

Mycgr3G52682 Mycgr3T
  
Location: 5664-9231

Mycgr3G52682\_Mycgr3T

Mycgr3G107072 Mycgr3
  
Location: 9331-13279

Mycgr3G107072\_Mycgr3

Mycgr3G34982 Mycgr3T
  
Location: 13379-15116

Mycgr3G34982\_Mycgr3T

Mycgr3G107069 Mycgr3
  
Location: 15216-17097

Mycgr3G107069\_Mycgr3

Mycgr3G32432 Mycgr3T
  
Location: 17197-19042

Mycgr3G32432\_Mycgr3T

Mycgr3G98385 Mycgr3T
  
Location: 19142-19898

Mycgr3G98385\_Mycgr3T

hypothetical protein
  
Accession: EME42893
  
Location: 226239-228242
  
 NCBI BlastP on this gene

EME42893

hypothetical protein
  
Accession: EME42894
  
Location: 229568-230574
  
 NCBI BlastP on this gene

EME42894

hypothetical protein
  
Accession: EME42895
  
Location: 231308-232759
  
 NCBI BlastP on this gene

EME42895

hypothetical protein
  
Accession: EME42896
  
Location: 234835-235788
  
 NCBI BlastP on this gene

EME42896

hypothetical protein
  
Accession: EME42898
  
Location: 236343-236617
  
 NCBI BlastP on this gene

EME42898

hypothetical protein
  
Accession: EME42899
  
Location: 239820-240920
  
  
**BlastP hit with Mycgr3G102276\_Mycgr3**
  
Percentage identity: 62 %
  
BlastP bit score: 450
  
Sequence coverage: 99 %
  
E-value: 3e-154
  
  
 NCBI BlastP on this gene

EME42899

hypothetical protein
  
Accession: EME42900
  
Location: 241815-245052
  
 NCBI BlastP on this gene

EME42900

hypothetical protein
  
Accession: EME42901
  
Location: 247088-248897
  
 NCBI BlastP on this gene

EME42901

hypothetical protein
  
Accession: EME42902
  
Location: 249247-249813
  
 NCBI BlastP on this gene

EME42902

hypothetical protein
  
Accession: EME42903
  
Location: 252680-254872
  
 NCBI BlastP on this gene

EME42903

Query: Architecture Search FASTA input

DS178270 : Puccinia graminis f. sp. tritici CRL 75-36-700-3 supercont2.9 genomic scaffold    Total score: 1.0     Cumulative Blast bit score: 446

Hit cluster cross-links:

Mycgr3G52686 Mycgr3T
  
Location: 0-861

Mycgr3G52686\_Mycgr3T

Mycgr3G102281 Mycgr3
  
Location: 961-1573

Mycgr3G102281\_Mycgr3

Mycgr3G89185 Mycgr3T
  
Location: 1673-2063

Mycgr3G89185\_Mycgr3T

Mycgr3G65725 Mycgr3T
  
Location: 2163-3612

Mycgr3G65725\_Mycgr3T

Mycgr3G102276 Mycgr3
  
Location: 3712-4801

Mycgr3G102276\_Mycgr3

Mycgr3G89189 Mycgr3T
  
Location: 4901-5564

Mycgr3G89189\_Mycgr3T

Mycgr3G52682 Mycgr3T
  
Location: 5664-9231

Mycgr3G52682\_Mycgr3T

Mycgr3G107072 Mycgr3
  
Location: 9331-13279

Mycgr3G107072\_Mycgr3

Mycgr3G34982 Mycgr3T
  
Location: 13379-15116

Mycgr3G34982\_Mycgr3T

Mycgr3G107069 Mycgr3
  
Location: 15216-17097

Mycgr3G107069\_Mycgr3

Mycgr3G32432 Mycgr3T
  
Location: 17197-19042

Mycgr3G32432\_Mycgr3T

Mycgr3G98385 Mycgr3T
  
Location: 19142-19898

Mycgr3G98385\_Mycgr3T

hypothetical protein
  
Accession: EFP78284
  
Location: 30827-34814
  
 NCBI BlastP on this gene

EFP78284

hypothetical protein
  
Accession: EFP78285
  
Location: 36141-37485
  
 NCBI BlastP on this gene

EFP78285

hypothetical protein
  
Accession: EFP78286
  
Location: 38388-39338
  
 NCBI BlastP on this gene

EFP78286

hypothetical protein
  
Accession: EFP78287
  
Location: 40656-43124
  
  
**BlastP hit with Mycgr3G34982\_Mycgr3T**
  
Percentage identity: 29 %
  
BlastP bit score: 224
  
Sequence coverage: 103 %
  
E-value: 3e-61
  
  
 NCBI BlastP on this gene

EFP78287

hypothetical protein
  
Accession: EFP78288
  
Location: 45228-47750
  
  
**BlastP hit with Mycgr3G34982\_Mycgr3T**
  
Percentage identity: 29 %
  
BlastP bit score: 222
  
Sequence coverage: 105 %
  
E-value: 2e-60
  
  
 NCBI BlastP on this gene

EFP78288

hypothetical protein
  
Accession: EFP78289
  
Location: 48837-49495
  
 NCBI BlastP on this gene

EFP78289

hypothetical protein
  
Accession: EFP78290
  
Location: 52616-54219
  
 NCBI BlastP on this gene

EFP78290

hypothetical protein
  
Accession: EFP78291
  
Location: 55239-56042
  
 NCBI BlastP on this gene

EFP78291

Query: Architecture Search FASTA input

CP000113 : Myxococcus xanthus DK 1622    Total score: 1.0     Cumulative Blast bit score: 446

Hit cluster cross-links:

Mycgr3G52686 Mycgr3T
  
Location: 0-861

Mycgr3G52686\_Mycgr3T

Mycgr3G102281 Mycgr3
  
Location: 961-1573

Mycgr3G102281\_Mycgr3

Mycgr3G89185 Mycgr3T
  
Location: 1673-2063

Mycgr3G89185\_Mycgr3T

Mycgr3G65725 Mycgr3T
  
Location: 2163-3612

Mycgr3G65725\_Mycgr3T

Mycgr3G102276 Mycgr3
  
Location: 3712-4801

Mycgr3G102276\_Mycgr3

Mycgr3G89189 Mycgr3T
  
Location: 4901-5564

Mycgr3G89189\_Mycgr3T

Mycgr3G52682 Mycgr3T
  
Location: 5664-9231

Mycgr3G52682\_Mycgr3T

Mycgr3G107072 Mycgr3
  
Location: 9331-13279

Mycgr3G107072\_Mycgr3

Mycgr3G34982 Mycgr3T
  
Location: 13379-15116

Mycgr3G34982\_Mycgr3T

Mycgr3G107069 Mycgr3
  
Location: 15216-17097

Mycgr3G107069\_Mycgr3

Mycgr3G32432 Mycgr3T
  
Location: 17197-19042

Mycgr3G32432\_Mycgr3T

Mycgr3G98385 Mycgr3T
  
Location: 19142-19898

Mycgr3G98385\_Mycgr3T

polyketide synthase
  
Accession: ABF91610
  
Location: 5611864-5627412
  
 NCBI BlastP on this gene

MXAN\_4527

polyketide synthase type I
  
Accession: ABF89696
  
Location: 5605427-5611867
  
 NCBI BlastP on this gene

MXAN\_4526

non-ribosomal peptide synthase MxaA
  
Accession: ABF90459
  
Location: 5600883-5605430
  
  
**BlastP hit with Mycgr3G107072\_Mycgr3**
  
Percentage identity: 34 %
  
BlastP bit score: 446
  
Sequence coverage: 74 %
  
E-value: 4e-129
  
  
 NCBI BlastP on this gene

MXAN\_4525

hypothetical protein
  
Accession: ABF90264
  
Location: 5600283-5600801
  
 NCBI BlastP on this gene

MXAN\_4524

putative lipoprotein
  
Accession: ABF86831
  
Location: 5598689-5599945
  
 NCBI BlastP on this gene

MXAN\_4523

hypothetical protein
  
Accession: ABF86081
  
Location: 5598558-5598668
  
 NCBI BlastP on this gene

MXAN\_4522

sulfatase family protein
  
Accession: ABF86036
  
Location: 5597314-5598186
  
 NCBI BlastP on this gene

MXAN\_4521

conserved domain protein
  
Accession: ABF87512
  
Location: 5596721-5597278
  
 NCBI BlastP on this gene

MXAN\_4520

hypothetical protein
  
Accession: ABF85867
  
Location: 5596617-5596769
  
 NCBI BlastP on this gene

MXAN\_4519

hypothetical protein
  
Accession: ABF92805
  
Location: 5596398-5596607
  
 NCBI BlastP on this gene

MXAN\_4518

hypothetical protein
  
Accession: ABF92090
  
Location: 5595240-5596337
  
 NCBI BlastP on this gene

MXAN\_4517

hypothetical protein
  
Accession: ABF87888
  
Location: 5594957-5595172
  
 NCBI BlastP on this gene

MXAN\_4516

hypothetical protein
  
Accession: ABF91051
  
Location: 5594884-5595045
  
 NCBI BlastP on this gene

MXAN\_4515

metallophosphoesterase
  
Accession: ABF90019
  
Location: 5593616-5594743
  
 NCBI BlastP on this gene

MXAN\_4514

conserved hypothetical protein
  
Accession: ABF86208
  
Location: 5590872-5593619
  
 NCBI BlastP on this gene

MXAN\_4513

Query: Architecture Search FASTA input

CM001196 : Mycosphaerella graminicola IPO323 chromosome 1    Total score: 1.0     Cumulative Blast bit score: 445

Hit cluster cross-links:

Mycgr3G52686 Mycgr3T
  
Location: 0-861

Mycgr3G52686\_Mycgr3T

Mycgr3G102281 Mycgr3
  
Location: 961-1573

Mycgr3G102281\_Mycgr3

Mycgr3G89185 Mycgr3T
  
Location: 1673-2063

Mycgr3G89185\_Mycgr3T

Mycgr3G65725 Mycgr3T
  
Location: 2163-3612

Mycgr3G65725\_Mycgr3T

Mycgr3G102276 Mycgr3
  
Location: 3712-4801

Mycgr3G102276\_Mycgr3

Mycgr3G89189 Mycgr3T
  
Location: 4901-5564

Mycgr3G89189\_Mycgr3T

Mycgr3G52682 Mycgr3T
  
Location: 5664-9231

Mycgr3G52682\_Mycgr3T

Mycgr3G107072 Mycgr3
  
Location: 9331-13279

Mycgr3G107072\_Mycgr3

Mycgr3G34982 Mycgr3T
  
Location: 13379-15116

Mycgr3G34982\_Mycgr3T

Mycgr3G107069 Mycgr3
  
Location: 15216-17097

Mycgr3G107069\_Mycgr3

Mycgr3G32432 Mycgr3T
  
Location: 17197-19042

Mycgr3G32432\_Mycgr3T

Mycgr3G98385 Mycgr3T
  
Location: 19142-19898

Mycgr3G98385\_Mycgr3T

hypothetical protein
  
Accession: EGP90902
  
Location: 799763-800017
  
 NCBI BlastP on this gene

EGP90902

hypothetical protein
  
Accession: EGP92692
  
Location: 801154-801935
  
 NCBI BlastP on this gene

EGP92692

hypothetical protein
  
Accession: EGP92691
  
Location: 805666-808885
  
 NCBI BlastP on this gene

EGP92691

hypothetical protein
  
Accession: EGP90903
  
Location: 809724-810611
  
 NCBI BlastP on this gene

EGP90903

hypothetical protein
  
Accession: EGP90904
  
Location: 811094-812542
  
  
**BlastP hit with Mycgr3G102276\_Mycgr3**
  
Percentage identity: 64 %
  
BlastP bit score: 445
  
Sequence coverage: 97 %
  
E-value: 2e-152
  
  
 NCBI BlastP on this gene

EGP90904

hypothetical protein
  
Accession: EGP92690
  
Location: 813851-814237
  
 NCBI BlastP on this gene

EGP92690

hypothetical protein
  
Accession: EGP92689
  
Location: 822031-822555
  
 NCBI BlastP on this gene

EGP92689

hypothetical protein
  
Accession: EGP90905
  
Location: 822994-823287
  
 NCBI BlastP on this gene

EGP90905

Query: Architecture Search FASTA input

KB908548 : Setosphaeria turcica Et28A unplaced genomic scaffold SETTUscaffold\_16    Total score: 1.0     Cumulative Blast bit score: 442

Hit cluster cross-links:

Mycgr3G52686 Mycgr3T
  
Location: 0-861

Mycgr3G52686\_Mycgr3T

Mycgr3G102281 Mycgr3
  
Location: 961-1573

Mycgr3G102281\_Mycgr3

Mycgr3G89185 Mycgr3T
  
Location: 1673-2063

Mycgr3G89185\_Mycgr3T

Mycgr3G65725 Mycgr3T
  
Location: 2163-3612

Mycgr3G65725\_Mycgr3T

Mycgr3G102276 Mycgr3
  
Location: 3712-4801

Mycgr3G102276\_Mycgr3

Mycgr3G89189 Mycgr3T
  
Location: 4901-5564

Mycgr3G89189\_Mycgr3T

Mycgr3G52682 Mycgr3T
  
Location: 5664-9231

Mycgr3G52682\_Mycgr3T

Mycgr3G107072 Mycgr3
  
Location: 9331-13279

Mycgr3G107072\_Mycgr3

Mycgr3G34982 Mycgr3T
  
Location: 13379-15116

Mycgr3G34982\_Mycgr3T

Mycgr3G107069 Mycgr3
  
Location: 15216-17097

Mycgr3G107069\_Mycgr3

Mycgr3G32432 Mycgr3T
  
Location: 17197-19042

Mycgr3G32432\_Mycgr3T

Mycgr3G98385 Mycgr3T
  
Location: 19142-19898

Mycgr3G98385\_Mycgr3T

hypothetical protein
  
Accession: EOA88291
  
Location: 885761-887625
  
 NCBI BlastP on this gene

EOA88291

carbohydrate esterase family 5 protein
  
Accession: EOA88292
  
Location: 888019-889154
  
 NCBI BlastP on this gene

EOA88292

hypothetical protein
  
Accession: EOA88293
  
Location: 891896-893788
  
 NCBI BlastP on this gene

EOA88293

hypothetical protein
  
Accession: EOA88294
  
Location: 893940-894185
  
 NCBI BlastP on this gene

EOA88294

hypothetical protein
  
Accession: EOA88295
  
Location: 895588-896261
  
 NCBI BlastP on this gene

EOA88295

hypothetical protein
  
Accession: EOA88296
  
Location: 896793-898838
  
  
**BlastP hit with Mycgr3G107069\_Mycgr3**
  
Percentage identity: 38 %
  
BlastP bit score: 442
  
Sequence coverage: 107 %
  
E-value: 8e-143
  
  
 NCBI BlastP on this gene

EOA88296

Query: Architecture Search FASTA input

GL385398 : Gaeumannomyces graminis var. tritici R3-111a-1 unplaced genomic scaffold supercont2.4    Total score: 1.0     Cumulative Blast bit score: 442

Hit cluster cross-links:

Mycgr3G52686 Mycgr3T
  
Location: 0-861

Mycgr3G52686\_Mycgr3T

Mycgr3G102281 Mycgr3
  
Location: 961-1573

Mycgr3G102281\_Mycgr3

Mycgr3G89185 Mycgr3T
  
Location: 1673-2063

Mycgr3G89185\_Mycgr3T

Mycgr3G65725 Mycgr3T
  
Location: 2163-3612

Mycgr3G65725\_Mycgr3T

Mycgr3G102276 Mycgr3
  
Location: 3712-4801

Mycgr3G102276\_Mycgr3

Mycgr3G89189 Mycgr3T
  
Location: 4901-5564

Mycgr3G89189\_Mycgr3T

Mycgr3G52682 Mycgr3T
  
Location: 5664-9231

Mycgr3G52682\_Mycgr3T

Mycgr3G107072 Mycgr3
  
Location: 9331-13279

Mycgr3G107072\_Mycgr3

Mycgr3G34982 Mycgr3T
  
Location: 13379-15116

Mycgr3G34982\_Mycgr3T

Mycgr3G107069 Mycgr3
  
Location: 15216-17097

Mycgr3G107069\_Mycgr3

Mycgr3G32432 Mycgr3T
  
Location: 17197-19042

Mycgr3G32432\_Mycgr3T

Mycgr3G98385 Mycgr3T
  
Location: 19142-19898

Mycgr3G98385\_Mycgr3T

hypothetical protein
  
Accession: EJT73605
  
Location: 173164-173730
  
 NCBI BlastP on this gene

EJT73605

hypothetical protein
  
Accession: EJT73604
  
Location: 169877-172792
  
 NCBI BlastP on this gene

EJT73604

pentalenene synthase
  
Accession: EJT73603
  
Location: 168117-169508
  
 NCBI BlastP on this gene

EJT73603

hypothetical protein
  
Accession: EJT73602
  
Location: 164499-166525
  
 NCBI BlastP on this gene

EJT73602

ferric reductase
  
Accession: EJT73601
  
Location: 161024-163060
  
  
**BlastP hit with Mycgr3G107069\_Mycgr3**
  
Percentage identity: 38 %
  
BlastP bit score: 442
  
Sequence coverage: 106 %
  
E-value: 9e-143
  
  
 NCBI BlastP on this gene

EJT73601

hypothetical protein
  
Accession: EJT73600
  
Location: 159156-160500
  
 NCBI BlastP on this gene

EJT73600

hypothetical protein
  
Accession: EJT73599
  
Location: 155778-159115
  
 NCBI BlastP on this gene

EJT73599

hypothetical protein
  
Accession: EJT73598
  
Location: 153599-154629
  
 NCBI BlastP on this gene

EJT73598

hypothetical protein
  
Accession: EJT73597
  
Location: 152580-153105
  
 NCBI BlastP on this gene

EJT73597

hypothetical protein
  
Accession: EJT73596
  
Location: 151826-152198
  
 NCBI BlastP on this gene

EJT73596

hypothetical protein
  
Accession: EJT73595
  
Location: 149929-151431
  
 NCBI BlastP on this gene

EJT73595

hypothetical protein
  
Accession: EJT73594
  
Location: 149251-149744
  
 NCBI BlastP on this gene

EJT73594

Query: Architecture Search FASTA input

JH126405 : Cordyceps militaris CM01 unplaced genomic scaffold CCM\_S00007    Total score: 1.0     Cumulative Blast bit score: 441

Hit cluster cross-links:

Mycgr3G52686 Mycgr3T
  
Location: 0-861

Mycgr3G52686\_Mycgr3T

Mycgr3G102281 Mycgr3
  
Location: 961-1573

Mycgr3G102281\_Mycgr3

Mycgr3G89185 Mycgr3T
  
Location: 1673-2063

Mycgr3G89185\_Mycgr3T

Mycgr3G65725 Mycgr3T
  
Location: 2163-3612

Mycgr3G65725\_Mycgr3T

Mycgr3G102276 Mycgr3
  
Location: 3712-4801

Mycgr3G102276\_Mycgr3

Mycgr3G89189 Mycgr3T
  
Location: 4901-5564

Mycgr3G89189\_Mycgr3T

Mycgr3G52682 Mycgr3T
  
Location: 5664-9231

Mycgr3G52682\_Mycgr3T

Mycgr3G107072 Mycgr3
  
Location: 9331-13279

Mycgr3G107072\_Mycgr3

Mycgr3G34982 Mycgr3T
  
Location: 13379-15116

Mycgr3G34982\_Mycgr3T

Mycgr3G107069 Mycgr3
  
Location: 15216-17097

Mycgr3G107069\_Mycgr3

Mycgr3G32432 Mycgr3T
  
Location: 17197-19042

Mycgr3G32432\_Mycgr3T

Mycgr3G98385 Mycgr3T
  
Location: 19142-19898

Mycgr3G98385\_Mycgr3T

zinc-binding alcohol dehydrogenase, putative
  
Accession: EGX88899
  
Location: 2330487-2331649
  
  
**BlastP hit with Mycgr3G102276\_Mycgr3**
  
Percentage identity: 61 %
  
BlastP bit score: 441
  
Sequence coverage: 99 %
  
E-value: 1e-150
  
  
 NCBI BlastP on this gene

EGX88899

hypothetical protein
  
Accession: EGX88898
  
Location: 2323352-2325603
  
 NCBI BlastP on this gene

EGX88898

ankyrin repeat-containing domain
  
Accession: EGX88897
  
Location: 2316564-2318828
  
 NCBI BlastP on this gene

EGX88897

Query: Architecture Search FASTA input

DS027694 : Neosartorya fischeri NRRL 181 1099437636262 genomic scaffold    Total score: 1.0     Cumulative Blast bit score: 441

Hit cluster cross-links:

Mycgr3G52686 Mycgr3T
  
Location: 0-861

Mycgr3G52686\_Mycgr3T

Mycgr3G102281 Mycgr3
  
Location: 961-1573

Mycgr3G102281\_Mycgr3

Mycgr3G89185 Mycgr3T
  
Location: 1673-2063

Mycgr3G89185\_Mycgr3T

Mycgr3G65725 Mycgr3T
  
Location: 2163-3612

Mycgr3G65725\_Mycgr3T

Mycgr3G102276 Mycgr3
  
Location: 3712-4801

Mycgr3G102276\_Mycgr3

Mycgr3G89189 Mycgr3T
  
Location: 4901-5564

Mycgr3G89189\_Mycgr3T

Mycgr3G52682 Mycgr3T
  
Location: 5664-9231

Mycgr3G52682\_Mycgr3T

Mycgr3G107072 Mycgr3
  
Location: 9331-13279

Mycgr3G107072\_Mycgr3

Mycgr3G34982 Mycgr3T
  
Location: 13379-15116

Mycgr3G34982\_Mycgr3T

Mycgr3G107069 Mycgr3
  
Location: 15216-17097

Mycgr3G107069\_Mycgr3

Mycgr3G32432 Mycgr3T
  
Location: 17197-19042

Mycgr3G32432\_Mycgr3T

Mycgr3G98385 Mycgr3T
  
Location: 19142-19898

Mycgr3G98385\_Mycgr3T

conserved hypothetical protein
  
Accession: EAW19996
  
Location: 672504-673418
  
 NCBI BlastP on this gene

EAW19996

MFS transporter, putative
  
Accession: EAW19995
  
Location: 668057-671408
  
 NCBI BlastP on this gene

EAW19995

conserved hypothetical protein
  
Accession: EAW19994
  
Location: 666403-667028
  
 NCBI BlastP on this gene

EAW19994

C-4 methyl sterol oxidase, putative
  
Accession: EAW19993
  
Location: 664750-665750
  
 NCBI BlastP on this gene

EAW19993

conserved hypothetical protein
  
Accession: EAW19992
  
Location: 663956-664275
  
 NCBI BlastP on this gene

EAW19992

hypothetical protein
  
Accession: EAW19991
  
Location: 662754-663380
  
 NCBI BlastP on this gene

EAW19991

zinc-binding alcohol dehydrogenase, putative
  
Accession: EAW19990
  
Location: 659933-661126
  
  
**BlastP hit with Mycgr3G102276\_Mycgr3**
  
Percentage identity: 63 %
  
BlastP bit score: 441
  
Sequence coverage: 99 %
  
E-value: 1e-150
  
  
 NCBI BlastP on this gene

EAW19990

conserved hypothetical protein
  
Accession: EAW19989
  
Location: 659055-659883
  
 NCBI BlastP on this gene

EAW19989

UbiA prenyltransferase family protein
  
Accession: EAW19988
  
Location: 656971-658091
  
 NCBI BlastP on this gene

EAW19988

geranylgeranyl pyrophosphate synthetase AtmG, putative
  
Accession: EAW19987
  
Location: 654508-655741
  
 NCBI BlastP on this gene

EAW19987

conserved hypothetical protein
  
Accession: EAW19986
  
Location: 652832-653618
  
 NCBI BlastP on this gene

EAW19986

FAD binding domain protein
  
Accession: EAW19985
  
Location: 650224-651821
  
 NCBI BlastP on this gene

EAW19985

conserved hypothetical protein
  
Accession: EAW19984
  
Location: 648053-649412
  
 NCBI BlastP on this gene

EAW19984

Query: Architecture Search FASTA input

CM001197 : Mycosphaerella graminicola IPO323 chromosome 2    Total score: 1.0     Cumulative Blast bit score: 441

Hit cluster cross-links:

Mycgr3G52686 Mycgr3T
  
Location: 0-861

Mycgr3G52686\_Mycgr3T

Mycgr3G102281 Mycgr3
  
Location: 961-1573

Mycgr3G102281\_Mycgr3

Mycgr3G89185 Mycgr3T
  
Location: 1673-2063

Mycgr3G89185\_Mycgr3T

Mycgr3G65725 Mycgr3T
  
Location: 2163-3612

Mycgr3G65725\_Mycgr3T

Mycgr3G102276 Mycgr3
  
Location: 3712-4801

Mycgr3G102276\_Mycgr3

Mycgr3G89189 Mycgr3T
  
Location: 4901-5564

Mycgr3G89189\_Mycgr3T

Mycgr3G52682 Mycgr3T
  
Location: 5664-9231

Mycgr3G52682\_Mycgr3T

Mycgr3G107072 Mycgr3
  
Location: 9331-13279

Mycgr3G107072\_Mycgr3

Mycgr3G34982 Mycgr3T
  
Location: 13379-15116

Mycgr3G34982\_Mycgr3T

Mycgr3G107069 Mycgr3
  
Location: 15216-17097

Mycgr3G107069\_Mycgr3

Mycgr3G32432 Mycgr3T
  
Location: 17197-19042

Mycgr3G32432\_Mycgr3T

Mycgr3G98385 Mycgr3T
  
Location: 19142-19898

Mycgr3G98385\_Mycgr3T

hypothetical protein
  
Accession: EGP90796
  
Location: 89430-90215
  
 NCBI BlastP on this gene

EGP90796

hypothetical protein
  
Accession: EGP90795
  
Location: 90713-92091
  
 NCBI BlastP on this gene

EGP90795

hypothetical protein
  
Accession: EGP89673
  
Location: 93145-95400
  
 NCBI BlastP on this gene

EGP89673

hypothetical protein
  
Accession: EGP89674
  
Location: 95885-97982
  
 NCBI BlastP on this gene

EGP89674

hypothetical protein
  
Accession: EGP90794
  
Location: 98374-99028
  
 NCBI BlastP on this gene

EGP90794

putative FRE ferric reductase-like transmembrane component
  
Accession: EGP89675
  
Location: 99609-101786
  
  
**BlastP hit with Mycgr3G107069\_Mycgr3**
  
Percentage identity: 37 %
  
BlastP bit score: 441
  
Sequence coverage: 109 %
  
E-value: 3e-142
  
  
 NCBI BlastP on this gene

EGP89675

putative ABC transporter
  
Accession: EGP90793
  
Location: 103528-107876
  
 NCBI BlastP on this gene

EGP90793

Query: Architecture Search FASTA input

KE145356 : Glarea lozoyensis ATCC 20868 chromosome Unknown GLAREA13    Total score: 1.0     Cumulative Blast bit score: 438

Hit cluster cross-links:

Mycgr3G52686 Mycgr3T
  
Location: 0-861

Mycgr3G52686\_Mycgr3T

Mycgr3G102281 Mycgr3
  
Location: 961-1573

Mycgr3G102281\_Mycgr3

Mycgr3G89185 Mycgr3T
  
Location: 1673-2063

Mycgr3G89185\_Mycgr3T

Mycgr3G65725 Mycgr3T
  
Location: 2163-3612

Mycgr3G65725\_Mycgr3T

Mycgr3G102276 Mycgr3
  
Location: 3712-4801

Mycgr3G102276\_Mycgr3

Mycgr3G89189 Mycgr3T
  
Location: 4901-5564

Mycgr3G89189\_Mycgr3T

Mycgr3G52682 Mycgr3T
  
Location: 5664-9231

Mycgr3G52682\_Mycgr3T

Mycgr3G107072 Mycgr3
  
Location: 9331-13279

Mycgr3G107072\_Mycgr3

Mycgr3G34982 Mycgr3T
  
Location: 13379-15116

Mycgr3G34982\_Mycgr3T

Mycgr3G107069 Mycgr3
  
Location: 15216-17097

Mycgr3G107069\_Mycgr3

Mycgr3G32432 Mycgr3T
  
Location: 17197-19042

Mycgr3G32432\_Mycgr3T

Mycgr3G98385 Mycgr3T
  
Location: 19142-19898

Mycgr3G98385\_Mycgr3T

GroES-like protein
  
Accession: EPE34719
  
Location: 1389878-1391072
  
  
**BlastP hit with Mycgr3G102276\_Mycgr3**
  
Percentage identity: 62 %
  
BlastP bit score: 438
  
Sequence coverage: 98 %
  
E-value: 9e-150
  
  
 NCBI BlastP on this gene

EPE34719

beta and beta-prime subunits of DNA dependent RNA-polymerase
  
Accession: EPE34718
  
Location: 1385305-1389205
  
 NCBI BlastP on this gene

EPE34718

hypothetical protein
  
Accession: EPE34717
  
Location: 1384442-1384762
  
 NCBI BlastP on this gene

EPE34717

hypothetical protein
  
Accession: EPE34716
  
Location: 1383346-1384114
  
 NCBI BlastP on this gene

EPE34716

alpha/beta-Hydrolase
  
Accession: EPE34715
  
Location: 1381648-1382826
  
 NCBI BlastP on this gene

EPE34715

hypothetical protein
  
Accession: EPE34714
  
Location: 1377126-1380394
  
 NCBI BlastP on this gene

EPE34714

Query: Architecture Search FASTA input

KB446558 : Pseudocercospora fijiensis CIRAD86 unplaced genomic scaffold MYCFIscaffold\_4    Total score: 1.0     Cumulative Blast bit score: 438

Hit cluster cross-links:

Mycgr3G52686 Mycgr3T
  
Location: 0-861

Mycgr3G52686\_Mycgr3T

Mycgr3G102281 Mycgr3
  
Location: 961-1573

Mycgr3G102281\_Mycgr3

Mycgr3G89185 Mycgr3T
  
Location: 1673-2063

Mycgr3G89185\_Mycgr3T

Mycgr3G65725 Mycgr3T
  
Location: 2163-3612

Mycgr3G65725\_Mycgr3T

Mycgr3G102276 Mycgr3
  
Location: 3712-4801

Mycgr3G102276\_Mycgr3

Mycgr3G89189 Mycgr3T
  
Location: 4901-5564

Mycgr3G89189\_Mycgr3T

Mycgr3G52682 Mycgr3T
  
Location: 5664-9231

Mycgr3G52682\_Mycgr3T

Mycgr3G107072 Mycgr3
  
Location: 9331-13279

Mycgr3G107072\_Mycgr3

Mycgr3G34982 Mycgr3T
  
Location: 13379-15116

Mycgr3G34982\_Mycgr3T

Mycgr3G107069 Mycgr3
  
Location: 15216-17097

Mycgr3G107069\_Mycgr3

Mycgr3G32432 Mycgr3T
  
Location: 17197-19042

Mycgr3G32432\_Mycgr3T

Mycgr3G98385 Mycgr3T
  
Location: 19142-19898

Mycgr3G98385\_Mycgr3T

hypothetical protein
  
Accession: EME83119
  
Location: 2737050-2737988
  
 NCBI BlastP on this gene

EME83119

hypothetical protein
  
Accession: EME83118
  
Location: 2733466-2736458
  
 NCBI BlastP on this gene

EME83118

hypothetical protein
  
Accession: EME83117
  
Location: 2730736-2732218
  
 NCBI BlastP on this gene

EME83117

glycoside hydrolase family 28 protein
  
Accession: EME83116
  
Location: 2728877-2730277
  
 NCBI BlastP on this gene

EME83116

hypothetical protein
  
Accession: EME83115
  
Location: 2725659-2726833
  
  
**BlastP hit with Mycgr3G102276\_Mycgr3**
  
Percentage identity: 63 %
  
BlastP bit score: 438
  
Sequence coverage: 98 %
  
E-value: 1e-149
  
  
 NCBI BlastP on this gene

EME83115

hypothetical protein
  
Accession: EME83114
  
Location: 2723335-2725104
  
 NCBI BlastP on this gene

EME83114

hypothetical protein
  
Accession: EME83113
  
Location: 2721597-2722643
  
 NCBI BlastP on this gene

EME83113

hypothetical protein
  
Accession: EME83112
  
Location: 2720512-2720913
  
 NCBI BlastP on this gene

EME83112

hypothetical protein
  
Accession: EME83111
  
Location: 2719308-2719807
  
 NCBI BlastP on this gene

EME83111

hypothetical protein
  
Accession: EME83110
  
Location: 2716179-2717769
  
 NCBI BlastP on this gene

EME83110

hypothetical protein
  
Accession: EME83109
  
Location: 2714894-2715616
  
 NCBI BlastP on this gene

EME83109

hypothetical protein
  
Accession: EME83108
  
Location: 2710645-2714664
  
 NCBI BlastP on this gene

EME83108

Query: Architecture Search FASTA input

DS990637 : Ajellomyces capsulatus H88 unplaced genomic scaffold supercont1.2    Total score: 1.0     Cumulative Blast bit score: 436

Hit cluster cross-links:

Mycgr3G52686 Mycgr3T
  
Location: 0-861

Mycgr3G52686\_Mycgr3T

Mycgr3G102281 Mycgr3
  
Location: 961-1573

Mycgr3G102281\_Mycgr3

Mycgr3G89185 Mycgr3T
  
Location: 1673-2063

Mycgr3G89185\_Mycgr3T

Mycgr3G65725 Mycgr3T
  
Location: 2163-3612

Mycgr3G65725\_Mycgr3T

Mycgr3G102276 Mycgr3
  
Location: 3712-4801

Mycgr3G102276\_Mycgr3

Mycgr3G89189 Mycgr3T
  
Location: 4901-5564

Mycgr3G89189\_Mycgr3T

Mycgr3G52682 Mycgr3T
  
Location: 5664-9231

Mycgr3G52682\_Mycgr3T

Mycgr3G107072 Mycgr3
  
Location: 9331-13279

Mycgr3G107072\_Mycgr3

Mycgr3G34982 Mycgr3T
  
Location: 13379-15116

Mycgr3G34982\_Mycgr3T

Mycgr3G107069 Mycgr3
  
Location: 15216-17097

Mycgr3G107069\_Mycgr3

Mycgr3G32432 Mycgr3T
  
Location: 17197-19042

Mycgr3G32432\_Mycgr3T

Mycgr3G98385 Mycgr3T
  
Location: 19142-19898

Mycgr3G98385\_Mycgr3T

glycerol-3-phosphate O-acyltransferase
  
Accession: EGC43177
  
Location: 2464383-2466916
  
 NCBI BlastP on this gene

EGC43177

dehydrodolichyl diphosphate synthetase
  
Accession: EGC43178
  
Location: 2468623-2470066
  
 NCBI BlastP on this gene

EGC43178

saccharopine dehydrogenase
  
Accession: EGC43179
  
Location: 2470740-2472777
  
 NCBI BlastP on this gene

EGC43179

cupin domain-containing protein
  
Accession: EGC43180
  
Location: 2473292-2473864
  
 NCBI BlastP on this gene

EGC43180

glucose-methanol-choline oxidoreductase:GMC oxidoreductase
  
Accession: EGC43181
  
Location: 2474861-2477167
  
  
**BlastP hit with Mycgr3G34982\_Mycgr3T**
  
Percentage identity: 42 %
  
BlastP bit score: 436
  
Sequence coverage: 94 %
  
E-value: 1e-142
  
  
 NCBI BlastP on this gene

EGC43181

Query: Architecture Search FASTA input

KB644408 : Penicillium oxalicum 114-2 unplaced genomic scaffold scaffold\_1    Total score: 1.0     Cumulative Blast bit score: 434

Hit cluster cross-links:

Mycgr3G52686 Mycgr3T
  
Location: 0-861

Mycgr3G52686\_Mycgr3T

Mycgr3G102281 Mycgr3
  
Location: 961-1573

Mycgr3G102281\_Mycgr3

Mycgr3G89185 Mycgr3T
  
Location: 1673-2063

Mycgr3G89185\_Mycgr3T

Mycgr3G65725 Mycgr3T
  
Location: 2163-3612

Mycgr3G65725\_Mycgr3T

Mycgr3G102276 Mycgr3
  
Location: 3712-4801

Mycgr3G102276\_Mycgr3

Mycgr3G89189 Mycgr3T
  
Location: 4901-5564

Mycgr3G89189\_Mycgr3T

Mycgr3G52682 Mycgr3T
  
Location: 5664-9231

Mycgr3G52682\_Mycgr3T

Mycgr3G107072 Mycgr3
  
Location: 9331-13279

Mycgr3G107072\_Mycgr3

Mycgr3G34982 Mycgr3T
  
Location: 13379-15116

Mycgr3G34982\_Mycgr3T

Mycgr3G107069 Mycgr3
  
Location: 15216-17097

Mycgr3G107069\_Mycgr3

Mycgr3G32432 Mycgr3T
  
Location: 17197-19042

Mycgr3G32432\_Mycgr3T

Mycgr3G98385 Mycgr3T
  
Location: 19142-19898

Mycgr3G98385\_Mycgr3T

hypothetical protein
  
Accession: EPS26264
  
Location: 3643693-3645393
  
 NCBI BlastP on this gene

EPS26264

alpha-amylase Amy13A
  
Accession: EPS26265
  
Location: 3645573-3647950
  
 NCBI BlastP on this gene

EPS26265

hypothetical protein
  
Accession: EPS26266
  
Location: 3654136-3655289
  
  
**BlastP hit with Mycgr3G102276\_Mycgr3**
  
Percentage identity: 62 %
  
BlastP bit score: 435
  
Sequence coverage: 99 %
  
E-value: 2e-148
  
  
 NCBI BlastP on this gene

EPS26266

Query: Architecture Search FASTA input

DS499600 : Aspergillus fumigatus A1163 scf\_000007 genomic scaffold    Total score: 1.0     Cumulative Blast bit score: 434

Hit cluster cross-links:

Mycgr3G52686 Mycgr3T
  
Location: 0-861

Mycgr3G52686\_Mycgr3T

Mycgr3G102281 Mycgr3
  
Location: 961-1573

Mycgr3G102281\_Mycgr3

Mycgr3G89185 Mycgr3T
  
Location: 1673-2063

Mycgr3G89185\_Mycgr3T

Mycgr3G65725 Mycgr3T
  
Location: 2163-3612

Mycgr3G65725\_Mycgr3T

Mycgr3G102276 Mycgr3
  
Location: 3712-4801

Mycgr3G102276\_Mycgr3

Mycgr3G89189 Mycgr3T
  
Location: 4901-5564

Mycgr3G89189\_Mycgr3T

Mycgr3G52682 Mycgr3T
  
Location: 5664-9231

Mycgr3G52682\_Mycgr3T

Mycgr3G107072 Mycgr3
  
Location: 9331-13279

Mycgr3G107072\_Mycgr3

Mycgr3G34982 Mycgr3T
  
Location: 13379-15116

Mycgr3G34982\_Mycgr3T

Mycgr3G107069 Mycgr3
  
Location: 15216-17097

Mycgr3G107069\_Mycgr3

Mycgr3G32432 Mycgr3T
  
Location: 17197-19042

Mycgr3G32432\_Mycgr3T

Mycgr3G98385 Mycgr3T
  
Location: 19142-19898

Mycgr3G98385\_Mycgr3T

conserved hypothetical protein
  
Accession: EDP48961
  
Location: 1274403-1275319
  
 NCBI BlastP on this gene

EDP48961

trihydroxytoluene oxygenase
  
Accession: EDP48962
  
Location: 1276364-1277310
  
 NCBI BlastP on this gene

EDP48962

MFS transporter, putative
  
Accession: EDP48963
  
Location: 1278082-1279698
  
 NCBI BlastP on this gene

EDP48963

conserved hypothetical protein
  
Accession: EDP48964
  
Location: 1280702-1281401
  
 NCBI BlastP on this gene

EDP48964

C-4 methyl sterol oxidase, putative
  
Accession: EDP48965
  
Location: 1282062-1283062
  
 NCBI BlastP on this gene

EDP48965

zinc-binding alcohol dehydrogenase, putative
  
Accession: EDP48966
  
Location: 1286825-1288017
  
  
**BlastP hit with Mycgr3G102276\_Mycgr3**
  
Percentage identity: 62 %
  
BlastP bit score: 435
  
Sequence coverage: 99 %
  
E-value: 2e-148
  
  
 NCBI BlastP on this gene

EDP48966

Query: Architecture Search FASTA input

GL377303 : Schizophyllum commune H4-8 unplaced genomic scaffold SCHCOscaffold\_2    Total score: 1.0     Cumulative Blast bit score: 434

Hit cluster cross-links:

Mycgr3G52686 Mycgr3T
  
Location: 0-861

Mycgr3G52686\_Mycgr3T

Mycgr3G102281 Mycgr3
  
Location: 961-1573

Mycgr3G102281\_Mycgr3

Mycgr3G89185 Mycgr3T
  
Location: 1673-2063

Mycgr3G89185\_Mycgr3T

Mycgr3G65725 Mycgr3T
  
Location: 2163-3612

Mycgr3G65725\_Mycgr3T

Mycgr3G102276 Mycgr3
  
Location: 3712-4801

Mycgr3G102276\_Mycgr3

Mycgr3G89189 Mycgr3T
  
Location: 4901-5564

Mycgr3G89189\_Mycgr3T

Mycgr3G52682 Mycgr3T
  
Location: 5664-9231

Mycgr3G52682\_Mycgr3T

Mycgr3G107072 Mycgr3
  
Location: 9331-13279

Mycgr3G107072\_Mycgr3

Mycgr3G34982 Mycgr3T
  
Location: 13379-15116

Mycgr3G34982\_Mycgr3T

Mycgr3G107069 Mycgr3
  
Location: 15216-17097

Mycgr3G107069\_Mycgr3

Mycgr3G32432 Mycgr3T
  
Location: 17197-19042

Mycgr3G32432\_Mycgr3T

Mycgr3G98385 Mycgr3T
  
Location: 19142-19898

Mycgr3G98385\_Mycgr3T

hypothetical protein
  
Accession: EFJ00610
  
Location: 4322737-4324776
  
 NCBI BlastP on this gene

EFJ00610

hypothetical protein
  
Accession: EFJ01443
  
Location: 4325488-4326234
  
 NCBI BlastP on this gene

EFJ01443

hypothetical protein
  
Accession: EFJ00611
  
Location: 4326893-4328421
  
 NCBI BlastP on this gene

EFJ00611

hypothetical protein
  
Accession: EFJ00612
  
Location: 4328996-4330626
  
 NCBI BlastP on this gene

EFJ00612

hypothetical protein
  
Accession: EFJ00613
  
Location: 4331327-4332598
  
 NCBI BlastP on this gene

EFJ00613

hypothetical protein
  
Accession: EFJ00614
  
Location: 4333196-4335214
  
  
**BlastP hit with Mycgr3G32432\_Mycgr3T**
  
Percentage identity: 32 %
  
BlastP bit score: 202
  
Sequence coverage: 75 %
  
E-value: 7e-54
  
  
 NCBI BlastP on this gene

EFJ00614

hypothetical protein
  
Accession: EFJ00615
  
Location: 4336342-4338390
  
  
**BlastP hit with Mycgr3G32432\_Mycgr3T**
  
Percentage identity: 33 %
  
BlastP bit score: 232
  
Sequence coverage: 79 %
  
E-value: 9e-65
  
  
 NCBI BlastP on this gene

EFJ00615

expressed protein
  
Accession: EFJ00616
  
Location: 4339533-4340219
  
 NCBI BlastP on this gene

EFJ00616

hypothetical protein
  
Accession: EFJ00617
  
Location: 4342854-4343597
  
 NCBI BlastP on this gene

EFJ00617

hypothetical protein
  
Accession: EFJ01444
  
Location: 4343666-4344545
  
 NCBI BlastP on this gene

EFJ01444

hypothetical protein
  
Accession: EFJ00618
  
Location: 4345403-4346160
  
 NCBI BlastP on this gene

EFJ00618

glycoside hydrolase family 5 protein
  
Accession: EFJ00619
  
Location: 4346936-4349977
  
 NCBI BlastP on this gene

EFJ00619

Query: Architecture Search FASTA input

AM920431 : Penicillium chrysogenum Wisconsin 54-1255 complete genome, contig Pc00c16.    Total score: 1.0     Cumulative Blast bit score: 434

Hit cluster cross-links:

Mycgr3G52686 Mycgr3T
  
Location: 0-861

Mycgr3G52686\_Mycgr3T

Mycgr3G102281 Mycgr3
  
Location: 961-1573

Mycgr3G102281\_Mycgr3

Mycgr3G89185 Mycgr3T
  
Location: 1673-2063

Mycgr3G89185\_Mycgr3T

Mycgr3G65725 Mycgr3T
  
Location: 2163-3612

Mycgr3G65725\_Mycgr3T

Mycgr3G102276 Mycgr3
  
Location: 3712-4801

Mycgr3G102276\_Mycgr3

Mycgr3G89189 Mycgr3T
  
Location: 4901-5564

Mycgr3G89189\_Mycgr3T

Mycgr3G52682 Mycgr3T
  
Location: 5664-9231

Mycgr3G52682\_Mycgr3T

Mycgr3G107072 Mycgr3
  
Location: 9331-13279

Mycgr3G107072\_Mycgr3

Mycgr3G34982 Mycgr3T
  
Location: 13379-15116

Mycgr3G34982\_Mycgr3T

Mycgr3G107069 Mycgr3
  
Location: 15216-17097

Mycgr3G107069\_Mycgr3

Mycgr3G32432 Mycgr3T
  
Location: 17197-19042

Mycgr3G32432\_Mycgr3T

Mycgr3G98385 Mycgr3T
  
Location: 19142-19898

Mycgr3G98385\_Mycgr3T

not annotated
  
Accession: CAP92721
  
Location: 109350-111423
  
 NCBI BlastP on this gene

Pc16g00510

not annotated
  
Accession: CAP92720
  
Location: 107077-108823
  
 NCBI BlastP on this gene

Pc16g00500

unnamed
  
Accession: CAP92719
  
Location: 105607-106396
  
 NCBI BlastP on this gene

Pc16g00490

not annotated
  
Accession: CAP92718
  
Location: 104779-105400
  
 NCBI BlastP on this gene

Pc16g00480

unnamed
  
Accession: CAP92717
  
Location: 103308-104356
  
 NCBI BlastP on this gene

Pc16g00470

hypothetical protein
  
Accession: CAP92716
  
Location: 101051-102882
  
 NCBI BlastP on this gene

Pc16g00460

not annotated
  
Accession: CAP92715
  
Location: 99550-100697
  
  
**BlastP hit with Mycgr3G102276\_Mycgr3**
  
Percentage identity: 62 %
  
BlastP bit score: 434
  
Sequence coverage: 99 %
  
E-value: 7e-148
  
  
 NCBI BlastP on this gene

Pc16g00450

not annotated
  
Accession: CAP92714
  
Location: 97348-98151
  
 NCBI BlastP on this gene

Pc16g00440

not annotated
  
Accession: CAP92713
  
Location: 95191-96138
  
 NCBI BlastP on this gene

Pc16g00430

hypothetical protein
  
Accession: CAP92712
  
Location: 93753-94586
  
 NCBI BlastP on this gene

Pc16g00420

not annotated
  
Accession: Pc16g00410
  
Location: 90386-93205
  
 NCBI BlastP on this gene

Pc16g00410

unnamed
  
Accession: CAP92710
  
Location: 88167-89894
  
 NCBI BlastP on this gene

Pc16g00400

not annotated
  
Accession: CAP92709
  
Location: 86693-87762
  
 NCBI BlastP on this gene

Pc16g00390

Query: Architecture Search FASTA input

GL988032 : Chaetomium thermophilum var. thermophilum DSM 1495 unplaced genomic scaffold scf7180000...    Total score: 1.0     Cumulative Blast bit score: 432

Hit cluster cross-links:

Mycgr3G52686 Mycgr3T
  
Location: 0-861

Mycgr3G52686\_Mycgr3T

Mycgr3G102281 Mycgr3
  
Location: 961-1573

Mycgr3G102281\_Mycgr3

Mycgr3G89185 Mycgr3T
  
Location: 1673-2063

Mycgr3G89185\_Mycgr3T

Mycgr3G65725 Mycgr3T
  
Location: 2163-3612

Mycgr3G65725\_Mycgr3T

Mycgr3G102276 Mycgr3
  
Location: 3712-4801

Mycgr3G102276\_Mycgr3

Mycgr3G89189 Mycgr3T
  
Location: 4901-5564

Mycgr3G89189\_Mycgr3T

Mycgr3G52682 Mycgr3T
  
Location: 5664-9231

Mycgr3G52682\_Mycgr3T

Mycgr3G107072 Mycgr3
  
Location: 9331-13279

Mycgr3G107072\_Mycgr3

Mycgr3G34982 Mycgr3T
  
Location: 13379-15116

Mycgr3G34982\_Mycgr3T

Mycgr3G107069 Mycgr3
  
Location: 15216-17097

Mycgr3G107069\_Mycgr3

Mycgr3G32432 Mycgr3T
  
Location: 17197-19042

Mycgr3G32432\_Mycgr3T

Mycgr3G98385 Mycgr3T
  
Location: 19142-19898

Mycgr3G98385\_Mycgr3T

hypothetical protein
  
Accession: EGS23685
  
Location: 1301806-1303611
  
 NCBI BlastP on this gene

EGS23685

hypothetical protein
  
Accession: EGS23686
  
Location: 1305702-1306968
  
 NCBI BlastP on this gene

EGS23686

dehydrogenase-like protein
  
Accession: EGS23687
  
Location: 1308553-1310234
  
 NCBI BlastP on this gene

EGS23687

putative high affinity copper protein
  
Accession: EGS23688
  
Location: 1310379-1311040
  
 NCBI BlastP on this gene

EGS23688

putative FAD binding protein
  
Accession: EGS23689
  
Location: 1312431-1314353
  
  
**BlastP hit with Mycgr3G107069\_Mycgr3**
  
Percentage identity: 39 %
  
BlastP bit score: 433
  
Sequence coverage: 102 %
  
E-value: 7e-140
  
  
 NCBI BlastP on this gene

EGS23689

Query: Architecture Search FASTA input

KB446537 : Dothistroma septosporum NZE10 unplaced genomic scaffold DOTSEscaffold\_3    Total score: 1.0     Cumulative Blast bit score: 430

Hit cluster cross-links:

Mycgr3G52686 Mycgr3T
  
Location: 0-861

Mycgr3G52686\_Mycgr3T

Mycgr3G102281 Mycgr3
  
Location: 961-1573

Mycgr3G102281\_Mycgr3

Mycgr3G89185 Mycgr3T
  
Location: 1673-2063

Mycgr3G89185\_Mycgr3T

Mycgr3G65725 Mycgr3T
  
Location: 2163-3612

Mycgr3G65725\_Mycgr3T

Mycgr3G102276 Mycgr3
  
Location: 3712-4801

Mycgr3G102276\_Mycgr3

Mycgr3G89189 Mycgr3T
  
Location: 4901-5564

Mycgr3G89189\_Mycgr3T

Mycgr3G52682 Mycgr3T
  
Location: 5664-9231

Mycgr3G52682\_Mycgr3T

Mycgr3G107072 Mycgr3
  
Location: 9331-13279

Mycgr3G107072\_Mycgr3

Mycgr3G34982 Mycgr3T
  
Location: 13379-15116

Mycgr3G34982\_Mycgr3T

Mycgr3G107069 Mycgr3
  
Location: 15216-17097

Mycgr3G107069\_Mycgr3

Mycgr3G32432 Mycgr3T
  
Location: 17197-19042

Mycgr3G32432\_Mycgr3T

Mycgr3G98385 Mycgr3T
  
Location: 19142-19898

Mycgr3G98385\_Mycgr3T

hypothetical protein
  
Accession: EME46885
  
Location: 2584198-2585443
  
  
**BlastP hit with Mycgr3G102276\_Mycgr3**
  
Percentage identity: 61 %
  
BlastP bit score: 430
  
Sequence coverage: 97 %
  
E-value: 3e-146
  
  
 NCBI BlastP on this gene

EME46885

hypothetical protein
  
Accession: EME46884
  
Location: 2582971-2583138
  
 NCBI BlastP on this gene

EME46884

hypothetical protein
  
Accession: EME46883
  
Location: 2582588-2582911
  
 NCBI BlastP on this gene

EME46883

hypothetical protein
  
Accession: EME46882
  
Location: 2580682-2580990
  
 NCBI BlastP on this gene

EME46882

hypothetical protein
  
Accession: EME46881
  
Location: 2578352-2580235
  
 NCBI BlastP on this gene

EME46881

glycosyltransferase family 71 protein
  
Accession: EME46880
  
Location: 2575317-2576795
  
 NCBI BlastP on this gene

EME46880

hypothetical protein
  
Accession: EME46879
  
Location: 2574092-2574823
  
 NCBI BlastP on this gene

EME46879

hypothetical protein
  
Accession: EME46878
  
Location: 2571443-2572529
  
 NCBI BlastP on this gene

EME46878

Query: Architecture Search FASTA input

AP012319 : Actinoplanes missouriensis 431 DNA    Total score: 1.0     Cumulative Blast bit score: 428

Hit cluster cross-links:

Mycgr3G52686 Mycgr3T
  
Location: 0-861

Mycgr3G52686\_Mycgr3T

Mycgr3G102281 Mycgr3
  
Location: 961-1573

Mycgr3G102281\_Mycgr3

Mycgr3G89185 Mycgr3T
  
Location: 1673-2063

Mycgr3G89185\_Mycgr3T

Mycgr3G65725 Mycgr3T
  
Location: 2163-3612

Mycgr3G65725\_Mycgr3T

Mycgr3G102276 Mycgr3
  
Location: 3712-4801

Mycgr3G102276\_Mycgr3

Mycgr3G89189 Mycgr3T
  
Location: 4901-5564

Mycgr3G89189\_Mycgr3T

Mycgr3G52682 Mycgr3T
  
Location: 5664-9231

Mycgr3G52682\_Mycgr3T

Mycgr3G107072 Mycgr3
  
Location: 9331-13279

Mycgr3G107072\_Mycgr3

Mycgr3G34982 Mycgr3T
  
Location: 13379-15116

Mycgr3G34982\_Mycgr3T

Mycgr3G107069 Mycgr3
  
Location: 15216-17097

Mycgr3G107069\_Mycgr3

Mycgr3G32432 Mycgr3T
  
Location: 17197-19042

Mycgr3G32432\_Mycgr3T

Mycgr3G98385 Mycgr3T
  
Location: 19142-19898

Mycgr3G98385\_Mycgr3T

putative transcriptional regulator
  
Accession: BAL88450
  
Location: 3391646-3392929
  
 NCBI BlastP on this gene

AMIS\_32300

hypothetical protein
  
Accession: BAL88451
  
Location: 3393157-3393735
  
 NCBI BlastP on this gene

AMIS\_32310

putative NRPS
  
Accession: BAL88452
  
Location: 3394007-3402589
  
 NCBI BlastP on this gene

AMIS\_32320

putative NRPS
  
Accession: BAL88453
  
Location: 3402586-3407013
  
  
**BlastP hit with Mycgr3G107072\_Mycgr3**
  
Percentage identity: 33 %
  
BlastP bit score: 428
  
Sequence coverage: 73 %
  
E-value: 3e-123
  
  
 NCBI BlastP on this gene

AMIS\_32330

hypothetical protein
  
Accession: BAL88454
  
Location: 3407010-3407996
  
 NCBI BlastP on this gene

AMIS\_32340

putative O-methyltransferase
  
Accession: BAL88455
  
Location: 3407993-3408667
  
 NCBI BlastP on this gene

AMIS\_32350

putative MbtH-like protein
  
Accession: BAL88456
  
Location: 3408695-3408904
  
 NCBI BlastP on this gene

AMIS\_32360

hypothetical protein
  
Accession: BAL88457
  
Location: 3408938-3410416
  
 NCBI BlastP on this gene

AMIS\_32370

putative M28-family peptidase
  
Accession: BAL88458
  
Location: 3410441-3412711
  
 NCBI BlastP on this gene

AMIS\_32380

putative short-chain dehydrogenase
  
Accession: BAL88459
  
Location: 3412729-3413604
  
 NCBI BlastP on this gene

AMIS\_32390

putative transcriptional regulator
  
Accession: BAL88460
  
Location: 3413692-3415116
  
 NCBI BlastP on this gene

AMIS\_32400

hypothetical protein
  
Accession: BAL88461
  
Location: 3415297-3415977
  
 NCBI BlastP on this gene

AMIS\_32410

putative NRPS-related enzyme
  
Accession: BAL88462
  
Location: 3416555-3419143
  
 NCBI BlastP on this gene

AMIS\_32420

Query: Architecture Search FASTA input

JH795880 : Dacryopinax sp. DJM-731 SS1 chromosome Unknown DACRYscaffold\_26    Total score: 1.0     Cumulative Blast bit score: 427

Hit cluster cross-links:

Mycgr3G52686 Mycgr3T
  
Location: 0-861

Mycgr3G52686\_Mycgr3T

Mycgr3G102281 Mycgr3
  
Location: 961-1573

Mycgr3G102281\_Mycgr3

Mycgr3G89185 Mycgr3T
  
Location: 1673-2063

Mycgr3G89185\_Mycgr3T

Mycgr3G65725 Mycgr3T
  
Location: 2163-3612

Mycgr3G65725\_Mycgr3T

Mycgr3G102276 Mycgr3
  
Location: 3712-4801

Mycgr3G102276\_Mycgr3

Mycgr3G89189 Mycgr3T
  
Location: 4901-5564

Mycgr3G89189\_Mycgr3T

Mycgr3G52682 Mycgr3T
  
Location: 5664-9231

Mycgr3G52682\_Mycgr3T

Mycgr3G107072 Mycgr3
  
Location: 9331-13279

Mycgr3G107072\_Mycgr3

Mycgr3G34982 Mycgr3T
  
Location: 13379-15116

Mycgr3G34982\_Mycgr3T

Mycgr3G107069 Mycgr3
  
Location: 15216-17097

Mycgr3G107069\_Mycgr3

Mycgr3G32432 Mycgr3T
  
Location: 17197-19042

Mycgr3G32432\_Mycgr3T

Mycgr3G98385 Mycgr3T
  
Location: 19142-19898

Mycgr3G98385\_Mycgr3T

hypothetical protein
  
Accession: EJT96819
  
Location: 166377-167926
  
 NCBI BlastP on this gene

EJT96819

GMC oxidoreductase
  
Accession: EJT96820
  
Location: 168788-171828
  
  
**BlastP hit with Mycgr3G34982\_Mycgr3T**
  
Percentage identity: 28 %
  
BlastP bit score: 234
  
Sequence coverage: 99 %
  
E-value: 5e-65
  
  
 NCBI BlastP on this gene

EJT96820

hypothetical protein
  
Accession: EJT96821
  
Location: 173201-173365
  
 NCBI BlastP on this gene

EJT96821

hypothetical protein
  
Accession: EJT96822
  
Location: 173988-174461
  
 NCBI BlastP on this gene

EJT96822

YebC-like protein
  
Accession: EJT96823
  
Location: 174840-175679
  
 NCBI BlastP on this gene

EJT96823

hypothetical protein
  
Accession: EJT96824
  
Location: 175916-177835
  
 NCBI BlastP on this gene

EJT96824

hypothetical protein
  
Accession: EJT96825
  
Location: 188066-189650
  
 NCBI BlastP on this gene

EJT96825

hypothetical protein
  
Accession: EJT96826
  
Location: 191303-191719
  
 NCBI BlastP on this gene

EJT96826

hypothetical protein
  
Accession: EJT96827
  
Location: 193093-193542
  
 NCBI BlastP on this gene

EJT96827

Aldo/keto reductase
  
Accession: EJT96828
  
Location: 194960-195868
  
 NCBI BlastP on this gene

EJT96828

alcohol oxidase
  
Accession: EJT96829
  
Location: 196101-198406
  
  
**BlastP hit with Mycgr3G34982\_Mycgr3T**
  
Percentage identity: 28 %
  
BlastP bit score: 193
  
Sequence coverage: 94 %
  
E-value: 7e-51
  
  
 NCBI BlastP on this gene

EJT96829

Query: Architecture Search FASTA input

KB726020 : Colletotrichum orbiculare MAFF 240422 unplaced genomic scaffold Scaffold\_447    Total score: 1.0     Cumulative Blast bit score: 426

Hit cluster cross-links:

Mycgr3G52686 Mycgr3T
  
Location: 0-861

Mycgr3G52686\_Mycgr3T

Mycgr3G102281 Mycgr3
  
Location: 961-1573

Mycgr3G102281\_Mycgr3

Mycgr3G89185 Mycgr3T
  
Location: 1673-2063

Mycgr3G89185\_Mycgr3T

Mycgr3G65725 Mycgr3T
  
Location: 2163-3612

Mycgr3G65725\_Mycgr3T

Mycgr3G102276 Mycgr3
  
Location: 3712-4801

Mycgr3G102276\_Mycgr3

Mycgr3G89189 Mycgr3T
  
Location: 4901-5564

Mycgr3G89189\_Mycgr3T

Mycgr3G52682 Mycgr3T
  
Location: 5664-9231

Mycgr3G52682\_Mycgr3T

Mycgr3G107072 Mycgr3
  
Location: 9331-13279

Mycgr3G107072\_Mycgr3

Mycgr3G34982 Mycgr3T
  
Location: 13379-15116

Mycgr3G34982\_Mycgr3T

Mycgr3G107069 Mycgr3
  
Location: 15216-17097

Mycgr3G107069\_Mycgr3

Mycgr3G32432 Mycgr3T
  
Location: 17197-19042

Mycgr3G32432\_Mycgr3T

Mycgr3G98385 Mycgr3T
  
Location: 19142-19898

Mycgr3G98385\_Mycgr3T

glucose-methanol-choline oxidoreductase:gmc oxidoreductase
  
Accession: ENH79579
  
Location: 348871-351025
  
  
**BlastP hit with Mycgr3G34982\_Mycgr3T**
  
Percentage identity: 42 %
  
BlastP bit score: 426
  
Sequence coverage: 103 %
  
E-value: 3e-138
  
  
 NCBI BlastP on this gene

ENH79579

bZIP transcription factor
  
Accession: ENH79578
  
Location: 345984-347800
  
 NCBI BlastP on this gene

ENH79578

hypothetical protein
  
Accession: ENH79577
  
Location: 338644-340007
  
 NCBI BlastP on this gene

ENH79577

cyclopentanone -monooxygenase
  
Accession: ENH79576
  
Location: 336812-338534
  
 NCBI BlastP on this gene

ENH79576

Query: Architecture Search FASTA input

CM001234 : Magnaporthe oryzae 70-15 chromosome 4    Total score: 1.0     Cumulative Blast bit score: 424

Hit cluster cross-links:

Mycgr3G52686 Mycgr3T
  
Location: 0-861

Mycgr3G52686\_Mycgr3T

Mycgr3G102281 Mycgr3
  
Location: 961-1573

Mycgr3G102281\_Mycgr3

Mycgr3G89185 Mycgr3T
  
Location: 1673-2063

Mycgr3G89185\_Mycgr3T

Mycgr3G65725 Mycgr3T
  
Location: 2163-3612

Mycgr3G65725\_Mycgr3T

Mycgr3G102276 Mycgr3
  
Location: 3712-4801

Mycgr3G102276\_Mycgr3

Mycgr3G89189 Mycgr3T
  
Location: 4901-5564

Mycgr3G89189\_Mycgr3T

Mycgr3G52682 Mycgr3T
  
Location: 5664-9231

Mycgr3G52682\_Mycgr3T

Mycgr3G107072 Mycgr3
  
Location: 9331-13279

Mycgr3G107072\_Mycgr3

Mycgr3G34982 Mycgr3T
  
Location: 13379-15116

Mycgr3G34982\_Mycgr3T

Mycgr3G107069 Mycgr3
  
Location: 15216-17097

Mycgr3G107069\_Mycgr3

Mycgr3G32432 Mycgr3T
  
Location: 17197-19042

Mycgr3G32432\_Mycgr3T

Mycgr3G98385 Mycgr3T
  
Location: 19142-19898

Mycgr3G98385\_Mycgr3T

choline dehydrogenase
  
Accession: EHA49619
  
Location: 213819-215798
  
  
**BlastP hit with Mycgr3G34982\_Mycgr3T**
  
Percentage identity: 41 %
  
BlastP bit score: 424
  
Sequence coverage: 103 %
  
E-value: 2e-137
  
  
 NCBI BlastP on this gene

EHA49619

hypothetical protein
  
Accession: EHA49620
  
Location: 216006-216961
  
 NCBI BlastP on this gene

EHA49620

trichothecene 3-O-acetyltransferase
  
Accession: EHA49621
  
Location: 217769-219196
  
 NCBI BlastP on this gene

EHA49621

hypothetical protein
  
Accession: EHA49622
  
Location: 221590-222228
  
 NCBI BlastP on this gene

EHA49622

hypothetical protein
  
Accession: EHA49623
  
Location: 222885-223409
  
 NCBI BlastP on this gene

EHA49623

Query: Architecture Search FASTA input

CAIF01000017 : Wickerhamomyces ciferrii strain NRRL Y-1031 F-60-10    Total score: 1.0     Cumulative Blast bit score: 424

Hit cluster cross-links:

Mycgr3G52686 Mycgr3T
  
Location: 0-861

Mycgr3G52686\_Mycgr3T

Mycgr3G102281 Mycgr3
  
Location: 961-1573

Mycgr3G102281\_Mycgr3

Mycgr3G89185 Mycgr3T
  
Location: 1673-2063

Mycgr3G89185\_Mycgr3T

Mycgr3G65725 Mycgr3T
  
Location: 2163-3612

Mycgr3G65725\_Mycgr3T

Mycgr3G102276 Mycgr3
  
Location: 3712-4801

Mycgr3G102276\_Mycgr3

Mycgr3G89189 Mycgr3T
  
Location: 4901-5564

Mycgr3G89189\_Mycgr3T

Mycgr3G52682 Mycgr3T
  
Location: 5664-9231

Mycgr3G52682\_Mycgr3T

Mycgr3G107072 Mycgr3
  
Location: 9331-13279

Mycgr3G107072\_Mycgr3

Mycgr3G34982 Mycgr3T
  
Location: 13379-15116

Mycgr3G34982\_Mycgr3T

Mycgr3G107069 Mycgr3
  
Location: 15216-17097

Mycgr3G107069\_Mycgr3

Mycgr3G32432 Mycgr3T
  
Location: 17197-19042

Mycgr3G32432\_Mycgr3T

Mycgr3G98385 Mycgr3T
  
Location: 19142-19898

Mycgr3G98385\_Mycgr3T

Ferric reductase transmembrane component
  
Accession: CCH41320
  
Location: 17195-19318
  
  
**BlastP hit with Mycgr3G107069\_Mycgr3**
  
Percentage identity: 28 %
  
BlastP bit score: 150
  
Sequence coverage: 73 %
  
E-value: 3e-35
  
  
 NCBI BlastP on this gene

CCH41320

Ferric reductase transmembrane component
  
Accession: CCH41321
  
Location: 20158-22008
  
  
**BlastP hit with Mycgr3G107069\_Mycgr3**
  
Percentage identity: 26 %
  
BlastP bit score: 133
  
Sequence coverage: 62 %
  
E-value: 8e-30
  
  
 NCBI BlastP on this gene

CCH41321

Ferric reductase transmembrane component
  
Accession: CCH41322
  
Location: 28478-29368
  
 NCBI BlastP on this gene

CCH41322

Respiratory burst oxidase protein
  
Accession: CCH41323
  
Location: 29530-30753
  
 NCBI BlastP on this gene

CCH41323

Ferric reductase transmembrane component
  
Accession: CCH41324
  
Location: 32475-34667
  
 NCBI BlastP on this gene

CCH41324

Ferric reductase transmembrane component
  
Accession: CCH41325
  
Location: 35643-37775
  
  
**BlastP hit with Mycgr3G107069\_Mycgr3**
  
Percentage identity: 26 %
  
BlastP bit score: 141
  
Sequence coverage: 72 %
  
E-value: 4e-32
  
  
 NCBI BlastP on this gene

CCH41325

Lysine biosynthesis regulatory protein
  
Accession: CCH41326
  
Location: 38338-39966
  
 NCBI BlastP on this gene

CCH41326

Query: Architecture Search FASTA input

KB446546 : Dothistroma septosporum NZE10 unplaced genomic scaffold DOTSEscaffold\_12    Total score: 1.0     Cumulative Blast bit score: 423

Hit cluster cross-links:

Mycgr3G52686 Mycgr3T
  
Location: 0-861

Mycgr3G52686\_Mycgr3T

Mycgr3G102281 Mycgr3
  
Location: 961-1573

Mycgr3G102281\_Mycgr3

Mycgr3G89185 Mycgr3T
  
Location: 1673-2063

Mycgr3G89185\_Mycgr3T

Mycgr3G65725 Mycgr3T
  
Location: 2163-3612

Mycgr3G65725\_Mycgr3T

Mycgr3G102276 Mycgr3
  
Location: 3712-4801

Mycgr3G102276\_Mycgr3

Mycgr3G89189 Mycgr3T
  
Location: 4901-5564

Mycgr3G89189\_Mycgr3T

Mycgr3G52682 Mycgr3T
  
Location: 5664-9231

Mycgr3G52682\_Mycgr3T

Mycgr3G107072 Mycgr3
  
Location: 9331-13279

Mycgr3G107072\_Mycgr3

Mycgr3G34982 Mycgr3T
  
Location: 13379-15116

Mycgr3G34982\_Mycgr3T

Mycgr3G107069 Mycgr3
  
Location: 15216-17097

Mycgr3G107069\_Mycgr3

Mycgr3G32432 Mycgr3T
  
Location: 17197-19042

Mycgr3G32432\_Mycgr3T

Mycgr3G98385 Mycgr3T
  
Location: 19142-19898

Mycgr3G98385\_Mycgr3T

hypothetical protein
  
Accession: EME38766
  
Location: 364212-364559
  
 NCBI BlastP on this gene

EME38766

hypothetical protein
  
Accession: EME38765
  
Location: 362807-363967
  
 NCBI BlastP on this gene

EME38765

hypothetical protein
  
Accession: EME38764
  
Location: 360522-362108
  
 NCBI BlastP on this gene

EME38764

hypothetical protein
  
Accession: EME38763
  
Location: 359234-360187
  
 NCBI BlastP on this gene

EME38763

hypothetical protein
  
Accession: EME38762
  
Location: 357188-357565
  
 NCBI BlastP on this gene

EME38762

hypothetical protein
  
Accession: EME38761
  
Location: 356293-356532
  
 NCBI BlastP on this gene

EME38761

hypothetical protein
  
Accession: EME38760
  
Location: 354582-355358
  
 NCBI BlastP on this gene

EME38760

hypothetical protein
  
Accession: EME38759
  
Location: 351356-352531
  
  
**BlastP hit with Mycgr3G102276\_Mycgr3**
  
Percentage identity: 60 %
  
BlastP bit score: 423
  
Sequence coverage: 98 %
  
E-value: 6e-144
  
  
 NCBI BlastP on this gene

EME38759

hypothetical protein
  
Accession: EME38757
  
Location: 348297-350465
  
 NCBI BlastP on this gene

EME38757

hypothetical protein
  
Accession: EME38756
  
Location: 337477-341494
  
 NCBI BlastP on this gene

EME38756

Query: Architecture Search FASTA input

CP003008 : Myceliophthora thermophila ATCC 42464 chromosome 7    Total score: 1.0     Cumulative Blast bit score: 423

Hit cluster cross-links:

Mycgr3G52686 Mycgr3T
  
Location: 0-861

Mycgr3G52686\_Mycgr3T

Mycgr3G102281 Mycgr3
  
Location: 961-1573

Mycgr3G102281\_Mycgr3

Mycgr3G89185 Mycgr3T
  
Location: 1673-2063

Mycgr3G89185\_Mycgr3T

Mycgr3G65725 Mycgr3T
  
Location: 2163-3612

Mycgr3G65725\_Mycgr3T

Mycgr3G102276 Mycgr3
  
Location: 3712-4801

Mycgr3G102276\_Mycgr3

Mycgr3G89189 Mycgr3T
  
Location: 4901-5564

Mycgr3G89189\_Mycgr3T

Mycgr3G52682 Mycgr3T
  
Location: 5664-9231

Mycgr3G52682\_Mycgr3T

Mycgr3G107072 Mycgr3
  
Location: 9331-13279

Mycgr3G107072\_Mycgr3

Mycgr3G34982 Mycgr3T
  
Location: 13379-15116

Mycgr3G34982\_Mycgr3T

Mycgr3G107069 Mycgr3
  
Location: 15216-17097

Mycgr3G107069\_Mycgr3

Mycgr3G32432 Mycgr3T
  
Location: 17197-19042

Mycgr3G32432\_Mycgr3T

Mycgr3G98385 Mycgr3T
  
Location: 19142-19898

Mycgr3G98385\_Mycgr3T

hypothetical protein
  
Accession: AEO62153
  
Location: 4060162-4062303
  
 NCBI BlastP on this gene

MYCTH\_2313211

hypothetical protein
  
Accession: AEO62154
  
Location: 4063578-4065957
  
 NCBI BlastP on this gene

MYCTH\_2313212

hypothetical protein
  
Accession: AEO62155
  
Location: 4066295-4067077
  
 NCBI BlastP on this gene

MYCTH\_2313215

hypothetical protein
  
Accession: AEO62156
  
Location: 4067463-4068598
  
 NCBI BlastP on this gene

MYCTH\_2313216

hypothetical protein
  
Accession: AEO62157
  
Location: 4069022-4070609
  
 NCBI BlastP on this gene

MYCTH\_2313219

alcohol dehydrogenase-like protein
  
Accession: AEO62158
  
Location: 4073171-4074377
  
  
**BlastP hit with Mycgr3G102276\_Mycgr3**
  
Percentage identity: 60 %
  
BlastP bit score: 423
  
Sequence coverage: 98 %
  
E-value: 1e-143
  
  
 NCBI BlastP on this gene

MYCTH\_84302

Query: Architecture Search FASTA input

CM001200 : Mycosphaerella graminicola IPO323 chromosome 5    Total score: 1.0     Cumulative Blast bit score: 422

Hit cluster cross-links:

Mycgr3G52686 Mycgr3T
  
Location: 0-861

Mycgr3G52686\_Mycgr3T

Mycgr3G102281 Mycgr3
  
Location: 961-1573

Mycgr3G102281\_Mycgr3

Mycgr3G89185 Mycgr3T
  
Location: 1673-2063

Mycgr3G89185\_Mycgr3T

Mycgr3G65725 Mycgr3T
  
Location: 2163-3612

Mycgr3G65725\_Mycgr3T

Mycgr3G102276 Mycgr3
  
Location: 3712-4801

Mycgr3G102276\_Mycgr3

Mycgr3G89189 Mycgr3T
  
Location: 4901-5564

Mycgr3G89189\_Mycgr3T

Mycgr3G52682 Mycgr3T
  
Location: 5664-9231

Mycgr3G52682\_Mycgr3T

Mycgr3G107072 Mycgr3
  
Location: 9331-13279

Mycgr3G107072\_Mycgr3

Mycgr3G34982 Mycgr3T
  
Location: 13379-15116

Mycgr3G34982\_Mycgr3T

Mycgr3G107069 Mycgr3
  
Location: 15216-17097

Mycgr3G107069\_Mycgr3

Mycgr3G32432 Mycgr3T
  
Location: 17197-19042

Mycgr3G32432\_Mycgr3T

Mycgr3G98385 Mycgr3T
  
Location: 19142-19898

Mycgr3G98385\_Mycgr3T

Ca2+-modulated channel polycystin
  
Accession: EGP87475
  
Location: 2115406-2116746
  
 NCBI BlastP on this gene

EGP87475

hypothetical protein
  
Accession: EGP87474
  
Location: 2117984-2119276
  
 NCBI BlastP on this gene

EGP87474

hypothetical protein
  
Accession: EGP87473
  
Location: 2120074-2120504
  
 NCBI BlastP on this gene

EGP87473

hypothetical protein
  
Accession: EGP87292
  
Location: 2121081-2121640
  
 NCBI BlastP on this gene

EGP87292

hypothetical protein
  
Accession: EGP87293
  
Location: 2122695-2124864
  
 NCBI BlastP on this gene

EGP87293

hypothetical protein
  
Accession: EGP87294
  
Location: 2125529-2127494
  
 NCBI BlastP on this gene

EGP87294

hypothetical protein
  
Accession: EGP87295
  
Location: 2128452-2129711
  
  
**BlastP hit with Mycgr3G102276\_Mycgr3**
  
Percentage identity: 60 %
  
BlastP bit score: 422
  
Sequence coverage: 98 %
  
E-value: 2e-143
  
  
 NCBI BlastP on this gene

EGP87295

Query: Architecture Search FASTA input

GL988032 : Chaetomium thermophilum var. thermophilum DSM 1495 unplaced genomic scaffold scf7180000...    Total score: 1.0     Cumulative Blast bit score: 421

Hit cluster cross-links:

Mycgr3G52686 Mycgr3T
  
Location: 0-861

Mycgr3G52686\_Mycgr3T

Mycgr3G102281 Mycgr3
  
Location: 961-1573

Mycgr3G102281\_Mycgr3

Mycgr3G89185 Mycgr3T
  
Location: 1673-2063

Mycgr3G89185\_Mycgr3T

Mycgr3G65725 Mycgr3T
  
Location: 2163-3612

Mycgr3G65725\_Mycgr3T

Mycgr3G102276 Mycgr3
  
Location: 3712-4801

Mycgr3G102276\_Mycgr3

Mycgr3G89189 Mycgr3T
  
Location: 4901-5564

Mycgr3G89189\_Mycgr3T

Mycgr3G52682 Mycgr3T
  
Location: 5664-9231

Mycgr3G52682\_Mycgr3T

Mycgr3G107072 Mycgr3
  
Location: 9331-13279

Mycgr3G107072\_Mycgr3

Mycgr3G34982 Mycgr3T
  
Location: 13379-15116

Mycgr3G34982\_Mycgr3T

Mycgr3G107069 Mycgr3
  
Location: 15216-17097

Mycgr3G107069\_Mycgr3

Mycgr3G32432 Mycgr3T
  
Location: 17197-19042

Mycgr3G32432\_Mycgr3T

Mycgr3G98385 Mycgr3T
  
Location: 19142-19898

Mycgr3G98385\_Mycgr3T

hypothetical protein
  
Accession: EGS23376
  
Location: 43932-46094
  
 NCBI BlastP on this gene

EGS23376

putative tryptophan protein
  
Accession: EGS23375
  
Location: 40739-43093
  
 NCBI BlastP on this gene

EGS23375

hypothetical protein
  
Accession: EGS23374
  
Location: 39533-40243
  
 NCBI BlastP on this gene

EGS23374

hypothetical protein
  
Accession: EGS23373
  
Location: 37935-38378
  
 NCBI BlastP on this gene

EGS23373

putative aspartate protein
  
Accession: EGS23372
  
Location: 35965-37543
  
 NCBI BlastP on this gene

EGS23372

alcohol dehydrogenase-like protein
  
Accession: EGS23371
  
Location: 31435-32613
  
  
**BlastP hit with Mycgr3G102276\_Mycgr3**
  
Percentage identity: 60 %
  
BlastP bit score: 421
  
Sequence coverage: 99 %
  
E-value: 1e-142
  
  
 NCBI BlastP on this gene

EGS23371

hypothetical protein
  
Accession: EGS23370
  
Location: 26065-30705
  
 NCBI BlastP on this gene

EGS23370

hypothetical protein
  
Accession: EGS23369
  
Location: 22652-23047
  
 NCBI BlastP on this gene

EGS23369

hypothetical protein
  
Accession: EGS23368
  
Location: 19693-22083
  
 NCBI BlastP on this gene

EGS23368

Query: Architecture Search FASTA input

EQ963472 : Aspergillus flavus NRRL3357 scf\_1106286418772 genomic scaffold    Total score: 1.0     Cumulative Blast bit score: 421

Hit cluster cross-links:

Mycgr3G52686 Mycgr3T
  
Location: 0-861

Mycgr3G52686\_Mycgr3T

Mycgr3G102281 Mycgr3
  
Location: 961-1573

Mycgr3G102281\_Mycgr3

Mycgr3G89185 Mycgr3T
  
Location: 1673-2063

Mycgr3G89185\_Mycgr3T

Mycgr3G65725 Mycgr3T
  
Location: 2163-3612

Mycgr3G65725\_Mycgr3T

Mycgr3G102276 Mycgr3
  
Location: 3712-4801

Mycgr3G102276\_Mycgr3

Mycgr3G89189 Mycgr3T
  
Location: 4901-5564

Mycgr3G89189\_Mycgr3T

Mycgr3G52682 Mycgr3T
  
Location: 5664-9231

Mycgr3G52682\_Mycgr3T

Mycgr3G107072 Mycgr3
  
Location: 9331-13279

Mycgr3G107072\_Mycgr3

Mycgr3G34982 Mycgr3T
  
Location: 13379-15116

Mycgr3G34982\_Mycgr3T

Mycgr3G107069 Mycgr3
  
Location: 15216-17097

Mycgr3G107069\_Mycgr3

Mycgr3G32432 Mycgr3T
  
Location: 17197-19042

Mycgr3G32432\_Mycgr3T

Mycgr3G98385 Mycgr3T
  
Location: 19142-19898

Mycgr3G98385\_Mycgr3T

conserved hypothetical protein
  
Accession: EED57891
  
Location: 3673039-3673671
  
 NCBI BlastP on this gene

EED57891

hypothetical protein
  
Accession: EED57892
  
Location: 3673960-3674157
  
 NCBI BlastP on this gene

EED57892

Coatomer subunit alpha, putative
  
Accession: EED57893
  
Location: 3674411-3678461
  
 NCBI BlastP on this gene

EED57893

FAD dependent sulfhydryl oxidase Erv1, putative
  
Accession: EED57894
  
Location: 3678906-3679604
  
 NCBI BlastP on this gene

EED57894

transcriptional regulator, putative
  
Accession: EED57895
  
Location: 3679762-3681402
  
 NCBI BlastP on this gene

EED57895

AP-1 adaptor complex subunit beta, putative
  
Accession: EED57896
  
Location: 3682118-3684315
  
 NCBI BlastP on this gene

EED57896

alcohol dehydrogenase, putative
  
Accession: EED57897
  
Location: 3685451-3686704
  
  
**BlastP hit with Mycgr3G102276\_Mycgr3**
  
Percentage identity: 60 %
  
BlastP bit score: 421
  
Sequence coverage: 99 %
  
E-value: 8e-143
  
  
 NCBI BlastP on this gene

EED57897

Query: Architecture Search FASTA input

DS985216 : Verticillium albo-atrum VaMs.102 supercont1.3 genomic scaffold    Total score: 1.0     Cumulative Blast bit score: 421

Hit cluster cross-links:

Mycgr3G52686 Mycgr3T
  
Location: 0-861

Mycgr3G52686\_Mycgr3T

Mycgr3G102281 Mycgr3
  
Location: 961-1573

Mycgr3G102281\_Mycgr3

Mycgr3G89185 Mycgr3T
  
Location: 1673-2063

Mycgr3G89185\_Mycgr3T

Mycgr3G65725 Mycgr3T
  
Location: 2163-3612

Mycgr3G65725\_Mycgr3T

Mycgr3G102276 Mycgr3
  
Location: 3712-4801

Mycgr3G102276\_Mycgr3

Mycgr3G89189 Mycgr3T
  
Location: 4901-5564

Mycgr3G89189\_Mycgr3T

Mycgr3G52682 Mycgr3T
  
Location: 5664-9231

Mycgr3G52682\_Mycgr3T

Mycgr3G107072 Mycgr3
  
Location: 9331-13279

Mycgr3G107072\_Mycgr3

Mycgr3G34982 Mycgr3T
  
Location: 13379-15116

Mycgr3G34982\_Mycgr3T

Mycgr3G107069 Mycgr3
  
Location: 15216-17097

Mycgr3G107069\_Mycgr3

Mycgr3G32432 Mycgr3T
  
Location: 17197-19042

Mycgr3G32432\_Mycgr3T

Mycgr3G98385 Mycgr3T
  
Location: 19142-19898

Mycgr3G98385\_Mycgr3T

conserved hypothetical protein
  
Accession: EEY17026
  
Location: 2054685-2055353
  
 NCBI BlastP on this gene

EEY17026

chitosanase
  
Accession: EEY17027
  
Location: 2056411-2057308
  
 NCBI BlastP on this gene

EEY17027

predicted protein
  
Accession: EEY17028
  
Location: 2059007-2060849
  
 NCBI BlastP on this gene

EEY17028

high affinity copper transporter
  
Accession: EEY17029
  
Location: 2062667-2063361
  
 NCBI BlastP on this gene

EEY17029

ferric reductase transmembrane component 2
  
Accession: EEY17030
  
Location: 2064417-2066444
  
  
**BlastP hit with Mycgr3G107069\_Mycgr3**
  
Percentage identity: 38 %
  
BlastP bit score: 421
  
Sequence coverage: 102 %
  
E-value: 6e-135
  
  
 NCBI BlastP on this gene

EEY17030

Query: Architecture Search FASTA input

AP007175 : Aspergillus oryzae RIB40 DNA, SC010.    Total score: 1.0     Cumulative Blast bit score: 421

Hit cluster cross-links:

Mycgr3G52686 Mycgr3T
  
Location: 0-861

Mycgr3G52686\_Mycgr3T

Mycgr3G102281 Mycgr3
  
Location: 961-1573

Mycgr3G102281\_Mycgr3

Mycgr3G89185 Mycgr3T
  
Location: 1673-2063

Mycgr3G89185\_Mycgr3T

Mycgr3G65725 Mycgr3T
  
Location: 2163-3612

Mycgr3G65725\_Mycgr3T

Mycgr3G102276 Mycgr3
  
Location: 3712-4801

Mycgr3G102276\_Mycgr3

Mycgr3G89189 Mycgr3T
  
Location: 4901-5564

Mycgr3G89189\_Mycgr3T

Mycgr3G52682 Mycgr3T
  
Location: 5664-9231

Mycgr3G52682\_Mycgr3T

Mycgr3G107072 Mycgr3
  
Location: 9331-13279

Mycgr3G107072\_Mycgr3

Mycgr3G34982 Mycgr3T
  
Location: 13379-15116

Mycgr3G34982\_Mycgr3T

Mycgr3G107069 Mycgr3
  
Location: 15216-17097

Mycgr3G107069\_Mycgr3

Mycgr3G32432 Mycgr3T
  
Location: 17197-19042

Mycgr3G32432\_Mycgr3T

Mycgr3G98385 Mycgr3T
  
Location: 19142-19898

Mycgr3G98385\_Mycgr3T

not annotated
  
Accession: BAE66488
  
Location: 1736104-1736940
  
 NCBI BlastP on this gene

AO090010000663

not annotated
  
Accession: BAE66489
  
Location: 1737150-1737934
  
 NCBI BlastP on this gene

AO090010000664

not annotated
  
Accession: BAE66490
  
Location: 1740786-1741396
  
 NCBI BlastP on this gene

AO090010000666

not annotated
  
Accession: BAE66491
  
Location: 1741692-1742685
  
 NCBI BlastP on this gene

AO090010000667

not annotated
  
Accession: BAE66492
  
Location: 1745184-1746322
  
  
**BlastP hit with Mycgr3G102276\_Mycgr3**
  
Percentage identity: 60 %
  
BlastP bit score: 421
  
Sequence coverage: 99 %
  
E-value: 7e-143
  
  
 NCBI BlastP on this gene

AO090010000668

Query: Architecture Search FASTA input

AKHY01000175 : Aspergillus oryzae 3.042    Total score: 1.0     Cumulative Blast bit score: 421

Hit cluster cross-links:

Mycgr3G52686 Mycgr3T
  
Location: 0-861

Mycgr3G52686\_Mycgr3T

Mycgr3G102281 Mycgr3
  
Location: 961-1573

Mycgr3G102281\_Mycgr3

Mycgr3G89185 Mycgr3T
  
Location: 1673-2063

Mycgr3G89185\_Mycgr3T

Mycgr3G65725 Mycgr3T
  
Location: 2163-3612

Mycgr3G65725\_Mycgr3T

Mycgr3G102276 Mycgr3
  
Location: 3712-4801

Mycgr3G102276\_Mycgr3

Mycgr3G89189 Mycgr3T
  
Location: 4901-5564

Mycgr3G89189\_Mycgr3T

Mycgr3G52682 Mycgr3T
  
Location: 5664-9231

Mycgr3G52682\_Mycgr3T

Mycgr3G107072 Mycgr3
  
Location: 9331-13279

Mycgr3G107072\_Mycgr3

Mycgr3G34982 Mycgr3T
  
Location: 13379-15116

Mycgr3G34982\_Mycgr3T

Mycgr3G107069 Mycgr3
  
Location: 15216-17097

Mycgr3G107069\_Mycgr3

Mycgr3G32432 Mycgr3T
  
Location: 17197-19042

Mycgr3G32432\_Mycgr3T

Mycgr3G98385 Mycgr3T
  
Location: 19142-19898

Mycgr3G98385\_Mycgr3T

hypothetical protein
  
Accession: EIT75821
  
Location: 298981-301112
  
 NCBI BlastP on this gene

EIT75821

hypothetical protein
  
Accession: EIT75618
  
Location: 295828-296438
  
 NCBI BlastP on this gene

EIT75618

C-4 sterol methyl oxidase
  
Accession: EIT75499
  
Location: 294539-295532
  
 NCBI BlastP on this gene

EIT75499

alcohol dehydrogenase, class V
  
Accession: EIT75591
  
Location: 290905-292043
  
  
**BlastP hit with Mycgr3G102276\_Mycgr3**
  
Percentage identity: 60 %
  
BlastP bit score: 421
  
Sequence coverage: 99 %
  
E-value: 7e-143
  
  
 NCBI BlastP on this gene

EIT75591

hypothetical protein
  
Accession: EIT75732
  
Location: 290023-290846
  
 NCBI BlastP on this gene

EIT75732

hypothetical protein
  
Accession: EIT75815
  
Location: 287968-289631
  
 NCBI BlastP on this gene

EIT75815

hypothetical protein
  
Accession: EIT75655
  
Location: 286034-286825
  
 NCBI BlastP on this gene

EIT75655

D-alanine transfer protein
  
Accession: EIT75493
  
Location: 285062-285938
  
 NCBI BlastP on this gene

EIT75493

hypothetical protein
  
Accession: EIT75515
  
Location: 283673-284413
  
 NCBI BlastP on this gene

EIT75515

permease of the major facilitator superfamily
  
Accession: EIT75380
  
Location: 281538-283433
  
 NCBI BlastP on this gene

EIT75380

hypothetical protein
  
Accession: EIT75761
  
Location: 279693-280500
  
 NCBI BlastP on this gene

EIT75761

Query: Architecture Search FASTA input

JH668231 : Wallemia sebi CBS 633.66 unplaced genomic scaffold WALSEscaffold\_9    Total score: 1.0     Cumulative Blast bit score: 419

Hit cluster cross-links:

Mycgr3G52686 Mycgr3T
  
Location: 0-861

Mycgr3G52686\_Mycgr3T

Mycgr3G102281 Mycgr3
  
Location: 961-1573

Mycgr3G102281\_Mycgr3

Mycgr3G89185 Mycgr3T
  
Location: 1673-2063

Mycgr3G89185\_Mycgr3T

Mycgr3G65725 Mycgr3T
  
Location: 2163-3612

Mycgr3G65725\_Mycgr3T

Mycgr3G102276 Mycgr3
  
Location: 3712-4801

Mycgr3G102276\_Mycgr3

Mycgr3G89189 Mycgr3T
  
Location: 4901-5564

Mycgr3G89189\_Mycgr3T

Mycgr3G52682 Mycgr3T
  
Location: 5664-9231

Mycgr3G52682\_Mycgr3T

Mycgr3G107072 Mycgr3
  
Location: 9331-13279

Mycgr3G107072\_Mycgr3

Mycgr3G34982 Mycgr3T
  
Location: 13379-15116

Mycgr3G34982\_Mycgr3T

Mycgr3G107069 Mycgr3
  
Location: 15216-17097

Mycgr3G107069\_Mycgr3

Mycgr3G32432 Mycgr3T
  
Location: 17197-19042

Mycgr3G32432\_Mycgr3T

Mycgr3G98385 Mycgr3T
  
Location: 19142-19898

Mycgr3G98385\_Mycgr3T

hypothetical protein
  
Accession: EIM21612
  
Location: 111269-114238
  
 NCBI BlastP on this gene

EIM21612

ARM repeat-containing protein
  
Accession: EIM21611
  
Location: 104962-107328
  
 NCBI BlastP on this gene

EIM21611

hypothetical protein
  
Accession: EIM21610
  
Location: 103739-104902
  
 NCBI BlastP on this gene

EIM21610

alcohol oxidase
  
Accession: EIM21609
  
Location: 101069-103600
  
  
**BlastP hit with Mycgr3G34982\_Mycgr3T**
  
Percentage identity: 28 %
  
BlastP bit score: 212
  
Sequence coverage: 101 %
  
E-value: 4e-57
  
  
 NCBI BlastP on this gene

EIM21609

mitochondrial NAD-dependent isocitrate dehydrogenase subunit 2 precursor
  
Accession: EIM21608
  
Location: 99653-100897
  
 NCBI BlastP on this gene

EIM21608

alcohol oxidase
  
Accession: EIM21607
  
Location: 96708-99109
  
  
**BlastP hit with Mycgr3G34982\_Mycgr3T**
  
Percentage identity: 27 %
  
BlastP bit score: 207
  
Sequence coverage: 103 %
  
E-value: 6e-55
  
  
 NCBI BlastP on this gene

EIM21607

20S proteasome subunit
  
Accession: EIM21605
  
Location: 94747-95789
  
 NCBI BlastP on this gene

EIM21605

ENTH-domain-containing protein
  
Accession: EIM21604
  
Location: 93050-94725
  
 NCBI BlastP on this gene

EIM21604

hypothetical protein
  
Accession: EIM21603
  
Location: 90157-92423
  
 NCBI BlastP on this gene

EIM21603

pyrroline-5-carboxylate reductase
  
Accession: EIM21602
  
Location: 88829-90011
  
 NCBI BlastP on this gene

EIM21602

hypothetical protein
  
Accession: EIM21601
  
Location: 87644-88394
  
 NCBI BlastP on this gene

EIM21601

Query: Architecture Search FASTA input

GL629765 : Grosmannia clavigera kw1407 unplaced genomic scaffold GCSC\_140    Total score: 1.0     Cumulative Blast bit score: 419

Hit cluster cross-links:

Mycgr3G52686 Mycgr3T
  
Location: 0-861

Mycgr3G52686\_Mycgr3T

Mycgr3G102281 Mycgr3
  
Location: 961-1573

Mycgr3G102281\_Mycgr3

Mycgr3G89185 Mycgr3T
  
Location: 1673-2063

Mycgr3G89185\_Mycgr3T

Mycgr3G65725 Mycgr3T
  
Location: 2163-3612

Mycgr3G65725\_Mycgr3T

Mycgr3G102276 Mycgr3
  
Location: 3712-4801

Mycgr3G102276\_Mycgr3

Mycgr3G89189 Mycgr3T
  
Location: 4901-5564

Mycgr3G89189\_Mycgr3T

Mycgr3G52682 Mycgr3T
  
Location: 5664-9231

Mycgr3G52682\_Mycgr3T

Mycgr3G107072 Mycgr3
  
Location: 9331-13279

Mycgr3G107072\_Mycgr3

Mycgr3G34982 Mycgr3T
  
Location: 13379-15116

Mycgr3G34982\_Mycgr3T

Mycgr3G107069 Mycgr3
  
Location: 15216-17097

Mycgr3G107069\_Mycgr3

Mycgr3G32432 Mycgr3T
  
Location: 17197-19042

Mycgr3G32432\_Mycgr3T

Mycgr3G98385 Mycgr3T
  
Location: 19142-19898

Mycgr3G98385\_Mycgr3T

hypothetical protein
  
Accession: EFX03935
  
Location: 337697-338249
  
 NCBI BlastP on this gene

EFX03935

caib baif family enzyme
  
Accession: EFX04776
  
Location: 340238-344171
  
 NCBI BlastP on this gene

EFX04776

fructosyl amine: oxygen oxidoreductase
  
Accession: EFX03499
  
Location: 344456-345736
  
 NCBI BlastP on this gene

EFX03499

trypsin-like serine typically contains c-terminal pdz domain protein
  
Accession: EFX04206
  
Location: 346817-348467
  
 NCBI BlastP on this gene

EFX04206

zinc-binding alcohol dehydrogenase
  
Accession: EFX03836
  
Location: 350059-351195
  
  
**BlastP hit with Mycgr3G102276\_Mycgr3**
  
Percentage identity: 59 %
  
BlastP bit score: 419
  
Sequence coverage: 99 %
  
E-value: 3e-142
  
  
 NCBI BlastP on this gene

EFX03836

hypothetical protein
  
Accession: EFX04644
  
Location: 352992-357307
  
 NCBI BlastP on this gene

EFX04644

hypothetical protein
  
Accession: EFX03599
  
Location: 357846-359352
  
 NCBI BlastP on this gene

EFX03599

pre-mRNA splicing factor dim1
  
Accession: EFX04646
  
Location: 360118-360608
  
 NCBI BlastP on this gene

EFX04646

small nuclear ribonucleoprotein
  
Accession: EFX04427
  
Location: 361364-361827
  
 NCBI BlastP on this gene

EFX04427

hypothetical protein
  
Accession: EFX03712
  
Location: 362059-363763
  
 NCBI BlastP on this gene

EFX03712

Query: Architecture Search FASTA input

EQ963475 : Aspergillus flavus NRRL3357 scf\_1106286419142 genomic scaffold    Total score: 1.0     Cumulative Blast bit score: 419

Hit cluster cross-links:

Mycgr3G52686 Mycgr3T
  
Location: 0-861

Mycgr3G52686\_Mycgr3T

Mycgr3G102281 Mycgr3
  
Location: 961-1573

Mycgr3G102281\_Mycgr3

Mycgr3G89185 Mycgr3T
  
Location: 1673-2063

Mycgr3G89185\_Mycgr3T

Mycgr3G65725 Mycgr3T
  
Location: 2163-3612

Mycgr3G65725\_Mycgr3T

Mycgr3G102276 Mycgr3
  
Location: 3712-4801

Mycgr3G102276\_Mycgr3

Mycgr3G89189 Mycgr3T
  
Location: 4901-5564

Mycgr3G89189\_Mycgr3T

Mycgr3G52682 Mycgr3T
  
Location: 5664-9231

Mycgr3G52682\_Mycgr3T

Mycgr3G107072 Mycgr3
  
Location: 9331-13279

Mycgr3G107072\_Mycgr3

Mycgr3G34982 Mycgr3T
  
Location: 13379-15116

Mycgr3G34982\_Mycgr3T

Mycgr3G107069 Mycgr3
  
Location: 15216-17097

Mycgr3G107069\_Mycgr3

Mycgr3G32432 Mycgr3T
  
Location: 17197-19042

Mycgr3G32432\_Mycgr3T

Mycgr3G98385 Mycgr3T
  
Location: 19142-19898

Mycgr3G98385\_Mycgr3T

conserved hypothetical protein
  
Accession: EED53491
  
Location: 1183626-1184972
  
 NCBI BlastP on this gene

EED53491

cytochrome P450, putative
  
Accession: EED53492
  
Location: 1186835-1188895
  
 NCBI BlastP on this gene

EED53492

conserved hypothetical protein
  
Accession: EED53493
  
Location: 1189258-1192172
  
 NCBI BlastP on this gene

EED53493

aminotransferase GliI-like, putative
  
Accession: EED53494
  
Location: 1192475-1193986
  
 NCBI BlastP on this gene

EED53494

quinone oxidoreductase, putative
  
Accession: EED53495
  
Location: 1196685-1197858
  
  
**BlastP hit with Mycgr3G102276\_Mycgr3**
  
Percentage identity: 60 %
  
BlastP bit score: 419
  
Sequence coverage: 99 %
  
E-value: 4e-142
  
  
 NCBI BlastP on this gene

EED53495

ankyrin repeat protein
  
Accession: EED53496
  
Location: 1198170-1199790
  
 NCBI BlastP on this gene

EED53496

conserved hypothetical protein
  
Accession: EED53497
  
Location: 1199819-1200658
  
 NCBI BlastP on this gene

EED53497

hypothetical protein
  
Accession: EED53498
  
Location: 1201342-1201936
  
 NCBI BlastP on this gene

EED53498

conserved hypothetical protein
  
Accession: EED53499
  
Location: 1206283-1207858
  
 NCBI BlastP on this gene

EED53499

conserved hypothetical protein
  
Accession: EED53500
  
Location: 1208743-1210001
  
 NCBI BlastP on this gene

EED53500

Query: Architecture Search FASTA input

KB644409 : Penicillium oxalicum 114-2 unplaced genomic scaffold scaffold\_2    Total score: 1.0     Cumulative Blast bit score: 417

Hit cluster cross-links:

Mycgr3G52686 Mycgr3T
  
Location: 0-861

Mycgr3G52686\_Mycgr3T

Mycgr3G102281 Mycgr3
  
Location: 961-1573

Mycgr3G102281\_Mycgr3

Mycgr3G89185 Mycgr3T
  
Location: 1673-2063

Mycgr3G89185\_Mycgr3T

Mycgr3G65725 Mycgr3T
  
Location: 2163-3612

Mycgr3G65725\_Mycgr3T

Mycgr3G102276 Mycgr3
  
Location: 3712-4801

Mycgr3G102276\_Mycgr3

Mycgr3G89189 Mycgr3T
  
Location: 4901-5564

Mycgr3G89189\_Mycgr3T

Mycgr3G52682 Mycgr3T
  
Location: 5664-9231

Mycgr3G52682\_Mycgr3T

Mycgr3G107072 Mycgr3
  
Location: 9331-13279

Mycgr3G107072\_Mycgr3

Mycgr3G34982 Mycgr3T
  
Location: 13379-15116

Mycgr3G34982\_Mycgr3T

Mycgr3G107069 Mycgr3
  
Location: 15216-17097

Mycgr3G107069\_Mycgr3

Mycgr3G32432 Mycgr3T
  
Location: 17197-19042

Mycgr3G32432\_Mycgr3T

Mycgr3G98385 Mycgr3T
  
Location: 19142-19898

Mycgr3G98385\_Mycgr3T

hypothetical protein
  
Accession: EPS26789
  
Location: 1457690-1458457
  
 NCBI BlastP on this gene

EPS26789

hypothetical protein
  
Accession: EPS26790
  
Location: 1459004-1459553
  
 NCBI BlastP on this gene

EPS26790

hypothetical protein
  
Accession: EPS26791
  
Location: 1460397-1462885
  
 NCBI BlastP on this gene

EPS26791

hypothetical protein
  
Accession: EPS26792
  
Location: 1463752-1464862
  
 NCBI BlastP on this gene

EPS26792

hypothetical protein
  
Accession: EPS26793
  
Location: 1465050-1465520
  
 NCBI BlastP on this gene

EPS26793

hypothetical protein
  
Accession: EPS26794
  
Location: 1465821-1468461
  
 NCBI BlastP on this gene

EPS26794

hypothetical protein
  
Accession: EPS26795
  
Location: 1469498-1470725
  
  
**BlastP hit with Mycgr3G102276\_Mycgr3**
  
Percentage identity: 60 %
  
BlastP bit score: 417
  
Sequence coverage: 99 %
  
E-value: 3e-141
  
  
 NCBI BlastP on this gene

EPS26795

Query: Architecture Search FASTA input

AP007157 : Aspergillus oryzae RIB40 DNA, SC023.    Total score: 1.0     Cumulative Blast bit score: 417

Hit cluster cross-links:

Mycgr3G52686 Mycgr3T
  
Location: 0-861

Mycgr3G52686\_Mycgr3T

Mycgr3G102281 Mycgr3
  
Location: 961-1573

Mycgr3G102281\_Mycgr3

Mycgr3G89185 Mycgr3T
  
Location: 1673-2063

Mycgr3G89185\_Mycgr3T

Mycgr3G65725 Mycgr3T
  
Location: 2163-3612

Mycgr3G65725\_Mycgr3T

Mycgr3G102276 Mycgr3
  
Location: 3712-4801

Mycgr3G102276\_Mycgr3

Mycgr3G89189 Mycgr3T
  
Location: 4901-5564

Mycgr3G89189\_Mycgr3T

Mycgr3G52682 Mycgr3T
  
Location: 5664-9231

Mycgr3G52682\_Mycgr3T

Mycgr3G107072 Mycgr3
  
Location: 9331-13279

Mycgr3G107072\_Mycgr3

Mycgr3G34982 Mycgr3T
  
Location: 13379-15116

Mycgr3G34982\_Mycgr3T

Mycgr3G107069 Mycgr3
  
Location: 15216-17097

Mycgr3G107069\_Mycgr3

Mycgr3G32432 Mycgr3T
  
Location: 17197-19042

Mycgr3G32432\_Mycgr3T

Mycgr3G98385 Mycgr3T
  
Location: 19142-19898

Mycgr3G98385\_Mycgr3T

not annotated
  
Accession: BAE59000
  
Location: 1167214-1168560
  
 NCBI BlastP on this gene

AO090023000455

not annotated
  
Accession: BAE59001
  
Location: 1170838-1172488
  
 NCBI BlastP on this gene

AO090023000456

not annotated
  
Accession: BAE59002
  
Location: 1172850-1173800
  
 NCBI BlastP on this gene

AO090023000457

not annotated
  
Accession: BAE59003
  
Location: 1174739-1177580
  
 NCBI BlastP on this gene

AO090023000458

not annotated
  
Accession: BAE59004
  
Location: 1180272-1181445
  
  
**BlastP hit with Mycgr3G102276\_Mycgr3**
  
Percentage identity: 60 %
  
BlastP bit score: 417
  
Sequence coverage: 99 %
  
E-value: 2e-141
  
  
 NCBI BlastP on this gene

AO090023000460

not annotated
  
Accession: BAE59005
  
Location: 1181757-1184245
  
 NCBI BlastP on this gene

AO090023000461

not annotated
  
Accession: BAE59006
  
Location: 1189953-1191568
  
 NCBI BlastP on this gene

AO090023000463

not annotated
  
Accession: BAE59007
  
Location: 1192203-1193348
  
 NCBI BlastP on this gene

AO090023000464

Query: Architecture Search FASTA input

AP007151 : Aspergillus oryzae RIB40 DNA, SC005.    Total score: 1.0     Cumulative Blast bit score: 417

Hit cluster cross-links:

Mycgr3G52686 Mycgr3T
  
Location: 0-861

Mycgr3G52686\_Mycgr3T

Mycgr3G102281 Mycgr3
  
Location: 961-1573

Mycgr3G102281\_Mycgr3

Mycgr3G89185 Mycgr3T
  
Location: 1673-2063

Mycgr3G89185\_Mycgr3T

Mycgr3G65725 Mycgr3T
  
Location: 2163-3612

Mycgr3G65725\_Mycgr3T

Mycgr3G102276 Mycgr3
  
Location: 3712-4801

Mycgr3G102276\_Mycgr3

Mycgr3G89189 Mycgr3T
  
Location: 4901-5564

Mycgr3G89189\_Mycgr3T

Mycgr3G52682 Mycgr3T
  
Location: 5664-9231

Mycgr3G52682\_Mycgr3T

Mycgr3G107072 Mycgr3
  
Location: 9331-13279

Mycgr3G107072\_Mycgr3

Mycgr3G34982 Mycgr3T
  
Location: 13379-15116

Mycgr3G34982\_Mycgr3T

Mycgr3G107069 Mycgr3
  
Location: 15216-17097

Mycgr3G107069\_Mycgr3

Mycgr3G32432 Mycgr3T
  
Location: 17197-19042

Mycgr3G32432\_Mycgr3T

Mycgr3G98385 Mycgr3T
  
Location: 19142-19898

Mycgr3G98385\_Mycgr3T

not annotated
  
Accession: BAE56281
  
Location: 3615479-3619529
  
 NCBI BlastP on this gene

AO090005001354

not annotated
  
Accession: BAE56282
  
Location: 3620224-3620692
  
 NCBI BlastP on this gene

AO090005001355

not annotated
  
Accession: BAE56283
  
Location: 3621291-3622493
  
 NCBI BlastP on this gene

AO090005001356

not annotated
  
Accession: BAE56284
  
Location: 3623347-3625907
  
 NCBI BlastP on this gene

AO090005001357

not annotated
  
Accession: BAE56285
  
Location: 3627043-3628296
  
  
**BlastP hit with Mycgr3G102276\_Mycgr3**
  
Percentage identity: 59 %
  
BlastP bit score: 417
  
Sequence coverage: 99 %
  
E-value: 2e-141
  
  
 NCBI BlastP on this gene

AO090005001358

Query: Architecture Search FASTA input

201. :  AM920431 Penicillium chrysogenum Wisconsin 54-1255 complete genome, contig Pc00c16.     Total score: 1.0     Cumulative Blast bit score: 462

Mycgr3G52686 Mycgr3T
  
Location: 0-861
  
 NCBI BlastP on this gene

Mycgr3G52686\_Mycgr3T

Mycgr3G102281 Mycgr3
  
Location: 961-1573
  
 NCBI BlastP on this gene

Mycgr3G102281\_Mycgr3

Mycgr3G89185 Mycgr3T
  
Location: 1673-2063
  
 NCBI BlastP on this gene

Mycgr3G89185\_Mycgr3T

Mycgr3G65725 Mycgr3T
  
Location: 2163-3612
  
 NCBI BlastP on this gene

Mycgr3G65725\_Mycgr3T

Mycgr3G102276 Mycgr3
  
Location: 3712-4801
  
 NCBI BlastP on this gene

Mycgr3G102276\_Mycgr3

Mycgr3G89189 Mycgr3T
  
Location: 4901-5564
  
 NCBI BlastP on this gene

Mycgr3G89189\_Mycgr3T

Mycgr3G52682 Mycgr3T
  
Location: 5664-9231
  
 NCBI BlastP on this gene

Mycgr3G52682\_Mycgr3T

Mycgr3G107072 Mycgr3
  
Location: 9331-13279
  
 NCBI BlastP on this gene

Mycgr3G107072\_Mycgr3

Mycgr3G34982 Mycgr3T
  
Location: 13379-15116
  
 NCBI BlastP on this gene

Mycgr3G34982\_Mycgr3T

Mycgr3G107069 Mycgr3
  
Location: 15216-17097
  
 NCBI BlastP on this gene

Mycgr3G107069\_Mycgr3

Mycgr3G32432 Mycgr3T
  
Location: 17197-19042
  
 NCBI BlastP on this gene

Mycgr3G32432\_Mycgr3T

Mycgr3G98385 Mycgr3T
  
Location: 19142-19898
  
 NCBI BlastP on this gene

Mycgr3G98385\_Mycgr3T

not annotated
  
Accession: CAP93318
  
Location: 1499144-1500800
  
 NCBI BlastP on this gene

Pc16g06480

not annotated
  
Accession: CAP93317
  
Location: 1496693-1498924
  
 NCBI BlastP on this gene

Pc16g06470

hypothetical protein
  
Accession: CAP93316
  
Location: 1496160-1496588
  
 NCBI BlastP on this gene

Pc16g06460

not annotated
  
Accession: CAP93315
  
Location: 1492540-1493617
  
 NCBI BlastP on this gene

Pc16g06450

hypothetical protein
  
Accession: CAP93314
  
Location: 1490079-1490520
  
 NCBI BlastP on this gene

Pc16g06440

hypothetical protein
  
Accession: CAP93313
  
Location: 1489135-1489584
  
 NCBI BlastP on this gene

Pc16g06430

unnamed
  
Accession: CAP93312
  
Location: 1486682-1488721
  
 NCBI BlastP on this gene

Pc16g06420

hypothetical protein
  
Accession: CAP93311
  
Location: 1484802-1485429
  
 NCBI BlastP on this gene

Pc16g06410

not annotated
  
Accession: CAP93310
  
Location: 1483942-1484514
  
 NCBI BlastP on this gene

Pc16g06400

not annotated
  
Accession: CAP93309
  
Location: 1481588-1483540
  
  
**BlastP hit with Mycgr3G107069\_Mycgr3**
  
Percentage identity: 40 %
  
BlastP bit score: 462
  
Sequence coverage: 100 %
  
E-value: 5e-151
  
  
 NCBI BlastP on this gene

Pc16g06390

not annotated
  
Accession: CAP93308
  
Location: 1479091-1480851
  
 NCBI BlastP on this gene

Pc16g06380

unnamed
  
Accession: CAP93307
  
Location: 1476259-1478399
  
 NCBI BlastP on this gene

Pc16g06370

not annotated
  
Accession: CAP93306
  
Location: 1473535-1474860
  
 NCBI BlastP on this gene

Pc16g06360

unnamed
  
Accession: CAP93305
  
Location: 1470706-1471610
  
 NCBI BlastP on this gene

Pc16g06350

not annotated
  
Accession: CAP93304
  
Location: 1469323-1470437
  
 NCBI BlastP on this gene

Pc16g06340

hypothetical protein
  
Accession: CAP93303
  
Location: 1468588-1469016
  
 NCBI BlastP on this gene

Pc16g06330

not annotated
  
Accession: CAP93302
  
Location: 1466318-1467001
  
 NCBI BlastP on this gene

Pc16g06320

not annotated
  
Accession: CAP93301
  
Location: 1465202-1465830
  
 NCBI BlastP on this gene

Pc16g06310

202. :  GL385395 Gaeumannomyces graminis var. tritici R3-111a-1 unplaced genomic scaffold supercont2.1     Total score: 1.0     Cumulative Blast bit score: 461

hypothetical protein
  
Accession: EJT82167
  
Location: 6793406-6794724
  
 NCBI BlastP on this gene

EJT82167

hypothetical protein
  
Accession: EJT82168
  
Location: 6795203-6796906
  
 NCBI BlastP on this gene

EJT82168

hypothetical protein
  
Accession: EJT82169
  
Location: 6799243-6803077
  
 NCBI BlastP on this gene

EJT82169

hypothetical protein
  
Accession: EJT82170
  
Location: 6804421-6804789
  
 NCBI BlastP on this gene

EJT82170

oligopeptide transporter 2
  
Accession: EJT82171
  
Location: 6805724-6808756
  
 NCBI BlastP on this gene

EJT82171

NADP-dependent alcohol dehydrogenase 6
  
Accession: EJT82172
  
Location: 6810657-6811868
  
  
**BlastP hit with Mycgr3G102276\_Mycgr3**
  
Percentage identity: 61 %
  
BlastP bit score: 461
  
Sequence coverage: 99 %
  
E-value: 3e-158
  
  
 NCBI BlastP on this gene

EJT82172

hypothetical protein
  
Accession: EJT82173
  
Location: 6813295-6814057
  
 NCBI BlastP on this gene

EJT82173

hypothetical protein
  
Accession: EJT82174
  
Location: 6815075-6816808
  
 NCBI BlastP on this gene

EJT82174

hypothetical protein
  
Accession: EJT82175
  
Location: 6818556-6821952
  
 NCBI BlastP on this gene

EJT82175

hypothetical protein
  
Accession: EJT82176
  
Location: 6823240-6826141
  
 NCBI BlastP on this gene

EJT82176

hypothetical protein
  
Accession: EJT82177
  
Location: 6827169-6827777
  
 NCBI BlastP on this gene

EJT82177

203. :  KB445643 Cochliobolus sativus ND90Pr unplaced genomic scaffold COCSAscaffold\_7     Total score: 1.0     Cumulative Blast bit score: 459

hypothetical protein
  
Accession: EMD64396
  
Location: 1246184-1248649
  
 NCBI BlastP on this gene

EMD64396

hypothetical protein
  
Accession: EMD64395
  
Location: 1242621-1244524
  
 NCBI BlastP on this gene

EMD64395

carbohydrate esterase family 5 protein
  
Accession: EMD64394
  
Location: 1241182-1242309
  
 NCBI BlastP on this gene

EMD64394

hypothetical protein
  
Accession: EMD64393
  
Location: 1237229-1238832
  
 NCBI BlastP on this gene

EMD64393

hypothetical protein
  
Accession: EMD64392
  
Location: 1233718-1235665
  
 NCBI BlastP on this gene

EMD64392

hypothetical protein
  
Accession: EMD64391
  
Location: 1231839-1232497
  
 NCBI BlastP on this gene

EMD64391

hypothetical protein
  
Accession: EMD64390
  
Location: 1229281-1231314
  
  
**BlastP hit with Mycgr3G107069\_Mycgr3**
  
Percentage identity: 38 %
  
BlastP bit score: 459
  
Sequence coverage: 104 %
  
E-value: 2e-149
  
  
 NCBI BlastP on this gene

EMD64390

hypothetical protein
  
Accession: EMD64389
  
Location: 1227049-1228671
  
 NCBI BlastP on this gene

EMD64389

carbohydrate-binding module family 35 protein
  
Accession: EMD64388
  
Location: 1221798-1226464
  
 NCBI BlastP on this gene

EMD64388

glycoside hydrolase family 3 protein
  
Accession: EMD64387
  
Location: 1218750-1221089
  
 NCBI BlastP on this gene

EMD64387

hypothetical protein
  
Accession: EMD64386
  
Location: 1214310-1215501
  
 NCBI BlastP on this gene

EMD64386

204. :  AACD01000093 Aspergillus nidulans FGSC A4     Total score: 1.0     Cumulative Blast bit score: 459

hypothetical protein
  
Accession: EAA62508
  
Location: 250316-251557
  
 NCBI BlastP on this gene

EAA62508

hypothetical protein
  
Accession: EAA62509
  
Location: 251794-253021
  
 NCBI BlastP on this gene

EAA62509

hypothetical protein
  
Accession: EAA62510
  
Location: 253948-254933
  
 NCBI BlastP on this gene

EAA62510

hypothetical protein
  
Accession: EAA62511
  
Location: 255546-256217
  
 NCBI BlastP on this gene

EAA62511

hypothetical protein
  
Accession: EAA62512
  
Location: 256895-260575
  
 NCBI BlastP on this gene

EAA62512

hypothetical protein
  
Accession: EAA62513
  
Location: 261614-262385
  
 NCBI BlastP on this gene

EAA62513

hypothetical protein
  
Accession: EAA62514
  
Location: 265652-266799
  
 NCBI BlastP on this gene

EAA62514

hypothetical protein
  
Accession: EAA62515
  
Location: 267703-268853
  
  
**BlastP hit with Mycgr3G102276\_Mycgr3**
  
Percentage identity: 64 %
  
BlastP bit score: 459
  
Sequence coverage: 98 %
  
E-value: 6e-158
  
  
 NCBI BlastP on this gene

EAA62515

205. :  CH408033 Chaetomium globosum CBS 148.51 scaffold\_5 genomic scaffold     Total score: 1.0     Cumulative Blast bit score: 457

hypothetical protein
  
Accession: EAQ86698
  
Location: 3404753-3408736
  
  
**BlastP hit with Mycgr3G52682\_Mycgr3T**
  
Percentage identity: 37 %
  
BlastP bit score: 457
  
Sequence coverage: 69 %
  
E-value: 1e-137
  
  
 NCBI BlastP on this gene

EAQ86698

hypothetical protein
  
Accession: EAQ86697
  
Location: 3401198-3402406
  
 NCBI BlastP on this gene

EAQ86697

T-complex protein 1
  
Accession: EAQ86696
  
Location: 3398198-3400130
  
 NCBI BlastP on this gene

EAQ86696

hypothetical protein
  
Accession: EAQ86695
  
Location: 3397401-3398075
  
 NCBI BlastP on this gene

EAQ86695

hypothetical protein
  
Accession: EAQ86694
  
Location: 3394164-3396102
  
 NCBI BlastP on this gene

EAQ86694

predicted protein
  
Accession: EAQ86693
  
Location: 3391828-3393347
  
 NCBI BlastP on this gene

EAQ86693

206. :  KB733454 Bipolaris maydis ATCC 48331 unplaced genomic scaffold COCC4scaffold\_11     Total score: 1.0     Cumulative Blast bit score: 456

hypothetical protein
  
Accession: ENI05575
  
Location: 701342-703794
  
 NCBI BlastP on this gene

ENI05575

hypothetical protein
  
Accession: ENI05574
  
Location: 697758-699661
  
 NCBI BlastP on this gene

ENI05574

carbohydrate esterase family 5 protein
  
Accession: ENI05573
  
Location: 696265-697434
  
 NCBI BlastP on this gene

ENI05573

hypothetical protein
  
Accession: ENI05572
  
Location: 690765-692757
  
 NCBI BlastP on this gene

ENI05572

hypothetical protein
  
Accession: ENI05571
  
Location: 688888-689547
  
 NCBI BlastP on this gene

ENI05571

hypothetical protein
  
Accession: ENI05570
  
Location: 686330-688363
  
  
**BlastP hit with Mycgr3G107069\_Mycgr3**
  
Percentage identity: 38 %
  
BlastP bit score: 456
  
Sequence coverage: 104 %
  
E-value: 2e-148
  
  
 NCBI BlastP on this gene

ENI05570

hypothetical protein
  
Accession: ENI05569
  
Location: 684122-685714
  
 NCBI BlastP on this gene

ENI05569

carbohydrate-binding module family 35 protein
  
Accession: ENI05568
  
Location: 677735-682396
  
 NCBI BlastP on this gene

ENI05568

glycoside hydrolase family 3 protein
  
Accession: ENI05567
  
Location: 674693-677032
  
 NCBI BlastP on this gene

ENI05567

hypothetical protein
  
Accession: ENI05566
  
Location: 670097-671286
  
 NCBI BlastP on this gene

ENI05566

207. :  KB445580 Cochliobolus heterostrophus C5 unplaced genomic scaffold COCHEscaffold\_12     Total score: 1.0     Cumulative Blast bit score: 456

hypothetical protein
  
Accession: EMD88708
  
Location: 1141897-1144349
  
 NCBI BlastP on this gene

EMD88708

hypothetical protein
  
Accession: EMD88709
  
Location: 1146030-1147933
  
 NCBI BlastP on this gene

EMD88709

carbohydrate esterase family 5 protein
  
Accession: EMD88710
  
Location: 1148257-1149427
  
 NCBI BlastP on this gene

EMD88710

hypothetical protein
  
Accession: EMD88711
  
Location: 1152935-1154927
  
 NCBI BlastP on this gene

EMD88711

hypothetical protein
  
Accession: EMD88712
  
Location: 1156145-1156804
  
 NCBI BlastP on this gene

EMD88712

hypothetical protein
  
Accession: EMD88713
  
Location: 1157329-1159362
  
  
**BlastP hit with Mycgr3G107069\_Mycgr3**
  
Percentage identity: 38 %
  
BlastP bit score: 456
  
Sequence coverage: 104 %
  
E-value: 2e-148
  
  
 NCBI BlastP on this gene

EMD88713

208. :  DF126453 Aspergillus kawachii IFO 4308 DNA, contig: scaffold00007     Total score: 1.0     Cumulative Blast bit score: 456

conserved serine proline-rich protein
  
Accession: GAA85232
  
Location: 165718-168188
  
 NCBI BlastP on this gene

GAA85232

similar to An07g05420
  
Accession: GAA85233
  
Location: 169030-169365
  
 NCBI BlastP on this gene

GAA85233

hypothetical protein
  
Accession: GAA85234
  
Location: 170012-171069
  
 NCBI BlastP on this gene

GAA85234

short-chain dehydrogenase
  
Accession: GAA85235
  
Location: 172398-173174
  
 NCBI BlastP on this gene

GAA85235

similar to An07g05450
  
Accession: GAA85236
  
Location: 174135-174659
  
 NCBI BlastP on this gene

GAA85236

methyltransferase family protein
  
Accession: GAA85237
  
Location: 175211-176206
  
 NCBI BlastP on this gene

GAA85237

stage V sporulation protein k
  
Accession: GAA85238
  
Location: 177005-177824
  
 NCBI BlastP on this gene

GAA85238

hypothetical protein
  
Accession: GAA85239
  
Location: 178247-178941
  
 NCBI BlastP on this gene

GAA85239

ferric-chelate reductase
  
Accession: GAA85240
  
Location: 179188-181176
  
  
**BlastP hit with Mycgr3G107069\_Mycgr3**
  
Percentage identity: 41 %
  
BlastP bit score: 456
  
Sequence coverage: 106 %
  
E-value: 2e-148
  
  
 NCBI BlastP on this gene

GAA85240

209. :  CU633899 Podospora anserina S mat+ genomic DNA chromosome 1, supercontig 4.     Total score: 1.0     Cumulative Blast bit score: 456

not annotated
  
Accession: CAP67747
  
Location: 697446-700892
  
 NCBI BlastP on this gene

CAP67747

not annotated
  
Accession: CAP67748
  
Location: 701001-702607
  
 NCBI BlastP on this gene

CAP67748

not annotated
  
Accession: CAP67749
  
Location: 704205-706226
  
 NCBI BlastP on this gene

CAP67749

not annotated
  
Accession: CAP67750
  
Location: 707784-709349
  
 NCBI BlastP on this gene

CAP67750

not annotated
  
Accession: CAP67751
  
Location: 709764-710465
  
 NCBI BlastP on this gene

CAP67751

not annotated
  
Accession: CAP67752
  
Location: 711799-713760
  
  
**BlastP hit with Mycgr3G107069\_Mycgr3**
  
Percentage identity: 38 %
  
BlastP bit score: 456
  
Sequence coverage: 103 %
  
E-value: 2e-148
  
  
 NCBI BlastP on this gene

CAP67752

210. :  KB908570 Setosphaeria turcica Et28A unplaced genomic scaffold SETTUscaffold\_18     Total score: 1.0     Cumulative Blast bit score: 455

hypothetical protein
  
Accession: EOA87797
  
Location: 534968-536815
  
  
**BlastP hit with Mycgr3G107069\_Mycgr3**
  
Percentage identity: 41 %
  
BlastP bit score: 455
  
Sequence coverage: 95 %
  
E-value: 1e-148
  
  
 NCBI BlastP on this gene

EOA87797

hypothetical protein
  
Accession: EOA87796
  
Location: 534478-534888
  
 NCBI BlastP on this gene

EOA87796

hypothetical protein
  
Accession: EOA87795
  
Location: 533642-533821
  
 NCBI BlastP on this gene

EOA87795

hypothetical protein
  
Accession: EOA87794
  
Location: 532418-533065
  
 NCBI BlastP on this gene

EOA87794

hypothetical protein
  
Accession: EOA87793
  
Location: 529970-531338
  
 NCBI BlastP on this gene

EOA87793

hypothetical protein
  
Accession: EOA87792
  
Location: 524512-528896
  
 NCBI BlastP on this gene

EOA87792

hypothetical protein
  
Accession: EOA87791
  
Location: 521513-523816
  
 NCBI BlastP on this gene

EOA87791

hypothetical protein
  
Accession: EOA87790
  
Location: 518407-521280
  
 NCBI BlastP on this gene

EOA87790

211. :  KB725930 Colletotrichum orbiculare MAFF 240422 unplaced genomic scaffold Scaffold\_366     Total score: 1.0     Cumulative Blast bit score: 451

hypothetical protein
  
Accession: ENH82409
  
Location: 550887-551476
  
 NCBI BlastP on this gene

ENH82409

wsc domain-containing protein
  
Accession: ENH82408
  
Location: 540858-546223
  
 NCBI BlastP on this gene

ENH82408

cytochrome p450
  
Accession: ENH82407
  
Location: 538278-540096
  
 NCBI BlastP on this gene

ENH82407

high affinity copper transporter
  
Accession: ENH82406
  
Location: 537176-537780
  
 NCBI BlastP on this gene

ENH82406

ferric reductase transmembrane component 2
  
Accession: ENH82405
  
Location: 533798-535957
  
  
**BlastP hit with Mycgr3G107069\_Mycgr3**
  
Percentage identity: 40 %
  
BlastP bit score: 452
  
Sequence coverage: 102 %
  
E-value: 1e-146
  
  
 NCBI BlastP on this gene

ENH82405

dynamin family protein
  
Accession: ENH82404
  
Location: 529946-532460
  
 NCBI BlastP on this gene

ENH82404

metalloprotease mep1
  
Accession: ENH82403
  
Location: 527744-528718
  
 NCBI BlastP on this gene

ENH82403

hypothetical protein
  
Accession: ENH82402
  
Location: 526667-527108
  
 NCBI BlastP on this gene

ENH82402

platelet-activating factor acetylhydrolase precursor
  
Accession: ENH82401
  
Location: 524331-526171
  
 NCBI BlastP on this gene

ENH82401

fatty acid oxygenase
  
Accession: ENH82400
  
Location: 518936-522797
  
 NCBI BlastP on this gene

ENH82400

212. :  GL385395 Gaeumannomyces graminis var. tritici R3-111a-1 unplaced genomic scaffold supercont2.1     Total score: 1.0     Cumulative Blast bit score: 451

hypothetical protein
  
Accession: EJT82544
  
Location: 7871696-7872729
  
 NCBI BlastP on this gene

EJT82544

hypothetical protein
  
Accession: EJT82545
  
Location: 7873951-7875411
  
 NCBI BlastP on this gene

EJT82545

integral membrane protein
  
Accession: EJT82546
  
Location: 7876483-7877619
  
 NCBI BlastP on this gene

EJT82546

hypothetical protein
  
Accession: EJT82547
  
Location: 7880535-7882603
  
 NCBI BlastP on this gene

EJT82547

hypothetical protein
  
Accession: EJT82548
  
Location: 7883762-7885938
  
 NCBI BlastP on this gene

EJT82548

hypothetical protein
  
Accession: EJT82549
  
Location: 7888106-7889923
  
  
**BlastP hit with Mycgr3G65725\_Mycgr3T**
  
Percentage identity: 55 %
  
BlastP bit score: 452
  
Sequence coverage: 88 %
  
E-value: 7e-151
  
  
 NCBI BlastP on this gene

EJT82549

213. :  CM001233 Magnaporthe oryzae 70-15 chromosome 3     Total score: 1.0     Cumulative Blast bit score: 451

hypothetical protein
  
Accession: EHA53256
  
Location: 5962733-5963773
  
 NCBI BlastP on this gene

EHA53256

glutamyl-tRNA(Gln) amidotransferase subunit A
  
Accession: EHA53257
  
Location: 5963961-5966063
  
 NCBI BlastP on this gene

EHA53257

nonselective cation channel
  
Accession: EHA53258
  
Location: 5967077-5969391
  
 NCBI BlastP on this gene

EHA53258

hypothetical protein
  
Accession: EHA53259
  
Location: 5970031-5971771
  
 NCBI BlastP on this gene

EHA53259

phytanoyl-CoA dioxygenase
  
Accession: EHA53260
  
Location: 5972051-5973168
  
 NCBI BlastP on this gene

EHA53260

high affinity copper transporter
  
Accession: EHA53261
  
Location: 5973738-5974452
  
 NCBI BlastP on this gene

EHA53261

ferric reductase
  
Accession: EHA53262
  
Location: 5976475-5978571
  
  
**BlastP hit with Mycgr3G107069\_Mycgr3**
  
Percentage identity: 39 %
  
BlastP bit score: 452
  
Sequence coverage: 107 %
  
E-value: 2e-146
  
  
 NCBI BlastP on this gene

EHA53262

hypothetical protein
  
Accession: EHA53263
  
Location: 5979581-5979943
  
 NCBI BlastP on this gene

EHA53263

hypothetical protein
  
Accession: EHA53264
  
Location: 5982855-5983352
  
 NCBI BlastP on this gene

EHA53264

hypothetical protein
  
Accession: EHA53265
  
Location: 5984940-5985395
  
 NCBI BlastP on this gene

EHA53265

beta-fructofuranosidase
  
Accession: EHA53266
  
Location: 5986465-5988793
  
 NCBI BlastP on this gene

EHA53266

hypothetical protein
  
Accession: EHA53267
  
Location: 5990904-5992192
  
 NCBI BlastP on this gene

EHA53267

214. :  KB446540 Dothistroma septosporum NZE10 unplaced genomic scaffold DOTSEscaffold\_6     Total score: 1.0     Cumulative Blast bit score: 449

hypothetical protein
  
Accession: EME42891
  
Location: 221457-223365
  
 NCBI BlastP on this gene

EME42891

hypothetical protein
  
Accession: EME42892
  
Location: 224418-225737
  
 NCBI BlastP on this gene

EME42892

hypothetical protein
  
Accession: EME42893
  
Location: 226239-228242
  
 NCBI BlastP on this gene

EME42893

hypothetical protein
  
Accession: EME42894
  
Location: 229568-230574
  
 NCBI BlastP on this gene

EME42894

hypothetical protein
  
Accession: EME42895
  
Location: 231308-232759
  
 NCBI BlastP on this gene

EME42895

hypothetical protein
  
Accession: EME42896
  
Location: 234835-235788
  
 NCBI BlastP on this gene

EME42896

hypothetical protein
  
Accession: EME42898
  
Location: 236343-236617
  
 NCBI BlastP on this gene

EME42898

hypothetical protein
  
Accession: EME42899
  
Location: 239820-240920
  
  
**BlastP hit with Mycgr3G102276\_Mycgr3**
  
Percentage identity: 62 %
  
BlastP bit score: 450
  
Sequence coverage: 99 %
  
E-value: 3e-154
  
  
 NCBI BlastP on this gene

EME42899

hypothetical protein
  
Accession: EME42900
  
Location: 241815-245052
  
 NCBI BlastP on this gene

EME42900

hypothetical protein
  
Accession: EME42901
  
Location: 247088-248897
  
 NCBI BlastP on this gene

EME42901

hypothetical protein
  
Accession: EME42902
  
Location: 249247-249813
  
 NCBI BlastP on this gene

EME42902

hypothetical protein
  
Accession: EME42903
  
Location: 252680-254872
  
 NCBI BlastP on this gene

EME42903

hypothetical protein
  
Accession: EME42904
  
Location: 255514-257641
  
 NCBI BlastP on this gene

EME42904

215. :  DS178270 Puccinia graminis f. sp. tritici CRL 75-36-700-3 supercont2.9 genomic scaffold     Total score: 1.0     Cumulative Blast bit score: 446

hypothetical protein
  
Accession: EFP78283
  
Location: 28021-28972
  
 NCBI BlastP on this gene

EFP78283

hypothetical protein
  
Accession: EFP78284
  
Location: 30827-34814
  
 NCBI BlastP on this gene

EFP78284

hypothetical protein
  
Accession: EFP78285
  
Location: 36141-37485
  
 NCBI BlastP on this gene

EFP78285

hypothetical protein
  
Accession: EFP78286
  
Location: 38388-39338
  
 NCBI BlastP on this gene

EFP78286

hypothetical protein
  
Accession: EFP78287
  
Location: 40656-43124
  
  
**BlastP hit with Mycgr3G34982\_Mycgr3T**
  
Percentage identity: 29 %
  
BlastP bit score: 224
  
Sequence coverage: 103 %
  
E-value: 3e-61
  
  
 NCBI BlastP on this gene

EFP78287

hypothetical protein
  
Accession: EFP78288
  
Location: 45228-47750
  
  
**BlastP hit with Mycgr3G34982\_Mycgr3T**
  
Percentage identity: 29 %
  
BlastP bit score: 222
  
Sequence coverage: 105 %
  
E-value: 2e-60
  
  
 NCBI BlastP on this gene

EFP78288

hypothetical protein
  
Accession: EFP78289
  
Location: 48837-49495
  
 NCBI BlastP on this gene

EFP78289

hypothetical protein
  
Accession: EFP78290
  
Location: 52616-54219
  
 NCBI BlastP on this gene

EFP78290

hypothetical protein
  
Accession: EFP78291
  
Location: 55239-56042
  
 NCBI BlastP on this gene

EFP78291

hypothetical protein
  
Accession: EFP78292
  
Location: 57918-60200
  
 NCBI BlastP on this gene

EFP78292

216. :  CP000113 Myxococcus xanthus DK 1622     Total score: 1.0     Cumulative Blast bit score: 446

polyketide synthase
  
Accession: ABF91610
  
Location: 5611864-5627412
  
 NCBI BlastP on this gene

MXAN\_4527

polyketide synthase type I
  
Accession: ABF89696
  
Location: 5605427-5611867
  
 NCBI BlastP on this gene

MXAN\_4526

non-ribosomal peptide synthase MxaA
  
Accession: ABF90459
  
Location: 5600883-5605430
  
  
**BlastP hit with Mycgr3G107072\_Mycgr3**
  
Percentage identity: 34 %
  
BlastP bit score: 446
  
Sequence coverage: 74 %
  
E-value: 4e-129
  
  
 NCBI BlastP on this gene

MXAN\_4525

hypothetical protein
  
Accession: ABF90264
  
Location: 5600283-5600801
  
 NCBI BlastP on this gene

MXAN\_4524

putative lipoprotein
  
Accession: ABF86831
  
Location: 5598689-5599945
  
 NCBI BlastP on this gene

MXAN\_4523

hypothetical protein
  
Accession: ABF86081
  
Location: 5598558-5598668
  
 NCBI BlastP on this gene

MXAN\_4522

sulfatase family protein
  
Accession: ABF86036
  
Location: 5597314-5598186
  
 NCBI BlastP on this gene

MXAN\_4521

conserved domain protein
  
Accession: ABF87512
  
Location: 5596721-5597278
  
 NCBI BlastP on this gene

MXAN\_4520

hypothetical protein
  
Accession: ABF85867
  
Location: 5596617-5596769
  
 NCBI BlastP on this gene

MXAN\_4519

hypothetical protein
  
Accession: ABF92805
  
Location: 5596398-5596607
  
 NCBI BlastP on this gene

MXAN\_4518

hypothetical protein
  
Accession: ABF92090
  
Location: 5595240-5596337
  
 NCBI BlastP on this gene

MXAN\_4517

hypothetical protein
  
Accession: ABF87888
  
Location: 5594957-5595172
  
 NCBI BlastP on this gene

MXAN\_4516

hypothetical protein
  
Accession: ABF91051
  
Location: 5594884-5595045
  
 NCBI BlastP on this gene

MXAN\_4515

metallophosphoesterase
  
Accession: ABF90019
  
Location: 5593616-5594743
  
 NCBI BlastP on this gene

MXAN\_4514

conserved hypothetical protein
  
Accession: ABF86208
  
Location: 5590872-5593619
  
 NCBI BlastP on this gene

MXAN\_4513

RibD domain protein
  
Accession: ABF90277
  
Location: 5589134-5589841
  
 NCBI BlastP on this gene

MXAN\_4511

ATPase, AAA family
  
Accession: ABF86043
  
Location: 5587130-5589103
  
 NCBI BlastP on this gene

MXAN\_4510

hypothetical protein
  
Accession: ABF91047
  
Location: 5585346-5587133
  
 NCBI BlastP on this gene

MXAN\_4509

217. :  CM001196 Mycosphaerella graminicola IPO323 chromosome 1     Total score: 1.0     Cumulative Blast bit score: 445

hypothetical protein
  
Accession: EGP92693
  
Location: 793634-795322
  
 NCBI BlastP on this gene

EGP92693

hypothetical protein
  
Accession: EGP90901
  
Location: 795627-797345
  
 NCBI BlastP on this gene

EGP90901

hypothetical protein
  
Accession: EGP90902
  
Location: 799763-800017
  
 NCBI BlastP on this gene

EGP90902

hypothetical protein
  
Accession: EGP92692
  
Location: 801154-801935
  
 NCBI BlastP on this gene

EGP92692

hypothetical protein
  
Accession: EGP92691
  
Location: 805666-808885
  
 NCBI BlastP on this gene

EGP92691

hypothetical protein
  
Accession: EGP90903
  
Location: 809724-810611
  
 NCBI BlastP on this gene

EGP90903

hypothetical protein
  
Accession: EGP90904
  
Location: 811094-812542
  
  
**BlastP hit with Mycgr3G102276\_Mycgr3**
  
Percentage identity: 64 %
  
BlastP bit score: 445
  
Sequence coverage: 97 %
  
E-value: 2e-152
  
  
 NCBI BlastP on this gene

EGP90904

hypothetical protein
  
Accession: EGP92690
  
Location: 813851-814237
  
 NCBI BlastP on this gene

EGP92690

hypothetical protein
  
Accession: EGP92689
  
Location: 822031-822555
  
 NCBI BlastP on this gene

EGP92689

hypothetical protein
  
Accession: EGP90905
  
Location: 822994-823287
  
 NCBI BlastP on this gene

EGP90905

peptidase M28
  
Accession: EGP90906
  
Location: 826199-828657
  
 NCBI BlastP on this gene

EGP90906

218. :  KB908548 Setosphaeria turcica Et28A unplaced genomic scaffold SETTUscaffold\_16     Total score: 1.0     Cumulative Blast bit score: 442

hypothetical protein
  
Accession: EOA88290
  
Location: 881762-884148
  
 NCBI BlastP on this gene

EOA88290

hypothetical protein
  
Accession: EOA88291
  
Location: 885761-887625
  
 NCBI BlastP on this gene

EOA88291

carbohydrate esterase family 5 protein
  
Accession: EOA88292
  
Location: 888019-889154
  
 NCBI BlastP on this gene

EOA88292

hypothetical protein
  
Accession: EOA88293
  
Location: 891896-893788
  
 NCBI BlastP on this gene

EOA88293

hypothetical protein
  
Accession: EOA88294
  
Location: 893940-894185
  
 NCBI BlastP on this gene

EOA88294

hypothetical protein
  
Accession: EOA88295
  
Location: 895588-896261
  
 NCBI BlastP on this gene

EOA88295

hypothetical protein
  
Accession: EOA88296
  
Location: 896793-898838
  
  
**BlastP hit with Mycgr3G107069\_Mycgr3**
  
Percentage identity: 38 %
  
BlastP bit score: 442
  
Sequence coverage: 107 %
  
E-value: 8e-143
  
  
 NCBI BlastP on this gene

EOA88296

219. :  GL385398 Gaeumannomyces graminis var. tritici R3-111a-1 unplaced genomic scaffold supercont2.4     Total score: 1.0     Cumulative Blast bit score: 442

hypothetical protein
  
Accession: EJT73608
  
Location: 178976-183782
  
 NCBI BlastP on this gene

EJT73608

hypothetical protein
  
Accession: EJT73607
  
Location: 177123-178787
  
 NCBI BlastP on this gene

EJT73607

hypothetical protein
  
Accession: EJT73606
  
Location: 174701-176241
  
 NCBI BlastP on this gene

EJT73606

hypothetical protein
  
Accession: EJT73605
  
Location: 173164-173730
  
 NCBI BlastP on this gene

EJT73605

hypothetical protein
  
Accession: EJT73604
  
Location: 169877-172792
  
 NCBI BlastP on this gene

EJT73604

pentalenene synthase
  
Accession: EJT73603
  
Location: 168117-169508
  
 NCBI BlastP on this gene

EJT73603

hypothetical protein
  
Accession: EJT73602
  
Location: 164499-166525
  
 NCBI BlastP on this gene

EJT73602

ferric reductase
  
Accession: EJT73601
  
Location: 161024-163060
  
  
**BlastP hit with Mycgr3G107069\_Mycgr3**
  
Percentage identity: 38 %
  
BlastP bit score: 442
  
Sequence coverage: 106 %
  
E-value: 9e-143
  
  
 NCBI BlastP on this gene

EJT73601

hypothetical protein
  
Accession: EJT73600
  
Location: 159156-160500
  
 NCBI BlastP on this gene

EJT73600

hypothetical protein
  
Accession: EJT73599
  
Location: 155778-159115
  
 NCBI BlastP on this gene

EJT73599

hypothetical protein
  
Accession: EJT73598
  
Location: 153599-154629
  
 NCBI BlastP on this gene

EJT73598

hypothetical protein
  
Accession: EJT73597
  
Location: 152580-153105
  
 NCBI BlastP on this gene

EJT73597

hypothetical protein
  
Accession: EJT73596
  
Location: 151826-152198
  
 NCBI BlastP on this gene

EJT73596

hypothetical protein
  
Accession: EJT73595
  
Location: 149929-151431
  
 NCBI BlastP on this gene

EJT73595

hypothetical protein
  
Accession: EJT73594
  
Location: 149251-149744
  
 NCBI BlastP on this gene

EJT73594

hypothetical protein
  
Accession: EJT73593
  
Location: 147102-148752
  
 NCBI BlastP on this gene

EJT73593

hypothetical protein
  
Accession: EJT73592
  
Location: 146222-147036
  
 NCBI BlastP on this gene

EJT73592

hypothetical protein
  
Accession: EJT73591
  
Location: 145463-146053
  
 NCBI BlastP on this gene

EJT73591

hypothetical protein
  
Accession: EJT73590
  
Location: 144471-145330
  
 NCBI BlastP on this gene

EJT73590

220. :  JH126405 Cordyceps militaris CM01 unplaced genomic scaffold CCM\_S00007     Total score: 1.0     Cumulative Blast bit score: 441

zinc-binding alcohol dehydrogenase, putative
  
Accession: EGX88899
  
Location: 2330487-2331649
  
  
**BlastP hit with Mycgr3G102276\_Mycgr3**
  
Percentage identity: 61 %
  
BlastP bit score: 441
  
Sequence coverage: 99 %
  
E-value: 1e-150
  
  
 NCBI BlastP on this gene

EGX88899

hypothetical protein
  
Accession: EGX88898
  
Location: 2323352-2325603
  
 NCBI BlastP on this gene

EGX88898

ankyrin repeat-containing domain
  
Accession: EGX88897
  
Location: 2316564-2318828
  
 NCBI BlastP on this gene

EGX88897

alpha/beta hydrolase, putative
  
Accession: EGX88896
  
Location: 2314642-2316261
  
 NCBI BlastP on this gene

EGX88896

221. :  DS027694 Neosartorya fischeri NRRL 181 1099437636262 genomic scaffold     Total score: 1.0     Cumulative Blast bit score: 441

conserved hypothetical protein
  
Accession: EAW19998
  
Location: 676304-677260
  
 NCBI BlastP on this gene

EAW19998

hypothetical protein
  
Accession: EAW19997
  
Location: 674884-675357
  
 NCBI BlastP on this gene

EAW19997

conserved hypothetical protein
  
Accession: EAW19996
  
Location: 672504-673418
  
 NCBI BlastP on this gene

EAW19996

MFS transporter, putative
  
Accession: EAW19995
  
Location: 668057-671408
  
 NCBI BlastP on this gene

EAW19995

conserved hypothetical protein
  
Accession: EAW19994
  
Location: 666403-667028
  
 NCBI BlastP on this gene

EAW19994

C-4 methyl sterol oxidase, putative
  
Accession: EAW19993
  
Location: 664750-665750
  
 NCBI BlastP on this gene

EAW19993

conserved hypothetical protein
  
Accession: EAW19992
  
Location: 663956-664275
  
 NCBI BlastP on this gene

EAW19992

hypothetical protein
  
Accession: EAW19991
  
Location: 662754-663380
  
 NCBI BlastP on this gene

EAW19991

zinc-binding alcohol dehydrogenase, putative
  
Accession: EAW19990
  
Location: 659933-661126
  
  
**BlastP hit with Mycgr3G102276\_Mycgr3**
  
Percentage identity: 63 %
  
BlastP bit score: 441
  
Sequence coverage: 99 %
  
E-value: 1e-150
  
  
 NCBI BlastP on this gene

EAW19990

conserved hypothetical protein
  
Accession: EAW19989
  
Location: 659055-659883
  
 NCBI BlastP on this gene

EAW19989

UbiA prenyltransferase family protein
  
Accession: EAW19988
  
Location: 656971-658091
  
 NCBI BlastP on this gene

EAW19988

geranylgeranyl pyrophosphate synthetase AtmG, putative
  
Accession: EAW19987
  
Location: 654508-655741
  
 NCBI BlastP on this gene

EAW19987

conserved hypothetical protein
  
Accession: EAW19986
  
Location: 652832-653618
  
 NCBI BlastP on this gene

EAW19986

FAD binding domain protein
  
Accession: EAW19985
  
Location: 650224-651821
  
 NCBI BlastP on this gene

EAW19985

conserved hypothetical protein
  
Accession: EAW19984
  
Location: 648053-649412
  
 NCBI BlastP on this gene

EAW19984

polyketide synthase, putative
  
Accession: EAW19983
  
Location: 642104-647616
  
 NCBI BlastP on this gene

EAW19983

222. :  CM001197 Mycosphaerella graminicola IPO323 chromosome 2     Total score: 1.0     Cumulative Blast bit score: 441

hypothetical protein
  
Accession: EGP90797
  
Location: 83434-84270
  
 NCBI BlastP on this gene

EGP90797

hypothetical protein
  
Accession: EGP89671
  
Location: 84999-85341
  
 NCBI BlastP on this gene

EGP89671

hypothetical protein
  
Accession: EGP89672
  
Location: 86076-87190
  
 NCBI BlastP on this gene

EGP89672

hypothetical protein
  
Accession: EGP90796
  
Location: 89430-90215
  
 NCBI BlastP on this gene

EGP90796

hypothetical protein
  
Accession: EGP90795
  
Location: 90713-92091
  
 NCBI BlastP on this gene

EGP90795

hypothetical protein
  
Accession: EGP89673
  
Location: 93145-95400
  
 NCBI BlastP on this gene

EGP89673

hypothetical protein
  
Accession: EGP89674
  
Location: 95885-97982
  
 NCBI BlastP on this gene

EGP89674

hypothetical protein
  
Accession: EGP90794
  
Location: 98374-99028
  
 NCBI BlastP on this gene

EGP90794

putative FRE ferric reductase-like transmembrane component
  
Accession: EGP89675
  
Location: 99609-101786
  
  
**BlastP hit with Mycgr3G107069\_Mycgr3**
  
Percentage identity: 37 %
  
BlastP bit score: 441
  
Sequence coverage: 109 %
  
E-value: 3e-142
  
  
 NCBI BlastP on this gene

EGP89675

putative ABC transporter
  
Accession: EGP90793
  
Location: 103528-107876
  
 NCBI BlastP on this gene

EGP90793

223. :  KE145356 Glarea lozoyensis ATCC 20868 chromosome Unknown GLAREA13     Total score: 1.0     Cumulative Blast bit score: 438

GroES-like protein
  
Accession: EPE34719
  
Location: 1389878-1391072
  
  
**BlastP hit with Mycgr3G102276\_Mycgr3**
  
Percentage identity: 62 %
  
BlastP bit score: 438
  
Sequence coverage: 98 %
  
E-value: 9e-150
  
  
 NCBI BlastP on this gene

EPE34719

beta and beta-prime subunits of DNA dependent RNA-polymerase
  
Accession: EPE34718
  
Location: 1385305-1389205
  
 NCBI BlastP on this gene

EPE34718

hypothetical protein
  
Accession: EPE34717
  
Location: 1384442-1384762
  
 NCBI BlastP on this gene

EPE34717

hypothetical protein
  
Accession: EPE34716
  
Location: 1383346-1384114
  
 NCBI BlastP on this gene

EPE34716

alpha/beta-Hydrolase
  
Accession: EPE34715
  
Location: 1381648-1382826
  
 NCBI BlastP on this gene

EPE34715

hypothetical protein
  
Accession: EPE34714
  
Location: 1377126-1380394
  
 NCBI BlastP on this gene

EPE34714

hypothetical protein
  
Accession: EPE34713
  
Location: 1374266-1375255
  
 NCBI BlastP on this gene

EPE34713

hypothetical protein
  
Accession: EPE34712
  
Location: 1370293-1374138
  
 NCBI BlastP on this gene

EPE34712

224. :  KB446558 Pseudocercospora fijiensis CIRAD86 unplaced genomic scaffold MYCFIscaffold\_4     Total score: 1.0     Cumulative Blast bit score: 438

hypothetical protein
  
Accession: EME83122
  
Location: 2740932-2743078
  
 NCBI BlastP on this gene

EME83122

hypothetical protein
  
Accession: EME83121
  
Location: 2740281-2740826
  
 NCBI BlastP on this gene

EME83121

hypothetical protein
  
Accession: EME83120
  
Location: 2739066-2739694
  
 NCBI BlastP on this gene

EME83120

hypothetical protein
  
Accession: EME83119
  
Location: 2737050-2737988
  
 NCBI BlastP on this gene

EME83119

hypothetical protein
  
Accession: EME83118
  
Location: 2733466-2736458
  
 NCBI BlastP on this gene

EME83118

hypothetical protein
  
Accession: EME83117
  
Location: 2730736-2732218
  
 NCBI BlastP on this gene

EME83117

glycoside hydrolase family 28 protein
  
Accession: EME83116
  
Location: 2728877-2730277
  
 NCBI BlastP on this gene

EME83116

hypothetical protein
  
Accession: EME83115
  
Location: 2725659-2726833
  
  
**BlastP hit with Mycgr3G102276\_Mycgr3**
  
Percentage identity: 63 %
  
BlastP bit score: 438
  
Sequence coverage: 98 %
  
E-value: 1e-149
  
  
 NCBI BlastP on this gene

EME83115

hypothetical protein
  
Accession: EME83114
  
Location: 2723335-2725104
  
 NCBI BlastP on this gene

EME83114

hypothetical protein
  
Accession: EME83113
  
Location: 2721597-2722643
  
 NCBI BlastP on this gene

EME83113

hypothetical protein
  
Accession: EME83112
  
Location: 2720512-2720913
  
 NCBI BlastP on this gene

EME83112

hypothetical protein
  
Accession: EME83111
  
Location: 2719308-2719807
  
 NCBI BlastP on this gene

EME83111

hypothetical protein
  
Accession: EME83110
  
Location: 2716179-2717769
  
 NCBI BlastP on this gene

EME83110

hypothetical protein
  
Accession: EME83109
  
Location: 2714894-2715616
  
 NCBI BlastP on this gene

EME83109

hypothetical protein
  
Accession: EME83108
  
Location: 2710645-2714664
  
 NCBI BlastP on this gene

EME83108

hypothetical protein
  
Accession: EME83107
  
Location: 2706824-2709731
  
 NCBI BlastP on this gene

EME83107

225. :  DS990637 Ajellomyces capsulatus H88 unplaced genomic scaffold supercont1.2     Total score: 1.0     Cumulative Blast bit score: 436

conserved hypothetical protein
  
Accession: EGC43174
  
Location: 2458191-2459030
  
 NCBI BlastP on this gene

EGC43174

predicted protein
  
Accession: EGC43175
  
Location: 2460974-2461393
  
 NCBI BlastP on this gene

EGC43175

conserved hypothetical protein
  
Accession: EGC43176
  
Location: 2462191-2463128
  
 NCBI BlastP on this gene

EGC43176

glycerol-3-phosphate O-acyltransferase
  
Accession: EGC43177
  
Location: 2464383-2466916
  
 NCBI BlastP on this gene

EGC43177

dehydrodolichyl diphosphate synthetase
  
Accession: EGC43178
  
Location: 2468623-2470066
  
 NCBI BlastP on this gene

EGC43178

saccharopine dehydrogenase
  
Accession: EGC43179
  
Location: 2470740-2472777
  
 NCBI BlastP on this gene

EGC43179

cupin domain-containing protein
  
Accession: EGC43180
  
Location: 2473292-2473864
  
 NCBI BlastP on this gene

EGC43180

glucose-methanol-choline oxidoreductase:GMC oxidoreductase
  
Accession: EGC43181
  
Location: 2474861-2477167
  
  
**BlastP hit with Mycgr3G34982\_Mycgr3T**
  
Percentage identity: 42 %
  
BlastP bit score: 436
  
Sequence coverage: 94 %
  
E-value: 1e-142
  
  
 NCBI BlastP on this gene

EGC43181

226. :  KB644408 Penicillium oxalicum 114-2 unplaced genomic scaffold scaffold\_1     Total score: 1.0     Cumulative Blast bit score: 434

hypothetical protein
  
Accession: EPS26262
  
Location: 3636886-3638676
  
 NCBI BlastP on this gene

EPS26262

hypothetical protein
  
Accession: EPS26263
  
Location: 3639979-3641217
  
 NCBI BlastP on this gene

EPS26263

hypothetical protein
  
Accession: EPS26264
  
Location: 3643693-3645393
  
 NCBI BlastP on this gene

EPS26264

alpha-amylase Amy13A
  
Accession: EPS26265
  
Location: 3645573-3647950
  
 NCBI BlastP on this gene

EPS26265

hypothetical protein
  
Accession: EPS26266
  
Location: 3654136-3655289
  
  
**BlastP hit with Mycgr3G102276\_Mycgr3**
  
Percentage identity: 62 %
  
BlastP bit score: 435
  
Sequence coverage: 99 %
  
E-value: 2e-148
  
  
 NCBI BlastP on this gene

EPS26266

227. :  DS499600 Aspergillus fumigatus A1163 scf\_000007 genomic scaffold     Total score: 1.0     Cumulative Blast bit score: 434

glutathione S-transferase
  
Accession: EDP48959
  
Location: 1270051-1271002
  
 NCBI BlastP on this gene

EDP48959

hypothetical protein
  
Accession: EDP48960
  
Location: 1271560-1272346
  
 NCBI BlastP on this gene

EDP48960

conserved hypothetical protein
  
Accession: EDP48961
  
Location: 1274403-1275319
  
 NCBI BlastP on this gene

EDP48961

trihydroxytoluene oxygenase
  
Accession: EDP48962
  
Location: 1276364-1277310
  
 NCBI BlastP on this gene

EDP48962

MFS transporter, putative
  
Accession: EDP48963
  
Location: 1278082-1279698
  
 NCBI BlastP on this gene

EDP48963

conserved hypothetical protein
  
Accession: EDP48964
  
Location: 1280702-1281401
  
 NCBI BlastP on this gene

EDP48964

C-4 methyl sterol oxidase, putative
  
Accession: EDP48965
  
Location: 1282062-1283062
  
 NCBI BlastP on this gene

EDP48965

zinc-binding alcohol dehydrogenase, putative
  
Accession: EDP48966
  
Location: 1286825-1288017
  
  
**BlastP hit with Mycgr3G102276\_Mycgr3**
  
Percentage identity: 62 %
  
BlastP bit score: 435
  
Sequence coverage: 99 %
  
E-value: 2e-148
  
  
 NCBI BlastP on this gene

EDP48966

228. :  GL377303 Schizophyllum commune H4-8 unplaced genomic scaffold SCHCOscaffold\_2     Total score: 1.0     Cumulative Blast bit score: 434

hypothetical protein
  
Accession: EFJ01441
  
Location: 4315934-4318743
  
 NCBI BlastP on this gene

EFJ01441

hypothetical protein
  
Accession: EFJ01442
  
Location: 4320317-4321570
  
 NCBI BlastP on this gene

EFJ01442

hypothetical protein
  
Accession: EFJ00610
  
Location: 4322737-4324776
  
 NCBI BlastP on this gene

EFJ00610

hypothetical protein
  
Accession: EFJ01443
  
Location: 4325488-4326234
  
 NCBI BlastP on this gene

EFJ01443

hypothetical protein
  
Accession: EFJ00611
  
Location: 4326893-4328421
  
 NCBI BlastP on this gene

EFJ00611

hypothetical protein
  
Accession: EFJ00612
  
Location: 4328996-4330626
  
 NCBI BlastP on this gene

EFJ00612

hypothetical protein
  
Accession: EFJ00613
  
Location: 4331327-4332598
  
 NCBI BlastP on this gene

EFJ00613

hypothetical protein
  
Accession: EFJ00614
  
Location: 4333196-4335214
  
  
**BlastP hit with Mycgr3G32432\_Mycgr3T**
  
Percentage identity: 32 %
  
BlastP bit score: 202
  
Sequence coverage: 75 %
  
E-value: 7e-54
  
  
 NCBI BlastP on this gene

EFJ00614

hypothetical protein
  
Accession: EFJ00615
  
Location: 4336342-4338390
  
  
**BlastP hit with Mycgr3G32432\_Mycgr3T**
  
Percentage identity: 33 %
  
BlastP bit score: 232
  
Sequence coverage: 79 %
  
E-value: 9e-65
  
  
 NCBI BlastP on this gene

EFJ00615

expressed protein
  
Accession: EFJ00616
  
Location: 4339533-4340219
  
 NCBI BlastP on this gene

EFJ00616

hypothetical protein
  
Accession: EFJ00617
  
Location: 4342854-4343597
  
 NCBI BlastP on this gene

EFJ00617

hypothetical protein
  
Accession: EFJ01444
  
Location: 4343666-4344545
  
 NCBI BlastP on this gene

EFJ01444

hypothetical protein
  
Accession: EFJ00618
  
Location: 4345403-4346160
  
 NCBI BlastP on this gene

EFJ00618

glycoside hydrolase family 5 protein
  
Accession: EFJ00619
  
Location: 4346936-4349977
  
 NCBI BlastP on this gene

EFJ00619

expressed protein
  
Accession: EFJ00620
  
Location: 4350917-4351833
  
 NCBI BlastP on this gene

EFJ00620

expressed protein
  
Accession: EFJ00621
  
Location: 4352474-4353358
  
 NCBI BlastP on this gene

EFJ00621

229. :  AM920431 Penicillium chrysogenum Wisconsin 54-1255 complete genome, contig Pc00c16.     Total score: 1.0     Cumulative Blast bit score: 434

not annotated
  
Accession: CAP92724
  
Location: 117077-118711
  
 NCBI BlastP on this gene

Pc16g00540

not annotated
  
Accession: CAP92723
  
Location: 114680-116401
  
 NCBI BlastP on this gene

Pc16g00530

not annotated
  
Accession: CAP92722
  
Location: 113357-114283
  
 NCBI BlastP on this gene

Pc16g00520

not annotated
  
Accession: CAP92721
  
Location: 109350-111423
  
 NCBI BlastP on this gene

Pc16g00510

not annotated
  
Accession: CAP92720
  
Location: 107077-108823
  
 NCBI BlastP on this gene

Pc16g00500

unnamed
  
Accession: CAP92719
  
Location: 105607-106396
  
 NCBI BlastP on this gene

Pc16g00490

not annotated
  
Accession: CAP92718
  
Location: 104779-105400
  
 NCBI BlastP on this gene

Pc16g00480

unnamed
  
Accession: CAP92717
  
Location: 103308-104356
  
 NCBI BlastP on this gene

Pc16g00470

hypothetical protein
  
Accession: CAP92716
  
Location: 101051-102882
  
 NCBI BlastP on this gene

Pc16g00460

not annotated
  
Accession: CAP92715
  
Location: 99550-100697
  
  
**BlastP hit with Mycgr3G102276\_Mycgr3**
  
Percentage identity: 62 %
  
BlastP bit score: 434
  
Sequence coverage: 99 %
  
E-value: 7e-148
  
  
 NCBI BlastP on this gene

Pc16g00450

not annotated
  
Accession: CAP92714
  
Location: 97348-98151
  
 NCBI BlastP on this gene

Pc16g00440

not annotated
  
Accession: CAP92713
  
Location: 95191-96138
  
 NCBI BlastP on this gene

Pc16g00430

hypothetical protein
  
Accession: CAP92712
  
Location: 93753-94586
  
 NCBI BlastP on this gene

Pc16g00420

not annotated
  
Accession: Pc16g00410
  
Location: 90386-93205
  
 NCBI BlastP on this gene

Pc16g00410

unnamed
  
Accession: CAP92710
  
Location: 88167-89894
  
 NCBI BlastP on this gene

Pc16g00400

not annotated
  
Accession: CAP92709
  
Location: 86693-87762
  
 NCBI BlastP on this gene

Pc16g00390

not annotated
  
Accession: CAP92708
  
Location: 85399-86411
  
 NCBI BlastP on this gene

Pc16g00380

not annotated
  
Accession: CAP92707
  
Location: 79583-84934
  
 NCBI BlastP on this gene

Pc16g00370

230. :  GL988032 Chaetomium thermophilum var. thermophilum DSM 1495 unplaced genomic scaffold scf7180000...     Total score: 1.0     Cumulative Blast bit score: 432

hypothetical protein
  
Accession: EGS23685
  
Location: 1301806-1303611
  
 NCBI BlastP on this gene

EGS23685

hypothetical protein
  
Accession: EGS23686
  
Location: 1305702-1306968
  
 NCBI BlastP on this gene

EGS23686

dehydrogenase-like protein
  
Accession: EGS23687
  
Location: 1308553-1310234
  
 NCBI BlastP on this gene

EGS23687

putative high affinity copper protein
  
Accession: EGS23688
  
Location: 1310379-1311040
  
 NCBI BlastP on this gene

EGS23688

putative FAD binding protein
  
Accession: EGS23689
  
Location: 1312431-1314353
  
  
**BlastP hit with Mycgr3G107069\_Mycgr3**
  
Percentage identity: 39 %
  
BlastP bit score: 433
  
Sequence coverage: 102 %
  
E-value: 7e-140
  
  
 NCBI BlastP on this gene

EGS23689

231. :  KB446537 Dothistroma septosporum NZE10 unplaced genomic scaffold DOTSEscaffold\_3     Total score: 1.0     Cumulative Blast bit score: 430

hypothetical protein
  
Accession: EME46885
  
Location: 2584198-2585443
  
  
**BlastP hit with Mycgr3G102276\_Mycgr3**
  
Percentage identity: 61 %
  
BlastP bit score: 430
  
Sequence coverage: 97 %
  
E-value: 3e-146
  
  
 NCBI BlastP on this gene

EME46885

hypothetical protein
  
Accession: EME46884
  
Location: 2582971-2583138
  
 NCBI BlastP on this gene

EME46884

hypothetical protein
  
Accession: EME46883
  
Location: 2582588-2582911
  
 NCBI BlastP on this gene

EME46883

hypothetical protein
  
Accession: EME46882
  
Location: 2580682-2580990
  
 NCBI BlastP on this gene

EME46882

hypothetical protein
  
Accession: EME46881
  
Location: 2578352-2580235
  
 NCBI BlastP on this gene

EME46881

glycosyltransferase family 71 protein
  
Accession: EME46880
  
Location: 2575317-2576795
  
 NCBI BlastP on this gene

EME46880

hypothetical protein
  
Accession: EME46879
  
Location: 2574092-2574823
  
 NCBI BlastP on this gene

EME46879

hypothetical protein
  
Accession: EME46878
  
Location: 2571443-2572529
  
 NCBI BlastP on this gene

EME46878

hypothetical protein
  
Accession: EME46877
  
Location: 2568363-2570249
  
 NCBI BlastP on this gene

EME46877

hypothetical protein
  
Accession: EME46876
  
Location: 2567771-2568251
  
 NCBI BlastP on this gene

EME46876

232. :  AP012319 Actinoplanes missouriensis 431 DNA     Total score: 1.0     Cumulative Blast bit score: 428

putative MFS transporter
  
Accession: BAL88448
  
Location: 3387407-3388840
  
 NCBI BlastP on this gene

AMIS\_32280

putative ABC transporter ATP-binding protein
  
Accession: BAL88449
  
Location: 3388920-3391370
  
 NCBI BlastP on this gene

AMIS\_32290

putative transcriptional regulator
  
Accession: BAL88450
  
Location: 3391646-3392929
  
 NCBI BlastP on this gene

AMIS\_32300

hypothetical protein
  
Accession: BAL88451
  
Location: 3393157-3393735
  
 NCBI BlastP on this gene

AMIS\_32310

putative NRPS
  
Accession: BAL88452
  
Location: 3394007-3402589
  
 NCBI BlastP on this gene

AMIS\_32320

putative NRPS
  
Accession: BAL88453
  
Location: 3402586-3407013
  
  
**BlastP hit with Mycgr3G107072\_Mycgr3**
  
Percentage identity: 33 %
  
BlastP bit score: 428
  
Sequence coverage: 73 %
  
E-value: 3e-123
  
  
 NCBI BlastP on this gene

AMIS\_32330

hypothetical protein
  
Accession: BAL88454
  
Location: 3407010-3407996
  
 NCBI BlastP on this gene

AMIS\_32340

putative O-methyltransferase
  
Accession: BAL88455
  
Location: 3407993-3408667
  
 NCBI BlastP on this gene

AMIS\_32350

putative MbtH-like protein
  
Accession: BAL88456
  
Location: 3408695-3408904
  
 NCBI BlastP on this gene

AMIS\_32360

hypothetical protein
  
Accession: BAL88457
  
Location: 3408938-3410416
  
 NCBI BlastP on this gene

AMIS\_32370

putative M28-family peptidase
  
Accession: BAL88458
  
Location: 3410441-3412711
  
 NCBI BlastP on this gene

AMIS\_32380

putative short-chain dehydrogenase
  
Accession: BAL88459
  
Location: 3412729-3413604
  
 NCBI BlastP on this gene

AMIS\_32390

putative transcriptional regulator
  
Accession: BAL88460
  
Location: 3413692-3415116
  
 NCBI BlastP on this gene

AMIS\_32400

hypothetical protein
  
Accession: BAL88461
  
Location: 3415297-3415977
  
 NCBI BlastP on this gene

AMIS\_32410

putative NRPS-related enzyme
  
Accession: BAL88462
  
Location: 3416555-3419143
  
 NCBI BlastP on this gene

AMIS\_32420

putative tryptophan halogenase
  
Accession: BAL88463
  
Location: 3419140-3420606
  
 NCBI BlastP on this gene

AMIS\_32430

putative multicopper oxidase
  
Accession: BAL88464
  
Location: 3420639-3422108
  
 NCBI BlastP on this gene

AMIS\_32440

putative sodium/proton antiporter
  
Accession: BAL88465
  
Location: 3422108-3423385
  
 NCBI BlastP on this gene

AMIS\_32450

233. :  JH795880 Dacryopinax sp. DJM-731 SS1 chromosome Unknown DACRYscaffold\_26     Total score: 1.0     Cumulative Blast bit score: 427

hypothetical protein
  
Accession: EJT96819
  
Location: 166377-167926
  
 NCBI BlastP on this gene

EJT96819

GMC oxidoreductase
  
Accession: EJT96820
  
Location: 168788-171828
  
  
**BlastP hit with Mycgr3G34982\_Mycgr3T**
  
Percentage identity: 28 %
  
BlastP bit score: 234
  
Sequence coverage: 99 %
  
E-value: 5e-65
  
  
 NCBI BlastP on this gene

EJT96820

hypothetical protein
  
Accession: EJT96821
  
Location: 173201-173365
  
 NCBI BlastP on this gene

EJT96821

hypothetical protein
  
Accession: EJT96822
  
Location: 173988-174461
  
 NCBI BlastP on this gene

EJT96822

YebC-like protein
  
Accession: EJT96823
  
Location: 174840-175679
  
 NCBI BlastP on this gene

EJT96823

hypothetical protein
  
Accession: EJT96824
  
Location: 175916-177835
  
 NCBI BlastP on this gene

EJT96824

hypothetical protein
  
Accession: EJT96825
  
Location: 188066-189650
  
 NCBI BlastP on this gene

EJT96825

hypothetical protein
  
Accession: EJT96826
  
Location: 191303-191719
  
 NCBI BlastP on this gene

EJT96826

hypothetical protein
  
Accession: EJT96827
  
Location: 193093-193542
  
 NCBI BlastP on this gene

EJT96827

Aldo/keto reductase
  
Accession: EJT96828
  
Location: 194960-195868
  
 NCBI BlastP on this gene

EJT96828

alcohol oxidase
  
Accession: EJT96829
  
Location: 196101-198406
  
  
**BlastP hit with Mycgr3G34982\_Mycgr3T**
  
Percentage identity: 28 %
  
BlastP bit score: 193
  
Sequence coverage: 94 %
  
E-value: 7e-51
  
  
 NCBI BlastP on this gene

EJT96829

234. :  KB726020 Colletotrichum orbiculare MAFF 240422 unplaced genomic scaffold Scaffold\_447     Total score: 1.0     Cumulative Blast bit score: 426

glucose-methanol-choline oxidoreductase:gmc oxidoreductase
  
Accession: ENH79579
  
Location: 348871-351025
  
  
**BlastP hit with Mycgr3G34982\_Mycgr3T**
  
Percentage identity: 42 %
  
BlastP bit score: 426
  
Sequence coverage: 103 %
  
E-value: 3e-138
  
  
 NCBI BlastP on this gene

ENH79579

bZIP transcription factor
  
Accession: ENH79578
  
Location: 345984-347800
  
 NCBI BlastP on this gene

ENH79578

hypothetical protein
  
Accession: ENH79577
  
Location: 338644-340007
  
 NCBI BlastP on this gene

ENH79577

cyclopentanone -monooxygenase
  
Accession: ENH79576
  
Location: 336812-338534
  
 NCBI BlastP on this gene

ENH79576

235. :  CM001234 Magnaporthe oryzae 70-15 chromosome 4     Total score: 1.0     Cumulative Blast bit score: 424

choline dehydrogenase
  
Accession: EHA49619
  
Location: 213819-215798
  
  
**BlastP hit with Mycgr3G34982\_Mycgr3T**
  
Percentage identity: 41 %
  
BlastP bit score: 424
  
Sequence coverage: 103 %
  
E-value: 2e-137
  
  
 NCBI BlastP on this gene

EHA49619

hypothetical protein
  
Accession: EHA49620
  
Location: 216006-216961
  
 NCBI BlastP on this gene

EHA49620

trichothecene 3-O-acetyltransferase
  
Accession: EHA49621
  
Location: 217769-219196
  
 NCBI BlastP on this gene

EHA49621

hypothetical protein
  
Accession: EHA49622
  
Location: 221590-222228
  
 NCBI BlastP on this gene

EHA49622

hypothetical protein
  
Accession: EHA49623
  
Location: 222885-223409
  
 NCBI BlastP on this gene

EHA49623

hypothetical protein
  
Accession: EHA49624
  
Location: 230520-231249
  
 NCBI BlastP on this gene

EHA49624

hypothetical protein
  
Accession: EHA49625
  
Location: 231662-232084
  
 NCBI BlastP on this gene

EHA49625

236. :  CAIF01000017 Wickerhamomyces ciferrii strain NRRL Y-1031 F-60-10     Total score: 1.0     Cumulative Blast bit score: 424

Ubiquitin carboxyl-terminal hydrolase
  
Accession: CCH41317
  
Location: 10382-11077
  
 NCBI BlastP on this gene

CCH41317

Leukocyte receptor cluster member 8
  
Accession: CCH41318
  
Location: 11201-12517
  
 NCBI BlastP on this gene

CCH41318

hypothetical protein
  
Accession: CCH41319
  
Location: 12873-14171
  
 NCBI BlastP on this gene

CCH41319

Ferric reductase transmembrane component
  
Accession: CCH41320
  
Location: 17195-19318
  
  
**BlastP hit with Mycgr3G107069\_Mycgr3**
  
Percentage identity: 28 %
  
BlastP bit score: 150
  
Sequence coverage: 73 %
  
E-value: 3e-35
  
  
 NCBI BlastP on this gene

CCH41320

Ferric reductase transmembrane component
  
Accession: CCH41321
  
Location: 20158-22008
  
  
**BlastP hit with Mycgr3G107069\_Mycgr3**
  
Percentage identity: 26 %
  
BlastP bit score: 133
  
Sequence coverage: 62 %
  
E-value: 8e-30
  
  
 NCBI BlastP on this gene

CCH41321

Ferric reductase transmembrane component
  
Accession: CCH41322
  
Location: 28478-29368
  
 NCBI BlastP on this gene

CCH41322

Respiratory burst oxidase protein
  
Accession: CCH41323
  
Location: 29530-30753
  
 NCBI BlastP on this gene

CCH41323

Ferric reductase transmembrane component
  
Accession: CCH41324
  
Location: 32475-34667
  
 NCBI BlastP on this gene

CCH41324

Ferric reductase transmembrane component
  
Accession: CCH41325
  
Location: 35643-37775
  
  
**BlastP hit with Mycgr3G107069\_Mycgr3**
  
Percentage identity: 26 %
  
BlastP bit score: 141
  
Sequence coverage: 72 %
  
E-value: 4e-32
  
  
 NCBI BlastP on this gene

CCH41325

Lysine biosynthesis regulatory protein
  
Accession: CCH41326
  
Location: 38338-39966
  
 NCBI BlastP on this gene

CCH41326

Pantothenate transporter
  
Accession: CCH41327
  
Location: 40707-42317
  
 NCBI BlastP on this gene

CCH41327

hypothetical protein
  
Accession: CCH41328
  
Location: 42662-43573
  
 NCBI BlastP on this gene

CCH41328

Glycylpeptide N-tetradecanoyltransferase
  
Accession: CCH41329
  
Location: 43858-45189
  
 NCBI BlastP on this gene

CCH41329

237. :  KB446546 Dothistroma septosporum NZE10 unplaced genomic scaffold DOTSEscaffold\_12     Total score: 1.0     Cumulative Blast bit score: 423

hypothetical protein
  
Accession: EME38769
  
Location: 367659-368132
  
 NCBI BlastP on this gene

EME38769

hypothetical protein
  
Accession: EME38768
  
Location: 366100-367035
  
 NCBI BlastP on this gene

EME38768

hypothetical protein
  
Accession: EME38767
  
Location: 365103-365852
  
 NCBI BlastP on this gene

EME38767

hypothetical protein
  
Accession: EME38766
  
Location: 364212-364559
  
 NCBI BlastP on this gene

EME38766

hypothetical protein
  
Accession: EME38765
  
Location: 362807-363967
  
 NCBI BlastP on this gene

EME38765

hypothetical protein
  
Accession: EME38764
  
Location: 360522-362108
  
 NCBI BlastP on this gene

EME38764

hypothetical protein
  
Accession: EME38763
  
Location: 359234-360187
  
 NCBI BlastP on this gene

EME38763

hypothetical protein
  
Accession: EME38762
  
Location: 357188-357565
  
 NCBI BlastP on this gene

EME38762

hypothetical protein
  
Accession: EME38761
  
Location: 356293-356532
  
 NCBI BlastP on this gene

EME38761

hypothetical protein
  
Accession: EME38760
  
Location: 354582-355358
  
 NCBI BlastP on this gene

EME38760

hypothetical protein
  
Accession: EME38759
  
Location: 351356-352531
  
  
**BlastP hit with Mycgr3G102276\_Mycgr3**
  
Percentage identity: 60 %
  
BlastP bit score: 423
  
Sequence coverage: 98 %
  
E-value: 6e-144
  
  
 NCBI BlastP on this gene

EME38759

hypothetical protein
  
Accession: EME38757
  
Location: 348297-350465
  
 NCBI BlastP on this gene

EME38757

hypothetical protein
  
Accession: EME38756
  
Location: 337477-341494
  
 NCBI BlastP on this gene

EME38756

hypothetical protein
  
Accession: EME38755
  
Location: 332637-336560
  
 NCBI BlastP on this gene

EME38755

238. :  CP003008 Myceliophthora thermophila ATCC 42464 chromosome 7     Total score: 1.0     Cumulative Blast bit score: 423

hypothetical protein
  
Accession: AEO62151
  
Location: 4054986-4056940
  
 NCBI BlastP on this gene

MYCTH\_2313207

hypothetical protein
  
Accession: AEO62152
  
Location: 4057656-4058432
  
 NCBI BlastP on this gene

MYCTH\_2313210

hypothetical protein
  
Accession: AEO62153
  
Location: 4060162-4062303
  
 NCBI BlastP on this gene

MYCTH\_2313211

hypothetical protein
  
Accession: AEO62154
  
Location: 4063578-4065957
  
 NCBI BlastP on this gene

MYCTH\_2313212

hypothetical protein
  
Accession: AEO62155
  
Location: 4066295-4067077
  
 NCBI BlastP on this gene

MYCTH\_2313215

hypothetical protein
  
Accession: AEO62156
  
Location: 4067463-4068598
  
 NCBI BlastP on this gene

MYCTH\_2313216

hypothetical protein
  
Accession: AEO62157
  
Location: 4069022-4070609
  
 NCBI BlastP on this gene

MYCTH\_2313219

alcohol dehydrogenase-like protein
  
Accession: AEO62158
  
Location: 4073171-4074377
  
  
**BlastP hit with Mycgr3G102276\_Mycgr3**
  
Percentage identity: 60 %
  
BlastP bit score: 423
  
Sequence coverage: 98 %
  
E-value: 1e-143
  
  
 NCBI BlastP on this gene

MYCTH\_84302

239. :  CM001200 Mycosphaerella graminicola IPO323 chromosome 5     Total score: 1.0     Cumulative Blast bit score: 422

hypothetical protein
  
Accession: EGP87291
  
Location: 2113167-2113793
  
 NCBI BlastP on this gene

EGP87291

Ca2+-modulated channel polycystin
  
Accession: EGP87475
  
Location: 2115406-2116746
  
 NCBI BlastP on this gene

EGP87475

hypothetical protein
  
Accession: EGP87474
  
Location: 2117984-2119276
  
 NCBI BlastP on this gene

EGP87474

hypothetical protein
  
Accession: EGP87473
  
Location: 2120074-2120504
  
 NCBI BlastP on this gene

EGP87473

hypothetical protein
  
Accession: EGP87292
  
Location: 2121081-2121640
  
 NCBI BlastP on this gene

EGP87292

hypothetical protein
  
Accession: EGP87293
  
Location: 2122695-2124864
  
 NCBI BlastP on this gene

EGP87293

hypothetical protein
  
Accession: EGP87294
  
Location: 2125529-2127494
  
 NCBI BlastP on this gene

EGP87294

hypothetical protein
  
Accession: EGP87295
  
Location: 2128452-2129711
  
  
**BlastP hit with Mycgr3G102276\_Mycgr3**
  
Percentage identity: 60 %
  
BlastP bit score: 422
  
Sequence coverage: 98 %
  
E-value: 2e-143
  
  
 NCBI BlastP on this gene

EGP87295

240. :  GL988032 Chaetomium thermophilum var. thermophilum DSM 1495 unplaced genomic scaffold scf7180000...     Total score: 1.0     Cumulative Blast bit score: 421

ATP-dependent RNA helicase-like protein
  
Accession: EGS23377
  
Location: 48901-50683
  
 NCBI BlastP on this gene

EGS23377

hypothetical protein
  
Accession: EGS23376
  
Location: 43932-46094
  
 NCBI BlastP on this gene

EGS23376

putative tryptophan protein
  
Accession: EGS23375
  
Location: 40739-43093
  
 NCBI BlastP on this gene

EGS23375

hypothetical protein
  
Accession: EGS23374
  
Location: 39533-40243
  
 NCBI BlastP on this gene

EGS23374

hypothetical protein
  
Accession: EGS23373
  
Location: 37935-38378
  
 NCBI BlastP on this gene

EGS23373

putative aspartate protein
  
Accession: EGS23372
  
Location: 35965-37543
  
 NCBI BlastP on this gene

EGS23372

alcohol dehydrogenase-like protein
  
Accession: EGS23371
  
Location: 31435-32613
  
  
**BlastP hit with Mycgr3G102276\_Mycgr3**
  
Percentage identity: 60 %
  
BlastP bit score: 421
  
Sequence coverage: 99 %
  
E-value: 1e-142
  
  
 NCBI BlastP on this gene

EGS23371

hypothetical protein
  
Accession: EGS23370
  
Location: 26065-30705
  
 NCBI BlastP on this gene

EGS23370

hypothetical protein
  
Accession: EGS23369
  
Location: 22652-23047
  
 NCBI BlastP on this gene

EGS23369

hypothetical protein
  
Accession: EGS23368
  
Location: 19693-22083
  
 NCBI BlastP on this gene

EGS23368

endo-1,4-beta-mannosidase-like protein
  
Accession: EGS23367
  
Location: 14062-15433
  
 NCBI BlastP on this gene

EGS23367

241. :  EQ963472 Aspergillus flavus NRRL3357 scf\_1106286418772 genomic scaffold     Total score: 1.0     Cumulative Blast bit score: 421

conserved hypothetical protein
  
Accession: EED57889
  
Location: 3668691-3670253
  
 NCBI BlastP on this gene

EED57889

BTB domain transcription factor, putative
  
Accession: EED57890
  
Location: 3670944-3672745
  
 NCBI BlastP on this gene

EED57890

conserved hypothetical protein
  
Accession: EED57891
  
Location: 3673039-3673671
  
 NCBI BlastP on this gene

EED57891

hypothetical protein
  
Accession: EED57892
  
Location: 3673960-3674157
  
 NCBI BlastP on this gene

EED57892

Coatomer subunit alpha, putative
  
Accession: EED57893
  
Location: 3674411-3678461
  
 NCBI BlastP on this gene

EED57893

FAD dependent sulfhydryl oxidase Erv1, putative
  
Accession: EED57894
  
Location: 3678906-3679604
  
 NCBI BlastP on this gene

EED57894

transcriptional regulator, putative
  
Accession: EED57895
  
Location: 3679762-3681402
  
 NCBI BlastP on this gene

EED57895

AP-1 adaptor complex subunit beta, putative
  
Accession: EED57896
  
Location: 3682118-3684315
  
 NCBI BlastP on this gene

EED57896

alcohol dehydrogenase, putative
  
Accession: EED57897
  
Location: 3685451-3686704
  
  
**BlastP hit with Mycgr3G102276\_Mycgr3**
  
Percentage identity: 60 %
  
BlastP bit score: 421
  
Sequence coverage: 99 %
  
E-value: 8e-143
  
  
 NCBI BlastP on this gene

EED57897

242. :  DS985216 Verticillium albo-atrum VaMs.102 supercont1.3 genomic scaffold     Total score: 1.0     Cumulative Blast bit score: 421

aspartyl-tRNA synthetase
  
Accession: EEY17025
  
Location: 2048856-2051587
  
 NCBI BlastP on this gene

EEY17025

conserved hypothetical protein
  
Accession: EEY17026
  
Location: 2054685-2055353
  
 NCBI BlastP on this gene

EEY17026

chitosanase
  
Accession: EEY17027
  
Location: 2056411-2057308
  
 NCBI BlastP on this gene

EEY17027

predicted protein
  
Accession: EEY17028
  
Location: 2059007-2060849
  
 NCBI BlastP on this gene

EEY17028

high affinity copper transporter
  
Accession: EEY17029
  
Location: 2062667-2063361
  
 NCBI BlastP on this gene

EEY17029

ferric reductase transmembrane component 2
  
Accession: EEY17030
  
Location: 2064417-2066444
  
  
**BlastP hit with Mycgr3G107069\_Mycgr3**
  
Percentage identity: 38 %
  
BlastP bit score: 421
  
Sequence coverage: 102 %
  
E-value: 6e-135
  
  
 NCBI BlastP on this gene

EEY17030

243. :  AP007175 Aspergillus oryzae RIB40 DNA, SC010.     Total score: 1.0     Cumulative Blast bit score: 421

not annotated
  
Accession: BAE66487
  
Location: 1728925-1732493
  
 NCBI BlastP on this gene

AO090010000662

not annotated
  
Accession: BAE66488
  
Location: 1736104-1736940
  
 NCBI BlastP on this gene

AO090010000663

not annotated
  
Accession: BAE66489
  
Location: 1737150-1737934
  
 NCBI BlastP on this gene

AO090010000664

not annotated
  
Accession: BAE66490
  
Location: 1740786-1741396
  
 NCBI BlastP on this gene

AO090010000666

not annotated
  
Accession: BAE66491
  
Location: 1741692-1742685
  
 NCBI BlastP on this gene

AO090010000667

not annotated
  
Accession: BAE66492
  
Location: 1745184-1746322
  
  
**BlastP hit with Mycgr3G102276\_Mycgr3**
  
Percentage identity: 60 %
  
BlastP bit score: 421
  
Sequence coverage: 99 %
  
E-value: 7e-143
  
  
 NCBI BlastP on this gene

AO090010000668

244. :  AKHY01000175 Aspergillus oryzae 3.042     Total score: 1.0     Cumulative Blast bit score: 421

hypothetical protein
  
Accession: EIT75461
  
Location: 304702-308270
  
 NCBI BlastP on this gene

EIT75461

hypothetical protein
  
Accession: EIT75821
  
Location: 298981-301112
  
 NCBI BlastP on this gene

EIT75821

hypothetical protein
  
Accession: EIT75618
  
Location: 295828-296438
  
 NCBI BlastP on this gene

EIT75618

C-4 sterol methyl oxidase
  
Accession: EIT75499
  
Location: 294539-295532
  
 NCBI BlastP on this gene

EIT75499

alcohol dehydrogenase, class V
  
Accession: EIT75591
  
Location: 290905-292043
  
  
**BlastP hit with Mycgr3G102276\_Mycgr3**
  
Percentage identity: 60 %
  
BlastP bit score: 421
  
Sequence coverage: 99 %
  
E-value: 7e-143
  
  
 NCBI BlastP on this gene

EIT75591

hypothetical protein
  
Accession: EIT75732
  
Location: 290023-290846
  
 NCBI BlastP on this gene

EIT75732

hypothetical protein
  
Accession: EIT75815
  
Location: 287968-289631
  
 NCBI BlastP on this gene

EIT75815

hypothetical protein
  
Accession: EIT75655
  
Location: 286034-286825
  
 NCBI BlastP on this gene

EIT75655

D-alanine transfer protein
  
Accession: EIT75493
  
Location: 285062-285938
  
 NCBI BlastP on this gene

EIT75493

hypothetical protein
  
Accession: EIT75515
  
Location: 283673-284413
  
 NCBI BlastP on this gene

EIT75515

permease of the major facilitator superfamily
  
Accession: EIT75380
  
Location: 281538-283433
  
 NCBI BlastP on this gene

EIT75380

hypothetical protein
  
Accession: EIT75761
  
Location: 279693-280500
  
 NCBI BlastP on this gene

EIT75761

cytochrome protein
  
Accession: EIT75636
  
Location: 274598-276383
  
 NCBI BlastP on this gene

EIT75636

245. :  JH668231 Wallemia sebi CBS 633.66 unplaced genomic scaffold WALSEscaffold\_9     Total score: 1.0     Cumulative Blast bit score: 419

hypothetical protein
  
Accession: EIM21724
  
Location: 115728-118337
  
 NCBI BlastP on this gene

EIM21724

hypothetical protein
  
Accession: EIM21613
  
Location: 114252-115709
  
 NCBI BlastP on this gene

EIM21613

hypothetical protein
  
Accession: EIM21612
  
Location: 111269-114238
  
 NCBI BlastP on this gene

EIM21612

ARM repeat-containing protein
  
Accession: EIM21611
  
Location: 104962-107328
  
 NCBI BlastP on this gene

EIM21611

hypothetical protein
  
Accession: EIM21610
  
Location: 103739-104902
  
 NCBI BlastP on this gene

EIM21610

alcohol oxidase
  
Accession: EIM21609
  
Location: 101069-103600
  
  
**BlastP hit with Mycgr3G34982\_Mycgr3T**
  
Percentage identity: 28 %
  
BlastP bit score: 212
  
Sequence coverage: 101 %
  
E-value: 4e-57
  
  
 NCBI BlastP on this gene

EIM21609

mitochondrial NAD-dependent isocitrate dehydrogenase subunit 2 precursor
  
Accession: EIM21608
  
Location: 99653-100897
  
 NCBI BlastP on this gene

EIM21608

alcohol oxidase
  
Accession: EIM21607
  
Location: 96708-99109
  
  
**BlastP hit with Mycgr3G34982\_Mycgr3T**
  
Percentage identity: 27 %
  
BlastP bit score: 207
  
Sequence coverage: 103 %
  
E-value: 6e-55
  
  
 NCBI BlastP on this gene

EIM21607

20S proteasome subunit
  
Accession: EIM21605
  
Location: 94747-95789
  
 NCBI BlastP on this gene

EIM21605

ENTH-domain-containing protein
  
Accession: EIM21604
  
Location: 93050-94725
  
 NCBI BlastP on this gene

EIM21604

hypothetical protein
  
Accession: EIM21603
  
Location: 90157-92423
  
 NCBI BlastP on this gene

EIM21603

pyrroline-5-carboxylate reductase
  
Accession: EIM21602
  
Location: 88829-90011
  
 NCBI BlastP on this gene

EIM21602

hypothetical protein
  
Accession: EIM21601
  
Location: 87644-88394
  
 NCBI BlastP on this gene

EIM21601

vesicular-fusion protein SEC18
  
Accession: EIM21600
  
Location: 84664-87430
  
 NCBI BlastP on this gene

EIM21600

hypothetical protein
  
Accession: EIM21599
  
Location: 82622-84499
  
 NCBI BlastP on this gene

EIM21599

246. :  GL629765 Grosmannia clavigera kw1407 unplaced genomic scaffold GCSC\_140     Total score: 1.0     Cumulative Blast bit score: 419

benzoate 4-monooxygenase cytochrome p450
  
Accession: EFX04226
  
Location: 333244-335002
  
 NCBI BlastP on this gene

EFX04226

c6 zinc finger domain containing protein
  
Accession: EFX04070
  
Location: 335066-337366
  
 NCBI BlastP on this gene

EFX04070

hypothetical protein
  
Accession: EFX03935
  
Location: 337697-338249
  
 NCBI BlastP on this gene

EFX03935

caib baif family enzyme
  
Accession: EFX04776
  
Location: 340238-344171
  
 NCBI BlastP on this gene

EFX04776

fructosyl amine: oxygen oxidoreductase
  
Accession: EFX03499
  
Location: 344456-345736
  
 NCBI BlastP on this gene

EFX03499

trypsin-like serine typically contains c-terminal pdz domain protein
  
Accession: EFX04206
  
Location: 346817-348467
  
 NCBI BlastP on this gene

EFX04206

zinc-binding alcohol dehydrogenase
  
Accession: EFX03836
  
Location: 350059-351195
  
  
**BlastP hit with Mycgr3G102276\_Mycgr3**
  
Percentage identity: 59 %
  
BlastP bit score: 419
  
Sequence coverage: 99 %
  
E-value: 3e-142
  
  
 NCBI BlastP on this gene

EFX03836

hypothetical protein
  
Accession: EFX04644
  
Location: 352992-357307
  
 NCBI BlastP on this gene

EFX04644

hypothetical protein
  
Accession: EFX03599
  
Location: 357846-359352
  
 NCBI BlastP on this gene

EFX03599

pre-mRNA splicing factor dim1
  
Accession: EFX04646
  
Location: 360118-360608
  
 NCBI BlastP on this gene

EFX04646

small nuclear ribonucleoprotein
  
Accession: EFX04427
  
Location: 361364-361827
  
 NCBI BlastP on this gene

EFX04427

hypothetical protein
  
Accession: EFX03712
  
Location: 362059-363763
  
 NCBI BlastP on this gene

EFX03712

hypothetical protein
  
Accession: EFX03963
  
Location: 364019-365397
  
 NCBI BlastP on this gene

EFX03963

hypothetical protein
  
Accession: EFX04563
  
Location: 365914-366422
  
 NCBI BlastP on this gene

EFX04563

hypothetical protein
  
Accession: EFX04127
  
Location: 366544-366885
  
 NCBI BlastP on this gene

EFX04127

hypothetical protein
  
Accession: EFX04271
  
Location: 367417-368014
  
 NCBI BlastP on this gene

EFX04271

247. :  EQ963475 Aspergillus flavus NRRL3357 scf\_1106286419142 genomic scaffold     Total score: 1.0     Cumulative Blast bit score: 419

mitochondrial uncoupling protein, putative
  
Accession: EED53489
  
Location: 1179033-1180125
  
 NCBI BlastP on this gene

EED53489

conserved hypothetical protein
  
Accession: EED53490
  
Location: 1180417-1182188
  
 NCBI BlastP on this gene

EED53490

conserved hypothetical protein
  
Accession: EED53491
  
Location: 1183626-1184972
  
 NCBI BlastP on this gene

EED53491

cytochrome P450, putative
  
Accession: EED53492
  
Location: 1186835-1188895
  
 NCBI BlastP on this gene

EED53492

conserved hypothetical protein
  
Accession: EED53493
  
Location: 1189258-1192172
  
 NCBI BlastP on this gene

EED53493

aminotransferase GliI-like, putative
  
Accession: EED53494
  
Location: 1192475-1193986
  
 NCBI BlastP on this gene

EED53494

quinone oxidoreductase, putative
  
Accession: EED53495
  
Location: 1196685-1197858
  
  
**BlastP hit with Mycgr3G102276\_Mycgr3**
  
Percentage identity: 60 %
  
BlastP bit score: 419
  
Sequence coverage: 99 %
  
E-value: 4e-142
  
  
 NCBI BlastP on this gene

EED53495

ankyrin repeat protein
  
Accession: EED53496
  
Location: 1198170-1199790
  
 NCBI BlastP on this gene

EED53496

conserved hypothetical protein
  
Accession: EED53497
  
Location: 1199819-1200658
  
 NCBI BlastP on this gene

EED53497

hypothetical protein
  
Accession: EED53498
  
Location: 1201342-1201936
  
 NCBI BlastP on this gene

EED53498

conserved hypothetical protein
  
Accession: EED53499
  
Location: 1206283-1207858
  
 NCBI BlastP on this gene

EED53499

conserved hypothetical protein
  
Accession: EED53500
  
Location: 1208743-1210001
  
 NCBI BlastP on this gene

EED53500

amino acid permease, putative
  
Accession: EED53501
  
Location: 1210675-1212460
  
 NCBI BlastP on this gene

EED53501

hypothetical protein
  
Accession: EED53502
  
Location: 1213289-1213514
  
 NCBI BlastP on this gene

EED53502

248. :  KB644409 Penicillium oxalicum 114-2 unplaced genomic scaffold scaffold\_2     Total score: 1.0     Cumulative Blast bit score: 417

hypothetical protein
  
Accession: EPS26788
  
Location: 1453009-1457138
  
 NCBI BlastP on this gene

EPS26788

hypothetical protein
  
Accession: EPS26789
  
Location: 1457690-1458457
  
 NCBI BlastP on this gene

EPS26789

hypothetical protein
  
Accession: EPS26790
  
Location: 1459004-1459553
  
 NCBI BlastP on this gene

EPS26790

hypothetical protein
  
Accession: EPS26791
  
Location: 1460397-1462885
  
 NCBI BlastP on this gene

EPS26791

hypothetical protein
  
Accession: EPS26792
  
Location: 1463752-1464862
  
 NCBI BlastP on this gene

EPS26792

hypothetical protein
  
Accession: EPS26793
  
Location: 1465050-1465520
  
 NCBI BlastP on this gene

EPS26793

hypothetical protein
  
Accession: EPS26794
  
Location: 1465821-1468461
  
 NCBI BlastP on this gene

EPS26794

hypothetical protein
  
Accession: EPS26795
  
Location: 1469498-1470725
  
  
**BlastP hit with Mycgr3G102276\_Mycgr3**
  
Percentage identity: 60 %
  
BlastP bit score: 417
  
Sequence coverage: 99 %
  
E-value: 3e-141
  
  
 NCBI BlastP on this gene

EPS26795

249. :  AP007157 Aspergillus oryzae RIB40 DNA, SC023.     Total score: 1.0     Cumulative Blast bit score: 417

not annotated
  
Accession: BAE58999
  
Location: 1162623-1165206
  
 NCBI BlastP on this gene

AO090023000454

not annotated
  
Accession: BAE59000
  
Location: 1167214-1168560
  
 NCBI BlastP on this gene

AO090023000455

not annotated
  
Accession: BAE59001
  
Location: 1170838-1172488
  
 NCBI BlastP on this gene

AO090023000456

not annotated
  
Accession: BAE59002
  
Location: 1172850-1173800
  
 NCBI BlastP on this gene

AO090023000457

not annotated
  
Accession: BAE59003
  
Location: 1174739-1177580
  
 NCBI BlastP on this gene

AO090023000458

not annotated
  
Accession: BAE59004
  
Location: 1180272-1181445
  
  
**BlastP hit with Mycgr3G102276\_Mycgr3**
  
Percentage identity: 60 %
  
BlastP bit score: 417
  
Sequence coverage: 99 %
  
E-value: 2e-141
  
  
 NCBI BlastP on this gene

AO090023000460

not annotated
  
Accession: BAE59005
  
Location: 1181757-1184245
  
 NCBI BlastP on this gene

AO090023000461

not annotated
  
Accession: BAE59006
  
Location: 1189953-1191568
  
 NCBI BlastP on this gene

AO090023000463

not annotated
  
Accession: BAE59007
  
Location: 1192203-1193348
  
 NCBI BlastP on this gene

AO090023000464

not annotated
  
Accession: BAE59008
  
Location: 1193917-1195706
  
 NCBI BlastP on this gene

AO090023000465

250. :  AP007151 Aspergillus oryzae RIB40 DNA, SC005.     Total score: 1.0     Cumulative Blast bit score: 417

not annotated
  
Accession: BAE56278
  
Location: 3610443-3611322
  
 NCBI BlastP on this gene

AO090005001350

not annotated
  
Accession: BAE56279
  
Location: 3612227-3613814
  
 NCBI BlastP on this gene

AO090005001351

not annotated
  
Accession: BAE56280
  
Location: 3614108-3614740
  
 NCBI BlastP on this gene

AO090005001352

not annotated
  
Accession: BAE56281
  
Location: 3615479-3619529
  
 NCBI BlastP on this gene

AO090005001354

not annotated
  
Accession: BAE56282
  
Location: 3620224-3620692
  
 NCBI BlastP on this gene

AO090005001355

not annotated
  
Accession: BAE56283
  
Location: 3621291-3622493
  
 NCBI BlastP on this gene

AO090005001356

not annotated
  
Accession: BAE56284
  
Location: 3623347-3625907
  
 NCBI BlastP on this gene

AO090005001357

not annotated
  
Accession: BAE56285
  
Location: 3627043-3628296
  
  
**BlastP hit with Mycgr3G102276\_Mycgr3**
  
Percentage identity: 59 %
  
BlastP bit score: 417
  
Sequence coverage: 99 %
  
E-value: 2e-141
  
  
 NCBI BlastP on this gene

AO090005001358

Detecting sequence homology at the gene cluster level with MultiGeneBlast.
  
Marnix H. Medema, Rainer Breitling & Eriko Takano (2013)
  
*Molecular Biology and Evolution* , 30: 1218-1223.
