# Supplementary material for: In silico prediction and characterization of secondary metabolite biosynthetic gene clusters in the wheat pathogen Zymoseptoria tritici
Source: BMC Genomics. 2017 Aug 17;18:631. doi: 10.1186/s12864-017-3969-y (PMC5561558; doi:10.1186/s12864-017-3969-y)
Supplement: Supplementary file 1 — MultiGeneBLAST analysis of putative secondary metabolite clusters. All encoded amino acid sequences from genes residing in clusters predicted by AntiSMASH are given as FASTA file format. All output data from MultiGeneBLASTs are also provided. (ZIP 42911 kb) [file 12864_2017_3969_MOESM1_ESM.zip › Cluster MultiGene BLAST/out/Clusters_1_34/Cluster_10/displaypage1.xhtml]

xml version="1.0" encoding="UTF-8"?


Search Results
  
  
 Results pages: 1, 2, 3, 4, 5

**MultiGeneBlast hits**

Select gene cluster alignment
1. CM001197\_0 Mycosphaerella graminicola IPO323 chromosome 2, whole genome sh...
2. KB456266\_4 Mycosphaerella populorum SO2202 unplaced genomic scaffold SEPMU...
3. KB908844\_1 Setosphaeria turcica Et28A unplaced genomic scaffold SETTUscaff...
4. DS231623\_3 Pyrenophora tritici-repentis Pt-1C-BFP supercont1.9 genomic sca...
5. AHHD01000090\_0 Macrophomina phaseolina MS6, whole genome shotgun sequencin...
6. KB916121\_0 Neofusicoccum parvum UCRNP2 chromosome Unknown NP2\_03\_scaffold\_...
7. KB445561\_0 Baudoinia compniacensis UAMH 10762 unplaced genomic scaffold BA...
8. AFWA01000002\_0 Pneumocystis murina B123, whole genome shotgun sequencing p...
9. KB446555\_1 Pseudocercospora fijiensis CIRAD86 unplaced genomic scaffold MY...
10. KB456266\_1 Mycosphaerella populorum SO2202 unplaced genomic scaffold SEPM...
11. KB445561\_5 Baudoinia compniacensis UAMH 10762 unplaced genomic scaffold B...
12. ACFW01000030\_2 Coccidioides posadasii C735 delta SOWgp, whole genome shot...
13. GL636488\_2 Coccidioides posadasii str. Silveira unplaced genomic scaffold...
14. GG704916\_0 Coccidioides immitis RS genomic scaffold supercont3.6, whole g...
15. GG749414\_0 Ajellomyces dermatitidis ATCC 18188 genomic scaffold supercont...
16. EQ999973\_3 Ajellomyces dermatitidis ER-3 genomic scaffold supercont1.1, w...
17. GG663372\_0 Ajellomyces capsulatus G186AR genomic scaffold supercont2.10, ...
18. DS989822\_4 Arthroderma gypseum CBS 118893 supercont1.1 genomic scaffold, ...
19. DS990636\_0 Ajellomyces capsulatus H88 supercont1.1 genomic scaffold, whol...
20. DS499594\_0 Aspergillus fumigatus A1163 scf\_000001 genomic scaffold, whole...
21. GG700648\_0 Trichophyton rubrum CBS 118892 genomic scaffold supercont2.1, ...
22. GG698540\_0 Trichophyton tonsurans CBS 112818 genomic scaffold supercont1....
23. DS995745\_0 Trichophyton equinum CBS 127.97 supercont1.28 genomic scaffold...
24. DS027059\_0 Aspergillus clavatus NRRL 1 1099423829805 genomic scaffold, wh...
25. KB644410\_1 Penicillium oxalicum 114-2 unplaced genomic scaffold scaffold\_...
26. DS995899\_1 Penicillium marneffei ATCC 18224 scf\_1105668340764 genomic sca...
27. AAHF01000007\_0 Aspergillus fumigatus Af293, whole genome shotgun sequenci...
28. AKCU01000442\_0 Penicillium digitatum Pd1, whole genome shotgun sequencing...
29. AKCT01000236\_0 Penicillium digitatum PHI26, whole genome shotgun sequenci...
30. CH476655\_0 Ajellomyces capsulatus NAm1 scaffold\_1 genomic scaffold, whole...
31. GG657460\_0 Ajellomyces dermatitidis SLH14081 genomic scaffold supercont1....
32. DS027688\_2 Neosartorya fischeri NRRL 181 1099437636249 genomic scaffold, ...
33. AP007154\_0 Aspergillus oryzae RIB40 DNA, SC001.
34. DS995701\_2 Microsporum canis CBS 113480 supercont1.1 genomic scaffold, wh...
35. AM920428\_0 Penicillium chrysogenum Wisconsin 54-1255 complete genome, con...
36. EQ963481\_0 Aspergillus flavus NRRL3357 scf\_1106286419476 genomic scaffold...
37. AACD01000068\_0 Aspergillus nidulans FGSC A4, whole genome shotgun sequenc...
38. DF126471\_0 Aspergillus kawachii IFO 4308 DNA, contig: scaffold00025, whol...
39. ACYE01000197\_0 Trichophyton verrucosum HKI 0517, whole genome shotgun seq...
40. CH476619\_0 Uncinocarpus reesii 1704 scaffold\_5 genomic scaffold, whole ge...
41. ABSU01000003\_0 Arthroderma benhamiae CBS 112371, whole genome shotgun seq...
42. ACJE01000020\_0 Aspergillus niger ATCC 1015, whole genome shotgun sequenci...
43. AKHY01000202\_0 Aspergillus oryzae 3.042, whole genome shotgun sequencing ...
44. EQ962654\_1 Talaromyces stipitatus ATCC 10500 scf\_1105507295541 genomic sc...
45. DS572750\_4 Paracoccidioides brasiliensis Pb18 supercont1.1 genomic scaffo...
46. GL534607\_0 Pyrenophora teres f. teres 0-1 unplaced genomic scaffold scaff...
47. KB445649\_3 Cochliobolus sativus ND90Pr unplaced genomic scaffold COCSAsca...
48. DS544805\_5 Paracoccidioides brasiliensis Pb03 supercont1.3 genomic scaffo...
49. KB733455\_0 Bipolaris maydis ATCC 48331 unplaced genomic scaffold COCC4sca...
50. KB445579\_3 Cochliobolus heterostrophus C5 unplaced genomic scaffold COCHE...

Query: Architecture Search FASTA input

CM001197 : Mycosphaerella graminicola IPO323 chromosome 2    Total score: 14.0     Cumulative Blast bit score: 25829

Hit cluster cross-links:

Mycgr3G67791 Mycgr3T
  
Location: 0-1542

Mycgr3G67791\_Mycgr3T

Mycgr3G90406 Mycgr3T
  
Location: 1642-3973

Mycgr3G90406\_Mycgr3T

Mycgr3G67785 Mycgr3T
  
Location: 4073-7865

Mycgr3G67785\_Mycgr3T

Mycgr3G67795 Mycgr3T
  
Location: 7965-15249

Mycgr3G67795\_Mycgr3T

Mycgr3G67775 Mycgr3T
  
Location: 15349-16237

Mycgr3G67775\_Mycgr3T

Mycgr3G90404 Mycgr3T
  
Location: 16337-17246

Mycgr3G90404\_Mycgr3T

Mycgr3G36951 Mycgr3T
  
Location: 17346-30891

Mycgr3G36951\_Mycgr3T

Mycgr3G103034 Mycgr3
  
Location: 30991-32644

Mycgr3G103034\_Mycgr3

Mycgr3G31119 Mycgr3T
  
Location: 32744-32906

Mycgr3G31119\_Mycgr3T

Mycgr3G28587 Mycgr3T
  
Location: 33006-33489

Mycgr3G28587\_Mycgr3T

Mycgr3G98959 Mycgr3T
  
Location: 33589-35035

Mycgr3G98959\_Mycgr3T

Mycgr3G35447 Mycgr3T
  
Location: 35135-36443

Mycgr3G35447\_Mycgr3T

Mycgr3G84402 Mycgr3T
  
Location: 36543-37884

Mycgr3G84402\_Mycgr3T

Mycgr3G98961 Mycgr3T
  
Location: 37984-38884

Mycgr3G98961\_Mycgr3T

hypothetical protein
  
Accession: EGP89720
  
Location: 474074-474397
  
 NCBI BlastP on this gene

EGP89720

hypothetical protein
  
Accession: EGP90749
  
Location: 472679-473598
  
 NCBI BlastP on this gene

EGP90749

hypothetical protein
  
Accession: EGP89719
  
Location: 471658-471819
  
  
**BlastP hit with Mycgr3G31119\_Mycgr3T**
  
Percentage identity: 100 %
  
BlastP bit score: 110
  
Sequence coverage: 100 %
  
E-value: 2e-30
  
  
 NCBI BlastP on this gene

EGP89719

hypothetical protein
  
Accession: EGP89718
  
Location: 468869-471319
  
  
**BlastP hit with Mycgr3G90406\_Mycgr3T**
  
Percentage identity: 100 %
  
BlastP bit score: 1594
  
Sequence coverage: 99 %
  
E-value: 0.0
  
  
 NCBI BlastP on this gene

EGP89718

TOR1 phosphatidylinositol 3-kinase
  
Accession: EGP90750
  
Location: 461149-468432
  
  
**BlastP hit with Mycgr3G67795\_Mycgr3T**
  
Percentage identity: 100 %
  
BlastP bit score: 5049
  
Sequence coverage: 99 %
  
E-value: 0.0
  
  
 NCBI BlastP on this gene

EGP90750

hypothetical protein
  
Accession: EGP90751
  
Location: 459143-460416
  
  
**BlastP hit with Mycgr3G90404\_Mycgr3T**
  
Percentage identity: 100 %
  
BlastP bit score: 624
  
Sequence coverage: 99 %
  
E-value: 0.0
  
  
 NCBI BlastP on this gene

EGP90751

hypothetical protein
  
Accession: EGP90752
  
Location: 456769-458519
  
  
**BlastP hit with Mycgr3G67791\_Mycgr3T**
  
Percentage identity: 100 %
  
BlastP bit score: 1051
  
Sequence coverage: 99 %
  
E-value: 0.0
  
  
 NCBI BlastP on this gene

EGP90752

hypothetical protein
  
Accession: EGP89717
  
Location: 455916-456450
  
  
**BlastP hit with Mycgr3G28587\_Mycgr3T**
  
Percentage identity: 100 %
  
BlastP bit score: 337
  
Sequence coverage: 100 %
  
E-value: 5e-116
  
  
 NCBI BlastP on this gene

EGP89717

hypothetical protein
  
Accession: EGP90753
  
Location: 453825-455477
  
  
**BlastP hit with Mycgr3G103034\_Mycgr3**
  
Percentage identity: 100 %
  
BlastP bit score: 1143
  
Sequence coverage: 99 %
  
E-value: 0.0
  
  
 NCBI BlastP on this gene

EGP90753

putative Non-ribosomal peptide synthetase
  
Accession: EGP89716
  
Location: 439283-453243
  
  
**BlastP hit with Mycgr3G36951\_Mycgr3T**
  
Percentage identity: 100 %
  
BlastP bit score: 9323
  
Sequence coverage: 99 %
  
E-value: 0.0
  
  
 NCBI BlastP on this gene

EGP89716

putative ABC transporter
  
Accession: EGP90754
  
Location: 433742-437840
  
  
**BlastP hit with Mycgr3G67785\_Mycgr3T**
  
Percentage identity: 100 %
  
BlastP bit score: 2565
  
Sequence coverage: 99 %
  
E-value: 0.0
  
  
 NCBI BlastP on this gene

EGP90754

putative L-ornithine 5-monooxygenase
  
Accession: EGP90755
  
Location: 431119-432712
  
 NCBI BlastP on this gene

EGP90755

hypothetical protein
  
Accession: EGP89715
  
Location: 429154-431323
  
  
**BlastP hit with Mycgr3G84402\_Mycgr3T**
  
Percentage identity: 100 %
  
BlastP bit score: 912
  
Sequence coverage: 99 %
  
E-value: 0.0
  
  
 NCBI BlastP on this gene

EGP89715

hypothetical protein
  
Accession: EGP90756
  
Location: 427564-428871
  
  
**BlastP hit with Mycgr3G35447\_Mycgr3T**
  
Percentage identity: 100 %
  
BlastP bit score: 897
  
Sequence coverage: 99 %
  
E-value: 0.0
  
  
 NCBI BlastP on this gene

EGP90756

hypothetical protein
  
Accession: EGP90757
  
Location: 423720-424702
  
  
**BlastP hit with Mycgr3G98961\_Mycgr3T**
  
Percentage identity: 100 %
  
BlastP bit score: 605
  
Sequence coverage: 99 %
  
E-value: 0.0
  
  
 NCBI BlastP on this gene

EGP90757

hypothetical protein
  
Accession: EGP89714
  
Location: 422453-423453
  
  
**BlastP hit with Mycgr3G67775\_Mycgr3T**
  
Percentage identity: 100 %
  
BlastP bit score: 615
  
Sequence coverage: 99 %
  
E-value: 0.0
  
  
 NCBI BlastP on this gene

EGP89714

hypothetical protein
  
Accession: EGP90758
  
Location: 420584-422080
  
  
**BlastP hit with Mycgr3G98959\_Mycgr3T**
  
Percentage identity: 100 %
  
BlastP bit score: 1005
  
Sequence coverage: 99 %
  
E-value: 0.0
  
  
 NCBI BlastP on this gene

EGP90758

putative alpha-amylase
  
Accession: EGP89713
  
Location: 418479-420089
  
 NCBI BlastP on this gene

EGP89713

Query: Architecture Search FASTA input

KB456266 : Mycosphaerella populorum SO2202 unplaced genomic scaffold SEPMUscaffold\_7    Total score: 4.0     Cumulative Blast bit score: 5901

Hit cluster cross-links:

Mycgr3G67791 Mycgr3T
  
Location: 0-1542

Mycgr3G67791\_Mycgr3T

Mycgr3G90406 Mycgr3T
  
Location: 1642-3973

Mycgr3G90406\_Mycgr3T

Mycgr3G67785 Mycgr3T
  
Location: 4073-7865

Mycgr3G67785\_Mycgr3T

Mycgr3G67795 Mycgr3T
  
Location: 7965-15249

Mycgr3G67795\_Mycgr3T

Mycgr3G67775 Mycgr3T
  
Location: 15349-16237

Mycgr3G67775\_Mycgr3T

Mycgr3G90404 Mycgr3T
  
Location: 16337-17246

Mycgr3G90404\_Mycgr3T

Mycgr3G36951 Mycgr3T
  
Location: 17346-30891

Mycgr3G36951\_Mycgr3T

Mycgr3G103034 Mycgr3
  
Location: 30991-32644

Mycgr3G103034\_Mycgr3

Mycgr3G31119 Mycgr3T
  
Location: 32744-32906

Mycgr3G31119\_Mycgr3T

Mycgr3G28587 Mycgr3T
  
Location: 33006-33489

Mycgr3G28587\_Mycgr3T

Mycgr3G98959 Mycgr3T
  
Location: 33589-35035

Mycgr3G98959\_Mycgr3T

Mycgr3G35447 Mycgr3T
  
Location: 35135-36443

Mycgr3G35447\_Mycgr3T

Mycgr3G84402 Mycgr3T
  
Location: 36543-37884

Mycgr3G84402\_Mycgr3T

Mycgr3G98961 Mycgr3T
  
Location: 37984-38884

Mycgr3G98961\_Mycgr3T

Yip1-domain-containing protein
  
Accession: EMF11550
  
Location: 1786238-1787388
  
 NCBI BlastP on this gene

EMF11550

hypothetical protein
  
Accession: EMF11551
  
Location: 1788997-1789983
  
 NCBI BlastP on this gene

EMF11551

glycine dehydrogenase
  
Accession: EMF11552
  
Location: 1792812-1796067
  
 NCBI BlastP on this gene

EMF11552

hypothetical protein
  
Accession: EMF11553
  
Location: 1797353-1798648
  
 NCBI BlastP on this gene

EMF11553

FAD/NAD(P)-binding domain-containing protein
  
Accession: EMF11554
  
Location: 1799219-1800878
  
 NCBI BlastP on this gene

EMF11554

hypothetical protein
  
Accession: EMF11555
  
Location: 1801529-1804075
  
  
**BlastP hit with Mycgr3G90406\_Mycgr3T**
  
Percentage identity: 45 %
  
BlastP bit score: 612
  
Sequence coverage: 111 %
  
E-value: 0.0
  
  
 NCBI BlastP on this gene

EMF11555

phosphatidylinositol 3-kinase tor2
  
Accession: EMF11556
  
Location: 1804489-1811826
  
  
**BlastP hit with Mycgr3G67795\_Mycgr3T**
  
Percentage identity: 83 %
  
BlastP bit score: 4207
  
Sequence coverage: 100 %
  
E-value: 0.0
  
  
 NCBI BlastP on this gene

EMF11556

hypothetical protein
  
Accession: EMF11557
  
Location: 1812242-1812865
  
  
**BlastP hit with Mycgr3G28587\_Mycgr3T**
  
Percentage identity: 57 %
  
BlastP bit score: 188
  
Sequence coverage: 106 %
  
E-value: 6e-57
  
  
 NCBI BlastP on this gene

EMF11557

Phosphoacetylglucosamine mutase
  
Accession: EMF11558
  
Location: 1813410-1815074
  
  
**BlastP hit with Mycgr3G103034\_Mycgr3**
  
Percentage identity: 77 %
  
BlastP bit score: 894
  
Sequence coverage: 98 %
  
E-value: 0.0
  
  
 NCBI BlastP on this gene

EMF11558

hypothetical protein
  
Accession: EMF11559
  
Location: 1816628-1817539
  
 NCBI BlastP on this gene

EMF11559

AA permease-domain-containing protein
  
Accession: EMF11560
  
Location: 1818794-1821168
  
 NCBI BlastP on this gene

EMF11560

hypothetical protein
  
Accession: EMF11561
  
Location: 1821661-1821921
  
 NCBI BlastP on this gene

EMF11561

hypothetical protein
  
Accession: EMF11562
  
Location: 1823743-1825239
  
 NCBI BlastP on this gene

EMF11562

hypothetical protein
  
Accession: EMF11563
  
Location: 1825651-1827145
  
 NCBI BlastP on this gene

EMF11563

Query: Architecture Search FASTA input

KB908844 : Setosphaeria turcica Et28A unplaced genomic scaffold SETTUscaffold\_6    Total score: 4.0     Cumulative Blast bit score: 4848

Hit cluster cross-links:

Mycgr3G67791 Mycgr3T
  
Location: 0-1542

Mycgr3G67791\_Mycgr3T

Mycgr3G90406 Mycgr3T
  
Location: 1642-3973

Mycgr3G90406\_Mycgr3T

Mycgr3G67785 Mycgr3T
  
Location: 4073-7865

Mycgr3G67785\_Mycgr3T

Mycgr3G67795 Mycgr3T
  
Location: 7965-15249

Mycgr3G67795\_Mycgr3T

Mycgr3G67775 Mycgr3T
  
Location: 15349-16237

Mycgr3G67775\_Mycgr3T

Mycgr3G90404 Mycgr3T
  
Location: 16337-17246

Mycgr3G90404\_Mycgr3T

Mycgr3G36951 Mycgr3T
  
Location: 17346-30891

Mycgr3G36951\_Mycgr3T

Mycgr3G103034 Mycgr3
  
Location: 30991-32644

Mycgr3G103034\_Mycgr3

Mycgr3G31119 Mycgr3T
  
Location: 32744-32906

Mycgr3G31119\_Mycgr3T

Mycgr3G28587 Mycgr3T
  
Location: 33006-33489

Mycgr3G28587\_Mycgr3T

Mycgr3G98959 Mycgr3T
  
Location: 33589-35035

Mycgr3G98959\_Mycgr3T

Mycgr3G35447 Mycgr3T
  
Location: 35135-36443

Mycgr3G35447\_Mycgr3T

Mycgr3G84402 Mycgr3T
  
Location: 36543-37884

Mycgr3G84402\_Mycgr3T

Mycgr3G98961 Mycgr3T
  
Location: 37984-38884

Mycgr3G98961\_Mycgr3T

hypothetical protein
  
Accession: EOA82529
  
Location: 513444-517091
  
 NCBI BlastP on this gene

EOA82529

hypothetical protein
  
Accession: EOA82530
  
Location: 518070-520105
  
 NCBI BlastP on this gene

EOA82530

hypothetical protein
  
Accession: EOA82531
  
Location: 520523-527949
  
  
**BlastP hit with Mycgr3G67795\_Mycgr3T**
  
Percentage identity: 66 %
  
BlastP bit score: 3281
  
Sequence coverage: 100 %
  
E-value: 0.0
  
  
 NCBI BlastP on this gene

EOA82531

hypothetical protein
  
Accession: EOA82532
  
Location: 531082-532927
  
  
**BlastP hit with Mycgr3G67791\_Mycgr3T**
  
Percentage identity: 57 %
  
BlastP bit score: 546
  
Sequence coverage: 94 %
  
E-value: 0.0
  
  
 NCBI BlastP on this gene

EOA82532

hypothetical protein
  
Accession: EOA82533
  
Location: 533667-534620
  
 NCBI BlastP on this gene

EOA82533

hypothetical protein
  
Accession: EOA82534
  
Location: 534710-535960
  
 NCBI BlastP on this gene

EOA82534

hypothetical protein
  
Accession: EOA82535
  
Location: 537340-538626
  
 NCBI BlastP on this gene

EOA82535

hypothetical protein
  
Accession: EOA82536
  
Location: 539713-541267
  
 NCBI BlastP on this gene

EOA82536

hypothetical protein
  
Accession: EOA82537
  
Location: 541910-542549
  
 NCBI BlastP on this gene

EOA82537

hypothetical protein
  
Accession: EOA82538
  
Location: 542936-544541
  
  
**BlastP hit with Mycgr3G84402\_Mycgr3T**
  
Percentage identity: 71 %
  
BlastP bit score: 611
  
Sequence coverage: 93 %
  
E-value: 0.0
  
  
 NCBI BlastP on this gene

EOA82538

hypothetical protein
  
Accession: EOA82539
  
Location: 544868-546320
  
  
**BlastP hit with Mycgr3G35447\_Mycgr3T**
  
Percentage identity: 57 %
  
BlastP bit score: 410
  
Sequence coverage: 86 %
  
E-value: 2e-136
  
  
 NCBI BlastP on this gene

EOA82539

hypothetical protein
  
Accession: EOA82540
  
Location: 546424-547503
  
 NCBI BlastP on this gene

EOA82540

hypothetical protein
  
Accession: EOA82541
  
Location: 547956-548897
  
 NCBI BlastP on this gene

EOA82541

hypothetical protein
  
Accession: EOA82542
  
Location: 549500-550517
  
 NCBI BlastP on this gene

EOA82542

hypothetical protein
  
Accession: EOA82543
  
Location: 551938-553422
  
 NCBI BlastP on this gene

EOA82543

Query: Architecture Search FASTA input

DS231623 : Pyrenophora tritici-repentis Pt-1C-BFP supercont1.9 genomic scaffold    Total score: 4.0     Cumulative Blast bit score: 4821

Hit cluster cross-links:

Mycgr3G67791 Mycgr3T
  
Location: 0-1542

Mycgr3G67791\_Mycgr3T

Mycgr3G90406 Mycgr3T
  
Location: 1642-3973

Mycgr3G90406\_Mycgr3T

Mycgr3G67785 Mycgr3T
  
Location: 4073-7865

Mycgr3G67785\_Mycgr3T

Mycgr3G67795 Mycgr3T
  
Location: 7965-15249

Mycgr3G67795\_Mycgr3T

Mycgr3G67775 Mycgr3T
  
Location: 15349-16237

Mycgr3G67775\_Mycgr3T

Mycgr3G90404 Mycgr3T
  
Location: 16337-17246

Mycgr3G90404\_Mycgr3T

Mycgr3G36951 Mycgr3T
  
Location: 17346-30891

Mycgr3G36951\_Mycgr3T

Mycgr3G103034 Mycgr3
  
Location: 30991-32644

Mycgr3G103034\_Mycgr3

Mycgr3G31119 Mycgr3T
  
Location: 32744-32906

Mycgr3G31119\_Mycgr3T

Mycgr3G28587 Mycgr3T
  
Location: 33006-33489

Mycgr3G28587\_Mycgr3T

Mycgr3G98959 Mycgr3T
  
Location: 33589-35035

Mycgr3G98959\_Mycgr3T

Mycgr3G35447 Mycgr3T
  
Location: 35135-36443

Mycgr3G35447\_Mycgr3T

Mycgr3G84402 Mycgr3T
  
Location: 36543-37884

Mycgr3G84402\_Mycgr3T

Mycgr3G98961 Mycgr3T
  
Location: 37984-38884

Mycgr3G98961\_Mycgr3T

importin-7
  
Accession: EDU51463
  
Location: 1317811-1321370
  
 NCBI BlastP on this gene

EDU51463

conserved hypothetical protein
  
Accession: EDU51464
  
Location: 1322414-1324467
  
 NCBI BlastP on this gene

EDU51464

phosphatidylinositol 3-kinase tor2
  
Accession: EDU51465
  
Location: 1325109-1332510
  
  
**BlastP hit with Mycgr3G67795\_Mycgr3T**
  
Percentage identity: 66 %
  
BlastP bit score: 3265
  
Sequence coverage: 100 %
  
E-value: 0.0
  
  
 NCBI BlastP on this gene

EDU51465

conserved hypothetical protein
  
Accession: EDU51466
  
Location: 1334262-1336148
  
  
**BlastP hit with Mycgr3G67791\_Mycgr3T**
  
Percentage identity: 62 %
  
BlastP bit score: 533
  
Sequence coverage: 87 %
  
E-value: 0.0
  
  
 NCBI BlastP on this gene

EDU51466

conserved hypothetical protein
  
Accession: EDU51467
  
Location: 1337026-1338027
  
 NCBI BlastP on this gene

EDU51467

conserved hypothetical protein
  
Accession: EDU51468
  
Location: 1338190-1339416
  
 NCBI BlastP on this gene

EDU51468

N2,N2-dimethylguanosine tRNA methyltransferase
  
Accession: EDU51469
  
Location: 1339991-1342042
  
 NCBI BlastP on this gene

EDU51469

stress response protein Rds1
  
Accession: EDU51470
  
Location: 1343074-1344642
  
 NCBI BlastP on this gene

EDU51470

predicted protein
  
Accession: EDU51471
  
Location: 1347721-1348336
  
 NCBI BlastP on this gene

EDU51471

2-isopropylmalate synthase
  
Accession: EDU51472
  
Location: 1349467-1349966
  
 NCBI BlastP on this gene

EDU51472

hypothetical protein
  
Accession: EDU51473
  
Location: 1350531-1351144
  
 NCBI BlastP on this gene

EDU51473

ATP-dependent rRNA helicase rrp3
  
Accession: EDU51474
  
Location: 1353104-1354707
  
  
**BlastP hit with Mycgr3G84402\_Mycgr3T**
  
Percentage identity: 71 %
  
BlastP bit score: 624
  
Sequence coverage: 93 %
  
E-value: 0.0
  
  
 NCBI BlastP on this gene

EDU51474

ribosome biogenesis protein Ssf2
  
Accession: EDU51475
  
Location: 1354984-1356388
  
  
**BlastP hit with Mycgr3G35447\_Mycgr3T**
  
Percentage identity: 55 %
  
BlastP bit score: 399
  
Sequence coverage: 88 %
  
E-value: 5e-132
  
  
 NCBI BlastP on this gene

EDU51475

conserved hypothetical protein
  
Accession: EDU51476
  
Location: 1356494-1357565
  
 NCBI BlastP on this gene

EDU51476

adiponectin receptor protein 1
  
Accession: EDU51477
  
Location: 1358025-1358895
  
 NCBI BlastP on this gene

EDU51477

hypothetical protein
  
Accession: EDU51478
  
Location: 1359551-1360601
  
 NCBI BlastP on this gene

EDU51478

conserved hypothetical protein
  
Accession: EDU51479
  
Location: 1361804-1363186
  
 NCBI BlastP on this gene

EDU51479

Query: Architecture Search FASTA input

AHHD01000090 : Macrophomina phaseolina MS6    Total score: 4.0     Cumulative Blast bit score: 4639

Hit cluster cross-links:

Mycgr3G67791 Mycgr3T
  
Location: 0-1542

Mycgr3G67791\_Mycgr3T

Mycgr3G90406 Mycgr3T
  
Location: 1642-3973

Mycgr3G90406\_Mycgr3T

Mycgr3G67785 Mycgr3T
  
Location: 4073-7865

Mycgr3G67785\_Mycgr3T

Mycgr3G67795 Mycgr3T
  
Location: 7965-15249

Mycgr3G67795\_Mycgr3T

Mycgr3G67775 Mycgr3T
  
Location: 15349-16237

Mycgr3G67775\_Mycgr3T

Mycgr3G90404 Mycgr3T
  
Location: 16337-17246

Mycgr3G90404\_Mycgr3T

Mycgr3G36951 Mycgr3T
  
Location: 17346-30891

Mycgr3G36951\_Mycgr3T

Mycgr3G103034 Mycgr3
  
Location: 30991-32644

Mycgr3G103034\_Mycgr3

Mycgr3G31119 Mycgr3T
  
Location: 32744-32906

Mycgr3G31119\_Mycgr3T

Mycgr3G28587 Mycgr3T
  
Location: 33006-33489

Mycgr3G28587\_Mycgr3T

Mycgr3G98959 Mycgr3T
  
Location: 33589-35035

Mycgr3G98959\_Mycgr3T

Mycgr3G35447 Mycgr3T
  
Location: 35135-36443

Mycgr3G35447\_Mycgr3T

Mycgr3G84402 Mycgr3T
  
Location: 36543-37884

Mycgr3G84402\_Mycgr3T

Mycgr3G98961 Mycgr3T
  
Location: 37984-38884

Mycgr3G98961\_Mycgr3T

Ribosomal protein L7 eukaryotic
  
Accession: EKG20267
  
Location: 47599-49064
  
 NCBI BlastP on this gene

EKG20267

Protein of unknown function DUF3245
  
Accession: EKG20266
  
Location: 46448-47322
  
 NCBI BlastP on this gene

EKG20266

hypothetical protein
  
Accession: EKG20265
  
Location: 43674-46300
  
 NCBI BlastP on this gene

EKG20265

RNA helicase ATP-dependent DEAD-box conserved site
  
Accession: EKG20264
  
Location: 42127-43218
  
  
**BlastP hit with Mycgr3G84402\_Mycgr3T**
  
Percentage identity: 73 %
  
BlastP bit score: 526
  
Sequence coverage: 75 %
  
E-value: 0.0
  
  
 NCBI BlastP on this gene

EKG20264

hypothetical protein
  
Accession: EKG20263
  
Location: 40114-41558
  
  
**BlastP hit with Mycgr3G35447\_Mycgr3T**
  
Percentage identity: 59 %
  
BlastP bit score: 469
  
Sequence coverage: 101 %
  
E-value: 1e-159
  
  
 NCBI BlastP on this gene

EKG20263

hypothetical protein
  
Accession: EKG20262
  
Location: 37179-37543
  
 NCBI BlastP on this gene

EKG20262

hypothetical protein
  
Accession: EKG20261
  
Location: 33317-35887
  
 NCBI BlastP on this gene

EKG20261

Zinc finger PARP-type protein
  
Accession: EKG20260
  
Location: 30479-31998
  
 NCBI BlastP on this gene

EKG20260

hypothetical protein
  
Accession: EKG20259
  
Location: 28453-29989
  
 NCBI BlastP on this gene

EKG20259

hypothetical protein
  
Accession: EKG20258
  
Location: 25131-27331
  
 NCBI BlastP on this gene

EKG20258

hypothetical protein
  
Accession: EKG20257
  
Location: 21516-22825
  
 NCBI BlastP on this gene

EKG20257

SWAP/Surp
  
Accession: EKG20256
  
Location: 18668-21073
  
  
**BlastP hit with Mycgr3G90406\_Mycgr3T**
  
Percentage identity: 31 %
  
BlastP bit score: 271
  
Sequence coverage: 96 %
  
E-value: 1e-75
  
  
 NCBI BlastP on this gene

EKG20256

Phosphatidylinositol 3-/4-kinase catalytic
  
Accession: EKG20255
  
Location: 10791-18187
  
  
**BlastP hit with Mycgr3G67795\_Mycgr3T**
  
Percentage identity: 67 %
  
BlastP bit score: 3373
  
Sequence coverage: 101 %
  
E-value: 0.0
  
  
 NCBI BlastP on this gene

EKG20255

hypothetical protein
  
Accession: EKG20254
  
Location: 8850-9422
  
 NCBI BlastP on this gene

EKG20254

Autophagy-related protein 11
  
Accession: EKG20253
  
Location: 3895-8378
  
 NCBI BlastP on this gene

EKG20253

Query: Architecture Search FASTA input

KB916121 : Neofusicoccum parvum UCRNP2 chromosome Unknown NP2\_03\_scaffold\_483    Total score: 4.0     Cumulative Blast bit score: 4206

Hit cluster cross-links:

Mycgr3G67791 Mycgr3T
  
Location: 0-1542

Mycgr3G67791\_Mycgr3T

Mycgr3G90406 Mycgr3T
  
Location: 1642-3973

Mycgr3G90406\_Mycgr3T

Mycgr3G67785 Mycgr3T
  
Location: 4073-7865

Mycgr3G67785\_Mycgr3T

Mycgr3G67795 Mycgr3T
  
Location: 7965-15249

Mycgr3G67795\_Mycgr3T

Mycgr3G67775 Mycgr3T
  
Location: 15349-16237

Mycgr3G67775\_Mycgr3T

Mycgr3G90404 Mycgr3T
  
Location: 16337-17246

Mycgr3G90404\_Mycgr3T

Mycgr3G36951 Mycgr3T
  
Location: 17346-30891

Mycgr3G36951\_Mycgr3T

Mycgr3G103034 Mycgr3
  
Location: 30991-32644

Mycgr3G103034\_Mycgr3

Mycgr3G31119 Mycgr3T
  
Location: 32744-32906

Mycgr3G31119\_Mycgr3T

Mycgr3G28587 Mycgr3T
  
Location: 33006-33489

Mycgr3G28587\_Mycgr3T

Mycgr3G98959 Mycgr3T
  
Location: 33589-35035

Mycgr3G98959\_Mycgr3T

Mycgr3G35447 Mycgr3T
  
Location: 35135-36443

Mycgr3G35447\_Mycgr3T

Mycgr3G84402 Mycgr3T
  
Location: 36543-37884

Mycgr3G84402\_Mycgr3T

Mycgr3G98961 Mycgr3T
  
Location: 37984-38884

Mycgr3G98961\_Mycgr3T

putative 60s ribosomal protein l7 protein
  
Accession: EOD48936
  
Location: 12790-14241
  
 NCBI BlastP on this gene

EOD48936

putative ell complex subunit eap30 protein
  
Accession: EOD48941
  
Location: 14501-15379
  
 NCBI BlastP on this gene

EOD48941

putative eukaryotic translation initiation factor 3 subunit protein
  
Accession: EOD48932
  
Location: 15529-18204
  
 NCBI BlastP on this gene

EOD48932

putative atp-dependent rrna helicase rrp3 protein
  
Accession: EOD48947
  
Location: 18687-20171
  
  
**BlastP hit with Mycgr3G84402\_Mycgr3T**
  
Percentage identity: 71 %
  
BlastP bit score: 629
  
Sequence coverage: 94 %
  
E-value: 0.0
  
  
 NCBI BlastP on this gene

EOD48947

putative ribosome biogenesis protein
  
Accession: EOD48933
  
Location: 20334-21788
  
  
**BlastP hit with Mycgr3G35447\_Mycgr3T**
  
Percentage identity: 59 %
  
BlastP bit score: 476
  
Sequence coverage: 101 %
  
E-value: 3e-162
  
  
 NCBI BlastP on this gene

EOD48933

hypothetical protein
  
Accession: EOD48928
  
Location: 22166-22532
  
 NCBI BlastP on this gene

EOD48928

putative zf-parp-type zinc finger protein
  
Accession: EOD48938
  
Location: 27353-28837
  
 NCBI BlastP on this gene

EOD48938

hypothetical protein
  
Accession: EOD48929
  
Location: 31702-33888
  
 NCBI BlastP on this gene

EOD48929

putative coatamer subunit protein
  
Accession: EOD48944
  
Location: 39174-40445
  
  
**BlastP hit with Mycgr3G90406\_Mycgr3T**
  
Percentage identity: 37 %
  
BlastP bit score: 179
  
Sequence coverage: 33 %
  
E-value: 4e-46
  
  
 NCBI BlastP on this gene

EOD48944

putative phosphatidylinositol 3-kinase tor2 protein
  
Accession: EOD48942
  
Location: 40959-48353
  
  
**BlastP hit with Mycgr3G67795\_Mycgr3T**
  
Percentage identity: 69 %
  
BlastP bit score: 2922
  
Sequence coverage: 85 %
  
E-value: 0.0
  
  
 NCBI BlastP on this gene

EOD48942

hypothetical protein
  
Accession: EOD48931
  
Location: 49066-50067
  
 NCBI BlastP on this gene

EOD48931

putative autophagy-related protein 11 protein
  
Accession: EOD48937
  
Location: 50598-54683
  
 NCBI BlastP on this gene

EOD48937

Query: Architecture Search FASTA input

KB445561 : Baudoinia compniacensis UAMH 10762 unplaced genomic scaffold BAUCOscaffold\_12    Total score: 3.0     Cumulative Blast bit score: 4743

Hit cluster cross-links:

Mycgr3G67791 Mycgr3T
  
Location: 0-1542

Mycgr3G67791\_Mycgr3T

Mycgr3G90406 Mycgr3T
  
Location: 1642-3973

Mycgr3G90406\_Mycgr3T

Mycgr3G67785 Mycgr3T
  
Location: 4073-7865

Mycgr3G67785\_Mycgr3T

Mycgr3G67795 Mycgr3T
  
Location: 7965-15249

Mycgr3G67795\_Mycgr3T

Mycgr3G67775 Mycgr3T
  
Location: 15349-16237

Mycgr3G67775\_Mycgr3T

Mycgr3G90404 Mycgr3T
  
Location: 16337-17246

Mycgr3G90404\_Mycgr3T

Mycgr3G36951 Mycgr3T
  
Location: 17346-30891

Mycgr3G36951\_Mycgr3T

Mycgr3G103034 Mycgr3
  
Location: 30991-32644

Mycgr3G103034\_Mycgr3

Mycgr3G31119 Mycgr3T
  
Location: 32744-32906

Mycgr3G31119\_Mycgr3T

Mycgr3G28587 Mycgr3T
  
Location: 33006-33489

Mycgr3G28587\_Mycgr3T

Mycgr3G98959 Mycgr3T
  
Location: 33589-35035

Mycgr3G98959\_Mycgr3T

Mycgr3G35447 Mycgr3T
  
Location: 35135-36443

Mycgr3G35447\_Mycgr3T

Mycgr3G84402 Mycgr3T
  
Location: 36543-37884

Mycgr3G84402\_Mycgr3T

Mycgr3G98961 Mycgr3T
  
Location: 37984-38884

Mycgr3G98961\_Mycgr3T

hypothetical protein
  
Accession: EMC92570
  
Location: 26044-26731
  
 NCBI BlastP on this gene

EMC92570

hypothetical protein
  
Accession: EMC92571
  
Location: 27066-27971
  
 NCBI BlastP on this gene

EMC92571

hypothetical protein
  
Accession: EMC92572
  
Location: 28303-32011
  
 NCBI BlastP on this gene

EMC92572

hypothetical protein
  
Accession: EMC92573
  
Location: 32343-33077
  
 NCBI BlastP on this gene

EMC92573

hypothetical protein
  
Accession: EMC92574
  
Location: 33896-35380
  
 NCBI BlastP on this gene

EMC92574

hypothetical protein
  
Accession: EMC92575
  
Location: 35575-35838
  
 NCBI BlastP on this gene

EMC92575

hypothetical protein
  
Accession: EMC92576
  
Location: 36154-40320
  
  
**BlastP hit with Mycgr3G67785\_Mycgr3T**
  
Percentage identity: 50 %
  
BlastP bit score: 1278
  
Sequence coverage: 100 %
  
E-value: 0.0
  
  
 NCBI BlastP on this gene

EMC92576

hypothetical protein
  
Accession: EMC92577
  
Location: 41474-56633
  
  
**BlastP hit with Mycgr3G36951\_Mycgr3T**
  
Percentage identity: 43 %
  
BlastP bit score: 2759
  
Sequence coverage: 78 %
  
E-value: 0.0
  
  
 NCBI BlastP on this gene

EMC92577

hypothetical protein
  
Accession: EMC92578
  
Location: 57268-57495
  
 NCBI BlastP on this gene

EMC92578

hypothetical protein
  
Accession: EMC92579
  
Location: 57967-59685
  
  
**BlastP hit with Mycgr3G103034\_Mycgr3**
  
Percentage identity: 63 %
  
BlastP bit score: 706
  
Sequence coverage: 96 %
  
E-value: 0.0
  
  
 NCBI BlastP on this gene

EMC92579

hypothetical protein
  
Accession: EMC92580
  
Location: 60737-63110
  
 NCBI BlastP on this gene

EMC92580

hypothetical protein
  
Accession: EMC92581
  
Location: 63617-64963
  
 NCBI BlastP on this gene

EMC92581

hypothetical protein
  
Accession: EMC92582
  
Location: 65142-66611
  
 NCBI BlastP on this gene

EMC92582

hypothetical protein
  
Accession: EMC92583
  
Location: 66856-67243
  
 NCBI BlastP on this gene

EMC92583

hypothetical protein
  
Accession: EMC92584
  
Location: 67360-69525
  
 NCBI BlastP on this gene

EMC92584

Query: Architecture Search FASTA input

AFWA01000002 : Pneumocystis murina B123    Total score: 3.0     Cumulative Blast bit score: 3295

Hit cluster cross-links:

Mycgr3G67791 Mycgr3T
  
Location: 0-1542

Mycgr3G67791\_Mycgr3T

Mycgr3G90406 Mycgr3T
  
Location: 1642-3973

Mycgr3G90406\_Mycgr3T

Mycgr3G67785 Mycgr3T
  
Location: 4073-7865

Mycgr3G67785\_Mycgr3T

Mycgr3G67795 Mycgr3T
  
Location: 7965-15249

Mycgr3G67795\_Mycgr3T

Mycgr3G67775 Mycgr3T
  
Location: 15349-16237

Mycgr3G67775\_Mycgr3T

Mycgr3G90404 Mycgr3T
  
Location: 16337-17246

Mycgr3G90404\_Mycgr3T

Mycgr3G36951 Mycgr3T
  
Location: 17346-30891

Mycgr3G36951\_Mycgr3T

Mycgr3G103034 Mycgr3
  
Location: 30991-32644

Mycgr3G103034\_Mycgr3

Mycgr3G31119 Mycgr3T
  
Location: 32744-32906

Mycgr3G31119\_Mycgr3T

Mycgr3G28587 Mycgr3T
  
Location: 33006-33489

Mycgr3G28587\_Mycgr3T

Mycgr3G98959 Mycgr3T
  
Location: 33589-35035

Mycgr3G98959\_Mycgr3T

Mycgr3G35447 Mycgr3T
  
Location: 35135-36443

Mycgr3G35447\_Mycgr3T

Mycgr3G84402 Mycgr3T
  
Location: 36543-37884

Mycgr3G84402\_Mycgr3T

Mycgr3G98961 Mycgr3T
  
Location: 37984-38884

Mycgr3G98961\_Mycgr3T

hypothetical protein
  
Accession: EMR11326
  
Location: 167768-169071
  
 NCBI BlastP on this gene

EMR11326

hypothetical protein
  
Accession: EMR11325
  
Location: 166735-167608
  
 NCBI BlastP on this gene

EMR11325

hypothetical protein
  
Accession: EMR11324
  
Location: 164319-166021
  
  
**BlastP hit with Mycgr3G98959\_Mycgr3T**
  
Percentage identity: 53 %
  
BlastP bit score: 486
  
Sequence coverage: 86 %
  
E-value: 7e-165
  
  
 NCBI BlastP on this gene

EMR11324

hypothetical protein
  
Accession: EMR11323
  
Location: 163670-163953
  
 NCBI BlastP on this gene

EMR11323

hypothetical protein
  
Accession: EMR11322
  
Location: 161431-163607
  
 NCBI BlastP on this gene

EMR11322

hypothetical protein
  
Accession: EMR11321
  
Location: 157160-161339
  
 NCBI BlastP on this gene

EMR11321

CMGC/DYRK/YAK protein kinase
  
Accession: EMR11320
  
Location: 154460-156834
  
 NCBI BlastP on this gene

EMR11320

hypothetical protein
  
Accession: EMR11319
  
Location: 151537-154105
  
 NCBI BlastP on this gene

EMR11319

hypothetical protein
  
Accession: EMR11318
  
Location: 151020-151502
  
  
**BlastP hit with Mycgr3G28587\_Mycgr3T**
  
Percentage identity: 33 %
  
BlastP bit score: 51
  
Sequence coverage: 75 %
  
E-value: 7e-06
  
  
 NCBI BlastP on this gene

EMR11318

hypothetical protein
  
Accession: EMR11317
  
Location: 149558-150915
  
 NCBI BlastP on this gene

EMR11317

hypothetical protein
  
Accession: EMR11316
  
Location: 148984-149382
  
 NCBI BlastP on this gene

EMR11316

hypothetical protein
  
Accession: EMR11315
  
Location: 147315-148796
  
 NCBI BlastP on this gene

EMR11315

hypothetical protein
  
Accession: EMR11314
  
Location: 143890-147064
  
 NCBI BlastP on this gene

EMR11314

hypothetical protein
  
Accession: EMR11313
  
Location: 142749-143355
  
 NCBI BlastP on this gene

EMR11313

hypothetical protein
  
Accession: EMR11312
  
Location: 140814-142172
  
 NCBI BlastP on this gene

EMR11312

hypothetical protein
  
Accession: EMR11311
  
Location: 139530-140790
  
 NCBI BlastP on this gene

EMR11311

signal peptidase I
  
Accession: EMR11310
  
Location: 138630-139299
  
 NCBI BlastP on this gene

EMR11310

hypothetical protein
  
Accession: EMR11309
  
Location: 138052-138452
  
 NCBI BlastP on this gene

EMR11309

hypothetical protein
  
Accession: EMR11308
  
Location: 137711-137995
  
 NCBI BlastP on this gene

EMR11308

hypothetical protein
  
Accession: EMR11307
  
Location: 136640-137523
  
 NCBI BlastP on this gene

EMR11307

hypothetical protein
  
Accession: EMR11306
  
Location: 134219-136403
  
 NCBI BlastP on this gene

EMR11306

hypothetical protein
  
Accession: EMR11305
  
Location: 129142-134027
  
 NCBI BlastP on this gene

EMR11305

hypothetical protein
  
Accession: EMR11304
  
Location: 127256-129072
  
 NCBI BlastP on this gene

EMR11304

hypothetical protein
  
Accession: EMR11303
  
Location: 118666-126508
  
  
**BlastP hit with Mycgr3G67795\_Mycgr3T**
  
Percentage identity: 56 %
  
BlastP bit score: 2758
  
Sequence coverage: 100 %
  
E-value: 0.0
  
  
 NCBI BlastP on this gene

EMR11303

hypothetical protein
  
Accession: EMR11302
  
Location: 118138-118546
  
 NCBI BlastP on this gene

EMR11302

hypothetical protein
  
Accession: EMR11301
  
Location: 115258-117282
  
 NCBI BlastP on this gene

EMR11301

Query: Architecture Search FASTA input

KB446555 : Pseudocercospora fijiensis CIRAD86 unplaced genomic scaffold MYCFIscaffold\_1    Total score: 3.0     Cumulative Blast bit score: 1842

Hit cluster cross-links:

Mycgr3G67791 Mycgr3T
  
Location: 0-1542

Mycgr3G67791\_Mycgr3T

Mycgr3G90406 Mycgr3T
  
Location: 1642-3973

Mycgr3G90406\_Mycgr3T

Mycgr3G67785 Mycgr3T
  
Location: 4073-7865

Mycgr3G67785\_Mycgr3T

Mycgr3G67795 Mycgr3T
  
Location: 7965-15249

Mycgr3G67795\_Mycgr3T

Mycgr3G67775 Mycgr3T
  
Location: 15349-16237

Mycgr3G67775\_Mycgr3T

Mycgr3G90404 Mycgr3T
  
Location: 16337-17246

Mycgr3G90404\_Mycgr3T

Mycgr3G36951 Mycgr3T
  
Location: 17346-30891

Mycgr3G36951\_Mycgr3T

Mycgr3G103034 Mycgr3
  
Location: 30991-32644

Mycgr3G103034\_Mycgr3

Mycgr3G31119 Mycgr3T
  
Location: 32744-32906

Mycgr3G31119\_Mycgr3T

Mycgr3G28587 Mycgr3T
  
Location: 33006-33489

Mycgr3G28587\_Mycgr3T

Mycgr3G98959 Mycgr3T
  
Location: 33589-35035

Mycgr3G98959\_Mycgr3T

Mycgr3G35447 Mycgr3T
  
Location: 35135-36443

Mycgr3G35447\_Mycgr3T

Mycgr3G84402 Mycgr3T
  
Location: 36543-37884

Mycgr3G84402\_Mycgr3T

Mycgr3G98961 Mycgr3T
  
Location: 37984-38884

Mycgr3G98961\_Mycgr3T

hypothetical protein
  
Accession: EME88551
  
Location: 5960377-5963568
  
 NCBI BlastP on this gene

EME88551

hypothetical protein
  
Accession: EME88550
  
Location: 5957888-5959027
  
 NCBI BlastP on this gene

EME88550

hypothetical protein
  
Accession: EME88549
  
Location: 5956534-5956878
  
 NCBI BlastP on this gene

EME88549

hypothetical protein
  
Accession: EME88548
  
Location: 5954727-5955884
  
 NCBI BlastP on this gene

EME88548

hypothetical protein
  
Accession: EME88547
  
Location: 5952966-5953373
  
 NCBI BlastP on this gene

EME88547

hypothetical protein
  
Accession: EME88546
  
Location: 5948287-5949777
  
 NCBI BlastP on this gene

EME88546

hypothetical protein
  
Accession: EME88545
  
Location: 5945320-5948050
  
 NCBI BlastP on this gene

EME88545

hypothetical protein
  
Accession: EME88544
  
Location: 5942207-5945026
  
 NCBI BlastP on this gene

EME88544

hypothetical protein
  
Accession: EME88543
  
Location: 5940351-5941302
  
  
**BlastP hit with Mycgr3G98961\_Mycgr3T**
  
Percentage identity: 67 %
  
BlastP bit score: 407
  
Sequence coverage: 96 %
  
E-value: 3e-139
  
  
 NCBI BlastP on this gene

EME88543

hypothetical protein
  
Accession: EME88542
  
Location: 5937866-5939372
  
  
**BlastP hit with Mycgr3G98959\_Mycgr3T**
  
Percentage identity: 87 %
  
BlastP bit score: 888
  
Sequence coverage: 101 %
  
E-value: 0.0
  
  
 NCBI BlastP on this gene

EME88542

hypothetical protein
  
Accession: EME88541
  
Location: 5936388-5937658
  
  
**BlastP hit with Mycgr3G67775\_Mycgr3T**
  
Percentage identity: 87 %
  
BlastP bit score: 547
  
Sequence coverage: 98 %
  
E-value: 0.0
  
  
 NCBI BlastP on this gene

EME88541

hypothetical protein
  
Accession: EME88540
  
Location: 5933260-5933904
  
 NCBI BlastP on this gene

EME88540

hypothetical protein
  
Accession: EME88539
  
Location: 5930728-5932836
  
 NCBI BlastP on this gene

EME88539

Query: Architecture Search FASTA input

KB456266 : Mycosphaerella populorum SO2202 unplaced genomic scaffold SEPMUscaffold\_7    Total score: 3.0     Cumulative Blast bit score: 1807

Hit cluster cross-links:

Mycgr3G67791 Mycgr3T
  
Location: 0-1542

Mycgr3G67791\_Mycgr3T

Mycgr3G90406 Mycgr3T
  
Location: 1642-3973

Mycgr3G90406\_Mycgr3T

Mycgr3G67785 Mycgr3T
  
Location: 4073-7865

Mycgr3G67785\_Mycgr3T

Mycgr3G67795 Mycgr3T
  
Location: 7965-15249

Mycgr3G67795\_Mycgr3T

Mycgr3G67775 Mycgr3T
  
Location: 15349-16237

Mycgr3G67775\_Mycgr3T

Mycgr3G90404 Mycgr3T
  
Location: 16337-17246

Mycgr3G90404\_Mycgr3T

Mycgr3G36951 Mycgr3T
  
Location: 17346-30891

Mycgr3G36951\_Mycgr3T

Mycgr3G103034 Mycgr3
  
Location: 30991-32644

Mycgr3G103034\_Mycgr3

Mycgr3G31119 Mycgr3T
  
Location: 32744-32906

Mycgr3G31119\_Mycgr3T

Mycgr3G28587 Mycgr3T
  
Location: 33006-33489

Mycgr3G28587\_Mycgr3T

Mycgr3G98959 Mycgr3T
  
Location: 33589-35035

Mycgr3G98959\_Mycgr3T

Mycgr3G35447 Mycgr3T
  
Location: 35135-36443

Mycgr3G35447\_Mycgr3T

Mycgr3G84402 Mycgr3T
  
Location: 36543-37884

Mycgr3G84402\_Mycgr3T

Mycgr3G98961 Mycgr3T
  
Location: 37984-38884

Mycgr3G98961\_Mycgr3T

Inositol P-domain-containing protein
  
Accession: EMF11378
  
Location: 1239678-1240419
  
 NCBI BlastP on this gene

EMF11378

betaine lipid synthase
  
Accession: EMF11377
  
Location: 1236418-1239008
  
 NCBI BlastP on this gene

EMF11377

L-lactate dehydrogenase
  
Accession: EMF11376
  
Location: 1233361-1234998
  
 NCBI BlastP on this gene

EMF11376

hypothetical protein
  
Accession: EMF11375
  
Location: 1231813-1232418
  
 NCBI BlastP on this gene

EMF11375

hypothetical protein
  
Accession: EMF11374
  
Location: 1225916-1230769
  
 NCBI BlastP on this gene

EMF11374

cyclin domain protein
  
Accession: EMF11372
  
Location: 1220169-1221139
  
  
**BlastP hit with Mycgr3G98961\_Mycgr3T**
  
Percentage identity: 67 %
  
BlastP bit score: 407
  
Sequence coverage: 98 %
  
E-value: 2e-139
  
  
 NCBI BlastP on this gene

EMF11372

eukaryotic translation initiation factor 3
  
Accession: EMF11371
  
Location: 1217023-1218522
  
  
**BlastP hit with Mycgr3G98959\_Mycgr3T**
  
Percentage identity: 86 %
  
BlastP bit score: 862
  
Sequence coverage: 101 %
  
E-value: 0.0
  
  
 NCBI BlastP on this gene

EMF11371

carbon-nitrogen hydrolase
  
Accession: EMF11370
  
Location: 1215362-1216578
  
  
**BlastP hit with Mycgr3G67775\_Mycgr3T**
  
Percentage identity: 85 %
  
BlastP bit score: 538
  
Sequence coverage: 98 %
  
E-value: 0.0
  
  
 NCBI BlastP on this gene

EMF11370

Sugar tr-domain-containing protein
  
Accession: EMF11369
  
Location: 1211802-1214224
  
 NCBI BlastP on this gene

EMF11369

hypothetical protein
  
Accession: EMF11368
  
Location: 1210913-1211147
  
 NCBI BlastP on this gene

EMF11368

hypothetical protein
  
Accession: EMF11367
  
Location: 1210041-1210676
  
 NCBI BlastP on this gene

EMF11367

Trimethyllysine dioxygenase
  
Accession: EMF11366
  
Location: 1207798-1209733
  
 NCBI BlastP on this gene

EMF11366

amino acid permease
  
Accession: EMF11365
  
Location: 1205760-1207304
  
 NCBI BlastP on this gene

EMF11365

glycoside hydrolase family 51 protein
  
Accession: EMF11364
  
Location: 1202194-1204311
  
 NCBI BlastP on this gene

EMF11364

hypothetical protein
  
Accession: EMF11363
  
Location: 1200290-1201519
  
 NCBI BlastP on this gene

EMF11363

hypothetical protein
  
Accession: EMF11362
  
Location: 1198503-1200013
  
 NCBI BlastP on this gene

EMF11362

Query: Architecture Search FASTA input

KB445561 : Baudoinia compniacensis UAMH 10762 unplaced genomic scaffold BAUCOscaffold\_12    Total score: 3.0     Cumulative Blast bit score: 1777

Hit cluster cross-links:

Mycgr3G67791 Mycgr3T
  
Location: 0-1542

Mycgr3G67791\_Mycgr3T

Mycgr3G90406 Mycgr3T
  
Location: 1642-3973

Mycgr3G90406\_Mycgr3T

Mycgr3G67785 Mycgr3T
  
Location: 4073-7865

Mycgr3G67785\_Mycgr3T

Mycgr3G67795 Mycgr3T
  
Location: 7965-15249

Mycgr3G67795\_Mycgr3T

Mycgr3G67775 Mycgr3T
  
Location: 15349-16237

Mycgr3G67775\_Mycgr3T

Mycgr3G90404 Mycgr3T
  
Location: 16337-17246

Mycgr3G90404\_Mycgr3T

Mycgr3G36951 Mycgr3T
  
Location: 17346-30891

Mycgr3G36951\_Mycgr3T

Mycgr3G103034 Mycgr3
  
Location: 30991-32644

Mycgr3G103034\_Mycgr3

Mycgr3G31119 Mycgr3T
  
Location: 32744-32906

Mycgr3G31119\_Mycgr3T

Mycgr3G28587 Mycgr3T
  
Location: 33006-33489

Mycgr3G28587\_Mycgr3T

Mycgr3G98959 Mycgr3T
  
Location: 33589-35035

Mycgr3G98959\_Mycgr3T

Mycgr3G35447 Mycgr3T
  
Location: 35135-36443

Mycgr3G35447\_Mycgr3T

Mycgr3G84402 Mycgr3T
  
Location: 36543-37884

Mycgr3G84402\_Mycgr3T

Mycgr3G98961 Mycgr3T
  
Location: 37984-38884

Mycgr3G98961\_Mycgr3T

hypothetical protein
  
Accession: EMC93079
  
Location: 1092076-1092297
  
 NCBI BlastP on this gene

EMC93079

hypothetical protein
  
Accession: EMC93080
  
Location: 1093036-1093652
  
 NCBI BlastP on this gene

EMC93080

hypothetical protein
  
Accession: EMC93081
  
Location: 1094692-1096683
  
 NCBI BlastP on this gene

EMC93081

hypothetical protein
  
Accession: EMC93082
  
Location: 1097979-1098302
  
 NCBI BlastP on this gene

EMC93082

hypothetical protein
  
Accession: EMC93083
  
Location: 1099256-1100960
  
 NCBI BlastP on this gene

EMC93083

hypothetical protein
  
Accession: EMC93084
  
Location: 1101624-1102882
  
 NCBI BlastP on this gene

EMC93084

hypothetical protein
  
Accession: EMC93085
  
Location: 1104791-1105237
  
 NCBI BlastP on this gene

EMC93085

hypothetical protein
  
Accession: EMC93086
  
Location: 1105701-1106770
  
 NCBI BlastP on this gene

EMC93086

hypothetical protein
  
Accession: EMC93087
  
Location: 1107121-1108546
  
 NCBI BlastP on this gene

EMC93087

hypothetical protein
  
Accession: EMC93088
  
Location: 1108834-1109418
  
 NCBI BlastP on this gene

EMC93088

hypothetical protein
  
Accession: EMC93089
  
Location: 1109979-1110807
  
 NCBI BlastP on this gene

EMC93089

hypothetical protein
  
Accession: EMC93090
  
Location: 1111597-1112905
  
  
**BlastP hit with Mycgr3G67791\_Mycgr3T**
  
Percentage identity: 71 %
  
BlastP bit score: 548
  
Sequence coverage: 74 %
  
E-value: 0.0
  
  
 NCBI BlastP on this gene

EMC93090

hypothetical protein
  
Accession: EMC93091
  
Location: 1113353-1114768
  
  
**BlastP hit with Mycgr3G35447\_Mycgr3T**
  
Percentage identity: 65 %
  
BlastP bit score: 538
  
Sequence coverage: 97 %
  
E-value: 0.0
  
  
 NCBI BlastP on this gene

EMC93091

hypothetical protein
  
Accession: EMC93092
  
Location: 1115107-1116597
  
  
**BlastP hit with Mycgr3G84402\_Mycgr3T**
  
Percentage identity: 84 %
  
BlastP bit score: 691
  
Sequence coverage: 88 %
  
E-value: 0.0
  
  
 NCBI BlastP on this gene

EMC93092

hypothetical protein
  
Accession: EMC93093
  
Location: 1116877-1117773
  
 NCBI BlastP on this gene

EMC93093

hypothetical protein
  
Accession: EMC93094
  
Location: 1118125-1118596
  
 NCBI BlastP on this gene

EMC93094

hypothetical protein
  
Accession: EMC93095
  
Location: 1120088-1123319
  
 NCBI BlastP on this gene

EMC93095

carbohydrate-binding module family 32 protein
  
Accession: EMC93096
  
Location: 1124007-1127101
  
 NCBI BlastP on this gene

EMC93096

hypothetical protein
  
Accession: EMC93097
  
Location: 1127900-1129326
  
 NCBI BlastP on this gene

EMC93097

hypothetical protein
  
Accession: EMC93098
  
Location: 1129524-1130521
  
 NCBI BlastP on this gene

EMC93098

hypothetical protein
  
Accession: EMC93099
  
Location: 1130721-1135265
  
 NCBI BlastP on this gene

EMC93099

hypothetical protein
  
Accession: EMC93100
  
Location: 1135735-1137169
  
 NCBI BlastP on this gene

EMC93100

Query: Architecture Search FASTA input

ACFW01000030 : Coccidioides posadasii C735 delta SOWgp    Total score: 3.0     Cumulative Blast bit score: 1772

Hit cluster cross-links:

Mycgr3G67791 Mycgr3T
  
Location: 0-1542

Mycgr3G67791\_Mycgr3T

Mycgr3G90406 Mycgr3T
  
Location: 1642-3973

Mycgr3G90406\_Mycgr3T

Mycgr3G67785 Mycgr3T
  
Location: 4073-7865

Mycgr3G67785\_Mycgr3T

Mycgr3G67795 Mycgr3T
  
Location: 7965-15249

Mycgr3G67795\_Mycgr3T

Mycgr3G67775 Mycgr3T
  
Location: 15349-16237

Mycgr3G67775\_Mycgr3T

Mycgr3G90404 Mycgr3T
  
Location: 16337-17246

Mycgr3G90404\_Mycgr3T

Mycgr3G36951 Mycgr3T
  
Location: 17346-30891

Mycgr3G36951\_Mycgr3T

Mycgr3G103034 Mycgr3
  
Location: 30991-32644

Mycgr3G103034\_Mycgr3

Mycgr3G31119 Mycgr3T
  
Location: 32744-32906

Mycgr3G31119\_Mycgr3T

Mycgr3G28587 Mycgr3T
  
Location: 33006-33489

Mycgr3G28587\_Mycgr3T

Mycgr3G98959 Mycgr3T
  
Location: 33589-35035

Mycgr3G98959\_Mycgr3T

Mycgr3G35447 Mycgr3T
  
Location: 35135-36443

Mycgr3G35447\_Mycgr3T

Mycgr3G84402 Mycgr3T
  
Location: 36543-37884

Mycgr3G84402\_Mycgr3T

Mycgr3G98961 Mycgr3T
  
Location: 37984-38884

Mycgr3G98961\_Mycgr3T

hypothetical protein
  
Accession: EER26540
  
Location: 1817143-1819589
  
 NCBI BlastP on this gene

EER26540

26S protease regulatory subunit 6A, putative
  
Accession: EER26541
  
Location: 1820057-1821569
  
 NCBI BlastP on this gene

EER26541

Trimethyllysine dioxygenase, putative
  
Accession: EER26542
  
Location: 1822084-1824103
  
 NCBI BlastP on this gene

EER26542

MaoC like domain containing protein
  
Accession: EER26543
  
Location: 1827308-1828466
  
 NCBI BlastP on this gene

EER26543

protein arginine N-methyltransferase, putative
  
Accession: EER26544
  
Location: 1829177-1830606
  
 NCBI BlastP on this gene

EER26544

Per1-like family protein
  
Accession: EER26545
  
Location: 1831451-1832524
  
 NCBI BlastP on this gene

EER26545

phosphoglucomutase/phosphomannomutase, putative
  
Accession: EER26546
  
Location: 1833193-1835166
  
  
**BlastP hit with Mycgr3G103034\_Mycgr3**
  
Percentage identity: 57 %
  
BlastP bit score: 632
  
Sequence coverage: 98 %
  
E-value: 0.0
  
  
 NCBI BlastP on this gene

EER26546

ATP-dependent rRNA helicase RRP3, putative
  
Accession: EER26547
  
Location: 1835630-1837116
  
  
**BlastP hit with Mycgr3G84402\_Mycgr3T**
  
Percentage identity: 72 %
  
BlastP bit score: 665
  
Sequence coverage: 101 %
  
E-value: 0.0
  
  
 NCBI BlastP on this gene

EER26547

Brix domain containing protein
  
Accession: EER26548
  
Location: 1837398-1838773
  
  
**BlastP hit with Mycgr3G35447\_Mycgr3T**
  
Percentage identity: 55 %
  
BlastP bit score: 475
  
Sequence coverage: 101 %
  
E-value: 9e-162
  
  
 NCBI BlastP on this gene

EER26548

hypothetical protein
  
Accession: EER26549
  
Location: 1839017-1840262
  
 NCBI BlastP on this gene

EER26549

riboflavin synthase, alpha subunit family protein
  
Accession: EER26550
  
Location: 1840518-1841381
  
 NCBI BlastP on this gene

EER26550

CAIB/BAIF family protein
  
Accession: EER26551
  
Location: 1841715-1843363
  
 NCBI BlastP on this gene

EER26551

PHD-finger motif containing protein
  
Accession: EER26552
  
Location: 1843787-1845547
  
 NCBI BlastP on this gene

EER26552

SPFH domain / Band 7 family protein
  
Accession: EER26553
  
Location: 1847784-1849084
  
 NCBI BlastP on this gene

EER26553

hypothetical protein
  
Accession: EER26554
  
Location: 1851951-1852608
  
 NCBI BlastP on this gene

EER26554

60S ribosomal protein L27-B, putative
  
Accession: EER26555
  
Location: 1856163-1856984
  
 NCBI BlastP on this gene

EER26555

hypothetical protein
  
Accession: EER26556
  
Location: 1857703-1864198
  
 NCBI BlastP on this gene

EER26556

Query: Architecture Search FASTA input

GL636488 : Coccidioides posadasii str. Silveira unplaced genomic scaffold supercont2.3    Total score: 3.0     Cumulative Blast bit score: 1771

Hit cluster cross-links:

Mycgr3G67791 Mycgr3T
  
Location: 0-1542

Mycgr3G67791\_Mycgr3T

Mycgr3G90406 Mycgr3T
  
Location: 1642-3973

Mycgr3G90406\_Mycgr3T

Mycgr3G67785 Mycgr3T
  
Location: 4073-7865

Mycgr3G67785\_Mycgr3T

Mycgr3G67795 Mycgr3T
  
Location: 7965-15249

Mycgr3G67795\_Mycgr3T

Mycgr3G67775 Mycgr3T
  
Location: 15349-16237

Mycgr3G67775\_Mycgr3T

Mycgr3G90404 Mycgr3T
  
Location: 16337-17246

Mycgr3G90404\_Mycgr3T

Mycgr3G36951 Mycgr3T
  
Location: 17346-30891

Mycgr3G36951\_Mycgr3T

Mycgr3G103034 Mycgr3
  
Location: 30991-32644

Mycgr3G103034\_Mycgr3

Mycgr3G31119 Mycgr3T
  
Location: 32744-32906

Mycgr3G31119\_Mycgr3T

Mycgr3G28587 Mycgr3T
  
Location: 33006-33489

Mycgr3G28587\_Mycgr3T

Mycgr3G98959 Mycgr3T
  
Location: 33589-35035

Mycgr3G98959\_Mycgr3T

Mycgr3G35447 Mycgr3T
  
Location: 35135-36443

Mycgr3G35447\_Mycgr3T

Mycgr3G84402 Mycgr3T
  
Location: 36543-37884

Mycgr3G84402\_Mycgr3T

Mycgr3G98961 Mycgr3T
  
Location: 37984-38884

Mycgr3G98961\_Mycgr3T

conserved hypothetical protein
  
Accession: EFW20575
  
Location: 375459-377905
  
 NCBI BlastP on this gene

EFW20575

26S protease regulatory subunit 6A
  
Accession: EFW20576
  
Location: 378373-379885
  
 NCBI BlastP on this gene

EFW20576

trimethyllysine dioxygenase
  
Accession: EFW20577
  
Location: 380400-382419
  
 NCBI BlastP on this gene

EFW20577

predicted protein
  
Accession: EFW20578
  
Location: 383333-384218
  
 NCBI BlastP on this gene

EFW20578

predicted protein
  
Accession: EFW20579
  
Location: 384635-385318
  
 NCBI BlastP on this gene

EFW20579

peroxisomal dehydratase
  
Accession: EFW20580
  
Location: 385624-386783
  
 NCBI BlastP on this gene

EFW20580

histone-arginine methyltransferase
  
Accession: EFW20581
  
Location: 387494-388923
  
 NCBI BlastP on this gene

EFW20581

Mn2+ homeostasis protein
  
Accession: EFW20582
  
Location: 389649-390847
  
 NCBI BlastP on this gene

EFW20582

N-acetylglucosamine-phosphate mutase
  
Accession: EFW20583
  
Location: 391555-393528
  
  
**BlastP hit with Mycgr3G103034\_Mycgr3**
  
Percentage identity: 57 %
  
BlastP bit score: 632
  
Sequence coverage: 98 %
  
E-value: 0.0
  
  
 NCBI BlastP on this gene

EFW20583

ATP-dependent rRNA helicase RRP3
  
Accession: EFW20584
  
Location: 393992-395478
  
  
**BlastP hit with Mycgr3G84402\_Mycgr3T**
  
Percentage identity: 72 %
  
BlastP bit score: 664
  
Sequence coverage: 101 %
  
E-value: 0.0
  
  
 NCBI BlastP on this gene

EFW20584

ribosome biogenesis protein Ssf2
  
Accession: EFW20585
  
Location: 395756-397131
  
  
**BlastP hit with Mycgr3G35447\_Mycgr3T**
  
Percentage identity: 55 %
  
BlastP bit score: 475
  
Sequence coverage: 101 %
  
E-value: 9e-162
  
  
 NCBI BlastP on this gene

EFW20585

conserved hypothetical protein
  
Accession: EFW20586
  
Location: 397375-398620
  
 NCBI BlastP on this gene

EFW20586

riboflavin synthase subunit alpha
  
Accession: EFW20587
  
Location: 398876-399739
  
 NCBI BlastP on this gene

EFW20587

CAIB/BAIF family enzyme
  
Accession: EFW20588
  
Location: 400073-401721
  
 NCBI BlastP on this gene

EFW20588

hypothetical protein
  
Accession: EFW20589
  
Location: 402056-403905
  
 NCBI BlastP on this gene

EFW20589

stomatin family protein
  
Accession: EFW20590
  
Location: 406142-407441
  
 NCBI BlastP on this gene

EFW20590

predicted protein
  
Accession: EFW20591
  
Location: 407907-408359
  
 NCBI BlastP on this gene

EFW20591

conserved hypothetical protein
  
Accession: EFW20592
  
Location: 408489-409023
  
 NCBI BlastP on this gene

EFW20592

conserved hypothetical protein
  
Accession: EFW20593
  
Location: 410329-410962
  
 NCBI BlastP on this gene

EFW20593

predicted protein
  
Accession: EFW20594
  
Location: 411280-411726
  
 NCBI BlastP on this gene

EFW20594

hypothetical protein
  
Accession: EFW20595
  
Location: 413444-413629
  
 NCBI BlastP on this gene

EFW20595

60S ribosomal protein L27e
  
Accession: EFW20596
  
Location: 414535-415356
  
 NCBI BlastP on this gene

EFW20596

conserved hypothetical protein
  
Accession: EFW20597
  
Location: 416075-422505
  
 NCBI BlastP on this gene

EFW20597

Query: Architecture Search FASTA input

GG704916 : Coccidioides immitis RS genomic scaffold supercont3.6    Total score: 3.0     Cumulative Blast bit score: 1771

Hit cluster cross-links:

Mycgr3G67791 Mycgr3T
  
Location: 0-1542

Mycgr3G67791\_Mycgr3T

Mycgr3G90406 Mycgr3T
  
Location: 1642-3973

Mycgr3G90406\_Mycgr3T

Mycgr3G67785 Mycgr3T
  
Location: 4073-7865

Mycgr3G67785\_Mycgr3T

Mycgr3G67795 Mycgr3T
  
Location: 7965-15249

Mycgr3G67795\_Mycgr3T

Mycgr3G67775 Mycgr3T
  
Location: 15349-16237

Mycgr3G67775\_Mycgr3T

Mycgr3G90404 Mycgr3T
  
Location: 16337-17246

Mycgr3G90404\_Mycgr3T

Mycgr3G36951 Mycgr3T
  
Location: 17346-30891

Mycgr3G36951\_Mycgr3T

Mycgr3G103034 Mycgr3
  
Location: 30991-32644

Mycgr3G103034\_Mycgr3

Mycgr3G31119 Mycgr3T
  
Location: 32744-32906

Mycgr3G31119\_Mycgr3T

Mycgr3G28587 Mycgr3T
  
Location: 33006-33489

Mycgr3G28587\_Mycgr3T

Mycgr3G98959 Mycgr3T
  
Location: 33589-35035

Mycgr3G98959\_Mycgr3T

Mycgr3G35447 Mycgr3T
  
Location: 35135-36443

Mycgr3G35447\_Mycgr3T

Mycgr3G84402 Mycgr3T
  
Location: 36543-37884

Mycgr3G84402\_Mycgr3T

Mycgr3G98961 Mycgr3T
  
Location: 37984-38884

Mycgr3G98961\_Mycgr3T

hypothetical protein
  
Accession: EJB12070
  
Location: 1565730-1566396
  
 NCBI BlastP on this gene

EJB12070

trimethyllysine dioxygenase
  
Accession: EAS32649
  
Location: 1559464-1561484
  
 NCBI BlastP on this gene

EAS32649

hypothetical protein
  
Accession: EJB12069
  
Location: 1557719-1558805
  
 NCBI BlastP on this gene

EJB12069

hypothetical protein
  
Accession: EAS32646
  
Location: 1556439-1557216
  
 NCBI BlastP on this gene

EAS32646

hypothetical protein
  
Accession: EAS32645
  
Location: 1555184-1556042
  
 NCBI BlastP on this gene

EAS32645

peroxisomal dehydratase
  
Accession: EAS32644
  
Location: 1552602-1553760
  
 NCBI BlastP on this gene

EAS32644

HNRNP arginine N-methyltransferase, variant
  
Accession: EJB12068
  
Location: 1550469-1551832
  
 NCBI BlastP on this gene

EJB12068

Mn2+ homeostasis protein
  
Accession: EAS32641
  
Location: 1548538-1549736
  
 NCBI BlastP on this gene

EAS32641

N-acetylglucosamine-phosphate mutase
  
Accession: EAS32640
  
Location: 1545895-1547868
  
  
**BlastP hit with Mycgr3G103034\_Mycgr3**
  
Percentage identity: 57 %
  
BlastP bit score: 634
  
Sequence coverage: 98 %
  
E-value: 0.0
  
  
 NCBI BlastP on this gene

EAS32640

ATP-dependent rRNA helicase RRP3
  
Accession: EAS32639
  
Location: 1543945-1545431
  
  
**BlastP hit with Mycgr3G84402\_Mycgr3T**
  
Percentage identity: 72 %
  
BlastP bit score: 665
  
Sequence coverage: 101 %
  
E-value: 0.0
  
  
 NCBI BlastP on this gene

EAS32639

ribosome biogenesis protein Ssf2
  
Accession: EAS32638
  
Location: 1542294-1543669
  
  
**BlastP hit with Mycgr3G35447\_Mycgr3T**
  
Percentage identity: 55 %
  
BlastP bit score: 472
  
Sequence coverage: 101 %
  
E-value: 1e-160
  
  
 NCBI BlastP on this gene

EAS32638

hypothetical protein
  
Accession: EAS32637
  
Location: 1540805-1541759
  
 NCBI BlastP on this gene

EAS32637

riboflavin synthase, alpha subunit
  
Accession: EAS32636
  
Location: 1539686-1540549
  
 NCBI BlastP on this gene

EAS32636

CAIB/BAIF family enzyme
  
Accession: EAS32635
  
Location: 1537704-1539352
  
 NCBI BlastP on this gene

EAS32635

hypothetical protein
  
Accession: EAS32634
  
Location: 1535518-1537278
  
 NCBI BlastP on this gene

EAS32634

stomatin family protein
  
Accession: EAS32633
  
Location: 1532863-1534164
  
 NCBI BlastP on this gene

EAS32633

hypothetical protein
  
Accession: EAS32632
  
Location: 1531289-1531807
  
 NCBI BlastP on this gene

EAS32632

pathogenesis associated protein Cap20
  
Accession: EAS32631
  
Location: 1529336-1529993
  
 NCBI BlastP on this gene

EAS32631

hypothetical protein
  
Accession: EJB12066
  
Location: 1527603-1528013
  
 NCBI BlastP on this gene

EJB12066

60S ribosomal protein L27-B
  
Accession: EAS32629
  
Location: 1525033-1525856
  
 NCBI BlastP on this gene

EAS32629

Query: Architecture Search FASTA input

GG749414 : Ajellomyces dermatitidis ATCC 18188 genomic scaffold supercont1.8    Total score: 3.0     Cumulative Blast bit score: 1756

Hit cluster cross-links:

Mycgr3G67791 Mycgr3T
  
Location: 0-1542

Mycgr3G67791\_Mycgr3T

Mycgr3G90406 Mycgr3T
  
Location: 1642-3973

Mycgr3G90406\_Mycgr3T

Mycgr3G67785 Mycgr3T
  
Location: 4073-7865

Mycgr3G67785\_Mycgr3T

Mycgr3G67795 Mycgr3T
  
Location: 7965-15249

Mycgr3G67795\_Mycgr3T

Mycgr3G67775 Mycgr3T
  
Location: 15349-16237

Mycgr3G67775\_Mycgr3T

Mycgr3G90404 Mycgr3T
  
Location: 16337-17246

Mycgr3G90404\_Mycgr3T

Mycgr3G36951 Mycgr3T
  
Location: 17346-30891

Mycgr3G36951\_Mycgr3T

Mycgr3G103034 Mycgr3
  
Location: 30991-32644

Mycgr3G103034\_Mycgr3

Mycgr3G31119 Mycgr3T
  
Location: 32744-32906

Mycgr3G31119\_Mycgr3T

Mycgr3G28587 Mycgr3T
  
Location: 33006-33489

Mycgr3G28587\_Mycgr3T

Mycgr3G98959 Mycgr3T
  
Location: 33589-35035

Mycgr3G98959\_Mycgr3T

Mycgr3G35447 Mycgr3T
  
Location: 35135-36443

Mycgr3G35447\_Mycgr3T

Mycgr3G84402 Mycgr3T
  
Location: 36543-37884

Mycgr3G84402\_Mycgr3T

Mycgr3G98961 Mycgr3T
  
Location: 37984-38884

Mycgr3G98961\_Mycgr3T

hypothetical protein
  
Accession: EGE79446
  
Location: 925657-925975
  
 NCBI BlastP on this gene

EGE79446

trimethyllysine dioxygenase
  
Accession: EGE79447
  
Location: 927499-929800
  
 NCBI BlastP on this gene

EGE79447

peroxisomal dehydratase
  
Accession: EGE79448
  
Location: 931829-933212
  
 NCBI BlastP on this gene

EGE79448

hypothetical protein
  
Accession: EGE79449
  
Location: 936255-936733
  
 NCBI BlastP on this gene

EGE79449

hypothetical protein
  
Accession: EGE79450
  
Location: 936736-937491
  
 NCBI BlastP on this gene

EGE79450

N-acetylglucosamine-phosphate mutase
  
Accession: EGE79451
  
Location: 937989-940064
  
  
**BlastP hit with Mycgr3G103034\_Mycgr3**
  
Percentage identity: 56 %
  
BlastP bit score: 628
  
Sequence coverage: 98 %
  
E-value: 0.0
  
  
 NCBI BlastP on this gene

EGE79451

ATP-dependent rRNA helicase RRP3
  
Accession: EGE79452
  
Location: 940788-942319
  
  
**BlastP hit with Mycgr3G84402\_Mycgr3T**
  
Percentage identity: 77 %
  
BlastP bit score: 647
  
Sequence coverage: 91 %
  
E-value: 0.0
  
  
 NCBI BlastP on this gene

EGE79452

ribosome biogenesis protein Ssf2
  
Accession: EGE79453
  
Location: 942550-943963
  
  
**BlastP hit with Mycgr3G35447\_Mycgr3T**
  
Percentage identity: 58 %
  
BlastP bit score: 481
  
Sequence coverage: 96 %
  
E-value: 3e-164
  
  
 NCBI BlastP on this gene

EGE79453

riboflavin synthase subunit alpha
  
Accession: EGE79454
  
Location: 944346-945198
  
 NCBI BlastP on this gene

EGE79454

CAIB/BAIF family enzyme
  
Accession: EGE79455
  
Location: 945541-947352
  
 NCBI BlastP on this gene

EGE79455

Query: Architecture Search FASTA input

EQ999973 : Ajellomyces dermatitidis ER-3 genomic scaffold supercont1.1    Total score: 3.0     Cumulative Blast bit score: 1756

Hit cluster cross-links:

Mycgr3G67791 Mycgr3T
  
Location: 0-1542

Mycgr3G67791\_Mycgr3T

Mycgr3G90406 Mycgr3T
  
Location: 1642-3973

Mycgr3G90406\_Mycgr3T

Mycgr3G67785 Mycgr3T
  
Location: 4073-7865

Mycgr3G67785\_Mycgr3T

Mycgr3G67795 Mycgr3T
  
Location: 7965-15249

Mycgr3G67795\_Mycgr3T

Mycgr3G67775 Mycgr3T
  
Location: 15349-16237

Mycgr3G67775\_Mycgr3T

Mycgr3G90404 Mycgr3T
  
Location: 16337-17246

Mycgr3G90404\_Mycgr3T

Mycgr3G36951 Mycgr3T
  
Location: 17346-30891

Mycgr3G36951\_Mycgr3T

Mycgr3G103034 Mycgr3
  
Location: 30991-32644

Mycgr3G103034\_Mycgr3

Mycgr3G31119 Mycgr3T
  
Location: 32744-32906

Mycgr3G31119\_Mycgr3T

Mycgr3G28587 Mycgr3T
  
Location: 33006-33489

Mycgr3G28587\_Mycgr3T

Mycgr3G98959 Mycgr3T
  
Location: 33589-35035

Mycgr3G98959\_Mycgr3T

Mycgr3G35447 Mycgr3T
  
Location: 35135-36443

Mycgr3G35447\_Mycgr3T

Mycgr3G84402 Mycgr3T
  
Location: 36543-37884

Mycgr3G84402\_Mycgr3T

Mycgr3G98961 Mycgr3T
  
Location: 37984-38884

Mycgr3G98961\_Mycgr3T

predicted protein
  
Accession: EEQ83927
  
Location: 5776724-5777534
  
 NCBI BlastP on this gene

EEQ83927

conserved hypothetical protein
  
Accession: EEQ83928
  
Location: 5778730-5781253
  
 NCBI BlastP on this gene

EEQ83928

26S protease regulatory subunit 6A
  
Accession: EEQ83929
  
Location: 5782090-5783669
  
 NCBI BlastP on this gene

EEQ83929

trimethyllysine dioxygenase
  
Accession: EEQ83930
  
Location: 5784069-5786339
  
 NCBI BlastP on this gene

EEQ83930

peroxisomal dehydratase
  
Accession: EEQ83931
  
Location: 5788378-5789765
  
 NCBI BlastP on this gene

EEQ83931

HNRNP arginine N-methyltransferase
  
Accession: EEQ83932
  
Location: 5790520-5792138
  
 NCBI BlastP on this gene

EEQ83932

Mn2+ homeostasis protein
  
Accession: EEQ83933
  
Location: 5792815-5794051
  
 NCBI BlastP on this gene

EEQ83933

N-acetylglucosamine-phosphate mutase
  
Accession: EEQ83934
  
Location: 5794539-5796614
  
  
**BlastP hit with Mycgr3G103034\_Mycgr3**
  
Percentage identity: 56 %
  
BlastP bit score: 628
  
Sequence coverage: 98 %
  
E-value: 0.0
  
  
 NCBI BlastP on this gene

EEQ83934

ATP-dependent rRNA helicase RRP3
  
Accession: EEQ83935
  
Location: 5797347-5798878
  
  
**BlastP hit with Mycgr3G84402\_Mycgr3T**
  
Percentage identity: 77 %
  
BlastP bit score: 647
  
Sequence coverage: 91 %
  
E-value: 0.0
  
  
 NCBI BlastP on this gene

EEQ83935

ribosome biogenesis protein Ssf2
  
Accession: EEQ83936
  
Location: 5799109-5800522
  
  
**BlastP hit with Mycgr3G35447\_Mycgr3T**
  
Percentage identity: 58 %
  
BlastP bit score: 481
  
Sequence coverage: 94 %
  
E-value: 3e-164
  
  
 NCBI BlastP on this gene

EEQ83936

riboflavin synthase subunit alpha
  
Accession: EEQ83937
  
Location: 5800905-5801757
  
 NCBI BlastP on this gene

EEQ83937

CAIB/BAIF family enzyme
  
Accession: EEQ83938
  
Location: 5802100-5803911
  
 NCBI BlastP on this gene

EEQ83938

Query: Architecture Search FASTA input

GG663372 : Ajellomyces capsulatus G186AR genomic scaffold supercont2.10    Total score: 3.0     Cumulative Blast bit score: 1752

Hit cluster cross-links:

Mycgr3G67791 Mycgr3T
  
Location: 0-1542

Mycgr3G67791\_Mycgr3T

Mycgr3G90406 Mycgr3T
  
Location: 1642-3973

Mycgr3G90406\_Mycgr3T

Mycgr3G67785 Mycgr3T
  
Location: 4073-7865

Mycgr3G67785\_Mycgr3T

Mycgr3G67795 Mycgr3T
  
Location: 7965-15249

Mycgr3G67795\_Mycgr3T

Mycgr3G67775 Mycgr3T
  
Location: 15349-16237

Mycgr3G67775\_Mycgr3T

Mycgr3G90404 Mycgr3T
  
Location: 16337-17246

Mycgr3G90404\_Mycgr3T

Mycgr3G36951 Mycgr3T
  
Location: 17346-30891

Mycgr3G36951\_Mycgr3T

Mycgr3G103034 Mycgr3
  
Location: 30991-32644

Mycgr3G103034\_Mycgr3

Mycgr3G31119 Mycgr3T
  
Location: 32744-32906

Mycgr3G31119\_Mycgr3T

Mycgr3G28587 Mycgr3T
  
Location: 33006-33489

Mycgr3G28587\_Mycgr3T

Mycgr3G98959 Mycgr3T
  
Location: 33589-35035

Mycgr3G98959\_Mycgr3T

Mycgr3G35447 Mycgr3T
  
Location: 35135-36443

Mycgr3G35447\_Mycgr3T

Mycgr3G84402 Mycgr3T
  
Location: 36543-37884

Mycgr3G84402\_Mycgr3T

Mycgr3G98961 Mycgr3T
  
Location: 37984-38884

Mycgr3G98961\_Mycgr3T

serine/threonine-protein kinase sck1
  
Accession: EEH04859
  
Location: 463737-466737
  
 NCBI BlastP on this gene

EEH04859

conserved hypothetical protein
  
Accession: EEH04858
  
Location: 457429-459946
  
 NCBI BlastP on this gene

EEH04858

26S protease regulatory subunit
  
Accession: EEH04857
  
Location: 454926-456502
  
 NCBI BlastP on this gene

EEH04857

trimethyllysine dioxygenase
  
Accession: EEH04856
  
Location: 449718-454580
  
 NCBI BlastP on this gene

EEH04856

HNRNP arginine N-methyltransferase
  
Accession: EEH04855
  
Location: 447117-448972
  
 NCBI BlastP on this gene

EEH04855

PER1 precursor
  
Accession: EEH04854
  
Location: 445531-446758
  
 NCBI BlastP on this gene

EEH04854

N-acetylglucosamine-phosphate mutase
  
Accession: EEH04853
  
Location: 442948-445022
  
  
**BlastP hit with Mycgr3G103034\_Mycgr3**
  
Percentage identity: 55 %
  
BlastP bit score: 615
  
Sequence coverage: 100 %
  
E-value: 0.0
  
  
 NCBI BlastP on this gene

EEH04853

ATP-dependent rRNA helicase RRP3
  
Accession: EEH04852
  
Location: 440712-442246
  
  
**BlastP hit with Mycgr3G84402\_Mycgr3T**
  
Percentage identity: 73 %
  
BlastP bit score: 659
  
Sequence coverage: 96 %
  
E-value: 0.0
  
  
 NCBI BlastP on this gene

EEH04852

brix domain-containing protein c
  
Accession: EEH04851
  
Location: 439121-440520
  
  
**BlastP hit with Mycgr3G35447\_Mycgr3T**
  
Percentage identity: 56 %
  
BlastP bit score: 478
  
Sequence coverage: 101 %
  
E-value: 9e-163
  
  
 NCBI BlastP on this gene

EEH04851

riboflavin synthase
  
Accession: EEH04850
  
Location: 437946-438796
  
 NCBI BlastP on this gene

EEH04850

CAIB/BAIF family enzyme
  
Accession: EEH04849
  
Location: 436174-437688
  
 NCBI BlastP on this gene

EEH04849

conserved hypothetical protein
  
Accession: EEH04848
  
Location: 434313-435290
  
 NCBI BlastP on this gene

EEH04848

PHD finger containing protein Phf1
  
Accession: EEH04847
  
Location: 432087-433888
  
 NCBI BlastP on this gene

EEH04847

DUF887 domain-containing protein
  
Accession: EEH04846
  
Location: 429897-431199
  
 NCBI BlastP on this gene

EEH04846

conserved hypothetical protein
  
Accession: EEH04845
  
Location: 422928-429058
  
 NCBI BlastP on this gene

EEH04845

60S ribosomal protein L27A
  
Accession: EEH04844
  
Location: 421407-422237
  
 NCBI BlastP on this gene

EEH04844

Query: Architecture Search FASTA input

DS989822 : Arthroderma gypseum CBS 118893 supercont1.1 genomic scaffold    Total score: 3.0     Cumulative Blast bit score: 1716

Hit cluster cross-links:

Mycgr3G67791 Mycgr3T
  
Location: 0-1542

Mycgr3G67791\_Mycgr3T

Mycgr3G90406 Mycgr3T
  
Location: 1642-3973

Mycgr3G90406\_Mycgr3T

Mycgr3G67785 Mycgr3T
  
Location: 4073-7865

Mycgr3G67785\_Mycgr3T

Mycgr3G67795 Mycgr3T
  
Location: 7965-15249

Mycgr3G67795\_Mycgr3T

Mycgr3G67775 Mycgr3T
  
Location: 15349-16237

Mycgr3G67775\_Mycgr3T

Mycgr3G90404 Mycgr3T
  
Location: 16337-17246

Mycgr3G90404\_Mycgr3T

Mycgr3G36951 Mycgr3T
  
Location: 17346-30891

Mycgr3G36951\_Mycgr3T

Mycgr3G103034 Mycgr3
  
Location: 30991-32644

Mycgr3G103034\_Mycgr3

Mycgr3G31119 Mycgr3T
  
Location: 32744-32906

Mycgr3G31119\_Mycgr3T

Mycgr3G28587 Mycgr3T
  
Location: 33006-33489

Mycgr3G28587\_Mycgr3T

Mycgr3G98959 Mycgr3T
  
Location: 33589-35035

Mycgr3G98959\_Mycgr3T

Mycgr3G35447 Mycgr3T
  
Location: 35135-36443

Mycgr3G35447\_Mycgr3T

Mycgr3G84402 Mycgr3T
  
Location: 36543-37884

Mycgr3G84402\_Mycgr3T

Mycgr3G98961 Mycgr3T
  
Location: 37984-38884

Mycgr3G98961\_Mycgr3T

AGC/AKT protein kinase
  
Accession: EFQ98218
  
Location: 3416124-3419245
  
 NCBI BlastP on this gene

EFQ98218

hypothetical protein
  
Accession: EFQ98219
  
Location: 3420684-3421223
  
 NCBI BlastP on this gene

EFQ98219

hypothetical protein
  
Accession: EFQ98220
  
Location: 3421837-3424375
  
 NCBI BlastP on this gene

EFQ98220

26S protease regulatory subunit 6A
  
Accession: EFQ98221
  
Location: 3424941-3426467
  
 NCBI BlastP on this gene

EFQ98221

trimethyllysine dioxygenase
  
Accession: EFQ98222
  
Location: 3426900-3428834
  
 NCBI BlastP on this gene

EFQ98222

peroxisomal dehydratase
  
Accession: EFQ98223
  
Location: 3429272-3430448
  
 NCBI BlastP on this gene

EFQ98223

HNRNP arginine N-methyltransferase
  
Accession: EFQ98224
  
Location: 3431032-3432401
  
 NCBI BlastP on this gene

EFQ98224

hypothetical protein
  
Accession: EFQ98225
  
Location: 3432791-3433660
  
 NCBI BlastP on this gene

EFQ98225

PER1
  
Accession: EFQ98226
  
Location: 3434173-3435408
  
 NCBI BlastP on this gene

EFQ98226

phosphoacetylglucosamine mutase
  
Accession: EFQ98227
  
Location: 3435864-3437863
  
  
**BlastP hit with Mycgr3G103034\_Mycgr3**
  
Percentage identity: 55 %
  
BlastP bit score: 604
  
Sequence coverage: 100 %
  
E-value: 0.0
  
  
 NCBI BlastP on this gene

EFQ98227

ATP-dependent rRNA helicase RRP3
  
Accession: EFQ98228
  
Location: 3438432-3439938
  
  
**BlastP hit with Mycgr3G84402\_Mycgr3T**
  
Percentage identity: 72 %
  
BlastP bit score: 671
  
Sequence coverage: 100 %
  
E-value: 0.0
  
  
 NCBI BlastP on this gene

EFQ98228

ribosome biogenesis protein SSF1
  
Accession: EFQ98229
  
Location: 3440194-3441582
  
  
**BlastP hit with Mycgr3G35447\_Mycgr3T**
  
Percentage identity: 55 %
  
BlastP bit score: 441
  
Sequence coverage: 93 %
  
E-value: 1e-148
  
  
 NCBI BlastP on this gene

EFQ98229

riboflavin synthase subunit alpha
  
Accession: EFQ98230
  
Location: 3441891-3442731
  
 NCBI BlastP on this gene

EFQ98230

formyl-coenzyme A transferase
  
Accession: EFQ98231
  
Location: 3443082-3444753
  
 NCBI BlastP on this gene

EFQ98231

hypothetical protein
  
Accession: EFQ98232
  
Location: 3445318-3446913
  
 NCBI BlastP on this gene

EFQ98232

stomatin-2
  
Accession: EFQ98233
  
Location: 3447362-3448643
  
 NCBI BlastP on this gene

EFQ98233

CAP20
  
Accession: EFQ98234
  
Location: 3450440-3451038
  
 NCBI BlastP on this gene

EFQ98234

60S ribosomal protein L27-A
  
Accession: EFQ98235
  
Location: 3452952-3453839
  
 NCBI BlastP on this gene

EFQ98235

hypothetical protein
  
Accession: EFQ98236
  
Location: 3454522-3461853
  
 NCBI BlastP on this gene

EFQ98236

Query: Architecture Search FASTA input

DS990636 : Ajellomyces capsulatus H88 supercont1.1 genomic scaffold    Total score: 3.0     Cumulative Blast bit score: 1712

Hit cluster cross-links:

Mycgr3G67791 Mycgr3T
  
Location: 0-1542

Mycgr3G67791\_Mycgr3T

Mycgr3G90406 Mycgr3T
  
Location: 1642-3973

Mycgr3G90406\_Mycgr3T

Mycgr3G67785 Mycgr3T
  
Location: 4073-7865

Mycgr3G67785\_Mycgr3T

Mycgr3G67795 Mycgr3T
  
Location: 7965-15249

Mycgr3G67795\_Mycgr3T

Mycgr3G67775 Mycgr3T
  
Location: 15349-16237

Mycgr3G67775\_Mycgr3T

Mycgr3G90404 Mycgr3T
  
Location: 16337-17246

Mycgr3G90404\_Mycgr3T

Mycgr3G36951 Mycgr3T
  
Location: 17346-30891

Mycgr3G36951\_Mycgr3T

Mycgr3G103034 Mycgr3
  
Location: 30991-32644

Mycgr3G103034\_Mycgr3

Mycgr3G31119 Mycgr3T
  
Location: 32744-32906

Mycgr3G31119\_Mycgr3T

Mycgr3G28587 Mycgr3T
  
Location: 33006-33489

Mycgr3G28587\_Mycgr3T

Mycgr3G98959 Mycgr3T
  
Location: 33589-35035

Mycgr3G98959\_Mycgr3T

Mycgr3G35447 Mycgr3T
  
Location: 35135-36443

Mycgr3G35447\_Mycgr3T

Mycgr3G84402 Mycgr3T
  
Location: 36543-37884

Mycgr3G84402\_Mycgr3T

Mycgr3G98961 Mycgr3T
  
Location: 37984-38884

Mycgr3G98961\_Mycgr3T

serine/threonine protein kinase sck1
  
Accession: EGC40893
  
Location: 753900-756902
  
 NCBI BlastP on this gene

EGC40893

conserved hypothetical protein
  
Accession: EGC40892
  
Location: 747637-750154
  
 NCBI BlastP on this gene

EGC40892

26S protease regulatory subunit 6A-B
  
Accession: EGC40891
  
Location: 745134-746710
  
 NCBI BlastP on this gene

EGC40891

trimethyllysine dioxygenase
  
Accession: EGC40890
  
Location: 742570-744789
  
 NCBI BlastP on this gene

EGC40890

phospholipase/carboxylesterase
  
Accession: EGC40889
  
Location: 741729-742198
  
 NCBI BlastP on this gene

EGC40889

peroxisomal dehydratase
  
Accession: EGC40888
  
Location: 739951-741304
  
 NCBI BlastP on this gene

EGC40888

HNRNP arginine N-methyltransferase
  
Accession: EGC40887
  
Location: 737720-739254
  
 NCBI BlastP on this gene

EGC40887

Mn2+ homeostasis protein
  
Accession: EGC40886
  
Location: 735813-737040
  
 NCBI BlastP on this gene

EGC40886

N-acetylglucosamine-phosphate mutase
  
Accession: EGC40885
  
Location: 733222-735296
  
  
**BlastP hit with Mycgr3G103034\_Mycgr3**
  
Percentage identity: 54 %
  
BlastP bit score: 597
  
Sequence coverage: 101 %
  
E-value: 0.0
  
  
 NCBI BlastP on this gene

EGC40885

ATP-dependent rRNA helicase RRP3
  
Accession: EGC40884
  
Location: 730986-732520
  
  
**BlastP hit with Mycgr3G84402\_Mycgr3T**
  
Percentage identity: 73 %
  
BlastP bit score: 658
  
Sequence coverage: 96 %
  
E-value: 0.0
  
  
 NCBI BlastP on this gene

EGC40884

ribosome biogenesis protein Ssf2
  
Accession: EGC40883
  
Location: 729402-730717
  
  
**BlastP hit with Mycgr3G35447\_Mycgr3T**
  
Percentage identity: 56 %
  
BlastP bit score: 457
  
Sequence coverage: 94 %
  
E-value: 5e-155
  
  
 NCBI BlastP on this gene

EGC40883

riboflavin synthase
  
Accession: EGC40882
  
Location: 728228-729078
  
 NCBI BlastP on this gene

EGC40882

CAIB/BAIF family enzyme
  
Accession: EGC40881
  
Location: 726199-727970
  
 NCBI BlastP on this gene

EGC40881

Na+/H+ antiporter
  
Accession: EGC40880
  
Location: 724604-725581
  
 NCBI BlastP on this gene

EGC40880

PHD finger containing protein Phf1
  
Accession: EGC40879
  
Location: 722381-724181
  
 NCBI BlastP on this gene

EGC40879

DUF887 domain-containing protein
  
Accession: EGC40878
  
Location: 720166-721467
  
 NCBI BlastP on this gene

EGC40878

conserved hypothetical protein
  
Accession: EGC40877
  
Location: 713203-719333
  
 NCBI BlastP on this gene

EGC40877

60S ribosomal protein
  
Accession: EGC40876
  
Location: 711694-712514
  
 NCBI BlastP on this gene

EGC40876

Query: Architecture Search FASTA input

DS499594 : Aspergillus fumigatus A1163 scf\_000001 genomic scaffold    Total score: 3.0     Cumulative Blast bit score: 1697

Hit cluster cross-links:

Mycgr3G67791 Mycgr3T
  
Location: 0-1542

Mycgr3G67791\_Mycgr3T

Mycgr3G90406 Mycgr3T
  
Location: 1642-3973

Mycgr3G90406\_Mycgr3T

Mycgr3G67785 Mycgr3T
  
Location: 4073-7865

Mycgr3G67785\_Mycgr3T

Mycgr3G67795 Mycgr3T
  
Location: 7965-15249

Mycgr3G67795\_Mycgr3T

Mycgr3G67775 Mycgr3T
  
Location: 15349-16237

Mycgr3G67775\_Mycgr3T

Mycgr3G90404 Mycgr3T
  
Location: 16337-17246

Mycgr3G90404\_Mycgr3T

Mycgr3G36951 Mycgr3T
  
Location: 17346-30891

Mycgr3G36951\_Mycgr3T

Mycgr3G103034 Mycgr3
  
Location: 30991-32644

Mycgr3G103034\_Mycgr3

Mycgr3G31119 Mycgr3T
  
Location: 32744-32906

Mycgr3G31119\_Mycgr3T

Mycgr3G28587 Mycgr3T
  
Location: 33006-33489

Mycgr3G28587\_Mycgr3T

Mycgr3G98959 Mycgr3T
  
Location: 33589-35035

Mycgr3G98959\_Mycgr3T

Mycgr3G35447 Mycgr3T
  
Location: 35135-36443

Mycgr3G35447\_Mycgr3T

Mycgr3G84402 Mycgr3T
  
Location: 36543-37884

Mycgr3G84402\_Mycgr3T

Mycgr3G98961 Mycgr3T
  
Location: 37984-38884

Mycgr3G98961\_Mycgr3T

extracellular exo-polygalacturonase, putative
  
Accession: EDP55950
  
Location: 1848351-1849900
  
 NCBI BlastP on this gene

EDP55950

L-serine dehydratase, putative
  
Accession: EDP55951
  
Location: 1850025-1851524
  
 NCBI BlastP on this gene

EDP55951

conserved predicted protein
  
Accession: EDP55952
  
Location: 1855095-1857494
  
 NCBI BlastP on this gene

EDP55952

proteasome regulatory particle subunit Rpt5, putative
  
Accession: EDP55953
  
Location: 1858334-1859954
  
 NCBI BlastP on this gene

EDP55953

trimethyllysine dioxygenase, putative
  
Accession: EDP55954
  
Location: 1860353-1861724
  
 NCBI BlastP on this gene

EDP55954

histone H4 arginine methyltransferase RmtA
  
Accession: EDP55955
  
Location: 1863624-1864982
  
 NCBI BlastP on this gene

EDP55955

Mn2+ homeostasis protein (Per1), putative
  
Accession: EDP55956
  
Location: 1865644-1866825
  
 NCBI BlastP on this gene

EDP55956

N-acetylglucosamine-phosphate mutase
  
Accession: EDP55957
  
Location: 1867380-1869370
  
  
**BlastP hit with Mycgr3G103034\_Mycgr3**
  
Percentage identity: 54 %
  
BlastP bit score: 608
  
Sequence coverage: 101 %
  
E-value: 0.0
  
  
 NCBI BlastP on this gene

EDP55957

ATP-dependent RNA helicase , putative
  
Accession: EDP55958
  
Location: 1869784-1871298
  
  
**BlastP hit with Mycgr3G84402\_Mycgr3T**
  
Percentage identity: 73 %
  
BlastP bit score: 647
  
Sequence coverage: 92 %
  
E-value: 0.0
  
  
 NCBI BlastP on this gene

EDP55958

ribosome biogenesis protein Ssf2, putative
  
Accession: EDP55959
  
Location: 1871553-1872955
  
  
**BlastP hit with Mycgr3G35447\_Mycgr3T**
  
Percentage identity: 59 %
  
BlastP bit score: 443
  
Sequence coverage: 86 %
  
E-value: 4e-149
  
  
 NCBI BlastP on this gene

EDP55959

riboflavin synthase, alpha subunit
  
Accession: EDP55960
  
Location: 1873542-1874469
  
 NCBI BlastP on this gene

EDP55960

CAIB/BAIF family enzyme
  
Accession: EDP55961
  
Location: 1874794-1876418
  
 NCBI BlastP on this gene

EDP55961

PHD finger domain protein, putative
  
Accession: EDP55962
  
Location: 1876882-1878747
  
 NCBI BlastP on this gene

EDP55962

zinc metallopeptidase, putative
  
Accession: EDP55963
  
Location: 1879001-1880422
  
 NCBI BlastP on this gene

EDP55963

short-chain dehydrogenase/reductase family protein, putative
  
Accession: EDP55964
  
Location: 1880718-1882201
  
 NCBI BlastP on this gene

EDP55964

small nucleolar ribonucleoprotein complex subunit (SOF1), putative
  
Accession: EDP55965
  
Location: 1882384-1883814
  
 NCBI BlastP on this gene

EDP55965

proteasome regulatory particle subunit (RpnF), putative
  
Accession: EDP55966
  
Location: 1883878-1885550
  
 NCBI BlastP on this gene

EDP55966

SWI-SNF complex subunit (BAF60b), putative
  
Accession: EDP55967
  
Location: 1885958-1887617
  
 NCBI BlastP on this gene

EDP55967

DUF887 domain protein
  
Accession: EDP55968
  
Location: 1888049-1889306
  
 NCBI BlastP on this gene

EDP55968

conserved hypothetical protein
  
Accession: EDP55969
  
Location: 1890150-1896332
  
 NCBI BlastP on this gene

EDP55969

Query: Architecture Search FASTA input

GG700648 : Trichophyton rubrum CBS 118892 genomic scaffold supercont2.1    Total score: 3.0     Cumulative Blast bit score: 1697

Hit cluster cross-links:

Mycgr3G67791 Mycgr3T
  
Location: 0-1542

Mycgr3G67791\_Mycgr3T

Mycgr3G90406 Mycgr3T
  
Location: 1642-3973

Mycgr3G90406\_Mycgr3T

Mycgr3G67785 Mycgr3T
  
Location: 4073-7865

Mycgr3G67785\_Mycgr3T

Mycgr3G67795 Mycgr3T
  
Location: 7965-15249

Mycgr3G67795\_Mycgr3T

Mycgr3G67775 Mycgr3T
  
Location: 15349-16237

Mycgr3G67775\_Mycgr3T

Mycgr3G90404 Mycgr3T
  
Location: 16337-17246

Mycgr3G90404\_Mycgr3T

Mycgr3G36951 Mycgr3T
  
Location: 17346-30891

Mycgr3G36951\_Mycgr3T

Mycgr3G103034 Mycgr3
  
Location: 30991-32644

Mycgr3G103034\_Mycgr3

Mycgr3G31119 Mycgr3T
  
Location: 32744-32906

Mycgr3G31119\_Mycgr3T

Mycgr3G28587 Mycgr3T
  
Location: 33006-33489

Mycgr3G28587\_Mycgr3T

Mycgr3G98959 Mycgr3T
  
Location: 33589-35035

Mycgr3G98959\_Mycgr3T

Mycgr3G35447 Mycgr3T
  
Location: 35135-36443

Mycgr3G35447\_Mycgr3T

Mycgr3G84402 Mycgr3T
  
Location: 36543-37884

Mycgr3G84402\_Mycgr3T

Mycgr3G98961 Mycgr3T
  
Location: 37984-38884

Mycgr3G98961\_Mycgr3T

AGC/AKT protein kinase
  
Accession: EGD83979
  
Location: 670518-673706
  
 NCBI BlastP on this gene

EGD83979

hypothetical protein
  
Accession: EGD83978
  
Location: 665467-667999
  
 NCBI BlastP on this gene

EGD83978

26S protease regulatory subunit 6A
  
Accession: EGD83977
  
Location: 663406-664932
  
 NCBI BlastP on this gene

EGD83977

trimethyllysine dioxygenase
  
Accession: EGD83976
  
Location: 661047-662979
  
 NCBI BlastP on this gene

EGD83976

peroxisomal multifunctional enzyme type 2
  
Accession: EGD83975
  
Location: 659310-660464
  
 NCBI BlastP on this gene

EGD83975

HNRNP arginine N-methyltransferase
  
Accession: EGD83974
  
Location: 657336-658702
  
 NCBI BlastP on this gene

EGD83974

hypothetical protein
  
Accession: EGD83973
  
Location: 656022-656948
  
 NCBI BlastP on this gene

EGD83973

Mn2+ homeostasis protein
  
Accession: EGD83972
  
Location: 654276-655521
  
 NCBI BlastP on this gene

EGD83972

N-acetylglucosamine-phosphate mutase
  
Accession: EGD83971
  
Location: 651790-653795
  
  
**BlastP hit with Mycgr3G103034\_Mycgr3**
  
Percentage identity: 56 %
  
BlastP bit score: 609
  
Sequence coverage: 100 %
  
E-value: 0.0
  
  
 NCBI BlastP on this gene

EGD83971

ATP-dependent rRNA helicase RRP3
  
Accession: EGD83970
  
Location: 649734-651238
  
  
**BlastP hit with Mycgr3G84402\_Mycgr3T**
  
Percentage identity: 71 %
  
BlastP bit score: 667
  
Sequence coverage: 100 %
  
E-value: 0.0
  
  
 NCBI BlastP on this gene

EGD83970

ribosome biogenesis protein Ssf2
  
Accession: EGD83969
  
Location: 648061-649460
  
  
**BlastP hit with Mycgr3G35447\_Mycgr3T**
  
Percentage identity: 56 %
  
BlastP bit score: 421
  
Sequence coverage: 91 %
  
E-value: 1e-140
  
  
 NCBI BlastP on this gene

EGD83969

riboflavin synthase subunit alpha
  
Accession: EGD83968
  
Location: 646919-647754
  
 NCBI BlastP on this gene

EGD83968

hypothetical protein
  
Accession: EGD83967
  
Location: 644926-646587
  
 NCBI BlastP on this gene

EGD83967

hypothetical protein
  
Accession: EGD83966
  
Location: 642751-644349
  
 NCBI BlastP on this gene

EGD83966

stomatin family protein
  
Accession: EGD83965
  
Location: 641018-642307
  
 NCBI BlastP on this gene

EGD83965

hypothetical protein
  
Accession: EGD83964
  
Location: 638664-639265
  
 NCBI BlastP on this gene

EGD83964

60S ribosomal protein L27-A
  
Accession: EGD83963
  
Location: 635886-636936
  
 NCBI BlastP on this gene

EGD83963

Query: Architecture Search FASTA input

GG698540 : Trichophyton tonsurans CBS 112818 genomic scaffold supercont1.64    Total score: 3.0     Cumulative Blast bit score: 1697

Hit cluster cross-links:

Mycgr3G67791 Mycgr3T
  
Location: 0-1542

Mycgr3G67791\_Mycgr3T

Mycgr3G90406 Mycgr3T
  
Location: 1642-3973

Mycgr3G90406\_Mycgr3T

Mycgr3G67785 Mycgr3T
  
Location: 4073-7865

Mycgr3G67785\_Mycgr3T

Mycgr3G67795 Mycgr3T
  
Location: 7965-15249

Mycgr3G67795\_Mycgr3T

Mycgr3G67775 Mycgr3T
  
Location: 15349-16237

Mycgr3G67775\_Mycgr3T

Mycgr3G90404 Mycgr3T
  
Location: 16337-17246

Mycgr3G90404\_Mycgr3T

Mycgr3G36951 Mycgr3T
  
Location: 17346-30891

Mycgr3G36951\_Mycgr3T

Mycgr3G103034 Mycgr3
  
Location: 30991-32644

Mycgr3G103034\_Mycgr3

Mycgr3G31119 Mycgr3T
  
Location: 32744-32906

Mycgr3G31119\_Mycgr3T

Mycgr3G28587 Mycgr3T
  
Location: 33006-33489

Mycgr3G28587\_Mycgr3T

Mycgr3G98959 Mycgr3T
  
Location: 33589-35035

Mycgr3G98959\_Mycgr3T

Mycgr3G35447 Mycgr3T
  
Location: 35135-36443

Mycgr3G35447\_Mycgr3T

Mycgr3G84402 Mycgr3T
  
Location: 36543-37884

Mycgr3G84402\_Mycgr3T

Mycgr3G98961 Mycgr3T
  
Location: 37984-38884

Mycgr3G98961\_Mycgr3T

hypothetical protein
  
Accession: EGE00442
  
Location: 6620-8604
  
 NCBI BlastP on this gene

EGE00442

ATPase
  
Accession: EGE00443
  
Location: 9025-10550
  
 NCBI BlastP on this gene

EGE00443

trimethyllysine dioxygenase
  
Accession: EGE00444
  
Location: 11005-13044
  
 NCBI BlastP on this gene

EGE00444

peroxisomal dehydratase
  
Accession: EGE00445
  
Location: 13619-14784
  
 NCBI BlastP on this gene

EGE00445

HNRNP arginine N-methyltransferase
  
Accession: EGE00446
  
Location: 15429-16792
  
 NCBI BlastP on this gene

EGE00446

hypothetical protein
  
Accession: EGE00447
  
Location: 17179-18106
  
 NCBI BlastP on this gene

EGE00447

Mn2+ homeostasis protein
  
Accession: EGE00448
  
Location: 18432-19891
  
 NCBI BlastP on this gene

EGE00448

N-acetylglucosamine-phosphate mutase
  
Accession: EGE00449
  
Location: 20379-22382
  
  
**BlastP hit with Mycgr3G103034\_Mycgr3**
  
Percentage identity: 56 %
  
BlastP bit score: 609
  
Sequence coverage: 98 %
  
E-value: 0.0
  
  
 NCBI BlastP on this gene

EGE00449

ATP-dependent rRNA helicase RRP3
  
Accession: EGE00450
  
Location: 22937-24441
  
  
**BlastP hit with Mycgr3G84402\_Mycgr3T**
  
Percentage identity: 71 %
  
BlastP bit score: 668
  
Sequence coverage: 100 %
  
E-value: 0.0
  
  
 NCBI BlastP on this gene

EGE00450

ribosome biogenesis protein Ssf2
  
Accession: EGE00451
  
Location: 24714-26119
  
  
**BlastP hit with Mycgr3G35447\_Mycgr3T**
  
Percentage identity: 55 %
  
BlastP bit score: 420
  
Sequence coverage: 91 %
  
E-value: 3e-140
  
  
 NCBI BlastP on this gene

EGE00451

riboflavin synthase subunit alpha
  
Accession: EGE00452
  
Location: 26424-27258
  
 NCBI BlastP on this gene

EGE00452

hypothetical protein
  
Accession: EGE00453
  
Location: 27573-29237
  
 NCBI BlastP on this gene

EGE00453

hypothetical protein
  
Accession: EGE00454
  
Location: 29843-31441
  
 NCBI BlastP on this gene

EGE00454

stomatin family protein
  
Accession: EGE00455
  
Location: 31893-33178
  
 NCBI BlastP on this gene

EGE00455

hypothetical protein
  
Accession: EGE00456
  
Location: 34948-35553
  
 NCBI BlastP on this gene

EGE00456

60S ribosomal protein L27-A
  
Accession: EGE00457
  
Location: 37353-38241
  
 NCBI BlastP on this gene

EGE00457

hypothetical protein
  
Accession: EGE00458
  
Location: 38917-46254
  
 NCBI BlastP on this gene

EGE00458

Query: Architecture Search FASTA input

DS995745 : Trichophyton equinum CBS 127.97 supercont1.28 genomic scaffold    Total score: 3.0     Cumulative Blast bit score: 1697

Hit cluster cross-links:

Mycgr3G67791 Mycgr3T
  
Location: 0-1542

Mycgr3G67791\_Mycgr3T

Mycgr3G90406 Mycgr3T
  
Location: 1642-3973

Mycgr3G90406\_Mycgr3T

Mycgr3G67785 Mycgr3T
  
Location: 4073-7865

Mycgr3G67785\_Mycgr3T

Mycgr3G67795 Mycgr3T
  
Location: 7965-15249

Mycgr3G67795\_Mycgr3T

Mycgr3G67775 Mycgr3T
  
Location: 15349-16237

Mycgr3G67775\_Mycgr3T

Mycgr3G90404 Mycgr3T
  
Location: 16337-17246

Mycgr3G90404\_Mycgr3T

Mycgr3G36951 Mycgr3T
  
Location: 17346-30891

Mycgr3G36951\_Mycgr3T

Mycgr3G103034 Mycgr3
  
Location: 30991-32644

Mycgr3G103034\_Mycgr3

Mycgr3G31119 Mycgr3T
  
Location: 32744-32906

Mycgr3G31119\_Mycgr3T

Mycgr3G28587 Mycgr3T
  
Location: 33006-33489

Mycgr3G28587\_Mycgr3T

Mycgr3G98959 Mycgr3T
  
Location: 33589-35035

Mycgr3G98959\_Mycgr3T

Mycgr3G35447 Mycgr3T
  
Location: 35135-36443

Mycgr3G35447\_Mycgr3T

Mycgr3G84402 Mycgr3T
  
Location: 36543-37884

Mycgr3G84402\_Mycgr3T

Mycgr3G98961 Mycgr3T
  
Location: 37984-38884

Mycgr3G98961\_Mycgr3T

AGC/AKT protein kinase
  
Accession: EGE06138
  
Location: 44782-47721
  
 NCBI BlastP on this gene

EGE06138

hypothetical protein
  
Accession: EGE06137
  
Location: 39467-41212
  
 NCBI BlastP on this gene

EGE06137

26S protease regulatory subunit 6A
  
Accession: EGE06136
  
Location: 37405-38929
  
 NCBI BlastP on this gene

EGE06136

trimethyllysine dioxygenase
  
Accession: EGE06135
  
Location: 35035-36974
  
 NCBI BlastP on this gene

EGE06135

peroxisomal dehydratase
  
Accession: EGE06134
  
Location: 33296-34560
  
 NCBI BlastP on this gene

EGE06134

HNRNP arginine N-methyltransferase
  
Accession: EGE06133
  
Location: 31288-32651
  
 NCBI BlastP on this gene

EGE06133

hypothetical protein
  
Accession: EGE06132
  
Location: 30201-30906
  
 NCBI BlastP on this gene

EGE06132

PER1
  
Accession: EGE06131
  
Location: 28194-29427
  
 NCBI BlastP on this gene

EGE06131

phosphoacetylglucosamine mutase
  
Accession: EGE06130
  
Location: 25703-27706
  
  
**BlastP hit with Mycgr3G103034\_Mycgr3**
  
Percentage identity: 56 %
  
BlastP bit score: 609
  
Sequence coverage: 100 %
  
E-value: 0.0
  
  
 NCBI BlastP on this gene

EGE06130

ATP-dependent rRNA helicase RRP3
  
Accession: EGE06129
  
Location: 23644-25148
  
  
**BlastP hit with Mycgr3G84402\_Mycgr3T**
  
Percentage identity: 71 %
  
BlastP bit score: 668
  
Sequence coverage: 100 %
  
E-value: 0.0
  
  
 NCBI BlastP on this gene

EGE06129

ribosome biogenesis protein Ssf2
  
Accession: EGE06128
  
Location: 21966-23371
  
  
**BlastP hit with Mycgr3G35447\_Mycgr3T**
  
Percentage identity: 55 %
  
BlastP bit score: 420
  
Sequence coverage: 91 %
  
E-value: 3e-140
  
  
 NCBI BlastP on this gene

EGE06128

riboflavin synthase subunit alpha
  
Accession: EGE06127
  
Location: 20827-21661
  
 NCBI BlastP on this gene

EGE06127

formyl-coenzyme A transferase
  
Accession: EGE06126
  
Location: 18848-20512
  
 NCBI BlastP on this gene

EGE06126

hypothetical protein
  
Accession: EGE06125
  
Location: 16644-18242
  
 NCBI BlastP on this gene

EGE06125

stomatin family protein
  
Accession: EGE06124
  
Location: 14907-16192
  
 NCBI BlastP on this gene

EGE06124

CAP20
  
Accession: EGE06123
  
Location: 12533-13138
  
 NCBI BlastP on this gene

EGE06123

60S ribosomal protein L27-A
  
Accession: EGE06122
  
Location: 9844-10798
  
 NCBI BlastP on this gene

EGE06122

hypothetical protein
  
Accession: EGE06121
  
Location: 3955-9166
  
 NCBI BlastP on this gene

EGE06121

Query: Architecture Search FASTA input

DS027059 : Aspergillus clavatus NRRL 1 1099423829805 genomic scaffold    Total score: 3.0     Cumulative Blast bit score: 1695

Hit cluster cross-links:

Mycgr3G67791 Mycgr3T
  
Location: 0-1542

Mycgr3G67791\_Mycgr3T

Mycgr3G90406 Mycgr3T
  
Location: 1642-3973

Mycgr3G90406\_Mycgr3T

Mycgr3G67785 Mycgr3T
  
Location: 4073-7865

Mycgr3G67785\_Mycgr3T

Mycgr3G67795 Mycgr3T
  
Location: 7965-15249

Mycgr3G67795\_Mycgr3T

Mycgr3G67775 Mycgr3T
  
Location: 15349-16237

Mycgr3G67775\_Mycgr3T

Mycgr3G90404 Mycgr3T
  
Location: 16337-17246

Mycgr3G90404\_Mycgr3T

Mycgr3G36951 Mycgr3T
  
Location: 17346-30891

Mycgr3G36951\_Mycgr3T

Mycgr3G103034 Mycgr3
  
Location: 30991-32644

Mycgr3G103034\_Mycgr3

Mycgr3G31119 Mycgr3T
  
Location: 32744-32906

Mycgr3G31119\_Mycgr3T

Mycgr3G28587 Mycgr3T
  
Location: 33006-33489

Mycgr3G28587\_Mycgr3T

Mycgr3G98959 Mycgr3T
  
Location: 33589-35035

Mycgr3G98959\_Mycgr3T

Mycgr3G35447 Mycgr3T
  
Location: 35135-36443

Mycgr3G35447\_Mycgr3T

Mycgr3G84402 Mycgr3T
  
Location: 36543-37884

Mycgr3G84402\_Mycgr3T

Mycgr3G98961 Mycgr3T
  
Location: 37984-38884

Mycgr3G98961\_Mycgr3T

conserved hypothetical protein
  
Accession: EAW08095
  
Location: 3073971-3075233
  
 NCBI BlastP on this gene

EAW08095

conserved predicted protein
  
Accession: EAW08096
  
Location: 3078225-3080650
  
 NCBI BlastP on this gene

EAW08096

proteasome regulatory particle subunit Rpt5, putative
  
Accession: EAW08097
  
Location: 3081418-3083035
  
 NCBI BlastP on this gene

EAW08097

trimethyllysine dioxygenase, putative
  
Accession: EAW08098
  
Location: 3083436-3084409
  
 NCBI BlastP on this gene

EAW08098

hypothetical protein
  
Accession: EAW08099
  
Location: 3085080-3085864
  
 NCBI BlastP on this gene

EAW08099

protein arginine n-methyltransferase 1
  
Accession: EAW08100
  
Location: 3086856-3088218
  
 NCBI BlastP on this gene

EAW08100

Mn2+ homeostasis protein (Per1), putative
  
Accession: EAW08101
  
Location: 3089042-3090234
  
 NCBI BlastP on this gene

EAW08101

N-acetylglucosamine-phosphate mutase
  
Accession: EAW08102
  
Location: 3090867-3092885
  
  
**BlastP hit with Mycgr3G103034\_Mycgr3**
  
Percentage identity: 55 %
  
BlastP bit score: 598
  
Sequence coverage: 98 %
  
E-value: 0.0
  
  
 NCBI BlastP on this gene

EAW08102

ATP-dependent RNA helicase , putative
  
Accession: EAW08103
  
Location: 3093335-3094820
  
  
**BlastP hit with Mycgr3G84402\_Mycgr3T**
  
Percentage identity: 74 %
  
BlastP bit score: 647
  
Sequence coverage: 92 %
  
E-value: 0.0
  
  
 NCBI BlastP on this gene

EAW08103

ribosome biogenesis protein Ssf2, putative
  
Accession: EAW08104
  
Location: 3095073-3096475
  
  
**BlastP hit with Mycgr3G35447\_Mycgr3T**
  
Percentage identity: 56 %
  
BlastP bit score: 450
  
Sequence coverage: 101 %
  
E-value: 6e-152
  
  
 NCBI BlastP on this gene

EAW08104

riboflavin synthase, alpha subunit
  
Accession: EAW08105
  
Location: 3096972-3097881
  
 NCBI BlastP on this gene

EAW08105

CAIB/BAIF family enzyme
  
Accession: EAW08106
  
Location: 3098169-3099832
  
 NCBI BlastP on this gene

EAW08106

PHD finger domain protein, putative
  
Accession: EAW08107
  
Location: 3100218-3102005
  
 NCBI BlastP on this gene

EAW08107

zinc metallopeptidase, putative
  
Accession: EAW08108
  
Location: 3102279-3103703
  
 NCBI BlastP on this gene

EAW08108

short-chain dehydrogenase/reductase family protein, putative
  
Accession: EAW08109
  
Location: 3104293-3105478
  
 NCBI BlastP on this gene

EAW08109

small nucleolar ribonucleoprotein complex subunit (SOF1), putative
  
Accession: EAW08110
  
Location: 3105644-3107101
  
 NCBI BlastP on this gene

EAW08110

proteasome regulatory particle subunit (RpnF), putative
  
Accession: EAW08111
  
Location: 3107460-3108793
  
 NCBI BlastP on this gene

EAW08111

SWI-SNF complex subunit (BAF60b), putative
  
Accession: EAW08112
  
Location: 3109240-3110902
  
 NCBI BlastP on this gene

EAW08112

DUF887 domain protein
  
Accession: EAW08113
  
Location: 3111349-3112596
  
 NCBI BlastP on this gene

EAW08113

conserved hypothetical protein
  
Accession: EAW08114
  
Location: 3113410-3119867
  
 NCBI BlastP on this gene

EAW08114

Query: Architecture Search FASTA input

KB644410 : Penicillium oxalicum 114-2 unplaced genomic scaffold scaffold\_3    Total score: 3.0     Cumulative Blast bit score: 1693

Hit cluster cross-links:

Mycgr3G67791 Mycgr3T
  
Location: 0-1542

Mycgr3G67791\_Mycgr3T

Mycgr3G90406 Mycgr3T
  
Location: 1642-3973

Mycgr3G90406\_Mycgr3T

Mycgr3G67785 Mycgr3T
  
Location: 4073-7865

Mycgr3G67785\_Mycgr3T

Mycgr3G67795 Mycgr3T
  
Location: 7965-15249

Mycgr3G67795\_Mycgr3T

Mycgr3G67775 Mycgr3T
  
Location: 15349-16237

Mycgr3G67775\_Mycgr3T

Mycgr3G90404 Mycgr3T
  
Location: 16337-17246

Mycgr3G90404\_Mycgr3T

Mycgr3G36951 Mycgr3T
  
Location: 17346-30891

Mycgr3G36951\_Mycgr3T

Mycgr3G103034 Mycgr3
  
Location: 30991-32644

Mycgr3G103034\_Mycgr3

Mycgr3G31119 Mycgr3T
  
Location: 32744-32906

Mycgr3G31119\_Mycgr3T

Mycgr3G28587 Mycgr3T
  
Location: 33006-33489

Mycgr3G28587\_Mycgr3T

Mycgr3G98959 Mycgr3T
  
Location: 33589-35035

Mycgr3G98959\_Mycgr3T

Mycgr3G35447 Mycgr3T
  
Location: 35135-36443

Mycgr3G35447\_Mycgr3T

Mycgr3G84402 Mycgr3T
  
Location: 36543-37884

Mycgr3G84402\_Mycgr3T

Mycgr3G98961 Mycgr3T
  
Location: 37984-38884

Mycgr3G98961\_Mycgr3T

hypothetical protein
  
Accession: EPS27667
  
Location: 1587044-1588846
  
 NCBI BlastP on this gene

EPS27667

hypothetical protein
  
Accession: EPS27668
  
Location: 1589868-1591586
  
 NCBI BlastP on this gene

EPS27668

hypothetical protein
  
Accession: EPS27669
  
Location: 1592756-1594738
  
 NCBI BlastP on this gene

EPS27669

hypothetical protein
  
Accession: EPS27670
  
Location: 1595234-1595950
  
 NCBI BlastP on this gene

EPS27670

hypothetical protein
  
Accession: EPS27671
  
Location: 1596437-1597505
  
 NCBI BlastP on this gene

EPS27671

hypothetical protein
  
Accession: EPS27672
  
Location: 1598189-1599573
  
  
**BlastP hit with Mycgr3G35447\_Mycgr3T**
  
Percentage identity: 59 %
  
BlastP bit score: 462
  
Sequence coverage: 91 %
  
E-value: 2e-156
  
  
 NCBI BlastP on this gene

EPS27672

hypothetical protein
  
Accession: EPS27673
  
Location: 1599818-1601236
  
  
**BlastP hit with Mycgr3G84402\_Mycgr3T**
  
Percentage identity: 68 %
  
BlastP bit score: 634
  
Sequence coverage: 100 %
  
E-value: 0.0
  
  
 NCBI BlastP on this gene

EPS27673

hypothetical protein
  
Accession: EPS27674
  
Location: 1602056-1603645
  
 NCBI BlastP on this gene

EPS27674

hypothetical protein
  
Accession: EPS27675
  
Location: 1604080-1606165
  
 NCBI BlastP on this gene

EPS27675

hypothetical protein
  
Accession: EPS27676
  
Location: 1606579-1607757
  
 NCBI BlastP on this gene

EPS27676

hypothetical protein
  
Accession: EPS27677
  
Location: 1608362-1609814
  
 NCBI BlastP on this gene

EPS27677

hypothetical protein
  
Accession: EPS27678
  
Location: 1610525-1611034
  
 NCBI BlastP on this gene

EPS27678

hypothetical protein
  
Accession: EPS27679
  
Location: 1611801-1613004
  
 NCBI BlastP on this gene

EPS27679

hypothetical protein
  
Accession: EPS27680
  
Location: 1613488-1615521
  
  
**BlastP hit with Mycgr3G103034\_Mycgr3**
  
Percentage identity: 54 %
  
BlastP bit score: 597
  
Sequence coverage: 98 %
  
E-value: 0.0
  
  
 NCBI BlastP on this gene

EPS27680

hypothetical protein
  
Accession: EPS27681
  
Location: 1616256-1618202
  
 NCBI BlastP on this gene

EPS27681

hypothetical protein
  
Accession: EPS27682
  
Location: 1619064-1621476
  
 NCBI BlastP on this gene

EPS27682

hypothetical protein
  
Accession: EPS27683
  
Location: 1623124-1624257
  
 NCBI BlastP on this gene

EPS27683

hypothetical protein
  
Accession: EPS27684
  
Location: 1625643-1629058
  
 NCBI BlastP on this gene

EPS27684

Query: Architecture Search FASTA input

DS995899 : Penicillium marneffei ATCC 18224 scf\_1105668340764 genomic scaffold    Total score: 3.0     Cumulative Blast bit score: 1693

Hit cluster cross-links:

Mycgr3G67791 Mycgr3T
  
Location: 0-1542

Mycgr3G67791\_Mycgr3T

Mycgr3G90406 Mycgr3T
  
Location: 1642-3973

Mycgr3G90406\_Mycgr3T

Mycgr3G67785 Mycgr3T
  
Location: 4073-7865

Mycgr3G67785\_Mycgr3T

Mycgr3G67795 Mycgr3T
  
Location: 7965-15249

Mycgr3G67795\_Mycgr3T

Mycgr3G67775 Mycgr3T
  
Location: 15349-16237

Mycgr3G67775\_Mycgr3T

Mycgr3G90404 Mycgr3T
  
Location: 16337-17246

Mycgr3G90404\_Mycgr3T

Mycgr3G36951 Mycgr3T
  
Location: 17346-30891

Mycgr3G36951\_Mycgr3T

Mycgr3G103034 Mycgr3
  
Location: 30991-32644

Mycgr3G103034\_Mycgr3

Mycgr3G31119 Mycgr3T
  
Location: 32744-32906

Mycgr3G31119\_Mycgr3T

Mycgr3G28587 Mycgr3T
  
Location: 33006-33489

Mycgr3G28587\_Mycgr3T

Mycgr3G98959 Mycgr3T
  
Location: 33589-35035

Mycgr3G98959\_Mycgr3T

Mycgr3G35447 Mycgr3T
  
Location: 35135-36443

Mycgr3G35447\_Mycgr3T

Mycgr3G84402 Mycgr3T
  
Location: 36543-37884

Mycgr3G84402\_Mycgr3T

Mycgr3G98961 Mycgr3T
  
Location: 37984-38884

Mycgr3G98961\_Mycgr3T

cAMP-dependent protein kinase-like, putative
  
Accession: EEA27110
  
Location: 1271784-1274853
  
 NCBI BlastP on this gene

EEA27110

hypothetical protein
  
Accession: EEA27109
  
Location: 1266695-1269339
  
 NCBI BlastP on this gene

EEA27109

proteasome regulatory particle subunit Rpt5, putative
  
Accession: EEA27108
  
Location: 1264465-1265975
  
 NCBI BlastP on this gene

EEA27108

hypothetical protein
  
Accession: EEA27107
  
Location: 1263498-1264084
  
 NCBI BlastP on this gene

EEA27107

trimethyllysine dioxygenase TmlH, putative
  
Accession: EEA27106
  
Location: 1261822-1263317
  
 NCBI BlastP on this gene

EEA27106

peroxisomal dehydratase, putative
  
Accession: EEA27104
  
Location: 1260509-1261648
  
 NCBI BlastP on this gene

EEA27104

hypothetical protein
  
Accession: EEA27105
  
Location: 1258480-1260004
  
 NCBI BlastP on this gene

EEA27105

histone H4 arginine methyltransferase RmtA
  
Accession: EEA27101
  
Location: 1256558-1257930
  
 NCBI BlastP on this gene

EEA27101

Mn2+ homeostasis protein (Per1), putative
  
Accession: EEA27103
  
Location: 1254788-1255958
  
 NCBI BlastP on this gene

EEA27103

N-acetylglucosamine-phosphate mutase
  
Accession: EEA27100
  
Location: 1252382-1254387
  
  
**BlastP hit with Mycgr3G103034\_Mycgr3**
  
Percentage identity: 56 %
  
BlastP bit score: 626
  
Sequence coverage: 99 %
  
E-value: 0.0
  
  
 NCBI BlastP on this gene

EEA27100

ATP-dependent RNA helicase , putative
  
Accession: EEA27099
  
Location: 1250658-1252133
  
  
**BlastP hit with Mycgr3G84402\_Mycgr3T**
  
Percentage identity: 66 %
  
BlastP bit score: 613
  
Sequence coverage: 99 %
  
E-value: 0.0
  
  
 NCBI BlastP on this gene

EEA27099

ribosome biogenesis protein Ssf2, putative
  
Accession: EEA27098
  
Location: 1249051-1250439
  
  
**BlastP hit with Mycgr3G35447\_Mycgr3T**
  
Percentage identity: 60 %
  
BlastP bit score: 454
  
Sequence coverage: 91 %
  
E-value: 2e-153
  
  
 NCBI BlastP on this gene

EEA27098

riboflavin synthase, alpha subunit
  
Accession: EEA27097
  
Location: 1248124-1248949
  
 NCBI BlastP on this gene

EEA27097

CAIB/BAIF family enzyme
  
Accession: EEA27096
  
Location: 1246303-1247926
  
 NCBI BlastP on this gene

EEA27096

PHD finger domain protein, putative
  
Accession: EEA27095
  
Location: 1244326-1246008
  
 NCBI BlastP on this gene

EEA27095

short-chain dehydrogenase/reductase family protein, putative
  
Accession: EEA27094
  
Location: 1241280-1242465
  
 NCBI BlastP on this gene

EEA27094

small nucleolar ribonucleoprotein complex subunit (SOF1), putative
  
Accession: EEA27093
  
Location: 1239732-1241188
  
 NCBI BlastP on this gene

EEA27093

proteasome regulatory particle subunit (RpnF), putative
  
Accession: EEA27092
  
Location: 1238078-1239398
  
 NCBI BlastP on this gene

EEA27092

SWI-SNF complex subunit (BAF60b), putative
  
Accession: EEA27091
  
Location: 1236024-1237702
  
 NCBI BlastP on this gene

EEA27091

DUF887 domain protein
  
Accession: EEA27090
  
Location: 1234464-1235700
  
 NCBI BlastP on this gene

EEA27090

Query: Architecture Search FASTA input

AAHF01000007 : Aspergillus fumigatus Af293    Total score: 3.0     Cumulative Blast bit score: 1693

Hit cluster cross-links:

Mycgr3G67791 Mycgr3T
  
Location: 0-1542

Mycgr3G67791\_Mycgr3T

Mycgr3G90406 Mycgr3T
  
Location: 1642-3973

Mycgr3G90406\_Mycgr3T

Mycgr3G67785 Mycgr3T
  
Location: 4073-7865

Mycgr3G67785\_Mycgr3T

Mycgr3G67795 Mycgr3T
  
Location: 7965-15249

Mycgr3G67795\_Mycgr3T

Mycgr3G67775 Mycgr3T
  
Location: 15349-16237

Mycgr3G67775\_Mycgr3T

Mycgr3G90404 Mycgr3T
  
Location: 16337-17246

Mycgr3G90404\_Mycgr3T

Mycgr3G36951 Mycgr3T
  
Location: 17346-30891

Mycgr3G36951\_Mycgr3T

Mycgr3G103034 Mycgr3
  
Location: 30991-32644

Mycgr3G103034\_Mycgr3

Mycgr3G31119 Mycgr3T
  
Location: 32744-32906

Mycgr3G31119\_Mycgr3T

Mycgr3G28587 Mycgr3T
  
Location: 33006-33489

Mycgr3G28587\_Mycgr3T

Mycgr3G98959 Mycgr3T
  
Location: 33589-35035

Mycgr3G98959\_Mycgr3T

Mycgr3G35447 Mycgr3T
  
Location: 35135-36443

Mycgr3G35447\_Mycgr3T

Mycgr3G84402 Mycgr3T
  
Location: 36543-37884

Mycgr3G84402\_Mycgr3T

Mycgr3G98961 Mycgr3T
  
Location: 37984-38884

Mycgr3G98961\_Mycgr3T

extracellular exo-polygalacturonase, putative
  
Accession: EAL88325
  
Location: 1772681-1774230
  
 NCBI BlastP on this gene

EAL88325

L-serine dehydratase, putative
  
Accession: EAL88326
  
Location: 1774355-1775854
  
 NCBI BlastP on this gene

EAL88326

conserved predicted protein
  
Accession: EAL88327
  
Location: 1779425-1781824
  
 NCBI BlastP on this gene

EAL88327

proteasome regulatory particle subunit Rpt5, putative
  
Accession: EAL88328
  
Location: 1782664-1784284
  
 NCBI BlastP on this gene

EAL88328

trimethyllysine dioxygenase TmlH, putative
  
Accession: EAL88329
  
Location: 1784683-1786054
  
 NCBI BlastP on this gene

EAL88329

histone H4 arginine methyltransferase RmtA
  
Accession: EAL88330
  
Location: 1787954-1789312
  
 NCBI BlastP on this gene

EAL88330

Mn2+ homeostasis protein (Per1), putative
  
Accession: EAL88331
  
Location: 1789974-1791155
  
 NCBI BlastP on this gene

EAL88331

N-acetylglucosamine-phosphate mutase
  
Accession: EAL88332
  
Location: 1791710-1793700
  
  
**BlastP hit with Mycgr3G103034\_Mycgr3**
  
Percentage identity: 54 %
  
BlastP bit score: 604
  
Sequence coverage: 101 %
  
E-value: 0.0
  
  
 NCBI BlastP on this gene

EAL88332

ATP-dependent RNA helicase , putative
  
Accession: EAL88333
  
Location: 1794114-1795628
  
  
**BlastP hit with Mycgr3G84402\_Mycgr3T**
  
Percentage identity: 73 %
  
BlastP bit score: 645
  
Sequence coverage: 92 %
  
E-value: 0.0
  
  
 NCBI BlastP on this gene

EAL88333

ribosome biogenesis protein Ssf2, putative
  
Accession: EAL88334
  
Location: 1795883-1797285
  
  
**BlastP hit with Mycgr3G35447\_Mycgr3T**
  
Percentage identity: 59 %
  
BlastP bit score: 444
  
Sequence coverage: 86 %
  
E-value: 2e-149
  
  
 NCBI BlastP on this gene

EAL88334

riboflavin synthase, alpha subunit
  
Accession: EAL88335
  
Location: 1797872-1798799
  
 NCBI BlastP on this gene

EAL88335

CAIB/BAIF family enzyme
  
Accession: EAL88336
  
Location: 1799124-1800748
  
 NCBI BlastP on this gene

EAL88336

PHD finger domain protein, putative
  
Accession: EAL88337
  
Location: 1801212-1803077
  
 NCBI BlastP on this gene

EAL88337

zinc metallopeptidase, putative
  
Accession: EAL88338
  
Location: 1803331-1804530
  
 NCBI BlastP on this gene

EAL88338

short-chain dehydrogenase/reductase family protein, putative
  
Accession: EAL88339
  
Location: 1805046-1806529
  
 NCBI BlastP on this gene

EAL88339

small nucleolar ribonucleoprotein complex subunit (SOF1), putative
  
Accession: EAL88340
  
Location: 1806712-1808142
  
 NCBI BlastP on this gene

EAL88340

proteasome regulatory particle subunit (RpnF), putative
  
Accession: EAL88341
  
Location: 1808206-1809878
  
 NCBI BlastP on this gene

EAL88341

SWI-SNF complex subunit (BAF60b), putative
  
Accession: EAL88342
  
Location: 1810286-1811945
  
 NCBI BlastP on this gene

EAL88342

DUF887 domain protein
  
Accession: EAL88343
  
Location: 1812377-1813634
  
 NCBI BlastP on this gene

EAL88343

conserved hypothetical protein
  
Accession: EAL88344
  
Location: 1814478-1820660
  
 NCBI BlastP on this gene

EAL88344

Query: Architecture Search FASTA input

AKCU01000442 : Penicillium digitatum Pd1    Total score: 3.0     Cumulative Blast bit score: 1692

Hit cluster cross-links:

Mycgr3G67791 Mycgr3T
  
Location: 0-1542

Mycgr3G67791\_Mycgr3T

Mycgr3G90406 Mycgr3T
  
Location: 1642-3973

Mycgr3G90406\_Mycgr3T

Mycgr3G67785 Mycgr3T
  
Location: 4073-7865

Mycgr3G67785\_Mycgr3T

Mycgr3G67795 Mycgr3T
  
Location: 7965-15249

Mycgr3G67795\_Mycgr3T

Mycgr3G67775 Mycgr3T
  
Location: 15349-16237

Mycgr3G67775\_Mycgr3T

Mycgr3G90404 Mycgr3T
  
Location: 16337-17246

Mycgr3G90404\_Mycgr3T

Mycgr3G36951 Mycgr3T
  
Location: 17346-30891

Mycgr3G36951\_Mycgr3T

Mycgr3G103034 Mycgr3
  
Location: 30991-32644

Mycgr3G103034\_Mycgr3

Mycgr3G31119 Mycgr3T
  
Location: 32744-32906

Mycgr3G31119\_Mycgr3T

Mycgr3G28587 Mycgr3T
  
Location: 33006-33489

Mycgr3G28587\_Mycgr3T

Mycgr3G98959 Mycgr3T
  
Location: 33589-35035

Mycgr3G98959\_Mycgr3T

Mycgr3G35447 Mycgr3T
  
Location: 35135-36443

Mycgr3G35447\_Mycgr3T

Mycgr3G84402 Mycgr3T
  
Location: 36543-37884

Mycgr3G84402\_Mycgr3T

Mycgr3G98961 Mycgr3T
  
Location: 37984-38884

Mycgr3G98961\_Mycgr3T

hypothetical protein
  
Accession: EKV08241
  
Location: 24966-25838
  
 NCBI BlastP on this gene

EKV08241

hypothetical protein
  
Accession: EKV08240
  
Location: 19808-21292
  
 NCBI BlastP on this gene

EKV08240

Glutaminyl-tRNA synthetase
  
Accession: EKV08239
  
Location: 17253-19196
  
 NCBI BlastP on this gene

EKV08239

Proteasome regulatory particle subunit Rpt5, putative
  
Accession: EKV08238
  
Location: 15100-16677
  
 NCBI BlastP on this gene

EKV08238

Trimethyllysine dioxygenase TmlH, putative
  
Accession: EKV08237
  
Location: 13886-14828
  
 NCBI BlastP on this gene

EKV08237

Peroxisomal dehydratase
  
Accession: EKV08236
  
Location: 12191-12409
  
 NCBI BlastP on this gene

EKV08236

Peroxisomal dehydratase, putative
  
Accession: EKV08235
  
Location: 11350-11926
  
 NCBI BlastP on this gene

EKV08235

Histone H4 arginine methyltransferase RmtA
  
Accession: EKV08234
  
Location: 9377-10733
  
 NCBI BlastP on this gene

EKV08234

hypothetical protein
  
Accession: EKV08233
  
Location: 8496-8720
  
 NCBI BlastP on this gene

EKV08233

Mn2+ homeostasis protein (Per1), putative
  
Accession: EKV08232
  
Location: 7629-8348
  
 NCBI BlastP on this gene

EKV08232

N-acetylglucosamine-phosphate mutase
  
Accession: EKV08231
  
Location: 5167-7154
  
  
**BlastP hit with Mycgr3G103034\_Mycgr3**
  
Percentage identity: 54 %
  
BlastP bit score: 598
  
Sequence coverage: 98 %
  
E-value: 0.0
  
  
 NCBI BlastP on this gene

EKV08231

ATP-dependent RNA helicase , putative
  
Accession: EKV08230
  
Location: 3342-4879
  
  
**BlastP hit with Mycgr3G84402\_Mycgr3T**
  
Percentage identity: 70 %
  
BlastP bit score: 606
  
Sequence coverage: 91 %
  
E-value: 0.0
  
  
 NCBI BlastP on this gene

EKV08230

hypothetical protein
  
Accession: EKV08229
  
Location: 1744-3140
  
  
**BlastP hit with Mycgr3G35447\_Mycgr3T**
  
Percentage identity: 60 %
  
BlastP bit score: 488
  
Sequence coverage: 97 %
  
E-value: 1e-166
  
  
 NCBI BlastP on this gene

EKV08229

Query: Architecture Search FASTA input

AKCT01000236 : Penicillium digitatum PHI26    Total score: 3.0     Cumulative Blast bit score: 1692

Hit cluster cross-links:

Mycgr3G67791 Mycgr3T
  
Location: 0-1542

Mycgr3G67791\_Mycgr3T

Mycgr3G90406 Mycgr3T
  
Location: 1642-3973

Mycgr3G90406\_Mycgr3T

Mycgr3G67785 Mycgr3T
  
Location: 4073-7865

Mycgr3G67785\_Mycgr3T

Mycgr3G67795 Mycgr3T
  
Location: 7965-15249

Mycgr3G67795\_Mycgr3T

Mycgr3G67775 Mycgr3T
  
Location: 15349-16237

Mycgr3G67775\_Mycgr3T

Mycgr3G90404 Mycgr3T
  
Location: 16337-17246

Mycgr3G90404\_Mycgr3T

Mycgr3G36951 Mycgr3T
  
Location: 17346-30891

Mycgr3G36951\_Mycgr3T

Mycgr3G103034 Mycgr3
  
Location: 30991-32644

Mycgr3G103034\_Mycgr3

Mycgr3G31119 Mycgr3T
  
Location: 32744-32906

Mycgr3G31119\_Mycgr3T

Mycgr3G28587 Mycgr3T
  
Location: 33006-33489

Mycgr3G28587\_Mycgr3T

Mycgr3G98959 Mycgr3T
  
Location: 33589-35035

Mycgr3G98959\_Mycgr3T

Mycgr3G35447 Mycgr3T
  
Location: 35135-36443

Mycgr3G35447\_Mycgr3T

Mycgr3G84402 Mycgr3T
  
Location: 36543-37884

Mycgr3G84402\_Mycgr3T

Mycgr3G98961 Mycgr3T
  
Location: 37984-38884

Mycgr3G98961\_Mycgr3T

hypothetical protein
  
Accession: EKV09850
  
Location: 20025-21509
  
 NCBI BlastP on this gene

EKV09850

Glutaminyl-tRNA synthetase
  
Accession: EKV09849
  
Location: 17469-19412
  
 NCBI BlastP on this gene

EKV09849

Proteasome regulatory particle subunit Rpt5, putative
  
Accession: EKV09848
  
Location: 15316-16893
  
 NCBI BlastP on this gene

EKV09848

Trimethyllysine dioxygenase TmlH, putative
  
Accession: EKV09847
  
Location: 14102-15044
  
 NCBI BlastP on this gene

EKV09847

Peroxisomal dehydratase
  
Accession: EKV09846
  
Location: 12407-12625
  
 NCBI BlastP on this gene

EKV09846

Peroxisomal dehydratase, putative
  
Accession: EKV09845
  
Location: 11566-12142
  
 NCBI BlastP on this gene

EKV09845

Histone H4 arginine methyltransferase RmtA
  
Accession: EKV09844
  
Location: 9593-10949
  
 NCBI BlastP on this gene

EKV09844

hypothetical protein
  
Accession: EKV09843
  
Location: 8711-8935
  
 NCBI BlastP on this gene

EKV09843

Mn2+ homeostasis protein (Per1), putative
  
Accession: EKV09842
  
Location: 7844-8563
  
 NCBI BlastP on this gene

EKV09842

N-acetylglucosamine-phosphate mutase
  
Accession: EKV09841
  
Location: 5382-7369
  
  
**BlastP hit with Mycgr3G103034\_Mycgr3**
  
Percentage identity: 54 %
  
BlastP bit score: 598
  
Sequence coverage: 98 %
  
E-value: 0.0
  
  
 NCBI BlastP on this gene

EKV09841

ATP-dependent RNA helicase , putative
  
Accession: EKV09840
  
Location: 3557-5094
  
  
**BlastP hit with Mycgr3G84402\_Mycgr3T**
  
Percentage identity: 70 %
  
BlastP bit score: 606
  
Sequence coverage: 91 %
  
E-value: 0.0
  
  
 NCBI BlastP on this gene

EKV09840

hypothetical protein
  
Accession: EKV09839
  
Location: 1958-3354
  
  
**BlastP hit with Mycgr3G35447\_Mycgr3T**
  
Percentage identity: 60 %
  
BlastP bit score: 488
  
Sequence coverage: 97 %
  
E-value: 1e-166
  
  
 NCBI BlastP on this gene

EKV09839

Query: Architecture Search FASTA input

CH476655 : Ajellomyces capsulatus NAm1 scaffold\_1 genomic scaffold    Total score: 3.0     Cumulative Blast bit score: 1688

Hit cluster cross-links:

Mycgr3G67791 Mycgr3T
  
Location: 0-1542

Mycgr3G67791\_Mycgr3T

Mycgr3G90406 Mycgr3T
  
Location: 1642-3973

Mycgr3G90406\_Mycgr3T

Mycgr3G67785 Mycgr3T
  
Location: 4073-7865

Mycgr3G67785\_Mycgr3T

Mycgr3G67795 Mycgr3T
  
Location: 7965-15249

Mycgr3G67795\_Mycgr3T

Mycgr3G67775 Mycgr3T
  
Location: 15349-16237

Mycgr3G67775\_Mycgr3T

Mycgr3G90404 Mycgr3T
  
Location: 16337-17246

Mycgr3G90404\_Mycgr3T

Mycgr3G36951 Mycgr3T
  
Location: 17346-30891

Mycgr3G36951\_Mycgr3T

Mycgr3G103034 Mycgr3
  
Location: 30991-32644

Mycgr3G103034\_Mycgr3

Mycgr3G31119 Mycgr3T
  
Location: 32744-32906

Mycgr3G31119\_Mycgr3T

Mycgr3G28587 Mycgr3T
  
Location: 33006-33489

Mycgr3G28587\_Mycgr3T

Mycgr3G98959 Mycgr3T
  
Location: 33589-35035

Mycgr3G98959\_Mycgr3T

Mycgr3G35447 Mycgr3T
  
Location: 35135-36443

Mycgr3G35447\_Mycgr3T

Mycgr3G84402 Mycgr3T
  
Location: 36543-37884

Mycgr3G84402\_Mycgr3T

Mycgr3G98961 Mycgr3T
  
Location: 37984-38884

Mycgr3G98961\_Mycgr3T

serine/threonine-protein kinase sck1
  
Accession: EDN02207
  
Location: 221550-224564
  
 NCBI BlastP on this gene

EDN02207

predicted protein
  
Accession: EDN02206
  
Location: 215269-217786
  
 NCBI BlastP on this gene

EDN02206

26S protease regulatory subunit 6A
  
Accession: EDN02205
  
Location: 212766-214344
  
 NCBI BlastP on this gene

EDN02205

conserved hypothetical protein
  
Accession: EDN02204
  
Location: 210276-212353
  
 NCBI BlastP on this gene

EDN02204

peroxisomal dehydratase
  
Accession: EDN02203
  
Location: 207577-208930
  
 NCBI BlastP on this gene

EDN02203

HNRNP arginine N-methyltransferase
  
Accession: EDN02202
  
Location: 205462-207095
  
 NCBI BlastP on this gene

EDN02202

conserved hypothetical protein
  
Accession: EDN02201
  
Location: 203391-204961
  
 NCBI BlastP on this gene

EDN02201

hypothetical protein
  
Accession: EDN02200
  
Location: 200818-202890
  
  
**BlastP hit with Mycgr3G103034\_Mycgr3**
  
Percentage identity: 57 %
  
BlastP bit score: 635
  
Sequence coverage: 98 %
  
E-value: 0.0
  
  
 NCBI BlastP on this gene

EDN02200

ATP-dependent rRNA helicase RRP3
  
Accession: EDN02199
  
Location: 198577-200130
  
  
**BlastP hit with Mycgr3G84402\_Mycgr3T**
  
Percentage identity: 74 %
  
BlastP bit score: 660
  
Sequence coverage: 96 %
  
E-value: 0.0
  
  
 NCBI BlastP on this gene

EDN02199

conserved hypothetical protein
  
Accession: EDN02198
  
Location: 196979-198204
  
  
**BlastP hit with Mycgr3G35447\_Mycgr3T**
  
Percentage identity: 53 %
  
BlastP bit score: 394
  
Sequence coverage: 88 %
  
E-value: 1e-130
  
  
 NCBI BlastP on this gene

EDN02198

riboflavin synthase alpha chain
  
Accession: EDN02197
  
Location: 195808-196655
  
 NCBI BlastP on this gene

EDN02197

hypothetical protein
  
Accession: EDN02196
  
Location: 193744-195540
  
 NCBI BlastP on this gene

EDN02196

conserved hypothetical protein
  
Accession: EDN02195
  
Location: 191047-192833
  
 NCBI BlastP on this gene

EDN02195

predicted protein
  
Accession: EDN02194
  
Location: 188813-190324
  
 NCBI BlastP on this gene

EDN02194

conserved hypothetical protein
  
Accession: EDN02193
  
Location: 186677-187901
  
 NCBI BlastP on this gene

EDN02193

predicted protein
  
Accession: EDN02192
  
Location: 179592-185725
  
 NCBI BlastP on this gene

EDN02192

60S ribosomal protein L27-A
  
Accession: EDN02191
  
Location: 178071-178891
  
 NCBI BlastP on this gene

EDN02191

Query: Architecture Search FASTA input

GG657460 : Ajellomyces dermatitidis SLH14081 genomic scaffold supercont1.13    Total score: 3.0     Cumulative Blast bit score: 1686

Hit cluster cross-links:

Mycgr3G67791 Mycgr3T
  
Location: 0-1542

Mycgr3G67791\_Mycgr3T

Mycgr3G90406 Mycgr3T
  
Location: 1642-3973

Mycgr3G90406\_Mycgr3T

Mycgr3G67785 Mycgr3T
  
Location: 4073-7865

Mycgr3G67785\_Mycgr3T

Mycgr3G67795 Mycgr3T
  
Location: 7965-15249

Mycgr3G67795\_Mycgr3T

Mycgr3G67775 Mycgr3T
  
Location: 15349-16237

Mycgr3G67775\_Mycgr3T

Mycgr3G90404 Mycgr3T
  
Location: 16337-17246

Mycgr3G90404\_Mycgr3T

Mycgr3G36951 Mycgr3T
  
Location: 17346-30891

Mycgr3G36951\_Mycgr3T

Mycgr3G103034 Mycgr3
  
Location: 30991-32644

Mycgr3G103034\_Mycgr3

Mycgr3G31119 Mycgr3T
  
Location: 32744-32906

Mycgr3G31119\_Mycgr3T

Mycgr3G28587 Mycgr3T
  
Location: 33006-33489

Mycgr3G28587\_Mycgr3T

Mycgr3G98959 Mycgr3T
  
Location: 33589-35035

Mycgr3G98959\_Mycgr3T

Mycgr3G35447 Mycgr3T
  
Location: 35135-36443

Mycgr3G35447\_Mycgr3T

Mycgr3G84402 Mycgr3T
  
Location: 36543-37884

Mycgr3G84402\_Mycgr3T

Mycgr3G98961 Mycgr3T
  
Location: 37984-38884

Mycgr3G98961\_Mycgr3T

predicted protein
  
Accession: EEQ71142
  
Location: 719994-720804
  
 NCBI BlastP on this gene

EEQ71142

conserved hypothetical protein
  
Accession: EEQ71141
  
Location: 716279-718802
  
 NCBI BlastP on this gene

EEQ71141

26S protease regulatory subunit 6A
  
Accession: EEQ71140
  
Location: 713865-715442
  
 NCBI BlastP on this gene

EEQ71140

trimethyllysine dioxygenase TmlH
  
Accession: EEQ71139
  
Location: 711194-713464
  
 NCBI BlastP on this gene

EEQ71139

peroxisomal dehydratase
  
Accession: EEQ71138
  
Location: 707653-709039
  
 NCBI BlastP on this gene

EEQ71138

HNRNP arginine N-methyltransferase
  
Accession: EEQ71137
  
Location: 705273-706894
  
 NCBI BlastP on this gene

EEQ71137

Mn2+ homeostasis protein
  
Accession: EEQ71136
  
Location: 703348-704584
  
 NCBI BlastP on this gene

EEQ71136

N-acetylglucosamine-phosphate mutase
  
Accession: EEQ71135
  
Location: 700775-702850
  
  
**BlastP hit with Mycgr3G103034\_Mycgr3**
  
Percentage identity: 56 %
  
BlastP bit score: 628
  
Sequence coverage: 98 %
  
E-value: 0.0
  
  
 NCBI BlastP on this gene

EEQ71135

ATP-dependent rRNA helicase RRP3
  
Accession: EEQ71134
  
Location: 698509-700042
  
  
**BlastP hit with Mycgr3G84402\_Mycgr3T**
  
Percentage identity: 72 %
  
BlastP bit score: 577
  
Sequence coverage: 91 %
  
E-value: 0.0
  
  
 NCBI BlastP on this gene

EEQ71134

ribosome biogenesis protein Ssf2
  
Accession: EEQ71133
  
Location: 696867-698280
  
  
**BlastP hit with Mycgr3G35447\_Mycgr3T**
  
Percentage identity: 58 %
  
BlastP bit score: 481
  
Sequence coverage: 94 %
  
E-value: 3e-164
  
  
 NCBI BlastP on this gene

EEQ71133

riboflavin synthase subunit alpha
  
Accession: EEQ71132
  
Location: 695632-696484
  
 NCBI BlastP on this gene

EEQ71132

CAIB/BAIF family enzyme
  
Accession: EEQ71131
  
Location: 693478-695351
  
 NCBI BlastP on this gene

EEQ71131

Query: Architecture Search FASTA input

DS027688 : Neosartorya fischeri NRRL 181 1099437636249 genomic scaffold    Total score: 3.0     Cumulative Blast bit score: 1680

Hit cluster cross-links:

Mycgr3G67791 Mycgr3T
  
Location: 0-1542

Mycgr3G67791\_Mycgr3T

Mycgr3G90406 Mycgr3T
  
Location: 1642-3973

Mycgr3G90406\_Mycgr3T

Mycgr3G67785 Mycgr3T
  
Location: 4073-7865

Mycgr3G67785\_Mycgr3T

Mycgr3G67795 Mycgr3T
  
Location: 7965-15249

Mycgr3G67795\_Mycgr3T

Mycgr3G67775 Mycgr3T
  
Location: 15349-16237

Mycgr3G67775\_Mycgr3T

Mycgr3G90404 Mycgr3T
  
Location: 16337-17246

Mycgr3G90404\_Mycgr3T

Mycgr3G36951 Mycgr3T
  
Location: 17346-30891

Mycgr3G36951\_Mycgr3T

Mycgr3G103034 Mycgr3
  
Location: 30991-32644

Mycgr3G103034\_Mycgr3

Mycgr3G31119 Mycgr3T
  
Location: 32744-32906

Mycgr3G31119\_Mycgr3T

Mycgr3G28587 Mycgr3T
  
Location: 33006-33489

Mycgr3G28587\_Mycgr3T

Mycgr3G98959 Mycgr3T
  
Location: 33589-35035

Mycgr3G98959\_Mycgr3T

Mycgr3G35447 Mycgr3T
  
Location: 35135-36443

Mycgr3G35447\_Mycgr3T

Mycgr3G84402 Mycgr3T
  
Location: 36543-37884

Mycgr3G84402\_Mycgr3T

Mycgr3G98961 Mycgr3T
  
Location: 37984-38884

Mycgr3G98961\_Mycgr3T

L-serine dehydratase, putative
  
Accession: EAW23157
  
Location: 3228231-3229422
  
 NCBI BlastP on this gene

EAW23157

hypothetical protein
  
Accession: EAW23156
  
Location: 3226403-3226682
  
 NCBI BlastP on this gene

EAW23156

hypothetical protein
  
Accession: EAW23155
  
Location: 3224932-3225746
  
 NCBI BlastP on this gene

EAW23155

conserved predicted protein
  
Accession: EAW23154
  
Location: 3219527-3221925
  
 NCBI BlastP on this gene

EAW23154

proteasome regulatory particle subunit Rpt5, putative
  
Accession: EAW23153
  
Location: 3217077-3218693
  
 NCBI BlastP on this gene

EAW23153

trimethyllysine dioxygenase, putative
  
Accession: EAW23152
  
Location: 3214987-3216681
  
 NCBI BlastP on this gene

EAW23152

protein arginine n-methyltransferase 1
  
Accession: EAW23151
  
Location: 3212057-3213417
  
 NCBI BlastP on this gene

EAW23151

Mn2+ homeostasis protein (Per1), putative
  
Accession: EAW23150
  
Location: 3210205-3211384
  
 NCBI BlastP on this gene

EAW23150

N-acetylglucosamine-phosphate mutase
  
Accession: EAW23149
  
Location: 3207682-3209672
  
  
**BlastP hit with Mycgr3G103034\_Mycgr3**
  
Percentage identity: 55 %
  
BlastP bit score: 603
  
Sequence coverage: 98 %
  
E-value: 0.0
  
  
 NCBI BlastP on this gene

EAW23149

ATP-dependent RNA helicase , putative
  
Accession: EAW23148
  
Location: 3205754-3207267
  
  
**BlastP hit with Mycgr3G84402\_Mycgr3T**
  
Percentage identity: 73 %
  
BlastP bit score: 647
  
Sequence coverage: 92 %
  
E-value: 0.0
  
  
 NCBI BlastP on this gene

EAW23148

ribosome biogenesis protein Ssf2, putative
  
Accession: EAW23147
  
Location: 3204095-3205497
  
  
**BlastP hit with Mycgr3G35447\_Mycgr3T**
  
Percentage identity: 58 %
  
BlastP bit score: 431
  
Sequence coverage: 86 %
  
E-value: 2e-144
  
  
 NCBI BlastP on this gene

EAW23147

riboflavin synthase, alpha subunit
  
Accession: EAW23146
  
Location: 3202579-3203506
  
 NCBI BlastP on this gene

EAW23146

CAIB/BAIF family enzyme
  
Accession: EAW23145
  
Location: 3200633-3202294
  
 NCBI BlastP on this gene

EAW23145

PHD finger domain protein, putative
  
Accession: EAW23144
  
Location: 3198397-3200184
  
 NCBI BlastP on this gene

EAW23144

zinc metallopeptidase, putative
  
Accession: EAW23143
  
Location: 3196718-3198145
  
 NCBI BlastP on this gene

EAW23143

short-chain dehydrogenase/reductase family protein, putative
  
Accession: EAW23142
  
Location: 3194940-3196168
  
 NCBI BlastP on this gene

EAW23142

small nucleolar ribonucleoprotein complex subunit (SOF1), putative
  
Accession: EAW23141
  
Location: 3193327-3194758
  
 NCBI BlastP on this gene

EAW23141

proteasome regulatory particle subunit (RpnF), putative
  
Accession: EAW23140
  
Location: 3191598-3192937
  
 NCBI BlastP on this gene

EAW23140

SWI-SNF complex subunit (BAF60b), putative
  
Accession: EAW23139
  
Location: 3189513-3191179
  
 NCBI BlastP on this gene

EAW23139

conserved hypothetical protein
  
Accession: EAW23138
  
Location: 3187816-3189074
  
 NCBI BlastP on this gene

EAW23138

Query: Architecture Search FASTA input

AP007154 : Aspergillus oryzae RIB40 DNA, SC001.    Total score: 3.0     Cumulative Blast bit score: 1680

Hit cluster cross-links:

Mycgr3G67791 Mycgr3T
  
Location: 0-1542

Mycgr3G67791\_Mycgr3T

Mycgr3G90406 Mycgr3T
  
Location: 1642-3973

Mycgr3G90406\_Mycgr3T

Mycgr3G67785 Mycgr3T
  
Location: 4073-7865

Mycgr3G67785\_Mycgr3T

Mycgr3G67795 Mycgr3T
  
Location: 7965-15249

Mycgr3G67795\_Mycgr3T

Mycgr3G67775 Mycgr3T
  
Location: 15349-16237

Mycgr3G67775\_Mycgr3T

Mycgr3G90404 Mycgr3T
  
Location: 16337-17246

Mycgr3G90404\_Mycgr3T

Mycgr3G36951 Mycgr3T
  
Location: 17346-30891

Mycgr3G36951\_Mycgr3T

Mycgr3G103034 Mycgr3
  
Location: 30991-32644

Mycgr3G103034\_Mycgr3

Mycgr3G31119 Mycgr3T
  
Location: 32744-32906

Mycgr3G31119\_Mycgr3T

Mycgr3G28587 Mycgr3T
  
Location: 33006-33489

Mycgr3G28587\_Mycgr3T

Mycgr3G98959 Mycgr3T
  
Location: 33589-35035

Mycgr3G98959\_Mycgr3T

Mycgr3G35447 Mycgr3T
  
Location: 35135-36443

Mycgr3G35447\_Mycgr3T

Mycgr3G84402 Mycgr3T
  
Location: 36543-37884

Mycgr3G84402\_Mycgr3T

Mycgr3G98961 Mycgr3T
  
Location: 37984-38884

Mycgr3G98961\_Mycgr3T

not annotated
  
Accession: BAE56956
  
Location: 1096280-1098142
  
 NCBI BlastP on this gene

AO090001000437

not annotated
  
Accession: BAE56955
  
Location: 1094490-1095684
  
 NCBI BlastP on this gene

AO090001000436

not annotated
  
Accession: BAE56954
  
Location: 1093385-1093843
  
 NCBI BlastP on this gene

AO090001000435

not annotated
  
Accession: BAE56953
  
Location: 1089758-1091367
  
 NCBI BlastP on this gene

AO090001000434

not annotated
  
Accession: BAE56952
  
Location: 1088290-1089343
  
 NCBI BlastP on this gene

AO090001000433

not annotated
  
Accession: BAE56951
  
Location: 1083567-1084736
  
 NCBI BlastP on this gene

AO090001000431

not annotated
  
Accession: BAE56950
  
Location: 1079558-1082802
  
 NCBI BlastP on this gene

AO090001000430

not annotated
  
Accession: BAE56949
  
Location: 1076931-1078941
  
  
**BlastP hit with Mycgr3G103034\_Mycgr3**
  
Percentage identity: 54 %
  
BlastP bit score: 596
  
Sequence coverage: 98 %
  
E-value: 0.0
  
  
 NCBI BlastP on this gene

AO090001000429

not annotated
  
Accession: BAE56948
  
Location: 1074890-1076482
  
  
**BlastP hit with Mycgr3G84402\_Mycgr3T**
  
Percentage identity: 68 %
  
BlastP bit score: 637
  
Sequence coverage: 100 %
  
E-value: 0.0
  
  
 NCBI BlastP on this gene

AO090001000428

not annotated
  
Accession: BAE56947
  
Location: 1073220-1074636
  
  
**BlastP hit with Mycgr3G35447\_Mycgr3T**
  
Percentage identity: 60 %
  
BlastP bit score: 447
  
Sequence coverage: 87 %
  
E-value: 2e-150
  
  
 NCBI BlastP on this gene

AO090001000427

not annotated
  
Accession: BAE56946
  
Location: 1071908-1072790
  
 NCBI BlastP on this gene

AO090001000426

not annotated
  
Accession: BAE56945
  
Location: 1069718-1071375
  
 NCBI BlastP on this gene

AO090001000425

not annotated
  
Accession: BAE56944
  
Location: 1067533-1069320
  
 NCBI BlastP on this gene

AO090001000424

not annotated
  
Accession: BAE56943
  
Location: 1065533-1067310
  
 NCBI BlastP on this gene

AO090001000423

not annotated
  
Accession: BAE56942
  
Location: 1063181-1065463
  
 NCBI BlastP on this gene

AO090001000422

not annotated
  
Accession: BAE56941
  
Location: 1061749-1062510
  
 NCBI BlastP on this gene

AO090001000421

not annotated
  
Accession: BAE56940
  
Location: 1060347-1061522
  
 NCBI BlastP on this gene

AO090001000420

not annotated
  
Accession: BAE56939
  
Location: 1058199-1059486
  
 NCBI BlastP on this gene

AO090001000419

not annotated
  
Accession: BAE56938
  
Location: 1056618-1058071
  
 NCBI BlastP on this gene

AO090001000418

not annotated
  
Accession: BAE56937
  
Location: 1054954-1056286
  
 NCBI BlastP on this gene

AO090001000417

Query: Architecture Search FASTA input

DS995701 : Microsporum canis CBS 113480 supercont1.1 genomic scaffold    Total score: 3.0     Cumulative Blast bit score: 1679

Hit cluster cross-links:

Mycgr3G67791 Mycgr3T
  
Location: 0-1542

Mycgr3G67791\_Mycgr3T

Mycgr3G90406 Mycgr3T
  
Location: 1642-3973

Mycgr3G90406\_Mycgr3T

Mycgr3G67785 Mycgr3T
  
Location: 4073-7865

Mycgr3G67785\_Mycgr3T

Mycgr3G67795 Mycgr3T
  
Location: 7965-15249

Mycgr3G67795\_Mycgr3T

Mycgr3G67775 Mycgr3T
  
Location: 15349-16237

Mycgr3G67775\_Mycgr3T

Mycgr3G90404 Mycgr3T
  
Location: 16337-17246

Mycgr3G90404\_Mycgr3T

Mycgr3G36951 Mycgr3T
  
Location: 17346-30891

Mycgr3G36951\_Mycgr3T

Mycgr3G103034 Mycgr3
  
Location: 30991-32644

Mycgr3G103034\_Mycgr3

Mycgr3G31119 Mycgr3T
  
Location: 32744-32906

Mycgr3G31119\_Mycgr3T

Mycgr3G28587 Mycgr3T
  
Location: 33006-33489

Mycgr3G28587\_Mycgr3T

Mycgr3G98959 Mycgr3T
  
Location: 33589-35035

Mycgr3G98959\_Mycgr3T

Mycgr3G35447 Mycgr3T
  
Location: 35135-36443

Mycgr3G35447\_Mycgr3T

Mycgr3G84402 Mycgr3T
  
Location: 36543-37884

Mycgr3G84402\_Mycgr3T

Mycgr3G98961 Mycgr3T
  
Location: 37984-38884

Mycgr3G98961\_Mycgr3T

serine/threonine-protein kinase sck1
  
Accession: EEQ27824
  
Location: 1928641-1931675
  
 NCBI BlastP on this gene

EEQ27824

conserved hypothetical protein
  
Accession: EEQ27823
  
Location: 1924111-1926627
  
 NCBI BlastP on this gene

EEQ27823

26S protease regulatory subunit 6A
  
Accession: EEQ27822
  
Location: 1922028-1923581
  
 NCBI BlastP on this gene

EEQ27822

trimethyllysine dioxygenase
  
Accession: EEQ27821
  
Location: 1919673-1921642
  
 NCBI BlastP on this gene

EEQ27821

peroxisomal dehydratase
  
Accession: EEQ27820
  
Location: 1918064-1919238
  
 NCBI BlastP on this gene

EEQ27820

histone H4 arginine methyltransferase RmtA
  
Accession: EEQ27819
  
Location: 1916093-1917442
  
 NCBI BlastP on this gene

EEQ27819

conserved hypothetical protein
  
Accession: EEQ27818
  
Location: 1911058-1912271
  
 NCBI BlastP on this gene

EEQ27818

N-acetylglucosamine-phosphate mutase
  
Accession: EEQ27817
  
Location: 1908684-1910658
  
  
**BlastP hit with Mycgr3G103034\_Mycgr3**
  
Percentage identity: 53 %
  
BlastP bit score: 583
  
Sequence coverage: 102 %
  
E-value: 0.0
  
  
 NCBI BlastP on this gene

EEQ27817

ATP-dependent rRNA helicase RRP3
  
Accession: EEQ27816
  
Location: 1906600-1908105
  
  
**BlastP hit with Mycgr3G84402\_Mycgr3T**
  
Percentage identity: 75 %
  
BlastP bit score: 656
  
Sequence coverage: 91 %
  
E-value: 0.0
  
  
 NCBI BlastP on this gene

EEQ27816

ribosome biogenesis protein SSF1
  
Accession: EEQ27815
  
Location: 1904945-1906345
  
  
**BlastP hit with Mycgr3G35447\_Mycgr3T**
  
Percentage identity: 56 %
  
BlastP bit score: 440
  
Sequence coverage: 92 %
  
E-value: 5e-148
  
  
 NCBI BlastP on this gene

EEQ27815

riboflavin synthase alpha chain
  
Accession: EEQ27814
  
Location: 1903839-1904668
  
 NCBI BlastP on this gene

EEQ27814

formyl-coenzyme A transferase
  
Accession: EEQ27813
  
Location: 1901907-1903570
  
 NCBI BlastP on this gene

EEQ27813

conserved hypothetical protein
  
Accession: EEQ27812
  
Location: 1899760-1901379
  
 NCBI BlastP on this gene

EEQ27812

stomatin family protein
  
Accession: EEQ27811
  
Location: 1898023-1899304
  
 NCBI BlastP on this gene

EEQ27811

CAP20
  
Accession: EEQ27810
  
Location: 1895795-1896391
  
 NCBI BlastP on this gene

EEQ27810

60S ribosomal protein L27-A
  
Accession: EEQ27809
  
Location: 1893228-1894120
  
 NCBI BlastP on this gene

EEQ27809

conserved hypothetical protein
  
Accession: EEQ27808
  
Location: 1885400-1892590
  
 NCBI BlastP on this gene

EEQ27808

Query: Architecture Search FASTA input

AM920428 : Penicillium chrysogenum Wisconsin 54-1255 complete genome, contig Pc00c13.    Total score: 3.0     Cumulative Blast bit score: 1675

Hit cluster cross-links:

Mycgr3G67791 Mycgr3T
  
Location: 0-1542

Mycgr3G67791\_Mycgr3T

Mycgr3G90406 Mycgr3T
  
Location: 1642-3973

Mycgr3G90406\_Mycgr3T

Mycgr3G67785 Mycgr3T
  
Location: 4073-7865

Mycgr3G67785\_Mycgr3T

Mycgr3G67795 Mycgr3T
  
Location: 7965-15249

Mycgr3G67795\_Mycgr3T

Mycgr3G67775 Mycgr3T
  
Location: 15349-16237

Mycgr3G67775\_Mycgr3T

Mycgr3G90404 Mycgr3T
  
Location: 16337-17246

Mycgr3G90404\_Mycgr3T

Mycgr3G36951 Mycgr3T
  
Location: 17346-30891

Mycgr3G36951\_Mycgr3T

Mycgr3G103034 Mycgr3
  
Location: 30991-32644

Mycgr3G103034\_Mycgr3

Mycgr3G31119 Mycgr3T
  
Location: 32744-32906

Mycgr3G31119\_Mycgr3T

Mycgr3G28587 Mycgr3T
  
Location: 33006-33489

Mycgr3G28587\_Mycgr3T

Mycgr3G98959 Mycgr3T
  
Location: 33589-35035

Mycgr3G98959\_Mycgr3T

Mycgr3G35447 Mycgr3T
  
Location: 35135-36443

Mycgr3G35447\_Mycgr3T

Mycgr3G84402 Mycgr3T
  
Location: 36543-37884

Mycgr3G84402\_Mycgr3T

Mycgr3G98961 Mycgr3T
  
Location: 37984-38884

Mycgr3G98961\_Mycgr3T

hypothetical protein
  
Accession: CAP91334
  
Location: 614937-615948
  
 NCBI BlastP on this gene

Pc13g02650

not annotated
  
Accession: CAP91335
  
Location: 616696-617133
  
 NCBI BlastP on this gene

Pc13g02660

not annotated
  
Accession: CAP91336
  
Location: 618980-621352
  
 NCBI BlastP on this gene

Pc13g02670

not annotated
  
Accession: CAP91337
  
Location: 622004-623950
  
 NCBI BlastP on this gene

Pc13g02680

not annotated
  
Accession: CAP91338
  
Location: 624530-626107
  
 NCBI BlastP on this gene

Pc13g02690

not annotated
  
Accession: CAP91339
  
Location: 626394-628120
  
 NCBI BlastP on this gene

Pc13g02700

not annotated
  
Accession: CAP91340
  
Location: 628667-629828
  
 NCBI BlastP on this gene

Pc13g02710

not annotated
  
Accession: CAP91341
  
Location: 630476-631831
  
 NCBI BlastP on this gene

Pc13g02720

unnamed
  
Accession: CAP91342
  
Location: 632518-633683
  
 NCBI BlastP on this gene

Pc13g02730

not annotated
  
Accession: CAP91343
  
Location: 634098-636079
  
  
**BlastP hit with Mycgr3G103034\_Mycgr3**
  
Percentage identity: 54 %
  
BlastP bit score: 597
  
Sequence coverage: 98 %
  
E-value: 0.0
  
  
 NCBI BlastP on this gene

Pc13g02740

not annotated
  
Accession: CAP91344
  
Location: 636362-637899
  
  
**BlastP hit with Mycgr3G84402\_Mycgr3T**
  
Percentage identity: 71 %
  
BlastP bit score: 599
  
Sequence coverage: 88 %
  
E-value: 0.0
  
  
 NCBI BlastP on this gene

Pc13g02750

not annotated
  
Accession: CAP91345
  
Location: 638108-639501
  
  
**BlastP hit with Mycgr3G35447\_Mycgr3T**
  
Percentage identity: 60 %
  
BlastP bit score: 479
  
Sequence coverage: 95 %
  
E-value: 3e-163
  
  
 NCBI BlastP on this gene

Pc13g02760

not annotated
  
Accession: CAP91346
  
Location: 640039-640846
  
 NCBI BlastP on this gene

Pc13g02770

not annotated
  
Accession: CAP91347
  
Location: 641042-642690
  
 NCBI BlastP on this gene

Pc13g02780

not annotated
  
Accession: CAP91348
  
Location: 643313-644860
  
 NCBI BlastP on this gene

Pc13g02790

hypothetical protein
  
Accession: CAP91349
  
Location: 645084-646202
  
 NCBI BlastP on this gene

Pc13g02800

not annotated
  
Accession: CAP91350
  
Location: 646315-648881
  
 NCBI BlastP on this gene

Pc13g02810

not annotated
  
Accession: CAP91351
  
Location: 650582-651856
  
 NCBI BlastP on this gene

Pc13g02820

not annotated
  
Accession: CAP91352
  
Location: 652057-653457
  
 NCBI BlastP on this gene

Pc13g02830

not annotated
  
Accession: CAP91353
  
Location: 653681-655003
  
 NCBI BlastP on this gene

Pc13g02840

not annotated
  
Accession: CAP91354
  
Location: 655342-656939
  
 NCBI BlastP on this gene

Pc13g02850

not annotated
  
Accession: CAP91355
  
Location: 657372-658690
  
 NCBI BlastP on this gene

Pc13g02860

Query: Architecture Search FASTA input

EQ963481 : Aspergillus flavus NRRL3357 scf\_1106286419476 genomic scaffold    Total score: 3.0     Cumulative Blast bit score: 1670

Hit cluster cross-links:

Mycgr3G67791 Mycgr3T
  
Location: 0-1542

Mycgr3G67791\_Mycgr3T

Mycgr3G90406 Mycgr3T
  
Location: 1642-3973

Mycgr3G90406\_Mycgr3T

Mycgr3G67785 Mycgr3T
  
Location: 4073-7865

Mycgr3G67785\_Mycgr3T

Mycgr3G67795 Mycgr3T
  
Location: 7965-15249

Mycgr3G67795\_Mycgr3T

Mycgr3G67775 Mycgr3T
  
Location: 15349-16237

Mycgr3G67775\_Mycgr3T

Mycgr3G90404 Mycgr3T
  
Location: 16337-17246

Mycgr3G90404\_Mycgr3T

Mycgr3G36951 Mycgr3T
  
Location: 17346-30891

Mycgr3G36951\_Mycgr3T

Mycgr3G103034 Mycgr3
  
Location: 30991-32644

Mycgr3G103034\_Mycgr3

Mycgr3G31119 Mycgr3T
  
Location: 32744-32906

Mycgr3G31119\_Mycgr3T

Mycgr3G28587 Mycgr3T
  
Location: 33006-33489

Mycgr3G28587\_Mycgr3T

Mycgr3G98959 Mycgr3T
  
Location: 33589-35035

Mycgr3G98959\_Mycgr3T

Mycgr3G35447 Mycgr3T
  
Location: 35135-36443

Mycgr3G35447\_Mycgr3T

Mycgr3G84402 Mycgr3T
  
Location: 36543-37884

Mycgr3G84402\_Mycgr3T

Mycgr3G98961 Mycgr3T
  
Location: 37984-38884

Mycgr3G98961\_Mycgr3T

conserved predicted protein
  
Accession: EED48519
  
Location: 1117746-1119608
  
 NCBI BlastP on this gene

EED48519

conserved hypothetical protein
  
Accession: EED48518
  
Location: 1115956-1116300
  
 NCBI BlastP on this gene

EED48518

conserved hypothetical protein
  
Accession: EED48517
  
Location: 1114850-1115308
  
 NCBI BlastP on this gene

EED48517

proteasome regulatory particle subunit Rpt5, putative
  
Accession: EED48516
  
Location: 1111114-1112723
  
 NCBI BlastP on this gene

EED48516

trimethyllysine dioxygenase TmlH, putative
  
Accession: EED48515
  
Location: 1108759-1110682
  
 NCBI BlastP on this gene

EED48515

histone H4 arginine methyltransferase RmtA
  
Accession: EED48514
  
Location: 1102867-1104283
  
 NCBI BlastP on this gene

EED48514

Mn2+ homeostasis protein (Per1), putative
  
Accession: EED48513
  
Location: 1100874-1102061
  
 NCBI BlastP on this gene

EED48513

N-acetylglucosamine-phosphate mutase
  
Accession: EED48512
  
Location: 1098410-1100420
  
  
**BlastP hit with Mycgr3G103034\_Mycgr3**
  
Percentage identity: 56 %
  
BlastP bit score: 587
  
Sequence coverage: 91 %
  
E-value: 0.0
  
  
 NCBI BlastP on this gene

EED48512

ATP-dependent RNA helicase , putative
  
Accession: EED48511
  
Location: 1096369-1097961
  
  
**BlastP hit with Mycgr3G84402\_Mycgr3T**
  
Percentage identity: 68 %
  
BlastP bit score: 636
  
Sequence coverage: 100 %
  
E-value: 0.0
  
  
 NCBI BlastP on this gene

EED48511

ribosome biogenesis protein Ssf2, putative
  
Accession: EED48510
  
Location: 1094699-1096115
  
  
**BlastP hit with Mycgr3G35447\_Mycgr3T**
  
Percentage identity: 60 %
  
BlastP bit score: 447
  
Sequence coverage: 87 %
  
E-value: 2e-150
  
  
 NCBI BlastP on this gene

EED48510

riboflavin synthase, alpha subunit
  
Accession: EED48509
  
Location: 1093386-1094269
  
 NCBI BlastP on this gene

EED48509

CAIB/BAIF family enzyme
  
Accession: EED48508
  
Location: 1091196-1092853
  
 NCBI BlastP on this gene

EED48508

PHD finger domain protein, putative
  
Accession: EED48507
  
Location: 1089011-1090771
  
 NCBI BlastP on this gene

EED48507

zinc metallopeptidase, putative
  
Accession: EED48506
  
Location: 1087209-1088678
  
 NCBI BlastP on this gene

EED48506

conserved hypothetical protein
  
Accession: EED48505
  
Location: 1085850-1086929
  
 NCBI BlastP on this gene

EED48505

conserved hypothetical protein
  
Accession: EED48504
  
Location: 1083223-1083984
  
 NCBI BlastP on this gene

EED48504

cop9 signalosome complex subunit, putative
  
Accession: EED48503
  
Location: 1081429-1082996
  
 NCBI BlastP on this gene

EED48503

SWI-SNF complex subunit (BAF60b), putative
  
Accession: EED48502
  
Location: 1079681-1081079
  
 NCBI BlastP on this gene

EED48502

DUF887 domain protein
  
Accession: EED48501
  
Location: 1078330-1079049
  
 NCBI BlastP on this gene

EED48501

Query: Architecture Search FASTA input

AACD01000068 : Aspergillus nidulans FGSC A4    Total score: 3.0     Cumulative Blast bit score: 1668

Hit cluster cross-links:

Mycgr3G67791 Mycgr3T
  
Location: 0-1542

Mycgr3G67791\_Mycgr3T

Mycgr3G90406 Mycgr3T
  
Location: 1642-3973

Mycgr3G90406\_Mycgr3T

Mycgr3G67785 Mycgr3T
  
Location: 4073-7865

Mycgr3G67785\_Mycgr3T

Mycgr3G67795 Mycgr3T
  
Location: 7965-15249

Mycgr3G67795\_Mycgr3T

Mycgr3G67775 Mycgr3T
  
Location: 15349-16237

Mycgr3G67775\_Mycgr3T

Mycgr3G90404 Mycgr3T
  
Location: 16337-17246

Mycgr3G90404\_Mycgr3T

Mycgr3G36951 Mycgr3T
  
Location: 17346-30891

Mycgr3G36951\_Mycgr3T

Mycgr3G103034 Mycgr3
  
Location: 30991-32644

Mycgr3G103034\_Mycgr3

Mycgr3G31119 Mycgr3T
  
Location: 32744-32906

Mycgr3G31119\_Mycgr3T

Mycgr3G28587 Mycgr3T
  
Location: 33006-33489

Mycgr3G28587\_Mycgr3T

Mycgr3G98959 Mycgr3T
  
Location: 33589-35035

Mycgr3G98959\_Mycgr3T

Mycgr3G35447 Mycgr3T
  
Location: 35135-36443

Mycgr3G35447\_Mycgr3T

Mycgr3G84402 Mycgr3T
  
Location: 36543-37884

Mycgr3G84402\_Mycgr3T

Mycgr3G98961 Mycgr3T
  
Location: 37984-38884

Mycgr3G98961\_Mycgr3T

hypothetical protein
  
Accession: EAA59337
  
Location: 219164-222390
  
 NCBI BlastP on this gene

EAA59337

hypothetical protein
  
Accession: EAA59336
  
Location: 213922-216341
  
 NCBI BlastP on this gene

EAA59336

conserved hypothetical protein
  
Accession: EAA59335
  
Location: 210862-212441
  
 NCBI BlastP on this gene

EAA59335

hypothetical protein
  
Accession: EAA59334
  
Location: 204272-205445
  
 NCBI BlastP on this gene

EAA59334

hypothetical protein
  
Accession: EAA59333
  
Location: 201764-203760
  
  
**BlastP hit with Mycgr3G103034\_Mycgr3**
  
Percentage identity: 54 %
  
BlastP bit score: 595
  
Sequence coverage: 98 %
  
E-value: 0.0
  
  
 NCBI BlastP on this gene

EAA59333

hypothetical protein
  
Accession: EAA59332
  
Location: 199978-201422
  
  
**BlastP hit with Mycgr3G84402\_Mycgr3T**
  
Percentage identity: 66 %
  
BlastP bit score: 612
  
Sequence coverage: 99 %
  
E-value: 0.0
  
  
 NCBI BlastP on this gene

EAA59332

hypothetical protein
  
Accession: EAA59331
  
Location: 198380-199768
  
  
**BlastP hit with Mycgr3G35447\_Mycgr3T**
  
Percentage identity: 57 %
  
BlastP bit score: 461
  
Sequence coverage: 92 %
  
E-value: 4e-156
  
  
 NCBI BlastP on this gene

EAA59331

hypothetical protein
  
Accession: EAA59330
  
Location: 197155-198012
  
 NCBI BlastP on this gene

EAA59330

hypothetical protein
  
Accession: EAA59329
  
Location: 195109-196781
  
 NCBI BlastP on this gene

EAA59329

hypothetical protein
  
Accession: EAA59328
  
Location: 192464-194673
  
 NCBI BlastP on this gene

EAA59328

hypothetical protein
  
Accession: EAA59327
  
Location: 190773-192260
  
 NCBI BlastP on this gene

EAA59327

hypothetical protein
  
Accession: EAA59326
  
Location: 189144-190308
  
 NCBI BlastP on this gene

EAA59326

hypothetical protein
  
Accession: EAA59325
  
Location: 187532-188939
  
 NCBI BlastP on this gene

EAA59325

hypothetical protein
  
Accession: EAA59324
  
Location: 183790-187124
  
 NCBI BlastP on this gene

EAA59324

hypothetical protein
  
Accession: EAA59323
  
Location: 182114-183336
  
 NCBI BlastP on this gene

EAA59323

Query: Architecture Search FASTA input

DF126471 : Aspergillus kawachii IFO 4308 DNA, contig: scaffold00025    Total score: 3.0     Cumulative Blast bit score: 1665

Hit cluster cross-links:

Mycgr3G67791 Mycgr3T
  
Location: 0-1542

Mycgr3G67791\_Mycgr3T

Mycgr3G90406 Mycgr3T
  
Location: 1642-3973

Mycgr3G90406\_Mycgr3T

Mycgr3G67785 Mycgr3T
  
Location: 4073-7865

Mycgr3G67785\_Mycgr3T

Mycgr3G67795 Mycgr3T
  
Location: 7965-15249

Mycgr3G67795\_Mycgr3T

Mycgr3G67775 Mycgr3T
  
Location: 15349-16237

Mycgr3G67775\_Mycgr3T

Mycgr3G90404 Mycgr3T
  
Location: 16337-17246

Mycgr3G90404\_Mycgr3T

Mycgr3G36951 Mycgr3T
  
Location: 17346-30891

Mycgr3G36951\_Mycgr3T

Mycgr3G103034 Mycgr3
  
Location: 30991-32644

Mycgr3G103034\_Mycgr3

Mycgr3G31119 Mycgr3T
  
Location: 32744-32906

Mycgr3G31119\_Mycgr3T

Mycgr3G28587 Mycgr3T
  
Location: 33006-33489

Mycgr3G28587\_Mycgr3T

Mycgr3G98959 Mycgr3T
  
Location: 33589-35035

Mycgr3G98959\_Mycgr3T

Mycgr3G35447 Mycgr3T
  
Location: 35135-36443

Mycgr3G35447\_Mycgr3T

Mycgr3G84402 Mycgr3T
  
Location: 36543-37884

Mycgr3G84402\_Mycgr3T

Mycgr3G98961 Mycgr3T
  
Location: 37984-38884

Mycgr3G98961\_Mycgr3T

mitogen-activated protein kinase mpkC
  
Accession: GAA89999
  
Location: 171342-173129
  
 NCBI BlastP on this gene

GAA89999

similar to An18g05240
  
Accession: GAA89998
  
Location: 167262-168084
  
 NCBI BlastP on this gene

GAA89998

26S protease regulatory subunit 6A-B
  
Accession: GAA89997
  
Location: 162634-164185
  
 NCBI BlastP on this gene

GAA89997

trimethyllysine dioxygenase TmlH
  
Accession: GAA89996
  
Location: 158628-162260
  
 NCBI BlastP on this gene

GAA89996

HNRNP arginine N-methyltransferase
  
Accession: GAA89995
  
Location: 156490-157883
  
 NCBI BlastP on this gene

GAA89995

Mn2+ homeostasis protein
  
Accession: GAA89994
  
Location: 154416-155590
  
 NCBI BlastP on this gene

GAA89994

N-acetylglucosamine-phosphate mutase
  
Accession: GAA89993
  
Location: 151797-153814
  
  
**BlastP hit with Mycgr3G103034\_Mycgr3**
  
Percentage identity: 55 %
  
BlastP bit score: 613
  
Sequence coverage: 98 %
  
E-value: 0.0
  
  
 NCBI BlastP on this gene

GAA89993

ATP-dependent RNA helicase
  
Accession: GAA89992
  
Location: 149860-151425
  
  
**BlastP hit with Mycgr3G84402\_Mycgr3T**
  
Percentage identity: 72 %
  
BlastP bit score: 615
  
Sequence coverage: 88 %
  
E-value: 0.0
  
  
 NCBI BlastP on this gene

GAA89992

ribosome biogenesis protein Ssf2
  
Accession: GAA89991
  
Location: 148184-149599
  
  
**BlastP hit with Mycgr3G35447\_Mycgr3T**
  
Percentage identity: 59 %
  
BlastP bit score: 437
  
Sequence coverage: 91 %
  
E-value: 1e-146
  
  
 NCBI BlastP on this gene

GAA89991

riboflavin synthase, alpha subunit
  
Accession: GAA89990
  
Location: 146677-147668
  
 NCBI BlastP on this gene

GAA89990

CAIB/BAIF family enzyme
  
Accession: GAA89989
  
Location: 144709-146352
  
 NCBI BlastP on this gene

GAA89989

PHD finger domain protein
  
Accession: GAA89988
  
Location: 142268-144250
  
 NCBI BlastP on this gene

GAA89988

zinc metallopeptidase
  
Accession: GAA89987
  
Location: 140347-142049
  
 NCBI BlastP on this gene

GAA89987

short-chain dehydrogenase/reductase family protein
  
Accession: GAA89986
  
Location: 138732-140024
  
 NCBI BlastP on this gene

GAA89986

small nucleolar ribonucleoprotein complex subunit
  
Accession: GAA89985
  
Location: 137119-138578
  
 NCBI BlastP on this gene

GAA89985

SWI-SNF complex subunit (BAF60b)
  
Accession: GAA89984
  
Location: 133398-136768
  
 NCBI BlastP on this gene

GAA89984

DUF887 domain protein
  
Accession: GAA89983
  
Location: 131326-132557
  
 NCBI BlastP on this gene

GAA89983

Query: Architecture Search FASTA input

ACYE01000197 : Trichophyton verrucosum HKI 0517    Total score: 3.0     Cumulative Blast bit score: 1663

Hit cluster cross-links:

Mycgr3G67791 Mycgr3T
  
Location: 0-1542

Mycgr3G67791\_Mycgr3T

Mycgr3G90406 Mycgr3T
  
Location: 1642-3973

Mycgr3G90406\_Mycgr3T

Mycgr3G67785 Mycgr3T
  
Location: 4073-7865

Mycgr3G67785\_Mycgr3T

Mycgr3G67795 Mycgr3T
  
Location: 7965-15249

Mycgr3G67795\_Mycgr3T

Mycgr3G67775 Mycgr3T
  
Location: 15349-16237

Mycgr3G67775\_Mycgr3T

Mycgr3G90404 Mycgr3T
  
Location: 16337-17246

Mycgr3G90404\_Mycgr3T

Mycgr3G36951 Mycgr3T
  
Location: 17346-30891

Mycgr3G36951\_Mycgr3T

Mycgr3G103034 Mycgr3
  
Location: 30991-32644

Mycgr3G103034\_Mycgr3

Mycgr3G31119 Mycgr3T
  
Location: 32744-32906

Mycgr3G31119\_Mycgr3T

Mycgr3G28587 Mycgr3T
  
Location: 33006-33489

Mycgr3G28587\_Mycgr3T

Mycgr3G98959 Mycgr3T
  
Location: 33589-35035

Mycgr3G98959\_Mycgr3T

Mycgr3G35447 Mycgr3T
  
Location: 35135-36443

Mycgr3G35447\_Mycgr3T

Mycgr3G84402 Mycgr3T
  
Location: 36543-37884

Mycgr3G84402\_Mycgr3T

Mycgr3G98961 Mycgr3T
  
Location: 37984-38884

Mycgr3G98961\_Mycgr3T

conserved hypothetical protein
  
Accession: EFE41400
  
Location: 6281-8813
  
 NCBI BlastP on this gene

EFE41400

hypothetical protein
  
Accession: EFE41401
  
Location: 9350-10873
  
 NCBI BlastP on this gene

EFE41401

hypothetical protein
  
Accession: EFE41402
  
Location: 11433-12053
  
 NCBI BlastP on this gene

EFE41402

hypothetical protein
  
Accession: EFE41403
  
Location: 13852-15019
  
 NCBI BlastP on this gene

EFE41403

hypothetical protein
  
Accession: EFE41404
  
Location: 15620-16984
  
 NCBI BlastP on this gene

EFE41404

hypothetical protein
  
Accession: EFE41405
  
Location: 19343-20107
  
 NCBI BlastP on this gene

EFE41405

hypothetical protein
  
Accession: EFE41406
  
Location: 20895-22643
  
  
**BlastP hit with Mycgr3G103034\_Mycgr3**
  
Percentage identity: 56 %
  
BlastP bit score: 569
  
Sequence coverage: 93 %
  
E-value: 0.0
  
  
 NCBI BlastP on this gene

EFE41406

hypothetical protein
  
Accession: EFE41407
  
Location: 23202-24707
  
  
**BlastP hit with Mycgr3G84402\_Mycgr3T**
  
Percentage identity: 71 %
  
BlastP bit score: 670
  
Sequence coverage: 100 %
  
E-value: 0.0
  
  
 NCBI BlastP on this gene

EFE41407

hypothetical protein
  
Accession: EFE41408
  
Location: 24990-26389
  
  
**BlastP hit with Mycgr3G35447\_Mycgr3T**
  
Percentage identity: 55 %
  
BlastP bit score: 424
  
Sequence coverage: 93 %
  
E-value: 2e-141
  
  
 NCBI BlastP on this gene

EFE41408

hypothetical protein
  
Accession: EFE41409
  
Location: 26694-27323
  
 NCBI BlastP on this gene

EFE41409

hypothetical protein
  
Accession: EFE41410
  
Location: 27860-29525
  
 NCBI BlastP on this gene

EFE41410

PHD finger domain protein, putative
  
Accession: EFE41411
  
Location: 30130-31728
  
 NCBI BlastP on this gene

EFE41411

hypothetical protein
  
Accession: EFE41412
  
Location: 32265-33554
  
 NCBI BlastP on this gene

EFE41412

pathogenesis associated protein Cap20, putative
  
Accession: EFE41413
  
Location: 35300-35900
  
 NCBI BlastP on this gene

EFE41413

hypothetical protein
  
Accession: EFE41414
  
Location: 37833-38474
  
 NCBI BlastP on this gene

EFE41414

conserved hypothetical protein
  
Accession: EFE41415
  
Location: 39382-46543
  
 NCBI BlastP on this gene

EFE41415

Query: Architecture Search FASTA input

CH476619 : Uncinocarpus reesii 1704 scaffold\_5 genomic scaffold    Total score: 3.0     Cumulative Blast bit score: 1661

Hit cluster cross-links:

Mycgr3G67791 Mycgr3T
  
Location: 0-1542

Mycgr3G67791\_Mycgr3T

Mycgr3G90406 Mycgr3T
  
Location: 1642-3973

Mycgr3G90406\_Mycgr3T

Mycgr3G67785 Mycgr3T
  
Location: 4073-7865

Mycgr3G67785\_Mycgr3T

Mycgr3G67795 Mycgr3T
  
Location: 7965-15249

Mycgr3G67795\_Mycgr3T

Mycgr3G67775 Mycgr3T
  
Location: 15349-16237

Mycgr3G67775\_Mycgr3T

Mycgr3G90404 Mycgr3T
  
Location: 16337-17246

Mycgr3G90404\_Mycgr3T

Mycgr3G36951 Mycgr3T
  
Location: 17346-30891

Mycgr3G36951\_Mycgr3T

Mycgr3G103034 Mycgr3
  
Location: 30991-32644

Mycgr3G103034\_Mycgr3

Mycgr3G31119 Mycgr3T
  
Location: 32744-32906

Mycgr3G31119\_Mycgr3T

Mycgr3G28587 Mycgr3T
  
Location: 33006-33489

Mycgr3G28587\_Mycgr3T

Mycgr3G98959 Mycgr3T
  
Location: 33589-35035

Mycgr3G98959\_Mycgr3T

Mycgr3G35447 Mycgr3T
  
Location: 35135-36443

Mycgr3G35447\_Mycgr3T

Mycgr3G84402 Mycgr3T
  
Location: 36543-37884

Mycgr3G84402\_Mycgr3T

Mycgr3G98961 Mycgr3T
  
Location: 37984-38884

Mycgr3G98961\_Mycgr3T

serine/threonine-protein kinase SCH9
  
Accession: EEP82467
  
Location: 922030-924823
  
 NCBI BlastP on this gene

EEP82467

predicted protein
  
Accession: EEP82466
  
Location: 917680-921238
  
 NCBI BlastP on this gene

EEP82466

26S protease regulatory subunit 6A-B
  
Accession: EEP82465
  
Location: 915736-917248
  
 NCBI BlastP on this gene

EEP82465

predicted protein
  
Accession: EEP82464
  
Location: 913360-914385
  
 NCBI BlastP on this gene

EEP82464

conserved hypothetical protein
  
Accession: EEP82463
  
Location: 910764-911749
  
 NCBI BlastP on this gene

EEP82463

HNRNP arginine N-methyltransferase
  
Accession: EEP82462
  
Location: 908453-909824
  
 NCBI BlastP on this gene

EEP82462

conserved hypothetical protein
  
Accession: EEP82461
  
Location: 906522-907713
  
 NCBI BlastP on this gene

EEP82461

phosphoacetylglucosamine mutase
  
Accession: EEP82460
  
Location: 904015-905992
  
  
**BlastP hit with Mycgr3G103034\_Mycgr3**
  
Percentage identity: 51 %
  
BlastP bit score: 543
  
Sequence coverage: 99 %
  
E-value: 0.0
  
  
 NCBI BlastP on this gene

EEP82460

ATP-dependent rRNA helicase RRP3
  
Accession: EEP82459
  
Location: 902045-903519
  
  
**BlastP hit with Mycgr3G84402\_Mycgr3T**
  
Percentage identity: 72 %
  
BlastP bit score: 662
  
Sequence coverage: 101 %
  
E-value: 0.0
  
  
 NCBI BlastP on this gene

EEP82459

conserved hypothetical protein
  
Accession: EEP82458
  
Location: 900414-901794
  
  
**BlastP hit with Mycgr3G35447\_Mycgr3T**
  
Percentage identity: 55 %
  
BlastP bit score: 456
  
Sequence coverage: 102 %
  
E-value: 4e-154
  
  
 NCBI BlastP on this gene

EEP82458

riboflavin synthase alpha chain
  
Accession: EEP82457
  
Location: 899310-899703
  
 NCBI BlastP on this gene

EEP82457

hypothetical protein
  
Accession: EEP82456
  
Location: 897370-899007
  
 NCBI BlastP on this gene

EEP82456

predicted protein
  
Accession: EEP82455
  
Location: 895304-896524
  
 NCBI BlastP on this gene

EEP82455

conserved hypothetical protein
  
Accession: EEP82454
  
Location: 893625-894836
  
 NCBI BlastP on this gene

EEP82454

predicted protein
  
Accession: EEP82453
  
Location: 890106-890707
  
 NCBI BlastP on this gene

EEP82453

predicted protein
  
Accession: EEP82452
  
Location: 888984-889502
  
 NCBI BlastP on this gene

EEP82452

60S ribosomal protein L27-A
  
Accession: EEP82451
  
Location: 885971-887011
  
 NCBI BlastP on this gene

EEP82451

Query: Architecture Search FASTA input

ABSU01000003 : Arthroderma benhamiae CBS 112371    Total score: 3.0     Cumulative Blast bit score: 1661

Hit cluster cross-links:

Mycgr3G67791 Mycgr3T
  
Location: 0-1542

Mycgr3G67791\_Mycgr3T

Mycgr3G90406 Mycgr3T
  
Location: 1642-3973

Mycgr3G90406\_Mycgr3T

Mycgr3G67785 Mycgr3T
  
Location: 4073-7865

Mycgr3G67785\_Mycgr3T

Mycgr3G67795 Mycgr3T
  
Location: 7965-15249

Mycgr3G67795\_Mycgr3T

Mycgr3G67775 Mycgr3T
  
Location: 15349-16237

Mycgr3G67775\_Mycgr3T

Mycgr3G90404 Mycgr3T
  
Location: 16337-17246

Mycgr3G90404\_Mycgr3T

Mycgr3G36951 Mycgr3T
  
Location: 17346-30891

Mycgr3G36951\_Mycgr3T

Mycgr3G103034 Mycgr3
  
Location: 30991-32644

Mycgr3G103034\_Mycgr3

Mycgr3G31119 Mycgr3T
  
Location: 32744-32906

Mycgr3G31119\_Mycgr3T

Mycgr3G28587 Mycgr3T
  
Location: 33006-33489

Mycgr3G28587\_Mycgr3T

Mycgr3G98959 Mycgr3T
  
Location: 33589-35035

Mycgr3G98959\_Mycgr3T

Mycgr3G35447 Mycgr3T
  
Location: 35135-36443

Mycgr3G35447\_Mycgr3T

Mycgr3G84402 Mycgr3T
  
Location: 36543-37884

Mycgr3G84402\_Mycgr3T

Mycgr3G98961 Mycgr3T
  
Location: 37984-38884

Mycgr3G98961\_Mycgr3T

hypothetical protein
  
Accession: EFE35393
  
Location: 183000-186189
  
 NCBI BlastP on this gene

EFE35393

conserved predicted protein
  
Accession: EFE35392
  
Location: 177905-180437
  
 NCBI BlastP on this gene

EFE35392

hypothetical protein
  
Accession: EFE35391
  
Location: 175843-177365
  
 NCBI BlastP on this gene

EFE35391

hypothetical protein
  
Accession: EFE35390
  
Location: 174671-175291
  
 NCBI BlastP on this gene

EFE35390

hypothetical protein
  
Accession: EFE35389
  
Location: 171724-172889
  
 NCBI BlastP on this gene

EFE35389

hypothetical protein
  
Accession: EFE35388
  
Location: 169768-171116
  
 NCBI BlastP on this gene

EFE35388

hypothetical protein
  
Accession: EFE35387
  
Location: 166654-167472
  
 NCBI BlastP on this gene

EFE35387

hypothetical protein
  
Accession: EFE35386
  
Location: 164145-165892
  
  
**BlastP hit with Mycgr3G103034\_Mycgr3**
  
Percentage identity: 56 %
  
BlastP bit score: 571
  
Sequence coverage: 93 %
  
E-value: 0.0
  
  
 NCBI BlastP on this gene

EFE35386

hypothetical protein
  
Accession: EFE35385
  
Location: 162094-163598
  
  
**BlastP hit with Mycgr3G84402\_Mycgr3T**
  
Percentage identity: 71 %
  
BlastP bit score: 667
  
Sequence coverage: 100 %
  
E-value: 0.0
  
  
 NCBI BlastP on this gene

EFE35385

hypothetical protein
  
Accession: EFE35384
  
Location: 160414-161815
  
  
**BlastP hit with Mycgr3G35447\_Mycgr3T**
  
Percentage identity: 55 %
  
BlastP bit score: 423
  
Sequence coverage: 93 %
  
E-value: 3e-141
  
  
 NCBI BlastP on this gene

EFE35384

hypothetical protein
  
Accession: EFE35383
  
Location: 159483-160112
  
 NCBI BlastP on this gene

EFE35383

hypothetical protein
  
Accession: EFE35382
  
Location: 157320-158748
  
 NCBI BlastP on this gene

EFE35382

PHD finger domain protein, putative
  
Accession: EFE35381
  
Location: 155110-156708
  
 NCBI BlastP on this gene

EFE35381

hypothetical protein
  
Accession: EFE35380
  
Location: 153375-154664
  
 NCBI BlastP on this gene

EFE35380

pathogenesis associated protein Cap20, putative
  
Accession: EFE35379
  
Location: 151021-151622
  
 NCBI BlastP on this gene

EFE35379

hypothetical protein
  
Accession: EFE35378
  
Location: 148334-149098
  
 NCBI BlastP on this gene

EFE35378

Query: Architecture Search FASTA input

ACJE01000020 : Aspergillus niger ATCC 1015    Total score: 3.0     Cumulative Blast bit score: 1658

Hit cluster cross-links:

Mycgr3G67791 Mycgr3T
  
Location: 0-1542

Mycgr3G67791\_Mycgr3T

Mycgr3G90406 Mycgr3T
  
Location: 1642-3973

Mycgr3G90406\_Mycgr3T

Mycgr3G67785 Mycgr3T
  
Location: 4073-7865

Mycgr3G67785\_Mycgr3T

Mycgr3G67795 Mycgr3T
  
Location: 7965-15249

Mycgr3G67795\_Mycgr3T

Mycgr3G67775 Mycgr3T
  
Location: 15349-16237

Mycgr3G67775\_Mycgr3T

Mycgr3G90404 Mycgr3T
  
Location: 16337-17246

Mycgr3G90404\_Mycgr3T

Mycgr3G36951 Mycgr3T
  
Location: 17346-30891

Mycgr3G36951\_Mycgr3T

Mycgr3G103034 Mycgr3
  
Location: 30991-32644

Mycgr3G103034\_Mycgr3

Mycgr3G31119 Mycgr3T
  
Location: 32744-32906

Mycgr3G31119\_Mycgr3T

Mycgr3G28587 Mycgr3T
  
Location: 33006-33489

Mycgr3G28587\_Mycgr3T

Mycgr3G98959 Mycgr3T
  
Location: 33589-35035

Mycgr3G98959\_Mycgr3T

Mycgr3G35447 Mycgr3T
  
Location: 35135-36443

Mycgr3G35447\_Mycgr3T

Mycgr3G84402 Mycgr3T
  
Location: 36543-37884

Mycgr3G84402\_Mycgr3T

Mycgr3G98961 Mycgr3T
  
Location: 37984-38884

Mycgr3G98961\_Mycgr3T

mitogen-activated protein kinase
  
Accession: EHA19136
  
Location: 1226482-1227997
  
 NCBI BlastP on this gene

EHA19136

hypothetical protein
  
Accession: EHA19135
  
Location: 1221871-1223277
  
 NCBI BlastP on this gene

EHA19135

hypothetical protein
  
Accession: EHA19134
  
Location: 1217619-1219163
  
 NCBI BlastP on this gene

EHA19134

hypothetical protein
  
Accession: EHA19133
  
Location: 1216158-1217243
  
 NCBI BlastP on this gene

EHA19133

hypothetical protein
  
Accession: EHA19132
  
Location: 1213618-1214885
  
 NCBI BlastP on this gene

EHA19132

hypothetical protein
  
Accession: EHA19131
  
Location: 1211455-1212798
  
 NCBI BlastP on this gene

EHA19131

hypothetical protein
  
Accession: EHA19130
  
Location: 1209351-1210527
  
 NCBI BlastP on this gene

EHA19130

hypothetical protein
  
Accession: EHA19129
  
Location: 1206757-1208771
  
  
**BlastP hit with Mycgr3G103034\_Mycgr3**
  
Percentage identity: 54 %
  
BlastP bit score: 604
  
Sequence coverage: 99 %
  
E-value: 0.0
  
  
 NCBI BlastP on this gene

EHA19129

hypothetical protein
  
Accession: EHA19128
  
Location: 1204832-1206387
  
  
**BlastP hit with Mycgr3G84402\_Mycgr3T**
  
Percentage identity: 72 %
  
BlastP bit score: 621
  
Sequence coverage: 89 %
  
E-value: 0.0
  
  
 NCBI BlastP on this gene

EHA19128

hypothetical protein
  
Accession: EHA19127
  
Location: 1203155-1204585
  
  
**BlastP hit with Mycgr3G35447\_Mycgr3T**
  
Percentage identity: 59 %
  
BlastP bit score: 433
  
Sequence coverage: 91 %
  
E-value: 4e-145
  
  
 NCBI BlastP on this gene

EHA19127

hypothetical protein
  
Accession: EHA19126
  
Location: 1201686-1202655
  
 NCBI BlastP on this gene

EHA19126

hypothetical protein
  
Accession: EHA19125
  
Location: 1199739-1201378
  
 NCBI BlastP on this gene

EHA19125

hypothetical protein
  
Accession: EHA19124
  
Location: 1197299-1199284
  
 NCBI BlastP on this gene

EHA19124

hypothetical protein
  
Accession: EHA19123
  
Location: 1195691-1197076
  
 NCBI BlastP on this gene

EHA19123

hypothetical protein
  
Accession: EHA19122
  
Location: 1193827-1194948
  
 NCBI BlastP on this gene

EHA19122

hypothetical protein
  
Accession: EHA19121
  
Location: 1188433-1191816
  
 NCBI BlastP on this gene

EHA19121

hypothetical protein
  
Accession: EHA19120
  
Location: 1186370-1187603
  
 NCBI BlastP on this gene

EHA19120

Query: Architecture Search FASTA input

AKHY01000202 : Aspergillus oryzae 3.042    Total score: 3.0     Cumulative Blast bit score: 1654

Hit cluster cross-links:

Mycgr3G67791 Mycgr3T
  
Location: 0-1542

Mycgr3G67791\_Mycgr3T

Mycgr3G90406 Mycgr3T
  
Location: 1642-3973

Mycgr3G90406\_Mycgr3T

Mycgr3G67785 Mycgr3T
  
Location: 4073-7865

Mycgr3G67785\_Mycgr3T

Mycgr3G67795 Mycgr3T
  
Location: 7965-15249

Mycgr3G67795\_Mycgr3T

Mycgr3G67775 Mycgr3T
  
Location: 15349-16237

Mycgr3G67775\_Mycgr3T

Mycgr3G90404 Mycgr3T
  
Location: 16337-17246

Mycgr3G90404\_Mycgr3T

Mycgr3G36951 Mycgr3T
  
Location: 17346-30891

Mycgr3G36951\_Mycgr3T

Mycgr3G103034 Mycgr3
  
Location: 30991-32644

Mycgr3G103034\_Mycgr3

Mycgr3G31119 Mycgr3T
  
Location: 32744-32906

Mycgr3G31119\_Mycgr3T

Mycgr3G28587 Mycgr3T
  
Location: 33006-33489

Mycgr3G28587\_Mycgr3T

Mycgr3G98959 Mycgr3T
  
Location: 33589-35035

Mycgr3G98959\_Mycgr3T

Mycgr3G35447 Mycgr3T
  
Location: 35135-36443

Mycgr3G35447\_Mycgr3T

Mycgr3G84402 Mycgr3T
  
Location: 36543-37884

Mycgr3G84402\_Mycgr3T

Mycgr3G98961 Mycgr3T
  
Location: 37984-38884

Mycgr3G98961\_Mycgr3T

hypothetical protein
  
Accession: EIT73174
  
Location: 683276-685688
  
 NCBI BlastP on this gene

EIT73174

hypothetical protein
  
Accession: EIT73229
  
Location: 686284-687478
  
 NCBI BlastP on this gene

EIT73229

hypothetical protein
  
Accession: EIT73055
  
Location: 688126-688584
  
 NCBI BlastP on this gene

EIT73055

26S proteasome regulatory complex, ATPase RPT5
  
Accession: EIT73108
  
Location: 690602-692211
  
 NCBI BlastP on this gene

EIT73108

putative gamma-butyrobetaine,2-oxoglutarate dioxygenase
  
Accession: EIT73250
  
Location: 692643-694848
  
 NCBI BlastP on this gene

EIT73250

peroxisomal multifunctional beta-oxidation protein
  
Accession: EIT73278
  
Location: 697075-698244
  
 NCBI BlastP on this gene

EIT73278

protein arginine N-methyltransferase PRMT1
  
Accession: EIT73110
  
Location: 699009-700425
  
 NCBI BlastP on this gene

EIT73110

phosphoglucomutase/phosphomannomutase
  
Accession: EIT73157
  
Location: 702872-704882
  
  
**BlastP hit with Mycgr3G103034\_Mycgr3**
  
Percentage identity: 55 %
  
BlastP bit score: 571
  
Sequence coverage: 91 %
  
E-value: 0.0
  
  
 NCBI BlastP on this gene

EIT73157

ATP-dependent RNA helicase
  
Accession: EIT73155
  
Location: 705331-706923
  
  
**BlastP hit with Mycgr3G84402\_Mycgr3T**
  
Percentage identity: 68 %
  
BlastP bit score: 636
  
Sequence coverage: 100 %
  
E-value: 0.0
  
  
 NCBI BlastP on this gene

EIT73155

RNA-binding protein required for 60S ribosomal subunit biogenesis
  
Accession: EIT73217
  
Location: 707177-708593
  
  
**BlastP hit with Mycgr3G35447\_Mycgr3T**
  
Percentage identity: 60 %
  
BlastP bit score: 447
  
Sequence coverage: 87 %
  
E-value: 2e-150
  
  
 NCBI BlastP on this gene

EIT73217

riboflavin synthase alpha chain
  
Accession: EIT73165
  
Location: 709023-709906
  
 NCBI BlastP on this gene

EIT73165

putative L-carnitine dehydratase/alpha-methylacyl-CoA racemase
  
Accession: EIT73083
  
Location: 710439-712096
  
 NCBI BlastP on this gene

EIT73083

PHD finger domain protein
  
Accession: EIT73158
  
Location: 712494-714281
  
 NCBI BlastP on this gene

EIT73158

hypothetical protein
  
Accession: EIT73205
  
Location: 714504-716274
  
 NCBI BlastP on this gene

EIT73205

hypothetical protein
  
Accession: EIT73262
  
Location: 716344-718623
  
 NCBI BlastP on this gene

EIT73262

hypothetical protein
  
Accession: EIT73089
  
Location: 719295-720056
  
 NCBI BlastP on this gene

EIT73089

hypothetical protein
  
Accession: EIT73159
  
Location: 720283-721446
  
 NCBI BlastP on this gene

EIT73159

hydroxysteroid 17-beta dehydrogenase 11
  
Accession: EIT73130
  
Location: 722333-723614
  
 NCBI BlastP on this gene

EIT73130

Sof1-like rRNA processing protein
  
Accession: EIT73199
  
Location: 723742-725195
  
 NCBI BlastP on this gene

EIT73199

26S proteasome regulatory complex, subunit RPN6/PSMD11
  
Accession: EIT73212
  
Location: 725529-726861
  
 NCBI BlastP on this gene

EIT73212

SWI/SNF transcription activation complex subunit
  
Accession: EIT73270
  
Location: 727329-728839
  
 NCBI BlastP on this gene

EIT73270

Query: Architecture Search FASTA input

EQ962654 : Talaromyces stipitatus ATCC 10500 scf\_1105507295541 genomic scaffold    Total score: 3.0     Cumulative Blast bit score: 1643

Hit cluster cross-links:

Mycgr3G67791 Mycgr3T
  
Location: 0-1542

Mycgr3G67791\_Mycgr3T

Mycgr3G90406 Mycgr3T
  
Location: 1642-3973

Mycgr3G90406\_Mycgr3T

Mycgr3G67785 Mycgr3T
  
Location: 4073-7865

Mycgr3G67785\_Mycgr3T

Mycgr3G67795 Mycgr3T
  
Location: 7965-15249

Mycgr3G67795\_Mycgr3T

Mycgr3G67775 Mycgr3T
  
Location: 15349-16237

Mycgr3G67775\_Mycgr3T

Mycgr3G90404 Mycgr3T
  
Location: 16337-17246

Mycgr3G90404\_Mycgr3T

Mycgr3G36951 Mycgr3T
  
Location: 17346-30891

Mycgr3G36951\_Mycgr3T

Mycgr3G103034 Mycgr3
  
Location: 30991-32644

Mycgr3G103034\_Mycgr3

Mycgr3G31119 Mycgr3T
  
Location: 32744-32906

Mycgr3G31119\_Mycgr3T

Mycgr3G28587 Mycgr3T
  
Location: 33006-33489

Mycgr3G28587\_Mycgr3T

Mycgr3G98959 Mycgr3T
  
Location: 33589-35035

Mycgr3G98959\_Mycgr3T

Mycgr3G35447 Mycgr3T
  
Location: 35135-36443

Mycgr3G35447\_Mycgr3T

Mycgr3G84402 Mycgr3T
  
Location: 36543-37884

Mycgr3G84402\_Mycgr3T

Mycgr3G98961 Mycgr3T
  
Location: 37984-38884

Mycgr3G98961\_Mycgr3T

cAMP-dependent protein kinase-like, putative
  
Accession: EED19512
  
Location: 1298364-1301518
  
 NCBI BlastP on this gene

EED19512

conserved hypothetical protein
  
Accession: EED19511
  
Location: 1293561-1296219
  
 NCBI BlastP on this gene

EED19511

proteasome regulatory particle subunit Rpt5, putative
  
Accession: EED19510
  
Location: 1291527-1293045
  
 NCBI BlastP on this gene

EED19510

trimethyllysine dioxygenase TmlH, putative
  
Accession: EED19509
  
Location: 1288917-1291154
  
 NCBI BlastP on this gene

EED19509

peroxisomal dehydratase, putative
  
Accession: EED19508
  
Location: 1287567-1288719
  
 NCBI BlastP on this gene

EED19508

histone H4 arginine methyltransferase RmtA
  
Accession: EED19507
  
Location: 1285559-1286963
  
 NCBI BlastP on this gene

EED19507

Mn2 homeostasis protein (Per1), putative
  
Accession: EED19506
  
Location: 1283803-1284981
  
 NCBI BlastP on this gene

EED19506

N-acetylglucosamine-phosphate mutase
  
Accession: EED19505
  
Location: 1281462-1283449
  
  
**BlastP hit with Mycgr3G103034\_Mycgr3**
  
Percentage identity: 54 %
  
BlastP bit score: 596
  
Sequence coverage: 100 %
  
E-value: 0.0
  
  
 NCBI BlastP on this gene

EED19505

ATP-dependent RNA helicase , putative
  
Accession: EED19504
  
Location: 1279684-1281178
  
  
**BlastP hit with Mycgr3G84402\_Mycgr3T**
  
Percentage identity: 68 %
  
BlastP bit score: 613
  
Sequence coverage: 95 %
  
E-value: 0.0
  
  
 NCBI BlastP on this gene

EED19504

ribosome biogenesis protein Ssf2, putative
  
Accession: EED19503
  
Location: 1278041-1279437
  
  
**BlastP hit with Mycgr3G35447\_Mycgr3T**
  
Percentage identity: 59 %
  
BlastP bit score: 434
  
Sequence coverage: 91 %
  
E-value: 2e-145
  
  
 NCBI BlastP on this gene

EED19503

riboflavin synthase, alpha subunit
  
Accession: EED19502
  
Location: 1277100-1277917
  
 NCBI BlastP on this gene

EED19502

CAIB/BAIF family enzyme
  
Accession: EED19501
  
Location: 1275281-1276916
  
 NCBI BlastP on this gene

EED19501

PHD finger domain protein, putative
  
Accession: EED19500
  
Location: 1273327-1274997
  
 NCBI BlastP on this gene

EED19500

hypothetical protein
  
Accession: EED19499
  
Location: 1272337-1273074
  
 NCBI BlastP on this gene

EED19499

short-chain dehydrogenase/reductase family protein, putative
  
Accession: EED19498
  
Location: 1269714-1270912
  
 NCBI BlastP on this gene

EED19498

small nucleolar ribonucleoprotein complex subunit (SOF1), putative
  
Accession: EED19497
  
Location: 1268142-1269602
  
 NCBI BlastP on this gene

EED19497

proteasome regulatory particle subunit (RpnF), putative
  
Accession: EED19496
  
Location: 1266489-1267809
  
 NCBI BlastP on this gene

EED19496

conserved hypothetical protein
  
Accession: EED19495
  
Location: 1264485-1265077
  
 NCBI BlastP on this gene

EED19495

SWI-SNF complex subunit (BAF60b), putative
  
Accession: EED19494
  
Location: 1262214-1263884
  
 NCBI BlastP on this gene

EED19494

DUF887 domain protein
  
Accession: EED19493
  
Location: 1260539-1261769
  
 NCBI BlastP on this gene

EED19493

Query: Architecture Search FASTA input

DS572750 : Paracoccidioides brasiliensis Pb18 supercont1.1 genomic scaffold    Total score: 3.0     Cumulative Blast bit score: 1607

Hit cluster cross-links:

Mycgr3G67791 Mycgr3T
  
Location: 0-1542

Mycgr3G67791\_Mycgr3T

Mycgr3G90406 Mycgr3T
  
Location: 1642-3973

Mycgr3G90406\_Mycgr3T

Mycgr3G67785 Mycgr3T
  
Location: 4073-7865

Mycgr3G67785\_Mycgr3T

Mycgr3G67795 Mycgr3T
  
Location: 7965-15249

Mycgr3G67795\_Mycgr3T

Mycgr3G67775 Mycgr3T
  
Location: 15349-16237

Mycgr3G67775\_Mycgr3T

Mycgr3G90404 Mycgr3T
  
Location: 16337-17246

Mycgr3G90404\_Mycgr3T

Mycgr3G36951 Mycgr3T
  
Location: 17346-30891

Mycgr3G36951\_Mycgr3T

Mycgr3G103034 Mycgr3
  
Location: 30991-32644

Mycgr3G103034\_Mycgr3

Mycgr3G31119 Mycgr3T
  
Location: 32744-32906

Mycgr3G31119\_Mycgr3T

Mycgr3G28587 Mycgr3T
  
Location: 33006-33489

Mycgr3G28587\_Mycgr3T

Mycgr3G98959 Mycgr3T
  
Location: 33589-35035

Mycgr3G98959\_Mycgr3T

Mycgr3G35447 Mycgr3T
  
Location: 35135-36443

Mycgr3G35447\_Mycgr3T

Mycgr3G84402 Mycgr3T
  
Location: 36543-37884

Mycgr3G84402\_Mycgr3T

Mycgr3G98961 Mycgr3T
  
Location: 37984-38884

Mycgr3G98961\_Mycgr3T

conserved hypothetical protein
  
Accession: EEH44309
  
Location: 2018710-2021155
  
 NCBI BlastP on this gene

EEH44309

26S protease regulatory subunit 6A
  
Accession: EEH44310
  
Location: 2022010-2023580
  
 NCBI BlastP on this gene

EEH44310

trimethyllysine dioxygenase
  
Accession: EEH44311
  
Location: 2023747-2026297
  
 NCBI BlastP on this gene

EEH44311

peroxisomal dehydratase
  
Accession: EEH44312
  
Location: 2026791-2028127
  
 NCBI BlastP on this gene

EEH44312

HNRNP arginine N-methyltransferase
  
Accession: EEH44313
  
Location: 2028981-2030408
  
 NCBI BlastP on this gene

EEH44313

Mn2+ homeostasis protein (Per1)
  
Accession: EEH44314
  
Location: 2031141-2032379
  
 NCBI BlastP on this gene

EEH44314

phosphoacetylglucosamine mutase
  
Accession: EEH44315
  
Location: 2033854-2035236
  
  
**BlastP hit with Mycgr3G103034\_Mycgr3**
  
Percentage identity: 55 %
  
BlastP bit score: 482
  
Sequence coverage: 78 %
  
E-value: 8e-163
  
  
 NCBI BlastP on this gene

EEH44315

ATP-dependent rRNA helicase RRP3
  
Accession: EEH44316
  
Location: 2035931-2037448
  
  
**BlastP hit with Mycgr3G84402\_Mycgr3T**
  
Percentage identity: 75 %
  
BlastP bit score: 652
  
Sequence coverage: 97 %
  
E-value: 0.0
  
  
 NCBI BlastP on this gene

EEH44316

ribosome biogenesis protein SSF1
  
Accession: EEH44317
  
Location: 2037745-2039184
  
  
**BlastP hit with Mycgr3G35447\_Mycgr3T**
  
Percentage identity: 58 %
  
BlastP bit score: 473
  
Sequence coverage: 94 %
  
E-value: 9e-161
  
  
 NCBI BlastP on this gene

EEH44317

riboflavin synthase alpha chain
  
Accession: EEH44318
  
Location: 2039520-2040388
  
 NCBI BlastP on this gene

EEH44318

formyl-coenzyme A transferase
  
Accession: EEH44319
  
Location: 2040735-2042530
  
 NCBI BlastP on this gene

EEH44319

conserved hypothetical protein
  
Accession: EEH44320
  
Location: 2043049-2044893
  
 NCBI BlastP on this gene

EEH44320

DUF887 domain-containing protein
  
Accession: EEH44321
  
Location: 2045686-2047001
  
 NCBI BlastP on this gene

EEH44321

conserved hypothetical protein
  
Accession: EEH44322
  
Location: 2047978-2054139
  
 NCBI BlastP on this gene

EEH44322

60S ribosomal protein L27-A
  
Accession: EEH44323
  
Location: 2054793-2055576
  
 NCBI BlastP on this gene

EEH44323

predicted protein
  
Accession: EEH44324
  
Location: 2057481-2058054
  
 NCBI BlastP on this gene

EEH44324

Query: Architecture Search FASTA input

GL534607 : Pyrenophora teres f. teres 0-1 unplaced genomic scaffold scaffold\_190867    Total score: 3.0     Cumulative Blast bit score: 1578

Hit cluster cross-links:

Mycgr3G67791 Mycgr3T
  
Location: 0-1542

Mycgr3G67791\_Mycgr3T

Mycgr3G90406 Mycgr3T
  
Location: 1642-3973

Mycgr3G90406\_Mycgr3T

Mycgr3G67785 Mycgr3T
  
Location: 4073-7865

Mycgr3G67785\_Mycgr3T

Mycgr3G67795 Mycgr3T
  
Location: 7965-15249

Mycgr3G67795\_Mycgr3T

Mycgr3G67775 Mycgr3T
  
Location: 15349-16237

Mycgr3G67775\_Mycgr3T

Mycgr3G90404 Mycgr3T
  
Location: 16337-17246

Mycgr3G90404\_Mycgr3T

Mycgr3G36951 Mycgr3T
  
Location: 17346-30891

Mycgr3G36951\_Mycgr3T

Mycgr3G103034 Mycgr3
  
Location: 30991-32644

Mycgr3G103034\_Mycgr3

Mycgr3G31119 Mycgr3T
  
Location: 32744-32906

Mycgr3G31119\_Mycgr3T

Mycgr3G28587 Mycgr3T
  
Location: 33006-33489

Mycgr3G28587\_Mycgr3T

Mycgr3G98959 Mycgr3T
  
Location: 33589-35035

Mycgr3G98959\_Mycgr3T

Mycgr3G35447 Mycgr3T
  
Location: 35135-36443

Mycgr3G35447\_Mycgr3T

Mycgr3G84402 Mycgr3T
  
Location: 36543-37884

Mycgr3G84402\_Mycgr3T

Mycgr3G98961 Mycgr3T
  
Location: 37984-38884

Mycgr3G98961\_Mycgr3T

hypothetical protein
  
Accession: EFQ91772
  
Location: 1067-2976
  
  
**BlastP hit with Mycgr3G67791\_Mycgr3T**
  
Percentage identity: 64 %
  
BlastP bit score: 530
  
Sequence coverage: 83 %
  
E-value: 1e-179
  
  
 NCBI BlastP on this gene

EFQ91772

hypothetical protein
  
Accession: EFQ91773
  
Location: 3686-4861
  
 NCBI BlastP on this gene

EFQ91773

hypothetical protein
  
Accession: EFQ91774
  
Location: 5015-6241
  
 NCBI BlastP on this gene

EFQ91774

hypothetical protein
  
Accession: EFQ91775
  
Location: 6847-8898
  
 NCBI BlastP on this gene

EFQ91775

hypothetical protein
  
Accession: EFQ91776
  
Location: 9965-11538
  
 NCBI BlastP on this gene

EFQ91776

hypothetical protein
  
Accession: EFQ91777
  
Location: 13076-15425
  
 NCBI BlastP on this gene

EFQ91777

hypothetical protein
  
Accession: EFQ91778
  
Location: 16471-17517
  
 NCBI BlastP on this gene

EFQ91778

hypothetical protein
  
Accession: EFQ91779
  
Location: 17835-18453
  
 NCBI BlastP on this gene

EFQ91779

hypothetical protein
  
Accession: EFQ91780
  
Location: 20044-21644
  
  
**BlastP hit with Mycgr3G84402\_Mycgr3T**
  
Percentage identity: 71 %
  
BlastP bit score: 625
  
Sequence coverage: 93 %
  
E-value: 0.0
  
  
 NCBI BlastP on this gene

EFQ91780

hypothetical protein
  
Accession: EFQ91781
  
Location: 21922-23318
  
  
**BlastP hit with Mycgr3G35447\_Mycgr3T**
  
Percentage identity: 58 %
  
BlastP bit score: 423
  
Sequence coverage: 88 %
  
E-value: 3e-141
  
  
 NCBI BlastP on this gene

EFQ91781

hypothetical protein
  
Accession: EFQ91782
  
Location: 23443-24515
  
 NCBI BlastP on this gene

EFQ91782

hypothetical protein
  
Accession: EFQ91783
  
Location: 24979-25923
  
 NCBI BlastP on this gene

EFQ91783

hypothetical protein
  
Accession: EFQ91784
  
Location: 26443-27504
  
 NCBI BlastP on this gene

EFQ91784

hypothetical protein
  
Accession: EFQ91785
  
Location: 28729-30222
  
 NCBI BlastP on this gene

EFQ91785

hypothetical protein
  
Accession: EFQ91786
  
Location: 30917-31906
  
 NCBI BlastP on this gene

EFQ91786

hypothetical protein
  
Accession: EFQ91787
  
Location: 33370-34738
  
 NCBI BlastP on this gene

EFQ91787

Query: Architecture Search FASTA input

KB445649 : Cochliobolus sativus ND90Pr unplaced genomic scaffold COCSAscaffold\_13    Total score: 3.0     Cumulative Blast bit score: 1569

Hit cluster cross-links:

Mycgr3G67791 Mycgr3T
  
Location: 0-1542

Mycgr3G67791\_Mycgr3T

Mycgr3G90406 Mycgr3T
  
Location: 1642-3973

Mycgr3G90406\_Mycgr3T

Mycgr3G67785 Mycgr3T
  
Location: 4073-7865

Mycgr3G67785\_Mycgr3T

Mycgr3G67795 Mycgr3T
  
Location: 7965-15249

Mycgr3G67795\_Mycgr3T

Mycgr3G67775 Mycgr3T
  
Location: 15349-16237

Mycgr3G67775\_Mycgr3T

Mycgr3G90404 Mycgr3T
  
Location: 16337-17246

Mycgr3G90404\_Mycgr3T

Mycgr3G36951 Mycgr3T
  
Location: 17346-30891

Mycgr3G36951\_Mycgr3T

Mycgr3G103034 Mycgr3
  
Location: 30991-32644

Mycgr3G103034\_Mycgr3

Mycgr3G31119 Mycgr3T
  
Location: 32744-32906

Mycgr3G31119\_Mycgr3T

Mycgr3G28587 Mycgr3T
  
Location: 33006-33489

Mycgr3G28587\_Mycgr3T

Mycgr3G98959 Mycgr3T
  
Location: 33589-35035

Mycgr3G98959\_Mycgr3T

Mycgr3G35447 Mycgr3T
  
Location: 35135-36443

Mycgr3G35447\_Mycgr3T

Mycgr3G84402 Mycgr3T
  
Location: 36543-37884

Mycgr3G84402\_Mycgr3T

Mycgr3G98961 Mycgr3T
  
Location: 37984-38884

Mycgr3G98961\_Mycgr3T

hypothetical protein
  
Accession: EMD61125
  
Location: 1004553-1006333
  
 NCBI BlastP on this gene

EMD61125

hypothetical protein
  
Accession: EMD61124
  
Location: 1003654-1004130
  
 NCBI BlastP on this gene

EMD61124

hypothetical protein
  
Accession: EMD61123
  
Location: 1002481-1003494
  
 NCBI BlastP on this gene

EMD61123

hypothetical protein
  
Accession: EMD61122
  
Location: 998502-1002182
  
 NCBI BlastP on this gene

EMD61122

hypothetical protein
  
Accession: EMD61121
  
Location: 997097-997788
  
 NCBI BlastP on this gene

EMD61121

hypothetical protein
  
Accession: EMD61120
  
Location: 994634-996412
  
 NCBI BlastP on this gene

EMD61120

hypothetical protein
  
Accession: EMD61119
  
Location: 990115-991958
  
  
**BlastP hit with Mycgr3G67791\_Mycgr3T**
  
Percentage identity: 67 %
  
BlastP bit score: 520
  
Sequence coverage: 75 %
  
E-value: 6e-176
  
  
 NCBI BlastP on this gene

EMD61119

hypothetical protein
  
Accession: EMD61118
  
Location: 988263-989448
  
 NCBI BlastP on this gene

EMD61118

hypothetical protein
  
Accession: EMD61117
  
Location: 986977-988224
  
 NCBI BlastP on this gene

EMD61117

hypothetical protein
  
Accession: EMD61116
  
Location: 984353-986380
  
 NCBI BlastP on this gene

EMD61116

hypothetical protein
  
Accession: EMD61115
  
Location: 981749-983292
  
 NCBI BlastP on this gene

EMD61115

hypothetical protein
  
Accession: EMD61114
  
Location: 980710-981458
  
 NCBI BlastP on this gene

EMD61114

hypothetical protein
  
Accession: EMD61113
  
Location: 978499-980104
  
  
**BlastP hit with Mycgr3G84402\_Mycgr3T**
  
Percentage identity: 71 %
  
BlastP bit score: 618
  
Sequence coverage: 93 %
  
E-value: 0.0
  
  
 NCBI BlastP on this gene

EMD61113

hypothetical protein
  
Accession: EMD61112
  
Location: 976718-978172
  
  
**BlastP hit with Mycgr3G35447\_Mycgr3T**
  
Percentage identity: 58 %
  
BlastP bit score: 431
  
Sequence coverage: 87 %
  
E-value: 2e-144
  
  
 NCBI BlastP on this gene

EMD61112

hypothetical protein
  
Accession: EMD61111
  
Location: 975565-976615
  
 NCBI BlastP on this gene

EMD61111

hypothetical protein
  
Accession: EMD61110
  
Location: 974152-975093
  
 NCBI BlastP on this gene

EMD61110

hypothetical protein
  
Accession: EMD61109
  
Location: 972561-973566
  
 NCBI BlastP on this gene

EMD61109

hypothetical protein
  
Accession: EMD61108
  
Location: 972017-972355
  
 NCBI BlastP on this gene

EMD61108

hypothetical protein
  
Accession: EMD61107
  
Location: 970355-971809
  
 NCBI BlastP on this gene

EMD61107

hypothetical protein
  
Accession: EMD61106
  
Location: 966702-968366
  
 NCBI BlastP on this gene

EMD61106

hypothetical protein
  
Accession: EMD61105
  
Location: 964536-966125
  
 NCBI BlastP on this gene

EMD61105

hypothetical protein
  
Accession: EMD61104
  
Location: 963862-964410
  
 NCBI BlastP on this gene

EMD61104

hypothetical protein
  
Accession: EMD61103
  
Location: 961669-962435
  
 NCBI BlastP on this gene

EMD61103

Query: Architecture Search FASTA input

DS544805 : Paracoccidioides brasiliensis Pb03 supercont1.3 genomic scaffold    Total score: 3.0     Cumulative Blast bit score: 1568

Hit cluster cross-links:

Mycgr3G67791 Mycgr3T
  
Location: 0-1542

Mycgr3G67791\_Mycgr3T

Mycgr3G90406 Mycgr3T
  
Location: 1642-3973

Mycgr3G90406\_Mycgr3T

Mycgr3G67785 Mycgr3T
  
Location: 4073-7865

Mycgr3G67785\_Mycgr3T

Mycgr3G67795 Mycgr3T
  
Location: 7965-15249

Mycgr3G67795\_Mycgr3T

Mycgr3G67775 Mycgr3T
  
Location: 15349-16237

Mycgr3G67775\_Mycgr3T

Mycgr3G90404 Mycgr3T
  
Location: 16337-17246

Mycgr3G90404\_Mycgr3T

Mycgr3G36951 Mycgr3T
  
Location: 17346-30891

Mycgr3G36951\_Mycgr3T

Mycgr3G103034 Mycgr3
  
Location: 30991-32644

Mycgr3G103034\_Mycgr3

Mycgr3G31119 Mycgr3T
  
Location: 32744-32906

Mycgr3G31119\_Mycgr3T

Mycgr3G28587 Mycgr3T
  
Location: 33006-33489

Mycgr3G28587\_Mycgr3T

Mycgr3G98959 Mycgr3T
  
Location: 33589-35035

Mycgr3G98959\_Mycgr3T

Mycgr3G35447 Mycgr3T
  
Location: 35135-36443

Mycgr3G35447\_Mycgr3T

Mycgr3G84402 Mycgr3T
  
Location: 36543-37884

Mycgr3G84402\_Mycgr3T

Mycgr3G98961 Mycgr3T
  
Location: 37984-38884

Mycgr3G98961\_Mycgr3T

conserved hypothetical protein
  
Accession: EEH19938
  
Location: 1090569-1093014
  
 NCBI BlastP on this gene

EEH19938

26S protease regulatory subunit 6A
  
Accession: EEH19939
  
Location: 1093870-1095338
  
 NCBI BlastP on this gene

EEH19939

trimethyllysine dioxygenase
  
Accession: EEH19940
  
Location: 1095938-1096993
  
 NCBI BlastP on this gene

EEH19940

conserved hypothetical protein
  
Accession: EEH19941
  
Location: 1098647-1099843
  
 NCBI BlastP on this gene

EEH19941

arginine N-methyltransferase
  
Accession: EEH19942
  
Location: 1100128-1102261
  
 NCBI BlastP on this gene

EEH19942

conserved hypothetical protein
  
Accession: EEH19943
  
Location: 1102990-1104225
  
 NCBI BlastP on this gene

EEH19943

phosphoacetylglucosamine mutase
  
Accession: EEH19944
  
Location: 1105032-1107088
  
  
**BlastP hit with Mycgr3G103034\_Mycgr3**
  
Percentage identity: 54 %
  
BlastP bit score: 580
  
Sequence coverage: 99 %
  
E-value: 0.0
  
  
 NCBI BlastP on this gene

EEH19944

ATP-dependent rRNA helicase rrp3
  
Accession: EEH19945
  
Location: 1107783-1109304
  
  
**BlastP hit with Mycgr3G84402\_Mycgr3T**
  
Percentage identity: 65 %
  
BlastP bit score: 534
  
Sequence coverage: 97 %
  
E-value: 0.0
  
  
 NCBI BlastP on this gene

EEH19945

splicing factor
  
Accession: EEH19946
  
Location: 1109684-1111021
  
  
**BlastP hit with Mycgr3G35447\_Mycgr3T**
  
Percentage identity: 58 %
  
BlastP bit score: 454
  
Sequence coverage: 87 %
  
E-value: 5e-154
  
  
 NCBI BlastP on this gene

EEH19946

riboflavin synthase alpha chain
  
Accession: EEH19947
  
Location: 1111361-1112228
  
 NCBI BlastP on this gene

EEH19947

formyl-coenzyme A transferase
  
Accession: EEH19948
  
Location: 1112507-1114288
  
 NCBI BlastP on this gene

EEH19948

conserved hypothetical protein
  
Accession: EEH19949
  
Location: 1114800-1116626
  
 NCBI BlastP on this gene

EEH19949

DUF887 domain-containing protein
  
Accession: EEH19950
  
Location: 1117429-1118744
  
 NCBI BlastP on this gene

EEH19950

conserved hypothetical protein
  
Accession: EEH19951
  
Location: 1119719-1125815
  
 NCBI BlastP on this gene

EEH19951

60S ribosomal protein L27-B
  
Accession: EEH19952
  
Location: 1126626-1127259
  
 NCBI BlastP on this gene

EEH19952

predicted protein
  
Accession: EEH19953
  
Location: 1129144-1129816
  
 NCBI BlastP on this gene

EEH19953

Query: Architecture Search FASTA input

KB733455 : Bipolaris maydis ATCC 48331 unplaced genomic scaffold COCC4scaffold\_12    Total score: 3.0     Cumulative Blast bit score: 1562

Hit cluster cross-links:

Mycgr3G67791 Mycgr3T
  
Location: 0-1542

Mycgr3G67791\_Mycgr3T

Mycgr3G90406 Mycgr3T
  
Location: 1642-3973

Mycgr3G90406\_Mycgr3T

Mycgr3G67785 Mycgr3T
  
Location: 4073-7865

Mycgr3G67785\_Mycgr3T

Mycgr3G67795 Mycgr3T
  
Location: 7965-15249

Mycgr3G67795\_Mycgr3T

Mycgr3G67775 Mycgr3T
  
Location: 15349-16237

Mycgr3G67775\_Mycgr3T

Mycgr3G90404 Mycgr3T
  
Location: 16337-17246

Mycgr3G90404\_Mycgr3T

Mycgr3G36951 Mycgr3T
  
Location: 17346-30891

Mycgr3G36951\_Mycgr3T

Mycgr3G103034 Mycgr3
  
Location: 30991-32644

Mycgr3G103034\_Mycgr3

Mycgr3G31119 Mycgr3T
  
Location: 32744-32906

Mycgr3G31119\_Mycgr3T

Mycgr3G28587 Mycgr3T
  
Location: 33006-33489

Mycgr3G28587\_Mycgr3T

Mycgr3G98959 Mycgr3T
  
Location: 33589-35035

Mycgr3G98959\_Mycgr3T

Mycgr3G35447 Mycgr3T
  
Location: 35135-36443

Mycgr3G35447\_Mycgr3T

Mycgr3G84402 Mycgr3T
  
Location: 36543-37884

Mycgr3G84402\_Mycgr3T

Mycgr3G98961 Mycgr3T
  
Location: 37984-38884

Mycgr3G98961\_Mycgr3T

hypothetical protein
  
Accession: ENI04925
  
Location: 50783-52550
  
 NCBI BlastP on this gene

ENI04925

hypothetical protein
  
Accession: ENI04926
  
Location: 52974-53504
  
 NCBI BlastP on this gene

ENI04926

hypothetical protein
  
Accession: ENI04927
  
Location: 53539-54554
  
 NCBI BlastP on this gene

ENI04927

hypothetical protein
  
Accession: ENI04928
  
Location: 54858-58536
  
 NCBI BlastP on this gene

ENI04928

hypothetical protein
  
Accession: ENI04929
  
Location: 59210-59953
  
 NCBI BlastP on this gene

ENI04929

hypothetical protein
  
Accession: ENI04930
  
Location: 60670-62449
  
 NCBI BlastP on this gene

ENI04930

hypothetical protein
  
Accession: ENI04931
  
Location: 65788-67632
  
  
**BlastP hit with Mycgr3G67791\_Mycgr3T**
  
Percentage identity: 66 %
  
BlastP bit score: 513
  
Sequence coverage: 75 %
  
E-value: 3e-173
  
  
 NCBI BlastP on this gene

ENI04931

hypothetical protein
  
Accession: ENI04932
  
Location: 68518-69414
  
 NCBI BlastP on this gene

ENI04932

hypothetical protein
  
Accession: ENI04933
  
Location: 69530-70786
  
 NCBI BlastP on this gene

ENI04933

hypothetical protein
  
Accession: ENI04934
  
Location: 71409-73430
  
 NCBI BlastP on this gene

ENI04934

hypothetical protein
  
Accession: ENI04935
  
Location: 74517-76060
  
 NCBI BlastP on this gene

ENI04935

hypothetical protein
  
Accession: ENI04936
  
Location: 76404-77135
  
 NCBI BlastP on this gene

ENI04936

hypothetical protein
  
Accession: ENI04937
  
Location: 77766-79371
  
  
**BlastP hit with Mycgr3G84402\_Mycgr3T**
  
Percentage identity: 70 %
  
BlastP bit score: 618
  
Sequence coverage: 95 %
  
E-value: 0.0
  
  
 NCBI BlastP on this gene

ENI04937

hypothetical protein
  
Accession: ENI04938
  
Location: 79699-81162
  
  
**BlastP hit with Mycgr3G35447\_Mycgr3T**
  
Percentage identity: 58 %
  
BlastP bit score: 431
  
Sequence coverage: 88 %
  
E-value: 2e-144
  
  
 NCBI BlastP on this gene

ENI04938

hypothetical protein
  
Accession: ENI04939
  
Location: 81266-82306
  
 NCBI BlastP on this gene

ENI04939

hypothetical protein
  
Accession: ENI04940
  
Location: 82778-83719
  
 NCBI BlastP on this gene

ENI04940

hypothetical protein
  
Accession: ENI04941
  
Location: 84313-85318
  
 NCBI BlastP on this gene

ENI04941

hypothetical protein
  
Accession: ENI04942
  
Location: 86065-87297
  
 NCBI BlastP on this gene

ENI04942

hypothetical protein
  
Accession: ENI04943
  
Location: 89762-91428
  
 NCBI BlastP on this gene

ENI04943

hypothetical protein
  
Accession: ENI04944
  
Location: 91977-93553
  
 NCBI BlastP on this gene

ENI04944

hypothetical protein
  
Accession: ENI04945
  
Location: 93657-94205
  
 NCBI BlastP on this gene

ENI04945

Query: Architecture Search FASTA input

KB445579 : Cochliobolus heterostrophus C5 unplaced genomic scaffold COCHEscaffold\_11    Total score: 3.0     Cumulative Blast bit score: 1562

Hit cluster cross-links:

Mycgr3G67791 Mycgr3T
  
Location: 0-1542

Mycgr3G67791\_Mycgr3T

Mycgr3G90406 Mycgr3T
  
Location: 1642-3973

Mycgr3G90406\_Mycgr3T

Mycgr3G67785 Mycgr3T
  
Location: 4073-7865

Mycgr3G67785\_Mycgr3T

Mycgr3G67795 Mycgr3T
  
Location: 7965-15249

Mycgr3G67795\_Mycgr3T

Mycgr3G67775 Mycgr3T
  
Location: 15349-16237

Mycgr3G67775\_Mycgr3T

Mycgr3G90404 Mycgr3T
  
Location: 16337-17246

Mycgr3G90404\_Mycgr3T

Mycgr3G36951 Mycgr3T
  
Location: 17346-30891

Mycgr3G36951\_Mycgr3T

Mycgr3G103034 Mycgr3
  
Location: 30991-32644

Mycgr3G103034\_Mycgr3

Mycgr3G31119 Mycgr3T
  
Location: 32744-32906

Mycgr3G31119\_Mycgr3T

Mycgr3G28587 Mycgr3T
  
Location: 33006-33489

Mycgr3G28587\_Mycgr3T

Mycgr3G98959 Mycgr3T
  
Location: 33589-35035

Mycgr3G98959\_Mycgr3T

Mycgr3G35447 Mycgr3T
  
Location: 35135-36443

Mycgr3G35447\_Mycgr3T

Mycgr3G84402 Mycgr3T
  
Location: 36543-37884

Mycgr3G84402\_Mycgr3T

Mycgr3G98961 Mycgr3T
  
Location: 37984-38884

Mycgr3G98961\_Mycgr3T

hypothetical protein
  
Accession: EMD89358
  
Location: 953222-954989
  
 NCBI BlastP on this gene

EMD89358

hypothetical protein
  
Accession: EMD89357
  
Location: 952268-952798
  
 NCBI BlastP on this gene

EMD89357

hypothetical protein
  
Accession: EMD89356
  
Location: 951218-952233
  
 NCBI BlastP on this gene

EMD89356

hypothetical protein
  
Accession: EMD89355
  
Location: 947236-950914
  
 NCBI BlastP on this gene

EMD89355

hypothetical protein
  
Accession: EMD89354
  
Location: 945819-946562
  
 NCBI BlastP on this gene

EMD89354

hypothetical protein
  
Accession: EMD89353
  
Location: 943323-945102
  
 NCBI BlastP on this gene

EMD89353

hypothetical protein
  
Accession: EMD89352
  
Location: 938097-939941
  
  
**BlastP hit with Mycgr3G67791\_Mycgr3T**
  
Percentage identity: 66 %
  
BlastP bit score: 513
  
Sequence coverage: 75 %
  
E-value: 3e-173
  
  
 NCBI BlastP on this gene

EMD89352

hypothetical protein
  
Accession: EMD89351
  
Location: 936309-937346
  
 NCBI BlastP on this gene

EMD89351

hypothetical protein
  
Accession: EMD89350
  
Location: 934943-936199
  
 NCBI BlastP on this gene

EMD89350

hypothetical protein
  
Accession: EMD89349
  
Location: 932302-934320
  
 NCBI BlastP on this gene

EMD89349

hypothetical protein
  
Accession: EMD89348
  
Location: 929669-931212
  
 NCBI BlastP on this gene

EMD89348

hypothetical protein
  
Accession: EMD89347
  
Location: 928594-929325
  
 NCBI BlastP on this gene

EMD89347

hypothetical protein
  
Accession: EMD89346
  
Location: 926358-927963
  
  
**BlastP hit with Mycgr3G84402\_Mycgr3T**
  
Percentage identity: 70 %
  
BlastP bit score: 618
  
Sequence coverage: 95 %
  
E-value: 0.0
  
  
 NCBI BlastP on this gene

EMD89346

hypothetical protein
  
Accession: EMD89345
  
Location: 924567-926030
  
  
**BlastP hit with Mycgr3G35447\_Mycgr3T**
  
Percentage identity: 58 %
  
BlastP bit score: 431
  
Sequence coverage: 88 %
  
E-value: 2e-144
  
  
 NCBI BlastP on this gene

EMD89345

hypothetical protein
  
Accession: EMD89344
  
Location: 923423-924463
  
 NCBI BlastP on this gene

EMD89344

hypothetical protein
  
Accession: EMD89343
  
Location: 922010-922951
  
 NCBI BlastP on this gene

EMD89343

hypothetical protein
  
Accession: EMD89342
  
Location: 920411-921416
  
 NCBI BlastP on this gene

EMD89342

hypothetical protein
  
Accession: EMD89341
  
Location: 918432-919664
  
 NCBI BlastP on this gene

EMD89341

hypothetical protein
  
Accession: EMD89340
  
Location: 914245-915911
  
 NCBI BlastP on this gene

EMD89340

hypothetical protein
  
Accession: EMD89339
  
Location: 912120-913696
  
 NCBI BlastP on this gene

EMD89339

hypothetical protein
  
Accession: EMD89338
  
Location: 911468-912016
  
 NCBI BlastP on this gene

EMD89338

Query: Architecture Search FASTA input

1. :  CM001197 Mycosphaerella graminicola IPO323 chromosome 2     Total score: 14.0     Cumulative Blast bit score: 25829

Mycgr3G67791 Mycgr3T
  
Location: 0-1542
  
 NCBI BlastP on this gene

Mycgr3G67791\_Mycgr3T

Mycgr3G90406 Mycgr3T
  
Location: 1642-3973
  
 NCBI BlastP on this gene

Mycgr3G90406\_Mycgr3T

Mycgr3G67785 Mycgr3T
  
Location: 4073-7865
  
 NCBI BlastP on this gene

Mycgr3G67785\_Mycgr3T

Mycgr3G67795 Mycgr3T
  
Location: 7965-15249
  
 NCBI BlastP on this gene

Mycgr3G67795\_Mycgr3T

Mycgr3G67775 Mycgr3T
  
Location: 15349-16237
  
 NCBI BlastP on this gene

Mycgr3G67775\_Mycgr3T

Mycgr3G90404 Mycgr3T
  
Location: 16337-17246
  
 NCBI BlastP on this gene

Mycgr3G90404\_Mycgr3T

Mycgr3G36951 Mycgr3T
  
Location: 17346-30891
  
 NCBI BlastP on this gene

Mycgr3G36951\_Mycgr3T

Mycgr3G103034 Mycgr3
  
Location: 30991-32644
  
 NCBI BlastP on this gene

Mycgr3G103034\_Mycgr3

Mycgr3G31119 Mycgr3T
  
Location: 32744-32906
  
 NCBI BlastP on this gene

Mycgr3G31119\_Mycgr3T

Mycgr3G28587 Mycgr3T
  
Location: 33006-33489
  
 NCBI BlastP on this gene

Mycgr3G28587\_Mycgr3T

Mycgr3G98959 Mycgr3T
  
Location: 33589-35035
  
 NCBI BlastP on this gene

Mycgr3G98959\_Mycgr3T

Mycgr3G35447 Mycgr3T
  
Location: 35135-36443
  
 NCBI BlastP on this gene

Mycgr3G35447\_Mycgr3T

Mycgr3G84402 Mycgr3T
  
Location: 36543-37884
  
 NCBI BlastP on this gene

Mycgr3G84402\_Mycgr3T

Mycgr3G98961 Mycgr3T
  
Location: 37984-38884
  
 NCBI BlastP on this gene

Mycgr3G98961\_Mycgr3T

hypothetical protein
  
Accession: EGP89720
  
Location: 474074-474397
  
 NCBI BlastP on this gene

EGP89720

hypothetical protein
  
Accession: EGP90749
  
Location: 472679-473598
  
 NCBI BlastP on this gene

EGP90749

hypothetical protein
  
Accession: EGP89719
  
Location: 471658-471819
  
  
**BlastP hit with Mycgr3G31119\_Mycgr3T**
  
Percentage identity: 100 %
  
BlastP bit score: 110
  
Sequence coverage: 100 %
  
E-value: 2e-30
  
  
 NCBI BlastP on this gene

EGP89719

hypothetical protein
  
Accession: EGP89718
  
Location: 468869-471319
  
  
**BlastP hit with Mycgr3G90406\_Mycgr3T**
  
Percentage identity: 100 %
  
BlastP bit score: 1594
  
Sequence coverage: 99 %
  
E-value: 0.0
  
  
 NCBI BlastP on this gene

EGP89718

TOR1 phosphatidylinositol 3-kinase
  
Accession: EGP90750
  
Location: 461149-468432
  
  
**BlastP hit with Mycgr3G67795\_Mycgr3T**
  
Percentage identity: 100 %
  
BlastP bit score: 5049
  
Sequence coverage: 99 %
  
E-value: 0.0
  
  
 NCBI BlastP on this gene

EGP90750

hypothetical protein
  
Accession: EGP90751
  
Location: 459143-460416
  
  
**BlastP hit with Mycgr3G90404\_Mycgr3T**
  
Percentage identity: 100 %
  
BlastP bit score: 624
  
Sequence coverage: 99 %
  
E-value: 0.0
  
  
 NCBI BlastP on this gene

EGP90751

hypothetical protein
  
Accession: EGP90752
  
Location: 456769-458519
  
  
**BlastP hit with Mycgr3G67791\_Mycgr3T**
  
Percentage identity: 100 %
  
BlastP bit score: 1051
  
Sequence coverage: 99 %
  
E-value: 0.0
  
  
 NCBI BlastP on this gene

EGP90752

hypothetical protein
  
Accession: EGP89717
  
Location: 455916-456450
  
  
**BlastP hit with Mycgr3G28587\_Mycgr3T**
  
Percentage identity: 100 %
  
BlastP bit score: 337
  
Sequence coverage: 100 %
  
E-value: 5e-116
  
  
 NCBI BlastP on this gene

EGP89717

hypothetical protein
  
Accession: EGP90753
  
Location: 453825-455477
  
  
**BlastP hit with Mycgr3G103034\_Mycgr3**
  
Percentage identity: 100 %
  
BlastP bit score: 1143
  
Sequence coverage: 99 %
  
E-value: 0.0
  
  
 NCBI BlastP on this gene

EGP90753

putative Non-ribosomal peptide synthetase
  
Accession: EGP89716
  
Location: 439283-453243
  
  
**BlastP hit with Mycgr3G36951\_Mycgr3T**
  
Percentage identity: 100 %
  
BlastP bit score: 9323
  
Sequence coverage: 99 %
  
E-value: 0.0
  
  
 NCBI BlastP on this gene

EGP89716

putative ABC transporter
  
Accession: EGP90754
  
Location: 433742-437840
  
  
**BlastP hit with Mycgr3G67785\_Mycgr3T**
  
Percentage identity: 100 %
  
BlastP bit score: 2565
  
Sequence coverage: 99 %
  
E-value: 0.0
  
  
 NCBI BlastP on this gene

EGP90754

putative L-ornithine 5-monooxygenase
  
Accession: EGP90755
  
Location: 431119-432712
  
 NCBI BlastP on this gene

EGP90755

hypothetical protein
  
Accession: EGP89715
  
Location: 429154-431323
  
  
**BlastP hit with Mycgr3G84402\_Mycgr3T**
  
Percentage identity: 100 %
  
BlastP bit score: 912
  
Sequence coverage: 99 %
  
E-value: 0.0
  
  
 NCBI BlastP on this gene

EGP89715

hypothetical protein
  
Accession: EGP90756
  
Location: 427564-428871
  
  
**BlastP hit with Mycgr3G35447\_Mycgr3T**
  
Percentage identity: 100 %
  
BlastP bit score: 897
  
Sequence coverage: 99 %
  
E-value: 0.0
  
  
 NCBI BlastP on this gene

EGP90756

hypothetical protein
  
Accession: EGP90757
  
Location: 423720-424702
  
  
**BlastP hit with Mycgr3G98961\_Mycgr3T**
  
Percentage identity: 100 %
  
BlastP bit score: 605
  
Sequence coverage: 99 %
  
E-value: 0.0
  
  
 NCBI BlastP on this gene

EGP90757

hypothetical protein
  
Accession: EGP89714
  
Location: 422453-423453
  
  
**BlastP hit with Mycgr3G67775\_Mycgr3T**
  
Percentage identity: 100 %
  
BlastP bit score: 615
  
Sequence coverage: 99 %
  
E-value: 0.0
  
  
 NCBI BlastP on this gene

EGP89714

hypothetical protein
  
Accession: EGP90758
  
Location: 420584-422080
  
  
**BlastP hit with Mycgr3G98959\_Mycgr3T**
  
Percentage identity: 100 %
  
BlastP bit score: 1005
  
Sequence coverage: 99 %
  
E-value: 0.0
  
  
 NCBI BlastP on this gene

EGP90758

putative alpha-amylase
  
Accession: EGP89713
  
Location: 418479-420089
  
 NCBI BlastP on this gene

EGP89713

2. :  KB456266 Mycosphaerella populorum SO2202 unplaced genomic scaffold SEPMUscaffold\_7     Total score: 4.0     Cumulative Blast bit score: 5901

Cloroperoxidase
  
Accession: EMF11548
  
Location: 1782135-1783115
  
 NCBI BlastP on this gene

EMF11548

hypothetical protein
  
Accession: EMF11549
  
Location: 1783317-1783988
  
 NCBI BlastP on this gene

EMF11549

Yip1-domain-containing protein
  
Accession: EMF11550
  
Location: 1786238-1787388
  
 NCBI BlastP on this gene

EMF11550

hypothetical protein
  
Accession: EMF11551
  
Location: 1788997-1789983
  
 NCBI BlastP on this gene

EMF11551

glycine dehydrogenase
  
Accession: EMF11552
  
Location: 1792812-1796067
  
 NCBI BlastP on this gene

EMF11552

hypothetical protein
  
Accession: EMF11553
  
Location: 1797353-1798648
  
 NCBI BlastP on this gene

EMF11553

FAD/NAD(P)-binding domain-containing protein
  
Accession: EMF11554
  
Location: 1799219-1800878
  
 NCBI BlastP on this gene

EMF11554

hypothetical protein
  
Accession: EMF11555
  
Location: 1801529-1804075
  
  
**BlastP hit with Mycgr3G90406\_Mycgr3T**
  
Percentage identity: 45 %
  
BlastP bit score: 612
  
Sequence coverage: 111 %
  
E-value: 0.0
  
  
 NCBI BlastP on this gene

EMF11555

phosphatidylinositol 3-kinase tor2
  
Accession: EMF11556
  
Location: 1804489-1811826
  
  
**BlastP hit with Mycgr3G67795\_Mycgr3T**
  
Percentage identity: 83 %
  
BlastP bit score: 4207
  
Sequence coverage: 100 %
  
E-value: 0.0
  
  
 NCBI BlastP on this gene

EMF11556

hypothetical protein
  
Accession: EMF11557
  
Location: 1812242-1812865
  
  
**BlastP hit with Mycgr3G28587\_Mycgr3T**
  
Percentage identity: 57 %
  
BlastP bit score: 188
  
Sequence coverage: 106 %
  
E-value: 6e-57
  
  
 NCBI BlastP on this gene

EMF11557

Phosphoacetylglucosamine mutase
  
Accession: EMF11558
  
Location: 1813410-1815074
  
  
**BlastP hit with Mycgr3G103034\_Mycgr3**
  
Percentage identity: 77 %
  
BlastP bit score: 894
  
Sequence coverage: 98 %
  
E-value: 0.0
  
  
 NCBI BlastP on this gene

EMF11558

hypothetical protein
  
Accession: EMF11559
  
Location: 1816628-1817539
  
 NCBI BlastP on this gene

EMF11559

AA permease-domain-containing protein
  
Accession: EMF11560
  
Location: 1818794-1821168
  
 NCBI BlastP on this gene

EMF11560

hypothetical protein
  
Accession: EMF11561
  
Location: 1821661-1821921
  
 NCBI BlastP on this gene

EMF11561

hypothetical protein
  
Accession: EMF11562
  
Location: 1823743-1825239
  
 NCBI BlastP on this gene

EMF11562

hypothetical protein
  
Accession: EMF11563
  
Location: 1825651-1827145
  
 NCBI BlastP on this gene

EMF11563

kinase-like protein
  
Accession: EMF11564
  
Location: 1830272-1831391
  
 NCBI BlastP on this gene

EMF11564

hypothetical protein
  
Accession: EMF11565
  
Location: 1832186-1833088
  
 NCBI BlastP on this gene

EMF11565

hypothetical protein
  
Accession: EMF11566
  
Location: 1834099-1834521
  
 NCBI BlastP on this gene

EMF11566

hypothetical protein
  
Accession: EMF11567
  
Location: 1834987-1836027
  
 NCBI BlastP on this gene

EMF11567

3. :  KB908844 Setosphaeria turcica Et28A unplaced genomic scaffold SETTUscaffold\_6     Total score: 4.0     Cumulative Blast bit score: 4848

hypothetical protein
  
Accession: EOA82527
  
Location: 506202-507804
  
 NCBI BlastP on this gene

EOA82527

hypothetical protein
  
Accession: EOA82528
  
Location: 509609-511402
  
 NCBI BlastP on this gene

EOA82528

hypothetical protein
  
Accession: EOA82529
  
Location: 513444-517091
  
 NCBI BlastP on this gene

EOA82529

hypothetical protein
  
Accession: EOA82530
  
Location: 518070-520105
  
 NCBI BlastP on this gene

EOA82530

hypothetical protein
  
Accession: EOA82531
  
Location: 520523-527949
  
  
**BlastP hit with Mycgr3G67795\_Mycgr3T**
  
Percentage identity: 66 %
  
BlastP bit score: 3281
  
Sequence coverage: 100 %
  
E-value: 0.0
  
  
 NCBI BlastP on this gene

EOA82531

hypothetical protein
  
Accession: EOA82532
  
Location: 531082-532927
  
  
**BlastP hit with Mycgr3G67791\_Mycgr3T**
  
Percentage identity: 57 %
  
BlastP bit score: 546
  
Sequence coverage: 94 %
  
E-value: 0.0
  
  
 NCBI BlastP on this gene

EOA82532

hypothetical protein
  
Accession: EOA82533
  
Location: 533667-534620
  
 NCBI BlastP on this gene

EOA82533

hypothetical protein
  
Accession: EOA82534
  
Location: 534710-535960
  
 NCBI BlastP on this gene

EOA82534

hypothetical protein
  
Accession: EOA82535
  
Location: 537340-538626
  
 NCBI BlastP on this gene

EOA82535

hypothetical protein
  
Accession: EOA82536
  
Location: 539713-541267
  
 NCBI BlastP on this gene

EOA82536

hypothetical protein
  
Accession: EOA82537
  
Location: 541910-542549
  
 NCBI BlastP on this gene

EOA82537

hypothetical protein
  
Accession: EOA82538
  
Location: 542936-544541
  
  
**BlastP hit with Mycgr3G84402\_Mycgr3T**
  
Percentage identity: 71 %
  
BlastP bit score: 611
  
Sequence coverage: 93 %
  
E-value: 0.0
  
  
 NCBI BlastP on this gene

EOA82538

hypothetical protein
  
Accession: EOA82539
  
Location: 544868-546320
  
  
**BlastP hit with Mycgr3G35447\_Mycgr3T**
  
Percentage identity: 57 %
  
BlastP bit score: 410
  
Sequence coverage: 86 %
  
E-value: 2e-136
  
  
 NCBI BlastP on this gene

EOA82539

hypothetical protein
  
Accession: EOA82540
  
Location: 546424-547503
  
 NCBI BlastP on this gene

EOA82540

hypothetical protein
  
Accession: EOA82541
  
Location: 547956-548897
  
 NCBI BlastP on this gene

EOA82541

hypothetical protein
  
Accession: EOA82542
  
Location: 549500-550517
  
 NCBI BlastP on this gene

EOA82542

hypothetical protein
  
Accession: EOA82543
  
Location: 551938-553422
  
 NCBI BlastP on this gene

EOA82543

hypothetical protein
  
Accession: EOA82544
  
Location: 555801-557461
  
 NCBI BlastP on this gene

EOA82544

hypothetical protein
  
Accession: EOA82545
  
Location: 558284-559890
  
 NCBI BlastP on this gene

EOA82545

hypothetical protein
  
Accession: EOA82546
  
Location: 560251-560901
  
 NCBI BlastP on this gene

EOA82546

4. :  DS231623 Pyrenophora tritici-repentis Pt-1C-BFP supercont1.9 genomic scaffold     Total score: 4.0     Cumulative Blast bit score: 4821

3-hydroxybutyryl-CoA dehydrogenase
  
Accession: EDU51460
  
Location: 1311875-1312887
  
 NCBI BlastP on this gene

EDU51460

predicted protein
  
Accession: EDU51461
  
Location: 1313264-1314115
  
 NCBI BlastP on this gene

EDU51461

conserved hypothetical protein
  
Accession: EDU51462
  
Location: 1314781-1316274
  
 NCBI BlastP on this gene

EDU51462

importin-7
  
Accession: EDU51463
  
Location: 1317811-1321370
  
 NCBI BlastP on this gene

EDU51463

conserved hypothetical protein
  
Accession: EDU51464
  
Location: 1322414-1324467
  
 NCBI BlastP on this gene

EDU51464

phosphatidylinositol 3-kinase tor2
  
Accession: EDU51465
  
Location: 1325109-1332510
  
  
**BlastP hit with Mycgr3G67795\_Mycgr3T**
  
Percentage identity: 66 %
  
BlastP bit score: 3265
  
Sequence coverage: 100 %
  
E-value: 0.0
  
  
 NCBI BlastP on this gene

EDU51465

conserved hypothetical protein
  
Accession: EDU51466
  
Location: 1334262-1336148
  
  
**BlastP hit with Mycgr3G67791\_Mycgr3T**
  
Percentage identity: 62 %
  
BlastP bit score: 533
  
Sequence coverage: 87 %
  
E-value: 0.0
  
  
 NCBI BlastP on this gene

EDU51466

conserved hypothetical protein
  
Accession: EDU51467
  
Location: 1337026-1338027
  
 NCBI BlastP on this gene

EDU51467

conserved hypothetical protein
  
Accession: EDU51468
  
Location: 1338190-1339416
  
 NCBI BlastP on this gene

EDU51468

N2,N2-dimethylguanosine tRNA methyltransferase
  
Accession: EDU51469
  
Location: 1339991-1342042
  
 NCBI BlastP on this gene

EDU51469

stress response protein Rds1
  
Accession: EDU51470
  
Location: 1343074-1344642
  
 NCBI BlastP on this gene

EDU51470

predicted protein
  
Accession: EDU51471
  
Location: 1347721-1348336
  
 NCBI BlastP on this gene

EDU51471

2-isopropylmalate synthase
  
Accession: EDU51472
  
Location: 1349467-1349966
  
 NCBI BlastP on this gene

EDU51472

hypothetical protein
  
Accession: EDU51473
  
Location: 1350531-1351144
  
 NCBI BlastP on this gene

EDU51473

ATP-dependent rRNA helicase rrp3
  
Accession: EDU51474
  
Location: 1353104-1354707
  
  
**BlastP hit with Mycgr3G84402\_Mycgr3T**
  
Percentage identity: 71 %
  
BlastP bit score: 624
  
Sequence coverage: 93 %
  
E-value: 0.0
  
  
 NCBI BlastP on this gene

EDU51474

ribosome biogenesis protein Ssf2
  
Accession: EDU51475
  
Location: 1354984-1356388
  
  
**BlastP hit with Mycgr3G35447\_Mycgr3T**
  
Percentage identity: 55 %
  
BlastP bit score: 399
  
Sequence coverage: 88 %
  
E-value: 5e-132
  
  
 NCBI BlastP on this gene

EDU51475

conserved hypothetical protein
  
Accession: EDU51476
  
Location: 1356494-1357565
  
 NCBI BlastP on this gene

EDU51476

adiponectin receptor protein 1
  
Accession: EDU51477
  
Location: 1358025-1358895
  
 NCBI BlastP on this gene

EDU51477

hypothetical protein
  
Accession: EDU51478
  
Location: 1359551-1360601
  
 NCBI BlastP on this gene

EDU51478

conserved hypothetical protein
  
Accession: EDU51479
  
Location: 1361804-1363186
  
 NCBI BlastP on this gene

EDU51479

2-nitropropane dioxygenase precursor
  
Accession: EDU51480
  
Location: 1363982-1365079
  
 NCBI BlastP on this gene

EDU51480

N-acetylglucosaminyltransferase
  
Accession: EDU51481
  
Location: 1365656-1367194
  
 NCBI BlastP on this gene

EDU51481

biotin-[acetyl-CoA-carboxylaseligase
  
Accession: EDU51482
  
Location: 1368069-1369928
  
 NCBI BlastP on this gene

EDU51482

5. :  AHHD01000090 Macrophomina phaseolina MS6     Total score: 4.0     Cumulative Blast bit score: 4639

Ribosomal protein L7 eukaryotic
  
Accession: EKG20267
  
Location: 47599-49064
  
 NCBI BlastP on this gene

EKG20267

Protein of unknown function DUF3245
  
Accession: EKG20266
  
Location: 46448-47322
  
 NCBI BlastP on this gene

EKG20266

hypothetical protein
  
Accession: EKG20265
  
Location: 43674-46300
  
 NCBI BlastP on this gene

EKG20265

RNA helicase ATP-dependent DEAD-box conserved site
  
Accession: EKG20264
  
Location: 42127-43218
  
  
**BlastP hit with Mycgr3G84402\_Mycgr3T**
  
Percentage identity: 73 %
  
BlastP bit score: 526
  
Sequence coverage: 75 %
  
E-value: 0.0
  
  
 NCBI BlastP on this gene

EKG20264

hypothetical protein
  
Accession: EKG20263
  
Location: 40114-41558
  
  
**BlastP hit with Mycgr3G35447\_Mycgr3T**
  
Percentage identity: 59 %
  
BlastP bit score: 469
  
Sequence coverage: 101 %
  
E-value: 1e-159
  
  
 NCBI BlastP on this gene

EKG20263

hypothetical protein
  
Accession: EKG20262
  
Location: 37179-37543
  
 NCBI BlastP on this gene

EKG20262

hypothetical protein
  
Accession: EKG20261
  
Location: 33317-35887
  
 NCBI BlastP on this gene

EKG20261

Zinc finger PARP-type protein
  
Accession: EKG20260
  
Location: 30479-31998
  
 NCBI BlastP on this gene

EKG20260

hypothetical protein
  
Accession: EKG20259
  
Location: 28453-29989
  
 NCBI BlastP on this gene

EKG20259

hypothetical protein
  
Accession: EKG20258
  
Location: 25131-27331
  
 NCBI BlastP on this gene

EKG20258

hypothetical protein
  
Accession: EKG20257
  
Location: 21516-22825
  
 NCBI BlastP on this gene

EKG20257

SWAP/Surp
  
Accession: EKG20256
  
Location: 18668-21073
  
  
**BlastP hit with Mycgr3G90406\_Mycgr3T**
  
Percentage identity: 31 %
  
BlastP bit score: 271
  
Sequence coverage: 96 %
  
E-value: 1e-75
  
  
 NCBI BlastP on this gene

EKG20256

Phosphatidylinositol 3-/4-kinase catalytic
  
Accession: EKG20255
  
Location: 10791-18187
  
  
**BlastP hit with Mycgr3G67795\_Mycgr3T**
  
Percentage identity: 67 %
  
BlastP bit score: 3373
  
Sequence coverage: 101 %
  
E-value: 0.0
  
  
 NCBI BlastP on this gene

EKG20255

hypothetical protein
  
Accession: EKG20254
  
Location: 8850-9422
  
 NCBI BlastP on this gene

EKG20254

Autophagy-related protein 11
  
Accession: EKG20253
  
Location: 3895-8378
  
 NCBI BlastP on this gene

EKG20253

hypothetical protein
  
Accession: EKG20252
  
Location: 1900-2940
  
 NCBI BlastP on this gene

EKG20252

N2N2-dimethylguanosine tRNA methyltransferase
  
Accession: EKG20251
  
Location: 124-942
  
 NCBI BlastP on this gene

EKG20251

6. :  KB916121 Neofusicoccum parvum UCRNP2 chromosome Unknown NP2\_03\_scaffold\_483     Total score: 4.0     Cumulative Blast bit score: 4206

putative 60s ribosomal protein l7 protein
  
Accession: EOD48936
  
Location: 12790-14241
  
 NCBI BlastP on this gene

EOD48936

putative ell complex subunit eap30 protein
  
Accession: EOD48941
  
Location: 14501-15379
  
 NCBI BlastP on this gene

EOD48941

putative eukaryotic translation initiation factor 3 subunit protein
  
Accession: EOD48932
  
Location: 15529-18204
  
 NCBI BlastP on this gene

EOD48932

putative atp-dependent rrna helicase rrp3 protein
  
Accession: EOD48947
  
Location: 18687-20171
  
  
**BlastP hit with Mycgr3G84402\_Mycgr3T**
  
Percentage identity: 71 %
  
BlastP bit score: 629
  
Sequence coverage: 94 %
  
E-value: 0.0
  
  
 NCBI BlastP on this gene

EOD48947

putative ribosome biogenesis protein
  
Accession: EOD48933
  
Location: 20334-21788
  
  
**BlastP hit with Mycgr3G35447\_Mycgr3T**
  
Percentage identity: 59 %
  
BlastP bit score: 476
  
Sequence coverage: 101 %
  
E-value: 3e-162
  
  
 NCBI BlastP on this gene

EOD48933

hypothetical protein
  
Accession: EOD48928
  
Location: 22166-22532
  
 NCBI BlastP on this gene

EOD48928

putative zf-parp-type zinc finger protein
  
Accession: EOD48938
  
Location: 27353-28837
  
 NCBI BlastP on this gene

EOD48938

hypothetical protein
  
Accession: EOD48929
  
Location: 31702-33888
  
 NCBI BlastP on this gene

EOD48929

putative coatamer subunit protein
  
Accession: EOD48944
  
Location: 39174-40445
  
  
**BlastP hit with Mycgr3G90406\_Mycgr3T**
  
Percentage identity: 37 %
  
BlastP bit score: 179
  
Sequence coverage: 33 %
  
E-value: 4e-46
  
  
 NCBI BlastP on this gene

EOD48944

putative phosphatidylinositol 3-kinase tor2 protein
  
Accession: EOD48942
  
Location: 40959-48353
  
  
**BlastP hit with Mycgr3G67795\_Mycgr3T**
  
Percentage identity: 69 %
  
BlastP bit score: 2922
  
Sequence coverage: 85 %
  
E-value: 0.0
  
  
 NCBI BlastP on this gene

EOD48942

hypothetical protein
  
Accession: EOD48931
  
Location: 49066-50067
  
 NCBI BlastP on this gene

EOD48931

putative autophagy-related protein 11 protein
  
Accession: EOD48937
  
Location: 50598-54683
  
 NCBI BlastP on this gene

EOD48937

putative -dimethylguanosine trna methyltransferase protein
  
Accession: EOD48946
  
Location: 57420-59623
  
 NCBI BlastP on this gene

EOD48946

hypothetical protein
  
Accession: EOD48940
  
Location: 60370-61588
  
 NCBI BlastP on this gene

EOD48940

7. :  KB445561 Baudoinia compniacensis UAMH 10762 unplaced genomic scaffold BAUCOscaffold\_12     Total score: 3.0     Cumulative Blast bit score: 4743

hypothetical protein
  
Accession: EMC92566
  
Location: 19682-21370
  
 NCBI BlastP on this gene

EMC92566

hypothetical protein
  
Accession: EMC92567
  
Location: 21659-22099
  
 NCBI BlastP on this gene

EMC92567

hypothetical protein
  
Accession: EMC92568
  
Location: 22553-23578
  
 NCBI BlastP on this gene

EMC92568

hypothetical protein
  
Accession: EMC92569
  
Location: 25160-25747
  
 NCBI BlastP on this gene

EMC92569

hypothetical protein
  
Accession: EMC92570
  
Location: 26044-26731
  
 NCBI BlastP on this gene

EMC92570

hypothetical protein
  
Accession: EMC92571
  
Location: 27066-27971
  
 NCBI BlastP on this gene

EMC92571

hypothetical protein
  
Accession: EMC92572
  
Location: 28303-32011
  
 NCBI BlastP on this gene

EMC92572

hypothetical protein
  
Accession: EMC92573
  
Location: 32343-33077
  
 NCBI BlastP on this gene

EMC92573

hypothetical protein
  
Accession: EMC92574
  
Location: 33896-35380
  
 NCBI BlastP on this gene

EMC92574

hypothetical protein
  
Accession: EMC92575
  
Location: 35575-35838
  
 NCBI BlastP on this gene

EMC92575

hypothetical protein
  
Accession: EMC92576
  
Location: 36154-40320
  
  
**BlastP hit with Mycgr3G67785\_Mycgr3T**
  
Percentage identity: 50 %
  
BlastP bit score: 1278
  
Sequence coverage: 100 %
  
E-value: 0.0
  
  
 NCBI BlastP on this gene

EMC92576

hypothetical protein
  
Accession: EMC92577
  
Location: 41474-56633
  
  
**BlastP hit with Mycgr3G36951\_Mycgr3T**
  
Percentage identity: 43 %
  
BlastP bit score: 2759
  
Sequence coverage: 78 %
  
E-value: 0.0
  
  
 NCBI BlastP on this gene

EMC92577

hypothetical protein
  
Accession: EMC92578
  
Location: 57268-57495
  
 NCBI BlastP on this gene

EMC92578

hypothetical protein
  
Accession: EMC92579
  
Location: 57967-59685
  
  
**BlastP hit with Mycgr3G103034\_Mycgr3**
  
Percentage identity: 63 %
  
BlastP bit score: 706
  
Sequence coverage: 96 %
  
E-value: 0.0
  
  
 NCBI BlastP on this gene

EMC92579

hypothetical protein
  
Accession: EMC92580
  
Location: 60737-63110
  
 NCBI BlastP on this gene

EMC92580

hypothetical protein
  
Accession: EMC92581
  
Location: 63617-64963
  
 NCBI BlastP on this gene

EMC92581

hypothetical protein
  
Accession: EMC92582
  
Location: 65142-66611
  
 NCBI BlastP on this gene

EMC92582

hypothetical protein
  
Accession: EMC92583
  
Location: 66856-67243
  
 NCBI BlastP on this gene

EMC92583

hypothetical protein
  
Accession: EMC92584
  
Location: 67360-69525
  
 NCBI BlastP on this gene

EMC92584

hypothetical protein
  
Accession: EMC92585
  
Location: 71827-72441
  
 NCBI BlastP on this gene

EMC92585

hypothetical protein
  
Accession: EMC92586
  
Location: 72910-74354
  
 NCBI BlastP on this gene

EMC92586

hypothetical protein
  
Accession: EMC92587
  
Location: 75548-78061
  
 NCBI BlastP on this gene

EMC92587

8. :  AFWA01000002 Pneumocystis murina B123     Total score: 3.0     Cumulative Blast bit score: 3295

Mating-type switching protein swi1
  
Accession: EMR11327
  
Location: 169356-172968
  
 NCBI BlastP on this gene

EMR11327

hypothetical protein
  
Accession: EMR11326
  
Location: 167768-169071
  
 NCBI BlastP on this gene

EMR11326

hypothetical protein
  
Accession: EMR11325
  
Location: 166735-167608
  
 NCBI BlastP on this gene

EMR11325

hypothetical protein
  
Accession: EMR11324
  
Location: 164319-166021
  
  
**BlastP hit with Mycgr3G98959\_Mycgr3T**
  
Percentage identity: 53 %
  
BlastP bit score: 486
  
Sequence coverage: 86 %
  
E-value: 7e-165
  
  
 NCBI BlastP on this gene

EMR11324

hypothetical protein
  
Accession: EMR11323
  
Location: 163670-163953
  
 NCBI BlastP on this gene

EMR11323

hypothetical protein
  
Accession: EMR11322
  
Location: 161431-163607
  
 NCBI BlastP on this gene

EMR11322

hypothetical protein
  
Accession: EMR11321
  
Location: 157160-161339
  
 NCBI BlastP on this gene

EMR11321

CMGC/DYRK/YAK protein kinase
  
Accession: EMR11320
  
Location: 154460-156834
  
 NCBI BlastP on this gene

EMR11320

hypothetical protein
  
Accession: EMR11319
  
Location: 151537-154105
  
 NCBI BlastP on this gene

EMR11319

hypothetical protein
  
Accession: EMR11318
  
Location: 151020-151502
  
  
**BlastP hit with Mycgr3G28587\_Mycgr3T**
  
Percentage identity: 33 %
  
BlastP bit score: 51
  
Sequence coverage: 75 %
  
E-value: 7e-06
  
  
 NCBI BlastP on this gene

EMR11318

hypothetical protein
  
Accession: EMR11317
  
Location: 149558-150915
  
 NCBI BlastP on this gene

EMR11317

hypothetical protein
  
Accession: EMR11316
  
Location: 148984-149382
  
 NCBI BlastP on this gene

EMR11316

hypothetical protein
  
Accession: EMR11315
  
Location: 147315-148796
  
 NCBI BlastP on this gene

EMR11315

hypothetical protein
  
Accession: EMR11314
  
Location: 143890-147064
  
 NCBI BlastP on this gene

EMR11314

hypothetical protein
  
Accession: EMR11313
  
Location: 142749-143355
  
 NCBI BlastP on this gene

EMR11313

hypothetical protein
  
Accession: EMR11312
  
Location: 140814-142172
  
 NCBI BlastP on this gene

EMR11312

hypothetical protein
  
Accession: EMR11311
  
Location: 139530-140790
  
 NCBI BlastP on this gene

EMR11311

signal peptidase I
  
Accession: EMR11310
  
Location: 138630-139299
  
 NCBI BlastP on this gene

EMR11310

hypothetical protein
  
Accession: EMR11309
  
Location: 138052-138452
  
 NCBI BlastP on this gene

EMR11309

hypothetical protein
  
Accession: EMR11308
  
Location: 137711-137995
  
 NCBI BlastP on this gene

EMR11308

hypothetical protein
  
Accession: EMR11307
  
Location: 136640-137523
  
 NCBI BlastP on this gene

EMR11307

hypothetical protein
  
Accession: EMR11306
  
Location: 134219-136403
  
 NCBI BlastP on this gene

EMR11306

hypothetical protein
  
Accession: EMR11305
  
Location: 129142-134027
  
 NCBI BlastP on this gene

EMR11305

hypothetical protein
  
Accession: EMR11304
  
Location: 127256-129072
  
 NCBI BlastP on this gene

EMR11304

hypothetical protein
  
Accession: EMR11303
  
Location: 118666-126508
  
  
**BlastP hit with Mycgr3G67795\_Mycgr3T**
  
Percentage identity: 56 %
  
BlastP bit score: 2758
  
Sequence coverage: 100 %
  
E-value: 0.0
  
  
 NCBI BlastP on this gene

EMR11303

hypothetical protein
  
Accession: EMR11302
  
Location: 118138-118546
  
 NCBI BlastP on this gene

EMR11302

hypothetical protein
  
Accession: EMR11301
  
Location: 115258-117282
  
 NCBI BlastP on this gene

EMR11301

hypothetical protein
  
Accession: EMR11300
  
Location: 112375-114906
  
 NCBI BlastP on this gene

EMR11300

9. :  KB446555 Pseudocercospora fijiensis CIRAD86 unplaced genomic scaffold MYCFIscaffold\_1     Total score: 3.0     Cumulative Blast bit score: 1842

hypothetical protein
  
Accession: EME88551
  
Location: 5960377-5963568
  
 NCBI BlastP on this gene

EME88551

hypothetical protein
  
Accession: EME88550
  
Location: 5957888-5959027
  
 NCBI BlastP on this gene

EME88550

hypothetical protein
  
Accession: EME88549
  
Location: 5956534-5956878
  
 NCBI BlastP on this gene

EME88549

hypothetical protein
  
Accession: EME88548
  
Location: 5954727-5955884
  
 NCBI BlastP on this gene

EME88548

hypothetical protein
  
Accession: EME88547
  
Location: 5952966-5953373
  
 NCBI BlastP on this gene

EME88547

hypothetical protein
  
Accession: EME88546
  
Location: 5948287-5949777
  
 NCBI BlastP on this gene

EME88546

hypothetical protein
  
Accession: EME88545
  
Location: 5945320-5948050
  
 NCBI BlastP on this gene

EME88545

hypothetical protein
  
Accession: EME88544
  
Location: 5942207-5945026
  
 NCBI BlastP on this gene

EME88544

hypothetical protein
  
Accession: EME88543
  
Location: 5940351-5941302
  
  
**BlastP hit with Mycgr3G98961\_Mycgr3T**
  
Percentage identity: 67 %
  
BlastP bit score: 407
  
Sequence coverage: 96 %
  
E-value: 3e-139
  
  
 NCBI BlastP on this gene

EME88543

hypothetical protein
  
Accession: EME88542
  
Location: 5937866-5939372
  
  
**BlastP hit with Mycgr3G98959\_Mycgr3T**
  
Percentage identity: 87 %
  
BlastP bit score: 888
  
Sequence coverage: 101 %
  
E-value: 0.0
  
  
 NCBI BlastP on this gene

EME88542

hypothetical protein
  
Accession: EME88541
  
Location: 5936388-5937658
  
  
**BlastP hit with Mycgr3G67775\_Mycgr3T**
  
Percentage identity: 87 %
  
BlastP bit score: 547
  
Sequence coverage: 98 %
  
E-value: 0.0
  
  
 NCBI BlastP on this gene

EME88541

hypothetical protein
  
Accession: EME88540
  
Location: 5933260-5933904
  
 NCBI BlastP on this gene

EME88540

hypothetical protein
  
Accession: EME88539
  
Location: 5930728-5932836
  
 NCBI BlastP on this gene

EME88539

10. :  KB456266 Mycosphaerella populorum SO2202 unplaced genomic scaffold SEPMUscaffold\_7     Total score: 3.0     Cumulative Blast bit score: 1807

hypothetical protein
  
Accession: EMF11379
  
Location: 1240913-1242832
  
 NCBI BlastP on this gene

EMF11379

Inositol P-domain-containing protein
  
Accession: EMF11378
  
Location: 1239678-1240419
  
 NCBI BlastP on this gene

EMF11378

betaine lipid synthase
  
Accession: EMF11377
  
Location: 1236418-1239008
  
 NCBI BlastP on this gene

EMF11377

L-lactate dehydrogenase
  
Accession: EMF11376
  
Location: 1233361-1234998
  
 NCBI BlastP on this gene

EMF11376

hypothetical protein
  
Accession: EMF11375
  
Location: 1231813-1232418
  
 NCBI BlastP on this gene

EMF11375

hypothetical protein
  
Accession: EMF11374
  
Location: 1225916-1230769
  
 NCBI BlastP on this gene

EMF11374

cyclin domain protein
  
Accession: EMF11372
  
Location: 1220169-1221139
  
  
**BlastP hit with Mycgr3G98961\_Mycgr3T**
  
Percentage identity: 67 %
  
BlastP bit score: 407
  
Sequence coverage: 98 %
  
E-value: 2e-139
  
  
 NCBI BlastP on this gene

EMF11372

eukaryotic translation initiation factor 3
  
Accession: EMF11371
  
Location: 1217023-1218522
  
  
**BlastP hit with Mycgr3G98959\_Mycgr3T**
  
Percentage identity: 86 %
  
BlastP bit score: 862
  
Sequence coverage: 101 %
  
E-value: 0.0
  
  
 NCBI BlastP on this gene

EMF11371

carbon-nitrogen hydrolase
  
Accession: EMF11370
  
Location: 1215362-1216578
  
  
**BlastP hit with Mycgr3G67775\_Mycgr3T**
  
Percentage identity: 85 %
  
BlastP bit score: 538
  
Sequence coverage: 98 %
  
E-value: 0.0
  
  
 NCBI BlastP on this gene

EMF11370

Sugar tr-domain-containing protein
  
Accession: EMF11369
  
Location: 1211802-1214224
  
 NCBI BlastP on this gene

EMF11369

hypothetical protein
  
Accession: EMF11368
  
Location: 1210913-1211147
  
 NCBI BlastP on this gene

EMF11368

hypothetical protein
  
Accession: EMF11367
  
Location: 1210041-1210676
  
 NCBI BlastP on this gene

EMF11367

Trimethyllysine dioxygenase
  
Accession: EMF11366
  
Location: 1207798-1209733
  
 NCBI BlastP on this gene

EMF11366

amino acid permease
  
Accession: EMF11365
  
Location: 1205760-1207304
  
 NCBI BlastP on this gene

EMF11365

glycoside hydrolase family 51 protein
  
Accession: EMF11364
  
Location: 1202194-1204311
  
 NCBI BlastP on this gene

EMF11364

hypothetical protein
  
Accession: EMF11363
  
Location: 1200290-1201519
  
 NCBI BlastP on this gene

EMF11363

hypothetical protein
  
Accession: EMF11362
  
Location: 1198503-1200013
  
 NCBI BlastP on this gene

EMF11362

11. :  KB445561 Baudoinia compniacensis UAMH 10762 unplaced genomic scaffold BAUCOscaffold\_12     Total score: 3.0     Cumulative Blast bit score: 1777

hypothetical protein
  
Accession: EMC93079
  
Location: 1092076-1092297
  
 NCBI BlastP on this gene

EMC93079

hypothetical protein
  
Accession: EMC93080
  
Location: 1093036-1093652
  
 NCBI BlastP on this gene

EMC93080

hypothetical protein
  
Accession: EMC93081
  
Location: 1094692-1096683
  
 NCBI BlastP on this gene

EMC93081

hypothetical protein
  
Accession: EMC93082
  
Location: 1097979-1098302
  
 NCBI BlastP on this gene

EMC93082

hypothetical protein
  
Accession: EMC93083
  
Location: 1099256-1100960
  
 NCBI BlastP on this gene

EMC93083

hypothetical protein
  
Accession: EMC93084
  
Location: 1101624-1102882
  
 NCBI BlastP on this gene

EMC93084

hypothetical protein
  
Accession: EMC93085
  
Location: 1104791-1105237
  
 NCBI BlastP on this gene

EMC93085

hypothetical protein
  
Accession: EMC93086
  
Location: 1105701-1106770
  
 NCBI BlastP on this gene

EMC93086

hypothetical protein
  
Accession: EMC93087
  
Location: 1107121-1108546
  
 NCBI BlastP on this gene

EMC93087

hypothetical protein
  
Accession: EMC93088
  
Location: 1108834-1109418
  
 NCBI BlastP on this gene

EMC93088

hypothetical protein
  
Accession: EMC93089
  
Location: 1109979-1110807
  
 NCBI BlastP on this gene

EMC93089

hypothetical protein
  
Accession: EMC93090
  
Location: 1111597-1112905
  
  
**BlastP hit with Mycgr3G67791\_Mycgr3T**
  
Percentage identity: 71 %
  
BlastP bit score: 548
  
Sequence coverage: 74 %
  
E-value: 0.0
  
  
 NCBI BlastP on this gene

EMC93090

hypothetical protein
  
Accession: EMC93091
  
Location: 1113353-1114768
  
  
**BlastP hit with Mycgr3G35447\_Mycgr3T**
  
Percentage identity: 65 %
  
BlastP bit score: 538
  
Sequence coverage: 97 %
  
E-value: 0.0
  
  
 NCBI BlastP on this gene

EMC93091

hypothetical protein
  
Accession: EMC93092
  
Location: 1115107-1116597
  
  
**BlastP hit with Mycgr3G84402\_Mycgr3T**
  
Percentage identity: 84 %
  
BlastP bit score: 691
  
Sequence coverage: 88 %
  
E-value: 0.0
  
  
 NCBI BlastP on this gene

EMC93092

hypothetical protein
  
Accession: EMC93093
  
Location: 1116877-1117773
  
 NCBI BlastP on this gene

EMC93093

hypothetical protein
  
Accession: EMC93094
  
Location: 1118125-1118596
  
 NCBI BlastP on this gene

EMC93094

hypothetical protein
  
Accession: EMC93095
  
Location: 1120088-1123319
  
 NCBI BlastP on this gene

EMC93095

carbohydrate-binding module family 32 protein
  
Accession: EMC93096
  
Location: 1124007-1127101
  
 NCBI BlastP on this gene

EMC93096

hypothetical protein
  
Accession: EMC93097
  
Location: 1127900-1129326
  
 NCBI BlastP on this gene

EMC93097

hypothetical protein
  
Accession: EMC93098
  
Location: 1129524-1130521
  
 NCBI BlastP on this gene

EMC93098

hypothetical protein
  
Accession: EMC93099
  
Location: 1130721-1135265
  
 NCBI BlastP on this gene

EMC93099

hypothetical protein
  
Accession: EMC93100
  
Location: 1135735-1137169
  
 NCBI BlastP on this gene

EMC93100

12. :  ACFW01000030 Coccidioides posadasii C735 delta SOWgp     Total score: 3.0     Cumulative Blast bit score: 1772

hypothetical protein
  
Accession: EER26540
  
Location: 1817143-1819589
  
 NCBI BlastP on this gene

EER26540

26S protease regulatory subunit 6A, putative
  
Accession: EER26541
  
Location: 1820057-1821569
  
 NCBI BlastP on this gene

EER26541

Trimethyllysine dioxygenase, putative
  
Accession: EER26542
  
Location: 1822084-1824103
  
 NCBI BlastP on this gene

EER26542

MaoC like domain containing protein
  
Accession: EER26543
  
Location: 1827308-1828466
  
 NCBI BlastP on this gene

EER26543

protein arginine N-methyltransferase, putative
  
Accession: EER26544
  
Location: 1829177-1830606
  
 NCBI BlastP on this gene

EER26544

Per1-like family protein
  
Accession: EER26545
  
Location: 1831451-1832524
  
 NCBI BlastP on this gene

EER26545

phosphoglucomutase/phosphomannomutase, putative
  
Accession: EER26546
  
Location: 1833193-1835166
  
  
**BlastP hit with Mycgr3G103034\_Mycgr3**
  
Percentage identity: 57 %
  
BlastP bit score: 632
  
Sequence coverage: 98 %
  
E-value: 0.0
  
  
 NCBI BlastP on this gene

EER26546

ATP-dependent rRNA helicase RRP3, putative
  
Accession: EER26547
  
Location: 1835630-1837116
  
  
**BlastP hit with Mycgr3G84402\_Mycgr3T**
  
Percentage identity: 72 %
  
BlastP bit score: 665
  
Sequence coverage: 101 %
  
E-value: 0.0
  
  
 NCBI BlastP on this gene

EER26547

Brix domain containing protein
  
Accession: EER26548
  
Location: 1837398-1838773
  
  
**BlastP hit with Mycgr3G35447\_Mycgr3T**
  
Percentage identity: 55 %
  
BlastP bit score: 475
  
Sequence coverage: 101 %
  
E-value: 9e-162
  
  
 NCBI BlastP on this gene

EER26548

hypothetical protein
  
Accession: EER26549
  
Location: 1839017-1840262
  
 NCBI BlastP on this gene

EER26549

riboflavin synthase, alpha subunit family protein
  
Accession: EER26550
  
Location: 1840518-1841381
  
 NCBI BlastP on this gene

EER26550

CAIB/BAIF family protein
  
Accession: EER26551
  
Location: 1841715-1843363
  
 NCBI BlastP on this gene

EER26551

PHD-finger motif containing protein
  
Accession: EER26552
  
Location: 1843787-1845547
  
 NCBI BlastP on this gene

EER26552

SPFH domain / Band 7 family protein
  
Accession: EER26553
  
Location: 1847784-1849084
  
 NCBI BlastP on this gene

EER26553

hypothetical protein
  
Accession: EER26554
  
Location: 1851951-1852608
  
 NCBI BlastP on this gene

EER26554

60S ribosomal protein L27-B, putative
  
Accession: EER26555
  
Location: 1856163-1856984
  
 NCBI BlastP on this gene

EER26555

hypothetical protein
  
Accession: EER26556
  
Location: 1857703-1864198
  
 NCBI BlastP on this gene

EER26556

13. :  GL636488 Coccidioides posadasii str. Silveira unplaced genomic scaffold supercont2.3     Total score: 3.0     Cumulative Blast bit score: 1771

conserved hypothetical protein
  
Accession: EFW20575
  
Location: 375459-377905
  
 NCBI BlastP on this gene

EFW20575

26S protease regulatory subunit 6A
  
Accession: EFW20576
  
Location: 378373-379885
  
 NCBI BlastP on this gene

EFW20576

trimethyllysine dioxygenase
  
Accession: EFW20577
  
Location: 380400-382419
  
 NCBI BlastP on this gene

EFW20577

predicted protein
  
Accession: EFW20578
  
Location: 383333-384218
  
 NCBI BlastP on this gene

EFW20578

predicted protein
  
Accession: EFW20579
  
Location: 384635-385318
  
 NCBI BlastP on this gene

EFW20579

peroxisomal dehydratase
  
Accession: EFW20580
  
Location: 385624-386783
  
 NCBI BlastP on this gene

EFW20580

histone-arginine methyltransferase
  
Accession: EFW20581
  
Location: 387494-388923
  
 NCBI BlastP on this gene

EFW20581

Mn2+ homeostasis protein
  
Accession: EFW20582
  
Location: 389649-390847
  
 NCBI BlastP on this gene

EFW20582

N-acetylglucosamine-phosphate mutase
  
Accession: EFW20583
  
Location: 391555-393528
  
  
**BlastP hit with Mycgr3G103034\_Mycgr3**
  
Percentage identity: 57 %
  
BlastP bit score: 632
  
Sequence coverage: 98 %
  
E-value: 0.0
  
  
 NCBI BlastP on this gene

EFW20583

ATP-dependent rRNA helicase RRP3
  
Accession: EFW20584
  
Location: 393992-395478
  
  
**BlastP hit with Mycgr3G84402\_Mycgr3T**
  
Percentage identity: 72 %
  
BlastP bit score: 664
  
Sequence coverage: 101 %
  
E-value: 0.0
  
  
 NCBI BlastP on this gene

EFW20584

ribosome biogenesis protein Ssf2
  
Accession: EFW20585
  
Location: 395756-397131
  
  
**BlastP hit with Mycgr3G35447\_Mycgr3T**
  
Percentage identity: 55 %
  
BlastP bit score: 475
  
Sequence coverage: 101 %
  
E-value: 9e-162
  
  
 NCBI BlastP on this gene

EFW20585

conserved hypothetical protein
  
Accession: EFW20586
  
Location: 397375-398620
  
 NCBI BlastP on this gene

EFW20586

riboflavin synthase subunit alpha
  
Accession: EFW20587
  
Location: 398876-399739
  
 NCBI BlastP on this gene

EFW20587

CAIB/BAIF family enzyme
  
Accession: EFW20588
  
Location: 400073-401721
  
 NCBI BlastP on this gene

EFW20588

hypothetical protein
  
Accession: EFW20589
  
Location: 402056-403905
  
 NCBI BlastP on this gene

EFW20589

stomatin family protein
  
Accession: EFW20590
  
Location: 406142-407441
  
 NCBI BlastP on this gene

EFW20590

predicted protein
  
Accession: EFW20591
  
Location: 407907-408359
  
 NCBI BlastP on this gene

EFW20591

conserved hypothetical protein
  
Accession: EFW20592
  
Location: 408489-409023
  
 NCBI BlastP on this gene

EFW20592

conserved hypothetical protein
  
Accession: EFW20593
  
Location: 410329-410962
  
 NCBI BlastP on this gene

EFW20593

predicted protein
  
Accession: EFW20594
  
Location: 411280-411726
  
 NCBI BlastP on this gene

EFW20594

hypothetical protein
  
Accession: EFW20595
  
Location: 413444-413629
  
 NCBI BlastP on this gene

EFW20595

60S ribosomal protein L27e
  
Accession: EFW20596
  
Location: 414535-415356
  
 NCBI BlastP on this gene

EFW20596

conserved hypothetical protein
  
Accession: EFW20597
  
Location: 416075-422505
  
 NCBI BlastP on this gene

EFW20597

14. :  GG704916 Coccidioides immitis RS genomic scaffold supercont3.6     Total score: 3.0     Cumulative Blast bit score: 1771

hypothetical protein
  
Accession: EJB12070
  
Location: 1565730-1566396
  
 NCBI BlastP on this gene

EJB12070

trimethyllysine dioxygenase
  
Accession: EAS32649
  
Location: 1559464-1561484
  
 NCBI BlastP on this gene

EAS32649

hypothetical protein
  
Accession: EJB12069
  
Location: 1557719-1558805
  
 NCBI BlastP on this gene

EJB12069

hypothetical protein
  
Accession: EAS32646
  
Location: 1556439-1557216
  
 NCBI BlastP on this gene

EAS32646

hypothetical protein
  
Accession: EAS32645
  
Location: 1555184-1556042
  
 NCBI BlastP on this gene

EAS32645

peroxisomal dehydratase
  
Accession: EAS32644
  
Location: 1552602-1553760
  
 NCBI BlastP on this gene

EAS32644

HNRNP arginine N-methyltransferase, variant
  
Accession: EJB12068
  
Location: 1550469-1551832
  
 NCBI BlastP on this gene

EJB12068

Mn2+ homeostasis protein
  
Accession: EAS32641
  
Location: 1548538-1549736
  
 NCBI BlastP on this gene

EAS32641

N-acetylglucosamine-phosphate mutase
  
Accession: EAS32640
  
Location: 1545895-1547868
  
  
**BlastP hit with Mycgr3G103034\_Mycgr3**
  
Percentage identity: 57 %
  
BlastP bit score: 634
  
Sequence coverage: 98 %
  
E-value: 0.0
  
  
 NCBI BlastP on this gene

EAS32640

ATP-dependent rRNA helicase RRP3
  
Accession: EAS32639
  
Location: 1543945-1545431
  
  
**BlastP hit with Mycgr3G84402\_Mycgr3T**
  
Percentage identity: 72 %
  
BlastP bit score: 665
  
Sequence coverage: 101 %
  
E-value: 0.0
  
  
 NCBI BlastP on this gene

EAS32639

ribosome biogenesis protein Ssf2
  
Accession: EAS32638
  
Location: 1542294-1543669
  
  
**BlastP hit with Mycgr3G35447\_Mycgr3T**
  
Percentage identity: 55 %
  
BlastP bit score: 472
  
Sequence coverage: 101 %
  
E-value: 1e-160
  
  
 NCBI BlastP on this gene

EAS32638

hypothetical protein
  
Accession: EAS32637
  
Location: 1540805-1541759
  
 NCBI BlastP on this gene

EAS32637

riboflavin synthase, alpha subunit
  
Accession: EAS32636
  
Location: 1539686-1540549
  
 NCBI BlastP on this gene

EAS32636

CAIB/BAIF family enzyme
  
Accession: EAS32635
  
Location: 1537704-1539352
  
 NCBI BlastP on this gene

EAS32635

hypothetical protein
  
Accession: EAS32634
  
Location: 1535518-1537278
  
 NCBI BlastP on this gene

EAS32634

stomatin family protein
  
Accession: EAS32633
  
Location: 1532863-1534164
  
 NCBI BlastP on this gene

EAS32633

hypothetical protein
  
Accession: EAS32632
  
Location: 1531289-1531807
  
 NCBI BlastP on this gene

EAS32632

pathogenesis associated protein Cap20
  
Accession: EAS32631
  
Location: 1529336-1529993
  
 NCBI BlastP on this gene

EAS32631

hypothetical protein
  
Accession: EJB12066
  
Location: 1527603-1528013
  
 NCBI BlastP on this gene

EJB12066

60S ribosomal protein L27-B
  
Accession: EAS32629
  
Location: 1525033-1525856
  
 NCBI BlastP on this gene

EAS32629

15. :  GG749414 Ajellomyces dermatitidis ATCC 18188 genomic scaffold supercont1.8     Total score: 3.0     Cumulative Blast bit score: 1756

hypothetical protein
  
Accession: EGE79446
  
Location: 925657-925975
  
 NCBI BlastP on this gene

EGE79446

trimethyllysine dioxygenase
  
Accession: EGE79447
  
Location: 927499-929800
  
 NCBI BlastP on this gene

EGE79447

peroxisomal dehydratase
  
Accession: EGE79448
  
Location: 931829-933212
  
 NCBI BlastP on this gene

EGE79448

hypothetical protein
  
Accession: EGE79449
  
Location: 936255-936733
  
 NCBI BlastP on this gene

EGE79449

hypothetical protein
  
Accession: EGE79450
  
Location: 936736-937491
  
 NCBI BlastP on this gene

EGE79450

N-acetylglucosamine-phosphate mutase
  
Accession: EGE79451
  
Location: 937989-940064
  
  
**BlastP hit with Mycgr3G103034\_Mycgr3**
  
Percentage identity: 56 %
  
BlastP bit score: 628
  
Sequence coverage: 98 %
  
E-value: 0.0
  
  
 NCBI BlastP on this gene

EGE79451

ATP-dependent rRNA helicase RRP3
  
Accession: EGE79452
  
Location: 940788-942319
  
  
**BlastP hit with Mycgr3G84402\_Mycgr3T**
  
Percentage identity: 77 %
  
BlastP bit score: 647
  
Sequence coverage: 91 %
  
E-value: 0.0
  
  
 NCBI BlastP on this gene

EGE79452

ribosome biogenesis protein Ssf2
  
Accession: EGE79453
  
Location: 942550-943963
  
  
**BlastP hit with Mycgr3G35447\_Mycgr3T**
  
Percentage identity: 58 %
  
BlastP bit score: 481
  
Sequence coverage: 96 %
  
E-value: 3e-164
  
  
 NCBI BlastP on this gene

EGE79453

riboflavin synthase subunit alpha
  
Accession: EGE79454
  
Location: 944346-945198
  
 NCBI BlastP on this gene

EGE79454

CAIB/BAIF family enzyme
  
Accession: EGE79455
  
Location: 945541-947352
  
 NCBI BlastP on this gene

EGE79455

16. :  EQ999973 Ajellomyces dermatitidis ER-3 genomic scaffold supercont1.1     Total score: 3.0     Cumulative Blast bit score: 1756

predicted protein
  
Accession: EEQ83927
  
Location: 5776724-5777534
  
 NCBI BlastP on this gene

EEQ83927

conserved hypothetical protein
  
Accession: EEQ83928
  
Location: 5778730-5781253
  
 NCBI BlastP on this gene

EEQ83928

26S protease regulatory subunit 6A
  
Accession: EEQ83929
  
Location: 5782090-5783669
  
 NCBI BlastP on this gene

EEQ83929

trimethyllysine dioxygenase
  
Accession: EEQ83930
  
Location: 5784069-5786339
  
 NCBI BlastP on this gene

EEQ83930

peroxisomal dehydratase
  
Accession: EEQ83931
  
Location: 5788378-5789765
  
 NCBI BlastP on this gene

EEQ83931

HNRNP arginine N-methyltransferase
  
Accession: EEQ83932
  
Location: 5790520-5792138
  
 NCBI BlastP on this gene

EEQ83932

Mn2+ homeostasis protein
  
Accession: EEQ83933
  
Location: 5792815-5794051
  
 NCBI BlastP on this gene

EEQ83933

N-acetylglucosamine-phosphate mutase
  
Accession: EEQ83934
  
Location: 5794539-5796614
  
  
**BlastP hit with Mycgr3G103034\_Mycgr3**
  
Percentage identity: 56 %
  
BlastP bit score: 628
  
Sequence coverage: 98 %
  
E-value: 0.0
  
  
 NCBI BlastP on this gene

EEQ83934

ATP-dependent rRNA helicase RRP3
  
Accession: EEQ83935
  
Location: 5797347-5798878
  
  
**BlastP hit with Mycgr3G84402\_Mycgr3T**
  
Percentage identity: 77 %
  
BlastP bit score: 647
  
Sequence coverage: 91 %
  
E-value: 0.0
  
  
 NCBI BlastP on this gene

EEQ83935

ribosome biogenesis protein Ssf2
  
Accession: EEQ83936
  
Location: 5799109-5800522
  
  
**BlastP hit with Mycgr3G35447\_Mycgr3T**
  
Percentage identity: 58 %
  
BlastP bit score: 481
  
Sequence coverage: 94 %
  
E-value: 3e-164
  
  
 NCBI BlastP on this gene

EEQ83936

riboflavin synthase subunit alpha
  
Accession: EEQ83937
  
Location: 5800905-5801757
  
 NCBI BlastP on this gene

EEQ83937

CAIB/BAIF family enzyme
  
Accession: EEQ83938
  
Location: 5802100-5803911
  
 NCBI BlastP on this gene

EEQ83938

17. :  GG663372 Ajellomyces capsulatus G186AR genomic scaffold supercont2.10     Total score: 3.0     Cumulative Blast bit score: 1752

serine/threonine-protein kinase sck1
  
Accession: EEH04859
  
Location: 463737-466737
  
 NCBI BlastP on this gene

EEH04859

conserved hypothetical protein
  
Accession: EEH04858
  
Location: 457429-459946
  
 NCBI BlastP on this gene

EEH04858

26S protease regulatory subunit
  
Accession: EEH04857
  
Location: 454926-456502
  
 NCBI BlastP on this gene

EEH04857

trimethyllysine dioxygenase
  
Accession: EEH04856
  
Location: 449718-454580
  
 NCBI BlastP on this gene

EEH04856

HNRNP arginine N-methyltransferase
  
Accession: EEH04855
  
Location: 447117-448972
  
 NCBI BlastP on this gene

EEH04855

PER1 precursor
  
Accession: EEH04854
  
Location: 445531-446758
  
 NCBI BlastP on this gene

EEH04854

N-acetylglucosamine-phosphate mutase
  
Accession: EEH04853
  
Location: 442948-445022
  
  
**BlastP hit with Mycgr3G103034\_Mycgr3**
  
Percentage identity: 55 %
  
BlastP bit score: 615
  
Sequence coverage: 100 %
  
E-value: 0.0
  
  
 NCBI BlastP on this gene

EEH04853

ATP-dependent rRNA helicase RRP3
  
Accession: EEH04852
  
Location: 440712-442246
  
  
**BlastP hit with Mycgr3G84402\_Mycgr3T**
  
Percentage identity: 73 %
  
BlastP bit score: 659
  
Sequence coverage: 96 %
  
E-value: 0.0
  
  
 NCBI BlastP on this gene

EEH04852

brix domain-containing protein c
  
Accession: EEH04851
  
Location: 439121-440520
  
  
**BlastP hit with Mycgr3G35447\_Mycgr3T**
  
Percentage identity: 56 %
  
BlastP bit score: 478
  
Sequence coverage: 101 %
  
E-value: 9e-163
  
  
 NCBI BlastP on this gene

EEH04851

riboflavin synthase
  
Accession: EEH04850
  
Location: 437946-438796
  
 NCBI BlastP on this gene

EEH04850

CAIB/BAIF family enzyme
  
Accession: EEH04849
  
Location: 436174-437688
  
 NCBI BlastP on this gene

EEH04849

conserved hypothetical protein
  
Accession: EEH04848
  
Location: 434313-435290
  
 NCBI BlastP on this gene

EEH04848

PHD finger containing protein Phf1
  
Accession: EEH04847
  
Location: 432087-433888
  
 NCBI BlastP on this gene

EEH04847

DUF887 domain-containing protein
  
Accession: EEH04846
  
Location: 429897-431199
  
 NCBI BlastP on this gene

EEH04846

conserved hypothetical protein
  
Accession: EEH04845
  
Location: 422928-429058
  
 NCBI BlastP on this gene

EEH04845

60S ribosomal protein L27A
  
Accession: EEH04844
  
Location: 421407-422237
  
 NCBI BlastP on this gene

EEH04844

18. :  DS989822 Arthroderma gypseum CBS 118893 supercont1.1 genomic scaffold     Total score: 3.0     Cumulative Blast bit score: 1716

AGC/AKT protein kinase
  
Accession: EFQ98218
  
Location: 3416124-3419245
  
 NCBI BlastP on this gene

EFQ98218

hypothetical protein
  
Accession: EFQ98219
  
Location: 3420684-3421223
  
 NCBI BlastP on this gene

EFQ98219

hypothetical protein
  
Accession: EFQ98220
  
Location: 3421837-3424375
  
 NCBI BlastP on this gene

EFQ98220

26S protease regulatory subunit 6A
  
Accession: EFQ98221
  
Location: 3424941-3426467
  
 NCBI BlastP on this gene

EFQ98221

trimethyllysine dioxygenase
  
Accession: EFQ98222
  
Location: 3426900-3428834
  
 NCBI BlastP on this gene

EFQ98222

peroxisomal dehydratase
  
Accession: EFQ98223
  
Location: 3429272-3430448
  
 NCBI BlastP on this gene

EFQ98223

HNRNP arginine N-methyltransferase
  
Accession: EFQ98224
  
Location: 3431032-3432401
  
 NCBI BlastP on this gene

EFQ98224

hypothetical protein
  
Accession: EFQ98225
  
Location: 3432791-3433660
  
 NCBI BlastP on this gene

EFQ98225

PER1
  
Accession: EFQ98226
  
Location: 3434173-3435408
  
 NCBI BlastP on this gene

EFQ98226

phosphoacetylglucosamine mutase
  
Accession: EFQ98227
  
Location: 3435864-3437863
  
  
**BlastP hit with Mycgr3G103034\_Mycgr3**
  
Percentage identity: 55 %
  
BlastP bit score: 604
  
Sequence coverage: 100 %
  
E-value: 0.0
  
  
 NCBI BlastP on this gene

EFQ98227

ATP-dependent rRNA helicase RRP3
  
Accession: EFQ98228
  
Location: 3438432-3439938
  
  
**BlastP hit with Mycgr3G84402\_Mycgr3T**
  
Percentage identity: 72 %
  
BlastP bit score: 671
  
Sequence coverage: 100 %
  
E-value: 0.0
  
  
 NCBI BlastP on this gene

EFQ98228

ribosome biogenesis protein SSF1
  
Accession: EFQ98229
  
Location: 3440194-3441582
  
  
**BlastP hit with Mycgr3G35447\_Mycgr3T**
  
Percentage identity: 55 %
  
BlastP bit score: 441
  
Sequence coverage: 93 %
  
E-value: 1e-148
  
  
 NCBI BlastP on this gene

EFQ98229

riboflavin synthase subunit alpha
  
Accession: EFQ98230
  
Location: 3441891-3442731
  
 NCBI BlastP on this gene

EFQ98230

formyl-coenzyme A transferase
  
Accession: EFQ98231
  
Location: 3443082-3444753
  
 NCBI BlastP on this gene

EFQ98231

hypothetical protein
  
Accession: EFQ98232
  
Location: 3445318-3446913
  
 NCBI BlastP on this gene

EFQ98232

stomatin-2
  
Accession: EFQ98233
  
Location: 3447362-3448643
  
 NCBI BlastP on this gene

EFQ98233

CAP20
  
Accession: EFQ98234
  
Location: 3450440-3451038
  
 NCBI BlastP on this gene

EFQ98234

60S ribosomal protein L27-A
  
Accession: EFQ98235
  
Location: 3452952-3453839
  
 NCBI BlastP on this gene

EFQ98235

hypothetical protein
  
Accession: EFQ98236
  
Location: 3454522-3461853
  
 NCBI BlastP on this gene

EFQ98236

19. :  DS990636 Ajellomyces capsulatus H88 supercont1.1 genomic scaffold     Total score: 3.0     Cumulative Blast bit score: 1712

serine/threonine protein kinase sck1
  
Accession: EGC40893
  
Location: 753900-756902
  
 NCBI BlastP on this gene

EGC40893

conserved hypothetical protein
  
Accession: EGC40892
  
Location: 747637-750154
  
 NCBI BlastP on this gene

EGC40892

26S protease regulatory subunit 6A-B
  
Accession: EGC40891
  
Location: 745134-746710
  
 NCBI BlastP on this gene

EGC40891

trimethyllysine dioxygenase
  
Accession: EGC40890
  
Location: 742570-744789
  
 NCBI BlastP on this gene

EGC40890

phospholipase/carboxylesterase
  
Accession: EGC40889
  
Location: 741729-742198
  
 NCBI BlastP on this gene

EGC40889

peroxisomal dehydratase
  
Accession: EGC40888
  
Location: 739951-741304
  
 NCBI BlastP on this gene

EGC40888

HNRNP arginine N-methyltransferase
  
Accession: EGC40887
  
Location: 737720-739254
  
 NCBI BlastP on this gene

EGC40887

Mn2+ homeostasis protein
  
Accession: EGC40886
  
Location: 735813-737040
  
 NCBI BlastP on this gene

EGC40886

N-acetylglucosamine-phosphate mutase
  
Accession: EGC40885
  
Location: 733222-735296
  
  
**BlastP hit with Mycgr3G103034\_Mycgr3**
  
Percentage identity: 54 %
  
BlastP bit score: 597
  
Sequence coverage: 101 %
  
E-value: 0.0
  
  
 NCBI BlastP on this gene

EGC40885

ATP-dependent rRNA helicase RRP3
  
Accession: EGC40884
  
Location: 730986-732520
  
  
**BlastP hit with Mycgr3G84402\_Mycgr3T**
  
Percentage identity: 73 %
  
BlastP bit score: 658
  
Sequence coverage: 96 %
  
E-value: 0.0
  
  
 NCBI BlastP on this gene

EGC40884

ribosome biogenesis protein Ssf2
  
Accession: EGC40883
  
Location: 729402-730717
  
  
**BlastP hit with Mycgr3G35447\_Mycgr3T**
  
Percentage identity: 56 %
  
BlastP bit score: 457
  
Sequence coverage: 94 %
  
E-value: 5e-155
  
  
 NCBI BlastP on this gene

EGC40883

riboflavin synthase
  
Accession: EGC40882
  
Location: 728228-729078
  
 NCBI BlastP on this gene

EGC40882

CAIB/BAIF family enzyme
  
Accession: EGC40881
  
Location: 726199-727970
  
 NCBI BlastP on this gene

EGC40881

Na+/H+ antiporter
  
Accession: EGC40880
  
Location: 724604-725581
  
 NCBI BlastP on this gene

EGC40880

PHD finger containing protein Phf1
  
Accession: EGC40879
  
Location: 722381-724181
  
 NCBI BlastP on this gene

EGC40879

DUF887 domain-containing protein
  
Accession: EGC40878
  
Location: 720166-721467
  
 NCBI BlastP on this gene

EGC40878

conserved hypothetical protein
  
Accession: EGC40877
  
Location: 713203-719333
  
 NCBI BlastP on this gene

EGC40877

60S ribosomal protein
  
Accession: EGC40876
  
Location: 711694-712514
  
 NCBI BlastP on this gene

EGC40876

20. :  DS499594 Aspergillus fumigatus A1163 scf\_000001 genomic scaffold     Total score: 3.0     Cumulative Blast bit score: 1697

extracellular exo-polygalacturonase, putative
  
Accession: EDP55950
  
Location: 1848351-1849900
  
 NCBI BlastP on this gene

EDP55950

L-serine dehydratase, putative
  
Accession: EDP55951
  
Location: 1850025-1851524
  
 NCBI BlastP on this gene

EDP55951

conserved predicted protein
  
Accession: EDP55952
  
Location: 1855095-1857494
  
 NCBI BlastP on this gene

EDP55952

proteasome regulatory particle subunit Rpt5, putative
  
Accession: EDP55953
  
Location: 1858334-1859954
  
 NCBI BlastP on this gene

EDP55953

trimethyllysine dioxygenase, putative
  
Accession: EDP55954
  
Location: 1860353-1861724
  
 NCBI BlastP on this gene

EDP55954

histone H4 arginine methyltransferase RmtA
  
Accession: EDP55955
  
Location: 1863624-1864982
  
 NCBI BlastP on this gene

EDP55955

Mn2+ homeostasis protein (Per1), putative
  
Accession: EDP55956
  
Location: 1865644-1866825
  
 NCBI BlastP on this gene

EDP55956

N-acetylglucosamine-phosphate mutase
  
Accession: EDP55957
  
Location: 1867380-1869370
  
  
**BlastP hit with Mycgr3G103034\_Mycgr3**
  
Percentage identity: 54 %
  
BlastP bit score: 608
  
Sequence coverage: 101 %
  
E-value: 0.0
  
  
 NCBI BlastP on this gene

EDP55957

ATP-dependent RNA helicase , putative
  
Accession: EDP55958
  
Location: 1869784-1871298
  
  
**BlastP hit with Mycgr3G84402\_Mycgr3T**
  
Percentage identity: 73 %
  
BlastP bit score: 647
  
Sequence coverage: 92 %
  
E-value: 0.0
  
  
 NCBI BlastP on this gene

EDP55958

ribosome biogenesis protein Ssf2, putative
  
Accession: EDP55959
  
Location: 1871553-1872955
  
  
**BlastP hit with Mycgr3G35447\_Mycgr3T**
  
Percentage identity: 59 %
  
BlastP bit score: 443
  
Sequence coverage: 86 %
  
E-value: 4e-149
  
  
 NCBI BlastP on this gene

EDP55959

riboflavin synthase, alpha subunit
  
Accession: EDP55960
  
Location: 1873542-1874469
  
 NCBI BlastP on this gene

EDP55960

CAIB/BAIF family enzyme
  
Accession: EDP55961
  
Location: 1874794-1876418
  
 NCBI BlastP on this gene

EDP55961

PHD finger domain protein, putative
  
Accession: EDP55962
  
Location: 1876882-1878747
  
 NCBI BlastP on this gene

EDP55962

zinc metallopeptidase, putative
  
Accession: EDP55963
  
Location: 1879001-1880422
  
 NCBI BlastP on this gene

EDP55963

short-chain dehydrogenase/reductase family protein, putative
  
Accession: EDP55964
  
Location: 1880718-1882201
  
 NCBI BlastP on this gene

EDP55964

small nucleolar ribonucleoprotein complex subunit (SOF1), putative
  
Accession: EDP55965
  
Location: 1882384-1883814
  
 NCBI BlastP on this gene

EDP55965

proteasome regulatory particle subunit (RpnF), putative
  
Accession: EDP55966
  
Location: 1883878-1885550
  
 NCBI BlastP on this gene

EDP55966

SWI-SNF complex subunit (BAF60b), putative
  
Accession: EDP55967
  
Location: 1885958-1887617
  
 NCBI BlastP on this gene

EDP55967

DUF887 domain protein
  
Accession: EDP55968
  
Location: 1888049-1889306
  
 NCBI BlastP on this gene

EDP55968

conserved hypothetical protein
  
Accession: EDP55969
  
Location: 1890150-1896332
  
 NCBI BlastP on this gene

EDP55969

21. :  GG700648 Trichophyton rubrum CBS 118892 genomic scaffold supercont2.1     Total score: 3.0     Cumulative Blast bit score: 1697

AGC/AKT protein kinase
  
Accession: EGD83979
  
Location: 670518-673706
  
 NCBI BlastP on this gene

EGD83979

hypothetical protein
  
Accession: EGD83978
  
Location: 665467-667999
  
 NCBI BlastP on this gene

EGD83978

26S protease regulatory subunit 6A
  
Accession: EGD83977
  
Location: 663406-664932
  
 NCBI BlastP on this gene

EGD83977

trimethyllysine dioxygenase
  
Accession: EGD83976
  
Location: 661047-662979
  
 NCBI BlastP on this gene

EGD83976

peroxisomal multifunctional enzyme type 2
  
Accession: EGD83975
  
Location: 659310-660464
  
 NCBI BlastP on this gene

EGD83975

HNRNP arginine N-methyltransferase
  
Accession: EGD83974
  
Location: 657336-658702
  
 NCBI BlastP on this gene

EGD83974

hypothetical protein
  
Accession: EGD83973
  
Location: 656022-656948
  
 NCBI BlastP on this gene

EGD83973

Mn2+ homeostasis protein
  
Accession: EGD83972
  
Location: 654276-655521
  
 NCBI BlastP on this gene

EGD83972

N-acetylglucosamine-phosphate mutase
  
Accession: EGD83971
  
Location: 651790-653795
  
  
**BlastP hit with Mycgr3G103034\_Mycgr3**
  
Percentage identity: 56 %
  
BlastP bit score: 609
  
Sequence coverage: 100 %
  
E-value: 0.0
  
  
 NCBI BlastP on this gene

EGD83971

ATP-dependent rRNA helicase RRP3
  
Accession: EGD83970
  
Location: 649734-651238
  
  
**BlastP hit with Mycgr3G84402\_Mycgr3T**
  
Percentage identity: 71 %
  
BlastP bit score: 667
  
Sequence coverage: 100 %
  
E-value: 0.0
  
  
 NCBI BlastP on this gene

EGD83970

ribosome biogenesis protein Ssf2
  
Accession: EGD83969
  
Location: 648061-649460
  
  
**BlastP hit with Mycgr3G35447\_Mycgr3T**
  
Percentage identity: 56 %
  
BlastP bit score: 421
  
Sequence coverage: 91 %
  
E-value: 1e-140
  
  
 NCBI BlastP on this gene

EGD83969

riboflavin synthase subunit alpha
  
Accession: EGD83968
  
Location: 646919-647754
  
 NCBI BlastP on this gene

EGD83968

hypothetical protein
  
Accession: EGD83967
  
Location: 644926-646587
  
 NCBI BlastP on this gene

EGD83967

hypothetical protein
  
Accession: EGD83966
  
Location: 642751-644349
  
 NCBI BlastP on this gene

EGD83966

stomatin family protein
  
Accession: EGD83965
  
Location: 641018-642307
  
 NCBI BlastP on this gene

EGD83965

hypothetical protein
  
Accession: EGD83964
  
Location: 638664-639265
  
 NCBI BlastP on this gene

EGD83964

60S ribosomal protein L27-A
  
Accession: EGD83963
  
Location: 635886-636936
  
 NCBI BlastP on this gene

EGD83963

22. :  GG698540 Trichophyton tonsurans CBS 112818 genomic scaffold supercont1.64     Total score: 3.0     Cumulative Blast bit score: 1697

hypothetical protein
  
Accession: EGE00442
  
Location: 6620-8604
  
 NCBI BlastP on this gene

EGE00442

ATPase
  
Accession: EGE00443
  
Location: 9025-10550
  
 NCBI BlastP on this gene

EGE00443

trimethyllysine dioxygenase
  
Accession: EGE00444
  
Location: 11005-13044
  
 NCBI BlastP on this gene

EGE00444

peroxisomal dehydratase
  
Accession: EGE00445
  
Location: 13619-14784
  
 NCBI BlastP on this gene

EGE00445

HNRNP arginine N-methyltransferase
  
Accession: EGE00446
  
Location: 15429-16792
  
 NCBI BlastP on this gene

EGE00446

hypothetical protein
  
Accession: EGE00447
  
Location: 17179-18106
  
 NCBI BlastP on this gene

EGE00447

Mn2+ homeostasis protein
  
Accession: EGE00448
  
Location: 18432-19891
  
 NCBI BlastP on this gene

EGE00448

N-acetylglucosamine-phosphate mutase
  
Accession: EGE00449
  
Location: 20379-22382
  
  
**BlastP hit with Mycgr3G103034\_Mycgr3**
  
Percentage identity: 56 %
  
BlastP bit score: 609
  
Sequence coverage: 98 %
  
E-value: 0.0
  
  
 NCBI BlastP on this gene

EGE00449

ATP-dependent rRNA helicase RRP3
  
Accession: EGE00450
  
Location: 22937-24441
  
  
**BlastP hit with Mycgr3G84402\_Mycgr3T**
  
Percentage identity: 71 %
  
BlastP bit score: 668
  
Sequence coverage: 100 %
  
E-value: 0.0
  
  
 NCBI BlastP on this gene

EGE00450

ribosome biogenesis protein Ssf2
  
Accession: EGE00451
  
Location: 24714-26119
  
  
**BlastP hit with Mycgr3G35447\_Mycgr3T**
  
Percentage identity: 55 %
  
BlastP bit score: 420
  
Sequence coverage: 91 %
  
E-value: 3e-140
  
  
 NCBI BlastP on this gene

EGE00451

riboflavin synthase subunit alpha
  
Accession: EGE00452
  
Location: 26424-27258
  
 NCBI BlastP on this gene

EGE00452

hypothetical protein
  
Accession: EGE00453
  
Location: 27573-29237
  
 NCBI BlastP on this gene

EGE00453

hypothetical protein
  
Accession: EGE00454
  
Location: 29843-31441
  
 NCBI BlastP on this gene

EGE00454

stomatin family protein
  
Accession: EGE00455
  
Location: 31893-33178
  
 NCBI BlastP on this gene

EGE00455

hypothetical protein
  
Accession: EGE00456
  
Location: 34948-35553
  
 NCBI BlastP on this gene

EGE00456

60S ribosomal protein L27-A
  
Accession: EGE00457
  
Location: 37353-38241
  
 NCBI BlastP on this gene

EGE00457

hypothetical protein
  
Accession: EGE00458
  
Location: 38917-46254
  
 NCBI BlastP on this gene

EGE00458

23. :  DS995745 Trichophyton equinum CBS 127.97 supercont1.28 genomic scaffold     Total score: 3.0     Cumulative Blast bit score: 1697

AGC/AKT protein kinase
  
Accession: EGE06138
  
Location: 44782-47721
  
 NCBI BlastP on this gene

EGE06138

hypothetical protein
  
Accession: EGE06137
  
Location: 39467-41212
  
 NCBI BlastP on this gene

EGE06137

26S protease regulatory subunit 6A
  
Accession: EGE06136
  
Location: 37405-38929
  
 NCBI BlastP on this gene

EGE06136

trimethyllysine dioxygenase
  
Accession: EGE06135
  
Location: 35035-36974
  
 NCBI BlastP on this gene

EGE06135

peroxisomal dehydratase
  
Accession: EGE06134
  
Location: 33296-34560
  
 NCBI BlastP on this gene

EGE06134

HNRNP arginine N-methyltransferase
  
Accession: EGE06133
  
Location: 31288-32651
  
 NCBI BlastP on this gene

EGE06133

hypothetical protein
  
Accession: EGE06132
  
Location: 30201-30906
  
 NCBI BlastP on this gene

EGE06132

PER1
  
Accession: EGE06131
  
Location: 28194-29427
  
 NCBI BlastP on this gene

EGE06131

phosphoacetylglucosamine mutase
  
Accession: EGE06130
  
Location: 25703-27706
  
  
**BlastP hit with Mycgr3G103034\_Mycgr3**
  
Percentage identity: 56 %
  
BlastP bit score: 609
  
Sequence coverage: 100 %
  
E-value: 0.0
  
  
 NCBI BlastP on this gene

EGE06130

ATP-dependent rRNA helicase RRP3
  
Accession: EGE06129
  
Location: 23644-25148
  
  
**BlastP hit with Mycgr3G84402\_Mycgr3T**
  
Percentage identity: 71 %
  
BlastP bit score: 668
  
Sequence coverage: 100 %
  
E-value: 0.0
  
  
 NCBI BlastP on this gene

EGE06129

ribosome biogenesis protein Ssf2
  
Accession: EGE06128
  
Location: 21966-23371
  
  
**BlastP hit with Mycgr3G35447\_Mycgr3T**
  
Percentage identity: 55 %
  
BlastP bit score: 420
  
Sequence coverage: 91 %
  
E-value: 3e-140
  
  
 NCBI BlastP on this gene

EGE06128

riboflavin synthase subunit alpha
  
Accession: EGE06127
  
Location: 20827-21661
  
 NCBI BlastP on this gene

EGE06127

formyl-coenzyme A transferase
  
Accession: EGE06126
  
Location: 18848-20512
  
 NCBI BlastP on this gene

EGE06126

hypothetical protein
  
Accession: EGE06125
  
Location: 16644-18242
  
 NCBI BlastP on this gene

EGE06125

stomatin family protein
  
Accession: EGE06124
  
Location: 14907-16192
  
 NCBI BlastP on this gene

EGE06124

CAP20
  
Accession: EGE06123
  
Location: 12533-13138
  
 NCBI BlastP on this gene

EGE06123

60S ribosomal protein L27-A
  
Accession: EGE06122
  
Location: 9844-10798
  
 NCBI BlastP on this gene

EGE06122

hypothetical protein
  
Accession: EGE06121
  
Location: 3955-9166
  
 NCBI BlastP on this gene

EGE06121

24. :  DS027059 Aspergillus clavatus NRRL 1 1099423829805 genomic scaffold     Total score: 3.0     Cumulative Blast bit score: 1695

conserved hypothetical protein
  
Accession: EAW08095
  
Location: 3073971-3075233
  
 NCBI BlastP on this gene

EAW08095

conserved predicted protein
  
Accession: EAW08096
  
Location: 3078225-3080650
  
 NCBI BlastP on this gene

EAW08096

proteasome regulatory particle subunit Rpt5, putative
  
Accession: EAW08097
  
Location: 3081418-3083035
  
 NCBI BlastP on this gene

EAW08097

trimethyllysine dioxygenase, putative
  
Accession: EAW08098
  
Location: 3083436-3084409
  
 NCBI BlastP on this gene

EAW08098

hypothetical protein
  
Accession: EAW08099
  
Location: 3085080-3085864
  
 NCBI BlastP on this gene

EAW08099

protein arginine n-methyltransferase 1
  
Accession: EAW08100
  
Location: 3086856-3088218
  
 NCBI BlastP on this gene

EAW08100

Mn2+ homeostasis protein (Per1), putative
  
Accession: EAW08101
  
Location: 3089042-3090234
  
 NCBI BlastP on this gene

EAW08101

N-acetylglucosamine-phosphate mutase
  
Accession: EAW08102
  
Location: 3090867-3092885
  
  
**BlastP hit with Mycgr3G103034\_Mycgr3**
  
Percentage identity: 55 %
  
BlastP bit score: 598
  
Sequence coverage: 98 %
  
E-value: 0.0
  
  
 NCBI BlastP on this gene

EAW08102

ATP-dependent RNA helicase , putative
  
Accession: EAW08103
  
Location: 3093335-3094820
  
  
**BlastP hit with Mycgr3G84402\_Mycgr3T**
  
Percentage identity: 74 %
  
BlastP bit score: 647
  
Sequence coverage: 92 %
  
E-value: 0.0
  
  
 NCBI BlastP on this gene

EAW08103

ribosome biogenesis protein Ssf2, putative
  
Accession: EAW08104
  
Location: 3095073-3096475
  
  
**BlastP hit with Mycgr3G35447\_Mycgr3T**
  
Percentage identity: 56 %
  
BlastP bit score: 450
  
Sequence coverage: 101 %
  
E-value: 6e-152
  
  
 NCBI BlastP on this gene

EAW08104

riboflavin synthase, alpha subunit
  
Accession: EAW08105
  
Location: 3096972-3097881
  
 NCBI BlastP on this gene

EAW08105

CAIB/BAIF family enzyme
  
Accession: EAW08106
  
Location: 3098169-3099832
  
 NCBI BlastP on this gene

EAW08106

PHD finger domain protein, putative
  
Accession: EAW08107
  
Location: 3100218-3102005
  
 NCBI BlastP on this gene

EAW08107

zinc metallopeptidase, putative
  
Accession: EAW08108
  
Location: 3102279-3103703
  
 NCBI BlastP on this gene

EAW08108

short-chain dehydrogenase/reductase family protein, putative
  
Accession: EAW08109
  
Location: 3104293-3105478
  
 NCBI BlastP on this gene

EAW08109

small nucleolar ribonucleoprotein complex subunit (SOF1), putative
  
Accession: EAW08110
  
Location: 3105644-3107101
  
 NCBI BlastP on this gene

EAW08110

proteasome regulatory particle subunit (RpnF), putative
  
Accession: EAW08111
  
Location: 3107460-3108793
  
 NCBI BlastP on this gene

EAW08111

SWI-SNF complex subunit (BAF60b), putative
  
Accession: EAW08112
  
Location: 3109240-3110902
  
 NCBI BlastP on this gene

EAW08112

DUF887 domain protein
  
Accession: EAW08113
  
Location: 3111349-3112596
  
 NCBI BlastP on this gene

EAW08113

conserved hypothetical protein
  
Accession: EAW08114
  
Location: 3113410-3119867
  
 NCBI BlastP on this gene

EAW08114

25. :  KB644410 Penicillium oxalicum 114-2 unplaced genomic scaffold scaffold\_3     Total score: 3.0     Cumulative Blast bit score: 1693

hypothetical protein
  
Accession: EPS27667
  
Location: 1587044-1588846
  
 NCBI BlastP on this gene

EPS27667

hypothetical protein
  
Accession: EPS27668
  
Location: 1589868-1591586
  
 NCBI BlastP on this gene

EPS27668

hypothetical protein
  
Accession: EPS27669
  
Location: 1592756-1594738
  
 NCBI BlastP on this gene

EPS27669

hypothetical protein
  
Accession: EPS27670
  
Location: 1595234-1595950
  
 NCBI BlastP on this gene

EPS27670

hypothetical protein
  
Accession: EPS27671
  
Location: 1596437-1597505
  
 NCBI BlastP on this gene

EPS27671

hypothetical protein
  
Accession: EPS27672
  
Location: 1598189-1599573
  
  
**BlastP hit with Mycgr3G35447\_Mycgr3T**
  
Percentage identity: 59 %
  
BlastP bit score: 462
  
Sequence coverage: 91 %
  
E-value: 2e-156
  
  
 NCBI BlastP on this gene

EPS27672

hypothetical protein
  
Accession: EPS27673
  
Location: 1599818-1601236
  
  
**BlastP hit with Mycgr3G84402\_Mycgr3T**
  
Percentage identity: 68 %
  
BlastP bit score: 634
  
Sequence coverage: 100 %
  
E-value: 0.0
  
  
 NCBI BlastP on this gene

EPS27673

hypothetical protein
  
Accession: EPS27674
  
Location: 1602056-1603645
  
 NCBI BlastP on this gene

EPS27674

hypothetical protein
  
Accession: EPS27675
  
Location: 1604080-1606165
  
 NCBI BlastP on this gene

EPS27675

hypothetical protein
  
Accession: EPS27676
  
Location: 1606579-1607757
  
 NCBI BlastP on this gene

EPS27676

hypothetical protein
  
Accession: EPS27677
  
Location: 1608362-1609814
  
 NCBI BlastP on this gene

EPS27677

hypothetical protein
  
Accession: EPS27678
  
Location: 1610525-1611034
  
 NCBI BlastP on this gene

EPS27678

hypothetical protein
  
Accession: EPS27679
  
Location: 1611801-1613004
  
 NCBI BlastP on this gene

EPS27679

hypothetical protein
  
Accession: EPS27680
  
Location: 1613488-1615521
  
  
**BlastP hit with Mycgr3G103034\_Mycgr3**
  
Percentage identity: 54 %
  
BlastP bit score: 597
  
Sequence coverage: 98 %
  
E-value: 0.0
  
  
 NCBI BlastP on this gene

EPS27680

hypothetical protein
  
Accession: EPS27681
  
Location: 1616256-1618202
  
 NCBI BlastP on this gene

EPS27681

hypothetical protein
  
Accession: EPS27682
  
Location: 1619064-1621476
  
 NCBI BlastP on this gene

EPS27682

hypothetical protein
  
Accession: EPS27683
  
Location: 1623124-1624257
  
 NCBI BlastP on this gene

EPS27683

hypothetical protein
  
Accession: EPS27684
  
Location: 1625643-1629058
  
 NCBI BlastP on this gene

EPS27684

hypothetical protein
  
Accession: EPS27685
  
Location: 1632974-1634882
  
 NCBI BlastP on this gene

EPS27685

26. :  DS995899 Penicillium marneffei ATCC 18224 scf\_1105668340764 genomic scaffold     Total score: 3.0     Cumulative Blast bit score: 1693

cAMP-dependent protein kinase-like, putative
  
Accession: EEA27110
  
Location: 1271784-1274853
  
 NCBI BlastP on this gene

EEA27110

hypothetical protein
  
Accession: EEA27109
  
Location: 1266695-1269339
  
 NCBI BlastP on this gene

EEA27109

proteasome regulatory particle subunit Rpt5, putative
  
Accession: EEA27108
  
Location: 1264465-1265975
  
 NCBI BlastP on this gene

EEA27108

hypothetical protein
  
Accession: EEA27107
  
Location: 1263498-1264084
  
 NCBI BlastP on this gene

EEA27107

trimethyllysine dioxygenase TmlH, putative
  
Accession: EEA27106
  
Location: 1261822-1263317
  
 NCBI BlastP on this gene

EEA27106

peroxisomal dehydratase, putative
  
Accession: EEA27104
  
Location: 1260509-1261648
  
 NCBI BlastP on this gene

EEA27104

hypothetical protein
  
Accession: EEA27105
  
Location: 1258480-1260004
  
 NCBI BlastP on this gene

EEA27105

histone H4 arginine methyltransferase RmtA
  
Accession: EEA27101
  
Location: 1256558-1257930
  
 NCBI BlastP on this gene

EEA27101

Mn2+ homeostasis protein (Per1), putative
  
Accession: EEA27103
  
Location: 1254788-1255958
  
 NCBI BlastP on this gene

EEA27103

N-acetylglucosamine-phosphate mutase
  
Accession: EEA27100
  
Location: 1252382-1254387
  
  
**BlastP hit with Mycgr3G103034\_Mycgr3**
  
Percentage identity: 56 %
  
BlastP bit score: 626
  
Sequence coverage: 99 %
  
E-value: 0.0
  
  
 NCBI BlastP on this gene

EEA27100

ATP-dependent RNA helicase , putative
  
Accession: EEA27099
  
Location: 1250658-1252133
  
  
**BlastP hit with Mycgr3G84402\_Mycgr3T**
  
Percentage identity: 66 %
  
BlastP bit score: 613
  
Sequence coverage: 99 %
  
E-value: 0.0
  
  
 NCBI BlastP on this gene

EEA27099

ribosome biogenesis protein Ssf2, putative
  
Accession: EEA27098
  
Location: 1249051-1250439
  
  
**BlastP hit with Mycgr3G35447\_Mycgr3T**
  
Percentage identity: 60 %
  
BlastP bit score: 454
  
Sequence coverage: 91 %
  
E-value: 2e-153
  
  
 NCBI BlastP on this gene

EEA27098

riboflavin synthase, alpha subunit
  
Accession: EEA27097
  
Location: 1248124-1248949
  
 NCBI BlastP on this gene

EEA27097

CAIB/BAIF family enzyme
  
Accession: EEA27096
  
Location: 1246303-1247926
  
 NCBI BlastP on this gene

EEA27096

PHD finger domain protein, putative
  
Accession: EEA27095
  
Location: 1244326-1246008
  
 NCBI BlastP on this gene

EEA27095

short-chain dehydrogenase/reductase family protein, putative
  
Accession: EEA27094
  
Location: 1241280-1242465
  
 NCBI BlastP on this gene

EEA27094

small nucleolar ribonucleoprotein complex subunit (SOF1), putative
  
Accession: EEA27093
  
Location: 1239732-1241188
  
 NCBI BlastP on this gene

EEA27093

proteasome regulatory particle subunit (RpnF), putative
  
Accession: EEA27092
  
Location: 1238078-1239398
  
 NCBI BlastP on this gene

EEA27092

SWI-SNF complex subunit (BAF60b), putative
  
Accession: EEA27091
  
Location: 1236024-1237702
  
 NCBI BlastP on this gene

EEA27091

DUF887 domain protein
  
Accession: EEA27090
  
Location: 1234464-1235700
  
 NCBI BlastP on this gene

EEA27090

27. :  AAHF01000007 Aspergillus fumigatus Af293     Total score: 3.0     Cumulative Blast bit score: 1693

extracellular exo-polygalacturonase, putative
  
Accession: EAL88325
  
Location: 1772681-1774230
  
 NCBI BlastP on this gene

EAL88325

L-serine dehydratase, putative
  
Accession: EAL88326
  
Location: 1774355-1775854
  
 NCBI BlastP on this gene

EAL88326

conserved predicted protein
  
Accession: EAL88327
  
Location: 1779425-1781824
  
 NCBI BlastP on this gene

EAL88327

proteasome regulatory particle subunit Rpt5, putative
  
Accession: EAL88328
  
Location: 1782664-1784284
  
 NCBI BlastP on this gene

EAL88328

trimethyllysine dioxygenase TmlH, putative
  
Accession: EAL88329
  
Location: 1784683-1786054
  
 NCBI BlastP on this gene

EAL88329

histone H4 arginine methyltransferase RmtA
  
Accession: EAL88330
  
Location: 1787954-1789312
  
 NCBI BlastP on this gene

EAL88330

Mn2+ homeostasis protein (Per1), putative
  
Accession: EAL88331
  
Location: 1789974-1791155
  
 NCBI BlastP on this gene

EAL88331

N-acetylglucosamine-phosphate mutase
  
Accession: EAL88332
  
Location: 1791710-1793700
  
  
**BlastP hit with Mycgr3G103034\_Mycgr3**
  
Percentage identity: 54 %
  
BlastP bit score: 604
  
Sequence coverage: 101 %
  
E-value: 0.0
  
  
 NCBI BlastP on this gene

EAL88332

ATP-dependent RNA helicase , putative
  
Accession: EAL88333
  
Location: 1794114-1795628
  
  
**BlastP hit with Mycgr3G84402\_Mycgr3T**
  
Percentage identity: 73 %
  
BlastP bit score: 645
  
Sequence coverage: 92 %
  
E-value: 0.0
  
  
 NCBI BlastP on this gene

EAL88333

ribosome biogenesis protein Ssf2, putative
  
Accession: EAL88334
  
Location: 1795883-1797285
  
  
**BlastP hit with Mycgr3G35447\_Mycgr3T**
  
Percentage identity: 59 %
  
BlastP bit score: 444
  
Sequence coverage: 86 %
  
E-value: 2e-149
  
  
 NCBI BlastP on this gene

EAL88334

riboflavin synthase, alpha subunit
  
Accession: EAL88335
  
Location: 1797872-1798799
  
 NCBI BlastP on this gene

EAL88335

CAIB/BAIF family enzyme
  
Accession: EAL88336
  
Location: 1799124-1800748
  
 NCBI BlastP on this gene

EAL88336

PHD finger domain protein, putative
  
Accession: EAL88337
  
Location: 1801212-1803077
  
 NCBI BlastP on this gene

EAL88337

zinc metallopeptidase, putative
  
Accession: EAL88338
  
Location: 1803331-1804530
  
 NCBI BlastP on this gene

EAL88338

short-chain dehydrogenase/reductase family protein, putative
  
Accession: EAL88339
  
Location: 1805046-1806529
  
 NCBI BlastP on this gene

EAL88339

small nucleolar ribonucleoprotein complex subunit (SOF1), putative
  
Accession: EAL88340
  
Location: 1806712-1808142
  
 NCBI BlastP on this gene

EAL88340

proteasome regulatory particle subunit (RpnF), putative
  
Accession: EAL88341
  
Location: 1808206-1809878
  
 NCBI BlastP on this gene

EAL88341

SWI-SNF complex subunit (BAF60b), putative
  
Accession: EAL88342
  
Location: 1810286-1811945
  
 NCBI BlastP on this gene

EAL88342

DUF887 domain protein
  
Accession: EAL88343
  
Location: 1812377-1813634
  
 NCBI BlastP on this gene

EAL88343

conserved hypothetical protein
  
Accession: EAL88344
  
Location: 1814478-1820660
  
 NCBI BlastP on this gene

EAL88344

28. :  AKCU01000442 Penicillium digitatum Pd1     Total score: 3.0     Cumulative Blast bit score: 1692

hypothetical protein
  
Accession: EKV08241
  
Location: 24966-25838
  
 NCBI BlastP on this gene

EKV08241

hypothetical protein
  
Accession: EKV08240
  
Location: 19808-21292
  
 NCBI BlastP on this gene

EKV08240

Glutaminyl-tRNA synthetase
  
Accession: EKV08239
  
Location: 17253-19196
  
 NCBI BlastP on this gene

EKV08239

Proteasome regulatory particle subunit Rpt5, putative
  
Accession: EKV08238
  
Location: 15100-16677
  
 NCBI BlastP on this gene

EKV08238

Trimethyllysine dioxygenase TmlH, putative
  
Accession: EKV08237
  
Location: 13886-14828
  
 NCBI BlastP on this gene

EKV08237

Peroxisomal dehydratase
  
Accession: EKV08236
  
Location: 12191-12409
  
 NCBI BlastP on this gene

EKV08236

Peroxisomal dehydratase, putative
  
Accession: EKV08235
  
Location: 11350-11926
  
 NCBI BlastP on this gene

EKV08235

Histone H4 arginine methyltransferase RmtA
  
Accession: EKV08234
  
Location: 9377-10733
  
 NCBI BlastP on this gene

EKV08234

hypothetical protein
  
Accession: EKV08233
  
Location: 8496-8720
  
 NCBI BlastP on this gene

EKV08233

Mn2+ homeostasis protein (Per1), putative
  
Accession: EKV08232
  
Location: 7629-8348
  
 NCBI BlastP on this gene

EKV08232

N-acetylglucosamine-phosphate mutase
  
Accession: EKV08231
  
Location: 5167-7154
  
  
**BlastP hit with Mycgr3G103034\_Mycgr3**
  
Percentage identity: 54 %
  
BlastP bit score: 598
  
Sequence coverage: 98 %
  
E-value: 0.0
  
  
 NCBI BlastP on this gene

EKV08231

ATP-dependent RNA helicase , putative
  
Accession: EKV08230
  
Location: 3342-4879
  
  
**BlastP hit with Mycgr3G84402\_Mycgr3T**
  
Percentage identity: 70 %
  
BlastP bit score: 606
  
Sequence coverage: 91 %
  
E-value: 0.0
  
  
 NCBI BlastP on this gene

EKV08230

hypothetical protein
  
Accession: EKV08229
  
Location: 1744-3140
  
  
**BlastP hit with Mycgr3G35447\_Mycgr3T**
  
Percentage identity: 60 %
  
BlastP bit score: 488
  
Sequence coverage: 97 %
  
E-value: 1e-166
  
  
 NCBI BlastP on this gene

EKV08229

29. :  AKCT01000236 Penicillium digitatum PHI26     Total score: 3.0     Cumulative Blast bit score: 1692

hypothetical protein
  
Accession: EKV09850
  
Location: 20025-21509
  
 NCBI BlastP on this gene

EKV09850

Glutaminyl-tRNA synthetase
  
Accession: EKV09849
  
Location: 17469-19412
  
 NCBI BlastP on this gene

EKV09849

Proteasome regulatory particle subunit Rpt5, putative
  
Accession: EKV09848
  
Location: 15316-16893
  
 NCBI BlastP on this gene

EKV09848

Trimethyllysine dioxygenase TmlH, putative
  
Accession: EKV09847
  
Location: 14102-15044
  
 NCBI BlastP on this gene

EKV09847

Peroxisomal dehydratase
  
Accession: EKV09846
  
Location: 12407-12625
  
 NCBI BlastP on this gene

EKV09846

Peroxisomal dehydratase, putative
  
Accession: EKV09845
  
Location: 11566-12142
  
 NCBI BlastP on this gene

EKV09845

Histone H4 arginine methyltransferase RmtA
  
Accession: EKV09844
  
Location: 9593-10949
  
 NCBI BlastP on this gene

EKV09844

hypothetical protein
  
Accession: EKV09843
  
Location: 8711-8935
  
 NCBI BlastP on this gene

EKV09843

Mn2+ homeostasis protein (Per1), putative
  
Accession: EKV09842
  
Location: 7844-8563
  
 NCBI BlastP on this gene

EKV09842

N-acetylglucosamine-phosphate mutase
  
Accession: EKV09841
  
Location: 5382-7369
  
  
**BlastP hit with Mycgr3G103034\_Mycgr3**
  
Percentage identity: 54 %
  
BlastP bit score: 598
  
Sequence coverage: 98 %
  
E-value: 0.0
  
  
 NCBI BlastP on this gene

EKV09841

ATP-dependent RNA helicase , putative
  
Accession: EKV09840
  
Location: 3557-5094
  
  
**BlastP hit with Mycgr3G84402\_Mycgr3T**
  
Percentage identity: 70 %
  
BlastP bit score: 606
  
Sequence coverage: 91 %
  
E-value: 0.0
  
  
 NCBI BlastP on this gene

EKV09840

hypothetical protein
  
Accession: EKV09839
  
Location: 1958-3354
  
  
**BlastP hit with Mycgr3G35447\_Mycgr3T**
  
Percentage identity: 60 %
  
BlastP bit score: 488
  
Sequence coverage: 97 %
  
E-value: 1e-166
  
  
 NCBI BlastP on this gene

EKV09839

30. :  CH476655 Ajellomyces capsulatus NAm1 scaffold\_1 genomic scaffold     Total score: 3.0     Cumulative Blast bit score: 1688

serine/threonine-protein kinase sck1
  
Accession: EDN02207
  
Location: 221550-224564
  
 NCBI BlastP on this gene

EDN02207

predicted protein
  
Accession: EDN02206
  
Location: 215269-217786
  
 NCBI BlastP on this gene

EDN02206

26S protease regulatory subunit 6A
  
Accession: EDN02205
  
Location: 212766-214344
  
 NCBI BlastP on this gene

EDN02205

conserved hypothetical protein
  
Accession: EDN02204
  
Location: 210276-212353
  
 NCBI BlastP on this gene

EDN02204

peroxisomal dehydratase
  
Accession: EDN02203
  
Location: 207577-208930
  
 NCBI BlastP on this gene

EDN02203

HNRNP arginine N-methyltransferase
  
Accession: EDN02202
  
Location: 205462-207095
  
 NCBI BlastP on this gene

EDN02202

conserved hypothetical protein
  
Accession: EDN02201
  
Location: 203391-204961
  
 NCBI BlastP on this gene

EDN02201

hypothetical protein
  
Accession: EDN02200
  
Location: 200818-202890
  
  
**BlastP hit with Mycgr3G103034\_Mycgr3**
  
Percentage identity: 57 %
  
BlastP bit score: 635
  
Sequence coverage: 98 %
  
E-value: 0.0
  
  
 NCBI BlastP on this gene

EDN02200

ATP-dependent rRNA helicase RRP3
  
Accession: EDN02199
  
Location: 198577-200130
  
  
**BlastP hit with Mycgr3G84402\_Mycgr3T**
  
Percentage identity: 74 %
  
BlastP bit score: 660
  
Sequence coverage: 96 %
  
E-value: 0.0
  
  
 NCBI BlastP on this gene

EDN02199

conserved hypothetical protein
  
Accession: EDN02198
  
Location: 196979-198204
  
  
**BlastP hit with Mycgr3G35447\_Mycgr3T**
  
Percentage identity: 53 %
  
BlastP bit score: 394
  
Sequence coverage: 88 %
  
E-value: 1e-130
  
  
 NCBI BlastP on this gene

EDN02198

riboflavin synthase alpha chain
  
Accession: EDN02197
  
Location: 195808-196655
  
 NCBI BlastP on this gene

EDN02197

hypothetical protein
  
Accession: EDN02196
  
Location: 193744-195540
  
 NCBI BlastP on this gene

EDN02196

conserved hypothetical protein
  
Accession: EDN02195
  
Location: 191047-192833
  
 NCBI BlastP on this gene

EDN02195

predicted protein
  
Accession: EDN02194
  
Location: 188813-190324
  
 NCBI BlastP on this gene

EDN02194

conserved hypothetical protein
  
Accession: EDN02193
  
Location: 186677-187901
  
 NCBI BlastP on this gene

EDN02193

predicted protein
  
Accession: EDN02192
  
Location: 179592-185725
  
 NCBI BlastP on this gene

EDN02192

60S ribosomal protein L27-A
  
Accession: EDN02191
  
Location: 178071-178891
  
 NCBI BlastP on this gene

EDN02191

31. :  GG657460 Ajellomyces dermatitidis SLH14081 genomic scaffold supercont1.13     Total score: 3.0     Cumulative Blast bit score: 1686

predicted protein
  
Accession: EEQ71142
  
Location: 719994-720804
  
 NCBI BlastP on this gene

EEQ71142

conserved hypothetical protein
  
Accession: EEQ71141
  
Location: 716279-718802
  
 NCBI BlastP on this gene

EEQ71141

26S protease regulatory subunit 6A
  
Accession: EEQ71140
  
Location: 713865-715442
  
 NCBI BlastP on this gene

EEQ71140

trimethyllysine dioxygenase TmlH
  
Accession: EEQ71139
  
Location: 711194-713464
  
 NCBI BlastP on this gene

EEQ71139

peroxisomal dehydratase
  
Accession: EEQ71138
  
Location: 707653-709039
  
 NCBI BlastP on this gene

EEQ71138

HNRNP arginine N-methyltransferase
  
Accession: EEQ71137
  
Location: 705273-706894
  
 NCBI BlastP on this gene

EEQ71137

Mn2+ homeostasis protein
  
Accession: EEQ71136
  
Location: 703348-704584
  
 NCBI BlastP on this gene

EEQ71136

N-acetylglucosamine-phosphate mutase
  
Accession: EEQ71135
  
Location: 700775-702850
  
  
**BlastP hit with Mycgr3G103034\_Mycgr3**
  
Percentage identity: 56 %
  
BlastP bit score: 628
  
Sequence coverage: 98 %
  
E-value: 0.0
  
  
 NCBI BlastP on this gene

EEQ71135

ATP-dependent rRNA helicase RRP3
  
Accession: EEQ71134
  
Location: 698509-700042
  
  
**BlastP hit with Mycgr3G84402\_Mycgr3T**
  
Percentage identity: 72 %
  
BlastP bit score: 577
  
Sequence coverage: 91 %
  
E-value: 0.0
  
  
 NCBI BlastP on this gene

EEQ71134

ribosome biogenesis protein Ssf2
  
Accession: EEQ71133
  
Location: 696867-698280
  
  
**BlastP hit with Mycgr3G35447\_Mycgr3T**
  
Percentage identity: 58 %
  
BlastP bit score: 481
  
Sequence coverage: 94 %
  
E-value: 3e-164
  
  
 NCBI BlastP on this gene

EEQ71133

riboflavin synthase subunit alpha
  
Accession: EEQ71132
  
Location: 695632-696484
  
 NCBI BlastP on this gene

EEQ71132

CAIB/BAIF family enzyme
  
Accession: EEQ71131
  
Location: 693478-695351
  
 NCBI BlastP on this gene

EEQ71131

32. :  DS027688 Neosartorya fischeri NRRL 181 1099437636249 genomic scaffold     Total score: 3.0     Cumulative Blast bit score: 1680

L-serine dehydratase, putative
  
Accession: EAW23157
  
Location: 3228231-3229422
  
 NCBI BlastP on this gene

EAW23157

hypothetical protein
  
Accession: EAW23156
  
Location: 3226403-3226682
  
 NCBI BlastP on this gene

EAW23156

hypothetical protein
  
Accession: EAW23155
  
Location: 3224932-3225746
  
 NCBI BlastP on this gene

EAW23155

conserved predicted protein
  
Accession: EAW23154
  
Location: 3219527-3221925
  
 NCBI BlastP on this gene

EAW23154

proteasome regulatory particle subunit Rpt5, putative
  
Accession: EAW23153
  
Location: 3217077-3218693
  
 NCBI BlastP on this gene

EAW23153

trimethyllysine dioxygenase, putative
  
Accession: EAW23152
  
Location: 3214987-3216681
  
 NCBI BlastP on this gene

EAW23152

protein arginine n-methyltransferase 1
  
Accession: EAW23151
  
Location: 3212057-3213417
  
 NCBI BlastP on this gene

EAW23151

Mn2+ homeostasis protein (Per1), putative
  
Accession: EAW23150
  
Location: 3210205-3211384
  
 NCBI BlastP on this gene

EAW23150

N-acetylglucosamine-phosphate mutase
  
Accession: EAW23149
  
Location: 3207682-3209672
  
  
**BlastP hit with Mycgr3G103034\_Mycgr3**
  
Percentage identity: 55 %
  
BlastP bit score: 603
  
Sequence coverage: 98 %
  
E-value: 0.0
  
  
 NCBI BlastP on this gene

EAW23149

ATP-dependent RNA helicase , putative
  
Accession: EAW23148
  
Location: 3205754-3207267
  
  
**BlastP hit with Mycgr3G84402\_Mycgr3T**
  
Percentage identity: 73 %
  
BlastP bit score: 647
  
Sequence coverage: 92 %
  
E-value: 0.0
  
  
 NCBI BlastP on this gene

EAW23148

ribosome biogenesis protein Ssf2, putative
  
Accession: EAW23147
  
Location: 3204095-3205497
  
  
**BlastP hit with Mycgr3G35447\_Mycgr3T**
  
Percentage identity: 58 %
  
BlastP bit score: 431
  
Sequence coverage: 86 %
  
E-value: 2e-144
  
  
 NCBI BlastP on this gene

EAW23147

riboflavin synthase, alpha subunit
  
Accession: EAW23146
  
Location: 3202579-3203506
  
 NCBI BlastP on this gene

EAW23146

CAIB/BAIF family enzyme
  
Accession: EAW23145
  
Location: 3200633-3202294
  
 NCBI BlastP on this gene

EAW23145

PHD finger domain protein, putative
  
Accession: EAW23144
  
Location: 3198397-3200184
  
 NCBI BlastP on this gene

EAW23144

zinc metallopeptidase, putative
  
Accession: EAW23143
  
Location: 3196718-3198145
  
 NCBI BlastP on this gene

EAW23143

short-chain dehydrogenase/reductase family protein, putative
  
Accession: EAW23142
  
Location: 3194940-3196168
  
 NCBI BlastP on this gene

EAW23142

small nucleolar ribonucleoprotein complex subunit (SOF1), putative
  
Accession: EAW23141
  
Location: 3193327-3194758
  
 NCBI BlastP on this gene

EAW23141

proteasome regulatory particle subunit (RpnF), putative
  
Accession: EAW23140
  
Location: 3191598-3192937
  
 NCBI BlastP on this gene

EAW23140

SWI-SNF complex subunit (BAF60b), putative
  
Accession: EAW23139
  
Location: 3189513-3191179
  
 NCBI BlastP on this gene

EAW23139

conserved hypothetical protein
  
Accession: EAW23138
  
Location: 3187816-3189074
  
 NCBI BlastP on this gene

EAW23138

33. :  AP007154 Aspergillus oryzae RIB40 DNA, SC001.     Total score: 3.0     Cumulative Blast bit score: 1680

not annotated
  
Accession: BAE56956
  
Location: 1096280-1098142
  
 NCBI BlastP on this gene

AO090001000437

not annotated
  
Accession: BAE56955
  
Location: 1094490-1095684
  
 NCBI BlastP on this gene

AO090001000436

not annotated
  
Accession: BAE56954
  
Location: 1093385-1093843
  
 NCBI BlastP on this gene

AO090001000435

not annotated
  
Accession: BAE56953
  
Location: 1089758-1091367
  
 NCBI BlastP on this gene

AO090001000434

not annotated
  
Accession: BAE56952
  
Location: 1088290-1089343
  
 NCBI BlastP on this gene

AO090001000433

not annotated
  
Accession: BAE56951
  
Location: 1083567-1084736
  
 NCBI BlastP on this gene

AO090001000431

not annotated
  
Accession: BAE56950
  
Location: 1079558-1082802
  
 NCBI BlastP on this gene

AO090001000430

not annotated
  
Accession: BAE56949
  
Location: 1076931-1078941
  
  
**BlastP hit with Mycgr3G103034\_Mycgr3**
  
Percentage identity: 54 %
  
BlastP bit score: 596
  
Sequence coverage: 98 %
  
E-value: 0.0
  
  
 NCBI BlastP on this gene

AO090001000429

not annotated
  
Accession: BAE56948
  
Location: 1074890-1076482
  
  
**BlastP hit with Mycgr3G84402\_Mycgr3T**
  
Percentage identity: 68 %
  
BlastP bit score: 637
  
Sequence coverage: 100 %
  
E-value: 0.0
  
  
 NCBI BlastP on this gene

AO090001000428

not annotated
  
Accession: BAE56947
  
Location: 1073220-1074636
  
  
**BlastP hit with Mycgr3G35447\_Mycgr3T**
  
Percentage identity: 60 %
  
BlastP bit score: 447
  
Sequence coverage: 87 %
  
E-value: 2e-150
  
  
 NCBI BlastP on this gene

AO090001000427

not annotated
  
Accession: BAE56946
  
Location: 1071908-1072790
  
 NCBI BlastP on this gene

AO090001000426

not annotated
  
Accession: BAE56945
  
Location: 1069718-1071375
  
 NCBI BlastP on this gene

AO090001000425

not annotated
  
Accession: BAE56944
  
Location: 1067533-1069320
  
 NCBI BlastP on this gene

AO090001000424

not annotated
  
Accession: BAE56943
  
Location: 1065533-1067310
  
 NCBI BlastP on this gene

AO090001000423

not annotated
  
Accession: BAE56942
  
Location: 1063181-1065463
  
 NCBI BlastP on this gene

AO090001000422

not annotated
  
Accession: BAE56941
  
Location: 1061749-1062510
  
 NCBI BlastP on this gene

AO090001000421

not annotated
  
Accession: BAE56940
  
Location: 1060347-1061522
  
 NCBI BlastP on this gene

AO090001000420

not annotated
  
Accession: BAE56939
  
Location: 1058199-1059486
  
 NCBI BlastP on this gene

AO090001000419

not annotated
  
Accession: BAE56938
  
Location: 1056618-1058071
  
 NCBI BlastP on this gene

AO090001000418

not annotated
  
Accession: BAE56937
  
Location: 1054954-1056286
  
 NCBI BlastP on this gene

AO090001000417

34. :  DS995701 Microsporum canis CBS 113480 supercont1.1 genomic scaffold     Total score: 3.0     Cumulative Blast bit score: 1679

serine/threonine-protein kinase sck1
  
Accession: EEQ27824
  
Location: 1928641-1931675
  
 NCBI BlastP on this gene

EEQ27824

conserved hypothetical protein
  
Accession: EEQ27823
  
Location: 1924111-1926627
  
 NCBI BlastP on this gene

EEQ27823

26S protease regulatory subunit 6A
  
Accession: EEQ27822
  
Location: 1922028-1923581
  
 NCBI BlastP on this gene

EEQ27822

trimethyllysine dioxygenase
  
Accession: EEQ27821
  
Location: 1919673-1921642
  
 NCBI BlastP on this gene

EEQ27821

peroxisomal dehydratase
  
Accession: EEQ27820
  
Location: 1918064-1919238
  
 NCBI BlastP on this gene

EEQ27820

histone H4 arginine methyltransferase RmtA
  
Accession: EEQ27819
  
Location: 1916093-1917442
  
 NCBI BlastP on this gene

EEQ27819

conserved hypothetical protein
  
Accession: EEQ27818
  
Location: 1911058-1912271
  
 NCBI BlastP on this gene

EEQ27818

N-acetylglucosamine-phosphate mutase
  
Accession: EEQ27817
  
Location: 1908684-1910658
  
  
**BlastP hit with Mycgr3G103034\_Mycgr3**
  
Percentage identity: 53 %
  
BlastP bit score: 583
  
Sequence coverage: 102 %
  
E-value: 0.0
  
  
 NCBI BlastP on this gene

EEQ27817

ATP-dependent rRNA helicase RRP3
  
Accession: EEQ27816
  
Location: 1906600-1908105
  
  
**BlastP hit with Mycgr3G84402\_Mycgr3T**
  
Percentage identity: 75 %
  
BlastP bit score: 656
  
Sequence coverage: 91 %
  
E-value: 0.0
  
  
 NCBI BlastP on this gene

EEQ27816

ribosome biogenesis protein SSF1
  
Accession: EEQ27815
  
Location: 1904945-1906345
  
  
**BlastP hit with Mycgr3G35447\_Mycgr3T**
  
Percentage identity: 56 %
  
BlastP bit score: 440
  
Sequence coverage: 92 %
  
E-value: 5e-148
  
  
 NCBI BlastP on this gene

EEQ27815

riboflavin synthase alpha chain
  
Accession: EEQ27814
  
Location: 1903839-1904668
  
 NCBI BlastP on this gene

EEQ27814

formyl-coenzyme A transferase
  
Accession: EEQ27813
  
Location: 1901907-1903570
  
 NCBI BlastP on this gene

EEQ27813

conserved hypothetical protein
  
Accession: EEQ27812
  
Location: 1899760-1901379
  
 NCBI BlastP on this gene

EEQ27812

stomatin family protein
  
Accession: EEQ27811
  
Location: 1898023-1899304
  
 NCBI BlastP on this gene

EEQ27811

CAP20
  
Accession: EEQ27810
  
Location: 1895795-1896391
  
 NCBI BlastP on this gene

EEQ27810

60S ribosomal protein L27-A
  
Accession: EEQ27809
  
Location: 1893228-1894120
  
 NCBI BlastP on this gene

EEQ27809

conserved hypothetical protein
  
Accession: EEQ27808
  
Location: 1885400-1892590
  
 NCBI BlastP on this gene

EEQ27808

35. :  AM920428 Penicillium chrysogenum Wisconsin 54-1255 complete genome, contig Pc00c13.     Total score: 3.0     Cumulative Blast bit score: 1675

hypothetical protein
  
Accession: CAP91334
  
Location: 614937-615948
  
 NCBI BlastP on this gene

Pc13g02650

not annotated
  
Accession: CAP91335
  
Location: 616696-617133
  
 NCBI BlastP on this gene

Pc13g02660

not annotated
  
Accession: CAP91336
  
Location: 618980-621352
  
 NCBI BlastP on this gene

Pc13g02670

not annotated
  
Accession: CAP91337
  
Location: 622004-623950
  
 NCBI BlastP on this gene

Pc13g02680

not annotated
  
Accession: CAP91338
  
Location: 624530-626107
  
 NCBI BlastP on this gene

Pc13g02690

not annotated
  
Accession: CAP91339
  
Location: 626394-628120
  
 NCBI BlastP on this gene

Pc13g02700

not annotated
  
Accession: CAP91340
  
Location: 628667-629828
  
 NCBI BlastP on this gene

Pc13g02710

not annotated
  
Accession: CAP91341
  
Location: 630476-631831
  
 NCBI BlastP on this gene

Pc13g02720

unnamed
  
Accession: CAP91342
  
Location: 632518-633683
  
 NCBI BlastP on this gene

Pc13g02730

not annotated
  
Accession: CAP91343
  
Location: 634098-636079
  
  
**BlastP hit with Mycgr3G103034\_Mycgr3**
  
Percentage identity: 54 %
  
BlastP bit score: 597
  
Sequence coverage: 98 %
  
E-value: 0.0
  
  
 NCBI BlastP on this gene

Pc13g02740

not annotated
  
Accession: CAP91344
  
Location: 636362-637899
  
  
**BlastP hit with Mycgr3G84402\_Mycgr3T**
  
Percentage identity: 71 %
  
BlastP bit score: 599
  
Sequence coverage: 88 %
  
E-value: 0.0
  
  
 NCBI BlastP on this gene

Pc13g02750

not annotated
  
Accession: CAP91345
  
Location: 638108-639501
  
  
**BlastP hit with Mycgr3G35447\_Mycgr3T**
  
Percentage identity: 60 %
  
BlastP bit score: 479
  
Sequence coverage: 95 %
  
E-value: 3e-163
  
  
 NCBI BlastP on this gene

Pc13g02760

not annotated
  
Accession: CAP91346
  
Location: 640039-640846
  
 NCBI BlastP on this gene

Pc13g02770

not annotated
  
Accession: CAP91347
  
Location: 641042-642690
  
 NCBI BlastP on this gene

Pc13g02780

not annotated
  
Accession: CAP91348
  
Location: 643313-644860
  
 NCBI BlastP on this gene

Pc13g02790

hypothetical protein
  
Accession: CAP91349
  
Location: 645084-646202
  
 NCBI BlastP on this gene

Pc13g02800

not annotated
  
Accession: CAP91350
  
Location: 646315-648881
  
 NCBI BlastP on this gene

Pc13g02810

not annotated
  
Accession: CAP91351
  
Location: 650582-651856
  
 NCBI BlastP on this gene

Pc13g02820

not annotated
  
Accession: CAP91352
  
Location: 652057-653457
  
 NCBI BlastP on this gene

Pc13g02830

not annotated
  
Accession: CAP91353
  
Location: 653681-655003
  
 NCBI BlastP on this gene

Pc13g02840

not annotated
  
Accession: CAP91354
  
Location: 655342-656939
  
 NCBI BlastP on this gene

Pc13g02850

not annotated
  
Accession: CAP91355
  
Location: 657372-658690
  
 NCBI BlastP on this gene

Pc13g02860

not annotated
  
Accession: Pc13g02870
  
Location: 659500-661019
  
 NCBI BlastP on this gene

Pc13g02870

36. :  EQ963481 Aspergillus flavus NRRL3357 scf\_1106286419476 genomic scaffold     Total score: 3.0     Cumulative Blast bit score: 1670

conserved predicted protein
  
Accession: EED48519
  
Location: 1117746-1119608
  
 NCBI BlastP on this gene

EED48519

conserved hypothetical protein
  
Accession: EED48518
  
Location: 1115956-1116300
  
 NCBI BlastP on this gene

EED48518

conserved hypothetical protein
  
Accession: EED48517
  
Location: 1114850-1115308
  
 NCBI BlastP on this gene

EED48517

proteasome regulatory particle subunit Rpt5, putative
  
Accession: EED48516
  
Location: 1111114-1112723
  
 NCBI BlastP on this gene

EED48516

trimethyllysine dioxygenase TmlH, putative
  
Accession: EED48515
  
Location: 1108759-1110682
  
 NCBI BlastP on this gene

EED48515

histone H4 arginine methyltransferase RmtA
  
Accession: EED48514
  
Location: 1102867-1104283
  
 NCBI BlastP on this gene

EED48514

Mn2+ homeostasis protein (Per1), putative
  
Accession: EED48513
  
Location: 1100874-1102061
  
 NCBI BlastP on this gene

EED48513

N-acetylglucosamine-phosphate mutase
  
Accession: EED48512
  
Location: 1098410-1100420
  
  
**BlastP hit with Mycgr3G103034\_Mycgr3**
  
Percentage identity: 56 %
  
BlastP bit score: 587
  
Sequence coverage: 91 %
  
E-value: 0.0
  
  
 NCBI BlastP on this gene

EED48512

ATP-dependent RNA helicase , putative
  
Accession: EED48511
  
Location: 1096369-1097961
  
  
**BlastP hit with Mycgr3G84402\_Mycgr3T**
  
Percentage identity: 68 %
  
BlastP bit score: 636
  
Sequence coverage: 100 %
  
E-value: 0.0
  
  
 NCBI BlastP on this gene

EED48511

ribosome biogenesis protein Ssf2, putative
  
Accession: EED48510
  
Location: 1094699-1096115
  
  
**BlastP hit with Mycgr3G35447\_Mycgr3T**
  
Percentage identity: 60 %
  
BlastP bit score: 447
  
Sequence coverage: 87 %
  
E-value: 2e-150
  
  
 NCBI BlastP on this gene

EED48510

riboflavin synthase, alpha subunit
  
Accession: EED48509
  
Location: 1093386-1094269
  
 NCBI BlastP on this gene

EED48509

CAIB/BAIF family enzyme
  
Accession: EED48508
  
Location: 1091196-1092853
  
 NCBI BlastP on this gene

EED48508

PHD finger domain protein, putative
  
Accession: EED48507
  
Location: 1089011-1090771
  
 NCBI BlastP on this gene

EED48507

zinc metallopeptidase, putative
  
Accession: EED48506
  
Location: 1087209-1088678
  
 NCBI BlastP on this gene

EED48506

conserved hypothetical protein
  
Accession: EED48505
  
Location: 1085850-1086929
  
 NCBI BlastP on this gene

EED48505

conserved hypothetical protein
  
Accession: EED48504
  
Location: 1083223-1083984
  
 NCBI BlastP on this gene

EED48504

cop9 signalosome complex subunit, putative
  
Accession: EED48503
  
Location: 1081429-1082996
  
 NCBI BlastP on this gene

EED48503

SWI-SNF complex subunit (BAF60b), putative
  
Accession: EED48502
  
Location: 1079681-1081079
  
 NCBI BlastP on this gene

EED48502

DUF887 domain protein
  
Accession: EED48501
  
Location: 1078330-1079049
  
 NCBI BlastP on this gene

EED48501

37. :  AACD01000068 Aspergillus nidulans FGSC A4     Total score: 3.0     Cumulative Blast bit score: 1668

hypothetical protein
  
Accession: EAA59337
  
Location: 219164-222390
  
 NCBI BlastP on this gene

EAA59337

hypothetical protein
  
Accession: EAA59336
  
Location: 213922-216341
  
 NCBI BlastP on this gene

EAA59336

conserved hypothetical protein
  
Accession: EAA59335
  
Location: 210862-212441
  
 NCBI BlastP on this gene

EAA59335

hypothetical protein
  
Accession: EAA59334
  
Location: 204272-205445
  
 NCBI BlastP on this gene

EAA59334

hypothetical protein
  
Accession: EAA59333
  
Location: 201764-203760
  
  
**BlastP hit with Mycgr3G103034\_Mycgr3**
  
Percentage identity: 54 %
  
BlastP bit score: 595
  
Sequence coverage: 98 %
  
E-value: 0.0
  
  
 NCBI BlastP on this gene

EAA59333

hypothetical protein
  
Accession: EAA59332
  
Location: 199978-201422
  
  
**BlastP hit with Mycgr3G84402\_Mycgr3T**
  
Percentage identity: 66 %
  
BlastP bit score: 612
  
Sequence coverage: 99 %
  
E-value: 0.0
  
  
 NCBI BlastP on this gene

EAA59332

hypothetical protein
  
Accession: EAA59331
  
Location: 198380-199768
  
  
**BlastP hit with Mycgr3G35447\_Mycgr3T**
  
Percentage identity: 57 %
  
BlastP bit score: 461
  
Sequence coverage: 92 %
  
E-value: 4e-156
  
  
 NCBI BlastP on this gene

EAA59331

hypothetical protein
  
Accession: EAA59330
  
Location: 197155-198012
  
 NCBI BlastP on this gene

EAA59330

hypothetical protein
  
Accession: EAA59329
  
Location: 195109-196781
  
 NCBI BlastP on this gene

EAA59329

hypothetical protein
  
Accession: EAA59328
  
Location: 192464-194673
  
 NCBI BlastP on this gene

EAA59328

hypothetical protein
  
Accession: EAA59327
  
Location: 190773-192260
  
 NCBI BlastP on this gene

EAA59327

hypothetical protein
  
Accession: EAA59326
  
Location: 189144-190308
  
 NCBI BlastP on this gene

EAA59326

hypothetical protein
  
Accession: EAA59325
  
Location: 187532-188939
  
 NCBI BlastP on this gene

EAA59325

hypothetical protein
  
Accession: EAA59324
  
Location: 183790-187124
  
 NCBI BlastP on this gene

EAA59324

hypothetical protein
  
Accession: EAA59323
  
Location: 182114-183336
  
 NCBI BlastP on this gene

EAA59323

38. :  DF126471 Aspergillus kawachii IFO 4308 DNA, contig: scaffold00025     Total score: 3.0     Cumulative Blast bit score: 1665

mitogen-activated protein kinase mpkC
  
Accession: GAA89999
  
Location: 171342-173129
  
 NCBI BlastP on this gene

GAA89999

similar to An18g05240
  
Accession: GAA89998
  
Location: 167262-168084
  
 NCBI BlastP on this gene

GAA89998

26S protease regulatory subunit 6A-B
  
Accession: GAA89997
  
Location: 162634-164185
  
 NCBI BlastP on this gene

GAA89997

trimethyllysine dioxygenase TmlH
  
Accession: GAA89996
  
Location: 158628-162260
  
 NCBI BlastP on this gene

GAA89996

HNRNP arginine N-methyltransferase
  
Accession: GAA89995
  
Location: 156490-157883
  
 NCBI BlastP on this gene

GAA89995

Mn2+ homeostasis protein
  
Accession: GAA89994
  
Location: 154416-155590
  
 NCBI BlastP on this gene

GAA89994

N-acetylglucosamine-phosphate mutase
  
Accession: GAA89993
  
Location: 151797-153814
  
  
**BlastP hit with Mycgr3G103034\_Mycgr3**
  
Percentage identity: 55 %
  
BlastP bit score: 613
  
Sequence coverage: 98 %
  
E-value: 0.0
  
  
 NCBI BlastP on this gene

GAA89993

ATP-dependent RNA helicase
  
Accession: GAA89992
  
Location: 149860-151425
  
  
**BlastP hit with Mycgr3G84402\_Mycgr3T**
  
Percentage identity: 72 %
  
BlastP bit score: 615
  
Sequence coverage: 88 %
  
E-value: 0.0
  
  
 NCBI BlastP on this gene

GAA89992

ribosome biogenesis protein Ssf2
  
Accession: GAA89991
  
Location: 148184-149599
  
  
**BlastP hit with Mycgr3G35447\_Mycgr3T**
  
Percentage identity: 59 %
  
BlastP bit score: 437
  
Sequence coverage: 91 %
  
E-value: 1e-146
  
  
 NCBI BlastP on this gene

GAA89991

riboflavin synthase, alpha subunit
  
Accession: GAA89990
  
Location: 146677-147668
  
 NCBI BlastP on this gene

GAA89990

CAIB/BAIF family enzyme
  
Accession: GAA89989
  
Location: 144709-146352
  
 NCBI BlastP on this gene

GAA89989

PHD finger domain protein
  
Accession: GAA89988
  
Location: 142268-144250
  
 NCBI BlastP on this gene

GAA89988

zinc metallopeptidase
  
Accession: GAA89987
  
Location: 140347-142049
  
 NCBI BlastP on this gene

GAA89987

short-chain dehydrogenase/reductase family protein
  
Accession: GAA89986
  
Location: 138732-140024
  
 NCBI BlastP on this gene

GAA89986

small nucleolar ribonucleoprotein complex subunit
  
Accession: GAA89985
  
Location: 137119-138578
  
 NCBI BlastP on this gene

GAA89985

SWI-SNF complex subunit (BAF60b)
  
Accession: GAA89984
  
Location: 133398-136768
  
 NCBI BlastP on this gene

GAA89984

DUF887 domain protein
  
Accession: GAA89983
  
Location: 131326-132557
  
 NCBI BlastP on this gene

GAA89983

39. :  ACYE01000197 Trichophyton verrucosum HKI 0517     Total score: 3.0     Cumulative Blast bit score: 1663

conserved hypothetical protein
  
Accession: EFE41400
  
Location: 6281-8813
  
 NCBI BlastP on this gene

EFE41400

hypothetical protein
  
Accession: EFE41401
  
Location: 9350-10873
  
 NCBI BlastP on this gene

EFE41401

hypothetical protein
  
Accession: EFE41402
  
Location: 11433-12053
  
 NCBI BlastP on this gene

EFE41402

hypothetical protein
  
Accession: EFE41403
  
Location: 13852-15019
  
 NCBI BlastP on this gene

EFE41403

hypothetical protein
  
Accession: EFE41404
  
Location: 15620-16984
  
 NCBI BlastP on this gene

EFE41404

hypothetical protein
  
Accession: EFE41405
  
Location: 19343-20107
  
 NCBI BlastP on this gene

EFE41405

hypothetical protein
  
Accession: EFE41406
  
Location: 20895-22643
  
  
**BlastP hit with Mycgr3G103034\_Mycgr3**
  
Percentage identity: 56 %
  
BlastP bit score: 569
  
Sequence coverage: 93 %
  
E-value: 0.0
  
  
 NCBI BlastP on this gene

EFE41406

hypothetical protein
  
Accession: EFE41407
  
Location: 23202-24707
  
  
**BlastP hit with Mycgr3G84402\_Mycgr3T**
  
Percentage identity: 71 %
  
BlastP bit score: 670
  
Sequence coverage: 100 %
  
E-value: 0.0
  
  
 NCBI BlastP on this gene

EFE41407

hypothetical protein
  
Accession: EFE41408
  
Location: 24990-26389
  
  
**BlastP hit with Mycgr3G35447\_Mycgr3T**
  
Percentage identity: 55 %
  
BlastP bit score: 424
  
Sequence coverage: 93 %
  
E-value: 2e-141
  
  
 NCBI BlastP on this gene

EFE41408

hypothetical protein
  
Accession: EFE41409
  
Location: 26694-27323
  
 NCBI BlastP on this gene

EFE41409

hypothetical protein
  
Accession: EFE41410
  
Location: 27860-29525
  
 NCBI BlastP on this gene

EFE41410

PHD finger domain protein, putative
  
Accession: EFE41411
  
Location: 30130-31728
  
 NCBI BlastP on this gene

EFE41411

hypothetical protein
  
Accession: EFE41412
  
Location: 32265-33554
  
 NCBI BlastP on this gene

EFE41412

pathogenesis associated protein Cap20, putative
  
Accession: EFE41413
  
Location: 35300-35900
  
 NCBI BlastP on this gene

EFE41413

hypothetical protein
  
Accession: EFE41414
  
Location: 37833-38474
  
 NCBI BlastP on this gene

EFE41414

conserved hypothetical protein
  
Accession: EFE41415
  
Location: 39382-46543
  
 NCBI BlastP on this gene

EFE41415

40. :  CH476619 Uncinocarpus reesii 1704 scaffold\_5 genomic scaffold     Total score: 3.0     Cumulative Blast bit score: 1661

serine/threonine-protein kinase SCH9
  
Accession: EEP82467
  
Location: 922030-924823
  
 NCBI BlastP on this gene

EEP82467

predicted protein
  
Accession: EEP82466
  
Location: 917680-921238
  
 NCBI BlastP on this gene

EEP82466

26S protease regulatory subunit 6A-B
  
Accession: EEP82465
  
Location: 915736-917248
  
 NCBI BlastP on this gene

EEP82465

predicted protein
  
Accession: EEP82464
  
Location: 913360-914385
  
 NCBI BlastP on this gene

EEP82464

conserved hypothetical protein
  
Accession: EEP82463
  
Location: 910764-911749
  
 NCBI BlastP on this gene

EEP82463

HNRNP arginine N-methyltransferase
  
Accession: EEP82462
  
Location: 908453-909824
  
 NCBI BlastP on this gene

EEP82462

conserved hypothetical protein
  
Accession: EEP82461
  
Location: 906522-907713
  
 NCBI BlastP on this gene

EEP82461

phosphoacetylglucosamine mutase
  
Accession: EEP82460
  
Location: 904015-905992
  
  
**BlastP hit with Mycgr3G103034\_Mycgr3**
  
Percentage identity: 51 %
  
BlastP bit score: 543
  
Sequence coverage: 99 %
  
E-value: 0.0
  
  
 NCBI BlastP on this gene

EEP82460

ATP-dependent rRNA helicase RRP3
  
Accession: EEP82459
  
Location: 902045-903519
  
  
**BlastP hit with Mycgr3G84402\_Mycgr3T**
  
Percentage identity: 72 %
  
BlastP bit score: 662
  
Sequence coverage: 101 %
  
E-value: 0.0
  
  
 NCBI BlastP on this gene

EEP82459

conserved hypothetical protein
  
Accession: EEP82458
  
Location: 900414-901794
  
  
**BlastP hit with Mycgr3G35447\_Mycgr3T**
  
Percentage identity: 55 %
  
BlastP bit score: 456
  
Sequence coverage: 102 %
  
E-value: 4e-154
  
  
 NCBI BlastP on this gene

EEP82458

riboflavin synthase alpha chain
  
Accession: EEP82457
  
Location: 899310-899703
  
 NCBI BlastP on this gene

EEP82457

hypothetical protein
  
Accession: EEP82456
  
Location: 897370-899007
  
 NCBI BlastP on this gene

EEP82456

predicted protein
  
Accession: EEP82455
  
Location: 895304-896524
  
 NCBI BlastP on this gene

EEP82455

conserved hypothetical protein
  
Accession: EEP82454
  
Location: 893625-894836
  
 NCBI BlastP on this gene

EEP82454

predicted protein
  
Accession: EEP82453
  
Location: 890106-890707
  
 NCBI BlastP on this gene

EEP82453

predicted protein
  
Accession: EEP82452
  
Location: 888984-889502
  
 NCBI BlastP on this gene

EEP82452

60S ribosomal protein L27-A
  
Accession: EEP82451
  
Location: 885971-887011
  
 NCBI BlastP on this gene

EEP82451

41. :  ABSU01000003 Arthroderma benhamiae CBS 112371     Total score: 3.0     Cumulative Blast bit score: 1661

hypothetical protein
  
Accession: EFE35393
  
Location: 183000-186189
  
 NCBI BlastP on this gene

EFE35393

conserved predicted protein
  
Accession: EFE35392
  
Location: 177905-180437
  
 NCBI BlastP on this gene

EFE35392

hypothetical protein
  
Accession: EFE35391
  
Location: 175843-177365
  
 NCBI BlastP on this gene

EFE35391

hypothetical protein
  
Accession: EFE35390
  
Location: 174671-175291
  
 NCBI BlastP on this gene

EFE35390

hypothetical protein
  
Accession: EFE35389
  
Location: 171724-172889
  
 NCBI BlastP on this gene

EFE35389

hypothetical protein
  
Accession: EFE35388
  
Location: 169768-171116
  
 NCBI BlastP on this gene

EFE35388

hypothetical protein
  
Accession: EFE35387
  
Location: 166654-167472
  
 NCBI BlastP on this gene

EFE35387

hypothetical protein
  
Accession: EFE35386
  
Location: 164145-165892
  
  
**BlastP hit with Mycgr3G103034\_Mycgr3**
  
Percentage identity: 56 %
  
BlastP bit score: 571
  
Sequence coverage: 93 %
  
E-value: 0.0
  
  
 NCBI BlastP on this gene

EFE35386

hypothetical protein
  
Accession: EFE35385
  
Location: 162094-163598
  
  
**BlastP hit with Mycgr3G84402\_Mycgr3T**
  
Percentage identity: 71 %
  
BlastP bit score: 667
  
Sequence coverage: 100 %
  
E-value: 0.0
  
  
 NCBI BlastP on this gene

EFE35385

hypothetical protein
  
Accession: EFE35384
  
Location: 160414-161815
  
  
**BlastP hit with Mycgr3G35447\_Mycgr3T**
  
Percentage identity: 55 %
  
BlastP bit score: 423
  
Sequence coverage: 93 %
  
E-value: 3e-141
  
  
 NCBI BlastP on this gene

EFE35384

hypothetical protein
  
Accession: EFE35383
  
Location: 159483-160112
  
 NCBI BlastP on this gene

EFE35383

hypothetical protein
  
Accession: EFE35382
  
Location: 157320-158748
  
 NCBI BlastP on this gene

EFE35382

PHD finger domain protein, putative
  
Accession: EFE35381
  
Location: 155110-156708
  
 NCBI BlastP on this gene

EFE35381

hypothetical protein
  
Accession: EFE35380
  
Location: 153375-154664
  
 NCBI BlastP on this gene

EFE35380

pathogenesis associated protein Cap20, putative
  
Accession: EFE35379
  
Location: 151021-151622
  
 NCBI BlastP on this gene

EFE35379

hypothetical protein
  
Accession: EFE35378
  
Location: 148334-149098
  
 NCBI BlastP on this gene

EFE35378

42. :  ACJE01000020 Aspergillus niger ATCC 1015     Total score: 3.0     Cumulative Blast bit score: 1658

mitogen-activated protein kinase
  
Accession: EHA19136
  
Location: 1226482-1227997
  
 NCBI BlastP on this gene

EHA19136

hypothetical protein
  
Accession: EHA19135
  
Location: 1221871-1223277
  
 NCBI BlastP on this gene

EHA19135

hypothetical protein
  
Accession: EHA19134
  
Location: 1217619-1219163
  
 NCBI BlastP on this gene

EHA19134

hypothetical protein
  
Accession: EHA19133
  
Location: 1216158-1217243
  
 NCBI BlastP on this gene

EHA19133

hypothetical protein
  
Accession: EHA19132
  
Location: 1213618-1214885
  
 NCBI BlastP on this gene

EHA19132

hypothetical protein
  
Accession: EHA19131
  
Location: 1211455-1212798
  
 NCBI BlastP on this gene

EHA19131

hypothetical protein
  
Accession: EHA19130
  
Location: 1209351-1210527
  
 NCBI BlastP on this gene

EHA19130

hypothetical protein
  
Accession: EHA19129
  
Location: 1206757-1208771
  
  
**BlastP hit with Mycgr3G103034\_Mycgr3**
  
Percentage identity: 54 %
  
BlastP bit score: 604
  
Sequence coverage: 99 %
  
E-value: 0.0
  
  
 NCBI BlastP on this gene

EHA19129

hypothetical protein
  
Accession: EHA19128
  
Location: 1204832-1206387
  
  
**BlastP hit with Mycgr3G84402\_Mycgr3T**
  
Percentage identity: 72 %
  
BlastP bit score: 621
  
Sequence coverage: 89 %
  
E-value: 0.0
  
  
 NCBI BlastP on this gene

EHA19128

hypothetical protein
  
Accession: EHA19127
  
Location: 1203155-1204585
  
  
**BlastP hit with Mycgr3G35447\_Mycgr3T**
  
Percentage identity: 59 %
  
BlastP bit score: 433
  
Sequence coverage: 91 %
  
E-value: 4e-145
  
  
 NCBI BlastP on this gene

EHA19127

hypothetical protein
  
Accession: EHA19126
  
Location: 1201686-1202655
  
 NCBI BlastP on this gene

EHA19126

hypothetical protein
  
Accession: EHA19125
  
Location: 1199739-1201378
  
 NCBI BlastP on this gene

EHA19125

hypothetical protein
  
Accession: EHA19124
  
Location: 1197299-1199284
  
 NCBI BlastP on this gene

EHA19124

hypothetical protein
  
Accession: EHA19123
  
Location: 1195691-1197076
  
 NCBI BlastP on this gene

EHA19123

hypothetical protein
  
Accession: EHA19122
  
Location: 1193827-1194948
  
 NCBI BlastP on this gene

EHA19122

hypothetical protein
  
Accession: EHA19121
  
Location: 1188433-1191816
  
 NCBI BlastP on this gene

EHA19121

hypothetical protein
  
Accession: EHA19120
  
Location: 1186370-1187603
  
 NCBI BlastP on this gene

EHA19120

43. :  AKHY01000202 Aspergillus oryzae 3.042     Total score: 3.0     Cumulative Blast bit score: 1654

hypothetical protein
  
Accession: EIT73174
  
Location: 683276-685688
  
 NCBI BlastP on this gene

EIT73174

hypothetical protein
  
Accession: EIT73229
  
Location: 686284-687478
  
 NCBI BlastP on this gene

EIT73229

hypothetical protein
  
Accession: EIT73055
  
Location: 688126-688584
  
 NCBI BlastP on this gene

EIT73055

26S proteasome regulatory complex, ATPase RPT5
  
Accession: EIT73108
  
Location: 690602-692211
  
 NCBI BlastP on this gene

EIT73108

putative gamma-butyrobetaine,2-oxoglutarate dioxygenase
  
Accession: EIT73250
  
Location: 692643-694848
  
 NCBI BlastP on this gene

EIT73250

peroxisomal multifunctional beta-oxidation protein
  
Accession: EIT73278
  
Location: 697075-698244
  
 NCBI BlastP on this gene

EIT73278

protein arginine N-methyltransferase PRMT1
  
Accession: EIT73110
  
Location: 699009-700425
  
 NCBI BlastP on this gene

EIT73110

phosphoglucomutase/phosphomannomutase
  
Accession: EIT73157
  
Location: 702872-704882
  
  
**BlastP hit with Mycgr3G103034\_Mycgr3**
  
Percentage identity: 55 %
  
BlastP bit score: 571
  
Sequence coverage: 91 %
  
E-value: 0.0
  
  
 NCBI BlastP on this gene

EIT73157

ATP-dependent RNA helicase
  
Accession: EIT73155
  
Location: 705331-706923
  
  
**BlastP hit with Mycgr3G84402\_Mycgr3T**
  
Percentage identity: 68 %
  
BlastP bit score: 636
  
Sequence coverage: 100 %
  
E-value: 0.0
  
  
 NCBI BlastP on this gene

EIT73155

RNA-binding protein required for 60S ribosomal subunit biogenesis
  
Accession: EIT73217
  
Location: 707177-708593
  
  
**BlastP hit with Mycgr3G35447\_Mycgr3T**
  
Percentage identity: 60 %
  
BlastP bit score: 447
  
Sequence coverage: 87 %
  
E-value: 2e-150
  
  
 NCBI BlastP on this gene

EIT73217

riboflavin synthase alpha chain
  
Accession: EIT73165
  
Location: 709023-709906
  
 NCBI BlastP on this gene

EIT73165

putative L-carnitine dehydratase/alpha-methylacyl-CoA racemase
  
Accession: EIT73083
  
Location: 710439-712096
  
 NCBI BlastP on this gene

EIT73083

PHD finger domain protein
  
Accession: EIT73158
  
Location: 712494-714281
  
 NCBI BlastP on this gene

EIT73158

hypothetical protein
  
Accession: EIT73205
  
Location: 714504-716274
  
 NCBI BlastP on this gene

EIT73205

hypothetical protein
  
Accession: EIT73262
  
Location: 716344-718623
  
 NCBI BlastP on this gene

EIT73262

hypothetical protein
  
Accession: EIT73089
  
Location: 719295-720056
  
 NCBI BlastP on this gene

EIT73089

hypothetical protein
  
Accession: EIT73159
  
Location: 720283-721446
  
 NCBI BlastP on this gene

EIT73159

hydroxysteroid 17-beta dehydrogenase 11
  
Accession: EIT73130
  
Location: 722333-723614
  
 NCBI BlastP on this gene

EIT73130

Sof1-like rRNA processing protein
  
Accession: EIT73199
  
Location: 723742-725195
  
 NCBI BlastP on this gene

EIT73199

26S proteasome regulatory complex, subunit RPN6/PSMD11
  
Accession: EIT73212
  
Location: 725529-726861
  
 NCBI BlastP on this gene

EIT73212

SWI/SNF transcription activation complex subunit
  
Accession: EIT73270
  
Location: 727329-728839
  
 NCBI BlastP on this gene

EIT73270

44. :  EQ962654 Talaromyces stipitatus ATCC 10500 scf\_1105507295541 genomic scaffold     Total score: 3.0     Cumulative Blast bit score: 1643

cAMP-dependent protein kinase-like, putative
  
Accession: EED19512
  
Location: 1298364-1301518
  
 NCBI BlastP on this gene

EED19512

conserved hypothetical protein
  
Accession: EED19511
  
Location: 1293561-1296219
  
 NCBI BlastP on this gene

EED19511

proteasome regulatory particle subunit Rpt5, putative
  
Accession: EED19510
  
Location: 1291527-1293045
  
 NCBI BlastP on this gene

EED19510

trimethyllysine dioxygenase TmlH, putative
  
Accession: EED19509
  
Location: 1288917-1291154
  
 NCBI BlastP on this gene

EED19509

peroxisomal dehydratase, putative
  
Accession: EED19508
  
Location: 1287567-1288719
  
 NCBI BlastP on this gene

EED19508

histone H4 arginine methyltransferase RmtA
  
Accession: EED19507
  
Location: 1285559-1286963
  
 NCBI BlastP on this gene

EED19507

Mn2 homeostasis protein (Per1), putative
  
Accession: EED19506
  
Location: 1283803-1284981
  
 NCBI BlastP on this gene

EED19506

N-acetylglucosamine-phosphate mutase
  
Accession: EED19505
  
Location: 1281462-1283449
  
  
**BlastP hit with Mycgr3G103034\_Mycgr3**
  
Percentage identity: 54 %
  
BlastP bit score: 596
  
Sequence coverage: 100 %
  
E-value: 0.0
  
  
 NCBI BlastP on this gene

EED19505

ATP-dependent RNA helicase , putative
  
Accession: EED19504
  
Location: 1279684-1281178
  
  
**BlastP hit with Mycgr3G84402\_Mycgr3T**
  
Percentage identity: 68 %
  
BlastP bit score: 613
  
Sequence coverage: 95 %
  
E-value: 0.0
  
  
 NCBI BlastP on this gene

EED19504

ribosome biogenesis protein Ssf2, putative
  
Accession: EED19503
  
Location: 1278041-1279437
  
  
**BlastP hit with Mycgr3G35447\_Mycgr3T**
  
Percentage identity: 59 %
  
BlastP bit score: 434
  
Sequence coverage: 91 %
  
E-value: 2e-145
  
  
 NCBI BlastP on this gene

EED19503

riboflavin synthase, alpha subunit
  
Accession: EED19502
  
Location: 1277100-1277917
  
 NCBI BlastP on this gene

EED19502

CAIB/BAIF family enzyme
  
Accession: EED19501
  
Location: 1275281-1276916
  
 NCBI BlastP on this gene

EED19501

PHD finger domain protein, putative
  
Accession: EED19500
  
Location: 1273327-1274997
  
 NCBI BlastP on this gene

EED19500

hypothetical protein
  
Accession: EED19499
  
Location: 1272337-1273074
  
 NCBI BlastP on this gene

EED19499

short-chain dehydrogenase/reductase family protein, putative
  
Accession: EED19498
  
Location: 1269714-1270912
  
 NCBI BlastP on this gene

EED19498

small nucleolar ribonucleoprotein complex subunit (SOF1), putative
  
Accession: EED19497
  
Location: 1268142-1269602
  
 NCBI BlastP on this gene

EED19497

proteasome regulatory particle subunit (RpnF), putative
  
Accession: EED19496
  
Location: 1266489-1267809
  
 NCBI BlastP on this gene

EED19496

conserved hypothetical protein
  
Accession: EED19495
  
Location: 1264485-1265077
  
 NCBI BlastP on this gene

EED19495

SWI-SNF complex subunit (BAF60b), putative
  
Accession: EED19494
  
Location: 1262214-1263884
  
 NCBI BlastP on this gene

EED19494

DUF887 domain protein
  
Accession: EED19493
  
Location: 1260539-1261769
  
 NCBI BlastP on this gene

EED19493

45. :  DS572750 Paracoccidioides brasiliensis Pb18 supercont1.1 genomic scaffold     Total score: 3.0     Cumulative Blast bit score: 1607

conserved hypothetical protein
  
Accession: EEH44309
  
Location: 2018710-2021155
  
 NCBI BlastP on this gene

EEH44309

26S protease regulatory subunit 6A
  
Accession: EEH44310
  
Location: 2022010-2023580
  
 NCBI BlastP on this gene

EEH44310

trimethyllysine dioxygenase
  
Accession: EEH44311
  
Location: 2023747-2026297
  
 NCBI BlastP on this gene

EEH44311

peroxisomal dehydratase
  
Accession: EEH44312
  
Location: 2026791-2028127
  
 NCBI BlastP on this gene

EEH44312

HNRNP arginine N-methyltransferase
  
Accession: EEH44313
  
Location: 2028981-2030408
  
 NCBI BlastP on this gene

EEH44313

Mn2+ homeostasis protein (Per1)
  
Accession: EEH44314
  
Location: 2031141-2032379
  
 NCBI BlastP on this gene

EEH44314

phosphoacetylglucosamine mutase
  
Accession: EEH44315
  
Location: 2033854-2035236
  
  
**BlastP hit with Mycgr3G103034\_Mycgr3**
  
Percentage identity: 55 %
  
BlastP bit score: 482
  
Sequence coverage: 78 %
  
E-value: 8e-163
  
  
 NCBI BlastP on this gene

EEH44315

ATP-dependent rRNA helicase RRP3
  
Accession: EEH44316
  
Location: 2035931-2037448
  
  
**BlastP hit with Mycgr3G84402\_Mycgr3T**
  
Percentage identity: 75 %
  
BlastP bit score: 652
  
Sequence coverage: 97 %
  
E-value: 0.0
  
  
 NCBI BlastP on this gene

EEH44316

ribosome biogenesis protein SSF1
  
Accession: EEH44317
  
Location: 2037745-2039184
  
  
**BlastP hit with Mycgr3G35447\_Mycgr3T**
  
Percentage identity: 58 %
  
BlastP bit score: 473
  
Sequence coverage: 94 %
  
E-value: 9e-161
  
  
 NCBI BlastP on this gene

EEH44317

riboflavin synthase alpha chain
  
Accession: EEH44318
  
Location: 2039520-2040388
  
 NCBI BlastP on this gene

EEH44318

formyl-coenzyme A transferase
  
Accession: EEH44319
  
Location: 2040735-2042530
  
 NCBI BlastP on this gene

EEH44319

conserved hypothetical protein
  
Accession: EEH44320
  
Location: 2043049-2044893
  
 NCBI BlastP on this gene

EEH44320

DUF887 domain-containing protein
  
Accession: EEH44321
  
Location: 2045686-2047001
  
 NCBI BlastP on this gene

EEH44321

conserved hypothetical protein
  
Accession: EEH44322
  
Location: 2047978-2054139
  
 NCBI BlastP on this gene

EEH44322

60S ribosomal protein L27-A
  
Accession: EEH44323
  
Location: 2054793-2055576
  
 NCBI BlastP on this gene

EEH44323

predicted protein
  
Accession: EEH44324
  
Location: 2057481-2058054
  
 NCBI BlastP on this gene

EEH44324

46. :  GL534607 Pyrenophora teres f. teres 0-1 unplaced genomic scaffold scaffold\_190867     Total score: 3.0     Cumulative Blast bit score: 1578

hypothetical protein
  
Accession: EFQ91772
  
Location: 1067-2976
  
  
**BlastP hit with Mycgr3G67791\_Mycgr3T**
  
Percentage identity: 64 %
  
BlastP bit score: 530
  
Sequence coverage: 83 %
  
E-value: 1e-179
  
  
 NCBI BlastP on this gene

EFQ91772

hypothetical protein
  
Accession: EFQ91773
  
Location: 3686-4861
  
 NCBI BlastP on this gene

EFQ91773

hypothetical protein
  
Accession: EFQ91774
  
Location: 5015-6241
  
 NCBI BlastP on this gene

EFQ91774

hypothetical protein
  
Accession: EFQ91775
  
Location: 6847-8898
  
 NCBI BlastP on this gene

EFQ91775

hypothetical protein
  
Accession: EFQ91776
  
Location: 9965-11538
  
 NCBI BlastP on this gene

EFQ91776

hypothetical protein
  
Accession: EFQ91777
  
Location: 13076-15425
  
 NCBI BlastP on this gene

EFQ91777

hypothetical protein
  
Accession: EFQ91778
  
Location: 16471-17517
  
 NCBI BlastP on this gene

EFQ91778

hypothetical protein
  
Accession: EFQ91779
  
Location: 17835-18453
  
 NCBI BlastP on this gene

EFQ91779

hypothetical protein
  
Accession: EFQ91780
  
Location: 20044-21644
  
  
**BlastP hit with Mycgr3G84402\_Mycgr3T**
  
Percentage identity: 71 %
  
BlastP bit score: 625
  
Sequence coverage: 93 %
  
E-value: 0.0
  
  
 NCBI BlastP on this gene

EFQ91780

hypothetical protein
  
Accession: EFQ91781
  
Location: 21922-23318
  
  
**BlastP hit with Mycgr3G35447\_Mycgr3T**
  
Percentage identity: 58 %
  
BlastP bit score: 423
  
Sequence coverage: 88 %
  
E-value: 3e-141
  
  
 NCBI BlastP on this gene

EFQ91781

hypothetical protein
  
Accession: EFQ91782
  
Location: 23443-24515
  
 NCBI BlastP on this gene

EFQ91782

hypothetical protein
  
Accession: EFQ91783
  
Location: 24979-25923
  
 NCBI BlastP on this gene

EFQ91783

hypothetical protein
  
Accession: EFQ91784
  
Location: 26443-27504
  
 NCBI BlastP on this gene

EFQ91784

hypothetical protein
  
Accession: EFQ91785
  
Location: 28729-30222
  
 NCBI BlastP on this gene

EFQ91785

hypothetical protein
  
Accession: EFQ91786
  
Location: 30917-31906
  
 NCBI BlastP on this gene

EFQ91786

hypothetical protein
  
Accession: EFQ91787
  
Location: 33370-34738
  
 NCBI BlastP on this gene

EFQ91787

hypothetical protein
  
Accession: EFQ91788
  
Location: 35781-38024
  
 NCBI BlastP on this gene

EFQ91788

hypothetical protein
  
Accession: EFQ91789
  
Location: 38951-40348
  
 NCBI BlastP on this gene

EFQ91789

47. :  KB445649 Cochliobolus sativus ND90Pr unplaced genomic scaffold COCSAscaffold\_13     Total score: 3.0     Cumulative Blast bit score: 1569

hypothetical protein
  
Accession: EMD61128
  
Location: 1011238-1012726
  
 NCBI BlastP on this gene

EMD61128

hypothetical protein
  
Accession: EMD61127
  
Location: 1009492-1009844
  
 NCBI BlastP on this gene

EMD61127

hypothetical protein
  
Accession: EMD61126
  
Location: 1007189-1008399
  
 NCBI BlastP on this gene

EMD61126

hypothetical protein
  
Accession: EMD61125
  
Location: 1004553-1006333
  
 NCBI BlastP on this gene

EMD61125

hypothetical protein
  
Accession: EMD61124
  
Location: 1003654-1004130
  
 NCBI BlastP on this gene

EMD61124

hypothetical protein
  
Accession: EMD61123
  
Location: 1002481-1003494
  
 NCBI BlastP on this gene

EMD61123

hypothetical protein
  
Accession: EMD61122
  
Location: 998502-1002182
  
 NCBI BlastP on this gene

EMD61122

hypothetical protein
  
Accession: EMD61121
  
Location: 997097-997788
  
 NCBI BlastP on this gene

EMD61121

hypothetical protein
  
Accession: EMD61120
  
Location: 994634-996412
  
 NCBI BlastP on this gene

EMD61120

hypothetical protein
  
Accession: EMD61119
  
Location: 990115-991958
  
  
**BlastP hit with Mycgr3G67791\_Mycgr3T**
  
Percentage identity: 67 %
  
BlastP bit score: 520
  
Sequence coverage: 75 %
  
E-value: 6e-176
  
  
 NCBI BlastP on this gene

EMD61119

hypothetical protein
  
Accession: EMD61118
  
Location: 988263-989448
  
 NCBI BlastP on this gene

EMD61118

hypothetical protein
  
Accession: EMD61117
  
Location: 986977-988224
  
 NCBI BlastP on this gene

EMD61117

hypothetical protein
  
Accession: EMD61116
  
Location: 984353-986380
  
 NCBI BlastP on this gene

EMD61116

hypothetical protein
  
Accession: EMD61115
  
Location: 981749-983292
  
 NCBI BlastP on this gene

EMD61115

hypothetical protein
  
Accession: EMD61114
  
Location: 980710-981458
  
 NCBI BlastP on this gene

EMD61114

hypothetical protein
  
Accession: EMD61113
  
Location: 978499-980104
  
  
**BlastP hit with Mycgr3G84402\_Mycgr3T**
  
Percentage identity: 71 %
  
BlastP bit score: 618
  
Sequence coverage: 93 %
  
E-value: 0.0
  
  
 NCBI BlastP on this gene

EMD61113

hypothetical protein
  
Accession: EMD61112
  
Location: 976718-978172
  
  
**BlastP hit with Mycgr3G35447\_Mycgr3T**
  
Percentage identity: 58 %
  
BlastP bit score: 431
  
Sequence coverage: 87 %
  
E-value: 2e-144
  
  
 NCBI BlastP on this gene

EMD61112

hypothetical protein
  
Accession: EMD61111
  
Location: 975565-976615
  
 NCBI BlastP on this gene

EMD61111

hypothetical protein
  
Accession: EMD61110
  
Location: 974152-975093
  
 NCBI BlastP on this gene

EMD61110

hypothetical protein
  
Accession: EMD61109
  
Location: 972561-973566
  
 NCBI BlastP on this gene

EMD61109

hypothetical protein
  
Accession: EMD61108
  
Location: 972017-972355
  
 NCBI BlastP on this gene

EMD61108

hypothetical protein
  
Accession: EMD61107
  
Location: 970355-971809
  
 NCBI BlastP on this gene

EMD61107

hypothetical protein
  
Accession: EMD61106
  
Location: 966702-968366
  
 NCBI BlastP on this gene

EMD61106

hypothetical protein
  
Accession: EMD61105
  
Location: 964536-966125
  
 NCBI BlastP on this gene

EMD61105

hypothetical protein
  
Accession: EMD61104
  
Location: 963862-964410
  
 NCBI BlastP on this gene

EMD61104

hypothetical protein
  
Accession: EMD61103
  
Location: 961669-962435
  
 NCBI BlastP on this gene

EMD61103

hypothetical protein
  
Accession: EMD61102
  
Location: 958584-961348
  
 NCBI BlastP on this gene

EMD61102

48. :  DS544805 Paracoccidioides brasiliensis Pb03 supercont1.3 genomic scaffold     Total score: 3.0     Cumulative Blast bit score: 1568

conserved hypothetical protein
  
Accession: EEH19938
  
Location: 1090569-1093014
  
 NCBI BlastP on this gene

EEH19938

26S protease regulatory subunit 6A
  
Accession: EEH19939
  
Location: 1093870-1095338
  
 NCBI BlastP on this gene

EEH19939

trimethyllysine dioxygenase
  
Accession: EEH19940
  
Location: 1095938-1096993
  
 NCBI BlastP on this gene

EEH19940

conserved hypothetical protein
  
Accession: EEH19941
  
Location: 1098647-1099843
  
 NCBI BlastP on this gene

EEH19941

arginine N-methyltransferase
  
Accession: EEH19942
  
Location: 1100128-1102261
  
 NCBI BlastP on this gene

EEH19942

conserved hypothetical protein
  
Accession: EEH19943
  
Location: 1102990-1104225
  
 NCBI BlastP on this gene

EEH19943

phosphoacetylglucosamine mutase
  
Accession: EEH19944
  
Location: 1105032-1107088
  
  
**BlastP hit with Mycgr3G103034\_Mycgr3**
  
Percentage identity: 54 %
  
BlastP bit score: 580
  
Sequence coverage: 99 %
  
E-value: 0.0
  
  
 NCBI BlastP on this gene

EEH19944

ATP-dependent rRNA helicase rrp3
  
Accession: EEH19945
  
Location: 1107783-1109304
  
  
**BlastP hit with Mycgr3G84402\_Mycgr3T**
  
Percentage identity: 65 %
  
BlastP bit score: 534
  
Sequence coverage: 97 %
  
E-value: 0.0
  
  
 NCBI BlastP on this gene

EEH19945

splicing factor
  
Accession: EEH19946
  
Location: 1109684-1111021
  
  
**BlastP hit with Mycgr3G35447\_Mycgr3T**
  
Percentage identity: 58 %
  
BlastP bit score: 454
  
Sequence coverage: 87 %
  
E-value: 5e-154
  
  
 NCBI BlastP on this gene

EEH19946

riboflavin synthase alpha chain
  
Accession: EEH19947
  
Location: 1111361-1112228
  
 NCBI BlastP on this gene

EEH19947

formyl-coenzyme A transferase
  
Accession: EEH19948
  
Location: 1112507-1114288
  
 NCBI BlastP on this gene

EEH19948

conserved hypothetical protein
  
Accession: EEH19949
  
Location: 1114800-1116626
  
 NCBI BlastP on this gene

EEH19949

DUF887 domain-containing protein
  
Accession: EEH19950
  
Location: 1117429-1118744
  
 NCBI BlastP on this gene

EEH19950

conserved hypothetical protein
  
Accession: EEH19951
  
Location: 1119719-1125815
  
 NCBI BlastP on this gene

EEH19951

60S ribosomal protein L27-B
  
Accession: EEH19952
  
Location: 1126626-1127259
  
 NCBI BlastP on this gene

EEH19952

predicted protein
  
Accession: EEH19953
  
Location: 1129144-1129816
  
 NCBI BlastP on this gene

EEH19953

49. :  KB733455 Bipolaris maydis ATCC 48331 unplaced genomic scaffold COCC4scaffold\_12     Total score: 3.0     Cumulative Blast bit score: 1562

hypothetical protein
  
Accession: ENI04923
  
Location: 47305-47658
  
 NCBI BlastP on this gene

ENI04923

hypothetical protein
  
Accession: ENI04924
  
Location: 48752-49947
  
 NCBI BlastP on this gene

ENI04924

hypothetical protein
  
Accession: ENI04925
  
Location: 50783-52550
  
 NCBI BlastP on this gene

ENI04925

hypothetical protein
  
Accession: ENI04926
  
Location: 52974-53504
  
 NCBI BlastP on this gene

ENI04926

hypothetical protein
  
Accession: ENI04927
  
Location: 53539-54554
  
 NCBI BlastP on this gene

ENI04927

hypothetical protein
  
Accession: ENI04928
  
Location: 54858-58536
  
 NCBI BlastP on this gene

ENI04928

hypothetical protein
  
Accession: ENI04929
  
Location: 59210-59953
  
 NCBI BlastP on this gene

ENI04929

hypothetical protein
  
Accession: ENI04930
  
Location: 60670-62449
  
 NCBI BlastP on this gene

ENI04930

hypothetical protein
  
Accession: ENI04931
  
Location: 65788-67632
  
  
**BlastP hit with Mycgr3G67791\_Mycgr3T**
  
Percentage identity: 66 %
  
BlastP bit score: 513
  
Sequence coverage: 75 %
  
E-value: 3e-173
  
  
 NCBI BlastP on this gene

ENI04931

hypothetical protein
  
Accession: ENI04932
  
Location: 68518-69414
  
 NCBI BlastP on this gene

ENI04932

hypothetical protein
  
Accession: ENI04933
  
Location: 69530-70786
  
 NCBI BlastP on this gene

ENI04933

hypothetical protein
  
Accession: ENI04934
  
Location: 71409-73430
  
 NCBI BlastP on this gene

ENI04934

hypothetical protein
  
Accession: ENI04935
  
Location: 74517-76060
  
 NCBI BlastP on this gene

ENI04935

hypothetical protein
  
Accession: ENI04936
  
Location: 76404-77135
  
 NCBI BlastP on this gene

ENI04936

hypothetical protein
  
Accession: ENI04937
  
Location: 77766-79371
  
  
**BlastP hit with Mycgr3G84402\_Mycgr3T**
  
Percentage identity: 70 %
  
BlastP bit score: 618
  
Sequence coverage: 95 %
  
E-value: 0.0
  
  
 NCBI BlastP on this gene

ENI04937

hypothetical protein
  
Accession: ENI04938
  
Location: 79699-81162
  
  
**BlastP hit with Mycgr3G35447\_Mycgr3T**
  
Percentage identity: 58 %
  
BlastP bit score: 431
  
Sequence coverage: 88 %
  
E-value: 2e-144
  
  
 NCBI BlastP on this gene

ENI04938

hypothetical protein
  
Accession: ENI04939
  
Location: 81266-82306
  
 NCBI BlastP on this gene

ENI04939

hypothetical protein
  
Accession: ENI04940
  
Location: 82778-83719
  
 NCBI BlastP on this gene

ENI04940

hypothetical protein
  
Accession: ENI04941
  
Location: 84313-85318
  
 NCBI BlastP on this gene

ENI04941

hypothetical protein
  
Accession: ENI04942
  
Location: 86065-87297
  
 NCBI BlastP on this gene

ENI04942

hypothetical protein
  
Accession: ENI04943
  
Location: 89762-91428
  
 NCBI BlastP on this gene

ENI04943

hypothetical protein
  
Accession: ENI04944
  
Location: 91977-93553
  
 NCBI BlastP on this gene

ENI04944

hypothetical protein
  
Accession: ENI04945
  
Location: 93657-94205
  
 NCBI BlastP on this gene

ENI04945

hypothetical protein
  
Accession: ENI04946
  
Location: 95683-96449
  
 NCBI BlastP on this gene

ENI04946

hypothetical protein
  
Accession: ENI04947
  
Location: 96804-99568
  
 NCBI BlastP on this gene

ENI04947

hypothetical protein
  
Accession: ENI04948
  
Location: 100551-102287
  
 NCBI BlastP on this gene

ENI04948

50. :  KB445579 Cochliobolus heterostrophus C5 unplaced genomic scaffold COCHEscaffold\_11     Total score: 3.0     Cumulative Blast bit score: 1562

hypothetical protein
  
Accession: EMD89361
  
Location: 959693-960070
  
 NCBI BlastP on this gene

EMD89361

hypothetical protein
  
Accession: EMD89360
  
Location: 958114-958467
  
 NCBI BlastP on this gene

EMD89360

hypothetical protein
  
Accession: EMD89359
  
Location: 955825-957020
  
 NCBI BlastP on this gene

EMD89359

hypothetical protein
  
Accession: EMD89358
  
Location: 953222-954989
  
 NCBI BlastP on this gene

EMD89358

hypothetical protein
  
Accession: EMD89357
  
Location: 952268-952798
  
 NCBI BlastP on this gene

EMD89357

hypothetical protein
  
Accession: EMD89356
  
Location: 951218-952233
  
 NCBI BlastP on this gene

EMD89356

hypothetical protein
  
Accession: EMD89355
  
Location: 947236-950914
  
 NCBI BlastP on this gene

EMD89355

hypothetical protein
  
Accession: EMD89354
  
Location: 945819-946562
  
 NCBI BlastP on this gene

EMD89354

hypothetical protein
  
Accession: EMD89353
  
Location: 943323-945102
  
 NCBI BlastP on this gene

EMD89353

hypothetical protein
  
Accession: EMD89352
  
Location: 938097-939941
  
  
**BlastP hit with Mycgr3G67791\_Mycgr3T**
  
Percentage identity: 66 %
  
BlastP bit score: 513
  
Sequence coverage: 75 %
  
E-value: 3e-173
  
  
 NCBI BlastP on this gene

EMD89352

hypothetical protein
  
Accession: EMD89351
  
Location: 936309-937346
  
 NCBI BlastP on this gene

EMD89351

hypothetical protein
  
Accession: EMD89350
  
Location: 934943-936199
  
 NCBI BlastP on this gene

EMD89350

hypothetical protein
  
Accession: EMD89349
  
Location: 932302-934320
  
 NCBI BlastP on this gene

EMD89349

hypothetical protein
  
Accession: EMD89348
  
Location: 929669-931212
  
 NCBI BlastP on this gene

EMD89348

hypothetical protein
  
Accession: EMD89347
  
Location: 928594-929325
  
 NCBI BlastP on this gene

EMD89347

hypothetical protein
  
Accession: EMD89346
  
Location: 926358-927963
  
  
**BlastP hit with Mycgr3G84402\_Mycgr3T**
  
Percentage identity: 70 %
  
BlastP bit score: 618
  
Sequence coverage: 95 %
  
E-value: 0.0
  
  
 NCBI BlastP on this gene

EMD89346

hypothetical protein
  
Accession: EMD89345
  
Location: 924567-926030
  
  
**BlastP hit with Mycgr3G35447\_Mycgr3T**
  
Percentage identity: 58 %
  
BlastP bit score: 431
  
Sequence coverage: 88 %
  
E-value: 2e-144
  
  
 NCBI BlastP on this gene

EMD89345

hypothetical protein
  
Accession: EMD89344
  
Location: 923423-924463
  
 NCBI BlastP on this gene

EMD89344

hypothetical protein
  
Accession: EMD89343
  
Location: 922010-922951
  
 NCBI BlastP on this gene

EMD89343

hypothetical protein
  
Accession: EMD89342
  
Location: 920411-921416
  
 NCBI BlastP on this gene

EMD89342

hypothetical protein
  
Accession: EMD89341
  
Location: 918432-919664
  
 NCBI BlastP on this gene

EMD89341

hypothetical protein
  
Accession: EMD89340
  
Location: 914245-915911
  
 NCBI BlastP on this gene

EMD89340

hypothetical protein
  
Accession: EMD89339
  
Location: 912120-913696
  
 NCBI BlastP on this gene

EMD89339

hypothetical protein
  
Accession: EMD89338
  
Location: 911468-912016
  
 NCBI BlastP on this gene

EMD89338

hypothetical protein
  
Accession: EMD89337
  
Location: 909224-909990
  
 NCBI BlastP on this gene

EMD89337

hypothetical protein
  
Accession: EMD89336
  
Location: 906564-908869
  
 NCBI BlastP on this gene

EMD89336

Detecting sequence homology at the gene cluster level with MultiGeneBlast.
  
Marnix H. Medema, Rainer Breitling & Eriko Takano (2013)
  
*Molecular Biology and Evolution* , 30: 1218-1223.
