# Supplementary material for: In silico prediction and characterization of secondary metabolite biosynthetic gene clusters in the wheat pathogen Zymoseptoria tritici
Source: BMC Genomics. 2017 Aug 17;18:631. doi: 10.1186/s12864-017-3969-y (PMC5561558; doi:10.1186/s12864-017-3969-y)
Supplement: Supplementary file 1 — MultiGeneBLAST analysis of putative secondary metabolite clusters. All encoded amino acid sequences from genes residing in clusters predicted by AntiSMASH are given as FASTA file format. All output data from MultiGeneBLASTs are also provided. (ZIP 42911 kb) [file 12864_2017_3969_MOESM1_ESM.zip › Cluster MultiGene BLAST/out/Clusters_1_34/Cluster_10/displaypage2.xhtml]

xml version="1.0" encoding="UTF-8"?


Search Results
  
  
 Results pages: 1, 2, 3, 4, 5

**MultiGeneBlast hits**

Select gene cluster alignment
51. KE145370\_1 Glarea lozoyensis ATCC 20868 chromosome Unknown GLAREA6, whole...
52. FQ790359\_0 Botryotinia fuckeliana T4 SupSuperContig\_114\_320\_122\_1 genomic...
53. AFWA01000017\_0 Pneumocystis murina B123, whole genome shotgun sequencing ...
54. CH476628\_0 Sclerotinia sclerotiorum 1980 scaffold\_8 genomic scaffold, who...
55. JH921451\_1 Marssonina brunnea f. sp. 'multigermtubi' MB\_m1 unplaced genom...
56. KB708068\_0 Botryotinia fuckeliana BcDW1 unplaced genomic scaffold Scaffol...
57. FP929137\_1 Leptosphaeria maculans JN3 lm\_SuperContig\_10\_v2 genomic superc...
58. KB446542\_6 Dothistroma septosporum NZE10 unplaced genomic scaffold DOTSEs...
59. KB456266\_3 Mycosphaerella populorum SO2202 unplaced genomic scaffold SEPM...
60. KB446542\_5 Dothistroma septosporum NZE10 unplaced genomic scaffold DOTSEs...
61. KB446566\_0 Pseudocercospora fijiensis CIRAD86 unplaced genomic scaffold M...
62. KB446566\_1 Pseudocercospora fijiensis CIRAD86 unplaced genomic scaffold M...
63. DS027696\_1 Neosartorya fischeri NRRL 181 1099437636264 genomic scaffold, ...
64. DS499595\_0 Aspergillus fumigatus A1163 scf\_000002 genomic scaffold, whole...
65. AAHF01000001\_0 Aspergillus fumigatus Af293, whole genome shotgun sequenci...
66. DS027045\_0 Aspergillus clavatus NRRL 1 1099423829791 genomic scaffold, wh...
67. ACJE01000013\_2 Aspergillus niger ATCC 1015, whole genome shotgun sequenci...
68. AM270369\_0 Aspergillus niger contig An16c0170, genomic contig.
69. DF126452\_2 Aspergillus kawachii IFO 4308 DNA, contig: scaffold00006, whol...
70. AM920437\_1 Penicillium chrysogenum Wisconsin 54-1255 complete genome, con...
71. CH476615\_1 Uncinocarpus reesii 1704 scaffold\_1 genomic scaffold, whole ge...
72. AKCU01000427\_0 Penicillium digitatum Pd1, whole genome shotgun sequencing...
73. AKCT01000128\_0 Penicillium digitatum PHI26, whole genome shotgun sequenci...
74. GG700648\_4 Trichophyton rubrum CBS 118892 genomic scaffold supercont2.1, ...
75. DS995701\_0 Microsporum canis CBS 113480 supercont1.1 genomic scaffold, wh...
76. ABSU01000003\_3 Arthroderma benhamiae CBS 112371, whole genome shotgun seq...
77. DS995728\_0 Trichophyton equinum CBS 127.97 supercont1.11 genomic scaffold...
78. DS989822\_0 Arthroderma gypseum CBS 118893 supercont1.1 genomic scaffold, ...
79. ACFW01000009\_0 Coccidioides posadasii C735 delta SOWgp, whole genome shot...
80. GG704914\_0 Coccidioides immitis RS genomic scaffold supercont3.4, whole g...
81. GG698484\_0 Trichophyton tonsurans CBS 112818 genomic scaffold supercont1....
82. U85909\_0 Aureobasidium pullulans cosmid pPSR-22 hydroxylase, multidrug re...
83. ADOT01000056\_0 Arthrobotrys oligospora ATCC 24927, whole genome shotgun s...
84. FQ790277\_0 Botryotinia fuckeliana T4 SuperContig\_379\_1 genomic supercontig.
85. AHHD01000089\_0 Macrophomina phaseolina MS6, whole genome shotgun sequenci...
86. KB916185\_0 Neofusicoccum parvum UCRNP2 chromosome Unknown NP2\_03\_scaffold...
87. JH767570\_0 Coniosporium apollinis CBS 100218 chromosome Unknown supercont...
88. JH921428\_0 Marssonina brunnea f. sp. 'multigermtubi' MB\_m1 unplaced genom...
89. KB908844\_5 Setosphaeria turcica Et28A unplaced genomic scaffold SETTUscaf...
90. CAKM01000256\_0 Pneumocystis jirovecii strain SE8, whole genome shotgun se...
91. KB445589\_0 Cochliobolus heterostrophus C5 unplaced genomic scaffold COCHE...
92. KB733486\_0 Bipolaris maydis ATCC 48331 unplaced genomic scaffold COCC4sca...
93. KB707804\_0 Botryotinia fuckeliana BcDW1 unplaced genomic scaffold Scaffol...
94. KB445657\_0 Cochliobolus sativus ND90Pr unplaced genomic scaffold COCSAsca...
95. FP929137\_0 Leptosphaeria maculans JN3 lm\_SuperContig\_10\_v2 genomic superc...
96. GL535377\_0 Pyrenophora teres f. teres 0-1 unplaced genomic scaffold scaff...
97. CH445327\_0 Phaeosphaeria nodorum SN15 scaffold\_3, whole genome shotgun se...
98. GL988041\_0 Chaetomium thermophilum var. thermophilum DSM 1495 unplaced ge...
99. DS231623\_0 Pyrenophora tritici-repentis Pt-1C-BFP supercont1.9 genomic sc...
100. AGUE01000061\_0 Glarea lozoyensis 74030, whole genome shotgun sequencing ...

Query: Architecture Search FASTA input

KE145370 : Glarea lozoyensis ATCC 20868 chromosome Unknown GLAREA6    Total score: 3.0     Cumulative Blast bit score: 1309

Hit cluster cross-links:

Mycgr3G67791 Mycgr3T
  
Location: 0-1542

Mycgr3G67791\_Mycgr3T

Mycgr3G90406 Mycgr3T
  
Location: 1642-3973

Mycgr3G90406\_Mycgr3T

Mycgr3G67785 Mycgr3T
  
Location: 4073-7865

Mycgr3G67785\_Mycgr3T

Mycgr3G67795 Mycgr3T
  
Location: 7965-15249

Mycgr3G67795\_Mycgr3T

Mycgr3G67775 Mycgr3T
  
Location: 15349-16237

Mycgr3G67775\_Mycgr3T

Mycgr3G90404 Mycgr3T
  
Location: 16337-17246

Mycgr3G90404\_Mycgr3T

Mycgr3G36951 Mycgr3T
  
Location: 17346-30891

Mycgr3G36951\_Mycgr3T

Mycgr3G103034 Mycgr3
  
Location: 30991-32644

Mycgr3G103034\_Mycgr3

Mycgr3G31119 Mycgr3T
  
Location: 32744-32906

Mycgr3G31119\_Mycgr3T

Mycgr3G28587 Mycgr3T
  
Location: 33006-33489

Mycgr3G28587\_Mycgr3T

Mycgr3G98959 Mycgr3T
  
Location: 33589-35035

Mycgr3G98959\_Mycgr3T

Mycgr3G35447 Mycgr3T
  
Location: 35135-36443

Mycgr3G35447\_Mycgr3T

Mycgr3G84402 Mycgr3T
  
Location: 36543-37884

Mycgr3G84402\_Mycgr3T

Mycgr3G98961 Mycgr3T
  
Location: 37984-38884

Mycgr3G98961\_Mycgr3T

(Trans)glycosidase
  
Accession: EPE26861
  
Location: 1394387-1397815
  
 NCBI BlastP on this gene

EPE26861

Riboflavin synthase
  
Accession: EPE26860
  
Location: 1393019-1393765
  
 NCBI BlastP on this gene

EPE26860

hypothetical protein
  
Accession: EPE26859
  
Location: 1391893-1392132
  
 NCBI BlastP on this gene

EPE26859

Mss4-like protein
  
Accession: EPE26858
  
Location: 1390442-1391029
  
 NCBI BlastP on this gene

EPE26858

Cloroperoxidase
  
Accession: EPE26857
  
Location: 1387949-1389344
  
 NCBI BlastP on this gene

EPE26857

FYVE/PHD zinc finger
  
Accession: EPE26856
  
Location: 1385409-1386917
  
 NCBI BlastP on this gene

EPE26856

CoA-transferase family III (CaiB/BaiF)
  
Accession: EPE26855
  
Location: 1383331-1384904
  
 NCBI BlastP on this gene

EPE26855

hypothetical protein
  
Accession: EPE26854
  
Location: 1380937-1381852
  
 NCBI BlastP on this gene

EPE26854

hypothetical protein
  
Accession: EPE26853
  
Location: 1379796-1380386
  
 NCBI BlastP on this gene

EPE26853

hypothetical protein
  
Accession: EPE26852
  
Location: 1377511-1378911
  
  
**BlastP hit with Mycgr3G35447\_Mycgr3T**
  
Percentage identity: 57 %
  
BlastP bit score: 496
  
Sequence coverage: 103 %
  
E-value: 5e-170
  
  
 NCBI BlastP on this gene

EPE26852

P-loop containing nucleoside triphosphate hydrolase
  
Accession: EPE26851
  
Location: 1375781-1377289
  
  
**BlastP hit with Mycgr3G84402\_Mycgr3T**
  
Percentage identity: 72 %
  
BlastP bit score: 670
  
Sequence coverage: 102 %
  
E-value: 0.0
  
  
 NCBI BlastP on this gene

EPE26851

hypothetical protein
  
Accession: EPE26850
  
Location: 1373623-1374447
  
 NCBI BlastP on this gene

EPE26850

alpha/beta-Hydrolase
  
Accession: EPE26849
  
Location: 1372005-1373321
  
 NCBI BlastP on this gene

EPE26849

hypothetical protein
  
Accession: EPE26848
  
Location: 1370777-1371395
  
  
**BlastP hit with Mycgr3G28587\_Mycgr3T**
  
Percentage identity: 49 %
  
BlastP bit score: 143
  
Sequence coverage: 100 %
  
E-value: 7e-40
  
  
 NCBI BlastP on this gene

EPE26848

P-loop containing nucleoside triphosphate hydrolase
  
Accession: EPE26847
  
Location: 1369207-1370120
  
 NCBI BlastP on this gene

EPE26847

hypothetical protein
  
Accession: EPE26846
  
Location: 1364073-1367792
  
 NCBI BlastP on this gene

EPE26846

NAD(P)-binding Rossmann-fold containing protein
  
Accession: EPE26845
  
Location: 1361328-1362310
  
 NCBI BlastP on this gene

EPE26845

hypothetical protein
  
Accession: EPE26844
  
Location: 1360156-1360785
  
 NCBI BlastP on this gene

EPE26844

hypothetical protein
  
Accession: EPE26843
  
Location: 1357121-1357936
  
 NCBI BlastP on this gene

EPE26843

Thiamin diphosphate-binding fold (THDP-binding)
  
Accession: EPE26842
  
Location: 1352946-1355238
  
 NCBI BlastP on this gene

EPE26842

Query: Architecture Search FASTA input

FQ790359 : Botryotinia fuckeliana T4 SupSuperContig\_114\_320\_122\_1 genomic supercontig.    Total score: 3.0     Cumulative Blast bit score: 1286

Hit cluster cross-links:

Mycgr3G67791 Mycgr3T
  
Location: 0-1542

Mycgr3G67791\_Mycgr3T

Mycgr3G90406 Mycgr3T
  
Location: 1642-3973

Mycgr3G90406\_Mycgr3T

Mycgr3G67785 Mycgr3T
  
Location: 4073-7865

Mycgr3G67785\_Mycgr3T

Mycgr3G67795 Mycgr3T
  
Location: 7965-15249

Mycgr3G67795\_Mycgr3T

Mycgr3G67775 Mycgr3T
  
Location: 15349-16237

Mycgr3G67775\_Mycgr3T

Mycgr3G90404 Mycgr3T
  
Location: 16337-17246

Mycgr3G90404\_Mycgr3T

Mycgr3G36951 Mycgr3T
  
Location: 17346-30891

Mycgr3G36951\_Mycgr3T

Mycgr3G103034 Mycgr3
  
Location: 30991-32644

Mycgr3G103034\_Mycgr3

Mycgr3G31119 Mycgr3T
  
Location: 32744-32906

Mycgr3G31119\_Mycgr3T

Mycgr3G28587 Mycgr3T
  
Location: 33006-33489

Mycgr3G28587\_Mycgr3T

Mycgr3G98959 Mycgr3T
  
Location: 33589-35035

Mycgr3G98959\_Mycgr3T

Mycgr3G35447 Mycgr3T
  
Location: 35135-36443

Mycgr3G35447\_Mycgr3T

Mycgr3G84402 Mycgr3T
  
Location: 36543-37884

Mycgr3G84402\_Mycgr3T

Mycgr3G98961 Mycgr3T
  
Location: 37984-38884

Mycgr3G98961\_Mycgr3T

hypothetical protein
  
Accession: CCD56341
  
Location: 647660-647995
  
 NCBI BlastP on this gene

BofuT4\_P149510.1

hypothetical protein
  
Accession: CCD56340
  
Location: 644879-647048
  
 NCBI BlastP on this gene

BofuT4\_P149500.1

hypothetical protein
  
Accession: CCD56339
  
Location: 641338-641580
  
 NCBI BlastP on this gene

BofuT4\_uP149490.1

similar to siderophore biosynthesis
  
Accession: CCD56338
  
Location: 639542-640599
  
 NCBI BlastP on this gene

BofuT4\_P149480.1

hypothetical protein
  
Accession: CCD56337
  
Location: 638348-639167
  
  
**BlastP hit with Mycgr3G28587\_Mycgr3T**
  
Percentage identity: 51 %
  
BlastP bit score: 148
  
Sequence coverage: 99 %
  
E-value: 1e-41
  
  
 NCBI BlastP on this gene

BofuT4\_P149470.1

similar to GTP-binding protein rho2
  
Accession: CCD56336
  
Location: 636089-637084
  
 NCBI BlastP on this gene

BofuT4\_P149460.1

hypothetical protein
  
Accession: CCD56335
  
Location: 628910-632583
  
 NCBI BlastP on this gene

BofuT4\_P149450.1

hypothetical protein
  
Accession: CCD56334
  
Location: 627642-627791
  
 NCBI BlastP on this gene

BofuT4\_uP149440.1

predicted protein
  
Accession: CCD56333
  
Location: 626861-627016
  
 NCBI BlastP on this gene

BofuT4\_uP149430.1

predicted protein
  
Accession: CCD56332
  
Location: 625876-626739
  
 NCBI BlastP on this gene

BofuT4\_P149420.1

hypothetical protein
  
Accession: CCD56331
  
Location: 623016-624587
  
 NCBI BlastP on this gene

BofuT4\_P149410.1

similar to ser/Thr protein phosphatase superfamily
  
Accession: CCD56330
  
Location: 621006-621899
  
 NCBI BlastP on this gene

BofuT4\_P149400.1

hypothetical protein
  
Accession: CCD56329
  
Location: 618281-620087
  
 NCBI BlastP on this gene

BofuT4\_P149390.1

similar to caib/baif family protein
  
Accession: CCD56328
  
Location: 616311-618023
  
 NCBI BlastP on this gene

BofuT4\_P149380.1

similar to ATP-dependent rRNA helicase rrp3
  
Accession: CCD56327
  
Location: 614670-616177
  
  
**BlastP hit with Mycgr3G84402\_Mycgr3T**
  
Percentage identity: 71 %
  
BlastP bit score: 660
  
Sequence coverage: 103 %
  
E-value: 0.0
  
  
 NCBI BlastP on this gene

BofuT4\_P149370.1

similar to ribosome biogenesis protein Ssf2
  
Accession: CCD56326
  
Location: 612997-614396
  
  
**BlastP hit with Mycgr3G35447\_Mycgr3T**
  
Percentage identity: 62 %
  
BlastP bit score: 478
  
Sequence coverage: 91 %
  
E-value: 1e-162
  
  
 NCBI BlastP on this gene

BofuT4\_P149360.1

predicted protein
  
Accession: CCD56325
  
Location: 611236-611580
  
 NCBI BlastP on this gene

BofuT4\_P149350.1

hypothetical protein
  
Accession: CCD56324
  
Location: 607674-610070
  
 NCBI BlastP on this gene

BofuT4\_P149340.1

similar to U1biquitin-specific peptidase
  
Accession: CCD56323
  
Location: 605002-607008
  
 NCBI BlastP on this gene

BofuT4\_P149330.1

hypothetical protein
  
Accession: CCD56322
  
Location: 603428-604393
  
 NCBI BlastP on this gene

BofuT4\_P149320.1

Query: Architecture Search FASTA input

AFWA01000017 : Pneumocystis murina B123    Total score: 3.0     Cumulative Blast bit score: 1276

Hit cluster cross-links:

Mycgr3G67791 Mycgr3T
  
Location: 0-1542

Mycgr3G67791\_Mycgr3T

Mycgr3G90406 Mycgr3T
  
Location: 1642-3973

Mycgr3G90406\_Mycgr3T

Mycgr3G67785 Mycgr3T
  
Location: 4073-7865

Mycgr3G67785\_Mycgr3T

Mycgr3G67795 Mycgr3T
  
Location: 7965-15249

Mycgr3G67795\_Mycgr3T

Mycgr3G67775 Mycgr3T
  
Location: 15349-16237

Mycgr3G67775\_Mycgr3T

Mycgr3G90404 Mycgr3T
  
Location: 16337-17246

Mycgr3G90404\_Mycgr3T

Mycgr3G36951 Mycgr3T
  
Location: 17346-30891

Mycgr3G36951\_Mycgr3T

Mycgr3G103034 Mycgr3
  
Location: 30991-32644

Mycgr3G103034\_Mycgr3

Mycgr3G31119 Mycgr3T
  
Location: 32744-32906

Mycgr3G31119\_Mycgr3T

Mycgr3G28587 Mycgr3T
  
Location: 33006-33489

Mycgr3G28587\_Mycgr3T

Mycgr3G98959 Mycgr3T
  
Location: 33589-35035

Mycgr3G98959\_Mycgr3T

Mycgr3G35447 Mycgr3T
  
Location: 35135-36443

Mycgr3G35447\_Mycgr3T

Mycgr3G84402 Mycgr3T
  
Location: 36543-37884

Mycgr3G84402\_Mycgr3T

Mycgr3G98961 Mycgr3T
  
Location: 37984-38884

Mycgr3G98961\_Mycgr3T

hypothetical protein
  
Accession: EMR08120
  
Location: 222330-224159
  
 NCBI BlastP on this gene

EMR08120

hypothetical protein
  
Accession: EMR08119
  
Location: 219170-221707
  
 NCBI BlastP on this gene

EMR08119

hypothetical protein
  
Accession: EMR08118
  
Location: 212613-219018
  
 NCBI BlastP on this gene

EMR08118

hypothetical protein
  
Accession: EMR08117
  
Location: 209957-211795
  
 NCBI BlastP on this gene

EMR08117

hypothetical protein
  
Accession: EMR08116
  
Location: 207756-209538
  
 NCBI BlastP on this gene

EMR08116

hypothetical protein
  
Accession: EMR08115
  
Location: 205220-207140
  
  
**BlastP hit with Mycgr3G103034\_Mycgr3**
  
Percentage identity: 45 %
  
BlastP bit score: 473
  
Sequence coverage: 95 %
  
E-value: 5e-158
  
  
 NCBI BlastP on this gene

EMR08115

hypothetical protein
  
Accession: EMR08114
  
Location: 204078-205098
  
 NCBI BlastP on this gene

EMR08114

AGC/AKT protein kinase
  
Accession: EMR08113
  
Location: 201258-203629
  
 NCBI BlastP on this gene

EMR08113

riboflavin synthase, alpha subunit
  
Accession: EMR08112
  
Location: 200421-201110
  
 NCBI BlastP on this gene

EMR08112

hypothetical protein
  
Accession: EMR08111
  
Location: 198786-200354
  
  
**BlastP hit with Mycgr3G84402\_Mycgr3T**
  
Percentage identity: 62 %
  
BlastP bit score: 543
  
Sequence coverage: 90 %
  
E-value: 0.0
  
  
 NCBI BlastP on this gene

EMR08111

hypothetical protein
  
Accession: EMR08110
  
Location: 197570-198709
  
  
**BlastP hit with Mycgr3G35447\_Mycgr3T**
  
Percentage identity: 44 %
  
BlastP bit score: 260
  
Sequence coverage: 77 %
  
E-value: 1e-79
  
  
 NCBI BlastP on this gene

EMR08110

hypothetical protein
  
Accession: EMR08109
  
Location: 195736-197131
  
 NCBI BlastP on this gene

EMR08109

hypothetical protein
  
Accession: EMR08108
  
Location: 193855-195104
  
 NCBI BlastP on this gene

EMR08108

hypothetical protein
  
Accession: EMR08107
  
Location: 189871-191963
  
 NCBI BlastP on this gene

EMR08107

hypothetical protein
  
Accession: EMR08106
  
Location: 188500-189655
  
 NCBI BlastP on this gene

EMR08106

hypothetical protein
  
Accession: EMR08105
  
Location: 185523-188101
  
 NCBI BlastP on this gene

EMR08105

hypothetical protein
  
Accession: EMR08104
  
Location: 183980-184642
  
 NCBI BlastP on this gene

EMR08104

hypothetical protein, variant
  
Accession: EMR08103
  
Location: 181221-182078
  
 NCBI BlastP on this gene

EMR08103

hypothetical protein
  
Accession: EMR08102
  
Location: 181221-182490
  
 NCBI BlastP on this gene

EMR08102

hypothetical protein
  
Accession: EMR08101
  
Location: 179013-181054
  
 NCBI BlastP on this gene

EMR08101

Query: Architecture Search FASTA input

CH476628 : Sclerotinia sclerotiorum 1980 scaffold\_8 genomic scaffold    Total score: 3.0     Cumulative Blast bit score: 1242

Hit cluster cross-links:

Mycgr3G67791 Mycgr3T
  
Location: 0-1542

Mycgr3G67791\_Mycgr3T

Mycgr3G90406 Mycgr3T
  
Location: 1642-3973

Mycgr3G90406\_Mycgr3T

Mycgr3G67785 Mycgr3T
  
Location: 4073-7865

Mycgr3G67785\_Mycgr3T

Mycgr3G67795 Mycgr3T
  
Location: 7965-15249

Mycgr3G67795\_Mycgr3T

Mycgr3G67775 Mycgr3T
  
Location: 15349-16237

Mycgr3G67775\_Mycgr3T

Mycgr3G90404 Mycgr3T
  
Location: 16337-17246

Mycgr3G90404\_Mycgr3T

Mycgr3G36951 Mycgr3T
  
Location: 17346-30891

Mycgr3G36951\_Mycgr3T

Mycgr3G103034 Mycgr3
  
Location: 30991-32644

Mycgr3G103034\_Mycgr3

Mycgr3G31119 Mycgr3T
  
Location: 32744-32906

Mycgr3G31119\_Mycgr3T

Mycgr3G28587 Mycgr3T
  
Location: 33006-33489

Mycgr3G28587\_Mycgr3T

Mycgr3G98959 Mycgr3T
  
Location: 33589-35035

Mycgr3G98959\_Mycgr3T

Mycgr3G35447 Mycgr3T
  
Location: 35135-36443

Mycgr3G35447\_Mycgr3T

Mycgr3G84402 Mycgr3T
  
Location: 36543-37884

Mycgr3G84402\_Mycgr3T

Mycgr3G98961 Mycgr3T
  
Location: 37984-38884

Mycgr3G98961\_Mycgr3T

hypothetical protein
  
Accession: EDO04075
  
Location: 640662-642581
  
 NCBI BlastP on this gene

EDO04075

predicted protein
  
Accession: EDO04076
  
Location: 643303-644266
  
 NCBI BlastP on this gene

EDO04076

predicted protein
  
Accession: EDO04077
  
Location: 644578-644791
  
 NCBI BlastP on this gene

EDO04077

hypothetical protein
  
Accession: EDO04078
  
Location: 645403-646699
  
 NCBI BlastP on this gene

EDO04078

hypothetical protein
  
Accession: EDO04079
  
Location: 648149-649418
  
 NCBI BlastP on this gene

EDO04079

hypothetical protein
  
Accession: EDO04080
  
Location: 650282-652591
  
 NCBI BlastP on this gene

EDO04080

predicted protein
  
Accession: EDO04081
  
Location: 655026-655217
  
 NCBI BlastP on this gene

EDO04081

predicted protein
  
Accession: EDO04082
  
Location: 655630-655815
  
 NCBI BlastP on this gene

EDO04082

hypothetical protein
  
Accession: EDO04083
  
Location: 656768-658170
  
  
**BlastP hit with Mycgr3G35447\_Mycgr3T**
  
Percentage identity: 61 %
  
BlastP bit score: 471
  
Sequence coverage: 91 %
  
E-value: 6e-160
  
  
 NCBI BlastP on this gene

EDO04083

conserved hypothetical protein
  
Accession: EDO04084
  
Location: 658462-659957
  
  
**BlastP hit with Mycgr3G84402\_Mycgr3T**
  
Percentage identity: 78 %
  
BlastP bit score: 657
  
Sequence coverage: 89 %
  
E-value: 0.0
  
  
 NCBI BlastP on this gene

EDO04084

hypothetical protein
  
Accession: EDO04085
  
Location: 660223-663824
  
 NCBI BlastP on this gene

EDO04085

hypothetical protein
  
Accession: EDO04086
  
Location: 665821-666867
  
 NCBI BlastP on this gene

EDO04086

hypothetical protein
  
Accession: EDO04087
  
Location: 667228-668328
  
  
**BlastP hit with Mycgr3G28587\_Mycgr3T**
  
Percentage identity: 45 %
  
BlastP bit score: 114
  
Sequence coverage: 85 %
  
E-value: 8e-29
  
  
 NCBI BlastP on this gene

EDO04087

hypothetical protein
  
Accession: EDO04088
  
Location: 669408-670413
  
 NCBI BlastP on this gene

EDO04088

hypothetical protein
  
Accession: EDO04089
  
Location: 673063-676779
  
 NCBI BlastP on this gene

EDO04089

predicted protein
  
Accession: EDO04090
  
Location: 678041-678217
  
 NCBI BlastP on this gene

EDO04090

predicted protein
  
Accession: EDO04091
  
Location: 678284-678858
  
 NCBI BlastP on this gene

EDO04091

hypothetical protein
  
Accession: EDO04092
  
Location: 680620-682071
  
 NCBI BlastP on this gene

EDO04092

hypothetical protein
  
Accession: EDO04093
  
Location: 682700-684252
  
 NCBI BlastP on this gene

EDO04093

Query: Architecture Search FASTA input

JH921451 : Marssonina brunnea f. sp. 'multigermtubi' MB\_m1 unplaced genomic scaffold M6\_S00024    Total score: 3.0     Cumulative Blast bit score: 1205

Hit cluster cross-links:

Mycgr3G67791 Mycgr3T
  
Location: 0-1542

Mycgr3G67791\_Mycgr3T

Mycgr3G90406 Mycgr3T
  
Location: 1642-3973

Mycgr3G90406\_Mycgr3T

Mycgr3G67785 Mycgr3T
  
Location: 4073-7865

Mycgr3G67785\_Mycgr3T

Mycgr3G67795 Mycgr3T
  
Location: 7965-15249

Mycgr3G67795\_Mycgr3T

Mycgr3G67775 Mycgr3T
  
Location: 15349-16237

Mycgr3G67775\_Mycgr3T

Mycgr3G90404 Mycgr3T
  
Location: 16337-17246

Mycgr3G90404\_Mycgr3T

Mycgr3G36951 Mycgr3T
  
Location: 17346-30891

Mycgr3G36951\_Mycgr3T

Mycgr3G103034 Mycgr3
  
Location: 30991-32644

Mycgr3G103034\_Mycgr3

Mycgr3G31119 Mycgr3T
  
Location: 32744-32906

Mycgr3G31119\_Mycgr3T

Mycgr3G28587 Mycgr3T
  
Location: 33006-33489

Mycgr3G28587\_Mycgr3T

Mycgr3G98959 Mycgr3T
  
Location: 33589-35035

Mycgr3G98959\_Mycgr3T

Mycgr3G35447 Mycgr3T
  
Location: 35135-36443

Mycgr3G35447\_Mycgr3T

Mycgr3G84402 Mycgr3T
  
Location: 36543-37884

Mycgr3G84402\_Mycgr3T

Mycgr3G98961 Mycgr3T
  
Location: 37984-38884

Mycgr3G98961\_Mycgr3T

cyclin
  
Accession: EKD13183
  
Location: 271965-273458
  
 NCBI BlastP on this gene

EKD13183

hypothetical protein
  
Accession: EKD13182
  
Location: 270981-271550
  
 NCBI BlastP on this gene

EKD13182

hypothetical protein
  
Accession: EKD13181
  
Location: 268924-270896
  
 NCBI BlastP on this gene

EKD13181

hypothetical protein
  
Accession: EKD13180
  
Location: 265357-266286
  
 NCBI BlastP on this gene

EKD13180

hypothetical protein
  
Accession: EKD13179
  
Location: 262163-262950
  
 NCBI BlastP on this gene

EKD13179

RING finger domain protein
  
Accession: EKD13178
  
Location: 260911-261667
  
  
**BlastP hit with Mycgr3G28587\_Mycgr3T**
  
Percentage identity: 48 %
  
BlastP bit score: 114
  
Sequence coverage: 101 %
  
E-value: 1e-28
  
  
 NCBI BlastP on this gene

EKD13178

GTP-binding protein rho2
  
Accession: EKD13177
  
Location: 258921-259894
  
 NCBI BlastP on this gene

EKD13177

stress response protein NST1
  
Accession: EKD13176
  
Location: 253980-257640
  
 NCBI BlastP on this gene

EKD13176

hypothetical protein
  
Accession: EKD13175
  
Location: 251794-252723
  
 NCBI BlastP on this gene

EKD13175

hypothetical protein
  
Accession: EKD13174
  
Location: 246072-247975
  
 NCBI BlastP on this gene

EKD13174

ribosome biogenesis protein
  
Accession: EKD13173
  
Location: 244283-245702
  
  
**BlastP hit with Mycgr3G35447\_Mycgr3T**
  
Percentage identity: 59 %
  
BlastP bit score: 483
  
Sequence coverage: 101 %
  
E-value: 2e-164
  
  
 NCBI BlastP on this gene

EKD13173

ATP-dependent rRNA helicase RRP3
  
Accession: EKD13172
  
Location: 242552-244092
  
  
**BlastP hit with Mycgr3G84402\_Mycgr3T**
  
Percentage identity: 73 %
  
BlastP bit score: 608
  
Sequence coverage: 89 %
  
E-value: 0.0
  
  
 NCBI BlastP on this gene

EKD13172

hypothetical protein
  
Accession: EKD13171
  
Location: 240927-241295
  
 NCBI BlastP on this gene

EKD13171

prolyl-tRNA synthetase
  
Accession: EKD13170
  
Location: 238569-240670
  
 NCBI BlastP on this gene

EKD13170

CCAAT-box-binding transcription factor
  
Accession: EKD13169
  
Location: 234490-237908
  
 NCBI BlastP on this gene

EKD13169

hypothetical protein
  
Accession: EKD13168
  
Location: 233170-234112
  
 NCBI BlastP on this gene

EKD13168

adenylyl-sulfate kinase
  
Accession: EKD13167
  
Location: 229496-232024
  
 NCBI BlastP on this gene

EKD13167

Query: Architecture Search FASTA input

KB708068 : Botryotinia fuckeliana BcDW1 unplaced genomic scaffold Scaffold\_396    Total score: 3.0     Cumulative Blast bit score: 1193

Hit cluster cross-links:

Mycgr3G67791 Mycgr3T
  
Location: 0-1542

Mycgr3G67791\_Mycgr3T

Mycgr3G90406 Mycgr3T
  
Location: 1642-3973

Mycgr3G90406\_Mycgr3T

Mycgr3G67785 Mycgr3T
  
Location: 4073-7865

Mycgr3G67785\_Mycgr3T

Mycgr3G67795 Mycgr3T
  
Location: 7965-15249

Mycgr3G67795\_Mycgr3T

Mycgr3G67775 Mycgr3T
  
Location: 15349-16237

Mycgr3G67775\_Mycgr3T

Mycgr3G90404 Mycgr3T
  
Location: 16337-17246

Mycgr3G90404\_Mycgr3T

Mycgr3G36951 Mycgr3T
  
Location: 17346-30891

Mycgr3G36951\_Mycgr3T

Mycgr3G103034 Mycgr3
  
Location: 30991-32644

Mycgr3G103034\_Mycgr3

Mycgr3G31119 Mycgr3T
  
Location: 32744-32906

Mycgr3G31119\_Mycgr3T

Mycgr3G28587 Mycgr3T
  
Location: 33006-33489

Mycgr3G28587\_Mycgr3T

Mycgr3G98959 Mycgr3T
  
Location: 33589-35035

Mycgr3G98959\_Mycgr3T

Mycgr3G35447 Mycgr3T
  
Location: 35135-36443

Mycgr3G35447\_Mycgr3T

Mycgr3G84402 Mycgr3T
  
Location: 36543-37884

Mycgr3G84402\_Mycgr3T

Mycgr3G98961 Mycgr3T
  
Location: 37984-38884

Mycgr3G98961\_Mycgr3T

hypothetical protein
  
Accession: EMR81738
  
Location: 261668-263479
  
 NCBI BlastP on this gene

EMR81738

putative c2h2-like zinc finger protein
  
Accession: EMR81737
  
Location: 258562-259293
  
 NCBI BlastP on this gene

EMR81737

putative siderophore biosynthesis lipase esterase protein
  
Accession: EMR81736
  
Location: 256551-257608
  
 NCBI BlastP on this gene

EMR81736

hypothetical protein
  
Accession: EMR81735
  
Location: 255150-256175
  
  
**BlastP hit with Mycgr3G28587\_Mycgr3T**
  
Percentage identity: 56 %
  
BlastP bit score: 55
  
Sequence coverage: 34 %
  
E-value: 2e-07
  
  
 NCBI BlastP on this gene

EMR81735

putative rho gtpase rho protein
  
Accession: EMR81734
  
Location: 253097-254092
  
 NCBI BlastP on this gene

EMR81734

putative stress response protein nst1 protein
  
Accession: EMR81733
  
Location: 246519-250227
  
 NCBI BlastP on this gene

EMR81733

putative integral membrane protein
  
Accession: EMR81732
  
Location: 240885-241750
  
 NCBI BlastP on this gene

EMR81732

putative ser thr protein phosphatase superfamily protein
  
Accession: EMR81731
  
Location: 238878-239771
  
 NCBI BlastP on this gene

EMR81731

putative phd finger containing protein phf1 protein
  
Accession: EMR81730
  
Location: 236425-237959
  
 NCBI BlastP on this gene

EMR81730

putative formyl-coenzyme a transferase protein
  
Accession: EMR81729
  
Location: 234341-235895
  
 NCBI BlastP on this gene

EMR81729

putative atp-dependent rrna helicase rrp3 protein
  
Accession: EMR81728
  
Location: 232542-234049
  
  
**BlastP hit with Mycgr3G84402\_Mycgr3T**
  
Percentage identity: 71 %
  
BlastP bit score: 660
  
Sequence coverage: 103 %
  
E-value: 0.0
  
  
 NCBI BlastP on this gene

EMR81728

putative ribosome biogenesis protein ssf1 protein
  
Accession: EMR81727
  
Location: 230869-232268
  
  
**BlastP hit with Mycgr3G35447\_Mycgr3T**
  
Percentage identity: 62 %
  
BlastP bit score: 478
  
Sequence coverage: 91 %
  
E-value: 1e-162
  
  
 NCBI BlastP on this gene

EMR81727

hypothetical protein
  
Accession: EMR81726
  
Location: 225539-227935
  
 NCBI BlastP on this gene

EMR81726

putative ubiquitin carboxyl-terminal hydrolase protein
  
Accession: EMR81725
  
Location: 222867-224873
  
 NCBI BlastP on this gene

EMR81725

Query: Architecture Search FASTA input

FP929137 : Leptosphaeria maculans JN3 lm\_SuperContig\_10\_v2 genomic supercontig    Total score: 3.0     Cumulative Blast bit score: 739

Hit cluster cross-links:

Mycgr3G67791 Mycgr3T
  
Location: 0-1542

Mycgr3G67791\_Mycgr3T

Mycgr3G90406 Mycgr3T
  
Location: 1642-3973

Mycgr3G90406\_Mycgr3T

Mycgr3G67785 Mycgr3T
  
Location: 4073-7865

Mycgr3G67785\_Mycgr3T

Mycgr3G67795 Mycgr3T
  
Location: 7965-15249

Mycgr3G67795\_Mycgr3T

Mycgr3G67775 Mycgr3T
  
Location: 15349-16237

Mycgr3G67775\_Mycgr3T

Mycgr3G90404 Mycgr3T
  
Location: 16337-17246

Mycgr3G90404\_Mycgr3T

Mycgr3G36951 Mycgr3T
  
Location: 17346-30891

Mycgr3G36951\_Mycgr3T

Mycgr3G103034 Mycgr3
  
Location: 30991-32644

Mycgr3G103034\_Mycgr3

Mycgr3G31119 Mycgr3T
  
Location: 32744-32906

Mycgr3G31119\_Mycgr3T

Mycgr3G28587 Mycgr3T
  
Location: 33006-33489

Mycgr3G28587\_Mycgr3T

Mycgr3G98959 Mycgr3T
  
Location: 33589-35035

Mycgr3G98959\_Mycgr3T

Mycgr3G35447 Mycgr3T
  
Location: 35135-36443

Mycgr3G35447\_Mycgr3T

Mycgr3G84402 Mycgr3T
  
Location: 36543-37884

Mycgr3G84402\_Mycgr3T

Mycgr3G98961 Mycgr3T
  
Location: 37984-38884

Mycgr3G98961\_Mycgr3T

predicted protein
  
Accession: CBX99904
  
Location: 865926-866473
  
 NCBI BlastP on this gene

LEMA\_P074930.1

predicted protein
  
Accession: CBX99905
  
Location: 866690-867234
  
 NCBI BlastP on this gene

LEMA\_P074940.1

predicted protein
  
Accession: CBX99906
  
Location: 867664-868193
  
 NCBI BlastP on this gene

LEMA\_P074950.1

predicted protein
  
Accession: CBX99907
  
Location: 868472-869227
  
  
**BlastP hit with Mycgr3G28587\_Mycgr3T**
  
Percentage identity: 74 %
  
BlastP bit score: 76
  
Sequence coverage: 36 %
  
E-value: 3e-14
  
  
 NCBI BlastP on this gene

LEMA\_P074960.1

similar to GTP-binding protein RHO-1 protein
  
Accession: CBX99908
  
Location: 870397-871545
  
 NCBI BlastP on this gene

LEMA\_P074970.1

hypothetical protein
  
Accession: CBX99909
  
Location: 873693-876858
  
 NCBI BlastP on this gene

LEMA\_P074980.1

similar to N-acetylglucosamine-phosphate mutase
  
Accession: CBX99910
  
Location: 877974-879904
  
  
**BlastP hit with Mycgr3G103034\_Mycgr3**
  
Percentage identity: 54 %
  
BlastP bit score: 594
  
Sequence coverage: 97 %
  
E-value: 0.0
  
  
 NCBI BlastP on this gene

LEMA\_P074990.1

similar to DUF221 domain-containing protein
  
Accession: CBX99911
  
Location: 882042-885034
  
 NCBI BlastP on this gene

LEMA\_P075000.1

predicted protein
  
Accession: CBX99912
  
Location: 886467-886834
  
 NCBI BlastP on this gene

LEMA\_uP075010.1

hypothetical protein
  
Accession: CBX99913
  
Location: 888253-889352
  
 NCBI BlastP on this gene

LEMA\_P075020.1

predicted protein
  
Accession: CBX99914
  
Location: 889963-890146
  
 NCBI BlastP on this gene

LEMA\_uP075030.1

predicted protein
  
Accession: CBX99915
  
Location: 892773-892931
  
 NCBI BlastP on this gene

LEMA\_uP075040.1

predicted protein
  
Accession: CBX99916
  
Location: 893265-893632
  
 NCBI BlastP on this gene

LEMA\_uP075050.1

predicted protein
  
Accession: CBX99917
  
Location: 894839-897481
  
 NCBI BlastP on this gene

LEMA\_P075060.1

predicted protein
  
Accession: CBX99918
  
Location: 897806-898789
  
 NCBI BlastP on this gene

LEMA\_P075070.1

predicted protein
  
Accession: CBX99919
  
Location: 901323-902667
  
 NCBI BlastP on this gene

LEMA\_P075080.1

hypothetical protein
  
Accession: CBX99920
  
Location: 904042-906207
  
  
**BlastP hit with Mycgr3G90406\_Mycgr3T**
  
Percentage identity: 26 %
  
BlastP bit score: 69
  
Sequence coverage: 23 %
  
E-value: 5e-09
  
  
 NCBI BlastP on this gene

LEMA\_P075090.1

hypothetical protein
  
Accession: CBX99921
  
Location: 907205-909011
  
 NCBI BlastP on this gene

LEMA\_P075100.1

Query: Architecture Search FASTA input

KB446542 : Dothistroma septosporum NZE10 unplaced genomic scaffold DOTSEscaffold\_8    Total score: 2.0     Cumulative Blast bit score: 5340

Hit cluster cross-links:

Mycgr3G67791 Mycgr3T
  
Location: 0-1542

Mycgr3G67791\_Mycgr3T

Mycgr3G90406 Mycgr3T
  
Location: 1642-3973

Mycgr3G90406\_Mycgr3T

Mycgr3G67785 Mycgr3T
  
Location: 4073-7865

Mycgr3G67785\_Mycgr3T

Mycgr3G67795 Mycgr3T
  
Location: 7965-15249

Mycgr3G67795\_Mycgr3T

Mycgr3G67775 Mycgr3T
  
Location: 15349-16237

Mycgr3G67775\_Mycgr3T

Mycgr3G90404 Mycgr3T
  
Location: 16337-17246

Mycgr3G90404\_Mycgr3T

Mycgr3G36951 Mycgr3T
  
Location: 17346-30891

Mycgr3G36951\_Mycgr3T

Mycgr3G103034 Mycgr3
  
Location: 30991-32644

Mycgr3G103034\_Mycgr3

Mycgr3G31119 Mycgr3T
  
Location: 32744-32906

Mycgr3G31119\_Mycgr3T

Mycgr3G28587 Mycgr3T
  
Location: 33006-33489

Mycgr3G28587\_Mycgr3T

Mycgr3G98959 Mycgr3T
  
Location: 33589-35035

Mycgr3G98959\_Mycgr3T

Mycgr3G35447 Mycgr3T
  
Location: 35135-36443

Mycgr3G35447\_Mycgr3T

Mycgr3G84402 Mycgr3T
  
Location: 36543-37884

Mycgr3G84402\_Mycgr3T

Mycgr3G98961 Mycgr3T
  
Location: 37984-38884

Mycgr3G98961\_Mycgr3T

glycoside hydrolase family 43 protein
  
Accession: EME41694
  
Location: 1326279-1327413
  
 NCBI BlastP on this gene

EME41694

hypothetical protein
  
Accession: EME41695
  
Location: 1329262-1329861
  
 NCBI BlastP on this gene

EME41695

hypothetical protein
  
Accession: EME41696
  
Location: 1331123-1331417
  
 NCBI BlastP on this gene

EME41696

hypothetical protein
  
Accession: EME41697
  
Location: 1333402-1334617
  
 NCBI BlastP on this gene

EME41697

hypothetical protein
  
Accession: EME41698
  
Location: 1334915-1335880
  
 NCBI BlastP on this gene

EME41698

hypothetical protein
  
Accession: EME41699
  
Location: 1336762-1337446
  
 NCBI BlastP on this gene

EME41699

non-ribosomal peptide synthetase-like protein
  
Accession: EME41700
  
Location: 1338920-1353680
  
  
**BlastP hit with Mycgr3G36951\_Mycgr3T**
  
Percentage identity: 51 %
  
BlastP bit score: 3545
  
Sequence coverage: 76 %
  
E-value: 0.0
  
  
 NCBI BlastP on this gene

EME41700

hypothetical protein
  
Accession: EME41701
  
Location: 1355325-1359405
  
  
**BlastP hit with Mycgr3G67785\_Mycgr3T**
  
Percentage identity: 69 %
  
BlastP bit score: 1795
  
Sequence coverage: 100 %
  
E-value: 0.0
  
  
 NCBI BlastP on this gene

EME41701

hypothetical protein
  
Accession: EME41702
  
Location: 1360459-1362032
  
 NCBI BlastP on this gene

EME41702

hypothetical protein
  
Accession: EME41703
  
Location: 1363655-1364053
  
 NCBI BlastP on this gene

EME41703

hypothetical protein
  
Accession: EME41704
  
Location: 1365956-1366207
  
 NCBI BlastP on this gene

EME41704

hypothetical protein
  
Accession: EME41705
  
Location: 1366605-1367807
  
 NCBI BlastP on this gene

EME41705

hypothetical protein
  
Accession: EME41706
  
Location: 1368462-1372073
  
 NCBI BlastP on this gene

EME41706

Query: Architecture Search FASTA input

KB456266 : Mycosphaerella populorum SO2202 unplaced genomic scaffold SEPMUscaffold\_7    Total score: 2.0     Cumulative Blast bit score: 5225

Hit cluster cross-links:

Mycgr3G67791 Mycgr3T
  
Location: 0-1542

Mycgr3G67791\_Mycgr3T

Mycgr3G90406 Mycgr3T
  
Location: 1642-3973

Mycgr3G90406\_Mycgr3T

Mycgr3G67785 Mycgr3T
  
Location: 4073-7865

Mycgr3G67785\_Mycgr3T

Mycgr3G67795 Mycgr3T
  
Location: 7965-15249

Mycgr3G67795\_Mycgr3T

Mycgr3G67775 Mycgr3T
  
Location: 15349-16237

Mycgr3G67775\_Mycgr3T

Mycgr3G90404 Mycgr3T
  
Location: 16337-17246

Mycgr3G90404\_Mycgr3T

Mycgr3G36951 Mycgr3T
  
Location: 17346-30891

Mycgr3G36951\_Mycgr3T

Mycgr3G103034 Mycgr3
  
Location: 30991-32644

Mycgr3G103034\_Mycgr3

Mycgr3G31119 Mycgr3T
  
Location: 32744-32906

Mycgr3G31119\_Mycgr3T

Mycgr3G28587 Mycgr3T
  
Location: 33006-33489

Mycgr3G28587\_Mycgr3T

Mycgr3G98959 Mycgr3T
  
Location: 33589-35035

Mycgr3G98959\_Mycgr3T

Mycgr3G35447 Mycgr3T
  
Location: 35135-36443

Mycgr3G35447\_Mycgr3T

Mycgr3G84402 Mycgr3T
  
Location: 36543-37884

Mycgr3G84402\_Mycgr3T

Mycgr3G98961 Mycgr3T
  
Location: 37984-38884

Mycgr3G98961\_Mycgr3T

timeless-domain-containing protein
  
Accession: EMF11463
  
Location: 1491062-1494763
  
 NCBI BlastP on this gene

EMF11463

3HCDH N-domain-containing protein
  
Accession: EMF11464
  
Location: 1495065-1496419
  
 NCBI BlastP on this gene

EMF11464

hypothetical protein
  
Accession: EMF11465
  
Location: 1497078-1498041
  
 NCBI BlastP on this gene

EMF11465

hypothetical protein
  
Accession: EMF11466
  
Location: 1498279-1499301
  
 NCBI BlastP on this gene

EMF11466

hydroxylase
  
Accession: EMF11467
  
Location: 1502653-1504258
  
 NCBI BlastP on this gene

EMF11467

multidrug resistance-like protein
  
Accession: EMF11468
  
Location: 1505460-1509410
  
  
**BlastP hit with Mycgr3G67785\_Mycgr3T**
  
Percentage identity: 66 %
  
BlastP bit score: 1719
  
Sequence coverage: 101 %
  
E-value: 0.0
  
  
 NCBI BlastP on this gene

EMF11468

peptide synthetase
  
Accession: EMF11469
  
Location: 1511725-1526534
  
  
**BlastP hit with Mycgr3G36951\_Mycgr3T**
  
Percentage identity: 50 %
  
BlastP bit score: 3506
  
Sequence coverage: 77 %
  
E-value: 0.0
  
  
 NCBI BlastP on this gene

EMF11469

TPT-domain-containing protein
  
Accession: EMF11470
  
Location: 1527568-1529162
  
 NCBI BlastP on this gene

EMF11470

hypothetical protein
  
Accession: EMF11471
  
Location: 1529869-1531596
  
 NCBI BlastP on this gene

EMF11471

hypothetical protein
  
Accession: EMF11472
  
Location: 1531789-1533762
  
 NCBI BlastP on this gene

EMF11472

nitrate transporter CrnA
  
Accession: EMF11473
  
Location: 1535969-1537634
  
 NCBI BlastP on this gene

EMF11473

Query: Architecture Search FASTA input

KB446542 : Dothistroma septosporum NZE10 unplaced genomic scaffold DOTSEscaffold\_8    Total score: 2.0     Cumulative Blast bit score: 5215

Hit cluster cross-links:

Mycgr3G67791 Mycgr3T
  
Location: 0-1542

Mycgr3G67791\_Mycgr3T

Mycgr3G90406 Mycgr3T
  
Location: 1642-3973

Mycgr3G90406\_Mycgr3T

Mycgr3G67785 Mycgr3T
  
Location: 4073-7865

Mycgr3G67785\_Mycgr3T

Mycgr3G67795 Mycgr3T
  
Location: 7965-15249

Mycgr3G67795\_Mycgr3T

Mycgr3G67775 Mycgr3T
  
Location: 15349-16237

Mycgr3G67775\_Mycgr3T

Mycgr3G90404 Mycgr3T
  
Location: 16337-17246

Mycgr3G90404\_Mycgr3T

Mycgr3G36951 Mycgr3T
  
Location: 17346-30891

Mycgr3G36951\_Mycgr3T

Mycgr3G103034 Mycgr3
  
Location: 30991-32644

Mycgr3G103034\_Mycgr3

Mycgr3G31119 Mycgr3T
  
Location: 32744-32906

Mycgr3G31119\_Mycgr3T

Mycgr3G28587 Mycgr3T
  
Location: 33006-33489

Mycgr3G28587\_Mycgr3T

Mycgr3G98959 Mycgr3T
  
Location: 33589-35035

Mycgr3G98959\_Mycgr3T

Mycgr3G35447 Mycgr3T
  
Location: 35135-36443

Mycgr3G35447\_Mycgr3T

Mycgr3G84402 Mycgr3T
  
Location: 36543-37884

Mycgr3G84402\_Mycgr3T

Mycgr3G98961 Mycgr3T
  
Location: 37984-38884

Mycgr3G98961\_Mycgr3T

hypothetical protein
  
Accession: EME41608
  
Location: 1113435-1113626
  
 NCBI BlastP on this gene

EME41608

hypothetical protein
  
Accession: EME41609
  
Location: 1115501-1116935
  
 NCBI BlastP on this gene

EME41609

hypothetical protein
  
Accession: EME41610
  
Location: 1117740-1118039
  
 NCBI BlastP on this gene

EME41610

hypothetical protein
  
Accession: EME41611
  
Location: 1118421-1119251
  
 NCBI BlastP on this gene

EME41611

hypothetical protein
  
Accession: EME41612
  
Location: 1120539-1120775
  
 NCBI BlastP on this gene

EME41612

hypothetical protein
  
Accession: EME41613
  
Location: 1121646-1123271
  
 NCBI BlastP on this gene

EME41613

glycoside hydrolase family 78 protein
  
Accession: EME41614
  
Location: 1123851-1126076
  
 NCBI BlastP on this gene

EME41614

hypothetical protein
  
Accession: EME41615
  
Location: 1126662-1127513
  
 NCBI BlastP on this gene

EME41615

hypothetical protein
  
Accession: EME41616
  
Location: 1129486-1129732
  
 NCBI BlastP on this gene

EME41616

hypothetical protein
  
Accession: EME41617
  
Location: 1129792-1132263
  
  
**BlastP hit with Mycgr3G90406\_Mycgr3T**
  
Percentage identity: 51 %
  
BlastP bit score: 713
  
Sequence coverage: 108 %
  
E-value: 0.0
  
  
 NCBI BlastP on this gene

EME41617

hypothetical protein
  
Accession: EME41618
  
Location: 1132564-1139865
  
  
**BlastP hit with Mycgr3G67795\_Mycgr3T**
  
Percentage identity: 88 %
  
BlastP bit score: 4502
  
Sequence coverage: 100 %
  
E-value: 0.0
  
  
 NCBI BlastP on this gene

EME41618

hypothetical protein
  
Accession: EME41619
  
Location: 1140547-1141539
  
 NCBI BlastP on this gene

EME41619

C6 transcription factor-like protein
  
Accession: EME41620
  
Location: 1142797-1145066
  
 NCBI BlastP on this gene

EME41620

hypothetical protein
  
Accession: EME41621
  
Location: 1147061-1148194
  
 NCBI BlastP on this gene

EME41621

hypothetical protein
  
Accession: EME41622
  
Location: 1149388-1149687
  
 NCBI BlastP on this gene

EME41622

hypothetical protein
  
Accession: EME41623
  
Location: 1150320-1150922
  
 NCBI BlastP on this gene

EME41623

hypothetical protein
  
Accession: EME41624
  
Location: 1151465-1151982
  
 NCBI BlastP on this gene

EME41624

hypothetical protein
  
Accession: EME41625
  
Location: 1152789-1153627
  
 NCBI BlastP on this gene

EME41625

hypothetical protein
  
Accession: EME41626
  
Location: 1154005-1155544
  
 NCBI BlastP on this gene

EME41626

hypothetical protein
  
Accession: EME41627
  
Location: 1156723-1158438
  
 NCBI BlastP on this gene

EME41627

Query: Architecture Search FASTA input

KB446566 : Pseudocercospora fijiensis CIRAD86 unplaced genomic scaffold MYCFIscaffold\_12    Total score: 2.0     Cumulative Blast bit score: 4985

Hit cluster cross-links:

Mycgr3G67791 Mycgr3T
  
Location: 0-1542

Mycgr3G67791\_Mycgr3T

Mycgr3G90406 Mycgr3T
  
Location: 1642-3973

Mycgr3G90406\_Mycgr3T

Mycgr3G67785 Mycgr3T
  
Location: 4073-7865

Mycgr3G67785\_Mycgr3T

Mycgr3G67795 Mycgr3T
  
Location: 7965-15249

Mycgr3G67795\_Mycgr3T

Mycgr3G67775 Mycgr3T
  
Location: 15349-16237

Mycgr3G67775\_Mycgr3T

Mycgr3G90404 Mycgr3T
  
Location: 16337-17246

Mycgr3G90404\_Mycgr3T

Mycgr3G36951 Mycgr3T
  
Location: 17346-30891

Mycgr3G36951\_Mycgr3T

Mycgr3G103034 Mycgr3
  
Location: 30991-32644

Mycgr3G103034\_Mycgr3

Mycgr3G31119 Mycgr3T
  
Location: 32744-32906

Mycgr3G31119\_Mycgr3T

Mycgr3G28587 Mycgr3T
  
Location: 33006-33489

Mycgr3G28587\_Mycgr3T

Mycgr3G98959 Mycgr3T
  
Location: 33589-35035

Mycgr3G98959\_Mycgr3T

Mycgr3G35447 Mycgr3T
  
Location: 35135-36443

Mycgr3G35447\_Mycgr3T

Mycgr3G84402 Mycgr3T
  
Location: 36543-37884

Mycgr3G84402\_Mycgr3T

Mycgr3G98961 Mycgr3T
  
Location: 37984-38884

Mycgr3G98961\_Mycgr3T

hypothetical protein
  
Accession: EME77235
  
Location: 171939-175580
  
 NCBI BlastP on this gene

EME77235

hypothetical protein
  
Accession: EME77236
  
Location: 176123-177181
  
 NCBI BlastP on this gene

EME77236

hypothetical protein
  
Accession: EME77237
  
Location: 178439-179328
  
 NCBI BlastP on this gene

EME77237

hypothetical protein
  
Accession: EME77238
  
Location: 180357-184409
  
 NCBI BlastP on this gene

EME77238

hypothetical protein
  
Accession: EME77239
  
Location: 184392-186065
  
 NCBI BlastP on this gene

EME77239

ABC transporter, ABC-B family, MDR type
  
Accession: EME77240
  
Location: 187109-191177
  
  
**BlastP hit with Mycgr3G67785\_Mycgr3T**
  
Percentage identity: 64 %
  
BlastP bit score: 1653
  
Sequence coverage: 98 %
  
E-value: 0.0
  
  
 NCBI BlastP on this gene

EME77240

hypothetical protein
  
Accession: EME77241
  
Location: 192291-206966
  
  
**BlastP hit with Mycgr3G36951\_Mycgr3T**
  
Percentage identity: 48 %
  
BlastP bit score: 3332
  
Sequence coverage: 76 %
  
E-value: 0.0
  
  
 NCBI BlastP on this gene

EME77241

carbohydrate esterase family 9 protein
  
Accession: EME77242
  
Location: 208189-209508
  
 NCBI BlastP on this gene

EME77242

glycoside hydrolase family 3 protein
  
Accession: EME77243
  
Location: 210347-213450
  
 NCBI BlastP on this gene

EME77243

hypothetical protein
  
Accession: EME77244
  
Location: 216553-217101
  
 NCBI BlastP on this gene

EME77244

Query: Architecture Search FASTA input

KB446566 : Pseudocercospora fijiensis CIRAD86 unplaced genomic scaffold MYCFIscaffold\_12    Total score: 2.0     Cumulative Blast bit score: 4443

Hit cluster cross-links:

Mycgr3G67791 Mycgr3T
  
Location: 0-1542

Mycgr3G67791\_Mycgr3T

Mycgr3G90406 Mycgr3T
  
Location: 1642-3973

Mycgr3G90406\_Mycgr3T

Mycgr3G67785 Mycgr3T
  
Location: 4073-7865

Mycgr3G67785\_Mycgr3T

Mycgr3G67795 Mycgr3T
  
Location: 7965-15249

Mycgr3G67795\_Mycgr3T

Mycgr3G67775 Mycgr3T
  
Location: 15349-16237

Mycgr3G67775\_Mycgr3T

Mycgr3G90404 Mycgr3T
  
Location: 16337-17246

Mycgr3G90404\_Mycgr3T

Mycgr3G36951 Mycgr3T
  
Location: 17346-30891

Mycgr3G36951\_Mycgr3T

Mycgr3G103034 Mycgr3
  
Location: 30991-32644

Mycgr3G103034\_Mycgr3

Mycgr3G31119 Mycgr3T
  
Location: 32744-32906

Mycgr3G31119\_Mycgr3T

Mycgr3G28587 Mycgr3T
  
Location: 33006-33489

Mycgr3G28587\_Mycgr3T

Mycgr3G98959 Mycgr3T
  
Location: 33589-35035

Mycgr3G98959\_Mycgr3T

Mycgr3G35447 Mycgr3T
  
Location: 35135-36443

Mycgr3G35447\_Mycgr3T

Mycgr3G84402 Mycgr3T
  
Location: 36543-37884

Mycgr3G84402\_Mycgr3T

Mycgr3G98961 Mycgr3T
  
Location: 37984-38884

Mycgr3G98961\_Mycgr3T

hypothetical protein
  
Accession: EME77371
  
Location: 936380-937201
  
 NCBI BlastP on this gene

EME77371

hypothetical protein
  
Accession: EME77372
  
Location: 938799-941387
  
 NCBI BlastP on this gene

EME77372

hypothetical protein
  
Accession: EME77373
  
Location: 942550-945648
  
 NCBI BlastP on this gene

EME77373

hypothetical protein
  
Accession: EME77374
  
Location: 946401-947795
  
 NCBI BlastP on this gene

EME77374

hypothetical protein
  
Accession: EME77375
  
Location: 949865-951562
  
 NCBI BlastP on this gene

EME77375

hypothetical protein
  
Accession: EME77376
  
Location: 954705-954960
  
  
**BlastP hit with Mycgr3G90406\_Mycgr3T**
  
Percentage identity: 59 %
  
BlastP bit score: 73
  
Sequence coverage: 11 %
  
E-value: 3e-12
  
  
 NCBI BlastP on this gene

EME77376

hypothetical protein
  
Accession: EME77377
  
Location: 954984-955358
  
 NCBI BlastP on this gene

EME77377

phosphatidylinositol 3-kinase tor2
  
Accession: EME77378
  
Location: 955417-962775
  
  
**BlastP hit with Mycgr3G67795\_Mycgr3T**
  
Percentage identity: 85 %
  
BlastP bit score: 4370
  
Sequence coverage: 101 %
  
E-value: 0.0
  
  
 NCBI BlastP on this gene

EME77378

hypothetical protein
  
Accession: EME77379
  
Location: 963166-965354
  
 NCBI BlastP on this gene

EME77379

hypothetical protein
  
Accession: EME77380
  
Location: 965775-967148
  
 NCBI BlastP on this gene

EME77380

hypothetical protein
  
Accession: EME77381
  
Location: 967712-968608
  
 NCBI BlastP on this gene

EME77381

hypothetical protein
  
Accession: EME77382
  
Location: 968868-969207
  
 NCBI BlastP on this gene

EME77382

hypothetical protein
  
Accession: EME77383
  
Location: 969599-971245
  
 NCBI BlastP on this gene

EME77383

hypothetical protein
  
Accession: EME77384
  
Location: 972220-973722
  
 NCBI BlastP on this gene

EME77384

hypothetical protein
  
Accession: EME77385
  
Location: 973802-974758
  
 NCBI BlastP on this gene

EME77385

hypothetical protein
  
Accession: EME77386
  
Location: 976957-979710
  
 NCBI BlastP on this gene

EME77386

hypothetical protein
  
Accession: EME77387
  
Location: 980361-984296
  
 NCBI BlastP on this gene

EME77387

Query: Architecture Search FASTA input

DS027696 : Neosartorya fischeri NRRL 181 1099437636264 genomic scaffold    Total score: 2.0     Cumulative Blast bit score: 3830

Hit cluster cross-links:

Mycgr3G67791 Mycgr3T
  
Location: 0-1542

Mycgr3G67791\_Mycgr3T

Mycgr3G90406 Mycgr3T
  
Location: 1642-3973

Mycgr3G90406\_Mycgr3T

Mycgr3G67785 Mycgr3T
  
Location: 4073-7865

Mycgr3G67785\_Mycgr3T

Mycgr3G67795 Mycgr3T
  
Location: 7965-15249

Mycgr3G67795\_Mycgr3T

Mycgr3G67775 Mycgr3T
  
Location: 15349-16237

Mycgr3G67775\_Mycgr3T

Mycgr3G90404 Mycgr3T
  
Location: 16337-17246

Mycgr3G90404\_Mycgr3T

Mycgr3G36951 Mycgr3T
  
Location: 17346-30891

Mycgr3G36951\_Mycgr3T

Mycgr3G103034 Mycgr3
  
Location: 30991-32644

Mycgr3G103034\_Mycgr3

Mycgr3G31119 Mycgr3T
  
Location: 32744-32906

Mycgr3G31119\_Mycgr3T

Mycgr3G28587 Mycgr3T
  
Location: 33006-33489

Mycgr3G28587\_Mycgr3T

Mycgr3G98959 Mycgr3T
  
Location: 33589-35035

Mycgr3G98959\_Mycgr3T

Mycgr3G35447 Mycgr3T
  
Location: 35135-36443

Mycgr3G35447\_Mycgr3T

Mycgr3G84402 Mycgr3T
  
Location: 36543-37884

Mycgr3G84402\_Mycgr3T

Mycgr3G98961 Mycgr3T
  
Location: 37984-38884

Mycgr3G98961\_Mycgr3T

fungal specific transcription factor, putative
  
Accession: EAW18624
  
Location: 4022615-4024116
  
 NCBI BlastP on this gene

EAW18624

eukaryotic translation initiation factor 3 subunit EifCl, putative
  
Accession: EAW18623
  
Location: 4018954-4020528
  
  
**BlastP hit with Mycgr3G98959\_Mycgr3T**
  
Percentage identity: 75 %
  
BlastP bit score: 744
  
Sequence coverage: 100 %
  
E-value: 0.0
  
  
 NCBI BlastP on this gene

EAW18623

iron-sulfur cluster assembly accessory protein Isa2, putative
  
Accession: EAW18622
  
Location: 4017055-4018198
  
 NCBI BlastP on this gene

EAW18622

kynureninase
  
Accession: EAW18621
  
Location: 4015011-4016679
  
 NCBI BlastP on this gene

EAW18621

conserved hypothetical protein
  
Accession: EAW18620
  
Location: 4014101-4014818
  
 NCBI BlastP on this gene

EAW18620

AP-2 adaptor complex subunit beta, putative
  
Accession: EAW18619
  
Location: 4010802-4013492
  
 NCBI BlastP on this gene

EAW18619

conserved hypothetical protein
  
Accession: EAW18618
  
Location: 4008967-4010393
  
 NCBI BlastP on this gene

EAW18618

SH3 domain protein
  
Accession: EAW18617
  
Location: 4004511-4008312
  
 NCBI BlastP on this gene

EAW18617

conserved hypothetical protein
  
Accession: EAW18616
  
Location: 4002677-4003657
  
 NCBI BlastP on this gene

EAW18616

40S ribosomal protein S17, putative
  
Accession: EAW18615
  
Location: 4001562-4002200
  
 NCBI BlastP on this gene

EAW18615

conserved hypothetical protein
  
Accession: EAW18614
  
Location: 3999703-4001121
  
 NCBI BlastP on this gene

EAW18614

ketoreductase
  
Accession: EAW18613
  
Location: 3998139-3999396
  
 NCBI BlastP on this gene

EAW18613

TOR pathway phosphatidylinositol 3-kinase TorA, putative
  
Accession: EAW18612
  
Location: 3988834-3996176
  
  
**BlastP hit with Mycgr3G67795\_Mycgr3T**
  
Percentage identity: 62 %
  
BlastP bit score: 3086
  
Sequence coverage: 100 %
  
E-value: 0.0
  
  
 NCBI BlastP on this gene

EAW18612

conserved hypothetical protein
  
Accession: EAW18611
  
Location: 3986457-3988266
  
 NCBI BlastP on this gene

EAW18611

conserved hypothetical protein
  
Accession: EAW18610
  
Location: 3985026-3985997
  
 NCBI BlastP on this gene

EAW18610

NAD binding Rossmann fold oxidoreductase, putative
  
Accession: EAW18609
  
Location: 3982477-3983605
  
 NCBI BlastP on this gene

EAW18609

Query: Architecture Search FASTA input

DS499595 : Aspergillus fumigatus A1163 scf\_000002 genomic scaffold    Total score: 2.0     Cumulative Blast bit score: 3827

Hit cluster cross-links:

Mycgr3G67791 Mycgr3T
  
Location: 0-1542

Mycgr3G67791\_Mycgr3T

Mycgr3G90406 Mycgr3T
  
Location: 1642-3973

Mycgr3G90406\_Mycgr3T

Mycgr3G67785 Mycgr3T
  
Location: 4073-7865

Mycgr3G67785\_Mycgr3T

Mycgr3G67795 Mycgr3T
  
Location: 7965-15249

Mycgr3G67795\_Mycgr3T

Mycgr3G67775 Mycgr3T
  
Location: 15349-16237

Mycgr3G67775\_Mycgr3T

Mycgr3G90404 Mycgr3T
  
Location: 16337-17246

Mycgr3G90404\_Mycgr3T

Mycgr3G36951 Mycgr3T
  
Location: 17346-30891

Mycgr3G36951\_Mycgr3T

Mycgr3G103034 Mycgr3
  
Location: 30991-32644

Mycgr3G103034\_Mycgr3

Mycgr3G31119 Mycgr3T
  
Location: 32744-32906

Mycgr3G31119\_Mycgr3T

Mycgr3G28587 Mycgr3T
  
Location: 33006-33489

Mycgr3G28587\_Mycgr3T

Mycgr3G98959 Mycgr3T
  
Location: 33589-35035

Mycgr3G98959\_Mycgr3T

Mycgr3G35447 Mycgr3T
  
Location: 35135-36443

Mycgr3G35447\_Mycgr3T

Mycgr3G84402 Mycgr3T
  
Location: 36543-37884

Mycgr3G84402\_Mycgr3T

Mycgr3G98961 Mycgr3T
  
Location: 37984-38884

Mycgr3G98961\_Mycgr3T

C6 transcription factor, putative
  
Accession: EDP54562
  
Location: 2633984-2636333
  
 NCBI BlastP on this gene

EDP54562

hypothetical protein
  
Accession: EDP54561
  
Location: 2633495-2633934
  
 NCBI BlastP on this gene

EDP54561

eukaryotic translation initiation factor 3 subunit EifCl, putative
  
Accession: EDP54560
  
Location: 2631139-2632713
  
  
**BlastP hit with Mycgr3G98959\_Mycgr3T**
  
Percentage identity: 75 %
  
BlastP bit score: 746
  
Sequence coverage: 100 %
  
E-value: 0.0
  
  
 NCBI BlastP on this gene

EDP54560

iron-sulfur cluster assembly accessory protein Isa2, putative
  
Accession: EDP54559
  
Location: 2629361-2630387
  
 NCBI BlastP on this gene

EDP54559

kynureninase
  
Accession: EDP54558
  
Location: 2625368-2627027
  
 NCBI BlastP on this gene

EDP54558

ER membrane DUF1077 domain protein, putative
  
Accession: EDP54557
  
Location: 2624482-2625198
  
 NCBI BlastP on this gene

EDP54557

AP-2 adaptor complex subunit beta, putative
  
Accession: EDP54556
  
Location: 2621193-2623579
  
 NCBI BlastP on this gene

EDP54556

conserved hypothetical protein
  
Accession: EDP54555
  
Location: 2619376-2620786
  
 NCBI BlastP on this gene

EDP54555

SH3 domain protein
  
Accession: EDP54554
  
Location: 2614933-2618726
  
 NCBI BlastP on this gene

EDP54554

DUF408 domain protein
  
Accession: EDP54553
  
Location: 2613107-2614087
  
 NCBI BlastP on this gene

EDP54553

40S ribosomal protein S17, putative
  
Accession: EDP54552
  
Location: 2611678-2612634
  
 NCBI BlastP on this gene

EDP54552

conserved hypothetical protein
  
Accession: EDP54551
  
Location: 2610136-2611551
  
 NCBI BlastP on this gene

EDP54551

ketoreductase
  
Accession: EDP54550
  
Location: 2608558-2609823
  
 NCBI BlastP on this gene

EDP54550

TOR pathway phosphatidylinositol 3-kinase TorA, putative
  
Accession: EDP54549
  
Location: 2599279-2606624
  
  
**BlastP hit with Mycgr3G67795\_Mycgr3T**
  
Percentage identity: 62 %
  
BlastP bit score: 3081
  
Sequence coverage: 100 %
  
E-value: 0.0
  
  
 NCBI BlastP on this gene

EDP54549

conserved hypothetical protein
  
Accession: EDP54548
  
Location: 2596906-2598716
  
 NCBI BlastP on this gene

EDP54548

conserved hypothetical protein
  
Accession: EDP54547
  
Location: 2595474-2596424
  
 NCBI BlastP on this gene

EDP54547

NAD binding Rossmann fold oxidoreductase, putative
  
Accession: EDP54546
  
Location: 2592970-2594125
  
 NCBI BlastP on this gene

EDP54546

Query: Architecture Search FASTA input

AAHF01000001 : Aspergillus fumigatus Af293    Total score: 2.0     Cumulative Blast bit score: 3827

Hit cluster cross-links:

Mycgr3G67791 Mycgr3T
  
Location: 0-1542

Mycgr3G67791\_Mycgr3T

Mycgr3G90406 Mycgr3T
  
Location: 1642-3973

Mycgr3G90406\_Mycgr3T

Mycgr3G67785 Mycgr3T
  
Location: 4073-7865

Mycgr3G67785\_Mycgr3T

Mycgr3G67795 Mycgr3T
  
Location: 7965-15249

Mycgr3G67795\_Mycgr3T

Mycgr3G67775 Mycgr3T
  
Location: 15349-16237

Mycgr3G67775\_Mycgr3T

Mycgr3G90404 Mycgr3T
  
Location: 16337-17246

Mycgr3G90404\_Mycgr3T

Mycgr3G36951 Mycgr3T
  
Location: 17346-30891

Mycgr3G36951\_Mycgr3T

Mycgr3G103034 Mycgr3
  
Location: 30991-32644

Mycgr3G103034\_Mycgr3

Mycgr3G31119 Mycgr3T
  
Location: 32744-32906

Mycgr3G31119\_Mycgr3T

Mycgr3G28587 Mycgr3T
  
Location: 33006-33489

Mycgr3G28587\_Mycgr3T

Mycgr3G98959 Mycgr3T
  
Location: 33589-35035

Mycgr3G98959\_Mycgr3T

Mycgr3G35447 Mycgr3T
  
Location: 35135-36443

Mycgr3G35447\_Mycgr3T

Mycgr3G84402 Mycgr3T
  
Location: 36543-37884

Mycgr3G84402\_Mycgr3T

Mycgr3G98961 Mycgr3T
  
Location: 37984-38884

Mycgr3G98961\_Mycgr3T

C6 transcription factor, putative
  
Accession: EAL93335
  
Location: 788948-791297
  
 NCBI BlastP on this gene

EAL93335

hypothetical protein
  
Accession: EAL93334
  
Location: 788459-788898
  
 NCBI BlastP on this gene

EAL93334

eukaryotic translation initiation factor 3 subunit EifCl, putative
  
Accession: EAL93333
  
Location: 786103-787677
  
  
**BlastP hit with Mycgr3G98959\_Mycgr3T**
  
Percentage identity: 75 %
  
BlastP bit score: 746
  
Sequence coverage: 100 %
  
E-value: 0.0
  
  
 NCBI BlastP on this gene

EAL93333

iron-sulfur cluster assembly accessory protein Isa2, putative
  
Accession: EAL93332
  
Location: 784325-785351
  
 NCBI BlastP on this gene

EAL93332

kynureninase
  
Accession: EAL93331
  
Location: 780332-781991
  
 NCBI BlastP on this gene

EAL93331

ER membrane DUF1077 domain protein, putative
  
Accession: EAL93330
  
Location: 779446-780162
  
 NCBI BlastP on this gene

EAL93330

AP-2 adaptor complex subunit beta, putative
  
Accession: EAL93329
  
Location: 776157-778543
  
 NCBI BlastP on this gene

EAL93329

conserved hypothetical protein
  
Accession: EAL93328
  
Location: 774340-775750
  
 NCBI BlastP on this gene

EAL93328

SH3 domain protein
  
Accession: EAL93327
  
Location: 769897-773690
  
 NCBI BlastP on this gene

EAL93327

DUF408 domain protein
  
Accession: EAL93326
  
Location: 768072-769052
  
 NCBI BlastP on this gene

EAL93326

40S ribosomal protein S17, putative
  
Accession: EAL93325
  
Location: 766643-767599
  
 NCBI BlastP on this gene

EAL93325

conserved hypothetical protein
  
Accession: EAL93324
  
Location: 765101-766516
  
 NCBI BlastP on this gene

EAL93324

ketoreductase
  
Accession: EAL93323
  
Location: 763523-764788
  
 NCBI BlastP on this gene

EAL93323

TOR pathway phosphatidylinositol 3-kinase TorA, putative
  
Accession: EAL93322
  
Location: 754244-761589
  
  
**BlastP hit with Mycgr3G67795\_Mycgr3T**
  
Percentage identity: 62 %
  
BlastP bit score: 3081
  
Sequence coverage: 100 %
  
E-value: 0.0
  
  
 NCBI BlastP on this gene

EAL93322

conserved hypothetical protein
  
Accession: EAL93321
  
Location: 751871-753681
  
 NCBI BlastP on this gene

EAL93321

conserved hypothetical protein
  
Accession: EAL93320
  
Location: 750439-751389
  
 NCBI BlastP on this gene

EAL93320

NAD binding Rossmann fold oxidoreductase, putative
  
Accession: EAL93319
  
Location: 747935-749090
  
 NCBI BlastP on this gene

EAL93319

Query: Architecture Search FASTA input

DS027045 : Aspergillus clavatus NRRL 1 1099423829791 genomic scaffold    Total score: 2.0     Cumulative Blast bit score: 3803

Hit cluster cross-links:

Mycgr3G67791 Mycgr3T
  
Location: 0-1542

Mycgr3G67791\_Mycgr3T

Mycgr3G90406 Mycgr3T
  
Location: 1642-3973

Mycgr3G90406\_Mycgr3T

Mycgr3G67785 Mycgr3T
  
Location: 4073-7865

Mycgr3G67785\_Mycgr3T

Mycgr3G67795 Mycgr3T
  
Location: 7965-15249

Mycgr3G67795\_Mycgr3T

Mycgr3G67775 Mycgr3T
  
Location: 15349-16237

Mycgr3G67775\_Mycgr3T

Mycgr3G90404 Mycgr3T
  
Location: 16337-17246

Mycgr3G90404\_Mycgr3T

Mycgr3G36951 Mycgr3T
  
Location: 17346-30891

Mycgr3G36951\_Mycgr3T

Mycgr3G103034 Mycgr3
  
Location: 30991-32644

Mycgr3G103034\_Mycgr3

Mycgr3G31119 Mycgr3T
  
Location: 32744-32906

Mycgr3G31119\_Mycgr3T

Mycgr3G28587 Mycgr3T
  
Location: 33006-33489

Mycgr3G28587\_Mycgr3T

Mycgr3G98959 Mycgr3T
  
Location: 33589-35035

Mycgr3G98959\_Mycgr3T

Mycgr3G35447 Mycgr3T
  
Location: 35135-36443

Mycgr3G35447\_Mycgr3T

Mycgr3G84402 Mycgr3T
  
Location: 36543-37884

Mycgr3G84402\_Mycgr3T

Mycgr3G98961 Mycgr3T
  
Location: 37984-38884

Mycgr3G98961\_Mycgr3T

fungal specific transcription factor domain protein
  
Accession: EAW13913
  
Location: 488013-489527
  
 NCBI BlastP on this gene

EAW13913

hypothetical protein
  
Accession: EAW13912
  
Location: 487540-487935
  
 NCBI BlastP on this gene

EAW13912

eukaryotic translation initiation factor 3 subunit EifCl, putative
  
Accession: EAW13911
  
Location: 484523-486104
  
  
**BlastP hit with Mycgr3G98959\_Mycgr3T**
  
Percentage identity: 75 %
  
BlastP bit score: 747
  
Sequence coverage: 100 %
  
E-value: 0.0
  
  
 NCBI BlastP on this gene

EAW13911

iron-sulfur cluster assembly accessory protein Isa2, putative
  
Accession: EAW13910
  
Location: 482605-483732
  
 NCBI BlastP on this gene

EAW13910

kynureninase
  
Accession: EAW13909
  
Location: 480574-482231
  
 NCBI BlastP on this gene

EAW13909

ER membrane DUF1077 domain protein, putative
  
Accession: EAW13908
  
Location: 479686-480396
  
 NCBI BlastP on this gene

EAW13908

AP-2 adaptor complex subunit beta, putative
  
Accession: EAW13907
  
Location: 476355-479044
  
 NCBI BlastP on this gene

EAW13907

conserved hypothetical protein
  
Accession: EAW13906
  
Location: 474641-475974
  
 NCBI BlastP on this gene

EAW13906

SH3 domain protein
  
Accession: EAW13905
  
Location: 470160-473923
  
 NCBI BlastP on this gene

EAW13905

DUF408 domain protein
  
Accession: EAW13904
  
Location: 468313-469281
  
 NCBI BlastP on this gene

EAW13904

40S ribosomal protein S17, putative
  
Accession: EAW13903
  
Location: 467235-467874
  
 NCBI BlastP on this gene

EAW13903

conserved hypothetical protein
  
Accession: EAW13902
  
Location: 465421-466845
  
 NCBI BlastP on this gene

EAW13902

ketoreductase
  
Accession: EAW13901
  
Location: 463861-465126
  
 NCBI BlastP on this gene

EAW13901

TOR pathway phosphatidylinositol 3-kinase TorA, putative
  
Accession: EAW13900
  
Location: 454403-461762
  
  
**BlastP hit with Mycgr3G67795\_Mycgr3T**
  
Percentage identity: 62 %
  
BlastP bit score: 3057
  
Sequence coverage: 100 %
  
E-value: 0.0
  
  
 NCBI BlastP on this gene

EAW13900

conserved hypothetical protein
  
Accession: EAW13899
  
Location: 451935-453759
  
 NCBI BlastP on this gene

EAW13899

conserved hypothetical protein
  
Accession: EAW13898
  
Location: 450367-451518
  
 NCBI BlastP on this gene

EAW13898

NAD binding Rossmann fold oxidoreductase, putative
  
Accession: EAW13897
  
Location: 447723-448871
  
 NCBI BlastP on this gene

EAW13897

Query: Architecture Search FASTA input

ACJE01000013 : Aspergillus niger ATCC 1015    Total score: 2.0     Cumulative Blast bit score: 3803

Hit cluster cross-links:

Mycgr3G67791 Mycgr3T
  
Location: 0-1542

Mycgr3G67791\_Mycgr3T

Mycgr3G90406 Mycgr3T
  
Location: 1642-3973

Mycgr3G90406\_Mycgr3T

Mycgr3G67785 Mycgr3T
  
Location: 4073-7865

Mycgr3G67785\_Mycgr3T

Mycgr3G67795 Mycgr3T
  
Location: 7965-15249

Mycgr3G67795\_Mycgr3T

Mycgr3G67775 Mycgr3T
  
Location: 15349-16237

Mycgr3G67775\_Mycgr3T

Mycgr3G90404 Mycgr3T
  
Location: 16337-17246

Mycgr3G90404\_Mycgr3T

Mycgr3G36951 Mycgr3T
  
Location: 17346-30891

Mycgr3G36951\_Mycgr3T

Mycgr3G103034 Mycgr3
  
Location: 30991-32644

Mycgr3G103034\_Mycgr3

Mycgr3G31119 Mycgr3T
  
Location: 32744-32906

Mycgr3G31119\_Mycgr3T

Mycgr3G28587 Mycgr3T
  
Location: 33006-33489

Mycgr3G28587\_Mycgr3T

Mycgr3G98959 Mycgr3T
  
Location: 33589-35035

Mycgr3G98959\_Mycgr3T

Mycgr3G35447 Mycgr3T
  
Location: 35135-36443

Mycgr3G35447\_Mycgr3T

Mycgr3G84402 Mycgr3T
  
Location: 36543-37884

Mycgr3G84402\_Mycgr3T

Mycgr3G98961 Mycgr3T
  
Location: 37984-38884

Mycgr3G98961\_Mycgr3T

hypothetical protein
  
Accession: EHA22042
  
Location: 1350049-1351014
  
 NCBI BlastP on this gene

EHA22042

hypothetical protein
  
Accession: EHA22043
  
Location: 1351496-1352531
  
 NCBI BlastP on this gene

EHA22043

hypothetical protein
  
Accession: EHA22044
  
Location: 1352736-1354050
  
 NCBI BlastP on this gene

EHA22044

hypothetical protein
  
Accession: EHA22045
  
Location: 1354670-1355503
  
 NCBI BlastP on this gene

EHA22045

hypothetical protein
  
Accession: EHA22046
  
Location: 1356357-1357939
  
  
**BlastP hit with Mycgr3G98959\_Mycgr3T**
  
Percentage identity: 75 %
  
BlastP bit score: 753
  
Sequence coverage: 100 %
  
E-value: 0.0
  
  
 NCBI BlastP on this gene

EHA22046

hypothetical protein
  
Accession: EHA22047
  
Location: 1358752-1359854
  
 NCBI BlastP on this gene

EHA22047

hypothetical protein
  
Accession: EHA22048
  
Location: 1360225-1361856
  
 NCBI BlastP on this gene

EHA22048

hypothetical protein
  
Accession: EHA22049
  
Location: 1362222-1362950
  
 NCBI BlastP on this gene

EHA22049

hypothetical protein
  
Accession: EHA22050
  
Location: 1363842-1366206
  
 NCBI BlastP on this gene

EHA22050

hypothetical protein
  
Accession: EHA22051
  
Location: 1366544-1367851
  
 NCBI BlastP on this gene

EHA22051

hypothetical protein
  
Accession: EHA22052
  
Location: 1368333-1372083
  
 NCBI BlastP on this gene

EHA22052

hypothetical protein
  
Accession: EHA22053
  
Location: 1373216-1374217
  
 NCBI BlastP on this gene

EHA22053

hypothetical protein
  
Accession: EHA22054
  
Location: 1374618-1375347
  
 NCBI BlastP on this gene

EHA22054

hypothetical protein
  
Accession: EHA22055
  
Location: 1375763-1377136
  
 NCBI BlastP on this gene

EHA22055

hypothetical protein
  
Accession: EHA22056
  
Location: 1377380-1378650
  
 NCBI BlastP on this gene

EHA22056

TorA protein
  
Accession: EHA22057
  
Location: 1380238-1387581
  
  
**BlastP hit with Mycgr3G67795\_Mycgr3T**
  
Percentage identity: 62 %
  
BlastP bit score: 3051
  
Sequence coverage: 100 %
  
E-value: 0.0
  
  
 NCBI BlastP on this gene

EHA22057

hypothetical protein
  
Accession: EHA22058
  
Location: 1388170-1389980
  
 NCBI BlastP on this gene

EHA22058

hypothetical protein
  
Accession: EHA22059
  
Location: 1391544-1392677
  
 NCBI BlastP on this gene

EHA22059

hypothetical protein
  
Accession: EHA22060
  
Location: 1393654-1394659
  
 NCBI BlastP on this gene

EHA22060

Query: Architecture Search FASTA input

AM270369 : Aspergillus niger contig An16c0170, genomic contig.    Total score: 2.0     Cumulative Blast bit score: 3801

Hit cluster cross-links:

Mycgr3G67791 Mycgr3T
  
Location: 0-1542

Mycgr3G67791\_Mycgr3T

Mycgr3G90406 Mycgr3T
  
Location: 1642-3973

Mycgr3G90406\_Mycgr3T

Mycgr3G67785 Mycgr3T
  
Location: 4073-7865

Mycgr3G67785\_Mycgr3T

Mycgr3G67795 Mycgr3T
  
Location: 7965-15249

Mycgr3G67795\_Mycgr3T

Mycgr3G67775 Mycgr3T
  
Location: 15349-16237

Mycgr3G67775\_Mycgr3T

Mycgr3G90404 Mycgr3T
  
Location: 16337-17246

Mycgr3G90404\_Mycgr3T

Mycgr3G36951 Mycgr3T
  
Location: 17346-30891

Mycgr3G36951\_Mycgr3T

Mycgr3G103034 Mycgr3
  
Location: 30991-32644

Mycgr3G103034\_Mycgr3

Mycgr3G31119 Mycgr3T
  
Location: 32744-32906

Mycgr3G31119\_Mycgr3T

Mycgr3G28587 Mycgr3T
  
Location: 33006-33489

Mycgr3G28587\_Mycgr3T

Mycgr3G98959 Mycgr3T
  
Location: 33589-35035

Mycgr3G98959\_Mycgr3T

Mycgr3G35447 Mycgr3T
  
Location: 35135-36443

Mycgr3G35447\_Mycgr3T

Mycgr3G84402 Mycgr3T
  
Location: 36543-37884

Mycgr3G84402\_Mycgr3T

Mycgr3G98961 Mycgr3T
  
Location: 37984-38884

Mycgr3G98961\_Mycgr3T

not annotated
  
Accession: CAK42884
  
Location: 77-1238
  
 NCBI BlastP on this gene

An16g04540

not annotated
  
Accession: CAK42885
  
Location: 1315-2353
  
 NCBI BlastP on this gene

An16g04550

not annotated
  
Accession: CAK42886
  
Location: 2790-4069
  
 NCBI BlastP on this gene

An16g04560

not annotated
  
Accession: CAK42887
  
Location: 4461-6118
  
 NCBI BlastP on this gene

An16g04570

not annotated
  
Accession: CAK42888
  
Location: 6179-7761
  
  
**BlastP hit with Mycgr3G98959\_Mycgr3T**
  
Percentage identity: 75 %
  
BlastP bit score: 753
  
Sequence coverage: 100 %
  
E-value: 0.0
  
  
 NCBI BlastP on this gene

An16g04580

not annotated
  
Accession: CAK42889
  
Location: 7917-8339
  
 NCBI BlastP on this gene

An16g04590

not annotated
  
Accession: CAK42890
  
Location: 8539-9794
  
 NCBI BlastP on this gene

An16g04600

not annotated
  
Accession: CAK42891
  
Location: 10051-11837
  
 NCBI BlastP on this gene

An16g04630

not annotated
  
Accession: CAK42892
  
Location: 12049-12777
  
 NCBI BlastP on this gene

An16g04640

not annotated
  
Accession: CAK42893
  
Location: 13350-16215
  
 NCBI BlastP on this gene

An16g04650

not annotated
  
Accession: CAK42894
  
Location: 16372-17679
  
 NCBI BlastP on this gene

An16g04660

not annotated
  
Accession: CAK42895
  
Location: 18164-21905
  
 NCBI BlastP on this gene

An16g04670

not annotated
  
Accession: CAK42896
  
Location: 23038-24039
  
 NCBI BlastP on this gene

An16g04680

not annotated
  
Accession: CAK42897
  
Location: 24443-25081
  
 NCBI BlastP on this gene

An16g04690

not annotated
  
Accession: CAK42898
  
Location: 25583-26950
  
 NCBI BlastP on this gene

An16g04700

unnamed
  
Accession: CAK42899
  
Location: 27203-28473
  
 NCBI BlastP on this gene

An16g04710

not annotated
  
Accession: CAK42900
  
Location: 30059-37405
  
  
**BlastP hit with Mycgr3G67795\_Mycgr3T**
  
Percentage identity: 62 %
  
BlastP bit score: 3048
  
Sequence coverage: 100 %
  
E-value: 0.0
  
  
 NCBI BlastP on this gene

An16g04720

not annotated
  
Accession: CAK42901
  
Location: 37991-39801
  
 NCBI BlastP on this gene

An16g04730

not annotated
  
Accession: CAK42902
  
Location: 41363-42496
  
 NCBI BlastP on this gene

An16g04750

not annotated
  
Accession: CAK42903
  
Location: 43470-44475
  
 NCBI BlastP on this gene

An16g04760

Query: Architecture Search FASTA input

DF126452 : Aspergillus kawachii IFO 4308 DNA, contig: scaffold00006    Total score: 2.0     Cumulative Blast bit score: 3800

Hit cluster cross-links:

Mycgr3G67791 Mycgr3T
  
Location: 0-1542

Mycgr3G67791\_Mycgr3T

Mycgr3G90406 Mycgr3T
  
Location: 1642-3973

Mycgr3G90406\_Mycgr3T

Mycgr3G67785 Mycgr3T
  
Location: 4073-7865

Mycgr3G67785\_Mycgr3T

Mycgr3G67795 Mycgr3T
  
Location: 7965-15249

Mycgr3G67795\_Mycgr3T

Mycgr3G67775 Mycgr3T
  
Location: 15349-16237

Mycgr3G67775\_Mycgr3T

Mycgr3G90404 Mycgr3T
  
Location: 16337-17246

Mycgr3G90404\_Mycgr3T

Mycgr3G36951 Mycgr3T
  
Location: 17346-30891

Mycgr3G36951\_Mycgr3T

Mycgr3G103034 Mycgr3
  
Location: 30991-32644

Mycgr3G103034\_Mycgr3

Mycgr3G31119 Mycgr3T
  
Location: 32744-32906

Mycgr3G31119\_Mycgr3T

Mycgr3G28587 Mycgr3T
  
Location: 33006-33489

Mycgr3G28587\_Mycgr3T

Mycgr3G98959 Mycgr3T
  
Location: 33589-35035

Mycgr3G98959\_Mycgr3T

Mycgr3G35447 Mycgr3T
  
Location: 35135-36443

Mycgr3G35447\_Mycgr3T

Mycgr3G84402 Mycgr3T
  
Location: 36543-37884

Mycgr3G84402\_Mycgr3T

Mycgr3G98961 Mycgr3T
  
Location: 37984-38884

Mycgr3G98961\_Mycgr3T

haemolysin-III channel protein Izh2
  
Accession: GAA85030
  
Location: 1025464-1026429
  
 NCBI BlastP on this gene

GAA85030

similar to An16g04550
  
Accession: GAA85031
  
Location: 1026913-1027948
  
 NCBI BlastP on this gene

GAA85031

hypothetical protein
  
Accession: GAA85032
  
Location: 1028147-1029660
  
 NCBI BlastP on this gene

GAA85032

clathrin-coated vesicle protein
  
Accession: GAA85033
  
Location: 1030082-1030922
  
 NCBI BlastP on this gene

GAA85033

eukaryotic translation initiation factor 3 subunit 6-interacting protein
  
Accession: GAA85034
  
Location: 1031780-1033362
  
  
**BlastP hit with Mycgr3G98959\_Mycgr3T**
  
Percentage identity: 76 %
  
BlastP bit score: 754
  
Sequence coverage: 100 %
  
E-value: 0.0
  
  
 NCBI BlastP on this gene

GAA85034

iron-sulfur cluster assembly accessory protein Isa2
  
Accession: GAA85035
  
Location: 1034160-1035318
  
 NCBI BlastP on this gene

GAA85035

kynureninase
  
Accession: GAA85036
  
Location: 1035693-1037324
  
 NCBI BlastP on this gene

GAA85036

ER membrane DUF1077 domain protein
  
Accession: GAA85037
  
Location: 1037661-1038394
  
 NCBI BlastP on this gene

GAA85037

AP-2 adaptor complex subunit beta
  
Accession: GAA85038
  
Location: 1039653-1042015
  
 NCBI BlastP on this gene

GAA85038

pre-mRNA-splicing factor CWC25
  
Accession: GAA85039
  
Location: 1042380-1043702
  
 NCBI BlastP on this gene

GAA85039

SH3 domain protein
  
Accession: GAA85040
  
Location: 1044186-1047926
  
 NCBI BlastP on this gene

GAA85040

DUF408 domain protein
  
Accession: GAA85041
  
Location: 1049082-1050086
  
 NCBI BlastP on this gene

GAA85041

40S ribosomal protein S17
  
Accession: GAA85042
  
Location: 1050497-1051141
  
 NCBI BlastP on this gene

GAA85042

hypothetical protein
  
Accession: GAA85043
  
Location: 1051628-1052995
  
 NCBI BlastP on this gene

GAA85043

ketoreductase
  
Accession: GAA85044
  
Location: 1053254-1054524
  
 NCBI BlastP on this gene

GAA85044

phosphatidylinositol 3-kinase Tor2
  
Accession: GAA85045
  
Location: 1056148-1063485
  
  
**BlastP hit with Mycgr3G67795\_Mycgr3T**
  
Percentage identity: 62 %
  
BlastP bit score: 3046
  
Sequence coverage: 100 %
  
E-value: 0.0
  
  
 NCBI BlastP on this gene

GAA85045

similar to An16g04730
  
Accession: GAA85046
  
Location: 1064079-1065890
  
 NCBI BlastP on this gene

GAA85046

hypothetical protein
  
Accession: GAA85047
  
Location: 1068712-1070542
  
 NCBI BlastP on this gene

GAA85047

Query: Architecture Search FASTA input

AM920437 : Penicillium chrysogenum Wisconsin 54-1255 complete genome, contig Pc00c22.    Total score: 2.0     Cumulative Blast bit score: 3794

Hit cluster cross-links:

Mycgr3G67791 Mycgr3T
  
Location: 0-1542

Mycgr3G67791\_Mycgr3T

Mycgr3G90406 Mycgr3T
  
Location: 1642-3973

Mycgr3G90406\_Mycgr3T

Mycgr3G67785 Mycgr3T
  
Location: 4073-7865

Mycgr3G67785\_Mycgr3T

Mycgr3G67795 Mycgr3T
  
Location: 7965-15249

Mycgr3G67795\_Mycgr3T

Mycgr3G67775 Mycgr3T
  
Location: 15349-16237

Mycgr3G67775\_Mycgr3T

Mycgr3G90404 Mycgr3T
  
Location: 16337-17246

Mycgr3G90404\_Mycgr3T

Mycgr3G36951 Mycgr3T
  
Location: 17346-30891

Mycgr3G36951\_Mycgr3T

Mycgr3G103034 Mycgr3
  
Location: 30991-32644

Mycgr3G103034\_Mycgr3

Mycgr3G31119 Mycgr3T
  
Location: 32744-32906

Mycgr3G31119\_Mycgr3T

Mycgr3G28587 Mycgr3T
  
Location: 33006-33489

Mycgr3G28587\_Mycgr3T

Mycgr3G98959 Mycgr3T
  
Location: 33589-35035

Mycgr3G98959\_Mycgr3T

Mycgr3G35447 Mycgr3T
  
Location: 35135-36443

Mycgr3G35447\_Mycgr3T

Mycgr3G84402 Mycgr3T
  
Location: 36543-37884

Mycgr3G84402\_Mycgr3T

Mycgr3G98961 Mycgr3T
  
Location: 37984-38884

Mycgr3G98961\_Mycgr3T

not annotated
  
Accession: CAP99333
  
Location: 4830117-4833915
  
 NCBI BlastP on this gene

Pc22g20450

not annotated
  
Accession: CAP99334
  
Location: 4834848-4835810
  
 NCBI BlastP on this gene

Pc22g20460

not annotated
  
Accession: CAP99335
  
Location: 4836321-4836864
  
 NCBI BlastP on this gene

Pc22g20470

unnamed
  
Accession: CAP99336
  
Location: 4837326-4838825
  
 NCBI BlastP on this gene

Pc22g20480

not annotated
  
Accession: CAP99337
  
Location: 4839043-4840260
  
 NCBI BlastP on this gene

Pc22g20490

not annotated
  
Accession: CAP99338
  
Location: 4841236-4848502
  
  
**BlastP hit with Mycgr3G67795\_Mycgr3T**
  
Percentage identity: 61 %
  
BlastP bit score: 3072
  
Sequence coverage: 100 %
  
E-value: 0.0
  
  
 NCBI BlastP on this gene

Pc22g20500

not annotated
  
Accession: CAP99339
  
Location: 4848929-4850283
  
 NCBI BlastP on this gene

Pc22g20510

unnamed
  
Accession: CAP99340
  
Location: 4851578-4852680
  
 NCBI BlastP on this gene

Pc22g20520

not annotated
  
Accession: CAP99341
  
Location: 4852951-4854208
  
 NCBI BlastP on this gene

Pc22g20530

hypothetical protein
  
Accession: CAP99342
  
Location: 4854304-4855773
  
 NCBI BlastP on this gene

Pc22g20540

not annotated
  
Accession: CAP99343
  
Location: 4856733-4858370
  
 NCBI BlastP on this gene

Pc22g20550

not annotated
  
Accession: CAP99344
  
Location: 4858947-4860055
  
 NCBI BlastP on this gene

Pc22g20560

not annotated
  
Accession: CAP99345
  
Location: 4860380-4862017
  
 NCBI BlastP on this gene

Pc22g20570

not annotated
  
Accession: CAP99346
  
Location: 4862354-4863963
  
 NCBI BlastP on this gene

Pc22g20580

not annotated
  
Accession: CAP99347
  
Location: 4864204-4864879
  
 NCBI BlastP on this gene

Pc22g20590

not annotated
  
Accession: CAP99348
  
Location: 4865348-4868074
  
 NCBI BlastP on this gene

Pc22g20600

not annotated
  
Accession: CAP99349
  
Location: 4868724-4870286
  
  
**BlastP hit with Mycgr3G98959\_Mycgr3T**
  
Percentage identity: 72 %
  
BlastP bit score: 722
  
Sequence coverage: 100 %
  
E-value: 0.0
  
  
 NCBI BlastP on this gene

Pc22g20610

not annotated
  
Accession: CAP99350
  
Location: 4871553-4873810
  
 NCBI BlastP on this gene

Pc22g20620

Query: Architecture Search FASTA input

CH476615 : Uncinocarpus reesii 1704 scaffold\_1 genomic scaffold    Total score: 2.0     Cumulative Blast bit score: 3786

Hit cluster cross-links:

Mycgr3G67791 Mycgr3T
  
Location: 0-1542

Mycgr3G67791\_Mycgr3T

Mycgr3G90406 Mycgr3T
  
Location: 1642-3973

Mycgr3G90406\_Mycgr3T

Mycgr3G67785 Mycgr3T
  
Location: 4073-7865

Mycgr3G67785\_Mycgr3T

Mycgr3G67795 Mycgr3T
  
Location: 7965-15249

Mycgr3G67795\_Mycgr3T

Mycgr3G67775 Mycgr3T
  
Location: 15349-16237

Mycgr3G67775\_Mycgr3T

Mycgr3G90404 Mycgr3T
  
Location: 16337-17246

Mycgr3G90404\_Mycgr3T

Mycgr3G36951 Mycgr3T
  
Location: 17346-30891

Mycgr3G36951\_Mycgr3T

Mycgr3G103034 Mycgr3
  
Location: 30991-32644

Mycgr3G103034\_Mycgr3

Mycgr3G31119 Mycgr3T
  
Location: 32744-32906

Mycgr3G31119\_Mycgr3T

Mycgr3G28587 Mycgr3T
  
Location: 33006-33489

Mycgr3G28587\_Mycgr3T

Mycgr3G98959 Mycgr3T
  
Location: 33589-35035

Mycgr3G98959\_Mycgr3T

Mycgr3G35447 Mycgr3T
  
Location: 35135-36443

Mycgr3G35447\_Mycgr3T

Mycgr3G84402 Mycgr3T
  
Location: 36543-37884

Mycgr3G84402\_Mycgr3T

Mycgr3G98961 Mycgr3T
  
Location: 37984-38884

Mycgr3G98961\_Mycgr3T

prephenate dehydrogenase
  
Accession: EEP76747
  
Location: 4109553-4111531
  
 NCBI BlastP on this gene

EEP76747

predicted protein
  
Accession: EEP76748
  
Location: 4113305-4114625
  
 NCBI BlastP on this gene

EEP76748

eukaryotic translation initiation factor 3 subunit 6-interacting protein
  
Accession: EEP76749
  
Location: 4117780-4119307
  
  
**BlastP hit with Mycgr3G98959\_Mycgr3T**
  
Percentage identity: 76 %
  
BlastP bit score: 768
  
Sequence coverage: 100 %
  
E-value: 0.0
  
  
 NCBI BlastP on this gene

EEP76749

hypothetical protein
  
Accession: EEP76750
  
Location: 4119885-4120908
  
 NCBI BlastP on this gene

EEP76750

predicted protein
  
Accession: EEP76751
  
Location: 4121247-4121915
  
 NCBI BlastP on this gene

EEP76751

predicted protein
  
Accession: EEP76752
  
Location: 4122516-4123410
  
 NCBI BlastP on this gene

EEP76752

kynureninase
  
Accession: EEP76753
  
Location: 4124090-4125709
  
 NCBI BlastP on this gene

EEP76753

conserved hypothetical protein
  
Accession: EEP76754
  
Location: 4126816-4128404
  
 NCBI BlastP on this gene

EEP76754

conserved hypothetical protein
  
Accession: EEP76755
  
Location: 4129627-4132222
  
 NCBI BlastP on this gene

EEP76755

predicted protein
  
Accession: EEP76756
  
Location: 4132815-4136524
  
 NCBI BlastP on this gene

EEP76756

predicted protein
  
Accession: EEP76757
  
Location: 4137147-4138154
  
 NCBI BlastP on this gene

EEP76757

40S ribosomal protein S17
  
Accession: EEP76758
  
Location: 4138552-4139194
  
 NCBI BlastP on this gene

EEP76758

predicted protein
  
Accession: EEP76759
  
Location: 4139460-4140440
  
 NCBI BlastP on this gene

EEP76759

phosphatidylinositol 3-kinase tor2
  
Accession: EEP76760
  
Location: 4141174-4148639
  
  
**BlastP hit with Mycgr3G67795\_Mycgr3T**
  
Percentage identity: 60 %
  
BlastP bit score: 3018
  
Sequence coverage: 101 %
  
E-value: 0.0
  
  
 NCBI BlastP on this gene

EEP76760

conserved hypothetical protein
  
Accession: EEP76761
  
Location: 4150834-4152592
  
 NCBI BlastP on this gene

EEP76761

Query: Architecture Search FASTA input

AKCU01000427 : Penicillium digitatum Pd1    Total score: 2.0     Cumulative Blast bit score: 3778

Hit cluster cross-links:

Mycgr3G67791 Mycgr3T
  
Location: 0-1542

Mycgr3G67791\_Mycgr3T

Mycgr3G90406 Mycgr3T
  
Location: 1642-3973

Mycgr3G90406\_Mycgr3T

Mycgr3G67785 Mycgr3T
  
Location: 4073-7865

Mycgr3G67785\_Mycgr3T

Mycgr3G67795 Mycgr3T
  
Location: 7965-15249

Mycgr3G67795\_Mycgr3T

Mycgr3G67775 Mycgr3T
  
Location: 15349-16237

Mycgr3G67775\_Mycgr3T

Mycgr3G90404 Mycgr3T
  
Location: 16337-17246

Mycgr3G90404\_Mycgr3T

Mycgr3G36951 Mycgr3T
  
Location: 17346-30891

Mycgr3G36951\_Mycgr3T

Mycgr3G103034 Mycgr3
  
Location: 30991-32644

Mycgr3G103034\_Mycgr3

Mycgr3G31119 Mycgr3T
  
Location: 32744-32906

Mycgr3G31119\_Mycgr3T

Mycgr3G28587 Mycgr3T
  
Location: 33006-33489

Mycgr3G28587\_Mycgr3T

Mycgr3G98959 Mycgr3T
  
Location: 33589-35035

Mycgr3G98959\_Mycgr3T

Mycgr3G35447 Mycgr3T
  
Location: 35135-36443

Mycgr3G35447\_Mycgr3T

Mycgr3G84402 Mycgr3T
  
Location: 36543-37884

Mycgr3G84402\_Mycgr3T

Mycgr3G98961 Mycgr3T
  
Location: 37984-38884

Mycgr3G98961\_Mycgr3T

C6 transcription factor, putative
  
Accession: EKV09458
  
Location: 1161-2077
  
 NCBI BlastP on this gene

EKV09458

Eukaryotic translation initiation factor 3 subunit L
  
Accession: EKV09459
  
Location: 3056-4621
  
  
**BlastP hit with Mycgr3G98959\_Mycgr3T**
  
Percentage identity: 73 %
  
BlastP bit score: 726
  
Sequence coverage: 100 %
  
E-value: 0.0
  
  
 NCBI BlastP on this gene

EKV09459

AP-2 adaptor complex subunit beta, putative
  
Accession: EKV09460
  
Location: 5311-7767
  
 NCBI BlastP on this gene

EKV09460

hypothetical protein
  
Accession: EKV09461
  
Location: 8489-9038
  
 NCBI BlastP on this gene

EKV09461

MFS transporter, putative
  
Accession: EKV09462
  
Location: 9375-12979
  
 NCBI BlastP on this gene

EKV09462

Iron-sulfur cluster assembly accessory protein Isa2, putative
  
Accession: EKV09463
  
Location: 13354-14408
  
 NCBI BlastP on this gene

EKV09463

hypothetical protein
  
Accession: EKV09464
  
Location: 16588-16725
  
 NCBI BlastP on this gene

EKV09464

Tor
  
Accession: EKV09465
  
Location: 16769-25844
  
  
**BlastP hit with Mycgr3G67795\_Mycgr3T**
  
Percentage identity: 61 %
  
BlastP bit score: 3052
  
Sequence coverage: 100 %
  
E-value: 0.0
  
  
 NCBI BlastP on this gene

EKV09465

Ketoreductase
  
Accession: EKV09466
  
Location: 27145-28370
  
 NCBI BlastP on this gene

EKV09466

hypothetical protein
  
Accession: EKV09467
  
Location: 28682-30091
  
 NCBI BlastP on this gene

EKV09467

40S ribosomal protein S17, putative
  
Accession: EKV09468
  
Location: 30526-31070
  
 NCBI BlastP on this gene

EKV09468

hypothetical protein
  
Accession: EKV09469
  
Location: 31582-32544
  
 NCBI BlastP on this gene

EKV09469

hypothetical protein
  
Accession: EKV09470
  
Location: 33526-37271
  
 NCBI BlastP on this gene

EKV09470

Query: Architecture Search FASTA input

AKCT01000128 : Penicillium digitatum PHI26    Total score: 2.0     Cumulative Blast bit score: 3778

Hit cluster cross-links:

Mycgr3G67791 Mycgr3T
  
Location: 0-1542

Mycgr3G67791\_Mycgr3T

Mycgr3G90406 Mycgr3T
  
Location: 1642-3973

Mycgr3G90406\_Mycgr3T

Mycgr3G67785 Mycgr3T
  
Location: 4073-7865

Mycgr3G67785\_Mycgr3T

Mycgr3G67795 Mycgr3T
  
Location: 7965-15249

Mycgr3G67795\_Mycgr3T

Mycgr3G67775 Mycgr3T
  
Location: 15349-16237

Mycgr3G67775\_Mycgr3T

Mycgr3G90404 Mycgr3T
  
Location: 16337-17246

Mycgr3G90404\_Mycgr3T

Mycgr3G36951 Mycgr3T
  
Location: 17346-30891

Mycgr3G36951\_Mycgr3T

Mycgr3G103034 Mycgr3
  
Location: 30991-32644

Mycgr3G103034\_Mycgr3

Mycgr3G31119 Mycgr3T
  
Location: 32744-32906

Mycgr3G31119\_Mycgr3T

Mycgr3G28587 Mycgr3T
  
Location: 33006-33489

Mycgr3G28587\_Mycgr3T

Mycgr3G98959 Mycgr3T
  
Location: 33589-35035

Mycgr3G98959\_Mycgr3T

Mycgr3G35447 Mycgr3T
  
Location: 35135-36443

Mycgr3G35447\_Mycgr3T

Mycgr3G84402 Mycgr3T
  
Location: 36543-37884

Mycgr3G84402\_Mycgr3T

Mycgr3G98961 Mycgr3T
  
Location: 37984-38884

Mycgr3G98961\_Mycgr3T

Branched-chain-amino-acid aminotransferase
  
Accession: EKV14903
  
Location: 130069-131387
  
 NCBI BlastP on this gene

EKV14903

C6 transcription factor, putative
  
Accession: EKV14904
  
Location: 138817-139733
  
 NCBI BlastP on this gene

EKV14904

Eukaryotic translation initiation factor 3 subunit L
  
Accession: EKV14905
  
Location: 140712-142277
  
  
**BlastP hit with Mycgr3G98959\_Mycgr3T**
  
Percentage identity: 73 %
  
BlastP bit score: 726
  
Sequence coverage: 100 %
  
E-value: 0.0
  
  
 NCBI BlastP on this gene

EKV14905

AP-2 adaptor complex subunit beta, putative
  
Accession: EKV14906
  
Location: 142933-145389
  
 NCBI BlastP on this gene

EKV14906

hypothetical protein
  
Accession: EKV14907
  
Location: 146111-146660
  
 NCBI BlastP on this gene

EKV14907

MFS transporter, putative
  
Accession: EKV14908
  
Location: 146997-150601
  
 NCBI BlastP on this gene

EKV14908

Iron-sulfur cluster assembly accessory protein Isa2, putative
  
Accession: EKV14909
  
Location: 150976-152030
  
 NCBI BlastP on this gene

EKV14909

hypothetical protein
  
Accession: EKV14910
  
Location: 154210-154347
  
 NCBI BlastP on this gene

EKV14910

Tor
  
Accession: EKV14911
  
Location: 154391-163466
  
  
**BlastP hit with Mycgr3G67795\_Mycgr3T**
  
Percentage identity: 61 %
  
BlastP bit score: 3052
  
Sequence coverage: 100 %
  
E-value: 0.0
  
  
 NCBI BlastP on this gene

EKV14911

Ketoreductase
  
Accession: EKV14912
  
Location: 164767-165992
  
 NCBI BlastP on this gene

EKV14912

hypothetical protein
  
Accession: EKV14913
  
Location: 166304-167713
  
 NCBI BlastP on this gene

EKV14913

40S ribosomal protein S17, putative
  
Accession: EKV14914
  
Location: 168148-168692
  
 NCBI BlastP on this gene

EKV14914

hypothetical protein
  
Accession: EKV14915
  
Location: 169204-170166
  
 NCBI BlastP on this gene

EKV14915

hypothetical protein
  
Accession: EKV14916
  
Location: 171150-174895
  
 NCBI BlastP on this gene

EKV14916

Query: Architecture Search FASTA input

GG700648 : Trichophyton rubrum CBS 118892 genomic scaffold supercont2.1    Total score: 2.0     Cumulative Blast bit score: 3771

Hit cluster cross-links:

Mycgr3G67791 Mycgr3T
  
Location: 0-1542

Mycgr3G67791\_Mycgr3T

Mycgr3G90406 Mycgr3T
  
Location: 1642-3973

Mycgr3G90406\_Mycgr3T

Mycgr3G67785 Mycgr3T
  
Location: 4073-7865

Mycgr3G67785\_Mycgr3T

Mycgr3G67795 Mycgr3T
  
Location: 7965-15249

Mycgr3G67795\_Mycgr3T

Mycgr3G67775 Mycgr3T
  
Location: 15349-16237

Mycgr3G67775\_Mycgr3T

Mycgr3G90404 Mycgr3T
  
Location: 16337-17246

Mycgr3G90404\_Mycgr3T

Mycgr3G36951 Mycgr3T
  
Location: 17346-30891

Mycgr3G36951\_Mycgr3T

Mycgr3G103034 Mycgr3
  
Location: 30991-32644

Mycgr3G103034\_Mycgr3

Mycgr3G31119 Mycgr3T
  
Location: 32744-32906

Mycgr3G31119\_Mycgr3T

Mycgr3G28587 Mycgr3T
  
Location: 33006-33489

Mycgr3G28587\_Mycgr3T

Mycgr3G98959 Mycgr3T
  
Location: 33589-35035

Mycgr3G98959\_Mycgr3T

Mycgr3G35447 Mycgr3T
  
Location: 35135-36443

Mycgr3G35447\_Mycgr3T

Mycgr3G84402 Mycgr3T
  
Location: 36543-37884

Mycgr3G84402\_Mycgr3T

Mycgr3G98961 Mycgr3T
  
Location: 37984-38884

Mycgr3G98961\_Mycgr3T

hypothetical protein
  
Accession: EGD84357
  
Location: 1649441-1650791
  
 NCBI BlastP on this gene

EGD84357

eukaryotic translation initiation factor 3
  
Accession: EGD84358
  
Location: 1655988-1657493
  
  
**BlastP hit with Mycgr3G98959\_Mycgr3T**
  
Percentage identity: 74 %
  
BlastP bit score: 738
  
Sequence coverage: 100 %
  
E-value: 0.0
  
  
 NCBI BlastP on this gene

EGD84358

iron-sulfur cluster assembly accessory protein
  
Accession: EGD84359
  
Location: 1658097-1659083
  
 NCBI BlastP on this gene

EGD84359

hypothetical protein
  
Accession: EGD84360
  
Location: 1659625-1660173
  
 NCBI BlastP on this gene

EGD84360

metallo-beta-lactamase
  
Accession: EGD84361
  
Location: 1660435-1661734
  
 NCBI BlastP on this gene

EGD84361

kynureninase
  
Accession: EGD84362
  
Location: 1661953-1663454
  
 NCBI BlastP on this gene

EGD84362

hypothetical protein
  
Accession: EGD84363
  
Location: 1663564-1664268
  
 NCBI BlastP on this gene

EGD84363

hypothetical protein
  
Accession: EGD84364
  
Location: 1665131-1665444
  
 NCBI BlastP on this gene

EGD84364

hypothetical protein
  
Accession: EGD84365
  
Location: 1666716-1667051
  
 NCBI BlastP on this gene

EGD84365

integral membrane protein
  
Accession: EGD84366
  
Location: 1667158-1668043
  
 NCBI BlastP on this gene

EGD84366

AP-2 adaptor complex subunit beta
  
Accession: EGD84367
  
Location: 1669207-1671636
  
 NCBI BlastP on this gene

EGD84367

SH3 domain-containing protein
  
Accession: EGD84368
  
Location: 1672158-1675731
  
 NCBI BlastP on this gene

EGD84368

hypothetical protein
  
Accession: EGD84369
  
Location: 1676450-1677466
  
 NCBI BlastP on this gene

EGD84369

40S ribosomal protein S17
  
Accession: EGD84370
  
Location: 1677863-1678512
  
 NCBI BlastP on this gene

EGD84370

hypothetical protein
  
Accession: EGD84371
  
Location: 1678822-1680120
  
 NCBI BlastP on this gene

EGD84371

phosphatidylinositol 3-kinase
  
Accession: EGD84372
  
Location: 1680503-1687920
  
  
**BlastP hit with Mycgr3G67795\_Mycgr3T**
  
Percentage identity: 61 %
  
BlastP bit score: 3034
  
Sequence coverage: 99 %
  
E-value: 0.0
  
  
 NCBI BlastP on this gene

EGD84372

hypothetical protein
  
Accession: EGD84373
  
Location: 1688480-1690284
  
 NCBI BlastP on this gene

EGD84373

hypothetical protein
  
Accession: EGD84374
  
Location: 1691495-1692556
  
 NCBI BlastP on this gene

EGD84374

Query: Architecture Search FASTA input

DS995701 : Microsporum canis CBS 113480 supercont1.1 genomic scaffold    Total score: 2.0     Cumulative Blast bit score: 3771

Hit cluster cross-links:

Mycgr3G67791 Mycgr3T
  
Location: 0-1542

Mycgr3G67791\_Mycgr3T

Mycgr3G90406 Mycgr3T
  
Location: 1642-3973

Mycgr3G90406\_Mycgr3T

Mycgr3G67785 Mycgr3T
  
Location: 4073-7865

Mycgr3G67785\_Mycgr3T

Mycgr3G67795 Mycgr3T
  
Location: 7965-15249

Mycgr3G67795\_Mycgr3T

Mycgr3G67775 Mycgr3T
  
Location: 15349-16237

Mycgr3G67775\_Mycgr3T

Mycgr3G90404 Mycgr3T
  
Location: 16337-17246

Mycgr3G90404\_Mycgr3T

Mycgr3G36951 Mycgr3T
  
Location: 17346-30891

Mycgr3G36951\_Mycgr3T

Mycgr3G103034 Mycgr3
  
Location: 30991-32644

Mycgr3G103034\_Mycgr3

Mycgr3G31119 Mycgr3T
  
Location: 32744-32906

Mycgr3G31119\_Mycgr3T

Mycgr3G28587 Mycgr3T
  
Location: 33006-33489

Mycgr3G28587\_Mycgr3T

Mycgr3G98959 Mycgr3T
  
Location: 33589-35035

Mycgr3G98959\_Mycgr3T

Mycgr3G35447 Mycgr3T
  
Location: 35135-36443

Mycgr3G35447\_Mycgr3T

Mycgr3G84402 Mycgr3T
  
Location: 36543-37884

Mycgr3G84402\_Mycgr3T

Mycgr3G98961 Mycgr3T
  
Location: 37984-38884

Mycgr3G98961\_Mycgr3T

integral membrane protein
  
Accession: EEQ27315
  
Location: 521551-522842
  
 NCBI BlastP on this gene

EEQ27315

conserved hypothetical protein
  
Accession: EEQ27316
  
Location: 524791-526589
  
 NCBI BlastP on this gene

EEQ27316

eukaryotic translation initiation factor 3
  
Accession: EEQ27317
  
Location: 527295-528799
  
  
**BlastP hit with Mycgr3G98959\_Mycgr3T**
  
Percentage identity: 75 %
  
BlastP bit score: 740
  
Sequence coverage: 100 %
  
E-value: 0.0
  
  
 NCBI BlastP on this gene

EEQ27317

HesB/YadR/YfhF
  
Accession: EEQ27318
  
Location: 529329-530340
  
 NCBI BlastP on this gene

EEQ27318

hemerythrin HHE cation binding domain-containing protein
  
Accession: EEQ27319
  
Location: 530996-531641
  
 NCBI BlastP on this gene

EEQ27319

metallo-beta-lactamase superfamily protein
  
Accession: EEQ27320
  
Location: 531801-533084
  
 NCBI BlastP on this gene

EEQ27320

kynureninase
  
Accession: EEQ27321
  
Location: 533288-534786
  
 NCBI BlastP on this gene

EEQ27321

DUF1077 family protein
  
Accession: EEQ27322
  
Location: 534874-535563
  
 NCBI BlastP on this gene

EEQ27322

predicted protein
  
Accession: EEQ27323
  
Location: 536104-536691
  
 NCBI BlastP on this gene

EEQ27323

adaptor protein complex AP-1
  
Accession: EEQ27324
  
Location: 536910-539508
  
 NCBI BlastP on this gene

EEQ27324

SH3 domain-containing protein
  
Accession: EEQ27325
  
Location: 539990-543534
  
 NCBI BlastP on this gene

EEQ27325

DUF408 domain-containing protein
  
Accession: EEQ27326
  
Location: 544204-545226
  
 NCBI BlastP on this gene

EEQ27326

40S ribosomal protein S17
  
Accession: EEQ27327
  
Location: 545629-546289
  
 NCBI BlastP on this gene

EEQ27327

conserved hypothetical protein
  
Accession: EEQ27328
  
Location: 546577-547875
  
 NCBI BlastP on this gene

EEQ27328

phosphatidylinositol 3-kinase tor2
  
Accession: EEQ27329
  
Location: 548242-555644
  
  
**BlastP hit with Mycgr3G67795\_Mycgr3T**
  
Percentage identity: 61 %
  
BlastP bit score: 3032
  
Sequence coverage: 100 %
  
E-value: 0.0
  
  
 NCBI BlastP on this gene

EEQ27329

conserved hypothetical protein
  
Accession: EEQ27330
  
Location: 556176-557993
  
 NCBI BlastP on this gene

EEQ27330

conserved hypothetical protein
  
Accession: EEQ27331
  
Location: 558314-559230
  
 NCBI BlastP on this gene

EEQ27331

conserved hypothetical protein
  
Accession: EEQ27332
  
Location: 559457-560491
  
 NCBI BlastP on this gene

EEQ27332

inositol oxygenase 1
  
Accession: EEQ27333
  
Location: 561444-562438
  
 NCBI BlastP on this gene

EEQ27333

Query: Architecture Search FASTA input

ABSU01000003 : Arthroderma benhamiae CBS 112371    Total score: 2.0     Cumulative Blast bit score: 3769

Hit cluster cross-links:

Mycgr3G67791 Mycgr3T
  
Location: 0-1542

Mycgr3G67791\_Mycgr3T

Mycgr3G90406 Mycgr3T
  
Location: 1642-3973

Mycgr3G90406\_Mycgr3T

Mycgr3G67785 Mycgr3T
  
Location: 4073-7865

Mycgr3G67785\_Mycgr3T

Mycgr3G67795 Mycgr3T
  
Location: 7965-15249

Mycgr3G67795\_Mycgr3T

Mycgr3G67775 Mycgr3T
  
Location: 15349-16237

Mycgr3G67775\_Mycgr3T

Mycgr3G90404 Mycgr3T
  
Location: 16337-17246

Mycgr3G90404\_Mycgr3T

Mycgr3G36951 Mycgr3T
  
Location: 17346-30891

Mycgr3G36951\_Mycgr3T

Mycgr3G103034 Mycgr3
  
Location: 30991-32644

Mycgr3G103034\_Mycgr3

Mycgr3G31119 Mycgr3T
  
Location: 32744-32906

Mycgr3G31119\_Mycgr3T

Mycgr3G28587 Mycgr3T
  
Location: 33006-33489

Mycgr3G28587\_Mycgr3T

Mycgr3G98959 Mycgr3T
  
Location: 33589-35035

Mycgr3G98959\_Mycgr3T

Mycgr3G35447 Mycgr3T
  
Location: 35135-36443

Mycgr3G35447\_Mycgr3T

Mycgr3G84402 Mycgr3T
  
Location: 36543-37884

Mycgr3G84402\_Mycgr3T

Mycgr3G98961 Mycgr3T
  
Location: 37984-38884

Mycgr3G98961\_Mycgr3T

cellobiose dehydrogenase, putative
  
Accession: EFE35740
  
Location: 1139178-1140491
  
 NCBI BlastP on this gene

EFE35740

conserved hypothetical protein
  
Accession: EFE35741
  
Location: 1142824-1144097
  
 NCBI BlastP on this gene

EFE35741

hypothetical protein
  
Accession: EFE35742
  
Location: 1144767-1146273
  
  
**BlastP hit with Mycgr3G98959\_Mycgr3T**
  
Percentage identity: 75 %
  
BlastP bit score: 739
  
Sequence coverage: 100 %
  
E-value: 0.0
  
  
 NCBI BlastP on this gene

EFE35742

hypothetical protein
  
Accession: EFE35743
  
Location: 1146856-1147841
  
 NCBI BlastP on this gene

EFE35743

hypothetical protein
  
Accession: EFE35744
  
Location: 1148421-1148972
  
 NCBI BlastP on this gene

EFE35744

metallo-beta-lactamase superfamily protein
  
Accession: EFE35745
  
Location: 1149238-1150539
  
 NCBI BlastP on this gene

EFE35745

kynureninase, putative
  
Accession: EFE35746
  
Location: 1150757-1152259
  
 NCBI BlastP on this gene

EFE35746

hypothetical protein
  
Accession: EFE35747
  
Location: 1152496-1153072
  
 NCBI BlastP on this gene

EFE35747

integral membrane protein
  
Accession: EFE35748
  
Location: 1155229-1156549
  
 NCBI BlastP on this gene

EFE35748

hypothetical protein
  
Accession: EFE35749
  
Location: 1157730-1160164
  
 NCBI BlastP on this gene

EFE35749

hypothetical protein
  
Accession: EFE35750
  
Location: 1160705-1164280
  
 NCBI BlastP on this gene

EFE35750

DUF408 domain protein
  
Accession: EFE35751
  
Location: 1165030-1166046
  
 NCBI BlastP on this gene

EFE35751

hypothetical protein
  
Accession: EFE35752
  
Location: 1166446-1167095
  
 NCBI BlastP on this gene

EFE35752

conserved hypothetical protein
  
Accession: EFE35753
  
Location: 1167397-1168795
  
 NCBI BlastP on this gene

EFE35753

hypothetical protein
  
Accession: EFE35754
  
Location: 1169084-1176494
  
  
**BlastP hit with Mycgr3G67795\_Mycgr3T**
  
Percentage identity: 61 %
  
BlastP bit score: 3030
  
Sequence coverage: 100 %
  
E-value: 0.0
  
  
 NCBI BlastP on this gene

EFE35754

conserved hypothetical protein
  
Accession: EFE35755
  
Location: 1177074-1178878
  
 NCBI BlastP on this gene

EFE35755

hypothetical protein
  
Accession: EFE35756
  
Location: 1179984-1181051
  
 NCBI BlastP on this gene

EFE35756

Query: Architecture Search FASTA input

DS995728 : Trichophyton equinum CBS 127.97 supercont1.11 genomic scaffold    Total score: 2.0     Cumulative Blast bit score: 3768

Hit cluster cross-links:

Mycgr3G67791 Mycgr3T
  
Location: 0-1542

Mycgr3G67791\_Mycgr3T

Mycgr3G90406 Mycgr3T
  
Location: 1642-3973

Mycgr3G90406\_Mycgr3T

Mycgr3G67785 Mycgr3T
  
Location: 4073-7865

Mycgr3G67785\_Mycgr3T

Mycgr3G67795 Mycgr3T
  
Location: 7965-15249

Mycgr3G67795\_Mycgr3T

Mycgr3G67775 Mycgr3T
  
Location: 15349-16237

Mycgr3G67775\_Mycgr3T

Mycgr3G90404 Mycgr3T
  
Location: 16337-17246

Mycgr3G90404\_Mycgr3T

Mycgr3G36951 Mycgr3T
  
Location: 17346-30891

Mycgr3G36951\_Mycgr3T

Mycgr3G103034 Mycgr3
  
Location: 30991-32644

Mycgr3G103034\_Mycgr3

Mycgr3G31119 Mycgr3T
  
Location: 32744-32906

Mycgr3G31119\_Mycgr3T

Mycgr3G28587 Mycgr3T
  
Location: 33006-33489

Mycgr3G28587\_Mycgr3T

Mycgr3G98959 Mycgr3T
  
Location: 33589-35035

Mycgr3G98959\_Mycgr3T

Mycgr3G35447 Mycgr3T
  
Location: 35135-36443

Mycgr3G35447\_Mycgr3T

Mycgr3G84402 Mycgr3T
  
Location: 36543-37884

Mycgr3G84402\_Mycgr3T

Mycgr3G98961 Mycgr3T
  
Location: 37984-38884

Mycgr3G98961\_Mycgr3T

cellobiose dehydrogenase
  
Accession: EGE03566
  
Location: 134013-135325
  
 NCBI BlastP on this gene

EGE03566

hypothetical protein
  
Accession: EGE03567
  
Location: 137100-138861
  
 NCBI BlastP on this gene

EGE03567

eukaryotic translation initiation factor 3
  
Accession: EGE03568
  
Location: 139554-141060
  
  
**BlastP hit with Mycgr3G98959\_Mycgr3T**
  
Percentage identity: 75 %
  
BlastP bit score: 739
  
Sequence coverage: 100 %
  
E-value: 0.0
  
  
 NCBI BlastP on this gene

EGE03568

iron-sulfur cluster assembly accessory protein I
  
Accession: EGE03569
  
Location: 141628-142612
  
 NCBI BlastP on this gene

EGE03569

HHE domain-containing protein
  
Accession: EGE03570
  
Location: 143207-143758
  
 NCBI BlastP on this gene

EGE03570

hypothetical protein
  
Accession: EGE03571
  
Location: 144508-145299
  
 NCBI BlastP on this gene

EGE03571

kynureninase
  
Accession: EGE03572
  
Location: 145506-147007
  
 NCBI BlastP on this gene

EGE03572

ER membrane DUF1077 domain-containing protein
  
Accession: EGE03573
  
Location: 147118-147825
  
 NCBI BlastP on this gene

EGE03573

hypothetical protein
  
Accession: EGE03574
  
Location: 148236-149696
  
 NCBI BlastP on this gene

EGE03574

integral membrane protein
  
Accession: EGE03575
  
Location: 150976-152307
  
 NCBI BlastP on this gene

EGE03575

hypothetical protein
  
Accession: EGE03576
  
Location: 152852-153148
  
 NCBI BlastP on this gene

EGE03576

AP-2 complex subunit beta
  
Accession: EGE03577
  
Location: 154530-156961
  
 NCBI BlastP on this gene

EGE03577

SH3 domain-containing protein
  
Accession: EGE03578
  
Location: 157477-160950
  
 NCBI BlastP on this gene

EGE03578

DUF408 domain-containing protein
  
Accession: EGE03579
  
Location: 161709-162725
  
 NCBI BlastP on this gene

EGE03579

40S ribosomal protein S17
  
Accession: EGE03580
  
Location: 163129-163780
  
 NCBI BlastP on this gene

EGE03580

hypothetical protein
  
Accession: EGE03581
  
Location: 164068-165366
  
 NCBI BlastP on this gene

EGE03581

phosphatidylinositol 3-kinase tor2
  
Accession: EGE03582
  
Location: 165755-173168
  
  
**BlastP hit with Mycgr3G67795\_Mycgr3T**
  
Percentage identity: 61 %
  
BlastP bit score: 3029
  
Sequence coverage: 100 %
  
E-value: 0.0
  
  
 NCBI BlastP on this gene

EGE03582

hypothetical protein
  
Accession: EGE03583
  
Location: 173740-175543
  
 NCBI BlastP on this gene

EGE03583

hypothetical protein
  
Accession: EGE03584
  
Location: 176486-177547
  
 NCBI BlastP on this gene

EGE03584

Query: Architecture Search FASTA input

DS989822 : Arthroderma gypseum CBS 118893 supercont1.1 genomic scaffold    Total score: 2.0     Cumulative Blast bit score: 3764

Hit cluster cross-links:

Mycgr3G67791 Mycgr3T
  
Location: 0-1542

Mycgr3G67791\_Mycgr3T

Mycgr3G90406 Mycgr3T
  
Location: 1642-3973

Mycgr3G90406\_Mycgr3T

Mycgr3G67785 Mycgr3T
  
Location: 4073-7865

Mycgr3G67785\_Mycgr3T

Mycgr3G67795 Mycgr3T
  
Location: 7965-15249

Mycgr3G67795\_Mycgr3T

Mycgr3G67775 Mycgr3T
  
Location: 15349-16237

Mycgr3G67775\_Mycgr3T

Mycgr3G90404 Mycgr3T
  
Location: 16337-17246

Mycgr3G90404\_Mycgr3T

Mycgr3G36951 Mycgr3T
  
Location: 17346-30891

Mycgr3G36951\_Mycgr3T

Mycgr3G103034 Mycgr3
  
Location: 30991-32644

Mycgr3G103034\_Mycgr3

Mycgr3G31119 Mycgr3T
  
Location: 32744-32906

Mycgr3G31119\_Mycgr3T

Mycgr3G28587 Mycgr3T
  
Location: 33006-33489

Mycgr3G28587\_Mycgr3T

Mycgr3G98959 Mycgr3T
  
Location: 33589-35035

Mycgr3G98959\_Mycgr3T

Mycgr3G35447 Mycgr3T
  
Location: 35135-36443

Mycgr3G35447\_Mycgr3T

Mycgr3G84402 Mycgr3T
  
Location: 36543-37884

Mycgr3G84402\_Mycgr3T

Mycgr3G98961 Mycgr3T
  
Location: 37984-38884

Mycgr3G98961\_Mycgr3T

integral membrane protein
  
Accession: EFQ97735
  
Location: 2041632-2042939
  
 NCBI BlastP on this gene

EFQ97735

hypothetical protein
  
Accession: EFQ97736
  
Location: 2044570-2046349
  
 NCBI BlastP on this gene

EFQ97736

eukaryotic translation initiation factor 3
  
Accession: EFQ97737
  
Location: 2047075-2048582
  
  
**BlastP hit with Mycgr3G98959\_Mycgr3T**
  
Percentage identity: 74 %
  
BlastP bit score: 738
  
Sequence coverage: 100 %
  
E-value: 0.0
  
  
 NCBI BlastP on this gene

EFQ97737

iron-binding protein erpA
  
Accession: EFQ97738
  
Location: 2049144-2050151
  
 NCBI BlastP on this gene

EFQ97738

HHE domain-containing protein
  
Accession: EFQ97739
  
Location: 2050715-2051266
  
 NCBI BlastP on this gene

EFQ97739

metallo-beta-lactamase superfamily protein
  
Accession: EFQ97740
  
Location: 2051531-2052837
  
 NCBI BlastP on this gene

EFQ97740

kynureninase
  
Accession: EFQ97741
  
Location: 2053070-2054581
  
 NCBI BlastP on this gene

EFQ97741

hypothetical protein
  
Accession: EFQ97742
  
Location: 2054690-2055383
  
 NCBI BlastP on this gene

EFQ97742

sterigmatocystin 8-O-methyltransferase
  
Accession: EFQ97743
  
Location: 2055769-2057233
  
 NCBI BlastP on this gene

EFQ97743

integral membrane protein
  
Accession: EFQ97744
  
Location: 2058522-2059826
  
 NCBI BlastP on this gene

EFQ97744

AP-2 complex subunit beta
  
Accession: EFQ97745
  
Location: 2061039-2063484
  
 NCBI BlastP on this gene

EFQ97745

SH3 domain-containing protein
  
Accession: EFQ97746
  
Location: 2064040-2067606
  
 NCBI BlastP on this gene

EFQ97746

DUF408 domain-containing protein
  
Accession: EFQ97747
  
Location: 2068374-2069402
  
 NCBI BlastP on this gene

EFQ97747

40S ribosomal protein S17
  
Accession: EFQ97748
  
Location: 2069815-2070465
  
 NCBI BlastP on this gene

EFQ97748

hypothetical protein
  
Accession: EFQ97749
  
Location: 2070740-2072038
  
 NCBI BlastP on this gene

EFQ97749

phosphatidylinositol 3-kinase tor2
  
Accession: EFQ97750
  
Location: 2072423-2079838
  
  
**BlastP hit with Mycgr3G67795\_Mycgr3T**
  
Percentage identity: 61 %
  
BlastP bit score: 3026
  
Sequence coverage: 100 %
  
E-value: 0.0
  
  
 NCBI BlastP on this gene

EFQ97750

hypothetical protein
  
Accession: EFQ97751
  
Location: 2080406-2082225
  
 NCBI BlastP on this gene

EFQ97751

hypothetical protein
  
Accession: EFQ97752
  
Location: 2082909-2084394
  
 NCBI BlastP on this gene

EFQ97752

hypothetical protein
  
Accession: EFQ97753
  
Location: 2084890-2086878
  
 NCBI BlastP on this gene

EFQ97753

Query: Architecture Search FASTA input

ACFW01000009 : Coccidioides posadasii C735 delta SOWgp    Total score: 2.0     Cumulative Blast bit score: 3755

Hit cluster cross-links:

Mycgr3G67791 Mycgr3T
  
Location: 0-1542

Mycgr3G67791\_Mycgr3T

Mycgr3G90406 Mycgr3T
  
Location: 1642-3973

Mycgr3G90406\_Mycgr3T

Mycgr3G67785 Mycgr3T
  
Location: 4073-7865

Mycgr3G67785\_Mycgr3T

Mycgr3G67795 Mycgr3T
  
Location: 7965-15249

Mycgr3G67795\_Mycgr3T

Mycgr3G67775 Mycgr3T
  
Location: 15349-16237

Mycgr3G67775\_Mycgr3T

Mycgr3G90404 Mycgr3T
  
Location: 16337-17246

Mycgr3G90404\_Mycgr3T

Mycgr3G36951 Mycgr3T
  
Location: 17346-30891

Mycgr3G36951\_Mycgr3T

Mycgr3G103034 Mycgr3
  
Location: 30991-32644

Mycgr3G103034\_Mycgr3

Mycgr3G31119 Mycgr3T
  
Location: 32744-32906

Mycgr3G31119\_Mycgr3T

Mycgr3G28587 Mycgr3T
  
Location: 33006-33489

Mycgr3G28587\_Mycgr3T

Mycgr3G98959 Mycgr3T
  
Location: 33589-35035

Mycgr3G98959\_Mycgr3T

Mycgr3G35447 Mycgr3T
  
Location: 35135-36443

Mycgr3G35447\_Mycgr3T

Mycgr3G84402 Mycgr3T
  
Location: 36543-37884

Mycgr3G84402\_Mycgr3T

Mycgr3G98961 Mycgr3T
  
Location: 37984-38884

Mycgr3G98961\_Mycgr3T

hypothetical protein
  
Accession: EER29698
  
Location: 2133755-2135096
  
 NCBI BlastP on this gene

EER29698

hypothetical protein
  
Accession: EER29697
  
Location: 2130456-2131906
  
 NCBI BlastP on this gene

EER29697

eukaryotic translation initiation factor 3 subunit 6-interacting protein, putative
  
Accession: EER29696
  
Location: 2128089-2129651
  
  
**BlastP hit with Mycgr3G98959\_Mycgr3T**
  
Percentage identity: 72 %
  
BlastP bit score: 736
  
Sequence coverage: 101 %
  
E-value: 0.0
  
  
 NCBI BlastP on this gene

EER29696

Iron-sulfur cluster assembly accessory family protein
  
Accession: EER29695
  
Location: 2126142-2127446
  
 NCBI BlastP on this gene

EER29695

hypothetical protein
  
Accession: EER29694
  
Location: 2125032-2125700
  
 NCBI BlastP on this gene

EER29694

metallo-beta-lactamase superfamily protein
  
Accession: EER29693
  
Location: 2123030-2124275
  
 NCBI BlastP on this gene

EER29693

Kynureninase , putative
  
Accession: EER29692
  
Location: 2120824-2122482
  
 NCBI BlastP on this gene

EER29692

hypothetical protein
  
Accession: EER29691
  
Location: 2117476-2118065
  
 NCBI BlastP on this gene

EER29691

AP-1 complex subunit beta-1, putative
  
Accession: EER29690
  
Location: 2114417-2117033
  
 NCBI BlastP on this gene

EER29690

SH3 domain containing protein
  
Accession: EER29689
  
Location: 2110132-2113783
  
 NCBI BlastP on this gene

EER29689

hypothetical protein
  
Accession: EER29688
  
Location: 2108455-2109468
  
 NCBI BlastP on this gene

EER29688

40S ribosomal protein S17, putative
  
Accession: EER29687
  
Location: 2107381-2108073
  
 NCBI BlastP on this gene

EER29687

hypothetical protein
  
Accession: EER29686
  
Location: 2105742-2107031
  
 NCBI BlastP on this gene

EER29686

Phosphatidylinositol 3- and 4-kinase family protein
  
Accession: EER29685
  
Location: 2097821-2105306
  
  
**BlastP hit with Mycgr3G67795\_Mycgr3T**
  
Percentage identity: 61 %
  
BlastP bit score: 3019
  
Sequence coverage: 100 %
  
E-value: 0.0
  
  
 NCBI BlastP on this gene

EER29685

hypothetical protein
  
Accession: EER29684
  
Location: 2095036-2096811
  
 NCBI BlastP on this gene

EER29684

hypothetical protein
  
Accession: EER29683
  
Location: 2093525-2094659
  
 NCBI BlastP on this gene

EER29683

Query: Architecture Search FASTA input

GG704914 : Coccidioides immitis RS genomic scaffold supercont3.4    Total score: 2.0     Cumulative Blast bit score: 3752

Hit cluster cross-links:

Mycgr3G67791 Mycgr3T
  
Location: 0-1542

Mycgr3G67791\_Mycgr3T

Mycgr3G90406 Mycgr3T
  
Location: 1642-3973

Mycgr3G90406\_Mycgr3T

Mycgr3G67785 Mycgr3T
  
Location: 4073-7865

Mycgr3G67785\_Mycgr3T

Mycgr3G67795 Mycgr3T
  
Location: 7965-15249

Mycgr3G67795\_Mycgr3T

Mycgr3G67775 Mycgr3T
  
Location: 15349-16237

Mycgr3G67775\_Mycgr3T

Mycgr3G90404 Mycgr3T
  
Location: 16337-17246

Mycgr3G90404\_Mycgr3T

Mycgr3G36951 Mycgr3T
  
Location: 17346-30891

Mycgr3G36951\_Mycgr3T

Mycgr3G103034 Mycgr3
  
Location: 30991-32644

Mycgr3G103034\_Mycgr3

Mycgr3G31119 Mycgr3T
  
Location: 32744-32906

Mycgr3G31119\_Mycgr3T

Mycgr3G28587 Mycgr3T
  
Location: 33006-33489

Mycgr3G28587\_Mycgr3T

Mycgr3G98959 Mycgr3T
  
Location: 33589-35035

Mycgr3G98959\_Mycgr3T

Mycgr3G35447 Mycgr3T
  
Location: 35135-36443

Mycgr3G35447\_Mycgr3T

Mycgr3G84402 Mycgr3T
  
Location: 36543-37884

Mycgr3G84402\_Mycgr3T

Mycgr3G98961 Mycgr3T
  
Location: 37984-38884

Mycgr3G98961\_Mycgr3T

hypothetical protein
  
Accession: EAS33514
  
Location: 310738-311388
  
 NCBI BlastP on this gene

EAS33514

cellobiose dehydrogenase
  
Accession: EAS33515
  
Location: 311775-313117
  
 NCBI BlastP on this gene

EAS33515

hypothetical protein
  
Accession: EJB11397
  
Location: 314681-316370
  
 NCBI BlastP on this gene

EJB11397

eukaryotic translation initiation factor 3 subunit L
  
Accession: EAS33518
  
Location: 317179-318741
  
  
**BlastP hit with Mycgr3G98959\_Mycgr3T**
  
Percentage identity: 71 %
  
BlastP bit score: 734
  
Sequence coverage: 101 %
  
E-value: 0.0
  
  
 NCBI BlastP on this gene

EAS33518

iron-sulfur cluster assembly accessory protein
  
Accession: EAS33519
  
Location: 319389-320679
  
 NCBI BlastP on this gene

EAS33519

HHE domain-containing protein
  
Accession: EAS33520
  
Location: 321113-321781
  
 NCBI BlastP on this gene

EAS33520

metallo-beta-lactamase superfamily protein
  
Accession: EAS33521
  
Location: 322528-323773
  
 NCBI BlastP on this gene

EAS33521

kynureninase
  
Accession: EAS33522
  
Location: 324322-325981
  
 NCBI BlastP on this gene

EAS33522

hypothetical protein
  
Accession: EAS33523
  
Location: 327192-327622
  
 NCBI BlastP on this gene

EAS33523

hypothetical protein
  
Accession: EAS33524
  
Location: 328684-329368
  
 NCBI BlastP on this gene

EAS33524

AP-2 adaptor complex subunit beta
  
Accession: EAS33525
  
Location: 329811-332427
  
 NCBI BlastP on this gene

EAS33525

SH3 domain-containing protein
  
Accession: EAS33526
  
Location: 333064-336706
  
 NCBI BlastP on this gene

EAS33526

hypothetical protein
  
Accession: EAS33527
  
Location: 337369-338385
  
 NCBI BlastP on this gene

EAS33527

40S ribosomal protein S17
  
Accession: EAS33528
  
Location: 338762-339443
  
 NCBI BlastP on this gene

EAS33528

hypothetical protein
  
Accession: EAS33529
  
Location: 339790-341079
  
 NCBI BlastP on this gene

EAS33529

phosphatidylinositol 3-kinase tor2
  
Accession: EAS33530
  
Location: 341513-349001
  
  
**BlastP hit with Mycgr3G67795\_Mycgr3T**
  
Percentage identity: 61 %
  
BlastP bit score: 3018
  
Sequence coverage: 101 %
  
E-value: 0.0
  
  
 NCBI BlastP on this gene

EAS33530

hypothetical protein
  
Accession: EAS33531
  
Location: 350014-351789
  
 NCBI BlastP on this gene

EAS33531

hypothetical protein
  
Accession: EAS33532
  
Location: 352177-353338
  
 NCBI BlastP on this gene

EAS33532

Query: Architecture Search FASTA input

GG698484 : Trichophyton tonsurans CBS 112818 genomic scaffold supercont1.8    Total score: 2.0     Cumulative Blast bit score: 3705

Hit cluster cross-links:

Mycgr3G67791 Mycgr3T
  
Location: 0-1542

Mycgr3G67791\_Mycgr3T

Mycgr3G90406 Mycgr3T
  
Location: 1642-3973

Mycgr3G90406\_Mycgr3T

Mycgr3G67785 Mycgr3T
  
Location: 4073-7865

Mycgr3G67785\_Mycgr3T

Mycgr3G67795 Mycgr3T
  
Location: 7965-15249

Mycgr3G67795\_Mycgr3T

Mycgr3G67775 Mycgr3T
  
Location: 15349-16237

Mycgr3G67775\_Mycgr3T

Mycgr3G90404 Mycgr3T
  
Location: 16337-17246

Mycgr3G90404\_Mycgr3T

Mycgr3G36951 Mycgr3T
  
Location: 17346-30891

Mycgr3G36951\_Mycgr3T

Mycgr3G103034 Mycgr3
  
Location: 30991-32644

Mycgr3G103034\_Mycgr3

Mycgr3G31119 Mycgr3T
  
Location: 32744-32906

Mycgr3G31119\_Mycgr3T

Mycgr3G28587 Mycgr3T
  
Location: 33006-33489

Mycgr3G28587\_Mycgr3T

Mycgr3G98959 Mycgr3T
  
Location: 33589-35035

Mycgr3G98959\_Mycgr3T

Mycgr3G35447 Mycgr3T
  
Location: 35135-36443

Mycgr3G35447\_Mycgr3T

Mycgr3G84402 Mycgr3T
  
Location: 36543-37884

Mycgr3G84402\_Mycgr3T

Mycgr3G98961 Mycgr3T
  
Location: 37984-38884

Mycgr3G98961\_Mycgr3T

hypothetical protein
  
Accession: EGD94649
  
Location: 134986-136298
  
 NCBI BlastP on this gene

EGD94649

hypothetical protein
  
Accession: EGD94650
  
Location: 138071-139832
  
 NCBI BlastP on this gene

EGD94650

eukaryotic translation initiation factor 3
  
Accession: EGD94651
  
Location: 140525-142031
  
  
**BlastP hit with Mycgr3G98959\_Mycgr3T**
  
Percentage identity: 75 %
  
BlastP bit score: 739
  
Sequence coverage: 100 %
  
E-value: 0.0
  
  
 NCBI BlastP on this gene

EGD94651

iron-sulfur cluster assembly accessory protein
  
Accession: EGD94652
  
Location: 142600-143580
  
 NCBI BlastP on this gene

EGD94652

hypothetical protein
  
Accession: EGD94653
  
Location: 144175-144726
  
 NCBI BlastP on this gene

EGD94653

metallo-beta-lactamase
  
Accession: EGD94654
  
Location: 144989-146285
  
 NCBI BlastP on this gene

EGD94654

kynureninase
  
Accession: EGD94655
  
Location: 146492-147993
  
 NCBI BlastP on this gene

EGD94655

hypothetical protein
  
Accession: EGD94656
  
Location: 148104-148811
  
 NCBI BlastP on this gene

EGD94656

hypothetical protein
  
Accession: EGD94657
  
Location: 149222-150682
  
 NCBI BlastP on this gene

EGD94657

integral membrane protein
  
Accession: EGD94658
  
Location: 151967-153298
  
 NCBI BlastP on this gene

EGD94658

hypothetical protein
  
Accession: EGD94659
  
Location: 153843-154179
  
 NCBI BlastP on this gene

EGD94659

AP-2 adaptor complex subunit beta
  
Accession: EGD94660
  
Location: 155687-157953
  
 NCBI BlastP on this gene

EGD94660

SH3 domain-containing protein
  
Accession: EGD94661
  
Location: 158469-161994
  
 NCBI BlastP on this gene

EGD94661

hypothetical protein
  
Accession: EGD94662
  
Location: 163029-163838
  
 NCBI BlastP on this gene

EGD94662

40S ribosomal protein S17
  
Accession: EGD94663
  
Location: 164242-164893
  
 NCBI BlastP on this gene

EGD94663

hypothetical protein
  
Accession: EGD94664
  
Location: 165181-166479
  
 NCBI BlastP on this gene

EGD94664

phosphatidylinositol 3-kinase
  
Accession: EGD94665
  
Location: 166869-174281
  
  
**BlastP hit with Mycgr3G67795\_Mycgr3T**
  
Percentage identity: 60 %
  
BlastP bit score: 2966
  
Sequence coverage: 100 %
  
E-value: 0.0
  
  
 NCBI BlastP on this gene

EGD94665

hypothetical protein
  
Accession: EGD94666
  
Location: 174854-176657
  
 NCBI BlastP on this gene

EGD94666

hypothetical protein
  
Accession: EGD94667
  
Location: 177598-178658
  
 NCBI BlastP on this gene

EGD94667

Query: Architecture Search FASTA input

U85909 : Aureobasidium pullulans cosmid pPSR-22 hydroxylase, multidrug resistance-like protein (...    Total score: 2.0     Cumulative Blast bit score: 3624

Hit cluster cross-links:

Mycgr3G67791 Mycgr3T
  
Location: 0-1542

Mycgr3G67791\_Mycgr3T

Mycgr3G90406 Mycgr3T
  
Location: 1642-3973

Mycgr3G90406\_Mycgr3T

Mycgr3G67785 Mycgr3T
  
Location: 4073-7865

Mycgr3G67785\_Mycgr3T

Mycgr3G67795 Mycgr3T
  
Location: 7965-15249

Mycgr3G67795\_Mycgr3T

Mycgr3G67775 Mycgr3T
  
Location: 15349-16237

Mycgr3G67775\_Mycgr3T

Mycgr3G90404 Mycgr3T
  
Location: 16337-17246

Mycgr3G90404\_Mycgr3T

Mycgr3G36951 Mycgr3T
  
Location: 17346-30891

Mycgr3G36951\_Mycgr3T

Mycgr3G103034 Mycgr3
  
Location: 30991-32644

Mycgr3G103034\_Mycgr3

Mycgr3G31119 Mycgr3T
  
Location: 32744-32906

Mycgr3G31119\_Mycgr3T

Mycgr3G28587 Mycgr3T
  
Location: 33006-33489

Mycgr3G28587\_Mycgr3T

Mycgr3G98959 Mycgr3T
  
Location: 33589-35035

Mycgr3G98959\_Mycgr3T

Mycgr3G35447 Mycgr3T
  
Location: 35135-36443

Mycgr3G35447\_Mycgr3T

Mycgr3G84402 Mycgr3T
  
Location: 36543-37884

Mycgr3G84402\_Mycgr3T

Mycgr3G98961 Mycgr3T
  
Location: 37984-38884

Mycgr3G98961\_Mycgr3T

hydroxylase
  
Accession: AAD00579
  
Location: 5312-6769
  
 NCBI BlastP on this gene

AAD00579

multidrug resistance-like protein
  
Accession: AAD00580
  
Location: 8090-11998
  
  
**BlastP hit with Mycgr3G67785\_Mycgr3T**
  
Percentage identity: 49 %
  
BlastP bit score: 1204
  
Sequence coverage: 101 %
  
E-value: 0.0
  
  
 NCBI BlastP on this gene

ApMDR1

peptide synthetase
  
Accession: AAD00581
  
Location: 13308-28158
  
  
**BlastP hit with Mycgr3G36951\_Mycgr3T**
  
Percentage identity: 38 %
  
BlastP bit score: 2420
  
Sequence coverage: 77 %
  
E-value: 0.0
  
  
 NCBI BlastP on this gene

AAD00581

Query: Architecture Search FASTA input

ADOT01000056 : Arthrobotrys oligospora ATCC 24927    Total score: 2.0     Cumulative Blast bit score: 3623

Hit cluster cross-links:

Mycgr3G67791 Mycgr3T
  
Location: 0-1542

Mycgr3G67791\_Mycgr3T

Mycgr3G90406 Mycgr3T
  
Location: 1642-3973

Mycgr3G90406\_Mycgr3T

Mycgr3G67785 Mycgr3T
  
Location: 4073-7865

Mycgr3G67785\_Mycgr3T

Mycgr3G67795 Mycgr3T
  
Location: 7965-15249

Mycgr3G67795\_Mycgr3T

Mycgr3G67775 Mycgr3T
  
Location: 15349-16237

Mycgr3G67775\_Mycgr3T

Mycgr3G90404 Mycgr3T
  
Location: 16337-17246

Mycgr3G90404\_Mycgr3T

Mycgr3G36951 Mycgr3T
  
Location: 17346-30891

Mycgr3G36951\_Mycgr3T

Mycgr3G103034 Mycgr3
  
Location: 30991-32644

Mycgr3G103034\_Mycgr3

Mycgr3G31119 Mycgr3T
  
Location: 32744-32906

Mycgr3G31119\_Mycgr3T

Mycgr3G28587 Mycgr3T
  
Location: 33006-33489

Mycgr3G28587\_Mycgr3T

Mycgr3G98959 Mycgr3T
  
Location: 33589-35035

Mycgr3G98959\_Mycgr3T

Mycgr3G35447 Mycgr3T
  
Location: 35135-36443

Mycgr3G35447\_Mycgr3T

Mycgr3G84402 Mycgr3T
  
Location: 36543-37884

Mycgr3G84402\_Mycgr3T

Mycgr3G98961 Mycgr3T
  
Location: 37984-38884

Mycgr3G98961\_Mycgr3T

hypothetical protein
  
Accession: EGX52507
  
Location: 11608-15484
  
 NCBI BlastP on this gene

EGX52507

hypothetical protein
  
Accession: EGX52508
  
Location: 16050-17037
  
 NCBI BlastP on this gene

EGX52508

hypothetical protein
  
Accession: EGX52509
  
Location: 17223-19331
  
 NCBI BlastP on this gene

EGX52509

hypothetical protein
  
Accession: EGX52510
  
Location: 20067-23001
  
 NCBI BlastP on this gene

EGX52510

hypothetical protein
  
Accession: EGX52511
  
Location: 24008-25748
  
  
**BlastP hit with Mycgr3G98959\_Mycgr3T**
  
Percentage identity: 64 %
  
BlastP bit score: 624
  
Sequence coverage: 98 %
  
E-value: 0.0
  
  
 NCBI BlastP on this gene

EGX52511

hypothetical protein
  
Accession: EGX52512
  
Location: 25994-27090
  
 NCBI BlastP on this gene

EGX52512

hypothetical protein
  
Accession: EGX52513
  
Location: 27941-28270
  
 NCBI BlastP on this gene

EGX52513

hypothetical protein
  
Accession: EGX52514
  
Location: 31105-32084
  
 NCBI BlastP on this gene

EGX52514

hypothetical protein
  
Accession: EGX52515
  
Location: 32559-32903
  
 NCBI BlastP on this gene

EGX52515

hypothetical protein
  
Accession: EGX52516
  
Location: 34085-36897
  
 NCBI BlastP on this gene

EGX52516

hypothetical protein
  
Accession: EGX52517
  
Location: 37826-40737
  
 NCBI BlastP on this gene

EGX52517

hypothetical protein
  
Accession: EGX52518
  
Location: 41365-49649
  
  
**BlastP hit with Mycgr3G67795\_Mycgr3T**
  
Percentage identity: 62 %
  
BlastP bit score: 2999
  
Sequence coverage: 100 %
  
E-value: 0.0
  
  
 NCBI BlastP on this gene

EGX52518

hypothetical protein
  
Accession: EGX52519
  
Location: 50117-51907
  
 NCBI BlastP on this gene

EGX52519

hypothetical protein
  
Accession: EGX52520
  
Location: 52010-56035
  
 NCBI BlastP on this gene

EGX52520

hypothetical protein
  
Accession: EGX52521
  
Location: 56840-59621
  
 NCBI BlastP on this gene

EGX52521

Query: Architecture Search FASTA input

FQ790277 : Botryotinia fuckeliana T4 SuperContig\_379\_1 genomic supercontig.    Total score: 2.0     Cumulative Blast bit score: 3377

Hit cluster cross-links:

Mycgr3G67791 Mycgr3T
  
Location: 0-1542

Mycgr3G67791\_Mycgr3T

Mycgr3G90406 Mycgr3T
  
Location: 1642-3973

Mycgr3G90406\_Mycgr3T

Mycgr3G67785 Mycgr3T
  
Location: 4073-7865

Mycgr3G67785\_Mycgr3T

Mycgr3G67795 Mycgr3T
  
Location: 7965-15249

Mycgr3G67795\_Mycgr3T

Mycgr3G67775 Mycgr3T
  
Location: 15349-16237

Mycgr3G67775\_Mycgr3T

Mycgr3G90404 Mycgr3T
  
Location: 16337-17246

Mycgr3G90404\_Mycgr3T

Mycgr3G36951 Mycgr3T
  
Location: 17346-30891

Mycgr3G36951\_Mycgr3T

Mycgr3G103034 Mycgr3
  
Location: 30991-32644

Mycgr3G103034\_Mycgr3

Mycgr3G31119 Mycgr3T
  
Location: 32744-32906

Mycgr3G31119\_Mycgr3T

Mycgr3G28587 Mycgr3T
  
Location: 33006-33489

Mycgr3G28587\_Mycgr3T

Mycgr3G98959 Mycgr3T
  
Location: 33589-35035

Mycgr3G98959\_Mycgr3T

Mycgr3G35447 Mycgr3T
  
Location: 35135-36443

Mycgr3G35447\_Mycgr3T

Mycgr3G84402 Mycgr3T
  
Location: 36543-37884

Mycgr3G84402\_Mycgr3T

Mycgr3G98961 Mycgr3T
  
Location: 37984-38884

Mycgr3G98961\_Mycgr3T

hypothetical protein
  
Accession: CCD45288
  
Location: 20035-21345
  
 NCBI BlastP on this gene

BofuT4\_P119940.1

hypothetical protein
  
Accession: CCD45289
  
Location: 21757-22388
  
 NCBI BlastP on this gene

BofuT4\_P119950.1

BcYVC1, Ca2+ channel protein, partial sequence
  
Accession: CCD45290
  
Location: 22451-23806
  
 NCBI BlastP on this gene

BofuT4\_P119960.1

BcYVC1, Ca2+ channel protein, partial sequence
  
Accession: CCD45291
  
Location: 24490-24945
  
 NCBI BlastP on this gene

BofuT4\_P119970.1

similar to l-ornithine 5-monooxygenase
  
Accession: CCD45292
  
Location: 28830-30466
  
 NCBI BlastP on this gene

BofuT4\_P119980.1

predicted protein
  
Accession: CCD45293
  
Location: 31845-32284
  
 NCBI BlastP on this gene

BofuT4\_uP119990.1

similar to ABC transporter
  
Accession: CCD45294
  
Location: 33266-37575
  
  
**BlastP hit with Mycgr3G67785\_Mycgr3T**
  
Percentage identity: 43 %
  
BlastP bit score: 980
  
Sequence coverage: 98 %
  
E-value: 0.0
  
  
 NCBI BlastP on this gene

BofuT4\_P120000.1

BcNRPS2, nonribosomal peptide synthetase, partial sequence
  
Accession: CCD45295
  
Location: 39844-43181
  
  
**BlastP hit with Mycgr3G36951\_Mycgr3T**
  
Percentage identity: 40 %
  
BlastP bit score: 706
  
Sequence coverage: 23 %
  
E-value: 0.0
  
  
 NCBI BlastP on this gene

BofuT4\_P120010.1

BcNRPS2, nonribosomal peptide synthetase, partial sequence
  
Accession: CCD45296
  
Location: 44232-52768
  
  
**BlastP hit with Mycgr3G36951\_Mycgr3T**
  
Percentage identity: 35 %
  
BlastP bit score: 1691
  
Sequence coverage: 64 %
  
E-value: 0.0
  
  
 NCBI BlastP on this gene

BofuT4\_P120020.1

hypothetical protein
  
Accession: CCD45297
  
Location: 53142-55183
  
 NCBI BlastP on this gene

BofuT4\_P120030.1

similar to C2HC5 finger protein
  
Accession: CCD45298
  
Location: 56018-57694
  
 NCBI BlastP on this gene

BofuT4\_P120040.1

similar to TPR domain-containing protein
  
Accession: CCD45299
  
Location: 57994-60876
  
 NCBI BlastP on this gene

BofuT4\_P120050.1

similar to GYF domain-containing protein
  
Accession: CCD45300
  
Location: 61369-66203
  
 NCBI BlastP on this gene

BofuT4\_P120060.1

Query: Architecture Search FASTA input

AHHD01000089 : Macrophomina phaseolina MS6    Total score: 2.0     Cumulative Blast bit score: 3072

Hit cluster cross-links:

Mycgr3G67791 Mycgr3T
  
Location: 0-1542

Mycgr3G67791\_Mycgr3T

Mycgr3G90406 Mycgr3T
  
Location: 1642-3973

Mycgr3G90406\_Mycgr3T

Mycgr3G67785 Mycgr3T
  
Location: 4073-7865

Mycgr3G67785\_Mycgr3T

Mycgr3G67795 Mycgr3T
  
Location: 7965-15249

Mycgr3G67795\_Mycgr3T

Mycgr3G67775 Mycgr3T
  
Location: 15349-16237

Mycgr3G67775\_Mycgr3T

Mycgr3G90404 Mycgr3T
  
Location: 16337-17246

Mycgr3G90404\_Mycgr3T

Mycgr3G36951 Mycgr3T
  
Location: 17346-30891

Mycgr3G36951\_Mycgr3T

Mycgr3G103034 Mycgr3
  
Location: 30991-32644

Mycgr3G103034\_Mycgr3

Mycgr3G31119 Mycgr3T
  
Location: 32744-32906

Mycgr3G31119\_Mycgr3T

Mycgr3G28587 Mycgr3T
  
Location: 33006-33489

Mycgr3G28587\_Mycgr3T

Mycgr3G98959 Mycgr3T
  
Location: 33589-35035

Mycgr3G98959\_Mycgr3T

Mycgr3G35447 Mycgr3T
  
Location: 35135-36443

Mycgr3G35447\_Mycgr3T

Mycgr3G84402 Mycgr3T
  
Location: 36543-37884

Mycgr3G84402\_Mycgr3T

Mycgr3G98961 Mycgr3T
  
Location: 37984-38884

Mycgr3G98961\_Mycgr3T

Major facilitator superfamily
  
Accession: EKG20334
  
Location: 29963-31651
  
 NCBI BlastP on this gene

EKG20334

F-box domain cyclin-like protein
  
Accession: EKG20335
  
Location: 32276-33556
  
 NCBI BlastP on this gene

EKG20335

hypothetical protein
  
Accession: EKG20336
  
Location: 34267-35757
  
 NCBI BlastP on this gene

EKG20336

Endoribonuclease L-PSP
  
Accession: EKG20337
  
Location: 37177-37527
  
 NCBI BlastP on this gene

EKG20337

hypothetical protein
  
Accession: EKG20338
  
Location: 38043-39374
  
 NCBI BlastP on this gene

EKG20338

hypothetical protein
  
Accession: EKG20339
  
Location: 39462-40438
  
 NCBI BlastP on this gene

EKG20339

AMP-dependent synthetase/ligase
  
Accession: EKG20340
  
Location: 40729-57750
  
  
**BlastP hit with Mycgr3G36951\_Mycgr3T**
  
Percentage identity: 38 %
  
BlastP bit score: 1925
  
Sequence coverage: 62 %
  
E-value: 0.0
  
  
 NCBI BlastP on this gene

EKG20340

Putative ABC transporter protein
  
Accession: EKG20341
  
Location: 59120-63149
  
  
**BlastP hit with Mycgr3G67785\_Mycgr3T**
  
Percentage identity: 47 %
  
BlastP bit score: 1147
  
Sequence coverage: 101 %
  
E-value: 0.0
  
  
 NCBI BlastP on this gene

EKG20341

FAD-dependent pyridine nucleotide-disulfide oxidoreductase
  
Accession: EKG20342
  
Location: 64827-66760
  
 NCBI BlastP on this gene

EKG20342

Alpha-D-phosphohexomutase superfamily
  
Accession: EKG20343
  
Location: 71832-73733
  
 NCBI BlastP on this gene

EKG20343

Query: Architecture Search FASTA input

KB916185 : Neofusicoccum parvum UCRNP2 chromosome Unknown NP2\_03\_scaffold\_547    Total score: 2.0     Cumulative Blast bit score: 3029

Hit cluster cross-links:

Mycgr3G67791 Mycgr3T
  
Location: 0-1542

Mycgr3G67791\_Mycgr3T

Mycgr3G90406 Mycgr3T
  
Location: 1642-3973

Mycgr3G90406\_Mycgr3T

Mycgr3G67785 Mycgr3T
  
Location: 4073-7865

Mycgr3G67785\_Mycgr3T

Mycgr3G67795 Mycgr3T
  
Location: 7965-15249

Mycgr3G67795\_Mycgr3T

Mycgr3G67775 Mycgr3T
  
Location: 15349-16237

Mycgr3G67775\_Mycgr3T

Mycgr3G90404 Mycgr3T
  
Location: 16337-17246

Mycgr3G90404\_Mycgr3T

Mycgr3G36951 Mycgr3T
  
Location: 17346-30891

Mycgr3G36951\_Mycgr3T

Mycgr3G103034 Mycgr3
  
Location: 30991-32644

Mycgr3G103034\_Mycgr3

Mycgr3G31119 Mycgr3T
  
Location: 32744-32906

Mycgr3G31119\_Mycgr3T

Mycgr3G28587 Mycgr3T
  
Location: 33006-33489

Mycgr3G28587\_Mycgr3T

Mycgr3G98959 Mycgr3T
  
Location: 33589-35035

Mycgr3G98959\_Mycgr3T

Mycgr3G35447 Mycgr3T
  
Location: 35135-36443

Mycgr3G35447\_Mycgr3T

Mycgr3G84402 Mycgr3T
  
Location: 36543-37884

Mycgr3G84402\_Mycgr3T

Mycgr3G98961 Mycgr3T
  
Location: 37984-38884

Mycgr3G98961\_Mycgr3T

hypothetical protein
  
Accession: EOD48662
  
Location: 44857-47148
  
 NCBI BlastP on this gene

EOD48662

putative l-psp endoribonuclease family protein
  
Accession: EOD48634
  
Location: 49279-49742
  
 NCBI BlastP on this gene

EOD48634

hypothetical protein
  
Accession: EOD48661
  
Location: 53367-54101
  
 NCBI BlastP on this gene

EOD48661

putative nonribosomal peptide synthetase 2 protein
  
Accession: EOD48651
  
Location: 54514-63750
  
  
**BlastP hit with Mycgr3G36951\_Mycgr3T**
  
Percentage identity: 39 %
  
BlastP bit score: 1933
  
Sequence coverage: 60 %
  
E-value: 0.0
  
  
 NCBI BlastP on this gene

EOD48651

putative nonribosomal siderophore peptide synthase protein
  
Accession: EOD48642
  
Location: 65978-68784
  
 NCBI BlastP on this gene

EOD48642

putative abc multidrug transporter mdr1 protein
  
Accession: EOD48645
  
Location: 73006-76896
  
  
**BlastP hit with Mycgr3G67785\_Mycgr3T**
  
Percentage identity: 46 %
  
BlastP bit score: 1096
  
Sequence coverage: 101 %
  
E-value: 0.0
  
  
 NCBI BlastP on this gene

EOD48645

putative l-ornithine n5-oxygenase protein
  
Accession: EOD48640
  
Location: 78529-80395
  
 NCBI BlastP on this gene

EOD48640

putative phosphoglucomutase protein
  
Accession: EOD48671
  
Location: 85500-87501
  
 NCBI BlastP on this gene

EOD48671

Query: Architecture Search FASTA input

JH767570 : Coniosporium apollinis CBS 100218 chromosome Unknown supercont1.17    Total score: 2.0     Cumulative Blast bit score: 3012

Hit cluster cross-links:

Mycgr3G67791 Mycgr3T
  
Location: 0-1542

Mycgr3G67791\_Mycgr3T

Mycgr3G90406 Mycgr3T
  
Location: 1642-3973

Mycgr3G90406\_Mycgr3T

Mycgr3G67785 Mycgr3T
  
Location: 4073-7865

Mycgr3G67785\_Mycgr3T

Mycgr3G67795 Mycgr3T
  
Location: 7965-15249

Mycgr3G67795\_Mycgr3T

Mycgr3G67775 Mycgr3T
  
Location: 15349-16237

Mycgr3G67775\_Mycgr3T

Mycgr3G90404 Mycgr3T
  
Location: 16337-17246

Mycgr3G90404\_Mycgr3T

Mycgr3G36951 Mycgr3T
  
Location: 17346-30891

Mycgr3G36951\_Mycgr3T

Mycgr3G103034 Mycgr3
  
Location: 30991-32644

Mycgr3G103034\_Mycgr3

Mycgr3G31119 Mycgr3T
  
Location: 32744-32906

Mycgr3G31119\_Mycgr3T

Mycgr3G28587 Mycgr3T
  
Location: 33006-33489

Mycgr3G28587\_Mycgr3T

Mycgr3G98959 Mycgr3T
  
Location: 33589-35035

Mycgr3G98959\_Mycgr3T

Mycgr3G35447 Mycgr3T
  
Location: 35135-36443

Mycgr3G35447\_Mycgr3T

Mycgr3G84402 Mycgr3T
  
Location: 36543-37884

Mycgr3G84402\_Mycgr3T

Mycgr3G98961 Mycgr3T
  
Location: 37984-38884

Mycgr3G98961\_Mycgr3T

pyruvate dehydrogenase E1 component subunit beta
  
Accession: EON64845
  
Location: 343875-345126
  
 NCBI BlastP on this gene

EON64845

CMGC/CDK protein kinase
  
Accession: EON64846
  
Location: 345656-347110
  
 NCBI BlastP on this gene

EON64846

hypothetical protein
  
Accession: EON64847
  
Location: 347262-348923
  
 NCBI BlastP on this gene

EON64847

hypothetical protein
  
Accession: EON64848
  
Location: 349166-351548
  
 NCBI BlastP on this gene

EON64848

hypothetical protein
  
Accession: EON64849
  
Location: 351861-354006
  
 NCBI BlastP on this gene

EON64849

hypothetical protein
  
Accession: EON64850
  
Location: 354228-355535
  
 NCBI BlastP on this gene

EON64850

hypothetical protein
  
Accession: EON64851
  
Location: 355806-372334
  
  
**BlastP hit with Mycgr3G36951\_Mycgr3T**
  
Percentage identity: 39 %
  
BlastP bit score: 1881
  
Sequence coverage: 60 %
  
E-value: 0.0
  
  
 NCBI BlastP on this gene

EON64851

hypothetical protein
  
Accession: EON64852
  
Location: 372507-372839
  
 NCBI BlastP on this gene

EON64852

hypothetical protein
  
Accession: EON64853
  
Location: 374125-378186
  
  
**BlastP hit with Mycgr3G67785\_Mycgr3T**
  
Percentage identity: 47 %
  
BlastP bit score: 1131
  
Sequence coverage: 100 %
  
E-value: 0.0
  
  
 NCBI BlastP on this gene

EON64853

hypothetical protein
  
Accession: EON64854
  
Location: 379628-381389
  
 NCBI BlastP on this gene

EON64854

hypothetical protein
  
Accession: EON64855
  
Location: 381707-382330
  
 NCBI BlastP on this gene

EON64855

DNA polymerase alpha subunit A
  
Accession: EON64856
  
Location: 382679-387334
  
 NCBI BlastP on this gene

EON64856

phosphoglucomutase
  
Accession: EON64857
  
Location: 387769-389768
  
 NCBI BlastP on this gene

EON64857

Query: Architecture Search FASTA input

JH921428 : Marssonina brunnea f. sp. 'multigermtubi' MB\_m1 unplaced genomic scaffold M6\_S00001    Total score: 2.0     Cumulative Blast bit score: 2914

Hit cluster cross-links:

Mycgr3G67791 Mycgr3T
  
Location: 0-1542

Mycgr3G67791\_Mycgr3T

Mycgr3G90406 Mycgr3T
  
Location: 1642-3973

Mycgr3G90406\_Mycgr3T

Mycgr3G67785 Mycgr3T
  
Location: 4073-7865

Mycgr3G67785\_Mycgr3T

Mycgr3G67795 Mycgr3T
  
Location: 7965-15249

Mycgr3G67795\_Mycgr3T

Mycgr3G67775 Mycgr3T
  
Location: 15349-16237

Mycgr3G67775\_Mycgr3T

Mycgr3G90404 Mycgr3T
  
Location: 16337-17246

Mycgr3G90404\_Mycgr3T

Mycgr3G36951 Mycgr3T
  
Location: 17346-30891

Mycgr3G36951\_Mycgr3T

Mycgr3G103034 Mycgr3
  
Location: 30991-32644

Mycgr3G103034\_Mycgr3

Mycgr3G31119 Mycgr3T
  
Location: 32744-32906

Mycgr3G31119\_Mycgr3T

Mycgr3G28587 Mycgr3T
  
Location: 33006-33489

Mycgr3G28587\_Mycgr3T

Mycgr3G98959 Mycgr3T
  
Location: 33589-35035

Mycgr3G98959\_Mycgr3T

Mycgr3G35447 Mycgr3T
  
Location: 35135-36443

Mycgr3G35447\_Mycgr3T

Mycgr3G84402 Mycgr3T
  
Location: 36543-37884

Mycgr3G84402\_Mycgr3T

Mycgr3G98961 Mycgr3T
  
Location: 37984-38884

Mycgr3G98961\_Mycgr3T

Swi6
  
Accession: EKD21645
  
Location: 4399505-4402231
  
 NCBI BlastP on this gene

EKD21645

hypothetical protein
  
Accession: EKD21646
  
Location: 4402858-4404396
  
 NCBI BlastP on this gene

EKD21646

gcn5-related n-acetyltransferase
  
Accession: EKD21647
  
Location: 4404535-4405256
  
 NCBI BlastP on this gene

EKD21647

hypothetical protein
  
Accession: EKD21648
  
Location: 4406332-4407994
  
 NCBI BlastP on this gene

EKD21648

hypothetical protein
  
Accession: EKD21649
  
Location: 4408497-4409583
  
 NCBI BlastP on this gene

EKD21649

leptomycin B resistance protein pmd1
  
Accession: EKD21650
  
Location: 4410599-4416118
  
  
**BlastP hit with Mycgr3G67785\_Mycgr3T**
  
Percentage identity: 43 %
  
BlastP bit score: 947
  
Sequence coverage: 99 %
  
E-value: 0.0
  
  
 NCBI BlastP on this gene

EKD21650

peptide synthetase
  
Accession: EKD21651
  
Location: 4416760-4431762
  
  
**BlastP hit with Mycgr3G36951\_Mycgr3T**
  
Percentage identity: 35 %
  
BlastP bit score: 1967
  
Sequence coverage: 76 %
  
E-value: 0.0
  
  
 NCBI BlastP on this gene

EKD21651

TPR domain-containing protein
  
Accession: EKD21652
  
Location: 4432248-4435301
  
 NCBI BlastP on this gene

EKD21652

GYF domain protein
  
Accession: EKD21653
  
Location: 4435755-4440399
  
 NCBI BlastP on this gene

EKD21653

hypothetical protein
  
Accession: EKD21654
  
Location: 4442173-4443389
  
 NCBI BlastP on this gene

EKD21654

Query: Architecture Search FASTA input

KB908844 : Setosphaeria turcica Et28A unplaced genomic scaffold SETTUscaffold\_6    Total score: 2.0     Cumulative Blast bit score: 2847

Hit cluster cross-links:

Mycgr3G67791 Mycgr3T
  
Location: 0-1542

Mycgr3G67791\_Mycgr3T

Mycgr3G90406 Mycgr3T
  
Location: 1642-3973

Mycgr3G90406\_Mycgr3T

Mycgr3G67785 Mycgr3T
  
Location: 4073-7865

Mycgr3G67785\_Mycgr3T

Mycgr3G67795 Mycgr3T
  
Location: 7965-15249

Mycgr3G67795\_Mycgr3T

Mycgr3G67775 Mycgr3T
  
Location: 15349-16237

Mycgr3G67775\_Mycgr3T

Mycgr3G90404 Mycgr3T
  
Location: 16337-17246

Mycgr3G90404\_Mycgr3T

Mycgr3G36951 Mycgr3T
  
Location: 17346-30891

Mycgr3G36951\_Mycgr3T

Mycgr3G103034 Mycgr3
  
Location: 30991-32644

Mycgr3G103034\_Mycgr3

Mycgr3G31119 Mycgr3T
  
Location: 32744-32906

Mycgr3G31119\_Mycgr3T

Mycgr3G28587 Mycgr3T
  
Location: 33006-33489

Mycgr3G28587\_Mycgr3T

Mycgr3G98959 Mycgr3T
  
Location: 33589-35035

Mycgr3G98959\_Mycgr3T

Mycgr3G35447 Mycgr3T
  
Location: 35135-36443

Mycgr3G35447\_Mycgr3T

Mycgr3G84402 Mycgr3T
  
Location: 36543-37884

Mycgr3G84402\_Mycgr3T

Mycgr3G98961 Mycgr3T
  
Location: 37984-38884

Mycgr3G98961\_Mycgr3T

hypothetical protein
  
Accession: EOA82994
  
Location: 2064190-2066580
  
 NCBI BlastP on this gene

EOA82994

hypothetical protein
  
Accession: EOA82995
  
Location: 2068660-2069922
  
 NCBI BlastP on this gene

EOA82995

hypothetical protein
  
Accession: EOA82996
  
Location: 2070087-2070890
  
 NCBI BlastP on this gene

EOA82996

hypothetical protein
  
Accession: EOA82997
  
Location: 2071715-2073118
  
 NCBI BlastP on this gene

EOA82997

hypothetical protein
  
Accession: EOA82998
  
Location: 2073881-2074297
  
 NCBI BlastP on this gene

EOA82998

hypothetical protein
  
Accession: EOA82999
  
Location: 2075371-2076692
  
 NCBI BlastP on this gene

EOA82999

hypothetical protein
  
Accession: EOA83000
  
Location: 2077032-2093337
  
  
**BlastP hit with Mycgr3G36951\_Mycgr3T**
  
Percentage identity: 37 %
  
BlastP bit score: 1758
  
Sequence coverage: 61 %
  
E-value: 0.0
  
  
 NCBI BlastP on this gene

EOA83000

hypothetical protein
  
Accession: EOA83001
  
Location: 2095043-2099543
  
  
**BlastP hit with Mycgr3G67785\_Mycgr3T**
  
Percentage identity: 46 %
  
BlastP bit score: 1089
  
Sequence coverage: 99 %
  
E-value: 0.0
  
  
 NCBI BlastP on this gene

EOA83001

hypothetical protein
  
Accession: EOA83002
  
Location: 2101280-2103001
  
 NCBI BlastP on this gene

EOA83002

hypothetical protein
  
Accession: EOA83003
  
Location: 2104240-2107252
  
 NCBI BlastP on this gene

EOA83003

hypothetical protein
  
Accession: EOA83004
  
Location: 2108215-2110964
  
 NCBI BlastP on this gene

EOA83004

Query: Architecture Search FASTA input

CAKM01000256 : Pneumocystis jirovecii strain SE8    Total score: 2.0     Cumulative Blast bit score: 2840

Hit cluster cross-links:

Mycgr3G67791 Mycgr3T
  
Location: 0-1542

Mycgr3G67791\_Mycgr3T

Mycgr3G90406 Mycgr3T
  
Location: 1642-3973

Mycgr3G90406\_Mycgr3T

Mycgr3G67785 Mycgr3T
  
Location: 4073-7865

Mycgr3G67785\_Mycgr3T

Mycgr3G67795 Mycgr3T
  
Location: 7965-15249

Mycgr3G67795\_Mycgr3T

Mycgr3G67775 Mycgr3T
  
Location: 15349-16237

Mycgr3G67775\_Mycgr3T

Mycgr3G90404 Mycgr3T
  
Location: 16337-17246

Mycgr3G90404\_Mycgr3T

Mycgr3G36951 Mycgr3T
  
Location: 17346-30891

Mycgr3G36951\_Mycgr3T

Mycgr3G103034 Mycgr3
  
Location: 30991-32644

Mycgr3G103034\_Mycgr3

Mycgr3G31119 Mycgr3T
  
Location: 32744-32906

Mycgr3G31119\_Mycgr3T

Mycgr3G28587 Mycgr3T
  
Location: 33006-33489

Mycgr3G28587\_Mycgr3T

Mycgr3G98959 Mycgr3T
  
Location: 33589-35035

Mycgr3G98959\_Mycgr3T

Mycgr3G35447 Mycgr3T
  
Location: 35135-36443

Mycgr3G35447\_Mycgr3T

Mycgr3G84402 Mycgr3T
  
Location: 36543-37884

Mycgr3G84402\_Mycgr3T

Mycgr3G98961 Mycgr3T
  
Location: 37984-38884

Mycgr3G98961\_Mycgr3T

not annotated
  
Accession: CCJ30564
  
Location: 71432-71758
  
  
**BlastP hit with Mycgr3G28587\_Mycgr3T**
  
Percentage identity: 31 %
  
BlastP bit score: 52
  
Sequence coverage: 75 %
  
E-value: 2e-06
  
  
 NCBI BlastP on this gene

CCJ30564

not annotated
  
Accession: CCJ30563
  
Location: 70347-70828
  
 NCBI BlastP on this gene

CCJ30563

not annotated
  
Accession: CCJ30562
  
Location: 69602-70141
  
 NCBI BlastP on this gene

CCJ30562

not annotated
  
Accession: CCJ30561
  
Location: 68032-69567
  
 NCBI BlastP on this gene

CCJ30561

not annotated
  
Accession: CCJ30560
  
Location: 65116-65412
  
 NCBI BlastP on this gene

CCJ30560

not annotated
  
Accession: CCJ30559
  
Location: 64688-67860
  
 NCBI BlastP on this gene

CCJ30559

not annotated
  
Accession: CCJ30558
  
Location: 63532-64376
  
 NCBI BlastP on this gene

CCJ30558

not annotated
  
Accession: CCJ30557
  
Location: 61812-62792
  
 NCBI BlastP on this gene

CCJ30557

not annotated
  
Accession: CCJ30556
  
Location: 60474-61511
  
 NCBI BlastP on this gene

CCJ30556

not annotated
  
Accession: CCJ30555
  
Location: 59421-60046
  
 NCBI BlastP on this gene

CCJ30555

not annotated
  
Accession: CCJ30554
  
Location: 58447-59203
  
 NCBI BlastP on this gene

CCJ30554

not annotated
  
Accession: CCJ30553
  
Location: 57354-58244
  
 NCBI BlastP on this gene

CCJ30553

not annotated
  
Accession: CCJ30552
  
Location: 49849-54784
  
 NCBI BlastP on this gene

CCJ30552

not annotated
  
Accession: CCJ30551
  
Location: 48456-49854
  
 NCBI BlastP on this gene

CCJ30551

not annotated
  
Accession: CCJ30550
  
Location: 47969-48406
  
 NCBI BlastP on this gene

CCJ30550

not annotated
  
Accession: CCJ30549
  
Location: 39399-47210
  
  
**BlastP hit with Mycgr3G67795\_Mycgr3T**
  
Percentage identity: 57 %
  
BlastP bit score: 2788
  
Sequence coverage: 100 %
  
E-value: 0.0
  
  
 NCBI BlastP on this gene

CCJ30549

not annotated
  
Accession: CCJ30548
  
Location: 38753-39258
  
 NCBI BlastP on this gene

CCJ30548

not annotated
  
Accession: CCJ30547
  
Location: 37471-37950
  
 NCBI BlastP on this gene

CCJ30547

not annotated
  
Accession: CCJ30546
  
Location: 36080-37315
  
 NCBI BlastP on this gene

CCJ30546

not annotated
  
Accession: CCJ30545
  
Location: 32764-35485
  
 NCBI BlastP on this gene

CCJ30545

Query: Architecture Search FASTA input

KB445589 : Cochliobolus heterostrophus C5 unplaced genomic scaffold COCHEscaffold\_21    Total score: 2.0     Cumulative Blast bit score: 2832

Hit cluster cross-links:

Mycgr3G67791 Mycgr3T
  
Location: 0-1542

Mycgr3G67791\_Mycgr3T

Mycgr3G90406 Mycgr3T
  
Location: 1642-3973

Mycgr3G90406\_Mycgr3T

Mycgr3G67785 Mycgr3T
  
Location: 4073-7865

Mycgr3G67785\_Mycgr3T

Mycgr3G67795 Mycgr3T
  
Location: 7965-15249

Mycgr3G67795\_Mycgr3T

Mycgr3G67775 Mycgr3T
  
Location: 15349-16237

Mycgr3G67775\_Mycgr3T

Mycgr3G90404 Mycgr3T
  
Location: 16337-17246

Mycgr3G90404\_Mycgr3T

Mycgr3G36951 Mycgr3T
  
Location: 17346-30891

Mycgr3G36951\_Mycgr3T

Mycgr3G103034 Mycgr3
  
Location: 30991-32644

Mycgr3G103034\_Mycgr3

Mycgr3G31119 Mycgr3T
  
Location: 32744-32906

Mycgr3G31119\_Mycgr3T

Mycgr3G28587 Mycgr3T
  
Location: 33006-33489

Mycgr3G28587\_Mycgr3T

Mycgr3G98959 Mycgr3T
  
Location: 33589-35035

Mycgr3G98959\_Mycgr3T

Mycgr3G35447 Mycgr3T
  
Location: 35135-36443

Mycgr3G35447\_Mycgr3T

Mycgr3G84402 Mycgr3T
  
Location: 36543-37884

Mycgr3G84402\_Mycgr3T

Mycgr3G98961 Mycgr3T
  
Location: 37984-38884

Mycgr3G98961\_Mycgr3T

hypothetical protein
  
Accession: EMD85428
  
Location: 131729-134466
  
 NCBI BlastP on this gene

EMD85428

hypothetical protein
  
Accession: EMD85429
  
Location: 134793-134957
  
 NCBI BlastP on this gene

EMD85429

hypothetical protein
  
Accession: EMD85430
  
Location: 135466-138463
  
 NCBI BlastP on this gene

EMD85430

hypothetical protein
  
Accession: EMD85431
  
Location: 139533-141286
  
 NCBI BlastP on this gene

EMD85431

hypothetical protein
  
Accession: EMD85432
  
Location: 143120-147425
  
  
**BlastP hit with Mycgr3G67785\_Mycgr3T**
  
Percentage identity: 46 %
  
BlastP bit score: 1088
  
Sequence coverage: 99 %
  
E-value: 0.0
  
  
 NCBI BlastP on this gene

EMD85432

hypothetical protein
  
Accession: EMD85433
  
Location: 149004-165126
  
  
**BlastP hit with Mycgr3G36951\_Mycgr3T**
  
Percentage identity: 37 %
  
BlastP bit score: 1744
  
Sequence coverage: 61 %
  
E-value: 0.0
  
  
 NCBI BlastP on this gene

EMD85433

hypothetical protein
  
Accession: EMD85434
  
Location: 165724-167044
  
 NCBI BlastP on this gene

EMD85434

hypothetical protein
  
Accession: EMD85435
  
Location: 167935-168553
  
 NCBI BlastP on this gene

EMD85435

hypothetical protein
  
Accession: EMD85436
  
Location: 169419-170834
  
 NCBI BlastP on this gene

EMD85436

hypothetical protein
  
Accession: EMD85437
  
Location: 171686-172507
  
 NCBI BlastP on this gene

EMD85437

hypothetical protein
  
Accession: EMD85438
  
Location: 172747-174237
  
 NCBI BlastP on this gene

EMD85438

hypothetical protein
  
Accession: EMD85439
  
Location: 175802-178190
  
 NCBI BlastP on this gene

EMD85439

Query: Architecture Search FASTA input

KB733486 : Bipolaris maydis ATCC 48331 unplaced genomic scaffold COCC4scaffold\_43    Total score: 2.0     Cumulative Blast bit score: 2829

Hit cluster cross-links:

Mycgr3G67791 Mycgr3T
  
Location: 0-1542

Mycgr3G67791\_Mycgr3T

Mycgr3G90406 Mycgr3T
  
Location: 1642-3973

Mycgr3G90406\_Mycgr3T

Mycgr3G67785 Mycgr3T
  
Location: 4073-7865

Mycgr3G67785\_Mycgr3T

Mycgr3G67795 Mycgr3T
  
Location: 7965-15249

Mycgr3G67795\_Mycgr3T

Mycgr3G67775 Mycgr3T
  
Location: 15349-16237

Mycgr3G67775\_Mycgr3T

Mycgr3G90404 Mycgr3T
  
Location: 16337-17246

Mycgr3G90404\_Mycgr3T

Mycgr3G36951 Mycgr3T
  
Location: 17346-30891

Mycgr3G36951\_Mycgr3T

Mycgr3G103034 Mycgr3
  
Location: 30991-32644

Mycgr3G103034\_Mycgr3

Mycgr3G31119 Mycgr3T
  
Location: 32744-32906

Mycgr3G31119\_Mycgr3T

Mycgr3G28587 Mycgr3T
  
Location: 33006-33489

Mycgr3G28587\_Mycgr3T

Mycgr3G98959 Mycgr3T
  
Location: 33589-35035

Mycgr3G98959\_Mycgr3T

Mycgr3G35447 Mycgr3T
  
Location: 35135-36443

Mycgr3G35447\_Mycgr3T

Mycgr3G84402 Mycgr3T
  
Location: 36543-37884

Mycgr3G84402\_Mycgr3T

Mycgr3G98961 Mycgr3T
  
Location: 37984-38884

Mycgr3G98961\_Mycgr3T

hypothetical protein
  
Accession: ENH99437
  
Location: 96335-99072
  
 NCBI BlastP on this gene

ENH99437

hypothetical protein
  
Accession: ENH99438
  
Location: 99399-99563
  
 NCBI BlastP on this gene

ENH99438

hypothetical protein
  
Accession: ENH99439
  
Location: 100072-103069
  
 NCBI BlastP on this gene

ENH99439

hypothetical protein
  
Accession: ENH99440
  
Location: 104139-105892
  
 NCBI BlastP on this gene

ENH99440

hypothetical protein
  
Accession: ENH99441
  
Location: 107723-112028
  
  
**BlastP hit with Mycgr3G67785\_Mycgr3T**
  
Percentage identity: 46 %
  
BlastP bit score: 1088
  
Sequence coverage: 99 %
  
E-value: 0.0
  
  
 NCBI BlastP on this gene

ENH99441

hypothetical protein
  
Accession: ENH99442
  
Location: 113580-129927
  
  
**BlastP hit with Mycgr3G36951\_Mycgr3T**
  
Percentage identity: 38 %
  
BlastP bit score: 1741
  
Sequence coverage: 59 %
  
E-value: 0.0
  
  
 NCBI BlastP on this gene

ENH99442

hypothetical protein
  
Accession: ENH99443
  
Location: 130327-131647
  
 NCBI BlastP on this gene

ENH99443

hypothetical protein
  
Accession: ENH99444
  
Location: 132538-133156
  
 NCBI BlastP on this gene

ENH99444

hypothetical protein
  
Accession: ENH99445
  
Location: 134022-135437
  
 NCBI BlastP on this gene

ENH99445

hypothetical protein
  
Accession: ENH99446
  
Location: 136289-137110
  
 NCBI BlastP on this gene

ENH99446

hypothetical protein
  
Accession: ENH99447
  
Location: 137350-138840
  
 NCBI BlastP on this gene

ENH99447

hypothetical protein
  
Accession: ENH99448
  
Location: 140405-142793
  
 NCBI BlastP on this gene

ENH99448

Query: Architecture Search FASTA input

KB707804 : Botryotinia fuckeliana BcDW1 unplaced genomic scaffold Scaffold\_132    Total score: 2.0     Cumulative Blast bit score: 2825

Hit cluster cross-links:

Mycgr3G67791 Mycgr3T
  
Location: 0-1542

Mycgr3G67791\_Mycgr3T

Mycgr3G90406 Mycgr3T
  
Location: 1642-3973

Mycgr3G90406\_Mycgr3T

Mycgr3G67785 Mycgr3T
  
Location: 4073-7865

Mycgr3G67785\_Mycgr3T

Mycgr3G67795 Mycgr3T
  
Location: 7965-15249

Mycgr3G67795\_Mycgr3T

Mycgr3G67775 Mycgr3T
  
Location: 15349-16237

Mycgr3G67775\_Mycgr3T

Mycgr3G90404 Mycgr3T
  
Location: 16337-17246

Mycgr3G90404\_Mycgr3T

Mycgr3G36951 Mycgr3T
  
Location: 17346-30891

Mycgr3G36951\_Mycgr3T

Mycgr3G103034 Mycgr3
  
Location: 30991-32644

Mycgr3G103034\_Mycgr3

Mycgr3G31119 Mycgr3T
  
Location: 32744-32906

Mycgr3G31119\_Mycgr3T

Mycgr3G28587 Mycgr3T
  
Location: 33006-33489

Mycgr3G28587\_Mycgr3T

Mycgr3G98959 Mycgr3T
  
Location: 33589-35035

Mycgr3G98959\_Mycgr3T

Mycgr3G35447 Mycgr3T
  
Location: 35135-36443

Mycgr3G35447\_Mycgr3T

Mycgr3G84402 Mycgr3T
  
Location: 36543-37884

Mycgr3G84402\_Mycgr3T

Mycgr3G98961 Mycgr3T
  
Location: 37984-38884

Mycgr3G98961\_Mycgr3T

putative potassium ion channel yvc1 protein
  
Accession: EMR87932
  
Location: 192229-194468
  
 NCBI BlastP on this gene

EMR87932

putative l-ornithine n5-oxygenase protein
  
Accession: EMR87933
  
Location: 199423-201059
  
 NCBI BlastP on this gene

EMR87933

putative multidrug resistance protein 1 protein
  
Accession: EMR87934
  
Location: 203854-208175
  
  
**BlastP hit with Mycgr3G67785\_Mycgr3T**
  
Percentage identity: 43 %
  
BlastP bit score: 978
  
Sequence coverage: 99 %
  
E-value: 0.0
  
  
 NCBI BlastP on this gene

EMR87934

putative nonribosomal siderophore peptide synthase protein
  
Accession: EMR87935
  
Location: 210434-225357
  
  
**BlastP hit with Mycgr3G36951\_Mycgr3T**
  
Percentage identity: 34 %
  
BlastP bit score: 1847
  
Sequence coverage: 77 %
  
E-value: 0.0
  
  
 NCBI BlastP on this gene

EMR87935

putative c2hc5 finger protein
  
Accession: EMR87936
  
Location: 226192-227868
  
 NCBI BlastP on this gene

EMR87936

putative tpr repeat-containing protein
  
Accession: EMR87937
  
Location: 228170-231052
  
 NCBI BlastP on this gene

EMR87937

putative gyf domain-containing protein
  
Accession: EMR87938
  
Location: 231545-236379
  
 NCBI BlastP on this gene

EMR87938

Query: Architecture Search FASTA input

KB445657 : Cochliobolus sativus ND90Pr unplaced genomic scaffold COCSAscaffold\_21    Total score: 2.0     Cumulative Blast bit score: 2808

Hit cluster cross-links:

Mycgr3G67791 Mycgr3T
  
Location: 0-1542

Mycgr3G67791\_Mycgr3T

Mycgr3G90406 Mycgr3T
  
Location: 1642-3973

Mycgr3G90406\_Mycgr3T

Mycgr3G67785 Mycgr3T
  
Location: 4073-7865

Mycgr3G67785\_Mycgr3T

Mycgr3G67795 Mycgr3T
  
Location: 7965-15249

Mycgr3G67795\_Mycgr3T

Mycgr3G67775 Mycgr3T
  
Location: 15349-16237

Mycgr3G67775\_Mycgr3T

Mycgr3G90404 Mycgr3T
  
Location: 16337-17246

Mycgr3G90404\_Mycgr3T

Mycgr3G36951 Mycgr3T
  
Location: 17346-30891

Mycgr3G36951\_Mycgr3T

Mycgr3G103034 Mycgr3
  
Location: 30991-32644

Mycgr3G103034\_Mycgr3

Mycgr3G31119 Mycgr3T
  
Location: 32744-32906

Mycgr3G31119\_Mycgr3T

Mycgr3G28587 Mycgr3T
  
Location: 33006-33489

Mycgr3G28587\_Mycgr3T

Mycgr3G98959 Mycgr3T
  
Location: 33589-35035

Mycgr3G98959\_Mycgr3T

Mycgr3G35447 Mycgr3T
  
Location: 35135-36443

Mycgr3G35447\_Mycgr3T

Mycgr3G84402 Mycgr3T
  
Location: 36543-37884

Mycgr3G84402\_Mycgr3T

Mycgr3G98961 Mycgr3T
  
Location: 37984-38884

Mycgr3G98961\_Mycgr3T

hypothetical protein
  
Accession: EMD58313
  
Location: 59939-62327
  
 NCBI BlastP on this gene

EMD58313

hypothetical protein
  
Accession: EMD58314
  
Location: 64250-65602
  
 NCBI BlastP on this gene

EMD58314

hypothetical protein
  
Accession: EMD58315
  
Location: 65813-66634
  
 NCBI BlastP on this gene

EMD58315

hypothetical protein
  
Accession: EMD58316
  
Location: 67479-68894
  
 NCBI BlastP on this gene

EMD58316

hypothetical protein
  
Accession: EMD58317
  
Location: 69737-70359
  
 NCBI BlastP on this gene

EMD58317

hypothetical protein
  
Accession: EMD58318
  
Location: 71242-72588
  
 NCBI BlastP on this gene

EMD58318

hypothetical protein
  
Accession: EMD58319
  
Location: 72932-89242
  
  
**BlastP hit with Mycgr3G36951\_Mycgr3T**
  
Percentage identity: 37 %
  
BlastP bit score: 1726
  
Sequence coverage: 59 %
  
E-value: 0.0
  
  
 NCBI BlastP on this gene

EMD58319

hypothetical protein
  
Accession: EMD58320
  
Location: 90823-95148
  
  
**BlastP hit with Mycgr3G67785\_Mycgr3T**
  
Percentage identity: 46 %
  
BlastP bit score: 1082
  
Sequence coverage: 100 %
  
E-value: 0.0
  
  
 NCBI BlastP on this gene

EMD58320

hypothetical protein
  
Accession: EMD58321
  
Location: 96888-98625
  
 NCBI BlastP on this gene

EMD58321

hypothetical protein
  
Accession: EMD58322
  
Location: 99768-102764
  
 NCBI BlastP on this gene

EMD58322

hypothetical protein
  
Accession: EMD58323
  
Location: 103756-106492
  
 NCBI BlastP on this gene

EMD58323

Query: Architecture Search FASTA input

FP929137 : Leptosphaeria maculans JN3 lm\_SuperContig\_10\_v2 genomic supercontig    Total score: 2.0     Cumulative Blast bit score: 2804

Hit cluster cross-links:

Mycgr3G67791 Mycgr3T
  
Location: 0-1542

Mycgr3G67791\_Mycgr3T

Mycgr3G90406 Mycgr3T
  
Location: 1642-3973

Mycgr3G90406\_Mycgr3T

Mycgr3G67785 Mycgr3T
  
Location: 4073-7865

Mycgr3G67785\_Mycgr3T

Mycgr3G67795 Mycgr3T
  
Location: 7965-15249

Mycgr3G67795\_Mycgr3T

Mycgr3G67775 Mycgr3T
  
Location: 15349-16237

Mycgr3G67775\_Mycgr3T

Mycgr3G90404 Mycgr3T
  
Location: 16337-17246

Mycgr3G90404\_Mycgr3T

Mycgr3G36951 Mycgr3T
  
Location: 17346-30891

Mycgr3G36951\_Mycgr3T

Mycgr3G103034 Mycgr3
  
Location: 30991-32644

Mycgr3G103034\_Mycgr3

Mycgr3G31119 Mycgr3T
  
Location: 32744-32906

Mycgr3G31119\_Mycgr3T

Mycgr3G28587 Mycgr3T
  
Location: 33006-33489

Mycgr3G28587\_Mycgr3T

Mycgr3G98959 Mycgr3T
  
Location: 33589-35035

Mycgr3G98959\_Mycgr3T

Mycgr3G35447 Mycgr3T
  
Location: 35135-36443

Mycgr3G35447\_Mycgr3T

Mycgr3G84402 Mycgr3T
  
Location: 36543-37884

Mycgr3G84402\_Mycgr3T

Mycgr3G98961 Mycgr3T
  
Location: 37984-38884

Mycgr3G98961\_Mycgr3T

hypothetical protein
  
Accession: CBX99726
  
Location: 244043-244684
  
 NCBI BlastP on this gene

LEMA\_P073150.1

predicted protein
  
Accession: CBX99727
  
Location: 244889-245643
  
 NCBI BlastP on this gene

LEMA\_P073160.1

predicted protein
  
Accession: CBX99728
  
Location: 246849-247064
  
 NCBI BlastP on this gene

LEMA\_uP073170.1

hypothetical protein
  
Accession: CBX99729
  
Location: 248541-252269
  
 NCBI BlastP on this gene

LEMA\_P073180.1

predicted protein
  
Accession: CBX99730
  
Location: 253886-254377
  
 NCBI BlastP on this gene

LEMA\_P073190.1

similar to nonribosomal peptide synthetase 2
  
Accession: CBX99731
  
Location: 254953-271464
  
  
**BlastP hit with Mycgr3G36951\_Mycgr3T**
  
Percentage identity: 37 %
  
BlastP bit score: 1734
  
Sequence coverage: 62 %
  
E-value: 0.0
  
  
 NCBI BlastP on this gene

LEMA\_P073200.1

similar to multidrug resistance protein 1
  
Accession: CBX99732
  
Location: 273017-277841
  
  
**BlastP hit with Mycgr3G67785\_Mycgr3T**
  
Percentage identity: 45 %
  
BlastP bit score: 1070
  
Sequence coverage: 103 %
  
E-value: 0.0
  
  
 NCBI BlastP on this gene

LEMA\_P073210.1

hypothetical protein
  
Accession: CBX99733
  
Location: 279081-281250
  
 NCBI BlastP on this gene

LEMA\_P073220.1

predicted protein
  
Accession: CBX99734
  
Location: 281497-281760
  
 NCBI BlastP on this gene

LEMA\_P073230.1

hypothetical protein
  
Accession: CBX99735
  
Location: 282546-285647
  
 NCBI BlastP on this gene

LEMA\_P073240.1

similar to eukaryotic translation initiation factor 3 subunit 8
  
Accession: CBX99736
  
Location: 286642-289527
  
 NCBI BlastP on this gene

LEMA\_P073250.1

Query: Architecture Search FASTA input

GL535377 : Pyrenophora teres f. teres 0-1 unplaced genomic scaffold scaffold\_191648    Total score: 2.0     Cumulative Blast bit score: 2760

Hit cluster cross-links:

Mycgr3G67791 Mycgr3T
  
Location: 0-1542

Mycgr3G67791\_Mycgr3T

Mycgr3G90406 Mycgr3T
  
Location: 1642-3973

Mycgr3G90406\_Mycgr3T

Mycgr3G67785 Mycgr3T
  
Location: 4073-7865

Mycgr3G67785\_Mycgr3T

Mycgr3G67795 Mycgr3T
  
Location: 7965-15249

Mycgr3G67795\_Mycgr3T

Mycgr3G67775 Mycgr3T
  
Location: 15349-16237

Mycgr3G67775\_Mycgr3T

Mycgr3G90404 Mycgr3T
  
Location: 16337-17246

Mycgr3G90404\_Mycgr3T

Mycgr3G36951 Mycgr3T
  
Location: 17346-30891

Mycgr3G36951\_Mycgr3T

Mycgr3G103034 Mycgr3
  
Location: 30991-32644

Mycgr3G103034\_Mycgr3

Mycgr3G31119 Mycgr3T
  
Location: 32744-32906

Mycgr3G31119\_Mycgr3T

Mycgr3G28587 Mycgr3T
  
Location: 33006-33489

Mycgr3G28587\_Mycgr3T

Mycgr3G98959 Mycgr3T
  
Location: 33589-35035

Mycgr3G98959\_Mycgr3T

Mycgr3G35447 Mycgr3T
  
Location: 35135-36443

Mycgr3G35447\_Mycgr3T

Mycgr3G84402 Mycgr3T
  
Location: 36543-37884

Mycgr3G84402\_Mycgr3T

Mycgr3G98961 Mycgr3T
  
Location: 37984-38884

Mycgr3G98961\_Mycgr3T

hypothetical protein
  
Accession: EFQ90039
  
Location: 345-16619
  
  
**BlastP hit with Mycgr3G36951\_Mycgr3T**
  
Percentage identity: 36 %
  
BlastP bit score: 1696
  
Sequence coverage: 60 %
  
E-value: 0.0
  
  
 NCBI BlastP on this gene

EFQ90039

hypothetical protein
  
Accession: EFQ90040
  
Location: 18180-22429
  
  
**BlastP hit with Mycgr3G67785\_Mycgr3T**
  
Percentage identity: 46 %
  
BlastP bit score: 1064
  
Sequence coverage: 101 %
  
E-value: 0.0
  
  
 NCBI BlastP on this gene

EFQ90040

hypothetical protein
  
Accession: EFQ90041
  
Location: 23863-25578
  
 NCBI BlastP on this gene

EFQ90041

hypothetical protein
  
Accession: EFQ90042
  
Location: 26622-29560
  
 NCBI BlastP on this gene

EFQ90042

Query: Architecture Search FASTA input

CH445327 : Phaeosphaeria nodorum SN15 scaffold\_3    Total score: 2.0     Cumulative Blast bit score: 2639

Hit cluster cross-links:

Mycgr3G67791 Mycgr3T
  
Location: 0-1542

Mycgr3G67791\_Mycgr3T

Mycgr3G90406 Mycgr3T
  
Location: 1642-3973

Mycgr3G90406\_Mycgr3T

Mycgr3G67785 Mycgr3T
  
Location: 4073-7865

Mycgr3G67785\_Mycgr3T

Mycgr3G67795 Mycgr3T
  
Location: 7965-15249

Mycgr3G67795\_Mycgr3T

Mycgr3G67775 Mycgr3T
  
Location: 15349-16237

Mycgr3G67775\_Mycgr3T

Mycgr3G90404 Mycgr3T
  
Location: 16337-17246

Mycgr3G90404\_Mycgr3T

Mycgr3G36951 Mycgr3T
  
Location: 17346-30891

Mycgr3G36951\_Mycgr3T

Mycgr3G103034 Mycgr3
  
Location: 30991-32644

Mycgr3G103034\_Mycgr3

Mycgr3G31119 Mycgr3T
  
Location: 32744-32906

Mycgr3G31119\_Mycgr3T

Mycgr3G28587 Mycgr3T
  
Location: 33006-33489

Mycgr3G28587\_Mycgr3T

Mycgr3G98959 Mycgr3T
  
Location: 33589-35035

Mycgr3G98959\_Mycgr3T

Mycgr3G35447 Mycgr3T
  
Location: 35135-36443

Mycgr3G35447\_Mycgr3T

Mycgr3G84402 Mycgr3T
  
Location: 36543-37884

Mycgr3G84402\_Mycgr3T

Mycgr3G98961 Mycgr3T
  
Location: 37984-38884

Mycgr3G98961\_Mycgr3T

hypothetical protein
  
Accession: EAT90339
  
Location: 616053-618542
  
 NCBI BlastP on this gene

EAT90339

hypothetical protein
  
Accession: EAT90340
  
Location: 618889-619751
  
 NCBI BlastP on this gene

EAT90340

hypothetical protein
  
Accession: EAT90341
  
Location: 621365-622403
  
 NCBI BlastP on this gene

EAT90341

hypothetical protein
  
Accession: EAT90342
  
Location: 622566-623249
  
 NCBI BlastP on this gene

EAT90342

hypothetical protein
  
Accession: EAT90343
  
Location: 624280-624858
  
 NCBI BlastP on this gene

EAT90343

hypothetical protein
  
Accession: EAT90344
  
Location: 625692-627063
  
 NCBI BlastP on this gene

EAT90344

hypothetical protein
  
Accession: EAT90345
  
Location: 627653-628171
  
 NCBI BlastP on this gene

EAT90345

hypothetical protein
  
Accession: EAT90346
  
Location: 628329-642276
  
  
**BlastP hit with Mycgr3G36951\_Mycgr3T**
  
Percentage identity: 36 %
  
BlastP bit score: 1677
  
Sequence coverage: 61 %
  
E-value: 0.0
  
  
 NCBI BlastP on this gene

EAT90346

hypothetical protein
  
Accession: EAT90347
  
Location: 646941-650670
  
  
**BlastP hit with Mycgr3G67785\_Mycgr3T**
  
Percentage identity: 45 %
  
BlastP bit score: 962
  
Sequence coverage: 90 %
  
E-value: 0.0
  
  
 NCBI BlastP on this gene

EAT90347

hypothetical protein
  
Accession: EAT90348
  
Location: 651226-651518
  
 NCBI BlastP on this gene

EAT90348

hypothetical protein
  
Accession: EAT90349
  
Location: 651951-653586
  
 NCBI BlastP on this gene

EAT90349

hypothetical protein
  
Accession: EAT90350
  
Location: 654701-657659
  
 NCBI BlastP on this gene

EAT90350

hypothetical protein
  
Accession: EAT90351
  
Location: 658718-661459
  
 NCBI BlastP on this gene

EAT90351

Query: Architecture Search FASTA input

GL988041 : Chaetomium thermophilum var. thermophilum DSM 1495 unplaced genomic scaffold scf7180000...    Total score: 2.0     Cumulative Blast bit score: 1846

Hit cluster cross-links:

Mycgr3G67791 Mycgr3T
  
Location: 0-1542

Mycgr3G67791\_Mycgr3T

Mycgr3G90406 Mycgr3T
  
Location: 1642-3973

Mycgr3G90406\_Mycgr3T

Mycgr3G67785 Mycgr3T
  
Location: 4073-7865

Mycgr3G67785\_Mycgr3T

Mycgr3G67795 Mycgr3T
  
Location: 7965-15249

Mycgr3G67795\_Mycgr3T

Mycgr3G67775 Mycgr3T
  
Location: 15349-16237

Mycgr3G67775\_Mycgr3T

Mycgr3G90404 Mycgr3T
  
Location: 16337-17246

Mycgr3G90404\_Mycgr3T

Mycgr3G36951 Mycgr3T
  
Location: 17346-30891

Mycgr3G36951\_Mycgr3T

Mycgr3G103034 Mycgr3
  
Location: 30991-32644

Mycgr3G103034\_Mycgr3

Mycgr3G31119 Mycgr3T
  
Location: 32744-32906

Mycgr3G31119\_Mycgr3T

Mycgr3G28587 Mycgr3T
  
Location: 33006-33489

Mycgr3G28587\_Mycgr3T

Mycgr3G98959 Mycgr3T
  
Location: 33589-35035

Mycgr3G98959\_Mycgr3T

Mycgr3G35447 Mycgr3T
  
Location: 35135-36443

Mycgr3G35447\_Mycgr3T

Mycgr3G84402 Mycgr3T
  
Location: 36543-37884

Mycgr3G84402\_Mycgr3T

Mycgr3G98961 Mycgr3T
  
Location: 37984-38884

Mycgr3G98961\_Mycgr3T

40S ribosomal protein S7-like protein
  
Accession: EGS20299
  
Location: 43430-44357
  
 NCBI BlastP on this gene

EGS20299

NADP-dependent alcohol dehydrogenase-like protein
  
Accession: EGS20298
  
Location: 40406-43119
  
 NCBI BlastP on this gene

EGS20298

hypothetical protein
  
Accession: EGS20297
  
Location: 38382-40308
  
 NCBI BlastP on this gene

EGS20297

putative malic acid transport protein
  
Accession: EGS20296
  
Location: 36137-37615
  
  
**BlastP hit with Mycgr3G67791\_Mycgr3T**
  
Percentage identity: 31 %
  
BlastP bit score: 223
  
Sequence coverage: 84 %
  
E-value: 1e-62
  
  
 NCBI BlastP on this gene

EGS20296

hypothetical protein
  
Accession: EGS20295
  
Location: 34043-35937
  
 NCBI BlastP on this gene

EGS20295

hypothetical protein
  
Accession: EGS20294
  
Location: 31475-33265
  
 NCBI BlastP on this gene

EGS20294

hypothetical protein
  
Accession: EGS20293
  
Location: 27302-28434
  
 NCBI BlastP on this gene

EGS20293

putative L-ornithine protein
  
Accession: EGS20292
  
Location: 22656-24251
  
 NCBI BlastP on this gene

EGS20292

nonribosomal peptide synthase-like protein
  
Accession: EGS20291
  
Location: 6067-19404
  
  
**BlastP hit with Mycgr3G36951\_Mycgr3T**
  
Percentage identity: 33 %
  
BlastP bit score: 1623
  
Sequence coverage: 73 %
  
E-value: 0.0
  
  
 NCBI BlastP on this gene

EGS20291

hypothetical protein
  
Accession: EGS20290
  
Location: 1255-3822
  
 NCBI BlastP on this gene

EGS20290

Query: Architecture Search FASTA input

DS231623 : Pyrenophora tritici-repentis Pt-1C-BFP supercont1.9 genomic scaffold    Total score: 2.0     Cumulative Blast bit score: 1776

Hit cluster cross-links:

Mycgr3G67791 Mycgr3T
  
Location: 0-1542

Mycgr3G67791\_Mycgr3T

Mycgr3G90406 Mycgr3T
  
Location: 1642-3973

Mycgr3G90406\_Mycgr3T

Mycgr3G67785 Mycgr3T
  
Location: 4073-7865

Mycgr3G67785\_Mycgr3T

Mycgr3G67795 Mycgr3T
  
Location: 7965-15249

Mycgr3G67795\_Mycgr3T

Mycgr3G67775 Mycgr3T
  
Location: 15349-16237

Mycgr3G67775\_Mycgr3T

Mycgr3G90404 Mycgr3T
  
Location: 16337-17246

Mycgr3G90404\_Mycgr3T

Mycgr3G36951 Mycgr3T
  
Location: 17346-30891

Mycgr3G36951\_Mycgr3T

Mycgr3G103034 Mycgr3
  
Location: 30991-32644

Mycgr3G103034\_Mycgr3

Mycgr3G31119 Mycgr3T
  
Location: 32744-32906

Mycgr3G31119\_Mycgr3T

Mycgr3G28587 Mycgr3T
  
Location: 33006-33489

Mycgr3G28587\_Mycgr3T

Mycgr3G98959 Mycgr3T
  
Location: 33589-35035

Mycgr3G98959\_Mycgr3T

Mycgr3G35447 Mycgr3T
  
Location: 35135-36443

Mycgr3G35447\_Mycgr3T

Mycgr3G84402 Mycgr3T
  
Location: 36543-37884

Mycgr3G84402\_Mycgr3T

Mycgr3G98961 Mycgr3T
  
Location: 37984-38884

Mycgr3G98961\_Mycgr3T

eukaryotic translation initiation factor 3 subunit C
  
Accession: EDU51191
  
Location: 601037-603782
  
 NCBI BlastP on this gene

EDU51191

serine/threonine-protein kinase minibrain
  
Accession: EDU51192
  
Location: 604727-607662
  
 NCBI BlastP on this gene

EDU51192

L-ornithine 5-monooxygenase (L-ornithine N(5)-oxygenase)
  
Accession: EDU51193
  
Location: 608720-610433
  
 NCBI BlastP on this gene

EDU51193

leptomycin B resistance protein pmd1
  
Accession: EDU51194
  
Location: 611856-616104
  
  
**BlastP hit with Mycgr3G67785\_Mycgr3T**
  
Percentage identity: 45 %
  
BlastP bit score: 1084
  
Sequence coverage: 101 %
  
E-value: 0.0
  
  
 NCBI BlastP on this gene

EDU51194

predicted protein
  
Accession: EDU51195
  
Location: 618297-618473
  
 NCBI BlastP on this gene

EDU51195

nonribosomal peptide synthetase 2
  
Accession: EDU51196
  
Location: 623920-624054
  
 NCBI BlastP on this gene

EDU51196

gramicidin S synthetase 1
  
Accession: EDU51197
  
Location: 627120-631429
  
  
**BlastP hit with Mycgr3G36951\_Mycgr3T**
  
Percentage identity: 51 %
  
BlastP bit score: 692
  
Sequence coverage: 15 %
  
E-value: 0.0
  
  
 NCBI BlastP on this gene

EDU51197

peptide synthetase
  
Accession: EDU51198
  
Location: 632022-633701
  
 NCBI BlastP on this gene

EDU51198

phosphatidic acid phosphatase beta
  
Accession: EDU51199
  
Location: 634081-635463
  
 NCBI BlastP on this gene

EDU51199

conserved hypothetical protein
  
Accession: EDU51200
  
Location: 636611-637207
  
 NCBI BlastP on this gene

EDU51200

AAR2 domain containing protein
  
Accession: EDU51201
  
Location: 638148-639578
  
 NCBI BlastP on this gene

EDU51201

vacuolar-sorting protein SNF8
  
Accession: EDU51202
  
Location: 640530-641360
  
 NCBI BlastP on this gene

EDU51202

predicted protein
  
Accession: EDU51203
  
Location: 641598-644747
  
 NCBI BlastP on this gene

EDU51203

Query: Architecture Search FASTA input

AGUE01000061 : Glarea lozoyensis 74030    Total score: 2.0     Cumulative Blast bit score: 1467

Hit cluster cross-links:

Mycgr3G67791 Mycgr3T
  
Location: 0-1542

Mycgr3G67791\_Mycgr3T

Mycgr3G90406 Mycgr3T
  
Location: 1642-3973

Mycgr3G90406\_Mycgr3T

Mycgr3G67785 Mycgr3T
  
Location: 4073-7865

Mycgr3G67785\_Mycgr3T

Mycgr3G67795 Mycgr3T
  
Location: 7965-15249

Mycgr3G67795\_Mycgr3T

Mycgr3G67775 Mycgr3T
  
Location: 15349-16237

Mycgr3G67775\_Mycgr3T

Mycgr3G90404 Mycgr3T
  
Location: 16337-17246

Mycgr3G90404\_Mycgr3T

Mycgr3G36951 Mycgr3T
  
Location: 17346-30891

Mycgr3G36951\_Mycgr3T

Mycgr3G103034 Mycgr3
  
Location: 30991-32644

Mycgr3G103034\_Mycgr3

Mycgr3G31119 Mycgr3T
  
Location: 32744-32906

Mycgr3G31119\_Mycgr3T

Mycgr3G28587 Mycgr3T
  
Location: 33006-33489

Mycgr3G28587\_Mycgr3T

Mycgr3G98959 Mycgr3T
  
Location: 33589-35035

Mycgr3G98959\_Mycgr3T

Mycgr3G35447 Mycgr3T
  
Location: 35135-36443

Mycgr3G35447\_Mycgr3T

Mycgr3G84402 Mycgr3T
  
Location: 36543-37884

Mycgr3G84402\_Mycgr3T

Mycgr3G98961 Mycgr3T
  
Location: 37984-38884

Mycgr3G98961\_Mycgr3T

putative Hydroxamate-type ferrichrome siderophore peptide synthetase
  
Accession: EHL01089
  
Location: 124523-132259
  
  
**BlastP hit with Mycgr3G36951\_Mycgr3T**
  
Percentage identity: 32 %
  
BlastP bit score: 781
  
Sequence coverage: 35 %
  
E-value: 0.0
  
  
 NCBI BlastP on this gene

EHL01089

putative aspergillopepsin A-like aspartic endopeptidase
  
Accession: EHL01088
  
Location: 120196-121625
  
 NCBI BlastP on this gene

EHL01088

hypothetical protein
  
Accession: EHL01087
  
Location: 116144-116620
  
 NCBI BlastP on this gene

EHL01087

putative Complement C1q tumor necrosis factor-related protein 9
  
Accession: EHL01086
  
Location: 104749-105733
  
 NCBI BlastP on this gene

EHL01086

putative Collagen alpha-1(XVI) chain
  
Accession: EHL01085
  
Location: 103627-104684
  
 NCBI BlastP on this gene

EHL01085

putative Leptomycin B resistance protein pmd1
  
Accession: EHL01084
  
Location: 96599-101168
  
  
**BlastP hit with Mycgr3G67785\_Mycgr3T**
  
Percentage identity: 34 %
  
BlastP bit score: 686
  
Sequence coverage: 100 %
  
E-value: 0.0
  
  
 NCBI BlastP on this gene

EHL01084

hypothetical protein
  
Accession: EHL01083
  
Location: 94178-95394
  
 NCBI BlastP on this gene

EHL01083

putative HC-toxin efflux carrier TOXA
  
Accession: EHL01082
  
Location: 91536-93546
  
 NCBI BlastP on this gene

EHL01082

Query: Architecture Search FASTA input

51. :  KE145370 Glarea lozoyensis ATCC 20868 chromosome Unknown GLAREA6     Total score: 3.0     Cumulative Blast bit score: 1309

Mycgr3G67791 Mycgr3T
  
Location: 0-1542
  
 NCBI BlastP on this gene

Mycgr3G67791\_Mycgr3T

Mycgr3G90406 Mycgr3T
  
Location: 1642-3973
  
 NCBI BlastP on this gene

Mycgr3G90406\_Mycgr3T

Mycgr3G67785 Mycgr3T
  
Location: 4073-7865
  
 NCBI BlastP on this gene

Mycgr3G67785\_Mycgr3T

Mycgr3G67795 Mycgr3T
  
Location: 7965-15249
  
 NCBI BlastP on this gene

Mycgr3G67795\_Mycgr3T

Mycgr3G67775 Mycgr3T
  
Location: 15349-16237
  
 NCBI BlastP on this gene

Mycgr3G67775\_Mycgr3T

Mycgr3G90404 Mycgr3T
  
Location: 16337-17246
  
 NCBI BlastP on this gene

Mycgr3G90404\_Mycgr3T

Mycgr3G36951 Mycgr3T
  
Location: 17346-30891
  
 NCBI BlastP on this gene

Mycgr3G36951\_Mycgr3T

Mycgr3G103034 Mycgr3
  
Location: 30991-32644
  
 NCBI BlastP on this gene

Mycgr3G103034\_Mycgr3

Mycgr3G31119 Mycgr3T
  
Location: 32744-32906
  
 NCBI BlastP on this gene

Mycgr3G31119\_Mycgr3T

Mycgr3G28587 Mycgr3T
  
Location: 33006-33489
  
 NCBI BlastP on this gene

Mycgr3G28587\_Mycgr3T

Mycgr3G98959 Mycgr3T
  
Location: 33589-35035
  
 NCBI BlastP on this gene

Mycgr3G98959\_Mycgr3T

Mycgr3G35447 Mycgr3T
  
Location: 35135-36443
  
 NCBI BlastP on this gene

Mycgr3G35447\_Mycgr3T

Mycgr3G84402 Mycgr3T
  
Location: 36543-37884
  
 NCBI BlastP on this gene

Mycgr3G84402\_Mycgr3T

Mycgr3G98961 Mycgr3T
  
Location: 37984-38884
  
 NCBI BlastP on this gene

Mycgr3G98961\_Mycgr3T

(Trans)glycosidase
  
Accession: EPE26861
  
Location: 1394387-1397815
  
 NCBI BlastP on this gene

EPE26861

Riboflavin synthase
  
Accession: EPE26860
  
Location: 1393019-1393765
  
 NCBI BlastP on this gene

EPE26860

hypothetical protein
  
Accession: EPE26859
  
Location: 1391893-1392132
  
 NCBI BlastP on this gene

EPE26859

Mss4-like protein
  
Accession: EPE26858
  
Location: 1390442-1391029
  
 NCBI BlastP on this gene

EPE26858

Cloroperoxidase
  
Accession: EPE26857
  
Location: 1387949-1389344
  
 NCBI BlastP on this gene

EPE26857

FYVE/PHD zinc finger
  
Accession: EPE26856
  
Location: 1385409-1386917
  
 NCBI BlastP on this gene

EPE26856

CoA-transferase family III (CaiB/BaiF)
  
Accession: EPE26855
  
Location: 1383331-1384904
  
 NCBI BlastP on this gene

EPE26855

hypothetical protein
  
Accession: EPE26854
  
Location: 1380937-1381852
  
 NCBI BlastP on this gene

EPE26854

hypothetical protein
  
Accession: EPE26853
  
Location: 1379796-1380386
  
 NCBI BlastP on this gene

EPE26853

hypothetical protein
  
Accession: EPE26852
  
Location: 1377511-1378911
  
  
**BlastP hit with Mycgr3G35447\_Mycgr3T**
  
Percentage identity: 57 %
  
BlastP bit score: 496
  
Sequence coverage: 103 %
  
E-value: 5e-170
  
  
 NCBI BlastP on this gene

EPE26852

P-loop containing nucleoside triphosphate hydrolase
  
Accession: EPE26851
  
Location: 1375781-1377289
  
  
**BlastP hit with Mycgr3G84402\_Mycgr3T**
  
Percentage identity: 72 %
  
BlastP bit score: 670
  
Sequence coverage: 102 %
  
E-value: 0.0
  
  
 NCBI BlastP on this gene

EPE26851

hypothetical protein
  
Accession: EPE26850
  
Location: 1373623-1374447
  
 NCBI BlastP on this gene

EPE26850

alpha/beta-Hydrolase
  
Accession: EPE26849
  
Location: 1372005-1373321
  
 NCBI BlastP on this gene

EPE26849

hypothetical protein
  
Accession: EPE26848
  
Location: 1370777-1371395
  
  
**BlastP hit with Mycgr3G28587\_Mycgr3T**
  
Percentage identity: 49 %
  
BlastP bit score: 143
  
Sequence coverage: 100 %
  
E-value: 7e-40
  
  
 NCBI BlastP on this gene

EPE26848

P-loop containing nucleoside triphosphate hydrolase
  
Accession: EPE26847
  
Location: 1369207-1370120
  
 NCBI BlastP on this gene

EPE26847

hypothetical protein
  
Accession: EPE26846
  
Location: 1364073-1367792
  
 NCBI BlastP on this gene

EPE26846

NAD(P)-binding Rossmann-fold containing protein
  
Accession: EPE26845
  
Location: 1361328-1362310
  
 NCBI BlastP on this gene

EPE26845

hypothetical protein
  
Accession: EPE26844
  
Location: 1360156-1360785
  
 NCBI BlastP on this gene

EPE26844

hypothetical protein
  
Accession: EPE26843
  
Location: 1357121-1357936
  
 NCBI BlastP on this gene

EPE26843

Thiamin diphosphate-binding fold (THDP-binding)
  
Accession: EPE26842
  
Location: 1352946-1355238
  
 NCBI BlastP on this gene

EPE26842

52. :  FQ790359 Botryotinia fuckeliana T4 SupSuperContig\_114\_320\_122\_1 genomic supercontig.     Total score: 3.0     Cumulative Blast bit score: 1286

hypothetical protein
  
Accession: CCD56341
  
Location: 647660-647995
  
 NCBI BlastP on this gene

BofuT4\_P149510.1

hypothetical protein
  
Accession: CCD56340
  
Location: 644879-647048
  
 NCBI BlastP on this gene

BofuT4\_P149500.1

hypothetical protein
  
Accession: CCD56339
  
Location: 641338-641580
  
 NCBI BlastP on this gene

BofuT4\_uP149490.1

similar to siderophore biosynthesis
  
Accession: CCD56338
  
Location: 639542-640599
  
 NCBI BlastP on this gene

BofuT4\_P149480.1

hypothetical protein
  
Accession: CCD56337
  
Location: 638348-639167
  
  
**BlastP hit with Mycgr3G28587\_Mycgr3T**
  
Percentage identity: 51 %
  
BlastP bit score: 148
  
Sequence coverage: 99 %
  
E-value: 1e-41
  
  
 NCBI BlastP on this gene

BofuT4\_P149470.1

similar to GTP-binding protein rho2
  
Accession: CCD56336
  
Location: 636089-637084
  
 NCBI BlastP on this gene

BofuT4\_P149460.1

hypothetical protein
  
Accession: CCD56335
  
Location: 628910-632583
  
 NCBI BlastP on this gene

BofuT4\_P149450.1

hypothetical protein
  
Accession: CCD56334
  
Location: 627642-627791
  
 NCBI BlastP on this gene

BofuT4\_uP149440.1

predicted protein
  
Accession: CCD56333
  
Location: 626861-627016
  
 NCBI BlastP on this gene

BofuT4\_uP149430.1

predicted protein
  
Accession: CCD56332
  
Location: 625876-626739
  
 NCBI BlastP on this gene

BofuT4\_P149420.1

hypothetical protein
  
Accession: CCD56331
  
Location: 623016-624587
  
 NCBI BlastP on this gene

BofuT4\_P149410.1

similar to ser/Thr protein phosphatase superfamily
  
Accession: CCD56330
  
Location: 621006-621899
  
 NCBI BlastP on this gene

BofuT4\_P149400.1

hypothetical protein
  
Accession: CCD56329
  
Location: 618281-620087
  
 NCBI BlastP on this gene

BofuT4\_P149390.1

similar to caib/baif family protein
  
Accession: CCD56328
  
Location: 616311-618023
  
 NCBI BlastP on this gene

BofuT4\_P149380.1

similar to ATP-dependent rRNA helicase rrp3
  
Accession: CCD56327
  
Location: 614670-616177
  
  
**BlastP hit with Mycgr3G84402\_Mycgr3T**
  
Percentage identity: 71 %
  
BlastP bit score: 660
  
Sequence coverage: 103 %
  
E-value: 0.0
  
  
 NCBI BlastP on this gene

BofuT4\_P149370.1

similar to ribosome biogenesis protein Ssf2
  
Accession: CCD56326
  
Location: 612997-614396
  
  
**BlastP hit with Mycgr3G35447\_Mycgr3T**
  
Percentage identity: 62 %
  
BlastP bit score: 478
  
Sequence coverage: 91 %
  
E-value: 1e-162
  
  
 NCBI BlastP on this gene

BofuT4\_P149360.1

predicted protein
  
Accession: CCD56325
  
Location: 611236-611580
  
 NCBI BlastP on this gene

BofuT4\_P149350.1

hypothetical protein
  
Accession: CCD56324
  
Location: 607674-610070
  
 NCBI BlastP on this gene

BofuT4\_P149340.1

similar to U1biquitin-specific peptidase
  
Accession: CCD56323
  
Location: 605002-607008
  
 NCBI BlastP on this gene

BofuT4\_P149330.1

hypothetical protein
  
Accession: CCD56322
  
Location: 603428-604393
  
 NCBI BlastP on this gene

BofuT4\_P149320.1

53. :  AFWA01000017 Pneumocystis murina B123     Total score: 3.0     Cumulative Blast bit score: 1276

hypothetical protein
  
Accession: EMR08120
  
Location: 222330-224159
  
 NCBI BlastP on this gene

EMR08120

hypothetical protein
  
Accession: EMR08119
  
Location: 219170-221707
  
 NCBI BlastP on this gene

EMR08119

hypothetical protein
  
Accession: EMR08118
  
Location: 212613-219018
  
 NCBI BlastP on this gene

EMR08118

hypothetical protein
  
Accession: EMR08117
  
Location: 209957-211795
  
 NCBI BlastP on this gene

EMR08117

hypothetical protein
  
Accession: EMR08116
  
Location: 207756-209538
  
 NCBI BlastP on this gene

EMR08116

hypothetical protein
  
Accession: EMR08115
  
Location: 205220-207140
  
  
**BlastP hit with Mycgr3G103034\_Mycgr3**
  
Percentage identity: 45 %
  
BlastP bit score: 473
  
Sequence coverage: 95 %
  
E-value: 5e-158
  
  
 NCBI BlastP on this gene

EMR08115

hypothetical protein
  
Accession: EMR08114
  
Location: 204078-205098
  
 NCBI BlastP on this gene

EMR08114

AGC/AKT protein kinase
  
Accession: EMR08113
  
Location: 201258-203629
  
 NCBI BlastP on this gene

EMR08113

riboflavin synthase, alpha subunit
  
Accession: EMR08112
  
Location: 200421-201110
  
 NCBI BlastP on this gene

EMR08112

hypothetical protein
  
Accession: EMR08111
  
Location: 198786-200354
  
  
**BlastP hit with Mycgr3G84402\_Mycgr3T**
  
Percentage identity: 62 %
  
BlastP bit score: 543
  
Sequence coverage: 90 %
  
E-value: 0.0
  
  
 NCBI BlastP on this gene

EMR08111

hypothetical protein
  
Accession: EMR08110
  
Location: 197570-198709
  
  
**BlastP hit with Mycgr3G35447\_Mycgr3T**
  
Percentage identity: 44 %
  
BlastP bit score: 260
  
Sequence coverage: 77 %
  
E-value: 1e-79
  
  
 NCBI BlastP on this gene

EMR08110

hypothetical protein
  
Accession: EMR08109
  
Location: 195736-197131
  
 NCBI BlastP on this gene

EMR08109

hypothetical protein
  
Accession: EMR08108
  
Location: 193855-195104
  
 NCBI BlastP on this gene

EMR08108

hypothetical protein
  
Accession: EMR08107
  
Location: 189871-191963
  
 NCBI BlastP on this gene

EMR08107

hypothetical protein
  
Accession: EMR08106
  
Location: 188500-189655
  
 NCBI BlastP on this gene

EMR08106

hypothetical protein
  
Accession: EMR08105
  
Location: 185523-188101
  
 NCBI BlastP on this gene

EMR08105

hypothetical protein
  
Accession: EMR08104
  
Location: 183980-184642
  
 NCBI BlastP on this gene

EMR08104

hypothetical protein, variant
  
Accession: EMR08103
  
Location: 181221-182078
  
 NCBI BlastP on this gene

EMR08103

hypothetical protein
  
Accession: EMR08102
  
Location: 181221-182490
  
 NCBI BlastP on this gene

EMR08102

hypothetical protein
  
Accession: EMR08101
  
Location: 179013-181054
  
 NCBI BlastP on this gene

EMR08101

54. :  CH476628 Sclerotinia sclerotiorum 1980 scaffold\_8 genomic scaffold     Total score: 3.0     Cumulative Blast bit score: 1242

hypothetical protein
  
Accession: EDO04075
  
Location: 640662-642581
  
 NCBI BlastP on this gene

EDO04075

predicted protein
  
Accession: EDO04076
  
Location: 643303-644266
  
 NCBI BlastP on this gene

EDO04076

predicted protein
  
Accession: EDO04077
  
Location: 644578-644791
  
 NCBI BlastP on this gene

EDO04077

hypothetical protein
  
Accession: EDO04078
  
Location: 645403-646699
  
 NCBI BlastP on this gene

EDO04078

hypothetical protein
  
Accession: EDO04079
  
Location: 648149-649418
  
 NCBI BlastP on this gene

EDO04079

hypothetical protein
  
Accession: EDO04080
  
Location: 650282-652591
  
 NCBI BlastP on this gene

EDO04080

predicted protein
  
Accession: EDO04081
  
Location: 655026-655217
  
 NCBI BlastP on this gene

EDO04081

predicted protein
  
Accession: EDO04082
  
Location: 655630-655815
  
 NCBI BlastP on this gene

EDO04082

hypothetical protein
  
Accession: EDO04083
  
Location: 656768-658170
  
  
**BlastP hit with Mycgr3G35447\_Mycgr3T**
  
Percentage identity: 61 %
  
BlastP bit score: 471
  
Sequence coverage: 91 %
  
E-value: 6e-160
  
  
 NCBI BlastP on this gene

EDO04083

conserved hypothetical protein
  
Accession: EDO04084
  
Location: 658462-659957
  
  
**BlastP hit with Mycgr3G84402\_Mycgr3T**
  
Percentage identity: 78 %
  
BlastP bit score: 657
  
Sequence coverage: 89 %
  
E-value: 0.0
  
  
 NCBI BlastP on this gene

EDO04084

hypothetical protein
  
Accession: EDO04085
  
Location: 660223-663824
  
 NCBI BlastP on this gene

EDO04085

hypothetical protein
  
Accession: EDO04086
  
Location: 665821-666867
  
 NCBI BlastP on this gene

EDO04086

hypothetical protein
  
Accession: EDO04087
  
Location: 667228-668328
  
  
**BlastP hit with Mycgr3G28587\_Mycgr3T**
  
Percentage identity: 45 %
  
BlastP bit score: 114
  
Sequence coverage: 85 %
  
E-value: 8e-29
  
  
 NCBI BlastP on this gene

EDO04087

hypothetical protein
  
Accession: EDO04088
  
Location: 669408-670413
  
 NCBI BlastP on this gene

EDO04088

hypothetical protein
  
Accession: EDO04089
  
Location: 673063-676779
  
 NCBI BlastP on this gene

EDO04089

predicted protein
  
Accession: EDO04090
  
Location: 678041-678217
  
 NCBI BlastP on this gene

EDO04090

predicted protein
  
Accession: EDO04091
  
Location: 678284-678858
  
 NCBI BlastP on this gene

EDO04091

hypothetical protein
  
Accession: EDO04092
  
Location: 680620-682071
  
 NCBI BlastP on this gene

EDO04092

hypothetical protein
  
Accession: EDO04093
  
Location: 682700-684252
  
 NCBI BlastP on this gene

EDO04093

55. :  JH921451 Marssonina brunnea f. sp. 'multigermtubi' MB\_m1 unplaced genomic scaffold M6\_S00024     Total score: 3.0     Cumulative Blast bit score: 1205

cyclin
  
Accession: EKD13183
  
Location: 271965-273458
  
 NCBI BlastP on this gene

EKD13183

hypothetical protein
  
Accession: EKD13182
  
Location: 270981-271550
  
 NCBI BlastP on this gene

EKD13182

hypothetical protein
  
Accession: EKD13181
  
Location: 268924-270896
  
 NCBI BlastP on this gene

EKD13181

hypothetical protein
  
Accession: EKD13180
  
Location: 265357-266286
  
 NCBI BlastP on this gene

EKD13180

hypothetical protein
  
Accession: EKD13179
  
Location: 262163-262950
  
 NCBI BlastP on this gene

EKD13179

RING finger domain protein
  
Accession: EKD13178
  
Location: 260911-261667
  
  
**BlastP hit with Mycgr3G28587\_Mycgr3T**
  
Percentage identity: 48 %
  
BlastP bit score: 114
  
Sequence coverage: 101 %
  
E-value: 1e-28
  
  
 NCBI BlastP on this gene

EKD13178

GTP-binding protein rho2
  
Accession: EKD13177
  
Location: 258921-259894
  
 NCBI BlastP on this gene

EKD13177

stress response protein NST1
  
Accession: EKD13176
  
Location: 253980-257640
  
 NCBI BlastP on this gene

EKD13176

hypothetical protein
  
Accession: EKD13175
  
Location: 251794-252723
  
 NCBI BlastP on this gene

EKD13175

hypothetical protein
  
Accession: EKD13174
  
Location: 246072-247975
  
 NCBI BlastP on this gene

EKD13174

ribosome biogenesis protein
  
Accession: EKD13173
  
Location: 244283-245702
  
  
**BlastP hit with Mycgr3G35447\_Mycgr3T**
  
Percentage identity: 59 %
  
BlastP bit score: 483
  
Sequence coverage: 101 %
  
E-value: 2e-164
  
  
 NCBI BlastP on this gene

EKD13173

ATP-dependent rRNA helicase RRP3
  
Accession: EKD13172
  
Location: 242552-244092
  
  
**BlastP hit with Mycgr3G84402\_Mycgr3T**
  
Percentage identity: 73 %
  
BlastP bit score: 608
  
Sequence coverage: 89 %
  
E-value: 0.0
  
  
 NCBI BlastP on this gene

EKD13172

hypothetical protein
  
Accession: EKD13171
  
Location: 240927-241295
  
 NCBI BlastP on this gene

EKD13171

prolyl-tRNA synthetase
  
Accession: EKD13170
  
Location: 238569-240670
  
 NCBI BlastP on this gene

EKD13170

CCAAT-box-binding transcription factor
  
Accession: EKD13169
  
Location: 234490-237908
  
 NCBI BlastP on this gene

EKD13169

hypothetical protein
  
Accession: EKD13168
  
Location: 233170-234112
  
 NCBI BlastP on this gene

EKD13168

adenylyl-sulfate kinase
  
Accession: EKD13167
  
Location: 229496-232024
  
 NCBI BlastP on this gene

EKD13167

56. :  KB708068 Botryotinia fuckeliana BcDW1 unplaced genomic scaffold Scaffold\_396     Total score: 3.0     Cumulative Blast bit score: 1193

hypothetical protein
  
Accession: EMR81738
  
Location: 261668-263479
  
 NCBI BlastP on this gene

EMR81738

putative c2h2-like zinc finger protein
  
Accession: EMR81737
  
Location: 258562-259293
  
 NCBI BlastP on this gene

EMR81737

putative siderophore biosynthesis lipase esterase protein
  
Accession: EMR81736
  
Location: 256551-257608
  
 NCBI BlastP on this gene

EMR81736

hypothetical protein
  
Accession: EMR81735
  
Location: 255150-256175
  
  
**BlastP hit with Mycgr3G28587\_Mycgr3T**
  
Percentage identity: 56 %
  
BlastP bit score: 55
  
Sequence coverage: 34 %
  
E-value: 2e-07
  
  
 NCBI BlastP on this gene

EMR81735

putative rho gtpase rho protein
  
Accession: EMR81734
  
Location: 253097-254092
  
 NCBI BlastP on this gene

EMR81734

putative stress response protein nst1 protein
  
Accession: EMR81733
  
Location: 246519-250227
  
 NCBI BlastP on this gene

EMR81733

putative integral membrane protein
  
Accession: EMR81732
  
Location: 240885-241750
  
 NCBI BlastP on this gene

EMR81732

putative ser thr protein phosphatase superfamily protein
  
Accession: EMR81731
  
Location: 238878-239771
  
 NCBI BlastP on this gene

EMR81731

putative phd finger containing protein phf1 protein
  
Accession: EMR81730
  
Location: 236425-237959
  
 NCBI BlastP on this gene

EMR81730

putative formyl-coenzyme a transferase protein
  
Accession: EMR81729
  
Location: 234341-235895
  
 NCBI BlastP on this gene

EMR81729

putative atp-dependent rrna helicase rrp3 protein
  
Accession: EMR81728
  
Location: 232542-234049
  
  
**BlastP hit with Mycgr3G84402\_Mycgr3T**
  
Percentage identity: 71 %
  
BlastP bit score: 660
  
Sequence coverage: 103 %
  
E-value: 0.0
  
  
 NCBI BlastP on this gene

EMR81728

putative ribosome biogenesis protein ssf1 protein
  
Accession: EMR81727
  
Location: 230869-232268
  
  
**BlastP hit with Mycgr3G35447\_Mycgr3T**
  
Percentage identity: 62 %
  
BlastP bit score: 478
  
Sequence coverage: 91 %
  
E-value: 1e-162
  
  
 NCBI BlastP on this gene

EMR81727

hypothetical protein
  
Accession: EMR81726
  
Location: 225539-227935
  
 NCBI BlastP on this gene

EMR81726

putative ubiquitin carboxyl-terminal hydrolase protein
  
Accession: EMR81725
  
Location: 222867-224873
  
 NCBI BlastP on this gene

EMR81725

57. :  FP929137 Leptosphaeria maculans JN3 lm\_SuperContig\_10\_v2 genomic supercontig     Total score: 3.0     Cumulative Blast bit score: 739

predicted protein
  
Accession: CBX99904
  
Location: 865926-866473
  
 NCBI BlastP on this gene

LEMA\_P074930.1

predicted protein
  
Accession: CBX99905
  
Location: 866690-867234
  
 NCBI BlastP on this gene

LEMA\_P074940.1

predicted protein
  
Accession: CBX99906
  
Location: 867664-868193
  
 NCBI BlastP on this gene

LEMA\_P074950.1

predicted protein
  
Accession: CBX99907
  
Location: 868472-869227
  
  
**BlastP hit with Mycgr3G28587\_Mycgr3T**
  
Percentage identity: 74 %
  
BlastP bit score: 76
  
Sequence coverage: 36 %
  
E-value: 3e-14
  
  
 NCBI BlastP on this gene

LEMA\_P074960.1

similar to GTP-binding protein RHO-1 protein
  
Accession: CBX99908
  
Location: 870397-871545
  
 NCBI BlastP on this gene

LEMA\_P074970.1

hypothetical protein
  
Accession: CBX99909
  
Location: 873693-876858
  
 NCBI BlastP on this gene

LEMA\_P074980.1

similar to N-acetylglucosamine-phosphate mutase
  
Accession: CBX99910
  
Location: 877974-879904
  
  
**BlastP hit with Mycgr3G103034\_Mycgr3**
  
Percentage identity: 54 %
  
BlastP bit score: 594
  
Sequence coverage: 97 %
  
E-value: 0.0
  
  
 NCBI BlastP on this gene

LEMA\_P074990.1

similar to DUF221 domain-containing protein
  
Accession: CBX99911
  
Location: 882042-885034
  
 NCBI BlastP on this gene

LEMA\_P075000.1

predicted protein
  
Accession: CBX99912
  
Location: 886467-886834
  
 NCBI BlastP on this gene

LEMA\_uP075010.1

hypothetical protein
  
Accession: CBX99913
  
Location: 888253-889352
  
 NCBI BlastP on this gene

LEMA\_P075020.1

predicted protein
  
Accession: CBX99914
  
Location: 889963-890146
  
 NCBI BlastP on this gene

LEMA\_uP075030.1

predicted protein
  
Accession: CBX99915
  
Location: 892773-892931
  
 NCBI BlastP on this gene

LEMA\_uP075040.1

predicted protein
  
Accession: CBX99916
  
Location: 893265-893632
  
 NCBI BlastP on this gene

LEMA\_uP075050.1

predicted protein
  
Accession: CBX99917
  
Location: 894839-897481
  
 NCBI BlastP on this gene

LEMA\_P075060.1

predicted protein
  
Accession: CBX99918
  
Location: 897806-898789
  
 NCBI BlastP on this gene

LEMA\_P075070.1

predicted protein
  
Accession: CBX99919
  
Location: 901323-902667
  
 NCBI BlastP on this gene

LEMA\_P075080.1

hypothetical protein
  
Accession: CBX99920
  
Location: 904042-906207
  
  
**BlastP hit with Mycgr3G90406\_Mycgr3T**
  
Percentage identity: 26 %
  
BlastP bit score: 69
  
Sequence coverage: 23 %
  
E-value: 5e-09
  
  
 NCBI BlastP on this gene

LEMA\_P075090.1

hypothetical protein
  
Accession: CBX99921
  
Location: 907205-909011
  
 NCBI BlastP on this gene

LEMA\_P075100.1

58. :  KB446542 Dothistroma septosporum NZE10 unplaced genomic scaffold DOTSEscaffold\_8     Total score: 2.0     Cumulative Blast bit score: 5340

glycoside hydrolase family 43 protein
  
Accession: EME41694
  
Location: 1326279-1327413
  
 NCBI BlastP on this gene

EME41694

hypothetical protein
  
Accession: EME41695
  
Location: 1329262-1329861
  
 NCBI BlastP on this gene

EME41695

hypothetical protein
  
Accession: EME41696
  
Location: 1331123-1331417
  
 NCBI BlastP on this gene

EME41696

hypothetical protein
  
Accession: EME41697
  
Location: 1333402-1334617
  
 NCBI BlastP on this gene

EME41697

hypothetical protein
  
Accession: EME41698
  
Location: 1334915-1335880
  
 NCBI BlastP on this gene

EME41698

hypothetical protein
  
Accession: EME41699
  
Location: 1336762-1337446
  
 NCBI BlastP on this gene

EME41699

non-ribosomal peptide synthetase-like protein
  
Accession: EME41700
  
Location: 1338920-1353680
  
  
**BlastP hit with Mycgr3G36951\_Mycgr3T**
  
Percentage identity: 51 %
  
BlastP bit score: 3545
  
Sequence coverage: 76 %
  
E-value: 0.0
  
  
 NCBI BlastP on this gene

EME41700

hypothetical protein
  
Accession: EME41701
  
Location: 1355325-1359405
  
  
**BlastP hit with Mycgr3G67785\_Mycgr3T**
  
Percentage identity: 69 %
  
BlastP bit score: 1795
  
Sequence coverage: 100 %
  
E-value: 0.0
  
  
 NCBI BlastP on this gene

EME41701

hypothetical protein
  
Accession: EME41702
  
Location: 1360459-1362032
  
 NCBI BlastP on this gene

EME41702

hypothetical protein
  
Accession: EME41703
  
Location: 1363655-1364053
  
 NCBI BlastP on this gene

EME41703

hypothetical protein
  
Accession: EME41704
  
Location: 1365956-1366207
  
 NCBI BlastP on this gene

EME41704

hypothetical protein
  
Accession: EME41705
  
Location: 1366605-1367807
  
 NCBI BlastP on this gene

EME41705

hypothetical protein
  
Accession: EME41706
  
Location: 1368462-1372073
  
 NCBI BlastP on this gene

EME41706

59. :  KB456266 Mycosphaerella populorum SO2202 unplaced genomic scaffold SEPMUscaffold\_7     Total score: 2.0     Cumulative Blast bit score: 5225

timeless-domain-containing protein
  
Accession: EMF11463
  
Location: 1491062-1494763
  
 NCBI BlastP on this gene

EMF11463

3HCDH N-domain-containing protein
  
Accession: EMF11464
  
Location: 1495065-1496419
  
 NCBI BlastP on this gene

EMF11464

hypothetical protein
  
Accession: EMF11465
  
Location: 1497078-1498041
  
 NCBI BlastP on this gene

EMF11465

hypothetical protein
  
Accession: EMF11466
  
Location: 1498279-1499301
  
 NCBI BlastP on this gene

EMF11466

hydroxylase
  
Accession: EMF11467
  
Location: 1502653-1504258
  
 NCBI BlastP on this gene

EMF11467

multidrug resistance-like protein
  
Accession: EMF11468
  
Location: 1505460-1509410
  
  
**BlastP hit with Mycgr3G67785\_Mycgr3T**
  
Percentage identity: 66 %
  
BlastP bit score: 1719
  
Sequence coverage: 101 %
  
E-value: 0.0
  
  
 NCBI BlastP on this gene

EMF11468

peptide synthetase
  
Accession: EMF11469
  
Location: 1511725-1526534
  
  
**BlastP hit with Mycgr3G36951\_Mycgr3T**
  
Percentage identity: 50 %
  
BlastP bit score: 3506
  
Sequence coverage: 77 %
  
E-value: 0.0
  
  
 NCBI BlastP on this gene

EMF11469

TPT-domain-containing protein
  
Accession: EMF11470
  
Location: 1527568-1529162
  
 NCBI BlastP on this gene

EMF11470

hypothetical protein
  
Accession: EMF11471
  
Location: 1529869-1531596
  
 NCBI BlastP on this gene

EMF11471

hypothetical protein
  
Accession: EMF11472
  
Location: 1531789-1533762
  
 NCBI BlastP on this gene

EMF11472

nitrate transporter CrnA
  
Accession: EMF11473
  
Location: 1535969-1537634
  
 NCBI BlastP on this gene

EMF11473

60. :  KB446542 Dothistroma septosporum NZE10 unplaced genomic scaffold DOTSEscaffold\_8     Total score: 2.0     Cumulative Blast bit score: 5215

hypothetical protein
  
Accession: EME41608
  
Location: 1113435-1113626
  
 NCBI BlastP on this gene

EME41608

hypothetical protein
  
Accession: EME41609
  
Location: 1115501-1116935
  
 NCBI BlastP on this gene

EME41609

hypothetical protein
  
Accession: EME41610
  
Location: 1117740-1118039
  
 NCBI BlastP on this gene

EME41610

hypothetical protein
  
Accession: EME41611
  
Location: 1118421-1119251
  
 NCBI BlastP on this gene

EME41611

hypothetical protein
  
Accession: EME41612
  
Location: 1120539-1120775
  
 NCBI BlastP on this gene

EME41612

hypothetical protein
  
Accession: EME41613
  
Location: 1121646-1123271
  
 NCBI BlastP on this gene

EME41613

glycoside hydrolase family 78 protein
  
Accession: EME41614
  
Location: 1123851-1126076
  
 NCBI BlastP on this gene

EME41614

hypothetical protein
  
Accession: EME41615
  
Location: 1126662-1127513
  
 NCBI BlastP on this gene

EME41615

hypothetical protein
  
Accession: EME41616
  
Location: 1129486-1129732
  
 NCBI BlastP on this gene

EME41616

hypothetical protein
  
Accession: EME41617
  
Location: 1129792-1132263
  
  
**BlastP hit with Mycgr3G90406\_Mycgr3T**
  
Percentage identity: 51 %
  
BlastP bit score: 713
  
Sequence coverage: 108 %
  
E-value: 0.0
  
  
 NCBI BlastP on this gene

EME41617

hypothetical protein
  
Accession: EME41618
  
Location: 1132564-1139865
  
  
**BlastP hit with Mycgr3G67795\_Mycgr3T**
  
Percentage identity: 88 %
  
BlastP bit score: 4502
  
Sequence coverage: 100 %
  
E-value: 0.0
  
  
 NCBI BlastP on this gene

EME41618

hypothetical protein
  
Accession: EME41619
  
Location: 1140547-1141539
  
 NCBI BlastP on this gene

EME41619

C6 transcription factor-like protein
  
Accession: EME41620
  
Location: 1142797-1145066
  
 NCBI BlastP on this gene

EME41620

hypothetical protein
  
Accession: EME41621
  
Location: 1147061-1148194
  
 NCBI BlastP on this gene

EME41621

hypothetical protein
  
Accession: EME41622
  
Location: 1149388-1149687
  
 NCBI BlastP on this gene

EME41622

hypothetical protein
  
Accession: EME41623
  
Location: 1150320-1150922
  
 NCBI BlastP on this gene

EME41623

hypothetical protein
  
Accession: EME41624
  
Location: 1151465-1151982
  
 NCBI BlastP on this gene

EME41624

hypothetical protein
  
Accession: EME41625
  
Location: 1152789-1153627
  
 NCBI BlastP on this gene

EME41625

hypothetical protein
  
Accession: EME41626
  
Location: 1154005-1155544
  
 NCBI BlastP on this gene

EME41626

hypothetical protein
  
Accession: EME41627
  
Location: 1156723-1158438
  
 NCBI BlastP on this gene

EME41627

61. :  KB446566 Pseudocercospora fijiensis CIRAD86 unplaced genomic scaffold MYCFIscaffold\_12     Total score: 2.0     Cumulative Blast bit score: 4985

hypothetical protein
  
Accession: EME77235
  
Location: 171939-175580
  
 NCBI BlastP on this gene

EME77235

hypothetical protein
  
Accession: EME77236
  
Location: 176123-177181
  
 NCBI BlastP on this gene

EME77236

hypothetical protein
  
Accession: EME77237
  
Location: 178439-179328
  
 NCBI BlastP on this gene

EME77237

hypothetical protein
  
Accession: EME77238
  
Location: 180357-184409
  
 NCBI BlastP on this gene

EME77238

hypothetical protein
  
Accession: EME77239
  
Location: 184392-186065
  
 NCBI BlastP on this gene

EME77239

ABC transporter, ABC-B family, MDR type
  
Accession: EME77240
  
Location: 187109-191177
  
  
**BlastP hit with Mycgr3G67785\_Mycgr3T**
  
Percentage identity: 64 %
  
BlastP bit score: 1653
  
Sequence coverage: 98 %
  
E-value: 0.0
  
  
 NCBI BlastP on this gene

EME77240

hypothetical protein
  
Accession: EME77241
  
Location: 192291-206966
  
  
**BlastP hit with Mycgr3G36951\_Mycgr3T**
  
Percentage identity: 48 %
  
BlastP bit score: 3332
  
Sequence coverage: 76 %
  
E-value: 0.0
  
  
 NCBI BlastP on this gene

EME77241

carbohydrate esterase family 9 protein
  
Accession: EME77242
  
Location: 208189-209508
  
 NCBI BlastP on this gene

EME77242

glycoside hydrolase family 3 protein
  
Accession: EME77243
  
Location: 210347-213450
  
 NCBI BlastP on this gene

EME77243

hypothetical protein
  
Accession: EME77244
  
Location: 216553-217101
  
 NCBI BlastP on this gene

EME77244

62. :  KB446566 Pseudocercospora fijiensis CIRAD86 unplaced genomic scaffold MYCFIscaffold\_12     Total score: 2.0     Cumulative Blast bit score: 4443

hypothetical protein
  
Accession: EME77371
  
Location: 936380-937201
  
 NCBI BlastP on this gene

EME77371

hypothetical protein
  
Accession: EME77372
  
Location: 938799-941387
  
 NCBI BlastP on this gene

EME77372

hypothetical protein
  
Accession: EME77373
  
Location: 942550-945648
  
 NCBI BlastP on this gene

EME77373

hypothetical protein
  
Accession: EME77374
  
Location: 946401-947795
  
 NCBI BlastP on this gene

EME77374

hypothetical protein
  
Accession: EME77375
  
Location: 949865-951562
  
 NCBI BlastP on this gene

EME77375

hypothetical protein
  
Accession: EME77376
  
Location: 954705-954960
  
  
**BlastP hit with Mycgr3G90406\_Mycgr3T**
  
Percentage identity: 59 %
  
BlastP bit score: 73
  
Sequence coverage: 11 %
  
E-value: 3e-12
  
  
 NCBI BlastP on this gene

EME77376

hypothetical protein
  
Accession: EME77377
  
Location: 954984-955358
  
 NCBI BlastP on this gene

EME77377

phosphatidylinositol 3-kinase tor2
  
Accession: EME77378
  
Location: 955417-962775
  
  
**BlastP hit with Mycgr3G67795\_Mycgr3T**
  
Percentage identity: 85 %
  
BlastP bit score: 4370
  
Sequence coverage: 101 %
  
E-value: 0.0
  
  
 NCBI BlastP on this gene

EME77378

hypothetical protein
  
Accession: EME77379
  
Location: 963166-965354
  
 NCBI BlastP on this gene

EME77379

hypothetical protein
  
Accession: EME77380
  
Location: 965775-967148
  
 NCBI BlastP on this gene

EME77380

hypothetical protein
  
Accession: EME77381
  
Location: 967712-968608
  
 NCBI BlastP on this gene

EME77381

hypothetical protein
  
Accession: EME77382
  
Location: 968868-969207
  
 NCBI BlastP on this gene

EME77382

hypothetical protein
  
Accession: EME77383
  
Location: 969599-971245
  
 NCBI BlastP on this gene

EME77383

hypothetical protein
  
Accession: EME77384
  
Location: 972220-973722
  
 NCBI BlastP on this gene

EME77384

hypothetical protein
  
Accession: EME77385
  
Location: 973802-974758
  
 NCBI BlastP on this gene

EME77385

hypothetical protein
  
Accession: EME77386
  
Location: 976957-979710
  
 NCBI BlastP on this gene

EME77386

hypothetical protein
  
Accession: EME77387
  
Location: 980361-984296
  
 NCBI BlastP on this gene

EME77387

63. :  DS027696 Neosartorya fischeri NRRL 181 1099437636264 genomic scaffold     Total score: 2.0     Cumulative Blast bit score: 3830

fungal specific transcription factor, putative
  
Accession: EAW18624
  
Location: 4022615-4024116
  
 NCBI BlastP on this gene

EAW18624

eukaryotic translation initiation factor 3 subunit EifCl, putative
  
Accession: EAW18623
  
Location: 4018954-4020528
  
  
**BlastP hit with Mycgr3G98959\_Mycgr3T**
  
Percentage identity: 75 %
  
BlastP bit score: 744
  
Sequence coverage: 100 %
  
E-value: 0.0
  
  
 NCBI BlastP on this gene

EAW18623

iron-sulfur cluster assembly accessory protein Isa2, putative
  
Accession: EAW18622
  
Location: 4017055-4018198
  
 NCBI BlastP on this gene

EAW18622

kynureninase
  
Accession: EAW18621
  
Location: 4015011-4016679
  
 NCBI BlastP on this gene

EAW18621

conserved hypothetical protein
  
Accession: EAW18620
  
Location: 4014101-4014818
  
 NCBI BlastP on this gene

EAW18620

AP-2 adaptor complex subunit beta, putative
  
Accession: EAW18619
  
Location: 4010802-4013492
  
 NCBI BlastP on this gene

EAW18619

conserved hypothetical protein
  
Accession: EAW18618
  
Location: 4008967-4010393
  
 NCBI BlastP on this gene

EAW18618

SH3 domain protein
  
Accession: EAW18617
  
Location: 4004511-4008312
  
 NCBI BlastP on this gene

EAW18617

conserved hypothetical protein
  
Accession: EAW18616
  
Location: 4002677-4003657
  
 NCBI BlastP on this gene

EAW18616

40S ribosomal protein S17, putative
  
Accession: EAW18615
  
Location: 4001562-4002200
  
 NCBI BlastP on this gene

EAW18615

conserved hypothetical protein
  
Accession: EAW18614
  
Location: 3999703-4001121
  
 NCBI BlastP on this gene

EAW18614

ketoreductase
  
Accession: EAW18613
  
Location: 3998139-3999396
  
 NCBI BlastP on this gene

EAW18613

TOR pathway phosphatidylinositol 3-kinase TorA, putative
  
Accession: EAW18612
  
Location: 3988834-3996176
  
  
**BlastP hit with Mycgr3G67795\_Mycgr3T**
  
Percentage identity: 62 %
  
BlastP bit score: 3086
  
Sequence coverage: 100 %
  
E-value: 0.0
  
  
 NCBI BlastP on this gene

EAW18612

conserved hypothetical protein
  
Accession: EAW18611
  
Location: 3986457-3988266
  
 NCBI BlastP on this gene

EAW18611

conserved hypothetical protein
  
Accession: EAW18610
  
Location: 3985026-3985997
  
 NCBI BlastP on this gene

EAW18610

NAD binding Rossmann fold oxidoreductase, putative
  
Accession: EAW18609
  
Location: 3982477-3983605
  
 NCBI BlastP on this gene

EAW18609

64. :  DS499595 Aspergillus fumigatus A1163 scf\_000002 genomic scaffold     Total score: 2.0     Cumulative Blast bit score: 3827

C6 transcription factor, putative
  
Accession: EDP54562
  
Location: 2633984-2636333
  
 NCBI BlastP on this gene

EDP54562

hypothetical protein
  
Accession: EDP54561
  
Location: 2633495-2633934
  
 NCBI BlastP on this gene

EDP54561

eukaryotic translation initiation factor 3 subunit EifCl, putative
  
Accession: EDP54560
  
Location: 2631139-2632713
  
  
**BlastP hit with Mycgr3G98959\_Mycgr3T**
  
Percentage identity: 75 %
  
BlastP bit score: 746
  
Sequence coverage: 100 %
  
E-value: 0.0
  
  
 NCBI BlastP on this gene

EDP54560

iron-sulfur cluster assembly accessory protein Isa2, putative
  
Accession: EDP54559
  
Location: 2629361-2630387
  
 NCBI BlastP on this gene

EDP54559

kynureninase
  
Accession: EDP54558
  
Location: 2625368-2627027
  
 NCBI BlastP on this gene

EDP54558

ER membrane DUF1077 domain protein, putative
  
Accession: EDP54557
  
Location: 2624482-2625198
  
 NCBI BlastP on this gene

EDP54557

AP-2 adaptor complex subunit beta, putative
  
Accession: EDP54556
  
Location: 2621193-2623579
  
 NCBI BlastP on this gene

EDP54556

conserved hypothetical protein
  
Accession: EDP54555
  
Location: 2619376-2620786
  
 NCBI BlastP on this gene

EDP54555

SH3 domain protein
  
Accession: EDP54554
  
Location: 2614933-2618726
  
 NCBI BlastP on this gene

EDP54554

DUF408 domain protein
  
Accession: EDP54553
  
Location: 2613107-2614087
  
 NCBI BlastP on this gene

EDP54553

40S ribosomal protein S17, putative
  
Accession: EDP54552
  
Location: 2611678-2612634
  
 NCBI BlastP on this gene

EDP54552

conserved hypothetical protein
  
Accession: EDP54551
  
Location: 2610136-2611551
  
 NCBI BlastP on this gene

EDP54551

ketoreductase
  
Accession: EDP54550
  
Location: 2608558-2609823
  
 NCBI BlastP on this gene

EDP54550

TOR pathway phosphatidylinositol 3-kinase TorA, putative
  
Accession: EDP54549
  
Location: 2599279-2606624
  
  
**BlastP hit with Mycgr3G67795\_Mycgr3T**
  
Percentage identity: 62 %
  
BlastP bit score: 3081
  
Sequence coverage: 100 %
  
E-value: 0.0
  
  
 NCBI BlastP on this gene

EDP54549

conserved hypothetical protein
  
Accession: EDP54548
  
Location: 2596906-2598716
  
 NCBI BlastP on this gene

EDP54548

conserved hypothetical protein
  
Accession: EDP54547
  
Location: 2595474-2596424
  
 NCBI BlastP on this gene

EDP54547

NAD binding Rossmann fold oxidoreductase, putative
  
Accession: EDP54546
  
Location: 2592970-2594125
  
 NCBI BlastP on this gene

EDP54546

65. :  AAHF01000001 Aspergillus fumigatus Af293     Total score: 2.0     Cumulative Blast bit score: 3827

C6 transcription factor, putative
  
Accession: EAL93335
  
Location: 788948-791297
  
 NCBI BlastP on this gene

EAL93335

hypothetical protein
  
Accession: EAL93334
  
Location: 788459-788898
  
 NCBI BlastP on this gene

EAL93334

eukaryotic translation initiation factor 3 subunit EifCl, putative
  
Accession: EAL93333
  
Location: 786103-787677
  
  
**BlastP hit with Mycgr3G98959\_Mycgr3T**
  
Percentage identity: 75 %
  
BlastP bit score: 746
  
Sequence coverage: 100 %
  
E-value: 0.0
  
  
 NCBI BlastP on this gene

EAL93333

iron-sulfur cluster assembly accessory protein Isa2, putative
  
Accession: EAL93332
  
Location: 784325-785351
  
 NCBI BlastP on this gene

EAL93332

kynureninase
  
Accession: EAL93331
  
Location: 780332-781991
  
 NCBI BlastP on this gene

EAL93331

ER membrane DUF1077 domain protein, putative
  
Accession: EAL93330
  
Location: 779446-780162
  
 NCBI BlastP on this gene

EAL93330

AP-2 adaptor complex subunit beta, putative
  
Accession: EAL93329
  
Location: 776157-778543
  
 NCBI BlastP on this gene

EAL93329

conserved hypothetical protein
  
Accession: EAL93328
  
Location: 774340-775750
  
 NCBI BlastP on this gene

EAL93328

SH3 domain protein
  
Accession: EAL93327
  
Location: 769897-773690
  
 NCBI BlastP on this gene

EAL93327

DUF408 domain protein
  
Accession: EAL93326
  
Location: 768072-769052
  
 NCBI BlastP on this gene

EAL93326

40S ribosomal protein S17, putative
  
Accession: EAL93325
  
Location: 766643-767599
  
 NCBI BlastP on this gene

EAL93325

conserved hypothetical protein
  
Accession: EAL93324
  
Location: 765101-766516
  
 NCBI BlastP on this gene

EAL93324

ketoreductase
  
Accession: EAL93323
  
Location: 763523-764788
  
 NCBI BlastP on this gene

EAL93323

TOR pathway phosphatidylinositol 3-kinase TorA, putative
  
Accession: EAL93322
  
Location: 754244-761589
  
  
**BlastP hit with Mycgr3G67795\_Mycgr3T**
  
Percentage identity: 62 %
  
BlastP bit score: 3081
  
Sequence coverage: 100 %
  
E-value: 0.0
  
  
 NCBI BlastP on this gene

EAL93322

conserved hypothetical protein
  
Accession: EAL93321
  
Location: 751871-753681
  
 NCBI BlastP on this gene

EAL93321

conserved hypothetical protein
  
Accession: EAL93320
  
Location: 750439-751389
  
 NCBI BlastP on this gene

EAL93320

NAD binding Rossmann fold oxidoreductase, putative
  
Accession: EAL93319
  
Location: 747935-749090
  
 NCBI BlastP on this gene

EAL93319

66. :  DS027045 Aspergillus clavatus NRRL 1 1099423829791 genomic scaffold     Total score: 2.0     Cumulative Blast bit score: 3803

fungal specific transcription factor domain protein
  
Accession: EAW13913
  
Location: 488013-489527
  
 NCBI BlastP on this gene

EAW13913

hypothetical protein
  
Accession: EAW13912
  
Location: 487540-487935
  
 NCBI BlastP on this gene

EAW13912

eukaryotic translation initiation factor 3 subunit EifCl, putative
  
Accession: EAW13911
  
Location: 484523-486104
  
  
**BlastP hit with Mycgr3G98959\_Mycgr3T**
  
Percentage identity: 75 %
  
BlastP bit score: 747
  
Sequence coverage: 100 %
  
E-value: 0.0
  
  
 NCBI BlastP on this gene

EAW13911

iron-sulfur cluster assembly accessory protein Isa2, putative
  
Accession: EAW13910
  
Location: 482605-483732
  
 NCBI BlastP on this gene

EAW13910

kynureninase
  
Accession: EAW13909
  
Location: 480574-482231
  
 NCBI BlastP on this gene

EAW13909

ER membrane DUF1077 domain protein, putative
  
Accession: EAW13908
  
Location: 479686-480396
  
 NCBI BlastP on this gene

EAW13908

AP-2 adaptor complex subunit beta, putative
  
Accession: EAW13907
  
Location: 476355-479044
  
 NCBI BlastP on this gene

EAW13907

conserved hypothetical protein
  
Accession: EAW13906
  
Location: 474641-475974
  
 NCBI BlastP on this gene

EAW13906

SH3 domain protein
  
Accession: EAW13905
  
Location: 470160-473923
  
 NCBI BlastP on this gene

EAW13905

DUF408 domain protein
  
Accession: EAW13904
  
Location: 468313-469281
  
 NCBI BlastP on this gene

EAW13904

40S ribosomal protein S17, putative
  
Accession: EAW13903
  
Location: 467235-467874
  
 NCBI BlastP on this gene

EAW13903

conserved hypothetical protein
  
Accession: EAW13902
  
Location: 465421-466845
  
 NCBI BlastP on this gene

EAW13902

ketoreductase
  
Accession: EAW13901
  
Location: 463861-465126
  
 NCBI BlastP on this gene

EAW13901

TOR pathway phosphatidylinositol 3-kinase TorA, putative
  
Accession: EAW13900
  
Location: 454403-461762
  
  
**BlastP hit with Mycgr3G67795\_Mycgr3T**
  
Percentage identity: 62 %
  
BlastP bit score: 3057
  
Sequence coverage: 100 %
  
E-value: 0.0
  
  
 NCBI BlastP on this gene

EAW13900

conserved hypothetical protein
  
Accession: EAW13899
  
Location: 451935-453759
  
 NCBI BlastP on this gene

EAW13899

conserved hypothetical protein
  
Accession: EAW13898
  
Location: 450367-451518
  
 NCBI BlastP on this gene

EAW13898

NAD binding Rossmann fold oxidoreductase, putative
  
Accession: EAW13897
  
Location: 447723-448871
  
 NCBI BlastP on this gene

EAW13897

67. :  ACJE01000013 Aspergillus niger ATCC 1015     Total score: 2.0     Cumulative Blast bit score: 3803

hypothetical protein
  
Accession: EHA22042
  
Location: 1350049-1351014
  
 NCBI BlastP on this gene

EHA22042

hypothetical protein
  
Accession: EHA22043
  
Location: 1351496-1352531
  
 NCBI BlastP on this gene

EHA22043

hypothetical protein
  
Accession: EHA22044
  
Location: 1352736-1354050
  
 NCBI BlastP on this gene

EHA22044

hypothetical protein
  
Accession: EHA22045
  
Location: 1354670-1355503
  
 NCBI BlastP on this gene

EHA22045

hypothetical protein
  
Accession: EHA22046
  
Location: 1356357-1357939
  
  
**BlastP hit with Mycgr3G98959\_Mycgr3T**
  
Percentage identity: 75 %
  
BlastP bit score: 753
  
Sequence coverage: 100 %
  
E-value: 0.0
  
  
 NCBI BlastP on this gene

EHA22046

hypothetical protein
  
Accession: EHA22047
  
Location: 1358752-1359854
  
 NCBI BlastP on this gene

EHA22047

hypothetical protein
  
Accession: EHA22048
  
Location: 1360225-1361856
  
 NCBI BlastP on this gene

EHA22048

hypothetical protein
  
Accession: EHA22049
  
Location: 1362222-1362950
  
 NCBI BlastP on this gene

EHA22049

hypothetical protein
  
Accession: EHA22050
  
Location: 1363842-1366206
  
 NCBI BlastP on this gene

EHA22050

hypothetical protein
  
Accession: EHA22051
  
Location: 1366544-1367851
  
 NCBI BlastP on this gene

EHA22051

hypothetical protein
  
Accession: EHA22052
  
Location: 1368333-1372083
  
 NCBI BlastP on this gene

EHA22052

hypothetical protein
  
Accession: EHA22053
  
Location: 1373216-1374217
  
 NCBI BlastP on this gene

EHA22053

hypothetical protein
  
Accession: EHA22054
  
Location: 1374618-1375347
  
 NCBI BlastP on this gene

EHA22054

hypothetical protein
  
Accession: EHA22055
  
Location: 1375763-1377136
  
 NCBI BlastP on this gene

EHA22055

hypothetical protein
  
Accession: EHA22056
  
Location: 1377380-1378650
  
 NCBI BlastP on this gene

EHA22056

TorA protein
  
Accession: EHA22057
  
Location: 1380238-1387581
  
  
**BlastP hit with Mycgr3G67795\_Mycgr3T**
  
Percentage identity: 62 %
  
BlastP bit score: 3051
  
Sequence coverage: 100 %
  
E-value: 0.0
  
  
 NCBI BlastP on this gene

EHA22057

hypothetical protein
  
Accession: EHA22058
  
Location: 1388170-1389980
  
 NCBI BlastP on this gene

EHA22058

hypothetical protein
  
Accession: EHA22059
  
Location: 1391544-1392677
  
 NCBI BlastP on this gene

EHA22059

hypothetical protein
  
Accession: EHA22060
  
Location: 1393654-1394659
  
 NCBI BlastP on this gene

EHA22060

68. :  AM270369 Aspergillus niger contig An16c0170, genomic contig.     Total score: 2.0     Cumulative Blast bit score: 3801

not annotated
  
Accession: CAK42884
  
Location: 77-1238
  
 NCBI BlastP on this gene

An16g04540

not annotated
  
Accession: CAK42885
  
Location: 1315-2353
  
 NCBI BlastP on this gene

An16g04550

not annotated
  
Accession: CAK42886
  
Location: 2790-4069
  
 NCBI BlastP on this gene

An16g04560

not annotated
  
Accession: CAK42887
  
Location: 4461-6118
  
 NCBI BlastP on this gene

An16g04570

not annotated
  
Accession: CAK42888
  
Location: 6179-7761
  
  
**BlastP hit with Mycgr3G98959\_Mycgr3T**
  
Percentage identity: 75 %
  
BlastP bit score: 753
  
Sequence coverage: 100 %
  
E-value: 0.0
  
  
 NCBI BlastP on this gene

An16g04580

not annotated
  
Accession: CAK42889
  
Location: 7917-8339
  
 NCBI BlastP on this gene

An16g04590

not annotated
  
Accession: CAK42890
  
Location: 8539-9794
  
 NCBI BlastP on this gene

An16g04600

not annotated
  
Accession: CAK42891
  
Location: 10051-11837
  
 NCBI BlastP on this gene

An16g04630

not annotated
  
Accession: CAK42892
  
Location: 12049-12777
  
 NCBI BlastP on this gene

An16g04640

not annotated
  
Accession: CAK42893
  
Location: 13350-16215
  
 NCBI BlastP on this gene

An16g04650

not annotated
  
Accession: CAK42894
  
Location: 16372-17679
  
 NCBI BlastP on this gene

An16g04660

not annotated
  
Accession: CAK42895
  
Location: 18164-21905
  
 NCBI BlastP on this gene

An16g04670

not annotated
  
Accession: CAK42896
  
Location: 23038-24039
  
 NCBI BlastP on this gene

An16g04680

not annotated
  
Accession: CAK42897
  
Location: 24443-25081
  
 NCBI BlastP on this gene

An16g04690

not annotated
  
Accession: CAK42898
  
Location: 25583-26950
  
 NCBI BlastP on this gene

An16g04700

unnamed
  
Accession: CAK42899
  
Location: 27203-28473
  
 NCBI BlastP on this gene

An16g04710

not annotated
  
Accession: CAK42900
  
Location: 30059-37405
  
  
**BlastP hit with Mycgr3G67795\_Mycgr3T**
  
Percentage identity: 62 %
  
BlastP bit score: 3048
  
Sequence coverage: 100 %
  
E-value: 0.0
  
  
 NCBI BlastP on this gene

An16g04720

not annotated
  
Accession: CAK42901
  
Location: 37991-39801
  
 NCBI BlastP on this gene

An16g04730

not annotated
  
Accession: CAK42902
  
Location: 41363-42496
  
 NCBI BlastP on this gene

An16g04750

not annotated
  
Accession: CAK42903
  
Location: 43470-44475
  
 NCBI BlastP on this gene

An16g04760

69. :  DF126452 Aspergillus kawachii IFO 4308 DNA, contig: scaffold00006     Total score: 2.0     Cumulative Blast bit score: 3800

haemolysin-III channel protein Izh2
  
Accession: GAA85030
  
Location: 1025464-1026429
  
 NCBI BlastP on this gene

GAA85030

similar to An16g04550
  
Accession: GAA85031
  
Location: 1026913-1027948
  
 NCBI BlastP on this gene

GAA85031

hypothetical protein
  
Accession: GAA85032
  
Location: 1028147-1029660
  
 NCBI BlastP on this gene

GAA85032

clathrin-coated vesicle protein
  
Accession: GAA85033
  
Location: 1030082-1030922
  
 NCBI BlastP on this gene

GAA85033

eukaryotic translation initiation factor 3 subunit 6-interacting protein
  
Accession: GAA85034
  
Location: 1031780-1033362
  
  
**BlastP hit with Mycgr3G98959\_Mycgr3T**
  
Percentage identity: 76 %
  
BlastP bit score: 754
  
Sequence coverage: 100 %
  
E-value: 0.0
  
  
 NCBI BlastP on this gene

GAA85034

iron-sulfur cluster assembly accessory protein Isa2
  
Accession: GAA85035
  
Location: 1034160-1035318
  
 NCBI BlastP on this gene

GAA85035

kynureninase
  
Accession: GAA85036
  
Location: 1035693-1037324
  
 NCBI BlastP on this gene

GAA85036

ER membrane DUF1077 domain protein
  
Accession: GAA85037
  
Location: 1037661-1038394
  
 NCBI BlastP on this gene

GAA85037

AP-2 adaptor complex subunit beta
  
Accession: GAA85038
  
Location: 1039653-1042015
  
 NCBI BlastP on this gene

GAA85038

pre-mRNA-splicing factor CWC25
  
Accession: GAA85039
  
Location: 1042380-1043702
  
 NCBI BlastP on this gene

GAA85039

SH3 domain protein
  
Accession: GAA85040
  
Location: 1044186-1047926
  
 NCBI BlastP on this gene

GAA85040

DUF408 domain protein
  
Accession: GAA85041
  
Location: 1049082-1050086
  
 NCBI BlastP on this gene

GAA85041

40S ribosomal protein S17
  
Accession: GAA85042
  
Location: 1050497-1051141
  
 NCBI BlastP on this gene

GAA85042

hypothetical protein
  
Accession: GAA85043
  
Location: 1051628-1052995
  
 NCBI BlastP on this gene

GAA85043

ketoreductase
  
Accession: GAA85044
  
Location: 1053254-1054524
  
 NCBI BlastP on this gene

GAA85044

phosphatidylinositol 3-kinase Tor2
  
Accession: GAA85045
  
Location: 1056148-1063485
  
  
**BlastP hit with Mycgr3G67795\_Mycgr3T**
  
Percentage identity: 62 %
  
BlastP bit score: 3046
  
Sequence coverage: 100 %
  
E-value: 0.0
  
  
 NCBI BlastP on this gene

GAA85045

similar to An16g04730
  
Accession: GAA85046
  
Location: 1064079-1065890
  
 NCBI BlastP on this gene

GAA85046

hypothetical protein
  
Accession: GAA85047
  
Location: 1068712-1070542
  
 NCBI BlastP on this gene

GAA85047

70. :  AM920437 Penicillium chrysogenum Wisconsin 54-1255 complete genome, contig Pc00c22.     Total score: 2.0     Cumulative Blast bit score: 3794

not annotated
  
Accession: CAP99333
  
Location: 4830117-4833915
  
 NCBI BlastP on this gene

Pc22g20450

not annotated
  
Accession: CAP99334
  
Location: 4834848-4835810
  
 NCBI BlastP on this gene

Pc22g20460

not annotated
  
Accession: CAP99335
  
Location: 4836321-4836864
  
 NCBI BlastP on this gene

Pc22g20470

unnamed
  
Accession: CAP99336
  
Location: 4837326-4838825
  
 NCBI BlastP on this gene

Pc22g20480

not annotated
  
Accession: CAP99337
  
Location: 4839043-4840260
  
 NCBI BlastP on this gene

Pc22g20490

not annotated
  
Accession: CAP99338
  
Location: 4841236-4848502
  
  
**BlastP hit with Mycgr3G67795\_Mycgr3T**
  
Percentage identity: 61 %
  
BlastP bit score: 3072
  
Sequence coverage: 100 %
  
E-value: 0.0
  
  
 NCBI BlastP on this gene

Pc22g20500

not annotated
  
Accession: CAP99339
  
Location: 4848929-4850283
  
 NCBI BlastP on this gene

Pc22g20510

unnamed
  
Accession: CAP99340
  
Location: 4851578-4852680
  
 NCBI BlastP on this gene

Pc22g20520

not annotated
  
Accession: CAP99341
  
Location: 4852951-4854208
  
 NCBI BlastP on this gene

Pc22g20530

hypothetical protein
  
Accession: CAP99342
  
Location: 4854304-4855773
  
 NCBI BlastP on this gene

Pc22g20540

not annotated
  
Accession: CAP99343
  
Location: 4856733-4858370
  
 NCBI BlastP on this gene

Pc22g20550

not annotated
  
Accession: CAP99344
  
Location: 4858947-4860055
  
 NCBI BlastP on this gene

Pc22g20560

not annotated
  
Accession: CAP99345
  
Location: 4860380-4862017
  
 NCBI BlastP on this gene

Pc22g20570

not annotated
  
Accession: CAP99346
  
Location: 4862354-4863963
  
 NCBI BlastP on this gene

Pc22g20580

not annotated
  
Accession: CAP99347
  
Location: 4864204-4864879
  
 NCBI BlastP on this gene

Pc22g20590

not annotated
  
Accession: CAP99348
  
Location: 4865348-4868074
  
 NCBI BlastP on this gene

Pc22g20600

not annotated
  
Accession: CAP99349
  
Location: 4868724-4870286
  
  
**BlastP hit with Mycgr3G98959\_Mycgr3T**
  
Percentage identity: 72 %
  
BlastP bit score: 722
  
Sequence coverage: 100 %
  
E-value: 0.0
  
  
 NCBI BlastP on this gene

Pc22g20610

not annotated
  
Accession: CAP99350
  
Location: 4871553-4873810
  
 NCBI BlastP on this gene

Pc22g20620

71. :  CH476615 Uncinocarpus reesii 1704 scaffold\_1 genomic scaffold     Total score: 2.0     Cumulative Blast bit score: 3786

prephenate dehydrogenase
  
Accession: EEP76747
  
Location: 4109553-4111531
  
 NCBI BlastP on this gene

EEP76747

predicted protein
  
Accession: EEP76748
  
Location: 4113305-4114625
  
 NCBI BlastP on this gene

EEP76748

eukaryotic translation initiation factor 3 subunit 6-interacting protein
  
Accession: EEP76749
  
Location: 4117780-4119307
  
  
**BlastP hit with Mycgr3G98959\_Mycgr3T**
  
Percentage identity: 76 %
  
BlastP bit score: 768
  
Sequence coverage: 100 %
  
E-value: 0.0
  
  
 NCBI BlastP on this gene

EEP76749

hypothetical protein
  
Accession: EEP76750
  
Location: 4119885-4120908
  
 NCBI BlastP on this gene

EEP76750

predicted protein
  
Accession: EEP76751
  
Location: 4121247-4121915
  
 NCBI BlastP on this gene

EEP76751

predicted protein
  
Accession: EEP76752
  
Location: 4122516-4123410
  
 NCBI BlastP on this gene

EEP76752

kynureninase
  
Accession: EEP76753
  
Location: 4124090-4125709
  
 NCBI BlastP on this gene

EEP76753

conserved hypothetical protein
  
Accession: EEP76754
  
Location: 4126816-4128404
  
 NCBI BlastP on this gene

EEP76754

conserved hypothetical protein
  
Accession: EEP76755
  
Location: 4129627-4132222
  
 NCBI BlastP on this gene

EEP76755

predicted protein
  
Accession: EEP76756
  
Location: 4132815-4136524
  
 NCBI BlastP on this gene

EEP76756

predicted protein
  
Accession: EEP76757
  
Location: 4137147-4138154
  
 NCBI BlastP on this gene

EEP76757

40S ribosomal protein S17
  
Accession: EEP76758
  
Location: 4138552-4139194
  
 NCBI BlastP on this gene

EEP76758

predicted protein
  
Accession: EEP76759
  
Location: 4139460-4140440
  
 NCBI BlastP on this gene

EEP76759

phosphatidylinositol 3-kinase tor2
  
Accession: EEP76760
  
Location: 4141174-4148639
  
  
**BlastP hit with Mycgr3G67795\_Mycgr3T**
  
Percentage identity: 60 %
  
BlastP bit score: 3018
  
Sequence coverage: 101 %
  
E-value: 0.0
  
  
 NCBI BlastP on this gene

EEP76760

conserved hypothetical protein
  
Accession: EEP76761
  
Location: 4150834-4152592
  
 NCBI BlastP on this gene

EEP76761

72. :  AKCU01000427 Penicillium digitatum Pd1     Total score: 2.0     Cumulative Blast bit score: 3778

C6 transcription factor, putative
  
Accession: EKV09458
  
Location: 1161-2077
  
 NCBI BlastP on this gene

EKV09458

Eukaryotic translation initiation factor 3 subunit L
  
Accession: EKV09459
  
Location: 3056-4621
  
  
**BlastP hit with Mycgr3G98959\_Mycgr3T**
  
Percentage identity: 73 %
  
BlastP bit score: 726
  
Sequence coverage: 100 %
  
E-value: 0.0
  
  
 NCBI BlastP on this gene

EKV09459

AP-2 adaptor complex subunit beta, putative
  
Accession: EKV09460
  
Location: 5311-7767
  
 NCBI BlastP on this gene

EKV09460

hypothetical protein
  
Accession: EKV09461
  
Location: 8489-9038
  
 NCBI BlastP on this gene

EKV09461

MFS transporter, putative
  
Accession: EKV09462
  
Location: 9375-12979
  
 NCBI BlastP on this gene

EKV09462

Iron-sulfur cluster assembly accessory protein Isa2, putative
  
Accession: EKV09463
  
Location: 13354-14408
  
 NCBI BlastP on this gene

EKV09463

hypothetical protein
  
Accession: EKV09464
  
Location: 16588-16725
  
 NCBI BlastP on this gene

EKV09464

Tor
  
Accession: EKV09465
  
Location: 16769-25844
  
  
**BlastP hit with Mycgr3G67795\_Mycgr3T**
  
Percentage identity: 61 %
  
BlastP bit score: 3052
  
Sequence coverage: 100 %
  
E-value: 0.0
  
  
 NCBI BlastP on this gene

EKV09465

Ketoreductase
  
Accession: EKV09466
  
Location: 27145-28370
  
 NCBI BlastP on this gene

EKV09466

hypothetical protein
  
Accession: EKV09467
  
Location: 28682-30091
  
 NCBI BlastP on this gene

EKV09467

40S ribosomal protein S17, putative
  
Accession: EKV09468
  
Location: 30526-31070
  
 NCBI BlastP on this gene

EKV09468

hypothetical protein
  
Accession: EKV09469
  
Location: 31582-32544
  
 NCBI BlastP on this gene

EKV09469

hypothetical protein
  
Accession: EKV09470
  
Location: 33526-37271
  
 NCBI BlastP on this gene

EKV09470

73. :  AKCT01000128 Penicillium digitatum PHI26     Total score: 2.0     Cumulative Blast bit score: 3778

Branched-chain-amino-acid aminotransferase
  
Accession: EKV14903
  
Location: 130069-131387
  
 NCBI BlastP on this gene

EKV14903

C6 transcription factor, putative
  
Accession: EKV14904
  
Location: 138817-139733
  
 NCBI BlastP on this gene

EKV14904

Eukaryotic translation initiation factor 3 subunit L
  
Accession: EKV14905
  
Location: 140712-142277
  
  
**BlastP hit with Mycgr3G98959\_Mycgr3T**
  
Percentage identity: 73 %
  
BlastP bit score: 726
  
Sequence coverage: 100 %
  
E-value: 0.0
  
  
 NCBI BlastP on this gene

EKV14905

AP-2 adaptor complex subunit beta, putative
  
Accession: EKV14906
  
Location: 142933-145389
  
 NCBI BlastP on this gene

EKV14906

hypothetical protein
  
Accession: EKV14907
  
Location: 146111-146660
  
 NCBI BlastP on this gene

EKV14907

MFS transporter, putative
  
Accession: EKV14908
  
Location: 146997-150601
  
 NCBI BlastP on this gene

EKV14908

Iron-sulfur cluster assembly accessory protein Isa2, putative
  
Accession: EKV14909
  
Location: 150976-152030
  
 NCBI BlastP on this gene

EKV14909

hypothetical protein
  
Accession: EKV14910
  
Location: 154210-154347
  
 NCBI BlastP on this gene

EKV14910

Tor
  
Accession: EKV14911
  
Location: 154391-163466
  
  
**BlastP hit with Mycgr3G67795\_Mycgr3T**
  
Percentage identity: 61 %
  
BlastP bit score: 3052
  
Sequence coverage: 100 %
  
E-value: 0.0
  
  
 NCBI BlastP on this gene

EKV14911

Ketoreductase
  
Accession: EKV14912
  
Location: 164767-165992
  
 NCBI BlastP on this gene

EKV14912

hypothetical protein
  
Accession: EKV14913
  
Location: 166304-167713
  
 NCBI BlastP on this gene

EKV14913

40S ribosomal protein S17, putative
  
Accession: EKV14914
  
Location: 168148-168692
  
 NCBI BlastP on this gene

EKV14914

hypothetical protein
  
Accession: EKV14915
  
Location: 169204-170166
  
 NCBI BlastP on this gene

EKV14915

hypothetical protein
  
Accession: EKV14916
  
Location: 171150-174895
  
 NCBI BlastP on this gene

EKV14916

74. :  GG700648 Trichophyton rubrum CBS 118892 genomic scaffold supercont2.1     Total score: 2.0     Cumulative Blast bit score: 3771

hypothetical protein
  
Accession: EGD84357
  
Location: 1649441-1650791
  
 NCBI BlastP on this gene

EGD84357

eukaryotic translation initiation factor 3
  
Accession: EGD84358
  
Location: 1655988-1657493
  
  
**BlastP hit with Mycgr3G98959\_Mycgr3T**
  
Percentage identity: 74 %
  
BlastP bit score: 738
  
Sequence coverage: 100 %
  
E-value: 0.0
  
  
 NCBI BlastP on this gene

EGD84358

iron-sulfur cluster assembly accessory protein
  
Accession: EGD84359
  
Location: 1658097-1659083
  
 NCBI BlastP on this gene

EGD84359

hypothetical protein
  
Accession: EGD84360
  
Location: 1659625-1660173
  
 NCBI BlastP on this gene

EGD84360

metallo-beta-lactamase
  
Accession: EGD84361
  
Location: 1660435-1661734
  
 NCBI BlastP on this gene

EGD84361

kynureninase
  
Accession: EGD84362
  
Location: 1661953-1663454
  
 NCBI BlastP on this gene

EGD84362

hypothetical protein
  
Accession: EGD84363
  
Location: 1663564-1664268
  
 NCBI BlastP on this gene

EGD84363

hypothetical protein
  
Accession: EGD84364
  
Location: 1665131-1665444
  
 NCBI BlastP on this gene

EGD84364

hypothetical protein
  
Accession: EGD84365
  
Location: 1666716-1667051
  
 NCBI BlastP on this gene

EGD84365

integral membrane protein
  
Accession: EGD84366
  
Location: 1667158-1668043
  
 NCBI BlastP on this gene

EGD84366

AP-2 adaptor complex subunit beta
  
Accession: EGD84367
  
Location: 1669207-1671636
  
 NCBI BlastP on this gene

EGD84367

SH3 domain-containing protein
  
Accession: EGD84368
  
Location: 1672158-1675731
  
 NCBI BlastP on this gene

EGD84368

hypothetical protein
  
Accession: EGD84369
  
Location: 1676450-1677466
  
 NCBI BlastP on this gene

EGD84369

40S ribosomal protein S17
  
Accession: EGD84370
  
Location: 1677863-1678512
  
 NCBI BlastP on this gene

EGD84370

hypothetical protein
  
Accession: EGD84371
  
Location: 1678822-1680120
  
 NCBI BlastP on this gene

EGD84371

phosphatidylinositol 3-kinase
  
Accession: EGD84372
  
Location: 1680503-1687920
  
  
**BlastP hit with Mycgr3G67795\_Mycgr3T**
  
Percentage identity: 61 %
  
BlastP bit score: 3034
  
Sequence coverage: 99 %
  
E-value: 0.0
  
  
 NCBI BlastP on this gene

EGD84372

hypothetical protein
  
Accession: EGD84373
  
Location: 1688480-1690284
  
 NCBI BlastP on this gene

EGD84373

hypothetical protein
  
Accession: EGD84374
  
Location: 1691495-1692556
  
 NCBI BlastP on this gene

EGD84374

75. :  DS995701 Microsporum canis CBS 113480 supercont1.1 genomic scaffold     Total score: 2.0     Cumulative Blast bit score: 3771

integral membrane protein
  
Accession: EEQ27315
  
Location: 521551-522842
  
 NCBI BlastP on this gene

EEQ27315

conserved hypothetical protein
  
Accession: EEQ27316
  
Location: 524791-526589
  
 NCBI BlastP on this gene

EEQ27316

eukaryotic translation initiation factor 3
  
Accession: EEQ27317
  
Location: 527295-528799
  
  
**BlastP hit with Mycgr3G98959\_Mycgr3T**
  
Percentage identity: 75 %
  
BlastP bit score: 740
  
Sequence coverage: 100 %
  
E-value: 0.0
  
  
 NCBI BlastP on this gene

EEQ27317

HesB/YadR/YfhF
  
Accession: EEQ27318
  
Location: 529329-530340
  
 NCBI BlastP on this gene

EEQ27318

hemerythrin HHE cation binding domain-containing protein
  
Accession: EEQ27319
  
Location: 530996-531641
  
 NCBI BlastP on this gene

EEQ27319

metallo-beta-lactamase superfamily protein
  
Accession: EEQ27320
  
Location: 531801-533084
  
 NCBI BlastP on this gene

EEQ27320

kynureninase
  
Accession: EEQ27321
  
Location: 533288-534786
  
 NCBI BlastP on this gene

EEQ27321

DUF1077 family protein
  
Accession: EEQ27322
  
Location: 534874-535563
  
 NCBI BlastP on this gene

EEQ27322

predicted protein
  
Accession: EEQ27323
  
Location: 536104-536691
  
 NCBI BlastP on this gene

EEQ27323

adaptor protein complex AP-1
  
Accession: EEQ27324
  
Location: 536910-539508
  
 NCBI BlastP on this gene

EEQ27324

SH3 domain-containing protein
  
Accession: EEQ27325
  
Location: 539990-543534
  
 NCBI BlastP on this gene

EEQ27325

DUF408 domain-containing protein
  
Accession: EEQ27326
  
Location: 544204-545226
  
 NCBI BlastP on this gene

EEQ27326

40S ribosomal protein S17
  
Accession: EEQ27327
  
Location: 545629-546289
  
 NCBI BlastP on this gene

EEQ27327

conserved hypothetical protein
  
Accession: EEQ27328
  
Location: 546577-547875
  
 NCBI BlastP on this gene

EEQ27328

phosphatidylinositol 3-kinase tor2
  
Accession: EEQ27329
  
Location: 548242-555644
  
  
**BlastP hit with Mycgr3G67795\_Mycgr3T**
  
Percentage identity: 61 %
  
BlastP bit score: 3032
  
Sequence coverage: 100 %
  
E-value: 0.0
  
  
 NCBI BlastP on this gene

EEQ27329

conserved hypothetical protein
  
Accession: EEQ27330
  
Location: 556176-557993
  
 NCBI BlastP on this gene

EEQ27330

conserved hypothetical protein
  
Accession: EEQ27331
  
Location: 558314-559230
  
 NCBI BlastP on this gene

EEQ27331

conserved hypothetical protein
  
Accession: EEQ27332
  
Location: 559457-560491
  
 NCBI BlastP on this gene

EEQ27332

inositol oxygenase 1
  
Accession: EEQ27333
  
Location: 561444-562438
  
 NCBI BlastP on this gene

EEQ27333

76. :  ABSU01000003 Arthroderma benhamiae CBS 112371     Total score: 2.0     Cumulative Blast bit score: 3769

cellobiose dehydrogenase, putative
  
Accession: EFE35740
  
Location: 1139178-1140491
  
 NCBI BlastP on this gene

EFE35740

conserved hypothetical protein
  
Accession: EFE35741
  
Location: 1142824-1144097
  
 NCBI BlastP on this gene

EFE35741

hypothetical protein
  
Accession: EFE35742
  
Location: 1144767-1146273
  
  
**BlastP hit with Mycgr3G98959\_Mycgr3T**
  
Percentage identity: 75 %
  
BlastP bit score: 739
  
Sequence coverage: 100 %
  
E-value: 0.0
  
  
 NCBI BlastP on this gene

EFE35742

hypothetical protein
  
Accession: EFE35743
  
Location: 1146856-1147841
  
 NCBI BlastP on this gene

EFE35743

hypothetical protein
  
Accession: EFE35744
  
Location: 1148421-1148972
  
 NCBI BlastP on this gene

EFE35744

metallo-beta-lactamase superfamily protein
  
Accession: EFE35745
  
Location: 1149238-1150539
  
 NCBI BlastP on this gene

EFE35745

kynureninase, putative
  
Accession: EFE35746
  
Location: 1150757-1152259
  
 NCBI BlastP on this gene

EFE35746

hypothetical protein
  
Accession: EFE35747
  
Location: 1152496-1153072
  
 NCBI BlastP on this gene

EFE35747

integral membrane protein
  
Accession: EFE35748
  
Location: 1155229-1156549
  
 NCBI BlastP on this gene

EFE35748

hypothetical protein
  
Accession: EFE35749
  
Location: 1157730-1160164
  
 NCBI BlastP on this gene

EFE35749

hypothetical protein
  
Accession: EFE35750
  
Location: 1160705-1164280
  
 NCBI BlastP on this gene

EFE35750

DUF408 domain protein
  
Accession: EFE35751
  
Location: 1165030-1166046
  
 NCBI BlastP on this gene

EFE35751

hypothetical protein
  
Accession: EFE35752
  
Location: 1166446-1167095
  
 NCBI BlastP on this gene

EFE35752

conserved hypothetical protein
  
Accession: EFE35753
  
Location: 1167397-1168795
  
 NCBI BlastP on this gene

EFE35753

hypothetical protein
  
Accession: EFE35754
  
Location: 1169084-1176494
  
  
**BlastP hit with Mycgr3G67795\_Mycgr3T**
  
Percentage identity: 61 %
  
BlastP bit score: 3030
  
Sequence coverage: 100 %
  
E-value: 0.0
  
  
 NCBI BlastP on this gene

EFE35754

conserved hypothetical protein
  
Accession: EFE35755
  
Location: 1177074-1178878
  
 NCBI BlastP on this gene

EFE35755

hypothetical protein
  
Accession: EFE35756
  
Location: 1179984-1181051
  
 NCBI BlastP on this gene

EFE35756

77. :  DS995728 Trichophyton equinum CBS 127.97 supercont1.11 genomic scaffold     Total score: 2.0     Cumulative Blast bit score: 3768

cellobiose dehydrogenase
  
Accession: EGE03566
  
Location: 134013-135325
  
 NCBI BlastP on this gene

EGE03566

hypothetical protein
  
Accession: EGE03567
  
Location: 137100-138861
  
 NCBI BlastP on this gene

EGE03567

eukaryotic translation initiation factor 3
  
Accession: EGE03568
  
Location: 139554-141060
  
  
**BlastP hit with Mycgr3G98959\_Mycgr3T**
  
Percentage identity: 75 %
  
BlastP bit score: 739
  
Sequence coverage: 100 %
  
E-value: 0.0
  
  
 NCBI BlastP on this gene

EGE03568

iron-sulfur cluster assembly accessory protein I
  
Accession: EGE03569
  
Location: 141628-142612
  
 NCBI BlastP on this gene

EGE03569

HHE domain-containing protein
  
Accession: EGE03570
  
Location: 143207-143758
  
 NCBI BlastP on this gene

EGE03570

hypothetical protein
  
Accession: EGE03571
  
Location: 144508-145299
  
 NCBI BlastP on this gene

EGE03571

kynureninase
  
Accession: EGE03572
  
Location: 145506-147007
  
 NCBI BlastP on this gene

EGE03572

ER membrane DUF1077 domain-containing protein
  
Accession: EGE03573
  
Location: 147118-147825
  
 NCBI BlastP on this gene

EGE03573

hypothetical protein
  
Accession: EGE03574
  
Location: 148236-149696
  
 NCBI BlastP on this gene

EGE03574

integral membrane protein
  
Accession: EGE03575
  
Location: 150976-152307
  
 NCBI BlastP on this gene

EGE03575

hypothetical protein
  
Accession: EGE03576
  
Location: 152852-153148
  
 NCBI BlastP on this gene

EGE03576

AP-2 complex subunit beta
  
Accession: EGE03577
  
Location: 154530-156961
  
 NCBI BlastP on this gene

EGE03577

SH3 domain-containing protein
  
Accession: EGE03578
  
Location: 157477-160950
  
 NCBI BlastP on this gene

EGE03578

DUF408 domain-containing protein
  
Accession: EGE03579
  
Location: 161709-162725
  
 NCBI BlastP on this gene

EGE03579

40S ribosomal protein S17
  
Accession: EGE03580
  
Location: 163129-163780
  
 NCBI BlastP on this gene

EGE03580

hypothetical protein
  
Accession: EGE03581
  
Location: 164068-165366
  
 NCBI BlastP on this gene

EGE03581

phosphatidylinositol 3-kinase tor2
  
Accession: EGE03582
  
Location: 165755-173168
  
  
**BlastP hit with Mycgr3G67795\_Mycgr3T**
  
Percentage identity: 61 %
  
BlastP bit score: 3029
  
Sequence coverage: 100 %
  
E-value: 0.0
  
  
 NCBI BlastP on this gene

EGE03582

hypothetical protein
  
Accession: EGE03583
  
Location: 173740-175543
  
 NCBI BlastP on this gene

EGE03583

hypothetical protein
  
Accession: EGE03584
  
Location: 176486-177547
  
 NCBI BlastP on this gene

EGE03584

78. :  DS989822 Arthroderma gypseum CBS 118893 supercont1.1 genomic scaffold     Total score: 2.0     Cumulative Blast bit score: 3764

integral membrane protein
  
Accession: EFQ97735
  
Location: 2041632-2042939
  
 NCBI BlastP on this gene

EFQ97735

hypothetical protein
  
Accession: EFQ97736
  
Location: 2044570-2046349
  
 NCBI BlastP on this gene

EFQ97736

eukaryotic translation initiation factor 3
  
Accession: EFQ97737
  
Location: 2047075-2048582
  
  
**BlastP hit with Mycgr3G98959\_Mycgr3T**
  
Percentage identity: 74 %
  
BlastP bit score: 738
  
Sequence coverage: 100 %
  
E-value: 0.0
  
  
 NCBI BlastP on this gene

EFQ97737

iron-binding protein erpA
  
Accession: EFQ97738
  
Location: 2049144-2050151
  
 NCBI BlastP on this gene

EFQ97738

HHE domain-containing protein
  
Accession: EFQ97739
  
Location: 2050715-2051266
  
 NCBI BlastP on this gene

EFQ97739

metallo-beta-lactamase superfamily protein
  
Accession: EFQ97740
  
Location: 2051531-2052837
  
 NCBI BlastP on this gene

EFQ97740

kynureninase
  
Accession: EFQ97741
  
Location: 2053070-2054581
  
 NCBI BlastP on this gene

EFQ97741

hypothetical protein
  
Accession: EFQ97742
  
Location: 2054690-2055383
  
 NCBI BlastP on this gene

EFQ97742

sterigmatocystin 8-O-methyltransferase
  
Accession: EFQ97743
  
Location: 2055769-2057233
  
 NCBI BlastP on this gene

EFQ97743

integral membrane protein
  
Accession: EFQ97744
  
Location: 2058522-2059826
  
 NCBI BlastP on this gene

EFQ97744

AP-2 complex subunit beta
  
Accession: EFQ97745
  
Location: 2061039-2063484
  
 NCBI BlastP on this gene

EFQ97745

SH3 domain-containing protein
  
Accession: EFQ97746
  
Location: 2064040-2067606
  
 NCBI BlastP on this gene

EFQ97746

DUF408 domain-containing protein
  
Accession: EFQ97747
  
Location: 2068374-2069402
  
 NCBI BlastP on this gene

EFQ97747

40S ribosomal protein S17
  
Accession: EFQ97748
  
Location: 2069815-2070465
  
 NCBI BlastP on this gene

EFQ97748

hypothetical protein
  
Accession: EFQ97749
  
Location: 2070740-2072038
  
 NCBI BlastP on this gene

EFQ97749

phosphatidylinositol 3-kinase tor2
  
Accession: EFQ97750
  
Location: 2072423-2079838
  
  
**BlastP hit with Mycgr3G67795\_Mycgr3T**
  
Percentage identity: 61 %
  
BlastP bit score: 3026
  
Sequence coverage: 100 %
  
E-value: 0.0
  
  
 NCBI BlastP on this gene

EFQ97750

hypothetical protein
  
Accession: EFQ97751
  
Location: 2080406-2082225
  
 NCBI BlastP on this gene

EFQ97751

hypothetical protein
  
Accession: EFQ97752
  
Location: 2082909-2084394
  
 NCBI BlastP on this gene

EFQ97752

hypothetical protein
  
Accession: EFQ97753
  
Location: 2084890-2086878
  
 NCBI BlastP on this gene

EFQ97753

79. :  ACFW01000009 Coccidioides posadasii C735 delta SOWgp     Total score: 2.0     Cumulative Blast bit score: 3755

hypothetical protein
  
Accession: EER29698
  
Location: 2133755-2135096
  
 NCBI BlastP on this gene

EER29698

hypothetical protein
  
Accession: EER29697
  
Location: 2130456-2131906
  
 NCBI BlastP on this gene

EER29697

eukaryotic translation initiation factor 3 subunit 6-interacting protein, putative
  
Accession: EER29696
  
Location: 2128089-2129651
  
  
**BlastP hit with Mycgr3G98959\_Mycgr3T**
  
Percentage identity: 72 %
  
BlastP bit score: 736
  
Sequence coverage: 101 %
  
E-value: 0.0
  
  
 NCBI BlastP on this gene

EER29696

Iron-sulfur cluster assembly accessory family protein
  
Accession: EER29695
  
Location: 2126142-2127446
  
 NCBI BlastP on this gene

EER29695

hypothetical protein
  
Accession: EER29694
  
Location: 2125032-2125700
  
 NCBI BlastP on this gene

EER29694

metallo-beta-lactamase superfamily protein
  
Accession: EER29693
  
Location: 2123030-2124275
  
 NCBI BlastP on this gene

EER29693

Kynureninase , putative
  
Accession: EER29692
  
Location: 2120824-2122482
  
 NCBI BlastP on this gene

EER29692

hypothetical protein
  
Accession: EER29691
  
Location: 2117476-2118065
  
 NCBI BlastP on this gene

EER29691

AP-1 complex subunit beta-1, putative
  
Accession: EER29690
  
Location: 2114417-2117033
  
 NCBI BlastP on this gene

EER29690

SH3 domain containing protein
  
Accession: EER29689
  
Location: 2110132-2113783
  
 NCBI BlastP on this gene

EER29689

hypothetical protein
  
Accession: EER29688
  
Location: 2108455-2109468
  
 NCBI BlastP on this gene

EER29688

40S ribosomal protein S17, putative
  
Accession: EER29687
  
Location: 2107381-2108073
  
 NCBI BlastP on this gene

EER29687

hypothetical protein
  
Accession: EER29686
  
Location: 2105742-2107031
  
 NCBI BlastP on this gene

EER29686

Phosphatidylinositol 3- and 4-kinase family protein
  
Accession: EER29685
  
Location: 2097821-2105306
  
  
**BlastP hit with Mycgr3G67795\_Mycgr3T**
  
Percentage identity: 61 %
  
BlastP bit score: 3019
  
Sequence coverage: 100 %
  
E-value: 0.0
  
  
 NCBI BlastP on this gene

EER29685

hypothetical protein
  
Accession: EER29684
  
Location: 2095036-2096811
  
 NCBI BlastP on this gene

EER29684

hypothetical protein
  
Accession: EER29683
  
Location: 2093525-2094659
  
 NCBI BlastP on this gene

EER29683

80. :  GG704914 Coccidioides immitis RS genomic scaffold supercont3.4     Total score: 2.0     Cumulative Blast bit score: 3752

hypothetical protein
  
Accession: EAS33514
  
Location: 310738-311388
  
 NCBI BlastP on this gene

EAS33514

cellobiose dehydrogenase
  
Accession: EAS33515
  
Location: 311775-313117
  
 NCBI BlastP on this gene

EAS33515

hypothetical protein
  
Accession: EJB11397
  
Location: 314681-316370
  
 NCBI BlastP on this gene

EJB11397

eukaryotic translation initiation factor 3 subunit L
  
Accession: EAS33518
  
Location: 317179-318741
  
  
**BlastP hit with Mycgr3G98959\_Mycgr3T**
  
Percentage identity: 71 %
  
BlastP bit score: 734
  
Sequence coverage: 101 %
  
E-value: 0.0
  
  
 NCBI BlastP on this gene

EAS33518

iron-sulfur cluster assembly accessory protein
  
Accession: EAS33519
  
Location: 319389-320679
  
 NCBI BlastP on this gene

EAS33519

HHE domain-containing protein
  
Accession: EAS33520
  
Location: 321113-321781
  
 NCBI BlastP on this gene

EAS33520

metallo-beta-lactamase superfamily protein
  
Accession: EAS33521
  
Location: 322528-323773
  
 NCBI BlastP on this gene

EAS33521

kynureninase
  
Accession: EAS33522
  
Location: 324322-325981
  
 NCBI BlastP on this gene

EAS33522

hypothetical protein
  
Accession: EAS33523
  
Location: 327192-327622
  
 NCBI BlastP on this gene

EAS33523

hypothetical protein
  
Accession: EAS33524
  
Location: 328684-329368
  
 NCBI BlastP on this gene

EAS33524

AP-2 adaptor complex subunit beta
  
Accession: EAS33525
  
Location: 329811-332427
  
 NCBI BlastP on this gene

EAS33525

SH3 domain-containing protein
  
Accession: EAS33526
  
Location: 333064-336706
  
 NCBI BlastP on this gene

EAS33526

hypothetical protein
  
Accession: EAS33527
  
Location: 337369-338385
  
 NCBI BlastP on this gene

EAS33527

40S ribosomal protein S17
  
Accession: EAS33528
  
Location: 338762-339443
  
 NCBI BlastP on this gene

EAS33528

hypothetical protein
  
Accession: EAS33529
  
Location: 339790-341079
  
 NCBI BlastP on this gene

EAS33529

phosphatidylinositol 3-kinase tor2
  
Accession: EAS33530
  
Location: 341513-349001
  
  
**BlastP hit with Mycgr3G67795\_Mycgr3T**
  
Percentage identity: 61 %
  
BlastP bit score: 3018
  
Sequence coverage: 101 %
  
E-value: 0.0
  
  
 NCBI BlastP on this gene

EAS33530

hypothetical protein
  
Accession: EAS33531
  
Location: 350014-351789
  
 NCBI BlastP on this gene

EAS33531

hypothetical protein
  
Accession: EAS33532
  
Location: 352177-353338
  
 NCBI BlastP on this gene

EAS33532

81. :  GG698484 Trichophyton tonsurans CBS 112818 genomic scaffold supercont1.8     Total score: 2.0     Cumulative Blast bit score: 3705

hypothetical protein
  
Accession: EGD94649
  
Location: 134986-136298
  
 NCBI BlastP on this gene

EGD94649

hypothetical protein
  
Accession: EGD94650
  
Location: 138071-139832
  
 NCBI BlastP on this gene

EGD94650

eukaryotic translation initiation factor 3
  
Accession: EGD94651
  
Location: 140525-142031
  
  
**BlastP hit with Mycgr3G98959\_Mycgr3T**
  
Percentage identity: 75 %
  
BlastP bit score: 739
  
Sequence coverage: 100 %
  
E-value: 0.0
  
  
 NCBI BlastP on this gene

EGD94651

iron-sulfur cluster assembly accessory protein
  
Accession: EGD94652
  
Location: 142600-143580
  
 NCBI BlastP on this gene

EGD94652

hypothetical protein
  
Accession: EGD94653
  
Location: 144175-144726
  
 NCBI BlastP on this gene

EGD94653

metallo-beta-lactamase
  
Accession: EGD94654
  
Location: 144989-146285
  
 NCBI BlastP on this gene

EGD94654

kynureninase
  
Accession: EGD94655
  
Location: 146492-147993
  
 NCBI BlastP on this gene

EGD94655

hypothetical protein
  
Accession: EGD94656
  
Location: 148104-148811
  
 NCBI BlastP on this gene

EGD94656

hypothetical protein
  
Accession: EGD94657
  
Location: 149222-150682
  
 NCBI BlastP on this gene

EGD94657

integral membrane protein
  
Accession: EGD94658
  
Location: 151967-153298
  
 NCBI BlastP on this gene

EGD94658

hypothetical protein
  
Accession: EGD94659
  
Location: 153843-154179
  
 NCBI BlastP on this gene

EGD94659

AP-2 adaptor complex subunit beta
  
Accession: EGD94660
  
Location: 155687-157953
  
 NCBI BlastP on this gene

EGD94660

SH3 domain-containing protein
  
Accession: EGD94661
  
Location: 158469-161994
  
 NCBI BlastP on this gene

EGD94661

hypothetical protein
  
Accession: EGD94662
  
Location: 163029-163838
  
 NCBI BlastP on this gene

EGD94662

40S ribosomal protein S17
  
Accession: EGD94663
  
Location: 164242-164893
  
 NCBI BlastP on this gene

EGD94663

hypothetical protein
  
Accession: EGD94664
  
Location: 165181-166479
  
 NCBI BlastP on this gene

EGD94664

phosphatidylinositol 3-kinase
  
Accession: EGD94665
  
Location: 166869-174281
  
  
**BlastP hit with Mycgr3G67795\_Mycgr3T**
  
Percentage identity: 60 %
  
BlastP bit score: 2966
  
Sequence coverage: 100 %
  
E-value: 0.0
  
  
 NCBI BlastP on this gene

EGD94665

hypothetical protein
  
Accession: EGD94666
  
Location: 174854-176657
  
 NCBI BlastP on this gene

EGD94666

hypothetical protein
  
Accession: EGD94667
  
Location: 177598-178658
  
 NCBI BlastP on this gene

EGD94667

82. :  U85909 Aureobasidium pullulans cosmid pPSR-22 hydroxylase, multidrug resistance-like protein (...     Total score: 2.0     Cumulative Blast bit score: 3624

hydroxylase
  
Accession: AAD00579
  
Location: 5312-6769
  
 NCBI BlastP on this gene

AAD00579

multidrug resistance-like protein
  
Accession: AAD00580
  
Location: 8090-11998
  
  
**BlastP hit with Mycgr3G67785\_Mycgr3T**
  
Percentage identity: 49 %
  
BlastP bit score: 1204
  
Sequence coverage: 101 %
  
E-value: 0.0
  
  
 NCBI BlastP on this gene

ApMDR1

peptide synthetase
  
Accession: AAD00581
  
Location: 13308-28158
  
  
**BlastP hit with Mycgr3G36951\_Mycgr3T**
  
Percentage identity: 38 %
  
BlastP bit score: 2420
  
Sequence coverage: 77 %
  
E-value: 0.0
  
  
 NCBI BlastP on this gene

AAD00581

83. :  ADOT01000056 Arthrobotrys oligospora ATCC 24927     Total score: 2.0     Cumulative Blast bit score: 3623

hypothetical protein
  
Accession: EGX52507
  
Location: 11608-15484
  
 NCBI BlastP on this gene

EGX52507

hypothetical protein
  
Accession: EGX52508
  
Location: 16050-17037
  
 NCBI BlastP on this gene

EGX52508

hypothetical protein
  
Accession: EGX52509
  
Location: 17223-19331
  
 NCBI BlastP on this gene

EGX52509

hypothetical protein
  
Accession: EGX52510
  
Location: 20067-23001
  
 NCBI BlastP on this gene

EGX52510

hypothetical protein
  
Accession: EGX52511
  
Location: 24008-25748
  
  
**BlastP hit with Mycgr3G98959\_Mycgr3T**
  
Percentage identity: 64 %
  
BlastP bit score: 624
  
Sequence coverage: 98 %
  
E-value: 0.0
  
  
 NCBI BlastP on this gene

EGX52511

hypothetical protein
  
Accession: EGX52512
  
Location: 25994-27090
  
 NCBI BlastP on this gene

EGX52512

hypothetical protein
  
Accession: EGX52513
  
Location: 27941-28270
  
 NCBI BlastP on this gene

EGX52513

hypothetical protein
  
Accession: EGX52514
  
Location: 31105-32084
  
 NCBI BlastP on this gene

EGX52514

hypothetical protein
  
Accession: EGX52515
  
Location: 32559-32903
  
 NCBI BlastP on this gene

EGX52515

hypothetical protein
  
Accession: EGX52516
  
Location: 34085-36897
  
 NCBI BlastP on this gene

EGX52516

hypothetical protein
  
Accession: EGX52517
  
Location: 37826-40737
  
 NCBI BlastP on this gene

EGX52517

hypothetical protein
  
Accession: EGX52518
  
Location: 41365-49649
  
  
**BlastP hit with Mycgr3G67795\_Mycgr3T**
  
Percentage identity: 62 %
  
BlastP bit score: 2999
  
Sequence coverage: 100 %
  
E-value: 0.0
  
  
 NCBI BlastP on this gene

EGX52518

hypothetical protein
  
Accession: EGX52519
  
Location: 50117-51907
  
 NCBI BlastP on this gene

EGX52519

hypothetical protein
  
Accession: EGX52520
  
Location: 52010-56035
  
 NCBI BlastP on this gene

EGX52520

hypothetical protein
  
Accession: EGX52521
  
Location: 56840-59621
  
 NCBI BlastP on this gene

EGX52521

84. :  FQ790277 Botryotinia fuckeliana T4 SuperContig\_379\_1 genomic supercontig.     Total score: 2.0     Cumulative Blast bit score: 3377

hypothetical protein
  
Accession: CCD45288
  
Location: 20035-21345
  
 NCBI BlastP on this gene

BofuT4\_P119940.1

hypothetical protein
  
Accession: CCD45289
  
Location: 21757-22388
  
 NCBI BlastP on this gene

BofuT4\_P119950.1

BcYVC1, Ca2+ channel protein, partial sequence
  
Accession: CCD45290
  
Location: 22451-23806
  
 NCBI BlastP on this gene

BofuT4\_P119960.1

BcYVC1, Ca2+ channel protein, partial sequence
  
Accession: CCD45291
  
Location: 24490-24945
  
 NCBI BlastP on this gene

BofuT4\_P119970.1

similar to l-ornithine 5-monooxygenase
  
Accession: CCD45292
  
Location: 28830-30466
  
 NCBI BlastP on this gene

BofuT4\_P119980.1

predicted protein
  
Accession: CCD45293
  
Location: 31845-32284
  
 NCBI BlastP on this gene

BofuT4\_uP119990.1

similar to ABC transporter
  
Accession: CCD45294
  
Location: 33266-37575
  
  
**BlastP hit with Mycgr3G67785\_Mycgr3T**
  
Percentage identity: 43 %
  
BlastP bit score: 980
  
Sequence coverage: 98 %
  
E-value: 0.0
  
  
 NCBI BlastP on this gene

BofuT4\_P120000.1

BcNRPS2, nonribosomal peptide synthetase, partial sequence
  
Accession: CCD45295
  
Location: 39844-43181
  
  
**BlastP hit with Mycgr3G36951\_Mycgr3T**
  
Percentage identity: 40 %
  
BlastP bit score: 706
  
Sequence coverage: 23 %
  
E-value: 0.0
  
  
 NCBI BlastP on this gene

BofuT4\_P120010.1

BcNRPS2, nonribosomal peptide synthetase, partial sequence
  
Accession: CCD45296
  
Location: 44232-52768
  
  
**BlastP hit with Mycgr3G36951\_Mycgr3T**
  
Percentage identity: 35 %
  
BlastP bit score: 1691
  
Sequence coverage: 64 %
  
E-value: 0.0
  
  
 NCBI BlastP on this gene

BofuT4\_P120020.1

hypothetical protein
  
Accession: CCD45297
  
Location: 53142-55183
  
 NCBI BlastP on this gene

BofuT4\_P120030.1

similar to C2HC5 finger protein
  
Accession: CCD45298
  
Location: 56018-57694
  
 NCBI BlastP on this gene

BofuT4\_P120040.1

similar to TPR domain-containing protein
  
Accession: CCD45299
  
Location: 57994-60876
  
 NCBI BlastP on this gene

BofuT4\_P120050.1

similar to GYF domain-containing protein
  
Accession: CCD45300
  
Location: 61369-66203
  
 NCBI BlastP on this gene

BofuT4\_P120060.1

85. :  AHHD01000089 Macrophomina phaseolina MS6     Total score: 2.0     Cumulative Blast bit score: 3072

Major facilitator superfamily
  
Accession: EKG20334
  
Location: 29963-31651
  
 NCBI BlastP on this gene

EKG20334

F-box domain cyclin-like protein
  
Accession: EKG20335
  
Location: 32276-33556
  
 NCBI BlastP on this gene

EKG20335

hypothetical protein
  
Accession: EKG20336
  
Location: 34267-35757
  
 NCBI BlastP on this gene

EKG20336

Endoribonuclease L-PSP
  
Accession: EKG20337
  
Location: 37177-37527
  
 NCBI BlastP on this gene

EKG20337

hypothetical protein
  
Accession: EKG20338
  
Location: 38043-39374
  
 NCBI BlastP on this gene

EKG20338

hypothetical protein
  
Accession: EKG20339
  
Location: 39462-40438
  
 NCBI BlastP on this gene

EKG20339

AMP-dependent synthetase/ligase
  
Accession: EKG20340
  
Location: 40729-57750
  
  
**BlastP hit with Mycgr3G36951\_Mycgr3T**
  
Percentage identity: 38 %
  
BlastP bit score: 1925
  
Sequence coverage: 62 %
  
E-value: 0.0
  
  
 NCBI BlastP on this gene

EKG20340

Putative ABC transporter protein
  
Accession: EKG20341
  
Location: 59120-63149
  
  
**BlastP hit with Mycgr3G67785\_Mycgr3T**
  
Percentage identity: 47 %
  
BlastP bit score: 1147
  
Sequence coverage: 101 %
  
E-value: 0.0
  
  
 NCBI BlastP on this gene

EKG20341

FAD-dependent pyridine nucleotide-disulfide oxidoreductase
  
Accession: EKG20342
  
Location: 64827-66760
  
 NCBI BlastP on this gene

EKG20342

Alpha-D-phosphohexomutase superfamily
  
Accession: EKG20343
  
Location: 71832-73733
  
 NCBI BlastP on this gene

EKG20343

86. :  KB916185 Neofusicoccum parvum UCRNP2 chromosome Unknown NP2\_03\_scaffold\_547     Total score: 2.0     Cumulative Blast bit score: 3029

hypothetical protein
  
Accession: EOD48662
  
Location: 44857-47148
  
 NCBI BlastP on this gene

EOD48662

putative l-psp endoribonuclease family protein
  
Accession: EOD48634
  
Location: 49279-49742
  
 NCBI BlastP on this gene

EOD48634

hypothetical protein
  
Accession: EOD48661
  
Location: 53367-54101
  
 NCBI BlastP on this gene

EOD48661

putative nonribosomal peptide synthetase 2 protein
  
Accession: EOD48651
  
Location: 54514-63750
  
  
**BlastP hit with Mycgr3G36951\_Mycgr3T**
  
Percentage identity: 39 %
  
BlastP bit score: 1933
  
Sequence coverage: 60 %
  
E-value: 0.0
  
  
 NCBI BlastP on this gene

EOD48651

putative nonribosomal siderophore peptide synthase protein
  
Accession: EOD48642
  
Location: 65978-68784
  
 NCBI BlastP on this gene

EOD48642

putative abc multidrug transporter mdr1 protein
  
Accession: EOD48645
  
Location: 73006-76896
  
  
**BlastP hit with Mycgr3G67785\_Mycgr3T**
  
Percentage identity: 46 %
  
BlastP bit score: 1096
  
Sequence coverage: 101 %
  
E-value: 0.0
  
  
 NCBI BlastP on this gene

EOD48645

putative l-ornithine n5-oxygenase protein
  
Accession: EOD48640
  
Location: 78529-80395
  
 NCBI BlastP on this gene

EOD48640

putative phosphoglucomutase protein
  
Accession: EOD48671
  
Location: 85500-87501
  
 NCBI BlastP on this gene

EOD48671

87. :  JH767570 Coniosporium apollinis CBS 100218 chromosome Unknown supercont1.17     Total score: 2.0     Cumulative Blast bit score: 3012

pyruvate dehydrogenase E1 component subunit beta
  
Accession: EON64845
  
Location: 343875-345126
  
 NCBI BlastP on this gene

EON64845

CMGC/CDK protein kinase
  
Accession: EON64846
  
Location: 345656-347110
  
 NCBI BlastP on this gene

EON64846

hypothetical protein
  
Accession: EON64847
  
Location: 347262-348923
  
 NCBI BlastP on this gene

EON64847

hypothetical protein
  
Accession: EON64848
  
Location: 349166-351548
  
 NCBI BlastP on this gene

EON64848

hypothetical protein
  
Accession: EON64849
  
Location: 351861-354006
  
 NCBI BlastP on this gene

EON64849

hypothetical protein
  
Accession: EON64850
  
Location: 354228-355535
  
 NCBI BlastP on this gene

EON64850

hypothetical protein
  
Accession: EON64851
  
Location: 355806-372334
  
  
**BlastP hit with Mycgr3G36951\_Mycgr3T**
  
Percentage identity: 39 %
  
BlastP bit score: 1881
  
Sequence coverage: 60 %
  
E-value: 0.0
  
  
 NCBI BlastP on this gene

EON64851

hypothetical protein
  
Accession: EON64852
  
Location: 372507-372839
  
 NCBI BlastP on this gene

EON64852

hypothetical protein
  
Accession: EON64853
  
Location: 374125-378186
  
  
**BlastP hit with Mycgr3G67785\_Mycgr3T**
  
Percentage identity: 47 %
  
BlastP bit score: 1131
  
Sequence coverage: 100 %
  
E-value: 0.0
  
  
 NCBI BlastP on this gene

EON64853

hypothetical protein
  
Accession: EON64854
  
Location: 379628-381389
  
 NCBI BlastP on this gene

EON64854

hypothetical protein
  
Accession: EON64855
  
Location: 381707-382330
  
 NCBI BlastP on this gene

EON64855

DNA polymerase alpha subunit A
  
Accession: EON64856
  
Location: 382679-387334
  
 NCBI BlastP on this gene

EON64856

phosphoglucomutase
  
Accession: EON64857
  
Location: 387769-389768
  
 NCBI BlastP on this gene

EON64857

88. :  JH921428 Marssonina brunnea f. sp. 'multigermtubi' MB\_m1 unplaced genomic scaffold M6\_S00001     Total score: 2.0     Cumulative Blast bit score: 2914

Swi6
  
Accession: EKD21645
  
Location: 4399505-4402231
  
 NCBI BlastP on this gene

EKD21645

hypothetical protein
  
Accession: EKD21646
  
Location: 4402858-4404396
  
 NCBI BlastP on this gene

EKD21646

gcn5-related n-acetyltransferase
  
Accession: EKD21647
  
Location: 4404535-4405256
  
 NCBI BlastP on this gene

EKD21647

hypothetical protein
  
Accession: EKD21648
  
Location: 4406332-4407994
  
 NCBI BlastP on this gene

EKD21648

hypothetical protein
  
Accession: EKD21649
  
Location: 4408497-4409583
  
 NCBI BlastP on this gene

EKD21649

leptomycin B resistance protein pmd1
  
Accession: EKD21650
  
Location: 4410599-4416118
  
  
**BlastP hit with Mycgr3G67785\_Mycgr3T**
  
Percentage identity: 43 %
  
BlastP bit score: 947
  
Sequence coverage: 99 %
  
E-value: 0.0
  
  
 NCBI BlastP on this gene

EKD21650

peptide synthetase
  
Accession: EKD21651
  
Location: 4416760-4431762
  
  
**BlastP hit with Mycgr3G36951\_Mycgr3T**
  
Percentage identity: 35 %
  
BlastP bit score: 1967
  
Sequence coverage: 76 %
  
E-value: 0.0
  
  
 NCBI BlastP on this gene

EKD21651

TPR domain-containing protein
  
Accession: EKD21652
  
Location: 4432248-4435301
  
 NCBI BlastP on this gene

EKD21652

GYF domain protein
  
Accession: EKD21653
  
Location: 4435755-4440399
  
 NCBI BlastP on this gene

EKD21653

hypothetical protein
  
Accession: EKD21654
  
Location: 4442173-4443389
  
 NCBI BlastP on this gene

EKD21654

89. :  KB908844 Setosphaeria turcica Et28A unplaced genomic scaffold SETTUscaffold\_6     Total score: 2.0     Cumulative Blast bit score: 2847

hypothetical protein
  
Accession: EOA82994
  
Location: 2064190-2066580
  
 NCBI BlastP on this gene

EOA82994

hypothetical protein
  
Accession: EOA82995
  
Location: 2068660-2069922
  
 NCBI BlastP on this gene

EOA82995

hypothetical protein
  
Accession: EOA82996
  
Location: 2070087-2070890
  
 NCBI BlastP on this gene

EOA82996

hypothetical protein
  
Accession: EOA82997
  
Location: 2071715-2073118
  
 NCBI BlastP on this gene

EOA82997

hypothetical protein
  
Accession: EOA82998
  
Location: 2073881-2074297
  
 NCBI BlastP on this gene

EOA82998

hypothetical protein
  
Accession: EOA82999
  
Location: 2075371-2076692
  
 NCBI BlastP on this gene

EOA82999

hypothetical protein
  
Accession: EOA83000
  
Location: 2077032-2093337
  
  
**BlastP hit with Mycgr3G36951\_Mycgr3T**
  
Percentage identity: 37 %
  
BlastP bit score: 1758
  
Sequence coverage: 61 %
  
E-value: 0.0
  
  
 NCBI BlastP on this gene

EOA83000

hypothetical protein
  
Accession: EOA83001
  
Location: 2095043-2099543
  
  
**BlastP hit with Mycgr3G67785\_Mycgr3T**
  
Percentage identity: 46 %
  
BlastP bit score: 1089
  
Sequence coverage: 99 %
  
E-value: 0.0
  
  
 NCBI BlastP on this gene

EOA83001

hypothetical protein
  
Accession: EOA83002
  
Location: 2101280-2103001
  
 NCBI BlastP on this gene

EOA83002

hypothetical protein
  
Accession: EOA83003
  
Location: 2104240-2107252
  
 NCBI BlastP on this gene

EOA83003

hypothetical protein
  
Accession: EOA83004
  
Location: 2108215-2110964
  
 NCBI BlastP on this gene

EOA83004

90. :  CAKM01000256 Pneumocystis jirovecii strain SE8     Total score: 2.0     Cumulative Blast bit score: 2840

not annotated
  
Accession: CCJ30564
  
Location: 71432-71758
  
  
**BlastP hit with Mycgr3G28587\_Mycgr3T**
  
Percentage identity: 31 %
  
BlastP bit score: 52
  
Sequence coverage: 75 %
  
E-value: 2e-06
  
  
 NCBI BlastP on this gene

CCJ30564

not annotated
  
Accession: CCJ30563
  
Location: 70347-70828
  
 NCBI BlastP on this gene

CCJ30563

not annotated
  
Accession: CCJ30562
  
Location: 69602-70141
  
 NCBI BlastP on this gene

CCJ30562

not annotated
  
Accession: CCJ30561
  
Location: 68032-69567
  
 NCBI BlastP on this gene

CCJ30561

not annotated
  
Accession: CCJ30560
  
Location: 65116-65412
  
 NCBI BlastP on this gene

CCJ30560

not annotated
  
Accession: CCJ30559
  
Location: 64688-67860
  
 NCBI BlastP on this gene

CCJ30559

not annotated
  
Accession: CCJ30558
  
Location: 63532-64376
  
 NCBI BlastP on this gene

CCJ30558

not annotated
  
Accession: CCJ30557
  
Location: 61812-62792
  
 NCBI BlastP on this gene

CCJ30557

not annotated
  
Accession: CCJ30556
  
Location: 60474-61511
  
 NCBI BlastP on this gene

CCJ30556

not annotated
  
Accession: CCJ30555
  
Location: 59421-60046
  
 NCBI BlastP on this gene

CCJ30555

not annotated
  
Accession: CCJ30554
  
Location: 58447-59203
  
 NCBI BlastP on this gene

CCJ30554

not annotated
  
Accession: CCJ30553
  
Location: 57354-58244
  
 NCBI BlastP on this gene

CCJ30553

not annotated
  
Accession: CCJ30552
  
Location: 49849-54784
  
 NCBI BlastP on this gene

CCJ30552

not annotated
  
Accession: CCJ30551
  
Location: 48456-49854
  
 NCBI BlastP on this gene

CCJ30551

not annotated
  
Accession: CCJ30550
  
Location: 47969-48406
  
 NCBI BlastP on this gene

CCJ30550

not annotated
  
Accession: CCJ30549
  
Location: 39399-47210
  
  
**BlastP hit with Mycgr3G67795\_Mycgr3T**
  
Percentage identity: 57 %
  
BlastP bit score: 2788
  
Sequence coverage: 100 %
  
E-value: 0.0
  
  
 NCBI BlastP on this gene

CCJ30549

not annotated
  
Accession: CCJ30548
  
Location: 38753-39258
  
 NCBI BlastP on this gene

CCJ30548

not annotated
  
Accession: CCJ30547
  
Location: 37471-37950
  
 NCBI BlastP on this gene

CCJ30547

not annotated
  
Accession: CCJ30546
  
Location: 36080-37315
  
 NCBI BlastP on this gene

CCJ30546

not annotated
  
Accession: CCJ30545
  
Location: 32764-35485
  
 NCBI BlastP on this gene

CCJ30545

91. :  KB445589 Cochliobolus heterostrophus C5 unplaced genomic scaffold COCHEscaffold\_21     Total score: 2.0     Cumulative Blast bit score: 2832

hypothetical protein
  
Accession: EMD85428
  
Location: 131729-134466
  
 NCBI BlastP on this gene

EMD85428

hypothetical protein
  
Accession: EMD85429
  
Location: 134793-134957
  
 NCBI BlastP on this gene

EMD85429

hypothetical protein
  
Accession: EMD85430
  
Location: 135466-138463
  
 NCBI BlastP on this gene

EMD85430

hypothetical protein
  
Accession: EMD85431
  
Location: 139533-141286
  
 NCBI BlastP on this gene

EMD85431

hypothetical protein
  
Accession: EMD85432
  
Location: 143120-147425
  
  
**BlastP hit with Mycgr3G67785\_Mycgr3T**
  
Percentage identity: 46 %
  
BlastP bit score: 1088
  
Sequence coverage: 99 %
  
E-value: 0.0
  
  
 NCBI BlastP on this gene

EMD85432

hypothetical protein
  
Accession: EMD85433
  
Location: 149004-165126
  
  
**BlastP hit with Mycgr3G36951\_Mycgr3T**
  
Percentage identity: 37 %
  
BlastP bit score: 1744
  
Sequence coverage: 61 %
  
E-value: 0.0
  
  
 NCBI BlastP on this gene

EMD85433

hypothetical protein
  
Accession: EMD85434
  
Location: 165724-167044
  
 NCBI BlastP on this gene

EMD85434

hypothetical protein
  
Accession: EMD85435
  
Location: 167935-168553
  
 NCBI BlastP on this gene

EMD85435

hypothetical protein
  
Accession: EMD85436
  
Location: 169419-170834
  
 NCBI BlastP on this gene

EMD85436

hypothetical protein
  
Accession: EMD85437
  
Location: 171686-172507
  
 NCBI BlastP on this gene

EMD85437

hypothetical protein
  
Accession: EMD85438
  
Location: 172747-174237
  
 NCBI BlastP on this gene

EMD85438

hypothetical protein
  
Accession: EMD85439
  
Location: 175802-178190
  
 NCBI BlastP on this gene

EMD85439

92. :  KB733486 Bipolaris maydis ATCC 48331 unplaced genomic scaffold COCC4scaffold\_43     Total score: 2.0     Cumulative Blast bit score: 2829

hypothetical protein
  
Accession: ENH99437
  
Location: 96335-99072
  
 NCBI BlastP on this gene

ENH99437

hypothetical protein
  
Accession: ENH99438
  
Location: 99399-99563
  
 NCBI BlastP on this gene

ENH99438

hypothetical protein
  
Accession: ENH99439
  
Location: 100072-103069
  
 NCBI BlastP on this gene

ENH99439

hypothetical protein
  
Accession: ENH99440
  
Location: 104139-105892
  
 NCBI BlastP on this gene

ENH99440

hypothetical protein
  
Accession: ENH99441
  
Location: 107723-112028
  
  
**BlastP hit with Mycgr3G67785\_Mycgr3T**
  
Percentage identity: 46 %
  
BlastP bit score: 1088
  
Sequence coverage: 99 %
  
E-value: 0.0
  
  
 NCBI BlastP on this gene

ENH99441

hypothetical protein
  
Accession: ENH99442
  
Location: 113580-129927
  
  
**BlastP hit with Mycgr3G36951\_Mycgr3T**
  
Percentage identity: 38 %
  
BlastP bit score: 1741
  
Sequence coverage: 59 %
  
E-value: 0.0
  
  
 NCBI BlastP on this gene

ENH99442

hypothetical protein
  
Accession: ENH99443
  
Location: 130327-131647
  
 NCBI BlastP on this gene

ENH99443

hypothetical protein
  
Accession: ENH99444
  
Location: 132538-133156
  
 NCBI BlastP on this gene

ENH99444

hypothetical protein
  
Accession: ENH99445
  
Location: 134022-135437
  
 NCBI BlastP on this gene

ENH99445

hypothetical protein
  
Accession: ENH99446
  
Location: 136289-137110
  
 NCBI BlastP on this gene

ENH99446

hypothetical protein
  
Accession: ENH99447
  
Location: 137350-138840
  
 NCBI BlastP on this gene

ENH99447

hypothetical protein
  
Accession: ENH99448
  
Location: 140405-142793
  
 NCBI BlastP on this gene

ENH99448

93. :  KB707804 Botryotinia fuckeliana BcDW1 unplaced genomic scaffold Scaffold\_132     Total score: 2.0     Cumulative Blast bit score: 2825

putative potassium ion channel yvc1 protein
  
Accession: EMR87932
  
Location: 192229-194468
  
 NCBI BlastP on this gene

EMR87932

putative l-ornithine n5-oxygenase protein
  
Accession: EMR87933
  
Location: 199423-201059
  
 NCBI BlastP on this gene

EMR87933

putative multidrug resistance protein 1 protein
  
Accession: EMR87934
  
Location: 203854-208175
  
  
**BlastP hit with Mycgr3G67785\_Mycgr3T**
  
Percentage identity: 43 %
  
BlastP bit score: 978
  
Sequence coverage: 99 %
  
E-value: 0.0
  
  
 NCBI BlastP on this gene

EMR87934

putative nonribosomal siderophore peptide synthase protein
  
Accession: EMR87935
  
Location: 210434-225357
  
  
**BlastP hit with Mycgr3G36951\_Mycgr3T**
  
Percentage identity: 34 %
  
BlastP bit score: 1847
  
Sequence coverage: 77 %
  
E-value: 0.0
  
  
 NCBI BlastP on this gene

EMR87935

putative c2hc5 finger protein
  
Accession: EMR87936
  
Location: 226192-227868
  
 NCBI BlastP on this gene

EMR87936

putative tpr repeat-containing protein
  
Accession: EMR87937
  
Location: 228170-231052
  
 NCBI BlastP on this gene

EMR87937

putative gyf domain-containing protein
  
Accession: EMR87938
  
Location: 231545-236379
  
 NCBI BlastP on this gene

EMR87938

94. :  KB445657 Cochliobolus sativus ND90Pr unplaced genomic scaffold COCSAscaffold\_21     Total score: 2.0     Cumulative Blast bit score: 2808

hypothetical protein
  
Accession: EMD58313
  
Location: 59939-62327
  
 NCBI BlastP on this gene

EMD58313

hypothetical protein
  
Accession: EMD58314
  
Location: 64250-65602
  
 NCBI BlastP on this gene

EMD58314

hypothetical protein
  
Accession: EMD58315
  
Location: 65813-66634
  
 NCBI BlastP on this gene

EMD58315

hypothetical protein
  
Accession: EMD58316
  
Location: 67479-68894
  
 NCBI BlastP on this gene

EMD58316

hypothetical protein
  
Accession: EMD58317
  
Location: 69737-70359
  
 NCBI BlastP on this gene

EMD58317

hypothetical protein
  
Accession: EMD58318
  
Location: 71242-72588
  
 NCBI BlastP on this gene

EMD58318

hypothetical protein
  
Accession: EMD58319
  
Location: 72932-89242
  
  
**BlastP hit with Mycgr3G36951\_Mycgr3T**
  
Percentage identity: 37 %
  
BlastP bit score: 1726
  
Sequence coverage: 59 %
  
E-value: 0.0
  
  
 NCBI BlastP on this gene

EMD58319

hypothetical protein
  
Accession: EMD58320
  
Location: 90823-95148
  
  
**BlastP hit with Mycgr3G67785\_Mycgr3T**
  
Percentage identity: 46 %
  
BlastP bit score: 1082
  
Sequence coverage: 100 %
  
E-value: 0.0
  
  
 NCBI BlastP on this gene

EMD58320

hypothetical protein
  
Accession: EMD58321
  
Location: 96888-98625
  
 NCBI BlastP on this gene

EMD58321

hypothetical protein
  
Accession: EMD58322
  
Location: 99768-102764
  
 NCBI BlastP on this gene

EMD58322

hypothetical protein
  
Accession: EMD58323
  
Location: 103756-106492
  
 NCBI BlastP on this gene

EMD58323

95. :  FP929137 Leptosphaeria maculans JN3 lm\_SuperContig\_10\_v2 genomic supercontig     Total score: 2.0     Cumulative Blast bit score: 2804

hypothetical protein
  
Accession: CBX99726
  
Location: 244043-244684
  
 NCBI BlastP on this gene

LEMA\_P073150.1

predicted protein
  
Accession: CBX99727
  
Location: 244889-245643
  
 NCBI BlastP on this gene

LEMA\_P073160.1

predicted protein
  
Accession: CBX99728
  
Location: 246849-247064
  
 NCBI BlastP on this gene

LEMA\_uP073170.1

hypothetical protein
  
Accession: CBX99729
  
Location: 248541-252269
  
 NCBI BlastP on this gene

LEMA\_P073180.1

predicted protein
  
Accession: CBX99730
  
Location: 253886-254377
  
 NCBI BlastP on this gene

LEMA\_P073190.1

similar to nonribosomal peptide synthetase 2
  
Accession: CBX99731
  
Location: 254953-271464
  
  
**BlastP hit with Mycgr3G36951\_Mycgr3T**
  
Percentage identity: 37 %
  
BlastP bit score: 1734
  
Sequence coverage: 62 %
  
E-value: 0.0
  
  
 NCBI BlastP on this gene

LEMA\_P073200.1

similar to multidrug resistance protein 1
  
Accession: CBX99732
  
Location: 273017-277841
  
  
**BlastP hit with Mycgr3G67785\_Mycgr3T**
  
Percentage identity: 45 %
  
BlastP bit score: 1070
  
Sequence coverage: 103 %
  
E-value: 0.0
  
  
 NCBI BlastP on this gene

LEMA\_P073210.1

hypothetical protein
  
Accession: CBX99733
  
Location: 279081-281250
  
 NCBI BlastP on this gene

LEMA\_P073220.1

predicted protein
  
Accession: CBX99734
  
Location: 281497-281760
  
 NCBI BlastP on this gene

LEMA\_P073230.1

hypothetical protein
  
Accession: CBX99735
  
Location: 282546-285647
  
 NCBI BlastP on this gene

LEMA\_P073240.1

similar to eukaryotic translation initiation factor 3 subunit 8
  
Accession: CBX99736
  
Location: 286642-289527
  
 NCBI BlastP on this gene

LEMA\_P073250.1

96. :  GL535377 Pyrenophora teres f. teres 0-1 unplaced genomic scaffold scaffold\_191648     Total score: 2.0     Cumulative Blast bit score: 2760

hypothetical protein
  
Accession: EFQ90039
  
Location: 345-16619
  
  
**BlastP hit with Mycgr3G36951\_Mycgr3T**
  
Percentage identity: 36 %
  
BlastP bit score: 1696
  
Sequence coverage: 60 %
  
E-value: 0.0
  
  
 NCBI BlastP on this gene

EFQ90039

hypothetical protein
  
Accession: EFQ90040
  
Location: 18180-22429
  
  
**BlastP hit with Mycgr3G67785\_Mycgr3T**
  
Percentage identity: 46 %
  
BlastP bit score: 1064
  
Sequence coverage: 101 %
  
E-value: 0.0
  
  
 NCBI BlastP on this gene

EFQ90040

hypothetical protein
  
Accession: EFQ90041
  
Location: 23863-25578
  
 NCBI BlastP on this gene

EFQ90041

hypothetical protein
  
Accession: EFQ90042
  
Location: 26622-29560
  
 NCBI BlastP on this gene

EFQ90042

97. :  CH445327 Phaeosphaeria nodorum SN15 scaffold\_3     Total score: 2.0     Cumulative Blast bit score: 2639

hypothetical protein
  
Accession: EAT90339
  
Location: 616053-618542
  
 NCBI BlastP on this gene

EAT90339

hypothetical protein
  
Accession: EAT90340
  
Location: 618889-619751
  
 NCBI BlastP on this gene

EAT90340

hypothetical protein
  
Accession: EAT90341
  
Location: 621365-622403
  
 NCBI BlastP on this gene

EAT90341

hypothetical protein
  
Accession: EAT90342
  
Location: 622566-623249
  
 NCBI BlastP on this gene

EAT90342

hypothetical protein
  
Accession: EAT90343
  
Location: 624280-624858
  
 NCBI BlastP on this gene

EAT90343

hypothetical protein
  
Accession: EAT90344
  
Location: 625692-627063
  
 NCBI BlastP on this gene

EAT90344

hypothetical protein
  
Accession: EAT90345
  
Location: 627653-628171
  
 NCBI BlastP on this gene

EAT90345

hypothetical protein
  
Accession: EAT90346
  
Location: 628329-642276
  
  
**BlastP hit with Mycgr3G36951\_Mycgr3T**
  
Percentage identity: 36 %
  
BlastP bit score: 1677
  
Sequence coverage: 61 %
  
E-value: 0.0
  
  
 NCBI BlastP on this gene

EAT90346

hypothetical protein
  
Accession: EAT90347
  
Location: 646941-650670
  
  
**BlastP hit with Mycgr3G67785\_Mycgr3T**
  
Percentage identity: 45 %
  
BlastP bit score: 962
  
Sequence coverage: 90 %
  
E-value: 0.0
  
  
 NCBI BlastP on this gene

EAT90347

hypothetical protein
  
Accession: EAT90348
  
Location: 651226-651518
  
 NCBI BlastP on this gene

EAT90348

hypothetical protein
  
Accession: EAT90349
  
Location: 651951-653586
  
 NCBI BlastP on this gene

EAT90349

hypothetical protein
  
Accession: EAT90350
  
Location: 654701-657659
  
 NCBI BlastP on this gene

EAT90350

hypothetical protein
  
Accession: EAT90351
  
Location: 658718-661459
  
 NCBI BlastP on this gene

EAT90351

98. :  GL988041 Chaetomium thermophilum var. thermophilum DSM 1495 unplaced genomic scaffold scf7180000...     Total score: 2.0     Cumulative Blast bit score: 1846

40S ribosomal protein S7-like protein
  
Accession: EGS20299
  
Location: 43430-44357
  
 NCBI BlastP on this gene

EGS20299

NADP-dependent alcohol dehydrogenase-like protein
  
Accession: EGS20298
  
Location: 40406-43119
  
 NCBI BlastP on this gene

EGS20298

hypothetical protein
  
Accession: EGS20297
  
Location: 38382-40308
  
 NCBI BlastP on this gene

EGS20297

putative malic acid transport protein
  
Accession: EGS20296
  
Location: 36137-37615
  
  
**BlastP hit with Mycgr3G67791\_Mycgr3T**
  
Percentage identity: 31 %
  
BlastP bit score: 223
  
Sequence coverage: 84 %
  
E-value: 1e-62
  
  
 NCBI BlastP on this gene

EGS20296

hypothetical protein
  
Accession: EGS20295
  
Location: 34043-35937
  
 NCBI BlastP on this gene

EGS20295

hypothetical protein
  
Accession: EGS20294
  
Location: 31475-33265
  
 NCBI BlastP on this gene

EGS20294

hypothetical protein
  
Accession: EGS20293
  
Location: 27302-28434
  
 NCBI BlastP on this gene

EGS20293

putative L-ornithine protein
  
Accession: EGS20292
  
Location: 22656-24251
  
 NCBI BlastP on this gene

EGS20292

nonribosomal peptide synthase-like protein
  
Accession: EGS20291
  
Location: 6067-19404
  
  
**BlastP hit with Mycgr3G36951\_Mycgr3T**
  
Percentage identity: 33 %
  
BlastP bit score: 1623
  
Sequence coverage: 73 %
  
E-value: 0.0
  
  
 NCBI BlastP on this gene

EGS20291

hypothetical protein
  
Accession: EGS20290
  
Location: 1255-3822
  
 NCBI BlastP on this gene

EGS20290

99. :  DS231623 Pyrenophora tritici-repentis Pt-1C-BFP supercont1.9 genomic scaffold     Total score: 2.0     Cumulative Blast bit score: 1776

eukaryotic translation initiation factor 3 subunit C
  
Accession: EDU51191
  
Location: 601037-603782
  
 NCBI BlastP on this gene

EDU51191

serine/threonine-protein kinase minibrain
  
Accession: EDU51192
  
Location: 604727-607662
  
 NCBI BlastP on this gene

EDU51192

L-ornithine 5-monooxygenase (L-ornithine N(5)-oxygenase)
  
Accession: EDU51193
  
Location: 608720-610433
  
 NCBI BlastP on this gene

EDU51193

leptomycin B resistance protein pmd1
  
Accession: EDU51194
  
Location: 611856-616104
  
  
**BlastP hit with Mycgr3G67785\_Mycgr3T**
  
Percentage identity: 45 %
  
BlastP bit score: 1084
  
Sequence coverage: 101 %
  
E-value: 0.0
  
  
 NCBI BlastP on this gene

EDU51194

predicted protein
  
Accession: EDU51195
  
Location: 618297-618473
  
 NCBI BlastP on this gene

EDU51195

nonribosomal peptide synthetase 2
  
Accession: EDU51196
  
Location: 623920-624054
  
 NCBI BlastP on this gene

EDU51196

gramicidin S synthetase 1
  
Accession: EDU51197
  
Location: 627120-631429
  
  
**BlastP hit with Mycgr3G36951\_Mycgr3T**
  
Percentage identity: 51 %
  
BlastP bit score: 692
  
Sequence coverage: 15 %
  
E-value: 0.0
  
  
 NCBI BlastP on this gene

EDU51197

peptide synthetase
  
Accession: EDU51198
  
Location: 632022-633701
  
 NCBI BlastP on this gene

EDU51198

phosphatidic acid phosphatase beta
  
Accession: EDU51199
  
Location: 634081-635463
  
 NCBI BlastP on this gene

EDU51199

conserved hypothetical protein
  
Accession: EDU51200
  
Location: 636611-637207
  
 NCBI BlastP on this gene

EDU51200

AAR2 domain containing protein
  
Accession: EDU51201
  
Location: 638148-639578
  
 NCBI BlastP on this gene

EDU51201

vacuolar-sorting protein SNF8
  
Accession: EDU51202
  
Location: 640530-641360
  
 NCBI BlastP on this gene

EDU51202

predicted protein
  
Accession: EDU51203
  
Location: 641598-644747
  
 NCBI BlastP on this gene

EDU51203

100. :  AGUE01000061 Glarea lozoyensis 74030     Total score: 2.0     Cumulative Blast bit score: 1467

putative Hydroxamate-type ferrichrome siderophore peptide synthetase
  
Accession: EHL01089
  
Location: 124523-132259
  
  
**BlastP hit with Mycgr3G36951\_Mycgr3T**
  
Percentage identity: 32 %
  
BlastP bit score: 781
  
Sequence coverage: 35 %
  
E-value: 0.0
  
  
 NCBI BlastP on this gene

EHL01089

putative aspergillopepsin A-like aspartic endopeptidase
  
Accession: EHL01088
  
Location: 120196-121625
  
 NCBI BlastP on this gene

EHL01088

hypothetical protein
  
Accession: EHL01087
  
Location: 116144-116620
  
 NCBI BlastP on this gene

EHL01087

putative Complement C1q tumor necrosis factor-related protein 9
  
Accession: EHL01086
  
Location: 104749-105733
  
 NCBI BlastP on this gene

EHL01086

putative Collagen alpha-1(XVI) chain
  
Accession: EHL01085
  
Location: 103627-104684
  
 NCBI BlastP on this gene

EHL01085

putative Leptomycin B resistance protein pmd1
  
Accession: EHL01084
  
Location: 96599-101168
  
  
**BlastP hit with Mycgr3G67785\_Mycgr3T**
  
Percentage identity: 34 %
  
BlastP bit score: 686
  
Sequence coverage: 100 %
  
E-value: 0.0
  
  
 NCBI BlastP on this gene

EHL01084

hypothetical protein
  
Accession: EHL01083
  
Location: 94178-95394
  
 NCBI BlastP on this gene

EHL01083

putative HC-toxin efflux carrier TOXA
  
Accession: EHL01082
  
Location: 91536-93546
  
 NCBI BlastP on this gene

EHL01082

Detecting sequence homology at the gene cluster level with MultiGeneBlast.
  
Marnix H. Medema, Rainer Breitling & Eriko Takano (2013)
  
*Molecular Biology and Evolution* , 30: 1218-1223.
