# Supplementary material for: In silico prediction and characterization of secondary metabolite biosynthetic gene clusters in the wheat pathogen Zymoseptoria tritici
Source: BMC Genomics. 2017 Aug 17;18:631. doi: 10.1186/s12864-017-3969-y (PMC5561558; doi:10.1186/s12864-017-3969-y)
Supplement: Supplementary file 1 — MultiGeneBLAST analysis of putative secondary metabolite clusters. All encoded amino acid sequences from genes residing in clusters predicted by AntiSMASH are given as FASTA file format. All output data from MultiGeneBLASTs are also provided. (ZIP 42911 kb) [file 12864_2017_3969_MOESM1_ESM.zip › Cluster MultiGene BLAST/out/Clusters_1_34/Cluster_10/displaypage3.xhtml]

xml version="1.0" encoding="UTF-8"?


Search Results
  
  
 Results pages: 1, 2, 3, 4, 5

**MultiGeneBlast hits**

Select gene cluster alignment
101. KB446542\_3 Dothistroma septosporum NZE10 unplaced genomic scaffold DOTSE...
102. KB445561\_1 Baudoinia compniacensis UAMH 10762 unplaced genomic scaffold ...
103. KB446566\_2 Pseudocercospora fijiensis CIRAD86 unplaced genomic scaffold ...
104. KB456266\_0 Mycosphaerella populorum SO2202 unplaced genomic scaffold SEP...
105. JH767573\_1 Coniosporium apollinis CBS 100218 chromosome Unknown supercon...
106. AHHD01000099\_0 Macrophomina phaseolina MS6, whole genome shotgun sequenc...
107. GL536348\_0 Pyrenophora teres f. teres 0-1 unplaced genomic scaffold scaf...
108. DS231623\_1 Pyrenophora tritici-repentis Pt-1C-BFP supercont1.9 genomic s...
109. KB733455\_2 Bipolaris maydis ATCC 48331 unplaced genomic scaffold COCC4sc...
110. KB445579\_1 Cochliobolus heterostrophus C5 unplaced genomic scaffold COCH...
111. KB445649\_1 Cochliobolus sativus ND90Pr unplaced genomic scaffold COCSAsc...
112. KB916388\_0 Neofusicoccum parvum UCRNP2 chromosome Unknown NP2\_03\_scaffol...
113. KB908844\_3 Setosphaeria turcica Et28A unplaced genomic scaffold SETTUsca...
114. KB446542\_1 Dothistroma septosporum NZE10 unplaced genomic scaffold DOTSE...
115. FP929137\_2 Leptosphaeria maculans JN3 lm\_SuperContig\_10\_v2 genomic super...
116. GL573222\_0 Geomyces destructans 20631-21 unplaced genomic scaffold super...
117. KB446555\_2 Pseudocercospora fijiensis CIRAD86 unplaced genomic scaffold ...
118. KB725774\_0 Colletotrichum orbiculare MAFF 240422 unplaced genomic scaffo...
119. AFNW01000108\_1 Fusarium pseudograminearum CS3096, whole genome shotgun s...
120. GG698928\_0 Nectria haematococca mpVI 77-13-4 chromosome 6 genomic scaffo...
121. KB726989\_0 Fusarium oxysporum f. sp. cubense race 4 unplaced genomic sca...
122. KE375219\_0 Blumeria graminis f. sp. tritici 96224 unplaced genomic scaff...
123. CAUH01001323\_0 Blumeria graminis f. sp. hordei DH14, whole genome shotgu...
124. HF679031\_2 Fusarium fujikuroi IMI 58289 draft genome, chromosome FFUJ\_ch...
125. CAGA01000008\_1 Claviceps purpurea 20.1, whole genome shotgun sequencing ...
126. JH767570\_1 Coniosporium apollinis CBS 100218 chromosome Unknown supercon...
127. CP003010\_2 Thielavia terrestris NRRL 8126 chromosome 2, complete sequence.
128. DS572814\_2 Paracoccidioides brasiliensis Pb01 supercont1.4 genomic scaff...
129. GL988041\_4 Chaetomium thermophilum var. thermophilum DSM 1495 unplaced g...
130. JH725173\_0 Beauveria bassiana ARSEF 2860 unplaced genomic scaffold BBA\_S...
131. GG697432\_0 Glomerella graminicola M1.001 genomic scaffold supercont1.102...
132. CH408032\_0 Chaetomium globosum CBS 148.51 scaffold\_4 genomic scaffold, w...
133. AFQF01002695\_0 Fusarium oxysporum Fo5176, whole genome shotgun sequencin...
134. CP003007\_2 Myceliophthora thermophila ATCC 42464 chromosome 6, complete ...
135. KB707406\_0 Eutypa lata UCREL1 unplaced genomic scaffold EL1\_03\_scaffold\_...
136. CU638744\_0 Podospora anserina S mat+ genomic DNA chromosome 6, supercont...
137. GL698748\_0 Metarhizium anisopliae ARSEF 23 unplaced genomic scaffold Scf...
138. GL985056\_2 Trichoderma reesei QM6a unplaced genomic scaffold TRIREscaffo...
139. ABDF02000004\_0 Trichoderma virens Gv29-8, whole genome shotgun sequencin...
140. CACQ02001212\_0 Colletotrichum higginsianum strain IMI 349063, whole geno...
141. GL698510\_0 Metarhizium acridum CQMa 102 unplaced genomic scaffold Scf\_04...
142. AMYD01001882\_0 Colletotrichum gloeosporioides Cg-14, whole genome shotgu...
143. AABX02000020\_0 Neurospora crassa OR74A, whole genome shotgun sequencing ...
144. GL891303\_0 Neurospora tetrasperma FGSC 2508 unplaced genomic scaffold NE...
145. GL891217\_0 Neurospora tetrasperma FGSC 2509 unplaced genomic scaffold NE...
146. KB020987\_0 Colletotrichum gloeosporioides Nara gc5 unplaced genomic scaf...
147. CH445336\_5 Phaeosphaeria nodorum SN15 scaffold\_12, whole genome shotgun ...
148. ABDG02000017\_2 Trichoderma atroviride IMI 206040, whole genome shotgun s...
149. JH126405\_0 Cordyceps militaris CM01 unplaced genomic scaffold CCM\_S00007...
150. GL385396\_3 Gaeumannomyces graminis var. tritici R3-111a-1 unplaced genom...

Query: Architecture Search FASTA input

KB446542 : Dothistroma septosporum NZE10 unplaced genomic scaffold DOTSEscaffold\_8    Total score: 2.0     Cumulative Blast bit score: 1403

Hit cluster cross-links:

Mycgr3G67791 Mycgr3T
  
Location: 0-1542

Mycgr3G67791\_Mycgr3T

Mycgr3G90406 Mycgr3T
  
Location: 1642-3973

Mycgr3G90406\_Mycgr3T

Mycgr3G67785 Mycgr3T
  
Location: 4073-7865

Mycgr3G67785\_Mycgr3T

Mycgr3G67795 Mycgr3T
  
Location: 7965-15249

Mycgr3G67795\_Mycgr3T

Mycgr3G67775 Mycgr3T
  
Location: 15349-16237

Mycgr3G67775\_Mycgr3T

Mycgr3G90404 Mycgr3T
  
Location: 16337-17246

Mycgr3G90404\_Mycgr3T

Mycgr3G36951 Mycgr3T
  
Location: 17346-30891

Mycgr3G36951\_Mycgr3T

Mycgr3G103034 Mycgr3
  
Location: 30991-32644

Mycgr3G103034\_Mycgr3

Mycgr3G31119 Mycgr3T
  
Location: 32744-32906

Mycgr3G31119\_Mycgr3T

Mycgr3G28587 Mycgr3T
  
Location: 33006-33489

Mycgr3G28587\_Mycgr3T

Mycgr3G98959 Mycgr3T
  
Location: 33589-35035

Mycgr3G98959\_Mycgr3T

Mycgr3G35447 Mycgr3T
  
Location: 35135-36443

Mycgr3G35447\_Mycgr3T

Mycgr3G84402 Mycgr3T
  
Location: 36543-37884

Mycgr3G84402\_Mycgr3T

Mycgr3G98961 Mycgr3T
  
Location: 37984-38884

Mycgr3G98961\_Mycgr3T

hypothetical protein
  
Accession: EME41272
  
Location: 349025-350310
  
 NCBI BlastP on this gene

EME41272

hypothetical protein
  
Accession: EME41273
  
Location: 350944-352110
  
 NCBI BlastP on this gene

EME41273

hypothetical protein
  
Accession: EME41274
  
Location: 352712-353245
  
 NCBI BlastP on this gene

EME41274

hypothetical protein
  
Accession: EME41275
  
Location: 354563-356878
  
 NCBI BlastP on this gene

EME41275

hypothetical protein
  
Accession: EME41276
  
Location: 358612-361134
  
 NCBI BlastP on this gene

EME41276

hypothetical protein
  
Accession: EME41277
  
Location: 362493-365000
  
 NCBI BlastP on this gene

EME41277

hypothetical protein
  
Accession: EME41278
  
Location: 366099-367262
  
 NCBI BlastP on this gene

EME41278

hypothetical protein
  
Accession: EME41279
  
Location: 367579-369120
  
  
**BlastP hit with Mycgr3G84402\_Mycgr3T**
  
Percentage identity: 89 %
  
BlastP bit score: 752
  
Sequence coverage: 91 %
  
E-value: 0.0
  
  
 NCBI BlastP on this gene

EME41279

hypothetical protein
  
Accession: EME41280
  
Location: 369314-370714
  
  
**BlastP hit with Mycgr3G35447\_Mycgr3T**
  
Percentage identity: 74 %
  
BlastP bit score: 651
  
Sequence coverage: 96 %
  
E-value: 0.0
  
  
 NCBI BlastP on this gene

EME41280

hypothetical protein
  
Accession: EME41281
  
Location: 373239-373854
  
 NCBI BlastP on this gene

EME41281

hypothetical protein
  
Accession: EME41283
  
Location: 376445-378245
  
 NCBI BlastP on this gene

EME41283

hypothetical protein
  
Accession: EME41284
  
Location: 379550-380364
  
 NCBI BlastP on this gene

EME41284

hypothetical protein
  
Accession: EME41285
  
Location: 381721-382374
  
 NCBI BlastP on this gene

EME41285

carbohydrate-binding module family 14 protein
  
Accession: EME41286
  
Location: 384698-385195
  
 NCBI BlastP on this gene

EME41286

hypothetical protein
  
Accession: EME41287
  
Location: 385453-389313
  
 NCBI BlastP on this gene

EME41287

hypothetical protein
  
Accession: EME41288
  
Location: 389953-390141
  
 NCBI BlastP on this gene

EME41288

Query: Architecture Search FASTA input

KB445561 : Baudoinia compniacensis UAMH 10762 unplaced genomic scaffold BAUCOscaffold\_12    Total score: 2.0     Cumulative Blast bit score: 1354

Hit cluster cross-links:

Mycgr3G67791 Mycgr3T
  
Location: 0-1542

Mycgr3G67791\_Mycgr3T

Mycgr3G90406 Mycgr3T
  
Location: 1642-3973

Mycgr3G90406\_Mycgr3T

Mycgr3G67785 Mycgr3T
  
Location: 4073-7865

Mycgr3G67785\_Mycgr3T

Mycgr3G67795 Mycgr3T
  
Location: 7965-15249

Mycgr3G67795\_Mycgr3T

Mycgr3G67775 Mycgr3T
  
Location: 15349-16237

Mycgr3G67775\_Mycgr3T

Mycgr3G90404 Mycgr3T
  
Location: 16337-17246

Mycgr3G90404\_Mycgr3T

Mycgr3G36951 Mycgr3T
  
Location: 17346-30891

Mycgr3G36951\_Mycgr3T

Mycgr3G103034 Mycgr3
  
Location: 30991-32644

Mycgr3G103034\_Mycgr3

Mycgr3G31119 Mycgr3T
  
Location: 32744-32906

Mycgr3G31119\_Mycgr3T

Mycgr3G28587 Mycgr3T
  
Location: 33006-33489

Mycgr3G28587\_Mycgr3T

Mycgr3G98959 Mycgr3T
  
Location: 33589-35035

Mycgr3G98959\_Mycgr3T

Mycgr3G35447 Mycgr3T
  
Location: 35135-36443

Mycgr3G35447\_Mycgr3T

Mycgr3G84402 Mycgr3T
  
Location: 36543-37884

Mycgr3G84402\_Mycgr3T

Mycgr3G98961 Mycgr3T
  
Location: 37984-38884

Mycgr3G98961\_Mycgr3T

hypothetical protein
  
Accession: EMC92627
  
Location: 153228-154769
  
 NCBI BlastP on this gene

EMC92627

hypothetical protein
  
Accession: EMC92628
  
Location: 156325-157119
  
 NCBI BlastP on this gene

EMC92628

hypothetical protein
  
Accession: EMC92629
  
Location: 157441-158089
  
 NCBI BlastP on this gene

EMC92629

hypothetical protein
  
Accession: EMC92630
  
Location: 158225-159192
  
 NCBI BlastP on this gene

EMC92630

hypothetical protein
  
Accession: EMC92631
  
Location: 160175-163936
  
 NCBI BlastP on this gene

EMC92631

hypothetical protein
  
Accession: EMC92632
  
Location: 165289-167097
  
 NCBI BlastP on this gene

EMC92632

glycosyltransferase family 71 protein
  
Accession: EMC92633
  
Location: 168375-169706
  
 NCBI BlastP on this gene

EMC92633

hypothetical protein
  
Accession: EMC92634
  
Location: 170127-170726
  
 NCBI BlastP on this gene

EMC92634

hypothetical protein
  
Accession: EMC92635
  
Location: 170801-171691
  
  
**BlastP hit with Mycgr3G67775\_Mycgr3T**
  
Percentage identity: 78 %
  
BlastP bit score: 483
  
Sequence coverage: 100 %
  
E-value: 2e-169
  
  
 NCBI BlastP on this gene

EMC92635

hypothetical protein
  
Accession: EMC92636
  
Location: 172150-173656
  
  
**BlastP hit with Mycgr3G98959\_Mycgr3T**
  
Percentage identity: 86 %
  
BlastP bit score: 871
  
Sequence coverage: 101 %
  
E-value: 0.0
  
  
 NCBI BlastP on this gene

EMC92636

hypothetical protein
  
Accession: EMC92637
  
Location: 174470-174703
  
 NCBI BlastP on this gene

EMC92637

hypothetical protein
  
Accession: EMC92638
  
Location: 174993-175280
  
 NCBI BlastP on this gene

EMC92638

hypothetical protein
  
Accession: EMC92639
  
Location: 176369-176638
  
 NCBI BlastP on this gene

EMC92639

hypothetical protein
  
Accession: EMC92640
  
Location: 177769-178137
  
 NCBI BlastP on this gene

EMC92640

hypothetical protein
  
Accession: EMC92641
  
Location: 179803-180665
  
 NCBI BlastP on this gene

EMC92641

hypothetical protein
  
Accession: EMC92642
  
Location: 180927-181490
  
 NCBI BlastP on this gene

EMC92642

hypothetical protein
  
Accession: EMC92643
  
Location: 181769-182424
  
 NCBI BlastP on this gene

EMC92643

hypothetical protein
  
Accession: EMC92644
  
Location: 182899-183996
  
 NCBI BlastP on this gene

EMC92644

hypothetical protein
  
Accession: EMC92645
  
Location: 184773-185060
  
 NCBI BlastP on this gene

EMC92645

hypothetical protein
  
Accession: EMC92646
  
Location: 185782-187573
  
 NCBI BlastP on this gene

EMC92646

hypothetical protein
  
Accession: EMC92647
  
Location: 187710-189691
  
 NCBI BlastP on this gene

EMC92647

hypothetical protein
  
Accession: EMC92648
  
Location: 189716-189871
  
 NCBI BlastP on this gene

EMC92648

hypothetical protein
  
Accession: EMC92649
  
Location: 191920-192923
  
 NCBI BlastP on this gene

EMC92649

Query: Architecture Search FASTA input

KB446566 : Pseudocercospora fijiensis CIRAD86 unplaced genomic scaffold MYCFIscaffold\_12    Total score: 2.0     Cumulative Blast bit score: 1322

Hit cluster cross-links:

Mycgr3G67791 Mycgr3T
  
Location: 0-1542

Mycgr3G67791\_Mycgr3T

Mycgr3G90406 Mycgr3T
  
Location: 1642-3973

Mycgr3G90406\_Mycgr3T

Mycgr3G67785 Mycgr3T
  
Location: 4073-7865

Mycgr3G67785\_Mycgr3T

Mycgr3G67795 Mycgr3T
  
Location: 7965-15249

Mycgr3G67795\_Mycgr3T

Mycgr3G67775 Mycgr3T
  
Location: 15349-16237

Mycgr3G67775\_Mycgr3T

Mycgr3G90404 Mycgr3T
  
Location: 16337-17246

Mycgr3G90404\_Mycgr3T

Mycgr3G36951 Mycgr3T
  
Location: 17346-30891

Mycgr3G36951\_Mycgr3T

Mycgr3G103034 Mycgr3
  
Location: 30991-32644

Mycgr3G103034\_Mycgr3

Mycgr3G31119 Mycgr3T
  
Location: 32744-32906

Mycgr3G31119\_Mycgr3T

Mycgr3G28587 Mycgr3T
  
Location: 33006-33489

Mycgr3G28587\_Mycgr3T

Mycgr3G98959 Mycgr3T
  
Location: 33589-35035

Mycgr3G98959\_Mycgr3T

Mycgr3G35447 Mycgr3T
  
Location: 35135-36443

Mycgr3G35447\_Mycgr3T

Mycgr3G84402 Mycgr3T
  
Location: 36543-37884

Mycgr3G84402\_Mycgr3T

Mycgr3G98961 Mycgr3T
  
Location: 37984-38884

Mycgr3G98961\_Mycgr3T

hypothetical protein
  
Accession: EME77405
  
Location: 1150927-1152879
  
 NCBI BlastP on this gene

EME77405

hypothetical protein
  
Accession: EME77406
  
Location: 1153296-1154701
  
 NCBI BlastP on this gene

EME77406

hypothetical protein
  
Accession: EME77407
  
Location: 1155393-1156790
  
 NCBI BlastP on this gene

EME77407

hypothetical protein
  
Accession: EME77408
  
Location: 1156960-1158511
  
 NCBI BlastP on this gene

EME77408

hypothetical protein
  
Accession: EME77409
  
Location: 1159267-1160631
  
  
**BlastP hit with Mycgr3G35447\_Mycgr3T**
  
Percentage identity: 71 %
  
BlastP bit score: 578
  
Sequence coverage: 90 %
  
E-value: 0.0
  
  
 NCBI BlastP on this gene

EME77409

hypothetical protein
  
Accession: EME77410
  
Location: 1160871-1162367
  
  
**BlastP hit with Mycgr3G84402\_Mycgr3T**
  
Percentage identity: 88 %
  
BlastP bit score: 744
  
Sequence coverage: 89 %
  
E-value: 0.0
  
  
 NCBI BlastP on this gene

EME77410

hypothetical protein
  
Accession: EME77411
  
Location: 1163031-1163869
  
 NCBI BlastP on this gene

EME77411

hypothetical protein
  
Accession: EME77413
  
Location: 1164854-1169444
  
 NCBI BlastP on this gene

EME77413

hypothetical protein
  
Accession: EME77414
  
Location: 1170792-1171562
  
 NCBI BlastP on this gene

EME77414

hypothetical protein
  
Accession: EME77415
  
Location: 1180053-1181913
  
 NCBI BlastP on this gene

EME77415

hypothetical protein
  
Accession: EME77416
  
Location: 1181886-1183973
  
 NCBI BlastP on this gene

EME77416

Query: Architecture Search FASTA input

KB456266 : Mycosphaerella populorum SO2202 unplaced genomic scaffold SEPMUscaffold\_7    Total score: 2.0     Cumulative Blast bit score: 1291

Hit cluster cross-links:

Mycgr3G67791 Mycgr3T
  
Location: 0-1542

Mycgr3G67791\_Mycgr3T

Mycgr3G90406 Mycgr3T
  
Location: 1642-3973

Mycgr3G90406\_Mycgr3T

Mycgr3G67785 Mycgr3T
  
Location: 4073-7865

Mycgr3G67785\_Mycgr3T

Mycgr3G67795 Mycgr3T
  
Location: 7965-15249

Mycgr3G67795\_Mycgr3T

Mycgr3G67775 Mycgr3T
  
Location: 15349-16237

Mycgr3G67775\_Mycgr3T

Mycgr3G90404 Mycgr3T
  
Location: 16337-17246

Mycgr3G90404\_Mycgr3T

Mycgr3G36951 Mycgr3T
  
Location: 17346-30891

Mycgr3G36951\_Mycgr3T

Mycgr3G103034 Mycgr3
  
Location: 30991-32644

Mycgr3G103034\_Mycgr3

Mycgr3G31119 Mycgr3T
  
Location: 32744-32906

Mycgr3G31119\_Mycgr3T

Mycgr3G28587 Mycgr3T
  
Location: 33006-33489

Mycgr3G28587\_Mycgr3T

Mycgr3G98959 Mycgr3T
  
Location: 33589-35035

Mycgr3G98959\_Mycgr3T

Mycgr3G35447 Mycgr3T
  
Location: 35135-36443

Mycgr3G35447\_Mycgr3T

Mycgr3G84402 Mycgr3T
  
Location: 36543-37884

Mycgr3G84402\_Mycgr3T

Mycgr3G98961 Mycgr3T
  
Location: 37984-38884

Mycgr3G98961\_Mycgr3T

hypothetical protein
  
Accession: EMF10998
  
Location: 68986-69657
  
 NCBI BlastP on this gene

EMF10998

hypothetical protein
  
Accession: EMF11000
  
Location: 69971-70756
  
 NCBI BlastP on this gene

EMF11000

Zn-dependent exopeptidase
  
Accession: EMF11001
  
Location: 71360-74176
  
 NCBI BlastP on this gene

EMF11001

hypothetical protein
  
Accession: EMF11002
  
Location: 74782-74937
  
 NCBI BlastP on this gene

EMF11002

hypothetical protein
  
Accession: EMF11003
  
Location: 76987-77259
  
 NCBI BlastP on this gene

EMF11003

acetyl-CoA synthetase-like protein
  
Accession: EMF11004
  
Location: 79093-80918
  
 NCBI BlastP on this gene

EMF11004

hypothetical protein
  
Accession: EMF11005
  
Location: 81812-83290
  
 NCBI BlastP on this gene

EMF11005

hypothetical protein
  
Accession: EMF11006
  
Location: 83563-84188
  
 NCBI BlastP on this gene

EMF11006

ATP-dependent rRNA helicase RRP3
  
Accession: EMF11008
  
Location: 86541-88091
  
  
**BlastP hit with Mycgr3G84402\_Mycgr3T**
  
Percentage identity: 87 %
  
BlastP bit score: 734
  
Sequence coverage: 89 %
  
E-value: 0.0
  
  
 NCBI BlastP on this gene

EMF11008

subtilisin-like protein
  
Accession: EMF11009
  
Location: 90389-93305
  
 NCBI BlastP on this gene

EMF11009

Brix-domain-containing protein
  
Accession: EMF11010
  
Location: 93602-95044
  
  
**BlastP hit with Mycgr3G35447\_Mycgr3T**
  
Percentage identity: 63 %
  
BlastP bit score: 557
  
Sequence coverage: 106 %
  
E-value: 0.0
  
  
 NCBI BlastP on this gene

EMF11010

RNA polymerase II transcription factor B subunit 2
  
Accession: EMF11011
  
Location: 98125-99648
  
 NCBI BlastP on this gene

EMF11011

Peptidase S9-domain-containing protein
  
Accession: EMF11013
  
Location: 100362-102585
  
 NCBI BlastP on this gene

EMF11013

hypothetical protein
  
Accession: EMF11014
  
Location: 102971-104359
  
 NCBI BlastP on this gene

EMF11014

ClpP/crotonase
  
Accession: EMF11015
  
Location: 105134-106038
  
 NCBI BlastP on this gene

EMF11015

ribosomal protein L13e
  
Accession: EMF11016
  
Location: 106559-107479
  
 NCBI BlastP on this gene

EMF11016

DNA polymerase alpha catalytic subunit
  
Accession: EMF11017
  
Location: 107802-112316
  
 NCBI BlastP on this gene

EMF11017

Query: Architecture Search FASTA input

JH767573 : Coniosporium apollinis CBS 100218 chromosome Unknown supercont1.20    Total score: 2.0     Cumulative Blast bit score: 1240

Hit cluster cross-links:

Mycgr3G67791 Mycgr3T
  
Location: 0-1542

Mycgr3G67791\_Mycgr3T

Mycgr3G90406 Mycgr3T
  
Location: 1642-3973

Mycgr3G90406\_Mycgr3T

Mycgr3G67785 Mycgr3T
  
Location: 4073-7865

Mycgr3G67785\_Mycgr3T

Mycgr3G67795 Mycgr3T
  
Location: 7965-15249

Mycgr3G67795\_Mycgr3T

Mycgr3G67775 Mycgr3T
  
Location: 15349-16237

Mycgr3G67775\_Mycgr3T

Mycgr3G90404 Mycgr3T
  
Location: 16337-17246

Mycgr3G90404\_Mycgr3T

Mycgr3G36951 Mycgr3T
  
Location: 17346-30891

Mycgr3G36951\_Mycgr3T

Mycgr3G103034 Mycgr3
  
Location: 30991-32644

Mycgr3G103034\_Mycgr3

Mycgr3G31119 Mycgr3T
  
Location: 32744-32906

Mycgr3G31119\_Mycgr3T

Mycgr3G28587 Mycgr3T
  
Location: 33006-33489

Mycgr3G28587\_Mycgr3T

Mycgr3G98959 Mycgr3T
  
Location: 33589-35035

Mycgr3G98959\_Mycgr3T

Mycgr3G35447 Mycgr3T
  
Location: 35135-36443

Mycgr3G35447\_Mycgr3T

Mycgr3G84402 Mycgr3T
  
Location: 36543-37884

Mycgr3G84402\_Mycgr3T

Mycgr3G98961 Mycgr3T
  
Location: 37984-38884

Mycgr3G98961\_Mycgr3T

hypothetical protein
  
Accession: EON65317
  
Location: 218777-219148
  
 NCBI BlastP on this gene

EON65317

hypothetical protein
  
Accession: EON65318
  
Location: 220746-221750
  
 NCBI BlastP on this gene

EON65318

hypothetical protein
  
Accession: EON65319
  
Location: 222122-223034
  
 NCBI BlastP on this gene

EON65319

hypothetical protein
  
Accession: EON65320
  
Location: 223425-223968
  
 NCBI BlastP on this gene

EON65320

hypothetical protein
  
Accession: EON65321
  
Location: 224255-226042
  
 NCBI BlastP on this gene

EON65321

hypothetical protein
  
Accession: EON65322
  
Location: 228497-228997
  
 NCBI BlastP on this gene

EON65322

hypothetical protein
  
Accession: EON65323
  
Location: 232889-233113
  
 NCBI BlastP on this gene

EON65323

hypothetical protein
  
Accession: EON65324
  
Location: 233710-235134
  
 NCBI BlastP on this gene

EON65324

hypothetical protein
  
Accession: EON65325
  
Location: 235688-236708
  
 NCBI BlastP on this gene

EON65325

eukaryotic translation initiation factor 3 subunit L
  
Accession: EON65326
  
Location: 237320-238896
  
  
**BlastP hit with Mycgr3G98959\_Mycgr3T**
  
Percentage identity: 80 %
  
BlastP bit score: 797
  
Sequence coverage: 97 %
  
E-value: 0.0
  
  
 NCBI BlastP on this gene

EON65326

hypothetical protein
  
Accession: EON65327
  
Location: 239207-240352
  
  
**BlastP hit with Mycgr3G67775\_Mycgr3T**
  
Percentage identity: 72 %
  
BlastP bit score: 443
  
Sequence coverage: 98 %
  
E-value: 2e-153
  
  
 NCBI BlastP on this gene

EON65327

hypothetical protein
  
Accession: EON65328
  
Location: 240921-242592
  
 NCBI BlastP on this gene

EON65328

hypothetical protein
  
Accession: EON65329
  
Location: 242832-244392
  
 NCBI BlastP on this gene

EON65329

hypothetical protein
  
Accession: EON65330
  
Location: 245157-248462
  
 NCBI BlastP on this gene

EON65330

hypothetical protein
  
Accession: EON65331
  
Location: 249235-250015
  
 NCBI BlastP on this gene

EON65331

hypothetical protein
  
Accession: EON65332
  
Location: 253636-256040
  
 NCBI BlastP on this gene

EON65332

hypothetical protein
  
Accession: EON65333
  
Location: 256333-257304
  
 NCBI BlastP on this gene

EON65333

hypothetical protein
  
Accession: EON65334
  
Location: 257516-258580
  
 NCBI BlastP on this gene

EON65334

Query: Architecture Search FASTA input

AHHD01000099 : Macrophomina phaseolina MS6    Total score: 2.0     Cumulative Blast bit score: 1223

Hit cluster cross-links:

Mycgr3G67791 Mycgr3T
  
Location: 0-1542

Mycgr3G67791\_Mycgr3T

Mycgr3G90406 Mycgr3T
  
Location: 1642-3973

Mycgr3G90406\_Mycgr3T

Mycgr3G67785 Mycgr3T
  
Location: 4073-7865

Mycgr3G67785\_Mycgr3T

Mycgr3G67795 Mycgr3T
  
Location: 7965-15249

Mycgr3G67795\_Mycgr3T

Mycgr3G67775 Mycgr3T
  
Location: 15349-16237

Mycgr3G67775\_Mycgr3T

Mycgr3G90404 Mycgr3T
  
Location: 16337-17246

Mycgr3G90404\_Mycgr3T

Mycgr3G36951 Mycgr3T
  
Location: 17346-30891

Mycgr3G36951\_Mycgr3T

Mycgr3G103034 Mycgr3
  
Location: 30991-32644

Mycgr3G103034\_Mycgr3

Mycgr3G31119 Mycgr3T
  
Location: 32744-32906

Mycgr3G31119\_Mycgr3T

Mycgr3G28587 Mycgr3T
  
Location: 33006-33489

Mycgr3G28587\_Mycgr3T

Mycgr3G98959 Mycgr3T
  
Location: 33589-35035

Mycgr3G98959\_Mycgr3T

Mycgr3G35447 Mycgr3T
  
Location: 35135-36443

Mycgr3G35447\_Mycgr3T

Mycgr3G84402 Mycgr3T
  
Location: 36543-37884

Mycgr3G84402\_Mycgr3T

Mycgr3G98961 Mycgr3T
  
Location: 37984-38884

Mycgr3G98961\_Mycgr3T

Mitochondrial genome maintenance MGM101
  
Accession: EKG20014
  
Location: 24832-25770
  
 NCBI BlastP on this gene

EKG20014

Carbohydrate kinase FGGY
  
Accession: EKG20015
  
Location: 26291-28195
  
 NCBI BlastP on this gene

EKG20015

hypothetical protein
  
Accession: EKG20016
  
Location: 29018-29649
  
 NCBI BlastP on this gene

EKG20016

Ras GTPase
  
Accession: EKG20017
  
Location: 30901-32130
  
 NCBI BlastP on this gene

EKG20017

FMN-dependent dehydrogenase
  
Accession: EKG20018
  
Location: 33155-34894
  
 NCBI BlastP on this gene

EKG20018

Short-chain dehydrogenase/reductase SDR
  
Accession: EKG20019
  
Location: 35387-35785
  
 NCBI BlastP on this gene

EKG20019

Cytochrome c heme lyase
  
Accession: EKG20020
  
Location: 36977-38013
  
 NCBI BlastP on this gene

EKG20020

Major facilitator superfamily
  
Accession: EKG20021
  
Location: 39981-41970
  
 NCBI BlastP on this gene

EKG20021

Translation initiation factor 3 complex subunit L
  
Accession: EKG20022
  
Location: 43691-45245
  
  
**BlastP hit with Mycgr3G98959\_Mycgr3T**
  
Percentage identity: 81 %
  
BlastP bit score: 793
  
Sequence coverage: 96 %
  
E-value: 0.0
  
  
 NCBI BlastP on this gene

EKG20022

Nitrilase/cyanide hydratase and apolipoprotein N-acyltransferase
  
Accession: EKG20023
  
Location: 45506-46639
  
  
**BlastP hit with Mycgr3G67775\_Mycgr3T**
  
Percentage identity: 69 %
  
BlastP bit score: 430
  
Sequence coverage: 99 %
  
E-value: 2e-148
  
  
 NCBI BlastP on this gene

EKG20023

Fungal lignin peroxidase
  
Accession: EKG20024
  
Location: 46935-48206
  
 NCBI BlastP on this gene

EKG20024

Cytochrome P450
  
Accession: EKG20025
  
Location: 50439-52161
  
 NCBI BlastP on this gene

EKG20025

Alanine racemase
  
Accession: EKG20026
  
Location: 53864-54799
  
 NCBI BlastP on this gene

EKG20026

hypothetical protein
  
Accession: EKG20027
  
Location: 56261-57263
  
 NCBI BlastP on this gene

EKG20027

Six-hairpin glycosidase-like protein
  
Accession: EKG20028
  
Location: 58594-60690
  
 NCBI BlastP on this gene

EKG20028

hypothetical protein
  
Accession: EKG20029
  
Location: 61766-62485
  
 NCBI BlastP on this gene

EKG20029

hypothetical protein
  
Accession: EKG20030
  
Location: 63027-65191
  
 NCBI BlastP on this gene

EKG20030

Query: Architecture Search FASTA input

GL536348 : Pyrenophora teres f. teres 0-1 unplaced genomic scaffold scaffold\_192633    Total score: 2.0     Cumulative Blast bit score: 1216

Hit cluster cross-links:

Mycgr3G67791 Mycgr3T
  
Location: 0-1542

Mycgr3G67791\_Mycgr3T

Mycgr3G90406 Mycgr3T
  
Location: 1642-3973

Mycgr3G90406\_Mycgr3T

Mycgr3G67785 Mycgr3T
  
Location: 4073-7865

Mycgr3G67785\_Mycgr3T

Mycgr3G67795 Mycgr3T
  
Location: 7965-15249

Mycgr3G67795\_Mycgr3T

Mycgr3G67775 Mycgr3T
  
Location: 15349-16237

Mycgr3G67775\_Mycgr3T

Mycgr3G90404 Mycgr3T
  
Location: 16337-17246

Mycgr3G90404\_Mycgr3T

Mycgr3G36951 Mycgr3T
  
Location: 17346-30891

Mycgr3G36951\_Mycgr3T

Mycgr3G103034 Mycgr3
  
Location: 30991-32644

Mycgr3G103034\_Mycgr3

Mycgr3G31119 Mycgr3T
  
Location: 32744-32906

Mycgr3G31119\_Mycgr3T

Mycgr3G28587 Mycgr3T
  
Location: 33006-33489

Mycgr3G28587\_Mycgr3T

Mycgr3G98959 Mycgr3T
  
Location: 33589-35035

Mycgr3G98959\_Mycgr3T

Mycgr3G35447 Mycgr3T
  
Location: 35135-36443

Mycgr3G35447\_Mycgr3T

Mycgr3G84402 Mycgr3T
  
Location: 36543-37884

Mycgr3G84402\_Mycgr3T

Mycgr3G98961 Mycgr3T
  
Location: 37984-38884

Mycgr3G98961\_Mycgr3T

hypothetical protein
  
Accession: EFQ88473
  
Location: 33232-34913
  
 NCBI BlastP on this gene

EFQ88473

hypothetical protein
  
Accession: EFQ88472
  
Location: 31254-32777
  
 NCBI BlastP on this gene

EFQ88472

hypothetical protein
  
Accession: EFQ88471
  
Location: 28491-29709
  
 NCBI BlastP on this gene

EFQ88471

hypothetical protein
  
Accession: EFQ88470
  
Location: 26867-27564
  
 NCBI BlastP on this gene

EFQ88470

hypothetical protein
  
Accession: EFQ88469
  
Location: 22498-25857
  
 NCBI BlastP on this gene

EFQ88469

hypothetical protein
  
Accession: EFQ88468
  
Location: 20447-22046
  
 NCBI BlastP on this gene

EFQ88468

hypothetical protein
  
Accession: EFQ88467
  
Location: 18528-20059
  
 NCBI BlastP on this gene

EFQ88467

hypothetical protein
  
Accession: EFQ88466
  
Location: 16942-17906
  
  
**BlastP hit with Mycgr3G67775\_Mycgr3T**
  
Percentage identity: 66 %
  
BlastP bit score: 418
  
Sequence coverage: 99 %
  
E-value: 1e-143
  
  
 NCBI BlastP on this gene

EFQ88466

hypothetical protein
  
Accession: EFQ88465
  
Location: 16200-16688
  
 NCBI BlastP on this gene

EFQ88465

hypothetical protein
  
Accession: EFQ88464
  
Location: 14088-15629
  
  
**BlastP hit with Mycgr3G98959\_Mycgr3T**
  
Percentage identity: 80 %
  
BlastP bit score: 798
  
Sequence coverage: 98 %
  
E-value: 0.0
  
  
 NCBI BlastP on this gene

EFQ88464

hypothetical protein
  
Accession: EFQ88463
  
Location: 12369-13232
  
 NCBI BlastP on this gene

EFQ88463

hypothetical protein
  
Accession: EFQ88462
  
Location: 8314-10080
  
 NCBI BlastP on this gene

EFQ88462

hypothetical protein
  
Accession: EFQ88461
  
Location: 7396-7932
  
 NCBI BlastP on this gene

EFQ88461

hypothetical protein
  
Accession: EFQ88460
  
Location: 6337-7186
  
 NCBI BlastP on this gene

EFQ88460

hypothetical protein
  
Accession: EFQ88459
  
Location: 4951-5886
  
 NCBI BlastP on this gene

EFQ88459

hypothetical protein
  
Accession: EFQ88458
  
Location: 3256-4298
  
 NCBI BlastP on this gene

EFQ88458

hypothetical protein
  
Accession: EFQ88457
  
Location: 58-2303
  
 NCBI BlastP on this gene

EFQ88457

Query: Architecture Search FASTA input

DS231623 : Pyrenophora tritici-repentis Pt-1C-BFP supercont1.9 genomic scaffold    Total score: 2.0     Cumulative Blast bit score: 1216

Hit cluster cross-links:

Mycgr3G67791 Mycgr3T
  
Location: 0-1542

Mycgr3G67791\_Mycgr3T

Mycgr3G90406 Mycgr3T
  
Location: 1642-3973

Mycgr3G90406\_Mycgr3T

Mycgr3G67785 Mycgr3T
  
Location: 4073-7865

Mycgr3G67785\_Mycgr3T

Mycgr3G67795 Mycgr3T
  
Location: 7965-15249

Mycgr3G67795\_Mycgr3T

Mycgr3G67775 Mycgr3T
  
Location: 15349-16237

Mycgr3G67775\_Mycgr3T

Mycgr3G90404 Mycgr3T
  
Location: 16337-17246

Mycgr3G90404\_Mycgr3T

Mycgr3G36951 Mycgr3T
  
Location: 17346-30891

Mycgr3G36951\_Mycgr3T

Mycgr3G103034 Mycgr3
  
Location: 30991-32644

Mycgr3G103034\_Mycgr3

Mycgr3G31119 Mycgr3T
  
Location: 32744-32906

Mycgr3G31119\_Mycgr3T

Mycgr3G28587 Mycgr3T
  
Location: 33006-33489

Mycgr3G28587\_Mycgr3T

Mycgr3G98959 Mycgr3T
  
Location: 33589-35035

Mycgr3G98959\_Mycgr3T

Mycgr3G35447 Mycgr3T
  
Location: 35135-36443

Mycgr3G35447\_Mycgr3T

Mycgr3G84402 Mycgr3T
  
Location: 36543-37884

Mycgr3G84402\_Mycgr3T

Mycgr3G98961 Mycgr3T
  
Location: 37984-38884

Mycgr3G98961\_Mycgr3T

conserved hypothetical protein
  
Accession: EDU51254
  
Location: 766697-767270
  
 NCBI BlastP on this gene

EDU51254

ubiquitin-conjugating enzyme E2 6
  
Accession: EDU51253
  
Location: 764589-765419
  
 NCBI BlastP on this gene

EDU51253

hypothetical protein
  
Accession: EDU51252
  
Location: 762548-764229
  
 NCBI BlastP on this gene

EDU51252

glucooligosaccharide oxidase
  
Accession: EDU51251
  
Location: 760544-762074
  
 NCBI BlastP on this gene

EDU51251

6-phosphogluconolactonase
  
Accession: EDU51250
  
Location: 757828-759046
  
 NCBI BlastP on this gene

EDU51250

predicted protein
  
Accession: EDU51249
  
Location: 756211-756902
  
 NCBI BlastP on this gene

EDU51249

oligopeptide transporter 4
  
Accession: EDU51248
  
Location: 751931-755281
  
 NCBI BlastP on this gene

EDU51248

26S protease regulatory subunit 8
  
Accession: EDU51247
  
Location: 749944-751459
  
 NCBI BlastP on this gene

EDU51247

conserved hypothetical protein
  
Accession: EDU51246
  
Location: 747901-749484
  
 NCBI BlastP on this gene

EDU51246

hypothetical protein
  
Accession: EDU51245
  
Location: 746315-747279
  
  
**BlastP hit with Mycgr3G67775\_Mycgr3T**
  
Percentage identity: 67 %
  
BlastP bit score: 418
  
Sequence coverage: 99 %
  
E-value: 1e-143
  
  
 NCBI BlastP on this gene

EDU51245

conserved hypothetical protein
  
Accession: EDU51244
  
Location: 745574-746062
  
 NCBI BlastP on this gene

EDU51244

eukaryotic translation initiation factor 3
  
Accession: EDU51243
  
Location: 743454-744995
  
  
**BlastP hit with Mycgr3G98959\_Mycgr3T**
  
Percentage identity: 80 %
  
BlastP bit score: 798
  
Sequence coverage: 98 %
  
E-value: 0.0
  
  
 NCBI BlastP on this gene

EDU51243

conserved hypothetical protein
  
Accession: EDU51242
  
Location: 741629-742465
  
 NCBI BlastP on this gene

EDU51242

conserved hypothetical protein
  
Accession: EDU51241
  
Location: 737429-739186
  
 NCBI BlastP on this gene

EDU51241

conserved hypothetical protein
  
Accession: EDU51240
  
Location: 736596-737052
  
 NCBI BlastP on this gene

EDU51240

conserved hypothetical protein
  
Accession: EDU51239
  
Location: 735458-736307
  
 NCBI BlastP on this gene

EDU51239

predicted protein
  
Accession: EDU51238
  
Location: 732469-735023
  
 NCBI BlastP on this gene

EDU51238

hypothetical protein
  
Accession: EDU51237
  
Location: 729373-731678
  
 NCBI BlastP on this gene

EDU51237

transcriptional coactivator/pterin dehydratase
  
Accession: EDU51236
  
Location: 727128-727481
  
 NCBI BlastP on this gene

EDU51236

conserved hypothetical protein
  
Accession: EDU51235
  
Location: 724484-725474
  
 NCBI BlastP on this gene

EDU51235

Query: Architecture Search FASTA input

KB733455 : Bipolaris maydis ATCC 48331 unplaced genomic scaffold COCC4scaffold\_12    Total score: 2.0     Cumulative Blast bit score: 1213

Hit cluster cross-links:

Mycgr3G67791 Mycgr3T
  
Location: 0-1542

Mycgr3G67791\_Mycgr3T

Mycgr3G90406 Mycgr3T
  
Location: 1642-3973

Mycgr3G90406\_Mycgr3T

Mycgr3G67785 Mycgr3T
  
Location: 4073-7865

Mycgr3G67785\_Mycgr3T

Mycgr3G67795 Mycgr3T
  
Location: 7965-15249

Mycgr3G67795\_Mycgr3T

Mycgr3G67775 Mycgr3T
  
Location: 15349-16237

Mycgr3G67775\_Mycgr3T

Mycgr3G90404 Mycgr3T
  
Location: 16337-17246

Mycgr3G90404\_Mycgr3T

Mycgr3G36951 Mycgr3T
  
Location: 17346-30891

Mycgr3G36951\_Mycgr3T

Mycgr3G103034 Mycgr3
  
Location: 30991-32644

Mycgr3G103034\_Mycgr3

Mycgr3G31119 Mycgr3T
  
Location: 32744-32906

Mycgr3G31119\_Mycgr3T

Mycgr3G28587 Mycgr3T
  
Location: 33006-33489

Mycgr3G28587\_Mycgr3T

Mycgr3G98959 Mycgr3T
  
Location: 33589-35035

Mycgr3G98959\_Mycgr3T

Mycgr3G35447 Mycgr3T
  
Location: 35135-36443

Mycgr3G35447\_Mycgr3T

Mycgr3G84402 Mycgr3T
  
Location: 36543-37884

Mycgr3G84402\_Mycgr3T

Mycgr3G98961 Mycgr3T
  
Location: 37984-38884

Mycgr3G98961\_Mycgr3T

hypothetical protein
  
Accession: ENI05137
  
Location: 586211-586381
  
 NCBI BlastP on this gene

ENI05137

hypothetical protein
  
Accession: ENI05138
  
Location: 587166-587999
  
 NCBI BlastP on this gene

ENI05138

hypothetical protein
  
Accession: ENI05139
  
Location: 588391-590084
  
 NCBI BlastP on this gene

ENI05139

hypothetical protein
  
Accession: ENI05140
  
Location: 590280-591831
  
 NCBI BlastP on this gene

ENI05140

hypothetical protein
  
Accession: ENI05141
  
Location: 592810-594034
  
 NCBI BlastP on this gene

ENI05141

hypothetical protein
  
Accession: ENI05142
  
Location: 595097-595822
  
 NCBI BlastP on this gene

ENI05142

hypothetical protein
  
Accession: ENI05143
  
Location: 596533-597210
  
 NCBI BlastP on this gene

ENI05143

hypothetical protein
  
Accession: ENI05144
  
Location: 597700-601061
  
 NCBI BlastP on this gene

ENI05144

hypothetical protein
  
Accession: ENI05145
  
Location: 601549-603071
  
 NCBI BlastP on this gene

ENI05145

hypothetical protein
  
Accession: ENI05146
  
Location: 603575-605091
  
 NCBI BlastP on this gene

ENI05146

hypothetical protein
  
Accession: ENI05147
  
Location: 605731-606818
  
  
**BlastP hit with Mycgr3G67775\_Mycgr3T**
  
Percentage identity: 67 %
  
BlastP bit score: 417
  
Sequence coverage: 98 %
  
E-value: 2e-143
  
  
 NCBI BlastP on this gene

ENI05147

hypothetical protein
  
Accession: ENI05148
  
Location: 607093-607590
  
 NCBI BlastP on this gene

ENI05148

hypothetical protein
  
Accession: ENI05149
  
Location: 608213-609703
  
  
**BlastP hit with Mycgr3G98959\_Mycgr3T**
  
Percentage identity: 78 %
  
BlastP bit score: 796
  
Sequence coverage: 99 %
  
E-value: 0.0
  
  
 NCBI BlastP on this gene

ENI05149

hypothetical protein
  
Accession: ENI05150
  
Location: 610002-610272
  
 NCBI BlastP on this gene

ENI05150

hypothetical protein
  
Accession: ENI05151
  
Location: 610583-611751
  
 NCBI BlastP on this gene

ENI05151

hypothetical protein
  
Accession: ENI05152
  
Location: 612298-612915
  
 NCBI BlastP on this gene

ENI05152

hypothetical protein
  
Accession: ENI05153
  
Location: 613197-614951
  
 NCBI BlastP on this gene

ENI05153

hypothetical protein
  
Accession: ENI05154
  
Location: 615286-615828
  
 NCBI BlastP on this gene

ENI05154

hypothetical protein
  
Accession: ENI05155
  
Location: 616034-616903
  
 NCBI BlastP on this gene

ENI05155

hypothetical protein
  
Accession: ENI05156
  
Location: 617378-618315
  
 NCBI BlastP on this gene

ENI05156

hypothetical protein
  
Accession: ENI05157
  
Location: 618566-619578
  
 NCBI BlastP on this gene

ENI05157

glycosyltransferase family 69 protein
  
Accession: ENI05158
  
Location: 620226-621642
  
 NCBI BlastP on this gene

ENI05158

hypothetical protein
  
Accession: ENI05159
  
Location: 622854-624683
  
 NCBI BlastP on this gene

ENI05159

hypothetical protein
  
Accession: ENI05160
  
Location: 625268-627489
  
 NCBI BlastP on this gene

ENI05160

hypothetical protein
  
Accession: ENI05161
  
Location: 627897-629252
  
 NCBI BlastP on this gene

ENI05161

Query: Architecture Search FASTA input

KB445579 : Cochliobolus heterostrophus C5 unplaced genomic scaffold COCHEscaffold\_11    Total score: 2.0     Cumulative Blast bit score: 1213

Hit cluster cross-links:

Mycgr3G67791 Mycgr3T
  
Location: 0-1542

Mycgr3G67791\_Mycgr3T

Mycgr3G90406 Mycgr3T
  
Location: 1642-3973

Mycgr3G90406\_Mycgr3T

Mycgr3G67785 Mycgr3T
  
Location: 4073-7865

Mycgr3G67785\_Mycgr3T

Mycgr3G67795 Mycgr3T
  
Location: 7965-15249

Mycgr3G67795\_Mycgr3T

Mycgr3G67775 Mycgr3T
  
Location: 15349-16237

Mycgr3G67775\_Mycgr3T

Mycgr3G90404 Mycgr3T
  
Location: 16337-17246

Mycgr3G90404\_Mycgr3T

Mycgr3G36951 Mycgr3T
  
Location: 17346-30891

Mycgr3G36951\_Mycgr3T

Mycgr3G103034 Mycgr3
  
Location: 30991-32644

Mycgr3G103034\_Mycgr3

Mycgr3G31119 Mycgr3T
  
Location: 32744-32906

Mycgr3G31119\_Mycgr3T

Mycgr3G28587 Mycgr3T
  
Location: 33006-33489

Mycgr3G28587\_Mycgr3T

Mycgr3G98959 Mycgr3T
  
Location: 33589-35035

Mycgr3G98959\_Mycgr3T

Mycgr3G35447 Mycgr3T
  
Location: 35135-36443

Mycgr3G35447\_Mycgr3T

Mycgr3G84402 Mycgr3T
  
Location: 36543-37884

Mycgr3G84402\_Mycgr3T

Mycgr3G98961 Mycgr3T
  
Location: 37984-38884

Mycgr3G98961\_Mycgr3T

hypothetical protein
  
Accession: EMD89143
  
Location: 418688-418858
  
 NCBI BlastP on this gene

EMD89143

hypothetical protein
  
Accession: EMD89142
  
Location: 417065-417898
  
 NCBI BlastP on this gene

EMD89142

hypothetical protein
  
Accession: EMD89141
  
Location: 414980-416673
  
 NCBI BlastP on this gene

EMD89141

hypothetical protein
  
Accession: EMD89140
  
Location: 413233-414784
  
 NCBI BlastP on this gene

EMD89140

hypothetical protein
  
Accession: EMD89139
  
Location: 411030-412254
  
 NCBI BlastP on this gene

EMD89139

hypothetical protein
  
Accession: EMD89138
  
Location: 409242-409967
  
 NCBI BlastP on this gene

EMD89138

hypothetical protein
  
Accession: EMD89137
  
Location: 407854-408531
  
 NCBI BlastP on this gene

EMD89137

hypothetical protein
  
Accession: EMD89136
  
Location: 404249-407364
  
 NCBI BlastP on this gene

EMD89136

hypothetical protein
  
Accession: EMD89135
  
Location: 401993-403515
  
 NCBI BlastP on this gene

EMD89135

hypothetical protein
  
Accession: EMD89134
  
Location: 399973-401489
  
 NCBI BlastP on this gene

EMD89134

hypothetical protein
  
Accession: EMD89133
  
Location: 398246-399333
  
  
**BlastP hit with Mycgr3G67775\_Mycgr3T**
  
Percentage identity: 67 %
  
BlastP bit score: 417
  
Sequence coverage: 98 %
  
E-value: 2e-143
  
  
 NCBI BlastP on this gene

EMD89133

hypothetical protein
  
Accession: EMD89132
  
Location: 397474-397971
  
 NCBI BlastP on this gene

EMD89132

hypothetical protein
  
Accession: EMD89131
  
Location: 395361-396851
  
  
**BlastP hit with Mycgr3G98959\_Mycgr3T**
  
Percentage identity: 78 %
  
BlastP bit score: 796
  
Sequence coverage: 99 %
  
E-value: 0.0
  
  
 NCBI BlastP on this gene

EMD89131

hypothetical protein
  
Accession: EMD89130
  
Location: 394792-395062
  
 NCBI BlastP on this gene

EMD89130

hypothetical protein
  
Accession: EMD89129
  
Location: 393792-394481
  
 NCBI BlastP on this gene

EMD89129

hypothetical protein
  
Accession: EMD89128
  
Location: 392118-392735
  
 NCBI BlastP on this gene

EMD89128

hypothetical protein
  
Accession: EMD89127
  
Location: 390082-391836
  
 NCBI BlastP on this gene

EMD89127

hypothetical protein
  
Accession: EMD89126
  
Location: 389205-389747
  
 NCBI BlastP on this gene

EMD89126

hypothetical protein
  
Accession: EMD89125
  
Location: 388130-388999
  
 NCBI BlastP on this gene

EMD89125

hypothetical protein
  
Accession: EMD89124
  
Location: 386690-387655
  
 NCBI BlastP on this gene

EMD89124

hypothetical protein
  
Accession: EMD89123
  
Location: 385518-386439
  
 NCBI BlastP on this gene

EMD89123

glycosyltransferase family 69 protein
  
Accession: EMD89122
  
Location: 383363-384779
  
 NCBI BlastP on this gene

EMD89122

hypothetical protein
  
Accession: EMD89121
  
Location: 380322-382151
  
 NCBI BlastP on this gene

EMD89121

hypothetical protein
  
Accession: EMD89120
  
Location: 377516-379737
  
 NCBI BlastP on this gene

EMD89120

hypothetical protein
  
Accession: EMD89119
  
Location: 375753-377108
  
 NCBI BlastP on this gene

EMD89119

Query: Architecture Search FASTA input

KB445649 : Cochliobolus sativus ND90Pr unplaced genomic scaffold COCSAscaffold\_13    Total score: 2.0     Cumulative Blast bit score: 1211

Hit cluster cross-links:

Mycgr3G67791 Mycgr3T
  
Location: 0-1542

Mycgr3G67791\_Mycgr3T

Mycgr3G90406 Mycgr3T
  
Location: 1642-3973

Mycgr3G90406\_Mycgr3T

Mycgr3G67785 Mycgr3T
  
Location: 4073-7865

Mycgr3G67785\_Mycgr3T

Mycgr3G67795 Mycgr3T
  
Location: 7965-15249

Mycgr3G67795\_Mycgr3T

Mycgr3G67775 Mycgr3T
  
Location: 15349-16237

Mycgr3G67775\_Mycgr3T

Mycgr3G90404 Mycgr3T
  
Location: 16337-17246

Mycgr3G90404\_Mycgr3T

Mycgr3G36951 Mycgr3T
  
Location: 17346-30891

Mycgr3G36951\_Mycgr3T

Mycgr3G103034 Mycgr3
  
Location: 30991-32644

Mycgr3G103034\_Mycgr3

Mycgr3G31119 Mycgr3T
  
Location: 32744-32906

Mycgr3G31119\_Mycgr3T

Mycgr3G28587 Mycgr3T
  
Location: 33006-33489

Mycgr3G28587\_Mycgr3T

Mycgr3G98959 Mycgr3T
  
Location: 33589-35035

Mycgr3G98959\_Mycgr3T

Mycgr3G35447 Mycgr3T
  
Location: 35135-36443

Mycgr3G35447\_Mycgr3T

Mycgr3G84402 Mycgr3T
  
Location: 36543-37884

Mycgr3G84402\_Mycgr3T

Mycgr3G98961 Mycgr3T
  
Location: 37984-38884

Mycgr3G98961\_Mycgr3T

hypothetical protein
  
Accession: EMD60915
  
Location: 478696-478887
  
 NCBI BlastP on this gene

EMD60915

hypothetical protein
  
Accession: EMD60914
  
Location: 476339-477172
  
 NCBI BlastP on this gene

EMD60914

hypothetical protein
  
Accession: EMD60913
  
Location: 474258-475951
  
 NCBI BlastP on this gene

EMD60913

hypothetical protein
  
Accession: EMD60912
  
Location: 472515-474065
  
 NCBI BlastP on this gene

EMD60912

hypothetical protein
  
Accession: EMD60911
  
Location: 470290-471514
  
 NCBI BlastP on this gene

EMD60911

hypothetical protein
  
Accession: EMD60910
  
Location: 467314-468790
  
 NCBI BlastP on this gene

EMD60910

hypothetical protein
  
Accession: EMD60909
  
Location: 463467-466818
  
 NCBI BlastP on this gene

EMD60909

hypothetical protein
  
Accession: EMD60908
  
Location: 461453-462975
  
 NCBI BlastP on this gene

EMD60908

hypothetical protein
  
Accession: EMD60907
  
Location: 459450-460964
  
 NCBI BlastP on this gene

EMD60907

hypothetical protein
  
Accession: EMD60906
  
Location: 457736-458802
  
  
**BlastP hit with Mycgr3G67775\_Mycgr3T**
  
Percentage identity: 67 %
  
BlastP bit score: 414
  
Sequence coverage: 98 %
  
E-value: 3e-142
  
  
 NCBI BlastP on this gene

EMD60906

hypothetical protein
  
Accession: EMD60905
  
Location: 456975-457472
  
 NCBI BlastP on this gene

EMD60905

hypothetical protein
  
Accession: EMD60904
  
Location: 454877-456367
  
  
**BlastP hit with Mycgr3G98959\_Mycgr3T**
  
Percentage identity: 79 %
  
BlastP bit score: 797
  
Sequence coverage: 99 %
  
E-value: 0.0
  
  
 NCBI BlastP on this gene

EMD60904

hypothetical protein
  
Accession: EMD60903
  
Location: 454279-454578
  
 NCBI BlastP on this gene

EMD60903

hypothetical protein
  
Accession: EMD60902
  
Location: 453239-454037
  
 NCBI BlastP on this gene

EMD60902

hypothetical protein
  
Accession: EMD60901
  
Location: 451756-452339
  
 NCBI BlastP on this gene

EMD60901

hypothetical protein
  
Accession: EMD60900
  
Location: 449773-451512
  
 NCBI BlastP on this gene

EMD60900

hypothetical protein
  
Accession: EMD60899
  
Location: 448967-449439
  
 NCBI BlastP on this gene

EMD60899

hypothetical protein
  
Accession: EMD60898
  
Location: 447823-448691
  
 NCBI BlastP on this gene

EMD60898

hypothetical protein
  
Accession: EMD60897
  
Location: 446388-447348
  
 NCBI BlastP on this gene

EMD60897

hypothetical protein
  
Accession: EMD60896
  
Location: 445262-446184
  
 NCBI BlastP on this gene

EMD60896

glycosyltransferase family 69 protein
  
Accession: EMD60895
  
Location: 443054-444470
  
 NCBI BlastP on this gene

EMD60895

hypothetical protein
  
Accession: EMD60894
  
Location: 440049-441879
  
 NCBI BlastP on this gene

EMD60894

hypothetical protein
  
Accession: EMD60893
  
Location: 437263-439484
  
 NCBI BlastP on this gene

EMD60893

hypothetical protein
  
Accession: EMD60892
  
Location: 435504-436859
  
 NCBI BlastP on this gene

EMD60892

Query: Architecture Search FASTA input

KB916388 : Neofusicoccum parvum UCRNP2 chromosome Unknown NP2\_03\_scaffold\_750    Total score: 2.0     Cumulative Blast bit score: 1198

Hit cluster cross-links:

Mycgr3G67791 Mycgr3T
  
Location: 0-1542

Mycgr3G67791\_Mycgr3T

Mycgr3G90406 Mycgr3T
  
Location: 1642-3973

Mycgr3G90406\_Mycgr3T

Mycgr3G67785 Mycgr3T
  
Location: 4073-7865

Mycgr3G67785\_Mycgr3T

Mycgr3G67795 Mycgr3T
  
Location: 7965-15249

Mycgr3G67795\_Mycgr3T

Mycgr3G67775 Mycgr3T
  
Location: 15349-16237

Mycgr3G67775\_Mycgr3T

Mycgr3G90404 Mycgr3T
  
Location: 16337-17246

Mycgr3G90404\_Mycgr3T

Mycgr3G36951 Mycgr3T
  
Location: 17346-30891

Mycgr3G36951\_Mycgr3T

Mycgr3G103034 Mycgr3
  
Location: 30991-32644

Mycgr3G103034\_Mycgr3

Mycgr3G31119 Mycgr3T
  
Location: 32744-32906

Mycgr3G31119\_Mycgr3T

Mycgr3G28587 Mycgr3T
  
Location: 33006-33489

Mycgr3G28587\_Mycgr3T

Mycgr3G98959 Mycgr3T
  
Location: 33589-35035

Mycgr3G98959\_Mycgr3T

Mycgr3G35447 Mycgr3T
  
Location: 35135-36443

Mycgr3G35447\_Mycgr3T

Mycgr3G84402 Mycgr3T
  
Location: 36543-37884

Mycgr3G84402\_Mycgr3T

Mycgr3G98961 Mycgr3T
  
Location: 37984-38884

Mycgr3G98961\_Mycgr3T

hypothetical protein
  
Accession: EOD46861
  
Location: 4535-5430
  
 NCBI BlastP on this gene

EOD46861

putative family pyridoxal phosphate enzyme protein
  
Accession: EOD46859
  
Location: 6816-7797
  
 NCBI BlastP on this gene

EOD46859

putative ligninase h2 precursor protein
  
Accession: EOD46855
  
Location: 8977-10092
  
 NCBI BlastP on this gene

EOD46855

putative carbon-nitrogen family protein
  
Accession: EOD46854
  
Location: 10350-11439
  
  
**BlastP hit with Mycgr3G67775\_Mycgr3T**
  
Percentage identity: 72 %
  
BlastP bit score: 398
  
Sequence coverage: 93 %
  
E-value: 3e-136
  
  
 NCBI BlastP on this gene

EOD46854

putative eukaryotic translation initiation factor 3 protein
  
Accession: EOD46853
  
Location: 11773-13330
  
  
**BlastP hit with Mycgr3G98959\_Mycgr3T**
  
Percentage identity: 78 %
  
BlastP bit score: 800
  
Sequence coverage: 99 %
  
E-value: 0.0
  
  
 NCBI BlastP on this gene

EOD46853

putative ubiquitin-conjugating enzyme protein
  
Accession: EOD46857
  
Location: 15885-16706
  
 NCBI BlastP on this gene

EOD46857

putative outer membrane protein
  
Accession: EOD46860
  
Location: 17057-18888
  
 NCBI BlastP on this gene

EOD46860

putative major facilitator superfamily transporter protein
  
Accession: EOD46852
  
Location: 20758-22487
  
 NCBI BlastP on this gene

EOD46852

putative chromo domain-like protein
  
Accession: EOD46863
  
Location: 23402-24166
  
 NCBI BlastP on this gene

EOD46863

putative glycosyltransferase family 2 protein
  
Accession: EOD46858
  
Location: 24837-27467
  
 NCBI BlastP on this gene

EOD46858

putative alcohol dehydrogenase domain protein
  
Accession: EOD46862
  
Location: 28838-29926
  
 NCBI BlastP on this gene

EOD46862

Query: Architecture Search FASTA input

KB908844 : Setosphaeria turcica Et28A unplaced genomic scaffold SETTUscaffold\_6    Total score: 2.0     Cumulative Blast bit score: 1190

Hit cluster cross-links:

Mycgr3G67791 Mycgr3T
  
Location: 0-1542

Mycgr3G67791\_Mycgr3T

Mycgr3G90406 Mycgr3T
  
Location: 1642-3973

Mycgr3G90406\_Mycgr3T

Mycgr3G67785 Mycgr3T
  
Location: 4073-7865

Mycgr3G67785\_Mycgr3T

Mycgr3G67795 Mycgr3T
  
Location: 7965-15249

Mycgr3G67795\_Mycgr3T

Mycgr3G67775 Mycgr3T
  
Location: 15349-16237

Mycgr3G67775\_Mycgr3T

Mycgr3G90404 Mycgr3T
  
Location: 16337-17246

Mycgr3G90404\_Mycgr3T

Mycgr3G36951 Mycgr3T
  
Location: 17346-30891

Mycgr3G36951\_Mycgr3T

Mycgr3G103034 Mycgr3
  
Location: 30991-32644

Mycgr3G103034\_Mycgr3

Mycgr3G31119 Mycgr3T
  
Location: 32744-32906

Mycgr3G31119\_Mycgr3T

Mycgr3G28587 Mycgr3T
  
Location: 33006-33489

Mycgr3G28587\_Mycgr3T

Mycgr3G98959 Mycgr3T
  
Location: 33589-35035

Mycgr3G98959\_Mycgr3T

Mycgr3G35447 Mycgr3T
  
Location: 35135-36443

Mycgr3G35447\_Mycgr3T

Mycgr3G84402 Mycgr3T
  
Location: 36543-37884

Mycgr3G84402\_Mycgr3T

Mycgr3G98961 Mycgr3T
  
Location: 37984-38884

Mycgr3G98961\_Mycgr3T

hypothetical protein
  
Accession: EOA82714
  
Location: 1110971-1112664
  
 NCBI BlastP on this gene

EOA82714

hypothetical protein
  
Accession: EOA82713
  
Location: 1109035-1110581
  
 NCBI BlastP on this gene

EOA82713

hypothetical protein
  
Accession: EOA82712
  
Location: 1106885-1108110
  
 NCBI BlastP on this gene

EOA82712

hypothetical protein
  
Accession: EOA82711
  
Location: 1104057-1104263
  
 NCBI BlastP on this gene

EOA82711

hypothetical protein
  
Accession: EOA82710
  
Location: 1101260-1101972
  
 NCBI BlastP on this gene

EOA82710

hypothetical protein
  
Accession: EOA82709
  
Location: 1097519-1100699
  
 NCBI BlastP on this gene

EOA82709

hypothetical protein
  
Accession: EOA82708
  
Location: 1095233-1096741
  
 NCBI BlastP on this gene

EOA82708

hypothetical protein
  
Accession: EOA82707
  
Location: 1093268-1094755
  
 NCBI BlastP on this gene

EOA82707

hypothetical protein
  
Accession: EOA82706
  
Location: 1091694-1092799
  
  
**BlastP hit with Mycgr3G67775\_Mycgr3T**
  
Percentage identity: 65 %
  
BlastP bit score: 392
  
Sequence coverage: 99 %
  
E-value: 2e-133
  
  
 NCBI BlastP on this gene

EOA82706

hypothetical protein
  
Accession: EOA82705
  
Location: 1090922-1091401
  
 NCBI BlastP on this gene

EOA82705

hypothetical protein
  
Accession: EOA82704
  
Location: 1090633-1090839
  
 NCBI BlastP on this gene

EOA82704

hypothetical protein
  
Accession: EOA82703
  
Location: 1088505-1090073
  
  
**BlastP hit with Mycgr3G98959\_Mycgr3T**
  
Percentage identity: 79 %
  
BlastP bit score: 798
  
Sequence coverage: 99 %
  
E-value: 0.0
  
  
 NCBI BlastP on this gene

EOA82703

hypothetical protein
  
Accession: EOA82702
  
Location: 1086092-1087567
  
 NCBI BlastP on this gene

EOA82702

hypothetical protein
  
Accession: EOA82701
  
Location: 1084016-1084592
  
 NCBI BlastP on this gene

EOA82701

hypothetical protein
  
Accession: EOA82700
  
Location: 1080033-1083654
  
 NCBI BlastP on this gene

EOA82700

hypothetical protein
  
Accession: EOA82699
  
Location: 1078713-1079611
  
 NCBI BlastP on this gene

EOA82699

hypothetical protein
  
Accession: EOA82698
  
Location: 1077202-1078212
  
 NCBI BlastP on this gene

EOA82698

hypothetical protein
  
Accession: EOA82697
  
Location: 1074137-1076480
  
 NCBI BlastP on this gene

EOA82697

hypothetical protein
  
Accession: EOA82696
  
Location: 1070954-1071319
  
 NCBI BlastP on this gene

EOA82696

hypothetical protein
  
Accession: EOA82695
  
Location: 1068668-1069677
  
 NCBI BlastP on this gene

EOA82695

Query: Architecture Search FASTA input

KB446542 : Dothistroma septosporum NZE10 unplaced genomic scaffold DOTSEscaffold\_8    Total score: 2.0     Cumulative Blast bit score: 1168

Hit cluster cross-links:

Mycgr3G67791 Mycgr3T
  
Location: 0-1542

Mycgr3G67791\_Mycgr3T

Mycgr3G90406 Mycgr3T
  
Location: 1642-3973

Mycgr3G90406\_Mycgr3T

Mycgr3G67785 Mycgr3T
  
Location: 4073-7865

Mycgr3G67785\_Mycgr3T

Mycgr3G67795 Mycgr3T
  
Location: 7965-15249

Mycgr3G67795\_Mycgr3T

Mycgr3G67775 Mycgr3T
  
Location: 15349-16237

Mycgr3G67775\_Mycgr3T

Mycgr3G90404 Mycgr3T
  
Location: 16337-17246

Mycgr3G90404\_Mycgr3T

Mycgr3G36951 Mycgr3T
  
Location: 17346-30891

Mycgr3G36951\_Mycgr3T

Mycgr3G103034 Mycgr3
  
Location: 30991-32644

Mycgr3G103034\_Mycgr3

Mycgr3G31119 Mycgr3T
  
Location: 32744-32906

Mycgr3G31119\_Mycgr3T

Mycgr3G28587 Mycgr3T
  
Location: 33006-33489

Mycgr3G28587\_Mycgr3T

Mycgr3G98959 Mycgr3T
  
Location: 33589-35035

Mycgr3G98959\_Mycgr3T

Mycgr3G35447 Mycgr3T
  
Location: 35135-36443

Mycgr3G35447\_Mycgr3T

Mycgr3G84402 Mycgr3T
  
Location: 36543-37884

Mycgr3G84402\_Mycgr3T

Mycgr3G98961 Mycgr3T
  
Location: 37984-38884

Mycgr3G98961\_Mycgr3T

hypothetical protein
  
Accession: EME41143
  
Location: 45588-47045
  
 NCBI BlastP on this gene

EME41143

hypothetical protein
  
Accession: EME41144
  
Location: 48587-50944
  
 NCBI BlastP on this gene

EME41144

hypothetical protein
  
Accession: EME41145
  
Location: 51638-52564
  
 NCBI BlastP on this gene

EME41145

hypothetical protein
  
Accession: EME41146
  
Location: 52658-53671
  
 NCBI BlastP on this gene

EME41146

hypothetical protein
  
Accession: EME41147
  
Location: 55763-56281
  
 NCBI BlastP on this gene

EME41147

hypothetical protein
  
Accession: EME41148
  
Location: 57816-58352
  
 NCBI BlastP on this gene

EME41148

hypothetical protein
  
Accession: EME41149
  
Location: 59135-59722
  
 NCBI BlastP on this gene

EME41149

hypothetical protein
  
Accession: EME41150
  
Location: 60221-60966
  
  
**BlastP hit with Mycgr3G28587\_Mycgr3T**
  
Percentage identity: 67 %
  
BlastP bit score: 214
  
Sequence coverage: 98 %
  
E-value: 9e-67
  
  
 NCBI BlastP on this gene

EME41150

hypothetical protein
  
Accession: EME41151
  
Location: 61362-63005
  
  
**BlastP hit with Mycgr3G103034\_Mycgr3**
  
Percentage identity: 83 %
  
BlastP bit score: 954
  
Sequence coverage: 97 %
  
E-value: 0.0
  
  
 NCBI BlastP on this gene

EME41151

hypothetical protein
  
Accession: EME41152
  
Location: 63502-64094
  
 NCBI BlastP on this gene

EME41152

hypothetical protein
  
Accession: EME41153
  
Location: 64939-66574
  
 NCBI BlastP on this gene

EME41153

hypothetical protein
  
Accession: EME41154
  
Location: 67327-68742
  
 NCBI BlastP on this gene

EME41154

hypothetical protein
  
Accession: EME41155
  
Location: 69141-69779
  
 NCBI BlastP on this gene

EME41155

hypothetical protein
  
Accession: EME41156
  
Location: 71029-71316
  
 NCBI BlastP on this gene

EME41156

glycoside hydrolase family 17 protein
  
Accession: EME41157
  
Location: 71899-73441
  
 NCBI BlastP on this gene

EME41157

hypothetical protein
  
Accession: EME41158
  
Location: 75168-75954
  
 NCBI BlastP on this gene

EME41158

hypothetical protein
  
Accession: EME41159
  
Location: 76340-77360
  
 NCBI BlastP on this gene

EME41159

hypothetical protein
  
Accession: EME41160
  
Location: 78500-80092
  
 NCBI BlastP on this gene

EME41160

hypothetical protein
  
Accession: EME41161
  
Location: 80524-82301
  
 NCBI BlastP on this gene

EME41161

Query: Architecture Search FASTA input

FP929137 : Leptosphaeria maculans JN3 lm\_SuperContig\_10\_v2 genomic supercontig    Total score: 2.0     Cumulative Blast bit score: 1147

Hit cluster cross-links:

Mycgr3G67791 Mycgr3T
  
Location: 0-1542

Mycgr3G67791\_Mycgr3T

Mycgr3G90406 Mycgr3T
  
Location: 1642-3973

Mycgr3G90406\_Mycgr3T

Mycgr3G67785 Mycgr3T
  
Location: 4073-7865

Mycgr3G67785\_Mycgr3T

Mycgr3G67795 Mycgr3T
  
Location: 7965-15249

Mycgr3G67795\_Mycgr3T

Mycgr3G67775 Mycgr3T
  
Location: 15349-16237

Mycgr3G67775\_Mycgr3T

Mycgr3G90404 Mycgr3T
  
Location: 16337-17246

Mycgr3G90404\_Mycgr3T

Mycgr3G36951 Mycgr3T
  
Location: 17346-30891

Mycgr3G36951\_Mycgr3T

Mycgr3G103034 Mycgr3
  
Location: 30991-32644

Mycgr3G103034\_Mycgr3

Mycgr3G31119 Mycgr3T
  
Location: 32744-32906

Mycgr3G31119\_Mycgr3T

Mycgr3G28587 Mycgr3T
  
Location: 33006-33489

Mycgr3G28587\_Mycgr3T

Mycgr3G98959 Mycgr3T
  
Location: 33589-35035

Mycgr3G98959\_Mycgr3T

Mycgr3G35447 Mycgr3T
  
Location: 35135-36443

Mycgr3G35447\_Mycgr3T

Mycgr3G84402 Mycgr3T
  
Location: 36543-37884

Mycgr3G84402\_Mycgr3T

Mycgr3G98961 Mycgr3T
  
Location: 37984-38884

Mycgr3G98961\_Mycgr3T

similar to 50S ribosomal protein L13
  
Accession: CBX99973
  
Location: 1074652-1075286
  
 NCBI BlastP on this gene

LEMA\_P075620.1

similar to uricase (Urate oxidase)
  
Accession: CBX99974
  
Location: 1075727-1076734
  
 NCBI BlastP on this gene

LEMA\_P075630.1

predicted protein
  
Accession: CBX99975
  
Location: 1077007-1077357
  
 NCBI BlastP on this gene

LEMA\_P075640.1

similar to cytosolic regulator Pianissimo
  
Accession: CBX99976
  
Location: 1077750-1081780
  
 NCBI BlastP on this gene

LEMA\_P075650.1

predicted protein
  
Accession: CBX99977
  
Location: 1082390-1082554
  
 NCBI BlastP on this gene

LEMA\_uP075660.1

similar to ATP-dependent protease (CrgA)
  
Accession: CBX99978
  
Location: 1083701-1085657
  
 NCBI BlastP on this gene

LEMA\_P075670.1

hypothetical protein
  
Accession: CBX99979
  
Location: 1087383-1090859
  
 NCBI BlastP on this gene

LEMA\_P075680.1

hypothetical protein
  
Accession: CBX99980
  
Location: 1091407-1093172
  
 NCBI BlastP on this gene

LEMA\_P075690.1

hypothetical protein
  
Accession: CBX99981
  
Location: 1093422-1097357
  
  
**BlastP hit with Mycgr3G67775\_Mycgr3T**
  
Percentage identity: 57 %
  
BlastP bit score: 383
  
Sequence coverage: 114 %
  
E-value: 1e-123
  
  
 NCBI BlastP on this gene

LEMA\_P075700.1

hypothetical protein
  
Accession: CBX99982
  
Location: 1097601-1098086
  
 NCBI BlastP on this gene

LEMA\_P075710.1

hypothetical protein
  
Accession: CBX99983
  
Location: 1098830-1100267
  
  
**BlastP hit with Mycgr3G98959\_Mycgr3T**
  
Percentage identity: 81 %
  
BlastP bit score: 764
  
Sequence coverage: 91 %
  
E-value: 0.0
  
  
 NCBI BlastP on this gene

LEMA\_P075720.1

hypothetical protein
  
Accession: CBX99984
  
Location: 1101210-1102004
  
 NCBI BlastP on this gene

LEMA\_P075730.1

hypothetical protein
  
Accession: CBX99985
  
Location: 1102388-1104268
  
 NCBI BlastP on this gene

LEMA\_P075740.1

hypothetical protein
  
Accession: CBX99986
  
Location: 1104579-1105161
  
 NCBI BlastP on this gene

LEMA\_P075750.1

hypothetical protein
  
Accession: CBX99987
  
Location: 1105389-1106274
  
 NCBI BlastP on this gene

LEMA\_P075760.1

hypothetical protein
  
Accession: CBX99988
  
Location: 1106751-1107680
  
 NCBI BlastP on this gene

LEMA\_P075770.1

predicted protein
  
Accession: CBX99989
  
Location: 1108368-1109247
  
 NCBI BlastP on this gene

LEMA\_P075780.1

predicted protein
  
Accession: CBX99990
  
Location: 1109619-1109874
  
 NCBI BlastP on this gene

LEMA\_uP075790.1

similar to chromatin remodeling complex subunit (Arp8)
  
Accession: CBX99991
  
Location: 1109961-1112225
  
 NCBI BlastP on this gene

LEMA\_P075800.1

hypothetical protein
  
Accession: CBX99992
  
Location: 1113611-1114422
  
 NCBI BlastP on this gene

LEMA\_P075810.1

hypothetical protein
  
Accession: CBX99993
  
Location: 1114777-1115423
  
 NCBI BlastP on this gene

LEMA\_P075820.1

hypothetical protein
  
Accession: CBX99994
  
Location: 1116310-1118447
  
 NCBI BlastP on this gene

LEMA\_P075830.1

Query: Architecture Search FASTA input

GL573222 : Geomyces destructans 20631-21 unplaced genomic scaffold supercont1.54    Total score: 2.0     Cumulative Blast bit score: 1135

Hit cluster cross-links:

Mycgr3G67791 Mycgr3T
  
Location: 0-1542

Mycgr3G67791\_Mycgr3T

Mycgr3G90406 Mycgr3T
  
Location: 1642-3973

Mycgr3G90406\_Mycgr3T

Mycgr3G67785 Mycgr3T
  
Location: 4073-7865

Mycgr3G67785\_Mycgr3T

Mycgr3G67795 Mycgr3T
  
Location: 7965-15249

Mycgr3G67795\_Mycgr3T

Mycgr3G67775 Mycgr3T
  
Location: 15349-16237

Mycgr3G67775\_Mycgr3T

Mycgr3G90404 Mycgr3T
  
Location: 16337-17246

Mycgr3G90404\_Mycgr3T

Mycgr3G36951 Mycgr3T
  
Location: 17346-30891

Mycgr3G36951\_Mycgr3T

Mycgr3G103034 Mycgr3
  
Location: 30991-32644

Mycgr3G103034\_Mycgr3

Mycgr3G31119 Mycgr3T
  
Location: 32744-32906

Mycgr3G31119\_Mycgr3T

Mycgr3G28587 Mycgr3T
  
Location: 33006-33489

Mycgr3G28587\_Mycgr3T

Mycgr3G98959 Mycgr3T
  
Location: 33589-35035

Mycgr3G98959\_Mycgr3T

Mycgr3G35447 Mycgr3T
  
Location: 35135-36443

Mycgr3G35447\_Mycgr3T

Mycgr3G84402 Mycgr3T
  
Location: 36543-37884

Mycgr3G84402\_Mycgr3T

Mycgr3G98961 Mycgr3T
  
Location: 37984-38884

Mycgr3G98961\_Mycgr3T

protein arginine N-methyltransferase 1
  
Accession: ELR08690
  
Location: 748-1988
  
 NCBI BlastP on this gene

ELR08690

hypothetical protein
  
Accession: ELR08691
  
Location: 2434-3582
  
 NCBI BlastP on this gene

ELR08691

ATP-dependent rRNA helicase rrp3
  
Accession: ELR08692
  
Location: 3740-5246
  
  
**BlastP hit with Mycgr3G84402\_Mycgr3T**
  
Percentage identity: 74 %
  
BlastP bit score: 646
  
Sequence coverage: 94 %
  
E-value: 0.0
  
  
 NCBI BlastP on this gene

ELR08692

hypothetical protein
  
Accession: ELR08693
  
Location: 5436-6872
  
  
**BlastP hit with Mycgr3G35447\_Mycgr3T**
  
Percentage identity: 59 %
  
BlastP bit score: 489
  
Sequence coverage: 102 %
  
E-value: 7e-167
  
  
 NCBI BlastP on this gene

ELR08693

hypothetical protein
  
Accession: ELR08694
  
Location: 7716-9945
  
 NCBI BlastP on this gene

ELR08694

hypothetical protein
  
Accession: ELR08695
  
Location: 10360-13183
  
 NCBI BlastP on this gene

ELR08695

hypothetical protein
  
Accession: ELR08696
  
Location: 18305-18772
  
 NCBI BlastP on this gene

ELR08696

hypothetical protein
  
Accession: ELR08697
  
Location: 24134-24472
  
 NCBI BlastP on this gene

ELR08697

Query: Architecture Search FASTA input

KB446555 : Pseudocercospora fijiensis CIRAD86 unplaced genomic scaffold MYCFIscaffold\_1    Total score: 2.0     Cumulative Blast bit score: 1123

Hit cluster cross-links:

Mycgr3G67791 Mycgr3T
  
Location: 0-1542

Mycgr3G67791\_Mycgr3T

Mycgr3G90406 Mycgr3T
  
Location: 1642-3973

Mycgr3G90406\_Mycgr3T

Mycgr3G67785 Mycgr3T
  
Location: 4073-7865

Mycgr3G67785\_Mycgr3T

Mycgr3G67795 Mycgr3T
  
Location: 7965-15249

Mycgr3G67795\_Mycgr3T

Mycgr3G67775 Mycgr3T
  
Location: 15349-16237

Mycgr3G67775\_Mycgr3T

Mycgr3G90404 Mycgr3T
  
Location: 16337-17246

Mycgr3G90404\_Mycgr3T

Mycgr3G36951 Mycgr3T
  
Location: 17346-30891

Mycgr3G36951\_Mycgr3T

Mycgr3G103034 Mycgr3
  
Location: 30991-32644

Mycgr3G103034\_Mycgr3

Mycgr3G31119 Mycgr3T
  
Location: 32744-32906

Mycgr3G31119\_Mycgr3T

Mycgr3G28587 Mycgr3T
  
Location: 33006-33489

Mycgr3G28587\_Mycgr3T

Mycgr3G98959 Mycgr3T
  
Location: 33589-35035

Mycgr3G98959\_Mycgr3T

Mycgr3G35447 Mycgr3T
  
Location: 35135-36443

Mycgr3G35447\_Mycgr3T

Mycgr3G84402 Mycgr3T
  
Location: 36543-37884

Mycgr3G84402\_Mycgr3T

Mycgr3G98961 Mycgr3T
  
Location: 37984-38884

Mycgr3G98961\_Mycgr3T

hypothetical protein
  
Accession: EME88814
  
Location: 7467052-7469042
  
 NCBI BlastP on this gene

EME88814

hypothetical protein
  
Accession: EME88815
  
Location: 7472543-7472884
  
 NCBI BlastP on this gene

EME88815

hypothetical protein
  
Accession: EME88816
  
Location: 7475312-7475927
  
  
**BlastP hit with Mycgr3G28587\_Mycgr3T**
  
Percentage identity: 62 %
  
BlastP bit score: 199
  
Sequence coverage: 96 %
  
E-value: 2e-61
  
  
 NCBI BlastP on this gene

EME88816

hypothetical protein
  
Accession: EME88817
  
Location: 7476397-7478073
  
  
**BlastP hit with Mycgr3G103034\_Mycgr3**
  
Percentage identity: 79 %
  
BlastP bit score: 924
  
Sequence coverage: 97 %
  
E-value: 0.0
  
  
 NCBI BlastP on this gene

EME88817

hypothetical protein
  
Accession: EME88818
  
Location: 7478153-7478965
  
 NCBI BlastP on this gene

EME88818

hypothetical protein
  
Accession: EME88819
  
Location: 7479369-7479737
  
 NCBI BlastP on this gene

EME88819

hypothetical protein
  
Accession: EME88820
  
Location: 7480952-7481537
  
 NCBI BlastP on this gene

EME88820

hypothetical protein
  
Accession: EME88821
  
Location: 7483280-7484460
  
 NCBI BlastP on this gene

EME88821

hypothetical protein
  
Accession: EME88822
  
Location: 7487433-7488785
  
 NCBI BlastP on this gene

EME88822

hypothetical protein
  
Accession: EME88823
  
Location: 7489763-7493364
  
 NCBI BlastP on this gene

EME88823

hypothetical protein
  
Accession: EME88824
  
Location: 7494074-7495324
  
 NCBI BlastP on this gene

EME88824

hypothetical protein
  
Accession: EME88825
  
Location: 7496175-7497332
  
 NCBI BlastP on this gene

EME88825

hypothetical protein
  
Accession: EME88826
  
Location: 7497421-7497950
  
 NCBI BlastP on this gene

EME88826

hypothetical protein
  
Accession: EME88827
  
Location: 7497979-7499333
  
 NCBI BlastP on this gene

EME88827

Query: Architecture Search FASTA input

KB725774 : Colletotrichum orbiculare MAFF 240422 unplaced genomic scaffold Scaffold\_225    Total score: 2.0     Cumulative Blast bit score: 1109

Hit cluster cross-links:

Mycgr3G67791 Mycgr3T
  
Location: 0-1542

Mycgr3G67791\_Mycgr3T

Mycgr3G90406 Mycgr3T
  
Location: 1642-3973

Mycgr3G90406\_Mycgr3T

Mycgr3G67785 Mycgr3T
  
Location: 4073-7865

Mycgr3G67785\_Mycgr3T

Mycgr3G67795 Mycgr3T
  
Location: 7965-15249

Mycgr3G67795\_Mycgr3T

Mycgr3G67775 Mycgr3T
  
Location: 15349-16237

Mycgr3G67775\_Mycgr3T

Mycgr3G90404 Mycgr3T
  
Location: 16337-17246

Mycgr3G90404\_Mycgr3T

Mycgr3G36951 Mycgr3T
  
Location: 17346-30891

Mycgr3G36951\_Mycgr3T

Mycgr3G103034 Mycgr3
  
Location: 30991-32644

Mycgr3G103034\_Mycgr3

Mycgr3G31119 Mycgr3T
  
Location: 32744-32906

Mycgr3G31119\_Mycgr3T

Mycgr3G28587 Mycgr3T
  
Location: 33006-33489

Mycgr3G28587\_Mycgr3T

Mycgr3G98959 Mycgr3T
  
Location: 33589-35035

Mycgr3G98959\_Mycgr3T

Mycgr3G35447 Mycgr3T
  
Location: 35135-36443

Mycgr3G35447\_Mycgr3T

Mycgr3G84402 Mycgr3T
  
Location: 36543-37884

Mycgr3G84402\_Mycgr3T

Mycgr3G98961 Mycgr3T
  
Location: 37984-38884

Mycgr3G98961\_Mycgr3T

C6 transcription factor
  
Accession: ENH85445
  
Location: 4894-6218
  
 NCBI BlastP on this gene

ENH85445

phytanoyl-dioxygenase family protein
  
Accession: ENH85446
  
Location: 7596-8720
  
 NCBI BlastP on this gene

ENH85446

ribosome biogenesis protein ssf2
  
Accession: ENH85447
  
Location: 10211-11595
  
  
**BlastP hit with Mycgr3G35447\_Mycgr3T**
  
Percentage identity: 57 %
  
BlastP bit score: 472
  
Sequence coverage: 100 %
  
E-value: 1e-160
  
  
 NCBI BlastP on this gene

ENH85447

ATP-dependent rrna helicase rrp3
  
Accession: ENH85448
  
Location: 11919-13502
  
  
**BlastP hit with Mycgr3G84402\_Mycgr3T**
  
Percentage identity: 74 %
  
BlastP bit score: 637
  
Sequence coverage: 92 %
  
E-value: 0.0
  
  
 NCBI BlastP on this gene

ENH85448

hypothetical protein
  
Accession: ENH85449
  
Location: 16270-18072
  
 NCBI BlastP on this gene

ENH85449

kinesin family protein
  
Accession: ENH85450
  
Location: 21665-23848
  
 NCBI BlastP on this gene

ENH85450

transcription factor tfiiib complex subunit
  
Accession: ENH85451
  
Location: 24125-26323
  
 NCBI BlastP on this gene

ENH85451

hypothetical protein
  
Accession: ENH85452
  
Location: 27767-28395
  
 NCBI BlastP on this gene

ENH85452

hypothetical protein
  
Accession: ENH85453
  
Location: 28723-29599
  
 NCBI BlastP on this gene

ENH85453

microsomal signal peptidase 18 kda subunit
  
Accession: ENH85454
  
Location: 30546-31334
  
 NCBI BlastP on this gene

ENH85454

hypothetical protein
  
Accession: ENH85455
  
Location: 32649-33986
  
 NCBI BlastP on this gene

ENH85455

Query: Architecture Search FASTA input

AFNW01000108 : Fusarium pseudograminearum CS3096    Total score: 2.0     Cumulative Blast bit score: 1107

Hit cluster cross-links:

Mycgr3G67791 Mycgr3T
  
Location: 0-1542

Mycgr3G67791\_Mycgr3T

Mycgr3G90406 Mycgr3T
  
Location: 1642-3973

Mycgr3G90406\_Mycgr3T

Mycgr3G67785 Mycgr3T
  
Location: 4073-7865

Mycgr3G67785\_Mycgr3T

Mycgr3G67795 Mycgr3T
  
Location: 7965-15249

Mycgr3G67795\_Mycgr3T

Mycgr3G67775 Mycgr3T
  
Location: 15349-16237

Mycgr3G67775\_Mycgr3T

Mycgr3G90404 Mycgr3T
  
Location: 16337-17246

Mycgr3G90404\_Mycgr3T

Mycgr3G36951 Mycgr3T
  
Location: 17346-30891

Mycgr3G36951\_Mycgr3T

Mycgr3G103034 Mycgr3
  
Location: 30991-32644

Mycgr3G103034\_Mycgr3

Mycgr3G31119 Mycgr3T
  
Location: 32744-32906

Mycgr3G31119\_Mycgr3T

Mycgr3G28587 Mycgr3T
  
Location: 33006-33489

Mycgr3G28587\_Mycgr3T

Mycgr3G98959 Mycgr3T
  
Location: 33589-35035

Mycgr3G98959\_Mycgr3T

Mycgr3G35447 Mycgr3T
  
Location: 35135-36443

Mycgr3G35447\_Mycgr3T

Mycgr3G84402 Mycgr3T
  
Location: 36543-37884

Mycgr3G84402\_Mycgr3T

Mycgr3G98961 Mycgr3T
  
Location: 37984-38884

Mycgr3G98961\_Mycgr3T

hypothetical protein
  
Accession: EKJ74497
  
Location: 154714-157641
  
 NCBI BlastP on this gene

EKJ74497

hypothetical protein
  
Accession: EKJ74498
  
Location: 158717-159256
  
 NCBI BlastP on this gene

EKJ74498

hypothetical protein
  
Accession: EKJ74499
  
Location: 160262-162620
  
 NCBI BlastP on this gene

EKJ74499

hypothetical protein
  
Accession: EKJ74500
  
Location: 163127-169341
  
 NCBI BlastP on this gene

EKJ74500

hypothetical protein
  
Accession: EKJ74501
  
Location: 169599-170674
  
 NCBI BlastP on this gene

EKJ74501

hypothetical protein
  
Accession: EKJ74502
  
Location: 171582-173095
  
  
**BlastP hit with Mycgr3G84402\_Mycgr3T**
  
Percentage identity: 74 %
  
BlastP bit score: 630
  
Sequence coverage: 90 %
  
E-value: 0.0
  
  
 NCBI BlastP on this gene

EKJ74502

hypothetical protein
  
Accession: EKJ74503
  
Location: 173317-174731
  
  
**BlastP hit with Mycgr3G35447\_Mycgr3T**
  
Percentage identity: 59 %
  
BlastP bit score: 477
  
Sequence coverage: 101 %
  
E-value: 2e-162
  
  
 NCBI BlastP on this gene

EKJ74503

hypothetical protein
  
Accession: EKJ74504
  
Location: 175412-176909
  
 NCBI BlastP on this gene

EKJ74504

hypothetical protein
  
Accession: EKJ74505
  
Location: 183029-184451
  
 NCBI BlastP on this gene

EKJ74505

hypothetical protein
  
Accession: EKJ74506
  
Location: 185276-187230
  
 NCBI BlastP on this gene

EKJ74506

hypothetical protein
  
Accession: EKJ74507
  
Location: 188396-188722
  
 NCBI BlastP on this gene

EKJ74507

hypothetical protein
  
Accession: EKJ74508
  
Location: 189489-190139
  
 NCBI BlastP on this gene

EKJ74508

hypothetical protein
  
Accession: EKJ74509
  
Location: 193267-194877
  
 NCBI BlastP on this gene

EKJ74509

Query: Architecture Search FASTA input

GG698928 : Nectria haematococca mpVI 77-13-4 chromosome 6 genomic scaffold NECHAsca\_37\_chr6\_2\_0    Total score: 2.0     Cumulative Blast bit score: 1099

Hit cluster cross-links:

Mycgr3G67791 Mycgr3T
  
Location: 0-1542

Mycgr3G67791\_Mycgr3T

Mycgr3G90406 Mycgr3T
  
Location: 1642-3973

Mycgr3G90406\_Mycgr3T

Mycgr3G67785 Mycgr3T
  
Location: 4073-7865

Mycgr3G67785\_Mycgr3T

Mycgr3G67795 Mycgr3T
  
Location: 7965-15249

Mycgr3G67795\_Mycgr3T

Mycgr3G67775 Mycgr3T
  
Location: 15349-16237

Mycgr3G67775\_Mycgr3T

Mycgr3G90404 Mycgr3T
  
Location: 16337-17246

Mycgr3G90404\_Mycgr3T

Mycgr3G36951 Mycgr3T
  
Location: 17346-30891

Mycgr3G36951\_Mycgr3T

Mycgr3G103034 Mycgr3
  
Location: 30991-32644

Mycgr3G103034\_Mycgr3

Mycgr3G31119 Mycgr3T
  
Location: 32744-32906

Mycgr3G31119\_Mycgr3T

Mycgr3G28587 Mycgr3T
  
Location: 33006-33489

Mycgr3G28587\_Mycgr3T

Mycgr3G98959 Mycgr3T
  
Location: 33589-35035

Mycgr3G98959\_Mycgr3T

Mycgr3G35447 Mycgr3T
  
Location: 35135-36443

Mycgr3G35447\_Mycgr3T

Mycgr3G84402 Mycgr3T
  
Location: 36543-37884

Mycgr3G84402\_Mycgr3T

Mycgr3G98961 Mycgr3T
  
Location: 37984-38884

Mycgr3G98961\_Mycgr3T

hypothetical protein
  
Accession: EEU36455
  
Location: 189030-190028
  
 NCBI BlastP on this gene

EEU36455

predicted protein
  
Accession: EEU36528
  
Location: 191331-193247
  
 NCBI BlastP on this gene

EEU36528

hypothetical protein
  
Accession: EEU36529
  
Location: 195028-197121
  
 NCBI BlastP on this gene

EEU36529

hypothetical protein
  
Accession: EEU36456
  
Location: 197582-198688
  
 NCBI BlastP on this gene

EEU36456

hypothetical protein
  
Accession: EEU36457
  
Location: 198992-200484
  
 NCBI BlastP on this gene

EEU36457

hypothetical protein
  
Accession: EEU36530
  
Location: 200849-202959
  
 NCBI BlastP on this gene

EEU36530

hypothetical protein
  
Accession: EEU36531
  
Location: 203399-204909
  
 NCBI BlastP on this gene

EEU36531

predicted protein
  
Accession: EEU36532
  
Location: 205551-206965
  
  
**BlastP hit with Mycgr3G35447\_Mycgr3T**
  
Percentage identity: 59 %
  
BlastP bit score: 480
  
Sequence coverage: 101 %
  
E-value: 1e-163
  
  
 NCBI BlastP on this gene

EEU36532

predicted protein
  
Accession: EEU36458
  
Location: 207184-208708
  
  
**BlastP hit with Mycgr3G84402\_Mycgr3T**
  
Percentage identity: 72 %
  
BlastP bit score: 619
  
Sequence coverage: 90 %
  
E-value: 0.0
  
  
 NCBI BlastP on this gene

EEU36458

predicted protein
  
Accession: EEU36459
  
Location: 209551-210620
  
 NCBI BlastP on this gene

EEU36459

hypothetical protein
  
Accession: EEU36460
  
Location: 211663-212799
  
 NCBI BlastP on this gene

EEU36460

predicted protein
  
Accession: EEU36533
  
Location: 213026-219247
  
 NCBI BlastP on this gene

EEU36533

predicted protein
  
Accession: EEU36461
  
Location: 219715-222067
  
 NCBI BlastP on this gene

EEU36461

predicted protein
  
Accession: EEU36462
  
Location: 224479-225694
  
 NCBI BlastP on this gene

EEU36462

hypothetical protein
  
Accession: EEU36534
  
Location: 226075-226269
  
 NCBI BlastP on this gene

EEU36534

hypothetical protein
  
Accession: EEU36463
  
Location: 227289-227980
  
 NCBI BlastP on this gene

EEU36463

hypothetical protein
  
Accession: EEU36535
  
Location: 228010-228942
  
 NCBI BlastP on this gene

EEU36535

Query: Architecture Search FASTA input

KB726989 : Fusarium oxysporum f. sp. cubense race 4 unplaced genomic scaffold scaffold3    Total score: 2.0     Cumulative Blast bit score: 1094

Hit cluster cross-links:

Mycgr3G67791 Mycgr3T
  
Location: 0-1542

Mycgr3G67791\_Mycgr3T

Mycgr3G90406 Mycgr3T
  
Location: 1642-3973

Mycgr3G90406\_Mycgr3T

Mycgr3G67785 Mycgr3T
  
Location: 4073-7865

Mycgr3G67785\_Mycgr3T

Mycgr3G67795 Mycgr3T
  
Location: 7965-15249

Mycgr3G67795\_Mycgr3T

Mycgr3G67775 Mycgr3T
  
Location: 15349-16237

Mycgr3G67775\_Mycgr3T

Mycgr3G90404 Mycgr3T
  
Location: 16337-17246

Mycgr3G90404\_Mycgr3T

Mycgr3G36951 Mycgr3T
  
Location: 17346-30891

Mycgr3G36951\_Mycgr3T

Mycgr3G103034 Mycgr3
  
Location: 30991-32644

Mycgr3G103034\_Mycgr3

Mycgr3G31119 Mycgr3T
  
Location: 32744-32906

Mycgr3G31119\_Mycgr3T

Mycgr3G28587 Mycgr3T
  
Location: 33006-33489

Mycgr3G28587\_Mycgr3T

Mycgr3G98959 Mycgr3T
  
Location: 33589-35035

Mycgr3G98959\_Mycgr3T

Mycgr3G35447 Mycgr3T
  
Location: 35135-36443

Mycgr3G35447\_Mycgr3T

Mycgr3G84402 Mycgr3T
  
Location: 36543-37884

Mycgr3G84402\_Mycgr3T

Mycgr3G98961 Mycgr3T
  
Location: 37984-38884

Mycgr3G98961\_Mycgr3T

hypothetical protein
  
Accession: EMT65795
  
Location: 699025-700954
  
 NCBI BlastP on this gene

EMT65795

hypothetical protein
  
Accession: EMT65796
  
Location: 701931-702861
  
 NCBI BlastP on this gene

EMT65796

hypothetical protein
  
Accession: EMT65797
  
Location: 705037-705733
  
 NCBI BlastP on this gene

EMT65797

hypothetical protein
  
Accession: EMT65798
  
Location: 707664-709358
  
 NCBI BlastP on this gene

EMT65798

hypothetical protein
  
Accession: EMT65799
  
Location: 710358-711452
  
 NCBI BlastP on this gene

EMT65799

hypothetical protein
  
Accession: EMT65800
  
Location: 711951-714139
  
 NCBI BlastP on this gene

EMT65800

hypothetical protein
  
Accession: EMT65801
  
Location: 714578-716239
  
 NCBI BlastP on this gene

EMT65801

Protein phosphatase 2C like protein C10F6.17c
  
Accession: EMT65802
  
Location: 716780-718269
  
 NCBI BlastP on this gene

EMT65802

Brix domain-containing protein C1B9.03c
  
Accession: EMT65803
  
Location: 718813-721803
  
  
**BlastP hit with Mycgr3G35447\_Mycgr3T**
  
Percentage identity: 57 %
  
BlastP bit score: 465
  
Sequence coverage: 102 %
  
E-value: 9e-158
  
  
 NCBI BlastP on this gene

EMT65803

ATP-dependent rRNA helicase RRP3
  
Accession: EMT65804
  
Location: 722022-723528
  
  
**BlastP hit with Mycgr3G84402\_Mycgr3T**
  
Percentage identity: 73 %
  
BlastP bit score: 629
  
Sequence coverage: 91 %
  
E-value: 0.0
  
  
 NCBI BlastP on this gene

EMT65804

hypothetical protein
  
Accession: EMT65805
  
Location: 724417-725480
  
 NCBI BlastP on this gene

EMT65805

Histone transcription regulator 3 like protein
  
Accession: EMT65806
  
Location: 725800-726998
  
 NCBI BlastP on this gene

EMT65806

Histone transcription regulator 3 like protein
  
Accession: EMT65807
  
Location: 727919-734077
  
 NCBI BlastP on this gene

EMT65807

hypothetical protein
  
Accession: EMT65808
  
Location: 734589-736954
  
 NCBI BlastP on this gene

EMT65808

Ubiquitin-conjugating enzyme E2 14
  
Accession: EMT65809
  
Location: 738128-738657
  
 NCBI BlastP on this gene

EMT65809

GTPase-activating protein gyp3
  
Accession: EMT65810
  
Location: 740423-743380
  
 NCBI BlastP on this gene

EMT65810

Query: Architecture Search FASTA input

KE375219 : Blumeria graminis f. sp. tritici 96224 unplaced genomic scaffold Scaffold-92    Total score: 2.0     Cumulative Blast bit score: 1093

Hit cluster cross-links:

Mycgr3G67791 Mycgr3T
  
Location: 0-1542

Mycgr3G67791\_Mycgr3T

Mycgr3G90406 Mycgr3T
  
Location: 1642-3973

Mycgr3G90406\_Mycgr3T

Mycgr3G67785 Mycgr3T
  
Location: 4073-7865

Mycgr3G67785\_Mycgr3T

Mycgr3G67795 Mycgr3T
  
Location: 7965-15249

Mycgr3G67795\_Mycgr3T

Mycgr3G67775 Mycgr3T
  
Location: 15349-16237

Mycgr3G67775\_Mycgr3T

Mycgr3G90404 Mycgr3T
  
Location: 16337-17246

Mycgr3G90404\_Mycgr3T

Mycgr3G36951 Mycgr3T
  
Location: 17346-30891

Mycgr3G36951\_Mycgr3T

Mycgr3G103034 Mycgr3
  
Location: 30991-32644

Mycgr3G103034\_Mycgr3

Mycgr3G31119 Mycgr3T
  
Location: 32744-32906

Mycgr3G31119\_Mycgr3T

Mycgr3G28587 Mycgr3T
  
Location: 33006-33489

Mycgr3G28587\_Mycgr3T

Mycgr3G98959 Mycgr3T
  
Location: 33589-35035

Mycgr3G98959\_Mycgr3T

Mycgr3G35447 Mycgr3T
  
Location: 35135-36443

Mycgr3G35447\_Mycgr3T

Mycgr3G84402 Mycgr3T
  
Location: 36543-37884

Mycgr3G84402\_Mycgr3T

Mycgr3G98961 Mycgr3T
  
Location: 37984-38884

Mycgr3G98961\_Mycgr3T

hypothetical protein
  
Accession: EPQ61605
  
Location: 299889-301402
  
  
**BlastP hit with Mycgr3G84402\_Mycgr3T**
  
Percentage identity: 74 %
  
BlastP bit score: 634
  
Sequence coverage: 92 %
  
E-value: 0.0
  
  
 NCBI BlastP on this gene

EPQ61605

Constituent of 66S pre-ribosomal particles
  
Accession: EPQ61606
  
Location: 301622-303007
  
  
**BlastP hit with Mycgr3G35447\_Mycgr3T**
  
Percentage identity: 55 %
  
BlastP bit score: 459
  
Sequence coverage: 101 %
  
E-value: 1e-155
  
  
 NCBI BlastP on this gene

EPQ61606

hypothetical protein
  
Accession: EPQ61607
  
Location: 304099-305663
  
 NCBI BlastP on this gene

EPQ61607

Catalase
  
Accession: EPQ61608
  
Location: 312338-314743
  
 NCBI BlastP on this gene

EPQ61608

ATPase of the 19S regulatory particle of the 26S proteasome
  
Accession: EPQ61609
  
Location: 319859-321344
  
 NCBI BlastP on this gene

EPQ61609

Transcription factor TFIIB
  
Accession: EPQ61610
  
Location: 322091-323175
  
 NCBI BlastP on this gene

EPQ61610

Query: Architecture Search FASTA input

CAUH01001323 : Blumeria graminis f. sp. hordei DH14    Total score: 2.0     Cumulative Blast bit score: 1093

Hit cluster cross-links:

Mycgr3G67791 Mycgr3T
  
Location: 0-1542

Mycgr3G67791\_Mycgr3T

Mycgr3G90406 Mycgr3T
  
Location: 1642-3973

Mycgr3G90406\_Mycgr3T

Mycgr3G67785 Mycgr3T
  
Location: 4073-7865

Mycgr3G67785\_Mycgr3T

Mycgr3G67795 Mycgr3T
  
Location: 7965-15249

Mycgr3G67795\_Mycgr3T

Mycgr3G67775 Mycgr3T
  
Location: 15349-16237

Mycgr3G67775\_Mycgr3T

Mycgr3G90404 Mycgr3T
  
Location: 16337-17246

Mycgr3G90404\_Mycgr3T

Mycgr3G36951 Mycgr3T
  
Location: 17346-30891

Mycgr3G36951\_Mycgr3T

Mycgr3G103034 Mycgr3
  
Location: 30991-32644

Mycgr3G103034\_Mycgr3

Mycgr3G31119 Mycgr3T
  
Location: 32744-32906

Mycgr3G31119\_Mycgr3T

Mycgr3G28587 Mycgr3T
  
Location: 33006-33489

Mycgr3G28587\_Mycgr3T

Mycgr3G98959 Mycgr3T
  
Location: 33589-35035

Mycgr3G98959\_Mycgr3T

Mycgr3G35447 Mycgr3T
  
Location: 35135-36443

Mycgr3G35447\_Mycgr3T

Mycgr3G84402 Mycgr3T
  
Location: 36543-37884

Mycgr3G84402\_Mycgr3T

Mycgr3G98961 Mycgr3T
  
Location: 37984-38884

Mycgr3G98961\_Mycgr3T

hypothetical protein
  
Accession: CCU75429
  
Location: 297-1637
  
  
**BlastP hit with Mycgr3G35447\_Mycgr3T**
  
Percentage identity: 57 %
  
BlastP bit score: 458
  
Sequence coverage: 94 %
  
E-value: 4e-155
  
  
 NCBI BlastP on this gene

CCU75429

Putative ATP-dependent rRNA helicase
  
Accession: CCU75430
  
Location: 1856-3369
  
  
**BlastP hit with Mycgr3G84402\_Mycgr3T**
  
Percentage identity: 73 %
  
BlastP bit score: 635
  
Sequence coverage: 94 %
  
E-value: 0.0
  
  
 NCBI BlastP on this gene

CCU75430

Query: Architecture Search FASTA input

HF679031 : Fusarium fujikuroi IMI 58289 draft genome, chromosome FFUJ\_chr09.    Total score: 2.0     Cumulative Blast bit score: 1092

Hit cluster cross-links:

Mycgr3G67791 Mycgr3T
  
Location: 0-1542

Mycgr3G67791\_Mycgr3T

Mycgr3G90406 Mycgr3T
  
Location: 1642-3973

Mycgr3G90406\_Mycgr3T

Mycgr3G67785 Mycgr3T
  
Location: 4073-7865

Mycgr3G67785\_Mycgr3T

Mycgr3G67795 Mycgr3T
  
Location: 7965-15249

Mycgr3G67795\_Mycgr3T

Mycgr3G67775 Mycgr3T
  
Location: 15349-16237

Mycgr3G67775\_Mycgr3T

Mycgr3G90404 Mycgr3T
  
Location: 16337-17246

Mycgr3G90404\_Mycgr3T

Mycgr3G36951 Mycgr3T
  
Location: 17346-30891

Mycgr3G36951\_Mycgr3T

Mycgr3G103034 Mycgr3
  
Location: 30991-32644

Mycgr3G103034\_Mycgr3

Mycgr3G31119 Mycgr3T
  
Location: 32744-32906

Mycgr3G31119\_Mycgr3T

Mycgr3G28587 Mycgr3T
  
Location: 33006-33489

Mycgr3G28587\_Mycgr3T

Mycgr3G98959 Mycgr3T
  
Location: 33589-35035

Mycgr3G98959\_Mycgr3T

Mycgr3G35447 Mycgr3T
  
Location: 35135-36443

Mycgr3G35447\_Mycgr3T

Mycgr3G84402 Mycgr3T
  
Location: 36543-37884

Mycgr3G84402\_Mycgr3T

Mycgr3G98961 Mycgr3T
  
Location: 37984-38884

Mycgr3G98961\_Mycgr3T

related to RAB GTPase activator
  
Accession: CCT73724
  
Location: 2267697-2270642
  
 NCBI BlastP on this gene

FFUJ\_09458

probable ubiquitin-protein ligase UBC4
  
Accession: CCT73725
  
Location: 2272409-2272938
  
 NCBI BlastP on this gene

FFUJ\_09457

related to BUD7 protein
  
Accession: CCT73726
  
Location: 2273970-2276334
  
 NCBI BlastP on this gene

FFUJ\_09456

related to transcriptional corepressor HIR3
  
Accession: CCT73727
  
Location: 2276805-2283018
  
 NCBI BlastP on this gene

FFUJ\_09455

uncharacterized protein
  
Accession: CCT73728
  
Location: 2283346-2284404
  
 NCBI BlastP on this gene

FFUJ\_09454

probable DEAD box protein (putative RNA helicase)
  
Accession: CCT73729
  
Location: 2285361-2286864
  
  
**BlastP hit with Mycgr3G84402\_Mycgr3T**
  
Percentage identity: 73 %
  
BlastP bit score: 629
  
Sequence coverage: 91 %
  
E-value: 0.0
  
  
 NCBI BlastP on this gene

FFUJ\_09453

related to mating protein SSF1
  
Accession: CCT73730
  
Location: 2287084-2288497
  
  
**BlastP hit with Mycgr3G35447\_Mycgr3T**
  
Percentage identity: 57 %
  
BlastP bit score: 463
  
Sequence coverage: 102 %
  
E-value: 8e-157
  
  
 NCBI BlastP on this gene

FFUJ\_09452

related to Type 2C Protein Phosphatase
  
Accession: CCT73731
  
Location: 2289026-2290515
  
 NCBI BlastP on this gene

FFUJ\_09451

uncharacterized protein
  
Accession: CCT73732
  
Location: 2290886-2293081
  
 NCBI BlastP on this gene

FFUJ\_09450

related to glycerate-and formate-dehydrogenases
  
Accession: CCT73733
  
Location: 2293573-2294664
  
 NCBI BlastP on this gene

FFUJ\_09449

related to heterokaryon incompatibility protein (het-6OR allele)
  
Accession: CCT73734
  
Location: 2295473-2297530
  
 NCBI BlastP on this gene

FFUJ\_09448

related to integral membrane protein pth11
  
Accession: CCT73735
  
Location: 2299564-2300903
  
 NCBI BlastP on this gene

FFUJ\_09447

related to glu/asp-tRNA amidotransferase subunit A
  
Accession: CCT73736
  
Location: 2301959-2303888
  
 NCBI BlastP on this gene

FFUJ\_09446

uncharacterized protein
  
Accession: CCT73737
  
Location: 2304802-2305200
  
 NCBI BlastP on this gene

FFUJ\_09445

uncharacterized protein
  
Accession: CCT73738
  
Location: 2305618-2306076
  
 NCBI BlastP on this gene

FFUJ\_09444

uncharacterized protein
  
Accession: CCT73739
  
Location: 2306343-2308508
  
 NCBI BlastP on this gene

FFUJ\_09443

Query: Architecture Search FASTA input

CAGA01000008 : Claviceps purpurea 20.1    Total score: 2.0     Cumulative Blast bit score: 1092

Hit cluster cross-links:

Mycgr3G67791 Mycgr3T
  
Location: 0-1542

Mycgr3G67791\_Mycgr3T

Mycgr3G90406 Mycgr3T
  
Location: 1642-3973

Mycgr3G90406\_Mycgr3T

Mycgr3G67785 Mycgr3T
  
Location: 4073-7865

Mycgr3G67785\_Mycgr3T

Mycgr3G67795 Mycgr3T
  
Location: 7965-15249

Mycgr3G67795\_Mycgr3T

Mycgr3G67775 Mycgr3T
  
Location: 15349-16237

Mycgr3G67775\_Mycgr3T

Mycgr3G90404 Mycgr3T
  
Location: 16337-17246

Mycgr3G90404\_Mycgr3T

Mycgr3G36951 Mycgr3T
  
Location: 17346-30891

Mycgr3G36951\_Mycgr3T

Mycgr3G103034 Mycgr3
  
Location: 30991-32644

Mycgr3G103034\_Mycgr3

Mycgr3G31119 Mycgr3T
  
Location: 32744-32906

Mycgr3G31119\_Mycgr3T

Mycgr3G28587 Mycgr3T
  
Location: 33006-33489

Mycgr3G28587\_Mycgr3T

Mycgr3G98959 Mycgr3T
  
Location: 33589-35035

Mycgr3G98959\_Mycgr3T

Mycgr3G35447 Mycgr3T
  
Location: 35135-36443

Mycgr3G35447\_Mycgr3T

Mycgr3G84402 Mycgr3T
  
Location: 36543-37884

Mycgr3G84402\_Mycgr3T

Mycgr3G98961 Mycgr3T
  
Location: 37984-38884

Mycgr3G98961\_Mycgr3T

uncharacterized protein
  
Accession: CCE28432
  
Location: 653715-659429
  
 NCBI BlastP on this gene

CCE28432

probable signal peptidase (endopeptidase SP18)
  
Accession: CCE28433
  
Location: 660806-661535
  
 NCBI BlastP on this gene

CCE28433

uncharacterized protein
  
Accession: CCE28434
  
Location: 662038-662416
  
 NCBI BlastP on this gene

CCE28434

uncharacterized protein
  
Accession: CCE28435
  
Location: 662861-664112
  
 NCBI BlastP on this gene

CCE28435

related to mating protein SSF1
  
Accession: CCE28436
  
Location: 669366-670902
  
  
**BlastP hit with Mycgr3G35447\_Mycgr3T**
  
Percentage identity: 59 %
  
BlastP bit score: 471
  
Sequence coverage: 93 %
  
E-value: 9e-160
  
  
 NCBI BlastP on this gene

CCE28436

probable DEAD box protein (putative RNA helicase)
  
Accession: CCE28437
  
Location: 671539-673152
  
  
**BlastP hit with Mycgr3G84402\_Mycgr3T**
  
Percentage identity: 72 %
  
BlastP bit score: 621
  
Sequence coverage: 92 %
  
E-value: 0.0
  
  
 NCBI BlastP on this gene

CCE28437

related to transcriptional corepressor HIR3
  
Accession: CCE28438
  
Location: 673563-680608
  
 NCBI BlastP on this gene

CCE28438

related to BUD7 protein
  
Accession: CCE28439
  
Location: 684601-690537
  
 NCBI BlastP on this gene

CCE28439

uncharacterized protein
  
Accession: CCE28440
  
Location: 691385-692699
  
 NCBI BlastP on this gene

CCE28440

Query: Architecture Search FASTA input

JH767570 : Coniosporium apollinis CBS 100218 chromosome Unknown supercont1.17    Total score: 2.0     Cumulative Blast bit score: 1090

Hit cluster cross-links:

Mycgr3G67791 Mycgr3T
  
Location: 0-1542

Mycgr3G67791\_Mycgr3T

Mycgr3G90406 Mycgr3T
  
Location: 1642-3973

Mycgr3G90406\_Mycgr3T

Mycgr3G67785 Mycgr3T
  
Location: 4073-7865

Mycgr3G67785\_Mycgr3T

Mycgr3G67795 Mycgr3T
  
Location: 7965-15249

Mycgr3G67795\_Mycgr3T

Mycgr3G67775 Mycgr3T
  
Location: 15349-16237

Mycgr3G67775\_Mycgr3T

Mycgr3G90404 Mycgr3T
  
Location: 16337-17246

Mycgr3G90404\_Mycgr3T

Mycgr3G36951 Mycgr3T
  
Location: 17346-30891

Mycgr3G36951\_Mycgr3T

Mycgr3G103034 Mycgr3
  
Location: 30991-32644

Mycgr3G103034\_Mycgr3

Mycgr3G31119 Mycgr3T
  
Location: 32744-32906

Mycgr3G31119\_Mycgr3T

Mycgr3G28587 Mycgr3T
  
Location: 33006-33489

Mycgr3G28587\_Mycgr3T

Mycgr3G98959 Mycgr3T
  
Location: 33589-35035

Mycgr3G98959\_Mycgr3T

Mycgr3G35447 Mycgr3T
  
Location: 35135-36443

Mycgr3G35447\_Mycgr3T

Mycgr3G84402 Mycgr3T
  
Location: 36543-37884

Mycgr3G84402\_Mycgr3T

Mycgr3G98961 Mycgr3T
  
Location: 37984-38884

Mycgr3G98961\_Mycgr3T

hypothetical protein
  
Accession: EON64871
  
Location: 432594-434000
  
 NCBI BlastP on this gene

EON64871

hypothetical protein
  
Accession: EON64872
  
Location: 435829-437528
  
 NCBI BlastP on this gene

EON64872

betaine-aldehyde dehydrogenase
  
Accession: EON64873
  
Location: 438386-439894
  
 NCBI BlastP on this gene

EON64873

hypothetical protein
  
Accession: EON64874
  
Location: 441134-442534
  
 NCBI BlastP on this gene

EON64874

hypothetical protein
  
Accession: EON64875
  
Location: 442994-444396
  
 NCBI BlastP on this gene

EON64875

hypothetical protein
  
Accession: EON64876
  
Location: 445265-447159
  
 NCBI BlastP on this gene

EON64876

hypothetical protein
  
Accession: EON64877
  
Location: 448023-449093
  
 NCBI BlastP on this gene

EON64877

hypothetical protein
  
Accession: EON64878
  
Location: 449566-450570
  
 NCBI BlastP on this gene

EON64878

hypothetical protein
  
Accession: EON64879
  
Location: 451043-452502
  
  
**BlastP hit with Mycgr3G35447\_Mycgr3T**
  
Percentage identity: 61 %
  
BlastP bit score: 494
  
Sequence coverage: 96 %
  
E-value: 7e-169
  
  
 NCBI BlastP on this gene

EON64879

ATP-dependent rRNA helicase RRP3
  
Accession: EON64880
  
Location: 452703-454573
  
  
**BlastP hit with Mycgr3G84402\_Mycgr3T**
  
Percentage identity: 67 %
  
BlastP bit score: 596
  
Sequence coverage: 96 %
  
E-value: 0.0
  
  
 NCBI BlastP on this gene

EON64880

hypothetical protein
  
Accession: EON64881
  
Location: 456677-458207
  
 NCBI BlastP on this gene

EON64881

acetolactate synthase, large subunit, biosynthetic type
  
Accession: EON64882
  
Location: 458578-460791
  
 NCBI BlastP on this gene

EON64882

hypothetical protein
  
Accession: EON64883
  
Location: 461458-462339
  
 NCBI BlastP on this gene

EON64883

hypothetical protein
  
Accession: EON64884
  
Location: 462830-465869
  
 NCBI BlastP on this gene

EON64884

golgi apparatus membrane protein TVP18
  
Accession: EON64885
  
Location: 467401-468171
  
 NCBI BlastP on this gene

EON64885

nuclear transport factor 2
  
Accession: EON64886
  
Location: 468857-469591
  
 NCBI BlastP on this gene

EON64886

dihydroflavonol-4-reductase
  
Accession: EON64887
  
Location: 470308-471441
  
 NCBI BlastP on this gene

EON64887

succinyl-CoA ligase [GDP-forming] subunit beta, mitochondrial
  
Accession: EON64888
  
Location: 472071-473677
  
 NCBI BlastP on this gene

EON64888

hypothetical protein
  
Accession: EON64889
  
Location: 473874-474641
  
 NCBI BlastP on this gene

EON64889

Query: Architecture Search FASTA input

CP003010 : Thielavia terrestris NRRL 8126 chromosome 2    Total score: 2.0     Cumulative Blast bit score: 1090

Hit cluster cross-links:

Mycgr3G67791 Mycgr3T
  
Location: 0-1542

Mycgr3G67791\_Mycgr3T

Mycgr3G90406 Mycgr3T
  
Location: 1642-3973

Mycgr3G90406\_Mycgr3T

Mycgr3G67785 Mycgr3T
  
Location: 4073-7865

Mycgr3G67785\_Mycgr3T

Mycgr3G67795 Mycgr3T
  
Location: 7965-15249

Mycgr3G67795\_Mycgr3T

Mycgr3G67775 Mycgr3T
  
Location: 15349-16237

Mycgr3G67775\_Mycgr3T

Mycgr3G90404 Mycgr3T
  
Location: 16337-17246

Mycgr3G90404\_Mycgr3T

Mycgr3G36951 Mycgr3T
  
Location: 17346-30891

Mycgr3G36951\_Mycgr3T

Mycgr3G103034 Mycgr3
  
Location: 30991-32644

Mycgr3G103034\_Mycgr3

Mycgr3G31119 Mycgr3T
  
Location: 32744-32906

Mycgr3G31119\_Mycgr3T

Mycgr3G28587 Mycgr3T
  
Location: 33006-33489

Mycgr3G28587\_Mycgr3T

Mycgr3G98959 Mycgr3T
  
Location: 33589-35035

Mycgr3G98959\_Mycgr3T

Mycgr3G35447 Mycgr3T
  
Location: 35135-36443

Mycgr3G35447\_Mycgr3T

Mycgr3G84402 Mycgr3T
  
Location: 36543-37884

Mycgr3G84402\_Mycgr3T

Mycgr3G98961 Mycgr3T
  
Location: 37984-38884

Mycgr3G98961\_Mycgr3T

hypothetical protein
  
Accession: AEO66451
  
Location: 5811902-5813355
  
 NCBI BlastP on this gene

THITE\_2087927

hypothetical protein
  
Accession: AEO66452
  
Location: 5813844-5814124
  
 NCBI BlastP on this gene

THITE\_152050

hypothetical protein
  
Accession: AEO66453
  
Location: 5814444-5815139
  
 NCBI BlastP on this gene

THITE\_2044327

hypothetical protein
  
Accession: AEO66454
  
Location: 5817233-5820816
  
 NCBI BlastP on this gene

THITE\_2144016

glycoside hydrolase family 18 protein
  
Accession: AEO66455
  
Location: 5821722-5823366
  
 NCBI BlastP on this gene

THITE\_35493

hypothetical protein
  
Accession: AEO66456
  
Location: 5827717-5828775
  
 NCBI BlastP on this gene

THITE\_2128521

hypothetical protein
  
Accession: AEO66457
  
Location: 5830219-5830697
  
 NCBI BlastP on this gene

THITE\_2114558

hypothetical protein
  
Accession: AEO66458
  
Location: 5831396-5832863
  
  
**BlastP hit with Mycgr3G35447\_Mycgr3T**
  
Percentage identity: 56 %
  
BlastP bit score: 463
  
Sequence coverage: 102 %
  
E-value: 1e-156
  
  
 NCBI BlastP on this gene

THITE\_2114560

hypothetical protein
  
Accession: AEO66459
  
Location: 5833120-5834823
  
  
**BlastP hit with Mycgr3G84402\_Mycgr3T**
  
Percentage identity: 75 %
  
BlastP bit score: 627
  
Sequence coverage: 89 %
  
E-value: 0.0
  
  
 NCBI BlastP on this gene

THITE\_2114564

hypothetical protein
  
Accession: AEO66460
  
Location: 5835023-5837227
  
 NCBI BlastP on this gene

THITE\_2144021

hypothetical protein
  
Accession: AEO66461
  
Location: 5839789-5841096
  
 NCBI BlastP on this gene

THITE\_2114567

hypothetical protein
  
Accession: AEO66462
  
Location: 5842190-5843449
  
 NCBI BlastP on this gene

THITE\_2114569

hypothetical protein
  
Accession: AEO66463
  
Location: 5843915-5844154
  
 NCBI BlastP on this gene

THITE\_2114571

hypothetical protein
  
Accession: AEO66464
  
Location: 5844667-5845299
  
 NCBI BlastP on this gene

THITE\_2114572

hypothetical protein
  
Accession: AEO66465
  
Location: 5845528-5846547
  
 NCBI BlastP on this gene

THITE\_125923

hypothetical protein
  
Accession: AEO66466
  
Location: 5847316-5849756
  
 NCBI BlastP on this gene

THITE\_2114578

hypothetical protein
  
Accession: AEO66467
  
Location: 5850466-5856661
  
 NCBI BlastP on this gene

THITE\_110621

Query: Architecture Search FASTA input

DS572814 : Paracoccidioides brasiliensis Pb01 supercont1.4 genomic scaffold    Total score: 2.0     Cumulative Blast bit score: 1088

Hit cluster cross-links:

Mycgr3G67791 Mycgr3T
  
Location: 0-1542

Mycgr3G67791\_Mycgr3T

Mycgr3G90406 Mycgr3T
  
Location: 1642-3973

Mycgr3G90406\_Mycgr3T

Mycgr3G67785 Mycgr3T
  
Location: 4073-7865

Mycgr3G67785\_Mycgr3T

Mycgr3G67795 Mycgr3T
  
Location: 7965-15249

Mycgr3G67795\_Mycgr3T

Mycgr3G67775 Mycgr3T
  
Location: 15349-16237

Mycgr3G67775\_Mycgr3T

Mycgr3G90404 Mycgr3T
  
Location: 16337-17246

Mycgr3G90404\_Mycgr3T

Mycgr3G36951 Mycgr3T
  
Location: 17346-30891

Mycgr3G36951\_Mycgr3T

Mycgr3G103034 Mycgr3
  
Location: 30991-32644

Mycgr3G103034\_Mycgr3

Mycgr3G31119 Mycgr3T
  
Location: 32744-32906

Mycgr3G31119\_Mycgr3T

Mycgr3G28587 Mycgr3T
  
Location: 33006-33489

Mycgr3G28587\_Mycgr3T

Mycgr3G98959 Mycgr3T
  
Location: 33589-35035

Mycgr3G98959\_Mycgr3T

Mycgr3G35447 Mycgr3T
  
Location: 35135-36443

Mycgr3G35447\_Mycgr3T

Mycgr3G84402 Mycgr3T
  
Location: 36543-37884

Mycgr3G84402\_Mycgr3T

Mycgr3G98961 Mycgr3T
  
Location: 37984-38884

Mycgr3G98961\_Mycgr3T

conserved hypothetical protein
  
Accession: EEH39736
  
Location: 544545-547339
  
 NCBI BlastP on this gene

EEH39736

conserved hypothetical protein
  
Accession: EEH39737
  
Location: 548836-549732
  
 NCBI BlastP on this gene

EEH39737

conserved hypothetical protein
  
Accession: EEH39738
  
Location: 550393-552444
  
 NCBI BlastP on this gene

EEH39738

peroxisomal dehydratase
  
Accession: EEH39739
  
Location: 552949-554277
  
 NCBI BlastP on this gene

EEH39739

HNRNP arginine N-methyltransferase
  
Accession: EEH39740
  
Location: 555127-556297
  
 NCBI BlastP on this gene

EEH39740

Mn2+ homeostasis protein (Per1)
  
Accession: EEH39741
  
Location: 557299-558524
  
 NCBI BlastP on this gene

EEH39741

phosphoacetylglucosamine mutase
  
Accession: EEH39742
  
Location: 559301-561344
  
  
**BlastP hit with Mycgr3G103034\_Mycgr3**
  
Percentage identity: 56 %
  
BlastP bit score: 617
  
Sequence coverage: 98 %
  
E-value: 0.0
  
  
 NCBI BlastP on this gene

EEH39742

DEAD-box ATP-dependent RNA helicase
  
Accession: EEH39743
  
Location: 562019-562978
  
 NCBI BlastP on this gene

EEH39743

ribosome biogenesis protein SSF2
  
Accession: EEH39744
  
Location: 563829-565250
  
  
**BlastP hit with Mycgr3G35447\_Mycgr3T**
  
Percentage identity: 58 %
  
BlastP bit score: 471
  
Sequence coverage: 94 %
  
E-value: 6e-160
  
  
 NCBI BlastP on this gene

EEH39744

riboflavin synthase alpha chain
  
Accession: EEH39745
  
Location: 565681-566543
  
 NCBI BlastP on this gene

EEH39745

formyl-coenzyme A transferase
  
Accession: EEH39746
  
Location: 566844-568656
  
 NCBI BlastP on this gene

EEH39746

conserved hypothetical protein
  
Accession: EEH39747
  
Location: 569167-571020
  
 NCBI BlastP on this gene

EEH39747

DUF887 domain-containing protein
  
Accession: EEH39748
  
Location: 571803-573117
  
 NCBI BlastP on this gene

EEH39748

conserved hypothetical protein
  
Accession: EEH39749
  
Location: 574107-580197
  
 NCBI BlastP on this gene

EEH39749

60S ribosomal protein L27-A
  
Accession: EEH39750
  
Location: 580922-581705
  
 NCBI BlastP on this gene

EEH39750

Query: Architecture Search FASTA input

GL988041 : Chaetomium thermophilum var. thermophilum DSM 1495 unplaced genomic scaffold scf7180000...    Total score: 2.0     Cumulative Blast bit score: 1086

Hit cluster cross-links:

Mycgr3G67791 Mycgr3T
  
Location: 0-1542

Mycgr3G67791\_Mycgr3T

Mycgr3G90406 Mycgr3T
  
Location: 1642-3973

Mycgr3G90406\_Mycgr3T

Mycgr3G67785 Mycgr3T
  
Location: 4073-7865

Mycgr3G67785\_Mycgr3T

Mycgr3G67795 Mycgr3T
  
Location: 7965-15249

Mycgr3G67795\_Mycgr3T

Mycgr3G67775 Mycgr3T
  
Location: 15349-16237

Mycgr3G67775\_Mycgr3T

Mycgr3G90404 Mycgr3T
  
Location: 16337-17246

Mycgr3G90404\_Mycgr3T

Mycgr3G36951 Mycgr3T
  
Location: 17346-30891

Mycgr3G36951\_Mycgr3T

Mycgr3G103034 Mycgr3
  
Location: 30991-32644

Mycgr3G103034\_Mycgr3

Mycgr3G31119 Mycgr3T
  
Location: 32744-32906

Mycgr3G31119\_Mycgr3T

Mycgr3G28587 Mycgr3T
  
Location: 33006-33489

Mycgr3G28587\_Mycgr3T

Mycgr3G98959 Mycgr3T
  
Location: 33589-35035

Mycgr3G98959\_Mycgr3T

Mycgr3G35447 Mycgr3T
  
Location: 35135-36443

Mycgr3G35447\_Mycgr3T

Mycgr3G84402 Mycgr3T
  
Location: 36543-37884

Mycgr3G84402\_Mycgr3T

Mycgr3G98961 Mycgr3T
  
Location: 37984-38884

Mycgr3G98961\_Mycgr3T

hypothetical protein
  
Accession: EGS21722
  
Location: 5519429-5521812
  
 NCBI BlastP on this gene

EGS21722

reductase-like protein
  
Accession: EGS21723
  
Location: 5522684-5523702
  
 NCBI BlastP on this gene

EGS21723

hypothetical protein
  
Accession: EGS21724
  
Location: 5523844-5524470
  
 NCBI BlastP on this gene

EGS21724

hypothetical protein
  
Accession: EGS21725
  
Location: 5525019-5525330
  
 NCBI BlastP on this gene

EGS21725

putative ATP binding protein
  
Accession: EGS21726
  
Location: 5525618-5526780
  
 NCBI BlastP on this gene

EGS21726

hypothetical protein
  
Accession: EGS21727
  
Location: 5527862-5529143
  
 NCBI BlastP on this gene

EGS21727

hypothetical protein
  
Accession: EGS21728
  
Location: 5531034-5533307
  
 NCBI BlastP on this gene

EGS21728

ATP-dependent rRNA helicase rrp3-like protein
  
Accession: EGS21729
  
Location: 5533540-5535237
  
  
**BlastP hit with Mycgr3G84402\_Mycgr3T**
  
Percentage identity: 73 %
  
BlastP bit score: 635
  
Sequence coverage: 92 %
  
E-value: 0.0
  
  
 NCBI BlastP on this gene

EGS21729

hypothetical protein
  
Accession: EGS21730
  
Location: 5535480-5536975
  
  
**BlastP hit with Mycgr3G35447\_Mycgr3T**
  
Percentage identity: 53 %
  
BlastP bit score: 451
  
Sequence coverage: 104 %
  
E-value: 6e-152
  
  
 NCBI BlastP on this gene

EGS21730

hypothetical protein
  
Accession: EGS21731
  
Location: 5537355-5537872
  
 NCBI BlastP on this gene

EGS21731

hypothetical protein
  
Accession: EGS21732
  
Location: 5546294-5547626
  
 NCBI BlastP on this gene

EGS21732

hypothetical protein
  
Accession: EGS21733
  
Location: 5551428-5552237
  
 NCBI BlastP on this gene

EGS21733

hypothetical protein
  
Accession: EGS21734
  
Location: 5554093-5555609
  
 NCBI BlastP on this gene

EGS21734

Query: Architecture Search FASTA input

JH725173 : Beauveria bassiana ARSEF 2860 unplaced genomic scaffold BBA\_S00024    Total score: 2.0     Cumulative Blast bit score: 1081

Hit cluster cross-links:

Mycgr3G67791 Mycgr3T
  
Location: 0-1542

Mycgr3G67791\_Mycgr3T

Mycgr3G90406 Mycgr3T
  
Location: 1642-3973

Mycgr3G90406\_Mycgr3T

Mycgr3G67785 Mycgr3T
  
Location: 4073-7865

Mycgr3G67785\_Mycgr3T

Mycgr3G67795 Mycgr3T
  
Location: 7965-15249

Mycgr3G67795\_Mycgr3T

Mycgr3G67775 Mycgr3T
  
Location: 15349-16237

Mycgr3G67775\_Mycgr3T

Mycgr3G90404 Mycgr3T
  
Location: 16337-17246

Mycgr3G90404\_Mycgr3T

Mycgr3G36951 Mycgr3T
  
Location: 17346-30891

Mycgr3G36951\_Mycgr3T

Mycgr3G103034 Mycgr3
  
Location: 30991-32644

Mycgr3G103034\_Mycgr3

Mycgr3G31119 Mycgr3T
  
Location: 32744-32906

Mycgr3G31119\_Mycgr3T

Mycgr3G28587 Mycgr3T
  
Location: 33006-33489

Mycgr3G28587\_Mycgr3T

Mycgr3G98959 Mycgr3T
  
Location: 33589-35035

Mycgr3G98959\_Mycgr3T

Mycgr3G35447 Mycgr3T
  
Location: 35135-36443

Mycgr3G35447\_Mycgr3T

Mycgr3G84402 Mycgr3T
  
Location: 36543-37884

Mycgr3G84402\_Mycgr3T

Mycgr3G98961 Mycgr3T
  
Location: 37984-38884

Mycgr3G98961\_Mycgr3T

Brf1-like TBP-binding domain-containing protein
  
Accession: EJP63740
  
Location: 320989-323157
  
 NCBI BlastP on this gene

EJP63740

MFS transporter
  
Accession: EJP63741
  
Location: 323319-325411
  
 NCBI BlastP on this gene

EJP63741

hypothetical protein
  
Accession: EJP63742
  
Location: 328135-328514
  
 NCBI BlastP on this gene

EJP63742

general amidase GmdB
  
Accession: EJP63743
  
Location: 329413-331101
  
 NCBI BlastP on this gene

EJP63743

Glycoside hydrolase, catalytic core
  
Accession: EJP63744
  
Location: 331861-333474
  
 NCBI BlastP on this gene

EJP63744

protein phosphatase 2C
  
Accession: EJP63745
  
Location: 334220-335778
  
 NCBI BlastP on this gene

EJP63745

DNA-binding protein
  
Accession: EJP63746
  
Location: 336601-337269
  
 NCBI BlastP on this gene

EJP63746

calcineurin-like phosphoesterase, putative
  
Accession: EJP63747
  
Location: 337741-338682
  
 NCBI BlastP on this gene

EJP63747

ribosome biogenesis protein SSF1
  
Accession: EJP63748
  
Location: 339420-340793
  
  
**BlastP hit with Mycgr3G35447\_Mycgr3T**
  
Percentage identity: 57 %
  
BlastP bit score: 449
  
Sequence coverage: 93 %
  
E-value: 2e-151
  
  
 NCBI BlastP on this gene

EJP63748

DEAD/DEAH box helicase
  
Accession: EJP63749
  
Location: 341109-342592
  
  
**BlastP hit with Mycgr3G84402\_Mycgr3T**
  
Percentage identity: 72 %
  
BlastP bit score: 632
  
Sequence coverage: 91 %
  
E-value: 0.0
  
  
 NCBI BlastP on this gene

EJP63749

transcriptional corepressor, putative
  
Accession: EJP63750
  
Location: 343218-349818
  
 NCBI BlastP on this gene

EJP63750

bud site selection protein
  
Accession: EJP63751
  
Location: 350618-352940
  
 NCBI BlastP on this gene

EJP63751

XPA-binding protein
  
Accession: EJP63752
  
Location: 353434-354665
  
 NCBI BlastP on this gene

EJP63752

DNA-directed RNA polymerases I, II, and III subunit RPABC4
  
Accession: EJP63753
  
Location: 354939-355253
  
 NCBI BlastP on this gene

EJP63753

HIT domain-containing protein
  
Accession: EJP63754
  
Location: 356150-356734
  
 NCBI BlastP on this gene

EJP63754

glycerol dehydrogenase Gcy1
  
Accession: EJP63755
  
Location: 356902-358001
  
 NCBI BlastP on this gene

EJP63755

hypothetical protein
  
Accession: EJP63756
  
Location: 358448-358819
  
 NCBI BlastP on this gene

EJP63756

fungal specific transcription factor
  
Accession: EJP63757
  
Location: 359259-361351
  
 NCBI BlastP on this gene

EJP63757

kelch repeat protein
  
Accession: EJP63758
  
Location: 361771-362820
  
 NCBI BlastP on this gene

EJP63758

Query: Architecture Search FASTA input

GG697432 : Glomerella graminicola M1.001 genomic scaffold supercont1.102    Total score: 2.0     Cumulative Blast bit score: 1081

Hit cluster cross-links:

Mycgr3G67791 Mycgr3T
  
Location: 0-1542

Mycgr3G67791\_Mycgr3T

Mycgr3G90406 Mycgr3T
  
Location: 1642-3973

Mycgr3G90406\_Mycgr3T

Mycgr3G67785 Mycgr3T
  
Location: 4073-7865

Mycgr3G67785\_Mycgr3T

Mycgr3G67795 Mycgr3T
  
Location: 7965-15249

Mycgr3G67795\_Mycgr3T

Mycgr3G67775 Mycgr3T
  
Location: 15349-16237

Mycgr3G67775\_Mycgr3T

Mycgr3G90404 Mycgr3T
  
Location: 16337-17246

Mycgr3G90404\_Mycgr3T

Mycgr3G36951 Mycgr3T
  
Location: 17346-30891

Mycgr3G36951\_Mycgr3T

Mycgr3G103034 Mycgr3
  
Location: 30991-32644

Mycgr3G103034\_Mycgr3

Mycgr3G31119 Mycgr3T
  
Location: 32744-32906

Mycgr3G31119\_Mycgr3T

Mycgr3G28587 Mycgr3T
  
Location: 33006-33489

Mycgr3G28587\_Mycgr3T

Mycgr3G98959 Mycgr3T
  
Location: 33589-35035

Mycgr3G98959\_Mycgr3T

Mycgr3G35447 Mycgr3T
  
Location: 35135-36443

Mycgr3G35447\_Mycgr3T

Mycgr3G84402 Mycgr3T
  
Location: 36543-37884

Mycgr3G84402\_Mycgr3T

Mycgr3G98961 Mycgr3T
  
Location: 37984-38884

Mycgr3G98961\_Mycgr3T

hypothetical protein
  
Accession: EFQ36514
  
Location: 5526-6965
  
 NCBI BlastP on this gene

EFQ36514

hypothetical protein
  
Accession: EFQ36515
  
Location: 10306-10700
  
 NCBI BlastP on this gene

EFQ36515

brix domain-containing protein
  
Accession: EFQ36516
  
Location: 22988-24367
  
  
**BlastP hit with Mycgr3G35447\_Mycgr3T**
  
Percentage identity: 56 %
  
BlastP bit score: 464
  
Sequence coverage: 100 %
  
E-value: 2e-157
  
  
 NCBI BlastP on this gene

EFQ36516

DEAD/DEAH box helicase
  
Accession: EFQ36517
  
Location: 24653-27022
  
  
**BlastP hit with Mycgr3G84402\_Mycgr3T**
  
Percentage identity: 70 %
  
BlastP bit score: 617
  
Sequence coverage: 93 %
  
E-value: 0.0
  
  
 NCBI BlastP on this gene

EFQ36517

kinesin motor domain-containing protein
  
Accession: EFQ36518
  
Location: 27574-30124
  
 NCBI BlastP on this gene

EFQ36518

hypothetical protein
  
Accession: EFQ36519
  
Location: 32915-34703
  
 NCBI BlastP on this gene

EFQ36519

hypothetical protein
  
Accession: EFQ36520
  
Location: 35387-35893
  
 NCBI BlastP on this gene

EFQ36520

Brf1-like TBP-binding domain-containing protein
  
Accession: EFQ36521
  
Location: 38164-40374
  
 NCBI BlastP on this gene

EFQ36521

Query: Architecture Search FASTA input

CH408032 : Chaetomium globosum CBS 148.51 scaffold\_4 genomic scaffold    Total score: 2.0     Cumulative Blast bit score: 1080

Hit cluster cross-links:

Mycgr3G67791 Mycgr3T
  
Location: 0-1542

Mycgr3G67791\_Mycgr3T

Mycgr3G90406 Mycgr3T
  
Location: 1642-3973

Mycgr3G90406\_Mycgr3T

Mycgr3G67785 Mycgr3T
  
Location: 4073-7865

Mycgr3G67785\_Mycgr3T

Mycgr3G67795 Mycgr3T
  
Location: 7965-15249

Mycgr3G67795\_Mycgr3T

Mycgr3G67775 Mycgr3T
  
Location: 15349-16237

Mycgr3G67775\_Mycgr3T

Mycgr3G90404 Mycgr3T
  
Location: 16337-17246

Mycgr3G90404\_Mycgr3T

Mycgr3G36951 Mycgr3T
  
Location: 17346-30891

Mycgr3G36951\_Mycgr3T

Mycgr3G103034 Mycgr3
  
Location: 30991-32644

Mycgr3G103034\_Mycgr3

Mycgr3G31119 Mycgr3T
  
Location: 32744-32906

Mycgr3G31119\_Mycgr3T

Mycgr3G28587 Mycgr3T
  
Location: 33006-33489

Mycgr3G28587\_Mycgr3T

Mycgr3G98959 Mycgr3T
  
Location: 33589-35035

Mycgr3G98959\_Mycgr3T

Mycgr3G35447 Mycgr3T
  
Location: 35135-36443

Mycgr3G35447\_Mycgr3T

Mycgr3G84402 Mycgr3T
  
Location: 36543-37884

Mycgr3G84402\_Mycgr3T

Mycgr3G98961 Mycgr3T
  
Location: 37984-38884

Mycgr3G98961\_Mycgr3T

hypothetical protein
  
Accession: EAQ87662
  
Location: 1773941-1776936
  
 NCBI BlastP on this gene

EAQ87662

hypothetical protein
  
Accession: EAQ87663
  
Location: 1777707-1779007
  
 NCBI BlastP on this gene

EAQ87663

predicted protein
  
Accession: EAQ87664
  
Location: 1781664-1781839
  
 NCBI BlastP on this gene

EAQ87664

predicted protein
  
Accession: EAQ87665
  
Location: 1782374-1782958
  
 NCBI BlastP on this gene

EAQ87665

predicted protein
  
Accession: EAQ87666
  
Location: 1784874-1785335
  
 NCBI BlastP on this gene

EAQ87666

hypothetical protein
  
Accession: EAQ87667
  
Location: 1786202-1791274
  
 NCBI BlastP on this gene

EAQ87667

hypothetical protein
  
Accession: EAQ87668
  
Location: 1791889-1793352
  
  
**BlastP hit with Mycgr3G35447\_Mycgr3T**
  
Percentage identity: 54 %
  
BlastP bit score: 450
  
Sequence coverage: 102 %
  
E-value: 2e-151
  
  
 NCBI BlastP on this gene

EAQ87668

hypothetical protein
  
Accession: EAQ87669
  
Location: 1793606-1795308
  
  
**BlastP hit with Mycgr3G84402\_Mycgr3T**
  
Percentage identity: 73 %
  
BlastP bit score: 630
  
Sequence coverage: 93 %
  
E-value: 0.0
  
  
 NCBI BlastP on this gene

EAQ87669

hypothetical protein
  
Accession: EAQ87670
  
Location: 1795499-1797697
  
 NCBI BlastP on this gene

EAQ87670

hypothetical protein
  
Accession: EAQ87671
  
Location: 1798901-1800027
  
 NCBI BlastP on this gene

EAQ87671

hypothetical protein
  
Accession: EAQ87672
  
Location: 1801061-1802219
  
 NCBI BlastP on this gene

EAQ87672

hypothetical protein
  
Accession: EAQ87673
  
Location: 1802656-1802970
  
 NCBI BlastP on this gene

EAQ87673

hypothetical protein
  
Accession: EAQ87674
  
Location: 1804645-1805667
  
 NCBI BlastP on this gene

EAQ87674

conserved hypothetical protein
  
Accession: EAQ87675
  
Location: 1806474-1808908
  
 NCBI BlastP on this gene

EAQ87675

hypothetical protein
  
Accession: EAQ87676
  
Location: 1809452-1815702
  
 NCBI BlastP on this gene

EAQ87676

Query: Architecture Search FASTA input

AFQF01002695 : Fusarium oxysporum Fo5176    Total score: 2.0     Cumulative Blast bit score: 1077

Hit cluster cross-links:

Mycgr3G67791 Mycgr3T
  
Location: 0-1542

Mycgr3G67791\_Mycgr3T

Mycgr3G90406 Mycgr3T
  
Location: 1642-3973

Mycgr3G90406\_Mycgr3T

Mycgr3G67785 Mycgr3T
  
Location: 4073-7865

Mycgr3G67785\_Mycgr3T

Mycgr3G67795 Mycgr3T
  
Location: 7965-15249

Mycgr3G67795\_Mycgr3T

Mycgr3G67775 Mycgr3T
  
Location: 15349-16237

Mycgr3G67775\_Mycgr3T

Mycgr3G90404 Mycgr3T
  
Location: 16337-17246

Mycgr3G90404\_Mycgr3T

Mycgr3G36951 Mycgr3T
  
Location: 17346-30891

Mycgr3G36951\_Mycgr3T

Mycgr3G103034 Mycgr3
  
Location: 30991-32644

Mycgr3G103034\_Mycgr3

Mycgr3G31119 Mycgr3T
  
Location: 32744-32906

Mycgr3G31119\_Mycgr3T

Mycgr3G28587 Mycgr3T
  
Location: 33006-33489

Mycgr3G28587\_Mycgr3T

Mycgr3G98959 Mycgr3T
  
Location: 33589-35035

Mycgr3G98959\_Mycgr3T

Mycgr3G35447 Mycgr3T
  
Location: 35135-36443

Mycgr3G35447\_Mycgr3T

Mycgr3G84402 Mycgr3T
  
Location: 36543-37884

Mycgr3G84402\_Mycgr3T

Mycgr3G98961 Mycgr3T
  
Location: 37984-38884

Mycgr3G98961\_Mycgr3T

hypothetical protein
  
Accession: EGU79266
  
Location: 17231-20185
  
 NCBI BlastP on this gene

EGU79266

hypothetical protein
  
Accession: EGU79267
  
Location: 21968-22497
  
 NCBI BlastP on this gene

EGU79267

hypothetical protein
  
Accession: EGU79268
  
Location: 23645-25541
  
 NCBI BlastP on this gene

EGU79268

hypothetical protein
  
Accession: EGU79269
  
Location: 26521-32679
  
 NCBI BlastP on this gene

EGU79269

hypothetical protein
  
Accession: EGU79270
  
Location: 33001-34064
  
 NCBI BlastP on this gene

EGU79270

hypothetical protein
  
Accession: EGU79271
  
Location: 34935-36441
  
  
**BlastP hit with Mycgr3G84402\_Mycgr3T**
  
Percentage identity: 73 %
  
BlastP bit score: 629
  
Sequence coverage: 91 %
  
E-value: 0.0
  
  
 NCBI BlastP on this gene

EGU79271

hypothetical protein
  
Accession: EGU79272
  
Location: 36659-38071
  
  
**BlastP hit with Mycgr3G35447\_Mycgr3T**
  
Percentage identity: 56 %
  
BlastP bit score: 448
  
Sequence coverage: 100 %
  
E-value: 2e-151
  
  
 NCBI BlastP on this gene

EGU79272

hypothetical protein
  
Accession: EGU79273
  
Location: 38608-40097
  
 NCBI BlastP on this gene

EGU79273

hypothetical protein
  
Accession: EGU79274
  
Location: 40642-42183
  
 NCBI BlastP on this gene

EGU79274

hypothetical protein
  
Accession: EGU79275
  
Location: 43325-44419
  
 NCBI BlastP on this gene

EGU79275

hypothetical protein
  
Accession: EGU79276
  
Location: 45061-47112
  
 NCBI BlastP on this gene

EGU79276

hypothetical protein
  
Accession: EGU79277
  
Location: 49036-50372
  
 NCBI BlastP on this gene

EGU79277

hypothetical protein
  
Accession: EGU79278
  
Location: 51337-53266
  
 NCBI BlastP on this gene

EGU79278

Query: Architecture Search FASTA input

CP003007 : Myceliophthora thermophila ATCC 42464 chromosome 6    Total score: 2.0     Cumulative Blast bit score: 1074

Hit cluster cross-links:

Mycgr3G67791 Mycgr3T
  
Location: 0-1542

Mycgr3G67791\_Mycgr3T

Mycgr3G90406 Mycgr3T
  
Location: 1642-3973

Mycgr3G90406\_Mycgr3T

Mycgr3G67785 Mycgr3T
  
Location: 4073-7865

Mycgr3G67785\_Mycgr3T

Mycgr3G67795 Mycgr3T
  
Location: 7965-15249

Mycgr3G67795\_Mycgr3T

Mycgr3G67775 Mycgr3T
  
Location: 15349-16237

Mycgr3G67775\_Mycgr3T

Mycgr3G90404 Mycgr3T
  
Location: 16337-17246

Mycgr3G90404\_Mycgr3T

Mycgr3G36951 Mycgr3T
  
Location: 17346-30891

Mycgr3G36951\_Mycgr3T

Mycgr3G103034 Mycgr3
  
Location: 30991-32644

Mycgr3G103034\_Mycgr3

Mycgr3G31119 Mycgr3T
  
Location: 32744-32906

Mycgr3G31119\_Mycgr3T

Mycgr3G28587 Mycgr3T
  
Location: 33006-33489

Mycgr3G28587\_Mycgr3T

Mycgr3G98959 Mycgr3T
  
Location: 33589-35035

Mycgr3G98959\_Mycgr3T

Mycgr3G35447 Mycgr3T
  
Location: 35135-36443

Mycgr3G35447\_Mycgr3T

Mycgr3G84402 Mycgr3T
  
Location: 36543-37884

Mycgr3G84402\_Mycgr3T

Mycgr3G98961 Mycgr3T
  
Location: 37984-38884

Mycgr3G98961\_Mycgr3T

hypothetical protein
  
Accession: AEO60946
  
Location: 2530038-2531138
  
 NCBI BlastP on this gene

MYCTH\_2112941

hypothetical protein
  
Accession: AEO60947
  
Location: 2532883-2533455
  
 NCBI BlastP on this gene

MYCTH\_2310703

hypothetical protein
  
Accession: AEO60948
  
Location: 2535302-2535870
  
 NCBI BlastP on this gene

MYCTH\_60397

hypothetical protein
  
Accession: AEO60949
  
Location: 2536718-2538312
  
  
**BlastP hit with Mycgr3G35447\_Mycgr3T**
  
Percentage identity: 55 %
  
BlastP bit score: 443
  
Sequence coverage: 102 %
  
E-value: 4e-148
  
  
 NCBI BlastP on this gene

MYCTH\_2310708

hypothetical protein
  
Accession: AEO60950
  
Location: 2538437-2540186
  
  
**BlastP hit with Mycgr3G84402\_Mycgr3T**
  
Percentage identity: 74 %
  
BlastP bit score: 631
  
Sequence coverage: 92 %
  
E-value: 0.0
  
  
 NCBI BlastP on this gene

MYCTH\_2096529

hypothetical protein
  
Accession: AEO60951
  
Location: 2544273-2545544
  
 NCBI BlastP on this gene

MYCTH\_2310716

hypothetical protein
  
Accession: AEO60952
  
Location: 2546775-2548026
  
 NCBI BlastP on this gene

MYCTH\_113239

hypothetical protein
  
Accession: AEO60953
  
Location: 2549258-2549914
  
 NCBI BlastP on this gene

MYCTH\_37799

Aldo/keto reductase-like protein
  
Accession: AEO60954
  
Location: 2550203-2551227
  
 NCBI BlastP on this gene

MYCTH\_113236

Query: Architecture Search FASTA input

KB707406 : Eutypa lata UCREL1 unplaced genomic scaffold EL1\_03\_scaffold\_2068    Total score: 2.0     Cumulative Blast bit score: 1072

Hit cluster cross-links:

Mycgr3G67791 Mycgr3T
  
Location: 0-1542

Mycgr3G67791\_Mycgr3T

Mycgr3G90406 Mycgr3T
  
Location: 1642-3973

Mycgr3G90406\_Mycgr3T

Mycgr3G67785 Mycgr3T
  
Location: 4073-7865

Mycgr3G67785\_Mycgr3T

Mycgr3G67795 Mycgr3T
  
Location: 7965-15249

Mycgr3G67795\_Mycgr3T

Mycgr3G67775 Mycgr3T
  
Location: 15349-16237

Mycgr3G67775\_Mycgr3T

Mycgr3G90404 Mycgr3T
  
Location: 16337-17246

Mycgr3G90404\_Mycgr3T

Mycgr3G36951 Mycgr3T
  
Location: 17346-30891

Mycgr3G36951\_Mycgr3T

Mycgr3G103034 Mycgr3
  
Location: 30991-32644

Mycgr3G103034\_Mycgr3

Mycgr3G31119 Mycgr3T
  
Location: 32744-32906

Mycgr3G31119\_Mycgr3T

Mycgr3G28587 Mycgr3T
  
Location: 33006-33489

Mycgr3G28587\_Mycgr3T

Mycgr3G98959 Mycgr3T
  
Location: 33589-35035

Mycgr3G98959\_Mycgr3T

Mycgr3G35447 Mycgr3T
  
Location: 35135-36443

Mycgr3G35447\_Mycgr3T

Mycgr3G84402 Mycgr3T
  
Location: 36543-37884

Mycgr3G84402\_Mycgr3T

Mycgr3G98961 Mycgr3T
  
Location: 37984-38884

Mycgr3G98961\_Mycgr3T

hypothetical protein
  
Accession: EMR62663
  
Location: 1346-1849
  
 NCBI BlastP on this gene

EMR62663

hypothetical protein
  
Accession: EMR62666
  
Location: 3551-4426
  
 NCBI BlastP on this gene

EMR62666

hypothetical protein
  
Accession: EMR62668
  
Location: 5523-6890
  
 NCBI BlastP on this gene

EMR62668

putative salicylate hydroxylase protein
  
Accession: EMR62658
  
Location: 13388-14738
  
 NCBI BlastP on this gene

EMR62658

hypothetical protein
  
Accession: EMR62656
  
Location: 15857-16806
  
 NCBI BlastP on this gene

EMR62656

putative ribosome biogenesis protein ssf1 protein
  
Accession: EMR62660
  
Location: 17657-19256
  
  
**BlastP hit with Mycgr3G35447\_Mycgr3T**
  
Percentage identity: 51 %
  
BlastP bit score: 431
  
Sequence coverage: 106 %
  
E-value: 1e-143
  
  
 NCBI BlastP on this gene

EMR62660

putative atp-dependent rrna helicase rrp3 protein
  
Accession: EMR62664
  
Location: 19678-21431
  
  
**BlastP hit with Mycgr3G84402\_Mycgr3T**
  
Percentage identity: 70 %
  
BlastP bit score: 641
  
Sequence coverage: 100 %
  
E-value: 0.0
  
  
 NCBI BlastP on this gene

EMR62664

putative alpha-ketoglutarate dependent xanthine dioxygenase protein
  
Accession: EMR62657
  
Location: 23239-24684
  
 NCBI BlastP on this gene

EMR62657

putative c6 zinc finger domain containing protein
  
Accession: EMR62653
  
Location: 26050-27843
  
 NCBI BlastP on this gene

EMR62653

putative ncs1 allantoate transporter protein
  
Accession: EMR62661
  
Location: 29028-30860
  
 NCBI BlastP on this gene

EMR62661

putative leucoanthocyanidin dioxygenase protein
  
Accession: EMR62654
  
Location: 31513-32601
  
 NCBI BlastP on this gene

EMR62654

putative cytidine and deoxycytidylate deaminase zinc-binding region protein
  
Accession: EMR62652
  
Location: 32866-33674
  
 NCBI BlastP on this gene

EMR62652

putative alpha-ketoglutarate dependent xanthine dioxygenase protein
  
Accession: EMR62665
  
Location: 33910-35372
  
 NCBI BlastP on this gene

EMR62665

putative extracellular dioxygenase protein
  
Accession: EMR62650
  
Location: 38496-39353
  
 NCBI BlastP on this gene

EMR62650

putative short-chain dehydrogenase reductase family protein
  
Accession: EMR62659
  
Location: 40983-41933
  
 NCBI BlastP on this gene

EMR62659

Query: Architecture Search FASTA input

CU638744 : Podospora anserina S mat+ genomic DNA chromosome 6, supercontig 2.    Total score: 2.0     Cumulative Blast bit score: 1072

Hit cluster cross-links:

Mycgr3G67791 Mycgr3T
  
Location: 0-1542

Mycgr3G67791\_Mycgr3T

Mycgr3G90406 Mycgr3T
  
Location: 1642-3973

Mycgr3G90406\_Mycgr3T

Mycgr3G67785 Mycgr3T
  
Location: 4073-7865

Mycgr3G67785\_Mycgr3T

Mycgr3G67795 Mycgr3T
  
Location: 7965-15249

Mycgr3G67795\_Mycgr3T

Mycgr3G67775 Mycgr3T
  
Location: 15349-16237

Mycgr3G67775\_Mycgr3T

Mycgr3G90404 Mycgr3T
  
Location: 16337-17246

Mycgr3G90404\_Mycgr3T

Mycgr3G36951 Mycgr3T
  
Location: 17346-30891

Mycgr3G36951\_Mycgr3T

Mycgr3G103034 Mycgr3
  
Location: 30991-32644

Mycgr3G103034\_Mycgr3

Mycgr3G31119 Mycgr3T
  
Location: 32744-32906

Mycgr3G31119\_Mycgr3T

Mycgr3G28587 Mycgr3T
  
Location: 33006-33489

Mycgr3G28587\_Mycgr3T

Mycgr3G98959 Mycgr3T
  
Location: 33589-35035

Mycgr3G98959\_Mycgr3T

Mycgr3G35447 Mycgr3T
  
Location: 35135-36443

Mycgr3G35447\_Mycgr3T

Mycgr3G84402 Mycgr3T
  
Location: 36543-37884

Mycgr3G84402\_Mycgr3T

Mycgr3G98961 Mycgr3T
  
Location: 37984-38884

Mycgr3G98961\_Mycgr3T

not annotated
  
Accession: CAP71745
  
Location: 2597327-2597725
  
 NCBI BlastP on this gene

CAP71745

not annotated
  
Accession: CAP71746
  
Location: 2598101-2599590
  
 NCBI BlastP on this gene

CAP71746

not annotated
  
Accession: CAP71747
  
Location: 2601643-2603132
  
 NCBI BlastP on this gene

CAP71747

not annotated
  
Accession: CAP71748
  
Location: 2605945-2607247
  
 NCBI BlastP on this gene

CAP71748

not annotated
  
Accession: CAP71749
  
Location: 2609718-2610130
  
 NCBI BlastP on this gene

CAP71749

not annotated
  
Accession: CAP71750
  
Location: 2612569-2613339
  
 NCBI BlastP on this gene

CAP71750

not annotated
  
Accession: CAP71751
  
Location: 2614799-2616271
  
  
**BlastP hit with Mycgr3G35447\_Mycgr3T**
  
Percentage identity: 56 %
  
BlastP bit score: 452
  
Sequence coverage: 95 %
  
E-value: 7e-152
  
  
 NCBI BlastP on this gene

CAP71751

not annotated
  
Accession: CAP71752
  
Location: 2616546-2618125
  
  
**BlastP hit with Mycgr3G84402\_Mycgr3T**
  
Percentage identity: 66 %
  
BlastP bit score: 620
  
Sequence coverage: 103 %
  
E-value: 0.0
  
  
 NCBI BlastP on this gene

CAP71752

not annotated
  
Accession: CAP71753
  
Location: 2618385-2620523
  
 NCBI BlastP on this gene

CAP71753

not annotated
  
Accession: CAP71754
  
Location: 2622314-2623693
  
 NCBI BlastP on this gene

CAP71754

not annotated
  
Accession: CAP71755
  
Location: 2624984-2626105
  
 NCBI BlastP on this gene

CAP71755

not annotated
  
Accession: CAP71756
  
Location: 2626411-2626685
  
 NCBI BlastP on this gene

CAP71756

not annotated
  
Accession: CAP71757
  
Location: 2627182-2627820
  
 NCBI BlastP on this gene

CAP71757

not annotated
  
Accession: CAP71758
  
Location: 2628136-2629279
  
 NCBI BlastP on this gene

CAP71758

not annotated
  
Accession: CAP71759
  
Location: 2630035-2632357
  
 NCBI BlastP on this gene

CAP71759

not annotated
  
Accession: CAP71760
  
Location: 2633044-2638734
  
 NCBI BlastP on this gene

CAP71760

Query: Architecture Search FASTA input

GL698748 : Metarhizium anisopliae ARSEF 23 unplaced genomic scaffold Scf\_038    Total score: 2.0     Cumulative Blast bit score: 1071

Hit cluster cross-links:

Mycgr3G67791 Mycgr3T
  
Location: 0-1542

Mycgr3G67791\_Mycgr3T

Mycgr3G90406 Mycgr3T
  
Location: 1642-3973

Mycgr3G90406\_Mycgr3T

Mycgr3G67785 Mycgr3T
  
Location: 4073-7865

Mycgr3G67785\_Mycgr3T

Mycgr3G67795 Mycgr3T
  
Location: 7965-15249

Mycgr3G67795\_Mycgr3T

Mycgr3G67775 Mycgr3T
  
Location: 15349-16237

Mycgr3G67775\_Mycgr3T

Mycgr3G90404 Mycgr3T
  
Location: 16337-17246

Mycgr3G90404\_Mycgr3T

Mycgr3G36951 Mycgr3T
  
Location: 17346-30891

Mycgr3G36951\_Mycgr3T

Mycgr3G103034 Mycgr3
  
Location: 30991-32644

Mycgr3G103034\_Mycgr3

Mycgr3G31119 Mycgr3T
  
Location: 32744-32906

Mycgr3G31119\_Mycgr3T

Mycgr3G28587 Mycgr3T
  
Location: 33006-33489

Mycgr3G28587\_Mycgr3T

Mycgr3G98959 Mycgr3T
  
Location: 33589-35035

Mycgr3G98959\_Mycgr3T

Mycgr3G35447 Mycgr3T
  
Location: 35135-36443

Mycgr3G35447\_Mycgr3T

Mycgr3G84402 Mycgr3T
  
Location: 36543-37884

Mycgr3G84402\_Mycgr3T

Mycgr3G98961 Mycgr3T
  
Location: 37984-38884

Mycgr3G98961\_Mycgr3T

UbiD family decarboxylase
  
Accession: EFY95020
  
Location: 17474-19151
  
 NCBI BlastP on this gene

EFY95020

pyruvate dehydrogenase, putative
  
Accession: EFY95021
  
Location: 21597-23178
  
 NCBI BlastP on this gene

EFY95021

hypothetical protein
  
Accession: EFY95022
  
Location: 28217-28738
  
 NCBI BlastP on this gene

EFY95022

glycerate-and formate-dehydrogenase
  
Accession: EFY95023
  
Location: 30926-33516
  
 NCBI BlastP on this gene

EFY95023

DUF1338 domain protein
  
Accession: EFY95024
  
Location: 34616-36048
  
 NCBI BlastP on this gene

EFY95024

ribosome biogenesis protein Ssf2, putative
  
Accession: EFY95025
  
Location: 36163-37611
  
  
**BlastP hit with Mycgr3G35447\_Mycgr3T**
  
Percentage identity: 59 %
  
BlastP bit score: 457
  
Sequence coverage: 91 %
  
E-value: 1e-154
  
  
 NCBI BlastP on this gene

EFY95025

ATP-dependent rRNA helicase RRP3
  
Accession: EFY95026
  
Location: 37958-39454
  
  
**BlastP hit with Mycgr3G84402\_Mycgr3T**
  
Percentage identity: 70 %
  
BlastP bit score: 614
  
Sequence coverage: 95 %
  
E-value: 0.0
  
  
 NCBI BlastP on this gene

EFY95026

transcriptional corepressor
  
Accession: EFY95027
  
Location: 40152-46740
  
 NCBI BlastP on this gene

EFY95027

budding site selection protein
  
Accession: EFY95028
  
Location: 47362-49674
  
 NCBI BlastP on this gene

EFY95028

ATP binding protein, putative
  
Accession: EFY95029
  
Location: 50254-51502
  
 NCBI BlastP on this gene

EFY95029

hypothetical protein
  
Accession: EFY95030
  
Location: 51708-53944
  
 NCBI BlastP on this gene

EFY95030

cysteine-rich secreted protein
  
Accession: EFY95031
  
Location: 54562-55937
  
 NCBI BlastP on this gene

EFY95031

polyketide synthase
  
Accession: EFY95032
  
Location: 58057-59442
  
 NCBI BlastP on this gene

EFY95032

Query: Architecture Search FASTA input

GL985056 : Trichoderma reesei QM6a unplaced genomic scaffold TRIREscaffold\_1    Total score: 2.0     Cumulative Blast bit score: 1070

Hit cluster cross-links:

Mycgr3G67791 Mycgr3T
  
Location: 0-1542

Mycgr3G67791\_Mycgr3T

Mycgr3G90406 Mycgr3T
  
Location: 1642-3973

Mycgr3G90406\_Mycgr3T

Mycgr3G67785 Mycgr3T
  
Location: 4073-7865

Mycgr3G67785\_Mycgr3T

Mycgr3G67795 Mycgr3T
  
Location: 7965-15249

Mycgr3G67795\_Mycgr3T

Mycgr3G67775 Mycgr3T
  
Location: 15349-16237

Mycgr3G67775\_Mycgr3T

Mycgr3G90404 Mycgr3T
  
Location: 16337-17246

Mycgr3G90404\_Mycgr3T

Mycgr3G36951 Mycgr3T
  
Location: 17346-30891

Mycgr3G36951\_Mycgr3T

Mycgr3G103034 Mycgr3
  
Location: 30991-32644

Mycgr3G103034\_Mycgr3

Mycgr3G31119 Mycgr3T
  
Location: 32744-32906

Mycgr3G31119\_Mycgr3T

Mycgr3G28587 Mycgr3T
  
Location: 33006-33489

Mycgr3G28587\_Mycgr3T

Mycgr3G98959 Mycgr3T
  
Location: 33589-35035

Mycgr3G98959\_Mycgr3T

Mycgr3G35447 Mycgr3T
  
Location: 35135-36443

Mycgr3G35447\_Mycgr3T

Mycgr3G84402 Mycgr3T
  
Location: 36543-37884

Mycgr3G84402\_Mycgr3T

Mycgr3G98961 Mycgr3T
  
Location: 37984-38884

Mycgr3G98961\_Mycgr3T

predicted protein
  
Accession: EGR52938
  
Location: 2184066-2184786
  
 NCBI BlastP on this gene

EGR52938

RNA polymerase III transcription initiation factor B complex component
  
Accession: EGR52397
  
Location: 2186328-2188364
  
 NCBI BlastP on this gene

EGR52397

predicted protein
  
Accession: EGR52939
  
Location: 2188867-2190018
  
 NCBI BlastP on this gene

EGR52939

aspartate protease
  
Accession: EGR52940
  
Location: 2191952-2193253
  
 NCBI BlastP on this gene

EGR52940

predicted protein
  
Accession: EGR52398
  
Location: 2194795-2195720
  
 NCBI BlastP on this gene

EGR52398

predicted protein
  
Accession: EGR52941
  
Location: 2203596-2205056
  
  
**BlastP hit with Mycgr3G35447\_Mycgr3T**
  
Percentage identity: 58 %
  
BlastP bit score: 438
  
Sequence coverage: 91 %
  
E-value: 9e-147
  
  
 NCBI BlastP on this gene

EGR52941

predicted protein
  
Accession: EGR52399
  
Location: 2205691-2207210
  
  
**BlastP hit with Mycgr3G84402\_Mycgr3T**
  
Percentage identity: 71 %
  
BlastP bit score: 632
  
Sequence coverage: 93 %
  
E-value: 0.0
  
  
 NCBI BlastP on this gene

EGR52399

predicted protein
  
Accession: EGR52942
  
Location: 2207638-2213921
  
 NCBI BlastP on this gene

EGR52942

predicted protein
  
Accession: EGR52400
  
Location: 2214559-2216921
  
 NCBI BlastP on this gene

EGR52400

hypothetical protein
  
Accession: EGR52401
  
Location: 2217411-2218673
  
 NCBI BlastP on this gene

EGR52401

predicted protein
  
Accession: EGR52943
  
Location: 2219043-2219373
  
 NCBI BlastP on this gene

EGR52943

predicted protein
  
Accession: EGR52402
  
Location: 2220460-2221065
  
 NCBI BlastP on this gene

EGR52402

predicted protein
  
Accession: EGR52944
  
Location: 2221835-2222733
  
 NCBI BlastP on this gene

EGR52944

Query: Architecture Search FASTA input

ABDF02000004 : Trichoderma virens Gv29-8    Total score: 2.0     Cumulative Blast bit score: 1069

Hit cluster cross-links:

Mycgr3G67791 Mycgr3T
  
Location: 0-1542

Mycgr3G67791\_Mycgr3T

Mycgr3G90406 Mycgr3T
  
Location: 1642-3973

Mycgr3G90406\_Mycgr3T

Mycgr3G67785 Mycgr3T
  
Location: 4073-7865

Mycgr3G67785\_Mycgr3T

Mycgr3G67795 Mycgr3T
  
Location: 7965-15249

Mycgr3G67795\_Mycgr3T

Mycgr3G67775 Mycgr3T
  
Location: 15349-16237

Mycgr3G67775\_Mycgr3T

Mycgr3G90404 Mycgr3T
  
Location: 16337-17246

Mycgr3G90404\_Mycgr3T

Mycgr3G36951 Mycgr3T
  
Location: 17346-30891

Mycgr3G36951\_Mycgr3T

Mycgr3G103034 Mycgr3
  
Location: 30991-32644

Mycgr3G103034\_Mycgr3

Mycgr3G31119 Mycgr3T
  
Location: 32744-32906

Mycgr3G31119\_Mycgr3T

Mycgr3G28587 Mycgr3T
  
Location: 33006-33489

Mycgr3G28587\_Mycgr3T

Mycgr3G98959 Mycgr3T
  
Location: 33589-35035

Mycgr3G98959\_Mycgr3T

Mycgr3G35447 Mycgr3T
  
Location: 35135-36443

Mycgr3G35447\_Mycgr3T

Mycgr3G84402 Mycgr3T
  
Location: 36543-37884

Mycgr3G84402\_Mycgr3T

Mycgr3G98961 Mycgr3T
  
Location: 37984-38884

Mycgr3G98961\_Mycgr3T

hypothetical protein
  
Accession: EHK24236
  
Location: 162725-163258
  
 NCBI BlastP on this gene

EHK24236

hypothetical protein
  
Accession: EHK24237
  
Location: 164217-164527
  
 NCBI BlastP on this gene

EHK24237

hypothetical protein
  
Accession: EHK24238
  
Location: 164876-165961
  
 NCBI BlastP on this gene

EHK24238

hypothetical protein
  
Accession: EHK24239
  
Location: 166581-168975
  
 NCBI BlastP on this gene

EHK24239

hypothetical protein
  
Accession: EHK24240
  
Location: 169577-175801
  
 NCBI BlastP on this gene

EHK24240

hypothetical protein
  
Accession: EHK24241
  
Location: 176351-177862
  
  
**BlastP hit with Mycgr3G84402\_Mycgr3T**
  
Percentage identity: 74 %
  
BlastP bit score: 630
  
Sequence coverage: 87 %
  
E-value: 0.0
  
  
 NCBI BlastP on this gene

EHK24241

hypothetical protein
  
Accession: EHK24242
  
Location: 178371-179812
  
  
**BlastP hit with Mycgr3G35447\_Mycgr3T**
  
Percentage identity: 57 %
  
BlastP bit score: 439
  
Sequence coverage: 91 %
  
E-value: 2e-147
  
  
 NCBI BlastP on this gene

EHK24242

hypothetical protein
  
Accession: EHK24243
  
Location: 180068-181617
  
 NCBI BlastP on this gene

EHK24243

hypothetical protein
  
Accession: EHK24244
  
Location: 182325-182855
  
 NCBI BlastP on this gene

EHK24244

hypothetical protein
  
Accession: EHK24245
  
Location: 184297-186980
  
 NCBI BlastP on this gene

EHK24245

hypothetical protein
  
Accession: EHK24246
  
Location: 187551-188708
  
 NCBI BlastP on this gene

EHK24246

hypothetical protein
  
Accession: EHK24247
  
Location: 189597-190682
  
 NCBI BlastP on this gene

EHK24247

hypothetical protein
  
Accession: EHK24248
  
Location: 192389-192709
  
 NCBI BlastP on this gene

EHK24248

hypothetical protein
  
Accession: EHK24249
  
Location: 193528-194130
  
 NCBI BlastP on this gene

EHK24249

hypothetical protein
  
Accession: EHK24250
  
Location: 194941-195822
  
 NCBI BlastP on this gene

EHK24250

hypothetical protein
  
Accession: EHK24251
  
Location: 196459-196928
  
 NCBI BlastP on this gene

EHK24251

hypothetical protein
  
Accession: EHK24252
  
Location: 199593-200193
  
 NCBI BlastP on this gene

EHK24252

Query: Architecture Search FASTA input

CACQ02001212 : Colletotrichum higginsianum strain IMI 349063    Total score: 2.0     Cumulative Blast bit score: 1066

Hit cluster cross-links:

Mycgr3G67791 Mycgr3T
  
Location: 0-1542

Mycgr3G67791\_Mycgr3T

Mycgr3G90406 Mycgr3T
  
Location: 1642-3973

Mycgr3G90406\_Mycgr3T

Mycgr3G67785 Mycgr3T
  
Location: 4073-7865

Mycgr3G67785\_Mycgr3T

Mycgr3G67795 Mycgr3T
  
Location: 7965-15249

Mycgr3G67795\_Mycgr3T

Mycgr3G67775 Mycgr3T
  
Location: 15349-16237

Mycgr3G67775\_Mycgr3T

Mycgr3G90404 Mycgr3T
  
Location: 16337-17246

Mycgr3G90404\_Mycgr3T

Mycgr3G36951 Mycgr3T
  
Location: 17346-30891

Mycgr3G36951\_Mycgr3T

Mycgr3G103034 Mycgr3
  
Location: 30991-32644

Mycgr3G103034\_Mycgr3

Mycgr3G31119 Mycgr3T
  
Location: 32744-32906

Mycgr3G31119\_Mycgr3T

Mycgr3G28587 Mycgr3T
  
Location: 33006-33489

Mycgr3G28587\_Mycgr3T

Mycgr3G98959 Mycgr3T
  
Location: 33589-35035

Mycgr3G98959\_Mycgr3T

Mycgr3G35447 Mycgr3T
  
Location: 35135-36443

Mycgr3G35447\_Mycgr3T

Mycgr3G84402 Mycgr3T
  
Location: 36543-37884

Mycgr3G84402\_Mycgr3T

Mycgr3G98961 Mycgr3T
  
Location: 37984-38884

Mycgr3G98961\_Mycgr3T

hypothetical protein
  
Accession: CCF34614
  
Location: 1289-3745
  
 NCBI BlastP on this gene

CCF34614

C6 zinc finger protein
  
Accession: CCF34615
  
Location: 3957-5919
  
 NCBI BlastP on this gene

CCF34615

phytanoyl-CoA dioxygenase
  
Accession: CCF34616
  
Location: 6341-7444
  
 NCBI BlastP on this gene

CCF34616

brix domain-containing protein
  
Accession: CCF34617
  
Location: 8075-9454
  
  
**BlastP hit with Mycgr3G35447\_Mycgr3T**
  
Percentage identity: 55 %
  
BlastP bit score: 454
  
Sequence coverage: 100 %
  
E-value: 2e-153
  
  
 NCBI BlastP on this gene

CCF34617

ATP-dependent rRNA helicase RRP3
  
Accession: CCF34618
  
Location: 9785-12065
  
  
**BlastP hit with Mycgr3G84402\_Mycgr3T**
  
Percentage identity: 69 %
  
BlastP bit score: 612
  
Sequence coverage: 93 %
  
E-value: 0.0
  
  
 NCBI BlastP on this gene

CCF34618

kinesin motor domain-containing protein
  
Accession: CCF34619
  
Location: 12653-15210
  
 NCBI BlastP on this gene

CCF34619

Query: Architecture Search FASTA input

GL698510 : Metarhizium acridum CQMa 102 unplaced genomic scaffold Scf\_041    Total score: 2.0     Cumulative Blast bit score: 1064

Hit cluster cross-links:

Mycgr3G67791 Mycgr3T
  
Location: 0-1542

Mycgr3G67791\_Mycgr3T

Mycgr3G90406 Mycgr3T
  
Location: 1642-3973

Mycgr3G90406\_Mycgr3T

Mycgr3G67785 Mycgr3T
  
Location: 4073-7865

Mycgr3G67785\_Mycgr3T

Mycgr3G67795 Mycgr3T
  
Location: 7965-15249

Mycgr3G67795\_Mycgr3T

Mycgr3G67775 Mycgr3T
  
Location: 15349-16237

Mycgr3G67775\_Mycgr3T

Mycgr3G90404 Mycgr3T
  
Location: 16337-17246

Mycgr3G90404\_Mycgr3T

Mycgr3G36951 Mycgr3T
  
Location: 17346-30891

Mycgr3G36951\_Mycgr3T

Mycgr3G103034 Mycgr3
  
Location: 30991-32644

Mycgr3G103034\_Mycgr3

Mycgr3G31119 Mycgr3T
  
Location: 32744-32906

Mycgr3G31119\_Mycgr3T

Mycgr3G28587 Mycgr3T
  
Location: 33006-33489

Mycgr3G28587\_Mycgr3T

Mycgr3G98959 Mycgr3T
  
Location: 33589-35035

Mycgr3G98959\_Mycgr3T

Mycgr3G35447 Mycgr3T
  
Location: 35135-36443

Mycgr3G35447\_Mycgr3T

Mycgr3G84402 Mycgr3T
  
Location: 36543-37884

Mycgr3G84402\_Mycgr3T

Mycgr3G98961 Mycgr3T
  
Location: 37984-38884

Mycgr3G98961\_Mycgr3T

cysteine-rich secreted protein
  
Accession: EFY88538
  
Location: 193727-195102
  
 NCBI BlastP on this gene

EFY88538

metallothionein-I gene transcription activator
  
Accession: EFY88539
  
Location: 198515-198831
  
 NCBI BlastP on this gene

EFY88539

ATP binding protein, putative
  
Accession: EFY88540
  
Location: 199038-200261
  
 NCBI BlastP on this gene

EFY88540

budding site selection protein
  
Accession: EFY88541
  
Location: 200839-203150
  
 NCBI BlastP on this gene

EFY88541

transcriptional corepressor of histone genes (Hir3)
  
Accession: EFY88542
  
Location: 203761-210426
  
 NCBI BlastP on this gene

EFY88542

ATP-dependent rRNA helicase RRP3
  
Accession: EFY88543
  
Location: 211141-212634
  
  
**BlastP hit with Mycgr3G84402\_Mycgr3T**
  
Percentage identity: 71 %
  
BlastP bit score: 614
  
Sequence coverage: 91 %
  
E-value: 0.0
  
  
 NCBI BlastP on this gene

EFY88543

ribosome biogenesis protein Ssf2, putative
  
Accession: EFY88544
  
Location: 213010-214459
  
  
**BlastP hit with Mycgr3G35447\_Mycgr3T**
  
Percentage identity: 58 %
  
BlastP bit score: 450
  
Sequence coverage: 91 %
  
E-value: 9e-152
  
  
 NCBI BlastP on this gene

EFY88544

hypothetical protein
  
Accession: EFY88545
  
Location: 215156-215764
  
 NCBI BlastP on this gene

EFY88545

2-hydroxyacid dehydrogenase, putative
  
Accession: EFY88546
  
Location: 217035-220947
  
 NCBI BlastP on this gene

EFY88546

hypothetical protein
  
Accession: EFY88547
  
Location: 221541-222669
  
 NCBI BlastP on this gene

EFY88547

pyruvate dehydrogenase, putative
  
Accession: EFY88548
  
Location: 228073-230664
  
 NCBI BlastP on this gene

EFY88548

UbiD family decarboxylase
  
Accession: EFY88549
  
Location: 231826-233496
  
 NCBI BlastP on this gene

EFY88549

Query: Architecture Search FASTA input

AMYD01001882 : Colletotrichum gloeosporioides Cg-14    Total score: 2.0     Cumulative Blast bit score: 1064

Hit cluster cross-links:

Mycgr3G67791 Mycgr3T
  
Location: 0-1542

Mycgr3G67791\_Mycgr3T

Mycgr3G90406 Mycgr3T
  
Location: 1642-3973

Mycgr3G90406\_Mycgr3T

Mycgr3G67785 Mycgr3T
  
Location: 4073-7865

Mycgr3G67785\_Mycgr3T

Mycgr3G67795 Mycgr3T
  
Location: 7965-15249

Mycgr3G67795\_Mycgr3T

Mycgr3G67775 Mycgr3T
  
Location: 15349-16237

Mycgr3G67775\_Mycgr3T

Mycgr3G90404 Mycgr3T
  
Location: 16337-17246

Mycgr3G90404\_Mycgr3T

Mycgr3G36951 Mycgr3T
  
Location: 17346-30891

Mycgr3G36951\_Mycgr3T

Mycgr3G103034 Mycgr3
  
Location: 30991-32644

Mycgr3G103034\_Mycgr3

Mycgr3G31119 Mycgr3T
  
Location: 32744-32906

Mycgr3G31119\_Mycgr3T

Mycgr3G28587 Mycgr3T
  
Location: 33006-33489

Mycgr3G28587\_Mycgr3T

Mycgr3G98959 Mycgr3T
  
Location: 33589-35035

Mycgr3G98959\_Mycgr3T

Mycgr3G35447 Mycgr3T
  
Location: 35135-36443

Mycgr3G35447\_Mycgr3T

Mycgr3G84402 Mycgr3T
  
Location: 36543-37884

Mycgr3G84402\_Mycgr3T

Mycgr3G98961 Mycgr3T
  
Location: 37984-38884

Mycgr3G98961\_Mycgr3T

hypothetical protein
  
Accession: EQB51131
  
Location: 23595-25325
  
 NCBI BlastP on this gene

EQB51131

3-hydroxyacyl-CoA dehydrogenase
  
Accession: EQB51132
  
Location: 26335-27303
  
 NCBI BlastP on this gene

EQB51132

hypothetical protein
  
Accession: EQB51133
  
Location: 28219-28932
  
 NCBI BlastP on this gene

EQB51133

hypothetical protein
  
Accession: EQB51134
  
Location: 29099-30921
  
 NCBI BlastP on this gene

EQB51134

copper amine oxidase
  
Accession: EQB51135
  
Location: 32280-33719
  
 NCBI BlastP on this gene

EQB51135

phytanoyl-CoA dioxygenase
  
Accession: EQB51136
  
Location: 37589-38689
  
 NCBI BlastP on this gene

EQB51136

amino acid permease
  
Accession: EQB51137
  
Location: 40795-41335
  
 NCBI BlastP on this gene

EQB51137

brix domain-containing protein
  
Accession: EQB51138
  
Location: 42567-44066
  
  
**BlastP hit with Mycgr3G35447\_Mycgr3T**
  
Percentage identity: 55 %
  
BlastP bit score: 453
  
Sequence coverage: 100 %
  
E-value: 5e-153
  
  
 NCBI BlastP on this gene

EQB51138

hypothetical protein
  
Accession: EQB51139
  
Location: 44525-46247
  
  
**BlastP hit with Mycgr3G84402\_Mycgr3T**
  
Percentage identity: 69 %
  
BlastP bit score: 611
  
Sequence coverage: 94 %
  
E-value: 0.0
  
  
 NCBI BlastP on this gene

EQB51139

hypothetical protein
  
Accession: EQB51140
  
Location: 47709-48215
  
 NCBI BlastP on this gene

EQB51140

hypothetical protein
  
Accession: EQB51141
  
Location: 48887-50678
  
 NCBI BlastP on this gene

EQB51141

Query: Architecture Search FASTA input

AABX02000020 : Neurospora crassa OR74A    Total score: 2.0     Cumulative Blast bit score: 1064

Hit cluster cross-links:

Mycgr3G67791 Mycgr3T
  
Location: 0-1542

Mycgr3G67791\_Mycgr3T

Mycgr3G90406 Mycgr3T
  
Location: 1642-3973

Mycgr3G90406\_Mycgr3T

Mycgr3G67785 Mycgr3T
  
Location: 4073-7865

Mycgr3G67785\_Mycgr3T

Mycgr3G67795 Mycgr3T
  
Location: 7965-15249

Mycgr3G67795\_Mycgr3T

Mycgr3G67775 Mycgr3T
  
Location: 15349-16237

Mycgr3G67775\_Mycgr3T

Mycgr3G90404 Mycgr3T
  
Location: 16337-17246

Mycgr3G90404\_Mycgr3T

Mycgr3G36951 Mycgr3T
  
Location: 17346-30891

Mycgr3G36951\_Mycgr3T

Mycgr3G103034 Mycgr3
  
Location: 30991-32644

Mycgr3G103034\_Mycgr3

Mycgr3G31119 Mycgr3T
  
Location: 32744-32906

Mycgr3G31119\_Mycgr3T

Mycgr3G28587 Mycgr3T
  
Location: 33006-33489

Mycgr3G28587\_Mycgr3T

Mycgr3G98959 Mycgr3T
  
Location: 33589-35035

Mycgr3G98959\_Mycgr3T

Mycgr3G35447 Mycgr3T
  
Location: 35135-36443

Mycgr3G35447\_Mycgr3T

Mycgr3G84402 Mycgr3T
  
Location: 36543-37884

Mycgr3G84402\_Mycgr3T

Mycgr3G98961 Mycgr3T
  
Location: 37984-38884

Mycgr3G98961\_Mycgr3T

conserved hypothetical protein
  
Accession: EAA27686
  
Location: 55527-57951
  
 NCBI BlastP on this gene

EAA27686

hypothetical protein
  
Accession: EAA27685
  
Location: 58828-59881
  
 NCBI BlastP on this gene

EAA27685

hypothetical protein
  
Accession: EAA27684
  
Location: 60041-60745
  
 NCBI BlastP on this gene

EAA27684

predicted protein
  
Accession: EAA27683
  
Location: 61706-62020
  
 NCBI BlastP on this gene

EAA27683

hypothetical protein
  
Accession: EAA27682
  
Location: 62611-63885
  
 NCBI BlastP on this gene

EAA27682

conserved hypothetical protein
  
Accession: EAA27681
  
Location: 65403-66671
  
 NCBI BlastP on this gene

EAA27681

hypothetical protein
  
Accession: EAA27680
  
Location: 67852-70125
  
 NCBI BlastP on this gene

EAA27680

ATP-dependent rRNA helicase RRP3
  
Accession: EAA27679
  
Location: 70396-72093
  
  
**BlastP hit with Mycgr3G84402\_Mycgr3T**
  
Percentage identity: 73 %
  
BlastP bit score: 619
  
Sequence coverage: 90 %
  
E-value: 0.0
  
  
 NCBI BlastP on this gene

EAA27679

hypothetical protein
  
Accession: EAA27678
  
Location: 72454-73958
  
  
**BlastP hit with Mycgr3G35447\_Mycgr3T**
  
Percentage identity: 53 %
  
BlastP bit score: 445
  
Sequence coverage: 105 %
  
E-value: 2e-149
  
  
 NCBI BlastP on this gene

EAA27678

predicted protein
  
Accession: EAA27835
  
Location: 76023-76431
  
 NCBI BlastP on this gene

EAA27835

predicted protein
  
Accession: EAA27834
  
Location: 79736-80248
  
 NCBI BlastP on this gene

EAA27834

hypothetical protein
  
Accession: EAA27833
  
Location: 82503-84084
  
 NCBI BlastP on this gene

EAA27833

predicted protein
  
Accession: EAA27832
  
Location: 85919-87635
  
 NCBI BlastP on this gene

EAA27832

conserved hypothetical protein
  
Accession: EAA27831
  
Location: 89169-90921
  
 NCBI BlastP on this gene

EAA27831

predicted protein
  
Accession: EAA27830
  
Location: 91315-92525
  
 NCBI BlastP on this gene

EAA27830

Query: Architecture Search FASTA input

GL891303 : Neurospora tetrasperma FGSC 2508 unplaced genomic scaffold NEUTE1scaffold\_2    Total score: 2.0     Cumulative Blast bit score: 1063

Hit cluster cross-links:

Mycgr3G67791 Mycgr3T
  
Location: 0-1542

Mycgr3G67791\_Mycgr3T

Mycgr3G90406 Mycgr3T
  
Location: 1642-3973

Mycgr3G90406\_Mycgr3T

Mycgr3G67785 Mycgr3T
  
Location: 4073-7865

Mycgr3G67785\_Mycgr3T

Mycgr3G67795 Mycgr3T
  
Location: 7965-15249

Mycgr3G67795\_Mycgr3T

Mycgr3G67775 Mycgr3T
  
Location: 15349-16237

Mycgr3G67775\_Mycgr3T

Mycgr3G90404 Mycgr3T
  
Location: 16337-17246

Mycgr3G90404\_Mycgr3T

Mycgr3G36951 Mycgr3T
  
Location: 17346-30891

Mycgr3G36951\_Mycgr3T

Mycgr3G103034 Mycgr3
  
Location: 30991-32644

Mycgr3G103034\_Mycgr3

Mycgr3G31119 Mycgr3T
  
Location: 32744-32906

Mycgr3G31119\_Mycgr3T

Mycgr3G28587 Mycgr3T
  
Location: 33006-33489

Mycgr3G28587\_Mycgr3T

Mycgr3G98959 Mycgr3T
  
Location: 33589-35035

Mycgr3G98959\_Mycgr3T

Mycgr3G35447 Mycgr3T
  
Location: 35135-36443

Mycgr3G35447\_Mycgr3T

Mycgr3G84402 Mycgr3T
  
Location: 36543-37884

Mycgr3G84402\_Mycgr3T

Mycgr3G98961 Mycgr3T
  
Location: 37984-38884

Mycgr3G98961\_Mycgr3T

hypothetical protein
  
Accession: EGO59281
  
Location: 2957253-2959676
  
 NCBI BlastP on this gene

EGO59281

hypothetical protein
  
Accession: EGO59282
  
Location: 2960531-2961582
  
 NCBI BlastP on this gene

EGO59282

hypothetical protein
  
Accession: EGO59283
  
Location: 2962599-2963297
  
 NCBI BlastP on this gene

EGO59283

hypothetical protein
  
Accession: EGO59284
  
Location: 2964235-2964550
  
 NCBI BlastP on this gene

EGO59284

hypothetical protein
  
Accession: EGO59285
  
Location: 2965140-2966414
  
 NCBI BlastP on this gene

EGO59285

hypothetical protein
  
Accession: EGO59286
  
Location: 2967925-2969193
  
 NCBI BlastP on this gene

EGO59286

hypothetical protein
  
Accession: EGO59287
  
Location: 2970332-2972602
  
 NCBI BlastP on this gene

EGO59287

ATP-dependent rRNA helicase rrp-3
  
Accession: EGO59288
  
Location: 2972874-2974570
  
  
**BlastP hit with Mycgr3G84402\_Mycgr3T**
  
Percentage identity: 73 %
  
BlastP bit score: 619
  
Sequence coverage: 90 %
  
E-value: 0.0
  
  
 NCBI BlastP on this gene

EGO59288

hypothetical protein
  
Accession: EGO59289
  
Location: 2974932-2976436
  
  
**BlastP hit with Mycgr3G35447\_Mycgr3T**
  
Percentage identity: 54 %
  
BlastP bit score: 444
  
Sequence coverage: 105 %
  
E-value: 4e-149
  
  
 NCBI BlastP on this gene

EGO59289

hypothetical protein
  
Accession: EGO59290
  
Location: 2977877-2978176
  
 NCBI BlastP on this gene

EGO59290

hypothetical protein
  
Accession: EGO59291
  
Location: 2978426-2978833
  
 NCBI BlastP on this gene

EGO59291

hypothetical protein
  
Accession: EGO59292
  
Location: 2982139-2982651
  
 NCBI BlastP on this gene

EGO59292

hypothetical protein
  
Accession: EGO59293
  
Location: 2984883-2986464
  
 NCBI BlastP on this gene

EGO59293

hypothetical protein
  
Accession: EGO59294
  
Location: 2988305-2990020
  
 NCBI BlastP on this gene

EGO59294

hypothetical protein
  
Accession: EGO59295
  
Location: 2991589-2993295
  
 NCBI BlastP on this gene

EGO59295

hypothetical protein
  
Accession: EGO59296
  
Location: 2993710-2994920
  
 NCBI BlastP on this gene

EGO59296

Query: Architecture Search FASTA input

GL891217 : Neurospora tetrasperma FGSC 2509 unplaced genomic scaffold NEUTE2scaffold\_3    Total score: 2.0     Cumulative Blast bit score: 1063

Hit cluster cross-links:

Mycgr3G67791 Mycgr3T
  
Location: 0-1542

Mycgr3G67791\_Mycgr3T

Mycgr3G90406 Mycgr3T
  
Location: 1642-3973

Mycgr3G90406\_Mycgr3T

Mycgr3G67785 Mycgr3T
  
Location: 4073-7865

Mycgr3G67785\_Mycgr3T

Mycgr3G67795 Mycgr3T
  
Location: 7965-15249

Mycgr3G67795\_Mycgr3T

Mycgr3G67775 Mycgr3T
  
Location: 15349-16237

Mycgr3G67775\_Mycgr3T

Mycgr3G90404 Mycgr3T
  
Location: 16337-17246

Mycgr3G90404\_Mycgr3T

Mycgr3G36951 Mycgr3T
  
Location: 17346-30891

Mycgr3G36951\_Mycgr3T

Mycgr3G103034 Mycgr3
  
Location: 30991-32644

Mycgr3G103034\_Mycgr3

Mycgr3G31119 Mycgr3T
  
Location: 32744-32906

Mycgr3G31119\_Mycgr3T

Mycgr3G28587 Mycgr3T
  
Location: 33006-33489

Mycgr3G28587\_Mycgr3T

Mycgr3G98959 Mycgr3T
  
Location: 33589-35035

Mycgr3G98959\_Mycgr3T

Mycgr3G35447 Mycgr3T
  
Location: 35135-36443

Mycgr3G35447\_Mycgr3T

Mycgr3G84402 Mycgr3T
  
Location: 36543-37884

Mycgr3G84402\_Mycgr3T

Mycgr3G98961 Mycgr3T
  
Location: 37984-38884

Mycgr3G98961\_Mycgr3T

chaps-domain-containing protein
  
Accession: EGZ73402
  
Location: 2959489-2961912
  
 NCBI BlastP on this gene

EGZ73402

Aldo/keto reductase
  
Accession: EGZ73403
  
Location: 2962767-2963818
  
 NCBI BlastP on this gene

EGZ73403

HIT-like protein
  
Accession: EGZ73404
  
Location: 2964136-2964834
  
 NCBI BlastP on this gene

EGZ73404

hypothetical protein
  
Accession: EGZ73405
  
Location: 2965772-2966087
  
 NCBI BlastP on this gene

EGZ73405

hypothetical protein
  
Accession: EGZ73406
  
Location: 2966677-2967951
  
 NCBI BlastP on this gene

EGZ73406

hypothetical protein
  
Accession: EGZ73407
  
Location: 2969455-2970723
  
 NCBI BlastP on this gene

EGZ73407

hypothetical protein
  
Accession: EGZ73408
  
Location: 2971861-2974131
  
 NCBI BlastP on this gene

EGZ73408

ATP-dependent rRNA helicase rrp-3
  
Accession: EGZ73409
  
Location: 2974403-2976099
  
  
**BlastP hit with Mycgr3G84402\_Mycgr3T**
  
Percentage identity: 73 %
  
BlastP bit score: 619
  
Sequence coverage: 90 %
  
E-value: 0.0
  
  
 NCBI BlastP on this gene

EGZ73409

Brix-domain-containing protein
  
Accession: EGZ73410
  
Location: 2976461-2977965
  
  
**BlastP hit with Mycgr3G35447\_Mycgr3T**
  
Percentage identity: 54 %
  
BlastP bit score: 444
  
Sequence coverage: 105 %
  
E-value: 4e-149
  
  
 NCBI BlastP on this gene

EGZ73410

hypothetical protein
  
Accession: EGZ73411
  
Location: 2979403-2979702
  
 NCBI BlastP on this gene

EGZ73411

hypothetical protein
  
Accession: EGZ73412
  
Location: 2979949-2980356
  
 NCBI BlastP on this gene

EGZ73412

hypothetical protein
  
Accession: EGZ73413
  
Location: 2983662-2984174
  
 NCBI BlastP on this gene

EGZ73413

glycoside hydrolase
  
Accession: EGZ73414
  
Location: 2986406-2987987
  
 NCBI BlastP on this gene

EGZ73414

hypothetical protein
  
Accession: EGZ73415
  
Location: 2989828-2991543
  
 NCBI BlastP on this gene

EGZ73415

hypothetical protein
  
Accession: EGZ73416
  
Location: 2993112-2994818
  
 NCBI BlastP on this gene

EGZ73416

hypothetical protein
  
Accession: EGZ73417
  
Location: 2995233-2996443
  
 NCBI BlastP on this gene

EGZ73417

Query: Architecture Search FASTA input

KB020987 : Colletotrichum gloeosporioides Nara gc5 unplaced genomic scaffold scaffold586    Total score: 2.0     Cumulative Blast bit score: 1060

Hit cluster cross-links:

Mycgr3G67791 Mycgr3T
  
Location: 0-1542

Mycgr3G67791\_Mycgr3T

Mycgr3G90406 Mycgr3T
  
Location: 1642-3973

Mycgr3G90406\_Mycgr3T

Mycgr3G67785 Mycgr3T
  
Location: 4073-7865

Mycgr3G67785\_Mycgr3T

Mycgr3G67795 Mycgr3T
  
Location: 7965-15249

Mycgr3G67795\_Mycgr3T

Mycgr3G67775 Mycgr3T
  
Location: 15349-16237

Mycgr3G67775\_Mycgr3T

Mycgr3G90404 Mycgr3T
  
Location: 16337-17246

Mycgr3G90404\_Mycgr3T

Mycgr3G36951 Mycgr3T
  
Location: 17346-30891

Mycgr3G36951\_Mycgr3T

Mycgr3G103034 Mycgr3
  
Location: 30991-32644

Mycgr3G103034\_Mycgr3

Mycgr3G31119 Mycgr3T
  
Location: 32744-32906

Mycgr3G31119\_Mycgr3T

Mycgr3G28587 Mycgr3T
  
Location: 33006-33489

Mycgr3G28587\_Mycgr3T

Mycgr3G98959 Mycgr3T
  
Location: 33589-35035

Mycgr3G98959\_Mycgr3T

Mycgr3G35447 Mycgr3T
  
Location: 35135-36443

Mycgr3G35447\_Mycgr3T

Mycgr3G84402 Mycgr3T
  
Location: 36543-37884

Mycgr3G84402\_Mycgr3T

Mycgr3G98961 Mycgr3T
  
Location: 37984-38884

Mycgr3G98961\_Mycgr3T

microsomal signal peptidase 18 kda subunit
  
Accession: ELA27449
  
Location: 96541-97351
  
 NCBI BlastP on this gene

ELA27449

hypothetical protein
  
Accession: ELA27450
  
Location: 98212-99112
  
 NCBI BlastP on this gene

ELA27450

hypothetical protein
  
Accession: ELA27451
  
Location: 99531-100160
  
 NCBI BlastP on this gene

ELA27451

transcription factor tfiiib complex subunit brf1
  
Accession: ELA27452
  
Location: 102067-104313
  
 NCBI BlastP on this gene

ELA27452

kinesin family protein
  
Accession: ELA27453
  
Location: 104726-107286
  
 NCBI BlastP on this gene

ELA27453

hypothetical protein
  
Accession: ELA27454
  
Location: 110618-112409
  
 NCBI BlastP on this gene

ELA27454

antigenic cell wall
  
Accession: ELA27455
  
Location: 113082-113588
  
 NCBI BlastP on this gene

ELA27455

ATP-dependent rRNA helicase rrp3
  
Accession: ELA27456
  
Location: 115041-116764
  
  
**BlastP hit with Mycgr3G84402\_Mycgr3T**
  
Percentage identity: 69 %
  
BlastP bit score: 608
  
Sequence coverage: 94 %
  
E-value: 0.0
  
  
 NCBI BlastP on this gene

ELA27456

ribosome biogenesis protein
  
Accession: ELA27457
  
Location: 117211-118708
  
  
**BlastP hit with Mycgr3G35447\_Mycgr3T**
  
Percentage identity: 55 %
  
BlastP bit score: 452
  
Sequence coverage: 100 %
  
E-value: 9e-153
  
  
 NCBI BlastP on this gene

ELA27457

phytanoyl- dioxygenase family protein
  
Accession: ELA27458
  
Location: 122554-123656
  
 NCBI BlastP on this gene

ELA27458

copper amine oxidase
  
Accession: ELA27459
  
Location: 127530-129917
  
 NCBI BlastP on this gene

ELA27459

hypothetical protein
  
Accession: ELA27460
  
Location: 130123-131652
  
 NCBI BlastP on this gene

ELA27460

3-hydroxyacyl- dehyrogenase
  
Accession: ELA27461
  
Location: 132577-133290
  
 NCBI BlastP on this gene

ELA27461

3-hydroxyacyl- dehydrogenase
  
Accession: ELA27462
  
Location: 134159-135127
  
 NCBI BlastP on this gene

ELA27462

alpha methylacyl-CoA racemase, putative
  
Accession: ELA27463
  
Location: 136142-137872
  
 NCBI BlastP on this gene

ELA27463

Query: Architecture Search FASTA input

CH445336 : Phaeosphaeria nodorum SN15 scaffold\_12    Total score: 2.0     Cumulative Blast bit score: 1060

Hit cluster cross-links:

Mycgr3G67791 Mycgr3T
  
Location: 0-1542

Mycgr3G67791\_Mycgr3T

Mycgr3G90406 Mycgr3T
  
Location: 1642-3973

Mycgr3G90406\_Mycgr3T

Mycgr3G67785 Mycgr3T
  
Location: 4073-7865

Mycgr3G67785\_Mycgr3T

Mycgr3G67795 Mycgr3T
  
Location: 7965-15249

Mycgr3G67795\_Mycgr3T

Mycgr3G67775 Mycgr3T
  
Location: 15349-16237

Mycgr3G67775\_Mycgr3T

Mycgr3G90404 Mycgr3T
  
Location: 16337-17246

Mycgr3G90404\_Mycgr3T

Mycgr3G36951 Mycgr3T
  
Location: 17346-30891

Mycgr3G36951\_Mycgr3T

Mycgr3G103034 Mycgr3
  
Location: 30991-32644

Mycgr3G103034\_Mycgr3

Mycgr3G31119 Mycgr3T
  
Location: 32744-32906

Mycgr3G31119\_Mycgr3T

Mycgr3G28587 Mycgr3T
  
Location: 33006-33489

Mycgr3G28587\_Mycgr3T

Mycgr3G98959 Mycgr3T
  
Location: 33589-35035

Mycgr3G98959\_Mycgr3T

Mycgr3G35447 Mycgr3T
  
Location: 35135-36443

Mycgr3G35447\_Mycgr3T

Mycgr3G84402 Mycgr3T
  
Location: 36543-37884

Mycgr3G84402\_Mycgr3T

Mycgr3G98961 Mycgr3T
  
Location: 37984-38884

Mycgr3G98961\_Mycgr3T

hypothetical protein
  
Accession: EAT84604
  
Location: 917040-917522
  
 NCBI BlastP on this gene

EAT84604

hypothetical protein
  
Accession: EAT84603
  
Location: 914209-915338
  
 NCBI BlastP on this gene

EAT84603

hypothetical protein
  
Accession: EAT84602
  
Location: 911534-912481
  
 NCBI BlastP on this gene

EAT84602

hypothetical protein
  
Accession: EAT84601
  
Location: 910374-910488
  
 NCBI BlastP on this gene

EAT84601

hypothetical protein
  
Accession: EAT84600
  
Location: 907974-909869
  
 NCBI BlastP on this gene

EAT84600

hypothetical protein
  
Accession: EAT84599
  
Location: 904906-906937
  
 NCBI BlastP on this gene

EAT84599

hypothetical protein
  
Accession: EAT84598
  
Location: 901855-903030
  
 NCBI BlastP on this gene

EAT84598

hypothetical protein
  
Accession: EAT84597
  
Location: 899512-901131
  
 NCBI BlastP on this gene

EAT84597

hypothetical protein
  
Accession: EAT84596
  
Location: 897642-898904
  
  
**BlastP hit with Mycgr3G67775\_Mycgr3T**
  
Percentage identity: 59 %
  
BlastP bit score: 361
  
Sequence coverage: 104 %
  
E-value: 3e-121
  
  
 NCBI BlastP on this gene

EAT84596

hypothetical protein
  
Accession: EAT84595
  
Location: 896633-897127
  
 NCBI BlastP on this gene

EAT84595

hypothetical protein
  
Accession: EAT84594
  
Location: 894466-896045
  
  
**BlastP hit with Mycgr3G98959\_Mycgr3T**
  
Percentage identity: 79 %
  
BlastP bit score: 699
  
Sequence coverage: 86 %
  
E-value: 0.0
  
  
 NCBI BlastP on this gene

EAT84594

hypothetical protein
  
Accession: EAT84593
  
Location: 892835-893653
  
 NCBI BlastP on this gene

EAT84593

hypothetical protein
  
Accession: EAT84592
  
Location: 891727-892156
  
 NCBI BlastP on this gene

EAT84592

hypothetical protein
  
Accession: EAT84591
  
Location: 889945-891648
  
 NCBI BlastP on this gene

EAT84591

hypothetical protein
  
Accession: EAT84590
  
Location: 889078-889557
  
 NCBI BlastP on this gene

EAT84590

hypothetical protein
  
Accession: EAT84589
  
Location: 887997-889148
  
 NCBI BlastP on this gene

EAT84589

hypothetical protein
  
Accession: EAT84588
  
Location: 886503-886796
  
 NCBI BlastP on this gene

EAT84588

hypothetical protein
  
Accession: EDP89787
  
Location: 884559-885029
  
 NCBI BlastP on this gene

EDP89787

hypothetical protein
  
Accession: EDP89786
  
Location: 884208-884501
  
 NCBI BlastP on this gene

EDP89786

hypothetical protein
  
Accession: EAT84586
  
Location: 882318-883284
  
 NCBI BlastP on this gene

EAT84586

hypothetical protein
  
Accession: EAT84585
  
Location: 881230-882208
  
 NCBI BlastP on this gene

EAT84585

hypothetical protein
  
Accession: EAT84584
  
Location: 878237-879665
  
 NCBI BlastP on this gene

EAT84584

hypothetical protein
  
Accession: EAT84583
  
Location: 877128-877815
  
 NCBI BlastP on this gene

EAT84583

hypothetical protein
  
Accession: EAT84582
  
Location: 875487-876248
  
 NCBI BlastP on this gene

EAT84582

Query: Architecture Search FASTA input

ABDG02000017 : Trichoderma atroviride IMI 206040    Total score: 2.0     Cumulative Blast bit score: 1059

Hit cluster cross-links:

Mycgr3G67791 Mycgr3T
  
Location: 0-1542

Mycgr3G67791\_Mycgr3T

Mycgr3G90406 Mycgr3T
  
Location: 1642-3973

Mycgr3G90406\_Mycgr3T

Mycgr3G67785 Mycgr3T
  
Location: 4073-7865

Mycgr3G67785\_Mycgr3T

Mycgr3G67795 Mycgr3T
  
Location: 7965-15249

Mycgr3G67795\_Mycgr3T

Mycgr3G67775 Mycgr3T
  
Location: 15349-16237

Mycgr3G67775\_Mycgr3T

Mycgr3G90404 Mycgr3T
  
Location: 16337-17246

Mycgr3G90404\_Mycgr3T

Mycgr3G36951 Mycgr3T
  
Location: 17346-30891

Mycgr3G36951\_Mycgr3T

Mycgr3G103034 Mycgr3
  
Location: 30991-32644

Mycgr3G103034\_Mycgr3

Mycgr3G31119 Mycgr3T
  
Location: 32744-32906

Mycgr3G31119\_Mycgr3T

Mycgr3G28587 Mycgr3T
  
Location: 33006-33489

Mycgr3G28587\_Mycgr3T

Mycgr3G98959 Mycgr3T
  
Location: 33589-35035

Mycgr3G98959\_Mycgr3T

Mycgr3G35447 Mycgr3T
  
Location: 35135-36443

Mycgr3G35447\_Mycgr3T

Mycgr3G84402 Mycgr3T
  
Location: 36543-37884

Mycgr3G84402\_Mycgr3T

Mycgr3G98961 Mycgr3T
  
Location: 37984-38884

Mycgr3G98961\_Mycgr3T

hypothetical protein
  
Accession: EHK49251
  
Location: 1140747-1141183
  
 NCBI BlastP on this gene

EHK49251

hypothetical protein
  
Accession: EHK49252
  
Location: 1141628-1142538
  
 NCBI BlastP on this gene

EHK49252

hypothetical protein
  
Accession: EHK49253
  
Location: 1143613-1144102
  
 NCBI BlastP on this gene

EHK49253

hypothetical protein
  
Accession: EHK49254
  
Location: 1144454-1145107
  
 NCBI BlastP on this gene

EHK49254

hypothetical protein
  
Accession: EHK49255
  
Location: 1145926-1146236
  
 NCBI BlastP on this gene

EHK49255

hypothetical protein
  
Accession: EHK49256
  
Location: 1146650-1147895
  
 NCBI BlastP on this gene

EHK49256

hypothetical protein
  
Accession: EHK49257
  
Location: 1148282-1150685
  
 NCBI BlastP on this gene

EHK49257

hypothetical protein
  
Accession: EHK49258
  
Location: 1151293-1157612
  
 NCBI BlastP on this gene

EHK49258

hypothetical protein
  
Accession: EHK49259
  
Location: 1158257-1158742
  
 NCBI BlastP on this gene

EHK49259

hypothetical protein
  
Accession: EHK49260
  
Location: 1159122-1160638
  
  
**BlastP hit with Mycgr3G84402\_Mycgr3T**
  
Percentage identity: 73 %
  
BlastP bit score: 620
  
Sequence coverage: 87 %
  
E-value: 0.0
  
  
 NCBI BlastP on this gene

EHK49260

hypothetical protein
  
Accession: EHK49261
  
Location: 1161131-1162567
  
  
**BlastP hit with Mycgr3G35447\_Mycgr3T**
  
Percentage identity: 57 %
  
BlastP bit score: 439
  
Sequence coverage: 90 %
  
E-value: 1e-147
  
  
 NCBI BlastP on this gene

EHK49261

hypothetical protein
  
Accession: EHK49262
  
Location: 1162975-1163476
  
 NCBI BlastP on this gene

EHK49262

hypothetical protein
  
Accession: EHK49263
  
Location: 1164655-1166349
  
 NCBI BlastP on this gene

EHK49263

hypothetical protein
  
Accession: EHK49264
  
Location: 1167407-1169221
  
 NCBI BlastP on this gene

EHK49264

hypothetical protein
  
Accession: EHK49265
  
Location: 1169389-1170366
  
 NCBI BlastP on this gene

EHK49265

hypothetical protein
  
Accession: EHK49266
  
Location: 1171697-1173199
  
 NCBI BlastP on this gene

EHK49266

hypothetical protein
  
Accession: EHK49267
  
Location: 1175948-1177012
  
 NCBI BlastP on this gene

EHK49267

hypothetical protein
  
Accession: EHK49268
  
Location: 1178006-1179635
  
 NCBI BlastP on this gene

EHK49268

hypothetical protein
  
Accession: EHK49269
  
Location: 1180154-1183414
  
 NCBI BlastP on this gene

EHK49269

Query: Architecture Search FASTA input

JH126405 : Cordyceps militaris CM01 unplaced genomic scaffold CCM\_S00007    Total score: 2.0     Cumulative Blast bit score: 1058

Hit cluster cross-links:

Mycgr3G67791 Mycgr3T
  
Location: 0-1542

Mycgr3G67791\_Mycgr3T

Mycgr3G90406 Mycgr3T
  
Location: 1642-3973

Mycgr3G90406\_Mycgr3T

Mycgr3G67785 Mycgr3T
  
Location: 4073-7865

Mycgr3G67785\_Mycgr3T

Mycgr3G67795 Mycgr3T
  
Location: 7965-15249

Mycgr3G67795\_Mycgr3T

Mycgr3G67775 Mycgr3T
  
Location: 15349-16237

Mycgr3G67775\_Mycgr3T

Mycgr3G90404 Mycgr3T
  
Location: 16337-17246

Mycgr3G90404\_Mycgr3T

Mycgr3G36951 Mycgr3T
  
Location: 17346-30891

Mycgr3G36951\_Mycgr3T

Mycgr3G103034 Mycgr3
  
Location: 30991-32644

Mycgr3G103034\_Mycgr3

Mycgr3G31119 Mycgr3T
  
Location: 32744-32906

Mycgr3G31119\_Mycgr3T

Mycgr3G28587 Mycgr3T
  
Location: 33006-33489

Mycgr3G28587\_Mycgr3T

Mycgr3G98959 Mycgr3T
  
Location: 33589-35035

Mycgr3G98959\_Mycgr3T

Mycgr3G35447 Mycgr3T
  
Location: 35135-36443

Mycgr3G35447\_Mycgr3T

Mycgr3G84402 Mycgr3T
  
Location: 36543-37884

Mycgr3G84402\_Mycgr3T

Mycgr3G98961 Mycgr3T
  
Location: 37984-38884

Mycgr3G98961\_Mycgr3T

MFS transporter
  
Accession: EGX88308
  
Location: 428366-430296
  
 NCBI BlastP on this gene

EGX88308

hypothetical protein
  
Accession: EGX88309
  
Location: 437781-438897
  
 NCBI BlastP on this gene

EGX88309

amidase, putative
  
Accession: EGX88310
  
Location: 439053-440279
  
 NCBI BlastP on this gene

EGX88310

pyruvate dehydrogenase, putative
  
Accession: EGX88311
  
Location: 441951-443503
  
 NCBI BlastP on this gene

EGX88311

Protein kinase-like domain
  
Accession: EGX88312
  
Location: 444141-445646
  
 NCBI BlastP on this gene

EGX88312

ribosome biogenesis protein Ssf2, putative
  
Accession: EGX88313
  
Location: 446277-447641
  
  
**BlastP hit with Mycgr3G35447\_Mycgr3T**
  
Percentage identity: 57 %
  
BlastP bit score: 450
  
Sequence coverage: 93 %
  
E-value: 7e-152
  
  
 NCBI BlastP on this gene

EGX88313

ATP-dependent rRNA helicase RRP3
  
Accession: EGX88314
  
Location: 448132-449616
  
  
**BlastP hit with Mycgr3G84402\_Mycgr3T**
  
Percentage identity: 65 %
  
BlastP bit score: 608
  
Sequence coverage: 103 %
  
E-value: 0.0
  
  
 NCBI BlastP on this gene

EGX88314

transcriptional corepressor of histone (Hir3), putative
  
Accession: EGX88315
  
Location: 449883-456625
  
 NCBI BlastP on this gene

EGX88315

clathrin-coated vesiclec protein (Bud7), putative
  
Accession: EGX88316
  
Location: 457244-459558
  
 NCBI BlastP on this gene

EGX88316

ATP binding protein
  
Accession: EGX88317
  
Location: 460036-461265
  
 NCBI BlastP on this gene

EGX88317

metallothionein-I transcription activator
  
Accession: EGX88318
  
Location: 461522-461845
  
 NCBI BlastP on this gene

EGX88318

Histidine triad-like protein
  
Accession: EGX88319
  
Location: 462643-463224
  
 NCBI BlastP on this gene

EGX88319

Caldecrin precursor (Chymotrypsin C) isoform 3
  
Accession: EGX88320
  
Location: 463651-464971
  
 NCBI BlastP on this gene

EGX88320

hypothetical protein
  
Accession: EGX88321
  
Location: 465663-466389
  
 NCBI BlastP on this gene

EGX88321

hypothetical protein
  
Accession: EGX88322
  
Location: 466837-467323
  
 NCBI BlastP on this gene

EGX88322

high affinity methionine permease
  
Accession: EGX88323
  
Location: 468935-470955
  
 NCBI BlastP on this gene

EGX88323

Query: Architecture Search FASTA input

GL385396 : Gaeumannomyces graminis var. tritici R3-111a-1 unplaced genomic scaffold supercont2.2    Total score: 2.0     Cumulative Blast bit score: 1054

Hit cluster cross-links:

Mycgr3G67791 Mycgr3T
  
Location: 0-1542

Mycgr3G67791\_Mycgr3T

Mycgr3G90406 Mycgr3T
  
Location: 1642-3973

Mycgr3G90406\_Mycgr3T

Mycgr3G67785 Mycgr3T
  
Location: 4073-7865

Mycgr3G67785\_Mycgr3T

Mycgr3G67795 Mycgr3T
  
Location: 7965-15249

Mycgr3G67795\_Mycgr3T

Mycgr3G67775 Mycgr3T
  
Location: 15349-16237

Mycgr3G67775\_Mycgr3T

Mycgr3G90404 Mycgr3T
  
Location: 16337-17246

Mycgr3G90404\_Mycgr3T

Mycgr3G36951 Mycgr3T
  
Location: 17346-30891

Mycgr3G36951\_Mycgr3T

Mycgr3G103034 Mycgr3
  
Location: 30991-32644

Mycgr3G103034\_Mycgr3

Mycgr3G31119 Mycgr3T
  
Location: 32744-32906

Mycgr3G31119\_Mycgr3T

Mycgr3G28587 Mycgr3T
  
Location: 33006-33489

Mycgr3G28587\_Mycgr3T

Mycgr3G98959 Mycgr3T
  
Location: 33589-35035

Mycgr3G98959\_Mycgr3T

Mycgr3G35447 Mycgr3T
  
Location: 35135-36443

Mycgr3G35447\_Mycgr3T

Mycgr3G84402 Mycgr3T
  
Location: 36543-37884

Mycgr3G84402\_Mycgr3T

Mycgr3G98961 Mycgr3T
  
Location: 37984-38884

Mycgr3G98961\_Mycgr3T

ATPase NPA3
  
Accession: EJT78748
  
Location: 3803861-3805164
  
 NCBI BlastP on this gene

EJT78748

hypothetical protein
  
Accession: EJT78749
  
Location: 3805615-3806289
  
 NCBI BlastP on this gene

EJT78749

hypothetical protein
  
Accession: EJT78750
  
Location: 3806859-3807446
  
 NCBI BlastP on this gene

EJT78750

hypothetical protein
  
Accession: EJT78751
  
Location: 3808017-3809653
  
 NCBI BlastP on this gene

EJT78751

hypothetical protein
  
Accession: EJT78752
  
Location: 3811315-3812289
  
 NCBI BlastP on this gene

EJT78752

hypothetical protein
  
Accession: EJT78753
  
Location: 3812606-3813591
  
 NCBI BlastP on this gene

EJT78753

hypothetical protein
  
Accession: EJT78754
  
Location: 3814658-3817077
  
 NCBI BlastP on this gene

EJT78754

hypothetical protein
  
Accession: EJT78755
  
Location: 3817478-3817795
  
 NCBI BlastP on this gene

EJT78755

hypothetical protein
  
Accession: EJT78756
  
Location: 3818534-3819865
  
 NCBI BlastP on this gene

EJT78756

hypothetical protein
  
Accession: EJT78757
  
Location: 3820965-3821570
  
 NCBI BlastP on this gene

EJT78757

ATP-dependent rRNA helicase RRP3
  
Accession: EJT78758
  
Location: 3822053-3823781
  
  
**BlastP hit with Mycgr3G84402\_Mycgr3T**
  
Percentage identity: 72 %
  
BlastP bit score: 608
  
Sequence coverage: 89 %
  
E-value: 0.0
  
  
 NCBI BlastP on this gene

EJT78758

ribosome biogenesis protein SSF1
  
Accession: EJT78759
  
Location: 3824120-3825619
  
  
**BlastP hit with Mycgr3G35447\_Mycgr3T**
  
Percentage identity: 58 %
  
BlastP bit score: 446
  
Sequence coverage: 94 %
  
E-value: 5e-150
  
  
 NCBI BlastP on this gene

EJT78759

hypothetical protein
  
Accession: EJT78760
  
Location: 3826055-3827677
  
 NCBI BlastP on this gene

EJT78760

choline dehydrogenase
  
Accession: EJT78761
  
Location: 3827904-3829796
  
 NCBI BlastP on this gene

EJT78761

hypothetical protein
  
Accession: EJT78762
  
Location: 3831230-3832237
  
 NCBI BlastP on this gene

EJT78762

hypothetical protein
  
Accession: EJT78763
  
Location: 3833547-3835439
  
 NCBI BlastP on this gene

EJT78763

hypothetical protein
  
Accession: EJT78764
  
Location: 3835608-3837422
  
 NCBI BlastP on this gene

EJT78764

hypothetical protein
  
Accession: EJT78765
  
Location: 3838952-3839491
  
 NCBI BlastP on this gene

EJT78765

hypothetical protein
  
Accession: EJT78766
  
Location: 3840183-3841378
  
 NCBI BlastP on this gene

EJT78766

ABC transporter
  
Accession: EJT78767
  
Location: 3843884-3849165
  
 NCBI BlastP on this gene

EJT78767

Query: Architecture Search FASTA input

101. :  KB446542 Dothistroma septosporum NZE10 unplaced genomic scaffold DOTSEscaffold\_8     Total score: 2.0     Cumulative Blast bit score: 1403

Mycgr3G67791 Mycgr3T
  
Location: 0-1542
  
 NCBI BlastP on this gene

Mycgr3G67791\_Mycgr3T

Mycgr3G90406 Mycgr3T
  
Location: 1642-3973
  
 NCBI BlastP on this gene

Mycgr3G90406\_Mycgr3T

Mycgr3G67785 Mycgr3T
  
Location: 4073-7865
  
 NCBI BlastP on this gene

Mycgr3G67785\_Mycgr3T

Mycgr3G67795 Mycgr3T
  
Location: 7965-15249
  
 NCBI BlastP on this gene

Mycgr3G67795\_Mycgr3T

Mycgr3G67775 Mycgr3T
  
Location: 15349-16237
  
 NCBI BlastP on this gene

Mycgr3G67775\_Mycgr3T

Mycgr3G90404 Mycgr3T
  
Location: 16337-17246
  
 NCBI BlastP on this gene

Mycgr3G90404\_Mycgr3T

Mycgr3G36951 Mycgr3T
  
Location: 17346-30891
  
 NCBI BlastP on this gene

Mycgr3G36951\_Mycgr3T

Mycgr3G103034 Mycgr3
  
Location: 30991-32644
  
 NCBI BlastP on this gene

Mycgr3G103034\_Mycgr3

Mycgr3G31119 Mycgr3T
  
Location: 32744-32906
  
 NCBI BlastP on this gene

Mycgr3G31119\_Mycgr3T

Mycgr3G28587 Mycgr3T
  
Location: 33006-33489
  
 NCBI BlastP on this gene

Mycgr3G28587\_Mycgr3T

Mycgr3G98959 Mycgr3T
  
Location: 33589-35035
  
 NCBI BlastP on this gene

Mycgr3G98959\_Mycgr3T

Mycgr3G35447 Mycgr3T
  
Location: 35135-36443
  
 NCBI BlastP on this gene

Mycgr3G35447\_Mycgr3T

Mycgr3G84402 Mycgr3T
  
Location: 36543-37884
  
 NCBI BlastP on this gene

Mycgr3G84402\_Mycgr3T

Mycgr3G98961 Mycgr3T
  
Location: 37984-38884
  
 NCBI BlastP on this gene

Mycgr3G98961\_Mycgr3T

hypothetical protein
  
Accession: EME41272
  
Location: 349025-350310
  
 NCBI BlastP on this gene

EME41272

hypothetical protein
  
Accession: EME41273
  
Location: 350944-352110
  
 NCBI BlastP on this gene

EME41273

hypothetical protein
  
Accession: EME41274
  
Location: 352712-353245
  
 NCBI BlastP on this gene

EME41274

hypothetical protein
  
Accession: EME41275
  
Location: 354563-356878
  
 NCBI BlastP on this gene

EME41275

hypothetical protein
  
Accession: EME41276
  
Location: 358612-361134
  
 NCBI BlastP on this gene

EME41276

hypothetical protein
  
Accession: EME41277
  
Location: 362493-365000
  
 NCBI BlastP on this gene

EME41277

hypothetical protein
  
Accession: EME41278
  
Location: 366099-367262
  
 NCBI BlastP on this gene

EME41278

hypothetical protein
  
Accession: EME41279
  
Location: 367579-369120
  
  
**BlastP hit with Mycgr3G84402\_Mycgr3T**
  
Percentage identity: 89 %
  
BlastP bit score: 752
  
Sequence coverage: 91 %
  
E-value: 0.0
  
  
 NCBI BlastP on this gene

EME41279

hypothetical protein
  
Accession: EME41280
  
Location: 369314-370714
  
  
**BlastP hit with Mycgr3G35447\_Mycgr3T**
  
Percentage identity: 74 %
  
BlastP bit score: 651
  
Sequence coverage: 96 %
  
E-value: 0.0
  
  
 NCBI BlastP on this gene

EME41280

hypothetical protein
  
Accession: EME41281
  
Location: 373239-373854
  
 NCBI BlastP on this gene

EME41281

hypothetical protein
  
Accession: EME41283
  
Location: 376445-378245
  
 NCBI BlastP on this gene

EME41283

hypothetical protein
  
Accession: EME41284
  
Location: 379550-380364
  
 NCBI BlastP on this gene

EME41284

hypothetical protein
  
Accession: EME41285
  
Location: 381721-382374
  
 NCBI BlastP on this gene

EME41285

carbohydrate-binding module family 14 protein
  
Accession: EME41286
  
Location: 384698-385195
  
 NCBI BlastP on this gene

EME41286

hypothetical protein
  
Accession: EME41287
  
Location: 385453-389313
  
 NCBI BlastP on this gene

EME41287

hypothetical protein
  
Accession: EME41288
  
Location: 389953-390141
  
 NCBI BlastP on this gene

EME41288

102. :  KB445561 Baudoinia compniacensis UAMH 10762 unplaced genomic scaffold BAUCOscaffold\_12     Total score: 2.0     Cumulative Blast bit score: 1354

hypothetical protein
  
Accession: EMC92627
  
Location: 153228-154769
  
 NCBI BlastP on this gene

EMC92627

hypothetical protein
  
Accession: EMC92628
  
Location: 156325-157119
  
 NCBI BlastP on this gene

EMC92628

hypothetical protein
  
Accession: EMC92629
  
Location: 157441-158089
  
 NCBI BlastP on this gene

EMC92629

hypothetical protein
  
Accession: EMC92630
  
Location: 158225-159192
  
 NCBI BlastP on this gene

EMC92630

hypothetical protein
  
Accession: EMC92631
  
Location: 160175-163936
  
 NCBI BlastP on this gene

EMC92631

hypothetical protein
  
Accession: EMC92632
  
Location: 165289-167097
  
 NCBI BlastP on this gene

EMC92632

glycosyltransferase family 71 protein
  
Accession: EMC92633
  
Location: 168375-169706
  
 NCBI BlastP on this gene

EMC92633

hypothetical protein
  
Accession: EMC92634
  
Location: 170127-170726
  
 NCBI BlastP on this gene

EMC92634

hypothetical protein
  
Accession: EMC92635
  
Location: 170801-171691
  
  
**BlastP hit with Mycgr3G67775\_Mycgr3T**
  
Percentage identity: 78 %
  
BlastP bit score: 483
  
Sequence coverage: 100 %
  
E-value: 2e-169
  
  
 NCBI BlastP on this gene

EMC92635

hypothetical protein
  
Accession: EMC92636
  
Location: 172150-173656
  
  
**BlastP hit with Mycgr3G98959\_Mycgr3T**
  
Percentage identity: 86 %
  
BlastP bit score: 871
  
Sequence coverage: 101 %
  
E-value: 0.0
  
  
 NCBI BlastP on this gene

EMC92636

hypothetical protein
  
Accession: EMC92637
  
Location: 174470-174703
  
 NCBI BlastP on this gene

EMC92637

hypothetical protein
  
Accession: EMC92638
  
Location: 174993-175280
  
 NCBI BlastP on this gene

EMC92638

hypothetical protein
  
Accession: EMC92639
  
Location: 176369-176638
  
 NCBI BlastP on this gene

EMC92639

hypothetical protein
  
Accession: EMC92640
  
Location: 177769-178137
  
 NCBI BlastP on this gene

EMC92640

hypothetical protein
  
Accession: EMC92641
  
Location: 179803-180665
  
 NCBI BlastP on this gene

EMC92641

hypothetical protein
  
Accession: EMC92642
  
Location: 180927-181490
  
 NCBI BlastP on this gene

EMC92642

hypothetical protein
  
Accession: EMC92643
  
Location: 181769-182424
  
 NCBI BlastP on this gene

EMC92643

hypothetical protein
  
Accession: EMC92644
  
Location: 182899-183996
  
 NCBI BlastP on this gene

EMC92644

hypothetical protein
  
Accession: EMC92645
  
Location: 184773-185060
  
 NCBI BlastP on this gene

EMC92645

hypothetical protein
  
Accession: EMC92646
  
Location: 185782-187573
  
 NCBI BlastP on this gene

EMC92646

hypothetical protein
  
Accession: EMC92647
  
Location: 187710-189691
  
 NCBI BlastP on this gene

EMC92647

hypothetical protein
  
Accession: EMC92648
  
Location: 189716-189871
  
 NCBI BlastP on this gene

EMC92648

hypothetical protein
  
Accession: EMC92649
  
Location: 191920-192923
  
 NCBI BlastP on this gene

EMC92649

103. :  KB446566 Pseudocercospora fijiensis CIRAD86 unplaced genomic scaffold MYCFIscaffold\_12     Total score: 2.0     Cumulative Blast bit score: 1322

hypothetical protein
  
Accession: EME77405
  
Location: 1150927-1152879
  
 NCBI BlastP on this gene

EME77405

hypothetical protein
  
Accession: EME77406
  
Location: 1153296-1154701
  
 NCBI BlastP on this gene

EME77406

hypothetical protein
  
Accession: EME77407
  
Location: 1155393-1156790
  
 NCBI BlastP on this gene

EME77407

hypothetical protein
  
Accession: EME77408
  
Location: 1156960-1158511
  
 NCBI BlastP on this gene

EME77408

hypothetical protein
  
Accession: EME77409
  
Location: 1159267-1160631
  
  
**BlastP hit with Mycgr3G35447\_Mycgr3T**
  
Percentage identity: 71 %
  
BlastP bit score: 578
  
Sequence coverage: 90 %
  
E-value: 0.0
  
  
 NCBI BlastP on this gene

EME77409

hypothetical protein
  
Accession: EME77410
  
Location: 1160871-1162367
  
  
**BlastP hit with Mycgr3G84402\_Mycgr3T**
  
Percentage identity: 88 %
  
BlastP bit score: 744
  
Sequence coverage: 89 %
  
E-value: 0.0
  
  
 NCBI BlastP on this gene

EME77410

hypothetical protein
  
Accession: EME77411
  
Location: 1163031-1163869
  
 NCBI BlastP on this gene

EME77411

hypothetical protein
  
Accession: EME77413
  
Location: 1164854-1169444
  
 NCBI BlastP on this gene

EME77413

hypothetical protein
  
Accession: EME77414
  
Location: 1170792-1171562
  
 NCBI BlastP on this gene

EME77414

hypothetical protein
  
Accession: EME77415
  
Location: 1180053-1181913
  
 NCBI BlastP on this gene

EME77415

hypothetical protein
  
Accession: EME77416
  
Location: 1181886-1183973
  
 NCBI BlastP on this gene

EME77416

104. :  KB456266 Mycosphaerella populorum SO2202 unplaced genomic scaffold SEPMUscaffold\_7     Total score: 2.0     Cumulative Blast bit score: 1291

hypothetical protein
  
Accession: EMF10998
  
Location: 68986-69657
  
 NCBI BlastP on this gene

EMF10998

hypothetical protein
  
Accession: EMF11000
  
Location: 69971-70756
  
 NCBI BlastP on this gene

EMF11000

Zn-dependent exopeptidase
  
Accession: EMF11001
  
Location: 71360-74176
  
 NCBI BlastP on this gene

EMF11001

hypothetical protein
  
Accession: EMF11002
  
Location: 74782-74937
  
 NCBI BlastP on this gene

EMF11002

hypothetical protein
  
Accession: EMF11003
  
Location: 76987-77259
  
 NCBI BlastP on this gene

EMF11003

acetyl-CoA synthetase-like protein
  
Accession: EMF11004
  
Location: 79093-80918
  
 NCBI BlastP on this gene

EMF11004

hypothetical protein
  
Accession: EMF11005
  
Location: 81812-83290
  
 NCBI BlastP on this gene

EMF11005

hypothetical protein
  
Accession: EMF11006
  
Location: 83563-84188
  
 NCBI BlastP on this gene

EMF11006

ATP-dependent rRNA helicase RRP3
  
Accession: EMF11008
  
Location: 86541-88091
  
  
**BlastP hit with Mycgr3G84402\_Mycgr3T**
  
Percentage identity: 87 %
  
BlastP bit score: 734
  
Sequence coverage: 89 %
  
E-value: 0.0
  
  
 NCBI BlastP on this gene

EMF11008

subtilisin-like protein
  
Accession: EMF11009
  
Location: 90389-93305
  
 NCBI BlastP on this gene

EMF11009

Brix-domain-containing protein
  
Accession: EMF11010
  
Location: 93602-95044
  
  
**BlastP hit with Mycgr3G35447\_Mycgr3T**
  
Percentage identity: 63 %
  
BlastP bit score: 557
  
Sequence coverage: 106 %
  
E-value: 0.0
  
  
 NCBI BlastP on this gene

EMF11010

RNA polymerase II transcription factor B subunit 2
  
Accession: EMF11011
  
Location: 98125-99648
  
 NCBI BlastP on this gene

EMF11011

Peptidase S9-domain-containing protein
  
Accession: EMF11013
  
Location: 100362-102585
  
 NCBI BlastP on this gene

EMF11013

hypothetical protein
  
Accession: EMF11014
  
Location: 102971-104359
  
 NCBI BlastP on this gene

EMF11014

ClpP/crotonase
  
Accession: EMF11015
  
Location: 105134-106038
  
 NCBI BlastP on this gene

EMF11015

ribosomal protein L13e
  
Accession: EMF11016
  
Location: 106559-107479
  
 NCBI BlastP on this gene

EMF11016

DNA polymerase alpha catalytic subunit
  
Accession: EMF11017
  
Location: 107802-112316
  
 NCBI BlastP on this gene

EMF11017

105. :  JH767573 Coniosporium apollinis CBS 100218 chromosome Unknown supercont1.20     Total score: 2.0     Cumulative Blast bit score: 1240

hypothetical protein
  
Accession: EON65317
  
Location: 218777-219148
  
 NCBI BlastP on this gene

EON65317

hypothetical protein
  
Accession: EON65318
  
Location: 220746-221750
  
 NCBI BlastP on this gene

EON65318

hypothetical protein
  
Accession: EON65319
  
Location: 222122-223034
  
 NCBI BlastP on this gene

EON65319

hypothetical protein
  
Accession: EON65320
  
Location: 223425-223968
  
 NCBI BlastP on this gene

EON65320

hypothetical protein
  
Accession: EON65321
  
Location: 224255-226042
  
 NCBI BlastP on this gene

EON65321

hypothetical protein
  
Accession: EON65322
  
Location: 228497-228997
  
 NCBI BlastP on this gene

EON65322

hypothetical protein
  
Accession: EON65323
  
Location: 232889-233113
  
 NCBI BlastP on this gene

EON65323

hypothetical protein
  
Accession: EON65324
  
Location: 233710-235134
  
 NCBI BlastP on this gene

EON65324

hypothetical protein
  
Accession: EON65325
  
Location: 235688-236708
  
 NCBI BlastP on this gene

EON65325

eukaryotic translation initiation factor 3 subunit L
  
Accession: EON65326
  
Location: 237320-238896
  
  
**BlastP hit with Mycgr3G98959\_Mycgr3T**
  
Percentage identity: 80 %
  
BlastP bit score: 797
  
Sequence coverage: 97 %
  
E-value: 0.0
  
  
 NCBI BlastP on this gene

EON65326

hypothetical protein
  
Accession: EON65327
  
Location: 239207-240352
  
  
**BlastP hit with Mycgr3G67775\_Mycgr3T**
  
Percentage identity: 72 %
  
BlastP bit score: 443
  
Sequence coverage: 98 %
  
E-value: 2e-153
  
  
 NCBI BlastP on this gene

EON65327

hypothetical protein
  
Accession: EON65328
  
Location: 240921-242592
  
 NCBI BlastP on this gene

EON65328

hypothetical protein
  
Accession: EON65329
  
Location: 242832-244392
  
 NCBI BlastP on this gene

EON65329

hypothetical protein
  
Accession: EON65330
  
Location: 245157-248462
  
 NCBI BlastP on this gene

EON65330

hypothetical protein
  
Accession: EON65331
  
Location: 249235-250015
  
 NCBI BlastP on this gene

EON65331

hypothetical protein
  
Accession: EON65332
  
Location: 253636-256040
  
 NCBI BlastP on this gene

EON65332

hypothetical protein
  
Accession: EON65333
  
Location: 256333-257304
  
 NCBI BlastP on this gene

EON65333

hypothetical protein
  
Accession: EON65334
  
Location: 257516-258580
  
 NCBI BlastP on this gene

EON65334

106. :  AHHD01000099 Macrophomina phaseolina MS6     Total score: 2.0     Cumulative Blast bit score: 1223

Mitochondrial genome maintenance MGM101
  
Accession: EKG20014
  
Location: 24832-25770
  
 NCBI BlastP on this gene

EKG20014

Carbohydrate kinase FGGY
  
Accession: EKG20015
  
Location: 26291-28195
  
 NCBI BlastP on this gene

EKG20015

hypothetical protein
  
Accession: EKG20016
  
Location: 29018-29649
  
 NCBI BlastP on this gene

EKG20016

Ras GTPase
  
Accession: EKG20017
  
Location: 30901-32130
  
 NCBI BlastP on this gene

EKG20017

FMN-dependent dehydrogenase
  
Accession: EKG20018
  
Location: 33155-34894
  
 NCBI BlastP on this gene

EKG20018

Short-chain dehydrogenase/reductase SDR
  
Accession: EKG20019
  
Location: 35387-35785
  
 NCBI BlastP on this gene

EKG20019

Cytochrome c heme lyase
  
Accession: EKG20020
  
Location: 36977-38013
  
 NCBI BlastP on this gene

EKG20020

Major facilitator superfamily
  
Accession: EKG20021
  
Location: 39981-41970
  
 NCBI BlastP on this gene

EKG20021

Translation initiation factor 3 complex subunit L
  
Accession: EKG20022
  
Location: 43691-45245
  
  
**BlastP hit with Mycgr3G98959\_Mycgr3T**
  
Percentage identity: 81 %
  
BlastP bit score: 793
  
Sequence coverage: 96 %
  
E-value: 0.0
  
  
 NCBI BlastP on this gene

EKG20022

Nitrilase/cyanide hydratase and apolipoprotein N-acyltransferase
  
Accession: EKG20023
  
Location: 45506-46639
  
  
**BlastP hit with Mycgr3G67775\_Mycgr3T**
  
Percentage identity: 69 %
  
BlastP bit score: 430
  
Sequence coverage: 99 %
  
E-value: 2e-148
  
  
 NCBI BlastP on this gene

EKG20023

Fungal lignin peroxidase
  
Accession: EKG20024
  
Location: 46935-48206
  
 NCBI BlastP on this gene

EKG20024

Cytochrome P450
  
Accession: EKG20025
  
Location: 50439-52161
  
 NCBI BlastP on this gene

EKG20025

Alanine racemase
  
Accession: EKG20026
  
Location: 53864-54799
  
 NCBI BlastP on this gene

EKG20026

hypothetical protein
  
Accession: EKG20027
  
Location: 56261-57263
  
 NCBI BlastP on this gene

EKG20027

Six-hairpin glycosidase-like protein
  
Accession: EKG20028
  
Location: 58594-60690
  
 NCBI BlastP on this gene

EKG20028

hypothetical protein
  
Accession: EKG20029
  
Location: 61766-62485
  
 NCBI BlastP on this gene

EKG20029

hypothetical protein
  
Accession: EKG20030
  
Location: 63027-65191
  
 NCBI BlastP on this gene

EKG20030

107. :  GL536348 Pyrenophora teres f. teres 0-1 unplaced genomic scaffold scaffold\_192633     Total score: 2.0     Cumulative Blast bit score: 1216

hypothetical protein
  
Accession: EFQ88473
  
Location: 33232-34913
  
 NCBI BlastP on this gene

EFQ88473

hypothetical protein
  
Accession: EFQ88472
  
Location: 31254-32777
  
 NCBI BlastP on this gene

EFQ88472

hypothetical protein
  
Accession: EFQ88471
  
Location: 28491-29709
  
 NCBI BlastP on this gene

EFQ88471

hypothetical protein
  
Accession: EFQ88470
  
Location: 26867-27564
  
 NCBI BlastP on this gene

EFQ88470

hypothetical protein
  
Accession: EFQ88469
  
Location: 22498-25857
  
 NCBI BlastP on this gene

EFQ88469

hypothetical protein
  
Accession: EFQ88468
  
Location: 20447-22046
  
 NCBI BlastP on this gene

EFQ88468

hypothetical protein
  
Accession: EFQ88467
  
Location: 18528-20059
  
 NCBI BlastP on this gene

EFQ88467

hypothetical protein
  
Accession: EFQ88466
  
Location: 16942-17906
  
  
**BlastP hit with Mycgr3G67775\_Mycgr3T**
  
Percentage identity: 66 %
  
BlastP bit score: 418
  
Sequence coverage: 99 %
  
E-value: 1e-143
  
  
 NCBI BlastP on this gene

EFQ88466

hypothetical protein
  
Accession: EFQ88465
  
Location: 16200-16688
  
 NCBI BlastP on this gene

EFQ88465

hypothetical protein
  
Accession: EFQ88464
  
Location: 14088-15629
  
  
**BlastP hit with Mycgr3G98959\_Mycgr3T**
  
Percentage identity: 80 %
  
BlastP bit score: 798
  
Sequence coverage: 98 %
  
E-value: 0.0
  
  
 NCBI BlastP on this gene

EFQ88464

hypothetical protein
  
Accession: EFQ88463
  
Location: 12369-13232
  
 NCBI BlastP on this gene

EFQ88463

hypothetical protein
  
Accession: EFQ88462
  
Location: 8314-10080
  
 NCBI BlastP on this gene

EFQ88462

hypothetical protein
  
Accession: EFQ88461
  
Location: 7396-7932
  
 NCBI BlastP on this gene

EFQ88461

hypothetical protein
  
Accession: EFQ88460
  
Location: 6337-7186
  
 NCBI BlastP on this gene

EFQ88460

hypothetical protein
  
Accession: EFQ88459
  
Location: 4951-5886
  
 NCBI BlastP on this gene

EFQ88459

hypothetical protein
  
Accession: EFQ88458
  
Location: 3256-4298
  
 NCBI BlastP on this gene

EFQ88458

hypothetical protein
  
Accession: EFQ88457
  
Location: 58-2303
  
 NCBI BlastP on this gene

EFQ88457

108. :  DS231623 Pyrenophora tritici-repentis Pt-1C-BFP supercont1.9 genomic scaffold     Total score: 2.0     Cumulative Blast bit score: 1216

conserved hypothetical protein
  
Accession: EDU51254
  
Location: 766697-767270
  
 NCBI BlastP on this gene

EDU51254

ubiquitin-conjugating enzyme E2 6
  
Accession: EDU51253
  
Location: 764589-765419
  
 NCBI BlastP on this gene

EDU51253

hypothetical protein
  
Accession: EDU51252
  
Location: 762548-764229
  
 NCBI BlastP on this gene

EDU51252

glucooligosaccharide oxidase
  
Accession: EDU51251
  
Location: 760544-762074
  
 NCBI BlastP on this gene

EDU51251

6-phosphogluconolactonase
  
Accession: EDU51250
  
Location: 757828-759046
  
 NCBI BlastP on this gene

EDU51250

predicted protein
  
Accession: EDU51249
  
Location: 756211-756902
  
 NCBI BlastP on this gene

EDU51249

oligopeptide transporter 4
  
Accession: EDU51248
  
Location: 751931-755281
  
 NCBI BlastP on this gene

EDU51248

26S protease regulatory subunit 8
  
Accession: EDU51247
  
Location: 749944-751459
  
 NCBI BlastP on this gene

EDU51247

conserved hypothetical protein
  
Accession: EDU51246
  
Location: 747901-749484
  
 NCBI BlastP on this gene

EDU51246

hypothetical protein
  
Accession: EDU51245
  
Location: 746315-747279
  
  
**BlastP hit with Mycgr3G67775\_Mycgr3T**
  
Percentage identity: 67 %
  
BlastP bit score: 418
  
Sequence coverage: 99 %
  
E-value: 1e-143
  
  
 NCBI BlastP on this gene

EDU51245

conserved hypothetical protein
  
Accession: EDU51244
  
Location: 745574-746062
  
 NCBI BlastP on this gene

EDU51244

eukaryotic translation initiation factor 3
  
Accession: EDU51243
  
Location: 743454-744995
  
  
**BlastP hit with Mycgr3G98959\_Mycgr3T**
  
Percentage identity: 80 %
  
BlastP bit score: 798
  
Sequence coverage: 98 %
  
E-value: 0.0
  
  
 NCBI BlastP on this gene

EDU51243

conserved hypothetical protein
  
Accession: EDU51242
  
Location: 741629-742465
  
 NCBI BlastP on this gene

EDU51242

conserved hypothetical protein
  
Accession: EDU51241
  
Location: 737429-739186
  
 NCBI BlastP on this gene

EDU51241

conserved hypothetical protein
  
Accession: EDU51240
  
Location: 736596-737052
  
 NCBI BlastP on this gene

EDU51240

conserved hypothetical protein
  
Accession: EDU51239
  
Location: 735458-736307
  
 NCBI BlastP on this gene

EDU51239

predicted protein
  
Accession: EDU51238
  
Location: 732469-735023
  
 NCBI BlastP on this gene

EDU51238

hypothetical protein
  
Accession: EDU51237
  
Location: 729373-731678
  
 NCBI BlastP on this gene

EDU51237

transcriptional coactivator/pterin dehydratase
  
Accession: EDU51236
  
Location: 727128-727481
  
 NCBI BlastP on this gene

EDU51236

conserved hypothetical protein
  
Accession: EDU51235
  
Location: 724484-725474
  
 NCBI BlastP on this gene

EDU51235

109. :  KB733455 Bipolaris maydis ATCC 48331 unplaced genomic scaffold COCC4scaffold\_12     Total score: 2.0     Cumulative Blast bit score: 1213

hypothetical protein
  
Accession: ENI05137
  
Location: 586211-586381
  
 NCBI BlastP on this gene

ENI05137

hypothetical protein
  
Accession: ENI05138
  
Location: 587166-587999
  
 NCBI BlastP on this gene

ENI05138

hypothetical protein
  
Accession: ENI05139
  
Location: 588391-590084
  
 NCBI BlastP on this gene

ENI05139

hypothetical protein
  
Accession: ENI05140
  
Location: 590280-591831
  
 NCBI BlastP on this gene

ENI05140

hypothetical protein
  
Accession: ENI05141
  
Location: 592810-594034
  
 NCBI BlastP on this gene

ENI05141

hypothetical protein
  
Accession: ENI05142
  
Location: 595097-595822
  
 NCBI BlastP on this gene

ENI05142

hypothetical protein
  
Accession: ENI05143
  
Location: 596533-597210
  
 NCBI BlastP on this gene

ENI05143

hypothetical protein
  
Accession: ENI05144
  
Location: 597700-601061
  
 NCBI BlastP on this gene

ENI05144

hypothetical protein
  
Accession: ENI05145
  
Location: 601549-603071
  
 NCBI BlastP on this gene

ENI05145

hypothetical protein
  
Accession: ENI05146
  
Location: 603575-605091
  
 NCBI BlastP on this gene

ENI05146

hypothetical protein
  
Accession: ENI05147
  
Location: 605731-606818
  
  
**BlastP hit with Mycgr3G67775\_Mycgr3T**
  
Percentage identity: 67 %
  
BlastP bit score: 417
  
Sequence coverage: 98 %
  
E-value: 2e-143
  
  
 NCBI BlastP on this gene

ENI05147

hypothetical protein
  
Accession: ENI05148
  
Location: 607093-607590
  
 NCBI BlastP on this gene

ENI05148

hypothetical protein
  
Accession: ENI05149
  
Location: 608213-609703
  
  
**BlastP hit with Mycgr3G98959\_Mycgr3T**
  
Percentage identity: 78 %
  
BlastP bit score: 796
  
Sequence coverage: 99 %
  
E-value: 0.0
  
  
 NCBI BlastP on this gene

ENI05149

hypothetical protein
  
Accession: ENI05150
  
Location: 610002-610272
  
 NCBI BlastP on this gene

ENI05150

hypothetical protein
  
Accession: ENI05151
  
Location: 610583-611751
  
 NCBI BlastP on this gene

ENI05151

hypothetical protein
  
Accession: ENI05152
  
Location: 612298-612915
  
 NCBI BlastP on this gene

ENI05152

hypothetical protein
  
Accession: ENI05153
  
Location: 613197-614951
  
 NCBI BlastP on this gene

ENI05153

hypothetical protein
  
Accession: ENI05154
  
Location: 615286-615828
  
 NCBI BlastP on this gene

ENI05154

hypothetical protein
  
Accession: ENI05155
  
Location: 616034-616903
  
 NCBI BlastP on this gene

ENI05155

hypothetical protein
  
Accession: ENI05156
  
Location: 617378-618315
  
 NCBI BlastP on this gene

ENI05156

hypothetical protein
  
Accession: ENI05157
  
Location: 618566-619578
  
 NCBI BlastP on this gene

ENI05157

glycosyltransferase family 69 protein
  
Accession: ENI05158
  
Location: 620226-621642
  
 NCBI BlastP on this gene

ENI05158

hypothetical protein
  
Accession: ENI05159
  
Location: 622854-624683
  
 NCBI BlastP on this gene

ENI05159

hypothetical protein
  
Accession: ENI05160
  
Location: 625268-627489
  
 NCBI BlastP on this gene

ENI05160

hypothetical protein
  
Accession: ENI05161
  
Location: 627897-629252
  
 NCBI BlastP on this gene

ENI05161

110. :  KB445579 Cochliobolus heterostrophus C5 unplaced genomic scaffold COCHEscaffold\_11     Total score: 2.0     Cumulative Blast bit score: 1213

hypothetical protein
  
Accession: EMD89143
  
Location: 418688-418858
  
 NCBI BlastP on this gene

EMD89143

hypothetical protein
  
Accession: EMD89142
  
Location: 417065-417898
  
 NCBI BlastP on this gene

EMD89142

hypothetical protein
  
Accession: EMD89141
  
Location: 414980-416673
  
 NCBI BlastP on this gene

EMD89141

hypothetical protein
  
Accession: EMD89140
  
Location: 413233-414784
  
 NCBI BlastP on this gene

EMD89140

hypothetical protein
  
Accession: EMD89139
  
Location: 411030-412254
  
 NCBI BlastP on this gene

EMD89139

hypothetical protein
  
Accession: EMD89138
  
Location: 409242-409967
  
 NCBI BlastP on this gene

EMD89138

hypothetical protein
  
Accession: EMD89137
  
Location: 407854-408531
  
 NCBI BlastP on this gene

EMD89137

hypothetical protein
  
Accession: EMD89136
  
Location: 404249-407364
  
 NCBI BlastP on this gene

EMD89136

hypothetical protein
  
Accession: EMD89135
  
Location: 401993-403515
  
 NCBI BlastP on this gene

EMD89135

hypothetical protein
  
Accession: EMD89134
  
Location: 399973-401489
  
 NCBI BlastP on this gene

EMD89134

hypothetical protein
  
Accession: EMD89133
  
Location: 398246-399333
  
  
**BlastP hit with Mycgr3G67775\_Mycgr3T**
  
Percentage identity: 67 %
  
BlastP bit score: 417
  
Sequence coverage: 98 %
  
E-value: 2e-143
  
  
 NCBI BlastP on this gene

EMD89133

hypothetical protein
  
Accession: EMD89132
  
Location: 397474-397971
  
 NCBI BlastP on this gene

EMD89132

hypothetical protein
  
Accession: EMD89131
  
Location: 395361-396851
  
  
**BlastP hit with Mycgr3G98959\_Mycgr3T**
  
Percentage identity: 78 %
  
BlastP bit score: 796
  
Sequence coverage: 99 %
  
E-value: 0.0
  
  
 NCBI BlastP on this gene

EMD89131

hypothetical protein
  
Accession: EMD89130
  
Location: 394792-395062
  
 NCBI BlastP on this gene

EMD89130

hypothetical protein
  
Accession: EMD89129
  
Location: 393792-394481
  
 NCBI BlastP on this gene

EMD89129

hypothetical protein
  
Accession: EMD89128
  
Location: 392118-392735
  
 NCBI BlastP on this gene

EMD89128

hypothetical protein
  
Accession: EMD89127
  
Location: 390082-391836
  
 NCBI BlastP on this gene

EMD89127

hypothetical protein
  
Accession: EMD89126
  
Location: 389205-389747
  
 NCBI BlastP on this gene

EMD89126

hypothetical protein
  
Accession: EMD89125
  
Location: 388130-388999
  
 NCBI BlastP on this gene

EMD89125

hypothetical protein
  
Accession: EMD89124
  
Location: 386690-387655
  
 NCBI BlastP on this gene

EMD89124

hypothetical protein
  
Accession: EMD89123
  
Location: 385518-386439
  
 NCBI BlastP on this gene

EMD89123

glycosyltransferase family 69 protein
  
Accession: EMD89122
  
Location: 383363-384779
  
 NCBI BlastP on this gene

EMD89122

hypothetical protein
  
Accession: EMD89121
  
Location: 380322-382151
  
 NCBI BlastP on this gene

EMD89121

hypothetical protein
  
Accession: EMD89120
  
Location: 377516-379737
  
 NCBI BlastP on this gene

EMD89120

hypothetical protein
  
Accession: EMD89119
  
Location: 375753-377108
  
 NCBI BlastP on this gene

EMD89119

111. :  KB445649 Cochliobolus sativus ND90Pr unplaced genomic scaffold COCSAscaffold\_13     Total score: 2.0     Cumulative Blast bit score: 1211

hypothetical protein
  
Accession: EMD60915
  
Location: 478696-478887
  
 NCBI BlastP on this gene

EMD60915

hypothetical protein
  
Accession: EMD60914
  
Location: 476339-477172
  
 NCBI BlastP on this gene

EMD60914

hypothetical protein
  
Accession: EMD60913
  
Location: 474258-475951
  
 NCBI BlastP on this gene

EMD60913

hypothetical protein
  
Accession: EMD60912
  
Location: 472515-474065
  
 NCBI BlastP on this gene

EMD60912

hypothetical protein
  
Accession: EMD60911
  
Location: 470290-471514
  
 NCBI BlastP on this gene

EMD60911

hypothetical protein
  
Accession: EMD60910
  
Location: 467314-468790
  
 NCBI BlastP on this gene

EMD60910

hypothetical protein
  
Accession: EMD60909
  
Location: 463467-466818
  
 NCBI BlastP on this gene

EMD60909

hypothetical protein
  
Accession: EMD60908
  
Location: 461453-462975
  
 NCBI BlastP on this gene

EMD60908

hypothetical protein
  
Accession: EMD60907
  
Location: 459450-460964
  
 NCBI BlastP on this gene

EMD60907

hypothetical protein
  
Accession: EMD60906
  
Location: 457736-458802
  
  
**BlastP hit with Mycgr3G67775\_Mycgr3T**
  
Percentage identity: 67 %
  
BlastP bit score: 414
  
Sequence coverage: 98 %
  
E-value: 3e-142
  
  
 NCBI BlastP on this gene

EMD60906

hypothetical protein
  
Accession: EMD60905
  
Location: 456975-457472
  
 NCBI BlastP on this gene

EMD60905

hypothetical protein
  
Accession: EMD60904
  
Location: 454877-456367
  
  
**BlastP hit with Mycgr3G98959\_Mycgr3T**
  
Percentage identity: 79 %
  
BlastP bit score: 797
  
Sequence coverage: 99 %
  
E-value: 0.0
  
  
 NCBI BlastP on this gene

EMD60904

hypothetical protein
  
Accession: EMD60903
  
Location: 454279-454578
  
 NCBI BlastP on this gene

EMD60903

hypothetical protein
  
Accession: EMD60902
  
Location: 453239-454037
  
 NCBI BlastP on this gene

EMD60902

hypothetical protein
  
Accession: EMD60901
  
Location: 451756-452339
  
 NCBI BlastP on this gene

EMD60901

hypothetical protein
  
Accession: EMD60900
  
Location: 449773-451512
  
 NCBI BlastP on this gene

EMD60900

hypothetical protein
  
Accession: EMD60899
  
Location: 448967-449439
  
 NCBI BlastP on this gene

EMD60899

hypothetical protein
  
Accession: EMD60898
  
Location: 447823-448691
  
 NCBI BlastP on this gene

EMD60898

hypothetical protein
  
Accession: EMD60897
  
Location: 446388-447348
  
 NCBI BlastP on this gene

EMD60897

hypothetical protein
  
Accession: EMD60896
  
Location: 445262-446184
  
 NCBI BlastP on this gene

EMD60896

glycosyltransferase family 69 protein
  
Accession: EMD60895
  
Location: 443054-444470
  
 NCBI BlastP on this gene

EMD60895

hypothetical protein
  
Accession: EMD60894
  
Location: 440049-441879
  
 NCBI BlastP on this gene

EMD60894

hypothetical protein
  
Accession: EMD60893
  
Location: 437263-439484
  
 NCBI BlastP on this gene

EMD60893

hypothetical protein
  
Accession: EMD60892
  
Location: 435504-436859
  
 NCBI BlastP on this gene

EMD60892

112. :  KB916388 Neofusicoccum parvum UCRNP2 chromosome Unknown NP2\_03\_scaffold\_750     Total score: 2.0     Cumulative Blast bit score: 1198

hypothetical protein
  
Accession: EOD46861
  
Location: 4535-5430
  
 NCBI BlastP on this gene

EOD46861

putative family pyridoxal phosphate enzyme protein
  
Accession: EOD46859
  
Location: 6816-7797
  
 NCBI BlastP on this gene

EOD46859

putative ligninase h2 precursor protein
  
Accession: EOD46855
  
Location: 8977-10092
  
 NCBI BlastP on this gene

EOD46855

putative carbon-nitrogen family protein
  
Accession: EOD46854
  
Location: 10350-11439
  
  
**BlastP hit with Mycgr3G67775\_Mycgr3T**
  
Percentage identity: 72 %
  
BlastP bit score: 398
  
Sequence coverage: 93 %
  
E-value: 3e-136
  
  
 NCBI BlastP on this gene

EOD46854

putative eukaryotic translation initiation factor 3 protein
  
Accession: EOD46853
  
Location: 11773-13330
  
  
**BlastP hit with Mycgr3G98959\_Mycgr3T**
  
Percentage identity: 78 %
  
BlastP bit score: 800
  
Sequence coverage: 99 %
  
E-value: 0.0
  
  
 NCBI BlastP on this gene

EOD46853

putative ubiquitin-conjugating enzyme protein
  
Accession: EOD46857
  
Location: 15885-16706
  
 NCBI BlastP on this gene

EOD46857

putative outer membrane protein
  
Accession: EOD46860
  
Location: 17057-18888
  
 NCBI BlastP on this gene

EOD46860

putative major facilitator superfamily transporter protein
  
Accession: EOD46852
  
Location: 20758-22487
  
 NCBI BlastP on this gene

EOD46852

putative chromo domain-like protein
  
Accession: EOD46863
  
Location: 23402-24166
  
 NCBI BlastP on this gene

EOD46863

putative glycosyltransferase family 2 protein
  
Accession: EOD46858
  
Location: 24837-27467
  
 NCBI BlastP on this gene

EOD46858

putative alcohol dehydrogenase domain protein
  
Accession: EOD46862
  
Location: 28838-29926
  
 NCBI BlastP on this gene

EOD46862

113. :  KB908844 Setosphaeria turcica Et28A unplaced genomic scaffold SETTUscaffold\_6     Total score: 2.0     Cumulative Blast bit score: 1190

hypothetical protein
  
Accession: EOA82714
  
Location: 1110971-1112664
  
 NCBI BlastP on this gene

EOA82714

hypothetical protein
  
Accession: EOA82713
  
Location: 1109035-1110581
  
 NCBI BlastP on this gene

EOA82713

hypothetical protein
  
Accession: EOA82712
  
Location: 1106885-1108110
  
 NCBI BlastP on this gene

EOA82712

hypothetical protein
  
Accession: EOA82711
  
Location: 1104057-1104263
  
 NCBI BlastP on this gene

EOA82711

hypothetical protein
  
Accession: EOA82710
  
Location: 1101260-1101972
  
 NCBI BlastP on this gene

EOA82710

hypothetical protein
  
Accession: EOA82709
  
Location: 1097519-1100699
  
 NCBI BlastP on this gene

EOA82709

hypothetical protein
  
Accession: EOA82708
  
Location: 1095233-1096741
  
 NCBI BlastP on this gene

EOA82708

hypothetical protein
  
Accession: EOA82707
  
Location: 1093268-1094755
  
 NCBI BlastP on this gene

EOA82707

hypothetical protein
  
Accession: EOA82706
  
Location: 1091694-1092799
  
  
**BlastP hit with Mycgr3G67775\_Mycgr3T**
  
Percentage identity: 65 %
  
BlastP bit score: 392
  
Sequence coverage: 99 %
  
E-value: 2e-133
  
  
 NCBI BlastP on this gene

EOA82706

hypothetical protein
  
Accession: EOA82705
  
Location: 1090922-1091401
  
 NCBI BlastP on this gene

EOA82705

hypothetical protein
  
Accession: EOA82704
  
Location: 1090633-1090839
  
 NCBI BlastP on this gene

EOA82704

hypothetical protein
  
Accession: EOA82703
  
Location: 1088505-1090073
  
  
**BlastP hit with Mycgr3G98959\_Mycgr3T**
  
Percentage identity: 79 %
  
BlastP bit score: 798
  
Sequence coverage: 99 %
  
E-value: 0.0
  
  
 NCBI BlastP on this gene

EOA82703

hypothetical protein
  
Accession: EOA82702
  
Location: 1086092-1087567
  
 NCBI BlastP on this gene

EOA82702

hypothetical protein
  
Accession: EOA82701
  
Location: 1084016-1084592
  
 NCBI BlastP on this gene

EOA82701

hypothetical protein
  
Accession: EOA82700
  
Location: 1080033-1083654
  
 NCBI BlastP on this gene

EOA82700

hypothetical protein
  
Accession: EOA82699
  
Location: 1078713-1079611
  
 NCBI BlastP on this gene

EOA82699

hypothetical protein
  
Accession: EOA82698
  
Location: 1077202-1078212
  
 NCBI BlastP on this gene

EOA82698

hypothetical protein
  
Accession: EOA82697
  
Location: 1074137-1076480
  
 NCBI BlastP on this gene

EOA82697

hypothetical protein
  
Accession: EOA82696
  
Location: 1070954-1071319
  
 NCBI BlastP on this gene

EOA82696

hypothetical protein
  
Accession: EOA82695
  
Location: 1068668-1069677
  
 NCBI BlastP on this gene

EOA82695

114. :  KB446542 Dothistroma septosporum NZE10 unplaced genomic scaffold DOTSEscaffold\_8     Total score: 2.0     Cumulative Blast bit score: 1168

hypothetical protein
  
Accession: EME41143
  
Location: 45588-47045
  
 NCBI BlastP on this gene

EME41143

hypothetical protein
  
Accession: EME41144
  
Location: 48587-50944
  
 NCBI BlastP on this gene

EME41144

hypothetical protein
  
Accession: EME41145
  
Location: 51638-52564
  
 NCBI BlastP on this gene

EME41145

hypothetical protein
  
Accession: EME41146
  
Location: 52658-53671
  
 NCBI BlastP on this gene

EME41146

hypothetical protein
  
Accession: EME41147
  
Location: 55763-56281
  
 NCBI BlastP on this gene

EME41147

hypothetical protein
  
Accession: EME41148
  
Location: 57816-58352
  
 NCBI BlastP on this gene

EME41148

hypothetical protein
  
Accession: EME41149
  
Location: 59135-59722
  
 NCBI BlastP on this gene

EME41149

hypothetical protein
  
Accession: EME41150
  
Location: 60221-60966
  
  
**BlastP hit with Mycgr3G28587\_Mycgr3T**
  
Percentage identity: 67 %
  
BlastP bit score: 214
  
Sequence coverage: 98 %
  
E-value: 9e-67
  
  
 NCBI BlastP on this gene

EME41150

hypothetical protein
  
Accession: EME41151
  
Location: 61362-63005
  
  
**BlastP hit with Mycgr3G103034\_Mycgr3**
  
Percentage identity: 83 %
  
BlastP bit score: 954
  
Sequence coverage: 97 %
  
E-value: 0.0
  
  
 NCBI BlastP on this gene

EME41151

hypothetical protein
  
Accession: EME41152
  
Location: 63502-64094
  
 NCBI BlastP on this gene

EME41152

hypothetical protein
  
Accession: EME41153
  
Location: 64939-66574
  
 NCBI BlastP on this gene

EME41153

hypothetical protein
  
Accession: EME41154
  
Location: 67327-68742
  
 NCBI BlastP on this gene

EME41154

hypothetical protein
  
Accession: EME41155
  
Location: 69141-69779
  
 NCBI BlastP on this gene

EME41155

hypothetical protein
  
Accession: EME41156
  
Location: 71029-71316
  
 NCBI BlastP on this gene

EME41156

glycoside hydrolase family 17 protein
  
Accession: EME41157
  
Location: 71899-73441
  
 NCBI BlastP on this gene

EME41157

hypothetical protein
  
Accession: EME41158
  
Location: 75168-75954
  
 NCBI BlastP on this gene

EME41158

hypothetical protein
  
Accession: EME41159
  
Location: 76340-77360
  
 NCBI BlastP on this gene

EME41159

hypothetical protein
  
Accession: EME41160
  
Location: 78500-80092
  
 NCBI BlastP on this gene

EME41160

hypothetical protein
  
Accession: EME41161
  
Location: 80524-82301
  
 NCBI BlastP on this gene

EME41161

115. :  FP929137 Leptosphaeria maculans JN3 lm\_SuperContig\_10\_v2 genomic supercontig     Total score: 2.0     Cumulative Blast bit score: 1147

similar to 50S ribosomal protein L13
  
Accession: CBX99973
  
Location: 1074652-1075286
  
 NCBI BlastP on this gene

LEMA\_P075620.1

similar to uricase (Urate oxidase)
  
Accession: CBX99974
  
Location: 1075727-1076734
  
 NCBI BlastP on this gene

LEMA\_P075630.1

predicted protein
  
Accession: CBX99975
  
Location: 1077007-1077357
  
 NCBI BlastP on this gene

LEMA\_P075640.1

similar to cytosolic regulator Pianissimo
  
Accession: CBX99976
  
Location: 1077750-1081780
  
 NCBI BlastP on this gene

LEMA\_P075650.1

predicted protein
  
Accession: CBX99977
  
Location: 1082390-1082554
  
 NCBI BlastP on this gene

LEMA\_uP075660.1

similar to ATP-dependent protease (CrgA)
  
Accession: CBX99978
  
Location: 1083701-1085657
  
 NCBI BlastP on this gene

LEMA\_P075670.1

hypothetical protein
  
Accession: CBX99979
  
Location: 1087383-1090859
  
 NCBI BlastP on this gene

LEMA\_P075680.1

hypothetical protein
  
Accession: CBX99980
  
Location: 1091407-1093172
  
 NCBI BlastP on this gene

LEMA\_P075690.1

hypothetical protein
  
Accession: CBX99981
  
Location: 1093422-1097357
  
  
**BlastP hit with Mycgr3G67775\_Mycgr3T**
  
Percentage identity: 57 %
  
BlastP bit score: 383
  
Sequence coverage: 114 %
  
E-value: 1e-123
  
  
 NCBI BlastP on this gene

LEMA\_P075700.1

hypothetical protein
  
Accession: CBX99982
  
Location: 1097601-1098086
  
 NCBI BlastP on this gene

LEMA\_P075710.1

hypothetical protein
  
Accession: CBX99983
  
Location: 1098830-1100267
  
  
**BlastP hit with Mycgr3G98959\_Mycgr3T**
  
Percentage identity: 81 %
  
BlastP bit score: 764
  
Sequence coverage: 91 %
  
E-value: 0.0
  
  
 NCBI BlastP on this gene

LEMA\_P075720.1

hypothetical protein
  
Accession: CBX99984
  
Location: 1101210-1102004
  
 NCBI BlastP on this gene

LEMA\_P075730.1

hypothetical protein
  
Accession: CBX99985
  
Location: 1102388-1104268
  
 NCBI BlastP on this gene

LEMA\_P075740.1

hypothetical protein
  
Accession: CBX99986
  
Location: 1104579-1105161
  
 NCBI BlastP on this gene

LEMA\_P075750.1

hypothetical protein
  
Accession: CBX99987
  
Location: 1105389-1106274
  
 NCBI BlastP on this gene

LEMA\_P075760.1

hypothetical protein
  
Accession: CBX99988
  
Location: 1106751-1107680
  
 NCBI BlastP on this gene

LEMA\_P075770.1

predicted protein
  
Accession: CBX99989
  
Location: 1108368-1109247
  
 NCBI BlastP on this gene

LEMA\_P075780.1

predicted protein
  
Accession: CBX99990
  
Location: 1109619-1109874
  
 NCBI BlastP on this gene

LEMA\_uP075790.1

similar to chromatin remodeling complex subunit (Arp8)
  
Accession: CBX99991
  
Location: 1109961-1112225
  
 NCBI BlastP on this gene

LEMA\_P075800.1

hypothetical protein
  
Accession: CBX99992
  
Location: 1113611-1114422
  
 NCBI BlastP on this gene

LEMA\_P075810.1

hypothetical protein
  
Accession: CBX99993
  
Location: 1114777-1115423
  
 NCBI BlastP on this gene

LEMA\_P075820.1

hypothetical protein
  
Accession: CBX99994
  
Location: 1116310-1118447
  
 NCBI BlastP on this gene

LEMA\_P075830.1

116. :  GL573222 Geomyces destructans 20631-21 unplaced genomic scaffold supercont1.54     Total score: 2.0     Cumulative Blast bit score: 1135

protein arginine N-methyltransferase 1
  
Accession: ELR08690
  
Location: 748-1988
  
 NCBI BlastP on this gene

ELR08690

hypothetical protein
  
Accession: ELR08691
  
Location: 2434-3582
  
 NCBI BlastP on this gene

ELR08691

ATP-dependent rRNA helicase rrp3
  
Accession: ELR08692
  
Location: 3740-5246
  
  
**BlastP hit with Mycgr3G84402\_Mycgr3T**
  
Percentage identity: 74 %
  
BlastP bit score: 646
  
Sequence coverage: 94 %
  
E-value: 0.0
  
  
 NCBI BlastP on this gene

ELR08692

hypothetical protein
  
Accession: ELR08693
  
Location: 5436-6872
  
  
**BlastP hit with Mycgr3G35447\_Mycgr3T**
  
Percentage identity: 59 %
  
BlastP bit score: 489
  
Sequence coverage: 102 %
  
E-value: 7e-167
  
  
 NCBI BlastP on this gene

ELR08693

hypothetical protein
  
Accession: ELR08694
  
Location: 7716-9945
  
 NCBI BlastP on this gene

ELR08694

hypothetical protein
  
Accession: ELR08695
  
Location: 10360-13183
  
 NCBI BlastP on this gene

ELR08695

hypothetical protein
  
Accession: ELR08696
  
Location: 18305-18772
  
 NCBI BlastP on this gene

ELR08696

hypothetical protein
  
Accession: ELR08697
  
Location: 24134-24472
  
 NCBI BlastP on this gene

ELR08697

117. :  KB446555 Pseudocercospora fijiensis CIRAD86 unplaced genomic scaffold MYCFIscaffold\_1     Total score: 2.0     Cumulative Blast bit score: 1123

hypothetical protein
  
Accession: EME88814
  
Location: 7467052-7469042
  
 NCBI BlastP on this gene

EME88814

hypothetical protein
  
Accession: EME88815
  
Location: 7472543-7472884
  
 NCBI BlastP on this gene

EME88815

hypothetical protein
  
Accession: EME88816
  
Location: 7475312-7475927
  
  
**BlastP hit with Mycgr3G28587\_Mycgr3T**
  
Percentage identity: 62 %
  
BlastP bit score: 199
  
Sequence coverage: 96 %
  
E-value: 2e-61
  
  
 NCBI BlastP on this gene

EME88816

hypothetical protein
  
Accession: EME88817
  
Location: 7476397-7478073
  
  
**BlastP hit with Mycgr3G103034\_Mycgr3**
  
Percentage identity: 79 %
  
BlastP bit score: 924
  
Sequence coverage: 97 %
  
E-value: 0.0
  
  
 NCBI BlastP on this gene

EME88817

hypothetical protein
  
Accession: EME88818
  
Location: 7478153-7478965
  
 NCBI BlastP on this gene

EME88818

hypothetical protein
  
Accession: EME88819
  
Location: 7479369-7479737
  
 NCBI BlastP on this gene

EME88819

hypothetical protein
  
Accession: EME88820
  
Location: 7480952-7481537
  
 NCBI BlastP on this gene

EME88820

hypothetical protein
  
Accession: EME88821
  
Location: 7483280-7484460
  
 NCBI BlastP on this gene

EME88821

hypothetical protein
  
Accession: EME88822
  
Location: 7487433-7488785
  
 NCBI BlastP on this gene

EME88822

hypothetical protein
  
Accession: EME88823
  
Location: 7489763-7493364
  
 NCBI BlastP on this gene

EME88823

hypothetical protein
  
Accession: EME88824
  
Location: 7494074-7495324
  
 NCBI BlastP on this gene

EME88824

hypothetical protein
  
Accession: EME88825
  
Location: 7496175-7497332
  
 NCBI BlastP on this gene

EME88825

hypothetical protein
  
Accession: EME88826
  
Location: 7497421-7497950
  
 NCBI BlastP on this gene

EME88826

hypothetical protein
  
Accession: EME88827
  
Location: 7497979-7499333
  
 NCBI BlastP on this gene

EME88827

118. :  KB725774 Colletotrichum orbiculare MAFF 240422 unplaced genomic scaffold Scaffold\_225     Total score: 2.0     Cumulative Blast bit score: 1109

C6 transcription factor
  
Accession: ENH85445
  
Location: 4894-6218
  
 NCBI BlastP on this gene

ENH85445

phytanoyl-dioxygenase family protein
  
Accession: ENH85446
  
Location: 7596-8720
  
 NCBI BlastP on this gene

ENH85446

ribosome biogenesis protein ssf2
  
Accession: ENH85447
  
Location: 10211-11595
  
  
**BlastP hit with Mycgr3G35447\_Mycgr3T**
  
Percentage identity: 57 %
  
BlastP bit score: 472
  
Sequence coverage: 100 %
  
E-value: 1e-160
  
  
 NCBI BlastP on this gene

ENH85447

ATP-dependent rrna helicase rrp3
  
Accession: ENH85448
  
Location: 11919-13502
  
  
**BlastP hit with Mycgr3G84402\_Mycgr3T**
  
Percentage identity: 74 %
  
BlastP bit score: 637
  
Sequence coverage: 92 %
  
E-value: 0.0
  
  
 NCBI BlastP on this gene

ENH85448

hypothetical protein
  
Accession: ENH85449
  
Location: 16270-18072
  
 NCBI BlastP on this gene

ENH85449

kinesin family protein
  
Accession: ENH85450
  
Location: 21665-23848
  
 NCBI BlastP on this gene

ENH85450

transcription factor tfiiib complex subunit
  
Accession: ENH85451
  
Location: 24125-26323
  
 NCBI BlastP on this gene

ENH85451

hypothetical protein
  
Accession: ENH85452
  
Location: 27767-28395
  
 NCBI BlastP on this gene

ENH85452

hypothetical protein
  
Accession: ENH85453
  
Location: 28723-29599
  
 NCBI BlastP on this gene

ENH85453

microsomal signal peptidase 18 kda subunit
  
Accession: ENH85454
  
Location: 30546-31334
  
 NCBI BlastP on this gene

ENH85454

hypothetical protein
  
Accession: ENH85455
  
Location: 32649-33986
  
 NCBI BlastP on this gene

ENH85455

119. :  AFNW01000108 Fusarium pseudograminearum CS3096     Total score: 2.0     Cumulative Blast bit score: 1107

hypothetical protein
  
Accession: EKJ74497
  
Location: 154714-157641
  
 NCBI BlastP on this gene

EKJ74497

hypothetical protein
  
Accession: EKJ74498
  
Location: 158717-159256
  
 NCBI BlastP on this gene

EKJ74498

hypothetical protein
  
Accession: EKJ74499
  
Location: 160262-162620
  
 NCBI BlastP on this gene

EKJ74499

hypothetical protein
  
Accession: EKJ74500
  
Location: 163127-169341
  
 NCBI BlastP on this gene

EKJ74500

hypothetical protein
  
Accession: EKJ74501
  
Location: 169599-170674
  
 NCBI BlastP on this gene

EKJ74501

hypothetical protein
  
Accession: EKJ74502
  
Location: 171582-173095
  
  
**BlastP hit with Mycgr3G84402\_Mycgr3T**
  
Percentage identity: 74 %
  
BlastP bit score: 630
  
Sequence coverage: 90 %
  
E-value: 0.0
  
  
 NCBI BlastP on this gene

EKJ74502

hypothetical protein
  
Accession: EKJ74503
  
Location: 173317-174731
  
  
**BlastP hit with Mycgr3G35447\_Mycgr3T**
  
Percentage identity: 59 %
  
BlastP bit score: 477
  
Sequence coverage: 101 %
  
E-value: 2e-162
  
  
 NCBI BlastP on this gene

EKJ74503

hypothetical protein
  
Accession: EKJ74504
  
Location: 175412-176909
  
 NCBI BlastP on this gene

EKJ74504

hypothetical protein
  
Accession: EKJ74505
  
Location: 183029-184451
  
 NCBI BlastP on this gene

EKJ74505

hypothetical protein
  
Accession: EKJ74506
  
Location: 185276-187230
  
 NCBI BlastP on this gene

EKJ74506

hypothetical protein
  
Accession: EKJ74507
  
Location: 188396-188722
  
 NCBI BlastP on this gene

EKJ74507

hypothetical protein
  
Accession: EKJ74508
  
Location: 189489-190139
  
 NCBI BlastP on this gene

EKJ74508

hypothetical protein
  
Accession: EKJ74509
  
Location: 193267-194877
  
 NCBI BlastP on this gene

EKJ74509

120. :  GG698928 Nectria haematococca mpVI 77-13-4 chromosome 6 genomic scaffold NECHAsca\_37\_chr6\_2\_0     Total score: 2.0     Cumulative Blast bit score: 1099

hypothetical protein
  
Accession: EEU36455
  
Location: 189030-190028
  
 NCBI BlastP on this gene

EEU36455

predicted protein
  
Accession: EEU36528
  
Location: 191331-193247
  
 NCBI BlastP on this gene

EEU36528

hypothetical protein
  
Accession: EEU36529
  
Location: 195028-197121
  
 NCBI BlastP on this gene

EEU36529

hypothetical protein
  
Accession: EEU36456
  
Location: 197582-198688
  
 NCBI BlastP on this gene

EEU36456

hypothetical protein
  
Accession: EEU36457
  
Location: 198992-200484
  
 NCBI BlastP on this gene

EEU36457

hypothetical protein
  
Accession: EEU36530
  
Location: 200849-202959
  
 NCBI BlastP on this gene

EEU36530

hypothetical protein
  
Accession: EEU36531
  
Location: 203399-204909
  
 NCBI BlastP on this gene

EEU36531

predicted protein
  
Accession: EEU36532
  
Location: 205551-206965
  
  
**BlastP hit with Mycgr3G35447\_Mycgr3T**
  
Percentage identity: 59 %
  
BlastP bit score: 480
  
Sequence coverage: 101 %
  
E-value: 1e-163
  
  
 NCBI BlastP on this gene

EEU36532

predicted protein
  
Accession: EEU36458
  
Location: 207184-208708
  
  
**BlastP hit with Mycgr3G84402\_Mycgr3T**
  
Percentage identity: 72 %
  
BlastP bit score: 619
  
Sequence coverage: 90 %
  
E-value: 0.0
  
  
 NCBI BlastP on this gene

EEU36458

predicted protein
  
Accession: EEU36459
  
Location: 209551-210620
  
 NCBI BlastP on this gene

EEU36459

hypothetical protein
  
Accession: EEU36460
  
Location: 211663-212799
  
 NCBI BlastP on this gene

EEU36460

predicted protein
  
Accession: EEU36533
  
Location: 213026-219247
  
 NCBI BlastP on this gene

EEU36533

predicted protein
  
Accession: EEU36461
  
Location: 219715-222067
  
 NCBI BlastP on this gene

EEU36461

predicted protein
  
Accession: EEU36462
  
Location: 224479-225694
  
 NCBI BlastP on this gene

EEU36462

hypothetical protein
  
Accession: EEU36534
  
Location: 226075-226269
  
 NCBI BlastP on this gene

EEU36534

hypothetical protein
  
Accession: EEU36463
  
Location: 227289-227980
  
 NCBI BlastP on this gene

EEU36463

hypothetical protein
  
Accession: EEU36535
  
Location: 228010-228942
  
 NCBI BlastP on this gene

EEU36535

121. :  KB726989 Fusarium oxysporum f. sp. cubense race 4 unplaced genomic scaffold scaffold3     Total score: 2.0     Cumulative Blast bit score: 1094

hypothetical protein
  
Accession: EMT65795
  
Location: 699025-700954
  
 NCBI BlastP on this gene

EMT65795

hypothetical protein
  
Accession: EMT65796
  
Location: 701931-702861
  
 NCBI BlastP on this gene

EMT65796

hypothetical protein
  
Accession: EMT65797
  
Location: 705037-705733
  
 NCBI BlastP on this gene

EMT65797

hypothetical protein
  
Accession: EMT65798
  
Location: 707664-709358
  
 NCBI BlastP on this gene

EMT65798

hypothetical protein
  
Accession: EMT65799
  
Location: 710358-711452
  
 NCBI BlastP on this gene

EMT65799

hypothetical protein
  
Accession: EMT65800
  
Location: 711951-714139
  
 NCBI BlastP on this gene

EMT65800

hypothetical protein
  
Accession: EMT65801
  
Location: 714578-716239
  
 NCBI BlastP on this gene

EMT65801

Protein phosphatase 2C like protein C10F6.17c
  
Accession: EMT65802
  
Location: 716780-718269
  
 NCBI BlastP on this gene

EMT65802

Brix domain-containing protein C1B9.03c
  
Accession: EMT65803
  
Location: 718813-721803
  
  
**BlastP hit with Mycgr3G35447\_Mycgr3T**
  
Percentage identity: 57 %
  
BlastP bit score: 465
  
Sequence coverage: 102 %
  
E-value: 9e-158
  
  
 NCBI BlastP on this gene

EMT65803

ATP-dependent rRNA helicase RRP3
  
Accession: EMT65804
  
Location: 722022-723528
  
  
**BlastP hit with Mycgr3G84402\_Mycgr3T**
  
Percentage identity: 73 %
  
BlastP bit score: 629
  
Sequence coverage: 91 %
  
E-value: 0.0
  
  
 NCBI BlastP on this gene

EMT65804

hypothetical protein
  
Accession: EMT65805
  
Location: 724417-725480
  
 NCBI BlastP on this gene

EMT65805

Histone transcription regulator 3 like protein
  
Accession: EMT65806
  
Location: 725800-726998
  
 NCBI BlastP on this gene

EMT65806

Histone transcription regulator 3 like protein
  
Accession: EMT65807
  
Location: 727919-734077
  
 NCBI BlastP on this gene

EMT65807

hypothetical protein
  
Accession: EMT65808
  
Location: 734589-736954
  
 NCBI BlastP on this gene

EMT65808

Ubiquitin-conjugating enzyme E2 14
  
Accession: EMT65809
  
Location: 738128-738657
  
 NCBI BlastP on this gene

EMT65809

GTPase-activating protein gyp3
  
Accession: EMT65810
  
Location: 740423-743380
  
 NCBI BlastP on this gene

EMT65810

122. :  KE375219 Blumeria graminis f. sp. tritici 96224 unplaced genomic scaffold Scaffold-92     Total score: 2.0     Cumulative Blast bit score: 1093

hypothetical protein
  
Accession: EPQ61605
  
Location: 299889-301402
  
  
**BlastP hit with Mycgr3G84402\_Mycgr3T**
  
Percentage identity: 74 %
  
BlastP bit score: 634
  
Sequence coverage: 92 %
  
E-value: 0.0
  
  
 NCBI BlastP on this gene

EPQ61605

Constituent of 66S pre-ribosomal particles
  
Accession: EPQ61606
  
Location: 301622-303007
  
  
**BlastP hit with Mycgr3G35447\_Mycgr3T**
  
Percentage identity: 55 %
  
BlastP bit score: 459
  
Sequence coverage: 101 %
  
E-value: 1e-155
  
  
 NCBI BlastP on this gene

EPQ61606

hypothetical protein
  
Accession: EPQ61607
  
Location: 304099-305663
  
 NCBI BlastP on this gene

EPQ61607

Catalase
  
Accession: EPQ61608
  
Location: 312338-314743
  
 NCBI BlastP on this gene

EPQ61608

ATPase of the 19S regulatory particle of the 26S proteasome
  
Accession: EPQ61609
  
Location: 319859-321344
  
 NCBI BlastP on this gene

EPQ61609

Transcription factor TFIIB
  
Accession: EPQ61610
  
Location: 322091-323175
  
 NCBI BlastP on this gene

EPQ61610

123. :  CAUH01001323 Blumeria graminis f. sp. hordei DH14     Total score: 2.0     Cumulative Blast bit score: 1093

hypothetical protein
  
Accession: CCU75429
  
Location: 297-1637
  
  
**BlastP hit with Mycgr3G35447\_Mycgr3T**
  
Percentage identity: 57 %
  
BlastP bit score: 458
  
Sequence coverage: 94 %
  
E-value: 4e-155
  
  
 NCBI BlastP on this gene

CCU75429

Putative ATP-dependent rRNA helicase
  
Accession: CCU75430
  
Location: 1856-3369
  
  
**BlastP hit with Mycgr3G84402\_Mycgr3T**
  
Percentage identity: 73 %
  
BlastP bit score: 635
  
Sequence coverage: 94 %
  
E-value: 0.0
  
  
 NCBI BlastP on this gene

CCU75430

124. :  HF679031 Fusarium fujikuroi IMI 58289 draft genome, chromosome FFUJ\_chr09.     Total score: 2.0     Cumulative Blast bit score: 1092

related to RAB GTPase activator
  
Accession: CCT73724
  
Location: 2267697-2270642
  
 NCBI BlastP on this gene

FFUJ\_09458

probable ubiquitin-protein ligase UBC4
  
Accession: CCT73725
  
Location: 2272409-2272938
  
 NCBI BlastP on this gene

FFUJ\_09457

related to BUD7 protein
  
Accession: CCT73726
  
Location: 2273970-2276334
  
 NCBI BlastP on this gene

FFUJ\_09456

related to transcriptional corepressor HIR3
  
Accession: CCT73727
  
Location: 2276805-2283018
  
 NCBI BlastP on this gene

FFUJ\_09455

uncharacterized protein
  
Accession: CCT73728
  
Location: 2283346-2284404
  
 NCBI BlastP on this gene

FFUJ\_09454

probable DEAD box protein (putative RNA helicase)
  
Accession: CCT73729
  
Location: 2285361-2286864
  
  
**BlastP hit with Mycgr3G84402\_Mycgr3T**
  
Percentage identity: 73 %
  
BlastP bit score: 629
  
Sequence coverage: 91 %
  
E-value: 0.0
  
  
 NCBI BlastP on this gene

FFUJ\_09453

related to mating protein SSF1
  
Accession: CCT73730
  
Location: 2287084-2288497
  
  
**BlastP hit with Mycgr3G35447\_Mycgr3T**
  
Percentage identity: 57 %
  
BlastP bit score: 463
  
Sequence coverage: 102 %
  
E-value: 8e-157
  
  
 NCBI BlastP on this gene

FFUJ\_09452

related to Type 2C Protein Phosphatase
  
Accession: CCT73731
  
Location: 2289026-2290515
  
 NCBI BlastP on this gene

FFUJ\_09451

uncharacterized protein
  
Accession: CCT73732
  
Location: 2290886-2293081
  
 NCBI BlastP on this gene

FFUJ\_09450

related to glycerate-and formate-dehydrogenases
  
Accession: CCT73733
  
Location: 2293573-2294664
  
 NCBI BlastP on this gene

FFUJ\_09449

related to heterokaryon incompatibility protein (het-6OR allele)
  
Accession: CCT73734
  
Location: 2295473-2297530
  
 NCBI BlastP on this gene

FFUJ\_09448

related to integral membrane protein pth11
  
Accession: CCT73735
  
Location: 2299564-2300903
  
 NCBI BlastP on this gene

FFUJ\_09447

related to glu/asp-tRNA amidotransferase subunit A
  
Accession: CCT73736
  
Location: 2301959-2303888
  
 NCBI BlastP on this gene

FFUJ\_09446

uncharacterized protein
  
Accession: CCT73737
  
Location: 2304802-2305200
  
 NCBI BlastP on this gene

FFUJ\_09445

uncharacterized protein
  
Accession: CCT73738
  
Location: 2305618-2306076
  
 NCBI BlastP on this gene

FFUJ\_09444

uncharacterized protein
  
Accession: CCT73739
  
Location: 2306343-2308508
  
 NCBI BlastP on this gene

FFUJ\_09443

125. :  CAGA01000008 Claviceps purpurea 20.1     Total score: 2.0     Cumulative Blast bit score: 1092

uncharacterized protein
  
Accession: CCE28432
  
Location: 653715-659429
  
 NCBI BlastP on this gene

CCE28432

probable signal peptidase (endopeptidase SP18)
  
Accession: CCE28433
  
Location: 660806-661535
  
 NCBI BlastP on this gene

CCE28433

uncharacterized protein
  
Accession: CCE28434
  
Location: 662038-662416
  
 NCBI BlastP on this gene

CCE28434

uncharacterized protein
  
Accession: CCE28435
  
Location: 662861-664112
  
 NCBI BlastP on this gene

CCE28435

related to mating protein SSF1
  
Accession: CCE28436
  
Location: 669366-670902
  
  
**BlastP hit with Mycgr3G35447\_Mycgr3T**
  
Percentage identity: 59 %
  
BlastP bit score: 471
  
Sequence coverage: 93 %
  
E-value: 9e-160
  
  
 NCBI BlastP on this gene

CCE28436

probable DEAD box protein (putative RNA helicase)
  
Accession: CCE28437
  
Location: 671539-673152
  
  
**BlastP hit with Mycgr3G84402\_Mycgr3T**
  
Percentage identity: 72 %
  
BlastP bit score: 621
  
Sequence coverage: 92 %
  
E-value: 0.0
  
  
 NCBI BlastP on this gene

CCE28437

related to transcriptional corepressor HIR3
  
Accession: CCE28438
  
Location: 673563-680608
  
 NCBI BlastP on this gene

CCE28438

related to BUD7 protein
  
Accession: CCE28439
  
Location: 684601-690537
  
 NCBI BlastP on this gene

CCE28439

uncharacterized protein
  
Accession: CCE28440
  
Location: 691385-692699
  
 NCBI BlastP on this gene

CCE28440

126. :  JH767570 Coniosporium apollinis CBS 100218 chromosome Unknown supercont1.17     Total score: 2.0     Cumulative Blast bit score: 1090

hypothetical protein
  
Accession: EON64871
  
Location: 432594-434000
  
 NCBI BlastP on this gene

EON64871

hypothetical protein
  
Accession: EON64872
  
Location: 435829-437528
  
 NCBI BlastP on this gene

EON64872

betaine-aldehyde dehydrogenase
  
Accession: EON64873
  
Location: 438386-439894
  
 NCBI BlastP on this gene

EON64873

hypothetical protein
  
Accession: EON64874
  
Location: 441134-442534
  
 NCBI BlastP on this gene

EON64874

hypothetical protein
  
Accession: EON64875
  
Location: 442994-444396
  
 NCBI BlastP on this gene

EON64875

hypothetical protein
  
Accession: EON64876
  
Location: 445265-447159
  
 NCBI BlastP on this gene

EON64876

hypothetical protein
  
Accession: EON64877
  
Location: 448023-449093
  
 NCBI BlastP on this gene

EON64877

hypothetical protein
  
Accession: EON64878
  
Location: 449566-450570
  
 NCBI BlastP on this gene

EON64878

hypothetical protein
  
Accession: EON64879
  
Location: 451043-452502
  
  
**BlastP hit with Mycgr3G35447\_Mycgr3T**
  
Percentage identity: 61 %
  
BlastP bit score: 494
  
Sequence coverage: 96 %
  
E-value: 7e-169
  
  
 NCBI BlastP on this gene

EON64879

ATP-dependent rRNA helicase RRP3
  
Accession: EON64880
  
Location: 452703-454573
  
  
**BlastP hit with Mycgr3G84402\_Mycgr3T**
  
Percentage identity: 67 %
  
BlastP bit score: 596
  
Sequence coverage: 96 %
  
E-value: 0.0
  
  
 NCBI BlastP on this gene

EON64880

hypothetical protein
  
Accession: EON64881
  
Location: 456677-458207
  
 NCBI BlastP on this gene

EON64881

acetolactate synthase, large subunit, biosynthetic type
  
Accession: EON64882
  
Location: 458578-460791
  
 NCBI BlastP on this gene

EON64882

hypothetical protein
  
Accession: EON64883
  
Location: 461458-462339
  
 NCBI BlastP on this gene

EON64883

hypothetical protein
  
Accession: EON64884
  
Location: 462830-465869
  
 NCBI BlastP on this gene

EON64884

golgi apparatus membrane protein TVP18
  
Accession: EON64885
  
Location: 467401-468171
  
 NCBI BlastP on this gene

EON64885

nuclear transport factor 2
  
Accession: EON64886
  
Location: 468857-469591
  
 NCBI BlastP on this gene

EON64886

dihydroflavonol-4-reductase
  
Accession: EON64887
  
Location: 470308-471441
  
 NCBI BlastP on this gene

EON64887

succinyl-CoA ligase [GDP-forming] subunit beta, mitochondrial
  
Accession: EON64888
  
Location: 472071-473677
  
 NCBI BlastP on this gene

EON64888

hypothetical protein
  
Accession: EON64889
  
Location: 473874-474641
  
 NCBI BlastP on this gene

EON64889

127. :  CP003010 Thielavia terrestris NRRL 8126 chromosome 2     Total score: 2.0     Cumulative Blast bit score: 1090

hypothetical protein
  
Accession: AEO66451
  
Location: 5811902-5813355
  
 NCBI BlastP on this gene

THITE\_2087927

hypothetical protein
  
Accession: AEO66452
  
Location: 5813844-5814124
  
 NCBI BlastP on this gene

THITE\_152050

hypothetical protein
  
Accession: AEO66453
  
Location: 5814444-5815139
  
 NCBI BlastP on this gene

THITE\_2044327

hypothetical protein
  
Accession: AEO66454
  
Location: 5817233-5820816
  
 NCBI BlastP on this gene

THITE\_2144016

glycoside hydrolase family 18 protein
  
Accession: AEO66455
  
Location: 5821722-5823366
  
 NCBI BlastP on this gene

THITE\_35493

hypothetical protein
  
Accession: AEO66456
  
Location: 5827717-5828775
  
 NCBI BlastP on this gene

THITE\_2128521

hypothetical protein
  
Accession: AEO66457
  
Location: 5830219-5830697
  
 NCBI BlastP on this gene

THITE\_2114558

hypothetical protein
  
Accession: AEO66458
  
Location: 5831396-5832863
  
  
**BlastP hit with Mycgr3G35447\_Mycgr3T**
  
Percentage identity: 56 %
  
BlastP bit score: 463
  
Sequence coverage: 102 %
  
E-value: 1e-156
  
  
 NCBI BlastP on this gene

THITE\_2114560

hypothetical protein
  
Accession: AEO66459
  
Location: 5833120-5834823
  
  
**BlastP hit with Mycgr3G84402\_Mycgr3T**
  
Percentage identity: 75 %
  
BlastP bit score: 627
  
Sequence coverage: 89 %
  
E-value: 0.0
  
  
 NCBI BlastP on this gene

THITE\_2114564

hypothetical protein
  
Accession: AEO66460
  
Location: 5835023-5837227
  
 NCBI BlastP on this gene

THITE\_2144021

hypothetical protein
  
Accession: AEO66461
  
Location: 5839789-5841096
  
 NCBI BlastP on this gene

THITE\_2114567

hypothetical protein
  
Accession: AEO66462
  
Location: 5842190-5843449
  
 NCBI BlastP on this gene

THITE\_2114569

hypothetical protein
  
Accession: AEO66463
  
Location: 5843915-5844154
  
 NCBI BlastP on this gene

THITE\_2114571

hypothetical protein
  
Accession: AEO66464
  
Location: 5844667-5845299
  
 NCBI BlastP on this gene

THITE\_2114572

hypothetical protein
  
Accession: AEO66465
  
Location: 5845528-5846547
  
 NCBI BlastP on this gene

THITE\_125923

hypothetical protein
  
Accession: AEO66466
  
Location: 5847316-5849756
  
 NCBI BlastP on this gene

THITE\_2114578

hypothetical protein
  
Accession: AEO66467
  
Location: 5850466-5856661
  
 NCBI BlastP on this gene

THITE\_110621

128. :  DS572814 Paracoccidioides brasiliensis Pb01 supercont1.4 genomic scaffold     Total score: 2.0     Cumulative Blast bit score: 1088

conserved hypothetical protein
  
Accession: EEH39736
  
Location: 544545-547339
  
 NCBI BlastP on this gene

EEH39736

conserved hypothetical protein
  
Accession: EEH39737
  
Location: 548836-549732
  
 NCBI BlastP on this gene

EEH39737

conserved hypothetical protein
  
Accession: EEH39738
  
Location: 550393-552444
  
 NCBI BlastP on this gene

EEH39738

peroxisomal dehydratase
  
Accession: EEH39739
  
Location: 552949-554277
  
 NCBI BlastP on this gene

EEH39739

HNRNP arginine N-methyltransferase
  
Accession: EEH39740
  
Location: 555127-556297
  
 NCBI BlastP on this gene

EEH39740

Mn2+ homeostasis protein (Per1)
  
Accession: EEH39741
  
Location: 557299-558524
  
 NCBI BlastP on this gene

EEH39741

phosphoacetylglucosamine mutase
  
Accession: EEH39742
  
Location: 559301-561344
  
  
**BlastP hit with Mycgr3G103034\_Mycgr3**
  
Percentage identity: 56 %
  
BlastP bit score: 617
  
Sequence coverage: 98 %
  
E-value: 0.0
  
  
 NCBI BlastP on this gene

EEH39742

DEAD-box ATP-dependent RNA helicase
  
Accession: EEH39743
  
Location: 562019-562978
  
 NCBI BlastP on this gene

EEH39743

ribosome biogenesis protein SSF2
  
Accession: EEH39744
  
Location: 563829-565250
  
  
**BlastP hit with Mycgr3G35447\_Mycgr3T**
  
Percentage identity: 58 %
  
BlastP bit score: 471
  
Sequence coverage: 94 %
  
E-value: 6e-160
  
  
 NCBI BlastP on this gene

EEH39744

riboflavin synthase alpha chain
  
Accession: EEH39745
  
Location: 565681-566543
  
 NCBI BlastP on this gene

EEH39745

formyl-coenzyme A transferase
  
Accession: EEH39746
  
Location: 566844-568656
  
 NCBI BlastP on this gene

EEH39746

conserved hypothetical protein
  
Accession: EEH39747
  
Location: 569167-571020
  
 NCBI BlastP on this gene

EEH39747

DUF887 domain-containing protein
  
Accession: EEH39748
  
Location: 571803-573117
  
 NCBI BlastP on this gene

EEH39748

conserved hypothetical protein
  
Accession: EEH39749
  
Location: 574107-580197
  
 NCBI BlastP on this gene

EEH39749

60S ribosomal protein L27-A
  
Accession: EEH39750
  
Location: 580922-581705
  
 NCBI BlastP on this gene

EEH39750

129. :  GL988041 Chaetomium thermophilum var. thermophilum DSM 1495 unplaced genomic scaffold scf7180000...     Total score: 2.0     Cumulative Blast bit score: 1086

hypothetical protein
  
Accession: EGS21722
  
Location: 5519429-5521812
  
 NCBI BlastP on this gene

EGS21722

reductase-like protein
  
Accession: EGS21723
  
Location: 5522684-5523702
  
 NCBI BlastP on this gene

EGS21723

hypothetical protein
  
Accession: EGS21724
  
Location: 5523844-5524470
  
 NCBI BlastP on this gene

EGS21724

hypothetical protein
  
Accession: EGS21725
  
Location: 5525019-5525330
  
 NCBI BlastP on this gene

EGS21725

putative ATP binding protein
  
Accession: EGS21726
  
Location: 5525618-5526780
  
 NCBI BlastP on this gene

EGS21726

hypothetical protein
  
Accession: EGS21727
  
Location: 5527862-5529143
  
 NCBI BlastP on this gene

EGS21727

hypothetical protein
  
Accession: EGS21728
  
Location: 5531034-5533307
  
 NCBI BlastP on this gene

EGS21728

ATP-dependent rRNA helicase rrp3-like protein
  
Accession: EGS21729
  
Location: 5533540-5535237
  
  
**BlastP hit with Mycgr3G84402\_Mycgr3T**
  
Percentage identity: 73 %
  
BlastP bit score: 635
  
Sequence coverage: 92 %
  
E-value: 0.0
  
  
 NCBI BlastP on this gene

EGS21729

hypothetical protein
  
Accession: EGS21730
  
Location: 5535480-5536975
  
  
**BlastP hit with Mycgr3G35447\_Mycgr3T**
  
Percentage identity: 53 %
  
BlastP bit score: 451
  
Sequence coverage: 104 %
  
E-value: 6e-152
  
  
 NCBI BlastP on this gene

EGS21730

hypothetical protein
  
Accession: EGS21731
  
Location: 5537355-5537872
  
 NCBI BlastP on this gene

EGS21731

hypothetical protein
  
Accession: EGS21732
  
Location: 5546294-5547626
  
 NCBI BlastP on this gene

EGS21732

hypothetical protein
  
Accession: EGS21733
  
Location: 5551428-5552237
  
 NCBI BlastP on this gene

EGS21733

hypothetical protein
  
Accession: EGS21734
  
Location: 5554093-5555609
  
 NCBI BlastP on this gene

EGS21734

130. :  JH725173 Beauveria bassiana ARSEF 2860 unplaced genomic scaffold BBA\_S00024     Total score: 2.0     Cumulative Blast bit score: 1081

Brf1-like TBP-binding domain-containing protein
  
Accession: EJP63740
  
Location: 320989-323157
  
 NCBI BlastP on this gene

EJP63740

MFS transporter
  
Accession: EJP63741
  
Location: 323319-325411
  
 NCBI BlastP on this gene

EJP63741

hypothetical protein
  
Accession: EJP63742
  
Location: 328135-328514
  
 NCBI BlastP on this gene

EJP63742

general amidase GmdB
  
Accession: EJP63743
  
Location: 329413-331101
  
 NCBI BlastP on this gene

EJP63743

Glycoside hydrolase, catalytic core
  
Accession: EJP63744
  
Location: 331861-333474
  
 NCBI BlastP on this gene

EJP63744

protein phosphatase 2C
  
Accession: EJP63745
  
Location: 334220-335778
  
 NCBI BlastP on this gene

EJP63745

DNA-binding protein
  
Accession: EJP63746
  
Location: 336601-337269
  
 NCBI BlastP on this gene

EJP63746

calcineurin-like phosphoesterase, putative
  
Accession: EJP63747
  
Location: 337741-338682
  
 NCBI BlastP on this gene

EJP63747

ribosome biogenesis protein SSF1
  
Accession: EJP63748
  
Location: 339420-340793
  
  
**BlastP hit with Mycgr3G35447\_Mycgr3T**
  
Percentage identity: 57 %
  
BlastP bit score: 449
  
Sequence coverage: 93 %
  
E-value: 2e-151
  
  
 NCBI BlastP on this gene

EJP63748

DEAD/DEAH box helicase
  
Accession: EJP63749
  
Location: 341109-342592
  
  
**BlastP hit with Mycgr3G84402\_Mycgr3T**
  
Percentage identity: 72 %
  
BlastP bit score: 632
  
Sequence coverage: 91 %
  
E-value: 0.0
  
  
 NCBI BlastP on this gene

EJP63749

transcriptional corepressor, putative
  
Accession: EJP63750
  
Location: 343218-349818
  
 NCBI BlastP on this gene

EJP63750

bud site selection protein
  
Accession: EJP63751
  
Location: 350618-352940
  
 NCBI BlastP on this gene

EJP63751

XPA-binding protein
  
Accession: EJP63752
  
Location: 353434-354665
  
 NCBI BlastP on this gene

EJP63752

DNA-directed RNA polymerases I, II, and III subunit RPABC4
  
Accession: EJP63753
  
Location: 354939-355253
  
 NCBI BlastP on this gene

EJP63753

HIT domain-containing protein
  
Accession: EJP63754
  
Location: 356150-356734
  
 NCBI BlastP on this gene

EJP63754

glycerol dehydrogenase Gcy1
  
Accession: EJP63755
  
Location: 356902-358001
  
 NCBI BlastP on this gene

EJP63755

hypothetical protein
  
Accession: EJP63756
  
Location: 358448-358819
  
 NCBI BlastP on this gene

EJP63756

fungal specific transcription factor
  
Accession: EJP63757
  
Location: 359259-361351
  
 NCBI BlastP on this gene

EJP63757

kelch repeat protein
  
Accession: EJP63758
  
Location: 361771-362820
  
 NCBI BlastP on this gene

EJP63758

131. :  GG697432 Glomerella graminicola M1.001 genomic scaffold supercont1.102     Total score: 2.0     Cumulative Blast bit score: 1081

hypothetical protein
  
Accession: EFQ36514
  
Location: 5526-6965
  
 NCBI BlastP on this gene

EFQ36514

hypothetical protein
  
Accession: EFQ36515
  
Location: 10306-10700
  
 NCBI BlastP on this gene

EFQ36515

brix domain-containing protein
  
Accession: EFQ36516
  
Location: 22988-24367
  
  
**BlastP hit with Mycgr3G35447\_Mycgr3T**
  
Percentage identity: 56 %
  
BlastP bit score: 464
  
Sequence coverage: 100 %
  
E-value: 2e-157
  
  
 NCBI BlastP on this gene

EFQ36516

DEAD/DEAH box helicase
  
Accession: EFQ36517
  
Location: 24653-27022
  
  
**BlastP hit with Mycgr3G84402\_Mycgr3T**
  
Percentage identity: 70 %
  
BlastP bit score: 617
  
Sequence coverage: 93 %
  
E-value: 0.0
  
  
 NCBI BlastP on this gene

EFQ36517

kinesin motor domain-containing protein
  
Accession: EFQ36518
  
Location: 27574-30124
  
 NCBI BlastP on this gene

EFQ36518

hypothetical protein
  
Accession: EFQ36519
  
Location: 32915-34703
  
 NCBI BlastP on this gene

EFQ36519

hypothetical protein
  
Accession: EFQ36520
  
Location: 35387-35893
  
 NCBI BlastP on this gene

EFQ36520

Brf1-like TBP-binding domain-containing protein
  
Accession: EFQ36521
  
Location: 38164-40374
  
 NCBI BlastP on this gene

EFQ36521

132. :  CH408032 Chaetomium globosum CBS 148.51 scaffold\_4 genomic scaffold     Total score: 2.0     Cumulative Blast bit score: 1080

hypothetical protein
  
Accession: EAQ87662
  
Location: 1773941-1776936
  
 NCBI BlastP on this gene

EAQ87662

hypothetical protein
  
Accession: EAQ87663
  
Location: 1777707-1779007
  
 NCBI BlastP on this gene

EAQ87663

predicted protein
  
Accession: EAQ87664
  
Location: 1781664-1781839
  
 NCBI BlastP on this gene

EAQ87664

predicted protein
  
Accession: EAQ87665
  
Location: 1782374-1782958
  
 NCBI BlastP on this gene

EAQ87665

predicted protein
  
Accession: EAQ87666
  
Location: 1784874-1785335
  
 NCBI BlastP on this gene

EAQ87666

hypothetical protein
  
Accession: EAQ87667
  
Location: 1786202-1791274
  
 NCBI BlastP on this gene

EAQ87667

hypothetical protein
  
Accession: EAQ87668
  
Location: 1791889-1793352
  
  
**BlastP hit with Mycgr3G35447\_Mycgr3T**
  
Percentage identity: 54 %
  
BlastP bit score: 450
  
Sequence coverage: 102 %
  
E-value: 2e-151
  
  
 NCBI BlastP on this gene

EAQ87668

hypothetical protein
  
Accession: EAQ87669
  
Location: 1793606-1795308
  
  
**BlastP hit with Mycgr3G84402\_Mycgr3T**
  
Percentage identity: 73 %
  
BlastP bit score: 630
  
Sequence coverage: 93 %
  
E-value: 0.0
  
  
 NCBI BlastP on this gene

EAQ87669

hypothetical protein
  
Accession: EAQ87670
  
Location: 1795499-1797697
  
 NCBI BlastP on this gene

EAQ87670

hypothetical protein
  
Accession: EAQ87671
  
Location: 1798901-1800027
  
 NCBI BlastP on this gene

EAQ87671

hypothetical protein
  
Accession: EAQ87672
  
Location: 1801061-1802219
  
 NCBI BlastP on this gene

EAQ87672

hypothetical protein
  
Accession: EAQ87673
  
Location: 1802656-1802970
  
 NCBI BlastP on this gene

EAQ87673

hypothetical protein
  
Accession: EAQ87674
  
Location: 1804645-1805667
  
 NCBI BlastP on this gene

EAQ87674

conserved hypothetical protein
  
Accession: EAQ87675
  
Location: 1806474-1808908
  
 NCBI BlastP on this gene

EAQ87675

hypothetical protein
  
Accession: EAQ87676
  
Location: 1809452-1815702
  
 NCBI BlastP on this gene

EAQ87676

133. :  AFQF01002695 Fusarium oxysporum Fo5176     Total score: 2.0     Cumulative Blast bit score: 1077

hypothetical protein
  
Accession: EGU79266
  
Location: 17231-20185
  
 NCBI BlastP on this gene

EGU79266

hypothetical protein
  
Accession: EGU79267
  
Location: 21968-22497
  
 NCBI BlastP on this gene

EGU79267

hypothetical protein
  
Accession: EGU79268
  
Location: 23645-25541
  
 NCBI BlastP on this gene

EGU79268

hypothetical protein
  
Accession: EGU79269
  
Location: 26521-32679
  
 NCBI BlastP on this gene

EGU79269

hypothetical protein
  
Accession: EGU79270
  
Location: 33001-34064
  
 NCBI BlastP on this gene

EGU79270

hypothetical protein
  
Accession: EGU79271
  
Location: 34935-36441
  
  
**BlastP hit with Mycgr3G84402\_Mycgr3T**
  
Percentage identity: 73 %
  
BlastP bit score: 629
  
Sequence coverage: 91 %
  
E-value: 0.0
  
  
 NCBI BlastP on this gene

EGU79271

hypothetical protein
  
Accession: EGU79272
  
Location: 36659-38071
  
  
**BlastP hit with Mycgr3G35447\_Mycgr3T**
  
Percentage identity: 56 %
  
BlastP bit score: 448
  
Sequence coverage: 100 %
  
E-value: 2e-151
  
  
 NCBI BlastP on this gene

EGU79272

hypothetical protein
  
Accession: EGU79273
  
Location: 38608-40097
  
 NCBI BlastP on this gene

EGU79273

hypothetical protein
  
Accession: EGU79274
  
Location: 40642-42183
  
 NCBI BlastP on this gene

EGU79274

hypothetical protein
  
Accession: EGU79275
  
Location: 43325-44419
  
 NCBI BlastP on this gene

EGU79275

hypothetical protein
  
Accession: EGU79276
  
Location: 45061-47112
  
 NCBI BlastP on this gene

EGU79276

hypothetical protein
  
Accession: EGU79277
  
Location: 49036-50372
  
 NCBI BlastP on this gene

EGU79277

hypothetical protein
  
Accession: EGU79278
  
Location: 51337-53266
  
 NCBI BlastP on this gene

EGU79278

134. :  CP003007 Myceliophthora thermophila ATCC 42464 chromosome 6     Total score: 2.0     Cumulative Blast bit score: 1074

hypothetical protein
  
Accession: AEO60946
  
Location: 2530038-2531138
  
 NCBI BlastP on this gene

MYCTH\_2112941

hypothetical protein
  
Accession: AEO60947
  
Location: 2532883-2533455
  
 NCBI BlastP on this gene

MYCTH\_2310703

hypothetical protein
  
Accession: AEO60948
  
Location: 2535302-2535870
  
 NCBI BlastP on this gene

MYCTH\_60397

hypothetical protein
  
Accession: AEO60949
  
Location: 2536718-2538312
  
  
**BlastP hit with Mycgr3G35447\_Mycgr3T**
  
Percentage identity: 55 %
  
BlastP bit score: 443
  
Sequence coverage: 102 %
  
E-value: 4e-148
  
  
 NCBI BlastP on this gene

MYCTH\_2310708

hypothetical protein
  
Accession: AEO60950
  
Location: 2538437-2540186
  
  
**BlastP hit with Mycgr3G84402\_Mycgr3T**
  
Percentage identity: 74 %
  
BlastP bit score: 631
  
Sequence coverage: 92 %
  
E-value: 0.0
  
  
 NCBI BlastP on this gene

MYCTH\_2096529

hypothetical protein
  
Accession: AEO60951
  
Location: 2544273-2545544
  
 NCBI BlastP on this gene

MYCTH\_2310716

hypothetical protein
  
Accession: AEO60952
  
Location: 2546775-2548026
  
 NCBI BlastP on this gene

MYCTH\_113239

hypothetical protein
  
Accession: AEO60953
  
Location: 2549258-2549914
  
 NCBI BlastP on this gene

MYCTH\_37799

Aldo/keto reductase-like protein
  
Accession: AEO60954
  
Location: 2550203-2551227
  
 NCBI BlastP on this gene

MYCTH\_113236

135. :  KB707406 Eutypa lata UCREL1 unplaced genomic scaffold EL1\_03\_scaffold\_2068     Total score: 2.0     Cumulative Blast bit score: 1072

hypothetical protein
  
Accession: EMR62663
  
Location: 1346-1849
  
 NCBI BlastP on this gene

EMR62663

hypothetical protein
  
Accession: EMR62666
  
Location: 3551-4426
  
 NCBI BlastP on this gene

EMR62666

hypothetical protein
  
Accession: EMR62668
  
Location: 5523-6890
  
 NCBI BlastP on this gene

EMR62668

putative salicylate hydroxylase protein
  
Accession: EMR62658
  
Location: 13388-14738
  
 NCBI BlastP on this gene

EMR62658

hypothetical protein
  
Accession: EMR62656
  
Location: 15857-16806
  
 NCBI BlastP on this gene

EMR62656

putative ribosome biogenesis protein ssf1 protein
  
Accession: EMR62660
  
Location: 17657-19256
  
  
**BlastP hit with Mycgr3G35447\_Mycgr3T**
  
Percentage identity: 51 %
  
BlastP bit score: 431
  
Sequence coverage: 106 %
  
E-value: 1e-143
  
  
 NCBI BlastP on this gene

EMR62660

putative atp-dependent rrna helicase rrp3 protein
  
Accession: EMR62664
  
Location: 19678-21431
  
  
**BlastP hit with Mycgr3G84402\_Mycgr3T**
  
Percentage identity: 70 %
  
BlastP bit score: 641
  
Sequence coverage: 100 %
  
E-value: 0.0
  
  
 NCBI BlastP on this gene

EMR62664

putative alpha-ketoglutarate dependent xanthine dioxygenase protein
  
Accession: EMR62657
  
Location: 23239-24684
  
 NCBI BlastP on this gene

EMR62657

putative c6 zinc finger domain containing protein
  
Accession: EMR62653
  
Location: 26050-27843
  
 NCBI BlastP on this gene

EMR62653

putative ncs1 allantoate transporter protein
  
Accession: EMR62661
  
Location: 29028-30860
  
 NCBI BlastP on this gene

EMR62661

putative leucoanthocyanidin dioxygenase protein
  
Accession: EMR62654
  
Location: 31513-32601
  
 NCBI BlastP on this gene

EMR62654

putative cytidine and deoxycytidylate deaminase zinc-binding region protein
  
Accession: EMR62652
  
Location: 32866-33674
  
 NCBI BlastP on this gene

EMR62652

putative alpha-ketoglutarate dependent xanthine dioxygenase protein
  
Accession: EMR62665
  
Location: 33910-35372
  
 NCBI BlastP on this gene

EMR62665

putative extracellular dioxygenase protein
  
Accession: EMR62650
  
Location: 38496-39353
  
 NCBI BlastP on this gene

EMR62650

putative short-chain dehydrogenase reductase family protein
  
Accession: EMR62659
  
Location: 40983-41933
  
 NCBI BlastP on this gene

EMR62659

136. :  CU638744 Podospora anserina S mat+ genomic DNA chromosome 6, supercontig 2.     Total score: 2.0     Cumulative Blast bit score: 1072

not annotated
  
Accession: CAP71745
  
Location: 2597327-2597725
  
 NCBI BlastP on this gene

CAP71745

not annotated
  
Accession: CAP71746
  
Location: 2598101-2599590
  
 NCBI BlastP on this gene

CAP71746

not annotated
  
Accession: CAP71747
  
Location: 2601643-2603132
  
 NCBI BlastP on this gene

CAP71747

not annotated
  
Accession: CAP71748
  
Location: 2605945-2607247
  
 NCBI BlastP on this gene

CAP71748

not annotated
  
Accession: CAP71749
  
Location: 2609718-2610130
  
 NCBI BlastP on this gene

CAP71749

not annotated
  
Accession: CAP71750
  
Location: 2612569-2613339
  
 NCBI BlastP on this gene

CAP71750

not annotated
  
Accession: CAP71751
  
Location: 2614799-2616271
  
  
**BlastP hit with Mycgr3G35447\_Mycgr3T**
  
Percentage identity: 56 %
  
BlastP bit score: 452
  
Sequence coverage: 95 %
  
E-value: 7e-152
  
  
 NCBI BlastP on this gene

CAP71751

not annotated
  
Accession: CAP71752
  
Location: 2616546-2618125
  
  
**BlastP hit with Mycgr3G84402\_Mycgr3T**
  
Percentage identity: 66 %
  
BlastP bit score: 620
  
Sequence coverage: 103 %
  
E-value: 0.0
  
  
 NCBI BlastP on this gene

CAP71752

not annotated
  
Accession: CAP71753
  
Location: 2618385-2620523
  
 NCBI BlastP on this gene

CAP71753

not annotated
  
Accession: CAP71754
  
Location: 2622314-2623693
  
 NCBI BlastP on this gene

CAP71754

not annotated
  
Accession: CAP71755
  
Location: 2624984-2626105
  
 NCBI BlastP on this gene

CAP71755

not annotated
  
Accession: CAP71756
  
Location: 2626411-2626685
  
 NCBI BlastP on this gene

CAP71756

not annotated
  
Accession: CAP71757
  
Location: 2627182-2627820
  
 NCBI BlastP on this gene

CAP71757

not annotated
  
Accession: CAP71758
  
Location: 2628136-2629279
  
 NCBI BlastP on this gene

CAP71758

not annotated
  
Accession: CAP71759
  
Location: 2630035-2632357
  
 NCBI BlastP on this gene

CAP71759

not annotated
  
Accession: CAP71760
  
Location: 2633044-2638734
  
 NCBI BlastP on this gene

CAP71760

137. :  GL698748 Metarhizium anisopliae ARSEF 23 unplaced genomic scaffold Scf\_038     Total score: 2.0     Cumulative Blast bit score: 1071

UbiD family decarboxylase
  
Accession: EFY95020
  
Location: 17474-19151
  
 NCBI BlastP on this gene

EFY95020

pyruvate dehydrogenase, putative
  
Accession: EFY95021
  
Location: 21597-23178
  
 NCBI BlastP on this gene

EFY95021

hypothetical protein
  
Accession: EFY95022
  
Location: 28217-28738
  
 NCBI BlastP on this gene

EFY95022

glycerate-and formate-dehydrogenase
  
Accession: EFY95023
  
Location: 30926-33516
  
 NCBI BlastP on this gene

EFY95023

DUF1338 domain protein
  
Accession: EFY95024
  
Location: 34616-36048
  
 NCBI BlastP on this gene

EFY95024

ribosome biogenesis protein Ssf2, putative
  
Accession: EFY95025
  
Location: 36163-37611
  
  
**BlastP hit with Mycgr3G35447\_Mycgr3T**
  
Percentage identity: 59 %
  
BlastP bit score: 457
  
Sequence coverage: 91 %
  
E-value: 1e-154
  
  
 NCBI BlastP on this gene

EFY95025

ATP-dependent rRNA helicase RRP3
  
Accession: EFY95026
  
Location: 37958-39454
  
  
**BlastP hit with Mycgr3G84402\_Mycgr3T**
  
Percentage identity: 70 %
  
BlastP bit score: 614
  
Sequence coverage: 95 %
  
E-value: 0.0
  
  
 NCBI BlastP on this gene

EFY95026

transcriptional corepressor
  
Accession: EFY95027
  
Location: 40152-46740
  
 NCBI BlastP on this gene

EFY95027

budding site selection protein
  
Accession: EFY95028
  
Location: 47362-49674
  
 NCBI BlastP on this gene

EFY95028

ATP binding protein, putative
  
Accession: EFY95029
  
Location: 50254-51502
  
 NCBI BlastP on this gene

EFY95029

hypothetical protein
  
Accession: EFY95030
  
Location: 51708-53944
  
 NCBI BlastP on this gene

EFY95030

cysteine-rich secreted protein
  
Accession: EFY95031
  
Location: 54562-55937
  
 NCBI BlastP on this gene

EFY95031

polyketide synthase
  
Accession: EFY95032
  
Location: 58057-59442
  
 NCBI BlastP on this gene

EFY95032

138. :  GL985056 Trichoderma reesei QM6a unplaced genomic scaffold TRIREscaffold\_1     Total score: 2.0     Cumulative Blast bit score: 1070

predicted protein
  
Accession: EGR52938
  
Location: 2184066-2184786
  
 NCBI BlastP on this gene

EGR52938

RNA polymerase III transcription initiation factor B complex component
  
Accession: EGR52397
  
Location: 2186328-2188364
  
 NCBI BlastP on this gene

EGR52397

predicted protein
  
Accession: EGR52939
  
Location: 2188867-2190018
  
 NCBI BlastP on this gene

EGR52939

aspartate protease
  
Accession: EGR52940
  
Location: 2191952-2193253
  
 NCBI BlastP on this gene

EGR52940

predicted protein
  
Accession: EGR52398
  
Location: 2194795-2195720
  
 NCBI BlastP on this gene

EGR52398

predicted protein
  
Accession: EGR52941
  
Location: 2203596-2205056
  
  
**BlastP hit with Mycgr3G35447\_Mycgr3T**
  
Percentage identity: 58 %
  
BlastP bit score: 438
  
Sequence coverage: 91 %
  
E-value: 9e-147
  
  
 NCBI BlastP on this gene

EGR52941

predicted protein
  
Accession: EGR52399
  
Location: 2205691-2207210
  
  
**BlastP hit with Mycgr3G84402\_Mycgr3T**
  
Percentage identity: 71 %
  
BlastP bit score: 632
  
Sequence coverage: 93 %
  
E-value: 0.0
  
  
 NCBI BlastP on this gene

EGR52399

predicted protein
  
Accession: EGR52942
  
Location: 2207638-2213921
  
 NCBI BlastP on this gene

EGR52942

predicted protein
  
Accession: EGR52400
  
Location: 2214559-2216921
  
 NCBI BlastP on this gene

EGR52400

hypothetical protein
  
Accession: EGR52401
  
Location: 2217411-2218673
  
 NCBI BlastP on this gene

EGR52401

predicted protein
  
Accession: EGR52943
  
Location: 2219043-2219373
  
 NCBI BlastP on this gene

EGR52943

predicted protein
  
Accession: EGR52402
  
Location: 2220460-2221065
  
 NCBI BlastP on this gene

EGR52402

predicted protein
  
Accession: EGR52944
  
Location: 2221835-2222733
  
 NCBI BlastP on this gene

EGR52944

139. :  ABDF02000004 Trichoderma virens Gv29-8     Total score: 2.0     Cumulative Blast bit score: 1069

hypothetical protein
  
Accession: EHK24236
  
Location: 162725-163258
  
 NCBI BlastP on this gene

EHK24236

hypothetical protein
  
Accession: EHK24237
  
Location: 164217-164527
  
 NCBI BlastP on this gene

EHK24237

hypothetical protein
  
Accession: EHK24238
  
Location: 164876-165961
  
 NCBI BlastP on this gene

EHK24238

hypothetical protein
  
Accession: EHK24239
  
Location: 166581-168975
  
 NCBI BlastP on this gene

EHK24239

hypothetical protein
  
Accession: EHK24240
  
Location: 169577-175801
  
 NCBI BlastP on this gene

EHK24240

hypothetical protein
  
Accession: EHK24241
  
Location: 176351-177862
  
  
**BlastP hit with Mycgr3G84402\_Mycgr3T**
  
Percentage identity: 74 %
  
BlastP bit score: 630
  
Sequence coverage: 87 %
  
E-value: 0.0
  
  
 NCBI BlastP on this gene

EHK24241

hypothetical protein
  
Accession: EHK24242
  
Location: 178371-179812
  
  
**BlastP hit with Mycgr3G35447\_Mycgr3T**
  
Percentage identity: 57 %
  
BlastP bit score: 439
  
Sequence coverage: 91 %
  
E-value: 2e-147
  
  
 NCBI BlastP on this gene

EHK24242

hypothetical protein
  
Accession: EHK24243
  
Location: 180068-181617
  
 NCBI BlastP on this gene

EHK24243

hypothetical protein
  
Accession: EHK24244
  
Location: 182325-182855
  
 NCBI BlastP on this gene

EHK24244

hypothetical protein
  
Accession: EHK24245
  
Location: 184297-186980
  
 NCBI BlastP on this gene

EHK24245

hypothetical protein
  
Accession: EHK24246
  
Location: 187551-188708
  
 NCBI BlastP on this gene

EHK24246

hypothetical protein
  
Accession: EHK24247
  
Location: 189597-190682
  
 NCBI BlastP on this gene

EHK24247

hypothetical protein
  
Accession: EHK24248
  
Location: 192389-192709
  
 NCBI BlastP on this gene

EHK24248

hypothetical protein
  
Accession: EHK24249
  
Location: 193528-194130
  
 NCBI BlastP on this gene

EHK24249

hypothetical protein
  
Accession: EHK24250
  
Location: 194941-195822
  
 NCBI BlastP on this gene

EHK24250

hypothetical protein
  
Accession: EHK24251
  
Location: 196459-196928
  
 NCBI BlastP on this gene

EHK24251

hypothetical protein
  
Accession: EHK24252
  
Location: 199593-200193
  
 NCBI BlastP on this gene

EHK24252

140. :  CACQ02001212 Colletotrichum higginsianum strain IMI 349063     Total score: 2.0     Cumulative Blast bit score: 1066

hypothetical protein
  
Accession: CCF34614
  
Location: 1289-3745
  
 NCBI BlastP on this gene

CCF34614

C6 zinc finger protein
  
Accession: CCF34615
  
Location: 3957-5919
  
 NCBI BlastP on this gene

CCF34615

phytanoyl-CoA dioxygenase
  
Accession: CCF34616
  
Location: 6341-7444
  
 NCBI BlastP on this gene

CCF34616

brix domain-containing protein
  
Accession: CCF34617
  
Location: 8075-9454
  
  
**BlastP hit with Mycgr3G35447\_Mycgr3T**
  
Percentage identity: 55 %
  
BlastP bit score: 454
  
Sequence coverage: 100 %
  
E-value: 2e-153
  
  
 NCBI BlastP on this gene

CCF34617

ATP-dependent rRNA helicase RRP3
  
Accession: CCF34618
  
Location: 9785-12065
  
  
**BlastP hit with Mycgr3G84402\_Mycgr3T**
  
Percentage identity: 69 %
  
BlastP bit score: 612
  
Sequence coverage: 93 %
  
E-value: 0.0
  
  
 NCBI BlastP on this gene

CCF34618

kinesin motor domain-containing protein
  
Accession: CCF34619
  
Location: 12653-15210
  
 NCBI BlastP on this gene

CCF34619

141. :  GL698510 Metarhizium acridum CQMa 102 unplaced genomic scaffold Scf\_041     Total score: 2.0     Cumulative Blast bit score: 1064

cysteine-rich secreted protein
  
Accession: EFY88538
  
Location: 193727-195102
  
 NCBI BlastP on this gene

EFY88538

metallothionein-I gene transcription activator
  
Accession: EFY88539
  
Location: 198515-198831
  
 NCBI BlastP on this gene

EFY88539

ATP binding protein, putative
  
Accession: EFY88540
  
Location: 199038-200261
  
 NCBI BlastP on this gene

EFY88540

budding site selection protein
  
Accession: EFY88541
  
Location: 200839-203150
  
 NCBI BlastP on this gene

EFY88541

transcriptional corepressor of histone genes (Hir3)
  
Accession: EFY88542
  
Location: 203761-210426
  
 NCBI BlastP on this gene

EFY88542

ATP-dependent rRNA helicase RRP3
  
Accession: EFY88543
  
Location: 211141-212634
  
  
**BlastP hit with Mycgr3G84402\_Mycgr3T**
  
Percentage identity: 71 %
  
BlastP bit score: 614
  
Sequence coverage: 91 %
  
E-value: 0.0
  
  
 NCBI BlastP on this gene

EFY88543

ribosome biogenesis protein Ssf2, putative
  
Accession: EFY88544
  
Location: 213010-214459
  
  
**BlastP hit with Mycgr3G35447\_Mycgr3T**
  
Percentage identity: 58 %
  
BlastP bit score: 450
  
Sequence coverage: 91 %
  
E-value: 9e-152
  
  
 NCBI BlastP on this gene

EFY88544

hypothetical protein
  
Accession: EFY88545
  
Location: 215156-215764
  
 NCBI BlastP on this gene

EFY88545

2-hydroxyacid dehydrogenase, putative
  
Accession: EFY88546
  
Location: 217035-220947
  
 NCBI BlastP on this gene

EFY88546

hypothetical protein
  
Accession: EFY88547
  
Location: 221541-222669
  
 NCBI BlastP on this gene

EFY88547

pyruvate dehydrogenase, putative
  
Accession: EFY88548
  
Location: 228073-230664
  
 NCBI BlastP on this gene

EFY88548

UbiD family decarboxylase
  
Accession: EFY88549
  
Location: 231826-233496
  
 NCBI BlastP on this gene

EFY88549

142. :  AMYD01001882 Colletotrichum gloeosporioides Cg-14     Total score: 2.0     Cumulative Blast bit score: 1064

hypothetical protein
  
Accession: EQB51131
  
Location: 23595-25325
  
 NCBI BlastP on this gene

EQB51131

3-hydroxyacyl-CoA dehydrogenase
  
Accession: EQB51132
  
Location: 26335-27303
  
 NCBI BlastP on this gene

EQB51132

hypothetical protein
  
Accession: EQB51133
  
Location: 28219-28932
  
 NCBI BlastP on this gene

EQB51133

hypothetical protein
  
Accession: EQB51134
  
Location: 29099-30921
  
 NCBI BlastP on this gene

EQB51134

copper amine oxidase
  
Accession: EQB51135
  
Location: 32280-33719
  
 NCBI BlastP on this gene

EQB51135

phytanoyl-CoA dioxygenase
  
Accession: EQB51136
  
Location: 37589-38689
  
 NCBI BlastP on this gene

EQB51136

amino acid permease
  
Accession: EQB51137
  
Location: 40795-41335
  
 NCBI BlastP on this gene

EQB51137

brix domain-containing protein
  
Accession: EQB51138
  
Location: 42567-44066
  
  
**BlastP hit with Mycgr3G35447\_Mycgr3T**
  
Percentage identity: 55 %
  
BlastP bit score: 453
  
Sequence coverage: 100 %
  
E-value: 5e-153
  
  
 NCBI BlastP on this gene

EQB51138

hypothetical protein
  
Accession: EQB51139
  
Location: 44525-46247
  
  
**BlastP hit with Mycgr3G84402\_Mycgr3T**
  
Percentage identity: 69 %
  
BlastP bit score: 611
  
Sequence coverage: 94 %
  
E-value: 0.0
  
  
 NCBI BlastP on this gene

EQB51139

hypothetical protein
  
Accession: EQB51140
  
Location: 47709-48215
  
 NCBI BlastP on this gene

EQB51140

hypothetical protein
  
Accession: EQB51141
  
Location: 48887-50678
  
 NCBI BlastP on this gene

EQB51141

143. :  AABX02000020 Neurospora crassa OR74A     Total score: 2.0     Cumulative Blast bit score: 1064

conserved hypothetical protein
  
Accession: EAA27686
  
Location: 55527-57951
  
 NCBI BlastP on this gene

EAA27686

hypothetical protein
  
Accession: EAA27685
  
Location: 58828-59881
  
 NCBI BlastP on this gene

EAA27685

hypothetical protein
  
Accession: EAA27684
  
Location: 60041-60745
  
 NCBI BlastP on this gene

EAA27684

predicted protein
  
Accession: EAA27683
  
Location: 61706-62020
  
 NCBI BlastP on this gene

EAA27683

hypothetical protein
  
Accession: EAA27682
  
Location: 62611-63885
  
 NCBI BlastP on this gene

EAA27682

conserved hypothetical protein
  
Accession: EAA27681
  
Location: 65403-66671
  
 NCBI BlastP on this gene

EAA27681

hypothetical protein
  
Accession: EAA27680
  
Location: 67852-70125
  
 NCBI BlastP on this gene

EAA27680

ATP-dependent rRNA helicase RRP3
  
Accession: EAA27679
  
Location: 70396-72093
  
  
**BlastP hit with Mycgr3G84402\_Mycgr3T**
  
Percentage identity: 73 %
  
BlastP bit score: 619
  
Sequence coverage: 90 %
  
E-value: 0.0
  
  
 NCBI BlastP on this gene

EAA27679

hypothetical protein
  
Accession: EAA27678
  
Location: 72454-73958
  
  
**BlastP hit with Mycgr3G35447\_Mycgr3T**
  
Percentage identity: 53 %
  
BlastP bit score: 445
  
Sequence coverage: 105 %
  
E-value: 2e-149
  
  
 NCBI BlastP on this gene

EAA27678

predicted protein
  
Accession: EAA27835
  
Location: 76023-76431
  
 NCBI BlastP on this gene

EAA27835

predicted protein
  
Accession: EAA27834
  
Location: 79736-80248
  
 NCBI BlastP on this gene

EAA27834

hypothetical protein
  
Accession: EAA27833
  
Location: 82503-84084
  
 NCBI BlastP on this gene

EAA27833

predicted protein
  
Accession: EAA27832
  
Location: 85919-87635
  
 NCBI BlastP on this gene

EAA27832

conserved hypothetical protein
  
Accession: EAA27831
  
Location: 89169-90921
  
 NCBI BlastP on this gene

EAA27831

predicted protein
  
Accession: EAA27830
  
Location: 91315-92525
  
 NCBI BlastP on this gene

EAA27830

144. :  GL891303 Neurospora tetrasperma FGSC 2508 unplaced genomic scaffold NEUTE1scaffold\_2     Total score: 2.0     Cumulative Blast bit score: 1063

hypothetical protein
  
Accession: EGO59281
  
Location: 2957253-2959676
  
 NCBI BlastP on this gene

EGO59281

hypothetical protein
  
Accession: EGO59282
  
Location: 2960531-2961582
  
 NCBI BlastP on this gene

EGO59282

hypothetical protein
  
Accession: EGO59283
  
Location: 2962599-2963297
  
 NCBI BlastP on this gene

EGO59283

hypothetical protein
  
Accession: EGO59284
  
Location: 2964235-2964550
  
 NCBI BlastP on this gene

EGO59284

hypothetical protein
  
Accession: EGO59285
  
Location: 2965140-2966414
  
 NCBI BlastP on this gene

EGO59285

hypothetical protein
  
Accession: EGO59286
  
Location: 2967925-2969193
  
 NCBI BlastP on this gene

EGO59286

hypothetical protein
  
Accession: EGO59287
  
Location: 2970332-2972602
  
 NCBI BlastP on this gene

EGO59287

ATP-dependent rRNA helicase rrp-3
  
Accession: EGO59288
  
Location: 2972874-2974570
  
  
**BlastP hit with Mycgr3G84402\_Mycgr3T**
  
Percentage identity: 73 %
  
BlastP bit score: 619
  
Sequence coverage: 90 %
  
E-value: 0.0
  
  
 NCBI BlastP on this gene

EGO59288

hypothetical protein
  
Accession: EGO59289
  
Location: 2974932-2976436
  
  
**BlastP hit with Mycgr3G35447\_Mycgr3T**
  
Percentage identity: 54 %
  
BlastP bit score: 444
  
Sequence coverage: 105 %
  
E-value: 4e-149
  
  
 NCBI BlastP on this gene

EGO59289

hypothetical protein
  
Accession: EGO59290
  
Location: 2977877-2978176
  
 NCBI BlastP on this gene

EGO59290

hypothetical protein
  
Accession: EGO59291
  
Location: 2978426-2978833
  
 NCBI BlastP on this gene

EGO59291

hypothetical protein
  
Accession: EGO59292
  
Location: 2982139-2982651
  
 NCBI BlastP on this gene

EGO59292

hypothetical protein
  
Accession: EGO59293
  
Location: 2984883-2986464
  
 NCBI BlastP on this gene

EGO59293

hypothetical protein
  
Accession: EGO59294
  
Location: 2988305-2990020
  
 NCBI BlastP on this gene

EGO59294

hypothetical protein
  
Accession: EGO59295
  
Location: 2991589-2993295
  
 NCBI BlastP on this gene

EGO59295

hypothetical protein
  
Accession: EGO59296
  
Location: 2993710-2994920
  
 NCBI BlastP on this gene

EGO59296

145. :  GL891217 Neurospora tetrasperma FGSC 2509 unplaced genomic scaffold NEUTE2scaffold\_3     Total score: 2.0     Cumulative Blast bit score: 1063

chaps-domain-containing protein
  
Accession: EGZ73402
  
Location: 2959489-2961912
  
 NCBI BlastP on this gene

EGZ73402

Aldo/keto reductase
  
Accession: EGZ73403
  
Location: 2962767-2963818
  
 NCBI BlastP on this gene

EGZ73403

HIT-like protein
  
Accession: EGZ73404
  
Location: 2964136-2964834
  
 NCBI BlastP on this gene

EGZ73404

hypothetical protein
  
Accession: EGZ73405
  
Location: 2965772-2966087
  
 NCBI BlastP on this gene

EGZ73405

hypothetical protein
  
Accession: EGZ73406
  
Location: 2966677-2967951
  
 NCBI BlastP on this gene

EGZ73406

hypothetical protein
  
Accession: EGZ73407
  
Location: 2969455-2970723
  
 NCBI BlastP on this gene

EGZ73407

hypothetical protein
  
Accession: EGZ73408
  
Location: 2971861-2974131
  
 NCBI BlastP on this gene

EGZ73408

ATP-dependent rRNA helicase rrp-3
  
Accession: EGZ73409
  
Location: 2974403-2976099
  
  
**BlastP hit with Mycgr3G84402\_Mycgr3T**
  
Percentage identity: 73 %
  
BlastP bit score: 619
  
Sequence coverage: 90 %
  
E-value: 0.0
  
  
 NCBI BlastP on this gene

EGZ73409

Brix-domain-containing protein
  
Accession: EGZ73410
  
Location: 2976461-2977965
  
  
**BlastP hit with Mycgr3G35447\_Mycgr3T**
  
Percentage identity: 54 %
  
BlastP bit score: 444
  
Sequence coverage: 105 %
  
E-value: 4e-149
  
  
 NCBI BlastP on this gene

EGZ73410

hypothetical protein
  
Accession: EGZ73411
  
Location: 2979403-2979702
  
 NCBI BlastP on this gene

EGZ73411

hypothetical protein
  
Accession: EGZ73412
  
Location: 2979949-2980356
  
 NCBI BlastP on this gene

EGZ73412

hypothetical protein
  
Accession: EGZ73413
  
Location: 2983662-2984174
  
 NCBI BlastP on this gene

EGZ73413

glycoside hydrolase
  
Accession: EGZ73414
  
Location: 2986406-2987987
  
 NCBI BlastP on this gene

EGZ73414

hypothetical protein
  
Accession: EGZ73415
  
Location: 2989828-2991543
  
 NCBI BlastP on this gene

EGZ73415

hypothetical protein
  
Accession: EGZ73416
  
Location: 2993112-2994818
  
 NCBI BlastP on this gene

EGZ73416

hypothetical protein
  
Accession: EGZ73417
  
Location: 2995233-2996443
  
 NCBI BlastP on this gene

EGZ73417

146. :  KB020987 Colletotrichum gloeosporioides Nara gc5 unplaced genomic scaffold scaffold586     Total score: 2.0     Cumulative Blast bit score: 1060

microsomal signal peptidase 18 kda subunit
  
Accession: ELA27449
  
Location: 96541-97351
  
 NCBI BlastP on this gene

ELA27449

hypothetical protein
  
Accession: ELA27450
  
Location: 98212-99112
  
 NCBI BlastP on this gene

ELA27450

hypothetical protein
  
Accession: ELA27451
  
Location: 99531-100160
  
 NCBI BlastP on this gene

ELA27451

transcription factor tfiiib complex subunit brf1
  
Accession: ELA27452
  
Location: 102067-104313
  
 NCBI BlastP on this gene

ELA27452

kinesin family protein
  
Accession: ELA27453
  
Location: 104726-107286
  
 NCBI BlastP on this gene

ELA27453

hypothetical protein
  
Accession: ELA27454
  
Location: 110618-112409
  
 NCBI BlastP on this gene

ELA27454

antigenic cell wall
  
Accession: ELA27455
  
Location: 113082-113588
  
 NCBI BlastP on this gene

ELA27455

ATP-dependent rRNA helicase rrp3
  
Accession: ELA27456
  
Location: 115041-116764
  
  
**BlastP hit with Mycgr3G84402\_Mycgr3T**
  
Percentage identity: 69 %
  
BlastP bit score: 608
  
Sequence coverage: 94 %
  
E-value: 0.0
  
  
 NCBI BlastP on this gene

ELA27456

ribosome biogenesis protein
  
Accession: ELA27457
  
Location: 117211-118708
  
  
**BlastP hit with Mycgr3G35447\_Mycgr3T**
  
Percentage identity: 55 %
  
BlastP bit score: 452
  
Sequence coverage: 100 %
  
E-value: 9e-153
  
  
 NCBI BlastP on this gene

ELA27457

phytanoyl- dioxygenase family protein
  
Accession: ELA27458
  
Location: 122554-123656
  
 NCBI BlastP on this gene

ELA27458

copper amine oxidase
  
Accession: ELA27459
  
Location: 127530-129917
  
 NCBI BlastP on this gene

ELA27459

hypothetical protein
  
Accession: ELA27460
  
Location: 130123-131652
  
 NCBI BlastP on this gene

ELA27460

3-hydroxyacyl- dehyrogenase
  
Accession: ELA27461
  
Location: 132577-133290
  
 NCBI BlastP on this gene

ELA27461

3-hydroxyacyl- dehydrogenase
  
Accession: ELA27462
  
Location: 134159-135127
  
 NCBI BlastP on this gene

ELA27462

alpha methylacyl-CoA racemase, putative
  
Accession: ELA27463
  
Location: 136142-137872
  
 NCBI BlastP on this gene

ELA27463

147. :  CH445336 Phaeosphaeria nodorum SN15 scaffold\_12     Total score: 2.0     Cumulative Blast bit score: 1060

hypothetical protein
  
Accession: EAT84604
  
Location: 917040-917522
  
 NCBI BlastP on this gene

EAT84604

hypothetical protein
  
Accession: EAT84603
  
Location: 914209-915338
  
 NCBI BlastP on this gene

EAT84603

hypothetical protein
  
Accession: EAT84602
  
Location: 911534-912481
  
 NCBI BlastP on this gene

EAT84602

hypothetical protein
  
Accession: EAT84601
  
Location: 910374-910488
  
 NCBI BlastP on this gene

EAT84601

hypothetical protein
  
Accession: EAT84600
  
Location: 907974-909869
  
 NCBI BlastP on this gene

EAT84600

hypothetical protein
  
Accession: EAT84599
  
Location: 904906-906937
  
 NCBI BlastP on this gene

EAT84599

hypothetical protein
  
Accession: EAT84598
  
Location: 901855-903030
  
 NCBI BlastP on this gene

EAT84598

hypothetical protein
  
Accession: EAT84597
  
Location: 899512-901131
  
 NCBI BlastP on this gene

EAT84597

hypothetical protein
  
Accession: EAT84596
  
Location: 897642-898904
  
  
**BlastP hit with Mycgr3G67775\_Mycgr3T**
  
Percentage identity: 59 %
  
BlastP bit score: 361
  
Sequence coverage: 104 %
  
E-value: 3e-121
  
  
 NCBI BlastP on this gene

EAT84596

hypothetical protein
  
Accession: EAT84595
  
Location: 896633-897127
  
 NCBI BlastP on this gene

EAT84595

hypothetical protein
  
Accession: EAT84594
  
Location: 894466-896045
  
  
**BlastP hit with Mycgr3G98959\_Mycgr3T**
  
Percentage identity: 79 %
  
BlastP bit score: 699
  
Sequence coverage: 86 %
  
E-value: 0.0
  
  
 NCBI BlastP on this gene

EAT84594

hypothetical protein
  
Accession: EAT84593
  
Location: 892835-893653
  
 NCBI BlastP on this gene

EAT84593

hypothetical protein
  
Accession: EAT84592
  
Location: 891727-892156
  
 NCBI BlastP on this gene

EAT84592

hypothetical protein
  
Accession: EAT84591
  
Location: 889945-891648
  
 NCBI BlastP on this gene

EAT84591

hypothetical protein
  
Accession: EAT84590
  
Location: 889078-889557
  
 NCBI BlastP on this gene

EAT84590

hypothetical protein
  
Accession: EAT84589
  
Location: 887997-889148
  
 NCBI BlastP on this gene

EAT84589

hypothetical protein
  
Accession: EAT84588
  
Location: 886503-886796
  
 NCBI BlastP on this gene

EAT84588

hypothetical protein
  
Accession: EDP89787
  
Location: 884559-885029
  
 NCBI BlastP on this gene

EDP89787

hypothetical protein
  
Accession: EDP89786
  
Location: 884208-884501
  
 NCBI BlastP on this gene

EDP89786

hypothetical protein
  
Accession: EAT84586
  
Location: 882318-883284
  
 NCBI BlastP on this gene

EAT84586

hypothetical protein
  
Accession: EAT84585
  
Location: 881230-882208
  
 NCBI BlastP on this gene

EAT84585

hypothetical protein
  
Accession: EAT84584
  
Location: 878237-879665
  
 NCBI BlastP on this gene

EAT84584

hypothetical protein
  
Accession: EAT84583
  
Location: 877128-877815
  
 NCBI BlastP on this gene

EAT84583

hypothetical protein
  
Accession: EAT84582
  
Location: 875487-876248
  
 NCBI BlastP on this gene

EAT84582

148. :  ABDG02000017 Trichoderma atroviride IMI 206040     Total score: 2.0     Cumulative Blast bit score: 1059

hypothetical protein
  
Accession: EHK49251
  
Location: 1140747-1141183
  
 NCBI BlastP on this gene

EHK49251

hypothetical protein
  
Accession: EHK49252
  
Location: 1141628-1142538
  
 NCBI BlastP on this gene

EHK49252

hypothetical protein
  
Accession: EHK49253
  
Location: 1143613-1144102
  
 NCBI BlastP on this gene

EHK49253

hypothetical protein
  
Accession: EHK49254
  
Location: 1144454-1145107
  
 NCBI BlastP on this gene

EHK49254

hypothetical protein
  
Accession: EHK49255
  
Location: 1145926-1146236
  
 NCBI BlastP on this gene

EHK49255

hypothetical protein
  
Accession: EHK49256
  
Location: 1146650-1147895
  
 NCBI BlastP on this gene

EHK49256

hypothetical protein
  
Accession: EHK49257
  
Location: 1148282-1150685
  
 NCBI BlastP on this gene

EHK49257

hypothetical protein
  
Accession: EHK49258
  
Location: 1151293-1157612
  
 NCBI BlastP on this gene

EHK49258

hypothetical protein
  
Accession: EHK49259
  
Location: 1158257-1158742
  
 NCBI BlastP on this gene

EHK49259

hypothetical protein
  
Accession: EHK49260
  
Location: 1159122-1160638
  
  
**BlastP hit with Mycgr3G84402\_Mycgr3T**
  
Percentage identity: 73 %
  
BlastP bit score: 620
  
Sequence coverage: 87 %
  
E-value: 0.0
  
  
 NCBI BlastP on this gene

EHK49260

hypothetical protein
  
Accession: EHK49261
  
Location: 1161131-1162567
  
  
**BlastP hit with Mycgr3G35447\_Mycgr3T**
  
Percentage identity: 57 %
  
BlastP bit score: 439
  
Sequence coverage: 90 %
  
E-value: 1e-147
  
  
 NCBI BlastP on this gene

EHK49261

hypothetical protein
  
Accession: EHK49262
  
Location: 1162975-1163476
  
 NCBI BlastP on this gene

EHK49262

hypothetical protein
  
Accession: EHK49263
  
Location: 1164655-1166349
  
 NCBI BlastP on this gene

EHK49263

hypothetical protein
  
Accession: EHK49264
  
Location: 1167407-1169221
  
 NCBI BlastP on this gene

EHK49264

hypothetical protein
  
Accession: EHK49265
  
Location: 1169389-1170366
  
 NCBI BlastP on this gene

EHK49265

hypothetical protein
  
Accession: EHK49266
  
Location: 1171697-1173199
  
 NCBI BlastP on this gene

EHK49266

hypothetical protein
  
Accession: EHK49267
  
Location: 1175948-1177012
  
 NCBI BlastP on this gene

EHK49267

hypothetical protein
  
Accession: EHK49268
  
Location: 1178006-1179635
  
 NCBI BlastP on this gene

EHK49268

hypothetical protein
  
Accession: EHK49269
  
Location: 1180154-1183414
  
 NCBI BlastP on this gene

EHK49269

149. :  JH126405 Cordyceps militaris CM01 unplaced genomic scaffold CCM\_S00007     Total score: 2.0     Cumulative Blast bit score: 1058

MFS transporter
  
Accession: EGX88308
  
Location: 428366-430296
  
 NCBI BlastP on this gene

EGX88308

hypothetical protein
  
Accession: EGX88309
  
Location: 437781-438897
  
 NCBI BlastP on this gene

EGX88309

amidase, putative
  
Accession: EGX88310
  
Location: 439053-440279
  
 NCBI BlastP on this gene

EGX88310

pyruvate dehydrogenase, putative
  
Accession: EGX88311
  
Location: 441951-443503
  
 NCBI BlastP on this gene

EGX88311

Protein kinase-like domain
  
Accession: EGX88312
  
Location: 444141-445646
  
 NCBI BlastP on this gene

EGX88312

ribosome biogenesis protein Ssf2, putative
  
Accession: EGX88313
  
Location: 446277-447641
  
  
**BlastP hit with Mycgr3G35447\_Mycgr3T**
  
Percentage identity: 57 %
  
BlastP bit score: 450
  
Sequence coverage: 93 %
  
E-value: 7e-152
  
  
 NCBI BlastP on this gene

EGX88313

ATP-dependent rRNA helicase RRP3
  
Accession: EGX88314
  
Location: 448132-449616
  
  
**BlastP hit with Mycgr3G84402\_Mycgr3T**
  
Percentage identity: 65 %
  
BlastP bit score: 608
  
Sequence coverage: 103 %
  
E-value: 0.0
  
  
 NCBI BlastP on this gene

EGX88314

transcriptional corepressor of histone (Hir3), putative
  
Accession: EGX88315
  
Location: 449883-456625
  
 NCBI BlastP on this gene

EGX88315

clathrin-coated vesiclec protein (Bud7), putative
  
Accession: EGX88316
  
Location: 457244-459558
  
 NCBI BlastP on this gene

EGX88316

ATP binding protein
  
Accession: EGX88317
  
Location: 460036-461265
  
 NCBI BlastP on this gene

EGX88317

metallothionein-I transcription activator
  
Accession: EGX88318
  
Location: 461522-461845
  
 NCBI BlastP on this gene

EGX88318

Histidine triad-like protein
  
Accession: EGX88319
  
Location: 462643-463224
  
 NCBI BlastP on this gene

EGX88319

Caldecrin precursor (Chymotrypsin C) isoform 3
  
Accession: EGX88320
  
Location: 463651-464971
  
 NCBI BlastP on this gene

EGX88320

hypothetical protein
  
Accession: EGX88321
  
Location: 465663-466389
  
 NCBI BlastP on this gene

EGX88321

hypothetical protein
  
Accession: EGX88322
  
Location: 466837-467323
  
 NCBI BlastP on this gene

EGX88322

high affinity methionine permease
  
Accession: EGX88323
  
Location: 468935-470955
  
 NCBI BlastP on this gene

EGX88323

150. :  GL385396 Gaeumannomyces graminis var. tritici R3-111a-1 unplaced genomic scaffold supercont2.2     Total score: 2.0     Cumulative Blast bit score: 1054

ATPase NPA3
  
Accession: EJT78748
  
Location: 3803861-3805164
  
 NCBI BlastP on this gene

EJT78748

hypothetical protein
  
Accession: EJT78749
  
Location: 3805615-3806289
  
 NCBI BlastP on this gene

EJT78749

hypothetical protein
  
Accession: EJT78750
  
Location: 3806859-3807446
  
 NCBI BlastP on this gene

EJT78750

hypothetical protein
  
Accession: EJT78751
  
Location: 3808017-3809653
  
 NCBI BlastP on this gene

EJT78751

hypothetical protein
  
Accession: EJT78752
  
Location: 3811315-3812289
  
 NCBI BlastP on this gene

EJT78752

hypothetical protein
  
Accession: EJT78753
  
Location: 3812606-3813591
  
 NCBI BlastP on this gene

EJT78753

hypothetical protein
  
Accession: EJT78754
  
Location: 3814658-3817077
  
 NCBI BlastP on this gene

EJT78754

hypothetical protein
  
Accession: EJT78755
  
Location: 3817478-3817795
  
 NCBI BlastP on this gene

EJT78755

hypothetical protein
  
Accession: EJT78756
  
Location: 3818534-3819865
  
 NCBI BlastP on this gene

EJT78756

hypothetical protein
  
Accession: EJT78757
  
Location: 3820965-3821570
  
 NCBI BlastP on this gene

EJT78757

ATP-dependent rRNA helicase RRP3
  
Accession: EJT78758
  
Location: 3822053-3823781
  
  
**BlastP hit with Mycgr3G84402\_Mycgr3T**
  
Percentage identity: 72 %
  
BlastP bit score: 608
  
Sequence coverage: 89 %
  
E-value: 0.0
  
  
 NCBI BlastP on this gene

EJT78758

ribosome biogenesis protein SSF1
  
Accession: EJT78759
  
Location: 3824120-3825619
  
  
**BlastP hit with Mycgr3G35447\_Mycgr3T**
  
Percentage identity: 58 %
  
BlastP bit score: 446
  
Sequence coverage: 94 %
  
E-value: 5e-150
  
  
 NCBI BlastP on this gene

EJT78759

hypothetical protein
  
Accession: EJT78760
  
Location: 3826055-3827677
  
 NCBI BlastP on this gene

EJT78760

choline dehydrogenase
  
Accession: EJT78761
  
Location: 3827904-3829796
  
 NCBI BlastP on this gene

EJT78761

hypothetical protein
  
Accession: EJT78762
  
Location: 3831230-3832237
  
 NCBI BlastP on this gene

EJT78762

hypothetical protein
  
Accession: EJT78763
  
Location: 3833547-3835439
  
 NCBI BlastP on this gene

EJT78763

hypothetical protein
  
Accession: EJT78764
  
Location: 3835608-3837422
  
 NCBI BlastP on this gene

EJT78764

hypothetical protein
  
Accession: EJT78765
  
Location: 3838952-3839491
  
 NCBI BlastP on this gene

EJT78765

hypothetical protein
  
Accession: EJT78766
  
Location: 3840183-3841378
  
 NCBI BlastP on this gene

EJT78766

ABC transporter
  
Accession: EJT78767
  
Location: 3843884-3849165
  
 NCBI BlastP on this gene

EJT78767

Detecting sequence homology at the gene cluster level with MultiGeneBlast.
  
Marnix H. Medema, Rainer Breitling & Eriko Takano (2013)
  
*Molecular Biology and Evolution* , 30: 1218-1223.
