# Supplementary material for: In silico prediction and characterization of secondary metabolite biosynthetic gene clusters in the wheat pathogen Zymoseptoria tritici
Source: BMC Genomics. 2017 Aug 17;18:631. doi: 10.1186/s12864-017-3969-y (PMC5561558; doi:10.1186/s12864-017-3969-y)
Supplement: Supplementary file 1 — MultiGeneBLAST analysis of putative secondary metabolite clusters. All encoded amino acid sequences from genes residing in clusters predicted by AntiSMASH are given as FASTA file format. All output data from MultiGeneBLASTs are also provided. (ZIP 42911 kb) [file 12864_2017_3969_MOESM1_ESM.zip › Cluster MultiGene BLAST/out/Clusters_1_34/Cluster_10/displaypage4.xhtml]

xml version="1.0" encoding="UTF-8"?


Search Results
  
  
 Results pages: 1, 2, 3, 4, 5

**MultiGeneBlast hits**

Select gene cluster alignment
151. AM270408\_0 Aspergillus niger contig An18c0160, genomic contig.
152. CH445336\_0 Phaeosphaeria nodorum SN15 scaffold\_12, whole genome shotgun ...
153. KB730000\_0 Fusarium oxysporum f. sp. cubense race 1 unplaced genomic sca...
154. JH794139\_0 Magnaporthe oryzae P131 unplaced genomic scaffold P131\_scaffo...
155. DS572699\_1 Verticillium dahliae VdLs.17 supercont1.5 genomic scaffold, w...
156. CM001235\_2 Magnaporthe oryzae 70-15 chromosome 5, whole genome shotgun s...
157. DS985220\_0 Verticillium albo-atrum VaMs.102 supercont1.7 genomic scaffol...
158. JH793228\_0 Magnaporthe oryzae Y34 unplaced genomic scaffold Y34\_scaffold...
159. FP929065\_0 Leptosphaeria maculans JN3 lm\_SuperContig\_8\_v2 genomic superc...
160. CH476597\_0 Aspergillus terreus NIH2624 scaffold\_4 genomic scaffold, whol...
161. KB933264\_0 Togninia minima UCRPA7 unplaced genomic scaffold PA7\_03\_scaff...
162. CABT02000002\_2 Sordaria macrospora k-hell, whole genome shotgun sequenci...
163. FN430009\_0 Tuber melanosporum whole genome shotgun sequence assembly, sc...
164. JH226130\_1 Exophiala dermatitidis NIH/UT8656 unplaced genomic scaffold s...
165. CH991557\_0 Monosiga brevicollis MX1 MONBRscaffold\_16 genomic scaffold, w...
166. CR382127\_0 Yarrowia lipolytica CLIB122 chromosome A complete sequence.
167. KB445649\_5 Cochliobolus sativus ND90Pr unplaced genomic scaffold COCSAsc...
168. AHHD01000101\_0 Macrophomina phaseolina MS6, whole genome shotgun sequenc...
169. KB733482\_0 Bipolaris maydis ATCC 48331 unplaced genomic scaffold COCC4sc...
170. KB445579\_5 Cochliobolus heterostrophus C5 unplaced genomic scaffold COCH...
171. GL534835\_0 Pyrenophora teres f. teres 0-1 unplaced genomic scaffold scaf...
172. KB908844\_0 Setosphaeria turcica Et28A unplaced genomic scaffold SETTUsca...
173. JH767590\_0 Coniosporium apollinis CBS 100218 chromosome Unknown supercon...
174. KB916790\_0 Neofusicoccum parvum UCRNP2 chromosome Unknown NP2\_03\_scaffol...
175. CAIF01000186\_0 Wickerhamomyces ciferrii strain NRRL Y-1031 F-60-10, whol...
176. CH408161\_0 Pichia guilliermondii ATCC 6260 scaffold\_7 genomic scaffold, ...
177. CR382137\_0 Debaryomyces hansenii CBS767 chromosome E complete sequence.
178. CH445336\_2 Phaeosphaeria nodorum SN15 scaffold\_12, whole genome shotgun ...
179. HE605209\_0 Candida parapsilosis strain CDC317 annotated contig 006110.
180. FO082049\_0 Pichia sorbitophila strain CBS 7064 chromosome K complete seq...
181. FO082048\_0 Pichia sorbitophila strain CBS 7064 chromosome L complete seq...
182. DS231623\_4 Pyrenophora tritici-repentis Pt-1C-BFP supercont1.9 genomic s...
183. AOGT01000769\_0 Candida maltosa Xu316, whole genome shotgun sequencing pr...
184. GL996500\_0 Spathaspora passalidarum NRRL Y-27907 unplaced genomic scaffo...
185. GG692404\_0 Candida tropicalis MYA-3404 genomic scaffold supercont3.10, w...
186. AEOI01000005\_0 Ogataea parapolymorpha DL-1, whole genome shotgun sequenc...
187. HE681725\_0 Candida orthopsilosis Co 90-125, chromosome 7 draft sequence.
188. CH408079\_1 Clavispora lusitaniae ATCC 42720 scaffold\_4 genomic scaffold,...
189. FR839628\_1 Pichia pastoris CBS 7435 chromosome 1, complete replicon sequ...
190. FN392319\_1 Pichia pastoris GS115 chromosome 1, complete sequence.
191. FM992695\_0 Candida dubliniensis CD36 chromosome R, complete sequence.
192. KB733474\_0 Bipolaris maydis ATCC 48331 unplaced genomic scaffold COCC4sc...
193. FP929139\_1 Leptosphaeria maculans JN3 lm\_SuperContig\_0\_v2 genomic superc...
194. GL534459\_0 Pyrenophora teres f. teres 0-1 unplaced genomic scaffold scaf...
195. CU329670\_1 Schizosaccharomyces pombe chromosome I, complete sequence.
196. KB445561\_2 Baudoinia compniacensis UAMH 10762 unplaced genomic scaffold ...
197. ADOT01000171\_0 Arthrobotrys oligospora ATCC 24927, whole genome shotgun ...
198. JH971386\_0 Agaricus bisporus var. burnettii JB137-S8 unplaced genomic sc...
199. JH931606\_0 Agaricus bisporus var. bisporus H97 unplaced genomic scaffold...
200. DS547096\_1 Laccaria bicolor S238N-H82 LACBIscaffold\_6 genomic scaffold, ...

Query: Architecture Search FASTA input

AM270408 : Aspergillus niger contig An18c0160, genomic contig.    Total score: 2.0     Cumulative Blast bit score: 1054

Hit cluster cross-links:

Mycgr3G67791 Mycgr3T
  
Location: 0-1542

Mycgr3G67791\_Mycgr3T

Mycgr3G90406 Mycgr3T
  
Location: 1642-3973

Mycgr3G90406\_Mycgr3T

Mycgr3G67785 Mycgr3T
  
Location: 4073-7865

Mycgr3G67785\_Mycgr3T

Mycgr3G67795 Mycgr3T
  
Location: 7965-15249

Mycgr3G67795\_Mycgr3T

Mycgr3G67775 Mycgr3T
  
Location: 15349-16237

Mycgr3G67775\_Mycgr3T

Mycgr3G90404 Mycgr3T
  
Location: 16337-17246

Mycgr3G90404\_Mycgr3T

Mycgr3G36951 Mycgr3T
  
Location: 17346-30891

Mycgr3G36951\_Mycgr3T

Mycgr3G103034 Mycgr3
  
Location: 30991-32644

Mycgr3G103034\_Mycgr3

Mycgr3G31119 Mycgr3T
  
Location: 32744-32906

Mycgr3G31119\_Mycgr3T

Mycgr3G28587 Mycgr3T
  
Location: 33006-33489

Mycgr3G28587\_Mycgr3T

Mycgr3G98959 Mycgr3T
  
Location: 33589-35035

Mycgr3G98959\_Mycgr3T

Mycgr3G35447 Mycgr3T
  
Location: 35135-36443

Mycgr3G35447\_Mycgr3T

Mycgr3G84402 Mycgr3T
  
Location: 36543-37884

Mycgr3G84402\_Mycgr3T

Mycgr3G98961 Mycgr3T
  
Location: 37984-38884

Mycgr3G98961\_Mycgr3T

not annotated
  
Accession: CAK43303
  
Location: 100563-101796
  
 NCBI BlastP on this gene

An18g05050

not annotated
  
Accession: CAK43304
  
Location: 102626-104302
  
 NCBI BlastP on this gene

An18g05060

not annotated
  
Accession: CAK43305
  
Location: 104671-106009
  
 NCBI BlastP on this gene

An18g05070

not annotated
  
Accession: CAK43306
  
Location: 106360-107821
  
 NCBI BlastP on this gene

An18g05080

not annotated
  
Accession: CAK43307
  
Location: 107966-109231
  
 NCBI BlastP on this gene

An18g05090

not annotated
  
Accession: CAK43308
  
Location: 109884-111269
  
 NCBI BlastP on this gene

An18g05100

not annotated
  
Accession: CAK43309
  
Location: 111492-113477
  
 NCBI BlastP on this gene

An18g05110

not annotated
  
Accession: CAK43310
  
Location: 113932-115571
  
 NCBI BlastP on this gene

An18g05120

not annotated
  
Accession: CAK43311
  
Location: 115879-117189
  
 NCBI BlastP on this gene

An18g05130

not annotated
  
Accession: CAK43312
  
Location: 117348-118778
  
  
**BlastP hit with Mycgr3G35447\_Mycgr3T**
  
Percentage identity: 59 %
  
BlastP bit score: 433
  
Sequence coverage: 91 %
  
E-value: 4e-145
  
  
 NCBI BlastP on this gene

An18g05140

not annotated
  
Accession: CAK43313
  
Location: 119025-120580
  
  
**BlastP hit with Mycgr3G84402\_Mycgr3T**
  
Percentage identity: 72 %
  
BlastP bit score: 621
  
Sequence coverage: 89 %
  
E-value: 0.0
  
  
 NCBI BlastP on this gene

An18g05150

not annotated
  
Accession: CAK43314
  
Location: 120950-121588
  
 NCBI BlastP on this gene

An18g05160

Query: Architecture Search FASTA input

CH445336 : Phaeosphaeria nodorum SN15 scaffold\_12    Total score: 2.0     Cumulative Blast bit score: 1036

Hit cluster cross-links:

Mycgr3G67791 Mycgr3T
  
Location: 0-1542

Mycgr3G67791\_Mycgr3T

Mycgr3G90406 Mycgr3T
  
Location: 1642-3973

Mycgr3G90406\_Mycgr3T

Mycgr3G67785 Mycgr3T
  
Location: 4073-7865

Mycgr3G67785\_Mycgr3T

Mycgr3G67795 Mycgr3T
  
Location: 7965-15249

Mycgr3G67795\_Mycgr3T

Mycgr3G67775 Mycgr3T
  
Location: 15349-16237

Mycgr3G67775\_Mycgr3T

Mycgr3G90404 Mycgr3T
  
Location: 16337-17246

Mycgr3G90404\_Mycgr3T

Mycgr3G36951 Mycgr3T
  
Location: 17346-30891

Mycgr3G36951\_Mycgr3T

Mycgr3G103034 Mycgr3
  
Location: 30991-32644

Mycgr3G103034\_Mycgr3

Mycgr3G31119 Mycgr3T
  
Location: 32744-32906

Mycgr3G31119\_Mycgr3T

Mycgr3G28587 Mycgr3T
  
Location: 33006-33489

Mycgr3G28587\_Mycgr3T

Mycgr3G98959 Mycgr3T
  
Location: 33589-35035

Mycgr3G98959\_Mycgr3T

Mycgr3G35447 Mycgr3T
  
Location: 35135-36443

Mycgr3G35447\_Mycgr3T

Mycgr3G84402 Mycgr3T
  
Location: 36543-37884

Mycgr3G84402\_Mycgr3T

Mycgr3G98961 Mycgr3T
  
Location: 37984-38884

Mycgr3G98961\_Mycgr3T

hypothetical protein
  
Accession: EAT84171
  
Location: 55123-56908
  
 NCBI BlastP on this gene

EAT84171

hypothetical protein
  
Accession: EAT84172
  
Location: 58391-58835
  
 NCBI BlastP on this gene

EAT84172

hypothetical protein
  
Accession: EAT84173
  
Location: 59029-60261
  
 NCBI BlastP on this gene

EAT84173

hypothetical protein
  
Accession: EAT84174
  
Location: 61449-62148
  
 NCBI BlastP on this gene

EAT84174

hypothetical protein
  
Accession: EAT84175
  
Location: 63759-67162
  
 NCBI BlastP on this gene

EAT84175

hypothetical protein
  
Accession: EAT84176
  
Location: 70418-71425
  
 NCBI BlastP on this gene

EAT84176

hypothetical protein
  
Accession: EAT84177
  
Location: 71919-72960
  
 NCBI BlastP on this gene

EAT84177

hypothetical protein
  
Accession: EAT84178
  
Location: 73038-74678
  
  
**BlastP hit with Mycgr3G84402\_Mycgr3T**
  
Percentage identity: 73 %
  
BlastP bit score: 612
  
Sequence coverage: 89 %
  
E-value: 0.0
  
  
 NCBI BlastP on this gene

EAT84178

hypothetical protein
  
Accession: EAT84179
  
Location: 74857-76292
  
  
**BlastP hit with Mycgr3G35447\_Mycgr3T**
  
Percentage identity: 57 %
  
BlastP bit score: 424
  
Sequence coverage: 90 %
  
E-value: 2e-141
  
  
 NCBI BlastP on this gene

EAT84179

hypothetical protein
  
Accession: EAT84180
  
Location: 76387-81945
  
 NCBI BlastP on this gene

EAT84180

hypothetical protein
  
Accession: EAT84181
  
Location: 83219-84580
  
 NCBI BlastP on this gene

EAT84181

hypothetical protein
  
Accession: EAT84182
  
Location: 85100-85780
  
 NCBI BlastP on this gene

EAT84182

hypothetical protein
  
Accession: EAT84183
  
Location: 86722-87674
  
 NCBI BlastP on this gene

EAT84183

hypothetical protein
  
Accession: EAT84184
  
Location: 88892-89566
  
 NCBI BlastP on this gene

EAT84184

hypothetical protein
  
Accession: EAT84185
  
Location: 89872-90198
  
 NCBI BlastP on this gene

EAT84185

hypothetical protein
  
Accession: EAT84186
  
Location: 90955-91740
  
 NCBI BlastP on this gene

EAT84186

hypothetical protein
  
Accession: EAT84187
  
Location: 93525-94717
  
 NCBI BlastP on this gene

EAT84187

hypothetical protein
  
Accession: EAT84188
  
Location: 95609-96347
  
 NCBI BlastP on this gene

EAT84188

Query: Architecture Search FASTA input

KB730000 : Fusarium oxysporum f. sp. cubense race 1 unplaced genomic scaffold scaffold2    Total score: 2.0     Cumulative Blast bit score: 1035

Hit cluster cross-links:

Mycgr3G67791 Mycgr3T
  
Location: 0-1542

Mycgr3G67791\_Mycgr3T

Mycgr3G90406 Mycgr3T
  
Location: 1642-3973

Mycgr3G90406\_Mycgr3T

Mycgr3G67785 Mycgr3T
  
Location: 4073-7865

Mycgr3G67785\_Mycgr3T

Mycgr3G67795 Mycgr3T
  
Location: 7965-15249

Mycgr3G67795\_Mycgr3T

Mycgr3G67775 Mycgr3T
  
Location: 15349-16237

Mycgr3G67775\_Mycgr3T

Mycgr3G90404 Mycgr3T
  
Location: 16337-17246

Mycgr3G90404\_Mycgr3T

Mycgr3G36951 Mycgr3T
  
Location: 17346-30891

Mycgr3G36951\_Mycgr3T

Mycgr3G103034 Mycgr3
  
Location: 30991-32644

Mycgr3G103034\_Mycgr3

Mycgr3G31119 Mycgr3T
  
Location: 32744-32906

Mycgr3G31119\_Mycgr3T

Mycgr3G28587 Mycgr3T
  
Location: 33006-33489

Mycgr3G28587\_Mycgr3T

Mycgr3G98959 Mycgr3T
  
Location: 33589-35035

Mycgr3G98959\_Mycgr3T

Mycgr3G35447 Mycgr3T
  
Location: 35135-36443

Mycgr3G35447\_Mycgr3T

Mycgr3G84402 Mycgr3T
  
Location: 36543-37884

Mycgr3G84402\_Mycgr3T

Mycgr3G98961 Mycgr3T
  
Location: 37984-38884

Mycgr3G98961\_Mycgr3T

hypothetical protein
  
Accession: ENH74983
  
Location: 2-1414
  
 NCBI BlastP on this gene

ENH74983

hypothetical protein
  
Accession: ENH74984
  
Location: 2413-3507
  
 NCBI BlastP on this gene

ENH74984

hypothetical protein
  
Accession: ENH74985
  
Location: 4169-6148
  
 NCBI BlastP on this gene

ENH74985

Protein phosphatase 2C like protein C10F6.17c
  
Accession: ENH74986
  
Location: 6693-8182
  
 NCBI BlastP on this gene

ENH74986

Brix domain-containing protein C1B9.03c
  
Accession: ENH74987
  
Location: 8718-10097
  
  
**BlastP hit with Mycgr3G35447\_Mycgr3T**
  
Percentage identity: 52 %
  
BlastP bit score: 406
  
Sequence coverage: 102 %
  
E-value: 5e-135
  
  
 NCBI BlastP on this gene

ENH74987

ATP-dependent rRNA helicase RRP3
  
Accession: ENH74988
  
Location: 10316-11822
  
  
**BlastP hit with Mycgr3G84402\_Mycgr3T**
  
Percentage identity: 73 %
  
BlastP bit score: 629
  
Sequence coverage: 91 %
  
E-value: 0.0
  
  
 NCBI BlastP on this gene

ENH74988

hypothetical protein
  
Accession: ENH74989
  
Location: 12692-13755
  
 NCBI BlastP on this gene

ENH74989

Histone transcription regulator 3 like protein
  
Accession: ENH74990
  
Location: 14077-20235
  
 NCBI BlastP on this gene

ENH74990

hypothetical protein
  
Accession: ENH74991
  
Location: 20725-23089
  
 NCBI BlastP on this gene

ENH74991

Ubiquitin-conjugating enzyme E2 14
  
Accession: ENH74992
  
Location: 24266-24795
  
 NCBI BlastP on this gene

ENH74992

GTPase-activating protein gyp3
  
Accession: ENH74993
  
Location: 26578-29532
  
 NCBI BlastP on this gene

ENH74993

Query: Architecture Search FASTA input

JH794139 : Magnaporthe oryzae P131 unplaced genomic scaffold P131\_scaffold00389    Total score: 2.0     Cumulative Blast bit score: 1035

Hit cluster cross-links:

Mycgr3G67791 Mycgr3T
  
Location: 0-1542

Mycgr3G67791\_Mycgr3T

Mycgr3G90406 Mycgr3T
  
Location: 1642-3973

Mycgr3G90406\_Mycgr3T

Mycgr3G67785 Mycgr3T
  
Location: 4073-7865

Mycgr3G67785\_Mycgr3T

Mycgr3G67795 Mycgr3T
  
Location: 7965-15249

Mycgr3G67795\_Mycgr3T

Mycgr3G67775 Mycgr3T
  
Location: 15349-16237

Mycgr3G67775\_Mycgr3T

Mycgr3G90404 Mycgr3T
  
Location: 16337-17246

Mycgr3G90404\_Mycgr3T

Mycgr3G36951 Mycgr3T
  
Location: 17346-30891

Mycgr3G36951\_Mycgr3T

Mycgr3G103034 Mycgr3
  
Location: 30991-32644

Mycgr3G103034\_Mycgr3

Mycgr3G31119 Mycgr3T
  
Location: 32744-32906

Mycgr3G31119\_Mycgr3T

Mycgr3G28587 Mycgr3T
  
Location: 33006-33489

Mycgr3G28587\_Mycgr3T

Mycgr3G98959 Mycgr3T
  
Location: 33589-35035

Mycgr3G98959\_Mycgr3T

Mycgr3G35447 Mycgr3T
  
Location: 35135-36443

Mycgr3G35447\_Mycgr3T

Mycgr3G84402 Mycgr3T
  
Location: 36543-37884

Mycgr3G84402\_Mycgr3T

Mycgr3G98961 Mycgr3T
  
Location: 37984-38884

Mycgr3G98961\_Mycgr3T

hypothetical protein
  
Accession: ELQ66441
  
Location: 70250-74992
  
 NCBI BlastP on this gene

ELQ66441

mannosyl-oligosaccharide 1,2-alpha-mannosidase IB
  
Accession: ELQ66442
  
Location: 76305-77966
  
 NCBI BlastP on this gene

ELQ66442

lactose permease
  
Accession: ELQ66443
  
Location: 78395-80267
  
 NCBI BlastP on this gene

ELQ66443

42 kDa endochitinase
  
Accession: ELQ66444
  
Location: 81927-83443
  
 NCBI BlastP on this gene

ELQ66444

hypothetical protein
  
Accession: ELQ66445
  
Location: 87172-88372
  
 NCBI BlastP on this gene

ELQ66445

ATP-dependent rRNA helicase rrp3
  
Accession: ELQ66446
  
Location: 88941-90702
  
  
**BlastP hit with Mycgr3G84402\_Mycgr3T**
  
Percentage identity: 70 %
  
BlastP bit score: 601
  
Sequence coverage: 91 %
  
E-value: 0.0
  
  
 NCBI BlastP on this gene

ELQ66446

ribosome biogenesis protein SSF1
  
Accession: ELQ66447
  
Location: 91118-92603
  
  
**BlastP hit with Mycgr3G35447\_Mycgr3T**
  
Percentage identity: 57 %
  
BlastP bit score: 434
  
Sequence coverage: 94 %
  
E-value: 4e-145
  
  
 NCBI BlastP on this gene

ELQ66447

glycosyl hydrolase
  
Accession: ELQ66448
  
Location: 94710-97154
  
 NCBI BlastP on this gene

ELQ66448

Query: Architecture Search FASTA input

DS572699 : Verticillium dahliae VdLs.17 supercont1.5 genomic scaffold    Total score: 2.0     Cumulative Blast bit score: 1034

Hit cluster cross-links:

Mycgr3G67791 Mycgr3T
  
Location: 0-1542

Mycgr3G67791\_Mycgr3T

Mycgr3G90406 Mycgr3T
  
Location: 1642-3973

Mycgr3G90406\_Mycgr3T

Mycgr3G67785 Mycgr3T
  
Location: 4073-7865

Mycgr3G67785\_Mycgr3T

Mycgr3G67795 Mycgr3T
  
Location: 7965-15249

Mycgr3G67795\_Mycgr3T

Mycgr3G67775 Mycgr3T
  
Location: 15349-16237

Mycgr3G67775\_Mycgr3T

Mycgr3G90404 Mycgr3T
  
Location: 16337-17246

Mycgr3G90404\_Mycgr3T

Mycgr3G36951 Mycgr3T
  
Location: 17346-30891

Mycgr3G36951\_Mycgr3T

Mycgr3G103034 Mycgr3
  
Location: 30991-32644

Mycgr3G103034\_Mycgr3

Mycgr3G31119 Mycgr3T
  
Location: 32744-32906

Mycgr3G31119\_Mycgr3T

Mycgr3G28587 Mycgr3T
  
Location: 33006-33489

Mycgr3G28587\_Mycgr3T

Mycgr3G98959 Mycgr3T
  
Location: 33589-35035

Mycgr3G98959\_Mycgr3T

Mycgr3G35447 Mycgr3T
  
Location: 35135-36443

Mycgr3G35447\_Mycgr3T

Mycgr3G84402 Mycgr3T
  
Location: 36543-37884

Mycgr3G84402\_Mycgr3T

Mycgr3G98961 Mycgr3T
  
Location: 37984-38884

Mycgr3G98961\_Mycgr3T

yetA
  
Accession: EGY22096
  
Location: 1731837-1734614
  
 NCBI BlastP on this gene

EGY22096

allantoate permease
  
Accession: EGY22097
  
Location: 1736059-1738095
  
 NCBI BlastP on this gene

EGY22097

integral membrane protein
  
Accession: EGY22098
  
Location: 1739779-1741049
  
 NCBI BlastP on this gene

EGY22098

FAD binding domain-containing protein
  
Accession: EGY22099
  
Location: 1741279-1742763
  
 NCBI BlastP on this gene

EGY22099

hypothetical protein
  
Accession: EGY22100
  
Location: 1744545-1744862
  
 NCBI BlastP on this gene

EGY22100

hypothetical protein
  
Accession: EGY22101
  
Location: 1747495-1749299
  
 NCBI BlastP on this gene

EGY22101

ribosome biogenesis protein SSF1
  
Accession: EGY22102
  
Location: 1751309-1752758
  
  
**BlastP hit with Mycgr3G35447\_Mycgr3T**
  
Percentage identity: 52 %
  
BlastP bit score: 436
  
Sequence coverage: 103 %
  
E-value: 3e-146
  
  
 NCBI BlastP on this gene

EGY22102

ATP-dependent rRNA helicase rrp-3
  
Accession: EGY22103
  
Location: 1753081-1754867
  
  
**BlastP hit with Mycgr3G84402\_Mycgr3T**
  
Percentage identity: 71 %
  
BlastP bit score: 598
  
Sequence coverage: 89 %
  
E-value: 0.0
  
  
 NCBI BlastP on this gene

EGY22103

hypothetical protein
  
Accession: EGY22104
  
Location: 1755583-1756428
  
 NCBI BlastP on this gene

EGY22104

flavonol synthase
  
Accession: EGY22105
  
Location: 1758390-1759609
  
 NCBI BlastP on this gene

EGY22105

hypothetical protein
  
Accession: EGY22106
  
Location: 1759746-1761406
  
 NCBI BlastP on this gene

EGY22106

hypothetical protein
  
Accession: EGY22107
  
Location: 1763672-1764706
  
 NCBI BlastP on this gene

EGY22107

hypothetical protein
  
Accession: EGY22108
  
Location: 1765472-1766683
  
 NCBI BlastP on this gene

EGY22108

hypothetical protein
  
Accession: EGY22109
  
Location: 1767531-1770697
  
 NCBI BlastP on this gene

EGY22109

dimethylaniline monooxygenase
  
Accession: EGY22110
  
Location: 1771331-1772959
  
 NCBI BlastP on this gene

EGY22110

Query: Architecture Search FASTA input

CM001235 : Magnaporthe oryzae 70-15 chromosome 5    Total score: 2.0     Cumulative Blast bit score: 1033

Hit cluster cross-links:

Mycgr3G67791 Mycgr3T
  
Location: 0-1542

Mycgr3G67791\_Mycgr3T

Mycgr3G90406 Mycgr3T
  
Location: 1642-3973

Mycgr3G90406\_Mycgr3T

Mycgr3G67785 Mycgr3T
  
Location: 4073-7865

Mycgr3G67785\_Mycgr3T

Mycgr3G67795 Mycgr3T
  
Location: 7965-15249

Mycgr3G67795\_Mycgr3T

Mycgr3G67775 Mycgr3T
  
Location: 15349-16237

Mycgr3G67775\_Mycgr3T

Mycgr3G90404 Mycgr3T
  
Location: 16337-17246

Mycgr3G90404\_Mycgr3T

Mycgr3G36951 Mycgr3T
  
Location: 17346-30891

Mycgr3G36951\_Mycgr3T

Mycgr3G103034 Mycgr3
  
Location: 30991-32644

Mycgr3G103034\_Mycgr3

Mycgr3G31119 Mycgr3T
  
Location: 32744-32906

Mycgr3G31119\_Mycgr3T

Mycgr3G28587 Mycgr3T
  
Location: 33006-33489

Mycgr3G28587\_Mycgr3T

Mycgr3G98959 Mycgr3T
  
Location: 33589-35035

Mycgr3G98959\_Mycgr3T

Mycgr3G35447 Mycgr3T
  
Location: 35135-36443

Mycgr3G35447\_Mycgr3T

Mycgr3G84402 Mycgr3T
  
Location: 36543-37884

Mycgr3G84402\_Mycgr3T

Mycgr3G98961 Mycgr3T
  
Location: 37984-38884

Mycgr3G98961\_Mycgr3T

hypothetical protein
  
Accession: EHA49456
  
Location: 4143097-4143702
  
 NCBI BlastP on this gene

EHA49456

hypothetical protein
  
Accession: EHA49457
  
Location: 4144256-4145985
  
 NCBI BlastP on this gene

EHA49457

hypothetical protein
  
Accession: EHA49458
  
Location: 4147145-4147420
  
 NCBI BlastP on this gene

EHA49458

hypothetical protein
  
Accession: EHA49459
  
Location: 4148099-4149070
  
 NCBI BlastP on this gene

EHA49459

hypothetical protein
  
Accession: EHA49460
  
Location: 4149210-4151088
  
 NCBI BlastP on this gene

EHA49460

hypothetical protein
  
Accession: EHA49461
  
Location: 4152446-4153759
  
 NCBI BlastP on this gene

EHA49461

hypothetical protein
  
Accession: EHA49462
  
Location: 4154898-4155611
  
 NCBI BlastP on this gene

EHA49462

glycosyl hydrolase
  
Accession: EHA49463
  
Location: 4158931-4160807
  
 NCBI BlastP on this gene

EHA49463

ribosome biogenesis protein SSF1
  
Accession: EHA49464
  
Location: 4162914-4164399
  
  
**BlastP hit with Mycgr3G35447\_Mycgr3T**
  
Percentage identity: 57 %
  
BlastP bit score: 434
  
Sequence coverage: 94 %
  
E-value: 4e-145
  
  
 NCBI BlastP on this gene

EHA49464

ATP-dependent rRNA helicase RRP3
  
Accession: EHA49465
  
Location: 4164743-4166576
  
  
**BlastP hit with Mycgr3G84402\_Mycgr3T**
  
Percentage identity: 70 %
  
BlastP bit score: 599
  
Sequence coverage: 91 %
  
E-value: 0.0
  
  
 NCBI BlastP on this gene

EHA49465

hypothetical protein
  
Accession: EHA49466
  
Location: 4167145-4168344
  
 NCBI BlastP on this gene

EHA49466

hypothetical protein
  
Accession: EHA49467
  
Location: 4170304-4171256
  
 NCBI BlastP on this gene

EHA49467

endochitinase
  
Accession: EHA49468
  
Location: 4171908-4173424
  
 NCBI BlastP on this gene

EHA49468

lactose permease
  
Accession: EHA49469
  
Location: 4175088-4176965
  
 NCBI BlastP on this gene

EHA49469

mannosyl-oligosaccharide 1,2-alpha-mannosidase IB
  
Accession: EHA49470
  
Location: 4177394-4179055
  
 NCBI BlastP on this gene

EHA49470

hypothetical protein
  
Accession: EHA49471
  
Location: 4180174-4181049
  
 NCBI BlastP on this gene

EHA49471

hypothetical protein
  
Accession: EHA49472
  
Location: 4182465-4184917
  
 NCBI BlastP on this gene

EHA49472

hypothetical protein
  
Accession: EHA49473
  
Location: 4186055-4186624
  
 NCBI BlastP on this gene

EHA49473

Query: Architecture Search FASTA input

DS985220 : Verticillium albo-atrum VaMs.102 supercont1.7 genomic scaffold    Total score: 2.0     Cumulative Blast bit score: 1031

Hit cluster cross-links:

Mycgr3G67791 Mycgr3T
  
Location: 0-1542

Mycgr3G67791\_Mycgr3T

Mycgr3G90406 Mycgr3T
  
Location: 1642-3973

Mycgr3G90406\_Mycgr3T

Mycgr3G67785 Mycgr3T
  
Location: 4073-7865

Mycgr3G67785\_Mycgr3T

Mycgr3G67795 Mycgr3T
  
Location: 7965-15249

Mycgr3G67795\_Mycgr3T

Mycgr3G67775 Mycgr3T
  
Location: 15349-16237

Mycgr3G67775\_Mycgr3T

Mycgr3G90404 Mycgr3T
  
Location: 16337-17246

Mycgr3G90404\_Mycgr3T

Mycgr3G36951 Mycgr3T
  
Location: 17346-30891

Mycgr3G36951\_Mycgr3T

Mycgr3G103034 Mycgr3
  
Location: 30991-32644

Mycgr3G103034\_Mycgr3

Mycgr3G31119 Mycgr3T
  
Location: 32744-32906

Mycgr3G31119\_Mycgr3T

Mycgr3G28587 Mycgr3T
  
Location: 33006-33489

Mycgr3G28587\_Mycgr3T

Mycgr3G98959 Mycgr3T
  
Location: 33589-35035

Mycgr3G98959\_Mycgr3T

Mycgr3G35447 Mycgr3T
  
Location: 35135-36443

Mycgr3G35447\_Mycgr3T

Mycgr3G84402 Mycgr3T
  
Location: 36543-37884

Mycgr3G84402\_Mycgr3T

Mycgr3G98961 Mycgr3T
  
Location: 37984-38884

Mycgr3G98961\_Mycgr3T

allantoate permease
  
Accession: EEY20278
  
Location: 1738916-1740952
  
 NCBI BlastP on this gene

EEY20278

integral membrane protein
  
Accession: EEY20279
  
Location: 1742636-1743908
  
 NCBI BlastP on this gene

EEY20279

FAD binding domain-containing protein
  
Accession: EEY20280
  
Location: 1744117-1745609
  
 NCBI BlastP on this gene

EEY20280

conserved hypothetical protein
  
Accession: EEY20281
  
Location: 1747877-1749258
  
 NCBI BlastP on this gene

EEY20281

conserved hypothetical protein
  
Accession: EEY20282
  
Location: 1752604-1754410
  
 NCBI BlastP on this gene

EEY20282

ribosome biogenesis protein SSF1
  
Accession: EEY20283
  
Location: 1756393-1757842
  
  
**BlastP hit with Mycgr3G35447\_Mycgr3T**
  
Percentage identity: 55 %
  
BlastP bit score: 437
  
Sequence coverage: 96 %
  
E-value: 1e-146
  
  
 NCBI BlastP on this gene

EEY20283

ATP-dependent rRNA helicase RRP3
  
Accession: EEY20284
  
Location: 1758165-1759949
  
  
**BlastP hit with Mycgr3G84402\_Mycgr3T**
  
Percentage identity: 68 %
  
BlastP bit score: 594
  
Sequence coverage: 93 %
  
E-value: 0.0
  
  
 NCBI BlastP on this gene

EEY20284

conserved hypothetical protein
  
Accession: EEY20285
  
Location: 1760651-1761523
  
 NCBI BlastP on this gene

EEY20285

flavonol synthase
  
Accession: EEY20286
  
Location: 1763452-1764661
  
 NCBI BlastP on this gene

EEY20286

predicted protein
  
Accession: EEY20287
  
Location: 1764868-1765705
  
 NCBI BlastP on this gene

EEY20287

conserved hypothetical protein
  
Accession: EEY20288
  
Location: 1775945-1776217
  
 NCBI BlastP on this gene

EEY20288

conserved hypothetical protein
  
Accession: EEY20289
  
Location: 1779030-1782180
  
 NCBI BlastP on this gene

EEY20289

Query: Architecture Search FASTA input

JH793228 : Magnaporthe oryzae Y34 unplaced genomic scaffold Y34\_scaffold00073    Total score: 2.0     Cumulative Blast bit score: 1030

Hit cluster cross-links:

Mycgr3G67791 Mycgr3T
  
Location: 0-1542

Mycgr3G67791\_Mycgr3T

Mycgr3G90406 Mycgr3T
  
Location: 1642-3973

Mycgr3G90406\_Mycgr3T

Mycgr3G67785 Mycgr3T
  
Location: 4073-7865

Mycgr3G67785\_Mycgr3T

Mycgr3G67795 Mycgr3T
  
Location: 7965-15249

Mycgr3G67795\_Mycgr3T

Mycgr3G67775 Mycgr3T
  
Location: 15349-16237

Mycgr3G67775\_Mycgr3T

Mycgr3G90404 Mycgr3T
  
Location: 16337-17246

Mycgr3G90404\_Mycgr3T

Mycgr3G36951 Mycgr3T
  
Location: 17346-30891

Mycgr3G36951\_Mycgr3T

Mycgr3G103034 Mycgr3
  
Location: 30991-32644

Mycgr3G103034\_Mycgr3

Mycgr3G31119 Mycgr3T
  
Location: 32744-32906

Mycgr3G31119\_Mycgr3T

Mycgr3G28587 Mycgr3T
  
Location: 33006-33489

Mycgr3G28587\_Mycgr3T

Mycgr3G98959 Mycgr3T
  
Location: 33589-35035

Mycgr3G98959\_Mycgr3T

Mycgr3G35447 Mycgr3T
  
Location: 35135-36443

Mycgr3G35447\_Mycgr3T

Mycgr3G84402 Mycgr3T
  
Location: 36543-37884

Mycgr3G84402\_Mycgr3T

Mycgr3G98961 Mycgr3T
  
Location: 37984-38884

Mycgr3G98961\_Mycgr3T

hypothetical protein
  
Accession: ELQ44566
  
Location: 333-1025
  
 NCBI BlastP on this gene

ELQ44566

glycosyl hydrolase
  
Accession: ELQ44567
  
Location: 1353-3710
  
 NCBI BlastP on this gene

ELQ44567

ribosome biogenesis protein SSF1
  
Accession: ELQ44568
  
Location: 5904-7389
  
  
**BlastP hit with Mycgr3G35447\_Mycgr3T**
  
Percentage identity: 57 %
  
BlastP bit score: 434
  
Sequence coverage: 94 %
  
E-value: 4e-145
  
  
 NCBI BlastP on this gene

ELQ44568

ATP-dependent rRNA helicase rrp3
  
Accession: ELQ44569
  
Location: 7805-11334
  
  
**BlastP hit with Mycgr3G84402\_Mycgr3T**
  
Percentage identity: 70 %
  
BlastP bit score: 596
  
Sequence coverage: 91 %
  
E-value: 0.0
  
  
 NCBI BlastP on this gene

ELQ44569

hypothetical protein
  
Accession: ELQ44570
  
Location: 11952-13070
  
 NCBI BlastP on this gene

ELQ44570

42 kDa endochitinase
  
Accession: ELQ44571
  
Location: 14897-16413
  
 NCBI BlastP on this gene

ELQ44571

lactose permease
  
Accession: ELQ44572
  
Location: 18078-19949
  
 NCBI BlastP on this gene

ELQ44572

mannosyl-oligosaccharide 1,2-alpha-mannosidase IB
  
Accession: ELQ44573
  
Location: 20380-22041
  
 NCBI BlastP on this gene

ELQ44573

hypothetical protein
  
Accession: ELQ44574
  
Location: 23370-25364
  
 NCBI BlastP on this gene

ELQ44574

hypothetical protein
  
Accession: ELQ44575
  
Location: 25661-28113
  
 NCBI BlastP on this gene

ELQ44575

hypothetical protein
  
Accession: ELQ44576
  
Location: 29053-29139
  
 NCBI BlastP on this gene

ELQ44576

hypothetical protein
  
Accession: ELQ44577
  
Location: 29251-29820
  
 NCBI BlastP on this gene

ELQ44577

Query: Architecture Search FASTA input

FP929065 : Leptosphaeria maculans JN3 lm\_SuperContig\_8\_v2 genomic supercontig    Total score: 2.0     Cumulative Blast bit score: 1027

Hit cluster cross-links:

Mycgr3G67791 Mycgr3T
  
Location: 0-1542

Mycgr3G67791\_Mycgr3T

Mycgr3G90406 Mycgr3T
  
Location: 1642-3973

Mycgr3G90406\_Mycgr3T

Mycgr3G67785 Mycgr3T
  
Location: 4073-7865

Mycgr3G67785\_Mycgr3T

Mycgr3G67795 Mycgr3T
  
Location: 7965-15249

Mycgr3G67795\_Mycgr3T

Mycgr3G67775 Mycgr3T
  
Location: 15349-16237

Mycgr3G67775\_Mycgr3T

Mycgr3G90404 Mycgr3T
  
Location: 16337-17246

Mycgr3G90404\_Mycgr3T

Mycgr3G36951 Mycgr3T
  
Location: 17346-30891

Mycgr3G36951\_Mycgr3T

Mycgr3G103034 Mycgr3
  
Location: 30991-32644

Mycgr3G103034\_Mycgr3

Mycgr3G31119 Mycgr3T
  
Location: 32744-32906

Mycgr3G31119\_Mycgr3T

Mycgr3G28587 Mycgr3T
  
Location: 33006-33489

Mycgr3G28587\_Mycgr3T

Mycgr3G98959 Mycgr3T
  
Location: 33589-35035

Mycgr3G98959\_Mycgr3T

Mycgr3G35447 Mycgr3T
  
Location: 35135-36443

Mycgr3G35447\_Mycgr3T

Mycgr3G84402 Mycgr3T
  
Location: 36543-37884

Mycgr3G84402\_Mycgr3T

Mycgr3G98961 Mycgr3T
  
Location: 37984-38884

Mycgr3G98961\_Mycgr3T

predicted protein
  
Accession: CBX91007
  
Location: 1290671-1290998
  
 NCBI BlastP on this gene

LEMA\_uP060410.1

predicted protein
  
Accession: CBX91008
  
Location: 1292236-1293447
  
 NCBI BlastP on this gene

LEMA\_P060420.1

predicted protein
  
Accession: CBX91009
  
Location: 1295165-1295719
  
 NCBI BlastP on this gene

LEMA\_P060430.1

hypothetical protein
  
Accession: CBX91010
  
Location: 1296527-1297390
  
 NCBI BlastP on this gene

LEMA\_P060440.1

similar to monooxygenase FAD-binding
  
Accession: CBX91011
  
Location: 1299572-1300884
  
 NCBI BlastP on this gene

LEMA\_P060450.1

predicted protein
  
Accession: CBX91012
  
Location: 1301631-1302579
  
 NCBI BlastP on this gene

LEMA\_P060460.1

predicted protein
  
Accession: CBX91013
  
Location: 1303011-1303633
  
 NCBI BlastP on this gene

LEMA\_P060470.1

hypothetical protein
  
Accession: CBX91014
  
Location: 1304782-1305333
  
 NCBI BlastP on this gene

LEMA\_P060480.1

similar to hemolysin-III channel protein Izh2
  
Accession: CBX91015
  
Location: 1305774-1306775
  
 NCBI BlastP on this gene

LEMA\_P060490.1

hypothetical protein
  
Accession: CBX91016
  
Location: 1307370-1308406
  
 NCBI BlastP on this gene

LEMA\_P060500.1

hypothetical protein
  
Accession: CBX91017
  
Location: 1308642-1310064
  
  
**BlastP hit with Mycgr3G35447\_Mycgr3T**
  
Percentage identity: 57 %
  
BlastP bit score: 419
  
Sequence coverage: 88 %
  
E-value: 1e-139
  
  
 NCBI BlastP on this gene

LEMA\_P060510.1

hypothetical protein
  
Accession: CBX91018
  
Location: 1310237-1311836
  
  
**BlastP hit with Mycgr3G84402\_Mycgr3T**
  
Percentage identity: 72 %
  
BlastP bit score: 608
  
Sequence coverage: 89 %
  
E-value: 0.0
  
  
 NCBI BlastP on this gene

LEMA\_P060520.1

predicted protein
  
Accession: CBX91019
  
Location: 1312028-1312477
  
 NCBI BlastP on this gene

LEMA\_P060530.1

predicted protein
  
Accession: CBX91020
  
Location: 1312601-1313587
  
 NCBI BlastP on this gene

LEMA\_P060540.1

similar to MFS monocarboxylate transporter
  
Accession: CBX91021
  
Location: 1314496-1315983
  
 NCBI BlastP on this gene

LEMA\_P060550.1

hypothetical protein
  
Accession: CBX91022
  
Location: 1317923-1319474
  
 NCBI BlastP on this gene

LEMA\_P060560.1

similar to N2,N2-dimethylguanosine tRNA methyltransferase
  
Accession: CBX91023
  
Location: 1320527-1322605
  
 NCBI BlastP on this gene

LEMA\_P060570.1

hypothetical protein
  
Accession: CBX91024
  
Location: 1323243-1324529
  
 NCBI BlastP on this gene

LEMA\_P060580.1

predicted protein
  
Accession: CBX91025
  
Location: 1324699-1325970
  
 NCBI BlastP on this gene

LEMA\_P060590.1

hypothetical protein
  
Accession: CBX91026
  
Location: 1326777-1327980
  
 NCBI BlastP on this gene

LEMA\_P060600.1

hypothetical protein
  
Accession: CBX91027
  
Location: 1328240-1329784
  
 NCBI BlastP on this gene

LEMA\_P060610.1

hypothetical protein
  
Accession: CBX91028
  
Location: 1330283-1334106
  
 NCBI BlastP on this gene

LEMA\_P060620.1

Query: Architecture Search FASTA input

CH476597 : Aspergillus terreus NIH2624 scaffold\_4 genomic scaffold    Total score: 2.0     Cumulative Blast bit score: 1017

Hit cluster cross-links:

Mycgr3G67791 Mycgr3T
  
Location: 0-1542

Mycgr3G67791\_Mycgr3T

Mycgr3G90406 Mycgr3T
  
Location: 1642-3973

Mycgr3G90406\_Mycgr3T

Mycgr3G67785 Mycgr3T
  
Location: 4073-7865

Mycgr3G67785\_Mycgr3T

Mycgr3G67795 Mycgr3T
  
Location: 7965-15249

Mycgr3G67795\_Mycgr3T

Mycgr3G67775 Mycgr3T
  
Location: 15349-16237

Mycgr3G67775\_Mycgr3T

Mycgr3G90404 Mycgr3T
  
Location: 16337-17246

Mycgr3G90404\_Mycgr3T

Mycgr3G36951 Mycgr3T
  
Location: 17346-30891

Mycgr3G36951\_Mycgr3T

Mycgr3G103034 Mycgr3
  
Location: 30991-32644

Mycgr3G103034\_Mycgr3

Mycgr3G31119 Mycgr3T
  
Location: 32744-32906

Mycgr3G31119\_Mycgr3T

Mycgr3G28587 Mycgr3T
  
Location: 33006-33489

Mycgr3G28587\_Mycgr3T

Mycgr3G98959 Mycgr3T
  
Location: 33589-35035

Mycgr3G98959\_Mycgr3T

Mycgr3G35447 Mycgr3T
  
Location: 35135-36443

Mycgr3G35447\_Mycgr3T

Mycgr3G84402 Mycgr3T
  
Location: 36543-37884

Mycgr3G84402\_Mycgr3T

Mycgr3G98961 Mycgr3T
  
Location: 37984-38884

Mycgr3G98961\_Mycgr3T

serine/threonine-protein kinase sck1
  
Accession: EAU36256
  
Location: 546105-548797
  
 NCBI BlastP on this gene

EAU36256

conserved hypothetical protein
  
Accession: EAU36257
  
Location: 552309-554663
  
 NCBI BlastP on this gene

EAU36257

26S protease regulatory subunit 6A-B
  
Accession: EAU36258
  
Location: 555494-557087
  
 NCBI BlastP on this gene

EAU36258

hypothetical protein
  
Accession: EAU36259
  
Location: 557370-559414
  
 NCBI BlastP on this gene

EAU36259

conserved hypothetical protein
  
Accession: EAU36260
  
Location: 559798-560927
  
 NCBI BlastP on this gene

EAU36260

HNRNP arginine N-methyltransferase
  
Accession: EAU36261
  
Location: 561782-563135
  
 NCBI BlastP on this gene

EAU36261

conserved hypothetical protein
  
Accession: EAU36262
  
Location: 563922-565092
  
 NCBI BlastP on this gene

EAU36262

hypothetical protein
  
Accession: EAU36263
  
Location: 565654-567681
  
  
**BlastP hit with Mycgr3G103034\_Mycgr3**
  
Percentage identity: 52 %
  
BlastP bit score: 585
  
Sequence coverage: 103 %
  
E-value: 0.0
  
  
 NCBI BlastP on this gene

EAU36263

conserved hypothetical protein
  
Accession: EAU36264
  
Location: 568125-569544
  
  
**BlastP hit with Mycgr3G35447\_Mycgr3T**
  
Percentage identity: 59 %
  
BlastP bit score: 432
  
Sequence coverage: 88 %
  
E-value: 1e-144
  
  
 NCBI BlastP on this gene

EAU36264

riboflavin synthase alpha chain
  
Accession: EAU36265
  
Location: 569957-570585
  
 NCBI BlastP on this gene

EAU36265

hypothetical protein
  
Accession: EAU36266
  
Location: 570990-572623
  
 NCBI BlastP on this gene

EAU36266

conserved hypothetical protein
  
Accession: EAU36267
  
Location: 573339-574733
  
 NCBI BlastP on this gene

EAU36267

conserved hypothetical protein
  
Accession: EAU36268
  
Location: 574972-576509
  
 NCBI BlastP on this gene

EAU36268

conserved hypothetical protein
  
Accession: EAU36269
  
Location: 577325-577827
  
 NCBI BlastP on this gene

EAU36269

predicted protein
  
Accession: EAU36270
  
Location: 578549-579718
  
 NCBI BlastP on this gene

EAU36270

predicted protein
  
Accession: EAU36271
  
Location: 581042-581642
  
 NCBI BlastP on this gene

EAU36271

predicted protein
  
Accession: EAU36272
  
Location: 582134-582433
  
 NCBI BlastP on this gene

EAU36272

conserved hypothetical protein
  
Accession: EAU36273
  
Location: 582939-584980
  
 NCBI BlastP on this gene

EAU36273

predicted protein
  
Accession: EAU36274
  
Location: 585814-588011
  
 NCBI BlastP on this gene

EAU36274

conserved hypothetical protein
  
Accession: EAU36275
  
Location: 588848-590078
  
 NCBI BlastP on this gene

EAU36275

Query: Architecture Search FASTA input

KB933264 : Togninia minima UCRPA7 unplaced genomic scaffold PA7\_03\_scaffold\_496    Total score: 2.0     Cumulative Blast bit score: 1001

Hit cluster cross-links:

Mycgr3G67791 Mycgr3T
  
Location: 0-1542

Mycgr3G67791\_Mycgr3T

Mycgr3G90406 Mycgr3T
  
Location: 1642-3973

Mycgr3G90406\_Mycgr3T

Mycgr3G67785 Mycgr3T
  
Location: 4073-7865

Mycgr3G67785\_Mycgr3T

Mycgr3G67795 Mycgr3T
  
Location: 7965-15249

Mycgr3G67795\_Mycgr3T

Mycgr3G67775 Mycgr3T
  
Location: 15349-16237

Mycgr3G67775\_Mycgr3T

Mycgr3G90404 Mycgr3T
  
Location: 16337-17246

Mycgr3G90404\_Mycgr3T

Mycgr3G36951 Mycgr3T
  
Location: 17346-30891

Mycgr3G36951\_Mycgr3T

Mycgr3G103034 Mycgr3
  
Location: 30991-32644

Mycgr3G103034\_Mycgr3

Mycgr3G31119 Mycgr3T
  
Location: 32744-32906

Mycgr3G31119\_Mycgr3T

Mycgr3G28587 Mycgr3T
  
Location: 33006-33489

Mycgr3G28587\_Mycgr3T

Mycgr3G98959 Mycgr3T
  
Location: 33589-35035

Mycgr3G98959\_Mycgr3T

Mycgr3G35447 Mycgr3T
  
Location: 35135-36443

Mycgr3G35447\_Mycgr3T

Mycgr3G84402 Mycgr3T
  
Location: 36543-37884

Mycgr3G84402\_Mycgr3T

Mycgr3G98961 Mycgr3T
  
Location: 37984-38884

Mycgr3G98961\_Mycgr3T

putative sugar transporter protein
  
Accession: EON97545
  
Location: 1009100-1010623
  
 NCBI BlastP on this gene

EON97545

putative atp-dependent rrna helicase rrp3 protein
  
Accession: EON97455
  
Location: 1014440-1015794
  
  
**BlastP hit with Mycgr3G84402\_Mycgr3T**
  
Percentage identity: 73 %
  
BlastP bit score: 560
  
Sequence coverage: 80 %
  
E-value: 0.0
  
  
 NCBI BlastP on this gene

EON97455

putative ribosome biogenesis protein ssf1 protein
  
Accession: EON97642
  
Location: 1016288-1017743
  
  
**BlastP hit with Mycgr3G35447\_Mycgr3T**
  
Percentage identity: 54 %
  
BlastP bit score: 441
  
Sequence coverage: 103 %
  
E-value: 4e-148
  
  
 NCBI BlastP on this gene

EON97642

putative succinate dehydrogenase fumarate reductase flavoprotein subunit protein
  
Accession: EON97680
  
Location: 1026039-1028105
  
 NCBI BlastP on this gene

EON97680

hypothetical protein
  
Accession: EON97582
  
Location: 1033730-1034287
  
 NCBI BlastP on this gene

EON97582

Query: Architecture Search FASTA input

CABT02000002 : Sordaria macrospora k-hell    Total score: 2.0     Cumulative Blast bit score: 993

Hit cluster cross-links:

Mycgr3G67791 Mycgr3T
  
Location: 0-1542

Mycgr3G67791\_Mycgr3T

Mycgr3G90406 Mycgr3T
  
Location: 1642-3973

Mycgr3G90406\_Mycgr3T

Mycgr3G67785 Mycgr3T
  
Location: 4073-7865

Mycgr3G67785\_Mycgr3T

Mycgr3G67795 Mycgr3T
  
Location: 7965-15249

Mycgr3G67795\_Mycgr3T

Mycgr3G67775 Mycgr3T
  
Location: 15349-16237

Mycgr3G67775\_Mycgr3T

Mycgr3G90404 Mycgr3T
  
Location: 16337-17246

Mycgr3G90404\_Mycgr3T

Mycgr3G36951 Mycgr3T
  
Location: 17346-30891

Mycgr3G36951\_Mycgr3T

Mycgr3G103034 Mycgr3
  
Location: 30991-32644

Mycgr3G103034\_Mycgr3

Mycgr3G31119 Mycgr3T
  
Location: 32744-32906

Mycgr3G31119\_Mycgr3T

Mycgr3G28587 Mycgr3T
  
Location: 33006-33489

Mycgr3G28587\_Mycgr3T

Mycgr3G98959 Mycgr3T
  
Location: 33589-35035

Mycgr3G98959\_Mycgr3T

Mycgr3G35447 Mycgr3T
  
Location: 35135-36443

Mycgr3G35447\_Mycgr3T

Mycgr3G84402 Mycgr3T
  
Location: 36543-37884

Mycgr3G84402\_Mycgr3T

Mycgr3G98961 Mycgr3T
  
Location: 37984-38884

Mycgr3G98961\_Mycgr3T

not annotated
  
Accession: CCC07065
  
Location: 1757910-1760331
  
 NCBI BlastP on this gene

CCC07065

not annotated
  
Accession: CCC07066
  
Location: 1761071-1762217
  
 NCBI BlastP on this gene

CCC07066

not annotated
  
Accession: CCC07067
  
Location: 1762547-1763236
  
 NCBI BlastP on this gene

CCC07067

not annotated
  
Accession: CCC07068
  
Location: 1764182-1764513
  
 NCBI BlastP on this gene

CCC07068

not annotated
  
Accession: CCC07069
  
Location: 1765068-1766339
  
 NCBI BlastP on this gene

CCC07069

not annotated
  
Accession: CCC07070
  
Location: 1767792-1769066
  
 NCBI BlastP on this gene

CCC07070

not annotated
  
Accession: CCC07071
  
Location: 1771086-1773341
  
 NCBI BlastP on this gene

CCC07071

not annotated
  
Accession: CCC07072
  
Location: 1773641-1775339
  
  
**BlastP hit with Mycgr3G84402\_Mycgr3T**
  
Percentage identity: 67 %
  
BlastP bit score: 534
  
Sequence coverage: 88 %
  
E-value: 0.0
  
  
 NCBI BlastP on this gene

CCC07072

not annotated
  
Accession: CCC07073
  
Location: 1775693-1777189
  
  
**BlastP hit with Mycgr3G35447\_Mycgr3T**
  
Percentage identity: 54 %
  
BlastP bit score: 459
  
Sequence coverage: 103 %
  
E-value: 5e-155
  
  
 NCBI BlastP on this gene

CCC07073

not annotated
  
Accession: CCC07074
  
Location: 1779052-1779436
  
 NCBI BlastP on this gene

CCC07074

not annotated
  
Accession: CCC07075
  
Location: 1782982-1783425
  
 NCBI BlastP on this gene

CCC07075

not annotated
  
Accession: CCC07076
  
Location: 1785534-1787112
  
 NCBI BlastP on this gene

CCC07076

not annotated
  
Accession: CCC07077
  
Location: 1788998-1790740
  
 NCBI BlastP on this gene

CCC07077

not annotated
  
Accession: CCC07078
  
Location: 1792558-1794342
  
 NCBI BlastP on this gene

CCC07078

not annotated
  
Accession: CCC07079
  
Location: 1794689-1795929
  
 NCBI BlastP on this gene

CCC07079

Query: Architecture Search FASTA input

FN430009 : Tuber melanosporum whole genome shotgun sequence assembly, scaffold\_12, strain Mel28.    Total score: 2.0     Cumulative Blast bit score: 975

Hit cluster cross-links:

Mycgr3G67791 Mycgr3T
  
Location: 0-1542

Mycgr3G67791\_Mycgr3T

Mycgr3G90406 Mycgr3T
  
Location: 1642-3973

Mycgr3G90406\_Mycgr3T

Mycgr3G67785 Mycgr3T
  
Location: 4073-7865

Mycgr3G67785\_Mycgr3T

Mycgr3G67795 Mycgr3T
  
Location: 7965-15249

Mycgr3G67795\_Mycgr3T

Mycgr3G67775 Mycgr3T
  
Location: 15349-16237

Mycgr3G67775\_Mycgr3T

Mycgr3G90404 Mycgr3T
  
Location: 16337-17246

Mycgr3G90404\_Mycgr3T

Mycgr3G36951 Mycgr3T
  
Location: 17346-30891

Mycgr3G36951\_Mycgr3T

Mycgr3G103034 Mycgr3
  
Location: 30991-32644

Mycgr3G103034\_Mycgr3

Mycgr3G31119 Mycgr3T
  
Location: 32744-32906

Mycgr3G31119\_Mycgr3T

Mycgr3G28587 Mycgr3T
  
Location: 33006-33489

Mycgr3G28587\_Mycgr3T

Mycgr3G98959 Mycgr3T
  
Location: 33589-35035

Mycgr3G98959\_Mycgr3T

Mycgr3G35447 Mycgr3T
  
Location: 35135-36443

Mycgr3G35447\_Mycgr3T

Mycgr3G84402 Mycgr3T
  
Location: 36543-37884

Mycgr3G84402\_Mycgr3T

Mycgr3G98961 Mycgr3T
  
Location: 37984-38884

Mycgr3G98961\_Mycgr3T

not annotated
  
Accession: CAZ80070
  
Location: 560316-562116
  
 NCBI BlastP on this gene

CAZ80070

not annotated
  
Accession: CAZ80071
  
Location: 562680-563387
  
 NCBI BlastP on this gene

CAZ80071

not annotated
  
Accession: CAZ80072
  
Location: 563802-565127
  
 NCBI BlastP on this gene

CAZ80072

not annotated
  
Accession: CAZ80073
  
Location: 565352-567722
  
 NCBI BlastP on this gene

CAZ80073

not annotated
  
Accession: CAZ80074
  
Location: 568909-569753
  
 NCBI BlastP on this gene

CAZ80074

not annotated
  
Accession: CAZ80075
  
Location: 569964-571096
  
  
**BlastP hit with Mycgr3G67775\_Mycgr3T**
  
Percentage identity: 53 %
  
BlastP bit score: 288
  
Sequence coverage: 94 %
  
E-value: 1e-92
  
  
 NCBI BlastP on this gene

CAZ80075

not annotated
  
Accession: CAZ80076
  
Location: 571598-573427
  
  
**BlastP hit with Mycgr3G98959\_Mycgr3T**
  
Percentage identity: 67 %
  
BlastP bit score: 687
  
Sequence coverage: 100 %
  
E-value: 0.0
  
  
 NCBI BlastP on this gene

CAZ80076

Query: Architecture Search FASTA input

JH226130 : Exophiala dermatitidis NIH/UT8656 unplaced genomic scaffold supercont1.1    Total score: 2.0     Cumulative Blast bit score: 970

Hit cluster cross-links:

Mycgr3G67791 Mycgr3T
  
Location: 0-1542

Mycgr3G67791\_Mycgr3T

Mycgr3G90406 Mycgr3T
  
Location: 1642-3973

Mycgr3G90406\_Mycgr3T

Mycgr3G67785 Mycgr3T
  
Location: 4073-7865

Mycgr3G67785\_Mycgr3T

Mycgr3G67795 Mycgr3T
  
Location: 7965-15249

Mycgr3G67795\_Mycgr3T

Mycgr3G67775 Mycgr3T
  
Location: 15349-16237

Mycgr3G67775\_Mycgr3T

Mycgr3G90404 Mycgr3T
  
Location: 16337-17246

Mycgr3G90404\_Mycgr3T

Mycgr3G36951 Mycgr3T
  
Location: 17346-30891

Mycgr3G36951\_Mycgr3T

Mycgr3G103034 Mycgr3
  
Location: 30991-32644

Mycgr3G103034\_Mycgr3

Mycgr3G31119 Mycgr3T
  
Location: 32744-32906

Mycgr3G31119\_Mycgr3T

Mycgr3G28587 Mycgr3T
  
Location: 33006-33489

Mycgr3G28587\_Mycgr3T

Mycgr3G98959 Mycgr3T
  
Location: 33589-35035

Mycgr3G98959\_Mycgr3T

Mycgr3G35447 Mycgr3T
  
Location: 35135-36443

Mycgr3G35447\_Mycgr3T

Mycgr3G84402 Mycgr3T
  
Location: 36543-37884

Mycgr3G84402\_Mycgr3T

Mycgr3G98961 Mycgr3T
  
Location: 37984-38884

Mycgr3G98961\_Mycgr3T

deoxyribodipyrimidine photo-lyase
  
Accession: EHY53006
  
Location: 3393096-3394721
  
 NCBI BlastP on this gene

EHY53006

hypothetical protein
  
Accession: EHY53007
  
Location: 3395663-3397556
  
 NCBI BlastP on this gene

EHY53007

hypothetical protein
  
Accession: EHY53008
  
Location: 3398518-3399258
  
 NCBI BlastP on this gene

EHY53008

hypothetical protein
  
Accession: EHY53009
  
Location: 3399780-3400295
  
 NCBI BlastP on this gene

EHY53009

hypothetical protein
  
Accession: EHY53010
  
Location: 3401208-3402128
  
 NCBI BlastP on this gene

EHY53010

hypothetical protein
  
Accession: EHY53011
  
Location: 3403002-3404299
  
 NCBI BlastP on this gene

EHY53011

hypothetical protein
  
Accession: EHY53012
  
Location: 3404997-3406049
  
  
**BlastP hit with Mycgr3G67775\_Mycgr3T**
  
Percentage identity: 43 %
  
BlastP bit score: 266
  
Sequence coverage: 113 %
  
E-value: 1e-83
  
  
 NCBI BlastP on this gene

EHY53012

eukaryotic translation initiation factor 3 subunit L
  
Accession: EHY53013
  
Location: 3406729-3408102
  
  
**BlastP hit with Mycgr3G98959\_Mycgr3T**
  
Percentage identity: 71 %
  
BlastP bit score: 704
  
Sequence coverage: 97 %
  
E-value: 0.0
  
  
 NCBI BlastP on this gene

EHY53013

hypothetical protein
  
Accession: EHY53014
  
Location: 3408572-3410239
  
 NCBI BlastP on this gene

EHY53014

cytochrome c heme-lyase
  
Accession: EHY53015
  
Location: 3410821-3412034
  
 NCBI BlastP on this gene

EHY53015

hypothetical protein
  
Accession: EHY53016
  
Location: 3412451-3413098
  
 NCBI BlastP on this gene

EHY53016

hypothetical protein
  
Accession: EHY53017
  
Location: 3413106-3414300
  
 NCBI BlastP on this gene

EHY53017

ubiquitin-conjugating enzyme E2 J2
  
Accession: EHY53018
  
Location: 3415352-3416188
  
 NCBI BlastP on this gene

EHY53018

MFS transporter, SIT family, siderophore-iron:H+ symporter
  
Accession: EHY53019
  
Location: 3417691-3419555
  
 NCBI BlastP on this gene

EHY53019

outer membrane protein
  
Accession: EHY53020
  
Location: 3420085-3421683
  
 NCBI BlastP on this gene

EHY53020

hypothetical protein
  
Accession: EHY53021
  
Location: 3422345-3423405
  
 NCBI BlastP on this gene

EHY53021

hypothetical protein
  
Accession: EHY53022
  
Location: 3424286-3425571
  
 NCBI BlastP on this gene

EHY53022

beta-glucosidase
  
Accession: EHY53023
  
Location: 3427212-3429827
  
 NCBI BlastP on this gene

EHY53023

Query: Architecture Search FASTA input

CH991557 : Monosiga brevicollis MX1 MONBRscaffold\_16 genomic scaffold    Total score: 2.0     Cumulative Blast bit score: 924

Hit cluster cross-links:

Mycgr3G67791 Mycgr3T
  
Location: 0-1542

Mycgr3G67791\_Mycgr3T

Mycgr3G90406 Mycgr3T
  
Location: 1642-3973

Mycgr3G90406\_Mycgr3T

Mycgr3G67785 Mycgr3T
  
Location: 4073-7865

Mycgr3G67785\_Mycgr3T

Mycgr3G67795 Mycgr3T
  
Location: 7965-15249

Mycgr3G67795\_Mycgr3T

Mycgr3G67775 Mycgr3T
  
Location: 15349-16237

Mycgr3G67775\_Mycgr3T

Mycgr3G90404 Mycgr3T
  
Location: 16337-17246

Mycgr3G90404\_Mycgr3T

Mycgr3G36951 Mycgr3T
  
Location: 17346-30891

Mycgr3G36951\_Mycgr3T

Mycgr3G103034 Mycgr3
  
Location: 30991-32644

Mycgr3G103034\_Mycgr3

Mycgr3G31119 Mycgr3T
  
Location: 32744-32906

Mycgr3G31119\_Mycgr3T

Mycgr3G28587 Mycgr3T
  
Location: 33006-33489

Mycgr3G28587\_Mycgr3T

Mycgr3G98959 Mycgr3T
  
Location: 33589-35035

Mycgr3G98959\_Mycgr3T

Mycgr3G35447 Mycgr3T
  
Location: 35135-36443

Mycgr3G35447\_Mycgr3T

Mycgr3G84402 Mycgr3T
  
Location: 36543-37884

Mycgr3G84402\_Mycgr3T

Mycgr3G98961 Mycgr3T
  
Location: 37984-38884

Mycgr3G98961\_Mycgr3T

predicted protein
  
Accession: EDQ87859
  
Location: 241452-243687
  
 NCBI BlastP on this gene

EDQ87859

predicted protein
  
Accession: EDQ87753
  
Location: 233055-237534
  
 NCBI BlastP on this gene

EDQ87753

predicted protein
  
Accession: EDQ87858
  
Location: 230149-232853
  
  
**BlastP hit with Mycgr3G84402\_Mycgr3T**
  
Percentage identity: 61 %
  
BlastP bit score: 514
  
Sequence coverage: 88 %
  
E-value: 6e-176
  
  
 NCBI BlastP on this gene

EDQ87858

predicted protein
  
Accession: EDQ87752
  
Location: 228947-229366
  
 NCBI BlastP on this gene

EDQ87752

predicted protein
  
Accession: EDQ87857
  
Location: 225073-227734
  
 NCBI BlastP on this gene

EDQ87857

predicted protein
  
Accession: EDQ87751
  
Location: 223057-223898
  
 NCBI BlastP on this gene

EDQ87751

predicted protein
  
Accession: EDQ87856
  
Location: 214119-222306
  
 NCBI BlastP on this gene

EDQ87856

predicted protein
  
Accession: EDQ87750
  
Location: 211144-213619
  
 NCBI BlastP on this gene

EDQ87750

predicted protein
  
Accession: EDQ87855
  
Location: 206832-210646
  
  
**BlastP hit with Mycgr3G103034\_Mycgr3**
  
Percentage identity: 44 %
  
BlastP bit score: 410
  
Sequence coverage: 95 %
  
E-value: 1e-132
  
  
 NCBI BlastP on this gene

EDQ87855

predicted protein
  
Accession: EDQ87749
  
Location: 204037-206167
  
 NCBI BlastP on this gene

EDQ87749

predicted protein
  
Accession: EDQ87854
  
Location: 201722-203306
  
 NCBI BlastP on this gene

EDQ87854

predicted protein
  
Accession: EDQ87853
  
Location: 199484-200680
  
 NCBI BlastP on this gene

EDQ87853

predicted protein
  
Accession: EDQ87748
  
Location: 195327-198430
  
 NCBI BlastP on this gene

EDQ87748

Query: Architecture Search FASTA input

CR382127 : Yarrowia lipolytica CLIB122 chromosome A complete sequence.    Total score: 2.0     Cumulative Blast bit score: 841

Hit cluster cross-links:

Mycgr3G67791 Mycgr3T
  
Location: 0-1542

Mycgr3G67791\_Mycgr3T

Mycgr3G90406 Mycgr3T
  
Location: 1642-3973

Mycgr3G90406\_Mycgr3T

Mycgr3G67785 Mycgr3T
  
Location: 4073-7865

Mycgr3G67785\_Mycgr3T

Mycgr3G67795 Mycgr3T
  
Location: 7965-15249

Mycgr3G67795\_Mycgr3T

Mycgr3G67775 Mycgr3T
  
Location: 15349-16237

Mycgr3G67775\_Mycgr3T

Mycgr3G90404 Mycgr3T
  
Location: 16337-17246

Mycgr3G90404\_Mycgr3T

Mycgr3G36951 Mycgr3T
  
Location: 17346-30891

Mycgr3G36951\_Mycgr3T

Mycgr3G103034 Mycgr3
  
Location: 30991-32644

Mycgr3G103034\_Mycgr3

Mycgr3G31119 Mycgr3T
  
Location: 32744-32906

Mycgr3G31119\_Mycgr3T

Mycgr3G28587 Mycgr3T
  
Location: 33006-33489

Mycgr3G28587\_Mycgr3T

Mycgr3G98959 Mycgr3T
  
Location: 33589-35035

Mycgr3G98959\_Mycgr3T

Mycgr3G35447 Mycgr3T
  
Location: 35135-36443

Mycgr3G35447\_Mycgr3T

Mycgr3G84402 Mycgr3T
  
Location: 36543-37884

Mycgr3G84402\_Mycgr3T

Mycgr3G98961 Mycgr3T
  
Location: 37984-38884

Mycgr3G98961\_Mycgr3T

YALI0A12023p
  
Accession: CAG83928
  
Location: 1258715-1259593
  
 NCBI BlastP on this gene

YALI0\_A12023g

YALI0A12045p
  
Accession: CAG83929
  
Location: 1262156-1262653
  
 NCBI BlastP on this gene

YALI0\_A12045g

YALI0A12067p
  
Accession: CAG83930
  
Location: 1264497-1265117
  
 NCBI BlastP on this gene

YALI0\_A12067g

YALI0A12111p
  
Accession: CAG83932
  
Location: 1268323-1270002
  
 NCBI BlastP on this gene

YALI0\_A12111g

YALI0A12133p
  
Accession: CAG83933
  
Location: 1273269-1274711
  
  
**BlastP hit with Mycgr3G84402\_Mycgr3T**
  
Percentage identity: 64 %
  
BlastP bit score: 561
  
Sequence coverage: 89 %
  
E-value: 0.0
  
  
 NCBI BlastP on this gene

YALI0\_A12133g

YALI0A12155p
  
Accession: CAG83934
  
Location: 1275461-1276681
  
  
**BlastP hit with Mycgr3G35447\_Mycgr3T**
  
Percentage identity: 42 %
  
BlastP bit score: 280
  
Sequence coverage: 97 %
  
E-value: 4e-86
  
  
 NCBI BlastP on this gene

YALI0\_A12155g

YALI0A12177p
  
Accession: CAG83935
  
Location: 1276926-1278074
  
 NCBI BlastP on this gene

YALI0\_A12177g

YALI0A12199p
  
Accession: CAG83936
  
Location: 1278340-1278915
  
 NCBI BlastP on this gene

YALI0\_A12199g

YALI0A12221p
  
Accession: CAG83937
  
Location: 1280932-1281747
  
 NCBI BlastP on this gene

YALI0\_A12221g

YALI0A12243p
  
Accession: CAG83938
  
Location: 1282612-1283529
  
 NCBI BlastP on this gene

YALI0\_A12243g

YALI0A12265p
  
Accession: CAG83939
  
Location: 1283611-1284030
  
 NCBI BlastP on this gene

YALI0\_A12265g

YALI0A12287p
  
Accession: CAG83940
  
Location: 1284636-1287083
  
 NCBI BlastP on this gene

YALI0\_A12287g

YALI0A12353p
  
Accession: CAG83941
  
Location: 1288451-1289557
  
 NCBI BlastP on this gene

YALI0\_A12353g

YALI0A12419p
  
Accession: CAG83942
  
Location: 1294500-1294952
  
 NCBI BlastP on this gene

YALI0\_A12419g

YALI0A12441p
  
Accession: CAG83943
  
Location: 1295014-1296066
  
 NCBI BlastP on this gene

YALI0\_A12441g

Query: Architecture Search FASTA input

KB445649 : Cochliobolus sativus ND90Pr unplaced genomic scaffold COCSAscaffold\_13    Total score: 2.0     Cumulative Blast bit score: 820

Hit cluster cross-links:

Mycgr3G67791 Mycgr3T
  
Location: 0-1542

Mycgr3G67791\_Mycgr3T

Mycgr3G90406 Mycgr3T
  
Location: 1642-3973

Mycgr3G90406\_Mycgr3T

Mycgr3G67785 Mycgr3T
  
Location: 4073-7865

Mycgr3G67785\_Mycgr3T

Mycgr3G67795 Mycgr3T
  
Location: 7965-15249

Mycgr3G67795\_Mycgr3T

Mycgr3G67775 Mycgr3T
  
Location: 15349-16237

Mycgr3G67775\_Mycgr3T

Mycgr3G90404 Mycgr3T
  
Location: 16337-17246

Mycgr3G90404\_Mycgr3T

Mycgr3G36951 Mycgr3T
  
Location: 17346-30891

Mycgr3G36951\_Mycgr3T

Mycgr3G103034 Mycgr3
  
Location: 30991-32644

Mycgr3G103034\_Mycgr3

Mycgr3G31119 Mycgr3T
  
Location: 32744-32906

Mycgr3G31119\_Mycgr3T

Mycgr3G28587 Mycgr3T
  
Location: 33006-33489

Mycgr3G28587\_Mycgr3T

Mycgr3G98959 Mycgr3T
  
Location: 33589-35035

Mycgr3G98959\_Mycgr3T

Mycgr3G35447 Mycgr3T
  
Location: 35135-36443

Mycgr3G35447\_Mycgr3T

Mycgr3G84402 Mycgr3T
  
Location: 36543-37884

Mycgr3G84402\_Mycgr3T

Mycgr3G98961 Mycgr3T
  
Location: 37984-38884

Mycgr3G98961\_Mycgr3T

hypothetical protein
  
Accession: EMD61220
  
Location: 1249977-1250603
  
 NCBI BlastP on this gene

EMD61220

hypothetical protein
  
Accession: EMD61221
  
Location: 1250927-1251475
  
 NCBI BlastP on this gene

EMD61221

hypothetical protein
  
Accession: EMD61222
  
Location: 1252677-1253181
  
 NCBI BlastP on this gene

EMD61222

hypothetical protein
  
Accession: EMD61223
  
Location: 1253830-1254873
  
 NCBI BlastP on this gene

EMD61223

hypothetical protein
  
Accession: EMD61224
  
Location: 1255191-1257139
  
 NCBI BlastP on this gene

EMD61224

hypothetical protein
  
Accession: EMD61225
  
Location: 1257554-1259357
  
 NCBI BlastP on this gene

EMD61225

hypothetical protein
  
Accession: EMD61226
  
Location: 1259856-1261342
  
 NCBI BlastP on this gene

EMD61226

hypothetical protein
  
Accession: EMD61227
  
Location: 1262244-1264492
  
 NCBI BlastP on this gene

EMD61227

hypothetical protein
  
Accession: EMD61228
  
Location: 1264880-1265545
  
  
**BlastP hit with Mycgr3G28587\_Mycgr3T**
  
Percentage identity: 58 %
  
BlastP bit score: 191
  
Sequence coverage: 102 %
  
E-value: 4e-58
  
  
 NCBI BlastP on this gene

EMD61228

hypothetical protein
  
Accession: EMD61229
  
Location: 1266354-1267483
  
 NCBI BlastP on this gene

EMD61229

hypothetical protein
  
Accession: EMD61230
  
Location: 1267680-1267970
  
 NCBI BlastP on this gene

EMD61230

hypothetical protein
  
Accession: EMD61231
  
Location: 1268823-1272367
  
 NCBI BlastP on this gene

EMD61231

hypothetical protein
  
Accession: EMD61232
  
Location: 1273190-1275127
  
  
**BlastP hit with Mycgr3G103034\_Mycgr3**
  
Percentage identity: 56 %
  
BlastP bit score: 629
  
Sequence coverage: 97 %
  
E-value: 0.0
  
  
 NCBI BlastP on this gene

EMD61232

hypothetical protein
  
Accession: EMD61233
  
Location: 1276593-1279662
  
 NCBI BlastP on this gene

EMD61233

hypothetical protein
  
Accession: EMD61234
  
Location: 1282784-1283944
  
 NCBI BlastP on this gene

EMD61234

hypothetical protein
  
Accession: EMD61235
  
Location: 1284217-1285488
  
 NCBI BlastP on this gene

EMD61235

hypothetical protein
  
Accession: EMD61236
  
Location: 1286308-1287657
  
 NCBI BlastP on this gene

EMD61236

hypothetical protein
  
Accession: EMD61237
  
Location: 1288711-1289883
  
 NCBI BlastP on this gene

EMD61237

hypothetical protein
  
Accession: EMD61238
  
Location: 1290507-1291220
  
 NCBI BlastP on this gene

EMD61238

hypothetical protein
  
Accession: EMD61239
  
Location: 1291868-1293631
  
 NCBI BlastP on this gene

EMD61239

Query: Architecture Search FASTA input

AHHD01000101 : Macrophomina phaseolina MS6    Total score: 2.0     Cumulative Blast bit score: 812

Hit cluster cross-links:

Mycgr3G67791 Mycgr3T
  
Location: 0-1542

Mycgr3G67791\_Mycgr3T

Mycgr3G90406 Mycgr3T
  
Location: 1642-3973

Mycgr3G90406\_Mycgr3T

Mycgr3G67785 Mycgr3T
  
Location: 4073-7865

Mycgr3G67785\_Mycgr3T

Mycgr3G67795 Mycgr3T
  
Location: 7965-15249

Mycgr3G67795\_Mycgr3T

Mycgr3G67775 Mycgr3T
  
Location: 15349-16237

Mycgr3G67775\_Mycgr3T

Mycgr3G90404 Mycgr3T
  
Location: 16337-17246

Mycgr3G90404\_Mycgr3T

Mycgr3G36951 Mycgr3T
  
Location: 17346-30891

Mycgr3G36951\_Mycgr3T

Mycgr3G103034 Mycgr3
  
Location: 30991-32644

Mycgr3G103034\_Mycgr3

Mycgr3G31119 Mycgr3T
  
Location: 32744-32906

Mycgr3G31119\_Mycgr3T

Mycgr3G28587 Mycgr3T
  
Location: 33006-33489

Mycgr3G28587\_Mycgr3T

Mycgr3G98959 Mycgr3T
  
Location: 33589-35035

Mycgr3G98959\_Mycgr3T

Mycgr3G35447 Mycgr3T
  
Location: 35135-36443

Mycgr3G35447\_Mycgr3T

Mycgr3G84402 Mycgr3T
  
Location: 36543-37884

Mycgr3G84402\_Mycgr3T

Mycgr3G98961 Mycgr3T
  
Location: 37984-38884

Mycgr3G98961\_Mycgr3T

Cytochrome P450
  
Accession: EKG19908
  
Location: 129-1545
  
 NCBI BlastP on this gene

EKG19908

hypothetical protein
  
Accession: EKG19909
  
Location: 1607-2089
  
 NCBI BlastP on this gene

EKG19909

Short-chain dehydrogenase/reductase SDR
  
Accession: EKG19910
  
Location: 2927-4172
  
 NCBI BlastP on this gene

EKG19910

Ribosomal protein L15 conserved site
  
Accession: EKG19911
  
Location: 5724-6287
  
 NCBI BlastP on this gene

EKG19911

FeS cluster biogenesis
  
Accession: EKG19912
  
Location: 6602-6901
  
 NCBI BlastP on this gene

EKG19912

Aminotransferase class V/Cysteine desulfurase
  
Accession: EKG19913
  
Location: 7553-9127
  
 NCBI BlastP on this gene

EKG19913

stress response protein nst1
  
Accession: EKG19914
  
Location: 10960-14762
  
 NCBI BlastP on this gene

EKG19914

Ras GTPase
  
Accession: EKG19915
  
Location: 15764-16935
  
 NCBI BlastP on this gene

EKG19915

hypothetical protein
  
Accession: EKG19916
  
Location: 18083-18709
  
  
**BlastP hit with Mycgr3G28587\_Mycgr3T**
  
Percentage identity: 57 %
  
BlastP bit score: 187
  
Sequence coverage: 99 %
  
E-value: 7e-57
  
  
 NCBI BlastP on this gene

EKG19916

Alpha-D-phosphohexomutase
  
Accession: EKG19917
  
Location: 19169-21115
  
  
**BlastP hit with Mycgr3G103034\_Mycgr3**
  
Percentage identity: 57 %
  
BlastP bit score: 625
  
Sequence coverage: 98 %
  
E-value: 0.0
  
  
 NCBI BlastP on this gene

EKG19917

Inositol monophosphatase
  
Accession: EKG19918
  
Location: 21636-22638
  
 NCBI BlastP on this gene

EKG19918

hypothetical protein
  
Accession: EKG19919
  
Location: 25211-26209
  
 NCBI BlastP on this gene

EKG19919

Saccharopine dehydrogenase / Homospermidine synthase
  
Accession: EKG19920
  
Location: 27262-28245
  
 NCBI BlastP on this gene

EKG19920

hypothetical protein
  
Accession: EKG19921
  
Location: 28298-28795
  
 NCBI BlastP on this gene

EKG19921

hypothetical protein
  
Accession: EKG19922
  
Location: 30120-30536
  
 NCBI BlastP on this gene

EKG19922

hypothetical protein
  
Accession: EKG19923
  
Location: 30574-30921
  
 NCBI BlastP on this gene

EKG19923

Major intrinsic protein
  
Accession: EKG19924
  
Location: 33367-34736
  
 NCBI BlastP on this gene

EKG19924

Carbohydrate kinase FGGY
  
Accession: EKG19925
  
Location: 36360-38332
  
 NCBI BlastP on this gene

EKG19925

HMG-I/HMG-Y DNA-binding conserved site
  
Accession: EKG19926
  
Location: 39084-40128
  
 NCBI BlastP on this gene

EKG19926

Query: Architecture Search FASTA input

KB733482 : Bipolaris maydis ATCC 48331 unplaced genomic scaffold COCC4scaffold\_39    Total score: 2.0     Cumulative Blast bit score: 808

Hit cluster cross-links:

Mycgr3G67791 Mycgr3T
  
Location: 0-1542

Mycgr3G67791\_Mycgr3T

Mycgr3G90406 Mycgr3T
  
Location: 1642-3973

Mycgr3G90406\_Mycgr3T

Mycgr3G67785 Mycgr3T
  
Location: 4073-7865

Mycgr3G67785\_Mycgr3T

Mycgr3G67795 Mycgr3T
  
Location: 7965-15249

Mycgr3G67795\_Mycgr3T

Mycgr3G67775 Mycgr3T
  
Location: 15349-16237

Mycgr3G67775\_Mycgr3T

Mycgr3G90404 Mycgr3T
  
Location: 16337-17246

Mycgr3G90404\_Mycgr3T

Mycgr3G36951 Mycgr3T
  
Location: 17346-30891

Mycgr3G36951\_Mycgr3T

Mycgr3G103034 Mycgr3
  
Location: 30991-32644

Mycgr3G103034\_Mycgr3

Mycgr3G31119 Mycgr3T
  
Location: 32744-32906

Mycgr3G31119\_Mycgr3T

Mycgr3G28587 Mycgr3T
  
Location: 33006-33489

Mycgr3G28587\_Mycgr3T

Mycgr3G98959 Mycgr3T
  
Location: 33589-35035

Mycgr3G98959\_Mycgr3T

Mycgr3G35447 Mycgr3T
  
Location: 35135-36443

Mycgr3G35447\_Mycgr3T

Mycgr3G84402 Mycgr3T
  
Location: 36543-37884

Mycgr3G84402\_Mycgr3T

Mycgr3G98961 Mycgr3T
  
Location: 37984-38884

Mycgr3G98961\_Mycgr3T

hypothetical protein
  
Accession: ENH99718
  
Location: 66480-68672
  
 NCBI BlastP on this gene

ENH99718

hypothetical protein
  
Accession: ENH99719
  
Location: 69713-71190
  
 NCBI BlastP on this gene

ENH99719

hypothetical protein
  
Accession: ENH99720
  
Location: 71517-72065
  
 NCBI BlastP on this gene

ENH99720

hypothetical protein
  
Accession: ENH99721
  
Location: 73506-73673
  
 NCBI BlastP on this gene

ENH99721

hypothetical protein
  
Accession: ENH99722
  
Location: 74280-75327
  
 NCBI BlastP on this gene

ENH99722

hypothetical protein
  
Accession: ENH99723
  
Location: 75708-77657
  
 NCBI BlastP on this gene

ENH99723

hypothetical protein
  
Accession: ENH99724
  
Location: 78070-79873
  
 NCBI BlastP on this gene

ENH99724

hypothetical protein
  
Accession: ENH99725
  
Location: 80371-81854
  
 NCBI BlastP on this gene

ENH99725

hypothetical protein
  
Accession: ENH99726
  
Location: 82206-82628
  
 NCBI BlastP on this gene

ENH99726

hypothetical protein
  
Accession: ENH99727
  
Location: 82756-85004
  
 NCBI BlastP on this gene

ENH99727

hypothetical protein
  
Accession: ENH99728
  
Location: 85392-86075
  
  
**BlastP hit with Mycgr3G28587\_Mycgr3T**
  
Percentage identity: 56 %
  
BlastP bit score: 182
  
Sequence coverage: 102 %
  
E-value: 1e-54
  
  
 NCBI BlastP on this gene

ENH99728

hypothetical protein
  
Accession: ENH99729
  
Location: 86963-88075
  
 NCBI BlastP on this gene

ENH99729

hypothetical protein
  
Accession: ENH99730
  
Location: 89383-92932
  
 NCBI BlastP on this gene

ENH99730

hypothetical protein
  
Accession: ENH99731
  
Location: 93749-95700
  
  
**BlastP hit with Mycgr3G103034\_Mycgr3**
  
Percentage identity: 56 %
  
BlastP bit score: 626
  
Sequence coverage: 97 %
  
E-value: 0.0
  
  
 NCBI BlastP on this gene

ENH99731

hypothetical protein
  
Accession: ENH99732
  
Location: 97209-100326
  
 NCBI BlastP on this gene

ENH99732

hypothetical protein
  
Accession: ENH99733
  
Location: 102832-103275
  
 NCBI BlastP on this gene

ENH99733

hypothetical protein
  
Accession: ENH99734
  
Location: 103877-105037
  
 NCBI BlastP on this gene

ENH99734

hypothetical protein
  
Accession: ENH99735
  
Location: 105337-106632
  
 NCBI BlastP on this gene

ENH99735

hypothetical protein
  
Accession: ENH99736
  
Location: 107469-108840
  
 NCBI BlastP on this gene

ENH99736

hypothetical protein
  
Accession: ENH99737
  
Location: 109877-111067
  
 NCBI BlastP on this gene

ENH99737

hypothetical protein
  
Accession: ENH99738
  
Location: 111696-112406
  
 NCBI BlastP on this gene

ENH99738

Query: Architecture Search FASTA input

KB445579 : Cochliobolus heterostrophus C5 unplaced genomic scaffold COCHEscaffold\_11    Total score: 2.0     Cumulative Blast bit score: 808

Hit cluster cross-links:

Mycgr3G67791 Mycgr3T
  
Location: 0-1542

Mycgr3G67791\_Mycgr3T

Mycgr3G90406 Mycgr3T
  
Location: 1642-3973

Mycgr3G90406\_Mycgr3T

Mycgr3G67785 Mycgr3T
  
Location: 4073-7865

Mycgr3G67785\_Mycgr3T

Mycgr3G67795 Mycgr3T
  
Location: 7965-15249

Mycgr3G67795\_Mycgr3T

Mycgr3G67775 Mycgr3T
  
Location: 15349-16237

Mycgr3G67775\_Mycgr3T

Mycgr3G90404 Mycgr3T
  
Location: 16337-17246

Mycgr3G90404\_Mycgr3T

Mycgr3G36951 Mycgr3T
  
Location: 17346-30891

Mycgr3G36951\_Mycgr3T

Mycgr3G103034 Mycgr3
  
Location: 30991-32644

Mycgr3G103034\_Mycgr3

Mycgr3G31119 Mycgr3T
  
Location: 32744-32906

Mycgr3G31119\_Mycgr3T

Mycgr3G28587 Mycgr3T
  
Location: 33006-33489

Mycgr3G28587\_Mycgr3T

Mycgr3G98959 Mycgr3T
  
Location: 33589-35035

Mycgr3G98959\_Mycgr3T

Mycgr3G35447 Mycgr3T
  
Location: 35135-36443

Mycgr3G35447\_Mycgr3T

Mycgr3G84402 Mycgr3T
  
Location: 36543-37884

Mycgr3G84402\_Mycgr3T

Mycgr3G98961 Mycgr3T
  
Location: 37984-38884

Mycgr3G98961\_Mycgr3T

hypothetical protein
  
Accession: EMD89463
  
Location: 1245765-1247957
  
 NCBI BlastP on this gene

EMD89463

hypothetical protein
  
Accession: EMD89464
  
Location: 1248998-1250475
  
 NCBI BlastP on this gene

EMD89464

hypothetical protein
  
Accession: EMD89465
  
Location: 1250802-1251350
  
 NCBI BlastP on this gene

EMD89465

hypothetical protein
  
Accession: EMD89466
  
Location: 1252791-1252958
  
 NCBI BlastP on this gene

EMD89466

hypothetical protein
  
Accession: EMD89467
  
Location: 1253565-1254612
  
 NCBI BlastP on this gene

EMD89467

hypothetical protein
  
Accession: EMD89468
  
Location: 1254993-1256942
  
 NCBI BlastP on this gene

EMD89468

hypothetical protein
  
Accession: EMD89469
  
Location: 1257372-1259175
  
 NCBI BlastP on this gene

EMD89469

hypothetical protein
  
Accession: EMD89470
  
Location: 1259673-1261156
  
 NCBI BlastP on this gene

EMD89470

hypothetical protein
  
Accession: EMD89471
  
Location: 1261508-1261930
  
 NCBI BlastP on this gene

EMD89471

hypothetical protein
  
Accession: EMD89472
  
Location: 1262058-1264306
  
 NCBI BlastP on this gene

EMD89472

hypothetical protein
  
Accession: EMD89473
  
Location: 1264694-1265377
  
  
**BlastP hit with Mycgr3G28587\_Mycgr3T**
  
Percentage identity: 56 %
  
BlastP bit score: 182
  
Sequence coverage: 102 %
  
E-value: 1e-54
  
  
 NCBI BlastP on this gene

EMD89473

hypothetical protein
  
Accession: EMD89474
  
Location: 1266265-1267377
  
 NCBI BlastP on this gene

EMD89474

hypothetical protein
  
Accession: EMD89475
  
Location: 1268733-1272282
  
 NCBI BlastP on this gene

EMD89475

hypothetical protein
  
Accession: EMD89476
  
Location: 1273099-1275050
  
  
**BlastP hit with Mycgr3G103034\_Mycgr3**
  
Percentage identity: 56 %
  
BlastP bit score: 626
  
Sequence coverage: 97 %
  
E-value: 0.0
  
  
 NCBI BlastP on this gene

EMD89476

hypothetical protein
  
Accession: EMD89477
  
Location: 1276559-1279676
  
 NCBI BlastP on this gene

EMD89477

hypothetical protein
  
Accession: EMD89478
  
Location: 1282277-1282720
  
 NCBI BlastP on this gene

EMD89478

hypothetical protein
  
Accession: EMD89479
  
Location: 1283322-1284482
  
 NCBI BlastP on this gene

EMD89479

hypothetical protein
  
Accession: EMD89480
  
Location: 1284782-1286077
  
 NCBI BlastP on this gene

EMD89480

hypothetical protein
  
Accession: EMD89481
  
Location: 1286914-1288285
  
 NCBI BlastP on this gene

EMD89481

hypothetical protein
  
Accession: EMD89482
  
Location: 1289322-1290512
  
 NCBI BlastP on this gene

EMD89482

hypothetical protein
  
Accession: EMD89483
  
Location: 1291141-1291851
  
 NCBI BlastP on this gene

EMD89483

Query: Architecture Search FASTA input

GL534835 : Pyrenophora teres f. teres 0-1 unplaced genomic scaffold scaffold\_191097    Total score: 2.0     Cumulative Blast bit score: 799

Hit cluster cross-links:

Mycgr3G67791 Mycgr3T
  
Location: 0-1542

Mycgr3G67791\_Mycgr3T

Mycgr3G90406 Mycgr3T
  
Location: 1642-3973

Mycgr3G90406\_Mycgr3T

Mycgr3G67785 Mycgr3T
  
Location: 4073-7865

Mycgr3G67785\_Mycgr3T

Mycgr3G67795 Mycgr3T
  
Location: 7965-15249

Mycgr3G67795\_Mycgr3T

Mycgr3G67775 Mycgr3T
  
Location: 15349-16237

Mycgr3G67775\_Mycgr3T

Mycgr3G90404 Mycgr3T
  
Location: 16337-17246

Mycgr3G90404\_Mycgr3T

Mycgr3G36951 Mycgr3T
  
Location: 17346-30891

Mycgr3G36951\_Mycgr3T

Mycgr3G103034 Mycgr3
  
Location: 30991-32644

Mycgr3G103034\_Mycgr3

Mycgr3G31119 Mycgr3T
  
Location: 32744-32906

Mycgr3G31119\_Mycgr3T

Mycgr3G28587 Mycgr3T
  
Location: 33006-33489

Mycgr3G28587\_Mycgr3T

Mycgr3G98959 Mycgr3T
  
Location: 33589-35035

Mycgr3G98959\_Mycgr3T

Mycgr3G35447 Mycgr3T
  
Location: 35135-36443

Mycgr3G35447\_Mycgr3T

Mycgr3G84402 Mycgr3T
  
Location: 36543-37884

Mycgr3G84402\_Mycgr3T

Mycgr3G98961 Mycgr3T
  
Location: 37984-38884

Mycgr3G98961\_Mycgr3T

hypothetical protein
  
Accession: EFQ91313
  
Location: 23118-23839
  
  
**BlastP hit with Mycgr3G28587\_Mycgr3T**
  
Percentage identity: 59 %
  
BlastP bit score: 177
  
Sequence coverage: 96 %
  
E-value: 6e-53
  
  
 NCBI BlastP on this gene

EFQ91313

hypothetical protein
  
Accession: EFQ91312
  
Location: 21473-22595
  
 NCBI BlastP on this gene

EFQ91312

hypothetical protein
  
Accession: EFQ91311
  
Location: 16943-20397
  
 NCBI BlastP on this gene

EFQ91311

hypothetical protein
  
Accession: EFQ91310
  
Location: 14170-16118
  
  
**BlastP hit with Mycgr3G103034\_Mycgr3**
  
Percentage identity: 56 %
  
BlastP bit score: 622
  
Sequence coverage: 97 %
  
E-value: 0.0
  
  
 NCBI BlastP on this gene

EFQ91310

hypothetical protein
  
Accession: EFQ91309
  
Location: 9895-12875
  
 NCBI BlastP on this gene

EFQ91309

hypothetical protein
  
Accession: EFQ91308
  
Location: 6064-7212
  
 NCBI BlastP on this gene

EFQ91308

hypothetical protein
  
Accession: EFQ91307
  
Location: 4596-5849
  
 NCBI BlastP on this gene

EFQ91307

hypothetical protein
  
Accession: EFQ91306
  
Location: 3096-3815
  
 NCBI BlastP on this gene

EFQ91306

hypothetical protein
  
Accession: EFQ91305
  
Location: 688-1650
  
 NCBI BlastP on this gene

EFQ91305

Query: Architecture Search FASTA input

KB908844 : Setosphaeria turcica Et28A unplaced genomic scaffold SETTUscaffold\_6    Total score: 2.0     Cumulative Blast bit score: 791

Hit cluster cross-links:

Mycgr3G67791 Mycgr3T
  
Location: 0-1542

Mycgr3G67791\_Mycgr3T

Mycgr3G90406 Mycgr3T
  
Location: 1642-3973

Mycgr3G90406\_Mycgr3T

Mycgr3G67785 Mycgr3T
  
Location: 4073-7865

Mycgr3G67785\_Mycgr3T

Mycgr3G67795 Mycgr3T
  
Location: 7965-15249

Mycgr3G67795\_Mycgr3T

Mycgr3G67775 Mycgr3T
  
Location: 15349-16237

Mycgr3G67775\_Mycgr3T

Mycgr3G90404 Mycgr3T
  
Location: 16337-17246

Mycgr3G90404\_Mycgr3T

Mycgr3G36951 Mycgr3T
  
Location: 17346-30891

Mycgr3G36951\_Mycgr3T

Mycgr3G103034 Mycgr3
  
Location: 30991-32644

Mycgr3G103034\_Mycgr3

Mycgr3G31119 Mycgr3T
  
Location: 32744-32906

Mycgr3G31119\_Mycgr3T

Mycgr3G28587 Mycgr3T
  
Location: 33006-33489

Mycgr3G28587\_Mycgr3T

Mycgr3G98959 Mycgr3T
  
Location: 33589-35035

Mycgr3G98959\_Mycgr3T

Mycgr3G35447 Mycgr3T
  
Location: 35135-36443

Mycgr3G35447\_Mycgr3T

Mycgr3G84402 Mycgr3T
  
Location: 36543-37884

Mycgr3G84402\_Mycgr3T

Mycgr3G98961 Mycgr3T
  
Location: 37984-38884

Mycgr3G98961\_Mycgr3T

hypothetical protein
  
Accession: EOA82440
  
Location: 247550-249623
  
 NCBI BlastP on this gene

EOA82440

hypothetical protein
  
Accession: EOA82439
  
Location: 245953-246506
  
 NCBI BlastP on this gene

EOA82439

hypothetical protein
  
Accession: EOA82438
  
Location: 243992-244972
  
 NCBI BlastP on this gene

EOA82438

hypothetical protein
  
Accession: EOA82437
  
Location: 242013-243921
  
 NCBI BlastP on this gene

EOA82437

hypothetical protein
  
Accession: EOA82436
  
Location: 239812-241589
  
 NCBI BlastP on this gene

EOA82436

hypothetical protein
  
Accession: EOA82435
  
Location: 235797-237188
  
 NCBI BlastP on this gene

EOA82435

hypothetical protein
  
Accession: EOA82434
  
Location: 232886-235086
  
 NCBI BlastP on this gene

EOA82434

hypothetical protein
  
Accession: EOA82433
  
Location: 231903-232644
  
  
**BlastP hit with Mycgr3G28587\_Mycgr3T**
  
Percentage identity: 56 %
  
BlastP bit score: 168
  
Sequence coverage: 96 %
  
E-value: 4e-49
  
  
 NCBI BlastP on this gene

EOA82433

hypothetical protein
  
Accession: EOA82432
  
Location: 230033-231126
  
 NCBI BlastP on this gene

EOA82432

hypothetical protein
  
Accession: EOA82431
  
Location: 221801-224729
  
 NCBI BlastP on this gene

EOA82431

hypothetical protein
  
Accession: EOA82430
  
Location: 219034-220964
  
  
**BlastP hit with Mycgr3G103034\_Mycgr3**
  
Percentage identity: 55 %
  
BlastP bit score: 623
  
Sequence coverage: 97 %
  
E-value: 0.0
  
  
 NCBI BlastP on this gene

EOA82430

hypothetical protein
  
Accession: EOA82429
  
Location: 214860-217764
  
 NCBI BlastP on this gene

EOA82429

hypothetical protein
  
Accession: EOA82428
  
Location: 211344-212762
  
 NCBI BlastP on this gene

EOA82428

hypothetical protein
  
Accession: EOA82427
  
Location: 208618-209034
  
 NCBI BlastP on this gene

EOA82427

hypothetical protein
  
Accession: EOA82426
  
Location: 205783-206886
  
 NCBI BlastP on this gene

EOA82426

hypothetical protein
  
Accession: EOA82425
  
Location: 204494-205696
  
 NCBI BlastP on this gene

EOA82425

Query: Architecture Search FASTA input

JH767590 : Coniosporium apollinis CBS 100218 chromosome Unknown supercont1.37    Total score: 2.0     Cumulative Blast bit score: 783

Hit cluster cross-links:

Mycgr3G67791 Mycgr3T
  
Location: 0-1542

Mycgr3G67791\_Mycgr3T

Mycgr3G90406 Mycgr3T
  
Location: 1642-3973

Mycgr3G90406\_Mycgr3T

Mycgr3G67785 Mycgr3T
  
Location: 4073-7865

Mycgr3G67785\_Mycgr3T

Mycgr3G67795 Mycgr3T
  
Location: 7965-15249

Mycgr3G67795\_Mycgr3T

Mycgr3G67775 Mycgr3T
  
Location: 15349-16237

Mycgr3G67775\_Mycgr3T

Mycgr3G90404 Mycgr3T
  
Location: 16337-17246

Mycgr3G90404\_Mycgr3T

Mycgr3G36951 Mycgr3T
  
Location: 17346-30891

Mycgr3G36951\_Mycgr3T

Mycgr3G103034 Mycgr3
  
Location: 30991-32644

Mycgr3G103034\_Mycgr3

Mycgr3G31119 Mycgr3T
  
Location: 32744-32906

Mycgr3G31119\_Mycgr3T

Mycgr3G28587 Mycgr3T
  
Location: 33006-33489

Mycgr3G28587\_Mycgr3T

Mycgr3G98959 Mycgr3T
  
Location: 33589-35035

Mycgr3G98959\_Mycgr3T

Mycgr3G35447 Mycgr3T
  
Location: 35135-36443

Mycgr3G35447\_Mycgr3T

Mycgr3G84402 Mycgr3T
  
Location: 36543-37884

Mycgr3G84402\_Mycgr3T

Mycgr3G98961 Mycgr3T
  
Location: 37984-38884

Mycgr3G98961\_Mycgr3T

hypothetical protein
  
Accession: EON67768
  
Location: 121676-123090
  
 NCBI BlastP on this gene

EON67768

hypothetical protein
  
Accession: EON67769
  
Location: 124560-126986
  
 NCBI BlastP on this gene

EON67769

hypothetical protein
  
Accession: EON67770
  
Location: 127363-128994
  
 NCBI BlastP on this gene

EON67770

hypothetical protein
  
Accession: EON67771
  
Location: 130295-130642
  
 NCBI BlastP on this gene

EON67771

kynureninase
  
Accession: EON67772
  
Location: 131239-132941
  
 NCBI BlastP on this gene

EON67772

hypothetical protein
  
Accession: EON67773
  
Location: 133231-137011
  
 NCBI BlastP on this gene

EON67773

GTP-binding protein rho2
  
Accession: EON67774
  
Location: 138539-139235
  
 NCBI BlastP on this gene

EON67774

hypothetical protein
  
Accession: EON67775
  
Location: 139652-140309
  
  
**BlastP hit with Mycgr3G28587\_Mycgr3T**
  
Percentage identity: 61 %
  
BlastP bit score: 166
  
Sequence coverage: 97 %
  
E-value: 1e-48
  
  
 NCBI BlastP on this gene

EON67775

phosphoacetylglucosamine mutase
  
Accession: EON67776
  
Location: 140692-142587
  
  
**BlastP hit with Mycgr3G103034\_Mycgr3**
  
Percentage identity: 56 %
  
BlastP bit score: 617
  
Sequence coverage: 98 %
  
E-value: 0.0
  
  
 NCBI BlastP on this gene

EON67776

hypothetical protein
  
Accession: EON67777
  
Location: 143051-144481
  
 NCBI BlastP on this gene

EON67777

hypothetical protein
  
Accession: EON67778
  
Location: 145160-146155
  
 NCBI BlastP on this gene

EON67778

hypothetical protein
  
Accession: EON67779
  
Location: 146855-147868
  
 NCBI BlastP on this gene

EON67779

hypothetical protein
  
Accession: EON67780
  
Location: 148254-149202
  
 NCBI BlastP on this gene

EON67780

26S protease regulatory subunit 6A
  
Accession: EON67781
  
Location: 149651-151052
  
 NCBI BlastP on this gene

EON67781

hypothetical protein
  
Accession: EON67782
  
Location: 151891-152451
  
 NCBI BlastP on this gene

EON67782

hypothetical protein
  
Accession: EON67783
  
Location: 153191-156870
  
 NCBI BlastP on this gene

EON67783

hypothetical protein
  
Accession: EON67784
  
Location: 158408-159100
  
 NCBI BlastP on this gene

EON67784

hypothetical protein
  
Accession: EON67785
  
Location: 160008-161806
  
 NCBI BlastP on this gene

EON67785

lysophospholipase NTE1
  
Accession: EON67786
  
Location: 162441-167252
  
 NCBI BlastP on this gene

EON67786

Query: Architecture Search FASTA input

KB916790 : Neofusicoccum parvum UCRNP2 chromosome Unknown NP2\_03\_scaffold\_1152    Total score: 2.0     Cumulative Blast bit score: 775

Hit cluster cross-links:

Mycgr3G67791 Mycgr3T
  
Location: 0-1542

Mycgr3G67791\_Mycgr3T

Mycgr3G90406 Mycgr3T
  
Location: 1642-3973

Mycgr3G90406\_Mycgr3T

Mycgr3G67785 Mycgr3T
  
Location: 4073-7865

Mycgr3G67785\_Mycgr3T

Mycgr3G67795 Mycgr3T
  
Location: 7965-15249

Mycgr3G67795\_Mycgr3T

Mycgr3G67775 Mycgr3T
  
Location: 15349-16237

Mycgr3G67775\_Mycgr3T

Mycgr3G90404 Mycgr3T
  
Location: 16337-17246

Mycgr3G90404\_Mycgr3T

Mycgr3G36951 Mycgr3T
  
Location: 17346-30891

Mycgr3G36951\_Mycgr3T

Mycgr3G103034 Mycgr3
  
Location: 30991-32644

Mycgr3G103034\_Mycgr3

Mycgr3G31119 Mycgr3T
  
Location: 32744-32906

Mycgr3G31119\_Mycgr3T

Mycgr3G28587 Mycgr3T
  
Location: 33006-33489

Mycgr3G28587\_Mycgr3T

Mycgr3G98959 Mycgr3T
  
Location: 33589-35035

Mycgr3G98959\_Mycgr3T

Mycgr3G35447 Mycgr3T
  
Location: 35135-36443

Mycgr3G35447\_Mycgr3T

Mycgr3G84402 Mycgr3T
  
Location: 36543-37884

Mycgr3G84402\_Mycgr3T

Mycgr3G98961 Mycgr3T
  
Location: 37984-38884

Mycgr3G98961\_Mycgr3T

putative inositol kinase kinase protein
  
Accession: EOD43838
  
Location: 35101-36229
  
 NCBI BlastP on this gene

EOD43838

putative tripeptidyl-peptidase 1 protein
  
Accession: EOD43848
  
Location: 36625-38379
  
 NCBI BlastP on this gene

EOD43848

putative peptidase a1 protein
  
Accession: EOD43845
  
Location: 39307-40611
  
 NCBI BlastP on this gene

EOD43845

hypothetical protein
  
Accession: EOD43855
  
Location: 41735-42108
  
 NCBI BlastP on this gene

EOD43855

hypothetical protein
  
Accession: EOD43835
  
Location: 44088-45220
  
 NCBI BlastP on this gene

EOD43835

putative inositol monophosphatase protein
  
Accession: EOD43854
  
Location: 49536-50816
  
 NCBI BlastP on this gene

EOD43854

putative n-acetylglucosamine-phosphate mutase protein
  
Accession: EOD43852
  
Location: 51293-53266
  
  
**BlastP hit with Mycgr3G103034\_Mycgr3**
  
Percentage identity: 57 %
  
BlastP bit score: 619
  
Sequence coverage: 98 %
  
E-value: 0.0
  
  
 NCBI BlastP on this gene

EOD43852

putative ring finger domain protein
  
Accession: EOD43849
  
Location: 53970-54371
  
  
**BlastP hit with Mycgr3G28587\_Mycgr3T**
  
Percentage identity: 59 %
  
BlastP bit score: 156
  
Sequence coverage: 78 %
  
E-value: 2e-45
  
  
 NCBI BlastP on this gene

EOD43849

putative rho gtpase rho protein
  
Accession: EOD43851
  
Location: 55240-55812
  
 NCBI BlastP on this gene

EOD43851

putative stress response protein nst1 protein
  
Accession: EOD43843
  
Location: 57462-59659
  
 NCBI BlastP on this gene

EOD43843

putative kynureninase protein
  
Accession: EOD43858
  
Location: 62728-64288
  
 NCBI BlastP on this gene

EOD43858

putative 60s ribosomal protein l28 protein
  
Accession: EOD43857
  
Location: 65592-66557
  
 NCBI BlastP on this gene

EOD43857

Query: Architecture Search FASTA input

CAIF01000186 : Wickerhamomyces ciferrii strain NRRL Y-1031 F-60-10    Total score: 2.0     Cumulative Blast bit score: 773

Hit cluster cross-links:

Mycgr3G67791 Mycgr3T
  
Location: 0-1542

Mycgr3G67791\_Mycgr3T

Mycgr3G90406 Mycgr3T
  
Location: 1642-3973

Mycgr3G90406\_Mycgr3T

Mycgr3G67785 Mycgr3T
  
Location: 4073-7865

Mycgr3G67785\_Mycgr3T

Mycgr3G67795 Mycgr3T
  
Location: 7965-15249

Mycgr3G67795\_Mycgr3T

Mycgr3G67775 Mycgr3T
  
Location: 15349-16237

Mycgr3G67775\_Mycgr3T

Mycgr3G90404 Mycgr3T
  
Location: 16337-17246

Mycgr3G90404\_Mycgr3T

Mycgr3G36951 Mycgr3T
  
Location: 17346-30891

Mycgr3G36951\_Mycgr3T

Mycgr3G103034 Mycgr3
  
Location: 30991-32644

Mycgr3G103034\_Mycgr3

Mycgr3G31119 Mycgr3T
  
Location: 32744-32906

Mycgr3G31119\_Mycgr3T

Mycgr3G28587 Mycgr3T
  
Location: 33006-33489

Mycgr3G28587\_Mycgr3T

Mycgr3G98959 Mycgr3T
  
Location: 33589-35035

Mycgr3G98959\_Mycgr3T

Mycgr3G35447 Mycgr3T
  
Location: 35135-36443

Mycgr3G35447\_Mycgr3T

Mycgr3G84402 Mycgr3T
  
Location: 36543-37884

Mycgr3G84402\_Mycgr3T

Mycgr3G98961 Mycgr3T
  
Location: 37984-38884

Mycgr3G98961\_Mycgr3T

Transcription factor tau subunit
  
Accession: CCH45239
  
Location: 12315-13871
  
 NCBI BlastP on this gene

CCH45239

54S ribosomal protein L36, mitochondrial
  
Accession: CCH45240
  
Location: 14421-14975
  
 NCBI BlastP on this gene

CCH45240

glycyl-tRNA synthetase
  
Accession: CCH45241
  
Location: 16434-18410
  
 NCBI BlastP on this gene

CCH45241

Cytochrome B pre-mRNA-processing protein 6
  
Accession: CCH45242
  
Location: 18657-19082
  
 NCBI BlastP on this gene

CCH45242

U1 small nuclear ribonucleoprotein
  
Accession: CCH45243
  
Location: 19277-19975
  
 NCBI BlastP on this gene

CCH45243

Myosin-11
  
Accession: CCH45244
  
Location: 20215-23505
  
 NCBI BlastP on this gene

CCH45244

Uric acid-xanthine permease
  
Accession: CCH45245
  
Location: 23729-25491
  
 NCBI BlastP on this gene

CCH45245

Ubiquinone/menaquinone biosynthesis methyltransferase
  
Accession: CCH45246
  
Location: 28156-30522
  
 NCBI BlastP on this gene

CCH45246

ATP-dependent rRNA helicase RRP3
  
Accession: CCH45247
  
Location: 30750-32216
  
  
**BlastP hit with Mycgr3G84402\_Mycgr3T**
  
Percentage identity: 59 %
  
BlastP bit score: 486
  
Sequence coverage: 89 %
  
E-value: 5e-165
  
  
 NCBI BlastP on this gene

CCH45247

Suppressor of SWI4 1
  
Accession: CCH45248
  
Location: 32603-33823
  
  
**BlastP hit with Mycgr3G35447\_Mycgr3T**
  
Percentage identity: 43 %
  
BlastP bit score: 287
  
Sequence coverage: 81 %
  
E-value: 8e-89
  
  
 NCBI BlastP on this gene

CCH45248

E3 ubiquitin-protein ligase
  
Accession: CCH45249
  
Location: 34413-35219
  
 NCBI BlastP on this gene

CCH45249

Auxilin-like clathrin uncoating factor SWA2
  
Accession: CCH45250
  
Location: 36961-39438
  
 NCBI BlastP on this gene

CCH45250

RNA polymerase II transcription factor B subunit 1
  
Accession: CCH45251
  
Location: 39557-41299
  
 NCBI BlastP on this gene

CCH45251

Heat shock 70 kDa protein
  
Accession: CCH45252
  
Location: 41592-43226
  
 NCBI BlastP on this gene

CCH45252

putative secreted protein
  
Accession: CCH45253
  
Location: 43562-44620
  
 NCBI BlastP on this gene

CCH45253

Nucleoporin
  
Accession: CCH45254
  
Location: 44876-49852
  
 NCBI BlastP on this gene

CCH45254

Nucleoporin
  
Accession: CCH45255
  
Location: 49995-51811
  
 NCBI BlastP on this gene

CCH45255

hypothetical protein
  
Accession: CCH45256
  
Location: 52354-53094
  
 NCBI BlastP on this gene

CCH45256

MAP-ous protein 1
  
Accession: CCH45257
  
Location: 53331-57044
  
 NCBI BlastP on this gene

CCH45257

Query: Architecture Search FASTA input

CH408161 : Pichia guilliermondii ATCC 6260 scaffold\_7 genomic scaffold    Total score: 2.0     Cumulative Blast bit score: 772

Hit cluster cross-links:

Mycgr3G67791 Mycgr3T
  
Location: 0-1542

Mycgr3G67791\_Mycgr3T

Mycgr3G90406 Mycgr3T
  
Location: 1642-3973

Mycgr3G90406\_Mycgr3T

Mycgr3G67785 Mycgr3T
  
Location: 4073-7865

Mycgr3G67785\_Mycgr3T

Mycgr3G67795 Mycgr3T
  
Location: 7965-15249

Mycgr3G67795\_Mycgr3T

Mycgr3G67775 Mycgr3T
  
Location: 15349-16237

Mycgr3G67775\_Mycgr3T

Mycgr3G90404 Mycgr3T
  
Location: 16337-17246

Mycgr3G90404\_Mycgr3T

Mycgr3G36951 Mycgr3T
  
Location: 17346-30891

Mycgr3G36951\_Mycgr3T

Mycgr3G103034 Mycgr3
  
Location: 30991-32644

Mycgr3G103034\_Mycgr3

Mycgr3G31119 Mycgr3T
  
Location: 32744-32906

Mycgr3G31119\_Mycgr3T

Mycgr3G28587 Mycgr3T
  
Location: 33006-33489

Mycgr3G28587\_Mycgr3T

Mycgr3G98959 Mycgr3T
  
Location: 33589-35035

Mycgr3G98959\_Mycgr3T

Mycgr3G35447 Mycgr3T
  
Location: 35135-36443

Mycgr3G35447\_Mycgr3T

Mycgr3G84402 Mycgr3T
  
Location: 36543-37884

Mycgr3G84402\_Mycgr3T

Mycgr3G98961 Mycgr3T
  
Location: 37984-38884

Mycgr3G98961\_Mycgr3T

hypothetical protein
  
Accession: EDK41390
  
Location: 576330-578711
  
 NCBI BlastP on this gene

EDK41390

hypothetical protein
  
Accession: EDK41391
  
Location: 578767-579711
  
 NCBI BlastP on this gene

EDK41391

hypothetical protein
  
Accession: EDK41392
  
Location: 580595-581689
  
 NCBI BlastP on this gene

EDK41392

40S ribosomal protein S23
  
Accession: EDK41393
  
Location: 582042-582479
  
 NCBI BlastP on this gene

EDK41393

hypothetical protein
  
Accession: EDK41394
  
Location: 582868-583869
  
 NCBI BlastP on this gene

EDK41394

hypothetical protein
  
Accession: EDK41395
  
Location: 584438-585679
  
 NCBI BlastP on this gene

EDK41395

hypothetical protein
  
Accession: EDK41396
  
Location: 586042-587220
  
 NCBI BlastP on this gene

EDK41396

hypothetical protein
  
Accession: EDK41397
  
Location: 587613-588497
  
 NCBI BlastP on this gene

EDK41397

hypothetical protein
  
Accession: EDK41398
  
Location: 588645-589685
  
 NCBI BlastP on this gene

EDK41398

hypothetical protein
  
Accession: EDK41399
  
Location: 589847-591166
  
 NCBI BlastP on this gene

EDK41399

hypothetical protein
  
Accession: EDK41400
  
Location: 591805-592920
  
 NCBI BlastP on this gene

EDK41400

hypothetical protein
  
Accession: EDK41401
  
Location: 593168-594238
  
 NCBI BlastP on this gene

EDK41401

hypothetical protein
  
Accession: EDK41402
  
Location: 594294-594857
  
 NCBI BlastP on this gene

EDK41402

hypothetical protein
  
Accession: EDK41403
  
Location: 594884-596128
  
  
**BlastP hit with Mycgr3G35447\_Mycgr3T**
  
Percentage identity: 40 %
  
BlastP bit score: 274
  
Sequence coverage: 91 %
  
E-value: 9e-84
  
  
 NCBI BlastP on this gene

EDK41403

hypothetical protein
  
Accession: EDK41404
  
Location: 596402-597829
  
  
**BlastP hit with Mycgr3G84402\_Mycgr3T**
  
Percentage identity: 59 %
  
BlastP bit score: 498
  
Sequence coverage: 93 %
  
E-value: 4e-170
  
  
 NCBI BlastP on this gene

EDK41404

hypothetical protein
  
Accession: EDK41405
  
Location: 597864-598829
  
 NCBI BlastP on this gene

EDK41405

hypothetical protein
  
Accession: EDK41406
  
Location: 599290-599802
  
 NCBI BlastP on this gene

EDK41406

hypothetical protein
  
Accession: EDK41407
  
Location: 600107-602320
  
 NCBI BlastP on this gene

EDK41407

hypothetical protein
  
Accession: EDK41408
  
Location: 602394-603542
  
 NCBI BlastP on this gene

EDK41408

predicted protein
  
Accession: EDK41409
  
Location: 603874-604944
  
 NCBI BlastP on this gene

EDK41409

hypothetical protein
  
Accession: EDK41411
  
Location: 605793-608177
  
 NCBI BlastP on this gene

EDK41411

hypothetical protein
  
Accession: EDK41410
  
Location: 608146-608991
  
 NCBI BlastP on this gene

EDK41410

hypothetical protein
  
Accession: EDK41412
  
Location: 608978-610162
  
 NCBI BlastP on this gene

EDK41412

hypothetical protein
  
Accession: EDK41413
  
Location: 610188-611285
  
 NCBI BlastP on this gene

EDK41413

hypothetical protein
  
Accession: EDK41414
  
Location: 611509-615717
  
 NCBI BlastP on this gene

EDK41414

hypothetical protein
  
Accession: EDK41415
  
Location: 616052-616786
  
 NCBI BlastP on this gene

EDK41415

hypothetical protein
  
Accession: EDK41416
  
Location: 617293-618729
  
 NCBI BlastP on this gene

EDK41416

Query: Architecture Search FASTA input

CR382137 : Debaryomyces hansenii CBS767 chromosome E complete sequence.    Total score: 2.0     Cumulative Blast bit score: 767

Hit cluster cross-links:

Mycgr3G67791 Mycgr3T
  
Location: 0-1542

Mycgr3G67791\_Mycgr3T

Mycgr3G90406 Mycgr3T
  
Location: 1642-3973

Mycgr3G90406\_Mycgr3T

Mycgr3G67785 Mycgr3T
  
Location: 4073-7865

Mycgr3G67785\_Mycgr3T

Mycgr3G67795 Mycgr3T
  
Location: 7965-15249

Mycgr3G67795\_Mycgr3T

Mycgr3G67775 Mycgr3T
  
Location: 15349-16237

Mycgr3G67775\_Mycgr3T

Mycgr3G90404 Mycgr3T
  
Location: 16337-17246

Mycgr3G90404\_Mycgr3T

Mycgr3G36951 Mycgr3T
  
Location: 17346-30891

Mycgr3G36951\_Mycgr3T

Mycgr3G103034 Mycgr3
  
Location: 30991-32644

Mycgr3G103034\_Mycgr3

Mycgr3G31119 Mycgr3T
  
Location: 32744-32906

Mycgr3G31119\_Mycgr3T

Mycgr3G28587 Mycgr3T
  
Location: 33006-33489

Mycgr3G28587\_Mycgr3T

Mycgr3G98959 Mycgr3T
  
Location: 33589-35035

Mycgr3G98959\_Mycgr3T

Mycgr3G35447 Mycgr3T
  
Location: 35135-36443

Mycgr3G35447\_Mycgr3T

Mycgr3G84402 Mycgr3T
  
Location: 36543-37884

Mycgr3G84402\_Mycgr3T

Mycgr3G98961 Mycgr3T
  
Location: 37984-38884

Mycgr3G98961\_Mycgr3T

DEHA2E07832p
  
Accession: CAG87881
  
Location: 622567-623733
  
 NCBI BlastP on this gene

DEHA2E07832g

DEHA2E07854p
  
Accession: CAG87882
  
Location: 623868-624977
  
 NCBI BlastP on this gene

DEHA2E07854g

DEHA2E07876p
  
Accession: CAG87883
  
Location: 625457-626194
  
 NCBI BlastP on this gene

DEHA2E07876g

DEHA2E07898p
  
Accession: CAG87884
  
Location: 626420-628813
  
 NCBI BlastP on this gene

DEHA2E07898g

DEHA2E07920p
  
Accession: CAG87885
  
Location: 629107-629355
  
 NCBI BlastP on this gene

DEHA2E07920g

DEHA2E07942p
  
Accession: CAG87886
  
Location: 629658-632225
  
 NCBI BlastP on this gene

DEHA2E07942g

DEHA2E07964p
  
Accession: CAG87887
  
Location: 632741-633826
  
 NCBI BlastP on this gene

DEHA2E07964g

DEHA2E07986p
  
Accession: CAR65785
  
Location: 633997-636225
  
 NCBI BlastP on this gene

DEHA2E07986g

DEHA2E08008p
  
Accession: CAG87890
  
Location: 636306-636827
  
 NCBI BlastP on this gene

DEHA2E08008g

DEHA2E08030p
  
Accession: CAG87891
  
Location: 636946-638124
  
 NCBI BlastP on this gene

DEHA2E08030g

DEHA2E08052p
  
Accession: CAG87892
  
Location: 638390-639823
  
  
**BlastP hit with Mycgr3G84402\_Mycgr3T**
  
Percentage identity: 57 %
  
BlastP bit score: 497
  
Sequence coverage: 95 %
  
E-value: 8e-170
  
  
 NCBI BlastP on this gene

DEHA2E08052g

DEHA2E08074p
  
Accession: CAG87893
  
Location: 640025-641281
  
  
**BlastP hit with Mycgr3G35447\_Mycgr3T**
  
Percentage identity: 38 %
  
BlastP bit score: 270
  
Sequence coverage: 105 %
  
E-value: 5e-82
  
  
 NCBI BlastP on this gene

DEHA2E08074g

DEHA2E08096p
  
Accession: CAG87894
  
Location: 641304-641957
  
 NCBI BlastP on this gene

DEHA2E08096g

DEHA2E08118p
  
Accession: CAR65786
  
Location: 642045-643109
  
 NCBI BlastP on this gene

DEHA2E08118g

DEHA2E08140p
  
Accession: CAG87896
  
Location: 643323-644432
  
 NCBI BlastP on this gene

DEHA2E08140g

DEHA2E08162p
  
Accession: CAG87897
  
Location: 644878-645045
  
 NCBI BlastP on this gene

DEHA2E08162g

DEHA2E08184p
  
Accession: CAR65787
  
Location: 645571-646812
  
 NCBI BlastP on this gene

DEHA2E08184g

DEHA2E08206p
  
Accession: CAG87899
  
Location: 647084-648172
  
 NCBI BlastP on this gene

DEHA2E08206g

DEHA2E08228p
  
Accession: CAG87900
  
Location: 648490-649389
  
 NCBI BlastP on this gene

DEHA2E08228g

DEHA2E08250p
  
Accession: CAG87901
  
Location: 649567-651108
  
 NCBI BlastP on this gene

DEHA2E08250g

DEHA2E08294p
  
Accession: CAG87902
  
Location: 651533-652798
  
 NCBI BlastP on this gene

DEHA2E08294g

DEHA2E08316p
  
Accession: CAG87903
  
Location: 653081-654106
  
 NCBI BlastP on this gene

DEHA2E08316g

DEHA2E08338p
  
Accession: CAG87904
  
Location: 654634-655071
  
 NCBI BlastP on this gene

DEHA2E08338g

DEHA2E08360p
  
Accession: CAG87905
  
Location: 655377-656591
  
 NCBI BlastP on this gene

DEHA2E08360g

DEHA2E08382p
  
Accession: CAG87906
  
Location: 657206-658183
  
 NCBI BlastP on this gene

DEHA2E08382g

DEHA2E08404p
  
Accession: CAG87909
  
Location: 660813-660926
  
 NCBI BlastP on this gene

DEHA2E08404g

DEHA2E08426p
  
Accession: CAR65788
  
Location: 660947-661120
  
 NCBI BlastP on this gene

DEHA2E08426g

Query: Architecture Search FASTA input

CH445336 : Phaeosphaeria nodorum SN15 scaffold\_12    Total score: 2.0     Cumulative Blast bit score: 765

Hit cluster cross-links:

Mycgr3G67791 Mycgr3T
  
Location: 0-1542

Mycgr3G67791\_Mycgr3T

Mycgr3G90406 Mycgr3T
  
Location: 1642-3973

Mycgr3G90406\_Mycgr3T

Mycgr3G67785 Mycgr3T
  
Location: 4073-7865

Mycgr3G67785\_Mycgr3T

Mycgr3G67795 Mycgr3T
  
Location: 7965-15249

Mycgr3G67795\_Mycgr3T

Mycgr3G67775 Mycgr3T
  
Location: 15349-16237

Mycgr3G67775\_Mycgr3T

Mycgr3G90404 Mycgr3T
  
Location: 16337-17246

Mycgr3G90404\_Mycgr3T

Mycgr3G36951 Mycgr3T
  
Location: 17346-30891

Mycgr3G36951\_Mycgr3T

Mycgr3G103034 Mycgr3
  
Location: 30991-32644

Mycgr3G103034\_Mycgr3

Mycgr3G31119 Mycgr3T
  
Location: 32744-32906

Mycgr3G31119\_Mycgr3T

Mycgr3G28587 Mycgr3T
  
Location: 33006-33489

Mycgr3G28587\_Mycgr3T

Mycgr3G98959 Mycgr3T
  
Location: 33589-35035

Mycgr3G98959\_Mycgr3T

Mycgr3G35447 Mycgr3T
  
Location: 35135-36443

Mycgr3G35447\_Mycgr3T

Mycgr3G84402 Mycgr3T
  
Location: 36543-37884

Mycgr3G84402\_Mycgr3T

Mycgr3G98961 Mycgr3T
  
Location: 37984-38884

Mycgr3G98961\_Mycgr3T

hypothetical protein
  
Accession: EAT84352
  
Location: 401427-402866
  
 NCBI BlastP on this gene

EAT84352

hypothetical protein
  
Accession: EAT84351
  
Location: 397565-401273
  
 NCBI BlastP on this gene

EAT84351

hypothetical protein
  
Accession: EAT84350
  
Location: 394277-396410
  
 NCBI BlastP on this gene

EAT84350

hypothetical protein
  
Accession: EAT84349
  
Location: 391778-393680
  
 NCBI BlastP on this gene

EAT84349

hypothetical protein
  
Accession: EAT84348
  
Location: 389919-390751
  
 NCBI BlastP on this gene

EAT84348

hypothetical protein
  
Accession: EAT84347
  
Location: 386501-388900
  
 NCBI BlastP on this gene

EAT84347

hypothetical protein
  
Accession: EAT84346
  
Location: 386285-386499
  
 NCBI BlastP on this gene

EAT84346

hypothetical protein
  
Accession: EDP89783
  
Location: 385869-386030
  
 NCBI BlastP on this gene

EDP89783

hypothetical protein
  
Accession: EAT84345
  
Location: 385010-385698
  
  
**BlastP hit with Mycgr3G28587\_Mycgr3T**
  
Percentage identity: 57 %
  
BlastP bit score: 191
  
Sequence coverage: 103 %
  
E-value: 3e-58
  
  
 NCBI BlastP on this gene

EAT84345

hypothetical protein
  
Accession: EAT84344
  
Location: 383410-384550
  
 NCBI BlastP on this gene

EAT84344

hypothetical protein
  
Accession: EAT84343
  
Location: 378879-382416
  
 NCBI BlastP on this gene

EAT84343

hypothetical protein
  
Accession: EAT84342
  
Location: 375960-378077
  
  
**BlastP hit with Mycgr3G103034\_Mycgr3**
  
Percentage identity: 54 %
  
BlastP bit score: 574
  
Sequence coverage: 93 %
  
E-value: 0.0
  
  
 NCBI BlastP on this gene

EAT84342

hypothetical protein
  
Accession: EAT84340
  
Location: 374297-375393
  
 NCBI BlastP on this gene

EAT84340

hypothetical protein
  
Accession: EAT84339
  
Location: 372829-373736
  
 NCBI BlastP on this gene

EAT84339

hypothetical protein
  
Accession: EAT84338
  
Location: 371638-372407
  
 NCBI BlastP on this gene

EAT84338

hypothetical protein
  
Accession: EAT84337
  
Location: 371280-371550
  
 NCBI BlastP on this gene

EAT84337

hypothetical protein
  
Accession: EAT84336
  
Location: 370275-370979
  
 NCBI BlastP on this gene

EAT84336

hypothetical protein
  
Accession: EAT84335
  
Location: 368364-369314
  
 NCBI BlastP on this gene

EAT84335

hypothetical protein
  
Accession: EAT84334
  
Location: 367377-367971
  
 NCBI BlastP on this gene

EAT84334

hypothetical protein
  
Accession: EAT84333
  
Location: 366780-367286
  
 NCBI BlastP on this gene

EAT84333

hypothetical protein
  
Accession: EAT84332
  
Location: 364834-365841
  
 NCBI BlastP on this gene

EAT84332

hypothetical protein
  
Accession: EAT84331
  
Location: 363738-364251
  
 NCBI BlastP on this gene

EAT84331

hypothetical protein
  
Accession: EAT84330
  
Location: 361981-363276
  
 NCBI BlastP on this gene

EAT84330

hypothetical protein
  
Accession: EAT84329
  
Location: 360632-361322
  
 NCBI BlastP on this gene

EAT84329

hypothetical protein
  
Accession: EAT84328
  
Location: 359131-360155
  
 NCBI BlastP on this gene

EAT84328

Query: Architecture Search FASTA input

HE605209 : Candida parapsilosis strain CDC317 annotated contig 006110.    Total score: 2.0     Cumulative Blast bit score: 764

Hit cluster cross-links:

Mycgr3G67791 Mycgr3T
  
Location: 0-1542

Mycgr3G67791\_Mycgr3T

Mycgr3G90406 Mycgr3T
  
Location: 1642-3973

Mycgr3G90406\_Mycgr3T

Mycgr3G67785 Mycgr3T
  
Location: 4073-7865

Mycgr3G67785\_Mycgr3T

Mycgr3G67795 Mycgr3T
  
Location: 7965-15249

Mycgr3G67795\_Mycgr3T

Mycgr3G67775 Mycgr3T
  
Location: 15349-16237

Mycgr3G67775\_Mycgr3T

Mycgr3G90404 Mycgr3T
  
Location: 16337-17246

Mycgr3G90404\_Mycgr3T

Mycgr3G36951 Mycgr3T
  
Location: 17346-30891

Mycgr3G36951\_Mycgr3T

Mycgr3G103034 Mycgr3
  
Location: 30991-32644

Mycgr3G103034\_Mycgr3

Mycgr3G31119 Mycgr3T
  
Location: 32744-32906

Mycgr3G31119\_Mycgr3T

Mycgr3G28587 Mycgr3T
  
Location: 33006-33489

Mycgr3G28587\_Mycgr3T

Mycgr3G98959 Mycgr3T
  
Location: 33589-35035

Mycgr3G98959\_Mycgr3T

Mycgr3G35447 Mycgr3T
  
Location: 35135-36443

Mycgr3G35447\_Mycgr3T

Mycgr3G84402 Mycgr3T
  
Location: 36543-37884

Mycgr3G84402\_Mycgr3T

Mycgr3G98961 Mycgr3T
  
Location: 37984-38884

Mycgr3G98961\_Mycgr3T

hypothetical protein
  
Accession: CCE45104
  
Location: 239103-240593
  
 NCBI BlastP on this gene

CPAR2\_701080

hypothetical protein
  
Accession: CCE45105
  
Location: 241435-241629
  
 NCBI BlastP on this gene

CPAR2\_701090

hypothetical protein
  
Accession: CCE45106
  
Location: 241808-243844
  
 NCBI BlastP on this gene

CPAR2\_701100

hypothetical protein
  
Accession: CCE45107
  
Location: 243913-244899
  
 NCBI BlastP on this gene

CPAR2\_701110

hypothetical protein
  
Accession: CCE45108
  
Location: 244934-245788
  
 NCBI BlastP on this gene

CPAR2\_701120

hypothetical protein
  
Accession: CCE45109
  
Location: 246025-248001
  
 NCBI BlastP on this gene

CPAR2\_701130

hypothetical protein
  
Accession: CCE45110
  
Location: 248632-250554
  
 NCBI BlastP on this gene

CPAR2\_701140

hypothetical protein
  
Accession: CCE45111
  
Location: 252821-253981
  
 NCBI BlastP on this gene

CPAR2\_701150

hypothetical protein
  
Accession: CCE45112
  
Location: 254120-254653
  
 NCBI BlastP on this gene

CPAR2\_701160

hypothetical protein
  
Accession: CCE45113
  
Location: 254696-255964
  
  
**BlastP hit with Mycgr3G35447\_Mycgr3T**
  
Percentage identity: 37 %
  
BlastP bit score: 268
  
Sequence coverage: 105 %
  
E-value: 4e-81
  
  
 NCBI BlastP on this gene

CPAR2\_701170

hypothetical protein
  
Accession: CCE45114
  
Location: 256132-257568
  
  
**BlastP hit with Mycgr3G84402\_Mycgr3T**
  
Percentage identity: 56 %
  
BlastP bit score: 496
  
Sequence coverage: 97 %
  
E-value: 3e-169
  
  
 NCBI BlastP on this gene

CPAR2\_701180

hypothetical protein
  
Accession: CCE45115
  
Location: 257614-258537
  
 NCBI BlastP on this gene

CPAR2\_701190

hypothetical protein
  
Accession: CCE45116
  
Location: 258840-259385
  
 NCBI BlastP on this gene

CPAR2\_701200

hypothetical protein
  
Accession: CCE45117
  
Location: 259436-261595
  
 NCBI BlastP on this gene

CPAR2\_701210

hypothetical protein
  
Accession: CCE45118
  
Location: 261727-263232
  
 NCBI BlastP on this gene

CPAR2\_701220

hypothetical protein
  
Accession: CCE45119
  
Location: 265123-265917
  
 NCBI BlastP on this gene

CPAR2\_701230

hypothetical protein
  
Accession: CCE45120
  
Location: 265992-266825
  
 NCBI BlastP on this gene

CPAR2\_701240

hypothetical protein
  
Accession: CCE45121
  
Location: 267397-268089
  
 NCBI BlastP on this gene

CPAR2\_701250

hypothetical protein
  
Accession: CCE45122
  
Location: 268469-269494
  
 NCBI BlastP on this gene

CPAR2\_701260

hypothetical protein
  
Accession: CCE45123
  
Location: 269732-270307
  
 NCBI BlastP on this gene

CPAR2\_701270

hypothetical protein
  
Accession: CCE45124
  
Location: 270361-272058
  
 NCBI BlastP on this gene

CPAR2\_701280

hypothetical protein
  
Accession: CCE45125
  
Location: 272405-273310
  
 NCBI BlastP on this gene

CPAR2\_701290

hypothetical protein
  
Accession: CCE45126
  
Location: 273614-275023
  
 NCBI BlastP on this gene

CPAR2\_701300

hypothetical protein
  
Accession: CCE45127
  
Location: 275649-277082
  
 NCBI BlastP on this gene

CPAR2\_701310

hypothetical protein
  
Accession: CCE45128
  
Location: 277348-277824
  
 NCBI BlastP on this gene

CPAR2\_701320

Query: Architecture Search FASTA input

FO082049 : Pichia sorbitophila strain CBS 7064 chromosome K complete sequence.    Total score: 2.0     Cumulative Blast bit score: 761

Hit cluster cross-links:

Mycgr3G67791 Mycgr3T
  
Location: 0-1542

Mycgr3G67791\_Mycgr3T

Mycgr3G90406 Mycgr3T
  
Location: 1642-3973

Mycgr3G90406\_Mycgr3T

Mycgr3G67785 Mycgr3T
  
Location: 4073-7865

Mycgr3G67785\_Mycgr3T

Mycgr3G67795 Mycgr3T
  
Location: 7965-15249

Mycgr3G67795\_Mycgr3T

Mycgr3G67775 Mycgr3T
  
Location: 15349-16237

Mycgr3G67775\_Mycgr3T

Mycgr3G90404 Mycgr3T
  
Location: 16337-17246

Mycgr3G90404\_Mycgr3T

Mycgr3G36951 Mycgr3T
  
Location: 17346-30891

Mycgr3G36951\_Mycgr3T

Mycgr3G103034 Mycgr3
  
Location: 30991-32644

Mycgr3G103034\_Mycgr3

Mycgr3G31119 Mycgr3T
  
Location: 32744-32906

Mycgr3G31119\_Mycgr3T

Mycgr3G28587 Mycgr3T
  
Location: 33006-33489

Mycgr3G28587\_Mycgr3T

Mycgr3G98959 Mycgr3T
  
Location: 33589-35035

Mycgr3G98959\_Mycgr3T

Mycgr3G35447 Mycgr3T
  
Location: 35135-36443

Mycgr3G35447\_Mycgr3T

Mycgr3G84402 Mycgr3T
  
Location: 36543-37884

Mycgr3G84402\_Mycgr3T

Mycgr3G98961 Mycgr3T
  
Location: 37984-38884

Mycgr3G98961\_Mycgr3T

not annotated
  
Accession: CCE83668
  
Location: 1089670-1090653
  
 NCBI BlastP on this gene

Piso0\_004253

not annotated
  
Accession: CCE83669
  
Location: 1091289-1092629
  
 NCBI BlastP on this gene

Piso0\_004254

not annotated
  
Accession: CCE83670
  
Location: 1092877-1093314
  
 NCBI BlastP on this gene

Piso0\_004255

not annotated
  
Accession: CCE83671
  
Location: 1093741-1094766
  
 NCBI BlastP on this gene

Piso0\_004256

not annotated
  
Accession: CCE83672
  
Location: 1095440-1096843
  
 NCBI BlastP on this gene

Piso0\_004257

not annotated
  
Accession: CCE83673
  
Location: 1097184-1098083
  
 NCBI BlastP on this gene

Piso0\_004258

not annotated
  
Accession: CCE83674
  
Location: 1098730-1099812
  
 NCBI BlastP on this gene

Piso0\_004259

not annotated
  
Accession: CCE83675
  
Location: 1100352-1101539
  
 NCBI BlastP on this gene

Piso0\_004260

not annotated
  
Accession: CCE83676
  
Location: 1101804-1102202
  
 NCBI BlastP on this gene

Piso0\_004261

not annotated
  
Accession: CCE83677
  
Location: 1102667-1103170
  
 NCBI BlastP on this gene

Piso0\_004262

not annotated
  
Accession: CCE83678
  
Location: 1103638-1104750
  
 NCBI BlastP on this gene

Piso0\_004263

not annotated
  
Accession: CCE83679
  
Location: 1105232-1106287
  
 NCBI BlastP on this gene

Piso0\_004264

not annotated
  
Accession: CCE83680
  
Location: 1106529-1107218
  
 NCBI BlastP on this gene

Piso0\_004265

not annotated
  
Accession: CCE83681
  
Location: 1107282-1108544
  
  
**BlastP hit with Mycgr3G35447\_Mycgr3T**
  
Percentage identity: 38 %
  
BlastP bit score: 253
  
Sequence coverage: 91 %
  
E-value: 1e-75
  
  
 NCBI BlastP on this gene

Piso0\_004266

not annotated
  
Accession: CCE83682
  
Location: 1108792-1110294
  
  
**BlastP hit with Mycgr3G84402\_Mycgr3T**
  
Percentage identity: 57 %
  
BlastP bit score: 508
  
Sequence coverage: 98 %
  
E-value: 1e-173
  
  
 NCBI BlastP on this gene

Piso0\_004267

not annotated
  
Accession: CCE83683
  
Location: 1110510-1111547
  
 NCBI BlastP on this gene

Piso0\_004268

not annotated
  
Accession: CCE83684
  
Location: 1111712-1112230
  
 NCBI BlastP on this gene

Piso0\_004269

not annotated
  
Accession: CCE83685
  
Location: 1112280-1114505
  
 NCBI BlastP on this gene

Piso0\_004270

not annotated
  
Accession: CCE83686
  
Location: 1114622-1115665
  
 NCBI BlastP on this gene

Piso0\_004271

not annotated
  
Accession: CCE83687
  
Location: 1116164-1116706
  
 NCBI BlastP on this gene

Piso0\_004272

not annotated
  
Accession: CCE83688
  
Location: 1116920-1119361
  
 NCBI BlastP on this gene

Piso0\_004273

not annotated
  
Accession: CCE83689
  
Location: 1119615-1119863
  
 NCBI BlastP on this gene

Piso0\_004274

not annotated
  
Accession: CCE83690
  
Location: 1120183-1122507
  
 NCBI BlastP on this gene

Piso0\_004275

not annotated
  
Accession: CCE83691
  
Location: 1122685-1123422
  
 NCBI BlastP on this gene

Piso0\_004276

not annotated
  
Accession: CCE83692
  
Location: 1123947-1125089
  
 NCBI BlastP on this gene

Piso0\_004277

not annotated
  
Accession: CCE83693
  
Location: 1125291-1126403
  
 NCBI BlastP on this gene

Piso0\_004278

not annotated
  
Accession: CCE83694
  
Location: 1126559-1130920
  
 NCBI BlastP on this gene

Piso0\_004279

Query: Architecture Search FASTA input

FO082048 : Pichia sorbitophila strain CBS 7064 chromosome L complete sequence.    Total score: 2.0     Cumulative Blast bit score: 761

Hit cluster cross-links:

Mycgr3G67791 Mycgr3T
  
Location: 0-1542

Mycgr3G67791\_Mycgr3T

Mycgr3G90406 Mycgr3T
  
Location: 1642-3973

Mycgr3G90406\_Mycgr3T

Mycgr3G67785 Mycgr3T
  
Location: 4073-7865

Mycgr3G67785\_Mycgr3T

Mycgr3G67795 Mycgr3T
  
Location: 7965-15249

Mycgr3G67795\_Mycgr3T

Mycgr3G67775 Mycgr3T
  
Location: 15349-16237

Mycgr3G67775\_Mycgr3T

Mycgr3G90404 Mycgr3T
  
Location: 16337-17246

Mycgr3G90404\_Mycgr3T

Mycgr3G36951 Mycgr3T
  
Location: 17346-30891

Mycgr3G36951\_Mycgr3T

Mycgr3G103034 Mycgr3
  
Location: 30991-32644

Mycgr3G103034\_Mycgr3

Mycgr3G31119 Mycgr3T
  
Location: 32744-32906

Mycgr3G31119\_Mycgr3T

Mycgr3G28587 Mycgr3T
  
Location: 33006-33489

Mycgr3G28587\_Mycgr3T

Mycgr3G98959 Mycgr3T
  
Location: 33589-35035

Mycgr3G98959\_Mycgr3T

Mycgr3G35447 Mycgr3T
  
Location: 35135-36443

Mycgr3G35447\_Mycgr3T

Mycgr3G84402 Mycgr3T
  
Location: 36543-37884

Mycgr3G84402\_Mycgr3T

Mycgr3G98961 Mycgr3T
  
Location: 37984-38884

Mycgr3G98961\_Mycgr3T

not annotated
  
Accession: CCE84699
  
Location: 1089670-1090653
  
 NCBI BlastP on this gene

Piso0\_004253

not annotated
  
Accession: CCE84700
  
Location: 1091289-1092629
  
 NCBI BlastP on this gene

Piso0\_004254

not annotated
  
Accession: CCE84701
  
Location: 1092877-1093314
  
 NCBI BlastP on this gene

Piso0\_004255

not annotated
  
Accession: CCE84702
  
Location: 1093741-1094766
  
 NCBI BlastP on this gene

Piso0\_004256

not annotated
  
Accession: CCE84703
  
Location: 1095440-1096843
  
 NCBI BlastP on this gene

Piso0\_004257

not annotated
  
Accession: CCE84704
  
Location: 1097184-1098083
  
 NCBI BlastP on this gene

Piso0\_004258

not annotated
  
Accession: CCE84705
  
Location: 1098730-1099812
  
 NCBI BlastP on this gene

Piso0\_004259

not annotated
  
Accession: CCE84706
  
Location: 1100352-1101539
  
 NCBI BlastP on this gene

Piso0\_004260

not annotated
  
Accession: CCE84707
  
Location: 1101804-1102202
  
 NCBI BlastP on this gene

Piso0\_004261

not annotated
  
Accession: CCE84708
  
Location: 1102667-1103170
  
 NCBI BlastP on this gene

Piso0\_004262

not annotated
  
Accession: CCE84709
  
Location: 1103638-1104750
  
 NCBI BlastP on this gene

Piso0\_004263

not annotated
  
Accession: CCE84710
  
Location: 1105232-1106287
  
 NCBI BlastP on this gene

Piso0\_004264

not annotated
  
Accession: CCE84711
  
Location: 1106529-1107218
  
 NCBI BlastP on this gene

Piso0\_004265

not annotated
  
Accession: CCE84712
  
Location: 1107282-1108544
  
  
**BlastP hit with Mycgr3G35447\_Mycgr3T**
  
Percentage identity: 38 %
  
BlastP bit score: 253
  
Sequence coverage: 91 %
  
E-value: 1e-75
  
  
 NCBI BlastP on this gene

Piso0\_004266

not annotated
  
Accession: CCE84713
  
Location: 1108792-1110294
  
  
**BlastP hit with Mycgr3G84402\_Mycgr3T**
  
Percentage identity: 57 %
  
BlastP bit score: 508
  
Sequence coverage: 98 %
  
E-value: 1e-173
  
  
 NCBI BlastP on this gene

Piso0\_004267

not annotated
  
Accession: CCE84714
  
Location: 1110510-1111547
  
 NCBI BlastP on this gene

Piso0\_004268

not annotated
  
Accession: CCE84715
  
Location: 1111712-1112230
  
 NCBI BlastP on this gene

Piso0\_004269

not annotated
  
Accession: CCE84716
  
Location: 1112280-1114505
  
 NCBI BlastP on this gene

Piso0\_004270

not annotated
  
Accession: CCE84717
  
Location: 1114622-1115665
  
 NCBI BlastP on this gene

Piso0\_004271

not annotated
  
Accession: CCE84718
  
Location: 1116164-1116706
  
 NCBI BlastP on this gene

Piso0\_004272

not annotated
  
Accession: CCE84719
  
Location: 1116920-1119361
  
 NCBI BlastP on this gene

Piso0\_004273

not annotated
  
Accession: CCE84720
  
Location: 1119615-1119863
  
 NCBI BlastP on this gene

Piso0\_004274

not annotated
  
Accession: CCE84721
  
Location: 1120183-1122507
  
 NCBI BlastP on this gene

Piso0\_004275

not annotated
  
Accession: CCE84722
  
Location: 1122685-1123422
  
 NCBI BlastP on this gene

Piso0\_004276

not annotated
  
Accession: CCE84723
  
Location: 1123947-1125089
  
 NCBI BlastP on this gene

Piso0\_004277

not annotated
  
Accession: CCE84724
  
Location: 1125291-1126403
  
 NCBI BlastP on this gene

Piso0\_004278

not annotated
  
Accession: CCE84725
  
Location: 1126559-1130920
  
 NCBI BlastP on this gene

Piso0\_004279

Query: Architecture Search FASTA input

DS231623 : Pyrenophora tritici-repentis Pt-1C-BFP supercont1.9 genomic scaffold    Total score: 2.0     Cumulative Blast bit score: 761

Hit cluster cross-links:

Mycgr3G67791 Mycgr3T
  
Location: 0-1542

Mycgr3G67791\_Mycgr3T

Mycgr3G90406 Mycgr3T
  
Location: 1642-3973

Mycgr3G90406\_Mycgr3T

Mycgr3G67785 Mycgr3T
  
Location: 4073-7865

Mycgr3G67785\_Mycgr3T

Mycgr3G67795 Mycgr3T
  
Location: 7965-15249

Mycgr3G67795\_Mycgr3T

Mycgr3G67775 Mycgr3T
  
Location: 15349-16237

Mycgr3G67775\_Mycgr3T

Mycgr3G90404 Mycgr3T
  
Location: 16337-17246

Mycgr3G90404\_Mycgr3T

Mycgr3G36951 Mycgr3T
  
Location: 17346-30891

Mycgr3G36951\_Mycgr3T

Mycgr3G103034 Mycgr3
  
Location: 30991-32644

Mycgr3G103034\_Mycgr3

Mycgr3G31119 Mycgr3T
  
Location: 32744-32906

Mycgr3G31119\_Mycgr3T

Mycgr3G28587 Mycgr3T
  
Location: 33006-33489

Mycgr3G28587\_Mycgr3T

Mycgr3G98959 Mycgr3T
  
Location: 33589-35035

Mycgr3G98959\_Mycgr3T

Mycgr3G35447 Mycgr3T
  
Location: 35135-36443

Mycgr3G35447\_Mycgr3T

Mycgr3G84402 Mycgr3T
  
Location: 36543-37884

Mycgr3G84402\_Mycgr3T

Mycgr3G98961 Mycgr3T
  
Location: 37984-38884

Mycgr3G98961\_Mycgr3T

transcriptional regulator
  
Accession: EDU51512
  
Location: 1459783-1461298
  
 NCBI BlastP on this gene

EDU51512

predicted protein
  
Accession: EDU51513
  
Location: 1470425-1471339
  
 NCBI BlastP on this gene

EDU51513

conserved hypothetical protein
  
Accession: EDU51514
  
Location: 1474594-1475292
  
  
**BlastP hit with Mycgr3G28587\_Mycgr3T**
  
Percentage identity: 59 %
  
BlastP bit score: 176
  
Sequence coverage: 96 %
  
E-value: 2e-52
  
  
 NCBI BlastP on this gene

EDU51514

GTP-binding protein rho2 precursor
  
Accession: EDU51515
  
Location: 1475809-1476941
  
 NCBI BlastP on this gene

EDU51515

hypothetical protein
  
Accession: EDU51516
  
Location: 1477988-1481442
  
 NCBI BlastP on this gene

EDU51516

N-acetylglucosamine-phosphate mutase
  
Accession: EDU51517
  
Location: 1482242-1484186
  
  
**BlastP hit with Mycgr3G103034\_Mycgr3**
  
Percentage identity: 53 %
  
BlastP bit score: 585
  
Sequence coverage: 100 %
  
E-value: 0.0
  
  
 NCBI BlastP on this gene

EDU51517

hypothetical protein
  
Accession: EDU51518
  
Location: 1485544-1488523
  
 NCBI BlastP on this gene

EDU51518

conserved hypothetical protein
  
Accession: EDU51519
  
Location: 1491418-1492539
  
 NCBI BlastP on this gene

EDU51519

conserved hypothetical protein
  
Accession: EDU51520
  
Location: 1492715-1493962
  
 NCBI BlastP on this gene

EDU51520

conserved hypothetical protein
  
Accession: EDU51521
  
Location: 1494731-1495450
  
 NCBI BlastP on this gene

EDU51521

predicted protein
  
Accession: EDU51522
  
Location: 1496858-1497924
  
 NCBI BlastP on this gene

EDU51522

Query: Architecture Search FASTA input

AOGT01000769 : Candida maltosa Xu316    Total score: 2.0     Cumulative Blast bit score: 754

Hit cluster cross-links:

Mycgr3G67791 Mycgr3T
  
Location: 0-1542

Mycgr3G67791\_Mycgr3T

Mycgr3G90406 Mycgr3T
  
Location: 1642-3973

Mycgr3G90406\_Mycgr3T

Mycgr3G67785 Mycgr3T
  
Location: 4073-7865

Mycgr3G67785\_Mycgr3T

Mycgr3G67795 Mycgr3T
  
Location: 7965-15249

Mycgr3G67795\_Mycgr3T

Mycgr3G67775 Mycgr3T
  
Location: 15349-16237

Mycgr3G67775\_Mycgr3T

Mycgr3G90404 Mycgr3T
  
Location: 16337-17246

Mycgr3G90404\_Mycgr3T

Mycgr3G36951 Mycgr3T
  
Location: 17346-30891

Mycgr3G36951\_Mycgr3T

Mycgr3G103034 Mycgr3
  
Location: 30991-32644

Mycgr3G103034\_Mycgr3

Mycgr3G31119 Mycgr3T
  
Location: 32744-32906

Mycgr3G31119\_Mycgr3T

Mycgr3G28587 Mycgr3T
  
Location: 33006-33489

Mycgr3G28587\_Mycgr3T

Mycgr3G98959 Mycgr3T
  
Location: 33589-35035

Mycgr3G98959\_Mycgr3T

Mycgr3G35447 Mycgr3T
  
Location: 35135-36443

Mycgr3G35447\_Mycgr3T

Mycgr3G84402 Mycgr3T
  
Location: 36543-37884

Mycgr3G84402\_Mycgr3T

Mycgr3G98961 Mycgr3T
  
Location: 37984-38884

Mycgr3G98961\_Mycgr3T

hypothetical protein
  
Accession: EMG49263
  
Location: 122-1117
  
 NCBI BlastP on this gene

EMG49263

hypothetical protein
  
Accession: EMG49264
  
Location: 2244-3542
  
  
**BlastP hit with Mycgr3G35447\_Mycgr3T**
  
Percentage identity: 39 %
  
BlastP bit score: 259
  
Sequence coverage: 94 %
  
E-value: 9e-78
  
  
 NCBI BlastP on this gene

EMG49264

ATP-dependent rRNA helicase RRP3
  
Accession: EMG49265
  
Location: 3737-5176
  
  
**BlastP hit with Mycgr3G84402\_Mycgr3T**
  
Percentage identity: 58 %
  
BlastP bit score: 495
  
Sequence coverage: 94 %
  
E-value: 5e-169
  
  
 NCBI BlastP on this gene

EMG49265

hypothetical protein
  
Accession: EMG49266
  
Location: 5196-6233
  
 NCBI BlastP on this gene

EMG49266

hypothetical protein
  
Accession: EMG49267
  
Location: 6519-7091
  
 NCBI BlastP on this gene

EMG49267

hypothetical protein
  
Accession: EMG49268
  
Location: 7123-9315
  
 NCBI BlastP on this gene

EMG49268

hypothetical protein
  
Accession: EMG49269
  
Location: 9712-11679
  
 NCBI BlastP on this gene

EMG49269

Query: Architecture Search FASTA input

GL996500 : Spathaspora passalidarum NRRL Y-27907 unplaced genomic scaffold SPAPAscaffold\_2    Total score: 2.0     Cumulative Blast bit score: 753

Hit cluster cross-links:

Mycgr3G67791 Mycgr3T
  
Location: 0-1542

Mycgr3G67791\_Mycgr3T

Mycgr3G90406 Mycgr3T
  
Location: 1642-3973

Mycgr3G90406\_Mycgr3T

Mycgr3G67785 Mycgr3T
  
Location: 4073-7865

Mycgr3G67785\_Mycgr3T

Mycgr3G67795 Mycgr3T
  
Location: 7965-15249

Mycgr3G67795\_Mycgr3T

Mycgr3G67775 Mycgr3T
  
Location: 15349-16237

Mycgr3G67775\_Mycgr3T

Mycgr3G90404 Mycgr3T
  
Location: 16337-17246

Mycgr3G90404\_Mycgr3T

Mycgr3G36951 Mycgr3T
  
Location: 17346-30891

Mycgr3G36951\_Mycgr3T

Mycgr3G103034 Mycgr3
  
Location: 30991-32644

Mycgr3G103034\_Mycgr3

Mycgr3G31119 Mycgr3T
  
Location: 32744-32906

Mycgr3G31119\_Mycgr3T

Mycgr3G28587 Mycgr3T
  
Location: 33006-33489

Mycgr3G28587\_Mycgr3T

Mycgr3G98959 Mycgr3T
  
Location: 33589-35035

Mycgr3G98959\_Mycgr3T

Mycgr3G35447 Mycgr3T
  
Location: 35135-36443

Mycgr3G35447\_Mycgr3T

Mycgr3G84402 Mycgr3T
  
Location: 36543-37884

Mycgr3G84402\_Mycgr3T

Mycgr3G98961 Mycgr3T
  
Location: 37984-38884

Mycgr3G98961\_Mycgr3T

hypothetical protein
  
Accession: EGW33776
  
Location: 224959-226941
  
 NCBI BlastP on this gene

EGW33776

hypothetical protein
  
Accession: EGW33777
  
Location: 227093-228139
  
 NCBI BlastP on this gene

EGW33777

hypothetical protein
  
Accession: EGW33778
  
Location: 228185-229042
  
 NCBI BlastP on this gene

EGW33778

hypothetical protein
  
Accession: EGW33779
  
Location: 229192-230760
  
 NCBI BlastP on this gene

EGW33779

phospholipase B
  
Accession: EGW33780
  
Location: 231307-233049
  
 NCBI BlastP on this gene

EGW33780

hypothetical protein
  
Accession: EGW33781
  
Location: 234307-236441
  
 NCBI BlastP on this gene

EGW33781

hypothetical protein
  
Accession: EGW33782
  
Location: 237935-238584
  
 NCBI BlastP on this gene

EGW33782

hypothetical protein
  
Accession: EGW33783
  
Location: 240222-241310
  
 NCBI BlastP on this gene

EGW33783

hypothetical protein
  
Accession: EGW33784
  
Location: 244089-244607
  
 NCBI BlastP on this gene

EGW33784

hypothetical protein
  
Accession: EGW33785
  
Location: 244636-245820
  
  
**BlastP hit with Mycgr3G35447\_Mycgr3T**
  
Percentage identity: 38 %
  
BlastP bit score: 267
  
Sequence coverage: 96 %
  
E-value: 2e-81
  
  
 NCBI BlastP on this gene

EGW33785

ATP-dependent rRNA helicase RRP3
  
Accession: EGW33786
  
Location: 246009-247436
  
  
**BlastP hit with Mycgr3G84402\_Mycgr3T**
  
Percentage identity: 57 %
  
BlastP bit score: 486
  
Sequence coverage: 91 %
  
E-value: 3e-165
  
  
 NCBI BlastP on this gene

EGW33786

hypothetical protein
  
Accession: EGW33787
  
Location: 247488-248498
  
 NCBI BlastP on this gene

EGW33787

mediator of RNA polymerase II transcription subunit 21
  
Accession: EGW33788
  
Location: 248853-249362
  
 NCBI BlastP on this gene

EGW33788

hypothetical protein
  
Accession: EGW33789
  
Location: 249373-251448
  
 NCBI BlastP on this gene

EGW33789

hypothetical protein
  
Accession: EGW33790
  
Location: 260725-262110
  
 NCBI BlastP on this gene

EGW33790

hypothetical protein
  
Accession: EGW33792
  
Location: 263522-264295
  
 NCBI BlastP on this gene

EGW33792

hypothetical protein
  
Accession: EGW33793
  
Location: 264325-265224
  
 NCBI BlastP on this gene

EGW33793

hypothetical protein
  
Accession: EGW33794
  
Location: 265396-266982
  
 NCBI BlastP on this gene

EGW33794

Query: Architecture Search FASTA input

GG692404 : Candida tropicalis MYA-3404 genomic scaffold supercont3.10    Total score: 2.0     Cumulative Blast bit score: 750

Hit cluster cross-links:

Mycgr3G67791 Mycgr3T
  
Location: 0-1542

Mycgr3G67791\_Mycgr3T

Mycgr3G90406 Mycgr3T
  
Location: 1642-3973

Mycgr3G90406\_Mycgr3T

Mycgr3G67785 Mycgr3T
  
Location: 4073-7865

Mycgr3G67785\_Mycgr3T

Mycgr3G67795 Mycgr3T
  
Location: 7965-15249

Mycgr3G67795\_Mycgr3T

Mycgr3G67775 Mycgr3T
  
Location: 15349-16237

Mycgr3G67775\_Mycgr3T

Mycgr3G90404 Mycgr3T
  
Location: 16337-17246

Mycgr3G90404\_Mycgr3T

Mycgr3G36951 Mycgr3T
  
Location: 17346-30891

Mycgr3G36951\_Mycgr3T

Mycgr3G103034 Mycgr3
  
Location: 30991-32644

Mycgr3G103034\_Mycgr3

Mycgr3G31119 Mycgr3T
  
Location: 32744-32906

Mycgr3G31119\_Mycgr3T

Mycgr3G28587 Mycgr3T
  
Location: 33006-33489

Mycgr3G28587\_Mycgr3T

Mycgr3G98959 Mycgr3T
  
Location: 33589-35035

Mycgr3G98959\_Mycgr3T

Mycgr3G35447 Mycgr3T
  
Location: 35135-36443

Mycgr3G35447\_Mycgr3T

Mycgr3G84402 Mycgr3T
  
Location: 36543-37884

Mycgr3G84402\_Mycgr3T

Mycgr3G98961 Mycgr3T
  
Location: 37984-38884

Mycgr3G98961\_Mycgr3T

hypothetical protein
  
Accession: EER30475
  
Location: 219873-221486
  
 NCBI BlastP on this gene

EER30475

predicted protein
  
Accession: EER30476
  
Location: 221536-222159
  
 NCBI BlastP on this gene

EER30476

hypothetical protein
  
Accession: EER30477
  
Location: 223245-225509
  
 NCBI BlastP on this gene

EER30477

predicted protein
  
Accession: EER30478
  
Location: 225612-226841
  
 NCBI BlastP on this gene

EER30478

conserved hypothetical protein
  
Accession: EER30479
  
Location: 227280-229967
  
 NCBI BlastP on this gene

EER30479

D-arabinono-1,4-lactone oxidase
  
Accession: EER30480
  
Location: 230073-231746
  
 NCBI BlastP on this gene

EER30480

conserved hypothetical protein
  
Accession: EER30481
  
Location: 232817-235009
  
 NCBI BlastP on this gene

EER30481

conserved hypothetical protein
  
Accession: EER30482
  
Location: 235051-235638
  
 NCBI BlastP on this gene

EER30482

conserved hypothetical protein
  
Accession: EER30483
  
Location: 235884-236969
  
 NCBI BlastP on this gene

EER30483

ATP-dependent rRNA helicase RRP3
  
Accession: EER30484
  
Location: 237018-238469
  
  
**BlastP hit with Mycgr3G84402\_Mycgr3T**
  
Percentage identity: 58 %
  
BlastP bit score: 494
  
Sequence coverage: 93 %
  
E-value: 3e-168
  
  
 NCBI BlastP on this gene

EER30484

conserved hypothetical protein
  
Accession: EER30485
  
Location: 238676-239980
  
  
**BlastP hit with Mycgr3G35447\_Mycgr3T**
  
Percentage identity: 39 %
  
BlastP bit score: 256
  
Sequence coverage: 93 %
  
E-value: 1e-76
  
  
 NCBI BlastP on this gene

EER30485

predicted protein
  
Accession: EER30486
  
Location: 240016-240591
  
 NCBI BlastP on this gene

EER30486

hypothetical protein
  
Accession: EER30487
  
Location: 241551-242666
  
 NCBI BlastP on this gene

EER30487

conserved hypothetical protein
  
Accession: EER30488
  
Location: 244170-245231
  
 NCBI BlastP on this gene

EER30488

esterase D
  
Accession: EER30489
  
Location: 245245-246102
  
 NCBI BlastP on this gene

EER30489

lysophospholipase 1 precursor
  
Accession: EER30490
  
Location: 248062-249939
  
 NCBI BlastP on this gene

EER30490

hypothetical protein
  
Accession: EER30491
  
Location: 252139-253740
  
 NCBI BlastP on this gene

EER30491

predicted protein
  
Accession: EER30492
  
Location: 253869-255959
  
 NCBI BlastP on this gene

EER30492

predicted protein
  
Accession: EER30493
  
Location: 256281-256475
  
 NCBI BlastP on this gene

EER30493

hypothetical protein
  
Accession: EER30494
  
Location: 257504-258052
  
 NCBI BlastP on this gene

EER30494

predicted protein
  
Accession: EER30495
  
Location: 258650-259309
  
 NCBI BlastP on this gene

EER30495

prefoldin subunit 6
  
Accession: EER30496
  
Location: 259744-260091
  
 NCBI BlastP on this gene

EER30496

Query: Architecture Search FASTA input

AEOI01000005 : Ogataea parapolymorpha DL-1    Total score: 2.0     Cumulative Blast bit score: 750

Hit cluster cross-links:

Mycgr3G67791 Mycgr3T
  
Location: 0-1542

Mycgr3G67791\_Mycgr3T

Mycgr3G90406 Mycgr3T
  
Location: 1642-3973

Mycgr3G90406\_Mycgr3T

Mycgr3G67785 Mycgr3T
  
Location: 4073-7865

Mycgr3G67785\_Mycgr3T

Mycgr3G67795 Mycgr3T
  
Location: 7965-15249

Mycgr3G67795\_Mycgr3T

Mycgr3G67775 Mycgr3T
  
Location: 15349-16237

Mycgr3G67775\_Mycgr3T

Mycgr3G90404 Mycgr3T
  
Location: 16337-17246

Mycgr3G90404\_Mycgr3T

Mycgr3G36951 Mycgr3T
  
Location: 17346-30891

Mycgr3G36951\_Mycgr3T

Mycgr3G103034 Mycgr3
  
Location: 30991-32644

Mycgr3G103034\_Mycgr3

Mycgr3G31119 Mycgr3T
  
Location: 32744-32906

Mycgr3G31119\_Mycgr3T

Mycgr3G28587 Mycgr3T
  
Location: 33006-33489

Mycgr3G28587\_Mycgr3T

Mycgr3G98959 Mycgr3T
  
Location: 33589-35035

Mycgr3G98959\_Mycgr3T

Mycgr3G35447 Mycgr3T
  
Location: 35135-36443

Mycgr3G35447\_Mycgr3T

Mycgr3G84402 Mycgr3T
  
Location: 36543-37884

Mycgr3G84402\_Mycgr3T

Mycgr3G98961 Mycgr3T
  
Location: 37984-38884

Mycgr3G98961\_Mycgr3T

Cytosolic Fe-S cluster assembling factor CFD1
  
Accession: EFW97529
  
Location: 255193-255996
  
 NCBI BlastP on this gene

EFW97529

microsomal dipeptidase, putative
  
Accession: EFW97530
  
Location: 256046-257044
  
 NCBI BlastP on this gene

EFW97530

pantothenate transporter, putative
  
Accession: EFW97531
  
Location: 257409-258908
  
 NCBI BlastP on this gene

EFW97531

Flavin-containing monooxygenase
  
Accession: EFW97532
  
Location: 259030-260349
  
 NCBI BlastP on this gene

EFW97532

glycosyl transferase
  
Accession: EFW97533
  
Location: 262585-263526
  
 NCBI BlastP on this gene

EFW97533

hypothetical protein
  
Accession: EFW97534
  
Location: 263853-265568
  
 NCBI BlastP on this gene

EFW97534

putative kinase Pak1p
  
Accession: EFW97535
  
Location: 265640-267418
  
 NCBI BlastP on this gene

EFW97535

Dihydroxyacetone kinase
  
Accession: EFW97536
  
Location: 267460-269289
  
 NCBI BlastP on this gene

EFW97536

Autophagy-related protein 21
  
Accession: EFW97537
  
Location: 269313-270766
  
 NCBI BlastP on this gene

EFW97537

Subunit of the CCR4-NOT complex
  
Accession: EFW97538
  
Location: 270797-274299
  
 NCBI BlastP on this gene

EFW97538

hypothetical protein
  
Accession: EFW97539
  
Location: 274331-275487
  
  
**BlastP hit with Mycgr3G35447\_Mycgr3T**
  
Percentage identity: 43 %
  
BlastP bit score: 261
  
Sequence coverage: 85 %
  
E-value: 3e-79
  
  
 NCBI BlastP on this gene

EFW97539

ATP-dependent rRNA helicase, putative
  
Accession: EFW97540
  
Location: 275574-276986
  
  
**BlastP hit with Mycgr3G84402\_Mycgr3T**
  
Percentage identity: 54 %
  
BlastP bit score: 489
  
Sequence coverage: 97 %
  
E-value: 7e-167
  
  
 NCBI BlastP on this gene

EFW97540

pre-mRNA-splicing ATP-dependent RNA helicase PRP28
  
Accession: EFW97541
  
Location: 277068-278669
  
 NCBI BlastP on this gene

EFW97541

Protein involved in pre-mRNA splicing, component of a complex containing Cef1p
  
Accession: EFW97542
  
Location: 278673-279614
  
 NCBI BlastP on this gene

EFW97542

Fimbrin, actin-bundling protein
  
Accession: EFW97543
  
Location: 279741-286352
  
 NCBI BlastP on this gene

EFW97543

3-keto sterol reductase
  
Accession: EFW97544
  
Location: 286419-287474
  
 NCBI BlastP on this gene

EFW97544

mitogen activated protein kinase
  
Accession: EFW97545
  
Location: 287775-288941
  
 NCBI BlastP on this gene

EFW97545

monothiol glutaredoxin
  
Accession: EFW97546
  
Location: 289051-289938
  
 NCBI BlastP on this gene

EFW97546

hypothetical protein
  
Accession: EFW97547
  
Location: 289982-290680
  
 NCBI BlastP on this gene

EFW97547

Protein component of the small (40S) ribosomal subunit
  
Accession: EFW97548
  
Location: 290825-291467
  
 NCBI BlastP on this gene

EFW97548

DNA-binding protein
  
Accession: EFW97549
  
Location: 291673-293502
  
 NCBI BlastP on this gene

EFW97549

Fungal-specific transcription factor
  
Accession: EFW97550
  
Location: 294001-297156
  
 NCBI BlastP on this gene

EFW97550

Query: Architecture Search FASTA input

HE681725 : Candida orthopsilosis Co 90-125, chromosome 7 draft sequence.    Total score: 2.0     Cumulative Blast bit score: 749

Hit cluster cross-links:

Mycgr3G67791 Mycgr3T
  
Location: 0-1542

Mycgr3G67791\_Mycgr3T

Mycgr3G90406 Mycgr3T
  
Location: 1642-3973

Mycgr3G90406\_Mycgr3T

Mycgr3G67785 Mycgr3T
  
Location: 4073-7865

Mycgr3G67785\_Mycgr3T

Mycgr3G67795 Mycgr3T
  
Location: 7965-15249

Mycgr3G67795\_Mycgr3T

Mycgr3G67775 Mycgr3T
  
Location: 15349-16237

Mycgr3G67775\_Mycgr3T

Mycgr3G90404 Mycgr3T
  
Location: 16337-17246

Mycgr3G90404\_Mycgr3T

Mycgr3G36951 Mycgr3T
  
Location: 17346-30891

Mycgr3G36951\_Mycgr3T

Mycgr3G103034 Mycgr3
  
Location: 30991-32644

Mycgr3G103034\_Mycgr3

Mycgr3G31119 Mycgr3T
  
Location: 32744-32906

Mycgr3G31119\_Mycgr3T

Mycgr3G28587 Mycgr3T
  
Location: 33006-33489

Mycgr3G28587\_Mycgr3T

Mycgr3G98959 Mycgr3T
  
Location: 33589-35035

Mycgr3G98959\_Mycgr3T

Mycgr3G35447 Mycgr3T
  
Location: 35135-36443

Mycgr3G35447\_Mycgr3T

Mycgr3G84402 Mycgr3T
  
Location: 36543-37884

Mycgr3G84402\_Mycgr3T

Mycgr3G98961 Mycgr3T
  
Location: 37984-38884

Mycgr3G98961\_Mycgr3T

Cdc6 ATP-binding protein
  
Accession: CCG24811
  
Location: 247527-249005
  
 NCBI BlastP on this gene

CORT\_0G01240

hypothetical protein
  
Accession: CCG24812
  
Location: 249810-250004
  
 NCBI BlastP on this gene

CORT\_0G01245

Wal1 h
  
Accession: CCG24813
  
Location: 250199-252316
  
 NCBI BlastP on this gene

CORT\_0G01250

hypothetical protein
  
Accession: CCG24814
  
Location: 252380-253369
  
 NCBI BlastP on this gene

CORT\_0G01260

esterase
  
Accession: CCG24815
  
Location: 253406-254260
  
 NCBI BlastP on this gene

CORT\_0G01270

esterase
  
Accession: CCG24816
  
Location: 254658-255023
  
 NCBI BlastP on this gene

CORT\_0G01280

hypothetical protein
  
Accession: CCG24817
  
Location: 255874-257847
  
 NCBI BlastP on this gene

CORT\_0G01300

Plb3 GPI-anchored cell surface phospholipase B
  
Accession: CCG24818
  
Location: 258441-260363
  
 NCBI BlastP on this gene

CORT\_0G01310

Pcd1 protein
  
Accession: CCG24819
  
Location: 263415-264674
  
 NCBI BlastP on this gene

CORT\_0G01320

Vma22 protein
  
Accession: CCG24820
  
Location: 264710-265294
  
 NCBI BlastP on this gene

CORT\_0G01330

hypothetical protein
  
Accession: CCG24821
  
Location: 265351-266619
  
  
**BlastP hit with Mycgr3G35447\_Mycgr3T**
  
Percentage identity: 39 %
  
BlastP bit score: 260
  
Sequence coverage: 92 %
  
E-value: 3e-78
  
  
 NCBI BlastP on this gene

CORT\_0G01340

Rrp3 protein
  
Accession: CCG24822
  
Location: 266757-268202
  
  
**BlastP hit with Mycgr3G84402\_Mycgr3T**
  
Percentage identity: 57 %
  
BlastP bit score: 489
  
Sequence coverage: 92 %
  
E-value: 2e-166
  
  
 NCBI BlastP on this gene

CORT\_0G01350

Pib1 protein
  
Accession: CCG24823
  
Location: 268229-269146
  
 NCBI BlastP on this gene

CORT\_0G01360

Srb7 protein
  
Accession: CCG24824
  
Location: 269432-269977
  
 NCBI BlastP on this gene

CORT\_0G01370

Pmt5 protein mannosyltransferase (PMT)
  
Accession: CCG24825
  
Location: 270024-272183
  
 NCBI BlastP on this gene

CORT\_0G01380

Ssu1 protein
  
Accession: CCG24826
  
Location: 272309-273805
  
 NCBI BlastP on this gene

CORT\_0G01390

Cdg1 cysteine dioxygenases
  
Accession: CCG24827
  
Location: 275757-276557
  
 NCBI BlastP on this gene

CORT\_0G01400

phytanoyl-CoA dioxygenase family protein
  
Accession: CCG24828
  
Location: 276636-277517
  
 NCBI BlastP on this gene

CORT\_0G01410

Pet18 protein
  
Accession: CCG24829
  
Location: 278020-278712
  
 NCBI BlastP on this gene

CORT\_0G01420

Thi13 protein
  
Accession: CCG24830
  
Location: 279121-280146
  
 NCBI BlastP on this gene

CORT\_0G01430

Pho88 h
  
Accession: CCG24831
  
Location: 280395-280970
  
 NCBI BlastP on this gene

CORT\_0G01440

Rkm1 protein
  
Accession: CCG24832
  
Location: 281023-282720
  
 NCBI BlastP on this gene

CORT\_0G01450

Sco1 copper transporter
  
Accession: CCG24833
  
Location: 283034-283948
  
 NCBI BlastP on this gene

CORT\_0G01460

hypothetical protein
  
Accession: CCG24834
  
Location: 284269-285678
  
 NCBI BlastP on this gene

CORT\_0G01470

Cbp1 corticosteroid binding protein
  
Accession: CCG24835
  
Location: 286289-287722
  
 NCBI BlastP on this gene

CORT\_0G01480

hypothetical protein
  
Accession: CCG24836
  
Location: 287979-288443
  
 NCBI BlastP on this gene

CORT\_0G01490

Query: Architecture Search FASTA input

CH408079 : Clavispora lusitaniae ATCC 42720 scaffold\_4 genomic scaffold    Total score: 2.0     Cumulative Blast bit score: 741

Hit cluster cross-links:

Mycgr3G67791 Mycgr3T
  
Location: 0-1542

Mycgr3G67791\_Mycgr3T

Mycgr3G90406 Mycgr3T
  
Location: 1642-3973

Mycgr3G90406\_Mycgr3T

Mycgr3G67785 Mycgr3T
  
Location: 4073-7865

Mycgr3G67785\_Mycgr3T

Mycgr3G67795 Mycgr3T
  
Location: 7965-15249

Mycgr3G67795\_Mycgr3T

Mycgr3G67775 Mycgr3T
  
Location: 15349-16237

Mycgr3G67775\_Mycgr3T

Mycgr3G90404 Mycgr3T
  
Location: 16337-17246

Mycgr3G90404\_Mycgr3T

Mycgr3G36951 Mycgr3T
  
Location: 17346-30891

Mycgr3G36951\_Mycgr3T

Mycgr3G103034 Mycgr3
  
Location: 30991-32644

Mycgr3G103034\_Mycgr3

Mycgr3G31119 Mycgr3T
  
Location: 32744-32906

Mycgr3G31119\_Mycgr3T

Mycgr3G28587 Mycgr3T
  
Location: 33006-33489

Mycgr3G28587\_Mycgr3T

Mycgr3G98959 Mycgr3T
  
Location: 33589-35035

Mycgr3G98959\_Mycgr3T

Mycgr3G35447 Mycgr3T
  
Location: 35135-36443

Mycgr3G35447\_Mycgr3T

Mycgr3G84402 Mycgr3T
  
Location: 36543-37884

Mycgr3G84402\_Mycgr3T

Mycgr3G98961 Mycgr3T
  
Location: 37984-38884

Mycgr3G98961\_Mycgr3T

hypothetical protein
  
Accession: EEQ39212
  
Location: 306096-307127
  
 NCBI BlastP on this gene

EEQ39212

hypothetical protein
  
Accession: EEQ39213
  
Location: 307593-308849
  
 NCBI BlastP on this gene

EEQ39213

hypothetical protein
  
Accession: EEQ39214
  
Location: 308964-310451
  
 NCBI BlastP on this gene

EEQ39214

hypothetical protein
  
Accession: EEQ39215
  
Location: 310645-311526
  
 NCBI BlastP on this gene

EEQ39215

hypothetical protein
  
Accession: EEQ39216
  
Location: 312176-313228
  
 NCBI BlastP on this gene

EEQ39216

hypothetical protein
  
Accession: EEQ39217
  
Location: 314035-315639
  
 NCBI BlastP on this gene

EEQ39217

hypothetical protein
  
Accession: EEQ39218
  
Location: 314602-315420
  
 NCBI BlastP on this gene

EEQ39218

hypothetical protein
  
Accession: EEQ39219
  
Location: 317628-318842
  
 NCBI BlastP on this gene

EEQ39219

hypothetical protein
  
Accession: EEQ39220
  
Location: 320307-321221
  
 NCBI BlastP on this gene

EEQ39220

predicted protein
  
Accession: EEQ39221
  
Location: 323413-324273
  
 NCBI BlastP on this gene

EEQ39221

hypothetical protein
  
Accession: EEQ39222
  
Location: 324341-325573
  
  
**BlastP hit with Mycgr3G35447\_Mycgr3T**
  
Percentage identity: 38 %
  
BlastP bit score: 250
  
Sequence coverage: 90 %
  
E-value: 1e-74
  
  
 NCBI BlastP on this gene

EEQ39222

conserved hypothetical protein
  
Accession: EEQ39223
  
Location: 325881-327314
  
  
**BlastP hit with Mycgr3G84402\_Mycgr3T**
  
Percentage identity: 59 %
  
BlastP bit score: 491
  
Sequence coverage: 89 %
  
E-value: 3e-167
  
  
 NCBI BlastP on this gene

EEQ39223

hypothetical protein
  
Accession: EEQ39224
  
Location: 327512-328342
  
 NCBI BlastP on this gene

EEQ39224

hypothetical protein
  
Accession: EEQ39225
  
Location: 329065-329541
  
 NCBI BlastP on this gene

EEQ39225

hypothetical protein
  
Accession: EEQ39226
  
Location: 329613-331838
  
 NCBI BlastP on this gene

EEQ39226

hypothetical protein
  
Accession: EEQ39227
  
Location: 331954-332946
  
 NCBI BlastP on this gene

EEQ39227

predicted protein
  
Accession: EEQ39228
  
Location: 333567-334127
  
 NCBI BlastP on this gene

EEQ39228

predicted protein
  
Accession: EEQ39229
  
Location: 334000-335337
  
 NCBI BlastP on this gene

EEQ39229

40S ribosomal protein S27-B
  
Accession: EEQ39230
  
Location: 336824-337072
  
 NCBI BlastP on this gene

EEQ39230

hypothetical protein
  
Accession: EEQ39231
  
Location: 337795-340173
  
 NCBI BlastP on this gene

EEQ39231

hypothetical protein
  
Accession: EEQ39232
  
Location: 340242-341093
  
 NCBI BlastP on this gene

EEQ39232

hypothetical protein
  
Accession: EEQ39233
  
Location: 341780-342766
  
 NCBI BlastP on this gene

EEQ39233

hypothetical protein
  
Accession: EEQ39234
  
Location: 342130-342729
  
 NCBI BlastP on this gene

EEQ39234

hypothetical protein
  
Accession: EEQ39235
  
Location: 342835-343920
  
 NCBI BlastP on this gene

EEQ39235

hypothetical protein
  
Accession: EEQ39236
  
Location: 344616-348860
  
 NCBI BlastP on this gene

EEQ39236

Query: Architecture Search FASTA input

FR839628 : Pichia pastoris CBS 7435 chromosome 1    Total score: 2.0     Cumulative Blast bit score: 737

Hit cluster cross-links:

Mycgr3G67791 Mycgr3T
  
Location: 0-1542

Mycgr3G67791\_Mycgr3T

Mycgr3G90406 Mycgr3T
  
Location: 1642-3973

Mycgr3G90406\_Mycgr3T

Mycgr3G67785 Mycgr3T
  
Location: 4073-7865

Mycgr3G67785\_Mycgr3T

Mycgr3G67795 Mycgr3T
  
Location: 7965-15249

Mycgr3G67795\_Mycgr3T

Mycgr3G67775 Mycgr3T
  
Location: 15349-16237

Mycgr3G67775\_Mycgr3T

Mycgr3G90404 Mycgr3T
  
Location: 16337-17246

Mycgr3G90404\_Mycgr3T

Mycgr3G36951 Mycgr3T
  
Location: 17346-30891

Mycgr3G36951\_Mycgr3T

Mycgr3G103034 Mycgr3
  
Location: 30991-32644

Mycgr3G103034\_Mycgr3

Mycgr3G31119 Mycgr3T
  
Location: 32744-32906

Mycgr3G31119\_Mycgr3T

Mycgr3G28587 Mycgr3T
  
Location: 33006-33489

Mycgr3G28587\_Mycgr3T

Mycgr3G98959 Mycgr3T
  
Location: 33589-35035

Mycgr3G98959\_Mycgr3T

Mycgr3G35447 Mycgr3T
  
Location: 35135-36443

Mycgr3G35447\_Mycgr3T

Mycgr3G84402 Mycgr3T
  
Location: 36543-37884

Mycgr3G84402\_Mycgr3T

Mycgr3G98961 Mycgr3T
  
Location: 37984-38884

Mycgr3G98961\_Mycgr3T

Arsenical-resistance protein ACR3
  
Accession: CCA36748
  
Location: 1093439-1094710
  
 NCBI BlastP on this gene

ARR3

ribonucleoside-diphosphate reductase subunit M1
  
Accession: CCA36749
  
Location: 1095139-1097742
  
 NCBI BlastP on this gene

Rrm1

40S ribosomal protein S27
  
Accession: CCA36750
  
Location: 1098498-1098746
  
 NCBI BlastP on this gene

PP7435\_Chr1-0601

Uncharacterized transporter C11D3.18C
  
Accession: CCA36751
  
Location: 1099244-1100728
  
 NCBI BlastP on this gene

THI73

hypothetical protein
  
Accession: CCA36752
  
Location: 1100839-1101822
  
 NCBI BlastP on this gene

PP7435\_Chr1-0603

F-box protein YDR306C
  
Accession: CCA36753
  
Location: 1102300-1103532
  
 NCBI BlastP on this gene

PP7435\_Chr1-0604

dolichyl-phosphate-mannose-protein mannosyltransferase
  
Accession: CCA36754
  
Location: 1103612-1105963
  
 NCBI BlastP on this gene

PP7435\_Chr1-0605

Allantoate permease
  
Accession: CCA36755
  
Location: 1106543-1108054
  
 NCBI BlastP on this gene

PP7435\_Chr1-0606

Mediator of RNA polymerase II transcription subunit 21
  
Accession: CCA36756
  
Location: 1108117-1108533
  
 NCBI BlastP on this gene

PP7435\_Chr1-0607

Coiled-coil domain-containing protein 115
  
Accession: CCA36757
  
Location: 1108716-1109453
  
 NCBI BlastP on this gene

PP7435\_Chr1-0608

hypothetical protein
  
Accession: CCA36758
  
Location: 1109586-1110158
  
 NCBI BlastP on this gene

PP7435\_Chr1-0609

Suppressor of SWI4 1 homolog
  
Accession: CCA36759
  
Location: 1111559-1112785
  
  
**BlastP hit with Mycgr3G35447\_Mycgr3T**
  
Percentage identity: 37 %
  
BlastP bit score: 246
  
Sequence coverage: 94 %
  
E-value: 5e-73
  
  
 NCBI BlastP on this gene

PP7435\_Chr1-0610

ATP-dependent RNA helicase
  
Accession: CCA36760
  
Location: 1113141-1114577
  
  
**BlastP hit with Mycgr3G84402\_Mycgr3T**
  
Percentage identity: 57 %
  
BlastP bit score: 491
  
Sequence coverage: 93 %
  
E-value: 3e-167
  
  
 NCBI BlastP on this gene

RRP3

putative secreted protein
  
Accession: CCA36761
  
Location: 1114904-1116250
  
 NCBI BlastP on this gene

PP7435\_Chr1-0612

Coatomer subunit alpha
  
Accession: CCA36762
  
Location: 1116552-1120172
  
 NCBI BlastP on this gene

PP7435\_Chr1-0613

hypothetical protein
  
Accession: CCA36763
  
Location: 1120248-1121624
  
 NCBI BlastP on this gene

PP7435\_Chr1-0614

37S ribosomal protein rsm18, mitochondrial
  
Accession: CCA36764
  
Location: 1122150-1122605
  
 NCBI BlastP on this gene

PP7435\_Chr1-0615

[Pyruvate dehydrogenase [lipoamide]] kinase isozyme 2, mitochondrial
  
Accession: CCA36765
  
Location: 1122672-1123976
  
 NCBI BlastP on this gene

PDK2

Protein LDB17
  
Accession: CCA36766
  
Location: 1124356-1125672
  
 NCBI BlastP on this gene

PP7435\_Chr1-0617

hypothetical protein
  
Accession: CCA36767
  
Location: 1126150-1126811
  
 NCBI BlastP on this gene

PP7435\_Chr1-0618

26S proteasome non-ATPase regulatory subunit 12
  
Accession: CCA36768
  
Location: 1126923-1128248
  
 NCBI BlastP on this gene

PP7435\_Chr1-0619

High-affinity glucose transporter RGT2
  
Accession: CCA36769
  
Location: 1128727-1130838
  
 NCBI BlastP on this gene

SNF3

hypothetical protein
  
Accession: CCA36770
  
Location: 1131139-1132167
  
 NCBI BlastP on this gene

PP7435\_Chr1-0621

60S ribosomal protein L35
  
Accession: CCA36771
  
Location: 1132494-1133152
  
 NCBI BlastP on this gene

PP7435\_Chr1-0622

ubiquitin conjugation factor E4 B
  
Accession: CCA36772
  
Location: 1133245-1136109
  
 NCBI BlastP on this gene

UFD2

Query: Architecture Search FASTA input

FN392319 : Pichia pastoris GS115 chromosome 1    Total score: 2.0     Cumulative Blast bit score: 737

Hit cluster cross-links:

Mycgr3G67791 Mycgr3T
  
Location: 0-1542

Mycgr3G67791\_Mycgr3T

Mycgr3G90406 Mycgr3T
  
Location: 1642-3973

Mycgr3G90406\_Mycgr3T

Mycgr3G67785 Mycgr3T
  
Location: 4073-7865

Mycgr3G67785\_Mycgr3T

Mycgr3G67795 Mycgr3T
  
Location: 7965-15249

Mycgr3G67795\_Mycgr3T

Mycgr3G67775 Mycgr3T
  
Location: 15349-16237

Mycgr3G67775\_Mycgr3T

Mycgr3G90404 Mycgr3T
  
Location: 16337-17246

Mycgr3G90404\_Mycgr3T

Mycgr3G36951 Mycgr3T
  
Location: 17346-30891

Mycgr3G36951\_Mycgr3T

Mycgr3G103034 Mycgr3
  
Location: 30991-32644

Mycgr3G103034\_Mycgr3

Mycgr3G31119 Mycgr3T
  
Location: 32744-32906

Mycgr3G31119\_Mycgr3T

Mycgr3G28587 Mycgr3T
  
Location: 33006-33489

Mycgr3G28587\_Mycgr3T

Mycgr3G98959 Mycgr3T
  
Location: 33589-35035

Mycgr3G98959\_Mycgr3T

Mycgr3G35447 Mycgr3T
  
Location: 35135-36443

Mycgr3G35447\_Mycgr3T

Mycgr3G84402 Mycgr3T
  
Location: 36543-37884

Mycgr3G84402\_Mycgr3T

Mycgr3G98961 Mycgr3T
  
Location: 37984-38884

Mycgr3G98961\_Mycgr3T

Arsenite transporter of the plasma membrane, required for resistance to arsenic compounds
  
Accession: CAY67655
  
Location: 1093416-1094687
  
 NCBI BlastP on this gene

PAS\_chr1-1\_0281

One of two large regulatory subunits of ribonucleotide-diphosphate reductase
  
Accession: CAY67656
  
Location: 1095115-1097718
  
 NCBI BlastP on this gene

PAS\_chr1-1\_0282

Protein component of the small (40S) ribosomal subunit
  
Accession: CAY67657
  
Location: 1098474-1098722
  
 NCBI BlastP on this gene

PAS\_chr1-1\_0283

Putative plasma membrane permease proposed to be involved in carboxylic acid uptake
  
Accession: CAY67658
  
Location: 1099220-1100704
  
 NCBI BlastP on this gene

PAS\_chr1-1\_0284

hypothetical protein
  
Accession: CAY67659
  
Location: 1100815-1101798
  
 NCBI BlastP on this gene

PAS\_chr1-1\_0492

F-box protein of unknown function
  
Accession: CAY67660
  
Location: 1102276-1103508
  
 NCBI BlastP on this gene

PAS\_chr1-1\_0285

Protein O-mannosyltransferase, transfers mannose residues from dolichyl phosphate-D-mannose
  
Accession: CAY67661
  
Location: 1103714-1105939
  
 NCBI BlastP on this gene

PAS\_chr1-1\_0286

Allantoin permease
  
Accession: CAY67662
  
Location: 1106519-1108030
  
 NCBI BlastP on this gene

PAS\_chr1-1\_0287

Subunit of the RNA polymerase II mediator complex
  
Accession: CAY67663
  
Location: 1108093-1108509
  
 NCBI BlastP on this gene

PAS\_chr1-1\_0288

hypothetical protein
  
Accession: CAY67664
  
Location: 1108692-1109429
  
 NCBI BlastP on this gene

PAS\_chr1-1\_0493

Hypothetical protein
  
Accession: CAY67665
  
Location: 1109562-1110134
  
 NCBI BlastP on this gene

PAS\_chr1-1\_0289

RING-type ubiquitin ligase of the endosomal and vacuolar membranes
  
Accession: CAY67666
  
Location: 1110628-1111299
  
 NCBI BlastP on this gene

PAS\_chr1-1\_0290

Protein required for ribosomal large subunit maturation, functionally redundant with Ssf1p
  
Accession: CAY67667
  
Location: 1111535-1112761
  
  
**BlastP hit with Mycgr3G35447\_Mycgr3T**
  
Percentage identity: 37 %
  
BlastP bit score: 246
  
Sequence coverage: 94 %
  
E-value: 5e-73
  
  
 NCBI BlastP on this gene

PAS\_chr1-1\_0291

Protein involved in rRNA processing
  
Accession: CAY67668
  
Location: 1113117-1114553
  
  
**BlastP hit with Mycgr3G84402\_Mycgr3T**
  
Percentage identity: 57 %
  
BlastP bit score: 491
  
Sequence coverage: 93 %
  
E-value: 3e-167
  
  
 NCBI BlastP on this gene

PAS\_chr1-1\_0292

Cell wall protein that functions in the transfer of chitin to beta(1-6)glucan
  
Accession: CAY67669
  
Location: 1114881-1116227
  
 NCBI BlastP on this gene

PAS\_chr1-1\_0293

Alpha subunit of COPI vesicle coatomer complex
  
Accession: CAY67670
  
Location: 1116529-1120149
  
 NCBI BlastP on this gene

PAS\_chr1-1\_0294

JmjC domain family histone demethylase specific for H3-K36
  
Accession: CAY67671
  
Location: 1120225-1121799
  
 NCBI BlastP on this gene

PAS\_chr1-1\_0295

Mitochondrial ribosomal protein of the small subunit
  
Accession: CAY67672
  
Location: 1122127-1122582
  
 NCBI BlastP on this gene

PAS\_chr1-1\_0296

Mitochondrial protein kinase
  
Accession: CAY67673
  
Location: 1122649-1123953
  
 NCBI BlastP on this gene

PAS\_chr1-1\_0297

Protein of unknown function
  
Accession: CAY67674
  
Location: 1124333-1125649
  
 NCBI BlastP on this gene

PAS\_chr1-1\_0298

hypothetical protein
  
Accession: CAY67675
  
Location: 1126127-1126903
  
 NCBI BlastP on this gene

PAS\_chr1-1\_0495

Essential, non-ATPase regulatory subunit of the 26S proteasome lid
  
Accession: CAY67676
  
Location: 1126900-1128225
  
 NCBI BlastP on this gene

PAS\_chr1-1\_0299

Plasma membrane glucose sensor that regulates glucose transport
  
Accession: CAY67677
  
Location: 1128726-1130816
  
 NCBI BlastP on this gene

PAS\_chr1-1\_0300

Prenyltransferase, required for cell viability
  
Accession: CAY67678
  
Location: 1131117-1132145
  
 NCBI BlastP on this gene

PAS\_chr1-1\_0301

Protein component of the large (60S) ribosomal subunit, identical to Rpl35Ap
  
Accession: CAY67679
  
Location: 1132471-1133129
  
 NCBI BlastP on this gene

PAS\_chr1-1\_0302

Ubiquitin chain assembly factor (E4) that cooperates with a ubiquitin-activating enzyme (E1)
  
Accession: CAY67680
  
Location: 1133222-1136086
  
 NCBI BlastP on this gene

PAS\_chr1-1\_0303

Query: Architecture Search FASTA input

FM992695 : Candida dubliniensis CD36 chromosome R    Total score: 2.0     Cumulative Blast bit score: 734

Hit cluster cross-links:

Mycgr3G67791 Mycgr3T
  
Location: 0-1542

Mycgr3G67791\_Mycgr3T

Mycgr3G90406 Mycgr3T
  
Location: 1642-3973

Mycgr3G90406\_Mycgr3T

Mycgr3G67785 Mycgr3T
  
Location: 4073-7865

Mycgr3G67785\_Mycgr3T

Mycgr3G67795 Mycgr3T
  
Location: 7965-15249

Mycgr3G67795\_Mycgr3T

Mycgr3G67775 Mycgr3T
  
Location: 15349-16237

Mycgr3G67775\_Mycgr3T

Mycgr3G90404 Mycgr3T
  
Location: 16337-17246

Mycgr3G90404\_Mycgr3T

Mycgr3G36951 Mycgr3T
  
Location: 17346-30891

Mycgr3G36951\_Mycgr3T

Mycgr3G103034 Mycgr3
  
Location: 30991-32644

Mycgr3G103034\_Mycgr3

Mycgr3G31119 Mycgr3T
  
Location: 32744-32906

Mycgr3G31119\_Mycgr3T

Mycgr3G28587 Mycgr3T
  
Location: 33006-33489

Mycgr3G28587\_Mycgr3T

Mycgr3G98959 Mycgr3T
  
Location: 33589-35035

Mycgr3G98959\_Mycgr3T

Mycgr3G35447 Mycgr3T
  
Location: 35135-36443

Mycgr3G35447\_Mycgr3T

Mycgr3G84402 Mycgr3T
  
Location: 36543-37884

Mycgr3G84402\_Mycgr3T

Mycgr3G98961 Mycgr3T
  
Location: 37984-38884

Mycgr3G98961\_Mycgr3T

translation machinery-associated protein, putative
  
Accession: CAX40437
  
Location: 2016858-2017052
  
 NCBI BlastP on this gene

CD36\_34817

actin assembly factor, putative
  
Accession: CAX40438
  
Location: 2017378-2019402
  
 NCBI BlastP on this gene

CD36\_34820

conserved hypothetical protein
  
Accession: CAX40439
  
Location: 2019903-2021057
  
 NCBI BlastP on this gene

CD36\_34830

S-formylglutathione hydrolase, putative
  
Accession: CAX40440
  
Location: 2021082-2021975
  
 NCBI BlastP on this gene

CD36\_34840

sphingoid long-chain base transporter, putative
  
Accession: CAX40441
  
Location: 2022447-2023868
  
 NCBI BlastP on this gene

CD36\_34850

lysophospholipase precursor, putative
  
Accession: CAX40442
  
Location: 2027492-2029378
  
 NCBI BlastP on this gene

CD36\_34860

inorganic phosphate transporter, putative
  
Accession: CAX40443
  
Location: 2031680-2033359
  
 NCBI BlastP on this gene

CD36\_34870

peroxisomal coenzyme A diphosphatase, peroxisomal precursor, putative
  
Accession: CAX40444
  
Location: 2034327-2035466
  
 NCBI BlastP on this gene

CD36\_34880

conserved hypothetical protein
  
Accession: CAX40445
  
Location: 2035712-2036287
  
 NCBI BlastP on this gene

CD36\_34885

ribosome biogenesis protein, putative
  
Accession: CAX40446
  
Location: 2036376-2037725
  
  
**BlastP hit with Mycgr3G35447\_Mycgr3T**
  
Percentage identity: 37 %
  
BlastP bit score: 242
  
Sequence coverage: 94 %
  
E-value: 5e-71
  
  
 NCBI BlastP on this gene

CD36\_34890

ATP-dependent rRNA helicase, putative
  
Accession: CAX40447
  
Location: 2038023-2039522
  
  
**BlastP hit with Mycgr3G84402\_Mycgr3T**
  
Percentage identity: 60 %
  
BlastP bit score: 492
  
Sequence coverage: 90 %
  
E-value: 3e-167
  
  
 NCBI BlastP on this gene

CD36\_34900

phosphatidylinositol-3-phosphate-binding protein, putative
  
Accession: CAX40448
  
Location: 2039599-2040627
  
 NCBI BlastP on this gene

CD36\_34910

conserved hypothetical protein
  
Accession: CAX40449
  
Location: 2041334-2041921
  
 NCBI BlastP on this gene

CD36\_34915

dolichyl-phosphate-mannose-protein mannosyltransferase, putative
  
Accession: CAX40450
  
Location: 2041973-2044150
  
 NCBI BlastP on this gene

CD36\_34920

LPF family protein, putative
  
Accession: CAX40451
  
Location: 2046068-2048167
  
 NCBI BlastP on this gene

CD36\_34930

Query: Architecture Search FASTA input

KB733474 : Bipolaris maydis ATCC 48331 unplaced genomic scaffold COCC4scaffold\_31    Total score: 2.0     Cumulative Blast bit score: 630

Hit cluster cross-links:

Mycgr3G67791 Mycgr3T
  
Location: 0-1542

Mycgr3G67791\_Mycgr3T

Mycgr3G90406 Mycgr3T
  
Location: 1642-3973

Mycgr3G90406\_Mycgr3T

Mycgr3G67785 Mycgr3T
  
Location: 4073-7865

Mycgr3G67785\_Mycgr3T

Mycgr3G67795 Mycgr3T
  
Location: 7965-15249

Mycgr3G67795\_Mycgr3T

Mycgr3G67775 Mycgr3T
  
Location: 15349-16237

Mycgr3G67775\_Mycgr3T

Mycgr3G90404 Mycgr3T
  
Location: 16337-17246

Mycgr3G90404\_Mycgr3T

Mycgr3G36951 Mycgr3T
  
Location: 17346-30891

Mycgr3G36951\_Mycgr3T

Mycgr3G103034 Mycgr3
  
Location: 30991-32644

Mycgr3G103034\_Mycgr3

Mycgr3G31119 Mycgr3T
  
Location: 32744-32906

Mycgr3G31119\_Mycgr3T

Mycgr3G28587 Mycgr3T
  
Location: 33006-33489

Mycgr3G28587\_Mycgr3T

Mycgr3G98959 Mycgr3T
  
Location: 33589-35035

Mycgr3G98959\_Mycgr3T

Mycgr3G35447 Mycgr3T
  
Location: 35135-36443

Mycgr3G35447\_Mycgr3T

Mycgr3G84402 Mycgr3T
  
Location: 36543-37884

Mycgr3G84402\_Mycgr3T

Mycgr3G98961 Mycgr3T
  
Location: 37984-38884

Mycgr3G98961\_Mycgr3T

hypothetical protein
  
Accession: ENI00475
  
Location: 115603-116224
  
 NCBI BlastP on this gene

ENI00475

hypothetical protein
  
Accession: ENI00476
  
Location: 116583-116926
  
 NCBI BlastP on this gene

ENI00476

hypothetical protein
  
Accession: ENI00477
  
Location: 117252-139356
  
  
**BlastP hit with Mycgr3G36951\_Mycgr3T**
  
Percentage identity: 26 %
  
BlastP bit score: 397
  
Sequence coverage: 33 %
  
E-value: 5e-107
  
  
 NCBI BlastP on this gene

ENI00477

hypothetical protein
  
Accession: ENI00478
  
Location: 139861-140364
  
 NCBI BlastP on this gene

ENI00478

hypothetical protein
  
Accession: ENI00479
  
Location: 140945-142566
  
 NCBI BlastP on this gene

ENI00479

hypothetical protein
  
Accession: ENI00480
  
Location: 143102-143839
  
 NCBI BlastP on this gene

ENI00480

hypothetical protein
  
Accession: ENI00481
  
Location: 144594-145646
  
 NCBI BlastP on this gene

ENI00481

hypothetical protein
  
Accession: ENI00482
  
Location: 146544-147726
  
 NCBI BlastP on this gene

ENI00482

hypothetical protein
  
Accession: ENI00483
  
Location: 148458-150330
  
 NCBI BlastP on this gene

ENI00483

hypothetical protein
  
Accession: ENI00484
  
Location: 150611-153478
  
 NCBI BlastP on this gene

ENI00484

hypothetical protein
  
Accession: ENI00485
  
Location: 156675-158089
  
 NCBI BlastP on this gene

ENI00485

hypothetical protein
  
Accession: ENI00486
  
Location: 158150-158958
  
 NCBI BlastP on this gene

ENI00486

hypothetical protein
  
Accession: ENI00487
  
Location: 159593-160402
  
 NCBI BlastP on this gene

ENI00487

hypothetical protein
  
Accession: ENI00488
  
Location: 160650-161894
  
  
**BlastP hit with Mycgr3G67791\_Mycgr3T**
  
Percentage identity: 32 %
  
BlastP bit score: 233
  
Sequence coverage: 82 %
  
E-value: 3e-67
  
  
 NCBI BlastP on this gene

ENI00488

hypothetical protein
  
Accession: ENI00489
  
Location: 164062-164693
  
 NCBI BlastP on this gene

ENI00489

Query: Architecture Search FASTA input

FP929139 : Leptosphaeria maculans JN3 lm\_SuperContig\_0\_v2 genomic supercontig    Total score: 2.0     Cumulative Blast bit score: 607

Hit cluster cross-links:

Mycgr3G67791 Mycgr3T
  
Location: 0-1542

Mycgr3G67791\_Mycgr3T

Mycgr3G90406 Mycgr3T
  
Location: 1642-3973

Mycgr3G90406\_Mycgr3T

Mycgr3G67785 Mycgr3T
  
Location: 4073-7865

Mycgr3G67785\_Mycgr3T

Mycgr3G67795 Mycgr3T
  
Location: 7965-15249

Mycgr3G67795\_Mycgr3T

Mycgr3G67775 Mycgr3T
  
Location: 15349-16237

Mycgr3G67775\_Mycgr3T

Mycgr3G90404 Mycgr3T
  
Location: 16337-17246

Mycgr3G90404\_Mycgr3T

Mycgr3G36951 Mycgr3T
  
Location: 17346-30891

Mycgr3G36951\_Mycgr3T

Mycgr3G103034 Mycgr3
  
Location: 30991-32644

Mycgr3G103034\_Mycgr3

Mycgr3G31119 Mycgr3T
  
Location: 32744-32906

Mycgr3G31119\_Mycgr3T

Mycgr3G28587 Mycgr3T
  
Location: 33006-33489

Mycgr3G28587\_Mycgr3T

Mycgr3G98959 Mycgr3T
  
Location: 33589-35035

Mycgr3G98959\_Mycgr3T

Mycgr3G35447 Mycgr3T
  
Location: 35135-36443

Mycgr3G35447\_Mycgr3T

Mycgr3G84402 Mycgr3T
  
Location: 36543-37884

Mycgr3G84402\_Mycgr3T

Mycgr3G98961 Mycgr3T
  
Location: 37984-38884

Mycgr3G98961\_Mycgr3T

hypothetical protein
  
Accession: CBY02108
  
Location: 3089333-3090020
  
 NCBI BlastP on this gene

LEMA\_P008950.1

similar to C4-dicarboxylate transporter/malic acid transport protein
  
Accession: CBY02109
  
Location: 3092351-3093610
  
  
**BlastP hit with Mycgr3G67791\_Mycgr3T**
  
Percentage identity: 31 %
  
BlastP bit score: 211
  
Sequence coverage: 84 %
  
E-value: 4e-59
  
  
 NCBI BlastP on this gene

LEMA\_P008960.1

predicted protein
  
Accession: CBY02110
  
Location: 3094169-3094447
  
 NCBI BlastP on this gene

LEMA\_uP008970.1

hypothetical protein
  
Accession: CBY02111
  
Location: 3094894-3099025
  
 NCBI BlastP on this gene

LEMA\_P008980.1

similar to MFS transporter
  
Accession: CBY02112
  
Location: 3099159-3100841
  
 NCBI BlastP on this gene

LEMA\_P008990.1

predicted protein
  
Accession: CBY02113
  
Location: 3102156-3103294
  
 NCBI BlastP on this gene

LEMA\_P009000.1

predicted protein
  
Accession: CBY02114
  
Location: 3104087-3105211
  
 NCBI BlastP on this gene

LEMA\_P009010.1

similar to ABC multidrug transporter
  
Accession: CBY02115
  
Location: 3106079-3111642
  
 NCBI BlastP on this gene

LEMA\_P009020.1

predicted protein
  
Accession: CBY02116
  
Location: 3113323-3114354
  
 NCBI BlastP on this gene

LEMA\_P009030.1

similar to nonribosomal peptide synthase
  
Accession: CBY02117
  
Location: 3115242-3137649
  
  
**BlastP hit with Mycgr3G36951\_Mycgr3T**
  
Percentage identity: 26 %
  
BlastP bit score: 396
  
Sequence coverage: 32 %
  
E-value: 9e-107
  
  
 NCBI BlastP on this gene

LEMA\_P009040.1

similar to rhamnogalacturonate lyase
  
Accession: CBY02118
  
Location: 3138626-3140467
  
 NCBI BlastP on this gene

LEMA\_P009050.1

Query: Architecture Search FASTA input

GL534459 : Pyrenophora teres f. teres 0-1 unplaced genomic scaffold scaffold\_190719    Total score: 2.0     Cumulative Blast bit score: 584

Hit cluster cross-links:

Mycgr3G67791 Mycgr3T
  
Location: 0-1542

Mycgr3G67791\_Mycgr3T

Mycgr3G90406 Mycgr3T
  
Location: 1642-3973

Mycgr3G90406\_Mycgr3T

Mycgr3G67785 Mycgr3T
  
Location: 4073-7865

Mycgr3G67785\_Mycgr3T

Mycgr3G67795 Mycgr3T
  
Location: 7965-15249

Mycgr3G67795\_Mycgr3T

Mycgr3G67775 Mycgr3T
  
Location: 15349-16237

Mycgr3G67775\_Mycgr3T

Mycgr3G90404 Mycgr3T
  
Location: 16337-17246

Mycgr3G90404\_Mycgr3T

Mycgr3G36951 Mycgr3T
  
Location: 17346-30891

Mycgr3G36951\_Mycgr3T

Mycgr3G103034 Mycgr3
  
Location: 30991-32644

Mycgr3G103034\_Mycgr3

Mycgr3G31119 Mycgr3T
  
Location: 32744-32906

Mycgr3G31119\_Mycgr3T

Mycgr3G28587 Mycgr3T
  
Location: 33006-33489

Mycgr3G28587\_Mycgr3T

Mycgr3G98959 Mycgr3T
  
Location: 33589-35035

Mycgr3G98959\_Mycgr3T

Mycgr3G35447 Mycgr3T
  
Location: 35135-36443

Mycgr3G35447\_Mycgr3T

Mycgr3G84402 Mycgr3T
  
Location: 36543-37884

Mycgr3G84402\_Mycgr3T

Mycgr3G98961 Mycgr3T
  
Location: 37984-38884

Mycgr3G98961\_Mycgr3T

hypothetical protein
  
Accession: EFQ92067
  
Location: 6868-8987
  
 NCBI BlastP on this gene

EFQ92067

hypothetical protein
  
Accession: EFQ92068
  
Location: 9593-11135
  
 NCBI BlastP on this gene

EFQ92068

hypothetical protein
  
Accession: EFQ92069
  
Location: 11882-12418
  
 NCBI BlastP on this gene

EFQ92069

hypothetical protein
  
Accession: EFQ92070
  
Location: 15120-15701
  
 NCBI BlastP on this gene

EFQ92070

hypothetical protein
  
Accession: EFQ92071
  
Location: 17941-19050
  
  
**BlastP hit with Mycgr3G67791\_Mycgr3T**
  
Percentage identity: 32 %
  
BlastP bit score: 203
  
Sequence coverage: 69 %
  
E-value: 1e-56
  
  
 NCBI BlastP on this gene

EFQ92071

hypothetical protein
  
Accession: EFQ92072
  
Location: 19293-19652
  
 NCBI BlastP on this gene

EFQ92072

hypothetical protein
  
Accession: EFQ92073
  
Location: 19943-41977
  
  
**BlastP hit with Mycgr3G36951\_Mycgr3T**
  
Percentage identity: 26 %
  
BlastP bit score: 381
  
Sequence coverage: 34 %
  
E-value: 4e-102
  
  
 NCBI BlastP on this gene

EFQ92073

hypothetical protein
  
Accession: EFQ92074
  
Location: 46095-51051
  
 NCBI BlastP on this gene

EFQ92074

hypothetical protein
  
Accession: EFQ92075
  
Location: 51524-53825
  
 NCBI BlastP on this gene

EFQ92075

Query: Architecture Search FASTA input

CU329670 : Schizosaccharomyces pombe chromosome I    Total score: 2.0     Cumulative Blast bit score: 543

Hit cluster cross-links:

Mycgr3G67791 Mycgr3T
  
Location: 0-1542

Mycgr3G67791\_Mycgr3T

Mycgr3G90406 Mycgr3T
  
Location: 1642-3973

Mycgr3G90406\_Mycgr3T

Mycgr3G67785 Mycgr3T
  
Location: 4073-7865

Mycgr3G67785\_Mycgr3T

Mycgr3G67795 Mycgr3T
  
Location: 7965-15249

Mycgr3G67795\_Mycgr3T

Mycgr3G67775 Mycgr3T
  
Location: 15349-16237

Mycgr3G67775\_Mycgr3T

Mycgr3G90404 Mycgr3T
  
Location: 16337-17246

Mycgr3G90404\_Mycgr3T

Mycgr3G36951 Mycgr3T
  
Location: 17346-30891

Mycgr3G36951\_Mycgr3T

Mycgr3G103034 Mycgr3
  
Location: 30991-32644

Mycgr3G103034\_Mycgr3

Mycgr3G31119 Mycgr3T
  
Location: 32744-32906

Mycgr3G31119\_Mycgr3T

Mycgr3G28587 Mycgr3T
  
Location: 33006-33489

Mycgr3G28587\_Mycgr3T

Mycgr3G98959 Mycgr3T
  
Location: 33589-35035

Mycgr3G98959\_Mycgr3T

Mycgr3G35447 Mycgr3T
  
Location: 35135-36443

Mycgr3G35447\_Mycgr3T

Mycgr3G84402 Mycgr3T
  
Location: 36543-37884

Mycgr3G84402\_Mycgr3T

Mycgr3G98961 Mycgr3T
  
Location: 37984-38884

Mycgr3G98961\_Mycgr3T

ubiquitin C-terminal hydrolase, poly(A)-specific ribonuclease complex subunit Pan2 (predicted)
  
Accession: CAA91128
  
Location: 732880-736465
  
 NCBI BlastP on this gene

ubp13

sequence orphan
  
Accession: CAA91127
  
Location: 731938-732648
  
 NCBI BlastP on this gene

SPAC22G7.03

karyopherin Kap111 (predicted)
  
Accession: CAA91126
  
Location: 728420-731706
  
 NCBI BlastP on this gene

kap111

iron responsive transcriptional regulator, peptidase family (predicted)
  
Accession: CAB62423
  
Location: 726081-728076
  
 NCBI BlastP on this gene

SPAC22G7.01c

actin cortical patch component Lsb4 (predicted)
  
Accession: CAB62422
  
Location: 724289-725720
  
 NCBI BlastP on this gene

SPAPJ696.02

retromer complex subunit Vps17
  
Accession: CAB62421
  
Location: 721617-723425
  
 NCBI BlastP on this gene

vps17

NADPH-dependent diflavin oxidoreductase,
  
Accession: CAB36512
  
Location: 719447-721410
  
 NCBI BlastP on this gene

tah18

cyclin L family cyclin (predicted)
  
Accession: CAB36511
  
Location: 716940-717804
  
  
**BlastP hit with Mycgr3G98961\_Mycgr3T**
  
Percentage identity: 28 %
  
BlastP bit score: 84
  
Sequence coverage: 80 %
  
E-value: 6e-16
  
  
 NCBI BlastP on this gene

SPAC1296.05c

spore wall assembly protein (predicted)
  
Accession: CAB36510
  
Location: 715480-716621
  
 NCBI BlastP on this gene

mug65

serine carboxypeptidase Sxa2
  
Accession: CAB36509
  
Location: 712491-714014
  
 NCBI BlastP on this gene

sxa2

cytochrome c oxidase subunit IV (predicted)
  
Accession: CAB36508
  
Location: 711301-712150
  
 NCBI BlastP on this gene

cox4

phosphoacetylglucosamine mutase (predicted)
  
Accession: CAA91066
  
Location: 708055-710023
  
  
**BlastP hit with Mycgr3G103034\_Mycgr3**
  
Percentage identity: 46 %
  
BlastP bit score: 459
  
Sequence coverage: 95 %
  
E-value: 3e-152
  
  
 NCBI BlastP on this gene

SPAC1296.01c

transcription factor Atf31
  
Accession: CAA91067
  
Location: 706844-707473
  
 NCBI BlastP on this gene

atf31

ATP-dependent DNA helicase Rdh54
  
Accession: CAA91068
  
Location: 703789-706828
  
 NCBI BlastP on this gene

rdh54

AMP binding enzyme (predicted)
  
Accession: CAA91069
  
Location: 698033-702533
  
 NCBI BlastP on this gene

mug62

ADP-ribosylation factor Alp41
  
Accession: CAA91070
  
Location: 697291-698068
  
 NCBI BlastP on this gene

alp41

Lon protease homolog Lon1 (predicted)
  
Accession: CAA91071
  
Location: 693831-697034
  
 NCBI BlastP on this gene

lon1

GatB/YqeY domain protein
  
Accession: CBA11491
  
Location: 692567-693061
  
 NCBI BlastP on this gene

SPAC22F3.15

F0-ATPase subunit G (predicted)
  
Accession: CAA91072
  
Location: 691530-691984
  
 NCBI BlastP on this gene

atp20

Query: Architecture Search FASTA input

KB445561 : Baudoinia compniacensis UAMH 10762 unplaced genomic scaffold BAUCOscaffold\_12    Total score: 2.0     Cumulative Blast bit score: 524

Hit cluster cross-links:

Mycgr3G67791 Mycgr3T
  
Location: 0-1542

Mycgr3G67791\_Mycgr3T

Mycgr3G90406 Mycgr3T
  
Location: 1642-3973

Mycgr3G90406\_Mycgr3T

Mycgr3G67785 Mycgr3T
  
Location: 4073-7865

Mycgr3G67785\_Mycgr3T

Mycgr3G67795 Mycgr3T
  
Location: 7965-15249

Mycgr3G67795\_Mycgr3T

Mycgr3G67775 Mycgr3T
  
Location: 15349-16237

Mycgr3G67775\_Mycgr3T

Mycgr3G90404 Mycgr3T
  
Location: 16337-17246

Mycgr3G90404\_Mycgr3T

Mycgr3G36951 Mycgr3T
  
Location: 17346-30891

Mycgr3G36951\_Mycgr3T

Mycgr3G103034 Mycgr3
  
Location: 30991-32644

Mycgr3G103034\_Mycgr3

Mycgr3G31119 Mycgr3T
  
Location: 32744-32906

Mycgr3G31119\_Mycgr3T

Mycgr3G28587 Mycgr3T
  
Location: 33006-33489

Mycgr3G28587\_Mycgr3T

Mycgr3G98959 Mycgr3T
  
Location: 33589-35035

Mycgr3G98959\_Mycgr3T

Mycgr3G35447 Mycgr3T
  
Location: 35135-36443

Mycgr3G35447\_Mycgr3T

Mycgr3G84402 Mycgr3T
  
Location: 36543-37884

Mycgr3G84402\_Mycgr3T

Mycgr3G98961 Mycgr3T
  
Location: 37984-38884

Mycgr3G98961\_Mycgr3T

glycoside hydrolase family 16 protein
  
Accession: EMC92656
  
Location: 211429-212813
  
 NCBI BlastP on this gene

EMC92656

hypothetical protein
  
Accession: EMC92657
  
Location: 213466-213663
  
 NCBI BlastP on this gene

EMC92657

hypothetical protein
  
Accession: EMC92658
  
Location: 214005-218039
  
 NCBI BlastP on this gene

EMC92658

hypothetical protein
  
Accession: EMC92659
  
Location: 218563-218865
  
 NCBI BlastP on this gene

EMC92659

hypothetical protein
  
Accession: EMC92660
  
Location: 219891-220836
  
 NCBI BlastP on this gene

EMC92660

hypothetical protein
  
Accession: EMC92661
  
Location: 220955-221227
  
 NCBI BlastP on this gene

EMC92661

hypothetical protein
  
Accession: EMC92662
  
Location: 222551-223261
  
 NCBI BlastP on this gene

EMC92662

hypothetical protein
  
Accession: EMC92663
  
Location: 224544-224756
  
 NCBI BlastP on this gene

EMC92663

hypothetical protein
  
Accession: EMC92664
  
Location: 225388-226582
  
 NCBI BlastP on this gene

EMC92664

hypothetical protein
  
Accession: EMC92665
  
Location: 227251-228507
  
 NCBI BlastP on this gene

EMC92665

glycosyltransferase family 2 protein
  
Accession: EMC92666
  
Location: 228684-229451
  
 NCBI BlastP on this gene

EMC92666

hypothetical protein
  
Accession: EMC92667
  
Location: 229778-230711
  
  
**BlastP hit with Mycgr3G98961\_Mycgr3T**
  
Percentage identity: 59 %
  
BlastP bit score: 342
  
Sequence coverage: 96 %
  
E-value: 6e-114
  
  
 NCBI BlastP on this gene

EMC92667

hypothetical protein
  
Accession: EMC92668
  
Location: 232087-234101
  
  
**BlastP hit with Mycgr3G90404\_Mycgr3T**
  
Percentage identity: 56 %
  
BlastP bit score: 182
  
Sequence coverage: 49 %
  
E-value: 2e-49
  
  
 NCBI BlastP on this gene

EMC92668

hypothetical protein
  
Accession: EMC92669
  
Location: 236331-238115
  
 NCBI BlastP on this gene

EMC92669

hypothetical protein
  
Accession: EMC92670
  
Location: 238465-241030
  
 NCBI BlastP on this gene

EMC92670

hypothetical protein
  
Accession: EMC92671
  
Location: 241063-242474
  
 NCBI BlastP on this gene

EMC92671

hypothetical protein
  
Accession: EMC92672
  
Location: 242720-244525
  
 NCBI BlastP on this gene

EMC92672

hypothetical protein
  
Accession: EMC92673
  
Location: 245255-247660
  
 NCBI BlastP on this gene

EMC92673

hypothetical protein
  
Accession: EMC92674
  
Location: 249738-255662
  
 NCBI BlastP on this gene

EMC92674

Query: Architecture Search FASTA input

ADOT01000171 : Arthrobotrys oligospora ATCC 24927    Total score: 2.0     Cumulative Blast bit score: 342

Hit cluster cross-links:

Mycgr3G67791 Mycgr3T
  
Location: 0-1542

Mycgr3G67791\_Mycgr3T

Mycgr3G90406 Mycgr3T
  
Location: 1642-3973

Mycgr3G90406\_Mycgr3T

Mycgr3G67785 Mycgr3T
  
Location: 4073-7865

Mycgr3G67785\_Mycgr3T

Mycgr3G67795 Mycgr3T
  
Location: 7965-15249

Mycgr3G67795\_Mycgr3T

Mycgr3G67775 Mycgr3T
  
Location: 15349-16237

Mycgr3G67775\_Mycgr3T

Mycgr3G90404 Mycgr3T
  
Location: 16337-17246

Mycgr3G90404\_Mycgr3T

Mycgr3G36951 Mycgr3T
  
Location: 17346-30891

Mycgr3G36951\_Mycgr3T

Mycgr3G103034 Mycgr3
  
Location: 30991-32644

Mycgr3G103034\_Mycgr3

Mycgr3G31119 Mycgr3T
  
Location: 32744-32906

Mycgr3G31119\_Mycgr3T

Mycgr3G28587 Mycgr3T
  
Location: 33006-33489

Mycgr3G28587\_Mycgr3T

Mycgr3G98959 Mycgr3T
  
Location: 33589-35035

Mycgr3G98959\_Mycgr3T

Mycgr3G35447 Mycgr3T
  
Location: 35135-36443

Mycgr3G35447\_Mycgr3T

Mycgr3G84402 Mycgr3T
  
Location: 36543-37884

Mycgr3G84402\_Mycgr3T

Mycgr3G98961 Mycgr3T
  
Location: 37984-38884

Mycgr3G98961\_Mycgr3T

hypothetical protein
  
Accession: EGX47162
  
Location: 1439-1765
  
 NCBI BlastP on this gene

EGX47162

hypothetical protein
  
Accession: EGX47163
  
Location: 2811-4157
  
 NCBI BlastP on this gene

EGX47163

hypothetical protein
  
Accession: EGX47164
  
Location: 7787-10021
  
 NCBI BlastP on this gene

EGX47164

hypothetical protein
  
Accession: EGX47165
  
Location: 10653-12811
  
 NCBI BlastP on this gene

EGX47165

hypothetical protein
  
Accession: EGX47166
  
Location: 14003-15742
  
  
**BlastP hit with Mycgr3G90404\_Mycgr3T**
  
Percentage identity: 32 %
  
BlastP bit score: 110
  
Sequence coverage: 71 %
  
E-value: 7e-24
  
  
 NCBI BlastP on this gene

EGX47166

hypothetical protein
  
Accession: EGX47167
  
Location: 16427-17287
  
 NCBI BlastP on this gene

EGX47167

hypothetical protein
  
Accession: EGX47168
  
Location: 17821-18807
  
 NCBI BlastP on this gene

EGX47168

hypothetical protein
  
Accession: EGX47169
  
Location: 23161-24474
  
  
**BlastP hit with Mycgr3G67791\_Mycgr3T**
  
Percentage identity: 30 %
  
BlastP bit score: 232
  
Sequence coverage: 80 %
  
E-value: 1e-66
  
  
 NCBI BlastP on this gene

EGX47169

hypothetical protein
  
Accession: EGX47170
  
Location: 27256-28266
  
 NCBI BlastP on this gene

EGX47170

hypothetical protein
  
Accession: EGX47171
  
Location: 28472-29332
  
 NCBI BlastP on this gene

EGX47171

hypothetical protein
  
Accession: EGX47172
  
Location: 30077-31085
  
 NCBI BlastP on this gene

EGX47172

hypothetical protein
  
Accession: EGX47173
  
Location: 33465-34693
  
 NCBI BlastP on this gene

EGX47173

hypothetical protein
  
Accession: EGX47174
  
Location: 35576-38196
  
 NCBI BlastP on this gene

EGX47174

hypothetical protein
  
Accession: EGX47175
  
Location: 39610-40663
  
 NCBI BlastP on this gene

EGX47175

Query: Architecture Search FASTA input

JH971386 : Agaricus bisporus var. burnettii JB137-S8 unplaced genomic scaffold AGABI1scaffold\_2    Total score: 2.0     Cumulative Blast bit score: 318

Hit cluster cross-links:

Mycgr3G67791 Mycgr3T
  
Location: 0-1542

Mycgr3G67791\_Mycgr3T

Mycgr3G90406 Mycgr3T
  
Location: 1642-3973

Mycgr3G90406\_Mycgr3T

Mycgr3G67785 Mycgr3T
  
Location: 4073-7865

Mycgr3G67785\_Mycgr3T

Mycgr3G67795 Mycgr3T
  
Location: 7965-15249

Mycgr3G67795\_Mycgr3T

Mycgr3G67775 Mycgr3T
  
Location: 15349-16237

Mycgr3G67775\_Mycgr3T

Mycgr3G90404 Mycgr3T
  
Location: 16337-17246

Mycgr3G90404\_Mycgr3T

Mycgr3G36951 Mycgr3T
  
Location: 17346-30891

Mycgr3G36951\_Mycgr3T

Mycgr3G103034 Mycgr3
  
Location: 30991-32644

Mycgr3G103034\_Mycgr3

Mycgr3G31119 Mycgr3T
  
Location: 32744-32906

Mycgr3G31119\_Mycgr3T

Mycgr3G28587 Mycgr3T
  
Location: 33006-33489

Mycgr3G28587\_Mycgr3T

Mycgr3G98959 Mycgr3T
  
Location: 33589-35035

Mycgr3G98959\_Mycgr3T

Mycgr3G35447 Mycgr3T
  
Location: 35135-36443

Mycgr3G35447\_Mycgr3T

Mycgr3G84402 Mycgr3T
  
Location: 36543-37884

Mycgr3G84402\_Mycgr3T

Mycgr3G98961 Mycgr3T
  
Location: 37984-38884

Mycgr3G98961\_Mycgr3T

hypothetical protein
  
Accession: EKM82815
  
Location: 1448903-1449377
  
 NCBI BlastP on this gene

EKM82815

hypothetical protein
  
Accession: EKM82816
  
Location: 1449648-1450061
  
 NCBI BlastP on this gene

EKM82816

hypothetical protein
  
Accession: EKM82817
  
Location: 1450329-1452071
  
 NCBI BlastP on this gene

EKM82817

hypothetical protein
  
Accession: EKM82818
  
Location: 1454336-1456021
  
 NCBI BlastP on this gene

EKM82818

hypothetical protein
  
Accession: EKM82819
  
Location: 1456066-1460005
  
  
**BlastP hit with Mycgr3G98961\_Mycgr3T**
  
Percentage identity: 31 %
  
BlastP bit score: 100
  
Sequence coverage: 81 %
  
E-value: 4e-20
  
  
 NCBI BlastP on this gene

EKM82819

hypothetical protein
  
Accession: EKM82820
  
Location: 1460713-1461959
  
 NCBI BlastP on this gene

EKM82820

hypothetical protein
  
Accession: EKM82821
  
Location: 1462197-1463249
  
 NCBI BlastP on this gene

EKM82821

hypothetical protein
  
Accession: EKM82822
  
Location: 1464688-1467100
  
 NCBI BlastP on this gene

EKM82822

hypothetical protein
  
Accession: EKM82823
  
Location: 1469019-1473814
  
 NCBI BlastP on this gene

EKM82823

hypothetical protein
  
Accession: EKM82824
  
Location: 1476422-1476632
  
 NCBI BlastP on this gene

EKM82824

hypothetical protein
  
Accession: EKM82825
  
Location: 1476699-1477681
  
 NCBI BlastP on this gene

EKM82825

hypothetical protein
  
Accession: EKM82826
  
Location: 1478204-1481493
  
 NCBI BlastP on this gene

EKM82826

hypothetical protein
  
Accession: EKM82827
  
Location: 1481611-1482473
  
 NCBI BlastP on this gene

EKM82827

hypothetical protein
  
Accession: EKM82828
  
Location: 1482600-1484052
  
  
**BlastP hit with Mycgr3G35447\_Mycgr3T**
  
Percentage identity: 35 %
  
BlastP bit score: 218
  
Sequence coverage: 101 %
  
E-value: 3e-62
  
  
 NCBI BlastP on this gene

EKM82828

hypothetical protein
  
Accession: EKM82829
  
Location: 1484114-1484863
  
 NCBI BlastP on this gene

EKM82829

hypothetical protein
  
Accession: EKM82830
  
Location: 1485033-1486692
  
 NCBI BlastP on this gene

EKM82830

hypothetical protein
  
Accession: EKM82831
  
Location: 1487016-1488355
  
 NCBI BlastP on this gene

EKM82831

hypothetical protein
  
Accession: EKM82832
  
Location: 1488430-1489166
  
 NCBI BlastP on this gene

EKM82832

hypothetical protein
  
Accession: EKM82833
  
Location: 1490541-1491738
  
 NCBI BlastP on this gene

EKM82833

Query: Architecture Search FASTA input

JH931606 : Agaricus bisporus var. bisporus H97 unplaced genomic scaffold AGABI2scaffold\_2    Total score: 2.0     Cumulative Blast bit score: 317

Hit cluster cross-links:

Mycgr3G67791 Mycgr3T
  
Location: 0-1542

Mycgr3G67791\_Mycgr3T

Mycgr3G90406 Mycgr3T
  
Location: 1642-3973

Mycgr3G90406\_Mycgr3T

Mycgr3G67785 Mycgr3T
  
Location: 4073-7865

Mycgr3G67785\_Mycgr3T

Mycgr3G67795 Mycgr3T
  
Location: 7965-15249

Mycgr3G67795\_Mycgr3T

Mycgr3G67775 Mycgr3T
  
Location: 15349-16237

Mycgr3G67775\_Mycgr3T

Mycgr3G90404 Mycgr3T
  
Location: 16337-17246

Mycgr3G90404\_Mycgr3T

Mycgr3G36951 Mycgr3T
  
Location: 17346-30891

Mycgr3G36951\_Mycgr3T

Mycgr3G103034 Mycgr3
  
Location: 30991-32644

Mycgr3G103034\_Mycgr3

Mycgr3G31119 Mycgr3T
  
Location: 32744-32906

Mycgr3G31119\_Mycgr3T

Mycgr3G28587 Mycgr3T
  
Location: 33006-33489

Mycgr3G28587\_Mycgr3T

Mycgr3G98959 Mycgr3T
  
Location: 33589-35035

Mycgr3G98959\_Mycgr3T

Mycgr3G35447 Mycgr3T
  
Location: 35135-36443

Mycgr3G35447\_Mycgr3T

Mycgr3G84402 Mycgr3T
  
Location: 36543-37884

Mycgr3G84402\_Mycgr3T

Mycgr3G98961 Mycgr3T
  
Location: 37984-38884

Mycgr3G98961\_Mycgr3T

hypothetical protein
  
Accession: EKV50215
  
Location: 1493683-1494074
  
 NCBI BlastP on this gene

EKV50215

hypothetical protein
  
Accession: EKV50216
  
Location: 1494199-1494612
  
 NCBI BlastP on this gene

EKV50216

hypothetical protein
  
Accession: EKV50217
  
Location: 1494880-1496625
  
 NCBI BlastP on this gene

EKV50217

hypothetical protein
  
Accession: EKV50218
  
Location: 1499281-1500965
  
 NCBI BlastP on this gene

EKV50218

hypothetical protein
  
Accession: EKV50219
  
Location: 1501007-1504940
  
  
**BlastP hit with Mycgr3G98961\_Mycgr3T**
  
Percentage identity: 31 %
  
BlastP bit score: 100
  
Sequence coverage: 81 %
  
E-value: 4e-20
  
  
 NCBI BlastP on this gene

EKV50219

hypothetical protein
  
Accession: EKV50220
  
Location: 1505674-1507000
  
 NCBI BlastP on this gene

EKV50220

hypothetical protein
  
Accession: EKV50221
  
Location: 1507160-1508211
  
 NCBI BlastP on this gene

EKV50221

argonaute-like protein
  
Accession: EKV50222
  
Location: 1509662-1513281
  
 NCBI BlastP on this gene

EKV50222

hypothetical protein
  
Accession: EKV50223
  
Location: 1513995-1518790
  
 NCBI BlastP on this gene

EKV50223

hypothetical protein
  
Accession: EKV50224
  
Location: 1521507-1522922
  
 NCBI BlastP on this gene

EKV50224

hypothetical protein
  
Accession: EKV50225
  
Location: 1523012-1526301
  
 NCBI BlastP on this gene

EKV50225

hypothetical protein
  
Accession: EKV50226
  
Location: 1526648-1527280
  
 NCBI BlastP on this gene

EKV50226

hypothetical protein
  
Accession: EKV50227
  
Location: 1527407-1528859
  
  
**BlastP hit with Mycgr3G35447\_Mycgr3T**
  
Percentage identity: 35 %
  
BlastP bit score: 217
  
Sequence coverage: 102 %
  
E-value: 1e-61
  
  
 NCBI BlastP on this gene

EKV50227

hypothetical protein
  
Accession: EKV50228
  
Location: 1528933-1529682
  
 NCBI BlastP on this gene

EKV50228

hypothetical protein
  
Accession: EKV50229
  
Location: 1529835-1531499
  
 NCBI BlastP on this gene

EKV50229

hypothetical protein
  
Accession: EKV50230
  
Location: 1531955-1533165
  
 NCBI BlastP on this gene

EKV50230

hypothetical protein
  
Accession: EKV50231
  
Location: 1533240-1534841
  
 NCBI BlastP on this gene

EKV50231

hypothetical protein
  
Accession: EKV50232
  
Location: 1535352-1536549
  
 NCBI BlastP on this gene

EKV50232

Query: Architecture Search FASTA input

DS547096 : Laccaria bicolor S238N-H82 LACBIscaffold\_6 genomic scaffold    Total score: 2.0     Cumulative Blast bit score: 315

Hit cluster cross-links:

Mycgr3G67791 Mycgr3T
  
Location: 0-1542

Mycgr3G67791\_Mycgr3T

Mycgr3G90406 Mycgr3T
  
Location: 1642-3973

Mycgr3G90406\_Mycgr3T

Mycgr3G67785 Mycgr3T
  
Location: 4073-7865

Mycgr3G67785\_Mycgr3T

Mycgr3G67795 Mycgr3T
  
Location: 7965-15249

Mycgr3G67795\_Mycgr3T

Mycgr3G67775 Mycgr3T
  
Location: 15349-16237

Mycgr3G67775\_Mycgr3T

Mycgr3G90404 Mycgr3T
  
Location: 16337-17246

Mycgr3G90404\_Mycgr3T

Mycgr3G36951 Mycgr3T
  
Location: 17346-30891

Mycgr3G36951\_Mycgr3T

Mycgr3G103034 Mycgr3
  
Location: 30991-32644

Mycgr3G103034\_Mycgr3

Mycgr3G31119 Mycgr3T
  
Location: 32744-32906

Mycgr3G31119\_Mycgr3T

Mycgr3G28587 Mycgr3T
  
Location: 33006-33489

Mycgr3G28587\_Mycgr3T

Mycgr3G98959 Mycgr3T
  
Location: 33589-35035

Mycgr3G98959\_Mycgr3T

Mycgr3G35447 Mycgr3T
  
Location: 35135-36443

Mycgr3G35447\_Mycgr3T

Mycgr3G84402 Mycgr3T
  
Location: 36543-37884

Mycgr3G84402\_Mycgr3T

Mycgr3G98961 Mycgr3T
  
Location: 37984-38884

Mycgr3G98961\_Mycgr3T

predicted protein
  
Accession: EDR11327
  
Location: 1757062-1757434
  
 NCBI BlastP on this gene

EDR11327

predicted protein
  
Accession: EDR10983
  
Location: 1757572-1758817
  
 NCBI BlastP on this gene

EDR10983

predicted protein
  
Accession: EDR10984
  
Location: 1759565-1762637
  
 NCBI BlastP on this gene

EDR10984

predicted protein
  
Accession: EDR10985
  
Location: 1763185-1763693
  
 NCBI BlastP on this gene

EDR10985

predicted protein
  
Accession: EDR10986
  
Location: 1763845-1764825
  
 NCBI BlastP on this gene

EDR10986

predicted protein
  
Accession: EDR10987
  
Location: 1765102-1765525
  
 NCBI BlastP on this gene

EDR10987

predicted protein
  
Accession: EDR10988
  
Location: 1765649-1767270
  
 NCBI BlastP on this gene

EDR10988

predicted protein
  
Accession: EDR11328
  
Location: 1767737-1769360
  
 NCBI BlastP on this gene

EDR11328

predicted protein
  
Accession: EDR10989
  
Location: 1769448-1770358
  
  
**BlastP hit with Mycgr3G98961\_Mycgr3T**
  
Percentage identity: 29 %
  
BlastP bit score: 98
  
Sequence coverage: 89 %
  
E-value: 7e-21
  
  
 NCBI BlastP on this gene

EDR10989

predicted protein
  
Accession: EDR10990
  
Location: 1770650-1773278
  
 NCBI BlastP on this gene

EDR10990

predicted protein
  
Accession: EDR10991
  
Location: 1773510-1774455
  
 NCBI BlastP on this gene

EDR10991

ER-to-Golgi vesicle protein transport Sft2
  
Accession: EDR11329
  
Location: 1774529-1775496
  
 NCBI BlastP on this gene

EDR11329

predicted protein
  
Accession: EDR11330
  
Location: 1775794-1780521
  
 NCBI BlastP on this gene

EDR11330

predicted protein
  
Accession: EDR11331
  
Location: 1781071-1782384
  
 NCBI BlastP on this gene

EDR11331

predicted protein
  
Accession: EDR10992
  
Location: 1782522-1785928
  
 NCBI BlastP on this gene

EDR10992

predicted protein
  
Accession: EDR11332
  
Location: 1785983-1786838
  
 NCBI BlastP on this gene

EDR11332

predicted protein
  
Accession: EDR10993
  
Location: 1786885-1788321
  
  
**BlastP hit with Mycgr3G35447\_Mycgr3T**
  
Percentage identity: 36 %
  
BlastP bit score: 217
  
Sequence coverage: 105 %
  
E-value: 8e-62
  
  
 NCBI BlastP on this gene

EDR10993

predicted protein
  
Accession: EDR11333
  
Location: 1788346-1789107
  
 NCBI BlastP on this gene

EDR11333

predicted protein
  
Accession: EDR10994
  
Location: 1789221-1790809
  
 NCBI BlastP on this gene

EDR10994

predicted protein
  
Accession: EDR11334
  
Location: 1792967-1794161
  
 NCBI BlastP on this gene

EDR11334

predicted protein
  
Accession: EDR11335
  
Location: 1794664-1795810
  
 NCBI BlastP on this gene

EDR11335

predicted protein
  
Accession: EDR10995
  
Location: 1796053-1797456
  
 NCBI BlastP on this gene

EDR10995

predicted protein
  
Accession: EDR11336
  
Location: 1797481-1799199
  
 NCBI BlastP on this gene

EDR11336

20S proteasome subunit
  
Accession: EDR10996
  
Location: 1799285-1800512
  
 NCBI BlastP on this gene

EDR10996

Sec7-like domain is implicated in guanine nucleotide exchange function
  
Accession: EDR10997
  
Location: 1800764-1806242
  
 NCBI BlastP on this gene

EDR10997

Query: Architecture Search FASTA input

151. :  AM270408 Aspergillus niger contig An18c0160, genomic contig.     Total score: 2.0     Cumulative Blast bit score: 1054

Mycgr3G67791 Mycgr3T
  
Location: 0-1542
  
 NCBI BlastP on this gene

Mycgr3G67791\_Mycgr3T

Mycgr3G90406 Mycgr3T
  
Location: 1642-3973
  
 NCBI BlastP on this gene

Mycgr3G90406\_Mycgr3T

Mycgr3G67785 Mycgr3T
  
Location: 4073-7865
  
 NCBI BlastP on this gene

Mycgr3G67785\_Mycgr3T

Mycgr3G67795 Mycgr3T
  
Location: 7965-15249
  
 NCBI BlastP on this gene

Mycgr3G67795\_Mycgr3T

Mycgr3G67775 Mycgr3T
  
Location: 15349-16237
  
 NCBI BlastP on this gene

Mycgr3G67775\_Mycgr3T

Mycgr3G90404 Mycgr3T
  
Location: 16337-17246
  
 NCBI BlastP on this gene

Mycgr3G90404\_Mycgr3T

Mycgr3G36951 Mycgr3T
  
Location: 17346-30891
  
 NCBI BlastP on this gene

Mycgr3G36951\_Mycgr3T

Mycgr3G103034 Mycgr3
  
Location: 30991-32644
  
 NCBI BlastP on this gene

Mycgr3G103034\_Mycgr3

Mycgr3G31119 Mycgr3T
  
Location: 32744-32906
  
 NCBI BlastP on this gene

Mycgr3G31119\_Mycgr3T

Mycgr3G28587 Mycgr3T
  
Location: 33006-33489
  
 NCBI BlastP on this gene

Mycgr3G28587\_Mycgr3T

Mycgr3G98959 Mycgr3T
  
Location: 33589-35035
  
 NCBI BlastP on this gene

Mycgr3G98959\_Mycgr3T

Mycgr3G35447 Mycgr3T
  
Location: 35135-36443
  
 NCBI BlastP on this gene

Mycgr3G35447\_Mycgr3T

Mycgr3G84402 Mycgr3T
  
Location: 36543-37884
  
 NCBI BlastP on this gene

Mycgr3G84402\_Mycgr3T

Mycgr3G98961 Mycgr3T
  
Location: 37984-38884
  
 NCBI BlastP on this gene

Mycgr3G98961\_Mycgr3T

not annotated
  
Accession: CAK43303
  
Location: 100563-101796
  
 NCBI BlastP on this gene

An18g05050

not annotated
  
Accession: CAK43304
  
Location: 102626-104302
  
 NCBI BlastP on this gene

An18g05060

not annotated
  
Accession: CAK43305
  
Location: 104671-106009
  
 NCBI BlastP on this gene

An18g05070

not annotated
  
Accession: CAK43306
  
Location: 106360-107821
  
 NCBI BlastP on this gene

An18g05080

not annotated
  
Accession: CAK43307
  
Location: 107966-109231
  
 NCBI BlastP on this gene

An18g05090

not annotated
  
Accession: CAK43308
  
Location: 109884-111269
  
 NCBI BlastP on this gene

An18g05100

not annotated
  
Accession: CAK43309
  
Location: 111492-113477
  
 NCBI BlastP on this gene

An18g05110

not annotated
  
Accession: CAK43310
  
Location: 113932-115571
  
 NCBI BlastP on this gene

An18g05120

not annotated
  
Accession: CAK43311
  
Location: 115879-117189
  
 NCBI BlastP on this gene

An18g05130

not annotated
  
Accession: CAK43312
  
Location: 117348-118778
  
  
**BlastP hit with Mycgr3G35447\_Mycgr3T**
  
Percentage identity: 59 %
  
BlastP bit score: 433
  
Sequence coverage: 91 %
  
E-value: 4e-145
  
  
 NCBI BlastP on this gene

An18g05140

not annotated
  
Accession: CAK43313
  
Location: 119025-120580
  
  
**BlastP hit with Mycgr3G84402\_Mycgr3T**
  
Percentage identity: 72 %
  
BlastP bit score: 621
  
Sequence coverage: 89 %
  
E-value: 0.0
  
  
 NCBI BlastP on this gene

An18g05150

not annotated
  
Accession: CAK43314
  
Location: 120950-121588
  
 NCBI BlastP on this gene

An18g05160

152. :  CH445336 Phaeosphaeria nodorum SN15 scaffold\_12     Total score: 2.0     Cumulative Blast bit score: 1036

hypothetical protein
  
Accession: EAT84171
  
Location: 55123-56908
  
 NCBI BlastP on this gene

EAT84171

hypothetical protein
  
Accession: EAT84172
  
Location: 58391-58835
  
 NCBI BlastP on this gene

EAT84172

hypothetical protein
  
Accession: EAT84173
  
Location: 59029-60261
  
 NCBI BlastP on this gene

EAT84173

hypothetical protein
  
Accession: EAT84174
  
Location: 61449-62148
  
 NCBI BlastP on this gene

EAT84174

hypothetical protein
  
Accession: EAT84175
  
Location: 63759-67162
  
 NCBI BlastP on this gene

EAT84175

hypothetical protein
  
Accession: EAT84176
  
Location: 70418-71425
  
 NCBI BlastP on this gene

EAT84176

hypothetical protein
  
Accession: EAT84177
  
Location: 71919-72960
  
 NCBI BlastP on this gene

EAT84177

hypothetical protein
  
Accession: EAT84178
  
Location: 73038-74678
  
  
**BlastP hit with Mycgr3G84402\_Mycgr3T**
  
Percentage identity: 73 %
  
BlastP bit score: 612
  
Sequence coverage: 89 %
  
E-value: 0.0
  
  
 NCBI BlastP on this gene

EAT84178

hypothetical protein
  
Accession: EAT84179
  
Location: 74857-76292
  
  
**BlastP hit with Mycgr3G35447\_Mycgr3T**
  
Percentage identity: 57 %
  
BlastP bit score: 424
  
Sequence coverage: 90 %
  
E-value: 2e-141
  
  
 NCBI BlastP on this gene

EAT84179

hypothetical protein
  
Accession: EAT84180
  
Location: 76387-81945
  
 NCBI BlastP on this gene

EAT84180

hypothetical protein
  
Accession: EAT84181
  
Location: 83219-84580
  
 NCBI BlastP on this gene

EAT84181

hypothetical protein
  
Accession: EAT84182
  
Location: 85100-85780
  
 NCBI BlastP on this gene

EAT84182

hypothetical protein
  
Accession: EAT84183
  
Location: 86722-87674
  
 NCBI BlastP on this gene

EAT84183

hypothetical protein
  
Accession: EAT84184
  
Location: 88892-89566
  
 NCBI BlastP on this gene

EAT84184

hypothetical protein
  
Accession: EAT84185
  
Location: 89872-90198
  
 NCBI BlastP on this gene

EAT84185

hypothetical protein
  
Accession: EAT84186
  
Location: 90955-91740
  
 NCBI BlastP on this gene

EAT84186

hypothetical protein
  
Accession: EAT84187
  
Location: 93525-94717
  
 NCBI BlastP on this gene

EAT84187

hypothetical protein
  
Accession: EAT84188
  
Location: 95609-96347
  
 NCBI BlastP on this gene

EAT84188

153. :  KB730000 Fusarium oxysporum f. sp. cubense race 1 unplaced genomic scaffold scaffold2     Total score: 2.0     Cumulative Blast bit score: 1035

hypothetical protein
  
Accession: ENH74983
  
Location: 2-1414
  
 NCBI BlastP on this gene

ENH74983

hypothetical protein
  
Accession: ENH74984
  
Location: 2413-3507
  
 NCBI BlastP on this gene

ENH74984

hypothetical protein
  
Accession: ENH74985
  
Location: 4169-6148
  
 NCBI BlastP on this gene

ENH74985

Protein phosphatase 2C like protein C10F6.17c
  
Accession: ENH74986
  
Location: 6693-8182
  
 NCBI BlastP on this gene

ENH74986

Brix domain-containing protein C1B9.03c
  
Accession: ENH74987
  
Location: 8718-10097
  
  
**BlastP hit with Mycgr3G35447\_Mycgr3T**
  
Percentage identity: 52 %
  
BlastP bit score: 406
  
Sequence coverage: 102 %
  
E-value: 5e-135
  
  
 NCBI BlastP on this gene

ENH74987

ATP-dependent rRNA helicase RRP3
  
Accession: ENH74988
  
Location: 10316-11822
  
  
**BlastP hit with Mycgr3G84402\_Mycgr3T**
  
Percentage identity: 73 %
  
BlastP bit score: 629
  
Sequence coverage: 91 %
  
E-value: 0.0
  
  
 NCBI BlastP on this gene

ENH74988

hypothetical protein
  
Accession: ENH74989
  
Location: 12692-13755
  
 NCBI BlastP on this gene

ENH74989

Histone transcription regulator 3 like protein
  
Accession: ENH74990
  
Location: 14077-20235
  
 NCBI BlastP on this gene

ENH74990

hypothetical protein
  
Accession: ENH74991
  
Location: 20725-23089
  
 NCBI BlastP on this gene

ENH74991

Ubiquitin-conjugating enzyme E2 14
  
Accession: ENH74992
  
Location: 24266-24795
  
 NCBI BlastP on this gene

ENH74992

GTPase-activating protein gyp3
  
Accession: ENH74993
  
Location: 26578-29532
  
 NCBI BlastP on this gene

ENH74993

154. :  JH794139 Magnaporthe oryzae P131 unplaced genomic scaffold P131\_scaffold00389     Total score: 2.0     Cumulative Blast bit score: 1035

hypothetical protein
  
Accession: ELQ66441
  
Location: 70250-74992
  
 NCBI BlastP on this gene

ELQ66441

mannosyl-oligosaccharide 1,2-alpha-mannosidase IB
  
Accession: ELQ66442
  
Location: 76305-77966
  
 NCBI BlastP on this gene

ELQ66442

lactose permease
  
Accession: ELQ66443
  
Location: 78395-80267
  
 NCBI BlastP on this gene

ELQ66443

42 kDa endochitinase
  
Accession: ELQ66444
  
Location: 81927-83443
  
 NCBI BlastP on this gene

ELQ66444

hypothetical protein
  
Accession: ELQ66445
  
Location: 87172-88372
  
 NCBI BlastP on this gene

ELQ66445

ATP-dependent rRNA helicase rrp3
  
Accession: ELQ66446
  
Location: 88941-90702
  
  
**BlastP hit with Mycgr3G84402\_Mycgr3T**
  
Percentage identity: 70 %
  
BlastP bit score: 601
  
Sequence coverage: 91 %
  
E-value: 0.0
  
  
 NCBI BlastP on this gene

ELQ66446

ribosome biogenesis protein SSF1
  
Accession: ELQ66447
  
Location: 91118-92603
  
  
**BlastP hit with Mycgr3G35447\_Mycgr3T**
  
Percentage identity: 57 %
  
BlastP bit score: 434
  
Sequence coverage: 94 %
  
E-value: 4e-145
  
  
 NCBI BlastP on this gene

ELQ66447

glycosyl hydrolase
  
Accession: ELQ66448
  
Location: 94710-97154
  
 NCBI BlastP on this gene

ELQ66448

155. :  DS572699 Verticillium dahliae VdLs.17 supercont1.5 genomic scaffold     Total score: 2.0     Cumulative Blast bit score: 1034

yetA
  
Accession: EGY22096
  
Location: 1731837-1734614
  
 NCBI BlastP on this gene

EGY22096

allantoate permease
  
Accession: EGY22097
  
Location: 1736059-1738095
  
 NCBI BlastP on this gene

EGY22097

integral membrane protein
  
Accession: EGY22098
  
Location: 1739779-1741049
  
 NCBI BlastP on this gene

EGY22098

FAD binding domain-containing protein
  
Accession: EGY22099
  
Location: 1741279-1742763
  
 NCBI BlastP on this gene

EGY22099

hypothetical protein
  
Accession: EGY22100
  
Location: 1744545-1744862
  
 NCBI BlastP on this gene

EGY22100

hypothetical protein
  
Accession: EGY22101
  
Location: 1747495-1749299
  
 NCBI BlastP on this gene

EGY22101

ribosome biogenesis protein SSF1
  
Accession: EGY22102
  
Location: 1751309-1752758
  
  
**BlastP hit with Mycgr3G35447\_Mycgr3T**
  
Percentage identity: 52 %
  
BlastP bit score: 436
  
Sequence coverage: 103 %
  
E-value: 3e-146
  
  
 NCBI BlastP on this gene

EGY22102

ATP-dependent rRNA helicase rrp-3
  
Accession: EGY22103
  
Location: 1753081-1754867
  
  
**BlastP hit with Mycgr3G84402\_Mycgr3T**
  
Percentage identity: 71 %
  
BlastP bit score: 598
  
Sequence coverage: 89 %
  
E-value: 0.0
  
  
 NCBI BlastP on this gene

EGY22103

hypothetical protein
  
Accession: EGY22104
  
Location: 1755583-1756428
  
 NCBI BlastP on this gene

EGY22104

flavonol synthase
  
Accession: EGY22105
  
Location: 1758390-1759609
  
 NCBI BlastP on this gene

EGY22105

hypothetical protein
  
Accession: EGY22106
  
Location: 1759746-1761406
  
 NCBI BlastP on this gene

EGY22106

hypothetical protein
  
Accession: EGY22107
  
Location: 1763672-1764706
  
 NCBI BlastP on this gene

EGY22107

hypothetical protein
  
Accession: EGY22108
  
Location: 1765472-1766683
  
 NCBI BlastP on this gene

EGY22108

hypothetical protein
  
Accession: EGY22109
  
Location: 1767531-1770697
  
 NCBI BlastP on this gene

EGY22109

dimethylaniline monooxygenase
  
Accession: EGY22110
  
Location: 1771331-1772959
  
 NCBI BlastP on this gene

EGY22110

156. :  CM001235 Magnaporthe oryzae 70-15 chromosome 5     Total score: 2.0     Cumulative Blast bit score: 1033

hypothetical protein
  
Accession: EHA49456
  
Location: 4143097-4143702
  
 NCBI BlastP on this gene

EHA49456

hypothetical protein
  
Accession: EHA49457
  
Location: 4144256-4145985
  
 NCBI BlastP on this gene

EHA49457

hypothetical protein
  
Accession: EHA49458
  
Location: 4147145-4147420
  
 NCBI BlastP on this gene

EHA49458

hypothetical protein
  
Accession: EHA49459
  
Location: 4148099-4149070
  
 NCBI BlastP on this gene

EHA49459

hypothetical protein
  
Accession: EHA49460
  
Location: 4149210-4151088
  
 NCBI BlastP on this gene

EHA49460

hypothetical protein
  
Accession: EHA49461
  
Location: 4152446-4153759
  
 NCBI BlastP on this gene

EHA49461

hypothetical protein
  
Accession: EHA49462
  
Location: 4154898-4155611
  
 NCBI BlastP on this gene

EHA49462

glycosyl hydrolase
  
Accession: EHA49463
  
Location: 4158931-4160807
  
 NCBI BlastP on this gene

EHA49463

ribosome biogenesis protein SSF1
  
Accession: EHA49464
  
Location: 4162914-4164399
  
  
**BlastP hit with Mycgr3G35447\_Mycgr3T**
  
Percentage identity: 57 %
  
BlastP bit score: 434
  
Sequence coverage: 94 %
  
E-value: 4e-145
  
  
 NCBI BlastP on this gene

EHA49464

ATP-dependent rRNA helicase RRP3
  
Accession: EHA49465
  
Location: 4164743-4166576
  
  
**BlastP hit with Mycgr3G84402\_Mycgr3T**
  
Percentage identity: 70 %
  
BlastP bit score: 599
  
Sequence coverage: 91 %
  
E-value: 0.0
  
  
 NCBI BlastP on this gene

EHA49465

hypothetical protein
  
Accession: EHA49466
  
Location: 4167145-4168344
  
 NCBI BlastP on this gene

EHA49466

hypothetical protein
  
Accession: EHA49467
  
Location: 4170304-4171256
  
 NCBI BlastP on this gene

EHA49467

endochitinase
  
Accession: EHA49468
  
Location: 4171908-4173424
  
 NCBI BlastP on this gene

EHA49468

lactose permease
  
Accession: EHA49469
  
Location: 4175088-4176965
  
 NCBI BlastP on this gene

EHA49469

mannosyl-oligosaccharide 1,2-alpha-mannosidase IB
  
Accession: EHA49470
  
Location: 4177394-4179055
  
 NCBI BlastP on this gene

EHA49470

hypothetical protein
  
Accession: EHA49471
  
Location: 4180174-4181049
  
 NCBI BlastP on this gene

EHA49471

hypothetical protein
  
Accession: EHA49472
  
Location: 4182465-4184917
  
 NCBI BlastP on this gene

EHA49472

hypothetical protein
  
Accession: EHA49473
  
Location: 4186055-4186624
  
 NCBI BlastP on this gene

EHA49473

157. :  DS985220 Verticillium albo-atrum VaMs.102 supercont1.7 genomic scaffold     Total score: 2.0     Cumulative Blast bit score: 1031

allantoate permease
  
Accession: EEY20278
  
Location: 1738916-1740952
  
 NCBI BlastP on this gene

EEY20278

integral membrane protein
  
Accession: EEY20279
  
Location: 1742636-1743908
  
 NCBI BlastP on this gene

EEY20279

FAD binding domain-containing protein
  
Accession: EEY20280
  
Location: 1744117-1745609
  
 NCBI BlastP on this gene

EEY20280

conserved hypothetical protein
  
Accession: EEY20281
  
Location: 1747877-1749258
  
 NCBI BlastP on this gene

EEY20281

conserved hypothetical protein
  
Accession: EEY20282
  
Location: 1752604-1754410
  
 NCBI BlastP on this gene

EEY20282

ribosome biogenesis protein SSF1
  
Accession: EEY20283
  
Location: 1756393-1757842
  
  
**BlastP hit with Mycgr3G35447\_Mycgr3T**
  
Percentage identity: 55 %
  
BlastP bit score: 437
  
Sequence coverage: 96 %
  
E-value: 1e-146
  
  
 NCBI BlastP on this gene

EEY20283

ATP-dependent rRNA helicase RRP3
  
Accession: EEY20284
  
Location: 1758165-1759949
  
  
**BlastP hit with Mycgr3G84402\_Mycgr3T**
  
Percentage identity: 68 %
  
BlastP bit score: 594
  
Sequence coverage: 93 %
  
E-value: 0.0
  
  
 NCBI BlastP on this gene

EEY20284

conserved hypothetical protein
  
Accession: EEY20285
  
Location: 1760651-1761523
  
 NCBI BlastP on this gene

EEY20285

flavonol synthase
  
Accession: EEY20286
  
Location: 1763452-1764661
  
 NCBI BlastP on this gene

EEY20286

predicted protein
  
Accession: EEY20287
  
Location: 1764868-1765705
  
 NCBI BlastP on this gene

EEY20287

conserved hypothetical protein
  
Accession: EEY20288
  
Location: 1775945-1776217
  
 NCBI BlastP on this gene

EEY20288

conserved hypothetical protein
  
Accession: EEY20289
  
Location: 1779030-1782180
  
 NCBI BlastP on this gene

EEY20289

158. :  JH793228 Magnaporthe oryzae Y34 unplaced genomic scaffold Y34\_scaffold00073     Total score: 2.0     Cumulative Blast bit score: 1030

hypothetical protein
  
Accession: ELQ44566
  
Location: 333-1025
  
 NCBI BlastP on this gene

ELQ44566

glycosyl hydrolase
  
Accession: ELQ44567
  
Location: 1353-3710
  
 NCBI BlastP on this gene

ELQ44567

ribosome biogenesis protein SSF1
  
Accession: ELQ44568
  
Location: 5904-7389
  
  
**BlastP hit with Mycgr3G35447\_Mycgr3T**
  
Percentage identity: 57 %
  
BlastP bit score: 434
  
Sequence coverage: 94 %
  
E-value: 4e-145
  
  
 NCBI BlastP on this gene

ELQ44568

ATP-dependent rRNA helicase rrp3
  
Accession: ELQ44569
  
Location: 7805-11334
  
  
**BlastP hit with Mycgr3G84402\_Mycgr3T**
  
Percentage identity: 70 %
  
BlastP bit score: 596
  
Sequence coverage: 91 %
  
E-value: 0.0
  
  
 NCBI BlastP on this gene

ELQ44569

hypothetical protein
  
Accession: ELQ44570
  
Location: 11952-13070
  
 NCBI BlastP on this gene

ELQ44570

42 kDa endochitinase
  
Accession: ELQ44571
  
Location: 14897-16413
  
 NCBI BlastP on this gene

ELQ44571

lactose permease
  
Accession: ELQ44572
  
Location: 18078-19949
  
 NCBI BlastP on this gene

ELQ44572

mannosyl-oligosaccharide 1,2-alpha-mannosidase IB
  
Accession: ELQ44573
  
Location: 20380-22041
  
 NCBI BlastP on this gene

ELQ44573

hypothetical protein
  
Accession: ELQ44574
  
Location: 23370-25364
  
 NCBI BlastP on this gene

ELQ44574

hypothetical protein
  
Accession: ELQ44575
  
Location: 25661-28113
  
 NCBI BlastP on this gene

ELQ44575

hypothetical protein
  
Accession: ELQ44576
  
Location: 29053-29139
  
 NCBI BlastP on this gene

ELQ44576

hypothetical protein
  
Accession: ELQ44577
  
Location: 29251-29820
  
 NCBI BlastP on this gene

ELQ44577

159. :  FP929065 Leptosphaeria maculans JN3 lm\_SuperContig\_8\_v2 genomic supercontig     Total score: 2.0     Cumulative Blast bit score: 1027

predicted protein
  
Accession: CBX91007
  
Location: 1290671-1290998
  
 NCBI BlastP on this gene

LEMA\_uP060410.1

predicted protein
  
Accession: CBX91008
  
Location: 1292236-1293447
  
 NCBI BlastP on this gene

LEMA\_P060420.1

predicted protein
  
Accession: CBX91009
  
Location: 1295165-1295719
  
 NCBI BlastP on this gene

LEMA\_P060430.1

hypothetical protein
  
Accession: CBX91010
  
Location: 1296527-1297390
  
 NCBI BlastP on this gene

LEMA\_P060440.1

similar to monooxygenase FAD-binding
  
Accession: CBX91011
  
Location: 1299572-1300884
  
 NCBI BlastP on this gene

LEMA\_P060450.1

predicted protein
  
Accession: CBX91012
  
Location: 1301631-1302579
  
 NCBI BlastP on this gene

LEMA\_P060460.1

predicted protein
  
Accession: CBX91013
  
Location: 1303011-1303633
  
 NCBI BlastP on this gene

LEMA\_P060470.1

hypothetical protein
  
Accession: CBX91014
  
Location: 1304782-1305333
  
 NCBI BlastP on this gene

LEMA\_P060480.1

similar to hemolysin-III channel protein Izh2
  
Accession: CBX91015
  
Location: 1305774-1306775
  
 NCBI BlastP on this gene

LEMA\_P060490.1

hypothetical protein
  
Accession: CBX91016
  
Location: 1307370-1308406
  
 NCBI BlastP on this gene

LEMA\_P060500.1

hypothetical protein
  
Accession: CBX91017
  
Location: 1308642-1310064
  
  
**BlastP hit with Mycgr3G35447\_Mycgr3T**
  
Percentage identity: 57 %
  
BlastP bit score: 419
  
Sequence coverage: 88 %
  
E-value: 1e-139
  
  
 NCBI BlastP on this gene

LEMA\_P060510.1

hypothetical protein
  
Accession: CBX91018
  
Location: 1310237-1311836
  
  
**BlastP hit with Mycgr3G84402\_Mycgr3T**
  
Percentage identity: 72 %
  
BlastP bit score: 608
  
Sequence coverage: 89 %
  
E-value: 0.0
  
  
 NCBI BlastP on this gene

LEMA\_P060520.1

predicted protein
  
Accession: CBX91019
  
Location: 1312028-1312477
  
 NCBI BlastP on this gene

LEMA\_P060530.1

predicted protein
  
Accession: CBX91020
  
Location: 1312601-1313587
  
 NCBI BlastP on this gene

LEMA\_P060540.1

similar to MFS monocarboxylate transporter
  
Accession: CBX91021
  
Location: 1314496-1315983
  
 NCBI BlastP on this gene

LEMA\_P060550.1

hypothetical protein
  
Accession: CBX91022
  
Location: 1317923-1319474
  
 NCBI BlastP on this gene

LEMA\_P060560.1

similar to N2,N2-dimethylguanosine tRNA methyltransferase
  
Accession: CBX91023
  
Location: 1320527-1322605
  
 NCBI BlastP on this gene

LEMA\_P060570.1

hypothetical protein
  
Accession: CBX91024
  
Location: 1323243-1324529
  
 NCBI BlastP on this gene

LEMA\_P060580.1

predicted protein
  
Accession: CBX91025
  
Location: 1324699-1325970
  
 NCBI BlastP on this gene

LEMA\_P060590.1

hypothetical protein
  
Accession: CBX91026
  
Location: 1326777-1327980
  
 NCBI BlastP on this gene

LEMA\_P060600.1

hypothetical protein
  
Accession: CBX91027
  
Location: 1328240-1329784
  
 NCBI BlastP on this gene

LEMA\_P060610.1

hypothetical protein
  
Accession: CBX91028
  
Location: 1330283-1334106
  
 NCBI BlastP on this gene

LEMA\_P060620.1

160. :  CH476597 Aspergillus terreus NIH2624 scaffold\_4 genomic scaffold     Total score: 2.0     Cumulative Blast bit score: 1017

serine/threonine-protein kinase sck1
  
Accession: EAU36256
  
Location: 546105-548797
  
 NCBI BlastP on this gene

EAU36256

conserved hypothetical protein
  
Accession: EAU36257
  
Location: 552309-554663
  
 NCBI BlastP on this gene

EAU36257

26S protease regulatory subunit 6A-B
  
Accession: EAU36258
  
Location: 555494-557087
  
 NCBI BlastP on this gene

EAU36258

hypothetical protein
  
Accession: EAU36259
  
Location: 557370-559414
  
 NCBI BlastP on this gene

EAU36259

conserved hypothetical protein
  
Accession: EAU36260
  
Location: 559798-560927
  
 NCBI BlastP on this gene

EAU36260

HNRNP arginine N-methyltransferase
  
Accession: EAU36261
  
Location: 561782-563135
  
 NCBI BlastP on this gene

EAU36261

conserved hypothetical protein
  
Accession: EAU36262
  
Location: 563922-565092
  
 NCBI BlastP on this gene

EAU36262

hypothetical protein
  
Accession: EAU36263
  
Location: 565654-567681
  
  
**BlastP hit with Mycgr3G103034\_Mycgr3**
  
Percentage identity: 52 %
  
BlastP bit score: 585
  
Sequence coverage: 103 %
  
E-value: 0.0
  
  
 NCBI BlastP on this gene

EAU36263

conserved hypothetical protein
  
Accession: EAU36264
  
Location: 568125-569544
  
  
**BlastP hit with Mycgr3G35447\_Mycgr3T**
  
Percentage identity: 59 %
  
BlastP bit score: 432
  
Sequence coverage: 88 %
  
E-value: 1e-144
  
  
 NCBI BlastP on this gene

EAU36264

riboflavin synthase alpha chain
  
Accession: EAU36265
  
Location: 569957-570585
  
 NCBI BlastP on this gene

EAU36265

hypothetical protein
  
Accession: EAU36266
  
Location: 570990-572623
  
 NCBI BlastP on this gene

EAU36266

conserved hypothetical protein
  
Accession: EAU36267
  
Location: 573339-574733
  
 NCBI BlastP on this gene

EAU36267

conserved hypothetical protein
  
Accession: EAU36268
  
Location: 574972-576509
  
 NCBI BlastP on this gene

EAU36268

conserved hypothetical protein
  
Accession: EAU36269
  
Location: 577325-577827
  
 NCBI BlastP on this gene

EAU36269

predicted protein
  
Accession: EAU36270
  
Location: 578549-579718
  
 NCBI BlastP on this gene

EAU36270

predicted protein
  
Accession: EAU36271
  
Location: 581042-581642
  
 NCBI BlastP on this gene

EAU36271

predicted protein
  
Accession: EAU36272
  
Location: 582134-582433
  
 NCBI BlastP on this gene

EAU36272

conserved hypothetical protein
  
Accession: EAU36273
  
Location: 582939-584980
  
 NCBI BlastP on this gene

EAU36273

predicted protein
  
Accession: EAU36274
  
Location: 585814-588011
  
 NCBI BlastP on this gene

EAU36274

conserved hypothetical protein
  
Accession: EAU36275
  
Location: 588848-590078
  
 NCBI BlastP on this gene

EAU36275

161. :  KB933264 Togninia minima UCRPA7 unplaced genomic scaffold PA7\_03\_scaffold\_496     Total score: 2.0     Cumulative Blast bit score: 1001

putative sugar transporter protein
  
Accession: EON97545
  
Location: 1009100-1010623
  
 NCBI BlastP on this gene

EON97545

putative atp-dependent rrna helicase rrp3 protein
  
Accession: EON97455
  
Location: 1014440-1015794
  
  
**BlastP hit with Mycgr3G84402\_Mycgr3T**
  
Percentage identity: 73 %
  
BlastP bit score: 560
  
Sequence coverage: 80 %
  
E-value: 0.0
  
  
 NCBI BlastP on this gene

EON97455

putative ribosome biogenesis protein ssf1 protein
  
Accession: EON97642
  
Location: 1016288-1017743
  
  
**BlastP hit with Mycgr3G35447\_Mycgr3T**
  
Percentage identity: 54 %
  
BlastP bit score: 441
  
Sequence coverage: 103 %
  
E-value: 4e-148
  
  
 NCBI BlastP on this gene

EON97642

putative succinate dehydrogenase fumarate reductase flavoprotein subunit protein
  
Accession: EON97680
  
Location: 1026039-1028105
  
 NCBI BlastP on this gene

EON97680

hypothetical protein
  
Accession: EON97582
  
Location: 1033730-1034287
  
 NCBI BlastP on this gene

EON97582

162. :  CABT02000002 Sordaria macrospora k-hell     Total score: 2.0     Cumulative Blast bit score: 993

not annotated
  
Accession: CCC07065
  
Location: 1757910-1760331
  
 NCBI BlastP on this gene

CCC07065

not annotated
  
Accession: CCC07066
  
Location: 1761071-1762217
  
 NCBI BlastP on this gene

CCC07066

not annotated
  
Accession: CCC07067
  
Location: 1762547-1763236
  
 NCBI BlastP on this gene

CCC07067

not annotated
  
Accession: CCC07068
  
Location: 1764182-1764513
  
 NCBI BlastP on this gene

CCC07068

not annotated
  
Accession: CCC07069
  
Location: 1765068-1766339
  
 NCBI BlastP on this gene

CCC07069

not annotated
  
Accession: CCC07070
  
Location: 1767792-1769066
  
 NCBI BlastP on this gene

CCC07070

not annotated
  
Accession: CCC07071
  
Location: 1771086-1773341
  
 NCBI BlastP on this gene

CCC07071

not annotated
  
Accession: CCC07072
  
Location: 1773641-1775339
  
  
**BlastP hit with Mycgr3G84402\_Mycgr3T**
  
Percentage identity: 67 %
  
BlastP bit score: 534
  
Sequence coverage: 88 %
  
E-value: 0.0
  
  
 NCBI BlastP on this gene

CCC07072

not annotated
  
Accession: CCC07073
  
Location: 1775693-1777189
  
  
**BlastP hit with Mycgr3G35447\_Mycgr3T**
  
Percentage identity: 54 %
  
BlastP bit score: 459
  
Sequence coverage: 103 %
  
E-value: 5e-155
  
  
 NCBI BlastP on this gene

CCC07073

not annotated
  
Accession: CCC07074
  
Location: 1779052-1779436
  
 NCBI BlastP on this gene

CCC07074

not annotated
  
Accession: CCC07075
  
Location: 1782982-1783425
  
 NCBI BlastP on this gene

CCC07075

not annotated
  
Accession: CCC07076
  
Location: 1785534-1787112
  
 NCBI BlastP on this gene

CCC07076

not annotated
  
Accession: CCC07077
  
Location: 1788998-1790740
  
 NCBI BlastP on this gene

CCC07077

not annotated
  
Accession: CCC07078
  
Location: 1792558-1794342
  
 NCBI BlastP on this gene

CCC07078

not annotated
  
Accession: CCC07079
  
Location: 1794689-1795929
  
 NCBI BlastP on this gene

CCC07079

163. :  FN430009 Tuber melanosporum whole genome shotgun sequence assembly, scaffold\_12, strain Mel28.     Total score: 2.0     Cumulative Blast bit score: 975

not annotated
  
Accession: CAZ80070
  
Location: 560316-562116
  
 NCBI BlastP on this gene

CAZ80070

not annotated
  
Accession: CAZ80071
  
Location: 562680-563387
  
 NCBI BlastP on this gene

CAZ80071

not annotated
  
Accession: CAZ80072
  
Location: 563802-565127
  
 NCBI BlastP on this gene

CAZ80072

not annotated
  
Accession: CAZ80073
  
Location: 565352-567722
  
 NCBI BlastP on this gene

CAZ80073

not annotated
  
Accession: CAZ80074
  
Location: 568909-569753
  
 NCBI BlastP on this gene

CAZ80074

not annotated
  
Accession: CAZ80075
  
Location: 569964-571096
  
  
**BlastP hit with Mycgr3G67775\_Mycgr3T**
  
Percentage identity: 53 %
  
BlastP bit score: 288
  
Sequence coverage: 94 %
  
E-value: 1e-92
  
  
 NCBI BlastP on this gene

CAZ80075

not annotated
  
Accession: CAZ80076
  
Location: 571598-573427
  
  
**BlastP hit with Mycgr3G98959\_Mycgr3T**
  
Percentage identity: 67 %
  
BlastP bit score: 687
  
Sequence coverage: 100 %
  
E-value: 0.0
  
  
 NCBI BlastP on this gene

CAZ80076

164. :  JH226130 Exophiala dermatitidis NIH/UT8656 unplaced genomic scaffold supercont1.1     Total score: 2.0     Cumulative Blast bit score: 970

deoxyribodipyrimidine photo-lyase
  
Accession: EHY53006
  
Location: 3393096-3394721
  
 NCBI BlastP on this gene

EHY53006

hypothetical protein
  
Accession: EHY53007
  
Location: 3395663-3397556
  
 NCBI BlastP on this gene

EHY53007

hypothetical protein
  
Accession: EHY53008
  
Location: 3398518-3399258
  
 NCBI BlastP on this gene

EHY53008

hypothetical protein
  
Accession: EHY53009
  
Location: 3399780-3400295
  
 NCBI BlastP on this gene

EHY53009

hypothetical protein
  
Accession: EHY53010
  
Location: 3401208-3402128
  
 NCBI BlastP on this gene

EHY53010

hypothetical protein
  
Accession: EHY53011
  
Location: 3403002-3404299
  
 NCBI BlastP on this gene

EHY53011

hypothetical protein
  
Accession: EHY53012
  
Location: 3404997-3406049
  
  
**BlastP hit with Mycgr3G67775\_Mycgr3T**
  
Percentage identity: 43 %
  
BlastP bit score: 266
  
Sequence coverage: 113 %
  
E-value: 1e-83
  
  
 NCBI BlastP on this gene

EHY53012

eukaryotic translation initiation factor 3 subunit L
  
Accession: EHY53013
  
Location: 3406729-3408102
  
  
**BlastP hit with Mycgr3G98959\_Mycgr3T**
  
Percentage identity: 71 %
  
BlastP bit score: 704
  
Sequence coverage: 97 %
  
E-value: 0.0
  
  
 NCBI BlastP on this gene

EHY53013

hypothetical protein
  
Accession: EHY53014
  
Location: 3408572-3410239
  
 NCBI BlastP on this gene

EHY53014

cytochrome c heme-lyase
  
Accession: EHY53015
  
Location: 3410821-3412034
  
 NCBI BlastP on this gene

EHY53015

hypothetical protein
  
Accession: EHY53016
  
Location: 3412451-3413098
  
 NCBI BlastP on this gene

EHY53016

hypothetical protein
  
Accession: EHY53017
  
Location: 3413106-3414300
  
 NCBI BlastP on this gene

EHY53017

ubiquitin-conjugating enzyme E2 J2
  
Accession: EHY53018
  
Location: 3415352-3416188
  
 NCBI BlastP on this gene

EHY53018

MFS transporter, SIT family, siderophore-iron:H+ symporter
  
Accession: EHY53019
  
Location: 3417691-3419555
  
 NCBI BlastP on this gene

EHY53019

outer membrane protein
  
Accession: EHY53020
  
Location: 3420085-3421683
  
 NCBI BlastP on this gene

EHY53020

hypothetical protein
  
Accession: EHY53021
  
Location: 3422345-3423405
  
 NCBI BlastP on this gene

EHY53021

hypothetical protein
  
Accession: EHY53022
  
Location: 3424286-3425571
  
 NCBI BlastP on this gene

EHY53022

beta-glucosidase
  
Accession: EHY53023
  
Location: 3427212-3429827
  
 NCBI BlastP on this gene

EHY53023

165. :  CH991557 Monosiga brevicollis MX1 MONBRscaffold\_16 genomic scaffold     Total score: 2.0     Cumulative Blast bit score: 924

predicted protein
  
Accession: EDQ87859
  
Location: 241452-243687
  
 NCBI BlastP on this gene

EDQ87859

predicted protein
  
Accession: EDQ87753
  
Location: 233055-237534
  
 NCBI BlastP on this gene

EDQ87753

predicted protein
  
Accession: EDQ87858
  
Location: 230149-232853
  
  
**BlastP hit with Mycgr3G84402\_Mycgr3T**
  
Percentage identity: 61 %
  
BlastP bit score: 514
  
Sequence coverage: 88 %
  
E-value: 6e-176
  
  
 NCBI BlastP on this gene

EDQ87858

predicted protein
  
Accession: EDQ87752
  
Location: 228947-229366
  
 NCBI BlastP on this gene

EDQ87752

predicted protein
  
Accession: EDQ87857
  
Location: 225073-227734
  
 NCBI BlastP on this gene

EDQ87857

predicted protein
  
Accession: EDQ87751
  
Location: 223057-223898
  
 NCBI BlastP on this gene

EDQ87751

predicted protein
  
Accession: EDQ87856
  
Location: 214119-222306
  
 NCBI BlastP on this gene

EDQ87856

predicted protein
  
Accession: EDQ87750
  
Location: 211144-213619
  
 NCBI BlastP on this gene

EDQ87750

predicted protein
  
Accession: EDQ87855
  
Location: 206832-210646
  
  
**BlastP hit with Mycgr3G103034\_Mycgr3**
  
Percentage identity: 44 %
  
BlastP bit score: 410
  
Sequence coverage: 95 %
  
E-value: 1e-132
  
  
 NCBI BlastP on this gene

EDQ87855

predicted protein
  
Accession: EDQ87749
  
Location: 204037-206167
  
 NCBI BlastP on this gene

EDQ87749

predicted protein
  
Accession: EDQ87854
  
Location: 201722-203306
  
 NCBI BlastP on this gene

EDQ87854

predicted protein
  
Accession: EDQ87853
  
Location: 199484-200680
  
 NCBI BlastP on this gene

EDQ87853

predicted protein
  
Accession: EDQ87748
  
Location: 195327-198430
  
 NCBI BlastP on this gene

EDQ87748

166. :  CR382127 Yarrowia lipolytica CLIB122 chromosome A complete sequence.     Total score: 2.0     Cumulative Blast bit score: 841

YALI0A12023p
  
Accession: CAG83928
  
Location: 1258715-1259593
  
 NCBI BlastP on this gene

YALI0\_A12023g

YALI0A12045p
  
Accession: CAG83929
  
Location: 1262156-1262653
  
 NCBI BlastP on this gene

YALI0\_A12045g

YALI0A12067p
  
Accession: CAG83930
  
Location: 1264497-1265117
  
 NCBI BlastP on this gene

YALI0\_A12067g

YALI0A12111p
  
Accession: CAG83932
  
Location: 1268323-1270002
  
 NCBI BlastP on this gene

YALI0\_A12111g

YALI0A12133p
  
Accession: CAG83933
  
Location: 1273269-1274711
  
  
**BlastP hit with Mycgr3G84402\_Mycgr3T**
  
Percentage identity: 64 %
  
BlastP bit score: 561
  
Sequence coverage: 89 %
  
E-value: 0.0
  
  
 NCBI BlastP on this gene

YALI0\_A12133g

YALI0A12155p
  
Accession: CAG83934
  
Location: 1275461-1276681
  
  
**BlastP hit with Mycgr3G35447\_Mycgr3T**
  
Percentage identity: 42 %
  
BlastP bit score: 280
  
Sequence coverage: 97 %
  
E-value: 4e-86
  
  
 NCBI BlastP on this gene

YALI0\_A12155g

YALI0A12177p
  
Accession: CAG83935
  
Location: 1276926-1278074
  
 NCBI BlastP on this gene

YALI0\_A12177g

YALI0A12199p
  
Accession: CAG83936
  
Location: 1278340-1278915
  
 NCBI BlastP on this gene

YALI0\_A12199g

YALI0A12221p
  
Accession: CAG83937
  
Location: 1280932-1281747
  
 NCBI BlastP on this gene

YALI0\_A12221g

YALI0A12243p
  
Accession: CAG83938
  
Location: 1282612-1283529
  
 NCBI BlastP on this gene

YALI0\_A12243g

YALI0A12265p
  
Accession: CAG83939
  
Location: 1283611-1284030
  
 NCBI BlastP on this gene

YALI0\_A12265g

YALI0A12287p
  
Accession: CAG83940
  
Location: 1284636-1287083
  
 NCBI BlastP on this gene

YALI0\_A12287g

YALI0A12353p
  
Accession: CAG83941
  
Location: 1288451-1289557
  
 NCBI BlastP on this gene

YALI0\_A12353g

YALI0A12419p
  
Accession: CAG83942
  
Location: 1294500-1294952
  
 NCBI BlastP on this gene

YALI0\_A12419g

YALI0A12441p
  
Accession: CAG83943
  
Location: 1295014-1296066
  
 NCBI BlastP on this gene

YALI0\_A12441g

167. :  KB445649 Cochliobolus sativus ND90Pr unplaced genomic scaffold COCSAscaffold\_13     Total score: 2.0     Cumulative Blast bit score: 820

hypothetical protein
  
Accession: EMD61219
  
Location: 1245814-1247988
  
 NCBI BlastP on this gene

EMD61219

hypothetical protein
  
Accession: EMD61220
  
Location: 1249977-1250603
  
 NCBI BlastP on this gene

EMD61220

hypothetical protein
  
Accession: EMD61221
  
Location: 1250927-1251475
  
 NCBI BlastP on this gene

EMD61221

hypothetical protein
  
Accession: EMD61222
  
Location: 1252677-1253181
  
 NCBI BlastP on this gene

EMD61222

hypothetical protein
  
Accession: EMD61223
  
Location: 1253830-1254873
  
 NCBI BlastP on this gene

EMD61223

hypothetical protein
  
Accession: EMD61224
  
Location: 1255191-1257139
  
 NCBI BlastP on this gene

EMD61224

hypothetical protein
  
Accession: EMD61225
  
Location: 1257554-1259357
  
 NCBI BlastP on this gene

EMD61225

hypothetical protein
  
Accession: EMD61226
  
Location: 1259856-1261342
  
 NCBI BlastP on this gene

EMD61226

hypothetical protein
  
Accession: EMD61227
  
Location: 1262244-1264492
  
 NCBI BlastP on this gene

EMD61227

hypothetical protein
  
Accession: EMD61228
  
Location: 1264880-1265545
  
  
**BlastP hit with Mycgr3G28587\_Mycgr3T**
  
Percentage identity: 58 %
  
BlastP bit score: 191
  
Sequence coverage: 102 %
  
E-value: 4e-58
  
  
 NCBI BlastP on this gene

EMD61228

hypothetical protein
  
Accession: EMD61229
  
Location: 1266354-1267483
  
 NCBI BlastP on this gene

EMD61229

hypothetical protein
  
Accession: EMD61230
  
Location: 1267680-1267970
  
 NCBI BlastP on this gene

EMD61230

hypothetical protein
  
Accession: EMD61231
  
Location: 1268823-1272367
  
 NCBI BlastP on this gene

EMD61231

hypothetical protein
  
Accession: EMD61232
  
Location: 1273190-1275127
  
  
**BlastP hit with Mycgr3G103034\_Mycgr3**
  
Percentage identity: 56 %
  
BlastP bit score: 629
  
Sequence coverage: 97 %
  
E-value: 0.0
  
  
 NCBI BlastP on this gene

EMD61232

hypothetical protein
  
Accession: EMD61233
  
Location: 1276593-1279662
  
 NCBI BlastP on this gene

EMD61233

hypothetical protein
  
Accession: EMD61234
  
Location: 1282784-1283944
  
 NCBI BlastP on this gene

EMD61234

hypothetical protein
  
Accession: EMD61235
  
Location: 1284217-1285488
  
 NCBI BlastP on this gene

EMD61235

hypothetical protein
  
Accession: EMD61236
  
Location: 1286308-1287657
  
 NCBI BlastP on this gene

EMD61236

hypothetical protein
  
Accession: EMD61237
  
Location: 1288711-1289883
  
 NCBI BlastP on this gene

EMD61237

hypothetical protein
  
Accession: EMD61238
  
Location: 1290507-1291220
  
 NCBI BlastP on this gene

EMD61238

hypothetical protein
  
Accession: EMD61239
  
Location: 1291868-1293631
  
 NCBI BlastP on this gene

EMD61239

hypothetical protein
  
Accession: EMD61240
  
Location: 1294744-1296208
  
 NCBI BlastP on this gene

EMD61240

168. :  AHHD01000101 Macrophomina phaseolina MS6     Total score: 2.0     Cumulative Blast bit score: 812

Cytochrome P450
  
Accession: EKG19908
  
Location: 129-1545
  
 NCBI BlastP on this gene

EKG19908

hypothetical protein
  
Accession: EKG19909
  
Location: 1607-2089
  
 NCBI BlastP on this gene

EKG19909

Short-chain dehydrogenase/reductase SDR
  
Accession: EKG19910
  
Location: 2927-4172
  
 NCBI BlastP on this gene

EKG19910

Ribosomal protein L15 conserved site
  
Accession: EKG19911
  
Location: 5724-6287
  
 NCBI BlastP on this gene

EKG19911

FeS cluster biogenesis
  
Accession: EKG19912
  
Location: 6602-6901
  
 NCBI BlastP on this gene

EKG19912

Aminotransferase class V/Cysteine desulfurase
  
Accession: EKG19913
  
Location: 7553-9127
  
 NCBI BlastP on this gene

EKG19913

stress response protein nst1
  
Accession: EKG19914
  
Location: 10960-14762
  
 NCBI BlastP on this gene

EKG19914

Ras GTPase
  
Accession: EKG19915
  
Location: 15764-16935
  
 NCBI BlastP on this gene

EKG19915

hypothetical protein
  
Accession: EKG19916
  
Location: 18083-18709
  
  
**BlastP hit with Mycgr3G28587\_Mycgr3T**
  
Percentage identity: 57 %
  
BlastP bit score: 187
  
Sequence coverage: 99 %
  
E-value: 7e-57
  
  
 NCBI BlastP on this gene

EKG19916

Alpha-D-phosphohexomutase
  
Accession: EKG19917
  
Location: 19169-21115
  
  
**BlastP hit with Mycgr3G103034\_Mycgr3**
  
Percentage identity: 57 %
  
BlastP bit score: 625
  
Sequence coverage: 98 %
  
E-value: 0.0
  
  
 NCBI BlastP on this gene

EKG19917

Inositol monophosphatase
  
Accession: EKG19918
  
Location: 21636-22638
  
 NCBI BlastP on this gene

EKG19918

hypothetical protein
  
Accession: EKG19919
  
Location: 25211-26209
  
 NCBI BlastP on this gene

EKG19919

Saccharopine dehydrogenase / Homospermidine synthase
  
Accession: EKG19920
  
Location: 27262-28245
  
 NCBI BlastP on this gene

EKG19920

hypothetical protein
  
Accession: EKG19921
  
Location: 28298-28795
  
 NCBI BlastP on this gene

EKG19921

hypothetical protein
  
Accession: EKG19922
  
Location: 30120-30536
  
 NCBI BlastP on this gene

EKG19922

hypothetical protein
  
Accession: EKG19923
  
Location: 30574-30921
  
 NCBI BlastP on this gene

EKG19923

Major intrinsic protein
  
Accession: EKG19924
  
Location: 33367-34736
  
 NCBI BlastP on this gene

EKG19924

Carbohydrate kinase FGGY
  
Accession: EKG19925
  
Location: 36360-38332
  
 NCBI BlastP on this gene

EKG19925

HMG-I/HMG-Y DNA-binding conserved site
  
Accession: EKG19926
  
Location: 39084-40128
  
 NCBI BlastP on this gene

EKG19926

169. :  KB733482 Bipolaris maydis ATCC 48331 unplaced genomic scaffold COCC4scaffold\_39     Total score: 2.0     Cumulative Blast bit score: 808

hypothetical protein
  
Accession: ENH99718
  
Location: 66480-68672
  
 NCBI BlastP on this gene

ENH99718

hypothetical protein
  
Accession: ENH99719
  
Location: 69713-71190
  
 NCBI BlastP on this gene

ENH99719

hypothetical protein
  
Accession: ENH99720
  
Location: 71517-72065
  
 NCBI BlastP on this gene

ENH99720

hypothetical protein
  
Accession: ENH99721
  
Location: 73506-73673
  
 NCBI BlastP on this gene

ENH99721

hypothetical protein
  
Accession: ENH99722
  
Location: 74280-75327
  
 NCBI BlastP on this gene

ENH99722

hypothetical protein
  
Accession: ENH99723
  
Location: 75708-77657
  
 NCBI BlastP on this gene

ENH99723

hypothetical protein
  
Accession: ENH99724
  
Location: 78070-79873
  
 NCBI BlastP on this gene

ENH99724

hypothetical protein
  
Accession: ENH99725
  
Location: 80371-81854
  
 NCBI BlastP on this gene

ENH99725

hypothetical protein
  
Accession: ENH99726
  
Location: 82206-82628
  
 NCBI BlastP on this gene

ENH99726

hypothetical protein
  
Accession: ENH99727
  
Location: 82756-85004
  
 NCBI BlastP on this gene

ENH99727

hypothetical protein
  
Accession: ENH99728
  
Location: 85392-86075
  
  
**BlastP hit with Mycgr3G28587\_Mycgr3T**
  
Percentage identity: 56 %
  
BlastP bit score: 182
  
Sequence coverage: 102 %
  
E-value: 1e-54
  
  
 NCBI BlastP on this gene

ENH99728

hypothetical protein
  
Accession: ENH99729
  
Location: 86963-88075
  
 NCBI BlastP on this gene

ENH99729

hypothetical protein
  
Accession: ENH99730
  
Location: 89383-92932
  
 NCBI BlastP on this gene

ENH99730

hypothetical protein
  
Accession: ENH99731
  
Location: 93749-95700
  
  
**BlastP hit with Mycgr3G103034\_Mycgr3**
  
Percentage identity: 56 %
  
BlastP bit score: 626
  
Sequence coverage: 97 %
  
E-value: 0.0
  
  
 NCBI BlastP on this gene

ENH99731

hypothetical protein
  
Accession: ENH99732
  
Location: 97209-100326
  
 NCBI BlastP on this gene

ENH99732

hypothetical protein
  
Accession: ENH99733
  
Location: 102832-103275
  
 NCBI BlastP on this gene

ENH99733

hypothetical protein
  
Accession: ENH99734
  
Location: 103877-105037
  
 NCBI BlastP on this gene

ENH99734

hypothetical protein
  
Accession: ENH99735
  
Location: 105337-106632
  
 NCBI BlastP on this gene

ENH99735

hypothetical protein
  
Accession: ENH99736
  
Location: 107469-108840
  
 NCBI BlastP on this gene

ENH99736

hypothetical protein
  
Accession: ENH99737
  
Location: 109877-111067
  
 NCBI BlastP on this gene

ENH99737

hypothetical protein
  
Accession: ENH99738
  
Location: 111696-112406
  
 NCBI BlastP on this gene

ENH99738

hypothetical protein
  
Accession: ENH99739
  
Location: 113038-114638
  
 NCBI BlastP on this gene

ENH99739

170. :  KB445579 Cochliobolus heterostrophus C5 unplaced genomic scaffold COCHEscaffold\_11     Total score: 2.0     Cumulative Blast bit score: 808

hypothetical protein
  
Accession: EMD89463
  
Location: 1245765-1247957
  
 NCBI BlastP on this gene

EMD89463

hypothetical protein
  
Accession: EMD89464
  
Location: 1248998-1250475
  
 NCBI BlastP on this gene

EMD89464

hypothetical protein
  
Accession: EMD89465
  
Location: 1250802-1251350
  
 NCBI BlastP on this gene

EMD89465

hypothetical protein
  
Accession: EMD89466
  
Location: 1252791-1252958
  
 NCBI BlastP on this gene

EMD89466

hypothetical protein
  
Accession: EMD89467
  
Location: 1253565-1254612
  
 NCBI BlastP on this gene

EMD89467

hypothetical protein
  
Accession: EMD89468
  
Location: 1254993-1256942
  
 NCBI BlastP on this gene

EMD89468

hypothetical protein
  
Accession: EMD89469
  
Location: 1257372-1259175
  
 NCBI BlastP on this gene

EMD89469

hypothetical protein
  
Accession: EMD89470
  
Location: 1259673-1261156
  
 NCBI BlastP on this gene

EMD89470

hypothetical protein
  
Accession: EMD89471
  
Location: 1261508-1261930
  
 NCBI BlastP on this gene

EMD89471

hypothetical protein
  
Accession: EMD89472
  
Location: 1262058-1264306
  
 NCBI BlastP on this gene

EMD89472

hypothetical protein
  
Accession: EMD89473
  
Location: 1264694-1265377
  
  
**BlastP hit with Mycgr3G28587\_Mycgr3T**
  
Percentage identity: 56 %
  
BlastP bit score: 182
  
Sequence coverage: 102 %
  
E-value: 1e-54
  
  
 NCBI BlastP on this gene

EMD89473

hypothetical protein
  
Accession: EMD89474
  
Location: 1266265-1267377
  
 NCBI BlastP on this gene

EMD89474

hypothetical protein
  
Accession: EMD89475
  
Location: 1268733-1272282
  
 NCBI BlastP on this gene

EMD89475

hypothetical protein
  
Accession: EMD89476
  
Location: 1273099-1275050
  
  
**BlastP hit with Mycgr3G103034\_Mycgr3**
  
Percentage identity: 56 %
  
BlastP bit score: 626
  
Sequence coverage: 97 %
  
E-value: 0.0
  
  
 NCBI BlastP on this gene

EMD89476

hypothetical protein
  
Accession: EMD89477
  
Location: 1276559-1279676
  
 NCBI BlastP on this gene

EMD89477

hypothetical protein
  
Accession: EMD89478
  
Location: 1282277-1282720
  
 NCBI BlastP on this gene

EMD89478

hypothetical protein
  
Accession: EMD89479
  
Location: 1283322-1284482
  
 NCBI BlastP on this gene

EMD89479

hypothetical protein
  
Accession: EMD89480
  
Location: 1284782-1286077
  
 NCBI BlastP on this gene

EMD89480

hypothetical protein
  
Accession: EMD89481
  
Location: 1286914-1288285
  
 NCBI BlastP on this gene

EMD89481

hypothetical protein
  
Accession: EMD89482
  
Location: 1289322-1290512
  
 NCBI BlastP on this gene

EMD89482

hypothetical protein
  
Accession: EMD89483
  
Location: 1291141-1291851
  
 NCBI BlastP on this gene

EMD89483

hypothetical protein
  
Accession: EMD89484
  
Location: 1292483-1294083
  
 NCBI BlastP on this gene

EMD89484

171. :  GL534835 Pyrenophora teres f. teres 0-1 unplaced genomic scaffold scaffold\_191097     Total score: 2.0     Cumulative Blast bit score: 799

hypothetical protein
  
Accession: EFQ91313
  
Location: 23118-23839
  
  
**BlastP hit with Mycgr3G28587\_Mycgr3T**
  
Percentage identity: 59 %
  
BlastP bit score: 177
  
Sequence coverage: 96 %
  
E-value: 6e-53
  
  
 NCBI BlastP on this gene

EFQ91313

hypothetical protein
  
Accession: EFQ91312
  
Location: 21473-22595
  
 NCBI BlastP on this gene

EFQ91312

hypothetical protein
  
Accession: EFQ91311
  
Location: 16943-20397
  
 NCBI BlastP on this gene

EFQ91311

hypothetical protein
  
Accession: EFQ91310
  
Location: 14170-16118
  
  
**BlastP hit with Mycgr3G103034\_Mycgr3**
  
Percentage identity: 56 %
  
BlastP bit score: 622
  
Sequence coverage: 97 %
  
E-value: 0.0
  
  
 NCBI BlastP on this gene

EFQ91310

hypothetical protein
  
Accession: EFQ91309
  
Location: 9895-12875
  
 NCBI BlastP on this gene

EFQ91309

hypothetical protein
  
Accession: EFQ91308
  
Location: 6064-7212
  
 NCBI BlastP on this gene

EFQ91308

hypothetical protein
  
Accession: EFQ91307
  
Location: 4596-5849
  
 NCBI BlastP on this gene

EFQ91307

hypothetical protein
  
Accession: EFQ91306
  
Location: 3096-3815
  
 NCBI BlastP on this gene

EFQ91306

hypothetical protein
  
Accession: EFQ91305
  
Location: 688-1650
  
 NCBI BlastP on this gene

EFQ91305

172. :  KB908844 Setosphaeria turcica Et28A unplaced genomic scaffold SETTUscaffold\_6     Total score: 2.0     Cumulative Blast bit score: 791

hypothetical protein
  
Accession: EOA82441
  
Location: 250652-251728
  
 NCBI BlastP on this gene

EOA82441

hypothetical protein
  
Accession: EOA82440
  
Location: 247550-249623
  
 NCBI BlastP on this gene

EOA82440

hypothetical protein
  
Accession: EOA82439
  
Location: 245953-246506
  
 NCBI BlastP on this gene

EOA82439

hypothetical protein
  
Accession: EOA82438
  
Location: 243992-244972
  
 NCBI BlastP on this gene

EOA82438

hypothetical protein
  
Accession: EOA82437
  
Location: 242013-243921
  
 NCBI BlastP on this gene

EOA82437

hypothetical protein
  
Accession: EOA82436
  
Location: 239812-241589
  
 NCBI BlastP on this gene

EOA82436

hypothetical protein
  
Accession: EOA82435
  
Location: 235797-237188
  
 NCBI BlastP on this gene

EOA82435

hypothetical protein
  
Accession: EOA82434
  
Location: 232886-235086
  
 NCBI BlastP on this gene

EOA82434

hypothetical protein
  
Accession: EOA82433
  
Location: 231903-232644
  
  
**BlastP hit with Mycgr3G28587\_Mycgr3T**
  
Percentage identity: 56 %
  
BlastP bit score: 168
  
Sequence coverage: 96 %
  
E-value: 4e-49
  
  
 NCBI BlastP on this gene

EOA82433

hypothetical protein
  
Accession: EOA82432
  
Location: 230033-231126
  
 NCBI BlastP on this gene

EOA82432

hypothetical protein
  
Accession: EOA82431
  
Location: 221801-224729
  
 NCBI BlastP on this gene

EOA82431

hypothetical protein
  
Accession: EOA82430
  
Location: 219034-220964
  
  
**BlastP hit with Mycgr3G103034\_Mycgr3**
  
Percentage identity: 55 %
  
BlastP bit score: 623
  
Sequence coverage: 97 %
  
E-value: 0.0
  
  
 NCBI BlastP on this gene

EOA82430

hypothetical protein
  
Accession: EOA82429
  
Location: 214860-217764
  
 NCBI BlastP on this gene

EOA82429

hypothetical protein
  
Accession: EOA82428
  
Location: 211344-212762
  
 NCBI BlastP on this gene

EOA82428

hypothetical protein
  
Accession: EOA82427
  
Location: 208618-209034
  
 NCBI BlastP on this gene

EOA82427

hypothetical protein
  
Accession: EOA82426
  
Location: 205783-206886
  
 NCBI BlastP on this gene

EOA82426

hypothetical protein
  
Accession: EOA82425
  
Location: 204494-205696
  
 NCBI BlastP on this gene

EOA82425

hypothetical protein
  
Accession: EOA82424
  
Location: 203097-203792
  
 NCBI BlastP on this gene

EOA82424

hypothetical protein
  
Accession: EOA82423
  
Location: 200384-202614
  
 NCBI BlastP on this gene

EOA82423

173. :  JH767590 Coniosporium apollinis CBS 100218 chromosome Unknown supercont1.37     Total score: 2.0     Cumulative Blast bit score: 783

hypothetical protein
  
Accession: EON67768
  
Location: 121676-123090
  
 NCBI BlastP on this gene

EON67768

hypothetical protein
  
Accession: EON67769
  
Location: 124560-126986
  
 NCBI BlastP on this gene

EON67769

hypothetical protein
  
Accession: EON67770
  
Location: 127363-128994
  
 NCBI BlastP on this gene

EON67770

hypothetical protein
  
Accession: EON67771
  
Location: 130295-130642
  
 NCBI BlastP on this gene

EON67771

kynureninase
  
Accession: EON67772
  
Location: 131239-132941
  
 NCBI BlastP on this gene

EON67772

hypothetical protein
  
Accession: EON67773
  
Location: 133231-137011
  
 NCBI BlastP on this gene

EON67773

GTP-binding protein rho2
  
Accession: EON67774
  
Location: 138539-139235
  
 NCBI BlastP on this gene

EON67774

hypothetical protein
  
Accession: EON67775
  
Location: 139652-140309
  
  
**BlastP hit with Mycgr3G28587\_Mycgr3T**
  
Percentage identity: 61 %
  
BlastP bit score: 166
  
Sequence coverage: 97 %
  
E-value: 1e-48
  
  
 NCBI BlastP on this gene

EON67775

phosphoacetylglucosamine mutase
  
Accession: EON67776
  
Location: 140692-142587
  
  
**BlastP hit with Mycgr3G103034\_Mycgr3**
  
Percentage identity: 56 %
  
BlastP bit score: 617
  
Sequence coverage: 98 %
  
E-value: 0.0
  
  
 NCBI BlastP on this gene

EON67776

hypothetical protein
  
Accession: EON67777
  
Location: 143051-144481
  
 NCBI BlastP on this gene

EON67777

hypothetical protein
  
Accession: EON67778
  
Location: 145160-146155
  
 NCBI BlastP on this gene

EON67778

hypothetical protein
  
Accession: EON67779
  
Location: 146855-147868
  
 NCBI BlastP on this gene

EON67779

hypothetical protein
  
Accession: EON67780
  
Location: 148254-149202
  
 NCBI BlastP on this gene

EON67780

26S protease regulatory subunit 6A
  
Accession: EON67781
  
Location: 149651-151052
  
 NCBI BlastP on this gene

EON67781

hypothetical protein
  
Accession: EON67782
  
Location: 151891-152451
  
 NCBI BlastP on this gene

EON67782

hypothetical protein
  
Accession: EON67783
  
Location: 153191-156870
  
 NCBI BlastP on this gene

EON67783

hypothetical protein
  
Accession: EON67784
  
Location: 158408-159100
  
 NCBI BlastP on this gene

EON67784

hypothetical protein
  
Accession: EON67785
  
Location: 160008-161806
  
 NCBI BlastP on this gene

EON67785

lysophospholipase NTE1
  
Accession: EON67786
  
Location: 162441-167252
  
 NCBI BlastP on this gene

EON67786

174. :  KB916790 Neofusicoccum parvum UCRNP2 chromosome Unknown NP2\_03\_scaffold\_1152     Total score: 2.0     Cumulative Blast bit score: 775

putative inositol kinase kinase protein
  
Accession: EOD43838
  
Location: 35101-36229
  
 NCBI BlastP on this gene

EOD43838

putative tripeptidyl-peptidase 1 protein
  
Accession: EOD43848
  
Location: 36625-38379
  
 NCBI BlastP on this gene

EOD43848

putative peptidase a1 protein
  
Accession: EOD43845
  
Location: 39307-40611
  
 NCBI BlastP on this gene

EOD43845

hypothetical protein
  
Accession: EOD43855
  
Location: 41735-42108
  
 NCBI BlastP on this gene

EOD43855

hypothetical protein
  
Accession: EOD43835
  
Location: 44088-45220
  
 NCBI BlastP on this gene

EOD43835

putative inositol monophosphatase protein
  
Accession: EOD43854
  
Location: 49536-50816
  
 NCBI BlastP on this gene

EOD43854

putative n-acetylglucosamine-phosphate mutase protein
  
Accession: EOD43852
  
Location: 51293-53266
  
  
**BlastP hit with Mycgr3G103034\_Mycgr3**
  
Percentage identity: 57 %
  
BlastP bit score: 619
  
Sequence coverage: 98 %
  
E-value: 0.0
  
  
 NCBI BlastP on this gene

EOD43852

putative ring finger domain protein
  
Accession: EOD43849
  
Location: 53970-54371
  
  
**BlastP hit with Mycgr3G28587\_Mycgr3T**
  
Percentage identity: 59 %
  
BlastP bit score: 156
  
Sequence coverage: 78 %
  
E-value: 2e-45
  
  
 NCBI BlastP on this gene

EOD43849

putative rho gtpase rho protein
  
Accession: EOD43851
  
Location: 55240-55812
  
 NCBI BlastP on this gene

EOD43851

putative stress response protein nst1 protein
  
Accession: EOD43843
  
Location: 57462-59659
  
 NCBI BlastP on this gene

EOD43843

putative kynureninase protein
  
Accession: EOD43858
  
Location: 62728-64288
  
 NCBI BlastP on this gene

EOD43858

putative 60s ribosomal protein l28 protein
  
Accession: EOD43857
  
Location: 65592-66557
  
 NCBI BlastP on this gene

EOD43857

175. :  CAIF01000186 Wickerhamomyces ciferrii strain NRRL Y-1031 F-60-10     Total score: 2.0     Cumulative Blast bit score: 773

Transcription factor tau subunit
  
Accession: CCH45239
  
Location: 12315-13871
  
 NCBI BlastP on this gene

CCH45239

54S ribosomal protein L36, mitochondrial
  
Accession: CCH45240
  
Location: 14421-14975
  
 NCBI BlastP on this gene

CCH45240

glycyl-tRNA synthetase
  
Accession: CCH45241
  
Location: 16434-18410
  
 NCBI BlastP on this gene

CCH45241

Cytochrome B pre-mRNA-processing protein 6
  
Accession: CCH45242
  
Location: 18657-19082
  
 NCBI BlastP on this gene

CCH45242

U1 small nuclear ribonucleoprotein
  
Accession: CCH45243
  
Location: 19277-19975
  
 NCBI BlastP on this gene

CCH45243

Myosin-11
  
Accession: CCH45244
  
Location: 20215-23505
  
 NCBI BlastP on this gene

CCH45244

Uric acid-xanthine permease
  
Accession: CCH45245
  
Location: 23729-25491
  
 NCBI BlastP on this gene

CCH45245

Ubiquinone/menaquinone biosynthesis methyltransferase
  
Accession: CCH45246
  
Location: 28156-30522
  
 NCBI BlastP on this gene

CCH45246

ATP-dependent rRNA helicase RRP3
  
Accession: CCH45247
  
Location: 30750-32216
  
  
**BlastP hit with Mycgr3G84402\_Mycgr3T**
  
Percentage identity: 59 %
  
BlastP bit score: 486
  
Sequence coverage: 89 %
  
E-value: 5e-165
  
  
 NCBI BlastP on this gene

CCH45247

Suppressor of SWI4 1
  
Accession: CCH45248
  
Location: 32603-33823
  
  
**BlastP hit with Mycgr3G35447\_Mycgr3T**
  
Percentage identity: 43 %
  
BlastP bit score: 287
  
Sequence coverage: 81 %
  
E-value: 8e-89
  
  
 NCBI BlastP on this gene

CCH45248

E3 ubiquitin-protein ligase
  
Accession: CCH45249
  
Location: 34413-35219
  
 NCBI BlastP on this gene

CCH45249

Auxilin-like clathrin uncoating factor SWA2
  
Accession: CCH45250
  
Location: 36961-39438
  
 NCBI BlastP on this gene

CCH45250

RNA polymerase II transcription factor B subunit 1
  
Accession: CCH45251
  
Location: 39557-41299
  
 NCBI BlastP on this gene

CCH45251

Heat shock 70 kDa protein
  
Accession: CCH45252
  
Location: 41592-43226
  
 NCBI BlastP on this gene

CCH45252

putative secreted protein
  
Accession: CCH45253
  
Location: 43562-44620
  
 NCBI BlastP on this gene

CCH45253

Nucleoporin
  
Accession: CCH45254
  
Location: 44876-49852
  
 NCBI BlastP on this gene

CCH45254

Nucleoporin
  
Accession: CCH45255
  
Location: 49995-51811
  
 NCBI BlastP on this gene

CCH45255

hypothetical protein
  
Accession: CCH45256
  
Location: 52354-53094
  
 NCBI BlastP on this gene

CCH45256

MAP-ous protein 1
  
Accession: CCH45257
  
Location: 53331-57044
  
 NCBI BlastP on this gene

CCH45257

176. :  CH408161 Pichia guilliermondii ATCC 6260 scaffold\_7 genomic scaffold     Total score: 2.0     Cumulative Blast bit score: 772

hypothetical protein
  
Accession: EDK41390
  
Location: 576330-578711
  
 NCBI BlastP on this gene

EDK41390

hypothetical protein
  
Accession: EDK41391
  
Location: 578767-579711
  
 NCBI BlastP on this gene

EDK41391

hypothetical protein
  
Accession: EDK41392
  
Location: 580595-581689
  
 NCBI BlastP on this gene

EDK41392

40S ribosomal protein S23
  
Accession: EDK41393
  
Location: 582042-582479
  
 NCBI BlastP on this gene

EDK41393

hypothetical protein
  
Accession: EDK41394
  
Location: 582868-583869
  
 NCBI BlastP on this gene

EDK41394

hypothetical protein
  
Accession: EDK41395
  
Location: 584438-585679
  
 NCBI BlastP on this gene

EDK41395

hypothetical protein
  
Accession: EDK41396
  
Location: 586042-587220
  
 NCBI BlastP on this gene

EDK41396

hypothetical protein
  
Accession: EDK41397
  
Location: 587613-588497
  
 NCBI BlastP on this gene

EDK41397

hypothetical protein
  
Accession: EDK41398
  
Location: 588645-589685
  
 NCBI BlastP on this gene

EDK41398

hypothetical protein
  
Accession: EDK41399
  
Location: 589847-591166
  
 NCBI BlastP on this gene

EDK41399

hypothetical protein
  
Accession: EDK41400
  
Location: 591805-592920
  
 NCBI BlastP on this gene

EDK41400

hypothetical protein
  
Accession: EDK41401
  
Location: 593168-594238
  
 NCBI BlastP on this gene

EDK41401

hypothetical protein
  
Accession: EDK41402
  
Location: 594294-594857
  
 NCBI BlastP on this gene

EDK41402

hypothetical protein
  
Accession: EDK41403
  
Location: 594884-596128
  
  
**BlastP hit with Mycgr3G35447\_Mycgr3T**
  
Percentage identity: 40 %
  
BlastP bit score: 274
  
Sequence coverage: 91 %
  
E-value: 9e-84
  
  
 NCBI BlastP on this gene

EDK41403

hypothetical protein
  
Accession: EDK41404
  
Location: 596402-597829
  
  
**BlastP hit with Mycgr3G84402\_Mycgr3T**
  
Percentage identity: 59 %
  
BlastP bit score: 498
  
Sequence coverage: 93 %
  
E-value: 4e-170
  
  
 NCBI BlastP on this gene

EDK41404

hypothetical protein
  
Accession: EDK41405
  
Location: 597864-598829
  
 NCBI BlastP on this gene

EDK41405

hypothetical protein
  
Accession: EDK41406
  
Location: 599290-599802
  
 NCBI BlastP on this gene

EDK41406

hypothetical protein
  
Accession: EDK41407
  
Location: 600107-602320
  
 NCBI BlastP on this gene

EDK41407

hypothetical protein
  
Accession: EDK41408
  
Location: 602394-603542
  
 NCBI BlastP on this gene

EDK41408

predicted protein
  
Accession: EDK41409
  
Location: 603874-604944
  
 NCBI BlastP on this gene

EDK41409

hypothetical protein
  
Accession: EDK41411
  
Location: 605793-608177
  
 NCBI BlastP on this gene

EDK41411

hypothetical protein
  
Accession: EDK41410
  
Location: 608146-608991
  
 NCBI BlastP on this gene

EDK41410

hypothetical protein
  
Accession: EDK41412
  
Location: 608978-610162
  
 NCBI BlastP on this gene

EDK41412

hypothetical protein
  
Accession: EDK41413
  
Location: 610188-611285
  
 NCBI BlastP on this gene

EDK41413

hypothetical protein
  
Accession: EDK41414
  
Location: 611509-615717
  
 NCBI BlastP on this gene

EDK41414

hypothetical protein
  
Accession: EDK41415
  
Location: 616052-616786
  
 NCBI BlastP on this gene

EDK41415

hypothetical protein
  
Accession: EDK41416
  
Location: 617293-618729
  
 NCBI BlastP on this gene

EDK41416

177. :  CR382137 Debaryomyces hansenii CBS767 chromosome E complete sequence.     Total score: 2.0     Cumulative Blast bit score: 767

DEHA2E07832p
  
Accession: CAG87881
  
Location: 622567-623733
  
 NCBI BlastP on this gene

DEHA2E07832g

DEHA2E07854p
  
Accession: CAG87882
  
Location: 623868-624977
  
 NCBI BlastP on this gene

DEHA2E07854g

DEHA2E07876p
  
Accession: CAG87883
  
Location: 625457-626194
  
 NCBI BlastP on this gene

DEHA2E07876g

DEHA2E07898p
  
Accession: CAG87884
  
Location: 626420-628813
  
 NCBI BlastP on this gene

DEHA2E07898g

DEHA2E07920p
  
Accession: CAG87885
  
Location: 629107-629355
  
 NCBI BlastP on this gene

DEHA2E07920g

DEHA2E07942p
  
Accession: CAG87886
  
Location: 629658-632225
  
 NCBI BlastP on this gene

DEHA2E07942g

DEHA2E07964p
  
Accession: CAG87887
  
Location: 632741-633826
  
 NCBI BlastP on this gene

DEHA2E07964g

DEHA2E07986p
  
Accession: CAR65785
  
Location: 633997-636225
  
 NCBI BlastP on this gene

DEHA2E07986g

DEHA2E08008p
  
Accession: CAG87890
  
Location: 636306-636827
  
 NCBI BlastP on this gene

DEHA2E08008g

DEHA2E08030p
  
Accession: CAG87891
  
Location: 636946-638124
  
 NCBI BlastP on this gene

DEHA2E08030g

DEHA2E08052p
  
Accession: CAG87892
  
Location: 638390-639823
  
  
**BlastP hit with Mycgr3G84402\_Mycgr3T**
  
Percentage identity: 57 %
  
BlastP bit score: 497
  
Sequence coverage: 95 %
  
E-value: 8e-170
  
  
 NCBI BlastP on this gene

DEHA2E08052g

DEHA2E08074p
  
Accession: CAG87893
  
Location: 640025-641281
  
  
**BlastP hit with Mycgr3G35447\_Mycgr3T**
  
Percentage identity: 38 %
  
BlastP bit score: 270
  
Sequence coverage: 105 %
  
E-value: 5e-82
  
  
 NCBI BlastP on this gene

DEHA2E08074g

DEHA2E08096p
  
Accession: CAG87894
  
Location: 641304-641957
  
 NCBI BlastP on this gene

DEHA2E08096g

DEHA2E08118p
  
Accession: CAR65786
  
Location: 642045-643109
  
 NCBI BlastP on this gene

DEHA2E08118g

DEHA2E08140p
  
Accession: CAG87896
  
Location: 643323-644432
  
 NCBI BlastP on this gene

DEHA2E08140g

DEHA2E08162p
  
Accession: CAG87897
  
Location: 644878-645045
  
 NCBI BlastP on this gene

DEHA2E08162g

DEHA2E08184p
  
Accession: CAR65787
  
Location: 645571-646812
  
 NCBI BlastP on this gene

DEHA2E08184g

DEHA2E08206p
  
Accession: CAG87899
  
Location: 647084-648172
  
 NCBI BlastP on this gene

DEHA2E08206g

DEHA2E08228p
  
Accession: CAG87900
  
Location: 648490-649389
  
 NCBI BlastP on this gene

DEHA2E08228g

DEHA2E08250p
  
Accession: CAG87901
  
Location: 649567-651108
  
 NCBI BlastP on this gene

DEHA2E08250g

DEHA2E08294p
  
Accession: CAG87902
  
Location: 651533-652798
  
 NCBI BlastP on this gene

DEHA2E08294g

DEHA2E08316p
  
Accession: CAG87903
  
Location: 653081-654106
  
 NCBI BlastP on this gene

DEHA2E08316g

DEHA2E08338p
  
Accession: CAG87904
  
Location: 654634-655071
  
 NCBI BlastP on this gene

DEHA2E08338g

DEHA2E08360p
  
Accession: CAG87905
  
Location: 655377-656591
  
 NCBI BlastP on this gene

DEHA2E08360g

DEHA2E08382p
  
Accession: CAG87906
  
Location: 657206-658183
  
 NCBI BlastP on this gene

DEHA2E08382g

DEHA2E08404p
  
Accession: CAG87909
  
Location: 660813-660926
  
 NCBI BlastP on this gene

DEHA2E08404g

DEHA2E08426p
  
Accession: CAR65788
  
Location: 660947-661120
  
 NCBI BlastP on this gene

DEHA2E08426g

178. :  CH445336 Phaeosphaeria nodorum SN15 scaffold\_12     Total score: 2.0     Cumulative Blast bit score: 765

hypothetical protein
  
Accession: EAT84355
  
Location: 405658-407311
  
 NCBI BlastP on this gene

EAT84355

hypothetical protein
  
Accession: EAT84354
  
Location: 403301-405201
  
 NCBI BlastP on this gene

EAT84354

hypothetical protein
  
Accession: EAT84353
  
Location: 402962-403131
  
 NCBI BlastP on this gene

EAT84353

hypothetical protein
  
Accession: EAT84352
  
Location: 401427-402866
  
 NCBI BlastP on this gene

EAT84352

hypothetical protein
  
Accession: EAT84351
  
Location: 397565-401273
  
 NCBI BlastP on this gene

EAT84351

hypothetical protein
  
Accession: EAT84350
  
Location: 394277-396410
  
 NCBI BlastP on this gene

EAT84350

hypothetical protein
  
Accession: EAT84349
  
Location: 391778-393680
  
 NCBI BlastP on this gene

EAT84349

hypothetical protein
  
Accession: EAT84348
  
Location: 389919-390751
  
 NCBI BlastP on this gene

EAT84348

hypothetical protein
  
Accession: EAT84347
  
Location: 386501-388900
  
 NCBI BlastP on this gene

EAT84347

hypothetical protein
  
Accession: EAT84346
  
Location: 386285-386499
  
 NCBI BlastP on this gene

EAT84346

hypothetical protein
  
Accession: EDP89783
  
Location: 385869-386030
  
 NCBI BlastP on this gene

EDP89783

hypothetical protein
  
Accession: EAT84345
  
Location: 385010-385698
  
  
**BlastP hit with Mycgr3G28587\_Mycgr3T**
  
Percentage identity: 57 %
  
BlastP bit score: 191
  
Sequence coverage: 103 %
  
E-value: 3e-58
  
  
 NCBI BlastP on this gene

EAT84345

hypothetical protein
  
Accession: EAT84344
  
Location: 383410-384550
  
 NCBI BlastP on this gene

EAT84344

hypothetical protein
  
Accession: EAT84343
  
Location: 378879-382416
  
 NCBI BlastP on this gene

EAT84343

hypothetical protein
  
Accession: EAT84342
  
Location: 375960-378077
  
  
**BlastP hit with Mycgr3G103034\_Mycgr3**
  
Percentage identity: 54 %
  
BlastP bit score: 574
  
Sequence coverage: 93 %
  
E-value: 0.0
  
  
 NCBI BlastP on this gene

EAT84342

hypothetical protein
  
Accession: EAT84340
  
Location: 374297-375393
  
 NCBI BlastP on this gene

EAT84340

hypothetical protein
  
Accession: EAT84339
  
Location: 372829-373736
  
 NCBI BlastP on this gene

EAT84339

hypothetical protein
  
Accession: EAT84338
  
Location: 371638-372407
  
 NCBI BlastP on this gene

EAT84338

hypothetical protein
  
Accession: EAT84337
  
Location: 371280-371550
  
 NCBI BlastP on this gene

EAT84337

hypothetical protein
  
Accession: EAT84336
  
Location: 370275-370979
  
 NCBI BlastP on this gene

EAT84336

hypothetical protein
  
Accession: EAT84335
  
Location: 368364-369314
  
 NCBI BlastP on this gene

EAT84335

hypothetical protein
  
Accession: EAT84334
  
Location: 367377-367971
  
 NCBI BlastP on this gene

EAT84334

hypothetical protein
  
Accession: EAT84333
  
Location: 366780-367286
  
 NCBI BlastP on this gene

EAT84333

hypothetical protein
  
Accession: EAT84332
  
Location: 364834-365841
  
 NCBI BlastP on this gene

EAT84332

hypothetical protein
  
Accession: EAT84331
  
Location: 363738-364251
  
 NCBI BlastP on this gene

EAT84331

hypothetical protein
  
Accession: EAT84330
  
Location: 361981-363276
  
 NCBI BlastP on this gene

EAT84330

hypothetical protein
  
Accession: EAT84329
  
Location: 360632-361322
  
 NCBI BlastP on this gene

EAT84329

hypothetical protein
  
Accession: EAT84328
  
Location: 359131-360155
  
 NCBI BlastP on this gene

EAT84328

179. :  HE605209 Candida parapsilosis strain CDC317 annotated contig 006110.     Total score: 2.0     Cumulative Blast bit score: 764

hypothetical protein
  
Accession: CCE45104
  
Location: 239103-240593
  
 NCBI BlastP on this gene

CPAR2\_701080

hypothetical protein
  
Accession: CCE45105
  
Location: 241435-241629
  
 NCBI BlastP on this gene

CPAR2\_701090

hypothetical protein
  
Accession: CCE45106
  
Location: 241808-243844
  
 NCBI BlastP on this gene

CPAR2\_701100

hypothetical protein
  
Accession: CCE45107
  
Location: 243913-244899
  
 NCBI BlastP on this gene

CPAR2\_701110

hypothetical protein
  
Accession: CCE45108
  
Location: 244934-245788
  
 NCBI BlastP on this gene

CPAR2\_701120

hypothetical protein
  
Accession: CCE45109
  
Location: 246025-248001
  
 NCBI BlastP on this gene

CPAR2\_701130

hypothetical protein
  
Accession: CCE45110
  
Location: 248632-250554
  
 NCBI BlastP on this gene

CPAR2\_701140

hypothetical protein
  
Accession: CCE45111
  
Location: 252821-253981
  
 NCBI BlastP on this gene

CPAR2\_701150

hypothetical protein
  
Accession: CCE45112
  
Location: 254120-254653
  
 NCBI BlastP on this gene

CPAR2\_701160

hypothetical protein
  
Accession: CCE45113
  
Location: 254696-255964
  
  
**BlastP hit with Mycgr3G35447\_Mycgr3T**
  
Percentage identity: 37 %
  
BlastP bit score: 268
  
Sequence coverage: 105 %
  
E-value: 4e-81
  
  
 NCBI BlastP on this gene

CPAR2\_701170

hypothetical protein
  
Accession: CCE45114
  
Location: 256132-257568
  
  
**BlastP hit with Mycgr3G84402\_Mycgr3T**
  
Percentage identity: 56 %
  
BlastP bit score: 496
  
Sequence coverage: 97 %
  
E-value: 3e-169
  
  
 NCBI BlastP on this gene

CPAR2\_701180

hypothetical protein
  
Accession: CCE45115
  
Location: 257614-258537
  
 NCBI BlastP on this gene

CPAR2\_701190

hypothetical protein
  
Accession: CCE45116
  
Location: 258840-259385
  
 NCBI BlastP on this gene

CPAR2\_701200

hypothetical protein
  
Accession: CCE45117
  
Location: 259436-261595
  
 NCBI BlastP on this gene

CPAR2\_701210

hypothetical protein
  
Accession: CCE45118
  
Location: 261727-263232
  
 NCBI BlastP on this gene

CPAR2\_701220

hypothetical protein
  
Accession: CCE45119
  
Location: 265123-265917
  
 NCBI BlastP on this gene

CPAR2\_701230

hypothetical protein
  
Accession: CCE45120
  
Location: 265992-266825
  
 NCBI BlastP on this gene

CPAR2\_701240

hypothetical protein
  
Accession: CCE45121
  
Location: 267397-268089
  
 NCBI BlastP on this gene

CPAR2\_701250

hypothetical protein
  
Accession: CCE45122
  
Location: 268469-269494
  
 NCBI BlastP on this gene

CPAR2\_701260

hypothetical protein
  
Accession: CCE45123
  
Location: 269732-270307
  
 NCBI BlastP on this gene

CPAR2\_701270

hypothetical protein
  
Accession: CCE45124
  
Location: 270361-272058
  
 NCBI BlastP on this gene

CPAR2\_701280

hypothetical protein
  
Accession: CCE45125
  
Location: 272405-273310
  
 NCBI BlastP on this gene

CPAR2\_701290

hypothetical protein
  
Accession: CCE45126
  
Location: 273614-275023
  
 NCBI BlastP on this gene

CPAR2\_701300

hypothetical protein
  
Accession: CCE45127
  
Location: 275649-277082
  
 NCBI BlastP on this gene

CPAR2\_701310

hypothetical protein
  
Accession: CCE45128
  
Location: 277348-277824
  
 NCBI BlastP on this gene

CPAR2\_701320

180. :  FO082049 Pichia sorbitophila strain CBS 7064 chromosome K complete sequence.     Total score: 2.0     Cumulative Blast bit score: 761

not annotated
  
Accession: CCE83668
  
Location: 1089670-1090653
  
 NCBI BlastP on this gene

Piso0\_004253

not annotated
  
Accession: CCE83669
  
Location: 1091289-1092629
  
 NCBI BlastP on this gene

Piso0\_004254

not annotated
  
Accession: CCE83670
  
Location: 1092877-1093314
  
 NCBI BlastP on this gene

Piso0\_004255

not annotated
  
Accession: CCE83671
  
Location: 1093741-1094766
  
 NCBI BlastP on this gene

Piso0\_004256

not annotated
  
Accession: CCE83672
  
Location: 1095440-1096843
  
 NCBI BlastP on this gene

Piso0\_004257

not annotated
  
Accession: CCE83673
  
Location: 1097184-1098083
  
 NCBI BlastP on this gene

Piso0\_004258

not annotated
  
Accession: CCE83674
  
Location: 1098730-1099812
  
 NCBI BlastP on this gene

Piso0\_004259

not annotated
  
Accession: CCE83675
  
Location: 1100352-1101539
  
 NCBI BlastP on this gene

Piso0\_004260

not annotated
  
Accession: CCE83676
  
Location: 1101804-1102202
  
 NCBI BlastP on this gene

Piso0\_004261

not annotated
  
Accession: CCE83677
  
Location: 1102667-1103170
  
 NCBI BlastP on this gene

Piso0\_004262

not annotated
  
Accession: CCE83678
  
Location: 1103638-1104750
  
 NCBI BlastP on this gene

Piso0\_004263

not annotated
  
Accession: CCE83679
  
Location: 1105232-1106287
  
 NCBI BlastP on this gene

Piso0\_004264

not annotated
  
Accession: CCE83680
  
Location: 1106529-1107218
  
 NCBI BlastP on this gene

Piso0\_004265

not annotated
  
Accession: CCE83681
  
Location: 1107282-1108544
  
  
**BlastP hit with Mycgr3G35447\_Mycgr3T**
  
Percentage identity: 38 %
  
BlastP bit score: 253
  
Sequence coverage: 91 %
  
E-value: 1e-75
  
  
 NCBI BlastP on this gene

Piso0\_004266

not annotated
  
Accession: CCE83682
  
Location: 1108792-1110294
  
  
**BlastP hit with Mycgr3G84402\_Mycgr3T**
  
Percentage identity: 57 %
  
BlastP bit score: 508
  
Sequence coverage: 98 %
  
E-value: 1e-173
  
  
 NCBI BlastP on this gene

Piso0\_004267

not annotated
  
Accession: CCE83683
  
Location: 1110510-1111547
  
 NCBI BlastP on this gene

Piso0\_004268

not annotated
  
Accession: CCE83684
  
Location: 1111712-1112230
  
 NCBI BlastP on this gene

Piso0\_004269

not annotated
  
Accession: CCE83685
  
Location: 1112280-1114505
  
 NCBI BlastP on this gene

Piso0\_004270

not annotated
  
Accession: CCE83686
  
Location: 1114622-1115665
  
 NCBI BlastP on this gene

Piso0\_004271

not annotated
  
Accession: CCE83687
  
Location: 1116164-1116706
  
 NCBI BlastP on this gene

Piso0\_004272

not annotated
  
Accession: CCE83688
  
Location: 1116920-1119361
  
 NCBI BlastP on this gene

Piso0\_004273

not annotated
  
Accession: CCE83689
  
Location: 1119615-1119863
  
 NCBI BlastP on this gene

Piso0\_004274

not annotated
  
Accession: CCE83690
  
Location: 1120183-1122507
  
 NCBI BlastP on this gene

Piso0\_004275

not annotated
  
Accession: CCE83691
  
Location: 1122685-1123422
  
 NCBI BlastP on this gene

Piso0\_004276

not annotated
  
Accession: CCE83692
  
Location: 1123947-1125089
  
 NCBI BlastP on this gene

Piso0\_004277

not annotated
  
Accession: CCE83693
  
Location: 1125291-1126403
  
 NCBI BlastP on this gene

Piso0\_004278

not annotated
  
Accession: CCE83694
  
Location: 1126559-1130920
  
 NCBI BlastP on this gene

Piso0\_004279

181. :  FO082048 Pichia sorbitophila strain CBS 7064 chromosome L complete sequence.     Total score: 2.0     Cumulative Blast bit score: 761

not annotated
  
Accession: CCE84699
  
Location: 1089670-1090653
  
 NCBI BlastP on this gene

Piso0\_004253

not annotated
  
Accession: CCE84700
  
Location: 1091289-1092629
  
 NCBI BlastP on this gene

Piso0\_004254

not annotated
  
Accession: CCE84701
  
Location: 1092877-1093314
  
 NCBI BlastP on this gene

Piso0\_004255

not annotated
  
Accession: CCE84702
  
Location: 1093741-1094766
  
 NCBI BlastP on this gene

Piso0\_004256

not annotated
  
Accession: CCE84703
  
Location: 1095440-1096843
  
 NCBI BlastP on this gene

Piso0\_004257

not annotated
  
Accession: CCE84704
  
Location: 1097184-1098083
  
 NCBI BlastP on this gene

Piso0\_004258

not annotated
  
Accession: CCE84705
  
Location: 1098730-1099812
  
 NCBI BlastP on this gene

Piso0\_004259

not annotated
  
Accession: CCE84706
  
Location: 1100352-1101539
  
 NCBI BlastP on this gene

Piso0\_004260

not annotated
  
Accession: CCE84707
  
Location: 1101804-1102202
  
 NCBI BlastP on this gene

Piso0\_004261

not annotated
  
Accession: CCE84708
  
Location: 1102667-1103170
  
 NCBI BlastP on this gene

Piso0\_004262

not annotated
  
Accession: CCE84709
  
Location: 1103638-1104750
  
 NCBI BlastP on this gene

Piso0\_004263

not annotated
  
Accession: CCE84710
  
Location: 1105232-1106287
  
 NCBI BlastP on this gene

Piso0\_004264

not annotated
  
Accession: CCE84711
  
Location: 1106529-1107218
  
 NCBI BlastP on this gene

Piso0\_004265

not annotated
  
Accession: CCE84712
  
Location: 1107282-1108544
  
  
**BlastP hit with Mycgr3G35447\_Mycgr3T**
  
Percentage identity: 38 %
  
BlastP bit score: 253
  
Sequence coverage: 91 %
  
E-value: 1e-75
  
  
 NCBI BlastP on this gene

Piso0\_004266

not annotated
  
Accession: CCE84713
  
Location: 1108792-1110294
  
  
**BlastP hit with Mycgr3G84402\_Mycgr3T**
  
Percentage identity: 57 %
  
BlastP bit score: 508
  
Sequence coverage: 98 %
  
E-value: 1e-173
  
  
 NCBI BlastP on this gene

Piso0\_004267

not annotated
  
Accession: CCE84714
  
Location: 1110510-1111547
  
 NCBI BlastP on this gene

Piso0\_004268

not annotated
  
Accession: CCE84715
  
Location: 1111712-1112230
  
 NCBI BlastP on this gene

Piso0\_004269

not annotated
  
Accession: CCE84716
  
Location: 1112280-1114505
  
 NCBI BlastP on this gene

Piso0\_004270

not annotated
  
Accession: CCE84717
  
Location: 1114622-1115665
  
 NCBI BlastP on this gene

Piso0\_004271

not annotated
  
Accession: CCE84718
  
Location: 1116164-1116706
  
 NCBI BlastP on this gene

Piso0\_004272

not annotated
  
Accession: CCE84719
  
Location: 1116920-1119361
  
 NCBI BlastP on this gene

Piso0\_004273

not annotated
  
Accession: CCE84720
  
Location: 1119615-1119863
  
 NCBI BlastP on this gene

Piso0\_004274

not annotated
  
Accession: CCE84721
  
Location: 1120183-1122507
  
 NCBI BlastP on this gene

Piso0\_004275

not annotated
  
Accession: CCE84722
  
Location: 1122685-1123422
  
 NCBI BlastP on this gene

Piso0\_004276

not annotated
  
Accession: CCE84723
  
Location: 1123947-1125089
  
 NCBI BlastP on this gene

Piso0\_004277

not annotated
  
Accession: CCE84724
  
Location: 1125291-1126403
  
 NCBI BlastP on this gene

Piso0\_004278

not annotated
  
Accession: CCE84725
  
Location: 1126559-1130920
  
 NCBI BlastP on this gene

Piso0\_004279

182. :  DS231623 Pyrenophora tritici-repentis Pt-1C-BFP supercont1.9 genomic scaffold     Total score: 2.0     Cumulative Blast bit score: 761

transcriptional regulator
  
Accession: EDU51512
  
Location: 1459783-1461298
  
 NCBI BlastP on this gene

EDU51512

predicted protein
  
Accession: EDU51513
  
Location: 1470425-1471339
  
 NCBI BlastP on this gene

EDU51513

conserved hypothetical protein
  
Accession: EDU51514
  
Location: 1474594-1475292
  
  
**BlastP hit with Mycgr3G28587\_Mycgr3T**
  
Percentage identity: 59 %
  
BlastP bit score: 176
  
Sequence coverage: 96 %
  
E-value: 2e-52
  
  
 NCBI BlastP on this gene

EDU51514

GTP-binding protein rho2 precursor
  
Accession: EDU51515
  
Location: 1475809-1476941
  
 NCBI BlastP on this gene

EDU51515

hypothetical protein
  
Accession: EDU51516
  
Location: 1477988-1481442
  
 NCBI BlastP on this gene

EDU51516

N-acetylglucosamine-phosphate mutase
  
Accession: EDU51517
  
Location: 1482242-1484186
  
  
**BlastP hit with Mycgr3G103034\_Mycgr3**
  
Percentage identity: 53 %
  
BlastP bit score: 585
  
Sequence coverage: 100 %
  
E-value: 0.0
  
  
 NCBI BlastP on this gene

EDU51517

hypothetical protein
  
Accession: EDU51518
  
Location: 1485544-1488523
  
 NCBI BlastP on this gene

EDU51518

conserved hypothetical protein
  
Accession: EDU51519
  
Location: 1491418-1492539
  
 NCBI BlastP on this gene

EDU51519

conserved hypothetical protein
  
Accession: EDU51520
  
Location: 1492715-1493962
  
 NCBI BlastP on this gene

EDU51520

conserved hypothetical protein
  
Accession: EDU51521
  
Location: 1494731-1495450
  
 NCBI BlastP on this gene

EDU51521

predicted protein
  
Accession: EDU51522
  
Location: 1496858-1497924
  
 NCBI BlastP on this gene

EDU51522

alpha-ketoglutarate dependent xanthine dioxygenase
  
Accession: EDU51523
  
Location: 1502407-1503699
  
 NCBI BlastP on this gene

EDU51523

183. :  AOGT01000769 Candida maltosa Xu316     Total score: 2.0     Cumulative Blast bit score: 754

hypothetical protein
  
Accession: EMG49263
  
Location: 122-1117
  
 NCBI BlastP on this gene

EMG49263

hypothetical protein
  
Accession: EMG49264
  
Location: 2244-3542
  
  
**BlastP hit with Mycgr3G35447\_Mycgr3T**
  
Percentage identity: 39 %
  
BlastP bit score: 259
  
Sequence coverage: 94 %
  
E-value: 9e-78
  
  
 NCBI BlastP on this gene

EMG49264

ATP-dependent rRNA helicase RRP3
  
Accession: EMG49265
  
Location: 3737-5176
  
  
**BlastP hit with Mycgr3G84402\_Mycgr3T**
  
Percentage identity: 58 %
  
BlastP bit score: 495
  
Sequence coverage: 94 %
  
E-value: 5e-169
  
  
 NCBI BlastP on this gene

EMG49265

hypothetical protein
  
Accession: EMG49266
  
Location: 5196-6233
  
 NCBI BlastP on this gene

EMG49266

hypothetical protein
  
Accession: EMG49267
  
Location: 6519-7091
  
 NCBI BlastP on this gene

EMG49267

hypothetical protein
  
Accession: EMG49268
  
Location: 7123-9315
  
 NCBI BlastP on this gene

EMG49268

hypothetical protein
  
Accession: EMG49269
  
Location: 9712-11679
  
 NCBI BlastP on this gene

EMG49269

184. :  GL996500 Spathaspora passalidarum NRRL Y-27907 unplaced genomic scaffold SPAPAscaffold\_2     Total score: 2.0     Cumulative Blast bit score: 753

hypothetical protein
  
Accession: EGW33776
  
Location: 224959-226941
  
 NCBI BlastP on this gene

EGW33776

hypothetical protein
  
Accession: EGW33777
  
Location: 227093-228139
  
 NCBI BlastP on this gene

EGW33777

hypothetical protein
  
Accession: EGW33778
  
Location: 228185-229042
  
 NCBI BlastP on this gene

EGW33778

hypothetical protein
  
Accession: EGW33779
  
Location: 229192-230760
  
 NCBI BlastP on this gene

EGW33779

phospholipase B
  
Accession: EGW33780
  
Location: 231307-233049
  
 NCBI BlastP on this gene

EGW33780

hypothetical protein
  
Accession: EGW33781
  
Location: 234307-236441
  
 NCBI BlastP on this gene

EGW33781

hypothetical protein
  
Accession: EGW33782
  
Location: 237935-238584
  
 NCBI BlastP on this gene

EGW33782

hypothetical protein
  
Accession: EGW33783
  
Location: 240222-241310
  
 NCBI BlastP on this gene

EGW33783

hypothetical protein
  
Accession: EGW33784
  
Location: 244089-244607
  
 NCBI BlastP on this gene

EGW33784

hypothetical protein
  
Accession: EGW33785
  
Location: 244636-245820
  
  
**BlastP hit with Mycgr3G35447\_Mycgr3T**
  
Percentage identity: 38 %
  
BlastP bit score: 267
  
Sequence coverage: 96 %
  
E-value: 2e-81
  
  
 NCBI BlastP on this gene

EGW33785

ATP-dependent rRNA helicase RRP3
  
Accession: EGW33786
  
Location: 246009-247436
  
  
**BlastP hit with Mycgr3G84402\_Mycgr3T**
  
Percentage identity: 57 %
  
BlastP bit score: 486
  
Sequence coverage: 91 %
  
E-value: 3e-165
  
  
 NCBI BlastP on this gene

EGW33786

hypothetical protein
  
Accession: EGW33787
  
Location: 247488-248498
  
 NCBI BlastP on this gene

EGW33787

mediator of RNA polymerase II transcription subunit 21
  
Accession: EGW33788
  
Location: 248853-249362
  
 NCBI BlastP on this gene

EGW33788

hypothetical protein
  
Accession: EGW33789
  
Location: 249373-251448
  
 NCBI BlastP on this gene

EGW33789

hypothetical protein
  
Accession: EGW33790
  
Location: 260725-262110
  
 NCBI BlastP on this gene

EGW33790

hypothetical protein
  
Accession: EGW33792
  
Location: 263522-264295
  
 NCBI BlastP on this gene

EGW33792

hypothetical protein
  
Accession: EGW33793
  
Location: 264325-265224
  
 NCBI BlastP on this gene

EGW33793

hypothetical protein
  
Accession: EGW33794
  
Location: 265396-266982
  
 NCBI BlastP on this gene

EGW33794

185. :  GG692404 Candida tropicalis MYA-3404 genomic scaffold supercont3.10     Total score: 2.0     Cumulative Blast bit score: 750

hypothetical protein
  
Accession: EER30475
  
Location: 219873-221486
  
 NCBI BlastP on this gene

EER30475

predicted protein
  
Accession: EER30476
  
Location: 221536-222159
  
 NCBI BlastP on this gene

EER30476

hypothetical protein
  
Accession: EER30477
  
Location: 223245-225509
  
 NCBI BlastP on this gene

EER30477

predicted protein
  
Accession: EER30478
  
Location: 225612-226841
  
 NCBI BlastP on this gene

EER30478

conserved hypothetical protein
  
Accession: EER30479
  
Location: 227280-229967
  
 NCBI BlastP on this gene

EER30479

D-arabinono-1,4-lactone oxidase
  
Accession: EER30480
  
Location: 230073-231746
  
 NCBI BlastP on this gene

EER30480

conserved hypothetical protein
  
Accession: EER30481
  
Location: 232817-235009
  
 NCBI BlastP on this gene

EER30481

conserved hypothetical protein
  
Accession: EER30482
  
Location: 235051-235638
  
 NCBI BlastP on this gene

EER30482

conserved hypothetical protein
  
Accession: EER30483
  
Location: 235884-236969
  
 NCBI BlastP on this gene

EER30483

ATP-dependent rRNA helicase RRP3
  
Accession: EER30484
  
Location: 237018-238469
  
  
**BlastP hit with Mycgr3G84402\_Mycgr3T**
  
Percentage identity: 58 %
  
BlastP bit score: 494
  
Sequence coverage: 93 %
  
E-value: 3e-168
  
  
 NCBI BlastP on this gene

EER30484

conserved hypothetical protein
  
Accession: EER30485
  
Location: 238676-239980
  
  
**BlastP hit with Mycgr3G35447\_Mycgr3T**
  
Percentage identity: 39 %
  
BlastP bit score: 256
  
Sequence coverage: 93 %
  
E-value: 1e-76
  
  
 NCBI BlastP on this gene

EER30485

predicted protein
  
Accession: EER30486
  
Location: 240016-240591
  
 NCBI BlastP on this gene

EER30486

hypothetical protein
  
Accession: EER30487
  
Location: 241551-242666
  
 NCBI BlastP on this gene

EER30487

conserved hypothetical protein
  
Accession: EER30488
  
Location: 244170-245231
  
 NCBI BlastP on this gene

EER30488

esterase D
  
Accession: EER30489
  
Location: 245245-246102
  
 NCBI BlastP on this gene

EER30489

lysophospholipase 1 precursor
  
Accession: EER30490
  
Location: 248062-249939
  
 NCBI BlastP on this gene

EER30490

hypothetical protein
  
Accession: EER30491
  
Location: 252139-253740
  
 NCBI BlastP on this gene

EER30491

predicted protein
  
Accession: EER30492
  
Location: 253869-255959
  
 NCBI BlastP on this gene

EER30492

predicted protein
  
Accession: EER30493
  
Location: 256281-256475
  
 NCBI BlastP on this gene

EER30493

hypothetical protein
  
Accession: EER30494
  
Location: 257504-258052
  
 NCBI BlastP on this gene

EER30494

predicted protein
  
Accession: EER30495
  
Location: 258650-259309
  
 NCBI BlastP on this gene

EER30495

prefoldin subunit 6
  
Accession: EER30496
  
Location: 259744-260091
  
 NCBI BlastP on this gene

EER30496

186. :  AEOI01000005 Ogataea parapolymorpha DL-1     Total score: 2.0     Cumulative Blast bit score: 750

Cytosolic Fe-S cluster assembling factor CFD1
  
Accession: EFW97529
  
Location: 255193-255996
  
 NCBI BlastP on this gene

EFW97529

microsomal dipeptidase, putative
  
Accession: EFW97530
  
Location: 256046-257044
  
 NCBI BlastP on this gene

EFW97530

pantothenate transporter, putative
  
Accession: EFW97531
  
Location: 257409-258908
  
 NCBI BlastP on this gene

EFW97531

Flavin-containing monooxygenase
  
Accession: EFW97532
  
Location: 259030-260349
  
 NCBI BlastP on this gene

EFW97532

glycosyl transferase
  
Accession: EFW97533
  
Location: 262585-263526
  
 NCBI BlastP on this gene

EFW97533

hypothetical protein
  
Accession: EFW97534
  
Location: 263853-265568
  
 NCBI BlastP on this gene

EFW97534

putative kinase Pak1p
  
Accession: EFW97535
  
Location: 265640-267418
  
 NCBI BlastP on this gene

EFW97535

Dihydroxyacetone kinase
  
Accession: EFW97536
  
Location: 267460-269289
  
 NCBI BlastP on this gene

EFW97536

Autophagy-related protein 21
  
Accession: EFW97537
  
Location: 269313-270766
  
 NCBI BlastP on this gene

EFW97537

Subunit of the CCR4-NOT complex
  
Accession: EFW97538
  
Location: 270797-274299
  
 NCBI BlastP on this gene

EFW97538

hypothetical protein
  
Accession: EFW97539
  
Location: 274331-275487
  
  
**BlastP hit with Mycgr3G35447\_Mycgr3T**
  
Percentage identity: 43 %
  
BlastP bit score: 261
  
Sequence coverage: 85 %
  
E-value: 3e-79
  
  
 NCBI BlastP on this gene

EFW97539

ATP-dependent rRNA helicase, putative
  
Accession: EFW97540
  
Location: 275574-276986
  
  
**BlastP hit with Mycgr3G84402\_Mycgr3T**
  
Percentage identity: 54 %
  
BlastP bit score: 489
  
Sequence coverage: 97 %
  
E-value: 7e-167
  
  
 NCBI BlastP on this gene

EFW97540

pre-mRNA-splicing ATP-dependent RNA helicase PRP28
  
Accession: EFW97541
  
Location: 277068-278669
  
 NCBI BlastP on this gene

EFW97541

Protein involved in pre-mRNA splicing, component of a complex containing Cef1p
  
Accession: EFW97542
  
Location: 278673-279614
  
 NCBI BlastP on this gene

EFW97542

Fimbrin, actin-bundling protein
  
Accession: EFW97543
  
Location: 279741-286352
  
 NCBI BlastP on this gene

EFW97543

3-keto sterol reductase
  
Accession: EFW97544
  
Location: 286419-287474
  
 NCBI BlastP on this gene

EFW97544

mitogen activated protein kinase
  
Accession: EFW97545
  
Location: 287775-288941
  
 NCBI BlastP on this gene

EFW97545

monothiol glutaredoxin
  
Accession: EFW97546
  
Location: 289051-289938
  
 NCBI BlastP on this gene

EFW97546

hypothetical protein
  
Accession: EFW97547
  
Location: 289982-290680
  
 NCBI BlastP on this gene

EFW97547

Protein component of the small (40S) ribosomal subunit
  
Accession: EFW97548
  
Location: 290825-291467
  
 NCBI BlastP on this gene

EFW97548

DNA-binding protein
  
Accession: EFW97549
  
Location: 291673-293502
  
 NCBI BlastP on this gene

EFW97549

Fungal-specific transcription factor
  
Accession: EFW97550
  
Location: 294001-297156
  
 NCBI BlastP on this gene

EFW97550

187. :  HE681725 Candida orthopsilosis Co 90-125, chromosome 7 draft sequence.     Total score: 2.0     Cumulative Blast bit score: 749

Cdc6 ATP-binding protein
  
Accession: CCG24811
  
Location: 247527-249005
  
 NCBI BlastP on this gene

CORT\_0G01240

hypothetical protein
  
Accession: CCG24812
  
Location: 249810-250004
  
 NCBI BlastP on this gene

CORT\_0G01245

Wal1 h
  
Accession: CCG24813
  
Location: 250199-252316
  
 NCBI BlastP on this gene

CORT\_0G01250

hypothetical protein
  
Accession: CCG24814
  
Location: 252380-253369
  
 NCBI BlastP on this gene

CORT\_0G01260

esterase
  
Accession: CCG24815
  
Location: 253406-254260
  
 NCBI BlastP on this gene

CORT\_0G01270

esterase
  
Accession: CCG24816
  
Location: 254658-255023
  
 NCBI BlastP on this gene

CORT\_0G01280

hypothetical protein
  
Accession: CCG24817
  
Location: 255874-257847
  
 NCBI BlastP on this gene

CORT\_0G01300

Plb3 GPI-anchored cell surface phospholipase B
  
Accession: CCG24818
  
Location: 258441-260363
  
 NCBI BlastP on this gene

CORT\_0G01310

Pcd1 protein
  
Accession: CCG24819
  
Location: 263415-264674
  
 NCBI BlastP on this gene

CORT\_0G01320

Vma22 protein
  
Accession: CCG24820
  
Location: 264710-265294
  
 NCBI BlastP on this gene

CORT\_0G01330

hypothetical protein
  
Accession: CCG24821
  
Location: 265351-266619
  
  
**BlastP hit with Mycgr3G35447\_Mycgr3T**
  
Percentage identity: 39 %
  
BlastP bit score: 260
  
Sequence coverage: 92 %
  
E-value: 3e-78
  
  
 NCBI BlastP on this gene

CORT\_0G01340

Rrp3 protein
  
Accession: CCG24822
  
Location: 266757-268202
  
  
**BlastP hit with Mycgr3G84402\_Mycgr3T**
  
Percentage identity: 57 %
  
BlastP bit score: 489
  
Sequence coverage: 92 %
  
E-value: 2e-166
  
  
 NCBI BlastP on this gene

CORT\_0G01350

Pib1 protein
  
Accession: CCG24823
  
Location: 268229-269146
  
 NCBI BlastP on this gene

CORT\_0G01360

Srb7 protein
  
Accession: CCG24824
  
Location: 269432-269977
  
 NCBI BlastP on this gene

CORT\_0G01370

Pmt5 protein mannosyltransferase (PMT)
  
Accession: CCG24825
  
Location: 270024-272183
  
 NCBI BlastP on this gene

CORT\_0G01380

Ssu1 protein
  
Accession: CCG24826
  
Location: 272309-273805
  
 NCBI BlastP on this gene

CORT\_0G01390

Cdg1 cysteine dioxygenases
  
Accession: CCG24827
  
Location: 275757-276557
  
 NCBI BlastP on this gene

CORT\_0G01400

phytanoyl-CoA dioxygenase family protein
  
Accession: CCG24828
  
Location: 276636-277517
  
 NCBI BlastP on this gene

CORT\_0G01410

Pet18 protein
  
Accession: CCG24829
  
Location: 278020-278712
  
 NCBI BlastP on this gene

CORT\_0G01420

Thi13 protein
  
Accession: CCG24830
  
Location: 279121-280146
  
 NCBI BlastP on this gene

CORT\_0G01430

Pho88 h
  
Accession: CCG24831
  
Location: 280395-280970
  
 NCBI BlastP on this gene

CORT\_0G01440

Rkm1 protein
  
Accession: CCG24832
  
Location: 281023-282720
  
 NCBI BlastP on this gene

CORT\_0G01450

Sco1 copper transporter
  
Accession: CCG24833
  
Location: 283034-283948
  
 NCBI BlastP on this gene

CORT\_0G01460

hypothetical protein
  
Accession: CCG24834
  
Location: 284269-285678
  
 NCBI BlastP on this gene

CORT\_0G01470

Cbp1 corticosteroid binding protein
  
Accession: CCG24835
  
Location: 286289-287722
  
 NCBI BlastP on this gene

CORT\_0G01480

hypothetical protein
  
Accession: CCG24836
  
Location: 287979-288443
  
 NCBI BlastP on this gene

CORT\_0G01490

188. :  CH408079 Clavispora lusitaniae ATCC 42720 scaffold\_4 genomic scaffold     Total score: 2.0     Cumulative Blast bit score: 741

hypothetical protein
  
Accession: EEQ39212
  
Location: 306096-307127
  
 NCBI BlastP on this gene

EEQ39212

hypothetical protein
  
Accession: EEQ39213
  
Location: 307593-308849
  
 NCBI BlastP on this gene

EEQ39213

hypothetical protein
  
Accession: EEQ39214
  
Location: 308964-310451
  
 NCBI BlastP on this gene

EEQ39214

hypothetical protein
  
Accession: EEQ39215
  
Location: 310645-311526
  
 NCBI BlastP on this gene

EEQ39215

hypothetical protein
  
Accession: EEQ39216
  
Location: 312176-313228
  
 NCBI BlastP on this gene

EEQ39216

hypothetical protein
  
Accession: EEQ39217
  
Location: 314035-315639
  
 NCBI BlastP on this gene

EEQ39217

hypothetical protein
  
Accession: EEQ39218
  
Location: 314602-315420
  
 NCBI BlastP on this gene

EEQ39218

hypothetical protein
  
Accession: EEQ39219
  
Location: 317628-318842
  
 NCBI BlastP on this gene

EEQ39219

hypothetical protein
  
Accession: EEQ39220
  
Location: 320307-321221
  
 NCBI BlastP on this gene

EEQ39220

predicted protein
  
Accession: EEQ39221
  
Location: 323413-324273
  
 NCBI BlastP on this gene

EEQ39221

hypothetical protein
  
Accession: EEQ39222
  
Location: 324341-325573
  
  
**BlastP hit with Mycgr3G35447\_Mycgr3T**
  
Percentage identity: 38 %
  
BlastP bit score: 250
  
Sequence coverage: 90 %
  
E-value: 1e-74
  
  
 NCBI BlastP on this gene

EEQ39222

conserved hypothetical protein
  
Accession: EEQ39223
  
Location: 325881-327314
  
  
**BlastP hit with Mycgr3G84402\_Mycgr3T**
  
Percentage identity: 59 %
  
BlastP bit score: 491
  
Sequence coverage: 89 %
  
E-value: 3e-167
  
  
 NCBI BlastP on this gene

EEQ39223

hypothetical protein
  
Accession: EEQ39224
  
Location: 327512-328342
  
 NCBI BlastP on this gene

EEQ39224

hypothetical protein
  
Accession: EEQ39225
  
Location: 329065-329541
  
 NCBI BlastP on this gene

EEQ39225

hypothetical protein
  
Accession: EEQ39226
  
Location: 329613-331838
  
 NCBI BlastP on this gene

EEQ39226

hypothetical protein
  
Accession: EEQ39227
  
Location: 331954-332946
  
 NCBI BlastP on this gene

EEQ39227

predicted protein
  
Accession: EEQ39228
  
Location: 333567-334127
  
 NCBI BlastP on this gene

EEQ39228

predicted protein
  
Accession: EEQ39229
  
Location: 334000-335337
  
 NCBI BlastP on this gene

EEQ39229

40S ribosomal protein S27-B
  
Accession: EEQ39230
  
Location: 336824-337072
  
 NCBI BlastP on this gene

EEQ39230

hypothetical protein
  
Accession: EEQ39231
  
Location: 337795-340173
  
 NCBI BlastP on this gene

EEQ39231

hypothetical protein
  
Accession: EEQ39232
  
Location: 340242-341093
  
 NCBI BlastP on this gene

EEQ39232

hypothetical protein
  
Accession: EEQ39233
  
Location: 341780-342766
  
 NCBI BlastP on this gene

EEQ39233

hypothetical protein
  
Accession: EEQ39234
  
Location: 342130-342729
  
 NCBI BlastP on this gene

EEQ39234

hypothetical protein
  
Accession: EEQ39235
  
Location: 342835-343920
  
 NCBI BlastP on this gene

EEQ39235

hypothetical protein
  
Accession: EEQ39236
  
Location: 344616-348860
  
 NCBI BlastP on this gene

EEQ39236

189. :  FR839628 Pichia pastoris CBS 7435 chromosome 1     Total score: 2.0     Cumulative Blast bit score: 737

Arsenical-resistance protein ACR3
  
Accession: CCA36748
  
Location: 1093439-1094710
  
 NCBI BlastP on this gene

ARR3

ribonucleoside-diphosphate reductase subunit M1
  
Accession: CCA36749
  
Location: 1095139-1097742
  
 NCBI BlastP on this gene

Rrm1

40S ribosomal protein S27
  
Accession: CCA36750
  
Location: 1098498-1098746
  
 NCBI BlastP on this gene

PP7435\_Chr1-0601

Uncharacterized transporter C11D3.18C
  
Accession: CCA36751
  
Location: 1099244-1100728
  
 NCBI BlastP on this gene

THI73

hypothetical protein
  
Accession: CCA36752
  
Location: 1100839-1101822
  
 NCBI BlastP on this gene

PP7435\_Chr1-0603

F-box protein YDR306C
  
Accession: CCA36753
  
Location: 1102300-1103532
  
 NCBI BlastP on this gene

PP7435\_Chr1-0604

dolichyl-phosphate-mannose-protein mannosyltransferase
  
Accession: CCA36754
  
Location: 1103612-1105963
  
 NCBI BlastP on this gene

PP7435\_Chr1-0605

Allantoate permease
  
Accession: CCA36755
  
Location: 1106543-1108054
  
 NCBI BlastP on this gene

PP7435\_Chr1-0606

Mediator of RNA polymerase II transcription subunit 21
  
Accession: CCA36756
  
Location: 1108117-1108533
  
 NCBI BlastP on this gene

PP7435\_Chr1-0607

Coiled-coil domain-containing protein 115
  
Accession: CCA36757
  
Location: 1108716-1109453
  
 NCBI BlastP on this gene

PP7435\_Chr1-0608

hypothetical protein
  
Accession: CCA36758
  
Location: 1109586-1110158
  
 NCBI BlastP on this gene

PP7435\_Chr1-0609

Suppressor of SWI4 1 homolog
  
Accession: CCA36759
  
Location: 1111559-1112785
  
  
**BlastP hit with Mycgr3G35447\_Mycgr3T**
  
Percentage identity: 37 %
  
BlastP bit score: 246
  
Sequence coverage: 94 %
  
E-value: 5e-73
  
  
 NCBI BlastP on this gene

PP7435\_Chr1-0610

ATP-dependent RNA helicase
  
Accession: CCA36760
  
Location: 1113141-1114577
  
  
**BlastP hit with Mycgr3G84402\_Mycgr3T**
  
Percentage identity: 57 %
  
BlastP bit score: 491
  
Sequence coverage: 93 %
  
E-value: 3e-167
  
  
 NCBI BlastP on this gene

RRP3

putative secreted protein
  
Accession: CCA36761
  
Location: 1114904-1116250
  
 NCBI BlastP on this gene

PP7435\_Chr1-0612

Coatomer subunit alpha
  
Accession: CCA36762
  
Location: 1116552-1120172
  
 NCBI BlastP on this gene

PP7435\_Chr1-0613

hypothetical protein
  
Accession: CCA36763
  
Location: 1120248-1121624
  
 NCBI BlastP on this gene

PP7435\_Chr1-0614

37S ribosomal protein rsm18, mitochondrial
  
Accession: CCA36764
  
Location: 1122150-1122605
  
 NCBI BlastP on this gene

PP7435\_Chr1-0615

[Pyruvate dehydrogenase [lipoamide]] kinase isozyme 2, mitochondrial
  
Accession: CCA36765
  
Location: 1122672-1123976
  
 NCBI BlastP on this gene

PDK2

Protein LDB17
  
Accession: CCA36766
  
Location: 1124356-1125672
  
 NCBI BlastP on this gene

PP7435\_Chr1-0617

hypothetical protein
  
Accession: CCA36767
  
Location: 1126150-1126811
  
 NCBI BlastP on this gene

PP7435\_Chr1-0618

26S proteasome non-ATPase regulatory subunit 12
  
Accession: CCA36768
  
Location: 1126923-1128248
  
 NCBI BlastP on this gene

PP7435\_Chr1-0619

High-affinity glucose transporter RGT2
  
Accession: CCA36769
  
Location: 1128727-1130838
  
 NCBI BlastP on this gene

SNF3

hypothetical protein
  
Accession: CCA36770
  
Location: 1131139-1132167
  
 NCBI BlastP on this gene

PP7435\_Chr1-0621

60S ribosomal protein L35
  
Accession: CCA36771
  
Location: 1132494-1133152
  
 NCBI BlastP on this gene

PP7435\_Chr1-0622

ubiquitin conjugation factor E4 B
  
Accession: CCA36772
  
Location: 1133245-1136109
  
 NCBI BlastP on this gene

UFD2

190. :  FN392319 Pichia pastoris GS115 chromosome 1     Total score: 2.0     Cumulative Blast bit score: 737

Arsenite transporter of the plasma membrane, required for resistance to arsenic compounds
  
Accession: CAY67655
  
Location: 1093416-1094687
  
 NCBI BlastP on this gene

PAS\_chr1-1\_0281

One of two large regulatory subunits of ribonucleotide-diphosphate reductase
  
Accession: CAY67656
  
Location: 1095115-1097718
  
 NCBI BlastP on this gene

PAS\_chr1-1\_0282

Protein component of the small (40S) ribosomal subunit
  
Accession: CAY67657
  
Location: 1098474-1098722
  
 NCBI BlastP on this gene

PAS\_chr1-1\_0283

Putative plasma membrane permease proposed to be involved in carboxylic acid uptake
  
Accession: CAY67658
  
Location: 1099220-1100704
  
 NCBI BlastP on this gene

PAS\_chr1-1\_0284

hypothetical protein
  
Accession: CAY67659
  
Location: 1100815-1101798
  
 NCBI BlastP on this gene

PAS\_chr1-1\_0492

F-box protein of unknown function
  
Accession: CAY67660
  
Location: 1102276-1103508
  
 NCBI BlastP on this gene

PAS\_chr1-1\_0285

Protein O-mannosyltransferase, transfers mannose residues from dolichyl phosphate-D-mannose
  
Accession: CAY67661
  
Location: 1103714-1105939
  
 NCBI BlastP on this gene

PAS\_chr1-1\_0286

Allantoin permease
  
Accession: CAY67662
  
Location: 1106519-1108030
  
 NCBI BlastP on this gene

PAS\_chr1-1\_0287

Subunit of the RNA polymerase II mediator complex
  
Accession: CAY67663
  
Location: 1108093-1108509
  
 NCBI BlastP on this gene

PAS\_chr1-1\_0288

hypothetical protein
  
Accession: CAY67664
  
Location: 1108692-1109429
  
 NCBI BlastP on this gene

PAS\_chr1-1\_0493

Hypothetical protein
  
Accession: CAY67665
  
Location: 1109562-1110134
  
 NCBI BlastP on this gene

PAS\_chr1-1\_0289

RING-type ubiquitin ligase of the endosomal and vacuolar membranes
  
Accession: CAY67666
  
Location: 1110628-1111299
  
 NCBI BlastP on this gene

PAS\_chr1-1\_0290

Protein required for ribosomal large subunit maturation, functionally redundant with Ssf1p
  
Accession: CAY67667
  
Location: 1111535-1112761
  
  
**BlastP hit with Mycgr3G35447\_Mycgr3T**
  
Percentage identity: 37 %
  
BlastP bit score: 246
  
Sequence coverage: 94 %
  
E-value: 5e-73
  
  
 NCBI BlastP on this gene

PAS\_chr1-1\_0291

Protein involved in rRNA processing
  
Accession: CAY67668
  
Location: 1113117-1114553
  
  
**BlastP hit with Mycgr3G84402\_Mycgr3T**
  
Percentage identity: 57 %
  
BlastP bit score: 491
  
Sequence coverage: 93 %
  
E-value: 3e-167
  
  
 NCBI BlastP on this gene

PAS\_chr1-1\_0292

Cell wall protein that functions in the transfer of chitin to beta(1-6)glucan
  
Accession: CAY67669
  
Location: 1114881-1116227
  
 NCBI BlastP on this gene

PAS\_chr1-1\_0293

Alpha subunit of COPI vesicle coatomer complex
  
Accession: CAY67670
  
Location: 1116529-1120149
  
 NCBI BlastP on this gene

PAS\_chr1-1\_0294

JmjC domain family histone demethylase specific for H3-K36
  
Accession: CAY67671
  
Location: 1120225-1121799
  
 NCBI BlastP on this gene

PAS\_chr1-1\_0295

Mitochondrial ribosomal protein of the small subunit
  
Accession: CAY67672
  
Location: 1122127-1122582
  
 NCBI BlastP on this gene

PAS\_chr1-1\_0296

Mitochondrial protein kinase
  
Accession: CAY67673
  
Location: 1122649-1123953
  
 NCBI BlastP on this gene

PAS\_chr1-1\_0297

Protein of unknown function
  
Accession: CAY67674
  
Location: 1124333-1125649
  
 NCBI BlastP on this gene

PAS\_chr1-1\_0298

hypothetical protein
  
Accession: CAY67675
  
Location: 1126127-1126903
  
 NCBI BlastP on this gene

PAS\_chr1-1\_0495

Essential, non-ATPase regulatory subunit of the 26S proteasome lid
  
Accession: CAY67676
  
Location: 1126900-1128225
  
 NCBI BlastP on this gene

PAS\_chr1-1\_0299

Plasma membrane glucose sensor that regulates glucose transport
  
Accession: CAY67677
  
Location: 1128726-1130816
  
 NCBI BlastP on this gene

PAS\_chr1-1\_0300

Prenyltransferase, required for cell viability
  
Accession: CAY67678
  
Location: 1131117-1132145
  
 NCBI BlastP on this gene

PAS\_chr1-1\_0301

Protein component of the large (60S) ribosomal subunit, identical to Rpl35Ap
  
Accession: CAY67679
  
Location: 1132471-1133129
  
 NCBI BlastP on this gene

PAS\_chr1-1\_0302

Ubiquitin chain assembly factor (E4) that cooperates with a ubiquitin-activating enzyme (E1)
  
Accession: CAY67680
  
Location: 1133222-1136086
  
 NCBI BlastP on this gene

PAS\_chr1-1\_0303

191. :  FM992695 Candida dubliniensis CD36 chromosome R     Total score: 2.0     Cumulative Blast bit score: 734

translation machinery-associated protein, putative
  
Accession: CAX40437
  
Location: 2016858-2017052
  
 NCBI BlastP on this gene

CD36\_34817

actin assembly factor, putative
  
Accession: CAX40438
  
Location: 2017378-2019402
  
 NCBI BlastP on this gene

CD36\_34820

conserved hypothetical protein
  
Accession: CAX40439
  
Location: 2019903-2021057
  
 NCBI BlastP on this gene

CD36\_34830

S-formylglutathione hydrolase, putative
  
Accession: CAX40440
  
Location: 2021082-2021975
  
 NCBI BlastP on this gene

CD36\_34840

sphingoid long-chain base transporter, putative
  
Accession: CAX40441
  
Location: 2022447-2023868
  
 NCBI BlastP on this gene

CD36\_34850

lysophospholipase precursor, putative
  
Accession: CAX40442
  
Location: 2027492-2029378
  
 NCBI BlastP on this gene

CD36\_34860

inorganic phosphate transporter, putative
  
Accession: CAX40443
  
Location: 2031680-2033359
  
 NCBI BlastP on this gene

CD36\_34870

peroxisomal coenzyme A diphosphatase, peroxisomal precursor, putative
  
Accession: CAX40444
  
Location: 2034327-2035466
  
 NCBI BlastP on this gene

CD36\_34880

conserved hypothetical protein
  
Accession: CAX40445
  
Location: 2035712-2036287
  
 NCBI BlastP on this gene

CD36\_34885

ribosome biogenesis protein, putative
  
Accession: CAX40446
  
Location: 2036376-2037725
  
  
**BlastP hit with Mycgr3G35447\_Mycgr3T**
  
Percentage identity: 37 %
  
BlastP bit score: 242
  
Sequence coverage: 94 %
  
E-value: 5e-71
  
  
 NCBI BlastP on this gene

CD36\_34890

ATP-dependent rRNA helicase, putative
  
Accession: CAX40447
  
Location: 2038023-2039522
  
  
**BlastP hit with Mycgr3G84402\_Mycgr3T**
  
Percentage identity: 60 %
  
BlastP bit score: 492
  
Sequence coverage: 90 %
  
E-value: 3e-167
  
  
 NCBI BlastP on this gene

CD36\_34900

phosphatidylinositol-3-phosphate-binding protein, putative
  
Accession: CAX40448
  
Location: 2039599-2040627
  
 NCBI BlastP on this gene

CD36\_34910

conserved hypothetical protein
  
Accession: CAX40449
  
Location: 2041334-2041921
  
 NCBI BlastP on this gene

CD36\_34915

dolichyl-phosphate-mannose-protein mannosyltransferase, putative
  
Accession: CAX40450
  
Location: 2041973-2044150
  
 NCBI BlastP on this gene

CD36\_34920

LPF family protein, putative
  
Accession: CAX40451
  
Location: 2046068-2048167
  
 NCBI BlastP on this gene

CD36\_34930

192. :  KB733474 Bipolaris maydis ATCC 48331 unplaced genomic scaffold COCC4scaffold\_31     Total score: 2.0     Cumulative Blast bit score: 630

hypothetical protein
  
Accession: ENI00475
  
Location: 115603-116224
  
 NCBI BlastP on this gene

ENI00475

hypothetical protein
  
Accession: ENI00476
  
Location: 116583-116926
  
 NCBI BlastP on this gene

ENI00476

hypothetical protein
  
Accession: ENI00477
  
Location: 117252-139356
  
  
**BlastP hit with Mycgr3G36951\_Mycgr3T**
  
Percentage identity: 26 %
  
BlastP bit score: 397
  
Sequence coverage: 33 %
  
E-value: 5e-107
  
  
 NCBI BlastP on this gene

ENI00477

hypothetical protein
  
Accession: ENI00478
  
Location: 139861-140364
  
 NCBI BlastP on this gene

ENI00478

hypothetical protein
  
Accession: ENI00479
  
Location: 140945-142566
  
 NCBI BlastP on this gene

ENI00479

hypothetical protein
  
Accession: ENI00480
  
Location: 143102-143839
  
 NCBI BlastP on this gene

ENI00480

hypothetical protein
  
Accession: ENI00481
  
Location: 144594-145646
  
 NCBI BlastP on this gene

ENI00481

hypothetical protein
  
Accession: ENI00482
  
Location: 146544-147726
  
 NCBI BlastP on this gene

ENI00482

hypothetical protein
  
Accession: ENI00483
  
Location: 148458-150330
  
 NCBI BlastP on this gene

ENI00483

hypothetical protein
  
Accession: ENI00484
  
Location: 150611-153478
  
 NCBI BlastP on this gene

ENI00484

hypothetical protein
  
Accession: ENI00485
  
Location: 156675-158089
  
 NCBI BlastP on this gene

ENI00485

hypothetical protein
  
Accession: ENI00486
  
Location: 158150-158958
  
 NCBI BlastP on this gene

ENI00486

hypothetical protein
  
Accession: ENI00487
  
Location: 159593-160402
  
 NCBI BlastP on this gene

ENI00487

hypothetical protein
  
Accession: ENI00488
  
Location: 160650-161894
  
  
**BlastP hit with Mycgr3G67791\_Mycgr3T**
  
Percentage identity: 32 %
  
BlastP bit score: 233
  
Sequence coverage: 82 %
  
E-value: 3e-67
  
  
 NCBI BlastP on this gene

ENI00488

hypothetical protein
  
Accession: ENI00489
  
Location: 164062-164693
  
 NCBI BlastP on this gene

ENI00489

193. :  FP929139 Leptosphaeria maculans JN3 lm\_SuperContig\_0\_v2 genomic supercontig     Total score: 2.0     Cumulative Blast bit score: 607

hypothetical protein
  
Accession: CBY02108
  
Location: 3089333-3090020
  
 NCBI BlastP on this gene

LEMA\_P008950.1

similar to C4-dicarboxylate transporter/malic acid transport protein
  
Accession: CBY02109
  
Location: 3092351-3093610
  
  
**BlastP hit with Mycgr3G67791\_Mycgr3T**
  
Percentage identity: 31 %
  
BlastP bit score: 211
  
Sequence coverage: 84 %
  
E-value: 4e-59
  
  
 NCBI BlastP on this gene

LEMA\_P008960.1

predicted protein
  
Accession: CBY02110
  
Location: 3094169-3094447
  
 NCBI BlastP on this gene

LEMA\_uP008970.1

hypothetical protein
  
Accession: CBY02111
  
Location: 3094894-3099025
  
 NCBI BlastP on this gene

LEMA\_P008980.1

similar to MFS transporter
  
Accession: CBY02112
  
Location: 3099159-3100841
  
 NCBI BlastP on this gene

LEMA\_P008990.1

predicted protein
  
Accession: CBY02113
  
Location: 3102156-3103294
  
 NCBI BlastP on this gene

LEMA\_P009000.1

predicted protein
  
Accession: CBY02114
  
Location: 3104087-3105211
  
 NCBI BlastP on this gene

LEMA\_P009010.1

similar to ABC multidrug transporter
  
Accession: CBY02115
  
Location: 3106079-3111642
  
 NCBI BlastP on this gene

LEMA\_P009020.1

predicted protein
  
Accession: CBY02116
  
Location: 3113323-3114354
  
 NCBI BlastP on this gene

LEMA\_P009030.1

similar to nonribosomal peptide synthase
  
Accession: CBY02117
  
Location: 3115242-3137649
  
  
**BlastP hit with Mycgr3G36951\_Mycgr3T**
  
Percentage identity: 26 %
  
BlastP bit score: 396
  
Sequence coverage: 32 %
  
E-value: 9e-107
  
  
 NCBI BlastP on this gene

LEMA\_P009040.1

similar to rhamnogalacturonate lyase
  
Accession: CBY02118
  
Location: 3138626-3140467
  
 NCBI BlastP on this gene

LEMA\_P009050.1

194. :  GL534459 Pyrenophora teres f. teres 0-1 unplaced genomic scaffold scaffold\_190719     Total score: 2.0     Cumulative Blast bit score: 584

hypothetical protein
  
Accession: EFQ92066
  
Location: 5204-6562
  
 NCBI BlastP on this gene

EFQ92066

hypothetical protein
  
Accession: EFQ92067
  
Location: 6868-8987
  
 NCBI BlastP on this gene

EFQ92067

hypothetical protein
  
Accession: EFQ92068
  
Location: 9593-11135
  
 NCBI BlastP on this gene

EFQ92068

hypothetical protein
  
Accession: EFQ92069
  
Location: 11882-12418
  
 NCBI BlastP on this gene

EFQ92069

hypothetical protein
  
Accession: EFQ92070
  
Location: 15120-15701
  
 NCBI BlastP on this gene

EFQ92070

hypothetical protein
  
Accession: EFQ92071
  
Location: 17941-19050
  
  
**BlastP hit with Mycgr3G67791\_Mycgr3T**
  
Percentage identity: 32 %
  
BlastP bit score: 203
  
Sequence coverage: 69 %
  
E-value: 1e-56
  
  
 NCBI BlastP on this gene

EFQ92071

hypothetical protein
  
Accession: EFQ92072
  
Location: 19293-19652
  
 NCBI BlastP on this gene

EFQ92072

hypothetical protein
  
Accession: EFQ92073
  
Location: 19943-41977
  
  
**BlastP hit with Mycgr3G36951\_Mycgr3T**
  
Percentage identity: 26 %
  
BlastP bit score: 381
  
Sequence coverage: 34 %
  
E-value: 4e-102
  
  
 NCBI BlastP on this gene

EFQ92073

hypothetical protein
  
Accession: EFQ92074
  
Location: 46095-51051
  
 NCBI BlastP on this gene

EFQ92074

hypothetical protein
  
Accession: EFQ92075
  
Location: 51524-53825
  
 NCBI BlastP on this gene

EFQ92075

hypothetical protein
  
Accession: EFQ92076
  
Location: 54862-56502
  
 NCBI BlastP on this gene

EFQ92076

195. :  CU329670 Schizosaccharomyces pombe chromosome I     Total score: 2.0     Cumulative Blast bit score: 543

krr family protein (predicted)
  
Accession: CAA91129
  
Location: 736797-738720
  
 NCBI BlastP on this gene

SPAC22G7.05

ubiquitin C-terminal hydrolase, poly(A)-specific ribonuclease complex subunit Pan2 (predicted)
  
Accession: CAA91128
  
Location: 732880-736465
  
 NCBI BlastP on this gene

ubp13

sequence orphan
  
Accession: CAA91127
  
Location: 731938-732648
  
 NCBI BlastP on this gene

SPAC22G7.03

karyopherin Kap111 (predicted)
  
Accession: CAA91126
  
Location: 728420-731706
  
 NCBI BlastP on this gene

kap111

iron responsive transcriptional regulator, peptidase family (predicted)
  
Accession: CAB62423
  
Location: 726081-728076
  
 NCBI BlastP on this gene

SPAC22G7.01c

actin cortical patch component Lsb4 (predicted)
  
Accession: CAB62422
  
Location: 724289-725720
  
 NCBI BlastP on this gene

SPAPJ696.02

retromer complex subunit Vps17
  
Accession: CAB62421
  
Location: 721617-723425
  
 NCBI BlastP on this gene

vps17

NADPH-dependent diflavin oxidoreductase,
  
Accession: CAB36512
  
Location: 719447-721410
  
 NCBI BlastP on this gene

tah18

cyclin L family cyclin (predicted)
  
Accession: CAB36511
  
Location: 716940-717804
  
  
**BlastP hit with Mycgr3G98961\_Mycgr3T**
  
Percentage identity: 28 %
  
BlastP bit score: 84
  
Sequence coverage: 80 %
  
E-value: 6e-16
  
  
 NCBI BlastP on this gene

SPAC1296.05c

spore wall assembly protein (predicted)
  
Accession: CAB36510
  
Location: 715480-716621
  
 NCBI BlastP on this gene

mug65

serine carboxypeptidase Sxa2
  
Accession: CAB36509
  
Location: 712491-714014
  
 NCBI BlastP on this gene

sxa2

cytochrome c oxidase subunit IV (predicted)
  
Accession: CAB36508
  
Location: 711301-712150
  
 NCBI BlastP on this gene

cox4

phosphoacetylglucosamine mutase (predicted)
  
Accession: CAA91066
  
Location: 708055-710023
  
  
**BlastP hit with Mycgr3G103034\_Mycgr3**
  
Percentage identity: 46 %
  
BlastP bit score: 459
  
Sequence coverage: 95 %
  
E-value: 3e-152
  
  
 NCBI BlastP on this gene

SPAC1296.01c

transcription factor Atf31
  
Accession: CAA91067
  
Location: 706844-707473
  
 NCBI BlastP on this gene

atf31

ATP-dependent DNA helicase Rdh54
  
Accession: CAA91068
  
Location: 703789-706828
  
 NCBI BlastP on this gene

rdh54

AMP binding enzyme (predicted)
  
Accession: CAA91069
  
Location: 698033-702533
  
 NCBI BlastP on this gene

mug62

ADP-ribosylation factor Alp41
  
Accession: CAA91070
  
Location: 697291-698068
  
 NCBI BlastP on this gene

alp41

Lon protease homolog Lon1 (predicted)
  
Accession: CAA91071
  
Location: 693831-697034
  
 NCBI BlastP on this gene

lon1

GatB/YqeY domain protein
  
Accession: CBA11491
  
Location: 692567-693061
  
 NCBI BlastP on this gene

SPAC22F3.15

F0-ATPase subunit G (predicted)
  
Accession: CAA91072
  
Location: 691530-691984
  
 NCBI BlastP on this gene

atp20

ATP-dependent RNA helicase Rok1 (predicted)
  
Accession: CAA91073
  
Location: 689483-690928
  
 NCBI BlastP on this gene

rok1

196. :  KB445561 Baudoinia compniacensis UAMH 10762 unplaced genomic scaffold BAUCOscaffold\_12     Total score: 2.0     Cumulative Blast bit score: 524

glycoside hydrolase family 16 protein
  
Accession: EMC92656
  
Location: 211429-212813
  
 NCBI BlastP on this gene

EMC92656

hypothetical protein
  
Accession: EMC92657
  
Location: 213466-213663
  
 NCBI BlastP on this gene

EMC92657

hypothetical protein
  
Accession: EMC92658
  
Location: 214005-218039
  
 NCBI BlastP on this gene

EMC92658

hypothetical protein
  
Accession: EMC92659
  
Location: 218563-218865
  
 NCBI BlastP on this gene

EMC92659

hypothetical protein
  
Accession: EMC92660
  
Location: 219891-220836
  
 NCBI BlastP on this gene

EMC92660

hypothetical protein
  
Accession: EMC92661
  
Location: 220955-221227
  
 NCBI BlastP on this gene

EMC92661

hypothetical protein
  
Accession: EMC92662
  
Location: 222551-223261
  
 NCBI BlastP on this gene

EMC92662

hypothetical protein
  
Accession: EMC92663
  
Location: 224544-224756
  
 NCBI BlastP on this gene

EMC92663

hypothetical protein
  
Accession: EMC92664
  
Location: 225388-226582
  
 NCBI BlastP on this gene

EMC92664

hypothetical protein
  
Accession: EMC92665
  
Location: 227251-228507
  
 NCBI BlastP on this gene

EMC92665

glycosyltransferase family 2 protein
  
Accession: EMC92666
  
Location: 228684-229451
  
 NCBI BlastP on this gene

EMC92666

hypothetical protein
  
Accession: EMC92667
  
Location: 229778-230711
  
  
**BlastP hit with Mycgr3G98961\_Mycgr3T**
  
Percentage identity: 59 %
  
BlastP bit score: 342
  
Sequence coverage: 96 %
  
E-value: 6e-114
  
  
 NCBI BlastP on this gene

EMC92667

hypothetical protein
  
Accession: EMC92668
  
Location: 232087-234101
  
  
**BlastP hit with Mycgr3G90404\_Mycgr3T**
  
Percentage identity: 56 %
  
BlastP bit score: 182
  
Sequence coverage: 49 %
  
E-value: 2e-49
  
  
 NCBI BlastP on this gene

EMC92668

hypothetical protein
  
Accession: EMC92669
  
Location: 236331-238115
  
 NCBI BlastP on this gene

EMC92669

hypothetical protein
  
Accession: EMC92670
  
Location: 238465-241030
  
 NCBI BlastP on this gene

EMC92670

hypothetical protein
  
Accession: EMC92671
  
Location: 241063-242474
  
 NCBI BlastP on this gene

EMC92671

hypothetical protein
  
Accession: EMC92672
  
Location: 242720-244525
  
 NCBI BlastP on this gene

EMC92672

hypothetical protein
  
Accession: EMC92673
  
Location: 245255-247660
  
 NCBI BlastP on this gene

EMC92673

hypothetical protein
  
Accession: EMC92674
  
Location: 249738-255662
  
 NCBI BlastP on this gene

EMC92674

197. :  ADOT01000171 Arthrobotrys oligospora ATCC 24927     Total score: 2.0     Cumulative Blast bit score: 342

hypothetical protein
  
Accession: EGX47162
  
Location: 1439-1765
  
 NCBI BlastP on this gene

EGX47162

hypothetical protein
  
Accession: EGX47163
  
Location: 2811-4157
  
 NCBI BlastP on this gene

EGX47163

hypothetical protein
  
Accession: EGX47164
  
Location: 7787-10021
  
 NCBI BlastP on this gene

EGX47164

hypothetical protein
  
Accession: EGX47165
  
Location: 10653-12811
  
 NCBI BlastP on this gene

EGX47165

hypothetical protein
  
Accession: EGX47166
  
Location: 14003-15742
  
  
**BlastP hit with Mycgr3G90404\_Mycgr3T**
  
Percentage identity: 32 %
  
BlastP bit score: 110
  
Sequence coverage: 71 %
  
E-value: 7e-24
  
  
 NCBI BlastP on this gene

EGX47166

hypothetical protein
  
Accession: EGX47167
  
Location: 16427-17287
  
 NCBI BlastP on this gene

EGX47167

hypothetical protein
  
Accession: EGX47168
  
Location: 17821-18807
  
 NCBI BlastP on this gene

EGX47168

hypothetical protein
  
Accession: EGX47169
  
Location: 23161-24474
  
  
**BlastP hit with Mycgr3G67791\_Mycgr3T**
  
Percentage identity: 30 %
  
BlastP bit score: 232
  
Sequence coverage: 80 %
  
E-value: 1e-66
  
  
 NCBI BlastP on this gene

EGX47169

hypothetical protein
  
Accession: EGX47170
  
Location: 27256-28266
  
 NCBI BlastP on this gene

EGX47170

hypothetical protein
  
Accession: EGX47171
  
Location: 28472-29332
  
 NCBI BlastP on this gene

EGX47171

hypothetical protein
  
Accession: EGX47172
  
Location: 30077-31085
  
 NCBI BlastP on this gene

EGX47172

hypothetical protein
  
Accession: EGX47173
  
Location: 33465-34693
  
 NCBI BlastP on this gene

EGX47173

hypothetical protein
  
Accession: EGX47174
  
Location: 35576-38196
  
 NCBI BlastP on this gene

EGX47174

hypothetical protein
  
Accession: EGX47175
  
Location: 39610-40663
  
 NCBI BlastP on this gene

EGX47175

hypothetical protein
  
Accession: EGX47176
  
Location: 41803-43413
  
 NCBI BlastP on this gene

EGX47176

hypothetical protein
  
Accession: EGX47177
  
Location: 44200-45655
  
 NCBI BlastP on this gene

EGX47177

198. :  JH971386 Agaricus bisporus var. burnettii JB137-S8 unplaced genomic scaffold AGABI1scaffold\_2     Total score: 2.0     Cumulative Blast bit score: 318

hypothetical protein
  
Accession: EKM82815
  
Location: 1448903-1449377
  
 NCBI BlastP on this gene

EKM82815

hypothetical protein
  
Accession: EKM82816
  
Location: 1449648-1450061
  
 NCBI BlastP on this gene

EKM82816

hypothetical protein
  
Accession: EKM82817
  
Location: 1450329-1452071
  
 NCBI BlastP on this gene

EKM82817

hypothetical protein
  
Accession: EKM82818
  
Location: 1454336-1456021
  
 NCBI BlastP on this gene

EKM82818

hypothetical protein
  
Accession: EKM82819
  
Location: 1456066-1460005
  
  
**BlastP hit with Mycgr3G98961\_Mycgr3T**
  
Percentage identity: 31 %
  
BlastP bit score: 100
  
Sequence coverage: 81 %
  
E-value: 4e-20
  
  
 NCBI BlastP on this gene

EKM82819

hypothetical protein
  
Accession: EKM82820
  
Location: 1460713-1461959
  
 NCBI BlastP on this gene

EKM82820

hypothetical protein
  
Accession: EKM82821
  
Location: 1462197-1463249
  
 NCBI BlastP on this gene

EKM82821

hypothetical protein
  
Accession: EKM82822
  
Location: 1464688-1467100
  
 NCBI BlastP on this gene

EKM82822

hypothetical protein
  
Accession: EKM82823
  
Location: 1469019-1473814
  
 NCBI BlastP on this gene

EKM82823

hypothetical protein
  
Accession: EKM82824
  
Location: 1476422-1476632
  
 NCBI BlastP on this gene

EKM82824

hypothetical protein
  
Accession: EKM82825
  
Location: 1476699-1477681
  
 NCBI BlastP on this gene

EKM82825

hypothetical protein
  
Accession: EKM82826
  
Location: 1478204-1481493
  
 NCBI BlastP on this gene

EKM82826

hypothetical protein
  
Accession: EKM82827
  
Location: 1481611-1482473
  
 NCBI BlastP on this gene

EKM82827

hypothetical protein
  
Accession: EKM82828
  
Location: 1482600-1484052
  
  
**BlastP hit with Mycgr3G35447\_Mycgr3T**
  
Percentage identity: 35 %
  
BlastP bit score: 218
  
Sequence coverage: 101 %
  
E-value: 3e-62
  
  
 NCBI BlastP on this gene

EKM82828

hypothetical protein
  
Accession: EKM82829
  
Location: 1484114-1484863
  
 NCBI BlastP on this gene

EKM82829

hypothetical protein
  
Accession: EKM82830
  
Location: 1485033-1486692
  
 NCBI BlastP on this gene

EKM82830

hypothetical protein
  
Accession: EKM82831
  
Location: 1487016-1488355
  
 NCBI BlastP on this gene

EKM82831

hypothetical protein
  
Accession: EKM82832
  
Location: 1488430-1489166
  
 NCBI BlastP on this gene

EKM82832

hypothetical protein
  
Accession: EKM82833
  
Location: 1490541-1491738
  
 NCBI BlastP on this gene

EKM82833

hypothetical protein
  
Accession: EKM82834
  
Location: 1492443-1493640
  
 NCBI BlastP on this gene

EKM82834

hypothetical protein
  
Accession: EKM82835
  
Location: 1494077-1496907
  
 NCBI BlastP on this gene

EKM82835

199. :  JH931606 Agaricus bisporus var. bisporus H97 unplaced genomic scaffold AGABI2scaffold\_2     Total score: 2.0     Cumulative Blast bit score: 317

hypothetical protein
  
Accession: EKV50215
  
Location: 1493683-1494074
  
 NCBI BlastP on this gene

EKV50215

hypothetical protein
  
Accession: EKV50216
  
Location: 1494199-1494612
  
 NCBI BlastP on this gene

EKV50216

hypothetical protein
  
Accession: EKV50217
  
Location: 1494880-1496625
  
 NCBI BlastP on this gene

EKV50217

hypothetical protein
  
Accession: EKV50218
  
Location: 1499281-1500965
  
 NCBI BlastP on this gene

EKV50218

hypothetical protein
  
Accession: EKV50219
  
Location: 1501007-1504940
  
  
**BlastP hit with Mycgr3G98961\_Mycgr3T**
  
Percentage identity: 31 %
  
BlastP bit score: 100
  
Sequence coverage: 81 %
  
E-value: 4e-20
  
  
 NCBI BlastP on this gene

EKV50219

hypothetical protein
  
Accession: EKV50220
  
Location: 1505674-1507000
  
 NCBI BlastP on this gene

EKV50220

hypothetical protein
  
Accession: EKV50221
  
Location: 1507160-1508211
  
 NCBI BlastP on this gene

EKV50221

argonaute-like protein
  
Accession: EKV50222
  
Location: 1509662-1513281
  
 NCBI BlastP on this gene

EKV50222

hypothetical protein
  
Accession: EKV50223
  
Location: 1513995-1518790
  
 NCBI BlastP on this gene

EKV50223

hypothetical protein
  
Accession: EKV50224
  
Location: 1521507-1522922
  
 NCBI BlastP on this gene

EKV50224

hypothetical protein
  
Accession: EKV50225
  
Location: 1523012-1526301
  
 NCBI BlastP on this gene

EKV50225

hypothetical protein
  
Accession: EKV50226
  
Location: 1526648-1527280
  
 NCBI BlastP on this gene

EKV50226

hypothetical protein
  
Accession: EKV50227
  
Location: 1527407-1528859
  
  
**BlastP hit with Mycgr3G35447\_Mycgr3T**
  
Percentage identity: 35 %
  
BlastP bit score: 217
  
Sequence coverage: 102 %
  
E-value: 1e-61
  
  
 NCBI BlastP on this gene

EKV50227

hypothetical protein
  
Accession: EKV50228
  
Location: 1528933-1529682
  
 NCBI BlastP on this gene

EKV50228

hypothetical protein
  
Accession: EKV50229
  
Location: 1529835-1531499
  
 NCBI BlastP on this gene

EKV50229

hypothetical protein
  
Accession: EKV50230
  
Location: 1531955-1533165
  
 NCBI BlastP on this gene

EKV50230

hypothetical protein
  
Accession: EKV50231
  
Location: 1533240-1534841
  
 NCBI BlastP on this gene

EKV50231

hypothetical protein
  
Accession: EKV50232
  
Location: 1535352-1536549
  
 NCBI BlastP on this gene

EKV50232

hypothetical protein
  
Accession: EKV50233
  
Location: 1537254-1540113
  
 NCBI BlastP on this gene

EKV50233

200. :  DS547096 Laccaria bicolor S238N-H82 LACBIscaffold\_6 genomic scaffold     Total score: 2.0     Cumulative Blast bit score: 315

predicted protein
  
Accession: EDR11325
  
Location: 1750943-1753791
  
 NCBI BlastP on this gene

EDR11325

predicted protein
  
Accession: EDR11326
  
Location: 1754108-1755159
  
 NCBI BlastP on this gene

EDR11326

predicted protein
  
Accession: EDR10982
  
Location: 1755699-1756798
  
 NCBI BlastP on this gene

EDR10982

predicted protein
  
Accession: EDR11327
  
Location: 1757062-1757434
  
 NCBI BlastP on this gene

EDR11327

predicted protein
  
Accession: EDR10983
  
Location: 1757572-1758817
  
 NCBI BlastP on this gene

EDR10983

predicted protein
  
Accession: EDR10984
  
Location: 1759565-1762637
  
 NCBI BlastP on this gene

EDR10984

predicted protein
  
Accession: EDR10985
  
Location: 1763185-1763693
  
 NCBI BlastP on this gene

EDR10985

predicted protein
  
Accession: EDR10986
  
Location: 1763845-1764825
  
 NCBI BlastP on this gene

EDR10986

predicted protein
  
Accession: EDR10987
  
Location: 1765102-1765525
  
 NCBI BlastP on this gene

EDR10987

predicted protein
  
Accession: EDR10988
  
Location: 1765649-1767270
  
 NCBI BlastP on this gene

EDR10988

predicted protein
  
Accession: EDR11328
  
Location: 1767737-1769360
  
 NCBI BlastP on this gene

EDR11328

predicted protein
  
Accession: EDR10989
  
Location: 1769448-1770358
  
  
**BlastP hit with Mycgr3G98961\_Mycgr3T**
  
Percentage identity: 29 %
  
BlastP bit score: 98
  
Sequence coverage: 89 %
  
E-value: 7e-21
  
  
 NCBI BlastP on this gene

EDR10989

predicted protein
  
Accession: EDR10990
  
Location: 1770650-1773278
  
 NCBI BlastP on this gene

EDR10990

predicted protein
  
Accession: EDR10991
  
Location: 1773510-1774455
  
 NCBI BlastP on this gene

EDR10991

ER-to-Golgi vesicle protein transport Sft2
  
Accession: EDR11329
  
Location: 1774529-1775496
  
 NCBI BlastP on this gene

EDR11329

predicted protein
  
Accession: EDR11330
  
Location: 1775794-1780521
  
 NCBI BlastP on this gene

EDR11330

predicted protein
  
Accession: EDR11331
  
Location: 1781071-1782384
  
 NCBI BlastP on this gene

EDR11331

predicted protein
  
Accession: EDR10992
  
Location: 1782522-1785928
  
 NCBI BlastP on this gene

EDR10992

predicted protein
  
Accession: EDR11332
  
Location: 1785983-1786838
  
 NCBI BlastP on this gene

EDR11332

predicted protein
  
Accession: EDR10993
  
Location: 1786885-1788321
  
  
**BlastP hit with Mycgr3G35447\_Mycgr3T**
  
Percentage identity: 36 %
  
BlastP bit score: 217
  
Sequence coverage: 105 %
  
E-value: 8e-62
  
  
 NCBI BlastP on this gene

EDR10993

predicted protein
  
Accession: EDR11333
  
Location: 1788346-1789107
  
 NCBI BlastP on this gene

EDR11333

predicted protein
  
Accession: EDR10994
  
Location: 1789221-1790809
  
 NCBI BlastP on this gene

EDR10994

predicted protein
  
Accession: EDR11334
  
Location: 1792967-1794161
  
 NCBI BlastP on this gene

EDR11334

predicted protein
  
Accession: EDR11335
  
Location: 1794664-1795810
  
 NCBI BlastP on this gene

EDR11335

predicted protein
  
Accession: EDR10995
  
Location: 1796053-1797456
  
 NCBI BlastP on this gene

EDR10995

predicted protein
  
Accession: EDR11336
  
Location: 1797481-1799199
  
 NCBI BlastP on this gene

EDR11336

20S proteasome subunit
  
Accession: EDR10996
  
Location: 1799285-1800512
  
 NCBI BlastP on this gene

EDR10996

Sec7-like domain is implicated in guanine nucleotide exchange function
  
Accession: EDR10997
  
Location: 1800764-1806242
  
 NCBI BlastP on this gene

EDR10997

Detecting sequence homology at the gene cluster level with MultiGeneBlast.
  
Marnix H. Medema, Rainer Breitling & Eriko Takano (2013)
  
*Molecular Biology and Evolution* , 30: 1218-1223.
