# Supplementary material for: In silico prediction and characterization of secondary metabolite biosynthetic gene clusters in the wheat pathogen Zymoseptoria tritici
Source: BMC Genomics. 2017 Aug 17;18:631. doi: 10.1186/s12864-017-3969-y (PMC5561558; doi:10.1186/s12864-017-3969-y)
Supplement: Supplementary file 1 — MultiGeneBLAST analysis of putative secondary metabolite clusters. All encoded amino acid sequences from genes residing in clusters predicted by AntiSMASH are given as FASTA file format. All output data from MultiGeneBLASTs are also provided. (ZIP 42911 kb) [file 12864_2017_3969_MOESM1_ESM.zip › Cluster MultiGene BLAST/out/Clusters_1_34/Cluster_10/displaypage5.xhtml]

xml version="1.0" encoding="UTF-8"?


Search Results
  
  
 Results pages: 1, 2, 3, 4, 5

**MultiGeneBlast hits**

Select gene cluster alignment
201. JH971387\_0 Agaricus bisporus var. burnettii JB137-S8 unplaced genomic sc...
202. JH931610\_0 Agaricus bisporus var. bisporus H97 unplaced genomic scaffold...
203. KB445561\_3 Baudoinia compniacensis UAMH 10762 unplaced genomic scaffold ...
204. CM001880\_0 Theobroma cacao cultivar Matina 1-6 chromosome 2, whole genom...
205. CP002684\_0 Arabidopsis thaliana chromosome 1, complete sequence.
206. JH767573\_2 Coniosporium apollinis CBS 100218 chromosome Unknown supercon...
207. KB445649\_4 Cochliobolus sativus ND90Pr unplaced genomic scaffold COCSAsc...
208. KB445579\_4 Cochliobolus heterostrophus C5 unplaced genomic scaffold COCH...
209. CH476599\_0 Aspergillus terreus NIH2624 scaffold\_6 genomic scaffold, whol...
210. DS572813\_0 Paracoccidioides brasiliensis Pb01 supercont1.3 genomic scaff...
211. DS572750\_1 Paracoccidioides brasiliensis Pb18 supercont1.1 genomic scaff...
212. GG749410\_1 Ajellomyces dermatitidis ATCC 18188 genomic scaffold supercon...
213. DS544805\_2 Paracoccidioides brasiliensis Pb03 supercont1.3 genomic scaff...
214. GG663373\_0 Ajellomyces capsulatus G186AR genomic scaffold supercont2.11,...
215. EQ963476\_3 Aspergillus flavus NRRL3357 scf\_1106286417850 genomic scaffol...
216. AKHY01000140\_1 Aspergillus oryzae 3.042, whole genome shotgun sequencing...
217. EQ962654\_3 Talaromyces stipitatus ATCC 10500 scf\_1105507295541 genomic s...
218. DS990641\_0 Ajellomyces capsulatus H88 supercont1.6 genomic scaffold, who...
219. GG657464\_0 Ajellomyces dermatitidis SLH14081 genomic scaffold supercont1...
220. EQ999980\_0 Ajellomyces dermatitidis ER-3 genomic scaffold supercont1.8, ...
221. DS995904\_1 Penicillium marneffei ATCC 18224 scf\_1105668340738 genomic sc...
222. GG692427\_1 Ajellomyces capsulatus H143 genomic scaffold supercont2.9, wh...
223. AP007171\_2 Aspergillus oryzae RIB40 DNA, SC011.
224. JH921440\_1 Marssonina brunnea f. sp. 'multigermtubi' MB\_m1 unplaced geno...
225. KE145367\_1 Glarea lozoyensis ATCC 20868 chromosome Unknown GLAREA3, whol...
226. KB707916\_0 Botryotinia fuckeliana BcDW1 unplaced genomic scaffold Scaffo...
227. FQ790307\_1 Botryotinia fuckeliana T4 SuperContig\_19\_1 genomic supercontig.
228. KB644411\_0 Penicillium oxalicum 114-2 unplaced genomic scaffold scaffold...
229. CH445336\_4 Phaeosphaeria nodorum SN15 scaffold\_12, whole genome shotgun ...
230. ABDF02000005\_0 Trichoderma virens Gv29-8, whole genome shotgun sequencin...
231. ABDG02000025\_0 Trichoderma atroviride IMI 206040, whole genome shotgun s...
232. GL698476\_0 Metarhizium acridum CQMa 102 unplaced genomic scaffold Scf\_00...
233. HF679028\_4 Fusarium fujikuroi IMI 58289 draft genome, chromosome FFUJ\_ch...
234. JH226130\_0 Exophiala dermatitidis NIH/UT8656 unplaced genomic scaffold s...
235. KB020785\_0 Colletotrichum gloeosporioides Nara gc5 unplaced genomic scaf...
236. DS985228\_1 Verticillium albo-atrum VaMs.102 supercont1.15 genomic scaffo...
237. KB731260\_2 Fusarium oxysporum f. sp. cubense race 1 unplaced genomic sca...
238. GL891307\_2 Neurospora tetrasperma FGSC 2508 unplaced genomic scaffold NE...
239. GL988043\_2 Chaetomium thermophilum var. thermophilum DSM 1495 unplaced g...
240. GL891269\_1 Neurospora tetrasperma FGSC 2509 unplaced genomic scaffold NE...
241. JH126401\_2 Cordyceps militaris CM01 unplaced genomic scaffold CCM\_S00003...
242. CABT02000010\_0 Sordaria macrospora k-hell, whole genome shotgun sequenci...
243. CP003005\_2 Myceliophthora thermophila ATCC 42464 chromosome 4, complete ...
244. CP003009\_3 Thielavia terrestris NRRL 8126 chromosome 1, complete sequence.
245. CM001231\_4 Magnaporthe oryzae 70-15 chromosome 1, whole genome shotgun s...
246. GL385396\_0 Gaeumannomyces graminis var. tritici R3-111a-1 unplaced genom...
247. KE503206\_0 Schizosaccharomyces octosporus yFS286 unplaced genomic scaffo...
248. DS022226\_2 Schizosaccharomyces japonicus yFS275 supercont1.3 genomic sca...
249. KE546988\_3 Schizosaccharomyces cryophilus OY26 unplaced genomic scaffold...
250. CU329671\_0 Schizosaccharomyces pombe chromosome II, complete sequence.

Query: Architecture Search FASTA input

JH971387 : Agaricus bisporus var. burnettii JB137-S8 unplaced genomic scaffold AGABI1scaffold\_3    Total score: 1.0     Cumulative Blast bit score: 4601

Hit cluster cross-links:

Mycgr3G67791 Mycgr3T
  
Location: 0-1542

Mycgr3G67791\_Mycgr3T

Mycgr3G90406 Mycgr3T
  
Location: 1642-3973

Mycgr3G90406\_Mycgr3T

Mycgr3G67785 Mycgr3T
  
Location: 4073-7865

Mycgr3G67785\_Mycgr3T

Mycgr3G67795 Mycgr3T
  
Location: 7965-15249

Mycgr3G67795\_Mycgr3T

Mycgr3G67775 Mycgr3T
  
Location: 15349-16237

Mycgr3G67775\_Mycgr3T

Mycgr3G90404 Mycgr3T
  
Location: 16337-17246

Mycgr3G90404\_Mycgr3T

Mycgr3G36951 Mycgr3T
  
Location: 17346-30891

Mycgr3G36951\_Mycgr3T

Mycgr3G103034 Mycgr3
  
Location: 30991-32644

Mycgr3G103034\_Mycgr3

Mycgr3G31119 Mycgr3T
  
Location: 32744-32906

Mycgr3G31119\_Mycgr3T

Mycgr3G28587 Mycgr3T
  
Location: 33006-33489

Mycgr3G28587\_Mycgr3T

Mycgr3G98959 Mycgr3T
  
Location: 33589-35035

Mycgr3G98959\_Mycgr3T

Mycgr3G35447 Mycgr3T
  
Location: 35135-36443

Mycgr3G35447\_Mycgr3T

Mycgr3G84402 Mycgr3T
  
Location: 36543-37884

Mycgr3G84402\_Mycgr3T

Mycgr3G98961 Mycgr3T
  
Location: 37984-38884

Mycgr3G98961\_Mycgr3T

hypothetical protein
  
Accession: EKM81702
  
Location: 939805-942551
  
 NCBI BlastP on this gene

EKM81702

hypothetical protein
  
Accession: EKM81703
  
Location: 942695-951294
  
  
**BlastP hit with Mycgr3G67795\_Mycgr3T**
  
Percentage identity: 48 %
  
BlastP bit score: 2275
  
Sequence coverage: 100 %
  
E-value: 0.0
  
  
 NCBI BlastP on this gene

EKM81703

hypothetical protein
  
Accession: EKM81704
  
Location: 951900-954220
  
 NCBI BlastP on this gene

EKM81704

hypothetical protein
  
Accession: EKM81705
  
Location: 955336-957297
  
 NCBI BlastP on this gene

EKM81705

hypothetical protein
  
Accession: EKM81706
  
Location: 958013-959566
  
 NCBI BlastP on this gene

EKM81706

hypothetical protein
  
Accession: EKM81707
  
Location: 959723-961604
  
 NCBI BlastP on this gene

EKM81707

hypothetical protein
  
Accession: EKM81708
  
Location: 961953-964389
  
 NCBI BlastP on this gene

EKM81708

hypothetical protein
  
Accession: EKM81709
  
Location: 965277-966233
  
 NCBI BlastP on this gene

EKM81709

hypothetical protein
  
Accession: EKM81710
  
Location: 966379-967014
  
 NCBI BlastP on this gene

EKM81710

hypothetical protein
  
Accession: EKM81711
  
Location: 967235-969276
  
 NCBI BlastP on this gene

EKM81711

hypothetical protein
  
Accession: EKM81712
  
Location: 970960-974593
  
 NCBI BlastP on this gene

EKM81712

hypothetical protein
  
Accession: EKM81713
  
Location: 976048-984685
  
  
**BlastP hit with Mycgr3G67795\_Mycgr3T**
  
Percentage identity: 48 %
  
BlastP bit score: 2326
  
Sequence coverage: 100 %
  
E-value: 0.0
  
  
 NCBI BlastP on this gene

EKM81713

hypothetical protein
  
Accession: EKM81714
  
Location: 984985-986406
  
 NCBI BlastP on this gene

EKM81714

Query: Architecture Search FASTA input

JH931610 : Agaricus bisporus var. bisporus H97 unplaced genomic scaffold AGABI2scaffold\_6    Total score: 1.0     Cumulative Blast bit score: 4432

Hit cluster cross-links:

Mycgr3G67791 Mycgr3T
  
Location: 0-1542

Mycgr3G67791\_Mycgr3T

Mycgr3G90406 Mycgr3T
  
Location: 1642-3973

Mycgr3G90406\_Mycgr3T

Mycgr3G67785 Mycgr3T
  
Location: 4073-7865

Mycgr3G67785\_Mycgr3T

Mycgr3G67795 Mycgr3T
  
Location: 7965-15249

Mycgr3G67795\_Mycgr3T

Mycgr3G67775 Mycgr3T
  
Location: 15349-16237

Mycgr3G67775\_Mycgr3T

Mycgr3G90404 Mycgr3T
  
Location: 16337-17246

Mycgr3G90404\_Mycgr3T

Mycgr3G36951 Mycgr3T
  
Location: 17346-30891

Mycgr3G36951\_Mycgr3T

Mycgr3G103034 Mycgr3
  
Location: 30991-32644

Mycgr3G103034\_Mycgr3

Mycgr3G31119 Mycgr3T
  
Location: 32744-32906

Mycgr3G31119\_Mycgr3T

Mycgr3G28587 Mycgr3T
  
Location: 33006-33489

Mycgr3G28587\_Mycgr3T

Mycgr3G98959 Mycgr3T
  
Location: 33589-35035

Mycgr3G98959\_Mycgr3T

Mycgr3G35447 Mycgr3T
  
Location: 35135-36443

Mycgr3G35447\_Mycgr3T

Mycgr3G84402 Mycgr3T
  
Location: 36543-37884

Mycgr3G84402\_Mycgr3T

Mycgr3G98961 Mycgr3T
  
Location: 37984-38884

Mycgr3G98961\_Mycgr3T

hypothetical protein
  
Accession: EKV46504
  
Location: 932444-935191
  
 NCBI BlastP on this gene

EKV46504

hypothetical protein
  
Accession: EKV46505
  
Location: 935335-943750
  
  
**BlastP hit with Mycgr3G67795\_Mycgr3T**
  
Percentage identity: 47 %
  
BlastP bit score: 2205
  
Sequence coverage: 99 %
  
E-value: 0.0
  
  
 NCBI BlastP on this gene

EKV46505

hypothetical protein
  
Accession: EKV46506
  
Location: 944546-946866
  
 NCBI BlastP on this gene

EKV46506

hypothetical protein
  
Accession: EKV46507
  
Location: 947983-949945
  
 NCBI BlastP on this gene

EKV46507

hypothetical protein
  
Accession: EKV46508
  
Location: 950659-952208
  
 NCBI BlastP on this gene

EKV46508

hypothetical protein
  
Accession: EKV46509
  
Location: 952371-954254
  
 NCBI BlastP on this gene

EKV46509

hypothetical protein
  
Accession: EKV46510
  
Location: 954603-957037
  
 NCBI BlastP on this gene

EKV46510

hypothetical protein
  
Accession: EKV46511
  
Location: 957296-958866
  
 NCBI BlastP on this gene

EKV46511

hypothetical protein
  
Accession: EKV46512
  
Location: 959025-959660
  
 NCBI BlastP on this gene

EKV46512

hypothetical protein
  
Accession: EKV46513
  
Location: 959882-961922
  
 NCBI BlastP on this gene

EKV46513

hypothetical protein
  
Accession: EKV46514
  
Location: 963596-965540
  
 NCBI BlastP on this gene

EKV46514

hypothetical protein
  
Accession: EKV46515
  
Location: 966158-967240
  
 NCBI BlastP on this gene

EKV46515

hypothetical protein
  
Accession: EKV46516
  
Location: 968671-977303
  
  
**BlastP hit with Mycgr3G67795\_Mycgr3T**
  
Percentage identity: 47 %
  
BlastP bit score: 2227
  
Sequence coverage: 100 %
  
E-value: 0.0
  
  
 NCBI BlastP on this gene

EKV46516

hypothetical protein
  
Accession: EKV46517
  
Location: 977603-979026
  
 NCBI BlastP on this gene

EKV46517

Query: Architecture Search FASTA input

KB445561 : Baudoinia compniacensis UAMH 10762 unplaced genomic scaffold BAUCOscaffold\_12    Total score: 1.0     Cumulative Blast bit score: 4110

Hit cluster cross-links:

Mycgr3G67791 Mycgr3T
  
Location: 0-1542

Mycgr3G67791\_Mycgr3T

Mycgr3G90406 Mycgr3T
  
Location: 1642-3973

Mycgr3G90406\_Mycgr3T

Mycgr3G67785 Mycgr3T
  
Location: 4073-7865

Mycgr3G67785\_Mycgr3T

Mycgr3G67795 Mycgr3T
  
Location: 7965-15249

Mycgr3G67795\_Mycgr3T

Mycgr3G67775 Mycgr3T
  
Location: 15349-16237

Mycgr3G67775\_Mycgr3T

Mycgr3G90404 Mycgr3T
  
Location: 16337-17246

Mycgr3G90404\_Mycgr3T

Mycgr3G36951 Mycgr3T
  
Location: 17346-30891

Mycgr3G36951\_Mycgr3T

Mycgr3G103034 Mycgr3
  
Location: 30991-32644

Mycgr3G103034\_Mycgr3

Mycgr3G31119 Mycgr3T
  
Location: 32744-32906

Mycgr3G31119\_Mycgr3T

Mycgr3G28587 Mycgr3T
  
Location: 33006-33489

Mycgr3G28587\_Mycgr3T

Mycgr3G98959 Mycgr3T
  
Location: 33589-35035

Mycgr3G98959\_Mycgr3T

Mycgr3G35447 Mycgr3T
  
Location: 35135-36443

Mycgr3G35447\_Mycgr3T

Mycgr3G84402 Mycgr3T
  
Location: 36543-37884

Mycgr3G84402\_Mycgr3T

Mycgr3G98961 Mycgr3T
  
Location: 37984-38884

Mycgr3G98961\_Mycgr3T

hypothetical protein
  
Accession: EMC92720
  
Location: 363457-364234
  
 NCBI BlastP on this gene

EMC92720

hypothetical protein
  
Accession: EMC92721
  
Location: 364886-367775
  
 NCBI BlastP on this gene

EMC92721

hypothetical protein
  
Accession: EMC92722
  
Location: 368811-371612
  
 NCBI BlastP on this gene

EMC92722

hypothetical protein
  
Accession: EMC92723
  
Location: 373800-374824
  
 NCBI BlastP on this gene

EMC92723

hypothetical protein
  
Accession: EMC92724
  
Location: 376422-377997
  
 NCBI BlastP on this gene

EMC92724

hypothetical protein
  
Accession: EMC92725
  
Location: 378750-380891
  
 NCBI BlastP on this gene

EMC92725

hypothetical protein
  
Accession: EMC92726
  
Location: 381310-388614
  
  
**BlastP hit with Mycgr3G67795\_Mycgr3T**
  
Percentage identity: 82 %
  
BlastP bit score: 4110
  
Sequence coverage: 100 %
  
E-value: 0.0
  
  
 NCBI BlastP on this gene

EMC92726

hypothetical protein
  
Accession: EMC92727
  
Location: 389399-389978
  
 NCBI BlastP on this gene

EMC92727

hypothetical protein
  
Accession: EMC92728
  
Location: 390056-390821
  
 NCBI BlastP on this gene

EMC92728

hypothetical protein
  
Accession: EMC92729
  
Location: 391438-394138
  
 NCBI BlastP on this gene

EMC92729

hypothetical protein
  
Accession: EMC92730
  
Location: 396285-396938
  
 NCBI BlastP on this gene

EMC92730

hypothetical protein
  
Accession: EMC92731
  
Location: 397913-398519
  
 NCBI BlastP on this gene

EMC92731

hypothetical protein
  
Accession: EMC92732
  
Location: 398955-402315
  
 NCBI BlastP on this gene

EMC92732

hypothetical protein
  
Accession: EMC92733
  
Location: 402812-404569
  
 NCBI BlastP on this gene

EMC92733

hypothetical protein
  
Accession: EMC92734
  
Location: 405538-406134
  
 NCBI BlastP on this gene

EMC92734

Query: Architecture Search FASTA input

CM001880 : Theobroma cacao cultivar Matina 1-6 chromosome 2    Total score: 1.0     Cumulative Blast bit score: 3688

Hit cluster cross-links:

Mycgr3G67791 Mycgr3T
  
Location: 0-1542

Mycgr3G67791\_Mycgr3T

Mycgr3G90406 Mycgr3T
  
Location: 1642-3973

Mycgr3G90406\_Mycgr3T

Mycgr3G67785 Mycgr3T
  
Location: 4073-7865

Mycgr3G67785\_Mycgr3T

Mycgr3G67795 Mycgr3T
  
Location: 7965-15249

Mycgr3G67795\_Mycgr3T

Mycgr3G67775 Mycgr3T
  
Location: 15349-16237

Mycgr3G67775\_Mycgr3T

Mycgr3G90404 Mycgr3T
  
Location: 16337-17246

Mycgr3G90404\_Mycgr3T

Mycgr3G36951 Mycgr3T
  
Location: 17346-30891

Mycgr3G36951\_Mycgr3T

Mycgr3G103034 Mycgr3
  
Location: 30991-32644

Mycgr3G103034\_Mycgr3

Mycgr3G31119 Mycgr3T
  
Location: 32744-32906

Mycgr3G31119\_Mycgr3T

Mycgr3G28587 Mycgr3T
  
Location: 33006-33489

Mycgr3G28587\_Mycgr3T

Mycgr3G98959 Mycgr3T
  
Location: 33589-35035

Mycgr3G98959\_Mycgr3T

Mycgr3G35447 Mycgr3T
  
Location: 35135-36443

Mycgr3G35447\_Mycgr3T

Mycgr3G84402 Mycgr3T
  
Location: 36543-37884

Mycgr3G84402\_Mycgr3T

Mycgr3G98961 Mycgr3T
  
Location: 37984-38884

Mycgr3G98961\_Mycgr3T

S-adenosyl-L-methionine-dependent methyltransferases superfamily protein isoform 2
  
Accession: EOX98538
  
Location: 6125552-6127465
  
 NCBI BlastP on this gene

EOX98538

Uncharacterized protein isoform 2
  
Accession: EOX98536
  
Location: 6121047-6124174
  
 NCBI BlastP on this gene

EOX98536

Target of rapamycin isoform 3
  
Accession: EOX98534
  
Location: 6095522-6118875
  
  
**BlastP hit with Mycgr3G67795\_Mycgr3T**
  
Percentage identity: 42 %
  
BlastP bit score: 1816
  
Sequence coverage: 98 %
  
E-value: 0.0
  
  
 NCBI BlastP on this gene

EOX98534

Target of rapamycin isoform 1
  
Accession: EOX98533
  
Location: 6094651-6118875
  
  
**BlastP hit with Mycgr3G67795\_Mycgr3T**
  
Percentage identity: 42 %
  
BlastP bit score: 1872
  
Sequence coverage: 104 %
  
E-value: 0.0
  
  
 NCBI BlastP on this gene

EOX98533

Inositol 1,3,4-trisphosphate 5/6-kinase family protein isoform 4
  
Accession: EOX98531
  
Location: 6090406-6093687
  
 NCBI BlastP on this gene

EOX98531

Inositol 1,3,4-trisphosphate 5/6-kinase family protein isoform 1
  
Accession: EOX98529
  
Location: 6087530-6093687
  
 NCBI BlastP on this gene

EOX98529

Query: Architecture Search FASTA input

CP002684 : Arabidopsis thaliana chromosome 1    Total score: 1.0     Cumulative Blast bit score: 3658

Hit cluster cross-links:

Mycgr3G67791 Mycgr3T
  
Location: 0-1542

Mycgr3G67791\_Mycgr3T

Mycgr3G90406 Mycgr3T
  
Location: 1642-3973

Mycgr3G90406\_Mycgr3T

Mycgr3G67785 Mycgr3T
  
Location: 4073-7865

Mycgr3G67785\_Mycgr3T

Mycgr3G67795 Mycgr3T
  
Location: 7965-15249

Mycgr3G67795\_Mycgr3T

Mycgr3G67775 Mycgr3T
  
Location: 15349-16237

Mycgr3G67775\_Mycgr3T

Mycgr3G90404 Mycgr3T
  
Location: 16337-17246

Mycgr3G90404\_Mycgr3T

Mycgr3G36951 Mycgr3T
  
Location: 17346-30891

Mycgr3G36951\_Mycgr3T

Mycgr3G103034 Mycgr3
  
Location: 30991-32644

Mycgr3G103034\_Mycgr3

Mycgr3G31119 Mycgr3T
  
Location: 32744-32906

Mycgr3G31119\_Mycgr3T

Mycgr3G28587 Mycgr3T
  
Location: 33006-33489

Mycgr3G28587\_Mycgr3T

Mycgr3G98959 Mycgr3T
  
Location: 33589-35035

Mycgr3G98959\_Mycgr3T

Mycgr3G35447 Mycgr3T
  
Location: 35135-36443

Mycgr3G35447\_Mycgr3T

Mycgr3G84402 Mycgr3T
  
Location: 36543-37884

Mycgr3G84402\_Mycgr3T

Mycgr3G98961 Mycgr3T
  
Location: 37984-38884

Mycgr3G98961\_Mycgr3T

putative pathogenesis-related protein
  
Accession: AEE32513
  
Location: 18551186-18552446
  
 NCBI BlastP on this gene

AT1G50060

putative pathogenesis-related protein
  
Accession: AEE32512
  
Location: 18546165-18549046
  
 NCBI BlastP on this gene

AT1G50050

uncharacterized protein
  
Accession: AEE32511
  
Location: 18542236-18543823
  
 NCBI BlastP on this gene

AT1G50040

phosphatidylinositol 3-kinase family protein TOR
  
Accession: AEE32510
  
Location: 18522626-18539619
  
  
**BlastP hit with Mycgr3G67795\_Mycgr3T**
  
Percentage identity: 41 %
  
BlastP bit score: 1818
  
Sequence coverage: 104 %
  
E-value: 0.0
  
  
 NCBI BlastP on this gene

TOR

phosphatidylinositol 3-kinase family protein TOR
  
Accession: AEE32509
  
Location: 18522626-18539619
  
  
**BlastP hit with Mycgr3G67795\_Mycgr3T**
  
Percentage identity: 41 %
  
BlastP bit score: 1840
  
Sequence coverage: 105 %
  
E-value: 0.0
  
  
 NCBI BlastP on this gene

TOR

uncharacterized protein
  
Accession: AEE32508
  
Location: 18520144-18521600
  
 NCBI BlastP on this gene

AT1G50020

tubulin alpha-2 chain
  
Accession: AEE32507
  
Location: 18517737-18519729
  
 NCBI BlastP on this gene

TUA2

putative ribosomal RNA small subunit methyltransferase
  
Accession: AEE32506
  
Location: 18515595-18517244
  
 NCBI BlastP on this gene

AT1G50000

putative ribosomal RNA small subunit methyltransferase
  
Accession: AEE32505
  
Location: 18515183-18517244
  
 NCBI BlastP on this gene

AT1G50000

F-box protein
  
Accession: AEE32504
  
Location: 18513696-18514988
  
 NCBI BlastP on this gene

AT1G49990

DNA/RNA polymerases superfamily protein
  
Accession: AEE32503
  
Location: 18508026-18512111
  
 NCBI BlastP on this gene

AT1G49980

Query: Architecture Search FASTA input

JH767573 : Coniosporium apollinis CBS 100218 chromosome Unknown supercont1.20    Total score: 1.0     Cumulative Blast bit score: 3508

Hit cluster cross-links:

Mycgr3G67791 Mycgr3T
  
Location: 0-1542

Mycgr3G67791\_Mycgr3T

Mycgr3G90406 Mycgr3T
  
Location: 1642-3973

Mycgr3G90406\_Mycgr3T

Mycgr3G67785 Mycgr3T
  
Location: 4073-7865

Mycgr3G67785\_Mycgr3T

Mycgr3G67795 Mycgr3T
  
Location: 7965-15249

Mycgr3G67795\_Mycgr3T

Mycgr3G67775 Mycgr3T
  
Location: 15349-16237

Mycgr3G67775\_Mycgr3T

Mycgr3G90404 Mycgr3T
  
Location: 16337-17246

Mycgr3G90404\_Mycgr3T

Mycgr3G36951 Mycgr3T
  
Location: 17346-30891

Mycgr3G36951\_Mycgr3T

Mycgr3G103034 Mycgr3
  
Location: 30991-32644

Mycgr3G103034\_Mycgr3

Mycgr3G31119 Mycgr3T
  
Location: 32744-32906

Mycgr3G31119\_Mycgr3T

Mycgr3G28587 Mycgr3T
  
Location: 33006-33489

Mycgr3G28587\_Mycgr3T

Mycgr3G98959 Mycgr3T
  
Location: 33589-35035

Mycgr3G98959\_Mycgr3T

Mycgr3G35447 Mycgr3T
  
Location: 35135-36443

Mycgr3G35447\_Mycgr3T

Mycgr3G84402 Mycgr3T
  
Location: 36543-37884

Mycgr3G84402\_Mycgr3T

Mycgr3G98961 Mycgr3T
  
Location: 37984-38884

Mycgr3G98961\_Mycgr3T

FKBP12-rapamycin complex-associated protein
  
Accession: EON65353
  
Location: 306195-313744
  
  
**BlastP hit with Mycgr3G67795\_Mycgr3T**
  
Percentage identity: 70 %
  
BlastP bit score: 3508
  
Sequence coverage: 100 %
  
E-value: 0.0
  
  
 NCBI BlastP on this gene

EON65353

hypothetical protein
  
Accession: EON65352
  
Location: 304091-305197
  
 NCBI BlastP on this gene

EON65352

hypothetical protein
  
Accession: EON65351
  
Location: 300541-302976
  
 NCBI BlastP on this gene

EON65351

hypothetical protein
  
Accession: EON65350
  
Location: 299508-300138
  
 NCBI BlastP on this gene

EON65350

methylsterol monooxygenase
  
Accession: EON65349
  
Location: 297639-298854
  
 NCBI BlastP on this gene

EON65349

hypothetical protein
  
Accession: EON65348
  
Location: 294650-296903
  
 NCBI BlastP on this gene

EON65348

hypothetical protein
  
Accession: EON65347
  
Location: 291603-293328
  
 NCBI BlastP on this gene

EON65347

hypothetical protein
  
Accession: EON65346
  
Location: 289160-291049
  
 NCBI BlastP on this gene

EON65346

Query: Architecture Search FASTA input

KB445649 : Cochliobolus sativus ND90Pr unplaced genomic scaffold COCSAscaffold\_13    Total score: 1.0     Cumulative Blast bit score: 3284

Hit cluster cross-links:

Mycgr3G67791 Mycgr3T
  
Location: 0-1542

Mycgr3G67791\_Mycgr3T

Mycgr3G90406 Mycgr3T
  
Location: 1642-3973

Mycgr3G90406\_Mycgr3T

Mycgr3G67785 Mycgr3T
  
Location: 4073-7865

Mycgr3G67785\_Mycgr3T

Mycgr3G67795 Mycgr3T
  
Location: 7965-15249

Mycgr3G67795\_Mycgr3T

Mycgr3G67775 Mycgr3T
  
Location: 15349-16237

Mycgr3G67775\_Mycgr3T

Mycgr3G90404 Mycgr3T
  
Location: 16337-17246

Mycgr3G90404\_Mycgr3T

Mycgr3G36951 Mycgr3T
  
Location: 17346-30891

Mycgr3G36951\_Mycgr3T

Mycgr3G103034 Mycgr3
  
Location: 30991-32644

Mycgr3G103034\_Mycgr3

Mycgr3G31119 Mycgr3T
  
Location: 32744-32906

Mycgr3G31119\_Mycgr3T

Mycgr3G28587 Mycgr3T
  
Location: 33006-33489

Mycgr3G28587\_Mycgr3T

Mycgr3G98959 Mycgr3T
  
Location: 33589-35035

Mycgr3G98959\_Mycgr3T

Mycgr3G35447 Mycgr3T
  
Location: 35135-36443

Mycgr3G35447\_Mycgr3T

Mycgr3G84402 Mycgr3T
  
Location: 36543-37884

Mycgr3G84402\_Mycgr3T

Mycgr3G98961 Mycgr3T
  
Location: 37984-38884

Mycgr3G98961\_Mycgr3T

hypothetical protein
  
Accession: EMD61144
  
Location: 1053135-1054140
  
 NCBI BlastP on this gene

EMD61144

hypothetical protein
  
Accession: EMD61145
  
Location: 1054495-1056363
  
 NCBI BlastP on this gene

EMD61145

hypothetical protein
  
Accession: EMD61146
  
Location: 1057099-1058768
  
 NCBI BlastP on this gene

EMD61146

hypothetical protein
  
Accession: EMD61147
  
Location: 1060552-1062345
  
 NCBI BlastP on this gene

EMD61147

hypothetical protein
  
Accession: EMD61148
  
Location: 1063393-1063554
  
 NCBI BlastP on this gene

EMD61148

hypothetical protein
  
Accession: EMD61149
  
Location: 1064213-1067830
  
 NCBI BlastP on this gene

EMD61149

hypothetical protein
  
Accession: EMD61150
  
Location: 1068781-1070840
  
 NCBI BlastP on this gene

EMD61150

hypothetical protein
  
Accession: EMD61151
  
Location: 1071254-1078633
  
  
**BlastP hit with Mycgr3G67795\_Mycgr3T**
  
Percentage identity: 66 %
  
BlastP bit score: 3285
  
Sequence coverage: 100 %
  
E-value: 0.0
  
  
 NCBI BlastP on this gene

EMD61151

hypothetical protein
  
Accession: EMD61152
  
Location: 1079541-1081164
  
 NCBI BlastP on this gene

EMD61152

hypothetical protein
  
Accession: EMD61153
  
Location: 1081281-1082021
  
 NCBI BlastP on this gene

EMD61153

hypothetical protein
  
Accession: EMD61154
  
Location: 1082303-1086774
  
 NCBI BlastP on this gene

EMD61154

hypothetical protein
  
Accession: EMD61155
  
Location: 1087175-1088723
  
 NCBI BlastP on this gene

EMD61155

hypothetical protein
  
Accession: EMD61156
  
Location: 1089030-1089923
  
 NCBI BlastP on this gene

EMD61156

hypothetical protein
  
Accession: EMD61157
  
Location: 1090213-1092558
  
 NCBI BlastP on this gene

EMD61157

hypothetical protein
  
Accession: EMD61158
  
Location: 1095918-1096745
  
 NCBI BlastP on this gene

EMD61158

Query: Architecture Search FASTA input

KB445579 : Cochliobolus heterostrophus C5 unplaced genomic scaffold COCHEscaffold\_11    Total score: 1.0     Cumulative Blast bit score: 3284

Hit cluster cross-links:

Mycgr3G67791 Mycgr3T
  
Location: 0-1542

Mycgr3G67791\_Mycgr3T

Mycgr3G90406 Mycgr3T
  
Location: 1642-3973

Mycgr3G90406\_Mycgr3T

Mycgr3G67785 Mycgr3T
  
Location: 4073-7865

Mycgr3G67785\_Mycgr3T

Mycgr3G67795 Mycgr3T
  
Location: 7965-15249

Mycgr3G67795\_Mycgr3T

Mycgr3G67775 Mycgr3T
  
Location: 15349-16237

Mycgr3G67775\_Mycgr3T

Mycgr3G90404 Mycgr3T
  
Location: 16337-17246

Mycgr3G90404\_Mycgr3T

Mycgr3G36951 Mycgr3T
  
Location: 17346-30891

Mycgr3G36951\_Mycgr3T

Mycgr3G103034 Mycgr3
  
Location: 30991-32644

Mycgr3G103034\_Mycgr3

Mycgr3G31119 Mycgr3T
  
Location: 32744-32906

Mycgr3G31119\_Mycgr3T

Mycgr3G28587 Mycgr3T
  
Location: 33006-33489

Mycgr3G28587\_Mycgr3T

Mycgr3G98959 Mycgr3T
  
Location: 33589-35035

Mycgr3G98959\_Mycgr3T

Mycgr3G35447 Mycgr3T
  
Location: 35135-36443

Mycgr3G35447\_Mycgr3T

Mycgr3G84402 Mycgr3T
  
Location: 36543-37884

Mycgr3G84402\_Mycgr3T

Mycgr3G98961 Mycgr3T
  
Location: 37984-38884

Mycgr3G98961\_Mycgr3T

hypothetical protein
  
Accession: EMD89388
  
Location: 1028926-1030794
  
 NCBI BlastP on this gene

EMD89388

hypothetical protein
  
Accession: EMD89389
  
Location: 1031594-1033267
  
 NCBI BlastP on this gene

EMD89389

hypothetical protein
  
Accession: EMD89390
  
Location: 1035068-1036861
  
 NCBI BlastP on this gene

EMD89390

hypothetical protein
  
Accession: EMD89391
  
Location: 1040515-1044133
  
 NCBI BlastP on this gene

EMD89391

hypothetical protein
  
Accession: EMD89392
  
Location: 1045080-1047139
  
 NCBI BlastP on this gene

EMD89392

hypothetical protein
  
Accession: EMD89393
  
Location: 1047554-1054933
  
  
**BlastP hit with Mycgr3G67795\_Mycgr3T**
  
Percentage identity: 66 %
  
BlastP bit score: 3284
  
Sequence coverage: 100 %
  
E-value: 0.0
  
  
 NCBI BlastP on this gene

EMD89393

hypothetical protein
  
Accession: EMD89394
  
Location: 1055874-1057518
  
 NCBI BlastP on this gene

EMD89394

hypothetical protein
  
Accession: EMD89395
  
Location: 1057636-1058374
  
 NCBI BlastP on this gene

EMD89395

hypothetical protein
  
Accession: EMD89396
  
Location: 1058656-1063128
  
 NCBI BlastP on this gene

EMD89396

hypothetical protein
  
Accession: EMD89397
  
Location: 1063524-1065071
  
 NCBI BlastP on this gene

EMD89397

hypothetical protein
  
Accession: EMD89398
  
Location: 1065379-1066229
  
 NCBI BlastP on this gene

EMD89398

hypothetical protein
  
Accession: EMD89399
  
Location: 1066519-1068888
  
 NCBI BlastP on this gene

EMD89399

hypothetical protein
  
Accession: EMD89400
  
Location: 1069880-1070895
  
 NCBI BlastP on this gene

EMD89400

hypothetical protein
  
Accession: EMD89401
  
Location: 1071218-1071918
  
 NCBI BlastP on this gene

EMD89401

hypothetical protein
  
Accession: EMD89402
  
Location: 1072230-1073126
  
 NCBI BlastP on this gene

EMD89402

Query: Architecture Search FASTA input

CH476599 : Aspergillus terreus NIH2624 scaffold\_6 genomic scaffold    Total score: 1.0     Cumulative Blast bit score: 3099

Hit cluster cross-links:

Mycgr3G67791 Mycgr3T
  
Location: 0-1542

Mycgr3G67791\_Mycgr3T

Mycgr3G90406 Mycgr3T
  
Location: 1642-3973

Mycgr3G90406\_Mycgr3T

Mycgr3G67785 Mycgr3T
  
Location: 4073-7865

Mycgr3G67785\_Mycgr3T

Mycgr3G67795 Mycgr3T
  
Location: 7965-15249

Mycgr3G67795\_Mycgr3T

Mycgr3G67775 Mycgr3T
  
Location: 15349-16237

Mycgr3G67775\_Mycgr3T

Mycgr3G90404 Mycgr3T
  
Location: 16337-17246

Mycgr3G90404\_Mycgr3T

Mycgr3G36951 Mycgr3T
  
Location: 17346-30891

Mycgr3G36951\_Mycgr3T

Mycgr3G103034 Mycgr3
  
Location: 30991-32644

Mycgr3G103034\_Mycgr3

Mycgr3G31119 Mycgr3T
  
Location: 32744-32906

Mycgr3G31119\_Mycgr3T

Mycgr3G28587 Mycgr3T
  
Location: 33006-33489

Mycgr3G28587\_Mycgr3T

Mycgr3G98959 Mycgr3T
  
Location: 33589-35035

Mycgr3G98959\_Mycgr3T

Mycgr3G35447 Mycgr3T
  
Location: 35135-36443

Mycgr3G35447\_Mycgr3T

Mycgr3G84402 Mycgr3T
  
Location: 36543-37884

Mycgr3G84402\_Mycgr3T

Mycgr3G98961 Mycgr3T
  
Location: 37984-38884

Mycgr3G98961\_Mycgr3T

conserved hypothetical protein
  
Accession: EAU34917
  
Location: 624743-628497
  
 NCBI BlastP on this gene

EAU34917

predicted protein
  
Accession: EAU34916
  
Location: 622752-624053
  
 NCBI BlastP on this gene

EAU34916

predicted protein
  
Accession: EAU34915
  
Location: 619648-621138
  
 NCBI BlastP on this gene

EAU34915

predicted protein
  
Accession: EAU34914
  
Location: 617304-619090
  
 NCBI BlastP on this gene

EAU34914

conserved hypothetical protein
  
Accession: EAU34913
  
Location: 615562-616512
  
 NCBI BlastP on this gene

EAU34913

40S ribosomal protein S17
  
Accession: EAU34912
  
Location: 614582-615186
  
 NCBI BlastP on this gene

EAU34912

conserved hypothetical protein
  
Accession: EAU34911
  
Location: 612813-614180
  
 NCBI BlastP on this gene

EAU34911

conserved hypothetical protein
  
Accession: EAU34910
  
Location: 611322-612554
  
 NCBI BlastP on this gene

EAU34910

phosphatidylinositol 3-kinase tor2
  
Accession: EAU34909
  
Location: 602484-609814
  
  
**BlastP hit with Mycgr3G67795\_Mycgr3T**
  
Percentage identity: 63 %
  
BlastP bit score: 3099
  
Sequence coverage: 100 %
  
E-value: 0.0
  
  
 NCBI BlastP on this gene

EAU34909

conserved hypothetical protein
  
Accession: EAU34908
  
Location: 600284-602058
  
 NCBI BlastP on this gene

EAU34908

conserved hypothetical protein
  
Accession: EAU34907
  
Location: 597858-598978
  
 NCBI BlastP on this gene

EAU34907

predicted protein
  
Accession: EAU34906
  
Location: 595991-597310
  
 NCBI BlastP on this gene

EAU34906

conserved hypothetical protein
  
Accession: EAU34905
  
Location: 594706-595433
  
 NCBI BlastP on this gene

EAU34905

conserved hypothetical protein
  
Accession: EAU34904
  
Location: 590489-594179
  
 NCBI BlastP on this gene

EAU34904

inositol oxygenase 1
  
Accession: EAU34903
  
Location: 589034-589987
  
 NCBI BlastP on this gene

EAU34903

hypothetical protein
  
Accession: EAU34902
  
Location: 587005-588088
  
 NCBI BlastP on this gene

EAU34902

predicted protein
  
Accession: EAU34901
  
Location: 584309-586643
  
 NCBI BlastP on this gene

EAU34901

Query: Architecture Search FASTA input

DS572813 : Paracoccidioides brasiliensis Pb01 supercont1.3 genomic scaffold    Total score: 1.0     Cumulative Blast bit score: 3089

Hit cluster cross-links:

Mycgr3G67791 Mycgr3T
  
Location: 0-1542

Mycgr3G67791\_Mycgr3T

Mycgr3G90406 Mycgr3T
  
Location: 1642-3973

Mycgr3G90406\_Mycgr3T

Mycgr3G67785 Mycgr3T
  
Location: 4073-7865

Mycgr3G67785\_Mycgr3T

Mycgr3G67795 Mycgr3T
  
Location: 7965-15249

Mycgr3G67795\_Mycgr3T

Mycgr3G67775 Mycgr3T
  
Location: 15349-16237

Mycgr3G67775\_Mycgr3T

Mycgr3G90404 Mycgr3T
  
Location: 16337-17246

Mycgr3G90404\_Mycgr3T

Mycgr3G36951 Mycgr3T
  
Location: 17346-30891

Mycgr3G36951\_Mycgr3T

Mycgr3G103034 Mycgr3
  
Location: 30991-32644

Mycgr3G103034\_Mycgr3

Mycgr3G31119 Mycgr3T
  
Location: 32744-32906

Mycgr3G31119\_Mycgr3T

Mycgr3G28587 Mycgr3T
  
Location: 33006-33489

Mycgr3G28587\_Mycgr3T

Mycgr3G98959 Mycgr3T
  
Location: 33589-35035

Mycgr3G98959\_Mycgr3T

Mycgr3G35447 Mycgr3T
  
Location: 35135-36443

Mycgr3G35447\_Mycgr3T

Mycgr3G84402 Mycgr3T
  
Location: 36543-37884

Mycgr3G84402\_Mycgr3T

Mycgr3G98961 Mycgr3T
  
Location: 37984-38884

Mycgr3G98961\_Mycgr3T

AP-2 complex subunit beta
  
Accession: EEH38956
  
Location: 328470-331315
  
 NCBI BlastP on this gene

EEH38956

conserved hypothetical protein
  
Accession: EEH38955
  
Location: 327166-327582
  
 NCBI BlastP on this gene

EEH38955

predicted protein
  
Accession: EEH38954
  
Location: 325466-327057
  
 NCBI BlastP on this gene

EEH38954

SH3 domain-containing protein
  
Accession: EEH38953
  
Location: 321267-324952
  
 NCBI BlastP on this gene

EEH38953

conserved hypothetical protein
  
Accession: EEH38952
  
Location: 319053-320213
  
 NCBI BlastP on this gene

EEH38952

40S ribosomal protein S17
  
Accession: EEH38951
  
Location: 317722-318420
  
 NCBI BlastP on this gene

EEH38951

conserved hypothetical protein
  
Accession: EEH38950
  
Location: 315672-317045
  
 NCBI BlastP on this gene

EEH38950

predicted protein
  
Accession: EEH38949
  
Location: 313151-313988
  
 NCBI BlastP on this gene

EEH38949

phosphatidylinositol 3-kinase tor2
  
Accession: EEH38948
  
Location: 304639-312042
  
  
**BlastP hit with Mycgr3G67795\_Mycgr3T**
  
Percentage identity: 63 %
  
BlastP bit score: 3089
  
Sequence coverage: 99 %
  
E-value: 0.0
  
  
 NCBI BlastP on this gene

EEH38948

conserved hypothetical protein
  
Accession: EEH38947
  
Location: 301989-303837
  
 NCBI BlastP on this gene

EEH38947

predicted protein
  
Accession: EEH38946
  
Location: 300837-301388
  
 NCBI BlastP on this gene

EEH38946

fungal specific transcription factor domain-containing protein
  
Accession: EEH38945
  
Location: 298517-300784
  
 NCBI BlastP on this gene

EEH38945

conserved hypothetical protein
  
Accession: EEH38944
  
Location: 296546-297884
  
 NCBI BlastP on this gene

EEH38944

inositol oxygenase
  
Accession: EEH38943
  
Location: 294802-295846
  
 NCBI BlastP on this gene

EEH38943

predicted protein
  
Accession: EEH38942
  
Location: 289193-291549
  
 NCBI BlastP on this gene

EEH38942

Query: Architecture Search FASTA input

DS572750 : Paracoccidioides brasiliensis Pb18 supercont1.1 genomic scaffold    Total score: 1.0     Cumulative Blast bit score: 3088

Hit cluster cross-links:

Mycgr3G67791 Mycgr3T
  
Location: 0-1542

Mycgr3G67791\_Mycgr3T

Mycgr3G90406 Mycgr3T
  
Location: 1642-3973

Mycgr3G90406\_Mycgr3T

Mycgr3G67785 Mycgr3T
  
Location: 4073-7865

Mycgr3G67785\_Mycgr3T

Mycgr3G67795 Mycgr3T
  
Location: 7965-15249

Mycgr3G67795\_Mycgr3T

Mycgr3G67775 Mycgr3T
  
Location: 15349-16237

Mycgr3G67775\_Mycgr3T

Mycgr3G90404 Mycgr3T
  
Location: 16337-17246

Mycgr3G90404\_Mycgr3T

Mycgr3G36951 Mycgr3T
  
Location: 17346-30891

Mycgr3G36951\_Mycgr3T

Mycgr3G103034 Mycgr3
  
Location: 30991-32644

Mycgr3G103034\_Mycgr3

Mycgr3G31119 Mycgr3T
  
Location: 32744-32906

Mycgr3G31119\_Mycgr3T

Mycgr3G28587 Mycgr3T
  
Location: 33006-33489

Mycgr3G28587\_Mycgr3T

Mycgr3G98959 Mycgr3T
  
Location: 33589-35035

Mycgr3G98959\_Mycgr3T

Mycgr3G35447 Mycgr3T
  
Location: 35135-36443

Mycgr3G35447\_Mycgr3T

Mycgr3G84402 Mycgr3T
  
Location: 36543-37884

Mycgr3G84402\_Mycgr3T

Mycgr3G98961 Mycgr3T
  
Location: 37984-38884

Mycgr3G98961\_Mycgr3T

AP-2 complex subunit beta
  
Accession: EEH44061
  
Location: 1141409-1144108
  
 NCBI BlastP on this gene

EEH44061

conserved hypothetical protein
  
Accession: EEH44062
  
Location: 1144985-1145401
  
 NCBI BlastP on this gene

EEH44062

SH3 domain-containing protein
  
Accession: EEH44063
  
Location: 1147026-1150718
  
 NCBI BlastP on this gene

EEH44063

conserved hypothetical protein
  
Accession: EEH44064
  
Location: 1151726-1152910
  
 NCBI BlastP on this gene

EEH44064

40S ribosomal protein S17
  
Accession: EEH44065
  
Location: 1153486-1154177
  
 NCBI BlastP on this gene

EEH44065

conserved hypothetical protein
  
Accession: EEH44066
  
Location: 1154804-1156177
  
 NCBI BlastP on this gene

EEH44066

predicted protein
  
Accession: EEH44067
  
Location: 1157168-1158530
  
 NCBI BlastP on this gene

EEH44067

phosphatidylinositol 3-kinase tor2
  
Accession: EEH44068
  
Location: 1159696-1167099
  
  
**BlastP hit with Mycgr3G67795\_Mycgr3T**
  
Percentage identity: 63 %
  
BlastP bit score: 3088
  
Sequence coverage: 99 %
  
E-value: 0.0
  
  
 NCBI BlastP on this gene

EEH44068

conserved hypothetical protein
  
Accession: EEH44069
  
Location: 1167888-1170877
  
 NCBI BlastP on this gene

EEH44069

fungal specific transcription factor domain-containing protein
  
Accession: EEH44070
  
Location: 1170930-1173158
  
 NCBI BlastP on this gene

EEH44070

conserved hypothetical protein
  
Accession: EEH44071
  
Location: 1173867-1175031
  
 NCBI BlastP on this gene

EEH44071

inositol oxygenase
  
Accession: EEH44072
  
Location: 1175825-1176869
  
 NCBI BlastP on this gene

EEH44072

predicted protein
  
Accession: EEH44073
  
Location: 1178946-1181309
  
 NCBI BlastP on this gene

EEH44073

conserved hypothetical protein
  
Accession: EEH44074
  
Location: 1183140-1186877
  
 NCBI BlastP on this gene

EEH44074

Query: Architecture Search FASTA input

GG749410 : Ajellomyces dermatitidis ATCC 18188 genomic scaffold supercont1.4    Total score: 1.0     Cumulative Blast bit score: 3085

Hit cluster cross-links:

Mycgr3G67791 Mycgr3T
  
Location: 0-1542

Mycgr3G67791\_Mycgr3T

Mycgr3G90406 Mycgr3T
  
Location: 1642-3973

Mycgr3G90406\_Mycgr3T

Mycgr3G67785 Mycgr3T
  
Location: 4073-7865

Mycgr3G67785\_Mycgr3T

Mycgr3G67795 Mycgr3T
  
Location: 7965-15249

Mycgr3G67795\_Mycgr3T

Mycgr3G67775 Mycgr3T
  
Location: 15349-16237

Mycgr3G67775\_Mycgr3T

Mycgr3G90404 Mycgr3T
  
Location: 16337-17246

Mycgr3G90404\_Mycgr3T

Mycgr3G36951 Mycgr3T
  
Location: 17346-30891

Mycgr3G36951\_Mycgr3T

Mycgr3G103034 Mycgr3
  
Location: 30991-32644

Mycgr3G103034\_Mycgr3

Mycgr3G31119 Mycgr3T
  
Location: 32744-32906

Mycgr3G31119\_Mycgr3T

Mycgr3G28587 Mycgr3T
  
Location: 33006-33489

Mycgr3G28587\_Mycgr3T

Mycgr3G98959 Mycgr3T
  
Location: 33589-35035

Mycgr3G98959\_Mycgr3T

Mycgr3G35447 Mycgr3T
  
Location: 35135-36443

Mycgr3G35447\_Mycgr3T

Mycgr3G84402 Mycgr3T
  
Location: 36543-37884

Mycgr3G84402\_Mycgr3T

Mycgr3G98961 Mycgr3T
  
Location: 37984-38884

Mycgr3G98961\_Mycgr3T

kynureninase
  
Accession: EGE78575
  
Location: 487917-489666
  
 NCBI BlastP on this gene

EGE78575

AP-2 adaptor complex subunit beta
  
Accession: EGE78576
  
Location: 490176-492565
  
 NCBI BlastP on this gene

EGE78576

ER membrane DUF1077 domain-containing protein
  
Accession: EGE78577
  
Location: 493422-494229
  
 NCBI BlastP on this gene

EGE78577

MFS transporter
  
Accession: EGE78578
  
Location: 494300-495919
  
 NCBI BlastP on this gene

EGE78578

SH3 domain-containing protein
  
Accession: EGE78579
  
Location: 496847-500580
  
 NCBI BlastP on this gene

EGE78579

DUF408 domain-containing protein
  
Accession: EGE78580
  
Location: 501689-502861
  
 NCBI BlastP on this gene

EGE78580

40S ribosomal protein S17
  
Accession: EGE78581
  
Location: 503555-504208
  
 NCBI BlastP on this gene

EGE78581

hypothetical protein
  
Accession: EGE78582
  
Location: 504576-505979
  
 NCBI BlastP on this gene

EGE78582

TorA protein
  
Accession: EGE78583
  
Location: 506676-514087
  
  
**BlastP hit with Mycgr3G67795\_Mycgr3T**
  
Percentage identity: 63 %
  
BlastP bit score: 3085
  
Sequence coverage: 99 %
  
E-value: 0.0
  
  
 NCBI BlastP on this gene

EGE78583

Query: Architecture Search FASTA input

DS544805 : Paracoccidioides brasiliensis Pb03 supercont1.3 genomic scaffold    Total score: 1.0     Cumulative Blast bit score: 3085

Hit cluster cross-links:

Mycgr3G67791 Mycgr3T
  
Location: 0-1542

Mycgr3G67791\_Mycgr3T

Mycgr3G90406 Mycgr3T
  
Location: 1642-3973

Mycgr3G90406\_Mycgr3T

Mycgr3G67785 Mycgr3T
  
Location: 4073-7865

Mycgr3G67785\_Mycgr3T

Mycgr3G67795 Mycgr3T
  
Location: 7965-15249

Mycgr3G67795\_Mycgr3T

Mycgr3G67775 Mycgr3T
  
Location: 15349-16237

Mycgr3G67775\_Mycgr3T

Mycgr3G90404 Mycgr3T
  
Location: 16337-17246

Mycgr3G90404\_Mycgr3T

Mycgr3G36951 Mycgr3T
  
Location: 17346-30891

Mycgr3G36951\_Mycgr3T

Mycgr3G103034 Mycgr3
  
Location: 30991-32644

Mycgr3G103034\_Mycgr3

Mycgr3G31119 Mycgr3T
  
Location: 32744-32906

Mycgr3G31119\_Mycgr3T

Mycgr3G28587 Mycgr3T
  
Location: 33006-33489

Mycgr3G28587\_Mycgr3T

Mycgr3G98959 Mycgr3T
  
Location: 33589-35035

Mycgr3G98959\_Mycgr3T

Mycgr3G35447 Mycgr3T
  
Location: 35135-36443

Mycgr3G35447\_Mycgr3T

Mycgr3G84402 Mycgr3T
  
Location: 36543-37884

Mycgr3G84402\_Mycgr3T

Mycgr3G98961 Mycgr3T
  
Location: 37984-38884

Mycgr3G98961\_Mycgr3T

AP-2 complex subunit beta
  
Accession: EEH19707
  
Location: 235229-237977
  
 NCBI BlastP on this gene

EEH19707

conserved hypothetical protein
  
Accession: EEH19708
  
Location: 238480-239277
  
 NCBI BlastP on this gene

EEH19708

conserved hypothetical protein
  
Accession: EEH19709
  
Location: 240898-244590
  
 NCBI BlastP on this gene

EEH19709

conserved hypothetical protein
  
Accession: EEH19710
  
Location: 245646-246794
  
 NCBI BlastP on this gene

EEH19710

40S ribosomal protein S17
  
Accession: EEH19711
  
Location: 247367-248058
  
 NCBI BlastP on this gene

EEH19711

predicted protein
  
Accession: EEH19712
  
Location: 250533-251906
  
 NCBI BlastP on this gene

EEH19712

predicted protein
  
Accession: EEH19713
  
Location: 253300-254423
  
 NCBI BlastP on this gene

EEH19713

phosphatidylinositol 3-kinase tor2
  
Accession: EEH19714
  
Location: 255589-262992
  
  
**BlastP hit with Mycgr3G67795\_Mycgr3T**
  
Percentage identity: 62 %
  
BlastP bit score: 3085
  
Sequence coverage: 99 %
  
E-value: 0.0
  
  
 NCBI BlastP on this gene

EEH19714

conserved hypothetical protein
  
Accession: EEH19715
  
Location: 263780-265628
  
 NCBI BlastP on this gene

EEH19715

conserved hypothetical protein
  
Accession: EEH19716
  
Location: 266831-269077
  
 NCBI BlastP on this gene

EEH19716

conserved hypothetical protein
  
Accession: EEH19717
  
Location: 269779-270266
  
 NCBI BlastP on this gene

EEH19717

inositol oxygenase
  
Accession: EEH19718
  
Location: 271736-272780
  
 NCBI BlastP on this gene

EEH19718

predicted protein
  
Accession: EEH19719
  
Location: 274876-277239
  
 NCBI BlastP on this gene

EEH19719

conserved hypothetical protein
  
Accession: EEH19720
  
Location: 278910-282655
  
 NCBI BlastP on this gene

EEH19720

Query: Architecture Search FASTA input

GG663373 : Ajellomyces capsulatus G186AR genomic scaffold supercont2.11    Total score: 1.0     Cumulative Blast bit score: 3076

Hit cluster cross-links:

Mycgr3G67791 Mycgr3T
  
Location: 0-1542

Mycgr3G67791\_Mycgr3T

Mycgr3G90406 Mycgr3T
  
Location: 1642-3973

Mycgr3G90406\_Mycgr3T

Mycgr3G67785 Mycgr3T
  
Location: 4073-7865

Mycgr3G67785\_Mycgr3T

Mycgr3G67795 Mycgr3T
  
Location: 7965-15249

Mycgr3G67795\_Mycgr3T

Mycgr3G67775 Mycgr3T
  
Location: 15349-16237

Mycgr3G67775\_Mycgr3T

Mycgr3G90404 Mycgr3T
  
Location: 16337-17246

Mycgr3G90404\_Mycgr3T

Mycgr3G36951 Mycgr3T
  
Location: 17346-30891

Mycgr3G36951\_Mycgr3T

Mycgr3G103034 Mycgr3
  
Location: 30991-32644

Mycgr3G103034\_Mycgr3

Mycgr3G31119 Mycgr3T
  
Location: 32744-32906

Mycgr3G31119\_Mycgr3T

Mycgr3G28587 Mycgr3T
  
Location: 33006-33489

Mycgr3G28587\_Mycgr3T

Mycgr3G98959 Mycgr3T
  
Location: 33589-35035

Mycgr3G98959\_Mycgr3T

Mycgr3G35447 Mycgr3T
  
Location: 35135-36443

Mycgr3G35447\_Mycgr3T

Mycgr3G84402 Mycgr3T
  
Location: 36543-37884

Mycgr3G84402\_Mycgr3T

Mycgr3G98961 Mycgr3T
  
Location: 37984-38884

Mycgr3G98961\_Mycgr3T

adaptor protein complex AP-1
  
Accession: EEH04471
  
Location: 135725-138120
  
 NCBI BlastP on this gene

EEH04471

DUF1077 domain-containing protein
  
Accession: EEH04470
  
Location: 134204-134919
  
 NCBI BlastP on this gene

EEH04470

conserved hypothetical protein
  
Accession: EEH04469
  
Location: 132085-133869
  
 NCBI BlastP on this gene

EEH04469

SH3 domain-containing protein
  
Accession: EEH04468
  
Location: 125204-128950
  
 NCBI BlastP on this gene

EEH04468

predicted protein
  
Accession: EEH04467
  
Location: 124456-124902
  
 NCBI BlastP on this gene

EEH04467

DUF408 domain-containing protein
  
Accession: EEH04466
  
Location: 122980-124149
  
 NCBI BlastP on this gene

EEH04466

40S ribosomal protein S17
  
Accession: EEH04465
  
Location: 121646-122299
  
 NCBI BlastP on this gene

EEH04465

conserved hypothetical protein
  
Accession: EEH04464
  
Location: 119939-121342
  
 NCBI BlastP on this gene

EEH04464

conserved hypothetical protein
  
Accession: EEH04463
  
Location: 112016-119429
  
  
**BlastP hit with Mycgr3G67795\_Mycgr3T**
  
Percentage identity: 62 %
  
BlastP bit score: 3076
  
Sequence coverage: 99 %
  
E-value: 0.0
  
  
 NCBI BlastP on this gene

EEH04463

conserved hypothetical protein
  
Accession: EEH04462
  
Location: 109436-111280
  
 NCBI BlastP on this gene

EEH04462

conserved hypothetical protein
  
Accession: EEH04461
  
Location: 105630-106865
  
 NCBI BlastP on this gene

EEH04461

conserved hypothetical protein
  
Accession: EEH04460
  
Location: 101208-103705
  
 NCBI BlastP on this gene

EEH04460

conserved hypothetical protein
  
Accession: EEH04459
  
Location: 95710-99468
  
 NCBI BlastP on this gene

EEH04459

Query: Architecture Search FASTA input

EQ963476 : Aspergillus flavus NRRL3357 scf\_1106286417850 genomic scaffold    Total score: 1.0     Cumulative Blast bit score: 3076

Hit cluster cross-links:

Mycgr3G67791 Mycgr3T
  
Location: 0-1542

Mycgr3G67791\_Mycgr3T

Mycgr3G90406 Mycgr3T
  
Location: 1642-3973

Mycgr3G90406\_Mycgr3T

Mycgr3G67785 Mycgr3T
  
Location: 4073-7865

Mycgr3G67785\_Mycgr3T

Mycgr3G67795 Mycgr3T
  
Location: 7965-15249

Mycgr3G67795\_Mycgr3T

Mycgr3G67775 Mycgr3T
  
Location: 15349-16237

Mycgr3G67775\_Mycgr3T

Mycgr3G90404 Mycgr3T
  
Location: 16337-17246

Mycgr3G90404\_Mycgr3T

Mycgr3G36951 Mycgr3T
  
Location: 17346-30891

Mycgr3G36951\_Mycgr3T

Mycgr3G103034 Mycgr3
  
Location: 30991-32644

Mycgr3G103034\_Mycgr3

Mycgr3G31119 Mycgr3T
  
Location: 32744-32906

Mycgr3G31119\_Mycgr3T

Mycgr3G28587 Mycgr3T
  
Location: 33006-33489

Mycgr3G28587\_Mycgr3T

Mycgr3G98959 Mycgr3T
  
Location: 33589-35035

Mycgr3G98959\_Mycgr3T

Mycgr3G35447 Mycgr3T
  
Location: 35135-36443

Mycgr3G35447\_Mycgr3T

Mycgr3G84402 Mycgr3T
  
Location: 36543-37884

Mycgr3G84402\_Mycgr3T

Mycgr3G98961 Mycgr3T
  
Location: 37984-38884

Mycgr3G98961\_Mycgr3T

kynureninase
  
Accession: EED52724
  
Location: 1601582-1603215
  
 NCBI BlastP on this gene

EED52724

ER membrane DUF1077 domain protein, putative
  
Accession: EED52725
  
Location: 1603765-1604471
  
 NCBI BlastP on this gene

EED52725

AP-2 adaptor complex subunit beta, putative
  
Accession: EED52726
  
Location: 1605408-1607789
  
 NCBI BlastP on this gene

EED52726

pre-mRNA-splicing factor cwc25, putative
  
Accession: EED52727
  
Location: 1608437-1609381
  
 NCBI BlastP on this gene

EED52727

SH3 domain protein
  
Accession: EED52728
  
Location: 1610568-1611560
  
 NCBI BlastP on this gene

EED52728

SH3 domain protein
  
Accession: EED52729
  
Location: 1612503-1614416
  
 NCBI BlastP on this gene

EED52729

DUF408 domain protein
  
Accession: EED52730
  
Location: 1615233-1616210
  
 NCBI BlastP on this gene

EED52730

ketoreductase
  
Accession: EED52731
  
Location: 1617105-1618357
  
 NCBI BlastP on this gene

EED52731

conserved hypothetical protein
  
Accession: EED52732
  
Location: 1619002-1620396
  
 NCBI BlastP on this gene

EED52732

TOR pathway phosphatidylinositol 3-kinase TorA
  
Accession: EED52733
  
Location: 1621252-1628532
  
  
**BlastP hit with Mycgr3G67795\_Mycgr3T**
  
Percentage identity: 62 %
  
BlastP bit score: 3076
  
Sequence coverage: 100 %
  
E-value: 0.0
  
  
 NCBI BlastP on this gene

EED52733

Query: Architecture Search FASTA input

AKHY01000140 : Aspergillus oryzae 3.042    Total score: 1.0     Cumulative Blast bit score: 3075

Hit cluster cross-links:

Mycgr3G67791 Mycgr3T
  
Location: 0-1542

Mycgr3G67791\_Mycgr3T

Mycgr3G90406 Mycgr3T
  
Location: 1642-3973

Mycgr3G90406\_Mycgr3T

Mycgr3G67785 Mycgr3T
  
Location: 4073-7865

Mycgr3G67785\_Mycgr3T

Mycgr3G67795 Mycgr3T
  
Location: 7965-15249

Mycgr3G67795\_Mycgr3T

Mycgr3G67775 Mycgr3T
  
Location: 15349-16237

Mycgr3G67775\_Mycgr3T

Mycgr3G90404 Mycgr3T
  
Location: 16337-17246

Mycgr3G90404\_Mycgr3T

Mycgr3G36951 Mycgr3T
  
Location: 17346-30891

Mycgr3G36951\_Mycgr3T

Mycgr3G103034 Mycgr3
  
Location: 30991-32644

Mycgr3G103034\_Mycgr3

Mycgr3G31119 Mycgr3T
  
Location: 32744-32906

Mycgr3G31119\_Mycgr3T

Mycgr3G28587 Mycgr3T
  
Location: 33006-33489

Mycgr3G28587\_Mycgr3T

Mycgr3G98959 Mycgr3T
  
Location: 33589-35035

Mycgr3G98959\_Mycgr3T

Mycgr3G35447 Mycgr3T
  
Location: 35135-36443

Mycgr3G35447\_Mycgr3T

Mycgr3G84402 Mycgr3T
  
Location: 36543-37884

Mycgr3G84402\_Mycgr3T

Mycgr3G98961 Mycgr3T
  
Location: 37984-38884

Mycgr3G98961\_Mycgr3T

L-kynurenine hydrolase
  
Accession: EIT78181
  
Location: 31697-33330
  
 NCBI BlastP on this gene

EIT78181

putative membrane protein
  
Accession: EIT78189
  
Location: 33880-34586
  
 NCBI BlastP on this gene

EIT78189

vesicle coat complex AP-1/AP-2/AP-4, beta subunit
  
Accession: EIT78153
  
Location: 35199-37904
  
 NCBI BlastP on this gene

EIT78153

pre-mRNA-splicing factor cwc25
  
Accession: EIT78247
  
Location: 38552-39948
  
 NCBI BlastP on this gene

EIT78247

SH3 domain protein
  
Accession: EIT78248
  
Location: 40686-44454
  
 NCBI BlastP on this gene

EIT78248

hypothetical protein
  
Accession: EIT78192
  
Location: 45271-46248
  
 NCBI BlastP on this gene

EIT78192

flavonol reductase/cinnamoyl-CoA reductase
  
Accession: EIT78245
  
Location: 47147-48399
  
 NCBI BlastP on this gene

EIT78245

DNA-dependent protein kinase
  
Accession: EIT78158
  
Location: 51294-58574
  
  
**BlastP hit with Mycgr3G67795\_Mycgr3T**
  
Percentage identity: 62 %
  
BlastP bit score: 3075
  
Sequence coverage: 100 %
  
E-value: 0.0
  
  
 NCBI BlastP on this gene

EIT78158

Query: Architecture Search FASTA input

EQ962654 : Talaromyces stipitatus ATCC 10500 scf\_1105507295541 genomic scaffold    Total score: 1.0     Cumulative Blast bit score: 3074

Hit cluster cross-links:

Mycgr3G67791 Mycgr3T
  
Location: 0-1542

Mycgr3G67791\_Mycgr3T

Mycgr3G90406 Mycgr3T
  
Location: 1642-3973

Mycgr3G90406\_Mycgr3T

Mycgr3G67785 Mycgr3T
  
Location: 4073-7865

Mycgr3G67785\_Mycgr3T

Mycgr3G67795 Mycgr3T
  
Location: 7965-15249

Mycgr3G67795\_Mycgr3T

Mycgr3G67775 Mycgr3T
  
Location: 15349-16237

Mycgr3G67775\_Mycgr3T

Mycgr3G90404 Mycgr3T
  
Location: 16337-17246

Mycgr3G90404\_Mycgr3T

Mycgr3G36951 Mycgr3T
  
Location: 17346-30891

Mycgr3G36951\_Mycgr3T

Mycgr3G103034 Mycgr3
  
Location: 30991-32644

Mycgr3G103034\_Mycgr3

Mycgr3G31119 Mycgr3T
  
Location: 32744-32906

Mycgr3G31119\_Mycgr3T

Mycgr3G28587 Mycgr3T
  
Location: 33006-33489

Mycgr3G28587\_Mycgr3T

Mycgr3G98959 Mycgr3T
  
Location: 33589-35035

Mycgr3G98959\_Mycgr3T

Mycgr3G35447 Mycgr3T
  
Location: 35135-36443

Mycgr3G35447\_Mycgr3T

Mycgr3G84402 Mycgr3T
  
Location: 36543-37884

Mycgr3G84402\_Mycgr3T

Mycgr3G98961 Mycgr3T
  
Location: 37984-38884

Mycgr3G98961\_Mycgr3T

kynureninase
  
Accession: EED20516
  
Location: 3762851-3764574
  
 NCBI BlastP on this gene

EED20516

AP-2 adaptor complex subunit beta, putative
  
Accession: EED20515
  
Location: 3760118-3762696
  
 NCBI BlastP on this gene

EED20515

ER membrane DUF1077 domain protein, putative
  
Accession: EED20514
  
Location: 3758736-3759416
  
 NCBI BlastP on this gene

EED20514

florfenicol exporter, putative
  
Accession: EED20513
  
Location: 3756791-3758532
  
 NCBI BlastP on this gene

EED20513

SH3 domain protein
  
Accession: EED20512
  
Location: 3752353-3755811
  
 NCBI BlastP on this gene

EED20512

DUF408 domain protein
  
Accession: EED20511
  
Location: 3750454-3751580
  
 NCBI BlastP on this gene

EED20511

40S ribosomal protein S17, putative
  
Accession: EED20510
  
Location: 3749443-3750079
  
 NCBI BlastP on this gene

EED20510

conserved hypothetical protein
  
Accession: EED20509
  
Location: 3746242-3749071
  
 NCBI BlastP on this gene

EED20509

TOR pathway phosphatidylinositol 3-kinase TorA
  
Accession: EED20508
  
Location: 3737981-3745355
  
  
**BlastP hit with Mycgr3G67795\_Mycgr3T**
  
Percentage identity: 62 %
  
BlastP bit score: 3074
  
Sequence coverage: 100 %
  
E-value: 0.0
  
  
 NCBI BlastP on this gene

EED20508

conserved hypothetical protein
  
Accession: EED20507
  
Location: 3735711-3737531
  
 NCBI BlastP on this gene

EED20507

conserved hypothetical protein
  
Accession: EED20506
  
Location: 3733307-3735448
  
 NCBI BlastP on this gene

EED20506

hypothetical protein
  
Accession: EED20505
  
Location: 3729674-3730022
  
 NCBI BlastP on this gene

EED20505

hypothetical protein
  
Accession: EED20504
  
Location: 3728759-3729639
  
 NCBI BlastP on this gene

EED20504

glycerol dehydrogenase, putative
  
Accession: EED20503
  
Location: 3726280-3727382
  
 NCBI BlastP on this gene

EED20503

inositol oxygenase, putative
  
Accession: EED20502
  
Location: 3724490-3725614
  
 NCBI BlastP on this gene

EED20502

conserved hypothetical protein
  
Accession: EED20501
  
Location: 3723057-3724466
  
 NCBI BlastP on this gene

EED20501

Query: Architecture Search FASTA input

DS990641 : Ajellomyces capsulatus H88 supercont1.6 genomic scaffold    Total score: 1.0     Cumulative Blast bit score: 3074

Hit cluster cross-links:

Mycgr3G67791 Mycgr3T
  
Location: 0-1542

Mycgr3G67791\_Mycgr3T

Mycgr3G90406 Mycgr3T
  
Location: 1642-3973

Mycgr3G90406\_Mycgr3T

Mycgr3G67785 Mycgr3T
  
Location: 4073-7865

Mycgr3G67785\_Mycgr3T

Mycgr3G67795 Mycgr3T
  
Location: 7965-15249

Mycgr3G67795\_Mycgr3T

Mycgr3G67775 Mycgr3T
  
Location: 15349-16237

Mycgr3G67775\_Mycgr3T

Mycgr3G90404 Mycgr3T
  
Location: 16337-17246

Mycgr3G90404\_Mycgr3T

Mycgr3G36951 Mycgr3T
  
Location: 17346-30891

Mycgr3G36951\_Mycgr3T

Mycgr3G103034 Mycgr3
  
Location: 30991-32644

Mycgr3G103034\_Mycgr3

Mycgr3G31119 Mycgr3T
  
Location: 32744-32906

Mycgr3G31119\_Mycgr3T

Mycgr3G28587 Mycgr3T
  
Location: 33006-33489

Mycgr3G28587\_Mycgr3T

Mycgr3G98959 Mycgr3T
  
Location: 33589-35035

Mycgr3G98959\_Mycgr3T

Mycgr3G35447 Mycgr3T
  
Location: 35135-36443

Mycgr3G35447\_Mycgr3T

Mycgr3G84402 Mycgr3T
  
Location: 36543-37884

Mycgr3G84402\_Mycgr3T

Mycgr3G98961 Mycgr3T
  
Location: 37984-38884

Mycgr3G98961\_Mycgr3T

adaptor protein complex AP-1
  
Accession: EGC48538
  
Location: 1906951-1909346
  
 NCBI BlastP on this gene

EGC48538

DUF1077 domain-containing protein
  
Accession: EGC48537
  
Location: 1905378-1906146
  
 NCBI BlastP on this gene

EGC48537

MFS transporter
  
Accession: EGC48536
  
Location: 1903313-1905096
  
 NCBI BlastP on this gene

EGC48536

SH3 domain-containing protein
  
Accession: EGC48535
  
Location: 1896733-1900476
  
 NCBI BlastP on this gene

EGC48535

predicted protein
  
Accession: EGC48534
  
Location: 1895969-1896433
  
 NCBI BlastP on this gene

EGC48534

DUF408 domain-containing protein
  
Accession: EGC48533
  
Location: 1894514-1895656
  
 NCBI BlastP on this gene

EGC48533

40S ribosomal protein S17
  
Accession: EGC48532
  
Location: 1893145-1893797
  
 NCBI BlastP on this gene

EGC48532

conserved hypothetical protein
  
Accession: EGC48531
  
Location: 1891434-1892837
  
 NCBI BlastP on this gene

EGC48531

TorA protein
  
Accession: EGC48530
  
Location: 1883517-1890927
  
  
**BlastP hit with Mycgr3G67795\_Mycgr3T**
  
Percentage identity: 62 %
  
BlastP bit score: 3074
  
Sequence coverage: 99 %
  
E-value: 0.0
  
  
 NCBI BlastP on this gene

EGC48530

conserved hypothetical protein
  
Accession: EGC48529
  
Location: 1880964-1882808
  
 NCBI BlastP on this gene

EGC48529

predicted protein
  
Accession: EGC48528
  
Location: 1879778-1880437
  
 NCBI BlastP on this gene

EGC48528

conserved hypothetical protein
  
Accession: EGC48527
  
Location: 1877245-1878469
  
 NCBI BlastP on this gene

EGC48527

conserved hypothetical protein
  
Accession: EGC48526
  
Location: 1872829-1875326
  
 NCBI BlastP on this gene

EGC48526

conserved hypothetical protein
  
Accession: EGC48525
  
Location: 1867328-1871086
  
 NCBI BlastP on this gene

EGC48525

predicted protein
  
Accession: EGC48524
  
Location: 1865051-1865542
  
 NCBI BlastP on this gene

EGC48524

Query: Architecture Search FASTA input

GG657464 : Ajellomyces dermatitidis SLH14081 genomic scaffold supercont1.17    Total score: 1.0     Cumulative Blast bit score: 3069

Hit cluster cross-links:

Mycgr3G67791 Mycgr3T
  
Location: 0-1542

Mycgr3G67791\_Mycgr3T

Mycgr3G90406 Mycgr3T
  
Location: 1642-3973

Mycgr3G90406\_Mycgr3T

Mycgr3G67785 Mycgr3T
  
Location: 4073-7865

Mycgr3G67785\_Mycgr3T

Mycgr3G67795 Mycgr3T
  
Location: 7965-15249

Mycgr3G67795\_Mycgr3T

Mycgr3G67775 Mycgr3T
  
Location: 15349-16237

Mycgr3G67775\_Mycgr3T

Mycgr3G90404 Mycgr3T
  
Location: 16337-17246

Mycgr3G90404\_Mycgr3T

Mycgr3G36951 Mycgr3T
  
Location: 17346-30891

Mycgr3G36951\_Mycgr3T

Mycgr3G103034 Mycgr3
  
Location: 30991-32644

Mycgr3G103034\_Mycgr3

Mycgr3G31119 Mycgr3T
  
Location: 32744-32906

Mycgr3G31119\_Mycgr3T

Mycgr3G28587 Mycgr3T
  
Location: 33006-33489

Mycgr3G28587\_Mycgr3T

Mycgr3G98959 Mycgr3T
  
Location: 33589-35035

Mycgr3G98959\_Mycgr3T

Mycgr3G35447 Mycgr3T
  
Location: 35135-36443

Mycgr3G35447\_Mycgr3T

Mycgr3G84402 Mycgr3T
  
Location: 36543-37884

Mycgr3G84402\_Mycgr3T

Mycgr3G98961 Mycgr3T
  
Location: 37984-38884

Mycgr3G98961\_Mycgr3T

kynureninase
  
Accession: EEQ72089
  
Location: 723343-725092
  
 NCBI BlastP on this gene

EEQ72089

AP-2 adaptor complex subunit beta
  
Accession: EEQ72088
  
Location: 720440-722829
  
 NCBI BlastP on this gene

EEQ72088

ER membrane DUF1077 domain-containing protein
  
Accession: EEQ72087
  
Location: 718774-719581
  
 NCBI BlastP on this gene

EEQ72087

MFS multidrug transporter
  
Accession: EEQ72086
  
Location: 717083-718703
  
 NCBI BlastP on this gene

EEQ72086

conserved hypothetical protein
  
Accession: EEQ72085
  
Location: 712400-716133
  
 NCBI BlastP on this gene

EEQ72085

DUF408 domain-containing protein
  
Accession: EEQ72084
  
Location: 710104-711249
  
 NCBI BlastP on this gene

EEQ72084

40S ribosomal protein S17
  
Accession: EEQ72083
  
Location: 708774-709427
  
 NCBI BlastP on this gene

EEQ72083

conserved hypothetical protein
  
Accession: EEQ72082
  
Location: 707003-708406
  
 NCBI BlastP on this gene

EEQ72082

phosphatidylinositol 3-kinase tor2
  
Accession: EEQ72081
  
Location: 698894-706305
  
  
**BlastP hit with Mycgr3G67795\_Mycgr3T**
  
Percentage identity: 62 %
  
BlastP bit score: 3069
  
Sequence coverage: 99 %
  
E-value: 0.0
  
  
 NCBI BlastP on this gene

EEQ72081

conserved hypothetical protein
  
Accession: EEQ72080
  
Location: 696115-697946
  
 NCBI BlastP on this gene

EEQ72080

C6 transcription factor
  
Accession: EEQ72079
  
Location: 692956-694817
  
 NCBI BlastP on this gene

EEQ72079

methyltransferase type 11
  
Accession: EEQ72078
  
Location: 690899-692414
  
 NCBI BlastP on this gene

EEQ72078

inositol oxygenase
  
Accession: EEQ72077
  
Location: 688939-689971
  
 NCBI BlastP on this gene

EEQ72077

predicted protein
  
Accession: EEQ72076
  
Location: 688009-688504
  
 NCBI BlastP on this gene

EEQ72076

hypothetical protein
  
Accession: EEQ72075
  
Location: 685827-687311
  
 NCBI BlastP on this gene

EEQ72075

predicted protein
  
Accession: EEQ72074
  
Location: 684919-685509
  
 NCBI BlastP on this gene

EEQ72074

conserved hypothetical protein
  
Accession: EEQ72073
  
Location: 679389-683210
  
 NCBI BlastP on this gene

EEQ72073

Query: Architecture Search FASTA input

EQ999980 : Ajellomyces dermatitidis ER-3 genomic scaffold supercont1.8    Total score: 1.0     Cumulative Blast bit score: 3069

Hit cluster cross-links:

Mycgr3G67791 Mycgr3T
  
Location: 0-1542

Mycgr3G67791\_Mycgr3T

Mycgr3G90406 Mycgr3T
  
Location: 1642-3973

Mycgr3G90406\_Mycgr3T

Mycgr3G67785 Mycgr3T
  
Location: 4073-7865

Mycgr3G67785\_Mycgr3T

Mycgr3G67795 Mycgr3T
  
Location: 7965-15249

Mycgr3G67795\_Mycgr3T

Mycgr3G67775 Mycgr3T
  
Location: 15349-16237

Mycgr3G67775\_Mycgr3T

Mycgr3G90404 Mycgr3T
  
Location: 16337-17246

Mycgr3G90404\_Mycgr3T

Mycgr3G36951 Mycgr3T
  
Location: 17346-30891

Mycgr3G36951\_Mycgr3T

Mycgr3G103034 Mycgr3
  
Location: 30991-32644

Mycgr3G103034\_Mycgr3

Mycgr3G31119 Mycgr3T
  
Location: 32744-32906

Mycgr3G31119\_Mycgr3T

Mycgr3G28587 Mycgr3T
  
Location: 33006-33489

Mycgr3G28587\_Mycgr3T

Mycgr3G98959 Mycgr3T
  
Location: 33589-35035

Mycgr3G98959\_Mycgr3T

Mycgr3G35447 Mycgr3T
  
Location: 35135-36443

Mycgr3G35447\_Mycgr3T

Mycgr3G84402 Mycgr3T
  
Location: 36543-37884

Mycgr3G84402\_Mycgr3T

Mycgr3G98961 Mycgr3T
  
Location: 37984-38884

Mycgr3G98961\_Mycgr3T

kynureninase
  
Accession: EEQ92146
  
Location: 1537131-1538880
  
 NCBI BlastP on this gene

EEQ92146

AP-2 adaptor complex subunit beta
  
Accession: EEQ92145
  
Location: 1534232-1536621
  
 NCBI BlastP on this gene

EEQ92145

ER membrane DUF1077 domain-containing protein
  
Accession: EEQ92144
  
Location: 1532568-1533375
  
 NCBI BlastP on this gene

EEQ92144

MFS transporter
  
Accession: EEQ92143
  
Location: 1530878-1532497
  
 NCBI BlastP on this gene

EEQ92143

conserved hypothetical protein
  
Accession: EEQ92142
  
Location: 1526218-1529951
  
 NCBI BlastP on this gene

EEQ92142

DUF408 domain-containing protein
  
Accession: EEQ92141
  
Location: 1523939-1525072
  
 NCBI BlastP on this gene

EEQ92141

40S ribosomal protein S17
  
Accession: EEQ92140
  
Location: 1522592-1523245
  
 NCBI BlastP on this gene

EEQ92140

conserved hypothetical protein
  
Accession: EEQ92139
  
Location: 1520821-1522224
  
 NCBI BlastP on this gene

EEQ92139

phosphatidylinositol 3-kinase tor2
  
Accession: EEQ92138
  
Location: 1512709-1520120
  
  
**BlastP hit with Mycgr3G67795\_Mycgr3T**
  
Percentage identity: 62 %
  
BlastP bit score: 3069
  
Sequence coverage: 99 %
  
E-value: 0.0
  
  
 NCBI BlastP on this gene

EEQ92138

conserved hypothetical protein
  
Accession: EEQ92137
  
Location: 1509929-1511760
  
 NCBI BlastP on this gene

EEQ92137

C6 transcription factor
  
Accession: EEQ92136
  
Location: 1506769-1508630
  
 NCBI BlastP on this gene

EEQ92136

methyltransferase type 11
  
Accession: EEQ92135
  
Location: 1504704-1505952
  
 NCBI BlastP on this gene

EEQ92135

inositol oxygenase
  
Accession: EEQ92134
  
Location: 1502744-1503776
  
 NCBI BlastP on this gene

EEQ92134

predicted protein
  
Accession: EEQ92133
  
Location: 1501444-1502317
  
 NCBI BlastP on this gene

EEQ92133

hypothetical protein
  
Accession: EEQ92132
  
Location: 1499633-1501117
  
 NCBI BlastP on this gene

EEQ92132

predicted protein
  
Accession: EEQ92131
  
Location: 1498725-1499315
  
 NCBI BlastP on this gene

EEQ92131

conserved hypothetical protein
  
Accession: EEQ92130
  
Location: 1493207-1497027
  
 NCBI BlastP on this gene

EEQ92130

Query: Architecture Search FASTA input

DS995904 : Penicillium marneffei ATCC 18224 scf\_1105668340738 genomic scaffold    Total score: 1.0     Cumulative Blast bit score: 3068

Hit cluster cross-links:

Mycgr3G67791 Mycgr3T
  
Location: 0-1542

Mycgr3G67791\_Mycgr3T

Mycgr3G90406 Mycgr3T
  
Location: 1642-3973

Mycgr3G90406\_Mycgr3T

Mycgr3G67785 Mycgr3T
  
Location: 4073-7865

Mycgr3G67785\_Mycgr3T

Mycgr3G67795 Mycgr3T
  
Location: 7965-15249

Mycgr3G67795\_Mycgr3T

Mycgr3G67775 Mycgr3T
  
Location: 15349-16237

Mycgr3G67775\_Mycgr3T

Mycgr3G90404 Mycgr3T
  
Location: 16337-17246

Mycgr3G90404\_Mycgr3T

Mycgr3G36951 Mycgr3T
  
Location: 17346-30891

Mycgr3G36951\_Mycgr3T

Mycgr3G103034 Mycgr3
  
Location: 30991-32644

Mycgr3G103034\_Mycgr3

Mycgr3G31119 Mycgr3T
  
Location: 32744-32906

Mycgr3G31119\_Mycgr3T

Mycgr3G28587 Mycgr3T
  
Location: 33006-33489

Mycgr3G28587\_Mycgr3T

Mycgr3G98959 Mycgr3T
  
Location: 33589-35035

Mycgr3G98959\_Mycgr3T

Mycgr3G35447 Mycgr3T
  
Location: 35135-36443

Mycgr3G35447\_Mycgr3T

Mycgr3G84402 Mycgr3T
  
Location: 36543-37884

Mycgr3G84402\_Mycgr3T

Mycgr3G98961 Mycgr3T
  
Location: 37984-38884

Mycgr3G98961\_Mycgr3T

kynureninase
  
Accession: EEA20947
  
Location: 2407602-2408776
  
 NCBI BlastP on this gene

EEA20947

AP-2 adaptor complex subunit beta, putative
  
Accession: EEA20946
  
Location: 2404854-2407463
  
 NCBI BlastP on this gene

EEA20946

ER membrane DUF1077 domain protein, putative
  
Accession: EEA20945
  
Location: 2403473-2404150
  
 NCBI BlastP on this gene

EEA20945

conserved hypothetical protein
  
Accession: EEA20944
  
Location: 2401505-2403247
  
 NCBI BlastP on this gene

EEA20944

SH3 domain protein
  
Accession: EEA20943
  
Location: 2396965-2400510
  
 NCBI BlastP on this gene

EEA20943

DUF408 domain protein
  
Accession: EEA20941
  
Location: 2395222-2396154
  
 NCBI BlastP on this gene

EEA20941

40S ribosomal protein S17, putative
  
Accession: EEA20940
  
Location: 2394082-2394693
  
 NCBI BlastP on this gene

EEA20940

conserved hypothetical protein
  
Accession: EEA20939
  
Location: 2392377-2393684
  
 NCBI BlastP on this gene

EEA20939

ketoreductase
  
Accession: EEA20938
  
Location: 2390888-2392120
  
 NCBI BlastP on this gene

EEA20938

TOR pathway phosphatidylinositol 3-kinase TorA
  
Accession: EEA20937
  
Location: 2382686-2389997
  
  
**BlastP hit with Mycgr3G67795\_Mycgr3T**
  
Percentage identity: 62 %
  
BlastP bit score: 3068
  
Sequence coverage: 100 %
  
E-value: 0.0
  
  
 NCBI BlastP on this gene

EEA20937

conserved hypothetical protein
  
Accession: EEA20936
  
Location: 2380402-2382175
  
 NCBI BlastP on this gene

EEA20936

glycerol dehydrogenase, putative
  
Accession: EEA20935
  
Location: 2378368-2379506
  
 NCBI BlastP on this gene

EEA20935

inositol oxygenase, putative
  
Accession: EEA20934
  
Location: 2376267-2377286
  
 NCBI BlastP on this gene

EEA20934

conserved hypothetical protein
  
Accession: EEA20933
  
Location: 2374267-2375932
  
 NCBI BlastP on this gene

EEA20933

conserved hypothetical protein
  
Accession: EEA20932
  
Location: 2368957-2372523
  
 NCBI BlastP on this gene

EEA20932

Query: Architecture Search FASTA input

GG692427 : Ajellomyces capsulatus H143 genomic scaffold supercont2.9    Total score: 1.0     Cumulative Blast bit score: 3066

Hit cluster cross-links:

Mycgr3G67791 Mycgr3T
  
Location: 0-1542

Mycgr3G67791\_Mycgr3T

Mycgr3G90406 Mycgr3T
  
Location: 1642-3973

Mycgr3G90406\_Mycgr3T

Mycgr3G67785 Mycgr3T
  
Location: 4073-7865

Mycgr3G67785\_Mycgr3T

Mycgr3G67795 Mycgr3T
  
Location: 7965-15249

Mycgr3G67795\_Mycgr3T

Mycgr3G67775 Mycgr3T
  
Location: 15349-16237

Mycgr3G67775\_Mycgr3T

Mycgr3G90404 Mycgr3T
  
Location: 16337-17246

Mycgr3G90404\_Mycgr3T

Mycgr3G36951 Mycgr3T
  
Location: 17346-30891

Mycgr3G36951\_Mycgr3T

Mycgr3G103034 Mycgr3
  
Location: 30991-32644

Mycgr3G103034\_Mycgr3

Mycgr3G31119 Mycgr3T
  
Location: 32744-32906

Mycgr3G31119\_Mycgr3T

Mycgr3G28587 Mycgr3T
  
Location: 33006-33489

Mycgr3G28587\_Mycgr3T

Mycgr3G98959 Mycgr3T
  
Location: 33589-35035

Mycgr3G98959\_Mycgr3T

Mycgr3G35447 Mycgr3T
  
Location: 35135-36443

Mycgr3G35447\_Mycgr3T

Mycgr3G84402 Mycgr3T
  
Location: 36543-37884

Mycgr3G84402\_Mycgr3T

Mycgr3G98961 Mycgr3T
  
Location: 37984-38884

Mycgr3G98961\_Mycgr3T

adaptin
  
Accession: EER40250
  
Location: 793861-796256
  
 NCBI BlastP on this gene

EER40250

DUF1077 domain-containing protein
  
Accession: EER40251
  
Location: 797061-797829
  
 NCBI BlastP on this gene

EER40251

conserved hypothetical protein
  
Accession: EER40252
  
Location: 802704-803303
  
 NCBI BlastP on this gene

EER40252

hypothetical protein
  
Accession: EER40253
  
Location: 804428-805790
  
 NCBI BlastP on this gene

EER40253

predicted protein
  
Accession: EER40254
  
Location: 806900-807363
  
 NCBI BlastP on this gene

EER40254

DUF408 domain-containing protein
  
Accession: EER40255
  
Location: 807676-808818
  
 NCBI BlastP on this gene

EER40255

hypothetical protein
  
Accession: EER40256
  
Location: 809401-810184
  
 NCBI BlastP on this gene

EER40256

conserved hypothetical protein
  
Accession: EER40257
  
Location: 810489-811892
  
 NCBI BlastP on this gene

EER40257

TorA protein
  
Accession: EER40258
  
Location: 812399-819809
  
  
**BlastP hit with Mycgr3G67795\_Mycgr3T**
  
Percentage identity: 62 %
  
BlastP bit score: 3066
  
Sequence coverage: 99 %
  
E-value: 0.0
  
  
 NCBI BlastP on this gene

EER40258

Query: Architecture Search FASTA input

AP007171 : Aspergillus oryzae RIB40 DNA, SC011.    Total score: 1.0     Cumulative Blast bit score: 3054

Hit cluster cross-links:

Mycgr3G67791 Mycgr3T
  
Location: 0-1542

Mycgr3G67791\_Mycgr3T

Mycgr3G90406 Mycgr3T
  
Location: 1642-3973

Mycgr3G90406\_Mycgr3T

Mycgr3G67785 Mycgr3T
  
Location: 4073-7865

Mycgr3G67785\_Mycgr3T

Mycgr3G67795 Mycgr3T
  
Location: 7965-15249

Mycgr3G67795\_Mycgr3T

Mycgr3G67775 Mycgr3T
  
Location: 15349-16237

Mycgr3G67775\_Mycgr3T

Mycgr3G90404 Mycgr3T
  
Location: 16337-17246

Mycgr3G90404\_Mycgr3T

Mycgr3G36951 Mycgr3T
  
Location: 17346-30891

Mycgr3G36951\_Mycgr3T

Mycgr3G103034 Mycgr3
  
Location: 30991-32644

Mycgr3G103034\_Mycgr3

Mycgr3G31119 Mycgr3T
  
Location: 32744-32906

Mycgr3G31119\_Mycgr3T

Mycgr3G28587 Mycgr3T
  
Location: 33006-33489

Mycgr3G28587\_Mycgr3T

Mycgr3G98959 Mycgr3T
  
Location: 33589-35035

Mycgr3G98959\_Mycgr3T

Mycgr3G35447 Mycgr3T
  
Location: 35135-36443

Mycgr3G35447\_Mycgr3T

Mycgr3G84402 Mycgr3T
  
Location: 36543-37884

Mycgr3G84402\_Mycgr3T

Mycgr3G98961 Mycgr3T
  
Location: 37984-38884

Mycgr3G98961\_Mycgr3T

not annotated
  
Accession: BAE65077
  
Location: 1532825-1534458
  
 NCBI BlastP on this gene

AO090011000602

not annotated
  
Accession: BAE65078
  
Location: 1534887-1535714
  
 NCBI BlastP on this gene

AO090011000603

not annotated
  
Accession: BAE65079
  
Location: 1536327-1539032
  
 NCBI BlastP on this gene

AO090011000604

not annotated
  
Accession: BAE65080
  
Location: 1539680-1541076
  
 NCBI BlastP on this gene

AO090011000605

not annotated
  
Accession: BAE65081
  
Location: 1541814-1545582
  
 NCBI BlastP on this gene

AO090011000606

not annotated
  
Accession: BAE65082
  
Location: 1546399-1547376
  
 NCBI BlastP on this gene

AO090011000607

not annotated
  
Accession: BAE65083
  
Location: 1550520-1559702
  
  
**BlastP hit with Mycgr3G67795\_Mycgr3T**
  
Percentage identity: 63 %
  
BlastP bit score: 3054
  
Sequence coverage: 98 %
  
E-value: 0.0
  
  
 NCBI BlastP on this gene

AO090011000608

Query: Architecture Search FASTA input

JH921440 : Marssonina brunnea f. sp. 'multigermtubi' MB\_m1 unplaced genomic scaffold M6\_S00013    Total score: 1.0     Cumulative Blast bit score: 3051

Hit cluster cross-links:

Mycgr3G67791 Mycgr3T
  
Location: 0-1542

Mycgr3G67791\_Mycgr3T

Mycgr3G90406 Mycgr3T
  
Location: 1642-3973

Mycgr3G90406\_Mycgr3T

Mycgr3G67785 Mycgr3T
  
Location: 4073-7865

Mycgr3G67785\_Mycgr3T

Mycgr3G67795 Mycgr3T
  
Location: 7965-15249

Mycgr3G67795\_Mycgr3T

Mycgr3G67775 Mycgr3T
  
Location: 15349-16237

Mycgr3G67775\_Mycgr3T

Mycgr3G90404 Mycgr3T
  
Location: 16337-17246

Mycgr3G90404\_Mycgr3T

Mycgr3G36951 Mycgr3T
  
Location: 17346-30891

Mycgr3G36951\_Mycgr3T

Mycgr3G103034 Mycgr3
  
Location: 30991-32644

Mycgr3G103034\_Mycgr3

Mycgr3G31119 Mycgr3T
  
Location: 32744-32906

Mycgr3G31119\_Mycgr3T

Mycgr3G28587 Mycgr3T
  
Location: 33006-33489

Mycgr3G28587\_Mycgr3T

Mycgr3G98959 Mycgr3T
  
Location: 33589-35035

Mycgr3G98959\_Mycgr3T

Mycgr3G35447 Mycgr3T
  
Location: 35135-36443

Mycgr3G35447\_Mycgr3T

Mycgr3G84402 Mycgr3T
  
Location: 36543-37884

Mycgr3G84402\_Mycgr3T

Mycgr3G98961 Mycgr3T
  
Location: 37984-38884

Mycgr3G98961\_Mycgr3T

casein kinase II regulatory subunit
  
Accession: EKD15930
  
Location: 740405-742094
  
 NCBI BlastP on this gene

EKD15930

hypothetical protein
  
Accession: EKD15931
  
Location: 743091-747035
  
 NCBI BlastP on this gene

EKD15931

integral membrane protein
  
Accession: EKD15932
  
Location: 752425-752998
  
 NCBI BlastP on this gene

EKD15932

glucosidase 2 subunit beta precursor
  
Accession: EKD15933
  
Location: 755492-757856
  
 NCBI BlastP on this gene

EKD15933

DUF602 domain protein
  
Accession: EKD15934
  
Location: 758251-759117
  
 NCBI BlastP on this gene

EKD15934

FAT domain-containing protein
  
Accession: EKD15935
  
Location: 759438-766925
  
  
**BlastP hit with Mycgr3G67795\_Mycgr3T**
  
Percentage identity: 62 %
  
BlastP bit score: 3051
  
Sequence coverage: 101 %
  
E-value: 0.0
  
  
 NCBI BlastP on this gene

EKD15935

Query: Architecture Search FASTA input

KE145367 : Glarea lozoyensis ATCC 20868 chromosome Unknown GLAREA3    Total score: 1.0     Cumulative Blast bit score: 3042

Hit cluster cross-links:

Mycgr3G67791 Mycgr3T
  
Location: 0-1542

Mycgr3G67791\_Mycgr3T

Mycgr3G90406 Mycgr3T
  
Location: 1642-3973

Mycgr3G90406\_Mycgr3T

Mycgr3G67785 Mycgr3T
  
Location: 4073-7865

Mycgr3G67785\_Mycgr3T

Mycgr3G67795 Mycgr3T
  
Location: 7965-15249

Mycgr3G67795\_Mycgr3T

Mycgr3G67775 Mycgr3T
  
Location: 15349-16237

Mycgr3G67775\_Mycgr3T

Mycgr3G90404 Mycgr3T
  
Location: 16337-17246

Mycgr3G90404\_Mycgr3T

Mycgr3G36951 Mycgr3T
  
Location: 17346-30891

Mycgr3G36951\_Mycgr3T

Mycgr3G103034 Mycgr3
  
Location: 30991-32644

Mycgr3G103034\_Mycgr3

Mycgr3G31119 Mycgr3T
  
Location: 32744-32906

Mycgr3G31119\_Mycgr3T

Mycgr3G28587 Mycgr3T
  
Location: 33006-33489

Mycgr3G28587\_Mycgr3T

Mycgr3G98959 Mycgr3T
  
Location: 33589-35035

Mycgr3G98959\_Mycgr3T

Mycgr3G35447 Mycgr3T
  
Location: 35135-36443

Mycgr3G35447\_Mycgr3T

Mycgr3G84402 Mycgr3T
  
Location: 36543-37884

Mycgr3G84402\_Mycgr3T

Mycgr3G98961 Mycgr3T
  
Location: 37984-38884

Mycgr3G98961\_Mycgr3T

Mannose 6-phosphate receptor
  
Accession: EPE29047
  
Location: 562637-564469
  
 NCBI BlastP on this gene

EPE29047

hypothetical protein
  
Accession: EPE29048
  
Location: 565107-568928
  
 NCBI BlastP on this gene

EPE29048

hypothetical protein
  
Accession: EPE29049
  
Location: 569511-570177
  
 NCBI BlastP on this gene

EPE29049

hypothetical protein
  
Accession: EPE29050
  
Location: 577005-579430
  
 NCBI BlastP on this gene

EPE29050

hypothetical protein
  
Accession: EPE29051
  
Location: 581340-582296
  
 NCBI BlastP on this gene

EPE29051

ARM repeat-containing protein
  
Accession: EPE29052
  
Location: 582609-590120
  
  
**BlastP hit with Mycgr3G67795\_Mycgr3T**
  
Percentage identity: 62 %
  
BlastP bit score: 3042
  
Sequence coverage: 100 %
  
E-value: 0.0
  
  
 NCBI BlastP on this gene

EPE29052

Query: Architecture Search FASTA input

KB707916 : Botryotinia fuckeliana BcDW1 unplaced genomic scaffold Scaffold\_244    Total score: 1.0     Cumulative Blast bit score: 3017

Hit cluster cross-links:

Mycgr3G67791 Mycgr3T
  
Location: 0-1542

Mycgr3G67791\_Mycgr3T

Mycgr3G90406 Mycgr3T
  
Location: 1642-3973

Mycgr3G90406\_Mycgr3T

Mycgr3G67785 Mycgr3T
  
Location: 4073-7865

Mycgr3G67785\_Mycgr3T

Mycgr3G67795 Mycgr3T
  
Location: 7965-15249

Mycgr3G67795\_Mycgr3T

Mycgr3G67775 Mycgr3T
  
Location: 15349-16237

Mycgr3G67775\_Mycgr3T

Mycgr3G90404 Mycgr3T
  
Location: 16337-17246

Mycgr3G90404\_Mycgr3T

Mycgr3G36951 Mycgr3T
  
Location: 17346-30891

Mycgr3G36951\_Mycgr3T

Mycgr3G103034 Mycgr3
  
Location: 30991-32644

Mycgr3G103034\_Mycgr3

Mycgr3G31119 Mycgr3T
  
Location: 32744-32906

Mycgr3G31119\_Mycgr3T

Mycgr3G28587 Mycgr3T
  
Location: 33006-33489

Mycgr3G28587\_Mycgr3T

Mycgr3G98959 Mycgr3T
  
Location: 33589-35035

Mycgr3G98959\_Mycgr3T

Mycgr3G35447 Mycgr3T
  
Location: 35135-36443

Mycgr3G35447\_Mycgr3T

Mycgr3G84402 Mycgr3T
  
Location: 36543-37884

Mycgr3G84402\_Mycgr3T

Mycgr3G98961 Mycgr3T
  
Location: 37984-38884

Mycgr3G98961\_Mycgr3T

hypothetical protein
  
Accession: EMR85259
  
Location: 44678-45688
  
 NCBI BlastP on this gene

EMR85259

putative glucosidase 2 subunit beta protein
  
Accession: EMR85258
  
Location: 41925-43978
  
 NCBI BlastP on this gene

EMR85258

hypothetical protein
  
Accession: EMR85257
  
Location: 37192-41070
  
 NCBI BlastP on this gene

EMR85257

putative duf602 domain-containing protein
  
Accession: EMR85256
  
Location: 27516-28508
  
 NCBI BlastP on this gene

EMR85256

putative phosphatidylinositol 3-kinase tor2 protein
  
Accession: EMR85255
  
Location: 19482-26996
  
  
**BlastP hit with Mycgr3G67795\_Mycgr3T**
  
Percentage identity: 61 %
  
BlastP bit score: 3017
  
Sequence coverage: 100 %
  
E-value: 0.0
  
  
 NCBI BlastP on this gene

EMR85255

putative chaperone domain protein
  
Accession: EMR85254
  
Location: 17386-18306
  
 NCBI BlastP on this gene

EMR85254

putative siderophore iron transporter protein
  
Accession: EMR85253
  
Location: 14786-16736
  
 NCBI BlastP on this gene

EMR85253

putative paraben-hydrolyzing esterase precursor protein
  
Accession: EMR85252
  
Location: 11006-12648
  
 NCBI BlastP on this gene

EMR85252

hypothetical protein
  
Accession: EMR85251
  
Location: 8591-9657
  
 NCBI BlastP on this gene

EMR85251

putative transcription factor cys6 protein
  
Accession: EMR85250
  
Location: 6355-7395
  
 NCBI BlastP on this gene

EMR85250

putative glutathione s-transferase protein
  
Accession: EMR85249
  
Location: 4861-5346
  
 NCBI BlastP on this gene

EMR85249

putative 2-dehydropantoate 2-reductase protein
  
Accession: EMR85248
  
Location: 3113-4141
  
 NCBI BlastP on this gene

EMR85248

putative tannase subunit protein
  
Accession: EMR85247
  
Location: 2141-2356
  
 NCBI BlastP on this gene

EMR85247

Query: Architecture Search FASTA input

FQ790307 : Botryotinia fuckeliana T4 SuperContig\_19\_1 genomic supercontig.    Total score: 1.0     Cumulative Blast bit score: 3017

Hit cluster cross-links:

Mycgr3G67791 Mycgr3T
  
Location: 0-1542

Mycgr3G67791\_Mycgr3T

Mycgr3G90406 Mycgr3T
  
Location: 1642-3973

Mycgr3G90406\_Mycgr3T

Mycgr3G67785 Mycgr3T
  
Location: 4073-7865

Mycgr3G67785\_Mycgr3T

Mycgr3G67795 Mycgr3T
  
Location: 7965-15249

Mycgr3G67795\_Mycgr3T

Mycgr3G67775 Mycgr3T
  
Location: 15349-16237

Mycgr3G67775\_Mycgr3T

Mycgr3G90404 Mycgr3T
  
Location: 16337-17246

Mycgr3G90404\_Mycgr3T

Mycgr3G36951 Mycgr3T
  
Location: 17346-30891

Mycgr3G36951\_Mycgr3T

Mycgr3G103034 Mycgr3
  
Location: 30991-32644

Mycgr3G103034\_Mycgr3

Mycgr3G31119 Mycgr3T
  
Location: 32744-32906

Mycgr3G31119\_Mycgr3T

Mycgr3G28587 Mycgr3T
  
Location: 33006-33489

Mycgr3G28587\_Mycgr3T

Mycgr3G98959 Mycgr3T
  
Location: 33589-35035

Mycgr3G98959\_Mycgr3T

Mycgr3G35447 Mycgr3T
  
Location: 35135-36443

Mycgr3G35447\_Mycgr3T

Mycgr3G84402 Mycgr3T
  
Location: 36543-37884

Mycgr3G84402\_Mycgr3T

Mycgr3G98961 Mycgr3T
  
Location: 37984-38884

Mycgr3G98961\_Mycgr3T

hypothetical protein
  
Accession: CCD34327
  
Location: 94704-95714
  
 NCBI BlastP on this gene

BofuT4P19000007001

similar to protein kinase C substrate
  
Accession: CCD34328
  
Location: 96414-98467
  
 NCBI BlastP on this gene

BofuT4\_P027120.1

hypothetical protein
  
Accession: CCD34329
  
Location: 99322-103200
  
 NCBI BlastP on this gene

BofuT4\_P027130.1

hypothetical protein
  
Accession: CCD34330
  
Location: 106966-107127
  
 NCBI BlastP on this gene

BofuT4\_uP027140.1

predicted protein
  
Accession: CCD34331
  
Location: 109972-110618
  
 NCBI BlastP on this gene

BofuT4\_P027150.1

hypothetical protein
  
Accession: CCD34332
  
Location: 111884-113234
  
 NCBI BlastP on this gene

BofuT4\_P027160.1

similar to phosphatidylinositol 3-kinase tor2
  
Accession: CCD34333
  
Location: 113396-120910
  
  
**BlastP hit with Mycgr3G67795\_Mycgr3T**
  
Percentage identity: 61 %
  
BlastP bit score: 3017
  
Sequence coverage: 100 %
  
E-value: 0.0
  
  
 NCBI BlastP on this gene

BofuT4\_P027170.1

Query: Architecture Search FASTA input

KB644411 : Penicillium oxalicum 114-2 unplaced genomic scaffold scaffold\_4    Total score: 1.0     Cumulative Blast bit score: 2988

Hit cluster cross-links:

Mycgr3G67791 Mycgr3T
  
Location: 0-1542

Mycgr3G67791\_Mycgr3T

Mycgr3G90406 Mycgr3T
  
Location: 1642-3973

Mycgr3G90406\_Mycgr3T

Mycgr3G67785 Mycgr3T
  
Location: 4073-7865

Mycgr3G67785\_Mycgr3T

Mycgr3G67795 Mycgr3T
  
Location: 7965-15249

Mycgr3G67795\_Mycgr3T

Mycgr3G67775 Mycgr3T
  
Location: 15349-16237

Mycgr3G67775\_Mycgr3T

Mycgr3G90404 Mycgr3T
  
Location: 16337-17246

Mycgr3G90404\_Mycgr3T

Mycgr3G36951 Mycgr3T
  
Location: 17346-30891

Mycgr3G36951\_Mycgr3T

Mycgr3G103034 Mycgr3
  
Location: 30991-32644

Mycgr3G103034\_Mycgr3

Mycgr3G31119 Mycgr3T
  
Location: 32744-32906

Mycgr3G31119\_Mycgr3T

Mycgr3G28587 Mycgr3T
  
Location: 33006-33489

Mycgr3G28587\_Mycgr3T

Mycgr3G98959 Mycgr3T
  
Location: 33589-35035

Mycgr3G98959\_Mycgr3T

Mycgr3G35447 Mycgr3T
  
Location: 35135-36443

Mycgr3G35447\_Mycgr3T

Mycgr3G84402 Mycgr3T
  
Location: 36543-37884

Mycgr3G84402\_Mycgr3T

Mycgr3G98961 Mycgr3T
  
Location: 37984-38884

Mycgr3G98961\_Mycgr3T

hypothetical protein
  
Accession: EPS28850
  
Location: 1475338-1476459
  
 NCBI BlastP on this gene

EPS28850

hypothetical protein
  
Accession: EPS28849
  
Location: 1469180-1472940
  
 NCBI BlastP on this gene

EPS28849

hypothetical protein
  
Accession: EPS28848
  
Location: 1466475-1467446
  
 NCBI BlastP on this gene

EPS28848

hypothetical protein
  
Accession: EPS28847
  
Location: 1465401-1465968
  
 NCBI BlastP on this gene

EPS28847

hypothetical protein
  
Accession: EPS28846
  
Location: 1463235-1464659
  
 NCBI BlastP on this gene

EPS28846

hypothetical protein
  
Accession: EPS28845
  
Location: 1461612-1462881
  
 NCBI BlastP on this gene

EPS28845

hypothetical protein
  
Accession: EPS28844
  
Location: 1451506-1458895
  
  
**BlastP hit with Mycgr3G67795\_Mycgr3T**
  
Percentage identity: 60 %
  
BlastP bit score: 2988
  
Sequence coverage: 100 %
  
E-value: 0.0
  
  
 NCBI BlastP on this gene

EPS28844

hypothetical protein
  
Accession: EPS28843
  
Location: 1448733-1450621
  
 NCBI BlastP on this gene

EPS28843

hypothetical protein
  
Accession: EPS28842
  
Location: 1446499-1447452
  
 NCBI BlastP on this gene

EPS28842

hypothetical protein
  
Accession: EPS28841
  
Location: 1440708-1441670
  
 NCBI BlastP on this gene

EPS28841

hypothetical protein
  
Accession: EPS28840
  
Location: 1437791-1438978
  
 NCBI BlastP on this gene

EPS28840

hypothetical protein
  
Accession: EPS28839
  
Location: 1434401-1436878
  
 NCBI BlastP on this gene

EPS28839

Query: Architecture Search FASTA input

CH445336 : Phaeosphaeria nodorum SN15 scaffold\_12    Total score: 1.0     Cumulative Blast bit score: 2987

Hit cluster cross-links:

Mycgr3G67791 Mycgr3T
  
Location: 0-1542

Mycgr3G67791\_Mycgr3T

Mycgr3G90406 Mycgr3T
  
Location: 1642-3973

Mycgr3G90406\_Mycgr3T

Mycgr3G67785 Mycgr3T
  
Location: 4073-7865

Mycgr3G67785\_Mycgr3T

Mycgr3G67795 Mycgr3T
  
Location: 7965-15249

Mycgr3G67795\_Mycgr3T

Mycgr3G67775 Mycgr3T
  
Location: 15349-16237

Mycgr3G67775\_Mycgr3T

Mycgr3G90404 Mycgr3T
  
Location: 16337-17246

Mycgr3G90404\_Mycgr3T

Mycgr3G36951 Mycgr3T
  
Location: 17346-30891

Mycgr3G36951\_Mycgr3T

Mycgr3G103034 Mycgr3
  
Location: 30991-32644

Mycgr3G103034\_Mycgr3

Mycgr3G31119 Mycgr3T
  
Location: 32744-32906

Mycgr3G31119\_Mycgr3T

Mycgr3G28587 Mycgr3T
  
Location: 33006-33489

Mycgr3G28587\_Mycgr3T

Mycgr3G98959 Mycgr3T
  
Location: 33589-35035

Mycgr3G98959\_Mycgr3T

Mycgr3G35447 Mycgr3T
  
Location: 35135-36443

Mycgr3G35447\_Mycgr3T

Mycgr3G84402 Mycgr3T
  
Location: 36543-37884

Mycgr3G84402\_Mycgr3T

Mycgr3G98961 Mycgr3T
  
Location: 37984-38884

Mycgr3G98961\_Mycgr3T

hypothetical protein
  
Accession: EAT84514
  
Location: 736894-738688
  
 NCBI BlastP on this gene

EAT84514

hypothetical protein
  
Accession: EAT84515
  
Location: 739086-739871
  
 NCBI BlastP on this gene

EAT84515

hypothetical protein
  
Accession: EAT84516
  
Location: 740176-744201
  
 NCBI BlastP on this gene

EAT84516

hypothetical protein
  
Accession: EAT84518
  
Location: 745881-749668
  
 NCBI BlastP on this gene

EAT84518

hypothetical protein
  
Accession: EAT84519
  
Location: 749786-750073
  
 NCBI BlastP on this gene

EAT84519

hypothetical protein
  
Accession: EAT84520
  
Location: 750378-752609
  
 NCBI BlastP on this gene

EAT84520

hypothetical protein
  
Accession: EAT84522
  
Location: 753226-760586
  
  
**BlastP hit with Mycgr3G67795\_Mycgr3T**
  
Percentage identity: 67 %
  
BlastP bit score: 2987
  
Sequence coverage: 91 %
  
E-value: 0.0
  
  
 NCBI BlastP on this gene

EAT84522

hypothetical protein
  
Accession: EAT84523
  
Location: 761315-762679
  
 NCBI BlastP on this gene

EAT84523

hypothetical protein
  
Accession: EAT84524
  
Location: 766213-766611
  
 NCBI BlastP on this gene

EAT84524

hypothetical protein
  
Accession: EAT84525
  
Location: 766656-767614
  
 NCBI BlastP on this gene

EAT84525

hypothetical protein
  
Accession: EAT84526
  
Location: 768093-769623
  
 NCBI BlastP on this gene

EAT84526

hypothetical protein
  
Accession: EAT84527
  
Location: 770374-774862
  
 NCBI BlastP on this gene

EAT84527

hypothetical protein
  
Accession: EAT84528
  
Location: 775101-776516
  
 NCBI BlastP on this gene

EAT84528

hypothetical protein
  
Accession: EAT84529
  
Location: 775989-777279
  
 NCBI BlastP on this gene

EAT84529

hypothetical protein
  
Accession: EAT84530
  
Location: 777358-777966
  
 NCBI BlastP on this gene

EAT84530

Query: Architecture Search FASTA input

ABDF02000005 : Trichoderma virens Gv29-8    Total score: 1.0     Cumulative Blast bit score: 2985

Hit cluster cross-links:

Mycgr3G67791 Mycgr3T
  
Location: 0-1542

Mycgr3G67791\_Mycgr3T

Mycgr3G90406 Mycgr3T
  
Location: 1642-3973

Mycgr3G90406\_Mycgr3T

Mycgr3G67785 Mycgr3T
  
Location: 4073-7865

Mycgr3G67785\_Mycgr3T

Mycgr3G67795 Mycgr3T
  
Location: 7965-15249

Mycgr3G67795\_Mycgr3T

Mycgr3G67775 Mycgr3T
  
Location: 15349-16237

Mycgr3G67775\_Mycgr3T

Mycgr3G90404 Mycgr3T
  
Location: 16337-17246

Mycgr3G90404\_Mycgr3T

Mycgr3G36951 Mycgr3T
  
Location: 17346-30891

Mycgr3G36951\_Mycgr3T

Mycgr3G103034 Mycgr3
  
Location: 30991-32644

Mycgr3G103034\_Mycgr3

Mycgr3G31119 Mycgr3T
  
Location: 32744-32906

Mycgr3G31119\_Mycgr3T

Mycgr3G28587 Mycgr3T
  
Location: 33006-33489

Mycgr3G28587\_Mycgr3T

Mycgr3G98959 Mycgr3T
  
Location: 33589-35035

Mycgr3G98959\_Mycgr3T

Mycgr3G35447 Mycgr3T
  
Location: 35135-36443

Mycgr3G35447\_Mycgr3T

Mycgr3G84402 Mycgr3T
  
Location: 36543-37884

Mycgr3G84402\_Mycgr3T

Mycgr3G98961 Mycgr3T
  
Location: 37984-38884

Mycgr3G98961\_Mycgr3T

hypothetical protein
  
Accession: EHK23377
  
Location: 480599-482410
  
 NCBI BlastP on this gene

EHK23377

hypothetical protein
  
Accession: EHK23378
  
Location: 483472-483948
  
 NCBI BlastP on this gene

EHK23378

hypothetical protein
  
Accession: EHK23379
  
Location: 485856-487527
  
 NCBI BlastP on this gene

EHK23379

hypothetical protein
  
Accession: EHK23380
  
Location: 487946-490157
  
 NCBI BlastP on this gene

EHK23380

hypothetical protein
  
Accession: EHK23381
  
Location: 492806-493600
  
 NCBI BlastP on this gene

EHK23381

hypothetical protein
  
Accession: EHK23382
  
Location: 494284-501879
  
  
**BlastP hit with Mycgr3G67795\_Mycgr3T**
  
Percentage identity: 60 %
  
BlastP bit score: 2985
  
Sequence coverage: 100 %
  
E-value: 0.0
  
  
 NCBI BlastP on this gene

EHK23382

hypothetical protein
  
Accession: EHK23383
  
Location: 504240-504938
  
 NCBI BlastP on this gene

EHK23383

hypothetical protein
  
Accession: EHK23384
  
Location: 506569-508530
  
 NCBI BlastP on this gene

EHK23384

hypothetical protein
  
Accession: EHK23385
  
Location: 508905-510020
  
 NCBI BlastP on this gene

EHK23385

hypothetical protein
  
Accession: EHK23386
  
Location: 510982-513564
  
 NCBI BlastP on this gene

EHK23386

hypothetical protein
  
Accession: EHK23387
  
Location: 515730-518012
  
 NCBI BlastP on this gene

EHK23387

Query: Architecture Search FASTA input

ABDG02000025 : Trichoderma atroviride IMI 206040    Total score: 1.0     Cumulative Blast bit score: 2980

Hit cluster cross-links:

Mycgr3G67791 Mycgr3T
  
Location: 0-1542

Mycgr3G67791\_Mycgr3T

Mycgr3G90406 Mycgr3T
  
Location: 1642-3973

Mycgr3G90406\_Mycgr3T

Mycgr3G67785 Mycgr3T
  
Location: 4073-7865

Mycgr3G67785\_Mycgr3T

Mycgr3G67795 Mycgr3T
  
Location: 7965-15249

Mycgr3G67795\_Mycgr3T

Mycgr3G67775 Mycgr3T
  
Location: 15349-16237

Mycgr3G67775\_Mycgr3T

Mycgr3G90404 Mycgr3T
  
Location: 16337-17246

Mycgr3G90404\_Mycgr3T

Mycgr3G36951 Mycgr3T
  
Location: 17346-30891

Mycgr3G36951\_Mycgr3T

Mycgr3G103034 Mycgr3
  
Location: 30991-32644

Mycgr3G103034\_Mycgr3

Mycgr3G31119 Mycgr3T
  
Location: 32744-32906

Mycgr3G31119\_Mycgr3T

Mycgr3G28587 Mycgr3T
  
Location: 33006-33489

Mycgr3G28587\_Mycgr3T

Mycgr3G98959 Mycgr3T
  
Location: 33589-35035

Mycgr3G98959\_Mycgr3T

Mycgr3G35447 Mycgr3T
  
Location: 35135-36443

Mycgr3G35447\_Mycgr3T

Mycgr3G84402 Mycgr3T
  
Location: 36543-37884

Mycgr3G84402\_Mycgr3T

Mycgr3G98961 Mycgr3T
  
Location: 37984-38884

Mycgr3G98961\_Mycgr3T

hypothetical protein
  
Accession: EHK43811
  
Location: 493895-495681
  
 NCBI BlastP on this gene

EHK43811

hypothetical protein
  
Accession: EHK43812
  
Location: 496703-497170
  
 NCBI BlastP on this gene

EHK43812

hypothetical protein
  
Accession: EHK43813
  
Location: 498726-500417
  
 NCBI BlastP on this gene

EHK43813

hypothetical protein
  
Accession: EHK43814
  
Location: 501460-502778
  
 NCBI BlastP on this gene

EHK43814

hypothetical protein
  
Accession: EHK43815
  
Location: 503657-506039
  
 NCBI BlastP on this gene

EHK43815

hypothetical protein
  
Accession: EHK43816
  
Location: 507701-508095
  
 NCBI BlastP on this gene

EHK43816

hypothetical protein
  
Accession: EHK43817
  
Location: 508715-509509
  
 NCBI BlastP on this gene

EHK43817

hypothetical protein
  
Accession: EHK43818
  
Location: 510193-517811
  
  
**BlastP hit with Mycgr3G67795\_Mycgr3T**
  
Percentage identity: 60 %
  
BlastP bit score: 2980
  
Sequence coverage: 100 %
  
E-value: 0.0
  
  
 NCBI BlastP on this gene

EHK43818

hypothetical protein
  
Accession: EHK43819
  
Location: 520143-520862
  
 NCBI BlastP on this gene

EHK43819

hypothetical protein
  
Accession: EHK43820
  
Location: 522339-524223
  
 NCBI BlastP on this gene

EHK43820

hypothetical protein
  
Accession: EHK43821
  
Location: 524541-525692
  
 NCBI BlastP on this gene

EHK43821

hypothetical protein
  
Accession: EHK43822
  
Location: 526704-529260
  
 NCBI BlastP on this gene

EHK43822

hypothetical protein
  
Accession: EHK43823
  
Location: 531957-534221
  
 NCBI BlastP on this gene

EHK43823

Query: Architecture Search FASTA input

GL698476 : Metarhizium acridum CQMa 102 unplaced genomic scaffold Scf\_007    Total score: 1.0     Cumulative Blast bit score: 2969

Hit cluster cross-links:

Mycgr3G67791 Mycgr3T
  
Location: 0-1542

Mycgr3G67791\_Mycgr3T

Mycgr3G90406 Mycgr3T
  
Location: 1642-3973

Mycgr3G90406\_Mycgr3T

Mycgr3G67785 Mycgr3T
  
Location: 4073-7865

Mycgr3G67785\_Mycgr3T

Mycgr3G67795 Mycgr3T
  
Location: 7965-15249

Mycgr3G67795\_Mycgr3T

Mycgr3G67775 Mycgr3T
  
Location: 15349-16237

Mycgr3G67775\_Mycgr3T

Mycgr3G90404 Mycgr3T
  
Location: 16337-17246

Mycgr3G90404\_Mycgr3T

Mycgr3G36951 Mycgr3T
  
Location: 17346-30891

Mycgr3G36951\_Mycgr3T

Mycgr3G103034 Mycgr3
  
Location: 30991-32644

Mycgr3G103034\_Mycgr3

Mycgr3G31119 Mycgr3T
  
Location: 32744-32906

Mycgr3G31119\_Mycgr3T

Mycgr3G28587 Mycgr3T
  
Location: 33006-33489

Mycgr3G28587\_Mycgr3T

Mycgr3G98959 Mycgr3T
  
Location: 33589-35035

Mycgr3G98959\_Mycgr3T

Mycgr3G35447 Mycgr3T
  
Location: 35135-36443

Mycgr3G35447\_Mycgr3T

Mycgr3G84402 Mycgr3T
  
Location: 36543-37884

Mycgr3G84402\_Mycgr3T

Mycgr3G98961 Mycgr3T
  
Location: 37984-38884

Mycgr3G98961\_Mycgr3T

hypothetical protein
  
Accession: EFY92275
  
Location: 69167-70525
  
 NCBI BlastP on this gene

EFY92275

hypothetical protein
  
Accession: EFY92276
  
Location: 74732-75354
  
 NCBI BlastP on this gene

EFY92276

DOC family protein
  
Accession: EFY92277
  
Location: 75571-75912
  
 NCBI BlastP on this gene

EFY92277

hypothetical protein
  
Accession: EFY92278
  
Location: 80692-82985
  
 NCBI BlastP on this gene

EFY92278

DUF602 domain-containing protein
  
Accession: EFY92279
  
Location: 86568-87377
  
 NCBI BlastP on this gene

EFY92279

TOR kinase
  
Accession: EFY92280
  
Location: 88131-95605
  
  
**BlastP hit with Mycgr3G67795\_Mycgr3T**
  
Percentage identity: 59 %
  
BlastP bit score: 2969
  
Sequence coverage: 101 %
  
E-value: 0.0
  
  
 NCBI BlastP on this gene

EFY92280

ascus development protein 3
  
Accession: EFY92281
  
Location: 95971-97986
  
 NCBI BlastP on this gene

EFY92281

glucosidase 2 subunit beta precursor
  
Accession: EFY92282
  
Location: 98498-100398
  
 NCBI BlastP on this gene

EFY92282

hypothetical protein
  
Accession: EFY92283
  
Location: 100973-102124
  
 NCBI BlastP on this gene

EFY92283

hypothetical protein
  
Accession: EFY92284
  
Location: 103112-105667
  
 NCBI BlastP on this gene

EFY92284

hypothetical protein
  
Accession: EFY92285
  
Location: 108537-110176
  
 NCBI BlastP on this gene

EFY92285

ubiquitin fusion degradation protein (Ufd1), putative
  
Accession: EFY92286
  
Location: 111953-114226
  
 NCBI BlastP on this gene

EFY92286

Query: Architecture Search FASTA input

HF679028 : Fusarium fujikuroi IMI 58289 draft genome, chromosome FFUJ\_chr06.    Total score: 1.0     Cumulative Blast bit score: 2967

Hit cluster cross-links:

Mycgr3G67791 Mycgr3T
  
Location: 0-1542

Mycgr3G67791\_Mycgr3T

Mycgr3G90406 Mycgr3T
  
Location: 1642-3973

Mycgr3G90406\_Mycgr3T

Mycgr3G67785 Mycgr3T
  
Location: 4073-7865

Mycgr3G67785\_Mycgr3T

Mycgr3G67795 Mycgr3T
  
Location: 7965-15249

Mycgr3G67795\_Mycgr3T

Mycgr3G67775 Mycgr3T
  
Location: 15349-16237

Mycgr3G67775\_Mycgr3T

Mycgr3G90404 Mycgr3T
  
Location: 16337-17246

Mycgr3G90404\_Mycgr3T

Mycgr3G36951 Mycgr3T
  
Location: 17346-30891

Mycgr3G36951\_Mycgr3T

Mycgr3G103034 Mycgr3
  
Location: 30991-32644

Mycgr3G103034\_Mycgr3

Mycgr3G31119 Mycgr3T
  
Location: 32744-32906

Mycgr3G31119\_Mycgr3T

Mycgr3G28587 Mycgr3T
  
Location: 33006-33489

Mycgr3G28587\_Mycgr3T

Mycgr3G98959 Mycgr3T
  
Location: 33589-35035

Mycgr3G98959\_Mycgr3T

Mycgr3G35447 Mycgr3T
  
Location: 35135-36443

Mycgr3G35447\_Mycgr3T

Mycgr3G84402 Mycgr3T
  
Location: 36543-37884

Mycgr3G84402\_Mycgr3T

Mycgr3G98961 Mycgr3T
  
Location: 37984-38884

Mycgr3G98961\_Mycgr3T

related to heterokaryon incompatibility protein (het-6OR allele)
  
Accession: CCT70550
  
Location: 3811000-3814090
  
 NCBI BlastP on this gene

FFUJ\_06530

uncharacterized protein
  
Accession: CCT70551
  
Location: 3814215-3815876
  
 NCBI BlastP on this gene

FFUJ\_06531

uncharacterized protein
  
Accession: CCT70552
  
Location: 3816636-3818742
  
 NCBI BlastP on this gene

FFUJ\_06532

related to glutamate carboxypeptidase II
  
Accession: CCT70553
  
Location: 3819588-3822051
  
 NCBI BlastP on this gene

FFUJ\_06533

uncharacterized protein
  
Accession: CCT70554
  
Location: 3822750-3824676
  
 NCBI BlastP on this gene

FFUJ\_06534

uncharacterized protein
  
Accession: CCT70555
  
Location: 3825712-3827637
  
 NCBI BlastP on this gene

FFUJ\_06535

uncharacterized protein
  
Accession: CCT70556
  
Location: 3828315-3829094
  
 NCBI BlastP on this gene

FFUJ\_06536

related to 1-phosphatidylinositol 3-kinase
  
Accession: CCT70557
  
Location: 3829917-3837349
  
  
**BlastP hit with Mycgr3G67795\_Mycgr3T**
  
Percentage identity: 60 %
  
BlastP bit score: 2967
  
Sequence coverage: 101 %
  
E-value: 0.0
  
  
 NCBI BlastP on this gene

FFUJ\_06537

Query: Architecture Search FASTA input

JH226130 : Exophiala dermatitidis NIH/UT8656 unplaced genomic scaffold supercont1.1    Total score: 1.0     Cumulative Blast bit score: 2944

Hit cluster cross-links:

Mycgr3G67791 Mycgr3T
  
Location: 0-1542

Mycgr3G67791\_Mycgr3T

Mycgr3G90406 Mycgr3T
  
Location: 1642-3973

Mycgr3G90406\_Mycgr3T

Mycgr3G67785 Mycgr3T
  
Location: 4073-7865

Mycgr3G67785\_Mycgr3T

Mycgr3G67795 Mycgr3T
  
Location: 7965-15249

Mycgr3G67795\_Mycgr3T

Mycgr3G67775 Mycgr3T
  
Location: 15349-16237

Mycgr3G67775\_Mycgr3T

Mycgr3G90404 Mycgr3T
  
Location: 16337-17246

Mycgr3G90404\_Mycgr3T

Mycgr3G36951 Mycgr3T
  
Location: 17346-30891

Mycgr3G36951\_Mycgr3T

Mycgr3G103034 Mycgr3
  
Location: 30991-32644

Mycgr3G103034\_Mycgr3

Mycgr3G31119 Mycgr3T
  
Location: 32744-32906

Mycgr3G31119\_Mycgr3T

Mycgr3G28587 Mycgr3T
  
Location: 33006-33489

Mycgr3G28587\_Mycgr3T

Mycgr3G98959 Mycgr3T
  
Location: 33589-35035

Mycgr3G98959\_Mycgr3T

Mycgr3G35447 Mycgr3T
  
Location: 35135-36443

Mycgr3G35447\_Mycgr3T

Mycgr3G84402 Mycgr3T
  
Location: 36543-37884

Mycgr3G84402\_Mycgr3T

Mycgr3G98961 Mycgr3T
  
Location: 37984-38884

Mycgr3G98961\_Mycgr3T

hypothetical protein
  
Accession: EHY52968
  
Location: 3275321-3277908
  
 NCBI BlastP on this gene

EHY52968

hypothetical protein
  
Accession: EHY52969
  
Location: 3278708-3279868
  
 NCBI BlastP on this gene

EHY52969

hypothetical protein
  
Accession: EHY52970
  
Location: 3280489-3283019
  
 NCBI BlastP on this gene

EHY52970

hypothetical protein
  
Accession: EHY52971
  
Location: 3284361-3284744
  
 NCBI BlastP on this gene

EHY52971

ADP-ribosylglycohydrolase
  
Accession: EHY52972
  
Location: 3285271-3286308
  
 NCBI BlastP on this gene

EHY52972

hypothetical protein
  
Accession: EHY52973
  
Location: 3288749-3289630
  
 NCBI BlastP on this gene

EHY52973

DNA polymerase sigma subunit
  
Accession: EHY52974
  
Location: 3290507-3292709
  
 NCBI BlastP on this gene

EHY52974

FKBP12-rapamycin complex-associated protein
  
Accession: EHY52975
  
Location: 3294082-3301598
  
  
**BlastP hit with Mycgr3G67795\_Mycgr3T**
  
Percentage identity: 60 %
  
BlastP bit score: 2944
  
Sequence coverage: 100 %
  
E-value: 0.0
  
  
 NCBI BlastP on this gene

EHY52975

phospho-2-dehydro-3-deoxyheptonate aldolase, tyrosine-inhibited
  
Accession: EHY52976
  
Location: 3302250-3303386
  
 NCBI BlastP on this gene

EHY52976

hypothetical protein
  
Accession: EHY52977
  
Location: 3304313-3305203
  
 NCBI BlastP on this gene

EHY52977

hypothetical protein
  
Accession: EHY52978
  
Location: 3305490-3306482
  
 NCBI BlastP on this gene

EHY52978

hypothetical protein
  
Accession: EHY52979
  
Location: 3309672-3310484
  
 NCBI BlastP on this gene

EHY52979

hypothetical protein
  
Accession: EHY52980
  
Location: 3310878-3312380
  
 NCBI BlastP on this gene

EHY52980

hypothetical protein
  
Accession: EHY52981
  
Location: 3312853-3314227
  
 NCBI BlastP on this gene

EHY52981

hypothetical protein
  
Accession: EHY52982
  
Location: 3315060-3316210
  
 NCBI BlastP on this gene

EHY52982

hypothetical protein
  
Accession: EHY52983
  
Location: 3317185-3317503
  
 NCBI BlastP on this gene

EHY52983

glucan endo-1,3-beta-D-glucosidase
  
Accession: EHY52984
  
Location: 3319731-3321320
  
 NCBI BlastP on this gene

EHY52984

Query: Architecture Search FASTA input

KB020785 : Colletotrichum gloeosporioides Nara gc5 unplaced genomic scaffold scaffold403    Total score: 1.0     Cumulative Blast bit score: 2925

Hit cluster cross-links:

Mycgr3G67791 Mycgr3T
  
Location: 0-1542

Mycgr3G67791\_Mycgr3T

Mycgr3G90406 Mycgr3T
  
Location: 1642-3973

Mycgr3G90406\_Mycgr3T

Mycgr3G67785 Mycgr3T
  
Location: 4073-7865

Mycgr3G67785\_Mycgr3T

Mycgr3G67795 Mycgr3T
  
Location: 7965-15249

Mycgr3G67795\_Mycgr3T

Mycgr3G67775 Mycgr3T
  
Location: 15349-16237

Mycgr3G67775\_Mycgr3T

Mycgr3G90404 Mycgr3T
  
Location: 16337-17246

Mycgr3G90404\_Mycgr3T

Mycgr3G36951 Mycgr3T
  
Location: 17346-30891

Mycgr3G36951\_Mycgr3T

Mycgr3G103034 Mycgr3
  
Location: 30991-32644

Mycgr3G103034\_Mycgr3

Mycgr3G31119 Mycgr3T
  
Location: 32744-32906

Mycgr3G31119\_Mycgr3T

Mycgr3G28587 Mycgr3T
  
Location: 33006-33489

Mycgr3G28587\_Mycgr3T

Mycgr3G98959 Mycgr3T
  
Location: 33589-35035

Mycgr3G98959\_Mycgr3T

Mycgr3G35447 Mycgr3T
  
Location: 35135-36443

Mycgr3G35447\_Mycgr3T

Mycgr3G84402 Mycgr3T
  
Location: 36543-37884

Mycgr3G84402\_Mycgr3T

Mycgr3G98961 Mycgr3T
  
Location: 37984-38884

Mycgr3G98961\_Mycgr3T

hypothetical protein
  
Accession: ELA30756
  
Location: 140823-141864
  
 NCBI BlastP on this gene

ELA30756

hypothetical protein
  
Accession: ELA30755
  
Location: 140029-140456
  
 NCBI BlastP on this gene

ELA30755

hypothetical protein
  
Accession: ELA30754
  
Location: 138730-139747
  
 NCBI BlastP on this gene

ELA30754

oxidoreductase
  
Accession: ELA30753
  
Location: 135325-136617
  
 NCBI BlastP on this gene

ELA30753

hypothetical protein
  
Accession: ELA30752
  
Location: 134041-134373
  
 NCBI BlastP on this gene

ELA30752

GrpB domain protein
  
Accession: ELA30751
  
Location: 131422-132042
  
 NCBI BlastP on this gene

ELA30751

hypothetical protein
  
Accession: ELA30750
  
Location: 130127-131181
  
 NCBI BlastP on this gene

ELA30750

hypothetical protein
  
Accession: ELA30749
  
Location: 128594-129703
  
 NCBI BlastP on this gene

ELA30749

glucosidase 2 subunit beta precursor
  
Accession: ELA30748
  
Location: 126122-128005
  
 NCBI BlastP on this gene

ELA30748

duf602 domain-containing protein
  
Accession: ELA30747
  
Location: 124920-125759
  
 NCBI BlastP on this gene

ELA30747

phosphatidylinositol 3-kinase tor2
  
Accession: ELA30746
  
Location: 116523-123904
  
  
**BlastP hit with Mycgr3G67795\_Mycgr3T**
  
Percentage identity: 59 %
  
BlastP bit score: 2925
  
Sequence coverage: 99 %
  
E-value: 0.0
  
  
 NCBI BlastP on this gene

ELA30746

C6 zinc finger domain protein
  
Accession: ELA30745
  
Location: 112486-114592
  
 NCBI BlastP on this gene

ELA30745

hypothetical protein
  
Accession: ELA30744
  
Location: 109158-110980
  
 NCBI BlastP on this gene

ELA30744

MFS monosaccharide transporter
  
Accession: ELA30743
  
Location: 106371-108903
  
 NCBI BlastP on this gene

ELA30743

ankyrin repeat protein
  
Accession: ELA30742
  
Location: 102806-104497
  
 NCBI BlastP on this gene

ELA30742

hypothetical protein
  
Accession: ELA30741
  
Location: 101993-102421
  
 NCBI BlastP on this gene

ELA30741

duf895 domain membrane protein
  
Accession: ELA30740
  
Location: 99466-100952
  
 NCBI BlastP on this gene

ELA30740

Query: Architecture Search FASTA input

DS985228 : Verticillium albo-atrum VaMs.102 supercont1.15 genomic scaffold    Total score: 1.0     Cumulative Blast bit score: 2910

Hit cluster cross-links:

Mycgr3G67791 Mycgr3T
  
Location: 0-1542

Mycgr3G67791\_Mycgr3T

Mycgr3G90406 Mycgr3T
  
Location: 1642-3973

Mycgr3G90406\_Mycgr3T

Mycgr3G67785 Mycgr3T
  
Location: 4073-7865

Mycgr3G67785\_Mycgr3T

Mycgr3G67795 Mycgr3T
  
Location: 7965-15249

Mycgr3G67795\_Mycgr3T

Mycgr3G67775 Mycgr3T
  
Location: 15349-16237

Mycgr3G67775\_Mycgr3T

Mycgr3G90404 Mycgr3T
  
Location: 16337-17246

Mycgr3G90404\_Mycgr3T

Mycgr3G36951 Mycgr3T
  
Location: 17346-30891

Mycgr3G36951\_Mycgr3T

Mycgr3G103034 Mycgr3
  
Location: 30991-32644

Mycgr3G103034\_Mycgr3

Mycgr3G31119 Mycgr3T
  
Location: 32744-32906

Mycgr3G31119\_Mycgr3T

Mycgr3G28587 Mycgr3T
  
Location: 33006-33489

Mycgr3G28587\_Mycgr3T

Mycgr3G98959 Mycgr3T
  
Location: 33589-35035

Mycgr3G98959\_Mycgr3T

Mycgr3G35447 Mycgr3T
  
Location: 35135-36443

Mycgr3G35447\_Mycgr3T

Mycgr3G84402 Mycgr3T
  
Location: 36543-37884

Mycgr3G84402\_Mycgr3T

Mycgr3G98961 Mycgr3T
  
Location: 37984-38884

Mycgr3G98961\_Mycgr3T

conserved hypothetical protein
  
Accession: EEY23532
  
Location: 1017126-1018163
  
 NCBI BlastP on this gene

EEY23532

endoribonuclease L-PSP
  
Accession: EEY23533
  
Location: 1018520-1018998
  
 NCBI BlastP on this gene

EEY23533

high-affinity glucose transporter RGT2
  
Accession: EEY23534
  
Location: 1023472-1025294
  
 NCBI BlastP on this gene

EEY23534

oxidoreductase
  
Accession: EEY23535
  
Location: 1025616-1026875
  
 NCBI BlastP on this gene

EEY23535

bacterial leucyl aminopeptidase
  
Accession: EEY23536
  
Location: 1027413-1028668
  
 NCBI BlastP on this gene

EEY23536

conserved hypothetical protein
  
Accession: EEY23537
  
Location: 1029239-1030381
  
 NCBI BlastP on this gene

EEY23537

glucosidase 2 subunit beta
  
Accession: EEY23538
  
Location: 1030703-1032506
  
 NCBI BlastP on this gene

EEY23538

DUF602 domain-containing protein
  
Accession: EEY23539
  
Location: 1032634-1033449
  
 NCBI BlastP on this gene

EEY23539

phosphatidylinositol 3-kinase tor2
  
Accession: EEY23540
  
Location: 1034143-1041643
  
  
**BlastP hit with Mycgr3G67795\_Mycgr3T**
  
Percentage identity: 58 %
  
BlastP bit score: 2910
  
Sequence coverage: 101 %
  
E-value: 0.0
  
  
 NCBI BlastP on this gene

EEY23540

Query: Architecture Search FASTA input

KB731260 : Fusarium oxysporum f. sp. cubense race 1 unplaced genomic scaffold scaffold322    Total score: 1.0     Cumulative Blast bit score: 2907

Hit cluster cross-links:

Mycgr3G67791 Mycgr3T
  
Location: 0-1542

Mycgr3G67791\_Mycgr3T

Mycgr3G90406 Mycgr3T
  
Location: 1642-3973

Mycgr3G90406\_Mycgr3T

Mycgr3G67785 Mycgr3T
  
Location: 4073-7865

Mycgr3G67785\_Mycgr3T

Mycgr3G67795 Mycgr3T
  
Location: 7965-15249

Mycgr3G67795\_Mycgr3T

Mycgr3G67775 Mycgr3T
  
Location: 15349-16237

Mycgr3G67775\_Mycgr3T

Mycgr3G90404 Mycgr3T
  
Location: 16337-17246

Mycgr3G90404\_Mycgr3T

Mycgr3G36951 Mycgr3T
  
Location: 17346-30891

Mycgr3G36951\_Mycgr3T

Mycgr3G103034 Mycgr3
  
Location: 30991-32644

Mycgr3G103034\_Mycgr3

Mycgr3G31119 Mycgr3T
  
Location: 32744-32906

Mycgr3G31119\_Mycgr3T

Mycgr3G28587 Mycgr3T
  
Location: 33006-33489

Mycgr3G28587\_Mycgr3T

Mycgr3G98959 Mycgr3T
  
Location: 33589-35035

Mycgr3G98959\_Mycgr3T

Mycgr3G35447 Mycgr3T
  
Location: 35135-36443

Mycgr3G35447\_Mycgr3T

Mycgr3G84402 Mycgr3T
  
Location: 36543-37884

Mycgr3G84402\_Mycgr3T

Mycgr3G98961 Mycgr3T
  
Location: 37984-38884

Mycgr3G98961\_Mycgr3T

Vacuolar protein sorting-associated protein 70
  
Accession: ENH62246
  
Location: 1582801-1585267
  
 NCBI BlastP on this gene

ENH62246

L-gulonolactone oxidase
  
Accession: ENH62247
  
Location: 1588983-1590641
  
 NCBI BlastP on this gene

ENH62247

Putative mitochondrial chaperone BCS1-B
  
Accession: ENH62248
  
Location: 1590891-1592441
  
 NCBI BlastP on this gene

ENH62248

UPF0214 protein yfeW
  
Accession: ENH62249
  
Location: 1593058-1594985
  
 NCBI BlastP on this gene

ENH62249

Transcriptional regulatory protein moc3
  
Accession: ENH62250
  
Location: 1596042-1597969
  
 NCBI BlastP on this gene

ENH62250

UPF0549 protein C1D4.09c
  
Accession: ENH62251
  
Location: 1598600-1599379
  
 NCBI BlastP on this gene

ENH62251

Phosphatidylinositol 3-kinase tor2
  
Accession: ENH62252
  
Location: 1600209-1607605
  
  
**BlastP hit with Mycgr3G67795\_Mycgr3T**
  
Percentage identity: 59 %
  
BlastP bit score: 2907
  
Sequence coverage: 101 %
  
E-value: 0.0
  
  
 NCBI BlastP on this gene

ENH62252

Query: Architecture Search FASTA input

GL891307 : Neurospora tetrasperma FGSC 2508 unplaced genomic scaffold NEUTE1scaffold\_6    Total score: 1.0     Cumulative Blast bit score: 2890

Hit cluster cross-links:

Mycgr3G67791 Mycgr3T
  
Location: 0-1542

Mycgr3G67791\_Mycgr3T

Mycgr3G90406 Mycgr3T
  
Location: 1642-3973

Mycgr3G90406\_Mycgr3T

Mycgr3G67785 Mycgr3T
  
Location: 4073-7865

Mycgr3G67785\_Mycgr3T

Mycgr3G67795 Mycgr3T
  
Location: 7965-15249

Mycgr3G67795\_Mycgr3T

Mycgr3G67775 Mycgr3T
  
Location: 15349-16237

Mycgr3G67775\_Mycgr3T

Mycgr3G90404 Mycgr3T
  
Location: 16337-17246

Mycgr3G90404\_Mycgr3T

Mycgr3G36951 Mycgr3T
  
Location: 17346-30891

Mycgr3G36951\_Mycgr3T

Mycgr3G103034 Mycgr3
  
Location: 30991-32644

Mycgr3G103034\_Mycgr3

Mycgr3G31119 Mycgr3T
  
Location: 32744-32906

Mycgr3G31119\_Mycgr3T

Mycgr3G28587 Mycgr3T
  
Location: 33006-33489

Mycgr3G28587\_Mycgr3T

Mycgr3G98959 Mycgr3T
  
Location: 33589-35035

Mycgr3G98959\_Mycgr3T

Mycgr3G35447 Mycgr3T
  
Location: 35135-36443

Mycgr3G35447\_Mycgr3T

Mycgr3G84402 Mycgr3T
  
Location: 36543-37884

Mycgr3G84402\_Mycgr3T

Mycgr3G98961 Mycgr3T
  
Location: 37984-38884

Mycgr3G98961\_Mycgr3T

hypothetical protein
  
Accession: EGO54246
  
Location: 1305800-1307638
  
 NCBI BlastP on this gene

EGO54246

hypothetical protein
  
Accession: EGO54245
  
Location: 1301893-1305187
  
 NCBI BlastP on this gene

EGO54245

hypothetical protein
  
Accession: EGO54244
  
Location: 1301293-1301448
  
 NCBI BlastP on this gene

EGO54244

hypothetical protein
  
Accession: EGO54243
  
Location: 1297350-1300073
  
 NCBI BlastP on this gene

EGO54243

hypothetical protein
  
Accession: EGO54242
  
Location: 1295884-1297071
  
 NCBI BlastP on this gene

EGO54242

hypothetical protein
  
Accession: EGO54241
  
Location: 1293236-1295233
  
 NCBI BlastP on this gene

EGO54241

phosphatidylinositol 3-kinase tor2
  
Accession: EGO54240
  
Location: 1282635-1290514
  
  
**BlastP hit with Mycgr3G67795\_Mycgr3T**
  
Percentage identity: 59 %
  
BlastP bit score: 2890
  
Sequence coverage: 101 %
  
E-value: 0.0
  
  
 NCBI BlastP on this gene

EGO54240

hypothetical protein
  
Accession: EGO54239
  
Location: 1280950-1282213
  
 NCBI BlastP on this gene

EGO54239

hypothetical protein
  
Accession: EGO54238
  
Location: 1277587-1278941
  
 NCBI BlastP on this gene

EGO54238

hypothetical protein
  
Accession: EGO54237
  
Location: 1270261-1275212
  
 NCBI BlastP on this gene

EGO54237

hypothetical protein
  
Accession: EGO54236
  
Location: 1267913-1268969
  
 NCBI BlastP on this gene

EGO54236

hypothetical protein
  
Accession: EGO54235
  
Location: 1265451-1266683
  
 NCBI BlastP on this gene

EGO54235

hypothetical protein
  
Accession: EGO54234
  
Location: 1263792-1264901
  
 NCBI BlastP on this gene

EGO54234

Query: Architecture Search FASTA input

GL988043 : Chaetomium thermophilum var. thermophilum DSM 1495 unplaced genomic scaffold scf7180000...    Total score: 1.0     Cumulative Blast bit score: 2886

Hit cluster cross-links:

Mycgr3G67791 Mycgr3T
  
Location: 0-1542

Mycgr3G67791\_Mycgr3T

Mycgr3G90406 Mycgr3T
  
Location: 1642-3973

Mycgr3G90406\_Mycgr3T

Mycgr3G67785 Mycgr3T
  
Location: 4073-7865

Mycgr3G67785\_Mycgr3T

Mycgr3G67795 Mycgr3T
  
Location: 7965-15249

Mycgr3G67795\_Mycgr3T

Mycgr3G67775 Mycgr3T
  
Location: 15349-16237

Mycgr3G67775\_Mycgr3T

Mycgr3G90404 Mycgr3T
  
Location: 16337-17246

Mycgr3G90404\_Mycgr3T

Mycgr3G36951 Mycgr3T
  
Location: 17346-30891

Mycgr3G36951\_Mycgr3T

Mycgr3G103034 Mycgr3
  
Location: 30991-32644

Mycgr3G103034\_Mycgr3

Mycgr3G31119 Mycgr3T
  
Location: 32744-32906

Mycgr3G31119\_Mycgr3T

Mycgr3G28587 Mycgr3T
  
Location: 33006-33489

Mycgr3G28587\_Mycgr3T

Mycgr3G98959 Mycgr3T
  
Location: 33589-35035

Mycgr3G98959\_Mycgr3T

Mycgr3G35447 Mycgr3T
  
Location: 35135-36443

Mycgr3G35447\_Mycgr3T

Mycgr3G84402 Mycgr3T
  
Location: 36543-37884

Mycgr3G84402\_Mycgr3T

Mycgr3G98961 Mycgr3T
  
Location: 37984-38884

Mycgr3G98961\_Mycgr3T

phosphatidylinositol 3-kinase-like protein
  
Accession: EGS20131
  
Location: 2367045-2374826
  
  
**BlastP hit with Mycgr3G67795\_Mycgr3T**
  
Percentage identity: 58 %
  
BlastP bit score: 2886
  
Sequence coverage: 101 %
  
E-value: 0.0
  
  
 NCBI BlastP on this gene

EGS20131

Query: Architecture Search FASTA input

GL891269 : Neurospora tetrasperma FGSC 2509 unplaced genomic scaffold NEUTE2scaffold\_7    Total score: 1.0     Cumulative Blast bit score: 2885

Hit cluster cross-links:

Mycgr3G67791 Mycgr3T
  
Location: 0-1542

Mycgr3G67791\_Mycgr3T

Mycgr3G90406 Mycgr3T
  
Location: 1642-3973

Mycgr3G90406\_Mycgr3T

Mycgr3G67785 Mycgr3T
  
Location: 4073-7865

Mycgr3G67785\_Mycgr3T

Mycgr3G67795 Mycgr3T
  
Location: 7965-15249

Mycgr3G67795\_Mycgr3T

Mycgr3G67775 Mycgr3T
  
Location: 15349-16237

Mycgr3G67775\_Mycgr3T

Mycgr3G90404 Mycgr3T
  
Location: 16337-17246

Mycgr3G90404\_Mycgr3T

Mycgr3G36951 Mycgr3T
  
Location: 17346-30891

Mycgr3G36951\_Mycgr3T

Mycgr3G103034 Mycgr3
  
Location: 30991-32644

Mycgr3G103034\_Mycgr3

Mycgr3G31119 Mycgr3T
  
Location: 32744-32906

Mycgr3G31119\_Mycgr3T

Mycgr3G28587 Mycgr3T
  
Location: 33006-33489

Mycgr3G28587\_Mycgr3T

Mycgr3G98959 Mycgr3T
  
Location: 33589-35035

Mycgr3G98959\_Mycgr3T

Mycgr3G35447 Mycgr3T
  
Location: 35135-36443

Mycgr3G35447\_Mycgr3T

Mycgr3G84402 Mycgr3T
  
Location: 36543-37884

Mycgr3G84402\_Mycgr3T

Mycgr3G98961 Mycgr3T
  
Location: 37984-38884

Mycgr3G98961\_Mycgr3T

hypothetical protein
  
Accession: EGZ68320
  
Location: 2586194-2588032
  
 NCBI BlastP on this gene

EGZ68320

hypothetical protein
  
Accession: EGZ68321
  
Location: 2588658-2591940
  
 NCBI BlastP on this gene

EGZ68321

hypothetical protein
  
Accession: EGZ68322
  
Location: 2592385-2592540
  
 NCBI BlastP on this gene

EGZ68322

hypothetical protein
  
Accession: EGZ68323
  
Location: 2594054-2596481
  
 NCBI BlastP on this gene

EGZ68323

hypothetical protein
  
Accession: EGZ68324
  
Location: 2596760-2597947
  
 NCBI BlastP on this gene

EGZ68324

PRKCSH-domain-containing protein
  
Accession: EGZ68325
  
Location: 2598598-2600595
  
 NCBI BlastP on this gene

EGZ68325

phosphatidylinositol 3-kinase tor2
  
Accession: EGZ68326
  
Location: 2603317-2611196
  
  
**BlastP hit with Mycgr3G67795\_Mycgr3T**
  
Percentage identity: 59 %
  
BlastP bit score: 2885
  
Sequence coverage: 101 %
  
E-value: 0.0
  
  
 NCBI BlastP on this gene

EGZ68326

hypothetical protein
  
Accession: EGZ68327
  
Location: 2611618-2612881
  
 NCBI BlastP on this gene

EGZ68327

hypothetical protein
  
Accession: EGZ68328
  
Location: 2614877-2616244
  
 NCBI BlastP on this gene

EGZ68328

hypothetical protein
  
Accession: EGZ68329
  
Location: 2618617-2623568
  
 NCBI BlastP on this gene

EGZ68329

hypothetical protein
  
Accession: EGZ68330
  
Location: 2624860-2625916
  
 NCBI BlastP on this gene

EGZ68330

hypothetical protein
  
Accession: EGZ68331
  
Location: 2626374-2626571
  
 NCBI BlastP on this gene

EGZ68331

hypothetical protein
  
Accession: EGZ68332
  
Location: 2627146-2628378
  
 NCBI BlastP on this gene

EGZ68332

NAD(P)-binding protein
  
Accession: EGZ68333
  
Location: 2628928-2630035
  
 NCBI BlastP on this gene

EGZ68333

Query: Architecture Search FASTA input

JH126401 : Cordyceps militaris CM01 unplaced genomic scaffold CCM\_S00003    Total score: 1.0     Cumulative Blast bit score: 2871

Hit cluster cross-links:

Mycgr3G67791 Mycgr3T
  
Location: 0-1542

Mycgr3G67791\_Mycgr3T

Mycgr3G90406 Mycgr3T
  
Location: 1642-3973

Mycgr3G90406\_Mycgr3T

Mycgr3G67785 Mycgr3T
  
Location: 4073-7865

Mycgr3G67785\_Mycgr3T

Mycgr3G67795 Mycgr3T
  
Location: 7965-15249

Mycgr3G67795\_Mycgr3T

Mycgr3G67775 Mycgr3T
  
Location: 15349-16237

Mycgr3G67775\_Mycgr3T

Mycgr3G90404 Mycgr3T
  
Location: 16337-17246

Mycgr3G90404\_Mycgr3T

Mycgr3G36951 Mycgr3T
  
Location: 17346-30891

Mycgr3G36951\_Mycgr3T

Mycgr3G103034 Mycgr3
  
Location: 30991-32644

Mycgr3G103034\_Mycgr3

Mycgr3G31119 Mycgr3T
  
Location: 32744-32906

Mycgr3G31119\_Mycgr3T

Mycgr3G28587 Mycgr3T
  
Location: 33006-33489

Mycgr3G28587\_Mycgr3T

Mycgr3G98959 Mycgr3T
  
Location: 33589-35035

Mycgr3G98959\_Mycgr3T

Mycgr3G35447 Mycgr3T
  
Location: 35135-36443

Mycgr3G35447\_Mycgr3T

Mycgr3G84402 Mycgr3T
  
Location: 36543-37884

Mycgr3G84402\_Mycgr3T

Mycgr3G98961 Mycgr3T
  
Location: 37984-38884

Mycgr3G98961\_Mycgr3T

phosphatidylinositol 3-kinase tor2
  
Accession: EGX93526
  
Location: 4074259-4081703
  
  
**BlastP hit with Mycgr3G67795\_Mycgr3T**
  
Percentage identity: 58 %
  
BlastP bit score: 2871
  
Sequence coverage: 100 %
  
E-value: 0.0
  
  
 NCBI BlastP on this gene

EGX93526

MFS transporter, putative
  
Accession: EGX93525
  
Location: 4071305-4073166
  
 NCBI BlastP on this gene

EGX93525

short-chain dehydrogenase
  
Accession: EGX93524
  
Location: 4070458-4071261
  
 NCBI BlastP on this gene

EGX93524

hypothetical protein
  
Accession: EGX93523
  
Location: 4068231-4069916
  
 NCBI BlastP on this gene

EGX93523

sugar transporter, putative
  
Accession: EGX93522
  
Location: 4062780-4064479
  
 NCBI BlastP on this gene

EGX93522

amino acid transporter, putative
  
Accession: EGX93521
  
Location: 4058118-4060856
  
 NCBI BlastP on this gene

EGX93521

mitochondrial ribosomal protein S16
  
Accession: EGX93520
  
Location: 4057161-4057590
  
 NCBI BlastP on this gene

EGX93520

Query: Architecture Search FASTA input

CABT02000010 : Sordaria macrospora k-hell    Total score: 1.0     Cumulative Blast bit score: 2867

Hit cluster cross-links:

Mycgr3G67791 Mycgr3T
  
Location: 0-1542

Mycgr3G67791\_Mycgr3T

Mycgr3G90406 Mycgr3T
  
Location: 1642-3973

Mycgr3G90406\_Mycgr3T

Mycgr3G67785 Mycgr3T
  
Location: 4073-7865

Mycgr3G67785\_Mycgr3T

Mycgr3G67795 Mycgr3T
  
Location: 7965-15249

Mycgr3G67795\_Mycgr3T

Mycgr3G67775 Mycgr3T
  
Location: 15349-16237

Mycgr3G67775\_Mycgr3T

Mycgr3G90404 Mycgr3T
  
Location: 16337-17246

Mycgr3G90404\_Mycgr3T

Mycgr3G36951 Mycgr3T
  
Location: 17346-30891

Mycgr3G36951\_Mycgr3T

Mycgr3G103034 Mycgr3
  
Location: 30991-32644

Mycgr3G103034\_Mycgr3

Mycgr3G31119 Mycgr3T
  
Location: 32744-32906

Mycgr3G31119\_Mycgr3T

Mycgr3G28587 Mycgr3T
  
Location: 33006-33489

Mycgr3G28587\_Mycgr3T

Mycgr3G98959 Mycgr3T
  
Location: 33589-35035

Mycgr3G98959\_Mycgr3T

Mycgr3G35447 Mycgr3T
  
Location: 35135-36443

Mycgr3G35447\_Mycgr3T

Mycgr3G84402 Mycgr3T
  
Location: 36543-37884

Mycgr3G84402\_Mycgr3T

Mycgr3G98961 Mycgr3T
  
Location: 37984-38884

Mycgr3G98961\_Mycgr3T

not annotated
  
Accession: CCC09760
  
Location: 257888-259750
  
 NCBI BlastP on this gene

CCC09760

not annotated
  
Accession: CCC09761
  
Location: 260556-263940
  
 NCBI BlastP on this gene

CCC09761

not annotated
  
Accession: CCC09762
  
Location: 267730-270139
  
 NCBI BlastP on this gene

CCC09762

not annotated
  
Accession: CCC09763
  
Location: 270438-271664
  
 NCBI BlastP on this gene

CCC09763

not annotated
  
Accession: CCC09764
  
Location: 272270-274265
  
 NCBI BlastP on this gene

CCC09764

not annotated
  
Accession: CCC09765
  
Location: 274530-275519
  
 NCBI BlastP on this gene

CCC09765

not annotated
  
Accession: CCC09766
  
Location: 277345-285153
  
  
**BlastP hit with Mycgr3G67795\_Mycgr3T**
  
Percentage identity: 58 %
  
BlastP bit score: 2867
  
Sequence coverage: 103 %
  
E-value: 0.0
  
  
 NCBI BlastP on this gene

CCC09766

not annotated
  
Accession: CCC09767
  
Location: 286034-286351
  
 NCBI BlastP on this gene

CCC09767

not annotated
  
Accession: CCC09768
  
Location: 286590-287373
  
 NCBI BlastP on this gene

CCC09768

not annotated
  
Accession: CCC09769
  
Location: 290160-291519
  
 NCBI BlastP on this gene

CCC09769

not annotated
  
Accession: CCC09770
  
Location: 294709-297433
  
 NCBI BlastP on this gene

CCC09770

not annotated
  
Accession: CCC09771
  
Location: 297940-299325
  
 NCBI BlastP on this gene

CCC09771

not annotated
  
Accession: CCC09772
  
Location: 300663-301070
  
 NCBI BlastP on this gene

CCC09772

not annotated
  
Accession: CCC09773
  
Location: 301089-301663
  
 NCBI BlastP on this gene

CCC09773

Query: Architecture Search FASTA input

CP003005 : Myceliophthora thermophila ATCC 42464 chromosome 4    Total score: 1.0     Cumulative Blast bit score: 2860

Hit cluster cross-links:

Mycgr3G67791 Mycgr3T
  
Location: 0-1542

Mycgr3G67791\_Mycgr3T

Mycgr3G90406 Mycgr3T
  
Location: 1642-3973

Mycgr3G90406\_Mycgr3T

Mycgr3G67785 Mycgr3T
  
Location: 4073-7865

Mycgr3G67785\_Mycgr3T

Mycgr3G67795 Mycgr3T
  
Location: 7965-15249

Mycgr3G67795\_Mycgr3T

Mycgr3G67775 Mycgr3T
  
Location: 15349-16237

Mycgr3G67775\_Mycgr3T

Mycgr3G90404 Mycgr3T
  
Location: 16337-17246

Mycgr3G90404\_Mycgr3T

Mycgr3G36951 Mycgr3T
  
Location: 17346-30891

Mycgr3G36951\_Mycgr3T

Mycgr3G103034 Mycgr3
  
Location: 30991-32644

Mycgr3G103034\_Mycgr3

Mycgr3G31119 Mycgr3T
  
Location: 32744-32906

Mycgr3G31119\_Mycgr3T

Mycgr3G28587 Mycgr3T
  
Location: 33006-33489

Mycgr3G28587\_Mycgr3T

Mycgr3G98959 Mycgr3T
  
Location: 33589-35035

Mycgr3G98959\_Mycgr3T

Mycgr3G35447 Mycgr3T
  
Location: 35135-36443

Mycgr3G35447\_Mycgr3T

Mycgr3G84402 Mycgr3T
  
Location: 36543-37884

Mycgr3G84402\_Mycgr3T

Mycgr3G98961 Mycgr3T
  
Location: 37984-38884

Mycgr3G98961\_Mycgr3T

hypothetical protein
  
Accession: AEO59199
  
Location: 3401001-3403023
  
 NCBI BlastP on this gene

MYCTH\_2307276

hypothetical protein
  
Accession: AEO59200
  
Location: 3403622-3404749
  
 NCBI BlastP on this gene

MYCTH\_68944

hypothetical protein
  
Accession: AEO59201
  
Location: 3405142-3405689
  
 NCBI BlastP on this gene

MYCTH\_2307282

hypothetical protein
  
Accession: AEO59202
  
Location: 3405875-3406788
  
 NCBI BlastP on this gene

MYCTH\_2307284

hypothetical protein
  
Accession: AEO59203
  
Location: 3407536-3409188
  
 NCBI BlastP on this gene

MYCTH\_2307285

hypothetical protein
  
Accession: AEO59204
  
Location: 3409613-3411808
  
 NCBI BlastP on this gene

MYCTH\_2119478

hypothetical protein
  
Accession: AEO59205
  
Location: 3412406-3412588
  
 NCBI BlastP on this gene

MYCTH\_2307286

hypothetical protein
  
Accession: AEO59206
  
Location: 3412748-3413932
  
 NCBI BlastP on this gene

MYCTH\_2307287

hypothetical protein
  
Accession: AEO59207
  
Location: 3414496-3416390
  
 NCBI BlastP on this gene

MYCTH\_2307289

hypothetical protein
  
Accession: AEO59208
  
Location: 3416625-3417485
  
 NCBI BlastP on this gene

MYCTH\_2139768

hypothetical protein
  
Accession: AEO59209
  
Location: 3418360-3426313
  
  
**BlastP hit with Mycgr3G67795\_Mycgr3T**
  
Percentage identity: 58 %
  
BlastP bit score: 2860
  
Sequence coverage: 101 %
  
E-value: 0.0
  
  
 NCBI BlastP on this gene

MYCTH\_2307294

Query: Architecture Search FASTA input

CP003009 : Thielavia terrestris NRRL 8126 chromosome 1    Total score: 1.0     Cumulative Blast bit score: 2858

Hit cluster cross-links:

Mycgr3G67791 Mycgr3T
  
Location: 0-1542

Mycgr3G67791\_Mycgr3T

Mycgr3G90406 Mycgr3T
  
Location: 1642-3973

Mycgr3G90406\_Mycgr3T

Mycgr3G67785 Mycgr3T
  
Location: 4073-7865

Mycgr3G67785\_Mycgr3T

Mycgr3G67795 Mycgr3T
  
Location: 7965-15249

Mycgr3G67795\_Mycgr3T

Mycgr3G67775 Mycgr3T
  
Location: 15349-16237

Mycgr3G67775\_Mycgr3T

Mycgr3G90404 Mycgr3T
  
Location: 16337-17246

Mycgr3G90404\_Mycgr3T

Mycgr3G36951 Mycgr3T
  
Location: 17346-30891

Mycgr3G36951\_Mycgr3T

Mycgr3G103034 Mycgr3
  
Location: 30991-32644

Mycgr3G103034\_Mycgr3

Mycgr3G31119 Mycgr3T
  
Location: 32744-32906

Mycgr3G31119\_Mycgr3T

Mycgr3G28587 Mycgr3T
  
Location: 33006-33489

Mycgr3G28587\_Mycgr3T

Mycgr3G98959 Mycgr3T
  
Location: 33589-35035

Mycgr3G98959\_Mycgr3T

Mycgr3G35447 Mycgr3T
  
Location: 35135-36443

Mycgr3G35447\_Mycgr3T

Mycgr3G84402 Mycgr3T
  
Location: 36543-37884

Mycgr3G84402\_Mycgr3T

Mycgr3G98961 Mycgr3T
  
Location: 37984-38884

Mycgr3G98961\_Mycgr3T

hypothetical protein
  
Accession: AEO64489
  
Location: 8783335-8785351
  
 NCBI BlastP on this gene

THITE\_2110665

hypothetical protein
  
Accession: AEO64490
  
Location: 8785907-8787049
  
 NCBI BlastP on this gene

THITE\_74570

hypothetical protein
  
Accession: AEO64491
  
Location: 8787722-8789079
  
 NCBI BlastP on this gene

THITE\_2142374

hypothetical protein
  
Accession: AEO64492
  
Location: 8789639-8791252
  
 NCBI BlastP on this gene

THITE\_2110674

hypothetical protein
  
Accession: AEO64493
  
Location: 8791617-8792579
  
 NCBI BlastP on this gene

THITE\_2110676

hypothetical protein
  
Accession: AEO64494
  
Location: 8793245-8793454
  
 NCBI BlastP on this gene

THITE\_2110677

hypothetical protein
  
Accession: AEO64495
  
Location: 8794587-8794820
  
 NCBI BlastP on this gene

THITE\_2169703

hypothetical protein
  
Accession: AEO64496
  
Location: 8795259-8796452
  
 NCBI BlastP on this gene

THITE\_2110679

hypothetical protein
  
Accession: AEO64497
  
Location: 8796807-8798705
  
 NCBI BlastP on this gene

THITE\_2110681

hypothetical protein
  
Accession: AEO64498
  
Location: 8798971-8799846
  
 NCBI BlastP on this gene

THITE\_2110682

hypothetical protein
  
Accession: AEO64499
  
Location: 8800947-8809066
  
  
**BlastP hit with Mycgr3G67795\_Mycgr3T**
  
Percentage identity: 59 %
  
BlastP bit score: 2858
  
Sequence coverage: 100 %
  
E-value: 0.0
  
  
 NCBI BlastP on this gene

THITE\_2110683

Query: Architecture Search FASTA input

CM001231 : Magnaporthe oryzae 70-15 chromosome 1    Total score: 1.0     Cumulative Blast bit score: 2791

Hit cluster cross-links:

Mycgr3G67791 Mycgr3T
  
Location: 0-1542

Mycgr3G67791\_Mycgr3T

Mycgr3G90406 Mycgr3T
  
Location: 1642-3973

Mycgr3G90406\_Mycgr3T

Mycgr3G67785 Mycgr3T
  
Location: 4073-7865

Mycgr3G67785\_Mycgr3T

Mycgr3G67795 Mycgr3T
  
Location: 7965-15249

Mycgr3G67795\_Mycgr3T

Mycgr3G67775 Mycgr3T
  
Location: 15349-16237

Mycgr3G67775\_Mycgr3T

Mycgr3G90404 Mycgr3T
  
Location: 16337-17246

Mycgr3G90404\_Mycgr3T

Mycgr3G36951 Mycgr3T
  
Location: 17346-30891

Mycgr3G36951\_Mycgr3T

Mycgr3G103034 Mycgr3
  
Location: 30991-32644

Mycgr3G103034\_Mycgr3

Mycgr3G31119 Mycgr3T
  
Location: 32744-32906

Mycgr3G31119\_Mycgr3T

Mycgr3G28587 Mycgr3T
  
Location: 33006-33489

Mycgr3G28587\_Mycgr3T

Mycgr3G98959 Mycgr3T
  
Location: 33589-35035

Mycgr3G98959\_Mycgr3T

Mycgr3G35447 Mycgr3T
  
Location: 35135-36443

Mycgr3G35447\_Mycgr3T

Mycgr3G84402 Mycgr3T
  
Location: 36543-37884

Mycgr3G84402\_Mycgr3T

Mycgr3G98961 Mycgr3T
  
Location: 37984-38884

Mycgr3G98961\_Mycgr3T

beta-glucosidase 1
  
Accession: EHA57868
  
Location: 5803841-5806249
  
 NCBI BlastP on this gene

EHA57868

quinate permease
  
Accession: EHA57869
  
Location: 5806949-5808856
  
 NCBI BlastP on this gene

EHA57869

hypothetical protein
  
Accession: EHA57870
  
Location: 5811254-5812363
  
 NCBI BlastP on this gene

EHA57870

hypothetical protein
  
Accession: EHA57871
  
Location: 5812898-5814782
  
 NCBI BlastP on this gene

EHA57871

hypothetical protein
  
Accession: EHA57872
  
Location: 5814996-5815808
  
 NCBI BlastP on this gene

EHA57872

phosphatidylinositol 3-kinase tor2
  
Accession: EHA57873
  
Location: 5816682-5824299
  
  
**BlastP hit with Mycgr3G67795\_Mycgr3T**
  
Percentage identity: 57 %
  
BlastP bit score: 2791
  
Sequence coverage: 100 %
  
E-value: 0.0
  
  
 NCBI BlastP on this gene

EHA57873

Query: Architecture Search FASTA input

GL385396 : Gaeumannomyces graminis var. tritici R3-111a-1 unplaced genomic scaffold supercont2.2    Total score: 1.0     Cumulative Blast bit score: 2783

Hit cluster cross-links:

Mycgr3G67791 Mycgr3T
  
Location: 0-1542

Mycgr3G67791\_Mycgr3T

Mycgr3G90406 Mycgr3T
  
Location: 1642-3973

Mycgr3G90406\_Mycgr3T

Mycgr3G67785 Mycgr3T
  
Location: 4073-7865

Mycgr3G67785\_Mycgr3T

Mycgr3G67795 Mycgr3T
  
Location: 7965-15249

Mycgr3G67795\_Mycgr3T

Mycgr3G67775 Mycgr3T
  
Location: 15349-16237

Mycgr3G67775\_Mycgr3T

Mycgr3G90404 Mycgr3T
  
Location: 16337-17246

Mycgr3G90404\_Mycgr3T

Mycgr3G36951 Mycgr3T
  
Location: 17346-30891

Mycgr3G36951\_Mycgr3T

Mycgr3G103034 Mycgr3
  
Location: 30991-32644

Mycgr3G103034\_Mycgr3

Mycgr3G31119 Mycgr3T
  
Location: 32744-32906

Mycgr3G31119\_Mycgr3T

Mycgr3G28587 Mycgr3T
  
Location: 33006-33489

Mycgr3G28587\_Mycgr3T

Mycgr3G98959 Mycgr3T
  
Location: 33589-35035

Mycgr3G98959\_Mycgr3T

Mycgr3G35447 Mycgr3T
  
Location: 35135-36443

Mycgr3G35447\_Mycgr3T

Mycgr3G84402 Mycgr3T
  
Location: 36543-37884

Mycgr3G84402\_Mycgr3T

Mycgr3G98961 Mycgr3T
  
Location: 37984-38884

Mycgr3G98961\_Mycgr3T

hypothetical protein
  
Accession: EJT78056
  
Location: 1899476-1899823
  
 NCBI BlastP on this gene

EJT78056

hypothetical protein
  
Accession: EJT78055
  
Location: 1897893-1898153
  
 NCBI BlastP on this gene

EJT78055

hypothetical protein
  
Accession: EJT78054
  
Location: 1896219-1896596
  
 NCBI BlastP on this gene

EJT78054

hypothetical protein
  
Accession: EJT78053
  
Location: 1893532-1895921
  
 NCBI BlastP on this gene

EJT78053

hypothetical protein
  
Accession: EJT78052
  
Location: 1889818-1891844
  
 NCBI BlastP on this gene

EJT78052

hypothetical protein
  
Accession: EJT78051
  
Location: 1888521-1889654
  
 NCBI BlastP on this gene

EJT78051

hypothetical protein
  
Accession: EJT78050
  
Location: 1886043-1887901
  
 NCBI BlastP on this gene

EJT78050

hypothetical protein
  
Accession: EJT78049
  
Location: 1885014-1885814
  
 NCBI BlastP on this gene

EJT78049

phosphatidylinositol 3-kinase tor2
  
Accession: EJT78048
  
Location: 1876371-1884071
  
  
**BlastP hit with Mycgr3G67795\_Mycgr3T**
  
Percentage identity: 56 %
  
BlastP bit score: 2783
  
Sequence coverage: 102 %
  
E-value: 0.0
  
  
 NCBI BlastP on this gene

EJT78048

atypical/ABC1/ABC1-C protein kinase
  
Accession: EJT78047
  
Location: 1873320-1875692
  
 NCBI BlastP on this gene

EJT78047

cytochrome c heme lyase
  
Accession: EJT78046
  
Location: 1871671-1872823
  
 NCBI BlastP on this gene

EJT78046

hypothetical protein
  
Accession: EJT78045
  
Location: 1870656-1871319
  
 NCBI BlastP on this gene

EJT78045

hypothetical protein
  
Accession: EJT78044
  
Location: 1867689-1869392
  
 NCBI BlastP on this gene

EJT78044

hypothetical protein
  
Accession: EJT78043
  
Location: 1864470-1867207
  
 NCBI BlastP on this gene

EJT78043

hypothetical protein
  
Accession: EJT78042
  
Location: 1860495-1861942
  
 NCBI BlastP on this gene

EJT78042

hypothetical protein
  
Accession: EJT78041
  
Location: 1857808-1859332
  
 NCBI BlastP on this gene

EJT78041

Query: Architecture Search FASTA input

KE503206 : Schizosaccharomyces octosporus yFS286 unplaced genomic scaffold supercont6.1    Total score: 1.0     Cumulative Blast bit score: 2608

Hit cluster cross-links:

Mycgr3G67791 Mycgr3T
  
Location: 0-1542

Mycgr3G67791\_Mycgr3T

Mycgr3G90406 Mycgr3T
  
Location: 1642-3973

Mycgr3G90406\_Mycgr3T

Mycgr3G67785 Mycgr3T
  
Location: 4073-7865

Mycgr3G67785\_Mycgr3T

Mycgr3G67795 Mycgr3T
  
Location: 7965-15249

Mycgr3G67795\_Mycgr3T

Mycgr3G67775 Mycgr3T
  
Location: 15349-16237

Mycgr3G67775\_Mycgr3T

Mycgr3G90404 Mycgr3T
  
Location: 16337-17246

Mycgr3G90404\_Mycgr3T

Mycgr3G36951 Mycgr3T
  
Location: 17346-30891

Mycgr3G36951\_Mycgr3T

Mycgr3G103034 Mycgr3
  
Location: 30991-32644

Mycgr3G103034\_Mycgr3

Mycgr3G31119 Mycgr3T
  
Location: 32744-32906

Mycgr3G31119\_Mycgr3T

Mycgr3G28587 Mycgr3T
  
Location: 33006-33489

Mycgr3G28587\_Mycgr3T

Mycgr3G98959 Mycgr3T
  
Location: 33589-35035

Mycgr3G98959\_Mycgr3T

Mycgr3G35447 Mycgr3T
  
Location: 35135-36443

Mycgr3G35447\_Mycgr3T

Mycgr3G84402 Mycgr3T
  
Location: 36543-37884

Mycgr3G84402\_Mycgr3T

Mycgr3G98961 Mycgr3T
  
Location: 37984-38884

Mycgr3G98961\_Mycgr3T

eIF3e subunit Int6
  
Accession: EPX74339
  
Location: 2204488-2206092
  
 NCBI BlastP on this gene

EPX74339

oxysterol binding protein
  
Accession: EPX74340
  
Location: 2206759-2208351
  
 NCBI BlastP on this gene

EPX74340

enoyl reductase
  
Accession: EPX74341
  
Location: 2209069-2210065
  
 NCBI BlastP on this gene

EPX74341

glucan endo-1,3-alpha-glucosidase Agn2
  
Accession: EPX74342
  
Location: 2210671-2212074
  
 NCBI BlastP on this gene

EPX74342

squalene synthase Erg9
  
Accession: EPX74343
  
Location: 2212767-2214164
  
 NCBI BlastP on this gene

EPX74343

poly(A) polymerase Pla1
  
Accession: EPX74344
  
Location: 2214406-2216170
  
 NCBI BlastP on this gene

EPX74344

glutamyl-tRNA amidotransferase alpha subunit
  
Accession: EPX74345
  
Location: 2216517-2218012
  
 NCBI BlastP on this gene

EPX74345

complexed with Cdc5 protein Cwf11
  
Accession: EPX74346
  
Location: 2218240-2222058
  
 NCBI BlastP on this gene

EPX74346

phosphatidylinositol kinase Tor2
  
Accession: EPX74347
  
Location: 2222529-2229545
  
  
**BlastP hit with Mycgr3G67795\_Mycgr3T**
  
Percentage identity: 54 %
  
BlastP bit score: 2608
  
Sequence coverage: 99 %
  
E-value: 0.0
  
  
 NCBI BlastP on this gene

EPX74347

hypothetical protein
  
Accession: EPX74348
  
Location: 2230008-2232995
  
 NCBI BlastP on this gene

EPX74348

ATR checkpoint kinase Rad3
  
Accession: EPX74349
  
Location: 2233034-2240209
  
 NCBI BlastP on this gene

EPX74349

methionine sulfoxide
  
Accession: EPX74350
  
Location: 2241382-2241885
  
 NCBI BlastP on this gene

EPX74350

fungal protein
  
Accession: EPX74351
  
Location: 2242264-2243007
  
 NCBI BlastP on this gene

EPX74351

cortical anchoring factor for dynein Mcp5/Num1
  
Accession: EPX74352
  
Location: 2243997-2246858
  
 NCBI BlastP on this gene

EPX74352

DNA damage response protein
  
Accession: EPX74353
  
Location: 2247466-2250004
  
 NCBI BlastP on this gene

EPX74353

Query: Architecture Search FASTA input

DS022226 : Schizosaccharomyces japonicus yFS275 supercont1.3 genomic scaffold    Total score: 1.0     Cumulative Blast bit score: 2598

Hit cluster cross-links:

Mycgr3G67791 Mycgr3T
  
Location: 0-1542

Mycgr3G67791\_Mycgr3T

Mycgr3G90406 Mycgr3T
  
Location: 1642-3973

Mycgr3G90406\_Mycgr3T

Mycgr3G67785 Mycgr3T
  
Location: 4073-7865

Mycgr3G67785\_Mycgr3T

Mycgr3G67795 Mycgr3T
  
Location: 7965-15249

Mycgr3G67795\_Mycgr3T

Mycgr3G67775 Mycgr3T
  
Location: 15349-16237

Mycgr3G67775\_Mycgr3T

Mycgr3G90404 Mycgr3T
  
Location: 16337-17246

Mycgr3G90404\_Mycgr3T

Mycgr3G36951 Mycgr3T
  
Location: 17346-30891

Mycgr3G36951\_Mycgr3T

Mycgr3G103034 Mycgr3
  
Location: 30991-32644

Mycgr3G103034\_Mycgr3

Mycgr3G31119 Mycgr3T
  
Location: 32744-32906

Mycgr3G31119\_Mycgr3T

Mycgr3G28587 Mycgr3T
  
Location: 33006-33489

Mycgr3G28587\_Mycgr3T

Mycgr3G98959 Mycgr3T
  
Location: 33589-35035

Mycgr3G98959\_Mycgr3T

Mycgr3G35447 Mycgr3T
  
Location: 35135-36443

Mycgr3G35447\_Mycgr3T

Mycgr3G84402 Mycgr3T
  
Location: 36543-37884

Mycgr3G84402\_Mycgr3T

Mycgr3G98961 Mycgr3T
  
Location: 37984-38884

Mycgr3G98961\_Mycgr3T

transmembrane and coiled-coil domain-containing protein
  
Accession: EEB07618
  
Location: 1314495-1316201
  
 NCBI BlastP on this gene

EEB07618

btn1
  
Accession: EEB07619
  
Location: 1316682-1318016
  
 NCBI BlastP on this gene

EEB07619

synaptic glycoprotein SC2
  
Accession: EEB07620
  
Location: 1318406-1319537
  
 NCBI BlastP on this gene

EEB07620

glucan endo-1,3-alpha-glucosidase agn2
  
Accession: EEB07621
  
Location: 1320337-1321704
  
 NCBI BlastP on this gene

EEB07621

squalene synthetase
  
Accession: EEB07622
  
Location: 1322316-1323719
  
 NCBI BlastP on this gene

EEB07622

Poly(A) polymerase pla1
  
Accession: EEB07623
  
Location: 1324131-1326144
  
 NCBI BlastP on this gene

EEB07623

glutamyl-tRNA(Gln) amidotransferase subunit A
  
Accession: EEB07624
  
Location: 1326774-1328366
  
 NCBI BlastP on this gene

EEB07624

predicted protein
  
Accession: EEB07625
  
Location: 1329419-1333300
  
 NCBI BlastP on this gene

EEB07625

phosphatidylinositol kinase Tor2
  
Accession: EEB07626
  
Location: 1333747-1340757
  
  
**BlastP hit with Mycgr3G67795\_Mycgr3T**
  
Percentage identity: 53 %
  
BlastP bit score: 2598
  
Sequence coverage: 100 %
  
E-value: 0.0
  
  
 NCBI BlastP on this gene

EEB07626

Query: Architecture Search FASTA input

KE546988 : Schizosaccharomyces cryophilus OY26 unplaced genomic scaffold supercont4.1    Total score: 1.0     Cumulative Blast bit score: 2588

Hit cluster cross-links:

Mycgr3G67791 Mycgr3T
  
Location: 0-1542

Mycgr3G67791\_Mycgr3T

Mycgr3G90406 Mycgr3T
  
Location: 1642-3973

Mycgr3G90406\_Mycgr3T

Mycgr3G67785 Mycgr3T
  
Location: 4073-7865

Mycgr3G67785\_Mycgr3T

Mycgr3G67795 Mycgr3T
  
Location: 7965-15249

Mycgr3G67795\_Mycgr3T

Mycgr3G67775 Mycgr3T
  
Location: 15349-16237

Mycgr3G67775\_Mycgr3T

Mycgr3G90404 Mycgr3T
  
Location: 16337-17246

Mycgr3G90404\_Mycgr3T

Mycgr3G36951 Mycgr3T
  
Location: 17346-30891

Mycgr3G36951\_Mycgr3T

Mycgr3G103034 Mycgr3
  
Location: 30991-32644

Mycgr3G103034\_Mycgr3

Mycgr3G31119 Mycgr3T
  
Location: 32744-32906

Mycgr3G31119\_Mycgr3T

Mycgr3G28587 Mycgr3T
  
Location: 33006-33489

Mycgr3G28587\_Mycgr3T

Mycgr3G98959 Mycgr3T
  
Location: 33589-35035

Mycgr3G98959\_Mycgr3T

Mycgr3G35447 Mycgr3T
  
Location: 35135-36443

Mycgr3G35447\_Mycgr3T

Mycgr3G84402 Mycgr3T
  
Location: 36543-37884

Mycgr3G84402\_Mycgr3T

Mycgr3G98961 Mycgr3T
  
Location: 37984-38884

Mycgr3G98961\_Mycgr3T

phosphatidylinositol kinase Tor2
  
Accession: EPY54289
  
Location: 2784543-2791559
  
  
**BlastP hit with Mycgr3G67795\_Mycgr3T**
  
Percentage identity: 54 %
  
BlastP bit score: 2588
  
Sequence coverage: 99 %
  
E-value: 0.0
  
  
 NCBI BlastP on this gene

EPY54289

replication fork protection complex subunit Swi1
  
Accession: EPY54288
  
Location: 2781068-2784057
  
 NCBI BlastP on this gene

EPY54288

ATR checkpoint kinase Rad3
  
Accession: EPY54287
  
Location: 2773840-2781018
  
 NCBI BlastP on this gene

EPY54287

methionine sulfoxide
  
Accession: EPY54286
  
Location: 2772007-2772507
  
 NCBI BlastP on this gene

EPY54286

fungal protein
  
Accession: EPY54285
  
Location: 2770856-2771599
  
 NCBI BlastP on this gene

EPY54285

cortical anchoring factor for dynein Mcp5/Num1
  
Accession: EPY54284
  
Location: 2766780-2769644
  
 NCBI BlastP on this gene

EPY54284

Query: Architecture Search FASTA input

CU329671 : Schizosaccharomyces pombe chromosome II    Total score: 1.0     Cumulative Blast bit score: 2580

Hit cluster cross-links:

Mycgr3G67791 Mycgr3T
  
Location: 0-1542

Mycgr3G67791\_Mycgr3T

Mycgr3G90406 Mycgr3T
  
Location: 1642-3973

Mycgr3G90406\_Mycgr3T

Mycgr3G67785 Mycgr3T
  
Location: 4073-7865

Mycgr3G67785\_Mycgr3T

Mycgr3G67795 Mycgr3T
  
Location: 7965-15249

Mycgr3G67795\_Mycgr3T

Mycgr3G67775 Mycgr3T
  
Location: 15349-16237

Mycgr3G67775\_Mycgr3T

Mycgr3G90404 Mycgr3T
  
Location: 16337-17246

Mycgr3G90404\_Mycgr3T

Mycgr3G36951 Mycgr3T
  
Location: 17346-30891

Mycgr3G36951\_Mycgr3T

Mycgr3G103034 Mycgr3
  
Location: 30991-32644

Mycgr3G103034\_Mycgr3

Mycgr3G31119 Mycgr3T
  
Location: 32744-32906

Mycgr3G31119\_Mycgr3T

Mycgr3G28587 Mycgr3T
  
Location: 33006-33489

Mycgr3G28587\_Mycgr3T

Mycgr3G98959 Mycgr3T
  
Location: 33589-35035

Mycgr3G98959\_Mycgr3T

Mycgr3G35447 Mycgr3T
  
Location: 35135-36443

Mycgr3G35447\_Mycgr3T

Mycgr3G84402 Mycgr3T
  
Location: 36543-37884

Mycgr3G84402\_Mycgr3T

Mycgr3G98961 Mycgr3T
  
Location: 37984-38884

Mycgr3G98961\_Mycgr3T

eIF3e subunit Int6
  
Accession: CAA22813
  
Location: 938700-940261
  
 NCBI BlastP on this gene

int6

oxysterol binding protein (predicted)
  
Accession: CAA22812
  
Location: 936541-938091
  
 NCBI BlastP on this gene

SPBC646.08c

enoyl reductase (predicted)
  
Accession: CAA22811
  
Location: 934716-935603
  
 NCBI BlastP on this gene

SPBC646.07c

glucan endo-1,3-alpha-glucosidase Agn2
  
Accession: CAH58744
  
Location: 932781-934082
  
 NCBI BlastP on this gene

agn2

squalene synthase Erg9 (predicted)
  
Accession: CAA22809
  
Location: 931060-932442
  
 NCBI BlastP on this gene

erg9

poly(A) polymerase Pla1
  
Accession: CAA22808
  
Location: 928989-930812
  
 NCBI BlastP on this gene

pla1

mitochondrial glutamyl-tRNA amidotransferase alpha subunit (predicted)
  
Accession: CAA22807
  
Location: 926850-928358
  
 NCBI BlastP on this gene

SPBC646.03

complexed with Cdc5 protein Cwf11
  
Accession: CAA22806
  
Location: 922725-926579
  
 NCBI BlastP on this gene

cwf11

phosphatidylinositol kinase Tor2
  
Accession: CAB40167
  
Location: 915271-922284
  
  
**BlastP hit with Mycgr3G67795\_Mycgr3T**
  
Percentage identity: 53 %
  
BlastP bit score: 2580
  
Sequence coverage: 100 %
  
E-value: 0.0
  
  
 NCBI BlastP on this gene

tor2

replication fork protection complex subunit Swi1
  
Accession: CAB40166
  
Location: 911603-914593
  
 NCBI BlastP on this gene

swi1

ATR checkpoint kinase Rad3
  
Accession: CAB40165
  
Location: 904365-911525
  
 NCBI BlastP on this gene

rad3

methionine sulfoxide (predicted)
  
Accession: CAB40164
  
Location: 903156-903572
  
 NCBI BlastP on this gene

SPBC216.04c

conserved fungal protein
  
Accession: CAB40163
  
Location: 901942-902685
  
 NCBI BlastP on this gene

SPBC216.03

cortical anchoring factor for dynein Mcp5/Num1
  
Accession: CAB40162
  
Location: 898075-900981
  
 NCBI BlastP on this gene

mcp5

Query: Architecture Search FASTA input

201. :  JH971387 Agaricus bisporus var. burnettii JB137-S8 unplaced genomic scaffold AGABI1scaffold\_3     Total score: 1.0     Cumulative Blast bit score: 4601

Mycgr3G67791 Mycgr3T
  
Location: 0-1542
  
 NCBI BlastP on this gene

Mycgr3G67791\_Mycgr3T

Mycgr3G90406 Mycgr3T
  
Location: 1642-3973
  
 NCBI BlastP on this gene

Mycgr3G90406\_Mycgr3T

Mycgr3G67785 Mycgr3T
  
Location: 4073-7865
  
 NCBI BlastP on this gene

Mycgr3G67785\_Mycgr3T

Mycgr3G67795 Mycgr3T
  
Location: 7965-15249
  
 NCBI BlastP on this gene

Mycgr3G67795\_Mycgr3T

Mycgr3G67775 Mycgr3T
  
Location: 15349-16237
  
 NCBI BlastP on this gene

Mycgr3G67775\_Mycgr3T

Mycgr3G90404 Mycgr3T
  
Location: 16337-17246
  
 NCBI BlastP on this gene

Mycgr3G90404\_Mycgr3T

Mycgr3G36951 Mycgr3T
  
Location: 17346-30891
  
 NCBI BlastP on this gene

Mycgr3G36951\_Mycgr3T

Mycgr3G103034 Mycgr3
  
Location: 30991-32644
  
 NCBI BlastP on this gene

Mycgr3G103034\_Mycgr3

Mycgr3G31119 Mycgr3T
  
Location: 32744-32906
  
 NCBI BlastP on this gene

Mycgr3G31119\_Mycgr3T

Mycgr3G28587 Mycgr3T
  
Location: 33006-33489
  
 NCBI BlastP on this gene

Mycgr3G28587\_Mycgr3T

Mycgr3G98959 Mycgr3T
  
Location: 33589-35035
  
 NCBI BlastP on this gene

Mycgr3G98959\_Mycgr3T

Mycgr3G35447 Mycgr3T
  
Location: 35135-36443
  
 NCBI BlastP on this gene

Mycgr3G35447\_Mycgr3T

Mycgr3G84402 Mycgr3T
  
Location: 36543-37884
  
 NCBI BlastP on this gene

Mycgr3G84402\_Mycgr3T

Mycgr3G98961 Mycgr3T
  
Location: 37984-38884
  
 NCBI BlastP on this gene

Mycgr3G98961\_Mycgr3T

hypothetical protein
  
Accession: EKM81702
  
Location: 939805-942551
  
 NCBI BlastP on this gene

EKM81702

hypothetical protein
  
Accession: EKM81703
  
Location: 942695-951294
  
  
**BlastP hit with Mycgr3G67795\_Mycgr3T**
  
Percentage identity: 48 %
  
BlastP bit score: 2275
  
Sequence coverage: 100 %
  
E-value: 0.0
  
  
 NCBI BlastP on this gene

EKM81703

hypothetical protein
  
Accession: EKM81704
  
Location: 951900-954220
  
 NCBI BlastP on this gene

EKM81704

hypothetical protein
  
Accession: EKM81705
  
Location: 955336-957297
  
 NCBI BlastP on this gene

EKM81705

hypothetical protein
  
Accession: EKM81706
  
Location: 958013-959566
  
 NCBI BlastP on this gene

EKM81706

hypothetical protein
  
Accession: EKM81707
  
Location: 959723-961604
  
 NCBI BlastP on this gene

EKM81707

hypothetical protein
  
Accession: EKM81708
  
Location: 961953-964389
  
 NCBI BlastP on this gene

EKM81708

hypothetical protein
  
Accession: EKM81709
  
Location: 965277-966233
  
 NCBI BlastP on this gene

EKM81709

hypothetical protein
  
Accession: EKM81710
  
Location: 966379-967014
  
 NCBI BlastP on this gene

EKM81710

hypothetical protein
  
Accession: EKM81711
  
Location: 967235-969276
  
 NCBI BlastP on this gene

EKM81711

hypothetical protein
  
Accession: EKM81712
  
Location: 970960-974593
  
 NCBI BlastP on this gene

EKM81712

hypothetical protein
  
Accession: EKM81713
  
Location: 976048-984685
  
  
**BlastP hit with Mycgr3G67795\_Mycgr3T**
  
Percentage identity: 48 %
  
BlastP bit score: 2326
  
Sequence coverage: 100 %
  
E-value: 0.0
  
  
 NCBI BlastP on this gene

EKM81713

hypothetical protein
  
Accession: EKM81714
  
Location: 984985-986406
  
 NCBI BlastP on this gene

EKM81714

202. :  JH931610 Agaricus bisporus var. bisporus H97 unplaced genomic scaffold AGABI2scaffold\_6     Total score: 1.0     Cumulative Blast bit score: 4432

hypothetical protein
  
Accession: EKV46504
  
Location: 932444-935191
  
 NCBI BlastP on this gene

EKV46504

hypothetical protein
  
Accession: EKV46505
  
Location: 935335-943750
  
  
**BlastP hit with Mycgr3G67795\_Mycgr3T**
  
Percentage identity: 47 %
  
BlastP bit score: 2205
  
Sequence coverage: 99 %
  
E-value: 0.0
  
  
 NCBI BlastP on this gene

EKV46505

hypothetical protein
  
Accession: EKV46506
  
Location: 944546-946866
  
 NCBI BlastP on this gene

EKV46506

hypothetical protein
  
Accession: EKV46507
  
Location: 947983-949945
  
 NCBI BlastP on this gene

EKV46507

hypothetical protein
  
Accession: EKV46508
  
Location: 950659-952208
  
 NCBI BlastP on this gene

EKV46508

hypothetical protein
  
Accession: EKV46509
  
Location: 952371-954254
  
 NCBI BlastP on this gene

EKV46509

hypothetical protein
  
Accession: EKV46510
  
Location: 954603-957037
  
 NCBI BlastP on this gene

EKV46510

hypothetical protein
  
Accession: EKV46511
  
Location: 957296-958866
  
 NCBI BlastP on this gene

EKV46511

hypothetical protein
  
Accession: EKV46512
  
Location: 959025-959660
  
 NCBI BlastP on this gene

EKV46512

hypothetical protein
  
Accession: EKV46513
  
Location: 959882-961922
  
 NCBI BlastP on this gene

EKV46513

hypothetical protein
  
Accession: EKV46514
  
Location: 963596-965540
  
 NCBI BlastP on this gene

EKV46514

hypothetical protein
  
Accession: EKV46515
  
Location: 966158-967240
  
 NCBI BlastP on this gene

EKV46515

hypothetical protein
  
Accession: EKV46516
  
Location: 968671-977303
  
  
**BlastP hit with Mycgr3G67795\_Mycgr3T**
  
Percentage identity: 47 %
  
BlastP bit score: 2227
  
Sequence coverage: 100 %
  
E-value: 0.0
  
  
 NCBI BlastP on this gene

EKV46516

hypothetical protein
  
Accession: EKV46517
  
Location: 977603-979026
  
 NCBI BlastP on this gene

EKV46517

203. :  KB445561 Baudoinia compniacensis UAMH 10762 unplaced genomic scaffold BAUCOscaffold\_12     Total score: 1.0     Cumulative Blast bit score: 4110

hypothetical protein
  
Accession: EMC92720
  
Location: 363457-364234
  
 NCBI BlastP on this gene

EMC92720

hypothetical protein
  
Accession: EMC92721
  
Location: 364886-367775
  
 NCBI BlastP on this gene

EMC92721

hypothetical protein
  
Accession: EMC92722
  
Location: 368811-371612
  
 NCBI BlastP on this gene

EMC92722

hypothetical protein
  
Accession: EMC92723
  
Location: 373800-374824
  
 NCBI BlastP on this gene

EMC92723

hypothetical protein
  
Accession: EMC92724
  
Location: 376422-377997
  
 NCBI BlastP on this gene

EMC92724

hypothetical protein
  
Accession: EMC92725
  
Location: 378750-380891
  
 NCBI BlastP on this gene

EMC92725

hypothetical protein
  
Accession: EMC92726
  
Location: 381310-388614
  
  
**BlastP hit with Mycgr3G67795\_Mycgr3T**
  
Percentage identity: 82 %
  
BlastP bit score: 4110
  
Sequence coverage: 100 %
  
E-value: 0.0
  
  
 NCBI BlastP on this gene

EMC92726

hypothetical protein
  
Accession: EMC92727
  
Location: 389399-389978
  
 NCBI BlastP on this gene

EMC92727

hypothetical protein
  
Accession: EMC92728
  
Location: 390056-390821
  
 NCBI BlastP on this gene

EMC92728

hypothetical protein
  
Accession: EMC92729
  
Location: 391438-394138
  
 NCBI BlastP on this gene

EMC92729

hypothetical protein
  
Accession: EMC92730
  
Location: 396285-396938
  
 NCBI BlastP on this gene

EMC92730

hypothetical protein
  
Accession: EMC92731
  
Location: 397913-398519
  
 NCBI BlastP on this gene

EMC92731

hypothetical protein
  
Accession: EMC92732
  
Location: 398955-402315
  
 NCBI BlastP on this gene

EMC92732

hypothetical protein
  
Accession: EMC92733
  
Location: 402812-404569
  
 NCBI BlastP on this gene

EMC92733

hypothetical protein
  
Accession: EMC92734
  
Location: 405538-406134
  
 NCBI BlastP on this gene

EMC92734

hypothetical protein
  
Accession: EMC92735
  
Location: 407281-409981
  
 NCBI BlastP on this gene

EMC92735

204. :  CM001880 Theobroma cacao cultivar Matina 1-6 chromosome 2     Total score: 1.0     Cumulative Blast bit score: 3688

NAD(P)-binding Rossmann-fold superfamily protein isoform 8
  
Accession: EOX98546
  
Location: 6129758-6133689
  
 NCBI BlastP on this gene

EOX98546

S-adenosyl-L-methionine-dependent methyltransferases superfamily protein isoform 2
  
Accession: EOX98538
  
Location: 6125552-6127465
  
 NCBI BlastP on this gene

EOX98538

Uncharacterized protein isoform 2
  
Accession: EOX98536
  
Location: 6121047-6124174
  
 NCBI BlastP on this gene

EOX98536

Target of rapamycin isoform 3
  
Accession: EOX98534
  
Location: 6095522-6118875
  
  
**BlastP hit with Mycgr3G67795\_Mycgr3T**
  
Percentage identity: 42 %
  
BlastP bit score: 1816
  
Sequence coverage: 98 %
  
E-value: 0.0
  
  
 NCBI BlastP on this gene

EOX98534

Target of rapamycin isoform 1
  
Accession: EOX98533
  
Location: 6094651-6118875
  
  
**BlastP hit with Mycgr3G67795\_Mycgr3T**
  
Percentage identity: 42 %
  
BlastP bit score: 1872
  
Sequence coverage: 104 %
  
E-value: 0.0
  
  
 NCBI BlastP on this gene

EOX98533

Inositol 1,3,4-trisphosphate 5/6-kinase family protein isoform 4
  
Accession: EOX98531
  
Location: 6090406-6093687
  
 NCBI BlastP on this gene

EOX98531

Inositol 1,3,4-trisphosphate 5/6-kinase family protein isoform 1
  
Accession: EOX98529
  
Location: 6087530-6093687
  
 NCBI BlastP on this gene

EOX98529

205. :  CP002684 Arabidopsis thaliana chromosome 1     Total score: 1.0     Cumulative Blast bit score: 3658

uncharacterized protein
  
Accession: AEE32514
  
Location: 18554001-18554276
  
 NCBI BlastP on this gene

AT1G50080

putative pathogenesis-related protein
  
Accession: AEE32513
  
Location: 18551186-18552446
  
 NCBI BlastP on this gene

AT1G50060

putative pathogenesis-related protein
  
Accession: AEE32512
  
Location: 18546165-18549046
  
 NCBI BlastP on this gene

AT1G50050

uncharacterized protein
  
Accession: AEE32511
  
Location: 18542236-18543823
  
 NCBI BlastP on this gene

AT1G50040

phosphatidylinositol 3-kinase family protein TOR
  
Accession: AEE32510
  
Location: 18522626-18539619
  
  
**BlastP hit with Mycgr3G67795\_Mycgr3T**
  
Percentage identity: 41 %
  
BlastP bit score: 1818
  
Sequence coverage: 104 %
  
E-value: 0.0
  
  
 NCBI BlastP on this gene

TOR

phosphatidylinositol 3-kinase family protein TOR
  
Accession: AEE32509
  
Location: 18522626-18539619
  
  
**BlastP hit with Mycgr3G67795\_Mycgr3T**
  
Percentage identity: 41 %
  
BlastP bit score: 1840
  
Sequence coverage: 105 %
  
E-value: 0.0
  
  
 NCBI BlastP on this gene

TOR

uncharacterized protein
  
Accession: AEE32508
  
Location: 18520144-18521600
  
 NCBI BlastP on this gene

AT1G50020

tubulin alpha-2 chain
  
Accession: AEE32507
  
Location: 18517737-18519729
  
 NCBI BlastP on this gene

TUA2

putative ribosomal RNA small subunit methyltransferase
  
Accession: AEE32506
  
Location: 18515595-18517244
  
 NCBI BlastP on this gene

AT1G50000

putative ribosomal RNA small subunit methyltransferase
  
Accession: AEE32505
  
Location: 18515183-18517244
  
 NCBI BlastP on this gene

AT1G50000

F-box protein
  
Accession: AEE32504
  
Location: 18513696-18514988
  
 NCBI BlastP on this gene

AT1G49990

DNA/RNA polymerases superfamily protein
  
Accession: AEE32503
  
Location: 18508026-18512111
  
 NCBI BlastP on this gene

AT1G49980

206. :  JH767573 Coniosporium apollinis CBS 100218 chromosome Unknown supercont1.20     Total score: 1.0     Cumulative Blast bit score: 3508

FKBP12-rapamycin complex-associated protein
  
Accession: EON65353
  
Location: 306195-313744
  
  
**BlastP hit with Mycgr3G67795\_Mycgr3T**
  
Percentage identity: 70 %
  
BlastP bit score: 3508
  
Sequence coverage: 100 %
  
E-value: 0.0
  
  
 NCBI BlastP on this gene

EON65353

hypothetical protein
  
Accession: EON65352
  
Location: 304091-305197
  
 NCBI BlastP on this gene

EON65352

hypothetical protein
  
Accession: EON65351
  
Location: 300541-302976
  
 NCBI BlastP on this gene

EON65351

hypothetical protein
  
Accession: EON65350
  
Location: 299508-300138
  
 NCBI BlastP on this gene

EON65350

methylsterol monooxygenase
  
Accession: EON65349
  
Location: 297639-298854
  
 NCBI BlastP on this gene

EON65349

hypothetical protein
  
Accession: EON65348
  
Location: 294650-296903
  
 NCBI BlastP on this gene

EON65348

hypothetical protein
  
Accession: EON65347
  
Location: 291603-293328
  
 NCBI BlastP on this gene

EON65347

hypothetical protein
  
Accession: EON65346
  
Location: 289160-291049
  
 NCBI BlastP on this gene

EON65346

hypothetical protein
  
Accession: EON65345
  
Location: 286462-287683
  
 NCBI BlastP on this gene

EON65345

207. :  KB445649 Cochliobolus sativus ND90Pr unplaced genomic scaffold COCSAscaffold\_13     Total score: 1.0     Cumulative Blast bit score: 3284

hypothetical protein
  
Accession: EMD61144
  
Location: 1053135-1054140
  
 NCBI BlastP on this gene

EMD61144

hypothetical protein
  
Accession: EMD61145
  
Location: 1054495-1056363
  
 NCBI BlastP on this gene

EMD61145

hypothetical protein
  
Accession: EMD61146
  
Location: 1057099-1058768
  
 NCBI BlastP on this gene

EMD61146

hypothetical protein
  
Accession: EMD61147
  
Location: 1060552-1062345
  
 NCBI BlastP on this gene

EMD61147

hypothetical protein
  
Accession: EMD61148
  
Location: 1063393-1063554
  
 NCBI BlastP on this gene

EMD61148

hypothetical protein
  
Accession: EMD61149
  
Location: 1064213-1067830
  
 NCBI BlastP on this gene

EMD61149

hypothetical protein
  
Accession: EMD61150
  
Location: 1068781-1070840
  
 NCBI BlastP on this gene

EMD61150

hypothetical protein
  
Accession: EMD61151
  
Location: 1071254-1078633
  
  
**BlastP hit with Mycgr3G67795\_Mycgr3T**
  
Percentage identity: 66 %
  
BlastP bit score: 3285
  
Sequence coverage: 100 %
  
E-value: 0.0
  
  
 NCBI BlastP on this gene

EMD61151

hypothetical protein
  
Accession: EMD61152
  
Location: 1079541-1081164
  
 NCBI BlastP on this gene

EMD61152

hypothetical protein
  
Accession: EMD61153
  
Location: 1081281-1082021
  
 NCBI BlastP on this gene

EMD61153

hypothetical protein
  
Accession: EMD61154
  
Location: 1082303-1086774
  
 NCBI BlastP on this gene

EMD61154

hypothetical protein
  
Accession: EMD61155
  
Location: 1087175-1088723
  
 NCBI BlastP on this gene

EMD61155

hypothetical protein
  
Accession: EMD61156
  
Location: 1089030-1089923
  
 NCBI BlastP on this gene

EMD61156

hypothetical protein
  
Accession: EMD61157
  
Location: 1090213-1092558
  
 NCBI BlastP on this gene

EMD61157

hypothetical protein
  
Accession: EMD61158
  
Location: 1095918-1096745
  
 NCBI BlastP on this gene

EMD61158

hypothetical protein
  
Accession: EMD61159
  
Location: 1096961-1097883
  
 NCBI BlastP on this gene

EMD61159

hypothetical protein
  
Accession: EMD61160
  
Location: 1098337-1099468
  
 NCBI BlastP on this gene

EMD61160

208. :  KB445579 Cochliobolus heterostrophus C5 unplaced genomic scaffold COCHEscaffold\_11     Total score: 1.0     Cumulative Blast bit score: 3284

hypothetical protein
  
Accession: EMD89387
  
Location: 1027565-1028570
  
 NCBI BlastP on this gene

EMD89387

hypothetical protein
  
Accession: EMD89388
  
Location: 1028926-1030794
  
 NCBI BlastP on this gene

EMD89388

hypothetical protein
  
Accession: EMD89389
  
Location: 1031594-1033267
  
 NCBI BlastP on this gene

EMD89389

hypothetical protein
  
Accession: EMD89390
  
Location: 1035068-1036861
  
 NCBI BlastP on this gene

EMD89390

hypothetical protein
  
Accession: EMD89391
  
Location: 1040515-1044133
  
 NCBI BlastP on this gene

EMD89391

hypothetical protein
  
Accession: EMD89392
  
Location: 1045080-1047139
  
 NCBI BlastP on this gene

EMD89392

hypothetical protein
  
Accession: EMD89393
  
Location: 1047554-1054933
  
  
**BlastP hit with Mycgr3G67795\_Mycgr3T**
  
Percentage identity: 66 %
  
BlastP bit score: 3284
  
Sequence coverage: 100 %
  
E-value: 0.0
  
  
 NCBI BlastP on this gene

EMD89393

hypothetical protein
  
Accession: EMD89394
  
Location: 1055874-1057518
  
 NCBI BlastP on this gene

EMD89394

hypothetical protein
  
Accession: EMD89395
  
Location: 1057636-1058374
  
 NCBI BlastP on this gene

EMD89395

hypothetical protein
  
Accession: EMD89396
  
Location: 1058656-1063128
  
 NCBI BlastP on this gene

EMD89396

hypothetical protein
  
Accession: EMD89397
  
Location: 1063524-1065071
  
 NCBI BlastP on this gene

EMD89397

hypothetical protein
  
Accession: EMD89398
  
Location: 1065379-1066229
  
 NCBI BlastP on this gene

EMD89398

hypothetical protein
  
Accession: EMD89399
  
Location: 1066519-1068888
  
 NCBI BlastP on this gene

EMD89399

hypothetical protein
  
Accession: EMD89400
  
Location: 1069880-1070895
  
 NCBI BlastP on this gene

EMD89400

hypothetical protein
  
Accession: EMD89401
  
Location: 1071218-1071918
  
 NCBI BlastP on this gene

EMD89401

hypothetical protein
  
Accession: EMD89402
  
Location: 1072230-1073126
  
 NCBI BlastP on this gene

EMD89402

hypothetical protein
  
Accession: EMD89403
  
Location: 1073614-1075047
  
 NCBI BlastP on this gene

EMD89403

209. :  CH476599 Aspergillus terreus NIH2624 scaffold\_6 genomic scaffold     Total score: 1.0     Cumulative Blast bit score: 3099

conserved hypothetical protein
  
Accession: EAU34918
  
Location: 629392-630630
  
 NCBI BlastP on this gene

EAU34918

conserved hypothetical protein
  
Accession: EAU34917
  
Location: 624743-628497
  
 NCBI BlastP on this gene

EAU34917

predicted protein
  
Accession: EAU34916
  
Location: 622752-624053
  
 NCBI BlastP on this gene

EAU34916

predicted protein
  
Accession: EAU34915
  
Location: 619648-621138
  
 NCBI BlastP on this gene

EAU34915

predicted protein
  
Accession: EAU34914
  
Location: 617304-619090
  
 NCBI BlastP on this gene

EAU34914

conserved hypothetical protein
  
Accession: EAU34913
  
Location: 615562-616512
  
 NCBI BlastP on this gene

EAU34913

40S ribosomal protein S17
  
Accession: EAU34912
  
Location: 614582-615186
  
 NCBI BlastP on this gene

EAU34912

conserved hypothetical protein
  
Accession: EAU34911
  
Location: 612813-614180
  
 NCBI BlastP on this gene

EAU34911

conserved hypothetical protein
  
Accession: EAU34910
  
Location: 611322-612554
  
 NCBI BlastP on this gene

EAU34910

phosphatidylinositol 3-kinase tor2
  
Accession: EAU34909
  
Location: 602484-609814
  
  
**BlastP hit with Mycgr3G67795\_Mycgr3T**
  
Percentage identity: 63 %
  
BlastP bit score: 3099
  
Sequence coverage: 100 %
  
E-value: 0.0
  
  
 NCBI BlastP on this gene

EAU34909

conserved hypothetical protein
  
Accession: EAU34908
  
Location: 600284-602058
  
 NCBI BlastP on this gene

EAU34908

conserved hypothetical protein
  
Accession: EAU34907
  
Location: 597858-598978
  
 NCBI BlastP on this gene

EAU34907

predicted protein
  
Accession: EAU34906
  
Location: 595991-597310
  
 NCBI BlastP on this gene

EAU34906

conserved hypothetical protein
  
Accession: EAU34905
  
Location: 594706-595433
  
 NCBI BlastP on this gene

EAU34905

conserved hypothetical protein
  
Accession: EAU34904
  
Location: 590489-594179
  
 NCBI BlastP on this gene

EAU34904

inositol oxygenase 1
  
Accession: EAU34903
  
Location: 589034-589987
  
 NCBI BlastP on this gene

EAU34903

hypothetical protein
  
Accession: EAU34902
  
Location: 587005-588088
  
 NCBI BlastP on this gene

EAU34902

predicted protein
  
Accession: EAU34901
  
Location: 584309-586643
  
 NCBI BlastP on this gene

EAU34901

210. :  DS572813 Paracoccidioides brasiliensis Pb01 supercont1.3 genomic scaffold     Total score: 1.0     Cumulative Blast bit score: 3089

AP-2 complex subunit beta
  
Accession: EEH38956
  
Location: 328470-331315
  
 NCBI BlastP on this gene

EEH38956

conserved hypothetical protein
  
Accession: EEH38955
  
Location: 327166-327582
  
 NCBI BlastP on this gene

EEH38955

predicted protein
  
Accession: EEH38954
  
Location: 325466-327057
  
 NCBI BlastP on this gene

EEH38954

SH3 domain-containing protein
  
Accession: EEH38953
  
Location: 321267-324952
  
 NCBI BlastP on this gene

EEH38953

conserved hypothetical protein
  
Accession: EEH38952
  
Location: 319053-320213
  
 NCBI BlastP on this gene

EEH38952

40S ribosomal protein S17
  
Accession: EEH38951
  
Location: 317722-318420
  
 NCBI BlastP on this gene

EEH38951

conserved hypothetical protein
  
Accession: EEH38950
  
Location: 315672-317045
  
 NCBI BlastP on this gene

EEH38950

predicted protein
  
Accession: EEH38949
  
Location: 313151-313988
  
 NCBI BlastP on this gene

EEH38949

phosphatidylinositol 3-kinase tor2
  
Accession: EEH38948
  
Location: 304639-312042
  
  
**BlastP hit with Mycgr3G67795\_Mycgr3T**
  
Percentage identity: 63 %
  
BlastP bit score: 3089
  
Sequence coverage: 99 %
  
E-value: 0.0
  
  
 NCBI BlastP on this gene

EEH38948

conserved hypothetical protein
  
Accession: EEH38947
  
Location: 301989-303837
  
 NCBI BlastP on this gene

EEH38947

predicted protein
  
Accession: EEH38946
  
Location: 300837-301388
  
 NCBI BlastP on this gene

EEH38946

fungal specific transcription factor domain-containing protein
  
Accession: EEH38945
  
Location: 298517-300784
  
 NCBI BlastP on this gene

EEH38945

conserved hypothetical protein
  
Accession: EEH38944
  
Location: 296546-297884
  
 NCBI BlastP on this gene

EEH38944

inositol oxygenase
  
Accession: EEH38943
  
Location: 294802-295846
  
 NCBI BlastP on this gene

EEH38943

predicted protein
  
Accession: EEH38942
  
Location: 289193-291549
  
 NCBI BlastP on this gene

EEH38942

211. :  DS572750 Paracoccidioides brasiliensis Pb18 supercont1.1 genomic scaffold     Total score: 1.0     Cumulative Blast bit score: 3088

kynureninase
  
Accession: EEH44060
  
Location: 1138890-1140729
  
 NCBI BlastP on this gene

EEH44060

AP-2 complex subunit beta
  
Accession: EEH44061
  
Location: 1141409-1144108
  
 NCBI BlastP on this gene

EEH44061

conserved hypothetical protein
  
Accession: EEH44062
  
Location: 1144985-1145401
  
 NCBI BlastP on this gene

EEH44062

SH3 domain-containing protein
  
Accession: EEH44063
  
Location: 1147026-1150718
  
 NCBI BlastP on this gene

EEH44063

conserved hypothetical protein
  
Accession: EEH44064
  
Location: 1151726-1152910
  
 NCBI BlastP on this gene

EEH44064

40S ribosomal protein S17
  
Accession: EEH44065
  
Location: 1153486-1154177
  
 NCBI BlastP on this gene

EEH44065

conserved hypothetical protein
  
Accession: EEH44066
  
Location: 1154804-1156177
  
 NCBI BlastP on this gene

EEH44066

predicted protein
  
Accession: EEH44067
  
Location: 1157168-1158530
  
 NCBI BlastP on this gene

EEH44067

phosphatidylinositol 3-kinase tor2
  
Accession: EEH44068
  
Location: 1159696-1167099
  
  
**BlastP hit with Mycgr3G67795\_Mycgr3T**
  
Percentage identity: 63 %
  
BlastP bit score: 3088
  
Sequence coverage: 99 %
  
E-value: 0.0
  
  
 NCBI BlastP on this gene

EEH44068

conserved hypothetical protein
  
Accession: EEH44069
  
Location: 1167888-1170877
  
 NCBI BlastP on this gene

EEH44069

fungal specific transcription factor domain-containing protein
  
Accession: EEH44070
  
Location: 1170930-1173158
  
 NCBI BlastP on this gene

EEH44070

conserved hypothetical protein
  
Accession: EEH44071
  
Location: 1173867-1175031
  
 NCBI BlastP on this gene

EEH44071

inositol oxygenase
  
Accession: EEH44072
  
Location: 1175825-1176869
  
 NCBI BlastP on this gene

EEH44072

predicted protein
  
Accession: EEH44073
  
Location: 1178946-1181309
  
 NCBI BlastP on this gene

EEH44073

conserved hypothetical protein
  
Accession: EEH44074
  
Location: 1183140-1186877
  
 NCBI BlastP on this gene

EEH44074

212. :  GG749410 Ajellomyces dermatitidis ATCC 18188 genomic scaffold supercont1.4     Total score: 1.0     Cumulative Blast bit score: 3085

kynureninase
  
Accession: EGE78575
  
Location: 487917-489666
  
 NCBI BlastP on this gene

EGE78575

AP-2 adaptor complex subunit beta
  
Accession: EGE78576
  
Location: 490176-492565
  
 NCBI BlastP on this gene

EGE78576

ER membrane DUF1077 domain-containing protein
  
Accession: EGE78577
  
Location: 493422-494229
  
 NCBI BlastP on this gene

EGE78577

MFS transporter
  
Accession: EGE78578
  
Location: 494300-495919
  
 NCBI BlastP on this gene

EGE78578

SH3 domain-containing protein
  
Accession: EGE78579
  
Location: 496847-500580
  
 NCBI BlastP on this gene

EGE78579

DUF408 domain-containing protein
  
Accession: EGE78580
  
Location: 501689-502861
  
 NCBI BlastP on this gene

EGE78580

40S ribosomal protein S17
  
Accession: EGE78581
  
Location: 503555-504208
  
 NCBI BlastP on this gene

EGE78581

hypothetical protein
  
Accession: EGE78582
  
Location: 504576-505979
  
 NCBI BlastP on this gene

EGE78582

TorA protein
  
Accession: EGE78583
  
Location: 506676-514087
  
  
**BlastP hit with Mycgr3G67795\_Mycgr3T**
  
Percentage identity: 63 %
  
BlastP bit score: 3085
  
Sequence coverage: 99 %
  
E-value: 0.0
  
  
 NCBI BlastP on this gene

EGE78583

213. :  DS544805 Paracoccidioides brasiliensis Pb03 supercont1.3 genomic scaffold     Total score: 1.0     Cumulative Blast bit score: 3085

AP-2 complex subunit beta
  
Accession: EEH19707
  
Location: 235229-237977
  
 NCBI BlastP on this gene

EEH19707

conserved hypothetical protein
  
Accession: EEH19708
  
Location: 238480-239277
  
 NCBI BlastP on this gene

EEH19708

conserved hypothetical protein
  
Accession: EEH19709
  
Location: 240898-244590
  
 NCBI BlastP on this gene

EEH19709

conserved hypothetical protein
  
Accession: EEH19710
  
Location: 245646-246794
  
 NCBI BlastP on this gene

EEH19710

40S ribosomal protein S17
  
Accession: EEH19711
  
Location: 247367-248058
  
 NCBI BlastP on this gene

EEH19711

predicted protein
  
Accession: EEH19712
  
Location: 250533-251906
  
 NCBI BlastP on this gene

EEH19712

predicted protein
  
Accession: EEH19713
  
Location: 253300-254423
  
 NCBI BlastP on this gene

EEH19713

phosphatidylinositol 3-kinase tor2
  
Accession: EEH19714
  
Location: 255589-262992
  
  
**BlastP hit with Mycgr3G67795\_Mycgr3T**
  
Percentage identity: 62 %
  
BlastP bit score: 3085
  
Sequence coverage: 99 %
  
E-value: 0.0
  
  
 NCBI BlastP on this gene

EEH19714

conserved hypothetical protein
  
Accession: EEH19715
  
Location: 263780-265628
  
 NCBI BlastP on this gene

EEH19715

conserved hypothetical protein
  
Accession: EEH19716
  
Location: 266831-269077
  
 NCBI BlastP on this gene

EEH19716

conserved hypothetical protein
  
Accession: EEH19717
  
Location: 269779-270266
  
 NCBI BlastP on this gene

EEH19717

inositol oxygenase
  
Accession: EEH19718
  
Location: 271736-272780
  
 NCBI BlastP on this gene

EEH19718

predicted protein
  
Accession: EEH19719
  
Location: 274876-277239
  
 NCBI BlastP on this gene

EEH19719

conserved hypothetical protein
  
Accession: EEH19720
  
Location: 278910-282655
  
 NCBI BlastP on this gene

EEH19720

214. :  GG663373 Ajellomyces capsulatus G186AR genomic scaffold supercont2.11     Total score: 1.0     Cumulative Blast bit score: 3076

kynureninase
  
Accession: EEH04472
  
Location: 138518-140329
  
 NCBI BlastP on this gene

EEH04472

adaptor protein complex AP-1
  
Accession: EEH04471
  
Location: 135725-138120
  
 NCBI BlastP on this gene

EEH04471

DUF1077 domain-containing protein
  
Accession: EEH04470
  
Location: 134204-134919
  
 NCBI BlastP on this gene

EEH04470

conserved hypothetical protein
  
Accession: EEH04469
  
Location: 132085-133869
  
 NCBI BlastP on this gene

EEH04469

SH3 domain-containing protein
  
Accession: EEH04468
  
Location: 125204-128950
  
 NCBI BlastP on this gene

EEH04468

predicted protein
  
Accession: EEH04467
  
Location: 124456-124902
  
 NCBI BlastP on this gene

EEH04467

DUF408 domain-containing protein
  
Accession: EEH04466
  
Location: 122980-124149
  
 NCBI BlastP on this gene

EEH04466

40S ribosomal protein S17
  
Accession: EEH04465
  
Location: 121646-122299
  
 NCBI BlastP on this gene

EEH04465

conserved hypothetical protein
  
Accession: EEH04464
  
Location: 119939-121342
  
 NCBI BlastP on this gene

EEH04464

conserved hypothetical protein
  
Accession: EEH04463
  
Location: 112016-119429
  
  
**BlastP hit with Mycgr3G67795\_Mycgr3T**
  
Percentage identity: 62 %
  
BlastP bit score: 3076
  
Sequence coverage: 99 %
  
E-value: 0.0
  
  
 NCBI BlastP on this gene

EEH04463

conserved hypothetical protein
  
Accession: EEH04462
  
Location: 109436-111280
  
 NCBI BlastP on this gene

EEH04462

conserved hypothetical protein
  
Accession: EEH04461
  
Location: 105630-106865
  
 NCBI BlastP on this gene

EEH04461

conserved hypothetical protein
  
Accession: EEH04460
  
Location: 101208-103705
  
 NCBI BlastP on this gene

EEH04460

conserved hypothetical protein
  
Accession: EEH04459
  
Location: 95710-99468
  
 NCBI BlastP on this gene

EEH04459

predicted protein
  
Accession: EEH04458
  
Location: 92145-92609
  
 NCBI BlastP on this gene

EEH04458

215. :  EQ963476 Aspergillus flavus NRRL3357 scf\_1106286417850 genomic scaffold     Total score: 1.0     Cumulative Blast bit score: 3076

kynureninase
  
Accession: EED52724
  
Location: 1601582-1603215
  
 NCBI BlastP on this gene

EED52724

ER membrane DUF1077 domain protein, putative
  
Accession: EED52725
  
Location: 1603765-1604471
  
 NCBI BlastP on this gene

EED52725

AP-2 adaptor complex subunit beta, putative
  
Accession: EED52726
  
Location: 1605408-1607789
  
 NCBI BlastP on this gene

EED52726

pre-mRNA-splicing factor cwc25, putative
  
Accession: EED52727
  
Location: 1608437-1609381
  
 NCBI BlastP on this gene

EED52727

SH3 domain protein
  
Accession: EED52728
  
Location: 1610568-1611560
  
 NCBI BlastP on this gene

EED52728

SH3 domain protein
  
Accession: EED52729
  
Location: 1612503-1614416
  
 NCBI BlastP on this gene

EED52729

DUF408 domain protein
  
Accession: EED52730
  
Location: 1615233-1616210
  
 NCBI BlastP on this gene

EED52730

ketoreductase
  
Accession: EED52731
  
Location: 1617105-1618357
  
 NCBI BlastP on this gene

EED52731

conserved hypothetical protein
  
Accession: EED52732
  
Location: 1619002-1620396
  
 NCBI BlastP on this gene

EED52732

TOR pathway phosphatidylinositol 3-kinase TorA
  
Accession: EED52733
  
Location: 1621252-1628532
  
  
**BlastP hit with Mycgr3G67795\_Mycgr3T**
  
Percentage identity: 62 %
  
BlastP bit score: 3076
  
Sequence coverage: 100 %
  
E-value: 0.0
  
  
 NCBI BlastP on this gene

EED52733

216. :  AKHY01000140 Aspergillus oryzae 3.042     Total score: 1.0     Cumulative Blast bit score: 3075

L-kynurenine hydrolase
  
Accession: EIT78181
  
Location: 31697-33330
  
 NCBI BlastP on this gene

EIT78181

putative membrane protein
  
Accession: EIT78189
  
Location: 33880-34586
  
 NCBI BlastP on this gene

EIT78189

vesicle coat complex AP-1/AP-2/AP-4, beta subunit
  
Accession: EIT78153
  
Location: 35199-37904
  
 NCBI BlastP on this gene

EIT78153

pre-mRNA-splicing factor cwc25
  
Accession: EIT78247
  
Location: 38552-39948
  
 NCBI BlastP on this gene

EIT78247

SH3 domain protein
  
Accession: EIT78248
  
Location: 40686-44454
  
 NCBI BlastP on this gene

EIT78248

hypothetical protein
  
Accession: EIT78192
  
Location: 45271-46248
  
 NCBI BlastP on this gene

EIT78192

flavonol reductase/cinnamoyl-CoA reductase
  
Accession: EIT78245
  
Location: 47147-48399
  
 NCBI BlastP on this gene

EIT78245

DNA-dependent protein kinase
  
Accession: EIT78158
  
Location: 51294-58574
  
  
**BlastP hit with Mycgr3G67795\_Mycgr3T**
  
Percentage identity: 62 %
  
BlastP bit score: 3075
  
Sequence coverage: 100 %
  
E-value: 0.0
  
  
 NCBI BlastP on this gene

EIT78158

217. :  EQ962654 Talaromyces stipitatus ATCC 10500 scf\_1105507295541 genomic scaffold     Total score: 1.0     Cumulative Blast bit score: 3074

iron-sulfur cluster assembly accessory protein Isa2, putative
  
Accession: EED20517
  
Location: 3765046-3766074
  
 NCBI BlastP on this gene

EED20517

kynureninase
  
Accession: EED20516
  
Location: 3762851-3764574
  
 NCBI BlastP on this gene

EED20516

AP-2 adaptor complex subunit beta, putative
  
Accession: EED20515
  
Location: 3760118-3762696
  
 NCBI BlastP on this gene

EED20515

ER membrane DUF1077 domain protein, putative
  
Accession: EED20514
  
Location: 3758736-3759416
  
 NCBI BlastP on this gene

EED20514

florfenicol exporter, putative
  
Accession: EED20513
  
Location: 3756791-3758532
  
 NCBI BlastP on this gene

EED20513

SH3 domain protein
  
Accession: EED20512
  
Location: 3752353-3755811
  
 NCBI BlastP on this gene

EED20512

DUF408 domain protein
  
Accession: EED20511
  
Location: 3750454-3751580
  
 NCBI BlastP on this gene

EED20511

40S ribosomal protein S17, putative
  
Accession: EED20510
  
Location: 3749443-3750079
  
 NCBI BlastP on this gene

EED20510

conserved hypothetical protein
  
Accession: EED20509
  
Location: 3746242-3749071
  
 NCBI BlastP on this gene

EED20509

TOR pathway phosphatidylinositol 3-kinase TorA
  
Accession: EED20508
  
Location: 3737981-3745355
  
  
**BlastP hit with Mycgr3G67795\_Mycgr3T**
  
Percentage identity: 62 %
  
BlastP bit score: 3074
  
Sequence coverage: 100 %
  
E-value: 0.0
  
  
 NCBI BlastP on this gene

EED20508

conserved hypothetical protein
  
Accession: EED20507
  
Location: 3735711-3737531
  
 NCBI BlastP on this gene

EED20507

conserved hypothetical protein
  
Accession: EED20506
  
Location: 3733307-3735448
  
 NCBI BlastP on this gene

EED20506

hypothetical protein
  
Accession: EED20505
  
Location: 3729674-3730022
  
 NCBI BlastP on this gene

EED20505

hypothetical protein
  
Accession: EED20504
  
Location: 3728759-3729639
  
 NCBI BlastP on this gene

EED20504

glycerol dehydrogenase, putative
  
Accession: EED20503
  
Location: 3726280-3727382
  
 NCBI BlastP on this gene

EED20503

inositol oxygenase, putative
  
Accession: EED20502
  
Location: 3724490-3725614
  
 NCBI BlastP on this gene

EED20502

conserved hypothetical protein
  
Accession: EED20501
  
Location: 3723057-3724466
  
 NCBI BlastP on this gene

EED20501

218. :  DS990641 Ajellomyces capsulatus H88 supercont1.6 genomic scaffold     Total score: 1.0     Cumulative Blast bit score: 3074

kynureninase
  
Accession: EGC48539
  
Location: 1909566-1911558
  
 NCBI BlastP on this gene

EGC48539

adaptor protein complex AP-1
  
Accession: EGC48538
  
Location: 1906951-1909346
  
 NCBI BlastP on this gene

EGC48538

DUF1077 domain-containing protein
  
Accession: EGC48537
  
Location: 1905378-1906146
  
 NCBI BlastP on this gene

EGC48537

MFS transporter
  
Accession: EGC48536
  
Location: 1903313-1905096
  
 NCBI BlastP on this gene

EGC48536

SH3 domain-containing protein
  
Accession: EGC48535
  
Location: 1896733-1900476
  
 NCBI BlastP on this gene

EGC48535

predicted protein
  
Accession: EGC48534
  
Location: 1895969-1896433
  
 NCBI BlastP on this gene

EGC48534

DUF408 domain-containing protein
  
Accession: EGC48533
  
Location: 1894514-1895656
  
 NCBI BlastP on this gene

EGC48533

40S ribosomal protein S17
  
Accession: EGC48532
  
Location: 1893145-1893797
  
 NCBI BlastP on this gene

EGC48532

conserved hypothetical protein
  
Accession: EGC48531
  
Location: 1891434-1892837
  
 NCBI BlastP on this gene

EGC48531

TorA protein
  
Accession: EGC48530
  
Location: 1883517-1890927
  
  
**BlastP hit with Mycgr3G67795\_Mycgr3T**
  
Percentage identity: 62 %
  
BlastP bit score: 3074
  
Sequence coverage: 99 %
  
E-value: 0.0
  
  
 NCBI BlastP on this gene

EGC48530

conserved hypothetical protein
  
Accession: EGC48529
  
Location: 1880964-1882808
  
 NCBI BlastP on this gene

EGC48529

predicted protein
  
Accession: EGC48528
  
Location: 1879778-1880437
  
 NCBI BlastP on this gene

EGC48528

conserved hypothetical protein
  
Accession: EGC48527
  
Location: 1877245-1878469
  
 NCBI BlastP on this gene

EGC48527

conserved hypothetical protein
  
Accession: EGC48526
  
Location: 1872829-1875326
  
 NCBI BlastP on this gene

EGC48526

conserved hypothetical protein
  
Accession: EGC48525
  
Location: 1867328-1871086
  
 NCBI BlastP on this gene

EGC48525

predicted protein
  
Accession: EGC48524
  
Location: 1865051-1865542
  
 NCBI BlastP on this gene

EGC48524

predicted protein
  
Accession: EGC48523
  
Location: 1863763-1864375
  
 NCBI BlastP on this gene

EGC48523

219. :  GG657464 Ajellomyces dermatitidis SLH14081 genomic scaffold supercont1.17     Total score: 1.0     Cumulative Blast bit score: 3069

metallo-beta-lactamase superfamily protein
  
Accession: EEQ72090
  
Location: 725563-726903
  
 NCBI BlastP on this gene

EEQ72090

kynureninase
  
Accession: EEQ72089
  
Location: 723343-725092
  
 NCBI BlastP on this gene

EEQ72089

AP-2 adaptor complex subunit beta
  
Accession: EEQ72088
  
Location: 720440-722829
  
 NCBI BlastP on this gene

EEQ72088

ER membrane DUF1077 domain-containing protein
  
Accession: EEQ72087
  
Location: 718774-719581
  
 NCBI BlastP on this gene

EEQ72087

MFS multidrug transporter
  
Accession: EEQ72086
  
Location: 717083-718703
  
 NCBI BlastP on this gene

EEQ72086

conserved hypothetical protein
  
Accession: EEQ72085
  
Location: 712400-716133
  
 NCBI BlastP on this gene

EEQ72085

DUF408 domain-containing protein
  
Accession: EEQ72084
  
Location: 710104-711249
  
 NCBI BlastP on this gene

EEQ72084

40S ribosomal protein S17
  
Accession: EEQ72083
  
Location: 708774-709427
  
 NCBI BlastP on this gene

EEQ72083

conserved hypothetical protein
  
Accession: EEQ72082
  
Location: 707003-708406
  
 NCBI BlastP on this gene

EEQ72082

phosphatidylinositol 3-kinase tor2
  
Accession: EEQ72081
  
Location: 698894-706305
  
  
**BlastP hit with Mycgr3G67795\_Mycgr3T**
  
Percentage identity: 62 %
  
BlastP bit score: 3069
  
Sequence coverage: 99 %
  
E-value: 0.0
  
  
 NCBI BlastP on this gene

EEQ72081

conserved hypothetical protein
  
Accession: EEQ72080
  
Location: 696115-697946
  
 NCBI BlastP on this gene

EEQ72080

C6 transcription factor
  
Accession: EEQ72079
  
Location: 692956-694817
  
 NCBI BlastP on this gene

EEQ72079

methyltransferase type 11
  
Accession: EEQ72078
  
Location: 690899-692414
  
 NCBI BlastP on this gene

EEQ72078

inositol oxygenase
  
Accession: EEQ72077
  
Location: 688939-689971
  
 NCBI BlastP on this gene

EEQ72077

predicted protein
  
Accession: EEQ72076
  
Location: 688009-688504
  
 NCBI BlastP on this gene

EEQ72076

hypothetical protein
  
Accession: EEQ72075
  
Location: 685827-687311
  
 NCBI BlastP on this gene

EEQ72075

predicted protein
  
Accession: EEQ72074
  
Location: 684919-685509
  
 NCBI BlastP on this gene

EEQ72074

conserved hypothetical protein
  
Accession: EEQ72073
  
Location: 679389-683210
  
 NCBI BlastP on this gene

EEQ72073

220. :  EQ999980 Ajellomyces dermatitidis ER-3 genomic scaffold supercont1.8     Total score: 1.0     Cumulative Blast bit score: 3069

metallo-beta-lactamase superfamily protein
  
Accession: EEQ92147
  
Location: 1539278-1540618
  
 NCBI BlastP on this gene

EEQ92147

kynureninase
  
Accession: EEQ92146
  
Location: 1537131-1538880
  
 NCBI BlastP on this gene

EEQ92146

AP-2 adaptor complex subunit beta
  
Accession: EEQ92145
  
Location: 1534232-1536621
  
 NCBI BlastP on this gene

EEQ92145

ER membrane DUF1077 domain-containing protein
  
Accession: EEQ92144
  
Location: 1532568-1533375
  
 NCBI BlastP on this gene

EEQ92144

MFS transporter
  
Accession: EEQ92143
  
Location: 1530878-1532497
  
 NCBI BlastP on this gene

EEQ92143

conserved hypothetical protein
  
Accession: EEQ92142
  
Location: 1526218-1529951
  
 NCBI BlastP on this gene

EEQ92142

DUF408 domain-containing protein
  
Accession: EEQ92141
  
Location: 1523939-1525072
  
 NCBI BlastP on this gene

EEQ92141

40S ribosomal protein S17
  
Accession: EEQ92140
  
Location: 1522592-1523245
  
 NCBI BlastP on this gene

EEQ92140

conserved hypothetical protein
  
Accession: EEQ92139
  
Location: 1520821-1522224
  
 NCBI BlastP on this gene

EEQ92139

phosphatidylinositol 3-kinase tor2
  
Accession: EEQ92138
  
Location: 1512709-1520120
  
  
**BlastP hit with Mycgr3G67795\_Mycgr3T**
  
Percentage identity: 62 %
  
BlastP bit score: 3069
  
Sequence coverage: 99 %
  
E-value: 0.0
  
  
 NCBI BlastP on this gene

EEQ92138

conserved hypothetical protein
  
Accession: EEQ92137
  
Location: 1509929-1511760
  
 NCBI BlastP on this gene

EEQ92137

C6 transcription factor
  
Accession: EEQ92136
  
Location: 1506769-1508630
  
 NCBI BlastP on this gene

EEQ92136

methyltransferase type 11
  
Accession: EEQ92135
  
Location: 1504704-1505952
  
 NCBI BlastP on this gene

EEQ92135

inositol oxygenase
  
Accession: EEQ92134
  
Location: 1502744-1503776
  
 NCBI BlastP on this gene

EEQ92134

predicted protein
  
Accession: EEQ92133
  
Location: 1501444-1502317
  
 NCBI BlastP on this gene

EEQ92133

hypothetical protein
  
Accession: EEQ92132
  
Location: 1499633-1501117
  
 NCBI BlastP on this gene

EEQ92132

predicted protein
  
Accession: EEQ92131
  
Location: 1498725-1499315
  
 NCBI BlastP on this gene

EEQ92131

conserved hypothetical protein
  
Accession: EEQ92130
  
Location: 1493207-1497027
  
 NCBI BlastP on this gene

EEQ92130

221. :  DS995904 Penicillium marneffei ATCC 18224 scf\_1105668340738 genomic scaffold     Total score: 1.0     Cumulative Blast bit score: 3068

iron-sulfur cluster assembly accessory protein Isa2, putative
  
Accession: EEA20948
  
Location: 2409782-2412388
  
 NCBI BlastP on this gene

EEA20948

kynureninase
  
Accession: EEA20947
  
Location: 2407602-2408776
  
 NCBI BlastP on this gene

EEA20947

AP-2 adaptor complex subunit beta, putative
  
Accession: EEA20946
  
Location: 2404854-2407463
  
 NCBI BlastP on this gene

EEA20946

ER membrane DUF1077 domain protein, putative
  
Accession: EEA20945
  
Location: 2403473-2404150
  
 NCBI BlastP on this gene

EEA20945

conserved hypothetical protein
  
Accession: EEA20944
  
Location: 2401505-2403247
  
 NCBI BlastP on this gene

EEA20944

SH3 domain protein
  
Accession: EEA20943
  
Location: 2396965-2400510
  
 NCBI BlastP on this gene

EEA20943

DUF408 domain protein
  
Accession: EEA20941
  
Location: 2395222-2396154
  
 NCBI BlastP on this gene

EEA20941

40S ribosomal protein S17, putative
  
Accession: EEA20940
  
Location: 2394082-2394693
  
 NCBI BlastP on this gene

EEA20940

conserved hypothetical protein
  
Accession: EEA20939
  
Location: 2392377-2393684
  
 NCBI BlastP on this gene

EEA20939

ketoreductase
  
Accession: EEA20938
  
Location: 2390888-2392120
  
 NCBI BlastP on this gene

EEA20938

TOR pathway phosphatidylinositol 3-kinase TorA
  
Accession: EEA20937
  
Location: 2382686-2389997
  
  
**BlastP hit with Mycgr3G67795\_Mycgr3T**
  
Percentage identity: 62 %
  
BlastP bit score: 3068
  
Sequence coverage: 100 %
  
E-value: 0.0
  
  
 NCBI BlastP on this gene

EEA20937

conserved hypothetical protein
  
Accession: EEA20936
  
Location: 2380402-2382175
  
 NCBI BlastP on this gene

EEA20936

glycerol dehydrogenase, putative
  
Accession: EEA20935
  
Location: 2378368-2379506
  
 NCBI BlastP on this gene

EEA20935

inositol oxygenase, putative
  
Accession: EEA20934
  
Location: 2376267-2377286
  
 NCBI BlastP on this gene

EEA20934

conserved hypothetical protein
  
Accession: EEA20933
  
Location: 2374267-2375932
  
 NCBI BlastP on this gene

EEA20933

conserved hypothetical protein
  
Accession: EEA20932
  
Location: 2368957-2372523
  
 NCBI BlastP on this gene

EEA20932

222. :  GG692427 Ajellomyces capsulatus H143 genomic scaffold supercont2.9     Total score: 1.0     Cumulative Blast bit score: 3066

kynureninase
  
Accession: EER40249
  
Location: 791649-793641
  
 NCBI BlastP on this gene

EER40249

adaptin
  
Accession: EER40250
  
Location: 793861-796256
  
 NCBI BlastP on this gene

EER40250

DUF1077 domain-containing protein
  
Accession: EER40251
  
Location: 797061-797829
  
 NCBI BlastP on this gene

EER40251

conserved hypothetical protein
  
Accession: EER40252
  
Location: 802704-803303
  
 NCBI BlastP on this gene

EER40252

hypothetical protein
  
Accession: EER40253
  
Location: 804428-805790
  
 NCBI BlastP on this gene

EER40253

predicted protein
  
Accession: EER40254
  
Location: 806900-807363
  
 NCBI BlastP on this gene

EER40254

DUF408 domain-containing protein
  
Accession: EER40255
  
Location: 807676-808818
  
 NCBI BlastP on this gene

EER40255

hypothetical protein
  
Accession: EER40256
  
Location: 809401-810184
  
 NCBI BlastP on this gene

EER40256

conserved hypothetical protein
  
Accession: EER40257
  
Location: 810489-811892
  
 NCBI BlastP on this gene

EER40257

TorA protein
  
Accession: EER40258
  
Location: 812399-819809
  
  
**BlastP hit with Mycgr3G67795\_Mycgr3T**
  
Percentage identity: 62 %
  
BlastP bit score: 3066
  
Sequence coverage: 99 %
  
E-value: 0.0
  
  
 NCBI BlastP on this gene

EER40258

223. :  AP007171 Aspergillus oryzae RIB40 DNA, SC011.     Total score: 1.0     Cumulative Blast bit score: 3054

not annotated
  
Accession: BAE65077
  
Location: 1532825-1534458
  
 NCBI BlastP on this gene

AO090011000602

not annotated
  
Accession: BAE65078
  
Location: 1534887-1535714
  
 NCBI BlastP on this gene

AO090011000603

not annotated
  
Accession: BAE65079
  
Location: 1536327-1539032
  
 NCBI BlastP on this gene

AO090011000604

not annotated
  
Accession: BAE65080
  
Location: 1539680-1541076
  
 NCBI BlastP on this gene

AO090011000605

not annotated
  
Accession: BAE65081
  
Location: 1541814-1545582
  
 NCBI BlastP on this gene

AO090011000606

not annotated
  
Accession: BAE65082
  
Location: 1546399-1547376
  
 NCBI BlastP on this gene

AO090011000607

not annotated
  
Accession: BAE65083
  
Location: 1550520-1559702
  
  
**BlastP hit with Mycgr3G67795\_Mycgr3T**
  
Percentage identity: 63 %
  
BlastP bit score: 3054
  
Sequence coverage: 98 %
  
E-value: 0.0
  
  
 NCBI BlastP on this gene

AO090011000608

224. :  JH921440 Marssonina brunnea f. sp. 'multigermtubi' MB\_m1 unplaced genomic scaffold M6\_S00013     Total score: 1.0     Cumulative Blast bit score: 3051

casein kinase II regulatory subunit
  
Accession: EKD15930
  
Location: 740405-742094
  
 NCBI BlastP on this gene

EKD15930

hypothetical protein
  
Accession: EKD15931
  
Location: 743091-747035
  
 NCBI BlastP on this gene

EKD15931

integral membrane protein
  
Accession: EKD15932
  
Location: 752425-752998
  
 NCBI BlastP on this gene

EKD15932

glucosidase 2 subunit beta precursor
  
Accession: EKD15933
  
Location: 755492-757856
  
 NCBI BlastP on this gene

EKD15933

DUF602 domain protein
  
Accession: EKD15934
  
Location: 758251-759117
  
 NCBI BlastP on this gene

EKD15934

FAT domain-containing protein
  
Accession: EKD15935
  
Location: 759438-766925
  
  
**BlastP hit with Mycgr3G67795\_Mycgr3T**
  
Percentage identity: 62 %
  
BlastP bit score: 3051
  
Sequence coverage: 101 %
  
E-value: 0.0
  
  
 NCBI BlastP on this gene

EKD15935

225. :  KE145367 Glarea lozoyensis ATCC 20868 chromosome Unknown GLAREA3     Total score: 1.0     Cumulative Blast bit score: 3042

Mannose 6-phosphate receptor
  
Accession: EPE29047
  
Location: 562637-564469
  
 NCBI BlastP on this gene

EPE29047

hypothetical protein
  
Accession: EPE29048
  
Location: 565107-568928
  
 NCBI BlastP on this gene

EPE29048

hypothetical protein
  
Accession: EPE29049
  
Location: 569511-570177
  
 NCBI BlastP on this gene

EPE29049

hypothetical protein
  
Accession: EPE29050
  
Location: 577005-579430
  
 NCBI BlastP on this gene

EPE29050

hypothetical protein
  
Accession: EPE29051
  
Location: 581340-582296
  
 NCBI BlastP on this gene

EPE29051

ARM repeat-containing protein
  
Accession: EPE29052
  
Location: 582609-590120
  
  
**BlastP hit with Mycgr3G67795\_Mycgr3T**
  
Percentage identity: 62 %
  
BlastP bit score: 3042
  
Sequence coverage: 100 %
  
E-value: 0.0
  
  
 NCBI BlastP on this gene

EPE29052

226. :  KB707916 Botryotinia fuckeliana BcDW1 unplaced genomic scaffold Scaffold\_244     Total score: 1.0     Cumulative Blast bit score: 3017

putative glucose oxidase protein
  
Accession: EMR85260
  
Location: 46279-48255
  
 NCBI BlastP on this gene

EMR85260

hypothetical protein
  
Accession: EMR85259
  
Location: 44678-45688
  
 NCBI BlastP on this gene

EMR85259

putative glucosidase 2 subunit beta protein
  
Accession: EMR85258
  
Location: 41925-43978
  
 NCBI BlastP on this gene

EMR85258

hypothetical protein
  
Accession: EMR85257
  
Location: 37192-41070
  
 NCBI BlastP on this gene

EMR85257

putative duf602 domain-containing protein
  
Accession: EMR85256
  
Location: 27516-28508
  
 NCBI BlastP on this gene

EMR85256

putative phosphatidylinositol 3-kinase tor2 protein
  
Accession: EMR85255
  
Location: 19482-26996
  
  
**BlastP hit with Mycgr3G67795\_Mycgr3T**
  
Percentage identity: 61 %
  
BlastP bit score: 3017
  
Sequence coverage: 100 %
  
E-value: 0.0
  
  
 NCBI BlastP on this gene

EMR85255

putative chaperone domain protein
  
Accession: EMR85254
  
Location: 17386-18306
  
 NCBI BlastP on this gene

EMR85254

putative siderophore iron transporter protein
  
Accession: EMR85253
  
Location: 14786-16736
  
 NCBI BlastP on this gene

EMR85253

putative paraben-hydrolyzing esterase precursor protein
  
Accession: EMR85252
  
Location: 11006-12648
  
 NCBI BlastP on this gene

EMR85252

hypothetical protein
  
Accession: EMR85251
  
Location: 8591-9657
  
 NCBI BlastP on this gene

EMR85251

putative transcription factor cys6 protein
  
Accession: EMR85250
  
Location: 6355-7395
  
 NCBI BlastP on this gene

EMR85250

putative glutathione s-transferase protein
  
Accession: EMR85249
  
Location: 4861-5346
  
 NCBI BlastP on this gene

EMR85249

putative 2-dehydropantoate 2-reductase protein
  
Accession: EMR85248
  
Location: 3113-4141
  
 NCBI BlastP on this gene

EMR85248

putative tannase subunit protein
  
Accession: EMR85247
  
Location: 2141-2356
  
 NCBI BlastP on this gene

EMR85247

227. :  FQ790307 Botryotinia fuckeliana T4 SuperContig\_19\_1 genomic supercontig.     Total score: 1.0     Cumulative Blast bit score: 3017

hypothetical protein
  
Accession: CCD34327
  
Location: 94704-95714
  
 NCBI BlastP on this gene

BofuT4P19000007001

similar to protein kinase C substrate
  
Accession: CCD34328
  
Location: 96414-98467
  
 NCBI BlastP on this gene

BofuT4\_P027120.1

hypothetical protein
  
Accession: CCD34329
  
Location: 99322-103200
  
 NCBI BlastP on this gene

BofuT4\_P027130.1

hypothetical protein
  
Accession: CCD34330
  
Location: 106966-107127
  
 NCBI BlastP on this gene

BofuT4\_uP027140.1

predicted protein
  
Accession: CCD34331
  
Location: 109972-110618
  
 NCBI BlastP on this gene

BofuT4\_P027150.1

hypothetical protein
  
Accession: CCD34332
  
Location: 111884-113234
  
 NCBI BlastP on this gene

BofuT4\_P027160.1

similar to phosphatidylinositol 3-kinase tor2
  
Accession: CCD34333
  
Location: 113396-120910
  
  
**BlastP hit with Mycgr3G67795\_Mycgr3T**
  
Percentage identity: 61 %
  
BlastP bit score: 3017
  
Sequence coverage: 100 %
  
E-value: 0.0
  
  
 NCBI BlastP on this gene

BofuT4\_P027170.1

228. :  KB644411 Penicillium oxalicum 114-2 unplaced genomic scaffold scaffold\_4     Total score: 1.0     Cumulative Blast bit score: 2988

hypothetical protein
  
Accession: EPS28851
  
Location: 1477479-1480217
  
 NCBI BlastP on this gene

EPS28851

hypothetical protein
  
Accession: EPS28850
  
Location: 1475338-1476459
  
 NCBI BlastP on this gene

EPS28850

hypothetical protein
  
Accession: EPS28849
  
Location: 1469180-1472940
  
 NCBI BlastP on this gene

EPS28849

hypothetical protein
  
Accession: EPS28848
  
Location: 1466475-1467446
  
 NCBI BlastP on this gene

EPS28848

hypothetical protein
  
Accession: EPS28847
  
Location: 1465401-1465968
  
 NCBI BlastP on this gene

EPS28847

hypothetical protein
  
Accession: EPS28846
  
Location: 1463235-1464659
  
 NCBI BlastP on this gene

EPS28846

hypothetical protein
  
Accession: EPS28845
  
Location: 1461612-1462881
  
 NCBI BlastP on this gene

EPS28845

hypothetical protein
  
Accession: EPS28844
  
Location: 1451506-1458895
  
  
**BlastP hit with Mycgr3G67795\_Mycgr3T**
  
Percentage identity: 60 %
  
BlastP bit score: 2988
  
Sequence coverage: 100 %
  
E-value: 0.0
  
  
 NCBI BlastP on this gene

EPS28844

hypothetical protein
  
Accession: EPS28843
  
Location: 1448733-1450621
  
 NCBI BlastP on this gene

EPS28843

hypothetical protein
  
Accession: EPS28842
  
Location: 1446499-1447452
  
 NCBI BlastP on this gene

EPS28842

hypothetical protein
  
Accession: EPS28841
  
Location: 1440708-1441670
  
 NCBI BlastP on this gene

EPS28841

hypothetical protein
  
Accession: EPS28840
  
Location: 1437791-1438978
  
 NCBI BlastP on this gene

EPS28840

hypothetical protein
  
Accession: EPS28839
  
Location: 1434401-1436878
  
 NCBI BlastP on this gene

EPS28839

229. :  CH445336 Phaeosphaeria nodorum SN15 scaffold\_12     Total score: 1.0     Cumulative Blast bit score: 2987

hypothetical protein
  
Accession: EAT84514
  
Location: 736894-738688
  
 NCBI BlastP on this gene

EAT84514

hypothetical protein
  
Accession: EAT84515
  
Location: 739086-739871
  
 NCBI BlastP on this gene

EAT84515

hypothetical protein
  
Accession: EAT84516
  
Location: 740176-744201
  
 NCBI BlastP on this gene

EAT84516

hypothetical protein
  
Accession: EAT84518
  
Location: 745881-749668
  
 NCBI BlastP on this gene

EAT84518

hypothetical protein
  
Accession: EAT84519
  
Location: 749786-750073
  
 NCBI BlastP on this gene

EAT84519

hypothetical protein
  
Accession: EAT84520
  
Location: 750378-752609
  
 NCBI BlastP on this gene

EAT84520

hypothetical protein
  
Accession: EAT84522
  
Location: 753226-760586
  
  
**BlastP hit with Mycgr3G67795\_Mycgr3T**
  
Percentage identity: 67 %
  
BlastP bit score: 2987
  
Sequence coverage: 91 %
  
E-value: 0.0
  
  
 NCBI BlastP on this gene

EAT84522

hypothetical protein
  
Accession: EAT84523
  
Location: 761315-762679
  
 NCBI BlastP on this gene

EAT84523

hypothetical protein
  
Accession: EAT84524
  
Location: 766213-766611
  
 NCBI BlastP on this gene

EAT84524

hypothetical protein
  
Accession: EAT84525
  
Location: 766656-767614
  
 NCBI BlastP on this gene

EAT84525

hypothetical protein
  
Accession: EAT84526
  
Location: 768093-769623
  
 NCBI BlastP on this gene

EAT84526

hypothetical protein
  
Accession: EAT84527
  
Location: 770374-774862
  
 NCBI BlastP on this gene

EAT84527

hypothetical protein
  
Accession: EAT84528
  
Location: 775101-776516
  
 NCBI BlastP on this gene

EAT84528

hypothetical protein
  
Accession: EAT84529
  
Location: 775989-777279
  
 NCBI BlastP on this gene

EAT84529

hypothetical protein
  
Accession: EAT84530
  
Location: 777358-777966
  
 NCBI BlastP on this gene

EAT84530

hypothetical protein
  
Accession: EAT84531
  
Location: 779210-780784
  
 NCBI BlastP on this gene

EAT84531

230. :  ABDF02000005 Trichoderma virens Gv29-8     Total score: 1.0     Cumulative Blast bit score: 2985

hypothetical protein
  
Accession: EHK23377
  
Location: 480599-482410
  
 NCBI BlastP on this gene

EHK23377

hypothetical protein
  
Accession: EHK23378
  
Location: 483472-483948
  
 NCBI BlastP on this gene

EHK23378

hypothetical protein
  
Accession: EHK23379
  
Location: 485856-487527
  
 NCBI BlastP on this gene

EHK23379

hypothetical protein
  
Accession: EHK23380
  
Location: 487946-490157
  
 NCBI BlastP on this gene

EHK23380

hypothetical protein
  
Accession: EHK23381
  
Location: 492806-493600
  
 NCBI BlastP on this gene

EHK23381

hypothetical protein
  
Accession: EHK23382
  
Location: 494284-501879
  
  
**BlastP hit with Mycgr3G67795\_Mycgr3T**
  
Percentage identity: 60 %
  
BlastP bit score: 2985
  
Sequence coverage: 100 %
  
E-value: 0.0
  
  
 NCBI BlastP on this gene

EHK23382

hypothetical protein
  
Accession: EHK23383
  
Location: 504240-504938
  
 NCBI BlastP on this gene

EHK23383

hypothetical protein
  
Accession: EHK23384
  
Location: 506569-508530
  
 NCBI BlastP on this gene

EHK23384

hypothetical protein
  
Accession: EHK23385
  
Location: 508905-510020
  
 NCBI BlastP on this gene

EHK23385

hypothetical protein
  
Accession: EHK23386
  
Location: 510982-513564
  
 NCBI BlastP on this gene

EHK23386

hypothetical protein
  
Accession: EHK23387
  
Location: 515730-518012
  
 NCBI BlastP on this gene

EHK23387

hypothetical protein
  
Accession: EHK23388
  
Location: 520232-522210
  
 NCBI BlastP on this gene

EHK23388

231. :  ABDG02000025 Trichoderma atroviride IMI 206040     Total score: 1.0     Cumulative Blast bit score: 2980

hypothetical protein
  
Accession: EHK43811
  
Location: 493895-495681
  
 NCBI BlastP on this gene

EHK43811

hypothetical protein
  
Accession: EHK43812
  
Location: 496703-497170
  
 NCBI BlastP on this gene

EHK43812

hypothetical protein
  
Accession: EHK43813
  
Location: 498726-500417
  
 NCBI BlastP on this gene

EHK43813

hypothetical protein
  
Accession: EHK43814
  
Location: 501460-502778
  
 NCBI BlastP on this gene

EHK43814

hypothetical protein
  
Accession: EHK43815
  
Location: 503657-506039
  
 NCBI BlastP on this gene

EHK43815

hypothetical protein
  
Accession: EHK43816
  
Location: 507701-508095
  
 NCBI BlastP on this gene

EHK43816

hypothetical protein
  
Accession: EHK43817
  
Location: 508715-509509
  
 NCBI BlastP on this gene

EHK43817

hypothetical protein
  
Accession: EHK43818
  
Location: 510193-517811
  
  
**BlastP hit with Mycgr3G67795\_Mycgr3T**
  
Percentage identity: 60 %
  
BlastP bit score: 2980
  
Sequence coverage: 100 %
  
E-value: 0.0
  
  
 NCBI BlastP on this gene

EHK43818

hypothetical protein
  
Accession: EHK43819
  
Location: 520143-520862
  
 NCBI BlastP on this gene

EHK43819

hypothetical protein
  
Accession: EHK43820
  
Location: 522339-524223
  
 NCBI BlastP on this gene

EHK43820

hypothetical protein
  
Accession: EHK43821
  
Location: 524541-525692
  
 NCBI BlastP on this gene

EHK43821

hypothetical protein
  
Accession: EHK43822
  
Location: 526704-529260
  
 NCBI BlastP on this gene

EHK43822

hypothetical protein
  
Accession: EHK43823
  
Location: 531957-534221
  
 NCBI BlastP on this gene

EHK43823

hypothetical protein
  
Accession: EHK43824
  
Location: 536415-538374
  
 NCBI BlastP on this gene

EHK43824

232. :  GL698476 Metarhizium acridum CQMa 102 unplaced genomic scaffold Scf\_007     Total score: 1.0     Cumulative Blast bit score: 2969

hypothetical protein
  
Accession: EFY92275
  
Location: 69167-70525
  
 NCBI BlastP on this gene

EFY92275

hypothetical protein
  
Accession: EFY92276
  
Location: 74732-75354
  
 NCBI BlastP on this gene

EFY92276

DOC family protein
  
Accession: EFY92277
  
Location: 75571-75912
  
 NCBI BlastP on this gene

EFY92277

hypothetical protein
  
Accession: EFY92278
  
Location: 80692-82985
  
 NCBI BlastP on this gene

EFY92278

DUF602 domain-containing protein
  
Accession: EFY92279
  
Location: 86568-87377
  
 NCBI BlastP on this gene

EFY92279

TOR kinase
  
Accession: EFY92280
  
Location: 88131-95605
  
  
**BlastP hit with Mycgr3G67795\_Mycgr3T**
  
Percentage identity: 59 %
  
BlastP bit score: 2969
  
Sequence coverage: 101 %
  
E-value: 0.0
  
  
 NCBI BlastP on this gene

EFY92280

ascus development protein 3
  
Accession: EFY92281
  
Location: 95971-97986
  
 NCBI BlastP on this gene

EFY92281

glucosidase 2 subunit beta precursor
  
Accession: EFY92282
  
Location: 98498-100398
  
 NCBI BlastP on this gene

EFY92282

hypothetical protein
  
Accession: EFY92283
  
Location: 100973-102124
  
 NCBI BlastP on this gene

EFY92283

hypothetical protein
  
Accession: EFY92284
  
Location: 103112-105667
  
 NCBI BlastP on this gene

EFY92284

hypothetical protein
  
Accession: EFY92285
  
Location: 108537-110176
  
 NCBI BlastP on this gene

EFY92285

ubiquitin fusion degradation protein (Ufd1), putative
  
Accession: EFY92286
  
Location: 111953-114226
  
 NCBI BlastP on this gene

EFY92286

233. :  HF679028 Fusarium fujikuroi IMI 58289 draft genome, chromosome FFUJ\_chr06.     Total score: 1.0     Cumulative Blast bit score: 2967

related to heterokaryon incompatibility protein (het-6OR allele)
  
Accession: CCT70550
  
Location: 3811000-3814090
  
 NCBI BlastP on this gene

FFUJ\_06530

uncharacterized protein
  
Accession: CCT70551
  
Location: 3814215-3815876
  
 NCBI BlastP on this gene

FFUJ\_06531

uncharacterized protein
  
Accession: CCT70552
  
Location: 3816636-3818742
  
 NCBI BlastP on this gene

FFUJ\_06532

related to glutamate carboxypeptidase II
  
Accession: CCT70553
  
Location: 3819588-3822051
  
 NCBI BlastP on this gene

FFUJ\_06533

uncharacterized protein
  
Accession: CCT70554
  
Location: 3822750-3824676
  
 NCBI BlastP on this gene

FFUJ\_06534

uncharacterized protein
  
Accession: CCT70555
  
Location: 3825712-3827637
  
 NCBI BlastP on this gene

FFUJ\_06535

uncharacterized protein
  
Accession: CCT70556
  
Location: 3828315-3829094
  
 NCBI BlastP on this gene

FFUJ\_06536

related to 1-phosphatidylinositol 3-kinase
  
Accession: CCT70557
  
Location: 3829917-3837349
  
  
**BlastP hit with Mycgr3G67795\_Mycgr3T**
  
Percentage identity: 60 %
  
BlastP bit score: 2967
  
Sequence coverage: 101 %
  
E-value: 0.0
  
  
 NCBI BlastP on this gene

FFUJ\_06537

234. :  JH226130 Exophiala dermatitidis NIH/UT8656 unplaced genomic scaffold supercont1.1     Total score: 1.0     Cumulative Blast bit score: 2944

hypothetical protein
  
Accession: EHY52968
  
Location: 3275321-3277908
  
 NCBI BlastP on this gene

EHY52968

hypothetical protein
  
Accession: EHY52969
  
Location: 3278708-3279868
  
 NCBI BlastP on this gene

EHY52969

hypothetical protein
  
Accession: EHY52970
  
Location: 3280489-3283019
  
 NCBI BlastP on this gene

EHY52970

hypothetical protein
  
Accession: EHY52971
  
Location: 3284361-3284744
  
 NCBI BlastP on this gene

EHY52971

ADP-ribosylglycohydrolase
  
Accession: EHY52972
  
Location: 3285271-3286308
  
 NCBI BlastP on this gene

EHY52972

hypothetical protein
  
Accession: EHY52973
  
Location: 3288749-3289630
  
 NCBI BlastP on this gene

EHY52973

DNA polymerase sigma subunit
  
Accession: EHY52974
  
Location: 3290507-3292709
  
 NCBI BlastP on this gene

EHY52974

FKBP12-rapamycin complex-associated protein
  
Accession: EHY52975
  
Location: 3294082-3301598
  
  
**BlastP hit with Mycgr3G67795\_Mycgr3T**
  
Percentage identity: 60 %
  
BlastP bit score: 2944
  
Sequence coverage: 100 %
  
E-value: 0.0
  
  
 NCBI BlastP on this gene

EHY52975

phospho-2-dehydro-3-deoxyheptonate aldolase, tyrosine-inhibited
  
Accession: EHY52976
  
Location: 3302250-3303386
  
 NCBI BlastP on this gene

EHY52976

hypothetical protein
  
Accession: EHY52977
  
Location: 3304313-3305203
  
 NCBI BlastP on this gene

EHY52977

hypothetical protein
  
Accession: EHY52978
  
Location: 3305490-3306482
  
 NCBI BlastP on this gene

EHY52978

hypothetical protein
  
Accession: EHY52979
  
Location: 3309672-3310484
  
 NCBI BlastP on this gene

EHY52979

hypothetical protein
  
Accession: EHY52980
  
Location: 3310878-3312380
  
 NCBI BlastP on this gene

EHY52980

hypothetical protein
  
Accession: EHY52981
  
Location: 3312853-3314227
  
 NCBI BlastP on this gene

EHY52981

hypothetical protein
  
Accession: EHY52982
  
Location: 3315060-3316210
  
 NCBI BlastP on this gene

EHY52982

hypothetical protein
  
Accession: EHY52983
  
Location: 3317185-3317503
  
 NCBI BlastP on this gene

EHY52983

glucan endo-1,3-beta-D-glucosidase
  
Accession: EHY52984
  
Location: 3319731-3321320
  
 NCBI BlastP on this gene

EHY52984

235. :  KB020785 Colletotrichum gloeosporioides Nara gc5 unplaced genomic scaffold scaffold403     Total score: 1.0     Cumulative Blast bit score: 2925

hypothetical protein
  
Accession: ELA30756
  
Location: 140823-141864
  
 NCBI BlastP on this gene

ELA30756

hypothetical protein
  
Accession: ELA30755
  
Location: 140029-140456
  
 NCBI BlastP on this gene

ELA30755

hypothetical protein
  
Accession: ELA30754
  
Location: 138730-139747
  
 NCBI BlastP on this gene

ELA30754

oxidoreductase
  
Accession: ELA30753
  
Location: 135325-136617
  
 NCBI BlastP on this gene

ELA30753

hypothetical protein
  
Accession: ELA30752
  
Location: 134041-134373
  
 NCBI BlastP on this gene

ELA30752

GrpB domain protein
  
Accession: ELA30751
  
Location: 131422-132042
  
 NCBI BlastP on this gene

ELA30751

hypothetical protein
  
Accession: ELA30750
  
Location: 130127-131181
  
 NCBI BlastP on this gene

ELA30750

hypothetical protein
  
Accession: ELA30749
  
Location: 128594-129703
  
 NCBI BlastP on this gene

ELA30749

glucosidase 2 subunit beta precursor
  
Accession: ELA30748
  
Location: 126122-128005
  
 NCBI BlastP on this gene

ELA30748

duf602 domain-containing protein
  
Accession: ELA30747
  
Location: 124920-125759
  
 NCBI BlastP on this gene

ELA30747

phosphatidylinositol 3-kinase tor2
  
Accession: ELA30746
  
Location: 116523-123904
  
  
**BlastP hit with Mycgr3G67795\_Mycgr3T**
  
Percentage identity: 59 %
  
BlastP bit score: 2925
  
Sequence coverage: 99 %
  
E-value: 0.0
  
  
 NCBI BlastP on this gene

ELA30746

C6 zinc finger domain protein
  
Accession: ELA30745
  
Location: 112486-114592
  
 NCBI BlastP on this gene

ELA30745

hypothetical protein
  
Accession: ELA30744
  
Location: 109158-110980
  
 NCBI BlastP on this gene

ELA30744

MFS monosaccharide transporter
  
Accession: ELA30743
  
Location: 106371-108903
  
 NCBI BlastP on this gene

ELA30743

ankyrin repeat protein
  
Accession: ELA30742
  
Location: 102806-104497
  
 NCBI BlastP on this gene

ELA30742

hypothetical protein
  
Accession: ELA30741
  
Location: 101993-102421
  
 NCBI BlastP on this gene

ELA30741

duf895 domain membrane protein
  
Accession: ELA30740
  
Location: 99466-100952
  
 NCBI BlastP on this gene

ELA30740

236. :  DS985228 Verticillium albo-atrum VaMs.102 supercont1.15 genomic scaffold     Total score: 1.0     Cumulative Blast bit score: 2910

conserved hypothetical protein
  
Accession: EEY23532
  
Location: 1017126-1018163
  
 NCBI BlastP on this gene

EEY23532

endoribonuclease L-PSP
  
Accession: EEY23533
  
Location: 1018520-1018998
  
 NCBI BlastP on this gene

EEY23533

high-affinity glucose transporter RGT2
  
Accession: EEY23534
  
Location: 1023472-1025294
  
 NCBI BlastP on this gene

EEY23534

oxidoreductase
  
Accession: EEY23535
  
Location: 1025616-1026875
  
 NCBI BlastP on this gene

EEY23535

bacterial leucyl aminopeptidase
  
Accession: EEY23536
  
Location: 1027413-1028668
  
 NCBI BlastP on this gene

EEY23536

conserved hypothetical protein
  
Accession: EEY23537
  
Location: 1029239-1030381
  
 NCBI BlastP on this gene

EEY23537

glucosidase 2 subunit beta
  
Accession: EEY23538
  
Location: 1030703-1032506
  
 NCBI BlastP on this gene

EEY23538

DUF602 domain-containing protein
  
Accession: EEY23539
  
Location: 1032634-1033449
  
 NCBI BlastP on this gene

EEY23539

phosphatidylinositol 3-kinase tor2
  
Accession: EEY23540
  
Location: 1034143-1041643
  
  
**BlastP hit with Mycgr3G67795\_Mycgr3T**
  
Percentage identity: 58 %
  
BlastP bit score: 2910
  
Sequence coverage: 101 %
  
E-value: 0.0
  
  
 NCBI BlastP on this gene

EEY23540

237. :  KB731260 Fusarium oxysporum f. sp. cubense race 1 unplaced genomic scaffold scaffold322     Total score: 1.0     Cumulative Blast bit score: 2907

Vacuolar protein sorting-associated protein 70
  
Accession: ENH62246
  
Location: 1582801-1585267
  
 NCBI BlastP on this gene

ENH62246

L-gulonolactone oxidase
  
Accession: ENH62247
  
Location: 1588983-1590641
  
 NCBI BlastP on this gene

ENH62247

Putative mitochondrial chaperone BCS1-B
  
Accession: ENH62248
  
Location: 1590891-1592441
  
 NCBI BlastP on this gene

ENH62248

UPF0214 protein yfeW
  
Accession: ENH62249
  
Location: 1593058-1594985
  
 NCBI BlastP on this gene

ENH62249

Transcriptional regulatory protein moc3
  
Accession: ENH62250
  
Location: 1596042-1597969
  
 NCBI BlastP on this gene

ENH62250

UPF0549 protein C1D4.09c
  
Accession: ENH62251
  
Location: 1598600-1599379
  
 NCBI BlastP on this gene

ENH62251

Phosphatidylinositol 3-kinase tor2
  
Accession: ENH62252
  
Location: 1600209-1607605
  
  
**BlastP hit with Mycgr3G67795\_Mycgr3T**
  
Percentage identity: 59 %
  
BlastP bit score: 2907
  
Sequence coverage: 101 %
  
E-value: 0.0
  
  
 NCBI BlastP on this gene

ENH62252

238. :  GL891307 Neurospora tetrasperma FGSC 2508 unplaced genomic scaffold NEUTE1scaffold\_6     Total score: 1.0     Cumulative Blast bit score: 2890

hypothetical protein
  
Accession: EGO54247
  
Location: 1308776-1309997
  
 NCBI BlastP on this gene

EGO54247

hypothetical protein
  
Accession: EGO54246
  
Location: 1305800-1307638
  
 NCBI BlastP on this gene

EGO54246

hypothetical protein
  
Accession: EGO54245
  
Location: 1301893-1305187
  
 NCBI BlastP on this gene

EGO54245

hypothetical protein
  
Accession: EGO54244
  
Location: 1301293-1301448
  
 NCBI BlastP on this gene

EGO54244

hypothetical protein
  
Accession: EGO54243
  
Location: 1297350-1300073
  
 NCBI BlastP on this gene

EGO54243

hypothetical protein
  
Accession: EGO54242
  
Location: 1295884-1297071
  
 NCBI BlastP on this gene

EGO54242

hypothetical protein
  
Accession: EGO54241
  
Location: 1293236-1295233
  
 NCBI BlastP on this gene

EGO54241

phosphatidylinositol 3-kinase tor2
  
Accession: EGO54240
  
Location: 1282635-1290514
  
  
**BlastP hit with Mycgr3G67795\_Mycgr3T**
  
Percentage identity: 59 %
  
BlastP bit score: 2890
  
Sequence coverage: 101 %
  
E-value: 0.0
  
  
 NCBI BlastP on this gene

EGO54240

hypothetical protein
  
Accession: EGO54239
  
Location: 1280950-1282213
  
 NCBI BlastP on this gene

EGO54239

hypothetical protein
  
Accession: EGO54238
  
Location: 1277587-1278941
  
 NCBI BlastP on this gene

EGO54238

hypothetical protein
  
Accession: EGO54237
  
Location: 1270261-1275212
  
 NCBI BlastP on this gene

EGO54237

hypothetical protein
  
Accession: EGO54236
  
Location: 1267913-1268969
  
 NCBI BlastP on this gene

EGO54236

hypothetical protein
  
Accession: EGO54235
  
Location: 1265451-1266683
  
 NCBI BlastP on this gene

EGO54235

hypothetical protein
  
Accession: EGO54234
  
Location: 1263792-1264901
  
 NCBI BlastP on this gene

EGO54234

239. :  GL988043 Chaetomium thermophilum var. thermophilum DSM 1495 unplaced genomic scaffold scf7180000...     Total score: 1.0     Cumulative Blast bit score: 2886

phosphatidylinositol 3-kinase-like protein
  
Accession: EGS20131
  
Location: 2367045-2374826
  
  
**BlastP hit with Mycgr3G67795\_Mycgr3T**
  
Percentage identity: 58 %
  
BlastP bit score: 2886
  
Sequence coverage: 101 %
  
E-value: 0.0
  
  
 NCBI BlastP on this gene

EGS20131

240. :  GL891269 Neurospora tetrasperma FGSC 2509 unplaced genomic scaffold NEUTE2scaffold\_7     Total score: 1.0     Cumulative Blast bit score: 2885

hypothetical protein
  
Accession: EGZ68319
  
Location: 2583835-2585056
  
 NCBI BlastP on this gene

EGZ68319

hypothetical protein
  
Accession: EGZ68320
  
Location: 2586194-2588032
  
 NCBI BlastP on this gene

EGZ68320

hypothetical protein
  
Accession: EGZ68321
  
Location: 2588658-2591940
  
 NCBI BlastP on this gene

EGZ68321

hypothetical protein
  
Accession: EGZ68322
  
Location: 2592385-2592540
  
 NCBI BlastP on this gene

EGZ68322

hypothetical protein
  
Accession: EGZ68323
  
Location: 2594054-2596481
  
 NCBI BlastP on this gene

EGZ68323

hypothetical protein
  
Accession: EGZ68324
  
Location: 2596760-2597947
  
 NCBI BlastP on this gene

EGZ68324

PRKCSH-domain-containing protein
  
Accession: EGZ68325
  
Location: 2598598-2600595
  
 NCBI BlastP on this gene

EGZ68325

phosphatidylinositol 3-kinase tor2
  
Accession: EGZ68326
  
Location: 2603317-2611196
  
  
**BlastP hit with Mycgr3G67795\_Mycgr3T**
  
Percentage identity: 59 %
  
BlastP bit score: 2885
  
Sequence coverage: 101 %
  
E-value: 0.0
  
  
 NCBI BlastP on this gene

EGZ68326

hypothetical protein
  
Accession: EGZ68327
  
Location: 2611618-2612881
  
 NCBI BlastP on this gene

EGZ68327

hypothetical protein
  
Accession: EGZ68328
  
Location: 2614877-2616244
  
 NCBI BlastP on this gene

EGZ68328

hypothetical protein
  
Accession: EGZ68329
  
Location: 2618617-2623568
  
 NCBI BlastP on this gene

EGZ68329

hypothetical protein
  
Accession: EGZ68330
  
Location: 2624860-2625916
  
 NCBI BlastP on this gene

EGZ68330

hypothetical protein
  
Accession: EGZ68331
  
Location: 2626374-2626571
  
 NCBI BlastP on this gene

EGZ68331

hypothetical protein
  
Accession: EGZ68332
  
Location: 2627146-2628378
  
 NCBI BlastP on this gene

EGZ68332

NAD(P)-binding protein
  
Accession: EGZ68333
  
Location: 2628928-2630035
  
 NCBI BlastP on this gene

EGZ68333

hypothetical protein
  
Accession: EGZ68334
  
Location: 2630034-2631828
  
 NCBI BlastP on this gene

EGZ68334

241. :  JH126401 Cordyceps militaris CM01 unplaced genomic scaffold CCM\_S00003     Total score: 1.0     Cumulative Blast bit score: 2871

phosphatidylinositol 3-kinase tor2
  
Accession: EGX93526
  
Location: 4074259-4081703
  
  
**BlastP hit with Mycgr3G67795\_Mycgr3T**
  
Percentage identity: 58 %
  
BlastP bit score: 2871
  
Sequence coverage: 100 %
  
E-value: 0.0
  
  
 NCBI BlastP on this gene

EGX93526

MFS transporter, putative
  
Accession: EGX93525
  
Location: 4071305-4073166
  
 NCBI BlastP on this gene

EGX93525

short-chain dehydrogenase
  
Accession: EGX93524
  
Location: 4070458-4071261
  
 NCBI BlastP on this gene

EGX93524

hypothetical protein
  
Accession: EGX93523
  
Location: 4068231-4069916
  
 NCBI BlastP on this gene

EGX93523

sugar transporter, putative
  
Accession: EGX93522
  
Location: 4062780-4064479
  
 NCBI BlastP on this gene

EGX93522

amino acid transporter, putative
  
Accession: EGX93521
  
Location: 4058118-4060856
  
 NCBI BlastP on this gene

EGX93521

mitochondrial ribosomal protein S16
  
Accession: EGX93520
  
Location: 4057161-4057590
  
 NCBI BlastP on this gene

EGX93520

YjeF-related protein
  
Accession: EGX93519
  
Location: 4054361-4055635
  
 NCBI BlastP on this gene

EGX93519

242. :  CABT02000010 Sordaria macrospora k-hell     Total score: 1.0     Cumulative Blast bit score: 2867

not annotated
  
Accession: CCC09760
  
Location: 257888-259750
  
 NCBI BlastP on this gene

CCC09760

not annotated
  
Accession: CCC09761
  
Location: 260556-263940
  
 NCBI BlastP on this gene

CCC09761

not annotated
  
Accession: CCC09762
  
Location: 267730-270139
  
 NCBI BlastP on this gene

CCC09762

not annotated
  
Accession: CCC09763
  
Location: 270438-271664
  
 NCBI BlastP on this gene

CCC09763

not annotated
  
Accession: CCC09764
  
Location: 272270-274265
  
 NCBI BlastP on this gene

CCC09764

not annotated
  
Accession: CCC09765
  
Location: 274530-275519
  
 NCBI BlastP on this gene

CCC09765

not annotated
  
Accession: CCC09766
  
Location: 277345-285153
  
  
**BlastP hit with Mycgr3G67795\_Mycgr3T**
  
Percentage identity: 58 %
  
BlastP bit score: 2867
  
Sequence coverage: 103 %
  
E-value: 0.0
  
  
 NCBI BlastP on this gene

CCC09766

not annotated
  
Accession: CCC09767
  
Location: 286034-286351
  
 NCBI BlastP on this gene

CCC09767

not annotated
  
Accession: CCC09768
  
Location: 286590-287373
  
 NCBI BlastP on this gene

CCC09768

not annotated
  
Accession: CCC09769
  
Location: 290160-291519
  
 NCBI BlastP on this gene

CCC09769

not annotated
  
Accession: CCC09770
  
Location: 294709-297433
  
 NCBI BlastP on this gene

CCC09770

not annotated
  
Accession: CCC09771
  
Location: 297940-299325
  
 NCBI BlastP on this gene

CCC09771

not annotated
  
Accession: CCC09772
  
Location: 300663-301070
  
 NCBI BlastP on this gene

CCC09772

not annotated
  
Accession: CCC09773
  
Location: 301089-301663
  
 NCBI BlastP on this gene

CCC09773

not annotated
  
Accession: CCC09774
  
Location: 303903-306457
  
 NCBI BlastP on this gene

CCC09774

243. :  CP003005 Myceliophthora thermophila ATCC 42464 chromosome 4     Total score: 1.0     Cumulative Blast bit score: 2860

hypothetical protein
  
Accession: AEO59199
  
Location: 3401001-3403023
  
 NCBI BlastP on this gene

MYCTH\_2307276

hypothetical protein
  
Accession: AEO59200
  
Location: 3403622-3404749
  
 NCBI BlastP on this gene

MYCTH\_68944

hypothetical protein
  
Accession: AEO59201
  
Location: 3405142-3405689
  
 NCBI BlastP on this gene

MYCTH\_2307282

hypothetical protein
  
Accession: AEO59202
  
Location: 3405875-3406788
  
 NCBI BlastP on this gene

MYCTH\_2307284

hypothetical protein
  
Accession: AEO59203
  
Location: 3407536-3409188
  
 NCBI BlastP on this gene

MYCTH\_2307285

hypothetical protein
  
Accession: AEO59204
  
Location: 3409613-3411808
  
 NCBI BlastP on this gene

MYCTH\_2119478

hypothetical protein
  
Accession: AEO59205
  
Location: 3412406-3412588
  
 NCBI BlastP on this gene

MYCTH\_2307286

hypothetical protein
  
Accession: AEO59206
  
Location: 3412748-3413932
  
 NCBI BlastP on this gene

MYCTH\_2307287

hypothetical protein
  
Accession: AEO59207
  
Location: 3414496-3416390
  
 NCBI BlastP on this gene

MYCTH\_2307289

hypothetical protein
  
Accession: AEO59208
  
Location: 3416625-3417485
  
 NCBI BlastP on this gene

MYCTH\_2139768

hypothetical protein
  
Accession: AEO59209
  
Location: 3418360-3426313
  
  
**BlastP hit with Mycgr3G67795\_Mycgr3T**
  
Percentage identity: 58 %
  
BlastP bit score: 2860
  
Sequence coverage: 101 %
  
E-value: 0.0
  
  
 NCBI BlastP on this gene

MYCTH\_2307294

244. :  CP003009 Thielavia terrestris NRRL 8126 chromosome 1     Total score: 1.0     Cumulative Blast bit score: 2858

hypothetical protein
  
Accession: AEO64488
  
Location: 8782057-8782233
  
 NCBI BlastP on this gene

THITE\_2110662

hypothetical protein
  
Accession: AEO64489
  
Location: 8783335-8785351
  
 NCBI BlastP on this gene

THITE\_2110665

hypothetical protein
  
Accession: AEO64490
  
Location: 8785907-8787049
  
 NCBI BlastP on this gene

THITE\_74570

hypothetical protein
  
Accession: AEO64491
  
Location: 8787722-8789079
  
 NCBI BlastP on this gene

THITE\_2142374

hypothetical protein
  
Accession: AEO64492
  
Location: 8789639-8791252
  
 NCBI BlastP on this gene

THITE\_2110674

hypothetical protein
  
Accession: AEO64493
  
Location: 8791617-8792579
  
 NCBI BlastP on this gene

THITE\_2110676

hypothetical protein
  
Accession: AEO64494
  
Location: 8793245-8793454
  
 NCBI BlastP on this gene

THITE\_2110677

hypothetical protein
  
Accession: AEO64495
  
Location: 8794587-8794820
  
 NCBI BlastP on this gene

THITE\_2169703

hypothetical protein
  
Accession: AEO64496
  
Location: 8795259-8796452
  
 NCBI BlastP on this gene

THITE\_2110679

hypothetical protein
  
Accession: AEO64497
  
Location: 8796807-8798705
  
 NCBI BlastP on this gene

THITE\_2110681

hypothetical protein
  
Accession: AEO64498
  
Location: 8798971-8799846
  
 NCBI BlastP on this gene

THITE\_2110682

hypothetical protein
  
Accession: AEO64499
  
Location: 8800947-8809066
  
  
**BlastP hit with Mycgr3G67795\_Mycgr3T**
  
Percentage identity: 59 %
  
BlastP bit score: 2858
  
Sequence coverage: 100 %
  
E-value: 0.0
  
  
 NCBI BlastP on this gene

THITE\_2110683

245. :  CM001231 Magnaporthe oryzae 70-15 chromosome 1     Total score: 1.0     Cumulative Blast bit score: 2791

beta-glucosidase 1
  
Accession: EHA57868
  
Location: 5803841-5806249
  
 NCBI BlastP on this gene

EHA57868

quinate permease
  
Accession: EHA57869
  
Location: 5806949-5808856
  
 NCBI BlastP on this gene

EHA57869

hypothetical protein
  
Accession: EHA57870
  
Location: 5811254-5812363
  
 NCBI BlastP on this gene

EHA57870

hypothetical protein
  
Accession: EHA57871
  
Location: 5812898-5814782
  
 NCBI BlastP on this gene

EHA57871

hypothetical protein
  
Accession: EHA57872
  
Location: 5814996-5815808
  
 NCBI BlastP on this gene

EHA57872

phosphatidylinositol 3-kinase tor2
  
Accession: EHA57873
  
Location: 5816682-5824299
  
  
**BlastP hit with Mycgr3G67795\_Mycgr3T**
  
Percentage identity: 57 %
  
BlastP bit score: 2791
  
Sequence coverage: 100 %
  
E-value: 0.0
  
  
 NCBI BlastP on this gene

EHA57873

246. :  GL385396 Gaeumannomyces graminis var. tritici R3-111a-1 unplaced genomic scaffold supercont2.2     Total score: 1.0     Cumulative Blast bit score: 2783

hypothetical protein
  
Accession: EJT78057
  
Location: 1902638-1906339
  
 NCBI BlastP on this gene

EJT78057

hypothetical protein
  
Accession: EJT78056
  
Location: 1899476-1899823
  
 NCBI BlastP on this gene

EJT78056

hypothetical protein
  
Accession: EJT78055
  
Location: 1897893-1898153
  
 NCBI BlastP on this gene

EJT78055

hypothetical protein
  
Accession: EJT78054
  
Location: 1896219-1896596
  
 NCBI BlastP on this gene

EJT78054

hypothetical protein
  
Accession: EJT78053
  
Location: 1893532-1895921
  
 NCBI BlastP on this gene

EJT78053

hypothetical protein
  
Accession: EJT78052
  
Location: 1889818-1891844
  
 NCBI BlastP on this gene

EJT78052

hypothetical protein
  
Accession: EJT78051
  
Location: 1888521-1889654
  
 NCBI BlastP on this gene

EJT78051

hypothetical protein
  
Accession: EJT78050
  
Location: 1886043-1887901
  
 NCBI BlastP on this gene

EJT78050

hypothetical protein
  
Accession: EJT78049
  
Location: 1885014-1885814
  
 NCBI BlastP on this gene

EJT78049

phosphatidylinositol 3-kinase tor2
  
Accession: EJT78048
  
Location: 1876371-1884071
  
  
**BlastP hit with Mycgr3G67795\_Mycgr3T**
  
Percentage identity: 56 %
  
BlastP bit score: 2783
  
Sequence coverage: 102 %
  
E-value: 0.0
  
  
 NCBI BlastP on this gene

EJT78048

atypical/ABC1/ABC1-C protein kinase
  
Accession: EJT78047
  
Location: 1873320-1875692
  
 NCBI BlastP on this gene

EJT78047

cytochrome c heme lyase
  
Accession: EJT78046
  
Location: 1871671-1872823
  
 NCBI BlastP on this gene

EJT78046

hypothetical protein
  
Accession: EJT78045
  
Location: 1870656-1871319
  
 NCBI BlastP on this gene

EJT78045

hypothetical protein
  
Accession: EJT78044
  
Location: 1867689-1869392
  
 NCBI BlastP on this gene

EJT78044

hypothetical protein
  
Accession: EJT78043
  
Location: 1864470-1867207
  
 NCBI BlastP on this gene

EJT78043

hypothetical protein
  
Accession: EJT78042
  
Location: 1860495-1861942
  
 NCBI BlastP on this gene

EJT78042

hypothetical protein
  
Accession: EJT78041
  
Location: 1857808-1859332
  
 NCBI BlastP on this gene

EJT78041

hypothetical protein
  
Accession: EJT78040
  
Location: 1856783-1857439
  
 NCBI BlastP on this gene

EJT78040

247. :  KE503206 Schizosaccharomyces octosporus yFS286 unplaced genomic scaffold supercont6.1     Total score: 1.0     Cumulative Blast bit score: 2608

eIF3e subunit Int6
  
Accession: EPX74339
  
Location: 2204488-2206092
  
 NCBI BlastP on this gene

EPX74339

oxysterol binding protein
  
Accession: EPX74340
  
Location: 2206759-2208351
  
 NCBI BlastP on this gene

EPX74340

enoyl reductase
  
Accession: EPX74341
  
Location: 2209069-2210065
  
 NCBI BlastP on this gene

EPX74341

glucan endo-1,3-alpha-glucosidase Agn2
  
Accession: EPX74342
  
Location: 2210671-2212074
  
 NCBI BlastP on this gene

EPX74342

squalene synthase Erg9
  
Accession: EPX74343
  
Location: 2212767-2214164
  
 NCBI BlastP on this gene

EPX74343

poly(A) polymerase Pla1
  
Accession: EPX74344
  
Location: 2214406-2216170
  
 NCBI BlastP on this gene

EPX74344

glutamyl-tRNA amidotransferase alpha subunit
  
Accession: EPX74345
  
Location: 2216517-2218012
  
 NCBI BlastP on this gene

EPX74345

complexed with Cdc5 protein Cwf11
  
Accession: EPX74346
  
Location: 2218240-2222058
  
 NCBI BlastP on this gene

EPX74346

phosphatidylinositol kinase Tor2
  
Accession: EPX74347
  
Location: 2222529-2229545
  
  
**BlastP hit with Mycgr3G67795\_Mycgr3T**
  
Percentage identity: 54 %
  
BlastP bit score: 2608
  
Sequence coverage: 99 %
  
E-value: 0.0
  
  
 NCBI BlastP on this gene

EPX74347

hypothetical protein
  
Accession: EPX74348
  
Location: 2230008-2232995
  
 NCBI BlastP on this gene

EPX74348

ATR checkpoint kinase Rad3
  
Accession: EPX74349
  
Location: 2233034-2240209
  
 NCBI BlastP on this gene

EPX74349

methionine sulfoxide
  
Accession: EPX74350
  
Location: 2241382-2241885
  
 NCBI BlastP on this gene

EPX74350

fungal protein
  
Accession: EPX74351
  
Location: 2242264-2243007
  
 NCBI BlastP on this gene

EPX74351

cortical anchoring factor for dynein Mcp5/Num1
  
Accession: EPX74352
  
Location: 2243997-2246858
  
 NCBI BlastP on this gene

EPX74352

DNA damage response protein
  
Accession: EPX74353
  
Location: 2247466-2250004
  
 NCBI BlastP on this gene

EPX74353

248. :  DS022226 Schizosaccharomyces japonicus yFS275 supercont1.3 genomic scaffold     Total score: 1.0     Cumulative Blast bit score: 2598

transmembrane and coiled-coil domain-containing protein
  
Accession: EEB07618
  
Location: 1314495-1316201
  
 NCBI BlastP on this gene

EEB07618

btn1
  
Accession: EEB07619
  
Location: 1316682-1318016
  
 NCBI BlastP on this gene

EEB07619

synaptic glycoprotein SC2
  
Accession: EEB07620
  
Location: 1318406-1319537
  
 NCBI BlastP on this gene

EEB07620

glucan endo-1,3-alpha-glucosidase agn2
  
Accession: EEB07621
  
Location: 1320337-1321704
  
 NCBI BlastP on this gene

EEB07621

squalene synthetase
  
Accession: EEB07622
  
Location: 1322316-1323719
  
 NCBI BlastP on this gene

EEB07622

Poly(A) polymerase pla1
  
Accession: EEB07623
  
Location: 1324131-1326144
  
 NCBI BlastP on this gene

EEB07623

glutamyl-tRNA(Gln) amidotransferase subunit A
  
Accession: EEB07624
  
Location: 1326774-1328366
  
 NCBI BlastP on this gene

EEB07624

predicted protein
  
Accession: EEB07625
  
Location: 1329419-1333300
  
 NCBI BlastP on this gene

EEB07625

phosphatidylinositol kinase Tor2
  
Accession: EEB07626
  
Location: 1333747-1340757
  
  
**BlastP hit with Mycgr3G67795\_Mycgr3T**
  
Percentage identity: 53 %
  
BlastP bit score: 2598
  
Sequence coverage: 100 %
  
E-value: 0.0
  
  
 NCBI BlastP on this gene

EEB07626

249. :  KE546988 Schizosaccharomyces cryophilus OY26 unplaced genomic scaffold supercont4.1     Total score: 1.0     Cumulative Blast bit score: 2588

phosphatidylinositol kinase Tor2
  
Accession: EPY54289
  
Location: 2784543-2791559
  
  
**BlastP hit with Mycgr3G67795\_Mycgr3T**
  
Percentage identity: 54 %
  
BlastP bit score: 2588
  
Sequence coverage: 99 %
  
E-value: 0.0
  
  
 NCBI BlastP on this gene

EPY54289

replication fork protection complex subunit Swi1
  
Accession: EPY54288
  
Location: 2781068-2784057
  
 NCBI BlastP on this gene

EPY54288

ATR checkpoint kinase Rad3
  
Accession: EPY54287
  
Location: 2773840-2781018
  
 NCBI BlastP on this gene

EPY54287

methionine sulfoxide
  
Accession: EPY54286
  
Location: 2772007-2772507
  
 NCBI BlastP on this gene

EPY54286

fungal protein
  
Accession: EPY54285
  
Location: 2770856-2771599
  
 NCBI BlastP on this gene

EPY54285

cortical anchoring factor for dynein Mcp5/Num1
  
Accession: EPY54284
  
Location: 2766780-2769644
  
 NCBI BlastP on this gene

EPY54284

250. :  CU329671 Schizosaccharomyces pombe chromosome II     Total score: 1.0     Cumulative Blast bit score: 2580

U3 snoRNP protein Nop56 (predicted)
  
Accession: CAA22814
  
Location: 940825-942318
  
 NCBI BlastP on this gene

SPBC646.10c

eIF3e subunit Int6
  
Accession: CAA22813
  
Location: 938700-940261
  
 NCBI BlastP on this gene

int6

oxysterol binding protein (predicted)
  
Accession: CAA22812
  
Location: 936541-938091
  
 NCBI BlastP on this gene

SPBC646.08c

enoyl reductase (predicted)
  
Accession: CAA22811
  
Location: 934716-935603
  
 NCBI BlastP on this gene

SPBC646.07c

glucan endo-1,3-alpha-glucosidase Agn2
  
Accession: CAH58744
  
Location: 932781-934082
  
 NCBI BlastP on this gene

agn2

squalene synthase Erg9 (predicted)
  
Accession: CAA22809
  
Location: 931060-932442
  
 NCBI BlastP on this gene

erg9

poly(A) polymerase Pla1
  
Accession: CAA22808
  
Location: 928989-930812
  
 NCBI BlastP on this gene

pla1

mitochondrial glutamyl-tRNA amidotransferase alpha subunit (predicted)
  
Accession: CAA22807
  
Location: 926850-928358
  
 NCBI BlastP on this gene

SPBC646.03

complexed with Cdc5 protein Cwf11
  
Accession: CAA22806
  
Location: 922725-926579
  
 NCBI BlastP on this gene

cwf11

phosphatidylinositol kinase Tor2
  
Accession: CAB40167
  
Location: 915271-922284
  
  
**BlastP hit with Mycgr3G67795\_Mycgr3T**
  
Percentage identity: 53 %
  
BlastP bit score: 2580
  
Sequence coverage: 100 %
  
E-value: 0.0
  
  
 NCBI BlastP on this gene

tor2

replication fork protection complex subunit Swi1
  
Accession: CAB40166
  
Location: 911603-914593
  
 NCBI BlastP on this gene

swi1

ATR checkpoint kinase Rad3
  
Accession: CAB40165
  
Location: 904365-911525
  
 NCBI BlastP on this gene

rad3

methionine sulfoxide (predicted)
  
Accession: CAB40164
  
Location: 903156-903572
  
 NCBI BlastP on this gene

SPBC216.04c

conserved fungal protein
  
Accession: CAB40163
  
Location: 901942-902685
  
 NCBI BlastP on this gene

SPBC216.03

cortical anchoring factor for dynein Mcp5/Num1
  
Accession: CAB40162
  
Location: 898075-900981
  
 NCBI BlastP on this gene

mcp5

Detecting sequence homology at the gene cluster level with MultiGeneBlast.
  
Marnix H. Medema, Rainer Breitling & Eriko Takano (2013)
  
*Molecular Biology and Evolution* , 30: 1218-1223.
