# Supplementary material for: In silico prediction and characterization of secondary metabolite biosynthetic gene clusters in the wheat pathogen Zymoseptoria tritici
Source: BMC Genomics. 2017 Aug 17;18:631. doi: 10.1186/s12864-017-3969-y (PMC5561558; doi:10.1186/s12864-017-3969-y)
Supplement: Supplementary file 1 — MultiGeneBLAST analysis of putative secondary metabolite clusters. All encoded amino acid sequences from genes residing in clusters predicted by AntiSMASH are given as FASTA file format. All output data from MultiGeneBLASTs are also provided. (ZIP 42911 kb) [file 12864_2017_3969_MOESM1_ESM.zip › Cluster MultiGene BLAST/out/Clusters_1_34/Cluster_11/displaypage1.xhtml]

xml version="1.0" encoding="UTF-8"?


Search Results
  
  
 Results pages: 1, 2, 3, 4, 5

**MultiGeneBlast hits**

Select gene cluster alignment
1. CM001197\_1 Mycosphaerella graminicola IPO323 chromosome 2, whole genome sh...
2. AHHD01000300\_0 Macrophomina phaseolina MS6, whole genome shotgun sequencin...
3. DS995900\_1 Penicillium marneffei ATCC 18224 scf\_1105668340758 genomic scaf...
4. KB725800\_0 Colletotrichum orbiculare MAFF 240422 unplaced genomic scaffold...
5. GL698590\_0 Metarhizium acridum CQMa 102 unplaced genomic scaffold Scf\_121,...
6. GL891306\_0 Neurospora tetrasperma FGSC 2508 unplaced genomic scaffold NEUT...
7. GL891258\_0 Neurospora tetrasperma FGSC 2509 unplaced genomic scaffold NEUT...
8. CABT02000031\_0 Sordaria macrospora k-hell, whole genome shotgun sequencing...
9. CM001199\_0 Mycosphaerella graminicola IPO323 chromosome 4, whole genome sh...
10. AHHD01000518\_1 Macrophomina phaseolina MS6, whole genome shotgun sequenci...
11. KE145356\_1 Glarea lozoyensis ATCC 20868 chromosome Unknown GLAREA13, whol...
12. KB916208\_0 Neofusicoccum parvum UCRNP2 chromosome Unknown NP2\_03\_scaffold...
13. KB644410\_0 Penicillium oxalicum 114-2 unplaced genomic scaffold scaffold\_...
14. ABDF02000064\_0 Trichoderma virens Gv29-8, whole genome shotgun sequencing...
15. JH226133\_0 Exophiala dermatitidis NIH/UT8656 unplaced genomic scaffold su...
16. JX421684\_0 Emericella rugulosa strain NRRL 11440 hypothetical protein gen...
17. EQ962654\_5 Talaromyces stipitatus ATCC 10500 scf\_1105507295541 genomic sc...
18. AKHY01000111\_0 Aspergillus oryzae 3.042, whole genome shotgun sequencing ...
19. AB705455\_0 Aspergillus oryzae DNA, WYK-1 cluster, ORF1, ORF2, ORF3, wykN,...
20. AP007154\_0 Aspergillus oryzae RIB40 DNA, SC001.
21. ABDF02000068\_0 Trichoderma virens Gv29-8, whole genome shotgun sequencing...
22. EQ963479\_1 Aspergillus flavus NRRL3357 scf\_1106286418500 genomic scaffold...
23. AKCU01000056\_0 Penicillium digitatum Pd1, whole genome shotgun sequencing...
24. AKCT01000339\_0 Penicillium digitatum PHI26, whole genome shotgun sequenci...
25. AP007154\_1 Aspergillus oryzae RIB40 DNA, SC001.
26. GL698751\_0 Metarhizium anisopliae ARSEF 23 unplaced genomic scaffold Scf\_...
27. GL698592\_0 Metarhizium acridum CQMa 102 unplaced genomic scaffold Scf\_123...
28. CM001231\_1 Magnaporthe oryzae 70-15 chromosome 1, whole genome shotgun se...
29. CABT02000016\_0 Sordaria macrospora k-hell, whole genome shotgun sequencin...
30. CH445354\_0 Phaeosphaeria nodorum SN15 scaffold\_30, whole genome shotgun s...
31. KB708022\_0 Botryotinia fuckeliana BcDW1 unplaced genomic scaffold Scaffol...
32. FQ790270\_0 Botryotinia fuckeliana T4 SuperContig\_51\_1 genomic supercontig.
33. KB705649\_0 Eutypa lata UCREL1 unplaced genomic scaffold EL1\_03\_scaffold\_3...
34. AAHF01000003\_0 Aspergillus fumigatus Af293, whole genome shotgun sequenci...
35. DS499597\_4 Aspergillus fumigatus A1163 scf\_000004 genomic scaffold, whole...
36. ACJE01000008\_1 Aspergillus niger ATCC 1015, whole genome shotgun sequenci...
37. EQ963479\_0 Aspergillus flavus NRRL3357 scf\_1106286418500 genomic scaffold...
38. ABDF02000003\_3 Trichoderma virens Gv29-8, whole genome shotgun sequencing...
39. GL534459\_0 Pyrenophora teres f. teres 0-1 unplaced genomic scaffold scaff...
40. DS027058\_1 Aspergillus clavatus NRRL 1 1099423829804 genomic scaffold, wh...
41. KE145353\_0 Glarea lozoyensis ATCC 20868 chromosome Unknown GLAREA10, whol...
42. FP929139\_3 Leptosphaeria maculans JN3 lm\_SuperContig\_0\_v2 genomic superco...
43. CH445358\_0 Phaeosphaeria nodorum SN15 scaffold\_34, whole genome shotgun s...
44. KB908703\_0 Setosphaeria turcica Et28A unplaced genomic scaffold SETTUscaf...
45. KB733474\_0 Bipolaris maydis ATCC 48331 unplaced genomic scaffold COCC4sca...
46. KB445640\_0 Cochliobolus sativus ND90Pr unplaced genomic scaffold COCSAsca...
47. KB445571\_1 Cochliobolus heterostrophus C5 unplaced genomic scaffold COCHE...
48. DS231615\_2 Pyrenophora tritici-repentis Pt-1C-BFP supercont1.1 genomic sc...
49. JH126401\_0 Cordyceps militaris CM01 unplaced genomic scaffold CCM\_S00003,...
50. DS027684\_0 Neosartorya fischeri NRRL 181 1099437636244 genomic scaffold, ...

Query: Architecture Search FASTA input

CM001197 : Mycosphaerella graminicola IPO323 chromosome 2    Total score: 13.0     Cumulative Blast bit score: 19999

Hit cluster cross-links:

Mycgr3G36335 Mycgr3T
  
Location: 0-423

Mycgr3G36335\_Mycgr3T

Mycgr3G84494 Mycgr3T
  
Location: 523-2047

Mycgr3G84494\_Mycgr3T

Mycgr3G90558 Mycgr3T
  
Location: 2147-15296

Mycgr3G90558\_Mycgr3T

Mycgr3G68036 Mycgr3T
  
Location: 15396-16395

Mycgr3G68036\_Mycgr3T

Mycgr3G90561 Mycgr3T
  
Location: 16495-17134

Mycgr3G90561\_Mycgr3T

Mycgr3G35862 Mycgr3T
  
Location: 17234-18662

Mycgr3G35862\_Mycgr3T

Mycgr3G68030 Mycgr3T
  
Location: 18762-19722

Mycgr3G68030\_Mycgr3T

Mycgr3G36449 Mycgr3T
  
Location: 19822-21886

Mycgr3G36449\_Mycgr3T

Mycgr3G35528 Mycgr3T
  
Location: 21986-22844

Mycgr3G35528\_Mycgr3T

Mycgr3G35932 Mycgr3T
  
Location: 22944-24390

Mycgr3G35932\_Mycgr3T

Mycgr3G23761 Mycgr3T
  
Location: 24490-25825

Mycgr3G23761\_Mycgr3T

Mycgr3G35535 Mycgr3T
  
Location: 25925-26429

Mycgr3G35535\_Mycgr3T

Mycgr3G9942 Mycgr3T9
  
Location: 26529-30375

Mycgr3G9942\_Mycgr3T9

hypothetical protein
  
Accession: EGP90649
  
Location: 1057230-1058615
  
  
**BlastP hit with Mycgr3G23761\_Mycgr3T**
  
Percentage identity: 100 %
  
BlastP bit score: 880
  
Sequence coverage: 100 %
  
E-value: 0.0
  
  
 NCBI BlastP on this gene

EGP90649

putative ABC transporter
  
Accession: EGP90650
  
Location: 1048431-1053118
  
  
**BlastP hit with Mycgr3G9942\_Mycgr3T9**
  
Percentage identity: 99 %
  
BlastP bit score: 2607
  
Sequence coverage: 100 %
  
E-value: 0.0
  
  
 NCBI BlastP on this gene

EGP90650

hypothetical protein
  
Accession: EGP89809
  
Location: 1047744-1048299
  
  
**BlastP hit with Mycgr3G35535\_Mycgr3T**
  
Percentage identity: 100 %
  
BlastP bit score: 345
  
Sequence coverage: 99 %
  
E-value: 3e-119
  
  
 NCBI BlastP on this gene

EGP89809

hypothetical protein
  
Accession: EGP90651
  
Location: 1045245-1046984
  
  
**BlastP hit with Mycgr3G90561\_Mycgr3T**
  
Percentage identity: 100 %
  
BlastP bit score: 449
  
Sequence coverage: 99 %
  
E-value: 2e-158
  
  
 NCBI BlastP on this gene

EGP90651

hypothetical protein
  
Accession: EGP89808
  
Location: 1042943-1045006
  
  
**BlastP hit with Mycgr3G36449\_Mycgr3T**
  
Percentage identity: 100 %
  
BlastP bit score: 1430
  
Sequence coverage: 99 %
  
E-value: 0.0
  
  
 NCBI BlastP on this gene

EGP89808

hypothetical protein
  
Accession: EGP89807
  
Location: 1041170-1042286
  
  
**BlastP hit with Mycgr3G68036\_Mycgr3T**
  
Percentage identity: 100 %
  
BlastP bit score: 689
  
Sequence coverage: 99 %
  
E-value: 0.0
  
  
 NCBI BlastP on this gene

EGP89807

hypothetical protein
  
Accession: EGP90652
  
Location: 1026842-1040878
  
  
**BlastP hit with Mycgr3G90558\_Mycgr3T**
  
Percentage identity: 100 %
  
BlastP bit score: 9057
  
Sequence coverage: 99 %
  
E-value: 0.0
  
  
 NCBI BlastP on this gene

EGP90652

hypothetical protein
  
Accession: EGP90653
  
Location: 1025441-1026407
  
  
**BlastP hit with Mycgr3G35528\_Mycgr3T**
  
Percentage identity: 100 %
  
BlastP bit score: 601
  
Sequence coverage: 99 %
  
E-value: 0.0
  
  
 NCBI BlastP on this gene

EGP90653

hypothetical protein
  
Accession: EGP90654
  
Location: 1016623-1017902
  
  
**BlastP hit with Mycgr3G68030\_Mycgr3T**
  
Percentage identity: 100 %
  
BlastP bit score: 669
  
Sequence coverage: 99 %
  
E-value: 0.0
  
  
 NCBI BlastP on this gene

EGP90654

putative major facilitator superfamily transporter
  
Accession: EGP90655
  
Location: 1014611-1016256
  
  
**BlastP hit with Mycgr3G84494\_Mycgr3T**
  
Percentage identity: 100 %
  
BlastP bit score: 1020
  
Sequence coverage: 99 %
  
E-value: 0.0
  
  
 NCBI BlastP on this gene

EGP90655

hypothetical protein
  
Accession: EGP90656
  
Location: 1013898-1014320
  
  
**BlastP hit with Mycgr3G36335\_Mycgr3T**
  
Percentage identity: 100 %
  
BlastP bit score: 296
  
Sequence coverage: 100 %
  
E-value: 1e-100
  
  
 NCBI BlastP on this gene

EGP90656

hypothetical protein
  
Accession: EGP89806
  
Location: 1010594-1012246
  
  
**BlastP hit with Mycgr3G35932\_Mycgr3T**
  
Percentage identity: 100 %
  
BlastP bit score: 969
  
Sequence coverage: 99 %
  
E-value: 0.0
  
  
 NCBI BlastP on this gene

EGP89806

peptidase M24
  
Accession: EGP90657
  
Location: 1008626-1010053
  
  
**BlastP hit with Mycgr3G35862\_Mycgr3T**
  
Percentage identity: 100 %
  
BlastP bit score: 987
  
Sequence coverage: 99 %
  
E-value: 0.0
  
  
 NCBI BlastP on this gene

EGP90657

hypothetical protein
  
Accession: EGP90658
  
Location: 1007540-1007846
  
 NCBI BlastP on this gene

EGP90658

Query: Architecture Search FASTA input

AHHD01000300 : Macrophomina phaseolina MS6    Total score: 4.0     Cumulative Blast bit score: 3861

Hit cluster cross-links:

Mycgr3G36335 Mycgr3T
  
Location: 0-423

Mycgr3G36335\_Mycgr3T

Mycgr3G84494 Mycgr3T
  
Location: 523-2047

Mycgr3G84494\_Mycgr3T

Mycgr3G90558 Mycgr3T
  
Location: 2147-15296

Mycgr3G90558\_Mycgr3T

Mycgr3G68036 Mycgr3T
  
Location: 15396-16395

Mycgr3G68036\_Mycgr3T

Mycgr3G90561 Mycgr3T
  
Location: 16495-17134

Mycgr3G90561\_Mycgr3T

Mycgr3G35862 Mycgr3T
  
Location: 17234-18662

Mycgr3G35862\_Mycgr3T

Mycgr3G68030 Mycgr3T
  
Location: 18762-19722

Mycgr3G68030\_Mycgr3T

Mycgr3G36449 Mycgr3T
  
Location: 19822-21886

Mycgr3G36449\_Mycgr3T

Mycgr3G35528 Mycgr3T
  
Location: 21986-22844

Mycgr3G35528\_Mycgr3T

Mycgr3G35932 Mycgr3T
  
Location: 22944-24390

Mycgr3G35932\_Mycgr3T

Mycgr3G23761 Mycgr3T
  
Location: 24490-25825

Mycgr3G23761\_Mycgr3T

Mycgr3G35535 Mycgr3T
  
Location: 25925-26429

Mycgr3G35535\_Mycgr3T

Mycgr3G9942 Mycgr3T9
  
Location: 26529-30375

Mycgr3G9942\_Mycgr3T9

Methyltransferase type 12
  
Accession: EKG15382
  
Location: 99605-100735
  
 NCBI BlastP on this gene

EKG15382

Oxoglutarate/iron-dependent oxygenase
  
Accession: EKG15383
  
Location: 101307-102147
  
  
**BlastP hit with Mycgr3G68036\_Mycgr3T**
  
Percentage identity: 42 %
  
BlastP bit score: 200
  
Sequence coverage: 79 %
  
E-value: 2e-58
  
  
 NCBI BlastP on this gene

EKG15383

Pyruvate carboxyltransferase
  
Accession: EKG15384
  
Location: 104816-106785
  
 NCBI BlastP on this gene

EKG15384

Oxoglutarate/iron-dependent oxygenase
  
Accession: EKG15385
  
Location: 107583-108893
  
  
**BlastP hit with Mycgr3G68036\_Mycgr3T**
  
Percentage identity: 36 %
  
BlastP bit score: 208
  
Sequence coverage: 100 %
  
E-value: 8e-61
  
  
 NCBI BlastP on this gene

EKG15385

Cytochrome P450
  
Accession: EKG15386
  
Location: 110362-110748
  
 NCBI BlastP on this gene

EKG15386

hypothetical protein
  
Accession: EKG15387
  
Location: 112596-113745
  
  
**BlastP hit with Mycgr3G68036\_Mycgr3T**
  
Percentage identity: 36 %
  
BlastP bit score: 215
  
Sequence coverage: 100 %
  
E-value: 2e-63
  
  
 NCBI BlastP on this gene

EKG15387

Oxoglutarate/iron-dependent oxygenase
  
Accession: EKG15388
  
Location: 114631-115776
  
  
**BlastP hit with Mycgr3G68036\_Mycgr3T**
  
Percentage identity: 35 %
  
BlastP bit score: 205
  
Sequence coverage: 99 %
  
E-value: 7e-60
  
  
 NCBI BlastP on this gene

EKG15388

hypothetical protein
  
Accession: EKG15389
  
Location: 116467-117560
  
  
**BlastP hit with Mycgr3G35528\_Mycgr3T**
  
Percentage identity: 55 %
  
BlastP bit score: 351
  
Sequence coverage: 104 %
  
E-value: 4e-117
  
  
 NCBI BlastP on this gene

EKG15389

AMP-dependent synthetase/ligase
  
Accession: EKG15390
  
Location: 118157-131808
  
  
**BlastP hit with Mycgr3G90558\_Mycgr3T**
  
Percentage identity: 33 %
  
BlastP bit score: 1995
  
Sequence coverage: 91 %
  
E-value: 0.0
  
  
 NCBI BlastP on this gene

EKG15390

hypothetical protein
  
Accession: EKG15391
  
Location: 132135-133640
  
 NCBI BlastP on this gene

EKG15391

Putative ABC transporter protein
  
Accession: EKG15392
  
Location: 134184-136829
  
  
**BlastP hit with Mycgr3G9942\_Mycgr3T9**
  
Percentage identity: 44 %
  
BlastP bit score: 687
  
Sequence coverage: 66 %
  
E-value: 0.0
  
  
 NCBI BlastP on this gene

EKG15392

hypothetical protein
  
Accession: EKG15393
  
Location: 138474-139917
  
 NCBI BlastP on this gene

EKG15393

Query: Architecture Search FASTA input

DS995900 : Penicillium marneffei ATCC 18224 scf\_1105668340758 genomic scaffold    Total score: 3.0     Cumulative Blast bit score: 2176

Hit cluster cross-links:

Mycgr3G36335 Mycgr3T
  
Location: 0-423

Mycgr3G36335\_Mycgr3T

Mycgr3G84494 Mycgr3T
  
Location: 523-2047

Mycgr3G84494\_Mycgr3T

Mycgr3G90558 Mycgr3T
  
Location: 2147-15296

Mycgr3G90558\_Mycgr3T

Mycgr3G68036 Mycgr3T
  
Location: 15396-16395

Mycgr3G68036\_Mycgr3T

Mycgr3G90561 Mycgr3T
  
Location: 16495-17134

Mycgr3G90561\_Mycgr3T

Mycgr3G35862 Mycgr3T
  
Location: 17234-18662

Mycgr3G35862\_Mycgr3T

Mycgr3G68030 Mycgr3T
  
Location: 18762-19722

Mycgr3G68030\_Mycgr3T

Mycgr3G36449 Mycgr3T
  
Location: 19822-21886

Mycgr3G36449\_Mycgr3T

Mycgr3G35528 Mycgr3T
  
Location: 21986-22844

Mycgr3G35528\_Mycgr3T

Mycgr3G35932 Mycgr3T
  
Location: 22944-24390

Mycgr3G35932\_Mycgr3T

Mycgr3G23761 Mycgr3T
  
Location: 24490-25825

Mycgr3G23761\_Mycgr3T

Mycgr3G35535 Mycgr3T
  
Location: 25925-26429

Mycgr3G35535\_Mycgr3T

Mycgr3G9942 Mycgr3T9
  
Location: 26529-30375

Mycgr3G9942\_Mycgr3T9

C2H2 type conidiation transcription factor BrlA
  
Accession: EEA26461
  
Location: 3741472-3743116
  
 NCBI BlastP on this gene

EEA26461

MFS multidrug transporter, putative
  
Accession: EEA26460
  
Location: 3738110-3739881
  
  
**BlastP hit with Mycgr3G23761\_Mycgr3T**
  
Percentage identity: 30 %
  
BlastP bit score: 189
  
Sequence coverage: 103 %
  
E-value: 1e-50
  
  
 NCBI BlastP on this gene

EEA26460

ribonuclease T2, putative
  
Accession: EEA26459
  
Location: 3735722-3736694
  
 NCBI BlastP on this gene

EEA26459

Swr1p complex component (Swc5), putative
  
Accession: EEA26458
  
Location: 3734328-3735473
  
 NCBI BlastP on this gene

EEA26458

eukaryotic translation initiation factor subunit eIF2B-gamma, putative
  
Accession: EEA26457
  
Location: 3732004-3733875
  
 NCBI BlastP on this gene

EEA26457

conserved hypothetical protein
  
Accession: EEA26456
  
Location: 3725846-3728147
  
 NCBI BlastP on this gene

EEA26456

fatty acid elongase (Gig30), putative
  
Accession: EEA26455
  
Location: 3722380-3723948
  
 NCBI BlastP on this gene

EEA26455

DUF1275 domain protein
  
Accession: EEA26454
  
Location: 3720204-3721119
  
 NCBI BlastP on this gene

EEA26454

conserved hypothetical protein
  
Accession: EEA26453
  
Location: 3717117-3718784
  
 NCBI BlastP on this gene

EEA26453

leucoanthocyanidin dioxygenase, putative
  
Accession: EEA26452
  
Location: 3715799-3717026
  
  
**BlastP hit with Mycgr3G68036\_Mycgr3T**
  
Percentage identity: 34 %
  
BlastP bit score: 177
  
Sequence coverage: 102 %
  
E-value: 6e-49
  
  
 NCBI BlastP on this gene

EEA26452

nonribosomal peptide synthase, putative
  
Accession: EEA26451
  
Location: 3698541-3715320
  
  
**BlastP hit with Mycgr3G90558\_Mycgr3T**
  
Percentage identity: 30 %
  
BlastP bit score: 1810
  
Sequence coverage: 95 %
  
E-value: 0.0
  
  
 NCBI BlastP on this gene

EEA26451

conserved hypothetical protein
  
Accession: EEA26450
  
Location: 3696307-3698119
  
 NCBI BlastP on this gene

EEA26450

Query: Architecture Search FASTA input

KB725800 : Colletotrichum orbiculare MAFF 240422 unplaced genomic scaffold Scaffold\_249    Total score: 3.0     Cumulative Blast bit score: 1103

Hit cluster cross-links:

Mycgr3G36335 Mycgr3T
  
Location: 0-423

Mycgr3G36335\_Mycgr3T

Mycgr3G84494 Mycgr3T
  
Location: 523-2047

Mycgr3G84494\_Mycgr3T

Mycgr3G90558 Mycgr3T
  
Location: 2147-15296

Mycgr3G90558\_Mycgr3T

Mycgr3G68036 Mycgr3T
  
Location: 15396-16395

Mycgr3G68036\_Mycgr3T

Mycgr3G90561 Mycgr3T
  
Location: 16495-17134

Mycgr3G90561\_Mycgr3T

Mycgr3G35862 Mycgr3T
  
Location: 17234-18662

Mycgr3G35862\_Mycgr3T

Mycgr3G68030 Mycgr3T
  
Location: 18762-19722

Mycgr3G68030\_Mycgr3T

Mycgr3G36449 Mycgr3T
  
Location: 19822-21886

Mycgr3G36449\_Mycgr3T

Mycgr3G35528 Mycgr3T
  
Location: 21986-22844

Mycgr3G35528\_Mycgr3T

Mycgr3G35932 Mycgr3T
  
Location: 22944-24390

Mycgr3G35932\_Mycgr3T

Mycgr3G23761 Mycgr3T
  
Location: 24490-25825

Mycgr3G23761\_Mycgr3T

Mycgr3G35535 Mycgr3T
  
Location: 25925-26429

Mycgr3G35535\_Mycgr3T

Mycgr3G9942 Mycgr3T9
  
Location: 26529-30375

Mycgr3G9942\_Mycgr3T9

nicotianamine synthase
  
Accession: ENH84509
  
Location: 126302-127894
  
 NCBI BlastP on this gene

ENH84509

peptidase s41 family protein
  
Accession: ENH84508
  
Location: 122232-124633
  
  
**BlastP hit with Mycgr3G36449\_Mycgr3T**
  
Percentage identity: 28 %
  
BlastP bit score: 215
  
Sequence coverage: 98 %
  
E-value: 2e-56
  
  
 NCBI BlastP on this gene

ENH84508

hypothetical protein
  
Accession: ENH84507
  
Location: 120839-121794
  
 NCBI BlastP on this gene

ENH84507

hypothetical protein
  
Accession: ENH84506
  
Location: 119587-120063
  
 NCBI BlastP on this gene

ENH84506

peptide transporter mtd1
  
Accession: ENH84505
  
Location: 113032-115748
  
 NCBI BlastP on this gene

ENH84505

hypothetical protein
  
Accession: ENH84504
  
Location: 111995-112375
  
 NCBI BlastP on this gene

ENH84504

glycoside hydrolase family 16 protein
  
Accession: ENH84503
  
Location: 110438-111281
  
 NCBI BlastP on this gene

ENH84503

inosine uridine-preferring nucleoside hydrolase
  
Accession: ENH84502
  
Location: 107517-108576
  
 NCBI BlastP on this gene

ENH84502

hypothetical protein
  
Accession: ENH84501
  
Location: 104994-106160
  
 NCBI BlastP on this gene

ENH84501

inorganic phosphate transporter
  
Accession: ENH84500
  
Location: 100268-101495
  
  
**BlastP hit with Mycgr3G35932\_Mycgr3T**
  
Percentage identity: 59 %
  
BlastP bit score: 442
  
Sequence coverage: 73 %
  
E-value: 4e-149
  
  
 NCBI BlastP on this gene

ENH84500

xaa-pro dipeptidase
  
Accession: ENH84499
  
Location: 98149-99735
  
  
**BlastP hit with Mycgr3G35862\_Mycgr3T**
  
Percentage identity: 52 %
  
BlastP bit score: 446
  
Sequence coverage: 98 %
  
E-value: 7e-149
  
  
 NCBI BlastP on this gene

ENH84499

2og-fe oxygenase family
  
Accession: ENH84498
  
Location: 96807-97500
  
 NCBI BlastP on this gene

ENH84498

Query: Architecture Search FASTA input

GL698590 : Metarhizium acridum CQMa 102 unplaced genomic scaffold Scf\_121    Total score: 3.0     Cumulative Blast bit score: 533

Hit cluster cross-links:

Mycgr3G36335 Mycgr3T
  
Location: 0-423

Mycgr3G36335\_Mycgr3T

Mycgr3G84494 Mycgr3T
  
Location: 523-2047

Mycgr3G84494\_Mycgr3T

Mycgr3G90558 Mycgr3T
  
Location: 2147-15296

Mycgr3G90558\_Mycgr3T

Mycgr3G68036 Mycgr3T
  
Location: 15396-16395

Mycgr3G68036\_Mycgr3T

Mycgr3G90561 Mycgr3T
  
Location: 16495-17134

Mycgr3G90561\_Mycgr3T

Mycgr3G35862 Mycgr3T
  
Location: 17234-18662

Mycgr3G35862\_Mycgr3T

Mycgr3G68030 Mycgr3T
  
Location: 18762-19722

Mycgr3G68030\_Mycgr3T

Mycgr3G36449 Mycgr3T
  
Location: 19822-21886

Mycgr3G36449\_Mycgr3T

Mycgr3G35528 Mycgr3T
  
Location: 21986-22844

Mycgr3G35528\_Mycgr3T

Mycgr3G35932 Mycgr3T
  
Location: 22944-24390

Mycgr3G35932\_Mycgr3T

Mycgr3G23761 Mycgr3T
  
Location: 24490-25825

Mycgr3G23761\_Mycgr3T

Mycgr3G35535 Mycgr3T
  
Location: 25925-26429

Mycgr3G35535\_Mycgr3T

Mycgr3G9942 Mycgr3T9
  
Location: 26529-30375

Mycgr3G9942\_Mycgr3T9

MFS multidrug transporter, putative
  
Accession: EFY85106
  
Location: 73881-76640
  
  
**BlastP hit with Mycgr3G23761\_Mycgr3T**
  
Percentage identity: 28 %
  
BlastP bit score: 166
  
Sequence coverage: 100 %
  
E-value: 6e-42
  
  
 NCBI BlastP on this gene

EFY85106

hypothetical protein
  
Accession: EFY85105
  
Location: 71142-71903
  
 NCBI BlastP on this gene

EFY85105

hypothetical protein
  
Accession: EFY85104
  
Location: 69140-70107
  
  
**BlastP hit with Mycgr3G35535\_Mycgr3T**
  
Percentage identity: 27 %
  
BlastP bit score: 82
  
Sequence coverage: 98 %
  
E-value: 2e-16
  
  
 NCBI BlastP on this gene

EFY85104

hypothetical protein
  
Accession: EFY85103
  
Location: 67816-68178
  
 NCBI BlastP on this gene

EFY85103

FAD binding domain containing protein
  
Accession: EFY85102
  
Location: 66812-67108
  
 NCBI BlastP on this gene

EFY85102

lysine methyltransferase
  
Accession: EFY85101
  
Location: 64102-65038
  
 NCBI BlastP on this gene

EFY85101

amino acid adenylation domain protein
  
Accession: EFY85100
  
Location: 58057-63278
  
 NCBI BlastP on this gene

EFY85100

aminotriazole resistance protein, putative
  
Accession: EFY85099
  
Location: 54595-57329
  
 NCBI BlastP on this gene

EFY85099

cysteine hydrolase family protein
  
Accession: EFY85098
  
Location: 52388-53102
  
 NCBI BlastP on this gene

EFY85098

MFS multidrug transporter, putative
  
Accession: EFY85097
  
Location: 50402-51977
  
  
**BlastP hit with Mycgr3G84494\_Mycgr3T**
  
Percentage identity: 36 %
  
BlastP bit score: 285
  
Sequence coverage: 99 %
  
E-value: 5e-86
  
  
 NCBI BlastP on this gene

EFY85097

C6 finger domain protein, putative
  
Accession: EFY85096
  
Location: 49593-50045
  
 NCBI BlastP on this gene

EFY85096

serin endopeptidase
  
Accession: EFY85095
  
Location: 46489-46857
  
 NCBI BlastP on this gene

EFY85095

Query: Architecture Search FASTA input

GL891306 : Neurospora tetrasperma FGSC 2508 unplaced genomic scaffold NEUTE1scaffold\_5    Total score: 3.0     Cumulative Blast bit score: 346

Hit cluster cross-links:

Mycgr3G36335 Mycgr3T
  
Location: 0-423

Mycgr3G36335\_Mycgr3T

Mycgr3G84494 Mycgr3T
  
Location: 523-2047

Mycgr3G84494\_Mycgr3T

Mycgr3G90558 Mycgr3T
  
Location: 2147-15296

Mycgr3G90558\_Mycgr3T

Mycgr3G68036 Mycgr3T
  
Location: 15396-16395

Mycgr3G68036\_Mycgr3T

Mycgr3G90561 Mycgr3T
  
Location: 16495-17134

Mycgr3G90561\_Mycgr3T

Mycgr3G35862 Mycgr3T
  
Location: 17234-18662

Mycgr3G35862\_Mycgr3T

Mycgr3G68030 Mycgr3T
  
Location: 18762-19722

Mycgr3G68030\_Mycgr3T

Mycgr3G36449 Mycgr3T
  
Location: 19822-21886

Mycgr3G36449\_Mycgr3T

Mycgr3G35528 Mycgr3T
  
Location: 21986-22844

Mycgr3G35528\_Mycgr3T

Mycgr3G35932 Mycgr3T
  
Location: 22944-24390

Mycgr3G35932\_Mycgr3T

Mycgr3G23761 Mycgr3T
  
Location: 24490-25825

Mycgr3G23761\_Mycgr3T

Mycgr3G35535 Mycgr3T
  
Location: 25925-26429

Mycgr3G35535\_Mycgr3T

Mycgr3G9942 Mycgr3T9
  
Location: 26529-30375

Mycgr3G9942\_Mycgr3T9

hypothetical protein
  
Accession: EGO55878
  
Location: 3441217-3443271
  
 NCBI BlastP on this gene

EGO55878

hypothetical protein
  
Accession: EGO55877
  
Location: 3435150-3435687
  
 NCBI BlastP on this gene

EGO55877

hypothetical protein
  
Accession: EGO55876
  
Location: 3427287-3434018
  
  
**BlastP hit with Mycgr3G36449\_Mycgr3T**
  
Percentage identity: 29 %
  
BlastP bit score: 228
  
Sequence coverage: 103 %
  
E-value: 2e-59
  
  
 NCBI BlastP on this gene

EGO55876

hypothetical protein
  
Accession: EGO55875
  
Location: 3426198-3427131
  
 NCBI BlastP on this gene

EGO55875

hypothetical protein
  
Accession: EGO55874
  
Location: 3424535-3425542
  
 NCBI BlastP on this gene

EGO55874

hypothetical protein
  
Accession: EGO55873
  
Location: 3422882-3424055
  
  
**BlastP hit with Mycgr3G90561\_Mycgr3T**
  
Percentage identity: 32 %
  
BlastP bit score: 55
  
Sequence coverage: 65 %
  
E-value: 5e-06
  
  
 NCBI BlastP on this gene

EGO55873

hypothetical protein
  
Accession: EGO55872
  
Location: 3422546-3422749
  
 NCBI BlastP on this gene

EGO55872

hypothetical protein
  
Accession: EGO55871
  
Location: 3419753-3421548
  
 NCBI BlastP on this gene

EGO55871

hypothetical protein
  
Accession: EGO55870
  
Location: 3418242-3419266
  
  
**BlastP hit with Mycgr3G35535\_Mycgr3T**
  
Percentage identity: 32 %
  
BlastP bit score: 63
  
Sequence coverage: 87 %
  
E-value: 2e-09
  
  
 NCBI BlastP on this gene

EGO55870

hypothetical protein
  
Accession: EGO55869
  
Location: 3416039-3417003
  
 NCBI BlastP on this gene

EGO55869

hypothetical protein
  
Accession: EGO55868
  
Location: 3414462-3415267
  
 NCBI BlastP on this gene

EGO55868

hypothetical protein
  
Accession: EGO55867
  
Location: 3410314-3412455
  
 NCBI BlastP on this gene

EGO55867

hypothetical protein
  
Accession: EGO55866
  
Location: 3408757-3409844
  
 NCBI BlastP on this gene

EGO55866

Query: Architecture Search FASTA input

GL891258 : Neurospora tetrasperma FGSC 2509 unplaced genomic scaffold NEUTE2scaffold\_6    Total score: 3.0     Cumulative Blast bit score: 346

Hit cluster cross-links:

Mycgr3G36335 Mycgr3T
  
Location: 0-423

Mycgr3G36335\_Mycgr3T

Mycgr3G84494 Mycgr3T
  
Location: 523-2047

Mycgr3G84494\_Mycgr3T

Mycgr3G90558 Mycgr3T
  
Location: 2147-15296

Mycgr3G90558\_Mycgr3T

Mycgr3G68036 Mycgr3T
  
Location: 15396-16395

Mycgr3G68036\_Mycgr3T

Mycgr3G90561 Mycgr3T
  
Location: 16495-17134

Mycgr3G90561\_Mycgr3T

Mycgr3G35862 Mycgr3T
  
Location: 17234-18662

Mycgr3G35862\_Mycgr3T

Mycgr3G68030 Mycgr3T
  
Location: 18762-19722

Mycgr3G68030\_Mycgr3T

Mycgr3G36449 Mycgr3T
  
Location: 19822-21886

Mycgr3G36449\_Mycgr3T

Mycgr3G35528 Mycgr3T
  
Location: 21986-22844

Mycgr3G35528\_Mycgr3T

Mycgr3G35932 Mycgr3T
  
Location: 22944-24390

Mycgr3G35932\_Mycgr3T

Mycgr3G23761 Mycgr3T
  
Location: 24490-25825

Mycgr3G23761\_Mycgr3T

Mycgr3G35535 Mycgr3T
  
Location: 25925-26429

Mycgr3G35535\_Mycgr3T

Mycgr3G9942 Mycgr3T9
  
Location: 26529-30375

Mycgr3G9942\_Mycgr3T9

hypothetical protein
  
Accession: EGZ68864
  
Location: 533410-533947
  
 NCBI BlastP on this gene

EGZ68864

hypothetical protein
  
Accession: EGZ68865
  
Location: 534571-534952
  
 NCBI BlastP on this gene

EGZ68865

OPT-domain-containing protein
  
Accession: EGZ68866
  
Location: 535079-541810
  
  
**BlastP hit with Mycgr3G36449\_Mycgr3T**
  
Percentage identity: 29 %
  
BlastP bit score: 228
  
Sequence coverage: 103 %
  
E-value: 2e-59
  
  
 NCBI BlastP on this gene

EGZ68866

hypothetical protein
  
Accession: EGZ68867
  
Location: 541966-542899
  
 NCBI BlastP on this gene

EGZ68867

hypothetical protein
  
Accession: EGZ68868
  
Location: 543554-544561
  
 NCBI BlastP on this gene

EGZ68868

hypothetical protein
  
Accession: EGZ68869
  
Location: 545041-546214
  
  
**BlastP hit with Mycgr3G90561\_Mycgr3T**
  
Percentage identity: 32 %
  
BlastP bit score: 55
  
Sequence coverage: 65 %
  
E-value: 5e-06
  
  
 NCBI BlastP on this gene

EGZ68869

hypothetical protein
  
Accession: EGZ68870
  
Location: 546347-546550
  
 NCBI BlastP on this gene

EGZ68870

Di-copper centre-containing protein
  
Accession: EGZ68871
  
Location: 547548-549343
  
 NCBI BlastP on this gene

EGZ68871

hypothetical protein
  
Accession: EGZ68872
  
Location: 549830-550854
  
  
**BlastP hit with Mycgr3G35535\_Mycgr3T**
  
Percentage identity: 32 %
  
BlastP bit score: 63
  
Sequence coverage: 87 %
  
E-value: 2e-09
  
  
 NCBI BlastP on this gene

EGZ68872

hypothetical protein
  
Accession: EGZ68873
  
Location: 552093-553057
  
 NCBI BlastP on this gene

EGZ68873

hypothetical protein
  
Accession: EGZ68874
  
Location: 553829-554634
  
 NCBI BlastP on this gene

EGZ68874

hypothetical protein
  
Accession: EGZ68875
  
Location: 555421-555603
  
 NCBI BlastP on this gene

EGZ68875

hypothetical protein
  
Accession: EGZ68876
  
Location: 556639-558780
  
 NCBI BlastP on this gene

EGZ68876

S-adenosyl-L-methionine-dependent methyltransferase
  
Accession: EGZ68877
  
Location: 559250-560337
  
 NCBI BlastP on this gene

EGZ68877

Query: Architecture Search FASTA input

CABT02000031 : Sordaria macrospora k-hell    Total score: 3.0     Cumulative Blast bit score: 334

Hit cluster cross-links:

Mycgr3G36335 Mycgr3T
  
Location: 0-423

Mycgr3G36335\_Mycgr3T

Mycgr3G84494 Mycgr3T
  
Location: 523-2047

Mycgr3G84494\_Mycgr3T

Mycgr3G90558 Mycgr3T
  
Location: 2147-15296

Mycgr3G90558\_Mycgr3T

Mycgr3G68036 Mycgr3T
  
Location: 15396-16395

Mycgr3G68036\_Mycgr3T

Mycgr3G90561 Mycgr3T
  
Location: 16495-17134

Mycgr3G90561\_Mycgr3T

Mycgr3G35862 Mycgr3T
  
Location: 17234-18662

Mycgr3G35862\_Mycgr3T

Mycgr3G68030 Mycgr3T
  
Location: 18762-19722

Mycgr3G68030\_Mycgr3T

Mycgr3G36449 Mycgr3T
  
Location: 19822-21886

Mycgr3G36449\_Mycgr3T

Mycgr3G35528 Mycgr3T
  
Location: 21986-22844

Mycgr3G35528\_Mycgr3T

Mycgr3G35932 Mycgr3T
  
Location: 22944-24390

Mycgr3G35932\_Mycgr3T

Mycgr3G23761 Mycgr3T
  
Location: 24490-25825

Mycgr3G23761\_Mycgr3T

Mycgr3G35535 Mycgr3T
  
Location: 25925-26429

Mycgr3G35535\_Mycgr3T

Mycgr3G9942 Mycgr3T9
  
Location: 26529-30375

Mycgr3G9942\_Mycgr3T9

not annotated
  
Accession: CCC12843
  
Location: 299392-299764
  
 NCBI BlastP on this gene

CCC12843

not annotated
  
Accession: CCC12844
  
Location: 301258-303171
  
 NCBI BlastP on this gene

CCC12844

not annotated
  
Accession: CCC12845
  
Location: 307033-308146
  
  
**BlastP hit with Mycgr3G90561\_Mycgr3T**
  
Percentage identity: 31 %
  
BlastP bit score: 64
  
Sequence coverage: 57 %
  
E-value: 2e-09
  
  
 NCBI BlastP on this gene

CCC12845

not annotated
  
Accession: CCC12846
  
Location: 309006-311620
  
  
**BlastP hit with Mycgr3G36449\_Mycgr3T**
  
Percentage identity: 30 %
  
BlastP bit score: 203
  
Sequence coverage: 81 %
  
E-value: 1e-52
  
  
 NCBI BlastP on this gene

CCC12846

not annotated
  
Accession: CCC12847
  
Location: 312511-313490
  
 NCBI BlastP on this gene

CCC12847

not annotated
  
Accession: CCC12848
  
Location: 315394-316309
  
 NCBI BlastP on this gene

CCC12848

not annotated
  
Accession: CCC12849
  
Location: 317009-317992
  
 NCBI BlastP on this gene

CCC12849

not annotated
  
Accession: CCC12850
  
Location: 321399-323056
  
 NCBI BlastP on this gene

CCC12850

not annotated
  
Accession: CCC12851
  
Location: 323560-325025
  
  
**BlastP hit with Mycgr3G35535\_Mycgr3T**
  
Percentage identity: 33 %
  
BlastP bit score: 67
  
Sequence coverage: 87 %
  
E-value: 1e-10
  
  
 NCBI BlastP on this gene

CCC12851

not annotated
  
Accession: CCC12852
  
Location: 326172-326453
  
 NCBI BlastP on this gene

CCC12852

not annotated
  
Accession: CCC12853
  
Location: 327343-333820
  
 NCBI BlastP on this gene

CCC12853

Query: Architecture Search FASTA input

CM001199 : Mycosphaerella graminicola IPO323 chromosome 4    Total score: 3.0     Cumulative Blast bit score: 314

Hit cluster cross-links:

Mycgr3G36335 Mycgr3T
  
Location: 0-423

Mycgr3G36335\_Mycgr3T

Mycgr3G84494 Mycgr3T
  
Location: 523-2047

Mycgr3G84494\_Mycgr3T

Mycgr3G90558 Mycgr3T
  
Location: 2147-15296

Mycgr3G90558\_Mycgr3T

Mycgr3G68036 Mycgr3T
  
Location: 15396-16395

Mycgr3G68036\_Mycgr3T

Mycgr3G90561 Mycgr3T
  
Location: 16495-17134

Mycgr3G90561\_Mycgr3T

Mycgr3G35862 Mycgr3T
  
Location: 17234-18662

Mycgr3G35862\_Mycgr3T

Mycgr3G68030 Mycgr3T
  
Location: 18762-19722

Mycgr3G68030\_Mycgr3T

Mycgr3G36449 Mycgr3T
  
Location: 19822-21886

Mycgr3G36449\_Mycgr3T

Mycgr3G35528 Mycgr3T
  
Location: 21986-22844

Mycgr3G35528\_Mycgr3T

Mycgr3G35932 Mycgr3T
  
Location: 22944-24390

Mycgr3G35932\_Mycgr3T

Mycgr3G23761 Mycgr3T
  
Location: 24490-25825

Mycgr3G23761\_Mycgr3T

Mycgr3G35535 Mycgr3T
  
Location: 25925-26429

Mycgr3G35535\_Mycgr3T

Mycgr3G9942 Mycgr3T9
  
Location: 26529-30375

Mycgr3G9942\_Mycgr3T9

hypothetical protein
  
Accession: EGP88367
  
Location: 1668361-1669627
  
 NCBI BlastP on this gene

EGP88367

hypothetical protein
  
Accession: EGP87997
  
Location: 1670187-1673954
  
 NCBI BlastP on this gene

EGP87997

hypothetical protein
  
Accession: EGP87998
  
Location: 1674414-1674693
  
 NCBI BlastP on this gene

EGP87998

hypothetical protein
  
Accession: EGP88366
  
Location: 1674958-1676304
  
 NCBI BlastP on this gene

EGP88366

hypothetical protein
  
Accession: EGP88365
  
Location: 1677000-1678580
  
 NCBI BlastP on this gene

EGP88365

hypothetical protein
  
Accession: EGP88364
  
Location: 1681686-1683950
  
  
**BlastP hit with Mycgr3G36449\_Mycgr3T**
  
Percentage identity: 29 %
  
BlastP bit score: 188
  
Sequence coverage: 91 %
  
E-value: 5e-48
  
  
 NCBI BlastP on this gene

EGP88364

hypothetical protein
  
Accession: EGP87999
  
Location: 1684438-1685014
  
  
**BlastP hit with Mycgr3G90561\_Mycgr3T**
  
Percentage identity: 33 %
  
BlastP bit score: 59
  
Sequence coverage: 56 %
  
E-value: 2e-08
  
  
 NCBI BlastP on this gene

EGP87999

hypothetical protein
  
Accession: EGP88000
  
Location: 1686972-1687360
  
 NCBI BlastP on this gene

EGP88000

hypothetical protein
  
Accession: EGP88001
  
Location: 1688171-1689246
  
 NCBI BlastP on this gene

EGP88001

hypothetical protein
  
Accession: EGP88363
  
Location: 1689340-1690777
  
 NCBI BlastP on this gene

EGP88363

hypothetical protein
  
Accession: EGP88002
  
Location: 1691332-1691995
  
  
**BlastP hit with Mycgr3G35535\_Mycgr3T**
  
Percentage identity: 29 %
  
BlastP bit score: 67
  
Sequence coverage: 96 %
  
E-value: 3e-11
  
  
 NCBI BlastP on this gene

EGP88002

serine carboxypeptidase
  
Accession: EGP88362
  
Location: 1692026-1693749
  
 NCBI BlastP on this gene

EGP88362

putative major facilitator superfamily transporter
  
Accession: EGP88361
  
Location: 1694042-1695664
  
 NCBI BlastP on this gene

EGP88361

hypothetical protein
  
Accession: EGP88003
  
Location: 1696670-1697425
  
 NCBI BlastP on this gene

EGP88003

putative ABC transporter
  
Accession: EGP88360
  
Location: 1697477-1702334
  
 NCBI BlastP on this gene

EGP88360

Query: Architecture Search FASTA input

AHHD01000518 : Macrophomina phaseolina MS6    Total score: 2.0     Cumulative Blast bit score: 3574

Hit cluster cross-links:

Mycgr3G36335 Mycgr3T
  
Location: 0-423

Mycgr3G36335\_Mycgr3T

Mycgr3G84494 Mycgr3T
  
Location: 523-2047

Mycgr3G84494\_Mycgr3T

Mycgr3G90558 Mycgr3T
  
Location: 2147-15296

Mycgr3G90558\_Mycgr3T

Mycgr3G68036 Mycgr3T
  
Location: 15396-16395

Mycgr3G68036\_Mycgr3T

Mycgr3G90561 Mycgr3T
  
Location: 16495-17134

Mycgr3G90561\_Mycgr3T

Mycgr3G35862 Mycgr3T
  
Location: 17234-18662

Mycgr3G35862\_Mycgr3T

Mycgr3G68030 Mycgr3T
  
Location: 18762-19722

Mycgr3G68030\_Mycgr3T

Mycgr3G36449 Mycgr3T
  
Location: 19822-21886

Mycgr3G36449\_Mycgr3T

Mycgr3G35528 Mycgr3T
  
Location: 21986-22844

Mycgr3G35528\_Mycgr3T

Mycgr3G35932 Mycgr3T
  
Location: 22944-24390

Mycgr3G35932\_Mycgr3T

Mycgr3G23761 Mycgr3T
  
Location: 24490-25825

Mycgr3G23761\_Mycgr3T

Mycgr3G35535 Mycgr3T
  
Location: 25925-26429

Mycgr3G35535\_Mycgr3T

Mycgr3G9942 Mycgr3T9
  
Location: 26529-30375

Mycgr3G9942\_Mycgr3T9

Beta-ketoacyl synthase
  
Accession: EKG10413
  
Location: 153932-161880
  
 NCBI BlastP on this gene

EKG10413

AMP-dependent synthetase/ligase
  
Accession: EKG10414
  
Location: 162119-175583
  
  
**BlastP hit with Mycgr3G90558\_Mycgr3T**
  
Percentage identity: 30 %
  
BlastP bit score: 1716
  
Sequence coverage: 94 %
  
E-value: 0.0
  
  
 NCBI BlastP on this gene

EKG10414

Beta-lactamase-related protein
  
Accession: EKG10415
  
Location: 176349-177917
  
 NCBI BlastP on this gene

EKG10415

hypothetical protein
  
Accession: EKG10416
  
Location: 179610-179885
  
 NCBI BlastP on this gene

EKG10416

hypothetical protein
  
Accession: EKG10417
  
Location: 181032-181859
  
 NCBI BlastP on this gene

EKG10417

Putative ABC transporter protein
  
Accession: EKG10418
  
Location: 182930-188124
  
 NCBI BlastP on this gene

EKG10418

Major facilitator superfamily
  
Accession: EKG10419
  
Location: 188622-190195
  
  
**BlastP hit with Mycgr3G23761\_Mycgr3T**
  
Percentage identity: 34 %
  
BlastP bit score: 218
  
Sequence coverage: 100 %
  
E-value: 3e-61
  
  
 NCBI BlastP on this gene

EKG10419

Peptidase M20
  
Accession: EKG10420
  
Location: 190579-191983
  
 NCBI BlastP on this gene

EKG10420

Indoleamine 23-dioxygenase
  
Accession: EKG10421
  
Location: 192815-194101
  
 NCBI BlastP on this gene

EKG10421

AMP-dependent synthetase/ligase
  
Accession: EKG10422
  
Location: 195427-207108
  
  
**BlastP hit with Mycgr3G90558\_Mycgr3T**
  
Percentage identity: 32 %
  
BlastP bit score: 1641
  
Sequence coverage: 84 %
  
E-value: 0.0
  
  
 NCBI BlastP on this gene

EKG10422

nacht and ankyrin domain containing protein
  
Accession: EKG10423
  
Location: 208421-208990
  
 NCBI BlastP on this gene

EKG10423

nb-arc and ankyrin domain containing protein
  
Accession: EKG10424
  
Location: 209145-210651
  
 NCBI BlastP on this gene

EKG10424

Query: Architecture Search FASTA input

KE145356 : Glarea lozoyensis ATCC 20868 chromosome Unknown GLAREA13    Total score: 2.0     Cumulative Blast bit score: 2476

Hit cluster cross-links:

Mycgr3G36335 Mycgr3T
  
Location: 0-423

Mycgr3G36335\_Mycgr3T

Mycgr3G84494 Mycgr3T
  
Location: 523-2047

Mycgr3G84494\_Mycgr3T

Mycgr3G90558 Mycgr3T
  
Location: 2147-15296

Mycgr3G90558\_Mycgr3T

Mycgr3G68036 Mycgr3T
  
Location: 15396-16395

Mycgr3G68036\_Mycgr3T

Mycgr3G90561 Mycgr3T
  
Location: 16495-17134

Mycgr3G90561\_Mycgr3T

Mycgr3G35862 Mycgr3T
  
Location: 17234-18662

Mycgr3G35862\_Mycgr3T

Mycgr3G68030 Mycgr3T
  
Location: 18762-19722

Mycgr3G68030\_Mycgr3T

Mycgr3G36449 Mycgr3T
  
Location: 19822-21886

Mycgr3G36449\_Mycgr3T

Mycgr3G35528 Mycgr3T
  
Location: 21986-22844

Mycgr3G35528\_Mycgr3T

Mycgr3G35932 Mycgr3T
  
Location: 22944-24390

Mycgr3G35932\_Mycgr3T

Mycgr3G23761 Mycgr3T
  
Location: 24490-25825

Mycgr3G23761\_Mycgr3T

Mycgr3G35535 Mycgr3T
  
Location: 25925-26429

Mycgr3G35535\_Mycgr3T

Mycgr3G9942 Mycgr3T9
  
Location: 26529-30375

Mycgr3G9942\_Mycgr3T9

Thiolase-like protein
  
Accession: EPE34340
  
Location: 220862-228891
  
 NCBI BlastP on this gene

EPE34340

Acetyl-CoA synthetase-like protein
  
Accession: EPE34341
  
Location: 230546-252235
  
  
**BlastP hit with Mycgr3G90558\_Mycgr3T**
  
Percentage identity: 32 %
  
BlastP bit score: 1973
  
Sequence coverage: 90 %
  
E-value: 0.0
  
  
 NCBI BlastP on this gene

EPE34341

P-loop containing nucleoside triphosphate hydrolase
  
Accession: EPE34342
  
Location: 253841-258579
  
 NCBI BlastP on this gene

EPE34342

Aconitase iron-sulfur
  
Accession: EPE34343
  
Location: 258935-261970
  
 NCBI BlastP on this gene

EPE34343

Isocitrate/Isopropylmalate dehydrogenase-like protein
  
Accession: EPE34344
  
Location: 262418-263855
  
 NCBI BlastP on this gene

EPE34344

Aldolase
  
Accession: EPE34345
  
Location: 264293-266317
  
 NCBI BlastP on this gene

EPE34345

D-aminoacid aminotransferase-like PLP-dependent enzyme
  
Accession: EPE34346
  
Location: 267099-268369
  
 NCBI BlastP on this gene

EPE34346

Clavaminate synthase-like protein
  
Accession: EPE34347
  
Location: 268608-269705
  
  
**BlastP hit with Mycgr3G68036\_Mycgr3T**
  
Percentage identity: 38 %
  
BlastP bit score: 220
  
Sequence coverage: 100 %
  
E-value: 3e-65
  
  
 NCBI BlastP on this gene

EPE34347

Clavaminate synthase-like protein
  
Accession: EPE34348
  
Location: 270159-271199
  
 NCBI BlastP on this gene

EPE34348

Acetyl-CoA synthetase-like protein
  
Accession: EPE34349
  
Location: 271791-273838
  
 NCBI BlastP on this gene

EPE34349

Clavaminate synthase-like protein
  
Accession: EPE34350
  
Location: 275478-276584
  
  
**BlastP hit with Mycgr3G68036\_Mycgr3T**
  
Percentage identity: 44 %
  
BlastP bit score: 283
  
Sequence coverage: 100 %
  
E-value: 5e-90
  
  
 NCBI BlastP on this gene

EPE34350

hypothetical protein
  
Accession: EPE34351
  
Location: 277253-279400
  
 NCBI BlastP on this gene

EPE34351

Query: Architecture Search FASTA input

KB916208 : Neofusicoccum parvum UCRNP2 chromosome Unknown NP2\_03\_scaffold\_570    Total score: 2.0     Cumulative Blast bit score: 2293

Hit cluster cross-links:

Mycgr3G36335 Mycgr3T
  
Location: 0-423

Mycgr3G36335\_Mycgr3T

Mycgr3G84494 Mycgr3T
  
Location: 523-2047

Mycgr3G84494\_Mycgr3T

Mycgr3G90558 Mycgr3T
  
Location: 2147-15296

Mycgr3G90558\_Mycgr3T

Mycgr3G68036 Mycgr3T
  
Location: 15396-16395

Mycgr3G68036\_Mycgr3T

Mycgr3G90561 Mycgr3T
  
Location: 16495-17134

Mycgr3G90561\_Mycgr3T

Mycgr3G35862 Mycgr3T
  
Location: 17234-18662

Mycgr3G35862\_Mycgr3T

Mycgr3G68030 Mycgr3T
  
Location: 18762-19722

Mycgr3G68030\_Mycgr3T

Mycgr3G36449 Mycgr3T
  
Location: 19822-21886

Mycgr3G36449\_Mycgr3T

Mycgr3G35528 Mycgr3T
  
Location: 21986-22844

Mycgr3G35528\_Mycgr3T

Mycgr3G35932 Mycgr3T
  
Location: 22944-24390

Mycgr3G35932\_Mycgr3T

Mycgr3G23761 Mycgr3T
  
Location: 24490-25825

Mycgr3G23761\_Mycgr3T

Mycgr3G35535 Mycgr3T
  
Location: 25925-26429

Mycgr3G35535\_Mycgr3T

Mycgr3G9942 Mycgr3T9
  
Location: 26529-30375

Mycgr3G9942\_Mycgr3T9

putative nucleoside-diphosphate-sugar epimerase protein
  
Accession: EOD48378
  
Location: 29560-30412
  
 NCBI BlastP on this gene

EOD48378

hypothetical protein
  
Accession: EOD48380
  
Location: 31386-64262
  
  
**BlastP hit with Mycgr3G90558\_Mycgr3T**
  
Percentage identity: 34 %
  
BlastP bit score: 2061
  
Sequence coverage: 90 %
  
E-value: 0.0
  
  
 NCBI BlastP on this gene

EOD48380

hypothetical protein
  
Accession: EOD48394
  
Location: 64495-65070
  
 NCBI BlastP on this gene

EOD48394

putative benzoate 4-monooxygenase cytochrome p450 protein
  
Accession: EOD48395
  
Location: 66956-67705
  
 NCBI BlastP on this gene

EOD48395

putative gibberellin 2-oxidase protein
  
Accession: EOD48392
  
Location: 69282-70276
  
  
**BlastP hit with Mycgr3G68036\_Mycgr3T**
  
Percentage identity: 43 %
  
BlastP bit score: 232
  
Sequence coverage: 84 %
  
E-value: 2e-70
  
  
 NCBI BlastP on this gene

EOD48392

Query: Architecture Search FASTA input

KB644410 : Penicillium oxalicum 114-2 unplaced genomic scaffold scaffold\_3    Total score: 2.0     Cumulative Blast bit score: 2284

Hit cluster cross-links:

Mycgr3G36335 Mycgr3T
  
Location: 0-423

Mycgr3G36335\_Mycgr3T

Mycgr3G84494 Mycgr3T
  
Location: 523-2047

Mycgr3G84494\_Mycgr3T

Mycgr3G90558 Mycgr3T
  
Location: 2147-15296

Mycgr3G90558\_Mycgr3T

Mycgr3G68036 Mycgr3T
  
Location: 15396-16395

Mycgr3G68036\_Mycgr3T

Mycgr3G90561 Mycgr3T
  
Location: 16495-17134

Mycgr3G90561\_Mycgr3T

Mycgr3G35862 Mycgr3T
  
Location: 17234-18662

Mycgr3G35862\_Mycgr3T

Mycgr3G68030 Mycgr3T
  
Location: 18762-19722

Mycgr3G68030\_Mycgr3T

Mycgr3G36449 Mycgr3T
  
Location: 19822-21886

Mycgr3G36449\_Mycgr3T

Mycgr3G35528 Mycgr3T
  
Location: 21986-22844

Mycgr3G35528\_Mycgr3T

Mycgr3G35932 Mycgr3T
  
Location: 22944-24390

Mycgr3G35932\_Mycgr3T

Mycgr3G23761 Mycgr3T
  
Location: 24490-25825

Mycgr3G23761\_Mycgr3T

Mycgr3G35535 Mycgr3T
  
Location: 25925-26429

Mycgr3G35535\_Mycgr3T

Mycgr3G9942 Mycgr3T9
  
Location: 26529-30375

Mycgr3G9942\_Mycgr3T9

hypothetical protein
  
Accession: EPS27187
  
Location: 46855-51660
  
 NCBI BlastP on this gene

EPS27187

hypothetical protein
  
Accession: EPS27188
  
Location: 54296-77982
  
  
**BlastP hit with Mycgr3G90558\_Mycgr3T**
  
Percentage identity: 32 %
  
BlastP bit score: 2083
  
Sequence coverage: 94 %
  
E-value: 0.0
  
  
 NCBI BlastP on this gene

EPS27188

hypothetical protein
  
Accession: EPS27189
  
Location: 78526-79566
  
 NCBI BlastP on this gene

EPS27189

hypothetical protein
  
Accession: EPS27190
  
Location: 79827-80877
  
 NCBI BlastP on this gene

EPS27190

hypothetical protein
  
Accession: EPS27191
  
Location: 81151-82295
  
  
**BlastP hit with Mycgr3G68036\_Mycgr3T**
  
Percentage identity: 34 %
  
BlastP bit score: 201
  
Sequence coverage: 101 %
  
E-value: 8e-58
  
  
 NCBI BlastP on this gene

EPS27191

hypothetical protein
  
Accession: EPS27192
  
Location: 83418-88263
  
 NCBI BlastP on this gene

EPS27192

Query: Architecture Search FASTA input

ABDF02000064 : Trichoderma virens Gv29-8    Total score: 2.0     Cumulative Blast bit score: 2269

Hit cluster cross-links:

Mycgr3G36335 Mycgr3T
  
Location: 0-423

Mycgr3G36335\_Mycgr3T

Mycgr3G84494 Mycgr3T
  
Location: 523-2047

Mycgr3G84494\_Mycgr3T

Mycgr3G90558 Mycgr3T
  
Location: 2147-15296

Mycgr3G90558\_Mycgr3T

Mycgr3G68036 Mycgr3T
  
Location: 15396-16395

Mycgr3G68036\_Mycgr3T

Mycgr3G90561 Mycgr3T
  
Location: 16495-17134

Mycgr3G90561\_Mycgr3T

Mycgr3G35862 Mycgr3T
  
Location: 17234-18662

Mycgr3G35862\_Mycgr3T

Mycgr3G68030 Mycgr3T
  
Location: 18762-19722

Mycgr3G68030\_Mycgr3T

Mycgr3G36449 Mycgr3T
  
Location: 19822-21886

Mycgr3G36449\_Mycgr3T

Mycgr3G35528 Mycgr3T
  
Location: 21986-22844

Mycgr3G35528\_Mycgr3T

Mycgr3G35932 Mycgr3T
  
Location: 22944-24390

Mycgr3G35932\_Mycgr3T

Mycgr3G23761 Mycgr3T
  
Location: 24490-25825

Mycgr3G23761\_Mycgr3T

Mycgr3G35535 Mycgr3T
  
Location: 25925-26429

Mycgr3G35535\_Mycgr3T

Mycgr3G9942 Mycgr3T9
  
Location: 26529-30375

Mycgr3G9942\_Mycgr3T9

hypothetical protein
  
Accession: EHK21754
  
Location: 32704-34932
  
 NCBI BlastP on this gene

EHK21754

hypothetical protein
  
Accession: EHK21755
  
Location: 36259-37212
  
  
**BlastP hit with Mycgr3G35535\_Mycgr3T**
  
Percentage identity: 32 %
  
BlastP bit score: 82
  
Sequence coverage: 88 %
  
E-value: 3e-16
  
  
 NCBI BlastP on this gene

EHK21755

hypothetical protein
  
Accession: EHK21756
  
Location: 38429-41947
  
 NCBI BlastP on this gene

EHK21756

non-ribosomal peptide synthetase
  
Accession: EHK21757
  
Location: 44838-68695
  
  
**BlastP hit with Mycgr3G90558\_Mycgr3T**
  
Percentage identity: 32 %
  
BlastP bit score: 2187
  
Sequence coverage: 100 %
  
E-value: 0.0
  
  
 NCBI BlastP on this gene

EHK21757

hypothetical protein
  
Accession: EHK21758
  
Location: 69039-70872
  
 NCBI BlastP on this gene

EHK21758

Query: Architecture Search FASTA input

JH226133 : Exophiala dermatitidis NIH/UT8656 unplaced genomic scaffold supercont1.4    Total score: 2.0     Cumulative Blast bit score: 2237

Hit cluster cross-links:

Mycgr3G36335 Mycgr3T
  
Location: 0-423

Mycgr3G36335\_Mycgr3T

Mycgr3G84494 Mycgr3T
  
Location: 523-2047

Mycgr3G84494\_Mycgr3T

Mycgr3G90558 Mycgr3T
  
Location: 2147-15296

Mycgr3G90558\_Mycgr3T

Mycgr3G68036 Mycgr3T
  
Location: 15396-16395

Mycgr3G68036\_Mycgr3T

Mycgr3G90561 Mycgr3T
  
Location: 16495-17134

Mycgr3G90561\_Mycgr3T

Mycgr3G35862 Mycgr3T
  
Location: 17234-18662

Mycgr3G35862\_Mycgr3T

Mycgr3G68030 Mycgr3T
  
Location: 18762-19722

Mycgr3G68030\_Mycgr3T

Mycgr3G36449 Mycgr3T
  
Location: 19822-21886

Mycgr3G36449\_Mycgr3T

Mycgr3G35528 Mycgr3T
  
Location: 21986-22844

Mycgr3G35528\_Mycgr3T

Mycgr3G35932 Mycgr3T
  
Location: 22944-24390

Mycgr3G35932\_Mycgr3T

Mycgr3G23761 Mycgr3T
  
Location: 24490-25825

Mycgr3G23761\_Mycgr3T

Mycgr3G35535 Mycgr3T
  
Location: 25925-26429

Mycgr3G35535\_Mycgr3T

Mycgr3G9942 Mycgr3T9
  
Location: 26529-30375

Mycgr3G9942\_Mycgr3T9

threonine aldolase
  
Accession: EHY56732
  
Location: 1306036-1307049
  
 NCBI BlastP on this gene

EHY56732

pyrroline-5-carboxylate reductase
  
Accession: EHY56733
  
Location: 1307459-1308509
  
 NCBI BlastP on this gene

EHY56733

hypothetical protein
  
Accession: EHY56734
  
Location: 1308789-1310125
  
  
**BlastP hit with Mycgr3G68036\_Mycgr3T**
  
Percentage identity: 43 %
  
BlastP bit score: 265
  
Sequence coverage: 95 %
  
E-value: 4e-82
  
  
 NCBI BlastP on this gene

EHY56734

hypothetical protein
  
Accession: EHY56735
  
Location: 1311029-1312639
  
 NCBI BlastP on this gene

EHY56735

cytochrome P450 monooxygenase
  
Accession: EHY56736
  
Location: 1313166-1315196
  
 NCBI BlastP on this gene

EHY56736

hypothetical protein
  
Accession: EHY56737
  
Location: 1315624-1316570
  
 NCBI BlastP on this gene

EHY56737

branched-chain amino acid aminotransferase
  
Accession: EHY56740
  
Location: 1317129-1318512
  
 NCBI BlastP on this gene

EHY56740

hypothetical protein, variant
  
Accession: EHY56738
  
Location: 1321252-1322172
  
 NCBI BlastP on this gene

EHY56738

nonribosomal peptide synthase
  
Accession: EHY56742
  
Location: 1323346-1340944
  
  
**BlastP hit with Mycgr3G90558\_Mycgr3T**
  
Percentage identity: 32 %
  
BlastP bit score: 1972
  
Sequence coverage: 94 %
  
E-value: 0.0
  
  
 NCBI BlastP on this gene

EHY56742

Query: Architecture Search FASTA input

JX421684 : Emericella rugulosa strain NRRL 11440 hypothetical protein genes    Total score: 2.0     Cumulative Blast bit score: 2232

Hit cluster cross-links:

Mycgr3G36335 Mycgr3T
  
Location: 0-423

Mycgr3G36335\_Mycgr3T

Mycgr3G84494 Mycgr3T
  
Location: 523-2047

Mycgr3G84494\_Mycgr3T

Mycgr3G90558 Mycgr3T
  
Location: 2147-15296

Mycgr3G90558\_Mycgr3T

Mycgr3G68036 Mycgr3T
  
Location: 15396-16395

Mycgr3G68036\_Mycgr3T

Mycgr3G90561 Mycgr3T
  
Location: 16495-17134

Mycgr3G90561\_Mycgr3T

Mycgr3G35862 Mycgr3T
  
Location: 17234-18662

Mycgr3G35862\_Mycgr3T

Mycgr3G68030 Mycgr3T
  
Location: 18762-19722

Mycgr3G68030\_Mycgr3T

Mycgr3G36449 Mycgr3T
  
Location: 19822-21886

Mycgr3G36449\_Mycgr3T

Mycgr3G35528 Mycgr3T
  
Location: 21986-22844

Mycgr3G35528\_Mycgr3T

Mycgr3G35932 Mycgr3T
  
Location: 22944-24390

Mycgr3G35932\_Mycgr3T

Mycgr3G23761 Mycgr3T
  
Location: 24490-25825

Mycgr3G23761\_Mycgr3T

Mycgr3G35535 Mycgr3T
  
Location: 25925-26429

Mycgr3G35535\_Mycgr3T

Mycgr3G9942 Mycgr3T9
  
Location: 26529-30375

Mycgr3G9942\_Mycgr3T9

EcdF
  
Accession: AFT91384
  
Location: 37085-39059
  
 NCBI BlastP on this gene

ecdF

EcdA
  
Accession: AFT91378
  
Location: 41269-63178
  
  
**BlastP hit with Mycgr3G90558\_Mycgr3T**
  
Percentage identity: 33 %
  
BlastP bit score: 1989
  
Sequence coverage: 90 %
  
E-value: 0.0
  
  
 NCBI BlastP on this gene

ecdA

EcdG
  
Accession: AFT91379
  
Location: 63384-64533
  
 NCBI BlastP on this gene

ecdG

EcdH
  
Accession: AFT91389
  
Location: 64922-66488
  
 NCBI BlastP on this gene

ecdH

EcdI
  
Accession: AFT91380
  
Location: 66559-68482
  
 NCBI BlastP on this gene

ecdI

EcdJ
  
Accession: AFT91381
  
Location: 70014-72170
  
 NCBI BlastP on this gene

ecdJ

EcdK
  
Accession: AFT91382
  
Location: 72258-73376
  
  
**BlastP hit with Mycgr3G68036\_Mycgr3T**
  
Percentage identity: 39 %
  
BlastP bit score: 243
  
Sequence coverage: 100 %
  
E-value: 2e-74
  
  
 NCBI BlastP on this gene

ecdK

EcdL
  
Accession: AFT91383
  
Location: 73639-78856
  
 NCBI BlastP on this gene

ecdL

Query: Architecture Search FASTA input

EQ962654 : Talaromyces stipitatus ATCC 10500 scf\_1105507295541 genomic scaffold    Total score: 2.0     Cumulative Blast bit score: 2224

Hit cluster cross-links:

Mycgr3G36335 Mycgr3T
  
Location: 0-423

Mycgr3G36335\_Mycgr3T

Mycgr3G84494 Mycgr3T
  
Location: 523-2047

Mycgr3G84494\_Mycgr3T

Mycgr3G90558 Mycgr3T
  
Location: 2147-15296

Mycgr3G90558\_Mycgr3T

Mycgr3G68036 Mycgr3T
  
Location: 15396-16395

Mycgr3G68036\_Mycgr3T

Mycgr3G90561 Mycgr3T
  
Location: 16495-17134

Mycgr3G90561\_Mycgr3T

Mycgr3G35862 Mycgr3T
  
Location: 17234-18662

Mycgr3G35862\_Mycgr3T

Mycgr3G68030 Mycgr3T
  
Location: 18762-19722

Mycgr3G68030\_Mycgr3T

Mycgr3G36449 Mycgr3T
  
Location: 19822-21886

Mycgr3G36449\_Mycgr3T

Mycgr3G35528 Mycgr3T
  
Location: 21986-22844

Mycgr3G35528\_Mycgr3T

Mycgr3G35932 Mycgr3T
  
Location: 22944-24390

Mycgr3G35932\_Mycgr3T

Mycgr3G23761 Mycgr3T
  
Location: 24490-25825

Mycgr3G23761\_Mycgr3T

Mycgr3G35535 Mycgr3T
  
Location: 25925-26429

Mycgr3G35535\_Mycgr3T

Mycgr3G9942 Mycgr3T9
  
Location: 26529-30375

Mycgr3G9942\_Mycgr3T9

C2H2 transcription factor RfeC
  
Accession: EED20595
  
Location: 3957523-3959187
  
 NCBI BlastP on this gene

EED20595

nonribosomal peptide synthase, putative
  
Accession: EED20596
  
Location: 3960945-3978353
  
  
**BlastP hit with Mycgr3G90558\_Mycgr3T**
  
Percentage identity: 29 %
  
BlastP bit score: 1680
  
Sequence coverage: 93 %
  
E-value: 0.0
  
  
 NCBI BlastP on this gene

EED20596

conserved hypothetical protein
  
Accession: EED20597
  
Location: 3979116-3980249
  
 NCBI BlastP on this gene

EED20597

hypothetical protein
  
Accession: EED20598
  
Location: 3980449-3981543
  
 NCBI BlastP on this gene

EED20598

hypothetical protein
  
Accession: EED20599
  
Location: 3983545-3984828
  
 NCBI BlastP on this gene

EED20599

phenylalanine ammonia-lyase, putative
  
Accession: EED20600
  
Location: 3987229-3989468
  
 NCBI BlastP on this gene

EED20600

conserved hypothetical protein
  
Accession: EED20601
  
Location: 3990241-3991314
  
 NCBI BlastP on this gene

EED20601

multidrug resistance protein, putative
  
Accession: EED20602
  
Location: 3994840-3998734
  
  
**BlastP hit with Mycgr3G9942\_Mycgr3T9**
  
Percentage identity: 31 %
  
BlastP bit score: 544
  
Sequence coverage: 94 %
  
E-value: 3e-167
  
  
 NCBI BlastP on this gene

EED20602

hypothetical protein
  
Accession: EED20603
  
Location: 4001018-4002714
  
 NCBI BlastP on this gene

EED20603

Query: Architecture Search FASTA input

AKHY01000111 : Aspergillus oryzae 3.042    Total score: 2.0     Cumulative Blast bit score: 2025

Hit cluster cross-links:

Mycgr3G36335 Mycgr3T
  
Location: 0-423

Mycgr3G36335\_Mycgr3T

Mycgr3G84494 Mycgr3T
  
Location: 523-2047

Mycgr3G84494\_Mycgr3T

Mycgr3G90558 Mycgr3T
  
Location: 2147-15296

Mycgr3G90558\_Mycgr3T

Mycgr3G68036 Mycgr3T
  
Location: 15396-16395

Mycgr3G68036\_Mycgr3T

Mycgr3G90561 Mycgr3T
  
Location: 16495-17134

Mycgr3G90561\_Mycgr3T

Mycgr3G35862 Mycgr3T
  
Location: 17234-18662

Mycgr3G35862\_Mycgr3T

Mycgr3G68030 Mycgr3T
  
Location: 18762-19722

Mycgr3G68030\_Mycgr3T

Mycgr3G36449 Mycgr3T
  
Location: 19822-21886

Mycgr3G36449\_Mycgr3T

Mycgr3G35528 Mycgr3T
  
Location: 21986-22844

Mycgr3G35528\_Mycgr3T

Mycgr3G35932 Mycgr3T
  
Location: 22944-24390

Mycgr3G35932\_Mycgr3T

Mycgr3G23761 Mycgr3T
  
Location: 24490-25825

Mycgr3G23761\_Mycgr3T

Mycgr3G35535 Mycgr3T
  
Location: 25925-26429

Mycgr3G35535\_Mycgr3T

Mycgr3G9942 Mycgr3T9
  
Location: 26529-30375

Mycgr3G9942\_Mycgr3T9

hypothetical protein
  
Accession: EIT80948
  
Location: 218943-222267
  
 NCBI BlastP on this gene

EIT80948

non-ribosomal peptide synthetase module
  
Accession: EIT80956
  
Location: 207086-217298
  
  
**BlastP hit with Mycgr3G90558\_Mycgr3T**
  
Percentage identity: 33 %
  
BlastP bit score: 1469
  
Sequence coverage: 65 %
  
E-value: 0.0
  
  
 NCBI BlastP on this gene

EIT80956

2-polyprenyl-6-methoxyphenol hydroxylase
  
Accession: EIT81007
  
Location: 204388-206656
  
 NCBI BlastP on this gene

EIT81007

H+/oligopeptide symporter
  
Accession: EIT80986
  
Location: 201968-203897
  
 NCBI BlastP on this gene

EIT80986

isopenicillin N synthase
  
Accession: EIT80966
  
Location: 200164-201318
  
  
**BlastP hit with Mycgr3G68036\_Mycgr3T**
  
Percentage identity: 41 %
  
BlastP bit score: 283
  
Sequence coverage: 97 %
  
E-value: 1e-89
  
  
 NCBI BlastP on this gene

EIT80966

hypothetical protein
  
Accession: EIT80983
  
Location: 198406-199627
  
 NCBI BlastP on this gene

EIT80983

hypothetical protein
  
Accession: EIT80993
  
Location: 197423-198121
  
 NCBI BlastP on this gene

EIT80993

FAD-dependent oxidoreductase
  
Accession: EIT81016
  
Location: 195514-197025
  
 NCBI BlastP on this gene

EIT81016

H+/oligopeptide symporter
  
Accession: EIT80996
  
Location: 193354-195070
  
 NCBI BlastP on this gene

EIT80996

iron/ascorbate family oxidoreductase
  
Accession: EIT80991
  
Location: 191485-192644
  
  
**BlastP hit with Mycgr3G68036\_Mycgr3T**
  
Percentage identity: 42 %
  
BlastP bit score: 273
  
Sequence coverage: 100 %
  
E-value: 6e-86
  
  
 NCBI BlastP on this gene

EIT80991

hypothetical protein
  
Accession: EIT80971
  
Location: 189403-191058
  
 NCBI BlastP on this gene

EIT80971

putative hydrolase
  
Accession: EIT80961
  
Location: 186517-187317
  
 NCBI BlastP on this gene

EIT80961

Query: Architecture Search FASTA input

AB705455 : Aspergillus oryzae DNA, WYK-1 cluster, ORF1, ORF2, ORF3, wykN, wykA, wykE, wykG, wykH, ...    Total score: 2.0     Cumulative Blast bit score: 1999

Hit cluster cross-links:

Mycgr3G36335 Mycgr3T
  
Location: 0-423

Mycgr3G36335\_Mycgr3T

Mycgr3G84494 Mycgr3T
  
Location: 523-2047

Mycgr3G84494\_Mycgr3T

Mycgr3G90558 Mycgr3T
  
Location: 2147-15296

Mycgr3G90558\_Mycgr3T

Mycgr3G68036 Mycgr3T
  
Location: 15396-16395

Mycgr3G68036\_Mycgr3T

Mycgr3G90561 Mycgr3T
  
Location: 16495-17134

Mycgr3G90561\_Mycgr3T

Mycgr3G35862 Mycgr3T
  
Location: 17234-18662

Mycgr3G35862\_Mycgr3T

Mycgr3G68030 Mycgr3T
  
Location: 18762-19722

Mycgr3G68030\_Mycgr3T

Mycgr3G36449 Mycgr3T
  
Location: 19822-21886

Mycgr3G36449\_Mycgr3T

Mycgr3G35528 Mycgr3T
  
Location: 21986-22844

Mycgr3G35528\_Mycgr3T

Mycgr3G35932 Mycgr3T
  
Location: 22944-24390

Mycgr3G35932\_Mycgr3T

Mycgr3G23761 Mycgr3T
  
Location: 24490-25825

Mycgr3G23761\_Mycgr3T

Mycgr3G35535 Mycgr3T
  
Location: 25925-26429

Mycgr3G35535\_Mycgr3T

Mycgr3G9942 Mycgr3T9
  
Location: 26529-30375

Mycgr3G9942\_Mycgr3T9

hypothetical protein
  
Accession: BAM31240
  
Location: 1086-2976
  
 NCBI BlastP on this gene

BAM31240

hypothetical protein
  
Accession: BAM31241
  
Location: 3516-4695
  
 NCBI BlastP on this gene

BAM31241

non-ribosomal peptide synthase
  
Accession: BAM31242
  
Location: 6718-16248
  
  
**BlastP hit with Mycgr3G90558\_Mycgr3T**
  
Percentage identity: 33 %
  
BlastP bit score: 1467
  
Sequence coverage: 65 %
  
E-value: 0.0
  
  
 NCBI BlastP on this gene

wykN

hypothetical protein
  
Accession: BAM31243
  
Location: 16678-18946
  
 NCBI BlastP on this gene

wykA

oligopeptide transporter
  
Accession: BAM31244
  
Location: 19439-21368
  
 NCBI BlastP on this gene

wykE

gibberellin 2-oxidase
  
Accession: BAM31245
  
Location: 22018-23172
  
  
**BlastP hit with Mycgr3G68036\_Mycgr3T**
  
Percentage identity: 40 %
  
BlastP bit score: 280
  
Sequence coverage: 104 %
  
E-value: 4e-88
  
  
 NCBI BlastP on this gene

wykG

N-methyltransferase
  
Accession: BAM31246
  
Location: 23709-25007
  
 NCBI BlastP on this gene

wykH

hypothetical protein
  
Accession: BAM31247
  
Location: 25215-25913
  
 NCBI BlastP on this gene

wykI

fructosyl amino acid oxidase
  
Accession: BAM31248
  
Location: 26312-27823
  
 NCBI BlastP on this gene

wykB

oligopeptide transporter
  
Accession: BAM31249
  
Location: 28267-29983
  
 NCBI BlastP on this gene

wykF

iron/ascorbate family oxidoreductase
  
Accession: BAM31250
  
Location: 30693-31852
  
  
**BlastP hit with Mycgr3G68036\_Mycgr3T**
  
Percentage identity: 41 %
  
BlastP bit score: 252
  
Sequence coverage: 100 %
  
E-value: 9e-78
  
  
 NCBI BlastP on this gene

wykC

hypothetical protein
  
Accession: BAM31251
  
Location: 32279-33934
  
 NCBI BlastP on this gene

wykR

haloacid dehalogenase
  
Accession: BAM31252
  
Location: 36043-36843
  
 NCBI BlastP on this gene

wykD

Query: Architecture Search FASTA input

AP007154 : Aspergillus oryzae RIB40 DNA, SC001.    Total score: 2.0     Cumulative Blast bit score: 1995

Hit cluster cross-links:

Mycgr3G36335 Mycgr3T
  
Location: 0-423

Mycgr3G36335\_Mycgr3T

Mycgr3G84494 Mycgr3T
  
Location: 523-2047

Mycgr3G84494\_Mycgr3T

Mycgr3G90558 Mycgr3T
  
Location: 2147-15296

Mycgr3G90558\_Mycgr3T

Mycgr3G68036 Mycgr3T
  
Location: 15396-16395

Mycgr3G68036\_Mycgr3T

Mycgr3G90561 Mycgr3T
  
Location: 16495-17134

Mycgr3G90561\_Mycgr3T

Mycgr3G35862 Mycgr3T
  
Location: 17234-18662

Mycgr3G35862\_Mycgr3T

Mycgr3G68030 Mycgr3T
  
Location: 18762-19722

Mycgr3G68030\_Mycgr3T

Mycgr3G36449 Mycgr3T
  
Location: 19822-21886

Mycgr3G36449\_Mycgr3T

Mycgr3G35528 Mycgr3T
  
Location: 21986-22844

Mycgr3G35528\_Mycgr3T

Mycgr3G35932 Mycgr3T
  
Location: 22944-24390

Mycgr3G35932\_Mycgr3T

Mycgr3G23761 Mycgr3T
  
Location: 24490-25825

Mycgr3G23761\_Mycgr3T

Mycgr3G35535 Mycgr3T
  
Location: 25925-26429

Mycgr3G35535\_Mycgr3T

Mycgr3G9942 Mycgr3T9
  
Location: 26529-30375

Mycgr3G9942\_Mycgr3T9

not annotated
  
Accession: BAE56571
  
Location: 10406-12296
  
 NCBI BlastP on this gene

AO090001000007

not annotated
  
Accession: BAE56572
  
Location: 12836-14015
  
 NCBI BlastP on this gene

AO090001000008

not annotated
  
Accession: BAE56573
  
Location: 16084-25587
  
  
**BlastP hit with Mycgr3G90558\_Mycgr3T**
  
Percentage identity: 33 %
  
BlastP bit score: 1466
  
Sequence coverage: 65 %
  
E-value: 0.0
  
  
 NCBI BlastP on this gene

AO090001000009

not annotated
  
Accession: BAE56574
  
Location: 26017-28285
  
 NCBI BlastP on this gene

AO090001000010

not annotated
  
Accession: BAE56575
  
Location: 28776-30705
  
 NCBI BlastP on this gene

AO090001000011

not annotated
  
Accession: BAE56576
  
Location: 31355-32509
  
  
**BlastP hit with Mycgr3G68036\_Mycgr3T**
  
Percentage identity: 39 %
  
BlastP bit score: 278
  
Sequence coverage: 104 %
  
E-value: 4e-87
  
  
 NCBI BlastP on this gene

AO090001000012

not annotated
  
Accession: BAE56577
  
Location: 33046-34344
  
 NCBI BlastP on this gene

AO090001000013

not annotated
  
Accession: BAE56578
  
Location: 34552-35250
  
 NCBI BlastP on this gene

AO090001000014

not annotated
  
Accession: BAE56579
  
Location: 35648-37159
  
 NCBI BlastP on this gene

AO090001000015

not annotated
  
Accession: BAE56580
  
Location: 37603-39319
  
 NCBI BlastP on this gene

AO090001000016

not annotated
  
Accession: BAE56581
  
Location: 40029-41188
  
  
**BlastP hit with Mycgr3G68036\_Mycgr3T**
  
Percentage identity: 40 %
  
BlastP bit score: 251
  
Sequence coverage: 100 %
  
E-value: 2e-77
  
  
 NCBI BlastP on this gene

AO090001000017

not annotated
  
Accession: BAE56582
  
Location: 41615-43270
  
 NCBI BlastP on this gene

AO090001000018

not annotated
  
Accession: BAE56583
  
Location: 45356-46156
  
 NCBI BlastP on this gene

AO090001000019

Query: Architecture Search FASTA input

ABDF02000068 : Trichoderma virens Gv29-8    Total score: 2.0     Cumulative Blast bit score: 1982

Hit cluster cross-links:

Mycgr3G36335 Mycgr3T
  
Location: 0-423

Mycgr3G36335\_Mycgr3T

Mycgr3G84494 Mycgr3T
  
Location: 523-2047

Mycgr3G84494\_Mycgr3T

Mycgr3G90558 Mycgr3T
  
Location: 2147-15296

Mycgr3G90558\_Mycgr3T

Mycgr3G68036 Mycgr3T
  
Location: 15396-16395

Mycgr3G68036\_Mycgr3T

Mycgr3G90561 Mycgr3T
  
Location: 16495-17134

Mycgr3G90561\_Mycgr3T

Mycgr3G35862 Mycgr3T
  
Location: 17234-18662

Mycgr3G35862\_Mycgr3T

Mycgr3G68030 Mycgr3T
  
Location: 18762-19722

Mycgr3G68030\_Mycgr3T

Mycgr3G36449 Mycgr3T
  
Location: 19822-21886

Mycgr3G36449\_Mycgr3T

Mycgr3G35528 Mycgr3T
  
Location: 21986-22844

Mycgr3G35528\_Mycgr3T

Mycgr3G35932 Mycgr3T
  
Location: 22944-24390

Mycgr3G35932\_Mycgr3T

Mycgr3G23761 Mycgr3T
  
Location: 24490-25825

Mycgr3G23761\_Mycgr3T

Mycgr3G35535 Mycgr3T
  
Location: 25925-26429

Mycgr3G35535\_Mycgr3T

Mycgr3G9942 Mycgr3T9
  
Location: 26529-30375

Mycgr3G9942\_Mycgr3T9

hypothetical protein
  
Accession: EHK21618
  
Location: 33438-37601
  
 NCBI BlastP on this gene

EHK21618

hypothetical protein
  
Accession: EHK21619
  
Location: 38123-38941
  
 NCBI BlastP on this gene

EHK21619

hypothetical protein
  
Accession: EHK21620
  
Location: 39740-41413
  
 NCBI BlastP on this gene

EHK21620

hypothetical protein
  
Accession: EHK21621
  
Location: 41533-42745
  
  
**BlastP hit with Mycgr3G68036\_Mycgr3T**
  
Percentage identity: 33 %
  
BlastP bit score: 171
  
Sequence coverage: 100 %
  
E-value: 8e-47
  
  
 NCBI BlastP on this gene

EHK21621

hypothetical protein
  
Accession: EHK21622
  
Location: 43706-60495
  
  
**BlastP hit with Mycgr3G90558\_Mycgr3T**
  
Percentage identity: 30 %
  
BlastP bit score: 1811
  
Sequence coverage: 96 %
  
E-value: 0.0
  
  
 NCBI BlastP on this gene

EHK21622

hypothetical protein
  
Accession: EHK21623
  
Location: 61093-61764
  
 NCBI BlastP on this gene

EHK21623

hypothetical protein
  
Accession: EHK21624
  
Location: 62337-63842
  
 NCBI BlastP on this gene

EHK21624

hypothetical protein
  
Accession: EHK21625
  
Location: 64333-65256
  
 NCBI BlastP on this gene

EHK21625

hypothetical protein
  
Accession: EHK21626
  
Location: 66527-67970
  
 NCBI BlastP on this gene

EHK21626

hypothetical protein
  
Accession: EHK21627
  
Location: 68674-71030
  
 NCBI BlastP on this gene

EHK21627

Query: Architecture Search FASTA input

EQ963479 : Aspergillus flavus NRRL3357 scf\_1106286418500 genomic scaffold    Total score: 2.0     Cumulative Blast bit score: 1963

Hit cluster cross-links:

Mycgr3G36335 Mycgr3T
  
Location: 0-423

Mycgr3G36335\_Mycgr3T

Mycgr3G84494 Mycgr3T
  
Location: 523-2047

Mycgr3G84494\_Mycgr3T

Mycgr3G90558 Mycgr3T
  
Location: 2147-15296

Mycgr3G90558\_Mycgr3T

Mycgr3G68036 Mycgr3T
  
Location: 15396-16395

Mycgr3G68036\_Mycgr3T

Mycgr3G90561 Mycgr3T
  
Location: 16495-17134

Mycgr3G90561\_Mycgr3T

Mycgr3G35862 Mycgr3T
  
Location: 17234-18662

Mycgr3G35862\_Mycgr3T

Mycgr3G68030 Mycgr3T
  
Location: 18762-19722

Mycgr3G68030\_Mycgr3T

Mycgr3G36449 Mycgr3T
  
Location: 19822-21886

Mycgr3G36449\_Mycgr3T

Mycgr3G35528 Mycgr3T
  
Location: 21986-22844

Mycgr3G35528\_Mycgr3T

Mycgr3G35932 Mycgr3T
  
Location: 22944-24390

Mycgr3G35932\_Mycgr3T

Mycgr3G23761 Mycgr3T
  
Location: 24490-25825

Mycgr3G23761\_Mycgr3T

Mycgr3G35535 Mycgr3T
  
Location: 25925-26429

Mycgr3G35535\_Mycgr3T

Mycgr3G9942 Mycgr3T9
  
Location: 26529-30375

Mycgr3G9942\_Mycgr3T9

conserved hypothetical protein
  
Accession: EED49845
  
Location: 644708-645803
  
 NCBI BlastP on this gene

EED49845

beta-galactosidase, putative
  
Accession: EED49846
  
Location: 646616-649969
  
 NCBI BlastP on this gene

EED49846

pyrroline-5-carboxylate reductase, putative
  
Accession: EED49847
  
Location: 650393-651371
  
 NCBI BlastP on this gene

EED49847

P450 family sporulation-specific N-formyltyrosine oxidase Dit2
  
Accession: EED49848
  
Location: 652134-653630
  
 NCBI BlastP on this gene

EED49848

conserved hypothetical protein
  
Accession: EED49849
  
Location: 653979-655109
  
  
**BlastP hit with Mycgr3G68036\_Mycgr3T**
  
Percentage identity: 33 %
  
BlastP bit score: 157
  
Sequence coverage: 94 %
  
E-value: 2e-41
  
  
 NCBI BlastP on this gene

EED49849

nonribosomal peptide synthase, putative
  
Accession: EED49850
  
Location: 655460-671802
  
  
**BlastP hit with Mycgr3G90558\_Mycgr3T**
  
Percentage identity: 30 %
  
BlastP bit score: 1806
  
Sequence coverage: 95 %
  
E-value: 0.0
  
  
 NCBI BlastP on this gene

EED49850

alcohol dehydrogenase, putative
  
Accession: EED49851
  
Location: 673317-674393
  
 NCBI BlastP on this gene

EED49851

MSF multidrug transporter, putative
  
Accession: EED49852
  
Location: 676143-677540
  
 NCBI BlastP on this gene

EED49852

periplasmic beta-glucosidase precursor, putative
  
Accession: EED49853
  
Location: 678960-681518
  
 NCBI BlastP on this gene

EED49853

Query: Architecture Search FASTA input

AKCU01000056 : Penicillium digitatum Pd1    Total score: 2.0     Cumulative Blast bit score: 1951

Hit cluster cross-links:

Mycgr3G36335 Mycgr3T
  
Location: 0-423

Mycgr3G36335\_Mycgr3T

Mycgr3G84494 Mycgr3T
  
Location: 523-2047

Mycgr3G84494\_Mycgr3T

Mycgr3G90558 Mycgr3T
  
Location: 2147-15296

Mycgr3G90558\_Mycgr3T

Mycgr3G68036 Mycgr3T
  
Location: 15396-16395

Mycgr3G68036\_Mycgr3T

Mycgr3G90561 Mycgr3T
  
Location: 16495-17134

Mycgr3G90561\_Mycgr3T

Mycgr3G35862 Mycgr3T
  
Location: 17234-18662

Mycgr3G35862\_Mycgr3T

Mycgr3G68030 Mycgr3T
  
Location: 18762-19722

Mycgr3G68030\_Mycgr3T

Mycgr3G36449 Mycgr3T
  
Location: 19822-21886

Mycgr3G36449\_Mycgr3T

Mycgr3G35528 Mycgr3T
  
Location: 21986-22844

Mycgr3G35528\_Mycgr3T

Mycgr3G35932 Mycgr3T
  
Location: 22944-24390

Mycgr3G35932\_Mycgr3T

Mycgr3G23761 Mycgr3T
  
Location: 24490-25825

Mycgr3G23761\_Mycgr3T

Mycgr3G35535 Mycgr3T
  
Location: 25925-26429

Mycgr3G35535\_Mycgr3T

Mycgr3G9942 Mycgr3T9
  
Location: 26529-30375

Mycgr3G9942\_Mycgr3T9

hypothetical protein
  
Accession: EKV21422
  
Location: 3282-4446
  
  
**BlastP hit with Mycgr3G68036\_Mycgr3T**
  
Percentage identity: 33 %
  
BlastP bit score: 179
  
Sequence coverage: 99 %
  
E-value: 7e-50
  
  
 NCBI BlastP on this gene

EKV21422

HC-toxin synthetase
  
Accession: EKV21423
  
Location: 5834-21508
  
  
**BlastP hit with Mycgr3G90558\_Mycgr3T**
  
Percentage identity: 30 %
  
BlastP bit score: 1772
  
Sequence coverage: 96 %
  
E-value: 0.0
  
  
 NCBI BlastP on this gene

EKV21423

Query: Architecture Search FASTA input

AKCT01000339 : Penicillium digitatum PHI26    Total score: 2.0     Cumulative Blast bit score: 1951

Hit cluster cross-links:

Mycgr3G36335 Mycgr3T
  
Location: 0-423

Mycgr3G36335\_Mycgr3T

Mycgr3G84494 Mycgr3T
  
Location: 523-2047

Mycgr3G84494\_Mycgr3T

Mycgr3G90558 Mycgr3T
  
Location: 2147-15296

Mycgr3G90558\_Mycgr3T

Mycgr3G68036 Mycgr3T
  
Location: 15396-16395

Mycgr3G68036\_Mycgr3T

Mycgr3G90561 Mycgr3T
  
Location: 16495-17134

Mycgr3G90561\_Mycgr3T

Mycgr3G35862 Mycgr3T
  
Location: 17234-18662

Mycgr3G35862\_Mycgr3T

Mycgr3G68030 Mycgr3T
  
Location: 18762-19722

Mycgr3G68030\_Mycgr3T

Mycgr3G36449 Mycgr3T
  
Location: 19822-21886

Mycgr3G36449\_Mycgr3T

Mycgr3G35528 Mycgr3T
  
Location: 21986-22844

Mycgr3G35528\_Mycgr3T

Mycgr3G35932 Mycgr3T
  
Location: 22944-24390

Mycgr3G35932\_Mycgr3T

Mycgr3G23761 Mycgr3T
  
Location: 24490-25825

Mycgr3G23761\_Mycgr3T

Mycgr3G35535 Mycgr3T
  
Location: 25925-26429

Mycgr3G35535\_Mycgr3T

Mycgr3G9942 Mycgr3T9
  
Location: 26529-30375

Mycgr3G9942\_Mycgr3T9

hypothetical protein
  
Accession: EKV04332
  
Location: 3361-4525
  
  
**BlastP hit with Mycgr3G68036\_Mycgr3T**
  
Percentage identity: 33 %
  
BlastP bit score: 179
  
Sequence coverage: 99 %
  
E-value: 7e-50
  
  
 NCBI BlastP on this gene

EKV04332

HC-toxin synthetase
  
Accession: EKV04333
  
Location: 5913-21587
  
  
**BlastP hit with Mycgr3G90558\_Mycgr3T**
  
Percentage identity: 30 %
  
BlastP bit score: 1772
  
Sequence coverage: 96 %
  
E-value: 0.0
  
  
 NCBI BlastP on this gene

EKV04333

Cell wall protein, putative
  
Accession: EKV04334
  
Location: 25617-27974
  
 NCBI BlastP on this gene

EKV04334

Serine/arginine repetitive matrix protein 1
  
Accession: EKV04335
  
Location: 29198-30442
  
 NCBI BlastP on this gene

EKV04335

Query: Architecture Search FASTA input

AP007154 : Aspergillus oryzae RIB40 DNA, SC001.    Total score: 2.0     Cumulative Blast bit score: 1902

Hit cluster cross-links:

Mycgr3G36335 Mycgr3T
  
Location: 0-423

Mycgr3G36335\_Mycgr3T

Mycgr3G84494 Mycgr3T
  
Location: 523-2047

Mycgr3G84494\_Mycgr3T

Mycgr3G90558 Mycgr3T
  
Location: 2147-15296

Mycgr3G90558\_Mycgr3T

Mycgr3G68036 Mycgr3T
  
Location: 15396-16395

Mycgr3G68036\_Mycgr3T

Mycgr3G90561 Mycgr3T
  
Location: 16495-17134

Mycgr3G90561\_Mycgr3T

Mycgr3G35862 Mycgr3T
  
Location: 17234-18662

Mycgr3G35862\_Mycgr3T

Mycgr3G68030 Mycgr3T
  
Location: 18762-19722

Mycgr3G68030\_Mycgr3T

Mycgr3G36449 Mycgr3T
  
Location: 19822-21886

Mycgr3G36449\_Mycgr3T

Mycgr3G35528 Mycgr3T
  
Location: 21986-22844

Mycgr3G35528\_Mycgr3T

Mycgr3G35932 Mycgr3T
  
Location: 22944-24390

Mycgr3G35932\_Mycgr3T

Mycgr3G23761 Mycgr3T
  
Location: 24490-25825

Mycgr3G23761\_Mycgr3T

Mycgr3G35535 Mycgr3T
  
Location: 25925-26429

Mycgr3G35535\_Mycgr3T

Mycgr3G9942 Mycgr3T9
  
Location: 26529-30375

Mycgr3G9942\_Mycgr3T9

not annotated
  
Accession: BAE56796
  
Location: 628006-628813
  
 NCBI BlastP on this gene

AO090001000257

not annotated
  
Accession: BAE56797
  
Location: 629120-630472
  
 NCBI BlastP on this gene

AO090001000258

not annotated
  
Accession: BAE56798
  
Location: 631077-633254
  
 NCBI BlastP on this gene

AO090001000259

not annotated
  
Accession: BAE56799
  
Location: 633887-636916
  
 NCBI BlastP on this gene

AO090001000260

not annotated
  
Accession: BAE56800
  
Location: 637265-638397
  
  
**BlastP hit with Mycgr3G68036\_Mycgr3T**
  
Percentage identity: 30 %
  
BlastP bit score: 132
  
Sequence coverage: 101 %
  
E-value: 5e-32
  
  
 NCBI BlastP on this gene

AO090001000261

not annotated
  
Accession: BAE56801
  
Location: 638748-655090
  
  
**BlastP hit with Mycgr3G90558\_Mycgr3T**
  
Percentage identity: 30 %
  
BlastP bit score: 1771
  
Sequence coverage: 94 %
  
E-value: 0.0
  
  
 NCBI BlastP on this gene

AO090001000262

not annotated
  
Accession: BAE56802
  
Location: 656686-657222
  
 NCBI BlastP on this gene

AO090001000263

not annotated
  
Accession: BAE56803
  
Location: 657634-658704
  
 NCBI BlastP on this gene

AO090001000264

not annotated
  
Accession: BAE56804
  
Location: 659394-662514
  
 NCBI BlastP on this gene

AO090001000265

not annotated
  
Accession: BAE56805
  
Location: 663368-665832
  
 NCBI BlastP on this gene

AO090001000266

Query: Architecture Search FASTA input

GL698751 : Metarhizium anisopliae ARSEF 23 unplaced genomic scaffold Scf\_041    Total score: 2.0     Cumulative Blast bit score: 1884

Hit cluster cross-links:

Mycgr3G36335 Mycgr3T
  
Location: 0-423

Mycgr3G36335\_Mycgr3T

Mycgr3G84494 Mycgr3T
  
Location: 523-2047

Mycgr3G84494\_Mycgr3T

Mycgr3G90558 Mycgr3T
  
Location: 2147-15296

Mycgr3G90558\_Mycgr3T

Mycgr3G68036 Mycgr3T
  
Location: 15396-16395

Mycgr3G68036\_Mycgr3T

Mycgr3G90561 Mycgr3T
  
Location: 16495-17134

Mycgr3G90561\_Mycgr3T

Mycgr3G35862 Mycgr3T
  
Location: 17234-18662

Mycgr3G35862\_Mycgr3T

Mycgr3G68030 Mycgr3T
  
Location: 18762-19722

Mycgr3G68030\_Mycgr3T

Mycgr3G36449 Mycgr3T
  
Location: 19822-21886

Mycgr3G36449\_Mycgr3T

Mycgr3G35528 Mycgr3T
  
Location: 21986-22844

Mycgr3G35528\_Mycgr3T

Mycgr3G35932 Mycgr3T
  
Location: 22944-24390

Mycgr3G35932\_Mycgr3T

Mycgr3G23761 Mycgr3T
  
Location: 24490-25825

Mycgr3G23761\_Mycgr3T

Mycgr3G35535 Mycgr3T
  
Location: 25925-26429

Mycgr3G35535\_Mycgr3T

Mycgr3G9942 Mycgr3T9
  
Location: 26529-30375

Mycgr3G9942\_Mycgr3T9

hypothetical protein
  
Accession: EFY94863
  
Location: 125087-126906
  
 NCBI BlastP on this gene

EFY94863

hypothetical protein
  
Accession: EFY94862
  
Location: 122685-123819
  
  
**BlastP hit with Mycgr3G68036\_Mycgr3T**
  
Percentage identity: 39 %
  
BlastP bit score: 227
  
Sequence coverage: 98 %
  
E-value: 4e-68
  
  
 NCBI BlastP on this gene

EFY94862

aspartate aminotransferase, putative
  
Accession: EFY94861
  
Location: 120966-122255
  
 NCBI BlastP on this gene

EFY94861

phytanoyl-CoA dioxygenase family protein
  
Accession: EFY94860
  
Location: 118929-120014
  
 NCBI BlastP on this gene

EFY94860

nonribosomal peptide synthase, putative
  
Accession: EFY94859
  
Location: 89548-118063
  
  
**BlastP hit with Mycgr3G90558\_Mycgr3T**
  
Percentage identity: 32 %
  
BlastP bit score: 1658
  
Sequence coverage: 85 %
  
E-value: 0.0
  
  
 NCBI BlastP on this gene

EFY94859

glutamate decarboxylase, putative
  
Accession: EFY94858
  
Location: 86972-88570
  
 NCBI BlastP on this gene

EFY94858

Query: Architecture Search FASTA input

GL698592 : Metarhizium acridum CQMa 102 unplaced genomic scaffold Scf\_123    Total score: 2.0     Cumulative Blast bit score: 1867

Hit cluster cross-links:

Mycgr3G36335 Mycgr3T
  
Location: 0-423

Mycgr3G36335\_Mycgr3T

Mycgr3G84494 Mycgr3T
  
Location: 523-2047

Mycgr3G84494\_Mycgr3T

Mycgr3G90558 Mycgr3T
  
Location: 2147-15296

Mycgr3G90558\_Mycgr3T

Mycgr3G68036 Mycgr3T
  
Location: 15396-16395

Mycgr3G68036\_Mycgr3T

Mycgr3G90561 Mycgr3T
  
Location: 16495-17134

Mycgr3G90561\_Mycgr3T

Mycgr3G35862 Mycgr3T
  
Location: 17234-18662

Mycgr3G35862\_Mycgr3T

Mycgr3G68030 Mycgr3T
  
Location: 18762-19722

Mycgr3G68030\_Mycgr3T

Mycgr3G36449 Mycgr3T
  
Location: 19822-21886

Mycgr3G36449\_Mycgr3T

Mycgr3G35528 Mycgr3T
  
Location: 21986-22844

Mycgr3G35528\_Mycgr3T

Mycgr3G35932 Mycgr3T
  
Location: 22944-24390

Mycgr3G35932\_Mycgr3T

Mycgr3G23761 Mycgr3T
  
Location: 24490-25825

Mycgr3G23761\_Mycgr3T

Mycgr3G35535 Mycgr3T
  
Location: 25925-26429

Mycgr3G35535\_Mycgr3T

Mycgr3G9942 Mycgr3T9
  
Location: 26529-30375

Mycgr3G9942\_Mycgr3T9

hypothetical protein
  
Accession: EFY85056
  
Location: 54018-55153
  
  
**BlastP hit with Mycgr3G68036\_Mycgr3T**
  
Percentage identity: 36 %
  
BlastP bit score: 210
  
Sequence coverage: 104 %
  
E-value: 3e-61
  
  
 NCBI BlastP on this gene

EFY85056

aspartate aminotransferase, putative
  
Accession: EFY85055
  
Location: 52531-53820
  
 NCBI BlastP on this gene

EFY85055

phytanoyl-CoA dioxygenase family protein
  
Accession: EFY85054
  
Location: 50538-51622
  
 NCBI BlastP on this gene

EFY85054

nonribosomal peptide synthase, putative
  
Accession: EFY85053
  
Location: 21161-49675
  
  
**BlastP hit with Mycgr3G90558\_Mycgr3T**
  
Percentage identity: 31 %
  
BlastP bit score: 1657
  
Sequence coverage: 85 %
  
E-value: 0.0
  
  
 NCBI BlastP on this gene

EFY85053

glutamate decarboxylase, putative
  
Accession: EFY85052
  
Location: 18619-20208
  
 NCBI BlastP on this gene

EFY85052

Query: Architecture Search FASTA input

CM001231 : Magnaporthe oryzae 70-15 chromosome 1    Total score: 2.0     Cumulative Blast bit score: 1841

Hit cluster cross-links:

Mycgr3G36335 Mycgr3T
  
Location: 0-423

Mycgr3G36335\_Mycgr3T

Mycgr3G84494 Mycgr3T
  
Location: 523-2047

Mycgr3G84494\_Mycgr3T

Mycgr3G90558 Mycgr3T
  
Location: 2147-15296

Mycgr3G90558\_Mycgr3T

Mycgr3G68036 Mycgr3T
  
Location: 15396-16395

Mycgr3G68036\_Mycgr3T

Mycgr3G90561 Mycgr3T
  
Location: 16495-17134

Mycgr3G90561\_Mycgr3T

Mycgr3G35862 Mycgr3T
  
Location: 17234-18662

Mycgr3G35862\_Mycgr3T

Mycgr3G68030 Mycgr3T
  
Location: 18762-19722

Mycgr3G68030\_Mycgr3T

Mycgr3G36449 Mycgr3T
  
Location: 19822-21886

Mycgr3G36449\_Mycgr3T

Mycgr3G35528 Mycgr3T
  
Location: 21986-22844

Mycgr3G35528\_Mycgr3T

Mycgr3G35932 Mycgr3T
  
Location: 22944-24390

Mycgr3G35932\_Mycgr3T

Mycgr3G23761 Mycgr3T
  
Location: 24490-25825

Mycgr3G23761\_Mycgr3T

Mycgr3G35535 Mycgr3T
  
Location: 25925-26429

Mycgr3G35535\_Mycgr3T

Mycgr3G9942 Mycgr3T9
  
Location: 26529-30375

Mycgr3G9942\_Mycgr3T9

multidrug resistance protein 3
  
Accession: EHA56506
  
Location: 1491590-1495606
  
 NCBI BlastP on this gene

EHA56506

pyrroline-5-carboxylate reductase
  
Accession: EHA56507
  
Location: 1495633-1496612
  
 NCBI BlastP on this gene

EHA56507

hypothetical protein
  
Accession: EHA56508
  
Location: 1497150-1498350
  
  
**BlastP hit with Mycgr3G68036\_Mycgr3T**
  
Percentage identity: 32 %
  
BlastP bit score: 152
  
Sequence coverage: 94 %
  
E-value: 9e-40
  
  
 NCBI BlastP on this gene

EHA56508

tyrocidine synthetase 1
  
Accession: EHA56509
  
Location: 1499425-1515742
  
  
**BlastP hit with Mycgr3G90558\_Mycgr3T**
  
Percentage identity: 30 %
  
BlastP bit score: 1689
  
Sequence coverage: 97 %
  
E-value: 0.0
  
  
 NCBI BlastP on this gene

EHA56509

hypothetical protein
  
Accession: EHA56510
  
Location: 1517518-1517837
  
 NCBI BlastP on this gene

EHA56510

hypothetical protein
  
Accession: EHA56511
  
Location: 1517915-1518511
  
 NCBI BlastP on this gene

EHA56511

hypothetical protein
  
Accession: EHA56512
  
Location: 1518880-1519141
  
 NCBI BlastP on this gene

EHA56512

Query: Architecture Search FASTA input

CABT02000016 : Sordaria macrospora k-hell    Total score: 2.0     Cumulative Blast bit score: 1791

Hit cluster cross-links:

Mycgr3G36335 Mycgr3T
  
Location: 0-423

Mycgr3G36335\_Mycgr3T

Mycgr3G84494 Mycgr3T
  
Location: 523-2047

Mycgr3G84494\_Mycgr3T

Mycgr3G90558 Mycgr3T
  
Location: 2147-15296

Mycgr3G90558\_Mycgr3T

Mycgr3G68036 Mycgr3T
  
Location: 15396-16395

Mycgr3G68036\_Mycgr3T

Mycgr3G90561 Mycgr3T
  
Location: 16495-17134

Mycgr3G90561\_Mycgr3T

Mycgr3G35862 Mycgr3T
  
Location: 17234-18662

Mycgr3G35862\_Mycgr3T

Mycgr3G68030 Mycgr3T
  
Location: 18762-19722

Mycgr3G68030\_Mycgr3T

Mycgr3G36449 Mycgr3T
  
Location: 19822-21886

Mycgr3G36449\_Mycgr3T

Mycgr3G35528 Mycgr3T
  
Location: 21986-22844

Mycgr3G35528\_Mycgr3T

Mycgr3G35932 Mycgr3T
  
Location: 22944-24390

Mycgr3G35932\_Mycgr3T

Mycgr3G23761 Mycgr3T
  
Location: 24490-25825

Mycgr3G23761\_Mycgr3T

Mycgr3G35535 Mycgr3T
  
Location: 25925-26429

Mycgr3G35535\_Mycgr3T

Mycgr3G9942 Mycgr3T9
  
Location: 26529-30375

Mycgr3G9942\_Mycgr3T9

not annotated
  
Accession: CCC11019
  
Location: 24624-26564
  
 NCBI BlastP on this gene

CCC11019

not annotated
  
Accession: CCC11020
  
Location: 27490-29035
  
 NCBI BlastP on this gene

CCC11020

not annotated
  
Accession: CCC11021
  
Location: 30874-47685
  
  
**BlastP hit with Mycgr3G90558\_Mycgr3T**
  
Percentage identity: 29 %
  
BlastP bit score: 1648
  
Sequence coverage: 91 %
  
E-value: 0.0
  
  
 NCBI BlastP on this gene

CCC11021

not annotated
  
Accession: CCC11022
  
Location: 49016-50295
  
  
**BlastP hit with Mycgr3G68036\_Mycgr3T**
  
Percentage identity: 32 %
  
BlastP bit score: 144
  
Sequence coverage: 106 %
  
E-value: 3e-36
  
  
 NCBI BlastP on this gene

CCC11022

not annotated
  
Accession: CCC11023
  
Location: 50873-51959
  
 NCBI BlastP on this gene

CCC11023

not annotated
  
Accession: CCC11024
  
Location: 52779-54014
  
 NCBI BlastP on this gene

CCC11024

not annotated
  
Accession: CCC11025
  
Location: 54692-58972
  
 NCBI BlastP on this gene

CCC11025

Query: Architecture Search FASTA input

CH445354 : Phaeosphaeria nodorum SN15 scaffold\_30    Total score: 2.0     Cumulative Blast bit score: 1777

Hit cluster cross-links:

Mycgr3G36335 Mycgr3T
  
Location: 0-423

Mycgr3G36335\_Mycgr3T

Mycgr3G84494 Mycgr3T
  
Location: 523-2047

Mycgr3G84494\_Mycgr3T

Mycgr3G90558 Mycgr3T
  
Location: 2147-15296

Mycgr3G90558\_Mycgr3T

Mycgr3G68036 Mycgr3T
  
Location: 15396-16395

Mycgr3G68036\_Mycgr3T

Mycgr3G90561 Mycgr3T
  
Location: 16495-17134

Mycgr3G90561\_Mycgr3T

Mycgr3G35862 Mycgr3T
  
Location: 17234-18662

Mycgr3G35862\_Mycgr3T

Mycgr3G68030 Mycgr3T
  
Location: 18762-19722

Mycgr3G68030\_Mycgr3T

Mycgr3G36449 Mycgr3T
  
Location: 19822-21886

Mycgr3G36449\_Mycgr3T

Mycgr3G35528 Mycgr3T
  
Location: 21986-22844

Mycgr3G35528\_Mycgr3T

Mycgr3G35932 Mycgr3T
  
Location: 22944-24390

Mycgr3G35932\_Mycgr3T

Mycgr3G23761 Mycgr3T
  
Location: 24490-25825

Mycgr3G23761\_Mycgr3T

Mycgr3G35535 Mycgr3T
  
Location: 25925-26429

Mycgr3G35535\_Mycgr3T

Mycgr3G9942 Mycgr3T9
  
Location: 26529-30375

Mycgr3G9942\_Mycgr3T9

hypothetical protein
  
Accession: EAT78333
  
Location: 10465-10858
  
 NCBI BlastP on this gene

EAT78333

hypothetical protein
  
Accession: EAT78335
  
Location: 11280-28516
  
  
**BlastP hit with Mycgr3G90558\_Mycgr3T**
  
Percentage identity: 31 %
  
BlastP bit score: 1672
  
Sequence coverage: 87 %
  
E-value: 0.0
  
  
 NCBI BlastP on this gene

EAT78335

hypothetical protein
  
Accession: EAT78336
  
Location: 29936-31095
  
 NCBI BlastP on this gene

EAT78336

hypothetical protein
  
Accession: EAT78337
  
Location: 31876-36120
  
 NCBI BlastP on this gene

EAT78337

hypothetical protein
  
Accession: EAT78338
  
Location: 35569-36229
  
 NCBI BlastP on this gene

EAT78338

hypothetical protein
  
Accession: EAT78339
  
Location: 36363-36696
  
 NCBI BlastP on this gene

EAT78339

hypothetical protein
  
Accession: EAT78340
  
Location: 37127-38285
  
  
**BlastP hit with Mycgr3G35528\_Mycgr3T**
  
Percentage identity: 27 %
  
BlastP bit score: 105
  
Sequence coverage: 100 %
  
E-value: 3e-23
  
  
 NCBI BlastP on this gene

EAT78340

hypothetical protein
  
Accession: EAT78341
  
Location: 38557-39423
  
 NCBI BlastP on this gene

EAT78341

hypothetical protein
  
Accession: EAT78342
  
Location: 40103-41047
  
 NCBI BlastP on this gene

EAT78342

hypothetical protein
  
Accession: EAT78343
  
Location: 42153-43496
  
 NCBI BlastP on this gene

EAT78343

Query: Architecture Search FASTA input

KB708022 : Botryotinia fuckeliana BcDW1 unplaced genomic scaffold Scaffold\_350    Total score: 2.0     Cumulative Blast bit score: 1725

Hit cluster cross-links:

Mycgr3G36335 Mycgr3T
  
Location: 0-423

Mycgr3G36335\_Mycgr3T

Mycgr3G84494 Mycgr3T
  
Location: 523-2047

Mycgr3G84494\_Mycgr3T

Mycgr3G90558 Mycgr3T
  
Location: 2147-15296

Mycgr3G90558\_Mycgr3T

Mycgr3G68036 Mycgr3T
  
Location: 15396-16395

Mycgr3G68036\_Mycgr3T

Mycgr3G90561 Mycgr3T
  
Location: 16495-17134

Mycgr3G90561\_Mycgr3T

Mycgr3G35862 Mycgr3T
  
Location: 17234-18662

Mycgr3G35862\_Mycgr3T

Mycgr3G68030 Mycgr3T
  
Location: 18762-19722

Mycgr3G68030\_Mycgr3T

Mycgr3G36449 Mycgr3T
  
Location: 19822-21886

Mycgr3G36449\_Mycgr3T

Mycgr3G35528 Mycgr3T
  
Location: 21986-22844

Mycgr3G35528\_Mycgr3T

Mycgr3G35932 Mycgr3T
  
Location: 22944-24390

Mycgr3G35932\_Mycgr3T

Mycgr3G23761 Mycgr3T
  
Location: 24490-25825

Mycgr3G23761\_Mycgr3T

Mycgr3G35535 Mycgr3T
  
Location: 25925-26429

Mycgr3G35535\_Mycgr3T

Mycgr3G9942 Mycgr3T9
  
Location: 26529-30375

Mycgr3G9942\_Mycgr3T9

putative polyketide synthase protein
  
Accession: EMR82969
  
Location: 57703-65560
  
 NCBI BlastP on this gene

EMR82969

putative nonribosomal peptide protein
  
Accession: EMR82968
  
Location: 41327-54610
  
  
**BlastP hit with Mycgr3G90558\_Mycgr3T**
  
Percentage identity: 29 %
  
BlastP bit score: 1402
  
Sequence coverage: 75 %
  
E-value: 0.0
  
  
 NCBI BlastP on this gene

EMR82968

putative mfs multidrug protein
  
Accession: EMR82967
  
Location: 34997-36748
  
  
**BlastP hit with Mycgr3G84494\_Mycgr3T**
  
Percentage identity: 36 %
  
BlastP bit score: 323
  
Sequence coverage: 98 %
  
E-value: 1e-99
  
  
 NCBI BlastP on this gene

EMR82967

putative taurine catabolism dioxygenase protein
  
Accession: EMR82966
  
Location: 33199-34636
  
 NCBI BlastP on this gene

EMR82966

putative indoleamine -dioxygenase family protein
  
Accession: EMR82965
  
Location: 27803-29499
  
 NCBI BlastP on this gene

EMR82965

Query: Architecture Search FASTA input

FQ790270 : Botryotinia fuckeliana T4 SuperContig\_51\_1 genomic supercontig.    Total score: 2.0     Cumulative Blast bit score: 1725

Hit cluster cross-links:

Mycgr3G36335 Mycgr3T
  
Location: 0-423

Mycgr3G36335\_Mycgr3T

Mycgr3G84494 Mycgr3T
  
Location: 523-2047

Mycgr3G84494\_Mycgr3T

Mycgr3G90558 Mycgr3T
  
Location: 2147-15296

Mycgr3G90558\_Mycgr3T

Mycgr3G68036 Mycgr3T
  
Location: 15396-16395

Mycgr3G68036\_Mycgr3T

Mycgr3G90561 Mycgr3T
  
Location: 16495-17134

Mycgr3G90561\_Mycgr3T

Mycgr3G35862 Mycgr3T
  
Location: 17234-18662

Mycgr3G35862\_Mycgr3T

Mycgr3G68030 Mycgr3T
  
Location: 18762-19722

Mycgr3G68030\_Mycgr3T

Mycgr3G36449 Mycgr3T
  
Location: 19822-21886

Mycgr3G36449\_Mycgr3T

Mycgr3G35528 Mycgr3T
  
Location: 21986-22844

Mycgr3G35528\_Mycgr3T

Mycgr3G35932 Mycgr3T
  
Location: 22944-24390

Mycgr3G35932\_Mycgr3T

Mycgr3G23761 Mycgr3T
  
Location: 24490-25825

Mycgr3G23761\_Mycgr3T

Mycgr3G35535 Mycgr3T
  
Location: 25925-26429

Mycgr3G35535\_Mycgr3T

Mycgr3G9942 Mycgr3T9
  
Location: 26529-30375

Mycgr3G9942\_Mycgr3T9

BcPKS11, polyketide synthase
  
Accession: CCD44403
  
Location: 676697-684554
  
 NCBI BlastP on this gene

BofuT4\_P059830.1

BcNRPS9, nonribosomal peptide synthetase
  
Accession: CCD44402
  
Location: 655974-669254
  
  
**BlastP hit with Mycgr3G90558\_Mycgr3T**
  
Percentage identity: 29 %
  
BlastP bit score: 1404
  
Sequence coverage: 75 %
  
E-value: 0.0
  
  
 NCBI BlastP on this gene

BofuT4\_P059820.1

hypothetical protein
  
Accession: CCD44401
  
Location: 654409-655008
  
 NCBI BlastP on this gene

BofuT4\_P059810.1

hypothetical protein
  
Accession: CCD44400
  
Location: 652819-654265
  
 NCBI BlastP on this gene

BofuT4\_P059800.1

similar to MFS multidrug transporter
  
Accession: CCD44399
  
Location: 650095-651846
  
  
**BlastP hit with Mycgr3G84494\_Mycgr3T**
  
Percentage identity: 36 %
  
BlastP bit score: 321
  
Sequence coverage: 98 %
  
E-value: 5e-99
  
  
 NCBI BlastP on this gene

BofuT4\_P059790.1

similar to tfdA family taurine dioxygenase
  
Accession: CCD44398
  
Location: 648297-649734
  
 NCBI BlastP on this gene

BofuT4\_P059780.1

predicted protein
  
Accession: CCD44397
  
Location: 647201-647649
  
 NCBI BlastP on this gene

BofuT4\_uP059770.1

predicted protein
  
Accession: CCD44396
  
Location: 646896-647150
  
 NCBI BlastP on this gene

BofuT4\_uP059760.1

hypothetical protein
  
Accession: CCD44395
  
Location: 645196-645780
  
 NCBI BlastP on this gene

BofuT4\_P059750.1

similar to indoleamine 2,3-dioxygenase family protein
  
Accession: CCD44394
  
Location: 642934-644630
  
 NCBI BlastP on this gene

BofuT4\_P059740.1

Query: Architecture Search FASTA input

KB705649 : Eutypa lata UCREL1 unplaced genomic scaffold EL1\_03\_scaffold\_311    Total score: 2.0     Cumulative Blast bit score: 1680

Hit cluster cross-links:

Mycgr3G36335 Mycgr3T
  
Location: 0-423

Mycgr3G36335\_Mycgr3T

Mycgr3G84494 Mycgr3T
  
Location: 523-2047

Mycgr3G84494\_Mycgr3T

Mycgr3G90558 Mycgr3T
  
Location: 2147-15296

Mycgr3G90558\_Mycgr3T

Mycgr3G68036 Mycgr3T
  
Location: 15396-16395

Mycgr3G68036\_Mycgr3T

Mycgr3G90561 Mycgr3T
  
Location: 16495-17134

Mycgr3G90561\_Mycgr3T

Mycgr3G35862 Mycgr3T
  
Location: 17234-18662

Mycgr3G35862\_Mycgr3T

Mycgr3G68030 Mycgr3T
  
Location: 18762-19722

Mycgr3G68030\_Mycgr3T

Mycgr3G36449 Mycgr3T
  
Location: 19822-21886

Mycgr3G36449\_Mycgr3T

Mycgr3G35528 Mycgr3T
  
Location: 21986-22844

Mycgr3G35528\_Mycgr3T

Mycgr3G35932 Mycgr3T
  
Location: 22944-24390

Mycgr3G35932\_Mycgr3T

Mycgr3G23761 Mycgr3T
  
Location: 24490-25825

Mycgr3G23761\_Mycgr3T

Mycgr3G35535 Mycgr3T
  
Location: 25925-26429

Mycgr3G35535\_Mycgr3T

Mycgr3G9942 Mycgr3T9
  
Location: 26529-30375

Mycgr3G9942\_Mycgr3T9

putative alanyl-trna synthetase protein
  
Accession: EMR71361
  
Location: 71768-72680
  
 NCBI BlastP on this gene

EMR71361

putative signal transduction protein
  
Accession: EMR71343
  
Location: 68736-69999
  
  
**BlastP hit with Mycgr3G35528\_Mycgr3T**
  
Percentage identity: 28 %
  
BlastP bit score: 86
  
Sequence coverage: 87 %
  
E-value: 2e-16
  
  
 NCBI BlastP on this gene

EMR71343

putative mfs toxin efflux pump protein
  
Accession: EMR71349
  
Location: 65751-67745
  
 NCBI BlastP on this gene

EMR71349

putative duf323 domain-containing protein
  
Accession: EMR71335
  
Location: 62623-63886
  
 NCBI BlastP on this gene

EMR71335

putative duf323 domain-containing protein
  
Accession: EMR71366
  
Location: 59290-60476
  
 NCBI BlastP on this gene

EMR71366

hypothetical protein
  
Accession: EMR71346
  
Location: 30714-57959
  
  
**BlastP hit with Mycgr3G90558\_Mycgr3T**
  
Percentage identity: 33 %
  
BlastP bit score: 1595
  
Sequence coverage: 68 %
  
E-value: 0.0
  
  
 NCBI BlastP on this gene

EMR71346

hypothetical protein
  
Accession: EMR71345
  
Location: 28312-28929
  
 NCBI BlastP on this gene

EMR71345

Query: Architecture Search FASTA input

AAHF01000003 : Aspergillus fumigatus Af293    Total score: 2.0     Cumulative Blast bit score: 1649

Hit cluster cross-links:

Mycgr3G36335 Mycgr3T
  
Location: 0-423

Mycgr3G36335\_Mycgr3T

Mycgr3G84494 Mycgr3T
  
Location: 523-2047

Mycgr3G84494\_Mycgr3T

Mycgr3G90558 Mycgr3T
  
Location: 2147-15296

Mycgr3G90558\_Mycgr3T

Mycgr3G68036 Mycgr3T
  
Location: 15396-16395

Mycgr3G68036\_Mycgr3T

Mycgr3G90561 Mycgr3T
  
Location: 16495-17134

Mycgr3G90561\_Mycgr3T

Mycgr3G35862 Mycgr3T
  
Location: 17234-18662

Mycgr3G35862\_Mycgr3T

Mycgr3G68030 Mycgr3T
  
Location: 18762-19722

Mycgr3G68030\_Mycgr3T

Mycgr3G36449 Mycgr3T
  
Location: 19822-21886

Mycgr3G36449\_Mycgr3T

Mycgr3G35528 Mycgr3T
  
Location: 21986-22844

Mycgr3G35528\_Mycgr3T

Mycgr3G35932 Mycgr3T
  
Location: 22944-24390

Mycgr3G35932\_Mycgr3T

Mycgr3G23761 Mycgr3T
  
Location: 24490-25825

Mycgr3G23761\_Mycgr3T

Mycgr3G35535 Mycgr3T
  
Location: 25925-26429

Mycgr3G35535\_Mycgr3T

Mycgr3G9942 Mycgr3T9
  
Location: 26529-30375

Mycgr3G9942\_Mycgr3T9

ABC multidrug transporter, putative
  
Accession: EAL91343
  
Location: 635720-640633
  
 NCBI BlastP on this gene

EAL91343

nonribosomal peptide synthase, putative
  
Accession: EAL91342
  
Location: 608358-633905
  
  
**BlastP hit with Mycgr3G90558\_Mycgr3T**
  
Percentage identity: 29 %
  
BlastP bit score: 1428
  
Sequence coverage: 87 %
  
E-value: 0.0
  
  
 NCBI BlastP on this gene

EAL91342

MFS multidrug transporter, putative
  
Accession: EAL91341
  
Location: 605650-607205
  
  
**BlastP hit with Mycgr3G23761\_Mycgr3T**
  
Percentage identity: 37 %
  
BlastP bit score: 221
  
Sequence coverage: 99 %
  
E-value: 9e-63
  
  
 NCBI BlastP on this gene

EAL91341

hypothetical protein
  
Accession: EAL91340
  
Location: 602510-604110
  
 NCBI BlastP on this gene

EAL91340

Query: Architecture Search FASTA input

DS499597 : Aspergillus fumigatus A1163 scf\_000004 genomic scaffold    Total score: 2.0     Cumulative Blast bit score: 1610

Hit cluster cross-links:

Mycgr3G36335 Mycgr3T
  
Location: 0-423

Mycgr3G36335\_Mycgr3T

Mycgr3G84494 Mycgr3T
  
Location: 523-2047

Mycgr3G84494\_Mycgr3T

Mycgr3G90558 Mycgr3T
  
Location: 2147-15296

Mycgr3G90558\_Mycgr3T

Mycgr3G68036 Mycgr3T
  
Location: 15396-16395

Mycgr3G68036\_Mycgr3T

Mycgr3G90561 Mycgr3T
  
Location: 16495-17134

Mycgr3G90561\_Mycgr3T

Mycgr3G35862 Mycgr3T
  
Location: 17234-18662

Mycgr3G35862\_Mycgr3T

Mycgr3G68030 Mycgr3T
  
Location: 18762-19722

Mycgr3G68030\_Mycgr3T

Mycgr3G36449 Mycgr3T
  
Location: 19822-21886

Mycgr3G36449\_Mycgr3T

Mycgr3G35528 Mycgr3T
  
Location: 21986-22844

Mycgr3G35528\_Mycgr3T

Mycgr3G35932 Mycgr3T
  
Location: 22944-24390

Mycgr3G35932\_Mycgr3T

Mycgr3G23761 Mycgr3T
  
Location: 24490-25825

Mycgr3G23761\_Mycgr3T

Mycgr3G35535 Mycgr3T
  
Location: 25925-26429

Mycgr3G35535\_Mycgr3T

Mycgr3G9942 Mycgr3T9
  
Location: 26529-30375

Mycgr3G9942\_Mycgr3T9

nonribosomal peptide synthase, putative
  
Accession: EDP52010
  
Location: 3224060-3249608
  
  
**BlastP hit with Mycgr3G90558\_Mycgr3T**
  
Percentage identity: 29 %
  
BlastP bit score: 1390
  
Sequence coverage: 86 %
  
E-value: 0.0
  
  
 NCBI BlastP on this gene

EDP52010

MFS multidrug transporter, putative
  
Accession: EDP52011
  
Location: 3250761-3252316
  
  
**BlastP hit with Mycgr3G23761\_Mycgr3T**
  
Percentage identity: 36 %
  
BlastP bit score: 220
  
Sequence coverage: 99 %
  
E-value: 3e-62
  
  
 NCBI BlastP on this gene

EDP52011

hypothetical protein
  
Accession: EDP52012
  
Location: 3253856-3255456
  
 NCBI BlastP on this gene

EDP52012

Query: Architecture Search FASTA input

ACJE01000008 : Aspergillus niger ATCC 1015    Total score: 2.0     Cumulative Blast bit score: 1440

Hit cluster cross-links:

Mycgr3G36335 Mycgr3T
  
Location: 0-423

Mycgr3G36335\_Mycgr3T

Mycgr3G84494 Mycgr3T
  
Location: 523-2047

Mycgr3G84494\_Mycgr3T

Mycgr3G90558 Mycgr3T
  
Location: 2147-15296

Mycgr3G90558\_Mycgr3T

Mycgr3G68036 Mycgr3T
  
Location: 15396-16395

Mycgr3G68036\_Mycgr3T

Mycgr3G90561 Mycgr3T
  
Location: 16495-17134

Mycgr3G90561\_Mycgr3T

Mycgr3G35862 Mycgr3T
  
Location: 17234-18662

Mycgr3G35862\_Mycgr3T

Mycgr3G68030 Mycgr3T
  
Location: 18762-19722

Mycgr3G68030\_Mycgr3T

Mycgr3G36449 Mycgr3T
  
Location: 19822-21886

Mycgr3G36449\_Mycgr3T

Mycgr3G35528 Mycgr3T
  
Location: 21986-22844

Mycgr3G35528\_Mycgr3T

Mycgr3G35932 Mycgr3T
  
Location: 22944-24390

Mycgr3G35932\_Mycgr3T

Mycgr3G23761 Mycgr3T
  
Location: 24490-25825

Mycgr3G23761\_Mycgr3T

Mycgr3G35535 Mycgr3T
  
Location: 25925-26429

Mycgr3G35535\_Mycgr3T

Mycgr3G9942 Mycgr3T9
  
Location: 26529-30375

Mycgr3G9942\_Mycgr3T9

hypothetical protein
  
Accession: EHA24937
  
Location: 1800474-1801133
  
 NCBI BlastP on this gene

EHA24937

hypothetical protein
  
Accession: EHA24938
  
Location: 1803005-1816475
  
  
**BlastP hit with Mycgr3G90558\_Mycgr3T**
  
Percentage identity: 30 %
  
BlastP bit score: 1283
  
Sequence coverage: 70 %
  
E-value: 0.0
  
  
 NCBI BlastP on this gene

EHA24938

hypothetical protein
  
Accession: EHA24939
  
Location: 1824432-1825889
  
 NCBI BlastP on this gene

EHA24939

hypothetical protein
  
Accession: EHA24940
  
Location: 1827516-1828076
  
 NCBI BlastP on this gene

EHA24940

hypothetical protein
  
Accession: EHA24941
  
Location: 1828436-1830408
  
 NCBI BlastP on this gene

EHA24941

hypothetical protein
  
Accession: EHA24942
  
Location: 1830622-1832126
  
 NCBI BlastP on this gene

EHA24942

hypothetical protein
  
Accession: EHA24943
  
Location: 1832910-1834405
  
 NCBI BlastP on this gene

EHA24943

hypothetical protein
  
Accession: EHA24944
  
Location: 1834991-1836596
  
  
**BlastP hit with Mycgr3G23761\_Mycgr3T**
  
Percentage identity: 28 %
  
BlastP bit score: 157
  
Sequence coverage: 98 %
  
E-value: 8e-40
  
  
 NCBI BlastP on this gene

EHA24944

hypothetical protein
  
Accession: EHA24945
  
Location: 1837742-1838215
  
 NCBI BlastP on this gene

EHA24945

hypothetical protein
  
Accession: EHA24946
  
Location: 1838985-1839854
  
 NCBI BlastP on this gene

EHA24946

Query: Architecture Search FASTA input

EQ963479 : Aspergillus flavus NRRL3357 scf\_1106286418500 genomic scaffold    Total score: 2.0     Cumulative Blast bit score: 1418

Hit cluster cross-links:

Mycgr3G36335 Mycgr3T
  
Location: 0-423

Mycgr3G36335\_Mycgr3T

Mycgr3G84494 Mycgr3T
  
Location: 523-2047

Mycgr3G84494\_Mycgr3T

Mycgr3G90558 Mycgr3T
  
Location: 2147-15296

Mycgr3G90558\_Mycgr3T

Mycgr3G68036 Mycgr3T
  
Location: 15396-16395

Mycgr3G68036\_Mycgr3T

Mycgr3G90561 Mycgr3T
  
Location: 16495-17134

Mycgr3G90561\_Mycgr3T

Mycgr3G35862 Mycgr3T
  
Location: 17234-18662

Mycgr3G35862\_Mycgr3T

Mycgr3G68030 Mycgr3T
  
Location: 18762-19722

Mycgr3G68030\_Mycgr3T

Mycgr3G36449 Mycgr3T
  
Location: 19822-21886

Mycgr3G36449\_Mycgr3T

Mycgr3G35528 Mycgr3T
  
Location: 21986-22844

Mycgr3G35528\_Mycgr3T

Mycgr3G35932 Mycgr3T
  
Location: 22944-24390

Mycgr3G35932\_Mycgr3T

Mycgr3G23761 Mycgr3T
  
Location: 24490-25825

Mycgr3G23761\_Mycgr3T

Mycgr3G35535 Mycgr3T
  
Location: 25925-26429

Mycgr3G35535\_Mycgr3T

Mycgr3G9942 Mycgr3T9
  
Location: 26529-30375

Mycgr3G9942\_Mycgr3T9

conserved hypothetical protein
  
Accession: EED49601
  
Location: 19380-24147
  
 NCBI BlastP on this gene

EED49601

hypothetical protein
  
Accession: EED49602
  
Location: 25295-25750
  
 NCBI BlastP on this gene

EED49602

nonribosomal peptide synthase, putative
  
Accession: EED49603
  
Location: 29095-35391
  
  
**BlastP hit with Mycgr3G90558\_Mycgr3T**
  
Percentage identity: 33 %
  
BlastP bit score: 960
  
Sequence coverage: 44 %
  
E-value: 0.0
  
  
 NCBI BlastP on this gene

EED49603

phenol 2-monooxygenase, putative
  
Accession: EED49604
  
Location: 36311-37102
  
 NCBI BlastP on this gene

EED49604

oligopeptide transporter, putative
  
Accession: EED49605
  
Location: 38582-40510
  
 NCBI BlastP on this gene

EED49605

gibberellin 2-oxidase, putative
  
Accession: EED49606
  
Location: 41154-42314
  
  
**BlastP hit with Mycgr3G68036\_Mycgr3T**
  
Percentage identity: 40 %
  
BlastP bit score: 284
  
Sequence coverage: 102 %
  
E-value: 8e-90
  
  
 NCBI BlastP on this gene

EED49606

N-methyltransferase, putative
  
Accession: EED49607
  
Location: 42851-44016
  
 NCBI BlastP on this gene

EED49607

O-methyltransferase, putative
  
Accession: EED49608
  
Location: 44443-45186
  
 NCBI BlastP on this gene

EED49608

fructosyl amino acid oxidase, putative
  
Accession: EED49609
  
Location: 45453-46964
  
 NCBI BlastP on this gene

EED49609

hypothetical protein
  
Accession: EED49610
  
Location: 47408-47725
  
 NCBI BlastP on this gene

EED49610

oligopeptide transporter, putative
  
Accession: EED49611
  
Location: 47758-50991
  
  
**BlastP hit with Mycgr3G68036\_Mycgr3T**
  
Percentage identity: 43 %
  
BlastP bit score: 174
  
Sequence coverage: 60 %
  
E-value: 3e-46
  
  
 NCBI BlastP on this gene

EED49611

conserved hypothetical protein
  
Accession: EED49612
  
Location: 51418-53073
  
 NCBI BlastP on this gene

EED49612

conserved hypothetical protein
  
Accession: EED49613
  
Location: 55171-55971
  
 NCBI BlastP on this gene

EED49613

MFS transporter, putative
  
Accession: EED49614
  
Location: 57672-59455
  
 NCBI BlastP on this gene

EED49614

Query: Architecture Search FASTA input

ABDF02000003 : Trichoderma virens Gv29-8    Total score: 2.0     Cumulative Blast bit score: 1409

Hit cluster cross-links:

Mycgr3G36335 Mycgr3T
  
Location: 0-423

Mycgr3G36335\_Mycgr3T

Mycgr3G84494 Mycgr3T
  
Location: 523-2047

Mycgr3G84494\_Mycgr3T

Mycgr3G90558 Mycgr3T
  
Location: 2147-15296

Mycgr3G90558\_Mycgr3T

Mycgr3G68036 Mycgr3T
  
Location: 15396-16395

Mycgr3G68036\_Mycgr3T

Mycgr3G90561 Mycgr3T
  
Location: 16495-17134

Mycgr3G90561\_Mycgr3T

Mycgr3G35862 Mycgr3T
  
Location: 17234-18662

Mycgr3G35862\_Mycgr3T

Mycgr3G68030 Mycgr3T
  
Location: 18762-19722

Mycgr3G68030\_Mycgr3T

Mycgr3G36449 Mycgr3T
  
Location: 19822-21886

Mycgr3G36449\_Mycgr3T

Mycgr3G35528 Mycgr3T
  
Location: 21986-22844

Mycgr3G35528\_Mycgr3T

Mycgr3G35932 Mycgr3T
  
Location: 22944-24390

Mycgr3G35932\_Mycgr3T

Mycgr3G23761 Mycgr3T
  
Location: 24490-25825

Mycgr3G23761\_Mycgr3T

Mycgr3G35535 Mycgr3T
  
Location: 25925-26429

Mycgr3G35535\_Mycgr3T

Mycgr3G9942 Mycgr3T9
  
Location: 26529-30375

Mycgr3G9942\_Mycgr3T9

hypothetical protein
  
Accession: EHK25880
  
Location: 2517486-2518427
  
 NCBI BlastP on this gene

EHK25880

hypothetical protein
  
Accession: EHK25881
  
Location: 2519235-2520380
  
  
**BlastP hit with Mycgr3G68036\_Mycgr3T**
  
Percentage identity: 44 %
  
BlastP bit score: 264
  
Sequence coverage: 96 %
  
E-value: 2e-82
  
  
 NCBI BlastP on this gene

EHK25881

hypothetical protein
  
Accession: EHK25882
  
Location: 2520859-2521212
  
 NCBI BlastP on this gene

EHK25882

hypothetical protein
  
Accession: EHK25883
  
Location: 2523390-2525529
  
 NCBI BlastP on this gene

EHK25883

hypothetical protein
  
Accession: EHK25884
  
Location: 2526677-2528013
  
 NCBI BlastP on this gene

EHK25884

non-ribosomal peptide synthetase
  
Accession: EHK25885
  
Location: 2529796-2553890
  
  
**BlastP hit with Mycgr3G90558\_Mycgr3T**
  
Percentage identity: 29 %
  
BlastP bit score: 1145
  
Sequence coverage: 71 %
  
E-value: 0.0
  
  
 NCBI BlastP on this gene

EHK25885

hypothetical protein
  
Accession: EHK25886
  
Location: 2555537-2559929
  
 NCBI BlastP on this gene

EHK25886

Query: Architecture Search FASTA input

GL534459 : Pyrenophora teres f. teres 0-1 unplaced genomic scaffold scaffold\_190719    Total score: 2.0     Cumulative Blast bit score: 1362

Hit cluster cross-links:

Mycgr3G36335 Mycgr3T
  
Location: 0-423

Mycgr3G36335\_Mycgr3T

Mycgr3G84494 Mycgr3T
  
Location: 523-2047

Mycgr3G84494\_Mycgr3T

Mycgr3G90558 Mycgr3T
  
Location: 2147-15296

Mycgr3G90558\_Mycgr3T

Mycgr3G68036 Mycgr3T
  
Location: 15396-16395

Mycgr3G68036\_Mycgr3T

Mycgr3G90561 Mycgr3T
  
Location: 16495-17134

Mycgr3G90561\_Mycgr3T

Mycgr3G35862 Mycgr3T
  
Location: 17234-18662

Mycgr3G35862\_Mycgr3T

Mycgr3G68030 Mycgr3T
  
Location: 18762-19722

Mycgr3G68030\_Mycgr3T

Mycgr3G36449 Mycgr3T
  
Location: 19822-21886

Mycgr3G36449\_Mycgr3T

Mycgr3G35528 Mycgr3T
  
Location: 21986-22844

Mycgr3G35528\_Mycgr3T

Mycgr3G35932 Mycgr3T
  
Location: 22944-24390

Mycgr3G35932\_Mycgr3T

Mycgr3G23761 Mycgr3T
  
Location: 24490-25825

Mycgr3G23761\_Mycgr3T

Mycgr3G35535 Mycgr3T
  
Location: 25925-26429

Mycgr3G35535\_Mycgr3T

Mycgr3G9942 Mycgr3T9
  
Location: 26529-30375

Mycgr3G9942\_Mycgr3T9

hypothetical protein
  
Accession: EFQ92071
  
Location: 17941-19050
  
 NCBI BlastP on this gene

EFQ92071

hypothetical protein
  
Accession: EFQ92072
  
Location: 19293-19652
  
 NCBI BlastP on this gene

EFQ92072

hypothetical protein
  
Accession: EFQ92073
  
Location: 19943-41977
  
  
**BlastP hit with Mycgr3G90558\_Mycgr3T**
  
Percentage identity: 32 %
  
BlastP bit score: 1177
  
Sequence coverage: 60 %
  
E-value: 0.0
  
  
 NCBI BlastP on this gene

EFQ92073

hypothetical protein
  
Accession: EFQ92074
  
Location: 46095-51051
  
 NCBI BlastP on this gene

EFQ92074

hypothetical protein
  
Accession: EFQ92075
  
Location: 51524-53825
  
 NCBI BlastP on this gene

EFQ92075

hypothetical protein
  
Accession: EFQ92076
  
Location: 54862-56502
  
  
**BlastP hit with Mycgr3G23761\_Mycgr3T**
  
Percentage identity: 29 %
  
BlastP bit score: 185
  
Sequence coverage: 99 %
  
E-value: 6e-49
  
  
 NCBI BlastP on this gene

EFQ92076

hypothetical protein
  
Accession: EFQ92077
  
Location: 56907-60731
  
 NCBI BlastP on this gene

EFQ92077

Query: Architecture Search FASTA input

DS027058 : Aspergillus clavatus NRRL 1 1099423829804 genomic scaffold    Total score: 2.0     Cumulative Blast bit score: 1344

Hit cluster cross-links:

Mycgr3G36335 Mycgr3T
  
Location: 0-423

Mycgr3G36335\_Mycgr3T

Mycgr3G84494 Mycgr3T
  
Location: 523-2047

Mycgr3G84494\_Mycgr3T

Mycgr3G90558 Mycgr3T
  
Location: 2147-15296

Mycgr3G90558\_Mycgr3T

Mycgr3G68036 Mycgr3T
  
Location: 15396-16395

Mycgr3G68036\_Mycgr3T

Mycgr3G90561 Mycgr3T
  
Location: 16495-17134

Mycgr3G90561\_Mycgr3T

Mycgr3G35862 Mycgr3T
  
Location: 17234-18662

Mycgr3G35862\_Mycgr3T

Mycgr3G68030 Mycgr3T
  
Location: 18762-19722

Mycgr3G68030\_Mycgr3T

Mycgr3G36449 Mycgr3T
  
Location: 19822-21886

Mycgr3G36449\_Mycgr3T

Mycgr3G35528 Mycgr3T
  
Location: 21986-22844

Mycgr3G35528\_Mycgr3T

Mycgr3G35932 Mycgr3T
  
Location: 22944-24390

Mycgr3G35932\_Mycgr3T

Mycgr3G23761 Mycgr3T
  
Location: 24490-25825

Mycgr3G23761\_Mycgr3T

Mycgr3G35535 Mycgr3T
  
Location: 25925-26429

Mycgr3G35535\_Mycgr3T

Mycgr3G9942 Mycgr3T9
  
Location: 26529-30375

Mycgr3G9942\_Mycgr3T9

hypothetical protein
  
Accession: EAW08897
  
Location: 908623-909746
  
 NCBI BlastP on this gene

EAW08897

MFS transporter, putative
  
Accession: EAW08898
  
Location: 910122-911892
  
  
**BlastP hit with Mycgr3G23761\_Mycgr3T**
  
Percentage identity: 29 %
  
BlastP bit score: 177
  
Sequence coverage: 103 %
  
E-value: 4e-46
  
  
 NCBI BlastP on this gene

EAW08898

ABC transporter, putative
  
Accession: EAW08899
  
Location: 912958-918070
  
 NCBI BlastP on this gene

EAW08899

nonribosomal peptide synthase, putative
  
Accession: EAW08900
  
Location: 919134-944153
  
  
**BlastP hit with Mycgr3G90558\_Mycgr3T**
  
Percentage identity: 31 %
  
BlastP bit score: 1167
  
Sequence coverage: 58 %
  
E-value: 0.0
  
  
 NCBI BlastP on this gene

EAW08900

SRF-type transcription factor (Umc1), putative
  
Accession: EAW08901
  
Location: 946418-947301
  
 NCBI BlastP on this gene

EAW08901

Query: Architecture Search FASTA input

KE145353 : Glarea lozoyensis ATCC 20868 chromosome Unknown GLAREA10    Total score: 2.0     Cumulative Blast bit score: 1329

Hit cluster cross-links:

Mycgr3G36335 Mycgr3T
  
Location: 0-423

Mycgr3G36335\_Mycgr3T

Mycgr3G84494 Mycgr3T
  
Location: 523-2047

Mycgr3G84494\_Mycgr3T

Mycgr3G90558 Mycgr3T
  
Location: 2147-15296

Mycgr3G90558\_Mycgr3T

Mycgr3G68036 Mycgr3T
  
Location: 15396-16395

Mycgr3G68036\_Mycgr3T

Mycgr3G90561 Mycgr3T
  
Location: 16495-17134

Mycgr3G90561\_Mycgr3T

Mycgr3G35862 Mycgr3T
  
Location: 17234-18662

Mycgr3G35862\_Mycgr3T

Mycgr3G68030 Mycgr3T
  
Location: 18762-19722

Mycgr3G68030\_Mycgr3T

Mycgr3G36449 Mycgr3T
  
Location: 19822-21886

Mycgr3G36449\_Mycgr3T

Mycgr3G35528 Mycgr3T
  
Location: 21986-22844

Mycgr3G35528\_Mycgr3T

Mycgr3G35932 Mycgr3T
  
Location: 22944-24390

Mycgr3G35932\_Mycgr3T

Mycgr3G23761 Mycgr3T
  
Location: 24490-25825

Mycgr3G23761\_Mycgr3T

Mycgr3G35535 Mycgr3T
  
Location: 25925-26429

Mycgr3G35535\_Mycgr3T

Mycgr3G9942 Mycgr3T9
  
Location: 26529-30375

Mycgr3G9942\_Mycgr3T9

Flavoprotein
  
Accession: EPE35677
  
Location: 52299-53210
  
 NCBI BlastP on this gene

EPE35677

hypothetical protein
  
Accession: EPE35676
  
Location: 49479-51359
  
  
**BlastP hit with Mycgr3G36335\_Mycgr3T**
  
Percentage identity: 38 %
  
BlastP bit score: 81
  
Sequence coverage: 88 %
  
E-value: 3e-15
  
  
 NCBI BlastP on this gene

EPE35676

hypothetical protein
  
Accession: EPE35675
  
Location: 48184-49107
  
 NCBI BlastP on this gene

EPE35675

hypothetical protein
  
Accession: EPE35674
  
Location: 46413-47523
  
 NCBI BlastP on this gene

EPE35674

P-loop containing nucleoside triphosphate hydrolase
  
Accession: EPE35673
  
Location: 34754-40016
  
 NCBI BlastP on this gene

EPE35673

Acetyl-CoA synthetase-like protein
  
Accession: EPE35672
  
Location: 8439-32264
  
  
**BlastP hit with Mycgr3G90558\_Mycgr3T**
  
Percentage identity: 32 %
  
BlastP bit score: 1248
  
Sequence coverage: 60 %
  
E-value: 0.0
  
  
 NCBI BlastP on this gene

EPE35672

MFS general substrate transporter
  
Accession: EPE35671
  
Location: 6656-8300
  
 NCBI BlastP on this gene

EPE35671

Query: Architecture Search FASTA input

FP929139 : Leptosphaeria maculans JN3 lm\_SuperContig\_0\_v2 genomic supercontig    Total score: 2.0     Cumulative Blast bit score: 1327

Hit cluster cross-links:

Mycgr3G36335 Mycgr3T
  
Location: 0-423

Mycgr3G36335\_Mycgr3T

Mycgr3G84494 Mycgr3T
  
Location: 523-2047

Mycgr3G84494\_Mycgr3T

Mycgr3G90558 Mycgr3T
  
Location: 2147-15296

Mycgr3G90558\_Mycgr3T

Mycgr3G68036 Mycgr3T
  
Location: 15396-16395

Mycgr3G68036\_Mycgr3T

Mycgr3G90561 Mycgr3T
  
Location: 16495-17134

Mycgr3G90561\_Mycgr3T

Mycgr3G35862 Mycgr3T
  
Location: 17234-18662

Mycgr3G35862\_Mycgr3T

Mycgr3G68030 Mycgr3T
  
Location: 18762-19722

Mycgr3G68030\_Mycgr3T

Mycgr3G36449 Mycgr3T
  
Location: 19822-21886

Mycgr3G36449\_Mycgr3T

Mycgr3G35528 Mycgr3T
  
Location: 21986-22844

Mycgr3G35528\_Mycgr3T

Mycgr3G35932 Mycgr3T
  
Location: 22944-24390

Mycgr3G35932\_Mycgr3T

Mycgr3G23761 Mycgr3T
  
Location: 24490-25825

Mycgr3G23761\_Mycgr3T

Mycgr3G35535 Mycgr3T
  
Location: 25925-26429

Mycgr3G35535\_Mycgr3T

Mycgr3G9942 Mycgr3T9
  
Location: 26529-30375

Mycgr3G9942\_Mycgr3T9

hypothetical protein
  
Accession: CBY02111
  
Location: 3094894-3099025
  
 NCBI BlastP on this gene

LEMA\_P008980.1

similar to MFS transporter
  
Accession: CBY02112
  
Location: 3099159-3100841
  
  
**BlastP hit with Mycgr3G23761\_Mycgr3T**
  
Percentage identity: 29 %
  
BlastP bit score: 182
  
Sequence coverage: 101 %
  
E-value: 1e-47
  
  
 NCBI BlastP on this gene

LEMA\_P008990.1

predicted protein
  
Accession: CBY02113
  
Location: 3102156-3103294
  
 NCBI BlastP on this gene

LEMA\_P009000.1

predicted protein
  
Accession: CBY02114
  
Location: 3104087-3105211
  
 NCBI BlastP on this gene

LEMA\_P009010.1

similar to ABC multidrug transporter
  
Accession: CBY02115
  
Location: 3106079-3111642
  
 NCBI BlastP on this gene

LEMA\_P009020.1

predicted protein
  
Accession: CBY02116
  
Location: 3113323-3114354
  
 NCBI BlastP on this gene

LEMA\_P009030.1

similar to nonribosomal peptide synthase
  
Accession: CBY02117
  
Location: 3115242-3137649
  
  
**BlastP hit with Mycgr3G90558\_Mycgr3T**
  
Percentage identity: 30 %
  
BlastP bit score: 1145
  
Sequence coverage: 60 %
  
E-value: 0.0
  
  
 NCBI BlastP on this gene

LEMA\_P009040.1

similar to rhamnogalacturonate lyase
  
Accession: CBY02118
  
Location: 3138626-3140467
  
 NCBI BlastP on this gene

LEMA\_P009050.1

Query: Architecture Search FASTA input

CH445358 : Phaeosphaeria nodorum SN15 scaffold\_34    Total score: 2.0     Cumulative Blast bit score: 1313

Hit cluster cross-links:

Mycgr3G36335 Mycgr3T
  
Location: 0-423

Mycgr3G36335\_Mycgr3T

Mycgr3G84494 Mycgr3T
  
Location: 523-2047

Mycgr3G84494\_Mycgr3T

Mycgr3G90558 Mycgr3T
  
Location: 2147-15296

Mycgr3G90558\_Mycgr3T

Mycgr3G68036 Mycgr3T
  
Location: 15396-16395

Mycgr3G68036\_Mycgr3T

Mycgr3G90561 Mycgr3T
  
Location: 16495-17134

Mycgr3G90561\_Mycgr3T

Mycgr3G35862 Mycgr3T
  
Location: 17234-18662

Mycgr3G35862\_Mycgr3T

Mycgr3G68030 Mycgr3T
  
Location: 18762-19722

Mycgr3G68030\_Mycgr3T

Mycgr3G36449 Mycgr3T
  
Location: 19822-21886

Mycgr3G36449\_Mycgr3T

Mycgr3G35528 Mycgr3T
  
Location: 21986-22844

Mycgr3G35528\_Mycgr3T

Mycgr3G35932 Mycgr3T
  
Location: 22944-24390

Mycgr3G35932\_Mycgr3T

Mycgr3G23761 Mycgr3T
  
Location: 24490-25825

Mycgr3G23761\_Mycgr3T

Mycgr3G35535 Mycgr3T
  
Location: 25925-26429

Mycgr3G35535\_Mycgr3T

Mycgr3G9942 Mycgr3T9
  
Location: 26529-30375

Mycgr3G9942\_Mycgr3T9

hypothetical protein
  
Accession: EAT77683
  
Location: 47598-49195
  
 NCBI BlastP on this gene

EAT77683

hypothetical protein
  
Accession: EAT77684
  
Location: 49639-50109
  
 NCBI BlastP on this gene

EAT77684

hypothetical protein
  
Accession: EAT77686
  
Location: 50136-72414
  
  
**BlastP hit with Mycgr3G90558\_Mycgr3T**
  
Percentage identity: 31 %
  
BlastP bit score: 1145
  
Sequence coverage: 60 %
  
E-value: 0.0
  
  
 NCBI BlastP on this gene

EAT77686

hypothetical protein
  
Accession: EAT77687
  
Location: 73844-74615
  
 NCBI BlastP on this gene

EAT77687

hypothetical protein
  
Accession: EAT77688
  
Location: 76243-81672
  
 NCBI BlastP on this gene

EAT77688

hypothetical protein
  
Accession: EAT77689
  
Location: 82012-83425
  
 NCBI BlastP on this gene

EAT77689

hypothetical protein
  
Accession: EAT77690
  
Location: 83764-84099
  
 NCBI BlastP on this gene

EAT77690

hypothetical protein
  
Accession: EAT77691
  
Location: 84803-85585
  
 NCBI BlastP on this gene

EAT77691

hypothetical protein
  
Accession: EAT77693
  
Location: 86818-92717
  
  
**BlastP hit with Mycgr3G23761\_Mycgr3T**
  
Percentage identity: 29 %
  
BlastP bit score: 168
  
Sequence coverage: 98 %
  
E-value: 2e-41
  
  
 NCBI BlastP on this gene

EAT77693

hypothetical protein
  
Accession: EAT77694
  
Location: 93377-95285
  
 NCBI BlastP on this gene

EAT77694

Query: Architecture Search FASTA input

KB908703 : Setosphaeria turcica Et28A unplaced genomic scaffold SETTUscaffold\_3    Total score: 2.0     Cumulative Blast bit score: 1305

Hit cluster cross-links:

Mycgr3G36335 Mycgr3T
  
Location: 0-423

Mycgr3G36335\_Mycgr3T

Mycgr3G84494 Mycgr3T
  
Location: 523-2047

Mycgr3G84494\_Mycgr3T

Mycgr3G90558 Mycgr3T
  
Location: 2147-15296

Mycgr3G90558\_Mycgr3T

Mycgr3G68036 Mycgr3T
  
Location: 15396-16395

Mycgr3G68036\_Mycgr3T

Mycgr3G90561 Mycgr3T
  
Location: 16495-17134

Mycgr3G90561\_Mycgr3T

Mycgr3G35862 Mycgr3T
  
Location: 17234-18662

Mycgr3G35862\_Mycgr3T

Mycgr3G68030 Mycgr3T
  
Location: 18762-19722

Mycgr3G68030\_Mycgr3T

Mycgr3G36449 Mycgr3T
  
Location: 19822-21886

Mycgr3G36449\_Mycgr3T

Mycgr3G35528 Mycgr3T
  
Location: 21986-22844

Mycgr3G35528\_Mycgr3T

Mycgr3G35932 Mycgr3T
  
Location: 22944-24390

Mycgr3G35932\_Mycgr3T

Mycgr3G23761 Mycgr3T
  
Location: 24490-25825

Mycgr3G23761\_Mycgr3T

Mycgr3G35535 Mycgr3T
  
Location: 25925-26429

Mycgr3G35535\_Mycgr3T

Mycgr3G9942 Mycgr3T9
  
Location: 26529-30375

Mycgr3G9942\_Mycgr3T9

hypothetical protein
  
Accession: EOA84587
  
Location: 391594-393219
  
  
**BlastP hit with Mycgr3G23761\_Mycgr3T**
  
Percentage identity: 28 %
  
BlastP bit score: 185
  
Sequence coverage: 102 %
  
E-value: 6e-49
  
  
 NCBI BlastP on this gene

EOA84587

hypothetical protein
  
Accession: EOA84588
  
Location: 393670-397407
  
 NCBI BlastP on this gene

EOA84588

hypothetical protein
  
Accession: EOA84589
  
Location: 405042-406021
  
 NCBI BlastP on this gene

EOA84589

hypothetical protein
  
Accession: EOA84590
  
Location: 406616-411618
  
 NCBI BlastP on this gene

EOA84590

hypothetical protein
  
Accession: EOA84591
  
Location: 415602-437804
  
  
**BlastP hit with Mycgr3G90558\_Mycgr3T**
  
Percentage identity: 30 %
  
BlastP bit score: 1120
  
Sequence coverage: 61 %
  
E-value: 0.0
  
  
 NCBI BlastP on this gene

EOA84591

hypothetical protein
  
Accession: EOA84592
  
Location: 440079-441191
  
 NCBI BlastP on this gene

EOA84592

Query: Architecture Search FASTA input

KB733474 : Bipolaris maydis ATCC 48331 unplaced genomic scaffold COCC4scaffold\_31    Total score: 2.0     Cumulative Blast bit score: 1305

Hit cluster cross-links:

Mycgr3G36335 Mycgr3T
  
Location: 0-423

Mycgr3G36335\_Mycgr3T

Mycgr3G84494 Mycgr3T
  
Location: 523-2047

Mycgr3G84494\_Mycgr3T

Mycgr3G90558 Mycgr3T
  
Location: 2147-15296

Mycgr3G90558\_Mycgr3T

Mycgr3G68036 Mycgr3T
  
Location: 15396-16395

Mycgr3G68036\_Mycgr3T

Mycgr3G90561 Mycgr3T
  
Location: 16495-17134

Mycgr3G90561\_Mycgr3T

Mycgr3G35862 Mycgr3T
  
Location: 17234-18662

Mycgr3G35862\_Mycgr3T

Mycgr3G68030 Mycgr3T
  
Location: 18762-19722

Mycgr3G68030\_Mycgr3T

Mycgr3G36449 Mycgr3T
  
Location: 19822-21886

Mycgr3G36449\_Mycgr3T

Mycgr3G35528 Mycgr3T
  
Location: 21986-22844

Mycgr3G35528\_Mycgr3T

Mycgr3G35932 Mycgr3T
  
Location: 22944-24390

Mycgr3G35932\_Mycgr3T

Mycgr3G23761 Mycgr3T
  
Location: 24490-25825

Mycgr3G23761\_Mycgr3T

Mycgr3G35535 Mycgr3T
  
Location: 25925-26429

Mycgr3G35535\_Mycgr3T

Mycgr3G9942 Mycgr3T9
  
Location: 26529-30375

Mycgr3G9942\_Mycgr3T9

hypothetical protein
  
Accession: ENI00472
  
Location: 99522-101176
  
  
**BlastP hit with Mycgr3G23761\_Mycgr3T**
  
Percentage identity: 29 %
  
BlastP bit score: 187
  
Sequence coverage: 99 %
  
E-value: 1e-49
  
  
 NCBI BlastP on this gene

ENI00472

hypothetical protein
  
Accession: ENI00473
  
Location: 101574-105227
  
 NCBI BlastP on this gene

ENI00473

hypothetical protein
  
Accession: ENI00474
  
Location: 108230-113263
  
 NCBI BlastP on this gene

ENI00474

hypothetical protein
  
Accession: ENI00475
  
Location: 115603-116224
  
 NCBI BlastP on this gene

ENI00475

hypothetical protein
  
Accession: ENI00476
  
Location: 116583-116926
  
 NCBI BlastP on this gene

ENI00476

hypothetical protein
  
Accession: ENI00477
  
Location: 117252-139356
  
  
**BlastP hit with Mycgr3G90558\_Mycgr3T**
  
Percentage identity: 30 %
  
BlastP bit score: 1118
  
Sequence coverage: 60 %
  
E-value: 0.0
  
  
 NCBI BlastP on this gene

ENI00477

hypothetical protein
  
Accession: ENI00478
  
Location: 139861-140364
  
 NCBI BlastP on this gene

ENI00478

hypothetical protein
  
Accession: ENI00479
  
Location: 140945-142566
  
 NCBI BlastP on this gene

ENI00479

Query: Architecture Search FASTA input

KB445640 : Cochliobolus sativus ND90Pr unplaced genomic scaffold COCSAscaffold\_4    Total score: 2.0     Cumulative Blast bit score: 1301

Hit cluster cross-links:

Mycgr3G36335 Mycgr3T
  
Location: 0-423

Mycgr3G36335\_Mycgr3T

Mycgr3G84494 Mycgr3T
  
Location: 523-2047

Mycgr3G84494\_Mycgr3T

Mycgr3G90558 Mycgr3T
  
Location: 2147-15296

Mycgr3G90558\_Mycgr3T

Mycgr3G68036 Mycgr3T
  
Location: 15396-16395

Mycgr3G68036\_Mycgr3T

Mycgr3G90561 Mycgr3T
  
Location: 16495-17134

Mycgr3G90561\_Mycgr3T

Mycgr3G35862 Mycgr3T
  
Location: 17234-18662

Mycgr3G35862\_Mycgr3T

Mycgr3G68030 Mycgr3T
  
Location: 18762-19722

Mycgr3G68030\_Mycgr3T

Mycgr3G36449 Mycgr3T
  
Location: 19822-21886

Mycgr3G36449\_Mycgr3T

Mycgr3G35528 Mycgr3T
  
Location: 21986-22844

Mycgr3G35528\_Mycgr3T

Mycgr3G35932 Mycgr3T
  
Location: 22944-24390

Mycgr3G35932\_Mycgr3T

Mycgr3G23761 Mycgr3T
  
Location: 24490-25825

Mycgr3G23761\_Mycgr3T

Mycgr3G35535 Mycgr3T
  
Location: 25925-26429

Mycgr3G35535\_Mycgr3T

Mycgr3G9942 Mycgr3T9
  
Location: 26529-30375

Mycgr3G9942\_Mycgr3T9

hypothetical protein
  
Accession: EMD66842
  
Location: 2229995-2231649
  
  
**BlastP hit with Mycgr3G23761\_Mycgr3T**
  
Percentage identity: 28 %
  
BlastP bit score: 182
  
Sequence coverage: 101 %
  
E-value: 7e-48
  
  
 NCBI BlastP on this gene

EMD66842

hypothetical protein
  
Accession: EMD66841
  
Location: 2225944-2229621
  
 NCBI BlastP on this gene

EMD66841

hypothetical protein
  
Accession: EMD66840
  
Location: 2217996-2223025
  
 NCBI BlastP on this gene

EMD66840

hypothetical protein
  
Accession: EMD66839
  
Location: 2191843-2213946
  
  
**BlastP hit with Mycgr3G90558\_Mycgr3T**
  
Percentage identity: 31 %
  
BlastP bit score: 1119
  
Sequence coverage: 60 %
  
E-value: 0.0
  
  
 NCBI BlastP on this gene

EMD66839

hypothetical protein
  
Accession: EMD66838
  
Location: 2190492-2191338
  
 NCBI BlastP on this gene

EMD66838

hypothetical protein
  
Accession: EMD66837
  
Location: 2188294-2189924
  
 NCBI BlastP on this gene

EMD66837

Query: Architecture Search FASTA input

KB445571 : Cochliobolus heterostrophus C5 unplaced genomic scaffold COCHEscaffold\_3    Total score: 2.0     Cumulative Blast bit score: 1299

Hit cluster cross-links:

Mycgr3G36335 Mycgr3T
  
Location: 0-423

Mycgr3G36335\_Mycgr3T

Mycgr3G84494 Mycgr3T
  
Location: 523-2047

Mycgr3G84494\_Mycgr3T

Mycgr3G90558 Mycgr3T
  
Location: 2147-15296

Mycgr3G90558\_Mycgr3T

Mycgr3G68036 Mycgr3T
  
Location: 15396-16395

Mycgr3G68036\_Mycgr3T

Mycgr3G90561 Mycgr3T
  
Location: 16495-17134

Mycgr3G90561\_Mycgr3T

Mycgr3G35862 Mycgr3T
  
Location: 17234-18662

Mycgr3G35862\_Mycgr3T

Mycgr3G68030 Mycgr3T
  
Location: 18762-19722

Mycgr3G68030\_Mycgr3T

Mycgr3G36449 Mycgr3T
  
Location: 19822-21886

Mycgr3G36449\_Mycgr3T

Mycgr3G35528 Mycgr3T
  
Location: 21986-22844

Mycgr3G35528\_Mycgr3T

Mycgr3G35932 Mycgr3T
  
Location: 22944-24390

Mycgr3G35932\_Mycgr3T

Mycgr3G23761 Mycgr3T
  
Location: 24490-25825

Mycgr3G23761\_Mycgr3T

Mycgr3G35535 Mycgr3T
  
Location: 25925-26429

Mycgr3G35535\_Mycgr3T

Mycgr3G9942 Mycgr3T9
  
Location: 26529-30375

Mycgr3G9942\_Mycgr3T9

hypothetical protein
  
Accession: EMD95325
  
Location: 2256271-2257925
  
  
**BlastP hit with Mycgr3G23761\_Mycgr3T**
  
Percentage identity: 29 %
  
BlastP bit score: 187
  
Sequence coverage: 99 %
  
E-value: 1e-49
  
  
 NCBI BlastP on this gene

EMD95325

hypothetical protein
  
Accession: EMD95326
  
Location: 2258323-2261976
  
 NCBI BlastP on this gene

EMD95326

hypothetical protein
  
Accession: EMD95327
  
Location: 2264979-2270012
  
 NCBI BlastP on this gene

EMD95327

hypothetical protein
  
Accession: EMD95328
  
Location: 2272352-2272973
  
 NCBI BlastP on this gene

EMD95328

hypothetical protein
  
Accession: EMD95329
  
Location: 2273332-2273675
  
 NCBI BlastP on this gene

EMD95329

hypothetical protein
  
Accession: EMD95330
  
Location: 2274001-2296105
  
  
**BlastP hit with Mycgr3G90558\_Mycgr3T**
  
Percentage identity: 30 %
  
BlastP bit score: 1112
  
Sequence coverage: 60 %
  
E-value: 0.0
  
  
 NCBI BlastP on this gene

EMD95330

hypothetical protein
  
Accession: EMD95331
  
Location: 2296610-2297689
  
 NCBI BlastP on this gene

EMD95331

hypothetical protein
  
Accession: EMD95332
  
Location: 2298270-2299891
  
 NCBI BlastP on this gene

EMD95332

Query: Architecture Search FASTA input

DS231615 : Pyrenophora tritici-repentis Pt-1C-BFP supercont1.1 genomic scaffold    Total score: 2.0     Cumulative Blast bit score: 1270

Hit cluster cross-links:

Mycgr3G36335 Mycgr3T
  
Location: 0-423

Mycgr3G36335\_Mycgr3T

Mycgr3G84494 Mycgr3T
  
Location: 523-2047

Mycgr3G84494\_Mycgr3T

Mycgr3G90558 Mycgr3T
  
Location: 2147-15296

Mycgr3G90558\_Mycgr3T

Mycgr3G68036 Mycgr3T
  
Location: 15396-16395

Mycgr3G68036\_Mycgr3T

Mycgr3G90561 Mycgr3T
  
Location: 16495-17134

Mycgr3G90561\_Mycgr3T

Mycgr3G35862 Mycgr3T
  
Location: 17234-18662

Mycgr3G35862\_Mycgr3T

Mycgr3G68030 Mycgr3T
  
Location: 18762-19722

Mycgr3G68030\_Mycgr3T

Mycgr3G36449 Mycgr3T
  
Location: 19822-21886

Mycgr3G36449\_Mycgr3T

Mycgr3G35528 Mycgr3T
  
Location: 21986-22844

Mycgr3G35528\_Mycgr3T

Mycgr3G35932 Mycgr3T
  
Location: 22944-24390

Mycgr3G35932\_Mycgr3T

Mycgr3G23761 Mycgr3T
  
Location: 24490-25825

Mycgr3G23761\_Mycgr3T

Mycgr3G35535 Mycgr3T
  
Location: 25925-26429

Mycgr3G35535\_Mycgr3T

Mycgr3G9942 Mycgr3T9
  
Location: 26529-30375

Mycgr3G9942\_Mycgr3T9

predicted protein
  
Accession: EDU41234
  
Location: 5159779-5163591
  
 NCBI BlastP on this gene

EDU41234

conserved hypothetical protein
  
Accession: EDU41235
  
Location: 5163962-5165632
  
  
**BlastP hit with Mycgr3G23761\_Mycgr3T**
  
Percentage identity: 28 %
  
BlastP bit score: 188
  
Sequence coverage: 99 %
  
E-value: 5e-50
  
  
 NCBI BlastP on this gene

EDU41235

predicted protein
  
Accession: EDU41236
  
Location: 5168266-5168588
  
 NCBI BlastP on this gene

EDU41236

canalicular multispecific organic anion transporter 1
  
Accession: EDU41237
  
Location: 5170114-5175070
  
 NCBI BlastP on this gene

EDU41237

HC-toxin synthetase
  
Accession: EDU41238
  
Location: 5179158-5201187
  
  
**BlastP hit with Mycgr3G90558\_Mycgr3T**
  
Percentage identity: 30 %
  
BlastP bit score: 1082
  
Sequence coverage: 56 %
  
E-value: 0.0
  
  
 NCBI BlastP on this gene

EDU41238

predicted protein
  
Accession: EDU41239
  
Location: 5201465-5201704
  
 NCBI BlastP on this gene

EDU41239

conserved hypothetical protein
  
Accession: EDU41240
  
Location: 5202103-5203212
  
 NCBI BlastP on this gene

EDU41240

Query: Architecture Search FASTA input

JH126401 : Cordyceps militaris CM01 unplaced genomic scaffold CCM\_S00003    Total score: 2.0     Cumulative Blast bit score: 1221

Hit cluster cross-links:

Mycgr3G36335 Mycgr3T
  
Location: 0-423

Mycgr3G36335\_Mycgr3T

Mycgr3G84494 Mycgr3T
  
Location: 523-2047

Mycgr3G84494\_Mycgr3T

Mycgr3G90558 Mycgr3T
  
Location: 2147-15296

Mycgr3G90558\_Mycgr3T

Mycgr3G68036 Mycgr3T
  
Location: 15396-16395

Mycgr3G68036\_Mycgr3T

Mycgr3G90561 Mycgr3T
  
Location: 16495-17134

Mycgr3G90561\_Mycgr3T

Mycgr3G35862 Mycgr3T
  
Location: 17234-18662

Mycgr3G35862\_Mycgr3T

Mycgr3G68030 Mycgr3T
  
Location: 18762-19722

Mycgr3G68030\_Mycgr3T

Mycgr3G36449 Mycgr3T
  
Location: 19822-21886

Mycgr3G36449\_Mycgr3T

Mycgr3G35528 Mycgr3T
  
Location: 21986-22844

Mycgr3G35528\_Mycgr3T

Mycgr3G35932 Mycgr3T
  
Location: 22944-24390

Mycgr3G35932\_Mycgr3T

Mycgr3G23761 Mycgr3T
  
Location: 24490-25825

Mycgr3G23761\_Mycgr3T

Mycgr3G35535 Mycgr3T
  
Location: 25925-26429

Mycgr3G35535\_Mycgr3T

Mycgr3G9942 Mycgr3T9
  
Location: 26529-30375

Mycgr3G9942\_Mycgr3T9

MFS transporter, putative
  
Accession: EGX92719
  
Location: 1484321-1485939
  
 NCBI BlastP on this gene

EGX92719

20S proteasome maturation protein Ump1
  
Accession: EGX92720
  
Location: 1487230-1487691
  
 NCBI BlastP on this gene

EGX92720

Ubiquitin
  
Accession: EGX92721
  
Location: 1488487-1489345
  
 NCBI BlastP on this gene

EGX92721

AP-2 complex subunit beta
  
Accession: EGX92722
  
Location: 1491469-1493990
  
 NCBI BlastP on this gene

EGX92722

ER membrane DUF1077 domain protein, putative
  
Accession: EGX92723
  
Location: 1494610-1495298
  
 NCBI BlastP on this gene

EGX92723

Major facilitator superfamily transporter
  
Accession: EGX92724
  
Location: 1495422-1497080
  
  
**BlastP hit with Mycgr3G84494\_Mycgr3T**
  
Percentage identity: 40 %
  
BlastP bit score: 358
  
Sequence coverage: 91 %
  
E-value: 1e-113
  
  
 NCBI BlastP on this gene

EGX92724

WD40 repeat-like-containing domain
  
Accession: EGX92725
  
Location: 1498049-1500381
  
 NCBI BlastP on this gene

EGX92725

hypothetical protein
  
Accession: EGX92726
  
Location: 1501507-1503154
  
 NCBI BlastP on this gene

EGX92726

ABC bile acid transporter, putative
  
Accession: EGX92727
  
Location: 1504827-1509584
  
  
**BlastP hit with Mycgr3G9942\_Mycgr3T9**
  
Percentage identity: 37 %
  
BlastP bit score: 863
  
Sequence coverage: 102 %
  
E-value: 0.0
  
  
 NCBI BlastP on this gene

EGX92727

autophagy protein Apg6, putative
  
Accession: EGX92728
  
Location: 1510108-1511692
  
 NCBI BlastP on this gene

EGX92728

hypothetical protein
  
Accession: EGX92729
  
Location: 1512367-1515817
  
 NCBI BlastP on this gene

EGX92729

Casein kinase II, alpha chain (CK II alpha subunit)
  
Accession: EGX92730
  
Location: 1516848-1518238
  
 NCBI BlastP on this gene

EGX92730

hypothetical protein
  
Accession: EGX92731
  
Location: 1519020-1521133
  
 NCBI BlastP on this gene

EGX92731

Query: Architecture Search FASTA input

DS027684 : Neosartorya fischeri NRRL 181 1099437636244 genomic scaffold    Total score: 2.0     Cumulative Blast bit score: 1219

Hit cluster cross-links:

Mycgr3G36335 Mycgr3T
  
Location: 0-423

Mycgr3G36335\_Mycgr3T

Mycgr3G84494 Mycgr3T
  
Location: 523-2047

Mycgr3G84494\_Mycgr3T

Mycgr3G90558 Mycgr3T
  
Location: 2147-15296

Mycgr3G90558\_Mycgr3T

Mycgr3G68036 Mycgr3T
  
Location: 15396-16395

Mycgr3G68036\_Mycgr3T

Mycgr3G90561 Mycgr3T
  
Location: 16495-17134

Mycgr3G90561\_Mycgr3T

Mycgr3G35862 Mycgr3T
  
Location: 17234-18662

Mycgr3G35862\_Mycgr3T

Mycgr3G68030 Mycgr3T
  
Location: 18762-19722

Mycgr3G68030\_Mycgr3T

Mycgr3G36449 Mycgr3T
  
Location: 19822-21886

Mycgr3G36449\_Mycgr3T

Mycgr3G35528 Mycgr3T
  
Location: 21986-22844

Mycgr3G35528\_Mycgr3T

Mycgr3G35932 Mycgr3T
  
Location: 22944-24390

Mycgr3G35932\_Mycgr3T

Mycgr3G23761 Mycgr3T
  
Location: 24490-25825

Mycgr3G23761\_Mycgr3T

Mycgr3G35535 Mycgr3T
  
Location: 25925-26429

Mycgr3G35535\_Mycgr3T

Mycgr3G9942 Mycgr3T9
  
Location: 26529-30375

Mycgr3G9942\_Mycgr3T9

MFS transporter, putative
  
Accession: EAW25616
  
Location: 246469-248075
  
  
**BlastP hit with Mycgr3G23761\_Mycgr3T**
  
Percentage identity: 33 %
  
BlastP bit score: 194
  
Sequence coverage: 95 %
  
E-value: 3e-53
  
  
 NCBI BlastP on this gene

EAW25616

conserved hypothetical protein
  
Accession: EAW25615
  
Location: 244471-245438
  
 NCBI BlastP on this gene

EAW25615

taurine dioxygenase family protein
  
Accession: EAW25614
  
Location: 242902-244172
  
 NCBI BlastP on this gene

EAW25614

aminotransferase, putative
  
Accession: EAW25613
  
Location: 240751-242146
  
 NCBI BlastP on this gene

EAW25613

hypothetical protein
  
Accession: EAW25612
  
Location: 238597-240163
  
 NCBI BlastP on this gene

EAW25612

2OG-Fe(II) oxygenase family oxidoreductase, putative
  
Accession: EAW25611
  
Location: 237111-238231
  
 NCBI BlastP on this gene

EAW25611

HpcH/HpaI aldolase/citrate lyase family protein
  
Accession: EAW25610
  
Location: 235729-236770
  
 NCBI BlastP on this gene

EAW25610

AMP-binding enzyme, putative
  
Accession: EAW25609
  
Location: 232711-234705
  
 NCBI BlastP on this gene

EAW25609

ornithine aminotransferase
  
Accession: EAW25608
  
Location: 230880-232453
  
 NCBI BlastP on this gene

EAW25608

aspartate aminotransferase, putative
  
Accession: EAW25607
  
Location: 229446-230579
  
 NCBI BlastP on this gene

EAW25607

benzoate 4-monooxygenase cytochrome P450
  
Accession: EAW25606
  
Location: 227637-229193
  
 NCBI BlastP on this gene

EAW25606

nonribosomal peptide synthase, putative
  
Accession: EAW25605
  
Location: 215213-226875
  
  
**BlastP hit with Mycgr3G90558\_Mycgr3T**
  
Percentage identity: 32 %
  
BlastP bit score: 1025
  
Sequence coverage: 51 %
  
E-value: 0.0
  
  
 NCBI BlastP on this gene

EAW25605

cytochrome P450
  
Accession: EAW25604
  
Location: 212041-213688
  
 NCBI BlastP on this gene

EAW25604

Query: Architecture Search FASTA input

1. :  CM001197 Mycosphaerella graminicola IPO323 chromosome 2     Total score: 13.0     Cumulative Blast bit score: 19999

Mycgr3G36335 Mycgr3T
  
Location: 0-423
  
 NCBI BlastP on this gene

Mycgr3G36335\_Mycgr3T

Mycgr3G84494 Mycgr3T
  
Location: 523-2047
  
 NCBI BlastP on this gene

Mycgr3G84494\_Mycgr3T

Mycgr3G90558 Mycgr3T
  
Location: 2147-15296
  
 NCBI BlastP on this gene

Mycgr3G90558\_Mycgr3T

Mycgr3G68036 Mycgr3T
  
Location: 15396-16395
  
 NCBI BlastP on this gene

Mycgr3G68036\_Mycgr3T

Mycgr3G90561 Mycgr3T
  
Location: 16495-17134
  
 NCBI BlastP on this gene

Mycgr3G90561\_Mycgr3T

Mycgr3G35862 Mycgr3T
  
Location: 17234-18662
  
 NCBI BlastP on this gene

Mycgr3G35862\_Mycgr3T

Mycgr3G68030 Mycgr3T
  
Location: 18762-19722
  
 NCBI BlastP on this gene

Mycgr3G68030\_Mycgr3T

Mycgr3G36449 Mycgr3T
  
Location: 19822-21886
  
 NCBI BlastP on this gene

Mycgr3G36449\_Mycgr3T

Mycgr3G35528 Mycgr3T
  
Location: 21986-22844
  
 NCBI BlastP on this gene

Mycgr3G35528\_Mycgr3T

Mycgr3G35932 Mycgr3T
  
Location: 22944-24390
  
 NCBI BlastP on this gene

Mycgr3G35932\_Mycgr3T

Mycgr3G23761 Mycgr3T
  
Location: 24490-25825
  
 NCBI BlastP on this gene

Mycgr3G23761\_Mycgr3T

Mycgr3G35535 Mycgr3T
  
Location: 25925-26429
  
 NCBI BlastP on this gene

Mycgr3G35535\_Mycgr3T

Mycgr3G9942 Mycgr3T9
  
Location: 26529-30375
  
 NCBI BlastP on this gene

Mycgr3G9942\_Mycgr3T9

hypothetical protein
  
Accession: EGP90649
  
Location: 1057230-1058615
  
  
**BlastP hit with Mycgr3G23761\_Mycgr3T**
  
Percentage identity: 100 %
  
BlastP bit score: 880
  
Sequence coverage: 100 %
  
E-value: 0.0
  
  
 NCBI BlastP on this gene

EGP90649

putative ABC transporter
  
Accession: EGP90650
  
Location: 1048431-1053118
  
  
**BlastP hit with Mycgr3G9942\_Mycgr3T9**
  
Percentage identity: 99 %
  
BlastP bit score: 2607
  
Sequence coverage: 100 %
  
E-value: 0.0
  
  
 NCBI BlastP on this gene

EGP90650

hypothetical protein
  
Accession: EGP89809
  
Location: 1047744-1048299
  
  
**BlastP hit with Mycgr3G35535\_Mycgr3T**
  
Percentage identity: 100 %
  
BlastP bit score: 345
  
Sequence coverage: 99 %
  
E-value: 3e-119
  
  
 NCBI BlastP on this gene

EGP89809

hypothetical protein
  
Accession: EGP90651
  
Location: 1045245-1046984
  
  
**BlastP hit with Mycgr3G90561\_Mycgr3T**
  
Percentage identity: 100 %
  
BlastP bit score: 449
  
Sequence coverage: 99 %
  
E-value: 2e-158
  
  
 NCBI BlastP on this gene

EGP90651

hypothetical protein
  
Accession: EGP89808
  
Location: 1042943-1045006
  
  
**BlastP hit with Mycgr3G36449\_Mycgr3T**
  
Percentage identity: 100 %
  
BlastP bit score: 1430
  
Sequence coverage: 99 %
  
E-value: 0.0
  
  
 NCBI BlastP on this gene

EGP89808

hypothetical protein
  
Accession: EGP89807
  
Location: 1041170-1042286
  
  
**BlastP hit with Mycgr3G68036\_Mycgr3T**
  
Percentage identity: 100 %
  
BlastP bit score: 689
  
Sequence coverage: 99 %
  
E-value: 0.0
  
  
 NCBI BlastP on this gene

EGP89807

hypothetical protein
  
Accession: EGP90652
  
Location: 1026842-1040878
  
  
**BlastP hit with Mycgr3G90558\_Mycgr3T**
  
Percentage identity: 100 %
  
BlastP bit score: 9057
  
Sequence coverage: 99 %
  
E-value: 0.0
  
  
 NCBI BlastP on this gene

EGP90652

hypothetical protein
  
Accession: EGP90653
  
Location: 1025441-1026407
  
  
**BlastP hit with Mycgr3G35528\_Mycgr3T**
  
Percentage identity: 100 %
  
BlastP bit score: 601
  
Sequence coverage: 99 %
  
E-value: 0.0
  
  
 NCBI BlastP on this gene

EGP90653

hypothetical protein
  
Accession: EGP90654
  
Location: 1016623-1017902
  
  
**BlastP hit with Mycgr3G68030\_Mycgr3T**
  
Percentage identity: 100 %
  
BlastP bit score: 669
  
Sequence coverage: 99 %
  
E-value: 0.0
  
  
 NCBI BlastP on this gene

EGP90654

putative major facilitator superfamily transporter
  
Accession: EGP90655
  
Location: 1014611-1016256
  
  
**BlastP hit with Mycgr3G84494\_Mycgr3T**
  
Percentage identity: 100 %
  
BlastP bit score: 1020
  
Sequence coverage: 99 %
  
E-value: 0.0
  
  
 NCBI BlastP on this gene

EGP90655

hypothetical protein
  
Accession: EGP90656
  
Location: 1013898-1014320
  
  
**BlastP hit with Mycgr3G36335\_Mycgr3T**
  
Percentage identity: 100 %
  
BlastP bit score: 296
  
Sequence coverage: 100 %
  
E-value: 1e-100
  
  
 NCBI BlastP on this gene

EGP90656

hypothetical protein
  
Accession: EGP89806
  
Location: 1010594-1012246
  
  
**BlastP hit with Mycgr3G35932\_Mycgr3T**
  
Percentage identity: 100 %
  
BlastP bit score: 969
  
Sequence coverage: 99 %
  
E-value: 0.0
  
  
 NCBI BlastP on this gene

EGP89806

peptidase M24
  
Accession: EGP90657
  
Location: 1008626-1010053
  
  
**BlastP hit with Mycgr3G35862\_Mycgr3T**
  
Percentage identity: 100 %
  
BlastP bit score: 987
  
Sequence coverage: 99 %
  
E-value: 0.0
  
  
 NCBI BlastP on this gene

EGP90657

hypothetical protein
  
Accession: EGP90658
  
Location: 1007540-1007846
  
 NCBI BlastP on this gene

EGP90658

2. :  AHHD01000300 Macrophomina phaseolina MS6     Total score: 4.0     Cumulative Blast bit score: 3861

Ergosterol biosynthesis ERG4/ERG24
  
Accession: EKG15379
  
Location: 91436-92899
  
 NCBI BlastP on this gene

EKG15379

hypothetical protein
  
Accession: EKG15380
  
Location: 93893-94222
  
 NCBI BlastP on this gene

EKG15380

Sugar/inositol transporter
  
Accession: EKG15381
  
Location: 96305-98113
  
 NCBI BlastP on this gene

EKG15381

Methyltransferase type 12
  
Accession: EKG15382
  
Location: 99605-100735
  
 NCBI BlastP on this gene

EKG15382

Oxoglutarate/iron-dependent oxygenase
  
Accession: EKG15383
  
Location: 101307-102147
  
  
**BlastP hit with Mycgr3G68036\_Mycgr3T**
  
Percentage identity: 42 %
  
BlastP bit score: 200
  
Sequence coverage: 79 %
  
E-value: 2e-58
  
  
 NCBI BlastP on this gene

EKG15383

Pyruvate carboxyltransferase
  
Accession: EKG15384
  
Location: 104816-106785
  
 NCBI BlastP on this gene

EKG15384

Oxoglutarate/iron-dependent oxygenase
  
Accession: EKG15385
  
Location: 107583-108893
  
  
**BlastP hit with Mycgr3G68036\_Mycgr3T**
  
Percentage identity: 36 %
  
BlastP bit score: 208
  
Sequence coverage: 100 %
  
E-value: 8e-61
  
  
 NCBI BlastP on this gene

EKG15385

Cytochrome P450
  
Accession: EKG15386
  
Location: 110362-110748
  
 NCBI BlastP on this gene

EKG15386

hypothetical protein
  
Accession: EKG15387
  
Location: 112596-113745
  
  
**BlastP hit with Mycgr3G68036\_Mycgr3T**
  
Percentage identity: 36 %
  
BlastP bit score: 215
  
Sequence coverage: 100 %
  
E-value: 2e-63
  
  
 NCBI BlastP on this gene

EKG15387

Oxoglutarate/iron-dependent oxygenase
  
Accession: EKG15388
  
Location: 114631-115776
  
  
**BlastP hit with Mycgr3G68036\_Mycgr3T**
  
Percentage identity: 35 %
  
BlastP bit score: 205
  
Sequence coverage: 99 %
  
E-value: 7e-60
  
  
 NCBI BlastP on this gene

EKG15388

hypothetical protein
  
Accession: EKG15389
  
Location: 116467-117560
  
  
**BlastP hit with Mycgr3G35528\_Mycgr3T**
  
Percentage identity: 55 %
  
BlastP bit score: 351
  
Sequence coverage: 104 %
  
E-value: 4e-117
  
  
 NCBI BlastP on this gene

EKG15389

AMP-dependent synthetase/ligase
  
Accession: EKG15390
  
Location: 118157-131808
  
  
**BlastP hit with Mycgr3G90558\_Mycgr3T**
  
Percentage identity: 33 %
  
BlastP bit score: 1995
  
Sequence coverage: 91 %
  
E-value: 0.0
  
  
 NCBI BlastP on this gene

EKG15390

hypothetical protein
  
Accession: EKG15391
  
Location: 132135-133640
  
 NCBI BlastP on this gene

EKG15391

Putative ABC transporter protein
  
Accession: EKG15392
  
Location: 134184-136829
  
  
**BlastP hit with Mycgr3G9942\_Mycgr3T9**
  
Percentage identity: 44 %
  
BlastP bit score: 687
  
Sequence coverage: 66 %
  
E-value: 0.0
  
  
 NCBI BlastP on this gene

EKG15392

hypothetical protein
  
Accession: EKG15393
  
Location: 138474-139917
  
 NCBI BlastP on this gene

EKG15393

Major facilitator superfamily
  
Accession: EKG15394
  
Location: 145082-146582
  
 NCBI BlastP on this gene

EKG15394

3. :  DS995900 Penicillium marneffei ATCC 18224 scf\_1105668340758 genomic scaffold     Total score: 3.0     Cumulative Blast bit score: 2176

C2H2 type conidiation transcription factor BrlA
  
Accession: EEA26461
  
Location: 3741472-3743116
  
 NCBI BlastP on this gene

EEA26461

MFS multidrug transporter, putative
  
Accession: EEA26460
  
Location: 3738110-3739881
  
  
**BlastP hit with Mycgr3G23761\_Mycgr3T**
  
Percentage identity: 30 %
  
BlastP bit score: 189
  
Sequence coverage: 103 %
  
E-value: 1e-50
  
  
 NCBI BlastP on this gene

EEA26460

ribonuclease T2, putative
  
Accession: EEA26459
  
Location: 3735722-3736694
  
 NCBI BlastP on this gene

EEA26459

Swr1p complex component (Swc5), putative
  
Accession: EEA26458
  
Location: 3734328-3735473
  
 NCBI BlastP on this gene

EEA26458

eukaryotic translation initiation factor subunit eIF2B-gamma, putative
  
Accession: EEA26457
  
Location: 3732004-3733875
  
 NCBI BlastP on this gene

EEA26457

conserved hypothetical protein
  
Accession: EEA26456
  
Location: 3725846-3728147
  
 NCBI BlastP on this gene

EEA26456

fatty acid elongase (Gig30), putative
  
Accession: EEA26455
  
Location: 3722380-3723948
  
 NCBI BlastP on this gene

EEA26455

DUF1275 domain protein
  
Accession: EEA26454
  
Location: 3720204-3721119
  
 NCBI BlastP on this gene

EEA26454

conserved hypothetical protein
  
Accession: EEA26453
  
Location: 3717117-3718784
  
 NCBI BlastP on this gene

EEA26453

leucoanthocyanidin dioxygenase, putative
  
Accession: EEA26452
  
Location: 3715799-3717026
  
  
**BlastP hit with Mycgr3G68036\_Mycgr3T**
  
Percentage identity: 34 %
  
BlastP bit score: 177
  
Sequence coverage: 102 %
  
E-value: 6e-49
  
  
 NCBI BlastP on this gene

EEA26452

nonribosomal peptide synthase, putative
  
Accession: EEA26451
  
Location: 3698541-3715320
  
  
**BlastP hit with Mycgr3G90558\_Mycgr3T**
  
Percentage identity: 30 %
  
BlastP bit score: 1810
  
Sequence coverage: 95 %
  
E-value: 0.0
  
  
 NCBI BlastP on this gene

EEA26451

conserved hypothetical protein
  
Accession: EEA26450
  
Location: 3696307-3698119
  
 NCBI BlastP on this gene

EEA26450

conserved hypothetical protein
  
Accession: EEA26449
  
Location: 3694306-3695566
  
 NCBI BlastP on this gene

EEA26449

MFS polyamine transporter, putative
  
Accession: EEA26448
  
Location: 3691873-3693653
  
 NCBI BlastP on this gene

EEA26448

4. :  KB725800 Colletotrichum orbiculare MAFF 240422 unplaced genomic scaffold Scaffold\_249     Total score: 3.0     Cumulative Blast bit score: 1103

membrane protein
  
Accession: ENH84510
  
Location: 129549-131157
  
 NCBI BlastP on this gene

ENH84510

nicotianamine synthase
  
Accession: ENH84509
  
Location: 126302-127894
  
 NCBI BlastP on this gene

ENH84509

peptidase s41 family protein
  
Accession: ENH84508
  
Location: 122232-124633
  
  
**BlastP hit with Mycgr3G36449\_Mycgr3T**
  
Percentage identity: 28 %
  
BlastP bit score: 215
  
Sequence coverage: 98 %
  
E-value: 2e-56
  
  
 NCBI BlastP on this gene

ENH84508

hypothetical protein
  
Accession: ENH84507
  
Location: 120839-121794
  
 NCBI BlastP on this gene

ENH84507

hypothetical protein
  
Accession: ENH84506
  
Location: 119587-120063
  
 NCBI BlastP on this gene

ENH84506

peptide transporter mtd1
  
Accession: ENH84505
  
Location: 113032-115748
  
 NCBI BlastP on this gene

ENH84505

hypothetical protein
  
Accession: ENH84504
  
Location: 111995-112375
  
 NCBI BlastP on this gene

ENH84504

glycoside hydrolase family 16 protein
  
Accession: ENH84503
  
Location: 110438-111281
  
 NCBI BlastP on this gene

ENH84503

inosine uridine-preferring nucleoside hydrolase
  
Accession: ENH84502
  
Location: 107517-108576
  
 NCBI BlastP on this gene

ENH84502

hypothetical protein
  
Accession: ENH84501
  
Location: 104994-106160
  
 NCBI BlastP on this gene

ENH84501

inorganic phosphate transporter
  
Accession: ENH84500
  
Location: 100268-101495
  
  
**BlastP hit with Mycgr3G35932\_Mycgr3T**
  
Percentage identity: 59 %
  
BlastP bit score: 442
  
Sequence coverage: 73 %
  
E-value: 4e-149
  
  
 NCBI BlastP on this gene

ENH84500

xaa-pro dipeptidase
  
Accession: ENH84499
  
Location: 98149-99735
  
  
**BlastP hit with Mycgr3G35862\_Mycgr3T**
  
Percentage identity: 52 %
  
BlastP bit score: 446
  
Sequence coverage: 98 %
  
E-value: 7e-149
  
  
 NCBI BlastP on this gene

ENH84499

2og-fe oxygenase family
  
Accession: ENH84498
  
Location: 96807-97500
  
 NCBI BlastP on this gene

ENH84498

hypothetical protein
  
Accession: ENH84497
  
Location: 90826-92396
  
 NCBI BlastP on this gene

ENH84497

replication factor c subunit 3
  
Accession: ENH84496
  
Location: 88562-89846
  
 NCBI BlastP on this gene

ENH84496

maltose permease
  
Accession: ENH84495
  
Location: 84874-86768
  
 NCBI BlastP on this gene

ENH84495

5. :  GL698590 Metarhizium acridum CQMa 102 unplaced genomic scaffold Scf\_121     Total score: 3.0     Cumulative Blast bit score: 533

MFS transporter
  
Accession: EFY85108
  
Location: 87671-89557
  
 NCBI BlastP on this gene

EFY85108

hypothetical protein
  
Accession: EFY85107
  
Location: 85074-85552
  
 NCBI BlastP on this gene

EFY85107

MFS multidrug transporter, putative
  
Accession: EFY85106
  
Location: 73881-76640
  
  
**BlastP hit with Mycgr3G23761\_Mycgr3T**
  
Percentage identity: 28 %
  
BlastP bit score: 166
  
Sequence coverage: 100 %
  
E-value: 6e-42
  
  
 NCBI BlastP on this gene

EFY85106

hypothetical protein
  
Accession: EFY85105
  
Location: 71142-71903
  
 NCBI BlastP on this gene

EFY85105

hypothetical protein
  
Accession: EFY85104
  
Location: 69140-70107
  
  
**BlastP hit with Mycgr3G35535\_Mycgr3T**
  
Percentage identity: 27 %
  
BlastP bit score: 82
  
Sequence coverage: 98 %
  
E-value: 2e-16
  
  
 NCBI BlastP on this gene

EFY85104

hypothetical protein
  
Accession: EFY85103
  
Location: 67816-68178
  
 NCBI BlastP on this gene

EFY85103

FAD binding domain containing protein
  
Accession: EFY85102
  
Location: 66812-67108
  
 NCBI BlastP on this gene

EFY85102

lysine methyltransferase
  
Accession: EFY85101
  
Location: 64102-65038
  
 NCBI BlastP on this gene

EFY85101

amino acid adenylation domain protein
  
Accession: EFY85100
  
Location: 58057-63278
  
 NCBI BlastP on this gene

EFY85100

aminotriazole resistance protein, putative
  
Accession: EFY85099
  
Location: 54595-57329
  
 NCBI BlastP on this gene

EFY85099

cysteine hydrolase family protein
  
Accession: EFY85098
  
Location: 52388-53102
  
 NCBI BlastP on this gene

EFY85098

MFS multidrug transporter, putative
  
Accession: EFY85097
  
Location: 50402-51977
  
  
**BlastP hit with Mycgr3G84494\_Mycgr3T**
  
Percentage identity: 36 %
  
BlastP bit score: 285
  
Sequence coverage: 99 %
  
E-value: 5e-86
  
  
 NCBI BlastP on this gene

EFY85097

C6 finger domain protein, putative
  
Accession: EFY85096
  
Location: 49593-50045
  
 NCBI BlastP on this gene

EFY85096

serin endopeptidase
  
Accession: EFY85095
  
Location: 46489-46857
  
 NCBI BlastP on this gene

EFY85095

acetolactate synthase precursor
  
Accession: EFY85094
  
Location: 37856-40428
  
 NCBI BlastP on this gene

EFY85094

6. :  GL891306 Neurospora tetrasperma FGSC 2508 unplaced genomic scaffold NEUTE1scaffold\_5     Total score: 3.0     Cumulative Blast bit score: 346

peroxidase/catalase 2
  
Accession: EGO55882
  
Location: 3449949-3452210
  
 NCBI BlastP on this gene

EGO55882

hypothetical protein
  
Accession: EGO55881
  
Location: 3447467-3448192
  
 NCBI BlastP on this gene

EGO55881

hypothetical protein
  
Accession: EGO55880
  
Location: 3446474-3447424
  
 NCBI BlastP on this gene

EGO55880

hypothetical protein
  
Accession: EGO55879
  
Location: 3444688-3445426
  
 NCBI BlastP on this gene

EGO55879

hypothetical protein
  
Accession: EGO55878
  
Location: 3441217-3443271
  
 NCBI BlastP on this gene

EGO55878

hypothetical protein
  
Accession: EGO55877
  
Location: 3435150-3435687
  
 NCBI BlastP on this gene

EGO55877

hypothetical protein
  
Accession: EGO55876
  
Location: 3427287-3434018
  
  
**BlastP hit with Mycgr3G36449\_Mycgr3T**
  
Percentage identity: 29 %
  
BlastP bit score: 228
  
Sequence coverage: 103 %
  
E-value: 2e-59
  
  
 NCBI BlastP on this gene

EGO55876

hypothetical protein
  
Accession: EGO55875
  
Location: 3426198-3427131
  
 NCBI BlastP on this gene

EGO55875

hypothetical protein
  
Accession: EGO55874
  
Location: 3424535-3425542
  
 NCBI BlastP on this gene

EGO55874

hypothetical protein
  
Accession: EGO55873
  
Location: 3422882-3424055
  
  
**BlastP hit with Mycgr3G90561\_Mycgr3T**
  
Percentage identity: 32 %
  
BlastP bit score: 55
  
Sequence coverage: 65 %
  
E-value: 5e-06
  
  
 NCBI BlastP on this gene

EGO55873

hypothetical protein
  
Accession: EGO55872
  
Location: 3422546-3422749
  
 NCBI BlastP on this gene

EGO55872

hypothetical protein
  
Accession: EGO55871
  
Location: 3419753-3421548
  
 NCBI BlastP on this gene

EGO55871

hypothetical protein
  
Accession: EGO55870
  
Location: 3418242-3419266
  
  
**BlastP hit with Mycgr3G35535\_Mycgr3T**
  
Percentage identity: 32 %
  
BlastP bit score: 63
  
Sequence coverage: 87 %
  
E-value: 2e-09
  
  
 NCBI BlastP on this gene

EGO55870

hypothetical protein
  
Accession: EGO55869
  
Location: 3416039-3417003
  
 NCBI BlastP on this gene

EGO55869

hypothetical protein
  
Accession: EGO55868
  
Location: 3414462-3415267
  
 NCBI BlastP on this gene

EGO55868

hypothetical protein
  
Accession: EGO55867
  
Location: 3410314-3412455
  
 NCBI BlastP on this gene

EGO55867

hypothetical protein
  
Accession: EGO55866
  
Location: 3408757-3409844
  
 NCBI BlastP on this gene

EGO55866

hypothetical protein
  
Accession: EGO55865
  
Location: 3407519-3408184
  
 NCBI BlastP on this gene

EGO55865

hypothetical protein
  
Accession: EGO55864
  
Location: 3406238-3407296
  
 NCBI BlastP on this gene

EGO55864

hypothetical protein
  
Accession: EGO55863
  
Location: 3403836-3404545
  
 NCBI BlastP on this gene

EGO55863

hypothetical protein
  
Accession: EGO55862
  
Location: 3401820-3402610
  
 NCBI BlastP on this gene

EGO55862

hypothetical protein
  
Accession: EGO55861
  
Location: 3398800-3400153
  
 NCBI BlastP on this gene

EGO55861

7. :  GL891258 Neurospora tetrasperma FGSC 2509 unplaced genomic scaffold NEUTE2scaffold\_6     Total score: 3.0     Cumulative Blast bit score: 346

hypothetical protein
  
Accession: EGZ68860
  
Location: 515701-516635
  
 NCBI BlastP on this gene

EGZ68860

peroxidase/catalase 2
  
Accession: EGZ68861
  
Location: 519155-521416
  
 NCBI BlastP on this gene

EGZ68861

hypothetical protein
  
Accession: EGZ68862
  
Location: 523173-523898
  
 NCBI BlastP on this gene

EGZ68862

hypothetical protein
  
Accession: EGZ68863
  
Location: 523941-524891
  
 NCBI BlastP on this gene

EGZ68863

hypothetical protein
  
Accession: EGZ68864
  
Location: 533410-533947
  
 NCBI BlastP on this gene

EGZ68864

hypothetical protein
  
Accession: EGZ68865
  
Location: 534571-534952
  
 NCBI BlastP on this gene

EGZ68865

OPT-domain-containing protein
  
Accession: EGZ68866
  
Location: 535079-541810
  
  
**BlastP hit with Mycgr3G36449\_Mycgr3T**
  
Percentage identity: 29 %
  
BlastP bit score: 228
  
Sequence coverage: 103 %
  
E-value: 2e-59
  
  
 NCBI BlastP on this gene

EGZ68866

hypothetical protein
  
Accession: EGZ68867
  
Location: 541966-542899
  
 NCBI BlastP on this gene

EGZ68867

hypothetical protein
  
Accession: EGZ68868
  
Location: 543554-544561
  
 NCBI BlastP on this gene

EGZ68868

hypothetical protein
  
Accession: EGZ68869
  
Location: 545041-546214
  
  
**BlastP hit with Mycgr3G90561\_Mycgr3T**
  
Percentage identity: 32 %
  
BlastP bit score: 55
  
Sequence coverage: 65 %
  
E-value: 5e-06
  
  
 NCBI BlastP on this gene

EGZ68869

hypothetical protein
  
Accession: EGZ68870
  
Location: 546347-546550
  
 NCBI BlastP on this gene

EGZ68870

Di-copper centre-containing protein
  
Accession: EGZ68871
  
Location: 547548-549343
  
 NCBI BlastP on this gene

EGZ68871

hypothetical protein
  
Accession: EGZ68872
  
Location: 549830-550854
  
  
**BlastP hit with Mycgr3G35535\_Mycgr3T**
  
Percentage identity: 32 %
  
BlastP bit score: 63
  
Sequence coverage: 87 %
  
E-value: 2e-09
  
  
 NCBI BlastP on this gene

EGZ68872

hypothetical protein
  
Accession: EGZ68873
  
Location: 552093-553057
  
 NCBI BlastP on this gene

EGZ68873

hypothetical protein
  
Accession: EGZ68874
  
Location: 553829-554634
  
 NCBI BlastP on this gene

EGZ68874

hypothetical protein
  
Accession: EGZ68875
  
Location: 555421-555603
  
 NCBI BlastP on this gene

EGZ68875

hypothetical protein
  
Accession: EGZ68876
  
Location: 556639-558780
  
 NCBI BlastP on this gene

EGZ68876

S-adenosyl-L-methionine-dependent methyltransferase
  
Accession: EGZ68877
  
Location: 559250-560337
  
 NCBI BlastP on this gene

EGZ68877

hypothetical protein
  
Accession: EGZ68878
  
Location: 560910-561575
  
 NCBI BlastP on this gene

EGZ68878

hypothetical protein
  
Accession: EGZ68879
  
Location: 561798-563637
  
 NCBI BlastP on this gene

EGZ68879

hypothetical protein
  
Accession: EGZ68880
  
Location: 564547-565256
  
 NCBI BlastP on this gene

EGZ68880

hypothetical protein
  
Accession: EGZ68881
  
Location: 566482-567272
  
 NCBI BlastP on this gene

EGZ68881

hypothetical protein
  
Accession: EGZ68882
  
Location: 568881-570291
  
 NCBI BlastP on this gene

EGZ68882

8. :  CABT02000031 Sordaria macrospora k-hell     Total score: 3.0     Cumulative Blast bit score: 334

not annotated
  
Accession: CCC12839
  
Location: 288462-289399
  
 NCBI BlastP on this gene

CCC12839

not annotated
  
Accession: CCC12840
  
Location: 291805-294065
  
 NCBI BlastP on this gene

CCC12840

not annotated
  
Accession: CCC12841
  
Location: 296620-297288
  
 NCBI BlastP on this gene

CCC12841

not annotated
  
Accession: CCC12842
  
Location: 297637-298287
  
 NCBI BlastP on this gene

CCC12842

not annotated
  
Accession: CCC12843
  
Location: 299392-299764
  
 NCBI BlastP on this gene

CCC12843

not annotated
  
Accession: CCC12844
  
Location: 301258-303171
  
 NCBI BlastP on this gene

CCC12844

not annotated
  
Accession: CCC12845
  
Location: 307033-308146
  
  
**BlastP hit with Mycgr3G90561\_Mycgr3T**
  
Percentage identity: 31 %
  
BlastP bit score: 64
  
Sequence coverage: 57 %
  
E-value: 2e-09
  
  
 NCBI BlastP on this gene

CCC12845

not annotated
  
Accession: CCC12846
  
Location: 309006-311620
  
  
**BlastP hit with Mycgr3G36449\_Mycgr3T**
  
Percentage identity: 30 %
  
BlastP bit score: 203
  
Sequence coverage: 81 %
  
E-value: 1e-52
  
  
 NCBI BlastP on this gene

CCC12846

not annotated
  
Accession: CCC12847
  
Location: 312511-313490
  
 NCBI BlastP on this gene

CCC12847

not annotated
  
Accession: CCC12848
  
Location: 315394-316309
  
 NCBI BlastP on this gene

CCC12848

not annotated
  
Accession: CCC12849
  
Location: 317009-317992
  
 NCBI BlastP on this gene

CCC12849

not annotated
  
Accession: CCC12850
  
Location: 321399-323056
  
 NCBI BlastP on this gene

CCC12850

not annotated
  
Accession: CCC12851
  
Location: 323560-325025
  
  
**BlastP hit with Mycgr3G35535\_Mycgr3T**
  
Percentage identity: 33 %
  
BlastP bit score: 67
  
Sequence coverage: 87 %
  
E-value: 1e-10
  
  
 NCBI BlastP on this gene

CCC12851

not annotated
  
Accession: CCC12852
  
Location: 326172-326453
  
 NCBI BlastP on this gene

CCC12852

not annotated
  
Accession: CCC12853
  
Location: 327343-333820
  
 NCBI BlastP on this gene

CCC12853

not annotated
  
Accession: CCC12854
  
Location: 335862-337991
  
 NCBI BlastP on this gene

CCC12854

not annotated
  
Accession: CCC12855
  
Location: 340482-341091
  
 NCBI BlastP on this gene

CCC12855

not annotated
  
Accession: CCC12856
  
Location: 341495-343279
  
 NCBI BlastP on this gene

CCC12856

9. :  CM001199 Mycosphaerella graminicola IPO323 chromosome 4     Total score: 3.0     Cumulative Blast bit score: 314

hypothetical protein
  
Accession: EGP87995
  
Location: 1661805-1662587
  
 NCBI BlastP on this gene

EGP87995

oxalate decarboxylase, secreted
  
Accession: EGP88369
  
Location: 1662688-1664273
  
 NCBI BlastP on this gene

EGP88369

hypothetical protein
  
Accession: EGP87996
  
Location: 1665102-1665958
  
 NCBI BlastP on this gene

EGP87996

hypothetical protein
  
Accession: EGP88368
  
Location: 1666833-1667921
  
 NCBI BlastP on this gene

EGP88368

hypothetical protein
  
Accession: EGP88367
  
Location: 1668361-1669627
  
 NCBI BlastP on this gene

EGP88367

hypothetical protein
  
Accession: EGP87997
  
Location: 1670187-1673954
  
 NCBI BlastP on this gene

EGP87997

hypothetical protein
  
Accession: EGP87998
  
Location: 1674414-1674693
  
 NCBI BlastP on this gene

EGP87998

hypothetical protein
  
Accession: EGP88366
  
Location: 1674958-1676304
  
 NCBI BlastP on this gene

EGP88366

hypothetical protein
  
Accession: EGP88365
  
Location: 1677000-1678580
  
 NCBI BlastP on this gene

EGP88365

hypothetical protein
  
Accession: EGP88364
  
Location: 1681686-1683950
  
  
**BlastP hit with Mycgr3G36449\_Mycgr3T**
  
Percentage identity: 29 %
  
BlastP bit score: 188
  
Sequence coverage: 91 %
  
E-value: 5e-48
  
  
 NCBI BlastP on this gene

EGP88364

hypothetical protein
  
Accession: EGP87999
  
Location: 1684438-1685014
  
  
**BlastP hit with Mycgr3G90561\_Mycgr3T**
  
Percentage identity: 33 %
  
BlastP bit score: 59
  
Sequence coverage: 56 %
  
E-value: 2e-08
  
  
 NCBI BlastP on this gene

EGP87999

hypothetical protein
  
Accession: EGP88000
  
Location: 1686972-1687360
  
 NCBI BlastP on this gene

EGP88000

hypothetical protein
  
Accession: EGP88001
  
Location: 1688171-1689246
  
 NCBI BlastP on this gene

EGP88001

hypothetical protein
  
Accession: EGP88363
  
Location: 1689340-1690777
  
 NCBI BlastP on this gene

EGP88363

hypothetical protein
  
Accession: EGP88002
  
Location: 1691332-1691995
  
  
**BlastP hit with Mycgr3G35535\_Mycgr3T**
  
Percentage identity: 29 %
  
BlastP bit score: 67
  
Sequence coverage: 96 %
  
E-value: 3e-11
  
  
 NCBI BlastP on this gene

EGP88002

serine carboxypeptidase
  
Accession: EGP88362
  
Location: 1692026-1693749
  
 NCBI BlastP on this gene

EGP88362

putative major facilitator superfamily transporter
  
Accession: EGP88361
  
Location: 1694042-1695664
  
 NCBI BlastP on this gene

EGP88361

hypothetical protein
  
Accession: EGP88003
  
Location: 1696670-1697425
  
 NCBI BlastP on this gene

EGP88003

putative ABC transporter
  
Accession: EGP88360
  
Location: 1697477-1702334
  
 NCBI BlastP on this gene

EGP88360

hypothetical protein
  
Accession: EGP88004
  
Location: 1706050-1706713
  
 NCBI BlastP on this gene

EGP88004

Rho protein
  
Accession: EGP88359
  
Location: 1707820-1708511
  
 NCBI BlastP on this gene

EGP88359

ribose-phosphate pyrophosphokinase
  
Accession: EGP88005
  
Location: 1709061-1710587
  
 NCBI BlastP on this gene

EGP88005

hypothetical protein
  
Accession: EGP88358
  
Location: 1710939-1712260
  
 NCBI BlastP on this gene

EGP88358

10. :  AHHD01000518 Macrophomina phaseolina MS6     Total score: 2.0     Cumulative Blast bit score: 3574

Beta-ketoacyl synthase
  
Accession: EKG10413
  
Location: 153932-161880
  
 NCBI BlastP on this gene

EKG10413

AMP-dependent synthetase/ligase
  
Accession: EKG10414
  
Location: 162119-175583
  
  
**BlastP hit with Mycgr3G90558\_Mycgr3T**
  
Percentage identity: 30 %
  
BlastP bit score: 1716
  
Sequence coverage: 94 %
  
E-value: 0.0
  
  
 NCBI BlastP on this gene

EKG10414

Beta-lactamase-related protein
  
Accession: EKG10415
  
Location: 176349-177917
  
 NCBI BlastP on this gene

EKG10415

hypothetical protein
  
Accession: EKG10416
  
Location: 179610-179885
  
 NCBI BlastP on this gene

EKG10416

hypothetical protein
  
Accession: EKG10417
  
Location: 181032-181859
  
 NCBI BlastP on this gene

EKG10417

Putative ABC transporter protein
  
Accession: EKG10418
  
Location: 182930-188124
  
 NCBI BlastP on this gene

EKG10418

Major facilitator superfamily
  
Accession: EKG10419
  
Location: 188622-190195
  
  
**BlastP hit with Mycgr3G23761\_Mycgr3T**
  
Percentage identity: 34 %
  
BlastP bit score: 218
  
Sequence coverage: 100 %
  
E-value: 3e-61
  
  
 NCBI BlastP on this gene

EKG10419

Peptidase M20
  
Accession: EKG10420
  
Location: 190579-191983
  
 NCBI BlastP on this gene

EKG10420

Indoleamine 23-dioxygenase
  
Accession: EKG10421
  
Location: 192815-194101
  
 NCBI BlastP on this gene

EKG10421

AMP-dependent synthetase/ligase
  
Accession: EKG10422
  
Location: 195427-207108
  
  
**BlastP hit with Mycgr3G90558\_Mycgr3T**
  
Percentage identity: 32 %
  
BlastP bit score: 1641
  
Sequence coverage: 84 %
  
E-value: 0.0
  
  
 NCBI BlastP on this gene

EKG10422

nacht and ankyrin domain containing protein
  
Accession: EKG10423
  
Location: 208421-208990
  
 NCBI BlastP on this gene

EKG10423

nb-arc and ankyrin domain containing protein
  
Accession: EKG10424
  
Location: 209145-210651
  
 NCBI BlastP on this gene

EKG10424

11. :  KE145356 Glarea lozoyensis ATCC 20868 chromosome Unknown GLAREA13     Total score: 2.0     Cumulative Blast bit score: 2476

Thiolase-like protein
  
Accession: EPE34340
  
Location: 220862-228891
  
 NCBI BlastP on this gene

EPE34340

Acetyl-CoA synthetase-like protein
  
Accession: EPE34341
  
Location: 230546-252235
  
  
**BlastP hit with Mycgr3G90558\_Mycgr3T**
  
Percentage identity: 32 %
  
BlastP bit score: 1973
  
Sequence coverage: 90 %
  
E-value: 0.0
  
  
 NCBI BlastP on this gene

EPE34341

P-loop containing nucleoside triphosphate hydrolase
  
Accession: EPE34342
  
Location: 253841-258579
  
 NCBI BlastP on this gene

EPE34342

Aconitase iron-sulfur
  
Accession: EPE34343
  
Location: 258935-261970
  
 NCBI BlastP on this gene

EPE34343

Isocitrate/Isopropylmalate dehydrogenase-like protein
  
Accession: EPE34344
  
Location: 262418-263855
  
 NCBI BlastP on this gene

EPE34344

Aldolase
  
Accession: EPE34345
  
Location: 264293-266317
  
 NCBI BlastP on this gene

EPE34345

D-aminoacid aminotransferase-like PLP-dependent enzyme
  
Accession: EPE34346
  
Location: 267099-268369
  
 NCBI BlastP on this gene

EPE34346

Clavaminate synthase-like protein
  
Accession: EPE34347
  
Location: 268608-269705
  
  
**BlastP hit with Mycgr3G68036\_Mycgr3T**
  
Percentage identity: 38 %
  
BlastP bit score: 220
  
Sequence coverage: 100 %
  
E-value: 3e-65
  
  
 NCBI BlastP on this gene

EPE34347

Clavaminate synthase-like protein
  
Accession: EPE34348
  
Location: 270159-271199
  
 NCBI BlastP on this gene

EPE34348

Acetyl-CoA synthetase-like protein
  
Accession: EPE34349
  
Location: 271791-273838
  
 NCBI BlastP on this gene

EPE34349

Clavaminate synthase-like protein
  
Accession: EPE34350
  
Location: 275478-276584
  
  
**BlastP hit with Mycgr3G68036\_Mycgr3T**
  
Percentage identity: 44 %
  
BlastP bit score: 283
  
Sequence coverage: 100 %
  
E-value: 5e-90
  
  
 NCBI BlastP on this gene

EPE34350

hypothetical protein
  
Accession: EPE34351
  
Location: 277253-279400
  
 NCBI BlastP on this gene

EPE34351

12. :  KB916208 Neofusicoccum parvum UCRNP2 chromosome Unknown NP2\_03\_scaffold\_570     Total score: 2.0     Cumulative Blast bit score: 2293

putative fatty acid synthase alpha subunit protein
  
Accession: EOD48379
  
Location: 23767-26598
  
 NCBI BlastP on this gene

EOD48379

putative nucleoside-diphosphate-sugar epimerase protein
  
Accession: EOD48378
  
Location: 29560-30412
  
 NCBI BlastP on this gene

EOD48378

hypothetical protein
  
Accession: EOD48380
  
Location: 31386-64262
  
  
**BlastP hit with Mycgr3G90558\_Mycgr3T**
  
Percentage identity: 34 %
  
BlastP bit score: 2061
  
Sequence coverage: 90 %
  
E-value: 0.0
  
  
 NCBI BlastP on this gene

EOD48380

hypothetical protein
  
Accession: EOD48394
  
Location: 64495-65070
  
 NCBI BlastP on this gene

EOD48394

putative benzoate 4-monooxygenase cytochrome p450 protein
  
Accession: EOD48395
  
Location: 66956-67705
  
 NCBI BlastP on this gene

EOD48395

putative gibberellin 2-oxidase protein
  
Accession: EOD48392
  
Location: 69282-70276
  
  
**BlastP hit with Mycgr3G68036\_Mycgr3T**
  
Percentage identity: 43 %
  
BlastP bit score: 232
  
Sequence coverage: 84 %
  
E-value: 2e-70
  
  
 NCBI BlastP on this gene

EOD48392

hypothetical protein
  
Accession: EOD48385
  
Location: 74583-75797
  
 NCBI BlastP on this gene

EOD48385

hypothetical protein
  
Accession: EOD48384
  
Location: 76825-77843
  
 NCBI BlastP on this gene

EOD48384

13. :  KB644410 Penicillium oxalicum 114-2 unplaced genomic scaffold scaffold\_3     Total score: 2.0     Cumulative Blast bit score: 2284

hypothetical protein
  
Accession: EPS27187
  
Location: 46855-51660
  
 NCBI BlastP on this gene

EPS27187

hypothetical protein
  
Accession: EPS27188
  
Location: 54296-77982
  
  
**BlastP hit with Mycgr3G90558\_Mycgr3T**
  
Percentage identity: 32 %
  
BlastP bit score: 2083
  
Sequence coverage: 94 %
  
E-value: 0.0
  
  
 NCBI BlastP on this gene

EPS27188

hypothetical protein
  
Accession: EPS27189
  
Location: 78526-79566
  
 NCBI BlastP on this gene

EPS27189

hypothetical protein
  
Accession: EPS27190
  
Location: 79827-80877
  
 NCBI BlastP on this gene

EPS27190

hypothetical protein
  
Accession: EPS27191
  
Location: 81151-82295
  
  
**BlastP hit with Mycgr3G68036\_Mycgr3T**
  
Percentage identity: 34 %
  
BlastP bit score: 201
  
Sequence coverage: 101 %
  
E-value: 8e-58
  
  
 NCBI BlastP on this gene

EPS27191

hypothetical protein
  
Accession: EPS27192
  
Location: 83418-88263
  
 NCBI BlastP on this gene

EPS27192

hypothetical protein
  
Accession: EPS27193
  
Location: 89510-90586
  
 NCBI BlastP on this gene

EPS27193

hypothetical protein
  
Accession: EPS27194
  
Location: 92729-98836
  
 NCBI BlastP on this gene

EPS27194

14. :  ABDF02000064 Trichoderma virens Gv29-8     Total score: 2.0     Cumulative Blast bit score: 2269

hypothetical protein
  
Accession: EHK21751
  
Location: 25346-29939
  
 NCBI BlastP on this gene

EHK21751

hypothetical protein
  
Accession: EHK21752
  
Location: 30416-30739
  
 NCBI BlastP on this gene

EHK21752

hypothetical protein
  
Accession: EHK21753
  
Location: 30902-32168
  
 NCBI BlastP on this gene

EHK21753

hypothetical protein
  
Accession: EHK21754
  
Location: 32704-34932
  
 NCBI BlastP on this gene

EHK21754

hypothetical protein
  
Accession: EHK21755
  
Location: 36259-37212
  
  
**BlastP hit with Mycgr3G35535\_Mycgr3T**
  
Percentage identity: 32 %
  
BlastP bit score: 82
  
Sequence coverage: 88 %
  
E-value: 3e-16
  
  
 NCBI BlastP on this gene

EHK21755

hypothetical protein
  
Accession: EHK21756
  
Location: 38429-41947
  
 NCBI BlastP on this gene

EHK21756

non-ribosomal peptide synthetase
  
Accession: EHK21757
  
Location: 44838-68695
  
  
**BlastP hit with Mycgr3G90558\_Mycgr3T**
  
Percentage identity: 32 %
  
BlastP bit score: 2187
  
Sequence coverage: 100 %
  
E-value: 0.0
  
  
 NCBI BlastP on this gene

EHK21757

hypothetical protein
  
Accession: EHK21758
  
Location: 69039-70872
  
 NCBI BlastP on this gene

EHK21758

glycoside hydrolase family 18 protein
  
Accession: EHK21761
  
Location: 72658-79239
  
 NCBI BlastP on this gene

EHK21761

15. :  JH226133 Exophiala dermatitidis NIH/UT8656 unplaced genomic scaffold supercont1.4     Total score: 2.0     Cumulative Blast bit score: 2237

ABC multidrug transporter
  
Accession: EHY56729
  
Location: 1293075-1298326
  
 NCBI BlastP on this gene

EHY56729

fatty acid synthase subunit beta, yeast type, variant
  
Accession: EHY56730
  
Location: 1298864-1305352
  
 NCBI BlastP on this gene

EHY56730

threonine aldolase
  
Accession: EHY56732
  
Location: 1306036-1307049
  
 NCBI BlastP on this gene

EHY56732

pyrroline-5-carboxylate reductase
  
Accession: EHY56733
  
Location: 1307459-1308509
  
 NCBI BlastP on this gene

EHY56733

hypothetical protein
  
Accession: EHY56734
  
Location: 1308789-1310125
  
  
**BlastP hit with Mycgr3G68036\_Mycgr3T**
  
Percentage identity: 43 %
  
BlastP bit score: 265
  
Sequence coverage: 95 %
  
E-value: 4e-82
  
  
 NCBI BlastP on this gene

EHY56734

hypothetical protein
  
Accession: EHY56735
  
Location: 1311029-1312639
  
 NCBI BlastP on this gene

EHY56735

cytochrome P450 monooxygenase
  
Accession: EHY56736
  
Location: 1313166-1315196
  
 NCBI BlastP on this gene

EHY56736

hypothetical protein
  
Accession: EHY56737
  
Location: 1315624-1316570
  
 NCBI BlastP on this gene

EHY56737

branched-chain amino acid aminotransferase
  
Accession: EHY56740
  
Location: 1317129-1318512
  
 NCBI BlastP on this gene

EHY56740

hypothetical protein, variant
  
Accession: EHY56738
  
Location: 1321252-1322172
  
 NCBI BlastP on this gene

EHY56738

nonribosomal peptide synthase
  
Accession: EHY56742
  
Location: 1323346-1340944
  
  
**BlastP hit with Mycgr3G90558\_Mycgr3T**
  
Percentage identity: 32 %
  
BlastP bit score: 1972
  
Sequence coverage: 94 %
  
E-value: 0.0
  
  
 NCBI BlastP on this gene

EHY56742

hypothetical protein
  
Accession: EHY56743
  
Location: 1343770-1345089
  
 NCBI BlastP on this gene

EHY56743

phosphatidate phosphatase
  
Accession: EHY56744
  
Location: 1348206-1349533
  
 NCBI BlastP on this gene

EHY56744

hypothetical protein
  
Accession: EHY56745
  
Location: 1350853-1351155
  
 NCBI BlastP on this gene

EHY56745

3-oxoacyl-[acyl-carrier protein] reductase
  
Accession: EHY56746
  
Location: 1352338-1353324
  
 NCBI BlastP on this gene

EHY56746

16. :  JX421684 Emericella rugulosa strain NRRL 11440 hypothetical protein genes     Total score: 2.0     Cumulative Blast bit score: 2232

EcdC
  
Accession: AFT91386
  
Location: 28439-30467
  
 NCBI BlastP on this gene

ecdC

EcdD
  
Accession: AFT91387
  
Location: 31103-32936
  
 NCBI BlastP on this gene

ecdD

EcdE
  
Accession: AFT91388
  
Location: 34139-36306
  
 NCBI BlastP on this gene

ecdE

EcdF
  
Accession: AFT91384
  
Location: 37085-39059
  
 NCBI BlastP on this gene

ecdF

EcdA
  
Accession: AFT91378
  
Location: 41269-63178
  
  
**BlastP hit with Mycgr3G90558\_Mycgr3T**
  
Percentage identity: 33 %
  
BlastP bit score: 1989
  
Sequence coverage: 90 %
  
E-value: 0.0
  
  
 NCBI BlastP on this gene

ecdA

EcdG
  
Accession: AFT91379
  
Location: 63384-64533
  
 NCBI BlastP on this gene

ecdG

EcdH
  
Accession: AFT91389
  
Location: 64922-66488
  
 NCBI BlastP on this gene

ecdH

EcdI
  
Accession: AFT91380
  
Location: 66559-68482
  
 NCBI BlastP on this gene

ecdI

EcdJ
  
Accession: AFT91381
  
Location: 70014-72170
  
 NCBI BlastP on this gene

ecdJ

EcdK
  
Accession: AFT91382
  
Location: 72258-73376
  
  
**BlastP hit with Mycgr3G68036\_Mycgr3T**
  
Percentage identity: 39 %
  
BlastP bit score: 243
  
Sequence coverage: 100 %
  
E-value: 2e-74
  
  
 NCBI BlastP on this gene

ecdK

EcdL
  
Accession: AFT91383
  
Location: 73639-78856
  
 NCBI BlastP on this gene

ecdL

hypothetical protein
  
Accession: AFT91370
  
Location: 82060-88903
  
 NCBI BlastP on this gene

AFT91370

17. :  EQ962654 Talaromyces stipitatus ATCC 10500 scf\_1105507295541 genomic scaffold     Total score: 2.0     Cumulative Blast bit score: 2224

conserved hypothetical protein
  
Accession: EED20592
  
Location: 3951246-3953229
  
 NCBI BlastP on this gene

EED20592

C2H2 transcription factor RfeC
  
Accession: EED20595
  
Location: 3957523-3959187
  
 NCBI BlastP on this gene

EED20595

nonribosomal peptide synthase, putative
  
Accession: EED20596
  
Location: 3960945-3978353
  
  
**BlastP hit with Mycgr3G90558\_Mycgr3T**
  
Percentage identity: 29 %
  
BlastP bit score: 1680
  
Sequence coverage: 93 %
  
E-value: 0.0
  
  
 NCBI BlastP on this gene

EED20596

conserved hypothetical protein
  
Accession: EED20597
  
Location: 3979116-3980249
  
 NCBI BlastP on this gene

EED20597

hypothetical protein
  
Accession: EED20598
  
Location: 3980449-3981543
  
 NCBI BlastP on this gene

EED20598

hypothetical protein
  
Accession: EED20599
  
Location: 3983545-3984828
  
 NCBI BlastP on this gene

EED20599

phenylalanine ammonia-lyase, putative
  
Accession: EED20600
  
Location: 3987229-3989468
  
 NCBI BlastP on this gene

EED20600

conserved hypothetical protein
  
Accession: EED20601
  
Location: 3990241-3991314
  
 NCBI BlastP on this gene

EED20601

multidrug resistance protein, putative
  
Accession: EED20602
  
Location: 3994840-3998734
  
  
**BlastP hit with Mycgr3G9942\_Mycgr3T9**
  
Percentage identity: 31 %
  
BlastP bit score: 544
  
Sequence coverage: 94 %
  
E-value: 3e-167
  
  
 NCBI BlastP on this gene

EED20602

hypothetical protein
  
Accession: EED20603
  
Location: 4001018-4002714
  
 NCBI BlastP on this gene

EED20603

18. :  AKHY01000111 Aspergillus oryzae 3.042     Total score: 2.0     Cumulative Blast bit score: 2025

hypothetical protein
  
Accession: EIT80938
  
Location: 231577-232516
  
 NCBI BlastP on this gene

EIT80938

hypothetical protein
  
Accession: EIT81012
  
Location: 230123-231421
  
 NCBI BlastP on this gene

EIT81012

acyl-CoA synthetases (AMP-forming)/AMP-acid ligases II
  
Accession: EIT80997
  
Location: 226669-229617
  
 NCBI BlastP on this gene

EIT80997

hypothetical protein
  
Accession: EIT80934
  
Location: 222528-223346
  
 NCBI BlastP on this gene

EIT80934

hypothetical protein
  
Accession: EIT80948
  
Location: 218943-222267
  
 NCBI BlastP on this gene

EIT80948

non-ribosomal peptide synthetase module
  
Accession: EIT80956
  
Location: 207086-217298
  
  
**BlastP hit with Mycgr3G90558\_Mycgr3T**
  
Percentage identity: 33 %
  
BlastP bit score: 1469
  
Sequence coverage: 65 %
  
E-value: 0.0
  
  
 NCBI BlastP on this gene

EIT80956

2-polyprenyl-6-methoxyphenol hydroxylase
  
Accession: EIT81007
  
Location: 204388-206656
  
 NCBI BlastP on this gene

EIT81007

H+/oligopeptide symporter
  
Accession: EIT80986
  
Location: 201968-203897
  
 NCBI BlastP on this gene

EIT80986

isopenicillin N synthase
  
Accession: EIT80966
  
Location: 200164-201318
  
  
**BlastP hit with Mycgr3G68036\_Mycgr3T**
  
Percentage identity: 41 %
  
BlastP bit score: 283
  
Sequence coverage: 97 %
  
E-value: 1e-89
  
  
 NCBI BlastP on this gene

EIT80966

hypothetical protein
  
Accession: EIT80983
  
Location: 198406-199627
  
 NCBI BlastP on this gene

EIT80983

hypothetical protein
  
Accession: EIT80993
  
Location: 197423-198121
  
 NCBI BlastP on this gene

EIT80993

FAD-dependent oxidoreductase
  
Accession: EIT81016
  
Location: 195514-197025
  
 NCBI BlastP on this gene

EIT81016

H+/oligopeptide symporter
  
Accession: EIT80996
  
Location: 193354-195070
  
 NCBI BlastP on this gene

EIT80996

iron/ascorbate family oxidoreductase
  
Accession: EIT80991
  
Location: 191485-192644
  
  
**BlastP hit with Mycgr3G68036\_Mycgr3T**
  
Percentage identity: 42 %
  
BlastP bit score: 273
  
Sequence coverage: 100 %
  
E-value: 6e-86
  
  
 NCBI BlastP on this gene

EIT80991

hypothetical protein
  
Accession: EIT80971
  
Location: 189403-191058
  
 NCBI BlastP on this gene

EIT80971

putative hydrolase
  
Accession: EIT80961
  
Location: 186517-187317
  
 NCBI BlastP on this gene

EIT80961

permease of the major facilitator superfamily
  
Accession: EIT80984
  
Location: 182700-184483
  
 NCBI BlastP on this gene

EIT80984

multidrug/pheromone exporter, ABC superfamily
  
Accession: EIT80974
  
Location: 177256-181857
  
 NCBI BlastP on this gene

EIT80974

19. :  AB705455 Aspergillus oryzae DNA, WYK-1 cluster, ORF1, ORF2, ORF3, wykN, wykA, wykE, wykG, wykH, ...     Total score: 2.0     Cumulative Blast bit score: 1999

hypothetical protein
  
Accession: BAM31239
  
Location: 7-900
  
 NCBI BlastP on this gene

BAM31239

hypothetical protein
  
Accession: BAM31240
  
Location: 1086-2976
  
 NCBI BlastP on this gene

BAM31240

hypothetical protein
  
Accession: BAM31241
  
Location: 3516-4695
  
 NCBI BlastP on this gene

BAM31241

non-ribosomal peptide synthase
  
Accession: BAM31242
  
Location: 6718-16248
  
  
**BlastP hit with Mycgr3G90558\_Mycgr3T**
  
Percentage identity: 33 %
  
BlastP bit score: 1467
  
Sequence coverage: 65 %
  
E-value: 0.0
  
  
 NCBI BlastP on this gene

wykN

hypothetical protein
  
Accession: BAM31243
  
Location: 16678-18946
  
 NCBI BlastP on this gene

wykA

oligopeptide transporter
  
Accession: BAM31244
  
Location: 19439-21368
  
 NCBI BlastP on this gene

wykE

gibberellin 2-oxidase
  
Accession: BAM31245
  
Location: 22018-23172
  
  
**BlastP hit with Mycgr3G68036\_Mycgr3T**
  
Percentage identity: 40 %
  
BlastP bit score: 280
  
Sequence coverage: 104 %
  
E-value: 4e-88
  
  
 NCBI BlastP on this gene

wykG

N-methyltransferase
  
Accession: BAM31246
  
Location: 23709-25007
  
 NCBI BlastP on this gene

wykH

hypothetical protein
  
Accession: BAM31247
  
Location: 25215-25913
  
 NCBI BlastP on this gene

wykI

fructosyl amino acid oxidase
  
Accession: BAM31248
  
Location: 26312-27823
  
 NCBI BlastP on this gene

wykB

oligopeptide transporter
  
Accession: BAM31249
  
Location: 28267-29983
  
 NCBI BlastP on this gene

wykF

iron/ascorbate family oxidoreductase
  
Accession: BAM31250
  
Location: 30693-31852
  
  
**BlastP hit with Mycgr3G68036\_Mycgr3T**
  
Percentage identity: 41 %
  
BlastP bit score: 252
  
Sequence coverage: 100 %
  
E-value: 9e-78
  
  
 NCBI BlastP on this gene

wykC

hypothetical protein
  
Accession: BAM31251
  
Location: 32279-33934
  
 NCBI BlastP on this gene

wykR

haloacid dehalogenase
  
Accession: BAM31252
  
Location: 36043-36843
  
 NCBI BlastP on this gene

wykD

major facilitator superfamily transporter
  
Accession: BAM31253
  
Location: 38879-40662
  
 NCBI BlastP on this gene

BAM31253

multidrug/pheromone exporter
  
Accession: BAM31254
  
Location: 41505-46106
  
 NCBI BlastP on this gene

BAM31254

20. :  AP007154 Aspergillus oryzae RIB40 DNA, SC001.     Total score: 2.0     Cumulative Blast bit score: 1995

not annotated
  
Accession: BAE56567
  
Location: 350-1289
  
 NCBI BlastP on this gene

AO090001000001

not annotated
  
Accession: BAE56568
  
Location: 1445-2731
  
 NCBI BlastP on this gene

AO090001000002

not annotated
  
Accession: BAE56569
  
Location: 3284-6199
  
 NCBI BlastP on this gene

AO090001000003

not annotated
  
Accession: BAE56570
  
Location: 9327-10145
  
 NCBI BlastP on this gene

AO090001000006

not annotated
  
Accession: BAE56571
  
Location: 10406-12296
  
 NCBI BlastP on this gene

AO090001000007

not annotated
  
Accession: BAE56572
  
Location: 12836-14015
  
 NCBI BlastP on this gene

AO090001000008

not annotated
  
Accession: BAE56573
  
Location: 16084-25587
  
  
**BlastP hit with Mycgr3G90558\_Mycgr3T**
  
Percentage identity: 33 %
  
BlastP bit score: 1466
  
Sequence coverage: 65 %
  
E-value: 0.0
  
  
 NCBI BlastP on this gene

AO090001000009

not annotated
  
Accession: BAE56574
  
Location: 26017-28285
  
 NCBI BlastP on this gene

AO090001000010

not annotated
  
Accession: BAE56575
  
Location: 28776-30705
  
 NCBI BlastP on this gene

AO090001000011

not annotated
  
Accession: BAE56576
  
Location: 31355-32509
  
  
**BlastP hit with Mycgr3G68036\_Mycgr3T**
  
Percentage identity: 39 %
  
BlastP bit score: 278
  
Sequence coverage: 104 %
  
E-value: 4e-87
  
  
 NCBI BlastP on this gene

AO090001000012

not annotated
  
Accession: BAE56577
  
Location: 33046-34344
  
 NCBI BlastP on this gene

AO090001000013

not annotated
  
Accession: BAE56578
  
Location: 34552-35250
  
 NCBI BlastP on this gene

AO090001000014

not annotated
  
Accession: BAE56579
  
Location: 35648-37159
  
 NCBI BlastP on this gene

AO090001000015

not annotated
  
Accession: BAE56580
  
Location: 37603-39319
  
 NCBI BlastP on this gene

AO090001000016

not annotated
  
Accession: BAE56581
  
Location: 40029-41188
  
  
**BlastP hit with Mycgr3G68036\_Mycgr3T**
  
Percentage identity: 40 %
  
BlastP bit score: 251
  
Sequence coverage: 100 %
  
E-value: 2e-77
  
  
 NCBI BlastP on this gene

AO090001000017

not annotated
  
Accession: BAE56582
  
Location: 41615-43270
  
 NCBI BlastP on this gene

AO090001000018

not annotated
  
Accession: BAE56583
  
Location: 45356-46156
  
 NCBI BlastP on this gene

AO090001000019

not annotated
  
Accession: BAE56584
  
Location: 48190-49973
  
 NCBI BlastP on this gene

AO090001000020

not annotated
  
Accession: BAE56585
  
Location: 50816-55417
  
 NCBI BlastP on this gene

AO090001000021

21. :  ABDF02000068 Trichoderma virens Gv29-8     Total score: 2.0     Cumulative Blast bit score: 1982

hypothetical protein
  
Accession: EHK21614
  
Location: 24575-25528
  
 NCBI BlastP on this gene

EHK21614

hypothetical protein
  
Accession: EHK21615
  
Location: 26152-27246
  
 NCBI BlastP on this gene

EHK21615

hypothetical protein
  
Accession: EHK21616
  
Location: 28003-28302
  
 NCBI BlastP on this gene

EHK21616

hypothetical protein
  
Accession: EHK21617
  
Location: 31443-32198
  
 NCBI BlastP on this gene

EHK21617

hypothetical protein
  
Accession: EHK21618
  
Location: 33438-37601
  
 NCBI BlastP on this gene

EHK21618

hypothetical protein
  
Accession: EHK21619
  
Location: 38123-38941
  
 NCBI BlastP on this gene

EHK21619

hypothetical protein
  
Accession: EHK21620
  
Location: 39740-41413
  
 NCBI BlastP on this gene

EHK21620

hypothetical protein
  
Accession: EHK21621
  
Location: 41533-42745
  
  
**BlastP hit with Mycgr3G68036\_Mycgr3T**
  
Percentage identity: 33 %
  
BlastP bit score: 171
  
Sequence coverage: 100 %
  
E-value: 8e-47
  
  
 NCBI BlastP on this gene

EHK21621

hypothetical protein
  
Accession: EHK21622
  
Location: 43706-60495
  
  
**BlastP hit with Mycgr3G90558\_Mycgr3T**
  
Percentage identity: 30 %
  
BlastP bit score: 1811
  
Sequence coverage: 96 %
  
E-value: 0.0
  
  
 NCBI BlastP on this gene

EHK21622

hypothetical protein
  
Accession: EHK21623
  
Location: 61093-61764
  
 NCBI BlastP on this gene

EHK21623

hypothetical protein
  
Accession: EHK21624
  
Location: 62337-63842
  
 NCBI BlastP on this gene

EHK21624

hypothetical protein
  
Accession: EHK21625
  
Location: 64333-65256
  
 NCBI BlastP on this gene

EHK21625

hypothetical protein
  
Accession: EHK21626
  
Location: 66527-67970
  
 NCBI BlastP on this gene

EHK21626

hypothetical protein
  
Accession: EHK21627
  
Location: 68674-71030
  
 NCBI BlastP on this gene

EHK21627

hypothetical protein
  
Accession: EHK21628
  
Location: 71630-72946
  
 NCBI BlastP on this gene

EHK21628

hypothetical protein
  
Accession: EHK21629
  
Location: 73121-74691
  
 NCBI BlastP on this gene

EHK21629

glycoside hydrolase family 18 protein
  
Accession: EHK21641
  
Location: 75384-80045
  
 NCBI BlastP on this gene

EHK21641

22. :  EQ963479 Aspergillus flavus NRRL3357 scf\_1106286418500 genomic scaffold     Total score: 2.0     Cumulative Blast bit score: 1963

transcriptional activator protein acu-15, putative
  
Accession: EED49841
  
Location: 634955-637395
  
 NCBI BlastP on this gene

EED49841

short chain dehydrogenase family protein
  
Accession: EED49842
  
Location: 638029-638836
  
 NCBI BlastP on this gene

EED49842

mandelate racemase, putative
  
Accession: EED49843
  
Location: 639143-640185
  
 NCBI BlastP on this gene

EED49843

sugar transporter, putative
  
Accession: EED49844
  
Location: 641926-643669
  
 NCBI BlastP on this gene

EED49844

conserved hypothetical protein
  
Accession: EED49845
  
Location: 644708-645803
  
 NCBI BlastP on this gene

EED49845

beta-galactosidase, putative
  
Accession: EED49846
  
Location: 646616-649969
  
 NCBI BlastP on this gene

EED49846

pyrroline-5-carboxylate reductase, putative
  
Accession: EED49847
  
Location: 650393-651371
  
 NCBI BlastP on this gene

EED49847

P450 family sporulation-specific N-formyltyrosine oxidase Dit2
  
Accession: EED49848
  
Location: 652134-653630
  
 NCBI BlastP on this gene

EED49848

conserved hypothetical protein
  
Accession: EED49849
  
Location: 653979-655109
  
  
**BlastP hit with Mycgr3G68036\_Mycgr3T**
  
Percentage identity: 33 %
  
BlastP bit score: 157
  
Sequence coverage: 94 %
  
E-value: 2e-41
  
  
 NCBI BlastP on this gene

EED49849

nonribosomal peptide synthase, putative
  
Accession: EED49850
  
Location: 655460-671802
  
  
**BlastP hit with Mycgr3G90558\_Mycgr3T**
  
Percentage identity: 30 %
  
BlastP bit score: 1806
  
Sequence coverage: 95 %
  
E-value: 0.0
  
  
 NCBI BlastP on this gene

EED49850

alcohol dehydrogenase, putative
  
Accession: EED49851
  
Location: 673317-674393
  
 NCBI BlastP on this gene

EED49851

MSF multidrug transporter, putative
  
Accession: EED49852
  
Location: 676143-677540
  
 NCBI BlastP on this gene

EED49852

periplasmic beta-glucosidase precursor, putative
  
Accession: EED49853
  
Location: 678960-681518
  
 NCBI BlastP on this gene

EED49853

conserved hypothetical protein
  
Accession: EED49854
  
Location: 682176-685132
  
 NCBI BlastP on this gene

EED49854

oxidoreductase, FAD-binding, putative
  
Accession: EED49855
  
Location: 686534-688074
  
 NCBI BlastP on this gene

EED49855

geranylgeranyl pyrophosphate synthase, putative
  
Accession: EED49856
  
Location: 688382-690085
  
 NCBI BlastP on this gene

EED49856

23. :  AKCU01000056 Penicillium digitatum Pd1     Total score: 2.0     Cumulative Blast bit score: 1951

hypothetical protein
  
Accession: EKV21422
  
Location: 3282-4446
  
  
**BlastP hit with Mycgr3G68036\_Mycgr3T**
  
Percentage identity: 33 %
  
BlastP bit score: 179
  
Sequence coverage: 99 %
  
E-value: 7e-50
  
  
 NCBI BlastP on this gene

EKV21422

HC-toxin synthetase
  
Accession: EKV21423
  
Location: 5834-21508
  
  
**BlastP hit with Mycgr3G90558\_Mycgr3T**
  
Percentage identity: 30 %
  
BlastP bit score: 1772
  
Sequence coverage: 96 %
  
E-value: 0.0
  
  
 NCBI BlastP on this gene

EKV21423

24. :  AKCT01000339 Penicillium digitatum PHI26     Total score: 2.0     Cumulative Blast bit score: 1951

hypothetical protein
  
Accession: EKV04332
  
Location: 3361-4525
  
  
**BlastP hit with Mycgr3G68036\_Mycgr3T**
  
Percentage identity: 33 %
  
BlastP bit score: 179
  
Sequence coverage: 99 %
  
E-value: 7e-50
  
  
 NCBI BlastP on this gene

EKV04332

HC-toxin synthetase
  
Accession: EKV04333
  
Location: 5913-21587
  
  
**BlastP hit with Mycgr3G90558\_Mycgr3T**
  
Percentage identity: 30 %
  
BlastP bit score: 1772
  
Sequence coverage: 96 %
  
E-value: 0.0
  
  
 NCBI BlastP on this gene

EKV04333

Cell wall protein, putative
  
Accession: EKV04334
  
Location: 25617-27974
  
 NCBI BlastP on this gene

EKV04334

Serine/arginine repetitive matrix protein 1
  
Accession: EKV04335
  
Location: 29198-30442
  
 NCBI BlastP on this gene

EKV04335

Checkpoint protein kinase, putative
  
Accession: EKV04336
  
Location: 31499-34054
  
 NCBI BlastP on this gene

EKV04336

Ribosome biogenesis protein nsa2
  
Accession: EKV04337
  
Location: 34877-35931
  
 NCBI BlastP on this gene

EKV04337

Aconitate hydratase, putative
  
Accession: EKV04338
  
Location: 36371-37156
  
 NCBI BlastP on this gene

EKV04338

hypothetical protein
  
Accession: EKV04339
  
Location: 38105-38245
  
 NCBI BlastP on this gene

EKV04339

hypothetical protein
  
Accession: EKV04340
  
Location: 39425-39667
  
 NCBI BlastP on this gene

EKV04340

25. :  AP007154 Aspergillus oryzae RIB40 DNA, SC001.     Total score: 2.0     Cumulative Blast bit score: 1902

not annotated
  
Accession: BAE56792
  
Location: 618690-619367
  
 NCBI BlastP on this gene

AO090001000253

not annotated
  
Accession: BAE56793
  
Location: 621183-622491
  
 NCBI BlastP on this gene

AO090001000254

not annotated
  
Accession: BAE56794
  
Location: 623341-624168
  
 NCBI BlastP on this gene

AO090001000255

not annotated
  
Accession: BAE56795
  
Location: 626435-627372
  
 NCBI BlastP on this gene

AO090001000256

not annotated
  
Accession: BAE56796
  
Location: 628006-628813
  
 NCBI BlastP on this gene

AO090001000257

not annotated
  
Accession: BAE56797
  
Location: 629120-630472
  
 NCBI BlastP on this gene

AO090001000258

not annotated
  
Accession: BAE56798
  
Location: 631077-633254
  
 NCBI BlastP on this gene

AO090001000259

not annotated
  
Accession: BAE56799
  
Location: 633887-636916
  
 NCBI BlastP on this gene

AO090001000260

not annotated
  
Accession: BAE56800
  
Location: 637265-638397
  
  
**BlastP hit with Mycgr3G68036\_Mycgr3T**
  
Percentage identity: 30 %
  
BlastP bit score: 132
  
Sequence coverage: 101 %
  
E-value: 5e-32
  
  
 NCBI BlastP on this gene

AO090001000261

not annotated
  
Accession: BAE56801
  
Location: 638748-655090
  
  
**BlastP hit with Mycgr3G90558\_Mycgr3T**
  
Percentage identity: 30 %
  
BlastP bit score: 1771
  
Sequence coverage: 94 %
  
E-value: 0.0
  
  
 NCBI BlastP on this gene

AO090001000262

not annotated
  
Accession: BAE56802
  
Location: 656686-657222
  
 NCBI BlastP on this gene

AO090001000263

not annotated
  
Accession: BAE56803
  
Location: 657634-658704
  
 NCBI BlastP on this gene

AO090001000264

not annotated
  
Accession: BAE56804
  
Location: 659394-662514
  
 NCBI BlastP on this gene

AO090001000265

not annotated
  
Accession: BAE56805
  
Location: 663368-665832
  
 NCBI BlastP on this gene

AO090001000266

not annotated
  
Accession: BAE56806
  
Location: 666525-670738
  
 NCBI BlastP on this gene

AO090001000267

not annotated
  
Accession: BAE56807
  
Location: 671670-674458
  
 NCBI BlastP on this gene

AO090001000268

26. :  GL698751 Metarhizium anisopliae ARSEF 23 unplaced genomic scaffold Scf\_041     Total score: 2.0     Cumulative Blast bit score: 1884

Palmitoyltransferase AKR1
  
Accession: EFY94865
  
Location: 133072-135539
  
 NCBI BlastP on this gene

EFY94865

aspartic protease precursor
  
Accession: EFY94864
  
Location: 131424-132618
  
 NCBI BlastP on this gene

EFY94864

hypothetical protein
  
Accession: EFY94863
  
Location: 125087-126906
  
 NCBI BlastP on this gene

EFY94863

hypothetical protein
  
Accession: EFY94862
  
Location: 122685-123819
  
  
**BlastP hit with Mycgr3G68036\_Mycgr3T**
  
Percentage identity: 39 %
  
BlastP bit score: 227
  
Sequence coverage: 98 %
  
E-value: 4e-68
  
  
 NCBI BlastP on this gene

EFY94862

aspartate aminotransferase, putative
  
Accession: EFY94861
  
Location: 120966-122255
  
 NCBI BlastP on this gene

EFY94861

phytanoyl-CoA dioxygenase family protein
  
Accession: EFY94860
  
Location: 118929-120014
  
 NCBI BlastP on this gene

EFY94860

nonribosomal peptide synthase, putative
  
Accession: EFY94859
  
Location: 89548-118063
  
  
**BlastP hit with Mycgr3G90558\_Mycgr3T**
  
Percentage identity: 32 %
  
BlastP bit score: 1658
  
Sequence coverage: 85 %
  
E-value: 0.0
  
  
 NCBI BlastP on this gene

EFY94859

glutamate decarboxylase, putative
  
Accession: EFY94858
  
Location: 86972-88570
  
 NCBI BlastP on this gene

EFY94858

hypothetical protein
  
Accession: EFY94857
  
Location: 85915-86481
  
 NCBI BlastP on this gene

EFY94857

benzoate 4-monooxygenase cytochrome P450
  
Accession: EFY94856
  
Location: 80899-85061
  
 NCBI BlastP on this gene

EFY94856

alkylmercury lyase
  
Accession: EFY94855
  
Location: 79195-80052
  
 NCBI BlastP on this gene

EFY94855

27. :  GL698592 Metarhizium acridum CQMa 102 unplaced genomic scaffold Scf\_123     Total score: 2.0     Cumulative Blast bit score: 1867

MFS transporter, putative
  
Accession: EFY85057
  
Location: 59221-61017
  
 NCBI BlastP on this gene

EFY85057

hypothetical protein
  
Accession: EFY85056
  
Location: 54018-55153
  
  
**BlastP hit with Mycgr3G68036\_Mycgr3T**
  
Percentage identity: 36 %
  
BlastP bit score: 210
  
Sequence coverage: 104 %
  
E-value: 3e-61
  
  
 NCBI BlastP on this gene

EFY85056

aspartate aminotransferase, putative
  
Accession: EFY85055
  
Location: 52531-53820
  
 NCBI BlastP on this gene

EFY85055

phytanoyl-CoA dioxygenase family protein
  
Accession: EFY85054
  
Location: 50538-51622
  
 NCBI BlastP on this gene

EFY85054

nonribosomal peptide synthase, putative
  
Accession: EFY85053
  
Location: 21161-49675
  
  
**BlastP hit with Mycgr3G90558\_Mycgr3T**
  
Percentage identity: 31 %
  
BlastP bit score: 1657
  
Sequence coverage: 85 %
  
E-value: 0.0
  
  
 NCBI BlastP on this gene

EFY85053

glutamate decarboxylase, putative
  
Accession: EFY85052
  
Location: 18619-20208
  
 NCBI BlastP on this gene

EFY85052

thioesterase family protein
  
Accession: EFY85051
  
Location: 17067-17997
  
 NCBI BlastP on this gene

EFY85051

hypothetical protein
  
Accession: EFY85050
  
Location: 12490-12765
  
 NCBI BlastP on this gene

EFY85050

28. :  CM001231 Magnaporthe oryzae 70-15 chromosome 1     Total score: 2.0     Cumulative Blast bit score: 1841

hypothetical protein
  
Accession: EHA56502
  
Location: 1479067-1479762
  
 NCBI BlastP on this gene

EHA56502

hypothetical protein
  
Accession: EHA56503
  
Location: 1481622-1483740
  
 NCBI BlastP on this gene

EHA56503

hypothetical protein
  
Accession: EHA56504
  
Location: 1485342-1485838
  
 NCBI BlastP on this gene

EHA56504

candidapepsin
  
Accession: EHA56505
  
Location: 1487150-1488737
  
 NCBI BlastP on this gene

EHA56505

multidrug resistance protein 3
  
Accession: EHA56506
  
Location: 1491590-1495606
  
 NCBI BlastP on this gene

EHA56506

pyrroline-5-carboxylate reductase
  
Accession: EHA56507
  
Location: 1495633-1496612
  
 NCBI BlastP on this gene

EHA56507

hypothetical protein
  
Accession: EHA56508
  
Location: 1497150-1498350
  
  
**BlastP hit with Mycgr3G68036\_Mycgr3T**
  
Percentage identity: 32 %
  
BlastP bit score: 152
  
Sequence coverage: 94 %
  
E-value: 9e-40
  
  
 NCBI BlastP on this gene

EHA56508

tyrocidine synthetase 1
  
Accession: EHA56509
  
Location: 1499425-1515742
  
  
**BlastP hit with Mycgr3G90558\_Mycgr3T**
  
Percentage identity: 30 %
  
BlastP bit score: 1689
  
Sequence coverage: 97 %
  
E-value: 0.0
  
  
 NCBI BlastP on this gene

EHA56509

hypothetical protein
  
Accession: EHA56510
  
Location: 1517518-1517837
  
 NCBI BlastP on this gene

EHA56510

hypothetical protein
  
Accession: EHA56511
  
Location: 1517915-1518511
  
 NCBI BlastP on this gene

EHA56511

hypothetical protein
  
Accession: EHA56512
  
Location: 1518880-1519141
  
 NCBI BlastP on this gene

EHA56512

hypothetical protein
  
Accession: EHA56513
  
Location: 1532508-1533194
  
 NCBI BlastP on this gene

EHA56513

29. :  CABT02000016 Sordaria macrospora k-hell     Total score: 2.0     Cumulative Blast bit score: 1791

not annotated
  
Accession: CCC11017
  
Location: 15662-16663
  
 NCBI BlastP on this gene

CCC11017

not annotated
  
Accession: CCC11018
  
Location: 19340-20968
  
 NCBI BlastP on this gene

CCC11018

not annotated
  
Accession: CCC11019
  
Location: 24624-26564
  
 NCBI BlastP on this gene

CCC11019

not annotated
  
Accession: CCC11020
  
Location: 27490-29035
  
 NCBI BlastP on this gene

CCC11020

not annotated
  
Accession: CCC11021
  
Location: 30874-47685
  
  
**BlastP hit with Mycgr3G90558\_Mycgr3T**
  
Percentage identity: 29 %
  
BlastP bit score: 1648
  
Sequence coverage: 91 %
  
E-value: 0.0
  
  
 NCBI BlastP on this gene

CCC11021

not annotated
  
Accession: CCC11022
  
Location: 49016-50295
  
  
**BlastP hit with Mycgr3G68036\_Mycgr3T**
  
Percentage identity: 32 %
  
BlastP bit score: 144
  
Sequence coverage: 106 %
  
E-value: 3e-36
  
  
 NCBI BlastP on this gene

CCC11022

not annotated
  
Accession: CCC11023
  
Location: 50873-51959
  
 NCBI BlastP on this gene

CCC11023

not annotated
  
Accession: CCC11024
  
Location: 52779-54014
  
 NCBI BlastP on this gene

CCC11024

not annotated
  
Accession: CCC11025
  
Location: 54692-58972
  
 NCBI BlastP on this gene

CCC11025

not annotated
  
Accession: CCC11026
  
Location: 60491-61160
  
 NCBI BlastP on this gene

CCC11026

not annotated
  
Accession: CCC11027
  
Location: 61716-62505
  
 NCBI BlastP on this gene

CCC11027

not annotated
  
Accession: CCC11028
  
Location: 64060-64365
  
 NCBI BlastP on this gene

CCC11028

not annotated
  
Accession: CCC11029
  
Location: 64974-65644
  
 NCBI BlastP on this gene

CCC11029

not annotated
  
Accession: CCC11030
  
Location: 66779-67385
  
 NCBI BlastP on this gene

CCC11030

30. :  CH445354 Phaeosphaeria nodorum SN15 scaffold\_30     Total score: 2.0     Cumulative Blast bit score: 1777

hypothetical protein
  
Accession: EAT78333
  
Location: 10465-10858
  
 NCBI BlastP on this gene

EAT78333

hypothetical protein
  
Accession: EAT78335
  
Location: 11280-28516
  
  
**BlastP hit with Mycgr3G90558\_Mycgr3T**
  
Percentage identity: 31 %
  
BlastP bit score: 1672
  
Sequence coverage: 87 %
  
E-value: 0.0
  
  
 NCBI BlastP on this gene

EAT78335

hypothetical protein
  
Accession: EAT78336
  
Location: 29936-31095
  
 NCBI BlastP on this gene

EAT78336

hypothetical protein
  
Accession: EAT78337
  
Location: 31876-36120
  
 NCBI BlastP on this gene

EAT78337

hypothetical protein
  
Accession: EAT78338
  
Location: 35569-36229
  
 NCBI BlastP on this gene

EAT78338

hypothetical protein
  
Accession: EAT78339
  
Location: 36363-36696
  
 NCBI BlastP on this gene

EAT78339

hypothetical protein
  
Accession: EAT78340
  
Location: 37127-38285
  
  
**BlastP hit with Mycgr3G35528\_Mycgr3T**
  
Percentage identity: 27 %
  
BlastP bit score: 105
  
Sequence coverage: 100 %
  
E-value: 3e-23
  
  
 NCBI BlastP on this gene

EAT78340

hypothetical protein
  
Accession: EAT78341
  
Location: 38557-39423
  
 NCBI BlastP on this gene

EAT78341

hypothetical protein
  
Accession: EAT78342
  
Location: 40103-41047
  
 NCBI BlastP on this gene

EAT78342

hypothetical protein
  
Accession: EAT78343
  
Location: 42153-43496
  
 NCBI BlastP on this gene

EAT78343

hypothetical protein
  
Accession: EAT78344
  
Location: 44907-45880
  
 NCBI BlastP on this gene

EAT78344

hypothetical protein
  
Accession: EAT78345
  
Location: 46925-47539
  
 NCBI BlastP on this gene

EAT78345

hypothetical protein
  
Accession: EAT78346
  
Location: 47710-48210
  
 NCBI BlastP on this gene

EAT78346

hypothetical protein
  
Accession: EAT78347
  
Location: 48682-50160
  
 NCBI BlastP on this gene

EAT78347

hypothetical protein
  
Accession: EAT78348
  
Location: 50481-51200
  
 NCBI BlastP on this gene

EAT78348

31. :  KB708022 Botryotinia fuckeliana BcDW1 unplaced genomic scaffold Scaffold\_350     Total score: 2.0     Cumulative Blast bit score: 1725

putative prostacyclin synthase protein
  
Accession: EMR82970
  
Location: 69087-70421
  
 NCBI BlastP on this gene

EMR82970

putative polyketide synthase protein
  
Accession: EMR82969
  
Location: 57703-65560
  
 NCBI BlastP on this gene

EMR82969

putative nonribosomal peptide protein
  
Accession: EMR82968
  
Location: 41327-54610
  
  
**BlastP hit with Mycgr3G90558\_Mycgr3T**
  
Percentage identity: 29 %
  
BlastP bit score: 1402
  
Sequence coverage: 75 %
  
E-value: 0.0
  
  
 NCBI BlastP on this gene

EMR82968

putative mfs multidrug protein
  
Accession: EMR82967
  
Location: 34997-36748
  
  
**BlastP hit with Mycgr3G84494\_Mycgr3T**
  
Percentage identity: 36 %
  
BlastP bit score: 323
  
Sequence coverage: 98 %
  
E-value: 1e-99
  
  
 NCBI BlastP on this gene

EMR82967

putative taurine catabolism dioxygenase protein
  
Accession: EMR82966
  
Location: 33199-34636
  
 NCBI BlastP on this gene

EMR82966

putative indoleamine -dioxygenase family protein
  
Accession: EMR82965
  
Location: 27803-29499
  
 NCBI BlastP on this gene

EMR82965

hypothetical protein
  
Accession: EMR82964
  
Location: 24657-26105
  
 NCBI BlastP on this gene

EMR82964

putative monodehydroascorbate reductase protein
  
Accession: EMR82963
  
Location: 22282-24111
  
 NCBI BlastP on this gene

EMR82963

putative arginase family protein
  
Accession: EMR82962
  
Location: 20159-21558
  
 NCBI BlastP on this gene

EMR82962

putative calpain family cysteine protease protein
  
Accession: EMR82961
  
Location: 15875-19278
  
 NCBI BlastP on this gene

EMR82961

32. :  FQ790270 Botryotinia fuckeliana T4 SuperContig\_51\_1 genomic supercontig.     Total score: 2.0     Cumulative Blast bit score: 1725

similar to cytochrome P450
  
Accession: CCD44404
  
Location: 686014-687650
  
 NCBI BlastP on this gene

BofuT4\_P059840.1

BcPKS11, polyketide synthase
  
Accession: CCD44403
  
Location: 676697-684554
  
 NCBI BlastP on this gene

BofuT4\_P059830.1

BcNRPS9, nonribosomal peptide synthetase
  
Accession: CCD44402
  
Location: 655974-669254
  
  
**BlastP hit with Mycgr3G90558\_Mycgr3T**
  
Percentage identity: 29 %
  
BlastP bit score: 1404
  
Sequence coverage: 75 %
  
E-value: 0.0
  
  
 NCBI BlastP on this gene

BofuT4\_P059820.1

hypothetical protein
  
Accession: CCD44401
  
Location: 654409-655008
  
 NCBI BlastP on this gene

BofuT4\_P059810.1

hypothetical protein
  
Accession: CCD44400
  
Location: 652819-654265
  
 NCBI BlastP on this gene

BofuT4\_P059800.1

similar to MFS multidrug transporter
  
Accession: CCD44399
  
Location: 650095-651846
  
  
**BlastP hit with Mycgr3G84494\_Mycgr3T**
  
Percentage identity: 36 %
  
BlastP bit score: 321
  
Sequence coverage: 98 %
  
E-value: 5e-99
  
  
 NCBI BlastP on this gene

BofuT4\_P059790.1

similar to tfdA family taurine dioxygenase
  
Accession: CCD44398
  
Location: 648297-649734
  
 NCBI BlastP on this gene

BofuT4\_P059780.1

predicted protein
  
Accession: CCD44397
  
Location: 647201-647649
  
 NCBI BlastP on this gene

BofuT4\_uP059770.1

predicted protein
  
Accession: CCD44396
  
Location: 646896-647150
  
 NCBI BlastP on this gene

BofuT4\_uP059760.1

hypothetical protein
  
Accession: CCD44395
  
Location: 645196-645780
  
 NCBI BlastP on this gene

BofuT4\_P059750.1

similar to indoleamine 2,3-dioxygenase family protein
  
Accession: CCD44394
  
Location: 642934-644630
  
 NCBI BlastP on this gene

BofuT4\_P059740.1

hypothetical protein
  
Accession: CCD44393
  
Location: 639789-641276
  
 NCBI BlastP on this gene

BofuT4\_P059730.1

similar to apoptosis-inducing factor 3
  
Accession: CCD44392
  
Location: 637406-639235
  
 NCBI BlastP on this gene

BofuT4\_P059720.1

predicted protein
  
Accession: CCD44391
  
Location: 636886-637089
  
 NCBI BlastP on this gene

BofuT4\_uP059710.1

hypothetical protein
  
Accession: CCD44390
  
Location: 635277-636676
  
 NCBI BlastP on this gene

BofuT4\_P059700.1

similar to calpain
  
Accession: CCD44389
  
Location: 630993-634396
  
 NCBI BlastP on this gene

BofuT4\_P059690.1

33. :  KB705649 Eutypa lata UCREL1 unplaced genomic scaffold EL1\_03\_scaffold\_311     Total score: 2.0     Cumulative Blast bit score: 1680

putative fungal specific transcription factor protein
  
Accession: EMR71344
  
Location: 74987-76980
  
 NCBI BlastP on this gene

EMR71344

putative alanyl-trna synthetase protein
  
Accession: EMR71361
  
Location: 71768-72680
  
 NCBI BlastP on this gene

EMR71361

putative signal transduction protein
  
Accession: EMR71343
  
Location: 68736-69999
  
  
**BlastP hit with Mycgr3G35528\_Mycgr3T**
  
Percentage identity: 28 %
  
BlastP bit score: 86
  
Sequence coverage: 87 %
  
E-value: 2e-16
  
  
 NCBI BlastP on this gene

EMR71343

putative mfs toxin efflux pump protein
  
Accession: EMR71349
  
Location: 65751-67745
  
 NCBI BlastP on this gene

EMR71349

putative duf323 domain-containing protein
  
Accession: EMR71335
  
Location: 62623-63886
  
 NCBI BlastP on this gene

EMR71335

putative duf323 domain-containing protein
  
Accession: EMR71366
  
Location: 59290-60476
  
 NCBI BlastP on this gene

EMR71366

hypothetical protein
  
Accession: EMR71346
  
Location: 30714-57959
  
  
**BlastP hit with Mycgr3G90558\_Mycgr3T**
  
Percentage identity: 33 %
  
BlastP bit score: 1595
  
Sequence coverage: 68 %
  
E-value: 0.0
  
  
 NCBI BlastP on this gene

EMR71346

hypothetical protein
  
Accession: EMR71345
  
Location: 28312-28929
  
 NCBI BlastP on this gene

EMR71345

putative acyltransferase protein
  
Accession: EMR71327
  
Location: 26121-27812
  
 NCBI BlastP on this gene

EMR71327

34. :  AAHF01000003 Aspergillus fumigatus Af293     Total score: 2.0     Cumulative Blast bit score: 1649

dihydroxyacetone kinase (DakA), putative
  
Accession: EAL91346
  
Location: 646964-648980
  
 NCBI BlastP on this gene

EAL91346

conserved hypothetical protein
  
Accession: EAL91345
  
Location: 645131-645637
  
 NCBI BlastP on this gene

EAL91345

TPR domain protein
  
Accession: EAL91344
  
Location: 641247-643594
  
 NCBI BlastP on this gene

EAL91344

ABC multidrug transporter, putative
  
Accession: EAL91343
  
Location: 635720-640633
  
 NCBI BlastP on this gene

EAL91343

nonribosomal peptide synthase, putative
  
Accession: EAL91342
  
Location: 608358-633905
  
  
**BlastP hit with Mycgr3G90558\_Mycgr3T**
  
Percentage identity: 29 %
  
BlastP bit score: 1428
  
Sequence coverage: 87 %
  
E-value: 0.0
  
  
 NCBI BlastP on this gene

EAL91342

MFS multidrug transporter, putative
  
Accession: EAL91341
  
Location: 605650-607205
  
  
**BlastP hit with Mycgr3G23761\_Mycgr3T**
  
Percentage identity: 37 %
  
BlastP bit score: 221
  
Sequence coverage: 99 %
  
E-value: 9e-63
  
  
 NCBI BlastP on this gene

EAL91341

hypothetical protein
  
Accession: EAL91340
  
Location: 602510-604110
  
 NCBI BlastP on this gene

EAL91340

CCCH zinc finger DNA binding protein
  
Accession: EAL91339
  
Location: 600006-601542
  
 NCBI BlastP on this gene

EAL91339

metallo-beta-lactamase family protein
  
Accession: EAL91338
  
Location: 598303-599331
  
 NCBI BlastP on this gene

EAL91338

Kelch repeat protein
  
Accession: EAL91337
  
Location: 595313-596367
  
 NCBI BlastP on this gene

EAL91337

mitochondrial 3-hydroxyisobutyryl-CoA hydrolase, putative
  
Accession: EAL91336
  
Location: 593275-595128
  
 NCBI BlastP on this gene

EAL91336

50S ribosomal protein L4
  
Accession: EAL91335
  
Location: 592287-593045
  
 NCBI BlastP on this gene

EAL91335

35. :  DS499597 Aspergillus fumigatus A1163 scf\_000004 genomic scaffold     Total score: 2.0     Cumulative Blast bit score: 1610

TPR domain protein
  
Accession: EDP52007
  
Location: 3208511-3210858
  
 NCBI BlastP on this gene

EDP52007

hypothetical protein
  
Accession: EDP52008
  
Location: 3211822-3212947
  
 NCBI BlastP on this gene

EDP52008

ABC multidrug transporter, putative
  
Accession: EDP52009
  
Location: 3213295-3215291
  
 NCBI BlastP on this gene

EDP52009

nonribosomal peptide synthase, putative
  
Accession: EDP52010
  
Location: 3224060-3249608
  
  
**BlastP hit with Mycgr3G90558\_Mycgr3T**
  
Percentage identity: 29 %
  
BlastP bit score: 1390
  
Sequence coverage: 86 %
  
E-value: 0.0
  
  
 NCBI BlastP on this gene

EDP52010

MFS multidrug transporter, putative
  
Accession: EDP52011
  
Location: 3250761-3252316
  
  
**BlastP hit with Mycgr3G23761\_Mycgr3T**
  
Percentage identity: 36 %
  
BlastP bit score: 220
  
Sequence coverage: 99 %
  
E-value: 3e-62
  
  
 NCBI BlastP on this gene

EDP52011

hypothetical protein
  
Accession: EDP52012
  
Location: 3253856-3255456
  
 NCBI BlastP on this gene

EDP52012

CCCH zinc finger DNA binding protein
  
Accession: EDP52013
  
Location: 3256424-3257960
  
 NCBI BlastP on this gene

EDP52013

metallo-beta-lactamase family protein
  
Accession: EDP52014
  
Location: 3258635-3259663
  
 NCBI BlastP on this gene

EDP52014

Kelch repeat protein
  
Accession: EDP52015
  
Location: 3261597-3262651
  
 NCBI BlastP on this gene

EDP52015

mitochondrial 3-hydroxyisobutyryl-CoA hydrolase, putative
  
Accession: EDP52016
  
Location: 3262836-3264689
  
 NCBI BlastP on this gene

EDP52016

50S ribosomal protein L4
  
Accession: EDP52017
  
Location: 3264919-3265677
  
 NCBI BlastP on this gene

EDP52017

36. :  ACJE01000008 Aspergillus niger ATCC 1015     Total score: 2.0     Cumulative Blast bit score: 1440

hypothetical protein
  
Accession: EHA24934
  
Location: 1793033-1794270
  
 NCBI BlastP on this gene

EHA24934

hypothetical protein
  
Accession: EHA24935
  
Location: 1795436-1796461
  
 NCBI BlastP on this gene

EHA24935

hypothetical protein
  
Accession: EHA24936
  
Location: 1796858-1798643
  
 NCBI BlastP on this gene

EHA24936

hypothetical protein
  
Accession: EHA24937
  
Location: 1800474-1801133
  
 NCBI BlastP on this gene

EHA24937

hypothetical protein
  
Accession: EHA24938
  
Location: 1803005-1816475
  
  
**BlastP hit with Mycgr3G90558\_Mycgr3T**
  
Percentage identity: 30 %
  
BlastP bit score: 1283
  
Sequence coverage: 70 %
  
E-value: 0.0
  
  
 NCBI BlastP on this gene

EHA24938

hypothetical protein
  
Accession: EHA24939
  
Location: 1824432-1825889
  
 NCBI BlastP on this gene

EHA24939

hypothetical protein
  
Accession: EHA24940
  
Location: 1827516-1828076
  
 NCBI BlastP on this gene

EHA24940

hypothetical protein
  
Accession: EHA24941
  
Location: 1828436-1830408
  
 NCBI BlastP on this gene

EHA24941

hypothetical protein
  
Accession: EHA24942
  
Location: 1830622-1832126
  
 NCBI BlastP on this gene

EHA24942

hypothetical protein
  
Accession: EHA24943
  
Location: 1832910-1834405
  
 NCBI BlastP on this gene

EHA24943

hypothetical protein
  
Accession: EHA24944
  
Location: 1834991-1836596
  
  
**BlastP hit with Mycgr3G23761\_Mycgr3T**
  
Percentage identity: 28 %
  
BlastP bit score: 157
  
Sequence coverage: 98 %
  
E-value: 8e-40
  
  
 NCBI BlastP on this gene

EHA24944

hypothetical protein
  
Accession: EHA24945
  
Location: 1837742-1838215
  
 NCBI BlastP on this gene

EHA24945

hypothetical protein
  
Accession: EHA24946
  
Location: 1838985-1839854
  
 NCBI BlastP on this gene

EHA24946

hypothetical protein
  
Accession: EHA24947
  
Location: 1842830-1844045
  
 NCBI BlastP on this gene

EHA24947

37. :  EQ963479 Aspergillus flavus NRRL3357 scf\_1106286418500 genomic scaffold     Total score: 2.0     Cumulative Blast bit score: 1418

conserved hypothetical protein
  
Accession: EED49599
  
Location: 11878-13233
  
 NCBI BlastP on this gene

EED49599

hypothetical protein
  
Accession: EED49600
  
Location: 17276-19009
  
 NCBI BlastP on this gene

EED49600

conserved hypothetical protein
  
Accession: EED49601
  
Location: 19380-24147
  
 NCBI BlastP on this gene

EED49601

hypothetical protein
  
Accession: EED49602
  
Location: 25295-25750
  
 NCBI BlastP on this gene

EED49602

nonribosomal peptide synthase, putative
  
Accession: EED49603
  
Location: 29095-35391
  
  
**BlastP hit with Mycgr3G90558\_Mycgr3T**
  
Percentage identity: 33 %
  
BlastP bit score: 960
  
Sequence coverage: 44 %
  
E-value: 0.0
  
  
 NCBI BlastP on this gene

EED49603

phenol 2-monooxygenase, putative
  
Accession: EED49604
  
Location: 36311-37102
  
 NCBI BlastP on this gene

EED49604

oligopeptide transporter, putative
  
Accession: EED49605
  
Location: 38582-40510
  
 NCBI BlastP on this gene

EED49605

gibberellin 2-oxidase, putative
  
Accession: EED49606
  
Location: 41154-42314
  
  
**BlastP hit with Mycgr3G68036\_Mycgr3T**
  
Percentage identity: 40 %
  
BlastP bit score: 284
  
Sequence coverage: 102 %
  
E-value: 8e-90
  
  
 NCBI BlastP on this gene

EED49606

N-methyltransferase, putative
  
Accession: EED49607
  
Location: 42851-44016
  
 NCBI BlastP on this gene

EED49607

O-methyltransferase, putative
  
Accession: EED49608
  
Location: 44443-45186
  
 NCBI BlastP on this gene

EED49608

fructosyl amino acid oxidase, putative
  
Accession: EED49609
  
Location: 45453-46964
  
 NCBI BlastP on this gene

EED49609

hypothetical protein
  
Accession: EED49610
  
Location: 47408-47725
  
 NCBI BlastP on this gene

EED49610

oligopeptide transporter, putative
  
Accession: EED49611
  
Location: 47758-50991
  
  
**BlastP hit with Mycgr3G68036\_Mycgr3T**
  
Percentage identity: 43 %
  
BlastP bit score: 174
  
Sequence coverage: 60 %
  
E-value: 3e-46
  
  
 NCBI BlastP on this gene

EED49611

conserved hypothetical protein
  
Accession: EED49612
  
Location: 51418-53073
  
 NCBI BlastP on this gene

EED49612

conserved hypothetical protein
  
Accession: EED49613
  
Location: 55171-55971
  
 NCBI BlastP on this gene

EED49613

MFS transporter, putative
  
Accession: EED49614
  
Location: 57672-59455
  
 NCBI BlastP on this gene

EED49614

ABC multidrug transporter, putative
  
Accession: EED49615
  
Location: 60297-64898
  
 NCBI BlastP on this gene

EED49615

C6 transcription factor, putative
  
Accession: EED49616
  
Location: 65641-68076
  
 NCBI BlastP on this gene

EED49616

38. :  ABDF02000003 Trichoderma virens Gv29-8     Total score: 2.0     Cumulative Blast bit score: 1409

hypothetical protein
  
Accession: EHK25877
  
Location: 2508447-2510463
  
 NCBI BlastP on this gene

EHK25877

hypothetical protein
  
Accession: EHK25878
  
Location: 2513376-2514238
  
 NCBI BlastP on this gene

EHK25878

hypothetical protein
  
Accession: EHK25879
  
Location: 2514950-2516570
  
 NCBI BlastP on this gene

EHK25879

hypothetical protein
  
Accession: EHK25880
  
Location: 2517486-2518427
  
 NCBI BlastP on this gene

EHK25880

hypothetical protein
  
Accession: EHK25881
  
Location: 2519235-2520380
  
  
**BlastP hit with Mycgr3G68036\_Mycgr3T**
  
Percentage identity: 44 %
  
BlastP bit score: 264
  
Sequence coverage: 96 %
  
E-value: 2e-82
  
  
 NCBI BlastP on this gene

EHK25881

hypothetical protein
  
Accession: EHK25882
  
Location: 2520859-2521212
  
 NCBI BlastP on this gene

EHK25882

hypothetical protein
  
Accession: EHK25883
  
Location: 2523390-2525529
  
 NCBI BlastP on this gene

EHK25883

hypothetical protein
  
Accession: EHK25884
  
Location: 2526677-2528013
  
 NCBI BlastP on this gene

EHK25884

non-ribosomal peptide synthetase
  
Accession: EHK25885
  
Location: 2529796-2553890
  
  
**BlastP hit with Mycgr3G90558\_Mycgr3T**
  
Percentage identity: 29 %
  
BlastP bit score: 1145
  
Sequence coverage: 71 %
  
E-value: 0.0
  
  
 NCBI BlastP on this gene

EHK25885

hypothetical protein
  
Accession: EHK25886
  
Location: 2555537-2559929
  
 NCBI BlastP on this gene

EHK25886

hypothetical protein
  
Accession: EHK25887
  
Location: 2564033-2565609
  
 NCBI BlastP on this gene

EHK25887

39. :  GL534459 Pyrenophora teres f. teres 0-1 unplaced genomic scaffold scaffold\_190719     Total score: 2.0     Cumulative Blast bit score: 1362

hypothetical protein
  
Accession: EFQ92068
  
Location: 9593-11135
  
 NCBI BlastP on this gene

EFQ92068

hypothetical protein
  
Accession: EFQ92069
  
Location: 11882-12418
  
 NCBI BlastP on this gene

EFQ92069

hypothetical protein
  
Accession: EFQ92070
  
Location: 15120-15701
  
 NCBI BlastP on this gene

EFQ92070

hypothetical protein
  
Accession: EFQ92071
  
Location: 17941-19050
  
 NCBI BlastP on this gene

EFQ92071

hypothetical protein
  
Accession: EFQ92072
  
Location: 19293-19652
  
 NCBI BlastP on this gene

EFQ92072

hypothetical protein
  
Accession: EFQ92073
  
Location: 19943-41977
  
  
**BlastP hit with Mycgr3G90558\_Mycgr3T**
  
Percentage identity: 32 %
  
BlastP bit score: 1177
  
Sequence coverage: 60 %
  
E-value: 0.0
  
  
 NCBI BlastP on this gene

EFQ92073

hypothetical protein
  
Accession: EFQ92074
  
Location: 46095-51051
  
 NCBI BlastP on this gene

EFQ92074

hypothetical protein
  
Accession: EFQ92075
  
Location: 51524-53825
  
 NCBI BlastP on this gene

EFQ92075

hypothetical protein
  
Accession: EFQ92076
  
Location: 54862-56502
  
  
**BlastP hit with Mycgr3G23761\_Mycgr3T**
  
Percentage identity: 29 %
  
BlastP bit score: 185
  
Sequence coverage: 99 %
  
E-value: 6e-49
  
  
 NCBI BlastP on this gene

EFQ92076

hypothetical protein
  
Accession: EFQ92077
  
Location: 56907-60731
  
 NCBI BlastP on this gene

EFQ92077

hypothetical protein
  
Accession: EFQ92078
  
Location: 61317-63578
  
 NCBI BlastP on this gene

EFQ92078

hypothetical protein
  
Accession: EFQ92079
  
Location: 64286-64612
  
 NCBI BlastP on this gene

EFQ92079

hypothetical protein
  
Accession: EFQ92080
  
Location: 65538-66650
  
 NCBI BlastP on this gene

EFQ92080

40. :  DS027058 Aspergillus clavatus NRRL 1 1099423829804 genomic scaffold     Total score: 2.0     Cumulative Blast bit score: 1344

polyketide synthase, putative
  
Accession: EAW08895
  
Location: 895872-903983
  
 NCBI BlastP on this gene

EAW08895

DUF341 domain protein
  
Accession: EAW08896
  
Location: 905354-906239
  
 NCBI BlastP on this gene

EAW08896

hypothetical protein
  
Accession: EAW08897
  
Location: 908623-909746
  
 NCBI BlastP on this gene

EAW08897

MFS transporter, putative
  
Accession: EAW08898
  
Location: 910122-911892
  
  
**BlastP hit with Mycgr3G23761\_Mycgr3T**
  
Percentage identity: 29 %
  
BlastP bit score: 177
  
Sequence coverage: 103 %
  
E-value: 4e-46
  
  
 NCBI BlastP on this gene

EAW08898

ABC transporter, putative
  
Accession: EAW08899
  
Location: 912958-918070
  
 NCBI BlastP on this gene

EAW08899

nonribosomal peptide synthase, putative
  
Accession: EAW08900
  
Location: 919134-944153
  
  
**BlastP hit with Mycgr3G90558\_Mycgr3T**
  
Percentage identity: 31 %
  
BlastP bit score: 1167
  
Sequence coverage: 58 %
  
E-value: 0.0
  
  
 NCBI BlastP on this gene

EAW08900

SRF-type transcription factor (Umc1), putative
  
Accession: EAW08901
  
Location: 946418-947301
  
 NCBI BlastP on this gene

EAW08901

DUF803 domain protein
  
Accession: EAW08902
  
Location: 948473-950445
  
 NCBI BlastP on this gene

EAW08902

ATP synthase subunit E, putative
  
Accession: EAW08903
  
Location: 951055-951877
  
 NCBI BlastP on this gene

EAW08903

hypothetical protein
  
Accession: EAW08904
  
Location: 952346-954740
  
 NCBI BlastP on this gene

EAW08904

41. :  KE145353 Glarea lozoyensis ATCC 20868 chromosome Unknown GLAREA10     Total score: 2.0     Cumulative Blast bit score: 1329

hypothetical protein
  
Accession: EPE35678
  
Location: 54190-56357
  
 NCBI BlastP on this gene

EPE35678

Flavoprotein
  
Accession: EPE35677
  
Location: 52299-53210
  
 NCBI BlastP on this gene

EPE35677

hypothetical protein
  
Accession: EPE35676
  
Location: 49479-51359
  
  
**BlastP hit with Mycgr3G36335\_Mycgr3T**
  
Percentage identity: 38 %
  
BlastP bit score: 81
  
Sequence coverage: 88 %
  
E-value: 3e-15
  
  
 NCBI BlastP on this gene

EPE35676

hypothetical protein
  
Accession: EPE35675
  
Location: 48184-49107
  
 NCBI BlastP on this gene

EPE35675

hypothetical protein
  
Accession: EPE35674
  
Location: 46413-47523
  
 NCBI BlastP on this gene

EPE35674

P-loop containing nucleoside triphosphate hydrolase
  
Accession: EPE35673
  
Location: 34754-40016
  
 NCBI BlastP on this gene

EPE35673

Acetyl-CoA synthetase-like protein
  
Accession: EPE35672
  
Location: 8439-32264
  
  
**BlastP hit with Mycgr3G90558\_Mycgr3T**
  
Percentage identity: 32 %
  
BlastP bit score: 1248
  
Sequence coverage: 60 %
  
E-value: 0.0
  
  
 NCBI BlastP on this gene

EPE35672

MFS general substrate transporter
  
Accession: EPE35671
  
Location: 6656-8300
  
 NCBI BlastP on this gene

EPE35671

PA
  
Accession: EPE35670
  
Location: 1567-5933
  
 NCBI BlastP on this gene

EPE35670

42. :  FP929139 Leptosphaeria maculans JN3 lm\_SuperContig\_0\_v2 genomic supercontig     Total score: 2.0     Cumulative Blast bit score: 1327

similar to C4-dicarboxylate transporter/malic acid transport protein
  
Accession: CBY02109
  
Location: 3092351-3093610
  
 NCBI BlastP on this gene

LEMA\_P008960.1

predicted protein
  
Accession: CBY02110
  
Location: 3094169-3094447
  
 NCBI BlastP on this gene

LEMA\_uP008970.1

hypothetical protein
  
Accession: CBY02111
  
Location: 3094894-3099025
  
 NCBI BlastP on this gene

LEMA\_P008980.1

similar to MFS transporter
  
Accession: CBY02112
  
Location: 3099159-3100841
  
  
**BlastP hit with Mycgr3G23761\_Mycgr3T**
  
Percentage identity: 29 %
  
BlastP bit score: 182
  
Sequence coverage: 101 %
  
E-value: 1e-47
  
  
 NCBI BlastP on this gene

LEMA\_P008990.1

predicted protein
  
Accession: CBY02113
  
Location: 3102156-3103294
  
 NCBI BlastP on this gene

LEMA\_P009000.1

predicted protein
  
Accession: CBY02114
  
Location: 3104087-3105211
  
 NCBI BlastP on this gene

LEMA\_P009010.1

similar to ABC multidrug transporter
  
Accession: CBY02115
  
Location: 3106079-3111642
  
 NCBI BlastP on this gene

LEMA\_P009020.1

predicted protein
  
Accession: CBY02116
  
Location: 3113323-3114354
  
 NCBI BlastP on this gene

LEMA\_P009030.1

similar to nonribosomal peptide synthase
  
Accession: CBY02117
  
Location: 3115242-3137649
  
  
**BlastP hit with Mycgr3G90558\_Mycgr3T**
  
Percentage identity: 30 %
  
BlastP bit score: 1145
  
Sequence coverage: 60 %
  
E-value: 0.0
  
  
 NCBI BlastP on this gene

LEMA\_P009040.1

similar to rhamnogalacturonate lyase
  
Accession: CBY02118
  
Location: 3138626-3140467
  
 NCBI BlastP on this gene

LEMA\_P009050.1

predicted protein
  
Accession: CBY02119
  
Location: 3140576-3140854
  
 NCBI BlastP on this gene

LEMA\_uP009060.1

43. :  CH445358 Phaeosphaeria nodorum SN15 scaffold\_34     Total score: 2.0     Cumulative Blast bit score: 1313

hypothetical protein
  
Accession: EAT77680
  
Location: 43029-44369
  
 NCBI BlastP on this gene

EAT77680

hypothetical protein
  
Accession: EAT77682
  
Location: 44769-46857
  
 NCBI BlastP on this gene

EAT77682

hypothetical protein
  
Accession: EAT77683
  
Location: 47598-49195
  
 NCBI BlastP on this gene

EAT77683

hypothetical protein
  
Accession: EAT77684
  
Location: 49639-50109
  
 NCBI BlastP on this gene

EAT77684

hypothetical protein
  
Accession: EAT77686
  
Location: 50136-72414
  
  
**BlastP hit with Mycgr3G90558\_Mycgr3T**
  
Percentage identity: 31 %
  
BlastP bit score: 1145
  
Sequence coverage: 60 %
  
E-value: 0.0
  
  
 NCBI BlastP on this gene

EAT77686

hypothetical protein
  
Accession: EAT77687
  
Location: 73844-74615
  
 NCBI BlastP on this gene

EAT77687

hypothetical protein
  
Accession: EAT77688
  
Location: 76243-81672
  
 NCBI BlastP on this gene

EAT77688

hypothetical protein
  
Accession: EAT77689
  
Location: 82012-83425
  
 NCBI BlastP on this gene

EAT77689

hypothetical protein
  
Accession: EAT77690
  
Location: 83764-84099
  
 NCBI BlastP on this gene

EAT77690

hypothetical protein
  
Accession: EAT77691
  
Location: 84803-85585
  
 NCBI BlastP on this gene

EAT77691

hypothetical protein
  
Accession: EAT77693
  
Location: 86818-92717
  
  
**BlastP hit with Mycgr3G23761\_Mycgr3T**
  
Percentage identity: 29 %
  
BlastP bit score: 168
  
Sequence coverage: 98 %
  
E-value: 2e-41
  
  
 NCBI BlastP on this gene

EAT77693

hypothetical protein
  
Accession: EAT77694
  
Location: 93377-95285
  
 NCBI BlastP on this gene

EAT77694

44. :  KB908703 Setosphaeria turcica Et28A unplaced genomic scaffold SETTUscaffold\_3     Total score: 2.0     Cumulative Blast bit score: 1305

hypothetical protein
  
Accession: EOA84587
  
Location: 391594-393219
  
  
**BlastP hit with Mycgr3G23761\_Mycgr3T**
  
Percentage identity: 28 %
  
BlastP bit score: 185
  
Sequence coverage: 102 %
  
E-value: 6e-49
  
  
 NCBI BlastP on this gene

EOA84587

hypothetical protein
  
Accession: EOA84588
  
Location: 393670-397407
  
 NCBI BlastP on this gene

EOA84588

hypothetical protein
  
Accession: EOA84589
  
Location: 405042-406021
  
 NCBI BlastP on this gene

EOA84589

hypothetical protein
  
Accession: EOA84590
  
Location: 406616-411618
  
 NCBI BlastP on this gene

EOA84590

hypothetical protein
  
Accession: EOA84591
  
Location: 415602-437804
  
  
**BlastP hit with Mycgr3G90558\_Mycgr3T**
  
Percentage identity: 30 %
  
BlastP bit score: 1120
  
Sequence coverage: 61 %
  
E-value: 0.0
  
  
 NCBI BlastP on this gene

EOA84591

hypothetical protein
  
Accession: EOA84592
  
Location: 440079-441191
  
 NCBI BlastP on this gene

EOA84592

45. :  KB733474 Bipolaris maydis ATCC 48331 unplaced genomic scaffold COCC4scaffold\_31     Total score: 2.0     Cumulative Blast bit score: 1305

hypothetical protein
  
Accession: ENI00469
  
Location: 91305-92635
  
 NCBI BlastP on this gene

ENI00469

hypothetical protein
  
Accession: ENI00470
  
Location: 93277-94051
  
 NCBI BlastP on this gene

ENI00470

hypothetical protein
  
Accession: ENI00471
  
Location: 95703-96926
  
 NCBI BlastP on this gene

ENI00471

hypothetical protein
  
Accession: ENI00472
  
Location: 99522-101176
  
  
**BlastP hit with Mycgr3G23761\_Mycgr3T**
  
Percentage identity: 29 %
  
BlastP bit score: 187
  
Sequence coverage: 99 %
  
E-value: 1e-49
  
  
 NCBI BlastP on this gene

ENI00472

hypothetical protein
  
Accession: ENI00473
  
Location: 101574-105227
  
 NCBI BlastP on this gene

ENI00473

hypothetical protein
  
Accession: ENI00474
  
Location: 108230-113263
  
 NCBI BlastP on this gene

ENI00474

hypothetical protein
  
Accession: ENI00475
  
Location: 115603-116224
  
 NCBI BlastP on this gene

ENI00475

hypothetical protein
  
Accession: ENI00476
  
Location: 116583-116926
  
 NCBI BlastP on this gene

ENI00476

hypothetical protein
  
Accession: ENI00477
  
Location: 117252-139356
  
  
**BlastP hit with Mycgr3G90558\_Mycgr3T**
  
Percentage identity: 30 %
  
BlastP bit score: 1118
  
Sequence coverage: 60 %
  
E-value: 0.0
  
  
 NCBI BlastP on this gene

ENI00477

hypothetical protein
  
Accession: ENI00478
  
Location: 139861-140364
  
 NCBI BlastP on this gene

ENI00478

hypothetical protein
  
Accession: ENI00479
  
Location: 140945-142566
  
 NCBI BlastP on this gene

ENI00479

hypothetical protein
  
Accession: ENI00480
  
Location: 143102-143839
  
 NCBI BlastP on this gene

ENI00480

hypothetical protein
  
Accession: ENI00481
  
Location: 144594-145646
  
 NCBI BlastP on this gene

ENI00481

hypothetical protein
  
Accession: ENI00482
  
Location: 146544-147726
  
 NCBI BlastP on this gene

ENI00482

46. :  KB445640 Cochliobolus sativus ND90Pr unplaced genomic scaffold COCSAscaffold\_4     Total score: 2.0     Cumulative Blast bit score: 1301

hypothetical protein
  
Accession: EMD66843
  
Location: 2236054-2237387
  
 NCBI BlastP on this gene

EMD66843

hypothetical protein
  
Accession: EMD66842
  
Location: 2229995-2231649
  
  
**BlastP hit with Mycgr3G23761\_Mycgr3T**
  
Percentage identity: 28 %
  
BlastP bit score: 182
  
Sequence coverage: 101 %
  
E-value: 7e-48
  
  
 NCBI BlastP on this gene

EMD66842

hypothetical protein
  
Accession: EMD66841
  
Location: 2225944-2229621
  
 NCBI BlastP on this gene

EMD66841

hypothetical protein
  
Accession: EMD66840
  
Location: 2217996-2223025
  
 NCBI BlastP on this gene

EMD66840

hypothetical protein
  
Accession: EMD66839
  
Location: 2191843-2213946
  
  
**BlastP hit with Mycgr3G90558\_Mycgr3T**
  
Percentage identity: 31 %
  
BlastP bit score: 1119
  
Sequence coverage: 60 %
  
E-value: 0.0
  
  
 NCBI BlastP on this gene

EMD66839

hypothetical protein
  
Accession: EMD66838
  
Location: 2190492-2191338
  
 NCBI BlastP on this gene

EMD66838

hypothetical protein
  
Accession: EMD66837
  
Location: 2188294-2189924
  
 NCBI BlastP on this gene

EMD66837

hypothetical protein
  
Accession: EMD66836
  
Location: 2187022-2187765
  
 NCBI BlastP on this gene

EMD66836

hypothetical protein
  
Accession: EMD66835
  
Location: 2185539-2186603
  
 NCBI BlastP on this gene

EMD66835

hypothetical protein
  
Accession: EMD66834
  
Location: 2183986-2184573
  
 NCBI BlastP on this gene

EMD66834

47. :  KB445571 Cochliobolus heterostrophus C5 unplaced genomic scaffold COCHEscaffold\_3     Total score: 2.0     Cumulative Blast bit score: 1299

hypothetical protein
  
Accession: EMD95322
  
Location: 2248054-2249384
  
 NCBI BlastP on this gene

EMD95322

hypothetical protein
  
Accession: EMD95323
  
Location: 2249955-2250800
  
 NCBI BlastP on this gene

EMD95323

hypothetical protein
  
Accession: EMD95324
  
Location: 2252452-2253675
  
 NCBI BlastP on this gene

EMD95324

hypothetical protein
  
Accession: EMD95325
  
Location: 2256271-2257925
  
  
**BlastP hit with Mycgr3G23761\_Mycgr3T**
  
Percentage identity: 29 %
  
BlastP bit score: 187
  
Sequence coverage: 99 %
  
E-value: 1e-49
  
  
 NCBI BlastP on this gene

EMD95325

hypothetical protein
  
Accession: EMD95326
  
Location: 2258323-2261976
  
 NCBI BlastP on this gene

EMD95326

hypothetical protein
  
Accession: EMD95327
  
Location: 2264979-2270012
  
 NCBI BlastP on this gene

EMD95327

hypothetical protein
  
Accession: EMD95328
  
Location: 2272352-2272973
  
 NCBI BlastP on this gene

EMD95328

hypothetical protein
  
Accession: EMD95329
  
Location: 2273332-2273675
  
 NCBI BlastP on this gene

EMD95329

hypothetical protein
  
Accession: EMD95330
  
Location: 2274001-2296105
  
  
**BlastP hit with Mycgr3G90558\_Mycgr3T**
  
Percentage identity: 30 %
  
BlastP bit score: 1112
  
Sequence coverage: 60 %
  
E-value: 0.0
  
  
 NCBI BlastP on this gene

EMD95330

hypothetical protein
  
Accession: EMD95331
  
Location: 2296610-2297689
  
 NCBI BlastP on this gene

EMD95331

hypothetical protein
  
Accession: EMD95332
  
Location: 2298270-2299891
  
 NCBI BlastP on this gene

EMD95332

hypothetical protein
  
Accession: EMD95333
  
Location: 2300427-2300960
  
 NCBI BlastP on this gene

EMD95333

hypothetical protein
  
Accession: EMD95334
  
Location: 2301919-2302971
  
 NCBI BlastP on this gene

EMD95334

48. :  DS231615 Pyrenophora tritici-repentis Pt-1C-BFP supercont1.1 genomic scaffold     Total score: 2.0     Cumulative Blast bit score: 1270

hypothetical protein
  
Accession: EDU41232
  
Location: 5155908-5156234
  
 NCBI BlastP on this gene

EDU41232

glycosyl hydrolase
  
Accession: EDU41233
  
Location: 5156920-5159181
  
 NCBI BlastP on this gene

EDU41233

predicted protein
  
Accession: EDU41234
  
Location: 5159779-5163591
  
 NCBI BlastP on this gene

EDU41234

conserved hypothetical protein
  
Accession: EDU41235
  
Location: 5163962-5165632
  
  
**BlastP hit with Mycgr3G23761\_Mycgr3T**
  
Percentage identity: 28 %
  
BlastP bit score: 188
  
Sequence coverage: 99 %
  
E-value: 5e-50
  
  
 NCBI BlastP on this gene

EDU41235

predicted protein
  
Accession: EDU41236
  
Location: 5168266-5168588
  
 NCBI BlastP on this gene

EDU41236

canalicular multispecific organic anion transporter 1
  
Accession: EDU41237
  
Location: 5170114-5175070
  
 NCBI BlastP on this gene

EDU41237

HC-toxin synthetase
  
Accession: EDU41238
  
Location: 5179158-5201187
  
  
**BlastP hit with Mycgr3G90558\_Mycgr3T**
  
Percentage identity: 30 %
  
BlastP bit score: 1082
  
Sequence coverage: 56 %
  
E-value: 0.0
  
  
 NCBI BlastP on this gene

EDU41238

predicted protein
  
Accession: EDU41239
  
Location: 5201465-5201704
  
 NCBI BlastP on this gene

EDU41239

conserved hypothetical protein
  
Accession: EDU41240
  
Location: 5202103-5203212
  
 NCBI BlastP on this gene

EDU41240

conserved hypothetical protein
  
Accession: EDU41241
  
Location: 5205511-5206128
  
 NCBI BlastP on this gene

EDU41241

49. :  JH126401 Cordyceps militaris CM01 unplaced genomic scaffold CCM\_S00003     Total score: 2.0     Cumulative Blast bit score: 1221

hypothetical protein
  
Accession: EGX92716
  
Location: 1477725-1478590
  
 NCBI BlastP on this gene

EGX92716

alpha-N-acetylglucosaminidase, putative
  
Accession: EGX92717
  
Location: 1479410-1481811
  
 NCBI BlastP on this gene

EGX92717

ABC transporter-like protein
  
Accession: EGX92718
  
Location: 1482127-1484052
  
 NCBI BlastP on this gene

EGX92718

MFS transporter, putative
  
Accession: EGX92719
  
Location: 1484321-1485939
  
 NCBI BlastP on this gene

EGX92719

20S proteasome maturation protein Ump1
  
Accession: EGX92720
  
Location: 1487230-1487691
  
 NCBI BlastP on this gene

EGX92720

Ubiquitin
  
Accession: EGX92721
  
Location: 1488487-1489345
  
 NCBI BlastP on this gene

EGX92721

AP-2 complex subunit beta
  
Accession: EGX92722
  
Location: 1491469-1493990
  
 NCBI BlastP on this gene

EGX92722

ER membrane DUF1077 domain protein, putative
  
Accession: EGX92723
  
Location: 1494610-1495298
  
 NCBI BlastP on this gene

EGX92723

Major facilitator superfamily transporter
  
Accession: EGX92724
  
Location: 1495422-1497080
  
  
**BlastP hit with Mycgr3G84494\_Mycgr3T**
  
Percentage identity: 40 %
  
BlastP bit score: 358
  
Sequence coverage: 91 %
  
E-value: 1e-113
  
  
 NCBI BlastP on this gene

EGX92724

WD40 repeat-like-containing domain
  
Accession: EGX92725
  
Location: 1498049-1500381
  
 NCBI BlastP on this gene

EGX92725

hypothetical protein
  
Accession: EGX92726
  
Location: 1501507-1503154
  
 NCBI BlastP on this gene

EGX92726

ABC bile acid transporter, putative
  
Accession: EGX92727
  
Location: 1504827-1509584
  
  
**BlastP hit with Mycgr3G9942\_Mycgr3T9**
  
Percentage identity: 37 %
  
BlastP bit score: 863
  
Sequence coverage: 102 %
  
E-value: 0.0
  
  
 NCBI BlastP on this gene

EGX92727

autophagy protein Apg6, putative
  
Accession: EGX92728
  
Location: 1510108-1511692
  
 NCBI BlastP on this gene

EGX92728

hypothetical protein
  
Accession: EGX92729
  
Location: 1512367-1515817
  
 NCBI BlastP on this gene

EGX92729

Casein kinase II, alpha chain (CK II alpha subunit)
  
Accession: EGX92730
  
Location: 1516848-1518238
  
 NCBI BlastP on this gene

EGX92730

hypothetical protein
  
Accession: EGX92731
  
Location: 1519020-1521133
  
 NCBI BlastP on this gene

EGX92731

hypothetical protein
  
Accession: EGX92732
  
Location: 1521773-1523204
  
 NCBI BlastP on this gene

EGX92732

chromosome segregation protein
  
Accession: EGX92733
  
Location: 1525704-1529694
  
 NCBI BlastP on this gene

EGX92733

50. :  DS027684 Neosartorya fischeri NRRL 181 1099437636244 genomic scaffold     Total score: 2.0     Cumulative Blast bit score: 1219

LysM domain protein
  
Accession: EAW25618
  
Location: 253883-258780
  
 NCBI BlastP on this gene

EAW25618

conserved hypothetical protein
  
Accession: EAW25617
  
Location: 253006-253710
  
 NCBI BlastP on this gene

EAW25617

MFS transporter, putative
  
Accession: EAW25616
  
Location: 246469-248075
  
  
**BlastP hit with Mycgr3G23761\_Mycgr3T**
  
Percentage identity: 33 %
  
BlastP bit score: 194
  
Sequence coverage: 95 %
  
E-value: 3e-53
  
  
 NCBI BlastP on this gene

EAW25616

conserved hypothetical protein
  
Accession: EAW25615
  
Location: 244471-245438
  
 NCBI BlastP on this gene

EAW25615

taurine dioxygenase family protein
  
Accession: EAW25614
  
Location: 242902-244172
  
 NCBI BlastP on this gene

EAW25614

aminotransferase, putative
  
Accession: EAW25613
  
Location: 240751-242146
  
 NCBI BlastP on this gene

EAW25613

hypothetical protein
  
Accession: EAW25612
  
Location: 238597-240163
  
 NCBI BlastP on this gene

EAW25612

2OG-Fe(II) oxygenase family oxidoreductase, putative
  
Accession: EAW25611
  
Location: 237111-238231
  
 NCBI BlastP on this gene

EAW25611

HpcH/HpaI aldolase/citrate lyase family protein
  
Accession: EAW25610
  
Location: 235729-236770
  
 NCBI BlastP on this gene

EAW25610

AMP-binding enzyme, putative
  
Accession: EAW25609
  
Location: 232711-234705
  
 NCBI BlastP on this gene

EAW25609

ornithine aminotransferase
  
Accession: EAW25608
  
Location: 230880-232453
  
 NCBI BlastP on this gene

EAW25608

aspartate aminotransferase, putative
  
Accession: EAW25607
  
Location: 229446-230579
  
 NCBI BlastP on this gene

EAW25607

benzoate 4-monooxygenase cytochrome P450
  
Accession: EAW25606
  
Location: 227637-229193
  
 NCBI BlastP on this gene

EAW25606

nonribosomal peptide synthase, putative
  
Accession: EAW25605
  
Location: 215213-226875
  
  
**BlastP hit with Mycgr3G90558\_Mycgr3T**
  
Percentage identity: 32 %
  
BlastP bit score: 1025
  
Sequence coverage: 51 %
  
E-value: 0.0
  
  
 NCBI BlastP on this gene

EAW25605

cytochrome P450
  
Accession: EAW25604
  
Location: 212041-213688
  
 NCBI BlastP on this gene

EAW25604

conserved hypothetical protein
  
Accession: EAW25603
  
Location: 210420-211260
  
 NCBI BlastP on this gene

EAW25603

conserved hypothetical protein
  
Accession: EAW25602
  
Location: 208166-209104
  
 NCBI BlastP on this gene

EAW25602

hypothetical protein
  
Accession: EAW25601
  
Location: 207169-207876
  
 NCBI BlastP on this gene

EAW25601

Detecting sequence homology at the gene cluster level with MultiGeneBlast.
  
Marnix H. Medema, Rainer Breitling & Eriko Takano (2013)
  
*Molecular Biology and Evolution* , 30: 1218-1223.
