# Supplementary material for: In silico prediction and characterization of secondary metabolite biosynthetic gene clusters in the wheat pathogen Zymoseptoria tritici
Source: BMC Genomics. 2017 Aug 17;18:631. doi: 10.1186/s12864-017-3969-y (PMC5561558; doi:10.1186/s12864-017-3969-y)
Supplement: Supplementary file 1 — MultiGeneBLAST analysis of putative secondary metabolite clusters. All encoded amino acid sequences from genes residing in clusters predicted by AntiSMASH are given as FASTA file format. All output data from MultiGeneBLASTs are also provided. (ZIP 42911 kb) [file 12864_2017_3969_MOESM1_ESM.zip › Cluster MultiGene BLAST/out/Clusters_1_34/Cluster_11/displaypage2.xhtml]

xml version="1.0" encoding="UTF-8"?


Search Results
  
  
 Results pages: 1, 2, 3, 4, 5

**MultiGeneBlast hits**

Select gene cluster alignment
51. KB446555\_4 Pseudocercospora fijiensis CIRAD86 unplaced genomic scaffold M...
52. KB456266\_0 Mycosphaerella populorum SO2202 unplaced genomic scaffold SEPM...
53. JH725152\_0 Beauveria bassiana ARSEF 2860 unplaced genomic scaffold BBA\_S0...
54. AAHF01000006\_1 Aspergillus fumigatus Af293, whole genome shotgun sequenci...
55. DS499599\_1 Aspergillus fumigatus A1163 scf\_000006 genomic scaffold, whole...
56. DF126469\_0 Aspergillus kawachii IFO 4308 DNA, contig: scaffold00023, whol...
57. AM269994\_0 Aspergillus niger contig An01c0480, genomic contig.
58. ACJE01000004\_5 Aspergillus niger ATCC 1015, whole genome shotgun sequenci...
59. ABDF02000005\_0 Trichoderma virens Gv29-8, whole genome shotgun sequencing...
60. AMYD01004056\_0 Colletotrichum gloeosporioides Cg-14, whole genome shotgun...
61. GL629735\_1 Grosmannia clavigera kw1407 unplaced genomic scaffold GCSC\_113...
62. KB706559\_0 Eutypa lata UCREL1 unplaced genomic scaffold EL1\_03\_scaffold\_1...
63. GG697353\_0 Glomerella graminicola M1.001 genomic scaffold supercont1.23, ...
64. CACQ02006567\_0 Colletotrichum higginsianum strain IMI 349063, whole genom...
65. ABDG02000025\_0 Trichoderma atroviride IMI 206040, whole genome shotgun se...
66. GG698907\_3 Nectria haematococca mpVI 77-13-4 chromosome 12 genomic scaffo...
67. GL985063\_0 Trichoderma reesei QM6a unplaced genomic scaffold TRIREscaffol...
68. CH476594\_1 Aspergillus terreus NIH2624 scaffold\_1 genomic scaffold, whole...
69. KE148169\_1 Ophiostoma piceae UAMH 11346 chromosome Unknown scf24, whole g...
70. JH795082\_0 Magnaporthe oryzae P131 unplaced genomic scaffold P131\_scaffol...
71. JH793116\_0 Magnaporthe oryzae Y34 unplaced genomic scaffold Y34\_scaffold0...
72. CM001233\_4 Magnaporthe oryzae 70-15 chromosome 3, whole genome shotgun se...
73. KB020307\_0 Colletotrichum gloeosporioides Nara gc5 unplaced genomic scaff...
74. GL385398\_0 Gaeumannomyces graminis var. tritici R3-111a-1 unplaced genomi...
75. JH687384\_0 Stereum hirsutum FP-91666 SS1 unplaced genomic scaffold STEHIs...
76. AHHD01000414\_0 Macrophomina phaseolina MS6, whole genome shotgun sequenci...
77. CH445371\_1 Phaeosphaeria nodorum SN15 scaffold\_47, whole genome shotgun s...
78. GG704912\_0 Coccidioides immitis RS genomic scaffold supercont3.2, whole g...
79. DF126477\_0 Aspergillus kawachii IFO 4308 DNA, contig: scaffold00031, whol...
80. ACJE01000018\_0 Aspergillus niger ATCC 1015, whole genome shotgun sequenci...
81. ABDG02000016\_0 Trichoderma atroviride IMI 206040, whole genome shotgun se...
82. DF126457\_0 Aspergillus kawachii IFO 4308 DNA, contig: scaffold00011, whol...
83. ABDF02000063\_0 Trichoderma virens Gv29-8, whole genome shotgun sequencing...
84. DF126469\_1 Aspergillus kawachii IFO 4308 DNA, contig: scaffold00023, whol...
85. JH126402\_4 Cordyceps militaris CM01 unplaced genomic scaffold CCM\_S00004,...
86. KE145357\_0 Glarea lozoyensis ATCC 20868 chromosome Unknown GLAREA14, whol...
87. ACJE01000004\_4 Aspergillus niger ATCC 1015, whole genome shotgun sequenci...
88. GL890999\_0 Neurospora tetrasperma FGSC 2509 unplaced genomic scaffold NEU...
89. GL891382\_0 Neurospora tetrasperma FGSC 2508 unplaced genomic scaffold NEU...
90. AABX02000002\_0 Neurospora crassa OR74A, whole genome shotgun sequencing p...
91. GL636512\_1 Coccidioides posadasii str. Silveira unplaced genomic scaffold...
92. CP003011\_3 Thielavia terrestris NRRL 8126 chromosome 3, complete sequence.
93. AKHY01000098\_0 Aspergillus oryzae 3.042, whole genome shotgun sequencing ...
94. CP003014\_1 Thielavia terrestris NRRL 8126 chromosome 6, complete sequence.
95. AP007164\_1 Aspergillus oryzae RIB40 DNA, SC111.
96. GG698970\_0 Nectria haematococca mpVI 77-13-4 chromosome 10 genomic scaffo...
97. KE148146\_1 Ophiostoma piceae UAMH 11346 chromosome Unknown scf01, whole g...
98. ACFW01000025\_0 Coccidioides posadasii C735 delta SOWgp, whole genome shot...
99. EQ962654\_3 Talaromyces stipitatus ATCC 10500 scf\_1105507295541 genomic sc...
100. KE145368\_1 Glarea lozoyensis ATCC 20868 chromosome Unknown GLAREA4, whol...

Query: Architecture Search FASTA input

KB446555 : Pseudocercospora fijiensis CIRAD86 unplaced genomic scaffold MYCFIscaffold\_1    Total score: 2.0     Cumulative Blast bit score: 1216

Hit cluster cross-links:

Mycgr3G36335 Mycgr3T
  
Location: 0-423

Mycgr3G36335\_Mycgr3T

Mycgr3G84494 Mycgr3T
  
Location: 523-2047

Mycgr3G84494\_Mycgr3T

Mycgr3G90558 Mycgr3T
  
Location: 2147-15296

Mycgr3G90558\_Mycgr3T

Mycgr3G68036 Mycgr3T
  
Location: 15396-16395

Mycgr3G68036\_Mycgr3T

Mycgr3G90561 Mycgr3T
  
Location: 16495-17134

Mycgr3G90561\_Mycgr3T

Mycgr3G35862 Mycgr3T
  
Location: 17234-18662

Mycgr3G35862\_Mycgr3T

Mycgr3G68030 Mycgr3T
  
Location: 18762-19722

Mycgr3G68030\_Mycgr3T

Mycgr3G36449 Mycgr3T
  
Location: 19822-21886

Mycgr3G36449\_Mycgr3T

Mycgr3G35528 Mycgr3T
  
Location: 21986-22844

Mycgr3G35528\_Mycgr3T

Mycgr3G35932 Mycgr3T
  
Location: 22944-24390

Mycgr3G35932\_Mycgr3T

Mycgr3G23761 Mycgr3T
  
Location: 24490-25825

Mycgr3G23761\_Mycgr3T

Mycgr3G35535 Mycgr3T
  
Location: 25925-26429

Mycgr3G35535\_Mycgr3T

Mycgr3G9942 Mycgr3T9
  
Location: 26529-30375

Mycgr3G9942\_Mycgr3T9

hypothetical protein
  
Accession: EME89456
  
Location: 10582040-10583080
  
  
**BlastP hit with Mycgr3G68036\_Mycgr3T**
  
Percentage identity: 36 %
  
BlastP bit score: 177
  
Sequence coverage: 99 %
  
E-value: 1e-48
  
  
 NCBI BlastP on this gene

EME89456

hypothetical protein
  
Accession: EME89457
  
Location: 10583716-10598238
  
  
**BlastP hit with Mycgr3G90558\_Mycgr3T**
  
Percentage identity: 30 %
  
BlastP bit score: 1039
  
Sequence coverage: 54 %
  
E-value: 0.0
  
  
 NCBI BlastP on this gene

EME89457

hypothetical protein
  
Accession: EME89458
  
Location: 10598450-10599385
  
 NCBI BlastP on this gene

EME89458

hypothetical protein
  
Accession: EME89459
  
Location: 10602096-10602956
  
 NCBI BlastP on this gene

EME89459

Query: Architecture Search FASTA input

KB456266 : Mycosphaerella populorum SO2202 unplaced genomic scaffold SEPMUscaffold\_7    Total score: 2.0     Cumulative Blast bit score: 1208

Hit cluster cross-links:

Mycgr3G36335 Mycgr3T
  
Location: 0-423

Mycgr3G36335\_Mycgr3T

Mycgr3G84494 Mycgr3T
  
Location: 523-2047

Mycgr3G84494\_Mycgr3T

Mycgr3G90558 Mycgr3T
  
Location: 2147-15296

Mycgr3G90558\_Mycgr3T

Mycgr3G68036 Mycgr3T
  
Location: 15396-16395

Mycgr3G68036\_Mycgr3T

Mycgr3G90561 Mycgr3T
  
Location: 16495-17134

Mycgr3G90561\_Mycgr3T

Mycgr3G35862 Mycgr3T
  
Location: 17234-18662

Mycgr3G35862\_Mycgr3T

Mycgr3G68030 Mycgr3T
  
Location: 18762-19722

Mycgr3G68030\_Mycgr3T

Mycgr3G36449 Mycgr3T
  
Location: 19822-21886

Mycgr3G36449\_Mycgr3T

Mycgr3G35528 Mycgr3T
  
Location: 21986-22844

Mycgr3G35528\_Mycgr3T

Mycgr3G35932 Mycgr3T
  
Location: 22944-24390

Mycgr3G35932\_Mycgr3T

Mycgr3G23761 Mycgr3T
  
Location: 24490-25825

Mycgr3G23761\_Mycgr3T

Mycgr3G35535 Mycgr3T
  
Location: 25925-26429

Mycgr3G35535\_Mycgr3T

Mycgr3G9942 Mycgr3T9
  
Location: 26529-30375

Mycgr3G9942\_Mycgr3T9

hypothetical protein
  
Accession: EMF11590
  
Location: 1906885-1907175
  
 NCBI BlastP on this gene

EMF11590

coenzyme F420-dependent NADP oxidoreductase
  
Accession: EMF11591
  
Location: 1909364-1910552
  
 NCBI BlastP on this gene

EMF11591

hypothetical protein
  
Accession: EMF11592
  
Location: 1911756-1913818
  
 NCBI BlastP on this gene

EMF11592

Clavaminate synthase-like protein
  
Accession: EMF11593
  
Location: 1914406-1915464
  
  
**BlastP hit with Mycgr3G68036\_Mycgr3T**
  
Percentage identity: 35 %
  
BlastP bit score: 180
  
Sequence coverage: 104 %
  
E-value: 1e-49
  
  
 NCBI BlastP on this gene

EMF11593

acetyl-CoA synthetase-like protein
  
Accession: EMF11594
  
Location: 1916348-1931212
  
  
**BlastP hit with Mycgr3G90558\_Mycgr3T**
  
Percentage identity: 31 %
  
BlastP bit score: 1028
  
Sequence coverage: 49 %
  
E-value: 0.0
  
  
 NCBI BlastP on this gene

EMF11594

Bac surface Ag-domain-containing protein
  
Accession: EMF11595
  
Location: 1933056-1934728
  
 NCBI BlastP on this gene

EMF11595

hypothetical protein
  
Accession: EMF11597
  
Location: 1934929-1935828
  
 NCBI BlastP on this gene

EMF11597

glutathione S-transferase
  
Accession: EMF11598
  
Location: 1936246-1936968
  
 NCBI BlastP on this gene

EMF11598

hypothetical protein
  
Accession: EMF11599
  
Location: 1937840-1939067
  
 NCBI BlastP on this gene

EMF11599

Query: Architecture Search FASTA input

JH725152 : Beauveria bassiana ARSEF 2860 unplaced genomic scaffold BBA\_S00003    Total score: 2.0     Cumulative Blast bit score: 1196

Hit cluster cross-links:

Mycgr3G36335 Mycgr3T
  
Location: 0-423

Mycgr3G36335\_Mycgr3T

Mycgr3G84494 Mycgr3T
  
Location: 523-2047

Mycgr3G84494\_Mycgr3T

Mycgr3G90558 Mycgr3T
  
Location: 2147-15296

Mycgr3G90558\_Mycgr3T

Mycgr3G68036 Mycgr3T
  
Location: 15396-16395

Mycgr3G68036\_Mycgr3T

Mycgr3G90561 Mycgr3T
  
Location: 16495-17134

Mycgr3G90561\_Mycgr3T

Mycgr3G35862 Mycgr3T
  
Location: 17234-18662

Mycgr3G35862\_Mycgr3T

Mycgr3G68030 Mycgr3T
  
Location: 18762-19722

Mycgr3G68030\_Mycgr3T

Mycgr3G36449 Mycgr3T
  
Location: 19822-21886

Mycgr3G36449\_Mycgr3T

Mycgr3G35528 Mycgr3T
  
Location: 21986-22844

Mycgr3G35528\_Mycgr3T

Mycgr3G35932 Mycgr3T
  
Location: 22944-24390

Mycgr3G35932\_Mycgr3T

Mycgr3G23761 Mycgr3T
  
Location: 24490-25825

Mycgr3G23761\_Mycgr3T

Mycgr3G35535 Mycgr3T
  
Location: 25925-26429

Mycgr3G35535\_Mycgr3T

Mycgr3G9942 Mycgr3T9
  
Location: 26529-30375

Mycgr3G9942\_Mycgr3T9

hypothetical protein
  
Accession: EJP69797
  
Location: 1688362-1689759
  
 NCBI BlastP on this gene

EJP69797

Casein kinase II, alpha chain (CK II alpha subunit)
  
Accession: EJP69798
  
Location: 1690656-1692026
  
 NCBI BlastP on this gene

EJP69798

hypothetical protein
  
Accession: EJP69799
  
Location: 1693120-1696548
  
 NCBI BlastP on this gene

EJP69799

autophagy protein Apg6
  
Accession: EJP69800
  
Location: 1697247-1698833
  
 NCBI BlastP on this gene

EJP69800

ABC transporter
  
Accession: EJP69801
  
Location: 1699411-1704198
  
  
**BlastP hit with Mycgr3G9942\_Mycgr3T9**
  
Percentage identity: 36 %
  
BlastP bit score: 836
  
Sequence coverage: 106 %
  
E-value: 0.0
  
  
 NCBI BlastP on this gene

EJP69801

WD domain-containing protein
  
Accession: EJP69802
  
Location: 1709737-1712072
  
 NCBI BlastP on this gene

EJP69802

major facilitator superfamily transporter
  
Accession: EJP69803
  
Location: 1713365-1715011
  
  
**BlastP hit with Mycgr3G84494\_Mycgr3T**
  
Percentage identity: 40 %
  
BlastP bit score: 360
  
Sequence coverage: 93 %
  
E-value: 2e-114
  
  
 NCBI BlastP on this gene

EJP69803

transmembrane protein
  
Accession: EJP69804
  
Location: 1715124-1715812
  
 NCBI BlastP on this gene

EJP69804

AP-2 adaptor complex subunit beta
  
Accession: EJP69805
  
Location: 1716443-1718970
  
 NCBI BlastP on this gene

EJP69805

hypothetical protein
  
Accession: EJP69806
  
Location: 1721320-1722186
  
 NCBI BlastP on this gene

EJP69806

proteasome maturation factor UMP1
  
Accession: EJP69807
  
Location: 1723019-1723492
  
 NCBI BlastP on this gene

EJP69807

ABC transporter
  
Accession: EJP69808
  
Location: 1724638-1726656
  
 NCBI BlastP on this gene

EJP69808

Query: Architecture Search FASTA input

AAHF01000006 : Aspergillus fumigatus Af293    Total score: 2.0     Cumulative Blast bit score: 1106

Hit cluster cross-links:

Mycgr3G36335 Mycgr3T
  
Location: 0-423

Mycgr3G36335\_Mycgr3T

Mycgr3G84494 Mycgr3T
  
Location: 523-2047

Mycgr3G84494\_Mycgr3T

Mycgr3G90558 Mycgr3T
  
Location: 2147-15296

Mycgr3G90558\_Mycgr3T

Mycgr3G68036 Mycgr3T
  
Location: 15396-16395

Mycgr3G68036\_Mycgr3T

Mycgr3G90561 Mycgr3T
  
Location: 16495-17134

Mycgr3G90561\_Mycgr3T

Mycgr3G35862 Mycgr3T
  
Location: 17234-18662

Mycgr3G35862\_Mycgr3T

Mycgr3G68030 Mycgr3T
  
Location: 18762-19722

Mycgr3G68030\_Mycgr3T

Mycgr3G36449 Mycgr3T
  
Location: 19822-21886

Mycgr3G36449\_Mycgr3T

Mycgr3G35528 Mycgr3T
  
Location: 21986-22844

Mycgr3G35528\_Mycgr3T

Mycgr3G35932 Mycgr3T
  
Location: 22944-24390

Mycgr3G35932\_Mycgr3T

Mycgr3G23761 Mycgr3T
  
Location: 24490-25825

Mycgr3G23761\_Mycgr3T

Mycgr3G35535 Mycgr3T
  
Location: 25925-26429

Mycgr3G35535\_Mycgr3T

Mycgr3G9942 Mycgr3T9
  
Location: 26529-30375

Mycgr3G9942\_Mycgr3T9

nonribosomal peptide synthase, putative
  
Accession: EAL89046
  
Location: 1694970-1698884
  
 NCBI BlastP on this gene

EAL89046

MAK1-like monooxygenase, putative
  
Accession: EAL89047
  
Location: 1699285-1700651
  
 NCBI BlastP on this gene

EAL89047

FAD binding domain protein
  
Accession: EAL89048
  
Location: 1701480-1703303
  
 NCBI BlastP on this gene

EAL89048

nonribosomal peptide synthase, putative
  
Accession: EAL89049
  
Location: 1704693-1716682
  
  
**BlastP hit with Mycgr3G90558\_Mycgr3T**
  
Percentage identity: 31 %
  
BlastP bit score: 1031
  
Sequence coverage: 51 %
  
E-value: 0.0
  
  
 NCBI BlastP on this gene

EAL89049

HET domain protein
  
Accession: EAL89050
  
Location: 1719714-1721570
  
  
**BlastP hit with Mycgr3G36335\_Mycgr3T**
  
Percentage identity: 34 %
  
BlastP bit score: 75
  
Sequence coverage: 94 %
  
E-value: 5e-13
  
  
 NCBI BlastP on this gene

EAL89050

nitrilase family protein
  
Accession: EAL89051
  
Location: 1723473-1724328
  
 NCBI BlastP on this gene

EAL89051

salicylate synthetase, putative
  
Accession: EAL89052
  
Location: 1724938-1726340
  
 NCBI BlastP on this gene

EAL89052

BNR/Asp-box repeat domain protein
  
Accession: EAL89053
  
Location: 1726765-1727971
  
 NCBI BlastP on this gene

EAL89053

C6 transcription factor, putative
  
Accession: EAL89054
  
Location: 1729943-1732113
  
 NCBI BlastP on this gene

EAL89054

Query: Architecture Search FASTA input

DS499599 : Aspergillus fumigatus A1163 scf\_000006 genomic scaffold    Total score: 2.0     Cumulative Blast bit score: 1105

Hit cluster cross-links:

Mycgr3G36335 Mycgr3T
  
Location: 0-423

Mycgr3G36335\_Mycgr3T

Mycgr3G84494 Mycgr3T
  
Location: 523-2047

Mycgr3G84494\_Mycgr3T

Mycgr3G90558 Mycgr3T
  
Location: 2147-15296

Mycgr3G90558\_Mycgr3T

Mycgr3G68036 Mycgr3T
  
Location: 15396-16395

Mycgr3G68036\_Mycgr3T

Mycgr3G90561 Mycgr3T
  
Location: 16495-17134

Mycgr3G90561\_Mycgr3T

Mycgr3G35862 Mycgr3T
  
Location: 17234-18662

Mycgr3G35862\_Mycgr3T

Mycgr3G68030 Mycgr3T
  
Location: 18762-19722

Mycgr3G68030\_Mycgr3T

Mycgr3G36449 Mycgr3T
  
Location: 19822-21886

Mycgr3G36449\_Mycgr3T

Mycgr3G35528 Mycgr3T
  
Location: 21986-22844

Mycgr3G35528\_Mycgr3T

Mycgr3G35932 Mycgr3T
  
Location: 22944-24390

Mycgr3G35932\_Mycgr3T

Mycgr3G23761 Mycgr3T
  
Location: 24490-25825

Mycgr3G23761\_Mycgr3T

Mycgr3G35535 Mycgr3T
  
Location: 25925-26429

Mycgr3G35535\_Mycgr3T

Mycgr3G9942 Mycgr3T9
  
Location: 26529-30375

Mycgr3G9942\_Mycgr3T9

nonribosomal peptide synthase, putative
  
Accession: EDP49773
  
Location: 1701787-1705701
  
 NCBI BlastP on this gene

EDP49773

MAK1-like monooxygenase, putative
  
Accession: EDP49774
  
Location: 1706102-1707468
  
 NCBI BlastP on this gene

EDP49774

FAD binding domain protein
  
Accession: EDP49775
  
Location: 1708297-1710120
  
 NCBI BlastP on this gene

EDP49775

nonribosomal peptide synthase, putative
  
Accession: EDP49776
  
Location: 1711510-1723499
  
  
**BlastP hit with Mycgr3G90558\_Mycgr3T**
  
Percentage identity: 31 %
  
BlastP bit score: 1030
  
Sequence coverage: 51 %
  
E-value: 0.0
  
  
 NCBI BlastP on this gene

EDP49776

HET domain protein
  
Accession: EDP49777
  
Location: 1726523-1728379
  
  
**BlastP hit with Mycgr3G36335\_Mycgr3T**
  
Percentage identity: 34 %
  
BlastP bit score: 75
  
Sequence coverage: 94 %
  
E-value: 5e-13
  
  
 NCBI BlastP on this gene

EDP49777

salicylate synthetase, putative
  
Accession: EDP49778
  
Location: 1731150-1732552
  
 NCBI BlastP on this gene

EDP49778

BNR/Asp-box repeat domain protein
  
Accession: EDP49779
  
Location: 1732977-1734183
  
 NCBI BlastP on this gene

EDP49779

C6 transcription factor, putative
  
Accession: EDP49780
  
Location: 1736155-1738325
  
 NCBI BlastP on this gene

EDP49780

Query: Architecture Search FASTA input

DF126469 : Aspergillus kawachii IFO 4308 DNA, contig: scaffold00023    Total score: 2.0     Cumulative Blast bit score: 1073

Hit cluster cross-links:

Mycgr3G36335 Mycgr3T
  
Location: 0-423

Mycgr3G36335\_Mycgr3T

Mycgr3G84494 Mycgr3T
  
Location: 523-2047

Mycgr3G84494\_Mycgr3T

Mycgr3G90558 Mycgr3T
  
Location: 2147-15296

Mycgr3G90558\_Mycgr3T

Mycgr3G68036 Mycgr3T
  
Location: 15396-16395

Mycgr3G68036\_Mycgr3T

Mycgr3G90561 Mycgr3T
  
Location: 16495-17134

Mycgr3G90561\_Mycgr3T

Mycgr3G35862 Mycgr3T
  
Location: 17234-18662

Mycgr3G35862\_Mycgr3T

Mycgr3G68030 Mycgr3T
  
Location: 18762-19722

Mycgr3G68030\_Mycgr3T

Mycgr3G36449 Mycgr3T
  
Location: 19822-21886

Mycgr3G36449\_Mycgr3T

Mycgr3G35528 Mycgr3T
  
Location: 21986-22844

Mycgr3G35528\_Mycgr3T

Mycgr3G35932 Mycgr3T
  
Location: 22944-24390

Mycgr3G35932\_Mycgr3T

Mycgr3G23761 Mycgr3T
  
Location: 24490-25825

Mycgr3G23761\_Mycgr3T

Mycgr3G35535 Mycgr3T
  
Location: 25925-26429

Mycgr3G35535\_Mycgr3T

Mycgr3G9942 Mycgr3T9
  
Location: 26529-30375

Mycgr3G9942\_Mycgr3T9

similar to An01g14840
  
Accession: GAA89740
  
Location: 470873-471697
  
 NCBI BlastP on this gene

GAA89740

NRPS-like enzyme
  
Accession: GAA89741
  
Location: 471862-475655
  
 NCBI BlastP on this gene

GAA89741

integral membrane protein
  
Accession: GAA89742
  
Location: 476219-477031
  
 NCBI BlastP on this gene

GAA89742

aldehyde reductase I
  
Accession: GAA89743
  
Location: 477440-478478
  
 NCBI BlastP on this gene

GAA89743

similar to An01g14890
  
Accession: GAA89744
  
Location: 478734-479769
  
 NCBI BlastP on this gene

GAA89744

pantothenate transporter
  
Accession: GAA89745
  
Location: 481114-482860
  
 NCBI BlastP on this gene

GAA89745

C6 transcription factor
  
Accession: GAA89746
  
Location: 483090-485409
  
 NCBI BlastP on this gene

GAA89746

xaa-pro dipeptidase
  
Accession: GAA89747
  
Location: 486362-488217
  
  
**BlastP hit with Mycgr3G35862\_Mycgr3T**
  
Percentage identity: 57 %
  
BlastP bit score: 520
  
Sequence coverage: 98 %
  
E-value: 3e-178
  
  
 NCBI BlastP on this gene

GAA89747

major facilitator superfamily transporter
  
Accession: GAA89748
  
Location: 488662-490297
  
  
**BlastP hit with Mycgr3G35932\_Mycgr3T**
  
Percentage identity: 62 %
  
BlastP bit score: 553
  
Sequence coverage: 93 %
  
E-value: 0.0
  
  
 NCBI BlastP on this gene

GAA89748

ATP synthase F1
  
Accession: GAA89749
  
Location: 490956-493572
  
 NCBI BlastP on this gene

GAA89749

phosphoesterase superfamily protein
  
Accession: GAA89750
  
Location: 493890-495312
  
 NCBI BlastP on this gene

GAA89750

thermolabile L-asparaginase
  
Accession: GAA89751
  
Location: 496684-497769
  
 NCBI BlastP on this gene

GAA89751

L-asparaginase
  
Accession: GAA89752
  
Location: 498574-499824
  
 NCBI BlastP on this gene

GAA89752

similar to delta-12 fatty acid desaturase
  
Accession: GAA89753
  
Location: 500944-502379
  
 NCBI BlastP on this gene

GAA89753

hypothetical protein
  
Accession: GAA89754
  
Location: 502583-503129
  
 NCBI BlastP on this gene

GAA89754

similar to An01g14980
  
Accession: GAA89755
  
Location: 503336-504286
  
 NCBI BlastP on this gene

GAA89755

cytochrome P450 monooxygenase
  
Accession: GAA89756
  
Location: 505079-506686
  
 NCBI BlastP on this gene

GAA89756

Query: Architecture Search FASTA input

AM269994 : Aspergillus niger contig An01c0480, genomic contig.    Total score: 2.0     Cumulative Blast bit score: 1071

Hit cluster cross-links:

Mycgr3G36335 Mycgr3T
  
Location: 0-423

Mycgr3G36335\_Mycgr3T

Mycgr3G84494 Mycgr3T
  
Location: 523-2047

Mycgr3G84494\_Mycgr3T

Mycgr3G90558 Mycgr3T
  
Location: 2147-15296

Mycgr3G90558\_Mycgr3T

Mycgr3G68036 Mycgr3T
  
Location: 15396-16395

Mycgr3G68036\_Mycgr3T

Mycgr3G90561 Mycgr3T
  
Location: 16495-17134

Mycgr3G90561\_Mycgr3T

Mycgr3G35862 Mycgr3T
  
Location: 17234-18662

Mycgr3G35862\_Mycgr3T

Mycgr3G68030 Mycgr3T
  
Location: 18762-19722

Mycgr3G68030\_Mycgr3T

Mycgr3G36449 Mycgr3T
  
Location: 19822-21886

Mycgr3G36449\_Mycgr3T

Mycgr3G35528 Mycgr3T
  
Location: 21986-22844

Mycgr3G35528\_Mycgr3T

Mycgr3G35932 Mycgr3T
  
Location: 22944-24390

Mycgr3G35932\_Mycgr3T

Mycgr3G23761 Mycgr3T
  
Location: 24490-25825

Mycgr3G23761\_Mycgr3T

Mycgr3G35535 Mycgr3T
  
Location: 25925-26429

Mycgr3G35535\_Mycgr3T

Mycgr3G9942 Mycgr3T9
  
Location: 26529-30375

Mycgr3G9942\_Mycgr3T9

not annotated
  
Accession: CAK37440
  
Location: 1-1880
  
 NCBI BlastP on this gene

An01g14860

not annotated
  
Accession: CAK37441
  
Location: 2434-3246
  
 NCBI BlastP on this gene

An01g14870

not annotated
  
Accession: CAK37442
  
Location: 3650-4687
  
 NCBI BlastP on this gene

An01g14880

not annotated
  
Accession: CAK37443
  
Location: 4947-6028
  
 NCBI BlastP on this gene

An01g14890

not annotated
  
Accession: CAK37444
  
Location: 7310-9059
  
 NCBI BlastP on this gene

An01g14900

not annotated
  
Accession: CAK37445
  
Location: 9216-11622
  
 NCBI BlastP on this gene

An01g14910

not annotated
  
Accession: CAK37446
  
Location: 12564-14418
  
  
**BlastP hit with Mycgr3G35862\_Mycgr3T**
  
Percentage identity: 57 %
  
BlastP bit score: 520
  
Sequence coverage: 98 %
  
E-value: 6e-178
  
  
 NCBI BlastP on this gene

An01g14920

not annotated
  
Accession: CAK37447
  
Location: 14877-16515
  
  
**BlastP hit with Mycgr3G35932\_Mycgr3T**
  
Percentage identity: 61 %
  
BlastP bit score: 551
  
Sequence coverage: 93 %
  
E-value: 0.0
  
  
 NCBI BlastP on this gene

An01g14930

unnamed
  
Accession: CAK37448
  
Location: 17453-18882
  
 NCBI BlastP on this gene

An01g14940

not annotated
  
Accession: CAK37449
  
Location: 20279-21364
  
 NCBI BlastP on this gene

An01g14950

unnamed
  
Accession: CAK37450
  
Location: 22072-23325
  
 NCBI BlastP on this gene

An01g14960

not annotated
  
Accession: CAK37451
  
Location: 24588-25908
  
 NCBI BlastP on this gene

An01g14970

unnamed
  
Accession: CAK37452
  
Location: 26966-27938
  
 NCBI BlastP on this gene

An01g14980

not annotated
  
Accession: CAK37453
  
Location: 28827-30602
  
 NCBI BlastP on this gene

An01g14990

not annotated
  
Accession: CAK37454
  
Location: 32067-33856
  
 NCBI BlastP on this gene

An01g15000

Query: Architecture Search FASTA input

ACJE01000004 : Aspergillus niger ATCC 1015    Total score: 2.0     Cumulative Blast bit score: 1063

Hit cluster cross-links:

Mycgr3G36335 Mycgr3T
  
Location: 0-423

Mycgr3G36335\_Mycgr3T

Mycgr3G84494 Mycgr3T
  
Location: 523-2047

Mycgr3G84494\_Mycgr3T

Mycgr3G90558 Mycgr3T
  
Location: 2147-15296

Mycgr3G90558\_Mycgr3T

Mycgr3G68036 Mycgr3T
  
Location: 15396-16395

Mycgr3G68036\_Mycgr3T

Mycgr3G90561 Mycgr3T
  
Location: 16495-17134

Mycgr3G90561\_Mycgr3T

Mycgr3G35862 Mycgr3T
  
Location: 17234-18662

Mycgr3G35862\_Mycgr3T

Mycgr3G68030 Mycgr3T
  
Location: 18762-19722

Mycgr3G68030\_Mycgr3T

Mycgr3G36449 Mycgr3T
  
Location: 19822-21886

Mycgr3G36449\_Mycgr3T

Mycgr3G35528 Mycgr3T
  
Location: 21986-22844

Mycgr3G35528\_Mycgr3T

Mycgr3G35932 Mycgr3T
  
Location: 22944-24390

Mycgr3G35932\_Mycgr3T

Mycgr3G23761 Mycgr3T
  
Location: 24490-25825

Mycgr3G23761\_Mycgr3T

Mycgr3G35535 Mycgr3T
  
Location: 25925-26429

Mycgr3G35535\_Mycgr3T

Mycgr3G9942 Mycgr3T9
  
Location: 26529-30375

Mycgr3G9942\_Mycgr3T9

hypothetical protein
  
Accession: EHA27188
  
Location: 3699750-3700571
  
 NCBI BlastP on this gene

EHA27188

Hypothetical protein
  
Accession: EHA27189
  
Location: 3700765-3704529
  
 NCBI BlastP on this gene

EHA27189

hypothetical protein
  
Accession: EHA27190
  
Location: 3705079-3705891
  
 NCBI BlastP on this gene

EHA27190

alcohol dehydrogenase NADP+-dependent
  
Accession: EHA27191
  
Location: 3706296-3707333
  
 NCBI BlastP on this gene

EHA27191

hypothetical protein
  
Accession: EHA27192
  
Location: 3707589-3708669
  
 NCBI BlastP on this gene

EHA27192

hypothetical protein
  
Accession: EHA27193
  
Location: 3709068-3709716
  
 NCBI BlastP on this gene

EHA27193

hypothetical protein
  
Accession: EHA27194
  
Location: 3709951-3711700
  
 NCBI BlastP on this gene

EHA27194

hypothetical protein
  
Accession: EHA27195
  
Location: 3712005-3714263
  
 NCBI BlastP on this gene

EHA27195

hypothetical protein
  
Accession: EHA27196
  
Location: 3715209-3716833
  
  
**BlastP hit with Mycgr3G35862\_Mycgr3T**
  
Percentage identity: 61 %
  
BlastP bit score: 512
  
Sequence coverage: 89 %
  
E-value: 7e-176
  
  
 NCBI BlastP on this gene

EHA27196

hypothetical protein
  
Accession: EHA27197
  
Location: 3717518-3719156
  
  
**BlastP hit with Mycgr3G35932\_Mycgr3T**
  
Percentage identity: 61 %
  
BlastP bit score: 551
  
Sequence coverage: 93 %
  
E-value: 0.0
  
  
 NCBI BlastP on this gene

EHA27197

hypothetical protein
  
Accession: EHA27198
  
Location: 3720094-3721523
  
 NCBI BlastP on this gene

EHA27198

hypothetical protein
  
Accession: EHA27199
  
Location: 3722936-3724021
  
 NCBI BlastP on this gene

EHA27199

hypothetical protein
  
Accession: EHA27200
  
Location: 3724729-3725982
  
 NCBI BlastP on this gene

EHA27200

hypothetical protein
  
Accession: EHA27201
  
Location: 3727257-3728565
  
 NCBI BlastP on this gene

EHA27201

hypothetical protein
  
Accession: EHA27202
  
Location: 3728876-3729197
  
 NCBI BlastP on this gene

EHA27202

hypothetical protein
  
Accession: EHA27203
  
Location: 3729623-3730595
  
 NCBI BlastP on this gene

EHA27203

hypothetical protein
  
Accession: EHA27204
  
Location: 3731595-3732143
  
 NCBI BlastP on this gene

EHA27204

hypothetical protein
  
Accession: EHA27205
  
Location: 3734725-3736691
  
 NCBI BlastP on this gene

EHA27205

Query: Architecture Search FASTA input

ABDF02000005 : Trichoderma virens Gv29-8    Total score: 2.0     Cumulative Blast bit score: 1027

Hit cluster cross-links:

Mycgr3G36335 Mycgr3T
  
Location: 0-423

Mycgr3G36335\_Mycgr3T

Mycgr3G84494 Mycgr3T
  
Location: 523-2047

Mycgr3G84494\_Mycgr3T

Mycgr3G90558 Mycgr3T
  
Location: 2147-15296

Mycgr3G90558\_Mycgr3T

Mycgr3G68036 Mycgr3T
  
Location: 15396-16395

Mycgr3G68036\_Mycgr3T

Mycgr3G90561 Mycgr3T
  
Location: 16495-17134

Mycgr3G90561\_Mycgr3T

Mycgr3G35862 Mycgr3T
  
Location: 17234-18662

Mycgr3G35862\_Mycgr3T

Mycgr3G68030 Mycgr3T
  
Location: 18762-19722

Mycgr3G68030\_Mycgr3T

Mycgr3G36449 Mycgr3T
  
Location: 19822-21886

Mycgr3G36449\_Mycgr3T

Mycgr3G35528 Mycgr3T
  
Location: 21986-22844

Mycgr3G35528\_Mycgr3T

Mycgr3G35932 Mycgr3T
  
Location: 22944-24390

Mycgr3G35932\_Mycgr3T

Mycgr3G23761 Mycgr3T
  
Location: 24490-25825

Mycgr3G23761\_Mycgr3T

Mycgr3G35535 Mycgr3T
  
Location: 25925-26429

Mycgr3G35535\_Mycgr3T

Mycgr3G9942 Mycgr3T9
  
Location: 26529-30375

Mycgr3G9942\_Mycgr3T9

hypothetical protein
  
Accession: EHK23631
  
Location: 1322196-1322384
  
 NCBI BlastP on this gene

EHK23631

hypothetical protein
  
Accession: EHK23632
  
Location: 1324257-1326123
  
 NCBI BlastP on this gene

EHK23632

hypothetical protein
  
Accession: EHK23633
  
Location: 1326518-1328043
  
 NCBI BlastP on this gene

EHK23633

hypothetical protein
  
Accession: EHK23634
  
Location: 1328819-1329022
  
 NCBI BlastP on this gene

EHK23634

hypothetical protein
  
Accession: EHK23636
  
Location: 1330295-1330568
  
 NCBI BlastP on this gene

EHK23636

hypothetical protein
  
Accession: EHK23635
  
Location: 1330857-1331228
  
 NCBI BlastP on this gene

EHK23635

hypothetical protein
  
Accession: EHK23637
  
Location: 1333624-1334350
  
 NCBI BlastP on this gene

EHK23637

hypothetical protein
  
Accession: EHK23638
  
Location: 1336188-1337570
  
 NCBI BlastP on this gene

EHK23638

hypothetical protein
  
Accession: EHK23639
  
Location: 1337707-1339194
  
  
**BlastP hit with Mycgr3G35862\_Mycgr3T**
  
Percentage identity: 57 %
  
BlastP bit score: 479
  
Sequence coverage: 89 %
  
E-value: 4e-163
  
  
 NCBI BlastP on this gene

EHK23639

hypothetical protein
  
Accession: EHK23640
  
Location: 1340307-1342143
  
  
**BlastP hit with Mycgr3G35932\_Mycgr3T**
  
Percentage identity: 60 %
  
BlastP bit score: 548
  
Sequence coverage: 96 %
  
E-value: 0.0
  
  
 NCBI BlastP on this gene

EHK23640

hypothetical protein
  
Accession: EHK23641
  
Location: 1342800-1343885
  
 NCBI BlastP on this gene

EHK23641

hypothetical protein
  
Accession: EHK23642
  
Location: 1344418-1345576
  
 NCBI BlastP on this gene

EHK23642

hypothetical protein
  
Accession: EHK23643
  
Location: 1347079-1347426
  
 NCBI BlastP on this gene

EHK23643

hypothetical protein
  
Accession: EHK23644
  
Location: 1347614-1348491
  
 NCBI BlastP on this gene

EHK23644

hypothetical protein
  
Accession: EHK23645
  
Location: 1352155-1352346
  
 NCBI BlastP on this gene

EHK23645

hypothetical protein
  
Accession: EHK23646
  
Location: 1353182-1354585
  
 NCBI BlastP on this gene

EHK23646

hypothetical protein
  
Accession: EHK23647
  
Location: 1355007-1357151
  
 NCBI BlastP on this gene

EHK23647

Query: Architecture Search FASTA input

AMYD01004056 : Colletotrichum gloeosporioides Cg-14    Total score: 2.0     Cumulative Blast bit score: 1023

Hit cluster cross-links:

Mycgr3G36335 Mycgr3T
  
Location: 0-423

Mycgr3G36335\_Mycgr3T

Mycgr3G84494 Mycgr3T
  
Location: 523-2047

Mycgr3G84494\_Mycgr3T

Mycgr3G90558 Mycgr3T
  
Location: 2147-15296

Mycgr3G90558\_Mycgr3T

Mycgr3G68036 Mycgr3T
  
Location: 15396-16395

Mycgr3G68036\_Mycgr3T

Mycgr3G90561 Mycgr3T
  
Location: 16495-17134

Mycgr3G90561\_Mycgr3T

Mycgr3G35862 Mycgr3T
  
Location: 17234-18662

Mycgr3G35862\_Mycgr3T

Mycgr3G68030 Mycgr3T
  
Location: 18762-19722

Mycgr3G68030\_Mycgr3T

Mycgr3G36449 Mycgr3T
  
Location: 19822-21886

Mycgr3G36449\_Mycgr3T

Mycgr3G35528 Mycgr3T
  
Location: 21986-22844

Mycgr3G35528\_Mycgr3T

Mycgr3G35932 Mycgr3T
  
Location: 22944-24390

Mycgr3G35932\_Mycgr3T

Mycgr3G23761 Mycgr3T
  
Location: 24490-25825

Mycgr3G23761\_Mycgr3T

Mycgr3G35535 Mycgr3T
  
Location: 25925-26429

Mycgr3G35535\_Mycgr3T

Mycgr3G9942 Mycgr3T9
  
Location: 26529-30375

Mycgr3G9942\_Mycgr3T9

replication factor C
  
Accession: EQB44234
  
Location: 45-529
  
 NCBI BlastP on this gene

EQB44234

hypothetical protein
  
Accession: EQB44235
  
Location: 1595-3201
  
 NCBI BlastP on this gene

EQB44235

hypothetical protein
  
Accession: EQB44236
  
Location: 6623-7465
  
 NCBI BlastP on this gene

EQB44236

hypothetical protein
  
Accession: EQB44237
  
Location: 8020-9183
  
 NCBI BlastP on this gene

EQB44237

hypothetical protein
  
Accession: EQB44238
  
Location: 10293-10625
  
 NCBI BlastP on this gene

EQB44238

metallopeptidase family M24
  
Accession: EQB44239
  
Location: 11768-13371
  
  
**BlastP hit with Mycgr3G35862\_Mycgr3T**
  
Percentage identity: 50 %
  
BlastP bit score: 455
  
Sequence coverage: 104 %
  
E-value: 1e-152
  
  
 NCBI BlastP on this gene

EQB44239

major facilitator superfamily transporter
  
Accession: EQB44240
  
Location: 13928-15686
  
  
**BlastP hit with Mycgr3G35932\_Mycgr3T**
  
Percentage identity: 58 %
  
BlastP bit score: 568
  
Sequence coverage: 97 %
  
E-value: 0.0
  
  
 NCBI BlastP on this gene

EQB44240

hypothetical protein
  
Accession: EQB44241
  
Location: 16180-17556
  
 NCBI BlastP on this gene

EQB44241

hypothetical protein
  
Accession: EQB44242
  
Location: 18014-19368
  
 NCBI BlastP on this gene

EQB44242

OPT oligopeptide transporter
  
Accession: EQB44243
  
Location: 25401-28147
  
 NCBI BlastP on this gene

EQB44243

hypothetical protein
  
Accession: EQB44244
  
Location: 29109-30327
  
 NCBI BlastP on this gene

EQB44244

Query: Architecture Search FASTA input

GL629735 : Grosmannia clavigera kw1407 unplaced genomic scaffold GCSC\_113    Total score: 2.0     Cumulative Blast bit score: 1020

Hit cluster cross-links:

Mycgr3G36335 Mycgr3T
  
Location: 0-423

Mycgr3G36335\_Mycgr3T

Mycgr3G84494 Mycgr3T
  
Location: 523-2047

Mycgr3G84494\_Mycgr3T

Mycgr3G90558 Mycgr3T
  
Location: 2147-15296

Mycgr3G90558\_Mycgr3T

Mycgr3G68036 Mycgr3T
  
Location: 15396-16395

Mycgr3G68036\_Mycgr3T

Mycgr3G90561 Mycgr3T
  
Location: 16495-17134

Mycgr3G90561\_Mycgr3T

Mycgr3G35862 Mycgr3T
  
Location: 17234-18662

Mycgr3G35862\_Mycgr3T

Mycgr3G68030 Mycgr3T
  
Location: 18762-19722

Mycgr3G68030\_Mycgr3T

Mycgr3G36449 Mycgr3T
  
Location: 19822-21886

Mycgr3G36449\_Mycgr3T

Mycgr3G35528 Mycgr3T
  
Location: 21986-22844

Mycgr3G35528\_Mycgr3T

Mycgr3G35932 Mycgr3T
  
Location: 22944-24390

Mycgr3G35932\_Mycgr3T

Mycgr3G23761 Mycgr3T
  
Location: 24490-25825

Mycgr3G23761\_Mycgr3T

Mycgr3G35535 Mycgr3T
  
Location: 25925-26429

Mycgr3G35535\_Mycgr3T

Mycgr3G9942 Mycgr3T9
  
Location: 26529-30375

Mycgr3G9942\_Mycgr3T9

hypothetical protein
  
Accession: EFX05798
  
Location: 2054706-2056376
  
 NCBI BlastP on this gene

EFX05798

c6 zinc finger domain containing protein
  
Accession: EFX05925
  
Location: 2060011-2062203
  
 NCBI BlastP on this gene

EFX05925

maltose permease
  
Accession: EFX05667
  
Location: 2063221-2064819
  
 NCBI BlastP on this gene

EFX05667

f-box domain containing protein
  
Accession: EFX05886
  
Location: 2066765-2067826
  
 NCBI BlastP on this gene

EFX05886

hypothetical protein
  
Accession: EFX05594
  
Location: 2068140-2068898
  
 NCBI BlastP on this gene

EFX05594

proline dipeptidase
  
Accession: EFX05237
  
Location: 2069715-2071282
  
  
**BlastP hit with Mycgr3G35862\_Mycgr3T**
  
Percentage identity: 54 %
  
BlastP bit score: 440
  
Sequence coverage: 93 %
  
E-value: 1e-146
  
  
 NCBI BlastP on this gene

EFX05237

metabolite transport protein
  
Accession: EFX05486
  
Location: 2071928-2073606
  
  
**BlastP hit with Mycgr3G35932\_Mycgr3T**
  
Percentage identity: 59 %
  
BlastP bit score: 580
  
Sequence coverage: 97 %
  
E-value: 0.0
  
  
 NCBI BlastP on this gene

EFX05486

membrane copper amine oxidase
  
Accession: EFX05707
  
Location: 2073771-2076446
  
 NCBI BlastP on this gene

EFX05707

hypothetical protein
  
Accession: EFX05464
  
Location: 2077839-2078430
  
 NCBI BlastP on this gene

EFX05464

zinc alcohol dehydrogenase
  
Accession: EFX05548
  
Location: 2080614-2081753
  
 NCBI BlastP on this gene

EFX05548

cytochrome p450 monooxygenase
  
Accession: EFX05735
  
Location: 2082011-2082328
  
 NCBI BlastP on this gene

EFX05735

trihydroxytoluene oxygenase
  
Accession: EFX06133
  
Location: 2084222-2085454
  
 NCBI BlastP on this gene

EFX06133

magnesium dependent phosphatase
  
Accession: EFX05882
  
Location: 2086322-2087125
  
 NCBI BlastP on this gene

EFX05882

cellular retinaldehyde-binding/triple function protein
  
Accession: EFX06001
  
Location: 2087418-2089106
  
 NCBI BlastP on this gene

EFX06001

Query: Architecture Search FASTA input

KB706559 : Eutypa lata UCREL1 unplaced genomic scaffold EL1\_03\_scaffold\_1221    Total score: 2.0     Cumulative Blast bit score: 1014

Hit cluster cross-links:

Mycgr3G36335 Mycgr3T
  
Location: 0-423

Mycgr3G36335\_Mycgr3T

Mycgr3G84494 Mycgr3T
  
Location: 523-2047

Mycgr3G84494\_Mycgr3T

Mycgr3G90558 Mycgr3T
  
Location: 2147-15296

Mycgr3G90558\_Mycgr3T

Mycgr3G68036 Mycgr3T
  
Location: 15396-16395

Mycgr3G68036\_Mycgr3T

Mycgr3G90561 Mycgr3T
  
Location: 16495-17134

Mycgr3G90561\_Mycgr3T

Mycgr3G35862 Mycgr3T
  
Location: 17234-18662

Mycgr3G35862\_Mycgr3T

Mycgr3G68030 Mycgr3T
  
Location: 18762-19722

Mycgr3G68030\_Mycgr3T

Mycgr3G36449 Mycgr3T
  
Location: 19822-21886

Mycgr3G36449\_Mycgr3T

Mycgr3G35528 Mycgr3T
  
Location: 21986-22844

Mycgr3G35528\_Mycgr3T

Mycgr3G35932 Mycgr3T
  
Location: 22944-24390

Mycgr3G35932\_Mycgr3T

Mycgr3G23761 Mycgr3T
  
Location: 24490-25825

Mycgr3G23761\_Mycgr3T

Mycgr3G35535 Mycgr3T
  
Location: 25925-26429

Mycgr3G35535\_Mycgr3T

Mycgr3G9942 Mycgr3T9
  
Location: 26529-30375

Mycgr3G9942\_Mycgr3T9

hypothetical protein
  
Accession: EMR66895
  
Location: 60-977
  
 NCBI BlastP on this gene

EMR66895

putative aminopeptidase ypdf protein
  
Accession: EMR66901
  
Location: 3943-5678
  
  
**BlastP hit with Mycgr3G35862\_Mycgr3T**
  
Percentage identity: 48 %
  
BlastP bit score: 442
  
Sequence coverage: 101 %
  
E-value: 5e-147
  
  
 NCBI BlastP on this gene

EMR66901

putative metabolite transporter protein
  
Accession: EMR66900
  
Location: 6469-8315
  
  
**BlastP hit with Mycgr3G35932\_Mycgr3T**
  
Percentage identity: 58 %
  
BlastP bit score: 572
  
Sequence coverage: 97 %
  
E-value: 0.0
  
  
 NCBI BlastP on this gene

EMR66900

hypothetical protein
  
Accession: EMR66903
  
Location: 11649-12550
  
 NCBI BlastP on this gene

EMR66903

hypothetical protein
  
Accession: EMR66896
  
Location: 16852-18500
  
 NCBI BlastP on this gene

EMR66896

putative fad dependent protein
  
Accession: EMR66904
  
Location: 21723-23150
  
 NCBI BlastP on this gene

EMR66904

Query: Architecture Search FASTA input

GG697353 : Glomerella graminicola M1.001 genomic scaffold supercont1.23    Total score: 2.0     Cumulative Blast bit score: 1012

Hit cluster cross-links:

Mycgr3G36335 Mycgr3T
  
Location: 0-423

Mycgr3G36335\_Mycgr3T

Mycgr3G84494 Mycgr3T
  
Location: 523-2047

Mycgr3G84494\_Mycgr3T

Mycgr3G90558 Mycgr3T
  
Location: 2147-15296

Mycgr3G90558\_Mycgr3T

Mycgr3G68036 Mycgr3T
  
Location: 15396-16395

Mycgr3G68036\_Mycgr3T

Mycgr3G90561 Mycgr3T
  
Location: 16495-17134

Mycgr3G90561\_Mycgr3T

Mycgr3G35862 Mycgr3T
  
Location: 17234-18662

Mycgr3G35862\_Mycgr3T

Mycgr3G68030 Mycgr3T
  
Location: 18762-19722

Mycgr3G68030\_Mycgr3T

Mycgr3G36449 Mycgr3T
  
Location: 19822-21886

Mycgr3G36449\_Mycgr3T

Mycgr3G35528 Mycgr3T
  
Location: 21986-22844

Mycgr3G35528\_Mycgr3T

Mycgr3G35932 Mycgr3T
  
Location: 22944-24390

Mycgr3G35932\_Mycgr3T

Mycgr3G23761 Mycgr3T
  
Location: 24490-25825

Mycgr3G23761\_Mycgr3T

Mycgr3G35535 Mycgr3T
  
Location: 25925-26429

Mycgr3G35535\_Mycgr3T

Mycgr3G9942 Mycgr3T9
  
Location: 26529-30375

Mycgr3G9942\_Mycgr3T9

hypothetical protein
  
Accession: EFQ31085
  
Location: 188641-189078
  
 NCBI BlastP on this gene

EFQ31085

NAD-dependent 15-hydroxyprostaglandin dehydrogenase
  
Accession: EFQ31086
  
Location: 191149-191370
  
 NCBI BlastP on this gene

EFQ31086

hypothetical protein
  
Accession: EFQ31087
  
Location: 192603-192950
  
 NCBI BlastP on this gene

EFQ31087

hypothetical protein
  
Accession: EFQ31088
  
Location: 194525-194698
  
 NCBI BlastP on this gene

EFQ31088

OPT oligopeptide transporter
  
Accession: EFQ31089
  
Location: 199439-202165
  
 NCBI BlastP on this gene

EFQ31089

major facilitator superfamily transporter
  
Accession: EFQ31090
  
Location: 202561-204304
  
  
**BlastP hit with Mycgr3G35932\_Mycgr3T**
  
Percentage identity: 57 %
  
BlastP bit score: 560
  
Sequence coverage: 97 %
  
E-value: 0.0
  
  
 NCBI BlastP on this gene

EFQ31090

metallopeptidase family M24
  
Accession: EFQ31091
  
Location: 204855-206419
  
  
**BlastP hit with Mycgr3G35862\_Mycgr3T**
  
Percentage identity: 53 %
  
BlastP bit score: 452
  
Sequence coverage: 94 %
  
E-value: 8e-152
  
  
 NCBI BlastP on this gene

EFQ31091

2OG-Fe(II) oxygenase superfamily protein
  
Accession: EFQ31092
  
Location: 207090-207922
  
 NCBI BlastP on this gene

EFQ31092

hypothetical protein
  
Accession: EFQ31093
  
Location: 210526-212161
  
 NCBI BlastP on this gene

EFQ31093

replication factor C
  
Accession: EFQ31094
  
Location: 213155-214450
  
 NCBI BlastP on this gene

EFQ31094

hypothetical protein
  
Accession: EFQ31095
  
Location: 215007-215900
  
 NCBI BlastP on this gene

EFQ31095

hypothetical protein
  
Accession: EFQ31096
  
Location: 216130-217551
  
 NCBI BlastP on this gene

EFQ31096

Query: Architecture Search FASTA input

CACQ02006567 : Colletotrichum higginsianum strain IMI 349063    Total score: 2.0     Cumulative Blast bit score: 1001

Hit cluster cross-links:

Mycgr3G36335 Mycgr3T
  
Location: 0-423

Mycgr3G36335\_Mycgr3T

Mycgr3G84494 Mycgr3T
  
Location: 523-2047

Mycgr3G84494\_Mycgr3T

Mycgr3G90558 Mycgr3T
  
Location: 2147-15296

Mycgr3G90558\_Mycgr3T

Mycgr3G68036 Mycgr3T
  
Location: 15396-16395

Mycgr3G68036\_Mycgr3T

Mycgr3G90561 Mycgr3T
  
Location: 16495-17134

Mycgr3G90561\_Mycgr3T

Mycgr3G35862 Mycgr3T
  
Location: 17234-18662

Mycgr3G35862\_Mycgr3T

Mycgr3G68030 Mycgr3T
  
Location: 18762-19722

Mycgr3G68030\_Mycgr3T

Mycgr3G36449 Mycgr3T
  
Location: 19822-21886

Mycgr3G36449\_Mycgr3T

Mycgr3G35528 Mycgr3T
  
Location: 21986-22844

Mycgr3G35528\_Mycgr3T

Mycgr3G35932 Mycgr3T
  
Location: 22944-24390

Mycgr3G35932\_Mycgr3T

Mycgr3G23761 Mycgr3T
  
Location: 24490-25825

Mycgr3G23761\_Mycgr3T

Mycgr3G35535 Mycgr3T
  
Location: 25925-26429

Mycgr3G35535\_Mycgr3T

Mycgr3G9942 Mycgr3T9
  
Location: 26529-30375

Mycgr3G9942\_Mycgr3T9

2OG-Fe(II) oxygenase
  
Accession: CCF44025
  
Location: 379-1131
  
 NCBI BlastP on this gene

CCF44025

metallopeptidase family M24
  
Accession: CCF44026
  
Location: 1814-3422
  
  
**BlastP hit with Mycgr3G35862\_Mycgr3T**
  
Percentage identity: 51 %
  
BlastP bit score: 446
  
Sequence coverage: 97 %
  
E-value: 3e-149
  
  
 NCBI BlastP on this gene

CCF44026

major facilitator superfamily transporter
  
Accession: CCF44027
  
Location: 3967-5703
  
  
**BlastP hit with Mycgr3G35932\_Mycgr3T**
  
Percentage identity: 57 %
  
BlastP bit score: 555
  
Sequence coverage: 97 %
  
E-value: 0.0
  
  
 NCBI BlastP on this gene

CCF44027

OPT oligopeptide transporter
  
Accession: CCF44028
  
Location: 7602-10336
  
 NCBI BlastP on this gene

CCF44028

Query: Architecture Search FASTA input

ABDG02000025 : Trichoderma atroviride IMI 206040    Total score: 2.0     Cumulative Blast bit score: 1000

Hit cluster cross-links:

Mycgr3G36335 Mycgr3T
  
Location: 0-423

Mycgr3G36335\_Mycgr3T

Mycgr3G84494 Mycgr3T
  
Location: 523-2047

Mycgr3G84494\_Mycgr3T

Mycgr3G90558 Mycgr3T
  
Location: 2147-15296

Mycgr3G90558\_Mycgr3T

Mycgr3G68036 Mycgr3T
  
Location: 15396-16395

Mycgr3G68036\_Mycgr3T

Mycgr3G90561 Mycgr3T
  
Location: 16495-17134

Mycgr3G90561\_Mycgr3T

Mycgr3G35862 Mycgr3T
  
Location: 17234-18662

Mycgr3G35862\_Mycgr3T

Mycgr3G68030 Mycgr3T
  
Location: 18762-19722

Mycgr3G68030\_Mycgr3T

Mycgr3G36449 Mycgr3T
  
Location: 19822-21886

Mycgr3G36449\_Mycgr3T

Mycgr3G35528 Mycgr3T
  
Location: 21986-22844

Mycgr3G35528\_Mycgr3T

Mycgr3G35932 Mycgr3T
  
Location: 22944-24390

Mycgr3G35932\_Mycgr3T

Mycgr3G23761 Mycgr3T
  
Location: 24490-25825

Mycgr3G23761\_Mycgr3T

Mycgr3G35535 Mycgr3T
  
Location: 25925-26429

Mycgr3G35535\_Mycgr3T

Mycgr3G9942 Mycgr3T9
  
Location: 26529-30375

Mycgr3G9942\_Mycgr3T9

hypothetical protein
  
Accession: EHK44042
  
Location: 1300516-1303228
  
 NCBI BlastP on this gene

EHK44042

hypothetical protein
  
Accession: EHK44043
  
Location: 1304707-1306559
  
 NCBI BlastP on this gene

EHK44043

hypothetical protein
  
Accession: EHK44044
  
Location: 1307072-1308534
  
 NCBI BlastP on this gene

EHK44044

hypothetical protein
  
Accession: EHK44045
  
Location: 1314024-1314743
  
 NCBI BlastP on this gene

EHK44045

hypothetical protein
  
Accession: EHK44046
  
Location: 1316703-1317986
  
 NCBI BlastP on this gene

EHK44046

hypothetical protein
  
Accession: EHK44047
  
Location: 1318177-1319661
  
  
**BlastP hit with Mycgr3G35862\_Mycgr3T**
  
Percentage identity: 55 %
  
BlastP bit score: 457
  
Sequence coverage: 88 %
  
E-value: 3e-153
  
  
 NCBI BlastP on this gene

EHK44047

inorganic phosphate transporter
  
Accession: EHK44048
  
Location: 1320897-1322442
  
  
**BlastP hit with Mycgr3G35932\_Mycgr3T**
  
Percentage identity: 61 %
  
BlastP bit score: 543
  
Sequence coverage: 94 %
  
E-value: 0.0
  
  
 NCBI BlastP on this gene

EHK44048

hypothetical protein
  
Accession: EHK44049
  
Location: 1322907-1323413
  
 NCBI BlastP on this gene

EHK44049

hypothetical protein
  
Accession: EHK44050
  
Location: 1324646-1325794
  
 NCBI BlastP on this gene

EHK44050

hypothetical protein
  
Accession: EHK44051
  
Location: 1326327-1327510
  
 NCBI BlastP on this gene

EHK44051

hypothetical protein
  
Accession: EHK44053
  
Location: 1328700-1329046
  
 NCBI BlastP on this gene

EHK44053

hypothetical protein
  
Accession: EHK44052
  
Location: 1329291-1330148
  
 NCBI BlastP on this gene

EHK44052

hypothetical protein
  
Accession: EHK44054
  
Location: 1333548-1333736
  
 NCBI BlastP on this gene

EHK44054

hypothetical protein
  
Accession: EHK44055
  
Location: 1334434-1335837
  
 NCBI BlastP on this gene

EHK44055

hypothetical protein
  
Accession: EHK44056
  
Location: 1336290-1338452
  
 NCBI BlastP on this gene

EHK44056

Query: Architecture Search FASTA input

GG698907 : Nectria haematococca mpVI 77-13-4 chromosome 12 genomic scaffold NECHAsca\_15\_chr12\_5\_0    Total score: 2.0     Cumulative Blast bit score: 999

Hit cluster cross-links:

Mycgr3G36335 Mycgr3T
  
Location: 0-423

Mycgr3G36335\_Mycgr3T

Mycgr3G84494 Mycgr3T
  
Location: 523-2047

Mycgr3G84494\_Mycgr3T

Mycgr3G90558 Mycgr3T
  
Location: 2147-15296

Mycgr3G90558\_Mycgr3T

Mycgr3G68036 Mycgr3T
  
Location: 15396-16395

Mycgr3G68036\_Mycgr3T

Mycgr3G90561 Mycgr3T
  
Location: 16495-17134

Mycgr3G90561\_Mycgr3T

Mycgr3G35862 Mycgr3T
  
Location: 17234-18662

Mycgr3G35862\_Mycgr3T

Mycgr3G68030 Mycgr3T
  
Location: 18762-19722

Mycgr3G68030\_Mycgr3T

Mycgr3G36449 Mycgr3T
  
Location: 19822-21886

Mycgr3G36449\_Mycgr3T

Mycgr3G35528 Mycgr3T
  
Location: 21986-22844

Mycgr3G35528\_Mycgr3T

Mycgr3G35932 Mycgr3T
  
Location: 22944-24390

Mycgr3G35932\_Mycgr3T

Mycgr3G23761 Mycgr3T
  
Location: 24490-25825

Mycgr3G23761\_Mycgr3T

Mycgr3G35535 Mycgr3T
  
Location: 25925-26429

Mycgr3G35535\_Mycgr3T

Mycgr3G9942 Mycgr3T9
  
Location: 26529-30375

Mycgr3G9942\_Mycgr3T9

hypothetical protein
  
Accession: EEU41613
  
Location: 799109-801227
  
 NCBI BlastP on this gene

EEU41613

hypothetical protein
  
Accession: EEU41789
  
Location: 801592-803647
  
 NCBI BlastP on this gene

EEU41789

hypothetical protein
  
Accession: EEU41790
  
Location: 804927-806979
  
 NCBI BlastP on this gene

EEU41790

hypothetical protein
  
Accession: EEU41791
  
Location: 808798-810219
  
 NCBI BlastP on this gene

EEU41791

predicted protein
  
Accession: EEU41792
  
Location: 811273-812358
  
 NCBI BlastP on this gene

EEU41792

hypothetical protein
  
Accession: EEU41793
  
Location: 812880-813776
  
 NCBI BlastP on this gene

EEU41793

hypothetical protein
  
Accession: EEU41794
  
Location: 814246-815925
  
  
**BlastP hit with Mycgr3G35932\_Mycgr3T**
  
Percentage identity: 58 %
  
BlastP bit score: 557
  
Sequence coverage: 96 %
  
E-value: 0.0
  
  
 NCBI BlastP on this gene

EEU41794

hypothetical protein
  
Accession: EEU41614
  
Location: 816360-817817
  
  
**BlastP hit with Mycgr3G35862\_Mycgr3T**
  
Percentage identity: 53 %
  
BlastP bit score: 442
  
Sequence coverage: 92 %
  
E-value: 2e-148
  
  
 NCBI BlastP on this gene

EEU41614

hypothetical protein
  
Accession: EEU41795
  
Location: 818543-820374
  
 NCBI BlastP on this gene

EEU41795

hypothetical protein
  
Accession: EEU41615
  
Location: 820819-821604
  
 NCBI BlastP on this gene

EEU41615

predicted protein
  
Accession: EEU41616
  
Location: 822333-823358
  
 NCBI BlastP on this gene

EEU41616

hypothetical protein
  
Accession: EEU41617
  
Location: 824458-825915
  
 NCBI BlastP on this gene

EEU41617

hypothetical protein
  
Accession: EEU41618
  
Location: 826540-827752
  
 NCBI BlastP on this gene

EEU41618

predicted protein
  
Accession: EEU41619
  
Location: 830758-832728
  
 NCBI BlastP on this gene

EEU41619

Query: Architecture Search FASTA input

GL985063 : Trichoderma reesei QM6a unplaced genomic scaffold TRIREscaffold\_8    Total score: 2.0     Cumulative Blast bit score: 993

Hit cluster cross-links:

Mycgr3G36335 Mycgr3T
  
Location: 0-423

Mycgr3G36335\_Mycgr3T

Mycgr3G84494 Mycgr3T
  
Location: 523-2047

Mycgr3G84494\_Mycgr3T

Mycgr3G90558 Mycgr3T
  
Location: 2147-15296

Mycgr3G90558\_Mycgr3T

Mycgr3G68036 Mycgr3T
  
Location: 15396-16395

Mycgr3G68036\_Mycgr3T

Mycgr3G90561 Mycgr3T
  
Location: 16495-17134

Mycgr3G90561\_Mycgr3T

Mycgr3G35862 Mycgr3T
  
Location: 17234-18662

Mycgr3G35862\_Mycgr3T

Mycgr3G68030 Mycgr3T
  
Location: 18762-19722

Mycgr3G68030\_Mycgr3T

Mycgr3G36449 Mycgr3T
  
Location: 19822-21886

Mycgr3G36449\_Mycgr3T

Mycgr3G35528 Mycgr3T
  
Location: 21986-22844

Mycgr3G35528\_Mycgr3T

Mycgr3G35932 Mycgr3T
  
Location: 22944-24390

Mycgr3G35932\_Mycgr3T

Mycgr3G23761 Mycgr3T
  
Location: 24490-25825

Mycgr3G23761\_Mycgr3T

Mycgr3G35535 Mycgr3T
  
Location: 25925-26429

Mycgr3G35535\_Mycgr3T

Mycgr3G9942 Mycgr3T9
  
Location: 26529-30375

Mycgr3G9942\_Mycgr3T9

predicted protein
  
Accession: EGR49035
  
Location: 1301642-1304393
  
 NCBI BlastP on this gene

EGR49035

predicted protein
  
Accession: EGR49036
  
Location: 1306431-1308359
  
 NCBI BlastP on this gene

EGR49036

homogentisate 1,2-dioxygenase
  
Accession: EGR49238
  
Location: 1308848-1310312
  
 NCBI BlastP on this gene

EGR49238

predicted protein
  
Accession: EGR49037
  
Location: 1315040-1315765
  
 NCBI BlastP on this gene

EGR49037

predicted protein
  
Accession: EGR49038
  
Location: 1317928-1319364
  
 NCBI BlastP on this gene

EGR49038

Prolidase/Aminopeptidase P-like protein
  
Accession: EGR49239
  
Location: 1319539-1320924
  
  
**BlastP hit with Mycgr3G35862\_Mycgr3T**
  
Percentage identity: 53 %
  
BlastP bit score: 454
  
Sequence coverage: 96 %
  
E-value: 1e-152
  
  
 NCBI BlastP on this gene

EGR49239

predicted protein
  
Accession: EGR49039
  
Location: 1322450-1323991
  
  
**BlastP hit with Mycgr3G35932\_Mycgr3T**
  
Percentage identity: 60 %
  
BlastP bit score: 540
  
Sequence coverage: 94 %
  
E-value: 0.0
  
  
 NCBI BlastP on this gene

EGR49039

predicted protein
  
Accession: EGR49040
  
Location: 1324748-1326100
  
 NCBI BlastP on this gene

EGR49040

predicted protein
  
Accession: EGR49240
  
Location: 1326643-1327875
  
 NCBI BlastP on this gene

EGR49240

predicted protein
  
Accession: EGR49041
  
Location: 1330240-1330538
  
 NCBI BlastP on this gene

EGR49041

predicted protein
  
Accession: EGR49241
  
Location: 1331108-1332019
  
 NCBI BlastP on this gene

EGR49241

4-aminobutyrate aminotransferase-like protein
  
Accession: EGR49242
  
Location: 1336705-1338111
  
 NCBI BlastP on this gene

EGR49242

N-terminal binuclear Zn cluster-containing/DNA binding domain-containing protein
  
Accession: EGR49243
  
Location: 1338556-1340706
  
 NCBI BlastP on this gene

EGR49243

Query: Architecture Search FASTA input

CH476594 : Aspergillus terreus NIH2624 scaffold\_1 genomic scaffold    Total score: 2.0     Cumulative Blast bit score: 984

Hit cluster cross-links:

Mycgr3G36335 Mycgr3T
  
Location: 0-423

Mycgr3G36335\_Mycgr3T

Mycgr3G84494 Mycgr3T
  
Location: 523-2047

Mycgr3G84494\_Mycgr3T

Mycgr3G90558 Mycgr3T
  
Location: 2147-15296

Mycgr3G90558\_Mycgr3T

Mycgr3G68036 Mycgr3T
  
Location: 15396-16395

Mycgr3G68036\_Mycgr3T

Mycgr3G90561 Mycgr3T
  
Location: 16495-17134

Mycgr3G90561\_Mycgr3T

Mycgr3G35862 Mycgr3T
  
Location: 17234-18662

Mycgr3G35862\_Mycgr3T

Mycgr3G68030 Mycgr3T
  
Location: 18762-19722

Mycgr3G68030\_Mycgr3T

Mycgr3G36449 Mycgr3T
  
Location: 19822-21886

Mycgr3G36449\_Mycgr3T

Mycgr3G35528 Mycgr3T
  
Location: 21986-22844

Mycgr3G35528\_Mycgr3T

Mycgr3G35932 Mycgr3T
  
Location: 22944-24390

Mycgr3G35932\_Mycgr3T

Mycgr3G23761 Mycgr3T
  
Location: 24490-25825

Mycgr3G23761\_Mycgr3T

Mycgr3G35535 Mycgr3T
  
Location: 25925-26429

Mycgr3G35535\_Mycgr3T

Mycgr3G9942 Mycgr3T9
  
Location: 26529-30375

Mycgr3G9942\_Mycgr3T9

conserved hypothetical protein
  
Accession: EAU39598
  
Location: 2671218-2674001
  
 NCBI BlastP on this gene

EAU39598

predicted protein
  
Accession: EAU39599
  
Location: 2674566-2676546
  
 NCBI BlastP on this gene

EAU39599

conserved hypothetical protein
  
Accession: EAU39600
  
Location: 2676738-2677445
  
 NCBI BlastP on this gene

EAU39600

conserved hypothetical protein
  
Accession: EAU39601
  
Location: 2677883-2679823
  
 NCBI BlastP on this gene

EAU39601

predicted protein
  
Accession: EAU39602
  
Location: 2681905-2683400
  
 NCBI BlastP on this gene

EAU39602

fatty acid transporter protein
  
Accession: EAU39603
  
Location: 2684247-2686282
  
 NCBI BlastP on this gene

EAU39603

conserved hypothetical protein
  
Accession: EAU39604
  
Location: 2686794-2688433
  
  
**BlastP hit with Mycgr3G35932\_Mycgr3T**
  
Percentage identity: 62 %
  
BlastP bit score: 561
  
Sequence coverage: 92 %
  
E-value: 0.0
  
  
 NCBI BlastP on this gene

EAU39604

conserved hypothetical protein
  
Accession: EAU39605
  
Location: 2689114-2690766
  
  
**BlastP hit with Mycgr3G35862\_Mycgr3T**
  
Percentage identity: 54 %
  
BlastP bit score: 423
  
Sequence coverage: 89 %
  
E-value: 1e-140
  
  
 NCBI BlastP on this gene

EAU39605

predicted protein
  
Accession: EAU39606
  
Location: 2691423-2692919
  
 NCBI BlastP on this gene

EAU39606

conserved hypothetical protein
  
Accession: EAU39607
  
Location: 2693687-2695368
  
 NCBI BlastP on this gene

EAU39607

predicted protein
  
Accession: EAU39608
  
Location: 2697059-2698520
  
 NCBI BlastP on this gene

EAU39608

conserved hypothetical protein
  
Accession: EAU39609
  
Location: 2700069-2702716
  
 NCBI BlastP on this gene

EAU39609

predicted protein
  
Accession: EAU39610
  
Location: 2703919-2704716
  
 NCBI BlastP on this gene

EAU39610

conserved hypothetical protein
  
Accession: EAU39611
  
Location: 2705214-2705862
  
 NCBI BlastP on this gene

EAU39611

Query: Architecture Search FASTA input

KE148169 : Ophiostoma piceae UAMH 11346 chromosome Unknown scf24    Total score: 2.0     Cumulative Blast bit score: 980

Hit cluster cross-links:

Mycgr3G36335 Mycgr3T
  
Location: 0-423

Mycgr3G36335\_Mycgr3T

Mycgr3G84494 Mycgr3T
  
Location: 523-2047

Mycgr3G84494\_Mycgr3T

Mycgr3G90558 Mycgr3T
  
Location: 2147-15296

Mycgr3G90558\_Mycgr3T

Mycgr3G68036 Mycgr3T
  
Location: 15396-16395

Mycgr3G68036\_Mycgr3T

Mycgr3G90561 Mycgr3T
  
Location: 16495-17134

Mycgr3G90561\_Mycgr3T

Mycgr3G35862 Mycgr3T
  
Location: 17234-18662

Mycgr3G35862\_Mycgr3T

Mycgr3G68030 Mycgr3T
  
Location: 18762-19722

Mycgr3G68030\_Mycgr3T

Mycgr3G36449 Mycgr3T
  
Location: 19822-21886

Mycgr3G36449\_Mycgr3T

Mycgr3G35528 Mycgr3T
  
Location: 21986-22844

Mycgr3G35528\_Mycgr3T

Mycgr3G35932 Mycgr3T
  
Location: 22944-24390

Mycgr3G35932\_Mycgr3T

Mycgr3G23761 Mycgr3T
  
Location: 24490-25825

Mycgr3G23761\_Mycgr3T

Mycgr3G35535 Mycgr3T
  
Location: 25925-26429

Mycgr3G35535\_Mycgr3T

Mycgr3G9942 Mycgr3T9
  
Location: 26529-30375

Mycgr3G9942\_Mycgr3T9

phytase
  
Accession: EPE03310
  
Location: 375363-377484
  
 NCBI BlastP on this gene

EPE03310

acid phosphatase-like protein
  
Accession: EPE03311
  
Location: 378053-380356
  
 NCBI BlastP on this gene

EPE03311

translocation protein sec62
  
Accession: EPE03312
  
Location: 385697-387065
  
 NCBI BlastP on this gene

EPE03312

gcn5-related n-acetyltransferase
  
Accession: EPE03313
  
Location: 387465-388464
  
 NCBI BlastP on this gene

EPE03313

thymine dioxygenase
  
Accession: EPE03314
  
Location: 389225-390313
  
 NCBI BlastP on this gene

EPE03314

hexose transporter
  
Accession: EPE03315
  
Location: 390898-392605
  
 NCBI BlastP on this gene

EPE03315

major facilitator superfamily transporter
  
Accession: EPE03316
  
Location: 392793-394456
  
  
**BlastP hit with Mycgr3G35932\_Mycgr3T**
  
Percentage identity: 56 %
  
BlastP bit score: 555
  
Sequence coverage: 97 %
  
E-value: 0.0
  
  
 NCBI BlastP on this gene

EPE03316

xaa-pro dipeptidase
  
Accession: EPE03317
  
Location: 395500-397088
  
  
**BlastP hit with Mycgr3G35862\_Mycgr3T**
  
Percentage identity: 50 %
  
BlastP bit score: 425
  
Sequence coverage: 94 %
  
E-value: 2e-140
  
  
 NCBI BlastP on this gene

EPE03317

hypothetical protein
  
Accession: EPE03318
  
Location: 398367-399075
  
 NCBI BlastP on this gene

EPE03318

hypothetical protein
  
Accession: EPE03319
  
Location: 399911-401234
  
 NCBI BlastP on this gene

EPE03319

mfs transporter
  
Accession: EPE03320
  
Location: 402247-404076
  
 NCBI BlastP on this gene

EPE03320

helix-turn-helix-domain containing protein type
  
Accession: EPE03321
  
Location: 404300-404901
  
 NCBI BlastP on this gene

EPE03321

Query: Architecture Search FASTA input

JH795082 : Magnaporthe oryzae P131 unplaced genomic scaffold P131\_scaffold01190    Total score: 2.0     Cumulative Blast bit score: 979

Hit cluster cross-links:

Mycgr3G36335 Mycgr3T
  
Location: 0-423

Mycgr3G36335\_Mycgr3T

Mycgr3G84494 Mycgr3T
  
Location: 523-2047

Mycgr3G84494\_Mycgr3T

Mycgr3G90558 Mycgr3T
  
Location: 2147-15296

Mycgr3G90558\_Mycgr3T

Mycgr3G68036 Mycgr3T
  
Location: 15396-16395

Mycgr3G68036\_Mycgr3T

Mycgr3G90561 Mycgr3T
  
Location: 16495-17134

Mycgr3G90561\_Mycgr3T

Mycgr3G35862 Mycgr3T
  
Location: 17234-18662

Mycgr3G35862\_Mycgr3T

Mycgr3G68030 Mycgr3T
  
Location: 18762-19722

Mycgr3G68030\_Mycgr3T

Mycgr3G36449 Mycgr3T
  
Location: 19822-21886

Mycgr3G36449\_Mycgr3T

Mycgr3G35528 Mycgr3T
  
Location: 21986-22844

Mycgr3G35528\_Mycgr3T

Mycgr3G35932 Mycgr3T
  
Location: 22944-24390

Mycgr3G35932\_Mycgr3T

Mycgr3G23761 Mycgr3T
  
Location: 24490-25825

Mycgr3G23761\_Mycgr3T

Mycgr3G35535 Mycgr3T
  
Location: 25925-26429

Mycgr3G35535\_Mycgr3T

Mycgr3G9942 Mycgr3T9
  
Location: 26529-30375

Mycgr3G9942\_Mycgr3T9

hypothetical protein
  
Accession: ELQ61354
  
Location: 10118-10846
  
 NCBI BlastP on this gene

ELQ61354

surface protein 1
  
Accession: ELQ61355
  
Location: 11670-12110
  
 NCBI BlastP on this gene

ELQ61355

hypothetical protein
  
Accession: ELQ61356
  
Location: 12745-12840
  
 NCBI BlastP on this gene

ELQ61356

C-signal protein
  
Accession: ELQ61357
  
Location: 13358-14092
  
 NCBI BlastP on this gene

ELQ61357

hypothetical protein
  
Accession: ELQ61358
  
Location: 14632-15210
  
 NCBI BlastP on this gene

ELQ61358

benzoate 4-monooxygenase
  
Accession: ELQ61359
  
Location: 17324-19165
  
 NCBI BlastP on this gene

ELQ61359

hypothetical protein
  
Accession: ELQ61360
  
Location: 20652-21650
  
 NCBI BlastP on this gene

ELQ61360

hypothetical protein
  
Accession: ELQ61361
  
Location: 21863-22450
  
 NCBI BlastP on this gene

ELQ61361

DUF563 domain-containing protein
  
Accession: ELQ61362
  
Location: 22706-24501
  
 NCBI BlastP on this gene

ELQ61362

metabolite transporter
  
Accession: ELQ61363
  
Location: 25605-27119
  
  
**BlastP hit with Mycgr3G35932\_Mycgr3T**
  
Percentage identity: 57 %
  
BlastP bit score: 542
  
Sequence coverage: 99 %
  
E-value: 0.0
  
  
 NCBI BlastP on this gene

ELQ61363

aminopeptidase ypdF
  
Accession: ELQ61364
  
Location: 27788-29328
  
  
**BlastP hit with Mycgr3G35862\_Mycgr3T**
  
Percentage identity: 50 %
  
BlastP bit score: 437
  
Sequence coverage: 102 %
  
E-value: 9e-146
  
  
 NCBI BlastP on this gene

ELQ61364

hypothetical protein
  
Accession: ELQ61365
  
Location: 29580-31338
  
 NCBI BlastP on this gene

ELQ61365

Query: Architecture Search FASTA input

JH793116 : Magnaporthe oryzae Y34 unplaced genomic scaffold Y34\_scaffold00799    Total score: 2.0     Cumulative Blast bit score: 979

Hit cluster cross-links:

Mycgr3G36335 Mycgr3T
  
Location: 0-423

Mycgr3G36335\_Mycgr3T

Mycgr3G84494 Mycgr3T
  
Location: 523-2047

Mycgr3G84494\_Mycgr3T

Mycgr3G90558 Mycgr3T
  
Location: 2147-15296

Mycgr3G90558\_Mycgr3T

Mycgr3G68036 Mycgr3T
  
Location: 15396-16395

Mycgr3G68036\_Mycgr3T

Mycgr3G90561 Mycgr3T
  
Location: 16495-17134

Mycgr3G90561\_Mycgr3T

Mycgr3G35862 Mycgr3T
  
Location: 17234-18662

Mycgr3G35862\_Mycgr3T

Mycgr3G68030 Mycgr3T
  
Location: 18762-19722

Mycgr3G68030\_Mycgr3T

Mycgr3G36449 Mycgr3T
  
Location: 19822-21886

Mycgr3G36449\_Mycgr3T

Mycgr3G35528 Mycgr3T
  
Location: 21986-22844

Mycgr3G35528\_Mycgr3T

Mycgr3G35932 Mycgr3T
  
Location: 22944-24390

Mycgr3G35932\_Mycgr3T

Mycgr3G23761 Mycgr3T
  
Location: 24490-25825

Mycgr3G23761\_Mycgr3T

Mycgr3G35535 Mycgr3T
  
Location: 25925-26429

Mycgr3G35535\_Mycgr3T

Mycgr3G9942 Mycgr3T9
  
Location: 26529-30375

Mycgr3G9942\_Mycgr3T9

hypothetical protein
  
Accession: ELQ34082
  
Location: 11583-12311
  
 NCBI BlastP on this gene

ELQ34082

surface protein 1
  
Accession: ELQ34083
  
Location: 13135-13575
  
 NCBI BlastP on this gene

ELQ34083

hypothetical protein
  
Accession: ELQ34084
  
Location: 14210-14305
  
 NCBI BlastP on this gene

ELQ34084

C-signal protein
  
Accession: ELQ34085
  
Location: 14823-15557
  
 NCBI BlastP on this gene

ELQ34085

hypothetical protein
  
Accession: ELQ34086
  
Location: 16097-16675
  
 NCBI BlastP on this gene

ELQ34086

benzoate 4-monooxygenase
  
Accession: ELQ34087
  
Location: 18788-20629
  
 NCBI BlastP on this gene

ELQ34087

hypothetical protein
  
Accession: ELQ34088
  
Location: 22116-23114
  
 NCBI BlastP on this gene

ELQ34088

hypothetical protein
  
Accession: ELQ34089
  
Location: 23327-23914
  
 NCBI BlastP on this gene

ELQ34089

DUF563 domain-containing protein
  
Accession: ELQ34090
  
Location: 24170-25965
  
 NCBI BlastP on this gene

ELQ34090

metabolite transporter
  
Accession: ELQ34091
  
Location: 27069-28583
  
  
**BlastP hit with Mycgr3G35932\_Mycgr3T**
  
Percentage identity: 57 %
  
BlastP bit score: 542
  
Sequence coverage: 99 %
  
E-value: 0.0
  
  
 NCBI BlastP on this gene

ELQ34091

aminopeptidase ypdF
  
Accession: ELQ34092
  
Location: 29252-30792
  
  
**BlastP hit with Mycgr3G35862\_Mycgr3T**
  
Percentage identity: 50 %
  
BlastP bit score: 437
  
Sequence coverage: 102 %
  
E-value: 9e-146
  
  
 NCBI BlastP on this gene

ELQ34092

Query: Architecture Search FASTA input

CM001233 : Magnaporthe oryzae 70-15 chromosome 3    Total score: 2.0     Cumulative Blast bit score: 979

Hit cluster cross-links:

Mycgr3G36335 Mycgr3T
  
Location: 0-423

Mycgr3G36335\_Mycgr3T

Mycgr3G84494 Mycgr3T
  
Location: 523-2047

Mycgr3G84494\_Mycgr3T

Mycgr3G90558 Mycgr3T
  
Location: 2147-15296

Mycgr3G90558\_Mycgr3T

Mycgr3G68036 Mycgr3T
  
Location: 15396-16395

Mycgr3G68036\_Mycgr3T

Mycgr3G90561 Mycgr3T
  
Location: 16495-17134

Mycgr3G90561\_Mycgr3T

Mycgr3G35862 Mycgr3T
  
Location: 17234-18662

Mycgr3G35862\_Mycgr3T

Mycgr3G68030 Mycgr3T
  
Location: 18762-19722

Mycgr3G68030\_Mycgr3T

Mycgr3G36449 Mycgr3T
  
Location: 19822-21886

Mycgr3G36449\_Mycgr3T

Mycgr3G35528 Mycgr3T
  
Location: 21986-22844

Mycgr3G35528\_Mycgr3T

Mycgr3G35932 Mycgr3T
  
Location: 22944-24390

Mycgr3G35932\_Mycgr3T

Mycgr3G23761 Mycgr3T
  
Location: 24490-25825

Mycgr3G23761\_Mycgr3T

Mycgr3G35535 Mycgr3T
  
Location: 25925-26429

Mycgr3G35535\_Mycgr3T

Mycgr3G9942 Mycgr3T9
  
Location: 26529-30375

Mycgr3G9942\_Mycgr3T9

calcium-transporting ATPase 1
  
Accession: EHA53411
  
Location: 6424367-6428369
  
 NCBI BlastP on this gene

EHA53411

hypothetical protein
  
Accession: EHA53412
  
Location: 6428603-6429478
  
 NCBI BlastP on this gene

EHA53412

surface protein 1
  
Accession: EHA53413
  
Location: 6430155-6430595
  
 NCBI BlastP on this gene

EHA53413

C-signal protein
  
Accession: EHA53414
  
Location: 6431843-6432577
  
 NCBI BlastP on this gene

EHA53414

hypothetical protein
  
Accession: EHA53415
  
Location: 6433117-6433695
  
 NCBI BlastP on this gene

EHA53415

benzoate 4-monooxygenase
  
Accession: EHA53416
  
Location: 6435808-6437483
  
 NCBI BlastP on this gene

EHA53416

hypothetical protein
  
Accession: EHA53417
  
Location: 6440376-6441085
  
 NCBI BlastP on this gene

EHA53417

hypothetical protein
  
Accession: EHA53418
  
Location: 6441461-6443014
  
 NCBI BlastP on this gene

EHA53418

metabolite transporter
  
Accession: EHA53419
  
Location: 6444118-6445632
  
  
**BlastP hit with Mycgr3G35932\_Mycgr3T**
  
Percentage identity: 57 %
  
BlastP bit score: 542
  
Sequence coverage: 99 %
  
E-value: 0.0
  
  
 NCBI BlastP on this gene

EHA53419

aminopeptidase ypdF
  
Accession: EHA53420
  
Location: 6446301-6447841
  
  
**BlastP hit with Mycgr3G35862\_Mycgr3T**
  
Percentage identity: 50 %
  
BlastP bit score: 437
  
Sequence coverage: 102 %
  
E-value: 9e-146
  
  
 NCBI BlastP on this gene

EHA53420

cytochrome P450 monooxygenase
  
Accession: EHA53421
  
Location: 6448093-6449952
  
 NCBI BlastP on this gene

EHA53421

hypothetical protein
  
Accession: EHA53422
  
Location: 6452890-6453801
  
 NCBI BlastP on this gene

EHA53422

Query: Architecture Search FASTA input

KB020307 : Colletotrichum gloeosporioides Nara gc5 unplaced genomic scaffold scaffold1090    Total score: 2.0     Cumulative Blast bit score: 950

Hit cluster cross-links:

Mycgr3G36335 Mycgr3T
  
Location: 0-423

Mycgr3G36335\_Mycgr3T

Mycgr3G84494 Mycgr3T
  
Location: 523-2047

Mycgr3G84494\_Mycgr3T

Mycgr3G90558 Mycgr3T
  
Location: 2147-15296

Mycgr3G90558\_Mycgr3T

Mycgr3G68036 Mycgr3T
  
Location: 15396-16395

Mycgr3G68036\_Mycgr3T

Mycgr3G90561 Mycgr3T
  
Location: 16495-17134

Mycgr3G90561\_Mycgr3T

Mycgr3G35862 Mycgr3T
  
Location: 17234-18662

Mycgr3G35862\_Mycgr3T

Mycgr3G68030 Mycgr3T
  
Location: 18762-19722

Mycgr3G68030\_Mycgr3T

Mycgr3G36449 Mycgr3T
  
Location: 19822-21886

Mycgr3G36449\_Mycgr3T

Mycgr3G35528 Mycgr3T
  
Location: 21986-22844

Mycgr3G35528\_Mycgr3T

Mycgr3G35932 Mycgr3T
  
Location: 22944-24390

Mycgr3G35932\_Mycgr3T

Mycgr3G23761 Mycgr3T
  
Location: 24490-25825

Mycgr3G23761\_Mycgr3T

Mycgr3G35535 Mycgr3T
  
Location: 25925-26429

Mycgr3G35535\_Mycgr3T

Mycgr3G9942 Mycgr3T9
  
Location: 26529-30375

Mycgr3G9942\_Mycgr3T9

xaa-pro dipeptidase
  
Accession: ELA37828
  
Location: 2619-4128
  
  
**BlastP hit with Mycgr3G35862\_Mycgr3T**
  
Percentage identity: 50 %
  
BlastP bit score: 425
  
Sequence coverage: 96 %
  
E-value: 3e-141
  
  
 NCBI BlastP on this gene

ELA37828

metabolite transport
  
Accession: ELA37829
  
Location: 4767-6506
  
  
**BlastP hit with Mycgr3G35932\_Mycgr3T**
  
Percentage identity: 55 %
  
BlastP bit score: 525
  
Sequence coverage: 97 %
  
E-value: 8e-180
  
  
 NCBI BlastP on this gene

ELA37829

alkaline serine protease alp1
  
Accession: ELA37830
  
Location: 6997-8374
  
 NCBI BlastP on this gene

ELA37830

oligopeptide transporter 7
  
Accession: ELA37831
  
Location: 15241-17987
  
 NCBI BlastP on this gene

ELA37831

hypothetical protein
  
Accession: ELA37832
  
Location: 18442-20248
  
 NCBI BlastP on this gene

ELA37832

Query: Architecture Search FASTA input

GL385398 : Gaeumannomyces graminis var. tritici R3-111a-1 unplaced genomic scaffold supercont2.4    Total score: 2.0     Cumulative Blast bit score: 885

Hit cluster cross-links:

Mycgr3G36335 Mycgr3T
  
Location: 0-423

Mycgr3G36335\_Mycgr3T

Mycgr3G84494 Mycgr3T
  
Location: 523-2047

Mycgr3G84494\_Mycgr3T

Mycgr3G90558 Mycgr3T
  
Location: 2147-15296

Mycgr3G90558\_Mycgr3T

Mycgr3G68036 Mycgr3T
  
Location: 15396-16395

Mycgr3G68036\_Mycgr3T

Mycgr3G90561 Mycgr3T
  
Location: 16495-17134

Mycgr3G90561\_Mycgr3T

Mycgr3G35862 Mycgr3T
  
Location: 17234-18662

Mycgr3G35862\_Mycgr3T

Mycgr3G68030 Mycgr3T
  
Location: 18762-19722

Mycgr3G68030\_Mycgr3T

Mycgr3G36449 Mycgr3T
  
Location: 19822-21886

Mycgr3G36449\_Mycgr3T

Mycgr3G35528 Mycgr3T
  
Location: 21986-22844

Mycgr3G35528\_Mycgr3T

Mycgr3G35932 Mycgr3T
  
Location: 22944-24390

Mycgr3G35932\_Mycgr3T

Mycgr3G23761 Mycgr3T
  
Location: 24490-25825

Mycgr3G23761\_Mycgr3T

Mycgr3G35535 Mycgr3T
  
Location: 25925-26429

Mycgr3G35535\_Mycgr3T

Mycgr3G9942 Mycgr3T9
  
Location: 26529-30375

Mycgr3G9942\_Mycgr3T9

hypothetical protein
  
Accession: EJT73605
  
Location: 173164-173730
  
 NCBI BlastP on this gene

EJT73605

hypothetical protein
  
Accession: EJT73606
  
Location: 174701-176241
  
 NCBI BlastP on this gene

EJT73606

hypothetical protein
  
Accession: EJT73607
  
Location: 177123-178787
  
 NCBI BlastP on this gene

EJT73607

hypothetical protein
  
Accession: EJT73608
  
Location: 178976-183782
  
 NCBI BlastP on this gene

EJT73608

hypothetical protein
  
Accession: EJT73609
  
Location: 184865-186627
  
 NCBI BlastP on this gene

EJT73609

high-affinity nicotinic acid transporter
  
Accession: EJT73610
  
Location: 187563-189348
  
 NCBI BlastP on this gene

EJT73610

hypothetical protein
  
Accession: EJT73611
  
Location: 189451-191029
  
  
**BlastP hit with Mycgr3G35862\_Mycgr3T**
  
Percentage identity: 51 %
  
BlastP bit score: 385
  
Sequence coverage: 90 %
  
E-value: 1e-125
  
  
 NCBI BlastP on this gene

EJT73611

hypothetical protein
  
Accession: EJT73612
  
Location: 191759-193354
  
  
**BlastP hit with Mycgr3G35932\_Mycgr3T**
  
Percentage identity: 54 %
  
BlastP bit score: 500
  
Sequence coverage: 98 %
  
E-value: 6e-170
  
  
 NCBI BlastP on this gene

EJT73612

hypothetical protein
  
Accession: EJT73613
  
Location: 193664-194366
  
 NCBI BlastP on this gene

EJT73613

hypothetical protein
  
Accession: EJT73614
  
Location: 194596-196058
  
 NCBI BlastP on this gene

EJT73614

hypothetical protein
  
Accession: EJT73615
  
Location: 198190-198462
  
 NCBI BlastP on this gene

EJT73615

hypothetical protein
  
Accession: EJT73616
  
Location: 199075-199425
  
 NCBI BlastP on this gene

EJT73616

hypothetical protein
  
Accession: EJT73617
  
Location: 199463-202816
  
 NCBI BlastP on this gene

EJT73617

hypothetical protein
  
Accession: EJT73618
  
Location: 202829-203749
  
 NCBI BlastP on this gene

EJT73618

hypothetical protein
  
Accession: EJT73619
  
Location: 204532-208027
  
 NCBI BlastP on this gene

EJT73619

Query: Architecture Search FASTA input

JH687384 : Stereum hirsutum FP-91666 SS1 unplaced genomic scaffold STEHIscaffold\_6    Total score: 2.0     Cumulative Blast bit score: 810

Hit cluster cross-links:

Mycgr3G36335 Mycgr3T
  
Location: 0-423

Mycgr3G36335\_Mycgr3T

Mycgr3G84494 Mycgr3T
  
Location: 523-2047

Mycgr3G84494\_Mycgr3T

Mycgr3G90558 Mycgr3T
  
Location: 2147-15296

Mycgr3G90558\_Mycgr3T

Mycgr3G68036 Mycgr3T
  
Location: 15396-16395

Mycgr3G68036\_Mycgr3T

Mycgr3G90561 Mycgr3T
  
Location: 16495-17134

Mycgr3G90561\_Mycgr3T

Mycgr3G35862 Mycgr3T
  
Location: 17234-18662

Mycgr3G35862\_Mycgr3T

Mycgr3G68030 Mycgr3T
  
Location: 18762-19722

Mycgr3G68030\_Mycgr3T

Mycgr3G36449 Mycgr3T
  
Location: 19822-21886

Mycgr3G36449\_Mycgr3T

Mycgr3G35528 Mycgr3T
  
Location: 21986-22844

Mycgr3G35528\_Mycgr3T

Mycgr3G35932 Mycgr3T
  
Location: 22944-24390

Mycgr3G35932\_Mycgr3T

Mycgr3G23761 Mycgr3T
  
Location: 24490-25825

Mycgr3G23761\_Mycgr3T

Mycgr3G35535 Mycgr3T
  
Location: 25925-26429

Mycgr3G35535\_Mycgr3T

Mycgr3G9942 Mycgr3T9
  
Location: 26529-30375

Mycgr3G9942\_Mycgr3T9

hypothetical protein
  
Accession: EIM87173
  
Location: 637480-639821
  
 NCBI BlastP on this gene

EIM87173

acetate--CoA ligase
  
Accession: EIM87174
  
Location: 641463-643987
  
 NCBI BlastP on this gene

EIM87174

general substrate transporter
  
Accession: EIM87175
  
Location: 644789-647609
  
 NCBI BlastP on this gene

EIM87175

hypothetical protein
  
Accession: EIM87176
  
Location: 648244-649646
  
 NCBI BlastP on this gene

EIM87176

amidohydrolase 2
  
Accession: EIM87177
  
Location: 650074-651506
  
 NCBI BlastP on this gene

EIM87177

FMN-dependent alpha-hydroxy acid dehydrogenase
  
Accession: EIM87178
  
Location: 652182-653955
  
 NCBI BlastP on this gene

EIM87178

metabolite transport protein
  
Accession: EIM87179
  
Location: 654154-656222
  
  
**BlastP hit with Mycgr3G35932\_Mycgr3T**
  
Percentage identity: 46 %
  
BlastP bit score: 434
  
Sequence coverage: 94 %
  
E-value: 3e-144
  
  
 NCBI BlastP on this gene

EIM87179

Creatinase/aminopeptidase
  
Accession: EIM87180
  
Location: 656846-658647
  
  
**BlastP hit with Mycgr3G35862\_Mycgr3T**
  
Percentage identity: 45 %
  
BlastP bit score: 377
  
Sequence coverage: 103 %
  
E-value: 2e-121
  
  
 NCBI BlastP on this gene

EIM87180

hypothetical protein
  
Accession: EIM87181
  
Location: 658972-661819
  
 NCBI BlastP on this gene

EIM87181

beta-glucosidase
  
Accession: EIM87182
  
Location: 663119-666626
  
 NCBI BlastP on this gene

EIM87182

NAD-P-binding protein
  
Accession: EIM87183
  
Location: 667254-668685
  
 NCBI BlastP on this gene

EIM87183

hypothetical protein
  
Accession: EIM87184
  
Location: 669441-671332
  
 NCBI BlastP on this gene

EIM87184

general substrate transporter
  
Accession: EIM87185
  
Location: 672897-675078
  
 NCBI BlastP on this gene

EIM87185

Query: Architecture Search FASTA input

AHHD01000414 : Macrophomina phaseolina MS6    Total score: 2.0     Cumulative Blast bit score: 807

Hit cluster cross-links:

Mycgr3G36335 Mycgr3T
  
Location: 0-423

Mycgr3G36335\_Mycgr3T

Mycgr3G84494 Mycgr3T
  
Location: 523-2047

Mycgr3G84494\_Mycgr3T

Mycgr3G90558 Mycgr3T
  
Location: 2147-15296

Mycgr3G90558\_Mycgr3T

Mycgr3G68036 Mycgr3T
  
Location: 15396-16395

Mycgr3G68036\_Mycgr3T

Mycgr3G90561 Mycgr3T
  
Location: 16495-17134

Mycgr3G90561\_Mycgr3T

Mycgr3G35862 Mycgr3T
  
Location: 17234-18662

Mycgr3G35862\_Mycgr3T

Mycgr3G68030 Mycgr3T
  
Location: 18762-19722

Mycgr3G68030\_Mycgr3T

Mycgr3G36449 Mycgr3T
  
Location: 19822-21886

Mycgr3G36449\_Mycgr3T

Mycgr3G35528 Mycgr3T
  
Location: 21986-22844

Mycgr3G35528\_Mycgr3T

Mycgr3G35932 Mycgr3T
  
Location: 22944-24390

Mycgr3G35932\_Mycgr3T

Mycgr3G23761 Mycgr3T
  
Location: 24490-25825

Mycgr3G23761\_Mycgr3T

Mycgr3G35535 Mycgr3T
  
Location: 25925-26429

Mycgr3G35535\_Mycgr3T

Mycgr3G9942 Mycgr3T9
  
Location: 26529-30375

Mycgr3G9942\_Mycgr3T9

hypothetical protein
  
Accession: EKG13212
  
Location: 1590-2141
  
 NCBI BlastP on this gene

EKG13212

hypothetical protein
  
Accession: EKG13213
  
Location: 3646-5694
  
 NCBI BlastP on this gene

EKG13213

hypothetical protein
  
Accession: EKG13214
  
Location: 5706-6242
  
 NCBI BlastP on this gene

EKG13214

General substrate transporter
  
Accession: EKG13215
  
Location: 6601-7136
  
 NCBI BlastP on this gene

EKG13215

Glycoside hydrolase family 3
  
Accession: EKG13216
  
Location: 9578-11620
  
 NCBI BlastP on this gene

EKG13216

Glucose-methanol-choline oxidoreductase
  
Accession: EKG13217
  
Location: 12418-14071
  
 NCBI BlastP on this gene

EKG13217

Protein of unknown function DUF1445
  
Accession: EKG13218
  
Location: 15314-17102
  
  
**BlastP hit with Mycgr3G35932\_Mycgr3T**
  
Percentage identity: 59 %
  
BlastP bit score: 566
  
Sequence coverage: 97 %
  
E-value: 0.0
  
  
 NCBI BlastP on this gene

EKG13218

hypothetical protein
  
Accession: EKG13219
  
Location: 18339-19179
  
  
**BlastP hit with Mycgr3G35862\_Mycgr3T**
  
Percentage identity: 54 %
  
BlastP bit score: 241
  
Sequence coverage: 48 %
  
E-value: 5e-73
  
  
 NCBI BlastP on this gene

EKG13219

hypothetical protein
  
Accession: EKG13220
  
Location: 19553-20731
  
 NCBI BlastP on this gene

EKG13220

Monooxygenase FAD-binding protein
  
Accession: EKG13221
  
Location: 21763-24237
  
 NCBI BlastP on this gene

EKG13221

Winged helix-turn-helix transcription repressor DNA-binding protein
  
Accession: EKG13222
  
Location: 24504-25524
  
 NCBI BlastP on this gene

EKG13222

SANT domain DNA binding protein
  
Accession: EKG13223
  
Location: 26565-28072
  
 NCBI BlastP on this gene

EKG13223

AMP-dependent synthetase/ligase
  
Accession: EKG13224
  
Location: 30736-34163
  
 NCBI BlastP on this gene

EKG13224

Beta-ketoacyl synthase
  
Accession: EKG13225
  
Location: 34843-37108
  
 NCBI BlastP on this gene

EKG13225

Query: Architecture Search FASTA input

CH445371 : Phaeosphaeria nodorum SN15 scaffold\_47    Total score: 2.0     Cumulative Blast bit score: 578

Hit cluster cross-links:

Mycgr3G36335 Mycgr3T
  
Location: 0-423

Mycgr3G36335\_Mycgr3T

Mycgr3G84494 Mycgr3T
  
Location: 523-2047

Mycgr3G84494\_Mycgr3T

Mycgr3G90558 Mycgr3T
  
Location: 2147-15296

Mycgr3G90558\_Mycgr3T

Mycgr3G68036 Mycgr3T
  
Location: 15396-16395

Mycgr3G68036\_Mycgr3T

Mycgr3G90561 Mycgr3T
  
Location: 16495-17134

Mycgr3G90561\_Mycgr3T

Mycgr3G35862 Mycgr3T
  
Location: 17234-18662

Mycgr3G35862\_Mycgr3T

Mycgr3G68030 Mycgr3T
  
Location: 18762-19722

Mycgr3G68030\_Mycgr3T

Mycgr3G36449 Mycgr3T
  
Location: 19822-21886

Mycgr3G36449\_Mycgr3T

Mycgr3G35528 Mycgr3T
  
Location: 21986-22844

Mycgr3G35528\_Mycgr3T

Mycgr3G35932 Mycgr3T
  
Location: 22944-24390

Mycgr3G35932\_Mycgr3T

Mycgr3G23761 Mycgr3T
  
Location: 24490-25825

Mycgr3G23761\_Mycgr3T

Mycgr3G35535 Mycgr3T
  
Location: 25925-26429

Mycgr3G35535\_Mycgr3T

Mycgr3G9942 Mycgr3T9
  
Location: 26529-30375

Mycgr3G9942\_Mycgr3T9

hypothetical protein
  
Accession: EAT76238
  
Location: 107631-109080
  
 NCBI BlastP on this gene

EAT76238

hypothetical protein
  
Accession: EAT76239
  
Location: 110797-112141
  
 NCBI BlastP on this gene

EAT76239

hypothetical protein
  
Accession: EAT76240
  
Location: 114611-114900
  
 NCBI BlastP on this gene

EAT76240

hypothetical protein
  
Accession: EAT76241
  
Location: 115518-115955
  
 NCBI BlastP on this gene

EAT76241

hypothetical protein
  
Accession: EAT76242
  
Location: 116294-117822
  
  
**BlastP hit with Mycgr3G35932\_Mycgr3T**
  
Percentage identity: 57 %
  
BlastP bit score: 500
  
Sequence coverage: 92 %
  
E-value: 2e-170
  
  
 NCBI BlastP on this gene

EAT76242

hypothetical protein
  
Accession: EAT76244
  
Location: 119214-122068
  
 NCBI BlastP on this gene

EAT76244

hypothetical protein
  
Accession: EAT76245
  
Location: 122529-123072
  
 NCBI BlastP on this gene

EAT76245

hypothetical protein
  
Accession: EAT76246
  
Location: 126384-127274
  
 NCBI BlastP on this gene

EAT76246

hypothetical protein
  
Accession: EAT76247
  
Location: 127864-129039
  
 NCBI BlastP on this gene

EAT76247

hypothetical protein
  
Accession: EAT76248
  
Location: 129744-131356
  
 NCBI BlastP on this gene

EAT76248

hypothetical protein
  
Accession: EAT76249
  
Location: 131478-131681
  
 NCBI BlastP on this gene

EAT76249

hypothetical protein
  
Accession: EAT76250
  
Location: 131742-132731
  
 NCBI BlastP on this gene

EAT76250

hypothetical protein
  
Accession: EAT76251
  
Location: 133015-133433
  
 NCBI BlastP on this gene

EAT76251

hypothetical protein
  
Accession: EAT76252
  
Location: 133985-134915
  
 NCBI BlastP on this gene

EAT76252

hypothetical protein
  
Accession: EAT76253
  
Location: 136188-137033
  
  
**BlastP hit with Mycgr3G36335\_Mycgr3T**
  
Percentage identity: 38 %
  
BlastP bit score: 78
  
Sequence coverage: 97 %
  
E-value: 4e-15
  
  
 NCBI BlastP on this gene

EAT76253

Query: Architecture Search FASTA input

GG704912 : Coccidioides immitis RS genomic scaffold supercont3.2    Total score: 2.0     Cumulative Blast bit score: 576

Hit cluster cross-links:

Mycgr3G36335 Mycgr3T
  
Location: 0-423

Mycgr3G36335\_Mycgr3T

Mycgr3G84494 Mycgr3T
  
Location: 523-2047

Mycgr3G84494\_Mycgr3T

Mycgr3G90558 Mycgr3T
  
Location: 2147-15296

Mycgr3G90558\_Mycgr3T

Mycgr3G68036 Mycgr3T
  
Location: 15396-16395

Mycgr3G68036\_Mycgr3T

Mycgr3G90561 Mycgr3T
  
Location: 16495-17134

Mycgr3G90561\_Mycgr3T

Mycgr3G35862 Mycgr3T
  
Location: 17234-18662

Mycgr3G35862\_Mycgr3T

Mycgr3G68030 Mycgr3T
  
Location: 18762-19722

Mycgr3G68030\_Mycgr3T

Mycgr3G36449 Mycgr3T
  
Location: 19822-21886

Mycgr3G36449\_Mycgr3T

Mycgr3G35528 Mycgr3T
  
Location: 21986-22844

Mycgr3G35528\_Mycgr3T

Mycgr3G35932 Mycgr3T
  
Location: 22944-24390

Mycgr3G35932\_Mycgr3T

Mycgr3G23761 Mycgr3T
  
Location: 24490-25825

Mycgr3G23761\_Mycgr3T

Mycgr3G35535 Mycgr3T
  
Location: 25925-26429

Mycgr3G35535\_Mycgr3T

Mycgr3G9942 Mycgr3T9
  
Location: 26529-30375

Mycgr3G9942\_Mycgr3T9

hypothetical protein
  
Accession: EAS27569
  
Location: 90127-91470
  
 NCBI BlastP on this gene

EAS27569

hypothetical protein
  
Accession: EAS27568
  
Location: 92721-93723
  
 NCBI BlastP on this gene

EAS27568

hypothetical protein
  
Accession: EAS27565
  
Location: 96298-97892
  
 NCBI BlastP on this gene

EAS27565

benzoate 4-monooxygenase cytochrome P450
  
Accession: EAS27564
  
Location: 98685-100477
  
 NCBI BlastP on this gene

EAS27564

multidrug resistance protein
  
Accession: EAS27563
  
Location: 102717-104545
  
  
**BlastP hit with Mycgr3G84494\_Mycgr3T**
  
Percentage identity: 43 %
  
BlastP bit score: 394
  
Sequence coverage: 93 %
  
E-value: 9e-128
  
  
 NCBI BlastP on this gene

EAS27563

hypothetical protein
  
Accession: EAS27562
  
Location: 105365-110490
  
 NCBI BlastP on this gene

EAS27562

hypothetical protein
  
Accession: EAS27561
  
Location: 111533-113158
  
  
**BlastP hit with Mycgr3G68030\_Mycgr3T**
  
Percentage identity: 34 %
  
BlastP bit score: 182
  
Sequence coverage: 94 %
  
E-value: 5e-50
  
  
 NCBI BlastP on this gene

EAS27561

oxidoreductase
  
Accession: EAS27560
  
Location: 113602-114939
  
 NCBI BlastP on this gene

EAS27560

serine/threonine-protein phosphatase
  
Accession: EAS27558
  
Location: 116213-117636
  
 NCBI BlastP on this gene

EAS27558

high affinity copper transporter
  
Accession: EAS27557
  
Location: 119003-119921
  
 NCBI BlastP on this gene

EAS27557

hypothetical protein
  
Accession: EAS27556
  
Location: 121101-123078
  
 NCBI BlastP on this gene

EAS27556

flavodoxin and radical SAM domain-containing protein
  
Accession: EAS27553
  
Location: 124325-126768
  
 NCBI BlastP on this gene

EAS27553

Query: Architecture Search FASTA input

DF126477 : Aspergillus kawachii IFO 4308 DNA, contig: scaffold00031    Total score: 2.0     Cumulative Blast bit score: 556

Hit cluster cross-links:

Mycgr3G36335 Mycgr3T
  
Location: 0-423

Mycgr3G36335\_Mycgr3T

Mycgr3G84494 Mycgr3T
  
Location: 523-2047

Mycgr3G84494\_Mycgr3T

Mycgr3G90558 Mycgr3T
  
Location: 2147-15296

Mycgr3G90558\_Mycgr3T

Mycgr3G68036 Mycgr3T
  
Location: 15396-16395

Mycgr3G68036\_Mycgr3T

Mycgr3G90561 Mycgr3T
  
Location: 16495-17134

Mycgr3G90561\_Mycgr3T

Mycgr3G35862 Mycgr3T
  
Location: 17234-18662

Mycgr3G35862\_Mycgr3T

Mycgr3G68030 Mycgr3T
  
Location: 18762-19722

Mycgr3G68030\_Mycgr3T

Mycgr3G36449 Mycgr3T
  
Location: 19822-21886

Mycgr3G36449\_Mycgr3T

Mycgr3G35528 Mycgr3T
  
Location: 21986-22844

Mycgr3G35528\_Mycgr3T

Mycgr3G35932 Mycgr3T
  
Location: 22944-24390

Mycgr3G35932\_Mycgr3T

Mycgr3G23761 Mycgr3T
  
Location: 24490-25825

Mycgr3G23761\_Mycgr3T

Mycgr3G35535 Mycgr3T
  
Location: 25925-26429

Mycgr3G35535\_Mycgr3T

Mycgr3G9942 Mycgr3T9
  
Location: 26529-30375

Mycgr3G9942\_Mycgr3T9

cytochrome P450
  
Accession: GAA91065
  
Location: 333227-334876
  
 NCBI BlastP on this gene

GAA91065

MFS phospholipid transporter
  
Accession: GAA91064
  
Location: 329915-331699
  
  
**BlastP hit with Mycgr3G35932\_Mycgr3T**
  
Percentage identity: 34 %
  
BlastP bit score: 252
  
Sequence coverage: 95 %
  
E-value: 5e-74
  
  
 NCBI BlastP on this gene

GAA91064

similar to An04g09840
  
Accession: GAA91063
  
Location: 327654-329000
  
 NCBI BlastP on this gene

GAA91063

acetyltransferase
  
Accession: GAA91062
  
Location: 326219-326904
  
 NCBI BlastP on this gene

GAA91062

sarcosine oxidase
  
Accession: GAA91061
  
Location: 324550-325971
  
 NCBI BlastP on this gene

GAA91061

rieske [2Fe-2S] domain protein
  
Accession: GAA91060
  
Location: 322205-323539
  
 NCBI BlastP on this gene

GAA91060

fungal specific transcription factor
  
Accession: GAA91059
  
Location: 318353-320470
  
 NCBI BlastP on this gene

GAA91059

alpha/beta hydrolase fold protein
  
Accession: GAA91058
  
Location: 315968-316993
  
 NCBI BlastP on this gene

GAA91058

pathway-specific regulatory protein
  
Accession: GAA91057
  
Location: 312471-314759
  
 NCBI BlastP on this gene

GAA91057

tetrahydrofolylpolyglutamate synthase
  
Accession: GAA91056
  
Location: 310313-312099
  
 NCBI BlastP on this gene

GAA91056

MFS transporter
  
Accession: GAA91055
  
Location: 307842-309449
  
  
**BlastP hit with Mycgr3G84494\_Mycgr3T**
  
Percentage identity: 34 %
  
BlastP bit score: 304
  
Sequence coverage: 93 %
  
E-value: 4e-93
  
  
 NCBI BlastP on this gene

GAA91055

4-coumarate-CoA ligase 1
  
Accession: GAA91054
  
Location: 304817-306825
  
 NCBI BlastP on this gene

GAA91054

catabolic 3-dehydroquinase
  
Accession: GAA91053
  
Location: 302380-303154
  
 NCBI BlastP on this gene

GAA91053

carboxylesterase, type B
  
Accession: GAA91052
  
Location: 300339-302208
  
 NCBI BlastP on this gene

GAA91052

Query: Architecture Search FASTA input

ACJE01000018 : Aspergillus niger ATCC 1015    Total score: 2.0     Cumulative Blast bit score: 553

Hit cluster cross-links:

Mycgr3G36335 Mycgr3T
  
Location: 0-423

Mycgr3G36335\_Mycgr3T

Mycgr3G84494 Mycgr3T
  
Location: 523-2047

Mycgr3G84494\_Mycgr3T

Mycgr3G90558 Mycgr3T
  
Location: 2147-15296

Mycgr3G90558\_Mycgr3T

Mycgr3G68036 Mycgr3T
  
Location: 15396-16395

Mycgr3G68036\_Mycgr3T

Mycgr3G90561 Mycgr3T
  
Location: 16495-17134

Mycgr3G90561\_Mycgr3T

Mycgr3G35862 Mycgr3T
  
Location: 17234-18662

Mycgr3G35862\_Mycgr3T

Mycgr3G68030 Mycgr3T
  
Location: 18762-19722

Mycgr3G68030\_Mycgr3T

Mycgr3G36449 Mycgr3T
  
Location: 19822-21886

Mycgr3G36449\_Mycgr3T

Mycgr3G35528 Mycgr3T
  
Location: 21986-22844

Mycgr3G35528\_Mycgr3T

Mycgr3G35932 Mycgr3T
  
Location: 22944-24390

Mycgr3G35932\_Mycgr3T

Mycgr3G23761 Mycgr3T
  
Location: 24490-25825

Mycgr3G23761\_Mycgr3T

Mycgr3G35535 Mycgr3T
  
Location: 25925-26429

Mycgr3G35535\_Mycgr3T

Mycgr3G9942 Mycgr3T9
  
Location: 26529-30375

Mycgr3G9942\_Mycgr3T9

hypothetical protein
  
Accession: EHA20182
  
Location: 90355-92014
  
 NCBI BlastP on this gene

EHA20182

hypothetical protein
  
Accession: EHA20181
  
Location: 87017-88796
  
  
**BlastP hit with Mycgr3G35932\_Mycgr3T**
  
Percentage identity: 33 %
  
BlastP bit score: 256
  
Sequence coverage: 95 %
  
E-value: 2e-75
  
  
 NCBI BlastP on this gene

EHA20181

hypothetical protein
  
Accession: EHA20180
  
Location: 83268-83946
  
 NCBI BlastP on this gene

EHA20180

hypothetical protein
  
Accession: EHA20179
  
Location: 81545-82965
  
 NCBI BlastP on this gene

EHA20179

hypothetical protein
  
Accession: EHA20178
  
Location: 79206-80542
  
 NCBI BlastP on this gene

EHA20178

hypothetical protein
  
Accession: EHA20177
  
Location: 75596-77975
  
 NCBI BlastP on this gene

EHA20177

hypothetical protein
  
Accession: EHA20176
  
Location: 73429-74061
  
 NCBI BlastP on this gene

EHA20176

hypothetical protein
  
Accession: EHA20175
  
Location: 71142-71859
  
 NCBI BlastP on this gene

EHA20175

hypothetical protein
  
Accession: EHA20174
  
Location: 69785-71051
  
 NCBI BlastP on this gene

EHA20174

hypothetical protein
  
Accession: EHA20173
  
Location: 67587-69376
  
 NCBI BlastP on this gene

EHA20173

hypothetical protein
  
Accession: EHA20172
  
Location: 65139-66587
  
  
**BlastP hit with Mycgr3G84494\_Mycgr3T**
  
Percentage identity: 33 %
  
BlastP bit score: 297
  
Sequence coverage: 92 %
  
E-value: 3e-91
  
  
 NCBI BlastP on this gene

EHA20172

hypothetical protein
  
Accession: EHA20171
  
Location: 62087-64082
  
 NCBI BlastP on this gene

EHA20171

hypothetical protein
  
Accession: EHA20170
  
Location: 59747-60521
  
 NCBI BlastP on this gene

EHA20170

carboxylesterase
  
Accession: EHA20169
  
Location: 57703-59573
  
 NCBI BlastP on this gene

EHA20169

Query: Architecture Search FASTA input

ABDG02000016 : Trichoderma atroviride IMI 206040    Total score: 2.0     Cumulative Blast bit score: 550

Hit cluster cross-links:

Mycgr3G36335 Mycgr3T
  
Location: 0-423

Mycgr3G36335\_Mycgr3T

Mycgr3G84494 Mycgr3T
  
Location: 523-2047

Mycgr3G84494\_Mycgr3T

Mycgr3G90558 Mycgr3T
  
Location: 2147-15296

Mycgr3G90558\_Mycgr3T

Mycgr3G68036 Mycgr3T
  
Location: 15396-16395

Mycgr3G68036\_Mycgr3T

Mycgr3G90561 Mycgr3T
  
Location: 16495-17134

Mycgr3G90561\_Mycgr3T

Mycgr3G35862 Mycgr3T
  
Location: 17234-18662

Mycgr3G35862\_Mycgr3T

Mycgr3G68030 Mycgr3T
  
Location: 18762-19722

Mycgr3G68030\_Mycgr3T

Mycgr3G36449 Mycgr3T
  
Location: 19822-21886

Mycgr3G36449\_Mycgr3T

Mycgr3G35528 Mycgr3T
  
Location: 21986-22844

Mycgr3G35528\_Mycgr3T

Mycgr3G35932 Mycgr3T
  
Location: 22944-24390

Mycgr3G35932\_Mycgr3T

Mycgr3G23761 Mycgr3T
  
Location: 24490-25825

Mycgr3G23761\_Mycgr3T

Mycgr3G35535 Mycgr3T
  
Location: 25925-26429

Mycgr3G35535\_Mycgr3T

Mycgr3G9942 Mycgr3T9
  
Location: 26529-30375

Mycgr3G9942\_Mycgr3T9

hypothetical protein
  
Accession: EHK49378
  
Location: 795-2618
  
 NCBI BlastP on this gene

EHK49378

hypothetical protein
  
Accession: EHK49379
  
Location: 3221-4448
  
 NCBI BlastP on this gene

EHK49379

hypothetical protein
  
Accession: EHK49380
  
Location: 4762-6039
  
 NCBI BlastP on this gene

EHK49380

hypothetical protein
  
Accession: EHK49381
  
Location: 6216-7176
  
 NCBI BlastP on this gene

EHK49381

hypothetical protein
  
Accession: EHK49382
  
Location: 7964-9106
  
 NCBI BlastP on this gene

EHK49382

hypothetical protein
  
Accession: EHK49383
  
Location: 10312-11939
  
  
**BlastP hit with Mycgr3G68030\_Mycgr3T**
  
Percentage identity: 37 %
  
BlastP bit score: 218
  
Sequence coverage: 98 %
  
E-value: 7e-64
  
  
 NCBI BlastP on this gene

EHK49383

hypothetical protein
  
Accession: EHK49384
  
Location: 12598-17971
  
 NCBI BlastP on this gene

EHK49384

hypothetical protein
  
Accession: EHK49385
  
Location: 18878-20543
  
  
**BlastP hit with Mycgr3G84494\_Mycgr3T**
  
Percentage identity: 39 %
  
BlastP bit score: 332
  
Sequence coverage: 95 %
  
E-value: 3e-104
  
  
 NCBI BlastP on this gene

EHK49385

hypothetical protein
  
Accession: EHK49386
  
Location: 21054-22532
  
 NCBI BlastP on this gene

EHK49386

hypothetical protein
  
Accession: EHK49387
  
Location: 23147-24613
  
 NCBI BlastP on this gene

EHK49387

hypothetical protein
  
Accession: EHK49388
  
Location: 25266-26274
  
 NCBI BlastP on this gene

EHK49388

hypothetical protein
  
Accession: EHK49389
  
Location: 26340-28312
  
 NCBI BlastP on this gene

EHK49389

hypothetical protein
  
Accession: EHK49390
  
Location: 29278-31363
  
 NCBI BlastP on this gene

EHK49390

hypothetical protein
  
Accession: EHK49391
  
Location: 32444-34292
  
 NCBI BlastP on this gene

EHK49391

Query: Architecture Search FASTA input

DF126457 : Aspergillus kawachii IFO 4308 DNA, contig: scaffold00011    Total score: 2.0     Cumulative Blast bit score: 545

Hit cluster cross-links:

Mycgr3G36335 Mycgr3T
  
Location: 0-423

Mycgr3G36335\_Mycgr3T

Mycgr3G84494 Mycgr3T
  
Location: 523-2047

Mycgr3G84494\_Mycgr3T

Mycgr3G90558 Mycgr3T
  
Location: 2147-15296

Mycgr3G90558\_Mycgr3T

Mycgr3G68036 Mycgr3T
  
Location: 15396-16395

Mycgr3G68036\_Mycgr3T

Mycgr3G90561 Mycgr3T
  
Location: 16495-17134

Mycgr3G90561\_Mycgr3T

Mycgr3G35862 Mycgr3T
  
Location: 17234-18662

Mycgr3G35862\_Mycgr3T

Mycgr3G68030 Mycgr3T
  
Location: 18762-19722

Mycgr3G68030\_Mycgr3T

Mycgr3G36449 Mycgr3T
  
Location: 19822-21886

Mycgr3G36449\_Mycgr3T

Mycgr3G35528 Mycgr3T
  
Location: 21986-22844

Mycgr3G35528\_Mycgr3T

Mycgr3G35932 Mycgr3T
  
Location: 22944-24390

Mycgr3G35932\_Mycgr3T

Mycgr3G23761 Mycgr3T
  
Location: 24490-25825

Mycgr3G23761\_Mycgr3T

Mycgr3G35535 Mycgr3T
  
Location: 25925-26429

Mycgr3G35535\_Mycgr3T

Mycgr3G9942 Mycgr3T9
  
Location: 26529-30375

Mycgr3G9942\_Mycgr3T9

cytochrome P450
  
Accession: GAA86841
  
Location: 645617-647134
  
 NCBI BlastP on this gene

GAA86841

hypothetical protein
  
Accession: GAA86840
  
Location: 644287-645438
  
 NCBI BlastP on this gene

GAA86840

efflux pump antibiotic resistance protein
  
Accession: GAA86839
  
Location: 641295-643193
  
 NCBI BlastP on this gene

GAA86839

MFS transporter
  
Accession: GAA86838
  
Location: 637614-639169
  
  
**BlastP hit with Mycgr3G84494\_Mycgr3T**
  
Percentage identity: 41 %
  
BlastP bit score: 357
  
Sequence coverage: 96 %
  
E-value: 1e-113
  
  
 NCBI BlastP on this gene

GAA86838

similar to An01g14440
  
Accession: GAA86837
  
Location: 633824-636379
  
 NCBI BlastP on this gene

GAA86837

hypothetical protein
  
Accession: GAA86836
  
Location: 632930-633322
  
 NCBI BlastP on this gene

GAA86836

hypothetical protein
  
Accession: GAA86835
  
Location: 630902-632252
  
 NCBI BlastP on this gene

GAA86835

epoxide hydrolase
  
Accession: GAA86834
  
Location: 629672-630842
  
 NCBI BlastP on this gene

GAA86834

similar to An01g14410
  
Accession: GAA86833
  
Location: 628052-628697
  
 NCBI BlastP on this gene

GAA86833

hypothetical protein
  
Accession: GAA86832
  
Location: 626016-626788
  
 NCBI BlastP on this gene

GAA86832

hypothetical protein
  
Accession: GAA86831
  
Location: 621352-624783
  
 NCBI BlastP on this gene

GAA86831

hypothetical protein
  
Accession: GAA86830
  
Location: 617733-619057
  
  
**BlastP hit with Mycgr3G68030\_Mycgr3T**
  
Percentage identity: 35 %
  
BlastP bit score: 188
  
Sequence coverage: 93 %
  
E-value: 3e-53
  
  
 NCBI BlastP on this gene

GAA86830

C6 transcription factor RosA-like
  
Accession: GAA86829
  
Location: 611796-613687
  
 NCBI BlastP on this gene

GAA86829

Query: Architecture Search FASTA input

ABDF02000063 : Trichoderma virens Gv29-8    Total score: 2.0     Cumulative Blast bit score: 543

Hit cluster cross-links:

Mycgr3G36335 Mycgr3T
  
Location: 0-423

Mycgr3G36335\_Mycgr3T

Mycgr3G84494 Mycgr3T
  
Location: 523-2047

Mycgr3G84494\_Mycgr3T

Mycgr3G90558 Mycgr3T
  
Location: 2147-15296

Mycgr3G90558\_Mycgr3T

Mycgr3G68036 Mycgr3T
  
Location: 15396-16395

Mycgr3G68036\_Mycgr3T

Mycgr3G90561 Mycgr3T
  
Location: 16495-17134

Mycgr3G90561\_Mycgr3T

Mycgr3G35862 Mycgr3T
  
Location: 17234-18662

Mycgr3G35862\_Mycgr3T

Mycgr3G68030 Mycgr3T
  
Location: 18762-19722

Mycgr3G68030\_Mycgr3T

Mycgr3G36449 Mycgr3T
  
Location: 19822-21886

Mycgr3G36449\_Mycgr3T

Mycgr3G35528 Mycgr3T
  
Location: 21986-22844

Mycgr3G35528\_Mycgr3T

Mycgr3G35932 Mycgr3T
  
Location: 22944-24390

Mycgr3G35932\_Mycgr3T

Mycgr3G23761 Mycgr3T
  
Location: 24490-25825

Mycgr3G23761\_Mycgr3T

Mycgr3G35535 Mycgr3T
  
Location: 25925-26429

Mycgr3G35535\_Mycgr3T

Mycgr3G9942 Mycgr3T9
  
Location: 26529-30375

Mycgr3G9942\_Mycgr3T9

hypothetical protein
  
Accession: EHK21762
  
Location: 2800-4429
  
  
**BlastP hit with Mycgr3G68030\_Mycgr3T**
  
Percentage identity: 36 %
  
BlastP bit score: 219
  
Sequence coverage: 98 %
  
E-value: 2e-64
  
  
 NCBI BlastP on this gene

EHK21762

hypothetical protein
  
Accession: EHK21763
  
Location: 5324-13003
  
  
**BlastP hit with Mycgr3G84494\_Mycgr3T**
  
Percentage identity: 39 %
  
BlastP bit score: 324
  
Sequence coverage: 92 %
  
E-value: 5e-94
  
  
 NCBI BlastP on this gene

EHK21763

hypothetical protein
  
Accession: EHK21764
  
Location: 13701-14183
  
 NCBI BlastP on this gene

EHK21764

hypothetical protein
  
Accession: EHK21765
  
Location: 15642-15977
  
 NCBI BlastP on this gene

EHK21765

hypothetical protein
  
Accession: EHK21766
  
Location: 16808-17917
  
 NCBI BlastP on this gene

EHK21766

hypothetical protein
  
Accession: EHK21767
  
Location: 18418-18911
  
 NCBI BlastP on this gene

EHK21767

hypothetical protein
  
Accession: EHK21768
  
Location: 19161-20140
  
 NCBI BlastP on this gene

EHK21768

hypothetical protein
  
Accession: EHK21769
  
Location: 22221-23862
  
 NCBI BlastP on this gene

EHK21769

hypothetical protein
  
Accession: EHK21770
  
Location: 24775-26652
  
 NCBI BlastP on this gene

EHK21770

Query: Architecture Search FASTA input

DF126469 : Aspergillus kawachii IFO 4308 DNA, contig: scaffold00023    Total score: 2.0     Cumulative Blast bit score: 520

Hit cluster cross-links:

Mycgr3G36335 Mycgr3T
  
Location: 0-423

Mycgr3G36335\_Mycgr3T

Mycgr3G84494 Mycgr3T
  
Location: 523-2047

Mycgr3G84494\_Mycgr3T

Mycgr3G90558 Mycgr3T
  
Location: 2147-15296

Mycgr3G90558\_Mycgr3T

Mycgr3G68036 Mycgr3T
  
Location: 15396-16395

Mycgr3G68036\_Mycgr3T

Mycgr3G90561 Mycgr3T
  
Location: 16495-17134

Mycgr3G90561\_Mycgr3T

Mycgr3G35862 Mycgr3T
  
Location: 17234-18662

Mycgr3G35862\_Mycgr3T

Mycgr3G68030 Mycgr3T
  
Location: 18762-19722

Mycgr3G68030\_Mycgr3T

Mycgr3G36449 Mycgr3T
  
Location: 19822-21886

Mycgr3G36449\_Mycgr3T

Mycgr3G35528 Mycgr3T
  
Location: 21986-22844

Mycgr3G35528\_Mycgr3T

Mycgr3G35932 Mycgr3T
  
Location: 22944-24390

Mycgr3G35932\_Mycgr3T

Mycgr3G23761 Mycgr3T
  
Location: 24490-25825

Mycgr3G23761\_Mycgr3T

Mycgr3G35535 Mycgr3T
  
Location: 25925-26429

Mycgr3G35535\_Mycgr3T

Mycgr3G9942 Mycgr3T9
  
Location: 26529-30375

Mycgr3G9942\_Mycgr3T9

O-methyltransferase
  
Accession: GAA89758
  
Location: 513000-514412
  
 NCBI BlastP on this gene

GAA89758

similar to An01g15030
  
Accession: GAA89759
  
Location: 515045-515401
  
 NCBI BlastP on this gene

GAA89759

PQ loop repeat protein
  
Accession: GAA89760
  
Location: 518169-519418
  
 NCBI BlastP on this gene

GAA89760

similar to An01g15070
  
Accession: GAA89761
  
Location: 519428-520582
  
 NCBI BlastP on this gene

GAA89761

gibberellin 20-oxidase
  
Accession: GAA89762
  
Location: 521958-523116
  
  
**BlastP hit with Mycgr3G68036\_Mycgr3T**
  
Percentage identity: 36 %
  
BlastP bit score: 224
  
Sequence coverage: 98 %
  
E-value: 7e-67
  
  
 NCBI BlastP on this gene

GAA89762

cytochrome P450 family protein
  
Accession: GAA89763
  
Location: 523866-525665
  
 NCBI BlastP on this gene

GAA89763

hypothetical protein
  
Accession: GAA89764
  
Location: 525972-527164
  
 NCBI BlastP on this gene

GAA89764

drug resistance protein
  
Accession: GAA89765
  
Location: 528920-530578
  
 NCBI BlastP on this gene

GAA89765

similar to agmatinase
  
Accession: GAA89766
  
Location: 531121-532694
  
 NCBI BlastP on this gene

GAA89766

hypothetical protein
  
Accession: GAA89767
  
Location: 533085-535302
  
 NCBI BlastP on this gene

GAA89767

hypothetical protein
  
Accession: GAA89768
  
Location: 537445-538286
  
 NCBI BlastP on this gene

GAA89768

MFS transporter
  
Accession: GAA89769
  
Location: 539683-541303
  
  
**BlastP hit with Mycgr3G84494\_Mycgr3T**
  
Percentage identity: 38 %
  
BlastP bit score: 296
  
Sequence coverage: 91 %
  
E-value: 3e-90
  
  
 NCBI BlastP on this gene

GAA89769

Query: Architecture Search FASTA input

JH126402 : Cordyceps militaris CM01 unplaced genomic scaffold CCM\_S00004    Total score: 2.0     Cumulative Blast bit score: 519

Hit cluster cross-links:

Mycgr3G36335 Mycgr3T
  
Location: 0-423

Mycgr3G36335\_Mycgr3T

Mycgr3G84494 Mycgr3T
  
Location: 523-2047

Mycgr3G84494\_Mycgr3T

Mycgr3G90558 Mycgr3T
  
Location: 2147-15296

Mycgr3G90558\_Mycgr3T

Mycgr3G68036 Mycgr3T
  
Location: 15396-16395

Mycgr3G68036\_Mycgr3T

Mycgr3G90561 Mycgr3T
  
Location: 16495-17134

Mycgr3G90561\_Mycgr3T

Mycgr3G35862 Mycgr3T
  
Location: 17234-18662

Mycgr3G35862\_Mycgr3T

Mycgr3G68030 Mycgr3T
  
Location: 18762-19722

Mycgr3G68030\_Mycgr3T

Mycgr3G36449 Mycgr3T
  
Location: 19822-21886

Mycgr3G36449\_Mycgr3T

Mycgr3G35528 Mycgr3T
  
Location: 21986-22844

Mycgr3G35528\_Mycgr3T

Mycgr3G35932 Mycgr3T
  
Location: 22944-24390

Mycgr3G35932\_Mycgr3T

Mycgr3G23761 Mycgr3T
  
Location: 24490-25825

Mycgr3G23761\_Mycgr3T

Mycgr3G35535 Mycgr3T
  
Location: 25925-26429

Mycgr3G35535\_Mycgr3T

Mycgr3G9942 Mycgr3T9
  
Location: 26529-30375

Mycgr3G9942\_Mycgr3T9

hypothetical protein
  
Accession: EGX91978
  
Location: 3553193-3553798
  
 NCBI BlastP on this gene

EGX91978

hypothetical protein
  
Accession: EGX91979
  
Location: 3554657-3556069
  
 NCBI BlastP on this gene

EGX91979

hypothetical protein
  
Accession: EGX91980
  
Location: 3559814-3561070
  
 NCBI BlastP on this gene

EGX91980

nucleoside hydrolase, putative
  
Accession: EGX91981
  
Location: 3563495-3564989
  
 NCBI BlastP on this gene

EGX91981

small nuclear ribonucleoprotein Lsm8, putative
  
Accession: EGX91982
  
Location: 3565343-3565847
  
 NCBI BlastP on this gene

EGX91982

glycerate dehydrogenase
  
Accession: EGX91983
  
Location: 3567730-3568830
  
 NCBI BlastP on this gene

EGX91983

Major facilitator superfamily transporter
  
Accession: EGX91984
  
Location: 3569362-3571070
  
  
**BlastP hit with Mycgr3G84494\_Mycgr3T**
  
Percentage identity: 41 %
  
BlastP bit score: 373
  
Sequence coverage: 93 %
  
E-value: 7e-120
  
  
 NCBI BlastP on this gene

EGX91984

Fructosamine/Ketosamine-3-kinase
  
Accession: EGX91985
  
Location: 3571670-3572983
  
  
**BlastP hit with Mycgr3G68030\_Mycgr3T**
  
Percentage identity: 34 %
  
BlastP bit score: 146
  
Sequence coverage: 79 %
  
E-value: 4e-37
  
  
 NCBI BlastP on this gene

EGX91985

WD40 repeat-like-containing domain
  
Accession: EGX91986
  
Location: 3575281-3576834
  
 NCBI BlastP on this gene

EGX91986

serine/threonine-protein kinase psk1
  
Accession: EGX91987
  
Location: 3579582-3581162
  
 NCBI BlastP on this gene

EGX91987

hypothetical protein
  
Accession: EGX91988
  
Location: 3582182-3582480
  
 NCBI BlastP on this gene

EGX91988

ABC transporter, putative
  
Accession: EGX91989
  
Location: 3583734-3588295
  
 NCBI BlastP on this gene

EGX91989

Query: Architecture Search FASTA input

KE145357 : Glarea lozoyensis ATCC 20868 chromosome Unknown GLAREA14    Total score: 2.0     Cumulative Blast bit score: 516

Hit cluster cross-links:

Mycgr3G36335 Mycgr3T
  
Location: 0-423

Mycgr3G36335\_Mycgr3T

Mycgr3G84494 Mycgr3T
  
Location: 523-2047

Mycgr3G84494\_Mycgr3T

Mycgr3G90558 Mycgr3T
  
Location: 2147-15296

Mycgr3G90558\_Mycgr3T

Mycgr3G68036 Mycgr3T
  
Location: 15396-16395

Mycgr3G68036\_Mycgr3T

Mycgr3G90561 Mycgr3T
  
Location: 16495-17134

Mycgr3G90561\_Mycgr3T

Mycgr3G35862 Mycgr3T
  
Location: 17234-18662

Mycgr3G35862\_Mycgr3T

Mycgr3G68030 Mycgr3T
  
Location: 18762-19722

Mycgr3G68030\_Mycgr3T

Mycgr3G36449 Mycgr3T
  
Location: 19822-21886

Mycgr3G36449\_Mycgr3T

Mycgr3G35528 Mycgr3T
  
Location: 21986-22844

Mycgr3G35528\_Mycgr3T

Mycgr3G35932 Mycgr3T
  
Location: 22944-24390

Mycgr3G35932\_Mycgr3T

Mycgr3G23761 Mycgr3T
  
Location: 24490-25825

Mycgr3G23761\_Mycgr3T

Mycgr3G35535 Mycgr3T
  
Location: 25925-26429

Mycgr3G35535\_Mycgr3T

Mycgr3G9942 Mycgr3T9
  
Location: 26529-30375

Mycgr3G9942\_Mycgr3T9

hypothetical protein
  
Accession: EPE34174
  
Location: 1790489-1791000
  
 NCBI BlastP on this gene

EPE34174

hypothetical protein
  
Accession: EPE34173
  
Location: 1788575-1789378
  
 NCBI BlastP on this gene

EPE34173

NAD(P)-binding Rossmann-fold containing protein
  
Accession: EPE34172
  
Location: 1787293-1788300
  
 NCBI BlastP on this gene

EPE34172

alpha/beta-Hydrolase
  
Accession: EPE34171
  
Location: 1783351-1784391
  
 NCBI BlastP on this gene

EPE34171

hypothetical protein
  
Accession: EPE34170
  
Location: 1778666-1781147
  
 NCBI BlastP on this gene

EPE34170

hypothetical protein
  
Accession: EPE34169
  
Location: 1775741-1777084
  
  
**BlastP hit with Mycgr3G68030\_Mycgr3T**
  
Percentage identity: 39 %
  
BlastP bit score: 236
  
Sequence coverage: 99 %
  
E-value: 3e-71
  
  
 NCBI BlastP on this gene

EPE34169

hypothetical protein
  
Accession: EPE34168
  
Location: 1772167-1773514
  
 NCBI BlastP on this gene

EPE34168

ClpP/crotonase
  
Accession: EPE34167
  
Location: 1768874-1771803
  
  
**BlastP hit with Mycgr3G36449\_Mycgr3T**
  
Percentage identity: 31 %
  
BlastP bit score: 280
  
Sequence coverage: 97 %
  
E-value: 9e-79
  
  
 NCBI BlastP on this gene

EPE34167

hypothetical protein
  
Accession: EPE34166
  
Location: 1766512-1768629
  
 NCBI BlastP on this gene

EPE34166

Query: Architecture Search FASTA input

ACJE01000004 : Aspergillus niger ATCC 1015    Total score: 2.0     Cumulative Blast bit score: 516

Hit cluster cross-links:

Mycgr3G36335 Mycgr3T
  
Location: 0-423

Mycgr3G36335\_Mycgr3T

Mycgr3G84494 Mycgr3T
  
Location: 523-2047

Mycgr3G84494\_Mycgr3T

Mycgr3G90558 Mycgr3T
  
Location: 2147-15296

Mycgr3G90558\_Mycgr3T

Mycgr3G68036 Mycgr3T
  
Location: 15396-16395

Mycgr3G68036\_Mycgr3T

Mycgr3G90561 Mycgr3T
  
Location: 16495-17134

Mycgr3G90561\_Mycgr3T

Mycgr3G35862 Mycgr3T
  
Location: 17234-18662

Mycgr3G35862\_Mycgr3T

Mycgr3G68030 Mycgr3T
  
Location: 18762-19722

Mycgr3G68030\_Mycgr3T

Mycgr3G36449 Mycgr3T
  
Location: 19822-21886

Mycgr3G36449\_Mycgr3T

Mycgr3G35528 Mycgr3T
  
Location: 21986-22844

Mycgr3G35528\_Mycgr3T

Mycgr3G35932 Mycgr3T
  
Location: 22944-24390

Mycgr3G35932\_Mycgr3T

Mycgr3G23761 Mycgr3T
  
Location: 24490-25825

Mycgr3G23761\_Mycgr3T

Mycgr3G35535 Mycgr3T
  
Location: 25925-26429

Mycgr3G35535\_Mycgr3T

Mycgr3G9942 Mycgr3T9
  
Location: 26529-30375

Mycgr3G9942\_Mycgr3T9

hypothetical protein
  
Accession: EHA27160
  
Location: 3615826-3616088
  
 NCBI BlastP on this gene

EHA27160

hypothetical protein
  
Accession: EHA27159
  
Location: 3615345-3615809
  
 NCBI BlastP on this gene

EHA27159

hypothetical protein
  
Accession: EHA27158
  
Location: 3611921-3613797
  
 NCBI BlastP on this gene

EHA27158

hypothetical protein
  
Accession: EHA27157
  
Location: 3608239-3609812
  
  
**BlastP hit with Mycgr3G84494\_Mycgr3T**
  
Percentage identity: 39 %
  
BlastP bit score: 357
  
Sequence coverage: 98 %
  
E-value: 6e-114
  
  
 NCBI BlastP on this gene

EHA27157

hypothetical protein
  
Accession: EHA27156
  
Location: 3604383-3607043
  
 NCBI BlastP on this gene

EHA27156

hypothetical protein
  
Accession: EHA27155
  
Location: 3601685-3603137
  
 NCBI BlastP on this gene

EHA27155

hypothetical protein
  
Accession: EHA27154
  
Location: 3600419-3601587
  
 NCBI BlastP on this gene

EHA27154

hypothetical protein
  
Accession: EHA27153
  
Location: 3598843-3599524
  
 NCBI BlastP on this gene

EHA27153

hypothetical protein
  
Accession: EHA27152
  
Location: 3595921-3598141
  
 NCBI BlastP on this gene

EHA27152

hypothetical protein
  
Accession: EHA27151
  
Location: 3591317-3595554
  
 NCBI BlastP on this gene

EHA27151

hypothetical protein
  
Accession: EHA27150
  
Location: 3588642-3589373
  
  
**BlastP hit with Mycgr3G68030\_Mycgr3T**
  
Percentage identity: 37 %
  
BlastP bit score: 159
  
Sequence coverage: 72 %
  
E-value: 2e-43
  
  
 NCBI BlastP on this gene

EHA27150

hypothetical protein
  
Accession: EHA27149
  
Location: 3586780-3587957
  
 NCBI BlastP on this gene

EHA27149

hypothetical protein
  
Accession: EHA27148
  
Location: 3582514-3584396
  
 NCBI BlastP on this gene

EHA27148

Query: Architecture Search FASTA input

GL890999 : Neurospora tetrasperma FGSC 2509 unplaced genomic scaffold NEUTE2scaffold\_1    Total score: 2.0     Cumulative Blast bit score: 515

Hit cluster cross-links:

Mycgr3G36335 Mycgr3T
  
Location: 0-423

Mycgr3G36335\_Mycgr3T

Mycgr3G84494 Mycgr3T
  
Location: 523-2047

Mycgr3G84494\_Mycgr3T

Mycgr3G90558 Mycgr3T
  
Location: 2147-15296

Mycgr3G90558\_Mycgr3T

Mycgr3G68036 Mycgr3T
  
Location: 15396-16395

Mycgr3G68036\_Mycgr3T

Mycgr3G90561 Mycgr3T
  
Location: 16495-17134

Mycgr3G90561\_Mycgr3T

Mycgr3G35862 Mycgr3T
  
Location: 17234-18662

Mycgr3G35862\_Mycgr3T

Mycgr3G68030 Mycgr3T
  
Location: 18762-19722

Mycgr3G68030\_Mycgr3T

Mycgr3G36449 Mycgr3T
  
Location: 19822-21886

Mycgr3G36449\_Mycgr3T

Mycgr3G35528 Mycgr3T
  
Location: 21986-22844

Mycgr3G35528\_Mycgr3T

Mycgr3G35932 Mycgr3T
  
Location: 22944-24390

Mycgr3G35932\_Mycgr3T

Mycgr3G23761 Mycgr3T
  
Location: 24490-25825

Mycgr3G23761\_Mycgr3T

Mycgr3G35535 Mycgr3T
  
Location: 25925-26429

Mycgr3G35535\_Mycgr3T

Mycgr3G9942 Mycgr3T9
  
Location: 26529-30375

Mycgr3G9942\_Mycgr3T9

hypothetical protein
  
Accession: EGZ76907
  
Location: 2943352-2944208
  
 NCBI BlastP on this gene

EGZ76907

hypothetical protein
  
Accession: EGZ76908
  
Location: 2945421-2946242
  
 NCBI BlastP on this gene

EGZ76908

hypothetical protein
  
Accession: EGZ76909
  
Location: 2946828-2949802
  
 NCBI BlastP on this gene

EGZ76909

hypothetical protein
  
Accession: EGZ76910
  
Location: 2950540-2952871
  
 NCBI BlastP on this gene

EGZ76910

hypothetical protein
  
Accession: EGZ76911
  
Location: 2953852-2954781
  
 NCBI BlastP on this gene

EGZ76911

hypothetical protein
  
Accession: EGZ76912
  
Location: 2955994-2957159
  
 NCBI BlastP on this gene

EGZ76912

hypothetical protein
  
Accession: EGZ76913
  
Location: 2957808-2959014
  
  
**BlastP hit with Mycgr3G68030\_Mycgr3T**
  
Percentage identity: 33 %
  
BlastP bit score: 184
  
Sequence coverage: 92 %
  
E-value: 2e-51
  
  
 NCBI BlastP on this gene

EGZ76913

MFS general substrate transporter
  
Accession: EGZ76914
  
Location: 2959740-2963949
  
  
**BlastP hit with Mycgr3G84494\_Mycgr3T**
  
Percentage identity: 38 %
  
BlastP bit score: 331
  
Sequence coverage: 91 %
  
E-value: 5e-98
  
  
 NCBI BlastP on this gene

EGZ76914

hypothetical protein
  
Accession: EGZ76915
  
Location: 2964373-2964720
  
 NCBI BlastP on this gene

EGZ76915

hypothetical protein
  
Accession: EGZ76916
  
Location: 2967388-2967627
  
 NCBI BlastP on this gene

EGZ76916

YVTN repeat-like/Quinoprotein amine dehydrogenase
  
Accession: EGZ76917
  
Location: 2968826-2969850
  
 NCBI BlastP on this gene

EGZ76917

PUL-domain-containing protein
  
Accession: EGZ76918
  
Location: 2970674-2974023
  
 NCBI BlastP on this gene

EGZ76918

hypothetical protein
  
Accession: EGZ76919
  
Location: 2975019-2975986
  
 NCBI BlastP on this gene

EGZ76919

hypothetical protein
  
Accession: EGZ76920
  
Location: 2976900-2977972
  
 NCBI BlastP on this gene

EGZ76920

hypothetical protein
  
Accession: EGZ76921
  
Location: 2978495-2979520
  
 NCBI BlastP on this gene

EGZ76921

Query: Architecture Search FASTA input

GL891382 : Neurospora tetrasperma FGSC 2508 unplaced genomic scaffold NEUTE1scaffold\_81    Total score: 2.0     Cumulative Blast bit score: 511

Hit cluster cross-links:

Mycgr3G36335 Mycgr3T
  
Location: 0-423

Mycgr3G36335\_Mycgr3T

Mycgr3G84494 Mycgr3T
  
Location: 523-2047

Mycgr3G84494\_Mycgr3T

Mycgr3G90558 Mycgr3T
  
Location: 2147-15296

Mycgr3G90558\_Mycgr3T

Mycgr3G68036 Mycgr3T
  
Location: 15396-16395

Mycgr3G68036\_Mycgr3T

Mycgr3G90561 Mycgr3T
  
Location: 16495-17134

Mycgr3G90561\_Mycgr3T

Mycgr3G35862 Mycgr3T
  
Location: 17234-18662

Mycgr3G35862\_Mycgr3T

Mycgr3G68030 Mycgr3T
  
Location: 18762-19722

Mycgr3G68030\_Mycgr3T

Mycgr3G36449 Mycgr3T
  
Location: 19822-21886

Mycgr3G36449\_Mycgr3T

Mycgr3G35528 Mycgr3T
  
Location: 21986-22844

Mycgr3G35528\_Mycgr3T

Mycgr3G35932 Mycgr3T
  
Location: 22944-24390

Mycgr3G35932\_Mycgr3T

Mycgr3G23761 Mycgr3T
  
Location: 24490-25825

Mycgr3G23761\_Mycgr3T

Mycgr3G35535 Mycgr3T
  
Location: 25925-26429

Mycgr3G35535\_Mycgr3T

Mycgr3G9942 Mycgr3T9
  
Location: 26529-30375

Mycgr3G9942\_Mycgr3T9

hypothetical protein
  
Accession: EGO52096
  
Location: 3044117-3045058
  
 NCBI BlastP on this gene

EGO52096

hypothetical protein
  
Accession: EGO52097
  
Location: 3045328-3046188
  
 NCBI BlastP on this gene

EGO52097

hypothetical protein
  
Accession: EGO52098
  
Location: 3047336-3048157
  
 NCBI BlastP on this gene

EGO52098

hypothetical protein
  
Accession: EGO52099
  
Location: 3048737-3051712
  
 NCBI BlastP on this gene

EGO52099

hypothetical protein
  
Accession: EGO52100
  
Location: 3052442-3054781
  
 NCBI BlastP on this gene

EGO52100

hypothetical protein
  
Accession: EGO52101
  
Location: 3055756-3056853
  
 NCBI BlastP on this gene

EGO52101

hypothetical protein
  
Accession: EGO52102
  
Location: 3058002-3059167
  
 NCBI BlastP on this gene

EGO52102

hypothetical protein
  
Accession: EGO52103
  
Location: 3059814-3061021
  
  
**BlastP hit with Mycgr3G68030\_Mycgr3T**
  
Percentage identity: 33 %
  
BlastP bit score: 187
  
Sequence coverage: 92 %
  
E-value: 1e-52
  
  
 NCBI BlastP on this gene

EGO52103

hypothetical protein
  
Accession: EGO52104
  
Location: 3061603-3063500
  
  
**BlastP hit with Mycgr3G84494\_Mycgr3T**
  
Percentage identity: 37 %
  
BlastP bit score: 324
  
Sequence coverage: 93 %
  
E-value: 9e-101
  
  
 NCBI BlastP on this gene

EGO52104

hypothetical protein
  
Accession: EGO52105
  
Location: 3063901-3066042
  
 NCBI BlastP on this gene

EGO52105

hypothetical protein
  
Accession: EGO52106
  
Location: 3066453-3066800
  
 NCBI BlastP on this gene

EGO52106

hypothetical protein
  
Accession: EGO52107
  
Location: 3068821-3069060
  
 NCBI BlastP on this gene

EGO52107

hypothetical protein
  
Accession: EGO52108
  
Location: 3070254-3071278
  
 NCBI BlastP on this gene

EGO52108

hypothetical protein
  
Accession: EGO52109
  
Location: 3072092-3075450
  
 NCBI BlastP on this gene

EGO52109

hypothetical protein
  
Accession: EGO52110
  
Location: 3076443-3077375
  
 NCBI BlastP on this gene

EGO52110

hypothetical protein
  
Accession: EGO52111
  
Location: 3077934-3078786
  
 NCBI BlastP on this gene

EGO52111

hypothetical protein
  
Accession: EGO52112
  
Location: 3079291-3080316
  
 NCBI BlastP on this gene

EGO52112

Query: Architecture Search FASTA input

AABX02000002 : Neurospora crassa OR74A    Total score: 2.0     Cumulative Blast bit score: 510

Hit cluster cross-links:

Mycgr3G36335 Mycgr3T
  
Location: 0-423

Mycgr3G36335\_Mycgr3T

Mycgr3G84494 Mycgr3T
  
Location: 523-2047

Mycgr3G84494\_Mycgr3T

Mycgr3G90558 Mycgr3T
  
Location: 2147-15296

Mycgr3G90558\_Mycgr3T

Mycgr3G68036 Mycgr3T
  
Location: 15396-16395

Mycgr3G68036\_Mycgr3T

Mycgr3G90561 Mycgr3T
  
Location: 16495-17134

Mycgr3G90561\_Mycgr3T

Mycgr3G35862 Mycgr3T
  
Location: 17234-18662

Mycgr3G35862\_Mycgr3T

Mycgr3G68030 Mycgr3T
  
Location: 18762-19722

Mycgr3G68030\_Mycgr3T

Mycgr3G36449 Mycgr3T
  
Location: 19822-21886

Mycgr3G36449\_Mycgr3T

Mycgr3G35528 Mycgr3T
  
Location: 21986-22844

Mycgr3G35528\_Mycgr3T

Mycgr3G35932 Mycgr3T
  
Location: 22944-24390

Mycgr3G35932\_Mycgr3T

Mycgr3G23761 Mycgr3T
  
Location: 24490-25825

Mycgr3G23761\_Mycgr3T

Mycgr3G35535 Mycgr3T
  
Location: 25925-26429

Mycgr3G35535\_Mycgr3T

Mycgr3G9942 Mycgr3T9
  
Location: 26529-30375

Mycgr3G9942\_Mycgr3T9

predicted protein
  
Accession: EAA35548
  
Location: 1315043-1315864
  
 NCBI BlastP on this gene

EAA35548

predicted protein
  
Accession: EAA35549
  
Location: 1316451-1319426
  
 NCBI BlastP on this gene

EAA35549

predicted protein
  
Accession: EAA35550
  
Location: 1320154-1322492
  
 NCBI BlastP on this gene

EAA35550

predicted protein
  
Accession: EAA35551
  
Location: 1323454-1324389
  
 NCBI BlastP on this gene

EAA35551

predicted protein
  
Accession: EAA35552
  
Location: 1325544-1326709
  
 NCBI BlastP on this gene

EAA35552

conserved hypothetical protein
  
Accession: EAA35553
  
Location: 1327357-1328563
  
  
**BlastP hit with Mycgr3G68030\_Mycgr3T**
  
Percentage identity: 33 %
  
BlastP bit score: 186
  
Sequence coverage: 92 %
  
E-value: 4e-52
  
  
 NCBI BlastP on this gene

EAA35553

predicted protein
  
Accession: EAA35554
  
Location: 1329168-1332438
  
 NCBI BlastP on this gene

EAA35554

predicted protein
  
Accession: EAA35555
  
Location: 1334983-1336904
  
  
**BlastP hit with Mycgr3G84494\_Mycgr3T**
  
Percentage identity: 37 %
  
BlastP bit score: 324
  
Sequence coverage: 93 %
  
E-value: 1e-100
  
  
 NCBI BlastP on this gene

EAA35555

conserved hypothetical protein
  
Accession: EAA35556
  
Location: 1337316-1339469
  
 NCBI BlastP on this gene

EAA35556

predicted protein
  
Accession: EAA35557
  
Location: 1339909-1340265
  
 NCBI BlastP on this gene

EAA35557

hypothetical protein
  
Accession: EAA35558
  
Location: 1341980-1342219
  
 NCBI BlastP on this gene

EAA35558

conserved hypothetical protein
  
Accession: EAA35559
  
Location: 1343413-1344333
  
 NCBI BlastP on this gene

EAA35559

conserved hypothetical protein
  
Accession: EAA35560
  
Location: 1345206-1348551
  
 NCBI BlastP on this gene

EAA35560

conserved hypothetical protein
  
Accession: EAA35561
  
Location: 1349588-1350525
  
 NCBI BlastP on this gene

EAA35561

Query: Architecture Search FASTA input

GL636512 : Coccidioides posadasii str. Silveira unplaced genomic scaffold supercont2.27    Total score: 2.0     Cumulative Blast bit score: 508

Hit cluster cross-links:

Mycgr3G36335 Mycgr3T
  
Location: 0-423

Mycgr3G36335\_Mycgr3T

Mycgr3G84494 Mycgr3T
  
Location: 523-2047

Mycgr3G84494\_Mycgr3T

Mycgr3G90558 Mycgr3T
  
Location: 2147-15296

Mycgr3G90558\_Mycgr3T

Mycgr3G68036 Mycgr3T
  
Location: 15396-16395

Mycgr3G68036\_Mycgr3T

Mycgr3G90561 Mycgr3T
  
Location: 16495-17134

Mycgr3G90561\_Mycgr3T

Mycgr3G35862 Mycgr3T
  
Location: 17234-18662

Mycgr3G35862\_Mycgr3T

Mycgr3G68030 Mycgr3T
  
Location: 18762-19722

Mycgr3G68030\_Mycgr3T

Mycgr3G36449 Mycgr3T
  
Location: 19822-21886

Mycgr3G36449\_Mycgr3T

Mycgr3G35528 Mycgr3T
  
Location: 21986-22844

Mycgr3G35528\_Mycgr3T

Mycgr3G35932 Mycgr3T
  
Location: 22944-24390

Mycgr3G35932\_Mycgr3T

Mycgr3G23761 Mycgr3T
  
Location: 24490-25825

Mycgr3G23761\_Mycgr3T

Mycgr3G35535 Mycgr3T
  
Location: 25925-26429

Mycgr3G35535\_Mycgr3T

Mycgr3G9942 Mycgr3T9
  
Location: 26529-30375

Mycgr3G9942\_Mycgr3T9

flavodoxin and radical SAM domain-containing protein
  
Accession: EFW13713
  
Location: 214883-217323
  
 NCBI BlastP on this gene

EFW13713

conserved hypothetical protein
  
Accession: EFW13714
  
Location: 218517-220405
  
 NCBI BlastP on this gene

EFW13714

high affinity copper transporter
  
Accession: EFW13715
  
Location: 221678-222608
  
 NCBI BlastP on this gene

EFW13715

serine/threonine-protein phosphatase ppe1
  
Accession: EFW13716
  
Location: 223951-225370
  
 NCBI BlastP on this gene

EFW13716

predicted protein
  
Accession: EFW13717
  
Location: 225709-226286
  
 NCBI BlastP on this gene

EFW13717

conserved hypothetical protein
  
Accession: EFW13718
  
Location: 226779-227998
  
 NCBI BlastP on this gene

EFW13718

conserved hypothetical protein
  
Accession: EFW13719
  
Location: 228388-229642
  
  
**BlastP hit with Mycgr3G68030\_Mycgr3T**
  
Percentage identity: 31 %
  
BlastP bit score: 130
  
Sequence coverage: 78 %
  
E-value: 2e-31
  
  
 NCBI BlastP on this gene

EFW13719

conserved hypothetical protein
  
Accession: EFW13720
  
Location: 231570-235795
  
 NCBI BlastP on this gene

EFW13720

conserved hypothetical protein
  
Accession: EFW13721
  
Location: 236428-238249
  
  
**BlastP hit with Mycgr3G84494\_Mycgr3T**
  
Percentage identity: 42 %
  
BlastP bit score: 378
  
Sequence coverage: 92 %
  
E-value: 8e-122
  
  
 NCBI BlastP on this gene

EFW13721

predicted protein
  
Accession: EFW13722
  
Location: 239252-239635
  
 NCBI BlastP on this gene

EFW13722

conserved hypothetical protein
  
Accession: EFW13723
  
Location: 240468-242266
  
 NCBI BlastP on this gene

EFW13723

predicted protein
  
Accession: EFW13724
  
Location: 242473-242923
  
 NCBI BlastP on this gene

EFW13724

conserved hypothetical protein
  
Accession: EFW13725
  
Location: 243056-244497
  
 NCBI BlastP on this gene

EFW13725

conserved hypothetical protein
  
Accession: EFW13726
  
Location: 245429-245743
  
 NCBI BlastP on this gene

EFW13726

conserved hypothetical protein
  
Accession: EFW13727
  
Location: 247025-248027
  
 NCBI BlastP on this gene

EFW13727

conserved hypothetical protein
  
Accession: EFW13728
  
Location: 248446-249014
  
 NCBI BlastP on this gene

EFW13728

conserved hypothetical protein
  
Accession: EFW13729
  
Location: 249316-250659
  
 NCBI BlastP on this gene

EFW13729

Query: Architecture Search FASTA input

CP003011 : Thielavia terrestris NRRL 8126 chromosome 3    Total score: 2.0     Cumulative Blast bit score: 507

Hit cluster cross-links:

Mycgr3G36335 Mycgr3T
  
Location: 0-423

Mycgr3G36335\_Mycgr3T

Mycgr3G84494 Mycgr3T
  
Location: 523-2047

Mycgr3G84494\_Mycgr3T

Mycgr3G90558 Mycgr3T
  
Location: 2147-15296

Mycgr3G90558\_Mycgr3T

Mycgr3G68036 Mycgr3T
  
Location: 15396-16395

Mycgr3G68036\_Mycgr3T

Mycgr3G90561 Mycgr3T
  
Location: 16495-17134

Mycgr3G90561\_Mycgr3T

Mycgr3G35862 Mycgr3T
  
Location: 17234-18662

Mycgr3G35862\_Mycgr3T

Mycgr3G68030 Mycgr3T
  
Location: 18762-19722

Mycgr3G68030\_Mycgr3T

Mycgr3G36449 Mycgr3T
  
Location: 19822-21886

Mycgr3G36449\_Mycgr3T

Mycgr3G35528 Mycgr3T
  
Location: 21986-22844

Mycgr3G35528\_Mycgr3T

Mycgr3G35932 Mycgr3T
  
Location: 22944-24390

Mycgr3G35932\_Mycgr3T

Mycgr3G23761 Mycgr3T
  
Location: 24490-25825

Mycgr3G23761\_Mycgr3T

Mycgr3G35535 Mycgr3T
  
Location: 25925-26429

Mycgr3G35535\_Mycgr3T

Mycgr3G9942 Mycgr3T9
  
Location: 26529-30375

Mycgr3G9942\_Mycgr3T9

glycoside hydrolase family 3 protein
  
Accession: AEO68568
  
Location: 4748811-4751203
  
 NCBI BlastP on this gene

THITE\_2118136

glycoside hydrolase family 31 protein
  
Accession: AEO68569
  
Location: 4752246-4754695
  
 NCBI BlastP on this gene

THITE\_2118137

hypothetical protein
  
Accession: AEO68570
  
Location: 4755841-4757524
  
 NCBI BlastP on this gene

THITE\_2118138

glycoside hydrolase family 11 protein
  
Accession: AEO68571
  
Location: 4758136-4758928
  
 NCBI BlastP on this gene

THITE\_2050870

hypothetical protein
  
Accession: AEO68572
  
Location: 4761172-4762995
  
 NCBI BlastP on this gene

THITE\_2118139

hypothetical protein
  
Accession: AEO68573
  
Location: 4763368-4765029
  
  
**BlastP hit with Mycgr3G84494\_Mycgr3T**
  
Percentage identity: 36 %
  
BlastP bit score: 331
  
Sequence coverage: 96 %
  
E-value: 2e-103
  
  
 NCBI BlastP on this gene

THITE\_2118141

hypothetical protein
  
Accession: AEO68574
  
Location: 4766795-4771807
  
 NCBI BlastP on this gene

THITE\_2050713

hypothetical protein
  
Accession: AEO68575
  
Location: 4772705-4773820
  
  
**BlastP hit with Mycgr3G68030\_Mycgr3T**
  
Percentage identity: 35 %
  
BlastP bit score: 176
  
Sequence coverage: 89 %
  
E-value: 9e-49
  
  
 NCBI BlastP on this gene

THITE\_2118142

hypothetical protein
  
Accession: AEO68576
  
Location: 4775258-4776463
  
 NCBI BlastP on this gene

THITE\_2118143

Query: Architecture Search FASTA input

AKHY01000098 : Aspergillus oryzae 3.042    Total score: 2.0     Cumulative Blast bit score: 506

Hit cluster cross-links:

Mycgr3G36335 Mycgr3T
  
Location: 0-423

Mycgr3G36335\_Mycgr3T

Mycgr3G84494 Mycgr3T
  
Location: 523-2047

Mycgr3G84494\_Mycgr3T

Mycgr3G90558 Mycgr3T
  
Location: 2147-15296

Mycgr3G90558\_Mycgr3T

Mycgr3G68036 Mycgr3T
  
Location: 15396-16395

Mycgr3G68036\_Mycgr3T

Mycgr3G90561 Mycgr3T
  
Location: 16495-17134

Mycgr3G90561\_Mycgr3T

Mycgr3G35862 Mycgr3T
  
Location: 17234-18662

Mycgr3G35862\_Mycgr3T

Mycgr3G68030 Mycgr3T
  
Location: 18762-19722

Mycgr3G68030\_Mycgr3T

Mycgr3G36449 Mycgr3T
  
Location: 19822-21886

Mycgr3G36449\_Mycgr3T

Mycgr3G35528 Mycgr3T
  
Location: 21986-22844

Mycgr3G35528\_Mycgr3T

Mycgr3G35932 Mycgr3T
  
Location: 22944-24390

Mycgr3G35932\_Mycgr3T

Mycgr3G23761 Mycgr3T
  
Location: 24490-25825

Mycgr3G23761\_Mycgr3T

Mycgr3G35535 Mycgr3T
  
Location: 25925-26429

Mycgr3G35535\_Mycgr3T

Mycgr3G9942 Mycgr3T9
  
Location: 26529-30375

Mycgr3G9942\_Mycgr3T9

hypothetical protein
  
Accession: EIT81734
  
Location: 42924-43721
  
 NCBI BlastP on this gene

EIT81734

hypothetical protein
  
Accession: EIT81719
  
Location: 44452-46443
  
 NCBI BlastP on this gene

EIT81719

putative epimerase, PhzC/PhzF like protein
  
Accession: EIT81717
  
Location: 47486-48439
  
 NCBI BlastP on this gene

EIT81717

NADH dehydrogenase, FAD-containing subunit
  
Accession: EIT81709
  
Location: 49696-50910
  
 NCBI BlastP on this gene

EIT81709

hypothetical protein
  
Accession: EIT81770
  
Location: 52634-55024
  
  
**BlastP hit with Mycgr3G36449\_Mycgr3T**
  
Percentage identity: 37 %
  
BlastP bit score: 433
  
Sequence coverage: 98 %
  
E-value: 5e-138
  
  
 NCBI BlastP on this gene

EIT81770

hypothetical protein
  
Accession: EIT81775
  
Location: 56152-56559
  
 NCBI BlastP on this gene

EIT81775

hypothetical protein
  
Accession: EIT81756
  
Location: 56994-60196
  
  
**BlastP hit with Mycgr3G36335\_Mycgr3T**
  
Percentage identity: 33 %
  
BlastP bit score: 73
  
Sequence coverage: 89 %
  
E-value: 1e-12
  
  
 NCBI BlastP on this gene

EIT81756

serine racemase
  
Accession: EIT81753
  
Location: 60626-61891
  
 NCBI BlastP on this gene

EIT81753

Rad51 family DNA repair protein, putative
  
Accession: EIT81687
  
Location: 61981-63450
  
 NCBI BlastP on this gene

EIT81687

hypothetical protein
  
Accession: EIT81684
  
Location: 63810-64189
  
 NCBI BlastP on this gene

EIT81684

bromodomain associated domain protein
  
Accession: EIT81754
  
Location: 64391-65501
  
 NCBI BlastP on this gene

EIT81754

ubiquitin-protein ligase
  
Accession: EIT81713
  
Location: 67144-67937
  
 NCBI BlastP on this gene

EIT81713

chitin synthase/hyaluronan synthase
  
Accession: EIT81685
  
Location: 68496-71524
  
 NCBI BlastP on this gene

EIT81685

Query: Architecture Search FASTA input

CP003014 : Thielavia terrestris NRRL 8126 chromosome 6    Total score: 2.0     Cumulative Blast bit score: 498

Hit cluster cross-links:

Mycgr3G36335 Mycgr3T
  
Location: 0-423

Mycgr3G36335\_Mycgr3T

Mycgr3G84494 Mycgr3T
  
Location: 523-2047

Mycgr3G84494\_Mycgr3T

Mycgr3G90558 Mycgr3T
  
Location: 2147-15296

Mycgr3G90558\_Mycgr3T

Mycgr3G68036 Mycgr3T
  
Location: 15396-16395

Mycgr3G68036\_Mycgr3T

Mycgr3G90561 Mycgr3T
  
Location: 16495-17134

Mycgr3G90561\_Mycgr3T

Mycgr3G35862 Mycgr3T
  
Location: 17234-18662

Mycgr3G35862\_Mycgr3T

Mycgr3G68030 Mycgr3T
  
Location: 18762-19722

Mycgr3G68030\_Mycgr3T

Mycgr3G36449 Mycgr3T
  
Location: 19822-21886

Mycgr3G36449\_Mycgr3T

Mycgr3G35528 Mycgr3T
  
Location: 21986-22844

Mycgr3G35528\_Mycgr3T

Mycgr3G35932 Mycgr3T
  
Location: 22944-24390

Mycgr3G35932\_Mycgr3T

Mycgr3G23761 Mycgr3T
  
Location: 24490-25825

Mycgr3G23761\_Mycgr3T

Mycgr3G35535 Mycgr3T
  
Location: 25925-26429

Mycgr3G35535\_Mycgr3T

Mycgr3G9942 Mycgr3T9
  
Location: 26529-30375

Mycgr3G9942\_Mycgr3T9

hypothetical protein
  
Accession: AEO71475
  
Location: 1802735-1805504
  
 NCBI BlastP on this gene

THITE\_2092975

hypothetical protein
  
Accession: AEO71474
  
Location: 1797194-1798720
  
 NCBI BlastP on this gene

THITE\_2123869

hypothetical protein
  
Accession: AEO71473
  
Location: 1794832-1795989
  
 NCBI BlastP on this gene

THITE\_2123868

hypothetical protein
  
Accession: AEO71472
  
Location: 1789985-1793839
  
 NCBI BlastP on this gene

THITE\_2123867

hypothetical protein
  
Accession: AEO71471
  
Location: 1786469-1787770
  
  
**BlastP hit with Mycgr3G68030\_Mycgr3T**
  
Percentage identity: 30 %
  
BlastP bit score: 152
  
Sequence coverage: 93 %
  
E-value: 4e-39
  
  
 NCBI BlastP on this gene

THITE\_2123863

hypothetical protein
  
Accession: AEO71470
  
Location: 1783539-1785282
  
  
**BlastP hit with Mycgr3G84494\_Mycgr3T**
  
Percentage identity: 38 %
  
BlastP bit score: 346
  
Sequence coverage: 93 %
  
E-value: 2e-109
  
  
 NCBI BlastP on this gene

THITE\_2123861

hypothetical protein
  
Accession: AEO71469
  
Location: 1780862-1782591
  
 NCBI BlastP on this gene

THITE\_21761

hypothetical protein
  
Accession: AEO71468
  
Location: 1778162-1779902
  
 NCBI BlastP on this gene

THITE\_124439

hypothetical protein
  
Accession: AEO71467
  
Location: 1775553-1776580
  
 NCBI BlastP on this gene

THITE\_2148110

hypothetical protein
  
Accession: AEO71466
  
Location: 1773332-1774405
  
 NCBI BlastP on this gene

THITE\_2123858

hypothetical protein
  
Accession: AEO71465
  
Location: 1770100-1772121
  
 NCBI BlastP on this gene

THITE\_2123849

Query: Architecture Search FASTA input

AP007164 : Aspergillus oryzae RIB40 DNA, SC111.    Total score: 2.0     Cumulative Blast bit score: 493

Hit cluster cross-links:

Mycgr3G36335 Mycgr3T
  
Location: 0-423

Mycgr3G36335\_Mycgr3T

Mycgr3G84494 Mycgr3T
  
Location: 523-2047

Mycgr3G84494\_Mycgr3T

Mycgr3G90558 Mycgr3T
  
Location: 2147-15296

Mycgr3G90558\_Mycgr3T

Mycgr3G68036 Mycgr3T
  
Location: 15396-16395

Mycgr3G68036\_Mycgr3T

Mycgr3G90561 Mycgr3T
  
Location: 16495-17134

Mycgr3G90561\_Mycgr3T

Mycgr3G35862 Mycgr3T
  
Location: 17234-18662

Mycgr3G35862\_Mycgr3T

Mycgr3G68030 Mycgr3T
  
Location: 18762-19722

Mycgr3G68030\_Mycgr3T

Mycgr3G36449 Mycgr3T
  
Location: 19822-21886

Mycgr3G36449\_Mycgr3T

Mycgr3G35528 Mycgr3T
  
Location: 21986-22844

Mycgr3G35528\_Mycgr3T

Mycgr3G35932 Mycgr3T
  
Location: 22944-24390

Mycgr3G35932\_Mycgr3T

Mycgr3G23761 Mycgr3T
  
Location: 24490-25825

Mycgr3G23761\_Mycgr3T

Mycgr3G35535 Mycgr3T
  
Location: 25925-26429

Mycgr3G35535\_Mycgr3T

Mycgr3G9942 Mycgr3T9
  
Location: 26529-30375

Mycgr3G9942\_Mycgr3T9

not annotated
  
Accession: BAE62269
  
Location: 1526127-1526924
  
 NCBI BlastP on this gene

AO090701000575

not annotated
  
Accession: BAE62270
  
Location: 1527654-1529645
  
 NCBI BlastP on this gene

AO090701000576

not annotated
  
Accession: BAE62271
  
Location: 1530692-1531645
  
 NCBI BlastP on this gene

AO090701000577

not annotated
  
Accession: BAE62272
  
Location: 1532901-1534115
  
 NCBI BlastP on this gene

AO090701000578

not annotated
  
Accession: BAE62273
  
Location: 1535818-1538207
  
  
**BlastP hit with Mycgr3G36449\_Mycgr3T**
  
Percentage identity: 36 %
  
BlastP bit score: 421
  
Sequence coverage: 101 %
  
E-value: 9e-133
  
  
 NCBI BlastP on this gene

AO090701000579

not annotated
  
Accession: BAE62274
  
Location: 1539317-1539724
  
 NCBI BlastP on this gene

AO090701000580

not annotated
  
Accession: BAE62275
  
Location: 1541688-1543321
  
  
**BlastP hit with Mycgr3G36335\_Mycgr3T**
  
Percentage identity: 33 %
  
BlastP bit score: 72
  
Sequence coverage: 89 %
  
E-value: 3e-12
  
  
 NCBI BlastP on this gene

AO090701000581

not annotated
  
Accession: BAE62276
  
Location: 1543759-1545024
  
 NCBI BlastP on this gene

AO090701000582

not annotated
  
Accession: BAE62277
  
Location: 1545114-1546585
  
 NCBI BlastP on this gene

AO090701000583

not annotated
  
Accession: BAE62278
  
Location: 1546946-1547325
  
 NCBI BlastP on this gene

AO090701000585

not annotated
  
Accession: BAE62279
  
Location: 1547527-1548637
  
 NCBI BlastP on this gene

AO090701000586

not annotated
  
Accession: BAE62280
  
Location: 1550279-1551072
  
 NCBI BlastP on this gene

AO090701000588

not annotated
  
Accession: BAE62281
  
Location: 1551631-1554659
  
 NCBI BlastP on this gene

AO090701000589

not annotated
  
Accession: BAE62282
  
Location: 1554890-1556014
  
 NCBI BlastP on this gene

AO090701000590

Query: Architecture Search FASTA input

GG698970 : Nectria haematococca mpVI 77-13-4 chromosome 10 genomic scaffold NECHAsca\_82\_chr10\_2\_0    Total score: 2.0     Cumulative Blast bit score: 492

Hit cluster cross-links:

Mycgr3G36335 Mycgr3T
  
Location: 0-423

Mycgr3G36335\_Mycgr3T

Mycgr3G84494 Mycgr3T
  
Location: 523-2047

Mycgr3G84494\_Mycgr3T

Mycgr3G90558 Mycgr3T
  
Location: 2147-15296

Mycgr3G90558\_Mycgr3T

Mycgr3G68036 Mycgr3T
  
Location: 15396-16395

Mycgr3G68036\_Mycgr3T

Mycgr3G90561 Mycgr3T
  
Location: 16495-17134

Mycgr3G90561\_Mycgr3T

Mycgr3G35862 Mycgr3T
  
Location: 17234-18662

Mycgr3G35862\_Mycgr3T

Mycgr3G68030 Mycgr3T
  
Location: 18762-19722

Mycgr3G68030\_Mycgr3T

Mycgr3G36449 Mycgr3T
  
Location: 19822-21886

Mycgr3G36449\_Mycgr3T

Mycgr3G35528 Mycgr3T
  
Location: 21986-22844

Mycgr3G35528\_Mycgr3T

Mycgr3G35932 Mycgr3T
  
Location: 22944-24390

Mycgr3G35932\_Mycgr3T

Mycgr3G23761 Mycgr3T
  
Location: 24490-25825

Mycgr3G23761\_Mycgr3T

Mycgr3G35535 Mycgr3T
  
Location: 25925-26429

Mycgr3G35535\_Mycgr3T

Mycgr3G9942 Mycgr3T9
  
Location: 26529-30375

Mycgr3G9942\_Mycgr3T9

hypothetical protein
  
Accession: EEU34248
  
Location: 366370-368746
  
 NCBI BlastP on this gene

EEU34248

hypothetical protein
  
Accession: EEU34058
  
Location: 365225-365887
  
 NCBI BlastP on this gene

EEU34058

hypothetical protein
  
Accession: EEU34057
  
Location: 354382-359365
  
 NCBI BlastP on this gene

EEU34057

hypothetical protein
  
Accession: EEU34247
  
Location: 352084-353419
  
  
**BlastP hit with Mycgr3G68030\_Mycgr3T**
  
Percentage identity: 33 %
  
BlastP bit score: 173
  
Sequence coverage: 90 %
  
E-value: 4e-47
  
  
 NCBI BlastP on this gene

EEU34247

hypothetical protein
  
Accession: EEU34246
  
Location: 349586-351354
  
  
**BlastP hit with Mycgr3G84494\_Mycgr3T**
  
Percentage identity: 39 %
  
BlastP bit score: 319
  
Sequence coverage: 92 %
  
E-value: 3e-99
  
  
 NCBI BlastP on this gene

EEU34246

hypothetical protein
  
Accession: EEU34056
  
Location: 344614-349500
  
 NCBI BlastP on this gene

EEU34056

hypothetical protein
  
Accession: EEU34245
  
Location: 341601-342912
  
 NCBI BlastP on this gene

EEU34245

hypothetical protein
  
Accession: EEU34055
  
Location: 339793-341223
  
 NCBI BlastP on this gene

EEU34055

predicted protein
  
Accession: EEU34054
  
Location: 338824-339466
  
 NCBI BlastP on this gene

EEU34054

hypothetical protein
  
Accession: EEU34053
  
Location: 336806-338441
  
 NCBI BlastP on this gene

EEU34053

hypothetical protein
  
Accession: EEU34244
  
Location: 335450-336419
  
 NCBI BlastP on this gene

EEU34244

hypothetical protein
  
Accession: EEU34052
  
Location: 333312-334271
  
 NCBI BlastP on this gene

EEU34052

Query: Architecture Search FASTA input

KE148146 : Ophiostoma piceae UAMH 11346 chromosome Unknown scf01    Total score: 2.0     Cumulative Blast bit score: 471

Hit cluster cross-links:

Mycgr3G36335 Mycgr3T
  
Location: 0-423

Mycgr3G36335\_Mycgr3T

Mycgr3G84494 Mycgr3T
  
Location: 523-2047

Mycgr3G84494\_Mycgr3T

Mycgr3G90558 Mycgr3T
  
Location: 2147-15296

Mycgr3G90558\_Mycgr3T

Mycgr3G68036 Mycgr3T
  
Location: 15396-16395

Mycgr3G68036\_Mycgr3T

Mycgr3G90561 Mycgr3T
  
Location: 16495-17134

Mycgr3G90561\_Mycgr3T

Mycgr3G35862 Mycgr3T
  
Location: 17234-18662

Mycgr3G35862\_Mycgr3T

Mycgr3G68030 Mycgr3T
  
Location: 18762-19722

Mycgr3G68030\_Mycgr3T

Mycgr3G36449 Mycgr3T
  
Location: 19822-21886

Mycgr3G36449\_Mycgr3T

Mycgr3G35528 Mycgr3T
  
Location: 21986-22844

Mycgr3G35528\_Mycgr3T

Mycgr3G35932 Mycgr3T
  
Location: 22944-24390

Mycgr3G35932\_Mycgr3T

Mycgr3G23761 Mycgr3T
  
Location: 24490-25825

Mycgr3G23761\_Mycgr3T

Mycgr3G35535 Mycgr3T
  
Location: 25925-26429

Mycgr3G35535\_Mycgr3T

Mycgr3G9942 Mycgr3T9
  
Location: 26529-30375

Mycgr3G9942\_Mycgr3T9

lrp16 family protein
  
Accession: EPE10903
  
Location: 3968772-3969844
  
 NCBI BlastP on this gene

EPE10903

hypothetical protein
  
Accession: EPE10902
  
Location: 3968057-3968445
  
 NCBI BlastP on this gene

EPE10902

hypothetical protein
  
Accession: EPE10901
  
Location: 3965200-3965985
  
 NCBI BlastP on this gene

EPE10901

specific serine endopeptidase
  
Accession: EPE10900
  
Location: 3963028-3963771
  
 NCBI BlastP on this gene

EPE10900

hypothetical protein
  
Accession: EPE10899
  
Location: 3959732-3960935
  
  
**BlastP hit with Mycgr3G35535\_Mycgr3T**
  
Percentage identity: 28 %
  
BlastP bit score: 57
  
Sequence coverage: 86 %
  
E-value: 5e-07
  
  
 NCBI BlastP on this gene

EPE10899

tat pathway signal sequence
  
Accession: EPE10898
  
Location: 3958326-3959431
  
 NCBI BlastP on this gene

EPE10898

hypothetical protein
  
Accession: EPE10897
  
Location: 3956279-3957049
  
 NCBI BlastP on this gene

EPE10897

hypothetical protein
  
Accession: EPE10896
  
Location: 3954625-3955793
  
 NCBI BlastP on this gene

EPE10896

pyridine nucleotide-disulfide
  
Accession: EPE10895
  
Location: 3952470-3953825
  
 NCBI BlastP on this gene

EPE10895

peptidase s41 family protein
  
Accession: EPE10894
  
Location: 3945810-3948383
  
  
**BlastP hit with Mycgr3G36449\_Mycgr3T**
  
Percentage identity: 37 %
  
BlastP bit score: 414
  
Sequence coverage: 95 %
  
E-value: 4e-129
  
  
 NCBI BlastP on this gene

EPE10894

hypothetical protein
  
Accession: EPE10893
  
Location: 3944613-3945530
  
 NCBI BlastP on this gene

EPE10893

elongation factor 3
  
Accession: EPE10892
  
Location: 3936991-3940660
  
 NCBI BlastP on this gene

EPE10892

Query: Architecture Search FASTA input

ACFW01000025 : Coccidioides posadasii C735 delta SOWgp    Total score: 2.0     Cumulative Blast bit score: 470

Hit cluster cross-links:

Mycgr3G36335 Mycgr3T
  
Location: 0-423

Mycgr3G36335\_Mycgr3T

Mycgr3G84494 Mycgr3T
  
Location: 523-2047

Mycgr3G84494\_Mycgr3T

Mycgr3G90558 Mycgr3T
  
Location: 2147-15296

Mycgr3G90558\_Mycgr3T

Mycgr3G68036 Mycgr3T
  
Location: 15396-16395

Mycgr3G68036\_Mycgr3T

Mycgr3G90561 Mycgr3T
  
Location: 16495-17134

Mycgr3G90561\_Mycgr3T

Mycgr3G35862 Mycgr3T
  
Location: 17234-18662

Mycgr3G35862\_Mycgr3T

Mycgr3G68030 Mycgr3T
  
Location: 18762-19722

Mycgr3G68030\_Mycgr3T

Mycgr3G36449 Mycgr3T
  
Location: 19822-21886

Mycgr3G36449\_Mycgr3T

Mycgr3G35528 Mycgr3T
  
Location: 21986-22844

Mycgr3G35528\_Mycgr3T

Mycgr3G35932 Mycgr3T
  
Location: 22944-24390

Mycgr3G35932\_Mycgr3T

Mycgr3G23761 Mycgr3T
  
Location: 24490-25825

Mycgr3G23761\_Mycgr3T

Mycgr3G35535 Mycgr3T
  
Location: 25925-26429

Mycgr3G35535\_Mycgr3T

Mycgr3G9942 Mycgr3T9
  
Location: 26529-30375

Mycgr3G9942\_Mycgr3T9

PAN domain containing protein
  
Accession: EER26924
  
Location: 13629-14972
  
 NCBI BlastP on this gene

EER26924

prolyl 4-hydroxylase alpha subunit, putative
  
Accession: EER26925
  
Location: 16260-17262
  
 NCBI BlastP on this gene

EER26925

PAN domain containing protein
  
Accession: EER26926
  
Location: 19796-21237
  
 NCBI BlastP on this gene

EER26926

Cytochrome P450 family protein
  
Accession: EER26927
  
Location: 22027-23819
  
 NCBI BlastP on this gene

EER26927

Major Facilitator Superfamily protein
  
Accession: EER26928
  
Location: 26245-27875
  
  
**BlastP hit with Mycgr3G84494\_Mycgr3T**
  
Percentage identity: 45 %
  
BlastP bit score: 340
  
Sequence coverage: 76 %
  
E-value: 6e-108
  
  
 NCBI BlastP on this gene

EER26928

WD domain, G-beta repeat containing protein
  
Accession: EER26929
  
Location: 28508-33558
  
 NCBI BlastP on this gene

EER26929

conserved hypothetical protein
  
Accession: EER26930
  
Location: 34670-35927
  
  
**BlastP hit with Mycgr3G68030\_Mycgr3T**
  
Percentage identity: 31 %
  
BlastP bit score: 130
  
Sequence coverage: 78 %
  
E-value: 2e-31
  
  
 NCBI BlastP on this gene

EER26930

oxidoreductase, 2OG-Fe(II) oxygenase family protein
  
Accession: EER26931
  
Location: 36317-37425
  
 NCBI BlastP on this gene

EER26931

serine/threonine protein phosphatase ppe1, putative
  
Accession: EER26932
  
Location: 38883-40298
  
 NCBI BlastP on this gene

EER26932

Ctr copper transporter family protein
  
Accession: EER26933
  
Location: 41664-42595
  
 NCBI BlastP on this gene

EER26933

RNA recognition motif containing protein
  
Accession: EER26934
  
Location: 43858-45749
  
 NCBI BlastP on this gene

EER26934

radical SAM domain containing protein
  
Accession: EER26935
  
Location: 46995-49435
  
 NCBI BlastP on this gene

EER26935

Query: Architecture Search FASTA input

EQ962654 : Talaromyces stipitatus ATCC 10500 scf\_1105507295541 genomic scaffold    Total score: 2.0     Cumulative Blast bit score: 462

Hit cluster cross-links:

Mycgr3G36335 Mycgr3T
  
Location: 0-423

Mycgr3G36335\_Mycgr3T

Mycgr3G84494 Mycgr3T
  
Location: 523-2047

Mycgr3G84494\_Mycgr3T

Mycgr3G90558 Mycgr3T
  
Location: 2147-15296

Mycgr3G90558\_Mycgr3T

Mycgr3G68036 Mycgr3T
  
Location: 15396-16395

Mycgr3G68036\_Mycgr3T

Mycgr3G90561 Mycgr3T
  
Location: 16495-17134

Mycgr3G90561\_Mycgr3T

Mycgr3G35862 Mycgr3T
  
Location: 17234-18662

Mycgr3G35862\_Mycgr3T

Mycgr3G68030 Mycgr3T
  
Location: 18762-19722

Mycgr3G68030\_Mycgr3T

Mycgr3G36449 Mycgr3T
  
Location: 19822-21886

Mycgr3G36449\_Mycgr3T

Mycgr3G35528 Mycgr3T
  
Location: 21986-22844

Mycgr3G35528\_Mycgr3T

Mycgr3G35932 Mycgr3T
  
Location: 22944-24390

Mycgr3G35932\_Mycgr3T

Mycgr3G23761 Mycgr3T
  
Location: 24490-25825

Mycgr3G23761\_Mycgr3T

Mycgr3G35535 Mycgr3T
  
Location: 25925-26429

Mycgr3G35535\_Mycgr3T

Mycgr3G9942 Mycgr3T9
  
Location: 26529-30375

Mycgr3G9942\_Mycgr3T9

RTA1 domain protein, putative
  
Accession: EED19857
  
Location: 2116645-2117679
  
 NCBI BlastP on this gene

EED19857

MFS transporter, putative
  
Accession: EED19856
  
Location: 2113588-2115281
  
  
**BlastP hit with Mycgr3G84494\_Mycgr3T**
  
Percentage identity: 37 %
  
BlastP bit score: 286
  
Sequence coverage: 81 %
  
E-value: 1e-86
  
  
 NCBI BlastP on this gene

EED19856

conserved hypothetical protein
  
Accession: EED19855
  
Location: 2111239-2112423
  
 NCBI BlastP on this gene

EED19855

polysaccharide deacetylase (NodB), putative
  
Accession: EED19853
  
Location: 2110011-2110760
  
 NCBI BlastP on this gene

EED19853

conserved hypothetical protein
  
Accession: EED19852
  
Location: 2108747-2109502
  
 NCBI BlastP on this gene

EED19852

conserved hypothetical protein
  
Accession: EED19851
  
Location: 2106905-2108514
  
 NCBI BlastP on this gene

EED19851

hypothetical protein
  
Accession: EED19850
  
Location: 2105188-2106172
  
 NCBI BlastP on this gene

EED19850

hypothetical protein
  
Accession: EED19849
  
Location: 2104292-2105103
  
 NCBI BlastP on this gene

EED19849

flavin containing polyamine oxidase, putative
  
Accession: EED19848
  
Location: 2102232-2104138
  
 NCBI BlastP on this gene

EED19848

conserved hypothetical protein
  
Accession: EED19846
  
Location: 2095211-2098040
  
 NCBI BlastP on this gene

EED19846

alcohol dehydrogenase, putative
  
Accession: EED19845
  
Location: 2093232-2094353
  
 NCBI BlastP on this gene

EED19845

glucosamine 6-phosphate N-acetyltransferase, putative
  
Accession: EED19843
  
Location: 2092065-2092689
  
 NCBI BlastP on this gene

EED19843

MFS transporter, putative
  
Accession: EED19842
  
Location: 2089885-2091477
  
  
**BlastP hit with Mycgr3G23761\_Mycgr3T**
  
Percentage identity: 28 %
  
BlastP bit score: 176
  
Sequence coverage: 104 %
  
E-value: 9e-46
  
  
 NCBI BlastP on this gene

EED19842

efflux pump antibiotic resistance protein, putative
  
Accession: EED19841
  
Location: 2087281-2089207
  
 NCBI BlastP on this gene

EED19841

conserved hypothetical protein
  
Accession: EED19840
  
Location: 2085060-2086510
  
 NCBI BlastP on this gene

EED19840

Query: Architecture Search FASTA input

KE145368 : Glarea lozoyensis ATCC 20868 chromosome Unknown GLAREA4    Total score: 2.0     Cumulative Blast bit score: 449

Hit cluster cross-links:

Mycgr3G36335 Mycgr3T
  
Location: 0-423

Mycgr3G36335\_Mycgr3T

Mycgr3G84494 Mycgr3T
  
Location: 523-2047

Mycgr3G84494\_Mycgr3T

Mycgr3G90558 Mycgr3T
  
Location: 2147-15296

Mycgr3G90558\_Mycgr3T

Mycgr3G68036 Mycgr3T
  
Location: 15396-16395

Mycgr3G68036\_Mycgr3T

Mycgr3G90561 Mycgr3T
  
Location: 16495-17134

Mycgr3G90561\_Mycgr3T

Mycgr3G35862 Mycgr3T
  
Location: 17234-18662

Mycgr3G35862\_Mycgr3T

Mycgr3G68030 Mycgr3T
  
Location: 18762-19722

Mycgr3G68030\_Mycgr3T

Mycgr3G36449 Mycgr3T
  
Location: 19822-21886

Mycgr3G36449\_Mycgr3T

Mycgr3G35528 Mycgr3T
  
Location: 21986-22844

Mycgr3G35528\_Mycgr3T

Mycgr3G35932 Mycgr3T
  
Location: 22944-24390

Mycgr3G35932\_Mycgr3T

Mycgr3G23761 Mycgr3T
  
Location: 24490-25825

Mycgr3G23761\_Mycgr3T

Mycgr3G35535 Mycgr3T
  
Location: 25925-26429

Mycgr3G35535\_Mycgr3T

Mycgr3G9942 Mycgr3T9
  
Location: 26529-30375

Mycgr3G9942\_Mycgr3T9

Acetyl-CoA synthetase-like protein
  
Accession: EPE28435
  
Location: 588414-597802
  
 NCBI BlastP on this gene

EPE28435

Clavaminate synthase-like protein
  
Accession: EPE28436
  
Location: 598904-599991
  
 NCBI BlastP on this gene

EPE28436

alpha/beta-Hydrolase
  
Accession: EPE28437
  
Location: 601010-602224
  
 NCBI BlastP on this gene

EPE28437

Clavaminate synthase-like protein
  
Accession: EPE28438
  
Location: 603234-604432
  
  
**BlastP hit with Mycgr3G68036\_Mycgr3T**
  
Percentage identity: 42 %
  
BlastP bit score: 279
  
Sequence coverage: 105 %
  
E-value: 8e-88
  
  
 NCBI BlastP on this gene

EPE28438

Acetyl-CoA synthetase-like protein
  
Accession: EPE28439
  
Location: 605456-608072
  
 NCBI BlastP on this gene

EPE28439

Acetyl-CoA synthetase-like protein
  
Accession: EPE28440
  
Location: 608150-611569
  
 NCBI BlastP on this gene

EPE28440

alpha/beta-Hydrolase
  
Accession: EPE28441
  
Location: 612330-613430
  
 NCBI BlastP on this gene

EPE28441

MFS general substrate transporter
  
Accession: EPE28442
  
Location: 614165-615961
  
  
**BlastP hit with Mycgr3G23761\_Mycgr3T**
  
Percentage identity: 28 %
  
BlastP bit score: 170
  
Sequence coverage: 103 %
  
E-value: 1e-43
  
  
 NCBI BlastP on this gene

EPE28442

Query: Architecture Search FASTA input

51. :  KB446555 Pseudocercospora fijiensis CIRAD86 unplaced genomic scaffold MYCFIscaffold\_1     Total score: 2.0     Cumulative Blast bit score: 1216

Mycgr3G36335 Mycgr3T
  
Location: 0-423
  
 NCBI BlastP on this gene

Mycgr3G36335\_Mycgr3T

Mycgr3G84494 Mycgr3T
  
Location: 523-2047
  
 NCBI BlastP on this gene

Mycgr3G84494\_Mycgr3T

Mycgr3G90558 Mycgr3T
  
Location: 2147-15296
  
 NCBI BlastP on this gene

Mycgr3G90558\_Mycgr3T

Mycgr3G68036 Mycgr3T
  
Location: 15396-16395
  
 NCBI BlastP on this gene

Mycgr3G68036\_Mycgr3T

Mycgr3G90561 Mycgr3T
  
Location: 16495-17134
  
 NCBI BlastP on this gene

Mycgr3G90561\_Mycgr3T

Mycgr3G35862 Mycgr3T
  
Location: 17234-18662
  
 NCBI BlastP on this gene

Mycgr3G35862\_Mycgr3T

Mycgr3G68030 Mycgr3T
  
Location: 18762-19722
  
 NCBI BlastP on this gene

Mycgr3G68030\_Mycgr3T

Mycgr3G36449 Mycgr3T
  
Location: 19822-21886
  
 NCBI BlastP on this gene

Mycgr3G36449\_Mycgr3T

Mycgr3G35528 Mycgr3T
  
Location: 21986-22844
  
 NCBI BlastP on this gene

Mycgr3G35528\_Mycgr3T

Mycgr3G35932 Mycgr3T
  
Location: 22944-24390
  
 NCBI BlastP on this gene

Mycgr3G35932\_Mycgr3T

Mycgr3G23761 Mycgr3T
  
Location: 24490-25825
  
 NCBI BlastP on this gene

Mycgr3G23761\_Mycgr3T

Mycgr3G35535 Mycgr3T
  
Location: 25925-26429
  
 NCBI BlastP on this gene

Mycgr3G35535\_Mycgr3T

Mycgr3G9942 Mycgr3T9
  
Location: 26529-30375
  
 NCBI BlastP on this gene

Mycgr3G9942\_Mycgr3T9

hypothetical protein
  
Accession: EME89456
  
Location: 10582040-10583080
  
  
**BlastP hit with Mycgr3G68036\_Mycgr3T**
  
Percentage identity: 36 %
  
BlastP bit score: 177
  
Sequence coverage: 99 %
  
E-value: 1e-48
  
  
 NCBI BlastP on this gene

EME89456

hypothetical protein
  
Accession: EME89457
  
Location: 10583716-10598238
  
  
**BlastP hit with Mycgr3G90558\_Mycgr3T**
  
Percentage identity: 30 %
  
BlastP bit score: 1039
  
Sequence coverage: 54 %
  
E-value: 0.0
  
  
 NCBI BlastP on this gene

EME89457

hypothetical protein
  
Accession: EME89458
  
Location: 10598450-10599385
  
 NCBI BlastP on this gene

EME89458

hypothetical protein
  
Accession: EME89459
  
Location: 10602096-10602956
  
 NCBI BlastP on this gene

EME89459

52. :  KB456266 Mycosphaerella populorum SO2202 unplaced genomic scaffold SEPMUscaffold\_7     Total score: 2.0     Cumulative Blast bit score: 1208

hypothetical protein
  
Accession: EMF11590
  
Location: 1906885-1907175
  
 NCBI BlastP on this gene

EMF11590

coenzyme F420-dependent NADP oxidoreductase
  
Accession: EMF11591
  
Location: 1909364-1910552
  
 NCBI BlastP on this gene

EMF11591

hypothetical protein
  
Accession: EMF11592
  
Location: 1911756-1913818
  
 NCBI BlastP on this gene

EMF11592

Clavaminate synthase-like protein
  
Accession: EMF11593
  
Location: 1914406-1915464
  
  
**BlastP hit with Mycgr3G68036\_Mycgr3T**
  
Percentage identity: 35 %
  
BlastP bit score: 180
  
Sequence coverage: 104 %
  
E-value: 1e-49
  
  
 NCBI BlastP on this gene

EMF11593

acetyl-CoA synthetase-like protein
  
Accession: EMF11594
  
Location: 1916348-1931212
  
  
**BlastP hit with Mycgr3G90558\_Mycgr3T**
  
Percentage identity: 31 %
  
BlastP bit score: 1028
  
Sequence coverage: 49 %
  
E-value: 0.0
  
  
 NCBI BlastP on this gene

EMF11594

Bac surface Ag-domain-containing protein
  
Accession: EMF11595
  
Location: 1933056-1934728
  
 NCBI BlastP on this gene

EMF11595

hypothetical protein
  
Accession: EMF11597
  
Location: 1934929-1935828
  
 NCBI BlastP on this gene

EMF11597

glutathione S-transferase
  
Accession: EMF11598
  
Location: 1936246-1936968
  
 NCBI BlastP on this gene

EMF11598

hypothetical protein
  
Accession: EMF11599
  
Location: 1937840-1939067
  
 NCBI BlastP on this gene

EMF11599

53. :  JH725152 Beauveria bassiana ARSEF 2860 unplaced genomic scaffold BBA\_S00003     Total score: 2.0     Cumulative Blast bit score: 1196

hypothetical protein
  
Accession: EJP69797
  
Location: 1688362-1689759
  
 NCBI BlastP on this gene

EJP69797

Casein kinase II, alpha chain (CK II alpha subunit)
  
Accession: EJP69798
  
Location: 1690656-1692026
  
 NCBI BlastP on this gene

EJP69798

hypothetical protein
  
Accession: EJP69799
  
Location: 1693120-1696548
  
 NCBI BlastP on this gene

EJP69799

autophagy protein Apg6
  
Accession: EJP69800
  
Location: 1697247-1698833
  
 NCBI BlastP on this gene

EJP69800

ABC transporter
  
Accession: EJP69801
  
Location: 1699411-1704198
  
  
**BlastP hit with Mycgr3G9942\_Mycgr3T9**
  
Percentage identity: 36 %
  
BlastP bit score: 836
  
Sequence coverage: 106 %
  
E-value: 0.0
  
  
 NCBI BlastP on this gene

EJP69801

WD domain-containing protein
  
Accession: EJP69802
  
Location: 1709737-1712072
  
 NCBI BlastP on this gene

EJP69802

major facilitator superfamily transporter
  
Accession: EJP69803
  
Location: 1713365-1715011
  
  
**BlastP hit with Mycgr3G84494\_Mycgr3T**
  
Percentage identity: 40 %
  
BlastP bit score: 360
  
Sequence coverage: 93 %
  
E-value: 2e-114
  
  
 NCBI BlastP on this gene

EJP69803

transmembrane protein
  
Accession: EJP69804
  
Location: 1715124-1715812
  
 NCBI BlastP on this gene

EJP69804

AP-2 adaptor complex subunit beta
  
Accession: EJP69805
  
Location: 1716443-1718970
  
 NCBI BlastP on this gene

EJP69805

hypothetical protein
  
Accession: EJP69806
  
Location: 1721320-1722186
  
 NCBI BlastP on this gene

EJP69806

proteasome maturation factor UMP1
  
Accession: EJP69807
  
Location: 1723019-1723492
  
 NCBI BlastP on this gene

EJP69807

ABC transporter
  
Accession: EJP69808
  
Location: 1724638-1726656
  
 NCBI BlastP on this gene

EJP69808

54. :  AAHF01000006 Aspergillus fumigatus Af293     Total score: 2.0     Cumulative Blast bit score: 1106

nonribosomal peptide synthase, putative
  
Accession: EAL89046
  
Location: 1694970-1698884
  
 NCBI BlastP on this gene

EAL89046

MAK1-like monooxygenase, putative
  
Accession: EAL89047
  
Location: 1699285-1700651
  
 NCBI BlastP on this gene

EAL89047

FAD binding domain protein
  
Accession: EAL89048
  
Location: 1701480-1703303
  
 NCBI BlastP on this gene

EAL89048

nonribosomal peptide synthase, putative
  
Accession: EAL89049
  
Location: 1704693-1716682
  
  
**BlastP hit with Mycgr3G90558\_Mycgr3T**
  
Percentage identity: 31 %
  
BlastP bit score: 1031
  
Sequence coverage: 51 %
  
E-value: 0.0
  
  
 NCBI BlastP on this gene

EAL89049

HET domain protein
  
Accession: EAL89050
  
Location: 1719714-1721570
  
  
**BlastP hit with Mycgr3G36335\_Mycgr3T**
  
Percentage identity: 34 %
  
BlastP bit score: 75
  
Sequence coverage: 94 %
  
E-value: 5e-13
  
  
 NCBI BlastP on this gene

EAL89050

nitrilase family protein
  
Accession: EAL89051
  
Location: 1723473-1724328
  
 NCBI BlastP on this gene

EAL89051

salicylate synthetase, putative
  
Accession: EAL89052
  
Location: 1724938-1726340
  
 NCBI BlastP on this gene

EAL89052

BNR/Asp-box repeat domain protein
  
Accession: EAL89053
  
Location: 1726765-1727971
  
 NCBI BlastP on this gene

EAL89053

C6 transcription factor, putative
  
Accession: EAL89054
  
Location: 1729943-1732113
  
 NCBI BlastP on this gene

EAL89054

55. :  DS499599 Aspergillus fumigatus A1163 scf\_000006 genomic scaffold     Total score: 2.0     Cumulative Blast bit score: 1105

nonribosomal peptide synthase, putative
  
Accession: EDP49773
  
Location: 1701787-1705701
  
 NCBI BlastP on this gene

EDP49773

MAK1-like monooxygenase, putative
  
Accession: EDP49774
  
Location: 1706102-1707468
  
 NCBI BlastP on this gene

EDP49774

FAD binding domain protein
  
Accession: EDP49775
  
Location: 1708297-1710120
  
 NCBI BlastP on this gene

EDP49775

nonribosomal peptide synthase, putative
  
Accession: EDP49776
  
Location: 1711510-1723499
  
  
**BlastP hit with Mycgr3G90558\_Mycgr3T**
  
Percentage identity: 31 %
  
BlastP bit score: 1030
  
Sequence coverage: 51 %
  
E-value: 0.0
  
  
 NCBI BlastP on this gene

EDP49776

HET domain protein
  
Accession: EDP49777
  
Location: 1726523-1728379
  
  
**BlastP hit with Mycgr3G36335\_Mycgr3T**
  
Percentage identity: 34 %
  
BlastP bit score: 75
  
Sequence coverage: 94 %
  
E-value: 5e-13
  
  
 NCBI BlastP on this gene

EDP49777

salicylate synthetase, putative
  
Accession: EDP49778
  
Location: 1731150-1732552
  
 NCBI BlastP on this gene

EDP49778

BNR/Asp-box repeat domain protein
  
Accession: EDP49779
  
Location: 1732977-1734183
  
 NCBI BlastP on this gene

EDP49779

C6 transcription factor, putative
  
Accession: EDP49780
  
Location: 1736155-1738325
  
 NCBI BlastP on this gene

EDP49780

56. :  DF126469 Aspergillus kawachii IFO 4308 DNA, contig: scaffold00023     Total score: 2.0     Cumulative Blast bit score: 1073

similar to An01g14840
  
Accession: GAA89740
  
Location: 470873-471697
  
 NCBI BlastP on this gene

GAA89740

NRPS-like enzyme
  
Accession: GAA89741
  
Location: 471862-475655
  
 NCBI BlastP on this gene

GAA89741

integral membrane protein
  
Accession: GAA89742
  
Location: 476219-477031
  
 NCBI BlastP on this gene

GAA89742

aldehyde reductase I
  
Accession: GAA89743
  
Location: 477440-478478
  
 NCBI BlastP on this gene

GAA89743

similar to An01g14890
  
Accession: GAA89744
  
Location: 478734-479769
  
 NCBI BlastP on this gene

GAA89744

pantothenate transporter
  
Accession: GAA89745
  
Location: 481114-482860
  
 NCBI BlastP on this gene

GAA89745

C6 transcription factor
  
Accession: GAA89746
  
Location: 483090-485409
  
 NCBI BlastP on this gene

GAA89746

xaa-pro dipeptidase
  
Accession: GAA89747
  
Location: 486362-488217
  
  
**BlastP hit with Mycgr3G35862\_Mycgr3T**
  
Percentage identity: 57 %
  
BlastP bit score: 520
  
Sequence coverage: 98 %
  
E-value: 3e-178
  
  
 NCBI BlastP on this gene

GAA89747

major facilitator superfamily transporter
  
Accession: GAA89748
  
Location: 488662-490297
  
  
**BlastP hit with Mycgr3G35932\_Mycgr3T**
  
Percentage identity: 62 %
  
BlastP bit score: 553
  
Sequence coverage: 93 %
  
E-value: 0.0
  
  
 NCBI BlastP on this gene

GAA89748

ATP synthase F1
  
Accession: GAA89749
  
Location: 490956-493572
  
 NCBI BlastP on this gene

GAA89749

phosphoesterase superfamily protein
  
Accession: GAA89750
  
Location: 493890-495312
  
 NCBI BlastP on this gene

GAA89750

thermolabile L-asparaginase
  
Accession: GAA89751
  
Location: 496684-497769
  
 NCBI BlastP on this gene

GAA89751

L-asparaginase
  
Accession: GAA89752
  
Location: 498574-499824
  
 NCBI BlastP on this gene

GAA89752

similar to delta-12 fatty acid desaturase
  
Accession: GAA89753
  
Location: 500944-502379
  
 NCBI BlastP on this gene

GAA89753

hypothetical protein
  
Accession: GAA89754
  
Location: 502583-503129
  
 NCBI BlastP on this gene

GAA89754

similar to An01g14980
  
Accession: GAA89755
  
Location: 503336-504286
  
 NCBI BlastP on this gene

GAA89755

cytochrome P450 monooxygenase
  
Accession: GAA89756
  
Location: 505079-506686
  
 NCBI BlastP on this gene

GAA89756

57. :  AM269994 Aspergillus niger contig An01c0480, genomic contig.     Total score: 2.0     Cumulative Blast bit score: 1071

not annotated
  
Accession: CAK37440
  
Location: 1-1880
  
 NCBI BlastP on this gene

An01g14860

not annotated
  
Accession: CAK37441
  
Location: 2434-3246
  
 NCBI BlastP on this gene

An01g14870

not annotated
  
Accession: CAK37442
  
Location: 3650-4687
  
 NCBI BlastP on this gene

An01g14880

not annotated
  
Accession: CAK37443
  
Location: 4947-6028
  
 NCBI BlastP on this gene

An01g14890

not annotated
  
Accession: CAK37444
  
Location: 7310-9059
  
 NCBI BlastP on this gene

An01g14900

not annotated
  
Accession: CAK37445
  
Location: 9216-11622
  
 NCBI BlastP on this gene

An01g14910

not annotated
  
Accession: CAK37446
  
Location: 12564-14418
  
  
**BlastP hit with Mycgr3G35862\_Mycgr3T**
  
Percentage identity: 57 %
  
BlastP bit score: 520
  
Sequence coverage: 98 %
  
E-value: 6e-178
  
  
 NCBI BlastP on this gene

An01g14920

not annotated
  
Accession: CAK37447
  
Location: 14877-16515
  
  
**BlastP hit with Mycgr3G35932\_Mycgr3T**
  
Percentage identity: 61 %
  
BlastP bit score: 551
  
Sequence coverage: 93 %
  
E-value: 0.0
  
  
 NCBI BlastP on this gene

An01g14930

unnamed
  
Accession: CAK37448
  
Location: 17453-18882
  
 NCBI BlastP on this gene

An01g14940

not annotated
  
Accession: CAK37449
  
Location: 20279-21364
  
 NCBI BlastP on this gene

An01g14950

unnamed
  
Accession: CAK37450
  
Location: 22072-23325
  
 NCBI BlastP on this gene

An01g14960

not annotated
  
Accession: CAK37451
  
Location: 24588-25908
  
 NCBI BlastP on this gene

An01g14970

unnamed
  
Accession: CAK37452
  
Location: 26966-27938
  
 NCBI BlastP on this gene

An01g14980

not annotated
  
Accession: CAK37453
  
Location: 28827-30602
  
 NCBI BlastP on this gene

An01g14990

not annotated
  
Accession: CAK37454
  
Location: 32067-33856
  
 NCBI BlastP on this gene

An01g15000

58. :  ACJE01000004 Aspergillus niger ATCC 1015     Total score: 2.0     Cumulative Blast bit score: 1063

hypothetical protein
  
Accession: EHA27188
  
Location: 3699750-3700571
  
 NCBI BlastP on this gene

EHA27188

Hypothetical protein
  
Accession: EHA27189
  
Location: 3700765-3704529
  
 NCBI BlastP on this gene

EHA27189

hypothetical protein
  
Accession: EHA27190
  
Location: 3705079-3705891
  
 NCBI BlastP on this gene

EHA27190

alcohol dehydrogenase NADP+-dependent
  
Accession: EHA27191
  
Location: 3706296-3707333
  
 NCBI BlastP on this gene

EHA27191

hypothetical protein
  
Accession: EHA27192
  
Location: 3707589-3708669
  
 NCBI BlastP on this gene

EHA27192

hypothetical protein
  
Accession: EHA27193
  
Location: 3709068-3709716
  
 NCBI BlastP on this gene

EHA27193

hypothetical protein
  
Accession: EHA27194
  
Location: 3709951-3711700
  
 NCBI BlastP on this gene

EHA27194

hypothetical protein
  
Accession: EHA27195
  
Location: 3712005-3714263
  
 NCBI BlastP on this gene

EHA27195

hypothetical protein
  
Accession: EHA27196
  
Location: 3715209-3716833
  
  
**BlastP hit with Mycgr3G35862\_Mycgr3T**
  
Percentage identity: 61 %
  
BlastP bit score: 512
  
Sequence coverage: 89 %
  
E-value: 7e-176
  
  
 NCBI BlastP on this gene

EHA27196

hypothetical protein
  
Accession: EHA27197
  
Location: 3717518-3719156
  
  
**BlastP hit with Mycgr3G35932\_Mycgr3T**
  
Percentage identity: 61 %
  
BlastP bit score: 551
  
Sequence coverage: 93 %
  
E-value: 0.0
  
  
 NCBI BlastP on this gene

EHA27197

hypothetical protein
  
Accession: EHA27198
  
Location: 3720094-3721523
  
 NCBI BlastP on this gene

EHA27198

hypothetical protein
  
Accession: EHA27199
  
Location: 3722936-3724021
  
 NCBI BlastP on this gene

EHA27199

hypothetical protein
  
Accession: EHA27200
  
Location: 3724729-3725982
  
 NCBI BlastP on this gene

EHA27200

hypothetical protein
  
Accession: EHA27201
  
Location: 3727257-3728565
  
 NCBI BlastP on this gene

EHA27201

hypothetical protein
  
Accession: EHA27202
  
Location: 3728876-3729197
  
 NCBI BlastP on this gene

EHA27202

hypothetical protein
  
Accession: EHA27203
  
Location: 3729623-3730595
  
 NCBI BlastP on this gene

EHA27203

hypothetical protein
  
Accession: EHA27204
  
Location: 3731595-3732143
  
 NCBI BlastP on this gene

EHA27204

hypothetical protein
  
Accession: EHA27205
  
Location: 3734725-3736691
  
 NCBI BlastP on this gene

EHA27205

59. :  ABDF02000005 Trichoderma virens Gv29-8     Total score: 2.0     Cumulative Blast bit score: 1027

hypothetical protein
  
Accession: EHK23631
  
Location: 1322196-1322384
  
 NCBI BlastP on this gene

EHK23631

hypothetical protein
  
Accession: EHK23632
  
Location: 1324257-1326123
  
 NCBI BlastP on this gene

EHK23632

hypothetical protein
  
Accession: EHK23633
  
Location: 1326518-1328043
  
 NCBI BlastP on this gene

EHK23633

hypothetical protein
  
Accession: EHK23634
  
Location: 1328819-1329022
  
 NCBI BlastP on this gene

EHK23634

hypothetical protein
  
Accession: EHK23636
  
Location: 1330295-1330568
  
 NCBI BlastP on this gene

EHK23636

hypothetical protein
  
Accession: EHK23635
  
Location: 1330857-1331228
  
 NCBI BlastP on this gene

EHK23635

hypothetical protein
  
Accession: EHK23637
  
Location: 1333624-1334350
  
 NCBI BlastP on this gene

EHK23637

hypothetical protein
  
Accession: EHK23638
  
Location: 1336188-1337570
  
 NCBI BlastP on this gene

EHK23638

hypothetical protein
  
Accession: EHK23639
  
Location: 1337707-1339194
  
  
**BlastP hit with Mycgr3G35862\_Mycgr3T**
  
Percentage identity: 57 %
  
BlastP bit score: 479
  
Sequence coverage: 89 %
  
E-value: 4e-163
  
  
 NCBI BlastP on this gene

EHK23639

hypothetical protein
  
Accession: EHK23640
  
Location: 1340307-1342143
  
  
**BlastP hit with Mycgr3G35932\_Mycgr3T**
  
Percentage identity: 60 %
  
BlastP bit score: 548
  
Sequence coverage: 96 %
  
E-value: 0.0
  
  
 NCBI BlastP on this gene

EHK23640

hypothetical protein
  
Accession: EHK23641
  
Location: 1342800-1343885
  
 NCBI BlastP on this gene

EHK23641

hypothetical protein
  
Accession: EHK23642
  
Location: 1344418-1345576
  
 NCBI BlastP on this gene

EHK23642

hypothetical protein
  
Accession: EHK23643
  
Location: 1347079-1347426
  
 NCBI BlastP on this gene

EHK23643

hypothetical protein
  
Accession: EHK23644
  
Location: 1347614-1348491
  
 NCBI BlastP on this gene

EHK23644

hypothetical protein
  
Accession: EHK23645
  
Location: 1352155-1352346
  
 NCBI BlastP on this gene

EHK23645

hypothetical protein
  
Accession: EHK23646
  
Location: 1353182-1354585
  
 NCBI BlastP on this gene

EHK23646

hypothetical protein
  
Accession: EHK23647
  
Location: 1355007-1357151
  
 NCBI BlastP on this gene

EHK23647

60. :  AMYD01004056 Colletotrichum gloeosporioides Cg-14     Total score: 2.0     Cumulative Blast bit score: 1023

replication factor C
  
Accession: EQB44234
  
Location: 45-529
  
 NCBI BlastP on this gene

EQB44234

hypothetical protein
  
Accession: EQB44235
  
Location: 1595-3201
  
 NCBI BlastP on this gene

EQB44235

hypothetical protein
  
Accession: EQB44236
  
Location: 6623-7465
  
 NCBI BlastP on this gene

EQB44236

hypothetical protein
  
Accession: EQB44237
  
Location: 8020-9183
  
 NCBI BlastP on this gene

EQB44237

hypothetical protein
  
Accession: EQB44238
  
Location: 10293-10625
  
 NCBI BlastP on this gene

EQB44238

metallopeptidase family M24
  
Accession: EQB44239
  
Location: 11768-13371
  
  
**BlastP hit with Mycgr3G35862\_Mycgr3T**
  
Percentage identity: 50 %
  
BlastP bit score: 455
  
Sequence coverage: 104 %
  
E-value: 1e-152
  
  
 NCBI BlastP on this gene

EQB44239

major facilitator superfamily transporter
  
Accession: EQB44240
  
Location: 13928-15686
  
  
**BlastP hit with Mycgr3G35932\_Mycgr3T**
  
Percentage identity: 58 %
  
BlastP bit score: 568
  
Sequence coverage: 97 %
  
E-value: 0.0
  
  
 NCBI BlastP on this gene

EQB44240

hypothetical protein
  
Accession: EQB44241
  
Location: 16180-17556
  
 NCBI BlastP on this gene

EQB44241

hypothetical protein
  
Accession: EQB44242
  
Location: 18014-19368
  
 NCBI BlastP on this gene

EQB44242

OPT oligopeptide transporter
  
Accession: EQB44243
  
Location: 25401-28147
  
 NCBI BlastP on this gene

EQB44243

hypothetical protein
  
Accession: EQB44244
  
Location: 29109-30327
  
 NCBI BlastP on this gene

EQB44244

61. :  GL629735 Grosmannia clavigera kw1407 unplaced genomic scaffold GCSC\_113     Total score: 2.0     Cumulative Blast bit score: 1020

hypothetical protein
  
Accession: EFX05798
  
Location: 2054706-2056376
  
 NCBI BlastP on this gene

EFX05798

c6 zinc finger domain containing protein
  
Accession: EFX05925
  
Location: 2060011-2062203
  
 NCBI BlastP on this gene

EFX05925

maltose permease
  
Accession: EFX05667
  
Location: 2063221-2064819
  
 NCBI BlastP on this gene

EFX05667

f-box domain containing protein
  
Accession: EFX05886
  
Location: 2066765-2067826
  
 NCBI BlastP on this gene

EFX05886

hypothetical protein
  
Accession: EFX05594
  
Location: 2068140-2068898
  
 NCBI BlastP on this gene

EFX05594

proline dipeptidase
  
Accession: EFX05237
  
Location: 2069715-2071282
  
  
**BlastP hit with Mycgr3G35862\_Mycgr3T**
  
Percentage identity: 54 %
  
BlastP bit score: 440
  
Sequence coverage: 93 %
  
E-value: 1e-146
  
  
 NCBI BlastP on this gene

EFX05237

metabolite transport protein
  
Accession: EFX05486
  
Location: 2071928-2073606
  
  
**BlastP hit with Mycgr3G35932\_Mycgr3T**
  
Percentage identity: 59 %
  
BlastP bit score: 580
  
Sequence coverage: 97 %
  
E-value: 0.0
  
  
 NCBI BlastP on this gene

EFX05486

membrane copper amine oxidase
  
Accession: EFX05707
  
Location: 2073771-2076446
  
 NCBI BlastP on this gene

EFX05707

hypothetical protein
  
Accession: EFX05464
  
Location: 2077839-2078430
  
 NCBI BlastP on this gene

EFX05464

zinc alcohol dehydrogenase
  
Accession: EFX05548
  
Location: 2080614-2081753
  
 NCBI BlastP on this gene

EFX05548

cytochrome p450 monooxygenase
  
Accession: EFX05735
  
Location: 2082011-2082328
  
 NCBI BlastP on this gene

EFX05735

trihydroxytoluene oxygenase
  
Accession: EFX06133
  
Location: 2084222-2085454
  
 NCBI BlastP on this gene

EFX06133

magnesium dependent phosphatase
  
Accession: EFX05882
  
Location: 2086322-2087125
  
 NCBI BlastP on this gene

EFX05882

cellular retinaldehyde-binding/triple function protein
  
Accession: EFX06001
  
Location: 2087418-2089106
  
 NCBI BlastP on this gene

EFX06001

62. :  KB706559 Eutypa lata UCREL1 unplaced genomic scaffold EL1\_03\_scaffold\_1221     Total score: 2.0     Cumulative Blast bit score: 1014

hypothetical protein
  
Accession: EMR66895
  
Location: 60-977
  
 NCBI BlastP on this gene

EMR66895

putative aminopeptidase ypdf protein
  
Accession: EMR66901
  
Location: 3943-5678
  
  
**BlastP hit with Mycgr3G35862\_Mycgr3T**
  
Percentage identity: 48 %
  
BlastP bit score: 442
  
Sequence coverage: 101 %
  
E-value: 5e-147
  
  
 NCBI BlastP on this gene

EMR66901

putative metabolite transporter protein
  
Accession: EMR66900
  
Location: 6469-8315
  
  
**BlastP hit with Mycgr3G35932\_Mycgr3T**
  
Percentage identity: 58 %
  
BlastP bit score: 572
  
Sequence coverage: 97 %
  
E-value: 0.0
  
  
 NCBI BlastP on this gene

EMR66900

hypothetical protein
  
Accession: EMR66903
  
Location: 11649-12550
  
 NCBI BlastP on this gene

EMR66903

hypothetical protein
  
Accession: EMR66896
  
Location: 16852-18500
  
 NCBI BlastP on this gene

EMR66896

putative fad dependent protein
  
Accession: EMR66904
  
Location: 21723-23150
  
 NCBI BlastP on this gene

EMR66904

63. :  GG697353 Glomerella graminicola M1.001 genomic scaffold supercont1.23     Total score: 2.0     Cumulative Blast bit score: 1012

hypothetical protein
  
Accession: EFQ31085
  
Location: 188641-189078
  
 NCBI BlastP on this gene

EFQ31085

NAD-dependent 15-hydroxyprostaglandin dehydrogenase
  
Accession: EFQ31086
  
Location: 191149-191370
  
 NCBI BlastP on this gene

EFQ31086

hypothetical protein
  
Accession: EFQ31087
  
Location: 192603-192950
  
 NCBI BlastP on this gene

EFQ31087

hypothetical protein
  
Accession: EFQ31088
  
Location: 194525-194698
  
 NCBI BlastP on this gene

EFQ31088

OPT oligopeptide transporter
  
Accession: EFQ31089
  
Location: 199439-202165
  
 NCBI BlastP on this gene

EFQ31089

major facilitator superfamily transporter
  
Accession: EFQ31090
  
Location: 202561-204304
  
  
**BlastP hit with Mycgr3G35932\_Mycgr3T**
  
Percentage identity: 57 %
  
BlastP bit score: 560
  
Sequence coverage: 97 %
  
E-value: 0.0
  
  
 NCBI BlastP on this gene

EFQ31090

metallopeptidase family M24
  
Accession: EFQ31091
  
Location: 204855-206419
  
  
**BlastP hit with Mycgr3G35862\_Mycgr3T**
  
Percentage identity: 53 %
  
BlastP bit score: 452
  
Sequence coverage: 94 %
  
E-value: 8e-152
  
  
 NCBI BlastP on this gene

EFQ31091

2OG-Fe(II) oxygenase superfamily protein
  
Accession: EFQ31092
  
Location: 207090-207922
  
 NCBI BlastP on this gene

EFQ31092

hypothetical protein
  
Accession: EFQ31093
  
Location: 210526-212161
  
 NCBI BlastP on this gene

EFQ31093

replication factor C
  
Accession: EFQ31094
  
Location: 213155-214450
  
 NCBI BlastP on this gene

EFQ31094

hypothetical protein
  
Accession: EFQ31095
  
Location: 215007-215900
  
 NCBI BlastP on this gene

EFQ31095

hypothetical protein
  
Accession: EFQ31096
  
Location: 216130-217551
  
 NCBI BlastP on this gene

EFQ31096

64. :  CACQ02006567 Colletotrichum higginsianum strain IMI 349063     Total score: 2.0     Cumulative Blast bit score: 1001

2OG-Fe(II) oxygenase
  
Accession: CCF44025
  
Location: 379-1131
  
 NCBI BlastP on this gene

CCF44025

metallopeptidase family M24
  
Accession: CCF44026
  
Location: 1814-3422
  
  
**BlastP hit with Mycgr3G35862\_Mycgr3T**
  
Percentage identity: 51 %
  
BlastP bit score: 446
  
Sequence coverage: 97 %
  
E-value: 3e-149
  
  
 NCBI BlastP on this gene

CCF44026

major facilitator superfamily transporter
  
Accession: CCF44027
  
Location: 3967-5703
  
  
**BlastP hit with Mycgr3G35932\_Mycgr3T**
  
Percentage identity: 57 %
  
BlastP bit score: 555
  
Sequence coverage: 97 %
  
E-value: 0.0
  
  
 NCBI BlastP on this gene

CCF44027

OPT oligopeptide transporter
  
Accession: CCF44028
  
Location: 7602-10336
  
 NCBI BlastP on this gene

CCF44028

65. :  ABDG02000025 Trichoderma atroviride IMI 206040     Total score: 2.0     Cumulative Blast bit score: 1000

hypothetical protein
  
Accession: EHK44042
  
Location: 1300516-1303228
  
 NCBI BlastP on this gene

EHK44042

hypothetical protein
  
Accession: EHK44043
  
Location: 1304707-1306559
  
 NCBI BlastP on this gene

EHK44043

hypothetical protein
  
Accession: EHK44044
  
Location: 1307072-1308534
  
 NCBI BlastP on this gene

EHK44044

hypothetical protein
  
Accession: EHK44045
  
Location: 1314024-1314743
  
 NCBI BlastP on this gene

EHK44045

hypothetical protein
  
Accession: EHK44046
  
Location: 1316703-1317986
  
 NCBI BlastP on this gene

EHK44046

hypothetical protein
  
Accession: EHK44047
  
Location: 1318177-1319661
  
  
**BlastP hit with Mycgr3G35862\_Mycgr3T**
  
Percentage identity: 55 %
  
BlastP bit score: 457
  
Sequence coverage: 88 %
  
E-value: 3e-153
  
  
 NCBI BlastP on this gene

EHK44047

inorganic phosphate transporter
  
Accession: EHK44048
  
Location: 1320897-1322442
  
  
**BlastP hit with Mycgr3G35932\_Mycgr3T**
  
Percentage identity: 61 %
  
BlastP bit score: 543
  
Sequence coverage: 94 %
  
E-value: 0.0
  
  
 NCBI BlastP on this gene

EHK44048

hypothetical protein
  
Accession: EHK44049
  
Location: 1322907-1323413
  
 NCBI BlastP on this gene

EHK44049

hypothetical protein
  
Accession: EHK44050
  
Location: 1324646-1325794
  
 NCBI BlastP on this gene

EHK44050

hypothetical protein
  
Accession: EHK44051
  
Location: 1326327-1327510
  
 NCBI BlastP on this gene

EHK44051

hypothetical protein
  
Accession: EHK44053
  
Location: 1328700-1329046
  
 NCBI BlastP on this gene

EHK44053

hypothetical protein
  
Accession: EHK44052
  
Location: 1329291-1330148
  
 NCBI BlastP on this gene

EHK44052

hypothetical protein
  
Accession: EHK44054
  
Location: 1333548-1333736
  
 NCBI BlastP on this gene

EHK44054

hypothetical protein
  
Accession: EHK44055
  
Location: 1334434-1335837
  
 NCBI BlastP on this gene

EHK44055

hypothetical protein
  
Accession: EHK44056
  
Location: 1336290-1338452
  
 NCBI BlastP on this gene

EHK44056

66. :  GG698907 Nectria haematococca mpVI 77-13-4 chromosome 12 genomic scaffold NECHAsca\_15\_chr12\_5\_0     Total score: 2.0     Cumulative Blast bit score: 999

hypothetical protein
  
Accession: EEU41613
  
Location: 799109-801227
  
 NCBI BlastP on this gene

EEU41613

hypothetical protein
  
Accession: EEU41789
  
Location: 801592-803647
  
 NCBI BlastP on this gene

EEU41789

hypothetical protein
  
Accession: EEU41790
  
Location: 804927-806979
  
 NCBI BlastP on this gene

EEU41790

hypothetical protein
  
Accession: EEU41791
  
Location: 808798-810219
  
 NCBI BlastP on this gene

EEU41791

predicted protein
  
Accession: EEU41792
  
Location: 811273-812358
  
 NCBI BlastP on this gene

EEU41792

hypothetical protein
  
Accession: EEU41793
  
Location: 812880-813776
  
 NCBI BlastP on this gene

EEU41793

hypothetical protein
  
Accession: EEU41794
  
Location: 814246-815925
  
  
**BlastP hit with Mycgr3G35932\_Mycgr3T**
  
Percentage identity: 58 %
  
BlastP bit score: 557
  
Sequence coverage: 96 %
  
E-value: 0.0
  
  
 NCBI BlastP on this gene

EEU41794

hypothetical protein
  
Accession: EEU41614
  
Location: 816360-817817
  
  
**BlastP hit with Mycgr3G35862\_Mycgr3T**
  
Percentage identity: 53 %
  
BlastP bit score: 442
  
Sequence coverage: 92 %
  
E-value: 2e-148
  
  
 NCBI BlastP on this gene

EEU41614

hypothetical protein
  
Accession: EEU41795
  
Location: 818543-820374
  
 NCBI BlastP on this gene

EEU41795

hypothetical protein
  
Accession: EEU41615
  
Location: 820819-821604
  
 NCBI BlastP on this gene

EEU41615

predicted protein
  
Accession: EEU41616
  
Location: 822333-823358
  
 NCBI BlastP on this gene

EEU41616

hypothetical protein
  
Accession: EEU41617
  
Location: 824458-825915
  
 NCBI BlastP on this gene

EEU41617

hypothetical protein
  
Accession: EEU41618
  
Location: 826540-827752
  
 NCBI BlastP on this gene

EEU41618

predicted protein
  
Accession: EEU41619
  
Location: 830758-832728
  
 NCBI BlastP on this gene

EEU41619

67. :  GL985063 Trichoderma reesei QM6a unplaced genomic scaffold TRIREscaffold\_8     Total score: 2.0     Cumulative Blast bit score: 993

predicted protein
  
Accession: EGR49035
  
Location: 1301642-1304393
  
 NCBI BlastP on this gene

EGR49035

predicted protein
  
Accession: EGR49036
  
Location: 1306431-1308359
  
 NCBI BlastP on this gene

EGR49036

homogentisate 1,2-dioxygenase
  
Accession: EGR49238
  
Location: 1308848-1310312
  
 NCBI BlastP on this gene

EGR49238

predicted protein
  
Accession: EGR49037
  
Location: 1315040-1315765
  
 NCBI BlastP on this gene

EGR49037

predicted protein
  
Accession: EGR49038
  
Location: 1317928-1319364
  
 NCBI BlastP on this gene

EGR49038

Prolidase/Aminopeptidase P-like protein
  
Accession: EGR49239
  
Location: 1319539-1320924
  
  
**BlastP hit with Mycgr3G35862\_Mycgr3T**
  
Percentage identity: 53 %
  
BlastP bit score: 454
  
Sequence coverage: 96 %
  
E-value: 1e-152
  
  
 NCBI BlastP on this gene

EGR49239

predicted protein
  
Accession: EGR49039
  
Location: 1322450-1323991
  
  
**BlastP hit with Mycgr3G35932\_Mycgr3T**
  
Percentage identity: 60 %
  
BlastP bit score: 540
  
Sequence coverage: 94 %
  
E-value: 0.0
  
  
 NCBI BlastP on this gene

EGR49039

predicted protein
  
Accession: EGR49040
  
Location: 1324748-1326100
  
 NCBI BlastP on this gene

EGR49040

predicted protein
  
Accession: EGR49240
  
Location: 1326643-1327875
  
 NCBI BlastP on this gene

EGR49240

predicted protein
  
Accession: EGR49041
  
Location: 1330240-1330538
  
 NCBI BlastP on this gene

EGR49041

predicted protein
  
Accession: EGR49241
  
Location: 1331108-1332019
  
 NCBI BlastP on this gene

EGR49241

4-aminobutyrate aminotransferase-like protein
  
Accession: EGR49242
  
Location: 1336705-1338111
  
 NCBI BlastP on this gene

EGR49242

N-terminal binuclear Zn cluster-containing/DNA binding domain-containing protein
  
Accession: EGR49243
  
Location: 1338556-1340706
  
 NCBI BlastP on this gene

EGR49243

68. :  CH476594 Aspergillus terreus NIH2624 scaffold\_1 genomic scaffold     Total score: 2.0     Cumulative Blast bit score: 984

conserved hypothetical protein
  
Accession: EAU39598
  
Location: 2671218-2674001
  
 NCBI BlastP on this gene

EAU39598

predicted protein
  
Accession: EAU39599
  
Location: 2674566-2676546
  
 NCBI BlastP on this gene

EAU39599

conserved hypothetical protein
  
Accession: EAU39600
  
Location: 2676738-2677445
  
 NCBI BlastP on this gene

EAU39600

conserved hypothetical protein
  
Accession: EAU39601
  
Location: 2677883-2679823
  
 NCBI BlastP on this gene

EAU39601

predicted protein
  
Accession: EAU39602
  
Location: 2681905-2683400
  
 NCBI BlastP on this gene

EAU39602

fatty acid transporter protein
  
Accession: EAU39603
  
Location: 2684247-2686282
  
 NCBI BlastP on this gene

EAU39603

conserved hypothetical protein
  
Accession: EAU39604
  
Location: 2686794-2688433
  
  
**BlastP hit with Mycgr3G35932\_Mycgr3T**
  
Percentage identity: 62 %
  
BlastP bit score: 561
  
Sequence coverage: 92 %
  
E-value: 0.0
  
  
 NCBI BlastP on this gene

EAU39604

conserved hypothetical protein
  
Accession: EAU39605
  
Location: 2689114-2690766
  
  
**BlastP hit with Mycgr3G35862\_Mycgr3T**
  
Percentage identity: 54 %
  
BlastP bit score: 423
  
Sequence coverage: 89 %
  
E-value: 1e-140
  
  
 NCBI BlastP on this gene

EAU39605

predicted protein
  
Accession: EAU39606
  
Location: 2691423-2692919
  
 NCBI BlastP on this gene

EAU39606

conserved hypothetical protein
  
Accession: EAU39607
  
Location: 2693687-2695368
  
 NCBI BlastP on this gene

EAU39607

predicted protein
  
Accession: EAU39608
  
Location: 2697059-2698520
  
 NCBI BlastP on this gene

EAU39608

conserved hypothetical protein
  
Accession: EAU39609
  
Location: 2700069-2702716
  
 NCBI BlastP on this gene

EAU39609

predicted protein
  
Accession: EAU39610
  
Location: 2703919-2704716
  
 NCBI BlastP on this gene

EAU39610

conserved hypothetical protein
  
Accession: EAU39611
  
Location: 2705214-2705862
  
 NCBI BlastP on this gene

EAU39611

69. :  KE148169 Ophiostoma piceae UAMH 11346 chromosome Unknown scf24     Total score: 2.0     Cumulative Blast bit score: 980

phytase
  
Accession: EPE03310
  
Location: 375363-377484
  
 NCBI BlastP on this gene

EPE03310

acid phosphatase-like protein
  
Accession: EPE03311
  
Location: 378053-380356
  
 NCBI BlastP on this gene

EPE03311

translocation protein sec62
  
Accession: EPE03312
  
Location: 385697-387065
  
 NCBI BlastP on this gene

EPE03312

gcn5-related n-acetyltransferase
  
Accession: EPE03313
  
Location: 387465-388464
  
 NCBI BlastP on this gene

EPE03313

thymine dioxygenase
  
Accession: EPE03314
  
Location: 389225-390313
  
 NCBI BlastP on this gene

EPE03314

hexose transporter
  
Accession: EPE03315
  
Location: 390898-392605
  
 NCBI BlastP on this gene

EPE03315

major facilitator superfamily transporter
  
Accession: EPE03316
  
Location: 392793-394456
  
  
**BlastP hit with Mycgr3G35932\_Mycgr3T**
  
Percentage identity: 56 %
  
BlastP bit score: 555
  
Sequence coverage: 97 %
  
E-value: 0.0
  
  
 NCBI BlastP on this gene

EPE03316

xaa-pro dipeptidase
  
Accession: EPE03317
  
Location: 395500-397088
  
  
**BlastP hit with Mycgr3G35862\_Mycgr3T**
  
Percentage identity: 50 %
  
BlastP bit score: 425
  
Sequence coverage: 94 %
  
E-value: 2e-140
  
  
 NCBI BlastP on this gene

EPE03317

hypothetical protein
  
Accession: EPE03318
  
Location: 398367-399075
  
 NCBI BlastP on this gene

EPE03318

hypothetical protein
  
Accession: EPE03319
  
Location: 399911-401234
  
 NCBI BlastP on this gene

EPE03319

mfs transporter
  
Accession: EPE03320
  
Location: 402247-404076
  
 NCBI BlastP on this gene

EPE03320

helix-turn-helix-domain containing protein type
  
Accession: EPE03321
  
Location: 404300-404901
  
 NCBI BlastP on this gene

EPE03321

70. :  JH795082 Magnaporthe oryzae P131 unplaced genomic scaffold P131\_scaffold01190     Total score: 2.0     Cumulative Blast bit score: 979

hypothetical protein
  
Accession: ELQ61354
  
Location: 10118-10846
  
 NCBI BlastP on this gene

ELQ61354

surface protein 1
  
Accession: ELQ61355
  
Location: 11670-12110
  
 NCBI BlastP on this gene

ELQ61355

hypothetical protein
  
Accession: ELQ61356
  
Location: 12745-12840
  
 NCBI BlastP on this gene

ELQ61356

C-signal protein
  
Accession: ELQ61357
  
Location: 13358-14092
  
 NCBI BlastP on this gene

ELQ61357

hypothetical protein
  
Accession: ELQ61358
  
Location: 14632-15210
  
 NCBI BlastP on this gene

ELQ61358

benzoate 4-monooxygenase
  
Accession: ELQ61359
  
Location: 17324-19165
  
 NCBI BlastP on this gene

ELQ61359

hypothetical protein
  
Accession: ELQ61360
  
Location: 20652-21650
  
 NCBI BlastP on this gene

ELQ61360

hypothetical protein
  
Accession: ELQ61361
  
Location: 21863-22450
  
 NCBI BlastP on this gene

ELQ61361

DUF563 domain-containing protein
  
Accession: ELQ61362
  
Location: 22706-24501
  
 NCBI BlastP on this gene

ELQ61362

metabolite transporter
  
Accession: ELQ61363
  
Location: 25605-27119
  
  
**BlastP hit with Mycgr3G35932\_Mycgr3T**
  
Percentage identity: 57 %
  
BlastP bit score: 542
  
Sequence coverage: 99 %
  
E-value: 0.0
  
  
 NCBI BlastP on this gene

ELQ61363

aminopeptidase ypdF
  
Accession: ELQ61364
  
Location: 27788-29328
  
  
**BlastP hit with Mycgr3G35862\_Mycgr3T**
  
Percentage identity: 50 %
  
BlastP bit score: 437
  
Sequence coverage: 102 %
  
E-value: 9e-146
  
  
 NCBI BlastP on this gene

ELQ61364

hypothetical protein
  
Accession: ELQ61365
  
Location: 29580-31338
  
 NCBI BlastP on this gene

ELQ61365

71. :  JH793116 Magnaporthe oryzae Y34 unplaced genomic scaffold Y34\_scaffold00799     Total score: 2.0     Cumulative Blast bit score: 979

hypothetical protein
  
Accession: ELQ34082
  
Location: 11583-12311
  
 NCBI BlastP on this gene

ELQ34082

surface protein 1
  
Accession: ELQ34083
  
Location: 13135-13575
  
 NCBI BlastP on this gene

ELQ34083

hypothetical protein
  
Accession: ELQ34084
  
Location: 14210-14305
  
 NCBI BlastP on this gene

ELQ34084

C-signal protein
  
Accession: ELQ34085
  
Location: 14823-15557
  
 NCBI BlastP on this gene

ELQ34085

hypothetical protein
  
Accession: ELQ34086
  
Location: 16097-16675
  
 NCBI BlastP on this gene

ELQ34086

benzoate 4-monooxygenase
  
Accession: ELQ34087
  
Location: 18788-20629
  
 NCBI BlastP on this gene

ELQ34087

hypothetical protein
  
Accession: ELQ34088
  
Location: 22116-23114
  
 NCBI BlastP on this gene

ELQ34088

hypothetical protein
  
Accession: ELQ34089
  
Location: 23327-23914
  
 NCBI BlastP on this gene

ELQ34089

DUF563 domain-containing protein
  
Accession: ELQ34090
  
Location: 24170-25965
  
 NCBI BlastP on this gene

ELQ34090

metabolite transporter
  
Accession: ELQ34091
  
Location: 27069-28583
  
  
**BlastP hit with Mycgr3G35932\_Mycgr3T**
  
Percentage identity: 57 %
  
BlastP bit score: 542
  
Sequence coverage: 99 %
  
E-value: 0.0
  
  
 NCBI BlastP on this gene

ELQ34091

aminopeptidase ypdF
  
Accession: ELQ34092
  
Location: 29252-30792
  
  
**BlastP hit with Mycgr3G35862\_Mycgr3T**
  
Percentage identity: 50 %
  
BlastP bit score: 437
  
Sequence coverage: 102 %
  
E-value: 9e-146
  
  
 NCBI BlastP on this gene

ELQ34092

72. :  CM001233 Magnaporthe oryzae 70-15 chromosome 3     Total score: 2.0     Cumulative Blast bit score: 979

calcium-transporting ATPase 1
  
Accession: EHA53411
  
Location: 6424367-6428369
  
 NCBI BlastP on this gene

EHA53411

hypothetical protein
  
Accession: EHA53412
  
Location: 6428603-6429478
  
 NCBI BlastP on this gene

EHA53412

surface protein 1
  
Accession: EHA53413
  
Location: 6430155-6430595
  
 NCBI BlastP on this gene

EHA53413

C-signal protein
  
Accession: EHA53414
  
Location: 6431843-6432577
  
 NCBI BlastP on this gene

EHA53414

hypothetical protein
  
Accession: EHA53415
  
Location: 6433117-6433695
  
 NCBI BlastP on this gene

EHA53415

benzoate 4-monooxygenase
  
Accession: EHA53416
  
Location: 6435808-6437483
  
 NCBI BlastP on this gene

EHA53416

hypothetical protein
  
Accession: EHA53417
  
Location: 6440376-6441085
  
 NCBI BlastP on this gene

EHA53417

hypothetical protein
  
Accession: EHA53418
  
Location: 6441461-6443014
  
 NCBI BlastP on this gene

EHA53418

metabolite transporter
  
Accession: EHA53419
  
Location: 6444118-6445632
  
  
**BlastP hit with Mycgr3G35932\_Mycgr3T**
  
Percentage identity: 57 %
  
BlastP bit score: 542
  
Sequence coverage: 99 %
  
E-value: 0.0
  
  
 NCBI BlastP on this gene

EHA53419

aminopeptidase ypdF
  
Accession: EHA53420
  
Location: 6446301-6447841
  
  
**BlastP hit with Mycgr3G35862\_Mycgr3T**
  
Percentage identity: 50 %
  
BlastP bit score: 437
  
Sequence coverage: 102 %
  
E-value: 9e-146
  
  
 NCBI BlastP on this gene

EHA53420

cytochrome P450 monooxygenase
  
Accession: EHA53421
  
Location: 6448093-6449952
  
 NCBI BlastP on this gene

EHA53421

hypothetical protein
  
Accession: EHA53422
  
Location: 6452890-6453801
  
 NCBI BlastP on this gene

EHA53422

73. :  KB020307 Colletotrichum gloeosporioides Nara gc5 unplaced genomic scaffold scaffold1090     Total score: 2.0     Cumulative Blast bit score: 950

xaa-pro dipeptidase
  
Accession: ELA37828
  
Location: 2619-4128
  
  
**BlastP hit with Mycgr3G35862\_Mycgr3T**
  
Percentage identity: 50 %
  
BlastP bit score: 425
  
Sequence coverage: 96 %
  
E-value: 3e-141
  
  
 NCBI BlastP on this gene

ELA37828

metabolite transport
  
Accession: ELA37829
  
Location: 4767-6506
  
  
**BlastP hit with Mycgr3G35932\_Mycgr3T**
  
Percentage identity: 55 %
  
BlastP bit score: 525
  
Sequence coverage: 97 %
  
E-value: 8e-180
  
  
 NCBI BlastP on this gene

ELA37829

alkaline serine protease alp1
  
Accession: ELA37830
  
Location: 6997-8374
  
 NCBI BlastP on this gene

ELA37830

oligopeptide transporter 7
  
Accession: ELA37831
  
Location: 15241-17987
  
 NCBI BlastP on this gene

ELA37831

hypothetical protein
  
Accession: ELA37832
  
Location: 18442-20248
  
 NCBI BlastP on this gene

ELA37832

74. :  GL385398 Gaeumannomyces graminis var. tritici R3-111a-1 unplaced genomic scaffold supercont2.4     Total score: 2.0     Cumulative Blast bit score: 885

hypothetical protein
  
Accession: EJT73605
  
Location: 173164-173730
  
 NCBI BlastP on this gene

EJT73605

hypothetical protein
  
Accession: EJT73606
  
Location: 174701-176241
  
 NCBI BlastP on this gene

EJT73606

hypothetical protein
  
Accession: EJT73607
  
Location: 177123-178787
  
 NCBI BlastP on this gene

EJT73607

hypothetical protein
  
Accession: EJT73608
  
Location: 178976-183782
  
 NCBI BlastP on this gene

EJT73608

hypothetical protein
  
Accession: EJT73609
  
Location: 184865-186627
  
 NCBI BlastP on this gene

EJT73609

high-affinity nicotinic acid transporter
  
Accession: EJT73610
  
Location: 187563-189348
  
 NCBI BlastP on this gene

EJT73610

hypothetical protein
  
Accession: EJT73611
  
Location: 189451-191029
  
  
**BlastP hit with Mycgr3G35862\_Mycgr3T**
  
Percentage identity: 51 %
  
BlastP bit score: 385
  
Sequence coverage: 90 %
  
E-value: 1e-125
  
  
 NCBI BlastP on this gene

EJT73611

hypothetical protein
  
Accession: EJT73612
  
Location: 191759-193354
  
  
**BlastP hit with Mycgr3G35932\_Mycgr3T**
  
Percentage identity: 54 %
  
BlastP bit score: 500
  
Sequence coverage: 98 %
  
E-value: 6e-170
  
  
 NCBI BlastP on this gene

EJT73612

hypothetical protein
  
Accession: EJT73613
  
Location: 193664-194366
  
 NCBI BlastP on this gene

EJT73613

hypothetical protein
  
Accession: EJT73614
  
Location: 194596-196058
  
 NCBI BlastP on this gene

EJT73614

hypothetical protein
  
Accession: EJT73615
  
Location: 198190-198462
  
 NCBI BlastP on this gene

EJT73615

hypothetical protein
  
Accession: EJT73616
  
Location: 199075-199425
  
 NCBI BlastP on this gene

EJT73616

hypothetical protein
  
Accession: EJT73617
  
Location: 199463-202816
  
 NCBI BlastP on this gene

EJT73617

hypothetical protein
  
Accession: EJT73618
  
Location: 202829-203749
  
 NCBI BlastP on this gene

EJT73618

hypothetical protein
  
Accession: EJT73619
  
Location: 204532-208027
  
 NCBI BlastP on this gene

EJT73619

75. :  JH687384 Stereum hirsutum FP-91666 SS1 unplaced genomic scaffold STEHIscaffold\_6     Total score: 2.0     Cumulative Blast bit score: 810

hypothetical protein
  
Accession: EIM87173
  
Location: 637480-639821
  
 NCBI BlastP on this gene

EIM87173

acetate--CoA ligase
  
Accession: EIM87174
  
Location: 641463-643987
  
 NCBI BlastP on this gene

EIM87174

general substrate transporter
  
Accession: EIM87175
  
Location: 644789-647609
  
 NCBI BlastP on this gene

EIM87175

hypothetical protein
  
Accession: EIM87176
  
Location: 648244-649646
  
 NCBI BlastP on this gene

EIM87176

amidohydrolase 2
  
Accession: EIM87177
  
Location: 650074-651506
  
 NCBI BlastP on this gene

EIM87177

FMN-dependent alpha-hydroxy acid dehydrogenase
  
Accession: EIM87178
  
Location: 652182-653955
  
 NCBI BlastP on this gene

EIM87178

metabolite transport protein
  
Accession: EIM87179
  
Location: 654154-656222
  
  
**BlastP hit with Mycgr3G35932\_Mycgr3T**
  
Percentage identity: 46 %
  
BlastP bit score: 434
  
Sequence coverage: 94 %
  
E-value: 3e-144
  
  
 NCBI BlastP on this gene

EIM87179

Creatinase/aminopeptidase
  
Accession: EIM87180
  
Location: 656846-658647
  
  
**BlastP hit with Mycgr3G35862\_Mycgr3T**
  
Percentage identity: 45 %
  
BlastP bit score: 377
  
Sequence coverage: 103 %
  
E-value: 2e-121
  
  
 NCBI BlastP on this gene

EIM87180

hypothetical protein
  
Accession: EIM87181
  
Location: 658972-661819
  
 NCBI BlastP on this gene

EIM87181

beta-glucosidase
  
Accession: EIM87182
  
Location: 663119-666626
  
 NCBI BlastP on this gene

EIM87182

NAD-P-binding protein
  
Accession: EIM87183
  
Location: 667254-668685
  
 NCBI BlastP on this gene

EIM87183

hypothetical protein
  
Accession: EIM87184
  
Location: 669441-671332
  
 NCBI BlastP on this gene

EIM87184

general substrate transporter
  
Accession: EIM87185
  
Location: 672897-675078
  
 NCBI BlastP on this gene

EIM87185

76. :  AHHD01000414 Macrophomina phaseolina MS6     Total score: 2.0     Cumulative Blast bit score: 807

hypothetical protein
  
Accession: EKG13212
  
Location: 1590-2141
  
 NCBI BlastP on this gene

EKG13212

hypothetical protein
  
Accession: EKG13213
  
Location: 3646-5694
  
 NCBI BlastP on this gene

EKG13213

hypothetical protein
  
Accession: EKG13214
  
Location: 5706-6242
  
 NCBI BlastP on this gene

EKG13214

General substrate transporter
  
Accession: EKG13215
  
Location: 6601-7136
  
 NCBI BlastP on this gene

EKG13215

Glycoside hydrolase family 3
  
Accession: EKG13216
  
Location: 9578-11620
  
 NCBI BlastP on this gene

EKG13216

Glucose-methanol-choline oxidoreductase
  
Accession: EKG13217
  
Location: 12418-14071
  
 NCBI BlastP on this gene

EKG13217

Protein of unknown function DUF1445
  
Accession: EKG13218
  
Location: 15314-17102
  
  
**BlastP hit with Mycgr3G35932\_Mycgr3T**
  
Percentage identity: 59 %
  
BlastP bit score: 566
  
Sequence coverage: 97 %
  
E-value: 0.0
  
  
 NCBI BlastP on this gene

EKG13218

hypothetical protein
  
Accession: EKG13219
  
Location: 18339-19179
  
  
**BlastP hit with Mycgr3G35862\_Mycgr3T**
  
Percentage identity: 54 %
  
BlastP bit score: 241
  
Sequence coverage: 48 %
  
E-value: 5e-73
  
  
 NCBI BlastP on this gene

EKG13219

hypothetical protein
  
Accession: EKG13220
  
Location: 19553-20731
  
 NCBI BlastP on this gene

EKG13220

Monooxygenase FAD-binding protein
  
Accession: EKG13221
  
Location: 21763-24237
  
 NCBI BlastP on this gene

EKG13221

Winged helix-turn-helix transcription repressor DNA-binding protein
  
Accession: EKG13222
  
Location: 24504-25524
  
 NCBI BlastP on this gene

EKG13222

SANT domain DNA binding protein
  
Accession: EKG13223
  
Location: 26565-28072
  
 NCBI BlastP on this gene

EKG13223

AMP-dependent synthetase/ligase
  
Accession: EKG13224
  
Location: 30736-34163
  
 NCBI BlastP on this gene

EKG13224

Beta-ketoacyl synthase
  
Accession: EKG13225
  
Location: 34843-37108
  
 NCBI BlastP on this gene

EKG13225

77. :  CH445371 Phaeosphaeria nodorum SN15 scaffold\_47     Total score: 2.0     Cumulative Blast bit score: 578

hypothetical protein
  
Accession: EAT76238
  
Location: 107631-109080
  
 NCBI BlastP on this gene

EAT76238

hypothetical protein
  
Accession: EAT76239
  
Location: 110797-112141
  
 NCBI BlastP on this gene

EAT76239

hypothetical protein
  
Accession: EAT76240
  
Location: 114611-114900
  
 NCBI BlastP on this gene

EAT76240

hypothetical protein
  
Accession: EAT76241
  
Location: 115518-115955
  
 NCBI BlastP on this gene

EAT76241

hypothetical protein
  
Accession: EAT76242
  
Location: 116294-117822
  
  
**BlastP hit with Mycgr3G35932\_Mycgr3T**
  
Percentage identity: 57 %
  
BlastP bit score: 500
  
Sequence coverage: 92 %
  
E-value: 2e-170
  
  
 NCBI BlastP on this gene

EAT76242

hypothetical protein
  
Accession: EAT76244
  
Location: 119214-122068
  
 NCBI BlastP on this gene

EAT76244

hypothetical protein
  
Accession: EAT76245
  
Location: 122529-123072
  
 NCBI BlastP on this gene

EAT76245

hypothetical protein
  
Accession: EAT76246
  
Location: 126384-127274
  
 NCBI BlastP on this gene

EAT76246

hypothetical protein
  
Accession: EAT76247
  
Location: 127864-129039
  
 NCBI BlastP on this gene

EAT76247

hypothetical protein
  
Accession: EAT76248
  
Location: 129744-131356
  
 NCBI BlastP on this gene

EAT76248

hypothetical protein
  
Accession: EAT76249
  
Location: 131478-131681
  
 NCBI BlastP on this gene

EAT76249

hypothetical protein
  
Accession: EAT76250
  
Location: 131742-132731
  
 NCBI BlastP on this gene

EAT76250

hypothetical protein
  
Accession: EAT76251
  
Location: 133015-133433
  
 NCBI BlastP on this gene

EAT76251

hypothetical protein
  
Accession: EAT76252
  
Location: 133985-134915
  
 NCBI BlastP on this gene

EAT76252

hypothetical protein
  
Accession: EAT76253
  
Location: 136188-137033
  
  
**BlastP hit with Mycgr3G36335\_Mycgr3T**
  
Percentage identity: 38 %
  
BlastP bit score: 78
  
Sequence coverage: 97 %
  
E-value: 4e-15
  
  
 NCBI BlastP on this gene

EAT76253

78. :  GG704912 Coccidioides immitis RS genomic scaffold supercont3.2     Total score: 2.0     Cumulative Blast bit score: 576

hypothetical protein
  
Accession: EAS27569
  
Location: 90127-91470
  
 NCBI BlastP on this gene

EAS27569

hypothetical protein
  
Accession: EAS27568
  
Location: 92721-93723
  
 NCBI BlastP on this gene

EAS27568

hypothetical protein
  
Accession: EAS27565
  
Location: 96298-97892
  
 NCBI BlastP on this gene

EAS27565

benzoate 4-monooxygenase cytochrome P450
  
Accession: EAS27564
  
Location: 98685-100477
  
 NCBI BlastP on this gene

EAS27564

multidrug resistance protein
  
Accession: EAS27563
  
Location: 102717-104545
  
  
**BlastP hit with Mycgr3G84494\_Mycgr3T**
  
Percentage identity: 43 %
  
BlastP bit score: 394
  
Sequence coverage: 93 %
  
E-value: 9e-128
  
  
 NCBI BlastP on this gene

EAS27563

hypothetical protein
  
Accession: EAS27562
  
Location: 105365-110490
  
 NCBI BlastP on this gene

EAS27562

hypothetical protein
  
Accession: EAS27561
  
Location: 111533-113158
  
  
**BlastP hit with Mycgr3G68030\_Mycgr3T**
  
Percentage identity: 34 %
  
BlastP bit score: 182
  
Sequence coverage: 94 %
  
E-value: 5e-50
  
  
 NCBI BlastP on this gene

EAS27561

oxidoreductase
  
Accession: EAS27560
  
Location: 113602-114939
  
 NCBI BlastP on this gene

EAS27560

serine/threonine-protein phosphatase
  
Accession: EAS27558
  
Location: 116213-117636
  
 NCBI BlastP on this gene

EAS27558

high affinity copper transporter
  
Accession: EAS27557
  
Location: 119003-119921
  
 NCBI BlastP on this gene

EAS27557

hypothetical protein
  
Accession: EAS27556
  
Location: 121101-123078
  
 NCBI BlastP on this gene

EAS27556

flavodoxin and radical SAM domain-containing protein
  
Accession: EAS27553
  
Location: 124325-126768
  
 NCBI BlastP on this gene

EAS27553

79. :  DF126477 Aspergillus kawachii IFO 4308 DNA, contig: scaffold00031     Total score: 2.0     Cumulative Blast bit score: 556

cytochrome P450
  
Accession: GAA91065
  
Location: 333227-334876
  
 NCBI BlastP on this gene

GAA91065

MFS phospholipid transporter
  
Accession: GAA91064
  
Location: 329915-331699
  
  
**BlastP hit with Mycgr3G35932\_Mycgr3T**
  
Percentage identity: 34 %
  
BlastP bit score: 252
  
Sequence coverage: 95 %
  
E-value: 5e-74
  
  
 NCBI BlastP on this gene

GAA91064

similar to An04g09840
  
Accession: GAA91063
  
Location: 327654-329000
  
 NCBI BlastP on this gene

GAA91063

acetyltransferase
  
Accession: GAA91062
  
Location: 326219-326904
  
 NCBI BlastP on this gene

GAA91062

sarcosine oxidase
  
Accession: GAA91061
  
Location: 324550-325971
  
 NCBI BlastP on this gene

GAA91061

rieske [2Fe-2S] domain protein
  
Accession: GAA91060
  
Location: 322205-323539
  
 NCBI BlastP on this gene

GAA91060

fungal specific transcription factor
  
Accession: GAA91059
  
Location: 318353-320470
  
 NCBI BlastP on this gene

GAA91059

alpha/beta hydrolase fold protein
  
Accession: GAA91058
  
Location: 315968-316993
  
 NCBI BlastP on this gene

GAA91058

pathway-specific regulatory protein
  
Accession: GAA91057
  
Location: 312471-314759
  
 NCBI BlastP on this gene

GAA91057

tetrahydrofolylpolyglutamate synthase
  
Accession: GAA91056
  
Location: 310313-312099
  
 NCBI BlastP on this gene

GAA91056

MFS transporter
  
Accession: GAA91055
  
Location: 307842-309449
  
  
**BlastP hit with Mycgr3G84494\_Mycgr3T**
  
Percentage identity: 34 %
  
BlastP bit score: 304
  
Sequence coverage: 93 %
  
E-value: 4e-93
  
  
 NCBI BlastP on this gene

GAA91055

4-coumarate-CoA ligase 1
  
Accession: GAA91054
  
Location: 304817-306825
  
 NCBI BlastP on this gene

GAA91054

catabolic 3-dehydroquinase
  
Accession: GAA91053
  
Location: 302380-303154
  
 NCBI BlastP on this gene

GAA91053

carboxylesterase, type B
  
Accession: GAA91052
  
Location: 300339-302208
  
 NCBI BlastP on this gene

GAA91052

80. :  ACJE01000018 Aspergillus niger ATCC 1015     Total score: 2.0     Cumulative Blast bit score: 553

hypothetical protein
  
Accession: EHA20182
  
Location: 90355-92014
  
 NCBI BlastP on this gene

EHA20182

hypothetical protein
  
Accession: EHA20181
  
Location: 87017-88796
  
  
**BlastP hit with Mycgr3G35932\_Mycgr3T**
  
Percentage identity: 33 %
  
BlastP bit score: 256
  
Sequence coverage: 95 %
  
E-value: 2e-75
  
  
 NCBI BlastP on this gene

EHA20181

hypothetical protein
  
Accession: EHA20180
  
Location: 83268-83946
  
 NCBI BlastP on this gene

EHA20180

hypothetical protein
  
Accession: EHA20179
  
Location: 81545-82965
  
 NCBI BlastP on this gene

EHA20179

hypothetical protein
  
Accession: EHA20178
  
Location: 79206-80542
  
 NCBI BlastP on this gene

EHA20178

hypothetical protein
  
Accession: EHA20177
  
Location: 75596-77975
  
 NCBI BlastP on this gene

EHA20177

hypothetical protein
  
Accession: EHA20176
  
Location: 73429-74061
  
 NCBI BlastP on this gene

EHA20176

hypothetical protein
  
Accession: EHA20175
  
Location: 71142-71859
  
 NCBI BlastP on this gene

EHA20175

hypothetical protein
  
Accession: EHA20174
  
Location: 69785-71051
  
 NCBI BlastP on this gene

EHA20174

hypothetical protein
  
Accession: EHA20173
  
Location: 67587-69376
  
 NCBI BlastP on this gene

EHA20173

hypothetical protein
  
Accession: EHA20172
  
Location: 65139-66587
  
  
**BlastP hit with Mycgr3G84494\_Mycgr3T**
  
Percentage identity: 33 %
  
BlastP bit score: 297
  
Sequence coverage: 92 %
  
E-value: 3e-91
  
  
 NCBI BlastP on this gene

EHA20172

hypothetical protein
  
Accession: EHA20171
  
Location: 62087-64082
  
 NCBI BlastP on this gene

EHA20171

hypothetical protein
  
Accession: EHA20170
  
Location: 59747-60521
  
 NCBI BlastP on this gene

EHA20170

carboxylesterase
  
Accession: EHA20169
  
Location: 57703-59573
  
 NCBI BlastP on this gene

EHA20169

81. :  ABDG02000016 Trichoderma atroviride IMI 206040     Total score: 2.0     Cumulative Blast bit score: 550

hypothetical protein
  
Accession: EHK49378
  
Location: 795-2618
  
 NCBI BlastP on this gene

EHK49378

hypothetical protein
  
Accession: EHK49379
  
Location: 3221-4448
  
 NCBI BlastP on this gene

EHK49379

hypothetical protein
  
Accession: EHK49380
  
Location: 4762-6039
  
 NCBI BlastP on this gene

EHK49380

hypothetical protein
  
Accession: EHK49381
  
Location: 6216-7176
  
 NCBI BlastP on this gene

EHK49381

hypothetical protein
  
Accession: EHK49382
  
Location: 7964-9106
  
 NCBI BlastP on this gene

EHK49382

hypothetical protein
  
Accession: EHK49383
  
Location: 10312-11939
  
  
**BlastP hit with Mycgr3G68030\_Mycgr3T**
  
Percentage identity: 37 %
  
BlastP bit score: 218
  
Sequence coverage: 98 %
  
E-value: 7e-64
  
  
 NCBI BlastP on this gene

EHK49383

hypothetical protein
  
Accession: EHK49384
  
Location: 12598-17971
  
 NCBI BlastP on this gene

EHK49384

hypothetical protein
  
Accession: EHK49385
  
Location: 18878-20543
  
  
**BlastP hit with Mycgr3G84494\_Mycgr3T**
  
Percentage identity: 39 %
  
BlastP bit score: 332
  
Sequence coverage: 95 %
  
E-value: 3e-104
  
  
 NCBI BlastP on this gene

EHK49385

hypothetical protein
  
Accession: EHK49386
  
Location: 21054-22532
  
 NCBI BlastP on this gene

EHK49386

hypothetical protein
  
Accession: EHK49387
  
Location: 23147-24613
  
 NCBI BlastP on this gene

EHK49387

hypothetical protein
  
Accession: EHK49388
  
Location: 25266-26274
  
 NCBI BlastP on this gene

EHK49388

hypothetical protein
  
Accession: EHK49389
  
Location: 26340-28312
  
 NCBI BlastP on this gene

EHK49389

hypothetical protein
  
Accession: EHK49390
  
Location: 29278-31363
  
 NCBI BlastP on this gene

EHK49390

hypothetical protein
  
Accession: EHK49391
  
Location: 32444-34292
  
 NCBI BlastP on this gene

EHK49391

82. :  DF126457 Aspergillus kawachii IFO 4308 DNA, contig: scaffold00011     Total score: 2.0     Cumulative Blast bit score: 545

cytochrome P450
  
Accession: GAA86841
  
Location: 645617-647134
  
 NCBI BlastP on this gene

GAA86841

hypothetical protein
  
Accession: GAA86840
  
Location: 644287-645438
  
 NCBI BlastP on this gene

GAA86840

efflux pump antibiotic resistance protein
  
Accession: GAA86839
  
Location: 641295-643193
  
 NCBI BlastP on this gene

GAA86839

MFS transporter
  
Accession: GAA86838
  
Location: 637614-639169
  
  
**BlastP hit with Mycgr3G84494\_Mycgr3T**
  
Percentage identity: 41 %
  
BlastP bit score: 357
  
Sequence coverage: 96 %
  
E-value: 1e-113
  
  
 NCBI BlastP on this gene

GAA86838

similar to An01g14440
  
Accession: GAA86837
  
Location: 633824-636379
  
 NCBI BlastP on this gene

GAA86837

hypothetical protein
  
Accession: GAA86836
  
Location: 632930-633322
  
 NCBI BlastP on this gene

GAA86836

hypothetical protein
  
Accession: GAA86835
  
Location: 630902-632252
  
 NCBI BlastP on this gene

GAA86835

epoxide hydrolase
  
Accession: GAA86834
  
Location: 629672-630842
  
 NCBI BlastP on this gene

GAA86834

similar to An01g14410
  
Accession: GAA86833
  
Location: 628052-628697
  
 NCBI BlastP on this gene

GAA86833

hypothetical protein
  
Accession: GAA86832
  
Location: 626016-626788
  
 NCBI BlastP on this gene

GAA86832

hypothetical protein
  
Accession: GAA86831
  
Location: 621352-624783
  
 NCBI BlastP on this gene

GAA86831

hypothetical protein
  
Accession: GAA86830
  
Location: 617733-619057
  
  
**BlastP hit with Mycgr3G68030\_Mycgr3T**
  
Percentage identity: 35 %
  
BlastP bit score: 188
  
Sequence coverage: 93 %
  
E-value: 3e-53
  
  
 NCBI BlastP on this gene

GAA86830

C6 transcription factor RosA-like
  
Accession: GAA86829
  
Location: 611796-613687
  
 NCBI BlastP on this gene

GAA86829

83. :  ABDF02000063 Trichoderma virens Gv29-8     Total score: 2.0     Cumulative Blast bit score: 543

hypothetical protein
  
Accession: EHK21762
  
Location: 2800-4429
  
  
**BlastP hit with Mycgr3G68030\_Mycgr3T**
  
Percentage identity: 36 %
  
BlastP bit score: 219
  
Sequence coverage: 98 %
  
E-value: 2e-64
  
  
 NCBI BlastP on this gene

EHK21762

hypothetical protein
  
Accession: EHK21763
  
Location: 5324-13003
  
  
**BlastP hit with Mycgr3G84494\_Mycgr3T**
  
Percentage identity: 39 %
  
BlastP bit score: 324
  
Sequence coverage: 92 %
  
E-value: 5e-94
  
  
 NCBI BlastP on this gene

EHK21763

hypothetical protein
  
Accession: EHK21764
  
Location: 13701-14183
  
 NCBI BlastP on this gene

EHK21764

hypothetical protein
  
Accession: EHK21765
  
Location: 15642-15977
  
 NCBI BlastP on this gene

EHK21765

hypothetical protein
  
Accession: EHK21766
  
Location: 16808-17917
  
 NCBI BlastP on this gene

EHK21766

hypothetical protein
  
Accession: EHK21767
  
Location: 18418-18911
  
 NCBI BlastP on this gene

EHK21767

hypothetical protein
  
Accession: EHK21768
  
Location: 19161-20140
  
 NCBI BlastP on this gene

EHK21768

hypothetical protein
  
Accession: EHK21769
  
Location: 22221-23862
  
 NCBI BlastP on this gene

EHK21769

hypothetical protein
  
Accession: EHK21770
  
Location: 24775-26652
  
 NCBI BlastP on this gene

EHK21770

84. :  DF126469 Aspergillus kawachii IFO 4308 DNA, contig: scaffold00023     Total score: 2.0     Cumulative Blast bit score: 520

O-methyltransferase
  
Accession: GAA89758
  
Location: 513000-514412
  
 NCBI BlastP on this gene

GAA89758

similar to An01g15030
  
Accession: GAA89759
  
Location: 515045-515401
  
 NCBI BlastP on this gene

GAA89759

PQ loop repeat protein
  
Accession: GAA89760
  
Location: 518169-519418
  
 NCBI BlastP on this gene

GAA89760

similar to An01g15070
  
Accession: GAA89761
  
Location: 519428-520582
  
 NCBI BlastP on this gene

GAA89761

gibberellin 20-oxidase
  
Accession: GAA89762
  
Location: 521958-523116
  
  
**BlastP hit with Mycgr3G68036\_Mycgr3T**
  
Percentage identity: 36 %
  
BlastP bit score: 224
  
Sequence coverage: 98 %
  
E-value: 7e-67
  
  
 NCBI BlastP on this gene

GAA89762

cytochrome P450 family protein
  
Accession: GAA89763
  
Location: 523866-525665
  
 NCBI BlastP on this gene

GAA89763

hypothetical protein
  
Accession: GAA89764
  
Location: 525972-527164
  
 NCBI BlastP on this gene

GAA89764

drug resistance protein
  
Accession: GAA89765
  
Location: 528920-530578
  
 NCBI BlastP on this gene

GAA89765

similar to agmatinase
  
Accession: GAA89766
  
Location: 531121-532694
  
 NCBI BlastP on this gene

GAA89766

hypothetical protein
  
Accession: GAA89767
  
Location: 533085-535302
  
 NCBI BlastP on this gene

GAA89767

hypothetical protein
  
Accession: GAA89768
  
Location: 537445-538286
  
 NCBI BlastP on this gene

GAA89768

MFS transporter
  
Accession: GAA89769
  
Location: 539683-541303
  
  
**BlastP hit with Mycgr3G84494\_Mycgr3T**
  
Percentage identity: 38 %
  
BlastP bit score: 296
  
Sequence coverage: 91 %
  
E-value: 3e-90
  
  
 NCBI BlastP on this gene

GAA89769

85. :  JH126402 Cordyceps militaris CM01 unplaced genomic scaffold CCM\_S00004     Total score: 2.0     Cumulative Blast bit score: 519

hypothetical protein
  
Accession: EGX91978
  
Location: 3553193-3553798
  
 NCBI BlastP on this gene

EGX91978

hypothetical protein
  
Accession: EGX91979
  
Location: 3554657-3556069
  
 NCBI BlastP on this gene

EGX91979

hypothetical protein
  
Accession: EGX91980
  
Location: 3559814-3561070
  
 NCBI BlastP on this gene

EGX91980

nucleoside hydrolase, putative
  
Accession: EGX91981
  
Location: 3563495-3564989
  
 NCBI BlastP on this gene

EGX91981

small nuclear ribonucleoprotein Lsm8, putative
  
Accession: EGX91982
  
Location: 3565343-3565847
  
 NCBI BlastP on this gene

EGX91982

glycerate dehydrogenase
  
Accession: EGX91983
  
Location: 3567730-3568830
  
 NCBI BlastP on this gene

EGX91983

Major facilitator superfamily transporter
  
Accession: EGX91984
  
Location: 3569362-3571070
  
  
**BlastP hit with Mycgr3G84494\_Mycgr3T**
  
Percentage identity: 41 %
  
BlastP bit score: 373
  
Sequence coverage: 93 %
  
E-value: 7e-120
  
  
 NCBI BlastP on this gene

EGX91984

Fructosamine/Ketosamine-3-kinase
  
Accession: EGX91985
  
Location: 3571670-3572983
  
  
**BlastP hit with Mycgr3G68030\_Mycgr3T**
  
Percentage identity: 34 %
  
BlastP bit score: 146
  
Sequence coverage: 79 %
  
E-value: 4e-37
  
  
 NCBI BlastP on this gene

EGX91985

WD40 repeat-like-containing domain
  
Accession: EGX91986
  
Location: 3575281-3576834
  
 NCBI BlastP on this gene

EGX91986

serine/threonine-protein kinase psk1
  
Accession: EGX91987
  
Location: 3579582-3581162
  
 NCBI BlastP on this gene

EGX91987

hypothetical protein
  
Accession: EGX91988
  
Location: 3582182-3582480
  
 NCBI BlastP on this gene

EGX91988

ABC transporter, putative
  
Accession: EGX91989
  
Location: 3583734-3588295
  
 NCBI BlastP on this gene

EGX91989

86. :  KE145357 Glarea lozoyensis ATCC 20868 chromosome Unknown GLAREA14     Total score: 2.0     Cumulative Blast bit score: 516

hypothetical protein
  
Accession: EPE34174
  
Location: 1790489-1791000
  
 NCBI BlastP on this gene

EPE34174

hypothetical protein
  
Accession: EPE34173
  
Location: 1788575-1789378
  
 NCBI BlastP on this gene

EPE34173

NAD(P)-binding Rossmann-fold containing protein
  
Accession: EPE34172
  
Location: 1787293-1788300
  
 NCBI BlastP on this gene

EPE34172

alpha/beta-Hydrolase
  
Accession: EPE34171
  
Location: 1783351-1784391
  
 NCBI BlastP on this gene

EPE34171

hypothetical protein
  
Accession: EPE34170
  
Location: 1778666-1781147
  
 NCBI BlastP on this gene

EPE34170

hypothetical protein
  
Accession: EPE34169
  
Location: 1775741-1777084
  
  
**BlastP hit with Mycgr3G68030\_Mycgr3T**
  
Percentage identity: 39 %
  
BlastP bit score: 236
  
Sequence coverage: 99 %
  
E-value: 3e-71
  
  
 NCBI BlastP on this gene

EPE34169

hypothetical protein
  
Accession: EPE34168
  
Location: 1772167-1773514
  
 NCBI BlastP on this gene

EPE34168

ClpP/crotonase
  
Accession: EPE34167
  
Location: 1768874-1771803
  
  
**BlastP hit with Mycgr3G36449\_Mycgr3T**
  
Percentage identity: 31 %
  
BlastP bit score: 280
  
Sequence coverage: 97 %
  
E-value: 9e-79
  
  
 NCBI BlastP on this gene

EPE34167

hypothetical protein
  
Accession: EPE34166
  
Location: 1766512-1768629
  
 NCBI BlastP on this gene

EPE34166

87. :  ACJE01000004 Aspergillus niger ATCC 1015     Total score: 2.0     Cumulative Blast bit score: 516

hypothetical protein
  
Accession: EHA27160
  
Location: 3615826-3616088
  
 NCBI BlastP on this gene

EHA27160

hypothetical protein
  
Accession: EHA27159
  
Location: 3615345-3615809
  
 NCBI BlastP on this gene

EHA27159

hypothetical protein
  
Accession: EHA27158
  
Location: 3611921-3613797
  
 NCBI BlastP on this gene

EHA27158

hypothetical protein
  
Accession: EHA27157
  
Location: 3608239-3609812
  
  
**BlastP hit with Mycgr3G84494\_Mycgr3T**
  
Percentage identity: 39 %
  
BlastP bit score: 357
  
Sequence coverage: 98 %
  
E-value: 6e-114
  
  
 NCBI BlastP on this gene

EHA27157

hypothetical protein
  
Accession: EHA27156
  
Location: 3604383-3607043
  
 NCBI BlastP on this gene

EHA27156

hypothetical protein
  
Accession: EHA27155
  
Location: 3601685-3603137
  
 NCBI BlastP on this gene

EHA27155

hypothetical protein
  
Accession: EHA27154
  
Location: 3600419-3601587
  
 NCBI BlastP on this gene

EHA27154

hypothetical protein
  
Accession: EHA27153
  
Location: 3598843-3599524
  
 NCBI BlastP on this gene

EHA27153

hypothetical protein
  
Accession: EHA27152
  
Location: 3595921-3598141
  
 NCBI BlastP on this gene

EHA27152

hypothetical protein
  
Accession: EHA27151
  
Location: 3591317-3595554
  
 NCBI BlastP on this gene

EHA27151

hypothetical protein
  
Accession: EHA27150
  
Location: 3588642-3589373
  
  
**BlastP hit with Mycgr3G68030\_Mycgr3T**
  
Percentage identity: 37 %
  
BlastP bit score: 159
  
Sequence coverage: 72 %
  
E-value: 2e-43
  
  
 NCBI BlastP on this gene

EHA27150

hypothetical protein
  
Accession: EHA27149
  
Location: 3586780-3587957
  
 NCBI BlastP on this gene

EHA27149

hypothetical protein
  
Accession: EHA27148
  
Location: 3582514-3584396
  
 NCBI BlastP on this gene

EHA27148

88. :  GL890999 Neurospora tetrasperma FGSC 2509 unplaced genomic scaffold NEUTE2scaffold\_1     Total score: 2.0     Cumulative Blast bit score: 515

hypothetical protein
  
Accession: EGZ76907
  
Location: 2943352-2944208
  
 NCBI BlastP on this gene

EGZ76907

hypothetical protein
  
Accession: EGZ76908
  
Location: 2945421-2946242
  
 NCBI BlastP on this gene

EGZ76908

hypothetical protein
  
Accession: EGZ76909
  
Location: 2946828-2949802
  
 NCBI BlastP on this gene

EGZ76909

hypothetical protein
  
Accession: EGZ76910
  
Location: 2950540-2952871
  
 NCBI BlastP on this gene

EGZ76910

hypothetical protein
  
Accession: EGZ76911
  
Location: 2953852-2954781
  
 NCBI BlastP on this gene

EGZ76911

hypothetical protein
  
Accession: EGZ76912
  
Location: 2955994-2957159
  
 NCBI BlastP on this gene

EGZ76912

hypothetical protein
  
Accession: EGZ76913
  
Location: 2957808-2959014
  
  
**BlastP hit with Mycgr3G68030\_Mycgr3T**
  
Percentage identity: 33 %
  
BlastP bit score: 184
  
Sequence coverage: 92 %
  
E-value: 2e-51
  
  
 NCBI BlastP on this gene

EGZ76913

MFS general substrate transporter
  
Accession: EGZ76914
  
Location: 2959740-2963949
  
  
**BlastP hit with Mycgr3G84494\_Mycgr3T**
  
Percentage identity: 38 %
  
BlastP bit score: 331
  
Sequence coverage: 91 %
  
E-value: 5e-98
  
  
 NCBI BlastP on this gene

EGZ76914

hypothetical protein
  
Accession: EGZ76915
  
Location: 2964373-2964720
  
 NCBI BlastP on this gene

EGZ76915

hypothetical protein
  
Accession: EGZ76916
  
Location: 2967388-2967627
  
 NCBI BlastP on this gene

EGZ76916

YVTN repeat-like/Quinoprotein amine dehydrogenase
  
Accession: EGZ76917
  
Location: 2968826-2969850
  
 NCBI BlastP on this gene

EGZ76917

PUL-domain-containing protein
  
Accession: EGZ76918
  
Location: 2970674-2974023
  
 NCBI BlastP on this gene

EGZ76918

hypothetical protein
  
Accession: EGZ76919
  
Location: 2975019-2975986
  
 NCBI BlastP on this gene

EGZ76919

hypothetical protein
  
Accession: EGZ76920
  
Location: 2976900-2977972
  
 NCBI BlastP on this gene

EGZ76920

hypothetical protein
  
Accession: EGZ76921
  
Location: 2978495-2979520
  
 NCBI BlastP on this gene

EGZ76921

89. :  GL891382 Neurospora tetrasperma FGSC 2508 unplaced genomic scaffold NEUTE1scaffold\_81     Total score: 2.0     Cumulative Blast bit score: 511

hypothetical protein
  
Accession: EGO52096
  
Location: 3044117-3045058
  
 NCBI BlastP on this gene

EGO52096

hypothetical protein
  
Accession: EGO52097
  
Location: 3045328-3046188
  
 NCBI BlastP on this gene

EGO52097

hypothetical protein
  
Accession: EGO52098
  
Location: 3047336-3048157
  
 NCBI BlastP on this gene

EGO52098

hypothetical protein
  
Accession: EGO52099
  
Location: 3048737-3051712
  
 NCBI BlastP on this gene

EGO52099

hypothetical protein
  
Accession: EGO52100
  
Location: 3052442-3054781
  
 NCBI BlastP on this gene

EGO52100

hypothetical protein
  
Accession: EGO52101
  
Location: 3055756-3056853
  
 NCBI BlastP on this gene

EGO52101

hypothetical protein
  
Accession: EGO52102
  
Location: 3058002-3059167
  
 NCBI BlastP on this gene

EGO52102

hypothetical protein
  
Accession: EGO52103
  
Location: 3059814-3061021
  
  
**BlastP hit with Mycgr3G68030\_Mycgr3T**
  
Percentage identity: 33 %
  
BlastP bit score: 187
  
Sequence coverage: 92 %
  
E-value: 1e-52
  
  
 NCBI BlastP on this gene

EGO52103

hypothetical protein
  
Accession: EGO52104
  
Location: 3061603-3063500
  
  
**BlastP hit with Mycgr3G84494\_Mycgr3T**
  
Percentage identity: 37 %
  
BlastP bit score: 324
  
Sequence coverage: 93 %
  
E-value: 9e-101
  
  
 NCBI BlastP on this gene

EGO52104

hypothetical protein
  
Accession: EGO52105
  
Location: 3063901-3066042
  
 NCBI BlastP on this gene

EGO52105

hypothetical protein
  
Accession: EGO52106
  
Location: 3066453-3066800
  
 NCBI BlastP on this gene

EGO52106

hypothetical protein
  
Accession: EGO52107
  
Location: 3068821-3069060
  
 NCBI BlastP on this gene

EGO52107

hypothetical protein
  
Accession: EGO52108
  
Location: 3070254-3071278
  
 NCBI BlastP on this gene

EGO52108

hypothetical protein
  
Accession: EGO52109
  
Location: 3072092-3075450
  
 NCBI BlastP on this gene

EGO52109

hypothetical protein
  
Accession: EGO52110
  
Location: 3076443-3077375
  
 NCBI BlastP on this gene

EGO52110

hypothetical protein
  
Accession: EGO52111
  
Location: 3077934-3078786
  
 NCBI BlastP on this gene

EGO52111

hypothetical protein
  
Accession: EGO52112
  
Location: 3079291-3080316
  
 NCBI BlastP on this gene

EGO52112

90. :  AABX02000002 Neurospora crassa OR74A     Total score: 2.0     Cumulative Blast bit score: 510

predicted protein
  
Accession: EAA35548
  
Location: 1315043-1315864
  
 NCBI BlastP on this gene

EAA35548

predicted protein
  
Accession: EAA35549
  
Location: 1316451-1319426
  
 NCBI BlastP on this gene

EAA35549

predicted protein
  
Accession: EAA35550
  
Location: 1320154-1322492
  
 NCBI BlastP on this gene

EAA35550

predicted protein
  
Accession: EAA35551
  
Location: 1323454-1324389
  
 NCBI BlastP on this gene

EAA35551

predicted protein
  
Accession: EAA35552
  
Location: 1325544-1326709
  
 NCBI BlastP on this gene

EAA35552

conserved hypothetical protein
  
Accession: EAA35553
  
Location: 1327357-1328563
  
  
**BlastP hit with Mycgr3G68030\_Mycgr3T**
  
Percentage identity: 33 %
  
BlastP bit score: 186
  
Sequence coverage: 92 %
  
E-value: 4e-52
  
  
 NCBI BlastP on this gene

EAA35553

predicted protein
  
Accession: EAA35554
  
Location: 1329168-1332438
  
 NCBI BlastP on this gene

EAA35554

predicted protein
  
Accession: EAA35555
  
Location: 1334983-1336904
  
  
**BlastP hit with Mycgr3G84494\_Mycgr3T**
  
Percentage identity: 37 %
  
BlastP bit score: 324
  
Sequence coverage: 93 %
  
E-value: 1e-100
  
  
 NCBI BlastP on this gene

EAA35555

conserved hypothetical protein
  
Accession: EAA35556
  
Location: 1337316-1339469
  
 NCBI BlastP on this gene

EAA35556

predicted protein
  
Accession: EAA35557
  
Location: 1339909-1340265
  
 NCBI BlastP on this gene

EAA35557

hypothetical protein
  
Accession: EAA35558
  
Location: 1341980-1342219
  
 NCBI BlastP on this gene

EAA35558

conserved hypothetical protein
  
Accession: EAA35559
  
Location: 1343413-1344333
  
 NCBI BlastP on this gene

EAA35559

conserved hypothetical protein
  
Accession: EAA35560
  
Location: 1345206-1348551
  
 NCBI BlastP on this gene

EAA35560

conserved hypothetical protein
  
Accession: EAA35561
  
Location: 1349588-1350525
  
 NCBI BlastP on this gene

EAA35561

91. :  GL636512 Coccidioides posadasii str. Silveira unplaced genomic scaffold supercont2.27     Total score: 2.0     Cumulative Blast bit score: 508

flavodoxin and radical SAM domain-containing protein
  
Accession: EFW13713
  
Location: 214883-217323
  
 NCBI BlastP on this gene

EFW13713

conserved hypothetical protein
  
Accession: EFW13714
  
Location: 218517-220405
  
 NCBI BlastP on this gene

EFW13714

high affinity copper transporter
  
Accession: EFW13715
  
Location: 221678-222608
  
 NCBI BlastP on this gene

EFW13715

serine/threonine-protein phosphatase ppe1
  
Accession: EFW13716
  
Location: 223951-225370
  
 NCBI BlastP on this gene

EFW13716

predicted protein
  
Accession: EFW13717
  
Location: 225709-226286
  
 NCBI BlastP on this gene

EFW13717

conserved hypothetical protein
  
Accession: EFW13718
  
Location: 226779-227998
  
 NCBI BlastP on this gene

EFW13718

conserved hypothetical protein
  
Accession: EFW13719
  
Location: 228388-229642
  
  
**BlastP hit with Mycgr3G68030\_Mycgr3T**
  
Percentage identity: 31 %
  
BlastP bit score: 130
  
Sequence coverage: 78 %
  
E-value: 2e-31
  
  
 NCBI BlastP on this gene

EFW13719

conserved hypothetical protein
  
Accession: EFW13720
  
Location: 231570-235795
  
 NCBI BlastP on this gene

EFW13720

conserved hypothetical protein
  
Accession: EFW13721
  
Location: 236428-238249
  
  
**BlastP hit with Mycgr3G84494\_Mycgr3T**
  
Percentage identity: 42 %
  
BlastP bit score: 378
  
Sequence coverage: 92 %
  
E-value: 8e-122
  
  
 NCBI BlastP on this gene

EFW13721

predicted protein
  
Accession: EFW13722
  
Location: 239252-239635
  
 NCBI BlastP on this gene

EFW13722

conserved hypothetical protein
  
Accession: EFW13723
  
Location: 240468-242266
  
 NCBI BlastP on this gene

EFW13723

predicted protein
  
Accession: EFW13724
  
Location: 242473-242923
  
 NCBI BlastP on this gene

EFW13724

conserved hypothetical protein
  
Accession: EFW13725
  
Location: 243056-244497
  
 NCBI BlastP on this gene

EFW13725

conserved hypothetical protein
  
Accession: EFW13726
  
Location: 245429-245743
  
 NCBI BlastP on this gene

EFW13726

conserved hypothetical protein
  
Accession: EFW13727
  
Location: 247025-248027
  
 NCBI BlastP on this gene

EFW13727

conserved hypothetical protein
  
Accession: EFW13728
  
Location: 248446-249014
  
 NCBI BlastP on this gene

EFW13728

conserved hypothetical protein
  
Accession: EFW13729
  
Location: 249316-250659
  
 NCBI BlastP on this gene

EFW13729

92. :  CP003011 Thielavia terrestris NRRL 8126 chromosome 3     Total score: 2.0     Cumulative Blast bit score: 507

glycoside hydrolase family 3 protein
  
Accession: AEO68568
  
Location: 4748811-4751203
  
 NCBI BlastP on this gene

THITE\_2118136

glycoside hydrolase family 31 protein
  
Accession: AEO68569
  
Location: 4752246-4754695
  
 NCBI BlastP on this gene

THITE\_2118137

hypothetical protein
  
Accession: AEO68570
  
Location: 4755841-4757524
  
 NCBI BlastP on this gene

THITE\_2118138

glycoside hydrolase family 11 protein
  
Accession: AEO68571
  
Location: 4758136-4758928
  
 NCBI BlastP on this gene

THITE\_2050870

hypothetical protein
  
Accession: AEO68572
  
Location: 4761172-4762995
  
 NCBI BlastP on this gene

THITE\_2118139

hypothetical protein
  
Accession: AEO68573
  
Location: 4763368-4765029
  
  
**BlastP hit with Mycgr3G84494\_Mycgr3T**
  
Percentage identity: 36 %
  
BlastP bit score: 331
  
Sequence coverage: 96 %
  
E-value: 2e-103
  
  
 NCBI BlastP on this gene

THITE\_2118141

hypothetical protein
  
Accession: AEO68574
  
Location: 4766795-4771807
  
 NCBI BlastP on this gene

THITE\_2050713

hypothetical protein
  
Accession: AEO68575
  
Location: 4772705-4773820
  
  
**BlastP hit with Mycgr3G68030\_Mycgr3T**
  
Percentage identity: 35 %
  
BlastP bit score: 176
  
Sequence coverage: 89 %
  
E-value: 9e-49
  
  
 NCBI BlastP on this gene

THITE\_2118142

hypothetical protein
  
Accession: AEO68576
  
Location: 4775258-4776463
  
 NCBI BlastP on this gene

THITE\_2118143

93. :  AKHY01000098 Aspergillus oryzae 3.042     Total score: 2.0     Cumulative Blast bit score: 506

hypothetical protein
  
Accession: EIT81734
  
Location: 42924-43721
  
 NCBI BlastP on this gene

EIT81734

hypothetical protein
  
Accession: EIT81719
  
Location: 44452-46443
  
 NCBI BlastP on this gene

EIT81719

putative epimerase, PhzC/PhzF like protein
  
Accession: EIT81717
  
Location: 47486-48439
  
 NCBI BlastP on this gene

EIT81717

NADH dehydrogenase, FAD-containing subunit
  
Accession: EIT81709
  
Location: 49696-50910
  
 NCBI BlastP on this gene

EIT81709

hypothetical protein
  
Accession: EIT81770
  
Location: 52634-55024
  
  
**BlastP hit with Mycgr3G36449\_Mycgr3T**
  
Percentage identity: 37 %
  
BlastP bit score: 433
  
Sequence coverage: 98 %
  
E-value: 5e-138
  
  
 NCBI BlastP on this gene

EIT81770

hypothetical protein
  
Accession: EIT81775
  
Location: 56152-56559
  
 NCBI BlastP on this gene

EIT81775

hypothetical protein
  
Accession: EIT81756
  
Location: 56994-60196
  
  
**BlastP hit with Mycgr3G36335\_Mycgr3T**
  
Percentage identity: 33 %
  
BlastP bit score: 73
  
Sequence coverage: 89 %
  
E-value: 1e-12
  
  
 NCBI BlastP on this gene

EIT81756

serine racemase
  
Accession: EIT81753
  
Location: 60626-61891
  
 NCBI BlastP on this gene

EIT81753

Rad51 family DNA repair protein, putative
  
Accession: EIT81687
  
Location: 61981-63450
  
 NCBI BlastP on this gene

EIT81687

hypothetical protein
  
Accession: EIT81684
  
Location: 63810-64189
  
 NCBI BlastP on this gene

EIT81684

bromodomain associated domain protein
  
Accession: EIT81754
  
Location: 64391-65501
  
 NCBI BlastP on this gene

EIT81754

ubiquitin-protein ligase
  
Accession: EIT81713
  
Location: 67144-67937
  
 NCBI BlastP on this gene

EIT81713

chitin synthase/hyaluronan synthase
  
Accession: EIT81685
  
Location: 68496-71524
  
 NCBI BlastP on this gene

EIT81685

94. :  CP003014 Thielavia terrestris NRRL 8126 chromosome 6     Total score: 2.0     Cumulative Blast bit score: 498

hypothetical protein
  
Accession: AEO71475
  
Location: 1802735-1805504
  
 NCBI BlastP on this gene

THITE\_2092975

hypothetical protein
  
Accession: AEO71474
  
Location: 1797194-1798720
  
 NCBI BlastP on this gene

THITE\_2123869

hypothetical protein
  
Accession: AEO71473
  
Location: 1794832-1795989
  
 NCBI BlastP on this gene

THITE\_2123868

hypothetical protein
  
Accession: AEO71472
  
Location: 1789985-1793839
  
 NCBI BlastP on this gene

THITE\_2123867

hypothetical protein
  
Accession: AEO71471
  
Location: 1786469-1787770
  
  
**BlastP hit with Mycgr3G68030\_Mycgr3T**
  
Percentage identity: 30 %
  
BlastP bit score: 152
  
Sequence coverage: 93 %
  
E-value: 4e-39
  
  
 NCBI BlastP on this gene

THITE\_2123863

hypothetical protein
  
Accession: AEO71470
  
Location: 1783539-1785282
  
  
**BlastP hit with Mycgr3G84494\_Mycgr3T**
  
Percentage identity: 38 %
  
BlastP bit score: 346
  
Sequence coverage: 93 %
  
E-value: 2e-109
  
  
 NCBI BlastP on this gene

THITE\_2123861

hypothetical protein
  
Accession: AEO71469
  
Location: 1780862-1782591
  
 NCBI BlastP on this gene

THITE\_21761

hypothetical protein
  
Accession: AEO71468
  
Location: 1778162-1779902
  
 NCBI BlastP on this gene

THITE\_124439

hypothetical protein
  
Accession: AEO71467
  
Location: 1775553-1776580
  
 NCBI BlastP on this gene

THITE\_2148110

hypothetical protein
  
Accession: AEO71466
  
Location: 1773332-1774405
  
 NCBI BlastP on this gene

THITE\_2123858

hypothetical protein
  
Accession: AEO71465
  
Location: 1770100-1772121
  
 NCBI BlastP on this gene

THITE\_2123849

95. :  AP007164 Aspergillus oryzae RIB40 DNA, SC111.     Total score: 2.0     Cumulative Blast bit score: 493

not annotated
  
Accession: BAE62269
  
Location: 1526127-1526924
  
 NCBI BlastP on this gene

AO090701000575

not annotated
  
Accession: BAE62270
  
Location: 1527654-1529645
  
 NCBI BlastP on this gene

AO090701000576

not annotated
  
Accession: BAE62271
  
Location: 1530692-1531645
  
 NCBI BlastP on this gene

AO090701000577

not annotated
  
Accession: BAE62272
  
Location: 1532901-1534115
  
 NCBI BlastP on this gene

AO090701000578

not annotated
  
Accession: BAE62273
  
Location: 1535818-1538207
  
  
**BlastP hit with Mycgr3G36449\_Mycgr3T**
  
Percentage identity: 36 %
  
BlastP bit score: 421
  
Sequence coverage: 101 %
  
E-value: 9e-133
  
  
 NCBI BlastP on this gene

AO090701000579

not annotated
  
Accession: BAE62274
  
Location: 1539317-1539724
  
 NCBI BlastP on this gene

AO090701000580

not annotated
  
Accession: BAE62275
  
Location: 1541688-1543321
  
  
**BlastP hit with Mycgr3G36335\_Mycgr3T**
  
Percentage identity: 33 %
  
BlastP bit score: 72
  
Sequence coverage: 89 %
  
E-value: 3e-12
  
  
 NCBI BlastP on this gene

AO090701000581

not annotated
  
Accession: BAE62276
  
Location: 1543759-1545024
  
 NCBI BlastP on this gene

AO090701000582

not annotated
  
Accession: BAE62277
  
Location: 1545114-1546585
  
 NCBI BlastP on this gene

AO090701000583

not annotated
  
Accession: BAE62278
  
Location: 1546946-1547325
  
 NCBI BlastP on this gene

AO090701000585

not annotated
  
Accession: BAE62279
  
Location: 1547527-1548637
  
 NCBI BlastP on this gene

AO090701000586

not annotated
  
Accession: BAE62280
  
Location: 1550279-1551072
  
 NCBI BlastP on this gene

AO090701000588

not annotated
  
Accession: BAE62281
  
Location: 1551631-1554659
  
 NCBI BlastP on this gene

AO090701000589

not annotated
  
Accession: BAE62282
  
Location: 1554890-1556014
  
 NCBI BlastP on this gene

AO090701000590

96. :  GG698970 Nectria haematococca mpVI 77-13-4 chromosome 10 genomic scaffold NECHAsca\_82\_chr10\_2\_0     Total score: 2.0     Cumulative Blast bit score: 492

hypothetical protein
  
Accession: EEU34248
  
Location: 366370-368746
  
 NCBI BlastP on this gene

EEU34248

hypothetical protein
  
Accession: EEU34058
  
Location: 365225-365887
  
 NCBI BlastP on this gene

EEU34058

hypothetical protein
  
Accession: EEU34057
  
Location: 354382-359365
  
 NCBI BlastP on this gene

EEU34057

hypothetical protein
  
Accession: EEU34247
  
Location: 352084-353419
  
  
**BlastP hit with Mycgr3G68030\_Mycgr3T**
  
Percentage identity: 33 %
  
BlastP bit score: 173
  
Sequence coverage: 90 %
  
E-value: 4e-47
  
  
 NCBI BlastP on this gene

EEU34247

hypothetical protein
  
Accession: EEU34246
  
Location: 349586-351354
  
  
**BlastP hit with Mycgr3G84494\_Mycgr3T**
  
Percentage identity: 39 %
  
BlastP bit score: 319
  
Sequence coverage: 92 %
  
E-value: 3e-99
  
  
 NCBI BlastP on this gene

EEU34246

hypothetical protein
  
Accession: EEU34056
  
Location: 344614-349500
  
 NCBI BlastP on this gene

EEU34056

hypothetical protein
  
Accession: EEU34245
  
Location: 341601-342912
  
 NCBI BlastP on this gene

EEU34245

hypothetical protein
  
Accession: EEU34055
  
Location: 339793-341223
  
 NCBI BlastP on this gene

EEU34055

predicted protein
  
Accession: EEU34054
  
Location: 338824-339466
  
 NCBI BlastP on this gene

EEU34054

hypothetical protein
  
Accession: EEU34053
  
Location: 336806-338441
  
 NCBI BlastP on this gene

EEU34053

hypothetical protein
  
Accession: EEU34244
  
Location: 335450-336419
  
 NCBI BlastP on this gene

EEU34244

hypothetical protein
  
Accession: EEU34052
  
Location: 333312-334271
  
 NCBI BlastP on this gene

EEU34052

97. :  KE148146 Ophiostoma piceae UAMH 11346 chromosome Unknown scf01     Total score: 2.0     Cumulative Blast bit score: 471

lrp16 family protein
  
Accession: EPE10903
  
Location: 3968772-3969844
  
 NCBI BlastP on this gene

EPE10903

hypothetical protein
  
Accession: EPE10902
  
Location: 3968057-3968445
  
 NCBI BlastP on this gene

EPE10902

hypothetical protein
  
Accession: EPE10901
  
Location: 3965200-3965985
  
 NCBI BlastP on this gene

EPE10901

specific serine endopeptidase
  
Accession: EPE10900
  
Location: 3963028-3963771
  
 NCBI BlastP on this gene

EPE10900

hypothetical protein
  
Accession: EPE10899
  
Location: 3959732-3960935
  
  
**BlastP hit with Mycgr3G35535\_Mycgr3T**
  
Percentage identity: 28 %
  
BlastP bit score: 57
  
Sequence coverage: 86 %
  
E-value: 5e-07
  
  
 NCBI BlastP on this gene

EPE10899

tat pathway signal sequence
  
Accession: EPE10898
  
Location: 3958326-3959431
  
 NCBI BlastP on this gene

EPE10898

hypothetical protein
  
Accession: EPE10897
  
Location: 3956279-3957049
  
 NCBI BlastP on this gene

EPE10897

hypothetical protein
  
Accession: EPE10896
  
Location: 3954625-3955793
  
 NCBI BlastP on this gene

EPE10896

pyridine nucleotide-disulfide
  
Accession: EPE10895
  
Location: 3952470-3953825
  
 NCBI BlastP on this gene

EPE10895

peptidase s41 family protein
  
Accession: EPE10894
  
Location: 3945810-3948383
  
  
**BlastP hit with Mycgr3G36449\_Mycgr3T**
  
Percentage identity: 37 %
  
BlastP bit score: 414
  
Sequence coverage: 95 %
  
E-value: 4e-129
  
  
 NCBI BlastP on this gene

EPE10894

hypothetical protein
  
Accession: EPE10893
  
Location: 3944613-3945530
  
 NCBI BlastP on this gene

EPE10893

elongation factor 3
  
Accession: EPE10892
  
Location: 3936991-3940660
  
 NCBI BlastP on this gene

EPE10892

98. :  ACFW01000025 Coccidioides posadasii C735 delta SOWgp     Total score: 2.0     Cumulative Blast bit score: 470

PAN domain containing protein
  
Accession: EER26924
  
Location: 13629-14972
  
 NCBI BlastP on this gene

EER26924

prolyl 4-hydroxylase alpha subunit, putative
  
Accession: EER26925
  
Location: 16260-17262
  
 NCBI BlastP on this gene

EER26925

PAN domain containing protein
  
Accession: EER26926
  
Location: 19796-21237
  
 NCBI BlastP on this gene

EER26926

Cytochrome P450 family protein
  
Accession: EER26927
  
Location: 22027-23819
  
 NCBI BlastP on this gene

EER26927

Major Facilitator Superfamily protein
  
Accession: EER26928
  
Location: 26245-27875
  
  
**BlastP hit with Mycgr3G84494\_Mycgr3T**
  
Percentage identity: 45 %
  
BlastP bit score: 340
  
Sequence coverage: 76 %
  
E-value: 6e-108
  
  
 NCBI BlastP on this gene

EER26928

WD domain, G-beta repeat containing protein
  
Accession: EER26929
  
Location: 28508-33558
  
 NCBI BlastP on this gene

EER26929

conserved hypothetical protein
  
Accession: EER26930
  
Location: 34670-35927
  
  
**BlastP hit with Mycgr3G68030\_Mycgr3T**
  
Percentage identity: 31 %
  
BlastP bit score: 130
  
Sequence coverage: 78 %
  
E-value: 2e-31
  
  
 NCBI BlastP on this gene

EER26930

oxidoreductase, 2OG-Fe(II) oxygenase family protein
  
Accession: EER26931
  
Location: 36317-37425
  
 NCBI BlastP on this gene

EER26931

serine/threonine protein phosphatase ppe1, putative
  
Accession: EER26932
  
Location: 38883-40298
  
 NCBI BlastP on this gene

EER26932

Ctr copper transporter family protein
  
Accession: EER26933
  
Location: 41664-42595
  
 NCBI BlastP on this gene

EER26933

RNA recognition motif containing protein
  
Accession: EER26934
  
Location: 43858-45749
  
 NCBI BlastP on this gene

EER26934

radical SAM domain containing protein
  
Accession: EER26935
  
Location: 46995-49435
  
 NCBI BlastP on this gene

EER26935

99. :  EQ962654 Talaromyces stipitatus ATCC 10500 scf\_1105507295541 genomic scaffold     Total score: 2.0     Cumulative Blast bit score: 462

RTA1 domain protein, putative
  
Accession: EED19857
  
Location: 2116645-2117679
  
 NCBI BlastP on this gene

EED19857

MFS transporter, putative
  
Accession: EED19856
  
Location: 2113588-2115281
  
  
**BlastP hit with Mycgr3G84494\_Mycgr3T**
  
Percentage identity: 37 %
  
BlastP bit score: 286
  
Sequence coverage: 81 %
  
E-value: 1e-86
  
  
 NCBI BlastP on this gene

EED19856

conserved hypothetical protein
  
Accession: EED19855
  
Location: 2111239-2112423
  
 NCBI BlastP on this gene

EED19855

polysaccharide deacetylase (NodB), putative
  
Accession: EED19853
  
Location: 2110011-2110760
  
 NCBI BlastP on this gene

EED19853

conserved hypothetical protein
  
Accession: EED19852
  
Location: 2108747-2109502
  
 NCBI BlastP on this gene

EED19852

conserved hypothetical protein
  
Accession: EED19851
  
Location: 2106905-2108514
  
 NCBI BlastP on this gene

EED19851

hypothetical protein
  
Accession: EED19850
  
Location: 2105188-2106172
  
 NCBI BlastP on this gene

EED19850

hypothetical protein
  
Accession: EED19849
  
Location: 2104292-2105103
  
 NCBI BlastP on this gene

EED19849

flavin containing polyamine oxidase, putative
  
Accession: EED19848
  
Location: 2102232-2104138
  
 NCBI BlastP on this gene

EED19848

conserved hypothetical protein
  
Accession: EED19846
  
Location: 2095211-2098040
  
 NCBI BlastP on this gene

EED19846

alcohol dehydrogenase, putative
  
Accession: EED19845
  
Location: 2093232-2094353
  
 NCBI BlastP on this gene

EED19845

glucosamine 6-phosphate N-acetyltransferase, putative
  
Accession: EED19843
  
Location: 2092065-2092689
  
 NCBI BlastP on this gene

EED19843

MFS transporter, putative
  
Accession: EED19842
  
Location: 2089885-2091477
  
  
**BlastP hit with Mycgr3G23761\_Mycgr3T**
  
Percentage identity: 28 %
  
BlastP bit score: 176
  
Sequence coverage: 104 %
  
E-value: 9e-46
  
  
 NCBI BlastP on this gene

EED19842

efflux pump antibiotic resistance protein, putative
  
Accession: EED19841
  
Location: 2087281-2089207
  
 NCBI BlastP on this gene

EED19841

conserved hypothetical protein
  
Accession: EED19840
  
Location: 2085060-2086510
  
 NCBI BlastP on this gene

EED19840

100. :  KE145368 Glarea lozoyensis ATCC 20868 chromosome Unknown GLAREA4     Total score: 2.0     Cumulative Blast bit score: 449

Acetyl-CoA synthetase-like protein
  
Accession: EPE28435
  
Location: 588414-597802
  
 NCBI BlastP on this gene

EPE28435

Clavaminate synthase-like protein
  
Accession: EPE28436
  
Location: 598904-599991
  
 NCBI BlastP on this gene

EPE28436

alpha/beta-Hydrolase
  
Accession: EPE28437
  
Location: 601010-602224
  
 NCBI BlastP on this gene

EPE28437

Clavaminate synthase-like protein
  
Accession: EPE28438
  
Location: 603234-604432
  
  
**BlastP hit with Mycgr3G68036\_Mycgr3T**
  
Percentage identity: 42 %
  
BlastP bit score: 279
  
Sequence coverage: 105 %
  
E-value: 8e-88
  
  
 NCBI BlastP on this gene

EPE28438

Acetyl-CoA synthetase-like protein
  
Accession: EPE28439
  
Location: 605456-608072
  
 NCBI BlastP on this gene

EPE28439

Acetyl-CoA synthetase-like protein
  
Accession: EPE28440
  
Location: 608150-611569
  
 NCBI BlastP on this gene

EPE28440

alpha/beta-Hydrolase
  
Accession: EPE28441
  
Location: 612330-613430
  
 NCBI BlastP on this gene

EPE28441

MFS general substrate transporter
  
Accession: EPE28442
  
Location: 614165-615961
  
  
**BlastP hit with Mycgr3G23761\_Mycgr3T**
  
Percentage identity: 28 %
  
BlastP bit score: 170
  
Sequence coverage: 103 %
  
E-value: 1e-43
  
  
 NCBI BlastP on this gene

EPE28442

Detecting sequence homology at the gene cluster level with MultiGeneBlast.
  
Marnix H. Medema, Rainer Breitling & Eriko Takano (2013)
  
*Molecular Biology and Evolution* , 30: 1218-1223.
