# Supplementary material for: In silico prediction and characterization of secondary metabolite biosynthetic gene clusters in the wheat pathogen Zymoseptoria tritici
Source: BMC Genomics. 2017 Aug 17;18:631. doi: 10.1186/s12864-017-3969-y (PMC5561558; doi:10.1186/s12864-017-3969-y)
Supplement: Supplementary file 1 — MultiGeneBLAST analysis of putative secondary metabolite clusters. All encoded amino acid sequences from genes residing in clusters predicted by AntiSMASH are given as FASTA file format. All output data from MultiGeneBLASTs are also provided. (ZIP 42911 kb) [file 12864_2017_3969_MOESM1_ESM.zip › Cluster MultiGene BLAST/out/Clusters_1_34/Cluster_11/displaypage3.xhtml]

xml version="1.0" encoding="UTF-8"?


Search Results
  
  
 Results pages: 1, 2, 3, 4, 5

**MultiGeneBlast hits**

Select gene cluster alignment
101. JH687379\_0 Stereum hirsutum FP-91666 SS1 unplaced genomic scaffold STEHI...
102. JH711783\_0 Trametes versicolor FP-101664 SS1 unplaced genomic scaffold T...
103. CABT02000016\_1 Sordaria macrospora k-hell, whole genome shotgun sequenci...
104. KE504141\_0 Fomitopsis pinicola FP-58527 SS1 unplaced genomic scaffold FO...
105. JH725165\_0 Beauveria bassiana ARSEF 2860 unplaced genomic scaffold BBA\_S...
106. JH711573\_1 Coniophora puteana RWD-64-598 SS2 unplaced genomic scaffold C...
107. KB733487\_0 Bipolaris maydis ATCC 48331 unplaced genomic scaffold COCC4sc...
108. KB445592\_0 Cochliobolus heterostrophus C5 unplaced genomic scaffold COCH...
109. KB445791\_0 Ceriporiopsis subvermispora B unplaced genomic scaffold CERSU...
110. DS231622\_1 Pyrenophora tritici-repentis Pt-1C-BFP supercont1.8 genomic s...
111. GL945428\_0 Serpula lacrymans var. lacrymans S7.9 unplaced genomic scaffo...
112. KB469296\_0 Gloeophyllum trabeum ATCC 11539 unplaced genomic scaffold GLO...
113. JH719397\_0 Dichomitus squalens LYAD-421 SS1 unplaced genomic scaffold DI...
114. KB725728\_0 Colletotrichum orbiculare MAFF 240422 unplaced genomic scaffo...
115. GL698718\_0 Metarhizium anisopliae ARSEF 23 unplaced genomic scaffold Scf...
116. AMYD01000373\_0 Colletotrichum gloeosporioides Cg-14, whole genome shotgu...
117. EQ962656\_3 Talaromyces stipitatus ATCC 10500 scf\_1105507295549 genomic s...
118. KE145371\_2 Glarea lozoyensis ATCC 20868 chromosome Unknown GLAREA7, whol...
119. AABX02000023\_0 Neurospora crassa OR74A, whole genome shotgun sequencing ...
120. JH126400\_0 Cordyceps militaris CM01 unplaced genomic scaffold CCM\_S00002...
121. KB725679\_0 Colletotrichum orbiculare MAFF 240422 unplaced genomic scaffo...
122. CM001208\_0 Mycosphaerella graminicola IPO323 chromosome 13, whole genome...
123. KB644411\_2 Penicillium oxalicum 114-2 unplaced genomic scaffold scaffold...
124. GG697358\_1 Glomerella graminicola M1.001 genomic scaffold supercont1.28,...
125. JH795672\_0 Magnaporthe oryzae P131 unplaced genomic scaffold P131\_scaffo...
126. CACQ02003267\_0 Colletotrichum higginsianum strain IMI 349063, whole geno...
127. CM001234\_1 Magnaporthe oryzae 70-15 chromosome 4, whole genome shotgun s...
128. EQ962659\_0 Talaromyces stipitatus ATCC 10500 scf\_1105507295487 genomic s...
129. JH725221\_0 Beauveria bassiana ARSEF 2860 unplaced genomic scaffold BBA\_S...
130. DS995901\_1 Penicillium marneffei ATCC 18224 scf\_1105668340960 genomic sc...
131. DS995727\_0 Trichophyton equinum CBS 127.97 supercont1.10 genomic scaffol...
132. GG700650\_1 Trichophyton rubrum CBS 118892 genomic scaffold supercont2.3,...
133. GG698487\_1 Trichophyton tonsurans CBS 112818 genomic scaffold supercont1...
134. EQ962656\_1 Talaromyces stipitatus ATCC 10500 scf\_1105507295549 genomic s...
135. KB446559\_2 Pseudocercospora fijiensis CIRAD86 unplaced genomic scaffold ...
136. AP007166\_1 Aspergillus oryzae RIB40 DNA, SC113.
137. KB725822\_0 Colletotrichum orbiculare MAFF 240422 unplaced genomic scaffo...
138. AMYD01004223\_0 Colletotrichum gloeosporioides Cg-14, whole genome shotgu...
139. KB726072\_0 Colletotrichum orbiculare MAFF 240422 unplaced genomic scaffo...
140. DS985225\_1 Verticillium albo-atrum VaMs.102 supercont1.12 genomic scaffo...
141. JN186799\_0 Claviceps purpurea strain 20.1 ergot alkaloid biosynthetic ge...
142. CAGA01000020\_1 Claviceps purpurea 20.1, whole genome shotgun sequencing ...
143. KB933064\_0 Togninia minima UCRPA7 unplaced genomic scaffold PA7\_03\_scaff...
144. ACYE01000348\_0 Trichophyton verrucosum HKI 0517, whole genome shotgun se...
145. AHHD01000518\_0 Macrophomina phaseolina MS6, whole genome shotgun sequenc...
146. ABDF02000078\_0 Trichoderma virens Gv29-8, whole genome shotgun sequencin...
147. ABDF02000005\_1 Trichoderma virens Gv29-8, whole genome shotgun sequencin...
148. CAGA01000020\_0 Claviceps purpurea 20.1, whole genome shotgun sequencing ...
149. ABDG02000023\_0 Trichoderma atroviride IMI 206040, whole genome shotgun s...
150. JH126400\_6 Cordyceps militaris CM01 unplaced genomic scaffold CCM\_S00002...

Query: Architecture Search FASTA input

JH687379 : Stereum hirsutum FP-91666 SS1 unplaced genomic scaffold STEHIscaffold\_1    Total score: 2.0     Cumulative Blast bit score: 438

Hit cluster cross-links:

Mycgr3G36335 Mycgr3T
  
Location: 0-423

Mycgr3G36335\_Mycgr3T

Mycgr3G84494 Mycgr3T
  
Location: 523-2047

Mycgr3G84494\_Mycgr3T

Mycgr3G90558 Mycgr3T
  
Location: 2147-15296

Mycgr3G90558\_Mycgr3T

Mycgr3G68036 Mycgr3T
  
Location: 15396-16395

Mycgr3G68036\_Mycgr3T

Mycgr3G90561 Mycgr3T
  
Location: 16495-17134

Mycgr3G90561\_Mycgr3T

Mycgr3G35862 Mycgr3T
  
Location: 17234-18662

Mycgr3G35862\_Mycgr3T

Mycgr3G68030 Mycgr3T
  
Location: 18762-19722

Mycgr3G68030\_Mycgr3T

Mycgr3G36449 Mycgr3T
  
Location: 19822-21886

Mycgr3G36449\_Mycgr3T

Mycgr3G35528 Mycgr3T
  
Location: 21986-22844

Mycgr3G35528\_Mycgr3T

Mycgr3G35932 Mycgr3T
  
Location: 22944-24390

Mycgr3G35932\_Mycgr3T

Mycgr3G23761 Mycgr3T
  
Location: 24490-25825

Mycgr3G23761\_Mycgr3T

Mycgr3G35535 Mycgr3T
  
Location: 25925-26429

Mycgr3G35535\_Mycgr3T

Mycgr3G9942 Mycgr3T9
  
Location: 26529-30375

Mycgr3G9942\_Mycgr3T9

hypothetical protein
  
Accession: EIM92262
  
Location: 1784447-1785944
  
 NCBI BlastP on this gene

EIM92262

hypothetical protein
  
Accession: EIM92263
  
Location: 1786101-1786702
  
 NCBI BlastP on this gene

EIM92263

NAD-P-binding protein
  
Accession: EIM92264
  
Location: 1787143-1788391
  
 NCBI BlastP on this gene

EIM92264

MFS general substrate transporter
  
Accession: EIM92265
  
Location: 1788927-1790979
  
  
**BlastP hit with Mycgr3G84494\_Mycgr3T**
  
Percentage identity: 38 %
  
BlastP bit score: 333
  
Sequence coverage: 97 %
  
E-value: 2e-104
  
  
 NCBI BlastP on this gene

EIM92265

hypothetical protein
  
Accession: EIM92266
  
Location: 1792213-1792532
  
 NCBI BlastP on this gene

EIM92266

hypothetical protein
  
Accession: EIM92267
  
Location: 1795602-1796105
  
 NCBI BlastP on this gene

EIM92267

DNA-binding protein
  
Accession: EIM92268
  
Location: 1797274-1798657
  
 NCBI BlastP on this gene

EIM92268

lipid binding protein
  
Accession: EIM92269
  
Location: 1798969-1800822
  
 NCBI BlastP on this gene

EIM92269

hypothetical protein
  
Accession: EIM92270
  
Location: 1800966-1803093
  
 NCBI BlastP on this gene

EIM92270

hypothetical protein
  
Accession: EIM92271
  
Location: 1803161-1803490
  
 NCBI BlastP on this gene

EIM92271

DNA-dependent RNA polymerase II second largest subunit
  
Accession: EIM92272
  
Location: 1803715-1807973
  
 NCBI BlastP on this gene

EIM92272

hypothetical protein
  
Accession: EIM92273
  
Location: 1808387-1810121
  
 NCBI BlastP on this gene

EIM92273

hypothetical protein
  
Accession: EIM92274
  
Location: 1811201-1814495
  
 NCBI BlastP on this gene

EIM92274

fructosamine kinase PKL/CAK/FruK
  
Accession: EIM92275
  
Location: 1815835-1816981
  
  
**BlastP hit with Mycgr3G68030\_Mycgr3T**
  
Percentage identity: 32 %
  
BlastP bit score: 105
  
Sequence coverage: 82 %
  
E-value: 6e-23
  
  
 NCBI BlastP on this gene

EIM92275

ABC1-domain-containing protein
  
Accession: EIM92276
  
Location: 1817562-1819982
  
 NCBI BlastP on this gene

EIM92276

hypothetical protein
  
Accession: EIM92277
  
Location: 1820519-1821019
  
 NCBI BlastP on this gene

EIM92277

Query: Architecture Search FASTA input

JH711783 : Trametes versicolor FP-101664 SS1 unplaced genomic scaffold TRAVEscaffold\_1    Total score: 2.0     Cumulative Blast bit score: 429

Hit cluster cross-links:

Mycgr3G36335 Mycgr3T
  
Location: 0-423

Mycgr3G36335\_Mycgr3T

Mycgr3G84494 Mycgr3T
  
Location: 523-2047

Mycgr3G84494\_Mycgr3T

Mycgr3G90558 Mycgr3T
  
Location: 2147-15296

Mycgr3G90558\_Mycgr3T

Mycgr3G68036 Mycgr3T
  
Location: 15396-16395

Mycgr3G68036\_Mycgr3T

Mycgr3G90561 Mycgr3T
  
Location: 16495-17134

Mycgr3G90561\_Mycgr3T

Mycgr3G35862 Mycgr3T
  
Location: 17234-18662

Mycgr3G35862\_Mycgr3T

Mycgr3G68030 Mycgr3T
  
Location: 18762-19722

Mycgr3G68030\_Mycgr3T

Mycgr3G36449 Mycgr3T
  
Location: 19822-21886

Mycgr3G36449\_Mycgr3T

Mycgr3G35528 Mycgr3T
  
Location: 21986-22844

Mycgr3G35528\_Mycgr3T

Mycgr3G35932 Mycgr3T
  
Location: 22944-24390

Mycgr3G35932\_Mycgr3T

Mycgr3G23761 Mycgr3T
  
Location: 24490-25825

Mycgr3G23761\_Mycgr3T

Mycgr3G35535 Mycgr3T
  
Location: 25925-26429

Mycgr3G35535\_Mycgr3T

Mycgr3G9942 Mycgr3T9
  
Location: 26529-30375

Mycgr3G9942\_Mycgr3T9

ABC1-domain-containing protein
  
Accession: EIW64210
  
Location: 1580988-1583228
  
 NCBI BlastP on this gene

EIW64210

FMN-linked oxidoreductase
  
Accession: EIW64211
  
Location: 1583437-1585179
  
 NCBI BlastP on this gene

EIW64211

fructosamine-3-kinase
  
Accession: EIW64212
  
Location: 1585594-1586730
  
 NCBI BlastP on this gene

EIW64212

fructosamine kinase PKL/CAK/FruK
  
Accession: EIW64213
  
Location: 1587212-1588348
  
  
**BlastP hit with Mycgr3G68030\_Mycgr3T**
  
Percentage identity: 32 %
  
BlastP bit score: 112
  
Sequence coverage: 86 %
  
E-value: 3e-25
  
  
 NCBI BlastP on this gene

EIW64213

cellobiohydrolaseI
  
Accession: EIW64214
  
Location: 1588636-1590113
  
 NCBI BlastP on this gene

EIW64214

DNA-dependent RNA polymerase II second largest subunit
  
Accession: EIW64215
  
Location: 1592435-1596632
  
 NCBI BlastP on this gene

EIW64215

hypothetical protein
  
Accession: EIW64216
  
Location: 1596732-1599114
  
 NCBI BlastP on this gene

EIW64216

lipid binding protein
  
Accession: EIW64217
  
Location: 1599317-1601194
  
 NCBI BlastP on this gene

EIW64217

hypothetical protein
  
Accession: EIW64218
  
Location: 1603261-1607459
  
 NCBI BlastP on this gene

EIW64218

MFS general substrate transporter
  
Accession: EIW64219
  
Location: 1608053-1610107
  
  
**BlastP hit with Mycgr3G84494\_Mycgr3T**
  
Percentage identity: 37 %
  
BlastP bit score: 317
  
Sequence coverage: 94 %
  
E-value: 5e-98
  
  
 NCBI BlastP on this gene

EIW64219

hypothetical protein
  
Accession: EIW64220
  
Location: 1610552-1611917
  
 NCBI BlastP on this gene

EIW64220

hypothetical protein
  
Accession: EIW64221
  
Location: 1612134-1613111
  
 NCBI BlastP on this gene

EIW64221

Query: Architecture Search FASTA input

CABT02000016 : Sordaria macrospora k-hell    Total score: 2.0     Cumulative Blast bit score: 426

Hit cluster cross-links:

Mycgr3G36335 Mycgr3T
  
Location: 0-423

Mycgr3G36335\_Mycgr3T

Mycgr3G84494 Mycgr3T
  
Location: 523-2047

Mycgr3G84494\_Mycgr3T

Mycgr3G90558 Mycgr3T
  
Location: 2147-15296

Mycgr3G90558\_Mycgr3T

Mycgr3G68036 Mycgr3T
  
Location: 15396-16395

Mycgr3G68036\_Mycgr3T

Mycgr3G90561 Mycgr3T
  
Location: 16495-17134

Mycgr3G90561\_Mycgr3T

Mycgr3G35862 Mycgr3T
  
Location: 17234-18662

Mycgr3G35862\_Mycgr3T

Mycgr3G68030 Mycgr3T
  
Location: 18762-19722

Mycgr3G68030\_Mycgr3T

Mycgr3G36449 Mycgr3T
  
Location: 19822-21886

Mycgr3G36449\_Mycgr3T

Mycgr3G35528 Mycgr3T
  
Location: 21986-22844

Mycgr3G35528\_Mycgr3T

Mycgr3G35932 Mycgr3T
  
Location: 22944-24390

Mycgr3G35932\_Mycgr3T

Mycgr3G23761 Mycgr3T
  
Location: 24490-25825

Mycgr3G23761\_Mycgr3T

Mycgr3G35535 Mycgr3T
  
Location: 25925-26429

Mycgr3G35535\_Mycgr3T

Mycgr3G9942 Mycgr3T9
  
Location: 26529-30375

Mycgr3G9942\_Mycgr3T9

not annotated
  
Accession: CCC11156
  
Location: 570307-576885
  
 NCBI BlastP on this gene

CCC11156

not annotated
  
Accession: CCC11157
  
Location: 582480-583167
  
 NCBI BlastP on this gene

CCC11157

not annotated
  
Accession: CCC11158
  
Location: 584231-585887
  
  
**BlastP hit with Mycgr3G84494\_Mycgr3T**
  
Percentage identity: 40 %
  
BlastP bit score: 298
  
Sequence coverage: 87 %
  
E-value: 2e-91
  
  
 NCBI BlastP on this gene

CCC11158

not annotated
  
Accession: CCC11159
  
Location: 586069-587362
  
  
**BlastP hit with Mycgr3G68030\_Mycgr3T**
  
Percentage identity: 29 %
  
BlastP bit score: 128
  
Sequence coverage: 98 %
  
E-value: 2e-30
  
  
 NCBI BlastP on this gene

CCC11159

not annotated
  
Accession: CCC11160
  
Location: 587752-590878
  
 NCBI BlastP on this gene

CCC11160

not annotated
  
Accession: CCC11161
  
Location: 592101-593340
  
 NCBI BlastP on this gene

CCC11161

not annotated
  
Accession: CCC11162
  
Location: 595389-598861
  
 NCBI BlastP on this gene

CCC11162

not annotated
  
Accession: CCC11163
  
Location: 601173-603236
  
 NCBI BlastP on this gene

CCC11163

Query: Architecture Search FASTA input

KE504141 : Fomitopsis pinicola FP-58527 SS1 unplaced genomic scaffold FOMPIscaffold\_20    Total score: 2.0     Cumulative Blast bit score: 417

Hit cluster cross-links:

Mycgr3G36335 Mycgr3T
  
Location: 0-423

Mycgr3G36335\_Mycgr3T

Mycgr3G84494 Mycgr3T
  
Location: 523-2047

Mycgr3G84494\_Mycgr3T

Mycgr3G90558 Mycgr3T
  
Location: 2147-15296

Mycgr3G90558\_Mycgr3T

Mycgr3G68036 Mycgr3T
  
Location: 15396-16395

Mycgr3G68036\_Mycgr3T

Mycgr3G90561 Mycgr3T
  
Location: 16495-17134

Mycgr3G90561\_Mycgr3T

Mycgr3G35862 Mycgr3T
  
Location: 17234-18662

Mycgr3G35862\_Mycgr3T

Mycgr3G68030 Mycgr3T
  
Location: 18762-19722

Mycgr3G68030\_Mycgr3T

Mycgr3G36449 Mycgr3T
  
Location: 19822-21886

Mycgr3G36449\_Mycgr3T

Mycgr3G35528 Mycgr3T
  
Location: 21986-22844

Mycgr3G35528\_Mycgr3T

Mycgr3G35932 Mycgr3T
  
Location: 22944-24390

Mycgr3G35932\_Mycgr3T

Mycgr3G23761 Mycgr3T
  
Location: 24490-25825

Mycgr3G23761\_Mycgr3T

Mycgr3G35535 Mycgr3T
  
Location: 25925-26429

Mycgr3G35535\_Mycgr3T

Mycgr3G9942 Mycgr3T9
  
Location: 26529-30375

Mycgr3G9942\_Mycgr3T9

hypothetical protein
  
Accession: EPT01524
  
Location: 158582-162524
  
 NCBI BlastP on this gene

EPT01524

hypothetical protein
  
Accession: EPT01525
  
Location: 163260-163917
  
 NCBI BlastP on this gene

EPT01525

hypothetical protein
  
Accession: EPT01526
  
Location: 166057-168041
  
 NCBI BlastP on this gene

EPT01526

hypothetical protein
  
Accession: EPT01527
  
Location: 168288-169945
  
 NCBI BlastP on this gene

EPT01527

hypothetical protein
  
Accession: EPT01528
  
Location: 170338-171479
  
  
**BlastP hit with Mycgr3G68030\_Mycgr3T**
  
Percentage identity: 33 %
  
BlastP bit score: 114
  
Sequence coverage: 82 %
  
E-value: 9e-26
  
  
 NCBI BlastP on this gene

EPT01528

hypothetical protein
  
Accession: EPT01529
  
Location: 172019-176128
  
 NCBI BlastP on this gene

EPT01529

hypothetical protein
  
Accession: EPT01530
  
Location: 176232-178553
  
 NCBI BlastP on this gene

EPT01530

hypothetical protein
  
Accession: EPT01531
  
Location: 178755-180601
  
 NCBI BlastP on this gene

EPT01531

hypothetical protein
  
Accession: EPT01532
  
Location: 180890-181991
  
 NCBI BlastP on this gene

EPT01532

hypothetical protein
  
Accession: EPT01533
  
Location: 182457-186420
  
 NCBI BlastP on this gene

EPT01533

hypothetical protein
  
Accession: EPT01534
  
Location: 186803-188971
  
  
**BlastP hit with Mycgr3G84494\_Mycgr3T**
  
Percentage identity: 36 %
  
BlastP bit score: 303
  
Sequence coverage: 100 %
  
E-value: 2e-92
  
  
 NCBI BlastP on this gene

EPT01534

hypothetical protein
  
Accession: EPT01535
  
Location: 190359-191708
  
 NCBI BlastP on this gene

EPT01535

hypothetical protein
  
Accession: EPT01536
  
Location: 192160-192851
  
 NCBI BlastP on this gene

EPT01536

hypothetical protein
  
Accession: EPT01537
  
Location: 193791-197279
  
 NCBI BlastP on this gene

EPT01537

Query: Architecture Search FASTA input

JH725165 : Beauveria bassiana ARSEF 2860 unplaced genomic scaffold BBA\_S00016    Total score: 2.0     Cumulative Blast bit score: 413

Hit cluster cross-links:

Mycgr3G36335 Mycgr3T
  
Location: 0-423

Mycgr3G36335\_Mycgr3T

Mycgr3G84494 Mycgr3T
  
Location: 523-2047

Mycgr3G84494\_Mycgr3T

Mycgr3G90558 Mycgr3T
  
Location: 2147-15296

Mycgr3G90558\_Mycgr3T

Mycgr3G68036 Mycgr3T
  
Location: 15396-16395

Mycgr3G68036\_Mycgr3T

Mycgr3G90561 Mycgr3T
  
Location: 16495-17134

Mycgr3G90561\_Mycgr3T

Mycgr3G35862 Mycgr3T
  
Location: 17234-18662

Mycgr3G35862\_Mycgr3T

Mycgr3G68030 Mycgr3T
  
Location: 18762-19722

Mycgr3G68030\_Mycgr3T

Mycgr3G36449 Mycgr3T
  
Location: 19822-21886

Mycgr3G36449\_Mycgr3T

Mycgr3G35528 Mycgr3T
  
Location: 21986-22844

Mycgr3G35528\_Mycgr3T

Mycgr3G35932 Mycgr3T
  
Location: 22944-24390

Mycgr3G35932\_Mycgr3T

Mycgr3G23761 Mycgr3T
  
Location: 24490-25825

Mycgr3G23761\_Mycgr3T

Mycgr3G35535 Mycgr3T
  
Location: 25925-26429

Mycgr3G35535\_Mycgr3T

Mycgr3G9942 Mycgr3T9
  
Location: 26529-30375

Mycgr3G9942\_Mycgr3T9

hypothetical protein
  
Accession: EJP65059
  
Location: 79779-80695
  
 NCBI BlastP on this gene

EJP65059

CHCH domain-containing protein
  
Accession: EJP65060
  
Location: 81146-82401
  
 NCBI BlastP on this gene

EJP65060

thiamine pyrophosphokinase
  
Accession: EJP65061
  
Location: 82879-83824
  
 NCBI BlastP on this gene

EJP65061

vesicle transport V-SNARE protein
  
Accession: EJP65062
  
Location: 84054-84961
  
 NCBI BlastP on this gene

EJP65062

major facilitator superfamily transporter
  
Accession: EJP65063
  
Location: 86358-87920
  
  
**BlastP hit with Mycgr3G84494\_Mycgr3T**
  
Percentage identity: 35 %
  
BlastP bit score: 294
  
Sequence coverage: 97 %
  
E-value: 3e-89
  
  
 NCBI BlastP on this gene

EJP65063

Cytochrome P450 CYP528A4
  
Accession: EJP65064
  
Location: 88419-90218
  
 NCBI BlastP on this gene

EJP65064

putative peptide transporter
  
Accession: EJP65065
  
Location: 94713-96651
  
 NCBI BlastP on this gene

EJP65065

dihydrofolate reductase-thymidylate synthase
  
Accession: EJP65066
  
Location: 97381-98453
  
 NCBI BlastP on this gene

EJP65066

WD domain-containing protein
  
Accession: EJP65067
  
Location: 98738-101470
  
 NCBI BlastP on this gene

EJP65067

glycosyl hydrolase family 2
  
Accession: EJP65068
  
Location: 102539-105688
  
 NCBI BlastP on this gene

EJP65068

MFS transporter, putative
  
Accession: EJP65069
  
Location: 107233-109024
  
  
**BlastP hit with Mycgr3G23761\_Mycgr3T**
  
Percentage identity: 26 %
  
BlastP bit score: 119
  
Sequence coverage: 94 %
  
E-value: 4e-26
  
  
 NCBI BlastP on this gene

EJP65069

transposase-like protein
  
Accession: EJP65070
  
Location: 109573-110631
  
 NCBI BlastP on this gene

EJP65070

ABC transporter, putative
  
Accession: EJP65071
  
Location: 111811-115827
  
 NCBI BlastP on this gene

EJP65071

Query: Architecture Search FASTA input

JH711573 : Coniophora puteana RWD-64-598 SS2 unplaced genomic scaffold CONPUscaffold\_1    Total score: 2.0     Cumulative Blast bit score: 413

Hit cluster cross-links:

Mycgr3G36335 Mycgr3T
  
Location: 0-423

Mycgr3G36335\_Mycgr3T

Mycgr3G84494 Mycgr3T
  
Location: 523-2047

Mycgr3G84494\_Mycgr3T

Mycgr3G90558 Mycgr3T
  
Location: 2147-15296

Mycgr3G90558\_Mycgr3T

Mycgr3G68036 Mycgr3T
  
Location: 15396-16395

Mycgr3G68036\_Mycgr3T

Mycgr3G90561 Mycgr3T
  
Location: 16495-17134

Mycgr3G90561\_Mycgr3T

Mycgr3G35862 Mycgr3T
  
Location: 17234-18662

Mycgr3G35862\_Mycgr3T

Mycgr3G68030 Mycgr3T
  
Location: 18762-19722

Mycgr3G68030\_Mycgr3T

Mycgr3G36449 Mycgr3T
  
Location: 19822-21886

Mycgr3G36449\_Mycgr3T

Mycgr3G35528 Mycgr3T
  
Location: 21986-22844

Mycgr3G35528\_Mycgr3T

Mycgr3G35932 Mycgr3T
  
Location: 22944-24390

Mycgr3G35932\_Mycgr3T

Mycgr3G23761 Mycgr3T
  
Location: 24490-25825

Mycgr3G23761\_Mycgr3T

Mycgr3G35535 Mycgr3T
  
Location: 25925-26429

Mycgr3G35535\_Mycgr3T

Mycgr3G9942 Mycgr3T9
  
Location: 26529-30375

Mycgr3G9942\_Mycgr3T9

ANTH-domain-containing protein
  
Accession: EIW87058
  
Location: 3317809-3321590
  
 NCBI BlastP on this gene

EIW87058

hypothetical protein
  
Accession: EIW87057
  
Location: 3314947-3317460
  
 NCBI BlastP on this gene

EIW87057

hypothetical protein
  
Accession: EIW87056
  
Location: 3312967-3313815
  
 NCBI BlastP on this gene

EIW87056

hypothetical protein
  
Accession: EIW87055
  
Location: 3311288-3312705
  
 NCBI BlastP on this gene

EIW87055

NAD(P)-binding protein
  
Accession: EIW87054
  
Location: 3308663-3309876
  
 NCBI BlastP on this gene

EIW87054

MFS general substrate transporter
  
Accession: EIW87053
  
Location: 3306297-3308281
  
  
**BlastP hit with Mycgr3G84494\_Mycgr3T**
  
Percentage identity: 37 %
  
BlastP bit score: 312
  
Sequence coverage: 96 %
  
E-value: 2e-96
  
  
 NCBI BlastP on this gene

EIW87053

hypothetical protein
  
Accession: EIW87052
  
Location: 3305599-3306199
  
 NCBI BlastP on this gene

EIW87052

ABC1-domain-containing protein
  
Accession: EIW87051
  
Location: 3302090-3304477
  
 NCBI BlastP on this gene

EIW87051

NADH:flavin oxidoreductase 2
  
Accession: EIW87050
  
Location: 3299034-3300665
  
 NCBI BlastP on this gene

EIW87050

Ketosamine-3-kinase
  
Accession: EIW87049
  
Location: 3297431-3298543
  
  
**BlastP hit with Mycgr3G68030\_Mycgr3T**
  
Percentage identity: 30 %
  
BlastP bit score: 101
  
Sequence coverage: 80 %
  
E-value: 2e-21
  
  
 NCBI BlastP on this gene

EIW87049

DNA-dependent RNA polymerase II second largest subunit
  
Accession: EIW87048
  
Location: 3293066-3297152
  
 NCBI BlastP on this gene

EIW87048

hypothetical protein
  
Accession: EIW87047
  
Location: 3289723-3291539
  
 NCBI BlastP on this gene

EIW87047

protein PTM1
  
Accession: EIW87046
  
Location: 3287414-3289476
  
 NCBI BlastP on this gene

EIW87046

hypothetical protein
  
Accession: EIW87468
  
Location: 3285526-3286715
  
 NCBI BlastP on this gene

EIW87468

Query: Architecture Search FASTA input

KB733487 : Bipolaris maydis ATCC 48331 unplaced genomic scaffold COCC4scaffold\_44    Total score: 2.0     Cumulative Blast bit score: 396

Hit cluster cross-links:

Mycgr3G36335 Mycgr3T
  
Location: 0-423

Mycgr3G36335\_Mycgr3T

Mycgr3G84494 Mycgr3T
  
Location: 523-2047

Mycgr3G84494\_Mycgr3T

Mycgr3G90558 Mycgr3T
  
Location: 2147-15296

Mycgr3G90558\_Mycgr3T

Mycgr3G68036 Mycgr3T
  
Location: 15396-16395

Mycgr3G68036\_Mycgr3T

Mycgr3G90561 Mycgr3T
  
Location: 16495-17134

Mycgr3G90561\_Mycgr3T

Mycgr3G35862 Mycgr3T
  
Location: 17234-18662

Mycgr3G35862\_Mycgr3T

Mycgr3G68030 Mycgr3T
  
Location: 18762-19722

Mycgr3G68030\_Mycgr3T

Mycgr3G36449 Mycgr3T
  
Location: 19822-21886

Mycgr3G36449\_Mycgr3T

Mycgr3G35528 Mycgr3T
  
Location: 21986-22844

Mycgr3G35528\_Mycgr3T

Mycgr3G35932 Mycgr3T
  
Location: 22944-24390

Mycgr3G35932\_Mycgr3T

Mycgr3G23761 Mycgr3T
  
Location: 24490-25825

Mycgr3G23761\_Mycgr3T

Mycgr3G35535 Mycgr3T
  
Location: 25925-26429

Mycgr3G35535\_Mycgr3T

Mycgr3G9942 Mycgr3T9
  
Location: 26529-30375

Mycgr3G9942\_Mycgr3T9

hypothetical protein
  
Accession: ENH99366
  
Location: 85395-87482
  
 NCBI BlastP on this gene

ENH99366

hypothetical protein
  
Accession: ENH99367
  
Location: 88200-90273
  
 NCBI BlastP on this gene

ENH99367

hypothetical protein
  
Accession: ENH99368
  
Location: 90682-95272
  
 NCBI BlastP on this gene

ENH99368

hypothetical protein
  
Accession: ENH99369
  
Location: 97058-97387
  
 NCBI BlastP on this gene

ENH99369

hypothetical protein
  
Accession: ENH99370
  
Location: 97632-98482
  
 NCBI BlastP on this gene

ENH99370

hypothetical protein
  
Accession: ENH99371
  
Location: 98873-100570
  
  
**BlastP hit with Mycgr3G84494\_Mycgr3T**
  
Percentage identity: 37 %
  
BlastP bit score: 324
  
Sequence coverage: 97 %
  
E-value: 9e-101
  
  
 NCBI BlastP on this gene

ENH99371

hypothetical protein
  
Accession: ENH99372
  
Location: 101680-102603
  
 NCBI BlastP on this gene

ENH99372

hypothetical protein
  
Accession: ENH99373
  
Location: 103143-104558
  
 NCBI BlastP on this gene

ENH99373

hypothetical protein
  
Accession: ENH99374
  
Location: 106283-107143
  
 NCBI BlastP on this gene

ENH99374

hypothetical protein
  
Accession: ENH99375
  
Location: 107958-108389
  
  
**BlastP hit with Mycgr3G36335\_Mycgr3T**
  
Percentage identity: 34 %
  
BlastP bit score: 72
  
Sequence coverage: 95 %
  
E-value: 2e-13
  
  
 NCBI BlastP on this gene

ENH99375

hypothetical protein
  
Accession: ENH99376
  
Location: 109859-110737
  
 NCBI BlastP on this gene

ENH99376

hypothetical protein
  
Accession: ENH99377
  
Location: 111042-112083
  
 NCBI BlastP on this gene

ENH99377

hypothetical protein
  
Accession: ENH99378
  
Location: 112323-115681
  
 NCBI BlastP on this gene

ENH99378

hypothetical protein
  
Accession: ENH99379
  
Location: 116034-117259
  
 NCBI BlastP on this gene

ENH99379

hypothetical protein
  
Accession: ENH99380
  
Location: 117805-122600
  
 NCBI BlastP on this gene

ENH99380

Query: Architecture Search FASTA input

KB445592 : Cochliobolus heterostrophus C5 unplaced genomic scaffold COCHEscaffold\_24    Total score: 2.0     Cumulative Blast bit score: 396

Hit cluster cross-links:

Mycgr3G36335 Mycgr3T
  
Location: 0-423

Mycgr3G36335\_Mycgr3T

Mycgr3G84494 Mycgr3T
  
Location: 523-2047

Mycgr3G84494\_Mycgr3T

Mycgr3G90558 Mycgr3T
  
Location: 2147-15296

Mycgr3G90558\_Mycgr3T

Mycgr3G68036 Mycgr3T
  
Location: 15396-16395

Mycgr3G68036\_Mycgr3T

Mycgr3G90561 Mycgr3T
  
Location: 16495-17134

Mycgr3G90561\_Mycgr3T

Mycgr3G35862 Mycgr3T
  
Location: 17234-18662

Mycgr3G35862\_Mycgr3T

Mycgr3G68030 Mycgr3T
  
Location: 18762-19722

Mycgr3G68030\_Mycgr3T

Mycgr3G36449 Mycgr3T
  
Location: 19822-21886

Mycgr3G36449\_Mycgr3T

Mycgr3G35528 Mycgr3T
  
Location: 21986-22844

Mycgr3G35528\_Mycgr3T

Mycgr3G35932 Mycgr3T
  
Location: 22944-24390

Mycgr3G35932\_Mycgr3T

Mycgr3G23761 Mycgr3T
  
Location: 24490-25825

Mycgr3G23761\_Mycgr3T

Mycgr3G35535 Mycgr3T
  
Location: 25925-26429

Mycgr3G35535\_Mycgr3T

Mycgr3G9942 Mycgr3T9
  
Location: 26529-30375

Mycgr3G9942\_Mycgr3T9

hypothetical protein
  
Accession: EMD85161
  
Location: 57519-62314
  
 NCBI BlastP on this gene

EMD85161

hypothetical protein
  
Accession: EMD85162
  
Location: 62860-64085
  
 NCBI BlastP on this gene

EMD85162

hypothetical protein
  
Accession: EMD85163
  
Location: 64438-67796
  
 NCBI BlastP on this gene

EMD85163

hypothetical protein
  
Accession: EMD85164
  
Location: 68036-69077
  
 NCBI BlastP on this gene

EMD85164

hypothetical protein
  
Accession: EMD85165
  
Location: 69382-70260
  
 NCBI BlastP on this gene

EMD85165

hypothetical protein
  
Accession: EMD85166
  
Location: 71598-72158
  
  
**BlastP hit with Mycgr3G36335\_Mycgr3T**
  
Percentage identity: 34 %
  
BlastP bit score: 72
  
Sequence coverage: 95 %
  
E-value: 3e-13
  
  
 NCBI BlastP on this gene

EMD85166

hypothetical protein
  
Accession: EMD85167
  
Location: 72976-73836
  
 NCBI BlastP on this gene

EMD85167

hypothetical protein
  
Accession: EMD85168
  
Location: 75561-76976
  
 NCBI BlastP on this gene

EMD85168

hypothetical protein
  
Accession: EMD85169
  
Location: 77516-78439
  
 NCBI BlastP on this gene

EMD85169

hypothetical protein
  
Accession: EMD85170
  
Location: 79549-81246
  
  
**BlastP hit with Mycgr3G84494\_Mycgr3T**
  
Percentage identity: 37 %
  
BlastP bit score: 324
  
Sequence coverage: 97 %
  
E-value: 9e-101
  
  
 NCBI BlastP on this gene

EMD85170

hypothetical protein
  
Accession: EMD85171
  
Location: 81637-82487
  
 NCBI BlastP on this gene

EMD85171

hypothetical protein
  
Accession: EMD85172
  
Location: 82732-83061
  
 NCBI BlastP on this gene

EMD85172

hypothetical protein
  
Accession: EMD85173
  
Location: 84847-89437
  
 NCBI BlastP on this gene

EMD85173

hypothetical protein
  
Accession: EMD85174
  
Location: 89847-91920
  
 NCBI BlastP on this gene

EMD85174

hypothetical protein
  
Accession: EMD85175
  
Location: 92638-94725
  
 NCBI BlastP on this gene

EMD85175

Query: Architecture Search FASTA input

KB445791 : Ceriporiopsis subvermispora B unplaced genomic scaffold CERSUscaffold\_1    Total score: 2.0     Cumulative Blast bit score: 394

Hit cluster cross-links:

Mycgr3G36335 Mycgr3T
  
Location: 0-423

Mycgr3G36335\_Mycgr3T

Mycgr3G84494 Mycgr3T
  
Location: 523-2047

Mycgr3G84494\_Mycgr3T

Mycgr3G90558 Mycgr3T
  
Location: 2147-15296

Mycgr3G90558\_Mycgr3T

Mycgr3G68036 Mycgr3T
  
Location: 15396-16395

Mycgr3G68036\_Mycgr3T

Mycgr3G90561 Mycgr3T
  
Location: 16495-17134

Mycgr3G90561\_Mycgr3T

Mycgr3G35862 Mycgr3T
  
Location: 17234-18662

Mycgr3G35862\_Mycgr3T

Mycgr3G68030 Mycgr3T
  
Location: 18762-19722

Mycgr3G68030\_Mycgr3T

Mycgr3G36449 Mycgr3T
  
Location: 19822-21886

Mycgr3G36449\_Mycgr3T

Mycgr3G35528 Mycgr3T
  
Location: 21986-22844

Mycgr3G35528\_Mycgr3T

Mycgr3G35932 Mycgr3T
  
Location: 22944-24390

Mycgr3G35932\_Mycgr3T

Mycgr3G23761 Mycgr3T
  
Location: 24490-25825

Mycgr3G23761\_Mycgr3T

Mycgr3G35535 Mycgr3T
  
Location: 25925-26429

Mycgr3G35535\_Mycgr3T

Mycgr3G9942 Mycgr3T9
  
Location: 26529-30375

Mycgr3G9942\_Mycgr3T9

hypothetical protein
  
Accession: EMD41892
  
Location: 2327376-2328150
  
 NCBI BlastP on this gene

EMD41892

hypothetical protein
  
Accession: EMD41893
  
Location: 2328501-2329917
  
 NCBI BlastP on this gene

EMD41893

hypothetical protein
  
Accession: EMD41894
  
Location: 2331222-2333117
  
  
**BlastP hit with Mycgr3G84494\_Mycgr3T**
  
Percentage identity: 38 %
  
BlastP bit score: 290
  
Sequence coverage: 91 %
  
E-value: 2e-88
  
  
 NCBI BlastP on this gene

EMD41894

hypothetical protein
  
Accession: EMD41895
  
Location: 2333413-2337470
  
 NCBI BlastP on this gene

EMD41895

hypothetical protein
  
Accession: EMD41896
  
Location: 2338083-2338720
  
 NCBI BlastP on this gene

EMD41896

SNX4-like sorting nexin
  
Accession: EMD41897
  
Location: 2339162-2341025
  
 NCBI BlastP on this gene

EMD41897

hypothetical protein
  
Accession: EMD41898
  
Location: 2343719-2347914
  
 NCBI BlastP on this gene

EMD41898

hypothetical protein
  
Accession: EMD41899
  
Location: 2348343-2349484
  
  
**BlastP hit with Mycgr3G68030\_Mycgr3T**
  
Percentage identity: 29 %
  
BlastP bit score: 104
  
Sequence coverage: 87 %
  
E-value: 2e-22
  
  
 NCBI BlastP on this gene

EMD41899

hypothetical protein
  
Accession: EMD41900
  
Location: 2349880-2351518
  
 NCBI BlastP on this gene

EMD41900

hypothetical protein
  
Accession: EMD41901
  
Location: 2353791-2356182
  
 NCBI BlastP on this gene

EMD41901

glycosyltransferase family 8 protein
  
Accession: EMD41902
  
Location: 2357605-2361194
  
 NCBI BlastP on this gene

EMD41902

Query: Architecture Search FASTA input

DS231622 : Pyrenophora tritici-repentis Pt-1C-BFP supercont1.8 genomic scaffold    Total score: 2.0     Cumulative Blast bit score: 392

Hit cluster cross-links:

Mycgr3G36335 Mycgr3T
  
Location: 0-423

Mycgr3G36335\_Mycgr3T

Mycgr3G84494 Mycgr3T
  
Location: 523-2047

Mycgr3G84494\_Mycgr3T

Mycgr3G90558 Mycgr3T
  
Location: 2147-15296

Mycgr3G90558\_Mycgr3T

Mycgr3G68036 Mycgr3T
  
Location: 15396-16395

Mycgr3G68036\_Mycgr3T

Mycgr3G90561 Mycgr3T
  
Location: 16495-17134

Mycgr3G90561\_Mycgr3T

Mycgr3G35862 Mycgr3T
  
Location: 17234-18662

Mycgr3G35862\_Mycgr3T

Mycgr3G68030 Mycgr3T
  
Location: 18762-19722

Mycgr3G68030\_Mycgr3T

Mycgr3G36449 Mycgr3T
  
Location: 19822-21886

Mycgr3G36449\_Mycgr3T

Mycgr3G35528 Mycgr3T
  
Location: 21986-22844

Mycgr3G35528\_Mycgr3T

Mycgr3G35932 Mycgr3T
  
Location: 22944-24390

Mycgr3G35932\_Mycgr3T

Mycgr3G23761 Mycgr3T
  
Location: 24490-25825

Mycgr3G23761\_Mycgr3T

Mycgr3G35535 Mycgr3T
  
Location: 25925-26429

Mycgr3G35535\_Mycgr3T

Mycgr3G9942 Mycgr3T9
  
Location: 26529-30375

Mycgr3G9942\_Mycgr3T9

dTDP-D-glucose 4,6-dehydratase
  
Accession: EDU50629
  
Location: 551892-553396
  
 NCBI BlastP on this gene

EDU50629

predicted protein
  
Accession: EDU50630
  
Location: 553572-553727
  
 NCBI BlastP on this gene

EDU50630

conserved hypothetical protein
  
Accession: EDU50631
  
Location: 553758-555266
  
 NCBI BlastP on this gene

EDU50631

predicted protein
  
Accession: EDU50632
  
Location: 556132-557885
  
 NCBI BlastP on this gene

EDU50632

maltose permease MAL61
  
Accession: EDU50633
  
Location: 560596-562251
  
 NCBI BlastP on this gene

EDU50633

hypothetical protein
  
Accession: EDU50634
  
Location: 563659-564127
  
 NCBI BlastP on this gene

EDU50634

conserved hypothetical protein
  
Accession: EDU50635
  
Location: 564486-566859
  
  
**BlastP hit with Mycgr3G36335\_Mycgr3T**
  
Percentage identity: 38 %
  
BlastP bit score: 75
  
Sequence coverage: 81 %
  
E-value: 4e-13
  
  
 NCBI BlastP on this gene

EDU50635

citrinin biosynthesis oxydoreductase CtnB
  
Accession: EDU50636
  
Location: 566946-567809
  
 NCBI BlastP on this gene

EDU50636

threonine aldolase
  
Accession: EDU50637
  
Location: 569090-570447
  
 NCBI BlastP on this gene

EDU50637

conserved hypothetical protein
  
Accession: EDU50638
  
Location: 570966-571889
  
 NCBI BlastP on this gene

EDU50638

conserved hypothetical protein
  
Accession: EDU50639
  
Location: 573142-574914
  
  
**BlastP hit with Mycgr3G84494\_Mycgr3T**
  
Percentage identity: 37 %
  
BlastP bit score: 317
  
Sequence coverage: 95 %
  
E-value: 5e-98
  
  
 NCBI BlastP on this gene

EDU50639

conserved hypothetical protein
  
Accession: EDU50640
  
Location: 575028-575853
  
 NCBI BlastP on this gene

EDU50640

HET domain containing protein
  
Accession: EDU50641
  
Location: 577336-580369
  
 NCBI BlastP on this gene

EDU50641

conserved hypothetical protein
  
Accession: EDU50642
  
Location: 582166-583099
  
 NCBI BlastP on this gene

EDU50642

predicted protein
  
Accession: EDU50643
  
Location: 583383-584685
  
 NCBI BlastP on this gene

EDU50643

serine/threonine-protein kinase CBK1
  
Accession: EDU50644
  
Location: 585109-591059
  
 NCBI BlastP on this gene

EDU50644

Query: Architecture Search FASTA input

GL945428 : Serpula lacrymans var. lacrymans S7.9 unplaced genomic scaffold SERLAscaffold\_1    Total score: 2.0     Cumulative Blast bit score: 391

Hit cluster cross-links:

Mycgr3G36335 Mycgr3T
  
Location: 0-423

Mycgr3G36335\_Mycgr3T

Mycgr3G84494 Mycgr3T
  
Location: 523-2047

Mycgr3G84494\_Mycgr3T

Mycgr3G90558 Mycgr3T
  
Location: 2147-15296

Mycgr3G90558\_Mycgr3T

Mycgr3G68036 Mycgr3T
  
Location: 15396-16395

Mycgr3G68036\_Mycgr3T

Mycgr3G90561 Mycgr3T
  
Location: 16495-17134

Mycgr3G90561\_Mycgr3T

Mycgr3G35862 Mycgr3T
  
Location: 17234-18662

Mycgr3G35862\_Mycgr3T

Mycgr3G68030 Mycgr3T
  
Location: 18762-19722

Mycgr3G68030\_Mycgr3T

Mycgr3G36449 Mycgr3T
  
Location: 19822-21886

Mycgr3G36449\_Mycgr3T

Mycgr3G35528 Mycgr3T
  
Location: 21986-22844

Mycgr3G35528\_Mycgr3T

Mycgr3G35932 Mycgr3T
  
Location: 22944-24390

Mycgr3G35932\_Mycgr3T

Mycgr3G23761 Mycgr3T
  
Location: 24490-25825

Mycgr3G23761\_Mycgr3T

Mycgr3G35535 Mycgr3T
  
Location: 25925-26429

Mycgr3G35535\_Mycgr3T

Mycgr3G9942 Mycgr3T9
  
Location: 26529-30375

Mycgr3G9942\_Mycgr3T9

hypothetical protein
  
Accession: EGO31172
  
Location: 5413110-5413526
  
 NCBI BlastP on this gene

EGO31172

hypothetical protein
  
Accession: EGO31173
  
Location: 5413994-5414233
  
 NCBI BlastP on this gene

EGO31173

hypothetical protein
  
Accession: EGO31174
  
Location: 5414989-5415218
  
 NCBI BlastP on this gene

EGO31174

hypothetical protein
  
Accession: EGO31175
  
Location: 5415695-5416411
  
 NCBI BlastP on this gene

EGO31175

hypothetical protein
  
Accession: EGO31176
  
Location: 5419309-5419894
  
 NCBI BlastP on this gene

EGO31176

hypothetical protein
  
Accession: EGO31177
  
Location: 5420942-5421245
  
 NCBI BlastP on this gene

EGO31177

hypothetical protein
  
Accession: EGO31178
  
Location: 5422235-5423465
  
 NCBI BlastP on this gene

EGO31178

hypothetical protein
  
Accession: EGO31179
  
Location: 5423923-5426283
  
  
**BlastP hit with Mycgr3G84494\_Mycgr3T**
  
Percentage identity: 36 %
  
BlastP bit score: 293
  
Sequence coverage: 93 %
  
E-value: 8e-89
  
  
 NCBI BlastP on this gene

EGO31179

hypothetical protein
  
Accession: EGO31180
  
Location: 5426446-5427574
  
 NCBI BlastP on this gene

EGO31180

hypothetical protein
  
Accession: EGO31181
  
Location: 5427827-5429701
  
 NCBI BlastP on this gene

EGO31181

DNA-directed RNA polymerase II, subunit 2
  
Accession: EGO31287
  
Location: 5432505-5436551
  
 NCBI BlastP on this gene

EGO31287

hypothetical protein
  
Accession: EGO31182
  
Location: 5436971-5438114
  
  
**BlastP hit with Mycgr3G68030\_Mycgr3T**
  
Percentage identity: 31 %
  
BlastP bit score: 98
  
Sequence coverage: 86 %
  
E-value: 2e-20
  
  
 NCBI BlastP on this gene

EGO31182

hypothetical protein
  
Accession: EGO31183
  
Location: 5438670-5440322
  
 NCBI BlastP on this gene

EGO31183

hypothetical protein
  
Accession: EGO31184
  
Location: 5440786-5443161
  
 NCBI BlastP on this gene

EGO31184

hypothetical protein
  
Accession: EGO31185
  
Location: 5444207-5445012
  
 NCBI BlastP on this gene

EGO31185

hypothetical protein
  
Accession: EGO31186
  
Location: 5446724-5449097
  
 NCBI BlastP on this gene

EGO31186

Query: Architecture Search FASTA input

KB469296 : Gloeophyllum trabeum ATCC 11539 unplaced genomic scaffold GLOTRscaffold\_00001    Total score: 2.0     Cumulative Blast bit score: 387

Hit cluster cross-links:

Mycgr3G36335 Mycgr3T
  
Location: 0-423

Mycgr3G36335\_Mycgr3T

Mycgr3G84494 Mycgr3T
  
Location: 523-2047

Mycgr3G84494\_Mycgr3T

Mycgr3G90558 Mycgr3T
  
Location: 2147-15296

Mycgr3G90558\_Mycgr3T

Mycgr3G68036 Mycgr3T
  
Location: 15396-16395

Mycgr3G68036\_Mycgr3T

Mycgr3G90561 Mycgr3T
  
Location: 16495-17134

Mycgr3G90561\_Mycgr3T

Mycgr3G35862 Mycgr3T
  
Location: 17234-18662

Mycgr3G35862\_Mycgr3T

Mycgr3G68030 Mycgr3T
  
Location: 18762-19722

Mycgr3G68030\_Mycgr3T

Mycgr3G36449 Mycgr3T
  
Location: 19822-21886

Mycgr3G36449\_Mycgr3T

Mycgr3G35528 Mycgr3T
  
Location: 21986-22844

Mycgr3G35528\_Mycgr3T

Mycgr3G35932 Mycgr3T
  
Location: 22944-24390

Mycgr3G35932\_Mycgr3T

Mycgr3G23761 Mycgr3T
  
Location: 24490-25825

Mycgr3G23761\_Mycgr3T

Mycgr3G35535 Mycgr3T
  
Location: 25925-26429

Mycgr3G35535\_Mycgr3T

Mycgr3G9942 Mycgr3T9
  
Location: 26529-30375

Mycgr3G9942\_Mycgr3T9

hypothetical protein
  
Accession: EPQ60390
  
Location: 1410405-1411227
  
 NCBI BlastP on this gene

EPQ60390

hypothetical protein
  
Accession: EPQ60391
  
Location: 1411710-1413100
  
 NCBI BlastP on this gene

EPQ60391

hypothetical protein
  
Accession: EPQ60392
  
Location: 1413334-1413678
  
 NCBI BlastP on this gene

EPQ60392

MFS general substrate transporter
  
Accession: EPQ60393
  
Location: 1415347-1418149
  
 NCBI BlastP on this gene

EPQ60393

NAD P-binding protein
  
Accession: EPQ60394
  
Location: 1418681-1419904
  
 NCBI BlastP on this gene

EPQ60394

MFS general substrate transporter
  
Accession: EPQ60395
  
Location: 1420323-1422307
  
  
**BlastP hit with Mycgr3G84494\_Mycgr3T**
  
Percentage identity: 37 %
  
BlastP bit score: 286
  
Sequence coverage: 91 %
  
E-value: 3e-86
  
  
 NCBI BlastP on this gene

EPQ60395

DNA-binding protein
  
Accession: EPQ60396
  
Location: 1422713-1423847
  
 NCBI BlastP on this gene

EPQ60396

hypothetical protein
  
Accession: EPQ61430
  
Location: 1423918-1425748
  
 NCBI BlastP on this gene

EPQ61430

protein PTM1
  
Accession: EPQ60397
  
Location: 1425957-1428057
  
 NCBI BlastP on this gene

EPQ60397

hypothetical protein
  
Accession: EPQ60398
  
Location: 1428116-1428439
  
 NCBI BlastP on this gene

EPQ60398

hypothetical protein
  
Accession: EPQ61429
  
Location: 1428560-1432528
  
 NCBI BlastP on this gene

EPQ61429

fructosamine-3-kinase
  
Accession: EPQ60399
  
Location: 1433069-1434202
  
  
**BlastP hit with Mycgr3G68030\_Mycgr3T**
  
Percentage identity: 30 %
  
BlastP bit score: 101
  
Sequence coverage: 83 %
  
E-value: 1e-21
  
  
 NCBI BlastP on this gene

EPQ60399

hypothetical protein
  
Accession: EPQ60400
  
Location: 1434732-1436428
  
 NCBI BlastP on this gene

EPQ60400

hypothetical protein
  
Accession: EPQ60401
  
Location: 1436538-1439549
  
 NCBI BlastP on this gene

EPQ60401

NADH flavin oxidoreductase 1
  
Accession: EPQ60402
  
Location: 1439758-1441265
  
 NCBI BlastP on this gene

EPQ60402

ABC1-domain-containing protein
  
Accession: EPQ60403
  
Location: 1441593-1443859
  
 NCBI BlastP on this gene

EPQ60403

hypothetical protein
  
Accession: EPQ60404
  
Location: 1444738-1448285
  
 NCBI BlastP on this gene

EPQ60404

Query: Architecture Search FASTA input

JH719397 : Dichomitus squalens LYAD-421 SS1 unplaced genomic scaffold DICSQscaffold\_1    Total score: 2.0     Cumulative Blast bit score: 385

Hit cluster cross-links:

Mycgr3G36335 Mycgr3T
  
Location: 0-423

Mycgr3G36335\_Mycgr3T

Mycgr3G84494 Mycgr3T
  
Location: 523-2047

Mycgr3G84494\_Mycgr3T

Mycgr3G90558 Mycgr3T
  
Location: 2147-15296

Mycgr3G90558\_Mycgr3T

Mycgr3G68036 Mycgr3T
  
Location: 15396-16395

Mycgr3G68036\_Mycgr3T

Mycgr3G90561 Mycgr3T
  
Location: 16495-17134

Mycgr3G90561\_Mycgr3T

Mycgr3G35862 Mycgr3T
  
Location: 17234-18662

Mycgr3G35862\_Mycgr3T

Mycgr3G68030 Mycgr3T
  
Location: 18762-19722

Mycgr3G68030\_Mycgr3T

Mycgr3G36449 Mycgr3T
  
Location: 19822-21886

Mycgr3G36449\_Mycgr3T

Mycgr3G35528 Mycgr3T
  
Location: 21986-22844

Mycgr3G35528\_Mycgr3T

Mycgr3G35932 Mycgr3T
  
Location: 22944-24390

Mycgr3G35932\_Mycgr3T

Mycgr3G23761 Mycgr3T
  
Location: 24490-25825

Mycgr3G23761\_Mycgr3T

Mycgr3G35535 Mycgr3T
  
Location: 25925-26429

Mycgr3G35535\_Mycgr3T

Mycgr3G9942 Mycgr3T9
  
Location: 26529-30375

Mycgr3G9942\_Mycgr3T9

hypothetical protein
  
Accession: EJF66835
  
Location: 1321349-1324541
  
 NCBI BlastP on this gene

EJF66835

hypothetical protein
  
Accession: EJF66836
  
Location: 1327081-1327914
  
 NCBI BlastP on this gene

EJF66836

hypothetical protein
  
Accession: EJF66837
  
Location: 1328341-1329745
  
 NCBI BlastP on this gene

EJF66837

MFS general substrate transporter
  
Accession: EJF66838
  
Location: 1330684-1332431
  
  
**BlastP hit with Mycgr3G84494\_Mycgr3T**
  
Percentage identity: 37 %
  
BlastP bit score: 287
  
Sequence coverage: 89 %
  
E-value: 1e-87
  
  
 NCBI BlastP on this gene

EJF66838

hypothetical protein
  
Accession: EJF66839
  
Location: 1332810-1336949
  
 NCBI BlastP on this gene

EJF66839

DNA-binding protein
  
Accession: EJF66840
  
Location: 1337219-1338323
  
 NCBI BlastP on this gene

EJF66840

hypothetical protein
  
Accession: EJF66841
  
Location: 1338432-1340287
  
 NCBI BlastP on this gene

EJF66841

hypothetical protein
  
Accession: EJF66842
  
Location: 1340512-1342909
  
 NCBI BlastP on this gene

EJF66842

DNA-dependent RNA polymerase II second largest subunit
  
Accession: EJF66843
  
Location: 1343004-1347176
  
 NCBI BlastP on this gene

EJF66843

cellobiohydrolaseI
  
Accession: EJF66844
  
Location: 1349210-1350691
  
 NCBI BlastP on this gene

EJF66844

fructosamine kinase PKL/CAK/FruK
  
Accession: EJF66845
  
Location: 1351027-1352146
  
  
**BlastP hit with Mycgr3G68030\_Mycgr3T**
  
Percentage identity: 28 %
  
BlastP bit score: 98
  
Sequence coverage: 85 %
  
E-value: 2e-20
  
  
 NCBI BlastP on this gene

EJF66845

FMN-linked oxidoreductase
  
Accession: EJF66846
  
Location: 1352658-1354350
  
 NCBI BlastP on this gene

EJF66846

hypothetical protein
  
Accession: EJF66847
  
Location: 1354595-1354854
  
 NCBI BlastP on this gene

EJF66847

FMN-linked oxidoreductase
  
Accession: EJF66848
  
Location: 1355559-1357303
  
 NCBI BlastP on this gene

EJF66848

ABC1-domain-containing protein
  
Accession: EJF66849
  
Location: 1357553-1360030
  
 NCBI BlastP on this gene

EJF66849

Query: Architecture Search FASTA input

KB725728 : Colletotrichum orbiculare MAFF 240422 unplaced genomic scaffold Scaffold\_184    Total score: 2.0     Cumulative Blast bit score: 369

Hit cluster cross-links:

Mycgr3G36335 Mycgr3T
  
Location: 0-423

Mycgr3G36335\_Mycgr3T

Mycgr3G84494 Mycgr3T
  
Location: 523-2047

Mycgr3G84494\_Mycgr3T

Mycgr3G90558 Mycgr3T
  
Location: 2147-15296

Mycgr3G90558\_Mycgr3T

Mycgr3G68036 Mycgr3T
  
Location: 15396-16395

Mycgr3G68036\_Mycgr3T

Mycgr3G90561 Mycgr3T
  
Location: 16495-17134

Mycgr3G90561\_Mycgr3T

Mycgr3G35862 Mycgr3T
  
Location: 17234-18662

Mycgr3G35862\_Mycgr3T

Mycgr3G68030 Mycgr3T
  
Location: 18762-19722

Mycgr3G68030\_Mycgr3T

Mycgr3G36449 Mycgr3T
  
Location: 19822-21886

Mycgr3G36449\_Mycgr3T

Mycgr3G35528 Mycgr3T
  
Location: 21986-22844

Mycgr3G35528\_Mycgr3T

Mycgr3G35932 Mycgr3T
  
Location: 22944-24390

Mycgr3G35932\_Mycgr3T

Mycgr3G23761 Mycgr3T
  
Location: 24490-25825

Mycgr3G23761\_Mycgr3T

Mycgr3G35535 Mycgr3T
  
Location: 25925-26429

Mycgr3G35535\_Mycgr3T

Mycgr3G9942 Mycgr3T9
  
Location: 26529-30375

Mycgr3G9942\_Mycgr3T9

homoserine o-acetyltransferase
  
Accession: ENH86768
  
Location: 260923-262482
  
 NCBI BlastP on this gene

ENH86768

nudix family
  
Accession: ENH86769
  
Location: 262999-263940
  
 NCBI BlastP on this gene

ENH86769

ADP-ribose pyrophosphatase
  
Accession: ENH86770
  
Location: 263981-264723
  
 NCBI BlastP on this gene

ENH86770

MFS transporter
  
Accession: ENH86771
  
Location: 266416-268205
  
  
**BlastP hit with Mycgr3G23761\_Mycgr3T**
  
Percentage identity: 29 %
  
BlastP bit score: 172
  
Sequence coverage: 99 %
  
E-value: 1e-44
  
  
 NCBI BlastP on this gene

ENH86771

nad-dependent deacetylase sirtuin-2
  
Accession: ENH86772
  
Location: 269415-271010
  
 NCBI BlastP on this gene

ENH86772

hypothetical protein
  
Accession: ENH86773
  
Location: 283204-283596
  
 NCBI BlastP on this gene

ENH86773

pectate lyase
  
Accession: ENH86774
  
Location: 287976-288776
  
 NCBI BlastP on this gene

ENH86774

thymine dioxygenase
  
Accession: ENH86775
  
Location: 289560-290673
  
  
**BlastP hit with Mycgr3G68036\_Mycgr3T**
  
Percentage identity: 36 %
  
BlastP bit score: 197
  
Sequence coverage: 87 %
  
E-value: 1e-56
  
  
 NCBI BlastP on this gene

ENH86775

C6 transcription factor
  
Accession: ENH86776
  
Location: 291302-293758
  
 NCBI BlastP on this gene

ENH86776

FAD binding domain protein
  
Accession: ENH86777
  
Location: 293891-295405
  
 NCBI BlastP on this gene

ENH86777

Query: Architecture Search FASTA input

GL698718 : Metarhizium anisopliae ARSEF 23 unplaced genomic scaffold Scf\_008    Total score: 2.0     Cumulative Blast bit score: 359

Hit cluster cross-links:

Mycgr3G36335 Mycgr3T
  
Location: 0-423

Mycgr3G36335\_Mycgr3T

Mycgr3G84494 Mycgr3T
  
Location: 523-2047

Mycgr3G84494\_Mycgr3T

Mycgr3G90558 Mycgr3T
  
Location: 2147-15296

Mycgr3G90558\_Mycgr3T

Mycgr3G68036 Mycgr3T
  
Location: 15396-16395

Mycgr3G68036\_Mycgr3T

Mycgr3G90561 Mycgr3T
  
Location: 16495-17134

Mycgr3G90561\_Mycgr3T

Mycgr3G35862 Mycgr3T
  
Location: 17234-18662

Mycgr3G35862\_Mycgr3T

Mycgr3G68030 Mycgr3T
  
Location: 18762-19722

Mycgr3G68030\_Mycgr3T

Mycgr3G36449 Mycgr3T
  
Location: 19822-21886

Mycgr3G36449\_Mycgr3T

Mycgr3G35528 Mycgr3T
  
Location: 21986-22844

Mycgr3G35528\_Mycgr3T

Mycgr3G35932 Mycgr3T
  
Location: 22944-24390

Mycgr3G35932\_Mycgr3T

Mycgr3G23761 Mycgr3T
  
Location: 24490-25825

Mycgr3G23761\_Mycgr3T

Mycgr3G35535 Mycgr3T
  
Location: 25925-26429

Mycgr3G35535\_Mycgr3T

Mycgr3G9942 Mycgr3T9
  
Location: 26529-30375

Mycgr3G9942\_Mycgr3T9

QDE-2-interacting protein
  
Accession: EFY98892
  
Location: 1651502-1653146
  
 NCBI BlastP on this gene

EFY98892

mitochondrial DnaJ chaperone (Tim14), putative
  
Accession: EFY98891
  
Location: 1650747-1651217
  
 NCBI BlastP on this gene

EFY98891

hypothetical protein
  
Accession: EFY98890
  
Location: 1649745-1650357
  
 NCBI BlastP on this gene

EFY98890

autophagy protein (Atg22), putative
  
Accession: EFY98889
  
Location: 1647674-1649302
  
 NCBI BlastP on this gene

EFY98889

methyltransferase type 12
  
Accession: EFY98888
  
Location: 1645826-1646776
  
 NCBI BlastP on this gene

EFY98888

phosphatidylserine decarboxylase family protein
  
Accession: EFY98887
  
Location: 1643679-1645046
  
 NCBI BlastP on this gene

EFY98887

PRO1A C6 Zink-finger protein
  
Accession: EFY98886
  
Location: 1640838-1642567
  
 NCBI BlastP on this gene

EFY98886

hypothetical protein
  
Accession: EFY98885
  
Location: 1639635-1640086
  
 NCBI BlastP on this gene

EFY98885

hypothetical protein
  
Accession: EFY98884
  
Location: 1638077-1638505
  
 NCBI BlastP on this gene

EFY98884

peptidase S41 family protein
  
Accession: EFY98883
  
Location: 1635197-1637656
  
  
**BlastP hit with Mycgr3G36449\_Mycgr3T**
  
Percentage identity: 30 %
  
BlastP bit score: 235
  
Sequence coverage: 101 %
  
E-value: 2e-63
  
  
 NCBI BlastP on this gene

EFY98883

MFS transporter, putative
  
Accession: EFY98882
  
Location: 1631515-1633064
  
  
**BlastP hit with Mycgr3G23761\_Mycgr3T**
  
Percentage identity: 27 %
  
BlastP bit score: 124
  
Sequence coverage: 81 %
  
E-value: 3e-28
  
  
 NCBI BlastP on this gene

EFY98882

C2H2 finger domain protein, putative
  
Accession: EFY98881
  
Location: 1622647-1624071
  
 NCBI BlastP on this gene

EFY98881

hypothetical protein
  
Accession: EFY98880
  
Location: 1620377-1621557
  
 NCBI BlastP on this gene

EFY98880

Query: Architecture Search FASTA input

AMYD01000373 : Colletotrichum gloeosporioides Cg-14    Total score: 2.0     Cumulative Blast bit score: 333

Hit cluster cross-links:

Mycgr3G36335 Mycgr3T
  
Location: 0-423

Mycgr3G36335\_Mycgr3T

Mycgr3G84494 Mycgr3T
  
Location: 523-2047

Mycgr3G84494\_Mycgr3T

Mycgr3G90558 Mycgr3T
  
Location: 2147-15296

Mycgr3G90558\_Mycgr3T

Mycgr3G68036 Mycgr3T
  
Location: 15396-16395

Mycgr3G68036\_Mycgr3T

Mycgr3G90561 Mycgr3T
  
Location: 16495-17134

Mycgr3G90561\_Mycgr3T

Mycgr3G35862 Mycgr3T
  
Location: 17234-18662

Mycgr3G35862\_Mycgr3T

Mycgr3G68030 Mycgr3T
  
Location: 18762-19722

Mycgr3G68030\_Mycgr3T

Mycgr3G36449 Mycgr3T
  
Location: 19822-21886

Mycgr3G36449\_Mycgr3T

Mycgr3G35528 Mycgr3T
  
Location: 21986-22844

Mycgr3G35528\_Mycgr3T

Mycgr3G35932 Mycgr3T
  
Location: 22944-24390

Mycgr3G35932\_Mycgr3T

Mycgr3G23761 Mycgr3T
  
Location: 24490-25825

Mycgr3G23761\_Mycgr3T

Mycgr3G35535 Mycgr3T
  
Location: 25925-26429

Mycgr3G35535\_Mycgr3T

Mycgr3G9942 Mycgr3T9
  
Location: 26529-30375

Mycgr3G9942\_Mycgr3T9

hypothetical protein
  
Accession: EQB58029
  
Location: 4331-5174
  
  
**BlastP hit with Mycgr3G90561\_Mycgr3T**
  
Percentage identity: 34 %
  
BlastP bit score: 77
  
Sequence coverage: 55 %
  
E-value: 3e-14
  
  
 NCBI BlastP on this gene

EQB58029

hypothetical protein
  
Accession: EQB58030
  
Location: 6014-8158
  
 NCBI BlastP on this gene

EQB58030

hypothetical protein
  
Accession: EQB58031
  
Location: 8931-12158
  
 NCBI BlastP on this gene

EQB58031

peptidase S41 family protein
  
Accession: EQB58032
  
Location: 14606-16921
  
  
**BlastP hit with Mycgr3G36449\_Mycgr3T**
  
Percentage identity: 28 %
  
BlastP bit score: 256
  
Sequence coverage: 105 %
  
E-value: 4e-71
  
  
 NCBI BlastP on this gene

EQB58032

hypothetical protein
  
Accession: EQB58033
  
Location: 19535-19805
  
 NCBI BlastP on this gene

EQB58033

hypothetical protein
  
Accession: EQB58034
  
Location: 23459-25517
  
 NCBI BlastP on this gene

EQB58034

Query: Architecture Search FASTA input

EQ962656 : Talaromyces stipitatus ATCC 10500 scf\_1105507295549 genomic scaffold    Total score: 2.0     Cumulative Blast bit score: 323

Hit cluster cross-links:

Mycgr3G36335 Mycgr3T
  
Location: 0-423

Mycgr3G36335\_Mycgr3T

Mycgr3G84494 Mycgr3T
  
Location: 523-2047

Mycgr3G84494\_Mycgr3T

Mycgr3G90558 Mycgr3T
  
Location: 2147-15296

Mycgr3G90558\_Mycgr3T

Mycgr3G68036 Mycgr3T
  
Location: 15396-16395

Mycgr3G68036\_Mycgr3T

Mycgr3G90561 Mycgr3T
  
Location: 16495-17134

Mycgr3G90561\_Mycgr3T

Mycgr3G35862 Mycgr3T
  
Location: 17234-18662

Mycgr3G35862\_Mycgr3T

Mycgr3G68030 Mycgr3T
  
Location: 18762-19722

Mycgr3G68030\_Mycgr3T

Mycgr3G36449 Mycgr3T
  
Location: 19822-21886

Mycgr3G36449\_Mycgr3T

Mycgr3G35528 Mycgr3T
  
Location: 21986-22844

Mycgr3G35528\_Mycgr3T

Mycgr3G35932 Mycgr3T
  
Location: 22944-24390

Mycgr3G35932\_Mycgr3T

Mycgr3G23761 Mycgr3T
  
Location: 24490-25825

Mycgr3G23761\_Mycgr3T

Mycgr3G35535 Mycgr3T
  
Location: 25925-26429

Mycgr3G35535\_Mycgr3T

Mycgr3G9942 Mycgr3T9
  
Location: 26529-30375

Mycgr3G9942\_Mycgr3T9

short chain dehydrogenase/reductase, putative
  
Accession: EED17220
  
Location: 3873650-3874893
  
 NCBI BlastP on this gene

EED17220

hypothetical protein
  
Accession: EED17221
  
Location: 3880622-3881014
  
 NCBI BlastP on this gene

EED17221

conserved hypothetical protein
  
Accession: EED17222
  
Location: 3882261-3882781
  
 NCBI BlastP on this gene

EED17222

conserved hypothetical protein
  
Accession: EED17223
  
Location: 3883055-3884197
  
 NCBI BlastP on this gene

EED17223

hypothetical protein
  
Accession: EED17224
  
Location: 3884280-3886346
  
 NCBI BlastP on this gene

EED17224

multidrug resistance-associated protein, putative
  
Accession: EED17225
  
Location: 3886590-3887537
  
 NCBI BlastP on this gene

EED17225

adenylate cyclase, putative
  
Accession: EED17226
  
Location: 3889022-3890743
  
  
**BlastP hit with Mycgr3G23761\_Mycgr3T**
  
Percentage identity: 36 %
  
BlastP bit score: 247
  
Sequence coverage: 100 %
  
E-value: 2e-72
  
  
 NCBI BlastP on this gene

EED17226

conserved hypothetical protein
  
Accession: EED17227
  
Location: 3890946-3891405
  
 NCBI BlastP on this gene

EED17227

conserved hypothetical protein
  
Accession: EED17228
  
Location: 3892368-3893436
  
  
**BlastP hit with Mycgr3G35535\_Mycgr3T**
  
Percentage identity: 28 %
  
BlastP bit score: 76
  
Sequence coverage: 82 %
  
E-value: 4e-14
  
  
 NCBI BlastP on this gene

EED17228

hypothetical protein
  
Accession: EED17229
  
Location: 3893804-3894088
  
 NCBI BlastP on this gene

EED17229

Query: Architecture Search FASTA input

KE145371 : Glarea lozoyensis ATCC 20868 chromosome Unknown GLAREA7    Total score: 2.0     Cumulative Blast bit score: 316

Hit cluster cross-links:

Mycgr3G36335 Mycgr3T
  
Location: 0-423

Mycgr3G36335\_Mycgr3T

Mycgr3G84494 Mycgr3T
  
Location: 523-2047

Mycgr3G84494\_Mycgr3T

Mycgr3G90558 Mycgr3T
  
Location: 2147-15296

Mycgr3G90558\_Mycgr3T

Mycgr3G68036 Mycgr3T
  
Location: 15396-16395

Mycgr3G68036\_Mycgr3T

Mycgr3G90561 Mycgr3T
  
Location: 16495-17134

Mycgr3G90561\_Mycgr3T

Mycgr3G35862 Mycgr3T
  
Location: 17234-18662

Mycgr3G35862\_Mycgr3T

Mycgr3G68030 Mycgr3T
  
Location: 18762-19722

Mycgr3G68030\_Mycgr3T

Mycgr3G36449 Mycgr3T
  
Location: 19822-21886

Mycgr3G36449\_Mycgr3T

Mycgr3G35528 Mycgr3T
  
Location: 21986-22844

Mycgr3G35528\_Mycgr3T

Mycgr3G35932 Mycgr3T
  
Location: 22944-24390

Mycgr3G35932\_Mycgr3T

Mycgr3G23761 Mycgr3T
  
Location: 24490-25825

Mycgr3G23761\_Mycgr3T

Mycgr3G35535 Mycgr3T
  
Location: 25925-26429

Mycgr3G35535\_Mycgr3T

Mycgr3G9942 Mycgr3T9
  
Location: 26529-30375

Mycgr3G9942\_Mycgr3T9

Nucleic acid-binding protein
  
Accession: EPE26323
  
Location: 3180192-3183364
  
 NCBI BlastP on this gene

EPE26323

MFS general substrate transporter
  
Accession: EPE26322
  
Location: 3174631-3177033
  
 NCBI BlastP on this gene

EPE26322

alpha/beta-Hydrolase
  
Accession: EPE26321
  
Location: 3172044-3174118
  
 NCBI BlastP on this gene

EPE26321

Acyl-CoA N-acyltransferases (Nat)
  
Accession: EPE26320
  
Location: 3170813-3171427
  
 NCBI BlastP on this gene

EPE26320

hypothetical protein
  
Accession: EPE26319
  
Location: 3168739-3169948
  
 NCBI BlastP on this gene

EPE26319

hypothetical protein
  
Accession: EPE26318
  
Location: 3166706-3167739
  
  
**BlastP hit with Mycgr3G35535\_Mycgr3T**
  
Percentage identity: 31 %
  
BlastP bit score: 73
  
Sequence coverage: 83 %
  
E-value: 6e-13
  
  
 NCBI BlastP on this gene

EPE26318

WD40 repeat-like protein
  
Accession: EPE26317
  
Location: 3160953-3166211
  
 NCBI BlastP on this gene

EPE26317

hypothetical protein
  
Accession: EPE26316
  
Location: 3158917-3160343
  
  
**BlastP hit with Mycgr3G68030\_Mycgr3T**
  
Percentage identity: 39 %
  
BlastP bit score: 243
  
Sequence coverage: 98 %
  
E-value: 2e-73
  
  
 NCBI BlastP on this gene

EPE26316

Putative cyclase
  
Accession: EPE26315
  
Location: 3157491-3158610
  
 NCBI BlastP on this gene

EPE26315

P-loop containing nucleoside triphosphate hydrolase
  
Accession: EPE26314
  
Location: 3154777-3157192
  
 NCBI BlastP on this gene

EPE26314

RING/U-box
  
Accession: EPE26313
  
Location: 3153061-3154540
  
 NCBI BlastP on this gene

EPE26313

FAD/NAD(P)-binding protein
  
Accession: EPE26312
  
Location: 3150468-3151953
  
 NCBI BlastP on this gene

EPE26312

hypothetical protein
  
Accession: EPE26311
  
Location: 3149013-3150185
  
 NCBI BlastP on this gene

EPE26311

hypothetical protein
  
Accession: EPE26310
  
Location: 3146551-3147702
  
 NCBI BlastP on this gene

EPE26310

Query: Architecture Search FASTA input

AABX02000023 : Neurospora crassa OR74A    Total score: 2.0     Cumulative Blast bit score: 265

Hit cluster cross-links:

Mycgr3G36335 Mycgr3T
  
Location: 0-423

Mycgr3G36335\_Mycgr3T

Mycgr3G84494 Mycgr3T
  
Location: 523-2047

Mycgr3G84494\_Mycgr3T

Mycgr3G90558 Mycgr3T
  
Location: 2147-15296

Mycgr3G90558\_Mycgr3T

Mycgr3G68036 Mycgr3T
  
Location: 15396-16395

Mycgr3G68036\_Mycgr3T

Mycgr3G90561 Mycgr3T
  
Location: 16495-17134

Mycgr3G90561\_Mycgr3T

Mycgr3G35862 Mycgr3T
  
Location: 17234-18662

Mycgr3G35862\_Mycgr3T

Mycgr3G68030 Mycgr3T
  
Location: 18762-19722

Mycgr3G68030\_Mycgr3T

Mycgr3G36449 Mycgr3T
  
Location: 19822-21886

Mycgr3G36449\_Mycgr3T

Mycgr3G35528 Mycgr3T
  
Location: 21986-22844

Mycgr3G35528\_Mycgr3T

Mycgr3G35932 Mycgr3T
  
Location: 22944-24390

Mycgr3G35932\_Mycgr3T

Mycgr3G23761 Mycgr3T
  
Location: 24490-25825

Mycgr3G23761\_Mycgr3T

Mycgr3G35535 Mycgr3T
  
Location: 25925-26429

Mycgr3G35535\_Mycgr3T

Mycgr3G9942 Mycgr3T9
  
Location: 26529-30375

Mycgr3G9942\_Mycgr3T9

peroxidase/catalase 2
  
Accession: EAA30509
  
Location: 473805-476066
  
 NCBI BlastP on this gene

EAA30509

predicted protein
  
Accession: EAA30508
  
Location: 478183-478890
  
 NCBI BlastP on this gene

EAA30508

conserved hypothetical protein
  
Accession: EAA30507
  
Location: 480936-481719
  
 NCBI BlastP on this gene

EAA30507

predicted protein
  
Accession: EAA30506
  
Location: 483147-485198
  
 NCBI BlastP on this gene

EAA30506

conserved hypothetical protein
  
Accession: EAA30505
  
Location: 489516-490481
  
  
**BlastP hit with Mycgr3G90561\_Mycgr3T**
  
Percentage identity: 31 %
  
BlastP bit score: 66
  
Sequence coverage: 61 %
  
E-value: 3e-10
  
  
 NCBI BlastP on this gene

EAA30505

conserved hypothetical protein
  
Accession: EAA30504
  
Location: 491221-497912
  
  
**BlastP hit with Mycgr3G36449\_Mycgr3T**
  
Percentage identity: 30 %
  
BlastP bit score: 199
  
Sequence coverage: 82 %
  
E-value: 8e-50
  
  
 NCBI BlastP on this gene

EAA30504

hypothetical protein
  
Accession: EAA30503
  
Location: 499619-500617
  
 NCBI BlastP on this gene

EAA30503

predicted protein
  
Accession: EAA30502
  
Location: 502393-503130
  
 NCBI BlastP on this gene

EAA30502

conserved hypothetical protein
  
Accession: EAA30501
  
Location: 503676-505571
  
 NCBI BlastP on this gene

EAA30501

hypothetical protein
  
Accession: EAA30500
  
Location: 506702-507079
  
 NCBI BlastP on this gene

EAA30500

predicted protein
  
Accession: EAA30499
  
Location: 508590-509288
  
 NCBI BlastP on this gene

EAA30499

predicted protein
  
Accession: EAA30498
  
Location: 510042-510848
  
 NCBI BlastP on this gene

EAA30498

Query: Architecture Search FASTA input

JH126400 : Cordyceps militaris CM01 unplaced genomic scaffold CCM\_S00002    Total score: 2.0     Cumulative Blast bit score: 225

Hit cluster cross-links:

Mycgr3G36335 Mycgr3T
  
Location: 0-423

Mycgr3G36335\_Mycgr3T

Mycgr3G84494 Mycgr3T
  
Location: 523-2047

Mycgr3G84494\_Mycgr3T

Mycgr3G90558 Mycgr3T
  
Location: 2147-15296

Mycgr3G90558\_Mycgr3T

Mycgr3G68036 Mycgr3T
  
Location: 15396-16395

Mycgr3G68036\_Mycgr3T

Mycgr3G90561 Mycgr3T
  
Location: 16495-17134

Mycgr3G90561\_Mycgr3T

Mycgr3G35862 Mycgr3T
  
Location: 17234-18662

Mycgr3G35862\_Mycgr3T

Mycgr3G68030 Mycgr3T
  
Location: 18762-19722

Mycgr3G68030\_Mycgr3T

Mycgr3G36449 Mycgr3T
  
Location: 19822-21886

Mycgr3G36449\_Mycgr3T

Mycgr3G35528 Mycgr3T
  
Location: 21986-22844

Mycgr3G35528\_Mycgr3T

Mycgr3G35932 Mycgr3T
  
Location: 22944-24390

Mycgr3G35932\_Mycgr3T

Mycgr3G23761 Mycgr3T
  
Location: 24490-25825

Mycgr3G23761\_Mycgr3T

Mycgr3G35535 Mycgr3T
  
Location: 25925-26429

Mycgr3G35535\_Mycgr3T

Mycgr3G9942 Mycgr3T9
  
Location: 26529-30375

Mycgr3G9942\_Mycgr3T9

pyridoxamine phosphate oxidase family protein
  
Accession: EGX93785
  
Location: 353946-354611
  
 NCBI BlastP on this gene

EGX93785

NRPS-like enzyme, putative
  
Accession: EGX93786
  
Location: 355014-356476
  
 NCBI BlastP on this gene

EGX93786

dimethylaniline monooxygenase, putative
  
Accession: EGX93787
  
Location: 356715-358363
  
 NCBI BlastP on this gene

EGX93787

FAD dependent oxidoreductase
  
Accession: EGX93788
  
Location: 359341-360667
  
 NCBI BlastP on this gene

EGX93788

tyrosinase, putative
  
Accession: EGX93789
  
Location: 360785-362095
  
 NCBI BlastP on this gene

EGX93789

hypothetical protein
  
Accession: EGX93790
  
Location: 362590-363523
  
  
**BlastP hit with Mycgr3G90561\_Mycgr3T**
  
Percentage identity: 33 %
  
BlastP bit score: 55
  
Sequence coverage: 53 %
  
E-value: 4e-06
  
  
 NCBI BlastP on this gene

EGX93790

hypothetical protein
  
Accession: EGX93791
  
Location: 364171-364900
  
 NCBI BlastP on this gene

EGX93791

Cytochrome P450
  
Accession: EGX93792
  
Location: 365954-368259
  
 NCBI BlastP on this gene

EGX93792

C6 transcription factor, putative
  
Accession: EGX93793
  
Location: 369669-371644
  
 NCBI BlastP on this gene

EGX93793

hypothetical protein
  
Accession: EGX93794
  
Location: 371724-372671
  
 NCBI BlastP on this gene

EGX93794

glutathione S-transferase, putative
  
Accession: EGX93795
  
Location: 373004-373874
  
 NCBI BlastP on this gene

EGX93795

gamma-glutamyltranspeptidase
  
Accession: EGX93796
  
Location: 374715-376689
  
 NCBI BlastP on this gene

EGX93796

MFS multidrug transporter, putative
  
Accession: EGX93797
  
Location: 377774-379684
  
  
**BlastP hit with Mycgr3G23761\_Mycgr3T**
  
Percentage identity: 28 %
  
BlastP bit score: 171
  
Sequence coverage: 99 %
  
E-value: 2e-44
  
  
 NCBI BlastP on this gene

EGX93797

mannosyltransferase
  
Accession: EGX93798
  
Location: 382261-383963
  
 NCBI BlastP on this gene

EGX93798

serine/threonine protein kinase, putative
  
Accession: EGX93799
  
Location: 384871-390200
  
 NCBI BlastP on this gene

EGX93799

Query: Architecture Search FASTA input

KB725679 : Colletotrichum orbiculare MAFF 240422 unplaced genomic scaffold Scaffold\_14    Total score: 2.0     Cumulative Blast bit score: 221

Hit cluster cross-links:

Mycgr3G36335 Mycgr3T
  
Location: 0-423

Mycgr3G36335\_Mycgr3T

Mycgr3G84494 Mycgr3T
  
Location: 523-2047

Mycgr3G84494\_Mycgr3T

Mycgr3G90558 Mycgr3T
  
Location: 2147-15296

Mycgr3G90558\_Mycgr3T

Mycgr3G68036 Mycgr3T
  
Location: 15396-16395

Mycgr3G68036\_Mycgr3T

Mycgr3G90561 Mycgr3T
  
Location: 16495-17134

Mycgr3G90561\_Mycgr3T

Mycgr3G35862 Mycgr3T
  
Location: 17234-18662

Mycgr3G35862\_Mycgr3T

Mycgr3G68030 Mycgr3T
  
Location: 18762-19722

Mycgr3G68030\_Mycgr3T

Mycgr3G36449 Mycgr3T
  
Location: 19822-21886

Mycgr3G36449\_Mycgr3T

Mycgr3G35528 Mycgr3T
  
Location: 21986-22844

Mycgr3G35528\_Mycgr3T

Mycgr3G35932 Mycgr3T
  
Location: 22944-24390

Mycgr3G35932\_Mycgr3T

Mycgr3G23761 Mycgr3T
  
Location: 24490-25825

Mycgr3G23761\_Mycgr3T

Mycgr3G35535 Mycgr3T
  
Location: 25925-26429

Mycgr3G35535\_Mycgr3T

Mycgr3G9942 Mycgr3T9
  
Location: 26529-30375

Mycgr3G9942\_Mycgr3T9

MFS transporter
  
Accession: ENH87622
  
Location: 40036-41710
  
  
**BlastP hit with Mycgr3G23761\_Mycgr3T**
  
Percentage identity: 27 %
  
BlastP bit score: 163
  
Sequence coverage: 98 %
  
E-value: 5e-41
  
  
 NCBI BlastP on this gene

ENH87622

hypothetical protein
  
Accession: ENH87621
  
Location: 35791-38940
  
 NCBI BlastP on this gene

ENH87621

minor allergen alt a 7
  
Accession: ENH87620
  
Location: 34230-34872
  
 NCBI BlastP on this gene

ENH87620

nad dependent epimerase dehydratase
  
Accession: ENH87619
  
Location: 30372-31163
  
 NCBI BlastP on this gene

ENH87619

MFS monocarboxylate
  
Accession: ENH87618
  
Location: 28939-30288
  
 NCBI BlastP on this gene

ENH87618

hypothetical protein
  
Accession: ENH87617
  
Location: 25469-26495
  
  
**BlastP hit with Mycgr3G90561\_Mycgr3T**
  
Percentage identity: 33 %
  
BlastP bit score: 58
  
Sequence coverage: 49 %
  
E-value: 3e-07
  
  
 NCBI BlastP on this gene

ENH87617

hypothetical protein
  
Accession: ENH87616
  
Location: 23225-24679
  
 NCBI BlastP on this gene

ENH87616

RNA recognition motif-containing protein
  
Accession: ENH87615
  
Location: 17963-19325
  
 NCBI BlastP on this gene

ENH87615

40s ribosomal protein s8
  
Accession: ENH87614
  
Location: 16150-16638
  
 NCBI BlastP on this gene

ENH87614

Query: Architecture Search FASTA input

CM001208 : Mycosphaerella graminicola IPO323 chromosome 13    Total score: 2.0     Cumulative Blast bit score: 207

Hit cluster cross-links:

Mycgr3G36335 Mycgr3T
  
Location: 0-423

Mycgr3G36335\_Mycgr3T

Mycgr3G84494 Mycgr3T
  
Location: 523-2047

Mycgr3G84494\_Mycgr3T

Mycgr3G90558 Mycgr3T
  
Location: 2147-15296

Mycgr3G90558\_Mycgr3T

Mycgr3G68036 Mycgr3T
  
Location: 15396-16395

Mycgr3G68036\_Mycgr3T

Mycgr3G90561 Mycgr3T
  
Location: 16495-17134

Mycgr3G90561\_Mycgr3T

Mycgr3G35862 Mycgr3T
  
Location: 17234-18662

Mycgr3G35862\_Mycgr3T

Mycgr3G68030 Mycgr3T
  
Location: 18762-19722

Mycgr3G68030\_Mycgr3T

Mycgr3G36449 Mycgr3T
  
Location: 19822-21886

Mycgr3G36449\_Mycgr3T

Mycgr3G35528 Mycgr3T
  
Location: 21986-22844

Mycgr3G35528\_Mycgr3T

Mycgr3G35932 Mycgr3T
  
Location: 22944-24390

Mycgr3G35932\_Mycgr3T

Mycgr3G23761 Mycgr3T
  
Location: 24490-25825

Mycgr3G23761\_Mycgr3T

Mycgr3G35535 Mycgr3T
  
Location: 25925-26429

Mycgr3G35535\_Mycgr3T

Mycgr3G9942 Mycgr3T9
  
Location: 26529-30375

Mycgr3G9942\_Mycgr3T9

beta-1,3 glucanosyltransferase
  
Accession: EGP82603
  
Location: 810930-812364
  
 NCBI BlastP on this gene

EGP82603

hypothetical protein
  
Accession: EGP82604
  
Location: 813075-814166
  
 NCBI BlastP on this gene

EGP82604

hypothetical protein
  
Accession: EGP82605
  
Location: 816529-817518
  
 NCBI BlastP on this gene

EGP82605

hypothetical protein
  
Accession: EGP82606
  
Location: 818318-819852
  
 NCBI BlastP on this gene

EGP82606

hypothetical protein
  
Accession: EGP82607
  
Location: 820315-821325
  
  
**BlastP hit with Mycgr3G68030\_Mycgr3T**
  
Percentage identity: 32 %
  
BlastP bit score: 148
  
Sequence coverage: 96 %
  
E-value: 4e-38
  
  
 NCBI BlastP on this gene

EGP82607

hypothetical protein
  
Accession: EGP82691
  
Location: 822110-823005
  
 NCBI BlastP on this gene

EGP82691

hypothetical protein
  
Accession: EGP82690
  
Location: 823635-824970
  
 NCBI BlastP on this gene

EGP82690

hypothetical protein
  
Accession: EGP82689
  
Location: 826641-827711
  
 NCBI BlastP on this gene

EGP82689

hypothetical protein
  
Accession: EGP82608
  
Location: 828199-829995
  
 NCBI BlastP on this gene

EGP82608

hypothetical protein
  
Accession: EGP82609
  
Location: 831385-833550
  
 NCBI BlastP on this gene

EGP82609

hypothetical protein
  
Accession: EGP82610
  
Location: 836491-837157
  
  
**BlastP hit with Mycgr3G35535\_Mycgr3T**
  
Percentage identity: 27 %
  
BlastP bit score: 60
  
Sequence coverage: 72 %
  
E-value: 7e-09
  
  
 NCBI BlastP on this gene

EGP82610

hypothetical protein
  
Accession: EGP82688
  
Location: 838404-839936
  
 NCBI BlastP on this gene

EGP82688

hypothetical protein
  
Accession: EGP82687
  
Location: 840173-845299
  
 NCBI BlastP on this gene

EGP82687

putative alpha-glucosidase
  
Accession: EGP82611
  
Location: 846383-849673
  
 NCBI BlastP on this gene

EGP82611

Query: Architecture Search FASTA input

KB644411 : Penicillium oxalicum 114-2 unplaced genomic scaffold scaffold\_4    Total score: 2.0     Cumulative Blast bit score: 206

Hit cluster cross-links:

Mycgr3G36335 Mycgr3T
  
Location: 0-423

Mycgr3G36335\_Mycgr3T

Mycgr3G84494 Mycgr3T
  
Location: 523-2047

Mycgr3G84494\_Mycgr3T

Mycgr3G90558 Mycgr3T
  
Location: 2147-15296

Mycgr3G90558\_Mycgr3T

Mycgr3G68036 Mycgr3T
  
Location: 15396-16395

Mycgr3G68036\_Mycgr3T

Mycgr3G90561 Mycgr3T
  
Location: 16495-17134

Mycgr3G90561\_Mycgr3T

Mycgr3G35862 Mycgr3T
  
Location: 17234-18662

Mycgr3G35862\_Mycgr3T

Mycgr3G68030 Mycgr3T
  
Location: 18762-19722

Mycgr3G68030\_Mycgr3T

Mycgr3G36449 Mycgr3T
  
Location: 19822-21886

Mycgr3G36449\_Mycgr3T

Mycgr3G35528 Mycgr3T
  
Location: 21986-22844

Mycgr3G35528\_Mycgr3T

Mycgr3G35932 Mycgr3T
  
Location: 22944-24390

Mycgr3G35932\_Mycgr3T

Mycgr3G23761 Mycgr3T
  
Location: 24490-25825

Mycgr3G23761\_Mycgr3T

Mycgr3G35535 Mycgr3T
  
Location: 25925-26429

Mycgr3G35535\_Mycgr3T

Mycgr3G9942 Mycgr3T9
  
Location: 26529-30375

Mycgr3G9942\_Mycgr3T9

hypothetical protein
  
Accession: EPS28973
  
Location: 1809385-1811415
  
 NCBI BlastP on this gene

EPS28973

hypothetical protein
  
Accession: EPS28974
  
Location: 1813216-1814881
  
 NCBI BlastP on this gene

EPS28974

hypothetical protein
  
Accession: EPS28975
  
Location: 1815283-1820827
  
 NCBI BlastP on this gene

EPS28975

hypothetical protein
  
Accession: EPS28976
  
Location: 1822821-1823692
  
 NCBI BlastP on this gene

EPS28976

hypothetical protein
  
Accession: EPS28977
  
Location: 1824484-1825589
  
  
**BlastP hit with Mycgr3G35535\_Mycgr3T**
  
Percentage identity: 28 %
  
BlastP bit score: 55
  
Sequence coverage: 83 %
  
E-value: 1e-06
  
  
 NCBI BlastP on this gene

EPS28977

hypothetical protein
  
Accession: EPS28978
  
Location: 1827526-1828688
  
 NCBI BlastP on this gene

EPS28978

hypothetical protein
  
Accession: EPS28979
  
Location: 1828767-1830349
  
  
**BlastP hit with Mycgr3G23761\_Mycgr3T**
  
Percentage identity: 30 %
  
BlastP bit score: 152
  
Sequence coverage: 92 %
  
E-value: 2e-37
  
  
 NCBI BlastP on this gene

EPS28979

hypothetical protein
  
Accession: EPS28980
  
Location: 1831447-1841720
  
 NCBI BlastP on this gene

EPS28980

hypothetical protein
  
Accession: EPS28981
  
Location: 1842874-1843885
  
 NCBI BlastP on this gene

EPS28981

hypothetical protein
  
Accession: EPS28982
  
Location: 1844758-1845714
  
 NCBI BlastP on this gene

EPS28982

Query: Architecture Search FASTA input

GG697358 : Glomerella graminicola M1.001 genomic scaffold supercont1.28    Total score: 2.0     Cumulative Blast bit score: 206

Hit cluster cross-links:

Mycgr3G36335 Mycgr3T
  
Location: 0-423

Mycgr3G36335\_Mycgr3T

Mycgr3G84494 Mycgr3T
  
Location: 523-2047

Mycgr3G84494\_Mycgr3T

Mycgr3G90558 Mycgr3T
  
Location: 2147-15296

Mycgr3G90558\_Mycgr3T

Mycgr3G68036 Mycgr3T
  
Location: 15396-16395

Mycgr3G68036\_Mycgr3T

Mycgr3G90561 Mycgr3T
  
Location: 16495-17134

Mycgr3G90561\_Mycgr3T

Mycgr3G35862 Mycgr3T
  
Location: 17234-18662

Mycgr3G35862\_Mycgr3T

Mycgr3G68030 Mycgr3T
  
Location: 18762-19722

Mycgr3G68030\_Mycgr3T

Mycgr3G36449 Mycgr3T
  
Location: 19822-21886

Mycgr3G36449\_Mycgr3T

Mycgr3G35528 Mycgr3T
  
Location: 21986-22844

Mycgr3G35528\_Mycgr3T

Mycgr3G35932 Mycgr3T
  
Location: 22944-24390

Mycgr3G35932\_Mycgr3T

Mycgr3G23761 Mycgr3T
  
Location: 24490-25825

Mycgr3G23761\_Mycgr3T

Mycgr3G35535 Mycgr3T
  
Location: 25925-26429

Mycgr3G35535\_Mycgr3T

Mycgr3G9942 Mycgr3T9
  
Location: 26529-30375

Mycgr3G9942\_Mycgr3T9

hypothetical protein
  
Accession: EFQ31900
  
Location: 223013-223906
  
 NCBI BlastP on this gene

EFQ31900

major facilitator superfamily transporter
  
Accession: EFQ31901
  
Location: 229026-230733
  
  
**BlastP hit with Mycgr3G23761\_Mycgr3T**
  
Percentage identity: 27 %
  
BlastP bit score: 149
  
Sequence coverage: 97 %
  
E-value: 3e-36
  
  
 NCBI BlastP on this gene

EFQ31901

hypothetical protein
  
Accession: EFQ31902
  
Location: 232011-232988
  
 NCBI BlastP on this gene

EFQ31902

hypothetical protein
  
Accession: EFQ31903
  
Location: 234969-235898
  
 NCBI BlastP on this gene

EFQ31903

quinone oxidoreductase
  
Accession: EFQ31904
  
Location: 236769-237666
  
 NCBI BlastP on this gene

EFQ31904

major facilitator superfamily transporter
  
Accession: EFQ31905
  
Location: 240177-241544
  
 NCBI BlastP on this gene

EFQ31905

hypothetical protein
  
Accession: EFQ31906
  
Location: 243962-244959
  
  
**BlastP hit with Mycgr3G90561\_Mycgr3T**
  
Percentage identity: 33 %
  
BlastP bit score: 57
  
Sequence coverage: 50 %
  
E-value: 9e-07
  
  
 NCBI BlastP on this gene

EFQ31906

translation elongation factor G
  
Accession: EFQ31907
  
Location: 245549-248034
  
 NCBI BlastP on this gene

EFQ31907

hypothetical protein
  
Accession: EFQ31908
  
Location: 253023-254950
  
 NCBI BlastP on this gene

EFQ31908

Query: Architecture Search FASTA input

JH795672 : Magnaporthe oryzae P131 unplaced genomic scaffold P131\_scaffold00357    Total score: 2.0     Cumulative Blast bit score: 171

Hit cluster cross-links:

Mycgr3G36335 Mycgr3T
  
Location: 0-423

Mycgr3G36335\_Mycgr3T

Mycgr3G84494 Mycgr3T
  
Location: 523-2047

Mycgr3G84494\_Mycgr3T

Mycgr3G90558 Mycgr3T
  
Location: 2147-15296

Mycgr3G90558\_Mycgr3T

Mycgr3G68036 Mycgr3T
  
Location: 15396-16395

Mycgr3G68036\_Mycgr3T

Mycgr3G90561 Mycgr3T
  
Location: 16495-17134

Mycgr3G90561\_Mycgr3T

Mycgr3G35862 Mycgr3T
  
Location: 17234-18662

Mycgr3G35862\_Mycgr3T

Mycgr3G68030 Mycgr3T
  
Location: 18762-19722

Mycgr3G68030\_Mycgr3T

Mycgr3G36449 Mycgr3T
  
Location: 19822-21886

Mycgr3G36449\_Mycgr3T

Mycgr3G35528 Mycgr3T
  
Location: 21986-22844

Mycgr3G35528\_Mycgr3T

Mycgr3G35932 Mycgr3T
  
Location: 22944-24390

Mycgr3G35932\_Mycgr3T

Mycgr3G23761 Mycgr3T
  
Location: 24490-25825

Mycgr3G23761\_Mycgr3T

Mycgr3G35535 Mycgr3T
  
Location: 25925-26429

Mycgr3G35535\_Mycgr3T

Mycgr3G9942 Mycgr3T9
  
Location: 26529-30375

Mycgr3G9942\_Mycgr3T9

cyclohexanone 1,2-monooxygenase
  
Accession: ELQ66784
  
Location: 16302-20208
  
 NCBI BlastP on this gene

ELQ66784

averantin oxidoreductase
  
Accession: ELQ66783
  
Location: 13801-15633
  
 NCBI BlastP on this gene

ELQ66783

geranylgeranyl pyrophosphate synthetase
  
Accession: ELQ66782
  
Location: 9642-11919
  
 NCBI BlastP on this gene

ELQ66782

hypothetical protein
  
Accession: ELQ66781
  
Location: 7816-9346
  
 NCBI BlastP on this gene

ELQ66781

hypothetical protein
  
Accession: ELQ66780
  
Location: 5389-6425
  
 NCBI BlastP on this gene

ELQ66780

hypothetical protein
  
Accession: ELQ66779
  
Location: 2837-4322
  
  
**BlastP hit with Mycgr3G35535\_Mycgr3T**
  
Percentage identity: 30 %
  
BlastP bit score: 58
  
Sequence coverage: 85 %
  
E-value: 2e-07
  
  
 NCBI BlastP on this gene

ELQ66779

hypothetical protein
  
Accession: ELQ66778
  
Location: 49-1696
  
  
**BlastP hit with Mycgr3G90561\_Mycgr3T**
  
Percentage identity: 32 %
  
BlastP bit score: 58
  
Sequence coverage: 60 %
  
E-value: 3e-07
  
  
  
**BlastP hit with Mycgr3G35535\_Mycgr3T**
  
Percentage identity: 34 %
  
BlastP bit score: 55
  
Sequence coverage: 60 %
  
E-value: 2e-06
  
  
 NCBI BlastP on this gene

ELQ66778

Query: Architecture Search FASTA input

CACQ02003267 : Colletotrichum higginsianum strain IMI 349063    Total score: 2.0     Cumulative Blast bit score: 171

Hit cluster cross-links:

Mycgr3G36335 Mycgr3T
  
Location: 0-423

Mycgr3G36335\_Mycgr3T

Mycgr3G84494 Mycgr3T
  
Location: 523-2047

Mycgr3G84494\_Mycgr3T

Mycgr3G90558 Mycgr3T
  
Location: 2147-15296

Mycgr3G90558\_Mycgr3T

Mycgr3G68036 Mycgr3T
  
Location: 15396-16395

Mycgr3G68036\_Mycgr3T

Mycgr3G90561 Mycgr3T
  
Location: 16495-17134

Mycgr3G90561\_Mycgr3T

Mycgr3G35862 Mycgr3T
  
Location: 17234-18662

Mycgr3G35862\_Mycgr3T

Mycgr3G68030 Mycgr3T
  
Location: 18762-19722

Mycgr3G68030\_Mycgr3T

Mycgr3G36449 Mycgr3T
  
Location: 19822-21886

Mycgr3G36449\_Mycgr3T

Mycgr3G35528 Mycgr3T
  
Location: 21986-22844

Mycgr3G35528\_Mycgr3T

Mycgr3G35932 Mycgr3T
  
Location: 22944-24390

Mycgr3G35932\_Mycgr3T

Mycgr3G23761 Mycgr3T
  
Location: 24490-25825

Mycgr3G23761\_Mycgr3T

Mycgr3G35535 Mycgr3T
  
Location: 25925-26429

Mycgr3G35535\_Mycgr3T

Mycgr3G9942 Mycgr3T9
  
Location: 26529-30375

Mycgr3G9942\_Mycgr3T9

hypothetical protein
  
Accession: CCF39013
  
Location: 358-1133
  
  
**BlastP hit with Mycgr3G35535\_Mycgr3T**
  
Percentage identity: 33 %
  
BlastP bit score: 54
  
Sequence coverage: 55 %
  
E-value: 1e-06
  
  
 NCBI BlastP on this gene

CCF39013

hypothetical protein
  
Accession: CCF39014
  
Location: 1939-2995
  
  
**BlastP hit with Mycgr3G90561\_Mycgr3T**
  
Percentage identity: 29 %
  
BlastP bit score: 59
  
Sequence coverage: 58 %
  
E-value: 9e-08
  
  
  
**BlastP hit with Mycgr3G35535\_Mycgr3T**
  
Percentage identity: 33 %
  
BlastP bit score: 58
  
Sequence coverage: 71 %
  
E-value: 1e-07
  
  
 NCBI BlastP on this gene

CCF39014

major facilitator superfamily transporter
  
Accession: CCF39015
  
Location: 3137-4402
  
 NCBI BlastP on this gene

CCF39015

Query: Architecture Search FASTA input

CM001234 : Magnaporthe oryzae 70-15 chromosome 4    Total score: 2.0     Cumulative Blast bit score: 169

Hit cluster cross-links:

Mycgr3G36335 Mycgr3T
  
Location: 0-423

Mycgr3G36335\_Mycgr3T

Mycgr3G84494 Mycgr3T
  
Location: 523-2047

Mycgr3G84494\_Mycgr3T

Mycgr3G90558 Mycgr3T
  
Location: 2147-15296

Mycgr3G90558\_Mycgr3T

Mycgr3G68036 Mycgr3T
  
Location: 15396-16395

Mycgr3G68036\_Mycgr3T

Mycgr3G90561 Mycgr3T
  
Location: 16495-17134

Mycgr3G90561\_Mycgr3T

Mycgr3G35862 Mycgr3T
  
Location: 17234-18662

Mycgr3G35862\_Mycgr3T

Mycgr3G68030 Mycgr3T
  
Location: 18762-19722

Mycgr3G68030\_Mycgr3T

Mycgr3G36449 Mycgr3T
  
Location: 19822-21886

Mycgr3G36449\_Mycgr3T

Mycgr3G35528 Mycgr3T
  
Location: 21986-22844

Mycgr3G35528\_Mycgr3T

Mycgr3G35932 Mycgr3T
  
Location: 22944-24390

Mycgr3G35932\_Mycgr3T

Mycgr3G23761 Mycgr3T
  
Location: 24490-25825

Mycgr3G23761\_Mycgr3T

Mycgr3G35535 Mycgr3T
  
Location: 25925-26429

Mycgr3G35535\_Mycgr3T

Mycgr3G9942 Mycgr3T9
  
Location: 26529-30375

Mycgr3G9942\_Mycgr3T9

hypothetical protein
  
Accession: EHA50180
  
Location: 1879947-1881930
  
 NCBI BlastP on this gene

EHA50180

hypothetical protein
  
Accession: EHA50181
  
Location: 1882368-1884353
  
 NCBI BlastP on this gene

EHA50181

averantin oxidoreductase
  
Accession: EHA50182
  
Location: 1885022-1886854
  
 NCBI BlastP on this gene

EHA50182

geranylgeranyl pyrophosphate synthetase
  
Accession: EHA50183
  
Location: 1888737-1891014
  
 NCBI BlastP on this gene

EHA50183

hypothetical protein
  
Accession: EHA50184
  
Location: 1891369-1892899
  
 NCBI BlastP on this gene

EHA50184

hypothetical protein
  
Accession: EHA50185
  
Location: 1894293-1895948
  
 NCBI BlastP on this gene

EHA50185

hypothetical protein
  
Accession: EHA50186
  
Location: 1896428-1897912
  
  
**BlastP hit with Mycgr3G35535\_Mycgr3T**
  
Percentage identity: 32 %
  
BlastP bit score: 54
  
Sequence coverage: 88 %
  
E-value: 3e-06
  
  
 NCBI BlastP on this gene

EHA50186

hypothetical protein
  
Accession: EHA50187
  
Location: 1898998-1900055
  
  
**BlastP hit with Mycgr3G90561\_Mycgr3T**
  
Percentage identity: 32 %
  
BlastP bit score: 60
  
Sequence coverage: 60 %
  
E-value: 3e-08
  
  
  
**BlastP hit with Mycgr3G35535\_Mycgr3T**
  
Percentage identity: 29 %
  
BlastP bit score: 55
  
Sequence coverage: 89 %
  
E-value: 9e-07
  
  
 NCBI BlastP on this gene

EHA50187

hypothetical protein
  
Accession: EHA50188
  
Location: 1900153-1901975
  
 NCBI BlastP on this gene

EHA50188

hypothetical protein
  
Accession: EHA50189
  
Location: 1903830-1905624
  
 NCBI BlastP on this gene

EHA50189

3-oxoacyl-[acyl-carrier-protein] reductase
  
Accession: EHA50190
  
Location: 1905897-1906791
  
 NCBI BlastP on this gene

EHA50190

hypothetical protein
  
Accession: EHA50191
  
Location: 1907641-1909165
  
 NCBI BlastP on this gene

EHA50191

oxidoreductase
  
Accession: EHA50192
  
Location: 1910018-1910707
  
 NCBI BlastP on this gene

EHA50192

enoyl reductase
  
Accession: EHA50193
  
Location: 1911131-1912707
  
 NCBI BlastP on this gene

EHA50193

hypothetical protein
  
Accession: EHA50194
  
Location: 1913437-1914470
  
 NCBI BlastP on this gene

EHA50194

hypothetical protein
  
Accession: EHA50195
  
Location: 1914666-1916366
  
 NCBI BlastP on this gene

EHA50195

Query: Architecture Search FASTA input

EQ962659 : Talaromyces stipitatus ATCC 10500 scf\_1105507295487 genomic scaffold    Total score: 2.0     Cumulative Blast bit score: 163

Hit cluster cross-links:

Mycgr3G36335 Mycgr3T
  
Location: 0-423

Mycgr3G36335\_Mycgr3T

Mycgr3G84494 Mycgr3T
  
Location: 523-2047

Mycgr3G84494\_Mycgr3T

Mycgr3G90558 Mycgr3T
  
Location: 2147-15296

Mycgr3G90558\_Mycgr3T

Mycgr3G68036 Mycgr3T
  
Location: 15396-16395

Mycgr3G68036\_Mycgr3T

Mycgr3G90561 Mycgr3T
  
Location: 16495-17134

Mycgr3G90561\_Mycgr3T

Mycgr3G35862 Mycgr3T
  
Location: 17234-18662

Mycgr3G35862\_Mycgr3T

Mycgr3G68030 Mycgr3T
  
Location: 18762-19722

Mycgr3G68030\_Mycgr3T

Mycgr3G36449 Mycgr3T
  
Location: 19822-21886

Mycgr3G36449\_Mycgr3T

Mycgr3G35528 Mycgr3T
  
Location: 21986-22844

Mycgr3G35528\_Mycgr3T

Mycgr3G35932 Mycgr3T
  
Location: 22944-24390

Mycgr3G35932\_Mycgr3T

Mycgr3G23761 Mycgr3T
  
Location: 24490-25825

Mycgr3G23761\_Mycgr3T

Mycgr3G35535 Mycgr3T
  
Location: 25925-26429

Mycgr3G35535\_Mycgr3T

Mycgr3G9942 Mycgr3T9
  
Location: 26529-30375

Mycgr3G9942\_Mycgr3T9

conserved hypothetical protein
  
Accession: EED12765
  
Location: 235814-241656
  
 NCBI BlastP on this gene

EED12765

conserved hypothetical protein
  
Accession: EED12766
  
Location: 241897-243811
  
 NCBI BlastP on this gene

EED12766

hypothetical protein
  
Accession: EED12767
  
Location: 244213-244617
  
 NCBI BlastP on this gene

EED12767

glycosyltransferase family protein
  
Accession: EED12768
  
Location: 245419-246293
  
 NCBI BlastP on this gene

EED12768

conserved hypothetical protein
  
Accession: EED12769
  
Location: 246577-247508
  
 NCBI BlastP on this gene

EED12769

hypothetical protein
  
Accession: EED12770
  
Location: 247856-248232
  
 NCBI BlastP on this gene

EED12770

hypothetical protein
  
Accession: EED12771
  
Location: 249662-250581
  
  
**BlastP hit with Mycgr3G90561\_Mycgr3T**
  
Percentage identity: 38 %
  
BlastP bit score: 81
  
Sequence coverage: 52 %
  
E-value: 1e-15
  
  
 NCBI BlastP on this gene

EED12771

conserved hypothetical protein
  
Accession: EED12773
  
Location: 251163-252037
  
  
**BlastP hit with Mycgr3G35535\_Mycgr3T**
  
Percentage identity: 32 %
  
BlastP bit score: 82
  
Sequence coverage: 90 %
  
E-value: 2e-16
  
  
 NCBI BlastP on this gene

EED12773

O-methyltransferase, putative
  
Accession: EED12775
  
Location: 252152-253324
  
 NCBI BlastP on this gene

EED12775

integral membrane protein
  
Accession: EED12777
  
Location: 254477-255474
  
 NCBI BlastP on this gene

EED12777

monocarboxylate transporter, putative
  
Accession: EED12778
  
Location: 255866-257293
  
 NCBI BlastP on this gene

EED12778

short-chain dehydrogenase, putative
  
Accession: EED12779
  
Location: 257849-259147
  
 NCBI BlastP on this gene

EED12779

methionyl-tRNA formyltransferase, putative
  
Accession: EED12780
  
Location: 259706-260658
  
 NCBI BlastP on this gene

EED12780

hypothetical protein
  
Accession: EED12781
  
Location: 260882-261720
  
 NCBI BlastP on this gene

EED12781

hypothetical protein
  
Accession: EED12782
  
Location: 262964-263422
  
 NCBI BlastP on this gene

EED12782

hypothetical protein
  
Accession: EED12783
  
Location: 263884-264455
  
 NCBI BlastP on this gene

EED12783

hypothetical protein
  
Accession: EED12784
  
Location: 264953-265790
  
 NCBI BlastP on this gene

EED12784

conserved hypothetical protein
  
Accession: EED12785
  
Location: 267308-268945
  
 NCBI BlastP on this gene

EED12785

Query: Architecture Search FASTA input

JH725221 : Beauveria bassiana ARSEF 2860 unplaced genomic scaffold BBA\_S00072    Total score: 2.0     Cumulative Blast bit score: 160

Hit cluster cross-links:

Mycgr3G36335 Mycgr3T
  
Location: 0-423

Mycgr3G36335\_Mycgr3T

Mycgr3G84494 Mycgr3T
  
Location: 523-2047

Mycgr3G84494\_Mycgr3T

Mycgr3G90558 Mycgr3T
  
Location: 2147-15296

Mycgr3G90558\_Mycgr3T

Mycgr3G68036 Mycgr3T
  
Location: 15396-16395

Mycgr3G68036\_Mycgr3T

Mycgr3G90561 Mycgr3T
  
Location: 16495-17134

Mycgr3G90561\_Mycgr3T

Mycgr3G35862 Mycgr3T
  
Location: 17234-18662

Mycgr3G35862\_Mycgr3T

Mycgr3G68030 Mycgr3T
  
Location: 18762-19722

Mycgr3G68030\_Mycgr3T

Mycgr3G36449 Mycgr3T
  
Location: 19822-21886

Mycgr3G36449\_Mycgr3T

Mycgr3G35528 Mycgr3T
  
Location: 21986-22844

Mycgr3G35528\_Mycgr3T

Mycgr3G35932 Mycgr3T
  
Location: 22944-24390

Mycgr3G35932\_Mycgr3T

Mycgr3G23761 Mycgr3T
  
Location: 24490-25825

Mycgr3G23761\_Mycgr3T

Mycgr3G35535 Mycgr3T
  
Location: 25925-26429

Mycgr3G35535\_Mycgr3T

Mycgr3G9942 Mycgr3T9
  
Location: 26529-30375

Mycgr3G9942\_Mycgr3T9

peptidase family M28 family
  
Accession: EJP61169
  
Location: 18302-21415
  
 NCBI BlastP on this gene

EJP61169

hypothetical protein
  
Accession: EJP61170
  
Location: 21800-22126
  
 NCBI BlastP on this gene

EJP61170

HhH-GPD superfamily base excision DNA repair protein
  
Accession: EJP61171
  
Location: 25707-26942
  
 NCBI BlastP on this gene

EJP61171

ribosomal protein L11
  
Accession: EJP61172
  
Location: 27227-27806
  
 NCBI BlastP on this gene

EJP61172

O-methyltransferase, family 3
  
Accession: EJP61173
  
Location: 28788-29492
  
 NCBI BlastP on this gene

EJP61173

monocarboxylate permease-like protein
  
Accession: EJP61174
  
Location: 29731-31234
  
 NCBI BlastP on this gene

EJP61174

hypothetical protein
  
Accession: EJP61175
  
Location: 32494-33470
  
 NCBI BlastP on this gene

EJP61175

hypothetical protein
  
Accession: EJP61176
  
Location: 33936-34416
  
 NCBI BlastP on this gene

EJP61176

hypothetical protein
  
Accession: EJP61177
  
Location: 35260-36175
  
  
**BlastP hit with Mycgr3G90561\_Mycgr3T**
  
Percentage identity: 40 %
  
BlastP bit score: 81
  
Sequence coverage: 55 %
  
E-value: 1e-15
  
  
 NCBI BlastP on this gene

EJP61177

hypothetical protein
  
Accession: EJP61178
  
Location: 36487-37534
  
  
**BlastP hit with Mycgr3G35535\_Mycgr3T**
  
Percentage identity: 30 %
  
BlastP bit score: 79
  
Sequence coverage: 90 %
  
E-value: 6e-15
  
  
 NCBI BlastP on this gene

EJP61178

sterigmatocystin 8-O-methyltransferase
  
Accession: EJP61179
  
Location: 37688-39172
  
 NCBI BlastP on this gene

EJP61179

integral membrane protein
  
Accession: EJP61180
  
Location: 39894-41250
  
 NCBI BlastP on this gene

EJP61180

rRNA assembly protein Mis3, putative
  
Accession: EJP61181
  
Location: 42100-43062
  
 NCBI BlastP on this gene

EJP61181

hypothetical protein
  
Accession: EJP61182
  
Location: 44056-44369
  
 NCBI BlastP on this gene

EJP61182

oxidoreductase-like protein
  
Accession: EJP61183
  
Location: 44716-45891
  
 NCBI BlastP on this gene

EJP61183

YeeE/YedE family protein
  
Accession: EJP61184
  
Location: 46834-47907
  
 NCBI BlastP on this gene

EJP61184

endoribonuclease L-PSP
  
Accession: EJP61185
  
Location: 47975-48425
  
 NCBI BlastP on this gene

EJP61185

GatB/YqeY family protein
  
Accession: EJP61186
  
Location: 48746-49351
  
 NCBI BlastP on this gene

EJP61186

putative succinyl-CoA ligase
  
Accession: EJP61187
  
Location: 49975-51511
  
 NCBI BlastP on this gene

EJP61187

Query: Architecture Search FASTA input

DS995901 : Penicillium marneffei ATCC 18224 scf\_1105668340960 genomic scaffold    Total score: 2.0     Cumulative Blast bit score: 157

Hit cluster cross-links:

Mycgr3G36335 Mycgr3T
  
Location: 0-423

Mycgr3G36335\_Mycgr3T

Mycgr3G84494 Mycgr3T
  
Location: 523-2047

Mycgr3G84494\_Mycgr3T

Mycgr3G90558 Mycgr3T
  
Location: 2147-15296

Mycgr3G90558\_Mycgr3T

Mycgr3G68036 Mycgr3T
  
Location: 15396-16395

Mycgr3G68036\_Mycgr3T

Mycgr3G90561 Mycgr3T
  
Location: 16495-17134

Mycgr3G90561\_Mycgr3T

Mycgr3G35862 Mycgr3T
  
Location: 17234-18662

Mycgr3G35862\_Mycgr3T

Mycgr3G68030 Mycgr3T
  
Location: 18762-19722

Mycgr3G68030\_Mycgr3T

Mycgr3G36449 Mycgr3T
  
Location: 19822-21886

Mycgr3G36449\_Mycgr3T

Mycgr3G35528 Mycgr3T
  
Location: 21986-22844

Mycgr3G35528\_Mycgr3T

Mycgr3G35932 Mycgr3T
  
Location: 22944-24390

Mycgr3G35932\_Mycgr3T

Mycgr3G23761 Mycgr3T
  
Location: 24490-25825

Mycgr3G23761\_Mycgr3T

Mycgr3G35535 Mycgr3T
  
Location: 25925-26429

Mycgr3G35535\_Mycgr3T

Mycgr3G9942 Mycgr3T9
  
Location: 26529-30375

Mycgr3G9942\_Mycgr3T9

trehalose-phosphate synthase/phosphatase complex subunit Tps1, putative
  
Accession: EEA23879
  
Location: 628393-630120
  
 NCBI BlastP on this gene

EEA23879

hypothetical protein
  
Accession: EEA23880
  
Location: 630754-631674
  
 NCBI BlastP on this gene

EEA23880

AAA family ATPase, putative
  
Accession: EEA23881
  
Location: 631886-634539
  
 NCBI BlastP on this gene

EEA23881

mitochondrial carrier protein, putative
  
Accession: EEA23882
  
Location: 635958-637358
  
 NCBI BlastP on this gene

EEA23882

RING finger protein
  
Accession: EEA23883
  
Location: 638534-640238
  
 NCBI BlastP on this gene

EEA23883

hypothetical protein
  
Accession: EEA23885
  
Location: 642058-642332
  
 NCBI BlastP on this gene

EEA23885

O-methyltransferase, putative
  
Accession: EEA23886
  
Location: 642675-644041
  
 NCBI BlastP on this gene

EEA23886

conserved hypothetical protein
  
Accession: EEA23887
  
Location: 645056-645879
  
  
**BlastP hit with Mycgr3G90561\_Mycgr3T**
  
Percentage identity: 33 %
  
BlastP bit score: 79
  
Sequence coverage: 55 %
  
E-value: 1e-14
  
  
 NCBI BlastP on this gene

EEA23887

conserved hypothetical protein
  
Accession: EEA23888
  
Location: 645896-646952
  
  
**BlastP hit with Mycgr3G35535\_Mycgr3T**
  
Percentage identity: 28 %
  
BlastP bit score: 78
  
Sequence coverage: 97 %
  
E-value: 7e-15
  
  
 NCBI BlastP on this gene

EEA23888

conserved hypothetical protein
  
Accession: EEA23889
  
Location: 647670-649149
  
 NCBI BlastP on this gene

EEA23889

translation initiation factor eif-2b epsilon subunit, putative
  
Accession: EEA23890
  
Location: 649395-651739
  
 NCBI BlastP on this gene

EEA23890

stress response transcription factor SrrA/Skn7, putative
  
Accession: EEA23891
  
Location: 652988-655243
  
 NCBI BlastP on this gene

EEA23891

conserved hypothetical protein
  
Accession: EEA23892
  
Location: 657213-657527
  
 NCBI BlastP on this gene

EEA23892

conserved hypothetical protein
  
Accession: EEA23893
  
Location: 657545-658237
  
 NCBI BlastP on this gene

EEA23893

glutathione S-transferase, putative
  
Accession: EEA23894
  
Location: 659249-660159
  
 NCBI BlastP on this gene

EEA23894

ER membrane protein Wsc4, putative
  
Accession: EEA23895
  
Location: 661498-662858
  
 NCBI BlastP on this gene

EEA23895

Query: Architecture Search FASTA input

DS995727 : Trichophyton equinum CBS 127.97 supercont1.10 genomic scaffold    Total score: 2.0     Cumulative Blast bit score: 150

Hit cluster cross-links:

Mycgr3G36335 Mycgr3T
  
Location: 0-423

Mycgr3G36335\_Mycgr3T

Mycgr3G84494 Mycgr3T
  
Location: 523-2047

Mycgr3G84494\_Mycgr3T

Mycgr3G90558 Mycgr3T
  
Location: 2147-15296

Mycgr3G90558\_Mycgr3T

Mycgr3G68036 Mycgr3T
  
Location: 15396-16395

Mycgr3G68036\_Mycgr3T

Mycgr3G90561 Mycgr3T
  
Location: 16495-17134

Mycgr3G90561\_Mycgr3T

Mycgr3G35862 Mycgr3T
  
Location: 17234-18662

Mycgr3G35862\_Mycgr3T

Mycgr3G68030 Mycgr3T
  
Location: 18762-19722

Mycgr3G68030\_Mycgr3T

Mycgr3G36449 Mycgr3T
  
Location: 19822-21886

Mycgr3G36449\_Mycgr3T

Mycgr3G35528 Mycgr3T
  
Location: 21986-22844

Mycgr3G35528\_Mycgr3T

Mycgr3G35932 Mycgr3T
  
Location: 22944-24390

Mycgr3G35932\_Mycgr3T

Mycgr3G23761 Mycgr3T
  
Location: 24490-25825

Mycgr3G23761\_Mycgr3T

Mycgr3G35535 Mycgr3T
  
Location: 25925-26429

Mycgr3G35535\_Mycgr3T

Mycgr3G9942 Mycgr3T9
  
Location: 26529-30375

Mycgr3G9942\_Mycgr3T9

hypothetical protein
  
Accession: EGE03382
  
Location: 126921-127484
  
 NCBI BlastP on this gene

EGE03382

hypothetical protein
  
Accession: EGE03383
  
Location: 128830-130434
  
 NCBI BlastP on this gene

EGE03383

metalloprotease MEP3
  
Accession: EGE03384
  
Location: 130924-133233
  
 NCBI BlastP on this gene

EGE03384

integral membrane protein
  
Accession: EGE03385
  
Location: 134572-135863
  
 NCBI BlastP on this gene

EGE03385

MFS monocarboxylate transporter
  
Accession: EGE03386
  
Location: 137171-138630
  
 NCBI BlastP on this gene

EGE03386

hypothetical protein
  
Accession: EGE03387
  
Location: 138744-139665
  
 NCBI BlastP on this gene

EGE03387

hypothetical protein
  
Accession: EGE03388
  
Location: 140815-141460
  
 NCBI BlastP on this gene

EGE03388

hypothetical protein
  
Accession: EGE03389
  
Location: 141716-142631
  
  
**BlastP hit with Mycgr3G90561\_Mycgr3T**
  
Percentage identity: 37 %
  
BlastP bit score: 77
  
Sequence coverage: 54 %
  
E-value: 5e-14
  
  
 NCBI BlastP on this gene

EGE03389

hypothetical protein
  
Accession: EGE03390
  
Location: 143242-144256
  
  
**BlastP hit with Mycgr3G35535\_Mycgr3T**
  
Percentage identity: 33 %
  
BlastP bit score: 73
  
Sequence coverage: 69 %
  
E-value: 6e-13
  
  
 NCBI BlastP on this gene

EGE03390

hypothetical protein
  
Accession: EGE03391
  
Location: 144389-145813
  
 NCBI BlastP on this gene

EGE03391

hypothetical protein
  
Accession: EGE03392
  
Location: 146776-147870
  
 NCBI BlastP on this gene

EGE03392

carbonate dehydratase
  
Accession: EGE03393
  
Location: 149560-150499
  
 NCBI BlastP on this gene

EGE03393

hypothetical protein
  
Accession: EGE03394
  
Location: 150915-151733
  
 NCBI BlastP on this gene

EGE03394

alkaline phosphatase
  
Accession: EGE03395
  
Location: 152224-153874
  
 NCBI BlastP on this gene

EGE03395

hypothetical protein
  
Accession: EGE03396
  
Location: 154779-158467
  
 NCBI BlastP on this gene

EGE03396

hypothetical protein
  
Accession: EGE03397
  
Location: 159617-160560
  
 NCBI BlastP on this gene

EGE03397

Query: Architecture Search FASTA input

GG700650 : Trichophyton rubrum CBS 118892 genomic scaffold supercont2.3    Total score: 2.0     Cumulative Blast bit score: 147

Hit cluster cross-links:

Mycgr3G36335 Mycgr3T
  
Location: 0-423

Mycgr3G36335\_Mycgr3T

Mycgr3G84494 Mycgr3T
  
Location: 523-2047

Mycgr3G84494\_Mycgr3T

Mycgr3G90558 Mycgr3T
  
Location: 2147-15296

Mycgr3G90558\_Mycgr3T

Mycgr3G68036 Mycgr3T
  
Location: 15396-16395

Mycgr3G68036\_Mycgr3T

Mycgr3G90561 Mycgr3T
  
Location: 16495-17134

Mycgr3G90561\_Mycgr3T

Mycgr3G35862 Mycgr3T
  
Location: 17234-18662

Mycgr3G35862\_Mycgr3T

Mycgr3G68030 Mycgr3T
  
Location: 18762-19722

Mycgr3G68030\_Mycgr3T

Mycgr3G36449 Mycgr3T
  
Location: 19822-21886

Mycgr3G36449\_Mycgr3T

Mycgr3G35528 Mycgr3T
  
Location: 21986-22844

Mycgr3G35528\_Mycgr3T

Mycgr3G35932 Mycgr3T
  
Location: 22944-24390

Mycgr3G35932\_Mycgr3T

Mycgr3G23761 Mycgr3T
  
Location: 24490-25825

Mycgr3G23761\_Mycgr3T

Mycgr3G35535 Mycgr3T
  
Location: 25925-26429

Mycgr3G35535\_Mycgr3T

Mycgr3G9942 Mycgr3T9
  
Location: 26529-30375

Mycgr3G9942\_Mycgr3T9

metalloprotease
  
Accession: EGD86992
  
Location: 1391162-1392186
  
 NCBI BlastP on this gene

EGD86992

metalloprotease
  
Accession: EGD86993
  
Location: 1394694-1396934
  
 NCBI BlastP on this gene

EGD86993

integral membrane protein
  
Accession: EGD86994
  
Location: 1398292-1399597
  
 NCBI BlastP on this gene

EGD86994

hypothetical protein
  
Accession: EGD86995
  
Location: 1400881-1402333
  
 NCBI BlastP on this gene

EGD86995

hypothetical protein
  
Accession: EGD86996
  
Location: 1402447-1403362
  
 NCBI BlastP on this gene

EGD86996

hypothetical protein
  
Accession: EGD86997
  
Location: 1403735-1404069
  
 NCBI BlastP on this gene

EGD86997

hypothetical protein
  
Accession: EGD86998
  
Location: 1405302-1406213
  
  
**BlastP hit with Mycgr3G90561\_Mycgr3T**
  
Percentage identity: 40 %
  
BlastP bit score: 84
  
Sequence coverage: 54 %
  
E-value: 2e-16
  
  
 NCBI BlastP on this gene

EGD86998

hypothetical protein
  
Accession: EGD86999
  
Location: 1406824-1408089
  
  
**BlastP hit with Mycgr3G35535\_Mycgr3T**
  
Percentage identity: 28 %
  
BlastP bit score: 63
  
Sequence coverage: 72 %
  
E-value: 3e-09
  
  
 NCBI BlastP on this gene

EGD86999

hypothetical protein
  
Accession: EGD87000
  
Location: 1408623-1409654
  
 NCBI BlastP on this gene

EGD87000

hypothetical protein
  
Accession: EGD87001
  
Location: 1411704-1412639
  
 NCBI BlastP on this gene

EGD87001

hypothetical protein
  
Accession: EGD87002
  
Location: 1413000-1413848
  
 NCBI BlastP on this gene

EGD87002

alkaline phosphatase
  
Accession: EGD87003
  
Location: 1414326-1415970
  
 NCBI BlastP on this gene

EGD87003

hypothetical protein
  
Accession: EGD87004
  
Location: 1417237-1420614
  
 NCBI BlastP on this gene

EGD87004

transcriptional co-activator
  
Accession: EGD87005
  
Location: 1421147-1422608
  
 NCBI BlastP on this gene

EGD87005

pre-mRNA splicing factor prp1
  
Accession: EGD87006
  
Location: 1423093-1426108
  
 NCBI BlastP on this gene

EGD87006

Query: Architecture Search FASTA input

GG698487 : Trichophyton tonsurans CBS 112818 genomic scaffold supercont1.11    Total score: 2.0     Cumulative Blast bit score: 147

Hit cluster cross-links:

Mycgr3G36335 Mycgr3T
  
Location: 0-423

Mycgr3G36335\_Mycgr3T

Mycgr3G84494 Mycgr3T
  
Location: 523-2047

Mycgr3G84494\_Mycgr3T

Mycgr3G90558 Mycgr3T
  
Location: 2147-15296

Mycgr3G90558\_Mycgr3T

Mycgr3G68036 Mycgr3T
  
Location: 15396-16395

Mycgr3G68036\_Mycgr3T

Mycgr3G90561 Mycgr3T
  
Location: 16495-17134

Mycgr3G90561\_Mycgr3T

Mycgr3G35862 Mycgr3T
  
Location: 17234-18662

Mycgr3G35862\_Mycgr3T

Mycgr3G68030 Mycgr3T
  
Location: 18762-19722

Mycgr3G68030\_Mycgr3T

Mycgr3G36449 Mycgr3T
  
Location: 19822-21886

Mycgr3G36449\_Mycgr3T

Mycgr3G35528 Mycgr3T
  
Location: 21986-22844

Mycgr3G35528\_Mycgr3T

Mycgr3G35932 Mycgr3T
  
Location: 22944-24390

Mycgr3G35932\_Mycgr3T

Mycgr3G23761 Mycgr3T
  
Location: 24490-25825

Mycgr3G23761\_Mycgr3T

Mycgr3G35535 Mycgr3T
  
Location: 25925-26429

Mycgr3G35535\_Mycgr3T

Mycgr3G9942 Mycgr3T9
  
Location: 26529-30375

Mycgr3G9942\_Mycgr3T9

hypothetical protein
  
Accession: EGD95285
  
Location: 204690-205723
  
 NCBI BlastP on this gene

EGD95285

hypothetical protein
  
Accession: EGD95286
  
Location: 209745-210155
  
 NCBI BlastP on this gene

EGD95286

metalloprotease
  
Accession: EGD95287
  
Location: 210637-212887
  
 NCBI BlastP on this gene

EGD95287

integral membrane protein
  
Accession: EGD95288
  
Location: 214226-215516
  
 NCBI BlastP on this gene

EGD95288

hypothetical protein
  
Accession: EGD95289
  
Location: 216824-218283
  
 NCBI BlastP on this gene

EGD95289

hypothetical protein
  
Accession: EGD95290
  
Location: 218397-219318
  
 NCBI BlastP on this gene

EGD95290

hypothetical protein
  
Accession: EGD95291
  
Location: 219698-220035
  
 NCBI BlastP on this gene

EGD95291

hypothetical protein
  
Accession: EGD95292
  
Location: 221300-222215
  
  
**BlastP hit with Mycgr3G90561\_Mycgr3T**
  
Percentage identity: 37 %
  
BlastP bit score: 74
  
Sequence coverage: 54 %
  
E-value: 3e-13
  
  
 NCBI BlastP on this gene

EGD95292

hypothetical protein
  
Accession: EGD95293
  
Location: 222826-223840
  
  
**BlastP hit with Mycgr3G35535\_Mycgr3T**
  
Percentage identity: 33 %
  
BlastP bit score: 73
  
Sequence coverage: 69 %
  
E-value: 6e-13
  
  
 NCBI BlastP on this gene

EGD95293

hypothetical protein
  
Accession: EGD95294
  
Location: 223973-225397
  
 NCBI BlastP on this gene

EGD95294

hypothetical protein
  
Accession: EGD95295
  
Location: 226340-227434
  
 NCBI BlastP on this gene

EGD95295

hypothetical protein
  
Accession: EGD95296
  
Location: 235015-237946
  
 NCBI BlastP on this gene

EGD95296

hypothetical protein
  
Accession: EGD95297
  
Location: 238285-239224
  
 NCBI BlastP on this gene

EGD95297

hypothetical protein
  
Accession: EGD95298
  
Location: 239619-240458
  
 NCBI BlastP on this gene

EGD95298

Query: Architecture Search FASTA input

EQ962656 : Talaromyces stipitatus ATCC 10500 scf\_1105507295549 genomic scaffold    Total score: 2.0     Cumulative Blast bit score: 135

Hit cluster cross-links:

Mycgr3G36335 Mycgr3T
  
Location: 0-423

Mycgr3G36335\_Mycgr3T

Mycgr3G84494 Mycgr3T
  
Location: 523-2047

Mycgr3G84494\_Mycgr3T

Mycgr3G90558 Mycgr3T
  
Location: 2147-15296

Mycgr3G90558\_Mycgr3T

Mycgr3G68036 Mycgr3T
  
Location: 15396-16395

Mycgr3G68036\_Mycgr3T

Mycgr3G90561 Mycgr3T
  
Location: 16495-17134

Mycgr3G90561\_Mycgr3T

Mycgr3G35862 Mycgr3T
  
Location: 17234-18662

Mycgr3G35862\_Mycgr3T

Mycgr3G68030 Mycgr3T
  
Location: 18762-19722

Mycgr3G68030\_Mycgr3T

Mycgr3G36449 Mycgr3T
  
Location: 19822-21886

Mycgr3G36449\_Mycgr3T

Mycgr3G35528 Mycgr3T
  
Location: 21986-22844

Mycgr3G35528\_Mycgr3T

Mycgr3G35932 Mycgr3T
  
Location: 22944-24390

Mycgr3G35932\_Mycgr3T

Mycgr3G23761 Mycgr3T
  
Location: 24490-25825

Mycgr3G23761\_Mycgr3T

Mycgr3G35535 Mycgr3T
  
Location: 25925-26429

Mycgr3G35535\_Mycgr3T

Mycgr3G9942 Mycgr3T9
  
Location: 26529-30375

Mycgr3G9942\_Mycgr3T9

conserved hypothetical protein
  
Accession: EED16322
  
Location: 1494275-1496605
  
 NCBI BlastP on this gene

EED16322

hypothetical protein
  
Accession: EED16323
  
Location: 1497772-1498824
  
 NCBI BlastP on this gene

EED16323

hypothetical protein
  
Accession: EED16324
  
Location: 1506732-1507079
  
 NCBI BlastP on this gene

EED16324

hypothetical protein
  
Accession: EED16325
  
Location: 1507947-1508375
  
 NCBI BlastP on this gene

EED16325

O-methyltransferase, putative
  
Accession: EED16326
  
Location: 1511062-1512489
  
 NCBI BlastP on this gene

EED16326

conserved hypothetical protein
  
Accession: EED16327
  
Location: 1513295-1514127
  
  
**BlastP hit with Mycgr3G90561\_Mycgr3T**
  
Percentage identity: 38 %
  
BlastP bit score: 65
  
Sequence coverage: 38 %
  
E-value: 2e-10
  
  
 NCBI BlastP on this gene

EED16327

conserved hypothetical protein
  
Accession: EED16331
  
Location: 1514132-1515205
  
  
**BlastP hit with Mycgr3G35535\_Mycgr3T**
  
Percentage identity: 26 %
  
BlastP bit score: 70
  
Sequence coverage: 96 %
  
E-value: 4e-12
  
  
 NCBI BlastP on this gene

EED16331

conserved hypothetical protein
  
Accession: EED16333
  
Location: 1516161-1517236
  
 NCBI BlastP on this gene

EED16333

hypothetical protein
  
Accession: EED16335
  
Location: 1517560-1517970
  
 NCBI BlastP on this gene

EED16335

F-box domain protein
  
Accession: EED16336
  
Location: 1518631-1520140
  
 NCBI BlastP on this gene

EED16336

conserved hypothetical protein
  
Accession: EED16337
  
Location: 1520411-1521122
  
 NCBI BlastP on this gene

EED16337

pyruvate kinase
  
Accession: EED16338
  
Location: 1522274-1524287
  
 NCBI BlastP on this gene

EED16338

hypothetical protein
  
Accession: EED16339
  
Location: 1525816-1526229
  
 NCBI BlastP on this gene

EED16339

conserved hypothetical protein
  
Accession: EED16340
  
Location: 1526286-1528208
  
 NCBI BlastP on this gene

EED16340

1-phosphatidylinositol-3-phosphate 5-kinase (Fab1), putative
  
Accession: EED16341
  
Location: 1528608-1536061
  
 NCBI BlastP on this gene

EED16341

Query: Architecture Search FASTA input

KB446559 : Pseudocercospora fijiensis CIRAD86 unplaced genomic scaffold MYCFIscaffold\_5    Total score: 2.0     Cumulative Blast bit score: 126

Hit cluster cross-links:

Mycgr3G36335 Mycgr3T
  
Location: 0-423

Mycgr3G36335\_Mycgr3T

Mycgr3G84494 Mycgr3T
  
Location: 523-2047

Mycgr3G84494\_Mycgr3T

Mycgr3G90558 Mycgr3T
  
Location: 2147-15296

Mycgr3G90558\_Mycgr3T

Mycgr3G68036 Mycgr3T
  
Location: 15396-16395

Mycgr3G68036\_Mycgr3T

Mycgr3G90561 Mycgr3T
  
Location: 16495-17134

Mycgr3G90561\_Mycgr3T

Mycgr3G35862 Mycgr3T
  
Location: 17234-18662

Mycgr3G35862\_Mycgr3T

Mycgr3G68030 Mycgr3T
  
Location: 18762-19722

Mycgr3G68030\_Mycgr3T

Mycgr3G36449 Mycgr3T
  
Location: 19822-21886

Mycgr3G36449\_Mycgr3T

Mycgr3G35528 Mycgr3T
  
Location: 21986-22844

Mycgr3G35528\_Mycgr3T

Mycgr3G35932 Mycgr3T
  
Location: 22944-24390

Mycgr3G35932\_Mycgr3T

Mycgr3G23761 Mycgr3T
  
Location: 24490-25825

Mycgr3G23761\_Mycgr3T

Mycgr3G35535 Mycgr3T
  
Location: 25925-26429

Mycgr3G35535\_Mycgr3T

Mycgr3G9942 Mycgr3T9
  
Location: 26529-30375

Mycgr3G9942\_Mycgr3T9

hypothetical protein
  
Accession: EME81784
  
Location: 1028981-1029753
  
 NCBI BlastP on this gene

EME81784

hypothetical protein
  
Accession: EME81785
  
Location: 1030585-1031741
  
 NCBI BlastP on this gene

EME81785

hypothetical protein
  
Accession: EME81786
  
Location: 1032206-1032536
  
 NCBI BlastP on this gene

EME81786

hypothetical protein
  
Accession: EME81787
  
Location: 1033141-1035119
  
 NCBI BlastP on this gene

EME81787

putative ABC transporter
  
Accession: EME81788
  
Location: 1035593-1040346
  
 NCBI BlastP on this gene

EME81788

hypothetical protein
  
Accession: EME81789
  
Location: 1040361-1041389
  
 NCBI BlastP on this gene

EME81789

hypothetical protein
  
Accession: EME81790
  
Location: 1041669-1042653
  
  
**BlastP hit with Mycgr3G35535\_Mycgr3T**
  
Percentage identity: 27 %
  
BlastP bit score: 62
  
Sequence coverage: 94 %
  
E-value: 4e-09
  
  
 NCBI BlastP on this gene

EME81790

hypothetical protein
  
Accession: EME81791
  
Location: 1042886-1043959
  
 NCBI BlastP on this gene

EME81791

hypothetical protein
  
Accession: EME81792
  
Location: 1044597-1045734
  
 NCBI BlastP on this gene

EME81792

hypothetical protein
  
Accession: EME81793
  
Location: 1046727-1047115
  
 NCBI BlastP on this gene

EME81793

hypothetical protein
  
Accession: EME81794
  
Location: 1048638-1050149
  
 NCBI BlastP on this gene

EME81794

hypothetical protein
  
Accession: EME81795
  
Location: 1051175-1052011
  
  
**BlastP hit with Mycgr3G90561\_Mycgr3T**
  
Percentage identity: 34 %
  
BlastP bit score: 64
  
Sequence coverage: 57 %
  
E-value: 1e-09
  
  
 NCBI BlastP on this gene

EME81795

hypothetical protein
  
Accession: EME81796
  
Location: 1053650-1054216
  
 NCBI BlastP on this gene

EME81796

hypothetical protein
  
Accession: EME81797
  
Location: 1054887-1055420
  
 NCBI BlastP on this gene

EME81797

Query: Architecture Search FASTA input

AP007166 : Aspergillus oryzae RIB40 DNA, SC113.    Total score: 2.0     Cumulative Blast bit score: 119

Hit cluster cross-links:

Mycgr3G36335 Mycgr3T
  
Location: 0-423

Mycgr3G36335\_Mycgr3T

Mycgr3G84494 Mycgr3T
  
Location: 523-2047

Mycgr3G84494\_Mycgr3T

Mycgr3G90558 Mycgr3T
  
Location: 2147-15296

Mycgr3G90558\_Mycgr3T

Mycgr3G68036 Mycgr3T
  
Location: 15396-16395

Mycgr3G68036\_Mycgr3T

Mycgr3G90561 Mycgr3T
  
Location: 16495-17134

Mycgr3G90561\_Mycgr3T

Mycgr3G35862 Mycgr3T
  
Location: 17234-18662

Mycgr3G35862\_Mycgr3T

Mycgr3G68030 Mycgr3T
  
Location: 18762-19722

Mycgr3G68030\_Mycgr3T

Mycgr3G36449 Mycgr3T
  
Location: 19822-21886

Mycgr3G36449\_Mycgr3T

Mycgr3G35528 Mycgr3T
  
Location: 21986-22844

Mycgr3G35528\_Mycgr3T

Mycgr3G35932 Mycgr3T
  
Location: 22944-24390

Mycgr3G35932\_Mycgr3T

Mycgr3G23761 Mycgr3T
  
Location: 24490-25825

Mycgr3G23761\_Mycgr3T

Mycgr3G35535 Mycgr3T
  
Location: 25925-26429

Mycgr3G35535\_Mycgr3T

Mycgr3G9942 Mycgr3T9
  
Location: 26529-30375

Mycgr3G9942\_Mycgr3T9

not annotated
  
Accession: BAE63089
  
Location: 1415647-1416318
  
  
**BlastP hit with Mycgr3G90561\_Mycgr3T**
  
Percentage identity: 34 %
  
BlastP bit score: 60
  
Sequence coverage: 60 %
  
E-value: 1e-08
  
  
  
**BlastP hit with Mycgr3G35535\_Mycgr3T**
  
Percentage identity: 30 %
  
BlastP bit score: 60
  
Sequence coverage: 85 %
  
E-value: 5e-09
  
  
 NCBI BlastP on this gene

AO090113000025

not annotated
  
Accession: BAE63088
  
Location: 1414473-1415203
  
 NCBI BlastP on this gene

AO090113000024

not annotated
  
Accession: BAE63087
  
Location: 1411903-1413731
  
 NCBI BlastP on this gene

AO090113000022

not annotated
  
Accession: BAE63086
  
Location: 1408019-1411696
  
 NCBI BlastP on this gene

AO090113000021

not annotated
  
Accession: BAE63085
  
Location: 1405065-1406126
  
 NCBI BlastP on this gene

AO090113000020

not annotated
  
Accession: BAE63084
  
Location: 1401539-1404894
  
 NCBI BlastP on this gene

AO090113000019

not annotated
  
Accession: BAE63083
  
Location: 1399519-1400124
  
 NCBI BlastP on this gene

AO090113000017

not annotated
  
Accession: BAE63082
  
Location: 1397319-1398389
  
 NCBI BlastP on this gene

AO090113000016

Query: Architecture Search FASTA input

KB725822 : Colletotrichum orbiculare MAFF 240422 unplaced genomic scaffold Scaffold\_269    Total score: 2.0     Cumulative Blast bit score: 118

Hit cluster cross-links:

Mycgr3G36335 Mycgr3T
  
Location: 0-423

Mycgr3G36335\_Mycgr3T

Mycgr3G84494 Mycgr3T
  
Location: 523-2047

Mycgr3G84494\_Mycgr3T

Mycgr3G90558 Mycgr3T
  
Location: 2147-15296

Mycgr3G90558\_Mycgr3T

Mycgr3G68036 Mycgr3T
  
Location: 15396-16395

Mycgr3G68036\_Mycgr3T

Mycgr3G90561 Mycgr3T
  
Location: 16495-17134

Mycgr3G90561\_Mycgr3T

Mycgr3G35862 Mycgr3T
  
Location: 17234-18662

Mycgr3G35862\_Mycgr3T

Mycgr3G68030 Mycgr3T
  
Location: 18762-19722

Mycgr3G68030\_Mycgr3T

Mycgr3G36449 Mycgr3T
  
Location: 19822-21886

Mycgr3G36449\_Mycgr3T

Mycgr3G35528 Mycgr3T
  
Location: 21986-22844

Mycgr3G35528\_Mycgr3T

Mycgr3G35932 Mycgr3T
  
Location: 22944-24390

Mycgr3G35932\_Mycgr3T

Mycgr3G23761 Mycgr3T
  
Location: 24490-25825

Mycgr3G23761\_Mycgr3T

Mycgr3G35535 Mycgr3T
  
Location: 25925-26429

Mycgr3G35535\_Mycgr3T

Mycgr3G9942 Mycgr3T9
  
Location: 26529-30375

Mycgr3G9942\_Mycgr3T9

hypothetical protein
  
Accession: ENH84130
  
Location: 25850-26575
  
 NCBI BlastP on this gene

ENH84130

hypothetical protein
  
Accession: ENH84131
  
Location: 26242-26654
  
 NCBI BlastP on this gene

ENH84131

regulatory protein
  
Accession: ENH84132
  
Location: 29775-31586
  
 NCBI BlastP on this gene

ENH84132

hypothetical protein
  
Accession: ENH84133
  
Location: 32189-32642
  
 NCBI BlastP on this gene

ENH84133

hypothetical protein
  
Accession: ENH84134
  
Location: 33021-33374
  
 NCBI BlastP on this gene

ENH84134

hypothetical protein
  
Accession: ENH84135
  
Location: 34864-35949
  
 NCBI BlastP on this gene

ENH84135

hypothetical protein
  
Accession: ENH84136
  
Location: 36845-37870
  
  
**BlastP hit with Mycgr3G35535\_Mycgr3T**
  
Percentage identity: 28 %
  
BlastP bit score: 57
  
Sequence coverage: 93 %
  
E-value: 4e-07
  
  
 NCBI BlastP on this gene

ENH84136

hypothetical protein
  
Accession: ENH84137
  
Location: 38739-40924
  
 NCBI BlastP on this gene

ENH84137

hypothetical protein
  
Accession: ENH84138
  
Location: 41065-41940
  
 NCBI BlastP on this gene

ENH84138

hypothetical protein
  
Accession: ENH84139
  
Location: 42503-43354
  
  
**BlastP hit with Mycgr3G90561\_Mycgr3T**
  
Percentage identity: 29 %
  
BlastP bit score: 61
  
Sequence coverage: 61 %
  
E-value: 2e-08
  
  
 NCBI BlastP on this gene

ENH84139

hypothetical protein
  
Accession: ENH84140
  
Location: 43554-44156
  
 NCBI BlastP on this gene

ENH84140

hypothetical protein
  
Accession: ENH84141
  
Location: 45034-46541
  
 NCBI BlastP on this gene

ENH84141

c-4 methylsterol oxidase
  
Accession: ENH84142
  
Location: 46995-48103
  
 NCBI BlastP on this gene

ENH84142

hypothetical protein
  
Accession: ENH84143
  
Location: 48849-51157
  
 NCBI BlastP on this gene

ENH84143

siderophore iron transporter mirb
  
Accession: ENH84144
  
Location: 52036-53799
  
 NCBI BlastP on this gene

ENH84144

hypothetical protein
  
Accession: ENH84145
  
Location: 55381-56301
  
 NCBI BlastP on this gene

ENH84145

short-chain dehydrogenase
  
Accession: ENH84146
  
Location: 57030-58028
  
 NCBI BlastP on this gene

ENH84146

Query: Architecture Search FASTA input

AMYD01004223 : Colletotrichum gloeosporioides Cg-14    Total score: 2.0     Cumulative Blast bit score: 118

Hit cluster cross-links:

Mycgr3G36335 Mycgr3T
  
Location: 0-423

Mycgr3G36335\_Mycgr3T

Mycgr3G84494 Mycgr3T
  
Location: 523-2047

Mycgr3G84494\_Mycgr3T

Mycgr3G90558 Mycgr3T
  
Location: 2147-15296

Mycgr3G90558\_Mycgr3T

Mycgr3G68036 Mycgr3T
  
Location: 15396-16395

Mycgr3G68036\_Mycgr3T

Mycgr3G90561 Mycgr3T
  
Location: 16495-17134

Mycgr3G90561\_Mycgr3T

Mycgr3G35862 Mycgr3T
  
Location: 17234-18662

Mycgr3G35862\_Mycgr3T

Mycgr3G68030 Mycgr3T
  
Location: 18762-19722

Mycgr3G68030\_Mycgr3T

Mycgr3G36449 Mycgr3T
  
Location: 19822-21886

Mycgr3G36449\_Mycgr3T

Mycgr3G35528 Mycgr3T
  
Location: 21986-22844

Mycgr3G35528\_Mycgr3T

Mycgr3G35932 Mycgr3T
  
Location: 22944-24390

Mycgr3G35932\_Mycgr3T

Mycgr3G23761 Mycgr3T
  
Location: 24490-25825

Mycgr3G23761\_Mycgr3T

Mycgr3G35535 Mycgr3T
  
Location: 25925-26429

Mycgr3G35535\_Mycgr3T

Mycgr3G9942 Mycgr3T9
  
Location: 26529-30375

Mycgr3G9942\_Mycgr3T9

hypothetical protein
  
Accession: EQB43618
  
Location: 15399-16209
  
 NCBI BlastP on this gene

EQB43618

hypothetical protein
  
Accession: EQB43619
  
Location: 16545-17156
  
 NCBI BlastP on this gene

EQB43619

cytochrome P450
  
Accession: EQB43620
  
Location: 18467-20166
  
 NCBI BlastP on this gene

EQB43620

hypothetical protein
  
Accession: EQB43621
  
Location: 20280-21770
  
 NCBI BlastP on this gene

EQB43621

hypothetical protein
  
Accession: EQB43622
  
Location: 22170-23267
  
 NCBI BlastP on this gene

EQB43622

hypothetical protein
  
Accession: EQB43623
  
Location: 30505-31342
  
  
**BlastP hit with Mycgr3G90561\_Mycgr3T**
  
Percentage identity: 28 %
  
BlastP bit score: 62
  
Sequence coverage: 61 %
  
E-value: 4e-09
  
  
 NCBI BlastP on this gene

EQB43623

hypothetical protein
  
Accession: EQB43624
  
Location: 31920-32792
  
 NCBI BlastP on this gene

EQB43624

hypothetical protein
  
Accession: EQB43625
  
Location: 32914-35130
  
 NCBI BlastP on this gene

EQB43625

hypothetical protein
  
Accession: EQB43626
  
Location: 36026-37003
  
  
**BlastP hit with Mycgr3G35535\_Mycgr3T**
  
Percentage identity: 30 %
  
BlastP bit score: 56
  
Sequence coverage: 91 %
  
E-value: 6e-07
  
  
 NCBI BlastP on this gene

EQB43626

hypothetical protein
  
Accession: EQB43627
  
Location: 37883-38963
  
 NCBI BlastP on this gene

EQB43627

hypothetical protein
  
Accession: EQB43628
  
Location: 39377-40677
  
 NCBI BlastP on this gene

EQB43628

hypothetical protein
  
Accession: EQB43629
  
Location: 41022-41466
  
 NCBI BlastP on this gene

EQB43629

Query: Architecture Search FASTA input

KB726072 : Colletotrichum orbiculare MAFF 240422 unplaced genomic scaffold Scaffold\_494    Total score: 2.0     Cumulative Blast bit score: 111

Hit cluster cross-links:

Mycgr3G36335 Mycgr3T
  
Location: 0-423

Mycgr3G36335\_Mycgr3T

Mycgr3G84494 Mycgr3T
  
Location: 523-2047

Mycgr3G84494\_Mycgr3T

Mycgr3G90558 Mycgr3T
  
Location: 2147-15296

Mycgr3G90558\_Mycgr3T

Mycgr3G68036 Mycgr3T
  
Location: 15396-16395

Mycgr3G68036\_Mycgr3T

Mycgr3G90561 Mycgr3T
  
Location: 16495-17134

Mycgr3G90561\_Mycgr3T

Mycgr3G35862 Mycgr3T
  
Location: 17234-18662

Mycgr3G35862\_Mycgr3T

Mycgr3G68030 Mycgr3T
  
Location: 18762-19722

Mycgr3G68030\_Mycgr3T

Mycgr3G36449 Mycgr3T
  
Location: 19822-21886

Mycgr3G36449\_Mycgr3T

Mycgr3G35528 Mycgr3T
  
Location: 21986-22844

Mycgr3G35528\_Mycgr3T

Mycgr3G35932 Mycgr3T
  
Location: 22944-24390

Mycgr3G35932\_Mycgr3T

Mycgr3G23761 Mycgr3T
  
Location: 24490-25825

Mycgr3G23761\_Mycgr3T

Mycgr3G35535 Mycgr3T
  
Location: 25925-26429

Mycgr3G35535\_Mycgr3T

Mycgr3G9942 Mycgr3T9
  
Location: 26529-30375

Mycgr3G9942\_Mycgr3T9

cytochrome c peroxidase
  
Accession: ENH78169
  
Location: 444400-445601
  
 NCBI BlastP on this gene

ENH78169

hypothetical protein
  
Accession: ENH78170
  
Location: 447587-447862
  
 NCBI BlastP on this gene

ENH78170

tankyrase 1 binding protein isoform-like protein
  
Accession: ENH78171
  
Location: 448307-449008
  
 NCBI BlastP on this gene

ENH78171

hypothetical protein
  
Accession: ENH78172
  
Location: 450630-451400
  
 NCBI BlastP on this gene

ENH78172

hypothetical protein
  
Accession: ENH78173
  
Location: 451640-452503
  
 NCBI BlastP on this gene

ENH78173

hypothetical protein
  
Accession: ENH78174
  
Location: 454654-454977
  
 NCBI BlastP on this gene

ENH78174

hypothetical protein
  
Accession: ENH78175
  
Location: 456745-457907
  
 NCBI BlastP on this gene

ENH78175

hypothetical protein
  
Accession: ENH78176
  
Location: 458469-459573
  
  
**BlastP hit with Mycgr3G35535\_Mycgr3T**
  
Percentage identity: 27 %
  
BlastP bit score: 57
  
Sequence coverage: 85 %
  
E-value: 3e-07
  
  
 NCBI BlastP on this gene

ENH78176

tat pathway signal sequence
  
Accession: ENH78177
  
Location: 460565-461404
  
 NCBI BlastP on this gene

ENH78177

hypothetical protein
  
Accession: ENH78178
  
Location: 461850-462849
  
  
**BlastP hit with Mycgr3G90561\_Mycgr3T**
  
Percentage identity: 33 %
  
BlastP bit score: 55
  
Sequence coverage: 53 %
  
E-value: 3e-06
  
  
 NCBI BlastP on this gene

ENH78178

f1f0 atp synthase assembly protein
  
Accession: ENH78179
  
Location: 467309-468268
  
 NCBI BlastP on this gene

ENH78179

gas1-like protein
  
Accession: ENH78180
  
Location: 471418-472614
  
 NCBI BlastP on this gene

ENH78180

gcn5-related n-acetyltransferase
  
Accession: ENH78181
  
Location: 475610-476251
  
 NCBI BlastP on this gene

ENH78181

pyridine nucleotide-disulfide oxidoreductase
  
Accession: ENH78182
  
Location: 476454-477719
  
 NCBI BlastP on this gene

ENH78182

Query: Architecture Search FASTA input

DS985225 : Verticillium albo-atrum VaMs.102 supercont1.12 genomic scaffold    Total score: 1.0     Cumulative Blast bit score: 3093

Hit cluster cross-links:

Mycgr3G36335 Mycgr3T
  
Location: 0-423

Mycgr3G36335\_Mycgr3T

Mycgr3G84494 Mycgr3T
  
Location: 523-2047

Mycgr3G84494\_Mycgr3T

Mycgr3G90558 Mycgr3T
  
Location: 2147-15296

Mycgr3G90558\_Mycgr3T

Mycgr3G68036 Mycgr3T
  
Location: 15396-16395

Mycgr3G68036\_Mycgr3T

Mycgr3G90561 Mycgr3T
  
Location: 16495-17134

Mycgr3G90561\_Mycgr3T

Mycgr3G35862 Mycgr3T
  
Location: 17234-18662

Mycgr3G35862\_Mycgr3T

Mycgr3G68030 Mycgr3T
  
Location: 18762-19722

Mycgr3G68030\_Mycgr3T

Mycgr3G36449 Mycgr3T
  
Location: 19822-21886

Mycgr3G36449\_Mycgr3T

Mycgr3G35528 Mycgr3T
  
Location: 21986-22844

Mycgr3G35528\_Mycgr3T

Mycgr3G35932 Mycgr3T
  
Location: 22944-24390

Mycgr3G35932\_Mycgr3T

Mycgr3G23761 Mycgr3T
  
Location: 24490-25825

Mycgr3G23761\_Mycgr3T

Mycgr3G35535 Mycgr3T
  
Location: 25925-26429

Mycgr3G35535\_Mycgr3T

Mycgr3G9942 Mycgr3T9
  
Location: 26529-30375

Mycgr3G9942\_Mycgr3T9

phenyloxazoline synthase mbtB
  
Accession: EEY22273
  
Location: 498532-500877
  
 NCBI BlastP on this gene

EEY22273

HC-toxin synthetase
  
Accession: EEY22272
  
Location: 490133-497717
  
  
**BlastP hit with Mycgr3G90558\_Mycgr3T**
  
Percentage identity: 30 %
  
BlastP bit score: 1082
  
Sequence coverage: 57 %
  
E-value: 0.0
  
  
 NCBI BlastP on this gene

EEY22272

HC-toxin synthetase
  
Accession: EEY22271
  
Location: 482333-487978
  
  
**BlastP hit with Mycgr3G90558\_Mycgr3T**
  
Percentage identity: 35 %
  
BlastP bit score: 1007
  
Sequence coverage: 41 %
  
E-value: 0.0
  
  
 NCBI BlastP on this gene

EEY22271

HC-toxin synthetase
  
Accession: EEY22270
  
Location: 473021-482054
  
  
**BlastP hit with Mycgr3G90558\_Mycgr3T**
  
Percentage identity: 29 %
  
BlastP bit score: 1004
  
Sequence coverage: 61 %
  
E-value: 0.0
  
  
 NCBI BlastP on this gene

EEY22270

lectin
  
Accession: EEY22269
  
Location: 470997-472199
  
 NCBI BlastP on this gene

EEY22269

Query: Architecture Search FASTA input

JN186799 : Claviceps purpurea strain 20.1 ergot alkaloid biosynthetic gene cluster    Total score: 1.0     Cumulative Blast bit score: 2413

Hit cluster cross-links:

Mycgr3G36335 Mycgr3T
  
Location: 0-423

Mycgr3G36335\_Mycgr3T

Mycgr3G84494 Mycgr3T
  
Location: 523-2047

Mycgr3G84494\_Mycgr3T

Mycgr3G90558 Mycgr3T
  
Location: 2147-15296

Mycgr3G90558\_Mycgr3T

Mycgr3G68036 Mycgr3T
  
Location: 15396-16395

Mycgr3G68036\_Mycgr3T

Mycgr3G90561 Mycgr3T
  
Location: 16495-17134

Mycgr3G90561\_Mycgr3T

Mycgr3G35862 Mycgr3T
  
Location: 17234-18662

Mycgr3G35862\_Mycgr3T

Mycgr3G68030 Mycgr3T
  
Location: 18762-19722

Mycgr3G68030\_Mycgr3T

Mycgr3G36449 Mycgr3T
  
Location: 19822-21886

Mycgr3G36449\_Mycgr3T

Mycgr3G35528 Mycgr3T
  
Location: 21986-22844

Mycgr3G35528\_Mycgr3T

Mycgr3G35932 Mycgr3T
  
Location: 22944-24390

Mycgr3G35932\_Mycgr3T

Mycgr3G23761 Mycgr3T
  
Location: 24490-25825

Mycgr3G23761\_Mycgr3T

Mycgr3G35535 Mycgr3T
  
Location: 25925-26429

Mycgr3G35535\_Mycgr3T

Mycgr3G9942 Mycgr3T9
  
Location: 26529-30375

Mycgr3G9942\_Mycgr3T9

dimethylallyl tryptophan synthase
  
Accession: AET79188
  
Location: 33089-34554
  
 NCBI BlastP on this gene

dmaW

putative oxygenase
  
Accession: AET79182
  
Location: 35947-36891
  
 NCBI BlastP on this gene

easH1

lysergyl peptide synthetase subunit 1
  
Accession: AET79183
  
Location: 37705-48636
  
  
**BlastP hit with Mycgr3G90558\_Mycgr3T**
  
Percentage identity: 31 %
  
BlastP bit score: 1215
  
Sequence coverage: 68 %
  
E-value: 0.0
  
  
 NCBI BlastP on this gene

lpsA1

lysergyl peptide synthetase subunit 1
  
Accession: AET79184
  
Location: 52447-63381
  
  
**BlastP hit with Mycgr3G90558\_Mycgr3T**
  
Percentage identity: 30 %
  
BlastP bit score: 1198
  
Sequence coverage: 68 %
  
E-value: 0.0
  
  
 NCBI BlastP on this gene

lpsA2

hypothetical protein
  
Accession: AET79185
  
Location: 66909-67226
  
 NCBI BlastP on this gene

AET79185

Query: Architecture Search FASTA input

CAGA01000020 : Claviceps purpurea 20.1    Total score: 1.0     Cumulative Blast bit score: 2413

Hit cluster cross-links:

Mycgr3G36335 Mycgr3T
  
Location: 0-423

Mycgr3G36335\_Mycgr3T

Mycgr3G84494 Mycgr3T
  
Location: 523-2047

Mycgr3G84494\_Mycgr3T

Mycgr3G90558 Mycgr3T
  
Location: 2147-15296

Mycgr3G90558\_Mycgr3T

Mycgr3G68036 Mycgr3T
  
Location: 15396-16395

Mycgr3G68036\_Mycgr3T

Mycgr3G90561 Mycgr3T
  
Location: 16495-17134

Mycgr3G90561\_Mycgr3T

Mycgr3G35862 Mycgr3T
  
Location: 17234-18662

Mycgr3G35862\_Mycgr3T

Mycgr3G68030 Mycgr3T
  
Location: 18762-19722

Mycgr3G68030\_Mycgr3T

Mycgr3G36449 Mycgr3T
  
Location: 19822-21886

Mycgr3G36449\_Mycgr3T

Mycgr3G35528 Mycgr3T
  
Location: 21986-22844

Mycgr3G35528\_Mycgr3T

Mycgr3G35932 Mycgr3T
  
Location: 22944-24390

Mycgr3G35932\_Mycgr3T

Mycgr3G23761 Mycgr3T
  
Location: 24490-25825

Mycgr3G23761\_Mycgr3T

Mycgr3G35535 Mycgr3T
  
Location: 25925-26429

Mycgr3G35535\_Mycgr3T

Mycgr3G9942 Mycgr3T9
  
Location: 26529-30375

Mycgr3G9942\_Mycgr3T9

uncharacterized protein
  
Accession: CCE30228
  
Location: 263944-265409
  
 NCBI BlastP on this gene

CCE30228

uncharacterized protein
  
Accession: CCE30227
  
Location: 261607-262551
  
 NCBI BlastP on this gene

CCE30227

non-ribosomal peptide synthetase
  
Accession: CCE30226
  
Location: 249862-260793
  
  
**BlastP hit with Mycgr3G90558\_Mycgr3T**
  
Percentage identity: 31 %
  
BlastP bit score: 1215
  
Sequence coverage: 68 %
  
E-value: 0.0
  
  
 NCBI BlastP on this gene

CCE30226

non-ribosomal peptide synthetase
  
Accession: CCE30225
  
Location: 235117-246051
  
  
**BlastP hit with Mycgr3G90558\_Mycgr3T**
  
Percentage identity: 30 %
  
BlastP bit score: 1198
  
Sequence coverage: 68 %
  
E-value: 0.0
  
  
 NCBI BlastP on this gene

CCE30225

uncharacterized protein
  
Accession: CCE30224
  
Location: 231272-231589
  
 NCBI BlastP on this gene

CCE30224

Query: Architecture Search FASTA input

KB933064 : Togninia minima UCRPA7 unplaced genomic scaffold PA7\_03\_scaffold\_293    Total score: 1.0     Cumulative Blast bit score: 2295

Hit cluster cross-links:

Mycgr3G36335 Mycgr3T
  
Location: 0-423

Mycgr3G36335\_Mycgr3T

Mycgr3G84494 Mycgr3T
  
Location: 523-2047

Mycgr3G84494\_Mycgr3T

Mycgr3G90558 Mycgr3T
  
Location: 2147-15296

Mycgr3G90558\_Mycgr3T

Mycgr3G68036 Mycgr3T
  
Location: 15396-16395

Mycgr3G68036\_Mycgr3T

Mycgr3G90561 Mycgr3T
  
Location: 16495-17134

Mycgr3G90561\_Mycgr3T

Mycgr3G35862 Mycgr3T
  
Location: 17234-18662

Mycgr3G35862\_Mycgr3T

Mycgr3G68030 Mycgr3T
  
Location: 18762-19722

Mycgr3G68030\_Mycgr3T

Mycgr3G36449 Mycgr3T
  
Location: 19822-21886

Mycgr3G36449\_Mycgr3T

Mycgr3G35528 Mycgr3T
  
Location: 21986-22844

Mycgr3G35528\_Mycgr3T

Mycgr3G35932 Mycgr3T
  
Location: 22944-24390

Mycgr3G35932\_Mycgr3T

Mycgr3G23761 Mycgr3T
  
Location: 24490-25825

Mycgr3G23761\_Mycgr3T

Mycgr3G35535 Mycgr3T
  
Location: 25925-26429

Mycgr3G35535\_Mycgr3T

Mycgr3G9942 Mycgr3T9
  
Location: 26529-30375

Mycgr3G9942\_Mycgr3T9

hypothetical protein
  
Accession: EOO00601
  
Location: 147741-180254
  
  
**BlastP hit with Mycgr3G90558\_Mycgr3T**
  
Percentage identity: 30 %
  
BlastP bit score: 1164
  
Sequence coverage: 61 %
  
E-value: 0.0
  
  
 NCBI BlastP on this gene

EOO00601

putative alpha beta hydrolase protein
  
Accession: EOO00594
  
Location: 183798-184880
  
 NCBI BlastP on this gene

EOO00594

hypothetical protein
  
Accession: EOO00604
  
Location: 185818-213011
  
  
**BlastP hit with Mycgr3G90558\_Mycgr3T**
  
Percentage identity: 30 %
  
BlastP bit score: 1131
  
Sequence coverage: 61 %
  
E-value: 0.0
  
  
 NCBI BlastP on this gene

EOO00604

Query: Architecture Search FASTA input

ACYE01000348 : Trichophyton verrucosum HKI 0517    Total score: 1.0     Cumulative Blast bit score: 2244

Hit cluster cross-links:

Mycgr3G36335 Mycgr3T
  
Location: 0-423

Mycgr3G36335\_Mycgr3T

Mycgr3G84494 Mycgr3T
  
Location: 523-2047

Mycgr3G84494\_Mycgr3T

Mycgr3G90558 Mycgr3T
  
Location: 2147-15296

Mycgr3G90558\_Mycgr3T

Mycgr3G68036 Mycgr3T
  
Location: 15396-16395

Mycgr3G68036\_Mycgr3T

Mycgr3G90561 Mycgr3T
  
Location: 16495-17134

Mycgr3G90561\_Mycgr3T

Mycgr3G35862 Mycgr3T
  
Location: 17234-18662

Mycgr3G35862\_Mycgr3T

Mycgr3G68030 Mycgr3T
  
Location: 18762-19722

Mycgr3G68030\_Mycgr3T

Mycgr3G36449 Mycgr3T
  
Location: 19822-21886

Mycgr3G36449\_Mycgr3T

Mycgr3G35528 Mycgr3T
  
Location: 21986-22844

Mycgr3G35528\_Mycgr3T

Mycgr3G35932 Mycgr3T
  
Location: 22944-24390

Mycgr3G35932\_Mycgr3T

Mycgr3G23761 Mycgr3T
  
Location: 24490-25825

Mycgr3G23761\_Mycgr3T

Mycgr3G35535 Mycgr3T
  
Location: 25925-26429

Mycgr3G35535\_Mycgr3T

Mycgr3G9942 Mycgr3T9
  
Location: 26529-30375

Mycgr3G9942\_Mycgr3T9

hypothetical protein
  
Accession: EFE39141
  
Location: 40783-46991
  
 NCBI BlastP on this gene

EFE39141

hypothetical protein
  
Accession: EFE39140
  
Location: 37562-39509
  
 NCBI BlastP on this gene

EFE39140

nonribosomal peptide synthase, putative
  
Accession: EFE39139
  
Location: 25673-36486
  
  
**BlastP hit with Mycgr3G90558\_Mycgr3T**
  
Percentage identity: 28 %
  
BlastP bit score: 1218
  
Sequence coverage: 78 %
  
E-value: 0.0
  
  
 NCBI BlastP on this gene

EFE39139

hypothetical protein
  
Accession: EFE39138
  
Location: 17606-25327
  
  
**BlastP hit with Mycgr3G90558\_Mycgr3T**
  
Percentage identity: 30 %
  
BlastP bit score: 1026
  
Sequence coverage: 54 %
  
E-value: 0.0
  
  
 NCBI BlastP on this gene

EFE39138

hypothetical protein
  
Accession: EFE39137
  
Location: 16187-17057
  
 NCBI BlastP on this gene

EFE39137

hypothetical protein
  
Accession: EFE39136
  
Location: 14389-15686
  
 NCBI BlastP on this gene

EFE39136

hypothetical protein
  
Accession: EFE39135
  
Location: 12901-14095
  
 NCBI BlastP on this gene

EFE39135

hypothetical protein
  
Accession: EFE39134
  
Location: 12238-12588
  
 NCBI BlastP on this gene

EFE39134

hypothetical protein
  
Accession: EFE39133
  
Location: 11379-11954
  
 NCBI BlastP on this gene

EFE39133

hypothetical protein
  
Accession: EFE39132
  
Location: 10511-11189
  
 NCBI BlastP on this gene

EFE39132

hypothetical protein
  
Accession: EFE39131
  
Location: 8110-10143
  
 NCBI BlastP on this gene

EFE39131

Query: Architecture Search FASTA input

AHHD01000518 : Macrophomina phaseolina MS6    Total score: 1.0     Cumulative Blast bit score: 2110

Hit cluster cross-links:

Mycgr3G36335 Mycgr3T
  
Location: 0-423

Mycgr3G36335\_Mycgr3T

Mycgr3G84494 Mycgr3T
  
Location: 523-2047

Mycgr3G84494\_Mycgr3T

Mycgr3G90558 Mycgr3T
  
Location: 2147-15296

Mycgr3G90558\_Mycgr3T

Mycgr3G68036 Mycgr3T
  
Location: 15396-16395

Mycgr3G68036\_Mycgr3T

Mycgr3G90561 Mycgr3T
  
Location: 16495-17134

Mycgr3G90561\_Mycgr3T

Mycgr3G35862 Mycgr3T
  
Location: 17234-18662

Mycgr3G35862\_Mycgr3T

Mycgr3G68030 Mycgr3T
  
Location: 18762-19722

Mycgr3G68030\_Mycgr3T

Mycgr3G36449 Mycgr3T
  
Location: 19822-21886

Mycgr3G36449\_Mycgr3T

Mycgr3G35528 Mycgr3T
  
Location: 21986-22844

Mycgr3G35528\_Mycgr3T

Mycgr3G35932 Mycgr3T
  
Location: 22944-24390

Mycgr3G35932\_Mycgr3T

Mycgr3G23761 Mycgr3T
  
Location: 24490-25825

Mycgr3G23761\_Mycgr3T

Mycgr3G35535 Mycgr3T
  
Location: 25925-26429

Mycgr3G35535\_Mycgr3T

Mycgr3G9942 Mycgr3T9
  
Location: 26529-30375

Mycgr3G9942\_Mycgr3T9

nucleoside-diphosphate-sugarepimerase,putative
  
Accession: EKG10374
  
Location: 43709-44092
  
 NCBI BlastP on this gene

EKG10374

AMP-dependent synthetase/ligase
  
Accession: EKG10373
  
Location: 10381-42790
  
  
**BlastP hit with Mycgr3G90558\_Mycgr3T**
  
Percentage identity: 34 %
  
BlastP bit score: 2110
  
Sequence coverage: 90 %
  
E-value: 0.0
  
  
 NCBI BlastP on this gene

EKG10373

hypothetical protein
  
Accession: EKG10372
  
Location: 8617-9045
  
 NCBI BlastP on this gene

EKG10372

Query: Architecture Search FASTA input

ABDF02000078 : Trichoderma virens Gv29-8    Total score: 1.0     Cumulative Blast bit score: 2074

Hit cluster cross-links:

Mycgr3G36335 Mycgr3T
  
Location: 0-423

Mycgr3G36335\_Mycgr3T

Mycgr3G84494 Mycgr3T
  
Location: 523-2047

Mycgr3G84494\_Mycgr3T

Mycgr3G90558 Mycgr3T
  
Location: 2147-15296

Mycgr3G90558\_Mycgr3T

Mycgr3G68036 Mycgr3T
  
Location: 15396-16395

Mycgr3G68036\_Mycgr3T

Mycgr3G90561 Mycgr3T
  
Location: 16495-17134

Mycgr3G90561\_Mycgr3T

Mycgr3G35862 Mycgr3T
  
Location: 17234-18662

Mycgr3G35862\_Mycgr3T

Mycgr3G68030 Mycgr3T
  
Location: 18762-19722

Mycgr3G68030\_Mycgr3T

Mycgr3G36449 Mycgr3T
  
Location: 19822-21886

Mycgr3G36449\_Mycgr3T

Mycgr3G35528 Mycgr3T
  
Location: 21986-22844

Mycgr3G35528\_Mycgr3T

Mycgr3G35932 Mycgr3T
  
Location: 22944-24390

Mycgr3G35932\_Mycgr3T

Mycgr3G23761 Mycgr3T
  
Location: 24490-25825

Mycgr3G23761\_Mycgr3T

Mycgr3G35535 Mycgr3T
  
Location: 25925-26429

Mycgr3G35535\_Mycgr3T

Mycgr3G9942 Mycgr3T9
  
Location: 26529-30375

Mycgr3G9942\_Mycgr3T9

hypothetical protein
  
Accession: EHK20662
  
Location: 89989-91931
  
 NCBI BlastP on this gene

EHK20662

hypothetical protein
  
Accession: EHK20661
  
Location: 87545-88785
  
 NCBI BlastP on this gene

EHK20661

non-ribosomal peptide synthetase
  
Accession: EHK20800
  
Location: 60046-84786
  
  
**BlastP hit with Mycgr3G90558\_Mycgr3T**
  
Percentage identity: 32 %
  
BlastP bit score: 2074
  
Sequence coverage: 93 %
  
E-value: 0.0
  
  
 NCBI BlastP on this gene

EHK20800

hypothetical protein
  
Accession: EHK20660
  
Location: 57119-58261
  
 NCBI BlastP on this gene

EHK20660

hypothetical protein
  
Accession: EHK20659
  
Location: 55312-56073
  
 NCBI BlastP on this gene

EHK20659

Query: Architecture Search FASTA input

ABDF02000005 : Trichoderma virens Gv29-8    Total score: 1.0     Cumulative Blast bit score: 2042

Hit cluster cross-links:

Mycgr3G36335 Mycgr3T
  
Location: 0-423

Mycgr3G36335\_Mycgr3T

Mycgr3G84494 Mycgr3T
  
Location: 523-2047

Mycgr3G84494\_Mycgr3T

Mycgr3G90558 Mycgr3T
  
Location: 2147-15296

Mycgr3G90558\_Mycgr3T

Mycgr3G68036 Mycgr3T
  
Location: 15396-16395

Mycgr3G68036\_Mycgr3T

Mycgr3G90561 Mycgr3T
  
Location: 16495-17134

Mycgr3G90561\_Mycgr3T

Mycgr3G35862 Mycgr3T
  
Location: 17234-18662

Mycgr3G35862\_Mycgr3T

Mycgr3G68030 Mycgr3T
  
Location: 18762-19722

Mycgr3G68030\_Mycgr3T

Mycgr3G36449 Mycgr3T
  
Location: 19822-21886

Mycgr3G36449\_Mycgr3T

Mycgr3G35528 Mycgr3T
  
Location: 21986-22844

Mycgr3G35528\_Mycgr3T

Mycgr3G35932 Mycgr3T
  
Location: 22944-24390

Mycgr3G35932\_Mycgr3T

Mycgr3G23761 Mycgr3T
  
Location: 24490-25825

Mycgr3G23761\_Mycgr3T

Mycgr3G35535 Mycgr3T
  
Location: 25925-26429

Mycgr3G35535\_Mycgr3T

Mycgr3G9942 Mycgr3T9
  
Location: 26529-30375

Mycgr3G9942\_Mycgr3T9

non-ribosomal peptide synthetase
  
Accession: EHK23788
  
Location: 1830830-1880925
  
  
**BlastP hit with Mycgr3G90558\_Mycgr3T**
  
Percentage identity: 33 %
  
BlastP bit score: 2042
  
Sequence coverage: 89 %
  
E-value: 0.0
  
  
 NCBI BlastP on this gene

EHK23788

Query: Architecture Search FASTA input

CAGA01000020 : Claviceps purpurea 20.1    Total score: 1.0     Cumulative Blast bit score: 2029

Hit cluster cross-links:

Mycgr3G36335 Mycgr3T
  
Location: 0-423

Mycgr3G36335\_Mycgr3T

Mycgr3G84494 Mycgr3T
  
Location: 523-2047

Mycgr3G84494\_Mycgr3T

Mycgr3G90558 Mycgr3T
  
Location: 2147-15296

Mycgr3G90558\_Mycgr3T

Mycgr3G68036 Mycgr3T
  
Location: 15396-16395

Mycgr3G68036\_Mycgr3T

Mycgr3G90561 Mycgr3T
  
Location: 16495-17134

Mycgr3G90561\_Mycgr3T

Mycgr3G35862 Mycgr3T
  
Location: 17234-18662

Mycgr3G35862\_Mycgr3T

Mycgr3G68030 Mycgr3T
  
Location: 18762-19722

Mycgr3G68030\_Mycgr3T

Mycgr3G36449 Mycgr3T
  
Location: 19822-21886

Mycgr3G36449\_Mycgr3T

Mycgr3G35528 Mycgr3T
  
Location: 21986-22844

Mycgr3G35528\_Mycgr3T

Mycgr3G35932 Mycgr3T
  
Location: 22944-24390

Mycgr3G35932\_Mycgr3T

Mycgr3G23761 Mycgr3T
  
Location: 24490-25825

Mycgr3G23761\_Mycgr3T

Mycgr3G35535 Mycgr3T
  
Location: 25925-26429

Mycgr3G35535\_Mycgr3T

Mycgr3G9942 Mycgr3T9
  
Location: 26529-30375

Mycgr3G9942\_Mycgr3T9

related to benzoate 4-monooxygenase cytochrome P450
  
Accession: CCE30173
  
Location: 18893-20505
  
 NCBI BlastP on this gene

CCE30173

related to AM-toxin synthetase (AMT)
  
Accession: CCE30174
  
Location: 22433-28783
  
  
**BlastP hit with Mycgr3G90558\_Mycgr3T**
  
Percentage identity: 32 %
  
BlastP bit score: 1029
  
Sequence coverage: 48 %
  
E-value: 0.0
  
  
 NCBI BlastP on this gene

CCE30174

related to non-ribosomal peptide synthetase
  
Accession: CCE30175
  
Location: 28880-53423
  
  
**BlastP hit with Mycgr3G90558\_Mycgr3T**
  
Percentage identity: 31 %
  
BlastP bit score: 1001
  
Sequence coverage: 49 %
  
E-value: 0.0
  
  
 NCBI BlastP on this gene

CCE30175

uncharacterized protein
  
Accession: CCE30176
  
Location: 55022-55696
  
 NCBI BlastP on this gene

CCE30176

Query: Architecture Search FASTA input

ABDG02000023 : Trichoderma atroviride IMI 206040    Total score: 1.0     Cumulative Blast bit score: 2018

Hit cluster cross-links:

Mycgr3G36335 Mycgr3T
  
Location: 0-423

Mycgr3G36335\_Mycgr3T

Mycgr3G84494 Mycgr3T
  
Location: 523-2047

Mycgr3G84494\_Mycgr3T

Mycgr3G90558 Mycgr3T
  
Location: 2147-15296

Mycgr3G90558\_Mycgr3T

Mycgr3G68036 Mycgr3T
  
Location: 15396-16395

Mycgr3G68036\_Mycgr3T

Mycgr3G90561 Mycgr3T
  
Location: 16495-17134

Mycgr3G90561\_Mycgr3T

Mycgr3G35862 Mycgr3T
  
Location: 17234-18662

Mycgr3G35862\_Mycgr3T

Mycgr3G68030 Mycgr3T
  
Location: 18762-19722

Mycgr3G68030\_Mycgr3T

Mycgr3G36449 Mycgr3T
  
Location: 19822-21886

Mycgr3G36449\_Mycgr3T

Mycgr3G35528 Mycgr3T
  
Location: 21986-22844

Mycgr3G35528\_Mycgr3T

Mycgr3G35932 Mycgr3T
  
Location: 22944-24390

Mycgr3G35932\_Mycgr3T

Mycgr3G23761 Mycgr3T
  
Location: 24490-25825

Mycgr3G23761\_Mycgr3T

Mycgr3G35535 Mycgr3T
  
Location: 25925-26429

Mycgr3G35535\_Mycgr3T

Mycgr3G9942 Mycgr3T9
  
Location: 26529-30375

Mycgr3G9942\_Mycgr3T9

non-ribosomal peptide synthetase
  
Accession: EHK45804
  
Location: 857001-923302
  
  
**BlastP hit with Mycgr3G90558\_Mycgr3T**
  
Percentage identity: 33 %
  
BlastP bit score: 2018
  
Sequence coverage: 89 %
  
E-value: 0.0
  
  
 NCBI BlastP on this gene

EHK45804

hypothetical protein
  
Accession: EHK45803
  
Location: 854648-856598
  
 NCBI BlastP on this gene

EHK45803

Query: Architecture Search FASTA input

JH126400 : Cordyceps militaris CM01 unplaced genomic scaffold CCM\_S00002    Total score: 1.0     Cumulative Blast bit score: 1948

Hit cluster cross-links:

Mycgr3G36335 Mycgr3T
  
Location: 0-423

Mycgr3G36335\_Mycgr3T

Mycgr3G84494 Mycgr3T
  
Location: 523-2047

Mycgr3G84494\_Mycgr3T

Mycgr3G90558 Mycgr3T
  
Location: 2147-15296

Mycgr3G90558\_Mycgr3T

Mycgr3G68036 Mycgr3T
  
Location: 15396-16395

Mycgr3G68036\_Mycgr3T

Mycgr3G90561 Mycgr3T
  
Location: 16495-17134

Mycgr3G90561\_Mycgr3T

Mycgr3G35862 Mycgr3T
  
Location: 17234-18662

Mycgr3G35862\_Mycgr3T

Mycgr3G68030 Mycgr3T
  
Location: 18762-19722

Mycgr3G68030\_Mycgr3T

Mycgr3G36449 Mycgr3T
  
Location: 19822-21886

Mycgr3G36449\_Mycgr3T

Mycgr3G35528 Mycgr3T
  
Location: 21986-22844

Mycgr3G35528\_Mycgr3T

Mycgr3G35932 Mycgr3T
  
Location: 22944-24390

Mycgr3G35932\_Mycgr3T

Mycgr3G23761 Mycgr3T
  
Location: 24490-25825

Mycgr3G23761\_Mycgr3T

Mycgr3G35535 Mycgr3T
  
Location: 25925-26429

Mycgr3G35535\_Mycgr3T

Mycgr3G9942 Mycgr3T9
  
Location: 26529-30375

Mycgr3G9942\_Mycgr3T9

ABC transporter, transmembrane region, type 1
  
Accession: EGX94981
  
Location: 4201881-4206855
  
 NCBI BlastP on this gene

EGX94981

benzoate 4-monooxygenase cytochrome P450
  
Accession: EGX94982
  
Location: 4208732-4210344
  
 NCBI BlastP on this gene

EGX94982

non-ribosomal peptide synthase, putative
  
Accession: EGX94983
  
Location: 4212343-4225710
  
  
**BlastP hit with Mycgr3G90558\_Mycgr3T**
  
Percentage identity: 32 %
  
BlastP bit score: 1948
  
Sequence coverage: 91 %
  
E-value: 0.0
  
  
 NCBI BlastP on this gene

EGX94983

Query: Architecture Search FASTA input

101. :  JH687379 Stereum hirsutum FP-91666 SS1 unplaced genomic scaffold STEHIscaffold\_1     Total score: 2.0     Cumulative Blast bit score: 438

Mycgr3G36335 Mycgr3T
  
Location: 0-423
  
 NCBI BlastP on this gene

Mycgr3G36335\_Mycgr3T

Mycgr3G84494 Mycgr3T
  
Location: 523-2047
  
 NCBI BlastP on this gene

Mycgr3G84494\_Mycgr3T

Mycgr3G90558 Mycgr3T
  
Location: 2147-15296
  
 NCBI BlastP on this gene

Mycgr3G90558\_Mycgr3T

Mycgr3G68036 Mycgr3T
  
Location: 15396-16395
  
 NCBI BlastP on this gene

Mycgr3G68036\_Mycgr3T

Mycgr3G90561 Mycgr3T
  
Location: 16495-17134
  
 NCBI BlastP on this gene

Mycgr3G90561\_Mycgr3T

Mycgr3G35862 Mycgr3T
  
Location: 17234-18662
  
 NCBI BlastP on this gene

Mycgr3G35862\_Mycgr3T

Mycgr3G68030 Mycgr3T
  
Location: 18762-19722
  
 NCBI BlastP on this gene

Mycgr3G68030\_Mycgr3T

Mycgr3G36449 Mycgr3T
  
Location: 19822-21886
  
 NCBI BlastP on this gene

Mycgr3G36449\_Mycgr3T

Mycgr3G35528 Mycgr3T
  
Location: 21986-22844
  
 NCBI BlastP on this gene

Mycgr3G35528\_Mycgr3T

Mycgr3G35932 Mycgr3T
  
Location: 22944-24390
  
 NCBI BlastP on this gene

Mycgr3G35932\_Mycgr3T

Mycgr3G23761 Mycgr3T
  
Location: 24490-25825
  
 NCBI BlastP on this gene

Mycgr3G23761\_Mycgr3T

Mycgr3G35535 Mycgr3T
  
Location: 25925-26429
  
 NCBI BlastP on this gene

Mycgr3G35535\_Mycgr3T

Mycgr3G9942 Mycgr3T9
  
Location: 26529-30375
  
 NCBI BlastP on this gene

Mycgr3G9942\_Mycgr3T9

hypothetical protein
  
Accession: EIM92256
  
Location: 1771255-1775584
  
 NCBI BlastP on this gene

EIM92256

proteasome activator pa28 REG alpha/beta subunit
  
Accession: EIM92257
  
Location: 1775783-1777147
  
 NCBI BlastP on this gene

EIM92257

glutamate-rich WD repeat-containing protein
  
Accession: EIM92258
  
Location: 1777328-1778971
  
 NCBI BlastP on this gene

EIM92258

hypothetical protein
  
Accession: EIM92259
  
Location: 1779528-1780114
  
 NCBI BlastP on this gene

EIM92259

hypothetical protein
  
Accession: EIM92260
  
Location: 1780676-1782278
  
 NCBI BlastP on this gene

EIM92260

hypothetical protein
  
Accession: EIM92261
  
Location: 1783012-1784185
  
 NCBI BlastP on this gene

EIM92261

hypothetical protein
  
Accession: EIM92262
  
Location: 1784447-1785944
  
 NCBI BlastP on this gene

EIM92262

hypothetical protein
  
Accession: EIM92263
  
Location: 1786101-1786702
  
 NCBI BlastP on this gene

EIM92263

NAD-P-binding protein
  
Accession: EIM92264
  
Location: 1787143-1788391
  
 NCBI BlastP on this gene

EIM92264

MFS general substrate transporter
  
Accession: EIM92265
  
Location: 1788927-1790979
  
  
**BlastP hit with Mycgr3G84494\_Mycgr3T**
  
Percentage identity: 38 %
  
BlastP bit score: 333
  
Sequence coverage: 97 %
  
E-value: 2e-104
  
  
 NCBI BlastP on this gene

EIM92265

hypothetical protein
  
Accession: EIM92266
  
Location: 1792213-1792532
  
 NCBI BlastP on this gene

EIM92266

hypothetical protein
  
Accession: EIM92267
  
Location: 1795602-1796105
  
 NCBI BlastP on this gene

EIM92267

DNA-binding protein
  
Accession: EIM92268
  
Location: 1797274-1798657
  
 NCBI BlastP on this gene

EIM92268

lipid binding protein
  
Accession: EIM92269
  
Location: 1798969-1800822
  
 NCBI BlastP on this gene

EIM92269

hypothetical protein
  
Accession: EIM92270
  
Location: 1800966-1803093
  
 NCBI BlastP on this gene

EIM92270

hypothetical protein
  
Accession: EIM92271
  
Location: 1803161-1803490
  
 NCBI BlastP on this gene

EIM92271

DNA-dependent RNA polymerase II second largest subunit
  
Accession: EIM92272
  
Location: 1803715-1807973
  
 NCBI BlastP on this gene

EIM92272

hypothetical protein
  
Accession: EIM92273
  
Location: 1808387-1810121
  
 NCBI BlastP on this gene

EIM92273

hypothetical protein
  
Accession: EIM92274
  
Location: 1811201-1814495
  
 NCBI BlastP on this gene

EIM92274

fructosamine kinase PKL/CAK/FruK
  
Accession: EIM92275
  
Location: 1815835-1816981
  
  
**BlastP hit with Mycgr3G68030\_Mycgr3T**
  
Percentage identity: 32 %
  
BlastP bit score: 105
  
Sequence coverage: 82 %
  
E-value: 6e-23
  
  
 NCBI BlastP on this gene

EIM92275

ABC1-domain-containing protein
  
Accession: EIM92276
  
Location: 1817562-1819982
  
 NCBI BlastP on this gene

EIM92276

hypothetical protein
  
Accession: EIM92277
  
Location: 1820519-1821019
  
 NCBI BlastP on this gene

EIM92277

hypothetical protein
  
Accession: EIM92278
  
Location: 1821151-1821339
  
 NCBI BlastP on this gene

EIM92278

hypothetical protein
  
Accession: EIM92279
  
Location: 1821845-1826381
  
 NCBI BlastP on this gene

EIM92279

hypothetical protein
  
Accession: EIM92280
  
Location: 1827959-1829529
  
 NCBI BlastP on this gene

EIM92280

hypothetical protein
  
Accession: EIM92281
  
Location: 1831234-1833852
  
 NCBI BlastP on this gene

EIM92281

cytoskeleton assembly control protein
  
Accession: EIM92282
  
Location: 1835349-1839209
  
 NCBI BlastP on this gene

EIM92282

102. :  JH711783 Trametes versicolor FP-101664 SS1 unplaced genomic scaffold TRAVEscaffold\_1     Total score: 2.0     Cumulative Blast bit score: 429

WD40 repeat-like protein
  
Accession: EIW64206
  
Location: 1567606-1569822
  
 NCBI BlastP on this gene

EIW64206

Bax inhibitor family protein
  
Accession: EIW64207
  
Location: 1570233-1571509
  
 NCBI BlastP on this gene

EIW64207

Sec1-like protein
  
Accession: EIW64208
  
Location: 1571703-1574183
  
 NCBI BlastP on this gene

EIW64208

hypothetical protein
  
Accession: EIW64209
  
Location: 1575554-1580078
  
 NCBI BlastP on this gene

EIW64209

ABC1-domain-containing protein
  
Accession: EIW64210
  
Location: 1580988-1583228
  
 NCBI BlastP on this gene

EIW64210

FMN-linked oxidoreductase
  
Accession: EIW64211
  
Location: 1583437-1585179
  
 NCBI BlastP on this gene

EIW64211

fructosamine-3-kinase
  
Accession: EIW64212
  
Location: 1585594-1586730
  
 NCBI BlastP on this gene

EIW64212

fructosamine kinase PKL/CAK/FruK
  
Accession: EIW64213
  
Location: 1587212-1588348
  
  
**BlastP hit with Mycgr3G68030\_Mycgr3T**
  
Percentage identity: 32 %
  
BlastP bit score: 112
  
Sequence coverage: 86 %
  
E-value: 3e-25
  
  
 NCBI BlastP on this gene

EIW64213

cellobiohydrolaseI
  
Accession: EIW64214
  
Location: 1588636-1590113
  
 NCBI BlastP on this gene

EIW64214

DNA-dependent RNA polymerase II second largest subunit
  
Accession: EIW64215
  
Location: 1592435-1596632
  
 NCBI BlastP on this gene

EIW64215

hypothetical protein
  
Accession: EIW64216
  
Location: 1596732-1599114
  
 NCBI BlastP on this gene

EIW64216

lipid binding protein
  
Accession: EIW64217
  
Location: 1599317-1601194
  
 NCBI BlastP on this gene

EIW64217

hypothetical protein
  
Accession: EIW64218
  
Location: 1603261-1607459
  
 NCBI BlastP on this gene

EIW64218

MFS general substrate transporter
  
Accession: EIW64219
  
Location: 1608053-1610107
  
  
**BlastP hit with Mycgr3G84494\_Mycgr3T**
  
Percentage identity: 37 %
  
BlastP bit score: 317
  
Sequence coverage: 94 %
  
E-value: 5e-98
  
  
 NCBI BlastP on this gene

EIW64219

hypothetical protein
  
Accession: EIW64220
  
Location: 1610552-1611917
  
 NCBI BlastP on this gene

EIW64220

hypothetical protein
  
Accession: EIW64221
  
Location: 1612134-1613111
  
 NCBI BlastP on this gene

EIW64221

hypothetical protein
  
Accession: EIW64222
  
Location: 1616468-1619851
  
 NCBI BlastP on this gene

EIW64222

cytoskeleton assembly control protein
  
Accession: EIW64223
  
Location: 1620297-1624094
  
 NCBI BlastP on this gene

EIW64223

SURF1-domain-containing protein
  
Accession: EIW64224
  
Location: 1624452-1625395
  
 NCBI BlastP on this gene

EIW64224

hypothetical protein
  
Accession: EIW64225
  
Location: 1626027-1627024
  
 NCBI BlastP on this gene

EIW64225

galactose oxidase
  
Accession: EIW64226
  
Location: 1630072-1631289
  
 NCBI BlastP on this gene

EIW64226

103. :  CABT02000016 Sordaria macrospora k-hell     Total score: 2.0     Cumulative Blast bit score: 426

not annotated
  
Accession: CCC11154
  
Location: 565445-566800
  
 NCBI BlastP on this gene

CCC11154

not annotated
  
Accession: CCC11155
  
Location: 567222-568103
  
 NCBI BlastP on this gene

CCC11155

not annotated
  
Accession: CCC11156
  
Location: 570307-576885
  
 NCBI BlastP on this gene

CCC11156

not annotated
  
Accession: CCC11157
  
Location: 582480-583167
  
 NCBI BlastP on this gene

CCC11157

not annotated
  
Accession: CCC11158
  
Location: 584231-585887
  
  
**BlastP hit with Mycgr3G84494\_Mycgr3T**
  
Percentage identity: 40 %
  
BlastP bit score: 298
  
Sequence coverage: 87 %
  
E-value: 2e-91
  
  
 NCBI BlastP on this gene

CCC11158

not annotated
  
Accession: CCC11159
  
Location: 586069-587362
  
  
**BlastP hit with Mycgr3G68030\_Mycgr3T**
  
Percentage identity: 29 %
  
BlastP bit score: 128
  
Sequence coverage: 98 %
  
E-value: 2e-30
  
  
 NCBI BlastP on this gene

CCC11159

not annotated
  
Accession: CCC11160
  
Location: 587752-590878
  
 NCBI BlastP on this gene

CCC11160

not annotated
  
Accession: CCC11161
  
Location: 592101-593340
  
 NCBI BlastP on this gene

CCC11161

not annotated
  
Accession: CCC11162
  
Location: 595389-598861
  
 NCBI BlastP on this gene

CCC11162

not annotated
  
Accession: CCC11163
  
Location: 601173-603236
  
 NCBI BlastP on this gene

CCC11163

not annotated
  
Accession: CCC11164
  
Location: 605476-607227
  
 NCBI BlastP on this gene

CCC11164

104. :  KE504141 Fomitopsis pinicola FP-58527 SS1 unplaced genomic scaffold FOMPIscaffold\_20     Total score: 2.0     Cumulative Blast bit score: 417

hypothetical protein
  
Accession: EPT01521
  
Location: 151059-153341
  
 NCBI BlastP on this gene

EPT01521

hypothetical protein
  
Accession: EPT01522
  
Location: 154638-156572
  
 NCBI BlastP on this gene

EPT01522

hypothetical protein
  
Accession: EPT01523
  
Location: 157016-157901
  
 NCBI BlastP on this gene

EPT01523

hypothetical protein
  
Accession: EPT01524
  
Location: 158582-162524
  
 NCBI BlastP on this gene

EPT01524

hypothetical protein
  
Accession: EPT01525
  
Location: 163260-163917
  
 NCBI BlastP on this gene

EPT01525

hypothetical protein
  
Accession: EPT01526
  
Location: 166057-168041
  
 NCBI BlastP on this gene

EPT01526

hypothetical protein
  
Accession: EPT01527
  
Location: 168288-169945
  
 NCBI BlastP on this gene

EPT01527

hypothetical protein
  
Accession: EPT01528
  
Location: 170338-171479
  
  
**BlastP hit with Mycgr3G68030\_Mycgr3T**
  
Percentage identity: 33 %
  
BlastP bit score: 114
  
Sequence coverage: 82 %
  
E-value: 9e-26
  
  
 NCBI BlastP on this gene

EPT01528

hypothetical protein
  
Accession: EPT01529
  
Location: 172019-176128
  
 NCBI BlastP on this gene

EPT01529

hypothetical protein
  
Accession: EPT01530
  
Location: 176232-178553
  
 NCBI BlastP on this gene

EPT01530

hypothetical protein
  
Accession: EPT01531
  
Location: 178755-180601
  
 NCBI BlastP on this gene

EPT01531

hypothetical protein
  
Accession: EPT01532
  
Location: 180890-181991
  
 NCBI BlastP on this gene

EPT01532

hypothetical protein
  
Accession: EPT01533
  
Location: 182457-186420
  
 NCBI BlastP on this gene

EPT01533

hypothetical protein
  
Accession: EPT01534
  
Location: 186803-188971
  
  
**BlastP hit with Mycgr3G84494\_Mycgr3T**
  
Percentage identity: 36 %
  
BlastP bit score: 303
  
Sequence coverage: 100 %
  
E-value: 2e-92
  
  
 NCBI BlastP on this gene

EPT01534

hypothetical protein
  
Accession: EPT01535
  
Location: 190359-191708
  
 NCBI BlastP on this gene

EPT01535

hypothetical protein
  
Accession: EPT01536
  
Location: 192160-192851
  
 NCBI BlastP on this gene

EPT01536

hypothetical protein
  
Accession: EPT01537
  
Location: 193791-197279
  
 NCBI BlastP on this gene

EPT01537

hypothetical protein
  
Accession: EPT01538
  
Location: 199264-203050
  
 NCBI BlastP on this gene

EPT01538

hypothetical protein
  
Accession: EPT01539
  
Location: 203411-204364
  
 NCBI BlastP on this gene

EPT01539

hypothetical protein
  
Accession: EPT01540
  
Location: 207470-210373
  
 NCBI BlastP on this gene

EPT01540

105. :  JH725165 Beauveria bassiana ARSEF 2860 unplaced genomic scaffold BBA\_S00016     Total score: 2.0     Cumulative Blast bit score: 413

20beta-hydroxysteroid dehydrogenase
  
Accession: EJP65056
  
Location: 70640-71637
  
 NCBI BlastP on this gene

EJP65056

siderophore iron transporter
  
Accession: EJP65057
  
Location: 72246-74143
  
 NCBI BlastP on this gene

EJP65057

hypothetical protein
  
Accession: EJP65058
  
Location: 77769-79012
  
 NCBI BlastP on this gene

EJP65058

hypothetical protein
  
Accession: EJP65059
  
Location: 79779-80695
  
 NCBI BlastP on this gene

EJP65059

CHCH domain-containing protein
  
Accession: EJP65060
  
Location: 81146-82401
  
 NCBI BlastP on this gene

EJP65060

thiamine pyrophosphokinase
  
Accession: EJP65061
  
Location: 82879-83824
  
 NCBI BlastP on this gene

EJP65061

vesicle transport V-SNARE protein
  
Accession: EJP65062
  
Location: 84054-84961
  
 NCBI BlastP on this gene

EJP65062

major facilitator superfamily transporter
  
Accession: EJP65063
  
Location: 86358-87920
  
  
**BlastP hit with Mycgr3G84494\_Mycgr3T**
  
Percentage identity: 35 %
  
BlastP bit score: 294
  
Sequence coverage: 97 %
  
E-value: 3e-89
  
  
 NCBI BlastP on this gene

EJP65063

Cytochrome P450 CYP528A4
  
Accession: EJP65064
  
Location: 88419-90218
  
 NCBI BlastP on this gene

EJP65064

putative peptide transporter
  
Accession: EJP65065
  
Location: 94713-96651
  
 NCBI BlastP on this gene

EJP65065

dihydrofolate reductase-thymidylate synthase
  
Accession: EJP65066
  
Location: 97381-98453
  
 NCBI BlastP on this gene

EJP65066

WD domain-containing protein
  
Accession: EJP65067
  
Location: 98738-101470
  
 NCBI BlastP on this gene

EJP65067

glycosyl hydrolase family 2
  
Accession: EJP65068
  
Location: 102539-105688
  
 NCBI BlastP on this gene

EJP65068

MFS transporter, putative
  
Accession: EJP65069
  
Location: 107233-109024
  
  
**BlastP hit with Mycgr3G23761\_Mycgr3T**
  
Percentage identity: 26 %
  
BlastP bit score: 119
  
Sequence coverage: 94 %
  
E-value: 4e-26
  
  
 NCBI BlastP on this gene

EJP65069

transposase-like protein
  
Accession: EJP65070
  
Location: 109573-110631
  
 NCBI BlastP on this gene

EJP65070

ABC transporter, putative
  
Accession: EJP65071
  
Location: 111811-115827
  
 NCBI BlastP on this gene

EJP65071

hypothetical protein
  
Accession: EJP65072
  
Location: 122970-124271
  
 NCBI BlastP on this gene

EJP65072

LUC7 protein
  
Accession: EJP65073
  
Location: 124687-125580
  
 NCBI BlastP on this gene

EJP65073

GPI transamidase component GPI16
  
Accession: EJP65074
  
Location: 125978-127789
  
 NCBI BlastP on this gene

EJP65074

acetylornithine aminotransferase
  
Accession: EJP65075
  
Location: 128400-129797
  
 NCBI BlastP on this gene

EJP65075

106. :  JH711573 Coniophora puteana RWD-64-598 SS2 unplaced genomic scaffold CONPUscaffold\_1     Total score: 2.0     Cumulative Blast bit score: 413

hypothetical protein
  
Accession: EIW87061
  
Location: 3327116-3328676
  
 NCBI BlastP on this gene

EIW87061

hypothetical protein
  
Accession: EIW87060
  
Location: 3324480-3327008
  
 NCBI BlastP on this gene

EIW87060

mitochondrial protein required for respiration
  
Accession: EIW87059
  
Location: 3321761-3322833
  
 NCBI BlastP on this gene

EIW87059

ANTH-domain-containing protein
  
Accession: EIW87058
  
Location: 3317809-3321590
  
 NCBI BlastP on this gene

EIW87058

hypothetical protein
  
Accession: EIW87057
  
Location: 3314947-3317460
  
 NCBI BlastP on this gene

EIW87057

hypothetical protein
  
Accession: EIW87056
  
Location: 3312967-3313815
  
 NCBI BlastP on this gene

EIW87056

hypothetical protein
  
Accession: EIW87055
  
Location: 3311288-3312705
  
 NCBI BlastP on this gene

EIW87055

NAD(P)-binding protein
  
Accession: EIW87054
  
Location: 3308663-3309876
  
 NCBI BlastP on this gene

EIW87054

MFS general substrate transporter
  
Accession: EIW87053
  
Location: 3306297-3308281
  
  
**BlastP hit with Mycgr3G84494\_Mycgr3T**
  
Percentage identity: 37 %
  
BlastP bit score: 312
  
Sequence coverage: 96 %
  
E-value: 2e-96
  
  
 NCBI BlastP on this gene

EIW87053

hypothetical protein
  
Accession: EIW87052
  
Location: 3305599-3306199
  
 NCBI BlastP on this gene

EIW87052

ABC1-domain-containing protein
  
Accession: EIW87051
  
Location: 3302090-3304477
  
 NCBI BlastP on this gene

EIW87051

NADH:flavin oxidoreductase 2
  
Accession: EIW87050
  
Location: 3299034-3300665
  
 NCBI BlastP on this gene

EIW87050

Ketosamine-3-kinase
  
Accession: EIW87049
  
Location: 3297431-3298543
  
  
**BlastP hit with Mycgr3G68030\_Mycgr3T**
  
Percentage identity: 30 %
  
BlastP bit score: 101
  
Sequence coverage: 80 %
  
E-value: 2e-21
  
  
 NCBI BlastP on this gene

EIW87049

DNA-dependent RNA polymerase II second largest subunit
  
Accession: EIW87048
  
Location: 3293066-3297152
  
 NCBI BlastP on this gene

EIW87048

hypothetical protein
  
Accession: EIW87047
  
Location: 3289723-3291539
  
 NCBI BlastP on this gene

EIW87047

protein PTM1
  
Accession: EIW87046
  
Location: 3287414-3289476
  
 NCBI BlastP on this gene

EIW87046

hypothetical protein
  
Accession: EIW87468
  
Location: 3285526-3286715
  
 NCBI BlastP on this gene

EIW87468

glycosyltransferase family 8 protein
  
Accession: EIW87045
  
Location: 3281562-3284581
  
 NCBI BlastP on this gene

EIW87045

hypothetical protein
  
Accession: EIW87044
  
Location: 3280185-3280836
  
 NCBI BlastP on this gene

EIW87044

beta-flanking protein
  
Accession: EIW87043
  
Location: 3278146-3279049
  
 NCBI BlastP on this gene

EIW87043

107. :  KB733487 Bipolaris maydis ATCC 48331 unplaced genomic scaffold COCC4scaffold\_44     Total score: 2.0     Cumulative Blast bit score: 396

hypothetical protein
  
Accession: ENH99363
  
Location: 79119-80296
  
 NCBI BlastP on this gene

ENH99363

hypothetical protein
  
Accession: ENH99364
  
Location: 80866-81559
  
 NCBI BlastP on this gene

ENH99364

hypothetical protein
  
Accession: ENH99365
  
Location: 82541-84059
  
 NCBI BlastP on this gene

ENH99365

hypothetical protein
  
Accession: ENH99366
  
Location: 85395-87482
  
 NCBI BlastP on this gene

ENH99366

hypothetical protein
  
Accession: ENH99367
  
Location: 88200-90273
  
 NCBI BlastP on this gene

ENH99367

hypothetical protein
  
Accession: ENH99368
  
Location: 90682-95272
  
 NCBI BlastP on this gene

ENH99368

hypothetical protein
  
Accession: ENH99369
  
Location: 97058-97387
  
 NCBI BlastP on this gene

ENH99369

hypothetical protein
  
Accession: ENH99370
  
Location: 97632-98482
  
 NCBI BlastP on this gene

ENH99370

hypothetical protein
  
Accession: ENH99371
  
Location: 98873-100570
  
  
**BlastP hit with Mycgr3G84494\_Mycgr3T**
  
Percentage identity: 37 %
  
BlastP bit score: 324
  
Sequence coverage: 97 %
  
E-value: 9e-101
  
  
 NCBI BlastP on this gene

ENH99371

hypothetical protein
  
Accession: ENH99372
  
Location: 101680-102603
  
 NCBI BlastP on this gene

ENH99372

hypothetical protein
  
Accession: ENH99373
  
Location: 103143-104558
  
 NCBI BlastP on this gene

ENH99373

hypothetical protein
  
Accession: ENH99374
  
Location: 106283-107143
  
 NCBI BlastP on this gene

ENH99374

hypothetical protein
  
Accession: ENH99375
  
Location: 107958-108389
  
  
**BlastP hit with Mycgr3G36335\_Mycgr3T**
  
Percentage identity: 34 %
  
BlastP bit score: 72
  
Sequence coverage: 95 %
  
E-value: 2e-13
  
  
 NCBI BlastP on this gene

ENH99375

hypothetical protein
  
Accession: ENH99376
  
Location: 109859-110737
  
 NCBI BlastP on this gene

ENH99376

hypothetical protein
  
Accession: ENH99377
  
Location: 111042-112083
  
 NCBI BlastP on this gene

ENH99377

hypothetical protein
  
Accession: ENH99378
  
Location: 112323-115681
  
 NCBI BlastP on this gene

ENH99378

hypothetical protein
  
Accession: ENH99379
  
Location: 116034-117259
  
 NCBI BlastP on this gene

ENH99379

hypothetical protein
  
Accession: ENH99380
  
Location: 117805-122600
  
 NCBI BlastP on this gene

ENH99380

hypothetical protein
  
Accession: ENH99381
  
Location: 123264-126095
  
 NCBI BlastP on this gene

ENH99381

hypothetical protein
  
Accession: ENH99382
  
Location: 126540-128163
  
 NCBI BlastP on this gene

ENH99382

108. :  KB445592 Cochliobolus heterostrophus C5 unplaced genomic scaffold COCHEscaffold\_24     Total score: 2.0     Cumulative Blast bit score: 396

hypothetical protein
  
Accession: EMD85159
  
Location: 51952-53575
  
 NCBI BlastP on this gene

EMD85159

hypothetical protein
  
Accession: EMD85160
  
Location: 54020-56851
  
 NCBI BlastP on this gene

EMD85160

hypothetical protein
  
Accession: EMD85161
  
Location: 57519-62314
  
 NCBI BlastP on this gene

EMD85161

hypothetical protein
  
Accession: EMD85162
  
Location: 62860-64085
  
 NCBI BlastP on this gene

EMD85162

hypothetical protein
  
Accession: EMD85163
  
Location: 64438-67796
  
 NCBI BlastP on this gene

EMD85163

hypothetical protein
  
Accession: EMD85164
  
Location: 68036-69077
  
 NCBI BlastP on this gene

EMD85164

hypothetical protein
  
Accession: EMD85165
  
Location: 69382-70260
  
 NCBI BlastP on this gene

EMD85165

hypothetical protein
  
Accession: EMD85166
  
Location: 71598-72158
  
  
**BlastP hit with Mycgr3G36335\_Mycgr3T**
  
Percentage identity: 34 %
  
BlastP bit score: 72
  
Sequence coverage: 95 %
  
E-value: 3e-13
  
  
 NCBI BlastP on this gene

EMD85166

hypothetical protein
  
Accession: EMD85167
  
Location: 72976-73836
  
 NCBI BlastP on this gene

EMD85167

hypothetical protein
  
Accession: EMD85168
  
Location: 75561-76976
  
 NCBI BlastP on this gene

EMD85168

hypothetical protein
  
Accession: EMD85169
  
Location: 77516-78439
  
 NCBI BlastP on this gene

EMD85169

hypothetical protein
  
Accession: EMD85170
  
Location: 79549-81246
  
  
**BlastP hit with Mycgr3G84494\_Mycgr3T**
  
Percentage identity: 37 %
  
BlastP bit score: 324
  
Sequence coverage: 97 %
  
E-value: 9e-101
  
  
 NCBI BlastP on this gene

EMD85170

hypothetical protein
  
Accession: EMD85171
  
Location: 81637-82487
  
 NCBI BlastP on this gene

EMD85171

hypothetical protein
  
Accession: EMD85172
  
Location: 82732-83061
  
 NCBI BlastP on this gene

EMD85172

hypothetical protein
  
Accession: EMD85173
  
Location: 84847-89437
  
 NCBI BlastP on this gene

EMD85173

hypothetical protein
  
Accession: EMD85174
  
Location: 89847-91920
  
 NCBI BlastP on this gene

EMD85174

hypothetical protein
  
Accession: EMD85175
  
Location: 92638-94725
  
 NCBI BlastP on this gene

EMD85175

hypothetical protein
  
Accession: EMD85176
  
Location: 96061-97579
  
 NCBI BlastP on this gene

EMD85176

hypothetical protein
  
Accession: EMD85177
  
Location: 98613-99306
  
 NCBI BlastP on this gene

EMD85177

hypothetical protein
  
Accession: EMD85178
  
Location: 99876-101053
  
 NCBI BlastP on this gene

EMD85178

hypothetical protein
  
Accession: EMD85179
  
Location: 101139-101900
  
 NCBI BlastP on this gene

EMD85179

109. :  KB445791 Ceriporiopsis subvermispora B unplaced genomic scaffold CERSUscaffold\_1     Total score: 2.0     Cumulative Blast bit score: 394

hypothetical protein
  
Accession: EMD41889
  
Location: 2312945-2316247
  
 NCBI BlastP on this gene

EMD41889

hypothetical protein
  
Accession: EMD41890
  
Location: 2316872-2317923
  
 NCBI BlastP on this gene

EMD41890

hypothetical protein
  
Accession: EMD41891
  
Location: 2318241-2322016
  
 NCBI BlastP on this gene

EMD41891

hypothetical protein
  
Accession: EMD41892
  
Location: 2327376-2328150
  
 NCBI BlastP on this gene

EMD41892

hypothetical protein
  
Accession: EMD41893
  
Location: 2328501-2329917
  
 NCBI BlastP on this gene

EMD41893

hypothetical protein
  
Accession: EMD41894
  
Location: 2331222-2333117
  
  
**BlastP hit with Mycgr3G84494\_Mycgr3T**
  
Percentage identity: 38 %
  
BlastP bit score: 290
  
Sequence coverage: 91 %
  
E-value: 2e-88
  
  
 NCBI BlastP on this gene

EMD41894

hypothetical protein
  
Accession: EMD41895
  
Location: 2333413-2337470
  
 NCBI BlastP on this gene

EMD41895

hypothetical protein
  
Accession: EMD41896
  
Location: 2338083-2338720
  
 NCBI BlastP on this gene

EMD41896

SNX4-like sorting nexin
  
Accession: EMD41897
  
Location: 2339162-2341025
  
 NCBI BlastP on this gene

EMD41897

hypothetical protein
  
Accession: EMD41898
  
Location: 2343719-2347914
  
 NCBI BlastP on this gene

EMD41898

hypothetical protein
  
Accession: EMD41899
  
Location: 2348343-2349484
  
  
**BlastP hit with Mycgr3G68030\_Mycgr3T**
  
Percentage identity: 29 %
  
BlastP bit score: 104
  
Sequence coverage: 87 %
  
E-value: 2e-22
  
  
 NCBI BlastP on this gene

EMD41899

hypothetical protein
  
Accession: EMD41900
  
Location: 2349880-2351518
  
 NCBI BlastP on this gene

EMD41900

hypothetical protein
  
Accession: EMD41901
  
Location: 2353791-2356182
  
 NCBI BlastP on this gene

EMD41901

glycosyltransferase family 8 protein
  
Accession: EMD41902
  
Location: 2357605-2361194
  
 NCBI BlastP on this gene

EMD41902

hypothetical protein
  
Accession: EMD41903
  
Location: 2361741-2362531
  
 NCBI BlastP on this gene

EMD41903

hypothetical protein
  
Accession: EMD41904
  
Location: 2362737-2364756
  
 NCBI BlastP on this gene

EMD41904

hypothetical protein
  
Accession: EMD41905
  
Location: 2365455-2367764
  
 NCBI BlastP on this gene

EMD41905

beta-flanking protein
  
Accession: EMD41906
  
Location: 2368171-2369230
  
 NCBI BlastP on this gene

EMD41906

110. :  DS231622 Pyrenophora tritici-repentis Pt-1C-BFP supercont1.8 genomic scaffold     Total score: 2.0     Cumulative Blast bit score: 392

predicted protein
  
Accession: EDU50626
  
Location: 545056-545388
  
 NCBI BlastP on this gene

EDU50626

alpha-mannosidase
  
Accession: EDU50627
  
Location: 546043-549529
  
 NCBI BlastP on this gene

EDU50627

protein transport protein SEC9
  
Accession: EDU50628
  
Location: 549860-551242
  
 NCBI BlastP on this gene

EDU50628

dTDP-D-glucose 4,6-dehydratase
  
Accession: EDU50629
  
Location: 551892-553396
  
 NCBI BlastP on this gene

EDU50629

predicted protein
  
Accession: EDU50630
  
Location: 553572-553727
  
 NCBI BlastP on this gene

EDU50630

conserved hypothetical protein
  
Accession: EDU50631
  
Location: 553758-555266
  
 NCBI BlastP on this gene

EDU50631

predicted protein
  
Accession: EDU50632
  
Location: 556132-557885
  
 NCBI BlastP on this gene

EDU50632

maltose permease MAL61
  
Accession: EDU50633
  
Location: 560596-562251
  
 NCBI BlastP on this gene

EDU50633

hypothetical protein
  
Accession: EDU50634
  
Location: 563659-564127
  
 NCBI BlastP on this gene

EDU50634

conserved hypothetical protein
  
Accession: EDU50635
  
Location: 564486-566859
  
  
**BlastP hit with Mycgr3G36335\_Mycgr3T**
  
Percentage identity: 38 %
  
BlastP bit score: 75
  
Sequence coverage: 81 %
  
E-value: 4e-13
  
  
 NCBI BlastP on this gene

EDU50635

citrinin biosynthesis oxydoreductase CtnB
  
Accession: EDU50636
  
Location: 566946-567809
  
 NCBI BlastP on this gene

EDU50636

threonine aldolase
  
Accession: EDU50637
  
Location: 569090-570447
  
 NCBI BlastP on this gene

EDU50637

conserved hypothetical protein
  
Accession: EDU50638
  
Location: 570966-571889
  
 NCBI BlastP on this gene

EDU50638

conserved hypothetical protein
  
Accession: EDU50639
  
Location: 573142-574914
  
  
**BlastP hit with Mycgr3G84494\_Mycgr3T**
  
Percentage identity: 37 %
  
BlastP bit score: 317
  
Sequence coverage: 95 %
  
E-value: 5e-98
  
  
 NCBI BlastP on this gene

EDU50639

conserved hypothetical protein
  
Accession: EDU50640
  
Location: 575028-575853
  
 NCBI BlastP on this gene

EDU50640

HET domain containing protein
  
Accession: EDU50641
  
Location: 577336-580369
  
 NCBI BlastP on this gene

EDU50641

conserved hypothetical protein
  
Accession: EDU50642
  
Location: 582166-583099
  
 NCBI BlastP on this gene

EDU50642

predicted protein
  
Accession: EDU50643
  
Location: 583383-584685
  
 NCBI BlastP on this gene

EDU50643

serine/threonine-protein kinase CBK1
  
Accession: EDU50644
  
Location: 585109-591059
  
 NCBI BlastP on this gene

EDU50644

meiosis-specific serine/threonine-protein kinase mek1
  
Accession: EDU50645
  
Location: 592193-595297
  
 NCBI BlastP on this gene

EDU50645

111. :  GL945428 Serpula lacrymans var. lacrymans S7.9 unplaced genomic scaffold SERLAscaffold\_1     Total score: 2.0     Cumulative Blast bit score: 391

hypothetical protein
  
Accession: EGO31169
  
Location: 5404200-5405074
  
 NCBI BlastP on this gene

EGO31169

hypothetical protein
  
Accession: EGO31170
  
Location: 5405504-5406574
  
 NCBI BlastP on this gene

EGO31170

hypothetical protein
  
Accession: EGO31171
  
Location: 5406808-5410592
  
 NCBI BlastP on this gene

EGO31171

hypothetical protein
  
Accession: EGO31172
  
Location: 5413110-5413526
  
 NCBI BlastP on this gene

EGO31172

hypothetical protein
  
Accession: EGO31173
  
Location: 5413994-5414233
  
 NCBI BlastP on this gene

EGO31173

hypothetical protein
  
Accession: EGO31174
  
Location: 5414989-5415218
  
 NCBI BlastP on this gene

EGO31174

hypothetical protein
  
Accession: EGO31175
  
Location: 5415695-5416411
  
 NCBI BlastP on this gene

EGO31175

hypothetical protein
  
Accession: EGO31176
  
Location: 5419309-5419894
  
 NCBI BlastP on this gene

EGO31176

hypothetical protein
  
Accession: EGO31177
  
Location: 5420942-5421245
  
 NCBI BlastP on this gene

EGO31177

hypothetical protein
  
Accession: EGO31178
  
Location: 5422235-5423465
  
 NCBI BlastP on this gene

EGO31178

hypothetical protein
  
Accession: EGO31179
  
Location: 5423923-5426283
  
  
**BlastP hit with Mycgr3G84494\_Mycgr3T**
  
Percentage identity: 36 %
  
BlastP bit score: 293
  
Sequence coverage: 93 %
  
E-value: 8e-89
  
  
 NCBI BlastP on this gene

EGO31179

hypothetical protein
  
Accession: EGO31180
  
Location: 5426446-5427574
  
 NCBI BlastP on this gene

EGO31180

hypothetical protein
  
Accession: EGO31181
  
Location: 5427827-5429701
  
 NCBI BlastP on this gene

EGO31181

DNA-directed RNA polymerase II, subunit 2
  
Accession: EGO31287
  
Location: 5432505-5436551
  
 NCBI BlastP on this gene

EGO31287

hypothetical protein
  
Accession: EGO31182
  
Location: 5436971-5438114
  
  
**BlastP hit with Mycgr3G68030\_Mycgr3T**
  
Percentage identity: 31 %
  
BlastP bit score: 98
  
Sequence coverage: 86 %
  
E-value: 2e-20
  
  
 NCBI BlastP on this gene

EGO31182

hypothetical protein
  
Accession: EGO31183
  
Location: 5438670-5440322
  
 NCBI BlastP on this gene

EGO31183

hypothetical protein
  
Accession: EGO31184
  
Location: 5440786-5443161
  
 NCBI BlastP on this gene

EGO31184

hypothetical protein
  
Accession: EGO31185
  
Location: 5444207-5445012
  
 NCBI BlastP on this gene

EGO31185

hypothetical protein
  
Accession: EGO31186
  
Location: 5446724-5449097
  
 NCBI BlastP on this gene

EGO31186

hypothetical protein
  
Accession: EGO31187
  
Location: 5458107-5458608
  
 NCBI BlastP on this gene

EGO31187

112. :  KB469296 Gloeophyllum trabeum ATCC 11539 unplaced genomic scaffold GLOTRscaffold\_00001     Total score: 2.0     Cumulative Blast bit score: 387

hypothetical protein
  
Accession: EPQ60389
  
Location: 1405350-1405748
  
 NCBI BlastP on this gene

EPQ60389

hypothetical protein
  
Accession: EPQ60390
  
Location: 1410405-1411227
  
 NCBI BlastP on this gene

EPQ60390

hypothetical protein
  
Accession: EPQ60391
  
Location: 1411710-1413100
  
 NCBI BlastP on this gene

EPQ60391

hypothetical protein
  
Accession: EPQ60392
  
Location: 1413334-1413678
  
 NCBI BlastP on this gene

EPQ60392

MFS general substrate transporter
  
Accession: EPQ60393
  
Location: 1415347-1418149
  
 NCBI BlastP on this gene

EPQ60393

NAD P-binding protein
  
Accession: EPQ60394
  
Location: 1418681-1419904
  
 NCBI BlastP on this gene

EPQ60394

MFS general substrate transporter
  
Accession: EPQ60395
  
Location: 1420323-1422307
  
  
**BlastP hit with Mycgr3G84494\_Mycgr3T**
  
Percentage identity: 37 %
  
BlastP bit score: 286
  
Sequence coverage: 91 %
  
E-value: 3e-86
  
  
 NCBI BlastP on this gene

EPQ60395

DNA-binding protein
  
Accession: EPQ60396
  
Location: 1422713-1423847
  
 NCBI BlastP on this gene

EPQ60396

hypothetical protein
  
Accession: EPQ61430
  
Location: 1423918-1425748
  
 NCBI BlastP on this gene

EPQ61430

protein PTM1
  
Accession: EPQ60397
  
Location: 1425957-1428057
  
 NCBI BlastP on this gene

EPQ60397

hypothetical protein
  
Accession: EPQ60398
  
Location: 1428116-1428439
  
 NCBI BlastP on this gene

EPQ60398

hypothetical protein
  
Accession: EPQ61429
  
Location: 1428560-1432528
  
 NCBI BlastP on this gene

EPQ61429

fructosamine-3-kinase
  
Accession: EPQ60399
  
Location: 1433069-1434202
  
  
**BlastP hit with Mycgr3G68030\_Mycgr3T**
  
Percentage identity: 30 %
  
BlastP bit score: 101
  
Sequence coverage: 83 %
  
E-value: 1e-21
  
  
 NCBI BlastP on this gene

EPQ60399

hypothetical protein
  
Accession: EPQ60400
  
Location: 1434732-1436428
  
 NCBI BlastP on this gene

EPQ60400

hypothetical protein
  
Accession: EPQ60401
  
Location: 1436538-1439549
  
 NCBI BlastP on this gene

EPQ60401

NADH flavin oxidoreductase 1
  
Accession: EPQ60402
  
Location: 1439758-1441265
  
 NCBI BlastP on this gene

EPQ60402

ABC1-domain-containing protein
  
Accession: EPQ60403
  
Location: 1441593-1443859
  
 NCBI BlastP on this gene

EPQ60403

hypothetical protein
  
Accession: EPQ60404
  
Location: 1444738-1448285
  
 NCBI BlastP on this gene

EPQ60404

hypothetical protein
  
Accession: EPQ60405
  
Location: 1448810-1449676
  
 NCBI BlastP on this gene

EPQ60405

hypothetical protein
  
Accession: EPQ60406
  
Location: 1450325-1451300
  
 NCBI BlastP on this gene

EPQ60406

hypothetical protein
  
Accession: EPQ60407
  
Location: 1452956-1453958
  
 NCBI BlastP on this gene

EPQ60407

hypothetical protein
  
Accession: EPQ60408
  
Location: 1454187-1456475
  
 NCBI BlastP on this gene

EPQ60408

113. :  JH719397 Dichomitus squalens LYAD-421 SS1 unplaced genomic scaffold DICSQscaffold\_1     Total score: 2.0     Cumulative Blast bit score: 385

mitochondrial protein required for respiration
  
Accession: EJF66833
  
Location: 1315731-1316687
  
 NCBI BlastP on this gene

EJF66833

cytoskeleton assembly control protein
  
Accession: EJF66834
  
Location: 1316996-1320803
  
 NCBI BlastP on this gene

EJF66834

hypothetical protein
  
Accession: EJF66835
  
Location: 1321349-1324541
  
 NCBI BlastP on this gene

EJF66835

hypothetical protein
  
Accession: EJF66836
  
Location: 1327081-1327914
  
 NCBI BlastP on this gene

EJF66836

hypothetical protein
  
Accession: EJF66837
  
Location: 1328341-1329745
  
 NCBI BlastP on this gene

EJF66837

MFS general substrate transporter
  
Accession: EJF66838
  
Location: 1330684-1332431
  
  
**BlastP hit with Mycgr3G84494\_Mycgr3T**
  
Percentage identity: 37 %
  
BlastP bit score: 287
  
Sequence coverage: 89 %
  
E-value: 1e-87
  
  
 NCBI BlastP on this gene

EJF66838

hypothetical protein
  
Accession: EJF66839
  
Location: 1332810-1336949
  
 NCBI BlastP on this gene

EJF66839

DNA-binding protein
  
Accession: EJF66840
  
Location: 1337219-1338323
  
 NCBI BlastP on this gene

EJF66840

hypothetical protein
  
Accession: EJF66841
  
Location: 1338432-1340287
  
 NCBI BlastP on this gene

EJF66841

hypothetical protein
  
Accession: EJF66842
  
Location: 1340512-1342909
  
 NCBI BlastP on this gene

EJF66842

DNA-dependent RNA polymerase II second largest subunit
  
Accession: EJF66843
  
Location: 1343004-1347176
  
 NCBI BlastP on this gene

EJF66843

cellobiohydrolaseI
  
Accession: EJF66844
  
Location: 1349210-1350691
  
 NCBI BlastP on this gene

EJF66844

fructosamine kinase PKL/CAK/FruK
  
Accession: EJF66845
  
Location: 1351027-1352146
  
  
**BlastP hit with Mycgr3G68030\_Mycgr3T**
  
Percentage identity: 28 %
  
BlastP bit score: 98
  
Sequence coverage: 85 %
  
E-value: 2e-20
  
  
 NCBI BlastP on this gene

EJF66845

FMN-linked oxidoreductase
  
Accession: EJF66846
  
Location: 1352658-1354350
  
 NCBI BlastP on this gene

EJF66846

hypothetical protein
  
Accession: EJF66847
  
Location: 1354595-1354854
  
 NCBI BlastP on this gene

EJF66847

FMN-linked oxidoreductase
  
Accession: EJF66848
  
Location: 1355559-1357303
  
 NCBI BlastP on this gene

EJF66848

ABC1-domain-containing protein
  
Accession: EJF66849
  
Location: 1357553-1360030
  
 NCBI BlastP on this gene

EJF66849

hypothetical protein
  
Accession: EJF66850
  
Location: 1361179-1365037
  
 NCBI BlastP on this gene

EJF66850

hypothetical protein
  
Accession: EJF66851
  
Location: 1365855-1366697
  
 NCBI BlastP on this gene

EJF66851

MFS polyamine transporter
  
Accession: EJF66852
  
Location: 1367419-1369504
  
 NCBI BlastP on this gene

EJF66852

hypothetical protein
  
Accession: EJF66853
  
Location: 1370174-1371156
  
 NCBI BlastP on this gene

EJF66853

114. :  KB725728 Colletotrichum orbiculare MAFF 240422 unplaced genomic scaffold Scaffold\_184     Total score: 2.0     Cumulative Blast bit score: 369

benzoate 4-monooxygenase cytochrome p450
  
Accession: ENH86763
  
Location: 247998-249889
  
 NCBI BlastP on this gene

ENH86763

benzoate 4-monooxygenase cytochrome p450
  
Accession: ENH86764
  
Location: 250014-252043
  
 NCBI BlastP on this gene

ENH86764

aristolochene synthase
  
Accession: ENH86765
  
Location: 252681-253844
  
 NCBI BlastP on this gene

ENH86765

short chain dehydrogenase reductase family
  
Accession: ENH86766
  
Location: 254214-255191
  
 NCBI BlastP on this gene

ENH86766

hypothetical protein
  
Accession: ENH86767
  
Location: 259179-260252
  
 NCBI BlastP on this gene

ENH86767

homoserine o-acetyltransferase
  
Accession: ENH86768
  
Location: 260923-262482
  
 NCBI BlastP on this gene

ENH86768

nudix family
  
Accession: ENH86769
  
Location: 262999-263940
  
 NCBI BlastP on this gene

ENH86769

ADP-ribose pyrophosphatase
  
Accession: ENH86770
  
Location: 263981-264723
  
 NCBI BlastP on this gene

ENH86770

MFS transporter
  
Accession: ENH86771
  
Location: 266416-268205
  
  
**BlastP hit with Mycgr3G23761\_Mycgr3T**
  
Percentage identity: 29 %
  
BlastP bit score: 172
  
Sequence coverage: 99 %
  
E-value: 1e-44
  
  
 NCBI BlastP on this gene

ENH86771

nad-dependent deacetylase sirtuin-2
  
Accession: ENH86772
  
Location: 269415-271010
  
 NCBI BlastP on this gene

ENH86772

hypothetical protein
  
Accession: ENH86773
  
Location: 283204-283596
  
 NCBI BlastP on this gene

ENH86773

pectate lyase
  
Accession: ENH86774
  
Location: 287976-288776
  
 NCBI BlastP on this gene

ENH86774

thymine dioxygenase
  
Accession: ENH86775
  
Location: 289560-290673
  
  
**BlastP hit with Mycgr3G68036\_Mycgr3T**
  
Percentage identity: 36 %
  
BlastP bit score: 197
  
Sequence coverage: 87 %
  
E-value: 1e-56
  
  
 NCBI BlastP on this gene

ENH86775

C6 transcription factor
  
Accession: ENH86776
  
Location: 291302-293758
  
 NCBI BlastP on this gene

ENH86776

FAD binding domain protein
  
Accession: ENH86777
  
Location: 293891-295405
  
 NCBI BlastP on this gene

ENH86777

afln vera monooxygenase
  
Accession: ENH86778
  
Location: 296715-298466
  
 NCBI BlastP on this gene

ENH86778

hypothetical protein
  
Accession: ENH86779
  
Location: 300134-300755
  
 NCBI BlastP on this gene

ENH86779

fumarylacetoacetate hydrolase
  
Accession: ENH86780
  
Location: 301907-303790
  
 NCBI BlastP on this gene

ENH86780

amidohydrolase
  
Accession: ENH86781
  
Location: 305026-306378
  
 NCBI BlastP on this gene

ENH86781

polysaccharide deacetylase family protein
  
Accession: ENH86782
  
Location: 307567-309963
  
 NCBI BlastP on this gene

ENH86782

115. :  GL698718 Metarhizium anisopliae ARSEF 23 unplaced genomic scaffold Scf\_008     Total score: 2.0     Cumulative Blast bit score: 359

hypothetical protein
  
Accession: EFY98894
  
Location: 1656421-1656935
  
 NCBI BlastP on this gene

EFY98894

ATP-dependent DNA helicase II subunit 2
  
Accession: EFY98893
  
Location: 1653459-1655873
  
 NCBI BlastP on this gene

EFY98893

QDE-2-interacting protein
  
Accession: EFY98892
  
Location: 1651502-1653146
  
 NCBI BlastP on this gene

EFY98892

mitochondrial DnaJ chaperone (Tim14), putative
  
Accession: EFY98891
  
Location: 1650747-1651217
  
 NCBI BlastP on this gene

EFY98891

hypothetical protein
  
Accession: EFY98890
  
Location: 1649745-1650357
  
 NCBI BlastP on this gene

EFY98890

autophagy protein (Atg22), putative
  
Accession: EFY98889
  
Location: 1647674-1649302
  
 NCBI BlastP on this gene

EFY98889

methyltransferase type 12
  
Accession: EFY98888
  
Location: 1645826-1646776
  
 NCBI BlastP on this gene

EFY98888

phosphatidylserine decarboxylase family protein
  
Accession: EFY98887
  
Location: 1643679-1645046
  
 NCBI BlastP on this gene

EFY98887

PRO1A C6 Zink-finger protein
  
Accession: EFY98886
  
Location: 1640838-1642567
  
 NCBI BlastP on this gene

EFY98886

hypothetical protein
  
Accession: EFY98885
  
Location: 1639635-1640086
  
 NCBI BlastP on this gene

EFY98885

hypothetical protein
  
Accession: EFY98884
  
Location: 1638077-1638505
  
 NCBI BlastP on this gene

EFY98884

peptidase S41 family protein
  
Accession: EFY98883
  
Location: 1635197-1637656
  
  
**BlastP hit with Mycgr3G36449\_Mycgr3T**
  
Percentage identity: 30 %
  
BlastP bit score: 235
  
Sequence coverage: 101 %
  
E-value: 2e-63
  
  
 NCBI BlastP on this gene

EFY98883

MFS transporter, putative
  
Accession: EFY98882
  
Location: 1631515-1633064
  
  
**BlastP hit with Mycgr3G23761\_Mycgr3T**
  
Percentage identity: 27 %
  
BlastP bit score: 124
  
Sequence coverage: 81 %
  
E-value: 3e-28
  
  
 NCBI BlastP on this gene

EFY98882

C2H2 finger domain protein, putative
  
Accession: EFY98881
  
Location: 1622647-1624071
  
 NCBI BlastP on this gene

EFY98881

hypothetical protein
  
Accession: EFY98880
  
Location: 1620377-1621557
  
 NCBI BlastP on this gene

EFY98880

PHD finger domain-containing protein
  
Accession: EFY98879
  
Location: 1613363-1615738
  
 NCBI BlastP on this gene

EFY98879

116. :  AMYD01000373 Colletotrichum gloeosporioides Cg-14     Total score: 2.0     Cumulative Blast bit score: 333

hypothetical protein
  
Accession: EQB58029
  
Location: 4331-5174
  
  
**BlastP hit with Mycgr3G90561\_Mycgr3T**
  
Percentage identity: 34 %
  
BlastP bit score: 77
  
Sequence coverage: 55 %
  
E-value: 3e-14
  
  
 NCBI BlastP on this gene

EQB58029

hypothetical protein
  
Accession: EQB58030
  
Location: 6014-8158
  
 NCBI BlastP on this gene

EQB58030

hypothetical protein
  
Accession: EQB58031
  
Location: 8931-12158
  
 NCBI BlastP on this gene

EQB58031

peptidase S41 family protein
  
Accession: EQB58032
  
Location: 14606-16921
  
  
**BlastP hit with Mycgr3G36449\_Mycgr3T**
  
Percentage identity: 28 %
  
BlastP bit score: 256
  
Sequence coverage: 105 %
  
E-value: 4e-71
  
  
 NCBI BlastP on this gene

EQB58032

hypothetical protein
  
Accession: EQB58033
  
Location: 19535-19805
  
 NCBI BlastP on this gene

EQB58033

hypothetical protein
  
Accession: EQB58034
  
Location: 23459-25517
  
 NCBI BlastP on this gene

EQB58034

117. :  EQ962656 Talaromyces stipitatus ATCC 10500 scf\_1105507295549 genomic scaffold     Total score: 2.0     Cumulative Blast bit score: 323

conserved hypothetical protein
  
Accession: EED17219
  
Location: 3870314-3871599
  
 NCBI BlastP on this gene

EED17219

short chain dehydrogenase/reductase, putative
  
Accession: EED17220
  
Location: 3873650-3874893
  
 NCBI BlastP on this gene

EED17220

hypothetical protein
  
Accession: EED17221
  
Location: 3880622-3881014
  
 NCBI BlastP on this gene

EED17221

conserved hypothetical protein
  
Accession: EED17222
  
Location: 3882261-3882781
  
 NCBI BlastP on this gene

EED17222

conserved hypothetical protein
  
Accession: EED17223
  
Location: 3883055-3884197
  
 NCBI BlastP on this gene

EED17223

hypothetical protein
  
Accession: EED17224
  
Location: 3884280-3886346
  
 NCBI BlastP on this gene

EED17224

multidrug resistance-associated protein, putative
  
Accession: EED17225
  
Location: 3886590-3887537
  
 NCBI BlastP on this gene

EED17225

adenylate cyclase, putative
  
Accession: EED17226
  
Location: 3889022-3890743
  
  
**BlastP hit with Mycgr3G23761\_Mycgr3T**
  
Percentage identity: 36 %
  
BlastP bit score: 247
  
Sequence coverage: 100 %
  
E-value: 2e-72
  
  
 NCBI BlastP on this gene

EED17226

conserved hypothetical protein
  
Accession: EED17227
  
Location: 3890946-3891405
  
 NCBI BlastP on this gene

EED17227

conserved hypothetical protein
  
Accession: EED17228
  
Location: 3892368-3893436
  
  
**BlastP hit with Mycgr3G35535\_Mycgr3T**
  
Percentage identity: 28 %
  
BlastP bit score: 76
  
Sequence coverage: 82 %
  
E-value: 4e-14
  
  
 NCBI BlastP on this gene

EED17228

hypothetical protein
  
Accession: EED17229
  
Location: 3893804-3894088
  
 NCBI BlastP on this gene

EED17229

hypothetical protein
  
Accession: EED17230
  
Location: 3908985-3909590
  
 NCBI BlastP on this gene

EED17230

hypothetical protein
  
Accession: EED17231
  
Location: 3909972-3910514
  
 NCBI BlastP on this gene

EED17231

O-methyltransferase, putative
  
Accession: EED17232
  
Location: 3911067-3912497
  
 NCBI BlastP on this gene

EED17232

118. :  KE145371 Glarea lozoyensis ATCC 20868 chromosome Unknown GLAREA7     Total score: 2.0     Cumulative Blast bit score: 316

hypothetical protein
  
Accession: EPE26324
  
Location: 3186589-3188685
  
 NCBI BlastP on this gene

EPE26324

Nucleic acid-binding protein
  
Accession: EPE26323
  
Location: 3180192-3183364
  
 NCBI BlastP on this gene

EPE26323

MFS general substrate transporter
  
Accession: EPE26322
  
Location: 3174631-3177033
  
 NCBI BlastP on this gene

EPE26322

alpha/beta-Hydrolase
  
Accession: EPE26321
  
Location: 3172044-3174118
  
 NCBI BlastP on this gene

EPE26321

Acyl-CoA N-acyltransferases (Nat)
  
Accession: EPE26320
  
Location: 3170813-3171427
  
 NCBI BlastP on this gene

EPE26320

hypothetical protein
  
Accession: EPE26319
  
Location: 3168739-3169948
  
 NCBI BlastP on this gene

EPE26319

hypothetical protein
  
Accession: EPE26318
  
Location: 3166706-3167739
  
  
**BlastP hit with Mycgr3G35535\_Mycgr3T**
  
Percentage identity: 31 %
  
BlastP bit score: 73
  
Sequence coverage: 83 %
  
E-value: 6e-13
  
  
 NCBI BlastP on this gene

EPE26318

WD40 repeat-like protein
  
Accession: EPE26317
  
Location: 3160953-3166211
  
 NCBI BlastP on this gene

EPE26317

hypothetical protein
  
Accession: EPE26316
  
Location: 3158917-3160343
  
  
**BlastP hit with Mycgr3G68030\_Mycgr3T**
  
Percentage identity: 39 %
  
BlastP bit score: 243
  
Sequence coverage: 98 %
  
E-value: 2e-73
  
  
 NCBI BlastP on this gene

EPE26316

Putative cyclase
  
Accession: EPE26315
  
Location: 3157491-3158610
  
 NCBI BlastP on this gene

EPE26315

P-loop containing nucleoside triphosphate hydrolase
  
Accession: EPE26314
  
Location: 3154777-3157192
  
 NCBI BlastP on this gene

EPE26314

RING/U-box
  
Accession: EPE26313
  
Location: 3153061-3154540
  
 NCBI BlastP on this gene

EPE26313

FAD/NAD(P)-binding protein
  
Accession: EPE26312
  
Location: 3150468-3151953
  
 NCBI BlastP on this gene

EPE26312

hypothetical protein
  
Accession: EPE26311
  
Location: 3149013-3150185
  
 NCBI BlastP on this gene

EPE26311

hypothetical protein
  
Accession: EPE26310
  
Location: 3146551-3147702
  
 NCBI BlastP on this gene

EPE26310

MFS general substrate transporter
  
Accession: EPE26309
  
Location: 3142653-3144630
  
 NCBI BlastP on this gene

EPE26309

119. :  AABX02000023 Neurospora crassa OR74A     Total score: 2.0     Cumulative Blast bit score: 265

predicted protein
  
Accession: EAA30510
  
Location: 470280-471214
  
 NCBI BlastP on this gene

EAA30510

peroxidase/catalase 2
  
Accession: EAA30509
  
Location: 473805-476066
  
 NCBI BlastP on this gene

EAA30509

predicted protein
  
Accession: EAA30508
  
Location: 478183-478890
  
 NCBI BlastP on this gene

EAA30508

conserved hypothetical protein
  
Accession: EAA30507
  
Location: 480936-481719
  
 NCBI BlastP on this gene

EAA30507

predicted protein
  
Accession: EAA30506
  
Location: 483147-485198
  
 NCBI BlastP on this gene

EAA30506

conserved hypothetical protein
  
Accession: EAA30505
  
Location: 489516-490481
  
  
**BlastP hit with Mycgr3G90561\_Mycgr3T**
  
Percentage identity: 31 %
  
BlastP bit score: 66
  
Sequence coverage: 61 %
  
E-value: 3e-10
  
  
 NCBI BlastP on this gene

EAA30505

conserved hypothetical protein
  
Accession: EAA30504
  
Location: 491221-497912
  
  
**BlastP hit with Mycgr3G36449\_Mycgr3T**
  
Percentage identity: 30 %
  
BlastP bit score: 199
  
Sequence coverage: 82 %
  
E-value: 8e-50
  
  
 NCBI BlastP on this gene

EAA30504

hypothetical protein
  
Accession: EAA30503
  
Location: 499619-500617
  
 NCBI BlastP on this gene

EAA30503

predicted protein
  
Accession: EAA30502
  
Location: 502393-503130
  
 NCBI BlastP on this gene

EAA30502

conserved hypothetical protein
  
Accession: EAA30501
  
Location: 503676-505571
  
 NCBI BlastP on this gene

EAA30501

hypothetical protein
  
Accession: EAA30500
  
Location: 506702-507079
  
 NCBI BlastP on this gene

EAA30500

predicted protein
  
Accession: EAA30499
  
Location: 508590-509288
  
 NCBI BlastP on this gene

EAA30499

predicted protein
  
Accession: EAA30498
  
Location: 510042-510848
  
 NCBI BlastP on this gene

EAA30498

hypothetical protein
  
Accession: EAA30497
  
Location: 512322-514022
  
 NCBI BlastP on this gene

EAA30497

hypothetical protein
  
Accession: EAA30496
  
Location: 515792-517028
  
 NCBI BlastP on this gene

EAA30496

predicted protein
  
Accession: EAA30495
  
Location: 517497-518905
  
 NCBI BlastP on this gene

EAA30495

120. :  JH126400 Cordyceps militaris CM01 unplaced genomic scaffold CCM\_S00002     Total score: 2.0     Cumulative Blast bit score: 225

hypothetical protein
  
Accession: EGX93779
  
Location: 343208-344550
  
 NCBI BlastP on this gene

EGX93779

hypothetical protein
  
Accession: EGX93780
  
Location: 345372-345734
  
 NCBI BlastP on this gene

EGX93780

GTP cyclohydrolase I, putative
  
Accession: EGX93781
  
Location: 346053-346765
  
 NCBI BlastP on this gene

EGX93781

phosphoribosyl transferase
  
Accession: EGX93782
  
Location: 347266-350671
  
 NCBI BlastP on this gene

EGX93782

alpha/beta hydrolase fold-1
  
Accession: EGX93783
  
Location: 351006-351863
  
 NCBI BlastP on this gene

EGX93783

hypothetical protein
  
Accession: EGX93784
  
Location: 352632-353401
  
 NCBI BlastP on this gene

EGX93784

pyridoxamine phosphate oxidase family protein
  
Accession: EGX93785
  
Location: 353946-354611
  
 NCBI BlastP on this gene

EGX93785

NRPS-like enzyme, putative
  
Accession: EGX93786
  
Location: 355014-356476
  
 NCBI BlastP on this gene

EGX93786

dimethylaniline monooxygenase, putative
  
Accession: EGX93787
  
Location: 356715-358363
  
 NCBI BlastP on this gene

EGX93787

FAD dependent oxidoreductase
  
Accession: EGX93788
  
Location: 359341-360667
  
 NCBI BlastP on this gene

EGX93788

tyrosinase, putative
  
Accession: EGX93789
  
Location: 360785-362095
  
 NCBI BlastP on this gene

EGX93789

hypothetical protein
  
Accession: EGX93790
  
Location: 362590-363523
  
  
**BlastP hit with Mycgr3G90561\_Mycgr3T**
  
Percentage identity: 33 %
  
BlastP bit score: 55
  
Sequence coverage: 53 %
  
E-value: 4e-06
  
  
 NCBI BlastP on this gene

EGX93790

hypothetical protein
  
Accession: EGX93791
  
Location: 364171-364900
  
 NCBI BlastP on this gene

EGX93791

Cytochrome P450
  
Accession: EGX93792
  
Location: 365954-368259
  
 NCBI BlastP on this gene

EGX93792

C6 transcription factor, putative
  
Accession: EGX93793
  
Location: 369669-371644
  
 NCBI BlastP on this gene

EGX93793

hypothetical protein
  
Accession: EGX93794
  
Location: 371724-372671
  
 NCBI BlastP on this gene

EGX93794

glutathione S-transferase, putative
  
Accession: EGX93795
  
Location: 373004-373874
  
 NCBI BlastP on this gene

EGX93795

gamma-glutamyltranspeptidase
  
Accession: EGX93796
  
Location: 374715-376689
  
 NCBI BlastP on this gene

EGX93796

MFS multidrug transporter, putative
  
Accession: EGX93797
  
Location: 377774-379684
  
  
**BlastP hit with Mycgr3G23761\_Mycgr3T**
  
Percentage identity: 28 %
  
BlastP bit score: 171
  
Sequence coverage: 99 %
  
E-value: 2e-44
  
  
 NCBI BlastP on this gene

EGX93797

mannosyltransferase
  
Accession: EGX93798
  
Location: 382261-383963
  
 NCBI BlastP on this gene

EGX93798

serine/threonine protein kinase, putative
  
Accession: EGX93799
  
Location: 384871-390200
  
 NCBI BlastP on this gene

EGX93799

hypothetical protein
  
Accession: EGX93800
  
Location: 390983-391319
  
 NCBI BlastP on this gene

EGX93800

hypothetical protein
  
Accession: EGX93801
  
Location: 395166-396103
  
 NCBI BlastP on this gene

EGX93801

121. :  KB725679 Colletotrichum orbiculare MAFF 240422 unplaced genomic scaffold Scaffold\_14     Total score: 2.0     Cumulative Blast bit score: 221

MFS transporter
  
Accession: ENH87622
  
Location: 40036-41710
  
  
**BlastP hit with Mycgr3G23761\_Mycgr3T**
  
Percentage identity: 27 %
  
BlastP bit score: 163
  
Sequence coverage: 98 %
  
E-value: 5e-41
  
  
 NCBI BlastP on this gene

ENH87622

hypothetical protein
  
Accession: ENH87621
  
Location: 35791-38940
  
 NCBI BlastP on this gene

ENH87621

minor allergen alt a 7
  
Accession: ENH87620
  
Location: 34230-34872
  
 NCBI BlastP on this gene

ENH87620

nad dependent epimerase dehydratase
  
Accession: ENH87619
  
Location: 30372-31163
  
 NCBI BlastP on this gene

ENH87619

MFS monocarboxylate
  
Accession: ENH87618
  
Location: 28939-30288
  
 NCBI BlastP on this gene

ENH87618

hypothetical protein
  
Accession: ENH87617
  
Location: 25469-26495
  
  
**BlastP hit with Mycgr3G90561\_Mycgr3T**
  
Percentage identity: 33 %
  
BlastP bit score: 58
  
Sequence coverage: 49 %
  
E-value: 3e-07
  
  
 NCBI BlastP on this gene

ENH87617

hypothetical protein
  
Accession: ENH87616
  
Location: 23225-24679
  
 NCBI BlastP on this gene

ENH87616

RNA recognition motif-containing protein
  
Accession: ENH87615
  
Location: 17963-19325
  
 NCBI BlastP on this gene

ENH87615

40s ribosomal protein s8
  
Accession: ENH87614
  
Location: 16150-16638
  
 NCBI BlastP on this gene

ENH87614

hypothetical protein
  
Accession: ENH87613
  
Location: 13744-15700
  
 NCBI BlastP on this gene

ENH87613

isocitrate dehydrogenase
  
Accession: ENH87612
  
Location: 8607-9732
  
 NCBI BlastP on this gene

ENH87612

hypothetical protein
  
Accession: ENH87611
  
Location: 7643-7922
  
 NCBI BlastP on this gene

ENH87611

122. :  CM001208 Mycosphaerella graminicola IPO323 chromosome 13     Total score: 2.0     Cumulative Blast bit score: 207

hypothetical protein
  
Accession: EGP82692
  
Location: 802885-803322
  
 NCBI BlastP on this gene

EGP82692

hypothetical protein
  
Accession: EGP82602
  
Location: 808254-809339
  
 NCBI BlastP on this gene

EGP82602

beta-1,3 glucanosyltransferase
  
Accession: EGP82603
  
Location: 810930-812364
  
 NCBI BlastP on this gene

EGP82603

hypothetical protein
  
Accession: EGP82604
  
Location: 813075-814166
  
 NCBI BlastP on this gene

EGP82604

hypothetical protein
  
Accession: EGP82605
  
Location: 816529-817518
  
 NCBI BlastP on this gene

EGP82605

hypothetical protein
  
Accession: EGP82606
  
Location: 818318-819852
  
 NCBI BlastP on this gene

EGP82606

hypothetical protein
  
Accession: EGP82607
  
Location: 820315-821325
  
  
**BlastP hit with Mycgr3G68030\_Mycgr3T**
  
Percentage identity: 32 %
  
BlastP bit score: 148
  
Sequence coverage: 96 %
  
E-value: 4e-38
  
  
 NCBI BlastP on this gene

EGP82607

hypothetical protein
  
Accession: EGP82691
  
Location: 822110-823005
  
 NCBI BlastP on this gene

EGP82691

hypothetical protein
  
Accession: EGP82690
  
Location: 823635-824970
  
 NCBI BlastP on this gene

EGP82690

hypothetical protein
  
Accession: EGP82689
  
Location: 826641-827711
  
 NCBI BlastP on this gene

EGP82689

hypothetical protein
  
Accession: EGP82608
  
Location: 828199-829995
  
 NCBI BlastP on this gene

EGP82608

hypothetical protein
  
Accession: EGP82609
  
Location: 831385-833550
  
 NCBI BlastP on this gene

EGP82609

hypothetical protein
  
Accession: EGP82610
  
Location: 836491-837157
  
  
**BlastP hit with Mycgr3G35535\_Mycgr3T**
  
Percentage identity: 27 %
  
BlastP bit score: 60
  
Sequence coverage: 72 %
  
E-value: 7e-09
  
  
 NCBI BlastP on this gene

EGP82610

hypothetical protein
  
Accession: EGP82688
  
Location: 838404-839936
  
 NCBI BlastP on this gene

EGP82688

hypothetical protein
  
Accession: EGP82687
  
Location: 840173-845299
  
 NCBI BlastP on this gene

EGP82687

putative alpha-glucosidase
  
Accession: EGP82611
  
Location: 846383-849673
  
 NCBI BlastP on this gene

EGP82611

hypothetical protein
  
Accession: EGP82686
  
Location: 851050-851979
  
 NCBI BlastP on this gene

EGP82686

hypothetical protein
  
Accession: EGP82612
  
Location: 852487-853472
  
 NCBI BlastP on this gene

EGP82612

hypothetical protein
  
Accession: EGP82685
  
Location: 853982-854428
  
 NCBI BlastP on this gene

EGP82685

123. :  KB644411 Penicillium oxalicum 114-2 unplaced genomic scaffold scaffold\_4     Total score: 2.0     Cumulative Blast bit score: 206

hypothetical protein
  
Accession: EPS28971
  
Location: 1805768-1806767
  
 NCBI BlastP on this gene

EPS28971

hypothetical protein
  
Accession: EPS28972
  
Location: 1807661-1808426
  
 NCBI BlastP on this gene

EPS28972

hypothetical protein
  
Accession: EPS28973
  
Location: 1809385-1811415
  
 NCBI BlastP on this gene

EPS28973

hypothetical protein
  
Accession: EPS28974
  
Location: 1813216-1814881
  
 NCBI BlastP on this gene

EPS28974

hypothetical protein
  
Accession: EPS28975
  
Location: 1815283-1820827
  
 NCBI BlastP on this gene

EPS28975

hypothetical protein
  
Accession: EPS28976
  
Location: 1822821-1823692
  
 NCBI BlastP on this gene

EPS28976

hypothetical protein
  
Accession: EPS28977
  
Location: 1824484-1825589
  
  
**BlastP hit with Mycgr3G35535\_Mycgr3T**
  
Percentage identity: 28 %
  
BlastP bit score: 55
  
Sequence coverage: 83 %
  
E-value: 1e-06
  
  
 NCBI BlastP on this gene

EPS28977

hypothetical protein
  
Accession: EPS28978
  
Location: 1827526-1828688
  
 NCBI BlastP on this gene

EPS28978

hypothetical protein
  
Accession: EPS28979
  
Location: 1828767-1830349
  
  
**BlastP hit with Mycgr3G23761\_Mycgr3T**
  
Percentage identity: 30 %
  
BlastP bit score: 152
  
Sequence coverage: 92 %
  
E-value: 2e-37
  
  
 NCBI BlastP on this gene

EPS28979

hypothetical protein
  
Accession: EPS28980
  
Location: 1831447-1841720
  
 NCBI BlastP on this gene

EPS28980

hypothetical protein
  
Accession: EPS28981
  
Location: 1842874-1843885
  
 NCBI BlastP on this gene

EPS28981

hypothetical protein
  
Accession: EPS28982
  
Location: 1844758-1845714
  
 NCBI BlastP on this gene

EPS28982

hypothetical protein
  
Accession: EPS28983
  
Location: 1846161-1848301
  
 NCBI BlastP on this gene

EPS28983

hypothetical protein
  
Accession: EPS28984
  
Location: 1848875-1850461
  
 NCBI BlastP on this gene

EPS28984

124. :  GG697358 Glomerella graminicola M1.001 genomic scaffold supercont1.28     Total score: 2.0     Cumulative Blast bit score: 206

hypothetical protein
  
Accession: EFQ31896
  
Location: 210493-211153
  
 NCBI BlastP on this gene

EFQ31896

hypothetical protein
  
Accession: EFQ31897
  
Location: 211296-212360
  
 NCBI BlastP on this gene

EFQ31897

fungal specific transcription factor domain-containing protein
  
Accession: EFQ31898
  
Location: 213559-216201
  
 NCBI BlastP on this gene

EFQ31898

hypothetical protein
  
Accession: EFQ31899
  
Location: 217136-218506
  
 NCBI BlastP on this gene

EFQ31899

hypothetical protein
  
Accession: EFQ31900
  
Location: 223013-223906
  
 NCBI BlastP on this gene

EFQ31900

major facilitator superfamily transporter
  
Accession: EFQ31901
  
Location: 229026-230733
  
  
**BlastP hit with Mycgr3G23761\_Mycgr3T**
  
Percentage identity: 27 %
  
BlastP bit score: 149
  
Sequence coverage: 97 %
  
E-value: 3e-36
  
  
 NCBI BlastP on this gene

EFQ31901

hypothetical protein
  
Accession: EFQ31902
  
Location: 232011-232988
  
 NCBI BlastP on this gene

EFQ31902

hypothetical protein
  
Accession: EFQ31903
  
Location: 234969-235898
  
 NCBI BlastP on this gene

EFQ31903

quinone oxidoreductase
  
Accession: EFQ31904
  
Location: 236769-237666
  
 NCBI BlastP on this gene

EFQ31904

major facilitator superfamily transporter
  
Accession: EFQ31905
  
Location: 240177-241544
  
 NCBI BlastP on this gene

EFQ31905

hypothetical protein
  
Accession: EFQ31906
  
Location: 243962-244959
  
  
**BlastP hit with Mycgr3G90561\_Mycgr3T**
  
Percentage identity: 33 %
  
BlastP bit score: 57
  
Sequence coverage: 50 %
  
E-value: 9e-07
  
  
 NCBI BlastP on this gene

EFQ31906

translation elongation factor G
  
Accession: EFQ31907
  
Location: 245549-248034
  
 NCBI BlastP on this gene

EFQ31907

hypothetical protein
  
Accession: EFQ31908
  
Location: 253023-254950
  
 NCBI BlastP on this gene

EFQ31908

hypothetical protein
  
Accession: EFQ31909
  
Location: 255620-256111
  
 NCBI BlastP on this gene

EFQ31909

hypothetical protein
  
Accession: EFQ31910
  
Location: 257366-259105
  
 NCBI BlastP on this gene

EFQ31910

hypothetical protein
  
Accession: EFQ31911
  
Location: 259596-259961
  
 NCBI BlastP on this gene

EFQ31911

hypothetical protein
  
Accession: EFQ31912
  
Location: 260394-262444
  
 NCBI BlastP on this gene

EFQ31912

hypothetical protein
  
Accession: EFQ31913
  
Location: 263271-265405
  
 NCBI BlastP on this gene

EFQ31913

125. :  JH795672 Magnaporthe oryzae P131 unplaced genomic scaffold P131\_scaffold00357     Total score: 2.0     Cumulative Blast bit score: 171

hypothetical protein
  
Accession: ELQ66786
  
Location: 22372-24011
  
 NCBI BlastP on this gene

ELQ66786

methyltransferase
  
Accession: ELQ66785
  
Location: 20897-21670
  
 NCBI BlastP on this gene

ELQ66785

cyclohexanone 1,2-monooxygenase
  
Accession: ELQ66784
  
Location: 16302-20208
  
 NCBI BlastP on this gene

ELQ66784

averantin oxidoreductase
  
Accession: ELQ66783
  
Location: 13801-15633
  
 NCBI BlastP on this gene

ELQ66783

geranylgeranyl pyrophosphate synthetase
  
Accession: ELQ66782
  
Location: 9642-11919
  
 NCBI BlastP on this gene

ELQ66782

hypothetical protein
  
Accession: ELQ66781
  
Location: 7816-9346
  
 NCBI BlastP on this gene

ELQ66781

hypothetical protein
  
Accession: ELQ66780
  
Location: 5389-6425
  
 NCBI BlastP on this gene

ELQ66780

hypothetical protein
  
Accession: ELQ66779
  
Location: 2837-4322
  
  
**BlastP hit with Mycgr3G35535\_Mycgr3T**
  
Percentage identity: 30 %
  
BlastP bit score: 58
  
Sequence coverage: 85 %
  
E-value: 2e-07
  
  
 NCBI BlastP on this gene

ELQ66779

hypothetical protein
  
Accession: ELQ66778
  
Location: 49-1696
  
  
**BlastP hit with Mycgr3G90561\_Mycgr3T**
  
Percentage identity: 32 %
  
BlastP bit score: 58
  
Sequence coverage: 60 %
  
E-value: 3e-07
  
  
  
**BlastP hit with Mycgr3G35535\_Mycgr3T**
  
Percentage identity: 34 %
  
BlastP bit score: 55
  
Sequence coverage: 60 %
  
E-value: 2e-06
  
  
 NCBI BlastP on this gene

ELQ66778

126. :  CACQ02003267 Colletotrichum higginsianum strain IMI 349063     Total score: 2.0     Cumulative Blast bit score: 171

hypothetical protein
  
Accession: CCF39013
  
Location: 358-1133
  
  
**BlastP hit with Mycgr3G35535\_Mycgr3T**
  
Percentage identity: 33 %
  
BlastP bit score: 54
  
Sequence coverage: 55 %
  
E-value: 1e-06
  
  
 NCBI BlastP on this gene

CCF39013

hypothetical protein
  
Accession: CCF39014
  
Location: 1939-2995
  
  
**BlastP hit with Mycgr3G90561\_Mycgr3T**
  
Percentage identity: 29 %
  
BlastP bit score: 59
  
Sequence coverage: 58 %
  
E-value: 9e-08
  
  
  
**BlastP hit with Mycgr3G35535\_Mycgr3T**
  
Percentage identity: 33 %
  
BlastP bit score: 58
  
Sequence coverage: 71 %
  
E-value: 1e-07
  
  
 NCBI BlastP on this gene

CCF39014

major facilitator superfamily transporter
  
Accession: CCF39015
  
Location: 3137-4402
  
 NCBI BlastP on this gene

CCF39015

127. :  CM001234 Magnaporthe oryzae 70-15 chromosome 4     Total score: 2.0     Cumulative Blast bit score: 169

hypothetical protein
  
Accession: EHA50179
  
Location: 1876645-1878284
  
 NCBI BlastP on this gene

EHA50179

hypothetical protein
  
Accession: EHA50180
  
Location: 1879947-1881930
  
 NCBI BlastP on this gene

EHA50180

hypothetical protein
  
Accession: EHA50181
  
Location: 1882368-1884353
  
 NCBI BlastP on this gene

EHA50181

averantin oxidoreductase
  
Accession: EHA50182
  
Location: 1885022-1886854
  
 NCBI BlastP on this gene

EHA50182

geranylgeranyl pyrophosphate synthetase
  
Accession: EHA50183
  
Location: 1888737-1891014
  
 NCBI BlastP on this gene

EHA50183

hypothetical protein
  
Accession: EHA50184
  
Location: 1891369-1892899
  
 NCBI BlastP on this gene

EHA50184

hypothetical protein
  
Accession: EHA50185
  
Location: 1894293-1895948
  
 NCBI BlastP on this gene

EHA50185

hypothetical protein
  
Accession: EHA50186
  
Location: 1896428-1897912
  
  
**BlastP hit with Mycgr3G35535\_Mycgr3T**
  
Percentage identity: 32 %
  
BlastP bit score: 54
  
Sequence coverage: 88 %
  
E-value: 3e-06
  
  
 NCBI BlastP on this gene

EHA50186

hypothetical protein
  
Accession: EHA50187
  
Location: 1898998-1900055
  
  
**BlastP hit with Mycgr3G90561\_Mycgr3T**
  
Percentage identity: 32 %
  
BlastP bit score: 60
  
Sequence coverage: 60 %
  
E-value: 3e-08
  
  
  
**BlastP hit with Mycgr3G35535\_Mycgr3T**
  
Percentage identity: 29 %
  
BlastP bit score: 55
  
Sequence coverage: 89 %
  
E-value: 9e-07
  
  
 NCBI BlastP on this gene

EHA50187

hypothetical protein
  
Accession: EHA50188
  
Location: 1900153-1901975
  
 NCBI BlastP on this gene

EHA50188

hypothetical protein
  
Accession: EHA50189
  
Location: 1903830-1905624
  
 NCBI BlastP on this gene

EHA50189

3-oxoacyl-[acyl-carrier-protein] reductase
  
Accession: EHA50190
  
Location: 1905897-1906791
  
 NCBI BlastP on this gene

EHA50190

hypothetical protein
  
Accession: EHA50191
  
Location: 1907641-1909165
  
 NCBI BlastP on this gene

EHA50191

oxidoreductase
  
Accession: EHA50192
  
Location: 1910018-1910707
  
 NCBI BlastP on this gene

EHA50192

enoyl reductase
  
Accession: EHA50193
  
Location: 1911131-1912707
  
 NCBI BlastP on this gene

EHA50193

hypothetical protein
  
Accession: EHA50194
  
Location: 1913437-1914470
  
 NCBI BlastP on this gene

EHA50194

hypothetical protein
  
Accession: EHA50195
  
Location: 1914666-1916366
  
 NCBI BlastP on this gene

EHA50195

hypothetical protein
  
Accession: EHA50196
  
Location: 1917005-1918060
  
 NCBI BlastP on this gene

EHA50196

128. :  EQ962659 Talaromyces stipitatus ATCC 10500 scf\_1105507295487 genomic scaffold     Total score: 2.0     Cumulative Blast bit score: 163

hypothetical protein
  
Accession: EED12763
  
Location: 229880-230257
  
 NCBI BlastP on this gene

EED12763

hypothetical protein
  
Accession: EED12764
  
Location: 230591-232737
  
 NCBI BlastP on this gene

EED12764

conserved hypothetical protein
  
Accession: EED12765
  
Location: 235814-241656
  
 NCBI BlastP on this gene

EED12765

conserved hypothetical protein
  
Accession: EED12766
  
Location: 241897-243811
  
 NCBI BlastP on this gene

EED12766

hypothetical protein
  
Accession: EED12767
  
Location: 244213-244617
  
 NCBI BlastP on this gene

EED12767

glycosyltransferase family protein
  
Accession: EED12768
  
Location: 245419-246293
  
 NCBI BlastP on this gene

EED12768

conserved hypothetical protein
  
Accession: EED12769
  
Location: 246577-247508
  
 NCBI BlastP on this gene

EED12769

hypothetical protein
  
Accession: EED12770
  
Location: 247856-248232
  
 NCBI BlastP on this gene

EED12770

hypothetical protein
  
Accession: EED12771
  
Location: 249662-250581
  
  
**BlastP hit with Mycgr3G90561\_Mycgr3T**
  
Percentage identity: 38 %
  
BlastP bit score: 81
  
Sequence coverage: 52 %
  
E-value: 1e-15
  
  
 NCBI BlastP on this gene

EED12771

conserved hypothetical protein
  
Accession: EED12773
  
Location: 251163-252037
  
  
**BlastP hit with Mycgr3G35535\_Mycgr3T**
  
Percentage identity: 32 %
  
BlastP bit score: 82
  
Sequence coverage: 90 %
  
E-value: 2e-16
  
  
 NCBI BlastP on this gene

EED12773

O-methyltransferase, putative
  
Accession: EED12775
  
Location: 252152-253324
  
 NCBI BlastP on this gene

EED12775

integral membrane protein
  
Accession: EED12777
  
Location: 254477-255474
  
 NCBI BlastP on this gene

EED12777

monocarboxylate transporter, putative
  
Accession: EED12778
  
Location: 255866-257293
  
 NCBI BlastP on this gene

EED12778

short-chain dehydrogenase, putative
  
Accession: EED12779
  
Location: 257849-259147
  
 NCBI BlastP on this gene

EED12779

methionyl-tRNA formyltransferase, putative
  
Accession: EED12780
  
Location: 259706-260658
  
 NCBI BlastP on this gene

EED12780

hypothetical protein
  
Accession: EED12781
  
Location: 260882-261720
  
 NCBI BlastP on this gene

EED12781

hypothetical protein
  
Accession: EED12782
  
Location: 262964-263422
  
 NCBI BlastP on this gene

EED12782

hypothetical protein
  
Accession: EED12783
  
Location: 263884-264455
  
 NCBI BlastP on this gene

EED12783

hypothetical protein
  
Accession: EED12784
  
Location: 264953-265790
  
 NCBI BlastP on this gene

EED12784

conserved hypothetical protein
  
Accession: EED12785
  
Location: 267308-268945
  
 NCBI BlastP on this gene

EED12785

conserved hypothetical protein
  
Accession: EED12786
  
Location: 270594-272300
  
 NCBI BlastP on this gene

EED12786

129. :  JH725221 Beauveria bassiana ARSEF 2860 unplaced genomic scaffold BBA\_S00072     Total score: 2.0     Cumulative Blast bit score: 160

branchpoint-bridging protein
  
Accession: EJP61168
  
Location: 15655-17686
  
 NCBI BlastP on this gene

EJP61168

peptidase family M28 family
  
Accession: EJP61169
  
Location: 18302-21415
  
 NCBI BlastP on this gene

EJP61169

hypothetical protein
  
Accession: EJP61170
  
Location: 21800-22126
  
 NCBI BlastP on this gene

EJP61170

HhH-GPD superfamily base excision DNA repair protein
  
Accession: EJP61171
  
Location: 25707-26942
  
 NCBI BlastP on this gene

EJP61171

ribosomal protein L11
  
Accession: EJP61172
  
Location: 27227-27806
  
 NCBI BlastP on this gene

EJP61172

O-methyltransferase, family 3
  
Accession: EJP61173
  
Location: 28788-29492
  
 NCBI BlastP on this gene

EJP61173

monocarboxylate permease-like protein
  
Accession: EJP61174
  
Location: 29731-31234
  
 NCBI BlastP on this gene

EJP61174

hypothetical protein
  
Accession: EJP61175
  
Location: 32494-33470
  
 NCBI BlastP on this gene

EJP61175

hypothetical protein
  
Accession: EJP61176
  
Location: 33936-34416
  
 NCBI BlastP on this gene

EJP61176

hypothetical protein
  
Accession: EJP61177
  
Location: 35260-36175
  
  
**BlastP hit with Mycgr3G90561\_Mycgr3T**
  
Percentage identity: 40 %
  
BlastP bit score: 81
  
Sequence coverage: 55 %
  
E-value: 1e-15
  
  
 NCBI BlastP on this gene

EJP61177

hypothetical protein
  
Accession: EJP61178
  
Location: 36487-37534
  
  
**BlastP hit with Mycgr3G35535\_Mycgr3T**
  
Percentage identity: 30 %
  
BlastP bit score: 79
  
Sequence coverage: 90 %
  
E-value: 6e-15
  
  
 NCBI BlastP on this gene

EJP61178

sterigmatocystin 8-O-methyltransferase
  
Accession: EJP61179
  
Location: 37688-39172
  
 NCBI BlastP on this gene

EJP61179

integral membrane protein
  
Accession: EJP61180
  
Location: 39894-41250
  
 NCBI BlastP on this gene

EJP61180

rRNA assembly protein Mis3, putative
  
Accession: EJP61181
  
Location: 42100-43062
  
 NCBI BlastP on this gene

EJP61181

hypothetical protein
  
Accession: EJP61182
  
Location: 44056-44369
  
 NCBI BlastP on this gene

EJP61182

oxidoreductase-like protein
  
Accession: EJP61183
  
Location: 44716-45891
  
 NCBI BlastP on this gene

EJP61183

YeeE/YedE family protein
  
Accession: EJP61184
  
Location: 46834-47907
  
 NCBI BlastP on this gene

EJP61184

endoribonuclease L-PSP
  
Accession: EJP61185
  
Location: 47975-48425
  
 NCBI BlastP on this gene

EJP61185

GatB/YqeY family protein
  
Accession: EJP61186
  
Location: 48746-49351
  
 NCBI BlastP on this gene

EJP61186

putative succinyl-CoA ligase
  
Accession: EJP61187
  
Location: 49975-51511
  
 NCBI BlastP on this gene

EJP61187

130. :  DS995901 Penicillium marneffei ATCC 18224 scf\_1105668340960 genomic scaffold     Total score: 2.0     Cumulative Blast bit score: 157

metallo-beta-lactamase domain protein, putative
  
Accession: EEA23878
  
Location: 626270-627307
  
 NCBI BlastP on this gene

EEA23878

trehalose-phosphate synthase/phosphatase complex subunit Tps1, putative
  
Accession: EEA23879
  
Location: 628393-630120
  
 NCBI BlastP on this gene

EEA23879

hypothetical protein
  
Accession: EEA23880
  
Location: 630754-631674
  
 NCBI BlastP on this gene

EEA23880

AAA family ATPase, putative
  
Accession: EEA23881
  
Location: 631886-634539
  
 NCBI BlastP on this gene

EEA23881

mitochondrial carrier protein, putative
  
Accession: EEA23882
  
Location: 635958-637358
  
 NCBI BlastP on this gene

EEA23882

RING finger protein
  
Accession: EEA23883
  
Location: 638534-640238
  
 NCBI BlastP on this gene

EEA23883

hypothetical protein
  
Accession: EEA23885
  
Location: 642058-642332
  
 NCBI BlastP on this gene

EEA23885

O-methyltransferase, putative
  
Accession: EEA23886
  
Location: 642675-644041
  
 NCBI BlastP on this gene

EEA23886

conserved hypothetical protein
  
Accession: EEA23887
  
Location: 645056-645879
  
  
**BlastP hit with Mycgr3G90561\_Mycgr3T**
  
Percentage identity: 33 %
  
BlastP bit score: 79
  
Sequence coverage: 55 %
  
E-value: 1e-14
  
  
 NCBI BlastP on this gene

EEA23887

conserved hypothetical protein
  
Accession: EEA23888
  
Location: 645896-646952
  
  
**BlastP hit with Mycgr3G35535\_Mycgr3T**
  
Percentage identity: 28 %
  
BlastP bit score: 78
  
Sequence coverage: 97 %
  
E-value: 7e-15
  
  
 NCBI BlastP on this gene

EEA23888

conserved hypothetical protein
  
Accession: EEA23889
  
Location: 647670-649149
  
 NCBI BlastP on this gene

EEA23889

translation initiation factor eif-2b epsilon subunit, putative
  
Accession: EEA23890
  
Location: 649395-651739
  
 NCBI BlastP on this gene

EEA23890

stress response transcription factor SrrA/Skn7, putative
  
Accession: EEA23891
  
Location: 652988-655243
  
 NCBI BlastP on this gene

EEA23891

conserved hypothetical protein
  
Accession: EEA23892
  
Location: 657213-657527
  
 NCBI BlastP on this gene

EEA23892

conserved hypothetical protein
  
Accession: EEA23893
  
Location: 657545-658237
  
 NCBI BlastP on this gene

EEA23893

glutathione S-transferase, putative
  
Accession: EEA23894
  
Location: 659249-660159
  
 NCBI BlastP on this gene

EEA23894

ER membrane protein Wsc4, putative
  
Accession: EEA23895
  
Location: 661498-662858
  
 NCBI BlastP on this gene

EEA23895

hypothetical protein
  
Accession: EEA23896
  
Location: 665660-667039
  
 NCBI BlastP on this gene

EEA23896

131. :  DS995727 Trichophyton equinum CBS 127.97 supercont1.10 genomic scaffold     Total score: 2.0     Cumulative Blast bit score: 150

hypothetical protein
  
Accession: EGE03381
  
Location: 122614-123667
  
 NCBI BlastP on this gene

EGE03381

hypothetical protein
  
Accession: EGE03382
  
Location: 126921-127484
  
 NCBI BlastP on this gene

EGE03382

hypothetical protein
  
Accession: EGE03383
  
Location: 128830-130434
  
 NCBI BlastP on this gene

EGE03383

metalloprotease MEP3
  
Accession: EGE03384
  
Location: 130924-133233
  
 NCBI BlastP on this gene

EGE03384

integral membrane protein
  
Accession: EGE03385
  
Location: 134572-135863
  
 NCBI BlastP on this gene

EGE03385

MFS monocarboxylate transporter
  
Accession: EGE03386
  
Location: 137171-138630
  
 NCBI BlastP on this gene

EGE03386

hypothetical protein
  
Accession: EGE03387
  
Location: 138744-139665
  
 NCBI BlastP on this gene

EGE03387

hypothetical protein
  
Accession: EGE03388
  
Location: 140815-141460
  
 NCBI BlastP on this gene

EGE03388

hypothetical protein
  
Accession: EGE03389
  
Location: 141716-142631
  
  
**BlastP hit with Mycgr3G90561\_Mycgr3T**
  
Percentage identity: 37 %
  
BlastP bit score: 77
  
Sequence coverage: 54 %
  
E-value: 5e-14
  
  
 NCBI BlastP on this gene

EGE03389

hypothetical protein
  
Accession: EGE03390
  
Location: 143242-144256
  
  
**BlastP hit with Mycgr3G35535\_Mycgr3T**
  
Percentage identity: 33 %
  
BlastP bit score: 73
  
Sequence coverage: 69 %
  
E-value: 6e-13
  
  
 NCBI BlastP on this gene

EGE03390

hypothetical protein
  
Accession: EGE03391
  
Location: 144389-145813
  
 NCBI BlastP on this gene

EGE03391

hypothetical protein
  
Accession: EGE03392
  
Location: 146776-147870
  
 NCBI BlastP on this gene

EGE03392

carbonate dehydratase
  
Accession: EGE03393
  
Location: 149560-150499
  
 NCBI BlastP on this gene

EGE03393

hypothetical protein
  
Accession: EGE03394
  
Location: 150915-151733
  
 NCBI BlastP on this gene

EGE03394

alkaline phosphatase
  
Accession: EGE03395
  
Location: 152224-153874
  
 NCBI BlastP on this gene

EGE03395

hypothetical protein
  
Accession: EGE03396
  
Location: 154779-158467
  
 NCBI BlastP on this gene

EGE03396

hypothetical protein
  
Accession: EGE03397
  
Location: 159617-160560
  
 NCBI BlastP on this gene

EGE03397

pre-mRNA splicing factor
  
Accession: EGE03398
  
Location: 161081-164127
  
 NCBI BlastP on this gene

EGE03398

132. :  GG700650 Trichophyton rubrum CBS 118892 genomic scaffold supercont2.3     Total score: 2.0     Cumulative Blast bit score: 147

hypothetical protein
  
Accession: EGD86991
  
Location: 1385837-1386869
  
 NCBI BlastP on this gene

EGD86991

metalloprotease
  
Accession: EGD86992
  
Location: 1391162-1392186
  
 NCBI BlastP on this gene

EGD86992

metalloprotease
  
Accession: EGD86993
  
Location: 1394694-1396934
  
 NCBI BlastP on this gene

EGD86993

integral membrane protein
  
Accession: EGD86994
  
Location: 1398292-1399597
  
 NCBI BlastP on this gene

EGD86994

hypothetical protein
  
Accession: EGD86995
  
Location: 1400881-1402333
  
 NCBI BlastP on this gene

EGD86995

hypothetical protein
  
Accession: EGD86996
  
Location: 1402447-1403362
  
 NCBI BlastP on this gene

EGD86996

hypothetical protein
  
Accession: EGD86997
  
Location: 1403735-1404069
  
 NCBI BlastP on this gene

EGD86997

hypothetical protein
  
Accession: EGD86998
  
Location: 1405302-1406213
  
  
**BlastP hit with Mycgr3G90561\_Mycgr3T**
  
Percentage identity: 40 %
  
BlastP bit score: 84
  
Sequence coverage: 54 %
  
E-value: 2e-16
  
  
 NCBI BlastP on this gene

EGD86998

hypothetical protein
  
Accession: EGD86999
  
Location: 1406824-1408089
  
  
**BlastP hit with Mycgr3G35535\_Mycgr3T**
  
Percentage identity: 28 %
  
BlastP bit score: 63
  
Sequence coverage: 72 %
  
E-value: 3e-09
  
  
 NCBI BlastP on this gene

EGD86999

hypothetical protein
  
Accession: EGD87000
  
Location: 1408623-1409654
  
 NCBI BlastP on this gene

EGD87000

hypothetical protein
  
Accession: EGD87001
  
Location: 1411704-1412639
  
 NCBI BlastP on this gene

EGD87001

hypothetical protein
  
Accession: EGD87002
  
Location: 1413000-1413848
  
 NCBI BlastP on this gene

EGD87002

alkaline phosphatase
  
Accession: EGD87003
  
Location: 1414326-1415970
  
 NCBI BlastP on this gene

EGD87003

hypothetical protein
  
Accession: EGD87004
  
Location: 1417237-1420614
  
 NCBI BlastP on this gene

EGD87004

transcriptional co-activator
  
Accession: EGD87005
  
Location: 1421147-1422608
  
 NCBI BlastP on this gene

EGD87005

pre-mRNA splicing factor prp1
  
Accession: EGD87006
  
Location: 1423093-1426108
  
 NCBI BlastP on this gene

EGD87006

dimethyladenosine transferase dimethyltransferase
  
Accession: EGD87007
  
Location: 1426413-1427672
  
 NCBI BlastP on this gene

EGD87007

GTPase activating protein Gyp1
  
Accession: EGD87008
  
Location: 1427715-1429877
  
 NCBI BlastP on this gene

EGD87008

133. :  GG698487 Trichophyton tonsurans CBS 112818 genomic scaffold supercont1.11     Total score: 2.0     Cumulative Blast bit score: 147

hypothetical protein
  
Accession: EGD95284
  
Location: 202243-203296
  
 NCBI BlastP on this gene

EGD95284

hypothetical protein
  
Accession: EGD95285
  
Location: 204690-205723
  
 NCBI BlastP on this gene

EGD95285

hypothetical protein
  
Accession: EGD95286
  
Location: 209745-210155
  
 NCBI BlastP on this gene

EGD95286

metalloprotease
  
Accession: EGD95287
  
Location: 210637-212887
  
 NCBI BlastP on this gene

EGD95287

integral membrane protein
  
Accession: EGD95288
  
Location: 214226-215516
  
 NCBI BlastP on this gene

EGD95288

hypothetical protein
  
Accession: EGD95289
  
Location: 216824-218283
  
 NCBI BlastP on this gene

EGD95289

hypothetical protein
  
Accession: EGD95290
  
Location: 218397-219318
  
 NCBI BlastP on this gene

EGD95290

hypothetical protein
  
Accession: EGD95291
  
Location: 219698-220035
  
 NCBI BlastP on this gene

EGD95291

hypothetical protein
  
Accession: EGD95292
  
Location: 221300-222215
  
  
**BlastP hit with Mycgr3G90561\_Mycgr3T**
  
Percentage identity: 37 %
  
BlastP bit score: 74
  
Sequence coverage: 54 %
  
E-value: 3e-13
  
  
 NCBI BlastP on this gene

EGD95292

hypothetical protein
  
Accession: EGD95293
  
Location: 222826-223840
  
  
**BlastP hit with Mycgr3G35535\_Mycgr3T**
  
Percentage identity: 33 %
  
BlastP bit score: 73
  
Sequence coverage: 69 %
  
E-value: 6e-13
  
  
 NCBI BlastP on this gene

EGD95293

hypothetical protein
  
Accession: EGD95294
  
Location: 223973-225397
  
 NCBI BlastP on this gene

EGD95294

hypothetical protein
  
Accession: EGD95295
  
Location: 226340-227434
  
 NCBI BlastP on this gene

EGD95295

hypothetical protein
  
Accession: EGD95296
  
Location: 235015-237946
  
 NCBI BlastP on this gene

EGD95296

hypothetical protein
  
Accession: EGD95297
  
Location: 238285-239224
  
 NCBI BlastP on this gene

EGD95297

hypothetical protein
  
Accession: EGD95298
  
Location: 239619-240458
  
 NCBI BlastP on this gene

EGD95298

alkaline phosphatase
  
Accession: EGD95299
  
Location: 240949-242599
  
 NCBI BlastP on this gene

EGD95299

hypothetical protein
  
Accession: EGD95300
  
Location: 243504-247192
  
 NCBI BlastP on this gene

EGD95300

134. :  EQ962656 Talaromyces stipitatus ATCC 10500 scf\_1105507295549 genomic scaffold     Total score: 2.0     Cumulative Blast bit score: 135

conserved hypothetical protein
  
Accession: EED16322
  
Location: 1494275-1496605
  
 NCBI BlastP on this gene

EED16322

hypothetical protein
  
Accession: EED16323
  
Location: 1497772-1498824
  
 NCBI BlastP on this gene

EED16323

hypothetical protein
  
Accession: EED16324
  
Location: 1506732-1507079
  
 NCBI BlastP on this gene

EED16324

hypothetical protein
  
Accession: EED16325
  
Location: 1507947-1508375
  
 NCBI BlastP on this gene

EED16325

O-methyltransferase, putative
  
Accession: EED16326
  
Location: 1511062-1512489
  
 NCBI BlastP on this gene

EED16326

conserved hypothetical protein
  
Accession: EED16327
  
Location: 1513295-1514127
  
  
**BlastP hit with Mycgr3G90561\_Mycgr3T**
  
Percentage identity: 38 %
  
BlastP bit score: 65
  
Sequence coverage: 38 %
  
E-value: 2e-10
  
  
 NCBI BlastP on this gene

EED16327

conserved hypothetical protein
  
Accession: EED16331
  
Location: 1514132-1515205
  
  
**BlastP hit with Mycgr3G35535\_Mycgr3T**
  
Percentage identity: 26 %
  
BlastP bit score: 70
  
Sequence coverage: 96 %
  
E-value: 4e-12
  
  
 NCBI BlastP on this gene

EED16331

conserved hypothetical protein
  
Accession: EED16333
  
Location: 1516161-1517236
  
 NCBI BlastP on this gene

EED16333

hypothetical protein
  
Accession: EED16335
  
Location: 1517560-1517970
  
 NCBI BlastP on this gene

EED16335

F-box domain protein
  
Accession: EED16336
  
Location: 1518631-1520140
  
 NCBI BlastP on this gene

EED16336

conserved hypothetical protein
  
Accession: EED16337
  
Location: 1520411-1521122
  
 NCBI BlastP on this gene

EED16337

pyruvate kinase
  
Accession: EED16338
  
Location: 1522274-1524287
  
 NCBI BlastP on this gene

EED16338

hypothetical protein
  
Accession: EED16339
  
Location: 1525816-1526229
  
 NCBI BlastP on this gene

EED16339

conserved hypothetical protein
  
Accession: EED16340
  
Location: 1526286-1528208
  
 NCBI BlastP on this gene

EED16340

1-phosphatidylinositol-3-phosphate 5-kinase (Fab1), putative
  
Accession: EED16341
  
Location: 1528608-1536061
  
 NCBI BlastP on this gene

EED16341

135. :  KB446559 Pseudocercospora fijiensis CIRAD86 unplaced genomic scaffold MYCFIscaffold\_5     Total score: 2.0     Cumulative Blast bit score: 126

hypothetical protein
  
Accession: EME81782
  
Location: 1024548-1025697
  
 NCBI BlastP on this gene

EME81782

hypothetical protein
  
Accession: EME81783
  
Location: 1026086-1028394
  
 NCBI BlastP on this gene

EME81783

hypothetical protein
  
Accession: EME81784
  
Location: 1028981-1029753
  
 NCBI BlastP on this gene

EME81784

hypothetical protein
  
Accession: EME81785
  
Location: 1030585-1031741
  
 NCBI BlastP on this gene

EME81785

hypothetical protein
  
Accession: EME81786
  
Location: 1032206-1032536
  
 NCBI BlastP on this gene

EME81786

hypothetical protein
  
Accession: EME81787
  
Location: 1033141-1035119
  
 NCBI BlastP on this gene

EME81787

putative ABC transporter
  
Accession: EME81788
  
Location: 1035593-1040346
  
 NCBI BlastP on this gene

EME81788

hypothetical protein
  
Accession: EME81789
  
Location: 1040361-1041389
  
 NCBI BlastP on this gene

EME81789

hypothetical protein
  
Accession: EME81790
  
Location: 1041669-1042653
  
  
**BlastP hit with Mycgr3G35535\_Mycgr3T**
  
Percentage identity: 27 %
  
BlastP bit score: 62
  
Sequence coverage: 94 %
  
E-value: 4e-09
  
  
 NCBI BlastP on this gene

EME81790

hypothetical protein
  
Accession: EME81791
  
Location: 1042886-1043959
  
 NCBI BlastP on this gene

EME81791

hypothetical protein
  
Accession: EME81792
  
Location: 1044597-1045734
  
 NCBI BlastP on this gene

EME81792

hypothetical protein
  
Accession: EME81793
  
Location: 1046727-1047115
  
 NCBI BlastP on this gene

EME81793

hypothetical protein
  
Accession: EME81794
  
Location: 1048638-1050149
  
 NCBI BlastP on this gene

EME81794

hypothetical protein
  
Accession: EME81795
  
Location: 1051175-1052011
  
  
**BlastP hit with Mycgr3G90561\_Mycgr3T**
  
Percentage identity: 34 %
  
BlastP bit score: 64
  
Sequence coverage: 57 %
  
E-value: 1e-09
  
  
 NCBI BlastP on this gene

EME81795

hypothetical protein
  
Accession: EME81796
  
Location: 1053650-1054216
  
 NCBI BlastP on this gene

EME81796

hypothetical protein
  
Accession: EME81797
  
Location: 1054887-1055420
  
 NCBI BlastP on this gene

EME81797

136. :  AP007166 Aspergillus oryzae RIB40 DNA, SC113.     Total score: 2.0     Cumulative Blast bit score: 119

not annotated
  
Accession: BAE63089
  
Location: 1415647-1416318
  
  
**BlastP hit with Mycgr3G90561\_Mycgr3T**
  
Percentage identity: 34 %
  
BlastP bit score: 60
  
Sequence coverage: 60 %
  
E-value: 1e-08
  
  
  
**BlastP hit with Mycgr3G35535\_Mycgr3T**
  
Percentage identity: 30 %
  
BlastP bit score: 60
  
Sequence coverage: 85 %
  
E-value: 5e-09
  
  
 NCBI BlastP on this gene

AO090113000025

not annotated
  
Accession: BAE63088
  
Location: 1414473-1415203
  
 NCBI BlastP on this gene

AO090113000024

not annotated
  
Accession: BAE63087
  
Location: 1411903-1413731
  
 NCBI BlastP on this gene

AO090113000022

not annotated
  
Accession: BAE63086
  
Location: 1408019-1411696
  
 NCBI BlastP on this gene

AO090113000021

not annotated
  
Accession: BAE63085
  
Location: 1405065-1406126
  
 NCBI BlastP on this gene

AO090113000020

not annotated
  
Accession: BAE63084
  
Location: 1401539-1404894
  
 NCBI BlastP on this gene

AO090113000019

not annotated
  
Accession: BAE63083
  
Location: 1399519-1400124
  
 NCBI BlastP on this gene

AO090113000017

not annotated
  
Accession: BAE63082
  
Location: 1397319-1398389
  
 NCBI BlastP on this gene

AO090113000016

not annotated
  
Accession: BAE63081
  
Location: 1395669-1396273
  
 NCBI BlastP on this gene

AO090113000015

137. :  KB725822 Colletotrichum orbiculare MAFF 240422 unplaced genomic scaffold Scaffold\_269     Total score: 2.0     Cumulative Blast bit score: 118

carbohydrate esterase family 9 protein
  
Accession: ENH84127
  
Location: 18103-20537
  
 NCBI BlastP on this gene

ENH84127

hypothetical protein
  
Accession: ENH84128
  
Location: 21230-21457
  
 NCBI BlastP on this gene

ENH84128

hypothetical protein
  
Accession: ENH84129
  
Location: 21608-22228
  
 NCBI BlastP on this gene

ENH84129

hypothetical protein
  
Accession: ENH84130
  
Location: 25850-26575
  
 NCBI BlastP on this gene

ENH84130

hypothetical protein
  
Accession: ENH84131
  
Location: 26242-26654
  
 NCBI BlastP on this gene

ENH84131

regulatory protein
  
Accession: ENH84132
  
Location: 29775-31586
  
 NCBI BlastP on this gene

ENH84132

hypothetical protein
  
Accession: ENH84133
  
Location: 32189-32642
  
 NCBI BlastP on this gene

ENH84133

hypothetical protein
  
Accession: ENH84134
  
Location: 33021-33374
  
 NCBI BlastP on this gene

ENH84134

hypothetical protein
  
Accession: ENH84135
  
Location: 34864-35949
  
 NCBI BlastP on this gene

ENH84135

hypothetical protein
  
Accession: ENH84136
  
Location: 36845-37870
  
  
**BlastP hit with Mycgr3G35535\_Mycgr3T**
  
Percentage identity: 28 %
  
BlastP bit score: 57
  
Sequence coverage: 93 %
  
E-value: 4e-07
  
  
 NCBI BlastP on this gene

ENH84136

hypothetical protein
  
Accession: ENH84137
  
Location: 38739-40924
  
 NCBI BlastP on this gene

ENH84137

hypothetical protein
  
Accession: ENH84138
  
Location: 41065-41940
  
 NCBI BlastP on this gene

ENH84138

hypothetical protein
  
Accession: ENH84139
  
Location: 42503-43354
  
  
**BlastP hit with Mycgr3G90561\_Mycgr3T**
  
Percentage identity: 29 %
  
BlastP bit score: 61
  
Sequence coverage: 61 %
  
E-value: 2e-08
  
  
 NCBI BlastP on this gene

ENH84139

hypothetical protein
  
Accession: ENH84140
  
Location: 43554-44156
  
 NCBI BlastP on this gene

ENH84140

hypothetical protein
  
Accession: ENH84141
  
Location: 45034-46541
  
 NCBI BlastP on this gene

ENH84141

c-4 methylsterol oxidase
  
Accession: ENH84142
  
Location: 46995-48103
  
 NCBI BlastP on this gene

ENH84142

hypothetical protein
  
Accession: ENH84143
  
Location: 48849-51157
  
 NCBI BlastP on this gene

ENH84143

siderophore iron transporter mirb
  
Accession: ENH84144
  
Location: 52036-53799
  
 NCBI BlastP on this gene

ENH84144

hypothetical protein
  
Accession: ENH84145
  
Location: 55381-56301
  
 NCBI BlastP on this gene

ENH84145

short-chain dehydrogenase
  
Accession: ENH84146
  
Location: 57030-58028
  
 NCBI BlastP on this gene

ENH84146

hypothetical protein
  
Accession: ENH84147
  
Location: 59020-59409
  
 NCBI BlastP on this gene

ENH84147

hypothetical protein
  
Accession: ENH84148
  
Location: 59918-60767
  
 NCBI BlastP on this gene

ENH84148

neutral amino acid
  
Accession: ENH84149
  
Location: 62741-64481
  
 NCBI BlastP on this gene

ENH84149

138. :  AMYD01004223 Colletotrichum gloeosporioides Cg-14     Total score: 2.0     Cumulative Blast bit score: 118

glycosyl hydrolase family 18
  
Accession: EQB43615
  
Location: 12156-12557
  
 NCBI BlastP on this gene

EQB43615

hypothetical protein
  
Accession: EQB43616
  
Location: 12974-13249
  
 NCBI BlastP on this gene

EQB43616

hypothetical protein
  
Accession: EQB43617
  
Location: 13590-14311
  
 NCBI BlastP on this gene

EQB43617

hypothetical protein
  
Accession: EQB43618
  
Location: 15399-16209
  
 NCBI BlastP on this gene

EQB43618

hypothetical protein
  
Accession: EQB43619
  
Location: 16545-17156
  
 NCBI BlastP on this gene

EQB43619

cytochrome P450
  
Accession: EQB43620
  
Location: 18467-20166
  
 NCBI BlastP on this gene

EQB43620

hypothetical protein
  
Accession: EQB43621
  
Location: 20280-21770
  
 NCBI BlastP on this gene

EQB43621

hypothetical protein
  
Accession: EQB43622
  
Location: 22170-23267
  
 NCBI BlastP on this gene

EQB43622

hypothetical protein
  
Accession: EQB43623
  
Location: 30505-31342
  
  
**BlastP hit with Mycgr3G90561\_Mycgr3T**
  
Percentage identity: 28 %
  
BlastP bit score: 62
  
Sequence coverage: 61 %
  
E-value: 4e-09
  
  
 NCBI BlastP on this gene

EQB43623

hypothetical protein
  
Accession: EQB43624
  
Location: 31920-32792
  
 NCBI BlastP on this gene

EQB43624

hypothetical protein
  
Accession: EQB43625
  
Location: 32914-35130
  
 NCBI BlastP on this gene

EQB43625

hypothetical protein
  
Accession: EQB43626
  
Location: 36026-37003
  
  
**BlastP hit with Mycgr3G35535\_Mycgr3T**
  
Percentage identity: 30 %
  
BlastP bit score: 56
  
Sequence coverage: 91 %
  
E-value: 6e-07
  
  
 NCBI BlastP on this gene

EQB43626

hypothetical protein
  
Accession: EQB43627
  
Location: 37883-38963
  
 NCBI BlastP on this gene

EQB43627

hypothetical protein
  
Accession: EQB43628
  
Location: 39377-40677
  
 NCBI BlastP on this gene

EQB43628

hypothetical protein
  
Accession: EQB43629
  
Location: 41022-41466
  
 NCBI BlastP on this gene

EQB43629

139. :  KB726072 Colletotrichum orbiculare MAFF 240422 unplaced genomic scaffold Scaffold\_494     Total score: 2.0     Cumulative Blast bit score: 111

mRNA splicing protein
  
Accession: ENH78168
  
Location: 439137-441534
  
 NCBI BlastP on this gene

ENH78168

cytochrome c peroxidase
  
Accession: ENH78169
  
Location: 444400-445601
  
 NCBI BlastP on this gene

ENH78169

hypothetical protein
  
Accession: ENH78170
  
Location: 447587-447862
  
 NCBI BlastP on this gene

ENH78170

tankyrase 1 binding protein isoform-like protein
  
Accession: ENH78171
  
Location: 448307-449008
  
 NCBI BlastP on this gene

ENH78171

hypothetical protein
  
Accession: ENH78172
  
Location: 450630-451400
  
 NCBI BlastP on this gene

ENH78172

hypothetical protein
  
Accession: ENH78173
  
Location: 451640-452503
  
 NCBI BlastP on this gene

ENH78173

hypothetical protein
  
Accession: ENH78174
  
Location: 454654-454977
  
 NCBI BlastP on this gene

ENH78174

hypothetical protein
  
Accession: ENH78175
  
Location: 456745-457907
  
 NCBI BlastP on this gene

ENH78175

hypothetical protein
  
Accession: ENH78176
  
Location: 458469-459573
  
  
**BlastP hit with Mycgr3G35535\_Mycgr3T**
  
Percentage identity: 27 %
  
BlastP bit score: 57
  
Sequence coverage: 85 %
  
E-value: 3e-07
  
  
 NCBI BlastP on this gene

ENH78176

tat pathway signal sequence
  
Accession: ENH78177
  
Location: 460565-461404
  
 NCBI BlastP on this gene

ENH78177

hypothetical protein
  
Accession: ENH78178
  
Location: 461850-462849
  
  
**BlastP hit with Mycgr3G90561\_Mycgr3T**
  
Percentage identity: 33 %
  
BlastP bit score: 55
  
Sequence coverage: 53 %
  
E-value: 3e-06
  
  
 NCBI BlastP on this gene

ENH78178

f1f0 atp synthase assembly protein
  
Accession: ENH78179
  
Location: 467309-468268
  
 NCBI BlastP on this gene

ENH78179

gas1-like protein
  
Accession: ENH78180
  
Location: 471418-472614
  
 NCBI BlastP on this gene

ENH78180

gcn5-related n-acetyltransferase
  
Accession: ENH78181
  
Location: 475610-476251
  
 NCBI BlastP on this gene

ENH78181

pyridine nucleotide-disulfide oxidoreductase
  
Accession: ENH78182
  
Location: 476454-477719
  
 NCBI BlastP on this gene

ENH78182

140. :  DS985225 Verticillium albo-atrum VaMs.102 supercont1.12 genomic scaffold     Total score: 1.0     Cumulative Blast bit score: 3093

pantothenate transporter FEN2
  
Accession: EEY22277
  
Location: 516505-518980
  
 NCBI BlastP on this gene

EEY22277

lipase
  
Accession: EEY22276
  
Location: 514058-515716
  
 NCBI BlastP on this gene

EEY22276

canalicular multispecific organic anion transporter 2
  
Accession: EEY22275
  
Location: 509287-513726
  
 NCBI BlastP on this gene

EEY22275

fatty acid synthase beta subunit
  
Accession: EEY22274
  
Location: 505878-507620
  
 NCBI BlastP on this gene

EEY22274

phenyloxazoline synthase mbtB
  
Accession: EEY22273
  
Location: 498532-500877
  
 NCBI BlastP on this gene

EEY22273

HC-toxin synthetase
  
Accession: EEY22272
  
Location: 490133-497717
  
  
**BlastP hit with Mycgr3G90558\_Mycgr3T**
  
Percentage identity: 30 %
  
BlastP bit score: 1082
  
Sequence coverage: 57 %
  
E-value: 0.0
  
  
 NCBI BlastP on this gene

EEY22272

HC-toxin synthetase
  
Accession: EEY22271
  
Location: 482333-487978
  
  
**BlastP hit with Mycgr3G90558\_Mycgr3T**
  
Percentage identity: 35 %
  
BlastP bit score: 1007
  
Sequence coverage: 41 %
  
E-value: 0.0
  
  
 NCBI BlastP on this gene

EEY22271

HC-toxin synthetase
  
Accession: EEY22270
  
Location: 473021-482054
  
  
**BlastP hit with Mycgr3G90558\_Mycgr3T**
  
Percentage identity: 29 %
  
BlastP bit score: 1004
  
Sequence coverage: 61 %
  
E-value: 0.0
  
  
 NCBI BlastP on this gene

EEY22270

lectin
  
Accession: EEY22269
  
Location: 470997-472199
  
 NCBI BlastP on this gene

EEY22269

predicted protein
  
Accession: EEY22268
  
Location: 465352-467085
  
 NCBI BlastP on this gene

EEY22268

predicted protein
  
Accession: EEY22267
  
Location: 464682-464998
  
 NCBI BlastP on this gene

EEY22267

conserved hypothetical protein
  
Accession: EEY22266
  
Location: 462558-463538
  
 NCBI BlastP on this gene

EEY22266

GS1 protein
  
Accession: EEY22265
  
Location: 461311-462197
  
 NCBI BlastP on this gene

EEY22265

C6 zinc finger domain-containing protein
  
Accession: EEY22264
  
Location: 458919-460885
  
 NCBI BlastP on this gene

EEY22264

pantothenate synthetase
  
Accession: EEY22263
  
Location: 453949-455657
  
 NCBI BlastP on this gene

EEY22263

141. :  JN186799 Claviceps purpurea strain 20.1 ergot alkaloid biosynthetic gene cluster     Total score: 1.0     Cumulative Blast bit score: 2413

elymoclavine monooxygenase
  
Accession: AET79191
  
Location: 21324-23435
  
 NCBI BlastP on this gene

cloA

chanoclavine synthase catalase protein
  
Accession: AET79180
  
Location: 24095-25602
  
 NCBI BlastP on this gene

easC

chanoclavine-I dehydrogenase
  
Accession: AET79189
  
Location: 26331-27179
  
 NCBI BlastP on this gene

easD

chanoclavine-I synthase oxidoreductase
  
Accession: AET79192
  
Location: 27587-29490
  
 NCBI BlastP on this gene

easE

dimethylallyltryptophan N-methyltransferase
  
Accession: AET79190
  
Location: 29844-30993
  
 NCBI BlastP on this gene

easF

agroclavine dehydrogenase
  
Accession: AET79181
  
Location: 31353-32431
  
 NCBI BlastP on this gene

easG

dimethylallyl tryptophan synthase
  
Accession: AET79188
  
Location: 33089-34554
  
 NCBI BlastP on this gene

dmaW

putative oxygenase
  
Accession: AET79182
  
Location: 35947-36891
  
 NCBI BlastP on this gene

easH1

lysergyl peptide synthetase subunit 1
  
Accession: AET79183
  
Location: 37705-48636
  
  
**BlastP hit with Mycgr3G90558\_Mycgr3T**
  
Percentage identity: 31 %
  
BlastP bit score: 1215
  
Sequence coverage: 68 %
  
E-value: 0.0
  
  
 NCBI BlastP on this gene

lpsA1

lysergyl peptide synthetase subunit 1
  
Accession: AET79184
  
Location: 52447-63381
  
  
**BlastP hit with Mycgr3G90558\_Mycgr3T**
  
Percentage identity: 30 %
  
BlastP bit score: 1198
  
Sequence coverage: 68 %
  
E-value: 0.0
  
  
 NCBI BlastP on this gene

lpsA2

hypothetical protein
  
Accession: AET79185
  
Location: 66909-67226
  
 NCBI BlastP on this gene

AET79185

hypothetical protein
  
Accession: AET79186
  
Location: 69960-71064
  
 NCBI BlastP on this gene

AET79186

142. :  CAGA01000020 Claviceps purpurea 20.1     Total score: 1.0     Cumulative Blast bit score: 2413

related to non-ribosomal peptide synthetase
  
Accession: CCE30235
  
Location: 277495-281470
  
 NCBI BlastP on this gene

CCE30235

related to trichodiene oxygenase cytochrome P450
  
Accession: CCE30234
  
Location: 275063-277174
  
 NCBI BlastP on this gene

CCE30234

probable catalase isozyme P
  
Accession: CCE30233
  
Location: 272896-274403
  
 NCBI BlastP on this gene

CCE30233

related to D-arabinitol 2-dehydrogenase
  
Accession: CCE30232
  
Location: 271319-272167
  
 NCBI BlastP on this gene

CCE30232

probable isoamyl alcohol oxidase
  
Accession: CCE30231
  
Location: 269008-270510
  
 NCBI BlastP on this gene

CCE30231

uncharacterized protein
  
Accession: CCE30230
  
Location: 267505-268654
  
 NCBI BlastP on this gene

CCE30230

uncharacterized protein
  
Accession: CCE30229
  
Location: 266067-267145
  
 NCBI BlastP on this gene

CCE30229

uncharacterized protein
  
Accession: CCE30228
  
Location: 263944-265409
  
 NCBI BlastP on this gene

CCE30228

uncharacterized protein
  
Accession: CCE30227
  
Location: 261607-262551
  
 NCBI BlastP on this gene

CCE30227

non-ribosomal peptide synthetase
  
Accession: CCE30226
  
Location: 249862-260793
  
  
**BlastP hit with Mycgr3G90558\_Mycgr3T**
  
Percentage identity: 31 %
  
BlastP bit score: 1215
  
Sequence coverage: 68 %
  
E-value: 0.0
  
  
 NCBI BlastP on this gene

CCE30226

non-ribosomal peptide synthetase
  
Accession: CCE30225
  
Location: 235117-246051
  
  
**BlastP hit with Mycgr3G90558\_Mycgr3T**
  
Percentage identity: 30 %
  
BlastP bit score: 1198
  
Sequence coverage: 68 %
  
E-value: 0.0
  
  
 NCBI BlastP on this gene

CCE30225

uncharacterized protein
  
Accession: CCE30224
  
Location: 231272-231589
  
 NCBI BlastP on this gene

CCE30224

uncharacterized protein
  
Accession: CCE30223
  
Location: 227434-228538
  
 NCBI BlastP on this gene

CCE30223

uncharacterized protein
  
Accession: CCE30222
  
Location: 224705-225007
  
 NCBI BlastP on this gene

CCE30222

probable regulator of phosphatidylinositol-4-OH kinase protein
  
Accession: CCE30221
  
Location: 220814-221929
  
 NCBI BlastP on this gene

CCE30221

related to sna41 protein
  
Accession: CCE30220
  
Location: 215789-218621
  
 NCBI BlastP on this gene

CCE30220

143. :  KB933064 Togninia minima UCRPA7 unplaced genomic scaffold PA7\_03\_scaffold\_293     Total score: 1.0     Cumulative Blast bit score: 2295

hypothetical protein
  
Accession: EOO00601
  
Location: 147741-180254
  
  
**BlastP hit with Mycgr3G90558\_Mycgr3T**
  
Percentage identity: 30 %
  
BlastP bit score: 1164
  
Sequence coverage: 61 %
  
E-value: 0.0
  
  
 NCBI BlastP on this gene

EOO00601

putative alpha beta hydrolase protein
  
Accession: EOO00594
  
Location: 183798-184880
  
 NCBI BlastP on this gene

EOO00594

hypothetical protein
  
Accession: EOO00604
  
Location: 185818-213011
  
  
**BlastP hit with Mycgr3G90558\_Mycgr3T**
  
Percentage identity: 30 %
  
BlastP bit score: 1131
  
Sequence coverage: 61 %
  
E-value: 0.0
  
  
 NCBI BlastP on this gene

EOO00604

144. :  ACYE01000348 Trichophyton verrucosum HKI 0517     Total score: 1.0     Cumulative Blast bit score: 2244

hypothetical protein
  
Accession: EFE39144
  
Location: 52095-57201
  
 NCBI BlastP on this gene

EFE39144

hypothetical protein
  
Accession: EFE39143
  
Location: 49578-50967
  
 NCBI BlastP on this gene

EFE39143

hypothetical protein
  
Accession: EFE39142
  
Location: 48208-49318
  
 NCBI BlastP on this gene

EFE39142

hypothetical protein
  
Accession: EFE39141
  
Location: 40783-46991
  
 NCBI BlastP on this gene

EFE39141

hypothetical protein
  
Accession: EFE39140
  
Location: 37562-39509
  
 NCBI BlastP on this gene

EFE39140

nonribosomal peptide synthase, putative
  
Accession: EFE39139
  
Location: 25673-36486
  
  
**BlastP hit with Mycgr3G90558\_Mycgr3T**
  
Percentage identity: 28 %
  
BlastP bit score: 1218
  
Sequence coverage: 78 %
  
E-value: 0.0
  
  
 NCBI BlastP on this gene

EFE39139

hypothetical protein
  
Accession: EFE39138
  
Location: 17606-25327
  
  
**BlastP hit with Mycgr3G90558\_Mycgr3T**
  
Percentage identity: 30 %
  
BlastP bit score: 1026
  
Sequence coverage: 54 %
  
E-value: 0.0
  
  
 NCBI BlastP on this gene

EFE39138

hypothetical protein
  
Accession: EFE39137
  
Location: 16187-17057
  
 NCBI BlastP on this gene

EFE39137

hypothetical protein
  
Accession: EFE39136
  
Location: 14389-15686
  
 NCBI BlastP on this gene

EFE39136

hypothetical protein
  
Accession: EFE39135
  
Location: 12901-14095
  
 NCBI BlastP on this gene

EFE39135

hypothetical protein
  
Accession: EFE39134
  
Location: 12238-12588
  
 NCBI BlastP on this gene

EFE39134

hypothetical protein
  
Accession: EFE39133
  
Location: 11379-11954
  
 NCBI BlastP on this gene

EFE39133

hypothetical protein
  
Accession: EFE39132
  
Location: 10511-11189
  
 NCBI BlastP on this gene

EFE39132

hypothetical protein
  
Accession: EFE39131
  
Location: 8110-10143
  
 NCBI BlastP on this gene

EFE39131

hypothetical protein
  
Accession: EFE39130
  
Location: 5775-7898
  
 NCBI BlastP on this gene

EFE39130

GPI anchored CFEM domain protein
  
Accession: EFE39129
  
Location: 3398-4093
  
 NCBI BlastP on this gene

EFE39129

hypothetical protein
  
Accession: EFE39128
  
Location: 2978-3244
  
 NCBI BlastP on this gene

EFE39128

hypothetical protein
  
Accession: EFE39127
  
Location: 298-651
  
 NCBI BlastP on this gene

EFE39127

145. :  AHHD01000518 Macrophomina phaseolina MS6     Total score: 1.0     Cumulative Blast bit score: 2110

Putative ABC transporter protein
  
Accession: EKG10379
  
Location: 60871-62298
  
 NCBI BlastP on this gene

EKG10379

Putative ABC transporter protein
  
Accession: EKG10378
  
Location: 57105-59808
  
 NCBI BlastP on this gene

EKG10378

hypothetical protein
  
Accession: EKG10377
  
Location: 55249-56310
  
 NCBI BlastP on this gene

EKG10377

hypothetical protein
  
Accession: EKG10376
  
Location: 48258-53445
  
 NCBI BlastP on this gene

EKG10376

hypothetical protein
  
Accession: EKG10375
  
Location: 47726-48229
  
 NCBI BlastP on this gene

EKG10375

nucleoside-diphosphate-sugarepimerase,putative
  
Accession: EKG10374
  
Location: 43709-44092
  
 NCBI BlastP on this gene

EKG10374

AMP-dependent synthetase/ligase
  
Accession: EKG10373
  
Location: 10381-42790
  
  
**BlastP hit with Mycgr3G90558\_Mycgr3T**
  
Percentage identity: 34 %
  
BlastP bit score: 2110
  
Sequence coverage: 90 %
  
E-value: 0.0
  
  
 NCBI BlastP on this gene

EKG10373

hypothetical protein
  
Accession: EKG10372
  
Location: 8617-9045
  
 NCBI BlastP on this gene

EKG10372

hypothetical protein
  
Accession: EKG10371
  
Location: 5304-6784
  
 NCBI BlastP on this gene

EKG10371

Thioesterase superfamily
  
Accession: EKG10370
  
Location: 4698-5147
  
 NCBI BlastP on this gene

EKG10370

hypothetical protein
  
Accession: EKG10369
  
Location: 2071-2853
  
 NCBI BlastP on this gene

EKG10369

146. :  ABDF02000078 Trichoderma virens Gv29-8     Total score: 1.0     Cumulative Blast bit score: 2074

hypothetical protein
  
Accession: EHK20666
  
Location: 101576-102283
  
 NCBI BlastP on this gene

EHK20666

hypothetical protein
  
Accession: EHK20665
  
Location: 100187-101289
  
 NCBI BlastP on this gene

EHK20665

hypothetical protein
  
Accession: EHK20664
  
Location: 98207-99623
  
 NCBI BlastP on this gene

EHK20664

hypothetical protein
  
Accession: EHK20663
  
Location: 92780-94866
  
 NCBI BlastP on this gene

EHK20663

hypothetical protein
  
Accession: EHK20662
  
Location: 89989-91931
  
 NCBI BlastP on this gene

EHK20662

hypothetical protein
  
Accession: EHK20661
  
Location: 87545-88785
  
 NCBI BlastP on this gene

EHK20661

non-ribosomal peptide synthetase
  
Accession: EHK20800
  
Location: 60046-84786
  
  
**BlastP hit with Mycgr3G90558\_Mycgr3T**
  
Percentage identity: 32 %
  
BlastP bit score: 2074
  
Sequence coverage: 93 %
  
E-value: 0.0
  
  
 NCBI BlastP on this gene

EHK20800

hypothetical protein
  
Accession: EHK20660
  
Location: 57119-58261
  
 NCBI BlastP on this gene

EHK20660

hypothetical protein
  
Accession: EHK20659
  
Location: 55312-56073
  
 NCBI BlastP on this gene

EHK20659

hypothetical protein
  
Accession: EHK20658
  
Location: 52268-53523
  
 NCBI BlastP on this gene

EHK20658

hypothetical protein
  
Accession: EHK20657
  
Location: 48508-52024
  
 NCBI BlastP on this gene

EHK20657

hypothetical protein
  
Accession: EHK20656
  
Location: 46398-47588
  
 NCBI BlastP on this gene

EHK20656

147. :  ABDF02000005 Trichoderma virens Gv29-8     Total score: 1.0     Cumulative Blast bit score: 2042

hypothetical protein
  
Accession: EHK23786
  
Location: 1819841-1821002
  
 NCBI BlastP on this gene

EHK23786

hypothetical protein
  
Accession: EHK23787
  
Location: 1825427-1826455
  
 NCBI BlastP on this gene

EHK23787

non-ribosomal peptide synthetase
  
Accession: EHK23788
  
Location: 1830830-1880925
  
  
**BlastP hit with Mycgr3G90558\_Mycgr3T**
  
Percentage identity: 33 %
  
BlastP bit score: 2042
  
Sequence coverage: 89 %
  
E-value: 0.0
  
  
 NCBI BlastP on this gene

EHK23788

148. :  CAGA01000020 Claviceps purpurea 20.1     Total score: 1.0     Cumulative Blast bit score: 2029

uncharacterized protein
  
Accession: CCE30170
  
Location: 3315-3897
  
 NCBI BlastP on this gene

CCE30170

related to l-arginine:lysine amidinotransferase
  
Accession: CCE30171
  
Location: 9567-10631
  
 NCBI BlastP on this gene

CCE30171

related to multidrug resistance protein
  
Accession: CCE30172
  
Location: 11215-16579
  
 NCBI BlastP on this gene

CCE30172

related to benzoate 4-monooxygenase cytochrome P450
  
Accession: CCE30173
  
Location: 18893-20505
  
 NCBI BlastP on this gene

CCE30173

related to AM-toxin synthetase (AMT)
  
Accession: CCE30174
  
Location: 22433-28783
  
  
**BlastP hit with Mycgr3G90558\_Mycgr3T**
  
Percentage identity: 32 %
  
BlastP bit score: 1029
  
Sequence coverage: 48 %
  
E-value: 0.0
  
  
 NCBI BlastP on this gene

CCE30174

related to non-ribosomal peptide synthetase
  
Accession: CCE30175
  
Location: 28880-53423
  
  
**BlastP hit with Mycgr3G90558\_Mycgr3T**
  
Percentage identity: 31 %
  
BlastP bit score: 1001
  
Sequence coverage: 49 %
  
E-value: 0.0
  
  
 NCBI BlastP on this gene

CCE30175

uncharacterized protein
  
Accession: CCE30176
  
Location: 55022-55696
  
 NCBI BlastP on this gene

CCE30176

probable sterol glucosyltransferase
  
Accession: CCE30177
  
Location: 56722-59865
  
 NCBI BlastP on this gene

CCE30177

probable carboxypeptidase
  
Accession: CCE30178
  
Location: 60970-62976
  
 NCBI BlastP on this gene

CCE30178

related to PMR1-Ca++-transporting P-type ATPase located in Golgi
  
Accession: CCE30179
  
Location: 63245-66541
  
 NCBI BlastP on this gene

CCE30179

probable asparaginyl-tRNA-synthetase
  
Accession: CCE30180
  
Location: 70107-71902
  
 NCBI BlastP on this gene

CCE30180

galactose oxidase precursor [GAO]
  
Accession: CCE30181
  
Location: 73216-75243
  
 NCBI BlastP on this gene

CCE30181

149. :  ABDG02000023 Trichoderma atroviride IMI 206040     Total score: 1.0     Cumulative Blast bit score: 2018

non-ribosomal peptide synthetase
  
Accession: EHK45804
  
Location: 857001-923302
  
  
**BlastP hit with Mycgr3G90558\_Mycgr3T**
  
Percentage identity: 33 %
  
BlastP bit score: 2018
  
Sequence coverage: 89 %
  
E-value: 0.0
  
  
 NCBI BlastP on this gene

EHK45804

hypothetical protein
  
Accession: EHK45803
  
Location: 854648-856598
  
 NCBI BlastP on this gene

EHK45803

150. :  JH126400 Cordyceps militaris CM01 unplaced genomic scaffold CCM\_S00002     Total score: 1.0     Cumulative Blast bit score: 1948

hypothetical protein
  
Accession: EGX94979
  
Location: 4193829-4195376
  
 NCBI BlastP on this gene

EGX94979

pH signal transduction protein PalI, putative
  
Accession: EGX94980
  
Location: 4196859-4199773
  
 NCBI BlastP on this gene

EGX94980

ABC transporter, transmembrane region, type 1
  
Accession: EGX94981
  
Location: 4201881-4206855
  
 NCBI BlastP on this gene

EGX94981

benzoate 4-monooxygenase cytochrome P450
  
Accession: EGX94982
  
Location: 4208732-4210344
  
 NCBI BlastP on this gene

EGX94982

non-ribosomal peptide synthase, putative
  
Accession: EGX94983
  
Location: 4212343-4225710
  
  
**BlastP hit with Mycgr3G90558\_Mycgr3T**
  
Percentage identity: 32 %
  
BlastP bit score: 1948
  
Sequence coverage: 91 %
  
E-value: 0.0
  
  
 NCBI BlastP on this gene

EGX94983

Detecting sequence homology at the gene cluster level with MultiGeneBlast.
  
Marnix H. Medema, Rainer Breitling & Eriko Takano (2013)
  
*Molecular Biology and Evolution* , 30: 1218-1223.
