# Supplementary material for: In silico prediction and characterization of secondary metabolite biosynthetic gene clusters in the wheat pathogen Zymoseptoria tritici
Source: BMC Genomics. 2017 Aug 17;18:631. doi: 10.1186/s12864-017-3969-y (PMC5561558; doi:10.1186/s12864-017-3969-y)
Supplement: Supplementary file 1 — MultiGeneBLAST analysis of putative secondary metabolite clusters. All encoded amino acid sequences from genes residing in clusters predicted by AntiSMASH are given as FASTA file format. All output data from MultiGeneBLASTs are also provided. (ZIP 42911 kb) [file 12864_2017_3969_MOESM1_ESM.zip › Cluster MultiGene BLAST/out/Clusters_1_34/Cluster_11/displaypage4.xhtml]

xml version="1.0" encoding="UTF-8"?


Search Results
  
  
 Results pages: 1, 2, 3, 4, 5

**MultiGeneBlast hits**

Select gene cluster alignment
151. ACJE01000015\_0 Aspergillus niger ATCC 1015, whole genome shotgun sequenc...
152. CM001233\_3 Magnaporthe oryzae 70-15 chromosome 3, whole genome shotgun s...
153. JH921444\_0 Marssonina brunnea f. sp. 'multigermtubi' MB\_m1 unplaced geno...
154. GG698914\_2 Nectria haematococca mpVI 77-13-4 chromosome 7 genomic scaffo...
155. AACD01000135\_0 Aspergillus nidulans FGSC A4, whole genome shotgun sequen...
156. EQ962654\_1 Talaromyces stipitatus ATCC 10500 scf\_1105507295541 genomic s...
157. CH445338\_1 Phaeosphaeria nodorum SN15 scaffold\_14, whole genome shotgun ...
158. DS995900\_2 Penicillium marneffei ATCC 18224 scf\_1105668340758 genomic sc...
159. DF126466\_0 Aspergillus kawachii IFO 4308 DNA, contig: scaffold00020, who...
160. ACJE01000005\_0 Aspergillus niger ATCC 1015, whole genome shotgun sequenc...
161. KB644410\_3 Penicillium oxalicum 114-2 unplaced genomic scaffold scaffold...
162. AP007171\_0 Aspergillus oryzae RIB40 DNA, SC011.
163. DS995905\_1 Penicillium marneffei ATCC 18224 scf\_1105668340970 genomic sc...
164. EQ962658\_0 Talaromyces stipitatus ATCC 10500 scf\_1105507295511 genomic s...
165. DS989824\_1 Arthroderma gypseum CBS 118893 supercont1.3 genomic scaffold,...
166. DF126447\_1 Aspergillus kawachii IFO 4308 DNA, contig: scaffold00001, who...
167. KB020599\_0 Colletotrichum gloeosporioides Nara gc5 unplaced genomic scaf...
168. KB644408\_2 Penicillium oxalicum 114-2 unplaced genomic scaffold scaffold...
169. GG698902\_1 Nectria haematococca mpVI 77-13-4 chromosome 7 genomic scaffo...
170. AM920436\_1 Penicillium chrysogenum Wisconsin 54-1255 complete genome, co...
171. DS027698\_2 Neosartorya fischeri NRRL 181 1099437636266 genomic scaffold,...
172. CH408034\_1 Chaetomium globosum CBS 148.51 scaffold\_6 genomic scaffold, w...
173. CP003005\_1 Myceliophthora thermophila ATCC 42464 chromosome 4, complete ...
174. JH725160\_1 Beauveria bassiana ARSEF 2860 unplaced genomic scaffold BBA\_S...
175. EQ963476\_1 Aspergillus flavus NRRL3357 scf\_1106286417850 genomic scaffol...
176. HF679023\_4 Fusarium fujikuroi IMI 58289 draft genome, chromosome FFUJ\_ch...
177. CH476607\_0 Aspergillus terreus NIH2624 scaffold\_14 genomic scaffold, who...
178. CM001201\_0 Mycosphaerella graminicola IPO323 chromosome 6, whole genome ...
179. CH476595\_0 Aspergillus terreus NIH2624 scaffold\_2 genomic scaffold, whol...
180. EQ962656\_0 Talaromyces stipitatus ATCC 10500 scf\_1105507295549 genomic s...
181. CP003010\_0 Thielavia terrestris NRRL 8126 chromosome 2, complete sequence.
182. DS027045\_2 Aspergillus clavatus NRRL 1 1099423829791 genomic scaffold, w...
183. DS995703\_0 Microsporum canis CBS 113480 supercont1.3 genomic scaffold, w...
184. DS995903\_1 Penicillium marneffei ATCC 18224 scf\_1105668340984 genomic sc...
185. GG704912\_1 Coccidioides immitis RS genomic scaffold supercont3.2, whole ...
186. ACFW01000025\_1 Coccidioides posadasii C735 delta SOWgp, whole genome sho...
187. DS995708\_1 Microsporum canis CBS 113480 supercont1.8 genomic scaffold, w...
188. BABT02000122\_0 Mixia osmundae IAM 14324, whole genome shotgun sequencing...
189. GG700648\_1 Trichophyton rubrum CBS 118892 genomic scaffold supercont2.1,...
190. DS989822\_0 Arthroderma gypseum CBS 118893 supercont1.1 genomic scaffold,...
191. EQ963479\_2 Aspergillus flavus NRRL3357 scf\_1106286418500 genomic scaffol...
192. DS995905\_0 Penicillium marneffei ATCC 18224 scf\_1105668340970 genomic sc...
193. KB908866\_1 Setosphaeria turcica Et28A unplaced genomic scaffold SETTUsca...
194. ABSU01000002\_1 Arthroderma benhamiae CBS 112371, whole genome shotgun se...
195. CH476616\_1 Uncinocarpus reesii 1704 scaffold\_2 genomic scaffold, whole g...
196. CH408031\_3 Chaetomium globosum CBS 148.51 scaffold\_3 genomic scaffold, w...
197. KB456260\_1 Mycosphaerella populorum SO2202 unplaced genomic scaffold SEP...
198. JH687760\_0 Auricularia delicata TFB-10046 SS5 unplaced genomic scaffold ...
199. GG698487\_0 Trichophyton tonsurans CBS 112818 genomic scaffold supercont1...
200. HF679029\_2 Fusarium fujikuroi IMI 58289 draft genome, chromosome FFUJ\_ch...

Query: Architecture Search FASTA input

ACJE01000015 : Aspergillus niger ATCC 1015    Total score: 1.0     Cumulative Blast bit score: 1938

Hit cluster cross-links:

Mycgr3G36335 Mycgr3T
  
Location: 0-423

Mycgr3G36335\_Mycgr3T

Mycgr3G84494 Mycgr3T
  
Location: 523-2047

Mycgr3G84494\_Mycgr3T

Mycgr3G90558 Mycgr3T
  
Location: 2147-15296

Mycgr3G90558\_Mycgr3T

Mycgr3G68036 Mycgr3T
  
Location: 15396-16395

Mycgr3G68036\_Mycgr3T

Mycgr3G90561 Mycgr3T
  
Location: 16495-17134

Mycgr3G90561\_Mycgr3T

Mycgr3G35862 Mycgr3T
  
Location: 17234-18662

Mycgr3G35862\_Mycgr3T

Mycgr3G68030 Mycgr3T
  
Location: 18762-19722

Mycgr3G68030\_Mycgr3T

Mycgr3G36449 Mycgr3T
  
Location: 19822-21886

Mycgr3G36449\_Mycgr3T

Mycgr3G35528 Mycgr3T
  
Location: 21986-22844

Mycgr3G35528\_Mycgr3T

Mycgr3G35932 Mycgr3T
  
Location: 22944-24390

Mycgr3G35932\_Mycgr3T

Mycgr3G23761 Mycgr3T
  
Location: 24490-25825

Mycgr3G23761\_Mycgr3T

Mycgr3G35535 Mycgr3T
  
Location: 25925-26429

Mycgr3G35535\_Mycgr3T

Mycgr3G9942 Mycgr3T9
  
Location: 26529-30375

Mycgr3G9942\_Mycgr3T9

hypothetical protein
  
Accession: EHA20899
  
Location: 26109-27074
  
 NCBI BlastP on this gene

EHA20899

catalytic protein
  
Accession: EHA20898
  
Location: 23886-24785
  
 NCBI BlastP on this gene

EHA20898

hypothetical protein
  
Accession: EHA20897
  
Location: 21614-23486
  
 NCBI BlastP on this gene

EHA20897

hypothetical protein
  
Accession: EHA20896
  
Location: 3496-20418
  
  
**BlastP hit with Mycgr3G90558\_Mycgr3T**
  
Percentage identity: 33 %
  
BlastP bit score: 1938
  
Sequence coverage: 91 %
  
E-value: 0.0
  
  
 NCBI BlastP on this gene

EHA20896

hypothetical protein
  
Accession: EHA20895
  
Location: 1462-2048
  
 NCBI BlastP on this gene

EHA20895

Query: Architecture Search FASTA input

CM001233 : Magnaporthe oryzae 70-15 chromosome 3    Total score: 1.0     Cumulative Blast bit score: 1910

Hit cluster cross-links:

Mycgr3G36335 Mycgr3T
  
Location: 0-423

Mycgr3G36335\_Mycgr3T

Mycgr3G84494 Mycgr3T
  
Location: 523-2047

Mycgr3G84494\_Mycgr3T

Mycgr3G90558 Mycgr3T
  
Location: 2147-15296

Mycgr3G90558\_Mycgr3T

Mycgr3G68036 Mycgr3T
  
Location: 15396-16395

Mycgr3G68036\_Mycgr3T

Mycgr3G90561 Mycgr3T
  
Location: 16495-17134

Mycgr3G90561\_Mycgr3T

Mycgr3G35862 Mycgr3T
  
Location: 17234-18662

Mycgr3G35862\_Mycgr3T

Mycgr3G68030 Mycgr3T
  
Location: 18762-19722

Mycgr3G68030\_Mycgr3T

Mycgr3G36449 Mycgr3T
  
Location: 19822-21886

Mycgr3G36449\_Mycgr3T

Mycgr3G35528 Mycgr3T
  
Location: 21986-22844

Mycgr3G35528\_Mycgr3T

Mycgr3G35932 Mycgr3T
  
Location: 22944-24390

Mycgr3G35932\_Mycgr3T

Mycgr3G23761 Mycgr3T
  
Location: 24490-25825

Mycgr3G23761\_Mycgr3T

Mycgr3G35535 Mycgr3T
  
Location: 25925-26429

Mycgr3G35535\_Mycgr3T

Mycgr3G9942 Mycgr3T9
  
Location: 26529-30375

Mycgr3G9942\_Mycgr3T9

hypothetical protein
  
Accession: EHA53290
  
Location: 6064103-6064824
  
 NCBI BlastP on this gene

EHA53290

hypothetical protein
  
Accession: EHA53289
  
Location: 6062762-6063081
  
 NCBI BlastP on this gene

EHA53289

hypothetical protein
  
Accession: EHA53288
  
Location: 6058549-6060887
  
 NCBI BlastP on this gene

EHA53288

cyclic peptide synthetase
  
Accession: EHA53287
  
Location: 6042642-6057224
  
  
**BlastP hit with Mycgr3G90558\_Mycgr3T**
  
Percentage identity: 30 %
  
BlastP bit score: 1910
  
Sequence coverage: 104 %
  
E-value: 0.0
  
  
 NCBI BlastP on this gene

EHA53287

hypothetical protein
  
Accession: EHA53286
  
Location: 6040865-6042065
  
 NCBI BlastP on this gene

EHA53286

pisatin demethylase
  
Accession: EHA53285
  
Location: 6038316-6040200
  
 NCBI BlastP on this gene

EHA53285

hypothetical protein
  
Accession: EHA53284
  
Location: 6035067-6037648
  
 NCBI BlastP on this gene

EHA53284

hypothetical protein
  
Accession: EHA53283
  
Location: 6033397-6034083
  
 NCBI BlastP on this gene

EHA53283

hypothetical protein
  
Accession: EHA53282
  
Location: 6031230-6033015
  
 NCBI BlastP on this gene

EHA53282

Query: Architecture Search FASTA input

JH921444 : Marssonina brunnea f. sp. 'multigermtubi' MB\_m1 unplaced genomic scaffold M6\_S00017    Total score: 1.0     Cumulative Blast bit score: 1900

Hit cluster cross-links:

Mycgr3G36335 Mycgr3T
  
Location: 0-423

Mycgr3G36335\_Mycgr3T

Mycgr3G84494 Mycgr3T
  
Location: 523-2047

Mycgr3G84494\_Mycgr3T

Mycgr3G90558 Mycgr3T
  
Location: 2147-15296

Mycgr3G90558\_Mycgr3T

Mycgr3G68036 Mycgr3T
  
Location: 15396-16395

Mycgr3G68036\_Mycgr3T

Mycgr3G90561 Mycgr3T
  
Location: 16495-17134

Mycgr3G90561\_Mycgr3T

Mycgr3G35862 Mycgr3T
  
Location: 17234-18662

Mycgr3G35862\_Mycgr3T

Mycgr3G68030 Mycgr3T
  
Location: 18762-19722

Mycgr3G68030\_Mycgr3T

Mycgr3G36449 Mycgr3T
  
Location: 19822-21886

Mycgr3G36449\_Mycgr3T

Mycgr3G35528 Mycgr3T
  
Location: 21986-22844

Mycgr3G35528\_Mycgr3T

Mycgr3G35932 Mycgr3T
  
Location: 22944-24390

Mycgr3G35932\_Mycgr3T

Mycgr3G23761 Mycgr3T
  
Location: 24490-25825

Mycgr3G23761\_Mycgr3T

Mycgr3G35535 Mycgr3T
  
Location: 25925-26429

Mycgr3G35535\_Mycgr3T

Mycgr3G9942 Mycgr3T9
  
Location: 26529-30375

Mycgr3G9942\_Mycgr3T9

nonribosomal peptide synthase Pes1
  
Accession: EKD14741
  
Location: 170637-172109
  
 NCBI BlastP on this gene

EKD14741

nonribosomal peptide synthetase
  
Accession: EKD14740
  
Location: 150851-165484
  
  
**BlastP hit with Mycgr3G90558\_Mycgr3T**
  
Percentage identity: 32 %
  
BlastP bit score: 1900
  
Sequence coverage: 95 %
  
E-value: 0.0
  
  
 NCBI BlastP on this gene

EKD14740

NADH pyrophosphatase
  
Accession: EKD14739
  
Location: 148577-149953
  
 NCBI BlastP on this gene

EKD14739

DNA repair helicase rad25
  
Accession: EKD14738
  
Location: 144410-146985
  
 NCBI BlastP on this gene

EKD14738

zinc finger protein
  
Accession: EKD14737
  
Location: 143513-144109
  
 NCBI BlastP on this gene

EKD14737

alpha-type of subunit of 20S proteasome
  
Accession: EKD14736
  
Location: 142248-143245
  
 NCBI BlastP on this gene

EKD14736

anaphase control protein cut9
  
Accession: EKD14735
  
Location: 140012-142156
  
 NCBI BlastP on this gene

EKD14735

Query: Architecture Search FASTA input

GG698914 : Nectria haematococca mpVI 77-13-4 chromosome 7 genomic scaffold NECHAsca\_22\_chr7\_11\_0    Total score: 1.0     Cumulative Blast bit score: 1873

Hit cluster cross-links:

Mycgr3G36335 Mycgr3T
  
Location: 0-423

Mycgr3G36335\_Mycgr3T

Mycgr3G84494 Mycgr3T
  
Location: 523-2047

Mycgr3G84494\_Mycgr3T

Mycgr3G90558 Mycgr3T
  
Location: 2147-15296

Mycgr3G90558\_Mycgr3T

Mycgr3G68036 Mycgr3T
  
Location: 15396-16395

Mycgr3G68036\_Mycgr3T

Mycgr3G90561 Mycgr3T
  
Location: 16495-17134

Mycgr3G90561\_Mycgr3T

Mycgr3G35862 Mycgr3T
  
Location: 17234-18662

Mycgr3G35862\_Mycgr3T

Mycgr3G68030 Mycgr3T
  
Location: 18762-19722

Mycgr3G68030\_Mycgr3T

Mycgr3G36449 Mycgr3T
  
Location: 19822-21886

Mycgr3G36449\_Mycgr3T

Mycgr3G35528 Mycgr3T
  
Location: 21986-22844

Mycgr3G35528\_Mycgr3T

Mycgr3G35932 Mycgr3T
  
Location: 22944-24390

Mycgr3G35932\_Mycgr3T

Mycgr3G23761 Mycgr3T
  
Location: 24490-25825

Mycgr3G23761\_Mycgr3T

Mycgr3G35535 Mycgr3T
  
Location: 25925-26429

Mycgr3G35535\_Mycgr3T

Mycgr3G9942 Mycgr3T9
  
Location: 26529-30375

Mycgr3G9942\_Mycgr3T9

hypothetical protein
  
Accession: EEU38841
  
Location: 483175-501313
  
  
**BlastP hit with Mycgr3G90558\_Mycgr3T**
  
Percentage identity: 31 %
  
BlastP bit score: 1873
  
Sequence coverage: 102 %
  
E-value: 0.0
  
  
 NCBI BlastP on this gene

EEU38841

hypothetical protein
  
Accession: EEU38840
  
Location: 480313-481098
  
 NCBI BlastP on this gene

EEU38840

hypothetical protein
  
Accession: EEU38839
  
Location: 477949-479357
  
 NCBI BlastP on this gene

EEU38839

predicted protein
  
Accession: EEU38723
  
Location: 476075-477795
  
 NCBI BlastP on this gene

EEU38723

Query: Architecture Search FASTA input

AACD01000135 : Aspergillus nidulans FGSC A4    Total score: 1.0     Cumulative Blast bit score: 1831

Hit cluster cross-links:

Mycgr3G36335 Mycgr3T
  
Location: 0-423

Mycgr3G36335\_Mycgr3T

Mycgr3G84494 Mycgr3T
  
Location: 523-2047

Mycgr3G84494\_Mycgr3T

Mycgr3G90558 Mycgr3T
  
Location: 2147-15296

Mycgr3G90558\_Mycgr3T

Mycgr3G68036 Mycgr3T
  
Location: 15396-16395

Mycgr3G68036\_Mycgr3T

Mycgr3G90561 Mycgr3T
  
Location: 16495-17134

Mycgr3G90561\_Mycgr3T

Mycgr3G35862 Mycgr3T
  
Location: 17234-18662

Mycgr3G35862\_Mycgr3T

Mycgr3G68030 Mycgr3T
  
Location: 18762-19722

Mycgr3G68030\_Mycgr3T

Mycgr3G36449 Mycgr3T
  
Location: 19822-21886

Mycgr3G36449\_Mycgr3T

Mycgr3G35528 Mycgr3T
  
Location: 21986-22844

Mycgr3G35528\_Mycgr3T

Mycgr3G35932 Mycgr3T
  
Location: 22944-24390

Mycgr3G35932\_Mycgr3T

Mycgr3G23761 Mycgr3T
  
Location: 24490-25825

Mycgr3G23761\_Mycgr3T

Mycgr3G35535 Mycgr3T
  
Location: 25925-26429

Mycgr3G35535\_Mycgr3T

Mycgr3G9942 Mycgr3T9
  
Location: 26529-30375

Mycgr3G9942\_Mycgr3T9

hypothetical protein
  
Accession: EAA59536
  
Location: 31536-35783
  
 NCBI BlastP on this gene

EAA59536

hypothetical protein
  
Accession: EAA59537
  
Location: 36304-37005
  
 NCBI BlastP on this gene

EAA59537

hypothetical protein
  
Accession: EAA59538
  
Location: 38104-59914
  
  
**BlastP hit with Mycgr3G90558\_Mycgr3T**
  
Percentage identity: 31 %
  
BlastP bit score: 1831
  
Sequence coverage: 88 %
  
E-value: 0.0
  
  
 NCBI BlastP on this gene

EAA59538

hypothetical protein
  
Accession: EAA59539
  
Location: 60857-61446
  
 NCBI BlastP on this gene

EAA59539

hypothetical protein
  
Accession: EAA59540
  
Location: 64256-64882
  
 NCBI BlastP on this gene

EAA59540

hypothetical protein
  
Accession: EAA59541
  
Location: 65871-66613
  
 NCBI BlastP on this gene

EAA59541

Query: Architecture Search FASTA input

EQ962654 : Talaromyces stipitatus ATCC 10500 scf\_1105507295541 genomic scaffold    Total score: 1.0     Cumulative Blast bit score: 1735

Hit cluster cross-links:

Mycgr3G36335 Mycgr3T
  
Location: 0-423

Mycgr3G36335\_Mycgr3T

Mycgr3G84494 Mycgr3T
  
Location: 523-2047

Mycgr3G84494\_Mycgr3T

Mycgr3G90558 Mycgr3T
  
Location: 2147-15296

Mycgr3G90558\_Mycgr3T

Mycgr3G68036 Mycgr3T
  
Location: 15396-16395

Mycgr3G68036\_Mycgr3T

Mycgr3G90561 Mycgr3T
  
Location: 16495-17134

Mycgr3G90561\_Mycgr3T

Mycgr3G35862 Mycgr3T
  
Location: 17234-18662

Mycgr3G35862\_Mycgr3T

Mycgr3G68030 Mycgr3T
  
Location: 18762-19722

Mycgr3G68030\_Mycgr3T

Mycgr3G36449 Mycgr3T
  
Location: 19822-21886

Mycgr3G36449\_Mycgr3T

Mycgr3G35528 Mycgr3T
  
Location: 21986-22844

Mycgr3G35528\_Mycgr3T

Mycgr3G35932 Mycgr3T
  
Location: 22944-24390

Mycgr3G35932\_Mycgr3T

Mycgr3G23761 Mycgr3T
  
Location: 24490-25825

Mycgr3G23761\_Mycgr3T

Mycgr3G35535 Mycgr3T
  
Location: 25925-26429

Mycgr3G35535\_Mycgr3T

Mycgr3G9942 Mycgr3T9
  
Location: 26529-30375

Mycgr3G9942\_Mycgr3T9

conserved hypothetical protein
  
Accession: EED19136
  
Location: 349803-350748
  
 NCBI BlastP on this gene

EED19136

hypothetical protein
  
Accession: EED19137
  
Location: 351399-352530
  
 NCBI BlastP on this gene

EED19137

hypothetical protein
  
Accession: EED19138
  
Location: 351399-352313
  
 NCBI BlastP on this gene

EED19138

dipeptidyl peptidase IV, putative
  
Accession: EED19140
  
Location: 353373-355765
  
 NCBI BlastP on this gene

EED19140

conserved hypothetical protein
  
Accession: EED19141
  
Location: 356632-358299
  
 NCBI BlastP on this gene

EED19141

nonribosomal peptide synthase, putative
  
Accession: EED19142
  
Location: 359434-376386
  
  
**BlastP hit with Mycgr3G90558\_Mycgr3T**
  
Percentage identity: 30 %
  
BlastP bit score: 1735
  
Sequence coverage: 93 %
  
E-value: 0.0
  
  
 NCBI BlastP on this gene

EED19142

phenylalanine ammonia-lyase
  
Accession: EED19143
  
Location: 377097-379447
  
 NCBI BlastP on this gene

EED19143

conserved hypothetical protein
  
Accession: EED19144
  
Location: 381515-382225
  
 NCBI BlastP on this gene

EED19144

conserved hypothetical protein
  
Accession: EED19145
  
Location: 383084-384310
  
 NCBI BlastP on this gene

EED19145

Query: Architecture Search FASTA input

CH445338 : Phaeosphaeria nodorum SN15 scaffold\_14    Total score: 1.0     Cumulative Blast bit score: 1696

Hit cluster cross-links:

Mycgr3G36335 Mycgr3T
  
Location: 0-423

Mycgr3G36335\_Mycgr3T

Mycgr3G84494 Mycgr3T
  
Location: 523-2047

Mycgr3G84494\_Mycgr3T

Mycgr3G90558 Mycgr3T
  
Location: 2147-15296

Mycgr3G90558\_Mycgr3T

Mycgr3G68036 Mycgr3T
  
Location: 15396-16395

Mycgr3G68036\_Mycgr3T

Mycgr3G90561 Mycgr3T
  
Location: 16495-17134

Mycgr3G90561\_Mycgr3T

Mycgr3G35862 Mycgr3T
  
Location: 17234-18662

Mycgr3G35862\_Mycgr3T

Mycgr3G68030 Mycgr3T
  
Location: 18762-19722

Mycgr3G68030\_Mycgr3T

Mycgr3G36449 Mycgr3T
  
Location: 19822-21886

Mycgr3G36449\_Mycgr3T

Mycgr3G35528 Mycgr3T
  
Location: 21986-22844

Mycgr3G35528\_Mycgr3T

Mycgr3G35932 Mycgr3T
  
Location: 22944-24390

Mycgr3G35932\_Mycgr3T

Mycgr3G23761 Mycgr3T
  
Location: 24490-25825

Mycgr3G23761\_Mycgr3T

Mycgr3G35535 Mycgr3T
  
Location: 25925-26429

Mycgr3G35535\_Mycgr3T

Mycgr3G9942 Mycgr3T9
  
Location: 26529-30375

Mycgr3G9942\_Mycgr3T9

hypothetical protein
  
Accession: EAT83273
  
Location: 205186-220770
  
  
**BlastP hit with Mycgr3G90558\_Mycgr3T**
  
Percentage identity: 32 %
  
BlastP bit score: 1696
  
Sequence coverage: 80 %
  
E-value: 0.0
  
  
 NCBI BlastP on this gene

EAT83273

hypothetical protein
  
Accession: EAT83272
  
Location: 203852-204885
  
 NCBI BlastP on this gene

EAT83272

hypothetical protein
  
Accession: EAT83271
  
Location: 203680-203868
  
 NCBI BlastP on this gene

EAT83271

hypothetical protein
  
Accession: EAT83270
  
Location: 203076-203499
  
 NCBI BlastP on this gene

EAT83270

hypothetical protein
  
Accession: EAT83269
  
Location: 199620-201220
  
 NCBI BlastP on this gene

EAT83269

hypothetical protein
  
Accession: EAT83267
  
Location: 198439-199124
  
 NCBI BlastP on this gene

EAT83267

hypothetical protein
  
Accession: EAT83266
  
Location: 196806-198108
  
 NCBI BlastP on this gene

EAT83266

hypothetical protein
  
Accession: EAT83265
  
Location: 194214-196665
  
 NCBI BlastP on this gene

EAT83265

Query: Architecture Search FASTA input

DS995900 : Penicillium marneffei ATCC 18224 scf\_1105668340758 genomic scaffold    Total score: 1.0     Cumulative Blast bit score: 1665

Hit cluster cross-links:

Mycgr3G36335 Mycgr3T
  
Location: 0-423

Mycgr3G36335\_Mycgr3T

Mycgr3G84494 Mycgr3T
  
Location: 523-2047

Mycgr3G84494\_Mycgr3T

Mycgr3G90558 Mycgr3T
  
Location: 2147-15296

Mycgr3G90558\_Mycgr3T

Mycgr3G68036 Mycgr3T
  
Location: 15396-16395

Mycgr3G68036\_Mycgr3T

Mycgr3G90561 Mycgr3T
  
Location: 16495-17134

Mycgr3G90561\_Mycgr3T

Mycgr3G35862 Mycgr3T
  
Location: 17234-18662

Mycgr3G35862\_Mycgr3T

Mycgr3G68030 Mycgr3T
  
Location: 18762-19722

Mycgr3G68030\_Mycgr3T

Mycgr3G36449 Mycgr3T
  
Location: 19822-21886

Mycgr3G36449\_Mycgr3T

Mycgr3G35528 Mycgr3T
  
Location: 21986-22844

Mycgr3G35528\_Mycgr3T

Mycgr3G35932 Mycgr3T
  
Location: 22944-24390

Mycgr3G35932\_Mycgr3T

Mycgr3G23761 Mycgr3T
  
Location: 24490-25825

Mycgr3G23761\_Mycgr3T

Mycgr3G35535 Mycgr3T
  
Location: 25925-26429

Mycgr3G35535\_Mycgr3T

Mycgr3G9942 Mycgr3T9
  
Location: 26529-30375

Mycgr3G9942\_Mycgr3T9

hypothetical protein
  
Accession: EEA26482
  
Location: 3820130-3821233
  
 NCBI BlastP on this gene

EEA26482

conserved hypothetical protein
  
Accession: EEA26481
  
Location: 3818623-3819691
  
 NCBI BlastP on this gene

EEA26481

hypothetical protein
  
Accession: EEA26480
  
Location: 3816693-3817493
  
 NCBI BlastP on this gene

EEA26480

conserved hypothetical protein
  
Accession: EEA26479
  
Location: 3814528-3815743
  
 NCBI BlastP on this gene

EEA26479

nonribosomal peptide synthase, putative
  
Accession: EEA26478
  
Location: 3796470-3813422
  
  
**BlastP hit with Mycgr3G90558\_Mycgr3T**
  
Percentage identity: 30 %
  
BlastP bit score: 1665
  
Sequence coverage: 89 %
  
E-value: 0.0
  
  
 NCBI BlastP on this gene

EEA26478

hypothetical protein
  
Accession: EEA26477
  
Location: 3794009-3794591
  
 NCBI BlastP on this gene

EEA26477

ATP-dependent bile acid permease, putative
  
Accession: EEA26476
  
Location: 3787656-3792480
  
 NCBI BlastP on this gene

EEA26476

aminotransferase, class III
  
Accession: EEA26475
  
Location: 3786005-3787550
  
 NCBI BlastP on this gene

EEA26475

Query: Architecture Search FASTA input

DF126466 : Aspergillus kawachii IFO 4308 DNA, contig: scaffold00020    Total score: 1.0     Cumulative Blast bit score: 1640

Hit cluster cross-links:

Mycgr3G36335 Mycgr3T
  
Location: 0-423

Mycgr3G36335\_Mycgr3T

Mycgr3G84494 Mycgr3T
  
Location: 523-2047

Mycgr3G84494\_Mycgr3T

Mycgr3G90558 Mycgr3T
  
Location: 2147-15296

Mycgr3G90558\_Mycgr3T

Mycgr3G68036 Mycgr3T
  
Location: 15396-16395

Mycgr3G68036\_Mycgr3T

Mycgr3G90561 Mycgr3T
  
Location: 16495-17134

Mycgr3G90561\_Mycgr3T

Mycgr3G35862 Mycgr3T
  
Location: 17234-18662

Mycgr3G35862\_Mycgr3T

Mycgr3G68030 Mycgr3T
  
Location: 18762-19722

Mycgr3G68030\_Mycgr3T

Mycgr3G36449 Mycgr3T
  
Location: 19822-21886

Mycgr3G36449\_Mycgr3T

Mycgr3G35528 Mycgr3T
  
Location: 21986-22844

Mycgr3G35528\_Mycgr3T

Mycgr3G35932 Mycgr3T
  
Location: 22944-24390

Mycgr3G35932\_Mycgr3T

Mycgr3G23761 Mycgr3T
  
Location: 24490-25825

Mycgr3G23761\_Mycgr3T

Mycgr3G35535 Mycgr3T
  
Location: 25925-26429

Mycgr3G35535\_Mycgr3T

Mycgr3G9942 Mycgr3T9
  
Location: 26529-30375

Mycgr3G9942\_Mycgr3T9

canalicular multispecific organic anion transporter 1
  
Accession: GAA89112
  
Location: 259668-264641
  
 NCBI BlastP on this gene

GAA89112

subgroup IIIi aminotransferase
  
Accession: GAA89111
  
Location: 257542-258796
  
 NCBI BlastP on this gene

GAA89111

hypothetical protein
  
Accession: GAA89110
  
Location: 256091-256963
  
 NCBI BlastP on this gene

GAA89110

hypothetical protein
  
Accession: GAA89109
  
Location: 253720-254606
  
 NCBI BlastP on this gene

GAA89109

RTA1 domain protein
  
Accession: GAA89108
  
Location: 250888-251906
  
 NCBI BlastP on this gene

GAA89108

peptide synthetase
  
Accession: GAA89107
  
Location: 233716-250245
  
  
**BlastP hit with Mycgr3G90558\_Mycgr3T**
  
Percentage identity: 31 %
  
BlastP bit score: 1640
  
Sequence coverage: 87 %
  
E-value: 0.0
  
  
 NCBI BlastP on this gene

GAA89107

C6 zinc finger domain protein
  
Accession: GAA89106
  
Location: 230770-232394
  
 NCBI BlastP on this gene

GAA89106

integral membrane protein
  
Accession: GAA89105
  
Location: 229360-230259
  
 NCBI BlastP on this gene

GAA89105

lysophospholipase phospholipase B (Plb1)
  
Accession: GAA89104
  
Location: 226264-228301
  
 NCBI BlastP on this gene

GAA89104

hypothetical protein
  
Accession: GAA89103
  
Location: 223097-224906
  
 NCBI BlastP on this gene

GAA89103

Query: Architecture Search FASTA input

ACJE01000005 : Aspergillus niger ATCC 1015    Total score: 1.0     Cumulative Blast bit score: 1631

Hit cluster cross-links:

Mycgr3G36335 Mycgr3T
  
Location: 0-423

Mycgr3G36335\_Mycgr3T

Mycgr3G84494 Mycgr3T
  
Location: 523-2047

Mycgr3G84494\_Mycgr3T

Mycgr3G90558 Mycgr3T
  
Location: 2147-15296

Mycgr3G90558\_Mycgr3T

Mycgr3G68036 Mycgr3T
  
Location: 15396-16395

Mycgr3G68036\_Mycgr3T

Mycgr3G90561 Mycgr3T
  
Location: 16495-17134

Mycgr3G90561\_Mycgr3T

Mycgr3G35862 Mycgr3T
  
Location: 17234-18662

Mycgr3G35862\_Mycgr3T

Mycgr3G68030 Mycgr3T
  
Location: 18762-19722

Mycgr3G68030\_Mycgr3T

Mycgr3G36449 Mycgr3T
  
Location: 19822-21886

Mycgr3G36449\_Mycgr3T

Mycgr3G35528 Mycgr3T
  
Location: 21986-22844

Mycgr3G35528\_Mycgr3T

Mycgr3G35932 Mycgr3T
  
Location: 22944-24390

Mycgr3G35932\_Mycgr3T

Mycgr3G23761 Mycgr3T
  
Location: 24490-25825

Mycgr3G23761\_Mycgr3T

Mycgr3G35535 Mycgr3T
  
Location: 25925-26429

Mycgr3G35535\_Mycgr3T

Mycgr3G9942 Mycgr3T9
  
Location: 26529-30375

Mycgr3G9942\_Mycgr3T9

hypothetical protein
  
Accession: EHA25710
  
Location: 280936-282215
  
 NCBI BlastP on this gene

EHA25710

hypothetical protein
  
Accession: EHA25709
  
Location: 277076-277810
  
 NCBI BlastP on this gene

EHA25709

hypothetical protein
  
Accession: EHA25708
  
Location: 274287-275303
  
 NCBI BlastP on this gene

EHA25708

hypothetical protein
  
Accession: EHA25707
  
Location: 257395-273191
  
  
**BlastP hit with Mycgr3G90558\_Mycgr3T**
  
Percentage identity: 31 %
  
BlastP bit score: 1631
  
Sequence coverage: 86 %
  
E-value: 0.0
  
  
 NCBI BlastP on this gene

EHA25707

lysophospholipase
  
Accession: EHA25706
  
Location: 250212-252057
  
 NCBI BlastP on this gene

EHA25706

hypothetical protein
  
Accession: EHA25705
  
Location: 248119-249606
  
 NCBI BlastP on this gene

EHA25705

hypothetical protein
  
Accession: EHA25704
  
Location: 246807-247874
  
 NCBI BlastP on this gene

EHA25704

Query: Architecture Search FASTA input

KB644410 : Penicillium oxalicum 114-2 unplaced genomic scaffold scaffold\_3    Total score: 1.0     Cumulative Blast bit score: 1575

Hit cluster cross-links:

Mycgr3G36335 Mycgr3T
  
Location: 0-423

Mycgr3G36335\_Mycgr3T

Mycgr3G84494 Mycgr3T
  
Location: 523-2047

Mycgr3G84494\_Mycgr3T

Mycgr3G90558 Mycgr3T
  
Location: 2147-15296

Mycgr3G90558\_Mycgr3T

Mycgr3G68036 Mycgr3T
  
Location: 15396-16395

Mycgr3G68036\_Mycgr3T

Mycgr3G90561 Mycgr3T
  
Location: 16495-17134

Mycgr3G90561\_Mycgr3T

Mycgr3G35862 Mycgr3T
  
Location: 17234-18662

Mycgr3G35862\_Mycgr3T

Mycgr3G68030 Mycgr3T
  
Location: 18762-19722

Mycgr3G68030\_Mycgr3T

Mycgr3G36449 Mycgr3T
  
Location: 19822-21886

Mycgr3G36449\_Mycgr3T

Mycgr3G35528 Mycgr3T
  
Location: 21986-22844

Mycgr3G35528\_Mycgr3T

Mycgr3G35932 Mycgr3T
  
Location: 22944-24390

Mycgr3G35932\_Mycgr3T

Mycgr3G23761 Mycgr3T
  
Location: 24490-25825

Mycgr3G23761\_Mycgr3T

Mycgr3G35535 Mycgr3T
  
Location: 25925-26429

Mycgr3G35535\_Mycgr3T

Mycgr3G9942 Mycgr3T9
  
Location: 26529-30375

Mycgr3G9942\_Mycgr3T9

hypothetical protein
  
Accession: EPS27660
  
Location: 1561005-1562473
  
 NCBI BlastP on this gene

EPS27660

hypothetical protein
  
Accession: EPS27661
  
Location: 1562637-1563877
  
 NCBI BlastP on this gene

EPS27661

hypothetical protein
  
Accession: EPS27662
  
Location: 1564448-1565641
  
 NCBI BlastP on this gene

EPS27662

hypothetical protein
  
Accession: EPS27663
  
Location: 1566197-1567863
  
 NCBI BlastP on this gene

EPS27663

hypothetical protein
  
Accession: EPS27664
  
Location: 1568091-1568927
  
 NCBI BlastP on this gene

EPS27664

hypothetical protein
  
Accession: EPS27665
  
Location: 1569400-1571493
  
 NCBI BlastP on this gene

EPS27665

hypothetical protein
  
Accession: EPS27666
  
Location: 1573119-1586511
  
  
**BlastP hit with Mycgr3G90558\_Mycgr3T**
  
Percentage identity: 29 %
  
BlastP bit score: 1575
  
Sequence coverage: 95 %
  
E-value: 0.0
  
  
 NCBI BlastP on this gene

EPS27666

Query: Architecture Search FASTA input

AP007171 : Aspergillus oryzae RIB40 DNA, SC011.    Total score: 1.0     Cumulative Blast bit score: 1573

Hit cluster cross-links:

Mycgr3G36335 Mycgr3T
  
Location: 0-423

Mycgr3G36335\_Mycgr3T

Mycgr3G84494 Mycgr3T
  
Location: 523-2047

Mycgr3G84494\_Mycgr3T

Mycgr3G90558 Mycgr3T
  
Location: 2147-15296

Mycgr3G90558\_Mycgr3T

Mycgr3G68036 Mycgr3T
  
Location: 15396-16395

Mycgr3G68036\_Mycgr3T

Mycgr3G90561 Mycgr3T
  
Location: 16495-17134

Mycgr3G90561\_Mycgr3T

Mycgr3G35862 Mycgr3T
  
Location: 17234-18662

Mycgr3G35862\_Mycgr3T

Mycgr3G68030 Mycgr3T
  
Location: 18762-19722

Mycgr3G68030\_Mycgr3T

Mycgr3G36449 Mycgr3T
  
Location: 19822-21886

Mycgr3G36449\_Mycgr3T

Mycgr3G35528 Mycgr3T
  
Location: 21986-22844

Mycgr3G35528\_Mycgr3T

Mycgr3G35932 Mycgr3T
  
Location: 22944-24390

Mycgr3G35932\_Mycgr3T

Mycgr3G23761 Mycgr3T
  
Location: 24490-25825

Mycgr3G23761\_Mycgr3T

Mycgr3G35535 Mycgr3T
  
Location: 25925-26429

Mycgr3G35535\_Mycgr3T

Mycgr3G9942 Mycgr3T9
  
Location: 26529-30375

Mycgr3G9942\_Mycgr3T9

not annotated
  
Accession: BAE64607
  
Location: 149759-151615
  
 NCBI BlastP on this gene

AO090011000045

not annotated
  
Accession: BAE64606
  
Location: 147752-149100
  
 NCBI BlastP on this gene

AO090011000044

not annotated
  
Accession: BAE64605
  
Location: 121832-142723
  
  
**BlastP hit with Mycgr3G90558\_Mycgr3T**
  
Percentage identity: 31 %
  
BlastP bit score: 1573
  
Sequence coverage: 75 %
  
E-value: 0.0
  
  
 NCBI BlastP on this gene

AO090011000043

not annotated
  
Accession: BAE64604
  
Location: 118053-118754
  
 NCBI BlastP on this gene

AO090011000042

not annotated
  
Accession: BAE64603
  
Location: 117022-117666
  
 NCBI BlastP on this gene

AO090011000041

not annotated
  
Accession: BAE64602
  
Location: 109146-115780
  
 NCBI BlastP on this gene

AO090011000040

Query: Architecture Search FASTA input

DS995905 : Penicillium marneffei ATCC 18224 scf\_1105668340970 genomic scaffold    Total score: 1.0     Cumulative Blast bit score: 1560

Hit cluster cross-links:

Mycgr3G36335 Mycgr3T
  
Location: 0-423

Mycgr3G36335\_Mycgr3T

Mycgr3G84494 Mycgr3T
  
Location: 523-2047

Mycgr3G84494\_Mycgr3T

Mycgr3G90558 Mycgr3T
  
Location: 2147-15296

Mycgr3G90558\_Mycgr3T

Mycgr3G68036 Mycgr3T
  
Location: 15396-16395

Mycgr3G68036\_Mycgr3T

Mycgr3G90561 Mycgr3T
  
Location: 16495-17134

Mycgr3G90561\_Mycgr3T

Mycgr3G35862 Mycgr3T
  
Location: 17234-18662

Mycgr3G35862\_Mycgr3T

Mycgr3G68030 Mycgr3T
  
Location: 18762-19722

Mycgr3G68030\_Mycgr3T

Mycgr3G36449 Mycgr3T
  
Location: 19822-21886

Mycgr3G36449\_Mycgr3T

Mycgr3G35528 Mycgr3T
  
Location: 21986-22844

Mycgr3G35528\_Mycgr3T

Mycgr3G35932 Mycgr3T
  
Location: 22944-24390

Mycgr3G35932\_Mycgr3T

Mycgr3G23761 Mycgr3T
  
Location: 24490-25825

Mycgr3G23761\_Mycgr3T

Mycgr3G35535 Mycgr3T
  
Location: 25925-26429

Mycgr3G35535\_Mycgr3T

Mycgr3G9942 Mycgr3T9
  
Location: 26529-30375

Mycgr3G9942\_Mycgr3T9

polyketide synthase, putative
  
Accession: EEA19308
  
Location: 148921-156916
  
 NCBI BlastP on this gene

EEA19308

AMP dependent CoA ligase, putative
  
Accession: EEA19309
  
Location: 157488-159502
  
 NCBI BlastP on this gene

EEA19309

conserved hypothetical protein
  
Accession: EEA19310
  
Location: 160657-161581
  
 NCBI BlastP on this gene

EEA19310

nonribosomal peptide synthetase, putative
  
Accession: EEA19311
  
Location: 163263-175132
  
  
**BlastP hit with Mycgr3G90558\_Mycgr3T**
  
Percentage identity: 31 %
  
BlastP bit score: 1560
  
Sequence coverage: 78 %
  
E-value: 0.0
  
  
 NCBI BlastP on this gene

EEA19311

Query: Architecture Search FASTA input

EQ962658 : Talaromyces stipitatus ATCC 10500 scf\_1105507295511 genomic scaffold    Total score: 1.0     Cumulative Blast bit score: 1521

Hit cluster cross-links:

Mycgr3G36335 Mycgr3T
  
Location: 0-423

Mycgr3G36335\_Mycgr3T

Mycgr3G84494 Mycgr3T
  
Location: 523-2047

Mycgr3G84494\_Mycgr3T

Mycgr3G90558 Mycgr3T
  
Location: 2147-15296

Mycgr3G90558\_Mycgr3T

Mycgr3G68036 Mycgr3T
  
Location: 15396-16395

Mycgr3G68036\_Mycgr3T

Mycgr3G90561 Mycgr3T
  
Location: 16495-17134

Mycgr3G90561\_Mycgr3T

Mycgr3G35862 Mycgr3T
  
Location: 17234-18662

Mycgr3G35862\_Mycgr3T

Mycgr3G68030 Mycgr3T
  
Location: 18762-19722

Mycgr3G68030\_Mycgr3T

Mycgr3G36449 Mycgr3T
  
Location: 19822-21886

Mycgr3G36449\_Mycgr3T

Mycgr3G35528 Mycgr3T
  
Location: 21986-22844

Mycgr3G35528\_Mycgr3T

Mycgr3G35932 Mycgr3T
  
Location: 22944-24390

Mycgr3G35932\_Mycgr3T

Mycgr3G23761 Mycgr3T
  
Location: 24490-25825

Mycgr3G23761\_Mycgr3T

Mycgr3G35535 Mycgr3T
  
Location: 25925-26429

Mycgr3G35535\_Mycgr3T

Mycgr3G9942 Mycgr3T9
  
Location: 26529-30375

Mycgr3G9942\_Mycgr3T9

polyketide synthase, putative
  
Accession: EED13571
  
Location: 307442-315406
  
 NCBI BlastP on this gene

EED13571

AMP dependent ligase/synthetase, putative
  
Accession: EED13572
  
Location: 315905-317933
  
 NCBI BlastP on this gene

EED13572

conserved hypothetical protein
  
Accession: EED13573
  
Location: 319874-320777
  
 NCBI BlastP on this gene

EED13573

nonribosomal peptide synthase, putative
  
Accession: EED13574
  
Location: 321838-333741
  
  
**BlastP hit with Mycgr3G90558\_Mycgr3T**
  
Percentage identity: 30 %
  
BlastP bit score: 1521
  
Sequence coverage: 78 %
  
E-value: 0.0
  
  
 NCBI BlastP on this gene

EED13574

conserved hypothetical protein
  
Accession: EED13575
  
Location: 333968-335762
  
 NCBI BlastP on this gene

EED13575

hypothetical protein
  
Accession: EED13576
  
Location: 337349-338134
  
 NCBI BlastP on this gene

EED13576

choline monooxygenase, putative
  
Accession: EED13577
  
Location: 339621-340353
  
 NCBI BlastP on this gene

EED13577

mitochondrial inner membrane translocase subunit (TIM17), putative
  
Accession: EED13578
  
Location: 341185-341726
  
 NCBI BlastP on this gene

EED13578

ABC transporter, putative
  
Accession: EED13579
  
Location: 344680-345903
  
 NCBI BlastP on this gene

EED13579

Query: Architecture Search FASTA input

DS989824 : Arthroderma gypseum CBS 118893 supercont1.3 genomic scaffold    Total score: 1.0     Cumulative Blast bit score: 1515

Hit cluster cross-links:

Mycgr3G36335 Mycgr3T
  
Location: 0-423

Mycgr3G36335\_Mycgr3T

Mycgr3G84494 Mycgr3T
  
Location: 523-2047

Mycgr3G84494\_Mycgr3T

Mycgr3G90558 Mycgr3T
  
Location: 2147-15296

Mycgr3G90558\_Mycgr3T

Mycgr3G68036 Mycgr3T
  
Location: 15396-16395

Mycgr3G68036\_Mycgr3T

Mycgr3G90561 Mycgr3T
  
Location: 16495-17134

Mycgr3G90561\_Mycgr3T

Mycgr3G35862 Mycgr3T
  
Location: 17234-18662

Mycgr3G35862\_Mycgr3T

Mycgr3G68030 Mycgr3T
  
Location: 18762-19722

Mycgr3G68030\_Mycgr3T

Mycgr3G36449 Mycgr3T
  
Location: 19822-21886

Mycgr3G36449\_Mycgr3T

Mycgr3G35528 Mycgr3T
  
Location: 21986-22844

Mycgr3G35528\_Mycgr3T

Mycgr3G35932 Mycgr3T
  
Location: 22944-24390

Mycgr3G35932\_Mycgr3T

Mycgr3G23761 Mycgr3T
  
Location: 24490-25825

Mycgr3G23761\_Mycgr3T

Mycgr3G35535 Mycgr3T
  
Location: 25925-26429

Mycgr3G35535\_Mycgr3T

Mycgr3G9942 Mycgr3T9
  
Location: 26529-30375

Mycgr3G9942\_Mycgr3T9

1-aminocyclopropane-1-carboxylate synthase
  
Accession: EFR00818
  
Location: 1081844-1083133
  
 NCBI BlastP on this gene

EFR00818

hypothetical protein
  
Accession: EFR00817
  
Location: 1080219-1081337
  
 NCBI BlastP on this gene

EFR00817

hypothetical protein
  
Accession: EFR00816
  
Location: 1051142-1079626
  
  
**BlastP hit with Mycgr3G90558\_Mycgr3T**
  
Percentage identity: 30 %
  
BlastP bit score: 1515
  
Sequence coverage: 86 %
  
E-value: 0.0
  
  
 NCBI BlastP on this gene

EFR00816

enoyl-CoA hydratase/isomerase
  
Accession: EFR00815
  
Location: 1049443-1050270
  
 NCBI BlastP on this gene

EFR00815

hypothetical protein
  
Accession: EFR00814
  
Location: 1047703-1048296
  
 NCBI BlastP on this gene

EFR00814

Query: Architecture Search FASTA input

DF126447 : Aspergillus kawachii IFO 4308 DNA, contig: scaffold00001    Total score: 1.0     Cumulative Blast bit score: 1497

Hit cluster cross-links:

Mycgr3G36335 Mycgr3T
  
Location: 0-423

Mycgr3G36335\_Mycgr3T

Mycgr3G84494 Mycgr3T
  
Location: 523-2047

Mycgr3G84494\_Mycgr3T

Mycgr3G90558 Mycgr3T
  
Location: 2147-15296

Mycgr3G90558\_Mycgr3T

Mycgr3G68036 Mycgr3T
  
Location: 15396-16395

Mycgr3G68036\_Mycgr3T

Mycgr3G90561 Mycgr3T
  
Location: 16495-17134

Mycgr3G90561\_Mycgr3T

Mycgr3G35862 Mycgr3T
  
Location: 17234-18662

Mycgr3G35862\_Mycgr3T

Mycgr3G68030 Mycgr3T
  
Location: 18762-19722

Mycgr3G68030\_Mycgr3T

Mycgr3G36449 Mycgr3T
  
Location: 19822-21886

Mycgr3G36449\_Mycgr3T

Mycgr3G35528 Mycgr3T
  
Location: 21986-22844

Mycgr3G35528\_Mycgr3T

Mycgr3G35932 Mycgr3T
  
Location: 22944-24390

Mycgr3G35932\_Mycgr3T

Mycgr3G23761 Mycgr3T
  
Location: 24490-25825

Mycgr3G23761\_Mycgr3T

Mycgr3G35535 Mycgr3T
  
Location: 25925-26429

Mycgr3G35535\_Mycgr3T

Mycgr3G9942 Mycgr3T9
  
Location: 26529-30375

Mycgr3G9942\_Mycgr3T9

membrane protein TMS1
  
Accession: GAA82425
  
Location: 1791453-1793087
  
 NCBI BlastP on this gene

GAA82425

DNA mismatch repair protein
  
Accession: GAA82424
  
Location: 1787801-1791084
  
 NCBI BlastP on this gene

GAA82424

delta-1-pyrroline-5-carboxylate dehydrogenase, mitochondrial precursor
  
Accession: GAA82423
  
Location: 1785419-1787224
  
 NCBI BlastP on this gene

GAA82423

nonribosomal peptide synthetase
  
Accession: GAA82422
  
Location: 1766590-1784212
  
  
**BlastP hit with Mycgr3G90558\_Mycgr3T**
  
Percentage identity: 31 %
  
BlastP bit score: 1497
  
Sequence coverage: 75 %
  
E-value: 0.0
  
  
 NCBI BlastP on this gene

GAA82422

similar to An02g02310
  
Accession: GAA82421
  
Location: 1765177-1765725
  
 NCBI BlastP on this gene

GAA82421

chitin synthase
  
Accession: GAA82420
  
Location: 1759239-1764667
  
 NCBI BlastP on this gene

GAA82420

Query: Architecture Search FASTA input

KB020599 : Colletotrichum gloeosporioides Nara gc5 unplaced genomic scaffold scaffold236    Total score: 1.0     Cumulative Blast bit score: 1492

Hit cluster cross-links:

Mycgr3G36335 Mycgr3T
  
Location: 0-423

Mycgr3G36335\_Mycgr3T

Mycgr3G84494 Mycgr3T
  
Location: 523-2047

Mycgr3G84494\_Mycgr3T

Mycgr3G90558 Mycgr3T
  
Location: 2147-15296

Mycgr3G90558\_Mycgr3T

Mycgr3G68036 Mycgr3T
  
Location: 15396-16395

Mycgr3G68036\_Mycgr3T

Mycgr3G90561 Mycgr3T
  
Location: 16495-17134

Mycgr3G90561\_Mycgr3T

Mycgr3G35862 Mycgr3T
  
Location: 17234-18662

Mycgr3G35862\_Mycgr3T

Mycgr3G68030 Mycgr3T
  
Location: 18762-19722

Mycgr3G68030\_Mycgr3T

Mycgr3G36449 Mycgr3T
  
Location: 19822-21886

Mycgr3G36449\_Mycgr3T

Mycgr3G35528 Mycgr3T
  
Location: 21986-22844

Mycgr3G35528\_Mycgr3T

Mycgr3G35932 Mycgr3T
  
Location: 22944-24390

Mycgr3G35932\_Mycgr3T

Mycgr3G23761 Mycgr3T
  
Location: 24490-25825

Mycgr3G23761\_Mycgr3T

Mycgr3G35535 Mycgr3T
  
Location: 25925-26429

Mycgr3G35535\_Mycgr3T

Mycgr3G9942 Mycgr3T9
  
Location: 26529-30375

Mycgr3G9942\_Mycgr3T9

indole-3-acetate beta-glucosyltransferase
  
Accession: ELA34719
  
Location: 70327-72235
  
 NCBI BlastP on this gene

ELA34719

hypothetical protein
  
Accession: ELA34720
  
Location: 73245-74773
  
 NCBI BlastP on this gene

ELA34720

multidrug resistance-associated protein 5
  
Accession: ELA34721
  
Location: 75264-79454
  
  
**BlastP hit with Mycgr3G9942\_Mycgr3T9**
  
Percentage identity: 30 %
  
BlastP bit score: 541
  
Sequence coverage: 105 %
  
E-value: 3e-165
  
  
 NCBI BlastP on this gene

ELA34721

isoflavone reductase family protein
  
Accession: ELA34722
  
Location: 79918-81079
  
 NCBI BlastP on this gene

ELA34722

DnaJ domain-containing protein
  
Accession: ELA34723
  
Location: 81295-82582
  
 NCBI BlastP on this gene

ELA34723

hypothetical protein
  
Accession: ELA34724
  
Location: 82701-83054
  
 NCBI BlastP on this gene

ELA34724

fungal specific transcription factor
  
Accession: ELA34725
  
Location: 84688-86337
  
 NCBI BlastP on this gene

ELA34725

ABC1 domain containing protein
  
Accession: ELA34726
  
Location: 86664-88013
  
 NCBI BlastP on this gene

ELA34726

chromatin assembly factor 1 subunit
  
Accession: ELA34727
  
Location: 88531-90859
  
 NCBI BlastP on this gene

ELA34727

ABC bile acid
  
Accession: ELA34728
  
Location: 97327-102043
  
  
**BlastP hit with Mycgr3G9942\_Mycgr3T9**
  
Percentage identity: 41 %
  
BlastP bit score: 951
  
Sequence coverage: 100 %
  
E-value: 0.0
  
  
 NCBI BlastP on this gene

ELA34728

hypothetical protein
  
Accession: ELA34729
  
Location: 102873-103394
  
 NCBI BlastP on this gene

ELA34729

GNAT family
  
Accession: ELA34730
  
Location: 103849-104595
  
 NCBI BlastP on this gene

ELA34730

ATPase
  
Accession: ELA34731
  
Location: 104938-107436
  
 NCBI BlastP on this gene

ELA34731

Query: Architecture Search FASTA input

KB644408 : Penicillium oxalicum 114-2 unplaced genomic scaffold scaffold\_1    Total score: 1.0     Cumulative Blast bit score: 1487

Hit cluster cross-links:

Mycgr3G36335 Mycgr3T
  
Location: 0-423

Mycgr3G36335\_Mycgr3T

Mycgr3G84494 Mycgr3T
  
Location: 523-2047

Mycgr3G84494\_Mycgr3T

Mycgr3G90558 Mycgr3T
  
Location: 2147-15296

Mycgr3G90558\_Mycgr3T

Mycgr3G68036 Mycgr3T
  
Location: 15396-16395

Mycgr3G68036\_Mycgr3T

Mycgr3G90561 Mycgr3T
  
Location: 16495-17134

Mycgr3G90561\_Mycgr3T

Mycgr3G35862 Mycgr3T
  
Location: 17234-18662

Mycgr3G35862\_Mycgr3T

Mycgr3G68030 Mycgr3T
  
Location: 18762-19722

Mycgr3G68030\_Mycgr3T

Mycgr3G36449 Mycgr3T
  
Location: 19822-21886

Mycgr3G36449\_Mycgr3T

Mycgr3G35528 Mycgr3T
  
Location: 21986-22844

Mycgr3G35528\_Mycgr3T

Mycgr3G35932 Mycgr3T
  
Location: 22944-24390

Mycgr3G35932\_Mycgr3T

Mycgr3G23761 Mycgr3T
  
Location: 24490-25825

Mycgr3G23761\_Mycgr3T

Mycgr3G35535 Mycgr3T
  
Location: 25925-26429

Mycgr3G35535\_Mycgr3T

Mycgr3G9942 Mycgr3T9
  
Location: 26529-30375

Mycgr3G9942\_Mycgr3T9

hypothetical protein
  
Accession: EPS26276
  
Location: 3684930-3707027
  
  
**BlastP hit with Mycgr3G90558\_Mycgr3T**
  
Percentage identity: 31 %
  
BlastP bit score: 1487
  
Sequence coverage: 75 %
  
E-value: 0.0
  
  
 NCBI BlastP on this gene

EPS26276

hypothetical protein
  
Accession: EPS26275
  
Location: 3681004-3683324
  
 NCBI BlastP on this gene

EPS26275

hypothetical protein
  
Accession: EPS26274
  
Location: 3675933-3680327
  
 NCBI BlastP on this gene

EPS26274

Query: Architecture Search FASTA input

GG698902 : Nectria haematococca mpVI 77-13-4 chromosome 7 genomic scaffold NECHAsca\_9\_chr7\_10\_0    Total score: 1.0     Cumulative Blast bit score: 1487

Hit cluster cross-links:

Mycgr3G36335 Mycgr3T
  
Location: 0-423

Mycgr3G36335\_Mycgr3T

Mycgr3G84494 Mycgr3T
  
Location: 523-2047

Mycgr3G84494\_Mycgr3T

Mycgr3G90558 Mycgr3T
  
Location: 2147-15296

Mycgr3G90558\_Mycgr3T

Mycgr3G68036 Mycgr3T
  
Location: 15396-16395

Mycgr3G68036\_Mycgr3T

Mycgr3G90561 Mycgr3T
  
Location: 16495-17134

Mycgr3G90561\_Mycgr3T

Mycgr3G35862 Mycgr3T
  
Location: 17234-18662

Mycgr3G35862\_Mycgr3T

Mycgr3G68030 Mycgr3T
  
Location: 18762-19722

Mycgr3G68030\_Mycgr3T

Mycgr3G36449 Mycgr3T
  
Location: 19822-21886

Mycgr3G36449\_Mycgr3T

Mycgr3G35528 Mycgr3T
  
Location: 21986-22844

Mycgr3G35528\_Mycgr3T

Mycgr3G35932 Mycgr3T
  
Location: 22944-24390

Mycgr3G35932\_Mycgr3T

Mycgr3G23761 Mycgr3T
  
Location: 24490-25825

Mycgr3G23761\_Mycgr3T

Mycgr3G35535 Mycgr3T
  
Location: 25925-26429

Mycgr3G35535\_Mycgr3T

Mycgr3G9942 Mycgr3T9
  
Location: 26529-30375

Mycgr3G9942\_Mycgr3T9

hypothetical protein
  
Accession: EEU43517
  
Location: 1034605-1036248
  
 NCBI BlastP on this gene

EEU43517

hypothetical protein
  
Accession: EEU43518
  
Location: 1036669-1037589
  
 NCBI BlastP on this gene

EEU43518

predicted protein
  
Accession: EEU43519
  
Location: 1038778-1040459
  
 NCBI BlastP on this gene

EEU43519

hypothetical protein
  
Accession: EEU43765
  
Location: 1040666-1041748
  
 NCBI BlastP on this gene

EEU43765

hypothetical protein
  
Accession: EEU43766
  
Location: 1042756-1043906
  
 NCBI BlastP on this gene

EEU43766

hypothetical protein
  
Accession: EEU43520
  
Location: 1044999-1061802
  
  
**BlastP hit with Mycgr3G90558\_Mycgr3T**
  
Percentage identity: 29 %
  
BlastP bit score: 1487
  
Sequence coverage: 91 %
  
E-value: 0.0
  
  
 NCBI BlastP on this gene

EEU43520

Query: Architecture Search FASTA input

AM920436 : Penicillium chrysogenum Wisconsin 54-1255 complete genome, contig Pc00c21.    Total score: 1.0     Cumulative Blast bit score: 1481

Hit cluster cross-links:

Mycgr3G36335 Mycgr3T
  
Location: 0-423

Mycgr3G36335\_Mycgr3T

Mycgr3G84494 Mycgr3T
  
Location: 523-2047

Mycgr3G84494\_Mycgr3T

Mycgr3G90558 Mycgr3T
  
Location: 2147-15296

Mycgr3G90558\_Mycgr3T

Mycgr3G68036 Mycgr3T
  
Location: 15396-16395

Mycgr3G68036\_Mycgr3T

Mycgr3G90561 Mycgr3T
  
Location: 16495-17134

Mycgr3G90561\_Mycgr3T

Mycgr3G35862 Mycgr3T
  
Location: 17234-18662

Mycgr3G35862\_Mycgr3T

Mycgr3G68030 Mycgr3T
  
Location: 18762-19722

Mycgr3G68030\_Mycgr3T

Mycgr3G36449 Mycgr3T
  
Location: 19822-21886

Mycgr3G36449\_Mycgr3T

Mycgr3G35528 Mycgr3T
  
Location: 21986-22844

Mycgr3G35528\_Mycgr3T

Mycgr3G35932 Mycgr3T
  
Location: 22944-24390

Mycgr3G35932\_Mycgr3T

Mycgr3G23761 Mycgr3T
  
Location: 24490-25825

Mycgr3G23761\_Mycgr3T

Mycgr3G35535 Mycgr3T
  
Location: 25925-26429

Mycgr3G35535\_Mycgr3T

Mycgr3G9942 Mycgr3T9
  
Location: 26529-30375

Mycgr3G9942\_Mycgr3T9

not annotated
  
Accession: CAP95979
  
Location: 2562419-2563636
  
 NCBI BlastP on this gene

Pc21g10820

not annotated
  
Accession: CAP95978
  
Location: 2560518-2561503
  
 NCBI BlastP on this gene

Pc21g10810

not annotated
  
Accession: CAP95977
  
Location: 2558169-2559581
  
 NCBI BlastP on this gene

Pc21g10800

not annotated
  
Accession: CAP95976
  
Location: 2535141-2557271
  
  
**BlastP hit with Mycgr3G90558\_Mycgr3T**
  
Percentage identity: 31 %
  
BlastP bit score: 1481
  
Sequence coverage: 77 %
  
E-value: 0.0
  
  
 NCBI BlastP on this gene

Pc21g10790

not annotated
  
Accession: CAP95975
  
Location: 2532959-2533900
  
 NCBI BlastP on this gene

Pc21g10780

not annotated
  
Accession: CAP95974
  
Location: 2531335-2532375
  
 NCBI BlastP on this gene

Pc21g10770

hypothetical protein
  
Accession: CAP95973
  
Location: 2530746-2531288
  
 NCBI BlastP on this gene

Pc21g10760

not annotated
  
Accession: CAP95972
  
Location: 2529805-2530722
  
 NCBI BlastP on this gene

Pc21g10750

hypothetical protein
  
Accession: CAP95971
  
Location: 2527570-2529340
  
 NCBI BlastP on this gene

Pc21g10740

Query: Architecture Search FASTA input

DS027698 : Neosartorya fischeri NRRL 181 1099437636266 genomic scaffold    Total score: 1.0     Cumulative Blast bit score: 1470

Hit cluster cross-links:

Mycgr3G36335 Mycgr3T
  
Location: 0-423

Mycgr3G36335\_Mycgr3T

Mycgr3G84494 Mycgr3T
  
Location: 523-2047

Mycgr3G84494\_Mycgr3T

Mycgr3G90558 Mycgr3T
  
Location: 2147-15296

Mycgr3G90558\_Mycgr3T

Mycgr3G68036 Mycgr3T
  
Location: 15396-16395

Mycgr3G68036\_Mycgr3T

Mycgr3G90561 Mycgr3T
  
Location: 16495-17134

Mycgr3G90561\_Mycgr3T

Mycgr3G35862 Mycgr3T
  
Location: 17234-18662

Mycgr3G35862\_Mycgr3T

Mycgr3G68030 Mycgr3T
  
Location: 18762-19722

Mycgr3G68030\_Mycgr3T

Mycgr3G36449 Mycgr3T
  
Location: 19822-21886

Mycgr3G36449\_Mycgr3T

Mycgr3G35528 Mycgr3T
  
Location: 21986-22844

Mycgr3G35528\_Mycgr3T

Mycgr3G35932 Mycgr3T
  
Location: 22944-24390

Mycgr3G35932\_Mycgr3T

Mycgr3G23761 Mycgr3T
  
Location: 24490-25825

Mycgr3G23761\_Mycgr3T

Mycgr3G35535 Mycgr3T
  
Location: 25925-26429

Mycgr3G35535\_Mycgr3T

Mycgr3G9942 Mycgr3T9
  
Location: 26529-30375

Mycgr3G9942\_Mycgr3T9

O-methyltransferase, putative
  
Accession: EAW16450
  
Location: 3234458-3235347
  
 NCBI BlastP on this gene

EAW16450

nonribosomal peptide synthase, putative
  
Accession: EAW16449
  
Location: 3229840-3233259
  
 NCBI BlastP on this gene

EAW16449

2OG-Fe(II) oxygenase family oxidoreductase, putative
  
Accession: EAW16448
  
Location: 3226649-3228035
  
 NCBI BlastP on this gene

EAW16448

MAK1-like monooxygenase, putative
  
Accession: EAW16447
  
Location: 3224165-3226117
  
 NCBI BlastP on this gene

EAW16447

nonribosomal peptide synthase, putative
  
Accession: EAW16446
  
Location: 3213365-3223759
  
  
**BlastP hit with Mycgr3G90558\_Mycgr3T**
  
Percentage identity: 33 %
  
BlastP bit score: 1470
  
Sequence coverage: 69 %
  
E-value: 0.0
  
  
 NCBI BlastP on this gene

EAW16446

hypothetical protein
  
Accession: EAW16445
  
Location: 3209654-3210370
  
 NCBI BlastP on this gene

EAW16445

MFS sugar transporter, putative
  
Accession: EAW16444
  
Location: 3205917-3207756
  
 NCBI BlastP on this gene

EAW16444

bacterial alpha-L-rhamnosidase domain protein
  
Accession: EAW16443
  
Location: 3202318-3205008
  
 NCBI BlastP on this gene

EAW16443

C2H2 type zinc finger domain protein
  
Accession: EAW16442
  
Location: 3198632-3201421
  
 NCBI BlastP on this gene

EAW16442

Query: Architecture Search FASTA input

CH408034 : Chaetomium globosum CBS 148.51 scaffold\_6 genomic scaffold    Total score: 1.0     Cumulative Blast bit score: 1465

Hit cluster cross-links:

Mycgr3G36335 Mycgr3T
  
Location: 0-423

Mycgr3G36335\_Mycgr3T

Mycgr3G84494 Mycgr3T
  
Location: 523-2047

Mycgr3G84494\_Mycgr3T

Mycgr3G90558 Mycgr3T
  
Location: 2147-15296

Mycgr3G90558\_Mycgr3T

Mycgr3G68036 Mycgr3T
  
Location: 15396-16395

Mycgr3G68036\_Mycgr3T

Mycgr3G90561 Mycgr3T
  
Location: 16495-17134

Mycgr3G90561\_Mycgr3T

Mycgr3G35862 Mycgr3T
  
Location: 17234-18662

Mycgr3G35862\_Mycgr3T

Mycgr3G68030 Mycgr3T
  
Location: 18762-19722

Mycgr3G68030\_Mycgr3T

Mycgr3G36449 Mycgr3T
  
Location: 19822-21886

Mycgr3G36449\_Mycgr3T

Mycgr3G35528 Mycgr3T
  
Location: 21986-22844

Mycgr3G35528\_Mycgr3T

Mycgr3G35932 Mycgr3T
  
Location: 22944-24390

Mycgr3G35932\_Mycgr3T

Mycgr3G23761 Mycgr3T
  
Location: 24490-25825

Mycgr3G23761\_Mycgr3T

Mycgr3G35535 Mycgr3T
  
Location: 25925-26429

Mycgr3G35535\_Mycgr3T

Mycgr3G9942 Mycgr3T9
  
Location: 26529-30375

Mycgr3G9942\_Mycgr3T9

hypothetical protein
  
Accession: EAQ84525
  
Location: 607887-613428
  
 NCBI BlastP on this gene

EAQ84525

hypothetical protein
  
Accession: EAQ84526
  
Location: 616844-639592
  
  
**BlastP hit with Mycgr3G90558\_Mycgr3T**
  
Percentage identity: 31 %
  
BlastP bit score: 1465
  
Sequence coverage: 76 %
  
E-value: 0.0
  
  
 NCBI BlastP on this gene

EAQ84526

hypothetical protein
  
Accession: EAQ84527
  
Location: 640205-644342
  
 NCBI BlastP on this gene

EAQ84527

hypothetical protein
  
Accession: EAQ84528
  
Location: 644693-649154
  
 NCBI BlastP on this gene

EAQ84528

Query: Architecture Search FASTA input

CP003005 : Myceliophthora thermophila ATCC 42464 chromosome 4    Total score: 1.0     Cumulative Blast bit score: 1454

Hit cluster cross-links:

Mycgr3G36335 Mycgr3T
  
Location: 0-423

Mycgr3G36335\_Mycgr3T

Mycgr3G84494 Mycgr3T
  
Location: 523-2047

Mycgr3G84494\_Mycgr3T

Mycgr3G90558 Mycgr3T
  
Location: 2147-15296

Mycgr3G90558\_Mycgr3T

Mycgr3G68036 Mycgr3T
  
Location: 15396-16395

Mycgr3G68036\_Mycgr3T

Mycgr3G90561 Mycgr3T
  
Location: 16495-17134

Mycgr3G90561\_Mycgr3T

Mycgr3G35862 Mycgr3T
  
Location: 17234-18662

Mycgr3G35862\_Mycgr3T

Mycgr3G68030 Mycgr3T
  
Location: 18762-19722

Mycgr3G68030\_Mycgr3T

Mycgr3G36449 Mycgr3T
  
Location: 19822-21886

Mycgr3G36449\_Mycgr3T

Mycgr3G35528 Mycgr3T
  
Location: 21986-22844

Mycgr3G35528\_Mycgr3T

Mycgr3G35932 Mycgr3T
  
Location: 22944-24390

Mycgr3G35932\_Mycgr3T

Mycgr3G23761 Mycgr3T
  
Location: 24490-25825

Mycgr3G23761\_Mycgr3T

Mycgr3G35535 Mycgr3T
  
Location: 25925-26429

Mycgr3G35535\_Mycgr3T

Mycgr3G9942 Mycgr3T9
  
Location: 26529-30375

Mycgr3G9942\_Mycgr3T9

non-ribosomal peptide synthetase
  
Accession: AEO59325
  
Location: 3950426-3973722
  
  
**BlastP hit with Mycgr3G90558\_Mycgr3T**
  
Percentage identity: 31 %
  
BlastP bit score: 1454
  
Sequence coverage: 77 %
  
E-value: 0.0
  
  
 NCBI BlastP on this gene

MYCTH\_94652

hypothetical protein
  
Accession: AEO59324
  
Location: 3945573-3949459
  
 NCBI BlastP on this gene

MYCTH\_2128378

Query: Architecture Search FASTA input

JH725160 : Beauveria bassiana ARSEF 2860 unplaced genomic scaffold BBA\_S00011    Total score: 1.0     Cumulative Blast bit score: 1419

Hit cluster cross-links:

Mycgr3G36335 Mycgr3T
  
Location: 0-423

Mycgr3G36335\_Mycgr3T

Mycgr3G84494 Mycgr3T
  
Location: 523-2047

Mycgr3G84494\_Mycgr3T

Mycgr3G90558 Mycgr3T
  
Location: 2147-15296

Mycgr3G90558\_Mycgr3T

Mycgr3G68036 Mycgr3T
  
Location: 15396-16395

Mycgr3G68036\_Mycgr3T

Mycgr3G90561 Mycgr3T
  
Location: 16495-17134

Mycgr3G90561\_Mycgr3T

Mycgr3G35862 Mycgr3T
  
Location: 17234-18662

Mycgr3G35862\_Mycgr3T

Mycgr3G68030 Mycgr3T
  
Location: 18762-19722

Mycgr3G68030\_Mycgr3T

Mycgr3G36449 Mycgr3T
  
Location: 19822-21886

Mycgr3G36449\_Mycgr3T

Mycgr3G35528 Mycgr3T
  
Location: 21986-22844

Mycgr3G35528\_Mycgr3T

Mycgr3G35932 Mycgr3T
  
Location: 22944-24390

Mycgr3G35932\_Mycgr3T

Mycgr3G23761 Mycgr3T
  
Location: 24490-25825

Mycgr3G23761\_Mycgr3T

Mycgr3G35535 Mycgr3T
  
Location: 25925-26429

Mycgr3G35535\_Mycgr3T

Mycgr3G9942 Mycgr3T9
  
Location: 26529-30375

Mycgr3G9942\_Mycgr3T9

ABC transporter with duplicated ATPase domains
  
Accession: EJP66331
  
Location: 595296-597590
  
 NCBI BlastP on this gene

EJP66331

oxidoreductase, 2OG-Fe(II) oxygenase family
  
Accession: EJP66332
  
Location: 598424-599397
  
 NCBI BlastP on this gene

EJP66332

aspartate-tRNA ligase
  
Accession: EJP66333
  
Location: 600473-602169
  
 NCBI BlastP on this gene

EJP66333

peptide synthetase
  
Accession: EJP66334
  
Location: 604188-614168
  
  
**BlastP hit with Mycgr3G90558\_Mycgr3T**
  
Percentage identity: 33 %
  
BlastP bit score: 1419
  
Sequence coverage: 66 %
  
E-value: 0.0
  
  
 NCBI BlastP on this gene

EJP66334

Query: Architecture Search FASTA input

EQ963476 : Aspergillus flavus NRRL3357 scf\_1106286417850 genomic scaffold    Total score: 1.0     Cumulative Blast bit score: 1402

Hit cluster cross-links:

Mycgr3G36335 Mycgr3T
  
Location: 0-423

Mycgr3G36335\_Mycgr3T

Mycgr3G84494 Mycgr3T
  
Location: 523-2047

Mycgr3G84494\_Mycgr3T

Mycgr3G90558 Mycgr3T
  
Location: 2147-15296

Mycgr3G90558\_Mycgr3T

Mycgr3G68036 Mycgr3T
  
Location: 15396-16395

Mycgr3G68036\_Mycgr3T

Mycgr3G90561 Mycgr3T
  
Location: 16495-17134

Mycgr3G90561\_Mycgr3T

Mycgr3G35862 Mycgr3T
  
Location: 17234-18662

Mycgr3G35862\_Mycgr3T

Mycgr3G68030 Mycgr3T
  
Location: 18762-19722

Mycgr3G68030\_Mycgr3T

Mycgr3G36449 Mycgr3T
  
Location: 19822-21886

Mycgr3G36449\_Mycgr3T

Mycgr3G35528 Mycgr3T
  
Location: 21986-22844

Mycgr3G35528\_Mycgr3T

Mycgr3G35932 Mycgr3T
  
Location: 22944-24390

Mycgr3G35932\_Mycgr3T

Mycgr3G23761 Mycgr3T
  
Location: 24490-25825

Mycgr3G23761\_Mycgr3T

Mycgr3G35535 Mycgr3T
  
Location: 25925-26429

Mycgr3G35535\_Mycgr3T

Mycgr3G9942 Mycgr3T9
  
Location: 26529-30375

Mycgr3G9942\_Mycgr3T9

fatty acid synthase alpha subunit, putative
  
Accession: EED52163
  
Location: 162029-167698
  
 NCBI BlastP on this gene

EED52163

cytochrome P450, putative
  
Accession: EED52162
  
Location: 159465-161321
  
 NCBI BlastP on this gene

EED52162

branched-chain amino acid aminotransferase, putative
  
Accession: EED52161
  
Location: 156808-158806
  
 NCBI BlastP on this gene

EED52161

Ankyrin repeat protein
  
Accession: EED52160
  
Location: 155841-156723
  
 NCBI BlastP on this gene

EED52160

nonribosomal peptide synthase, putative
  
Accession: EED52159
  
Location: 143335-152441
  
  
**BlastP hit with Mycgr3G90558\_Mycgr3T**
  
Percentage identity: 32 %
  
BlastP bit score: 1402
  
Sequence coverage: 63 %
  
E-value: 0.0
  
  
 NCBI BlastP on this gene

EED52159

Query: Architecture Search FASTA input

HF679023 : Fusarium fujikuroi IMI 58289 draft genome, chromosome FFUJ\_chr01.    Total score: 1.0     Cumulative Blast bit score: 1299

Hit cluster cross-links:

Mycgr3G36335 Mycgr3T
  
Location: 0-423

Mycgr3G36335\_Mycgr3T

Mycgr3G84494 Mycgr3T
  
Location: 523-2047

Mycgr3G84494\_Mycgr3T

Mycgr3G90558 Mycgr3T
  
Location: 2147-15296

Mycgr3G90558\_Mycgr3T

Mycgr3G68036 Mycgr3T
  
Location: 15396-16395

Mycgr3G68036\_Mycgr3T

Mycgr3G90561 Mycgr3T
  
Location: 16495-17134

Mycgr3G90561\_Mycgr3T

Mycgr3G35862 Mycgr3T
  
Location: 17234-18662

Mycgr3G35862\_Mycgr3T

Mycgr3G68030 Mycgr3T
  
Location: 18762-19722

Mycgr3G68030\_Mycgr3T

Mycgr3G36449 Mycgr3T
  
Location: 19822-21886

Mycgr3G36449\_Mycgr3T

Mycgr3G35528 Mycgr3T
  
Location: 21986-22844

Mycgr3G35528\_Mycgr3T

Mycgr3G35932 Mycgr3T
  
Location: 22944-24390

Mycgr3G35932\_Mycgr3T

Mycgr3G23761 Mycgr3T
  
Location: 24490-25825

Mycgr3G23761\_Mycgr3T

Mycgr3G35535 Mycgr3T
  
Location: 25925-26429

Mycgr3G35535\_Mycgr3T

Mycgr3G9942 Mycgr3T9
  
Location: 26529-30375

Mycgr3G9942\_Mycgr3T9

related to benzoate-para-hydroxylase (cytochrome P450)
  
Accession: CCT63357
  
Location: 6523368-6525190
  
 NCBI BlastP on this gene

FFUJ\_00007

related to cytochrom P450
  
Accession: CCT63358
  
Location: 6526140-6527351
  
 NCBI BlastP on this gene

FFUJ\_00006

related to isoamyl alcohol oxidase
  
Accession: CCT63359
  
Location: 6527917-6529835
  
 NCBI BlastP on this gene

FFUJ\_00005

probable DHA14-like major facilitator; ABC transporter
  
Accession: CCT63472
  
Location: 6530784-6532704
  
 NCBI BlastP on this gene

FFUJ\_00004

non-ribosomal peptide synthetase
  
Accession: CCT63360
  
Location: 6533353-6548739
  
  
**BlastP hit with Mycgr3G90558\_Mycgr3T**
  
Percentage identity: 29 %
  
BlastP bit score: 1299
  
Sequence coverage: 82 %
  
E-value: 0.0
  
  
 NCBI BlastP on this gene

FFUJ\_00003

Query: Architecture Search FASTA input

CH476607 : Aspergillus terreus NIH2624 scaffold\_14 genomic scaffold    Total score: 1.0     Cumulative Blast bit score: 1292

Hit cluster cross-links:

Mycgr3G36335 Mycgr3T
  
Location: 0-423

Mycgr3G36335\_Mycgr3T

Mycgr3G84494 Mycgr3T
  
Location: 523-2047

Mycgr3G84494\_Mycgr3T

Mycgr3G90558 Mycgr3T
  
Location: 2147-15296

Mycgr3G90558\_Mycgr3T

Mycgr3G68036 Mycgr3T
  
Location: 15396-16395

Mycgr3G68036\_Mycgr3T

Mycgr3G90561 Mycgr3T
  
Location: 16495-17134

Mycgr3G90561\_Mycgr3T

Mycgr3G35862 Mycgr3T
  
Location: 17234-18662

Mycgr3G35862\_Mycgr3T

Mycgr3G68030 Mycgr3T
  
Location: 18762-19722

Mycgr3G68030\_Mycgr3T

Mycgr3G36449 Mycgr3T
  
Location: 19822-21886

Mycgr3G36449\_Mycgr3T

Mycgr3G35528 Mycgr3T
  
Location: 21986-22844

Mycgr3G35528\_Mycgr3T

Mycgr3G35932 Mycgr3T
  
Location: 22944-24390

Mycgr3G35932\_Mycgr3T

Mycgr3G23761 Mycgr3T
  
Location: 24490-25825

Mycgr3G23761\_Mycgr3T

Mycgr3G35535 Mycgr3T
  
Location: 25925-26429

Mycgr3G35535\_Mycgr3T

Mycgr3G9942 Mycgr3T9
  
Location: 26529-30375

Mycgr3G9942\_Mycgr3T9

predicted protein
  
Accession: EAU30153
  
Location: 23226-24087
  
 NCBI BlastP on this gene

EAU30153

conserved hypothetical protein
  
Accession: EAU30154
  
Location: 24722-25102
  
 NCBI BlastP on this gene

EAU30154

hypothetical protein
  
Accession: EAU30155
  
Location: 25629-29896
  
 NCBI BlastP on this gene

EAU30155

predicted protein
  
Accession: EAU30156
  
Location: 30730-46891
  
  
**BlastP hit with Mycgr3G90558\_Mycgr3T**
  
Percentage identity: 29 %
  
BlastP bit score: 1292
  
Sequence coverage: 75 %
  
E-value: 0.0
  
  
 NCBI BlastP on this gene

EAU30156

predicted protein
  
Accession: EAU30157
  
Location: 47091-48057
  
 NCBI BlastP on this gene

EAU30157

predicted protein
  
Accession: EAU30158
  
Location: 52158-52450
  
 NCBI BlastP on this gene

EAU30158

conserved hypothetical protein
  
Accession: EAU30159
  
Location: 53272-53667
  
 NCBI BlastP on this gene

EAU30159

predicted protein
  
Accession: EAU30160
  
Location: 54208-55650
  
 NCBI BlastP on this gene

EAU30160

Query: Architecture Search FASTA input

CM001201 : Mycosphaerella graminicola IPO323 chromosome 6    Total score: 1.0     Cumulative Blast bit score: 1290

Hit cluster cross-links:

Mycgr3G36335 Mycgr3T
  
Location: 0-423

Mycgr3G36335\_Mycgr3T

Mycgr3G84494 Mycgr3T
  
Location: 523-2047

Mycgr3G84494\_Mycgr3T

Mycgr3G90558 Mycgr3T
  
Location: 2147-15296

Mycgr3G90558\_Mycgr3T

Mycgr3G68036 Mycgr3T
  
Location: 15396-16395

Mycgr3G68036\_Mycgr3T

Mycgr3G90561 Mycgr3T
  
Location: 16495-17134

Mycgr3G90561\_Mycgr3T

Mycgr3G35862 Mycgr3T
  
Location: 17234-18662

Mycgr3G35862\_Mycgr3T

Mycgr3G68030 Mycgr3T
  
Location: 18762-19722

Mycgr3G68030\_Mycgr3T

Mycgr3G36449 Mycgr3T
  
Location: 19822-21886

Mycgr3G36449\_Mycgr3T

Mycgr3G35528 Mycgr3T
  
Location: 21986-22844

Mycgr3G35528\_Mycgr3T

Mycgr3G35932 Mycgr3T
  
Location: 22944-24390

Mycgr3G35932\_Mycgr3T

Mycgr3G23761 Mycgr3T
  
Location: 24490-25825

Mycgr3G23761\_Mycgr3T

Mycgr3G35535 Mycgr3T
  
Location: 25925-26429

Mycgr3G35535\_Mycgr3T

Mycgr3G9942 Mycgr3T9
  
Location: 26529-30375

Mycgr3G9942\_Mycgr3T9

hypothetical protein
  
Accession: EGP86941
  
Location: 346604-349414
  
 NCBI BlastP on this gene

EGP86941

hypothetical protein
  
Accession: EGP86942
  
Location: 344644-346260
  
 NCBI BlastP on this gene

EGP86942

hypothetical protein
  
Accession: EGP86334
  
Location: 340624-341098
  
 NCBI BlastP on this gene

EGP86334

hypothetical protein
  
Accession: EGP86333
  
Location: 338390-339841
  
 NCBI BlastP on this gene

EGP86333

hypothetical protein
  
Accession: EGP86943
  
Location: 336860-337712
  
 NCBI BlastP on this gene

EGP86943

hypothetical protein
  
Accession: EGP86944
  
Location: 323267-335569
  
  
**BlastP hit with Mycgr3G90558\_Mycgr3T**
  
Percentage identity: 30 %
  
BlastP bit score: 1290
  
Sequence coverage: 76 %
  
E-value: 0.0
  
  
 NCBI BlastP on this gene

EGP86944

putative major facilitator superfamily transporter
  
Accession: EGP86332
  
Location: 321309-322960
  
 NCBI BlastP on this gene

EGP86332

hypothetical protein
  
Accession: EGP86945
  
Location: 318326-320221
  
 NCBI BlastP on this gene

EGP86945

hypothetical protein
  
Accession: EGP86331
  
Location: 317169-318230
  
 NCBI BlastP on this gene

EGP86331

hypothetical protein
  
Accession: EGP86946
  
Location: 315201-316479
  
 NCBI BlastP on this gene

EGP86946

hypothetical protein
  
Accession: EGP86330
  
Location: 312759-315135
  
 NCBI BlastP on this gene

EGP86330

Query: Architecture Search FASTA input

CH476595 : Aspergillus terreus NIH2624 scaffold\_2 genomic scaffold    Total score: 1.0     Cumulative Blast bit score: 1254

Hit cluster cross-links:

Mycgr3G36335 Mycgr3T
  
Location: 0-423

Mycgr3G36335\_Mycgr3T

Mycgr3G84494 Mycgr3T
  
Location: 523-2047

Mycgr3G84494\_Mycgr3T

Mycgr3G90558 Mycgr3T
  
Location: 2147-15296

Mycgr3G90558\_Mycgr3T

Mycgr3G68036 Mycgr3T
  
Location: 15396-16395

Mycgr3G68036\_Mycgr3T

Mycgr3G90561 Mycgr3T
  
Location: 16495-17134

Mycgr3G90561\_Mycgr3T

Mycgr3G35862 Mycgr3T
  
Location: 17234-18662

Mycgr3G35862\_Mycgr3T

Mycgr3G68030 Mycgr3T
  
Location: 18762-19722

Mycgr3G68030\_Mycgr3T

Mycgr3G36449 Mycgr3T
  
Location: 19822-21886

Mycgr3G36449\_Mycgr3T

Mycgr3G35528 Mycgr3T
  
Location: 21986-22844

Mycgr3G35528\_Mycgr3T

Mycgr3G35932 Mycgr3T
  
Location: 22944-24390

Mycgr3G35932\_Mycgr3T

Mycgr3G23761 Mycgr3T
  
Location: 24490-25825

Mycgr3G23761\_Mycgr3T

Mycgr3G35535 Mycgr3T
  
Location: 25925-26429

Mycgr3G35535\_Mycgr3T

Mycgr3G9942 Mycgr3T9
  
Location: 26529-30375

Mycgr3G9942\_Mycgr3T9

predicted protein
  
Accession: EAU37756
  
Location: 53723-54964
  
 NCBI BlastP on this gene

EAU37756

conserved hypothetical protein
  
Accession: EAU37757
  
Location: 59937-60612
  
 NCBI BlastP on this gene

EAU37757

predicted protein
  
Accession: EAU37758
  
Location: 62794-63544
  
 NCBI BlastP on this gene

EAU37758

predicted protein
  
Accession: EAU37759
  
Location: 64751-79683
  
  
**BlastP hit with Mycgr3G90558\_Mycgr3T**
  
Percentage identity: 30 %
  
BlastP bit score: 1254
  
Sequence coverage: 68 %
  
E-value: 0.0
  
  
 NCBI BlastP on this gene

EAU37759

hypothetical protein
  
Accession: EAU37760
  
Location: 79875-81645
  
 NCBI BlastP on this gene

EAU37760

predicted protein
  
Accession: EAU37761
  
Location: 84323-85070
  
 NCBI BlastP on this gene

EAU37761

predicted protein
  
Accession: EAU37762
  
Location: 86513-87609
  
 NCBI BlastP on this gene

EAU37762

Query: Architecture Search FASTA input

EQ962656 : Talaromyces stipitatus ATCC 10500 scf\_1105507295549 genomic scaffold    Total score: 1.0     Cumulative Blast bit score: 1241

Hit cluster cross-links:

Mycgr3G36335 Mycgr3T
  
Location: 0-423

Mycgr3G36335\_Mycgr3T

Mycgr3G84494 Mycgr3T
  
Location: 523-2047

Mycgr3G84494\_Mycgr3T

Mycgr3G90558 Mycgr3T
  
Location: 2147-15296

Mycgr3G90558\_Mycgr3T

Mycgr3G68036 Mycgr3T
  
Location: 15396-16395

Mycgr3G68036\_Mycgr3T

Mycgr3G90561 Mycgr3T
  
Location: 16495-17134

Mycgr3G90561\_Mycgr3T

Mycgr3G35862 Mycgr3T
  
Location: 17234-18662

Mycgr3G35862\_Mycgr3T

Mycgr3G68030 Mycgr3T
  
Location: 18762-19722

Mycgr3G68030\_Mycgr3T

Mycgr3G36449 Mycgr3T
  
Location: 19822-21886

Mycgr3G36449\_Mycgr3T

Mycgr3G35528 Mycgr3T
  
Location: 21986-22844

Mycgr3G35528\_Mycgr3T

Mycgr3G35932 Mycgr3T
  
Location: 22944-24390

Mycgr3G35932\_Mycgr3T

Mycgr3G23761 Mycgr3T
  
Location: 24490-25825

Mycgr3G23761\_Mycgr3T

Mycgr3G35535 Mycgr3T
  
Location: 25925-26429

Mycgr3G35535\_Mycgr3T

Mycgr3G9942 Mycgr3T9
  
Location: 26529-30375

Mycgr3G9942\_Mycgr3T9

nonribosomal peptide synthase, putative
  
Accession: EED16046
  
Location: 757374-781575
  
  
**BlastP hit with Mycgr3G90558\_Mycgr3T**
  
Percentage identity: 31 %
  
BlastP bit score: 1241
  
Sequence coverage: 60 %
  
E-value: 0.0
  
  
 NCBI BlastP on this gene

EED16046

conserved hypothetical protein
  
Accession: EED16045
  
Location: 755698-757080
  
 NCBI BlastP on this gene

EED16045

C2H2 finger domain protein, putative
  
Accession: EED16044
  
Location: 749795-753334
  
 NCBI BlastP on this gene

EED16044

Query: Architecture Search FASTA input

CP003010 : Thielavia terrestris NRRL 8126 chromosome 2    Total score: 1.0     Cumulative Blast bit score: 1237

Hit cluster cross-links:

Mycgr3G36335 Mycgr3T
  
Location: 0-423

Mycgr3G36335\_Mycgr3T

Mycgr3G84494 Mycgr3T
  
Location: 523-2047

Mycgr3G84494\_Mycgr3T

Mycgr3G90558 Mycgr3T
  
Location: 2147-15296

Mycgr3G90558\_Mycgr3T

Mycgr3G68036 Mycgr3T
  
Location: 15396-16395

Mycgr3G68036\_Mycgr3T

Mycgr3G90561 Mycgr3T
  
Location: 16495-17134

Mycgr3G90561\_Mycgr3T

Mycgr3G35862 Mycgr3T
  
Location: 17234-18662

Mycgr3G35862\_Mycgr3T

Mycgr3G68030 Mycgr3T
  
Location: 18762-19722

Mycgr3G68030\_Mycgr3T

Mycgr3G36449 Mycgr3T
  
Location: 19822-21886

Mycgr3G36449\_Mycgr3T

Mycgr3G35528 Mycgr3T
  
Location: 21986-22844

Mycgr3G35528\_Mycgr3T

Mycgr3G35932 Mycgr3T
  
Location: 22944-24390

Mycgr3G35932\_Mycgr3T

Mycgr3G23761 Mycgr3T
  
Location: 24490-25825

Mycgr3G23761\_Mycgr3T

Mycgr3G35535 Mycgr3T
  
Location: 25925-26429

Mycgr3G35535\_Mycgr3T

Mycgr3G9942 Mycgr3T9
  
Location: 26529-30375

Mycgr3G9942\_Mycgr3T9

hypothetical protein
  
Accession: AEO64844
  
Location: 130631-136188
  
 NCBI BlastP on this gene

THITE\_35966

non-ribosomal peptide synthetase
  
Accession: AEO64845
  
Location: 138796-164185
  
  
**BlastP hit with Mycgr3G90558\_Mycgr3T**
  
Percentage identity: 32 %
  
BlastP bit score: 1237
  
Sequence coverage: 62 %
  
E-value: 0.0
  
  
 NCBI BlastP on this gene

THITE\_112015

hypothetical protein
  
Accession: AEO64846
  
Location: 165873-170651
  
 NCBI BlastP on this gene

THITE\_2111183

Query: Architecture Search FASTA input

DS027045 : Aspergillus clavatus NRRL 1 1099423829791 genomic scaffold    Total score: 1.0     Cumulative Blast bit score: 1234

Hit cluster cross-links:

Mycgr3G36335 Mycgr3T
  
Location: 0-423

Mycgr3G36335\_Mycgr3T

Mycgr3G84494 Mycgr3T
  
Location: 523-2047

Mycgr3G84494\_Mycgr3T

Mycgr3G90558 Mycgr3T
  
Location: 2147-15296

Mycgr3G90558\_Mycgr3T

Mycgr3G68036 Mycgr3T
  
Location: 15396-16395

Mycgr3G68036\_Mycgr3T

Mycgr3G90561 Mycgr3T
  
Location: 16495-17134

Mycgr3G90561\_Mycgr3T

Mycgr3G35862 Mycgr3T
  
Location: 17234-18662

Mycgr3G35862\_Mycgr3T

Mycgr3G68030 Mycgr3T
  
Location: 18762-19722

Mycgr3G68030\_Mycgr3T

Mycgr3G36449 Mycgr3T
  
Location: 19822-21886

Mycgr3G36449\_Mycgr3T

Mycgr3G35528 Mycgr3T
  
Location: 21986-22844

Mycgr3G35528\_Mycgr3T

Mycgr3G35932 Mycgr3T
  
Location: 22944-24390

Mycgr3G35932\_Mycgr3T

Mycgr3G23761 Mycgr3T
  
Location: 24490-25825

Mycgr3G23761\_Mycgr3T

Mycgr3G35535 Mycgr3T
  
Location: 25925-26429

Mycgr3G35535\_Mycgr3T

Mycgr3G9942 Mycgr3T9
  
Location: 26529-30375

Mycgr3G9942\_Mycgr3T9

Ankyrin repeat protein
  
Accession: EAW14631
  
Location: 2660593-2661873
  
 NCBI BlastP on this gene

EAW14631

conserved hypothetical protein
  
Accession: EAW14632
  
Location: 2662519-2665119
  
 NCBI BlastP on this gene

EAW14632

F5/8 type C domain protein
  
Accession: EAW14633
  
Location: 2666483-2668804
  
 NCBI BlastP on this gene

EAW14633

NADPH-dependent methylglyoxal reductase (D-lactaldehyde dehydrogenase, putative
  
Accession: EAW14634
  
Location: 2669395-2670715
  
 NCBI BlastP on this gene

EAW14634

conserved hypothetical protein
  
Accession: EAW14635
  
Location: 2672242-2673321
  
 NCBI BlastP on this gene

EAW14635

nonribosomal peptide synthase, putative
  
Accession: EAW14636
  
Location: 2673911-2684578
  
  
**BlastP hit with Mycgr3G90558\_Mycgr3T**
  
Percentage identity: 30 %
  
BlastP bit score: 1234
  
Sequence coverage: 73 %
  
E-value: 0.0
  
  
 NCBI BlastP on this gene

EAW14636

Query: Architecture Search FASTA input

DS995703 : Microsporum canis CBS 113480 supercont1.3 genomic scaffold    Total score: 1.0     Cumulative Blast bit score: 1230

Hit cluster cross-links:

Mycgr3G36335 Mycgr3T
  
Location: 0-423

Mycgr3G36335\_Mycgr3T

Mycgr3G84494 Mycgr3T
  
Location: 523-2047

Mycgr3G84494\_Mycgr3T

Mycgr3G90558 Mycgr3T
  
Location: 2147-15296

Mycgr3G90558\_Mycgr3T

Mycgr3G68036 Mycgr3T
  
Location: 15396-16395

Mycgr3G68036\_Mycgr3T

Mycgr3G90561 Mycgr3T
  
Location: 16495-17134

Mycgr3G90561\_Mycgr3T

Mycgr3G35862 Mycgr3T
  
Location: 17234-18662

Mycgr3G35862\_Mycgr3T

Mycgr3G68030 Mycgr3T
  
Location: 18762-19722

Mycgr3G68030\_Mycgr3T

Mycgr3G36449 Mycgr3T
  
Location: 19822-21886

Mycgr3G36449\_Mycgr3T

Mycgr3G35528 Mycgr3T
  
Location: 21986-22844

Mycgr3G35528\_Mycgr3T

Mycgr3G35932 Mycgr3T
  
Location: 22944-24390

Mycgr3G35932\_Mycgr3T

Mycgr3G23761 Mycgr3T
  
Location: 24490-25825

Mycgr3G23761\_Mycgr3T

Mycgr3G35535 Mycgr3T
  
Location: 25925-26429

Mycgr3G35535\_Mycgr3T

Mycgr3G9942 Mycgr3T9
  
Location: 26529-30375

Mycgr3G9942\_Mycgr3T9

nonribosomal peptide synthase
  
Accession: EEQ30108
  
Location: 224937-249327
  
  
**BlastP hit with Mycgr3G90558\_Mycgr3T**
  
Percentage identity: 31 %
  
BlastP bit score: 1230
  
Sequence coverage: 60 %
  
E-value: 0.0
  
  
 NCBI BlastP on this gene

EEQ30108

conserved hypothetical protein
  
Accession: EEQ30107
  
Location: 224112-224493
  
 NCBI BlastP on this gene

EEQ30107

GMP synthase
  
Accession: EEQ30106
  
Location: 222165-223871
  
 NCBI BlastP on this gene

EEQ30106

bcp1
  
Accession: EEQ30105
  
Location: 220837-221828
  
 NCBI BlastP on this gene

EEQ30105

chorismate mutase
  
Accession: EEQ30104
  
Location: 219676-220630
  
 NCBI BlastP on this gene

EEQ30104

Query: Architecture Search FASTA input

DS995903 : Penicillium marneffei ATCC 18224 scf\_1105668340984 genomic scaffold    Total score: 1.0     Cumulative Blast bit score: 1229

Hit cluster cross-links:

Mycgr3G36335 Mycgr3T
  
Location: 0-423

Mycgr3G36335\_Mycgr3T

Mycgr3G84494 Mycgr3T
  
Location: 523-2047

Mycgr3G84494\_Mycgr3T

Mycgr3G90558 Mycgr3T
  
Location: 2147-15296

Mycgr3G90558\_Mycgr3T

Mycgr3G68036 Mycgr3T
  
Location: 15396-16395

Mycgr3G68036\_Mycgr3T

Mycgr3G90561 Mycgr3T
  
Location: 16495-17134

Mycgr3G90561\_Mycgr3T

Mycgr3G35862 Mycgr3T
  
Location: 17234-18662

Mycgr3G35862\_Mycgr3T

Mycgr3G68030 Mycgr3T
  
Location: 18762-19722

Mycgr3G68030\_Mycgr3T

Mycgr3G36449 Mycgr3T
  
Location: 19822-21886

Mycgr3G36449\_Mycgr3T

Mycgr3G35528 Mycgr3T
  
Location: 21986-22844

Mycgr3G35528\_Mycgr3T

Mycgr3G35932 Mycgr3T
  
Location: 22944-24390

Mycgr3G35932\_Mycgr3T

Mycgr3G23761 Mycgr3T
  
Location: 24490-25825

Mycgr3G23761\_Mycgr3T

Mycgr3G35535 Mycgr3T
  
Location: 25925-26429

Mycgr3G35535\_Mycgr3T

Mycgr3G9942 Mycgr3T9
  
Location: 26529-30375

Mycgr3G9942\_Mycgr3T9

nonribosomal peptide synthase, putative
  
Accession: EEA22218
  
Location: 2703820-2728032
  
  
**BlastP hit with Mycgr3G90558\_Mycgr3T**
  
Percentage identity: 30 %
  
BlastP bit score: 1229
  
Sequence coverage: 61 %
  
E-value: 0.0
  
  
 NCBI BlastP on this gene

EEA22218

Query: Architecture Search FASTA input

GG704912 : Coccidioides immitis RS genomic scaffold supercont3.2    Total score: 1.0     Cumulative Blast bit score: 1228

Hit cluster cross-links:

Mycgr3G36335 Mycgr3T
  
Location: 0-423

Mycgr3G36335\_Mycgr3T

Mycgr3G84494 Mycgr3T
  
Location: 523-2047

Mycgr3G84494\_Mycgr3T

Mycgr3G90558 Mycgr3T
  
Location: 2147-15296

Mycgr3G90558\_Mycgr3T

Mycgr3G68036 Mycgr3T
  
Location: 15396-16395

Mycgr3G68036\_Mycgr3T

Mycgr3G90561 Mycgr3T
  
Location: 16495-17134

Mycgr3G90561\_Mycgr3T

Mycgr3G35862 Mycgr3T
  
Location: 17234-18662

Mycgr3G35862\_Mycgr3T

Mycgr3G68030 Mycgr3T
  
Location: 18762-19722

Mycgr3G68030\_Mycgr3T

Mycgr3G36449 Mycgr3T
  
Location: 19822-21886

Mycgr3G36449\_Mycgr3T

Mycgr3G35528 Mycgr3T
  
Location: 21986-22844

Mycgr3G35528\_Mycgr3T

Mycgr3G35932 Mycgr3T
  
Location: 22944-24390

Mycgr3G35932\_Mycgr3T

Mycgr3G23761 Mycgr3T
  
Location: 24490-25825

Mycgr3G23761\_Mycgr3T

Mycgr3G35535 Mycgr3T
  
Location: 25925-26429

Mycgr3G35535\_Mycgr3T

Mycgr3G9942 Mycgr3T9
  
Location: 26529-30375

Mycgr3G9942\_Mycgr3T9

ABC multidrug transporter
  
Accession: EAS27487
  
Location: 292393-297767
  
 NCBI BlastP on this gene

EAS27487

hypothetical protein
  
Accession: EJB10845
  
Location: 299197-299787
  
 NCBI BlastP on this gene

EJB10845

hypothetical protein
  
Accession: EAS27483
  
Location: 301509-302702
  
 NCBI BlastP on this gene

EAS27483

amino acid adenylation domain-containing protein
  
Accession: EAS27482
  
Location: 302986-327648
  
  
**BlastP hit with Mycgr3G90558\_Mycgr3T**
  
Percentage identity: 30 %
  
BlastP bit score: 1228
  
Sequence coverage: 61 %
  
E-value: 0.0
  
  
 NCBI BlastP on this gene

EAS27482

hypothetical protein
  
Accession: EAS27481
  
Location: 328316-328738
  
 NCBI BlastP on this gene

EAS27481

hypothetical protein
  
Accession: EAS27480
  
Location: 329270-330270
  
 NCBI BlastP on this gene

EAS27480

chorismate mutase
  
Accession: EAS27479
  
Location: 330399-331379
  
 NCBI BlastP on this gene

EAS27479

hypothetical protein
  
Accession: EAS27477
  
Location: 332794-334785
  
 NCBI BlastP on this gene

EAS27477

Query: Architecture Search FASTA input

ACFW01000025 : Coccidioides posadasii C735 delta SOWgp    Total score: 1.0     Cumulative Blast bit score: 1225

Hit cluster cross-links:

Mycgr3G36335 Mycgr3T
  
Location: 0-423

Mycgr3G36335\_Mycgr3T

Mycgr3G84494 Mycgr3T
  
Location: 523-2047

Mycgr3G84494\_Mycgr3T

Mycgr3G90558 Mycgr3T
  
Location: 2147-15296

Mycgr3G90558\_Mycgr3T

Mycgr3G68036 Mycgr3T
  
Location: 15396-16395

Mycgr3G68036\_Mycgr3T

Mycgr3G90561 Mycgr3T
  
Location: 16495-17134

Mycgr3G90561\_Mycgr3T

Mycgr3G35862 Mycgr3T
  
Location: 17234-18662

Mycgr3G35862\_Mycgr3T

Mycgr3G68030 Mycgr3T
  
Location: 18762-19722

Mycgr3G68030\_Mycgr3T

Mycgr3G36449 Mycgr3T
  
Location: 19822-21886

Mycgr3G36449\_Mycgr3T

Mycgr3G35528 Mycgr3T
  
Location: 21986-22844

Mycgr3G35528\_Mycgr3T

Mycgr3G35932 Mycgr3T
  
Location: 22944-24390

Mycgr3G35932\_Mycgr3T

Mycgr3G23761 Mycgr3T
  
Location: 24490-25825

Mycgr3G23761\_Mycgr3T

Mycgr3G35535 Mycgr3T
  
Location: 25925-26429

Mycgr3G35535\_Mycgr3T

Mycgr3G9942 Mycgr3T9
  
Location: 26529-30375

Mycgr3G9942\_Mycgr3T9

multidrug resistance protein MDR, putative
  
Accession: EER26974
  
Location: 199688-205066
  
 NCBI BlastP on this gene

EER26974

nonribosomal peptide synthetase, putative
  
Accession: EER26975
  
Location: 210338-234994
  
  
**BlastP hit with Mycgr3G90558\_Mycgr3T**
  
Percentage identity: 30 %
  
BlastP bit score: 1225
  
Sequence coverage: 61 %
  
E-value: 0.0
  
  
 NCBI BlastP on this gene

EER26975

hypothetical protein
  
Accession: EER26976
  
Location: 235707-236128
  
 NCBI BlastP on this gene

EER26976

hypothetical protein
  
Accession: EER26977
  
Location: 236661-237661
  
 NCBI BlastP on this gene

EER26977

chorismate mutase, putative
  
Accession: EER26978
  
Location: 237790-238769
  
 NCBI BlastP on this gene

EER26978

hypothetical protein
  
Accession: EER26979
  
Location: 240179-242170
  
 NCBI BlastP on this gene

EER26979

Query: Architecture Search FASTA input

DS995708 : Microsporum canis CBS 113480 supercont1.8 genomic scaffold    Total score: 1.0     Cumulative Blast bit score: 1220

Hit cluster cross-links:

Mycgr3G36335 Mycgr3T
  
Location: 0-423

Mycgr3G36335\_Mycgr3T

Mycgr3G84494 Mycgr3T
  
Location: 523-2047

Mycgr3G84494\_Mycgr3T

Mycgr3G90558 Mycgr3T
  
Location: 2147-15296

Mycgr3G90558\_Mycgr3T

Mycgr3G68036 Mycgr3T
  
Location: 15396-16395

Mycgr3G68036\_Mycgr3T

Mycgr3G90561 Mycgr3T
  
Location: 16495-17134

Mycgr3G90561\_Mycgr3T

Mycgr3G35862 Mycgr3T
  
Location: 17234-18662

Mycgr3G35862\_Mycgr3T

Mycgr3G68030 Mycgr3T
  
Location: 18762-19722

Mycgr3G68030\_Mycgr3T

Mycgr3G36449 Mycgr3T
  
Location: 19822-21886

Mycgr3G36449\_Mycgr3T

Mycgr3G35528 Mycgr3T
  
Location: 21986-22844

Mycgr3G35528\_Mycgr3T

Mycgr3G35932 Mycgr3T
  
Location: 22944-24390

Mycgr3G35932\_Mycgr3T

Mycgr3G23761 Mycgr3T
  
Location: 24490-25825

Mycgr3G23761\_Mycgr3T

Mycgr3G35535 Mycgr3T
  
Location: 25925-26429

Mycgr3G35535\_Mycgr3T

Mycgr3G9942 Mycgr3T9
  
Location: 26529-30375

Mycgr3G9942\_Mycgr3T9

fatty acid synthase beta subunit dehydratase
  
Accession: EEQ35620
  
Location: 1321574-1327901
  
 NCBI BlastP on this gene

EEQ35620

benzoate 4-monooxygenase cytochrome P450
  
Accession: EEQ35621
  
Location: 1329009-1330899
  
 NCBI BlastP on this gene

EEQ35621

peptide synthetase
  
Accession: EEQ35622
  
Location: 1332241-1351067
  
  
**BlastP hit with Mycgr3G90558\_Mycgr3T**
  
Percentage identity: 28 %
  
BlastP bit score: 1220
  
Sequence coverage: 76 %
  
E-value: 0.0
  
  
 NCBI BlastP on this gene

EEQ35622

Query: Architecture Search FASTA input

BABT02000122 : Mixia osmundae IAM 14324    Total score: 1.0     Cumulative Blast bit score: 1218

Hit cluster cross-links:

Mycgr3G36335 Mycgr3T
  
Location: 0-423

Mycgr3G36335\_Mycgr3T

Mycgr3G84494 Mycgr3T
  
Location: 523-2047

Mycgr3G84494\_Mycgr3T

Mycgr3G90558 Mycgr3T
  
Location: 2147-15296

Mycgr3G90558\_Mycgr3T

Mycgr3G68036 Mycgr3T
  
Location: 15396-16395

Mycgr3G68036\_Mycgr3T

Mycgr3G90561 Mycgr3T
  
Location: 16495-17134

Mycgr3G90561\_Mycgr3T

Mycgr3G35862 Mycgr3T
  
Location: 17234-18662

Mycgr3G35862\_Mycgr3T

Mycgr3G68030 Mycgr3T
  
Location: 18762-19722

Mycgr3G68030\_Mycgr3T

Mycgr3G36449 Mycgr3T
  
Location: 19822-21886

Mycgr3G36449\_Mycgr3T

Mycgr3G35528 Mycgr3T
  
Location: 21986-22844

Mycgr3G35528\_Mycgr3T

Mycgr3G35932 Mycgr3T
  
Location: 22944-24390

Mycgr3G35932\_Mycgr3T

Mycgr3G23761 Mycgr3T
  
Location: 24490-25825

Mycgr3G23761\_Mycgr3T

Mycgr3G35535 Mycgr3T
  
Location: 25925-26429

Mycgr3G35535\_Mycgr3T

Mycgr3G9942 Mycgr3T9
  
Location: 26529-30375

Mycgr3G9942\_Mycgr3T9

hypothetical protein
  
Accession: GAA97477
  
Location: 48246-51701
  
  
**BlastP hit with Mycgr3G9942\_Mycgr3T9**
  
Percentage identity: 34 %
  
BlastP bit score: 566
  
Sequence coverage: 81 %
  
E-value: 2e-177
  
  
 NCBI BlastP on this gene

GAA97477

hypothetical protein
  
Accession: GAA97476
  
Location: 48246-53394
  
  
**BlastP hit with Mycgr3G9942\_Mycgr3T9**
  
Percentage identity: 32 %
  
BlastP bit score: 652
  
Sequence coverage: 108 %
  
E-value: 0.0
  
  
 NCBI BlastP on this gene

GAA97476

hypothetical protein
  
Accession: GAA97475
  
Location: 46993-48203
  
 NCBI BlastP on this gene

GAA97475

hypothetical protein
  
Accession: GAA97474
  
Location: 42096-46762
  
 NCBI BlastP on this gene

GAA97474

hypothetical protein
  
Accession: GAA97473
  
Location: 39404-42042
  
 NCBI BlastP on this gene

GAA97473

hypothetical protein
  
Accession: GAA97472
  
Location: 38364-39168
  
 NCBI BlastP on this gene

GAA97472

hypothetical protein
  
Accession: GAA97471
  
Location: 35654-38246
  
 NCBI BlastP on this gene

GAA97471

hypothetical protein
  
Accession: GAA97470
  
Location: 29227-34241
  
 NCBI BlastP on this gene

GAA97470

Query: Architecture Search FASTA input

GG700648 : Trichophyton rubrum CBS 118892 genomic scaffold supercont2.1    Total score: 1.0     Cumulative Blast bit score: 1209

Hit cluster cross-links:

Mycgr3G36335 Mycgr3T
  
Location: 0-423

Mycgr3G36335\_Mycgr3T

Mycgr3G84494 Mycgr3T
  
Location: 523-2047

Mycgr3G84494\_Mycgr3T

Mycgr3G90558 Mycgr3T
  
Location: 2147-15296

Mycgr3G90558\_Mycgr3T

Mycgr3G68036 Mycgr3T
  
Location: 15396-16395

Mycgr3G68036\_Mycgr3T

Mycgr3G90561 Mycgr3T
  
Location: 16495-17134

Mycgr3G90561\_Mycgr3T

Mycgr3G35862 Mycgr3T
  
Location: 17234-18662

Mycgr3G35862\_Mycgr3T

Mycgr3G68030 Mycgr3T
  
Location: 18762-19722

Mycgr3G68030\_Mycgr3T

Mycgr3G36449 Mycgr3T
  
Location: 19822-21886

Mycgr3G36449\_Mycgr3T

Mycgr3G35528 Mycgr3T
  
Location: 21986-22844

Mycgr3G35528\_Mycgr3T

Mycgr3G35932 Mycgr3T
  
Location: 22944-24390

Mycgr3G35932\_Mycgr3T

Mycgr3G23761 Mycgr3T
  
Location: 24490-25825

Mycgr3G23761\_Mycgr3T

Mycgr3G35535 Mycgr3T
  
Location: 25925-26429

Mycgr3G35535\_Mycgr3T

Mycgr3G9942 Mycgr3T9
  
Location: 26529-30375

Mycgr3G9942\_Mycgr3T9

nonribosomal peptide synthase
  
Accession: EGD85168
  
Location: 3814046-3838539
  
  
**BlastP hit with Mycgr3G90558\_Mycgr3T**
  
Percentage identity: 31 %
  
BlastP bit score: 1209
  
Sequence coverage: 61 %
  
E-value: 0.0
  
  
 NCBI BlastP on this gene

EGD85168

Query: Architecture Search FASTA input

DS989822 : Arthroderma gypseum CBS 118893 supercont1.1 genomic scaffold    Total score: 1.0     Cumulative Blast bit score: 1200

Hit cluster cross-links:

Mycgr3G36335 Mycgr3T
  
Location: 0-423

Mycgr3G36335\_Mycgr3T

Mycgr3G84494 Mycgr3T
  
Location: 523-2047

Mycgr3G84494\_Mycgr3T

Mycgr3G90558 Mycgr3T
  
Location: 2147-15296

Mycgr3G90558\_Mycgr3T

Mycgr3G68036 Mycgr3T
  
Location: 15396-16395

Mycgr3G68036\_Mycgr3T

Mycgr3G90561 Mycgr3T
  
Location: 16495-17134

Mycgr3G90561\_Mycgr3T

Mycgr3G35862 Mycgr3T
  
Location: 17234-18662

Mycgr3G35862\_Mycgr3T

Mycgr3G68030 Mycgr3T
  
Location: 18762-19722

Mycgr3G68030\_Mycgr3T

Mycgr3G36449 Mycgr3T
  
Location: 19822-21886

Mycgr3G36449\_Mycgr3T

Mycgr3G35528 Mycgr3T
  
Location: 21986-22844

Mycgr3G35528\_Mycgr3T

Mycgr3G35932 Mycgr3T
  
Location: 22944-24390

Mycgr3G35932\_Mycgr3T

Mycgr3G23761 Mycgr3T
  
Location: 24490-25825

Mycgr3G23761\_Mycgr3T

Mycgr3G35535 Mycgr3T
  
Location: 25925-26429

Mycgr3G35535\_Mycgr3T

Mycgr3G9942 Mycgr3T9
  
Location: 26529-30375

Mycgr3G9942\_Mycgr3T9

hypothetical protein
  
Accession: EFQ97051
  
Location: 272653-273331
  
 NCBI BlastP on this gene

EFQ97051

hypothetical protein
  
Accession: EFQ97050
  
Location: 244064-268485
  
  
**BlastP hit with Mycgr3G90558\_Mycgr3T**
  
Percentage identity: 30 %
  
BlastP bit score: 1200
  
Sequence coverage: 60 %
  
E-value: 0.0
  
  
 NCBI BlastP on this gene

EFQ97050

hypothetical protein
  
Accession: EFQ97049
  
Location: 242865-243245
  
 NCBI BlastP on this gene

EFQ97049

GMP synthase
  
Accession: EFQ97048
  
Location: 240940-242635
  
 NCBI BlastP on this gene

EFQ97048

bcp1
  
Accession: EFQ97047
  
Location: 239570-240560
  
 NCBI BlastP on this gene

EFQ97047

chorismate mutase
  
Accession: EFQ97046
  
Location: 238404-239327
  
 NCBI BlastP on this gene

EFQ97046

Query: Architecture Search FASTA input

EQ963479 : Aspergillus flavus NRRL3357 scf\_1106286418500 genomic scaffold    Total score: 1.0     Cumulative Blast bit score: 1172

Hit cluster cross-links:

Mycgr3G36335 Mycgr3T
  
Location: 0-423

Mycgr3G36335\_Mycgr3T

Mycgr3G84494 Mycgr3T
  
Location: 523-2047

Mycgr3G84494\_Mycgr3T

Mycgr3G90558 Mycgr3T
  
Location: 2147-15296

Mycgr3G90558\_Mycgr3T

Mycgr3G68036 Mycgr3T
  
Location: 15396-16395

Mycgr3G68036\_Mycgr3T

Mycgr3G90561 Mycgr3T
  
Location: 16495-17134

Mycgr3G90561\_Mycgr3T

Mycgr3G35862 Mycgr3T
  
Location: 17234-18662

Mycgr3G35862\_Mycgr3T

Mycgr3G68030 Mycgr3T
  
Location: 18762-19722

Mycgr3G68030\_Mycgr3T

Mycgr3G36449 Mycgr3T
  
Location: 19822-21886

Mycgr3G36449\_Mycgr3T

Mycgr3G35528 Mycgr3T
  
Location: 21986-22844

Mycgr3G35528\_Mycgr3T

Mycgr3G35932 Mycgr3T
  
Location: 22944-24390

Mycgr3G35932\_Mycgr3T

Mycgr3G23761 Mycgr3T
  
Location: 24490-25825

Mycgr3G23761\_Mycgr3T

Mycgr3G35535 Mycgr3T
  
Location: 25925-26429

Mycgr3G35535\_Mycgr3T

Mycgr3G9942 Mycgr3T9
  
Location: 26529-30375

Mycgr3G9942\_Mycgr3T9

ABC multidrug transporter, putative
  
Accession: EED50110
  
Location: 1353107-1358412
  
 NCBI BlastP on this gene

EED50110

nonribosomal peptide synthase Pes1
  
Accession: EED50111
  
Location: 1362826-1378774
  
  
**BlastP hit with Mycgr3G90558\_Mycgr3T**
  
Percentage identity: 30 %
  
BlastP bit score: 1172
  
Sequence coverage: 60 %
  
E-value: 0.0
  
  
 NCBI BlastP on this gene

EED50111

Query: Architecture Search FASTA input

DS995905 : Penicillium marneffei ATCC 18224 scf\_1105668340970 genomic scaffold    Total score: 1.0     Cumulative Blast bit score: 1167

Hit cluster cross-links:

Mycgr3G36335 Mycgr3T
  
Location: 0-423

Mycgr3G36335\_Mycgr3T

Mycgr3G84494 Mycgr3T
  
Location: 523-2047

Mycgr3G84494\_Mycgr3T

Mycgr3G90558 Mycgr3T
  
Location: 2147-15296

Mycgr3G90558\_Mycgr3T

Mycgr3G68036 Mycgr3T
  
Location: 15396-16395

Mycgr3G68036\_Mycgr3T

Mycgr3G90561 Mycgr3T
  
Location: 16495-17134

Mycgr3G90561\_Mycgr3T

Mycgr3G35862 Mycgr3T
  
Location: 17234-18662

Mycgr3G35862\_Mycgr3T

Mycgr3G68030 Mycgr3T
  
Location: 18762-19722

Mycgr3G68030\_Mycgr3T

Mycgr3G36449 Mycgr3T
  
Location: 19822-21886

Mycgr3G36449\_Mycgr3T

Mycgr3G35528 Mycgr3T
  
Location: 21986-22844

Mycgr3G35528\_Mycgr3T

Mycgr3G35932 Mycgr3T
  
Location: 22944-24390

Mycgr3G35932\_Mycgr3T

Mycgr3G23761 Mycgr3T
  
Location: 24490-25825

Mycgr3G23761\_Mycgr3T

Mycgr3G35535 Mycgr3T
  
Location: 25925-26429

Mycgr3G35535\_Mycgr3T

Mycgr3G9942 Mycgr3T9
  
Location: 26529-30375

Mycgr3G9942\_Mycgr3T9

conserved hypothetical protein
  
Accession: EEA19298
  
Location: 96181-97575
  
 NCBI BlastP on this gene

EEA19298

conserved hypothetical protein
  
Accession: EEA19299
  
Location: 98719-128920
  
  
**BlastP hit with Mycgr3G90558\_Mycgr3T**
  
Percentage identity: 31 %
  
BlastP bit score: 1167
  
Sequence coverage: 61 %
  
E-value: 0.0
  
  
 NCBI BlastP on this gene

EEA19299

extracellular guanyl-specific ribonuclease RntA
  
Accession: EEA19300
  
Location: 129184-129687
  
 NCBI BlastP on this gene

EEA19300

Query: Architecture Search FASTA input

KB908866 : Setosphaeria turcica Et28A unplaced genomic scaffold SETTUscaffold\_8    Total score: 1.0     Cumulative Blast bit score: 1162

Hit cluster cross-links:

Mycgr3G36335 Mycgr3T
  
Location: 0-423

Mycgr3G36335\_Mycgr3T

Mycgr3G84494 Mycgr3T
  
Location: 523-2047

Mycgr3G84494\_Mycgr3T

Mycgr3G90558 Mycgr3T
  
Location: 2147-15296

Mycgr3G90558\_Mycgr3T

Mycgr3G68036 Mycgr3T
  
Location: 15396-16395

Mycgr3G68036\_Mycgr3T

Mycgr3G90561 Mycgr3T
  
Location: 16495-17134

Mycgr3G90561\_Mycgr3T

Mycgr3G35862 Mycgr3T
  
Location: 17234-18662

Mycgr3G35862\_Mycgr3T

Mycgr3G68030 Mycgr3T
  
Location: 18762-19722

Mycgr3G68030\_Mycgr3T

Mycgr3G36449 Mycgr3T
  
Location: 19822-21886

Mycgr3G36449\_Mycgr3T

Mycgr3G35528 Mycgr3T
  
Location: 21986-22844

Mycgr3G35528\_Mycgr3T

Mycgr3G35932 Mycgr3T
  
Location: 22944-24390

Mycgr3G35932\_Mycgr3T

Mycgr3G23761 Mycgr3T
  
Location: 24490-25825

Mycgr3G23761\_Mycgr3T

Mycgr3G35535 Mycgr3T
  
Location: 25925-26429

Mycgr3G35535\_Mycgr3T

Mycgr3G9942 Mycgr3T9
  
Location: 26529-30375

Mycgr3G9942\_Mycgr3T9

hypothetical protein
  
Accession: EOA81675
  
Location: 1642148-1646752
  
 NCBI BlastP on this gene

EOA81675

hypothetical protein
  
Accession: EOA81676
  
Location: 1647885-1649771
  
 NCBI BlastP on this gene

EOA81676

hypothetical protein
  
Accession: EOA81677
  
Location: 1650732-1669532
  
  
**BlastP hit with Mycgr3G90558\_Mycgr3T**
  
Percentage identity: 29 %
  
BlastP bit score: 1162
  
Sequence coverage: 77 %
  
E-value: 0.0
  
  
 NCBI BlastP on this gene

EOA81677

Query: Architecture Search FASTA input

ABSU01000002 : Arthroderma benhamiae CBS 112371    Total score: 1.0     Cumulative Blast bit score: 1155

Hit cluster cross-links:

Mycgr3G36335 Mycgr3T
  
Location: 0-423

Mycgr3G36335\_Mycgr3T

Mycgr3G84494 Mycgr3T
  
Location: 523-2047

Mycgr3G84494\_Mycgr3T

Mycgr3G90558 Mycgr3T
  
Location: 2147-15296

Mycgr3G90558\_Mycgr3T

Mycgr3G68036 Mycgr3T
  
Location: 15396-16395

Mycgr3G68036\_Mycgr3T

Mycgr3G90561 Mycgr3T
  
Location: 16495-17134

Mycgr3G90561\_Mycgr3T

Mycgr3G35862 Mycgr3T
  
Location: 17234-18662

Mycgr3G35862\_Mycgr3T

Mycgr3G68030 Mycgr3T
  
Location: 18762-19722

Mycgr3G68030\_Mycgr3T

Mycgr3G36449 Mycgr3T
  
Location: 19822-21886

Mycgr3G36449\_Mycgr3T

Mycgr3G35528 Mycgr3T
  
Location: 21986-22844

Mycgr3G35528\_Mycgr3T

Mycgr3G35932 Mycgr3T
  
Location: 22944-24390

Mycgr3G35932\_Mycgr3T

Mycgr3G23761 Mycgr3T
  
Location: 24490-25825

Mycgr3G23761\_Mycgr3T

Mycgr3G35535 Mycgr3T
  
Location: 25925-26429

Mycgr3G35535\_Mycgr3T

Mycgr3G9942 Mycgr3T9
  
Location: 26529-30375

Mycgr3G9942\_Mycgr3T9

hypothetical protein
  
Accession: EFE36189
  
Location: 1179294-1183108
  
 NCBI BlastP on this gene

EFE36189

conserved hypothetical protein
  
Accession: EFE36190
  
Location: 1183838-1184810
  
 NCBI BlastP on this gene

EFE36190

conserved hypothetical protein
  
Accession: EFE36191
  
Location: 1186773-1187742
  
 NCBI BlastP on this gene

EFE36191

universal stress protein family domain protein
  
Accession: EFE36192
  
Location: 1188281-1191606
  
 NCBI BlastP on this gene

EFE36192

nonribosomal peptide synthase, putative
  
Accession: EFE36193
  
Location: 1193042-1204848
  
  
**BlastP hit with Mycgr3G90558\_Mycgr3T**
  
Percentage identity: 33 %
  
BlastP bit score: 1155
  
Sequence coverage: 50 %
  
E-value: 0.0
  
  
 NCBI BlastP on this gene

EFE36193

Query: Architecture Search FASTA input

CH476616 : Uncinocarpus reesii 1704 scaffold\_2 genomic scaffold    Total score: 1.0     Cumulative Blast bit score: 1150

Hit cluster cross-links:

Mycgr3G36335 Mycgr3T
  
Location: 0-423

Mycgr3G36335\_Mycgr3T

Mycgr3G84494 Mycgr3T
  
Location: 523-2047

Mycgr3G84494\_Mycgr3T

Mycgr3G90558 Mycgr3T
  
Location: 2147-15296

Mycgr3G90558\_Mycgr3T

Mycgr3G68036 Mycgr3T
  
Location: 15396-16395

Mycgr3G68036\_Mycgr3T

Mycgr3G90561 Mycgr3T
  
Location: 16495-17134

Mycgr3G90561\_Mycgr3T

Mycgr3G35862 Mycgr3T
  
Location: 17234-18662

Mycgr3G35862\_Mycgr3T

Mycgr3G68030 Mycgr3T
  
Location: 18762-19722

Mycgr3G68030\_Mycgr3T

Mycgr3G36449 Mycgr3T
  
Location: 19822-21886

Mycgr3G36449\_Mycgr3T

Mycgr3G35528 Mycgr3T
  
Location: 21986-22844

Mycgr3G35528\_Mycgr3T

Mycgr3G35932 Mycgr3T
  
Location: 22944-24390

Mycgr3G35932\_Mycgr3T

Mycgr3G23761 Mycgr3T
  
Location: 24490-25825

Mycgr3G23761\_Mycgr3T

Mycgr3G35535 Mycgr3T
  
Location: 25925-26429

Mycgr3G35535\_Mycgr3T

Mycgr3G9942 Mycgr3T9
  
Location: 26529-30375

Mycgr3G9942\_Mycgr3T9

predicted protein
  
Accession: EEP79831
  
Location: 5054874-5072607
  
  
**BlastP hit with Mycgr3G90558\_Mycgr3T**
  
Percentage identity: 30 %
  
BlastP bit score: 1150
  
Sequence coverage: 62 %
  
E-value: 0.0
  
  
 NCBI BlastP on this gene

EEP79831

predicted protein
  
Accession: EEP79830
  
Location: 5047933-5054073
  
 NCBI BlastP on this gene

EEP79830

predicted protein
  
Accession: EEP79829
  
Location: 5047129-5047550
  
 NCBI BlastP on this gene

EEP79829

conserved hypothetical protein
  
Accession: EEP79828
  
Location: 5046040-5046509
  
 NCBI BlastP on this gene

EEP79828

Query: Architecture Search FASTA input

CH408031 : Chaetomium globosum CBS 148.51 scaffold\_3 genomic scaffold    Total score: 1.0     Cumulative Blast bit score: 1149

Hit cluster cross-links:

Mycgr3G36335 Mycgr3T
  
Location: 0-423

Mycgr3G36335\_Mycgr3T

Mycgr3G84494 Mycgr3T
  
Location: 523-2047

Mycgr3G84494\_Mycgr3T

Mycgr3G90558 Mycgr3T
  
Location: 2147-15296

Mycgr3G90558\_Mycgr3T

Mycgr3G68036 Mycgr3T
  
Location: 15396-16395

Mycgr3G68036\_Mycgr3T

Mycgr3G90561 Mycgr3T
  
Location: 16495-17134

Mycgr3G90561\_Mycgr3T

Mycgr3G35862 Mycgr3T
  
Location: 17234-18662

Mycgr3G35862\_Mycgr3T

Mycgr3G68030 Mycgr3T
  
Location: 18762-19722

Mycgr3G68030\_Mycgr3T

Mycgr3G36449 Mycgr3T
  
Location: 19822-21886

Mycgr3G36449\_Mycgr3T

Mycgr3G35528 Mycgr3T
  
Location: 21986-22844

Mycgr3G35528\_Mycgr3T

Mycgr3G35932 Mycgr3T
  
Location: 22944-24390

Mycgr3G35932\_Mycgr3T

Mycgr3G23761 Mycgr3T
  
Location: 24490-25825

Mycgr3G23761\_Mycgr3T

Mycgr3G35535 Mycgr3T
  
Location: 25925-26429

Mycgr3G35535\_Mycgr3T

Mycgr3G9942 Mycgr3T9
  
Location: 26529-30375

Mycgr3G9942\_Mycgr3T9

hypothetical protein
  
Accession: EAQ90170
  
Location: 4563155-4588278
  
  
**BlastP hit with Mycgr3G90558\_Mycgr3T**
  
Percentage identity: 30 %
  
BlastP bit score: 1149
  
Sequence coverage: 60 %
  
E-value: 0.0
  
  
 NCBI BlastP on this gene

EAQ90170

hypothetical protein
  
Accession: EAQ90169
  
Location: 4561980-4562956
  
 NCBI BlastP on this gene

EAQ90169

hypothetical protein
  
Accession: EAQ90168
  
Location: 4559739-4561425
  
 NCBI BlastP on this gene

EAQ90168

hypothetical protein
  
Accession: EAQ90167
  
Location: 4557012-4558886
  
 NCBI BlastP on this gene

EAQ90167

Query: Architecture Search FASTA input

KB456260 : Mycosphaerella populorum SO2202 unplaced genomic scaffold SEPMUscaffold\_1    Total score: 1.0     Cumulative Blast bit score: 1144

Hit cluster cross-links:

Mycgr3G36335 Mycgr3T
  
Location: 0-423

Mycgr3G36335\_Mycgr3T

Mycgr3G84494 Mycgr3T
  
Location: 523-2047

Mycgr3G84494\_Mycgr3T

Mycgr3G90558 Mycgr3T
  
Location: 2147-15296

Mycgr3G90558\_Mycgr3T

Mycgr3G68036 Mycgr3T
  
Location: 15396-16395

Mycgr3G68036\_Mycgr3T

Mycgr3G90561 Mycgr3T
  
Location: 16495-17134

Mycgr3G90561\_Mycgr3T

Mycgr3G35862 Mycgr3T
  
Location: 17234-18662

Mycgr3G35862\_Mycgr3T

Mycgr3G68030 Mycgr3T
  
Location: 18762-19722

Mycgr3G68030\_Mycgr3T

Mycgr3G36449 Mycgr3T
  
Location: 19822-21886

Mycgr3G36449\_Mycgr3T

Mycgr3G35528 Mycgr3T
  
Location: 21986-22844

Mycgr3G35528\_Mycgr3T

Mycgr3G35932 Mycgr3T
  
Location: 22944-24390

Mycgr3G35932\_Mycgr3T

Mycgr3G23761 Mycgr3T
  
Location: 24490-25825

Mycgr3G23761\_Mycgr3T

Mycgr3G35535 Mycgr3T
  
Location: 25925-26429

Mycgr3G35535\_Mycgr3T

Mycgr3G9942 Mycgr3T9
  
Location: 26529-30375

Mycgr3G9942\_Mycgr3T9

O-methyltransferase
  
Accession: EMF16953
  
Location: 2239892-2241831
  
 NCBI BlastP on this gene

EMF16953

cytochrome P450
  
Accession: EMF16952
  
Location: 2238905-2239818
  
 NCBI BlastP on this gene

EMF16952

hypothetical protein
  
Accession: EMF16951
  
Location: 2221896-2236653
  
  
**BlastP hit with Mycgr3G90558\_Mycgr3T**
  
Percentage identity: 26 %
  
BlastP bit score: 1144
  
Sequence coverage: 97 %
  
E-value: 0.0
  
  
 NCBI BlastP on this gene

EMF16951

ankyrin
  
Accession: EMF16950
  
Location: 2220779-2221752
  
 NCBI BlastP on this gene

EMF16950

hypothetical protein
  
Accession: EMF16949
  
Location: 2216714-2219503
  
 NCBI BlastP on this gene

EMF16949

kinase-like protein
  
Accession: EMF16948
  
Location: 2213720-2214571
  
 NCBI BlastP on this gene

EMF16948

MFS general substrate transporter
  
Accession: EMF16947
  
Location: 2203998-2212806
  
 NCBI BlastP on this gene

EMF16947

Query: Architecture Search FASTA input

JH687760 : Auricularia delicata TFB-10046 SS5 unplaced genomic scaffold AURDEscaffold\_28    Total score: 1.0     Cumulative Blast bit score: 1142

Hit cluster cross-links:

Mycgr3G36335 Mycgr3T
  
Location: 0-423

Mycgr3G36335\_Mycgr3T

Mycgr3G84494 Mycgr3T
  
Location: 523-2047

Mycgr3G84494\_Mycgr3T

Mycgr3G90558 Mycgr3T
  
Location: 2147-15296

Mycgr3G90558\_Mycgr3T

Mycgr3G68036 Mycgr3T
  
Location: 15396-16395

Mycgr3G68036\_Mycgr3T

Mycgr3G90561 Mycgr3T
  
Location: 16495-17134

Mycgr3G90561\_Mycgr3T

Mycgr3G35862 Mycgr3T
  
Location: 17234-18662

Mycgr3G35862\_Mycgr3T

Mycgr3G68030 Mycgr3T
  
Location: 18762-19722

Mycgr3G68030\_Mycgr3T

Mycgr3G36449 Mycgr3T
  
Location: 19822-21886

Mycgr3G36449\_Mycgr3T

Mycgr3G35528 Mycgr3T
  
Location: 21986-22844

Mycgr3G35528\_Mycgr3T

Mycgr3G35932 Mycgr3T
  
Location: 22944-24390

Mycgr3G35932\_Mycgr3T

Mycgr3G23761 Mycgr3T
  
Location: 24490-25825

Mycgr3G23761\_Mycgr3T

Mycgr3G35535 Mycgr3T
  
Location: 25925-26429

Mycgr3G35535\_Mycgr3T

Mycgr3G9942 Mycgr3T9
  
Location: 26529-30375

Mycgr3G9942\_Mycgr3T9

hypothetical protein
  
Accession: EJD46365
  
Location: 372574-373830
  
 NCBI BlastP on this gene

EJD46365

cytochrome P450
  
Accession: EJD46366
  
Location: 374185-375982
  
 NCBI BlastP on this gene

EJD46366

P-loop containing nucleoside triphosphate hydrolase protein
  
Accession: EJD46367
  
Location: 378047-384179
  
  
**BlastP hit with Mycgr3G9942\_Mycgr3T9**
  
Percentage identity: 30 %
  
BlastP bit score: 583
  
Sequence coverage: 109 %
  
E-value: 1e-178
  
  
 NCBI BlastP on this gene

EJD46367

hypothetical protein
  
Accession: EJD46368
  
Location: 384426-386450
  
 NCBI BlastP on this gene

EJD46368

hypothetical protein
  
Accession: EJD46369
  
Location: 386869-387313
  
 NCBI BlastP on this gene

EJD46369

ribonuclease H-like protein
  
Accession: EJD46370
  
Location: 388341-389036
  
 NCBI BlastP on this gene

EJD46370

P-loop containing nucleoside triphosphate hydrolase protein
  
Accession: EJD46371
  
Location: 397492-402252
  
  
**BlastP hit with Mycgr3G9942\_Mycgr3T9**
  
Percentage identity: 31 %
  
BlastP bit score: 559
  
Sequence coverage: 97 %
  
E-value: 3e-173
  
  
 NCBI BlastP on this gene

EJD46371

phospholipid-translocating P-type ATPase
  
Accession: EJD46372
  
Location: 403709-408500
  
 NCBI BlastP on this gene

EJD46372

Query: Architecture Search FASTA input

GG698487 : Trichophyton tonsurans CBS 112818 genomic scaffold supercont1.11    Total score: 1.0     Cumulative Blast bit score: 1140

Hit cluster cross-links:

Mycgr3G36335 Mycgr3T
  
Location: 0-423

Mycgr3G36335\_Mycgr3T

Mycgr3G84494 Mycgr3T
  
Location: 523-2047

Mycgr3G84494\_Mycgr3T

Mycgr3G90558 Mycgr3T
  
Location: 2147-15296

Mycgr3G90558\_Mycgr3T

Mycgr3G68036 Mycgr3T
  
Location: 15396-16395

Mycgr3G68036\_Mycgr3T

Mycgr3G90561 Mycgr3T
  
Location: 16495-17134

Mycgr3G90561\_Mycgr3T

Mycgr3G35862 Mycgr3T
  
Location: 17234-18662

Mycgr3G35862\_Mycgr3T

Mycgr3G68030 Mycgr3T
  
Location: 18762-19722

Mycgr3G68030\_Mycgr3T

Mycgr3G36449 Mycgr3T
  
Location: 19822-21886

Mycgr3G36449\_Mycgr3T

Mycgr3G35528 Mycgr3T
  
Location: 21986-22844

Mycgr3G35528\_Mycgr3T

Mycgr3G35932 Mycgr3T
  
Location: 22944-24390

Mycgr3G35932\_Mycgr3T

Mycgr3G23761 Mycgr3T
  
Location: 24490-25825

Mycgr3G23761\_Mycgr3T

Mycgr3G35535 Mycgr3T
  
Location: 25925-26429

Mycgr3G35535\_Mycgr3T

Mycgr3G9942 Mycgr3T9
  
Location: 26529-30375

Mycgr3G9942\_Mycgr3T9

DNA repair and recombination protein RAD26
  
Accession: EGD95238
  
Location: 70982-74796
  
 NCBI BlastP on this gene

EGD95238

hypothetical protein
  
Accession: EGD95237
  
Location: 69251-70226
  
 NCBI BlastP on this gene

EGD95237

hypothetical protein
  
Accession: EGD95236
  
Location: 66330-67518
  
 NCBI BlastP on this gene

EGD95236

hypothetical protein
  
Accession: EGD95235
  
Location: 63526-65605
  
 NCBI BlastP on this gene

EGD95235

nonribosomal peptide synthase
  
Accession: EGD95234
  
Location: 49179-60831
  
  
**BlastP hit with Mycgr3G90558\_Mycgr3T**
  
Percentage identity: 33 %
  
BlastP bit score: 1140
  
Sequence coverage: 49 %
  
E-value: 0.0
  
  
 NCBI BlastP on this gene

EGD95234

hypothetical protein
  
Accession: EGD95233
  
Location: 47996-48892
  
 NCBI BlastP on this gene

EGD95233

cmgc/cdk/pitslre protein kinase
  
Accession: EGD95232
  
Location: 44346-45950
  
 NCBI BlastP on this gene

EGD95232

superoxide dismutase copper chaperone Lys7
  
Accession: EGD95231
  
Location: 43123-44091
  
 NCBI BlastP on this gene

EGD95231

NADPH oxidase regulator NoxR
  
Accession: EGD95230
  
Location: 40584-42473
  
 NCBI BlastP on this gene

EGD95230

SNARE protein
  
Accession: EGD95229
  
Location: 38765-40116
  
 NCBI BlastP on this gene

EGD95229

hypothetical protein
  
Accession: EGD95228
  
Location: 37777-38475
  
 NCBI BlastP on this gene

EGD95228

Query: Architecture Search FASTA input

HF679029 : Fusarium fujikuroi IMI 58289 draft genome, chromosome FFUJ\_chr07.    Total score: 1.0     Cumulative Blast bit score: 1137

Hit cluster cross-links:

Mycgr3G36335 Mycgr3T
  
Location: 0-423

Mycgr3G36335\_Mycgr3T

Mycgr3G84494 Mycgr3T
  
Location: 523-2047

Mycgr3G84494\_Mycgr3T

Mycgr3G90558 Mycgr3T
  
Location: 2147-15296

Mycgr3G90558\_Mycgr3T

Mycgr3G68036 Mycgr3T
  
Location: 15396-16395

Mycgr3G68036\_Mycgr3T

Mycgr3G90561 Mycgr3T
  
Location: 16495-17134

Mycgr3G90561\_Mycgr3T

Mycgr3G35862 Mycgr3T
  
Location: 17234-18662

Mycgr3G35862\_Mycgr3T

Mycgr3G68030 Mycgr3T
  
Location: 18762-19722

Mycgr3G68030\_Mycgr3T

Mycgr3G36449 Mycgr3T
  
Location: 19822-21886

Mycgr3G36449\_Mycgr3T

Mycgr3G35528 Mycgr3T
  
Location: 21986-22844

Mycgr3G35528\_Mycgr3T

Mycgr3G35932 Mycgr3T
  
Location: 22944-24390

Mycgr3G35932\_Mycgr3T

Mycgr3G23761 Mycgr3T
  
Location: 24490-25825

Mycgr3G23761\_Mycgr3T

Mycgr3G35535 Mycgr3T
  
Location: 25925-26429

Mycgr3G35535\_Mycgr3T

Mycgr3G9942 Mycgr3T9
  
Location: 26529-30375

Mycgr3G9942\_Mycgr3T9

related to multidrug resistance protein
  
Accession: CCT71781
  
Location: 3137319-3142263
  
 NCBI BlastP on this gene

FFUJ\_08114

related to non-ribosomal peptide synthetase
  
Accession: CCT71782
  
Location: 3144734-3167581
  
  
**BlastP hit with Mycgr3G90558\_Mycgr3T**
  
Percentage identity: 31 %
  
BlastP bit score: 1137
  
Sequence coverage: 60 %
  
E-value: 0.0
  
  
 NCBI BlastP on this gene

FFUJ\_08113

Query: Architecture Search FASTA input

151. :  ACJE01000015 Aspergillus niger ATCC 1015     Total score: 1.0     Cumulative Blast bit score: 1938

Mycgr3G36335 Mycgr3T
  
Location: 0-423
  
 NCBI BlastP on this gene

Mycgr3G36335\_Mycgr3T

Mycgr3G84494 Mycgr3T
  
Location: 523-2047
  
 NCBI BlastP on this gene

Mycgr3G84494\_Mycgr3T

Mycgr3G90558 Mycgr3T
  
Location: 2147-15296
  
 NCBI BlastP on this gene

Mycgr3G90558\_Mycgr3T

Mycgr3G68036 Mycgr3T
  
Location: 15396-16395
  
 NCBI BlastP on this gene

Mycgr3G68036\_Mycgr3T

Mycgr3G90561 Mycgr3T
  
Location: 16495-17134
  
 NCBI BlastP on this gene

Mycgr3G90561\_Mycgr3T

Mycgr3G35862 Mycgr3T
  
Location: 17234-18662
  
 NCBI BlastP on this gene

Mycgr3G35862\_Mycgr3T

Mycgr3G68030 Mycgr3T
  
Location: 18762-19722
  
 NCBI BlastP on this gene

Mycgr3G68030\_Mycgr3T

Mycgr3G36449 Mycgr3T
  
Location: 19822-21886
  
 NCBI BlastP on this gene

Mycgr3G36449\_Mycgr3T

Mycgr3G35528 Mycgr3T
  
Location: 21986-22844
  
 NCBI BlastP on this gene

Mycgr3G35528\_Mycgr3T

Mycgr3G35932 Mycgr3T
  
Location: 22944-24390
  
 NCBI BlastP on this gene

Mycgr3G35932\_Mycgr3T

Mycgr3G23761 Mycgr3T
  
Location: 24490-25825
  
 NCBI BlastP on this gene

Mycgr3G23761\_Mycgr3T

Mycgr3G35535 Mycgr3T
  
Location: 25925-26429
  
 NCBI BlastP on this gene

Mycgr3G35535\_Mycgr3T

Mycgr3G9942 Mycgr3T9
  
Location: 26529-30375
  
 NCBI BlastP on this gene

Mycgr3G9942\_Mycgr3T9

hypothetical protein
  
Accession: EHA20899
  
Location: 26109-27074
  
 NCBI BlastP on this gene

EHA20899

catalytic protein
  
Accession: EHA20898
  
Location: 23886-24785
  
 NCBI BlastP on this gene

EHA20898

hypothetical protein
  
Accession: EHA20897
  
Location: 21614-23486
  
 NCBI BlastP on this gene

EHA20897

hypothetical protein
  
Accession: EHA20896
  
Location: 3496-20418
  
  
**BlastP hit with Mycgr3G90558\_Mycgr3T**
  
Percentage identity: 33 %
  
BlastP bit score: 1938
  
Sequence coverage: 91 %
  
E-value: 0.0
  
  
 NCBI BlastP on this gene

EHA20896

hypothetical protein
  
Accession: EHA20895
  
Location: 1462-2048
  
 NCBI BlastP on this gene

EHA20895

152. :  CM001233 Magnaporthe oryzae 70-15 chromosome 3     Total score: 1.0     Cumulative Blast bit score: 1910

hypothetical protein
  
Accession: EHA53290
  
Location: 6064103-6064824
  
 NCBI BlastP on this gene

EHA53290

hypothetical protein
  
Accession: EHA53289
  
Location: 6062762-6063081
  
 NCBI BlastP on this gene

EHA53289

hypothetical protein
  
Accession: EHA53288
  
Location: 6058549-6060887
  
 NCBI BlastP on this gene

EHA53288

cyclic peptide synthetase
  
Accession: EHA53287
  
Location: 6042642-6057224
  
  
**BlastP hit with Mycgr3G90558\_Mycgr3T**
  
Percentage identity: 30 %
  
BlastP bit score: 1910
  
Sequence coverage: 104 %
  
E-value: 0.0
  
  
 NCBI BlastP on this gene

EHA53287

hypothetical protein
  
Accession: EHA53286
  
Location: 6040865-6042065
  
 NCBI BlastP on this gene

EHA53286

pisatin demethylase
  
Accession: EHA53285
  
Location: 6038316-6040200
  
 NCBI BlastP on this gene

EHA53285

hypothetical protein
  
Accession: EHA53284
  
Location: 6035067-6037648
  
 NCBI BlastP on this gene

EHA53284

hypothetical protein
  
Accession: EHA53283
  
Location: 6033397-6034083
  
 NCBI BlastP on this gene

EHA53283

hypothetical protein
  
Accession: EHA53282
  
Location: 6031230-6033015
  
 NCBI BlastP on this gene

EHA53282

153. :  JH921444 Marssonina brunnea f. sp. 'multigermtubi' MB\_m1 unplaced genomic scaffold M6\_S00017     Total score: 1.0     Cumulative Blast bit score: 1900

nonribosomal peptide synthase Pes1
  
Accession: EKD14741
  
Location: 170637-172109
  
 NCBI BlastP on this gene

EKD14741

nonribosomal peptide synthetase
  
Accession: EKD14740
  
Location: 150851-165484
  
  
**BlastP hit with Mycgr3G90558\_Mycgr3T**
  
Percentage identity: 32 %
  
BlastP bit score: 1900
  
Sequence coverage: 95 %
  
E-value: 0.0
  
  
 NCBI BlastP on this gene

EKD14740

NADH pyrophosphatase
  
Accession: EKD14739
  
Location: 148577-149953
  
 NCBI BlastP on this gene

EKD14739

DNA repair helicase rad25
  
Accession: EKD14738
  
Location: 144410-146985
  
 NCBI BlastP on this gene

EKD14738

zinc finger protein
  
Accession: EKD14737
  
Location: 143513-144109
  
 NCBI BlastP on this gene

EKD14737

alpha-type of subunit of 20S proteasome
  
Accession: EKD14736
  
Location: 142248-143245
  
 NCBI BlastP on this gene

EKD14736

anaphase control protein cut9
  
Accession: EKD14735
  
Location: 140012-142156
  
 NCBI BlastP on this gene

EKD14735

154. :  GG698914 Nectria haematococca mpVI 77-13-4 chromosome 7 genomic scaffold NECHAsca\_22\_chr7\_11\_0     Total score: 1.0     Cumulative Blast bit score: 1873

hypothetical protein
  
Accession: EEU38841
  
Location: 483175-501313
  
  
**BlastP hit with Mycgr3G90558\_Mycgr3T**
  
Percentage identity: 31 %
  
BlastP bit score: 1873
  
Sequence coverage: 102 %
  
E-value: 0.0
  
  
 NCBI BlastP on this gene

EEU38841

hypothetical protein
  
Accession: EEU38840
  
Location: 480313-481098
  
 NCBI BlastP on this gene

EEU38840

hypothetical protein
  
Accession: EEU38839
  
Location: 477949-479357
  
 NCBI BlastP on this gene

EEU38839

predicted protein
  
Accession: EEU38723
  
Location: 476075-477795
  
 NCBI BlastP on this gene

EEU38723

155. :  AACD01000135 Aspergillus nidulans FGSC A4     Total score: 1.0     Cumulative Blast bit score: 1831

hypothetical protein
  
Accession: EAA59536
  
Location: 31536-35783
  
 NCBI BlastP on this gene

EAA59536

hypothetical protein
  
Accession: EAA59537
  
Location: 36304-37005
  
 NCBI BlastP on this gene

EAA59537

hypothetical protein
  
Accession: EAA59538
  
Location: 38104-59914
  
  
**BlastP hit with Mycgr3G90558\_Mycgr3T**
  
Percentage identity: 31 %
  
BlastP bit score: 1831
  
Sequence coverage: 88 %
  
E-value: 0.0
  
  
 NCBI BlastP on this gene

EAA59538

hypothetical protein
  
Accession: EAA59539
  
Location: 60857-61446
  
 NCBI BlastP on this gene

EAA59539

hypothetical protein
  
Accession: EAA59540
  
Location: 64256-64882
  
 NCBI BlastP on this gene

EAA59540

hypothetical protein
  
Accession: EAA59541
  
Location: 65871-66613
  
 NCBI BlastP on this gene

EAA59541

156. :  EQ962654 Talaromyces stipitatus ATCC 10500 scf\_1105507295541 genomic scaffold     Total score: 1.0     Cumulative Blast bit score: 1735

conserved hypothetical protein
  
Accession: EED19136
  
Location: 349803-350748
  
 NCBI BlastP on this gene

EED19136

hypothetical protein
  
Accession: EED19137
  
Location: 351399-352530
  
 NCBI BlastP on this gene

EED19137

hypothetical protein
  
Accession: EED19138
  
Location: 351399-352313
  
 NCBI BlastP on this gene

EED19138

dipeptidyl peptidase IV, putative
  
Accession: EED19140
  
Location: 353373-355765
  
 NCBI BlastP on this gene

EED19140

conserved hypothetical protein
  
Accession: EED19141
  
Location: 356632-358299
  
 NCBI BlastP on this gene

EED19141

nonribosomal peptide synthase, putative
  
Accession: EED19142
  
Location: 359434-376386
  
  
**BlastP hit with Mycgr3G90558\_Mycgr3T**
  
Percentage identity: 30 %
  
BlastP bit score: 1735
  
Sequence coverage: 93 %
  
E-value: 0.0
  
  
 NCBI BlastP on this gene

EED19142

phenylalanine ammonia-lyase
  
Accession: EED19143
  
Location: 377097-379447
  
 NCBI BlastP on this gene

EED19143

conserved hypothetical protein
  
Accession: EED19144
  
Location: 381515-382225
  
 NCBI BlastP on this gene

EED19144

conserved hypothetical protein
  
Accession: EED19145
  
Location: 383084-384310
  
 NCBI BlastP on this gene

EED19145

157. :  CH445338 Phaeosphaeria nodorum SN15 scaffold\_14     Total score: 1.0     Cumulative Blast bit score: 1696

hypothetical protein
  
Accession: EAT83273
  
Location: 205186-220770
  
  
**BlastP hit with Mycgr3G90558\_Mycgr3T**
  
Percentage identity: 32 %
  
BlastP bit score: 1696
  
Sequence coverage: 80 %
  
E-value: 0.0
  
  
 NCBI BlastP on this gene

EAT83273

hypothetical protein
  
Accession: EAT83272
  
Location: 203852-204885
  
 NCBI BlastP on this gene

EAT83272

hypothetical protein
  
Accession: EAT83271
  
Location: 203680-203868
  
 NCBI BlastP on this gene

EAT83271

hypothetical protein
  
Accession: EAT83270
  
Location: 203076-203499
  
 NCBI BlastP on this gene

EAT83270

hypothetical protein
  
Accession: EAT83269
  
Location: 199620-201220
  
 NCBI BlastP on this gene

EAT83269

hypothetical protein
  
Accession: EAT83267
  
Location: 198439-199124
  
 NCBI BlastP on this gene

EAT83267

hypothetical protein
  
Accession: EAT83266
  
Location: 196806-198108
  
 NCBI BlastP on this gene

EAT83266

hypothetical protein
  
Accession: EAT83265
  
Location: 194214-196665
  
 NCBI BlastP on this gene

EAT83265

158. :  DS995900 Penicillium marneffei ATCC 18224 scf\_1105668340758 genomic scaffold     Total score: 1.0     Cumulative Blast bit score: 1665

hypothetical protein
  
Accession: EEA26482
  
Location: 3820130-3821233
  
 NCBI BlastP on this gene

EEA26482

conserved hypothetical protein
  
Accession: EEA26481
  
Location: 3818623-3819691
  
 NCBI BlastP on this gene

EEA26481

hypothetical protein
  
Accession: EEA26480
  
Location: 3816693-3817493
  
 NCBI BlastP on this gene

EEA26480

conserved hypothetical protein
  
Accession: EEA26479
  
Location: 3814528-3815743
  
 NCBI BlastP on this gene

EEA26479

nonribosomal peptide synthase, putative
  
Accession: EEA26478
  
Location: 3796470-3813422
  
  
**BlastP hit with Mycgr3G90558\_Mycgr3T**
  
Percentage identity: 30 %
  
BlastP bit score: 1665
  
Sequence coverage: 89 %
  
E-value: 0.0
  
  
 NCBI BlastP on this gene

EEA26478

hypothetical protein
  
Accession: EEA26477
  
Location: 3794009-3794591
  
 NCBI BlastP on this gene

EEA26477

ATP-dependent bile acid permease, putative
  
Accession: EEA26476
  
Location: 3787656-3792480
  
 NCBI BlastP on this gene

EEA26476

aminotransferase, class III
  
Accession: EEA26475
  
Location: 3786005-3787550
  
 NCBI BlastP on this gene

EEA26475

159. :  DF126466 Aspergillus kawachii IFO 4308 DNA, contig: scaffold00020     Total score: 1.0     Cumulative Blast bit score: 1640

canalicular multispecific organic anion transporter 1
  
Accession: GAA89112
  
Location: 259668-264641
  
 NCBI BlastP on this gene

GAA89112

subgroup IIIi aminotransferase
  
Accession: GAA89111
  
Location: 257542-258796
  
 NCBI BlastP on this gene

GAA89111

hypothetical protein
  
Accession: GAA89110
  
Location: 256091-256963
  
 NCBI BlastP on this gene

GAA89110

hypothetical protein
  
Accession: GAA89109
  
Location: 253720-254606
  
 NCBI BlastP on this gene

GAA89109

RTA1 domain protein
  
Accession: GAA89108
  
Location: 250888-251906
  
 NCBI BlastP on this gene

GAA89108

peptide synthetase
  
Accession: GAA89107
  
Location: 233716-250245
  
  
**BlastP hit with Mycgr3G90558\_Mycgr3T**
  
Percentage identity: 31 %
  
BlastP bit score: 1640
  
Sequence coverage: 87 %
  
E-value: 0.0
  
  
 NCBI BlastP on this gene

GAA89107

C6 zinc finger domain protein
  
Accession: GAA89106
  
Location: 230770-232394
  
 NCBI BlastP on this gene

GAA89106

integral membrane protein
  
Accession: GAA89105
  
Location: 229360-230259
  
 NCBI BlastP on this gene

GAA89105

lysophospholipase phospholipase B (Plb1)
  
Accession: GAA89104
  
Location: 226264-228301
  
 NCBI BlastP on this gene

GAA89104

hypothetical protein
  
Accession: GAA89103
  
Location: 223097-224906
  
 NCBI BlastP on this gene

GAA89103

160. :  ACJE01000005 Aspergillus niger ATCC 1015     Total score: 1.0     Cumulative Blast bit score: 1631

hypothetical protein
  
Accession: EHA25710
  
Location: 280936-282215
  
 NCBI BlastP on this gene

EHA25710

hypothetical protein
  
Accession: EHA25709
  
Location: 277076-277810
  
 NCBI BlastP on this gene

EHA25709

hypothetical protein
  
Accession: EHA25708
  
Location: 274287-275303
  
 NCBI BlastP on this gene

EHA25708

hypothetical protein
  
Accession: EHA25707
  
Location: 257395-273191
  
  
**BlastP hit with Mycgr3G90558\_Mycgr3T**
  
Percentage identity: 31 %
  
BlastP bit score: 1631
  
Sequence coverage: 86 %
  
E-value: 0.0
  
  
 NCBI BlastP on this gene

EHA25707

lysophospholipase
  
Accession: EHA25706
  
Location: 250212-252057
  
 NCBI BlastP on this gene

EHA25706

hypothetical protein
  
Accession: EHA25705
  
Location: 248119-249606
  
 NCBI BlastP on this gene

EHA25705

hypothetical protein
  
Accession: EHA25704
  
Location: 246807-247874
  
 NCBI BlastP on this gene

EHA25704

161. :  KB644410 Penicillium oxalicum 114-2 unplaced genomic scaffold scaffold\_3     Total score: 1.0     Cumulative Blast bit score: 1575

hypothetical protein
  
Accession: EPS27660
  
Location: 1561005-1562473
  
 NCBI BlastP on this gene

EPS27660

hypothetical protein
  
Accession: EPS27661
  
Location: 1562637-1563877
  
 NCBI BlastP on this gene

EPS27661

hypothetical protein
  
Accession: EPS27662
  
Location: 1564448-1565641
  
 NCBI BlastP on this gene

EPS27662

hypothetical protein
  
Accession: EPS27663
  
Location: 1566197-1567863
  
 NCBI BlastP on this gene

EPS27663

hypothetical protein
  
Accession: EPS27664
  
Location: 1568091-1568927
  
 NCBI BlastP on this gene

EPS27664

hypothetical protein
  
Accession: EPS27665
  
Location: 1569400-1571493
  
 NCBI BlastP on this gene

EPS27665

hypothetical protein
  
Accession: EPS27666
  
Location: 1573119-1586511
  
  
**BlastP hit with Mycgr3G90558\_Mycgr3T**
  
Percentage identity: 29 %
  
BlastP bit score: 1575
  
Sequence coverage: 95 %
  
E-value: 0.0
  
  
 NCBI BlastP on this gene

EPS27666

162. :  AP007171 Aspergillus oryzae RIB40 DNA, SC011.     Total score: 1.0     Cumulative Blast bit score: 1573

not annotated
  
Accession: BAE64607
  
Location: 149759-151615
  
 NCBI BlastP on this gene

AO090011000045

not annotated
  
Accession: BAE64606
  
Location: 147752-149100
  
 NCBI BlastP on this gene

AO090011000044

not annotated
  
Accession: BAE64605
  
Location: 121832-142723
  
  
**BlastP hit with Mycgr3G90558\_Mycgr3T**
  
Percentage identity: 31 %
  
BlastP bit score: 1573
  
Sequence coverage: 75 %
  
E-value: 0.0
  
  
 NCBI BlastP on this gene

AO090011000043

not annotated
  
Accession: BAE64604
  
Location: 118053-118754
  
 NCBI BlastP on this gene

AO090011000042

not annotated
  
Accession: BAE64603
  
Location: 117022-117666
  
 NCBI BlastP on this gene

AO090011000041

not annotated
  
Accession: BAE64602
  
Location: 109146-115780
  
 NCBI BlastP on this gene

AO090011000040

163. :  DS995905 Penicillium marneffei ATCC 18224 scf\_1105668340970 genomic scaffold     Total score: 1.0     Cumulative Blast bit score: 1560

polyketide synthase, putative
  
Accession: EEA19308
  
Location: 148921-156916
  
 NCBI BlastP on this gene

EEA19308

AMP dependent CoA ligase, putative
  
Accession: EEA19309
  
Location: 157488-159502
  
 NCBI BlastP on this gene

EEA19309

conserved hypothetical protein
  
Accession: EEA19310
  
Location: 160657-161581
  
 NCBI BlastP on this gene

EEA19310

nonribosomal peptide synthetase, putative
  
Accession: EEA19311
  
Location: 163263-175132
  
  
**BlastP hit with Mycgr3G90558\_Mycgr3T**
  
Percentage identity: 31 %
  
BlastP bit score: 1560
  
Sequence coverage: 78 %
  
E-value: 0.0
  
  
 NCBI BlastP on this gene

EEA19311

164. :  EQ962658 Talaromyces stipitatus ATCC 10500 scf\_1105507295511 genomic scaffold     Total score: 1.0     Cumulative Blast bit score: 1521

polyketide synthase, putative
  
Accession: EED13571
  
Location: 307442-315406
  
 NCBI BlastP on this gene

EED13571

AMP dependent ligase/synthetase, putative
  
Accession: EED13572
  
Location: 315905-317933
  
 NCBI BlastP on this gene

EED13572

conserved hypothetical protein
  
Accession: EED13573
  
Location: 319874-320777
  
 NCBI BlastP on this gene

EED13573

nonribosomal peptide synthase, putative
  
Accession: EED13574
  
Location: 321838-333741
  
  
**BlastP hit with Mycgr3G90558\_Mycgr3T**
  
Percentage identity: 30 %
  
BlastP bit score: 1521
  
Sequence coverage: 78 %
  
E-value: 0.0
  
  
 NCBI BlastP on this gene

EED13574

conserved hypothetical protein
  
Accession: EED13575
  
Location: 333968-335762
  
 NCBI BlastP on this gene

EED13575

hypothetical protein
  
Accession: EED13576
  
Location: 337349-338134
  
 NCBI BlastP on this gene

EED13576

choline monooxygenase, putative
  
Accession: EED13577
  
Location: 339621-340353
  
 NCBI BlastP on this gene

EED13577

mitochondrial inner membrane translocase subunit (TIM17), putative
  
Accession: EED13578
  
Location: 341185-341726
  
 NCBI BlastP on this gene

EED13578

ABC transporter, putative
  
Accession: EED13579
  
Location: 344680-345903
  
 NCBI BlastP on this gene

EED13579

165. :  DS989824 Arthroderma gypseum CBS 118893 supercont1.3 genomic scaffold     Total score: 1.0     Cumulative Blast bit score: 1515

1-aminocyclopropane-1-carboxylate synthase
  
Accession: EFR00818
  
Location: 1081844-1083133
  
 NCBI BlastP on this gene

EFR00818

hypothetical protein
  
Accession: EFR00817
  
Location: 1080219-1081337
  
 NCBI BlastP on this gene

EFR00817

hypothetical protein
  
Accession: EFR00816
  
Location: 1051142-1079626
  
  
**BlastP hit with Mycgr3G90558\_Mycgr3T**
  
Percentage identity: 30 %
  
BlastP bit score: 1515
  
Sequence coverage: 86 %
  
E-value: 0.0
  
  
 NCBI BlastP on this gene

EFR00816

enoyl-CoA hydratase/isomerase
  
Accession: EFR00815
  
Location: 1049443-1050270
  
 NCBI BlastP on this gene

EFR00815

hypothetical protein
  
Accession: EFR00814
  
Location: 1047703-1048296
  
 NCBI BlastP on this gene

EFR00814

166. :  DF126447 Aspergillus kawachii IFO 4308 DNA, contig: scaffold00001     Total score: 1.0     Cumulative Blast bit score: 1497

membrane protein TMS1
  
Accession: GAA82425
  
Location: 1791453-1793087
  
 NCBI BlastP on this gene

GAA82425

DNA mismatch repair protein
  
Accession: GAA82424
  
Location: 1787801-1791084
  
 NCBI BlastP on this gene

GAA82424

delta-1-pyrroline-5-carboxylate dehydrogenase, mitochondrial precursor
  
Accession: GAA82423
  
Location: 1785419-1787224
  
 NCBI BlastP on this gene

GAA82423

nonribosomal peptide synthetase
  
Accession: GAA82422
  
Location: 1766590-1784212
  
  
**BlastP hit with Mycgr3G90558\_Mycgr3T**
  
Percentage identity: 31 %
  
BlastP bit score: 1497
  
Sequence coverage: 75 %
  
E-value: 0.0
  
  
 NCBI BlastP on this gene

GAA82422

similar to An02g02310
  
Accession: GAA82421
  
Location: 1765177-1765725
  
 NCBI BlastP on this gene

GAA82421

chitin synthase
  
Accession: GAA82420
  
Location: 1759239-1764667
  
 NCBI BlastP on this gene

GAA82420

167. :  KB020599 Colletotrichum gloeosporioides Nara gc5 unplaced genomic scaffold scaffold236     Total score: 1.0     Cumulative Blast bit score: 1492

indole-3-acetate beta-glucosyltransferase
  
Accession: ELA34719
  
Location: 70327-72235
  
 NCBI BlastP on this gene

ELA34719

hypothetical protein
  
Accession: ELA34720
  
Location: 73245-74773
  
 NCBI BlastP on this gene

ELA34720

multidrug resistance-associated protein 5
  
Accession: ELA34721
  
Location: 75264-79454
  
  
**BlastP hit with Mycgr3G9942\_Mycgr3T9**
  
Percentage identity: 30 %
  
BlastP bit score: 541
  
Sequence coverage: 105 %
  
E-value: 3e-165
  
  
 NCBI BlastP on this gene

ELA34721

isoflavone reductase family protein
  
Accession: ELA34722
  
Location: 79918-81079
  
 NCBI BlastP on this gene

ELA34722

DnaJ domain-containing protein
  
Accession: ELA34723
  
Location: 81295-82582
  
 NCBI BlastP on this gene

ELA34723

hypothetical protein
  
Accession: ELA34724
  
Location: 82701-83054
  
 NCBI BlastP on this gene

ELA34724

fungal specific transcription factor
  
Accession: ELA34725
  
Location: 84688-86337
  
 NCBI BlastP on this gene

ELA34725

ABC1 domain containing protein
  
Accession: ELA34726
  
Location: 86664-88013
  
 NCBI BlastP on this gene

ELA34726

chromatin assembly factor 1 subunit
  
Accession: ELA34727
  
Location: 88531-90859
  
 NCBI BlastP on this gene

ELA34727

ABC bile acid
  
Accession: ELA34728
  
Location: 97327-102043
  
  
**BlastP hit with Mycgr3G9942\_Mycgr3T9**
  
Percentage identity: 41 %
  
BlastP bit score: 951
  
Sequence coverage: 100 %
  
E-value: 0.0
  
  
 NCBI BlastP on this gene

ELA34728

hypothetical protein
  
Accession: ELA34729
  
Location: 102873-103394
  
 NCBI BlastP on this gene

ELA34729

GNAT family
  
Accession: ELA34730
  
Location: 103849-104595
  
 NCBI BlastP on this gene

ELA34730

ATPase
  
Accession: ELA34731
  
Location: 104938-107436
  
 NCBI BlastP on this gene

ELA34731

168. :  KB644408 Penicillium oxalicum 114-2 unplaced genomic scaffold scaffold\_1     Total score: 1.0     Cumulative Blast bit score: 1487

hypothetical protein
  
Accession: EPS26276
  
Location: 3684930-3707027
  
  
**BlastP hit with Mycgr3G90558\_Mycgr3T**
  
Percentage identity: 31 %
  
BlastP bit score: 1487
  
Sequence coverage: 75 %
  
E-value: 0.0
  
  
 NCBI BlastP on this gene

EPS26276

hypothetical protein
  
Accession: EPS26275
  
Location: 3681004-3683324
  
 NCBI BlastP on this gene

EPS26275

hypothetical protein
  
Accession: EPS26274
  
Location: 3675933-3680327
  
 NCBI BlastP on this gene

EPS26274

169. :  GG698902 Nectria haematococca mpVI 77-13-4 chromosome 7 genomic scaffold NECHAsca\_9\_chr7\_10\_0     Total score: 1.0     Cumulative Blast bit score: 1487

hypothetical protein
  
Accession: EEU43517
  
Location: 1034605-1036248
  
 NCBI BlastP on this gene

EEU43517

hypothetical protein
  
Accession: EEU43518
  
Location: 1036669-1037589
  
 NCBI BlastP on this gene

EEU43518

predicted protein
  
Accession: EEU43519
  
Location: 1038778-1040459
  
 NCBI BlastP on this gene

EEU43519

hypothetical protein
  
Accession: EEU43765
  
Location: 1040666-1041748
  
 NCBI BlastP on this gene

EEU43765

hypothetical protein
  
Accession: EEU43766
  
Location: 1042756-1043906
  
 NCBI BlastP on this gene

EEU43766

hypothetical protein
  
Accession: EEU43520
  
Location: 1044999-1061802
  
  
**BlastP hit with Mycgr3G90558\_Mycgr3T**
  
Percentage identity: 29 %
  
BlastP bit score: 1487
  
Sequence coverage: 91 %
  
E-value: 0.0
  
  
 NCBI BlastP on this gene

EEU43520

170. :  AM920436 Penicillium chrysogenum Wisconsin 54-1255 complete genome, contig Pc00c21.     Total score: 1.0     Cumulative Blast bit score: 1481

not annotated
  
Accession: CAP95979
  
Location: 2562419-2563636
  
 NCBI BlastP on this gene

Pc21g10820

not annotated
  
Accession: CAP95978
  
Location: 2560518-2561503
  
 NCBI BlastP on this gene

Pc21g10810

not annotated
  
Accession: CAP95977
  
Location: 2558169-2559581
  
 NCBI BlastP on this gene

Pc21g10800

not annotated
  
Accession: CAP95976
  
Location: 2535141-2557271
  
  
**BlastP hit with Mycgr3G90558\_Mycgr3T**
  
Percentage identity: 31 %
  
BlastP bit score: 1481
  
Sequence coverage: 77 %
  
E-value: 0.0
  
  
 NCBI BlastP on this gene

Pc21g10790

not annotated
  
Accession: CAP95975
  
Location: 2532959-2533900
  
 NCBI BlastP on this gene

Pc21g10780

not annotated
  
Accession: CAP95974
  
Location: 2531335-2532375
  
 NCBI BlastP on this gene

Pc21g10770

hypothetical protein
  
Accession: CAP95973
  
Location: 2530746-2531288
  
 NCBI BlastP on this gene

Pc21g10760

not annotated
  
Accession: CAP95972
  
Location: 2529805-2530722
  
 NCBI BlastP on this gene

Pc21g10750

hypothetical protein
  
Accession: CAP95971
  
Location: 2527570-2529340
  
 NCBI BlastP on this gene

Pc21g10740

171. :  DS027698 Neosartorya fischeri NRRL 181 1099437636266 genomic scaffold     Total score: 1.0     Cumulative Blast bit score: 1470

O-methyltransferase, putative
  
Accession: EAW16450
  
Location: 3234458-3235347
  
 NCBI BlastP on this gene

EAW16450

nonribosomal peptide synthase, putative
  
Accession: EAW16449
  
Location: 3229840-3233259
  
 NCBI BlastP on this gene

EAW16449

2OG-Fe(II) oxygenase family oxidoreductase, putative
  
Accession: EAW16448
  
Location: 3226649-3228035
  
 NCBI BlastP on this gene

EAW16448

MAK1-like monooxygenase, putative
  
Accession: EAW16447
  
Location: 3224165-3226117
  
 NCBI BlastP on this gene

EAW16447

nonribosomal peptide synthase, putative
  
Accession: EAW16446
  
Location: 3213365-3223759
  
  
**BlastP hit with Mycgr3G90558\_Mycgr3T**
  
Percentage identity: 33 %
  
BlastP bit score: 1470
  
Sequence coverage: 69 %
  
E-value: 0.0
  
  
 NCBI BlastP on this gene

EAW16446

hypothetical protein
  
Accession: EAW16445
  
Location: 3209654-3210370
  
 NCBI BlastP on this gene

EAW16445

MFS sugar transporter, putative
  
Accession: EAW16444
  
Location: 3205917-3207756
  
 NCBI BlastP on this gene

EAW16444

bacterial alpha-L-rhamnosidase domain protein
  
Accession: EAW16443
  
Location: 3202318-3205008
  
 NCBI BlastP on this gene

EAW16443

C2H2 type zinc finger domain protein
  
Accession: EAW16442
  
Location: 3198632-3201421
  
 NCBI BlastP on this gene

EAW16442

172. :  CH408034 Chaetomium globosum CBS 148.51 scaffold\_6 genomic scaffold     Total score: 1.0     Cumulative Blast bit score: 1465

hypothetical protein
  
Accession: EAQ84525
  
Location: 607887-613428
  
 NCBI BlastP on this gene

EAQ84525

hypothetical protein
  
Accession: EAQ84526
  
Location: 616844-639592
  
  
**BlastP hit with Mycgr3G90558\_Mycgr3T**
  
Percentage identity: 31 %
  
BlastP bit score: 1465
  
Sequence coverage: 76 %
  
E-value: 0.0
  
  
 NCBI BlastP on this gene

EAQ84526

hypothetical protein
  
Accession: EAQ84527
  
Location: 640205-644342
  
 NCBI BlastP on this gene

EAQ84527

hypothetical protein
  
Accession: EAQ84528
  
Location: 644693-649154
  
 NCBI BlastP on this gene

EAQ84528

173. :  CP003005 Myceliophthora thermophila ATCC 42464 chromosome 4     Total score: 1.0     Cumulative Blast bit score: 1454

non-ribosomal peptide synthetase
  
Accession: AEO59325
  
Location: 3950426-3973722
  
  
**BlastP hit with Mycgr3G90558\_Mycgr3T**
  
Percentage identity: 31 %
  
BlastP bit score: 1454
  
Sequence coverage: 77 %
  
E-value: 0.0
  
  
 NCBI BlastP on this gene

MYCTH\_94652

hypothetical protein
  
Accession: AEO59324
  
Location: 3945573-3949459
  
 NCBI BlastP on this gene

MYCTH\_2128378

174. :  JH725160 Beauveria bassiana ARSEF 2860 unplaced genomic scaffold BBA\_S00011     Total score: 1.0     Cumulative Blast bit score: 1419

ABC transporter with duplicated ATPase domains
  
Accession: EJP66331
  
Location: 595296-597590
  
 NCBI BlastP on this gene

EJP66331

oxidoreductase, 2OG-Fe(II) oxygenase family
  
Accession: EJP66332
  
Location: 598424-599397
  
 NCBI BlastP on this gene

EJP66332

aspartate-tRNA ligase
  
Accession: EJP66333
  
Location: 600473-602169
  
 NCBI BlastP on this gene

EJP66333

peptide synthetase
  
Accession: EJP66334
  
Location: 604188-614168
  
  
**BlastP hit with Mycgr3G90558\_Mycgr3T**
  
Percentage identity: 33 %
  
BlastP bit score: 1419
  
Sequence coverage: 66 %
  
E-value: 0.0
  
  
 NCBI BlastP on this gene

EJP66334

175. :  EQ963476 Aspergillus flavus NRRL3357 scf\_1106286417850 genomic scaffold     Total score: 1.0     Cumulative Blast bit score: 1402

fatty acid synthase alpha subunit, putative
  
Accession: EED52163
  
Location: 162029-167698
  
 NCBI BlastP on this gene

EED52163

cytochrome P450, putative
  
Accession: EED52162
  
Location: 159465-161321
  
 NCBI BlastP on this gene

EED52162

branched-chain amino acid aminotransferase, putative
  
Accession: EED52161
  
Location: 156808-158806
  
 NCBI BlastP on this gene

EED52161

Ankyrin repeat protein
  
Accession: EED52160
  
Location: 155841-156723
  
 NCBI BlastP on this gene

EED52160

nonribosomal peptide synthase, putative
  
Accession: EED52159
  
Location: 143335-152441
  
  
**BlastP hit with Mycgr3G90558\_Mycgr3T**
  
Percentage identity: 32 %
  
BlastP bit score: 1402
  
Sequence coverage: 63 %
  
E-value: 0.0
  
  
 NCBI BlastP on this gene

EED52159

176. :  HF679023 Fusarium fujikuroi IMI 58289 draft genome, chromosome FFUJ\_chr01.     Total score: 1.0     Cumulative Blast bit score: 1299

related to benzoate-para-hydroxylase (cytochrome P450)
  
Accession: CCT63357
  
Location: 6523368-6525190
  
 NCBI BlastP on this gene

FFUJ\_00007

related to cytochrom P450
  
Accession: CCT63358
  
Location: 6526140-6527351
  
 NCBI BlastP on this gene

FFUJ\_00006

related to isoamyl alcohol oxidase
  
Accession: CCT63359
  
Location: 6527917-6529835
  
 NCBI BlastP on this gene

FFUJ\_00005

probable DHA14-like major facilitator; ABC transporter
  
Accession: CCT63472
  
Location: 6530784-6532704
  
 NCBI BlastP on this gene

FFUJ\_00004

non-ribosomal peptide synthetase
  
Accession: CCT63360
  
Location: 6533353-6548739
  
  
**BlastP hit with Mycgr3G90558\_Mycgr3T**
  
Percentage identity: 29 %
  
BlastP bit score: 1299
  
Sequence coverage: 82 %
  
E-value: 0.0
  
  
 NCBI BlastP on this gene

FFUJ\_00003

177. :  CH476607 Aspergillus terreus NIH2624 scaffold\_14 genomic scaffold     Total score: 1.0     Cumulative Blast bit score: 1292

predicted protein
  
Accession: EAU30153
  
Location: 23226-24087
  
 NCBI BlastP on this gene

EAU30153

conserved hypothetical protein
  
Accession: EAU30154
  
Location: 24722-25102
  
 NCBI BlastP on this gene

EAU30154

hypothetical protein
  
Accession: EAU30155
  
Location: 25629-29896
  
 NCBI BlastP on this gene

EAU30155

predicted protein
  
Accession: EAU30156
  
Location: 30730-46891
  
  
**BlastP hit with Mycgr3G90558\_Mycgr3T**
  
Percentage identity: 29 %
  
BlastP bit score: 1292
  
Sequence coverage: 75 %
  
E-value: 0.0
  
  
 NCBI BlastP on this gene

EAU30156

predicted protein
  
Accession: EAU30157
  
Location: 47091-48057
  
 NCBI BlastP on this gene

EAU30157

predicted protein
  
Accession: EAU30158
  
Location: 52158-52450
  
 NCBI BlastP on this gene

EAU30158

conserved hypothetical protein
  
Accession: EAU30159
  
Location: 53272-53667
  
 NCBI BlastP on this gene

EAU30159

predicted protein
  
Accession: EAU30160
  
Location: 54208-55650
  
 NCBI BlastP on this gene

EAU30160

178. :  CM001201 Mycosphaerella graminicola IPO323 chromosome 6     Total score: 1.0     Cumulative Blast bit score: 1290

hypothetical protein
  
Accession: EGP86941
  
Location: 346604-349414
  
 NCBI BlastP on this gene

EGP86941

hypothetical protein
  
Accession: EGP86942
  
Location: 344644-346260
  
 NCBI BlastP on this gene

EGP86942

hypothetical protein
  
Accession: EGP86334
  
Location: 340624-341098
  
 NCBI BlastP on this gene

EGP86334

hypothetical protein
  
Accession: EGP86333
  
Location: 338390-339841
  
 NCBI BlastP on this gene

EGP86333

hypothetical protein
  
Accession: EGP86943
  
Location: 336860-337712
  
 NCBI BlastP on this gene

EGP86943

hypothetical protein
  
Accession: EGP86944
  
Location: 323267-335569
  
  
**BlastP hit with Mycgr3G90558\_Mycgr3T**
  
Percentage identity: 30 %
  
BlastP bit score: 1290
  
Sequence coverage: 76 %
  
E-value: 0.0
  
  
 NCBI BlastP on this gene

EGP86944

putative major facilitator superfamily transporter
  
Accession: EGP86332
  
Location: 321309-322960
  
 NCBI BlastP on this gene

EGP86332

hypothetical protein
  
Accession: EGP86945
  
Location: 318326-320221
  
 NCBI BlastP on this gene

EGP86945

hypothetical protein
  
Accession: EGP86331
  
Location: 317169-318230
  
 NCBI BlastP on this gene

EGP86331

hypothetical protein
  
Accession: EGP86946
  
Location: 315201-316479
  
 NCBI BlastP on this gene

EGP86946

hypothetical protein
  
Accession: EGP86330
  
Location: 312759-315135
  
 NCBI BlastP on this gene

EGP86330

179. :  CH476595 Aspergillus terreus NIH2624 scaffold\_2 genomic scaffold     Total score: 1.0     Cumulative Blast bit score: 1254

predicted protein
  
Accession: EAU37756
  
Location: 53723-54964
  
 NCBI BlastP on this gene

EAU37756

conserved hypothetical protein
  
Accession: EAU37757
  
Location: 59937-60612
  
 NCBI BlastP on this gene

EAU37757

predicted protein
  
Accession: EAU37758
  
Location: 62794-63544
  
 NCBI BlastP on this gene

EAU37758

predicted protein
  
Accession: EAU37759
  
Location: 64751-79683
  
  
**BlastP hit with Mycgr3G90558\_Mycgr3T**
  
Percentage identity: 30 %
  
BlastP bit score: 1254
  
Sequence coverage: 68 %
  
E-value: 0.0
  
  
 NCBI BlastP on this gene

EAU37759

hypothetical protein
  
Accession: EAU37760
  
Location: 79875-81645
  
 NCBI BlastP on this gene

EAU37760

predicted protein
  
Accession: EAU37761
  
Location: 84323-85070
  
 NCBI BlastP on this gene

EAU37761

predicted protein
  
Accession: EAU37762
  
Location: 86513-87609
  
 NCBI BlastP on this gene

EAU37762

180. :  EQ962656 Talaromyces stipitatus ATCC 10500 scf\_1105507295549 genomic scaffold     Total score: 1.0     Cumulative Blast bit score: 1241

nonribosomal peptide synthase, putative
  
Accession: EED16046
  
Location: 757374-781575
  
  
**BlastP hit with Mycgr3G90558\_Mycgr3T**
  
Percentage identity: 31 %
  
BlastP bit score: 1241
  
Sequence coverage: 60 %
  
E-value: 0.0
  
  
 NCBI BlastP on this gene

EED16046

conserved hypothetical protein
  
Accession: EED16045
  
Location: 755698-757080
  
 NCBI BlastP on this gene

EED16045

C2H2 finger domain protein, putative
  
Accession: EED16044
  
Location: 749795-753334
  
 NCBI BlastP on this gene

EED16044

181. :  CP003010 Thielavia terrestris NRRL 8126 chromosome 2     Total score: 1.0     Cumulative Blast bit score: 1237

hypothetical protein
  
Accession: AEO64844
  
Location: 130631-136188
  
 NCBI BlastP on this gene

THITE\_35966

non-ribosomal peptide synthetase
  
Accession: AEO64845
  
Location: 138796-164185
  
  
**BlastP hit with Mycgr3G90558\_Mycgr3T**
  
Percentage identity: 32 %
  
BlastP bit score: 1237
  
Sequence coverage: 62 %
  
E-value: 0.0
  
  
 NCBI BlastP on this gene

THITE\_112015

hypothetical protein
  
Accession: AEO64846
  
Location: 165873-170651
  
 NCBI BlastP on this gene

THITE\_2111183

182. :  DS027045 Aspergillus clavatus NRRL 1 1099423829791 genomic scaffold     Total score: 1.0     Cumulative Blast bit score: 1234

Ankyrin repeat protein
  
Accession: EAW14631
  
Location: 2660593-2661873
  
 NCBI BlastP on this gene

EAW14631

conserved hypothetical protein
  
Accession: EAW14632
  
Location: 2662519-2665119
  
 NCBI BlastP on this gene

EAW14632

F5/8 type C domain protein
  
Accession: EAW14633
  
Location: 2666483-2668804
  
 NCBI BlastP on this gene

EAW14633

NADPH-dependent methylglyoxal reductase (D-lactaldehyde dehydrogenase, putative
  
Accession: EAW14634
  
Location: 2669395-2670715
  
 NCBI BlastP on this gene

EAW14634

conserved hypothetical protein
  
Accession: EAW14635
  
Location: 2672242-2673321
  
 NCBI BlastP on this gene

EAW14635

nonribosomal peptide synthase, putative
  
Accession: EAW14636
  
Location: 2673911-2684578
  
  
**BlastP hit with Mycgr3G90558\_Mycgr3T**
  
Percentage identity: 30 %
  
BlastP bit score: 1234
  
Sequence coverage: 73 %
  
E-value: 0.0
  
  
 NCBI BlastP on this gene

EAW14636

183. :  DS995703 Microsporum canis CBS 113480 supercont1.3 genomic scaffold     Total score: 1.0     Cumulative Blast bit score: 1230

nonribosomal peptide synthase
  
Accession: EEQ30108
  
Location: 224937-249327
  
  
**BlastP hit with Mycgr3G90558\_Mycgr3T**
  
Percentage identity: 31 %
  
BlastP bit score: 1230
  
Sequence coverage: 60 %
  
E-value: 0.0
  
  
 NCBI BlastP on this gene

EEQ30108

conserved hypothetical protein
  
Accession: EEQ30107
  
Location: 224112-224493
  
 NCBI BlastP on this gene

EEQ30107

GMP synthase
  
Accession: EEQ30106
  
Location: 222165-223871
  
 NCBI BlastP on this gene

EEQ30106

bcp1
  
Accession: EEQ30105
  
Location: 220837-221828
  
 NCBI BlastP on this gene

EEQ30105

chorismate mutase
  
Accession: EEQ30104
  
Location: 219676-220630
  
 NCBI BlastP on this gene

EEQ30104

184. :  DS995903 Penicillium marneffei ATCC 18224 scf\_1105668340984 genomic scaffold     Total score: 1.0     Cumulative Blast bit score: 1229

nonribosomal peptide synthase, putative
  
Accession: EEA22218
  
Location: 2703820-2728032
  
  
**BlastP hit with Mycgr3G90558\_Mycgr3T**
  
Percentage identity: 30 %
  
BlastP bit score: 1229
  
Sequence coverage: 61 %
  
E-value: 0.0
  
  
 NCBI BlastP on this gene

EEA22218

185. :  GG704912 Coccidioides immitis RS genomic scaffold supercont3.2     Total score: 1.0     Cumulative Blast bit score: 1228

ABC multidrug transporter
  
Accession: EAS27487
  
Location: 292393-297767
  
 NCBI BlastP on this gene

EAS27487

hypothetical protein
  
Accession: EJB10845
  
Location: 299197-299787
  
 NCBI BlastP on this gene

EJB10845

hypothetical protein
  
Accession: EAS27483
  
Location: 301509-302702
  
 NCBI BlastP on this gene

EAS27483

amino acid adenylation domain-containing protein
  
Accession: EAS27482
  
Location: 302986-327648
  
  
**BlastP hit with Mycgr3G90558\_Mycgr3T**
  
Percentage identity: 30 %
  
BlastP bit score: 1228
  
Sequence coverage: 61 %
  
E-value: 0.0
  
  
 NCBI BlastP on this gene

EAS27482

hypothetical protein
  
Accession: EAS27481
  
Location: 328316-328738
  
 NCBI BlastP on this gene

EAS27481

hypothetical protein
  
Accession: EAS27480
  
Location: 329270-330270
  
 NCBI BlastP on this gene

EAS27480

chorismate mutase
  
Accession: EAS27479
  
Location: 330399-331379
  
 NCBI BlastP on this gene

EAS27479

hypothetical protein
  
Accession: EAS27477
  
Location: 332794-334785
  
 NCBI BlastP on this gene

EAS27477

186. :  ACFW01000025 Coccidioides posadasii C735 delta SOWgp     Total score: 1.0     Cumulative Blast bit score: 1225

multidrug resistance protein MDR, putative
  
Accession: EER26974
  
Location: 199688-205066
  
 NCBI BlastP on this gene

EER26974

nonribosomal peptide synthetase, putative
  
Accession: EER26975
  
Location: 210338-234994
  
  
**BlastP hit with Mycgr3G90558\_Mycgr3T**
  
Percentage identity: 30 %
  
BlastP bit score: 1225
  
Sequence coverage: 61 %
  
E-value: 0.0
  
  
 NCBI BlastP on this gene

EER26975

hypothetical protein
  
Accession: EER26976
  
Location: 235707-236128
  
 NCBI BlastP on this gene

EER26976

hypothetical protein
  
Accession: EER26977
  
Location: 236661-237661
  
 NCBI BlastP on this gene

EER26977

chorismate mutase, putative
  
Accession: EER26978
  
Location: 237790-238769
  
 NCBI BlastP on this gene

EER26978

hypothetical protein
  
Accession: EER26979
  
Location: 240179-242170
  
 NCBI BlastP on this gene

EER26979

187. :  DS995708 Microsporum canis CBS 113480 supercont1.8 genomic scaffold     Total score: 1.0     Cumulative Blast bit score: 1220

fatty acid synthase beta subunit dehydratase
  
Accession: EEQ35620
  
Location: 1321574-1327901
  
 NCBI BlastP on this gene

EEQ35620

benzoate 4-monooxygenase cytochrome P450
  
Accession: EEQ35621
  
Location: 1329009-1330899
  
 NCBI BlastP on this gene

EEQ35621

peptide synthetase
  
Accession: EEQ35622
  
Location: 1332241-1351067
  
  
**BlastP hit with Mycgr3G90558\_Mycgr3T**
  
Percentage identity: 28 %
  
BlastP bit score: 1220
  
Sequence coverage: 76 %
  
E-value: 0.0
  
  
 NCBI BlastP on this gene

EEQ35622

188. :  BABT02000122 Mixia osmundae IAM 14324     Total score: 1.0     Cumulative Blast bit score: 1218

hypothetical protein
  
Accession: GAA97477
  
Location: 48246-51701
  
  
**BlastP hit with Mycgr3G9942\_Mycgr3T9**
  
Percentage identity: 34 %
  
BlastP bit score: 566
  
Sequence coverage: 81 %
  
E-value: 2e-177
  
  
 NCBI BlastP on this gene

GAA97477

hypothetical protein
  
Accession: GAA97476
  
Location: 48246-53394
  
  
**BlastP hit with Mycgr3G9942\_Mycgr3T9**
  
Percentage identity: 32 %
  
BlastP bit score: 652
  
Sequence coverage: 108 %
  
E-value: 0.0
  
  
 NCBI BlastP on this gene

GAA97476

hypothetical protein
  
Accession: GAA97475
  
Location: 46993-48203
  
 NCBI BlastP on this gene

GAA97475

hypothetical protein
  
Accession: GAA97474
  
Location: 42096-46762
  
 NCBI BlastP on this gene

GAA97474

hypothetical protein
  
Accession: GAA97473
  
Location: 39404-42042
  
 NCBI BlastP on this gene

GAA97473

hypothetical protein
  
Accession: GAA97472
  
Location: 38364-39168
  
 NCBI BlastP on this gene

GAA97472

hypothetical protein
  
Accession: GAA97471
  
Location: 35654-38246
  
 NCBI BlastP on this gene

GAA97471

hypothetical protein
  
Accession: GAA97470
  
Location: 29227-34241
  
 NCBI BlastP on this gene

GAA97470

189. :  GG700648 Trichophyton rubrum CBS 118892 genomic scaffold supercont2.1     Total score: 1.0     Cumulative Blast bit score: 1209

nonribosomal peptide synthase
  
Accession: EGD85168
  
Location: 3814046-3838539
  
  
**BlastP hit with Mycgr3G90558\_Mycgr3T**
  
Percentage identity: 31 %
  
BlastP bit score: 1209
  
Sequence coverage: 61 %
  
E-value: 0.0
  
  
 NCBI BlastP on this gene

EGD85168

190. :  DS989822 Arthroderma gypseum CBS 118893 supercont1.1 genomic scaffold     Total score: 1.0     Cumulative Blast bit score: 1200

hypothetical protein
  
Accession: EFQ97051
  
Location: 272653-273331
  
 NCBI BlastP on this gene

EFQ97051

hypothetical protein
  
Accession: EFQ97050
  
Location: 244064-268485
  
  
**BlastP hit with Mycgr3G90558\_Mycgr3T**
  
Percentage identity: 30 %
  
BlastP bit score: 1200
  
Sequence coverage: 60 %
  
E-value: 0.0
  
  
 NCBI BlastP on this gene

EFQ97050

hypothetical protein
  
Accession: EFQ97049
  
Location: 242865-243245
  
 NCBI BlastP on this gene

EFQ97049

GMP synthase
  
Accession: EFQ97048
  
Location: 240940-242635
  
 NCBI BlastP on this gene

EFQ97048

bcp1
  
Accession: EFQ97047
  
Location: 239570-240560
  
 NCBI BlastP on this gene

EFQ97047

chorismate mutase
  
Accession: EFQ97046
  
Location: 238404-239327
  
 NCBI BlastP on this gene

EFQ97046

191. :  EQ963479 Aspergillus flavus NRRL3357 scf\_1106286418500 genomic scaffold     Total score: 1.0     Cumulative Blast bit score: 1172

ABC multidrug transporter, putative
  
Accession: EED50110
  
Location: 1353107-1358412
  
 NCBI BlastP on this gene

EED50110

nonribosomal peptide synthase Pes1
  
Accession: EED50111
  
Location: 1362826-1378774
  
  
**BlastP hit with Mycgr3G90558\_Mycgr3T**
  
Percentage identity: 30 %
  
BlastP bit score: 1172
  
Sequence coverage: 60 %
  
E-value: 0.0
  
  
 NCBI BlastP on this gene

EED50111

192. :  DS995905 Penicillium marneffei ATCC 18224 scf\_1105668340970 genomic scaffold     Total score: 1.0     Cumulative Blast bit score: 1167

conserved hypothetical protein
  
Accession: EEA19298
  
Location: 96181-97575
  
 NCBI BlastP on this gene

EEA19298

conserved hypothetical protein
  
Accession: EEA19299
  
Location: 98719-128920
  
  
**BlastP hit with Mycgr3G90558\_Mycgr3T**
  
Percentage identity: 31 %
  
BlastP bit score: 1167
  
Sequence coverage: 61 %
  
E-value: 0.0
  
  
 NCBI BlastP on this gene

EEA19299

extracellular guanyl-specific ribonuclease RntA
  
Accession: EEA19300
  
Location: 129184-129687
  
 NCBI BlastP on this gene

EEA19300

193. :  KB908866 Setosphaeria turcica Et28A unplaced genomic scaffold SETTUscaffold\_8     Total score: 1.0     Cumulative Blast bit score: 1162

hypothetical protein
  
Accession: EOA81675
  
Location: 1642148-1646752
  
 NCBI BlastP on this gene

EOA81675

hypothetical protein
  
Accession: EOA81676
  
Location: 1647885-1649771
  
 NCBI BlastP on this gene

EOA81676

hypothetical protein
  
Accession: EOA81677
  
Location: 1650732-1669532
  
  
**BlastP hit with Mycgr3G90558\_Mycgr3T**
  
Percentage identity: 29 %
  
BlastP bit score: 1162
  
Sequence coverage: 77 %
  
E-value: 0.0
  
  
 NCBI BlastP on this gene

EOA81677

194. :  ABSU01000002 Arthroderma benhamiae CBS 112371     Total score: 1.0     Cumulative Blast bit score: 1155

hypothetical protein
  
Accession: EFE36189
  
Location: 1179294-1183108
  
 NCBI BlastP on this gene

EFE36189

conserved hypothetical protein
  
Accession: EFE36190
  
Location: 1183838-1184810
  
 NCBI BlastP on this gene

EFE36190

conserved hypothetical protein
  
Accession: EFE36191
  
Location: 1186773-1187742
  
 NCBI BlastP on this gene

EFE36191

universal stress protein family domain protein
  
Accession: EFE36192
  
Location: 1188281-1191606
  
 NCBI BlastP on this gene

EFE36192

nonribosomal peptide synthase, putative
  
Accession: EFE36193
  
Location: 1193042-1204848
  
  
**BlastP hit with Mycgr3G90558\_Mycgr3T**
  
Percentage identity: 33 %
  
BlastP bit score: 1155
  
Sequence coverage: 50 %
  
E-value: 0.0
  
  
 NCBI BlastP on this gene

EFE36193

195. :  CH476616 Uncinocarpus reesii 1704 scaffold\_2 genomic scaffold     Total score: 1.0     Cumulative Blast bit score: 1150

predicted protein
  
Accession: EEP79831
  
Location: 5054874-5072607
  
  
**BlastP hit with Mycgr3G90558\_Mycgr3T**
  
Percentage identity: 30 %
  
BlastP bit score: 1150
  
Sequence coverage: 62 %
  
E-value: 0.0
  
  
 NCBI BlastP on this gene

EEP79831

predicted protein
  
Accession: EEP79830
  
Location: 5047933-5054073
  
 NCBI BlastP on this gene

EEP79830

predicted protein
  
Accession: EEP79829
  
Location: 5047129-5047550
  
 NCBI BlastP on this gene

EEP79829

conserved hypothetical protein
  
Accession: EEP79828
  
Location: 5046040-5046509
  
 NCBI BlastP on this gene

EEP79828

196. :  CH408031 Chaetomium globosum CBS 148.51 scaffold\_3 genomic scaffold     Total score: 1.0     Cumulative Blast bit score: 1149

hypothetical protein
  
Accession: EAQ90170
  
Location: 4563155-4588278
  
  
**BlastP hit with Mycgr3G90558\_Mycgr3T**
  
Percentage identity: 30 %
  
BlastP bit score: 1149
  
Sequence coverage: 60 %
  
E-value: 0.0
  
  
 NCBI BlastP on this gene

EAQ90170

hypothetical protein
  
Accession: EAQ90169
  
Location: 4561980-4562956
  
 NCBI BlastP on this gene

EAQ90169

hypothetical protein
  
Accession: EAQ90168
  
Location: 4559739-4561425
  
 NCBI BlastP on this gene

EAQ90168

hypothetical protein
  
Accession: EAQ90167
  
Location: 4557012-4558886
  
 NCBI BlastP on this gene

EAQ90167

197. :  KB456260 Mycosphaerella populorum SO2202 unplaced genomic scaffold SEPMUscaffold\_1     Total score: 1.0     Cumulative Blast bit score: 1144

O-methyltransferase
  
Accession: EMF16953
  
Location: 2239892-2241831
  
 NCBI BlastP on this gene

EMF16953

cytochrome P450
  
Accession: EMF16952
  
Location: 2238905-2239818
  
 NCBI BlastP on this gene

EMF16952

hypothetical protein
  
Accession: EMF16951
  
Location: 2221896-2236653
  
  
**BlastP hit with Mycgr3G90558\_Mycgr3T**
  
Percentage identity: 26 %
  
BlastP bit score: 1144
  
Sequence coverage: 97 %
  
E-value: 0.0
  
  
 NCBI BlastP on this gene

EMF16951

ankyrin
  
Accession: EMF16950
  
Location: 2220779-2221752
  
 NCBI BlastP on this gene

EMF16950

hypothetical protein
  
Accession: EMF16949
  
Location: 2216714-2219503
  
 NCBI BlastP on this gene

EMF16949

kinase-like protein
  
Accession: EMF16948
  
Location: 2213720-2214571
  
 NCBI BlastP on this gene

EMF16948

MFS general substrate transporter
  
Accession: EMF16947
  
Location: 2203998-2212806
  
 NCBI BlastP on this gene

EMF16947

198. :  JH687760 Auricularia delicata TFB-10046 SS5 unplaced genomic scaffold AURDEscaffold\_28     Total score: 1.0     Cumulative Blast bit score: 1142

hypothetical protein
  
Accession: EJD46365
  
Location: 372574-373830
  
 NCBI BlastP on this gene

EJD46365

cytochrome P450
  
Accession: EJD46366
  
Location: 374185-375982
  
 NCBI BlastP on this gene

EJD46366

P-loop containing nucleoside triphosphate hydrolase protein
  
Accession: EJD46367
  
Location: 378047-384179
  
  
**BlastP hit with Mycgr3G9942\_Mycgr3T9**
  
Percentage identity: 30 %
  
BlastP bit score: 583
  
Sequence coverage: 109 %
  
E-value: 1e-178
  
  
 NCBI BlastP on this gene

EJD46367

hypothetical protein
  
Accession: EJD46368
  
Location: 384426-386450
  
 NCBI BlastP on this gene

EJD46368

hypothetical protein
  
Accession: EJD46369
  
Location: 386869-387313
  
 NCBI BlastP on this gene

EJD46369

ribonuclease H-like protein
  
Accession: EJD46370
  
Location: 388341-389036
  
 NCBI BlastP on this gene

EJD46370

P-loop containing nucleoside triphosphate hydrolase protein
  
Accession: EJD46371
  
Location: 397492-402252
  
  
**BlastP hit with Mycgr3G9942\_Mycgr3T9**
  
Percentage identity: 31 %
  
BlastP bit score: 559
  
Sequence coverage: 97 %
  
E-value: 3e-173
  
  
 NCBI BlastP on this gene

EJD46371

phospholipid-translocating P-type ATPase
  
Accession: EJD46372
  
Location: 403709-408500
  
 NCBI BlastP on this gene

EJD46372

199. :  GG698487 Trichophyton tonsurans CBS 112818 genomic scaffold supercont1.11     Total score: 1.0     Cumulative Blast bit score: 1140

DNA repair and recombination protein RAD26
  
Accession: EGD95238
  
Location: 70982-74796
  
 NCBI BlastP on this gene

EGD95238

hypothetical protein
  
Accession: EGD95237
  
Location: 69251-70226
  
 NCBI BlastP on this gene

EGD95237

hypothetical protein
  
Accession: EGD95236
  
Location: 66330-67518
  
 NCBI BlastP on this gene

EGD95236

hypothetical protein
  
Accession: EGD95235
  
Location: 63526-65605
  
 NCBI BlastP on this gene

EGD95235

nonribosomal peptide synthase
  
Accession: EGD95234
  
Location: 49179-60831
  
  
**BlastP hit with Mycgr3G90558\_Mycgr3T**
  
Percentage identity: 33 %
  
BlastP bit score: 1140
  
Sequence coverage: 49 %
  
E-value: 0.0
  
  
 NCBI BlastP on this gene

EGD95234

hypothetical protein
  
Accession: EGD95233
  
Location: 47996-48892
  
 NCBI BlastP on this gene

EGD95233

cmgc/cdk/pitslre protein kinase
  
Accession: EGD95232
  
Location: 44346-45950
  
 NCBI BlastP on this gene

EGD95232

superoxide dismutase copper chaperone Lys7
  
Accession: EGD95231
  
Location: 43123-44091
  
 NCBI BlastP on this gene

EGD95231

NADPH oxidase regulator NoxR
  
Accession: EGD95230
  
Location: 40584-42473
  
 NCBI BlastP on this gene

EGD95230

SNARE protein
  
Accession: EGD95229
  
Location: 38765-40116
  
 NCBI BlastP on this gene

EGD95229

hypothetical protein
  
Accession: EGD95228
  
Location: 37777-38475
  
 NCBI BlastP on this gene

EGD95228

200. :  HF679029 Fusarium fujikuroi IMI 58289 draft genome, chromosome FFUJ\_chr07.     Total score: 1.0     Cumulative Blast bit score: 1137

related to multidrug resistance protein
  
Accession: CCT71781
  
Location: 3137319-3142263
  
 NCBI BlastP on this gene

FFUJ\_08114

related to non-ribosomal peptide synthetase
  
Accession: CCT71782
  
Location: 3144734-3167581
  
  
**BlastP hit with Mycgr3G90558\_Mycgr3T**
  
Percentage identity: 31 %
  
BlastP bit score: 1137
  
Sequence coverage: 60 %
  
E-value: 0.0
  
  
 NCBI BlastP on this gene

FFUJ\_08113

Detecting sequence homology at the gene cluster level with MultiGeneBlast.
  
Marnix H. Medema, Rainer Breitling & Eriko Takano (2013)
  
*Molecular Biology and Evolution* , 30: 1218-1223.
