# Supplementary material for: In silico prediction and characterization of secondary metabolite biosynthetic gene clusters in the wheat pathogen Zymoseptoria tritici
Source: BMC Genomics. 2017 Aug 17;18:631. doi: 10.1186/s12864-017-3969-y (PMC5561558; doi:10.1186/s12864-017-3969-y)
Supplement: Supplementary file 1 — MultiGeneBLAST analysis of putative secondary metabolite clusters. All encoded amino acid sequences from genes residing in clusters predicted by AntiSMASH are given as FASTA file format. All output data from MultiGeneBLASTs are also provided. (ZIP 42911 kb) [file 12864_2017_3969_MOESM1_ESM.zip › Cluster MultiGene BLAST/out/Clusters_1_34/Cluster_11/displaypage5.xhtml]

xml version="1.0" encoding="UTF-8"?


Search Results
  
  
 Results pages: 1, 2, 3, 4, 5

**MultiGeneBlast hits**

Select gene cluster alignment
201. GG700650\_0 Trichophyton rubrum CBS 118892 genomic scaffold supercont2.3,...
202. AM920431\_0 Penicillium chrysogenum Wisconsin 54-1255 complete genome, co...
203. DS178264\_0 Puccinia graminis f. sp. tritici CRL 75-36-700-3 supercont2.3...
204. DS499594\_1 Aspergillus fumigatus A1163 scf\_000001 genomic scaffold, whol...
205. AFNW01000079\_0 Fusarium pseudograminearum CS3096, whole genome shotgun s...
206. AAHF01000004\_0 Aspergillus fumigatus Af293, whole genome shotgun sequenc...
207. GL636512\_0 Coccidioides posadasii str. Silveira unplaced genomic scaffol...
208. DS027688\_1 Neosartorya fischeri NRRL 181 1099437636249 genomic scaffold,...
209. DS027059\_1 Aspergillus clavatus NRRL 1 1099423829805 genomic scaffold, w...
210. ACJE01000021\_1 Aspergillus niger ATCC 1015, whole genome shotgun sequenc...
211. DS027058\_0 Aspergillus clavatus NRRL 1 1099423829804 genomic scaffold, w...
212. ABDG02000022\_1 Trichoderma atroviride IMI 206040, whole genome shotgun s...
213. KB445641\_1 Cochliobolus sativus ND90Pr unplaced genomic scaffold COCSAsc...
214. CH476607\_1 Aspergillus terreus NIH2624 scaffold\_14 genomic scaffold, who...
215. KB908481\_0 Setosphaeria turcica Et28A unplaced genomic scaffold SETTUsca...
216. KB644414\_0 Penicillium oxalicum 114-2 unplaced genomic scaffold scaffold...
217. DS027698\_1 Neosartorya fischeri NRRL 181 1099437636266 genomic scaffold,...
218. AM270372\_1 Aspergillus niger contig An16c0200, genomic contig.
219. CH476594\_0 Aspergillus terreus NIH2624 scaffold\_1 genomic scaffold, whol...
220. JH126399\_3 Cordyceps militaris CM01 unplaced genomic scaffold CCM\_S00001...
221. DS995899\_2 Penicillium marneffei ATCC 18224 scf\_1105668340764 genomic sc...
222. ACJE01000015\_1 Aspergillus niger ATCC 1015, whole genome shotgun sequenc...
223. EQ962652\_1 Talaromyces stipitatus ATCC 10500 scf\_1105507295523 genomic s...
224. DF126447\_0 Aspergillus kawachii IFO 4308 DNA, contig: scaffold00001, who...
225. DS231635\_1 Pyrenophora tritici-repentis Pt-1C-BFP supercont1.21 genomic ...
226. GL891382\_1 Neurospora tetrasperma FGSC 2508 unplaced genomic scaffold NE...
227. GL890999\_1 Neurospora tetrasperma FGSC 2509 unplaced genomic scaffold NE...
228. DS989823\_1 Arthroderma gypseum CBS 118893 supercont1.2 genomic scaffold,...
229. GL636493\_1 Coccidioides posadasii str. Silveira unplaced genomic scaffol...
230. GG704913\_1 Coccidioides immitis RS genomic scaffold supercont3.3, whole ...
231. ACFW01000015\_1 Coccidioides posadasii C735 delta SOWgp, whole genome sho...
232. DS231635\_0 Pyrenophora tritici-repentis Pt-1C-BFP supercont1.21 genomic ...
233. CH408029\_4 Chaetomium globosum CBS 148.51 scaffold\_1 genomic scaffold, w...
234. JH725157\_0 Beauveria bassiana ARSEF 2860 unplaced genomic scaffold BBA\_S...
235. CU633438\_1 Podospora anserina S mat+ genomic DNA chromosome 1, supercont...
236. CH476633\_0 Sclerotinia sclerotiorum 1980 scaffold\_13 genomic scaffold, w...
237. CABT02000004\_1 Sordaria macrospora k-hell, whole genome shotgun sequenci...
238. GL385398\_1 Gaeumannomyces graminis var. tritici R3-111a-1 unplaced genom...
239. DS989824\_0 Arthroderma gypseum CBS 118893 supercont1.3 genomic scaffold,...
240. JH921428\_2 Marssonina brunnea f. sp. 'multigermtubi' MB\_m1 unplaced geno...
241. GG749415\_1 Ajellomyces dermatitidis ATCC 18188 genomic scaffold supercon...
242. EQ999975\_1 Ajellomyces dermatitidis ER-3 genomic scaffold supercont1.3, ...
243. GG657450\_2 Ajellomyces dermatitidis SLH14081 genomic scaffold supercont1...
244. DS995719\_1 Trichophyton equinum CBS 127.97 supercont1.2 genomic scaffold...
245. DS027694\_3 Neosartorya fischeri NRRL 181 1099437636262 genomic scaffold,...
246. CP003013\_1 Thielavia terrestris NRRL 8126 chromosome 5, complete sequence.
247. KE145369\_0 Glarea lozoyensis ATCC 20868 chromosome Unknown GLAREA5, whol...
248. DS995705\_2 Microsporum canis CBS 113480 supercont1.5 genomic scaffold, w...
249. CP003002\_0 Myceliophthora thermophila ATCC 42464 chromosome 1, complete ...
250. CM001234\_0 Magnaporthe oryzae 70-15 chromosome 4, whole genome shotgun s...

Query: Architecture Search FASTA input

GG700650 : Trichophyton rubrum CBS 118892 genomic scaffold supercont2.3    Total score: 1.0     Cumulative Blast bit score: 1130

Hit cluster cross-links:

Mycgr3G36335 Mycgr3T
  
Location: 0-423

Mycgr3G36335\_Mycgr3T

Mycgr3G84494 Mycgr3T
  
Location: 523-2047

Mycgr3G84494\_Mycgr3T

Mycgr3G90558 Mycgr3T
  
Location: 2147-15296

Mycgr3G90558\_Mycgr3T

Mycgr3G68036 Mycgr3T
  
Location: 15396-16395

Mycgr3G68036\_Mycgr3T

Mycgr3G90561 Mycgr3T
  
Location: 16495-17134

Mycgr3G90561\_Mycgr3T

Mycgr3G35862 Mycgr3T
  
Location: 17234-18662

Mycgr3G35862\_Mycgr3T

Mycgr3G68030 Mycgr3T
  
Location: 18762-19722

Mycgr3G68030\_Mycgr3T

Mycgr3G36449 Mycgr3T
  
Location: 19822-21886

Mycgr3G36449\_Mycgr3T

Mycgr3G35528 Mycgr3T
  
Location: 21986-22844

Mycgr3G35528\_Mycgr3T

Mycgr3G35932 Mycgr3T
  
Location: 22944-24390

Mycgr3G35932\_Mycgr3T

Mycgr3G23761 Mycgr3T
  
Location: 24490-25825

Mycgr3G23761\_Mycgr3T

Mycgr3G35535 Mycgr3T
  
Location: 25925-26429

Mycgr3G35535\_Mycgr3T

Mycgr3G9942 Mycgr3T9
  
Location: 26529-30375

Mycgr3G9942\_Mycgr3T9

DNA repair protein Rhp26/Rad26
  
Accession: EGD86946
  
Location: 1259341-1263154
  
 NCBI BlastP on this gene

EGD86946

hypothetical protein
  
Accession: EGD86945
  
Location: 1257629-1258607
  
 NCBI BlastP on this gene

EGD86945

hypothetical protein
  
Accession: EGD86944
  
Location: 1255899-1256357
  
 NCBI BlastP on this gene

EGD86944

hypothetical protein
  
Accession: EGD86943
  
Location: 1254685-1255871
  
 NCBI BlastP on this gene

EGD86943

hypothetical protein
  
Accession: EGD86942
  
Location: 1251887-1253967
  
 NCBI BlastP on this gene

EGD86942

hypothetical protein
  
Accession: EGD86941
  
Location: 1250318-1250581
  
 NCBI BlastP on this gene

EGD86941

nonribosomal peptide synthase
  
Accession: EGD86940
  
Location: 1237601-1249263
  
  
**BlastP hit with Mycgr3G90558\_Mycgr3T**
  
Percentage identity: 33 %
  
BlastP bit score: 1130
  
Sequence coverage: 49 %
  
E-value: 0.0
  
  
 NCBI BlastP on this gene

EGD86940

hypothetical protein
  
Accession: EGD86939
  
Location: 1236459-1237313
  
 NCBI BlastP on this gene

EGD86939

cmgc/cdk/pitslre protein kinase
  
Accession: EGD86938
  
Location: 1232728-1234316
  
 NCBI BlastP on this gene

EGD86938

homocitrate dehydratase
  
Accession: EGD86937
  
Location: 1231528-1232496
  
 NCBI BlastP on this gene

EGD86937

NADPH oxidase regulator NoxR
  
Accession: EGD86936
  
Location: 1229028-1230923
  
 NCBI BlastP on this gene

EGD86936

SNARE protein
  
Accession: EGD86935
  
Location: 1227314-1228552
  
 NCBI BlastP on this gene

EGD86935

hypothetical protein
  
Accession: EGD86934
  
Location: 1226339-1227031
  
 NCBI BlastP on this gene

EGD86934

Query: Architecture Search FASTA input

AM920431 : Penicillium chrysogenum Wisconsin 54-1255 complete genome, contig Pc00c16.    Total score: 1.0     Cumulative Blast bit score: 1111

Hit cluster cross-links:

Mycgr3G36335 Mycgr3T
  
Location: 0-423

Mycgr3G36335\_Mycgr3T

Mycgr3G84494 Mycgr3T
  
Location: 523-2047

Mycgr3G84494\_Mycgr3T

Mycgr3G90558 Mycgr3T
  
Location: 2147-15296

Mycgr3G90558\_Mycgr3T

Mycgr3G68036 Mycgr3T
  
Location: 15396-16395

Mycgr3G68036\_Mycgr3T

Mycgr3G90561 Mycgr3T
  
Location: 16495-17134

Mycgr3G90561\_Mycgr3T

Mycgr3G35862 Mycgr3T
  
Location: 17234-18662

Mycgr3G35862\_Mycgr3T

Mycgr3G68030 Mycgr3T
  
Location: 18762-19722

Mycgr3G68030\_Mycgr3T

Mycgr3G36449 Mycgr3T
  
Location: 19822-21886

Mycgr3G36449\_Mycgr3T

Mycgr3G35528 Mycgr3T
  
Location: 21986-22844

Mycgr3G35528\_Mycgr3T

Mycgr3G35932 Mycgr3T
  
Location: 22944-24390

Mycgr3G35932\_Mycgr3T

Mycgr3G23761 Mycgr3T
  
Location: 24490-25825

Mycgr3G23761\_Mycgr3T

Mycgr3G35535 Mycgr3T
  
Location: 25925-26429

Mycgr3G35535\_Mycgr3T

Mycgr3G9942 Mycgr3T9
  
Location: 26529-30375

Mycgr3G9942\_Mycgr3T9

not annotated
  
Accession: CAP93137
  
Location: 1072077-1076885
  
 NCBI BlastP on this gene

Pc16g04670

hypothetical protein
  
Accession: CAP93138
  
Location: 1079837-1080293
  
 NCBI BlastP on this gene

Pc16g04680

not annotated
  
Accession: CAP93139
  
Location: 1080827-1099362
  
  
**BlastP hit with Mycgr3G90558\_Mycgr3T**
  
Percentage identity: 30 %
  
BlastP bit score: 1111
  
Sequence coverage: 57 %
  
E-value: 0.0
  
  
 NCBI BlastP on this gene

Pc16g04690

not annotated
  
Accession: CAP93140
  
Location: 1099963-1101744
  
 NCBI BlastP on this gene

Pc16g04700

not annotated
  
Accession: CAP93141
  
Location: 1102388-1104800
  
 NCBI BlastP on this gene

Pc16g04710

not annotated
  
Accession: CAP93142
  
Location: 1105448-1105741
  
 NCBI BlastP on this gene

Pc16g04720

phosphoglycerate kinase pgkA-Penicillium chrysogenum
  
Accession: CAP93143
  
Location: 1106637-1108007
  
 NCBI BlastP on this gene

pgkA

Query: Architecture Search FASTA input

DS178264 : Puccinia graminis f. sp. tritici CRL 75-36-700-3 supercont2.3 genomic scaffold    Total score: 1.0     Cumulative Blast bit score: 1108

Hit cluster cross-links:

Mycgr3G36335 Mycgr3T
  
Location: 0-423

Mycgr3G36335\_Mycgr3T

Mycgr3G84494 Mycgr3T
  
Location: 523-2047

Mycgr3G84494\_Mycgr3T

Mycgr3G90558 Mycgr3T
  
Location: 2147-15296

Mycgr3G90558\_Mycgr3T

Mycgr3G68036 Mycgr3T
  
Location: 15396-16395

Mycgr3G68036\_Mycgr3T

Mycgr3G90561 Mycgr3T
  
Location: 16495-17134

Mycgr3G90561\_Mycgr3T

Mycgr3G35862 Mycgr3T
  
Location: 17234-18662

Mycgr3G35862\_Mycgr3T

Mycgr3G68030 Mycgr3T
  
Location: 18762-19722

Mycgr3G68030\_Mycgr3T

Mycgr3G36449 Mycgr3T
  
Location: 19822-21886

Mycgr3G36449\_Mycgr3T

Mycgr3G35528 Mycgr3T
  
Location: 21986-22844

Mycgr3G35528\_Mycgr3T

Mycgr3G35932 Mycgr3T
  
Location: 22944-24390

Mycgr3G35932\_Mycgr3T

Mycgr3G23761 Mycgr3T
  
Location: 24490-25825

Mycgr3G23761\_Mycgr3T

Mycgr3G35535 Mycgr3T
  
Location: 25925-26429

Mycgr3G35535\_Mycgr3T

Mycgr3G9942 Mycgr3T9
  
Location: 26529-30375

Mycgr3G9942\_Mycgr3T9

hypothetical protein
  
Accession: EFP75548
  
Location: 612485-615652
  
 NCBI BlastP on this gene

EFP75548

hypothetical protein
  
Accession: EFP75547
  
Location: 598074-605294
  
  
**BlastP hit with Mycgr3G9942\_Mycgr3T9**
  
Percentage identity: 32 %
  
BlastP bit score: 560
  
Sequence coverage: 95 %
  
E-value: 9e-169
  
  
 NCBI BlastP on this gene

EFP75547

hypothetical protein
  
Accession: EFP75545
  
Location: 589501-596385
  
  
**BlastP hit with Mycgr3G9942\_Mycgr3T9**
  
Percentage identity: 31 %
  
BlastP bit score: 548
  
Sequence coverage: 93 %
  
E-value: 7e-165
  
  
 NCBI BlastP on this gene

EFP75545

Query: Architecture Search FASTA input

DS499594 : Aspergillus fumigatus A1163 scf\_000001 genomic scaffold    Total score: 1.0     Cumulative Blast bit score: 1103

Hit cluster cross-links:

Mycgr3G36335 Mycgr3T
  
Location: 0-423

Mycgr3G36335\_Mycgr3T

Mycgr3G84494 Mycgr3T
  
Location: 523-2047

Mycgr3G84494\_Mycgr3T

Mycgr3G90558 Mycgr3T
  
Location: 2147-15296

Mycgr3G90558\_Mycgr3T

Mycgr3G68036 Mycgr3T
  
Location: 15396-16395

Mycgr3G68036\_Mycgr3T

Mycgr3G90561 Mycgr3T
  
Location: 16495-17134

Mycgr3G90561\_Mycgr3T

Mycgr3G35862 Mycgr3T
  
Location: 17234-18662

Mycgr3G35862\_Mycgr3T

Mycgr3G68030 Mycgr3T
  
Location: 18762-19722

Mycgr3G68030\_Mycgr3T

Mycgr3G36449 Mycgr3T
  
Location: 19822-21886

Mycgr3G36449\_Mycgr3T

Mycgr3G35528 Mycgr3T
  
Location: 21986-22844

Mycgr3G35528\_Mycgr3T

Mycgr3G35932 Mycgr3T
  
Location: 22944-24390

Mycgr3G35932\_Mycgr3T

Mycgr3G23761 Mycgr3T
  
Location: 24490-25825

Mycgr3G23761\_Mycgr3T

Mycgr3G35535 Mycgr3T
  
Location: 25925-26429

Mycgr3G35535\_Mycgr3T

Mycgr3G9942 Mycgr3T9
  
Location: 26529-30375

Mycgr3G9942\_Mycgr3T9

ABC multidrug transporter, putative
  
Accession: EDP56273
  
Location: 2773451-2778675
  
 NCBI BlastP on this gene

EDP56273

nonribosomal peptide synthase Pes1
  
Accession: EDP56272
  
Location: 2748720-2767908
  
  
**BlastP hit with Mycgr3G90558\_Mycgr3T**
  
Percentage identity: 30 %
  
BlastP bit score: 1103
  
Sequence coverage: 58 %
  
E-value: 0.0
  
  
 NCBI BlastP on this gene

EDP56272

MFS multidrug transporter, putative
  
Accession: EDP56271
  
Location: 2746314-2748233
  
 NCBI BlastP on this gene

EDP56271

conserved hypothetical protein
  
Accession: EDP56270
  
Location: 2742713-2745250
  
 NCBI BlastP on this gene

EDP56270

26 proteasome complex subunit Sem1, putative
  
Accession: EDP56269
  
Location: 2741238-2741707
  
 NCBI BlastP on this gene

EDP56269

phosphoglycerate kinase PgkA, putative
  
Accession: EDP56268
  
Location: 2739275-2740690
  
 NCBI BlastP on this gene

EDP56268

Query: Architecture Search FASTA input

AFNW01000079 : Fusarium pseudograminearum CS3096    Total score: 1.0     Cumulative Blast bit score: 1101

Hit cluster cross-links:

Mycgr3G36335 Mycgr3T
  
Location: 0-423

Mycgr3G36335\_Mycgr3T

Mycgr3G84494 Mycgr3T
  
Location: 523-2047

Mycgr3G84494\_Mycgr3T

Mycgr3G90558 Mycgr3T
  
Location: 2147-15296

Mycgr3G90558\_Mycgr3T

Mycgr3G68036 Mycgr3T
  
Location: 15396-16395

Mycgr3G68036\_Mycgr3T

Mycgr3G90561 Mycgr3T
  
Location: 16495-17134

Mycgr3G90561\_Mycgr3T

Mycgr3G35862 Mycgr3T
  
Location: 17234-18662

Mycgr3G35862\_Mycgr3T

Mycgr3G68030 Mycgr3T
  
Location: 18762-19722

Mycgr3G68030\_Mycgr3T

Mycgr3G36449 Mycgr3T
  
Location: 19822-21886

Mycgr3G36449\_Mycgr3T

Mycgr3G35528 Mycgr3T
  
Location: 21986-22844

Mycgr3G35528\_Mycgr3T

Mycgr3G35932 Mycgr3T
  
Location: 22944-24390

Mycgr3G35932\_Mycgr3T

Mycgr3G23761 Mycgr3T
  
Location: 24490-25825

Mycgr3G23761\_Mycgr3T

Mycgr3G35535 Mycgr3T
  
Location: 25925-26429

Mycgr3G35535\_Mycgr3T

Mycgr3G9942 Mycgr3T9
  
Location: 26529-30375

Mycgr3G9942\_Mycgr3T9

hypothetical protein
  
Accession: EKJ76035
  
Location: 159580-164507
  
 NCBI BlastP on this gene

EKJ76035

NPS4
  
Accession: EKJ76036
  
Location: 166952-189868
  
  
**BlastP hit with Mycgr3G90558\_Mycgr3T**
  
Percentage identity: 30 %
  
BlastP bit score: 1101
  
Sequence coverage: 60 %
  
E-value: 0.0
  
  
 NCBI BlastP on this gene

EKJ76036

hypothetical protein
  
Accession: EKJ76037
  
Location: 190443-193127
  
 NCBI BlastP on this gene

EKJ76037

hypothetical protein
  
Accession: EKJ76038
  
Location: 193784-194656
  
 NCBI BlastP on this gene

EKJ76038

hypothetical protein
  
Accession: EKJ76039
  
Location: 194770-195391
  
 NCBI BlastP on this gene

EKJ76039

hypothetical protein
  
Accession: EKJ76040
  
Location: 195721-197439
  
 NCBI BlastP on this gene

EKJ76040

Query: Architecture Search FASTA input

AAHF01000004 : Aspergillus fumigatus Af293    Total score: 1.0     Cumulative Blast bit score: 1100

Hit cluster cross-links:

Mycgr3G36335 Mycgr3T
  
Location: 0-423

Mycgr3G36335\_Mycgr3T

Mycgr3G84494 Mycgr3T
  
Location: 523-2047

Mycgr3G84494\_Mycgr3T

Mycgr3G90558 Mycgr3T
  
Location: 2147-15296

Mycgr3G90558\_Mycgr3T

Mycgr3G68036 Mycgr3T
  
Location: 15396-16395

Mycgr3G68036\_Mycgr3T

Mycgr3G90561 Mycgr3T
  
Location: 16495-17134

Mycgr3G90561\_Mycgr3T

Mycgr3G35862 Mycgr3T
  
Location: 17234-18662

Mycgr3G35862\_Mycgr3T

Mycgr3G68030 Mycgr3T
  
Location: 18762-19722

Mycgr3G68030\_Mycgr3T

Mycgr3G36449 Mycgr3T
  
Location: 19822-21886

Mycgr3G36449\_Mycgr3T

Mycgr3G35528 Mycgr3T
  
Location: 21986-22844

Mycgr3G35528\_Mycgr3T

Mycgr3G35932 Mycgr3T
  
Location: 22944-24390

Mycgr3G35932\_Mycgr3T

Mycgr3G23761 Mycgr3T
  
Location: 24490-25825

Mycgr3G23761\_Mycgr3T

Mycgr3G35535 Mycgr3T
  
Location: 25925-26429

Mycgr3G35535\_Mycgr3T

Mycgr3G9942 Mycgr3T9
  
Location: 26529-30375

Mycgr3G9942\_Mycgr3T9

ABC multidrug transporter, putative
  
Accession: EAL90367
  
Location: 453260-458484
  
 NCBI BlastP on this gene

EAL90367

nonribosomal peptide synthase Pes1
  
Accession: EAL90366
  
Location: 428528-447716
  
  
**BlastP hit with Mycgr3G90558\_Mycgr3T**
  
Percentage identity: 30 %
  
BlastP bit score: 1100
  
Sequence coverage: 58 %
  
E-value: 0.0
  
  
 NCBI BlastP on this gene

EAL90366

MFS multidrug transporter, putative
  
Accession: EAL90365
  
Location: 426122-428041
  
 NCBI BlastP on this gene

EAL90365

conserved hypothetical protein
  
Accession: EAL90364
  
Location: 422521-425058
  
 NCBI BlastP on this gene

EAL90364

26 proteasome complex subunit Sem1, putative
  
Accession: EBA27404
  
Location: 421046-421515
  
 NCBI BlastP on this gene

EBA27404

phosphoglycerate kinase PgkA, putative
  
Accession: EAL90363
  
Location: 419083-420498
  
 NCBI BlastP on this gene

EAL90363

Query: Architecture Search FASTA input

GL636512 : Coccidioides posadasii str. Silveira unplaced genomic scaffold supercont2.27    Total score: 1.0     Cumulative Blast bit score: 1096

Hit cluster cross-links:

Mycgr3G36335 Mycgr3T
  
Location: 0-423

Mycgr3G36335\_Mycgr3T

Mycgr3G84494 Mycgr3T
  
Location: 523-2047

Mycgr3G84494\_Mycgr3T

Mycgr3G90558 Mycgr3T
  
Location: 2147-15296

Mycgr3G90558\_Mycgr3T

Mycgr3G68036 Mycgr3T
  
Location: 15396-16395

Mycgr3G68036\_Mycgr3T

Mycgr3G90561 Mycgr3T
  
Location: 16495-17134

Mycgr3G90561\_Mycgr3T

Mycgr3G35862 Mycgr3T
  
Location: 17234-18662

Mycgr3G35862\_Mycgr3T

Mycgr3G68030 Mycgr3T
  
Location: 18762-19722

Mycgr3G68030\_Mycgr3T

Mycgr3G36449 Mycgr3T
  
Location: 19822-21886

Mycgr3G36449\_Mycgr3T

Mycgr3G35528 Mycgr3T
  
Location: 21986-22844

Mycgr3G35528\_Mycgr3T

Mycgr3G35932 Mycgr3T
  
Location: 22944-24390

Mycgr3G35932\_Mycgr3T

Mycgr3G23761 Mycgr3T
  
Location: 24490-25825

Mycgr3G23761\_Mycgr3T

Mycgr3G35535 Mycgr3T
  
Location: 25925-26429

Mycgr3G35535\_Mycgr3T

Mycgr3G9942 Mycgr3T9
  
Location: 26529-30375

Mycgr3G9942\_Mycgr3T9

multidrug resistance protein MDR
  
Accession: EFW13651
  
Location: 58766-64144
  
 NCBI BlastP on this gene

EFW13651

hypothetical protein
  
Accession: EFW13650
  
Location: 56706-57200
  
 NCBI BlastP on this gene

EFW13650

hypothetical protein
  
Accession: EFW13649
  
Location: 55335-55577
  
 NCBI BlastP on this gene

EFW13649

predicted protein
  
Accession: EFW13648
  
Location: 54558-54986
  
 NCBI BlastP on this gene

EFW13648

cyclic peptide synthetase
  
Accession: EFW13647
  
Location: 34393-54038
  
  
**BlastP hit with Mycgr3G90558\_Mycgr3T**
  
Percentage identity: 32 %
  
BlastP bit score: 1096
  
Sequence coverage: 49 %
  
E-value: 0.0
  
  
 NCBI BlastP on this gene

EFW13647

conserved hypothetical protein
  
Accession: EFW13646
  
Location: 28756-34324
  
 NCBI BlastP on this gene

EFW13646

conserved hypothetical protein
  
Accession: EFW13645
  
Location: 27610-28031
  
 NCBI BlastP on this gene

EFW13645

hypothetical protein
  
Accession: EFW13644
  
Location: 26080-27080
  
 NCBI BlastP on this gene

EFW13644

Query: Architecture Search FASTA input

DS027688 : Neosartorya fischeri NRRL 181 1099437636249 genomic scaffold    Total score: 1.0     Cumulative Blast bit score: 1085

Hit cluster cross-links:

Mycgr3G36335 Mycgr3T
  
Location: 0-423

Mycgr3G36335\_Mycgr3T

Mycgr3G84494 Mycgr3T
  
Location: 523-2047

Mycgr3G84494\_Mycgr3T

Mycgr3G90558 Mycgr3T
  
Location: 2147-15296

Mycgr3G90558\_Mycgr3T

Mycgr3G68036 Mycgr3T
  
Location: 15396-16395

Mycgr3G68036\_Mycgr3T

Mycgr3G90561 Mycgr3T
  
Location: 16495-17134

Mycgr3G90561\_Mycgr3T

Mycgr3G35862 Mycgr3T
  
Location: 17234-18662

Mycgr3G35862\_Mycgr3T

Mycgr3G68030 Mycgr3T
  
Location: 18762-19722

Mycgr3G68030\_Mycgr3T

Mycgr3G36449 Mycgr3T
  
Location: 19822-21886

Mycgr3G36449\_Mycgr3T

Mycgr3G35528 Mycgr3T
  
Location: 21986-22844

Mycgr3G35528\_Mycgr3T

Mycgr3G35932 Mycgr3T
  
Location: 22944-24390

Mycgr3G35932\_Mycgr3T

Mycgr3G23761 Mycgr3T
  
Location: 24490-25825

Mycgr3G23761\_Mycgr3T

Mycgr3G35535 Mycgr3T
  
Location: 25925-26429

Mycgr3G35535\_Mycgr3T

Mycgr3G9942 Mycgr3T9
  
Location: 26529-30375

Mycgr3G9942\_Mycgr3T9

ABC multidrug transporter, putative
  
Accession: EAW22835
  
Location: 2268452-2273675
  
 NCBI BlastP on this gene

EAW22835

nonribosomal peptide synthase Pes1
  
Accession: EAW22836
  
Location: 2279236-2298428
  
  
**BlastP hit with Mycgr3G90558\_Mycgr3T**
  
Percentage identity: 30 %
  
BlastP bit score: 1085
  
Sequence coverage: 57 %
  
E-value: 0.0
  
  
 NCBI BlastP on this gene

EAW22836

MFS multidrug transporter, putative
  
Accession: EAW22837
  
Location: 2298902-2300812
  
 NCBI BlastP on this gene

EAW22837

conserved hypothetical protein
  
Accession: EAW22838
  
Location: 2301824-2304364
  
 NCBI BlastP on this gene

EAW22838

26 proteasome complex subunit Sem1, putative
  
Accession: EAW22839
  
Location: 2305394-2305863
  
 NCBI BlastP on this gene

EAW22839

phosphoglycerate kinase PgkA, putative
  
Accession: EAW22840
  
Location: 2306413-2307828
  
 NCBI BlastP on this gene

EAW22840

Query: Architecture Search FASTA input

DS027059 : Aspergillus clavatus NRRL 1 1099423829805 genomic scaffold    Total score: 1.0     Cumulative Blast bit score: 1084

Hit cluster cross-links:

Mycgr3G36335 Mycgr3T
  
Location: 0-423

Mycgr3G36335\_Mycgr3T

Mycgr3G84494 Mycgr3T
  
Location: 523-2047

Mycgr3G84494\_Mycgr3T

Mycgr3G90558 Mycgr3T
  
Location: 2147-15296

Mycgr3G90558\_Mycgr3T

Mycgr3G68036 Mycgr3T
  
Location: 15396-16395

Mycgr3G68036\_Mycgr3T

Mycgr3G90561 Mycgr3T
  
Location: 16495-17134

Mycgr3G90561\_Mycgr3T

Mycgr3G35862 Mycgr3T
  
Location: 17234-18662

Mycgr3G35862\_Mycgr3T

Mycgr3G68030 Mycgr3T
  
Location: 18762-19722

Mycgr3G68030\_Mycgr3T

Mycgr3G36449 Mycgr3T
  
Location: 19822-21886

Mycgr3G36449\_Mycgr3T

Mycgr3G35528 Mycgr3T
  
Location: 21986-22844

Mycgr3G35528\_Mycgr3T

Mycgr3G35932 Mycgr3T
  
Location: 22944-24390

Mycgr3G35932\_Mycgr3T

Mycgr3G23761 Mycgr3T
  
Location: 24490-25825

Mycgr3G23761\_Mycgr3T

Mycgr3G35535 Mycgr3T
  
Location: 25925-26429

Mycgr3G35535\_Mycgr3T

Mycgr3G9942 Mycgr3T9
  
Location: 26529-30375

Mycgr3G9942\_Mycgr3T9

ABC multidrug transporter, putative
  
Accession: EAW07798
  
Location: 2085505-2090860
  
 NCBI BlastP on this gene

EAW07798

nonribosomal peptide synthase Pes1
  
Accession: EAW07799
  
Location: 2098342-2117521
  
  
**BlastP hit with Mycgr3G90558\_Mycgr3T**
  
Percentage identity: 30 %
  
BlastP bit score: 1084
  
Sequence coverage: 57 %
  
E-value: 0.0
  
  
 NCBI BlastP on this gene

EAW07799

MFS multidrug transporter, putative
  
Accession: EAW07800
  
Location: 2118086-2119998
  
 NCBI BlastP on this gene

EAW07800

conserved hypothetical protein
  
Accession: EAW07801
  
Location: 2121198-2123770
  
 NCBI BlastP on this gene

EAW07801

26 proteasome complex subunit Sem1, putative
  
Accession: EAW07802
  
Location: 2124852-2125379
  
 NCBI BlastP on this gene

EAW07802

Query: Architecture Search FASTA input

ACJE01000021 : Aspergillus niger ATCC 1015    Total score: 1.0     Cumulative Blast bit score: 1080

Hit cluster cross-links:

Mycgr3G36335 Mycgr3T
  
Location: 0-423

Mycgr3G36335\_Mycgr3T

Mycgr3G84494 Mycgr3T
  
Location: 523-2047

Mycgr3G84494\_Mycgr3T

Mycgr3G90558 Mycgr3T
  
Location: 2147-15296

Mycgr3G90558\_Mycgr3T

Mycgr3G68036 Mycgr3T
  
Location: 15396-16395

Mycgr3G68036\_Mycgr3T

Mycgr3G90561 Mycgr3T
  
Location: 16495-17134

Mycgr3G90561\_Mycgr3T

Mycgr3G35862 Mycgr3T
  
Location: 17234-18662

Mycgr3G35862\_Mycgr3T

Mycgr3G68030 Mycgr3T
  
Location: 18762-19722

Mycgr3G68030\_Mycgr3T

Mycgr3G36449 Mycgr3T
  
Location: 19822-21886

Mycgr3G36449\_Mycgr3T

Mycgr3G35528 Mycgr3T
  
Location: 21986-22844

Mycgr3G35528\_Mycgr3T

Mycgr3G35932 Mycgr3T
  
Location: 22944-24390

Mycgr3G35932\_Mycgr3T

Mycgr3G23761 Mycgr3T
  
Location: 24490-25825

Mycgr3G23761\_Mycgr3T

Mycgr3G35535 Mycgr3T
  
Location: 25925-26429

Mycgr3G35535\_Mycgr3T

Mycgr3G9942 Mycgr3T9
  
Location: 26529-30375

Mycgr3G9942\_Mycgr3T9

non-ribosomal peptide synthetase
  
Accession: EHA17890
  
Location: 517116-536436
  
  
**BlastP hit with Mycgr3G90558\_Mycgr3T**
  
Percentage identity: 30 %
  
BlastP bit score: 1080
  
Sequence coverage: 56 %
  
E-value: 0.0
  
  
 NCBI BlastP on this gene

EHA17890

hypothetical protein
  
Accession: EHA17889
  
Location: 514316-516011
  
 NCBI BlastP on this gene

EHA17889

hypothetical protein
  
Accession: EHA17888
  
Location: 510811-513267
  
 NCBI BlastP on this gene

EHA17888

Query: Architecture Search FASTA input

DS027058 : Aspergillus clavatus NRRL 1 1099423829804 genomic scaffold    Total score: 1.0     Cumulative Blast bit score: 1070

Hit cluster cross-links:

Mycgr3G36335 Mycgr3T
  
Location: 0-423

Mycgr3G36335\_Mycgr3T

Mycgr3G84494 Mycgr3T
  
Location: 523-2047

Mycgr3G84494\_Mycgr3T

Mycgr3G90558 Mycgr3T
  
Location: 2147-15296

Mycgr3G90558\_Mycgr3T

Mycgr3G68036 Mycgr3T
  
Location: 15396-16395

Mycgr3G68036\_Mycgr3T

Mycgr3G90561 Mycgr3T
  
Location: 16495-17134

Mycgr3G90561\_Mycgr3T

Mycgr3G35862 Mycgr3T
  
Location: 17234-18662

Mycgr3G35862\_Mycgr3T

Mycgr3G68030 Mycgr3T
  
Location: 18762-19722

Mycgr3G68030\_Mycgr3T

Mycgr3G36449 Mycgr3T
  
Location: 19822-21886

Mycgr3G36449\_Mycgr3T

Mycgr3G35528 Mycgr3T
  
Location: 21986-22844

Mycgr3G35528\_Mycgr3T

Mycgr3G35932 Mycgr3T
  
Location: 22944-24390

Mycgr3G35932\_Mycgr3T

Mycgr3G23761 Mycgr3T
  
Location: 24490-25825

Mycgr3G23761\_Mycgr3T

Mycgr3G35535 Mycgr3T
  
Location: 25925-26429

Mycgr3G35535\_Mycgr3T

Mycgr3G9942 Mycgr3T9
  
Location: 26529-30375

Mycgr3G9942\_Mycgr3T9

alpha/beta fold family hydrolase, putative
  
Accession: EAW08659
  
Location: 215078-216870
  
 NCBI BlastP on this gene

EAW08659

conserved hypothetical protein
  
Accession: EAW08660
  
Location: 217072-217840
  
 NCBI BlastP on this gene

EAW08660

NADH-ubiquinone oxidoreductase B14 subunit, putative
  
Accession: EAW08661
  
Location: 218268-219011
  
 NCBI BlastP on this gene

EAW08661

DNA-directed RNA polymerase I and III 14 KDA polypeptide
  
Accession: EAW08662
  
Location: 219413-220077
  
 NCBI BlastP on this gene

EAW08662

Cytochrome P450 oxidoreductase, putative
  
Accession: EAW08663
  
Location: 221060-223000
  
 NCBI BlastP on this gene

EAW08663

FAD binding domain protein
  
Accession: EAW08664
  
Location: 223526-225225
  
 NCBI BlastP on this gene

EAW08664

nonribosomal peptide synthase, putative
  
Accession: EAW08665
  
Location: 226895-238713
  
  
**BlastP hit with Mycgr3G90558\_Mycgr3T**
  
Percentage identity: 33 %
  
BlastP bit score: 1070
  
Sequence coverage: 50 %
  
E-value: 0.0
  
  
 NCBI BlastP on this gene

EAW08665

tyrosyl-DNA phosphodiesterase domain protein
  
Accession: EAW08666
  
Location: 239650-242172
  
 NCBI BlastP on this gene

EAW08666

ABC drug exporter AtrF
  
Accession: EAW08667
  
Location: 242469-247322
  
 NCBI BlastP on this gene

EAW08667

Query: Architecture Search FASTA input

ABDG02000022 : Trichoderma atroviride IMI 206040    Total score: 1.0     Cumulative Blast bit score: 1066

Hit cluster cross-links:

Mycgr3G36335 Mycgr3T
  
Location: 0-423

Mycgr3G36335\_Mycgr3T

Mycgr3G84494 Mycgr3T
  
Location: 523-2047

Mycgr3G84494\_Mycgr3T

Mycgr3G90558 Mycgr3T
  
Location: 2147-15296

Mycgr3G90558\_Mycgr3T

Mycgr3G68036 Mycgr3T
  
Location: 15396-16395

Mycgr3G68036\_Mycgr3T

Mycgr3G90561 Mycgr3T
  
Location: 16495-17134

Mycgr3G90561\_Mycgr3T

Mycgr3G35862 Mycgr3T
  
Location: 17234-18662

Mycgr3G35862\_Mycgr3T

Mycgr3G68030 Mycgr3T
  
Location: 18762-19722

Mycgr3G68030\_Mycgr3T

Mycgr3G36449 Mycgr3T
  
Location: 19822-21886

Mycgr3G36449\_Mycgr3T

Mycgr3G35528 Mycgr3T
  
Location: 21986-22844

Mycgr3G35528\_Mycgr3T

Mycgr3G35932 Mycgr3T
  
Location: 22944-24390

Mycgr3G35932\_Mycgr3T

Mycgr3G23761 Mycgr3T
  
Location: 24490-25825

Mycgr3G23761\_Mycgr3T

Mycgr3G35535 Mycgr3T
  
Location: 25925-26429

Mycgr3G35535\_Mycgr3T

Mycgr3G9942 Mycgr3T9
  
Location: 26529-30375

Mycgr3G9942\_Mycgr3T9

hypothetical protein
  
Accession: EHK46791
  
Location: 1857036-1858537
  
 NCBI BlastP on this gene

EHK46791

hypothetical protein
  
Accession: EHK46792
  
Location: 1863768-1866101
  
 NCBI BlastP on this gene

EHK46792

hypothetical protein
  
Accession: EHK46793
  
Location: 1866512-1868122
  
 NCBI BlastP on this gene

EHK46793

hypothetical protein
  
Accession: EHK46794
  
Location: 1868845-1870457
  
 NCBI BlastP on this gene

EHK46794

non-ribosomal peptide synthetase
  
Accession: EHK46795
  
Location: 1872713-1879288
  
  
**BlastP hit with Mycgr3G90558\_Mycgr3T**
  
Percentage identity: 31 %
  
BlastP bit score: 1066
  
Sequence coverage: 50 %
  
E-value: 0.0
  
  
 NCBI BlastP on this gene

EHK46795

Query: Architecture Search FASTA input

KB445641 : Cochliobolus sativus ND90Pr unplaced genomic scaffold COCSAscaffold\_5    Total score: 1.0     Cumulative Blast bit score: 1065

Hit cluster cross-links:

Mycgr3G36335 Mycgr3T
  
Location: 0-423

Mycgr3G36335\_Mycgr3T

Mycgr3G84494 Mycgr3T
  
Location: 523-2047

Mycgr3G84494\_Mycgr3T

Mycgr3G90558 Mycgr3T
  
Location: 2147-15296

Mycgr3G90558\_Mycgr3T

Mycgr3G68036 Mycgr3T
  
Location: 15396-16395

Mycgr3G68036\_Mycgr3T

Mycgr3G90561 Mycgr3T
  
Location: 16495-17134

Mycgr3G90561\_Mycgr3T

Mycgr3G35862 Mycgr3T
  
Location: 17234-18662

Mycgr3G35862\_Mycgr3T

Mycgr3G68030 Mycgr3T
  
Location: 18762-19722

Mycgr3G68030\_Mycgr3T

Mycgr3G36449 Mycgr3T
  
Location: 19822-21886

Mycgr3G36449\_Mycgr3T

Mycgr3G35528 Mycgr3T
  
Location: 21986-22844

Mycgr3G35528\_Mycgr3T

Mycgr3G35932 Mycgr3T
  
Location: 22944-24390

Mycgr3G35932\_Mycgr3T

Mycgr3G23761 Mycgr3T
  
Location: 24490-25825

Mycgr3G23761\_Mycgr3T

Mycgr3G35535 Mycgr3T
  
Location: 25925-26429

Mycgr3G35535\_Mycgr3T

Mycgr3G9942 Mycgr3T9
  
Location: 26529-30375

Mycgr3G9942\_Mycgr3T9

Non-ribosomal peptide synthetase NPS4
  
Accession: EMD66011
  
Location: 2046340-2057828
  
  
**BlastP hit with Mycgr3G90558\_Mycgr3T**
  
Percentage identity: 30 %
  
BlastP bit score: 1065
  
Sequence coverage: 58 %
  
E-value: 0.0
  
  
 NCBI BlastP on this gene

EMD66011

carbohydrate esterase family 3 protein
  
Accession: EMD66010
  
Location: 2043937-2044323
  
 NCBI BlastP on this gene

EMD66010

hypothetical protein
  
Accession: EMD66009
  
Location: 2039778-2042201
  
 NCBI BlastP on this gene

EMD66009

hypothetical protein
  
Accession: EMD66008
  
Location: 2037712-2038965
  
 NCBI BlastP on this gene

EMD66008

glycoside hydrolase family 128 protein
  
Accession: EMD66007
  
Location: 2036463-2037478
  
 NCBI BlastP on this gene

EMD66007

hypothetical protein
  
Accession: EMD66006
  
Location: 2034106-2035949
  
 NCBI BlastP on this gene

EMD66006

Query: Architecture Search FASTA input

CH476607 : Aspergillus terreus NIH2624 scaffold\_14 genomic scaffold    Total score: 1.0     Cumulative Blast bit score: 1051

Hit cluster cross-links:

Mycgr3G36335 Mycgr3T
  
Location: 0-423

Mycgr3G36335\_Mycgr3T

Mycgr3G84494 Mycgr3T
  
Location: 523-2047

Mycgr3G84494\_Mycgr3T

Mycgr3G90558 Mycgr3T
  
Location: 2147-15296

Mycgr3G90558\_Mycgr3T

Mycgr3G68036 Mycgr3T
  
Location: 15396-16395

Mycgr3G68036\_Mycgr3T

Mycgr3G90561 Mycgr3T
  
Location: 16495-17134

Mycgr3G90561\_Mycgr3T

Mycgr3G35862 Mycgr3T
  
Location: 17234-18662

Mycgr3G35862\_Mycgr3T

Mycgr3G68030 Mycgr3T
  
Location: 18762-19722

Mycgr3G68030\_Mycgr3T

Mycgr3G36449 Mycgr3T
  
Location: 19822-21886

Mycgr3G36449\_Mycgr3T

Mycgr3G35528 Mycgr3T
  
Location: 21986-22844

Mycgr3G35528\_Mycgr3T

Mycgr3G35932 Mycgr3T
  
Location: 22944-24390

Mycgr3G35932\_Mycgr3T

Mycgr3G23761 Mycgr3T
  
Location: 24490-25825

Mycgr3G23761\_Mycgr3T

Mycgr3G35535 Mycgr3T
  
Location: 25925-26429

Mycgr3G35535\_Mycgr3T

Mycgr3G9942 Mycgr3T9
  
Location: 26529-30375

Mycgr3G9942\_Mycgr3T9

predicted protein
  
Accession: EAU30205
  
Location: 177150-184269
  
 NCBI BlastP on this gene

EAU30205

phospho-2-dehydro-3-deoxyheptonate aldolase
  
Accession: EAU30204
  
Location: 173909-175164
  
 NCBI BlastP on this gene

EAU30204

predicted protein
  
Accession: EAU30203
  
Location: 171401-172939
  
 NCBI BlastP on this gene

EAU30203

predicted protein
  
Accession: EAU30202
  
Location: 168854-169367
  
 NCBI BlastP on this gene

EAU30202

predicted protein
  
Accession: EAU30201
  
Location: 159864-167038
  
  
**BlastP hit with Mycgr3G90558\_Mycgr3T**
  
Percentage identity: 35 %
  
BlastP bit score: 1051
  
Sequence coverage: 39 %
  
E-value: 0.0
  
  
 NCBI BlastP on this gene

EAU30201

predicted protein
  
Accession: EAU30200
  
Location: 158579-159091
  
 NCBI BlastP on this gene

EAU30200

conserved hypothetical protein
  
Accession: EAU30199
  
Location: 154486-156087
  
 NCBI BlastP on this gene

EAU30199

hypothetical protein
  
Accession: EAU30198
  
Location: 152315-154117
  
 NCBI BlastP on this gene

EAU30198

conserved hypothetical protein
  
Accession: EAU30197
  
Location: 150399-151728
  
 NCBI BlastP on this gene

EAU30197

predicted protein
  
Accession: EAU30196
  
Location: 147868-149424
  
 NCBI BlastP on this gene

EAU30196

predicted protein
  
Accession: EAU30195
  
Location: 144834-146218
  
 NCBI BlastP on this gene

EAU30195

Query: Architecture Search FASTA input

KB908481 : Setosphaeria turcica Et28A unplaced genomic scaffold SETTUscaffold\_1    Total score: 1.0     Cumulative Blast bit score: 1050

Hit cluster cross-links:

Mycgr3G36335 Mycgr3T
  
Location: 0-423

Mycgr3G36335\_Mycgr3T

Mycgr3G84494 Mycgr3T
  
Location: 523-2047

Mycgr3G84494\_Mycgr3T

Mycgr3G90558 Mycgr3T
  
Location: 2147-15296

Mycgr3G90558\_Mycgr3T

Mycgr3G68036 Mycgr3T
  
Location: 15396-16395

Mycgr3G68036\_Mycgr3T

Mycgr3G90561 Mycgr3T
  
Location: 16495-17134

Mycgr3G90561\_Mycgr3T

Mycgr3G35862 Mycgr3T
  
Location: 17234-18662

Mycgr3G35862\_Mycgr3T

Mycgr3G68030 Mycgr3T
  
Location: 18762-19722

Mycgr3G68030\_Mycgr3T

Mycgr3G36449 Mycgr3T
  
Location: 19822-21886

Mycgr3G36449\_Mycgr3T

Mycgr3G35528 Mycgr3T
  
Location: 21986-22844

Mycgr3G35528\_Mycgr3T

Mycgr3G35932 Mycgr3T
  
Location: 22944-24390

Mycgr3G35932\_Mycgr3T

Mycgr3G23761 Mycgr3T
  
Location: 24490-25825

Mycgr3G23761\_Mycgr3T

Mycgr3G35535 Mycgr3T
  
Location: 25925-26429

Mycgr3G35535\_Mycgr3T

Mycgr3G9942 Mycgr3T9
  
Location: 26529-30375

Mycgr3G9942\_Mycgr3T9

hypothetical protein
  
Accession: EOA91188
  
Location: 50789-52032
  
 NCBI BlastP on this gene

EOA91188

hypothetical protein
  
Accession: EOA91189
  
Location: 52502-53511
  
 NCBI BlastP on this gene

EOA91189

hypothetical protein
  
Accession: EOA91190
  
Location: 54718-56672
  
 NCBI BlastP on this gene

EOA91190

hypothetical protein
  
Accession: EOA91191
  
Location: 56874-57983
  
 NCBI BlastP on this gene

EOA91191

hypothetical protein
  
Accession: EOA91192
  
Location: 58490-59290
  
 NCBI BlastP on this gene

EOA91192

hypothetical protein
  
Accession: EOA91193
  
Location: 59827-61733
  
 NCBI BlastP on this gene

EOA91193

hypothetical protein
  
Accession: EOA91194
  
Location: 62373-76298
  
  
**BlastP hit with Mycgr3G90558\_Mycgr3T**
  
Percentage identity: 27 %
  
BlastP bit score: 1050
  
Sequence coverage: 76 %
  
E-value: 0.0
  
  
 NCBI BlastP on this gene

EOA91194

hypothetical protein
  
Accession: EOA91195
  
Location: 77252-78831
  
 NCBI BlastP on this gene

EOA91195

hypothetical protein
  
Accession: EOA91196
  
Location: 79515-80336
  
 NCBI BlastP on this gene

EOA91196

hypothetical protein
  
Accession: EOA91197
  
Location: 83395-84911
  
 NCBI BlastP on this gene

EOA91197

Query: Architecture Search FASTA input

KB644414 : Penicillium oxalicum 114-2 unplaced genomic scaffold scaffold\_7    Total score: 1.0     Cumulative Blast bit score: 1047

Hit cluster cross-links:

Mycgr3G36335 Mycgr3T
  
Location: 0-423

Mycgr3G36335\_Mycgr3T

Mycgr3G84494 Mycgr3T
  
Location: 523-2047

Mycgr3G84494\_Mycgr3T

Mycgr3G90558 Mycgr3T
  
Location: 2147-15296

Mycgr3G90558\_Mycgr3T

Mycgr3G68036 Mycgr3T
  
Location: 15396-16395

Mycgr3G68036\_Mycgr3T

Mycgr3G90561 Mycgr3T
  
Location: 16495-17134

Mycgr3G90561\_Mycgr3T

Mycgr3G35862 Mycgr3T
  
Location: 17234-18662

Mycgr3G35862\_Mycgr3T

Mycgr3G68030 Mycgr3T
  
Location: 18762-19722

Mycgr3G68030\_Mycgr3T

Mycgr3G36449 Mycgr3T
  
Location: 19822-21886

Mycgr3G36449\_Mycgr3T

Mycgr3G35528 Mycgr3T
  
Location: 21986-22844

Mycgr3G35528\_Mycgr3T

Mycgr3G35932 Mycgr3T
  
Location: 22944-24390

Mycgr3G35932\_Mycgr3T

Mycgr3G23761 Mycgr3T
  
Location: 24490-25825

Mycgr3G23761\_Mycgr3T

Mycgr3G35535 Mycgr3T
  
Location: 25925-26429

Mycgr3G35535\_Mycgr3T

Mycgr3G9942 Mycgr3T9
  
Location: 26529-30375

Mycgr3G9942\_Mycgr3T9

hypothetical protein
  
Accession: EPS32204
  
Location: 1773861-1778676
  
 NCBI BlastP on this gene

EPS32204

hypothetical protein
  
Accession: EPS32203
  
Location: 1753106-1771943
  
  
**BlastP hit with Mycgr3G90558\_Mycgr3T**
  
Percentage identity: 30 %
  
BlastP bit score: 1047
  
Sequence coverage: 57 %
  
E-value: 0.0
  
  
 NCBI BlastP on this gene

EPS32203

hypothetical protein
  
Accession: EPS32202
  
Location: 1750653-1752573
  
 NCBI BlastP on this gene

EPS32202

hypothetical protein
  
Accession: EPS32201
  
Location: 1747067-1749637
  
 NCBI BlastP on this gene

EPS32201

hypothetical protein
  
Accession: EPS32200
  
Location: 1745491-1745990
  
 NCBI BlastP on this gene

EPS32200

hypothetical protein
  
Accession: EPS32199
  
Location: 1745193-1745477
  
 NCBI BlastP on this gene

EPS32199

Query: Architecture Search FASTA input

DS027698 : Neosartorya fischeri NRRL 181 1099437636266 genomic scaffold    Total score: 1.0     Cumulative Blast bit score: 1034

Hit cluster cross-links:

Mycgr3G36335 Mycgr3T
  
Location: 0-423

Mycgr3G36335\_Mycgr3T

Mycgr3G84494 Mycgr3T
  
Location: 523-2047

Mycgr3G84494\_Mycgr3T

Mycgr3G90558 Mycgr3T
  
Location: 2147-15296

Mycgr3G90558\_Mycgr3T

Mycgr3G68036 Mycgr3T
  
Location: 15396-16395

Mycgr3G68036\_Mycgr3T

Mycgr3G90561 Mycgr3T
  
Location: 16495-17134

Mycgr3G90561\_Mycgr3T

Mycgr3G35862 Mycgr3T
  
Location: 17234-18662

Mycgr3G35862\_Mycgr3T

Mycgr3G68030 Mycgr3T
  
Location: 18762-19722

Mycgr3G68030\_Mycgr3T

Mycgr3G36449 Mycgr3T
  
Location: 19822-21886

Mycgr3G36449\_Mycgr3T

Mycgr3G35528 Mycgr3T
  
Location: 21986-22844

Mycgr3G35528\_Mycgr3T

Mycgr3G35932 Mycgr3T
  
Location: 22944-24390

Mycgr3G35932\_Mycgr3T

Mycgr3G23761 Mycgr3T
  
Location: 24490-25825

Mycgr3G23761\_Mycgr3T

Mycgr3G35535 Mycgr3T
  
Location: 25925-26429

Mycgr3G35535\_Mycgr3T

Mycgr3G9942 Mycgr3T9
  
Location: 26529-30375

Mycgr3G9942\_Mycgr3T9

membrane dipeptidase GliJ
  
Accession: EAW16185
  
Location: 2447979-2449297
  
 NCBI BlastP on this gene

EAW16185

aminotransferase, putative
  
Accession: EAW16184
  
Location: 2446215-2447639
  
 NCBI BlastP on this gene

EAW16184

C6 zinc finger domain protein
  
Accession: EAW16183
  
Location: 2444056-2445444
  
 NCBI BlastP on this gene

EAW16183

transferase family protein
  
Accession: EAW16182
  
Location: 2439622-2441151
  
 NCBI BlastP on this gene

EAW16182

dimethylallyl tryptophan synthase, putative
  
Accession: EAW16181
  
Location: 2437444-2438808
  
 NCBI BlastP on this gene

EAW16181

nonribosomal peptide synthase, putative
  
Accession: EAW16180
  
Location: 2428387-2435518
  
  
**BlastP hit with Mycgr3G90558\_Mycgr3T**
  
Percentage identity: 32 %
  
BlastP bit score: 1034
  
Sequence coverage: 50 %
  
E-value: 0.0
  
  
 NCBI BlastP on this gene

EAW16180

Ankyrin repeat protein
  
Accession: EAW16179
  
Location: 2425838-2427307
  
 NCBI BlastP on this gene

EAW16179

peptidase family M20/M25/M40 protein
  
Accession: EAW16178
  
Location: 2424023-2425333
  
 NCBI BlastP on this gene

EAW16178

zinc alcohol dehydrogenase, putative
  
Accession: EAW16177
  
Location: 2422985-2423977
  
 NCBI BlastP on this gene

EAW16177

conserved hypothetical protein
  
Accession: EAW16176
  
Location: 2421654-2422766
  
 NCBI BlastP on this gene

EAW16176

glycosyl hydrolases family 11 protein
  
Accession: EAW16175
  
Location: 2420062-2420872
  
 NCBI BlastP on this gene

EAW16175

RTA1 like protein
  
Accession: EAW16174
  
Location: 2418546-2419510
  
 NCBI BlastP on this gene

EAW16174

fungal cellulose binding domain protein
  
Accession: EAW16173
  
Location: 2416853-2417860
  
 NCBI BlastP on this gene

EAW16173

PAN domain
  
Accession: EAW16172
  
Location: 2414338-2415559
  
 NCBI BlastP on this gene

EAW16172

Query: Architecture Search FASTA input

AM270372 : Aspergillus niger contig An16c0200, genomic contig.    Total score: 1.0     Cumulative Blast bit score: 1025

Hit cluster cross-links:

Mycgr3G36335 Mycgr3T
  
Location: 0-423

Mycgr3G36335\_Mycgr3T

Mycgr3G84494 Mycgr3T
  
Location: 523-2047

Mycgr3G84494\_Mycgr3T

Mycgr3G90558 Mycgr3T
  
Location: 2147-15296

Mycgr3G90558\_Mycgr3T

Mycgr3G68036 Mycgr3T
  
Location: 15396-16395

Mycgr3G68036\_Mycgr3T

Mycgr3G90561 Mycgr3T
  
Location: 16495-17134

Mycgr3G90561\_Mycgr3T

Mycgr3G35862 Mycgr3T
  
Location: 17234-18662

Mycgr3G35862\_Mycgr3T

Mycgr3G68030 Mycgr3T
  
Location: 18762-19722

Mycgr3G68030\_Mycgr3T

Mycgr3G36449 Mycgr3T
  
Location: 19822-21886

Mycgr3G36449\_Mycgr3T

Mycgr3G35528 Mycgr3T
  
Location: 21986-22844

Mycgr3G35528\_Mycgr3T

Mycgr3G35932 Mycgr3T
  
Location: 22944-24390

Mycgr3G35932\_Mycgr3T

Mycgr3G23761 Mycgr3T
  
Location: 24490-25825

Mycgr3G23761\_Mycgr3T

Mycgr3G35535 Mycgr3T
  
Location: 25925-26429

Mycgr3G35535\_Mycgr3T

Mycgr3G9942 Mycgr3T9
  
Location: 26529-30375

Mycgr3G9942\_Mycgr3T9

not annotated
  
Accession: CAK47006
  
Location: 150350-171653
  
  
**BlastP hit with Mycgr3G90558\_Mycgr3T**
  
Percentage identity: 29 %
  
BlastP bit score: 1025
  
Sequence coverage: 61 %
  
E-value: 0.0
  
  
 NCBI BlastP on this gene

An16g06720

not annotated
  
Accession: CAK47005
  
Location: 144347-149491
  
 NCBI BlastP on this gene

An16g06710

hypothetical protein
  
Accession: CAK47004
  
Location: 143614-144249
  
 NCBI BlastP on this gene

An16g06700

Query: Architecture Search FASTA input

CH476594 : Aspergillus terreus NIH2624 scaffold\_1 genomic scaffold    Total score: 1.0     Cumulative Blast bit score: 1005

Hit cluster cross-links:

Mycgr3G36335 Mycgr3T
  
Location: 0-423

Mycgr3G36335\_Mycgr3T

Mycgr3G84494 Mycgr3T
  
Location: 523-2047

Mycgr3G84494\_Mycgr3T

Mycgr3G90558 Mycgr3T
  
Location: 2147-15296

Mycgr3G90558\_Mycgr3T

Mycgr3G68036 Mycgr3T
  
Location: 15396-16395

Mycgr3G68036\_Mycgr3T

Mycgr3G90561 Mycgr3T
  
Location: 16495-17134

Mycgr3G90561\_Mycgr3T

Mycgr3G35862 Mycgr3T
  
Location: 17234-18662

Mycgr3G35862\_Mycgr3T

Mycgr3G68030 Mycgr3T
  
Location: 18762-19722

Mycgr3G68030\_Mycgr3T

Mycgr3G36449 Mycgr3T
  
Location: 19822-21886

Mycgr3G36449\_Mycgr3T

Mycgr3G35528 Mycgr3T
  
Location: 21986-22844

Mycgr3G35528\_Mycgr3T

Mycgr3G35932 Mycgr3T
  
Location: 22944-24390

Mycgr3G35932\_Mycgr3T

Mycgr3G23761 Mycgr3T
  
Location: 24490-25825

Mycgr3G23761\_Mycgr3T

Mycgr3G35535 Mycgr3T
  
Location: 25925-26429

Mycgr3G35535\_Mycgr3T

Mycgr3G9942 Mycgr3T9
  
Location: 26529-30375

Mycgr3G9942\_Mycgr3T9

DNA repair protein RAD51
  
Accession: EAU38876
  
Location: 666272-667562
  
 NCBI BlastP on this gene

EAU38876

conserved hypothetical protein
  
Accession: EAU38875
  
Location: 661751-665805
  
 NCBI BlastP on this gene

EAU38875

conserved hypothetical protein
  
Accession: EAU38874
  
Location: 639632-657679
  
  
**BlastP hit with Mycgr3G90558\_Mycgr3T**
  
Percentage identity: 29 %
  
BlastP bit score: 1005
  
Sequence coverage: 57 %
  
E-value: 0.0
  
  
 NCBI BlastP on this gene

EAU38874

conserved hypothetical protein
  
Accession: EAU38873
  
Location: 637521-639429
  
 NCBI BlastP on this gene

EAU38873

conserved hypothetical protein
  
Accession: EAU38872
  
Location: 634130-636496
  
 NCBI BlastP on this gene

EAU38872

predicted protein
  
Accession: EAU38871
  
Location: 632828-633278
  
 NCBI BlastP on this gene

EAU38871

phosphoglycerate kinase
  
Accession: EAU38870
  
Location: 630782-632162
  
 NCBI BlastP on this gene

EAU38870

Query: Architecture Search FASTA input

JH126399 : Cordyceps militaris CM01 unplaced genomic scaffold CCM\_S00001    Total score: 1.0     Cumulative Blast bit score: 1001

Hit cluster cross-links:

Mycgr3G36335 Mycgr3T
  
Location: 0-423

Mycgr3G36335\_Mycgr3T

Mycgr3G84494 Mycgr3T
  
Location: 523-2047

Mycgr3G84494\_Mycgr3T

Mycgr3G90558 Mycgr3T
  
Location: 2147-15296

Mycgr3G90558\_Mycgr3T

Mycgr3G68036 Mycgr3T
  
Location: 15396-16395

Mycgr3G68036\_Mycgr3T

Mycgr3G90561 Mycgr3T
  
Location: 16495-17134

Mycgr3G90561\_Mycgr3T

Mycgr3G35862 Mycgr3T
  
Location: 17234-18662

Mycgr3G35862\_Mycgr3T

Mycgr3G68030 Mycgr3T
  
Location: 18762-19722

Mycgr3G68030\_Mycgr3T

Mycgr3G36449 Mycgr3T
  
Location: 19822-21886

Mycgr3G36449\_Mycgr3T

Mycgr3G35528 Mycgr3T
  
Location: 21986-22844

Mycgr3G35528\_Mycgr3T

Mycgr3G35932 Mycgr3T
  
Location: 22944-24390

Mycgr3G35932\_Mycgr3T

Mycgr3G23761 Mycgr3T
  
Location: 24490-25825

Mycgr3G23761\_Mycgr3T

Mycgr3G35535 Mycgr3T
  
Location: 25925-26429

Mycgr3G35535\_Mycgr3T

Mycgr3G9942 Mycgr3T9
  
Location: 26529-30375

Mycgr3G9942\_Mycgr3T9

polyketide synthase, putative
  
Accession: EGX96624
  
Location: 4117047-4125452
  
 NCBI BlastP on this gene

EGX96624

Transferase
  
Accession: EGX96625
  
Location: 4126586-4128043
  
 NCBI BlastP on this gene

EGX96625

AMP dependent CoA ligase, putative
  
Accession: EGX96626
  
Location: 4128455-4131193
  
 NCBI BlastP on this gene

EGX96626

non-ribosomal peptide synthase, putative
  
Accession: EGX96627
  
Location: 4131512-4145862
  
  
**BlastP hit with Mycgr3G90558\_Mycgr3T**
  
Percentage identity: 29 %
  
BlastP bit score: 1001
  
Sequence coverage: 61 %
  
E-value: 0.0
  
  
 NCBI BlastP on this gene

EGX96627

Vps51/Vps67
  
Accession: EGX96628
  
Location: 4151594-4152580
  
 NCBI BlastP on this gene

EGX96628

UPF0135 protein
  
Accession: EGX96629
  
Location: 4152771-4153817
  
 NCBI BlastP on this gene

EGX96629

BolA domain protein
  
Accession: EGX96630
  
Location: 4154413-4154783
  
 NCBI BlastP on this gene

EGX96630

AP-1 complex subunit beta-1
  
Accession: EGX96631
  
Location: 4155095-4157538
  
 NCBI BlastP on this gene

EGX96631

Query: Architecture Search FASTA input

DS995899 : Penicillium marneffei ATCC 18224 scf\_1105668340764 genomic scaffold    Total score: 1.0     Cumulative Blast bit score: 1001

Hit cluster cross-links:

Mycgr3G36335 Mycgr3T
  
Location: 0-423

Mycgr3G36335\_Mycgr3T

Mycgr3G84494 Mycgr3T
  
Location: 523-2047

Mycgr3G84494\_Mycgr3T

Mycgr3G90558 Mycgr3T
  
Location: 2147-15296

Mycgr3G90558\_Mycgr3T

Mycgr3G68036 Mycgr3T
  
Location: 15396-16395

Mycgr3G68036\_Mycgr3T

Mycgr3G90561 Mycgr3T
  
Location: 16495-17134

Mycgr3G90561\_Mycgr3T

Mycgr3G35862 Mycgr3T
  
Location: 17234-18662

Mycgr3G35862\_Mycgr3T

Mycgr3G68030 Mycgr3T
  
Location: 18762-19722

Mycgr3G68030\_Mycgr3T

Mycgr3G36449 Mycgr3T
  
Location: 19822-21886

Mycgr3G36449\_Mycgr3T

Mycgr3G35528 Mycgr3T
  
Location: 21986-22844

Mycgr3G35528\_Mycgr3T

Mycgr3G35932 Mycgr3T
  
Location: 22944-24390

Mycgr3G35932\_Mycgr3T

Mycgr3G23761 Mycgr3T
  
Location: 24490-25825

Mycgr3G23761\_Mycgr3T

Mycgr3G35535 Mycgr3T
  
Location: 25925-26429

Mycgr3G35535\_Mycgr3T

Mycgr3G9942 Mycgr3T9
  
Location: 26529-30375

Mycgr3G9942\_Mycgr3T9

thioredoxin TrxA
  
Accession: EEA28461
  
Location: 4718277-4719087
  
 NCBI BlastP on this gene

EEA28461

thioredoxin TrxA
  
Accession: EEA28462
  
Location: 4718707-4719087
  
 NCBI BlastP on this gene

EEA28462

MFS transporter, putative
  
Accession: EEA28463
  
Location: 4719255-4721108
  
 NCBI BlastP on this gene

EEA28463

acetoacetyl-CoA synthase
  
Accession: EEA28465
  
Location: 4723225-4724976
  
 NCBI BlastP on this gene

EEA28465

hypothetical protein
  
Accession: EEA28466
  
Location: 4725469-4726530
  
 NCBI BlastP on this gene

EEA28466

conserved hypothetical protein
  
Accession: EEA28467
  
Location: 4726551-4727605
  
 NCBI BlastP on this gene

EEA28467

D-amino acid oxidase
  
Accession: EEA28468
  
Location: 4728047-4729508
  
 NCBI BlastP on this gene

EEA28468

hypothetical protein
  
Accession: EEA28469
  
Location: 4729705-4730176
  
 NCBI BlastP on this gene

EEA28469

ABC bile acid transporter, putative
  
Accession: EEA28470
  
Location: 4732381-4737426
  
  
**BlastP hit with Mycgr3G9942\_Mycgr3T9**
  
Percentage identity: 42 %
  
BlastP bit score: 1001
  
Sequence coverage: 105 %
  
E-value: 0.0
  
  
 NCBI BlastP on this gene

EEA28470

protein RDR1, putative
  
Accession: EEA28471
  
Location: 4738017-4739743
  
 NCBI BlastP on this gene

EEA28471

isoflavone reductase family protein (CipA), putative
  
Accession: EEA28472
  
Location: 4740124-4741193
  
 NCBI BlastP on this gene

EEA28472

protein CCC1, putative
  
Accession: EEA28473
  
Location: 4741768-4742652
  
 NCBI BlastP on this gene

EEA28473

hypothetical protein
  
Accession: EEA28474
  
Location: 4743192-4743947
  
 NCBI BlastP on this gene

EEA28474

extracellular OTU-like cysteine protease, putative
  
Accession: EEA28475
  
Location: 4745393-4746973
  
 NCBI BlastP on this gene

EEA28475

polyglutamate biosynthesis protein, putative
  
Accession: EEA28476
  
Location: 4747353-4748624
  
 NCBI BlastP on this gene

EEA28476

Query: Architecture Search FASTA input

ACJE01000015 : Aspergillus niger ATCC 1015    Total score: 1.0     Cumulative Blast bit score: 999

Hit cluster cross-links:

Mycgr3G36335 Mycgr3T
  
Location: 0-423

Mycgr3G36335\_Mycgr3T

Mycgr3G84494 Mycgr3T
  
Location: 523-2047

Mycgr3G84494\_Mycgr3T

Mycgr3G90558 Mycgr3T
  
Location: 2147-15296

Mycgr3G90558\_Mycgr3T

Mycgr3G68036 Mycgr3T
  
Location: 15396-16395

Mycgr3G68036\_Mycgr3T

Mycgr3G90561 Mycgr3T
  
Location: 16495-17134

Mycgr3G90561\_Mycgr3T

Mycgr3G35862 Mycgr3T
  
Location: 17234-18662

Mycgr3G35862\_Mycgr3T

Mycgr3G68030 Mycgr3T
  
Location: 18762-19722

Mycgr3G68030\_Mycgr3T

Mycgr3G36449 Mycgr3T
  
Location: 19822-21886

Mycgr3G36449\_Mycgr3T

Mycgr3G35528 Mycgr3T
  
Location: 21986-22844

Mycgr3G35528\_Mycgr3T

Mycgr3G35932 Mycgr3T
  
Location: 22944-24390

Mycgr3G35932\_Mycgr3T

Mycgr3G23761 Mycgr3T
  
Location: 24490-25825

Mycgr3G23761\_Mycgr3T

Mycgr3G35535 Mycgr3T
  
Location: 25925-26429

Mycgr3G35535\_Mycgr3T

Mycgr3G9942 Mycgr3T9
  
Location: 26529-30375

Mycgr3G9942\_Mycgr3T9

hypothetical protein
  
Accession: EHA20955
  
Location: 183359-188638
  
  
**BlastP hit with Mycgr3G90558\_Mycgr3T**
  
Percentage identity: 36 %
  
BlastP bit score: 999
  
Sequence coverage: 39 %
  
E-value: 0.0
  
  
 NCBI BlastP on this gene

EHA20955

hypothetical protein
  
Accession: EHA20954
  
Location: 179596-181600
  
 NCBI BlastP on this gene

EHA20954

hypothetical protein
  
Accession: EHA20953
  
Location: 175966-176229
  
 NCBI BlastP on this gene

EHA20953

hypothetical protein
  
Accession: EHA20952
  
Location: 173588-174637
  
 NCBI BlastP on this gene

EHA20952

hypothetical protein
  
Accession: EHA20951
  
Location: 172034-172738
  
 NCBI BlastP on this gene

EHA20951

hypothetical protein
  
Accession: EHA20950
  
Location: 168918-170385
  
 NCBI BlastP on this gene

EHA20950

Query: Architecture Search FASTA input

EQ962652 : Talaromyces stipitatus ATCC 10500 scf\_1105507295523 genomic scaffold    Total score: 1.0     Cumulative Blast bit score: 993

Hit cluster cross-links:

Mycgr3G36335 Mycgr3T
  
Location: 0-423

Mycgr3G36335\_Mycgr3T

Mycgr3G84494 Mycgr3T
  
Location: 523-2047

Mycgr3G84494\_Mycgr3T

Mycgr3G90558 Mycgr3T
  
Location: 2147-15296

Mycgr3G90558\_Mycgr3T

Mycgr3G68036 Mycgr3T
  
Location: 15396-16395

Mycgr3G68036\_Mycgr3T

Mycgr3G90561 Mycgr3T
  
Location: 16495-17134

Mycgr3G90561\_Mycgr3T

Mycgr3G35862 Mycgr3T
  
Location: 17234-18662

Mycgr3G35862\_Mycgr3T

Mycgr3G68030 Mycgr3T
  
Location: 18762-19722

Mycgr3G68030\_Mycgr3T

Mycgr3G36449 Mycgr3T
  
Location: 19822-21886

Mycgr3G36449\_Mycgr3T

Mycgr3G35528 Mycgr3T
  
Location: 21986-22844

Mycgr3G35528\_Mycgr3T

Mycgr3G35932 Mycgr3T
  
Location: 22944-24390

Mycgr3G35932\_Mycgr3T

Mycgr3G23761 Mycgr3T
  
Location: 24490-25825

Mycgr3G23761\_Mycgr3T

Mycgr3G35535 Mycgr3T
  
Location: 25925-26429

Mycgr3G35535\_Mycgr3T

Mycgr3G9942 Mycgr3T9
  
Location: 26529-30375

Mycgr3G9942\_Mycgr3T9

MFS transporter, putative
  
Accession: EED23013
  
Location: 1496467-1498489
  
 NCBI BlastP on this gene

EED23013

GNAT family N-acetyltransferase, putative
  
Accession: EED23012
  
Location: 1493729-1494985
  
 NCBI BlastP on this gene

EED23012

acetoacetyl-CoA synthase
  
Accession: EED23011
  
Location: 1491319-1493640
  
 NCBI BlastP on this gene

EED23011

hypothetical protein
  
Accession: EED23010
  
Location: 1489779-1490843
  
 NCBI BlastP on this gene

EED23010

D-amino acid oxidase
  
Accession: EED23008
  
Location: 1486876-1488301
  
 NCBI BlastP on this gene

EED23008

hypothetical protein
  
Accession: EED23007
  
Location: 1484941-1486361
  
 NCBI BlastP on this gene

EED23007

conserved hypothetical protein
  
Accession: EED23006
  
Location: 1483248-1484785
  
 NCBI BlastP on this gene

EED23006

ABC bile acid transporter, putative
  
Accession: EED23005
  
Location: 1477712-1482719
  
  
**BlastP hit with Mycgr3G9942\_Mycgr3T9**
  
Percentage identity: 41 %
  
BlastP bit score: 993
  
Sequence coverage: 104 %
  
E-value: 0.0
  
  
 NCBI BlastP on this gene

EED23005

acetyl xylan esterase, putative
  
Accession: EED23004
  
Location: 1476426-1477439
  
 NCBI BlastP on this gene

EED23004

protein CCC1, putative
  
Accession: EED23003
  
Location: 1474337-1475217
  
 NCBI BlastP on this gene

EED23003

hypothetical protein
  
Accession: EED23002
  
Location: 1473100-1473843
  
 NCBI BlastP on this gene

EED23002

extracellular OTU-like cysteine protease, putative
  
Accession: EED23001
  
Location: 1470434-1472041
  
 NCBI BlastP on this gene

EED23001

polyglutamate biosynthesis protein, putative
  
Accession: EED23000
  
Location: 1468772-1470025
  
 NCBI BlastP on this gene

EED23000

conserved hypothetical protein
  
Accession: EED22999
  
Location: 1466031-1466899
  
 NCBI BlastP on this gene

EED22999

conserved hypothetical protein
  
Accession: EED22998
  
Location: 1464238-1465280
  
 NCBI BlastP on this gene

EED22998

fungal specific transcription factor, putative
  
Accession: EED22997
  
Location: 1462310-1463752
  
 NCBI BlastP on this gene

EED22997

Query: Architecture Search FASTA input

DF126447 : Aspergillus kawachii IFO 4308 DNA, contig: scaffold00001    Total score: 1.0     Cumulative Blast bit score: 991

Hit cluster cross-links:

Mycgr3G36335 Mycgr3T
  
Location: 0-423

Mycgr3G36335\_Mycgr3T

Mycgr3G84494 Mycgr3T
  
Location: 523-2047

Mycgr3G84494\_Mycgr3T

Mycgr3G90558 Mycgr3T
  
Location: 2147-15296

Mycgr3G90558\_Mycgr3T

Mycgr3G68036 Mycgr3T
  
Location: 15396-16395

Mycgr3G68036\_Mycgr3T

Mycgr3G90561 Mycgr3T
  
Location: 16495-17134

Mycgr3G90561\_Mycgr3T

Mycgr3G35862 Mycgr3T
  
Location: 17234-18662

Mycgr3G35862\_Mycgr3T

Mycgr3G68030 Mycgr3T
  
Location: 18762-19722

Mycgr3G68030\_Mycgr3T

Mycgr3G36449 Mycgr3T
  
Location: 19822-21886

Mycgr3G36449\_Mycgr3T

Mycgr3G35528 Mycgr3T
  
Location: 21986-22844

Mycgr3G35528\_Mycgr3T

Mycgr3G35932 Mycgr3T
  
Location: 22944-24390

Mycgr3G35932\_Mycgr3T

Mycgr3G23761 Mycgr3T
  
Location: 24490-25825

Mycgr3G23761\_Mycgr3T

Mycgr3G35535 Mycgr3T
  
Location: 25925-26429

Mycgr3G35535\_Mycgr3T

Mycgr3G9942 Mycgr3T9
  
Location: 26529-30375

Mycgr3G9942\_Mycgr3T9

SNF2 family helicase/ATPase
  
Accession: GAA81997
  
Location: 371314-374966
  
 NCBI BlastP on this gene

GAA81997

RNA methyltransferase, TrmH family
  
Accession: GAA81996
  
Location: 369305-370810
  
 NCBI BlastP on this gene

GAA81996

chromatin remodeling complex subunit
  
Accession: GAA81995
  
Location: 364333-368888
  
 NCBI BlastP on this gene

GAA81995

nonribosomal peptide synthase
  
Accession: GAA81994
  
Location: 353309-361301
  
  
**BlastP hit with Mycgr3G90558\_Mycgr3T**
  
Percentage identity: 32 %
  
BlastP bit score: 991
  
Sequence coverage: 49 %
  
E-value: 0.0
  
  
 NCBI BlastP on this gene

GAA81994

cytochrome P450 monooxygenase
  
Accession: GAA81993
  
Location: 349695-351097
  
 NCBI BlastP on this gene

GAA81993

C-x8-C-x5-C-x3-H type zinc finger protein
  
Accession: GAA81992
  
Location: 345470-347135
  
 NCBI BlastP on this gene

GAA81992

37S ribosomal protein Rsm24
  
Accession: GAA81991
  
Location: 343257-344487
  
 NCBI BlastP on this gene

GAA81991

glycosyltransferase family 28
  
Accession: GAA81990
  
Location: 342095-342912
  
 NCBI BlastP on this gene

GAA81990

actin-1
  
Accession: GAA81989
  
Location: 340171-341430
  
 NCBI BlastP on this gene

GAA81989

Query: Architecture Search FASTA input

DS231635 : Pyrenophora tritici-repentis Pt-1C-BFP supercont1.21 genomic scaffold    Total score: 1.0     Cumulative Blast bit score: 988

Hit cluster cross-links:

Mycgr3G36335 Mycgr3T
  
Location: 0-423

Mycgr3G36335\_Mycgr3T

Mycgr3G84494 Mycgr3T
  
Location: 523-2047

Mycgr3G84494\_Mycgr3T

Mycgr3G90558 Mycgr3T
  
Location: 2147-15296

Mycgr3G90558\_Mycgr3T

Mycgr3G68036 Mycgr3T
  
Location: 15396-16395

Mycgr3G68036\_Mycgr3T

Mycgr3G90561 Mycgr3T
  
Location: 16495-17134

Mycgr3G90561\_Mycgr3T

Mycgr3G35862 Mycgr3T
  
Location: 17234-18662

Mycgr3G35862\_Mycgr3T

Mycgr3G68030 Mycgr3T
  
Location: 18762-19722

Mycgr3G68030\_Mycgr3T

Mycgr3G36449 Mycgr3T
  
Location: 19822-21886

Mycgr3G36449\_Mycgr3T

Mycgr3G35528 Mycgr3T
  
Location: 21986-22844

Mycgr3G35528\_Mycgr3T

Mycgr3G35932 Mycgr3T
  
Location: 22944-24390

Mycgr3G35932\_Mycgr3T

Mycgr3G23761 Mycgr3T
  
Location: 24490-25825

Mycgr3G23761\_Mycgr3T

Mycgr3G35535 Mycgr3T
  
Location: 25925-26429

Mycgr3G35535\_Mycgr3T

Mycgr3G9942 Mycgr3T9
  
Location: 26529-30375

Mycgr3G9942\_Mycgr3T9

predicted protein
  
Accession: EDU45988
  
Location: 130378-131078
  
 NCBI BlastP on this gene

EDU45988

methyltransferase MppJ
  
Accession: EDU45989
  
Location: 135456-136478
  
 NCBI BlastP on this gene

EDU45989

branched-chain-amino-acid aminotransferase 5
  
Accession: EDU45990
  
Location: 137788-138976
  
 NCBI BlastP on this gene

EDU45990

predicted protein
  
Accession: EDU45991
  
Location: 142850-143154
  
 NCBI BlastP on this gene

EDU45991

HC-toxin synthetase
  
Accession: EDU45992
  
Location: 143880-150272
  
  
**BlastP hit with Mycgr3G90558\_Mycgr3T**
  
Percentage identity: 33 %
  
BlastP bit score: 988
  
Sequence coverage: 39 %
  
E-value: 0.0
  
  
 NCBI BlastP on this gene

EDU45992

Query: Architecture Search FASTA input

GL891382 : Neurospora tetrasperma FGSC 2508 unplaced genomic scaffold NEUTE1scaffold\_81    Total score: 1.0     Cumulative Blast bit score: 980

Hit cluster cross-links:

Mycgr3G36335 Mycgr3T
  
Location: 0-423

Mycgr3G36335\_Mycgr3T

Mycgr3G84494 Mycgr3T
  
Location: 523-2047

Mycgr3G84494\_Mycgr3T

Mycgr3G90558 Mycgr3T
  
Location: 2147-15296

Mycgr3G90558\_Mycgr3T

Mycgr3G68036 Mycgr3T
  
Location: 15396-16395

Mycgr3G68036\_Mycgr3T

Mycgr3G90561 Mycgr3T
  
Location: 16495-17134

Mycgr3G90561\_Mycgr3T

Mycgr3G35862 Mycgr3T
  
Location: 17234-18662

Mycgr3G35862\_Mycgr3T

Mycgr3G68030 Mycgr3T
  
Location: 18762-19722

Mycgr3G68030\_Mycgr3T

Mycgr3G36449 Mycgr3T
  
Location: 19822-21886

Mycgr3G36449\_Mycgr3T

Mycgr3G35528 Mycgr3T
  
Location: 21986-22844

Mycgr3G35528\_Mycgr3T

Mycgr3G35932 Mycgr3T
  
Location: 22944-24390

Mycgr3G35932\_Mycgr3T

Mycgr3G23761 Mycgr3T
  
Location: 24490-25825

Mycgr3G23761\_Mycgr3T

Mycgr3G35535 Mycgr3T
  
Location: 25925-26429

Mycgr3G35535\_Mycgr3T

Mycgr3G9942 Mycgr3T9
  
Location: 26529-30375

Mycgr3G9942\_Mycgr3T9

hypothetical protein
  
Accession: EGO52402
  
Location: 4241036-4242639
  
 NCBI BlastP on this gene

EGO52402

hypothetical protein
  
Accession: EGO52403
  
Location: 4243110-4244203
  
 NCBI BlastP on this gene

EGO52403

hypothetical protein
  
Accession: EGO52404
  
Location: 4244848-4246755
  
 NCBI BlastP on this gene

EGO52404

hypothetical protein
  
Accession: EGO52405
  
Location: 4248267-4250677
  
 NCBI BlastP on this gene

EGO52405

hypothetical protein
  
Accession: EGO52406
  
Location: 4252355-4253411
  
 NCBI BlastP on this gene

EGO52406

hypothetical protein
  
Accession: EGO52407
  
Location: 4256121-4260982
  
  
**BlastP hit with Mycgr3G9942\_Mycgr3T9**
  
Percentage identity: 40 %
  
BlastP bit score: 980
  
Sequence coverage: 107 %
  
E-value: 0.0
  
  
 NCBI BlastP on this gene

EGO52407

hypothetical protein
  
Accession: EGO52408
  
Location: 4262378-4263127
  
 NCBI BlastP on this gene

EGO52408

hypothetical protein
  
Accession: EGO52409
  
Location: 4263989-4265809
  
 NCBI BlastP on this gene

EGO52409

hypothetical protein
  
Accession: EGO52410
  
Location: 4268783-4269743
  
 NCBI BlastP on this gene

EGO52410

hypothetical protein
  
Accession: EGO52411
  
Location: 4273353-4273625
  
 NCBI BlastP on this gene

EGO52411

hypothetical protein
  
Accession: EGO52412
  
Location: 4274543-4275525
  
 NCBI BlastP on this gene

EGO52412

Query: Architecture Search FASTA input

GL890999 : Neurospora tetrasperma FGSC 2509 unplaced genomic scaffold NEUTE2scaffold\_1    Total score: 1.0     Cumulative Blast bit score: 979

Hit cluster cross-links:

Mycgr3G36335 Mycgr3T
  
Location: 0-423

Mycgr3G36335\_Mycgr3T

Mycgr3G84494 Mycgr3T
  
Location: 523-2047

Mycgr3G84494\_Mycgr3T

Mycgr3G90558 Mycgr3T
  
Location: 2147-15296

Mycgr3G90558\_Mycgr3T

Mycgr3G68036 Mycgr3T
  
Location: 15396-16395

Mycgr3G68036\_Mycgr3T

Mycgr3G90561 Mycgr3T
  
Location: 16495-17134

Mycgr3G90561\_Mycgr3T

Mycgr3G35862 Mycgr3T
  
Location: 17234-18662

Mycgr3G35862\_Mycgr3T

Mycgr3G68030 Mycgr3T
  
Location: 18762-19722

Mycgr3G68030\_Mycgr3T

Mycgr3G36449 Mycgr3T
  
Location: 19822-21886

Mycgr3G36449\_Mycgr3T

Mycgr3G35528 Mycgr3T
  
Location: 21986-22844

Mycgr3G35528\_Mycgr3T

Mycgr3G35932 Mycgr3T
  
Location: 22944-24390

Mycgr3G35932\_Mycgr3T

Mycgr3G23761 Mycgr3T
  
Location: 24490-25825

Mycgr3G23761\_Mycgr3T

Mycgr3G35535 Mycgr3T
  
Location: 25925-26429

Mycgr3G35535\_Mycgr3T

Mycgr3G9942 Mycgr3T9
  
Location: 26529-30375

Mycgr3G9942\_Mycgr3T9

DUF1776-domain-containing protein
  
Accession: EGZ77221
  
Location: 4048871-4050474
  
 NCBI BlastP on this gene

EGZ77221

S-adenosyl-L-methionine-dependent methyltransferase
  
Accession: EGZ77222
  
Location: 4050943-4052036
  
 NCBI BlastP on this gene

EGZ77222

amidase signature enzyme
  
Accession: EGZ77223
  
Location: 4052680-4054578
  
 NCBI BlastP on this gene

EGZ77223

WD40 repeat-like protein
  
Accession: EGZ77224
  
Location: 4056089-4058499
  
 NCBI BlastP on this gene

EGZ77224

hypothetical protein
  
Accession: EGZ77225
  
Location: 4060109-4061171
  
 NCBI BlastP on this gene

EGZ77225

P-loop containing nucleoside triphosphate hydrolase protein
  
Accession: EGZ77226
  
Location: 4064337-4069193
  
  
**BlastP hit with Mycgr3G9942\_Mycgr3T9**
  
Percentage identity: 40 %
  
BlastP bit score: 979
  
Sequence coverage: 107 %
  
E-value: 0.0
  
  
 NCBI BlastP on this gene

EGZ77226

cytidine deaminase
  
Accession: EGZ77227
  
Location: 4070610-4071405
  
 NCBI BlastP on this gene

EGZ77227

FYVE-domain-containing protein
  
Accession: EGZ77228
  
Location: 4072267-4074081
  
 NCBI BlastP on this gene

EGZ77228

NAD(P)-binding protein
  
Accession: EGZ77229
  
Location: 4077361-4078321
  
 NCBI BlastP on this gene

EGZ77229

hypothetical protein
  
Accession: EGZ77230
  
Location: 4080415-4080576
  
 NCBI BlastP on this gene

EGZ77230

hypothetical protein
  
Accession: EGZ77231
  
Location: 4081949-4082395
  
 NCBI BlastP on this gene

EGZ77231

mannose-P-dolichol utilization defect 1 protein
  
Accession: EGZ77232
  
Location: 4083265-4084247
  
 NCBI BlastP on this gene

EGZ77232

Query: Architecture Search FASTA input

DS989823 : Arthroderma gypseum CBS 118893 supercont1.2 genomic scaffold    Total score: 1.0     Cumulative Blast bit score: 978

Hit cluster cross-links:

Mycgr3G36335 Mycgr3T
  
Location: 0-423

Mycgr3G36335\_Mycgr3T

Mycgr3G84494 Mycgr3T
  
Location: 523-2047

Mycgr3G84494\_Mycgr3T

Mycgr3G90558 Mycgr3T
  
Location: 2147-15296

Mycgr3G90558\_Mycgr3T

Mycgr3G68036 Mycgr3T
  
Location: 15396-16395

Mycgr3G68036\_Mycgr3T

Mycgr3G90561 Mycgr3T
  
Location: 16495-17134

Mycgr3G90561\_Mycgr3T

Mycgr3G35862 Mycgr3T
  
Location: 17234-18662

Mycgr3G35862\_Mycgr3T

Mycgr3G68030 Mycgr3T
  
Location: 18762-19722

Mycgr3G68030\_Mycgr3T

Mycgr3G36449 Mycgr3T
  
Location: 19822-21886

Mycgr3G36449\_Mycgr3T

Mycgr3G35528 Mycgr3T
  
Location: 21986-22844

Mycgr3G35528\_Mycgr3T

Mycgr3G35932 Mycgr3T
  
Location: 22944-24390

Mycgr3G35932\_Mycgr3T

Mycgr3G23761 Mycgr3T
  
Location: 24490-25825

Mycgr3G23761\_Mycgr3T

Mycgr3G35535 Mycgr3T
  
Location: 25925-26429

Mycgr3G35535\_Mycgr3T

Mycgr3G9942 Mycgr3T9
  
Location: 26529-30375

Mycgr3G9942\_Mycgr3T9

DNA mismatch repair protein msh6
  
Accession: EFQ99811
  
Location: 2145237-2149061
  
 NCBI BlastP on this gene

EFQ99811

hypothetical protein
  
Accession: EFQ99810
  
Location: 2144351-2145043
  
 NCBI BlastP on this gene

EFQ99810

hypothetical protein
  
Accession: EFQ99809
  
Location: 2142922-2143782
  
 NCBI BlastP on this gene

EFQ99809

hypothetical protein
  
Accession: EFQ99808
  
Location: 2141191-2142107
  
 NCBI BlastP on this gene

EFQ99808

glutaminyl-peptide cyclotransferase
  
Accession: EFQ99807
  
Location: 2139525-2140703
  
 NCBI BlastP on this gene

EFQ99807

YeeE/YedE family integral membrane protein
  
Accession: EFQ99806
  
Location: 2137902-2138970
  
 NCBI BlastP on this gene

EFQ99806

hypothetical protein
  
Accession: EFQ99805
  
Location: 2135912-2137366
  
 NCBI BlastP on this gene

EFQ99805

hypothetical protein
  
Accession: EFQ99804
  
Location: 2132724-2135045
  
 NCBI BlastP on this gene

EFQ99804

canalicular multispecific organic anion transporter 1
  
Accession: EFQ99803
  
Location: 2126683-2131526
  
  
**BlastP hit with Mycgr3G9942\_Mycgr3T9**
  
Percentage identity: 40 %
  
BlastP bit score: 978
  
Sequence coverage: 105 %
  
E-value: 0.0
  
  
 NCBI BlastP on this gene

EFQ99803

PEP phosphonomutase
  
Accession: EFQ99802
  
Location: 2125619-2126392
  
 NCBI BlastP on this gene

EFQ99802

hypothetical protein
  
Accession: EFQ99801
  
Location: 2122780-2124397
  
 NCBI BlastP on this gene

EFQ99801

aspartyl-tRNA synthetase
  
Accession: EFQ99800
  
Location: 2118202-2120391
  
 NCBI BlastP on this gene

EFQ99800

FAD binding domain-containing protein
  
Accession: EFQ99799
  
Location: 2116010-2117811
  
 NCBI BlastP on this gene

EFQ99799

hypothetical protein
  
Accession: EFQ99798
  
Location: 2113996-2115453
  
 NCBI BlastP on this gene

EFQ99798

cycloheximide resistance protein
  
Accession: EFQ99797
  
Location: 2111505-2113497
  
 NCBI BlastP on this gene

EFQ99797

Query: Architecture Search FASTA input

GL636493 : Coccidioides posadasii str. Silveira unplaced genomic scaffold supercont2.8    Total score: 1.0     Cumulative Blast bit score: 972

Hit cluster cross-links:

Mycgr3G36335 Mycgr3T
  
Location: 0-423

Mycgr3G36335\_Mycgr3T

Mycgr3G84494 Mycgr3T
  
Location: 523-2047

Mycgr3G84494\_Mycgr3T

Mycgr3G90558 Mycgr3T
  
Location: 2147-15296

Mycgr3G90558\_Mycgr3T

Mycgr3G68036 Mycgr3T
  
Location: 15396-16395

Mycgr3G68036\_Mycgr3T

Mycgr3G90561 Mycgr3T
  
Location: 16495-17134

Mycgr3G90561\_Mycgr3T

Mycgr3G35862 Mycgr3T
  
Location: 17234-18662

Mycgr3G35862\_Mycgr3T

Mycgr3G68030 Mycgr3T
  
Location: 18762-19722

Mycgr3G68030\_Mycgr3T

Mycgr3G36449 Mycgr3T
  
Location: 19822-21886

Mycgr3G36449\_Mycgr3T

Mycgr3G35528 Mycgr3T
  
Location: 21986-22844

Mycgr3G35528\_Mycgr3T

Mycgr3G35932 Mycgr3T
  
Location: 22944-24390

Mycgr3G35932\_Mycgr3T

Mycgr3G23761 Mycgr3T
  
Location: 24490-25825

Mycgr3G23761\_Mycgr3T

Mycgr3G35535 Mycgr3T
  
Location: 25925-26429

Mycgr3G35535\_Mycgr3T

Mycgr3G9942 Mycgr3T9
  
Location: 26529-30375

Mycgr3G9942\_Mycgr3T9

ABC bile acid transporter
  
Accession: EFW17833
  
Location: 651400-656328
  
  
**BlastP hit with Mycgr3G9942\_Mycgr3T9**
  
Percentage identity: 40 %
  
BlastP bit score: 972
  
Sequence coverage: 108 %
  
E-value: 0.0
  
  
 NCBI BlastP on this gene

EFW17833

conserved hypothetical protein
  
Accession: EFW17832
  
Location: 650308-651099
  
 NCBI BlastP on this gene

EFW17832

hypothetical protein
  
Accession: EFW17831
  
Location: 649618-649782
  
 NCBI BlastP on this gene

EFW17831

aspartyl-tRNA synthetase
  
Accession: EFW17830
  
Location: 646908-649102
  
 NCBI BlastP on this gene

EFW17830

nuclear movement protein nudC
  
Accession: EFW17829
  
Location: 645850-646584
  
 NCBI BlastP on this gene

EFW17829

transcription elongation factor spt5
  
Accession: EFW17828
  
Location: 641784-645162
  
 NCBI BlastP on this gene

EFW17828

acyl-CoA dehydrogenase
  
Accession: EFW17827
  
Location: 638973-640871
  
 NCBI BlastP on this gene

EFW17827

26S proteasome regulatory subunit Rpn2
  
Accession: EFW17826
  
Location: 634031-637741
  
 NCBI BlastP on this gene

EFW17826

Query: Architecture Search FASTA input

GG704913 : Coccidioides immitis RS genomic scaffold supercont3.3    Total score: 1.0     Cumulative Blast bit score: 972

Hit cluster cross-links:

Mycgr3G36335 Mycgr3T
  
Location: 0-423

Mycgr3G36335\_Mycgr3T

Mycgr3G84494 Mycgr3T
  
Location: 523-2047

Mycgr3G84494\_Mycgr3T

Mycgr3G90558 Mycgr3T
  
Location: 2147-15296

Mycgr3G90558\_Mycgr3T

Mycgr3G68036 Mycgr3T
  
Location: 15396-16395

Mycgr3G68036\_Mycgr3T

Mycgr3G90561 Mycgr3T
  
Location: 16495-17134

Mycgr3G90561\_Mycgr3T

Mycgr3G35862 Mycgr3T
  
Location: 17234-18662

Mycgr3G35862\_Mycgr3T

Mycgr3G68030 Mycgr3T
  
Location: 18762-19722

Mycgr3G68030\_Mycgr3T

Mycgr3G36449 Mycgr3T
  
Location: 19822-21886

Mycgr3G36449\_Mycgr3T

Mycgr3G35528 Mycgr3T
  
Location: 21986-22844

Mycgr3G35528\_Mycgr3T

Mycgr3G35932 Mycgr3T
  
Location: 22944-24390

Mycgr3G35932\_Mycgr3T

Mycgr3G23761 Mycgr3T
  
Location: 24490-25825

Mycgr3G23761\_Mycgr3T

Mycgr3G35535 Mycgr3T
  
Location: 25925-26429

Mycgr3G35535\_Mycgr3T

Mycgr3G9942 Mycgr3T9
  
Location: 26529-30375

Mycgr3G9942\_Mycgr3T9

tyrosine-tRNA ligase
  
Accession: EAS27906
  
Location: 292654-294847
  
 NCBI BlastP on this gene

EAS27906

DNA mismatch repair protein msh6
  
Accession: EAS27907
  
Location: 288348-292194
  
 NCBI BlastP on this gene

EAS27907

arsenate reductase
  
Accession: EAS27908
  
Location: 287368-288026
  
 NCBI BlastP on this gene

EAS27908

hypothetical protein
  
Accession: EAS27909
  
Location: 285775-286666
  
 NCBI BlastP on this gene

EAS27909

hypothetical protein
  
Accession: EAS27910
  
Location: 284326-285254
  
 NCBI BlastP on this gene

EAS27910

glutaminyl cyclase
  
Accession: EAS27911
  
Location: 282694-284015
  
 NCBI BlastP on this gene

EAS27911

YeeE/YedE family integral membrane protein
  
Accession: EAS27912
  
Location: 281133-282220
  
 NCBI BlastP on this gene

EAS27912

hypothetical protein
  
Accession: EAS27914
  
Location: 278939-280341
  
 NCBI BlastP on this gene

EAS27914

hypothetical protein
  
Accession: EAS27915
  
Location: 278054-278650
  
 NCBI BlastP on this gene

EAS27915

ABC bile acid transporter
  
Accession: EAS27916
  
Location: 272536-277464
  
  
**BlastP hit with Mycgr3G9942\_Mycgr3T9**
  
Percentage identity: 40 %
  
BlastP bit score: 972
  
Sequence coverage: 107 %
  
E-value: 0.0
  
  
 NCBI BlastP on this gene

EAS27916

hypothetical protein
  
Accession: EAS27917
  
Location: 271456-272247
  
 NCBI BlastP on this gene

EAS27917

aspartate-tRNA ligase
  
Accession: EAS27918
  
Location: 268058-270251
  
 NCBI BlastP on this gene

EAS27918

nuclear movement protein nudC
  
Accession: EAS27919
  
Location: 267001-267734
  
 NCBI BlastP on this gene

EAS27919

transcription initiation protein spt5
  
Accession: EAS27920
  
Location: 262958-266327
  
 NCBI BlastP on this gene

EAS27920

acyl-CoA dehydrogenase
  
Accession: EAS27921
  
Location: 260134-262032
  
 NCBI BlastP on this gene

EAS27921

26S proteasome regulatory subunit Rpn2
  
Accession: EAS27922
  
Location: 255198-258908
  
 NCBI BlastP on this gene

EAS27922

Query: Architecture Search FASTA input

ACFW01000015 : Coccidioides posadasii C735 delta SOWgp    Total score: 1.0     Cumulative Blast bit score: 972

Hit cluster cross-links:

Mycgr3G36335 Mycgr3T
  
Location: 0-423

Mycgr3G36335\_Mycgr3T

Mycgr3G84494 Mycgr3T
  
Location: 523-2047

Mycgr3G84494\_Mycgr3T

Mycgr3G90558 Mycgr3T
  
Location: 2147-15296

Mycgr3G90558\_Mycgr3T

Mycgr3G68036 Mycgr3T
  
Location: 15396-16395

Mycgr3G68036\_Mycgr3T

Mycgr3G90561 Mycgr3T
  
Location: 16495-17134

Mycgr3G90561\_Mycgr3T

Mycgr3G35862 Mycgr3T
  
Location: 17234-18662

Mycgr3G35862\_Mycgr3T

Mycgr3G68030 Mycgr3T
  
Location: 18762-19722

Mycgr3G68030\_Mycgr3T

Mycgr3G36449 Mycgr3T
  
Location: 19822-21886

Mycgr3G36449\_Mycgr3T

Mycgr3G35528 Mycgr3T
  
Location: 21986-22844

Mycgr3G35528\_Mycgr3T

Mycgr3G35932 Mycgr3T
  
Location: 22944-24390

Mycgr3G35932\_Mycgr3T

Mycgr3G23761 Mycgr3T
  
Location: 24490-25825

Mycgr3G23761\_Mycgr3T

Mycgr3G35535 Mycgr3T
  
Location: 25925-26429

Mycgr3G35535\_Mycgr3T

Mycgr3G9942 Mycgr3T9
  
Location: 26529-30375

Mycgr3G9942\_Mycgr3T9

MutS domain III family protein
  
Accession: EER28474
  
Location: 947971-951820
  
 NCBI BlastP on this gene

EER28474

conserved hypothetical protein
  
Accession: EER28475
  
Location: 952142-952800
  
 NCBI BlastP on this gene

EER28475

hypothetical protein
  
Accession: EER28476
  
Location: 953501-954392
  
 NCBI BlastP on this gene

EER28476

hypothetical protein
  
Accession: EER28477
  
Location: 954912-955963
  
 NCBI BlastP on this gene

EER28477

Peptidase family M28 protein
  
Accession: EER28478
  
Location: 956160-957362
  
 NCBI BlastP on this gene

EER28478

YeeE/YedE family protein
  
Accession: EER28479
  
Location: 957956-959045
  
 NCBI BlastP on this gene

EER28479

hypothetical protein
  
Accession: EER28480
  
Location: 959883-961285
  
 NCBI BlastP on this gene

EER28480

ABC transporter family protein
  
Accession: EER28481
  
Location: 962786-967714
  
  
**BlastP hit with Mycgr3G9942\_Mycgr3T9**
  
Percentage identity: 40 %
  
BlastP bit score: 972
  
Sequence coverage: 108 %
  
E-value: 0.0
  
  
 NCBI BlastP on this gene

EER28481

carboxyphosphonoenolpyruvate mutase, putative
  
Accession: EER28482
  
Location: 968015-968806
  
 NCBI BlastP on this gene

EER28482

aspartyl-tRNA synthetase, putative
  
Accession: EER28483
  
Location: 970012-972206
  
 NCBI BlastP on this gene

EER28483

nuclear movement protein nudC, putative
  
Accession: EER28484
  
Location: 972530-973264
  
 NCBI BlastP on this gene

EER28484

KOW motif containing protein
  
Accession: EER28485
  
Location: 973952-977330
  
 NCBI BlastP on this gene

EER28485

Acyl-CoA dehydrogenase, C-terminal domain containing protein
  
Accession: EER28486
  
Location: 978247-980157
  
 NCBI BlastP on this gene

EER28486

26S proteasome non-ATPase regulatory subunit 1, putative
  
Accession: EER28487
  
Location: 981389-985099
  
 NCBI BlastP on this gene

EER28487

Query: Architecture Search FASTA input

DS231635 : Pyrenophora tritici-repentis Pt-1C-BFP supercont1.21 genomic scaffold    Total score: 1.0     Cumulative Blast bit score: 971

Hit cluster cross-links:

Mycgr3G36335 Mycgr3T
  
Location: 0-423

Mycgr3G36335\_Mycgr3T

Mycgr3G84494 Mycgr3T
  
Location: 523-2047

Mycgr3G84494\_Mycgr3T

Mycgr3G90558 Mycgr3T
  
Location: 2147-15296

Mycgr3G90558\_Mycgr3T

Mycgr3G68036 Mycgr3T
  
Location: 15396-16395

Mycgr3G68036\_Mycgr3T

Mycgr3G90561 Mycgr3T
  
Location: 16495-17134

Mycgr3G90561\_Mycgr3T

Mycgr3G35862 Mycgr3T
  
Location: 17234-18662

Mycgr3G35862\_Mycgr3T

Mycgr3G68030 Mycgr3T
  
Location: 18762-19722

Mycgr3G68030\_Mycgr3T

Mycgr3G36449 Mycgr3T
  
Location: 19822-21886

Mycgr3G36449\_Mycgr3T

Mycgr3G35528 Mycgr3T
  
Location: 21986-22844

Mycgr3G35528\_Mycgr3T

Mycgr3G35932 Mycgr3T
  
Location: 22944-24390

Mycgr3G35932\_Mycgr3T

Mycgr3G23761 Mycgr3T
  
Location: 24490-25825

Mycgr3G23761\_Mycgr3T

Mycgr3G35535 Mycgr3T
  
Location: 25925-26429

Mycgr3G35535\_Mycgr3T

Mycgr3G9942 Mycgr3T9
  
Location: 26529-30375

Mycgr3G9942\_Mycgr3T9

methyltransferase MppJ
  
Accession: EDU45978
  
Location: 46956-47978
  
 NCBI BlastP on this gene

EDU45978

branched-chain-amino-acid aminotransferase 5
  
Accession: EDU45977
  
Location: 44458-45646
  
 NCBI BlastP on this gene

EDU45977

cytochrome P450 11A1, mitochondrial precursor
  
Accession: EDU45976
  
Location: 42383-43398
  
 NCBI BlastP on this gene

EDU45976

predicted protein
  
Accession: EDU45975
  
Location: 40622-40926
  
 NCBI BlastP on this gene

EDU45975

HC-toxin synthetase
  
Accession: EDU45974
  
Location: 33505-39896
  
  
**BlastP hit with Mycgr3G90558\_Mycgr3T**
  
Percentage identity: 33 %
  
BlastP bit score: 971
  
Sequence coverage: 39 %
  
E-value: 0.0
  
  
 NCBI BlastP on this gene

EDU45974

cytochrome P450
  
Accession: EDU45973
  
Location: 25871-27460
  
 NCBI BlastP on this gene

EDU45973

predicted protein
  
Accession: EDU45972
  
Location: 23181-23746
  
 NCBI BlastP on this gene

EDU45972

predicted protein
  
Accession: EDU45971
  
Location: 21535-23146
  
 NCBI BlastP on this gene

EDU45971

Query: Architecture Search FASTA input

CH408029 : Chaetomium globosum CBS 148.51 scaffold\_1 genomic scaffold    Total score: 1.0     Cumulative Blast bit score: 970

Hit cluster cross-links:

Mycgr3G36335 Mycgr3T
  
Location: 0-423

Mycgr3G36335\_Mycgr3T

Mycgr3G84494 Mycgr3T
  
Location: 523-2047

Mycgr3G84494\_Mycgr3T

Mycgr3G90558 Mycgr3T
  
Location: 2147-15296

Mycgr3G90558\_Mycgr3T

Mycgr3G68036 Mycgr3T
  
Location: 15396-16395

Mycgr3G68036\_Mycgr3T

Mycgr3G90561 Mycgr3T
  
Location: 16495-17134

Mycgr3G90561\_Mycgr3T

Mycgr3G35862 Mycgr3T
  
Location: 17234-18662

Mycgr3G35862\_Mycgr3T

Mycgr3G68030 Mycgr3T
  
Location: 18762-19722

Mycgr3G68030\_Mycgr3T

Mycgr3G36449 Mycgr3T
  
Location: 19822-21886

Mycgr3G36449\_Mycgr3T

Mycgr3G35528 Mycgr3T
  
Location: 21986-22844

Mycgr3G35528\_Mycgr3T

Mycgr3G35932 Mycgr3T
  
Location: 22944-24390

Mycgr3G35932\_Mycgr3T

Mycgr3G23761 Mycgr3T
  
Location: 24490-25825

Mycgr3G23761\_Mycgr3T

Mycgr3G35535 Mycgr3T
  
Location: 25925-26429

Mycgr3G35535\_Mycgr3T

Mycgr3G9942 Mycgr3T9
  
Location: 26529-30375

Mycgr3G9942\_Mycgr3T9

hypothetical protein
  
Accession: EAQ93550
  
Location: 5511220-5516115
  
  
**BlastP hit with Mycgr3G9942\_Mycgr3T9**
  
Percentage identity: 40 %
  
BlastP bit score: 970
  
Sequence coverage: 106 %
  
E-value: 0.0
  
  
 NCBI BlastP on this gene

EAQ93550

hypothetical protein
  
Accession: EAQ93549
  
Location: 5506535-5509773
  
 NCBI BlastP on this gene

EAQ93549

hypothetical protein
  
Accession: EAQ93548
  
Location: 5502531-5504798
  
 NCBI BlastP on this gene

EAQ93548

hypothetical protein
  
Accession: EAQ93547
  
Location: 5498972-5500508
  
 NCBI BlastP on this gene

EAQ93547

hypothetical protein
  
Accession: EAQ93546
  
Location: 5496230-5498311
  
 NCBI BlastP on this gene

EAQ93546

Query: Architecture Search FASTA input

JH725157 : Beauveria bassiana ARSEF 2860 unplaced genomic scaffold BBA\_S00008    Total score: 1.0     Cumulative Blast bit score: 967

Hit cluster cross-links:

Mycgr3G36335 Mycgr3T
  
Location: 0-423

Mycgr3G36335\_Mycgr3T

Mycgr3G84494 Mycgr3T
  
Location: 523-2047

Mycgr3G84494\_Mycgr3T

Mycgr3G90558 Mycgr3T
  
Location: 2147-15296

Mycgr3G90558\_Mycgr3T

Mycgr3G68036 Mycgr3T
  
Location: 15396-16395

Mycgr3G68036\_Mycgr3T

Mycgr3G90561 Mycgr3T
  
Location: 16495-17134

Mycgr3G90561\_Mycgr3T

Mycgr3G35862 Mycgr3T
  
Location: 17234-18662

Mycgr3G35862\_Mycgr3T

Mycgr3G68030 Mycgr3T
  
Location: 18762-19722

Mycgr3G68030\_Mycgr3T

Mycgr3G36449 Mycgr3T
  
Location: 19822-21886

Mycgr3G36449\_Mycgr3T

Mycgr3G35528 Mycgr3T
  
Location: 21986-22844

Mycgr3G35528\_Mycgr3T

Mycgr3G35932 Mycgr3T
  
Location: 22944-24390

Mycgr3G35932\_Mycgr3T

Mycgr3G23761 Mycgr3T
  
Location: 24490-25825

Mycgr3G23761\_Mycgr3T

Mycgr3G35535 Mycgr3T
  
Location: 25925-26429

Mycgr3G35535\_Mycgr3T

Mycgr3G9942 Mycgr3T9
  
Location: 26529-30375

Mycgr3G9942\_Mycgr3T9

ABC bile acid transporter, putative
  
Accession: EJP67092
  
Location: 99092-104075
  
 NCBI BlastP on this gene

EJP67092

methyltransferase domain-containing protein
  
Accession: EJP67093
  
Location: 105176-106045
  
 NCBI BlastP on this gene

EJP67093

hypothetical protein
  
Accession: EJP67094
  
Location: 106606-107538
  
 NCBI BlastP on this gene

EJP67094

MFS transporter, putative
  
Accession: EJP67095
  
Location: 110045-111581
  
 NCBI BlastP on this gene

EJP67095

MFS transporter, putative
  
Accession: EJP67096
  
Location: 111704-113283
  
 NCBI BlastP on this gene

EJP67096

nonribosomal peptide synthase, putative
  
Accession: EJP67097
  
Location: 115213-127028
  
  
**BlastP hit with Mycgr3G90558\_Mycgr3T**
  
Percentage identity: 31 %
  
BlastP bit score: 968
  
Sequence coverage: 50 %
  
E-value: 0.0
  
  
 NCBI BlastP on this gene

EJP67097

heterokaryon incompatibility protein
  
Accession: EJP67098
  
Location: 127717-130000
  
 NCBI BlastP on this gene

EJP67098

hypothetical protein
  
Accession: EJP67099
  
Location: 130019-132156
  
 NCBI BlastP on this gene

EJP67099

Formyl transferase
  
Accession: EJP67100
  
Location: 133080-134023
  
 NCBI BlastP on this gene

EJP67100

NAD dependent epimerase/dehydratase
  
Accession: EJP67101
  
Location: 135336-136424
  
 NCBI BlastP on this gene

EJP67101

thymidylate kinase
  
Accession: EJP67102
  
Location: 137120-138030
  
 NCBI BlastP on this gene

EJP67102

Query: Architecture Search FASTA input

CU633438 : Podospora anserina S mat+ genomic DNA chromosome 1, supercontig 1.    Total score: 1.0     Cumulative Blast bit score: 966

Hit cluster cross-links:

Mycgr3G36335 Mycgr3T
  
Location: 0-423

Mycgr3G36335\_Mycgr3T

Mycgr3G84494 Mycgr3T
  
Location: 523-2047

Mycgr3G84494\_Mycgr3T

Mycgr3G90558 Mycgr3T
  
Location: 2147-15296

Mycgr3G90558\_Mycgr3T

Mycgr3G68036 Mycgr3T
  
Location: 15396-16395

Mycgr3G68036\_Mycgr3T

Mycgr3G90561 Mycgr3T
  
Location: 16495-17134

Mycgr3G90561\_Mycgr3T

Mycgr3G35862 Mycgr3T
  
Location: 17234-18662

Mycgr3G35862\_Mycgr3T

Mycgr3G68030 Mycgr3T
  
Location: 18762-19722

Mycgr3G68030\_Mycgr3T

Mycgr3G36449 Mycgr3T
  
Location: 19822-21886

Mycgr3G36449\_Mycgr3T

Mycgr3G35528 Mycgr3T
  
Location: 21986-22844

Mycgr3G35528\_Mycgr3T

Mycgr3G35932 Mycgr3T
  
Location: 22944-24390

Mycgr3G35932\_Mycgr3T

Mycgr3G23761 Mycgr3T
  
Location: 24490-25825

Mycgr3G23761\_Mycgr3T

Mycgr3G35535 Mycgr3T
  
Location: 25925-26429

Mycgr3G35535\_Mycgr3T

Mycgr3G9942 Mycgr3T9
  
Location: 26529-30375

Mycgr3G9942\_Mycgr3T9

not annotated
  
Accession: CAP60085
  
Location: 1446828-1450400
  
 NCBI BlastP on this gene

CAP60085

not annotated
  
Accession: CAP60086
  
Location: 1451450-1452882
  
 NCBI BlastP on this gene

CAP60086

not annotated
  
Accession: CAP60087
  
Location: 1453539-1455826
  
 NCBI BlastP on this gene

CAP60087

not annotated
  
Accession: CAP60088
  
Location: 1456439-1458310
  
 NCBI BlastP on this gene

CAP60088

not annotated
  
Accession: CAP60089
  
Location: 1459064-1461493
  
 NCBI BlastP on this gene

CAP60089

not annotated
  
Accession: CAP60090
  
Location: 1464194-1469041
  
  
**BlastP hit with Mycgr3G9942\_Mycgr3T9**
  
Percentage identity: 40 %
  
BlastP bit score: 966
  
Sequence coverage: 108 %
  
E-value: 0.0
  
  
 NCBI BlastP on this gene

CAP60090

Query: Architecture Search FASTA input

CH476633 : Sclerotinia sclerotiorum 1980 scaffold\_13 genomic scaffold    Total score: 1.0     Cumulative Blast bit score: 966

Hit cluster cross-links:

Mycgr3G36335 Mycgr3T
  
Location: 0-423

Mycgr3G36335\_Mycgr3T

Mycgr3G84494 Mycgr3T
  
Location: 523-2047

Mycgr3G84494\_Mycgr3T

Mycgr3G90558 Mycgr3T
  
Location: 2147-15296

Mycgr3G90558\_Mycgr3T

Mycgr3G68036 Mycgr3T
  
Location: 15396-16395

Mycgr3G68036\_Mycgr3T

Mycgr3G90561 Mycgr3T
  
Location: 16495-17134

Mycgr3G90561\_Mycgr3T

Mycgr3G35862 Mycgr3T
  
Location: 17234-18662

Mycgr3G35862\_Mycgr3T

Mycgr3G68030 Mycgr3T
  
Location: 18762-19722

Mycgr3G68030\_Mycgr3T

Mycgr3G36449 Mycgr3T
  
Location: 19822-21886

Mycgr3G36449\_Mycgr3T

Mycgr3G35528 Mycgr3T
  
Location: 21986-22844

Mycgr3G35528\_Mycgr3T

Mycgr3G35932 Mycgr3T
  
Location: 22944-24390

Mycgr3G35932\_Mycgr3T

Mycgr3G23761 Mycgr3T
  
Location: 24490-25825

Mycgr3G23761\_Mycgr3T

Mycgr3G35535 Mycgr3T
  
Location: 25925-26429

Mycgr3G35535\_Mycgr3T

Mycgr3G9942 Mycgr3T9
  
Location: 26529-30375

Mycgr3G9942\_Mycgr3T9

hypothetical protein
  
Accession: EDN93385
  
Location: 302259-303418
  
 NCBI BlastP on this gene

EDN93385

hypothetical protein
  
Accession: EDN93386
  
Location: 305578-308810
  
 NCBI BlastP on this gene

EDN93386

hypothetical protein
  
Accession: EDN93387
  
Location: 310883-313315
  
 NCBI BlastP on this gene

EDN93387

predicted protein
  
Accession: EDN93388
  
Location: 313893-314757
  
 NCBI BlastP on this gene

EDN93388

hypothetical protein
  
Accession: EDN93389
  
Location: 316763-321759
  
  
**BlastP hit with Mycgr3G9942\_Mycgr3T9**
  
Percentage identity: 40 %
  
BlastP bit score: 966
  
Sequence coverage: 105 %
  
E-value: 0.0
  
  
 NCBI BlastP on this gene

EDN93389

predicted protein
  
Accession: EDN93390
  
Location: 323128-324474
  
 NCBI BlastP on this gene

EDN93390

hypothetical protein
  
Accession: EDN93391
  
Location: 325754-326035
  
 NCBI BlastP on this gene

EDN93391

predicted protein
  
Accession: EDN93392
  
Location: 328150-328326
  
 NCBI BlastP on this gene

EDN93392

hypothetical protein
  
Accession: EDN93393
  
Location: 329546-329752
  
 NCBI BlastP on this gene

EDN93393

hypothetical protein
  
Accession: EDN93394
  
Location: 330222-334364
  
 NCBI BlastP on this gene

EDN93394

hypothetical protein
  
Accession: EDN93395
  
Location: 335306-340081
  
 NCBI BlastP on this gene

EDN93395

Query: Architecture Search FASTA input

CABT02000004 : Sordaria macrospora k-hell    Total score: 1.0     Cumulative Blast bit score: 966

Hit cluster cross-links:

Mycgr3G36335 Mycgr3T
  
Location: 0-423

Mycgr3G36335\_Mycgr3T

Mycgr3G84494 Mycgr3T
  
Location: 523-2047

Mycgr3G84494\_Mycgr3T

Mycgr3G90558 Mycgr3T
  
Location: 2147-15296

Mycgr3G90558\_Mycgr3T

Mycgr3G68036 Mycgr3T
  
Location: 15396-16395

Mycgr3G68036\_Mycgr3T

Mycgr3G90561 Mycgr3T
  
Location: 16495-17134

Mycgr3G90561\_Mycgr3T

Mycgr3G35862 Mycgr3T
  
Location: 17234-18662

Mycgr3G35862\_Mycgr3T

Mycgr3G68030 Mycgr3T
  
Location: 18762-19722

Mycgr3G68030\_Mycgr3T

Mycgr3G36449 Mycgr3T
  
Location: 19822-21886

Mycgr3G36449\_Mycgr3T

Mycgr3G35528 Mycgr3T
  
Location: 21986-22844

Mycgr3G35528\_Mycgr3T

Mycgr3G35932 Mycgr3T
  
Location: 22944-24390

Mycgr3G35932\_Mycgr3T

Mycgr3G23761 Mycgr3T
  
Location: 24490-25825

Mycgr3G23761\_Mycgr3T

Mycgr3G35535 Mycgr3T
  
Location: 25925-26429

Mycgr3G35535\_Mycgr3T

Mycgr3G9942 Mycgr3T9
  
Location: 26529-30375

Mycgr3G9942\_Mycgr3T9

not annotated
  
Accession: CCC08062
  
Location: 1555424-1557042
  
 NCBI BlastP on this gene

CCC08062

not annotated
  
Accession: CCC08063
  
Location: 1557521-1558624
  
 NCBI BlastP on this gene

CCC08063

not annotated
  
Accession: CCC08064
  
Location: 1559273-1561179
  
 NCBI BlastP on this gene

CCC08064

not annotated
  
Accession: CCC08065
  
Location: 1562691-1565088
  
 NCBI BlastP on this gene

CCC08065

not annotated
  
Accession: CCC08066
  
Location: 1567154-1567344
  
 NCBI BlastP on this gene

CCC08066

not annotated
  
Accession: CCC08067
  
Location: 1570493-1577429
  
  
**BlastP hit with Mycgr3G9942\_Mycgr3T9**
  
Percentage identity: 40 %
  
BlastP bit score: 966
  
Sequence coverage: 107 %
  
E-value: 0.0
  
  
 NCBI BlastP on this gene

CCC08067

Query: Architecture Search FASTA input

GL385398 : Gaeumannomyces graminis var. tritici R3-111a-1 unplaced genomic scaffold supercont2.4    Total score: 1.0     Cumulative Blast bit score: 959

Hit cluster cross-links:

Mycgr3G36335 Mycgr3T
  
Location: 0-423

Mycgr3G36335\_Mycgr3T

Mycgr3G84494 Mycgr3T
  
Location: 523-2047

Mycgr3G84494\_Mycgr3T

Mycgr3G90558 Mycgr3T
  
Location: 2147-15296

Mycgr3G90558\_Mycgr3T

Mycgr3G68036 Mycgr3T
  
Location: 15396-16395

Mycgr3G68036\_Mycgr3T

Mycgr3G90561 Mycgr3T
  
Location: 16495-17134

Mycgr3G90561\_Mycgr3T

Mycgr3G35862 Mycgr3T
  
Location: 17234-18662

Mycgr3G35862\_Mycgr3T

Mycgr3G68030 Mycgr3T
  
Location: 18762-19722

Mycgr3G68030\_Mycgr3T

Mycgr3G36449 Mycgr3T
  
Location: 19822-21886

Mycgr3G36449\_Mycgr3T

Mycgr3G35528 Mycgr3T
  
Location: 21986-22844

Mycgr3G35528\_Mycgr3T

Mycgr3G35932 Mycgr3T
  
Location: 22944-24390

Mycgr3G35932\_Mycgr3T

Mycgr3G23761 Mycgr3T
  
Location: 24490-25825

Mycgr3G23761\_Mycgr3T

Mycgr3G35535 Mycgr3T
  
Location: 25925-26429

Mycgr3G35535\_Mycgr3T

Mycgr3G9942 Mycgr3T9
  
Location: 26529-30375

Mycgr3G9942\_Mycgr3T9

carboxy-cis,cis-muconate cyclase
  
Accession: EJT74519
  
Location: 2854513-2855917
  
 NCBI BlastP on this gene

EJT74519

hypothetical protein
  
Accession: EJT74518
  
Location: 2852623-2854142
  
 NCBI BlastP on this gene

EJT74518

hypothetical protein
  
Accession: EJT74517
  
Location: 2849570-2850761
  
 NCBI BlastP on this gene

EJT74517

hypothetical protein
  
Accession: EJT74516
  
Location: 2848184-2849016
  
 NCBI BlastP on this gene

EJT74516

hypothetical protein
  
Accession: EJT74515
  
Location: 2844033-2846557
  
 NCBI BlastP on this gene

EJT74515

hypothetical protein
  
Accession: EJT74514
  
Location: 2835242-2840146
  
  
**BlastP hit with Mycgr3G9942\_Mycgr3T9**
  
Percentage identity: 39 %
  
BlastP bit score: 960
  
Sequence coverage: 109 %
  
E-value: 0.0
  
  
 NCBI BlastP on this gene

EJT74514

hypothetical protein
  
Accession: EJT74513
  
Location: 2830961-2833720
  
 NCBI BlastP on this gene

EJT74513

NADH-cytochrome b5 reductase 2
  
Accession: EJT74512
  
Location: 2829097-2830273
  
 NCBI BlastP on this gene

EJT74512

SH3 domain-containing protein
  
Accession: EJT74511
  
Location: 2826306-2828033
  
 NCBI BlastP on this gene

EJT74511

RNA polymerase II subunit A domain phosphatase SSU72
  
Accession: EJT74510
  
Location: 2821714-2822551
  
 NCBI BlastP on this gene

EJT74510

Query: Architecture Search FASTA input

DS989824 : Arthroderma gypseum CBS 118893 supercont1.3 genomic scaffold    Total score: 1.0     Cumulative Blast bit score: 959

Hit cluster cross-links:

Mycgr3G36335 Mycgr3T
  
Location: 0-423

Mycgr3G36335\_Mycgr3T

Mycgr3G84494 Mycgr3T
  
Location: 523-2047

Mycgr3G84494\_Mycgr3T

Mycgr3G90558 Mycgr3T
  
Location: 2147-15296

Mycgr3G90558\_Mycgr3T

Mycgr3G68036 Mycgr3T
  
Location: 15396-16395

Mycgr3G68036\_Mycgr3T

Mycgr3G90561 Mycgr3T
  
Location: 16495-17134

Mycgr3G90561\_Mycgr3T

Mycgr3G35862 Mycgr3T
  
Location: 17234-18662

Mycgr3G35862\_Mycgr3T

Mycgr3G68030 Mycgr3T
  
Location: 18762-19722

Mycgr3G68030\_Mycgr3T

Mycgr3G36449 Mycgr3T
  
Location: 19822-21886

Mycgr3G36449\_Mycgr3T

Mycgr3G35528 Mycgr3T
  
Location: 21986-22844

Mycgr3G35528\_Mycgr3T

Mycgr3G35932 Mycgr3T
  
Location: 22944-24390

Mycgr3G35932\_Mycgr3T

Mycgr3G23761 Mycgr3T
  
Location: 24490-25825

Mycgr3G23761\_Mycgr3T

Mycgr3G35535 Mycgr3T
  
Location: 25925-26429

Mycgr3G35535\_Mycgr3T

Mycgr3G9942 Mycgr3T9
  
Location: 26529-30375

Mycgr3G9942\_Mycgr3T9

hypothetical protein
  
Accession: EFR00568
  
Location: 383665-386208
  
 NCBI BlastP on this gene

EFR00568

hypothetical protein
  
Accession: EFR00569
  
Location: 387047-387985
  
 NCBI BlastP on this gene

EFR00569

hypothetical protein
  
Accession: EFR00570
  
Location: 389798-390613
  
 NCBI BlastP on this gene

EFR00570

hypothetical protein
  
Accession: EFR00571
  
Location: 391334-392384
  
 NCBI BlastP on this gene

EFR00571

nuclear distribution protein nudF
  
Accession: EFR00572
  
Location: 393048-394625
  
 NCBI BlastP on this gene

EFR00572

CMGC/SRPK protein kinase
  
Accession: EFR00573
  
Location: 395363-396734
  
 NCBI BlastP on this gene

EFR00573

hypothetical protein
  
Accession: EFR00574
  
Location: 397292-403135
  
  
**BlastP hit with Mycgr3G9942\_Mycgr3T9**
  
Percentage identity: 40 %
  
BlastP bit score: 960
  
Sequence coverage: 103 %
  
E-value: 0.0
  
  
 NCBI BlastP on this gene

EFR00574

averantin oxidoreductase
  
Accession: EFR00575
  
Location: 404522-406244
  
 NCBI BlastP on this gene

EFR00575

hypothetical protein
  
Accession: EFR00576
  
Location: 406769-408112
  
 NCBI BlastP on this gene

EFR00576

hypothetical protein
  
Accession: EFR00577
  
Location: 408338-412797
  
 NCBI BlastP on this gene

EFR00577

DUF543 domain-containing protein
  
Accession: EFR00578
  
Location: 413100-413627
  
 NCBI BlastP on this gene

EFR00578

hypothetical protein
  
Accession: EFR00579
  
Location: 414121-415983
  
 NCBI BlastP on this gene

EFR00579

hypothetical protein
  
Accession: EFR00580
  
Location: 416700-417808
  
 NCBI BlastP on this gene

EFR00580

Query: Architecture Search FASTA input

JH921428 : Marssonina brunnea f. sp. 'multigermtubi' MB\_m1 unplaced genomic scaffold M6\_S00001    Total score: 1.0     Cumulative Blast bit score: 959

Hit cluster cross-links:

Mycgr3G36335 Mycgr3T
  
Location: 0-423

Mycgr3G36335\_Mycgr3T

Mycgr3G84494 Mycgr3T
  
Location: 523-2047

Mycgr3G84494\_Mycgr3T

Mycgr3G90558 Mycgr3T
  
Location: 2147-15296

Mycgr3G90558\_Mycgr3T

Mycgr3G68036 Mycgr3T
  
Location: 15396-16395

Mycgr3G68036\_Mycgr3T

Mycgr3G90561 Mycgr3T
  
Location: 16495-17134

Mycgr3G90561\_Mycgr3T

Mycgr3G35862 Mycgr3T
  
Location: 17234-18662

Mycgr3G35862\_Mycgr3T

Mycgr3G68030 Mycgr3T
  
Location: 18762-19722

Mycgr3G68030\_Mycgr3T

Mycgr3G36449 Mycgr3T
  
Location: 19822-21886

Mycgr3G36449\_Mycgr3T

Mycgr3G35528 Mycgr3T
  
Location: 21986-22844

Mycgr3G35528\_Mycgr3T

Mycgr3G35932 Mycgr3T
  
Location: 22944-24390

Mycgr3G35932\_Mycgr3T

Mycgr3G23761 Mycgr3T
  
Location: 24490-25825

Mycgr3G23761\_Mycgr3T

Mycgr3G35535 Mycgr3T
  
Location: 25925-26429

Mycgr3G35535\_Mycgr3T

Mycgr3G9942 Mycgr3T9
  
Location: 26529-30375

Mycgr3G9942\_Mycgr3T9

zearalenone hydrolase
  
Accession: EKD21810
  
Location: 5100098-5100958
  
 NCBI BlastP on this gene

EKD21810

WD domain-containing protein
  
Accession: EKD21811
  
Location: 5105381-5107727
  
 NCBI BlastP on this gene

EKD21811

ABC transporter
  
Accession: EKD21812
  
Location: 5110233-5115163
  
  
**BlastP hit with Mycgr3G9942\_Mycgr3T9**
  
Percentage identity: 39 %
  
BlastP bit score: 959
  
Sequence coverage: 107 %
  
E-value: 0.0
  
  
 NCBI BlastP on this gene

EKD21812

Query: Architecture Search FASTA input

GG749415 : Ajellomyces dermatitidis ATCC 18188 genomic scaffold supercont1.9    Total score: 1.0     Cumulative Blast bit score: 959

Hit cluster cross-links:

Mycgr3G36335 Mycgr3T
  
Location: 0-423

Mycgr3G36335\_Mycgr3T

Mycgr3G84494 Mycgr3T
  
Location: 523-2047

Mycgr3G84494\_Mycgr3T

Mycgr3G90558 Mycgr3T
  
Location: 2147-15296

Mycgr3G90558\_Mycgr3T

Mycgr3G68036 Mycgr3T
  
Location: 15396-16395

Mycgr3G68036\_Mycgr3T

Mycgr3G90561 Mycgr3T
  
Location: 16495-17134

Mycgr3G90561\_Mycgr3T

Mycgr3G35862 Mycgr3T
  
Location: 17234-18662

Mycgr3G35862\_Mycgr3T

Mycgr3G68030 Mycgr3T
  
Location: 18762-19722

Mycgr3G68030\_Mycgr3T

Mycgr3G36449 Mycgr3T
  
Location: 19822-21886

Mycgr3G36449\_Mycgr3T

Mycgr3G35528 Mycgr3T
  
Location: 21986-22844

Mycgr3G35528\_Mycgr3T

Mycgr3G35932 Mycgr3T
  
Location: 22944-24390

Mycgr3G35932\_Mycgr3T

Mycgr3G23761 Mycgr3T
  
Location: 24490-25825

Mycgr3G23761\_Mycgr3T

Mycgr3G35535 Mycgr3T
  
Location: 25925-26429

Mycgr3G35535\_Mycgr3T

Mycgr3G9942 Mycgr3T9
  
Location: 26529-30375

Mycgr3G9942\_Mycgr3T9

DNA mismatch repair protein msh6
  
Accession: EGE79590
  
Location: 841320-845315
  
 NCBI BlastP on this gene

EGE79590

hypothetical protein
  
Accession: EGE79591
  
Location: 845667-846579
  
 NCBI BlastP on this gene

EGE79591

hypothetical protein
  
Accession: EGE79592
  
Location: 847506-848417
  
 NCBI BlastP on this gene

EGE79592

hypothetical protein
  
Accession: EGE79593
  
Location: 848906-849829
  
 NCBI BlastP on this gene

EGE79593

glutaminyl cyclase
  
Accession: EGE79594
  
Location: 850303-851732
  
 NCBI BlastP on this gene

EGE79594

YeeE/YedE family integral membrane protein
  
Accession: EGE79595
  
Location: 852350-853521
  
 NCBI BlastP on this gene

EGE79595

hypothetical protein
  
Accession: EGE79596
  
Location: 855396-856222
  
 NCBI BlastP on this gene

EGE79596

ABC bile acid transporter
  
Accession: EGE79597
  
Location: 857083-861945
  
  
**BlastP hit with Mycgr3G9942\_Mycgr3T9**
  
Percentage identity: 40 %
  
BlastP bit score: 959
  
Sequence coverage: 106 %
  
E-value: 0.0
  
  
 NCBI BlastP on this gene

EGE79597

Query: Architecture Search FASTA input

EQ999975 : Ajellomyces dermatitidis ER-3 genomic scaffold supercont1.3    Total score: 1.0     Cumulative Blast bit score: 959

Hit cluster cross-links:

Mycgr3G36335 Mycgr3T
  
Location: 0-423

Mycgr3G36335\_Mycgr3T

Mycgr3G84494 Mycgr3T
  
Location: 523-2047

Mycgr3G84494\_Mycgr3T

Mycgr3G90558 Mycgr3T
  
Location: 2147-15296

Mycgr3G90558\_Mycgr3T

Mycgr3G68036 Mycgr3T
  
Location: 15396-16395

Mycgr3G68036\_Mycgr3T

Mycgr3G90561 Mycgr3T
  
Location: 16495-17134

Mycgr3G90561\_Mycgr3T

Mycgr3G35862 Mycgr3T
  
Location: 17234-18662

Mycgr3G35862\_Mycgr3T

Mycgr3G68030 Mycgr3T
  
Location: 18762-19722

Mycgr3G68030\_Mycgr3T

Mycgr3G36449 Mycgr3T
  
Location: 19822-21886

Mycgr3G36449\_Mycgr3T

Mycgr3G35528 Mycgr3T
  
Location: 21986-22844

Mycgr3G35528\_Mycgr3T

Mycgr3G35932 Mycgr3T
  
Location: 22944-24390

Mycgr3G35932\_Mycgr3T

Mycgr3G23761 Mycgr3T
  
Location: 24490-25825

Mycgr3G23761\_Mycgr3T

Mycgr3G35535 Mycgr3T
  
Location: 25925-26429

Mycgr3G35535\_Mycgr3T

Mycgr3G9942 Mycgr3T9
  
Location: 26529-30375

Mycgr3G9942\_Mycgr3T9

DNA mismatch repair protein msh6
  
Accession: EEQ88009
  
Location: 4069178-4073173
  
 NCBI BlastP on this gene

EEQ88009

conserved hypothetical protein
  
Accession: EEQ88008
  
Location: 4067934-4068845
  
 NCBI BlastP on this gene

EEQ88008

conserved hypothetical protein
  
Accession: EEQ88007
  
Location: 4066096-4067007
  
 NCBI BlastP on this gene

EEQ88007

conserved hypothetical protein
  
Accession: EEQ88006
  
Location: 4064684-4065607
  
 NCBI BlastP on this gene

EEQ88006

glutaminyl cyclase
  
Accession: EEQ88005
  
Location: 4062774-4064210
  
 NCBI BlastP on this gene

EEQ88005

YeeE/YedE family integral membrane protein
  
Accession: EEQ88004
  
Location: 4060981-4062156
  
 NCBI BlastP on this gene

EEQ88004

hypothetical protein
  
Accession: EEQ88003
  
Location: 4058355-4059734
  
 NCBI BlastP on this gene

EEQ88003

ABC bile acid transporter
  
Accession: EEQ88002
  
Location: 4052784-4057646
  
  
**BlastP hit with Mycgr3G9942\_Mycgr3T9**
  
Percentage identity: 40 %
  
BlastP bit score: 959
  
Sequence coverage: 106 %
  
E-value: 0.0
  
  
 NCBI BlastP on this gene

EEQ88002

glucose-methanol-choline oxidoreductase
  
Accession: EEQ88001
  
Location: 4050457-4052271
  
 NCBI BlastP on this gene

EEQ88001

conserved hypothetical protein
  
Accession: EEQ88000
  
Location: 4047058-4048740
  
 NCBI BlastP on this gene

EEQ88000

PEP phosphonomutase
  
Accession: EEQ87999
  
Location: 4045752-4046552
  
 NCBI BlastP on this gene

EEQ87999

26S proteasome regulatory subunit Rpn2
  
Accession: EEQ87998
  
Location: 4041021-4044761
  
 NCBI BlastP on this gene

EEQ87998

acyl-CoA dehydrogenase
  
Accession: EEQ87997
  
Location: 4037585-4039551
  
 NCBI BlastP on this gene

EEQ87997

Query: Architecture Search FASTA input

GG657450 : Ajellomyces dermatitidis SLH14081 genomic scaffold supercont1.3    Total score: 1.0     Cumulative Blast bit score: 957

Hit cluster cross-links:

Mycgr3G36335 Mycgr3T
  
Location: 0-423

Mycgr3G36335\_Mycgr3T

Mycgr3G84494 Mycgr3T
  
Location: 523-2047

Mycgr3G84494\_Mycgr3T

Mycgr3G90558 Mycgr3T
  
Location: 2147-15296

Mycgr3G90558\_Mycgr3T

Mycgr3G68036 Mycgr3T
  
Location: 15396-16395

Mycgr3G68036\_Mycgr3T

Mycgr3G90561 Mycgr3T
  
Location: 16495-17134

Mycgr3G90561\_Mycgr3T

Mycgr3G35862 Mycgr3T
  
Location: 17234-18662

Mycgr3G35862\_Mycgr3T

Mycgr3G68030 Mycgr3T
  
Location: 18762-19722

Mycgr3G68030\_Mycgr3T

Mycgr3G36449 Mycgr3T
  
Location: 19822-21886

Mycgr3G36449\_Mycgr3T

Mycgr3G35528 Mycgr3T
  
Location: 21986-22844

Mycgr3G35528\_Mycgr3T

Mycgr3G35932 Mycgr3T
  
Location: 22944-24390

Mycgr3G35932\_Mycgr3T

Mycgr3G23761 Mycgr3T
  
Location: 24490-25825

Mycgr3G23761\_Mycgr3T

Mycgr3G35535 Mycgr3T
  
Location: 25925-26429

Mycgr3G35535\_Mycgr3T

Mycgr3G9942 Mycgr3T9
  
Location: 26529-30375

Mycgr3G9942\_Mycgr3T9

DNA mismatch repair protein msh6
  
Accession: EEQ75395
  
Location: 3884607-3888602
  
 NCBI BlastP on this gene

EEQ75395

conserved hypothetical protein
  
Accession: EEQ75396
  
Location: 3888955-3889866
  
 NCBI BlastP on this gene

EEQ75396

conserved hypothetical protein
  
Accession: EEQ75397
  
Location: 3890793-3891704
  
 NCBI BlastP on this gene

EEQ75397

conserved hypothetical protein
  
Accession: EEQ75398
  
Location: 3892193-3893116
  
 NCBI BlastP on this gene

EEQ75398

glutaminyl cyclase
  
Accession: EEQ75399
  
Location: 3893589-3895010
  
 NCBI BlastP on this gene

EEQ75399

YeeE/YedE family integral membrane protein
  
Accession: EEQ75400
  
Location: 3895628-3896799
  
 NCBI BlastP on this gene

EEQ75400

hypothetical protein
  
Accession: EEQ75401
  
Location: 3898592-3899142
  
 NCBI BlastP on this gene

EEQ75401

ABC bile acid transporter
  
Accession: EEQ75402
  
Location: 3900121-3904983
  
  
**BlastP hit with Mycgr3G9942\_Mycgr3T9**
  
Percentage identity: 40 %
  
BlastP bit score: 957
  
Sequence coverage: 106 %
  
E-value: 0.0
  
  
 NCBI BlastP on this gene

EEQ75402

Query: Architecture Search FASTA input

DS995719 : Trichophyton equinum CBS 127.97 supercont1.2 genomic scaffold    Total score: 1.0     Cumulative Blast bit score: 956

Hit cluster cross-links:

Mycgr3G36335 Mycgr3T
  
Location: 0-423

Mycgr3G36335\_Mycgr3T

Mycgr3G84494 Mycgr3T
  
Location: 523-2047

Mycgr3G84494\_Mycgr3T

Mycgr3G90558 Mycgr3T
  
Location: 2147-15296

Mycgr3G90558\_Mycgr3T

Mycgr3G68036 Mycgr3T
  
Location: 15396-16395

Mycgr3G68036\_Mycgr3T

Mycgr3G90561 Mycgr3T
  
Location: 16495-17134

Mycgr3G90561\_Mycgr3T

Mycgr3G35862 Mycgr3T
  
Location: 17234-18662

Mycgr3G35862\_Mycgr3T

Mycgr3G68030 Mycgr3T
  
Location: 18762-19722

Mycgr3G68030\_Mycgr3T

Mycgr3G36449 Mycgr3T
  
Location: 19822-21886

Mycgr3G36449\_Mycgr3T

Mycgr3G35528 Mycgr3T
  
Location: 21986-22844

Mycgr3G35528\_Mycgr3T

Mycgr3G35932 Mycgr3T
  
Location: 22944-24390

Mycgr3G35932\_Mycgr3T

Mycgr3G23761 Mycgr3T
  
Location: 24490-25825

Mycgr3G23761\_Mycgr3T

Mycgr3G35535 Mycgr3T
  
Location: 25925-26429

Mycgr3G35535\_Mycgr3T

Mycgr3G9942 Mycgr3T9
  
Location: 26529-30375

Mycgr3G9942\_Mycgr3T9

canalicular multispecific organic anion transporter 1
  
Accession: EGE01692
  
Location: 724146-729017
  
  
**BlastP hit with Mycgr3G9942\_Mycgr3T9**
  
Percentage identity: 40 %
  
BlastP bit score: 956
  
Sequence coverage: 105 %
  
E-value: 0.0
  
  
 NCBI BlastP on this gene

EGE01692

PEP phosphonomutase
  
Accession: EGE01691
  
Location: 723087-723860
  
 NCBI BlastP on this gene

EGE01691

hypothetical protein
  
Accession: EGE01690
  
Location: 720265-721911
  
 NCBI BlastP on this gene

EGE01690

hypothetical protein
  
Accession: EGE01689
  
Location: 719353-719667
  
 NCBI BlastP on this gene

EGE01689

aspartyl-tRNA synthetase
  
Accession: EGE01688
  
Location: 715676-717862
  
 NCBI BlastP on this gene

EGE01688

ABC transporter
  
Accession: EGE01687
  
Location: 711789-714117
  
 NCBI BlastP on this gene

EGE01687

C6 transcription factor
  
Accession: EGE01686
  
Location: 708874-710724
  
 NCBI BlastP on this gene

EGE01686

Query: Architecture Search FASTA input

DS027694 : Neosartorya fischeri NRRL 181 1099437636262 genomic scaffold    Total score: 1.0     Cumulative Blast bit score: 956

Hit cluster cross-links:

Mycgr3G36335 Mycgr3T
  
Location: 0-423

Mycgr3G36335\_Mycgr3T

Mycgr3G84494 Mycgr3T
  
Location: 523-2047

Mycgr3G84494\_Mycgr3T

Mycgr3G90558 Mycgr3T
  
Location: 2147-15296

Mycgr3G90558\_Mycgr3T

Mycgr3G68036 Mycgr3T
  
Location: 15396-16395

Mycgr3G68036\_Mycgr3T

Mycgr3G90561 Mycgr3T
  
Location: 16495-17134

Mycgr3G90561\_Mycgr3T

Mycgr3G35862 Mycgr3T
  
Location: 17234-18662

Mycgr3G35862\_Mycgr3T

Mycgr3G68030 Mycgr3T
  
Location: 18762-19722

Mycgr3G68030\_Mycgr3T

Mycgr3G36449 Mycgr3T
  
Location: 19822-21886

Mycgr3G36449\_Mycgr3T

Mycgr3G35528 Mycgr3T
  
Location: 21986-22844

Mycgr3G35528\_Mycgr3T

Mycgr3G35932 Mycgr3T
  
Location: 22944-24390

Mycgr3G35932\_Mycgr3T

Mycgr3G23761 Mycgr3T
  
Location: 24490-25825

Mycgr3G23761\_Mycgr3T

Mycgr3G35535 Mycgr3T
  
Location: 25925-26429

Mycgr3G35535\_Mycgr3T

Mycgr3G9942 Mycgr3T9
  
Location: 26529-30375

Mycgr3G9942\_Mycgr3T9

nonribosomal peptide synthase, putative
  
Accession: EAW20390
  
Location: 1857043-1863732
  
 NCBI BlastP on this gene

EAW20390

hypothetical protein
  
Accession: EAW20391
  
Location: 1864511-1865464
  
 NCBI BlastP on this gene

EAW20391

oligopeptide transporter
  
Accession: EAW20392
  
Location: 1865942-1867853
  
 NCBI BlastP on this gene

EAW20392

GNAT family acetyltransferase, putative
  
Accession: EAW20393
  
Location: 1868755-1869483
  
 NCBI BlastP on this gene

EAW20393

nonribosomal peptide synthase, putative
  
Accession: EAW20394
  
Location: 1870166-1877179
  
  
**BlastP hit with Mycgr3G90558\_Mycgr3T**
  
Percentage identity: 30 %
  
BlastP bit score: 956
  
Sequence coverage: 49 %
  
E-value: 0.0
  
  
 NCBI BlastP on this gene

EAW20394

Query: Architecture Search FASTA input

CP003013 : Thielavia terrestris NRRL 8126 chromosome 5    Total score: 1.0     Cumulative Blast bit score: 956

Hit cluster cross-links:

Mycgr3G36335 Mycgr3T
  
Location: 0-423

Mycgr3G36335\_Mycgr3T

Mycgr3G84494 Mycgr3T
  
Location: 523-2047

Mycgr3G84494\_Mycgr3T

Mycgr3G90558 Mycgr3T
  
Location: 2147-15296

Mycgr3G90558\_Mycgr3T

Mycgr3G68036 Mycgr3T
  
Location: 15396-16395

Mycgr3G68036\_Mycgr3T

Mycgr3G90561 Mycgr3T
  
Location: 16495-17134

Mycgr3G90561\_Mycgr3T

Mycgr3G35862 Mycgr3T
  
Location: 17234-18662

Mycgr3G35862\_Mycgr3T

Mycgr3G68030 Mycgr3T
  
Location: 18762-19722

Mycgr3G68030\_Mycgr3T

Mycgr3G36449 Mycgr3T
  
Location: 19822-21886

Mycgr3G36449\_Mycgr3T

Mycgr3G35528 Mycgr3T
  
Location: 21986-22844

Mycgr3G35528\_Mycgr3T

Mycgr3G35932 Mycgr3T
  
Location: 22944-24390

Mycgr3G35932\_Mycgr3T

Mycgr3G23761 Mycgr3T
  
Location: 24490-25825

Mycgr3G23761\_Mycgr3T

Mycgr3G35535 Mycgr3T
  
Location: 25925-26429

Mycgr3G35535\_Mycgr3T

Mycgr3G9942 Mycgr3T9
  
Location: 26529-30375

Mycgr3G9942\_Mycgr3T9

hypothetical protein
  
Accession: AEO70139
  
Location: 1308213-1313226
  
  
**BlastP hit with Mycgr3G9942\_Mycgr3T9**
  
Percentage identity: 39 %
  
BlastP bit score: 956
  
Sequence coverage: 107 %
  
E-value: 0.0
  
  
 NCBI BlastP on this gene

THITE\_45545

hypothetical protein
  
Accession: AEO70138
  
Location: 1304350-1307445
  
 NCBI BlastP on this gene

THITE\_115141

hypothetical protein
  
Accession: AEO70137
  
Location: 1301387-1302500
  
 NCBI BlastP on this gene

THITE\_2121138

hypothetical protein
  
Accession: AEO70136
  
Location: 1300479-1300873
  
 NCBI BlastP on this gene

THITE\_2121136

hypothetical protein
  
Accession: AEO70135
  
Location: 1299525-1300073
  
 NCBI BlastP on this gene

THITE\_2121135

hypothetical protein
  
Accession: AEO70134
  
Location: 1297889-1298979
  
 NCBI BlastP on this gene

THITE\_2121132

hypothetical protein
  
Accession: AEO70133
  
Location: 1295369-1297156
  
 NCBI BlastP on this gene

THITE\_2121126

hypothetical protein
  
Accession: AEO70132
  
Location: 1291015-1294063
  
 NCBI BlastP on this gene

THITE\_2036687

Query: Architecture Search FASTA input

KE145369 : Glarea lozoyensis ATCC 20868 chromosome Unknown GLAREA5    Total score: 1.0     Cumulative Blast bit score: 954

Hit cluster cross-links:

Mycgr3G36335 Mycgr3T
  
Location: 0-423

Mycgr3G36335\_Mycgr3T

Mycgr3G84494 Mycgr3T
  
Location: 523-2047

Mycgr3G84494\_Mycgr3T

Mycgr3G90558 Mycgr3T
  
Location: 2147-15296

Mycgr3G90558\_Mycgr3T

Mycgr3G68036 Mycgr3T
  
Location: 15396-16395

Mycgr3G68036\_Mycgr3T

Mycgr3G90561 Mycgr3T
  
Location: 16495-17134

Mycgr3G90561\_Mycgr3T

Mycgr3G35862 Mycgr3T
  
Location: 17234-18662

Mycgr3G35862\_Mycgr3T

Mycgr3G68030 Mycgr3T
  
Location: 18762-19722

Mycgr3G68030\_Mycgr3T

Mycgr3G36449 Mycgr3T
  
Location: 19822-21886

Mycgr3G36449\_Mycgr3T

Mycgr3G35528 Mycgr3T
  
Location: 21986-22844

Mycgr3G35528\_Mycgr3T

Mycgr3G35932 Mycgr3T
  
Location: 22944-24390

Mycgr3G35932\_Mycgr3T

Mycgr3G23761 Mycgr3T
  
Location: 24490-25825

Mycgr3G23761\_Mycgr3T

Mycgr3G35535 Mycgr3T
  
Location: 25925-26429

Mycgr3G35535\_Mycgr3T

Mycgr3G9942 Mycgr3T9
  
Location: 26529-30375

Mycgr3G9942\_Mycgr3T9

RING/U-box
  
Accession: EPE27474
  
Location: 221104-222513
  
 NCBI BlastP on this gene

EPE27474

MurD-like peptide ligase, catalytic
  
Accession: EPE27475
  
Location: 223882-225569
  
 NCBI BlastP on this gene

EPE27475

MurD-like peptide ligase, catalytic
  
Accession: EPE27476
  
Location: 225692-227401
  
 NCBI BlastP on this gene

EPE27476

hypothetical protein
  
Accession: EPE27477
  
Location: 227943-229024
  
 NCBI BlastP on this gene

EPE27477

hypothetical protein
  
Accession: EPE27478
  
Location: 229591-230415
  
 NCBI BlastP on this gene

EPE27478

WD40 repeat-like protein
  
Accession: EPE27479
  
Location: 232251-234707
  
 NCBI BlastP on this gene

EPE27479

P-loop containing nucleoside triphosphate hydrolase
  
Accession: EPE27480
  
Location: 237327-242317
  
  
**BlastP hit with Mycgr3G9942\_Mycgr3T9**
  
Percentage identity: 40 %
  
BlastP bit score: 954
  
Sequence coverage: 104 %
  
E-value: 0.0
  
  
 NCBI BlastP on this gene

EPE27480

hypothetical protein
  
Accession: EPE27481
  
Location: 247028-248058
  
 NCBI BlastP on this gene

EPE27481

hypothetical protein
  
Accession: EPE27482
  
Location: 249261-250138
  
 NCBI BlastP on this gene

EPE27482

hypothetical protein
  
Accession: EPE27483
  
Location: 252352-253869
  
 NCBI BlastP on this gene

EPE27483

(Trans)glycosidase
  
Accession: EPE27484
  
Location: 254258-255933
  
 NCBI BlastP on this gene

EPE27484

Cytochrome P450
  
Accession: EPE27485
  
Location: 256844-258483
  
 NCBI BlastP on this gene

EPE27485

Query: Architecture Search FASTA input

DS995705 : Microsporum canis CBS 113480 supercont1.5 genomic scaffold    Total score: 1.0     Cumulative Blast bit score: 954

Hit cluster cross-links:

Mycgr3G36335 Mycgr3T
  
Location: 0-423

Mycgr3G36335\_Mycgr3T

Mycgr3G84494 Mycgr3T
  
Location: 523-2047

Mycgr3G84494\_Mycgr3T

Mycgr3G90558 Mycgr3T
  
Location: 2147-15296

Mycgr3G90558\_Mycgr3T

Mycgr3G68036 Mycgr3T
  
Location: 15396-16395

Mycgr3G68036\_Mycgr3T

Mycgr3G90561 Mycgr3T
  
Location: 16495-17134

Mycgr3G90561\_Mycgr3T

Mycgr3G35862 Mycgr3T
  
Location: 17234-18662

Mycgr3G35862\_Mycgr3T

Mycgr3G68030 Mycgr3T
  
Location: 18762-19722

Mycgr3G68030\_Mycgr3T

Mycgr3G36449 Mycgr3T
  
Location: 19822-21886

Mycgr3G36449\_Mycgr3T

Mycgr3G35528 Mycgr3T
  
Location: 21986-22844

Mycgr3G35528\_Mycgr3T

Mycgr3G35932 Mycgr3T
  
Location: 22944-24390

Mycgr3G35932\_Mycgr3T

Mycgr3G23761 Mycgr3T
  
Location: 24490-25825

Mycgr3G23761\_Mycgr3T

Mycgr3G35535 Mycgr3T
  
Location: 25925-26429

Mycgr3G35535\_Mycgr3T

Mycgr3G9942 Mycgr3T9
  
Location: 26529-30375

Mycgr3G9942\_Mycgr3T9

DNA mismatch repair protein msh6
  
Accession: EEQ32901
  
Location: 1686508-1690353
  
 NCBI BlastP on this gene

EEQ32901

conserved hypothetical protein
  
Accession: EEQ32900
  
Location: 1685541-1686236
  
 NCBI BlastP on this gene

EEQ32900

predicted protein
  
Accession: EEQ32899
  
Location: 1684092-1685002
  
 NCBI BlastP on this gene

EEQ32899

conserved hypothetical protein
  
Accession: EEQ32898
  
Location: 1682343-1683261
  
 NCBI BlastP on this gene

EEQ32898

glutaminyl-peptide cyclotransferase
  
Accession: EEQ32897
  
Location: 1680522-1681875
  
 NCBI BlastP on this gene

EEQ32897

YeeE/YedE family integral membrane protein
  
Accession: EEQ32896
  
Location: 1679071-1680145
  
 NCBI BlastP on this gene

EEQ32896

conserved hypothetical protein
  
Accession: EEQ32895
  
Location: 1677699-1678911
  
 NCBI BlastP on this gene

EEQ32895

predicted protein
  
Accession: EEQ32894
  
Location: 1676686-1677164
  
 NCBI BlastP on this gene

EEQ32894

conserved hypothetical protein
  
Accession: EEQ32893
  
Location: 1674051-1676372
  
 NCBI BlastP on this gene

EEQ32893

multidrug resistance-associated protein 13
  
Accession: EEQ32892
  
Location: 1668271-1673135
  
  
**BlastP hit with Mycgr3G9942\_Mycgr3T9**
  
Percentage identity: 39 %
  
BlastP bit score: 954
  
Sequence coverage: 106 %
  
E-value: 0.0
  
  
 NCBI BlastP on this gene

EEQ32892

PEP phosphonomutase
  
Accession: EEQ32891
  
Location: 1667198-1667971
  
 NCBI BlastP on this gene

EEQ32891

conserved hypothetical protein
  
Accession: EEQ32890
  
Location: 1664447-1666114
  
 NCBI BlastP on this gene

EEQ32890

aspartyl-tRNA synthetase
  
Accession: EEQ32889
  
Location: 1660101-1662295
  
 NCBI BlastP on this gene

EEQ32889

ABC transporter family protein
  
Accession: EEQ32888
  
Location: 1655919-1658480
  
 NCBI BlastP on this gene

EEQ32888

conserved hypothetical protein
  
Accession: EEQ32887
  
Location: 1653426-1655261
  
 NCBI BlastP on this gene

EEQ32887

Query: Architecture Search FASTA input

CP003002 : Myceliophthora thermophila ATCC 42464 chromosome 1    Total score: 1.0     Cumulative Blast bit score: 952

Hit cluster cross-links:

Mycgr3G36335 Mycgr3T
  
Location: 0-423

Mycgr3G36335\_Mycgr3T

Mycgr3G84494 Mycgr3T
  
Location: 523-2047

Mycgr3G84494\_Mycgr3T

Mycgr3G90558 Mycgr3T
  
Location: 2147-15296

Mycgr3G90558\_Mycgr3T

Mycgr3G68036 Mycgr3T
  
Location: 15396-16395

Mycgr3G68036\_Mycgr3T

Mycgr3G90561 Mycgr3T
  
Location: 16495-17134

Mycgr3G90561\_Mycgr3T

Mycgr3G35862 Mycgr3T
  
Location: 17234-18662

Mycgr3G35862\_Mycgr3T

Mycgr3G68030 Mycgr3T
  
Location: 18762-19722

Mycgr3G68030\_Mycgr3T

Mycgr3G36449 Mycgr3T
  
Location: 19822-21886

Mycgr3G36449\_Mycgr3T

Mycgr3G35528 Mycgr3T
  
Location: 21986-22844

Mycgr3G35528\_Mycgr3T

Mycgr3G35932 Mycgr3T
  
Location: 22944-24390

Mycgr3G35932\_Mycgr3T

Mycgr3G23761 Mycgr3T
  
Location: 24490-25825

Mycgr3G23761\_Mycgr3T

Mycgr3G35535 Mycgr3T
  
Location: 25925-26429

Mycgr3G35535\_Mycgr3T

Mycgr3G9942 Mycgr3T9
  
Location: 26529-30375

Mycgr3G9942\_Mycgr3T9

hypothetical protein
  
Accession: AEO53315
  
Location: 1092433-1104807
  
 NCBI BlastP on this gene

MYCTH\_2122159

hypothetical protein
  
Accession: AEO53314
  
Location: 1090919-1091677
  
 NCBI BlastP on this gene

MYCTH\_16224

hypothetical protein
  
Accession: AEO53313
  
Location: 1088909-1090544
  
 NCBI BlastP on this gene

MYCTH\_2294459

hypothetical protein
  
Accession: AEO53312
  
Location: 1087351-1088424
  
 NCBI BlastP on this gene

MYCTH\_2054555

hypothetical protein
  
Accession: AEO53311
  
Location: 1085001-1086845
  
 NCBI BlastP on this gene

MYCTH\_2294455

hypothetical protein
  
Accession: AEO53310
  
Location: 1081441-1083916
  
 NCBI BlastP on this gene

MYCTH\_75762

hypothetical protein
  
Accession: AEO53309
  
Location: 1074987-1079876
  
  
**BlastP hit with Mycgr3G9942\_Mycgr3T9**
  
Percentage identity: 40 %
  
BlastP bit score: 952
  
Sequence coverage: 105 %
  
E-value: 0.0
  
  
 NCBI BlastP on this gene

MYCTH\_2294448

hypothetical protein
  
Accession: AEO53308
  
Location: 1070825-1073962
  
 NCBI BlastP on this gene

MYCTH\_42476

hypothetical protein
  
Accession: AEO53307
  
Location: 1066988-1069197
  
 NCBI BlastP on this gene

MYCTH\_2294446

hypothetical protein
  
Accession: AEO53306
  
Location: 1063559-1065049
  
 NCBI BlastP on this gene

MYCTH\_2041072

hypothetical protein
  
Accession: AEO53305
  
Location: 1060422-1062320
  
 NCBI BlastP on this gene

MYCTH\_2294445

Query: Architecture Search FASTA input

CM001234 : Magnaporthe oryzae 70-15 chromosome 4    Total score: 1.0     Cumulative Blast bit score: 948

Hit cluster cross-links:

Mycgr3G36335 Mycgr3T
  
Location: 0-423

Mycgr3G36335\_Mycgr3T

Mycgr3G84494 Mycgr3T
  
Location: 523-2047

Mycgr3G84494\_Mycgr3T

Mycgr3G90558 Mycgr3T
  
Location: 2147-15296

Mycgr3G90558\_Mycgr3T

Mycgr3G68036 Mycgr3T
  
Location: 15396-16395

Mycgr3G68036\_Mycgr3T

Mycgr3G90561 Mycgr3T
  
Location: 16495-17134

Mycgr3G90561\_Mycgr3T

Mycgr3G35862 Mycgr3T
  
Location: 17234-18662

Mycgr3G35862\_Mycgr3T

Mycgr3G68030 Mycgr3T
  
Location: 18762-19722

Mycgr3G68030\_Mycgr3T

Mycgr3G36449 Mycgr3T
  
Location: 19822-21886

Mycgr3G36449\_Mycgr3T

Mycgr3G35528 Mycgr3T
  
Location: 21986-22844

Mycgr3G35528\_Mycgr3T

Mycgr3G35932 Mycgr3T
  
Location: 22944-24390

Mycgr3G35932\_Mycgr3T

Mycgr3G23761 Mycgr3T
  
Location: 24490-25825

Mycgr3G23761\_Mycgr3T

Mycgr3G35535 Mycgr3T
  
Location: 25925-26429

Mycgr3G35535\_Mycgr3T

Mycgr3G9942 Mycgr3T9
  
Location: 26529-30375

Mycgr3G9942\_Mycgr3T9

RNA-binding protein 8A
  
Accession: EHA49820
  
Location: 859807-860393
  
 NCBI BlastP on this gene

EHA49820

hypothetical protein
  
Accession: EHA49821
  
Location: 861918-864894
  
 NCBI BlastP on this gene

EHA49821

F-box domain-containing protein
  
Accession: EHA49822
  
Location: 867434-869451
  
 NCBI BlastP on this gene

EHA49822

hypothetical protein
  
Accession: EHA49823
  
Location: 870313-872680
  
 NCBI BlastP on this gene

EHA49823

multidrug resistance-associated protein 1
  
Accession: EHA49824
  
Location: 875536-880315
  
  
**BlastP hit with Mycgr3G9942\_Mycgr3T9**
  
Percentage identity: 40 %
  
BlastP bit score: 948
  
Sequence coverage: 106 %
  
E-value: 0.0
  
  
 NCBI BlastP on this gene

EHA49824

Sad1-interacting factor 3
  
Accession: EHA49825
  
Location: 881155-883209
  
 NCBI BlastP on this gene

EHA49825

hypothetical protein
  
Accession: EHA49826
  
Location: 883801-884369
  
 NCBI BlastP on this gene

EHA49826

ribonuclease P/MRP protein subunit RPP1
  
Accession: EHA49827
  
Location: 885282-886226
  
 NCBI BlastP on this gene

EHA49827

hypothetical protein
  
Accession: EHA49828
  
Location: 886916-888440
  
 NCBI BlastP on this gene

EHA49828

hypothetical protein
  
Accession: EHA49829
  
Location: 890304-892130
  
 NCBI BlastP on this gene

EHA49829

hypothetical protein
  
Accession: EHA49830
  
Location: 892971-894056
  
 NCBI BlastP on this gene

EHA49830

histidyl-tRNA synthetase
  
Accession: EHA49831
  
Location: 894478-896767
  
 NCBI BlastP on this gene

EHA49831

Query: Architecture Search FASTA input

201. :  GG700650 Trichophyton rubrum CBS 118892 genomic scaffold supercont2.3     Total score: 1.0     Cumulative Blast bit score: 1130

Mycgr3G36335 Mycgr3T
  
Location: 0-423
  
 NCBI BlastP on this gene

Mycgr3G36335\_Mycgr3T

Mycgr3G84494 Mycgr3T
  
Location: 523-2047
  
 NCBI BlastP on this gene

Mycgr3G84494\_Mycgr3T

Mycgr3G90558 Mycgr3T
  
Location: 2147-15296
  
 NCBI BlastP on this gene

Mycgr3G90558\_Mycgr3T

Mycgr3G68036 Mycgr3T
  
Location: 15396-16395
  
 NCBI BlastP on this gene

Mycgr3G68036\_Mycgr3T

Mycgr3G90561 Mycgr3T
  
Location: 16495-17134
  
 NCBI BlastP on this gene

Mycgr3G90561\_Mycgr3T

Mycgr3G35862 Mycgr3T
  
Location: 17234-18662
  
 NCBI BlastP on this gene

Mycgr3G35862\_Mycgr3T

Mycgr3G68030 Mycgr3T
  
Location: 18762-19722
  
 NCBI BlastP on this gene

Mycgr3G68030\_Mycgr3T

Mycgr3G36449 Mycgr3T
  
Location: 19822-21886
  
 NCBI BlastP on this gene

Mycgr3G36449\_Mycgr3T

Mycgr3G35528 Mycgr3T
  
Location: 21986-22844
  
 NCBI BlastP on this gene

Mycgr3G35528\_Mycgr3T

Mycgr3G35932 Mycgr3T
  
Location: 22944-24390
  
 NCBI BlastP on this gene

Mycgr3G35932\_Mycgr3T

Mycgr3G23761 Mycgr3T
  
Location: 24490-25825
  
 NCBI BlastP on this gene

Mycgr3G23761\_Mycgr3T

Mycgr3G35535 Mycgr3T
  
Location: 25925-26429
  
 NCBI BlastP on this gene

Mycgr3G35535\_Mycgr3T

Mycgr3G9942 Mycgr3T9
  
Location: 26529-30375
  
 NCBI BlastP on this gene

Mycgr3G9942\_Mycgr3T9

DNA repair protein Rhp26/Rad26
  
Accession: EGD86946
  
Location: 1259341-1263154
  
 NCBI BlastP on this gene

EGD86946

hypothetical protein
  
Accession: EGD86945
  
Location: 1257629-1258607
  
 NCBI BlastP on this gene

EGD86945

hypothetical protein
  
Accession: EGD86944
  
Location: 1255899-1256357
  
 NCBI BlastP on this gene

EGD86944

hypothetical protein
  
Accession: EGD86943
  
Location: 1254685-1255871
  
 NCBI BlastP on this gene

EGD86943

hypothetical protein
  
Accession: EGD86942
  
Location: 1251887-1253967
  
 NCBI BlastP on this gene

EGD86942

hypothetical protein
  
Accession: EGD86941
  
Location: 1250318-1250581
  
 NCBI BlastP on this gene

EGD86941

nonribosomal peptide synthase
  
Accession: EGD86940
  
Location: 1237601-1249263
  
  
**BlastP hit with Mycgr3G90558\_Mycgr3T**
  
Percentage identity: 33 %
  
BlastP bit score: 1130
  
Sequence coverage: 49 %
  
E-value: 0.0
  
  
 NCBI BlastP on this gene

EGD86940

hypothetical protein
  
Accession: EGD86939
  
Location: 1236459-1237313
  
 NCBI BlastP on this gene

EGD86939

cmgc/cdk/pitslre protein kinase
  
Accession: EGD86938
  
Location: 1232728-1234316
  
 NCBI BlastP on this gene

EGD86938

homocitrate dehydratase
  
Accession: EGD86937
  
Location: 1231528-1232496
  
 NCBI BlastP on this gene

EGD86937

NADPH oxidase regulator NoxR
  
Accession: EGD86936
  
Location: 1229028-1230923
  
 NCBI BlastP on this gene

EGD86936

SNARE protein
  
Accession: EGD86935
  
Location: 1227314-1228552
  
 NCBI BlastP on this gene

EGD86935

hypothetical protein
  
Accession: EGD86934
  
Location: 1226339-1227031
  
 NCBI BlastP on this gene

EGD86934

202. :  AM920431 Penicillium chrysogenum Wisconsin 54-1255 complete genome, contig Pc00c16.     Total score: 1.0     Cumulative Blast bit score: 1111

not annotated
  
Accession: CAP93137
  
Location: 1072077-1076885
  
 NCBI BlastP on this gene

Pc16g04670

hypothetical protein
  
Accession: CAP93138
  
Location: 1079837-1080293
  
 NCBI BlastP on this gene

Pc16g04680

not annotated
  
Accession: CAP93139
  
Location: 1080827-1099362
  
  
**BlastP hit with Mycgr3G90558\_Mycgr3T**
  
Percentage identity: 30 %
  
BlastP bit score: 1111
  
Sequence coverage: 57 %
  
E-value: 0.0
  
  
 NCBI BlastP on this gene

Pc16g04690

not annotated
  
Accession: CAP93140
  
Location: 1099963-1101744
  
 NCBI BlastP on this gene

Pc16g04700

not annotated
  
Accession: CAP93141
  
Location: 1102388-1104800
  
 NCBI BlastP on this gene

Pc16g04710

not annotated
  
Accession: CAP93142
  
Location: 1105448-1105741
  
 NCBI BlastP on this gene

Pc16g04720

phosphoglycerate kinase pgkA-Penicillium chrysogenum
  
Accession: CAP93143
  
Location: 1106637-1108007
  
 NCBI BlastP on this gene

pgkA

203. :  DS178264 Puccinia graminis f. sp. tritici CRL 75-36-700-3 supercont2.3 genomic scaffold     Total score: 1.0     Cumulative Blast bit score: 1108

hypothetical protein
  
Accession: EFP75548
  
Location: 612485-615652
  
 NCBI BlastP on this gene

EFP75548

hypothetical protein
  
Accession: EFP75547
  
Location: 598074-605294
  
  
**BlastP hit with Mycgr3G9942\_Mycgr3T9**
  
Percentage identity: 32 %
  
BlastP bit score: 560
  
Sequence coverage: 95 %
  
E-value: 9e-169
  
  
 NCBI BlastP on this gene

EFP75547

hypothetical protein
  
Accession: EFP75545
  
Location: 589501-596385
  
  
**BlastP hit with Mycgr3G9942\_Mycgr3T9**
  
Percentage identity: 31 %
  
BlastP bit score: 548
  
Sequence coverage: 93 %
  
E-value: 7e-165
  
  
 NCBI BlastP on this gene

EFP75545

204. :  DS499594 Aspergillus fumigatus A1163 scf\_000001 genomic scaffold     Total score: 1.0     Cumulative Blast bit score: 1103

ABC multidrug transporter, putative
  
Accession: EDP56273
  
Location: 2773451-2778675
  
 NCBI BlastP on this gene

EDP56273

nonribosomal peptide synthase Pes1
  
Accession: EDP56272
  
Location: 2748720-2767908
  
  
**BlastP hit with Mycgr3G90558\_Mycgr3T**
  
Percentage identity: 30 %
  
BlastP bit score: 1103
  
Sequence coverage: 58 %
  
E-value: 0.0
  
  
 NCBI BlastP on this gene

EDP56272

MFS multidrug transporter, putative
  
Accession: EDP56271
  
Location: 2746314-2748233
  
 NCBI BlastP on this gene

EDP56271

conserved hypothetical protein
  
Accession: EDP56270
  
Location: 2742713-2745250
  
 NCBI BlastP on this gene

EDP56270

26 proteasome complex subunit Sem1, putative
  
Accession: EDP56269
  
Location: 2741238-2741707
  
 NCBI BlastP on this gene

EDP56269

phosphoglycerate kinase PgkA, putative
  
Accession: EDP56268
  
Location: 2739275-2740690
  
 NCBI BlastP on this gene

EDP56268

205. :  AFNW01000079 Fusarium pseudograminearum CS3096     Total score: 1.0     Cumulative Blast bit score: 1101

hypothetical protein
  
Accession: EKJ76035
  
Location: 159580-164507
  
 NCBI BlastP on this gene

EKJ76035

NPS4
  
Accession: EKJ76036
  
Location: 166952-189868
  
  
**BlastP hit with Mycgr3G90558\_Mycgr3T**
  
Percentage identity: 30 %
  
BlastP bit score: 1101
  
Sequence coverage: 60 %
  
E-value: 0.0
  
  
 NCBI BlastP on this gene

EKJ76036

hypothetical protein
  
Accession: EKJ76037
  
Location: 190443-193127
  
 NCBI BlastP on this gene

EKJ76037

hypothetical protein
  
Accession: EKJ76038
  
Location: 193784-194656
  
 NCBI BlastP on this gene

EKJ76038

hypothetical protein
  
Accession: EKJ76039
  
Location: 194770-195391
  
 NCBI BlastP on this gene

EKJ76039

hypothetical protein
  
Accession: EKJ76040
  
Location: 195721-197439
  
 NCBI BlastP on this gene

EKJ76040

206. :  AAHF01000004 Aspergillus fumigatus Af293     Total score: 1.0     Cumulative Blast bit score: 1100

ABC multidrug transporter, putative
  
Accession: EAL90367
  
Location: 453260-458484
  
 NCBI BlastP on this gene

EAL90367

nonribosomal peptide synthase Pes1
  
Accession: EAL90366
  
Location: 428528-447716
  
  
**BlastP hit with Mycgr3G90558\_Mycgr3T**
  
Percentage identity: 30 %
  
BlastP bit score: 1100
  
Sequence coverage: 58 %
  
E-value: 0.0
  
  
 NCBI BlastP on this gene

EAL90366

MFS multidrug transporter, putative
  
Accession: EAL90365
  
Location: 426122-428041
  
 NCBI BlastP on this gene

EAL90365

conserved hypothetical protein
  
Accession: EAL90364
  
Location: 422521-425058
  
 NCBI BlastP on this gene

EAL90364

26 proteasome complex subunit Sem1, putative
  
Accession: EBA27404
  
Location: 421046-421515
  
 NCBI BlastP on this gene

EBA27404

phosphoglycerate kinase PgkA, putative
  
Accession: EAL90363
  
Location: 419083-420498
  
 NCBI BlastP on this gene

EAL90363

207. :  GL636512 Coccidioides posadasii str. Silveira unplaced genomic scaffold supercont2.27     Total score: 1.0     Cumulative Blast bit score: 1096

multidrug resistance protein MDR
  
Accession: EFW13651
  
Location: 58766-64144
  
 NCBI BlastP on this gene

EFW13651

hypothetical protein
  
Accession: EFW13650
  
Location: 56706-57200
  
 NCBI BlastP on this gene

EFW13650

hypothetical protein
  
Accession: EFW13649
  
Location: 55335-55577
  
 NCBI BlastP on this gene

EFW13649

predicted protein
  
Accession: EFW13648
  
Location: 54558-54986
  
 NCBI BlastP on this gene

EFW13648

cyclic peptide synthetase
  
Accession: EFW13647
  
Location: 34393-54038
  
  
**BlastP hit with Mycgr3G90558\_Mycgr3T**
  
Percentage identity: 32 %
  
BlastP bit score: 1096
  
Sequence coverage: 49 %
  
E-value: 0.0
  
  
 NCBI BlastP on this gene

EFW13647

conserved hypothetical protein
  
Accession: EFW13646
  
Location: 28756-34324
  
 NCBI BlastP on this gene

EFW13646

conserved hypothetical protein
  
Accession: EFW13645
  
Location: 27610-28031
  
 NCBI BlastP on this gene

EFW13645

hypothetical protein
  
Accession: EFW13644
  
Location: 26080-27080
  
 NCBI BlastP on this gene

EFW13644

208. :  DS027688 Neosartorya fischeri NRRL 181 1099437636249 genomic scaffold     Total score: 1.0     Cumulative Blast bit score: 1085

ABC multidrug transporter, putative
  
Accession: EAW22835
  
Location: 2268452-2273675
  
 NCBI BlastP on this gene

EAW22835

nonribosomal peptide synthase Pes1
  
Accession: EAW22836
  
Location: 2279236-2298428
  
  
**BlastP hit with Mycgr3G90558\_Mycgr3T**
  
Percentage identity: 30 %
  
BlastP bit score: 1085
  
Sequence coverage: 57 %
  
E-value: 0.0
  
  
 NCBI BlastP on this gene

EAW22836

MFS multidrug transporter, putative
  
Accession: EAW22837
  
Location: 2298902-2300812
  
 NCBI BlastP on this gene

EAW22837

conserved hypothetical protein
  
Accession: EAW22838
  
Location: 2301824-2304364
  
 NCBI BlastP on this gene

EAW22838

26 proteasome complex subunit Sem1, putative
  
Accession: EAW22839
  
Location: 2305394-2305863
  
 NCBI BlastP on this gene

EAW22839

phosphoglycerate kinase PgkA, putative
  
Accession: EAW22840
  
Location: 2306413-2307828
  
 NCBI BlastP on this gene

EAW22840

209. :  DS027059 Aspergillus clavatus NRRL 1 1099423829805 genomic scaffold     Total score: 1.0     Cumulative Blast bit score: 1084

ABC multidrug transporter, putative
  
Accession: EAW07798
  
Location: 2085505-2090860
  
 NCBI BlastP on this gene

EAW07798

nonribosomal peptide synthase Pes1
  
Accession: EAW07799
  
Location: 2098342-2117521
  
  
**BlastP hit with Mycgr3G90558\_Mycgr3T**
  
Percentage identity: 30 %
  
BlastP bit score: 1084
  
Sequence coverage: 57 %
  
E-value: 0.0
  
  
 NCBI BlastP on this gene

EAW07799

MFS multidrug transporter, putative
  
Accession: EAW07800
  
Location: 2118086-2119998
  
 NCBI BlastP on this gene

EAW07800

conserved hypothetical protein
  
Accession: EAW07801
  
Location: 2121198-2123770
  
 NCBI BlastP on this gene

EAW07801

26 proteasome complex subunit Sem1, putative
  
Accession: EAW07802
  
Location: 2124852-2125379
  
 NCBI BlastP on this gene

EAW07802

210. :  ACJE01000021 Aspergillus niger ATCC 1015     Total score: 1.0     Cumulative Blast bit score: 1080

non-ribosomal peptide synthetase
  
Accession: EHA17890
  
Location: 517116-536436
  
  
**BlastP hit with Mycgr3G90558\_Mycgr3T**
  
Percentage identity: 30 %
  
BlastP bit score: 1080
  
Sequence coverage: 56 %
  
E-value: 0.0
  
  
 NCBI BlastP on this gene

EHA17890

hypothetical protein
  
Accession: EHA17889
  
Location: 514316-516011
  
 NCBI BlastP on this gene

EHA17889

hypothetical protein
  
Accession: EHA17888
  
Location: 510811-513267
  
 NCBI BlastP on this gene

EHA17888

211. :  DS027058 Aspergillus clavatus NRRL 1 1099423829804 genomic scaffold     Total score: 1.0     Cumulative Blast bit score: 1070

alpha/beta fold family hydrolase, putative
  
Accession: EAW08659
  
Location: 215078-216870
  
 NCBI BlastP on this gene

EAW08659

conserved hypothetical protein
  
Accession: EAW08660
  
Location: 217072-217840
  
 NCBI BlastP on this gene

EAW08660

NADH-ubiquinone oxidoreductase B14 subunit, putative
  
Accession: EAW08661
  
Location: 218268-219011
  
 NCBI BlastP on this gene

EAW08661

DNA-directed RNA polymerase I and III 14 KDA polypeptide
  
Accession: EAW08662
  
Location: 219413-220077
  
 NCBI BlastP on this gene

EAW08662

Cytochrome P450 oxidoreductase, putative
  
Accession: EAW08663
  
Location: 221060-223000
  
 NCBI BlastP on this gene

EAW08663

FAD binding domain protein
  
Accession: EAW08664
  
Location: 223526-225225
  
 NCBI BlastP on this gene

EAW08664

nonribosomal peptide synthase, putative
  
Accession: EAW08665
  
Location: 226895-238713
  
  
**BlastP hit with Mycgr3G90558\_Mycgr3T**
  
Percentage identity: 33 %
  
BlastP bit score: 1070
  
Sequence coverage: 50 %
  
E-value: 0.0
  
  
 NCBI BlastP on this gene

EAW08665

tyrosyl-DNA phosphodiesterase domain protein
  
Accession: EAW08666
  
Location: 239650-242172
  
 NCBI BlastP on this gene

EAW08666

ABC drug exporter AtrF
  
Accession: EAW08667
  
Location: 242469-247322
  
 NCBI BlastP on this gene

EAW08667

212. :  ABDG02000022 Trichoderma atroviride IMI 206040     Total score: 1.0     Cumulative Blast bit score: 1066

hypothetical protein
  
Accession: EHK46791
  
Location: 1857036-1858537
  
 NCBI BlastP on this gene

EHK46791

hypothetical protein
  
Accession: EHK46792
  
Location: 1863768-1866101
  
 NCBI BlastP on this gene

EHK46792

hypothetical protein
  
Accession: EHK46793
  
Location: 1866512-1868122
  
 NCBI BlastP on this gene

EHK46793

hypothetical protein
  
Accession: EHK46794
  
Location: 1868845-1870457
  
 NCBI BlastP on this gene

EHK46794

non-ribosomal peptide synthetase
  
Accession: EHK46795
  
Location: 1872713-1879288
  
  
**BlastP hit with Mycgr3G90558\_Mycgr3T**
  
Percentage identity: 31 %
  
BlastP bit score: 1066
  
Sequence coverage: 50 %
  
E-value: 0.0
  
  
 NCBI BlastP on this gene

EHK46795

213. :  KB445641 Cochliobolus sativus ND90Pr unplaced genomic scaffold COCSAscaffold\_5     Total score: 1.0     Cumulative Blast bit score: 1065

Non-ribosomal peptide synthetase NPS4
  
Accession: EMD66011
  
Location: 2046340-2057828
  
  
**BlastP hit with Mycgr3G90558\_Mycgr3T**
  
Percentage identity: 30 %
  
BlastP bit score: 1065
  
Sequence coverage: 58 %
  
E-value: 0.0
  
  
 NCBI BlastP on this gene

EMD66011

carbohydrate esterase family 3 protein
  
Accession: EMD66010
  
Location: 2043937-2044323
  
 NCBI BlastP on this gene

EMD66010

hypothetical protein
  
Accession: EMD66009
  
Location: 2039778-2042201
  
 NCBI BlastP on this gene

EMD66009

hypothetical protein
  
Accession: EMD66008
  
Location: 2037712-2038965
  
 NCBI BlastP on this gene

EMD66008

glycoside hydrolase family 128 protein
  
Accession: EMD66007
  
Location: 2036463-2037478
  
 NCBI BlastP on this gene

EMD66007

hypothetical protein
  
Accession: EMD66006
  
Location: 2034106-2035949
  
 NCBI BlastP on this gene

EMD66006

214. :  CH476607 Aspergillus terreus NIH2624 scaffold\_14 genomic scaffold     Total score: 1.0     Cumulative Blast bit score: 1051

predicted protein
  
Accession: EAU30205
  
Location: 177150-184269
  
 NCBI BlastP on this gene

EAU30205

phospho-2-dehydro-3-deoxyheptonate aldolase
  
Accession: EAU30204
  
Location: 173909-175164
  
 NCBI BlastP on this gene

EAU30204

predicted protein
  
Accession: EAU30203
  
Location: 171401-172939
  
 NCBI BlastP on this gene

EAU30203

predicted protein
  
Accession: EAU30202
  
Location: 168854-169367
  
 NCBI BlastP on this gene

EAU30202

predicted protein
  
Accession: EAU30201
  
Location: 159864-167038
  
  
**BlastP hit with Mycgr3G90558\_Mycgr3T**
  
Percentage identity: 35 %
  
BlastP bit score: 1051
  
Sequence coverage: 39 %
  
E-value: 0.0
  
  
 NCBI BlastP on this gene

EAU30201

predicted protein
  
Accession: EAU30200
  
Location: 158579-159091
  
 NCBI BlastP on this gene

EAU30200

conserved hypothetical protein
  
Accession: EAU30199
  
Location: 154486-156087
  
 NCBI BlastP on this gene

EAU30199

hypothetical protein
  
Accession: EAU30198
  
Location: 152315-154117
  
 NCBI BlastP on this gene

EAU30198

conserved hypothetical protein
  
Accession: EAU30197
  
Location: 150399-151728
  
 NCBI BlastP on this gene

EAU30197

predicted protein
  
Accession: EAU30196
  
Location: 147868-149424
  
 NCBI BlastP on this gene

EAU30196

predicted protein
  
Accession: EAU30195
  
Location: 144834-146218
  
 NCBI BlastP on this gene

EAU30195

215. :  KB908481 Setosphaeria turcica Et28A unplaced genomic scaffold SETTUscaffold\_1     Total score: 1.0     Cumulative Blast bit score: 1050

hypothetical protein
  
Accession: EOA91188
  
Location: 50789-52032
  
 NCBI BlastP on this gene

EOA91188

hypothetical protein
  
Accession: EOA91189
  
Location: 52502-53511
  
 NCBI BlastP on this gene

EOA91189

hypothetical protein
  
Accession: EOA91190
  
Location: 54718-56672
  
 NCBI BlastP on this gene

EOA91190

hypothetical protein
  
Accession: EOA91191
  
Location: 56874-57983
  
 NCBI BlastP on this gene

EOA91191

hypothetical protein
  
Accession: EOA91192
  
Location: 58490-59290
  
 NCBI BlastP on this gene

EOA91192

hypothetical protein
  
Accession: EOA91193
  
Location: 59827-61733
  
 NCBI BlastP on this gene

EOA91193

hypothetical protein
  
Accession: EOA91194
  
Location: 62373-76298
  
  
**BlastP hit with Mycgr3G90558\_Mycgr3T**
  
Percentage identity: 27 %
  
BlastP bit score: 1050
  
Sequence coverage: 76 %
  
E-value: 0.0
  
  
 NCBI BlastP on this gene

EOA91194

hypothetical protein
  
Accession: EOA91195
  
Location: 77252-78831
  
 NCBI BlastP on this gene

EOA91195

hypothetical protein
  
Accession: EOA91196
  
Location: 79515-80336
  
 NCBI BlastP on this gene

EOA91196

hypothetical protein
  
Accession: EOA91197
  
Location: 83395-84911
  
 NCBI BlastP on this gene

EOA91197

216. :  KB644414 Penicillium oxalicum 114-2 unplaced genomic scaffold scaffold\_7     Total score: 1.0     Cumulative Blast bit score: 1047

hypothetical protein
  
Accession: EPS32204
  
Location: 1773861-1778676
  
 NCBI BlastP on this gene

EPS32204

hypothetical protein
  
Accession: EPS32203
  
Location: 1753106-1771943
  
  
**BlastP hit with Mycgr3G90558\_Mycgr3T**
  
Percentage identity: 30 %
  
BlastP bit score: 1047
  
Sequence coverage: 57 %
  
E-value: 0.0
  
  
 NCBI BlastP on this gene

EPS32203

hypothetical protein
  
Accession: EPS32202
  
Location: 1750653-1752573
  
 NCBI BlastP on this gene

EPS32202

hypothetical protein
  
Accession: EPS32201
  
Location: 1747067-1749637
  
 NCBI BlastP on this gene

EPS32201

hypothetical protein
  
Accession: EPS32200
  
Location: 1745491-1745990
  
 NCBI BlastP on this gene

EPS32200

hypothetical protein
  
Accession: EPS32199
  
Location: 1745193-1745477
  
 NCBI BlastP on this gene

EPS32199

217. :  DS027698 Neosartorya fischeri NRRL 181 1099437636266 genomic scaffold     Total score: 1.0     Cumulative Blast bit score: 1034

membrane dipeptidase GliJ
  
Accession: EAW16185
  
Location: 2447979-2449297
  
 NCBI BlastP on this gene

EAW16185

aminotransferase, putative
  
Accession: EAW16184
  
Location: 2446215-2447639
  
 NCBI BlastP on this gene

EAW16184

C6 zinc finger domain protein
  
Accession: EAW16183
  
Location: 2444056-2445444
  
 NCBI BlastP on this gene

EAW16183

transferase family protein
  
Accession: EAW16182
  
Location: 2439622-2441151
  
 NCBI BlastP on this gene

EAW16182

dimethylallyl tryptophan synthase, putative
  
Accession: EAW16181
  
Location: 2437444-2438808
  
 NCBI BlastP on this gene

EAW16181

nonribosomal peptide synthase, putative
  
Accession: EAW16180
  
Location: 2428387-2435518
  
  
**BlastP hit with Mycgr3G90558\_Mycgr3T**
  
Percentage identity: 32 %
  
BlastP bit score: 1034
  
Sequence coverage: 50 %
  
E-value: 0.0
  
  
 NCBI BlastP on this gene

EAW16180

Ankyrin repeat protein
  
Accession: EAW16179
  
Location: 2425838-2427307
  
 NCBI BlastP on this gene

EAW16179

peptidase family M20/M25/M40 protein
  
Accession: EAW16178
  
Location: 2424023-2425333
  
 NCBI BlastP on this gene

EAW16178

zinc alcohol dehydrogenase, putative
  
Accession: EAW16177
  
Location: 2422985-2423977
  
 NCBI BlastP on this gene

EAW16177

conserved hypothetical protein
  
Accession: EAW16176
  
Location: 2421654-2422766
  
 NCBI BlastP on this gene

EAW16176

glycosyl hydrolases family 11 protein
  
Accession: EAW16175
  
Location: 2420062-2420872
  
 NCBI BlastP on this gene

EAW16175

RTA1 like protein
  
Accession: EAW16174
  
Location: 2418546-2419510
  
 NCBI BlastP on this gene

EAW16174

fungal cellulose binding domain protein
  
Accession: EAW16173
  
Location: 2416853-2417860
  
 NCBI BlastP on this gene

EAW16173

PAN domain
  
Accession: EAW16172
  
Location: 2414338-2415559
  
 NCBI BlastP on this gene

EAW16172

218. :  AM270372 Aspergillus niger contig An16c0200, genomic contig.     Total score: 1.0     Cumulative Blast bit score: 1025

not annotated
  
Accession: CAK47006
  
Location: 150350-171653
  
  
**BlastP hit with Mycgr3G90558\_Mycgr3T**
  
Percentage identity: 29 %
  
BlastP bit score: 1025
  
Sequence coverage: 61 %
  
E-value: 0.0
  
  
 NCBI BlastP on this gene

An16g06720

not annotated
  
Accession: CAK47005
  
Location: 144347-149491
  
 NCBI BlastP on this gene

An16g06710

hypothetical protein
  
Accession: CAK47004
  
Location: 143614-144249
  
 NCBI BlastP on this gene

An16g06700

219. :  CH476594 Aspergillus terreus NIH2624 scaffold\_1 genomic scaffold     Total score: 1.0     Cumulative Blast bit score: 1005

DNA repair protein RAD51
  
Accession: EAU38876
  
Location: 666272-667562
  
 NCBI BlastP on this gene

EAU38876

conserved hypothetical protein
  
Accession: EAU38875
  
Location: 661751-665805
  
 NCBI BlastP on this gene

EAU38875

conserved hypothetical protein
  
Accession: EAU38874
  
Location: 639632-657679
  
  
**BlastP hit with Mycgr3G90558\_Mycgr3T**
  
Percentage identity: 29 %
  
BlastP bit score: 1005
  
Sequence coverage: 57 %
  
E-value: 0.0
  
  
 NCBI BlastP on this gene

EAU38874

conserved hypothetical protein
  
Accession: EAU38873
  
Location: 637521-639429
  
 NCBI BlastP on this gene

EAU38873

conserved hypothetical protein
  
Accession: EAU38872
  
Location: 634130-636496
  
 NCBI BlastP on this gene

EAU38872

predicted protein
  
Accession: EAU38871
  
Location: 632828-633278
  
 NCBI BlastP on this gene

EAU38871

phosphoglycerate kinase
  
Accession: EAU38870
  
Location: 630782-632162
  
 NCBI BlastP on this gene

EAU38870

220. :  JH126399 Cordyceps militaris CM01 unplaced genomic scaffold CCM\_S00001     Total score: 1.0     Cumulative Blast bit score: 1001

polyketide synthase, putative
  
Accession: EGX96624
  
Location: 4117047-4125452
  
 NCBI BlastP on this gene

EGX96624

Transferase
  
Accession: EGX96625
  
Location: 4126586-4128043
  
 NCBI BlastP on this gene

EGX96625

AMP dependent CoA ligase, putative
  
Accession: EGX96626
  
Location: 4128455-4131193
  
 NCBI BlastP on this gene

EGX96626

non-ribosomal peptide synthase, putative
  
Accession: EGX96627
  
Location: 4131512-4145862
  
  
**BlastP hit with Mycgr3G90558\_Mycgr3T**
  
Percentage identity: 29 %
  
BlastP bit score: 1001
  
Sequence coverage: 61 %
  
E-value: 0.0
  
  
 NCBI BlastP on this gene

EGX96627

Vps51/Vps67
  
Accession: EGX96628
  
Location: 4151594-4152580
  
 NCBI BlastP on this gene

EGX96628

UPF0135 protein
  
Accession: EGX96629
  
Location: 4152771-4153817
  
 NCBI BlastP on this gene

EGX96629

BolA domain protein
  
Accession: EGX96630
  
Location: 4154413-4154783
  
 NCBI BlastP on this gene

EGX96630

AP-1 complex subunit beta-1
  
Accession: EGX96631
  
Location: 4155095-4157538
  
 NCBI BlastP on this gene

EGX96631

221. :  DS995899 Penicillium marneffei ATCC 18224 scf\_1105668340764 genomic scaffold     Total score: 1.0     Cumulative Blast bit score: 1001

thioredoxin TrxA
  
Accession: EEA28461
  
Location: 4718277-4719087
  
 NCBI BlastP on this gene

EEA28461

thioredoxin TrxA
  
Accession: EEA28462
  
Location: 4718707-4719087
  
 NCBI BlastP on this gene

EEA28462

MFS transporter, putative
  
Accession: EEA28463
  
Location: 4719255-4721108
  
 NCBI BlastP on this gene

EEA28463

acetoacetyl-CoA synthase
  
Accession: EEA28465
  
Location: 4723225-4724976
  
 NCBI BlastP on this gene

EEA28465

hypothetical protein
  
Accession: EEA28466
  
Location: 4725469-4726530
  
 NCBI BlastP on this gene

EEA28466

conserved hypothetical protein
  
Accession: EEA28467
  
Location: 4726551-4727605
  
 NCBI BlastP on this gene

EEA28467

D-amino acid oxidase
  
Accession: EEA28468
  
Location: 4728047-4729508
  
 NCBI BlastP on this gene

EEA28468

hypothetical protein
  
Accession: EEA28469
  
Location: 4729705-4730176
  
 NCBI BlastP on this gene

EEA28469

ABC bile acid transporter, putative
  
Accession: EEA28470
  
Location: 4732381-4737426
  
  
**BlastP hit with Mycgr3G9942\_Mycgr3T9**
  
Percentage identity: 42 %
  
BlastP bit score: 1001
  
Sequence coverage: 105 %
  
E-value: 0.0
  
  
 NCBI BlastP on this gene

EEA28470

protein RDR1, putative
  
Accession: EEA28471
  
Location: 4738017-4739743
  
 NCBI BlastP on this gene

EEA28471

isoflavone reductase family protein (CipA), putative
  
Accession: EEA28472
  
Location: 4740124-4741193
  
 NCBI BlastP on this gene

EEA28472

protein CCC1, putative
  
Accession: EEA28473
  
Location: 4741768-4742652
  
 NCBI BlastP on this gene

EEA28473

hypothetical protein
  
Accession: EEA28474
  
Location: 4743192-4743947
  
 NCBI BlastP on this gene

EEA28474

extracellular OTU-like cysteine protease, putative
  
Accession: EEA28475
  
Location: 4745393-4746973
  
 NCBI BlastP on this gene

EEA28475

polyglutamate biosynthesis protein, putative
  
Accession: EEA28476
  
Location: 4747353-4748624
  
 NCBI BlastP on this gene

EEA28476

222. :  ACJE01000015 Aspergillus niger ATCC 1015     Total score: 1.0     Cumulative Blast bit score: 999

hypothetical protein
  
Accession: EHA20955
  
Location: 183359-188638
  
  
**BlastP hit with Mycgr3G90558\_Mycgr3T**
  
Percentage identity: 36 %
  
BlastP bit score: 999
  
Sequence coverage: 39 %
  
E-value: 0.0
  
  
 NCBI BlastP on this gene

EHA20955

hypothetical protein
  
Accession: EHA20954
  
Location: 179596-181600
  
 NCBI BlastP on this gene

EHA20954

hypothetical protein
  
Accession: EHA20953
  
Location: 175966-176229
  
 NCBI BlastP on this gene

EHA20953

hypothetical protein
  
Accession: EHA20952
  
Location: 173588-174637
  
 NCBI BlastP on this gene

EHA20952

hypothetical protein
  
Accession: EHA20951
  
Location: 172034-172738
  
 NCBI BlastP on this gene

EHA20951

hypothetical protein
  
Accession: EHA20950
  
Location: 168918-170385
  
 NCBI BlastP on this gene

EHA20950

223. :  EQ962652 Talaromyces stipitatus ATCC 10500 scf\_1105507295523 genomic scaffold     Total score: 1.0     Cumulative Blast bit score: 993

MFS transporter, putative
  
Accession: EED23013
  
Location: 1496467-1498489
  
 NCBI BlastP on this gene

EED23013

GNAT family N-acetyltransferase, putative
  
Accession: EED23012
  
Location: 1493729-1494985
  
 NCBI BlastP on this gene

EED23012

acetoacetyl-CoA synthase
  
Accession: EED23011
  
Location: 1491319-1493640
  
 NCBI BlastP on this gene

EED23011

hypothetical protein
  
Accession: EED23010
  
Location: 1489779-1490843
  
 NCBI BlastP on this gene

EED23010

D-amino acid oxidase
  
Accession: EED23008
  
Location: 1486876-1488301
  
 NCBI BlastP on this gene

EED23008

hypothetical protein
  
Accession: EED23007
  
Location: 1484941-1486361
  
 NCBI BlastP on this gene

EED23007

conserved hypothetical protein
  
Accession: EED23006
  
Location: 1483248-1484785
  
 NCBI BlastP on this gene

EED23006

ABC bile acid transporter, putative
  
Accession: EED23005
  
Location: 1477712-1482719
  
  
**BlastP hit with Mycgr3G9942\_Mycgr3T9**
  
Percentage identity: 41 %
  
BlastP bit score: 993
  
Sequence coverage: 104 %
  
E-value: 0.0
  
  
 NCBI BlastP on this gene

EED23005

acetyl xylan esterase, putative
  
Accession: EED23004
  
Location: 1476426-1477439
  
 NCBI BlastP on this gene

EED23004

protein CCC1, putative
  
Accession: EED23003
  
Location: 1474337-1475217
  
 NCBI BlastP on this gene

EED23003

hypothetical protein
  
Accession: EED23002
  
Location: 1473100-1473843
  
 NCBI BlastP on this gene

EED23002

extracellular OTU-like cysteine protease, putative
  
Accession: EED23001
  
Location: 1470434-1472041
  
 NCBI BlastP on this gene

EED23001

polyglutamate biosynthesis protein, putative
  
Accession: EED23000
  
Location: 1468772-1470025
  
 NCBI BlastP on this gene

EED23000

conserved hypothetical protein
  
Accession: EED22999
  
Location: 1466031-1466899
  
 NCBI BlastP on this gene

EED22999

conserved hypothetical protein
  
Accession: EED22998
  
Location: 1464238-1465280
  
 NCBI BlastP on this gene

EED22998

fungal specific transcription factor, putative
  
Accession: EED22997
  
Location: 1462310-1463752
  
 NCBI BlastP on this gene

EED22997

224. :  DF126447 Aspergillus kawachii IFO 4308 DNA, contig: scaffold00001     Total score: 1.0     Cumulative Blast bit score: 991

SNF2 family helicase/ATPase
  
Accession: GAA81997
  
Location: 371314-374966
  
 NCBI BlastP on this gene

GAA81997

RNA methyltransferase, TrmH family
  
Accession: GAA81996
  
Location: 369305-370810
  
 NCBI BlastP on this gene

GAA81996

chromatin remodeling complex subunit
  
Accession: GAA81995
  
Location: 364333-368888
  
 NCBI BlastP on this gene

GAA81995

nonribosomal peptide synthase
  
Accession: GAA81994
  
Location: 353309-361301
  
  
**BlastP hit with Mycgr3G90558\_Mycgr3T**
  
Percentage identity: 32 %
  
BlastP bit score: 991
  
Sequence coverage: 49 %
  
E-value: 0.0
  
  
 NCBI BlastP on this gene

GAA81994

cytochrome P450 monooxygenase
  
Accession: GAA81993
  
Location: 349695-351097
  
 NCBI BlastP on this gene

GAA81993

C-x8-C-x5-C-x3-H type zinc finger protein
  
Accession: GAA81992
  
Location: 345470-347135
  
 NCBI BlastP on this gene

GAA81992

37S ribosomal protein Rsm24
  
Accession: GAA81991
  
Location: 343257-344487
  
 NCBI BlastP on this gene

GAA81991

glycosyltransferase family 28
  
Accession: GAA81990
  
Location: 342095-342912
  
 NCBI BlastP on this gene

GAA81990

actin-1
  
Accession: GAA81989
  
Location: 340171-341430
  
 NCBI BlastP on this gene

GAA81989

225. :  DS231635 Pyrenophora tritici-repentis Pt-1C-BFP supercont1.21 genomic scaffold     Total score: 1.0     Cumulative Blast bit score: 988

predicted protein
  
Accession: EDU45988
  
Location: 130378-131078
  
 NCBI BlastP on this gene

EDU45988

methyltransferase MppJ
  
Accession: EDU45989
  
Location: 135456-136478
  
 NCBI BlastP on this gene

EDU45989

branched-chain-amino-acid aminotransferase 5
  
Accession: EDU45990
  
Location: 137788-138976
  
 NCBI BlastP on this gene

EDU45990

predicted protein
  
Accession: EDU45991
  
Location: 142850-143154
  
 NCBI BlastP on this gene

EDU45991

HC-toxin synthetase
  
Accession: EDU45992
  
Location: 143880-150272
  
  
**BlastP hit with Mycgr3G90558\_Mycgr3T**
  
Percentage identity: 33 %
  
BlastP bit score: 988
  
Sequence coverage: 39 %
  
E-value: 0.0
  
  
 NCBI BlastP on this gene

EDU45992

226. :  GL891382 Neurospora tetrasperma FGSC 2508 unplaced genomic scaffold NEUTE1scaffold\_81     Total score: 1.0     Cumulative Blast bit score: 980

hypothetical protein
  
Accession: EGO52402
  
Location: 4241036-4242639
  
 NCBI BlastP on this gene

EGO52402

hypothetical protein
  
Accession: EGO52403
  
Location: 4243110-4244203
  
 NCBI BlastP on this gene

EGO52403

hypothetical protein
  
Accession: EGO52404
  
Location: 4244848-4246755
  
 NCBI BlastP on this gene

EGO52404

hypothetical protein
  
Accession: EGO52405
  
Location: 4248267-4250677
  
 NCBI BlastP on this gene

EGO52405

hypothetical protein
  
Accession: EGO52406
  
Location: 4252355-4253411
  
 NCBI BlastP on this gene

EGO52406

hypothetical protein
  
Accession: EGO52407
  
Location: 4256121-4260982
  
  
**BlastP hit with Mycgr3G9942\_Mycgr3T9**
  
Percentage identity: 40 %
  
BlastP bit score: 980
  
Sequence coverage: 107 %
  
E-value: 0.0
  
  
 NCBI BlastP on this gene

EGO52407

hypothetical protein
  
Accession: EGO52408
  
Location: 4262378-4263127
  
 NCBI BlastP on this gene

EGO52408

hypothetical protein
  
Accession: EGO52409
  
Location: 4263989-4265809
  
 NCBI BlastP on this gene

EGO52409

hypothetical protein
  
Accession: EGO52410
  
Location: 4268783-4269743
  
 NCBI BlastP on this gene

EGO52410

hypothetical protein
  
Accession: EGO52411
  
Location: 4273353-4273625
  
 NCBI BlastP on this gene

EGO52411

hypothetical protein
  
Accession: EGO52412
  
Location: 4274543-4275525
  
 NCBI BlastP on this gene

EGO52412

227. :  GL890999 Neurospora tetrasperma FGSC 2509 unplaced genomic scaffold NEUTE2scaffold\_1     Total score: 1.0     Cumulative Blast bit score: 979

DUF1776-domain-containing protein
  
Accession: EGZ77221
  
Location: 4048871-4050474
  
 NCBI BlastP on this gene

EGZ77221

S-adenosyl-L-methionine-dependent methyltransferase
  
Accession: EGZ77222
  
Location: 4050943-4052036
  
 NCBI BlastP on this gene

EGZ77222

amidase signature enzyme
  
Accession: EGZ77223
  
Location: 4052680-4054578
  
 NCBI BlastP on this gene

EGZ77223

WD40 repeat-like protein
  
Accession: EGZ77224
  
Location: 4056089-4058499
  
 NCBI BlastP on this gene

EGZ77224

hypothetical protein
  
Accession: EGZ77225
  
Location: 4060109-4061171
  
 NCBI BlastP on this gene

EGZ77225

P-loop containing nucleoside triphosphate hydrolase protein
  
Accession: EGZ77226
  
Location: 4064337-4069193
  
  
**BlastP hit with Mycgr3G9942\_Mycgr3T9**
  
Percentage identity: 40 %
  
BlastP bit score: 979
  
Sequence coverage: 107 %
  
E-value: 0.0
  
  
 NCBI BlastP on this gene

EGZ77226

cytidine deaminase
  
Accession: EGZ77227
  
Location: 4070610-4071405
  
 NCBI BlastP on this gene

EGZ77227

FYVE-domain-containing protein
  
Accession: EGZ77228
  
Location: 4072267-4074081
  
 NCBI BlastP on this gene

EGZ77228

NAD(P)-binding protein
  
Accession: EGZ77229
  
Location: 4077361-4078321
  
 NCBI BlastP on this gene

EGZ77229

hypothetical protein
  
Accession: EGZ77230
  
Location: 4080415-4080576
  
 NCBI BlastP on this gene

EGZ77230

hypothetical protein
  
Accession: EGZ77231
  
Location: 4081949-4082395
  
 NCBI BlastP on this gene

EGZ77231

mannose-P-dolichol utilization defect 1 protein
  
Accession: EGZ77232
  
Location: 4083265-4084247
  
 NCBI BlastP on this gene

EGZ77232

228. :  DS989823 Arthroderma gypseum CBS 118893 supercont1.2 genomic scaffold     Total score: 1.0     Cumulative Blast bit score: 978

DNA mismatch repair protein msh6
  
Accession: EFQ99811
  
Location: 2145237-2149061
  
 NCBI BlastP on this gene

EFQ99811

hypothetical protein
  
Accession: EFQ99810
  
Location: 2144351-2145043
  
 NCBI BlastP on this gene

EFQ99810

hypothetical protein
  
Accession: EFQ99809
  
Location: 2142922-2143782
  
 NCBI BlastP on this gene

EFQ99809

hypothetical protein
  
Accession: EFQ99808
  
Location: 2141191-2142107
  
 NCBI BlastP on this gene

EFQ99808

glutaminyl-peptide cyclotransferase
  
Accession: EFQ99807
  
Location: 2139525-2140703
  
 NCBI BlastP on this gene

EFQ99807

YeeE/YedE family integral membrane protein
  
Accession: EFQ99806
  
Location: 2137902-2138970
  
 NCBI BlastP on this gene

EFQ99806

hypothetical protein
  
Accession: EFQ99805
  
Location: 2135912-2137366
  
 NCBI BlastP on this gene

EFQ99805

hypothetical protein
  
Accession: EFQ99804
  
Location: 2132724-2135045
  
 NCBI BlastP on this gene

EFQ99804

canalicular multispecific organic anion transporter 1
  
Accession: EFQ99803
  
Location: 2126683-2131526
  
  
**BlastP hit with Mycgr3G9942\_Mycgr3T9**
  
Percentage identity: 40 %
  
BlastP bit score: 978
  
Sequence coverage: 105 %
  
E-value: 0.0
  
  
 NCBI BlastP on this gene

EFQ99803

PEP phosphonomutase
  
Accession: EFQ99802
  
Location: 2125619-2126392
  
 NCBI BlastP on this gene

EFQ99802

hypothetical protein
  
Accession: EFQ99801
  
Location: 2122780-2124397
  
 NCBI BlastP on this gene

EFQ99801

aspartyl-tRNA synthetase
  
Accession: EFQ99800
  
Location: 2118202-2120391
  
 NCBI BlastP on this gene

EFQ99800

FAD binding domain-containing protein
  
Accession: EFQ99799
  
Location: 2116010-2117811
  
 NCBI BlastP on this gene

EFQ99799

hypothetical protein
  
Accession: EFQ99798
  
Location: 2113996-2115453
  
 NCBI BlastP on this gene

EFQ99798

cycloheximide resistance protein
  
Accession: EFQ99797
  
Location: 2111505-2113497
  
 NCBI BlastP on this gene

EFQ99797

229. :  GL636493 Coccidioides posadasii str. Silveira unplaced genomic scaffold supercont2.8     Total score: 1.0     Cumulative Blast bit score: 972

ABC bile acid transporter
  
Accession: EFW17833
  
Location: 651400-656328
  
  
**BlastP hit with Mycgr3G9942\_Mycgr3T9**
  
Percentage identity: 40 %
  
BlastP bit score: 972
  
Sequence coverage: 108 %
  
E-value: 0.0
  
  
 NCBI BlastP on this gene

EFW17833

conserved hypothetical protein
  
Accession: EFW17832
  
Location: 650308-651099
  
 NCBI BlastP on this gene

EFW17832

hypothetical protein
  
Accession: EFW17831
  
Location: 649618-649782
  
 NCBI BlastP on this gene

EFW17831

aspartyl-tRNA synthetase
  
Accession: EFW17830
  
Location: 646908-649102
  
 NCBI BlastP on this gene

EFW17830

nuclear movement protein nudC
  
Accession: EFW17829
  
Location: 645850-646584
  
 NCBI BlastP on this gene

EFW17829

transcription elongation factor spt5
  
Accession: EFW17828
  
Location: 641784-645162
  
 NCBI BlastP on this gene

EFW17828

acyl-CoA dehydrogenase
  
Accession: EFW17827
  
Location: 638973-640871
  
 NCBI BlastP on this gene

EFW17827

26S proteasome regulatory subunit Rpn2
  
Accession: EFW17826
  
Location: 634031-637741
  
 NCBI BlastP on this gene

EFW17826

230. :  GG704913 Coccidioides immitis RS genomic scaffold supercont3.3     Total score: 1.0     Cumulative Blast bit score: 972

tyrosine-tRNA ligase
  
Accession: EAS27906
  
Location: 292654-294847
  
 NCBI BlastP on this gene

EAS27906

DNA mismatch repair protein msh6
  
Accession: EAS27907
  
Location: 288348-292194
  
 NCBI BlastP on this gene

EAS27907

arsenate reductase
  
Accession: EAS27908
  
Location: 287368-288026
  
 NCBI BlastP on this gene

EAS27908

hypothetical protein
  
Accession: EAS27909
  
Location: 285775-286666
  
 NCBI BlastP on this gene

EAS27909

hypothetical protein
  
Accession: EAS27910
  
Location: 284326-285254
  
 NCBI BlastP on this gene

EAS27910

glutaminyl cyclase
  
Accession: EAS27911
  
Location: 282694-284015
  
 NCBI BlastP on this gene

EAS27911

YeeE/YedE family integral membrane protein
  
Accession: EAS27912
  
Location: 281133-282220
  
 NCBI BlastP on this gene

EAS27912

hypothetical protein
  
Accession: EAS27914
  
Location: 278939-280341
  
 NCBI BlastP on this gene

EAS27914

hypothetical protein
  
Accession: EAS27915
  
Location: 278054-278650
  
 NCBI BlastP on this gene

EAS27915

ABC bile acid transporter
  
Accession: EAS27916
  
Location: 272536-277464
  
  
**BlastP hit with Mycgr3G9942\_Mycgr3T9**
  
Percentage identity: 40 %
  
BlastP bit score: 972
  
Sequence coverage: 107 %
  
E-value: 0.0
  
  
 NCBI BlastP on this gene

EAS27916

hypothetical protein
  
Accession: EAS27917
  
Location: 271456-272247
  
 NCBI BlastP on this gene

EAS27917

aspartate-tRNA ligase
  
Accession: EAS27918
  
Location: 268058-270251
  
 NCBI BlastP on this gene

EAS27918

nuclear movement protein nudC
  
Accession: EAS27919
  
Location: 267001-267734
  
 NCBI BlastP on this gene

EAS27919

transcription initiation protein spt5
  
Accession: EAS27920
  
Location: 262958-266327
  
 NCBI BlastP on this gene

EAS27920

acyl-CoA dehydrogenase
  
Accession: EAS27921
  
Location: 260134-262032
  
 NCBI BlastP on this gene

EAS27921

26S proteasome regulatory subunit Rpn2
  
Accession: EAS27922
  
Location: 255198-258908
  
 NCBI BlastP on this gene

EAS27922

231. :  ACFW01000015 Coccidioides posadasii C735 delta SOWgp     Total score: 1.0     Cumulative Blast bit score: 972

MutS domain III family protein
  
Accession: EER28474
  
Location: 947971-951820
  
 NCBI BlastP on this gene

EER28474

conserved hypothetical protein
  
Accession: EER28475
  
Location: 952142-952800
  
 NCBI BlastP on this gene

EER28475

hypothetical protein
  
Accession: EER28476
  
Location: 953501-954392
  
 NCBI BlastP on this gene

EER28476

hypothetical protein
  
Accession: EER28477
  
Location: 954912-955963
  
 NCBI BlastP on this gene

EER28477

Peptidase family M28 protein
  
Accession: EER28478
  
Location: 956160-957362
  
 NCBI BlastP on this gene

EER28478

YeeE/YedE family protein
  
Accession: EER28479
  
Location: 957956-959045
  
 NCBI BlastP on this gene

EER28479

hypothetical protein
  
Accession: EER28480
  
Location: 959883-961285
  
 NCBI BlastP on this gene

EER28480

ABC transporter family protein
  
Accession: EER28481
  
Location: 962786-967714
  
  
**BlastP hit with Mycgr3G9942\_Mycgr3T9**
  
Percentage identity: 40 %
  
BlastP bit score: 972
  
Sequence coverage: 108 %
  
E-value: 0.0
  
  
 NCBI BlastP on this gene

EER28481

carboxyphosphonoenolpyruvate mutase, putative
  
Accession: EER28482
  
Location: 968015-968806
  
 NCBI BlastP on this gene

EER28482

aspartyl-tRNA synthetase, putative
  
Accession: EER28483
  
Location: 970012-972206
  
 NCBI BlastP on this gene

EER28483

nuclear movement protein nudC, putative
  
Accession: EER28484
  
Location: 972530-973264
  
 NCBI BlastP on this gene

EER28484

KOW motif containing protein
  
Accession: EER28485
  
Location: 973952-977330
  
 NCBI BlastP on this gene

EER28485

Acyl-CoA dehydrogenase, C-terminal domain containing protein
  
Accession: EER28486
  
Location: 978247-980157
  
 NCBI BlastP on this gene

EER28486

26S proteasome non-ATPase regulatory subunit 1, putative
  
Accession: EER28487
  
Location: 981389-985099
  
 NCBI BlastP on this gene

EER28487

232. :  DS231635 Pyrenophora tritici-repentis Pt-1C-BFP supercont1.21 genomic scaffold     Total score: 1.0     Cumulative Blast bit score: 971

methyltransferase MppJ
  
Accession: EDU45978
  
Location: 46956-47978
  
 NCBI BlastP on this gene

EDU45978

branched-chain-amino-acid aminotransferase 5
  
Accession: EDU45977
  
Location: 44458-45646
  
 NCBI BlastP on this gene

EDU45977

cytochrome P450 11A1, mitochondrial precursor
  
Accession: EDU45976
  
Location: 42383-43398
  
 NCBI BlastP on this gene

EDU45976

predicted protein
  
Accession: EDU45975
  
Location: 40622-40926
  
 NCBI BlastP on this gene

EDU45975

HC-toxin synthetase
  
Accession: EDU45974
  
Location: 33505-39896
  
  
**BlastP hit with Mycgr3G90558\_Mycgr3T**
  
Percentage identity: 33 %
  
BlastP bit score: 971
  
Sequence coverage: 39 %
  
E-value: 0.0
  
  
 NCBI BlastP on this gene

EDU45974

cytochrome P450
  
Accession: EDU45973
  
Location: 25871-27460
  
 NCBI BlastP on this gene

EDU45973

predicted protein
  
Accession: EDU45972
  
Location: 23181-23746
  
 NCBI BlastP on this gene

EDU45972

predicted protein
  
Accession: EDU45971
  
Location: 21535-23146
  
 NCBI BlastP on this gene

EDU45971

233. :  CH408029 Chaetomium globosum CBS 148.51 scaffold\_1 genomic scaffold     Total score: 1.0     Cumulative Blast bit score: 970

hypothetical protein
  
Accession: EAQ93550
  
Location: 5511220-5516115
  
  
**BlastP hit with Mycgr3G9942\_Mycgr3T9**
  
Percentage identity: 40 %
  
BlastP bit score: 970
  
Sequence coverage: 106 %
  
E-value: 0.0
  
  
 NCBI BlastP on this gene

EAQ93550

hypothetical protein
  
Accession: EAQ93549
  
Location: 5506535-5509773
  
 NCBI BlastP on this gene

EAQ93549

hypothetical protein
  
Accession: EAQ93548
  
Location: 5502531-5504798
  
 NCBI BlastP on this gene

EAQ93548

hypothetical protein
  
Accession: EAQ93547
  
Location: 5498972-5500508
  
 NCBI BlastP on this gene

EAQ93547

hypothetical protein
  
Accession: EAQ93546
  
Location: 5496230-5498311
  
 NCBI BlastP on this gene

EAQ93546

234. :  JH725157 Beauveria bassiana ARSEF 2860 unplaced genomic scaffold BBA\_S00008     Total score: 1.0     Cumulative Blast bit score: 967

ABC bile acid transporter, putative
  
Accession: EJP67092
  
Location: 99092-104075
  
 NCBI BlastP on this gene

EJP67092

methyltransferase domain-containing protein
  
Accession: EJP67093
  
Location: 105176-106045
  
 NCBI BlastP on this gene

EJP67093

hypothetical protein
  
Accession: EJP67094
  
Location: 106606-107538
  
 NCBI BlastP on this gene

EJP67094

MFS transporter, putative
  
Accession: EJP67095
  
Location: 110045-111581
  
 NCBI BlastP on this gene

EJP67095

MFS transporter, putative
  
Accession: EJP67096
  
Location: 111704-113283
  
 NCBI BlastP on this gene

EJP67096

nonribosomal peptide synthase, putative
  
Accession: EJP67097
  
Location: 115213-127028
  
  
**BlastP hit with Mycgr3G90558\_Mycgr3T**
  
Percentage identity: 31 %
  
BlastP bit score: 968
  
Sequence coverage: 50 %
  
E-value: 0.0
  
  
 NCBI BlastP on this gene

EJP67097

heterokaryon incompatibility protein
  
Accession: EJP67098
  
Location: 127717-130000
  
 NCBI BlastP on this gene

EJP67098

hypothetical protein
  
Accession: EJP67099
  
Location: 130019-132156
  
 NCBI BlastP on this gene

EJP67099

Formyl transferase
  
Accession: EJP67100
  
Location: 133080-134023
  
 NCBI BlastP on this gene

EJP67100

NAD dependent epimerase/dehydratase
  
Accession: EJP67101
  
Location: 135336-136424
  
 NCBI BlastP on this gene

EJP67101

thymidylate kinase
  
Accession: EJP67102
  
Location: 137120-138030
  
 NCBI BlastP on this gene

EJP67102

235. :  CU633438 Podospora anserina S mat+ genomic DNA chromosome 1, supercontig 1.     Total score: 1.0     Cumulative Blast bit score: 966

not annotated
  
Accession: CAP60085
  
Location: 1446828-1450400
  
 NCBI BlastP on this gene

CAP60085

not annotated
  
Accession: CAP60086
  
Location: 1451450-1452882
  
 NCBI BlastP on this gene

CAP60086

not annotated
  
Accession: CAP60087
  
Location: 1453539-1455826
  
 NCBI BlastP on this gene

CAP60087

not annotated
  
Accession: CAP60088
  
Location: 1456439-1458310
  
 NCBI BlastP on this gene

CAP60088

not annotated
  
Accession: CAP60089
  
Location: 1459064-1461493
  
 NCBI BlastP on this gene

CAP60089

not annotated
  
Accession: CAP60090
  
Location: 1464194-1469041
  
  
**BlastP hit with Mycgr3G9942\_Mycgr3T9**
  
Percentage identity: 40 %
  
BlastP bit score: 966
  
Sequence coverage: 108 %
  
E-value: 0.0
  
  
 NCBI BlastP on this gene

CAP60090

236. :  CH476633 Sclerotinia sclerotiorum 1980 scaffold\_13 genomic scaffold     Total score: 1.0     Cumulative Blast bit score: 966

hypothetical protein
  
Accession: EDN93385
  
Location: 302259-303418
  
 NCBI BlastP on this gene

EDN93385

hypothetical protein
  
Accession: EDN93386
  
Location: 305578-308810
  
 NCBI BlastP on this gene

EDN93386

hypothetical protein
  
Accession: EDN93387
  
Location: 310883-313315
  
 NCBI BlastP on this gene

EDN93387

predicted protein
  
Accession: EDN93388
  
Location: 313893-314757
  
 NCBI BlastP on this gene

EDN93388

hypothetical protein
  
Accession: EDN93389
  
Location: 316763-321759
  
  
**BlastP hit with Mycgr3G9942\_Mycgr3T9**
  
Percentage identity: 40 %
  
BlastP bit score: 966
  
Sequence coverage: 105 %
  
E-value: 0.0
  
  
 NCBI BlastP on this gene

EDN93389

predicted protein
  
Accession: EDN93390
  
Location: 323128-324474
  
 NCBI BlastP on this gene

EDN93390

hypothetical protein
  
Accession: EDN93391
  
Location: 325754-326035
  
 NCBI BlastP on this gene

EDN93391

predicted protein
  
Accession: EDN93392
  
Location: 328150-328326
  
 NCBI BlastP on this gene

EDN93392

hypothetical protein
  
Accession: EDN93393
  
Location: 329546-329752
  
 NCBI BlastP on this gene

EDN93393

hypothetical protein
  
Accession: EDN93394
  
Location: 330222-334364
  
 NCBI BlastP on this gene

EDN93394

hypothetical protein
  
Accession: EDN93395
  
Location: 335306-340081
  
 NCBI BlastP on this gene

EDN93395

237. :  CABT02000004 Sordaria macrospora k-hell     Total score: 1.0     Cumulative Blast bit score: 966

not annotated
  
Accession: CCC08062
  
Location: 1555424-1557042
  
 NCBI BlastP on this gene

CCC08062

not annotated
  
Accession: CCC08063
  
Location: 1557521-1558624
  
 NCBI BlastP on this gene

CCC08063

not annotated
  
Accession: CCC08064
  
Location: 1559273-1561179
  
 NCBI BlastP on this gene

CCC08064

not annotated
  
Accession: CCC08065
  
Location: 1562691-1565088
  
 NCBI BlastP on this gene

CCC08065

not annotated
  
Accession: CCC08066
  
Location: 1567154-1567344
  
 NCBI BlastP on this gene

CCC08066

not annotated
  
Accession: CCC08067
  
Location: 1570493-1577429
  
  
**BlastP hit with Mycgr3G9942\_Mycgr3T9**
  
Percentage identity: 40 %
  
BlastP bit score: 966
  
Sequence coverage: 107 %
  
E-value: 0.0
  
  
 NCBI BlastP on this gene

CCC08067

238. :  GL385398 Gaeumannomyces graminis var. tritici R3-111a-1 unplaced genomic scaffold supercont2.4     Total score: 1.0     Cumulative Blast bit score: 959

carboxy-cis,cis-muconate cyclase
  
Accession: EJT74519
  
Location: 2854513-2855917
  
 NCBI BlastP on this gene

EJT74519

hypothetical protein
  
Accession: EJT74518
  
Location: 2852623-2854142
  
 NCBI BlastP on this gene

EJT74518

hypothetical protein
  
Accession: EJT74517
  
Location: 2849570-2850761
  
 NCBI BlastP on this gene

EJT74517

hypothetical protein
  
Accession: EJT74516
  
Location: 2848184-2849016
  
 NCBI BlastP on this gene

EJT74516

hypothetical protein
  
Accession: EJT74515
  
Location: 2844033-2846557
  
 NCBI BlastP on this gene

EJT74515

hypothetical protein
  
Accession: EJT74514
  
Location: 2835242-2840146
  
  
**BlastP hit with Mycgr3G9942\_Mycgr3T9**
  
Percentage identity: 39 %
  
BlastP bit score: 960
  
Sequence coverage: 109 %
  
E-value: 0.0
  
  
 NCBI BlastP on this gene

EJT74514

hypothetical protein
  
Accession: EJT74513
  
Location: 2830961-2833720
  
 NCBI BlastP on this gene

EJT74513

NADH-cytochrome b5 reductase 2
  
Accession: EJT74512
  
Location: 2829097-2830273
  
 NCBI BlastP on this gene

EJT74512

SH3 domain-containing protein
  
Accession: EJT74511
  
Location: 2826306-2828033
  
 NCBI BlastP on this gene

EJT74511

RNA polymerase II subunit A domain phosphatase SSU72
  
Accession: EJT74510
  
Location: 2821714-2822551
  
 NCBI BlastP on this gene

EJT74510

239. :  DS989824 Arthroderma gypseum CBS 118893 supercont1.3 genomic scaffold     Total score: 1.0     Cumulative Blast bit score: 959

hypothetical protein
  
Accession: EFR00568
  
Location: 383665-386208
  
 NCBI BlastP on this gene

EFR00568

hypothetical protein
  
Accession: EFR00569
  
Location: 387047-387985
  
 NCBI BlastP on this gene

EFR00569

hypothetical protein
  
Accession: EFR00570
  
Location: 389798-390613
  
 NCBI BlastP on this gene

EFR00570

hypothetical protein
  
Accession: EFR00571
  
Location: 391334-392384
  
 NCBI BlastP on this gene

EFR00571

nuclear distribution protein nudF
  
Accession: EFR00572
  
Location: 393048-394625
  
 NCBI BlastP on this gene

EFR00572

CMGC/SRPK protein kinase
  
Accession: EFR00573
  
Location: 395363-396734
  
 NCBI BlastP on this gene

EFR00573

hypothetical protein
  
Accession: EFR00574
  
Location: 397292-403135
  
  
**BlastP hit with Mycgr3G9942\_Mycgr3T9**
  
Percentage identity: 40 %
  
BlastP bit score: 960
  
Sequence coverage: 103 %
  
E-value: 0.0
  
  
 NCBI BlastP on this gene

EFR00574

averantin oxidoreductase
  
Accession: EFR00575
  
Location: 404522-406244
  
 NCBI BlastP on this gene

EFR00575

hypothetical protein
  
Accession: EFR00576
  
Location: 406769-408112
  
 NCBI BlastP on this gene

EFR00576

hypothetical protein
  
Accession: EFR00577
  
Location: 408338-412797
  
 NCBI BlastP on this gene

EFR00577

DUF543 domain-containing protein
  
Accession: EFR00578
  
Location: 413100-413627
  
 NCBI BlastP on this gene

EFR00578

hypothetical protein
  
Accession: EFR00579
  
Location: 414121-415983
  
 NCBI BlastP on this gene

EFR00579

hypothetical protein
  
Accession: EFR00580
  
Location: 416700-417808
  
 NCBI BlastP on this gene

EFR00580

240. :  JH921428 Marssonina brunnea f. sp. 'multigermtubi' MB\_m1 unplaced genomic scaffold M6\_S00001     Total score: 1.0     Cumulative Blast bit score: 959

zearalenone hydrolase
  
Accession: EKD21810
  
Location: 5100098-5100958
  
 NCBI BlastP on this gene

EKD21810

WD domain-containing protein
  
Accession: EKD21811
  
Location: 5105381-5107727
  
 NCBI BlastP on this gene

EKD21811

ABC transporter
  
Accession: EKD21812
  
Location: 5110233-5115163
  
  
**BlastP hit with Mycgr3G9942\_Mycgr3T9**
  
Percentage identity: 39 %
  
BlastP bit score: 959
  
Sequence coverage: 107 %
  
E-value: 0.0
  
  
 NCBI BlastP on this gene

EKD21812

241. :  GG749415 Ajellomyces dermatitidis ATCC 18188 genomic scaffold supercont1.9     Total score: 1.0     Cumulative Blast bit score: 959

DNA mismatch repair protein msh6
  
Accession: EGE79590
  
Location: 841320-845315
  
 NCBI BlastP on this gene

EGE79590

hypothetical protein
  
Accession: EGE79591
  
Location: 845667-846579
  
 NCBI BlastP on this gene

EGE79591

hypothetical protein
  
Accession: EGE79592
  
Location: 847506-848417
  
 NCBI BlastP on this gene

EGE79592

hypothetical protein
  
Accession: EGE79593
  
Location: 848906-849829
  
 NCBI BlastP on this gene

EGE79593

glutaminyl cyclase
  
Accession: EGE79594
  
Location: 850303-851732
  
 NCBI BlastP on this gene

EGE79594

YeeE/YedE family integral membrane protein
  
Accession: EGE79595
  
Location: 852350-853521
  
 NCBI BlastP on this gene

EGE79595

hypothetical protein
  
Accession: EGE79596
  
Location: 855396-856222
  
 NCBI BlastP on this gene

EGE79596

ABC bile acid transporter
  
Accession: EGE79597
  
Location: 857083-861945
  
  
**BlastP hit with Mycgr3G9942\_Mycgr3T9**
  
Percentage identity: 40 %
  
BlastP bit score: 959
  
Sequence coverage: 106 %
  
E-value: 0.0
  
  
 NCBI BlastP on this gene

EGE79597

242. :  EQ999975 Ajellomyces dermatitidis ER-3 genomic scaffold supercont1.3     Total score: 1.0     Cumulative Blast bit score: 959

DNA mismatch repair protein msh6
  
Accession: EEQ88009
  
Location: 4069178-4073173
  
 NCBI BlastP on this gene

EEQ88009

conserved hypothetical protein
  
Accession: EEQ88008
  
Location: 4067934-4068845
  
 NCBI BlastP on this gene

EEQ88008

conserved hypothetical protein
  
Accession: EEQ88007
  
Location: 4066096-4067007
  
 NCBI BlastP on this gene

EEQ88007

conserved hypothetical protein
  
Accession: EEQ88006
  
Location: 4064684-4065607
  
 NCBI BlastP on this gene

EEQ88006

glutaminyl cyclase
  
Accession: EEQ88005
  
Location: 4062774-4064210
  
 NCBI BlastP on this gene

EEQ88005

YeeE/YedE family integral membrane protein
  
Accession: EEQ88004
  
Location: 4060981-4062156
  
 NCBI BlastP on this gene

EEQ88004

hypothetical protein
  
Accession: EEQ88003
  
Location: 4058355-4059734
  
 NCBI BlastP on this gene

EEQ88003

ABC bile acid transporter
  
Accession: EEQ88002
  
Location: 4052784-4057646
  
  
**BlastP hit with Mycgr3G9942\_Mycgr3T9**
  
Percentage identity: 40 %
  
BlastP bit score: 959
  
Sequence coverage: 106 %
  
E-value: 0.0
  
  
 NCBI BlastP on this gene

EEQ88002

glucose-methanol-choline oxidoreductase
  
Accession: EEQ88001
  
Location: 4050457-4052271
  
 NCBI BlastP on this gene

EEQ88001

conserved hypothetical protein
  
Accession: EEQ88000
  
Location: 4047058-4048740
  
 NCBI BlastP on this gene

EEQ88000

PEP phosphonomutase
  
Accession: EEQ87999
  
Location: 4045752-4046552
  
 NCBI BlastP on this gene

EEQ87999

26S proteasome regulatory subunit Rpn2
  
Accession: EEQ87998
  
Location: 4041021-4044761
  
 NCBI BlastP on this gene

EEQ87998

acyl-CoA dehydrogenase
  
Accession: EEQ87997
  
Location: 4037585-4039551
  
 NCBI BlastP on this gene

EEQ87997

243. :  GG657450 Ajellomyces dermatitidis SLH14081 genomic scaffold supercont1.3     Total score: 1.0     Cumulative Blast bit score: 957

DNA mismatch repair protein msh6
  
Accession: EEQ75395
  
Location: 3884607-3888602
  
 NCBI BlastP on this gene

EEQ75395

conserved hypothetical protein
  
Accession: EEQ75396
  
Location: 3888955-3889866
  
 NCBI BlastP on this gene

EEQ75396

conserved hypothetical protein
  
Accession: EEQ75397
  
Location: 3890793-3891704
  
 NCBI BlastP on this gene

EEQ75397

conserved hypothetical protein
  
Accession: EEQ75398
  
Location: 3892193-3893116
  
 NCBI BlastP on this gene

EEQ75398

glutaminyl cyclase
  
Accession: EEQ75399
  
Location: 3893589-3895010
  
 NCBI BlastP on this gene

EEQ75399

YeeE/YedE family integral membrane protein
  
Accession: EEQ75400
  
Location: 3895628-3896799
  
 NCBI BlastP on this gene

EEQ75400

hypothetical protein
  
Accession: EEQ75401
  
Location: 3898592-3899142
  
 NCBI BlastP on this gene

EEQ75401

ABC bile acid transporter
  
Accession: EEQ75402
  
Location: 3900121-3904983
  
  
**BlastP hit with Mycgr3G9942\_Mycgr3T9**
  
Percentage identity: 40 %
  
BlastP bit score: 957
  
Sequence coverage: 106 %
  
E-value: 0.0
  
  
 NCBI BlastP on this gene

EEQ75402

244. :  DS995719 Trichophyton equinum CBS 127.97 supercont1.2 genomic scaffold     Total score: 1.0     Cumulative Blast bit score: 956

canalicular multispecific organic anion transporter 1
  
Accession: EGE01692
  
Location: 724146-729017
  
  
**BlastP hit with Mycgr3G9942\_Mycgr3T9**
  
Percentage identity: 40 %
  
BlastP bit score: 956
  
Sequence coverage: 105 %
  
E-value: 0.0
  
  
 NCBI BlastP on this gene

EGE01692

PEP phosphonomutase
  
Accession: EGE01691
  
Location: 723087-723860
  
 NCBI BlastP on this gene

EGE01691

hypothetical protein
  
Accession: EGE01690
  
Location: 720265-721911
  
 NCBI BlastP on this gene

EGE01690

hypothetical protein
  
Accession: EGE01689
  
Location: 719353-719667
  
 NCBI BlastP on this gene

EGE01689

aspartyl-tRNA synthetase
  
Accession: EGE01688
  
Location: 715676-717862
  
 NCBI BlastP on this gene

EGE01688

ABC transporter
  
Accession: EGE01687
  
Location: 711789-714117
  
 NCBI BlastP on this gene

EGE01687

C6 transcription factor
  
Accession: EGE01686
  
Location: 708874-710724
  
 NCBI BlastP on this gene

EGE01686

245. :  DS027694 Neosartorya fischeri NRRL 181 1099437636262 genomic scaffold     Total score: 1.0     Cumulative Blast bit score: 956

nonribosomal peptide synthase, putative
  
Accession: EAW20390
  
Location: 1857043-1863732
  
 NCBI BlastP on this gene

EAW20390

hypothetical protein
  
Accession: EAW20391
  
Location: 1864511-1865464
  
 NCBI BlastP on this gene

EAW20391

oligopeptide transporter
  
Accession: EAW20392
  
Location: 1865942-1867853
  
 NCBI BlastP on this gene

EAW20392

GNAT family acetyltransferase, putative
  
Accession: EAW20393
  
Location: 1868755-1869483
  
 NCBI BlastP on this gene

EAW20393

nonribosomal peptide synthase, putative
  
Accession: EAW20394
  
Location: 1870166-1877179
  
  
**BlastP hit with Mycgr3G90558\_Mycgr3T**
  
Percentage identity: 30 %
  
BlastP bit score: 956
  
Sequence coverage: 49 %
  
E-value: 0.0
  
  
 NCBI BlastP on this gene

EAW20394

246. :  CP003013 Thielavia terrestris NRRL 8126 chromosome 5     Total score: 1.0     Cumulative Blast bit score: 956

hypothetical protein
  
Accession: AEO70139
  
Location: 1308213-1313226
  
  
**BlastP hit with Mycgr3G9942\_Mycgr3T9**
  
Percentage identity: 39 %
  
BlastP bit score: 956
  
Sequence coverage: 107 %
  
E-value: 0.0
  
  
 NCBI BlastP on this gene

THITE\_45545

hypothetical protein
  
Accession: AEO70138
  
Location: 1304350-1307445
  
 NCBI BlastP on this gene

THITE\_115141

hypothetical protein
  
Accession: AEO70137
  
Location: 1301387-1302500
  
 NCBI BlastP on this gene

THITE\_2121138

hypothetical protein
  
Accession: AEO70136
  
Location: 1300479-1300873
  
 NCBI BlastP on this gene

THITE\_2121136

hypothetical protein
  
Accession: AEO70135
  
Location: 1299525-1300073
  
 NCBI BlastP on this gene

THITE\_2121135

hypothetical protein
  
Accession: AEO70134
  
Location: 1297889-1298979
  
 NCBI BlastP on this gene

THITE\_2121132

hypothetical protein
  
Accession: AEO70133
  
Location: 1295369-1297156
  
 NCBI BlastP on this gene

THITE\_2121126

hypothetical protein
  
Accession: AEO70132
  
Location: 1291015-1294063
  
 NCBI BlastP on this gene

THITE\_2036687

247. :  KE145369 Glarea lozoyensis ATCC 20868 chromosome Unknown GLAREA5     Total score: 1.0     Cumulative Blast bit score: 954

RING/U-box
  
Accession: EPE27474
  
Location: 221104-222513
  
 NCBI BlastP on this gene

EPE27474

MurD-like peptide ligase, catalytic
  
Accession: EPE27475
  
Location: 223882-225569
  
 NCBI BlastP on this gene

EPE27475

MurD-like peptide ligase, catalytic
  
Accession: EPE27476
  
Location: 225692-227401
  
 NCBI BlastP on this gene

EPE27476

hypothetical protein
  
Accession: EPE27477
  
Location: 227943-229024
  
 NCBI BlastP on this gene

EPE27477

hypothetical protein
  
Accession: EPE27478
  
Location: 229591-230415
  
 NCBI BlastP on this gene

EPE27478

WD40 repeat-like protein
  
Accession: EPE27479
  
Location: 232251-234707
  
 NCBI BlastP on this gene

EPE27479

P-loop containing nucleoside triphosphate hydrolase
  
Accession: EPE27480
  
Location: 237327-242317
  
  
**BlastP hit with Mycgr3G9942\_Mycgr3T9**
  
Percentage identity: 40 %
  
BlastP bit score: 954
  
Sequence coverage: 104 %
  
E-value: 0.0
  
  
 NCBI BlastP on this gene

EPE27480

hypothetical protein
  
Accession: EPE27481
  
Location: 247028-248058
  
 NCBI BlastP on this gene

EPE27481

hypothetical protein
  
Accession: EPE27482
  
Location: 249261-250138
  
 NCBI BlastP on this gene

EPE27482

hypothetical protein
  
Accession: EPE27483
  
Location: 252352-253869
  
 NCBI BlastP on this gene

EPE27483

(Trans)glycosidase
  
Accession: EPE27484
  
Location: 254258-255933
  
 NCBI BlastP on this gene

EPE27484

Cytochrome P450
  
Accession: EPE27485
  
Location: 256844-258483
  
 NCBI BlastP on this gene

EPE27485

248. :  DS995705 Microsporum canis CBS 113480 supercont1.5 genomic scaffold     Total score: 1.0     Cumulative Blast bit score: 954

DNA mismatch repair protein msh6
  
Accession: EEQ32901
  
Location: 1686508-1690353
  
 NCBI BlastP on this gene

EEQ32901

conserved hypothetical protein
  
Accession: EEQ32900
  
Location: 1685541-1686236
  
 NCBI BlastP on this gene

EEQ32900

predicted protein
  
Accession: EEQ32899
  
Location: 1684092-1685002
  
 NCBI BlastP on this gene

EEQ32899

conserved hypothetical protein
  
Accession: EEQ32898
  
Location: 1682343-1683261
  
 NCBI BlastP on this gene

EEQ32898

glutaminyl-peptide cyclotransferase
  
Accession: EEQ32897
  
Location: 1680522-1681875
  
 NCBI BlastP on this gene

EEQ32897

YeeE/YedE family integral membrane protein
  
Accession: EEQ32896
  
Location: 1679071-1680145
  
 NCBI BlastP on this gene

EEQ32896

conserved hypothetical protein
  
Accession: EEQ32895
  
Location: 1677699-1678911
  
 NCBI BlastP on this gene

EEQ32895

predicted protein
  
Accession: EEQ32894
  
Location: 1676686-1677164
  
 NCBI BlastP on this gene

EEQ32894

conserved hypothetical protein
  
Accession: EEQ32893
  
Location: 1674051-1676372
  
 NCBI BlastP on this gene

EEQ32893

multidrug resistance-associated protein 13
  
Accession: EEQ32892
  
Location: 1668271-1673135
  
  
**BlastP hit with Mycgr3G9942\_Mycgr3T9**
  
Percentage identity: 39 %
  
BlastP bit score: 954
  
Sequence coverage: 106 %
  
E-value: 0.0
  
  
 NCBI BlastP on this gene

EEQ32892

PEP phosphonomutase
  
Accession: EEQ32891
  
Location: 1667198-1667971
  
 NCBI BlastP on this gene

EEQ32891

conserved hypothetical protein
  
Accession: EEQ32890
  
Location: 1664447-1666114
  
 NCBI BlastP on this gene

EEQ32890

aspartyl-tRNA synthetase
  
Accession: EEQ32889
  
Location: 1660101-1662295
  
 NCBI BlastP on this gene

EEQ32889

ABC transporter family protein
  
Accession: EEQ32888
  
Location: 1655919-1658480
  
 NCBI BlastP on this gene

EEQ32888

conserved hypothetical protein
  
Accession: EEQ32887
  
Location: 1653426-1655261
  
 NCBI BlastP on this gene

EEQ32887

249. :  CP003002 Myceliophthora thermophila ATCC 42464 chromosome 1     Total score: 1.0     Cumulative Blast bit score: 952

hypothetical protein
  
Accession: AEO53315
  
Location: 1092433-1104807
  
 NCBI BlastP on this gene

MYCTH\_2122159

hypothetical protein
  
Accession: AEO53314
  
Location: 1090919-1091677
  
 NCBI BlastP on this gene

MYCTH\_16224

hypothetical protein
  
Accession: AEO53313
  
Location: 1088909-1090544
  
 NCBI BlastP on this gene

MYCTH\_2294459

hypothetical protein
  
Accession: AEO53312
  
Location: 1087351-1088424
  
 NCBI BlastP on this gene

MYCTH\_2054555

hypothetical protein
  
Accession: AEO53311
  
Location: 1085001-1086845
  
 NCBI BlastP on this gene

MYCTH\_2294455

hypothetical protein
  
Accession: AEO53310
  
Location: 1081441-1083916
  
 NCBI BlastP on this gene

MYCTH\_75762

hypothetical protein
  
Accession: AEO53309
  
Location: 1074987-1079876
  
  
**BlastP hit with Mycgr3G9942\_Mycgr3T9**
  
Percentage identity: 40 %
  
BlastP bit score: 952
  
Sequence coverage: 105 %
  
E-value: 0.0
  
  
 NCBI BlastP on this gene

MYCTH\_2294448

hypothetical protein
  
Accession: AEO53308
  
Location: 1070825-1073962
  
 NCBI BlastP on this gene

MYCTH\_42476

hypothetical protein
  
Accession: AEO53307
  
Location: 1066988-1069197
  
 NCBI BlastP on this gene

MYCTH\_2294446

hypothetical protein
  
Accession: AEO53306
  
Location: 1063559-1065049
  
 NCBI BlastP on this gene

MYCTH\_2041072

hypothetical protein
  
Accession: AEO53305
  
Location: 1060422-1062320
  
 NCBI BlastP on this gene

MYCTH\_2294445

250. :  CM001234 Magnaporthe oryzae 70-15 chromosome 4     Total score: 1.0     Cumulative Blast bit score: 948

RNA-binding protein 8A
  
Accession: EHA49820
  
Location: 859807-860393
  
 NCBI BlastP on this gene

EHA49820

hypothetical protein
  
Accession: EHA49821
  
Location: 861918-864894
  
 NCBI BlastP on this gene

EHA49821

F-box domain-containing protein
  
Accession: EHA49822
  
Location: 867434-869451
  
 NCBI BlastP on this gene

EHA49822

hypothetical protein
  
Accession: EHA49823
  
Location: 870313-872680
  
 NCBI BlastP on this gene

EHA49823

multidrug resistance-associated protein 1
  
Accession: EHA49824
  
Location: 875536-880315
  
  
**BlastP hit with Mycgr3G9942\_Mycgr3T9**
  
Percentage identity: 40 %
  
BlastP bit score: 948
  
Sequence coverage: 106 %
  
E-value: 0.0
  
  
 NCBI BlastP on this gene

EHA49824

Sad1-interacting factor 3
  
Accession: EHA49825
  
Location: 881155-883209
  
 NCBI BlastP on this gene

EHA49825

hypothetical protein
  
Accession: EHA49826
  
Location: 883801-884369
  
 NCBI BlastP on this gene

EHA49826

ribonuclease P/MRP protein subunit RPP1
  
Accession: EHA49827
  
Location: 885282-886226
  
 NCBI BlastP on this gene

EHA49827

hypothetical protein
  
Accession: EHA49828
  
Location: 886916-888440
  
 NCBI BlastP on this gene

EHA49828

hypothetical protein
  
Accession: EHA49829
  
Location: 890304-892130
  
 NCBI BlastP on this gene

EHA49829

hypothetical protein
  
Accession: EHA49830
  
Location: 892971-894056
  
 NCBI BlastP on this gene

EHA49830

histidyl-tRNA synthetase
  
Accession: EHA49831
  
Location: 894478-896767
  
 NCBI BlastP on this gene

EHA49831

Detecting sequence homology at the gene cluster level with MultiGeneBlast.
  
Marnix H. Medema, Rainer Breitling & Eriko Takano (2013)
  
*Molecular Biology and Evolution* , 30: 1218-1223.
