# Supplementary material for: In silico prediction and characterization of secondary metabolite biosynthetic gene clusters in the wheat pathogen Zymoseptoria tritici
Source: BMC Genomics. 2017 Aug 17;18:631. doi: 10.1186/s12864-017-3969-y (PMC5561558; doi:10.1186/s12864-017-3969-y)
Supplement: Supplementary file 1 — MultiGeneBLAST analysis of putative secondary metabolite clusters. All encoded amino acid sequences from genes residing in clusters predicted by AntiSMASH are given as FASTA file format. All output data from MultiGeneBLASTs are also provided. (ZIP 42911 kb) [file 12864_2017_3969_MOESM1_ESM.zip › Cluster MultiGene BLAST/out/Clusters_1_34/Cluster_12/displaypage1.xhtml]

xml version="1.0" encoding="UTF-8"?


Search Results
  
  
 Results pages: 1, 2, 3, 4, 5

**MultiGeneBlast hits**

Select gene cluster alignment
1. CM001197\_0 Mycosphaerella graminicola IPO323 chromosome 2, whole genome sh...
2. KB445561\_5 Baudoinia compniacensis UAMH 10762 unplaced genomic scaffold BA...
3. KB456266\_5 Mycosphaerella populorum SO2202 unplaced genomic scaffold SEPMU...
4. KB446542\_5 Dothistroma septosporum NZE10 unplaced genomic scaffold DOTSEsc...
5. DS995702\_5 Microsporum canis CBS 113480 supercont1.2 genomic scaffold, who...
6. DS989830\_3 Arthroderma gypseum CBS 118893 supercont1.9 genomic scaffold, w...
7. DS995737\_1 Trichophyton equinum CBS 127.97 supercont1.20 genomic scaffold,...
8. GG700663\_0 Trichophyton rubrum CBS 118892 genomic scaffold supercont2.16, ...
9. ABSU01000004\_3 Arthroderma benhamiae CBS 112371, whole genome shotgun sequ...
10. DS027048\_1 Aspergillus clavatus NRRL 1 1099423829794 genomic scaffold, wh...
11. DS027693\_1 Neosartorya fischeri NRRL 181 1099437636261 genomic scaffold, ...
12. DS990639\_5 Ajellomyces capsulatus H88 supercont1.4 genomic scaffold, whol...
13. GG663377\_0 Ajellomyces capsulatus G186AR genomic scaffold supercont2.15, ...
14. DS499603\_1 Aspergillus fumigatus A1163 scf\_000010 genomic scaffold, whole...
15. AAHF01000016\_2 Aspergillus fumigatus Af293, whole genome shotgun sequenci...
16. ACJE01000002\_1 Aspergillus niger ATCC 1015, whole genome shotgun sequenci...
17. DF126458\_2 Aspergillus kawachii IFO 4308 DNA, contig: scaffold00012, whol...
18. CH476596\_1 Aspergillus terreus NIH2624 scaffold\_3 genomic scaffold, whole...
19. AM270309\_1 Aspergillus niger contig An14c0010, genomic contig.
20. AACD01000117\_1 Aspergillus nidulans FGSC A4, whole genome shotgun sequenc...
21. KB446542\_0 Dothistroma septosporum NZE10 unplaced genomic scaffold DOTSEs...
22. GG692419\_0 Ajellomyces capsulatus H143 genomic scaffold supercont2.1, who...
23. GG698482\_2 Trichophyton tonsurans CBS 112818 genomic scaffold supercont1....
24. AFWA01000008\_0 Pneumocystis murina B123, whole genome shotgun sequencing ...
25. KB446542\_1 Dothistroma septosporum NZE10 unplaced genomic scaffold DOTSEs...
26. JH767569\_0 Coniosporium apollinis CBS 100218 chromosome Unknown supercont...
27. EQ962655\_0 Talaromyces stipitatus ATCC 10500 scf\_1105507295555 genomic sc...
28. GG700653\_3 Trichophyton rubrum CBS 118892 genomic scaffold supercont2.6, ...
29. GG698517\_0 Trichophyton tonsurans CBS 112818 genomic scaffold supercont1....
30. DS995901\_0 Penicillium marneffei ATCC 18224 scf\_1105668340960 genomic sca...
31. DS995702\_0 Microsporum canis CBS 113480 supercont1.2 genomic scaffold, wh...
32. DS995757\_1 Trichophyton equinum CBS 127.97 supercont1.40 genomic scaffold...
33. CH476663\_4 Ajellomyces capsulatus NAm1 scaffold\_9 genomic scaffold, whole...
34. ACYE01000016\_0 Trichophyton verrucosum HKI 0517, whole genome shotgun seq...
35. CH445336\_8 Phaeosphaeria nodorum SN15 scaffold\_12, whole genome shotgun s...
36. CH476615\_3 Uncinocarpus reesii 1704 scaffold\_1 genomic scaffold, whole ge...
37. KB915899\_0 Neofusicoccum parvum UCRNP2 chromosome Unknown NP2\_03\_scaffold...
38. KB446566\_2 Pseudocercospora fijiensis CIRAD86 unplaced genomic scaffold M...
39. KB456266\_2 Mycosphaerella populorum SO2202 unplaced genomic scaffold SEPM...
40. AABX02000026\_0 Neurospora crassa OR74A, whole genome shotgun sequencing p...
41. KB456266\_0 Mycosphaerella populorum SO2202 unplaced genomic scaffold SEPM...
42. AP007172\_2 Aspergillus oryzae RIB40 DNA, SC206.
43. AKHY01000145\_2 Aspergillus oryzae 3.042, whole genome shotgun sequencing ...
44. ACFW01000015\_4 Coccidioides posadasii C735 delta SOWgp, whole genome shot...
45. DS989828\_0 Arthroderma gypseum CBS 118893 supercont1.7 genomic scaffold, ...
46. GL636493\_2 Coccidioides posadasii str. Silveira unplaced genomic scaffold...
47. GG704913\_2 Coccidioides immitis RS genomic scaffold supercont3.3, whole g...
48. GL573321\_0 Geomyces destructans 20631-21 unplaced genomic scaffold superc...
49. DS231623\_0 Pyrenophora tritici-repentis Pt-1C-BFP supercont1.9 genomic sc...
50. ABSU01000041\_0 Arthroderma benhamiae CBS 112371, whole genome shotgun seq...

Query: Architecture Search FASTA input

CM001197 : Mycosphaerella graminicola IPO323 chromosome 2    Total score: 31.0     Cumulative Blast bit score: 27011

Hit cluster cross-links:

Mycgr3G90785 Mycgr3T
  
Location: 0-1047

Mycgr3G90785\_Mycgr3T

Mycgr3G103262 Mycgr3
  
Location: 1147-1390

Mycgr3G103262\_Mycgr3

Mycgr3G68458 Mycgr3T
  
Location: 1490-3602

Mycgr3G68458\_Mycgr3T

Mycgr3G99145 Mycgr3T
  
Location: 3702-4326

Mycgr3G99145\_Mycgr3T

Mycgr3G103274 Mycgr3
  
Location: 4426-4957

Mycgr3G103274\_Mycgr3

Mycgr3G103264 Mycgr3
  
Location: 5057-5390

Mycgr3G103264\_Mycgr3

Mycgr3G37570 Mycgr3T
  
Location: 5490-6006

Mycgr3G37570\_Mycgr3T

Mycgr3G108094 Mycgr3
  
Location: 6106-10555

Mycgr3G108094\_Mycgr3

Mycgr3G90786 Mycgr3T
  
Location: 10655-12080

Mycgr3G90786\_Mycgr3T

Mycgr3G68429 Mycgr3T
  
Location: 12180-13440

Mycgr3G68429\_Mycgr3T

Mycgr3G68421 Mycgr3T
  
Location: 13540-17086

Mycgr3G68421\_Mycgr3T

Mycgr3G90801 Mycgr3T
  
Location: 17186-18056

Mycgr3G90801\_Mycgr3T

Mycgr3G84646 Mycgr3T
  
Location: 18156-20235

Mycgr3G84646\_Mycgr3T

Mycgr3G68456 Mycgr3T
  
Location: 20335-21970

Mycgr3G68456\_Mycgr3T

Mycgr3G103270 Mycgr3
  
Location: 22070-22355

Mycgr3G103270\_Mycgr3

Mycgr3G90803 Mycgr3T
  
Location: 22455-23019

Mycgr3G90803\_Mycgr3T

Mycgr3G36941 Mycgr3T
  
Location: 23119-24064

Mycgr3G36941\_Mycgr3T

Mycgr3G25746 Mycgr3T
  
Location: 24164-25241

Mycgr3G25746\_Mycgr3T

Mycgr3G90788 Mycgr3T
  
Location: 25341-25803

Mycgr3G90788\_Mycgr3T

Mycgr3G103260 Mycgr3
  
Location: 25903-26635

Mycgr3G103260\_Mycgr3

Mycgr3G84644 Mycgr3T
  
Location: 26735-28457

Mycgr3G84644\_Mycgr3T

Mycgr3G29227 Mycgr3T
  
Location: 28557-28863

Mycgr3G29227\_Mycgr3T

Mycgr3G36271 Mycgr3T
  
Location: 28963-29854

Mycgr3G36271\_Mycgr3T

Mycgr3G68433 Mycgr3T
  
Location: 29954-33041

Mycgr3G68433\_Mycgr3T

Mycgr3G79452 Mycgr3T
  
Location: 33141-33399

Mycgr3G79452\_Mycgr3T

Mycgr3G55345 Mycgr3T
  
Location: 33499-34126

Mycgr3G55345\_Mycgr3T

Mycgr3G103278 Mycgr3
  
Location: 34226-35195

Mycgr3G103278\_Mycgr3

Mycgr3G84654 Mycgr3T
  
Location: 35295-36630

Mycgr3G84654\_Mycgr3T

Mycgr3G108090 Mycgr3
  
Location: 36730-37591

Mycgr3G108090\_Mycgr3

Mycgr3G21922 Mycgr3T
  
Location: 37691-39149

Mycgr3G21922\_Mycgr3T

Mycgr3G99148 Mycgr3T
  
Location: 39249-42819

Mycgr3G99148\_Mycgr3T

hypothetical protein
  
Accession: EGP90508
  
Location: 1932911-1933903
  
 NCBI BlastP on this gene

EGP90508

hypothetical protein
  
Accession: EGP90507
  
Location: 1934751-1938296
  
  
**BlastP hit with Mycgr3G68421\_Mycgr3T**
  
Percentage identity: 100 %
  
BlastP bit score: 2434
  
Sequence coverage: 99 %
  
E-value: 0.0
  
  
 NCBI BlastP on this gene

EGP90507

serine/threonine protein kinase
  
Accession: EGP89932
  
Location: 1940327-1941058
  
  
**BlastP hit with Mycgr3G103260\_Mycgr3**
  
Percentage identity: 100 %
  
BlastP bit score: 507
  
Sequence coverage: 100 %
  
E-value: 1e-180
  
  
 NCBI BlastP on this gene

EGP89932

hypothetical protein
  
Accession: EGP89933
  
Location: 1941249-1941491
  
  
**BlastP hit with Mycgr3G103262\_Mycgr3**
  
Percentage identity: 100 %
  
BlastP bit score: 163
  
Sequence coverage: 98 %
  
E-value: 3e-50
  
  
 NCBI BlastP on this gene

EGP89933

hypothetical protein
  
Accession: EGP90506
  
Location: 1947793-1949108
  
  
**BlastP hit with Mycgr3G68429\_Mycgr3T**
  
Percentage identity: 100 %
  
BlastP bit score: 872
  
Sequence coverage: 99 %
  
E-value: 0.0
  
  
 NCBI BlastP on this gene

EGP90506

hypothetical protein
  
Accession: EGP89934
  
Location: 1964576-1965321
  
  
**BlastP hit with Mycgr3G55345\_Mycgr3T**
  
Percentage identity: 100 %
  
BlastP bit score: 426
  
Sequence coverage: 99 %
  
E-value: 1e-149
  
  
 NCBI BlastP on this gene

EGP89934

hypothetical protein
  
Accession: EGP90505
  
Location: 1966036-1967143
  
  
**BlastP hit with Mycgr3G90785\_Mycgr3T**
  
Percentage identity: 100 %
  
BlastP bit score: 697
  
Sequence coverage: 99 %
  
E-value: 0.0
  
  
 NCBI BlastP on this gene

EGP90505

hypothetical protein
  
Accession: EGP90504
  
Location: 1968003-1969427
  
  
**BlastP hit with Mycgr3G90786\_Mycgr3T**
  
Percentage identity: 100 %
  
BlastP bit score: 963
  
Sequence coverage: 99 %
  
E-value: 0.0
  
  
 NCBI BlastP on this gene

EGP90504

hypothetical protein
  
Accession: EGP89935
  
Location: 1969994-1973326
  
  
**BlastP hit with Mycgr3G68433\_Mycgr3T**
  
Percentage identity: 100 %
  
BlastP bit score: 2045
  
Sequence coverage: 99 %
  
E-value: 0.0
  
  
 NCBI BlastP on this gene

EGP89935

hypothetical protein
  
Accession: EGP89936
  
Location: 1973872-1975059
  
  
**BlastP hit with Mycgr3G90788\_Mycgr3T**
  
Percentage identity: 100 %
  
BlastP bit score: 306
  
Sequence coverage: 99 %
  
E-value: 2e-104
  
  
 NCBI BlastP on this gene

EGP89936

hypothetical protein
  
Accession: EGP89937
  
Location: 1990316-1990621
  
  
**BlastP hit with Mycgr3G29227\_Mycgr3T**
  
Percentage identity: 100 %
  
BlastP bit score: 210
  
Sequence coverage: 100 %
  
E-value: 5e-68
  
  
 NCBI BlastP on this gene

EGP89937

hypothetical protein
  
Accession: EGP90503
  
Location: 1993131-1993519
  
  
**BlastP hit with Mycgr3G103264\_Mycgr3**
  
Percentage identity: 100 %
  
BlastP bit score: 226
  
Sequence coverage: 99 %
  
E-value: 4e-74
  
  
 NCBI BlastP on this gene

EGP90503

hypothetical protein
  
Accession: EGP89938
  
Location: 1993869-1994126
  
  
**BlastP hit with Mycgr3G79452\_Mycgr3T**
  
Percentage identity: 100 %
  
BlastP bit score: 176
  
Sequence coverage: 98 %
  
E-value: 6e-55
  
  
 NCBI BlastP on this gene

EGP89938

hypothetical protein
  
Accession: EGP90502
  
Location: 1997117-1998457
  
  
**BlastP hit with Mycgr3G108090\_Mycgr3**
  
Percentage identity: 100 %
  
BlastP bit score: 598
  
Sequence coverage: 99 %
  
E-value: 0.0
  
  
 NCBI BlastP on this gene

EGP90502

serine/threonine protein kinase, CMGC family
  
Accession: EGP89939
  
Location: 1999481-2001820
  
  
**BlastP hit with Mycgr3G84644\_Mycgr3T**
  
Percentage identity: 100 %
  
BlastP bit score: 1188
  
Sequence coverage: 99 %
  
E-value: 0.0
  
  
 NCBI BlastP on this gene

EGP89939

ERG25, C-4 methyl sterol oxidase
  
Accession: EGP89940
  
Location: 2003038-2003996
  
  
**BlastP hit with Mycgr3G36271\_Mycgr3T**
  
Percentage identity: 100 %
  
BlastP bit score: 617
  
Sequence coverage: 99 %
  
E-value: 0.0
  
  
 NCBI BlastP on this gene

EGP89940

hypothetical protein
  
Accession: EGP89941
  
Location: 2004703-2006781
  
  
**BlastP hit with Mycgr3G84646\_Mycgr3T**
  
Percentage identity: 100 %
  
BlastP bit score: 1396
  
Sequence coverage: 99 %
  
E-value: 0.0
  
  
 NCBI BlastP on this gene

EGP89941

hypothetical protein
  
Accession: EGP90501
  
Location: 2009481-2009765
  
  
**BlastP hit with Mycgr3G103270\_Mycgr3**
  
Percentage identity: 100 %
  
BlastP bit score: 184
  
Sequence coverage: 98 %
  
E-value: 4e-58
  
  
 NCBI BlastP on this gene

EGP90501

hypothetical protein
  
Accession: EGP89942
  
Location: 2010448-2011344
  
  
**BlastP hit with Mycgr3G99145\_Mycgr3T**
  
Percentage identity: 100 %
  
BlastP bit score: 424
  
Sequence coverage: 99 %
  
E-value: 3e-149
  
  
 NCBI BlastP on this gene

EGP89942

hypothetical protein
  
Accession: EGP90500
  
Location: 2011853-2013153
  
  
**BlastP hit with Mycgr3G25746\_Mycgr3T**
  
Percentage identity: 100 %
  
BlastP bit score: 729
  
Sequence coverage: 100 %
  
E-value: 0.0
  
  
 NCBI BlastP on this gene

EGP90500

hypothetical protein
  
Accession: EGP89943
  
Location: 2013492-2018012
  
  
**BlastP hit with Mycgr3G108094\_Mycgr3**
  
Percentage identity: 100 %
  
BlastP bit score: 2945
  
Sequence coverage: 99 %
  
E-value: 0.0
  
  
 NCBI BlastP on this gene

EGP89943

hypothetical protein
  
Accession: EGP89944
  
Location: 2018146-2018676
  
  
**BlastP hit with Mycgr3G103274\_Mycgr3**
  
Percentage identity: 100 %
  
BlastP bit score: 355
  
Sequence coverage: 99 %
  
E-value: 6e-123
  
  
 NCBI BlastP on this gene

EGP89944

hypothetical protein
  
Accession: EGP90499
  
Location: 2018850-2020307
  
  
**BlastP hit with Mycgr3G21922\_Mycgr3T**
  
Percentage identity: 100 %
  
BlastP bit score: 1018
  
Sequence coverage: 100 %
  
E-value: 0.0
  
  
 NCBI BlastP on this gene

EGP90499

hypothetical protein
  
Accession: EGP89945
  
Location: 2021780-2022748
  
  
**BlastP hit with Mycgr3G103278\_Mycgr3**
  
Percentage identity: 100 %
  
BlastP bit score: 655
  
Sequence coverage: 100 %
  
E-value: 0.0
  
  
 NCBI BlastP on this gene

EGP89945

hypothetical protein
  
Accession: EGP90498
  
Location: 2024313-2025478
  
  
**BlastP hit with Mycgr3G36941\_Mycgr3T**
  
Percentage identity: 100 %
  
BlastP bit score: 658
  
Sequence coverage: 99 %
  
E-value: 0.0
  
  
 NCBI BlastP on this gene

EGP90498

hypothetical protein
  
Accession: EGP90497
  
Location: 2027009-2027878
  
  
**BlastP hit with Mycgr3G90801\_Mycgr3T**
  
Percentage identity: 100 %
  
BlastP bit score: 560
  
Sequence coverage: 99 %
  
E-value: 0.0
  
  
 NCBI BlastP on this gene

EGP90497

hypothetical protein
  
Accession: EGP89946
  
Location: 2028620-2029135
  
  
**BlastP hit with Mycgr3G37570\_Mycgr3T**
  
Percentage identity: 100 %
  
BlastP bit score: 352
  
Sequence coverage: 99 %
  
E-value: 6e-122
  
  
 NCBI BlastP on this gene

EGP89946

hypothetical protein
  
Accession: EGP90496
  
Location: 2031715-2032371
  
  
**BlastP hit with Mycgr3G90803\_Mycgr3T**
  
Percentage identity: 100 %
  
BlastP bit score: 387
  
Sequence coverage: 99 %
  
E-value: 4e-135
  
  
 NCBI BlastP on this gene

EGP90496

large subunit of alpha-aminoadipate reductase
  
Accession: EGP90495
  
Location: 2032893-2036462
  
  
**BlastP hit with Mycgr3G99148\_Mycgr3T**
  
Percentage identity: 100 %
  
BlastP bit score: 2464
  
Sequence coverage: 99 %
  
E-value: 0.0
  
  
 NCBI BlastP on this gene

EGP90495

hypothetical protein
  
Accession: EGP89947
  
Location: 2038028-2039912
  
  
**BlastP hit with Mycgr3G68456\_Mycgr3T**
  
Percentage identity: 100 %
  
BlastP bit score: 1107
  
Sequence coverage: 99 %
  
E-value: 0.0
  
  
 NCBI BlastP on this gene

EGP89947

hypothetical protein
  
Accession: EGP89948
  
Location: 2040265-2042436
  
  
**BlastP hit with Mycgr3G68458\_Mycgr3T**
  
Percentage identity: 100 %
  
BlastP bit score: 1444
  
Sequence coverage: 99 %
  
E-value: 0.0
  
  
 NCBI BlastP on this gene

EGP89948

hypothetical protein
  
Accession: EGP90494
  
Location: 2044191-2046004
  
  
**BlastP hit with Mycgr3G84654\_Mycgr3T**
  
Percentage identity: 100 %
  
BlastP bit score: 905
  
Sequence coverage: 99 %
  
E-value: 0.0
  
  
 NCBI BlastP on this gene

EGP90494

hypothetical protein
  
Accession: EGP90493
  
Location: 2047154-2050036
  
 NCBI BlastP on this gene

EGP90493

Query: Architecture Search FASTA input

KB445561 : Baudoinia compniacensis UAMH 10762 unplaced genomic scaffold BAUCOscaffold\_12    Total score: 13.0     Cumulative Blast bit score: 6657

Hit cluster cross-links:

Mycgr3G90785 Mycgr3T
  
Location: 0-1047

Mycgr3G90785\_Mycgr3T

Mycgr3G103262 Mycgr3
  
Location: 1147-1390

Mycgr3G103262\_Mycgr3

Mycgr3G68458 Mycgr3T
  
Location: 1490-3602

Mycgr3G68458\_Mycgr3T

Mycgr3G99145 Mycgr3T
  
Location: 3702-4326

Mycgr3G99145\_Mycgr3T

Mycgr3G103274 Mycgr3
  
Location: 4426-4957

Mycgr3G103274\_Mycgr3

Mycgr3G103264 Mycgr3
  
Location: 5057-5390

Mycgr3G103264\_Mycgr3

Mycgr3G37570 Mycgr3T
  
Location: 5490-6006

Mycgr3G37570\_Mycgr3T

Mycgr3G108094 Mycgr3
  
Location: 6106-10555

Mycgr3G108094\_Mycgr3

Mycgr3G90786 Mycgr3T
  
Location: 10655-12080

Mycgr3G90786\_Mycgr3T

Mycgr3G68429 Mycgr3T
  
Location: 12180-13440

Mycgr3G68429\_Mycgr3T

Mycgr3G68421 Mycgr3T
  
Location: 13540-17086

Mycgr3G68421\_Mycgr3T

Mycgr3G90801 Mycgr3T
  
Location: 17186-18056

Mycgr3G90801\_Mycgr3T

Mycgr3G84646 Mycgr3T
  
Location: 18156-20235

Mycgr3G84646\_Mycgr3T

Mycgr3G68456 Mycgr3T
  
Location: 20335-21970

Mycgr3G68456\_Mycgr3T

Mycgr3G103270 Mycgr3
  
Location: 22070-22355

Mycgr3G103270\_Mycgr3

Mycgr3G90803 Mycgr3T
  
Location: 22455-23019

Mycgr3G90803\_Mycgr3T

Mycgr3G36941 Mycgr3T
  
Location: 23119-24064

Mycgr3G36941\_Mycgr3T

Mycgr3G25746 Mycgr3T
  
Location: 24164-25241

Mycgr3G25746\_Mycgr3T

Mycgr3G90788 Mycgr3T
  
Location: 25341-25803

Mycgr3G90788\_Mycgr3T

Mycgr3G103260 Mycgr3
  
Location: 25903-26635

Mycgr3G103260\_Mycgr3

Mycgr3G84644 Mycgr3T
  
Location: 26735-28457

Mycgr3G84644\_Mycgr3T

Mycgr3G29227 Mycgr3T
  
Location: 28557-28863

Mycgr3G29227\_Mycgr3T

Mycgr3G36271 Mycgr3T
  
Location: 28963-29854

Mycgr3G36271\_Mycgr3T

Mycgr3G68433 Mycgr3T
  
Location: 29954-33041

Mycgr3G68433\_Mycgr3T

Mycgr3G79452 Mycgr3T
  
Location: 33141-33399

Mycgr3G79452\_Mycgr3T

Mycgr3G55345 Mycgr3T
  
Location: 33499-34126

Mycgr3G55345\_Mycgr3T

Mycgr3G103278 Mycgr3
  
Location: 34226-35195

Mycgr3G103278\_Mycgr3

Mycgr3G84654 Mycgr3T
  
Location: 35295-36630

Mycgr3G84654\_Mycgr3T

Mycgr3G108090 Mycgr3
  
Location: 36730-37591

Mycgr3G108090\_Mycgr3

Mycgr3G21922 Mycgr3T
  
Location: 37691-39149

Mycgr3G21922\_Mycgr3T

Mycgr3G99148 Mycgr3T
  
Location: 39249-42819

Mycgr3G99148\_Mycgr3T

hypothetical protein
  
Accession: EMC92917
  
Location: 746930-747223
  
 NCBI BlastP on this gene

EMC92917

hypothetical protein
  
Accession: EMC92918
  
Location: 748912-749796
  
  
**BlastP hit with Mycgr3G84644\_Mycgr3T**
  
Percentage identity: 82 %
  
BlastP bit score: 427
  
Sequence coverage: 44 %
  
E-value: 1e-143
  
  
 NCBI BlastP on this gene

EMC92918

hypothetical protein
  
Accession: EMC92919
  
Location: 750623-751564
  
  
**BlastP hit with Mycgr3G36271\_Mycgr3T**
  
Percentage identity: 84 %
  
BlastP bit score: 536
  
Sequence coverage: 98 %
  
E-value: 0.0
  
  
 NCBI BlastP on this gene

EMC92919

hypothetical protein
  
Accession: EMC92920
  
Location: 751772-752266
  
 NCBI BlastP on this gene

EMC92920

hypothetical protein
  
Accession: EMC92921
  
Location: 752849-754867
  
  
**BlastP hit with Mycgr3G84646\_Mycgr3T**
  
Percentage identity: 58 %
  
BlastP bit score: 799
  
Sequence coverage: 100 %
  
E-value: 0.0
  
  
 NCBI BlastP on this gene

EMC92921

hypothetical protein
  
Accession: EMC92922
  
Location: 755414-756097
  
  
**BlastP hit with Mycgr3G99145\_Mycgr3T**
  
Percentage identity: 94 %
  
BlastP bit score: 351
  
Sequence coverage: 85 %
  
E-value: 4e-120
  
  
 NCBI BlastP on this gene

EMC92922

hypothetical protein
  
Accession: EMC92923
  
Location: 756468-756789
  
 NCBI BlastP on this gene

EMC92923

hypothetical protein
  
Accession: EMC92924
  
Location: 757239-759197
  
 NCBI BlastP on this gene

EMC92924

hypothetical protein
  
Accession: EMC92925
  
Location: 759549-760554
  
 NCBI BlastP on this gene

EMC92925

hypothetical protein
  
Accession: EMC92926
  
Location: 761452-762288
  
 NCBI BlastP on this gene

EMC92926

hypothetical protein
  
Accession: EMC92927
  
Location: 762724-763711
  
 NCBI BlastP on this gene

EMC92927

hypothetical protein
  
Accession: EMC92928
  
Location: 763922-764290
  
 NCBI BlastP on this gene

EMC92928

hypothetical protein
  
Accession: EMC92929
  
Location: 764443-765270
  
 NCBI BlastP on this gene

EMC92929

hypothetical protein
  
Accession: EMC92930
  
Location: 766266-767018
  
 NCBI BlastP on this gene

EMC92930

hypothetical protein
  
Accession: EMC92931
  
Location: 768215-768463
  
 NCBI BlastP on this gene

EMC92931

hypothetical protein
  
Accession: EMC92932
  
Location: 769147-770486
  
  
**BlastP hit with Mycgr3G68429\_Mycgr3T**
  
Percentage identity: 63 %
  
BlastP bit score: 540
  
Sequence coverage: 100 %
  
E-value: 0.0
  
  
 NCBI BlastP on this gene

EMC92932

hypothetical protein
  
Accession: EMC92933
  
Location: 772220-774835
  
  
**BlastP hit with Mycgr3G103260\_Mycgr3**
  
Percentage identity: 63 %
  
BlastP bit score: 315
  
Sequence coverage: 97 %
  
E-value: 4e-98
  
  
 NCBI BlastP on this gene

EMC92933

hypothetical protein
  
Accession: EMC92934
  
Location: 775348-776018
  
  
**BlastP hit with Mycgr3G55345\_Mycgr3T**
  
Percentage identity: 79 %
  
BlastP bit score: 335
  
Sequence coverage: 97 %
  
E-value: 7e-114
  
  
 NCBI BlastP on this gene

EMC92934

hypothetical protein
  
Accession: EMC92935
  
Location: 776363-776767
  
 NCBI BlastP on this gene

EMC92935

hypothetical protein
  
Accession: EMC92936
  
Location: 776998-779526
  
  
**BlastP hit with Mycgr3G68433\_Mycgr3T**
  
Percentage identity: 49 %
  
BlastP bit score: 752
  
Sequence coverage: 84 %
  
E-value: 0.0
  
  
 NCBI BlastP on this gene

EMC92936

hypothetical protein
  
Accession: EMC92937
  
Location: 780610-782097
  
  
**BlastP hit with Mycgr3G90786\_Mycgr3T**
  
Percentage identity: 43 %
  
BlastP bit score: 300
  
Sequence coverage: 108 %
  
E-value: 3e-92
  
  
 NCBI BlastP on this gene

EMC92937

hypothetical protein
  
Accession: EMC92938
  
Location: 782923-783147
  
 NCBI BlastP on this gene

EMC92938

hypothetical protein
  
Accession: EMC92939
  
Location: 783623-783857
  
 NCBI BlastP on this gene

EMC92939

hypothetical protein
  
Accession: EMC92940
  
Location: 784078-784239
  
 NCBI BlastP on this gene

EMC92940

hypothetical protein
  
Accession: EMC92941
  
Location: 784270-788650
  
  
**BlastP hit with Mycgr3G108094\_Mycgr3**
  
Percentage identity: 46 %
  
BlastP bit score: 1043
  
Sequence coverage: 102 %
  
E-value: 0.0
  
  
 NCBI BlastP on this gene

EMC92941

hypothetical protein
  
Accession: EMC92942
  
Location: 788933-790130
  
  
**BlastP hit with Mycgr3G25746\_Mycgr3T**
  
Percentage identity: 66 %
  
BlastP bit score: 422
  
Sequence coverage: 100 %
  
E-value: 8e-144
  
  
 NCBI BlastP on this gene

EMC92942

hypothetical protein
  
Accession: EMC92943
  
Location: 790375-792369
  
  
**BlastP hit with Mycgr3G21922\_Mycgr3T**
  
Percentage identity: 62 %
  
BlastP bit score: 617
  
Sequence coverage: 100 %
  
E-value: 0.0
  
  
 NCBI BlastP on this gene

EMC92943

hypothetical protein
  
Accession: EMC92944
  
Location: 793401-795434
  
  
**BlastP hit with Mycgr3G103278\_Mycgr3**
  
Percentage identity: 47 %
  
BlastP bit score: 220
  
Sequence coverage: 96 %
  
E-value: 2e-62
  
  
 NCBI BlastP on this gene

EMC92944

hypothetical protein
  
Accession: EMC92945
  
Location: 795893-796865
  
 NCBI BlastP on this gene

EMC92945

Query: Architecture Search FASTA input

KB456266 : Mycosphaerella populorum SO2202 unplaced genomic scaffold SEPMUscaffold\_7    Total score: 8.0     Cumulative Blast bit score: 4669

Hit cluster cross-links:

Mycgr3G90785 Mycgr3T
  
Location: 0-1047

Mycgr3G90785\_Mycgr3T

Mycgr3G103262 Mycgr3
  
Location: 1147-1390

Mycgr3G103262\_Mycgr3

Mycgr3G68458 Mycgr3T
  
Location: 1490-3602

Mycgr3G68458\_Mycgr3T

Mycgr3G99145 Mycgr3T
  
Location: 3702-4326

Mycgr3G99145\_Mycgr3T

Mycgr3G103274 Mycgr3
  
Location: 4426-4957

Mycgr3G103274\_Mycgr3

Mycgr3G103264 Mycgr3
  
Location: 5057-5390

Mycgr3G103264\_Mycgr3

Mycgr3G37570 Mycgr3T
  
Location: 5490-6006

Mycgr3G37570\_Mycgr3T

Mycgr3G108094 Mycgr3
  
Location: 6106-10555

Mycgr3G108094\_Mycgr3

Mycgr3G90786 Mycgr3T
  
Location: 10655-12080

Mycgr3G90786\_Mycgr3T

Mycgr3G68429 Mycgr3T
  
Location: 12180-13440

Mycgr3G68429\_Mycgr3T

Mycgr3G68421 Mycgr3T
  
Location: 13540-17086

Mycgr3G68421\_Mycgr3T

Mycgr3G90801 Mycgr3T
  
Location: 17186-18056

Mycgr3G90801\_Mycgr3T

Mycgr3G84646 Mycgr3T
  
Location: 18156-20235

Mycgr3G84646\_Mycgr3T

Mycgr3G68456 Mycgr3T
  
Location: 20335-21970

Mycgr3G68456\_Mycgr3T

Mycgr3G103270 Mycgr3
  
Location: 22070-22355

Mycgr3G103270\_Mycgr3

Mycgr3G90803 Mycgr3T
  
Location: 22455-23019

Mycgr3G90803\_Mycgr3T

Mycgr3G36941 Mycgr3T
  
Location: 23119-24064

Mycgr3G36941\_Mycgr3T

Mycgr3G25746 Mycgr3T
  
Location: 24164-25241

Mycgr3G25746\_Mycgr3T

Mycgr3G90788 Mycgr3T
  
Location: 25341-25803

Mycgr3G90788\_Mycgr3T

Mycgr3G103260 Mycgr3
  
Location: 25903-26635

Mycgr3G103260\_Mycgr3

Mycgr3G84644 Mycgr3T
  
Location: 26735-28457

Mycgr3G84644\_Mycgr3T

Mycgr3G29227 Mycgr3T
  
Location: 28557-28863

Mycgr3G29227\_Mycgr3T

Mycgr3G36271 Mycgr3T
  
Location: 28963-29854

Mycgr3G36271\_Mycgr3T

Mycgr3G68433 Mycgr3T
  
Location: 29954-33041

Mycgr3G68433\_Mycgr3T

Mycgr3G79452 Mycgr3T
  
Location: 33141-33399

Mycgr3G79452\_Mycgr3T

Mycgr3G55345 Mycgr3T
  
Location: 33499-34126

Mycgr3G55345\_Mycgr3T

Mycgr3G103278 Mycgr3
  
Location: 34226-35195

Mycgr3G103278\_Mycgr3

Mycgr3G84654 Mycgr3T
  
Location: 35295-36630

Mycgr3G84654\_Mycgr3T

Mycgr3G108090 Mycgr3
  
Location: 36730-37591

Mycgr3G108090\_Mycgr3

Mycgr3G21922 Mycgr3T
  
Location: 37691-39149

Mycgr3G21922\_Mycgr3T

Mycgr3G99148 Mycgr3T
  
Location: 39249-42819

Mycgr3G99148\_Mycgr3T

hypothetical protein
  
Accession: EMF11492
  
Location: 1597749-1598179
  
 NCBI BlastP on this gene

EMF11492

ERG2 and sigma1 receptor-like protein
  
Accession: EMF11494
  
Location: 1601907-1602756
  
 NCBI BlastP on this gene

EMF11494

hypothetical protein
  
Accession: EMF11495
  
Location: 1603407-1605065
  
 NCBI BlastP on this gene

EMF11495

cytochrome b5
  
Accession: EMF11496
  
Location: 1605926-1606546
  
 NCBI BlastP on this gene

EMF11496

DUF383-domain-containing protein
  
Accession: EMF11497
  
Location: 1607592-1608966
  
  
**BlastP hit with Mycgr3G25746\_Mycgr3T**
  
Percentage identity: 70 %
  
BlastP bit score: 478
  
Sequence coverage: 98 %
  
E-value: 1e-164
  
  
 NCBI BlastP on this gene

EMF11497

hypothetical protein
  
Accession: EMF11498
  
Location: 1609288-1611783
  
  
**BlastP hit with Mycgr3G108094\_Mycgr3**
  
Percentage identity: 40 %
  
BlastP bit score: 439
  
Sequence coverage: 59 %
  
E-value: 2e-131
  
  
 NCBI BlastP on this gene

EMF11498

hypothetical protein
  
Accession: EMF11499
  
Location: 1614525-1615380
  
 NCBI BlastP on this gene

EMF11499

serine protein kinase Sky1
  
Accession: EMF11500
  
Location: 1615798-1618154
  
  
**BlastP hit with Mycgr3G84644\_Mycgr3T**
  
Percentage identity: 77 %
  
BlastP bit score: 931
  
Sequence coverage: 108 %
  
E-value: 0.0
  
  
 NCBI BlastP on this gene

EMF11500

Sterol desat-domain-containing protein
  
Accession: EMF11501
  
Location: 1619457-1620630
  
  
**BlastP hit with Mycgr3G36271\_Mycgr3T**
  
Percentage identity: 84 %
  
BlastP bit score: 546
  
Sequence coverage: 99 %
  
E-value: 0.0
  
  
 NCBI BlastP on this gene

EMF11501

NCA2-domain-containing protein
  
Accession: EMF11502
  
Location: 1621072-1623141
  
  
**BlastP hit with Mycgr3G84646\_Mycgr3T**
  
Percentage identity: 63 %
  
BlastP bit score: 857
  
Sequence coverage: 101 %
  
E-value: 0.0
  
  
 NCBI BlastP on this gene

EMF11502

GTP-binding protein SAS1
  
Accession: EMF11503
  
Location: 1623864-1624769
  
  
**BlastP hit with Mycgr3G99145\_Mycgr3T**
  
Percentage identity: 93 %
  
BlastP bit score: 379
  
Sequence coverage: 99 %
  
E-value: 4e-131
  
  
 NCBI BlastP on this gene

EMF11503

Rap30/74 interaction domain-containing protein
  
Accession: EMF11504
  
Location: 1628045-1630111
  
  
**BlastP hit with Mycgr3G103278\_Mycgr3**
  
Percentage identity: 66 %
  
BlastP bit score: 376
  
Sequence coverage: 100 %
  
E-value: 1e-121
  
  
 NCBI BlastP on this gene

EMF11504

PAP/OAS1 substrate-binding domain-containing protein
  
Accession: EMF11505
  
Location: 1631495-1633393
  
  
**BlastP hit with Mycgr3G21922\_Mycgr3T**
  
Percentage identity: 66 %
  
BlastP bit score: 663
  
Sequence coverage: 102 %
  
E-value: 0.0
  
  
 NCBI BlastP on this gene

EMF11505

Peptidase M36-domain-containing protein
  
Accession: EMF11506
  
Location: 1634421-1636368
  
 NCBI BlastP on this gene

EMF11506

transcription initiation factor TFIID, TATA binding protein
  
Accession: EMF11507
  
Location: 1639543-1640431
  
 NCBI BlastP on this gene

EMF11507

glycosyltransferase family 2 protein
  
Accession: EMF11508
  
Location: 1641973-1642769
  
 NCBI BlastP on this gene

EMF11508

hypothetical protein
  
Accession: EMF11509
  
Location: 1644274-1645599
  
 NCBI BlastP on this gene

EMF11509

Query: Architecture Search FASTA input

KB446542 : Dothistroma septosporum NZE10 unplaced genomic scaffold DOTSEscaffold\_8    Total score: 6.0     Cumulative Blast bit score: 2535

Hit cluster cross-links:

Mycgr3G90785 Mycgr3T
  
Location: 0-1047

Mycgr3G90785\_Mycgr3T

Mycgr3G103262 Mycgr3
  
Location: 1147-1390

Mycgr3G103262\_Mycgr3

Mycgr3G68458 Mycgr3T
  
Location: 1490-3602

Mycgr3G68458\_Mycgr3T

Mycgr3G99145 Mycgr3T
  
Location: 3702-4326

Mycgr3G99145\_Mycgr3T

Mycgr3G103274 Mycgr3
  
Location: 4426-4957

Mycgr3G103274\_Mycgr3

Mycgr3G103264 Mycgr3
  
Location: 5057-5390

Mycgr3G103264\_Mycgr3

Mycgr3G37570 Mycgr3T
  
Location: 5490-6006

Mycgr3G37570\_Mycgr3T

Mycgr3G108094 Mycgr3
  
Location: 6106-10555

Mycgr3G108094\_Mycgr3

Mycgr3G90786 Mycgr3T
  
Location: 10655-12080

Mycgr3G90786\_Mycgr3T

Mycgr3G68429 Mycgr3T
  
Location: 12180-13440

Mycgr3G68429\_Mycgr3T

Mycgr3G68421 Mycgr3T
  
Location: 13540-17086

Mycgr3G68421\_Mycgr3T

Mycgr3G90801 Mycgr3T
  
Location: 17186-18056

Mycgr3G90801\_Mycgr3T

Mycgr3G84646 Mycgr3T
  
Location: 18156-20235

Mycgr3G84646\_Mycgr3T

Mycgr3G68456 Mycgr3T
  
Location: 20335-21970

Mycgr3G68456\_Mycgr3T

Mycgr3G103270 Mycgr3
  
Location: 22070-22355

Mycgr3G103270\_Mycgr3

Mycgr3G90803 Mycgr3T
  
Location: 22455-23019

Mycgr3G90803\_Mycgr3T

Mycgr3G36941 Mycgr3T
  
Location: 23119-24064

Mycgr3G36941\_Mycgr3T

Mycgr3G25746 Mycgr3T
  
Location: 24164-25241

Mycgr3G25746\_Mycgr3T

Mycgr3G90788 Mycgr3T
  
Location: 25341-25803

Mycgr3G90788\_Mycgr3T

Mycgr3G103260 Mycgr3
  
Location: 25903-26635

Mycgr3G103260\_Mycgr3

Mycgr3G84644 Mycgr3T
  
Location: 26735-28457

Mycgr3G84644\_Mycgr3T

Mycgr3G29227 Mycgr3T
  
Location: 28557-28863

Mycgr3G29227\_Mycgr3T

Mycgr3G36271 Mycgr3T
  
Location: 28963-29854

Mycgr3G36271\_Mycgr3T

Mycgr3G68433 Mycgr3T
  
Location: 29954-33041

Mycgr3G68433\_Mycgr3T

Mycgr3G79452 Mycgr3T
  
Location: 33141-33399

Mycgr3G79452\_Mycgr3T

Mycgr3G55345 Mycgr3T
  
Location: 33499-34126

Mycgr3G55345\_Mycgr3T

Mycgr3G103278 Mycgr3
  
Location: 34226-35195

Mycgr3G103278\_Mycgr3

Mycgr3G84654 Mycgr3T
  
Location: 35295-36630

Mycgr3G84654\_Mycgr3T

Mycgr3G108090 Mycgr3
  
Location: 36730-37591

Mycgr3G108090\_Mycgr3

Mycgr3G21922 Mycgr3T
  
Location: 37691-39149

Mycgr3G21922\_Mycgr3T

Mycgr3G99148 Mycgr3T
  
Location: 39249-42819

Mycgr3G99148\_Mycgr3T

hypothetical protein
  
Accession: EME41892
  
Location: 1791873-1793172
  
 NCBI BlastP on this gene

EME41892

hypothetical protein
  
Accession: EME41891
  
Location: 1789238-1790498
  
 NCBI BlastP on this gene

EME41891

hypothetical protein
  
Accession: EME41890
  
Location: 1787211-1788689
  
 NCBI BlastP on this gene

EME41890

hypothetical protein
  
Accession: EME41889
  
Location: 1785871-1786797
  
 NCBI BlastP on this gene

EME41889

hypothetical protein
  
Accession: EME41888
  
Location: 1784878-1785828
  
 NCBI BlastP on this gene

EME41888

hypothetical protein
  
Accession: EME41887
  
Location: 1781820-1783742
  
 NCBI BlastP on this gene

EME41887

hypothetical protein
  
Accession: EME41886
  
Location: 1778673-1781162
  
  
**BlastP hit with Mycgr3G103260\_Mycgr3**
  
Percentage identity: 75 %
  
BlastP bit score: 396
  
Sequence coverage: 99 %
  
E-value: 2e-129
  
  
 NCBI BlastP on this gene

EME41886

hypothetical protein
  
Accession: EME41885
  
Location: 1775136-1776935
  
 NCBI BlastP on this gene

EME41885

hypothetical protein
  
Accession: EME41884
  
Location: 1773061-1774345
  
  
**BlastP hit with Mycgr3G68429\_Mycgr3T**
  
Percentage identity: 70 %
  
BlastP bit score: 593
  
Sequence coverage: 100 %
  
E-value: 0.0
  
  
 NCBI BlastP on this gene

EME41884

hypothetical protein
  
Accession: EME41883
  
Location: 1769399-1769734
  
 NCBI BlastP on this gene

EME41883

hypothetical protein
  
Accession: EME41881
  
Location: 1766447-1767113
  
  
**BlastP hit with Mycgr3G55345\_Mycgr3T**
  
Percentage identity: 83 %
  
BlastP bit score: 344
  
Sequence coverage: 97 %
  
E-value: 1e-117
  
  
 NCBI BlastP on this gene

EME41881

hypothetical protein
  
Accession: EME41880
  
Location: 1762953-1765403
  
  
**BlastP hit with Mycgr3G68433\_Mycgr3T**
  
Percentage identity: 55 %
  
BlastP bit score: 797
  
Sequence coverage: 79 %
  
E-value: 0.0
  
  
 NCBI BlastP on this gene

EME41880

hypothetical protein
  
Accession: EME41879
  
Location: 1760080-1761552
  
  
**BlastP hit with Mycgr3G90786\_Mycgr3T**
  
Percentage identity: 46 %
  
BlastP bit score: 328
  
Sequence coverage: 104 %
  
E-value: 4e-103
  
  
 NCBI BlastP on this gene

EME41879

hypothetical protein
  
Accession: EME41878
  
Location: 1758555-1759749
  
  
**BlastP hit with Mycgr3G90785\_Mycgr3T**
  
Percentage identity: 28 %
  
BlastP bit score: 77
  
Sequence coverage: 90 %
  
E-value: 2e-12
  
  
 NCBI BlastP on this gene

EME41878

hypothetical protein
  
Accession: EME41877
  
Location: 1754753-1757221
  
 NCBI BlastP on this gene

EME41877

hypothetical protein
  
Accession: EME41876
  
Location: 1753089-1754326
  
 NCBI BlastP on this gene

EME41876

hypothetical protein
  
Accession: EME41875
  
Location: 1748999-1750299
  
 NCBI BlastP on this gene

EME41875

hypothetical protein
  
Accession: EME41874
  
Location: 1747881-1748288
  
 NCBI BlastP on this gene

EME41874

Query: Architecture Search FASTA input

DS995702 : Microsporum canis CBS 113480 supercont1.2 genomic scaffold    Total score: 6.0     Cumulative Blast bit score: 1898

Hit cluster cross-links:

Mycgr3G90785 Mycgr3T
  
Location: 0-1047

Mycgr3G90785\_Mycgr3T

Mycgr3G103262 Mycgr3
  
Location: 1147-1390

Mycgr3G103262\_Mycgr3

Mycgr3G68458 Mycgr3T
  
Location: 1490-3602

Mycgr3G68458\_Mycgr3T

Mycgr3G99145 Mycgr3T
  
Location: 3702-4326

Mycgr3G99145\_Mycgr3T

Mycgr3G103274 Mycgr3
  
Location: 4426-4957

Mycgr3G103274\_Mycgr3

Mycgr3G103264 Mycgr3
  
Location: 5057-5390

Mycgr3G103264\_Mycgr3

Mycgr3G37570 Mycgr3T
  
Location: 5490-6006

Mycgr3G37570\_Mycgr3T

Mycgr3G108094 Mycgr3
  
Location: 6106-10555

Mycgr3G108094\_Mycgr3

Mycgr3G90786 Mycgr3T
  
Location: 10655-12080

Mycgr3G90786\_Mycgr3T

Mycgr3G68429 Mycgr3T
  
Location: 12180-13440

Mycgr3G68429\_Mycgr3T

Mycgr3G68421 Mycgr3T
  
Location: 13540-17086

Mycgr3G68421\_Mycgr3T

Mycgr3G90801 Mycgr3T
  
Location: 17186-18056

Mycgr3G90801\_Mycgr3T

Mycgr3G84646 Mycgr3T
  
Location: 18156-20235

Mycgr3G84646\_Mycgr3T

Mycgr3G68456 Mycgr3T
  
Location: 20335-21970

Mycgr3G68456\_Mycgr3T

Mycgr3G103270 Mycgr3
  
Location: 22070-22355

Mycgr3G103270\_Mycgr3

Mycgr3G90803 Mycgr3T
  
Location: 22455-23019

Mycgr3G90803\_Mycgr3T

Mycgr3G36941 Mycgr3T
  
Location: 23119-24064

Mycgr3G36941\_Mycgr3T

Mycgr3G25746 Mycgr3T
  
Location: 24164-25241

Mycgr3G25746\_Mycgr3T

Mycgr3G90788 Mycgr3T
  
Location: 25341-25803

Mycgr3G90788\_Mycgr3T

Mycgr3G103260 Mycgr3
  
Location: 25903-26635

Mycgr3G103260\_Mycgr3

Mycgr3G84644 Mycgr3T
  
Location: 26735-28457

Mycgr3G84644\_Mycgr3T

Mycgr3G29227 Mycgr3T
  
Location: 28557-28863

Mycgr3G29227\_Mycgr3T

Mycgr3G36271 Mycgr3T
  
Location: 28963-29854

Mycgr3G36271\_Mycgr3T

Mycgr3G68433 Mycgr3T
  
Location: 29954-33041

Mycgr3G68433\_Mycgr3T

Mycgr3G79452 Mycgr3T
  
Location: 33141-33399

Mycgr3G79452\_Mycgr3T

Mycgr3G55345 Mycgr3T
  
Location: 33499-34126

Mycgr3G55345\_Mycgr3T

Mycgr3G103278 Mycgr3
  
Location: 34226-35195

Mycgr3G103278\_Mycgr3

Mycgr3G84654 Mycgr3T
  
Location: 35295-36630

Mycgr3G84654\_Mycgr3T

Mycgr3G108090 Mycgr3
  
Location: 36730-37591

Mycgr3G108090\_Mycgr3

Mycgr3G21922 Mycgr3T
  
Location: 37691-39149

Mycgr3G21922\_Mycgr3T

Mycgr3G99148 Mycgr3T
  
Location: 39249-42819

Mycgr3G99148\_Mycgr3T

UVSB PI-3 kinase
  
Accession: EEQ29989
  
Location: 3575186-3584568
  
 NCBI BlastP on this gene

EEQ29989

SR45
  
Accession: EEQ29988
  
Location: 3573901-3575161
  
 NCBI BlastP on this gene

EEQ29988

cell division cycle protein 48
  
Accession: EEQ29987
  
Location: 3570896-3573371
  
 NCBI BlastP on this gene

EEQ29987

C4-methylsterol oxidase
  
Accession: EEQ29986
  
Location: 3569019-3569939
  
  
**BlastP hit with Mycgr3G36271\_Mycgr3T**
  
Percentage identity: 80 %
  
BlastP bit score: 454
  
Sequence coverage: 87 %
  
E-value: 1e-158
  
  
 NCBI BlastP on this gene

EEQ29986

conserved hypothetical protein
  
Accession: EEQ29985
  
Location: 3565901-3568301
  
  
**BlastP hit with Mycgr3G84646\_Mycgr3T**
  
Percentage identity: 38 %
  
BlastP bit score: 440
  
Sequence coverage: 104 %
  
E-value: 1e-140
  
  
 NCBI BlastP on this gene

EEQ29985

GTP-binding protein SAS1
  
Accession: EEQ29984
  
Location: 3564332-3565152
  
  
**BlastP hit with Mycgr3G99145\_Mycgr3T**
  
Percentage identity: 81 %
  
BlastP bit score: 342
  
Sequence coverage: 99 %
  
E-value: 9e-117
  
  
 NCBI BlastP on this gene

EEQ29984

conserved hypothetical protein
  
Accession: EEQ29983
  
Location: 3562339-3563603
  
 NCBI BlastP on this gene

EEQ29983

predicted protein
  
Accession: EEQ29982
  
Location: 3561604-3562110
  
 NCBI BlastP on this gene

EEQ29982

rRNA-processing protein FCF1
  
Accession: EEQ29981
  
Location: 3559538-3560389
  
 NCBI BlastP on this gene

EEQ29981

conserved hypothetical protein
  
Accession: EEQ29980
  
Location: 3554045-3558791
  
 NCBI BlastP on this gene

EEQ29980

protein kinase domain-containing protein
  
Accession: EEQ29979
  
Location: 3552414-3553832
  
 NCBI BlastP on this gene

EEQ29979

myo-inositol-1-monophosphotase
  
Accession: EEQ29978
  
Location: 3551144-3552136
  
 NCBI BlastP on this gene

EEQ29978

HGH1
  
Accession: EEQ29977
  
Location: 3549365-3550700
  
  
**BlastP hit with Mycgr3G25746\_Mycgr3T**
  
Percentage identity: 54 %
  
BlastP bit score: 355
  
Sequence coverage: 100 %
  
E-value: 6e-117
  
  
 NCBI BlastP on this gene

EEQ29977

conserved hypothetical protein
  
Accession: EEQ29976
  
Location: 3547526-3549241
  
 NCBI BlastP on this gene

EEQ29976

conserved hypothetical protein
  
Accession: EEQ29975
  
Location: 3545836-3547500
  
 NCBI BlastP on this gene

EEQ29975

poly(A) polymerase Cid1
  
Accession: EEQ29974
  
Location: 3543991-3545715
  
  
**BlastP hit with Mycgr3G21922\_Mycgr3T**
  
Percentage identity: 42 %
  
BlastP bit score: 163
  
Sequence coverage: 45 %
  
E-value: 9e-41
  
  
 NCBI BlastP on this gene

EEQ29974

transcription initiation factor IIF subunit alpha
  
Accession: EEQ29973
  
Location: 3540814-3543069
  
  
**BlastP hit with Mycgr3G103278\_Mycgr3**
  
Percentage identity: 35 %
  
BlastP bit score: 144
  
Sequence coverage: 95 %
  
E-value: 6e-35
  
  
 NCBI BlastP on this gene

EEQ29973

IBR domain-containing protein
  
Accession: EEQ29972
  
Location: 3538764-3540189
  
 NCBI BlastP on this gene

EEQ29972

conserved hypothetical protein
  
Accession: EEQ29971
  
Location: 3536362-3538264
  
 NCBI BlastP on this gene

EEQ29971

conserved hypothetical protein
  
Accession: EEQ29970
  
Location: 3533380-3535421
  
 NCBI BlastP on this gene

EEQ29970

oligopeptide transporter
  
Accession: EEQ29969
  
Location: 3529672-3531669
  
 NCBI BlastP on this gene

EEQ29969

Query: Architecture Search FASTA input

DS989830 : Arthroderma gypseum CBS 118893 supercont1.9 genomic scaffold    Total score: 6.0     Cumulative Blast bit score: 1895

Hit cluster cross-links:

Mycgr3G90785 Mycgr3T
  
Location: 0-1047

Mycgr3G90785\_Mycgr3T

Mycgr3G103262 Mycgr3
  
Location: 1147-1390

Mycgr3G103262\_Mycgr3

Mycgr3G68458 Mycgr3T
  
Location: 1490-3602

Mycgr3G68458\_Mycgr3T

Mycgr3G99145 Mycgr3T
  
Location: 3702-4326

Mycgr3G99145\_Mycgr3T

Mycgr3G103274 Mycgr3
  
Location: 4426-4957

Mycgr3G103274\_Mycgr3

Mycgr3G103264 Mycgr3
  
Location: 5057-5390

Mycgr3G103264\_Mycgr3

Mycgr3G37570 Mycgr3T
  
Location: 5490-6006

Mycgr3G37570\_Mycgr3T

Mycgr3G108094 Mycgr3
  
Location: 6106-10555

Mycgr3G108094\_Mycgr3

Mycgr3G90786 Mycgr3T
  
Location: 10655-12080

Mycgr3G90786\_Mycgr3T

Mycgr3G68429 Mycgr3T
  
Location: 12180-13440

Mycgr3G68429\_Mycgr3T

Mycgr3G68421 Mycgr3T
  
Location: 13540-17086

Mycgr3G68421\_Mycgr3T

Mycgr3G90801 Mycgr3T
  
Location: 17186-18056

Mycgr3G90801\_Mycgr3T

Mycgr3G84646 Mycgr3T
  
Location: 18156-20235

Mycgr3G84646\_Mycgr3T

Mycgr3G68456 Mycgr3T
  
Location: 20335-21970

Mycgr3G68456\_Mycgr3T

Mycgr3G103270 Mycgr3
  
Location: 22070-22355

Mycgr3G103270\_Mycgr3

Mycgr3G90803 Mycgr3T
  
Location: 22455-23019

Mycgr3G90803\_Mycgr3T

Mycgr3G36941 Mycgr3T
  
Location: 23119-24064

Mycgr3G36941\_Mycgr3T

Mycgr3G25746 Mycgr3T
  
Location: 24164-25241

Mycgr3G25746\_Mycgr3T

Mycgr3G90788 Mycgr3T
  
Location: 25341-25803

Mycgr3G90788\_Mycgr3T

Mycgr3G103260 Mycgr3
  
Location: 25903-26635

Mycgr3G103260\_Mycgr3

Mycgr3G84644 Mycgr3T
  
Location: 26735-28457

Mycgr3G84644\_Mycgr3T

Mycgr3G29227 Mycgr3T
  
Location: 28557-28863

Mycgr3G29227\_Mycgr3T

Mycgr3G36271 Mycgr3T
  
Location: 28963-29854

Mycgr3G36271\_Mycgr3T

Mycgr3G68433 Mycgr3T
  
Location: 29954-33041

Mycgr3G68433\_Mycgr3T

Mycgr3G79452 Mycgr3T
  
Location: 33141-33399

Mycgr3G79452\_Mycgr3T

Mycgr3G55345 Mycgr3T
  
Location: 33499-34126

Mycgr3G55345\_Mycgr3T

Mycgr3G103278 Mycgr3
  
Location: 34226-35195

Mycgr3G103278\_Mycgr3

Mycgr3G84654 Mycgr3T
  
Location: 35295-36630

Mycgr3G84654\_Mycgr3T

Mycgr3G108090 Mycgr3
  
Location: 36730-37591

Mycgr3G108090\_Mycgr3

Mycgr3G21922 Mycgr3T
  
Location: 37691-39149

Mycgr3G21922\_Mycgr3T

Mycgr3G99148 Mycgr3T
  
Location: 39249-42819

Mycgr3G99148\_Mycgr3T

cortical actin cytoskeleton protein asp1
  
Accession: EFR05514
  
Location: 743793-748547
  
 NCBI BlastP on this gene

EFR05514

U3 small nucleolar RNA-associated protein 21
  
Accession: EFR05515
  
Location: 748945-752393
  
 NCBI BlastP on this gene

EFR05515

hypothetical protein
  
Accession: EFR05516
  
Location: 752941-753612
  
 NCBI BlastP on this gene

EFR05516

hypothetical protein
  
Accession: EFR05517
  
Location: 754992-756399
  
  
**BlastP hit with Mycgr3G25746\_Mycgr3T**
  
Percentage identity: 56 %
  
BlastP bit score: 356
  
Sequence coverage: 98 %
  
E-value: 3e-117
  
  
 NCBI BlastP on this gene

EFR05517

hypothetical protein
  
Accession: EFR05518
  
Location: 756406-757849
  
 NCBI BlastP on this gene

EFR05518

hypothetical protein
  
Accession: EFR05519
  
Location: 757972-759804
  
  
**BlastP hit with Mycgr3G21922\_Mycgr3T**
  
Percentage identity: 45 %
  
BlastP bit score: 172
  
Sequence coverage: 45 %
  
E-value: 1e-43
  
  
 NCBI BlastP on this gene

EFR05519

transcription initiation factor IIF subunit alpha
  
Accession: EFR05520
  
Location: 760831-763286
  
  
**BlastP hit with Mycgr3G103278\_Mycgr3**
  
Percentage identity: 34 %
  
BlastP bit score: 137
  
Sequence coverage: 95 %
  
E-value: 2e-32
  
  
 NCBI BlastP on this gene

EFR05520

inositol monophosphatase 2
  
Accession: EFR05521
  
Location: 764390-765422
  
 NCBI BlastP on this gene

EFR05521

hypothetical protein
  
Accession: EFR05522
  
Location: 767126-769170
  
 NCBI BlastP on this gene

EFR05522

glycogen synthase kinase mutation revertant
  
Accession: EFR05523
  
Location: 769703-774506
  
 NCBI BlastP on this gene

EFR05523

rRNA-processing protein FCF1
  
Accession: EFR05524
  
Location: 775185-776061
  
 NCBI BlastP on this gene

EFR05524

hypothetical protein
  
Accession: EFR05525
  
Location: 776359-777644
  
 NCBI BlastP on this gene

EFR05525

GTP-binding protein
  
Accession: EFR05526
  
Location: 778341-779161
  
  
**BlastP hit with Mycgr3G99145\_Mycgr3T**
  
Percentage identity: 81 %
  
BlastP bit score: 337
  
Sequence coverage: 100 %
  
E-value: 1e-114
  
  
 NCBI BlastP on this gene

EFR05526

ATPase 2 nuclear control
  
Accession: EFR05527
  
Location: 780098-782562
  
  
**BlastP hit with Mycgr3G84646\_Mycgr3T**
  
Percentage identity: 36 %
  
BlastP bit score: 435
  
Sequence coverage: 105 %
  
E-value: 2e-138
  
  
 NCBI BlastP on this gene

EFR05527

C-4 methylsterol oxidase
  
Accession: EFR05528
  
Location: 783421-784351
  
  
**BlastP hit with Mycgr3G36271\_Mycgr3T**
  
Percentage identity: 81 %
  
BlastP bit score: 458
  
Sequence coverage: 87 %
  
E-value: 4e-160
  
  
 NCBI BlastP on this gene

EFR05528

peroxisome biogenesis factor 1
  
Accession: EFR05529
  
Location: 785322-787774
  
 NCBI BlastP on this gene

EFR05529

hypothetical protein
  
Accession: EFR05530
  
Location: 788331-789566
  
 NCBI BlastP on this gene

EFR05530

kinase rad3
  
Accession: EFR05531
  
Location: 790004-798936
  
 NCBI BlastP on this gene

EFR05531

Query: Architecture Search FASTA input

DS995737 : Trichophyton equinum CBS 127.97 supercont1.20 genomic scaffold    Total score: 6.0     Cumulative Blast bit score: 1878

Hit cluster cross-links:

Mycgr3G90785 Mycgr3T
  
Location: 0-1047

Mycgr3G90785\_Mycgr3T

Mycgr3G103262 Mycgr3
  
Location: 1147-1390

Mycgr3G103262\_Mycgr3

Mycgr3G68458 Mycgr3T
  
Location: 1490-3602

Mycgr3G68458\_Mycgr3T

Mycgr3G99145 Mycgr3T
  
Location: 3702-4326

Mycgr3G99145\_Mycgr3T

Mycgr3G103274 Mycgr3
  
Location: 4426-4957

Mycgr3G103274\_Mycgr3

Mycgr3G103264 Mycgr3
  
Location: 5057-5390

Mycgr3G103264\_Mycgr3

Mycgr3G37570 Mycgr3T
  
Location: 5490-6006

Mycgr3G37570\_Mycgr3T

Mycgr3G108094 Mycgr3
  
Location: 6106-10555

Mycgr3G108094\_Mycgr3

Mycgr3G90786 Mycgr3T
  
Location: 10655-12080

Mycgr3G90786\_Mycgr3T

Mycgr3G68429 Mycgr3T
  
Location: 12180-13440

Mycgr3G68429\_Mycgr3T

Mycgr3G68421 Mycgr3T
  
Location: 13540-17086

Mycgr3G68421\_Mycgr3T

Mycgr3G90801 Mycgr3T
  
Location: 17186-18056

Mycgr3G90801\_Mycgr3T

Mycgr3G84646 Mycgr3T
  
Location: 18156-20235

Mycgr3G84646\_Mycgr3T

Mycgr3G68456 Mycgr3T
  
Location: 20335-21970

Mycgr3G68456\_Mycgr3T

Mycgr3G103270 Mycgr3
  
Location: 22070-22355

Mycgr3G103270\_Mycgr3

Mycgr3G90803 Mycgr3T
  
Location: 22455-23019

Mycgr3G90803\_Mycgr3T

Mycgr3G36941 Mycgr3T
  
Location: 23119-24064

Mycgr3G36941\_Mycgr3T

Mycgr3G25746 Mycgr3T
  
Location: 24164-25241

Mycgr3G25746\_Mycgr3T

Mycgr3G90788 Mycgr3T
  
Location: 25341-25803

Mycgr3G90788\_Mycgr3T

Mycgr3G103260 Mycgr3
  
Location: 25903-26635

Mycgr3G103260\_Mycgr3

Mycgr3G84644 Mycgr3T
  
Location: 26735-28457

Mycgr3G84644\_Mycgr3T

Mycgr3G29227 Mycgr3T
  
Location: 28557-28863

Mycgr3G29227\_Mycgr3T

Mycgr3G36271 Mycgr3T
  
Location: 28963-29854

Mycgr3G36271\_Mycgr3T

Mycgr3G68433 Mycgr3T
  
Location: 29954-33041

Mycgr3G68433\_Mycgr3T

Mycgr3G79452 Mycgr3T
  
Location: 33141-33399

Mycgr3G79452\_Mycgr3T

Mycgr3G55345 Mycgr3T
  
Location: 33499-34126

Mycgr3G55345\_Mycgr3T

Mycgr3G103278 Mycgr3
  
Location: 34226-35195

Mycgr3G103278\_Mycgr3

Mycgr3G84654 Mycgr3T
  
Location: 35295-36630

Mycgr3G84654\_Mycgr3T

Mycgr3G108090 Mycgr3
  
Location: 36730-37591

Mycgr3G108090\_Mycgr3

Mycgr3G21922 Mycgr3T
  
Location: 37691-39149

Mycgr3G21922\_Mycgr3T

Mycgr3G99148 Mycgr3T
  
Location: 39249-42819

Mycgr3G99148\_Mycgr3T

histidine acid phosphatase
  
Accession: EGE05137
  
Location: 282714-287154
  
 NCBI BlastP on this gene

EGE05137

snoRNA binding protein
  
Accession: EGE05138
  
Location: 287891-291336
  
 NCBI BlastP on this gene

EGE05138

hypothetical protein
  
Accession: EGE05139
  
Location: 291855-292526
  
 NCBI BlastP on this gene

EGE05139

hypothetical protein
  
Accession: EGE05140
  
Location: 293845-295242
  
  
**BlastP hit with Mycgr3G25746\_Mycgr3T**
  
Percentage identity: 56 %
  
BlastP bit score: 358
  
Sequence coverage: 98 %
  
E-value: 3e-118
  
  
 NCBI BlastP on this gene

EGE05140

hypothetical protein
  
Accession: EGE05141
  
Location: 295441-296701
  
 NCBI BlastP on this gene

EGE05141

poly(A) polymerase Cid1
  
Accession: EGE05142
  
Location: 296856-298709
  
  
**BlastP hit with Mycgr3G21922\_Mycgr3T**
  
Percentage identity: 43 %
  
BlastP bit score: 162
  
Sequence coverage: 45 %
  
E-value: 3e-40
  
  
 NCBI BlastP on this gene

EGE05142

transcription initiation factor IIF subunit alpha
  
Accession: EGE05143
  
Location: 299828-302276
  
  
**BlastP hit with Mycgr3G103278\_Mycgr3**
  
Percentage identity: 34 %
  
BlastP bit score: 137
  
Sequence coverage: 95 %
  
E-value: 2e-32
  
  
 NCBI BlastP on this gene

EGE05143

IBR finger domain-containing protein
  
Accession: EGE05144
  
Location: 302787-304182
  
 NCBI BlastP on this gene

EGE05144

hypothetical protein
  
Accession: EGE05145
  
Location: 304397-306408
  
 NCBI BlastP on this gene

EGE05145

myo inositol monophosphatase
  
Accession: EGE05146
  
Location: 307254-308279
  
 NCBI BlastP on this gene

EGE05146

Diacylglycerol kinase domain-containing protein
  
Accession: EGE05147
  
Location: 308482-313357
  
 NCBI BlastP on this gene

EGE05147

rRNA-processing protein FCF1
  
Accession: EGE05148
  
Location: 314078-314936
  
 NCBI BlastP on this gene

EGE05148

hypothetical protein
  
Accession: EGE05149
  
Location: 315142-316513
  
 NCBI BlastP on this gene

EGE05149

GTP-binding protein
  
Accession: EGE05150
  
Location: 317229-318098
  
  
**BlastP hit with Mycgr3G99145\_Mycgr3T**
  
Percentage identity: 81 %
  
BlastP bit score: 340
  
Sequence coverage: 100 %
  
E-value: 6e-116
  
  
 NCBI BlastP on this gene

EGE05150

hypothetical protein
  
Accession: EGE05151
  
Location: 319039-321515
  
  
**BlastP hit with Mycgr3G84646\_Mycgr3T**
  
Percentage identity: 37 %
  
BlastP bit score: 422
  
Sequence coverage: 108 %
  
E-value: 1e-133
  
  
 NCBI BlastP on this gene

EGE05151

C-4 methyl sterol oxidase Erg25
  
Accession: EGE05152
  
Location: 322274-323213
  
  
**BlastP hit with Mycgr3G36271\_Mycgr3T**
  
Percentage identity: 80 %
  
BlastP bit score: 459
  
Sequence coverage: 87 %
  
E-value: 2e-160
  
  
 NCBI BlastP on this gene

EGE05152

peroxisome biogenesis factor 1
  
Accession: EGE05153
  
Location: 324321-326828
  
 NCBI BlastP on this gene

EGE05153

hypothetical protein
  
Accession: EGE05154
  
Location: 327401-328693
  
 NCBI BlastP on this gene

EGE05154

hypothetical protein
  
Accession: EGE05155
  
Location: 329150-330380
  
 NCBI BlastP on this gene

EGE05155

hypothetical protein
  
Accession: EGE05156
  
Location: 330737-339828
  
 NCBI BlastP on this gene

EGE05156

Query: Architecture Search FASTA input

GG700663 : Trichophyton rubrum CBS 118892 genomic scaffold supercont2.16    Total score: 6.0     Cumulative Blast bit score: 1781

Hit cluster cross-links:

Mycgr3G90785 Mycgr3T
  
Location: 0-1047

Mycgr3G90785\_Mycgr3T

Mycgr3G103262 Mycgr3
  
Location: 1147-1390

Mycgr3G103262\_Mycgr3

Mycgr3G68458 Mycgr3T
  
Location: 1490-3602

Mycgr3G68458\_Mycgr3T

Mycgr3G99145 Mycgr3T
  
Location: 3702-4326

Mycgr3G99145\_Mycgr3T

Mycgr3G103274 Mycgr3
  
Location: 4426-4957

Mycgr3G103274\_Mycgr3

Mycgr3G103264 Mycgr3
  
Location: 5057-5390

Mycgr3G103264\_Mycgr3

Mycgr3G37570 Mycgr3T
  
Location: 5490-6006

Mycgr3G37570\_Mycgr3T

Mycgr3G108094 Mycgr3
  
Location: 6106-10555

Mycgr3G108094\_Mycgr3

Mycgr3G90786 Mycgr3T
  
Location: 10655-12080

Mycgr3G90786\_Mycgr3T

Mycgr3G68429 Mycgr3T
  
Location: 12180-13440

Mycgr3G68429\_Mycgr3T

Mycgr3G68421 Mycgr3T
  
Location: 13540-17086

Mycgr3G68421\_Mycgr3T

Mycgr3G90801 Mycgr3T
  
Location: 17186-18056

Mycgr3G90801\_Mycgr3T

Mycgr3G84646 Mycgr3T
  
Location: 18156-20235

Mycgr3G84646\_Mycgr3T

Mycgr3G68456 Mycgr3T
  
Location: 20335-21970

Mycgr3G68456\_Mycgr3T

Mycgr3G103270 Mycgr3
  
Location: 22070-22355

Mycgr3G103270\_Mycgr3

Mycgr3G90803 Mycgr3T
  
Location: 22455-23019

Mycgr3G90803\_Mycgr3T

Mycgr3G36941 Mycgr3T
  
Location: 23119-24064

Mycgr3G36941\_Mycgr3T

Mycgr3G25746 Mycgr3T
  
Location: 24164-25241

Mycgr3G25746\_Mycgr3T

Mycgr3G90788 Mycgr3T
  
Location: 25341-25803

Mycgr3G90788\_Mycgr3T

Mycgr3G103260 Mycgr3
  
Location: 25903-26635

Mycgr3G103260\_Mycgr3

Mycgr3G84644 Mycgr3T
  
Location: 26735-28457

Mycgr3G84644\_Mycgr3T

Mycgr3G29227 Mycgr3T
  
Location: 28557-28863

Mycgr3G29227\_Mycgr3T

Mycgr3G36271 Mycgr3T
  
Location: 28963-29854

Mycgr3G36271\_Mycgr3T

Mycgr3G68433 Mycgr3T
  
Location: 29954-33041

Mycgr3G68433\_Mycgr3T

Mycgr3G79452 Mycgr3T
  
Location: 33141-33399

Mycgr3G79452\_Mycgr3T

Mycgr3G55345 Mycgr3T
  
Location: 33499-34126

Mycgr3G55345\_Mycgr3T

Mycgr3G103278 Mycgr3
  
Location: 34226-35195

Mycgr3G103278\_Mycgr3

Mycgr3G84654 Mycgr3T
  
Location: 35295-36630

Mycgr3G84654\_Mycgr3T

Mycgr3G108090 Mycgr3
  
Location: 36730-37591

Mycgr3G108090\_Mycgr3

Mycgr3G21922 Mycgr3T
  
Location: 37691-39149

Mycgr3G21922\_Mycgr3T

Mycgr3G99148 Mycgr3T
  
Location: 39249-42819

Mycgr3G99148\_Mycgr3T

cortical actin cytoskeleton protein asp1
  
Accession: EGD92315
  
Location: 45760-50223
  
 NCBI BlastP on this gene

EGD92315

WD repeat containing protein 36
  
Accession: EGD92316
  
Location: 50955-54404
  
 NCBI BlastP on this gene

EGD92316

hypothetical protein
  
Accession: EGD92317
  
Location: 54907-55578
  
 NCBI BlastP on this gene

EGD92317

DNA-binding protein HGH1
  
Accession: EGD92318
  
Location: 56826-58221
  
  
**BlastP hit with Mycgr3G25746\_Mycgr3T**
  
Percentage identity: 56 %
  
BlastP bit score: 362
  
Sequence coverage: 98 %
  
E-value: 8e-120
  
  
 NCBI BlastP on this gene

EGD92318

hypothetical protein
  
Accession: EGD92319
  
Location: 59825-61666
  
  
**BlastP hit with Mycgr3G21922\_Mycgr3T**
  
Percentage identity: 42 %
  
BlastP bit score: 159
  
Sequence coverage: 45 %
  
E-value: 3e-39
  
  
 NCBI BlastP on this gene

EGD92319

transcription initiation factor IIF subunit alpha
  
Accession: EGD92320
  
Location: 62719-65155
  
  
**BlastP hit with Mycgr3G103278\_Mycgr3**
  
Percentage identity: 34 %
  
BlastP bit score: 137
  
Sequence coverage: 95 %
  
E-value: 2e-32
  
  
 NCBI BlastP on this gene

EGD92320

hypothetical protein
  
Accession: EGD92321
  
Location: 65659-67057
  
 NCBI BlastP on this gene

EGD92321

hypothetical protein
  
Accession: EGD92322
  
Location: 67292-69298
  
 NCBI BlastP on this gene

EGD92322

inositol monophosphatase
  
Accession: EGD92323
  
Location: 70432-71454
  
 NCBI BlastP on this gene

EGD92323

hypothetical protein
  
Accession: EGD92324
  
Location: 71671-73056
  
 NCBI BlastP on this gene

EGD92324

hypothetical protein
  
Accession: EGD92325
  
Location: 73253-76551
  
 NCBI BlastP on this gene

EGD92325

hypothetical protein
  
Accession: EGD92326
  
Location: 77248-78109
  
 NCBI BlastP on this gene

EGD92326

hypothetical protein
  
Accession: EGD92327
  
Location: 78405-79676
  
 NCBI BlastP on this gene

EGD92327

GTP-binding protein
  
Accession: EGD92328
  
Location: 80383-81210
  
  
**BlastP hit with Mycgr3G99145\_Mycgr3T**
  
Percentage identity: 81 %
  
BlastP bit score: 340
  
Sequence coverage: 100 %
  
E-value: 6e-116
  
  
 NCBI BlastP on this gene

EGD92328

hypothetical protein
  
Accession: EGD92329
  
Location: 82098-84632
  
  
**BlastP hit with Mycgr3G84646\_Mycgr3T**
  
Percentage identity: 33 %
  
BlastP bit score: 324
  
Sequence coverage: 110 %
  
E-value: 1e-96
  
  
 NCBI BlastP on this gene

EGD92329

C-4 methylsterol oxidase
  
Accession: EGD92330
  
Location: 85353-86284
  
  
**BlastP hit with Mycgr3G36271\_Mycgr3T**
  
Percentage identity: 80 %
  
BlastP bit score: 459
  
Sequence coverage: 87 %
  
E-value: 2e-160
  
  
 NCBI BlastP on this gene

EGD92330

AAA family ATPase
  
Accession: EGD92331
  
Location: 87376-89892
  
 NCBI BlastP on this gene

EGD92331

hypothetical protein
  
Accession: EGD92332
  
Location: 90063-91358
  
 NCBI BlastP on this gene

EGD92332

hypothetical protein
  
Accession: EGD92333
  
Location: 91798-93011
  
 NCBI BlastP on this gene

EGD92333

phosphatidylinositol 3
  
Accession: EGD92334
  
Location: 93373-102298
  
 NCBI BlastP on this gene

EGD92334

Query: Architecture Search FASTA input

ABSU01000004 : Arthroderma benhamiae CBS 112371    Total score: 6.0     Cumulative Blast bit score: 1689

Hit cluster cross-links:

Mycgr3G90785 Mycgr3T
  
Location: 0-1047

Mycgr3G90785\_Mycgr3T

Mycgr3G103262 Mycgr3
  
Location: 1147-1390

Mycgr3G103262\_Mycgr3

Mycgr3G68458 Mycgr3T
  
Location: 1490-3602

Mycgr3G68458\_Mycgr3T

Mycgr3G99145 Mycgr3T
  
Location: 3702-4326

Mycgr3G99145\_Mycgr3T

Mycgr3G103274 Mycgr3
  
Location: 4426-4957

Mycgr3G103274\_Mycgr3

Mycgr3G103264 Mycgr3
  
Location: 5057-5390

Mycgr3G103264\_Mycgr3

Mycgr3G37570 Mycgr3T
  
Location: 5490-6006

Mycgr3G37570\_Mycgr3T

Mycgr3G108094 Mycgr3
  
Location: 6106-10555

Mycgr3G108094\_Mycgr3

Mycgr3G90786 Mycgr3T
  
Location: 10655-12080

Mycgr3G90786\_Mycgr3T

Mycgr3G68429 Mycgr3T
  
Location: 12180-13440

Mycgr3G68429\_Mycgr3T

Mycgr3G68421 Mycgr3T
  
Location: 13540-17086

Mycgr3G68421\_Mycgr3T

Mycgr3G90801 Mycgr3T
  
Location: 17186-18056

Mycgr3G90801\_Mycgr3T

Mycgr3G84646 Mycgr3T
  
Location: 18156-20235

Mycgr3G84646\_Mycgr3T

Mycgr3G68456 Mycgr3T
  
Location: 20335-21970

Mycgr3G68456\_Mycgr3T

Mycgr3G103270 Mycgr3
  
Location: 22070-22355

Mycgr3G103270\_Mycgr3

Mycgr3G90803 Mycgr3T
  
Location: 22455-23019

Mycgr3G90803\_Mycgr3T

Mycgr3G36941 Mycgr3T
  
Location: 23119-24064

Mycgr3G36941\_Mycgr3T

Mycgr3G25746 Mycgr3T
  
Location: 24164-25241

Mycgr3G25746\_Mycgr3T

Mycgr3G90788 Mycgr3T
  
Location: 25341-25803

Mycgr3G90788\_Mycgr3T

Mycgr3G103260 Mycgr3
  
Location: 25903-26635

Mycgr3G103260\_Mycgr3

Mycgr3G84644 Mycgr3T
  
Location: 26735-28457

Mycgr3G84644\_Mycgr3T

Mycgr3G29227 Mycgr3T
  
Location: 28557-28863

Mycgr3G29227\_Mycgr3T

Mycgr3G36271 Mycgr3T
  
Location: 28963-29854

Mycgr3G36271\_Mycgr3T

Mycgr3G68433 Mycgr3T
  
Location: 29954-33041

Mycgr3G68433\_Mycgr3T

Mycgr3G79452 Mycgr3T
  
Location: 33141-33399

Mycgr3G79452\_Mycgr3T

Mycgr3G55345 Mycgr3T
  
Location: 33499-34126

Mycgr3G55345\_Mycgr3T

Mycgr3G103278 Mycgr3
  
Location: 34226-35195

Mycgr3G103278\_Mycgr3

Mycgr3G84654 Mycgr3T
  
Location: 35295-36630

Mycgr3G84654\_Mycgr3T

Mycgr3G108090 Mycgr3
  
Location: 36730-37591

Mycgr3G108090\_Mycgr3

Mycgr3G21922 Mycgr3T
  
Location: 37691-39149

Mycgr3G21922\_Mycgr3T

Mycgr3G99148 Mycgr3T
  
Location: 39249-42819

Mycgr3G99148\_Mycgr3T

inositol kinase kinase (UvsB), putative
  
Accession: EFE35072
  
Location: 579706-586898
  
 NCBI BlastP on this gene

EFE35072

hypothetical protein
  
Accession: EFE35073
  
Location: 592444-594957
  
 NCBI BlastP on this gene

EFE35073

C-4 methyl sterol oxidase, putative
  
Accession: EFE35074
  
Location: 596030-596569
  
  
**BlastP hit with Mycgr3G36271\_Mycgr3T**
  
Percentage identity: 81 %
  
BlastP bit score: 317
  
Sequence coverage: 60 %
  
E-value: 1e-105
  
  
 NCBI BlastP on this gene

EFE35074

hypothetical protein
  
Accession: EFE35075
  
Location: 596672-597335
  
 NCBI BlastP on this gene

EFE35075

hypothetical protein
  
Accession: EFE35076
  
Location: 597771-600294
  
  
**BlastP hit with Mycgr3G84646\_Mycgr3T**
  
Percentage identity: 36 %
  
BlastP bit score: 435
  
Sequence coverage: 112 %
  
E-value: 2e-138
  
  
 NCBI BlastP on this gene

EFE35076

hypothetical protein
  
Accession: EFE35077
  
Location: 600593-602062
  
  
**BlastP hit with Mycgr3G99145\_Mycgr3T**
  
Percentage identity: 79 %
  
BlastP bit score: 324
  
Sequence coverage: 97 %
  
E-value: 2e-106
  
  
 NCBI BlastP on this gene

EFE35077

hypothetical protein
  
Accession: EFE35078
  
Location: 602773-604044
  
 NCBI BlastP on this gene

EFE35078

hypothetical protein
  
Accession: EFE35079
  
Location: 605917-610776
  
 NCBI BlastP on this gene

EFE35079

inositol monophosphatase QutG, putative
  
Accession: EFE35080
  
Location: 611002-612005
  
 NCBI BlastP on this gene

EFE35080

hypothetical protein
  
Accession: EFE35081
  
Location: 612607-612774
  
 NCBI BlastP on this gene

EFE35081

hypothetical protein
  
Accession: EFE35082
  
Location: 613034-615061
  
 NCBI BlastP on this gene

EFE35082

RING finger protein
  
Accession: EFE35083
  
Location: 615295-616715
  
 NCBI BlastP on this gene

EFE35083

conserved hypothetical protein
  
Accession: EFE35084
  
Location: 617208-619592
  
  
**BlastP hit with Mycgr3G103278\_Mycgr3**
  
Percentage identity: 34 %
  
BlastP bit score: 138
  
Sequence coverage: 95 %
  
E-value: 1e-32
  
  
 NCBI BlastP on this gene

EFE35084

hypothetical protein
  
Accession: EFE35085
  
Location: 620699-622540
  
  
**BlastP hit with Mycgr3G21922\_Mycgr3T**
  
Percentage identity: 41 %
  
BlastP bit score: 157
  
Sequence coverage: 45 %
  
E-value: 2e-38
  
  
 NCBI BlastP on this gene

EFE35085

hypothetical protein
  
Accession: EFE35086
  
Location: 622689-625565
  
  
**BlastP hit with Mycgr3G25746\_Mycgr3T**
  
Percentage identity: 53 %
  
BlastP bit score: 319
  
Sequence coverage: 94 %
  
E-value: 3e-98
  
  
 NCBI BlastP on this gene

EFE35086

hypothetical protein
  
Accession: EFE35087
  
Location: 626864-627535
  
 NCBI BlastP on this gene

EFE35087

hypothetical protein
  
Accession: EFE35088
  
Location: 628047-631813
  
 NCBI BlastP on this gene

EFE35088

hypothetical protein
  
Accession: EFE35089
  
Location: 632206-636534
  
 NCBI BlastP on this gene

EFE35089

Query: Architecture Search FASTA input

DS027048 : Aspergillus clavatus NRRL 1 1099423829794 genomic scaffold    Total score: 5.0     Cumulative Blast bit score: 2729

Hit cluster cross-links:

Mycgr3G90785 Mycgr3T
  
Location: 0-1047

Mycgr3G90785\_Mycgr3T

Mycgr3G103262 Mycgr3
  
Location: 1147-1390

Mycgr3G103262\_Mycgr3

Mycgr3G68458 Mycgr3T
  
Location: 1490-3602

Mycgr3G68458\_Mycgr3T

Mycgr3G99145 Mycgr3T
  
Location: 3702-4326

Mycgr3G99145\_Mycgr3T

Mycgr3G103274 Mycgr3
  
Location: 4426-4957

Mycgr3G103274\_Mycgr3

Mycgr3G103264 Mycgr3
  
Location: 5057-5390

Mycgr3G103264\_Mycgr3

Mycgr3G37570 Mycgr3T
  
Location: 5490-6006

Mycgr3G37570\_Mycgr3T

Mycgr3G108094 Mycgr3
  
Location: 6106-10555

Mycgr3G108094\_Mycgr3

Mycgr3G90786 Mycgr3T
  
Location: 10655-12080

Mycgr3G90786\_Mycgr3T

Mycgr3G68429 Mycgr3T
  
Location: 12180-13440

Mycgr3G68429\_Mycgr3T

Mycgr3G68421 Mycgr3T
  
Location: 13540-17086

Mycgr3G68421\_Mycgr3T

Mycgr3G90801 Mycgr3T
  
Location: 17186-18056

Mycgr3G90801\_Mycgr3T

Mycgr3G84646 Mycgr3T
  
Location: 18156-20235

Mycgr3G84646\_Mycgr3T

Mycgr3G68456 Mycgr3T
  
Location: 20335-21970

Mycgr3G68456\_Mycgr3T

Mycgr3G103270 Mycgr3
  
Location: 22070-22355

Mycgr3G103270\_Mycgr3

Mycgr3G90803 Mycgr3T
  
Location: 22455-23019

Mycgr3G90803\_Mycgr3T

Mycgr3G36941 Mycgr3T
  
Location: 23119-24064

Mycgr3G36941\_Mycgr3T

Mycgr3G25746 Mycgr3T
  
Location: 24164-25241

Mycgr3G25746\_Mycgr3T

Mycgr3G90788 Mycgr3T
  
Location: 25341-25803

Mycgr3G90788\_Mycgr3T

Mycgr3G103260 Mycgr3
  
Location: 25903-26635

Mycgr3G103260\_Mycgr3

Mycgr3G84644 Mycgr3T
  
Location: 26735-28457

Mycgr3G84644\_Mycgr3T

Mycgr3G29227 Mycgr3T
  
Location: 28557-28863

Mycgr3G29227\_Mycgr3T

Mycgr3G36271 Mycgr3T
  
Location: 28963-29854

Mycgr3G36271\_Mycgr3T

Mycgr3G68433 Mycgr3T
  
Location: 29954-33041

Mycgr3G68433\_Mycgr3T

Mycgr3G79452 Mycgr3T
  
Location: 33141-33399

Mycgr3G79452\_Mycgr3T

Mycgr3G55345 Mycgr3T
  
Location: 33499-34126

Mycgr3G55345\_Mycgr3T

Mycgr3G103278 Mycgr3
  
Location: 34226-35195

Mycgr3G103278\_Mycgr3

Mycgr3G84654 Mycgr3T
  
Location: 35295-36630

Mycgr3G84654\_Mycgr3T

Mycgr3G108090 Mycgr3
  
Location: 36730-37591

Mycgr3G108090\_Mycgr3

Mycgr3G21922 Mycgr3T
  
Location: 37691-39149

Mycgr3G21922\_Mycgr3T

Mycgr3G99148 Mycgr3T
  
Location: 39249-42819

Mycgr3G99148\_Mycgr3T

conserved hypothetical protein
  
Accession: EAW13354
  
Location: 176049-177932
  
 NCBI BlastP on this gene

EAW13354

phenazine biosynthesis-like protein, putative
  
Accession: EAW13353
  
Location: 174677-175639
  
 NCBI BlastP on this gene

EAW13353

mating-type switching protein swi1
  
Accession: EAW13352
  
Location: 170325-174046
  
  
**BlastP hit with Mycgr3G68421\_Mycgr3T**
  
Percentage identity: 43 %
  
BlastP bit score: 939
  
Sequence coverage: 102 %
  
E-value: 0.0
  
  
 NCBI BlastP on this gene

EAW13352

3-hydroxybutyryl-CoA dehydrogenase, putative
  
Accession: EAW13351
  
Location: 168855-169955
  
 NCBI BlastP on this gene

EAW13351

IBR domain protein
  
Accession: EAW13350
  
Location: 167899-168616
  
 NCBI BlastP on this gene

EAW13350

hypothetical protein
  
Accession: EAW13349
  
Location: 167316-167759
  
 NCBI BlastP on this gene

EAW13349

actin family protein
  
Accession: EAW13348
  
Location: 164879-166376
  
 NCBI BlastP on this gene

EAW13348

conserved hypothetical protein
  
Accession: EAW13347
  
Location: 163395-164510
  
 NCBI BlastP on this gene

EAW13347

conserved leucine-rich repeat protein
  
Accession: EAW13346
  
Location: 160111-163092
  
  
**BlastP hit with Mycgr3G68433\_Mycgr3T**
  
Percentage identity: 38 %
  
BlastP bit score: 506
  
Sequence coverage: 88 %
  
E-value: 2e-158
  
  
 NCBI BlastP on this gene

EAW13346

conserved hypothetical protein
  
Accession: EAW13345
  
Location: 156962-158380
  
  
**BlastP hit with Mycgr3G90786\_Mycgr3T**
  
Percentage identity: 27 %
  
BlastP bit score: 102
  
Sequence coverage: 101 %
  
E-value: 3e-20
  
  
 NCBI BlastP on this gene

EAW13345

60S ribosomal protein L13
  
Accession: EAW13344
  
Location: 154881-156059
  
 NCBI BlastP on this gene

EAW13344

hypothetical protein
  
Accession: EAW13343
  
Location: 152268-154245
  
 NCBI BlastP on this gene

EAW13343

conserved hypothetical protein
  
Accession: EAW13342
  
Location: 151083-151910
  
 NCBI BlastP on this gene

EAW13342

conserved hypothetical protein
  
Accession: EAW13341
  
Location: 149876-150631
  
 NCBI BlastP on this gene

EAW13341

conserved hypothetical protein
  
Accession: EAW13340
  
Location: 147068-149187
  
 NCBI BlastP on this gene

EAW13340

conserved hypothetical protein
  
Accession: EAW13339
  
Location: 143053-143793
  
 NCBI BlastP on this gene

EAW13339

succinyl-CoA synthetase beta subunit, putative
  
Accession: EAW13338
  
Location: 140678-142488
  
 NCBI BlastP on this gene

EAW13338

short chain dehydrogenase/reductase, putative
  
Accession: EAW13337
  
Location: 138656-139913
  
 NCBI BlastP on this gene

EAW13337

C4-dicarboxylate/malic acid transporter, putative
  
Accession: EAW13336
  
Location: 135448-136970
  
 NCBI BlastP on this gene

EAW13336

conserved hypothetical protein
  
Accession: EAW13335
  
Location: 133567-134592
  
  
**BlastP hit with Mycgr3G55345\_Mycgr3T**
  
Percentage identity: 73 %
  
BlastP bit score: 305
  
Sequence coverage: 96 %
  
E-value: 6e-102
  
  
 NCBI BlastP on this gene

EAW13335

conserved hypothetical protein
  
Accession: EAW13334
  
Location: 132593-133016
  
 NCBI BlastP on this gene

EAW13334

conserved hypothetical protein
  
Accession: EAW13333
  
Location: 129593-131809
  
 NCBI BlastP on this gene

EAW13333

conserved hypothetical protein
  
Accession: EAW13332
  
Location: 128042-129344
  
 NCBI BlastP on this gene

EAW13332

ubiquinone biosynthesis protein, putative
  
Accession: EAW13331
  
Location: 125181-127404
  
  
**BlastP hit with Mycgr3G68458\_Mycgr3T**
  
Percentage identity: 65 %
  
BlastP bit score: 877
  
Sequence coverage: 91 %
  
E-value: 0.0
  
  
 NCBI BlastP on this gene

EAW13331

cytochrome C1 heme lyase
  
Accession: EAW13330
  
Location: 123256-124420
  
 NCBI BlastP on this gene

EAW13330

tachykinin family protein
  
Accession: EAW13329
  
Location: 120717-122712
  
 NCBI BlastP on this gene

EAW13329

Query: Architecture Search FASTA input

DS027693 : Neosartorya fischeri NRRL 181 1099437636261 genomic scaffold    Total score: 5.0     Cumulative Blast bit score: 2721

Hit cluster cross-links:

Mycgr3G90785 Mycgr3T
  
Location: 0-1047

Mycgr3G90785\_Mycgr3T

Mycgr3G103262 Mycgr3
  
Location: 1147-1390

Mycgr3G103262\_Mycgr3

Mycgr3G68458 Mycgr3T
  
Location: 1490-3602

Mycgr3G68458\_Mycgr3T

Mycgr3G99145 Mycgr3T
  
Location: 3702-4326

Mycgr3G99145\_Mycgr3T

Mycgr3G103274 Mycgr3
  
Location: 4426-4957

Mycgr3G103274\_Mycgr3

Mycgr3G103264 Mycgr3
  
Location: 5057-5390

Mycgr3G103264\_Mycgr3

Mycgr3G37570 Mycgr3T
  
Location: 5490-6006

Mycgr3G37570\_Mycgr3T

Mycgr3G108094 Mycgr3
  
Location: 6106-10555

Mycgr3G108094\_Mycgr3

Mycgr3G90786 Mycgr3T
  
Location: 10655-12080

Mycgr3G90786\_Mycgr3T

Mycgr3G68429 Mycgr3T
  
Location: 12180-13440

Mycgr3G68429\_Mycgr3T

Mycgr3G68421 Mycgr3T
  
Location: 13540-17086

Mycgr3G68421\_Mycgr3T

Mycgr3G90801 Mycgr3T
  
Location: 17186-18056

Mycgr3G90801\_Mycgr3T

Mycgr3G84646 Mycgr3T
  
Location: 18156-20235

Mycgr3G84646\_Mycgr3T

Mycgr3G68456 Mycgr3T
  
Location: 20335-21970

Mycgr3G68456\_Mycgr3T

Mycgr3G103270 Mycgr3
  
Location: 22070-22355

Mycgr3G103270\_Mycgr3

Mycgr3G90803 Mycgr3T
  
Location: 22455-23019

Mycgr3G90803\_Mycgr3T

Mycgr3G36941 Mycgr3T
  
Location: 23119-24064

Mycgr3G36941\_Mycgr3T

Mycgr3G25746 Mycgr3T
  
Location: 24164-25241

Mycgr3G25746\_Mycgr3T

Mycgr3G90788 Mycgr3T
  
Location: 25341-25803

Mycgr3G90788\_Mycgr3T

Mycgr3G103260 Mycgr3
  
Location: 25903-26635

Mycgr3G103260\_Mycgr3

Mycgr3G84644 Mycgr3T
  
Location: 26735-28457

Mycgr3G84644\_Mycgr3T

Mycgr3G29227 Mycgr3T
  
Location: 28557-28863

Mycgr3G29227\_Mycgr3T

Mycgr3G36271 Mycgr3T
  
Location: 28963-29854

Mycgr3G36271\_Mycgr3T

Mycgr3G68433 Mycgr3T
  
Location: 29954-33041

Mycgr3G68433\_Mycgr3T

Mycgr3G79452 Mycgr3T
  
Location: 33141-33399

Mycgr3G79452\_Mycgr3T

Mycgr3G55345 Mycgr3T
  
Location: 33499-34126

Mycgr3G55345\_Mycgr3T

Mycgr3G103278 Mycgr3
  
Location: 34226-35195

Mycgr3G103278\_Mycgr3

Mycgr3G84654 Mycgr3T
  
Location: 35295-36630

Mycgr3G84654\_Mycgr3T

Mycgr3G108090 Mycgr3
  
Location: 36730-37591

Mycgr3G108090\_Mycgr3

Mycgr3G21922 Mycgr3T
  
Location: 37691-39149

Mycgr3G21922\_Mycgr3T

Mycgr3G99148 Mycgr3T
  
Location: 39249-42819

Mycgr3G99148\_Mycgr3T

conserved hypothetical protein
  
Accession: EAW20468
  
Location: 190214-192097
  
 NCBI BlastP on this gene

EAW20468

phenazine biosynthesis-like protein, putative
  
Accession: EAW20467
  
Location: 188801-189763
  
 NCBI BlastP on this gene

EAW20467

mating-type switching protein swi1
  
Accession: EAW20466
  
Location: 184514-188211
  
  
**BlastP hit with Mycgr3G68421\_Mycgr3T**
  
Percentage identity: 44 %
  
BlastP bit score: 956
  
Sequence coverage: 102 %
  
E-value: 0.0
  
  
 NCBI BlastP on this gene

EAW20466

3-hydroxybutyryl-CoA dehydrogenase, putative
  
Accession: EAW20465
  
Location: 183048-184149
  
 NCBI BlastP on this gene

EAW20465

IBR domain protein
  
Accession: EAW20464
  
Location: 181919-182758
  
 NCBI BlastP on this gene

EAW20464

actin family protein
  
Accession: EAW20463
  
Location: 179141-180713
  
 NCBI BlastP on this gene

EAW20463

conserved hypothetical protein
  
Accession: EAW20462
  
Location: 177648-178760
  
 NCBI BlastP on this gene

EAW20462

conserved leucine-rich repeat protein
  
Accession: EAW20461
  
Location: 174353-177349
  
  
**BlastP hit with Mycgr3G68433\_Mycgr3T**
  
Percentage identity: 40 %
  
BlastP bit score: 509
  
Sequence coverage: 86 %
  
E-value: 2e-159
  
  
 NCBI BlastP on this gene

EAW20461

hypothetical protein
  
Accession: EAW20460
  
Location: 171045-172469
  
  
**BlastP hit with Mycgr3G90786\_Mycgr3T**
  
Percentage identity: 28 %
  
BlastP bit score: 92
  
Sequence coverage: 92 %
  
E-value: 8e-17
  
  
 NCBI BlastP on this gene

EAW20460

60S ribosomal protein L13
  
Accession: EAW20459
  
Location: 169018-170024
  
 NCBI BlastP on this gene

EAW20459

hypothetical protein
  
Accession: EAW20458
  
Location: 166362-168301
  
 NCBI BlastP on this gene

EAW20458

conserved hypothetical protein
  
Accession: EAW20457
  
Location: 165218-166015
  
 NCBI BlastP on this gene

EAW20457

conserved hypothetical protein
  
Accession: EAW20456
  
Location: 164002-164768
  
 NCBI BlastP on this gene

EAW20456

HSF-type DNA-binding domain protein
  
Accession: EAW20455
  
Location: 160986-163113
  
 NCBI BlastP on this gene

EAW20455

F-box domain protein
  
Accession: EAW20454
  
Location: 157279-158990
  
 NCBI BlastP on this gene

EAW20454

succinyl-CoA synthetase beta subunit, putative
  
Accession: EAW20453
  
Location: 154723-156523
  
 NCBI BlastP on this gene

EAW20453

short chain dehydrogenase/reductase, putative
  
Accession: EAW20452
  
Location: 153361-154186
  
 NCBI BlastP on this gene

EAW20452

C4-dicarboxylate/malic acid transporter, putative
  
Accession: EAW20451
  
Location: 149998-151511
  
 NCBI BlastP on this gene

EAW20451

conserved hypothetical protein
  
Accession: EAW20450
  
Location: 148168-149180
  
  
**BlastP hit with Mycgr3G55345\_Mycgr3T**
  
Percentage identity: 74 %
  
BlastP bit score: 306
  
Sequence coverage: 96 %
  
E-value: 1e-102
  
  
 NCBI BlastP on this gene

EAW20450

conserved hypothetical protein
  
Accession: EAW20449
  
Location: 147090-147507
  
 NCBI BlastP on this gene

EAW20449

conserved hypothetical protein
  
Accession: EAW20448
  
Location: 144116-146292
  
 NCBI BlastP on this gene

EAW20448

conserved hypothetical protein
  
Accession: EAW20447
  
Location: 142443-143738
  
 NCBI BlastP on this gene

EAW20447

ubiquinone biosynthesis protein, putative
  
Accession: EAW20446
  
Location: 139558-141772
  
  
**BlastP hit with Mycgr3G68458\_Mycgr3T**
  
Percentage identity: 65 %
  
BlastP bit score: 858
  
Sequence coverage: 93 %
  
E-value: 0.0
  
  
 NCBI BlastP on this gene

EAW20446

cytochrome C1 heme lyase
  
Accession: EAW20445
  
Location: 137634-138742
  
 NCBI BlastP on this gene

EAW20445

conserved hypothetical protein
  
Accession: EAW20444
  
Location: 135066-137094
  
 NCBI BlastP on this gene

EAW20444

Query: Architecture Search FASTA input

DS990639 : Ajellomyces capsulatus H88 supercont1.4 genomic scaffold    Total score: 5.0     Cumulative Blast bit score: 2681

Hit cluster cross-links:

Mycgr3G90785 Mycgr3T
  
Location: 0-1047

Mycgr3G90785\_Mycgr3T

Mycgr3G103262 Mycgr3
  
Location: 1147-1390

Mycgr3G103262\_Mycgr3

Mycgr3G68458 Mycgr3T
  
Location: 1490-3602

Mycgr3G68458\_Mycgr3T

Mycgr3G99145 Mycgr3T
  
Location: 3702-4326

Mycgr3G99145\_Mycgr3T

Mycgr3G103274 Mycgr3
  
Location: 4426-4957

Mycgr3G103274\_Mycgr3

Mycgr3G103264 Mycgr3
  
Location: 5057-5390

Mycgr3G103264\_Mycgr3

Mycgr3G37570 Mycgr3T
  
Location: 5490-6006

Mycgr3G37570\_Mycgr3T

Mycgr3G108094 Mycgr3
  
Location: 6106-10555

Mycgr3G108094\_Mycgr3

Mycgr3G90786 Mycgr3T
  
Location: 10655-12080

Mycgr3G90786\_Mycgr3T

Mycgr3G68429 Mycgr3T
  
Location: 12180-13440

Mycgr3G68429\_Mycgr3T

Mycgr3G68421 Mycgr3T
  
Location: 13540-17086

Mycgr3G68421\_Mycgr3T

Mycgr3G90801 Mycgr3T
  
Location: 17186-18056

Mycgr3G90801\_Mycgr3T

Mycgr3G84646 Mycgr3T
  
Location: 18156-20235

Mycgr3G84646\_Mycgr3T

Mycgr3G68456 Mycgr3T
  
Location: 20335-21970

Mycgr3G68456\_Mycgr3T

Mycgr3G103270 Mycgr3
  
Location: 22070-22355

Mycgr3G103270\_Mycgr3

Mycgr3G90803 Mycgr3T
  
Location: 22455-23019

Mycgr3G90803\_Mycgr3T

Mycgr3G36941 Mycgr3T
  
Location: 23119-24064

Mycgr3G36941\_Mycgr3T

Mycgr3G25746 Mycgr3T
  
Location: 24164-25241

Mycgr3G25746\_Mycgr3T

Mycgr3G90788 Mycgr3T
  
Location: 25341-25803

Mycgr3G90788\_Mycgr3T

Mycgr3G103260 Mycgr3
  
Location: 25903-26635

Mycgr3G103260\_Mycgr3

Mycgr3G84644 Mycgr3T
  
Location: 26735-28457

Mycgr3G84644\_Mycgr3T

Mycgr3G29227 Mycgr3T
  
Location: 28557-28863

Mycgr3G29227\_Mycgr3T

Mycgr3G36271 Mycgr3T
  
Location: 28963-29854

Mycgr3G36271\_Mycgr3T

Mycgr3G68433 Mycgr3T
  
Location: 29954-33041

Mycgr3G68433\_Mycgr3T

Mycgr3G79452 Mycgr3T
  
Location: 33141-33399

Mycgr3G79452\_Mycgr3T

Mycgr3G55345 Mycgr3T
  
Location: 33499-34126

Mycgr3G55345\_Mycgr3T

Mycgr3G103278 Mycgr3
  
Location: 34226-35195

Mycgr3G103278\_Mycgr3

Mycgr3G84654 Mycgr3T
  
Location: 35295-36630

Mycgr3G84654\_Mycgr3T

Mycgr3G108090 Mycgr3
  
Location: 36730-37591

Mycgr3G108090\_Mycgr3

Mycgr3G21922 Mycgr3T
  
Location: 37691-39149

Mycgr3G21922\_Mycgr3T

Mycgr3G99148 Mycgr3T
  
Location: 39249-42819

Mycgr3G99148\_Mycgr3T

conserved hypothetical protein
  
Accession: EGC45542
  
Location: 903700-905226
  
 NCBI BlastP on this gene

EGC45542

predicted protein
  
Accession: EGC45543
  
Location: 906080-906584
  
 NCBI BlastP on this gene

EGC45543

ubiquinone biosynthesis protein
  
Accession: EGC45544
  
Location: 907429-909740
  
  
**BlastP hit with Mycgr3G68458\_Mycgr3T**
  
Percentage identity: 62 %
  
BlastP bit score: 880
  
Sequence coverage: 102 %
  
E-value: 0.0
  
  
 NCBI BlastP on this gene

EGC45544

TDT malic acid transporter
  
Accession: EGC45545
  
Location: 910719-912469
  
 NCBI BlastP on this gene

EGC45545

carbonic anhydraes family protein
  
Accession: EGC45546
  
Location: 912876-913525
  
 NCBI BlastP on this gene

EGC45546

short chain dehydrogenase/reductase
  
Accession: EGC45547
  
Location: 915524-917287
  
 NCBI BlastP on this gene

EGC45547

succinyl-CoA ligase beta-chain
  
Accession: EGC45548
  
Location: 917993-919929
  
 NCBI BlastP on this gene

EGC45548

conserved hypothetical protein
  
Accession: EGC45549
  
Location: 920184-920980
  
 NCBI BlastP on this gene

EGC45549

HSF-type DNA-binding domain-containing protein
  
Accession: EGC45550
  
Location: 922697-925003
  
 NCBI BlastP on this gene

EGC45550

predicted protein
  
Accession: EGC45551
  
Location: 928286-929741
  
 NCBI BlastP on this gene

EGC45551

xanthine phosphoribosyltransferase
  
Accession: EGC45552
  
Location: 929816-931502
  
  
**BlastP hit with Mycgr3G55345\_Mycgr3T**
  
Percentage identity: 75 %
  
BlastP bit score: 318
  
Sequence coverage: 97 %
  
E-value: 2e-107
  
  
 NCBI BlastP on this gene

EGC45552

conserved hypothetical protein
  
Accession: EGC45553
  
Location: 932732-933295
  
 NCBI BlastP on this gene

EGC45553

GTP binding protein
  
Accession: EGC45554
  
Location: 934602-937106
  
 NCBI BlastP on this gene

EGC45554

conserved hypothetical protein
  
Accession: EGC45555
  
Location: 937652-938665
  
 NCBI BlastP on this gene

EGC45555

actin-like protein arp6
  
Accession: EGC45556
  
Location: 939520-941175
  
 NCBI BlastP on this gene

EGC45556

conserved hypothetical protein
  
Accession: EGC45557
  
Location: 942304-943593
  
 NCBI BlastP on this gene

EGC45557

leucine rich repeat domain-containing protein
  
Accession: EGC45558
  
Location: 944452-947559
  
  
**BlastP hit with Mycgr3G68433\_Mycgr3T**
  
Percentage identity: 38 %
  
BlastP bit score: 483
  
Sequence coverage: 99 %
  
E-value: 2e-149
  
  
 NCBI BlastP on this gene

EGC45558

conserved hypothetical protein
  
Accession: EGC45559
  
Location: 948809-950254
  
  
**BlastP hit with Mycgr3G90786\_Mycgr3T**
  
Percentage identity: 27 %
  
BlastP bit score: 89
  
Sequence coverage: 108 %
  
E-value: 6e-16
  
  
 NCBI BlastP on this gene

EGC45559

60S ribosomal protein
  
Accession: EGC45560
  
Location: 951211-952453
  
 NCBI BlastP on this gene

EGC45560

conserved hypothetical protein
  
Accession: EGC45561
  
Location: 953868-956039
  
 NCBI BlastP on this gene

EGC45561

CAMK family protein kinase
  
Accession: EGC45562
  
Location: 957411-958879
  
 NCBI BlastP on this gene

EGC45562

conserved hypothetical protein
  
Accession: EGC45563
  
Location: 959517-961000
  
 NCBI BlastP on this gene

EGC45563

enoyl-CoA hydratase
  
Accession: EGC45564
  
Location: 961311-962070
  
 NCBI BlastP on this gene

EGC45564

topoisomerase 1-associated factor
  
Accession: EGC45565
  
Location: 962874-966419
  
  
**BlastP hit with Mycgr3G68421\_Mycgr3T**
  
Percentage identity: 45 %
  
BlastP bit score: 911
  
Sequence coverage: 92 %
  
E-value: 0.0
  
  
 NCBI BlastP on this gene

EGC45565

conserved hypothetical protein
  
Accession: EGC45566
  
Location: 968246-970053
  
 NCBI BlastP on this gene

EGC45566

Query: Architecture Search FASTA input

GG663377 : Ajellomyces capsulatus G186AR genomic scaffold supercont2.15    Total score: 5.0     Cumulative Blast bit score: 2668

Hit cluster cross-links:

Mycgr3G90785 Mycgr3T
  
Location: 0-1047

Mycgr3G90785\_Mycgr3T

Mycgr3G103262 Mycgr3
  
Location: 1147-1390

Mycgr3G103262\_Mycgr3

Mycgr3G68458 Mycgr3T
  
Location: 1490-3602

Mycgr3G68458\_Mycgr3T

Mycgr3G99145 Mycgr3T
  
Location: 3702-4326

Mycgr3G99145\_Mycgr3T

Mycgr3G103274 Mycgr3
  
Location: 4426-4957

Mycgr3G103274\_Mycgr3

Mycgr3G103264 Mycgr3
  
Location: 5057-5390

Mycgr3G103264\_Mycgr3

Mycgr3G37570 Mycgr3T
  
Location: 5490-6006

Mycgr3G37570\_Mycgr3T

Mycgr3G108094 Mycgr3
  
Location: 6106-10555

Mycgr3G108094\_Mycgr3

Mycgr3G90786 Mycgr3T
  
Location: 10655-12080

Mycgr3G90786\_Mycgr3T

Mycgr3G68429 Mycgr3T
  
Location: 12180-13440

Mycgr3G68429\_Mycgr3T

Mycgr3G68421 Mycgr3T
  
Location: 13540-17086

Mycgr3G68421\_Mycgr3T

Mycgr3G90801 Mycgr3T
  
Location: 17186-18056

Mycgr3G90801\_Mycgr3T

Mycgr3G84646 Mycgr3T
  
Location: 18156-20235

Mycgr3G84646\_Mycgr3T

Mycgr3G68456 Mycgr3T
  
Location: 20335-21970

Mycgr3G68456\_Mycgr3T

Mycgr3G103270 Mycgr3
  
Location: 22070-22355

Mycgr3G103270\_Mycgr3

Mycgr3G90803 Mycgr3T
  
Location: 22455-23019

Mycgr3G90803\_Mycgr3T

Mycgr3G36941 Mycgr3T
  
Location: 23119-24064

Mycgr3G36941\_Mycgr3T

Mycgr3G25746 Mycgr3T
  
Location: 24164-25241

Mycgr3G25746\_Mycgr3T

Mycgr3G90788 Mycgr3T
  
Location: 25341-25803

Mycgr3G90788\_Mycgr3T

Mycgr3G103260 Mycgr3
  
Location: 25903-26635

Mycgr3G103260\_Mycgr3

Mycgr3G84644 Mycgr3T
  
Location: 26735-28457

Mycgr3G84644\_Mycgr3T

Mycgr3G29227 Mycgr3T
  
Location: 28557-28863

Mycgr3G29227\_Mycgr3T

Mycgr3G36271 Mycgr3T
  
Location: 28963-29854

Mycgr3G36271\_Mycgr3T

Mycgr3G68433 Mycgr3T
  
Location: 29954-33041

Mycgr3G68433\_Mycgr3T

Mycgr3G79452 Mycgr3T
  
Location: 33141-33399

Mycgr3G79452\_Mycgr3T

Mycgr3G55345 Mycgr3T
  
Location: 33499-34126

Mycgr3G55345\_Mycgr3T

Mycgr3G103278 Mycgr3
  
Location: 34226-35195

Mycgr3G103278\_Mycgr3

Mycgr3G84654 Mycgr3T
  
Location: 35295-36630

Mycgr3G84654\_Mycgr3T

Mycgr3G108090 Mycgr3
  
Location: 36730-37591

Mycgr3G108090\_Mycgr3

Mycgr3G21922 Mycgr3T
  
Location: 37691-39149

Mycgr3G21922\_Mycgr3T

Mycgr3G99148 Mycgr3T
  
Location: 39249-42819

Mycgr3G99148\_Mycgr3T

predicted protein
  
Accession: EEH03359
  
Location: 177511-178016
  
 NCBI BlastP on this gene

EEH03359

conserved hypothetical protein
  
Accession: EEH03358
  
Location: 174341-176653
  
  
**BlastP hit with Mycgr3G68458\_Mycgr3T**
  
Percentage identity: 62 %
  
BlastP bit score: 878
  
Sequence coverage: 102 %
  
E-value: 0.0
  
  
 NCBI BlastP on this gene

EEH03358

conserved hypothetical protein
  
Accession: EEH03357
  
Location: 171616-173376
  
 NCBI BlastP on this gene

EEH03357

carbonic anhydrase
  
Accession: EEH03356
  
Location: 170553-171191
  
 NCBI BlastP on this gene

EEH03356

conserved hypothetical protein
  
Accession: EEH03355
  
Location: 167171-168564
  
 NCBI BlastP on this gene

EEH03355

succinyl-CoA ligase beta-chain
  
Accession: EEH03354
  
Location: 164163-166099
  
 NCBI BlastP on this gene

EEH03354

conserved hypothetical protein
  
Accession: EEH03353
  
Location: 163100-163895
  
 NCBI BlastP on this gene

EEH03353

flocculation suppression protein
  
Accession: EEH03352
  
Location: 160563-161396
  
 NCBI BlastP on this gene

EEH03352

flocculation suppression protein
  
Accession: EEH03351
  
Location: 158769-159932
  
 NCBI BlastP on this gene

EEH03351

predicted protein
  
Accession: EEH03350
  
Location: 158039-158517
  
 NCBI BlastP on this gene

EEH03350

predicted protein
  
Accession: EEH03349
  
Location: 155639-157064
  
 NCBI BlastP on this gene

EEH03349

predicted protein
  
Accession: EEH03348
  
Location: 154750-155358
  
 NCBI BlastP on this gene

EEH03348

xanthine phosphoribosyltransferase
  
Accession: EEH03347
  
Location: 152472-154155
  
  
**BlastP hit with Mycgr3G55345\_Mycgr3T**
  
Percentage identity: 75 %
  
BlastP bit score: 318
  
Sequence coverage: 97 %
  
E-value: 2e-107
  
  
 NCBI BlastP on this gene

EEH03347

conserved hypothetical protein
  
Accession: EEH03346
  
Location: 151721-151864
  
 NCBI BlastP on this gene

EEH03346

conserved hypothetical protein
  
Accession: EEH03345
  
Location: 147129-149998
  
 NCBI BlastP on this gene

EEH03345

conserved hypothetical protein
  
Accession: EEH03344
  
Location: 145941-146954
  
 NCBI BlastP on this gene

EEH03344

actin-like protein arp6
  
Accession: EEH03343
  
Location: 143419-145074
  
 NCBI BlastP on this gene

EEH03343

conserved hypothetical protein
  
Accession: EEH03342
  
Location: 141132-142421
  
 NCBI BlastP on this gene

EEH03342

leucine rich repeat domain-containing protein
  
Accession: EEH03341
  
Location: 137153-140269
  
  
**BlastP hit with Mycgr3G68433\_Mycgr3T**
  
Percentage identity: 38 %
  
BlastP bit score: 486
  
Sequence coverage: 99 %
  
E-value: 1e-150
  
  
 NCBI BlastP on this gene

EEH03341

conserved hypothetical protein
  
Accession: EEH03340
  
Location: 134431-135891
  
  
**BlastP hit with Mycgr3G90786\_Mycgr3T**
  
Percentage identity: 27 %
  
BlastP bit score: 87
  
Sequence coverage: 109 %
  
E-value: 2e-15
  
  
 NCBI BlastP on this gene

EEH03340

60S ribosomal protein L13
  
Accession: EEH03339
  
Location: 132235-133477
  
 NCBI BlastP on this gene

EEH03339

conserved hypothetical protein
  
Accession: EEH03338
  
Location: 128668-130833
  
 NCBI BlastP on this gene

EEH03338

CAMK family protein kinase
  
Accession: EEH03337
  
Location: 125055-126742
  
 NCBI BlastP on this gene

EEH03337

hypothetical protein
  
Accession: EEH03336
  
Location: 122821-124323
  
 NCBI BlastP on this gene

EEH03336

enoyl-CoA hydratase
  
Accession: EEH03335
  
Location: 121063-122519
  
 NCBI BlastP on this gene

EEH03335

topoisomerase 1
  
Accession: EEH03334
  
Location: 117454-120999
  
  
**BlastP hit with Mycgr3G68421\_Mycgr3T**
  
Percentage identity: 45 %
  
BlastP bit score: 899
  
Sequence coverage: 92 %
  
E-value: 0.0
  
  
 NCBI BlastP on this gene

EEH03334

3-hydroxybutyryl CoA dehydrogenase
  
Accession: EEH03333
  
Location: 115880-117028
  
 NCBI BlastP on this gene

EEH03333

Query: Architecture Search FASTA input

DS499603 : Aspergillus fumigatus A1163 scf\_000010 genomic scaffold    Total score: 5.0     Cumulative Blast bit score: 2664

Hit cluster cross-links:

Mycgr3G90785 Mycgr3T
  
Location: 0-1047

Mycgr3G90785\_Mycgr3T

Mycgr3G103262 Mycgr3
  
Location: 1147-1390

Mycgr3G103262\_Mycgr3

Mycgr3G68458 Mycgr3T
  
Location: 1490-3602

Mycgr3G68458\_Mycgr3T

Mycgr3G99145 Mycgr3T
  
Location: 3702-4326

Mycgr3G99145\_Mycgr3T

Mycgr3G103274 Mycgr3
  
Location: 4426-4957

Mycgr3G103274\_Mycgr3

Mycgr3G103264 Mycgr3
  
Location: 5057-5390

Mycgr3G103264\_Mycgr3

Mycgr3G37570 Mycgr3T
  
Location: 5490-6006

Mycgr3G37570\_Mycgr3T

Mycgr3G108094 Mycgr3
  
Location: 6106-10555

Mycgr3G108094\_Mycgr3

Mycgr3G90786 Mycgr3T
  
Location: 10655-12080

Mycgr3G90786\_Mycgr3T

Mycgr3G68429 Mycgr3T
  
Location: 12180-13440

Mycgr3G68429\_Mycgr3T

Mycgr3G68421 Mycgr3T
  
Location: 13540-17086

Mycgr3G68421\_Mycgr3T

Mycgr3G90801 Mycgr3T
  
Location: 17186-18056

Mycgr3G90801\_Mycgr3T

Mycgr3G84646 Mycgr3T
  
Location: 18156-20235

Mycgr3G84646\_Mycgr3T

Mycgr3G68456 Mycgr3T
  
Location: 20335-21970

Mycgr3G68456\_Mycgr3T

Mycgr3G103270 Mycgr3
  
Location: 22070-22355

Mycgr3G103270\_Mycgr3

Mycgr3G90803 Mycgr3T
  
Location: 22455-23019

Mycgr3G90803\_Mycgr3T

Mycgr3G36941 Mycgr3T
  
Location: 23119-24064

Mycgr3G36941\_Mycgr3T

Mycgr3G25746 Mycgr3T
  
Location: 24164-25241

Mycgr3G25746\_Mycgr3T

Mycgr3G90788 Mycgr3T
  
Location: 25341-25803

Mycgr3G90788\_Mycgr3T

Mycgr3G103260 Mycgr3
  
Location: 25903-26635

Mycgr3G103260\_Mycgr3

Mycgr3G84644 Mycgr3T
  
Location: 26735-28457

Mycgr3G84644\_Mycgr3T

Mycgr3G29227 Mycgr3T
  
Location: 28557-28863

Mycgr3G29227\_Mycgr3T

Mycgr3G36271 Mycgr3T
  
Location: 28963-29854

Mycgr3G36271\_Mycgr3T

Mycgr3G68433 Mycgr3T
  
Location: 29954-33041

Mycgr3G68433\_Mycgr3T

Mycgr3G79452 Mycgr3T
  
Location: 33141-33399

Mycgr3G79452\_Mycgr3T

Mycgr3G55345 Mycgr3T
  
Location: 33499-34126

Mycgr3G55345\_Mycgr3T

Mycgr3G103278 Mycgr3
  
Location: 34226-35195

Mycgr3G103278\_Mycgr3

Mycgr3G84654 Mycgr3T
  
Location: 35295-36630

Mycgr3G84654\_Mycgr3T

Mycgr3G108090 Mycgr3
  
Location: 36730-37591

Mycgr3G108090\_Mycgr3

Mycgr3G21922 Mycgr3T
  
Location: 37691-39149

Mycgr3G21922\_Mycgr3T

Mycgr3G99148 Mycgr3T
  
Location: 39249-42819

Mycgr3G99148\_Mycgr3T

conserved hypothetical protein
  
Accession: EDP47264
  
Location: 131579-133468
  
 NCBI BlastP on this gene

EDP47264

phenazine biosynthesis-like protein, putative
  
Accession: EDP47263
  
Location: 130166-131128
  
 NCBI BlastP on this gene

EDP47263

DNA repair protein (Tof1), putative
  
Accession: EDP47262
  
Location: 125889-129586
  
  
**BlastP hit with Mycgr3G68421\_Mycgr3T**
  
Percentage identity: 44 %
  
BlastP bit score: 946
  
Sequence coverage: 102 %
  
E-value: 0.0
  
  
 NCBI BlastP on this gene

EDP47262

3-hydroxybutyryl-CoA dehydrogenase, putative
  
Accession: EDP47261
  
Location: 124424-125521
  
 NCBI BlastP on this gene

EDP47261

IBR domain protein
  
Accession: EDP47260
  
Location: 123032-124142
  
 NCBI BlastP on this gene

EDP47260

actin family protein
  
Accession: EDP47259
  
Location: 120565-122134
  
 NCBI BlastP on this gene

EDP47259

conserved hypothetical protein
  
Accession: EDP47258
  
Location: 119081-120193
  
 NCBI BlastP on this gene

EDP47258

conserved leucine-rich repeat protein
  
Accession: EDP47257
  
Location: 115786-118782
  
  
**BlastP hit with Mycgr3G68433\_Mycgr3T**
  
Percentage identity: 40 %
  
BlastP bit score: 518
  
Sequence coverage: 85 %
  
E-value: 3e-163
  
  
 NCBI BlastP on this gene

EDP47257

conserved hypothetical protein
  
Accession: EDP47256
  
Location: 112472-113896
  
  
**BlastP hit with Mycgr3G90786\_Mycgr3T**
  
Percentage identity: 27 %
  
BlastP bit score: 85
  
Sequence coverage: 92 %
  
E-value: 1e-14
  
  
 NCBI BlastP on this gene

EDP47256

60S ribosome protein L13, putative
  
Accession: EDP47255
  
Location: 110403-111410
  
 NCBI BlastP on this gene

EDP47255

hypothetical protein
  
Accession: EDP47254
  
Location: 107749-109685
  
 NCBI BlastP on this gene

EDP47254

conserved hypothetical protein
  
Accession: EDP47253
  
Location: 106620-107432
  
 NCBI BlastP on this gene

EDP47253

conserved hypothetical protein
  
Accession: EDP47252
  
Location: 105401-106167
  
 NCBI BlastP on this gene

EDP47252

hypothetical protein
  
Accession: EDP47251
  
Location: 102414-104531
  
 NCBI BlastP on this gene

EDP47251

F-box domain protein
  
Accession: EDP47250
  
Location: 98681-100389
  
 NCBI BlastP on this gene

EDP47250

succinyl-CoA synthetase beta subunit, putative
  
Accession: EDP47249
  
Location: 96126-97928
  
 NCBI BlastP on this gene

EDP47249

short chain dehydrogenase/reductase, putative
  
Accession: EDP47248
  
Location: 94373-95601
  
 NCBI BlastP on this gene

EDP47248

C4-dicarboxylate transporter/malic acid transport protein, putative
  
Accession: EDP47247
  
Location: 91516-92623
  
 NCBI BlastP on this gene

EDP47247

conserved hypothetical protein
  
Accession: EDP47246
  
Location: 89794-90510
  
  
**BlastP hit with Mycgr3G55345\_Mycgr3T**
  
Percentage identity: 76 %
  
BlastP bit score: 261
  
Sequence coverage: 81 %
  
E-value: 4e-85
  
  
 NCBI BlastP on this gene

EDP47246

GTP binding protein, putative
  
Accession: EDP47245
  
Location: 85712-87887
  
 NCBI BlastP on this gene

EDP47245

conserved hypothetical protein
  
Accession: EDP47244
  
Location: 84035-85330
  
 NCBI BlastP on this gene

EDP47244

hypothetical protein
  
Accession: EDP47243
  
Location: 83521-83805
  
 NCBI BlastP on this gene

EDP47243

ubiquinone biosynthesis protein, putative
  
Accession: EDP47242
  
Location: 81149-83363
  
  
**BlastP hit with Mycgr3G68458\_Mycgr3T**
  
Percentage identity: 65 %
  
BlastP bit score: 854
  
Sequence coverage: 93 %
  
E-value: 0.0
  
  
 NCBI BlastP on this gene

EDP47242

cytochrome C1 heme lyase
  
Accession: EDP47241
  
Location: 79225-80333
  
 NCBI BlastP on this gene

EDP47241

conserved hypothetical protein
  
Accession: EDP47240
  
Location: 76653-78656
  
 NCBI BlastP on this gene

EDP47240

Query: Architecture Search FASTA input

AAHF01000016 : Aspergillus fumigatus Af293    Total score: 5.0     Cumulative Blast bit score: 2664

Hit cluster cross-links:

Mycgr3G90785 Mycgr3T
  
Location: 0-1047

Mycgr3G90785\_Mycgr3T

Mycgr3G103262 Mycgr3
  
Location: 1147-1390

Mycgr3G103262\_Mycgr3

Mycgr3G68458 Mycgr3T
  
Location: 1490-3602

Mycgr3G68458\_Mycgr3T

Mycgr3G99145 Mycgr3T
  
Location: 3702-4326

Mycgr3G99145\_Mycgr3T

Mycgr3G103274 Mycgr3
  
Location: 4426-4957

Mycgr3G103274\_Mycgr3

Mycgr3G103264 Mycgr3
  
Location: 5057-5390

Mycgr3G103264\_Mycgr3

Mycgr3G37570 Mycgr3T
  
Location: 5490-6006

Mycgr3G37570\_Mycgr3T

Mycgr3G108094 Mycgr3
  
Location: 6106-10555

Mycgr3G108094\_Mycgr3

Mycgr3G90786 Mycgr3T
  
Location: 10655-12080

Mycgr3G90786\_Mycgr3T

Mycgr3G68429 Mycgr3T
  
Location: 12180-13440

Mycgr3G68429\_Mycgr3T

Mycgr3G68421 Mycgr3T
  
Location: 13540-17086

Mycgr3G68421\_Mycgr3T

Mycgr3G90801 Mycgr3T
  
Location: 17186-18056

Mycgr3G90801\_Mycgr3T

Mycgr3G84646 Mycgr3T
  
Location: 18156-20235

Mycgr3G84646\_Mycgr3T

Mycgr3G68456 Mycgr3T
  
Location: 20335-21970

Mycgr3G68456\_Mycgr3T

Mycgr3G103270 Mycgr3
  
Location: 22070-22355

Mycgr3G103270\_Mycgr3

Mycgr3G90803 Mycgr3T
  
Location: 22455-23019

Mycgr3G90803\_Mycgr3T

Mycgr3G36941 Mycgr3T
  
Location: 23119-24064

Mycgr3G36941\_Mycgr3T

Mycgr3G25746 Mycgr3T
  
Location: 24164-25241

Mycgr3G25746\_Mycgr3T

Mycgr3G90788 Mycgr3T
  
Location: 25341-25803

Mycgr3G90788\_Mycgr3T

Mycgr3G103260 Mycgr3
  
Location: 25903-26635

Mycgr3G103260\_Mycgr3

Mycgr3G84644 Mycgr3T
  
Location: 26735-28457

Mycgr3G84644\_Mycgr3T

Mycgr3G29227 Mycgr3T
  
Location: 28557-28863

Mycgr3G29227\_Mycgr3T

Mycgr3G36271 Mycgr3T
  
Location: 28963-29854

Mycgr3G36271\_Mycgr3T

Mycgr3G68433 Mycgr3T
  
Location: 29954-33041

Mycgr3G68433\_Mycgr3T

Mycgr3G79452 Mycgr3T
  
Location: 33141-33399

Mycgr3G79452\_Mycgr3T

Mycgr3G55345 Mycgr3T
  
Location: 33499-34126

Mycgr3G55345\_Mycgr3T

Mycgr3G103278 Mycgr3
  
Location: 34226-35195

Mycgr3G103278\_Mycgr3

Mycgr3G84654 Mycgr3T
  
Location: 35295-36630

Mycgr3G84654\_Mycgr3T

Mycgr3G108090 Mycgr3
  
Location: 36730-37591

Mycgr3G108090\_Mycgr3

Mycgr3G21922 Mycgr3T
  
Location: 37691-39149

Mycgr3G21922\_Mycgr3T

Mycgr3G99148 Mycgr3T
  
Location: 39249-42819

Mycgr3G99148\_Mycgr3T

conserved hypothetical protein
  
Accession: EAL84584
  
Location: 531189-533078
  
 NCBI BlastP on this gene

EAL84584

phenazine biosynthesis-like protein, putative
  
Accession: EAL84585
  
Location: 533529-534491
  
 NCBI BlastP on this gene

EAL84585

DNA repair protein (Tof1), putative
  
Accession: EAL84586
  
Location: 535071-538768
  
  
**BlastP hit with Mycgr3G68421\_Mycgr3T**
  
Percentage identity: 44 %
  
BlastP bit score: 946
  
Sequence coverage: 102 %
  
E-value: 0.0
  
  
 NCBI BlastP on this gene

EAL84586

3-hydroxybutyryl-CoA dehydrogenase, putative
  
Accession: EAL84588
  
Location: 539136-540233
  
 NCBI BlastP on this gene

EAL84588

IBR domain protein
  
Accession: EAL84589
  
Location: 540515-541625
  
 NCBI BlastP on this gene

EAL84589

actin family protein
  
Accession: EAL84590
  
Location: 542523-544092
  
 NCBI BlastP on this gene

EAL84590

conserved hypothetical protein
  
Accession: EAL84591
  
Location: 544464-545576
  
 NCBI BlastP on this gene

EAL84591

conserved leucine-rich repeat protein
  
Accession: EAL84592
  
Location: 545875-548871
  
  
**BlastP hit with Mycgr3G68433\_Mycgr3T**
  
Percentage identity: 40 %
  
BlastP bit score: 518
  
Sequence coverage: 85 %
  
E-value: 3e-163
  
  
 NCBI BlastP on this gene

EAL84592

conserved hypothetical protein
  
Accession: EAL84593
  
Location: 550761-552185
  
  
**BlastP hit with Mycgr3G90786\_Mycgr3T**
  
Percentage identity: 27 %
  
BlastP bit score: 85
  
Sequence coverage: 92 %
  
E-value: 1e-14
  
  
 NCBI BlastP on this gene

EAL84593

60S ribosomal protein L13
  
Accession: EAL84594
  
Location: 553247-554254
  
 NCBI BlastP on this gene

EAL84594

conserved hypothetical protein
  
Accession: EBA27195
  
Location: 554972-556908
  
 NCBI BlastP on this gene

EBA27195

conserved hypothetical protein
  
Accession: EAL84595
  
Location: 557225-558037
  
 NCBI BlastP on this gene

EAL84595

conserved hypothetical protein
  
Accession: EAL84596
  
Location: 558490-559256
  
 NCBI BlastP on this gene

EAL84596

flocculation suppression protein
  
Accession: EBA27196
  
Location: 560126-562243
  
 NCBI BlastP on this gene

EBA27196

F-box domain protein
  
Accession: EAL84599
  
Location: 564272-565980
  
 NCBI BlastP on this gene

EAL84599

succinyl-CoA synthetase beta subunit, putative
  
Accession: EAL84600
  
Location: 566733-568535
  
 NCBI BlastP on this gene

EAL84600

short chain dehydrogenase/reductase (Ayr1), putative
  
Accession: EAL84601
  
Location: 569060-570288
  
 NCBI BlastP on this gene

EAL84601

C4-dicarboxylate transporter/malic acid transport protein, putative
  
Accession: EAL84602
  
Location: 572038-573145
  
 NCBI BlastP on this gene

EAL84602

xanthine-guanine phosphoribosyl transferase Xpt1, putative
  
Accession: EAL84603
  
Location: 574150-574866
  
  
**BlastP hit with Mycgr3G55345\_Mycgr3T**
  
Percentage identity: 76 %
  
BlastP bit score: 261
  
Sequence coverage: 81 %
  
E-value: 4e-85
  
  
 NCBI BlastP on this gene

EAL84603

GTP binding protein, putative
  
Accession: EAL84604
  
Location: 576773-578948
  
 NCBI BlastP on this gene

EAL84604

conserved hypothetical protein
  
Accession: EAL84605
  
Location: 579330-580625
  
 NCBI BlastP on this gene

EAL84605

hypothetical protein
  
Accession: EAL84606
  
Location: 580855-581139
  
 NCBI BlastP on this gene

EAL84606

ubiquinone biosynthesis protein, putative
  
Accession: EAL84607
  
Location: 581297-583511
  
  
**BlastP hit with Mycgr3G68458\_Mycgr3T**
  
Percentage identity: 65 %
  
BlastP bit score: 854
  
Sequence coverage: 93 %
  
E-value: 0.0
  
  
 NCBI BlastP on this gene

EAL84607

cytochrome c heme lyase, putative
  
Accession: EBA27197
  
Location: 584327-585435
  
 NCBI BlastP on this gene

EBA27197

conserved hypothetical protein
  
Accession: EBA27198
  
Location: 586004-588007
  
 NCBI BlastP on this gene

EBA27198

Query: Architecture Search FASTA input

ACJE01000002 : Aspergillus niger ATCC 1015    Total score: 5.0     Cumulative Blast bit score: 2581

Hit cluster cross-links:

Mycgr3G90785 Mycgr3T
  
Location: 0-1047

Mycgr3G90785\_Mycgr3T

Mycgr3G103262 Mycgr3
  
Location: 1147-1390

Mycgr3G103262\_Mycgr3

Mycgr3G68458 Mycgr3T
  
Location: 1490-3602

Mycgr3G68458\_Mycgr3T

Mycgr3G99145 Mycgr3T
  
Location: 3702-4326

Mycgr3G99145\_Mycgr3T

Mycgr3G103274 Mycgr3
  
Location: 4426-4957

Mycgr3G103274\_Mycgr3

Mycgr3G103264 Mycgr3
  
Location: 5057-5390

Mycgr3G103264\_Mycgr3

Mycgr3G37570 Mycgr3T
  
Location: 5490-6006

Mycgr3G37570\_Mycgr3T

Mycgr3G108094 Mycgr3
  
Location: 6106-10555

Mycgr3G108094\_Mycgr3

Mycgr3G90786 Mycgr3T
  
Location: 10655-12080

Mycgr3G90786\_Mycgr3T

Mycgr3G68429 Mycgr3T
  
Location: 12180-13440

Mycgr3G68429\_Mycgr3T

Mycgr3G68421 Mycgr3T
  
Location: 13540-17086

Mycgr3G68421\_Mycgr3T

Mycgr3G90801 Mycgr3T
  
Location: 17186-18056

Mycgr3G90801\_Mycgr3T

Mycgr3G84646 Mycgr3T
  
Location: 18156-20235

Mycgr3G84646\_Mycgr3T

Mycgr3G68456 Mycgr3T
  
Location: 20335-21970

Mycgr3G68456\_Mycgr3T

Mycgr3G103270 Mycgr3
  
Location: 22070-22355

Mycgr3G103270\_Mycgr3

Mycgr3G90803 Mycgr3T
  
Location: 22455-23019

Mycgr3G90803\_Mycgr3T

Mycgr3G36941 Mycgr3T
  
Location: 23119-24064

Mycgr3G36941\_Mycgr3T

Mycgr3G25746 Mycgr3T
  
Location: 24164-25241

Mycgr3G25746\_Mycgr3T

Mycgr3G90788 Mycgr3T
  
Location: 25341-25803

Mycgr3G90788\_Mycgr3T

Mycgr3G103260 Mycgr3
  
Location: 25903-26635

Mycgr3G103260\_Mycgr3

Mycgr3G84644 Mycgr3T
  
Location: 26735-28457

Mycgr3G84644\_Mycgr3T

Mycgr3G29227 Mycgr3T
  
Location: 28557-28863

Mycgr3G29227\_Mycgr3T

Mycgr3G36271 Mycgr3T
  
Location: 28963-29854

Mycgr3G36271\_Mycgr3T

Mycgr3G68433 Mycgr3T
  
Location: 29954-33041

Mycgr3G68433\_Mycgr3T

Mycgr3G79452 Mycgr3T
  
Location: 33141-33399

Mycgr3G79452\_Mycgr3T

Mycgr3G55345 Mycgr3T
  
Location: 33499-34126

Mycgr3G55345\_Mycgr3T

Mycgr3G103278 Mycgr3
  
Location: 34226-35195

Mycgr3G103278\_Mycgr3

Mycgr3G84654 Mycgr3T
  
Location: 35295-36630

Mycgr3G84654\_Mycgr3T

Mycgr3G108090 Mycgr3
  
Location: 36730-37591

Mycgr3G108090\_Mycgr3

Mycgr3G21922 Mycgr3T
  
Location: 37691-39149

Mycgr3G21922\_Mycgr3T

Mycgr3G99148 Mycgr3T
  
Location: 39249-42819

Mycgr3G99148\_Mycgr3T

hypothetical protein
  
Accession: EHA27556
  
Location: 123431-124861
  
 NCBI BlastP on this gene

EHA27556

hypothetical protein
  
Accession: EHA27555
  
Location: 121767-122948
  
 NCBI BlastP on this gene

EHA27555

hypothetical protein
  
Accession: EHA27554
  
Location: 117321-120994
  
  
**BlastP hit with Mycgr3G68421\_Mycgr3T**
  
Percentage identity: 50 %
  
BlastP bit score: 800
  
Sequence coverage: 67 %
  
E-value: 0.0
  
  
 NCBI BlastP on this gene

EHA27554

hypothetical protein
  
Accession: EHA27553
  
Location: 115907-117009
  
 NCBI BlastP on this gene

EHA27553

hypothetical protein
  
Accession: EHA27552
  
Location: 111302-112918
  
 NCBI BlastP on this gene

EHA27552

hypothetical protein
  
Accession: EHA27551
  
Location: 109229-110335
  
 NCBI BlastP on this gene

EHA27551

hypothetical protein
  
Accession: EHA27550
  
Location: 105990-108974
  
  
**BlastP hit with Mycgr3G68433\_Mycgr3T**
  
Percentage identity: 38 %
  
BlastP bit score: 531
  
Sequence coverage: 102 %
  
E-value: 3e-168
  
  
 NCBI BlastP on this gene

EHA27550

hypothetical protein
  
Accession: EHA27549
  
Location: 102354-103793
  
  
**BlastP hit with Mycgr3G90786\_Mycgr3T**
  
Percentage identity: 27 %
  
BlastP bit score: 95
  
Sequence coverage: 100 %
  
E-value: 8e-18
  
  
 NCBI BlastP on this gene

EHA27549

hypothetical protein
  
Accession: EHA27548
  
Location: 97332-99241
  
 NCBI BlastP on this gene

EHA27548

hypothetical protein
  
Accession: EHA27547
  
Location: 96279-96888
  
 NCBI BlastP on this gene

EHA27547

hypothetical protein
  
Accession: EHA27546
  
Location: 94922-95759
  
 NCBI BlastP on this gene

EHA27546

hypothetical protein
  
Accession: EHA27545
  
Location: 92085-94200
  
 NCBI BlastP on this gene

EHA27545

hypothetical protein
  
Accession: EHA27544
  
Location: 88522-90156
  
 NCBI BlastP on this gene

EHA27544

hypothetical protein
  
Accession: EHA27543
  
Location: 86277-88075
  
 NCBI BlastP on this gene

EHA27543

dehydrogenase
  
Accession: EHA27542
  
Location: 84308-85505
  
 NCBI BlastP on this gene

EHA27542

hypothetical protein
  
Accession: EHA27541
  
Location: 80595-81822
  
 NCBI BlastP on this gene

EHA27541

hypothetical protein
  
Accession: EHA27540
  
Location: 78120-79162
  
  
**BlastP hit with Mycgr3G55345\_Mycgr3T**
  
Percentage identity: 73 %
  
BlastP bit score: 309
  
Sequence coverage: 96 %
  
E-value: 9e-104
  
  
 NCBI BlastP on this gene

EHA27540

hypothetical protein
  
Accession: EHA27539
  
Location: 76854-77332
  
 NCBI BlastP on this gene

EHA27539

hypothetical protein
  
Accession: EHA27538
  
Location: 73689-75923
  
 NCBI BlastP on this gene

EHA27538

hypothetical protein
  
Accession: EHA27537
  
Location: 70947-73136
  
  
**BlastP hit with Mycgr3G68458\_Mycgr3T**
  
Percentage identity: 59 %
  
BlastP bit score: 846
  
Sequence coverage: 101 %
  
E-value: 0.0
  
  
 NCBI BlastP on this gene

EHA27537

hypothetical protein
  
Accession: EHA27536
  
Location: 67344-70037
  
 NCBI BlastP on this gene

EHA27536

Query: Architecture Search FASTA input

DF126458 : Aspergillus kawachii IFO 4308 DNA, contig: scaffold00012    Total score: 5.0     Cumulative Blast bit score: 2541

Hit cluster cross-links:

Mycgr3G90785 Mycgr3T
  
Location: 0-1047

Mycgr3G90785\_Mycgr3T

Mycgr3G103262 Mycgr3
  
Location: 1147-1390

Mycgr3G103262\_Mycgr3

Mycgr3G68458 Mycgr3T
  
Location: 1490-3602

Mycgr3G68458\_Mycgr3T

Mycgr3G99145 Mycgr3T
  
Location: 3702-4326

Mycgr3G99145\_Mycgr3T

Mycgr3G103274 Mycgr3
  
Location: 4426-4957

Mycgr3G103274\_Mycgr3

Mycgr3G103264 Mycgr3
  
Location: 5057-5390

Mycgr3G103264\_Mycgr3

Mycgr3G37570 Mycgr3T
  
Location: 5490-6006

Mycgr3G37570\_Mycgr3T

Mycgr3G108094 Mycgr3
  
Location: 6106-10555

Mycgr3G108094\_Mycgr3

Mycgr3G90786 Mycgr3T
  
Location: 10655-12080

Mycgr3G90786\_Mycgr3T

Mycgr3G68429 Mycgr3T
  
Location: 12180-13440

Mycgr3G68429\_Mycgr3T

Mycgr3G68421 Mycgr3T
  
Location: 13540-17086

Mycgr3G68421\_Mycgr3T

Mycgr3G90801 Mycgr3T
  
Location: 17186-18056

Mycgr3G90801\_Mycgr3T

Mycgr3G84646 Mycgr3T
  
Location: 18156-20235

Mycgr3G84646\_Mycgr3T

Mycgr3G68456 Mycgr3T
  
Location: 20335-21970

Mycgr3G68456\_Mycgr3T

Mycgr3G103270 Mycgr3
  
Location: 22070-22355

Mycgr3G103270\_Mycgr3

Mycgr3G90803 Mycgr3T
  
Location: 22455-23019

Mycgr3G90803\_Mycgr3T

Mycgr3G36941 Mycgr3T
  
Location: 23119-24064

Mycgr3G36941\_Mycgr3T

Mycgr3G25746 Mycgr3T
  
Location: 24164-25241

Mycgr3G25746\_Mycgr3T

Mycgr3G90788 Mycgr3T
  
Location: 25341-25803

Mycgr3G90788\_Mycgr3T

Mycgr3G103260 Mycgr3
  
Location: 25903-26635

Mycgr3G103260\_Mycgr3

Mycgr3G84644 Mycgr3T
  
Location: 26735-28457

Mycgr3G84644\_Mycgr3T

Mycgr3G29227 Mycgr3T
  
Location: 28557-28863

Mycgr3G29227\_Mycgr3T

Mycgr3G36271 Mycgr3T
  
Location: 28963-29854

Mycgr3G36271\_Mycgr3T

Mycgr3G68433 Mycgr3T
  
Location: 29954-33041

Mycgr3G68433\_Mycgr3T

Mycgr3G79452 Mycgr3T
  
Location: 33141-33399

Mycgr3G79452\_Mycgr3T

Mycgr3G55345 Mycgr3T
  
Location: 33499-34126

Mycgr3G55345\_Mycgr3T

Mycgr3G103278 Mycgr3
  
Location: 34226-35195

Mycgr3G103278\_Mycgr3

Mycgr3G84654 Mycgr3T
  
Location: 35295-36630

Mycgr3G84654\_Mycgr3T

Mycgr3G108090 Mycgr3
  
Location: 36730-37591

Mycgr3G108090\_Mycgr3

Mycgr3G21922 Mycgr3T
  
Location: 37691-39149

Mycgr3G21922\_Mycgr3T

Mycgr3G99148 Mycgr3T
  
Location: 39249-42819

Mycgr3G99148\_Mycgr3T

similar to An14g00450
  
Accession: GAA87231
  
Location: 817680-818840
  
 NCBI BlastP on this gene

GAA87231

topoisomerase 1-associated factor 1
  
Accession: GAA87232
  
Location: 819592-822817
  
  
**BlastP hit with Mycgr3G68421\_Mycgr3T**
  
Percentage identity: 42 %
  
BlastP bit score: 762
  
Sequence coverage: 93 %
  
E-value: 0.0
  
  
 NCBI BlastP on this gene

GAA87232

3-hydroxybutyryl-CoA dehydrogenase
  
Accession: GAA87233
  
Location: 823634-824679
  
 NCBI BlastP on this gene

GAA87233

IBR domain protein
  
Accession: GAA87234
  
Location: 825172-826375
  
 NCBI BlastP on this gene

GAA87234

actin family protein
  
Accession: GAA87235
  
Location: 827906-829310
  
 NCBI BlastP on this gene

GAA87235

hypothetical protein
  
Accession: GAA87236
  
Location: 830235-831341
  
 NCBI BlastP on this gene

GAA87236

conserved leucine-rich repeat protein
  
Accession: GAA87237
  
Location: 831596-834580
  
  
**BlastP hit with Mycgr3G68433\_Mycgr3T**
  
Percentage identity: 39 %
  
BlastP bit score: 538
  
Sequence coverage: 97 %
  
E-value: 1e-170
  
  
 NCBI BlastP on this gene

GAA87237

similar to An14g00380
  
Accession: GAA87238
  
Location: 836754-838193
  
  
**BlastP hit with Mycgr3G90786\_Mycgr3T**
  
Percentage identity: 27 %
  
BlastP bit score: 91
  
Sequence coverage: 99 %
  
E-value: 1e-16
  
  
 NCBI BlastP on this gene

GAA87238

60S ribosomal protein L13
  
Accession: GAA87239
  
Location: 839179-840364
  
 NCBI BlastP on this gene

GAA87239

similar to An14g00360
  
Accession: GAA87240
  
Location: 841267-843181
  
 NCBI BlastP on this gene

GAA87240

hypothetical protein
  
Accession: GAA87241
  
Location: 843475-844245
  
 NCBI BlastP on this gene

GAA87241

similar to An14g00340
  
Accession: GAA87242
  
Location: 844743-845592
  
 NCBI BlastP on this gene

GAA87242

flocculation suppression protein
  
Accession: GAA87243
  
Location: 846311-848419
  
 NCBI BlastP on this gene

GAA87243

F-box domain protein
  
Accession: GAA87244
  
Location: 850228-852026
  
 NCBI BlastP on this gene

GAA87244

succinyl-CoA ligase beta-chain, mitochondrial precursor
  
Accession: GAA87245
  
Location: 852471-854263
  
 NCBI BlastP on this gene

GAA87245

short chain dehydrogenase/reductase
  
Accession: GAA87246
  
Location: 855035-856237
  
 NCBI BlastP on this gene

GAA87246

C4-dicarboxylate/malic acid transporter
  
Accession: GAA87247
  
Location: 858478-860116
  
 NCBI BlastP on this gene

GAA87247

xanthine phosphoribosyltransferase 1
  
Accession: GAA87248
  
Location: 861048-862076
  
  
**BlastP hit with Mycgr3G55345\_Mycgr3T**
  
Percentage identity: 73 %
  
BlastP bit score: 306
  
Sequence coverage: 96 %
  
E-value: 2e-102
  
  
 NCBI BlastP on this gene

GAA87248

hypothetical protein
  
Accession: GAA87249
  
Location: 862857-863348
  
 NCBI BlastP on this gene

GAA87249

ubiquinone biosynthesis protein
  
Accession: GAA87250
  
Location: 864291-869225
  
  
**BlastP hit with Mycgr3G68458\_Mycgr3T**
  
Percentage identity: 64 %
  
BlastP bit score: 844
  
Sequence coverage: 89 %
  
E-value: 0.0
  
  
 NCBI BlastP on this gene

GAA87250

cytochrome C1 heme lyase
  
Accession: GAA87251
  
Location: 870092-871207
  
 NCBI BlastP on this gene

GAA87251

Query: Architecture Search FASTA input

CH476596 : Aspergillus terreus NIH2624 scaffold\_3 genomic scaffold    Total score: 5.0     Cumulative Blast bit score: 2524

Hit cluster cross-links:

Mycgr3G90785 Mycgr3T
  
Location: 0-1047

Mycgr3G90785\_Mycgr3T

Mycgr3G103262 Mycgr3
  
Location: 1147-1390

Mycgr3G103262\_Mycgr3

Mycgr3G68458 Mycgr3T
  
Location: 1490-3602

Mycgr3G68458\_Mycgr3T

Mycgr3G99145 Mycgr3T
  
Location: 3702-4326

Mycgr3G99145\_Mycgr3T

Mycgr3G103274 Mycgr3
  
Location: 4426-4957

Mycgr3G103274\_Mycgr3

Mycgr3G103264 Mycgr3
  
Location: 5057-5390

Mycgr3G103264\_Mycgr3

Mycgr3G37570 Mycgr3T
  
Location: 5490-6006

Mycgr3G37570\_Mycgr3T

Mycgr3G108094 Mycgr3
  
Location: 6106-10555

Mycgr3G108094\_Mycgr3

Mycgr3G90786 Mycgr3T
  
Location: 10655-12080

Mycgr3G90786\_Mycgr3T

Mycgr3G68429 Mycgr3T
  
Location: 12180-13440

Mycgr3G68429\_Mycgr3T

Mycgr3G68421 Mycgr3T
  
Location: 13540-17086

Mycgr3G68421\_Mycgr3T

Mycgr3G90801 Mycgr3T
  
Location: 17186-18056

Mycgr3G90801\_Mycgr3T

Mycgr3G84646 Mycgr3T
  
Location: 18156-20235

Mycgr3G84646\_Mycgr3T

Mycgr3G68456 Mycgr3T
  
Location: 20335-21970

Mycgr3G68456\_Mycgr3T

Mycgr3G103270 Mycgr3
  
Location: 22070-22355

Mycgr3G103270\_Mycgr3

Mycgr3G90803 Mycgr3T
  
Location: 22455-23019

Mycgr3G90803\_Mycgr3T

Mycgr3G36941 Mycgr3T
  
Location: 23119-24064

Mycgr3G36941\_Mycgr3T

Mycgr3G25746 Mycgr3T
  
Location: 24164-25241

Mycgr3G25746\_Mycgr3T

Mycgr3G90788 Mycgr3T
  
Location: 25341-25803

Mycgr3G90788\_Mycgr3T

Mycgr3G103260 Mycgr3
  
Location: 25903-26635

Mycgr3G103260\_Mycgr3

Mycgr3G84644 Mycgr3T
  
Location: 26735-28457

Mycgr3G84644\_Mycgr3T

Mycgr3G29227 Mycgr3T
  
Location: 28557-28863

Mycgr3G29227\_Mycgr3T

Mycgr3G36271 Mycgr3T
  
Location: 28963-29854

Mycgr3G36271\_Mycgr3T

Mycgr3G68433 Mycgr3T
  
Location: 29954-33041

Mycgr3G68433\_Mycgr3T

Mycgr3G79452 Mycgr3T
  
Location: 33141-33399

Mycgr3G79452\_Mycgr3T

Mycgr3G55345 Mycgr3T
  
Location: 33499-34126

Mycgr3G55345\_Mycgr3T

Mycgr3G103278 Mycgr3
  
Location: 34226-35195

Mycgr3G103278\_Mycgr3

Mycgr3G84654 Mycgr3T
  
Location: 35295-36630

Mycgr3G84654\_Mycgr3T

Mycgr3G108090 Mycgr3
  
Location: 36730-37591

Mycgr3G108090\_Mycgr3

Mycgr3G21922 Mycgr3T
  
Location: 37691-39149

Mycgr3G21922\_Mycgr3T

Mycgr3G99148 Mycgr3T
  
Location: 39249-42819

Mycgr3G99148\_Mycgr3T

conserved hypothetical protein
  
Accession: EAU37694
  
Location: 2356492-2358033
  
 NCBI BlastP on this gene

EAU37694

predicted protein
  
Accession: EAU37695
  
Location: 2358426-2359325
  
 NCBI BlastP on this gene

EAU37695

predicted protein
  
Accession: EAU37696
  
Location: 2359474-2359824
  
 NCBI BlastP on this gene

EAU37696

hypothetical protein
  
Accession: EAU37697
  
Location: 2361177-2364761
  
  
**BlastP hit with Mycgr3G68421\_Mycgr3T**
  
Percentage identity: 42 %
  
BlastP bit score: 862
  
Sequence coverage: 101 %
  
E-value: 0.0
  
  
 NCBI BlastP on this gene

EAU37697

hypothetical protein
  
Accession: EAU37698
  
Location: 2365103-2367599
  
 NCBI BlastP on this gene

EAU37698

conserved hypothetical protein
  
Accession: EAU37699
  
Location: 2368140-2370718
  
  
**BlastP hit with Mycgr3G68433\_Mycgr3T**
  
Percentage identity: 39 %
  
BlastP bit score: 417
  
Sequence coverage: 72 %
  
E-value: 1e-126
  
  
 NCBI BlastP on this gene

EAU37699

conserved hypothetical protein
  
Accession: EAU37700
  
Location: 2372691-2374121
  
  
**BlastP hit with Mycgr3G90786\_Mycgr3T**
  
Percentage identity: 27 %
  
BlastP bit score: 84
  
Sequence coverage: 99 %
  
E-value: 2e-14
  
  
 NCBI BlastP on this gene

EAU37700

60S ribosomal protein L13
  
Accession: EAU37701
  
Location: 2374714-2375785
  
 NCBI BlastP on this gene

EAU37701

predicted protein
  
Accession: EAU37702
  
Location: 2376426-2377803
  
 NCBI BlastP on this gene

EAU37702

conserved hypothetical protein
  
Accession: EAU37703
  
Location: 2378723-2380273
  
 NCBI BlastP on this gene

EAU37703

conserved hypothetical protein
  
Accession: EAU37704
  
Location: 2380723-2382754
  
 NCBI BlastP on this gene

EAU37704

predicted protein
  
Accession: EAU37705
  
Location: 2383268-2384894
  
 NCBI BlastP on this gene

EAU37705

succinyl-CoA ligase beta-chain, mitochondrial precursor
  
Accession: EAU37706
  
Location: 2385368-2387082
  
 NCBI BlastP on this gene

EAU37706

predicted protein
  
Accession: EAU37707
  
Location: 2387787-2388969
  
 NCBI BlastP on this gene

EAU37707

predicted protein
  
Accession: EAU37708
  
Location: 2390953-2392153
  
 NCBI BlastP on this gene

EAU37708

xanthine phosphoribosyltransferase 1
  
Accession: EAU37709
  
Location: 2392975-2394014
  
  
**BlastP hit with Mycgr3G55345\_Mycgr3T**
  
Percentage identity: 72 %
  
BlastP bit score: 303
  
Sequence coverage: 97 %
  
E-value: 3e-101
  
  
 NCBI BlastP on this gene

EAU37709

conserved hypothetical protein
  
Accession: EAU37710
  
Location: 2394481-2394881
  
 NCBI BlastP on this gene

EAU37710

conserved hypothetical protein
  
Accession: EAU37711
  
Location: 2395508-2397740
  
 NCBI BlastP on this gene

EAU37711

predicted protein
  
Accession: EAU37712
  
Location: 2397838-2399114
  
 NCBI BlastP on this gene

EAU37712

conserved hypothetical protein
  
Accession: EAU37713
  
Location: 2399834-2402002
  
  
**BlastP hit with Mycgr3G68458\_Mycgr3T**
  
Percentage identity: 60 %
  
BlastP bit score: 858
  
Sequence coverage: 100 %
  
E-value: 0.0
  
  
 NCBI BlastP on this gene

EAU37713

cytochrome c heme lyase
  
Accession: EAU37714
  
Location: 2402457-2403561
  
 NCBI BlastP on this gene

EAU37714

conserved hypothetical protein
  
Accession: EAU37715
  
Location: 2403895-2405773
  
 NCBI BlastP on this gene

EAU37715

Query: Architecture Search FASTA input

AM270309 : Aspergillus niger contig An14c0010, genomic contig.    Total score: 5.0     Cumulative Blast bit score: 2522

Hit cluster cross-links:

Mycgr3G90785 Mycgr3T
  
Location: 0-1047

Mycgr3G90785\_Mycgr3T

Mycgr3G103262 Mycgr3
  
Location: 1147-1390

Mycgr3G103262\_Mycgr3

Mycgr3G68458 Mycgr3T
  
Location: 1490-3602

Mycgr3G68458\_Mycgr3T

Mycgr3G99145 Mycgr3T
  
Location: 3702-4326

Mycgr3G99145\_Mycgr3T

Mycgr3G103274 Mycgr3
  
Location: 4426-4957

Mycgr3G103274\_Mycgr3

Mycgr3G103264 Mycgr3
  
Location: 5057-5390

Mycgr3G103264\_Mycgr3

Mycgr3G37570 Mycgr3T
  
Location: 5490-6006

Mycgr3G37570\_Mycgr3T

Mycgr3G108094 Mycgr3
  
Location: 6106-10555

Mycgr3G108094\_Mycgr3

Mycgr3G90786 Mycgr3T
  
Location: 10655-12080

Mycgr3G90786\_Mycgr3T

Mycgr3G68429 Mycgr3T
  
Location: 12180-13440

Mycgr3G68429\_Mycgr3T

Mycgr3G68421 Mycgr3T
  
Location: 13540-17086

Mycgr3G68421\_Mycgr3T

Mycgr3G90801 Mycgr3T
  
Location: 17186-18056

Mycgr3G90801\_Mycgr3T

Mycgr3G84646 Mycgr3T
  
Location: 18156-20235

Mycgr3G84646\_Mycgr3T

Mycgr3G68456 Mycgr3T
  
Location: 20335-21970

Mycgr3G68456\_Mycgr3T

Mycgr3G103270 Mycgr3
  
Location: 22070-22355

Mycgr3G103270\_Mycgr3

Mycgr3G90803 Mycgr3T
  
Location: 22455-23019

Mycgr3G90803\_Mycgr3T

Mycgr3G36941 Mycgr3T
  
Location: 23119-24064

Mycgr3G36941\_Mycgr3T

Mycgr3G25746 Mycgr3T
  
Location: 24164-25241

Mycgr3G25746\_Mycgr3T

Mycgr3G90788 Mycgr3T
  
Location: 25341-25803

Mycgr3G90788\_Mycgr3T

Mycgr3G103260 Mycgr3
  
Location: 25903-26635

Mycgr3G103260\_Mycgr3

Mycgr3G84644 Mycgr3T
  
Location: 26735-28457

Mycgr3G84644\_Mycgr3T

Mycgr3G29227 Mycgr3T
  
Location: 28557-28863

Mycgr3G29227\_Mycgr3T

Mycgr3G36271 Mycgr3T
  
Location: 28963-29854

Mycgr3G36271\_Mycgr3T

Mycgr3G68433 Mycgr3T
  
Location: 29954-33041

Mycgr3G68433\_Mycgr3T

Mycgr3G79452 Mycgr3T
  
Location: 33141-33399

Mycgr3G79452\_Mycgr3T

Mycgr3G55345 Mycgr3T
  
Location: 33499-34126

Mycgr3G55345\_Mycgr3T

Mycgr3G103278 Mycgr3
  
Location: 34226-35195

Mycgr3G103278\_Mycgr3

Mycgr3G84654 Mycgr3T
  
Location: 35295-36630

Mycgr3G84654\_Mycgr3T

Mycgr3G108090 Mycgr3
  
Location: 36730-37591

Mycgr3G108090\_Mycgr3

Mycgr3G21922 Mycgr3T
  
Location: 37691-39149

Mycgr3G21922\_Mycgr3T

Mycgr3G99148 Mycgr3T
  
Location: 39249-42819

Mycgr3G99148\_Mycgr3T

not annotated
  
Accession: CAK41845
  
Location: 117140-118570
  
 NCBI BlastP on this gene

An14g00460

not annotated
  
Accession: CAK41844
  
Location: 115383-116657
  
 NCBI BlastP on this gene

An14g00450

not annotated
  
Accession: CAK41843
  
Location: 111030-114703
  
  
**BlastP hit with Mycgr3G68421\_Mycgr3T**
  
Percentage identity: 49 %
  
BlastP bit score: 806
  
Sequence coverage: 70 %
  
E-value: 0.0
  
  
 NCBI BlastP on this gene

An14g00440

unnamed
  
Accession: CAK41842
  
Location: 109616-110718
  
 NCBI BlastP on this gene

An14g00430

not annotated
  
Accession: CAK41841
  
Location: 107933-109128
  
 NCBI BlastP on this gene

An14g00420

not annotated
  
Accession: CAK41840
  
Location: 105012-106628
  
 NCBI BlastP on this gene

An14g00410

unnamed
  
Accession: CAK41839
  
Location: 102941-104047
  
 NCBI BlastP on this gene

An14g00400

not annotated
  
Accession: CAK41838
  
Location: 99702-102686
  
  
**BlastP hit with Mycgr3G68433\_Mycgr3T**
  
Percentage identity: 38 %
  
BlastP bit score: 532
  
Sequence coverage: 102 %
  
E-value: 2e-168
  
  
 NCBI BlastP on this gene

An14g00390

not annotated
  
Accession: CAK41837
  
Location: 96066-97505
  
  
**BlastP hit with Mycgr3G90786\_Mycgr3T**
  
Percentage identity: 27 %
  
BlastP bit score: 94
  
Sequence coverage: 100 %
  
E-value: 9e-18
  
  
 NCBI BlastP on this gene

An14g00380

not annotated
  
Accession: CAK41836
  
Location: 93856-95040
  
 NCBI BlastP on this gene

An14g00370

hypothetical protein
  
Accession: CAK41835
  
Location: 91190-92955
  
 NCBI BlastP on this gene

An14g00360

not annotated
  
Accession: CAK41834
  
Location: 89981-90751
  
 NCBI BlastP on this gene

An14g00350

not annotated
  
Accession: CAK41833
  
Location: 88637-89471
  
 NCBI BlastP on this gene

An14g00340

not annotated
  
Accession: CAK41832
  
Location: 85803-87915
  
 NCBI BlastP on this gene

An14g00330

hypothetical protein
  
Accession: CAK41831
  
Location: 82278-84103
  
 NCBI BlastP on this gene

An14g00320

not annotated
  
Accession: CAK41830
  
Location: 80033-81831
  
 NCBI BlastP on this gene

An14g00310

not annotated
  
Accession: CAK41829
  
Location: 78064-79349
  
 NCBI BlastP on this gene

An14g00300

not annotated
  
Accession: CAK41828
  
Location: 73942-75347
  
 NCBI BlastP on this gene

An14g00290

not annotated
  
Accession: CAK41827
  
Location: 71876-72918
  
  
**BlastP hit with Mycgr3G55345\_Mycgr3T**
  
Percentage identity: 73 %
  
BlastP bit score: 309
  
Sequence coverage: 96 %
  
E-value: 9e-104
  
  
 NCBI BlastP on this gene

An14g00280

not annotated
  
Accession: CAK41826
  
Location: 70607-71085
  
 NCBI BlastP on this gene

An14g00270

not annotated
  
Accession: CAK41825
  
Location: 67446-69719
  
 NCBI BlastP on this gene

An14g00260

not annotated
  
Accession: CAK41824
  
Location: 64903-66713
  
  
**BlastP hit with Mycgr3G68458\_Mycgr3T**
  
Percentage identity: 64 %
  
BlastP bit score: 781
  
Sequence coverage: 81 %
  
E-value: 0.0
  
  
 NCBI BlastP on this gene

An14g00250

not annotated
  
Accession: CAK41823
  
Location: 62665-63797
  
 NCBI BlastP on this gene

An14g00240

Query: Architecture Search FASTA input

AACD01000117 : Aspergillus nidulans FGSC A4    Total score: 5.0     Cumulative Blast bit score: 2513

Hit cluster cross-links:

Mycgr3G90785 Mycgr3T
  
Location: 0-1047

Mycgr3G90785\_Mycgr3T

Mycgr3G103262 Mycgr3
  
Location: 1147-1390

Mycgr3G103262\_Mycgr3

Mycgr3G68458 Mycgr3T
  
Location: 1490-3602

Mycgr3G68458\_Mycgr3T

Mycgr3G99145 Mycgr3T
  
Location: 3702-4326

Mycgr3G99145\_Mycgr3T

Mycgr3G103274 Mycgr3
  
Location: 4426-4957

Mycgr3G103274\_Mycgr3

Mycgr3G103264 Mycgr3
  
Location: 5057-5390

Mycgr3G103264\_Mycgr3

Mycgr3G37570 Mycgr3T
  
Location: 5490-6006

Mycgr3G37570\_Mycgr3T

Mycgr3G108094 Mycgr3
  
Location: 6106-10555

Mycgr3G108094\_Mycgr3

Mycgr3G90786 Mycgr3T
  
Location: 10655-12080

Mycgr3G90786\_Mycgr3T

Mycgr3G68429 Mycgr3T
  
Location: 12180-13440

Mycgr3G68429\_Mycgr3T

Mycgr3G68421 Mycgr3T
  
Location: 13540-17086

Mycgr3G68421\_Mycgr3T

Mycgr3G90801 Mycgr3T
  
Location: 17186-18056

Mycgr3G90801\_Mycgr3T

Mycgr3G84646 Mycgr3T
  
Location: 18156-20235

Mycgr3G84646\_Mycgr3T

Mycgr3G68456 Mycgr3T
  
Location: 20335-21970

Mycgr3G68456\_Mycgr3T

Mycgr3G103270 Mycgr3
  
Location: 22070-22355

Mycgr3G103270\_Mycgr3

Mycgr3G90803 Mycgr3T
  
Location: 22455-23019

Mycgr3G90803\_Mycgr3T

Mycgr3G36941 Mycgr3T
  
Location: 23119-24064

Mycgr3G36941\_Mycgr3T

Mycgr3G25746 Mycgr3T
  
Location: 24164-25241

Mycgr3G25746\_Mycgr3T

Mycgr3G90788 Mycgr3T
  
Location: 25341-25803

Mycgr3G90788\_Mycgr3T

Mycgr3G103260 Mycgr3
  
Location: 25903-26635

Mycgr3G103260\_Mycgr3

Mycgr3G84644 Mycgr3T
  
Location: 26735-28457

Mycgr3G84644\_Mycgr3T

Mycgr3G29227 Mycgr3T
  
Location: 28557-28863

Mycgr3G29227\_Mycgr3T

Mycgr3G36271 Mycgr3T
  
Location: 28963-29854

Mycgr3G36271\_Mycgr3T

Mycgr3G68433 Mycgr3T
  
Location: 29954-33041

Mycgr3G68433\_Mycgr3T

Mycgr3G79452 Mycgr3T
  
Location: 33141-33399

Mycgr3G79452\_Mycgr3T

Mycgr3G55345 Mycgr3T
  
Location: 33499-34126

Mycgr3G55345\_Mycgr3T

Mycgr3G103278 Mycgr3
  
Location: 34226-35195

Mycgr3G103278\_Mycgr3

Mycgr3G84654 Mycgr3T
  
Location: 35295-36630

Mycgr3G84654\_Mycgr3T

Mycgr3G108090 Mycgr3
  
Location: 36730-37591

Mycgr3G108090\_Mycgr3

Mycgr3G21922 Mycgr3T
  
Location: 37691-39149

Mycgr3G21922\_Mycgr3T

Mycgr3G99148 Mycgr3T
  
Location: 39249-42819

Mycgr3G99148\_Mycgr3T

hypothetical protein
  
Accession: EAA61656
  
Location: 111973-115043
  
 NCBI BlastP on this gene

EAA61656

hypothetical protein
  
Accession: EAA61655
  
Location: 109886-111462
  
 NCBI BlastP on this gene

EAA61655

hypothetical protein
  
Accession: EAA61654
  
Location: 107614-109049
  
 NCBI BlastP on this gene

EAA61654

hypothetical protein
  
Accession: EAA61653
  
Location: 104009-107673
  
  
**BlastP hit with Mycgr3G68421\_Mycgr3T**
  
Percentage identity: 51 %
  
BlastP bit score: 826
  
Sequence coverage: 67 %
  
E-value: 0.0
  
  
 NCBI BlastP on this gene

EAA61653

hypothetical protein
  
Accession: EAA61652
  
Location: 102502-103599
  
 NCBI BlastP on this gene

EAA61652

hypothetical protein
  
Accession: EAA61651
  
Location: 99437-102355
  
  
**BlastP hit with Mycgr3G68433\_Mycgr3T**
  
Percentage identity: 38 %
  
BlastP bit score: 498
  
Sequence coverage: 85 %
  
E-value: 6e-156
  
  
 NCBI BlastP on this gene

EAA61651

hypothetical protein
  
Accession: EAA61650
  
Location: 96577-98638
  
  
**BlastP hit with Mycgr3G90786\_Mycgr3T**
  
Percentage identity: 29 %
  
BlastP bit score: 112
  
Sequence coverage: 97 %
  
E-value: 1e-23
  
  
 NCBI BlastP on this gene

EAA61650

hypothetical protein
  
Accession: EAA61649
  
Location: 94654-95748
  
 NCBI BlastP on this gene

EAA61649

predicted protein
  
Accession: EAA61648
  
Location: 92363-94234
  
 NCBI BlastP on this gene

EAA61648

hypothetical protein
  
Accession: EAA61647
  
Location: 91213-91989
  
 NCBI BlastP on this gene

EAA61647

hypothetical protein
  
Accession: EAA61646
  
Location: 88101-89798
  
 NCBI BlastP on this gene

EAA61646

predicted protein
  
Accession: EAA61645
  
Location: 85947-87438
  
 NCBI BlastP on this gene

EAA61645

hypothetical protein
  
Accession: EAA61644
  
Location: 83128-85108
  
 NCBI BlastP on this gene

EAA61644

predicted protein
  
Accession: EAA61643
  
Location: 81872-82605
  
 NCBI BlastP on this gene

EAA61643

hypothetical protein
  
Accession: EAA61642
  
Location: 79404-81608
  
  
**BlastP hit with Mycgr3G68458\_Mycgr3T**
  
Percentage identity: 59 %
  
BlastP bit score: 778
  
Sequence coverage: 95 %
  
E-value: 0.0
  
  
 NCBI BlastP on this gene

EAA61642

predicted protein
  
Accession: EAA61641
  
Location: 78060-78609
  
 NCBI BlastP on this gene

EAA61641

predicted protein
  
Accession: EAA61640
  
Location: 75260-76368
  
 NCBI BlastP on this gene

EAA61640

hypothetical protein
  
Accession: EAA61639
  
Location: 73911-74807
  
  
**BlastP hit with Mycgr3G55345\_Mycgr3T**
  
Percentage identity: 73 %
  
BlastP bit score: 299
  
Sequence coverage: 97 %
  
E-value: 7e-100
  
  
 NCBI BlastP on this gene

EAA61639

hypothetical protein
  
Accession: EAA61638
  
Location: 73029-73435
  
 NCBI BlastP on this gene

EAA61638

hypothetical protein
  
Accession: EAA61637
  
Location: 68762-72373
  
 NCBI BlastP on this gene

EAA61637

hypothetical protein
  
Accession: EAA61636
  
Location: 65902-68067
  
 NCBI BlastP on this gene

EAA61636

Query: Architecture Search FASTA input

KB446542 : Dothistroma septosporum NZE10 unplaced genomic scaffold DOTSEscaffold\_8    Total score: 5.0     Cumulative Blast bit score: 2454

Hit cluster cross-links:

Mycgr3G90785 Mycgr3T
  
Location: 0-1047

Mycgr3G90785\_Mycgr3T

Mycgr3G103262 Mycgr3
  
Location: 1147-1390

Mycgr3G103262\_Mycgr3

Mycgr3G68458 Mycgr3T
  
Location: 1490-3602

Mycgr3G68458\_Mycgr3T

Mycgr3G99145 Mycgr3T
  
Location: 3702-4326

Mycgr3G99145\_Mycgr3T

Mycgr3G103274 Mycgr3
  
Location: 4426-4957

Mycgr3G103274\_Mycgr3

Mycgr3G103264 Mycgr3
  
Location: 5057-5390

Mycgr3G103264\_Mycgr3

Mycgr3G37570 Mycgr3T
  
Location: 5490-6006

Mycgr3G37570\_Mycgr3T

Mycgr3G108094 Mycgr3
  
Location: 6106-10555

Mycgr3G108094\_Mycgr3

Mycgr3G90786 Mycgr3T
  
Location: 10655-12080

Mycgr3G90786\_Mycgr3T

Mycgr3G68429 Mycgr3T
  
Location: 12180-13440

Mycgr3G68429\_Mycgr3T

Mycgr3G68421 Mycgr3T
  
Location: 13540-17086

Mycgr3G68421\_Mycgr3T

Mycgr3G90801 Mycgr3T
  
Location: 17186-18056

Mycgr3G90801\_Mycgr3T

Mycgr3G84646 Mycgr3T
  
Location: 18156-20235

Mycgr3G84646\_Mycgr3T

Mycgr3G68456 Mycgr3T
  
Location: 20335-21970

Mycgr3G68456\_Mycgr3T

Mycgr3G103270 Mycgr3
  
Location: 22070-22355

Mycgr3G103270\_Mycgr3

Mycgr3G90803 Mycgr3T
  
Location: 22455-23019

Mycgr3G90803\_Mycgr3T

Mycgr3G36941 Mycgr3T
  
Location: 23119-24064

Mycgr3G36941\_Mycgr3T

Mycgr3G25746 Mycgr3T
  
Location: 24164-25241

Mycgr3G25746\_Mycgr3T

Mycgr3G90788 Mycgr3T
  
Location: 25341-25803

Mycgr3G90788\_Mycgr3T

Mycgr3G103260 Mycgr3
  
Location: 25903-26635

Mycgr3G103260\_Mycgr3

Mycgr3G84644 Mycgr3T
  
Location: 26735-28457

Mycgr3G84644\_Mycgr3T

Mycgr3G29227 Mycgr3T
  
Location: 28557-28863

Mycgr3G29227\_Mycgr3T

Mycgr3G36271 Mycgr3T
  
Location: 28963-29854

Mycgr3G36271\_Mycgr3T

Mycgr3G68433 Mycgr3T
  
Location: 29954-33041

Mycgr3G68433\_Mycgr3T

Mycgr3G79452 Mycgr3T
  
Location: 33141-33399

Mycgr3G79452\_Mycgr3T

Mycgr3G55345 Mycgr3T
  
Location: 33499-34126

Mycgr3G55345\_Mycgr3T

Mycgr3G103278 Mycgr3
  
Location: 34226-35195

Mycgr3G103278\_Mycgr3

Mycgr3G84654 Mycgr3T
  
Location: 35295-36630

Mycgr3G84654\_Mycgr3T

Mycgr3G108090 Mycgr3
  
Location: 36730-37591

Mycgr3G108090\_Mycgr3

Mycgr3G21922 Mycgr3T
  
Location: 37691-39149

Mycgr3G21922\_Mycgr3T

Mycgr3G99148 Mycgr3T
  
Location: 39249-42819

Mycgr3G99148\_Mycgr3T

hypothetical protein
  
Accession: EME41280
  
Location: 369314-370714
  
 NCBI BlastP on this gene

EME41280

hypothetical protein
  
Accession: EME41279
  
Location: 367579-369120
  
 NCBI BlastP on this gene

EME41279

hypothetical protein
  
Accession: EME41278
  
Location: 366099-367262
  
 NCBI BlastP on this gene

EME41278

hypothetical protein
  
Accession: EME41277
  
Location: 362493-365000
  
  
**BlastP hit with Mycgr3G21922\_Mycgr3T**
  
Percentage identity: 65 %
  
BlastP bit score: 663
  
Sequence coverage: 101 %
  
E-value: 0.0
  
  
 NCBI BlastP on this gene

EME41277

hypothetical protein
  
Accession: EME41276
  
Location: 358612-361134
  
  
**BlastP hit with Mycgr3G84644\_Mycgr3T**
  
Percentage identity: 75 %
  
BlastP bit score: 902
  
Sequence coverage: 108 %
  
E-value: 0.0
  
  
 NCBI BlastP on this gene

EME41276

hypothetical protein
  
Accession: EME41275
  
Location: 354563-356878
  
  
**BlastP hit with Mycgr3G108094\_Mycgr3**
  
Percentage identity: 46 %
  
BlastP bit score: 224
  
Sequence coverage: 19 %
  
E-value: 2e-57
  
  
 NCBI BlastP on this gene

EME41275

hypothetical protein
  
Accession: EME41274
  
Location: 352712-353245
  
 NCBI BlastP on this gene

EME41274

hypothetical protein
  
Accession: EME41273
  
Location: 350944-352110
  
  
**BlastP hit with Mycgr3G25746\_Mycgr3T**
  
Percentage identity: 68 %
  
BlastP bit score: 470
  
Sequence coverage: 100 %
  
E-value: 3e-162
  
  
 NCBI BlastP on this gene

EME41273

hypothetical protein
  
Accession: EME41272
  
Location: 349025-350310
  
 NCBI BlastP on this gene

EME41272

hypothetical protein
  
Accession: EME41271
  
Location: 346658-347699
  
 NCBI BlastP on this gene

EME41271

hypothetical protein
  
Accession: EME41270
  
Location: 345202-346254
  
 NCBI BlastP on this gene

EME41270

hypothetical protein
  
Accession: EME41269
  
Location: 343657-344277
  
 NCBI BlastP on this gene

EME41269

hypothetical protein
  
Accession: EME41268
  
Location: 340840-343116
  
 NCBI BlastP on this gene

EME41268

hypothetical protein
  
Accession: EME41267
  
Location: 338082-340201
  
 NCBI BlastP on this gene

EME41267

hypothetical protein
  
Accession: EME41266
  
Location: 336426-337541
  
 NCBI BlastP on this gene

EME41266

hypothetical protein
  
Accession: EME41265
  
Location: 332699-335686
  
 NCBI BlastP on this gene

EME41265

hypothetical protein
  
Accession: EME41264
  
Location: 330645-331181
  
  
**BlastP hit with Mycgr3G37570\_Mycgr3T**
  
Percentage identity: 57 %
  
BlastP bit score: 195
  
Sequence coverage: 96 %
  
E-value: 7e-60
  
  
 NCBI BlastP on this gene

EME41264

hypothetical protein
  
Accession: EME41263
  
Location: 328790-329509
  
 NCBI BlastP on this gene

EME41263

hypothetical protein
  
Accession: EME41262
  
Location: 325489-327385
  
 NCBI BlastP on this gene

EME41262

hypothetical protein
  
Accession: EME41260
  
Location: 324713-325282
  
 NCBI BlastP on this gene

EME41260

Query: Architecture Search FASTA input

GG692419 : Ajellomyces capsulatus H143 genomic scaffold supercont2.1    Total score: 5.0     Cumulative Blast bit score: 1946

Hit cluster cross-links:

Mycgr3G90785 Mycgr3T
  
Location: 0-1047

Mycgr3G90785\_Mycgr3T

Mycgr3G103262 Mycgr3
  
Location: 1147-1390

Mycgr3G103262\_Mycgr3

Mycgr3G68458 Mycgr3T
  
Location: 1490-3602

Mycgr3G68458\_Mycgr3T

Mycgr3G99145 Mycgr3T
  
Location: 3702-4326

Mycgr3G99145\_Mycgr3T

Mycgr3G103274 Mycgr3
  
Location: 4426-4957

Mycgr3G103274\_Mycgr3

Mycgr3G103264 Mycgr3
  
Location: 5057-5390

Mycgr3G103264\_Mycgr3

Mycgr3G37570 Mycgr3T
  
Location: 5490-6006

Mycgr3G37570\_Mycgr3T

Mycgr3G108094 Mycgr3
  
Location: 6106-10555

Mycgr3G108094\_Mycgr3

Mycgr3G90786 Mycgr3T
  
Location: 10655-12080

Mycgr3G90786\_Mycgr3T

Mycgr3G68429 Mycgr3T
  
Location: 12180-13440

Mycgr3G68429\_Mycgr3T

Mycgr3G68421 Mycgr3T
  
Location: 13540-17086

Mycgr3G68421\_Mycgr3T

Mycgr3G90801 Mycgr3T
  
Location: 17186-18056

Mycgr3G90801\_Mycgr3T

Mycgr3G84646 Mycgr3T
  
Location: 18156-20235

Mycgr3G84646\_Mycgr3T

Mycgr3G68456 Mycgr3T
  
Location: 20335-21970

Mycgr3G68456\_Mycgr3T

Mycgr3G103270 Mycgr3
  
Location: 22070-22355

Mycgr3G103270\_Mycgr3

Mycgr3G90803 Mycgr3T
  
Location: 22455-23019

Mycgr3G90803\_Mycgr3T

Mycgr3G36941 Mycgr3T
  
Location: 23119-24064

Mycgr3G36941\_Mycgr3T

Mycgr3G25746 Mycgr3T
  
Location: 24164-25241

Mycgr3G25746\_Mycgr3T

Mycgr3G90788 Mycgr3T
  
Location: 25341-25803

Mycgr3G90788\_Mycgr3T

Mycgr3G103260 Mycgr3
  
Location: 25903-26635

Mycgr3G103260\_Mycgr3

Mycgr3G84644 Mycgr3T
  
Location: 26735-28457

Mycgr3G84644\_Mycgr3T

Mycgr3G29227 Mycgr3T
  
Location: 28557-28863

Mycgr3G29227\_Mycgr3T

Mycgr3G36271 Mycgr3T
  
Location: 28963-29854

Mycgr3G36271\_Mycgr3T

Mycgr3G68433 Mycgr3T
  
Location: 29954-33041

Mycgr3G68433\_Mycgr3T

Mycgr3G79452 Mycgr3T
  
Location: 33141-33399

Mycgr3G79452\_Mycgr3T

Mycgr3G55345 Mycgr3T
  
Location: 33499-34126

Mycgr3G55345\_Mycgr3T

Mycgr3G103278 Mycgr3
  
Location: 34226-35195

Mycgr3G103278\_Mycgr3

Mycgr3G84654 Mycgr3T
  
Location: 35295-36630

Mycgr3G84654\_Mycgr3T

Mycgr3G108090 Mycgr3
  
Location: 36730-37591

Mycgr3G108090\_Mycgr3

Mycgr3G21922 Mycgr3T
  
Location: 37691-39149

Mycgr3G21922\_Mycgr3T

Mycgr3G99148 Mycgr3T
  
Location: 39249-42819

Mycgr3G99148\_Mycgr3T

topoisomerase I
  
Accession: EER44756
  
Location: 1522955-1523470
  
  
**BlastP hit with Mycgr3G68421\_Mycgr3T**
  
Percentage identity: 55 %
  
BlastP bit score: 102
  
Sequence coverage: 7 %
  
E-value: 1e-21
  
  
 NCBI BlastP on this gene

EER44756

topoisomerase 1-associated factor 1
  
Accession: EER44757
  
Location: 1524200-1526885
  
  
**BlastP hit with Mycgr3G68421\_Mycgr3T**
  
Percentage identity: 42 %
  
BlastP bit score: 531
  
Sequence coverage: 62 %
  
E-value: 7e-169
  
  
 NCBI BlastP on this gene

EER44757

conserved hypothetical protein
  
Accession: EER44759
  
Location: 1528507-1529959
  
 NCBI BlastP on this gene

EER44759

CAMK family protein kinase
  
Accession: EER44760
  
Location: 1530448-1532062
  
 NCBI BlastP on this gene

EER44760

conserved hypothetical protein
  
Accession: EER44761
  
Location: 1533424-1535632
  
 NCBI BlastP on this gene

EER44761

60S ribosomal protein L13
  
Accession: EER44762
  
Location: 1537074-1538316
  
 NCBI BlastP on this gene

EER44762

conserved hypothetical protein
  
Accession: EER44763
  
Location: 1540182-1540916
  
  
**BlastP hit with Mycgr3G90786\_Mycgr3T**
  
Percentage identity: 31 %
  
BlastP bit score: 58
  
Sequence coverage: 39 %
  
E-value: 1e-06
  
  
 NCBI BlastP on this gene

EER44763

leucine rich repeat domain-containing protein
  
Accession: EER44764
  
Location: 1541966-1545062
  
  
**BlastP hit with Mycgr3G68433\_Mycgr3T**
  
Percentage identity: 34 %
  
BlastP bit score: 392
  
Sequence coverage: 99 %
  
E-value: 7e-116
  
  
 NCBI BlastP on this gene

EER44764

conserved hypothetical protein
  
Accession: EER44765
  
Location: 1545904-1547193
  
 NCBI BlastP on this gene

EER44765

actin-like protein
  
Accession: EER44766
  
Location: 1548312-1549967
  
 NCBI BlastP on this gene

EER44766

conserved hypothetical protein
  
Accession: EER44767
  
Location: 1550822-1551835
  
 NCBI BlastP on this gene

EER44767

GTP binding protein
  
Accession: EER44768
  
Location: 1552381-1554875
  
 NCBI BlastP on this gene

EER44768

xanthine phosphoribosyltransferase
  
Accession: EER44769
  
Location: 1557337-1558243
  
  
**BlastP hit with Mycgr3G55345\_Mycgr3T**
  
Percentage identity: 75 %
  
BlastP bit score: 170
  
Sequence coverage: 49 %
  
E-value: 2e-50
  
  
 NCBI BlastP on this gene

EER44769

HSF-type DNA-binding domain-containing protein
  
Accession: EER44770
  
Location: 1563861-1566430
  
 NCBI BlastP on this gene

EER44770

conserved hypothetical protein
  
Accession: EER44771
  
Location: 1567946-1568742
  
 NCBI BlastP on this gene

EER44771

succinyl-CoA ligase beta-chain
  
Accession: EER44772
  
Location: 1569006-1570942
  
 NCBI BlastP on this gene

EER44772

short chain dehydrogenase/reductase
  
Accession: EER44773
  
Location: 1572036-1573418
  
 NCBI BlastP on this gene

EER44773

carbonic anhydrase
  
Accession: EER44774
  
Location: 1575394-1576040
  
 NCBI BlastP on this gene

EER44774

mitochondrion protein
  
Accession: EER44775
  
Location: 1576742-1577020
  
 NCBI BlastP on this gene

EER44775

ubiquinone biosynthesis protein
  
Accession: EER44776
  
Location: 1577170-1580472
  
  
**BlastP hit with Mycgr3G68458\_Mycgr3T**
  
Percentage identity: 61 %
  
BlastP bit score: 693
  
Sequence coverage: 81 %
  
E-value: 0.0
  
  
 NCBI BlastP on this gene

EER44776

conserved hypothetical protein
  
Accession: EER44777
  
Location: 1581389-1582914
  
 NCBI BlastP on this gene

EER44777

Query: Architecture Search FASTA input

GG698482 : Trichophyton tonsurans CBS 112818 genomic scaffold supercont1.6    Total score: 5.0     Cumulative Blast bit score: 1521

Hit cluster cross-links:

Mycgr3G90785 Mycgr3T
  
Location: 0-1047

Mycgr3G90785\_Mycgr3T

Mycgr3G103262 Mycgr3
  
Location: 1147-1390

Mycgr3G103262\_Mycgr3

Mycgr3G68458 Mycgr3T
  
Location: 1490-3602

Mycgr3G68458\_Mycgr3T

Mycgr3G99145 Mycgr3T
  
Location: 3702-4326

Mycgr3G99145\_Mycgr3T

Mycgr3G103274 Mycgr3
  
Location: 4426-4957

Mycgr3G103274\_Mycgr3

Mycgr3G103264 Mycgr3
  
Location: 5057-5390

Mycgr3G103264\_Mycgr3

Mycgr3G37570 Mycgr3T
  
Location: 5490-6006

Mycgr3G37570\_Mycgr3T

Mycgr3G108094 Mycgr3
  
Location: 6106-10555

Mycgr3G108094\_Mycgr3

Mycgr3G90786 Mycgr3T
  
Location: 10655-12080

Mycgr3G90786\_Mycgr3T

Mycgr3G68429 Mycgr3T
  
Location: 12180-13440

Mycgr3G68429\_Mycgr3T

Mycgr3G68421 Mycgr3T
  
Location: 13540-17086

Mycgr3G68421\_Mycgr3T

Mycgr3G90801 Mycgr3T
  
Location: 17186-18056

Mycgr3G90801\_Mycgr3T

Mycgr3G84646 Mycgr3T
  
Location: 18156-20235

Mycgr3G84646\_Mycgr3T

Mycgr3G68456 Mycgr3T
  
Location: 20335-21970

Mycgr3G68456\_Mycgr3T

Mycgr3G103270 Mycgr3
  
Location: 22070-22355

Mycgr3G103270\_Mycgr3

Mycgr3G90803 Mycgr3T
  
Location: 22455-23019

Mycgr3G90803\_Mycgr3T

Mycgr3G36941 Mycgr3T
  
Location: 23119-24064

Mycgr3G36941\_Mycgr3T

Mycgr3G25746 Mycgr3T
  
Location: 24164-25241

Mycgr3G25746\_Mycgr3T

Mycgr3G90788 Mycgr3T
  
Location: 25341-25803

Mycgr3G90788\_Mycgr3T

Mycgr3G103260 Mycgr3
  
Location: 25903-26635

Mycgr3G103260\_Mycgr3

Mycgr3G84644 Mycgr3T
  
Location: 26735-28457

Mycgr3G84644\_Mycgr3T

Mycgr3G29227 Mycgr3T
  
Location: 28557-28863

Mycgr3G29227\_Mycgr3T

Mycgr3G36271 Mycgr3T
  
Location: 28963-29854

Mycgr3G36271\_Mycgr3T

Mycgr3G68433 Mycgr3T
  
Location: 29954-33041

Mycgr3G68433\_Mycgr3T

Mycgr3G79452 Mycgr3T
  
Location: 33141-33399

Mycgr3G79452\_Mycgr3T

Mycgr3G55345 Mycgr3T
  
Location: 33499-34126

Mycgr3G55345\_Mycgr3T

Mycgr3G103278 Mycgr3
  
Location: 34226-35195

Mycgr3G103278\_Mycgr3

Mycgr3G84654 Mycgr3T
  
Location: 35295-36630

Mycgr3G84654\_Mycgr3T

Mycgr3G108090 Mycgr3
  
Location: 36730-37591

Mycgr3G108090\_Mycgr3

Mycgr3G21922 Mycgr3T
  
Location: 37691-39149

Mycgr3G21922\_Mycgr3T

Mycgr3G99148 Mycgr3T
  
Location: 39249-42819

Mycgr3G99148\_Mycgr3T

cortical actin cytoskeleton protein asp1
  
Accession: EGD94297
  
Location: 563618-568058
  
 NCBI BlastP on this gene

EGD94297

WD repeat containing protein 36
  
Accession: EGD94298
  
Location: 568796-572241
  
 NCBI BlastP on this gene

EGD94298

hypothetical protein
  
Accession: EGD94299
  
Location: 572760-573431
  
 NCBI BlastP on this gene

EGD94299

DNA-binding protein HGH1
  
Accession: EGD94300
  
Location: 574764-576164
  
  
**BlastP hit with Mycgr3G25746\_Mycgr3T**
  
Percentage identity: 56 %
  
BlastP bit score: 358
  
Sequence coverage: 98 %
  
E-value: 3e-118
  
  
 NCBI BlastP on this gene

EGD94300

hypothetical protein
  
Accession: EGD94301
  
Location: 576363-577623
  
 NCBI BlastP on this gene

EGD94301

hypothetical protein
  
Accession: EGD94302
  
Location: 577778-579631
  
  
**BlastP hit with Mycgr3G21922\_Mycgr3T**
  
Percentage identity: 44 %
  
BlastP bit score: 162
  
Sequence coverage: 45 %
  
E-value: 3e-40
  
  
 NCBI BlastP on this gene

EGD94302

transcription initiation factor IIF subunit alpha
  
Accession: EGD94303
  
Location: 580750-583198
  
  
**BlastP hit with Mycgr3G103278\_Mycgr3**
  
Percentage identity: 34 %
  
BlastP bit score: 137
  
Sequence coverage: 95 %
  
E-value: 2e-32
  
  
 NCBI BlastP on this gene

EGD94303

hypothetical protein
  
Accession: EGD94304
  
Location: 583709-585104
  
 NCBI BlastP on this gene

EGD94304

hypothetical protein
  
Accession: EGD94305
  
Location: 585319-587330
  
 NCBI BlastP on this gene

EGD94305

inositol monophosphatase
  
Accession: EGD94306
  
Location: 588136-589161
  
 NCBI BlastP on this gene

EGD94306

hypothetical protein
  
Accession: EGD94307
  
Location: 589365-594240
  
 NCBI BlastP on this gene

EGD94307

hypothetical protein
  
Accession: EGD94308
  
Location: 594961-595819
  
 NCBI BlastP on this gene

EGD94308

hypothetical protein
  
Accession: EGD94309
  
Location: 596298-597293
  
 NCBI BlastP on this gene

EGD94309

GTP-binding protein
  
Accession: EGD94310
  
Location: 598010-598544
  
 NCBI BlastP on this gene

EGD94310

hypothetical protein
  
Accession: EGD94311
  
Location: 599817-602307
  
  
**BlastP hit with Mycgr3G84646\_Mycgr3T**
  
Percentage identity: 36 %
  
BlastP bit score: 406
  
Sequence coverage: 110 %
  
E-value: 3e-127
  
  
 NCBI BlastP on this gene

EGD94311

C-4 methylsterol oxidase
  
Accession: EGD94312
  
Location: 603068-604004
  
  
**BlastP hit with Mycgr3G36271\_Mycgr3T**
  
Percentage identity: 80 %
  
BlastP bit score: 458
  
Sequence coverage: 87 %
  
E-value: 6e-160
  
  
 NCBI BlastP on this gene

EGD94312

AAA family ATPase
  
Accession: EGD94313
  
Location: 604992-607505
  
 NCBI BlastP on this gene

EGD94313

hypothetical protein
  
Accession: EGD94314
  
Location: 608078-609370
  
 NCBI BlastP on this gene

EGD94314

hypothetical protein
  
Accession: EGD94315
  
Location: 609827-611058
  
 NCBI BlastP on this gene

EGD94315

Atypical/PIKK/ATR protein kinase
  
Accession: EGD94316
  
Location: 611415-620368
  
 NCBI BlastP on this gene

EGD94316

Query: Architecture Search FASTA input

AFWA01000008 : Pneumocystis murina B123    Total score: 5.0     Cumulative Blast bit score: 1431

Hit cluster cross-links:

Mycgr3G90785 Mycgr3T
  
Location: 0-1047

Mycgr3G90785\_Mycgr3T

Mycgr3G103262 Mycgr3
  
Location: 1147-1390

Mycgr3G103262\_Mycgr3

Mycgr3G68458 Mycgr3T
  
Location: 1490-3602

Mycgr3G68458\_Mycgr3T

Mycgr3G99145 Mycgr3T
  
Location: 3702-4326

Mycgr3G99145\_Mycgr3T

Mycgr3G103274 Mycgr3
  
Location: 4426-4957

Mycgr3G103274\_Mycgr3

Mycgr3G103264 Mycgr3
  
Location: 5057-5390

Mycgr3G103264\_Mycgr3

Mycgr3G37570 Mycgr3T
  
Location: 5490-6006

Mycgr3G37570\_Mycgr3T

Mycgr3G108094 Mycgr3
  
Location: 6106-10555

Mycgr3G108094\_Mycgr3

Mycgr3G90786 Mycgr3T
  
Location: 10655-12080

Mycgr3G90786\_Mycgr3T

Mycgr3G68429 Mycgr3T
  
Location: 12180-13440

Mycgr3G68429\_Mycgr3T

Mycgr3G68421 Mycgr3T
  
Location: 13540-17086

Mycgr3G68421\_Mycgr3T

Mycgr3G90801 Mycgr3T
  
Location: 17186-18056

Mycgr3G90801\_Mycgr3T

Mycgr3G84646 Mycgr3T
  
Location: 18156-20235

Mycgr3G84646\_Mycgr3T

Mycgr3G68456 Mycgr3T
  
Location: 20335-21970

Mycgr3G68456\_Mycgr3T

Mycgr3G103270 Mycgr3
  
Location: 22070-22355

Mycgr3G103270\_Mycgr3

Mycgr3G90803 Mycgr3T
  
Location: 22455-23019

Mycgr3G90803\_Mycgr3T

Mycgr3G36941 Mycgr3T
  
Location: 23119-24064

Mycgr3G36941\_Mycgr3T

Mycgr3G25746 Mycgr3T
  
Location: 24164-25241

Mycgr3G25746\_Mycgr3T

Mycgr3G90788 Mycgr3T
  
Location: 25341-25803

Mycgr3G90788\_Mycgr3T

Mycgr3G103260 Mycgr3
  
Location: 25903-26635

Mycgr3G103260\_Mycgr3

Mycgr3G84644 Mycgr3T
  
Location: 26735-28457

Mycgr3G84644\_Mycgr3T

Mycgr3G29227 Mycgr3T
  
Location: 28557-28863

Mycgr3G29227\_Mycgr3T

Mycgr3G36271 Mycgr3T
  
Location: 28963-29854

Mycgr3G36271\_Mycgr3T

Mycgr3G68433 Mycgr3T
  
Location: 29954-33041

Mycgr3G68433\_Mycgr3T

Mycgr3G79452 Mycgr3T
  
Location: 33141-33399

Mycgr3G79452\_Mycgr3T

Mycgr3G55345 Mycgr3T
  
Location: 33499-34126

Mycgr3G55345\_Mycgr3T

Mycgr3G103278 Mycgr3
  
Location: 34226-35195

Mycgr3G103278\_Mycgr3

Mycgr3G84654 Mycgr3T
  
Location: 35295-36630

Mycgr3G84654\_Mycgr3T

Mycgr3G108090 Mycgr3
  
Location: 36730-37591

Mycgr3G108090\_Mycgr3

Mycgr3G21922 Mycgr3T
  
Location: 37691-39149

Mycgr3G21922\_Mycgr3T

Mycgr3G99148 Mycgr3T
  
Location: 39249-42819

Mycgr3G99148\_Mycgr3T

hypothetical protein
  
Accession: EMR09838
  
Location: 344297-345661
  
 NCBI BlastP on this gene

EMR09838

hypothetical protein
  
Accession: EMR09837
  
Location: 343411-344094
  
 NCBI BlastP on this gene

EMR09837

hypothetical protein
  
Accession: EMR09836
  
Location: 341288-343054
  
  
**BlastP hit with Mycgr3G84646\_Mycgr3T**
  
Percentage identity: 29 %
  
BlastP bit score: 283
  
Sequence coverage: 87 %
  
E-value: 1e-82
  
  
 NCBI BlastP on this gene

EMR09836

hypothetical protein
  
Accession: EMR09835
  
Location: 340074-340971
  
  
**BlastP hit with Mycgr3G99145\_Mycgr3T**
  
Percentage identity: 71 %
  
BlastP bit score: 280
  
Sequence coverage: 93 %
  
E-value: 3e-92
  
  
 NCBI BlastP on this gene

EMR09835

hypothetical protein
  
Accession: EMR09834
  
Location: 338111-339289
  
 NCBI BlastP on this gene

EMR09834

hypothetical protein
  
Accession: EMR09833
  
Location: 333272-336782
  
 NCBI BlastP on this gene

EMR09833

hypothetical protein
  
Accession: EMR09832
  
Location: 332078-332531
  
 NCBI BlastP on this gene

EMR09832

hypothetical protein
  
Accession: EMR09831
  
Location: 330954-332006
  
 NCBI BlastP on this gene

EMR09831

hypothetical protein
  
Accession: EMR09830
  
Location: 327745-330852
  
 NCBI BlastP on this gene

EMR09830

YggS family pyridoxal phosphate enzyme
  
Accession: EMR09829
  
Location: 326684-327691
  
 NCBI BlastP on this gene

EMR09829

hypothetical protein
  
Accession: EMR09828
  
Location: 324874-325959
  
 NCBI BlastP on this gene

EMR09828

hypothetical protein
  
Accession: EMR09827
  
Location: 323213-324351
  
 NCBI BlastP on this gene

EMR09827

CMGC/SRPK protein kinase
  
Accession: EMR09826
  
Location: 320682-322603
  
  
**BlastP hit with Mycgr3G84644\_Mycgr3T**
  
Percentage identity: 56 %
  
BlastP bit score: 555
  
Sequence coverage: 83 %
  
E-value: 0.0
  
  
 NCBI BlastP on this gene

EMR09826

hypothetical protein
  
Accession: EMR09825
  
Location: 319145-320430
  
 NCBI BlastP on this gene

EMR09825

hypothetical protein
  
Accession: EMR09824
  
Location: 316369-318730
  
 NCBI BlastP on this gene

EMR09824

hypothetical protein
  
Accession: EMR09823
  
Location: 315779-316083
  
 NCBI BlastP on this gene

EMR09823

hypothetical protein
  
Accession: EMR09822
  
Location: 314886-315352
  
 NCBI BlastP on this gene

EMR09822

hypothetical protein
  
Accession: EMR09821
  
Location: 302993-314700
  
 NCBI BlastP on this gene

EMR09821

hypothetical protein
  
Accession: EMR09820
  
Location: 301618-302651
  
 NCBI BlastP on this gene

EMR09820

hypothetical protein
  
Accession: EMR09819
  
Location: 300227-301526
  
 NCBI BlastP on this gene

EMR09819

hypothetical protein
  
Accession: EMR09818
  
Location: 299577-299965
  
 NCBI BlastP on this gene

EMR09818

hypothetical protein
  
Accession: EMR09817
  
Location: 298548-299560
  
 NCBI BlastP on this gene

EMR09817

hypothetical protein
  
Accession: EMR09816
  
Location: 296067-298351
  
 NCBI BlastP on this gene

EMR09816

hypothetical protein
  
Accession: EMR09815
  
Location: 294281-295927
  
  
**BlastP hit with Mycgr3G21922\_Mycgr3T**
  
Percentage identity: 45 %
  
BlastP bit score: 207
  
Sequence coverage: 51 %
  
E-value: 2e-57
  
  
 NCBI BlastP on this gene

EMR09815

hypothetical protein
  
Accession: EMR09814
  
Location: 291961-294032
  
  
**BlastP hit with Mycgr3G103278\_Mycgr3**
  
Percentage identity: 33 %
  
BlastP bit score: 106
  
Sequence coverage: 73 %
  
E-value: 2e-22
  
  
 NCBI BlastP on this gene

EMR09814

hypothetical protein
  
Accession: EMR09813
  
Location: 289651-291466
  
 NCBI BlastP on this gene

EMR09813

hypothetical protein
  
Accession: EMR09812
  
Location: 288930-289477
  
 NCBI BlastP on this gene

EMR09812

Query: Architecture Search FASTA input

KB446542 : Dothistroma septosporum NZE10 unplaced genomic scaffold DOTSEscaffold\_8    Total score: 4.0     Cumulative Blast bit score: 3753

Hit cluster cross-links:

Mycgr3G90785 Mycgr3T
  
Location: 0-1047

Mycgr3G90785\_Mycgr3T

Mycgr3G103262 Mycgr3
  
Location: 1147-1390

Mycgr3G103262\_Mycgr3

Mycgr3G68458 Mycgr3T
  
Location: 1490-3602

Mycgr3G68458\_Mycgr3T

Mycgr3G99145 Mycgr3T
  
Location: 3702-4326

Mycgr3G99145\_Mycgr3T

Mycgr3G103274 Mycgr3
  
Location: 4426-4957

Mycgr3G103274\_Mycgr3

Mycgr3G103264 Mycgr3
  
Location: 5057-5390

Mycgr3G103264\_Mycgr3

Mycgr3G37570 Mycgr3T
  
Location: 5490-6006

Mycgr3G37570\_Mycgr3T

Mycgr3G108094 Mycgr3
  
Location: 6106-10555

Mycgr3G108094\_Mycgr3

Mycgr3G90786 Mycgr3T
  
Location: 10655-12080

Mycgr3G90786\_Mycgr3T

Mycgr3G68429 Mycgr3T
  
Location: 12180-13440

Mycgr3G68429\_Mycgr3T

Mycgr3G68421 Mycgr3T
  
Location: 13540-17086

Mycgr3G68421\_Mycgr3T

Mycgr3G90801 Mycgr3T
  
Location: 17186-18056

Mycgr3G90801\_Mycgr3T

Mycgr3G84646 Mycgr3T
  
Location: 18156-20235

Mycgr3G84646\_Mycgr3T

Mycgr3G68456 Mycgr3T
  
Location: 20335-21970

Mycgr3G68456\_Mycgr3T

Mycgr3G103270 Mycgr3
  
Location: 22070-22355

Mycgr3G103270\_Mycgr3

Mycgr3G90803 Mycgr3T
  
Location: 22455-23019

Mycgr3G90803\_Mycgr3T

Mycgr3G36941 Mycgr3T
  
Location: 23119-24064

Mycgr3G36941\_Mycgr3T

Mycgr3G25746 Mycgr3T
  
Location: 24164-25241

Mycgr3G25746\_Mycgr3T

Mycgr3G90788 Mycgr3T
  
Location: 25341-25803

Mycgr3G90788\_Mycgr3T

Mycgr3G103260 Mycgr3
  
Location: 25903-26635

Mycgr3G103260\_Mycgr3

Mycgr3G84644 Mycgr3T
  
Location: 26735-28457

Mycgr3G84644\_Mycgr3T

Mycgr3G29227 Mycgr3T
  
Location: 28557-28863

Mycgr3G29227\_Mycgr3T

Mycgr3G36271 Mycgr3T
  
Location: 28963-29854

Mycgr3G36271\_Mycgr3T

Mycgr3G68433 Mycgr3T
  
Location: 29954-33041

Mycgr3G68433\_Mycgr3T

Mycgr3G79452 Mycgr3T
  
Location: 33141-33399

Mycgr3G79452\_Mycgr3T

Mycgr3G55345 Mycgr3T
  
Location: 33499-34126

Mycgr3G55345\_Mycgr3T

Mycgr3G103278 Mycgr3
  
Location: 34226-35195

Mycgr3G103278\_Mycgr3

Mycgr3G84654 Mycgr3T
  
Location: 35295-36630

Mycgr3G84654\_Mycgr3T

Mycgr3G108090 Mycgr3
  
Location: 36730-37591

Mycgr3G108090\_Mycgr3

Mycgr3G21922 Mycgr3T
  
Location: 37691-39149

Mycgr3G21922\_Mycgr3T

Mycgr3G99148 Mycgr3T
  
Location: 39249-42819

Mycgr3G99148\_Mycgr3T

hypothetical protein
  
Accession: EME41309
  
Location: 429505-430392
  
 NCBI BlastP on this gene

EME41309

hypothetical protein
  
Accession: EME41308
  
Location: 428548-428958
  
 NCBI BlastP on this gene

EME41308

hypothetical protein
  
Accession: EME41307
  
Location: 425577-426959
  
 NCBI BlastP on this gene

EME41307

hypothetical protein
  
Accession: EME41306
  
Location: 423992-424501
  
 NCBI BlastP on this gene

EME41306

hypothetical protein
  
Accession: EME41305
  
Location: 422848-423696
  
 NCBI BlastP on this gene

EME41305

hypothetical protein
  
Accession: EME41304
  
Location: 419775-421452
  
 NCBI BlastP on this gene

EME41304

hypothetical protein
  
Accession: EME41303
  
Location: 418489-419181
  
 NCBI BlastP on this gene

EME41303

hypothetical protein
  
Accession: EME41302
  
Location: 416345-416914
  
 NCBI BlastP on this gene

EME41302

hypothetical protein
  
Accession: EME41301
  
Location: 411717-415283
  
  
**BlastP hit with Mycgr3G99148\_Mycgr3T**
  
Percentage identity: 77 %
  
BlastP bit score: 1932
  
Sequence coverage: 100 %
  
E-value: 0.0
  
  
 NCBI BlastP on this gene

EME41301

hypothetical protein
  
Accession: EME41300
  
Location: 409872-410108
  
 NCBI BlastP on this gene

EME41300

hypothetical protein
  
Accession: EME41299
  
Location: 409150-409605
  
 NCBI BlastP on this gene

EME41299

hypothetical protein
  
Accession: EME41297
  
Location: 406475-407591
  
  
**BlastP hit with Mycgr3G36271\_Mycgr3T**
  
Percentage identity: 90 %
  
BlastP bit score: 551
  
Sequence coverage: 95 %
  
E-value: 0.0
  
  
 NCBI BlastP on this gene

EME41297

hypothetical protein
  
Accession: EME41296
  
Location: 403896-405887
  
  
**BlastP hit with Mycgr3G84646\_Mycgr3T**
  
Percentage identity: 67 %
  
BlastP bit score: 909
  
Sequence coverage: 99 %
  
E-value: 0.0
  
  
 NCBI BlastP on this gene

EME41296

hypothetical protein
  
Accession: EME41295
  
Location: 402292-403161
  
  
**BlastP hit with Mycgr3G99145\_Mycgr3T**
  
Percentage identity: 94 %
  
BlastP bit score: 362
  
Sequence coverage: 99 %
  
E-value: 1e-124
  
  
 NCBI BlastP on this gene

EME41295

hypothetical protein
  
Accession: EME41294
  
Location: 400341-401651
  
 NCBI BlastP on this gene

EME41294

hypothetical protein
  
Accession: EME41293
  
Location: 398757-399194
  
 NCBI BlastP on this gene

EME41293

hypothetical protein
  
Accession: EME41292
  
Location: 397639-398157
  
 NCBI BlastP on this gene

EME41292

hypothetical protein
  
Accession: EME41291
  
Location: 395606-397453
  
 NCBI BlastP on this gene

EME41291

hypothetical protein
  
Accession: EME41290
  
Location: 393487-395319
  
 NCBI BlastP on this gene

EME41290

hypothetical protein
  
Accession: EME41289
  
Location: 390879-393181
  
 NCBI BlastP on this gene

EME41289

hypothetical protein
  
Accession: EME41288
  
Location: 389953-390141
  
 NCBI BlastP on this gene

EME41288

hypothetical protein
  
Accession: EME41287
  
Location: 385453-389313
  
 NCBI BlastP on this gene

EME41287

carbohydrate-binding module family 14 protein
  
Accession: EME41286
  
Location: 384698-385195
  
 NCBI BlastP on this gene

EME41286

Query: Architecture Search FASTA input

JH767569 : Coniosporium apollinis CBS 100218 chromosome Unknown supercont1.16    Total score: 4.0     Cumulative Blast bit score: 2235

Hit cluster cross-links:

Mycgr3G90785 Mycgr3T
  
Location: 0-1047

Mycgr3G90785\_Mycgr3T

Mycgr3G103262 Mycgr3
  
Location: 1147-1390

Mycgr3G103262\_Mycgr3

Mycgr3G68458 Mycgr3T
  
Location: 1490-3602

Mycgr3G68458\_Mycgr3T

Mycgr3G99145 Mycgr3T
  
Location: 3702-4326

Mycgr3G99145\_Mycgr3T

Mycgr3G103274 Mycgr3
  
Location: 4426-4957

Mycgr3G103274\_Mycgr3

Mycgr3G103264 Mycgr3
  
Location: 5057-5390

Mycgr3G103264\_Mycgr3

Mycgr3G37570 Mycgr3T
  
Location: 5490-6006

Mycgr3G37570\_Mycgr3T

Mycgr3G108094 Mycgr3
  
Location: 6106-10555

Mycgr3G108094\_Mycgr3

Mycgr3G90786 Mycgr3T
  
Location: 10655-12080

Mycgr3G90786\_Mycgr3T

Mycgr3G68429 Mycgr3T
  
Location: 12180-13440

Mycgr3G68429\_Mycgr3T

Mycgr3G68421 Mycgr3T
  
Location: 13540-17086

Mycgr3G68421\_Mycgr3T

Mycgr3G90801 Mycgr3T
  
Location: 17186-18056

Mycgr3G90801\_Mycgr3T

Mycgr3G84646 Mycgr3T
  
Location: 18156-20235

Mycgr3G84646\_Mycgr3T

Mycgr3G68456 Mycgr3T
  
Location: 20335-21970

Mycgr3G68456\_Mycgr3T

Mycgr3G103270 Mycgr3
  
Location: 22070-22355

Mycgr3G103270\_Mycgr3

Mycgr3G90803 Mycgr3T
  
Location: 22455-23019

Mycgr3G90803\_Mycgr3T

Mycgr3G36941 Mycgr3T
  
Location: 23119-24064

Mycgr3G36941\_Mycgr3T

Mycgr3G25746 Mycgr3T
  
Location: 24164-25241

Mycgr3G25746\_Mycgr3T

Mycgr3G90788 Mycgr3T
  
Location: 25341-25803

Mycgr3G90788\_Mycgr3T

Mycgr3G103260 Mycgr3
  
Location: 25903-26635

Mycgr3G103260\_Mycgr3

Mycgr3G84644 Mycgr3T
  
Location: 26735-28457

Mycgr3G84644\_Mycgr3T

Mycgr3G29227 Mycgr3T
  
Location: 28557-28863

Mycgr3G29227\_Mycgr3T

Mycgr3G36271 Mycgr3T
  
Location: 28963-29854

Mycgr3G36271\_Mycgr3T

Mycgr3G68433 Mycgr3T
  
Location: 29954-33041

Mycgr3G68433\_Mycgr3T

Mycgr3G79452 Mycgr3T
  
Location: 33141-33399

Mycgr3G79452\_Mycgr3T

Mycgr3G55345 Mycgr3T
  
Location: 33499-34126

Mycgr3G55345\_Mycgr3T

Mycgr3G103278 Mycgr3
  
Location: 34226-35195

Mycgr3G103278\_Mycgr3

Mycgr3G84654 Mycgr3T
  
Location: 35295-36630

Mycgr3G84654\_Mycgr3T

Mycgr3G108090 Mycgr3
  
Location: 36730-37591

Mycgr3G108090\_Mycgr3

Mycgr3G21922 Mycgr3T
  
Location: 37691-39149

Mycgr3G21922\_Mycgr3T

Mycgr3G99148 Mycgr3T
  
Location: 39249-42819

Mycgr3G99148\_Mycgr3T

hypothetical protein
  
Accession: EON64685
  
Location: 454328-455287
  
 NCBI BlastP on this gene

EON64685

hypothetical protein
  
Accession: EON64684
  
Location: 452518-454016
  
 NCBI BlastP on this gene

EON64684

N-acetylglucosamine-6-phosphate deacetylase
  
Accession: EON64683
  
Location: 450921-452316
  
 NCBI BlastP on this gene

EON64683

beta-N-acetylglucosaminidase
  
Accession: EON64682
  
Location: 447202-450043
  
 NCBI BlastP on this gene

EON64682

hypothetical protein
  
Accession: EON64681
  
Location: 444645-446283
  
  
**BlastP hit with Mycgr3G84654\_Mycgr3T**
  
Percentage identity: 64 %
  
BlastP bit score: 405
  
Sequence coverage: 72 %
  
E-value: 4e-133
  
  
 NCBI BlastP on this gene

EON64681

hypothetical protein
  
Accession: EON64680
  
Location: 441263-442943
  
 NCBI BlastP on this gene

EON64680

hypothetical protein
  
Accession: EON64679
  
Location: 437536-439202
  
 NCBI BlastP on this gene

EON64679

glucosamine-6-phosphate isomerase
  
Accession: EON64678
  
Location: 435033-436622
  
 NCBI BlastP on this gene

EON64678

hypothetical protein
  
Accession: EON64677
  
Location: 432203-434438
  
  
**BlastP hit with Mycgr3G84646\_Mycgr3T**
  
Percentage identity: 45 %
  
BlastP bit score: 560
  
Sequence coverage: 100 %
  
E-value: 0.0
  
  
 NCBI BlastP on this gene

EON64677

GTP-binding protein ypt2
  
Accession: EON64676
  
Location: 430634-431547
  
  
**BlastP hit with Mycgr3G99145\_Mycgr3T**
  
Percentage identity: 89 %
  
BlastP bit score: 318
  
Sequence coverage: 81 %
  
E-value: 5e-107
  
  
 NCBI BlastP on this gene

EON64676

hypothetical protein
  
Accession: EON64675
  
Location: 426708-428282
  
 NCBI BlastP on this gene

EON64675

hypothetical protein
  
Accession: EON64674
  
Location: 422698-424608
  
 NCBI BlastP on this gene

EON64674

hypothetical protein
  
Accession: EON64673
  
Location: 419331-421270
  
 NCBI BlastP on this gene

EON64673

Atypical/ABC1/ABC1-C protein kinase
  
Accession: EON64672
  
Location: 416681-418930
  
  
**BlastP hit with Mycgr3G68458\_Mycgr3T**
  
Percentage identity: 72 %
  
BlastP bit score: 952
  
Sequence coverage: 91 %
  
E-value: 0.0
  
  
 NCBI BlastP on this gene

EON64672

hypothetical protein
  
Accession: EON64671
  
Location: 415036-416445
  
 NCBI BlastP on this gene

EON64671

hypothetical protein
  
Accession: EON64670
  
Location: 413256-414308
  
 NCBI BlastP on this gene

EON64670

hypothetical protein
  
Accession: EON64669
  
Location: 410572-411720
  
 NCBI BlastP on this gene

EON64669

hypothetical protein
  
Accession: EON64668
  
Location: 408737-409298
  
 NCBI BlastP on this gene

EON64668

hypothetical protein
  
Accession: EON64667
  
Location: 407267-408416
  
 NCBI BlastP on this gene

EON64667

Query: Architecture Search FASTA input

EQ962655 : Talaromyces stipitatus ATCC 10500 scf\_1105507295555 genomic scaffold    Total score: 4.0     Cumulative Blast bit score: 1804

Hit cluster cross-links:

Mycgr3G90785 Mycgr3T
  
Location: 0-1047

Mycgr3G90785\_Mycgr3T

Mycgr3G103262 Mycgr3
  
Location: 1147-1390

Mycgr3G103262\_Mycgr3

Mycgr3G68458 Mycgr3T
  
Location: 1490-3602

Mycgr3G68458\_Mycgr3T

Mycgr3G99145 Mycgr3T
  
Location: 3702-4326

Mycgr3G99145\_Mycgr3T

Mycgr3G103274 Mycgr3
  
Location: 4426-4957

Mycgr3G103274\_Mycgr3

Mycgr3G103264 Mycgr3
  
Location: 5057-5390

Mycgr3G103264\_Mycgr3

Mycgr3G37570 Mycgr3T
  
Location: 5490-6006

Mycgr3G37570\_Mycgr3T

Mycgr3G108094 Mycgr3
  
Location: 6106-10555

Mycgr3G108094\_Mycgr3

Mycgr3G90786 Mycgr3T
  
Location: 10655-12080

Mycgr3G90786\_Mycgr3T

Mycgr3G68429 Mycgr3T
  
Location: 12180-13440

Mycgr3G68429\_Mycgr3T

Mycgr3G68421 Mycgr3T
  
Location: 13540-17086

Mycgr3G68421\_Mycgr3T

Mycgr3G90801 Mycgr3T
  
Location: 17186-18056

Mycgr3G90801\_Mycgr3T

Mycgr3G84646 Mycgr3T
  
Location: 18156-20235

Mycgr3G84646\_Mycgr3T

Mycgr3G68456 Mycgr3T
  
Location: 20335-21970

Mycgr3G68456\_Mycgr3T

Mycgr3G103270 Mycgr3
  
Location: 22070-22355

Mycgr3G103270\_Mycgr3

Mycgr3G90803 Mycgr3T
  
Location: 22455-23019

Mycgr3G90803\_Mycgr3T

Mycgr3G36941 Mycgr3T
  
Location: 23119-24064

Mycgr3G36941\_Mycgr3T

Mycgr3G25746 Mycgr3T
  
Location: 24164-25241

Mycgr3G25746\_Mycgr3T

Mycgr3G90788 Mycgr3T
  
Location: 25341-25803

Mycgr3G90788\_Mycgr3T

Mycgr3G103260 Mycgr3
  
Location: 25903-26635

Mycgr3G103260\_Mycgr3

Mycgr3G84644 Mycgr3T
  
Location: 26735-28457

Mycgr3G84644\_Mycgr3T

Mycgr3G29227 Mycgr3T
  
Location: 28557-28863

Mycgr3G29227\_Mycgr3T

Mycgr3G36271 Mycgr3T
  
Location: 28963-29854

Mycgr3G36271\_Mycgr3T

Mycgr3G68433 Mycgr3T
  
Location: 29954-33041

Mycgr3G68433\_Mycgr3T

Mycgr3G79452 Mycgr3T
  
Location: 33141-33399

Mycgr3G79452\_Mycgr3T

Mycgr3G55345 Mycgr3T
  
Location: 33499-34126

Mycgr3G55345\_Mycgr3T

Mycgr3G103278 Mycgr3
  
Location: 34226-35195

Mycgr3G103278\_Mycgr3

Mycgr3G84654 Mycgr3T
  
Location: 35295-36630

Mycgr3G84654\_Mycgr3T

Mycgr3G108090 Mycgr3
  
Location: 36730-37591

Mycgr3G108090\_Mycgr3

Mycgr3G21922 Mycgr3T
  
Location: 37691-39149

Mycgr3G21922\_Mycgr3T

Mycgr3G99148 Mycgr3T
  
Location: 39249-42819

Mycgr3G99148\_Mycgr3T

benzoate 4-monooxygenase cytochrome P450, putative
  
Accession: EED18355
  
Location: 2695865-2697365
  
 NCBI BlastP on this gene

EED18355

GABA permease, putative
  
Accession: EED18354
  
Location: 2693594-2695406
  
 NCBI BlastP on this gene

EED18354

succinyl-CoA synthetase beta subunit, putative
  
Accession: EED18353
  
Location: 2691211-2693002
  
 NCBI BlastP on this gene

EED18353

short chain dehydrogenase/reductase (Ayr1), putative
  
Accession: EED18352
  
Location: 2689586-2690728
  
 NCBI BlastP on this gene

EED18352

xanthine-guanine phosphoribosyl transferase Xpt1, putative
  
Accession: EED18351
  
Location: 2687893-2688722
  
  
**BlastP hit with Mycgr3G55345\_Mycgr3T**
  
Percentage identity: 76 %
  
BlastP bit score: 313
  
Sequence coverage: 95 %
  
E-value: 2e-105
  
  
 NCBI BlastP on this gene

EED18351

conserved hypothetical protein
  
Accession: EED18350
  
Location: 2687089-2687491
  
 NCBI BlastP on this gene

EED18350

GTP binding protein, putative
  
Accession: EED18349
  
Location: 2683901-2686219
  
 NCBI BlastP on this gene

EED18349

conserved hypothetical protein
  
Accession: EED18347
  
Location: 2680568-2682591
  
 NCBI BlastP on this gene

EED18347

60S ribosomal protein L13
  
Accession: EED18346
  
Location: 2679128-2680230
  
 NCBI BlastP on this gene

EED18346

conserved hypothetical protein
  
Accession: EED18345
  
Location: 2677077-2678582
  
  
**BlastP hit with Mycgr3G90786\_Mycgr3T**
  
Percentage identity: 26 %
  
BlastP bit score: 85
  
Sequence coverage: 96 %
  
E-value: 1e-14
  
  
 NCBI BlastP on this gene

EED18345

hypothetical protein
  
Accession: EED18344
  
Location: 2674380-2674665
  
 NCBI BlastP on this gene

EED18344

conserved hypothetical protein
  
Accession: EED18343
  
Location: 2673584-2674193
  
 NCBI BlastP on this gene

EED18343

conserved hypothetical protein
  
Accession: EED18342
  
Location: 2672210-2673161
  
 NCBI BlastP on this gene

EED18342

nucleoside-diphosphate-sugar epimerase family protein
  
Accession: EED18341
  
Location: 2670976-2671935
  
 NCBI BlastP on this gene

EED18341

conserved hypothetical protein
  
Accession: EED18340
  
Location: 2668154-2669519
  
 NCBI BlastP on this gene

EED18340

conserved leucine-rich repeat protein
  
Accession: EED18339
  
Location: 2664385-2667402
  
  
**BlastP hit with Mycgr3G68433\_Mycgr3T**
  
Percentage identity: 37 %
  
BlastP bit score: 479
  
Sequence coverage: 99 %
  
E-value: 4e-148
  
  
 NCBI BlastP on this gene

EED18339

conserved hypothetical protein
  
Accession: EED18338
  
Location: 2663191-2664285
  
 NCBI BlastP on this gene

EED18338

actin family protein
  
Accession: EED18337
  
Location: 2660918-2662484
  
 NCBI BlastP on this gene

EED18337

3-hydroxybutyryl-CoA dehydrogenase, putative
  
Accession: EED18336
  
Location: 2659535-2660622
  
 NCBI BlastP on this gene

EED18336

DNA repair protein (Tof1), putative
  
Accession: EED18335
  
Location: 2655541-2659192
  
  
**BlastP hit with Mycgr3G68421\_Mycgr3T**
  
Percentage identity: 46 %
  
BlastP bit score: 927
  
Sequence coverage: 91 %
  
E-value: 0.0
  
  
 NCBI BlastP on this gene

EED18335

phenazine biosynthesis-like protein, putative
  
Accession: EED18334
  
Location: 2654181-2655387
  
 NCBI BlastP on this gene

EED18334

TFIIH and nucleotide excision repair factor 3 complexes subunit (Tfb2), putative
  
Accession: EED18333
  
Location: 2652501-2654059
  
 NCBI BlastP on this gene

EED18333

PaaI thioesterase family protein, putative
  
Accession: EED18331
  
Location: 2651145-2652195
  
 NCBI BlastP on this gene

EED18331

DEAD helicases superfamily protein (Aquarius), putative
  
Accession: EED18330
  
Location: 2646359-2650750
  
 NCBI BlastP on this gene

EED18330

Query: Architecture Search FASTA input

GG700653 : Trichophyton rubrum CBS 118892 genomic scaffold supercont2.6    Total score: 4.0     Cumulative Blast bit score: 1801

Hit cluster cross-links:

Mycgr3G90785 Mycgr3T
  
Location: 0-1047

Mycgr3G90785\_Mycgr3T

Mycgr3G103262 Mycgr3
  
Location: 1147-1390

Mycgr3G103262\_Mycgr3

Mycgr3G68458 Mycgr3T
  
Location: 1490-3602

Mycgr3G68458\_Mycgr3T

Mycgr3G99145 Mycgr3T
  
Location: 3702-4326

Mycgr3G99145\_Mycgr3T

Mycgr3G103274 Mycgr3
  
Location: 4426-4957

Mycgr3G103274\_Mycgr3

Mycgr3G103264 Mycgr3
  
Location: 5057-5390

Mycgr3G103264\_Mycgr3

Mycgr3G37570 Mycgr3T
  
Location: 5490-6006

Mycgr3G37570\_Mycgr3T

Mycgr3G108094 Mycgr3
  
Location: 6106-10555

Mycgr3G108094\_Mycgr3

Mycgr3G90786 Mycgr3T
  
Location: 10655-12080

Mycgr3G90786\_Mycgr3T

Mycgr3G68429 Mycgr3T
  
Location: 12180-13440

Mycgr3G68429\_Mycgr3T

Mycgr3G68421 Mycgr3T
  
Location: 13540-17086

Mycgr3G68421\_Mycgr3T

Mycgr3G90801 Mycgr3T
  
Location: 17186-18056

Mycgr3G90801\_Mycgr3T

Mycgr3G84646 Mycgr3T
  
Location: 18156-20235

Mycgr3G84646\_Mycgr3T

Mycgr3G68456 Mycgr3T
  
Location: 20335-21970

Mycgr3G68456\_Mycgr3T

Mycgr3G103270 Mycgr3
  
Location: 22070-22355

Mycgr3G103270\_Mycgr3

Mycgr3G90803 Mycgr3T
  
Location: 22455-23019

Mycgr3G90803\_Mycgr3T

Mycgr3G36941 Mycgr3T
  
Location: 23119-24064

Mycgr3G36941\_Mycgr3T

Mycgr3G25746 Mycgr3T
  
Location: 24164-25241

Mycgr3G25746\_Mycgr3T

Mycgr3G90788 Mycgr3T
  
Location: 25341-25803

Mycgr3G90788\_Mycgr3T

Mycgr3G103260 Mycgr3
  
Location: 25903-26635

Mycgr3G103260\_Mycgr3

Mycgr3G84644 Mycgr3T
  
Location: 26735-28457

Mycgr3G84644\_Mycgr3T

Mycgr3G29227 Mycgr3T
  
Location: 28557-28863

Mycgr3G29227\_Mycgr3T

Mycgr3G36271 Mycgr3T
  
Location: 28963-29854

Mycgr3G36271\_Mycgr3T

Mycgr3G68433 Mycgr3T
  
Location: 29954-33041

Mycgr3G68433\_Mycgr3T

Mycgr3G79452 Mycgr3T
  
Location: 33141-33399

Mycgr3G79452\_Mycgr3T

Mycgr3G55345 Mycgr3T
  
Location: 33499-34126

Mycgr3G55345\_Mycgr3T

Mycgr3G103278 Mycgr3
  
Location: 34226-35195

Mycgr3G103278\_Mycgr3

Mycgr3G84654 Mycgr3T
  
Location: 35295-36630

Mycgr3G84654\_Mycgr3T

Mycgr3G108090 Mycgr3
  
Location: 36730-37591

Mycgr3G108090\_Mycgr3

Mycgr3G21922 Mycgr3T
  
Location: 37691-39149

Mycgr3G21922\_Mycgr3T

Mycgr3G99148 Mycgr3T
  
Location: 39249-42819

Mycgr3G99148\_Mycgr3T

hypothetical protein
  
Accession: EGD89452
  
Location: 764875-765729
  
 NCBI BlastP on this gene

EGD89452

hypothetical protein
  
Accession: EGD89451
  
Location: 762580-763388
  
 NCBI BlastP on this gene

EGD89451

hypothetical protein
  
Accession: EGD89450
  
Location: 761182-761983
  
 NCBI BlastP on this gene

EGD89450

hypothetical protein
  
Accession: EGD89449
  
Location: 760043-760524
  
 NCBI BlastP on this gene

EGD89449

xanthine phosphoribosyltransferase
  
Accession: EGD89448
  
Location: 756499-757550
  
  
**BlastP hit with Mycgr3G55345\_Mycgr3T**
  
Percentage identity: 78 %
  
BlastP bit score: 318
  
Sequence coverage: 96 %
  
E-value: 4e-107
  
  
 NCBI BlastP on this gene

EGD89448

dolichyl-phosphate mannosyltransferase polypeptide 3
  
Accession: EGD89447
  
Location: 755534-755982
  
 NCBI BlastP on this gene

EGD89447

hypothetical protein
  
Accession: EGD89446
  
Location: 751570-753874
  
 NCBI BlastP on this gene

EGD89446

hypothetical protein
  
Accession: EGD89445
  
Location: 750389-751228
  
 NCBI BlastP on this gene

EGD89445

hypothetical protein
  
Accession: EGD89444
  
Location: 748078-750047
  
 NCBI BlastP on this gene

EGD89444

hypothetical protein
  
Accession: EGD89443
  
Location: 746451-747575
  
 NCBI BlastP on this gene

EGD89443

hypothetical protein
  
Accession: EGD89442
  
Location: 744152-745702
  
  
**BlastP hit with Mycgr3G90786\_Mycgr3T**
  
Percentage identity: 28 %
  
BlastP bit score: 74
  
Sequence coverage: 91 %
  
E-value: 3e-11
  
  
 NCBI BlastP on this gene

EGD89442

hypothetical protein
  
Accession: EGD89441
  
Location: 740036-743068
  
  
**BlastP hit with Mycgr3G68433\_Mycgr3T**
  
Percentage identity: 38 %
  
BlastP bit score: 472
  
Sequence coverage: 87 %
  
E-value: 1e-145
  
  
 NCBI BlastP on this gene

EGD89441

hypothetical protein
  
Accession: EGD89440
  
Location: 738686-739816
  
 NCBI BlastP on this gene

EGD89440

actin
  
Accession: EGD89439
  
Location: 736803-738398
  
 NCBI BlastP on this gene

EGD89439

hypothetical protein
  
Accession: EGD89438
  
Location: 734492-735095
  
 NCBI BlastP on this gene

EGD89438

3-hydroxybutyryl CoA dehydrogenase
  
Accession: EGD89437
  
Location: 731263-732371
  
 NCBI BlastP on this gene

EGD89437

topoisomerase 1-associated factor 1
  
Accession: EGD89436
  
Location: 727160-730848
  
  
**BlastP hit with Mycgr3G68421\_Mycgr3T**
  
Percentage identity: 44 %
  
BlastP bit score: 937
  
Sequence coverage: 102 %
  
E-value: 0.0
  
  
 NCBI BlastP on this gene

EGD89436

hypothetical protein
  
Accession: EGD89435
  
Location: 725916-726905
  
 NCBI BlastP on this gene

EGD89435

hypothetical protein
  
Accession: EGD89434
  
Location: 723993-725512
  
 NCBI BlastP on this gene

EGD89434

TFIIH and nucleotide excision repair factor 3 complexes subunit Tfb2
  
Accession: EGD89433
  
Location: 722031-723599
  
 NCBI BlastP on this gene

EGD89433

hypothetical protein
  
Accession: EGD89432
  
Location: 719216-721266
  
 NCBI BlastP on this gene

EGD89432

Query: Architecture Search FASTA input

GG698517 : Trichophyton tonsurans CBS 112818 genomic scaffold supercont1.41    Total score: 4.0     Cumulative Blast bit score: 1801

Hit cluster cross-links:

Mycgr3G90785 Mycgr3T
  
Location: 0-1047

Mycgr3G90785\_Mycgr3T

Mycgr3G103262 Mycgr3
  
Location: 1147-1390

Mycgr3G103262\_Mycgr3

Mycgr3G68458 Mycgr3T
  
Location: 1490-3602

Mycgr3G68458\_Mycgr3T

Mycgr3G99145 Mycgr3T
  
Location: 3702-4326

Mycgr3G99145\_Mycgr3T

Mycgr3G103274 Mycgr3
  
Location: 4426-4957

Mycgr3G103274\_Mycgr3

Mycgr3G103264 Mycgr3
  
Location: 5057-5390

Mycgr3G103264\_Mycgr3

Mycgr3G37570 Mycgr3T
  
Location: 5490-6006

Mycgr3G37570\_Mycgr3T

Mycgr3G108094 Mycgr3
  
Location: 6106-10555

Mycgr3G108094\_Mycgr3

Mycgr3G90786 Mycgr3T
  
Location: 10655-12080

Mycgr3G90786\_Mycgr3T

Mycgr3G68429 Mycgr3T
  
Location: 12180-13440

Mycgr3G68429\_Mycgr3T

Mycgr3G68421 Mycgr3T
  
Location: 13540-17086

Mycgr3G68421\_Mycgr3T

Mycgr3G90801 Mycgr3T
  
Location: 17186-18056

Mycgr3G90801\_Mycgr3T

Mycgr3G84646 Mycgr3T
  
Location: 18156-20235

Mycgr3G84646\_Mycgr3T

Mycgr3G68456 Mycgr3T
  
Location: 20335-21970

Mycgr3G68456\_Mycgr3T

Mycgr3G103270 Mycgr3
  
Location: 22070-22355

Mycgr3G103270\_Mycgr3

Mycgr3G90803 Mycgr3T
  
Location: 22455-23019

Mycgr3G90803\_Mycgr3T

Mycgr3G36941 Mycgr3T
  
Location: 23119-24064

Mycgr3G36941\_Mycgr3T

Mycgr3G25746 Mycgr3T
  
Location: 24164-25241

Mycgr3G25746\_Mycgr3T

Mycgr3G90788 Mycgr3T
  
Location: 25341-25803

Mycgr3G90788\_Mycgr3T

Mycgr3G103260 Mycgr3
  
Location: 25903-26635

Mycgr3G103260\_Mycgr3

Mycgr3G84644 Mycgr3T
  
Location: 26735-28457

Mycgr3G84644\_Mycgr3T

Mycgr3G29227 Mycgr3T
  
Location: 28557-28863

Mycgr3G29227\_Mycgr3T

Mycgr3G36271 Mycgr3T
  
Location: 28963-29854

Mycgr3G36271\_Mycgr3T

Mycgr3G68433 Mycgr3T
  
Location: 29954-33041

Mycgr3G68433\_Mycgr3T

Mycgr3G79452 Mycgr3T
  
Location: 33141-33399

Mycgr3G79452\_Mycgr3T

Mycgr3G55345 Mycgr3T
  
Location: 33499-34126

Mycgr3G55345\_Mycgr3T

Mycgr3G103278 Mycgr3
  
Location: 34226-35195

Mycgr3G103278\_Mycgr3

Mycgr3G84654 Mycgr3T
  
Location: 35295-36630

Mycgr3G84654\_Mycgr3T

Mycgr3G108090 Mycgr3
  
Location: 36730-37591

Mycgr3G108090\_Mycgr3

Mycgr3G21922 Mycgr3T
  
Location: 37691-39149

Mycgr3G21922\_Mycgr3T

Mycgr3G99148 Mycgr3T
  
Location: 39249-42819

Mycgr3G99148\_Mycgr3T

hypothetical protein
  
Accession: EGD98996
  
Location: 42818-44907
  
 NCBI BlastP on this gene

EGD98996

hypothetical protein
  
Accession: EGD98995
  
Location: 41820-42185
  
 NCBI BlastP on this gene

EGD98995

hypothetical protein
  
Accession: EGD98994
  
Location: 38016-38613
  
 NCBI BlastP on this gene

EGD98994

hypoxanthine guanine phosphoribosyltransferase
  
Accession: EGD98993
  
Location: 36471-37511
  
  
**BlastP hit with Mycgr3G55345\_Mycgr3T**
  
Percentage identity: 78 %
  
BlastP bit score: 318
  
Sequence coverage: 96 %
  
E-value: 2e-107
  
  
 NCBI BlastP on this gene

EGD98993

hypothetical protein
  
Accession: EGD98992
  
Location: 35488-35936
  
 NCBI BlastP on this gene

EGD98992

hypothetical protein
  
Accession: EGD98991
  
Location: 31496-33812
  
 NCBI BlastP on this gene

EGD98991

hypothetical protein
  
Accession: EGD98990
  
Location: 30297-31163
  
 NCBI BlastP on this gene

EGD98990

hypothetical protein
  
Accession: EGD98989
  
Location: 27990-29959
  
 NCBI BlastP on this gene

EGD98989

60S ribosomal protein L13
  
Accession: EGD98988
  
Location: 26337-27469
  
 NCBI BlastP on this gene

EGD98988

hypothetical protein
  
Accession: EGD98987
  
Location: 24039-25589
  
  
**BlastP hit with Mycgr3G90786\_Mycgr3T**
  
Percentage identity: 27 %
  
BlastP bit score: 64
  
Sequence coverage: 81 %
  
E-value: 4e-08
  
  
 NCBI BlastP on this gene

EGD98987

hypothetical protein
  
Accession: EGD98986
  
Location: 19903-22938
  
  
**BlastP hit with Mycgr3G68433\_Mycgr3T**
  
Percentage identity: 38 %
  
BlastP bit score: 491
  
Sequence coverage: 88 %
  
E-value: 9e-153
  
  
 NCBI BlastP on this gene

EGD98986

hypothetical protein
  
Accession: EGD98985
  
Location: 18551-19690
  
 NCBI BlastP on this gene

EGD98985

actin
  
Accession: EGD98984
  
Location: 16644-18239
  
 NCBI BlastP on this gene

EGD98984

hypothetical protein
  
Accession: EGD98983
  
Location: 15907-16283
  
 NCBI BlastP on this gene

EGD98983

hypothetical protein
  
Accession: EGD98982
  
Location: 14331-14927
  
 NCBI BlastP on this gene

EGD98982

3-hydroxybutyryl CoA dehydrogenase
  
Accession: EGD98981
  
Location: 11040-12150
  
 NCBI BlastP on this gene

EGD98981

topoisomerase 1-associated factor 1
  
Accession: EGD98980
  
Location: 6993-10670
  
  
**BlastP hit with Mycgr3G68421\_Mycgr3T**
  
Percentage identity: 44 %
  
BlastP bit score: 928
  
Sequence coverage: 101 %
  
E-value: 0.0
  
  
 NCBI BlastP on this gene

EGD98980

hypothetical protein
  
Accession: EGD98979
  
Location: 5762-6751
  
 NCBI BlastP on this gene

EGD98979

hypothetical protein
  
Accession: EGD98978
  
Location: 3881-5382
  
 NCBI BlastP on this gene

EGD98978

TFIIH and nucleotide excision repair factor 3 complexes subunit Tfb2
  
Accession: EGD98977
  
Location: 1897-3481
  
 NCBI BlastP on this gene

EGD98977

hypothetical protein
  
Accession: EGD98976
  
Location: 82-1147
  
 NCBI BlastP on this gene

EGD98976

Query: Architecture Search FASTA input

DS995901 : Penicillium marneffei ATCC 18224 scf\_1105668340960 genomic scaffold    Total score: 4.0     Cumulative Blast bit score: 1784

Hit cluster cross-links:

Mycgr3G90785 Mycgr3T
  
Location: 0-1047

Mycgr3G90785\_Mycgr3T

Mycgr3G103262 Mycgr3
  
Location: 1147-1390

Mycgr3G103262\_Mycgr3

Mycgr3G68458 Mycgr3T
  
Location: 1490-3602

Mycgr3G68458\_Mycgr3T

Mycgr3G99145 Mycgr3T
  
Location: 3702-4326

Mycgr3G99145\_Mycgr3T

Mycgr3G103274 Mycgr3
  
Location: 4426-4957

Mycgr3G103274\_Mycgr3

Mycgr3G103264 Mycgr3
  
Location: 5057-5390

Mycgr3G103264\_Mycgr3

Mycgr3G37570 Mycgr3T
  
Location: 5490-6006

Mycgr3G37570\_Mycgr3T

Mycgr3G108094 Mycgr3
  
Location: 6106-10555

Mycgr3G108094\_Mycgr3

Mycgr3G90786 Mycgr3T
  
Location: 10655-12080

Mycgr3G90786\_Mycgr3T

Mycgr3G68429 Mycgr3T
  
Location: 12180-13440

Mycgr3G68429\_Mycgr3T

Mycgr3G68421 Mycgr3T
  
Location: 13540-17086

Mycgr3G68421\_Mycgr3T

Mycgr3G90801 Mycgr3T
  
Location: 17186-18056

Mycgr3G90801\_Mycgr3T

Mycgr3G84646 Mycgr3T
  
Location: 18156-20235

Mycgr3G84646\_Mycgr3T

Mycgr3G68456 Mycgr3T
  
Location: 20335-21970

Mycgr3G68456\_Mycgr3T

Mycgr3G103270 Mycgr3
  
Location: 22070-22355

Mycgr3G103270\_Mycgr3

Mycgr3G90803 Mycgr3T
  
Location: 22455-23019

Mycgr3G90803\_Mycgr3T

Mycgr3G36941 Mycgr3T
  
Location: 23119-24064

Mycgr3G36941\_Mycgr3T

Mycgr3G25746 Mycgr3T
  
Location: 24164-25241

Mycgr3G25746\_Mycgr3T

Mycgr3G90788 Mycgr3T
  
Location: 25341-25803

Mycgr3G90788\_Mycgr3T

Mycgr3G103260 Mycgr3
  
Location: 25903-26635

Mycgr3G103260\_Mycgr3

Mycgr3G84644 Mycgr3T
  
Location: 26735-28457

Mycgr3G84644\_Mycgr3T

Mycgr3G29227 Mycgr3T
  
Location: 28557-28863

Mycgr3G29227\_Mycgr3T

Mycgr3G36271 Mycgr3T
  
Location: 28963-29854

Mycgr3G36271\_Mycgr3T

Mycgr3G68433 Mycgr3T
  
Location: 29954-33041

Mycgr3G68433\_Mycgr3T

Mycgr3G79452 Mycgr3T
  
Location: 33141-33399

Mycgr3G79452\_Mycgr3T

Mycgr3G55345 Mycgr3T
  
Location: 33499-34126

Mycgr3G55345\_Mycgr3T

Mycgr3G103278 Mycgr3
  
Location: 34226-35195

Mycgr3G103278\_Mycgr3

Mycgr3G84654 Mycgr3T
  
Location: 35295-36630

Mycgr3G84654\_Mycgr3T

Mycgr3G108090 Mycgr3
  
Location: 36730-37591

Mycgr3G108090\_Mycgr3

Mycgr3G21922 Mycgr3T
  
Location: 37691-39149

Mycgr3G21922\_Mycgr3T

Mycgr3G99148 Mycgr3T
  
Location: 39249-42819

Mycgr3G99148\_Mycgr3T

glutamyl-tRNA(gln) amidotransferase subunit A, putative
  
Accession: EEA24592
  
Location: 2525170-2526954
  
 NCBI BlastP on this gene

EEA24592

benzoate 4-monooxygenase cytochrome P450, putative
  
Accession: EEA24591
  
Location: 2523545-2525047
  
 NCBI BlastP on this gene

EEA24591

GABA permease, putative
  
Accession: EEA24590
  
Location: 2521044-2522871
  
 NCBI BlastP on this gene

EEA24590

succinyl-CoA synthetase beta subunit, putative
  
Accession: EEA24589
  
Location: 2518728-2520510
  
 NCBI BlastP on this gene

EEA24589

short chain dehydrogenase/reductase (Ayr1), putative
  
Accession: EEA24588
  
Location: 2517061-2518233
  
 NCBI BlastP on this gene

EEA24588

xanthine-guanine phosphoribosyl transferase Xpt1, putative
  
Accession: EEA24587
  
Location: 2515226-2516059
  
  
**BlastP hit with Mycgr3G55345\_Mycgr3T**
  
Percentage identity: 77 %
  
BlastP bit score: 309
  
Sequence coverage: 95 %
  
E-value: 1e-103
  
  
 NCBI BlastP on this gene

EEA24587

conserved hypothetical protein
  
Accession: EEA24586
  
Location: 2514478-2514813
  
 NCBI BlastP on this gene

EEA24586

conserved hypothetical protein
  
Accession: EEA24585
  
Location: 2514381-2514813
  
 NCBI BlastP on this gene

EEA24585

GTP binding protein, putative
  
Accession: EEA24584
  
Location: 2511061-2513428
  
 NCBI BlastP on this gene

EEA24584

conserved hypothetical protein
  
Accession: EEA24582
  
Location: 2507753-2509746
  
 NCBI BlastP on this gene

EEA24582

60S ribosomal protein L13
  
Accession: EEA24581
  
Location: 2506170-2507350
  
 NCBI BlastP on this gene

EEA24581

conserved hypothetical protein
  
Accession: EEA24580
  
Location: 2504060-2505586
  
  
**BlastP hit with Mycgr3G90786\_Mycgr3T**
  
Percentage identity: 26 %
  
BlastP bit score: 79
  
Sequence coverage: 102 %
  
E-value: 8e-13
  
  
 NCBI BlastP on this gene

EEA24580

conserved leucine-rich repeat protein
  
Accession: EEA24579
  
Location: 2499185-2502202
  
  
**BlastP hit with Mycgr3G68433\_Mycgr3T**
  
Percentage identity: 37 %
  
BlastP bit score: 470
  
Sequence coverage: 87 %
  
E-value: 3e-145
  
  
 NCBI BlastP on this gene

EEA24579

conserved hypothetical protein
  
Accession: EEA24578
  
Location: 2497917-2499041
  
 NCBI BlastP on this gene

EEA24578

actin family protein
  
Accession: EEA24577
  
Location: 2495241-2496818
  
 NCBI BlastP on this gene

EEA24577

3-hydroxybutyryl-CoA dehydrogenase, putative
  
Accession: EEA24576
  
Location: 2493862-2494949
  
 NCBI BlastP on this gene

EEA24576

DNA repair protein (Tof1), putative
  
Accession: EEA24575
  
Location: 2489756-2493436
  
  
**BlastP hit with Mycgr3G68421\_Mycgr3T**
  
Percentage identity: 43 %
  
BlastP bit score: 926
  
Sequence coverage: 102 %
  
E-value: 0.0
  
  
 NCBI BlastP on this gene

EEA24575

phenazine biosynthesis-like protein, putative
  
Accession: EEA24574
  
Location: 2488371-2489342
  
 NCBI BlastP on this gene

EEA24574

TFIIH and nucleotide excision repair factor 3 complexes subunit (Tfb2), putative
  
Accession: EEA24573
  
Location: 2486867-2488235
  
 NCBI BlastP on this gene

EEA24573

PaaI thioesterase family protein, putative
  
Accession: EEA24571
  
Location: 2485308-2486351
  
 NCBI BlastP on this gene

EEA24571

DEAD helicases superfamily protein (Aquarius), putative
  
Accession: EEA24570
  
Location: 2480643-2485025
  
 NCBI BlastP on this gene

EEA24570

THO complex subunit Tho1, putative
  
Accession: EEA24569
  
Location: 2477955-2480238
  
 NCBI BlastP on this gene

EEA24569

Query: Architecture Search FASTA input

DS995702 : Microsporum canis CBS 113480 supercont1.2 genomic scaffold    Total score: 4.0     Cumulative Blast bit score: 1773

Hit cluster cross-links:

Mycgr3G90785 Mycgr3T
  
Location: 0-1047

Mycgr3G90785\_Mycgr3T

Mycgr3G103262 Mycgr3
  
Location: 1147-1390

Mycgr3G103262\_Mycgr3

Mycgr3G68458 Mycgr3T
  
Location: 1490-3602

Mycgr3G68458\_Mycgr3T

Mycgr3G99145 Mycgr3T
  
Location: 3702-4326

Mycgr3G99145\_Mycgr3T

Mycgr3G103274 Mycgr3
  
Location: 4426-4957

Mycgr3G103274\_Mycgr3

Mycgr3G103264 Mycgr3
  
Location: 5057-5390

Mycgr3G103264\_Mycgr3

Mycgr3G37570 Mycgr3T
  
Location: 5490-6006

Mycgr3G37570\_Mycgr3T

Mycgr3G108094 Mycgr3
  
Location: 6106-10555

Mycgr3G108094\_Mycgr3

Mycgr3G90786 Mycgr3T
  
Location: 10655-12080

Mycgr3G90786\_Mycgr3T

Mycgr3G68429 Mycgr3T
  
Location: 12180-13440

Mycgr3G68429\_Mycgr3T

Mycgr3G68421 Mycgr3T
  
Location: 13540-17086

Mycgr3G68421\_Mycgr3T

Mycgr3G90801 Mycgr3T
  
Location: 17186-18056

Mycgr3G90801\_Mycgr3T

Mycgr3G84646 Mycgr3T
  
Location: 18156-20235

Mycgr3G84646\_Mycgr3T

Mycgr3G68456 Mycgr3T
  
Location: 20335-21970

Mycgr3G68456\_Mycgr3T

Mycgr3G103270 Mycgr3
  
Location: 22070-22355

Mycgr3G103270\_Mycgr3

Mycgr3G90803 Mycgr3T
  
Location: 22455-23019

Mycgr3G90803\_Mycgr3T

Mycgr3G36941 Mycgr3T
  
Location: 23119-24064

Mycgr3G36941\_Mycgr3T

Mycgr3G25746 Mycgr3T
  
Location: 24164-25241

Mycgr3G25746\_Mycgr3T

Mycgr3G90788 Mycgr3T
  
Location: 25341-25803

Mycgr3G90788\_Mycgr3T

Mycgr3G103260 Mycgr3
  
Location: 25903-26635

Mycgr3G103260\_Mycgr3

Mycgr3G84644 Mycgr3T
  
Location: 26735-28457

Mycgr3G84644\_Mycgr3T

Mycgr3G29227 Mycgr3T
  
Location: 28557-28863

Mycgr3G29227\_Mycgr3T

Mycgr3G36271 Mycgr3T
  
Location: 28963-29854

Mycgr3G36271\_Mycgr3T

Mycgr3G68433 Mycgr3T
  
Location: 29954-33041

Mycgr3G68433\_Mycgr3T

Mycgr3G79452 Mycgr3T
  
Location: 33141-33399

Mycgr3G79452\_Mycgr3T

Mycgr3G55345 Mycgr3T
  
Location: 33499-34126

Mycgr3G55345\_Mycgr3T

Mycgr3G103278 Mycgr3
  
Location: 34226-35195

Mycgr3G103278\_Mycgr3

Mycgr3G84654 Mycgr3T
  
Location: 35295-36630

Mycgr3G84654\_Mycgr3T

Mycgr3G108090 Mycgr3
  
Location: 36730-37591

Mycgr3G108090\_Mycgr3

Mycgr3G21922 Mycgr3T
  
Location: 37691-39149

Mycgr3G21922\_Mycgr3T

Mycgr3G99148 Mycgr3T
  
Location: 39249-42819

Mycgr3G99148\_Mycgr3T

predicted protein
  
Accession: EEQ29514
  
Location: 2343140-2344126
  
 NCBI BlastP on this gene

EEQ29514

xanthine phosphoribosyltransferase 1
  
Accession: EEQ29513
  
Location: 2341693-2342706
  
  
**BlastP hit with Mycgr3G55345\_Mycgr3T**
  
Percentage identity: 76 %
  
BlastP bit score: 316
  
Sequence coverage: 98 %
  
E-value: 1e-106
  
  
 NCBI BlastP on this gene

EEQ29513

conserved hypothetical protein
  
Accession: EEQ29512
  
Location: 2340632-2341108
  
 NCBI BlastP on this gene

EEQ29512

predicted protein
  
Accession: EEQ29511
  
Location: 2338554-2340288
  
 NCBI BlastP on this gene

EEQ29511

predicted protein
  
Accession: EEQ29510
  
Location: 2336205-2337335
  
 NCBI BlastP on this gene

EEQ29510

predicted protein
  
Accession: EEQ29509
  
Location: 2334650-2335993
  
 NCBI BlastP on this gene

EEQ29509

conserved hypothetical protein
  
Accession: EEQ29508
  
Location: 2330385-2332733
  
 NCBI BlastP on this gene

EEQ29508

conserved hypothetical protein
  
Accession: EEQ29507
  
Location: 2329150-2330071
  
 NCBI BlastP on this gene

EEQ29507

conserved hypothetical protein
  
Accession: EEQ29506
  
Location: 2326954-2328917
  
 NCBI BlastP on this gene

EEQ29506

60S ribosomal protein L13
  
Accession: EEQ29505
  
Location: 2325569-2326446
  
 NCBI BlastP on this gene

EEQ29505

conserved hypothetical protein
  
Accession: EEQ29504
  
Location: 2323124-2324577
  
  
**BlastP hit with Mycgr3G90786\_Mycgr3T**
  
Percentage identity: 27 %
  
BlastP bit score: 85
  
Sequence coverage: 88 %
  
E-value: 1e-14
  
  
 NCBI BlastP on this gene

EEQ29504

leucine-rich repeat-containing protein 40
  
Accession: EEQ29503
  
Location: 2319253-2322279
  
  
**BlastP hit with Mycgr3G68433\_Mycgr3T**
  
Percentage identity: 37 %
  
BlastP bit score: 449
  
Sequence coverage: 88 %
  
E-value: 3e-137
  
  
 NCBI BlastP on this gene

EEQ29503

conserved hypothetical protein
  
Accession: EEQ29502
  
Location: 2317949-2319064
  
 NCBI BlastP on this gene

EEQ29502

alcohol dehydrogenase
  
Accession: EEQ29501
  
Location: 2316445-2317635
  
 NCBI BlastP on this gene

EEQ29501

actin family protein
  
Accession: EEQ29500
  
Location: 2313908-2315679
  
 NCBI BlastP on this gene

EEQ29500

predicted protein
  
Accession: EEQ29499
  
Location: 2312461-2313359
  
 NCBI BlastP on this gene

EEQ29499

predicted protein
  
Accession: EEQ29498
  
Location: 2311692-2312215
  
 NCBI BlastP on this gene

EEQ29498

IBR domain-containing protein
  
Accession: EEQ29497
  
Location: 2308284-2310594
  
 NCBI BlastP on this gene

EEQ29497

fatty acid oxidation complex subunit alpha
  
Accession: EEQ29496
  
Location: 2306763-2307865
  
 NCBI BlastP on this gene

EEQ29496

topoisomerase 1-associated factor 1
  
Accession: EEQ29495
  
Location: 2302711-2306394
  
  
**BlastP hit with Mycgr3G68421\_Mycgr3T**
  
Percentage identity: 43 %
  
BlastP bit score: 923
  
Sequence coverage: 102 %
  
E-value: 0.0
  
  
 NCBI BlastP on this gene

EEQ29495

phenazine biosynthesis protein
  
Accession: EEQ29494
  
Location: 2301448-2302419
  
 NCBI BlastP on this gene

EEQ29494

conserved hypothetical protein
  
Accession: EEQ29493
  
Location: 2299571-2300679
  
 NCBI BlastP on this gene

EEQ29493

RNA polymerase II transcription factor B subunit 2
  
Accession: EEQ29492
  
Location: 2297575-2299145
  
 NCBI BlastP on this gene

EEQ29492

Query: Architecture Search FASTA input

DS995757 : Trichophyton equinum CBS 127.97 supercont1.40 genomic scaffold    Total score: 4.0     Cumulative Blast bit score: 1737

Hit cluster cross-links:

Mycgr3G90785 Mycgr3T
  
Location: 0-1047

Mycgr3G90785\_Mycgr3T

Mycgr3G103262 Mycgr3
  
Location: 1147-1390

Mycgr3G103262\_Mycgr3

Mycgr3G68458 Mycgr3T
  
Location: 1490-3602

Mycgr3G68458\_Mycgr3T

Mycgr3G99145 Mycgr3T
  
Location: 3702-4326

Mycgr3G99145\_Mycgr3T

Mycgr3G103274 Mycgr3
  
Location: 4426-4957

Mycgr3G103274\_Mycgr3

Mycgr3G103264 Mycgr3
  
Location: 5057-5390

Mycgr3G103264\_Mycgr3

Mycgr3G37570 Mycgr3T
  
Location: 5490-6006

Mycgr3G37570\_Mycgr3T

Mycgr3G108094 Mycgr3
  
Location: 6106-10555

Mycgr3G108094\_Mycgr3

Mycgr3G90786 Mycgr3T
  
Location: 10655-12080

Mycgr3G90786\_Mycgr3T

Mycgr3G68429 Mycgr3T
  
Location: 12180-13440

Mycgr3G68429\_Mycgr3T

Mycgr3G68421 Mycgr3T
  
Location: 13540-17086

Mycgr3G68421\_Mycgr3T

Mycgr3G90801 Mycgr3T
  
Location: 17186-18056

Mycgr3G90801\_Mycgr3T

Mycgr3G84646 Mycgr3T
  
Location: 18156-20235

Mycgr3G84646\_Mycgr3T

Mycgr3G68456 Mycgr3T
  
Location: 20335-21970

Mycgr3G68456\_Mycgr3T

Mycgr3G103270 Mycgr3
  
Location: 22070-22355

Mycgr3G103270\_Mycgr3

Mycgr3G90803 Mycgr3T
  
Location: 22455-23019

Mycgr3G90803\_Mycgr3T

Mycgr3G36941 Mycgr3T
  
Location: 23119-24064

Mycgr3G36941\_Mycgr3T

Mycgr3G25746 Mycgr3T
  
Location: 24164-25241

Mycgr3G25746\_Mycgr3T

Mycgr3G90788 Mycgr3T
  
Location: 25341-25803

Mycgr3G90788\_Mycgr3T

Mycgr3G103260 Mycgr3
  
Location: 25903-26635

Mycgr3G103260\_Mycgr3

Mycgr3G84644 Mycgr3T
  
Location: 26735-28457

Mycgr3G84644\_Mycgr3T

Mycgr3G29227 Mycgr3T
  
Location: 28557-28863

Mycgr3G29227\_Mycgr3T

Mycgr3G36271 Mycgr3T
  
Location: 28963-29854

Mycgr3G36271\_Mycgr3T

Mycgr3G68433 Mycgr3T
  
Location: 29954-33041

Mycgr3G68433\_Mycgr3T

Mycgr3G79452 Mycgr3T
  
Location: 33141-33399

Mycgr3G79452\_Mycgr3T

Mycgr3G55345 Mycgr3T
  
Location: 33499-34126

Mycgr3G55345\_Mycgr3T

Mycgr3G103278 Mycgr3
  
Location: 34226-35195

Mycgr3G103278\_Mycgr3

Mycgr3G84654 Mycgr3T
  
Location: 35295-36630

Mycgr3G84654\_Mycgr3T

Mycgr3G108090 Mycgr3
  
Location: 36730-37591

Mycgr3G108090\_Mycgr3

Mycgr3G21922 Mycgr3T
  
Location: 37691-39149

Mycgr3G21922\_Mycgr3T

Mycgr3G99148 Mycgr3T
  
Location: 39249-42819

Mycgr3G99148\_Mycgr3T

hypothetical protein
  
Accession: EGE07216
  
Location: 72205-74109
  
 NCBI BlastP on this gene

EGE07216

hypothetical protein
  
Accession: EGE07217
  
Location: 74745-75276
  
 NCBI BlastP on this gene

EGE07217

hypothetical protein
  
Accession: EGE07218
  
Location: 78320-78917
  
 NCBI BlastP on this gene

EGE07218

xanthine phosphoribosyltransferase
  
Accession: EGE07219
  
Location: 79422-80462
  
  
**BlastP hit with Mycgr3G55345\_Mycgr3T**
  
Percentage identity: 78 %
  
BlastP bit score: 318
  
Sequence coverage: 96 %
  
E-value: 2e-107
  
  
 NCBI BlastP on this gene

EGE07219

hypothetical protein
  
Accession: EGE07220
  
Location: 80997-81445
  
 NCBI BlastP on this gene

EGE07220

GTP binding protein
  
Accession: EGE07221
  
Location: 83121-85437
  
 NCBI BlastP on this gene

EGE07221

hypothetical protein
  
Accession: EGE07222
  
Location: 85770-86636
  
 NCBI BlastP on this gene

EGE07222

hypothetical protein
  
Accession: EGE07223
  
Location: 87285-88950
  
 NCBI BlastP on this gene

EGE07223

60S ribosomal protein L13
  
Accession: EGE07224
  
Location: 89471-90603
  
 NCBI BlastP on this gene

EGE07224

hypothetical protein
  
Accession: EGE07225
  
Location: 91351-92901
  
  
**BlastP hit with Mycgr3G90786\_Mycgr3T**
  
Percentage identity: 27 %
  
BlastP bit score: 65
  
Sequence coverage: 81 %
  
E-value: 4e-08
  
  
 NCBI BlastP on this gene

EGE07225

leucine rich repeat containing protein
  
Accession: EGE07226
  
Location: 94003-97036
  
  
**BlastP hit with Mycgr3G68433\_Mycgr3T**
  
Percentage identity: 40 %
  
BlastP bit score: 417
  
Sequence coverage: 71 %
  
E-value: 2e-125
  
  
 NCBI BlastP on this gene

EGE07226

hypothetical protein
  
Accession: EGE07227
  
Location: 97249-98388
  
 NCBI BlastP on this gene

EGE07227

actin-like protein arp6
  
Accession: EGE07228
  
Location: 98698-100293
  
 NCBI BlastP on this gene

EGE07228

hypothetical protein
  
Accession: EGE07229
  
Location: 100654-101030
  
 NCBI BlastP on this gene

EGE07229

IBR domain containing protein
  
Accession: EGE07230
  
Location: 102010-104349
  
 NCBI BlastP on this gene

EGE07230

fatty acid oxidation complex subunit alpha
  
Accession: EGE07231
  
Location: 104801-105911
  
 NCBI BlastP on this gene

EGE07231

topoisomerase 1-associated factor 1
  
Accession: EGE07232
  
Location: 106280-109959
  
  
**BlastP hit with Mycgr3G68421\_Mycgr3T**
  
Percentage identity: 44 %
  
BlastP bit score: 937
  
Sequence coverage: 101 %
  
E-value: 0.0
  
  
 NCBI BlastP on this gene

EGE07232

phenazine biosynthesis protein
  
Accession: EGE07233
  
Location: 110202-111191
  
 NCBI BlastP on this gene

EGE07233

hypothetical protein
  
Accession: EGE07234
  
Location: 111571-113072
  
 NCBI BlastP on this gene

EGE07234

transcription factor Tfb2
  
Accession: EGE07235
  
Location: 113490-115059
  
 NCBI BlastP on this gene

EGE07235

NCS1 nucleoside transporter
  
Accession: EGE07236
  
Location: 115809-117845
  
 NCBI BlastP on this gene

EGE07236

Query: Architecture Search FASTA input

CH476663 : Ajellomyces capsulatus NAm1 scaffold\_9 genomic scaffold    Total score: 4.0     Cumulative Blast bit score: 1646

Hit cluster cross-links:

Mycgr3G90785 Mycgr3T
  
Location: 0-1047

Mycgr3G90785\_Mycgr3T

Mycgr3G103262 Mycgr3
  
Location: 1147-1390

Mycgr3G103262\_Mycgr3

Mycgr3G68458 Mycgr3T
  
Location: 1490-3602

Mycgr3G68458\_Mycgr3T

Mycgr3G99145 Mycgr3T
  
Location: 3702-4326

Mycgr3G99145\_Mycgr3T

Mycgr3G103274 Mycgr3
  
Location: 4426-4957

Mycgr3G103274\_Mycgr3

Mycgr3G103264 Mycgr3
  
Location: 5057-5390

Mycgr3G103264\_Mycgr3

Mycgr3G37570 Mycgr3T
  
Location: 5490-6006

Mycgr3G37570\_Mycgr3T

Mycgr3G108094 Mycgr3
  
Location: 6106-10555

Mycgr3G108094\_Mycgr3

Mycgr3G90786 Mycgr3T
  
Location: 10655-12080

Mycgr3G90786\_Mycgr3T

Mycgr3G68429 Mycgr3T
  
Location: 12180-13440

Mycgr3G68429\_Mycgr3T

Mycgr3G68421 Mycgr3T
  
Location: 13540-17086

Mycgr3G68421\_Mycgr3T

Mycgr3G90801 Mycgr3T
  
Location: 17186-18056

Mycgr3G90801\_Mycgr3T

Mycgr3G84646 Mycgr3T
  
Location: 18156-20235

Mycgr3G84646\_Mycgr3T

Mycgr3G68456 Mycgr3T
  
Location: 20335-21970

Mycgr3G68456\_Mycgr3T

Mycgr3G103270 Mycgr3
  
Location: 22070-22355

Mycgr3G103270\_Mycgr3

Mycgr3G90803 Mycgr3T
  
Location: 22455-23019

Mycgr3G90803\_Mycgr3T

Mycgr3G36941 Mycgr3T
  
Location: 23119-24064

Mycgr3G36941\_Mycgr3T

Mycgr3G25746 Mycgr3T
  
Location: 24164-25241

Mycgr3G25746\_Mycgr3T

Mycgr3G90788 Mycgr3T
  
Location: 25341-25803

Mycgr3G90788\_Mycgr3T

Mycgr3G103260 Mycgr3
  
Location: 25903-26635

Mycgr3G103260\_Mycgr3

Mycgr3G84644 Mycgr3T
  
Location: 26735-28457

Mycgr3G84644\_Mycgr3T

Mycgr3G29227 Mycgr3T
  
Location: 28557-28863

Mycgr3G29227\_Mycgr3T

Mycgr3G36271 Mycgr3T
  
Location: 28963-29854

Mycgr3G36271\_Mycgr3T

Mycgr3G68433 Mycgr3T
  
Location: 29954-33041

Mycgr3G68433\_Mycgr3T

Mycgr3G79452 Mycgr3T
  
Location: 33141-33399

Mycgr3G79452\_Mycgr3T

Mycgr3G55345 Mycgr3T
  
Location: 33499-34126

Mycgr3G55345\_Mycgr3T

Mycgr3G103278 Mycgr3
  
Location: 34226-35195

Mycgr3G103278\_Mycgr3

Mycgr3G84654 Mycgr3T
  
Location: 35295-36630

Mycgr3G84654\_Mycgr3T

Mycgr3G108090 Mycgr3
  
Location: 36730-37591

Mycgr3G108090\_Mycgr3

Mycgr3G21922 Mycgr3T
  
Location: 37691-39149

Mycgr3G21922\_Mycgr3T

Mycgr3G99148 Mycgr3T
  
Location: 39249-42819

Mycgr3G99148\_Mycgr3T

predicted protein
  
Accession: EDN11239
  
Location: 722221-723746
  
 NCBI BlastP on this gene

EDN11239

conserved hypothetical protein
  
Accession: EDN11240
  
Location: 726067-728377
  
  
**BlastP hit with Mycgr3G68458\_Mycgr3T**
  
Percentage identity: 62 %
  
BlastP bit score: 866
  
Sequence coverage: 102 %
  
E-value: 0.0
  
  
 NCBI BlastP on this gene

EDN11240

predicted protein
  
Accession: EDN11241
  
Location: 729340-731074
  
 NCBI BlastP on this gene

EDN11241

conserved hypothetical protein
  
Accession: EDN11242
  
Location: 731489-732144
  
 NCBI BlastP on this gene

EDN11242

predicted protein
  
Accession: EDN11243
  
Location: 734215-735587
  
 NCBI BlastP on this gene

EDN11243

succinyl-CoA ligase beta-chain, mitochondrial precursor
  
Accession: EDN11244
  
Location: 736654-738591
  
 NCBI BlastP on this gene

EDN11244

predicted protein
  
Accession: EDN11245
  
Location: 738862-739655
  
 NCBI BlastP on this gene

EDN11245

predicted protein
  
Accession: EDN11246
  
Location: 741418-743706
  
 NCBI BlastP on this gene

EDN11246

predicted protein
  
Accession: EDN11247
  
Location: 748815-750137
  
  
**BlastP hit with Mycgr3G55345\_Mycgr3T**
  
Percentage identity: 74 %
  
BlastP bit score: 265
  
Sequence coverage: 82 %
  
E-value: 5e-87
  
  
 NCBI BlastP on this gene

EDN11247

predicted protein
  
Accession: EDN11248
  
Location: 750783-750926
  
 NCBI BlastP on this gene

EDN11248

predicted protein
  
Accession: EDN11249
  
Location: 752765-755270
  
 NCBI BlastP on this gene

EDN11249

conserved hypothetical protein
  
Accession: EDN11250
  
Location: 755834-756847
  
 NCBI BlastP on this gene

EDN11250

conserved hypothetical protein
  
Accession: EDN11251
  
Location: 757705-759358
  
 NCBI BlastP on this gene

EDN11251

predicted protein
  
Accession: EDN11252
  
Location: 760340-761629
  
 NCBI BlastP on this gene

EDN11252

predicted protein
  
Accession: EDN11253
  
Location: 762359-764368
  
  
**BlastP hit with Mycgr3G68433\_Mycgr3T**
  
Percentage identity: 44 %
  
BlastP bit score: 426
  
Sequence coverage: 61 %
  
E-value: 2e-132
  
  
 NCBI BlastP on this gene

EDN11253

predicted protein
  
Accession: EDN11254
  
Location: 766845-768302
  
  
**BlastP hit with Mycgr3G90786\_Mycgr3T**
  
Percentage identity: 27 %
  
BlastP bit score: 89
  
Sequence coverage: 109 %
  
E-value: 9e-16
  
  
 NCBI BlastP on this gene

EDN11254

hypothetical protein
  
Accession: EDN11255
  
Location: 769361-770494
  
 NCBI BlastP on this gene

EDN11255

Query: Architecture Search FASTA input

ACYE01000016 : Trichophyton verrucosum HKI 0517    Total score: 4.0     Cumulative Blast bit score: 1602

Hit cluster cross-links:

Mycgr3G90785 Mycgr3T
  
Location: 0-1047

Mycgr3G90785\_Mycgr3T

Mycgr3G103262 Mycgr3
  
Location: 1147-1390

Mycgr3G103262\_Mycgr3

Mycgr3G68458 Mycgr3T
  
Location: 1490-3602

Mycgr3G68458\_Mycgr3T

Mycgr3G99145 Mycgr3T
  
Location: 3702-4326

Mycgr3G99145\_Mycgr3T

Mycgr3G103274 Mycgr3
  
Location: 4426-4957

Mycgr3G103274\_Mycgr3

Mycgr3G103264 Mycgr3
  
Location: 5057-5390

Mycgr3G103264\_Mycgr3

Mycgr3G37570 Mycgr3T
  
Location: 5490-6006

Mycgr3G37570\_Mycgr3T

Mycgr3G108094 Mycgr3
  
Location: 6106-10555

Mycgr3G108094\_Mycgr3

Mycgr3G90786 Mycgr3T
  
Location: 10655-12080

Mycgr3G90786\_Mycgr3T

Mycgr3G68429 Mycgr3T
  
Location: 12180-13440

Mycgr3G68429\_Mycgr3T

Mycgr3G68421 Mycgr3T
  
Location: 13540-17086

Mycgr3G68421\_Mycgr3T

Mycgr3G90801 Mycgr3T
  
Location: 17186-18056

Mycgr3G90801\_Mycgr3T

Mycgr3G84646 Mycgr3T
  
Location: 18156-20235

Mycgr3G84646\_Mycgr3T

Mycgr3G68456 Mycgr3T
  
Location: 20335-21970

Mycgr3G68456\_Mycgr3T

Mycgr3G103270 Mycgr3
  
Location: 22070-22355

Mycgr3G103270\_Mycgr3

Mycgr3G90803 Mycgr3T
  
Location: 22455-23019

Mycgr3G90803\_Mycgr3T

Mycgr3G36941 Mycgr3T
  
Location: 23119-24064

Mycgr3G36941\_Mycgr3T

Mycgr3G25746 Mycgr3T
  
Location: 24164-25241

Mycgr3G25746\_Mycgr3T

Mycgr3G90788 Mycgr3T
  
Location: 25341-25803

Mycgr3G90788\_Mycgr3T

Mycgr3G103260 Mycgr3
  
Location: 25903-26635

Mycgr3G103260\_Mycgr3

Mycgr3G84644 Mycgr3T
  
Location: 26735-28457

Mycgr3G84644\_Mycgr3T

Mycgr3G29227 Mycgr3T
  
Location: 28557-28863

Mycgr3G29227\_Mycgr3T

Mycgr3G36271 Mycgr3T
  
Location: 28963-29854

Mycgr3G36271\_Mycgr3T

Mycgr3G68433 Mycgr3T
  
Location: 29954-33041

Mycgr3G68433\_Mycgr3T

Mycgr3G79452 Mycgr3T
  
Location: 33141-33399

Mycgr3G79452\_Mycgr3T

Mycgr3G55345 Mycgr3T
  
Location: 33499-34126

Mycgr3G55345\_Mycgr3T

Mycgr3G103278 Mycgr3
  
Location: 34226-35195

Mycgr3G103278\_Mycgr3

Mycgr3G84654 Mycgr3T
  
Location: 35295-36630

Mycgr3G84654\_Mycgr3T

Mycgr3G108090 Mycgr3
  
Location: 36730-37591

Mycgr3G108090\_Mycgr3

Mycgr3G21922 Mycgr3T
  
Location: 37691-39149

Mycgr3G21922\_Mycgr3T

Mycgr3G99148 Mycgr3T
  
Location: 39249-42819

Mycgr3G99148\_Mycgr3T

hypothetical protein
  
Accession: EFE44918
  
Location: 73736-75064
  
 NCBI BlastP on this gene

EFE44918

hypothetical protein
  
Accession: EFE44917
  
Location: 70615-72648
  
 NCBI BlastP on this gene

EFE44917

hypothetical protein
  
Accession: EFE44916
  
Location: 69607-69945
  
 NCBI BlastP on this gene

EFE44916

hypothetical protein
  
Accession: EFE44915
  
Location: 64144-65169
  
  
**BlastP hit with Mycgr3G55345\_Mycgr3T**
  
Percentage identity: 66 %
  
BlastP bit score: 280
  
Sequence coverage: 107 %
  
E-value: 2e-91
  
  
 NCBI BlastP on this gene

EFE44915

hypothetical protein
  
Accession: EFE44914
  
Location: 60994-61446
  
 NCBI BlastP on this gene

EFE44914

GTP binding protein, putative
  
Accession: EFE44913
  
Location: 59221-60776
  
 NCBI BlastP on this gene

EFE44913

hypothetical protein
  
Accession: EFE44912
  
Location: 57553-58794
  
 NCBI BlastP on this gene

EFE44912

conserved hypothetical protein
  
Accession: EFE44911
  
Location: 55627-57259
  
 NCBI BlastP on this gene

EFE44911

hypothetical protein
  
Accession: EFE44910
  
Location: 54199-55121
  
 NCBI BlastP on this gene

EFE44910

conserved hypothetical protein
  
Accession: EFE44909
  
Location: 51709-53259
  
  
**BlastP hit with Mycgr3G90786\_Mycgr3T**
  
Percentage identity: 27 %
  
BlastP bit score: 71
  
Sequence coverage: 90 %
  
E-value: 3e-10
  
  
 NCBI BlastP on this gene

EFE44909

hypothetical protein
  
Accession: EFE44908
  
Location: 50940-51517
  
 NCBI BlastP on this gene

EFE44908

conserved leucine-rich repeat protein
  
Accession: EFE44907
  
Location: 47568-50009
  
  
**BlastP hit with Mycgr3G68433\_Mycgr3T**
  
Percentage identity: 41 %
  
BlastP bit score: 457
  
Sequence coverage: 71 %
  
E-value: 5e-142
  
  
 NCBI BlastP on this gene

EFE44907

conserved hypothetical protein
  
Accession: EFE44906
  
Location: 46215-47342
  
 NCBI BlastP on this gene

EFE44906

hypothetical protein
  
Accession: EFE44905
  
Location: 44682-45931
  
 NCBI BlastP on this gene

EFE44905

hypothetical protein
  
Accession: EFE44904
  
Location: 40336-42673
  
 NCBI BlastP on this gene

EFE44904

3-hydroxyacyl-CoA dehydrogenase, putative
  
Accession: EFE44903
  
Location: 38800-39908
  
 NCBI BlastP on this gene

EFE44903

hypothetical protein
  
Accession: EFE44902
  
Location: 34712-37914
  
  
**BlastP hit with Mycgr3G68421\_Mycgr3T**
  
Percentage identity: 42 %
  
BlastP bit score: 794
  
Sequence coverage: 92 %
  
E-value: 0.0
  
  
 NCBI BlastP on this gene

EFE44902

hypothetical protein
  
Accession: EFE44901
  
Location: 33467-34456
  
 NCBI BlastP on this gene

EFE44901

hypothetical protein
  
Accession: EFE44900
  
Location: 31535-33055
  
 NCBI BlastP on this gene

EFE44900

hypothetical protein
  
Accession: EFE44899
  
Location: 29972-31132
  
 NCBI BlastP on this gene

EFE44899

hypothetical protein
  
Accession: EFE44898
  
Location: 26798-28843
  
 NCBI BlastP on this gene

EFE44898

Query: Architecture Search FASTA input

CH445336 : Phaeosphaeria nodorum SN15 scaffold\_12    Total score: 4.0     Cumulative Blast bit score: 1444

Hit cluster cross-links:

Mycgr3G90785 Mycgr3T
  
Location: 0-1047

Mycgr3G90785\_Mycgr3T

Mycgr3G103262 Mycgr3
  
Location: 1147-1390

Mycgr3G103262\_Mycgr3

Mycgr3G68458 Mycgr3T
  
Location: 1490-3602

Mycgr3G68458\_Mycgr3T

Mycgr3G99145 Mycgr3T
  
Location: 3702-4326

Mycgr3G99145\_Mycgr3T

Mycgr3G103274 Mycgr3
  
Location: 4426-4957

Mycgr3G103274\_Mycgr3

Mycgr3G103264 Mycgr3
  
Location: 5057-5390

Mycgr3G103264\_Mycgr3

Mycgr3G37570 Mycgr3T
  
Location: 5490-6006

Mycgr3G37570\_Mycgr3T

Mycgr3G108094 Mycgr3
  
Location: 6106-10555

Mycgr3G108094\_Mycgr3

Mycgr3G90786 Mycgr3T
  
Location: 10655-12080

Mycgr3G90786\_Mycgr3T

Mycgr3G68429 Mycgr3T
  
Location: 12180-13440

Mycgr3G68429\_Mycgr3T

Mycgr3G68421 Mycgr3T
  
Location: 13540-17086

Mycgr3G68421\_Mycgr3T

Mycgr3G90801 Mycgr3T
  
Location: 17186-18056

Mycgr3G90801\_Mycgr3T

Mycgr3G84646 Mycgr3T
  
Location: 18156-20235

Mycgr3G84646\_Mycgr3T

Mycgr3G68456 Mycgr3T
  
Location: 20335-21970

Mycgr3G68456\_Mycgr3T

Mycgr3G103270 Mycgr3
  
Location: 22070-22355

Mycgr3G103270\_Mycgr3

Mycgr3G90803 Mycgr3T
  
Location: 22455-23019

Mycgr3G90803\_Mycgr3T

Mycgr3G36941 Mycgr3T
  
Location: 23119-24064

Mycgr3G36941\_Mycgr3T

Mycgr3G25746 Mycgr3T
  
Location: 24164-25241

Mycgr3G25746\_Mycgr3T

Mycgr3G90788 Mycgr3T
  
Location: 25341-25803

Mycgr3G90788\_Mycgr3T

Mycgr3G103260 Mycgr3
  
Location: 25903-26635

Mycgr3G103260\_Mycgr3

Mycgr3G84644 Mycgr3T
  
Location: 26735-28457

Mycgr3G84644\_Mycgr3T

Mycgr3G29227 Mycgr3T
  
Location: 28557-28863

Mycgr3G29227\_Mycgr3T

Mycgr3G36271 Mycgr3T
  
Location: 28963-29854

Mycgr3G36271\_Mycgr3T

Mycgr3G68433 Mycgr3T
  
Location: 29954-33041

Mycgr3G68433\_Mycgr3T

Mycgr3G79452 Mycgr3T
  
Location: 33141-33399

Mycgr3G79452\_Mycgr3T

Mycgr3G55345 Mycgr3T
  
Location: 33499-34126

Mycgr3G55345\_Mycgr3T

Mycgr3G103278 Mycgr3
  
Location: 34226-35195

Mycgr3G103278\_Mycgr3

Mycgr3G84654 Mycgr3T
  
Location: 35295-36630

Mycgr3G84654\_Mycgr3T

Mycgr3G108090 Mycgr3
  
Location: 36730-37591

Mycgr3G108090\_Mycgr3

Mycgr3G21922 Mycgr3T
  
Location: 37691-39149

Mycgr3G21922\_Mycgr3T

Mycgr3G99148 Mycgr3T
  
Location: 39249-42819

Mycgr3G99148\_Mycgr3T

hypothetical protein
  
Accession: EAT84692
  
Location: 1115517-1116456
  
 NCBI BlastP on this gene

EAT84692

hypothetical protein
  
Accession: EAT84691
  
Location: 1114403-1114813
  
 NCBI BlastP on this gene

EAT84691

hypothetical protein
  
Accession: EAT84690
  
Location: 1111656-1112692
  
 NCBI BlastP on this gene

EAT84690

hypothetical protein
  
Accession: EAT84689
  
Location: 1106430-1109984
  
 NCBI BlastP on this gene

EAT84689

hypothetical protein
  
Accession: EAT84688
  
Location: 1104256-1105479
  
 NCBI BlastP on this gene

EAT84688

hypothetical protein
  
Accession: EAT84687
  
Location: 1102935-1103222
  
 NCBI BlastP on this gene

EAT84687

hypothetical protein
  
Accession: EAT84686
  
Location: 1102438-1102748
  
 NCBI BlastP on this gene

EAT84686

hypothetical protein
  
Accession: EAT84685
  
Location: 1100690-1102000
  
  
**BlastP hit with Mycgr3G25746\_Mycgr3T**
  
Percentage identity: 55 %
  
BlastP bit score: 390
  
Sequence coverage: 102 %
  
E-value: 2e-130
  
  
 NCBI BlastP on this gene

EAT84685

hypothetical protein
  
Accession: EAT84684
  
Location: 1099289-1100603
  
 NCBI BlastP on this gene

EAT84684

hypothetical protein
  
Accession: EAT84683
  
Location: 1096252-1098351
  
  
**BlastP hit with Mycgr3G103278\_Mycgr3**
  
Percentage identity: 35 %
  
BlastP bit score: 108
  
Sequence coverage: 95 %
  
E-value: 8e-23
  
  
 NCBI BlastP on this gene

EAT84683

hypothetical protein
  
Accession: EAT84682
  
Location: 1093540-1095557
  
  
**BlastP hit with Mycgr3G21922\_Mycgr3T**
  
Percentage identity: 49 %
  
BlastP bit score: 456
  
Sequence coverage: 107 %
  
E-value: 1e-150
  
  
 NCBI BlastP on this gene

EAT84682

hypothetical protein
  
Accession: EAT84681
  
Location: 1091993-1092784
  
 NCBI BlastP on this gene

EAT84681

hypothetical protein
  
Accession: EAT84680
  
Location: 1090178-1091105
  
  
**BlastP hit with Mycgr3G36271\_Mycgr3T**
  
Percentage identity: 79 %
  
BlastP bit score: 490
  
Sequence coverage: 97 %
  
E-value: 3e-172
  
  
 NCBI BlastP on this gene

EAT84680

hypothetical protein
  
Accession: EAT84679
  
Location: 1088187-1089601
  
 NCBI BlastP on this gene

EAT84679

hypothetical protein
  
Accession: EAT84678
  
Location: 1086384-1087474
  
 NCBI BlastP on this gene

EAT84678

hypothetical protein
  
Accession: EAT84677
  
Location: 1083184-1083337
  
 NCBI BlastP on this gene

EAT84677

hypothetical protein
  
Accession: EAT84676
  
Location: 1081843-1083161
  
 NCBI BlastP on this gene

EAT84676

hypothetical protein
  
Accession: EAT84675
  
Location: 1077513-1081212
  
 NCBI BlastP on this gene

EAT84675

hypothetical protein
  
Accession: EAT84674
  
Location: 1075513-1076995
  
 NCBI BlastP on this gene

EAT84674

hypothetical protein
  
Accession: EAT84673
  
Location: 1073026-1074691
  
 NCBI BlastP on this gene

EAT84673

hypothetical protein
  
Accession: EAT84672
  
Location: 1072187-1072595
  
 NCBI BlastP on this gene

EAT84672

Query: Architecture Search FASTA input

CH476615 : Uncinocarpus reesii 1704 scaffold\_1 genomic scaffold    Total score: 4.0     Cumulative Blast bit score: 1427

Hit cluster cross-links:

Mycgr3G90785 Mycgr3T
  
Location: 0-1047

Mycgr3G90785\_Mycgr3T

Mycgr3G103262 Mycgr3
  
Location: 1147-1390

Mycgr3G103262\_Mycgr3

Mycgr3G68458 Mycgr3T
  
Location: 1490-3602

Mycgr3G68458\_Mycgr3T

Mycgr3G99145 Mycgr3T
  
Location: 3702-4326

Mycgr3G99145\_Mycgr3T

Mycgr3G103274 Mycgr3
  
Location: 4426-4957

Mycgr3G103274\_Mycgr3

Mycgr3G103264 Mycgr3
  
Location: 5057-5390

Mycgr3G103264\_Mycgr3

Mycgr3G37570 Mycgr3T
  
Location: 5490-6006

Mycgr3G37570\_Mycgr3T

Mycgr3G108094 Mycgr3
  
Location: 6106-10555

Mycgr3G108094\_Mycgr3

Mycgr3G90786 Mycgr3T
  
Location: 10655-12080

Mycgr3G90786\_Mycgr3T

Mycgr3G68429 Mycgr3T
  
Location: 12180-13440

Mycgr3G68429\_Mycgr3T

Mycgr3G68421 Mycgr3T
  
Location: 13540-17086

Mycgr3G68421\_Mycgr3T

Mycgr3G90801 Mycgr3T
  
Location: 17186-18056

Mycgr3G90801\_Mycgr3T

Mycgr3G84646 Mycgr3T
  
Location: 18156-20235

Mycgr3G84646\_Mycgr3T

Mycgr3G68456 Mycgr3T
  
Location: 20335-21970

Mycgr3G68456\_Mycgr3T

Mycgr3G103270 Mycgr3
  
Location: 22070-22355

Mycgr3G103270\_Mycgr3

Mycgr3G90803 Mycgr3T
  
Location: 22455-23019

Mycgr3G90803\_Mycgr3T

Mycgr3G36941 Mycgr3T
  
Location: 23119-24064

Mycgr3G36941\_Mycgr3T

Mycgr3G25746 Mycgr3T
  
Location: 24164-25241

Mycgr3G25746\_Mycgr3T

Mycgr3G90788 Mycgr3T
  
Location: 25341-25803

Mycgr3G90788\_Mycgr3T

Mycgr3G103260 Mycgr3
  
Location: 25903-26635

Mycgr3G103260\_Mycgr3

Mycgr3G84644 Mycgr3T
  
Location: 26735-28457

Mycgr3G84644\_Mycgr3T

Mycgr3G29227 Mycgr3T
  
Location: 28557-28863

Mycgr3G29227\_Mycgr3T

Mycgr3G36271 Mycgr3T
  
Location: 28963-29854

Mycgr3G36271\_Mycgr3T

Mycgr3G68433 Mycgr3T
  
Location: 29954-33041

Mycgr3G68433\_Mycgr3T

Mycgr3G79452 Mycgr3T
  
Location: 33141-33399

Mycgr3G79452\_Mycgr3T

Mycgr3G55345 Mycgr3T
  
Location: 33499-34126

Mycgr3G55345\_Mycgr3T

Mycgr3G103278 Mycgr3
  
Location: 34226-35195

Mycgr3G103278\_Mycgr3

Mycgr3G84654 Mycgr3T
  
Location: 35295-36630

Mycgr3G84654\_Mycgr3T

Mycgr3G108090 Mycgr3
  
Location: 36730-37591

Mycgr3G108090\_Mycgr3

Mycgr3G21922 Mycgr3T
  
Location: 37691-39149

Mycgr3G21922\_Mycgr3T

Mycgr3G99148 Mycgr3T
  
Location: 39249-42819

Mycgr3G99148\_Mycgr3T

conserved hypothetical protein
  
Accession: EEP77764
  
Location: 6938643-6941297
  
 NCBI BlastP on this gene

EEP77764

predicted protein
  
Accession: EEP77765
  
Location: 6941919-6942506
  
 NCBI BlastP on this gene

EEP77765

xanthine phosphoribosyltransferase 1
  
Accession: EEP77766
  
Location: 6943354-6944347
  
  
**BlastP hit with Mycgr3G55345\_Mycgr3T**
  
Percentage identity: 69 %
  
BlastP bit score: 279
  
Sequence coverage: 96 %
  
E-value: 4e-92
  
  
 NCBI BlastP on this gene

EEP77766

predicted protein
  
Accession: EEP77767
  
Location: 6945012-6945431
  
 NCBI BlastP on this gene

EEP77767

predicted protein
  
Accession: EEP77768
  
Location: 6946423-6948118
  
 NCBI BlastP on this gene

EEP77768

predicted protein
  
Accession: EEP77769
  
Location: 6948857-6950235
  
 NCBI BlastP on this gene

EEP77769

predicted protein
  
Accession: EEP77770
  
Location: 6951821-6954223
  
 NCBI BlastP on this gene

EEP77770

predicted protein
  
Accession: EEP77771
  
Location: 6954565-6955383
  
 NCBI BlastP on this gene

EEP77771

predicted protein
  
Accession: EEP77772
  
Location: 6955636-6957595
  
 NCBI BlastP on this gene

EEP77772

60S ribosomal protein L13
  
Accession: EEP77773
  
Location: 6958180-6959272
  
 NCBI BlastP on this gene

EEP77773

conserved hypothetical protein
  
Accession: EEP77774
  
Location: 6959824-6961113
  
  
**BlastP hit with Mycgr3G90786\_Mycgr3T**
  
Percentage identity: 30 %
  
BlastP bit score: 103
  
Sequence coverage: 96 %
  
E-value: 5e-21
  
  
 NCBI BlastP on this gene

EEP77774

predicted protein
  
Accession: EEP77775
  
Location: 6962111-6964380
  
  
**BlastP hit with Mycgr3G68433\_Mycgr3T**
  
Percentage identity: 45 %
  
BlastP bit score: 328
  
Sequence coverage: 41 %
  
E-value: 4e-95
  
  
 NCBI BlastP on this gene

EEP77775

predicted protein
  
Accession: EEP77776
  
Location: 6965440-6966609
  
 NCBI BlastP on this gene

EEP77776

conserved hypothetical protein
  
Accession: EEP77777
  
Location: 6967621-6969210
  
 NCBI BlastP on this gene

EEP77777

predicted protein
  
Accession: EEP77778
  
Location: 6970337-6972752
  
 NCBI BlastP on this gene

EEP77778

hypothetical protein
  
Accession: EEP77779
  
Location: 6973421-6974831
  
 NCBI BlastP on this gene

EEP77779

hypothetical protein
  
Accession: EEP77780
  
Location: 6975500-6978560
  
  
**BlastP hit with Mycgr3G68421\_Mycgr3T**
  
Percentage identity: 42 %
  
BlastP bit score: 717
  
Sequence coverage: 89 %
  
E-value: 0.0
  
  
 NCBI BlastP on this gene

EEP77780

predicted protein
  
Accession: EEP77781
  
Location: 6978908-6979867
  
 NCBI BlastP on this gene

EEP77781

predicted protein
  
Accession: EEP77782
  
Location: 6982600-6983654
  
 NCBI BlastP on this gene

EEP77782

conserved hypothetical protein
  
Accession: EEP77783
  
Location: 6984291-6985541
  
 NCBI BlastP on this gene

EEP77783

Query: Architecture Search FASTA input

KB915899 : Neofusicoccum parvum UCRNP2 chromosome Unknown NP2\_03\_scaffold\_261    Total score: 4.0     Cumulative Blast bit score: 1306

Hit cluster cross-links:

Mycgr3G90785 Mycgr3T
  
Location: 0-1047

Mycgr3G90785\_Mycgr3T

Mycgr3G103262 Mycgr3
  
Location: 1147-1390

Mycgr3G103262\_Mycgr3

Mycgr3G68458 Mycgr3T
  
Location: 1490-3602

Mycgr3G68458\_Mycgr3T

Mycgr3G99145 Mycgr3T
  
Location: 3702-4326

Mycgr3G99145\_Mycgr3T

Mycgr3G103274 Mycgr3
  
Location: 4426-4957

Mycgr3G103274\_Mycgr3

Mycgr3G103264 Mycgr3
  
Location: 5057-5390

Mycgr3G103264\_Mycgr3

Mycgr3G37570 Mycgr3T
  
Location: 5490-6006

Mycgr3G37570\_Mycgr3T

Mycgr3G108094 Mycgr3
  
Location: 6106-10555

Mycgr3G108094\_Mycgr3

Mycgr3G90786 Mycgr3T
  
Location: 10655-12080

Mycgr3G90786\_Mycgr3T

Mycgr3G68429 Mycgr3T
  
Location: 12180-13440

Mycgr3G68429\_Mycgr3T

Mycgr3G68421 Mycgr3T
  
Location: 13540-17086

Mycgr3G68421\_Mycgr3T

Mycgr3G90801 Mycgr3T
  
Location: 17186-18056

Mycgr3G90801\_Mycgr3T

Mycgr3G84646 Mycgr3T
  
Location: 18156-20235

Mycgr3G84646\_Mycgr3T

Mycgr3G68456 Mycgr3T
  
Location: 20335-21970

Mycgr3G68456\_Mycgr3T

Mycgr3G103270 Mycgr3
  
Location: 22070-22355

Mycgr3G103270\_Mycgr3

Mycgr3G90803 Mycgr3T
  
Location: 22455-23019

Mycgr3G90803\_Mycgr3T

Mycgr3G36941 Mycgr3T
  
Location: 23119-24064

Mycgr3G36941\_Mycgr3T

Mycgr3G25746 Mycgr3T
  
Location: 24164-25241

Mycgr3G25746\_Mycgr3T

Mycgr3G90788 Mycgr3T
  
Location: 25341-25803

Mycgr3G90788\_Mycgr3T

Mycgr3G103260 Mycgr3
  
Location: 25903-26635

Mycgr3G103260\_Mycgr3

Mycgr3G84644 Mycgr3T
  
Location: 26735-28457

Mycgr3G84644\_Mycgr3T

Mycgr3G29227 Mycgr3T
  
Location: 28557-28863

Mycgr3G29227\_Mycgr3T

Mycgr3G36271 Mycgr3T
  
Location: 28963-29854

Mycgr3G36271\_Mycgr3T

Mycgr3G68433 Mycgr3T
  
Location: 29954-33041

Mycgr3G68433\_Mycgr3T

Mycgr3G79452 Mycgr3T
  
Location: 33141-33399

Mycgr3G79452\_Mycgr3T

Mycgr3G55345 Mycgr3T
  
Location: 33499-34126

Mycgr3G55345\_Mycgr3T

Mycgr3G103278 Mycgr3
  
Location: 34226-35195

Mycgr3G103278\_Mycgr3

Mycgr3G84654 Mycgr3T
  
Location: 35295-36630

Mycgr3G84654\_Mycgr3T

Mycgr3G108090 Mycgr3
  
Location: 36730-37591

Mycgr3G108090\_Mycgr3

Mycgr3G21922 Mycgr3T
  
Location: 37691-39149

Mycgr3G21922\_Mycgr3T

Mycgr3G99148 Mycgr3T
  
Location: 39249-42819

Mycgr3G99148\_Mycgr3T

putative gtpase activating protein
  
Accession: EOD51068
  
Location: 23029-25101
  
 NCBI BlastP on this gene

EOD51068

putative atp synthase regulation protein nca2 protein
  
Accession: EOD51078
  
Location: 26733-29016
  
  
**BlastP hit with Mycgr3G84646\_Mycgr3T**
  
Percentage identity: 46 %
  
BlastP bit score: 627
  
Sequence coverage: 103 %
  
E-value: 0.0
  
  
 NCBI BlastP on this gene

EOD51078

putative rab gtpase protein
  
Accession: EOD51074
  
Location: 29809-30933
  
  
**BlastP hit with Mycgr3G99145\_Mycgr3T**
  
Percentage identity: 82 %
  
BlastP bit score: 362
  
Sequence coverage: 107 %
  
E-value: 2e-124
  
  
 NCBI BlastP on this gene

EOD51074

putative major facilitator superfamily transporter protein
  
Accession: EOD51077
  
Location: 36613-37879
  
 NCBI BlastP on this gene

EOD51077

putative stress response protein rds1 protein
  
Accession: EOD51073
  
Location: 39638-41131
  
 NCBI BlastP on this gene

EOD51073

putative beta-ig-h3 fasciclin protein
  
Accession: EOD51071
  
Location: 42701-43675
  
 NCBI BlastP on this gene

EOD51071

putative cation efflux family protein family protein
  
Accession: EOD51061
  
Location: 44678-46204
  
 NCBI BlastP on this gene

EOD51061

hypothetical protein
  
Accession: EOD51070
  
Location: 46531-47700
  
 NCBI BlastP on this gene

EOD51070

putative gnat family protein
  
Accession: EOD51057
  
Location: 48863-49782
  
 NCBI BlastP on this gene

EOD51057

hypothetical protein
  
Accession: EOD51069
  
Location: 49996-52015
  
 NCBI BlastP on this gene

EOD51069

putative dna-directed rna polymerase iii subunit rpc7 protein
  
Accession: EOD51060
  
Location: 52540-53614
  
 NCBI BlastP on this gene

EOD51060

putative conserved leucine-rich repeat protein
  
Accession: EOD51065
  
Location: 54303-56134
  
  
**BlastP hit with Mycgr3G68433\_Mycgr3T**
  
Percentage identity: 45 %
  
BlastP bit score: 244
  
Sequence coverage: 31 %
  
E-value: 3e-68
  
  
 NCBI BlastP on this gene

EOD51065

putative mn2+ homeostasis protein
  
Accession: EOD51072
  
Location: 58321-62096
  
  
**BlastP hit with Mycgr3G90786\_Mycgr3T**
  
Percentage identity: 33 %
  
BlastP bit score: 73
  
Sequence coverage: 41 %
  
E-value: 1e-10
  
  
 NCBI BlastP on this gene

EOD51072

putative protein kinase protein
  
Accession: EOD51075
  
Location: 64315-66934
  
 NCBI BlastP on this gene

EOD51075

Query: Architecture Search FASTA input

KB446566 : Pseudocercospora fijiensis CIRAD86 unplaced genomic scaffold MYCFIscaffold\_12    Total score: 3.0     Cumulative Blast bit score: 2502

Hit cluster cross-links:

Mycgr3G90785 Mycgr3T
  
Location: 0-1047

Mycgr3G90785\_Mycgr3T

Mycgr3G103262 Mycgr3
  
Location: 1147-1390

Mycgr3G103262\_Mycgr3

Mycgr3G68458 Mycgr3T
  
Location: 1490-3602

Mycgr3G68458\_Mycgr3T

Mycgr3G99145 Mycgr3T
  
Location: 3702-4326

Mycgr3G99145\_Mycgr3T

Mycgr3G103274 Mycgr3
  
Location: 4426-4957

Mycgr3G103274\_Mycgr3

Mycgr3G103264 Mycgr3
  
Location: 5057-5390

Mycgr3G103264\_Mycgr3

Mycgr3G37570 Mycgr3T
  
Location: 5490-6006

Mycgr3G37570\_Mycgr3T

Mycgr3G108094 Mycgr3
  
Location: 6106-10555

Mycgr3G108094\_Mycgr3

Mycgr3G90786 Mycgr3T
  
Location: 10655-12080

Mycgr3G90786\_Mycgr3T

Mycgr3G68429 Mycgr3T
  
Location: 12180-13440

Mycgr3G68429\_Mycgr3T

Mycgr3G68421 Mycgr3T
  
Location: 13540-17086

Mycgr3G68421\_Mycgr3T

Mycgr3G90801 Mycgr3T
  
Location: 17186-18056

Mycgr3G90801\_Mycgr3T

Mycgr3G84646 Mycgr3T
  
Location: 18156-20235

Mycgr3G84646\_Mycgr3T

Mycgr3G68456 Mycgr3T
  
Location: 20335-21970

Mycgr3G68456\_Mycgr3T

Mycgr3G103270 Mycgr3
  
Location: 22070-22355

Mycgr3G103270\_Mycgr3

Mycgr3G90803 Mycgr3T
  
Location: 22455-23019

Mycgr3G90803\_Mycgr3T

Mycgr3G36941 Mycgr3T
  
Location: 23119-24064

Mycgr3G36941\_Mycgr3T

Mycgr3G25746 Mycgr3T
  
Location: 24164-25241

Mycgr3G25746\_Mycgr3T

Mycgr3G90788 Mycgr3T
  
Location: 25341-25803

Mycgr3G90788\_Mycgr3T

Mycgr3G103260 Mycgr3
  
Location: 25903-26635

Mycgr3G103260\_Mycgr3

Mycgr3G84644 Mycgr3T
  
Location: 26735-28457

Mycgr3G84644\_Mycgr3T

Mycgr3G29227 Mycgr3T
  
Location: 28557-28863

Mycgr3G29227\_Mycgr3T

Mycgr3G36271 Mycgr3T
  
Location: 28963-29854

Mycgr3G36271\_Mycgr3T

Mycgr3G68433 Mycgr3T
  
Location: 29954-33041

Mycgr3G68433\_Mycgr3T

Mycgr3G79452 Mycgr3T
  
Location: 33141-33399

Mycgr3G79452\_Mycgr3T

Mycgr3G55345 Mycgr3T
  
Location: 33499-34126

Mycgr3G55345\_Mycgr3T

Mycgr3G103278 Mycgr3
  
Location: 34226-35195

Mycgr3G103278\_Mycgr3

Mycgr3G84654 Mycgr3T
  
Location: 35295-36630

Mycgr3G84654\_Mycgr3T

Mycgr3G108090 Mycgr3
  
Location: 36730-37591

Mycgr3G108090\_Mycgr3

Mycgr3G21922 Mycgr3T
  
Location: 37691-39149

Mycgr3G21922\_Mycgr3T

Mycgr3G99148 Mycgr3T
  
Location: 39249-42819

Mycgr3G99148\_Mycgr3T

hypothetical protein
  
Accession: EME77349
  
Location: 816486-818781
  
 NCBI BlastP on this gene

EME77349

hypothetical protein
  
Accession: EME77350
  
Location: 824278-824604
  
 NCBI BlastP on this gene

EME77350

hypothetical protein
  
Accession: EME77351
  
Location: 824636-825178
  
 NCBI BlastP on this gene

EME77351

hypothetical protein
  
Accession: EME77352
  
Location: 825186-826985
  
  
**BlastP hit with Mycgr3G84654\_Mycgr3T**
  
Percentage identity: 84 %
  
BlastP bit score: 578
  
Sequence coverage: 74 %
  
E-value: 0.0
  
  
 NCBI BlastP on this gene

EME77352

hypothetical protein
  
Accession: EME77353
  
Location: 827105-827617
  
 NCBI BlastP on this gene

EME77353

hypothetical protein
  
Accession: EME77354
  
Location: 829186-830820
  
 NCBI BlastP on this gene

EME77354

hypothetical protein
  
Accession: EME77355
  
Location: 841580-843175
  
 NCBI BlastP on this gene

EME77355

hypothetical protein
  
Accession: EME77356
  
Location: 844760-846028
  
 NCBI BlastP on this gene

EME77356

hypothetical protein
  
Accession: EME77357
  
Location: 846832-849159
  
  
**BlastP hit with Mycgr3G68456\_Mycgr3T**
  
Percentage identity: 76 %
  
BlastP bit score: 820
  
Sequence coverage: 96 %
  
E-value: 0.0
  
  
 NCBI BlastP on this gene

EME77357

hypothetical protein
  
Accession: EME77358
  
Location: 849893-850859
  
 NCBI BlastP on this gene

EME77358

hypothetical protein
  
Accession: EME77359
  
Location: 851200-852839
  
 NCBI BlastP on this gene

EME77359

hypothetical protein
  
Accession: EME77360
  
Location: 854122-857859
  
  
**BlastP hit with Mycgr3G68458\_Mycgr3T**
  
Percentage identity: 76 %
  
BlastP bit score: 1104
  
Sequence coverage: 99 %
  
E-value: 0.0
  
  
 NCBI BlastP on this gene

EME77360

hypothetical protein
  
Accession: EME77361
  
Location: 857914-862221
  
 NCBI BlastP on this gene

EME77361

Query: Architecture Search FASTA input

KB456266 : Mycosphaerella populorum SO2202 unplaced genomic scaffold SEPMUscaffold\_7    Total score: 3.0     Cumulative Blast bit score: 2466

Hit cluster cross-links:

Mycgr3G90785 Mycgr3T
  
Location: 0-1047

Mycgr3G90785\_Mycgr3T

Mycgr3G103262 Mycgr3
  
Location: 1147-1390

Mycgr3G103262\_Mycgr3

Mycgr3G68458 Mycgr3T
  
Location: 1490-3602

Mycgr3G68458\_Mycgr3T

Mycgr3G99145 Mycgr3T
  
Location: 3702-4326

Mycgr3G99145\_Mycgr3T

Mycgr3G103274 Mycgr3
  
Location: 4426-4957

Mycgr3G103274\_Mycgr3

Mycgr3G103264 Mycgr3
  
Location: 5057-5390

Mycgr3G103264\_Mycgr3

Mycgr3G37570 Mycgr3T
  
Location: 5490-6006

Mycgr3G37570\_Mycgr3T

Mycgr3G108094 Mycgr3
  
Location: 6106-10555

Mycgr3G108094\_Mycgr3

Mycgr3G90786 Mycgr3T
  
Location: 10655-12080

Mycgr3G90786\_Mycgr3T

Mycgr3G68429 Mycgr3T
  
Location: 12180-13440

Mycgr3G68429\_Mycgr3T

Mycgr3G68421 Mycgr3T
  
Location: 13540-17086

Mycgr3G68421\_Mycgr3T

Mycgr3G90801 Mycgr3T
  
Location: 17186-18056

Mycgr3G90801\_Mycgr3T

Mycgr3G84646 Mycgr3T
  
Location: 18156-20235

Mycgr3G84646\_Mycgr3T

Mycgr3G68456 Mycgr3T
  
Location: 20335-21970

Mycgr3G68456\_Mycgr3T

Mycgr3G103270 Mycgr3
  
Location: 22070-22355

Mycgr3G103270\_Mycgr3

Mycgr3G90803 Mycgr3T
  
Location: 22455-23019

Mycgr3G90803\_Mycgr3T

Mycgr3G36941 Mycgr3T
  
Location: 23119-24064

Mycgr3G36941\_Mycgr3T

Mycgr3G25746 Mycgr3T
  
Location: 24164-25241

Mycgr3G25746\_Mycgr3T

Mycgr3G90788 Mycgr3T
  
Location: 25341-25803

Mycgr3G90788\_Mycgr3T

Mycgr3G103260 Mycgr3
  
Location: 25903-26635

Mycgr3G103260\_Mycgr3

Mycgr3G84644 Mycgr3T
  
Location: 26735-28457

Mycgr3G84644\_Mycgr3T

Mycgr3G29227 Mycgr3T
  
Location: 28557-28863

Mycgr3G29227\_Mycgr3T

Mycgr3G36271 Mycgr3T
  
Location: 28963-29854

Mycgr3G36271\_Mycgr3T

Mycgr3G68433 Mycgr3T
  
Location: 29954-33041

Mycgr3G68433\_Mycgr3T

Mycgr3G79452 Mycgr3T
  
Location: 33141-33399

Mycgr3G79452\_Mycgr3T

Mycgr3G55345 Mycgr3T
  
Location: 33499-34126

Mycgr3G55345\_Mycgr3T

Mycgr3G103278 Mycgr3
  
Location: 34226-35195

Mycgr3G103278\_Mycgr3

Mycgr3G84654 Mycgr3T
  
Location: 35295-36630

Mycgr3G84654\_Mycgr3T

Mycgr3G108090 Mycgr3
  
Location: 36730-37591

Mycgr3G108090\_Mycgr3

Mycgr3G21922 Mycgr3T
  
Location: 37691-39149

Mycgr3G21922\_Mycgr3T

Mycgr3G99148 Mycgr3T
  
Location: 39249-42819

Mycgr3G99148\_Mycgr3T

NRPS-like enzyme
  
Accession: EMF11166
  
Location: 553284-557222
  
 NCBI BlastP on this gene

EMF11166

exonuclease family protein
  
Accession: EMF11167
  
Location: 558053-558637
  
 NCBI BlastP on this gene

EMF11167

NAD(P)-binding protein
  
Accession: EMF11168
  
Location: 559130-560716
  
 NCBI BlastP on this gene

EMF11168

NAD(P)-binding protein
  
Accession: EMF11169
  
Location: 561138-562259
  
 NCBI BlastP on this gene

EMF11169

WSC-domain-containing protein
  
Accession: EMF11170
  
Location: 564079-564736
  
 NCBI BlastP on this gene

EMF11170

autophagy protein
  
Accession: EMF11171
  
Location: 565731-568041
  
  
**BlastP hit with Mycgr3G68456\_Mycgr3T**
  
Percentage identity: 80 %
  
BlastP bit score: 839
  
Sequence coverage: 95 %
  
E-value: 0.0
  
  
 NCBI BlastP on this gene

EMF11171

Glucosamine iso-domain-containing protein
  
Accession: EMF11172
  
Location: 568891-570187
  
 NCBI BlastP on this gene

EMF11172

MFS general substrate transporter
  
Accession: EMF11173
  
Location: 571888-573548
  
 NCBI BlastP on this gene

EMF11173

hexokinase-1
  
Accession: EMF11174
  
Location: 576247-577851
  
 NCBI BlastP on this gene

EMF11174

p53-like transcription factor
  
Accession: EMF11175
  
Location: 579165-580925
  
  
**BlastP hit with Mycgr3G84654\_Mycgr3T**
  
Percentage identity: 80 %
  
BlastP bit score: 537
  
Sequence coverage: 75 %
  
E-value: 0.0
  
  
 NCBI BlastP on this gene

EMF11175

glycoside hydrolase family 3 protein
  
Accession: EMF11176
  
Location: 582024-585188
  
 NCBI BlastP on this gene

EMF11176

carbohydrate esterase family 9 protein
  
Accession: EMF11177
  
Location: 586293-587609
  
 NCBI BlastP on this gene

EMF11177

ABC1-domain-containing protein
  
Accession: EMF11178
  
Location: 588012-590186
  
  
**BlastP hit with Mycgr3G68458\_Mycgr3T**
  
Percentage identity: 75 %
  
BlastP bit score: 1090
  
Sequence coverage: 100 %
  
E-value: 0.0
  
  
 NCBI BlastP on this gene

EMF11178

hypothetical protein
  
Accession: EMF11179
  
Location: 590721-593577
  
 NCBI BlastP on this gene

EMF11179

hypothetical protein
  
Accession: EMF11180
  
Location: 594567-597043
  
 NCBI BlastP on this gene

EMF11180

amino acid transporter
  
Accession: EMF11181
  
Location: 597627-599732
  
 NCBI BlastP on this gene

EMF11181

caleosin domain-containing protein
  
Accession: EMF11182
  
Location: 599924-600816
  
 NCBI BlastP on this gene

EMF11182

hypothetical protein
  
Accession: EMF11183
  
Location: 601423-604052
  
 NCBI BlastP on this gene

EMF11183

Query: Architecture Search FASTA input

AABX02000026 : Neurospora crassa OR74A    Total score: 3.0     Cumulative Blast bit score: 1676

Hit cluster cross-links:

Mycgr3G90785 Mycgr3T
  
Location: 0-1047

Mycgr3G90785\_Mycgr3T

Mycgr3G103262 Mycgr3
  
Location: 1147-1390

Mycgr3G103262\_Mycgr3

Mycgr3G68458 Mycgr3T
  
Location: 1490-3602

Mycgr3G68458\_Mycgr3T

Mycgr3G99145 Mycgr3T
  
Location: 3702-4326

Mycgr3G99145\_Mycgr3T

Mycgr3G103274 Mycgr3
  
Location: 4426-4957

Mycgr3G103274\_Mycgr3

Mycgr3G103264 Mycgr3
  
Location: 5057-5390

Mycgr3G103264\_Mycgr3

Mycgr3G37570 Mycgr3T
  
Location: 5490-6006

Mycgr3G37570\_Mycgr3T

Mycgr3G108094 Mycgr3
  
Location: 6106-10555

Mycgr3G108094\_Mycgr3

Mycgr3G90786 Mycgr3T
  
Location: 10655-12080

Mycgr3G90786\_Mycgr3T

Mycgr3G68429 Mycgr3T
  
Location: 12180-13440

Mycgr3G68429\_Mycgr3T

Mycgr3G68421 Mycgr3T
  
Location: 13540-17086

Mycgr3G68421\_Mycgr3T

Mycgr3G90801 Mycgr3T
  
Location: 17186-18056

Mycgr3G90801\_Mycgr3T

Mycgr3G84646 Mycgr3T
  
Location: 18156-20235

Mycgr3G84646\_Mycgr3T

Mycgr3G68456 Mycgr3T
  
Location: 20335-21970

Mycgr3G68456\_Mycgr3T

Mycgr3G103270 Mycgr3
  
Location: 22070-22355

Mycgr3G103270\_Mycgr3

Mycgr3G90803 Mycgr3T
  
Location: 22455-23019

Mycgr3G90803\_Mycgr3T

Mycgr3G36941 Mycgr3T
  
Location: 23119-24064

Mycgr3G36941\_Mycgr3T

Mycgr3G25746 Mycgr3T
  
Location: 24164-25241

Mycgr3G25746\_Mycgr3T

Mycgr3G90788 Mycgr3T
  
Location: 25341-25803

Mycgr3G90788\_Mycgr3T

Mycgr3G103260 Mycgr3
  
Location: 25903-26635

Mycgr3G103260\_Mycgr3

Mycgr3G84644 Mycgr3T
  
Location: 26735-28457

Mycgr3G84644\_Mycgr3T

Mycgr3G29227 Mycgr3T
  
Location: 28557-28863

Mycgr3G29227\_Mycgr3T

Mycgr3G36271 Mycgr3T
  
Location: 28963-29854

Mycgr3G36271\_Mycgr3T

Mycgr3G68433 Mycgr3T
  
Location: 29954-33041

Mycgr3G68433\_Mycgr3T

Mycgr3G79452 Mycgr3T
  
Location: 33141-33399

Mycgr3G79452\_Mycgr3T

Mycgr3G55345 Mycgr3T
  
Location: 33499-34126

Mycgr3G55345\_Mycgr3T

Mycgr3G103278 Mycgr3
  
Location: 34226-35195

Mycgr3G103278\_Mycgr3

Mycgr3G84654 Mycgr3T
  
Location: 35295-36630

Mycgr3G84654\_Mycgr3T

Mycgr3G108090 Mycgr3
  
Location: 36730-37591

Mycgr3G108090\_Mycgr3

Mycgr3G21922 Mycgr3T
  
Location: 37691-39149

Mycgr3G21922\_Mycgr3T

Mycgr3G99148 Mycgr3T
  
Location: 39249-42819

Mycgr3G99148\_Mycgr3T

GTP-binding protein SAS1
  
Accession: EAA33006
  
Location: 6477-7435
  
  
**BlastP hit with Mycgr3G99145\_Mycgr3T**
  
Percentage identity: 88 %
  
BlastP bit score: 355
  
Sequence coverage: 92 %
  
E-value: 1e-121
  
  
 NCBI BlastP on this gene

EAA33006

conserved hypothetical protein
  
Accession: EAA33005
  
Location: 9398-11483
  
  
**BlastP hit with Mycgr3G84646\_Mycgr3T**
  
Percentage identity: 42 %
  
BlastP bit score: 481
  
Sequence coverage: 94 %
  
E-value: 2e-157
  
  
 NCBI BlastP on this gene

EAA33005

C-4 methylsterol oxidase, variant
  
Accession: EAA33004
  
Location: 13935-15069
  
  
**BlastP hit with Mycgr3G36271\_Mycgr3T**
  
Percentage identity: 71 %
  
BlastP bit score: 436
  
Sequence coverage: 94 %
  
E-value: 9e-151
  
  
 NCBI BlastP on this gene

EAA33004

C-4 methylsterol oxidase
  
Accession: EDO65110
  
Location: 14225-15069
  
  
**BlastP hit with Mycgr3G36271\_Mycgr3T**
  
Percentage identity: 76 %
  
BlastP bit score: 404
  
Sequence coverage: 82 %
  
E-value: 1e-138
  
  
 NCBI BlastP on this gene

EDO65110

predicted protein
  
Accession: EAA33003
  
Location: 15655-17099
  
 NCBI BlastP on this gene

EAA33003

predicted protein
  
Accession: EAA33002
  
Location: 17940-19389
  
 NCBI BlastP on this gene

EAA33002

hypothetical protein
  
Accession: EAA33001
  
Location: 22269-23221
  
 NCBI BlastP on this gene

EAA33001

hypothetical protein
  
Accession: EAA33000
  
Location: 25014-27569
  
 NCBI BlastP on this gene

EAA33000

profilin
  
Accession: EAA32999
  
Location: 30516-31597
  
 NCBI BlastP on this gene

EAA32999

conserved hypothetical protein
  
Accession: EAA32998
  
Location: 32388-33272
  
 NCBI BlastP on this gene

EAA32998

conserved hypothetical protein
  
Accession: EAA32997
  
Location: 34444-35076
  
 NCBI BlastP on this gene

EAA32997

Query: Architecture Search FASTA input

KB456266 : Mycosphaerella populorum SO2202 unplaced genomic scaffold SEPMUscaffold\_7    Total score: 3.0     Cumulative Blast bit score: 1544

Hit cluster cross-links:

Mycgr3G90785 Mycgr3T
  
Location: 0-1047

Mycgr3G90785\_Mycgr3T

Mycgr3G103262 Mycgr3
  
Location: 1147-1390

Mycgr3G103262\_Mycgr3

Mycgr3G68458 Mycgr3T
  
Location: 1490-3602

Mycgr3G68458\_Mycgr3T

Mycgr3G99145 Mycgr3T
  
Location: 3702-4326

Mycgr3G99145\_Mycgr3T

Mycgr3G103274 Mycgr3
  
Location: 4426-4957

Mycgr3G103274\_Mycgr3

Mycgr3G103264 Mycgr3
  
Location: 5057-5390

Mycgr3G103264\_Mycgr3

Mycgr3G37570 Mycgr3T
  
Location: 5490-6006

Mycgr3G37570\_Mycgr3T

Mycgr3G108094 Mycgr3
  
Location: 6106-10555

Mycgr3G108094\_Mycgr3

Mycgr3G90786 Mycgr3T
  
Location: 10655-12080

Mycgr3G90786\_Mycgr3T

Mycgr3G68429 Mycgr3T
  
Location: 12180-13440

Mycgr3G68429\_Mycgr3T

Mycgr3G68421 Mycgr3T
  
Location: 13540-17086

Mycgr3G68421\_Mycgr3T

Mycgr3G90801 Mycgr3T
  
Location: 17186-18056

Mycgr3G90801\_Mycgr3T

Mycgr3G84646 Mycgr3T
  
Location: 18156-20235

Mycgr3G84646\_Mycgr3T

Mycgr3G68456 Mycgr3T
  
Location: 20335-21970

Mycgr3G68456\_Mycgr3T

Mycgr3G103270 Mycgr3
  
Location: 22070-22355

Mycgr3G103270\_Mycgr3

Mycgr3G90803 Mycgr3T
  
Location: 22455-23019

Mycgr3G90803\_Mycgr3T

Mycgr3G36941 Mycgr3T
  
Location: 23119-24064

Mycgr3G36941\_Mycgr3T

Mycgr3G25746 Mycgr3T
  
Location: 24164-25241

Mycgr3G25746\_Mycgr3T

Mycgr3G90788 Mycgr3T
  
Location: 25341-25803

Mycgr3G90788\_Mycgr3T

Mycgr3G103260 Mycgr3
  
Location: 25903-26635

Mycgr3G103260\_Mycgr3

Mycgr3G84644 Mycgr3T
  
Location: 26735-28457

Mycgr3G84644\_Mycgr3T

Mycgr3G29227 Mycgr3T
  
Location: 28557-28863

Mycgr3G29227\_Mycgr3T

Mycgr3G36271 Mycgr3T
  
Location: 28963-29854

Mycgr3G36271\_Mycgr3T

Mycgr3G68433 Mycgr3T
  
Location: 29954-33041

Mycgr3G68433\_Mycgr3T

Mycgr3G79452 Mycgr3T
  
Location: 33141-33399

Mycgr3G79452\_Mycgr3T

Mycgr3G55345 Mycgr3T
  
Location: 33499-34126

Mycgr3G55345\_Mycgr3T

Mycgr3G103278 Mycgr3
  
Location: 34226-35195

Mycgr3G103278\_Mycgr3

Mycgr3G84654 Mycgr3T
  
Location: 35295-36630

Mycgr3G84654\_Mycgr3T

Mycgr3G108090 Mycgr3
  
Location: 36730-37591

Mycgr3G108090\_Mycgr3

Mycgr3G21922 Mycgr3T
  
Location: 37691-39149

Mycgr3G21922\_Mycgr3T

Mycgr3G99148 Mycgr3T
  
Location: 39249-42819

Mycgr3G99148\_Mycgr3T

hypoxanthine guanine phosphoribosyltransferase
  
Accession: EMF10976
  
Location: 24136-24961
  
  
**BlastP hit with Mycgr3G55345\_Mycgr3T**
  
Percentage identity: 76 %
  
BlastP bit score: 333
  
Sequence coverage: 97 %
  
E-value: 2e-113
  
  
 NCBI BlastP on this gene

EMF10976

L domain-like protein
  
Accession: EMF10977
  
Location: 25526-28276
  
  
**BlastP hit with Mycgr3G68433\_Mycgr3T**
  
Percentage identity: 55 %
  
BlastP bit score: 898
  
Sequence coverage: 90 %
  
E-value: 0.0
  
  
 NCBI BlastP on this gene

EMF10977

hypothetical protein
  
Accession: EMF10978
  
Location: 29512-30864
  
  
**BlastP hit with Mycgr3G90786\_Mycgr3T**
  
Percentage identity: 46 %
  
BlastP bit score: 313
  
Sequence coverage: 99 %
  
E-value: 5e-98
  
  
 NCBI BlastP on this gene

EMF10978

hypothetical protein
  
Accession: EMF10979
  
Location: 31412-32726
  
 NCBI BlastP on this gene

EMF10979

S-adenosyl-L-methionine-dependent methyltransferase
  
Accession: EMF10980
  
Location: 34541-35614
  
 NCBI BlastP on this gene

EMF10980

hypothetical protein
  
Accession: EMF10982
  
Location: 36428-38453
  
 NCBI BlastP on this gene

EMF10982

hypothetical protein
  
Accession: EMF10983
  
Location: 39093-41212
  
 NCBI BlastP on this gene

EMF10983

hypothetical protein
  
Accession: EMF10984
  
Location: 42365-42733
  
 NCBI BlastP on this gene

EMF10984

hypothetical protein
  
Accession: EMF10985
  
Location: 44412-45140
  
 NCBI BlastP on this gene

EMF10985

ankyrin
  
Accession: EMF10986
  
Location: 45886-46938
  
 NCBI BlastP on this gene

EMF10986

hypothetical protein
  
Accession: EMF10987
  
Location: 47212-48735
  
 NCBI BlastP on this gene

EMF10987

hypothetical protein
  
Accession: EMF10988
  
Location: 49727-50947
  
 NCBI BlastP on this gene

EMF10988

Query: Architecture Search FASTA input

AP007172 : Aspergillus oryzae RIB40 DNA, SC206.    Total score: 3.0     Cumulative Blast bit score: 1522

Hit cluster cross-links:

Mycgr3G90785 Mycgr3T
  
Location: 0-1047

Mycgr3G90785\_Mycgr3T

Mycgr3G103262 Mycgr3
  
Location: 1147-1390

Mycgr3G103262\_Mycgr3

Mycgr3G68458 Mycgr3T
  
Location: 1490-3602

Mycgr3G68458\_Mycgr3T

Mycgr3G99145 Mycgr3T
  
Location: 3702-4326

Mycgr3G99145\_Mycgr3T

Mycgr3G103274 Mycgr3
  
Location: 4426-4957

Mycgr3G103274\_Mycgr3

Mycgr3G103264 Mycgr3
  
Location: 5057-5390

Mycgr3G103264\_Mycgr3

Mycgr3G37570 Mycgr3T
  
Location: 5490-6006

Mycgr3G37570\_Mycgr3T

Mycgr3G108094 Mycgr3
  
Location: 6106-10555

Mycgr3G108094\_Mycgr3

Mycgr3G90786 Mycgr3T
  
Location: 10655-12080

Mycgr3G90786\_Mycgr3T

Mycgr3G68429 Mycgr3T
  
Location: 12180-13440

Mycgr3G68429\_Mycgr3T

Mycgr3G68421 Mycgr3T
  
Location: 13540-17086

Mycgr3G68421\_Mycgr3T

Mycgr3G90801 Mycgr3T
  
Location: 17186-18056

Mycgr3G90801\_Mycgr3T

Mycgr3G84646 Mycgr3T
  
Location: 18156-20235

Mycgr3G84646\_Mycgr3T

Mycgr3G68456 Mycgr3T
  
Location: 20335-21970

Mycgr3G68456\_Mycgr3T

Mycgr3G103270 Mycgr3
  
Location: 22070-22355

Mycgr3G103270\_Mycgr3

Mycgr3G90803 Mycgr3T
  
Location: 22455-23019

Mycgr3G90803\_Mycgr3T

Mycgr3G36941 Mycgr3T
  
Location: 23119-24064

Mycgr3G36941\_Mycgr3T

Mycgr3G25746 Mycgr3T
  
Location: 24164-25241

Mycgr3G25746\_Mycgr3T

Mycgr3G90788 Mycgr3T
  
Location: 25341-25803

Mycgr3G90788\_Mycgr3T

Mycgr3G103260 Mycgr3
  
Location: 25903-26635

Mycgr3G103260\_Mycgr3

Mycgr3G84644 Mycgr3T
  
Location: 26735-28457

Mycgr3G84644\_Mycgr3T

Mycgr3G29227 Mycgr3T
  
Location: 28557-28863

Mycgr3G29227\_Mycgr3T

Mycgr3G36271 Mycgr3T
  
Location: 28963-29854

Mycgr3G36271\_Mycgr3T

Mycgr3G68433 Mycgr3T
  
Location: 29954-33041

Mycgr3G68433\_Mycgr3T

Mycgr3G79452 Mycgr3T
  
Location: 33141-33399

Mycgr3G79452\_Mycgr3T

Mycgr3G55345 Mycgr3T
  
Location: 33499-34126

Mycgr3G55345\_Mycgr3T

Mycgr3G103278 Mycgr3
  
Location: 34226-35195

Mycgr3G103278\_Mycgr3

Mycgr3G84654 Mycgr3T
  
Location: 35295-36630

Mycgr3G84654\_Mycgr3T

Mycgr3G108090 Mycgr3
  
Location: 36730-37591

Mycgr3G108090\_Mycgr3

Mycgr3G21922 Mycgr3T
  
Location: 37691-39149

Mycgr3G21922\_Mycgr3T

Mycgr3G99148 Mycgr3T
  
Location: 39249-42819

Mycgr3G99148\_Mycgr3T

not annotated
  
Accession: BAE65414
  
Location: 99018-99840
  
 NCBI BlastP on this gene

AO090206000042

not annotated
  
Accession: BAE65415
  
Location: 100010-101129
  
 NCBI BlastP on this gene

AO090206000043

not annotated
  
Accession: BAE65416
  
Location: 101848-102677
  
 NCBI BlastP on this gene

AO090206000044

not annotated
  
Accession: BAE65417
  
Location: 103211-105760
  
 NCBI BlastP on this gene

AO090206000045

not annotated
  
Accession: BAE65418
  
Location: 107211-108107
  
 NCBI BlastP on this gene

AO090206000046

not annotated
  
Accession: BAE65419
  
Location: 110806-111912
  
 NCBI BlastP on this gene

AO090206000047

not annotated
  
Accession: BAE65420
  
Location: 112848-114287
  
  
**BlastP hit with Mycgr3G90786\_Mycgr3T**
  
Percentage identity: 26 %
  
BlastP bit score: 98
  
Sequence coverage: 99 %
  
E-value: 7e-19
  
  
 NCBI BlastP on this gene

AO090206000048

not annotated
  
Accession: BAE65421
  
Location: 116497-119442
  
  
**BlastP hit with Mycgr3G68433\_Mycgr3T**
  
Percentage identity: 41 %
  
BlastP bit score: 484
  
Sequence coverage: 85 %
  
E-value: 3e-150
  
  
 NCBI BlastP on this gene

AO090206000049

not annotated
  
Accession: BAE65422
  
Location: 119711-120847
  
 NCBI BlastP on this gene

AO090206000050

not annotated
  
Accession: BAE65423
  
Location: 121682-123259
  
 NCBI BlastP on this gene

AO090206000051

not annotated
  
Accession: BAE65424
  
Location: 125116-125477
  
 NCBI BlastP on this gene

AO090206000052

not annotated
  
Accession: BAE65425
  
Location: 125834-126948
  
 NCBI BlastP on this gene

AO090206000053

not annotated
  
Accession: BAE65426
  
Location: 127275-130935
  
  
**BlastP hit with Mycgr3G68421\_Mycgr3T**
  
Percentage identity: 44 %
  
BlastP bit score: 940
  
Sequence coverage: 101 %
  
E-value: 0.0
  
  
 NCBI BlastP on this gene

AO090206000054

not annotated
  
Accession: BAE65427
  
Location: 134646-136215
  
 NCBI BlastP on this gene

AO090206000056

not annotated
  
Accession: BAE65428
  
Location: 136476-137675
  
 NCBI BlastP on this gene

AO090206000057

not annotated
  
Accession: BAE65429
  
Location: 138112-142544
  
 NCBI BlastP on this gene

AO090206000058

not annotated
  
Accession: BAE65430
  
Location: 142812-145169
  
 NCBI BlastP on this gene

AO090206000059

Query: Architecture Search FASTA input

AKHY01000145 : Aspergillus oryzae 3.042    Total score: 3.0     Cumulative Blast bit score: 1522

Hit cluster cross-links:

Mycgr3G90785 Mycgr3T
  
Location: 0-1047

Mycgr3G90785\_Mycgr3T

Mycgr3G103262 Mycgr3
  
Location: 1147-1390

Mycgr3G103262\_Mycgr3

Mycgr3G68458 Mycgr3T
  
Location: 1490-3602

Mycgr3G68458\_Mycgr3T

Mycgr3G99145 Mycgr3T
  
Location: 3702-4326

Mycgr3G99145\_Mycgr3T

Mycgr3G103274 Mycgr3
  
Location: 4426-4957

Mycgr3G103274\_Mycgr3

Mycgr3G103264 Mycgr3
  
Location: 5057-5390

Mycgr3G103264\_Mycgr3

Mycgr3G37570 Mycgr3T
  
Location: 5490-6006

Mycgr3G37570\_Mycgr3T

Mycgr3G108094 Mycgr3
  
Location: 6106-10555

Mycgr3G108094\_Mycgr3

Mycgr3G90786 Mycgr3T
  
Location: 10655-12080

Mycgr3G90786\_Mycgr3T

Mycgr3G68429 Mycgr3T
  
Location: 12180-13440

Mycgr3G68429\_Mycgr3T

Mycgr3G68421 Mycgr3T
  
Location: 13540-17086

Mycgr3G68421\_Mycgr3T

Mycgr3G90801 Mycgr3T
  
Location: 17186-18056

Mycgr3G90801\_Mycgr3T

Mycgr3G84646 Mycgr3T
  
Location: 18156-20235

Mycgr3G84646\_Mycgr3T

Mycgr3G68456 Mycgr3T
  
Location: 20335-21970

Mycgr3G68456\_Mycgr3T

Mycgr3G103270 Mycgr3
  
Location: 22070-22355

Mycgr3G103270\_Mycgr3

Mycgr3G90803 Mycgr3T
  
Location: 22455-23019

Mycgr3G90803\_Mycgr3T

Mycgr3G36941 Mycgr3T
  
Location: 23119-24064

Mycgr3G36941\_Mycgr3T

Mycgr3G25746 Mycgr3T
  
Location: 24164-25241

Mycgr3G25746\_Mycgr3T

Mycgr3G90788 Mycgr3T
  
Location: 25341-25803

Mycgr3G90788\_Mycgr3T

Mycgr3G103260 Mycgr3
  
Location: 25903-26635

Mycgr3G103260\_Mycgr3

Mycgr3G84644 Mycgr3T
  
Location: 26735-28457

Mycgr3G84644\_Mycgr3T

Mycgr3G29227 Mycgr3T
  
Location: 28557-28863

Mycgr3G29227\_Mycgr3T

Mycgr3G36271 Mycgr3T
  
Location: 28963-29854

Mycgr3G36271\_Mycgr3T

Mycgr3G68433 Mycgr3T
  
Location: 29954-33041

Mycgr3G68433\_Mycgr3T

Mycgr3G79452 Mycgr3T
  
Location: 33141-33399

Mycgr3G79452\_Mycgr3T

Mycgr3G55345 Mycgr3T
  
Location: 33499-34126

Mycgr3G55345\_Mycgr3T

Mycgr3G103278 Mycgr3
  
Location: 34226-35195

Mycgr3G103278\_Mycgr3

Mycgr3G84654 Mycgr3T
  
Location: 35295-36630

Mycgr3G84654\_Mycgr3T

Mycgr3G108090 Mycgr3
  
Location: 36730-37591

Mycgr3G108090\_Mycgr3

Mycgr3G21922 Mycgr3T
  
Location: 37691-39149

Mycgr3G21922\_Mycgr3T

Mycgr3G99148 Mycgr3T
  
Location: 39249-42819

Mycgr3G99148\_Mycgr3T

hypothetical protein
  
Accession: EIT77735
  
Location: 98019-100130
  
 NCBI BlastP on this gene

EIT77735

hypothetical protein
  
Accession: EIT77713
  
Location: 100849-101678
  
 NCBI BlastP on this gene

EIT77713

ATP-dependent RNA helicase A
  
Accession: EIT77726
  
Location: 102212-104760
  
 NCBI BlastP on this gene

EIT77726

hypothetical protein
  
Accession: EIT77730
  
Location: 106211-107107
  
 NCBI BlastP on this gene

EIT77730

60S Ribosomal protein
  
Accession: EIT77712
  
Location: 109806-110912
  
 NCBI BlastP on this gene

EIT77712

hypothetical protein
  
Accession: EIT77754
  
Location: 111848-113287
  
  
**BlastP hit with Mycgr3G90786\_Mycgr3T**
  
Percentage identity: 26 %
  
BlastP bit score: 98
  
Sequence coverage: 99 %
  
E-value: 7e-19
  
  
 NCBI BlastP on this gene

EIT77754

leucine-rich repeat protein
  
Accession: EIT77750
  
Location: 115497-118442
  
  
**BlastP hit with Mycgr3G68433\_Mycgr3T**
  
Percentage identity: 41 %
  
BlastP bit score: 484
  
Sequence coverage: 85 %
  
E-value: 3e-150
  
  
 NCBI BlastP on this gene

EIT77750

hypothetical protein
  
Accession: EIT77738
  
Location: 118711-119847
  
 NCBI BlastP on this gene

EIT77738

actin-related protein - Arp6p
  
Accession: EIT77734
  
Location: 120682-122259
  
 NCBI BlastP on this gene

EIT77734

hypothetical protein
  
Accession: EIT77732
  
Location: 124116-124477
  
 NCBI BlastP on this gene

EIT77732

3-hydroxyacyl-CoA dehydrogenase
  
Accession: EIT77749
  
Location: 124834-125948
  
 NCBI BlastP on this gene

EIT77749

DNA topoisomerase I-interacting protein
  
Accession: EIT77739
  
Location: 126275-129935
  
  
**BlastP hit with Mycgr3G68421\_Mycgr3T**
  
Percentage identity: 44 %
  
BlastP bit score: 940
  
Sequence coverage: 101 %
  
E-value: 0.0
  
  
 NCBI BlastP on this gene

EIT77739

RNA polymerase II transcription initiation protein
  
Accession: EIT77745
  
Location: 133616-135185
  
 NCBI BlastP on this gene

EIT77745

DEAD box containing helicase
  
Accession: EIT77743
  
Location: 137082-141538
  
 NCBI BlastP on this gene

EIT77743

nuclear matrix protein
  
Accession: EIT77751
  
Location: 141782-144142
  
 NCBI BlastP on this gene

EIT77751

Query: Architecture Search FASTA input

ACFW01000015 : Coccidioides posadasii C735 delta SOWgp    Total score: 3.0     Cumulative Blast bit score: 1483

Hit cluster cross-links:

Mycgr3G90785 Mycgr3T
  
Location: 0-1047

Mycgr3G90785\_Mycgr3T

Mycgr3G103262 Mycgr3
  
Location: 1147-1390

Mycgr3G103262\_Mycgr3

Mycgr3G68458 Mycgr3T
  
Location: 1490-3602

Mycgr3G68458\_Mycgr3T

Mycgr3G99145 Mycgr3T
  
Location: 3702-4326

Mycgr3G99145\_Mycgr3T

Mycgr3G103274 Mycgr3
  
Location: 4426-4957

Mycgr3G103274\_Mycgr3

Mycgr3G103264 Mycgr3
  
Location: 5057-5390

Mycgr3G103264\_Mycgr3

Mycgr3G37570 Mycgr3T
  
Location: 5490-6006

Mycgr3G37570\_Mycgr3T

Mycgr3G108094 Mycgr3
  
Location: 6106-10555

Mycgr3G108094\_Mycgr3

Mycgr3G90786 Mycgr3T
  
Location: 10655-12080

Mycgr3G90786\_Mycgr3T

Mycgr3G68429 Mycgr3T
  
Location: 12180-13440

Mycgr3G68429\_Mycgr3T

Mycgr3G68421 Mycgr3T
  
Location: 13540-17086

Mycgr3G68421\_Mycgr3T

Mycgr3G90801 Mycgr3T
  
Location: 17186-18056

Mycgr3G90801\_Mycgr3T

Mycgr3G84646 Mycgr3T
  
Location: 18156-20235

Mycgr3G84646\_Mycgr3T

Mycgr3G68456 Mycgr3T
  
Location: 20335-21970

Mycgr3G68456\_Mycgr3T

Mycgr3G103270 Mycgr3
  
Location: 22070-22355

Mycgr3G103270\_Mycgr3

Mycgr3G90803 Mycgr3T
  
Location: 22455-23019

Mycgr3G90803\_Mycgr3T

Mycgr3G36941 Mycgr3T
  
Location: 23119-24064

Mycgr3G36941\_Mycgr3T

Mycgr3G25746 Mycgr3T
  
Location: 24164-25241

Mycgr3G25746\_Mycgr3T

Mycgr3G90788 Mycgr3T
  
Location: 25341-25803

Mycgr3G90788\_Mycgr3T

Mycgr3G103260 Mycgr3
  
Location: 25903-26635

Mycgr3G103260\_Mycgr3

Mycgr3G84644 Mycgr3T
  
Location: 26735-28457

Mycgr3G84644\_Mycgr3T

Mycgr3G29227 Mycgr3T
  
Location: 28557-28863

Mycgr3G29227\_Mycgr3T

Mycgr3G36271 Mycgr3T
  
Location: 28963-29854

Mycgr3G36271\_Mycgr3T

Mycgr3G68433 Mycgr3T
  
Location: 29954-33041

Mycgr3G68433\_Mycgr3T

Mycgr3G79452 Mycgr3T
  
Location: 33141-33399

Mycgr3G79452\_Mycgr3T

Mycgr3G55345 Mycgr3T
  
Location: 33499-34126

Mycgr3G55345\_Mycgr3T

Mycgr3G103278 Mycgr3
  
Location: 34226-35195

Mycgr3G103278\_Mycgr3

Mycgr3G84654 Mycgr3T
  
Location: 35295-36630

Mycgr3G84654\_Mycgr3T

Mycgr3G108090 Mycgr3
  
Location: 36730-37591

Mycgr3G108090\_Mycgr3

Mycgr3G21922 Mycgr3T
  
Location: 37691-39149

Mycgr3G21922\_Mycgr3T

Mycgr3G99148 Mycgr3T
  
Location: 39249-42819

Mycgr3G99148\_Mycgr3T

Deuterolysin metalloprotease family protein
  
Accession: EER28352
  
Location: 526622-527770
  
 NCBI BlastP on this gene

EER28352

hypothetical protein
  
Accession: EER28353
  
Location: 531595-533980
  
 NCBI BlastP on this gene

EER28353

hypothetical protein
  
Accession: EER28354
  
Location: 534399-535208
  
 NCBI BlastP on this gene

EER28354

hypothetical protein
  
Accession: EER28355
  
Location: 535482-537453
  
 NCBI BlastP on this gene

EER28355

60S ribosomal protein L13, putative
  
Accession: EER28356
  
Location: 538177-539301
  
 NCBI BlastP on this gene

EER28356

hypothetical protein
  
Accession: EER28357
  
Location: 539956-541413
  
  
**BlastP hit with Mycgr3G90786\_Mycgr3T**
  
Percentage identity: 32 %
  
BlastP bit score: 116
  
Sequence coverage: 98 %
  
E-value: 5e-25
  
  
 NCBI BlastP on this gene

EER28357

Leucine Rich Repeat family protein
  
Accession: EER28358
  
Location: 542407-545343
  
  
**BlastP hit with Mycgr3G68433\_Mycgr3T**
  
Percentage identity: 42 %
  
BlastP bit score: 447
  
Sequence coverage: 60 %
  
E-value: 1e-136
  
  
 NCBI BlastP on this gene

EER28358

hypothetical protein
  
Accession: EER28359
  
Location: 546186-547376
  
 NCBI BlastP on this gene

EER28359

Actin family protein
  
Accession: EER28360
  
Location: 548525-550111
  
 NCBI BlastP on this gene

EER28360

C3HC4 type (RING finger) zinc finger containing protein
  
Accession: EER28361
  
Location: 553164-555588
  
 NCBI BlastP on this gene

EER28361

3-hydroxybutyryl-CoA dehydrogenase, putative
  
Accession: EER28362
  
Location: 556337-557445
  
 NCBI BlastP on this gene

EER28362

Timeless protein
  
Accession: EER28363
  
Location: 557899-561586
  
  
**BlastP hit with Mycgr3G68421\_Mycgr3T**
  
Percentage identity: 46 %
  
BlastP bit score: 920
  
Sequence coverage: 92 %
  
E-value: 0.0
  
  
 NCBI BlastP on this gene

EER28363

phenazine biosynthesis family protein
  
Accession: EER28364
  
Location: 562151-563119
  
 NCBI BlastP on this gene

EER28364

hypothetical protein
  
Accession: EER28365
  
Location: 563459-565312
  
 NCBI BlastP on this gene

EER28365

Transcription factor tfb2 family protein
  
Accession: EER28366
  
Location: 565534-567121
  
 NCBI BlastP on this gene

EER28366

thioesterase family protein
  
Accession: EER28367
  
Location: 567367-568663
  
 NCBI BlastP on this gene

EER28367

hypothetical protein
  
Accession: EER28368
  
Location: 569013-573421
  
 NCBI BlastP on this gene

EER28368

hypothetical protein
  
Accession: EER28369
  
Location: 573703-575944
  
 NCBI BlastP on this gene

EER28369

Query: Architecture Search FASTA input

DS989828 : Arthroderma gypseum CBS 118893 supercont1.7 genomic scaffold    Total score: 3.0     Cumulative Blast bit score: 1482

Hit cluster cross-links:

Mycgr3G90785 Mycgr3T
  
Location: 0-1047

Mycgr3G90785\_Mycgr3T

Mycgr3G103262 Mycgr3
  
Location: 1147-1390

Mycgr3G103262\_Mycgr3

Mycgr3G68458 Mycgr3T
  
Location: 1490-3602

Mycgr3G68458\_Mycgr3T

Mycgr3G99145 Mycgr3T
  
Location: 3702-4326

Mycgr3G99145\_Mycgr3T

Mycgr3G103274 Mycgr3
  
Location: 4426-4957

Mycgr3G103274\_Mycgr3

Mycgr3G103264 Mycgr3
  
Location: 5057-5390

Mycgr3G103264\_Mycgr3

Mycgr3G37570 Mycgr3T
  
Location: 5490-6006

Mycgr3G37570\_Mycgr3T

Mycgr3G108094 Mycgr3
  
Location: 6106-10555

Mycgr3G108094\_Mycgr3

Mycgr3G90786 Mycgr3T
  
Location: 10655-12080

Mycgr3G90786\_Mycgr3T

Mycgr3G68429 Mycgr3T
  
Location: 12180-13440

Mycgr3G68429\_Mycgr3T

Mycgr3G68421 Mycgr3T
  
Location: 13540-17086

Mycgr3G68421\_Mycgr3T

Mycgr3G90801 Mycgr3T
  
Location: 17186-18056

Mycgr3G90801\_Mycgr3T

Mycgr3G84646 Mycgr3T
  
Location: 18156-20235

Mycgr3G84646\_Mycgr3T

Mycgr3G68456 Mycgr3T
  
Location: 20335-21970

Mycgr3G68456\_Mycgr3T

Mycgr3G103270 Mycgr3
  
Location: 22070-22355

Mycgr3G103270\_Mycgr3

Mycgr3G90803 Mycgr3T
  
Location: 22455-23019

Mycgr3G90803\_Mycgr3T

Mycgr3G36941 Mycgr3T
  
Location: 23119-24064

Mycgr3G36941\_Mycgr3T

Mycgr3G25746 Mycgr3T
  
Location: 24164-25241

Mycgr3G25746\_Mycgr3T

Mycgr3G90788 Mycgr3T
  
Location: 25341-25803

Mycgr3G90788\_Mycgr3T

Mycgr3G103260 Mycgr3
  
Location: 25903-26635

Mycgr3G103260\_Mycgr3

Mycgr3G84644 Mycgr3T
  
Location: 26735-28457

Mycgr3G84644\_Mycgr3T

Mycgr3G29227 Mycgr3T
  
Location: 28557-28863

Mycgr3G29227\_Mycgr3T

Mycgr3G36271 Mycgr3T
  
Location: 28963-29854

Mycgr3G36271\_Mycgr3T

Mycgr3G68433 Mycgr3T
  
Location: 29954-33041

Mycgr3G68433\_Mycgr3T

Mycgr3G79452 Mycgr3T
  
Location: 33141-33399

Mycgr3G79452\_Mycgr3T

Mycgr3G55345 Mycgr3T
  
Location: 33499-34126

Mycgr3G55345\_Mycgr3T

Mycgr3G103278 Mycgr3
  
Location: 34226-35195

Mycgr3G103278\_Mycgr3

Mycgr3G84654 Mycgr3T
  
Location: 35295-36630

Mycgr3G84654\_Mycgr3T

Mycgr3G108090 Mycgr3
  
Location: 36730-37591

Mycgr3G108090\_Mycgr3

Mycgr3G21922 Mycgr3T
  
Location: 37691-39149

Mycgr3G21922\_Mycgr3T

Mycgr3G99148 Mycgr3T
  
Location: 39249-42819

Mycgr3G99148\_Mycgr3T

lovastatin nonaketide synthase
  
Accession: EFR04320
  
Location: 103213-110432
  
 NCBI BlastP on this gene

EFR04320

hypothetical protein
  
Accession: EFR04319
  
Location: 102186-102638
  
 NCBI BlastP on this gene

EFR04319

hypothetical protein
  
Accession: EFR04318
  
Location: 97944-100266
  
 NCBI BlastP on this gene

EFR04318

hypothetical protein
  
Accession: EFR04317
  
Location: 96678-97610
  
 NCBI BlastP on this gene

EFR04317

hypothetical protein
  
Accession: EFR04316
  
Location: 94443-96408
  
 NCBI BlastP on this gene

EFR04316

hypothetical protein
  
Accession: EFR04315
  
Location: 92795-93926
  
 NCBI BlastP on this gene

EFR04315

hypothetical protein
  
Accession: EFR04314
  
Location: 90475-92025
  
  
**BlastP hit with Mycgr3G90786\_Mycgr3T**
  
Percentage identity: 26 %
  
BlastP bit score: 74
  
Sequence coverage: 100 %
  
E-value: 4e-11
  
  
 NCBI BlastP on this gene

EFR04314

leucine-rich repeat-containing protein 40
  
Accession: EFR04313
  
Location: 86272-89301
  
  
**BlastP hit with Mycgr3G68433\_Mycgr3T**
  
Percentage identity: 37 %
  
BlastP bit score: 470
  
Sequence coverage: 89 %
  
E-value: 7e-145
  
  
 NCBI BlastP on this gene

EFR04313

hypothetical protein
  
Accession: EFR04312
  
Location: 84933-86063
  
 NCBI BlastP on this gene

EFR04312

actin
  
Accession: EFR04311
  
Location: 83030-84632
  
 NCBI BlastP on this gene

EFR04311

IBR domain-containing protein
  
Accession: EFR04310
  
Location: 79030-81351
  
 NCBI BlastP on this gene

EFR04310

fatty acid oxidation complex subunit alpha
  
Accession: EFR04309
  
Location: 77561-78667
  
 NCBI BlastP on this gene

EFR04309

topoisomerase 1-associated factor 1
  
Accession: EFR04308
  
Location: 73498-77180
  
  
**BlastP hit with Mycgr3G68421\_Mycgr3T**
  
Percentage identity: 43 %
  
BlastP bit score: 938
  
Sequence coverage: 102 %
  
E-value: 0.0
  
  
 NCBI BlastP on this gene

EFR04308

phenazine biosynthesis protein
  
Accession: EFR04307
  
Location: 72287-73276
  
 NCBI BlastP on this gene

EFR04307

hypothetical protein
  
Accession: EFR04306
  
Location: 70353-71878
  
 NCBI BlastP on this gene

EFR04306

TFIIH basal transcription factor complex p52 subunit
  
Accession: EFR04305
  
Location: 68394-69970
  
 NCBI BlastP on this gene

EFR04305

allantoin permease
  
Accession: EFR04304
  
Location: 65648-67713
  
 NCBI BlastP on this gene

EFR04304

PaaI-thioesterase
  
Accession: EFR04303
  
Location: 63080-64353
  
 NCBI BlastP on this gene

EFR04303

intron-binding protein aquarius
  
Accession: EFR04302
  
Location: 58171-62628
  
 NCBI BlastP on this gene

EFR04302

Query: Architecture Search FASTA input

GL636493 : Coccidioides posadasii str. Silveira unplaced genomic scaffold supercont2.8    Total score: 3.0     Cumulative Blast bit score: 1474

Hit cluster cross-links:

Mycgr3G90785 Mycgr3T
  
Location: 0-1047

Mycgr3G90785\_Mycgr3T

Mycgr3G103262 Mycgr3
  
Location: 1147-1390

Mycgr3G103262\_Mycgr3

Mycgr3G68458 Mycgr3T
  
Location: 1490-3602

Mycgr3G68458\_Mycgr3T

Mycgr3G99145 Mycgr3T
  
Location: 3702-4326

Mycgr3G99145\_Mycgr3T

Mycgr3G103274 Mycgr3
  
Location: 4426-4957

Mycgr3G103274\_Mycgr3

Mycgr3G103264 Mycgr3
  
Location: 5057-5390

Mycgr3G103264\_Mycgr3

Mycgr3G37570 Mycgr3T
  
Location: 5490-6006

Mycgr3G37570\_Mycgr3T

Mycgr3G108094 Mycgr3
  
Location: 6106-10555

Mycgr3G108094\_Mycgr3

Mycgr3G90786 Mycgr3T
  
Location: 10655-12080

Mycgr3G90786\_Mycgr3T

Mycgr3G68429 Mycgr3T
  
Location: 12180-13440

Mycgr3G68429\_Mycgr3T

Mycgr3G68421 Mycgr3T
  
Location: 13540-17086

Mycgr3G68421\_Mycgr3T

Mycgr3G90801 Mycgr3T
  
Location: 17186-18056

Mycgr3G90801\_Mycgr3T

Mycgr3G84646 Mycgr3T
  
Location: 18156-20235

Mycgr3G84646\_Mycgr3T

Mycgr3G68456 Mycgr3T
  
Location: 20335-21970

Mycgr3G68456\_Mycgr3T

Mycgr3G103270 Mycgr3
  
Location: 22070-22355

Mycgr3G103270\_Mycgr3

Mycgr3G90803 Mycgr3T
  
Location: 22455-23019

Mycgr3G90803\_Mycgr3T

Mycgr3G36941 Mycgr3T
  
Location: 23119-24064

Mycgr3G36941\_Mycgr3T

Mycgr3G25746 Mycgr3T
  
Location: 24164-25241

Mycgr3G25746\_Mycgr3T

Mycgr3G90788 Mycgr3T
  
Location: 25341-25803

Mycgr3G90788\_Mycgr3T

Mycgr3G103260 Mycgr3
  
Location: 25903-26635

Mycgr3G103260\_Mycgr3

Mycgr3G84644 Mycgr3T
  
Location: 26735-28457

Mycgr3G84644\_Mycgr3T

Mycgr3G29227 Mycgr3T
  
Location: 28557-28863

Mycgr3G29227\_Mycgr3T

Mycgr3G36271 Mycgr3T
  
Location: 28963-29854

Mycgr3G36271\_Mycgr3T

Mycgr3G68433 Mycgr3T
  
Location: 29954-33041

Mycgr3G68433\_Mycgr3T

Mycgr3G79452 Mycgr3T
  
Location: 33141-33399

Mycgr3G79452\_Mycgr3T

Mycgr3G55345 Mycgr3T
  
Location: 33499-34126

Mycgr3G55345\_Mycgr3T

Mycgr3G103278 Mycgr3
  
Location: 34226-35195

Mycgr3G103278\_Mycgr3

Mycgr3G84654 Mycgr3T
  
Location: 35295-36630

Mycgr3G84654\_Mycgr3T

Mycgr3G108090 Mycgr3
  
Location: 36730-37591

Mycgr3G108090\_Mycgr3

Mycgr3G21922 Mycgr3T
  
Location: 37691-39149

Mycgr3G21922\_Mycgr3T

Mycgr3G99148 Mycgr3T
  
Location: 39249-42819

Mycgr3G99148\_Mycgr3T

neutral protease 2
  
Accession: EFW18010
  
Location: 1073815-1074993
  
 NCBI BlastP on this gene

EFW18010

conserved hypothetical protein
  
Accession: EFW18009
  
Location: 1072552-1073535
  
 NCBI BlastP on this gene

EFW18009

conserved hypothetical protein
  
Accession: EFW18008
  
Location: 1071521-1071984
  
 NCBI BlastP on this gene

EFW18008

conserved hypothetical protein
  
Accession: EFW18007
  
Location: 1071008-1071509
  
 NCBI BlastP on this gene

EFW18007

conserved hypothetical protein
  
Accession: EFW18006
  
Location: 1067591-1069976
  
 NCBI BlastP on this gene

EFW18006

conserved hypothetical protein
  
Accession: EFW18005
  
Location: 1066363-1067172
  
 NCBI BlastP on this gene

EFW18005

conserved hypothetical protein
  
Accession: EFW18004
  
Location: 1064118-1066089
  
 NCBI BlastP on this gene

EFW18004

60S ribosomal protein L13
  
Accession: EFW18003
  
Location: 1062269-1063393
  
 NCBI BlastP on this gene

EFW18003

conserved hypothetical protein
  
Accession: EFW18002
  
Location: 1060152-1061743
  
  
**BlastP hit with Mycgr3G90786\_Mycgr3T**
  
Percentage identity: 32 %
  
BlastP bit score: 106
  
Sequence coverage: 65 %
  
E-value: 6e-22
  
  
 NCBI BlastP on this gene

EFW18002

leucine rich repeat protein
  
Accession: EFW18001
  
Location: 1056221-1059157
  
  
**BlastP hit with Mycgr3G68433\_Mycgr3T**
  
Percentage identity: 42 %
  
BlastP bit score: 448
  
Sequence coverage: 60 %
  
E-value: 1e-136
  
  
 NCBI BlastP on this gene

EFW18001

conserved hypothetical protein
  
Accession: EFW18000
  
Location: 1054187-1055377
  
 NCBI BlastP on this gene

EFW18000

actin
  
Accession: EFW17999
  
Location: 1051446-1053032
  
 NCBI BlastP on this gene

EFW17999

predicted protein
  
Accession: EFW17998
  
Location: 1050435-1050799
  
 NCBI BlastP on this gene

EFW17998

hypothetical protein
  
Accession: EFW17997
  
Location: 1045992-1048416
  
 NCBI BlastP on this gene

EFW17997

3-hydroxybutyryl CoA dehydrogenase
  
Accession: EFW17995
  
Location: 1044135-1045243
  
 NCBI BlastP on this gene

EFW17995

predicted protein
  
Accession: EFW17996
  
Location: 1043754-1044053
  
 NCBI BlastP on this gene

EFW17996

topoisomerase 1-associated factor 1
  
Accession: EFW17994
  
Location: 1039994-1043681
  
  
**BlastP hit with Mycgr3G68421\_Mycgr3T**
  
Percentage identity: 46 %
  
BlastP bit score: 920
  
Sequence coverage: 92 %
  
E-value: 0.0
  
  
 NCBI BlastP on this gene

EFW17994

conserved hypothetical protein
  
Accession: EFW17993
  
Location: 1038461-1039429
  
 NCBI BlastP on this gene

EFW17993

conserved hypothetical protein
  
Accession: EFW17992
  
Location: 1036253-1038105
  
 NCBI BlastP on this gene

EFW17992

TFIIH and nucleotide excision repair factor 3 complexes subunit
  
Accession: EFW17991
  
Location: 1034444-1036031
  
 NCBI BlastP on this gene

EFW17991

hypothetical protein
  
Accession: EFW17990
  
Location: 1032904-1034198
  
 NCBI BlastP on this gene

EFW17990

DEAD box helicase
  
Accession: EFW17989
  
Location: 1028167-1032554
  
 NCBI BlastP on this gene

EFW17989

nuclear matrix protein
  
Accession: EFW17988
  
Location: 1025620-1027861
  
 NCBI BlastP on this gene

EFW17988

Query: Architecture Search FASTA input

GG704913 : Coccidioides immitis RS genomic scaffold supercont3.3    Total score: 3.0     Cumulative Blast bit score: 1470

Hit cluster cross-links:

Mycgr3G90785 Mycgr3T
  
Location: 0-1047

Mycgr3G90785\_Mycgr3T

Mycgr3G103262 Mycgr3
  
Location: 1147-1390

Mycgr3G103262\_Mycgr3

Mycgr3G68458 Mycgr3T
  
Location: 1490-3602

Mycgr3G68458\_Mycgr3T

Mycgr3G99145 Mycgr3T
  
Location: 3702-4326

Mycgr3G99145\_Mycgr3T

Mycgr3G103274 Mycgr3
  
Location: 4426-4957

Mycgr3G103274\_Mycgr3

Mycgr3G103264 Mycgr3
  
Location: 5057-5390

Mycgr3G103264\_Mycgr3

Mycgr3G37570 Mycgr3T
  
Location: 5490-6006

Mycgr3G37570\_Mycgr3T

Mycgr3G108094 Mycgr3
  
Location: 6106-10555

Mycgr3G108094\_Mycgr3

Mycgr3G90786 Mycgr3T
  
Location: 10655-12080

Mycgr3G90786\_Mycgr3T

Mycgr3G68429 Mycgr3T
  
Location: 12180-13440

Mycgr3G68429\_Mycgr3T

Mycgr3G68421 Mycgr3T
  
Location: 13540-17086

Mycgr3G68421\_Mycgr3T

Mycgr3G90801 Mycgr3T
  
Location: 17186-18056

Mycgr3G90801\_Mycgr3T

Mycgr3G84646 Mycgr3T
  
Location: 18156-20235

Mycgr3G84646\_Mycgr3T

Mycgr3G68456 Mycgr3T
  
Location: 20335-21970

Mycgr3G68456\_Mycgr3T

Mycgr3G103270 Mycgr3
  
Location: 22070-22355

Mycgr3G103270\_Mycgr3

Mycgr3G90803 Mycgr3T
  
Location: 22455-23019

Mycgr3G90803\_Mycgr3T

Mycgr3G36941 Mycgr3T
  
Location: 23119-24064

Mycgr3G36941\_Mycgr3T

Mycgr3G25746 Mycgr3T
  
Location: 24164-25241

Mycgr3G25746\_Mycgr3T

Mycgr3G90788 Mycgr3T
  
Location: 25341-25803

Mycgr3G90788\_Mycgr3T

Mycgr3G103260 Mycgr3
  
Location: 25903-26635

Mycgr3G103260\_Mycgr3

Mycgr3G84644 Mycgr3T
  
Location: 26735-28457

Mycgr3G84644\_Mycgr3T

Mycgr3G29227 Mycgr3T
  
Location: 28557-28863

Mycgr3G29227\_Mycgr3T

Mycgr3G36271 Mycgr3T
  
Location: 28963-29854

Mycgr3G36271\_Mycgr3T

Mycgr3G68433 Mycgr3T
  
Location: 29954-33041

Mycgr3G68433\_Mycgr3T

Mycgr3G79452 Mycgr3T
  
Location: 33141-33399

Mycgr3G79452\_Mycgr3T

Mycgr3G55345 Mycgr3T
  
Location: 33499-34126

Mycgr3G55345\_Mycgr3T

Mycgr3G103278 Mycgr3
  
Location: 34226-35195

Mycgr3G103278\_Mycgr3

Mycgr3G84654 Mycgr3T
  
Location: 35295-36630

Mycgr3G84654\_Mycgr3T

Mycgr3G108090 Mycgr3
  
Location: 36730-37591

Mycgr3G108090\_Mycgr3

Mycgr3G21922 Mycgr3T
  
Location: 37691-39149

Mycgr3G21922\_Mycgr3T

Mycgr3G99148 Mycgr3T
  
Location: 39249-42819

Mycgr3G99148\_Mycgr3T

metalloproteinase 7
  
Accession: EAS30154
  
Location: 814935-816128
  
 NCBI BlastP on this gene

EAS30154

hypothetical protein
  
Accession: EAS30155
  
Location: 813687-814670
  
 NCBI BlastP on this gene

EAS30155

hypothetical protein
  
Accession: EAS30156
  
Location: 812758-813119
  
 NCBI BlastP on this gene

EAS30156

GTP binding protein
  
Accession: EAS30158
  
Location: 808725-811110
  
 NCBI BlastP on this gene

EAS30158

hypothetical protein
  
Accession: EAS30159
  
Location: 807497-808306
  
 NCBI BlastP on this gene

EAS30159

hypothetical protein
  
Accession: EAS30160
  
Location: 805252-807223
  
 NCBI BlastP on this gene

EAS30160

60S ribosomal protein L13E
  
Accession: EAS30161
  
Location: 803402-804526
  
 NCBI BlastP on this gene

EAS30161

hypothetical protein
  
Accession: EAS30162
  
Location: 801244-802710
  
  
**BlastP hit with Mycgr3G90786\_Mycgr3T**
  
Percentage identity: 30 %
  
BlastP bit score: 113
  
Sequence coverage: 103 %
  
E-value: 5e-24
  
  
 NCBI BlastP on this gene

EAS30162

hypothetical protein
  
Accession: EAS30163
  
Location: 797309-800245
  
  
**BlastP hit with Mycgr3G68433\_Mycgr3T**
  
Percentage identity: 42 %
  
BlastP bit score: 447
  
Sequence coverage: 60 %
  
E-value: 2e-136
  
  
 NCBI BlastP on this gene

EAS30163

hypothetical protein
  
Accession: EAS30164
  
Location: 795275-796465
  
 NCBI BlastP on this gene

EAS30164

actin-like protein arp6
  
Accession: EAS30165
  
Location: 792530-794116
  
 NCBI BlastP on this gene

EAS30165

IBR domain-containing protein, variant
  
Accession: EJB11161
  
Location: 788312-789499
  
 NCBI BlastP on this gene

EJB11161

IBR domain-containing protein
  
Accession: EJB11160
  
Location: 787075-789499
  
 NCBI BlastP on this gene

EJB11160

3-hydroxybutyryl CoA dehydrogenase
  
Accession: EAS30167
  
Location: 785227-786327
  
 NCBI BlastP on this gene

EAS30167

topoisomerase 1-associated factor 1
  
Accession: EAS30169
  
Location: 781084-784771
  
  
**BlastP hit with Mycgr3G68421\_Mycgr3T**
  
Percentage identity: 43 %
  
BlastP bit score: 910
  
Sequence coverage: 105 %
  
E-value: 0.0
  
  
 NCBI BlastP on this gene

EAS30169

PhzF family phenazine biosynthesis protein
  
Accession: EAS30170
  
Location: 779542-780510
  
 NCBI BlastP on this gene

EAS30170

hypothetical protein
  
Accession: EAS30171
  
Location: 777337-779191
  
 NCBI BlastP on this gene

EAS30171

transcription factor Tfb2
  
Accession: EAS30172
  
Location: 775528-777115
  
 NCBI BlastP on this gene

EAS30172

hypothetical protein
  
Accession: EAS30173
  
Location: 773969-775281
  
 NCBI BlastP on this gene

EAS30173

DEAD helicase superfamily protein
  
Accession: EAS30174
  
Location: 769208-773619
  
 NCBI BlastP on this gene

EAS30174

nuclear matrix protein
  
Accession: EAS30175
  
Location: 766692-768926
  
 NCBI BlastP on this gene

EAS30175

Query: Architecture Search FASTA input

GL573321 : Geomyces destructans 20631-21 unplaced genomic scaffold supercont1.153    Total score: 3.0     Cumulative Blast bit score: 1433

Hit cluster cross-links:

Mycgr3G90785 Mycgr3T
  
Location: 0-1047

Mycgr3G90785\_Mycgr3T

Mycgr3G103262 Mycgr3
  
Location: 1147-1390

Mycgr3G103262\_Mycgr3

Mycgr3G68458 Mycgr3T
  
Location: 1490-3602

Mycgr3G68458\_Mycgr3T

Mycgr3G99145 Mycgr3T
  
Location: 3702-4326

Mycgr3G99145\_Mycgr3T

Mycgr3G103274 Mycgr3
  
Location: 4426-4957

Mycgr3G103274\_Mycgr3

Mycgr3G103264 Mycgr3
  
Location: 5057-5390

Mycgr3G103264\_Mycgr3

Mycgr3G37570 Mycgr3T
  
Location: 5490-6006

Mycgr3G37570\_Mycgr3T

Mycgr3G108094 Mycgr3
  
Location: 6106-10555

Mycgr3G108094\_Mycgr3

Mycgr3G90786 Mycgr3T
  
Location: 10655-12080

Mycgr3G90786\_Mycgr3T

Mycgr3G68429 Mycgr3T
  
Location: 12180-13440

Mycgr3G68429\_Mycgr3T

Mycgr3G68421 Mycgr3T
  
Location: 13540-17086

Mycgr3G68421\_Mycgr3T

Mycgr3G90801 Mycgr3T
  
Location: 17186-18056

Mycgr3G90801\_Mycgr3T

Mycgr3G84646 Mycgr3T
  
Location: 18156-20235

Mycgr3G84646\_Mycgr3T

Mycgr3G68456 Mycgr3T
  
Location: 20335-21970

Mycgr3G68456\_Mycgr3T

Mycgr3G103270 Mycgr3
  
Location: 22070-22355

Mycgr3G103270\_Mycgr3

Mycgr3G90803 Mycgr3T
  
Location: 22455-23019

Mycgr3G90803\_Mycgr3T

Mycgr3G36941 Mycgr3T
  
Location: 23119-24064

Mycgr3G36941\_Mycgr3T

Mycgr3G25746 Mycgr3T
  
Location: 24164-25241

Mycgr3G25746\_Mycgr3T

Mycgr3G90788 Mycgr3T
  
Location: 25341-25803

Mycgr3G90788\_Mycgr3T

Mycgr3G103260 Mycgr3
  
Location: 25903-26635

Mycgr3G103260\_Mycgr3

Mycgr3G84644 Mycgr3T
  
Location: 26735-28457

Mycgr3G84644\_Mycgr3T

Mycgr3G29227 Mycgr3T
  
Location: 28557-28863

Mycgr3G29227\_Mycgr3T

Mycgr3G36271 Mycgr3T
  
Location: 28963-29854

Mycgr3G36271\_Mycgr3T

Mycgr3G68433 Mycgr3T
  
Location: 29954-33041

Mycgr3G68433\_Mycgr3T

Mycgr3G79452 Mycgr3T
  
Location: 33141-33399

Mycgr3G79452\_Mycgr3T

Mycgr3G55345 Mycgr3T
  
Location: 33499-34126

Mycgr3G55345\_Mycgr3T

Mycgr3G103278 Mycgr3
  
Location: 34226-35195

Mycgr3G103278\_Mycgr3

Mycgr3G84654 Mycgr3T
  
Location: 35295-36630

Mycgr3G84654\_Mycgr3T

Mycgr3G108090 Mycgr3
  
Location: 36730-37591

Mycgr3G108090\_Mycgr3

Mycgr3G21922 Mycgr3T
  
Location: 37691-39149

Mycgr3G21922\_Mycgr3T

Mycgr3G99148 Mycgr3T
  
Location: 39249-42819

Mycgr3G99148\_Mycgr3T

hypothetical protein
  
Accession: ELR03639
  
Location: 26252-27803
  
 NCBI BlastP on this gene

ELR03639

hypothetical protein
  
Accession: ELR03640
  
Location: 28836-30239
  
 NCBI BlastP on this gene

ELR03640

hypothetical protein
  
Accession: ELR03641
  
Location: 33561-34841
  
 NCBI BlastP on this gene

ELR03641

hypothetical protein
  
Accession: ELR03642
  
Location: 35109-37438
  
 NCBI BlastP on this gene

ELR03642

hypothetical protein
  
Accession: ELR03643
  
Location: 37808-38590
  
 NCBI BlastP on this gene

ELR03643

hypothetical protein
  
Accession: ELR03644
  
Location: 39348-39765
  
 NCBI BlastP on this gene

ELR03644

methylsterol monooxygenase
  
Accession: ELR03645
  
Location: 40953-42102
  
  
**BlastP hit with Mycgr3G36271\_Mycgr3T**
  
Percentage identity: 75 %
  
BlastP bit score: 458
  
Sequence coverage: 93 %
  
E-value: 2e-159
  
  
 NCBI BlastP on this gene

ELR03645

hypothetical protein
  
Accession: ELR03646
  
Location: 42856-45122
  
  
**BlastP hit with Mycgr3G84646\_Mycgr3T**
  
Percentage identity: 47 %
  
BlastP bit score: 613
  
Sequence coverage: 102 %
  
E-value: 0.0
  
  
 NCBI BlastP on this gene

ELR03646

hypothetical protein
  
Accession: ELR03647
  
Location: 45517-47113
  
 NCBI BlastP on this gene

ELR03647

hypothetical protein
  
Accession: ELR03648
  
Location: 48024-48958
  
  
**BlastP hit with Mycgr3G99145\_Mycgr3T**
  
Percentage identity: 85 %
  
BlastP bit score: 362
  
Sequence coverage: 99 %
  
E-value: 5e-124
  
  
 NCBI BlastP on this gene

ELR03648

hypothetical protein, variant
  
Accession: ELR03649
  
Location: 58465-59643
  
 NCBI BlastP on this gene

ELR03649

hypothetical protein
  
Accession: ELR03651
  
Location: 61045-62454
  
 NCBI BlastP on this gene

ELR03651

hypothetical protein
  
Accession: ELR03652
  
Location: 65687-68206
  
 NCBI BlastP on this gene

ELR03652

Query: Architecture Search FASTA input

DS231623 : Pyrenophora tritici-repentis Pt-1C-BFP supercont1.9 genomic scaffold    Total score: 3.0     Cumulative Blast bit score: 1414

Hit cluster cross-links:

Mycgr3G90785 Mycgr3T
  
Location: 0-1047

Mycgr3G90785\_Mycgr3T

Mycgr3G103262 Mycgr3
  
Location: 1147-1390

Mycgr3G103262\_Mycgr3

Mycgr3G68458 Mycgr3T
  
Location: 1490-3602

Mycgr3G68458\_Mycgr3T

Mycgr3G99145 Mycgr3T
  
Location: 3702-4326

Mycgr3G99145\_Mycgr3T

Mycgr3G103274 Mycgr3
  
Location: 4426-4957

Mycgr3G103274\_Mycgr3

Mycgr3G103264 Mycgr3
  
Location: 5057-5390

Mycgr3G103264\_Mycgr3

Mycgr3G37570 Mycgr3T
  
Location: 5490-6006

Mycgr3G37570\_Mycgr3T

Mycgr3G108094 Mycgr3
  
Location: 6106-10555

Mycgr3G108094\_Mycgr3

Mycgr3G90786 Mycgr3T
  
Location: 10655-12080

Mycgr3G90786\_Mycgr3T

Mycgr3G68429 Mycgr3T
  
Location: 12180-13440

Mycgr3G68429\_Mycgr3T

Mycgr3G68421 Mycgr3T
  
Location: 13540-17086

Mycgr3G68421\_Mycgr3T

Mycgr3G90801 Mycgr3T
  
Location: 17186-18056

Mycgr3G90801\_Mycgr3T

Mycgr3G84646 Mycgr3T
  
Location: 18156-20235

Mycgr3G84646\_Mycgr3T

Mycgr3G68456 Mycgr3T
  
Location: 20335-21970

Mycgr3G68456\_Mycgr3T

Mycgr3G103270 Mycgr3
  
Location: 22070-22355

Mycgr3G103270\_Mycgr3

Mycgr3G90803 Mycgr3T
  
Location: 22455-23019

Mycgr3G90803\_Mycgr3T

Mycgr3G36941 Mycgr3T
  
Location: 23119-24064

Mycgr3G36941\_Mycgr3T

Mycgr3G25746 Mycgr3T
  
Location: 24164-25241

Mycgr3G25746\_Mycgr3T

Mycgr3G90788 Mycgr3T
  
Location: 25341-25803

Mycgr3G90788\_Mycgr3T

Mycgr3G103260 Mycgr3
  
Location: 25903-26635

Mycgr3G103260\_Mycgr3

Mycgr3G84644 Mycgr3T
  
Location: 26735-28457

Mycgr3G84644\_Mycgr3T

Mycgr3G29227 Mycgr3T
  
Location: 28557-28863

Mycgr3G29227\_Mycgr3T

Mycgr3G36271 Mycgr3T
  
Location: 28963-29854

Mycgr3G36271\_Mycgr3T

Mycgr3G68433 Mycgr3T
  
Location: 29954-33041

Mycgr3G68433\_Mycgr3T

Mycgr3G79452 Mycgr3T
  
Location: 33141-33399

Mycgr3G79452\_Mycgr3T

Mycgr3G55345 Mycgr3T
  
Location: 33499-34126

Mycgr3G55345\_Mycgr3T

Mycgr3G103278 Mycgr3
  
Location: 34226-35195

Mycgr3G103278\_Mycgr3

Mycgr3G84654 Mycgr3T
  
Location: 35295-36630

Mycgr3G84654\_Mycgr3T

Mycgr3G108090 Mycgr3
  
Location: 36730-37591

Mycgr3G108090\_Mycgr3

Mycgr3G21922 Mycgr3T
  
Location: 37691-39149

Mycgr3G21922\_Mycgr3T

Mycgr3G99148 Mycgr3T
  
Location: 39249-42819

Mycgr3G99148\_Mycgr3T

hypothetical protein
  
Accession: EDU51065
  
Location: 164683-168286
  
 NCBI BlastP on this gene

EDU51065

conserved hypothetical protein
  
Accession: EDU51066
  
Location: 168652-169254
  
 NCBI BlastP on this gene

EDU51066

conserved hypothetical protein
  
Accession: EDU51067
  
Location: 169290-170287
  
 NCBI BlastP on this gene

EDU51067

L-lactate dehydrogenase
  
Accession: EDU51068
  
Location: 170677-172258
  
 NCBI BlastP on this gene

EDU51068

GTP-binding protein yptV5
  
Accession: EDU51069
  
Location: 173226-174364
  
 NCBI BlastP on this gene

EDU51069

N-acetylglucosamine-6-phosphate deacetylase
  
Accession: EDU51070
  
Location: 175005-176318
  
 NCBI BlastP on this gene

EDU51070

beta-hexosaminidase
  
Accession: EDU51071
  
Location: 176949-180017
  
 NCBI BlastP on this gene

EDU51071

acid phosphatase (PhoG)
  
Accession: EDU51072
  
Location: 181236-182889
  
  
**BlastP hit with Mycgr3G84654\_Mycgr3T**
  
Percentage identity: 50 %
  
BlastP bit score: 432
  
Sequence coverage: 114 %
  
E-value: 3e-143
  
  
 NCBI BlastP on this gene

EDU51072

hexokinase-1
  
Accession: EDU51073
  
Location: 186026-187794
  
 NCBI BlastP on this gene

EDU51073

hypothetical protein
  
Accession: EDU51074
  
Location: 189459-191084
  
 NCBI BlastP on this gene

EDU51074

glucosamine-6-phosphate deaminase
  
Accession: EDU51075
  
Location: 192422-193727
  
 NCBI BlastP on this gene

EDU51075

hypothetical protein
  
Accession: EDU51076
  
Location: 194311-196328
  
  
**BlastP hit with Mycgr3G84646\_Mycgr3T**
  
Percentage identity: 48 %
  
BlastP bit score: 619
  
Sequence coverage: 99 %
  
E-value: 0.0
  
  
 NCBI BlastP on this gene

EDU51076

GTP-binding protein SAS1
  
Accession: EDU51077
  
Location: 196809-197639
  
  
**BlastP hit with Mycgr3G99145\_Mycgr3T**
  
Percentage identity: 86 %
  
BlastP bit score: 363
  
Sequence coverage: 99 %
  
E-value: 3e-125
  
  
 NCBI BlastP on this gene

EDU51077

hypothetical protein
  
Accession: EDU51078
  
Location: 198707-199242
  
 NCBI BlastP on this gene

EDU51078

protein kinase rad3
  
Accession: EDU51079
  
Location: 200354-208154
  
 NCBI BlastP on this gene

EDU51079

cytochrome c oxidase-assembly factor cox-16, mitochondrial precursor
  
Accession: EDU51080
  
Location: 208548-208950
  
 NCBI BlastP on this gene

EDU51080

conserved hypothetical protein
  
Accession: EDU51081
  
Location: 209579-213187
  
 NCBI BlastP on this gene

EDU51081

Query: Architecture Search FASTA input

ABSU01000041 : Arthroderma benhamiae CBS 112371    Total score: 3.0     Cumulative Blast bit score: 1403

Hit cluster cross-links:

Mycgr3G90785 Mycgr3T
  
Location: 0-1047

Mycgr3G90785\_Mycgr3T

Mycgr3G103262 Mycgr3
  
Location: 1147-1390

Mycgr3G103262\_Mycgr3

Mycgr3G68458 Mycgr3T
  
Location: 1490-3602

Mycgr3G68458\_Mycgr3T

Mycgr3G99145 Mycgr3T
  
Location: 3702-4326

Mycgr3G99145\_Mycgr3T

Mycgr3G103274 Mycgr3
  
Location: 4426-4957

Mycgr3G103274\_Mycgr3

Mycgr3G103264 Mycgr3
  
Location: 5057-5390

Mycgr3G103264\_Mycgr3

Mycgr3G37570 Mycgr3T
  
Location: 5490-6006

Mycgr3G37570\_Mycgr3T

Mycgr3G108094 Mycgr3
  
Location: 6106-10555

Mycgr3G108094\_Mycgr3

Mycgr3G90786 Mycgr3T
  
Location: 10655-12080

Mycgr3G90786\_Mycgr3T

Mycgr3G68429 Mycgr3T
  
Location: 12180-13440

Mycgr3G68429\_Mycgr3T

Mycgr3G68421 Mycgr3T
  
Location: 13540-17086

Mycgr3G68421\_Mycgr3T

Mycgr3G90801 Mycgr3T
  
Location: 17186-18056

Mycgr3G90801\_Mycgr3T

Mycgr3G84646 Mycgr3T
  
Location: 18156-20235

Mycgr3G84646\_Mycgr3T

Mycgr3G68456 Mycgr3T
  
Location: 20335-21970

Mycgr3G68456\_Mycgr3T

Mycgr3G103270 Mycgr3
  
Location: 22070-22355

Mycgr3G103270\_Mycgr3

Mycgr3G90803 Mycgr3T
  
Location: 22455-23019

Mycgr3G90803\_Mycgr3T

Mycgr3G36941 Mycgr3T
  
Location: 23119-24064

Mycgr3G36941\_Mycgr3T

Mycgr3G25746 Mycgr3T
  
Location: 24164-25241

Mycgr3G25746\_Mycgr3T

Mycgr3G90788 Mycgr3T
  
Location: 25341-25803

Mycgr3G90788\_Mycgr3T

Mycgr3G103260 Mycgr3
  
Location: 25903-26635

Mycgr3G103260\_Mycgr3

Mycgr3G84644 Mycgr3T
  
Location: 26735-28457

Mycgr3G84644\_Mycgr3T

Mycgr3G29227 Mycgr3T
  
Location: 28557-28863

Mycgr3G29227\_Mycgr3T

Mycgr3G36271 Mycgr3T
  
Location: 28963-29854

Mycgr3G36271\_Mycgr3T

Mycgr3G68433 Mycgr3T
  
Location: 29954-33041

Mycgr3G68433\_Mycgr3T

Mycgr3G79452 Mycgr3T
  
Location: 33141-33399

Mycgr3G79452\_Mycgr3T

Mycgr3G55345 Mycgr3T
  
Location: 33499-34126

Mycgr3G55345\_Mycgr3T

Mycgr3G103278 Mycgr3
  
Location: 34226-35195

Mycgr3G103278\_Mycgr3

Mycgr3G84654 Mycgr3T
  
Location: 35295-36630

Mycgr3G84654\_Mycgr3T

Mycgr3G108090 Mycgr3
  
Location: 36730-37591

Mycgr3G108090\_Mycgr3

Mycgr3G21922 Mycgr3T
  
Location: 37691-39149

Mycgr3G21922\_Mycgr3T

Mycgr3G99148 Mycgr3T
  
Location: 39249-42819

Mycgr3G99148\_Mycgr3T

hypothetical protein
  
Accession: EFE29419
  
Location: 31008-33536
  
 NCBI BlastP on this gene

EFE29419

hypothetical protein
  
Accession: EFE29418
  
Location: 28059-29696
  
 NCBI BlastP on this gene

EFE29418

hypothetical protein
  
Accession: EFE29417
  
Location: 26810-27286
  
 NCBI BlastP on this gene

EFE29417

GTP binding protein, putative
  
Accession: EFE29416
  
Location: 24693-26353
  
 NCBI BlastP on this gene

EFE29416

hypothetical protein
  
Accession: EFE29415
  
Location: 23506-24369
  
 NCBI BlastP on this gene

EFE29415

conserved hypothetical protein
  
Accession: EFE29414
  
Location: 20848-23175
  
 NCBI BlastP on this gene

EFE29414

hypothetical protein
  
Accession: EFE29413
  
Location: 19778-20697
  
 NCBI BlastP on this gene

EFE29413

conserved hypothetical protein
  
Accession: EFE29412
  
Location: 17284-18834
  
  
**BlastP hit with Mycgr3G90786\_Mycgr3T**
  
Percentage identity: 28 %
  
BlastP bit score: 75
  
Sequence coverage: 88 %
  
E-value: 2e-11
  
  
 NCBI BlastP on this gene

EFE29412

hypothetical protein
  
Accession: EFE29411
  
Location: 16515-17092
  
 NCBI BlastP on this gene

EFE29411

conserved leucine-rich repeat protein
  
Accession: EFE29410
  
Location: 13142-15544
  
  
**BlastP hit with Mycgr3G68433\_Mycgr3T**
  
Percentage identity: 41 %
  
BlastP bit score: 456
  
Sequence coverage: 71 %
  
E-value: 6e-142
  
  
 NCBI BlastP on this gene

EFE29410

conserved hypothetical protein
  
Accession: EFE29409
  
Location: 11788-12915
  
 NCBI BlastP on this gene

EFE29409

hypothetical protein
  
Accession: EFE29408
  
Location: 10259-11610
  
 NCBI BlastP on this gene

EFE29408

hypothetical protein
  
Accession: EFE29407
  
Location: 5653-8232
  
 NCBI BlastP on this gene

EFE29407

3-hydroxyacyl-CoA dehydrogenase, putative
  
Accession: EFE29406
  
Location: 4345-5453
  
 NCBI BlastP on this gene

EFE29406

hypothetical protein
  
Accession: EFE29405
  
Location: 256-3946
  
  
**BlastP hit with Mycgr3G68421\_Mycgr3T**
  
Percentage identity: 42 %
  
BlastP bit score: 872
  
Sequence coverage: 101 %
  
E-value: 0.0
  
  
 NCBI BlastP on this gene

EFE29405

Query: Architecture Search FASTA input

1. :  CM001197 Mycosphaerella graminicola IPO323 chromosome 2     Total score: 31.0     Cumulative Blast bit score: 27011

Mycgr3G90785 Mycgr3T
  
Location: 0-1047
  
 NCBI BlastP on this gene

Mycgr3G90785\_Mycgr3T

Mycgr3G103262 Mycgr3
  
Location: 1147-1390
  
 NCBI BlastP on this gene

Mycgr3G103262\_Mycgr3

Mycgr3G68458 Mycgr3T
  
Location: 1490-3602
  
 NCBI BlastP on this gene

Mycgr3G68458\_Mycgr3T

Mycgr3G99145 Mycgr3T
  
Location: 3702-4326
  
 NCBI BlastP on this gene

Mycgr3G99145\_Mycgr3T

Mycgr3G103274 Mycgr3
  
Location: 4426-4957
  
 NCBI BlastP on this gene

Mycgr3G103274\_Mycgr3

Mycgr3G103264 Mycgr3
  
Location: 5057-5390
  
 NCBI BlastP on this gene

Mycgr3G103264\_Mycgr3

Mycgr3G37570 Mycgr3T
  
Location: 5490-6006
  
 NCBI BlastP on this gene

Mycgr3G37570\_Mycgr3T

Mycgr3G108094 Mycgr3
  
Location: 6106-10555
  
 NCBI BlastP on this gene

Mycgr3G108094\_Mycgr3

Mycgr3G90786 Mycgr3T
  
Location: 10655-12080
  
 NCBI BlastP on this gene

Mycgr3G90786\_Mycgr3T

Mycgr3G68429 Mycgr3T
  
Location: 12180-13440
  
 NCBI BlastP on this gene

Mycgr3G68429\_Mycgr3T

Mycgr3G68421 Mycgr3T
  
Location: 13540-17086
  
 NCBI BlastP on this gene

Mycgr3G68421\_Mycgr3T

Mycgr3G90801 Mycgr3T
  
Location: 17186-18056
  
 NCBI BlastP on this gene

Mycgr3G90801\_Mycgr3T

Mycgr3G84646 Mycgr3T
  
Location: 18156-20235
  
 NCBI BlastP on this gene

Mycgr3G84646\_Mycgr3T

Mycgr3G68456 Mycgr3T
  
Location: 20335-21970
  
 NCBI BlastP on this gene

Mycgr3G68456\_Mycgr3T

Mycgr3G103270 Mycgr3
  
Location: 22070-22355
  
 NCBI BlastP on this gene

Mycgr3G103270\_Mycgr3

Mycgr3G90803 Mycgr3T
  
Location: 22455-23019
  
 NCBI BlastP on this gene

Mycgr3G90803\_Mycgr3T

Mycgr3G36941 Mycgr3T
  
Location: 23119-24064
  
 NCBI BlastP on this gene

Mycgr3G36941\_Mycgr3T

Mycgr3G25746 Mycgr3T
  
Location: 24164-25241
  
 NCBI BlastP on this gene

Mycgr3G25746\_Mycgr3T

Mycgr3G90788 Mycgr3T
  
Location: 25341-25803
  
 NCBI BlastP on this gene

Mycgr3G90788\_Mycgr3T

Mycgr3G103260 Mycgr3
  
Location: 25903-26635
  
 NCBI BlastP on this gene

Mycgr3G103260\_Mycgr3

Mycgr3G84644 Mycgr3T
  
Location: 26735-28457
  
 NCBI BlastP on this gene

Mycgr3G84644\_Mycgr3T

Mycgr3G29227 Mycgr3T
  
Location: 28557-28863
  
 NCBI BlastP on this gene

Mycgr3G29227\_Mycgr3T

Mycgr3G36271 Mycgr3T
  
Location: 28963-29854
  
 NCBI BlastP on this gene

Mycgr3G36271\_Mycgr3T

Mycgr3G68433 Mycgr3T
  
Location: 29954-33041
  
 NCBI BlastP on this gene

Mycgr3G68433\_Mycgr3T

Mycgr3G79452 Mycgr3T
  
Location: 33141-33399
  
 NCBI BlastP on this gene

Mycgr3G79452\_Mycgr3T

Mycgr3G55345 Mycgr3T
  
Location: 33499-34126
  
 NCBI BlastP on this gene

Mycgr3G55345\_Mycgr3T

Mycgr3G103278 Mycgr3
  
Location: 34226-35195
  
 NCBI BlastP on this gene

Mycgr3G103278\_Mycgr3

Mycgr3G84654 Mycgr3T
  
Location: 35295-36630
  
 NCBI BlastP on this gene

Mycgr3G84654\_Mycgr3T

Mycgr3G108090 Mycgr3
  
Location: 36730-37591
  
 NCBI BlastP on this gene

Mycgr3G108090\_Mycgr3

Mycgr3G21922 Mycgr3T
  
Location: 37691-39149
  
 NCBI BlastP on this gene

Mycgr3G21922\_Mycgr3T

Mycgr3G99148 Mycgr3T
  
Location: 39249-42819
  
 NCBI BlastP on this gene

Mycgr3G99148\_Mycgr3T

hypothetical protein
  
Accession: EGP90508
  
Location: 1932911-1933903
  
 NCBI BlastP on this gene

EGP90508

hypothetical protein
  
Accession: EGP90507
  
Location: 1934751-1938296
  
  
**BlastP hit with Mycgr3G68421\_Mycgr3T**
  
Percentage identity: 100 %
  
BlastP bit score: 2434
  
Sequence coverage: 99 %
  
E-value: 0.0
  
  
 NCBI BlastP on this gene

EGP90507

serine/threonine protein kinase
  
Accession: EGP89932
  
Location: 1940327-1941058
  
  
**BlastP hit with Mycgr3G103260\_Mycgr3**
  
Percentage identity: 100 %
  
BlastP bit score: 507
  
Sequence coverage: 100 %
  
E-value: 1e-180
  
  
 NCBI BlastP on this gene

EGP89932

hypothetical protein
  
Accession: EGP89933
  
Location: 1941249-1941491
  
  
**BlastP hit with Mycgr3G103262\_Mycgr3**
  
Percentage identity: 100 %
  
BlastP bit score: 163
  
Sequence coverage: 98 %
  
E-value: 3e-50
  
  
 NCBI BlastP on this gene

EGP89933

hypothetical protein
  
Accession: EGP90506
  
Location: 1947793-1949108
  
  
**BlastP hit with Mycgr3G68429\_Mycgr3T**
  
Percentage identity: 100 %
  
BlastP bit score: 872
  
Sequence coverage: 99 %
  
E-value: 0.0
  
  
 NCBI BlastP on this gene

EGP90506

hypothetical protein
  
Accession: EGP89934
  
Location: 1964576-1965321
  
  
**BlastP hit with Mycgr3G55345\_Mycgr3T**
  
Percentage identity: 100 %
  
BlastP bit score: 426
  
Sequence coverage: 99 %
  
E-value: 1e-149
  
  
 NCBI BlastP on this gene

EGP89934

hypothetical protein
  
Accession: EGP90505
  
Location: 1966036-1967143
  
  
**BlastP hit with Mycgr3G90785\_Mycgr3T**
  
Percentage identity: 100 %
  
BlastP bit score: 697
  
Sequence coverage: 99 %
  
E-value: 0.0
  
  
 NCBI BlastP on this gene

EGP90505

hypothetical protein
  
Accession: EGP90504
  
Location: 1968003-1969427
  
  
**BlastP hit with Mycgr3G90786\_Mycgr3T**
  
Percentage identity: 100 %
  
BlastP bit score: 963
  
Sequence coverage: 99 %
  
E-value: 0.0
  
  
 NCBI BlastP on this gene

EGP90504

hypothetical protein
  
Accession: EGP89935
  
Location: 1969994-1973326
  
  
**BlastP hit with Mycgr3G68433\_Mycgr3T**
  
Percentage identity: 100 %
  
BlastP bit score: 2045
  
Sequence coverage: 99 %
  
E-value: 0.0
  
  
 NCBI BlastP on this gene

EGP89935

hypothetical protein
  
Accession: EGP89936
  
Location: 1973872-1975059
  
  
**BlastP hit with Mycgr3G90788\_Mycgr3T**
  
Percentage identity: 100 %
  
BlastP bit score: 306
  
Sequence coverage: 99 %
  
E-value: 2e-104
  
  
 NCBI BlastP on this gene

EGP89936

hypothetical protein
  
Accession: EGP89937
  
Location: 1990316-1990621
  
  
**BlastP hit with Mycgr3G29227\_Mycgr3T**
  
Percentage identity: 100 %
  
BlastP bit score: 210
  
Sequence coverage: 100 %
  
E-value: 5e-68
  
  
 NCBI BlastP on this gene

EGP89937

hypothetical protein
  
Accession: EGP90503
  
Location: 1993131-1993519
  
  
**BlastP hit with Mycgr3G103264\_Mycgr3**
  
Percentage identity: 100 %
  
BlastP bit score: 226
  
Sequence coverage: 99 %
  
E-value: 4e-74
  
  
 NCBI BlastP on this gene

EGP90503

hypothetical protein
  
Accession: EGP89938
  
Location: 1993869-1994126
  
  
**BlastP hit with Mycgr3G79452\_Mycgr3T**
  
Percentage identity: 100 %
  
BlastP bit score: 176
  
Sequence coverage: 98 %
  
E-value: 6e-55
  
  
 NCBI BlastP on this gene

EGP89938

hypothetical protein
  
Accession: EGP90502
  
Location: 1997117-1998457
  
  
**BlastP hit with Mycgr3G108090\_Mycgr3**
  
Percentage identity: 100 %
  
BlastP bit score: 598
  
Sequence coverage: 99 %
  
E-value: 0.0
  
  
 NCBI BlastP on this gene

EGP90502

serine/threonine protein kinase, CMGC family
  
Accession: EGP89939
  
Location: 1999481-2001820
  
  
**BlastP hit with Mycgr3G84644\_Mycgr3T**
  
Percentage identity: 100 %
  
BlastP bit score: 1188
  
Sequence coverage: 99 %
  
E-value: 0.0
  
  
 NCBI BlastP on this gene

EGP89939

ERG25, C-4 methyl sterol oxidase
  
Accession: EGP89940
  
Location: 2003038-2003996
  
  
**BlastP hit with Mycgr3G36271\_Mycgr3T**
  
Percentage identity: 100 %
  
BlastP bit score: 617
  
Sequence coverage: 99 %
  
E-value: 0.0
  
  
 NCBI BlastP on this gene

EGP89940

hypothetical protein
  
Accession: EGP89941
  
Location: 2004703-2006781
  
  
**BlastP hit with Mycgr3G84646\_Mycgr3T**
  
Percentage identity: 100 %
  
BlastP bit score: 1396
  
Sequence coverage: 99 %
  
E-value: 0.0
  
  
 NCBI BlastP on this gene

EGP89941

hypothetical protein
  
Accession: EGP90501
  
Location: 2009481-2009765
  
  
**BlastP hit with Mycgr3G103270\_Mycgr3**
  
Percentage identity: 100 %
  
BlastP bit score: 184
  
Sequence coverage: 98 %
  
E-value: 4e-58
  
  
 NCBI BlastP on this gene

EGP90501

hypothetical protein
  
Accession: EGP89942
  
Location: 2010448-2011344
  
  
**BlastP hit with Mycgr3G99145\_Mycgr3T**
  
Percentage identity: 100 %
  
BlastP bit score: 424
  
Sequence coverage: 99 %
  
E-value: 3e-149
  
  
 NCBI BlastP on this gene

EGP89942

hypothetical protein
  
Accession: EGP90500
  
Location: 2011853-2013153
  
  
**BlastP hit with Mycgr3G25746\_Mycgr3T**
  
Percentage identity: 100 %
  
BlastP bit score: 729
  
Sequence coverage: 100 %
  
E-value: 0.0
  
  
 NCBI BlastP on this gene

EGP90500

hypothetical protein
  
Accession: EGP89943
  
Location: 2013492-2018012
  
  
**BlastP hit with Mycgr3G108094\_Mycgr3**
  
Percentage identity: 100 %
  
BlastP bit score: 2945
  
Sequence coverage: 99 %
  
E-value: 0.0
  
  
 NCBI BlastP on this gene

EGP89943

hypothetical protein
  
Accession: EGP89944
  
Location: 2018146-2018676
  
  
**BlastP hit with Mycgr3G103274\_Mycgr3**
  
Percentage identity: 100 %
  
BlastP bit score: 355
  
Sequence coverage: 99 %
  
E-value: 6e-123
  
  
 NCBI BlastP on this gene

EGP89944

hypothetical protein
  
Accession: EGP90499
  
Location: 2018850-2020307
  
  
**BlastP hit with Mycgr3G21922\_Mycgr3T**
  
Percentage identity: 100 %
  
BlastP bit score: 1018
  
Sequence coverage: 100 %
  
E-value: 0.0
  
  
 NCBI BlastP on this gene

EGP90499

hypothetical protein
  
Accession: EGP89945
  
Location: 2021780-2022748
  
  
**BlastP hit with Mycgr3G103278\_Mycgr3**
  
Percentage identity: 100 %
  
BlastP bit score: 655
  
Sequence coverage: 100 %
  
E-value: 0.0
  
  
 NCBI BlastP on this gene

EGP89945

hypothetical protein
  
Accession: EGP90498
  
Location: 2024313-2025478
  
  
**BlastP hit with Mycgr3G36941\_Mycgr3T**
  
Percentage identity: 100 %
  
BlastP bit score: 658
  
Sequence coverage: 99 %
  
E-value: 0.0
  
  
 NCBI BlastP on this gene

EGP90498

hypothetical protein
  
Accession: EGP90497
  
Location: 2027009-2027878
  
  
**BlastP hit with Mycgr3G90801\_Mycgr3T**
  
Percentage identity: 100 %
  
BlastP bit score: 560
  
Sequence coverage: 99 %
  
E-value: 0.0
  
  
 NCBI BlastP on this gene

EGP90497

hypothetical protein
  
Accession: EGP89946
  
Location: 2028620-2029135
  
  
**BlastP hit with Mycgr3G37570\_Mycgr3T**
  
Percentage identity: 100 %
  
BlastP bit score: 352
  
Sequence coverage: 99 %
  
E-value: 6e-122
  
  
 NCBI BlastP on this gene

EGP89946

hypothetical protein
  
Accession: EGP90496
  
Location: 2031715-2032371
  
  
**BlastP hit with Mycgr3G90803\_Mycgr3T**
  
Percentage identity: 100 %
  
BlastP bit score: 387
  
Sequence coverage: 99 %
  
E-value: 4e-135
  
  
 NCBI BlastP on this gene

EGP90496

large subunit of alpha-aminoadipate reductase
  
Accession: EGP90495
  
Location: 2032893-2036462
  
  
**BlastP hit with Mycgr3G99148\_Mycgr3T**
  
Percentage identity: 100 %
  
BlastP bit score: 2464
  
Sequence coverage: 99 %
  
E-value: 0.0
  
  
 NCBI BlastP on this gene

EGP90495

hypothetical protein
  
Accession: EGP89947
  
Location: 2038028-2039912
  
  
**BlastP hit with Mycgr3G68456\_Mycgr3T**
  
Percentage identity: 100 %
  
BlastP bit score: 1107
  
Sequence coverage: 99 %
  
E-value: 0.0
  
  
 NCBI BlastP on this gene

EGP89947

hypothetical protein
  
Accession: EGP89948
  
Location: 2040265-2042436
  
  
**BlastP hit with Mycgr3G68458\_Mycgr3T**
  
Percentage identity: 100 %
  
BlastP bit score: 1444
  
Sequence coverage: 99 %
  
E-value: 0.0
  
  
 NCBI BlastP on this gene

EGP89948

hypothetical protein
  
Accession: EGP90494
  
Location: 2044191-2046004
  
  
**BlastP hit with Mycgr3G84654\_Mycgr3T**
  
Percentage identity: 100 %
  
BlastP bit score: 905
  
Sequence coverage: 99 %
  
E-value: 0.0
  
  
 NCBI BlastP on this gene

EGP90494

hypothetical protein
  
Accession: EGP90493
  
Location: 2047154-2050036
  
 NCBI BlastP on this gene

EGP90493

2. :  KB445561 Baudoinia compniacensis UAMH 10762 unplaced genomic scaffold BAUCOscaffold\_12     Total score: 13.0     Cumulative Blast bit score: 6657

hypothetical protein
  
Accession: EMC92909
  
Location: 729503-730849
  
 NCBI BlastP on this gene

EMC92909

glycoside hydrolase family 11 protein
  
Accession: EMC92910
  
Location: 732389-732906
  
 NCBI BlastP on this gene

EMC92910

hypothetical protein
  
Accession: EMC92911
  
Location: 733433-733888
  
 NCBI BlastP on this gene

EMC92911

hypothetical protein
  
Accession: EMC92912
  
Location: 734078-735085
  
 NCBI BlastP on this gene

EMC92912

hypothetical protein
  
Accession: EMC92913
  
Location: 735345-737903
  
 NCBI BlastP on this gene

EMC92913

glycosyltransferase family 2 protein
  
Accession: EMC92914
  
Location: 738940-740440
  
 NCBI BlastP on this gene

EMC92914

hypothetical protein
  
Accession: EMC92915
  
Location: 741790-743457
  
 NCBI BlastP on this gene

EMC92915

hypothetical protein
  
Accession: EMC92916
  
Location: 744209-744818
  
 NCBI BlastP on this gene

EMC92916

hypothetical protein
  
Accession: EMC92917
  
Location: 746930-747223
  
 NCBI BlastP on this gene

EMC92917

hypothetical protein
  
Accession: EMC92918
  
Location: 748912-749796
  
  
**BlastP hit with Mycgr3G84644\_Mycgr3T**
  
Percentage identity: 82 %
  
BlastP bit score: 427
  
Sequence coverage: 44 %
  
E-value: 1e-143
  
  
 NCBI BlastP on this gene

EMC92918

hypothetical protein
  
Accession: EMC92919
  
Location: 750623-751564
  
  
**BlastP hit with Mycgr3G36271\_Mycgr3T**
  
Percentage identity: 84 %
  
BlastP bit score: 536
  
Sequence coverage: 98 %
  
E-value: 0.0
  
  
 NCBI BlastP on this gene

EMC92919

hypothetical protein
  
Accession: EMC92920
  
Location: 751772-752266
  
 NCBI BlastP on this gene

EMC92920

hypothetical protein
  
Accession: EMC92921
  
Location: 752849-754867
  
  
**BlastP hit with Mycgr3G84646\_Mycgr3T**
  
Percentage identity: 58 %
  
BlastP bit score: 799
  
Sequence coverage: 100 %
  
E-value: 0.0
  
  
 NCBI BlastP on this gene

EMC92921

hypothetical protein
  
Accession: EMC92922
  
Location: 755414-756097
  
  
**BlastP hit with Mycgr3G99145\_Mycgr3T**
  
Percentage identity: 94 %
  
BlastP bit score: 351
  
Sequence coverage: 85 %
  
E-value: 4e-120
  
  
 NCBI BlastP on this gene

EMC92922

hypothetical protein
  
Accession: EMC92923
  
Location: 756468-756789
  
 NCBI BlastP on this gene

EMC92923

hypothetical protein
  
Accession: EMC92924
  
Location: 757239-759197
  
 NCBI BlastP on this gene

EMC92924

hypothetical protein
  
Accession: EMC92925
  
Location: 759549-760554
  
 NCBI BlastP on this gene

EMC92925

hypothetical protein
  
Accession: EMC92926
  
Location: 761452-762288
  
 NCBI BlastP on this gene

EMC92926

hypothetical protein
  
Accession: EMC92927
  
Location: 762724-763711
  
 NCBI BlastP on this gene

EMC92927

hypothetical protein
  
Accession: EMC92928
  
Location: 763922-764290
  
 NCBI BlastP on this gene

EMC92928

hypothetical protein
  
Accession: EMC92929
  
Location: 764443-765270
  
 NCBI BlastP on this gene

EMC92929

hypothetical protein
  
Accession: EMC92930
  
Location: 766266-767018
  
 NCBI BlastP on this gene

EMC92930

hypothetical protein
  
Accession: EMC92931
  
Location: 768215-768463
  
 NCBI BlastP on this gene

EMC92931

hypothetical protein
  
Accession: EMC92932
  
Location: 769147-770486
  
  
**BlastP hit with Mycgr3G68429\_Mycgr3T**
  
Percentage identity: 63 %
  
BlastP bit score: 540
  
Sequence coverage: 100 %
  
E-value: 0.0
  
  
 NCBI BlastP on this gene

EMC92932

hypothetical protein
  
Accession: EMC92933
  
Location: 772220-774835
  
  
**BlastP hit with Mycgr3G103260\_Mycgr3**
  
Percentage identity: 63 %
  
BlastP bit score: 315
  
Sequence coverage: 97 %
  
E-value: 4e-98
  
  
 NCBI BlastP on this gene

EMC92933

hypothetical protein
  
Accession: EMC92934
  
Location: 775348-776018
  
  
**BlastP hit with Mycgr3G55345\_Mycgr3T**
  
Percentage identity: 79 %
  
BlastP bit score: 335
  
Sequence coverage: 97 %
  
E-value: 7e-114
  
  
 NCBI BlastP on this gene

EMC92934

hypothetical protein
  
Accession: EMC92935
  
Location: 776363-776767
  
 NCBI BlastP on this gene

EMC92935

hypothetical protein
  
Accession: EMC92936
  
Location: 776998-779526
  
  
**BlastP hit with Mycgr3G68433\_Mycgr3T**
  
Percentage identity: 49 %
  
BlastP bit score: 752
  
Sequence coverage: 84 %
  
E-value: 0.0
  
  
 NCBI BlastP on this gene

EMC92936

hypothetical protein
  
Accession: EMC92937
  
Location: 780610-782097
  
  
**BlastP hit with Mycgr3G90786\_Mycgr3T**
  
Percentage identity: 43 %
  
BlastP bit score: 300
  
Sequence coverage: 108 %
  
E-value: 3e-92
  
  
 NCBI BlastP on this gene

EMC92937

hypothetical protein
  
Accession: EMC92938
  
Location: 782923-783147
  
 NCBI BlastP on this gene

EMC92938

hypothetical protein
  
Accession: EMC92939
  
Location: 783623-783857
  
 NCBI BlastP on this gene

EMC92939

hypothetical protein
  
Accession: EMC92940
  
Location: 784078-784239
  
 NCBI BlastP on this gene

EMC92940

hypothetical protein
  
Accession: EMC92941
  
Location: 784270-788650
  
  
**BlastP hit with Mycgr3G108094\_Mycgr3**
  
Percentage identity: 46 %
  
BlastP bit score: 1043
  
Sequence coverage: 102 %
  
E-value: 0.0
  
  
 NCBI BlastP on this gene

EMC92941

hypothetical protein
  
Accession: EMC92942
  
Location: 788933-790130
  
  
**BlastP hit with Mycgr3G25746\_Mycgr3T**
  
Percentage identity: 66 %
  
BlastP bit score: 422
  
Sequence coverage: 100 %
  
E-value: 8e-144
  
  
 NCBI BlastP on this gene

EMC92942

hypothetical protein
  
Accession: EMC92943
  
Location: 790375-792369
  
  
**BlastP hit with Mycgr3G21922\_Mycgr3T**
  
Percentage identity: 62 %
  
BlastP bit score: 617
  
Sequence coverage: 100 %
  
E-value: 0.0
  
  
 NCBI BlastP on this gene

EMC92943

hypothetical protein
  
Accession: EMC92944
  
Location: 793401-795434
  
  
**BlastP hit with Mycgr3G103278\_Mycgr3**
  
Percentage identity: 47 %
  
BlastP bit score: 220
  
Sequence coverage: 96 %
  
E-value: 2e-62
  
  
 NCBI BlastP on this gene

EMC92944

hypothetical protein
  
Accession: EMC92945
  
Location: 795893-796865
  
 NCBI BlastP on this gene

EMC92945

hypothetical protein
  
Accession: EMC92946
  
Location: 798441-799070
  
 NCBI BlastP on this gene

EMC92946

hypothetical protein
  
Accession: EMC92947
  
Location: 800808-803474
  
 NCBI BlastP on this gene

EMC92947

hypothetical protein
  
Accession: EMC92948
  
Location: 804993-806041
  
 NCBI BlastP on this gene

EMC92948

hypothetical protein
  
Accession: EMC92949
  
Location: 806244-806873
  
 NCBI BlastP on this gene

EMC92949

hypothetical protein
  
Accession: EMC92950
  
Location: 808169-809099
  
 NCBI BlastP on this gene

EMC92950

hypothetical protein
  
Accession: EMC92951
  
Location: 809467-810243
  
 NCBI BlastP on this gene

EMC92951

hypothetical protein
  
Accession: EMC92952
  
Location: 811366-813977
  
 NCBI BlastP on this gene

EMC92952

hypothetical protein
  
Accession: EMC92953
  
Location: 814350-815029
  
 NCBI BlastP on this gene

EMC92953

3. :  KB456266 Mycosphaerella populorum SO2202 unplaced genomic scaffold SEPMUscaffold\_7     Total score: 8.0     Cumulative Blast bit score: 4669

hypothetical protein
  
Accession: EMF11490
  
Location: 1589447-1591198
  
 NCBI BlastP on this gene

EMF11490

hypothetical protein
  
Accession: EMF11491
  
Location: 1591570-1593436
  
 NCBI BlastP on this gene

EMF11491

hypothetical protein
  
Accession: EMF11492
  
Location: 1597749-1598179
  
 NCBI BlastP on this gene

EMF11492

ERG2 and sigma1 receptor-like protein
  
Accession: EMF11494
  
Location: 1601907-1602756
  
 NCBI BlastP on this gene

EMF11494

hypothetical protein
  
Accession: EMF11495
  
Location: 1603407-1605065
  
 NCBI BlastP on this gene

EMF11495

cytochrome b5
  
Accession: EMF11496
  
Location: 1605926-1606546
  
 NCBI BlastP on this gene

EMF11496

DUF383-domain-containing protein
  
Accession: EMF11497
  
Location: 1607592-1608966
  
  
**BlastP hit with Mycgr3G25746\_Mycgr3T**
  
Percentage identity: 70 %
  
BlastP bit score: 478
  
Sequence coverage: 98 %
  
E-value: 1e-164
  
  
 NCBI BlastP on this gene

EMF11497

hypothetical protein
  
Accession: EMF11498
  
Location: 1609288-1611783
  
  
**BlastP hit with Mycgr3G108094\_Mycgr3**
  
Percentage identity: 40 %
  
BlastP bit score: 439
  
Sequence coverage: 59 %
  
E-value: 2e-131
  
  
 NCBI BlastP on this gene

EMF11498

hypothetical protein
  
Accession: EMF11499
  
Location: 1614525-1615380
  
 NCBI BlastP on this gene

EMF11499

serine protein kinase Sky1
  
Accession: EMF11500
  
Location: 1615798-1618154
  
  
**BlastP hit with Mycgr3G84644\_Mycgr3T**
  
Percentage identity: 77 %
  
BlastP bit score: 931
  
Sequence coverage: 108 %
  
E-value: 0.0
  
  
 NCBI BlastP on this gene

EMF11500

Sterol desat-domain-containing protein
  
Accession: EMF11501
  
Location: 1619457-1620630
  
  
**BlastP hit with Mycgr3G36271\_Mycgr3T**
  
Percentage identity: 84 %
  
BlastP bit score: 546
  
Sequence coverage: 99 %
  
E-value: 0.0
  
  
 NCBI BlastP on this gene

EMF11501

NCA2-domain-containing protein
  
Accession: EMF11502
  
Location: 1621072-1623141
  
  
**BlastP hit with Mycgr3G84646\_Mycgr3T**
  
Percentage identity: 63 %
  
BlastP bit score: 857
  
Sequence coverage: 101 %
  
E-value: 0.0
  
  
 NCBI BlastP on this gene

EMF11502

GTP-binding protein SAS1
  
Accession: EMF11503
  
Location: 1623864-1624769
  
  
**BlastP hit with Mycgr3G99145\_Mycgr3T**
  
Percentage identity: 93 %
  
BlastP bit score: 379
  
Sequence coverage: 99 %
  
E-value: 4e-131
  
  
 NCBI BlastP on this gene

EMF11503

Rap30/74 interaction domain-containing protein
  
Accession: EMF11504
  
Location: 1628045-1630111
  
  
**BlastP hit with Mycgr3G103278\_Mycgr3**
  
Percentage identity: 66 %
  
BlastP bit score: 376
  
Sequence coverage: 100 %
  
E-value: 1e-121
  
  
 NCBI BlastP on this gene

EMF11504

PAP/OAS1 substrate-binding domain-containing protein
  
Accession: EMF11505
  
Location: 1631495-1633393
  
  
**BlastP hit with Mycgr3G21922\_Mycgr3T**
  
Percentage identity: 66 %
  
BlastP bit score: 663
  
Sequence coverage: 102 %
  
E-value: 0.0
  
  
 NCBI BlastP on this gene

EMF11505

Peptidase M36-domain-containing protein
  
Accession: EMF11506
  
Location: 1634421-1636368
  
 NCBI BlastP on this gene

EMF11506

transcription initiation factor TFIID, TATA binding protein
  
Accession: EMF11507
  
Location: 1639543-1640431
  
 NCBI BlastP on this gene

EMF11507

glycosyltransferase family 2 protein
  
Accession: EMF11508
  
Location: 1641973-1642769
  
 NCBI BlastP on this gene

EMF11508

hypothetical protein
  
Accession: EMF11509
  
Location: 1644274-1645599
  
 NCBI BlastP on this gene

EMF11509

hypothetical protein
  
Accession: EMF11510
  
Location: 1646173-1648335
  
 NCBI BlastP on this gene

EMF11510

cat eye syndrome critical region protein 5 precursor
  
Accession: EMF11511
  
Location: 1649553-1651078
  
 NCBI BlastP on this gene

EMF11511

hypothetical protein
  
Accession: EMF11512
  
Location: 1651109-1651483
  
 NCBI BlastP on this gene

EMF11512

4. :  KB446542 Dothistroma septosporum NZE10 unplaced genomic scaffold DOTSEscaffold\_8     Total score: 6.0     Cumulative Blast bit score: 2535

hypothetical protein
  
Accession: EME41895
  
Location: 1799715-1800677
  
 NCBI BlastP on this gene

EME41895

hypothetical protein
  
Accession: EME41894
  
Location: 1796467-1798050
  
 NCBI BlastP on this gene

EME41894

hypothetical protein
  
Accession: EME41893
  
Location: 1794389-1795843
  
 NCBI BlastP on this gene

EME41893

hypothetical protein
  
Accession: EME41892
  
Location: 1791873-1793172
  
 NCBI BlastP on this gene

EME41892

hypothetical protein
  
Accession: EME41891
  
Location: 1789238-1790498
  
 NCBI BlastP on this gene

EME41891

hypothetical protein
  
Accession: EME41890
  
Location: 1787211-1788689
  
 NCBI BlastP on this gene

EME41890

hypothetical protein
  
Accession: EME41889
  
Location: 1785871-1786797
  
 NCBI BlastP on this gene

EME41889

hypothetical protein
  
Accession: EME41888
  
Location: 1784878-1785828
  
 NCBI BlastP on this gene

EME41888

hypothetical protein
  
Accession: EME41887
  
Location: 1781820-1783742
  
 NCBI BlastP on this gene

EME41887

hypothetical protein
  
Accession: EME41886
  
Location: 1778673-1781162
  
  
**BlastP hit with Mycgr3G103260\_Mycgr3**
  
Percentage identity: 75 %
  
BlastP bit score: 396
  
Sequence coverage: 99 %
  
E-value: 2e-129
  
  
 NCBI BlastP on this gene

EME41886

hypothetical protein
  
Accession: EME41885
  
Location: 1775136-1776935
  
 NCBI BlastP on this gene

EME41885

hypothetical protein
  
Accession: EME41884
  
Location: 1773061-1774345
  
  
**BlastP hit with Mycgr3G68429\_Mycgr3T**
  
Percentage identity: 70 %
  
BlastP bit score: 593
  
Sequence coverage: 100 %
  
E-value: 0.0
  
  
 NCBI BlastP on this gene

EME41884

hypothetical protein
  
Accession: EME41883
  
Location: 1769399-1769734
  
 NCBI BlastP on this gene

EME41883

hypothetical protein
  
Accession: EME41881
  
Location: 1766447-1767113
  
  
**BlastP hit with Mycgr3G55345\_Mycgr3T**
  
Percentage identity: 83 %
  
BlastP bit score: 344
  
Sequence coverage: 97 %
  
E-value: 1e-117
  
  
 NCBI BlastP on this gene

EME41881

hypothetical protein
  
Accession: EME41880
  
Location: 1762953-1765403
  
  
**BlastP hit with Mycgr3G68433\_Mycgr3T**
  
Percentage identity: 55 %
  
BlastP bit score: 797
  
Sequence coverage: 79 %
  
E-value: 0.0
  
  
 NCBI BlastP on this gene

EME41880

hypothetical protein
  
Accession: EME41879
  
Location: 1760080-1761552
  
  
**BlastP hit with Mycgr3G90786\_Mycgr3T**
  
Percentage identity: 46 %
  
BlastP bit score: 328
  
Sequence coverage: 104 %
  
E-value: 4e-103
  
  
 NCBI BlastP on this gene

EME41879

hypothetical protein
  
Accession: EME41878
  
Location: 1758555-1759749
  
  
**BlastP hit with Mycgr3G90785\_Mycgr3T**
  
Percentage identity: 28 %
  
BlastP bit score: 77
  
Sequence coverage: 90 %
  
E-value: 2e-12
  
  
 NCBI BlastP on this gene

EME41878

hypothetical protein
  
Accession: EME41877
  
Location: 1754753-1757221
  
 NCBI BlastP on this gene

EME41877

hypothetical protein
  
Accession: EME41876
  
Location: 1753089-1754326
  
 NCBI BlastP on this gene

EME41876

hypothetical protein
  
Accession: EME41875
  
Location: 1748999-1750299
  
 NCBI BlastP on this gene

EME41875

hypothetical protein
  
Accession: EME41874
  
Location: 1747881-1748288
  
 NCBI BlastP on this gene

EME41874

hypothetical protein
  
Accession: EME41873
  
Location: 1744902-1745204
  
 NCBI BlastP on this gene

EME41873

hypothetical protein
  
Accession: EME41872
  
Location: 1744312-1744539
  
 NCBI BlastP on this gene

EME41872

hypothetical protein
  
Accession: EME41871
  
Location: 1741019-1743263
  
 NCBI BlastP on this gene

EME41871

hypothetical protein
  
Accession: EME41870
  
Location: 1739781-1740192
  
 NCBI BlastP on this gene

EME41870

5. :  DS995702 Microsporum canis CBS 113480 supercont1.2 genomic scaffold     Total score: 6.0     Cumulative Blast bit score: 1898

RNA transport protein 2
  
Accession: EEQ29993
  
Location: 3589178-3590939
  
 NCBI BlastP on this gene

EEQ29993

chromosome segregation in meiosis protein 3
  
Accession: EEQ29992
  
Location: 3587240-3588291
  
 NCBI BlastP on this gene

EEQ29992

NADH-ubiquinone oxidoreductase 21 kDa subunit
  
Accession: EEQ29991
  
Location: 3586025-3586929
  
 NCBI BlastP on this gene

EEQ29991

tRNA specific adenosine deaminase
  
Accession: EEQ29990
  
Location: 3585012-3585851
  
 NCBI BlastP on this gene

EEQ29990

UVSB PI-3 kinase
  
Accession: EEQ29989
  
Location: 3575186-3584568
  
 NCBI BlastP on this gene

EEQ29989

SR45
  
Accession: EEQ29988
  
Location: 3573901-3575161
  
 NCBI BlastP on this gene

EEQ29988

cell division cycle protein 48
  
Accession: EEQ29987
  
Location: 3570896-3573371
  
 NCBI BlastP on this gene

EEQ29987

C4-methylsterol oxidase
  
Accession: EEQ29986
  
Location: 3569019-3569939
  
  
**BlastP hit with Mycgr3G36271\_Mycgr3T**
  
Percentage identity: 80 %
  
BlastP bit score: 454
  
Sequence coverage: 87 %
  
E-value: 1e-158
  
  
 NCBI BlastP on this gene

EEQ29986

conserved hypothetical protein
  
Accession: EEQ29985
  
Location: 3565901-3568301
  
  
**BlastP hit with Mycgr3G84646\_Mycgr3T**
  
Percentage identity: 38 %
  
BlastP bit score: 440
  
Sequence coverage: 104 %
  
E-value: 1e-140
  
  
 NCBI BlastP on this gene

EEQ29985

GTP-binding protein SAS1
  
Accession: EEQ29984
  
Location: 3564332-3565152
  
  
**BlastP hit with Mycgr3G99145\_Mycgr3T**
  
Percentage identity: 81 %
  
BlastP bit score: 342
  
Sequence coverage: 99 %
  
E-value: 9e-117
  
  
 NCBI BlastP on this gene

EEQ29984

conserved hypothetical protein
  
Accession: EEQ29983
  
Location: 3562339-3563603
  
 NCBI BlastP on this gene

EEQ29983

predicted protein
  
Accession: EEQ29982
  
Location: 3561604-3562110
  
 NCBI BlastP on this gene

EEQ29982

rRNA-processing protein FCF1
  
Accession: EEQ29981
  
Location: 3559538-3560389
  
 NCBI BlastP on this gene

EEQ29981

conserved hypothetical protein
  
Accession: EEQ29980
  
Location: 3554045-3558791
  
 NCBI BlastP on this gene

EEQ29980

protein kinase domain-containing protein
  
Accession: EEQ29979
  
Location: 3552414-3553832
  
 NCBI BlastP on this gene

EEQ29979

myo-inositol-1-monophosphotase
  
Accession: EEQ29978
  
Location: 3551144-3552136
  
 NCBI BlastP on this gene

EEQ29978

HGH1
  
Accession: EEQ29977
  
Location: 3549365-3550700
  
  
**BlastP hit with Mycgr3G25746\_Mycgr3T**
  
Percentage identity: 54 %
  
BlastP bit score: 355
  
Sequence coverage: 100 %
  
E-value: 6e-117
  
  
 NCBI BlastP on this gene

EEQ29977

conserved hypothetical protein
  
Accession: EEQ29976
  
Location: 3547526-3549241
  
 NCBI BlastP on this gene

EEQ29976

conserved hypothetical protein
  
Accession: EEQ29975
  
Location: 3545836-3547500
  
 NCBI BlastP on this gene

EEQ29975

poly(A) polymerase Cid1
  
Accession: EEQ29974
  
Location: 3543991-3545715
  
  
**BlastP hit with Mycgr3G21922\_Mycgr3T**
  
Percentage identity: 42 %
  
BlastP bit score: 163
  
Sequence coverage: 45 %
  
E-value: 9e-41
  
  
 NCBI BlastP on this gene

EEQ29974

transcription initiation factor IIF subunit alpha
  
Accession: EEQ29973
  
Location: 3540814-3543069
  
  
**BlastP hit with Mycgr3G103278\_Mycgr3**
  
Percentage identity: 35 %
  
BlastP bit score: 144
  
Sequence coverage: 95 %
  
E-value: 6e-35
  
  
 NCBI BlastP on this gene

EEQ29973

IBR domain-containing protein
  
Accession: EEQ29972
  
Location: 3538764-3540189
  
 NCBI BlastP on this gene

EEQ29972

conserved hypothetical protein
  
Accession: EEQ29971
  
Location: 3536362-3538264
  
 NCBI BlastP on this gene

EEQ29971

conserved hypothetical protein
  
Accession: EEQ29970
  
Location: 3533380-3535421
  
 NCBI BlastP on this gene

EEQ29970

oligopeptide transporter
  
Accession: EEQ29969
  
Location: 3529672-3531669
  
 NCBI BlastP on this gene

EEQ29969

predicted protein
  
Accession: EEQ29968
  
Location: 3527853-3528746
  
 NCBI BlastP on this gene

EEQ29968

conserved hypothetical protein
  
Accession: EEQ29967
  
Location: 3523416-3524072
  
 NCBI BlastP on this gene

EEQ29967

6. :  DS989830 Arthroderma gypseum CBS 118893 supercont1.9 genomic scaffold     Total score: 6.0     Cumulative Blast bit score: 1895

fatty acid synthase subunit beta
  
Accession: EFR05513
  
Location: 735348-741685
  
 NCBI BlastP on this gene

EFR05513

cortical actin cytoskeleton protein asp1
  
Accession: EFR05514
  
Location: 743793-748547
  
 NCBI BlastP on this gene

EFR05514

U3 small nucleolar RNA-associated protein 21
  
Accession: EFR05515
  
Location: 748945-752393
  
 NCBI BlastP on this gene

EFR05515

hypothetical protein
  
Accession: EFR05516
  
Location: 752941-753612
  
 NCBI BlastP on this gene

EFR05516

hypothetical protein
  
Accession: EFR05517
  
Location: 754992-756399
  
  
**BlastP hit with Mycgr3G25746\_Mycgr3T**
  
Percentage identity: 56 %
  
BlastP bit score: 356
  
Sequence coverage: 98 %
  
E-value: 3e-117
  
  
 NCBI BlastP on this gene

EFR05517

hypothetical protein
  
Accession: EFR05518
  
Location: 756406-757849
  
 NCBI BlastP on this gene

EFR05518

hypothetical protein
  
Accession: EFR05519
  
Location: 757972-759804
  
  
**BlastP hit with Mycgr3G21922\_Mycgr3T**
  
Percentage identity: 45 %
  
BlastP bit score: 172
  
Sequence coverage: 45 %
  
E-value: 1e-43
  
  
 NCBI BlastP on this gene

EFR05519

transcription initiation factor IIF subunit alpha
  
Accession: EFR05520
  
Location: 760831-763286
  
  
**BlastP hit with Mycgr3G103278\_Mycgr3**
  
Percentage identity: 34 %
  
BlastP bit score: 137
  
Sequence coverage: 95 %
  
E-value: 2e-32
  
  
 NCBI BlastP on this gene

EFR05520

inositol monophosphatase 2
  
Accession: EFR05521
  
Location: 764390-765422
  
 NCBI BlastP on this gene

EFR05521

hypothetical protein
  
Accession: EFR05522
  
Location: 767126-769170
  
 NCBI BlastP on this gene

EFR05522

glycogen synthase kinase mutation revertant
  
Accession: EFR05523
  
Location: 769703-774506
  
 NCBI BlastP on this gene

EFR05523

rRNA-processing protein FCF1
  
Accession: EFR05524
  
Location: 775185-776061
  
 NCBI BlastP on this gene

EFR05524

hypothetical protein
  
Accession: EFR05525
  
Location: 776359-777644
  
 NCBI BlastP on this gene

EFR05525

GTP-binding protein
  
Accession: EFR05526
  
Location: 778341-779161
  
  
**BlastP hit with Mycgr3G99145\_Mycgr3T**
  
Percentage identity: 81 %
  
BlastP bit score: 337
  
Sequence coverage: 100 %
  
E-value: 1e-114
  
  
 NCBI BlastP on this gene

EFR05526

ATPase 2 nuclear control
  
Accession: EFR05527
  
Location: 780098-782562
  
  
**BlastP hit with Mycgr3G84646\_Mycgr3T**
  
Percentage identity: 36 %
  
BlastP bit score: 435
  
Sequence coverage: 105 %
  
E-value: 2e-138
  
  
 NCBI BlastP on this gene

EFR05527

C-4 methylsterol oxidase
  
Accession: EFR05528
  
Location: 783421-784351
  
  
**BlastP hit with Mycgr3G36271\_Mycgr3T**
  
Percentage identity: 81 %
  
BlastP bit score: 458
  
Sequence coverage: 87 %
  
E-value: 4e-160
  
  
 NCBI BlastP on this gene

EFR05528

peroxisome biogenesis factor 1
  
Accession: EFR05529
  
Location: 785322-787774
  
 NCBI BlastP on this gene

EFR05529

hypothetical protein
  
Accession: EFR05530
  
Location: 788331-789566
  
 NCBI BlastP on this gene

EFR05530

kinase rad3
  
Accession: EFR05531
  
Location: 790004-798936
  
 NCBI BlastP on this gene

EFR05531

tRNA-specific adenosine deaminase subunit TAD2
  
Accession: EFR05532
  
Location: 799425-800255
  
 NCBI BlastP on this gene

EFR05532

NADH-ubiquinone oxidoreductase subunit
  
Accession: EFR05533
  
Location: 800438-801311
  
 NCBI BlastP on this gene

EFR05533

chromosome segregation in meiosis protein 3
  
Accession: EFR05534
  
Location: 801636-802714
  
 NCBI BlastP on this gene

EFR05534

RNA transporter 2
  
Accession: EFR05535
  
Location: 803445-805210
  
 NCBI BlastP on this gene

EFR05535

7. :  DS995737 Trichophyton equinum CBS 127.97 supercont1.20 genomic scaffold     Total score: 6.0     Cumulative Blast bit score: 1878

fatty acid synthase beta subunit dehydratase
  
Accession: EGE05135
  
Location: 274683-280682
  
 NCBI BlastP on this gene

EGE05135

hypothetical protein
  
Accession: EGE05136
  
Location: 281396-281733
  
 NCBI BlastP on this gene

EGE05136

histidine acid phosphatase
  
Accession: EGE05137
  
Location: 282714-287154
  
 NCBI BlastP on this gene

EGE05137

snoRNA binding protein
  
Accession: EGE05138
  
Location: 287891-291336
  
 NCBI BlastP on this gene

EGE05138

hypothetical protein
  
Accession: EGE05139
  
Location: 291855-292526
  
 NCBI BlastP on this gene

EGE05139

hypothetical protein
  
Accession: EGE05140
  
Location: 293845-295242
  
  
**BlastP hit with Mycgr3G25746\_Mycgr3T**
  
Percentage identity: 56 %
  
BlastP bit score: 358
  
Sequence coverage: 98 %
  
E-value: 3e-118
  
  
 NCBI BlastP on this gene

EGE05140

hypothetical protein
  
Accession: EGE05141
  
Location: 295441-296701
  
 NCBI BlastP on this gene

EGE05141

poly(A) polymerase Cid1
  
Accession: EGE05142
  
Location: 296856-298709
  
  
**BlastP hit with Mycgr3G21922\_Mycgr3T**
  
Percentage identity: 43 %
  
BlastP bit score: 162
  
Sequence coverage: 45 %
  
E-value: 3e-40
  
  
 NCBI BlastP on this gene

EGE05142

transcription initiation factor IIF subunit alpha
  
Accession: EGE05143
  
Location: 299828-302276
  
  
**BlastP hit with Mycgr3G103278\_Mycgr3**
  
Percentage identity: 34 %
  
BlastP bit score: 137
  
Sequence coverage: 95 %
  
E-value: 2e-32
  
  
 NCBI BlastP on this gene

EGE05143

IBR finger domain-containing protein
  
Accession: EGE05144
  
Location: 302787-304182
  
 NCBI BlastP on this gene

EGE05144

hypothetical protein
  
Accession: EGE05145
  
Location: 304397-306408
  
 NCBI BlastP on this gene

EGE05145

myo inositol monophosphatase
  
Accession: EGE05146
  
Location: 307254-308279
  
 NCBI BlastP on this gene

EGE05146

Diacylglycerol kinase domain-containing protein
  
Accession: EGE05147
  
Location: 308482-313357
  
 NCBI BlastP on this gene

EGE05147

rRNA-processing protein FCF1
  
Accession: EGE05148
  
Location: 314078-314936
  
 NCBI BlastP on this gene

EGE05148

hypothetical protein
  
Accession: EGE05149
  
Location: 315142-316513
  
 NCBI BlastP on this gene

EGE05149

GTP-binding protein
  
Accession: EGE05150
  
Location: 317229-318098
  
  
**BlastP hit with Mycgr3G99145\_Mycgr3T**
  
Percentage identity: 81 %
  
BlastP bit score: 340
  
Sequence coverage: 100 %
  
E-value: 6e-116
  
  
 NCBI BlastP on this gene

EGE05150

hypothetical protein
  
Accession: EGE05151
  
Location: 319039-321515
  
  
**BlastP hit with Mycgr3G84646\_Mycgr3T**
  
Percentage identity: 37 %
  
BlastP bit score: 422
  
Sequence coverage: 108 %
  
E-value: 1e-133
  
  
 NCBI BlastP on this gene

EGE05151

C-4 methyl sterol oxidase Erg25
  
Accession: EGE05152
  
Location: 322274-323213
  
  
**BlastP hit with Mycgr3G36271\_Mycgr3T**
  
Percentage identity: 80 %
  
BlastP bit score: 459
  
Sequence coverage: 87 %
  
E-value: 2e-160
  
  
 NCBI BlastP on this gene

EGE05152

peroxisome biogenesis factor 1
  
Accession: EGE05153
  
Location: 324321-326828
  
 NCBI BlastP on this gene

EGE05153

hypothetical protein
  
Accession: EGE05154
  
Location: 327401-328693
  
 NCBI BlastP on this gene

EGE05154

hypothetical protein
  
Accession: EGE05155
  
Location: 329150-330380
  
 NCBI BlastP on this gene

EGE05155

hypothetical protein
  
Accession: EGE05156
  
Location: 330737-339828
  
 NCBI BlastP on this gene

EGE05156

cytidine and deoxycytidylate deaminase
  
Accession: EGE05157
  
Location: 340347-341166
  
 NCBI BlastP on this gene

EGE05157

NADH-ubiquinone oxidoreductase 21 kDa subunit
  
Accession: EGE05158
  
Location: 341273-342153
  
 NCBI BlastP on this gene

EGE05158

chromosome segregation in meiosis protein 3
  
Accession: EGE05159
  
Location: 342486-343539
  
 NCBI BlastP on this gene

EGE05159

8. :  GG700663 Trichophyton rubrum CBS 118892 genomic scaffold supercont2.16     Total score: 6.0     Cumulative Blast bit score: 1781

fatty acid synthase beta subunit dehydratase
  
Accession: EGD92313
  
Location: 37378-43714
  
 NCBI BlastP on this gene

EGD92313

hypothetical protein
  
Accession: EGD92314
  
Location: 44437-44777
  
 NCBI BlastP on this gene

EGD92314

cortical actin cytoskeleton protein asp1
  
Accession: EGD92315
  
Location: 45760-50223
  
 NCBI BlastP on this gene

EGD92315

WD repeat containing protein 36
  
Accession: EGD92316
  
Location: 50955-54404
  
 NCBI BlastP on this gene

EGD92316

hypothetical protein
  
Accession: EGD92317
  
Location: 54907-55578
  
 NCBI BlastP on this gene

EGD92317

DNA-binding protein HGH1
  
Accession: EGD92318
  
Location: 56826-58221
  
  
**BlastP hit with Mycgr3G25746\_Mycgr3T**
  
Percentage identity: 56 %
  
BlastP bit score: 362
  
Sequence coverage: 98 %
  
E-value: 8e-120
  
  
 NCBI BlastP on this gene

EGD92318

hypothetical protein
  
Accession: EGD92319
  
Location: 59825-61666
  
  
**BlastP hit with Mycgr3G21922\_Mycgr3T**
  
Percentage identity: 42 %
  
BlastP bit score: 159
  
Sequence coverage: 45 %
  
E-value: 3e-39
  
  
 NCBI BlastP on this gene

EGD92319

transcription initiation factor IIF subunit alpha
  
Accession: EGD92320
  
Location: 62719-65155
  
  
**BlastP hit with Mycgr3G103278\_Mycgr3**
  
Percentage identity: 34 %
  
BlastP bit score: 137
  
Sequence coverage: 95 %
  
E-value: 2e-32
  
  
 NCBI BlastP on this gene

EGD92320

hypothetical protein
  
Accession: EGD92321
  
Location: 65659-67057
  
 NCBI BlastP on this gene

EGD92321

hypothetical protein
  
Accession: EGD92322
  
Location: 67292-69298
  
 NCBI BlastP on this gene

EGD92322

inositol monophosphatase
  
Accession: EGD92323
  
Location: 70432-71454
  
 NCBI BlastP on this gene

EGD92323

hypothetical protein
  
Accession: EGD92324
  
Location: 71671-73056
  
 NCBI BlastP on this gene

EGD92324

hypothetical protein
  
Accession: EGD92325
  
Location: 73253-76551
  
 NCBI BlastP on this gene

EGD92325

hypothetical protein
  
Accession: EGD92326
  
Location: 77248-78109
  
 NCBI BlastP on this gene

EGD92326

hypothetical protein
  
Accession: EGD92327
  
Location: 78405-79676
  
 NCBI BlastP on this gene

EGD92327

GTP-binding protein
  
Accession: EGD92328
  
Location: 80383-81210
  
  
**BlastP hit with Mycgr3G99145\_Mycgr3T**
  
Percentage identity: 81 %
  
BlastP bit score: 340
  
Sequence coverage: 100 %
  
E-value: 6e-116
  
  
 NCBI BlastP on this gene

EGD92328

hypothetical protein
  
Accession: EGD92329
  
Location: 82098-84632
  
  
**BlastP hit with Mycgr3G84646\_Mycgr3T**
  
Percentage identity: 33 %
  
BlastP bit score: 324
  
Sequence coverage: 110 %
  
E-value: 1e-96
  
  
 NCBI BlastP on this gene

EGD92329

C-4 methylsterol oxidase
  
Accession: EGD92330
  
Location: 85353-86284
  
  
**BlastP hit with Mycgr3G36271\_Mycgr3T**
  
Percentage identity: 80 %
  
BlastP bit score: 459
  
Sequence coverage: 87 %
  
E-value: 2e-160
  
  
 NCBI BlastP on this gene

EGD92330

AAA family ATPase
  
Accession: EGD92331
  
Location: 87376-89892
  
 NCBI BlastP on this gene

EGD92331

hypothetical protein
  
Accession: EGD92332
  
Location: 90063-91358
  
 NCBI BlastP on this gene

EGD92332

hypothetical protein
  
Accession: EGD92333
  
Location: 91798-93011
  
 NCBI BlastP on this gene

EGD92333

phosphatidylinositol 3
  
Accession: EGD92334
  
Location: 93373-102298
  
 NCBI BlastP on this gene

EGD92334

tRNA-specific adenosine deaminase subunit TAD2
  
Accession: EGD92335
  
Location: 102796-103636
  
 NCBI BlastP on this gene

EGD92335

NADH-ubiquinone oxidoreductase 21 kDa subunit
  
Accession: EGD92336
  
Location: 103744-104608
  
 NCBI BlastP on this gene

EGD92336

replication fork protection component Swi3
  
Accession: EGD92337
  
Location: 104931-106157
  
 NCBI BlastP on this gene

EGD92337

9. :  ABSU01000004 Arthroderma benhamiae CBS 112371     Total score: 6.0     Cumulative Blast bit score: 1689

inositol kinase kinase (UvsB), putative
  
Accession: EFE35072
  
Location: 579706-586898
  
 NCBI BlastP on this gene

EFE35072

hypothetical protein
  
Accession: EFE35073
  
Location: 592444-594957
  
 NCBI BlastP on this gene

EFE35073

C-4 methyl sterol oxidase, putative
  
Accession: EFE35074
  
Location: 596030-596569
  
  
**BlastP hit with Mycgr3G36271\_Mycgr3T**
  
Percentage identity: 81 %
  
BlastP bit score: 317
  
Sequence coverage: 60 %
  
E-value: 1e-105
  
  
 NCBI BlastP on this gene

EFE35074

hypothetical protein
  
Accession: EFE35075
  
Location: 596672-597335
  
 NCBI BlastP on this gene

EFE35075

hypothetical protein
  
Accession: EFE35076
  
Location: 597771-600294
  
  
**BlastP hit with Mycgr3G84646\_Mycgr3T**
  
Percentage identity: 36 %
  
BlastP bit score: 435
  
Sequence coverage: 112 %
  
E-value: 2e-138
  
  
 NCBI BlastP on this gene

EFE35076

hypothetical protein
  
Accession: EFE35077
  
Location: 600593-602062
  
  
**BlastP hit with Mycgr3G99145\_Mycgr3T**
  
Percentage identity: 79 %
  
BlastP bit score: 324
  
Sequence coverage: 97 %
  
E-value: 2e-106
  
  
 NCBI BlastP on this gene

EFE35077

hypothetical protein
  
Accession: EFE35078
  
Location: 602773-604044
  
 NCBI BlastP on this gene

EFE35078

hypothetical protein
  
Accession: EFE35079
  
Location: 605917-610776
  
 NCBI BlastP on this gene

EFE35079

inositol monophosphatase QutG, putative
  
Accession: EFE35080
  
Location: 611002-612005
  
 NCBI BlastP on this gene

EFE35080

hypothetical protein
  
Accession: EFE35081
  
Location: 612607-612774
  
 NCBI BlastP on this gene

EFE35081

hypothetical protein
  
Accession: EFE35082
  
Location: 613034-615061
  
 NCBI BlastP on this gene

EFE35082

RING finger protein
  
Accession: EFE35083
  
Location: 615295-616715
  
 NCBI BlastP on this gene

EFE35083

conserved hypothetical protein
  
Accession: EFE35084
  
Location: 617208-619592
  
  
**BlastP hit with Mycgr3G103278\_Mycgr3**
  
Percentage identity: 34 %
  
BlastP bit score: 138
  
Sequence coverage: 95 %
  
E-value: 1e-32
  
  
 NCBI BlastP on this gene

EFE35084

hypothetical protein
  
Accession: EFE35085
  
Location: 620699-622540
  
  
**BlastP hit with Mycgr3G21922\_Mycgr3T**
  
Percentage identity: 41 %
  
BlastP bit score: 157
  
Sequence coverage: 45 %
  
E-value: 2e-38
  
  
 NCBI BlastP on this gene

EFE35085

hypothetical protein
  
Accession: EFE35086
  
Location: 622689-625565
  
  
**BlastP hit with Mycgr3G25746\_Mycgr3T**
  
Percentage identity: 53 %
  
BlastP bit score: 319
  
Sequence coverage: 94 %
  
E-value: 3e-98
  
  
 NCBI BlastP on this gene

EFE35086

hypothetical protein
  
Accession: EFE35087
  
Location: 626864-627535
  
 NCBI BlastP on this gene

EFE35087

hypothetical protein
  
Accession: EFE35088
  
Location: 628047-631813
  
 NCBI BlastP on this gene

EFE35088

hypothetical protein
  
Accession: EFE35089
  
Location: 632206-636534
  
 NCBI BlastP on this gene

EFE35089

hypothetical protein
  
Accession: EFE35090
  
Location: 638768-645103
  
 NCBI BlastP on this gene

EFE35090

10. :  DS027048 Aspergillus clavatus NRRL 1 1099423829794 genomic scaffold     Total score: 5.0     Cumulative Blast bit score: 2729

nuclear matrix protein
  
Accession: EAW13358
  
Location: 186516-188924
  
 NCBI BlastP on this gene

EAW13358

DEAD helicases superfamily protein (Aquarius), putative
  
Accession: EAW13357
  
Location: 181858-186302
  
 NCBI BlastP on this gene

EAW13357

hypothetical protein
  
Accession: EAW13356
  
Location: 180762-181387
  
 NCBI BlastP on this gene

EAW13356

TFIIH and nucleotide excision repair factor 3 complexes subunit (Tfb2), putative
  
Accession: EAW13355
  
Location: 178418-179964
  
 NCBI BlastP on this gene

EAW13355

conserved hypothetical protein
  
Accession: EAW13354
  
Location: 176049-177932
  
 NCBI BlastP on this gene

EAW13354

phenazine biosynthesis-like protein, putative
  
Accession: EAW13353
  
Location: 174677-175639
  
 NCBI BlastP on this gene

EAW13353

mating-type switching protein swi1
  
Accession: EAW13352
  
Location: 170325-174046
  
  
**BlastP hit with Mycgr3G68421\_Mycgr3T**
  
Percentage identity: 43 %
  
BlastP bit score: 939
  
Sequence coverage: 102 %
  
E-value: 0.0
  
  
 NCBI BlastP on this gene

EAW13352

3-hydroxybutyryl-CoA dehydrogenase, putative
  
Accession: EAW13351
  
Location: 168855-169955
  
 NCBI BlastP on this gene

EAW13351

IBR domain protein
  
Accession: EAW13350
  
Location: 167899-168616
  
 NCBI BlastP on this gene

EAW13350

hypothetical protein
  
Accession: EAW13349
  
Location: 167316-167759
  
 NCBI BlastP on this gene

EAW13349

actin family protein
  
Accession: EAW13348
  
Location: 164879-166376
  
 NCBI BlastP on this gene

EAW13348

conserved hypothetical protein
  
Accession: EAW13347
  
Location: 163395-164510
  
 NCBI BlastP on this gene

EAW13347

conserved leucine-rich repeat protein
  
Accession: EAW13346
  
Location: 160111-163092
  
  
**BlastP hit with Mycgr3G68433\_Mycgr3T**
  
Percentage identity: 38 %
  
BlastP bit score: 506
  
Sequence coverage: 88 %
  
E-value: 2e-158
  
  
 NCBI BlastP on this gene

EAW13346

conserved hypothetical protein
  
Accession: EAW13345
  
Location: 156962-158380
  
  
**BlastP hit with Mycgr3G90786\_Mycgr3T**
  
Percentage identity: 27 %
  
BlastP bit score: 102
  
Sequence coverage: 101 %
  
E-value: 3e-20
  
  
 NCBI BlastP on this gene

EAW13345

60S ribosomal protein L13
  
Accession: EAW13344
  
Location: 154881-156059
  
 NCBI BlastP on this gene

EAW13344

hypothetical protein
  
Accession: EAW13343
  
Location: 152268-154245
  
 NCBI BlastP on this gene

EAW13343

conserved hypothetical protein
  
Accession: EAW13342
  
Location: 151083-151910
  
 NCBI BlastP on this gene

EAW13342

conserved hypothetical protein
  
Accession: EAW13341
  
Location: 149876-150631
  
 NCBI BlastP on this gene

EAW13341

conserved hypothetical protein
  
Accession: EAW13340
  
Location: 147068-149187
  
 NCBI BlastP on this gene

EAW13340

conserved hypothetical protein
  
Accession: EAW13339
  
Location: 143053-143793
  
 NCBI BlastP on this gene

EAW13339

succinyl-CoA synthetase beta subunit, putative
  
Accession: EAW13338
  
Location: 140678-142488
  
 NCBI BlastP on this gene

EAW13338

short chain dehydrogenase/reductase, putative
  
Accession: EAW13337
  
Location: 138656-139913
  
 NCBI BlastP on this gene

EAW13337

C4-dicarboxylate/malic acid transporter, putative
  
Accession: EAW13336
  
Location: 135448-136970
  
 NCBI BlastP on this gene

EAW13336

conserved hypothetical protein
  
Accession: EAW13335
  
Location: 133567-134592
  
  
**BlastP hit with Mycgr3G55345\_Mycgr3T**
  
Percentage identity: 73 %
  
BlastP bit score: 305
  
Sequence coverage: 96 %
  
E-value: 6e-102
  
  
 NCBI BlastP on this gene

EAW13335

conserved hypothetical protein
  
Accession: EAW13334
  
Location: 132593-133016
  
 NCBI BlastP on this gene

EAW13334

conserved hypothetical protein
  
Accession: EAW13333
  
Location: 129593-131809
  
 NCBI BlastP on this gene

EAW13333

conserved hypothetical protein
  
Accession: EAW13332
  
Location: 128042-129344
  
 NCBI BlastP on this gene

EAW13332

ubiquinone biosynthesis protein, putative
  
Accession: EAW13331
  
Location: 125181-127404
  
  
**BlastP hit with Mycgr3G68458\_Mycgr3T**
  
Percentage identity: 65 %
  
BlastP bit score: 877
  
Sequence coverage: 91 %
  
E-value: 0.0
  
  
 NCBI BlastP on this gene

EAW13331

cytochrome C1 heme lyase
  
Accession: EAW13330
  
Location: 123256-124420
  
 NCBI BlastP on this gene

EAW13330

tachykinin family protein
  
Accession: EAW13329
  
Location: 120717-122712
  
 NCBI BlastP on this gene

EAW13329

ubiquitin-conjugating enzyme Ubc6, putative
  
Accession: EAW13328
  
Location: 118032-119008
  
 NCBI BlastP on this gene

EAW13328

outer membrane protein, OMP85 family
  
Accession: EAW13327
  
Location: 115914-117681
  
 NCBI BlastP on this gene

EAW13327

Golgi membrane protein, putative
  
Accession: EAW13326
  
Location: 114478-115537
  
 NCBI BlastP on this gene

EAW13326

ubiquitin fusion degradation protein (Ufd1), putative
  
Accession: EAW13325
  
Location: 111692-114070
  
 NCBI BlastP on this gene

EAW13325

conserved hypothetical protein
  
Accession: EAW13324
  
Location: 109073-110626
  
 NCBI BlastP on this gene

EAW13324

11. :  DS027693 Neosartorya fischeri NRRL 181 1099437636261 genomic scaffold     Total score: 5.0     Cumulative Blast bit score: 2721

THO complex subunit Tho1, putative
  
Accession: EAW20472
  
Location: 200640-203046
  
 NCBI BlastP on this gene

EAW20472

DEAD helicases superfamily protein (Aquarius), putative
  
Accession: EAW20471
  
Location: 195985-200381
  
 NCBI BlastP on this gene

EAW20471

predicted protein
  
Accession: EAW20470
  
Location: 194433-195544
  
 NCBI BlastP on this gene

EAW20470

TFIIH and nucleotide excision repair factor 3 complexes subunit (Tfb2), putative
  
Accession: EAW20469
  
Location: 192580-194139
  
 NCBI BlastP on this gene

EAW20469

conserved hypothetical protein
  
Accession: EAW20468
  
Location: 190214-192097
  
 NCBI BlastP on this gene

EAW20468

phenazine biosynthesis-like protein, putative
  
Accession: EAW20467
  
Location: 188801-189763
  
 NCBI BlastP on this gene

EAW20467

mating-type switching protein swi1
  
Accession: EAW20466
  
Location: 184514-188211
  
  
**BlastP hit with Mycgr3G68421\_Mycgr3T**
  
Percentage identity: 44 %
  
BlastP bit score: 956
  
Sequence coverage: 102 %
  
E-value: 0.0
  
  
 NCBI BlastP on this gene

EAW20466

3-hydroxybutyryl-CoA dehydrogenase, putative
  
Accession: EAW20465
  
Location: 183048-184149
  
 NCBI BlastP on this gene

EAW20465

IBR domain protein
  
Accession: EAW20464
  
Location: 181919-182758
  
 NCBI BlastP on this gene

EAW20464

actin family protein
  
Accession: EAW20463
  
Location: 179141-180713
  
 NCBI BlastP on this gene

EAW20463

conserved hypothetical protein
  
Accession: EAW20462
  
Location: 177648-178760
  
 NCBI BlastP on this gene

EAW20462

conserved leucine-rich repeat protein
  
Accession: EAW20461
  
Location: 174353-177349
  
  
**BlastP hit with Mycgr3G68433\_Mycgr3T**
  
Percentage identity: 40 %
  
BlastP bit score: 509
  
Sequence coverage: 86 %
  
E-value: 2e-159
  
  
 NCBI BlastP on this gene

EAW20461

hypothetical protein
  
Accession: EAW20460
  
Location: 171045-172469
  
  
**BlastP hit with Mycgr3G90786\_Mycgr3T**
  
Percentage identity: 28 %
  
BlastP bit score: 92
  
Sequence coverage: 92 %
  
E-value: 8e-17
  
  
 NCBI BlastP on this gene

EAW20460

60S ribosomal protein L13
  
Accession: EAW20459
  
Location: 169018-170024
  
 NCBI BlastP on this gene

EAW20459

hypothetical protein
  
Accession: EAW20458
  
Location: 166362-168301
  
 NCBI BlastP on this gene

EAW20458

conserved hypothetical protein
  
Accession: EAW20457
  
Location: 165218-166015
  
 NCBI BlastP on this gene

EAW20457

conserved hypothetical protein
  
Accession: EAW20456
  
Location: 164002-164768
  
 NCBI BlastP on this gene

EAW20456

HSF-type DNA-binding domain protein
  
Accession: EAW20455
  
Location: 160986-163113
  
 NCBI BlastP on this gene

EAW20455

F-box domain protein
  
Accession: EAW20454
  
Location: 157279-158990
  
 NCBI BlastP on this gene

EAW20454

succinyl-CoA synthetase beta subunit, putative
  
Accession: EAW20453
  
Location: 154723-156523
  
 NCBI BlastP on this gene

EAW20453

short chain dehydrogenase/reductase, putative
  
Accession: EAW20452
  
Location: 153361-154186
  
 NCBI BlastP on this gene

EAW20452

C4-dicarboxylate/malic acid transporter, putative
  
Accession: EAW20451
  
Location: 149998-151511
  
 NCBI BlastP on this gene

EAW20451

conserved hypothetical protein
  
Accession: EAW20450
  
Location: 148168-149180
  
  
**BlastP hit with Mycgr3G55345\_Mycgr3T**
  
Percentage identity: 74 %
  
BlastP bit score: 306
  
Sequence coverage: 96 %
  
E-value: 1e-102
  
  
 NCBI BlastP on this gene

EAW20450

conserved hypothetical protein
  
Accession: EAW20449
  
Location: 147090-147507
  
 NCBI BlastP on this gene

EAW20449

conserved hypothetical protein
  
Accession: EAW20448
  
Location: 144116-146292
  
 NCBI BlastP on this gene

EAW20448

conserved hypothetical protein
  
Accession: EAW20447
  
Location: 142443-143738
  
 NCBI BlastP on this gene

EAW20447

ubiquinone biosynthesis protein, putative
  
Accession: EAW20446
  
Location: 139558-141772
  
  
**BlastP hit with Mycgr3G68458\_Mycgr3T**
  
Percentage identity: 65 %
  
BlastP bit score: 858
  
Sequence coverage: 93 %
  
E-value: 0.0
  
  
 NCBI BlastP on this gene

EAW20446

cytochrome C1 heme lyase
  
Accession: EAW20445
  
Location: 137634-138742
  
 NCBI BlastP on this gene

EAW20445

conserved hypothetical protein
  
Accession: EAW20444
  
Location: 135066-137094
  
 NCBI BlastP on this gene

EAW20444

ubiquitin-conjugating enzyme Ubc6, putative
  
Accession: EAW20443
  
Location: 132296-133248
  
 NCBI BlastP on this gene

EAW20443

outer membrane protein, OMP85 family
  
Accession: EAW20442
  
Location: 130175-131952
  
 NCBI BlastP on this gene

EAW20442

Golgi membrane protein, putative
  
Accession: EAW20441
  
Location: 128814-129860
  
 NCBI BlastP on this gene

EAW20441

ubiquitin fusion degradation protein (Ufd1), putative
  
Accession: EAW20440
  
Location: 126087-128480
  
 NCBI BlastP on this gene

EAW20440

conserved hypothetical protein
  
Accession: EAW20439
  
Location: 123451-125455
  
 NCBI BlastP on this gene

EAW20439

12. :  DS990639 Ajellomyces capsulatus H88 supercont1.4 genomic scaffold     Total score: 5.0     Cumulative Blast bit score: 2681

ubiquitin-conjugating enzyme E2
  
Accession: EGC45537
  
Location: 890102-891116
  
 NCBI BlastP on this gene

EGC45537

conserved hypothetical protein
  
Accession: EGC45538
  
Location: 891549-892984
  
 NCBI BlastP on this gene

EGC45538

conserved hypothetical protein
  
Accession: EGC45539
  
Location: 893579-894745
  
 NCBI BlastP on this gene

EGC45539

tachykinin family protein
  
Accession: EGC45540
  
Location: 897773-900544
  
 NCBI BlastP on this gene

EGC45540

cytochrome c heme lyase
  
Accession: EGC45541
  
Location: 901318-902499
  
 NCBI BlastP on this gene

EGC45541

conserved hypothetical protein
  
Accession: EGC45542
  
Location: 903700-905226
  
 NCBI BlastP on this gene

EGC45542

predicted protein
  
Accession: EGC45543
  
Location: 906080-906584
  
 NCBI BlastP on this gene

EGC45543

ubiquinone biosynthesis protein
  
Accession: EGC45544
  
Location: 907429-909740
  
  
**BlastP hit with Mycgr3G68458\_Mycgr3T**
  
Percentage identity: 62 %
  
BlastP bit score: 880
  
Sequence coverage: 102 %
  
E-value: 0.0
  
  
 NCBI BlastP on this gene

EGC45544

TDT malic acid transporter
  
Accession: EGC45545
  
Location: 910719-912469
  
 NCBI BlastP on this gene

EGC45545

carbonic anhydraes family protein
  
Accession: EGC45546
  
Location: 912876-913525
  
 NCBI BlastP on this gene

EGC45546

short chain dehydrogenase/reductase
  
Accession: EGC45547
  
Location: 915524-917287
  
 NCBI BlastP on this gene

EGC45547

succinyl-CoA ligase beta-chain
  
Accession: EGC45548
  
Location: 917993-919929
  
 NCBI BlastP on this gene

EGC45548

conserved hypothetical protein
  
Accession: EGC45549
  
Location: 920184-920980
  
 NCBI BlastP on this gene

EGC45549

HSF-type DNA-binding domain-containing protein
  
Accession: EGC45550
  
Location: 922697-925003
  
 NCBI BlastP on this gene

EGC45550

predicted protein
  
Accession: EGC45551
  
Location: 928286-929741
  
 NCBI BlastP on this gene

EGC45551

xanthine phosphoribosyltransferase
  
Accession: EGC45552
  
Location: 929816-931502
  
  
**BlastP hit with Mycgr3G55345\_Mycgr3T**
  
Percentage identity: 75 %
  
BlastP bit score: 318
  
Sequence coverage: 97 %
  
E-value: 2e-107
  
  
 NCBI BlastP on this gene

EGC45552

conserved hypothetical protein
  
Accession: EGC45553
  
Location: 932732-933295
  
 NCBI BlastP on this gene

EGC45553

GTP binding protein
  
Accession: EGC45554
  
Location: 934602-937106
  
 NCBI BlastP on this gene

EGC45554

conserved hypothetical protein
  
Accession: EGC45555
  
Location: 937652-938665
  
 NCBI BlastP on this gene

EGC45555

actin-like protein arp6
  
Accession: EGC45556
  
Location: 939520-941175
  
 NCBI BlastP on this gene

EGC45556

conserved hypothetical protein
  
Accession: EGC45557
  
Location: 942304-943593
  
 NCBI BlastP on this gene

EGC45557

leucine rich repeat domain-containing protein
  
Accession: EGC45558
  
Location: 944452-947559
  
  
**BlastP hit with Mycgr3G68433\_Mycgr3T**
  
Percentage identity: 38 %
  
BlastP bit score: 483
  
Sequence coverage: 99 %
  
E-value: 2e-149
  
  
 NCBI BlastP on this gene

EGC45558

conserved hypothetical protein
  
Accession: EGC45559
  
Location: 948809-950254
  
  
**BlastP hit with Mycgr3G90786\_Mycgr3T**
  
Percentage identity: 27 %
  
BlastP bit score: 89
  
Sequence coverage: 108 %
  
E-value: 6e-16
  
  
 NCBI BlastP on this gene

EGC45559

60S ribosomal protein
  
Accession: EGC45560
  
Location: 951211-952453
  
 NCBI BlastP on this gene

EGC45560

conserved hypothetical protein
  
Accession: EGC45561
  
Location: 953868-956039
  
 NCBI BlastP on this gene

EGC45561

CAMK family protein kinase
  
Accession: EGC45562
  
Location: 957411-958879
  
 NCBI BlastP on this gene

EGC45562

conserved hypothetical protein
  
Accession: EGC45563
  
Location: 959517-961000
  
 NCBI BlastP on this gene

EGC45563

enoyl-CoA hydratase
  
Accession: EGC45564
  
Location: 961311-962070
  
 NCBI BlastP on this gene

EGC45564

topoisomerase 1-associated factor
  
Accession: EGC45565
  
Location: 962874-966419
  
  
**BlastP hit with Mycgr3G68421\_Mycgr3T**
  
Percentage identity: 45 %
  
BlastP bit score: 911
  
Sequence coverage: 92 %
  
E-value: 0.0
  
  
 NCBI BlastP on this gene

EGC45565

conserved hypothetical protein
  
Accession: EGC45566
  
Location: 968246-970053
  
 NCBI BlastP on this gene

EGC45566

predicted protein
  
Accession: EGC45567
  
Location: 970829-971930
  
 NCBI BlastP on this gene

EGC45567

s-adenosylmethionine (SAM)-dependent methyltransferase
  
Accession: EGC45568
  
Location: 975682-976701
  
 NCBI BlastP on this gene

EGC45568

metallopeptidase
  
Accession: EGC45569
  
Location: 978037-981296
  
 NCBI BlastP on this gene

EGC45569

conserved hypothetical protein
  
Accession: EGC45570
  
Location: 982266-983955
  
 NCBI BlastP on this gene

EGC45570

2-nitropropane dioxygenase
  
Accession: EGC45571
  
Location: 984501-985541
  
 NCBI BlastP on this gene

EGC45571

13. :  GG663377 Ajellomyces capsulatus G186AR genomic scaffold supercont2.15     Total score: 5.0     Cumulative Blast bit score: 2668

outer membrane protein
  
Accession: EEH03366
  
Location: 196519-198364
  
 NCBI BlastP on this gene

EEH03366

ubiquitin-conjugating enzyme
  
Accession: EEH03365
  
Location: 195182-196193
  
 NCBI BlastP on this gene

EEH03365

conserved hypothetical protein
  
Accession: EEH03364
  
Location: 192484-194686
  
 NCBI BlastP on this gene

EEH03364

conserved hypothetical protein
  
Accession: EEH03363
  
Location: 191052-191865
  
 NCBI BlastP on this gene

EEH03363

conserved hypothetical protein
  
Accession: EEH03362
  
Location: 189788-190915
  
 NCBI BlastP on this gene

EEH03362

tachykinin family protein
  
Accession: EEH03361
  
Location: 183892-186671
  
 NCBI BlastP on this gene

EEH03361

cytochrome c heme lyase
  
Accession: EEH03360
  
Location: 181919-183086
  
 NCBI BlastP on this gene

EEH03360

predicted protein
  
Accession: EEH03359
  
Location: 177511-178016
  
 NCBI BlastP on this gene

EEH03359

conserved hypothetical protein
  
Accession: EEH03358
  
Location: 174341-176653
  
  
**BlastP hit with Mycgr3G68458\_Mycgr3T**
  
Percentage identity: 62 %
  
BlastP bit score: 878
  
Sequence coverage: 102 %
  
E-value: 0.0
  
  
 NCBI BlastP on this gene

EEH03358

conserved hypothetical protein
  
Accession: EEH03357
  
Location: 171616-173376
  
 NCBI BlastP on this gene

EEH03357

carbonic anhydrase
  
Accession: EEH03356
  
Location: 170553-171191
  
 NCBI BlastP on this gene

EEH03356

conserved hypothetical protein
  
Accession: EEH03355
  
Location: 167171-168564
  
 NCBI BlastP on this gene

EEH03355

succinyl-CoA ligase beta-chain
  
Accession: EEH03354
  
Location: 164163-166099
  
 NCBI BlastP on this gene

EEH03354

conserved hypothetical protein
  
Accession: EEH03353
  
Location: 163100-163895
  
 NCBI BlastP on this gene

EEH03353

flocculation suppression protein
  
Accession: EEH03352
  
Location: 160563-161396
  
 NCBI BlastP on this gene

EEH03352

flocculation suppression protein
  
Accession: EEH03351
  
Location: 158769-159932
  
 NCBI BlastP on this gene

EEH03351

predicted protein
  
Accession: EEH03350
  
Location: 158039-158517
  
 NCBI BlastP on this gene

EEH03350

predicted protein
  
Accession: EEH03349
  
Location: 155639-157064
  
 NCBI BlastP on this gene

EEH03349

predicted protein
  
Accession: EEH03348
  
Location: 154750-155358
  
 NCBI BlastP on this gene

EEH03348

xanthine phosphoribosyltransferase
  
Accession: EEH03347
  
Location: 152472-154155
  
  
**BlastP hit with Mycgr3G55345\_Mycgr3T**
  
Percentage identity: 75 %
  
BlastP bit score: 318
  
Sequence coverage: 97 %
  
E-value: 2e-107
  
  
 NCBI BlastP on this gene

EEH03347

conserved hypothetical protein
  
Accession: EEH03346
  
Location: 151721-151864
  
 NCBI BlastP on this gene

EEH03346

conserved hypothetical protein
  
Accession: EEH03345
  
Location: 147129-149998
  
 NCBI BlastP on this gene

EEH03345

conserved hypothetical protein
  
Accession: EEH03344
  
Location: 145941-146954
  
 NCBI BlastP on this gene

EEH03344

actin-like protein arp6
  
Accession: EEH03343
  
Location: 143419-145074
  
 NCBI BlastP on this gene

EEH03343

conserved hypothetical protein
  
Accession: EEH03342
  
Location: 141132-142421
  
 NCBI BlastP on this gene

EEH03342

leucine rich repeat domain-containing protein
  
Accession: EEH03341
  
Location: 137153-140269
  
  
**BlastP hit with Mycgr3G68433\_Mycgr3T**
  
Percentage identity: 38 %
  
BlastP bit score: 486
  
Sequence coverage: 99 %
  
E-value: 1e-150
  
  
 NCBI BlastP on this gene

EEH03341

conserved hypothetical protein
  
Accession: EEH03340
  
Location: 134431-135891
  
  
**BlastP hit with Mycgr3G90786\_Mycgr3T**
  
Percentage identity: 27 %
  
BlastP bit score: 87
  
Sequence coverage: 109 %
  
E-value: 2e-15
  
  
 NCBI BlastP on this gene

EEH03340

60S ribosomal protein L13
  
Accession: EEH03339
  
Location: 132235-133477
  
 NCBI BlastP on this gene

EEH03339

conserved hypothetical protein
  
Accession: EEH03338
  
Location: 128668-130833
  
 NCBI BlastP on this gene

EEH03338

CAMK family protein kinase
  
Accession: EEH03337
  
Location: 125055-126742
  
 NCBI BlastP on this gene

EEH03337

hypothetical protein
  
Accession: EEH03336
  
Location: 122821-124323
  
 NCBI BlastP on this gene

EEH03336

enoyl-CoA hydratase
  
Accession: EEH03335
  
Location: 121063-122519
  
 NCBI BlastP on this gene

EEH03335

topoisomerase 1
  
Accession: EEH03334
  
Location: 117454-120999
  
  
**BlastP hit with Mycgr3G68421\_Mycgr3T**
  
Percentage identity: 45 %
  
BlastP bit score: 899
  
Sequence coverage: 92 %
  
E-value: 0.0
  
  
 NCBI BlastP on this gene

EEH03334

3-hydroxybutyryl CoA dehydrogenase
  
Accession: EEH03333
  
Location: 115880-117028
  
 NCBI BlastP on this gene

EEH03333

IBR domain-containing protein
  
Accession: EEH03332
  
Location: 112977-114660
  
 NCBI BlastP on this gene

EEH03332

predicted protein
  
Accession: EEH03331
  
Location: 112256-112780
  
 NCBI BlastP on this gene

EEH03331

conserved hypothetical protein
  
Accession: EEH03330
  
Location: 110250-110840
  
 NCBI BlastP on this gene

EEH03330

conserved hypothetical protein
  
Accession: EEH03329
  
Location: 108154-109681
  
 NCBI BlastP on this gene

EEH03329

phenazine biosynthesis PhzC/PhzF protein
  
Accession: EEH03328
  
Location: 106485-107483
  
 NCBI BlastP on this gene

EEH03328

conserved hypothetical protein
  
Accession: EEH03327
  
Location: 104214-105884
  
 NCBI BlastP on this gene

EEH03327

RNA pol II transcription initiation subunit
  
Accession: EEH03326
  
Location: 102023-103614
  
 NCBI BlastP on this gene

EEH03326

predicted protein
  
Accession: EEH03325
  
Location: 100449-101681
  
 NCBI BlastP on this gene

EEH03325

14. :  DS499603 Aspergillus fumigatus A1163 scf\_000010 genomic scaffold     Total score: 5.0     Cumulative Blast bit score: 2664

THO complex subunit Tho1, putative
  
Accession: EDP47268
  
Location: 142021-144427
  
 NCBI BlastP on this gene

EDP47268

DEAD helicases superfamily protein (Aquarius), putative
  
Accession: EDP47267
  
Location: 137369-141790
  
 NCBI BlastP on this gene

EDP47267

PaaI thioesterase family protein
  
Accession: EDP47266
  
Location: 135815-136928
  
 NCBI BlastP on this gene

EDP47266

TFIIH and nucleotide excision repair factor 3 complexes subunit (Tfb2), putative
  
Accession: EDP47265
  
Location: 133955-135514
  
 NCBI BlastP on this gene

EDP47265

conserved hypothetical protein
  
Accession: EDP47264
  
Location: 131579-133468
  
 NCBI BlastP on this gene

EDP47264

phenazine biosynthesis-like protein, putative
  
Accession: EDP47263
  
Location: 130166-131128
  
 NCBI BlastP on this gene

EDP47263

DNA repair protein (Tof1), putative
  
Accession: EDP47262
  
Location: 125889-129586
  
  
**BlastP hit with Mycgr3G68421\_Mycgr3T**
  
Percentage identity: 44 %
  
BlastP bit score: 946
  
Sequence coverage: 102 %
  
E-value: 0.0
  
  
 NCBI BlastP on this gene

EDP47262

3-hydroxybutyryl-CoA dehydrogenase, putative
  
Accession: EDP47261
  
Location: 124424-125521
  
 NCBI BlastP on this gene

EDP47261

IBR domain protein
  
Accession: EDP47260
  
Location: 123032-124142
  
 NCBI BlastP on this gene

EDP47260

actin family protein
  
Accession: EDP47259
  
Location: 120565-122134
  
 NCBI BlastP on this gene

EDP47259

conserved hypothetical protein
  
Accession: EDP47258
  
Location: 119081-120193
  
 NCBI BlastP on this gene

EDP47258

conserved leucine-rich repeat protein
  
Accession: EDP47257
  
Location: 115786-118782
  
  
**BlastP hit with Mycgr3G68433\_Mycgr3T**
  
Percentage identity: 40 %
  
BlastP bit score: 518
  
Sequence coverage: 85 %
  
E-value: 3e-163
  
  
 NCBI BlastP on this gene

EDP47257

conserved hypothetical protein
  
Accession: EDP47256
  
Location: 112472-113896
  
  
**BlastP hit with Mycgr3G90786\_Mycgr3T**
  
Percentage identity: 27 %
  
BlastP bit score: 85
  
Sequence coverage: 92 %
  
E-value: 1e-14
  
  
 NCBI BlastP on this gene

EDP47256

60S ribosome protein L13, putative
  
Accession: EDP47255
  
Location: 110403-111410
  
 NCBI BlastP on this gene

EDP47255

hypothetical protein
  
Accession: EDP47254
  
Location: 107749-109685
  
 NCBI BlastP on this gene

EDP47254

conserved hypothetical protein
  
Accession: EDP47253
  
Location: 106620-107432
  
 NCBI BlastP on this gene

EDP47253

conserved hypothetical protein
  
Accession: EDP47252
  
Location: 105401-106167
  
 NCBI BlastP on this gene

EDP47252

hypothetical protein
  
Accession: EDP47251
  
Location: 102414-104531
  
 NCBI BlastP on this gene

EDP47251

F-box domain protein
  
Accession: EDP47250
  
Location: 98681-100389
  
 NCBI BlastP on this gene

EDP47250

succinyl-CoA synthetase beta subunit, putative
  
Accession: EDP47249
  
Location: 96126-97928
  
 NCBI BlastP on this gene

EDP47249

short chain dehydrogenase/reductase, putative
  
Accession: EDP47248
  
Location: 94373-95601
  
 NCBI BlastP on this gene

EDP47248

C4-dicarboxylate transporter/malic acid transport protein, putative
  
Accession: EDP47247
  
Location: 91516-92623
  
 NCBI BlastP on this gene

EDP47247

conserved hypothetical protein
  
Accession: EDP47246
  
Location: 89794-90510
  
  
**BlastP hit with Mycgr3G55345\_Mycgr3T**
  
Percentage identity: 76 %
  
BlastP bit score: 261
  
Sequence coverage: 81 %
  
E-value: 4e-85
  
  
 NCBI BlastP on this gene

EDP47246

GTP binding protein, putative
  
Accession: EDP47245
  
Location: 85712-87887
  
 NCBI BlastP on this gene

EDP47245

conserved hypothetical protein
  
Accession: EDP47244
  
Location: 84035-85330
  
 NCBI BlastP on this gene

EDP47244

hypothetical protein
  
Accession: EDP47243
  
Location: 83521-83805
  
 NCBI BlastP on this gene

EDP47243

ubiquinone biosynthesis protein, putative
  
Accession: EDP47242
  
Location: 81149-83363
  
  
**BlastP hit with Mycgr3G68458\_Mycgr3T**
  
Percentage identity: 65 %
  
BlastP bit score: 854
  
Sequence coverage: 93 %
  
E-value: 0.0
  
  
 NCBI BlastP on this gene

EDP47242

cytochrome C1 heme lyase
  
Accession: EDP47241
  
Location: 79225-80333
  
 NCBI BlastP on this gene

EDP47241

conserved hypothetical protein
  
Accession: EDP47240
  
Location: 76653-78656
  
 NCBI BlastP on this gene

EDP47240

ubiquitin-conjugating enzyme Ubc6, putative
  
Accession: EDP47239
  
Location: 73842-74795
  
 NCBI BlastP on this gene

EDP47239

outer membrane protein, OMP85 family
  
Accession: EDP47238
  
Location: 71722-73585
  
 NCBI BlastP on this gene

EDP47238

Golgi membrane protein, putative
  
Accession: EDP47237
  
Location: 70365-71412
  
 NCBI BlastP on this gene

EDP47237

ubiquitin fusion degradation protein (Ufd1), putative
  
Accession: EDP47236
  
Location: 67640-70027
  
 NCBI BlastP on this gene

EDP47236

conserved hypothetical protein
  
Accession: EDP47235
  
Location: 64994-66613
  
 NCBI BlastP on this gene

EDP47235

15. :  AAHF01000016 Aspergillus fumigatus Af293     Total score: 5.0     Cumulative Blast bit score: 2664

THO complex subunit Tho1, putative
  
Accession: EAL84580
  
Location: 520230-522636
  
 NCBI BlastP on this gene

EAL84580

DEAD helicases superfamily protein (Aquarius), putative
  
Accession: EAL84582
  
Location: 522867-527288
  
 NCBI BlastP on this gene

EAL84582

PaaI thioesterase family protein, putative
  
Accession: EBA27194
  
Location: 527729-528842
  
 NCBI BlastP on this gene

EBA27194

TFIIH and nucleotide excision repair factor 3 complexes subunit (Tfb2), putative
  
Accession: EAL84583
  
Location: 529143-530702
  
 NCBI BlastP on this gene

EAL84583

conserved hypothetical protein
  
Accession: EAL84584
  
Location: 531189-533078
  
 NCBI BlastP on this gene

EAL84584

phenazine biosynthesis-like protein, putative
  
Accession: EAL84585
  
Location: 533529-534491
  
 NCBI BlastP on this gene

EAL84585

DNA repair protein (Tof1), putative
  
Accession: EAL84586
  
Location: 535071-538768
  
  
**BlastP hit with Mycgr3G68421\_Mycgr3T**
  
Percentage identity: 44 %
  
BlastP bit score: 946
  
Sequence coverage: 102 %
  
E-value: 0.0
  
  
 NCBI BlastP on this gene

EAL84586

3-hydroxybutyryl-CoA dehydrogenase, putative
  
Accession: EAL84588
  
Location: 539136-540233
  
 NCBI BlastP on this gene

EAL84588

IBR domain protein
  
Accession: EAL84589
  
Location: 540515-541625
  
 NCBI BlastP on this gene

EAL84589

actin family protein
  
Accession: EAL84590
  
Location: 542523-544092
  
 NCBI BlastP on this gene

EAL84590

conserved hypothetical protein
  
Accession: EAL84591
  
Location: 544464-545576
  
 NCBI BlastP on this gene

EAL84591

conserved leucine-rich repeat protein
  
Accession: EAL84592
  
Location: 545875-548871
  
  
**BlastP hit with Mycgr3G68433\_Mycgr3T**
  
Percentage identity: 40 %
  
BlastP bit score: 518
  
Sequence coverage: 85 %
  
E-value: 3e-163
  
  
 NCBI BlastP on this gene

EAL84592

conserved hypothetical protein
  
Accession: EAL84593
  
Location: 550761-552185
  
  
**BlastP hit with Mycgr3G90786\_Mycgr3T**
  
Percentage identity: 27 %
  
BlastP bit score: 85
  
Sequence coverage: 92 %
  
E-value: 1e-14
  
  
 NCBI BlastP on this gene

EAL84593

60S ribosomal protein L13
  
Accession: EAL84594
  
Location: 553247-554254
  
 NCBI BlastP on this gene

EAL84594

conserved hypothetical protein
  
Accession: EBA27195
  
Location: 554972-556908
  
 NCBI BlastP on this gene

EBA27195

conserved hypothetical protein
  
Accession: EAL84595
  
Location: 557225-558037
  
 NCBI BlastP on this gene

EAL84595

conserved hypothetical protein
  
Accession: EAL84596
  
Location: 558490-559256
  
 NCBI BlastP on this gene

EAL84596

flocculation suppression protein
  
Accession: EBA27196
  
Location: 560126-562243
  
 NCBI BlastP on this gene

EBA27196

F-box domain protein
  
Accession: EAL84599
  
Location: 564272-565980
  
 NCBI BlastP on this gene

EAL84599

succinyl-CoA synthetase beta subunit, putative
  
Accession: EAL84600
  
Location: 566733-568535
  
 NCBI BlastP on this gene

EAL84600

short chain dehydrogenase/reductase (Ayr1), putative
  
Accession: EAL84601
  
Location: 569060-570288
  
 NCBI BlastP on this gene

EAL84601

C4-dicarboxylate transporter/malic acid transport protein, putative
  
Accession: EAL84602
  
Location: 572038-573145
  
 NCBI BlastP on this gene

EAL84602

xanthine-guanine phosphoribosyl transferase Xpt1, putative
  
Accession: EAL84603
  
Location: 574150-574866
  
  
**BlastP hit with Mycgr3G55345\_Mycgr3T**
  
Percentage identity: 76 %
  
BlastP bit score: 261
  
Sequence coverage: 81 %
  
E-value: 4e-85
  
  
 NCBI BlastP on this gene

EAL84603

GTP binding protein, putative
  
Accession: EAL84604
  
Location: 576773-578948
  
 NCBI BlastP on this gene

EAL84604

conserved hypothetical protein
  
Accession: EAL84605
  
Location: 579330-580625
  
 NCBI BlastP on this gene

EAL84605

hypothetical protein
  
Accession: EAL84606
  
Location: 580855-581139
  
 NCBI BlastP on this gene

EAL84606

ubiquinone biosynthesis protein, putative
  
Accession: EAL84607
  
Location: 581297-583511
  
  
**BlastP hit with Mycgr3G68458\_Mycgr3T**
  
Percentage identity: 65 %
  
BlastP bit score: 854
  
Sequence coverage: 93 %
  
E-value: 0.0
  
  
 NCBI BlastP on this gene

EAL84607

cytochrome c heme lyase, putative
  
Accession: EBA27197
  
Location: 584327-585435
  
 NCBI BlastP on this gene

EBA27197

conserved hypothetical protein
  
Accession: EBA27198
  
Location: 586004-588007
  
 NCBI BlastP on this gene

EBA27198

ubiquitin-conjugating enzyme Ubc6, putative
  
Accession: EBA27199
  
Location: 589740-590820
  
 NCBI BlastP on this gene

EBA27199

mitochondrial outer membrane protein (Sam50), putative
  
Accession: EAL84610
  
Location: 591077-592940
  
 NCBI BlastP on this gene

EAL84610

Golgi membrane protein, putative
  
Accession: EAL84611
  
Location: 593250-594297
  
 NCBI BlastP on this gene

EAL84611

ubiquitin fusion degradation protein (Ufd1), putative
  
Accession: EAL84612
  
Location: 594635-597022
  
 NCBI BlastP on this gene

EAL84612

conserved hypothetical protein
  
Accession: EAL84613
  
Location: 598049-599668
  
 NCBI BlastP on this gene

EAL84613

proteasome regulatory particle subunit Rpt6, putative
  
Accession: EAL84614
  
Location: 602763-604113
  
 NCBI BlastP on this gene

EAL84614

16. :  ACJE01000002 Aspergillus niger ATCC 1015     Total score: 5.0     Cumulative Blast bit score: 2581

hypothetical protein
  
Accession: EHA27560
  
Location: 140857-141547
  
 NCBI BlastP on this gene

EHA27560

hypothetical protein
  
Accession: EHA27559
  
Location: 136035-138412
  
 NCBI BlastP on this gene

EHA27559

Hypothetical protein
  
Accession: EHA27558
  
Location: 131320-135761
  
 NCBI BlastP on this gene

EHA27558

transcription factor Tfb2
  
Accession: EHA27557
  
Location: 127841-129402
  
 NCBI BlastP on this gene

EHA27557

hypothetical protein
  
Accession: EHA27556
  
Location: 123431-124861
  
 NCBI BlastP on this gene

EHA27556

hypothetical protein
  
Accession: EHA27555
  
Location: 121767-122948
  
 NCBI BlastP on this gene

EHA27555

hypothetical protein
  
Accession: EHA27554
  
Location: 117321-120994
  
  
**BlastP hit with Mycgr3G68421\_Mycgr3T**
  
Percentage identity: 50 %
  
BlastP bit score: 800
  
Sequence coverage: 67 %
  
E-value: 0.0
  
  
 NCBI BlastP on this gene

EHA27554

hypothetical protein
  
Accession: EHA27553
  
Location: 115907-117009
  
 NCBI BlastP on this gene

EHA27553

hypothetical protein
  
Accession: EHA27552
  
Location: 111302-112918
  
 NCBI BlastP on this gene

EHA27552

hypothetical protein
  
Accession: EHA27551
  
Location: 109229-110335
  
 NCBI BlastP on this gene

EHA27551

hypothetical protein
  
Accession: EHA27550
  
Location: 105990-108974
  
  
**BlastP hit with Mycgr3G68433\_Mycgr3T**
  
Percentage identity: 38 %
  
BlastP bit score: 531
  
Sequence coverage: 102 %
  
E-value: 3e-168
  
  
 NCBI BlastP on this gene

EHA27550

hypothetical protein
  
Accession: EHA27549
  
Location: 102354-103793
  
  
**BlastP hit with Mycgr3G90786\_Mycgr3T**
  
Percentage identity: 27 %
  
BlastP bit score: 95
  
Sequence coverage: 100 %
  
E-value: 8e-18
  
  
 NCBI BlastP on this gene

EHA27549

hypothetical protein
  
Accession: EHA27548
  
Location: 97332-99241
  
 NCBI BlastP on this gene

EHA27548

hypothetical protein
  
Accession: EHA27547
  
Location: 96279-96888
  
 NCBI BlastP on this gene

EHA27547

hypothetical protein
  
Accession: EHA27546
  
Location: 94922-95759
  
 NCBI BlastP on this gene

EHA27546

hypothetical protein
  
Accession: EHA27545
  
Location: 92085-94200
  
 NCBI BlastP on this gene

EHA27545

hypothetical protein
  
Accession: EHA27544
  
Location: 88522-90156
  
 NCBI BlastP on this gene

EHA27544

hypothetical protein
  
Accession: EHA27543
  
Location: 86277-88075
  
 NCBI BlastP on this gene

EHA27543

dehydrogenase
  
Accession: EHA27542
  
Location: 84308-85505
  
 NCBI BlastP on this gene

EHA27542

hypothetical protein
  
Accession: EHA27541
  
Location: 80595-81822
  
 NCBI BlastP on this gene

EHA27541

hypothetical protein
  
Accession: EHA27540
  
Location: 78120-79162
  
  
**BlastP hit with Mycgr3G55345\_Mycgr3T**
  
Percentage identity: 73 %
  
BlastP bit score: 309
  
Sequence coverage: 96 %
  
E-value: 9e-104
  
  
 NCBI BlastP on this gene

EHA27540

hypothetical protein
  
Accession: EHA27539
  
Location: 76854-77332
  
 NCBI BlastP on this gene

EHA27539

hypothetical protein
  
Accession: EHA27538
  
Location: 73689-75923
  
 NCBI BlastP on this gene

EHA27538

hypothetical protein
  
Accession: EHA27537
  
Location: 70947-73136
  
  
**BlastP hit with Mycgr3G68458\_Mycgr3T**
  
Percentage identity: 59 %
  
BlastP bit score: 846
  
Sequence coverage: 101 %
  
E-value: 0.0
  
  
 NCBI BlastP on this gene

EHA27537

hypothetical protein
  
Accession: EHA27536
  
Location: 67344-70037
  
 NCBI BlastP on this gene

EHA27536

hypothetical protein
  
Accession: EHA27535
  
Location: 63383-66631
  
 NCBI BlastP on this gene

EHA27535

hypothetical protein
  
Accession: EHA27534
  
Location: 58076-59643
  
 NCBI BlastP on this gene

EHA27534

hypothetical protein
  
Accession: EHA27533
  
Location: 52499-53831
  
 NCBI BlastP on this gene

EHA27533

17. :  DF126458 Aspergillus kawachii IFO 4308 DNA, contig: scaffold00012     Total score: 5.0     Cumulative Blast bit score: 2541

thioesterase family protein
  
Accession: GAA87226
  
Location: 803880-805058
  
 NCBI BlastP on this gene

GAA87226

TFIIH and nucleotide excision repair factor 3 complexes subunit
  
Accession: GAA87227
  
Location: 805335-806896
  
 NCBI BlastP on this gene

GAA87227

fibronectin type III domain protein
  
Accession: GAA87228
  
Location: 808947-812889
  
 NCBI BlastP on this gene

GAA87228

hypothetical protein
  
Accession: GAA87229
  
Location: 814723-815558
  
 NCBI BlastP on this gene

GAA87229

hypothetical protein
  
Accession: GAA87230
  
Location: 816029-816924
  
 NCBI BlastP on this gene

GAA87230

similar to An14g00450
  
Accession: GAA87231
  
Location: 817680-818840
  
 NCBI BlastP on this gene

GAA87231

topoisomerase 1-associated factor 1
  
Accession: GAA87232
  
Location: 819592-822817
  
  
**BlastP hit with Mycgr3G68421\_Mycgr3T**
  
Percentage identity: 42 %
  
BlastP bit score: 762
  
Sequence coverage: 93 %
  
E-value: 0.0
  
  
 NCBI BlastP on this gene

GAA87232

3-hydroxybutyryl-CoA dehydrogenase
  
Accession: GAA87233
  
Location: 823634-824679
  
 NCBI BlastP on this gene

GAA87233

IBR domain protein
  
Accession: GAA87234
  
Location: 825172-826375
  
 NCBI BlastP on this gene

GAA87234

actin family protein
  
Accession: GAA87235
  
Location: 827906-829310
  
 NCBI BlastP on this gene

GAA87235

hypothetical protein
  
Accession: GAA87236
  
Location: 830235-831341
  
 NCBI BlastP on this gene

GAA87236

conserved leucine-rich repeat protein
  
Accession: GAA87237
  
Location: 831596-834580
  
  
**BlastP hit with Mycgr3G68433\_Mycgr3T**
  
Percentage identity: 39 %
  
BlastP bit score: 538
  
Sequence coverage: 97 %
  
E-value: 1e-170
  
  
 NCBI BlastP on this gene

GAA87237

similar to An14g00380
  
Accession: GAA87238
  
Location: 836754-838193
  
  
**BlastP hit with Mycgr3G90786\_Mycgr3T**
  
Percentage identity: 27 %
  
BlastP bit score: 91
  
Sequence coverage: 99 %
  
E-value: 1e-16
  
  
 NCBI BlastP on this gene

GAA87238

60S ribosomal protein L13
  
Accession: GAA87239
  
Location: 839179-840364
  
 NCBI BlastP on this gene

GAA87239

similar to An14g00360
  
Accession: GAA87240
  
Location: 841267-843181
  
 NCBI BlastP on this gene

GAA87240

hypothetical protein
  
Accession: GAA87241
  
Location: 843475-844245
  
 NCBI BlastP on this gene

GAA87241

similar to An14g00340
  
Accession: GAA87242
  
Location: 844743-845592
  
 NCBI BlastP on this gene

GAA87242

flocculation suppression protein
  
Accession: GAA87243
  
Location: 846311-848419
  
 NCBI BlastP on this gene

GAA87243

F-box domain protein
  
Accession: GAA87244
  
Location: 850228-852026
  
 NCBI BlastP on this gene

GAA87244

succinyl-CoA ligase beta-chain, mitochondrial precursor
  
Accession: GAA87245
  
Location: 852471-854263
  
 NCBI BlastP on this gene

GAA87245

short chain dehydrogenase/reductase
  
Accession: GAA87246
  
Location: 855035-856237
  
 NCBI BlastP on this gene

GAA87246

C4-dicarboxylate/malic acid transporter
  
Accession: GAA87247
  
Location: 858478-860116
  
 NCBI BlastP on this gene

GAA87247

xanthine phosphoribosyltransferase 1
  
Accession: GAA87248
  
Location: 861048-862076
  
  
**BlastP hit with Mycgr3G55345\_Mycgr3T**
  
Percentage identity: 73 %
  
BlastP bit score: 306
  
Sequence coverage: 96 %
  
E-value: 2e-102
  
  
 NCBI BlastP on this gene

GAA87248

hypothetical protein
  
Accession: GAA87249
  
Location: 862857-863348
  
 NCBI BlastP on this gene

GAA87249

ubiquinone biosynthesis protein
  
Accession: GAA87250
  
Location: 864291-869225
  
  
**BlastP hit with Mycgr3G68458\_Mycgr3T**
  
Percentage identity: 64 %
  
BlastP bit score: 844
  
Sequence coverage: 89 %
  
E-value: 0.0
  
  
 NCBI BlastP on this gene

GAA87250

cytochrome C1 heme lyase
  
Accession: GAA87251
  
Location: 870092-871207
  
 NCBI BlastP on this gene

GAA87251

ubiquitin-conjugating enzyme Ubc6
  
Accession: GAA87252
  
Location: 872095-873053
  
 NCBI BlastP on this gene

GAA87252

outer membrane protein, OMP85 family
  
Accession: GAA87253
  
Location: 873463-875256
  
 NCBI BlastP on this gene

GAA87253

golgi membrane protein
  
Accession: GAA87254
  
Location: 875680-876737
  
 NCBI BlastP on this gene

GAA87254

ubiquitin fusion degradation protein
  
Accession: GAA87255
  
Location: 877045-879360
  
 NCBI BlastP on this gene

GAA87255

ubiquitin fusion degradation protein
  
Accession: GAA87256
  
Location: 880014-881923
  
 NCBI BlastP on this gene

GAA87256

proteasome regulatory particle subunit Rpt6
  
Accession: GAA87257
  
Location: 886227-887561
  
 NCBI BlastP on this gene

GAA87257

calcium permease family membrane transporter
  
Accession: GAA87258
  
Location: 888719-892332
  
 NCBI BlastP on this gene

GAA87258

18. :  CH476596 Aspergillus terreus NIH2624 scaffold\_3 genomic scaffold     Total score: 5.0     Cumulative Blast bit score: 2524

conserved hypothetical protein
  
Accession: EAU37690
  
Location: 2345455-2347771
  
 NCBI BlastP on this gene

EAU37690

hypothetical protein
  
Accession: EAU37691
  
Location: 2348045-2351973
  
 NCBI BlastP on this gene

EAU37691

predicted protein
  
Accession: EAU37692
  
Location: 2352690-2353190
  
 NCBI BlastP on this gene

EAU37692

predicted protein
  
Accession: EAU37693
  
Location: 2354188-2355119
  
 NCBI BlastP on this gene

EAU37693

conserved hypothetical protein
  
Accession: EAU37694
  
Location: 2356492-2358033
  
 NCBI BlastP on this gene

EAU37694

predicted protein
  
Accession: EAU37695
  
Location: 2358426-2359325
  
 NCBI BlastP on this gene

EAU37695

predicted protein
  
Accession: EAU37696
  
Location: 2359474-2359824
  
 NCBI BlastP on this gene

EAU37696

hypothetical protein
  
Accession: EAU37697
  
Location: 2361177-2364761
  
  
**BlastP hit with Mycgr3G68421\_Mycgr3T**
  
Percentage identity: 42 %
  
BlastP bit score: 862
  
Sequence coverage: 101 %
  
E-value: 0.0
  
  
 NCBI BlastP on this gene

EAU37697

hypothetical protein
  
Accession: EAU37698
  
Location: 2365103-2367599
  
 NCBI BlastP on this gene

EAU37698

conserved hypothetical protein
  
Accession: EAU37699
  
Location: 2368140-2370718
  
  
**BlastP hit with Mycgr3G68433\_Mycgr3T**
  
Percentage identity: 39 %
  
BlastP bit score: 417
  
Sequence coverage: 72 %
  
E-value: 1e-126
  
  
 NCBI BlastP on this gene

EAU37699

conserved hypothetical protein
  
Accession: EAU37700
  
Location: 2372691-2374121
  
  
**BlastP hit with Mycgr3G90786\_Mycgr3T**
  
Percentage identity: 27 %
  
BlastP bit score: 84
  
Sequence coverage: 99 %
  
E-value: 2e-14
  
  
 NCBI BlastP on this gene

EAU37700

60S ribosomal protein L13
  
Accession: EAU37701
  
Location: 2374714-2375785
  
 NCBI BlastP on this gene

EAU37701

predicted protein
  
Accession: EAU37702
  
Location: 2376426-2377803
  
 NCBI BlastP on this gene

EAU37702

conserved hypothetical protein
  
Accession: EAU37703
  
Location: 2378723-2380273
  
 NCBI BlastP on this gene

EAU37703

conserved hypothetical protein
  
Accession: EAU37704
  
Location: 2380723-2382754
  
 NCBI BlastP on this gene

EAU37704

predicted protein
  
Accession: EAU37705
  
Location: 2383268-2384894
  
 NCBI BlastP on this gene

EAU37705

succinyl-CoA ligase beta-chain, mitochondrial precursor
  
Accession: EAU37706
  
Location: 2385368-2387082
  
 NCBI BlastP on this gene

EAU37706

predicted protein
  
Accession: EAU37707
  
Location: 2387787-2388969
  
 NCBI BlastP on this gene

EAU37707

predicted protein
  
Accession: EAU37708
  
Location: 2390953-2392153
  
 NCBI BlastP on this gene

EAU37708

xanthine phosphoribosyltransferase 1
  
Accession: EAU37709
  
Location: 2392975-2394014
  
  
**BlastP hit with Mycgr3G55345\_Mycgr3T**
  
Percentage identity: 72 %
  
BlastP bit score: 303
  
Sequence coverage: 97 %
  
E-value: 3e-101
  
  
 NCBI BlastP on this gene

EAU37709

conserved hypothetical protein
  
Accession: EAU37710
  
Location: 2394481-2394881
  
 NCBI BlastP on this gene

EAU37710

conserved hypothetical protein
  
Accession: EAU37711
  
Location: 2395508-2397740
  
 NCBI BlastP on this gene

EAU37711

predicted protein
  
Accession: EAU37712
  
Location: 2397838-2399114
  
 NCBI BlastP on this gene

EAU37712

conserved hypothetical protein
  
Accession: EAU37713
  
Location: 2399834-2402002
  
  
**BlastP hit with Mycgr3G68458\_Mycgr3T**
  
Percentage identity: 60 %
  
BlastP bit score: 858
  
Sequence coverage: 100 %
  
E-value: 0.0
  
  
 NCBI BlastP on this gene

EAU37713

cytochrome c heme lyase
  
Accession: EAU37714
  
Location: 2402457-2403561
  
 NCBI BlastP on this gene

EAU37714

conserved hypothetical protein
  
Accession: EAU37715
  
Location: 2403895-2405773
  
 NCBI BlastP on this gene

EAU37715

ubiquitin-conjugating enzyme E2 6
  
Accession: EAU37716
  
Location: 2406806-2407746
  
 NCBI BlastP on this gene

EAU37716

hypothetical protein
  
Accession: EAU37717
  
Location: 2407832-2409824
  
 NCBI BlastP on this gene

EAU37717

conserved hypothetical protein
  
Accession: EAU37718
  
Location: 2410514-2413619
  
 NCBI BlastP on this gene

EAU37718

conserved hypothetical protein
  
Accession: EAU37719
  
Location: 2414136-2415811
  
 NCBI BlastP on this gene

EAU37719

26S protease regulatory subunit 8
  
Accession: EAU37720
  
Location: 2417813-2419110
  
 NCBI BlastP on this gene

EAU37720

conserved hypothetical protein
  
Accession: EAU37721
  
Location: 2420043-2423553
  
 NCBI BlastP on this gene

EAU37721

19. :  AM270309 Aspergillus niger contig An14c0010, genomic contig.     Total score: 5.0     Cumulative Blast bit score: 2522

not annotated
  
Accession: CAK41850
  
Location: 129753-132130
  
 NCBI BlastP on this gene

An14g00510

not annotated
  
Accession: CAK41849
  
Location: 125038-129479
  
 NCBI BlastP on this gene

An14g00500

not annotated
  
Accession: CAK41848
  
Location: 123379-124579
  
 NCBI BlastP on this gene

An14g00490

not annotated
  
Accession: CAK41847
  
Location: 121555-123116
  
 NCBI BlastP on this gene

An14g00480

not annotated
  
Accession: CAK41846
  
Location: 120443-121184
  
 NCBI BlastP on this gene

An14g00470

not annotated
  
Accession: CAK41845
  
Location: 117140-118570
  
 NCBI BlastP on this gene

An14g00460

not annotated
  
Accession: CAK41844
  
Location: 115383-116657
  
 NCBI BlastP on this gene

An14g00450

not annotated
  
Accession: CAK41843
  
Location: 111030-114703
  
  
**BlastP hit with Mycgr3G68421\_Mycgr3T**
  
Percentage identity: 49 %
  
BlastP bit score: 806
  
Sequence coverage: 70 %
  
E-value: 0.0
  
  
 NCBI BlastP on this gene

An14g00440

unnamed
  
Accession: CAK41842
  
Location: 109616-110718
  
 NCBI BlastP on this gene

An14g00430

not annotated
  
Accession: CAK41841
  
Location: 107933-109128
  
 NCBI BlastP on this gene

An14g00420

not annotated
  
Accession: CAK41840
  
Location: 105012-106628
  
 NCBI BlastP on this gene

An14g00410

unnamed
  
Accession: CAK41839
  
Location: 102941-104047
  
 NCBI BlastP on this gene

An14g00400

not annotated
  
Accession: CAK41838
  
Location: 99702-102686
  
  
**BlastP hit with Mycgr3G68433\_Mycgr3T**
  
Percentage identity: 38 %
  
BlastP bit score: 532
  
Sequence coverage: 102 %
  
E-value: 2e-168
  
  
 NCBI BlastP on this gene

An14g00390

not annotated
  
Accession: CAK41837
  
Location: 96066-97505
  
  
**BlastP hit with Mycgr3G90786\_Mycgr3T**
  
Percentage identity: 27 %
  
BlastP bit score: 94
  
Sequence coverage: 100 %
  
E-value: 9e-18
  
  
 NCBI BlastP on this gene

An14g00380

not annotated
  
Accession: CAK41836
  
Location: 93856-95040
  
 NCBI BlastP on this gene

An14g00370

hypothetical protein
  
Accession: CAK41835
  
Location: 91190-92955
  
 NCBI BlastP on this gene

An14g00360

not annotated
  
Accession: CAK41834
  
Location: 89981-90751
  
 NCBI BlastP on this gene

An14g00350

not annotated
  
Accession: CAK41833
  
Location: 88637-89471
  
 NCBI BlastP on this gene

An14g00340

not annotated
  
Accession: CAK41832
  
Location: 85803-87915
  
 NCBI BlastP on this gene

An14g00330

hypothetical protein
  
Accession: CAK41831
  
Location: 82278-84103
  
 NCBI BlastP on this gene

An14g00320

not annotated
  
Accession: CAK41830
  
Location: 80033-81831
  
 NCBI BlastP on this gene

An14g00310

not annotated
  
Accession: CAK41829
  
Location: 78064-79349
  
 NCBI BlastP on this gene

An14g00300

not annotated
  
Accession: CAK41828
  
Location: 73942-75347
  
 NCBI BlastP on this gene

An14g00290

not annotated
  
Accession: CAK41827
  
Location: 71876-72918
  
  
**BlastP hit with Mycgr3G55345\_Mycgr3T**
  
Percentage identity: 73 %
  
BlastP bit score: 309
  
Sequence coverage: 96 %
  
E-value: 9e-104
  
  
 NCBI BlastP on this gene

An14g00280

not annotated
  
Accession: CAK41826
  
Location: 70607-71085
  
 NCBI BlastP on this gene

An14g00270

not annotated
  
Accession: CAK41825
  
Location: 67446-69719
  
 NCBI BlastP on this gene

An14g00260

not annotated
  
Accession: CAK41824
  
Location: 64903-66713
  
  
**BlastP hit with Mycgr3G68458\_Mycgr3T**
  
Percentage identity: 64 %
  
BlastP bit score: 781
  
Sequence coverage: 81 %
  
E-value: 0.0
  
  
 NCBI BlastP on this gene

An14g00250

not annotated
  
Accession: CAK41823
  
Location: 62665-63797
  
 NCBI BlastP on this gene

An14g00240

not annotated
  
Accession: CAK41822
  
Location: 60773-61732
  
 NCBI BlastP on this gene

An14g00230

not annotated
  
Accession: CAK41821
  
Location: 58578-60366
  
 NCBI BlastP on this gene

An14g00220

not annotated
  
Accession: CAK41820
  
Location: 57118-58171
  
 NCBI BlastP on this gene

An14g00210

not annotated
  
Accession: CAK41819
  
Location: 54413-56740
  
 NCBI BlastP on this gene

An14g00200

unnamed
  
Accession: CAK41818
  
Location: 51811-53781
  
 NCBI BlastP on this gene

An14g00190

not annotated
  
Accession: CAK41817
  
Location: 46230-47563
  
 NCBI BlastP on this gene

An14g00180

20. :  AACD01000117 Aspergillus nidulans FGSC A4     Total score: 5.0     Cumulative Blast bit score: 2513

hypothetical protein
  
Accession: EAA61660
  
Location: 127023-130698
  
 NCBI BlastP on this gene

EAA61660

hypothetical protein
  
Accession: EAA61659
  
Location: 123726-126031
  
 NCBI BlastP on this gene

EAA61659

hypothetical protein
  
Accession: EAA61658
  
Location: 116361-118295
  
 NCBI BlastP on this gene

EAA61658

hypothetical protein
  
Accession: EAA61657
  
Location: 115108-115551
  
 NCBI BlastP on this gene

EAA61657

hypothetical protein
  
Accession: EAA61656
  
Location: 111973-115043
  
 NCBI BlastP on this gene

EAA61656

hypothetical protein
  
Accession: EAA61655
  
Location: 109886-111462
  
 NCBI BlastP on this gene

EAA61655

hypothetical protein
  
Accession: EAA61654
  
Location: 107614-109049
  
 NCBI BlastP on this gene

EAA61654

hypothetical protein
  
Accession: EAA61653
  
Location: 104009-107673
  
  
**BlastP hit with Mycgr3G68421\_Mycgr3T**
  
Percentage identity: 51 %
  
BlastP bit score: 826
  
Sequence coverage: 67 %
  
E-value: 0.0
  
  
 NCBI BlastP on this gene

EAA61653

hypothetical protein
  
Accession: EAA61652
  
Location: 102502-103599
  
 NCBI BlastP on this gene

EAA61652

hypothetical protein
  
Accession: EAA61651
  
Location: 99437-102355
  
  
**BlastP hit with Mycgr3G68433\_Mycgr3T**
  
Percentage identity: 38 %
  
BlastP bit score: 498
  
Sequence coverage: 85 %
  
E-value: 6e-156
  
  
 NCBI BlastP on this gene

EAA61651

hypothetical protein
  
Accession: EAA61650
  
Location: 96577-98638
  
  
**BlastP hit with Mycgr3G90786\_Mycgr3T**
  
Percentage identity: 29 %
  
BlastP bit score: 112
  
Sequence coverage: 97 %
  
E-value: 1e-23
  
  
 NCBI BlastP on this gene

EAA61650

hypothetical protein
  
Accession: EAA61649
  
Location: 94654-95748
  
 NCBI BlastP on this gene

EAA61649

predicted protein
  
Accession: EAA61648
  
Location: 92363-94234
  
 NCBI BlastP on this gene

EAA61648

hypothetical protein
  
Accession: EAA61647
  
Location: 91213-91989
  
 NCBI BlastP on this gene

EAA61647

hypothetical protein
  
Accession: EAA61646
  
Location: 88101-89798
  
 NCBI BlastP on this gene

EAA61646

predicted protein
  
Accession: EAA61645
  
Location: 85947-87438
  
 NCBI BlastP on this gene

EAA61645

hypothetical protein
  
Accession: EAA61644
  
Location: 83128-85108
  
 NCBI BlastP on this gene

EAA61644

predicted protein
  
Accession: EAA61643
  
Location: 81872-82605
  
 NCBI BlastP on this gene

EAA61643

hypothetical protein
  
Accession: EAA61642
  
Location: 79404-81608
  
  
**BlastP hit with Mycgr3G68458\_Mycgr3T**
  
Percentage identity: 59 %
  
BlastP bit score: 778
  
Sequence coverage: 95 %
  
E-value: 0.0
  
  
 NCBI BlastP on this gene

EAA61642

predicted protein
  
Accession: EAA61641
  
Location: 78060-78609
  
 NCBI BlastP on this gene

EAA61641

predicted protein
  
Accession: EAA61640
  
Location: 75260-76368
  
 NCBI BlastP on this gene

EAA61640

hypothetical protein
  
Accession: EAA61639
  
Location: 73911-74807
  
  
**BlastP hit with Mycgr3G55345\_Mycgr3T**
  
Percentage identity: 73 %
  
BlastP bit score: 299
  
Sequence coverage: 97 %
  
E-value: 7e-100
  
  
 NCBI BlastP on this gene

EAA61639

hypothetical protein
  
Accession: EAA61638
  
Location: 73029-73435
  
 NCBI BlastP on this gene

EAA61638

hypothetical protein
  
Accession: EAA61637
  
Location: 68762-72373
  
 NCBI BlastP on this gene

EAA61637

hypothetical protein
  
Accession: EAA61636
  
Location: 65902-68067
  
 NCBI BlastP on this gene

EAA61636

hypothetical protein
  
Accession: EAA61635
  
Location: 62297-65302
  
 NCBI BlastP on this gene

EAA61635

conserved hypothetical protein
  
Accession: EAA61634
  
Location: 60392-61691
  
 NCBI BlastP on this gene

EAA61634

hypothetical protein
  
Accession: EAA61633
  
Location: 54066-58697
  
 NCBI BlastP on this gene

EAA61633

21. :  KB446542 Dothistroma septosporum NZE10 unplaced genomic scaffold DOTSEscaffold\_8     Total score: 5.0     Cumulative Blast bit score: 2454

carbohydrate-binding module family 14 protein
  
Accession: EME41286
  
Location: 384698-385195
  
 NCBI BlastP on this gene

EME41286

hypothetical protein
  
Accession: EME41285
  
Location: 381721-382374
  
 NCBI BlastP on this gene

EME41285

hypothetical protein
  
Accession: EME41284
  
Location: 379550-380364
  
 NCBI BlastP on this gene

EME41284

hypothetical protein
  
Accession: EME41283
  
Location: 376445-378245
  
 NCBI BlastP on this gene

EME41283

hypothetical protein
  
Accession: EME41281
  
Location: 373239-373854
  
 NCBI BlastP on this gene

EME41281

hypothetical protein
  
Accession: EME41280
  
Location: 369314-370714
  
 NCBI BlastP on this gene

EME41280

hypothetical protein
  
Accession: EME41279
  
Location: 367579-369120
  
 NCBI BlastP on this gene

EME41279

hypothetical protein
  
Accession: EME41278
  
Location: 366099-367262
  
 NCBI BlastP on this gene

EME41278

hypothetical protein
  
Accession: EME41277
  
Location: 362493-365000
  
  
**BlastP hit with Mycgr3G21922\_Mycgr3T**
  
Percentage identity: 65 %
  
BlastP bit score: 663
  
Sequence coverage: 101 %
  
E-value: 0.0
  
  
 NCBI BlastP on this gene

EME41277

hypothetical protein
  
Accession: EME41276
  
Location: 358612-361134
  
  
**BlastP hit with Mycgr3G84644\_Mycgr3T**
  
Percentage identity: 75 %
  
BlastP bit score: 902
  
Sequence coverage: 108 %
  
E-value: 0.0
  
  
 NCBI BlastP on this gene

EME41276

hypothetical protein
  
Accession: EME41275
  
Location: 354563-356878
  
  
**BlastP hit with Mycgr3G108094\_Mycgr3**
  
Percentage identity: 46 %
  
BlastP bit score: 224
  
Sequence coverage: 19 %
  
E-value: 2e-57
  
  
 NCBI BlastP on this gene

EME41275

hypothetical protein
  
Accession: EME41274
  
Location: 352712-353245
  
 NCBI BlastP on this gene

EME41274

hypothetical protein
  
Accession: EME41273
  
Location: 350944-352110
  
  
**BlastP hit with Mycgr3G25746\_Mycgr3T**
  
Percentage identity: 68 %
  
BlastP bit score: 470
  
Sequence coverage: 100 %
  
E-value: 3e-162
  
  
 NCBI BlastP on this gene

EME41273

hypothetical protein
  
Accession: EME41272
  
Location: 349025-350310
  
 NCBI BlastP on this gene

EME41272

hypothetical protein
  
Accession: EME41271
  
Location: 346658-347699
  
 NCBI BlastP on this gene

EME41271

hypothetical protein
  
Accession: EME41270
  
Location: 345202-346254
  
 NCBI BlastP on this gene

EME41270

hypothetical protein
  
Accession: EME41269
  
Location: 343657-344277
  
 NCBI BlastP on this gene

EME41269

hypothetical protein
  
Accession: EME41268
  
Location: 340840-343116
  
 NCBI BlastP on this gene

EME41268

hypothetical protein
  
Accession: EME41267
  
Location: 338082-340201
  
 NCBI BlastP on this gene

EME41267

hypothetical protein
  
Accession: EME41266
  
Location: 336426-337541
  
 NCBI BlastP on this gene

EME41266

hypothetical protein
  
Accession: EME41265
  
Location: 332699-335686
  
 NCBI BlastP on this gene

EME41265

hypothetical protein
  
Accession: EME41264
  
Location: 330645-331181
  
  
**BlastP hit with Mycgr3G37570\_Mycgr3T**
  
Percentage identity: 57 %
  
BlastP bit score: 195
  
Sequence coverage: 96 %
  
E-value: 7e-60
  
  
 NCBI BlastP on this gene

EME41264

hypothetical protein
  
Accession: EME41263
  
Location: 328790-329509
  
 NCBI BlastP on this gene

EME41263

hypothetical protein
  
Accession: EME41262
  
Location: 325489-327385
  
 NCBI BlastP on this gene

EME41262

hypothetical protein
  
Accession: EME41260
  
Location: 324713-325282
  
 NCBI BlastP on this gene

EME41260

hypothetical protein
  
Accession: EME41259
  
Location: 320318-321659
  
 NCBI BlastP on this gene

EME41259

hypothetical protein
  
Accession: EME41257
  
Location: 319144-319638
  
 NCBI BlastP on this gene

EME41257

hypothetical protein
  
Accession: EME41256
  
Location: 315316-316306
  
 NCBI BlastP on this gene

EME41256

hypothetical protein
  
Accession: EME41255
  
Location: 314521-314781
  
 NCBI BlastP on this gene

EME41255

hypothetical protein
  
Accession: EME41254
  
Location: 312295-313434
  
 NCBI BlastP on this gene

EME41254

22. :  GG692419 Ajellomyces capsulatus H143 genomic scaffold supercont2.1     Total score: 5.0     Cumulative Blast bit score: 1946

topoisomerase I
  
Accession: EER44756
  
Location: 1522955-1523470
  
  
**BlastP hit with Mycgr3G68421\_Mycgr3T**
  
Percentage identity: 55 %
  
BlastP bit score: 102
  
Sequence coverage: 7 %
  
E-value: 1e-21
  
  
 NCBI BlastP on this gene

EER44756

topoisomerase 1-associated factor 1
  
Accession: EER44757
  
Location: 1524200-1526885
  
  
**BlastP hit with Mycgr3G68421\_Mycgr3T**
  
Percentage identity: 42 %
  
BlastP bit score: 531
  
Sequence coverage: 62 %
  
E-value: 7e-169
  
  
 NCBI BlastP on this gene

EER44757

conserved hypothetical protein
  
Accession: EER44759
  
Location: 1528507-1529959
  
 NCBI BlastP on this gene

EER44759

CAMK family protein kinase
  
Accession: EER44760
  
Location: 1530448-1532062
  
 NCBI BlastP on this gene

EER44760

conserved hypothetical protein
  
Accession: EER44761
  
Location: 1533424-1535632
  
 NCBI BlastP on this gene

EER44761

60S ribosomal protein L13
  
Accession: EER44762
  
Location: 1537074-1538316
  
 NCBI BlastP on this gene

EER44762

conserved hypothetical protein
  
Accession: EER44763
  
Location: 1540182-1540916
  
  
**BlastP hit with Mycgr3G90786\_Mycgr3T**
  
Percentage identity: 31 %
  
BlastP bit score: 58
  
Sequence coverage: 39 %
  
E-value: 1e-06
  
  
 NCBI BlastP on this gene

EER44763

leucine rich repeat domain-containing protein
  
Accession: EER44764
  
Location: 1541966-1545062
  
  
**BlastP hit with Mycgr3G68433\_Mycgr3T**
  
Percentage identity: 34 %
  
BlastP bit score: 392
  
Sequence coverage: 99 %
  
E-value: 7e-116
  
  
 NCBI BlastP on this gene

EER44764

conserved hypothetical protein
  
Accession: EER44765
  
Location: 1545904-1547193
  
 NCBI BlastP on this gene

EER44765

actin-like protein
  
Accession: EER44766
  
Location: 1548312-1549967
  
 NCBI BlastP on this gene

EER44766

conserved hypothetical protein
  
Accession: EER44767
  
Location: 1550822-1551835
  
 NCBI BlastP on this gene

EER44767

GTP binding protein
  
Accession: EER44768
  
Location: 1552381-1554875
  
 NCBI BlastP on this gene

EER44768

xanthine phosphoribosyltransferase
  
Accession: EER44769
  
Location: 1557337-1558243
  
  
**BlastP hit with Mycgr3G55345\_Mycgr3T**
  
Percentage identity: 75 %
  
BlastP bit score: 170
  
Sequence coverage: 49 %
  
E-value: 2e-50
  
  
 NCBI BlastP on this gene

EER44769

HSF-type DNA-binding domain-containing protein
  
Accession: EER44770
  
Location: 1563861-1566430
  
 NCBI BlastP on this gene

EER44770

conserved hypothetical protein
  
Accession: EER44771
  
Location: 1567946-1568742
  
 NCBI BlastP on this gene

EER44771

succinyl-CoA ligase beta-chain
  
Accession: EER44772
  
Location: 1569006-1570942
  
 NCBI BlastP on this gene

EER44772

short chain dehydrogenase/reductase
  
Accession: EER44773
  
Location: 1572036-1573418
  
 NCBI BlastP on this gene

EER44773

carbonic anhydrase
  
Accession: EER44774
  
Location: 1575394-1576040
  
 NCBI BlastP on this gene

EER44774

mitochondrion protein
  
Accession: EER44775
  
Location: 1576742-1577020
  
 NCBI BlastP on this gene

EER44775

ubiquinone biosynthesis protein
  
Accession: EER44776
  
Location: 1577170-1580472
  
  
**BlastP hit with Mycgr3G68458\_Mycgr3T**
  
Percentage identity: 61 %
  
BlastP bit score: 693
  
Sequence coverage: 81 %
  
E-value: 0.0
  
  
 NCBI BlastP on this gene

EER44776

conserved hypothetical protein
  
Accession: EER44777
  
Location: 1581389-1582914
  
 NCBI BlastP on this gene

EER44777

cytochrome c heme lyase
  
Accession: EER44778
  
Location: 1584115-1585296
  
 NCBI BlastP on this gene

EER44778

tachykinin family protein
  
Accession: EER44779
  
Location: 1586135-1588903
  
 NCBI BlastP on this gene

EER44779

conserved hypothetical protein
  
Accession: EER44780
  
Location: 1592620-1593090
  
 NCBI BlastP on this gene

EER44780

conserved hypothetical protein
  
Accession: EER44781
  
Location: 1593685-1595120
  
 NCBI BlastP on this gene

EER44781

ubiquitin-conjugating enzyme E2
  
Accession: EER44782
  
Location: 1595553-1596567
  
 NCBI BlastP on this gene

EER44782

outer membrane protein
  
Accession: EER44783
  
Location: 1596887-1599516
  
 NCBI BlastP on this gene

EER44783

23. :  GG698482 Trichophyton tonsurans CBS 112818 genomic scaffold supercont1.6     Total score: 5.0     Cumulative Blast bit score: 1521

fatty acid synthase beta subunit dehydratase
  
Accession: EGD94295
  
Location: 555259-561597
  
 NCBI BlastP on this gene

EGD94295

hypothetical protein
  
Accession: EGD94296
  
Location: 562311-562648
  
 NCBI BlastP on this gene

EGD94296

cortical actin cytoskeleton protein asp1
  
Accession: EGD94297
  
Location: 563618-568058
  
 NCBI BlastP on this gene

EGD94297

WD repeat containing protein 36
  
Accession: EGD94298
  
Location: 568796-572241
  
 NCBI BlastP on this gene

EGD94298

hypothetical protein
  
Accession: EGD94299
  
Location: 572760-573431
  
 NCBI BlastP on this gene

EGD94299

DNA-binding protein HGH1
  
Accession: EGD94300
  
Location: 574764-576164
  
  
**BlastP hit with Mycgr3G25746\_Mycgr3T**
  
Percentage identity: 56 %
  
BlastP bit score: 358
  
Sequence coverage: 98 %
  
E-value: 3e-118
  
  
 NCBI BlastP on this gene

EGD94300

hypothetical protein
  
Accession: EGD94301
  
Location: 576363-577623
  
 NCBI BlastP on this gene

EGD94301

hypothetical protein
  
Accession: EGD94302
  
Location: 577778-579631
  
  
**BlastP hit with Mycgr3G21922\_Mycgr3T**
  
Percentage identity: 44 %
  
BlastP bit score: 162
  
Sequence coverage: 45 %
  
E-value: 3e-40
  
  
 NCBI BlastP on this gene

EGD94302

transcription initiation factor IIF subunit alpha
  
Accession: EGD94303
  
Location: 580750-583198
  
  
**BlastP hit with Mycgr3G103278\_Mycgr3**
  
Percentage identity: 34 %
  
BlastP bit score: 137
  
Sequence coverage: 95 %
  
E-value: 2e-32
  
  
 NCBI BlastP on this gene

EGD94303

hypothetical protein
  
Accession: EGD94304
  
Location: 583709-585104
  
 NCBI BlastP on this gene

EGD94304

hypothetical protein
  
Accession: EGD94305
  
Location: 585319-587330
  
 NCBI BlastP on this gene

EGD94305

inositol monophosphatase
  
Accession: EGD94306
  
Location: 588136-589161
  
 NCBI BlastP on this gene

EGD94306

hypothetical protein
  
Accession: EGD94307
  
Location: 589365-594240
  
 NCBI BlastP on this gene

EGD94307

hypothetical protein
  
Accession: EGD94308
  
Location: 594961-595819
  
 NCBI BlastP on this gene

EGD94308

hypothetical protein
  
Accession: EGD94309
  
Location: 596298-597293
  
 NCBI BlastP on this gene

EGD94309

GTP-binding protein
  
Accession: EGD94310
  
Location: 598010-598544
  
 NCBI BlastP on this gene

EGD94310

hypothetical protein
  
Accession: EGD94311
  
Location: 599817-602307
  
  
**BlastP hit with Mycgr3G84646\_Mycgr3T**
  
Percentage identity: 36 %
  
BlastP bit score: 406
  
Sequence coverage: 110 %
  
E-value: 3e-127
  
  
 NCBI BlastP on this gene

EGD94311

C-4 methylsterol oxidase
  
Accession: EGD94312
  
Location: 603068-604004
  
  
**BlastP hit with Mycgr3G36271\_Mycgr3T**
  
Percentage identity: 80 %
  
BlastP bit score: 458
  
Sequence coverage: 87 %
  
E-value: 6e-160
  
  
 NCBI BlastP on this gene

EGD94312

AAA family ATPase
  
Accession: EGD94313
  
Location: 604992-607505
  
 NCBI BlastP on this gene

EGD94313

hypothetical protein
  
Accession: EGD94314
  
Location: 608078-609370
  
 NCBI BlastP on this gene

EGD94314

hypothetical protein
  
Accession: EGD94315
  
Location: 609827-611058
  
 NCBI BlastP on this gene

EGD94315

Atypical/PIKK/ATR protein kinase
  
Accession: EGD94316
  
Location: 611415-620368
  
 NCBI BlastP on this gene

EGD94316

tRNA-specific adenosine deaminase subunit
  
Accession: EGD94317
  
Location: 620887-621706
  
 NCBI BlastP on this gene

EGD94317

NADH-ubiquinone oxidoreductase 21 kDa subunit
  
Accession: EGD94318
  
Location: 621813-622693
  
 NCBI BlastP on this gene

EGD94318

replication fork protection component Swi3
  
Accession: EGD94319
  
Location: 623026-624079
  
 NCBI BlastP on this gene

EGD94319

24. :  AFWA01000008 Pneumocystis murina B123     Total score: 5.0     Cumulative Blast bit score: 1431

hypothetical protein
  
Accession: EMR09846
  
Location: 361153-362515
  
 NCBI BlastP on this gene

EMR09846

ribosomal RNA large subunit methyltransferase J
  
Accession: EMR09845
  
Location: 359940-360982
  
 NCBI BlastP on this gene

EMR09845

hypothetical protein
  
Accession: EMR09844
  
Location: 359151-359765
  
 NCBI BlastP on this gene

EMR09844

hypothetical protein
  
Accession: EMR09843
  
Location: 358495-358964
  
 NCBI BlastP on this gene

EMR09843

glycine dehydrogenase
  
Accession: EMR09842
  
Location: 354367-357543
  
 NCBI BlastP on this gene

EMR09842

leucyl-tRNA synthetase
  
Accession: EMR09841
  
Location: 349100-352484
  
 NCBI BlastP on this gene

EMR09841

hypothetical protein
  
Accession: EMR09840
  
Location: 347423-348918
  
 NCBI BlastP on this gene

EMR09840

hypothetical protein
  
Accession: EMR09839
  
Location: 345846-347102
  
 NCBI BlastP on this gene

EMR09839

hypothetical protein
  
Accession: EMR09838
  
Location: 344297-345661
  
 NCBI BlastP on this gene

EMR09838

hypothetical protein
  
Accession: EMR09837
  
Location: 343411-344094
  
 NCBI BlastP on this gene

EMR09837

hypothetical protein
  
Accession: EMR09836
  
Location: 341288-343054
  
  
**BlastP hit with Mycgr3G84646\_Mycgr3T**
  
Percentage identity: 29 %
  
BlastP bit score: 283
  
Sequence coverage: 87 %
  
E-value: 1e-82
  
  
 NCBI BlastP on this gene

EMR09836

hypothetical protein
  
Accession: EMR09835
  
Location: 340074-340971
  
  
**BlastP hit with Mycgr3G99145\_Mycgr3T**
  
Percentage identity: 71 %
  
BlastP bit score: 280
  
Sequence coverage: 93 %
  
E-value: 3e-92
  
  
 NCBI BlastP on this gene

EMR09835

hypothetical protein
  
Accession: EMR09834
  
Location: 338111-339289
  
 NCBI BlastP on this gene

EMR09834

hypothetical protein
  
Accession: EMR09833
  
Location: 333272-336782
  
 NCBI BlastP on this gene

EMR09833

hypothetical protein
  
Accession: EMR09832
  
Location: 332078-332531
  
 NCBI BlastP on this gene

EMR09832

hypothetical protein
  
Accession: EMR09831
  
Location: 330954-332006
  
 NCBI BlastP on this gene

EMR09831

hypothetical protein
  
Accession: EMR09830
  
Location: 327745-330852
  
 NCBI BlastP on this gene

EMR09830

YggS family pyridoxal phosphate enzyme
  
Accession: EMR09829
  
Location: 326684-327691
  
 NCBI BlastP on this gene

EMR09829

hypothetical protein
  
Accession: EMR09828
  
Location: 324874-325959
  
 NCBI BlastP on this gene

EMR09828

hypothetical protein
  
Accession: EMR09827
  
Location: 323213-324351
  
 NCBI BlastP on this gene

EMR09827

CMGC/SRPK protein kinase
  
Accession: EMR09826
  
Location: 320682-322603
  
  
**BlastP hit with Mycgr3G84644\_Mycgr3T**
  
Percentage identity: 56 %
  
BlastP bit score: 555
  
Sequence coverage: 83 %
  
E-value: 0.0
  
  
 NCBI BlastP on this gene

EMR09826

hypothetical protein
  
Accession: EMR09825
  
Location: 319145-320430
  
 NCBI BlastP on this gene

EMR09825

hypothetical protein
  
Accession: EMR09824
  
Location: 316369-318730
  
 NCBI BlastP on this gene

EMR09824

hypothetical protein
  
Accession: EMR09823
  
Location: 315779-316083
  
 NCBI BlastP on this gene

EMR09823

hypothetical protein
  
Accession: EMR09822
  
Location: 314886-315352
  
 NCBI BlastP on this gene

EMR09822

hypothetical protein
  
Accession: EMR09821
  
Location: 302993-314700
  
 NCBI BlastP on this gene

EMR09821

hypothetical protein
  
Accession: EMR09820
  
Location: 301618-302651
  
 NCBI BlastP on this gene

EMR09820

hypothetical protein
  
Accession: EMR09819
  
Location: 300227-301526
  
 NCBI BlastP on this gene

EMR09819

hypothetical protein
  
Accession: EMR09818
  
Location: 299577-299965
  
 NCBI BlastP on this gene

EMR09818

hypothetical protein
  
Accession: EMR09817
  
Location: 298548-299560
  
 NCBI BlastP on this gene

EMR09817

hypothetical protein
  
Accession: EMR09816
  
Location: 296067-298351
  
 NCBI BlastP on this gene

EMR09816

hypothetical protein
  
Accession: EMR09815
  
Location: 294281-295927
  
  
**BlastP hit with Mycgr3G21922\_Mycgr3T**
  
Percentage identity: 45 %
  
BlastP bit score: 207
  
Sequence coverage: 51 %
  
E-value: 2e-57
  
  
 NCBI BlastP on this gene

EMR09815

hypothetical protein
  
Accession: EMR09814
  
Location: 291961-294032
  
  
**BlastP hit with Mycgr3G103278\_Mycgr3**
  
Percentage identity: 33 %
  
BlastP bit score: 106
  
Sequence coverage: 73 %
  
E-value: 2e-22
  
  
 NCBI BlastP on this gene

EMR09814

hypothetical protein
  
Accession: EMR09813
  
Location: 289651-291466
  
 NCBI BlastP on this gene

EMR09813

hypothetical protein
  
Accession: EMR09812
  
Location: 288930-289477
  
 NCBI BlastP on this gene

EMR09812

hypothetical protein
  
Accession: EMR09811
  
Location: 287758-288467
  
 NCBI BlastP on this gene

EMR09811

hypothetical protein
  
Accession: EMR09810
  
Location: 284073-287275
  
 NCBI BlastP on this gene

EMR09810

hypothetical protein
  
Accession: EMR09809
  
Location: 283616-283981
  
 NCBI BlastP on this gene

EMR09809

hypothetical protein
  
Accession: EMR09808
  
Location: 281873-283339
  
 NCBI BlastP on this gene

EMR09808

hypothetical protein
  
Accession: EMR09807
  
Location: 278291-281503
  
 NCBI BlastP on this gene

EMR09807

hypothetical protein
  
Accession: EMR09806
  
Location: 277711-278140
  
 NCBI BlastP on this gene

EMR09806

5-formyltetrahydrofolate cyclo-ligase
  
Accession: EMR09805
  
Location: 277147-277683
  
 NCBI BlastP on this gene

EMR09805

hypothetical protein
  
Accession: EMR09804
  
Location: 275875-276512
  
 NCBI BlastP on this gene

EMR09804

hypothetical protein
  
Accession: EMR09803
  
Location: 274443-275077
  
 NCBI BlastP on this gene

EMR09803

V-type ATPase, F subunit
  
Accession: EMR09802
  
Location: 273740-274323
  
 NCBI BlastP on this gene

EMR09802

hypothetical protein
  
Accession: EMR09801
  
Location: 272227-273620
  
 NCBI BlastP on this gene

EMR09801

25. :  KB446542 Dothistroma septosporum NZE10 unplaced genomic scaffold DOTSEscaffold\_8     Total score: 4.0     Cumulative Blast bit score: 3753

hypothetical protein
  
Accession: EME41309
  
Location: 429505-430392
  
 NCBI BlastP on this gene

EME41309

hypothetical protein
  
Accession: EME41308
  
Location: 428548-428958
  
 NCBI BlastP on this gene

EME41308

hypothetical protein
  
Accession: EME41307
  
Location: 425577-426959
  
 NCBI BlastP on this gene

EME41307

hypothetical protein
  
Accession: EME41306
  
Location: 423992-424501
  
 NCBI BlastP on this gene

EME41306

hypothetical protein
  
Accession: EME41305
  
Location: 422848-423696
  
 NCBI BlastP on this gene

EME41305

hypothetical protein
  
Accession: EME41304
  
Location: 419775-421452
  
 NCBI BlastP on this gene

EME41304

hypothetical protein
  
Accession: EME41303
  
Location: 418489-419181
  
 NCBI BlastP on this gene

EME41303

hypothetical protein
  
Accession: EME41302
  
Location: 416345-416914
  
 NCBI BlastP on this gene

EME41302

hypothetical protein
  
Accession: EME41301
  
Location: 411717-415283
  
  
**BlastP hit with Mycgr3G99148\_Mycgr3T**
  
Percentage identity: 77 %
  
BlastP bit score: 1932
  
Sequence coverage: 100 %
  
E-value: 0.0
  
  
 NCBI BlastP on this gene

EME41301

hypothetical protein
  
Accession: EME41300
  
Location: 409872-410108
  
 NCBI BlastP on this gene

EME41300

hypothetical protein
  
Accession: EME41299
  
Location: 409150-409605
  
 NCBI BlastP on this gene

EME41299

hypothetical protein
  
Accession: EME41297
  
Location: 406475-407591
  
  
**BlastP hit with Mycgr3G36271\_Mycgr3T**
  
Percentage identity: 90 %
  
BlastP bit score: 551
  
Sequence coverage: 95 %
  
E-value: 0.0
  
  
 NCBI BlastP on this gene

EME41297

hypothetical protein
  
Accession: EME41296
  
Location: 403896-405887
  
  
**BlastP hit with Mycgr3G84646\_Mycgr3T**
  
Percentage identity: 67 %
  
BlastP bit score: 909
  
Sequence coverage: 99 %
  
E-value: 0.0
  
  
 NCBI BlastP on this gene

EME41296

hypothetical protein
  
Accession: EME41295
  
Location: 402292-403161
  
  
**BlastP hit with Mycgr3G99145\_Mycgr3T**
  
Percentage identity: 94 %
  
BlastP bit score: 362
  
Sequence coverage: 99 %
  
E-value: 1e-124
  
  
 NCBI BlastP on this gene

EME41295

hypothetical protein
  
Accession: EME41294
  
Location: 400341-401651
  
 NCBI BlastP on this gene

EME41294

hypothetical protein
  
Accession: EME41293
  
Location: 398757-399194
  
 NCBI BlastP on this gene

EME41293

hypothetical protein
  
Accession: EME41292
  
Location: 397639-398157
  
 NCBI BlastP on this gene

EME41292

hypothetical protein
  
Accession: EME41291
  
Location: 395606-397453
  
 NCBI BlastP on this gene

EME41291

hypothetical protein
[truncated: 84,457 more chars]
