# Supplementary material for: In silico prediction and characterization of secondary metabolite biosynthetic gene clusters in the wheat pathogen Zymoseptoria tritici
Source: BMC Genomics. 2017 Aug 17;18:631. doi: 10.1186/s12864-017-3969-y (PMC5561558; doi:10.1186/s12864-017-3969-y)
Supplement: Supplementary file 1 — MultiGeneBLAST analysis of putative secondary metabolite clusters. All encoded amino acid sequences from genes residing in clusters predicted by AntiSMASH are given as FASTA file format. All output data from MultiGeneBLASTs are also provided. (ZIP 42911 kb) [file 12864_2017_3969_MOESM1_ESM.zip › Cluster MultiGene BLAST/out/Clusters_1_34/Cluster_12/displaypage2.xhtml]

xml version="1.0" encoding="UTF-8"?


Search Results
  
  
 Results pages: 1, 2, 3, 4, 5

**MultiGeneBlast hits**

Select gene cluster alignment
51. KB020429\_1 Colletotrichum gloeosporioides Nara gc5 unplaced genomic scaff...
52. DS572751\_0 Paracoccidioides brasiliensis Pb18 supercont1.2 genomic scaffo...
53. KB726077\_1 Colletotrichum orbiculare MAFF 240422 unplaced genomic scaffol...
54. KB733455\_6 Bipolaris maydis ATCC 48331 unplaced genomic scaffold COCC4sca...
55. KB445579\_0 Cochliobolus heterostrophus C5 unplaced genomic scaffold COCHE...
56. KB445649\_0 Cochliobolus sativus ND90Pr unplaced genomic scaffold COCSAsca...
57. GG697338\_0 Glomerella graminicola M1.001 genomic scaffold supercont1.8, w...
58. KB644412\_3 Penicillium oxalicum 114-2 unplaced genomic scaffold scaffold\_...
59. CP003002\_4 Myceliophthora thermophila ATCC 42464 chromosome 1, complete s...
60. KB707370\_0 Eutypa lata UCREL1 unplaced genomic scaffold EL1\_03\_scaffold\_2...
61. KB446566\_3 Pseudocercospora fijiensis CIRAD86 unplaced genomic scaffold M...
62. FP929137\_3 Leptosphaeria maculans JN3 lm\_SuperContig\_10\_v2 genomic superc...
63. GG749488\_1 Ajellomyces dermatitidis ATCC 18188 genomic scaffold supercont...
64. GG657483\_1 Ajellomyces dermatitidis SLH14081 genomic scaffold supercont1....
65. EQ999987\_1 Ajellomyces dermatitidis ER-3 genomic scaffold supercont1.15, ...
66. JH794989\_0 Magnaporthe oryzae P131 unplaced genomic scaffold P131\_scaffol...
67. JH793752\_0 Magnaporthe oryzae Y34 unplaced genomic scaffold Y34\_scaffold0...
68. CM001233\_0 Magnaporthe oryzae 70-15 chromosome 3, whole genome shotgun se...
69. KB908844\_8 Setosphaeria turcica Et28A unplaced genomic scaffold SETTUscaf...
70. GG704913\_5 Coccidioides immitis RS genomic scaffold supercont3.3, whole g...
71. GL385398\_1 Gaeumannomyces graminis var. tritici R3-111a-1 unplaced genomi...
72. GL636502\_0 Coccidioides posadasii str. Silveira unplaced genomic scaffold...
73. ACFW01000015\_1 Coccidioides posadasii C735 delta SOWgp, whole genome shot...
74. KB446566\_1 Pseudocercospora fijiensis CIRAD86 unplaced genomic scaffold M...
75. CABT02000002\_1 Sordaria macrospora k-hell, whole genome shotgun sequencin...
76. DS572750\_2 Paracoccidioides brasiliensis Pb18 supercont1.1 genomic scaffo...
77. GL891303\_3 Neurospora tetrasperma FGSC 2508 unplaced genomic scaffold NEU...
78. GL891217\_3 Neurospora tetrasperma FGSC 2509 unplaced genomic scaffold NEU...
79. KE148177\_0 Ophiostoma piceae UAMH 11346 chromosome Unknown scf32, whole g...
80. CH408032\_1 Chaetomium globosum CBS 148.51 scaffold\_4 genomic scaffold, wh...
81. GL629769\_0 Grosmannia clavigera kw1407 unplaced genomic scaffold GCSC\_144...
82. CH476615\_1 Uncinocarpus reesii 1704 scaffold\_1 genomic scaffold, whole ge...
83. AM920428\_0 Penicillium chrysogenum Wisconsin 54-1255 complete genome, con...
84. CH476663\_3 Ajellomyces capsulatus NAm1 scaffold\_9 genomic scaffold, whole...
85. KB933328\_0 Togninia minima UCRPA7 unplaced genomic scaffold PA7\_03\_scaffo...
86. EQ963487\_0 Aspergillus flavus NRRL3357 scf\_1106286419368 genomic scaffold...
87. FN430379\_0 Tuber melanosporum whole genome shotgun sequence assembly, sca...
88. AP007172\_0 Aspergillus oryzae RIB40 DNA, SC206.
89. AKHY01000145\_0 Aspergillus oryzae 3.042, whole genome shotgun sequencing ...
90. KB644412\_4 Penicillium oxalicum 114-2 unplaced genomic scaffold scaffold\_...
91. DS499603\_0 Aspergillus fumigatus A1163 scf\_000010 genomic scaffold, whole...
92. CH476596\_2 Aspergillus terreus NIH2624 scaffold\_3 genomic scaffold, whole...
93. ACYE01000064\_0 Trichophyton verrucosum HKI 0517, whole genome shotgun seq...
94. JH767573\_2 Coniosporium apollinis CBS 100218 chromosome Unknown supercont...
95. AKCU01000109\_0 Penicillium digitatum Pd1, whole genome shotgun sequencing...
96. AKCT01000108\_0 Penicillium digitatum PHI26, whole genome shotgun sequenci...
97. GL573178\_0 Geomyces destructans 20631-21 unplaced genomic scaffold superc...
98. DS027693\_0 Neosartorya fischeri NRRL 181 1099437636261 genomic scaffold, ...
99. FM992691\_1 Candida dubliniensis CD36 chromosome 4, complete sequence.
100. KB733455\_3 Bipolaris maydis ATCC 48331 unplaced genomic scaffold COCC4sc...

Query: Architecture Search FASTA input

KB020429 : Colletotrichum gloeosporioides Nara gc5 unplaced genomic scaffold scaffold120    Total score: 3.0     Cumulative Blast bit score: 1376

Hit cluster cross-links:

Mycgr3G90785 Mycgr3T
  
Location: 0-1047

Mycgr3G90785\_Mycgr3T

Mycgr3G103262 Mycgr3
  
Location: 1147-1390

Mycgr3G103262\_Mycgr3

Mycgr3G68458 Mycgr3T
  
Location: 1490-3602

Mycgr3G68458\_Mycgr3T

Mycgr3G99145 Mycgr3T
  
Location: 3702-4326

Mycgr3G99145\_Mycgr3T

Mycgr3G103274 Mycgr3
  
Location: 4426-4957

Mycgr3G103274\_Mycgr3

Mycgr3G103264 Mycgr3
  
Location: 5057-5390

Mycgr3G103264\_Mycgr3

Mycgr3G37570 Mycgr3T
  
Location: 5490-6006

Mycgr3G37570\_Mycgr3T

Mycgr3G108094 Mycgr3
  
Location: 6106-10555

Mycgr3G108094\_Mycgr3

Mycgr3G90786 Mycgr3T
  
Location: 10655-12080

Mycgr3G90786\_Mycgr3T

Mycgr3G68429 Mycgr3T
  
Location: 12180-13440

Mycgr3G68429\_Mycgr3T

Mycgr3G68421 Mycgr3T
  
Location: 13540-17086

Mycgr3G68421\_Mycgr3T

Mycgr3G90801 Mycgr3T
  
Location: 17186-18056

Mycgr3G90801\_Mycgr3T

Mycgr3G84646 Mycgr3T
  
Location: 18156-20235

Mycgr3G84646\_Mycgr3T

Mycgr3G68456 Mycgr3T
  
Location: 20335-21970

Mycgr3G68456\_Mycgr3T

Mycgr3G103270 Mycgr3
  
Location: 22070-22355

Mycgr3G103270\_Mycgr3

Mycgr3G90803 Mycgr3T
  
Location: 22455-23019

Mycgr3G90803\_Mycgr3T

Mycgr3G36941 Mycgr3T
  
Location: 23119-24064

Mycgr3G36941\_Mycgr3T

Mycgr3G25746 Mycgr3T
  
Location: 24164-25241

Mycgr3G25746\_Mycgr3T

Mycgr3G90788 Mycgr3T
  
Location: 25341-25803

Mycgr3G90788\_Mycgr3T

Mycgr3G103260 Mycgr3
  
Location: 25903-26635

Mycgr3G103260\_Mycgr3

Mycgr3G84644 Mycgr3T
  
Location: 26735-28457

Mycgr3G84644\_Mycgr3T

Mycgr3G29227 Mycgr3T
  
Location: 28557-28863

Mycgr3G29227\_Mycgr3T

Mycgr3G36271 Mycgr3T
  
Location: 28963-29854

Mycgr3G36271\_Mycgr3T

Mycgr3G68433 Mycgr3T
  
Location: 29954-33041

Mycgr3G68433\_Mycgr3T

Mycgr3G79452 Mycgr3T
  
Location: 33141-33399

Mycgr3G79452\_Mycgr3T

Mycgr3G55345 Mycgr3T
  
Location: 33499-34126

Mycgr3G55345\_Mycgr3T

Mycgr3G103278 Mycgr3
  
Location: 34226-35195

Mycgr3G103278\_Mycgr3

Mycgr3G84654 Mycgr3T
  
Location: 35295-36630

Mycgr3G84654\_Mycgr3T

Mycgr3G108090 Mycgr3
  
Location: 36730-37591

Mycgr3G108090\_Mycgr3

Mycgr3G21922 Mycgr3T
  
Location: 37691-39149

Mycgr3G21922\_Mycgr3T

Mycgr3G99148 Mycgr3T
  
Location: 39249-42819

Mycgr3G99148\_Mycgr3T

pterin-4-alpha-carbinolamine dehydratase family protein
  
Accession: ELA37624
  
Location: 339976-340622
  
 NCBI BlastP on this gene

ELA37624

c-4 sterol methyl oxidase
  
Accession: ELA37623
  
Location: 338544-339653
  
  
**BlastP hit with Mycgr3G36271\_Mycgr3T**
  
Percentage identity: 73 %
  
BlastP bit score: 459
  
Sequence coverage: 96 %
  
E-value: 2e-159
  
  
 NCBI BlastP on this gene

ELA37623

ATP synthase regulation protein nca2
  
Accession: ELA37622
  
Location: 334342-336458
  
  
**BlastP hit with Mycgr3G84646\_Mycgr3T**
  
Percentage identity: 44 %
  
BlastP bit score: 561
  
Sequence coverage: 100 %
  
E-value: 0.0
  
  
 NCBI BlastP on this gene

ELA37622

rab GTPase
  
Accession: ELA37621
  
Location: 332139-333057
  
  
**BlastP hit with Mycgr3G99145\_Mycgr3T**
  
Percentage identity: 84 %
  
BlastP bit score: 356
  
Sequence coverage: 99 %
  
E-value: 2e-122
  
  
 NCBI BlastP on this gene

ELA37621

hypothetical protein
  
Accession: ELA37620
  
Location: 330179-331558
  
 NCBI BlastP on this gene

ELA37620

AAA family ATPase
  
Accession: ELA37619
  
Location: 327476-329002
  
 NCBI BlastP on this gene

ELA37619

AAA family ATPase
  
Accession: ELA37618
  
Location: 326398-327024
  
 NCBI BlastP on this gene

ELA37618

squalene epoxidase
  
Accession: ELA37617
  
Location: 323383-324869
  
 NCBI BlastP on this gene

ELA37617

dolichyl-phosphate beta-glucosyltransferase
  
Accession: ELA37616
  
Location: 321339-322654
  
 NCBI BlastP on this gene

ELA37616

hypothetical protein
  
Accession: ELA37615
  
Location: 316724-318128
  
 NCBI BlastP on this gene

ELA37615

Query: Architecture Search FASTA input

DS572751 : Paracoccidioides brasiliensis Pb18 supercont1.2 genomic scaffold    Total score: 3.0     Cumulative Blast bit score: 1369

Hit cluster cross-links:

Mycgr3G90785 Mycgr3T
  
Location: 0-1047

Mycgr3G90785\_Mycgr3T

Mycgr3G103262 Mycgr3
  
Location: 1147-1390

Mycgr3G103262\_Mycgr3

Mycgr3G68458 Mycgr3T
  
Location: 1490-3602

Mycgr3G68458\_Mycgr3T

Mycgr3G99145 Mycgr3T
  
Location: 3702-4326

Mycgr3G99145\_Mycgr3T

Mycgr3G103274 Mycgr3
  
Location: 4426-4957

Mycgr3G103274\_Mycgr3

Mycgr3G103264 Mycgr3
  
Location: 5057-5390

Mycgr3G103264\_Mycgr3

Mycgr3G37570 Mycgr3T
  
Location: 5490-6006

Mycgr3G37570\_Mycgr3T

Mycgr3G108094 Mycgr3
  
Location: 6106-10555

Mycgr3G108094\_Mycgr3

Mycgr3G90786 Mycgr3T
  
Location: 10655-12080

Mycgr3G90786\_Mycgr3T

Mycgr3G68429 Mycgr3T
  
Location: 12180-13440

Mycgr3G68429\_Mycgr3T

Mycgr3G68421 Mycgr3T
  
Location: 13540-17086

Mycgr3G68421\_Mycgr3T

Mycgr3G90801 Mycgr3T
  
Location: 17186-18056

Mycgr3G90801\_Mycgr3T

Mycgr3G84646 Mycgr3T
  
Location: 18156-20235

Mycgr3G84646\_Mycgr3T

Mycgr3G68456 Mycgr3T
  
Location: 20335-21970

Mycgr3G68456\_Mycgr3T

Mycgr3G103270 Mycgr3
  
Location: 22070-22355

Mycgr3G103270\_Mycgr3

Mycgr3G90803 Mycgr3T
  
Location: 22455-23019

Mycgr3G90803\_Mycgr3T

Mycgr3G36941 Mycgr3T
  
Location: 23119-24064

Mycgr3G36941\_Mycgr3T

Mycgr3G25746 Mycgr3T
  
Location: 24164-25241

Mycgr3G25746\_Mycgr3T

Mycgr3G90788 Mycgr3T
  
Location: 25341-25803

Mycgr3G90788\_Mycgr3T

Mycgr3G103260 Mycgr3
  
Location: 25903-26635

Mycgr3G103260\_Mycgr3

Mycgr3G84644 Mycgr3T
  
Location: 26735-28457

Mycgr3G84644\_Mycgr3T

Mycgr3G29227 Mycgr3T
  
Location: 28557-28863

Mycgr3G29227\_Mycgr3T

Mycgr3G36271 Mycgr3T
  
Location: 28963-29854

Mycgr3G36271\_Mycgr3T

Mycgr3G68433 Mycgr3T
  
Location: 29954-33041

Mycgr3G68433\_Mycgr3T

Mycgr3G79452 Mycgr3T
  
Location: 33141-33399

Mycgr3G79452\_Mycgr3T

Mycgr3G55345 Mycgr3T
  
Location: 33499-34126

Mycgr3G55345\_Mycgr3T

Mycgr3G103278 Mycgr3
  
Location: 34226-35195

Mycgr3G103278\_Mycgr3

Mycgr3G84654 Mycgr3T
  
Location: 35295-36630

Mycgr3G84654\_Mycgr3T

Mycgr3G108090 Mycgr3
  
Location: 36730-37591

Mycgr3G108090\_Mycgr3

Mycgr3G21922 Mycgr3T
  
Location: 37691-39149

Mycgr3G21922\_Mycgr3T

Mycgr3G99148 Mycgr3T
  
Location: 39249-42819

Mycgr3G99148\_Mycgr3T

conserved hypothetical protein
  
Accession: EEH45067
  
Location: 103715-106192
  
 NCBI BlastP on this gene

EEH45067

conserved hypothetical protein
  
Accession: EEH45068
  
Location: 106837-107862
  
 NCBI BlastP on this gene

EEH45068

conserved hypothetical protein
  
Accession: EEH45069
  
Location: 108236-110470
  
 NCBI BlastP on this gene

EEH45069

conserved hypothetical protein
  
Accession: EEH45070
  
Location: 111937-113233
  
 NCBI BlastP on this gene

EEH45070

conserved hypothetical protein
  
Accession: EEH45071
  
Location: 114154-115518
  
  
**BlastP hit with Mycgr3G90786\_Mycgr3T**
  
Percentage identity: 28 %
  
BlastP bit score: 84
  
Sequence coverage: 103 %
  
E-value: 2e-14
  
  
 NCBI BlastP on this gene

EEH45071

leucine-rich repeat-containing protein
  
Accession: EEH45072
  
Location: 116921-119959
  
  
**BlastP hit with Mycgr3G68433\_Mycgr3T**
  
Percentage identity: 37 %
  
BlastP bit score: 479
  
Sequence coverage: 95 %
  
E-value: 5e-148
  
  
 NCBI BlastP on this gene

EEH45072

conserved hypothetical protein
  
Accession: EEH45073
  
Location: 120585-121826
  
 NCBI BlastP on this gene

EEH45073

predicted protein
  
Accession: EEH45074
  
Location: 122284-123125
  
 NCBI BlastP on this gene

EEH45074

actin family protein
  
Accession: EEH45075
  
Location: 124434-126084
  
 NCBI BlastP on this gene

EEH45075

IBR domain-containing protein
  
Accession: EEH45076
  
Location: 129354-131723
  
 NCBI BlastP on this gene

EEH45076

predicted protein
  
Accession: EEH45077
  
Location: 132662-134098
  
 NCBI BlastP on this gene

EEH45077

3-hydroxybutyryl-CoA dehydrogenase
  
Accession: EEH45078
  
Location: 134531-135690
  
 NCBI BlastP on this gene

EEH45078

topoisomerase 1-associated factor 1
  
Accession: EEH45079
  
Location: 136062-139873
  
  
**BlastP hit with Mycgr3G68421\_Mycgr3T**
  
Percentage identity: 41 %
  
BlastP bit score: 806
  
Sequence coverage: 103 %
  
E-value: 0.0
  
  
 NCBI BlastP on this gene

EEH45079

predicted protein
  
Accession: EEH45080
  
Location: 140910-141266
  
 NCBI BlastP on this gene

EEH45080

conserved hypothetical protein
  
Accession: EEH45081
  
Location: 141749-143525
  
 NCBI BlastP on this gene

EEH45081

TFIIH basal transcription factor complex p52 subunit
  
Accession: EEH45083
  
Location: 144111-145699
  
 NCBI BlastP on this gene

EEH45083

predicted protein
  
Accession: EEH45082
  
Location: 145711-147251
  
 NCBI BlastP on this gene

EEH45082

intron-binding protein aquarius
  
Accession: EEH45084
  
Location: 147936-152389
  
 NCBI BlastP on this gene

EEH45084

Query: Architecture Search FASTA input

KB726077 : Colletotrichum orbiculare MAFF 240422 unplaced genomic scaffold Scaffold\_499    Total score: 3.0     Cumulative Blast bit score: 1368

Hit cluster cross-links:

Mycgr3G90785 Mycgr3T
  
Location: 0-1047

Mycgr3G90785\_Mycgr3T

Mycgr3G103262 Mycgr3
  
Location: 1147-1390

Mycgr3G103262\_Mycgr3

Mycgr3G68458 Mycgr3T
  
Location: 1490-3602

Mycgr3G68458\_Mycgr3T

Mycgr3G99145 Mycgr3T
  
Location: 3702-4326

Mycgr3G99145\_Mycgr3T

Mycgr3G103274 Mycgr3
  
Location: 4426-4957

Mycgr3G103274\_Mycgr3

Mycgr3G103264 Mycgr3
  
Location: 5057-5390

Mycgr3G103264\_Mycgr3

Mycgr3G37570 Mycgr3T
  
Location: 5490-6006

Mycgr3G37570\_Mycgr3T

Mycgr3G108094 Mycgr3
  
Location: 6106-10555

Mycgr3G108094\_Mycgr3

Mycgr3G90786 Mycgr3T
  
Location: 10655-12080

Mycgr3G90786\_Mycgr3T

Mycgr3G68429 Mycgr3T
  
Location: 12180-13440

Mycgr3G68429\_Mycgr3T

Mycgr3G68421 Mycgr3T
  
Location: 13540-17086

Mycgr3G68421\_Mycgr3T

Mycgr3G90801 Mycgr3T
  
Location: 17186-18056

Mycgr3G90801\_Mycgr3T

Mycgr3G84646 Mycgr3T
  
Location: 18156-20235

Mycgr3G84646\_Mycgr3T

Mycgr3G68456 Mycgr3T
  
Location: 20335-21970

Mycgr3G68456\_Mycgr3T

Mycgr3G103270 Mycgr3
  
Location: 22070-22355

Mycgr3G103270\_Mycgr3

Mycgr3G90803 Mycgr3T
  
Location: 22455-23019

Mycgr3G90803\_Mycgr3T

Mycgr3G36941 Mycgr3T
  
Location: 23119-24064

Mycgr3G36941\_Mycgr3T

Mycgr3G25746 Mycgr3T
  
Location: 24164-25241

Mycgr3G25746\_Mycgr3T

Mycgr3G90788 Mycgr3T
  
Location: 25341-25803

Mycgr3G90788\_Mycgr3T

Mycgr3G103260 Mycgr3
  
Location: 25903-26635

Mycgr3G103260\_Mycgr3

Mycgr3G84644 Mycgr3T
  
Location: 26735-28457

Mycgr3G84644\_Mycgr3T

Mycgr3G29227 Mycgr3T
  
Location: 28557-28863

Mycgr3G29227\_Mycgr3T

Mycgr3G36271 Mycgr3T
  
Location: 28963-29854

Mycgr3G36271\_Mycgr3T

Mycgr3G68433 Mycgr3T
  
Location: 29954-33041

Mycgr3G68433\_Mycgr3T

Mycgr3G79452 Mycgr3T
  
Location: 33141-33399

Mycgr3G79452\_Mycgr3T

Mycgr3G55345 Mycgr3T
  
Location: 33499-34126

Mycgr3G55345\_Mycgr3T

Mycgr3G103278 Mycgr3
  
Location: 34226-35195

Mycgr3G103278\_Mycgr3

Mycgr3G84654 Mycgr3T
  
Location: 35295-36630

Mycgr3G84654\_Mycgr3T

Mycgr3G108090 Mycgr3
  
Location: 36730-37591

Mycgr3G108090\_Mycgr3

Mycgr3G21922 Mycgr3T
  
Location: 37691-39149

Mycgr3G21922\_Mycgr3T

Mycgr3G99148 Mycgr3T
  
Location: 39249-42819

Mycgr3G99148\_Mycgr3T

hypothetical protein
  
Accession: ENH77775
  
Location: 1043553-1044227
  
 NCBI BlastP on this gene

ENH77775

WD repeat protein
  
Accession: ENH77776
  
Location: 1051744-1056440
  
 NCBI BlastP on this gene

ENH77776

ubiquitin conjugating enzyme
  
Accession: ENH77777
  
Location: 1057406-1058048
  
 NCBI BlastP on this gene

ENH77777

hypothetical protein
  
Accession: ENH77778
  
Location: 1058980-1059363
  
 NCBI BlastP on this gene

ENH77778

amidohydrolase
  
Accession: ENH77779
  
Location: 1060502-1061884
  
 NCBI BlastP on this gene

ENH77779

pterin-4-alpha-carbinolamine dehydratase family protein
  
Accession: ENH77780
  
Location: 1061998-1062472
  
 NCBI BlastP on this gene

ENH77780

c-4 methyl sterol oxidase
  
Accession: ENH77781
  
Location: 1062926-1064064
  
  
**BlastP hit with Mycgr3G36271\_Mycgr3T**
  
Percentage identity: 74 %
  
BlastP bit score: 462
  
Sequence coverage: 96 %
  
E-value: 5e-161
  
  
 NCBI BlastP on this gene

ENH77781

ATP synthase regulation protein nca2
  
Accession: ENH77782
  
Location: 1066304-1068496
  
  
**BlastP hit with Mycgr3G84646\_Mycgr3T**
  
Percentage identity: 43 %
  
BlastP bit score: 551
  
Sequence coverage: 102 %
  
E-value: 0.0
  
  
 NCBI BlastP on this gene

ENH77782

RAB GTPase
  
Accession: ENH77783
  
Location: 1070215-1071103
  
  
**BlastP hit with Mycgr3G99145\_Mycgr3T**
  
Percentage identity: 83 %
  
BlastP bit score: 355
  
Sequence coverage: 99 %
  
E-value: 5e-122
  
  
 NCBI BlastP on this gene

ENH77783

hypothetical protein
  
Accession: ENH77784
  
Location: 1071880-1073508
  
 NCBI BlastP on this gene

ENH77784

AAA family ATPase
  
Accession: ENH77785
  
Location: 1074599-1076912
  
 NCBI BlastP on this gene

ENH77785

squalene epoxidase
  
Accession: ENH77786
  
Location: 1078768-1080267
  
 NCBI BlastP on this gene

ENH77786

C6 zinc finger domain protein
  
Accession: ENH77787
  
Location: 1081064-1082314
  
 NCBI BlastP on this gene

ENH77787

ABC multidrug transporter
  
Accession: ENH77788
  
Location: 1083794-1088145
  
 NCBI BlastP on this gene

ENH77788

hypothetical protein
  
Accession: ENH77789
  
Location: 1089088-1089954
  
 NCBI BlastP on this gene

ENH77789

nonribosomal peptide synthetase
  
Accession: ENH77790
  
Location: 1090911-1094250
  
 NCBI BlastP on this gene

ENH77790

Query: Architecture Search FASTA input

KB733455 : Bipolaris maydis ATCC 48331 unplaced genomic scaffold COCC4scaffold\_12    Total score: 3.0     Cumulative Blast bit score: 1366

Hit cluster cross-links:

Mycgr3G90785 Mycgr3T
  
Location: 0-1047

Mycgr3G90785\_Mycgr3T

Mycgr3G103262 Mycgr3
  
Location: 1147-1390

Mycgr3G103262\_Mycgr3

Mycgr3G68458 Mycgr3T
  
Location: 1490-3602

Mycgr3G68458\_Mycgr3T

Mycgr3G99145 Mycgr3T
  
Location: 3702-4326

Mycgr3G99145\_Mycgr3T

Mycgr3G103274 Mycgr3
  
Location: 4426-4957

Mycgr3G103274\_Mycgr3

Mycgr3G103264 Mycgr3
  
Location: 5057-5390

Mycgr3G103264\_Mycgr3

Mycgr3G37570 Mycgr3T
  
Location: 5490-6006

Mycgr3G37570\_Mycgr3T

Mycgr3G108094 Mycgr3
  
Location: 6106-10555

Mycgr3G108094\_Mycgr3

Mycgr3G90786 Mycgr3T
  
Location: 10655-12080

Mycgr3G90786\_Mycgr3T

Mycgr3G68429 Mycgr3T
  
Location: 12180-13440

Mycgr3G68429\_Mycgr3T

Mycgr3G68421 Mycgr3T
  
Location: 13540-17086

Mycgr3G68421\_Mycgr3T

Mycgr3G90801 Mycgr3T
  
Location: 17186-18056

Mycgr3G90801\_Mycgr3T

Mycgr3G84646 Mycgr3T
  
Location: 18156-20235

Mycgr3G84646\_Mycgr3T

Mycgr3G68456 Mycgr3T
  
Location: 20335-21970

Mycgr3G68456\_Mycgr3T

Mycgr3G103270 Mycgr3
  
Location: 22070-22355

Mycgr3G103270\_Mycgr3

Mycgr3G90803 Mycgr3T
  
Location: 22455-23019

Mycgr3G90803\_Mycgr3T

Mycgr3G36941 Mycgr3T
  
Location: 23119-24064

Mycgr3G36941\_Mycgr3T

Mycgr3G25746 Mycgr3T
  
Location: 24164-25241

Mycgr3G25746\_Mycgr3T

Mycgr3G90788 Mycgr3T
  
Location: 25341-25803

Mycgr3G90788\_Mycgr3T

Mycgr3G103260 Mycgr3
  
Location: 25903-26635

Mycgr3G103260\_Mycgr3

Mycgr3G84644 Mycgr3T
  
Location: 26735-28457

Mycgr3G84644\_Mycgr3T

Mycgr3G29227 Mycgr3T
  
Location: 28557-28863

Mycgr3G29227\_Mycgr3T

Mycgr3G36271 Mycgr3T
  
Location: 28963-29854

Mycgr3G36271\_Mycgr3T

Mycgr3G68433 Mycgr3T
  
Location: 29954-33041

Mycgr3G68433\_Mycgr3T

Mycgr3G79452 Mycgr3T
  
Location: 33141-33399

Mycgr3G79452\_Mycgr3T

Mycgr3G55345 Mycgr3T
  
Location: 33499-34126

Mycgr3G55345\_Mycgr3T

Mycgr3G103278 Mycgr3
  
Location: 34226-35195

Mycgr3G103278\_Mycgr3

Mycgr3G84654 Mycgr3T
  
Location: 35295-36630

Mycgr3G84654\_Mycgr3T

Mycgr3G108090 Mycgr3
  
Location: 36730-37591

Mycgr3G108090\_Mycgr3

Mycgr3G21922 Mycgr3T
  
Location: 37691-39149

Mycgr3G21922\_Mycgr3T

Mycgr3G99148 Mycgr3T
  
Location: 39249-42819

Mycgr3G99148\_Mycgr3T

hypothetical protein
  
Accession: ENI05253
  
Location: 879556-883208
  
 NCBI BlastP on this gene

ENI05253

hypothetical protein
  
Accession: ENI05252
  
Location: 878521-879144
  
 NCBI BlastP on this gene

ENI05252

hypothetical protein
  
Accession: ENI05251
  
Location: 877466-878471
  
 NCBI BlastP on this gene

ENI05251

hypothetical protein
  
Accession: ENI05250
  
Location: 875499-877086
  
 NCBI BlastP on this gene

ENI05250

hypothetical protein
  
Accession: ENI05249
  
Location: 873558-874579
  
 NCBI BlastP on this gene

ENI05249

hypothetical protein
  
Accession: ENI05248
  
Location: 873258-873428
  
 NCBI BlastP on this gene

ENI05248

carbohydrate esterase family 9 protein
  
Accession: ENI05247
  
Location: 871739-873152
  
 NCBI BlastP on this gene

ENI05247

glycoside hydrolase family 3 protein
  
Accession: ENI05246
  
Location: 868116-871171
  
 NCBI BlastP on this gene

ENI05246

hypothetical protein
  
Accession: ENI05245
  
Location: 865240-866902
  
  
**BlastP hit with Mycgr3G84654\_Mycgr3T**
  
Percentage identity: 58 %
  
BlastP bit score: 395
  
Sequence coverage: 81 %
  
E-value: 5e-129
  
  
 NCBI BlastP on this gene

ENI05245

hypothetical protein
  
Accession: ENI05244
  
Location: 861018-862793
  
 NCBI BlastP on this gene

ENI05244

hypothetical protein
  
Accession: ENI05243
  
Location: 852143-853762
  
 NCBI BlastP on this gene

ENI05243

hypothetical protein
  
Accession: ENI05242
  
Location: 849230-850723
  
 NCBI BlastP on this gene

ENI05242

hypothetical protein
  
Accession: ENI05241
  
Location: 846575-848596
  
  
**BlastP hit with Mycgr3G84646\_Mycgr3T**
  
Percentage identity: 47 %
  
BlastP bit score: 607
  
Sequence coverage: 99 %
  
E-value: 0.0
  
  
 NCBI BlastP on this gene

ENI05241

hypothetical protein
  
Accession: ENI05240
  
Location: 845189-846053
  
  
**BlastP hit with Mycgr3G99145\_Mycgr3T**
  
Percentage identity: 86 %
  
BlastP bit score: 364
  
Sequence coverage: 99 %
  
E-value: 2e-125
  
  
 NCBI BlastP on this gene

ENI05240

hypothetical protein
  
Accession: ENI05239
  
Location: 836794-844658
  
 NCBI BlastP on this gene

ENI05239

hypothetical protein
  
Accession: ENI05238
  
Location: 835855-836258
  
 NCBI BlastP on this gene

ENI05238

hypothetical protein
  
Accession: ENI05237
  
Location: 832412-834819
  
 NCBI BlastP on this gene

ENI05237

Query: Architecture Search FASTA input

KB445579 : Cochliobolus heterostrophus C5 unplaced genomic scaffold COCHEscaffold\_11    Total score: 3.0     Cumulative Blast bit score: 1366

Hit cluster cross-links:

Mycgr3G90785 Mycgr3T
  
Location: 0-1047

Mycgr3G90785\_Mycgr3T

Mycgr3G103262 Mycgr3
  
Location: 1147-1390

Mycgr3G103262\_Mycgr3

Mycgr3G68458 Mycgr3T
  
Location: 1490-3602

Mycgr3G68458\_Mycgr3T

Mycgr3G99145 Mycgr3T
  
Location: 3702-4326

Mycgr3G99145\_Mycgr3T

Mycgr3G103274 Mycgr3
  
Location: 4426-4957

Mycgr3G103274\_Mycgr3

Mycgr3G103264 Mycgr3
  
Location: 5057-5390

Mycgr3G103264\_Mycgr3

Mycgr3G37570 Mycgr3T
  
Location: 5490-6006

Mycgr3G37570\_Mycgr3T

Mycgr3G108094 Mycgr3
  
Location: 6106-10555

Mycgr3G108094\_Mycgr3

Mycgr3G90786 Mycgr3T
  
Location: 10655-12080

Mycgr3G90786\_Mycgr3T

Mycgr3G68429 Mycgr3T
  
Location: 12180-13440

Mycgr3G68429\_Mycgr3T

Mycgr3G68421 Mycgr3T
  
Location: 13540-17086

Mycgr3G68421\_Mycgr3T

Mycgr3G90801 Mycgr3T
  
Location: 17186-18056

Mycgr3G90801\_Mycgr3T

Mycgr3G84646 Mycgr3T
  
Location: 18156-20235

Mycgr3G84646\_Mycgr3T

Mycgr3G68456 Mycgr3T
  
Location: 20335-21970

Mycgr3G68456\_Mycgr3T

Mycgr3G103270 Mycgr3
  
Location: 22070-22355

Mycgr3G103270\_Mycgr3

Mycgr3G90803 Mycgr3T
  
Location: 22455-23019

Mycgr3G90803\_Mycgr3T

Mycgr3G36941 Mycgr3T
  
Location: 23119-24064

Mycgr3G36941\_Mycgr3T

Mycgr3G25746 Mycgr3T
  
Location: 24164-25241

Mycgr3G25746\_Mycgr3T

Mycgr3G90788 Mycgr3T
  
Location: 25341-25803

Mycgr3G90788\_Mycgr3T

Mycgr3G103260 Mycgr3
  
Location: 25903-26635

Mycgr3G103260\_Mycgr3

Mycgr3G84644 Mycgr3T
  
Location: 26735-28457

Mycgr3G84644\_Mycgr3T

Mycgr3G29227 Mycgr3T
  
Location: 28557-28863

Mycgr3G29227\_Mycgr3T

Mycgr3G36271 Mycgr3T
  
Location: 28963-29854

Mycgr3G36271\_Mycgr3T

Mycgr3G68433 Mycgr3T
  
Location: 29954-33041

Mycgr3G68433\_Mycgr3T

Mycgr3G79452 Mycgr3T
  
Location: 33141-33399

Mycgr3G79452\_Mycgr3T

Mycgr3G55345 Mycgr3T
  
Location: 33499-34126

Mycgr3G55345\_Mycgr3T

Mycgr3G103278 Mycgr3
  
Location: 34226-35195

Mycgr3G103278\_Mycgr3

Mycgr3G84654 Mycgr3T
  
Location: 35295-36630

Mycgr3G84654\_Mycgr3T

Mycgr3G108090 Mycgr3
  
Location: 36730-37591

Mycgr3G108090\_Mycgr3

Mycgr3G21922 Mycgr3T
  
Location: 37691-39149

Mycgr3G21922\_Mycgr3T

Mycgr3G99148 Mycgr3T
  
Location: 39249-42819

Mycgr3G99148\_Mycgr3T

hypothetical protein
  
Accession: EMD89026
  
Location: 121449-125101
  
 NCBI BlastP on this gene

EMD89026

hypothetical protein
  
Accession: EMD89027
  
Location: 125513-126136
  
 NCBI BlastP on this gene

EMD89027

hypothetical protein
  
Accession: EMD89028
  
Location: 126186-127191
  
 NCBI BlastP on this gene

EMD89028

hypothetical protein
  
Accession: EMD89029
  
Location: 127571-129158
  
 NCBI BlastP on this gene

EMD89029

hypothetical protein
  
Accession: EMD89030
  
Location: 130078-131099
  
 NCBI BlastP on this gene

EMD89030

hypothetical protein
  
Accession: EMD89031
  
Location: 131230-131400
  
 NCBI BlastP on this gene

EMD89031

carbohydrate esterase family 9 protein
  
Accession: EMD89032
  
Location: 131606-132919
  
 NCBI BlastP on this gene

EMD89032

glycoside hydrolase family 3 protein
  
Accession: EMD89033
  
Location: 133487-136542
  
 NCBI BlastP on this gene

EMD89033

hypothetical protein
  
Accession: EMD89034
  
Location: 137756-139418
  
  
**BlastP hit with Mycgr3G84654\_Mycgr3T**
  
Percentage identity: 58 %
  
BlastP bit score: 395
  
Sequence coverage: 81 %
  
E-value: 5e-129
  
  
 NCBI BlastP on this gene

EMD89034

hypothetical protein
  
Accession: EMD89035
  
Location: 141865-143640
  
 NCBI BlastP on this gene

EMD89035

hypothetical protein
  
Accession: EMD89036
  
Location: 149496-150482
  
 NCBI BlastP on this gene

EMD89036

hypothetical protein
  
Accession: EMD89037
  
Location: 151080-152699
  
 NCBI BlastP on this gene

EMD89037

hypothetical protein
  
Accession: EMD89038
  
Location: 154119-155612
  
 NCBI BlastP on this gene

EMD89038

hypothetical protein
  
Accession: EMD89039
  
Location: 156246-158267
  
  
**BlastP hit with Mycgr3G84646\_Mycgr3T**
  
Percentage identity: 47 %
  
BlastP bit score: 607
  
Sequence coverage: 99 %
  
E-value: 0.0
  
  
 NCBI BlastP on this gene

EMD89039

hypothetical protein
  
Accession: EMD89040
  
Location: 158789-159653
  
  
**BlastP hit with Mycgr3G99145\_Mycgr3T**
  
Percentage identity: 86 %
  
BlastP bit score: 364
  
Sequence coverage: 99 %
  
E-value: 2e-125
  
  
 NCBI BlastP on this gene

EMD89040

hypothetical protein
  
Accession: EMD89041
  
Location: 160184-168048
  
 NCBI BlastP on this gene

EMD89041

hypothetical protein
  
Accession: EMD89042
  
Location: 168584-168987
  
 NCBI BlastP on this gene

EMD89042

hypothetical protein
  
Accession: EMD89043
  
Location: 170023-172430
  
 NCBI BlastP on this gene

EMD89043

Query: Architecture Search FASTA input

KB445649 : Cochliobolus sativus ND90Pr unplaced genomic scaffold COCSAscaffold\_13    Total score: 3.0     Cumulative Blast bit score: 1364

Hit cluster cross-links:

Mycgr3G90785 Mycgr3T
  
Location: 0-1047

Mycgr3G90785\_Mycgr3T

Mycgr3G103262 Mycgr3
  
Location: 1147-1390

Mycgr3G103262\_Mycgr3

Mycgr3G68458 Mycgr3T
  
Location: 1490-3602

Mycgr3G68458\_Mycgr3T

Mycgr3G99145 Mycgr3T
  
Location: 3702-4326

Mycgr3G99145\_Mycgr3T

Mycgr3G103274 Mycgr3
  
Location: 4426-4957

Mycgr3G103274\_Mycgr3

Mycgr3G103264 Mycgr3
  
Location: 5057-5390

Mycgr3G103264\_Mycgr3

Mycgr3G37570 Mycgr3T
  
Location: 5490-6006

Mycgr3G37570\_Mycgr3T

Mycgr3G108094 Mycgr3
  
Location: 6106-10555

Mycgr3G108094\_Mycgr3

Mycgr3G90786 Mycgr3T
  
Location: 10655-12080

Mycgr3G90786\_Mycgr3T

Mycgr3G68429 Mycgr3T
  
Location: 12180-13440

Mycgr3G68429\_Mycgr3T

Mycgr3G68421 Mycgr3T
  
Location: 13540-17086

Mycgr3G68421\_Mycgr3T

Mycgr3G90801 Mycgr3T
  
Location: 17186-18056

Mycgr3G90801\_Mycgr3T

Mycgr3G84646 Mycgr3T
  
Location: 18156-20235

Mycgr3G84646\_Mycgr3T

Mycgr3G68456 Mycgr3T
  
Location: 20335-21970

Mycgr3G68456\_Mycgr3T

Mycgr3G103270 Mycgr3
  
Location: 22070-22355

Mycgr3G103270\_Mycgr3

Mycgr3G90803 Mycgr3T
  
Location: 22455-23019

Mycgr3G90803\_Mycgr3T

Mycgr3G36941 Mycgr3T
  
Location: 23119-24064

Mycgr3G36941\_Mycgr3T

Mycgr3G25746 Mycgr3T
  
Location: 24164-25241

Mycgr3G25746\_Mycgr3T

Mycgr3G90788 Mycgr3T
  
Location: 25341-25803

Mycgr3G90788\_Mycgr3T

Mycgr3G103260 Mycgr3
  
Location: 25903-26635

Mycgr3G103260\_Mycgr3

Mycgr3G84644 Mycgr3T
  
Location: 26735-28457

Mycgr3G84644\_Mycgr3T

Mycgr3G29227 Mycgr3T
  
Location: 28557-28863

Mycgr3G29227\_Mycgr3T

Mycgr3G36271 Mycgr3T
  
Location: 28963-29854

Mycgr3G36271\_Mycgr3T

Mycgr3G68433 Mycgr3T
  
Location: 29954-33041

Mycgr3G68433\_Mycgr3T

Mycgr3G79452 Mycgr3T
  
Location: 33141-33399

Mycgr3G79452\_Mycgr3T

Mycgr3G55345 Mycgr3T
  
Location: 33499-34126

Mycgr3G55345\_Mycgr3T

Mycgr3G103278 Mycgr3
  
Location: 34226-35195

Mycgr3G103278\_Mycgr3

Mycgr3G84654 Mycgr3T
  
Location: 35295-36630

Mycgr3G84654\_Mycgr3T

Mycgr3G108090 Mycgr3
  
Location: 36730-37591

Mycgr3G108090\_Mycgr3

Mycgr3G21922 Mycgr3T
  
Location: 37691-39149

Mycgr3G21922\_Mycgr3T

Mycgr3G99148 Mycgr3T
  
Location: 39249-42819

Mycgr3G99148\_Mycgr3T

hypothetical protein
  
Accession: EMD60800
  
Location: 187265-190746
  
 NCBI BlastP on this gene

EMD60800

hypothetical protein
  
Accession: EMD60801
  
Location: 191317-191940
  
 NCBI BlastP on this gene

EMD60801

hypothetical protein
  
Accession: EMD60802
  
Location: 191990-192994
  
 NCBI BlastP on this gene

EMD60802

hypothetical protein
  
Accession: EMD60803
  
Location: 193668-195253
  
 NCBI BlastP on this gene

EMD60803

hypothetical protein
  
Accession: EMD60804
  
Location: 196158-197179
  
 NCBI BlastP on this gene

EMD60804

carbohydrate esterase family 9 protein
  
Accession: EMD60805
  
Location: 197676-198971
  
 NCBI BlastP on this gene

EMD60805

glycoside hydrolase family 3 protein
  
Accession: EMD60806
  
Location: 199560-202617
  
 NCBI BlastP on this gene

EMD60806

hypothetical protein
  
Accession: EMD60807
  
Location: 203814-205476
  
  
**BlastP hit with Mycgr3G84654\_Mycgr3T**
  
Percentage identity: 58 %
  
BlastP bit score: 395
  
Sequence coverage: 81 %
  
E-value: 7e-129
  
  
 NCBI BlastP on this gene

EMD60807

hypothetical protein
  
Accession: EMD60808
  
Location: 208333-210108
  
 NCBI BlastP on this gene

EMD60808

hypothetical protein
  
Accession: EMD60809
  
Location: 211684-213303
  
 NCBI BlastP on this gene

EMD60809

hypothetical protein
  
Accession: EMD60810
  
Location: 214787-216136
  
 NCBI BlastP on this gene

EMD60810

hypothetical protein
  
Accession: EMD60811
  
Location: 216775-218796
  
  
**BlastP hit with Mycgr3G84646\_Mycgr3T**
  
Percentage identity: 47 %
  
BlastP bit score: 605
  
Sequence coverage: 99 %
  
E-value: 0.0
  
  
 NCBI BlastP on this gene

EMD60811

hypothetical protein
  
Accession: EMD60812
  
Location: 219317-220181
  
  
**BlastP hit with Mycgr3G99145\_Mycgr3T**
  
Percentage identity: 86 %
  
BlastP bit score: 364
  
Sequence coverage: 99 %
  
E-value: 2e-125
  
  
 NCBI BlastP on this gene

EMD60812

hypothetical protein
  
Accession: EMD60813
  
Location: 220698-228562
  
 NCBI BlastP on this gene

EMD60813

hypothetical protein
  
Accession: EMD60814
  
Location: 229102-229505
  
 NCBI BlastP on this gene

EMD60814

hypothetical protein
  
Accession: EMD60815
  
Location: 230541-232948
  
 NCBI BlastP on this gene

EMD60815

hypothetical protein
  
Accession: EMD60816
  
Location: 235133-236736
  
 NCBI BlastP on this gene

EMD60816

Query: Architecture Search FASTA input

GG697338 : Glomerella graminicola M1.001 genomic scaffold supercont1.8    Total score: 3.0     Cumulative Blast bit score: 1362

Hit cluster cross-links:

Mycgr3G90785 Mycgr3T
  
Location: 0-1047

Mycgr3G90785\_Mycgr3T

Mycgr3G103262 Mycgr3
  
Location: 1147-1390

Mycgr3G103262\_Mycgr3

Mycgr3G68458 Mycgr3T
  
Location: 1490-3602

Mycgr3G68458\_Mycgr3T

Mycgr3G99145 Mycgr3T
  
Location: 3702-4326

Mycgr3G99145\_Mycgr3T

Mycgr3G103274 Mycgr3
  
Location: 4426-4957

Mycgr3G103274\_Mycgr3

Mycgr3G103264 Mycgr3
  
Location: 5057-5390

Mycgr3G103264\_Mycgr3

Mycgr3G37570 Mycgr3T
  
Location: 5490-6006

Mycgr3G37570\_Mycgr3T

Mycgr3G108094 Mycgr3
  
Location: 6106-10555

Mycgr3G108094\_Mycgr3

Mycgr3G90786 Mycgr3T
  
Location: 10655-12080

Mycgr3G90786\_Mycgr3T

Mycgr3G68429 Mycgr3T
  
Location: 12180-13440

Mycgr3G68429\_Mycgr3T

Mycgr3G68421 Mycgr3T
  
Location: 13540-17086

Mycgr3G68421\_Mycgr3T

Mycgr3G90801 Mycgr3T
  
Location: 17186-18056

Mycgr3G90801\_Mycgr3T

Mycgr3G84646 Mycgr3T
  
Location: 18156-20235

Mycgr3G84646\_Mycgr3T

Mycgr3G68456 Mycgr3T
  
Location: 20335-21970

Mycgr3G68456\_Mycgr3T

Mycgr3G103270 Mycgr3
  
Location: 22070-22355

Mycgr3G103270\_Mycgr3

Mycgr3G90803 Mycgr3T
  
Location: 22455-23019

Mycgr3G90803\_Mycgr3T

Mycgr3G36941 Mycgr3T
  
Location: 23119-24064

Mycgr3G36941\_Mycgr3T

Mycgr3G25746 Mycgr3T
  
Location: 24164-25241

Mycgr3G25746\_Mycgr3T

Mycgr3G90788 Mycgr3T
  
Location: 25341-25803

Mycgr3G90788\_Mycgr3T

Mycgr3G103260 Mycgr3
  
Location: 25903-26635

Mycgr3G103260\_Mycgr3

Mycgr3G84644 Mycgr3T
  
Location: 26735-28457

Mycgr3G84644\_Mycgr3T

Mycgr3G29227 Mycgr3T
  
Location: 28557-28863

Mycgr3G29227\_Mycgr3T

Mycgr3G36271 Mycgr3T
  
Location: 28963-29854

Mycgr3G36271\_Mycgr3T

Mycgr3G68433 Mycgr3T
  
Location: 29954-33041

Mycgr3G68433\_Mycgr3T

Mycgr3G79452 Mycgr3T
  
Location: 33141-33399

Mycgr3G79452\_Mycgr3T

Mycgr3G55345 Mycgr3T
  
Location: 33499-34126

Mycgr3G55345\_Mycgr3T

Mycgr3G103278 Mycgr3
  
Location: 34226-35195

Mycgr3G103278\_Mycgr3

Mycgr3G84654 Mycgr3T
  
Location: 35295-36630

Mycgr3G84654\_Mycgr3T

Mycgr3G108090 Mycgr3
  
Location: 36730-37591

Mycgr3G108090\_Mycgr3

Mycgr3G21922 Mycgr3T
  
Location: 37691-39149

Mycgr3G21922\_Mycgr3T

Mycgr3G99148 Mycgr3T
  
Location: 39249-42819

Mycgr3G99148\_Mycgr3T

profilin
  
Accession: EFQ27668
  
Location: 477127-477885
  
 NCBI BlastP on this gene

EFQ27668

Got1-like family protein
  
Accession: EFQ27669
  
Location: 478408-479180
  
 NCBI BlastP on this gene

EFQ27669

hypothetical protein
  
Accession: EFQ27670
  
Location: 480482-481096
  
 NCBI BlastP on this gene

EFQ27670

DASH complex subunit Dad4
  
Accession: EFQ27671
  
Location: 481424-481796
  
 NCBI BlastP on this gene

EFQ27671

actin
  
Accession: EFQ27672
  
Location: 482379-484864
  
 NCBI BlastP on this gene

EFQ27672

bZIP transcription factor
  
Accession: EFQ27673
  
Location: 485480-486365
  
 NCBI BlastP on this gene

EFQ27673

amidohydrolase
  
Accession: EFQ27674
  
Location: 491008-492046
  
 NCBI BlastP on this gene

EFQ27674

pterin 4 alpha carbinolamine dehydratase
  
Accession: EFQ27675
  
Location: 493360-494119
  
 NCBI BlastP on this gene

EFQ27675

fatty acid hydroxylase superfamily protein
  
Accession: EFQ27676
  
Location: 494661-495796
  
  
**BlastP hit with Mycgr3G36271\_Mycgr3T**
  
Percentage identity: 74 %
  
BlastP bit score: 461
  
Sequence coverage: 96 %
  
E-value: 2e-160
  
  
 NCBI BlastP on this gene

EFQ27676

ATP synthase regulation protein NCA2
  
Accession: EFQ27677
  
Location: 498128-500231
  
  
**BlastP hit with Mycgr3G84646\_Mycgr3T**
  
Percentage identity: 43 %
  
BlastP bit score: 546
  
Sequence coverage: 101 %
  
E-value: 0.0
  
  
 NCBI BlastP on this gene

EFQ27677

Ras family protein
  
Accession: EFQ27678
  
Location: 501788-502706
  
  
**BlastP hit with Mycgr3G99145\_Mycgr3T**
  
Percentage identity: 84 %
  
BlastP bit score: 355
  
Sequence coverage: 99 %
  
E-value: 1e-121
  
  
 NCBI BlastP on this gene

EFQ27678

hypothetical protein
  
Accession: EFQ27679
  
Location: 503250-505030
  
 NCBI BlastP on this gene

EFQ27679

ATPase
  
Accession: EFQ27680
  
Location: 505936-508329
  
 NCBI BlastP on this gene

EFQ27680

squalene epoxidase
  
Accession: EFQ27681
  
Location: 510106-511611
  
 NCBI BlastP on this gene

EFQ27681

glycosyl transferase family 2
  
Accession: EFQ27682
  
Location: 513442-514798
  
 NCBI BlastP on this gene

EFQ27682

hypothetical protein
  
Accession: EFQ27683
  
Location: 518101-519495
  
 NCBI BlastP on this gene

EFQ27683

Query: Architecture Search FASTA input

KB644412 : Penicillium oxalicum 114-2 unplaced genomic scaffold scaffold\_5    Total score: 3.0     Cumulative Blast bit score: 1358

Hit cluster cross-links:

Mycgr3G90785 Mycgr3T
  
Location: 0-1047

Mycgr3G90785\_Mycgr3T

Mycgr3G103262 Mycgr3
  
Location: 1147-1390

Mycgr3G103262\_Mycgr3

Mycgr3G68458 Mycgr3T
  
Location: 1490-3602

Mycgr3G68458\_Mycgr3T

Mycgr3G99145 Mycgr3T
  
Location: 3702-4326

Mycgr3G99145\_Mycgr3T

Mycgr3G103274 Mycgr3
  
Location: 4426-4957

Mycgr3G103274\_Mycgr3

Mycgr3G103264 Mycgr3
  
Location: 5057-5390

Mycgr3G103264\_Mycgr3

Mycgr3G37570 Mycgr3T
  
Location: 5490-6006

Mycgr3G37570\_Mycgr3T

Mycgr3G108094 Mycgr3
  
Location: 6106-10555

Mycgr3G108094\_Mycgr3

Mycgr3G90786 Mycgr3T
  
Location: 10655-12080

Mycgr3G90786\_Mycgr3T

Mycgr3G68429 Mycgr3T
  
Location: 12180-13440

Mycgr3G68429\_Mycgr3T

Mycgr3G68421 Mycgr3T
  
Location: 13540-17086

Mycgr3G68421\_Mycgr3T

Mycgr3G90801 Mycgr3T
  
Location: 17186-18056

Mycgr3G90801\_Mycgr3T

Mycgr3G84646 Mycgr3T
  
Location: 18156-20235

Mycgr3G84646\_Mycgr3T

Mycgr3G68456 Mycgr3T
  
Location: 20335-21970

Mycgr3G68456\_Mycgr3T

Mycgr3G103270 Mycgr3
  
Location: 22070-22355

Mycgr3G103270\_Mycgr3

Mycgr3G90803 Mycgr3T
  
Location: 22455-23019

Mycgr3G90803\_Mycgr3T

Mycgr3G36941 Mycgr3T
  
Location: 23119-24064

Mycgr3G36941\_Mycgr3T

Mycgr3G25746 Mycgr3T
  
Location: 24164-25241

Mycgr3G25746\_Mycgr3T

Mycgr3G90788 Mycgr3T
  
Location: 25341-25803

Mycgr3G90788\_Mycgr3T

Mycgr3G103260 Mycgr3
  
Location: 25903-26635

Mycgr3G103260\_Mycgr3

Mycgr3G84644 Mycgr3T
  
Location: 26735-28457

Mycgr3G84644\_Mycgr3T

Mycgr3G29227 Mycgr3T
  
Location: 28557-28863

Mycgr3G29227\_Mycgr3T

Mycgr3G36271 Mycgr3T
  
Location: 28963-29854

Mycgr3G36271\_Mycgr3T

Mycgr3G68433 Mycgr3T
  
Location: 29954-33041

Mycgr3G68433\_Mycgr3T

Mycgr3G79452 Mycgr3T
  
Location: 33141-33399

Mycgr3G79452\_Mycgr3T

Mycgr3G55345 Mycgr3T
  
Location: 33499-34126

Mycgr3G55345\_Mycgr3T

Mycgr3G103278 Mycgr3
  
Location: 34226-35195

Mycgr3G103278\_Mycgr3

Mycgr3G84654 Mycgr3T
  
Location: 35295-36630

Mycgr3G84654\_Mycgr3T

Mycgr3G108090 Mycgr3
  
Location: 36730-37591

Mycgr3G108090\_Mycgr3

Mycgr3G21922 Mycgr3T
  
Location: 37691-39149

Mycgr3G21922\_Mycgr3T

Mycgr3G99148 Mycgr3T
  
Location: 39249-42819

Mycgr3G99148\_Mycgr3T

hypothetical protein
  
Accession: EPS30815
  
Location: 3829129-3829935
  
 NCBI BlastP on this gene

EPS30815

hypothetical protein
  
Accession: EPS30816
  
Location: 3830099-3832030
  
 NCBI BlastP on this gene

EPS30816

hypothetical protein
  
Accession: EPS30817
  
Location: 3835368-3837000
  
 NCBI BlastP on this gene

EPS30817

hypothetical protein
  
Accession: EPS30818
  
Location: 3837647-3838235
  
 NCBI BlastP on this gene

EPS30818

hypothetical protein
  
Accession: EPS30819
  
Location: 3840318-3841460
  
 NCBI BlastP on this gene

EPS30819

hypothetical protein
  
Accession: EPS30820
  
Location: 3841744-3844827
  
  
**BlastP hit with Mycgr3G68433\_Mycgr3T**
  
Percentage identity: 37 %
  
BlastP bit score: 459
  
Sequence coverage: 88 %
  
E-value: 2e-140
  
  
 NCBI BlastP on this gene

EPS30820

hypothetical protein
  
Accession: EPS30821
  
Location: 3847235-3848524
  
  
**BlastP hit with Mycgr3G90786\_Mycgr3T**
  
Percentage identity: 29 %
  
BlastP bit score: 63
  
Sequence coverage: 45 %
  
E-value: 7e-08
  
  
 NCBI BlastP on this gene

EPS30821

hypothetical protein
  
Accession: EPS30822
  
Location: 3849825-3851058
  
 NCBI BlastP on this gene

EPS30822

hypothetical protein
  
Accession: EPS30823
  
Location: 3854324-3854545
  
 NCBI BlastP on this gene

EPS30823

hypothetical protein
  
Accession: EPS30824
  
Location: 3856811-3857342
  
 NCBI BlastP on this gene

EPS30824

hypothetical protein
  
Accession: EPS30825
  
Location: 3858415-3859480
  
 NCBI BlastP on this gene

EPS30825

hypothetical protein
  
Accession: EPS30826
  
Location: 3861186-3862494
  
 NCBI BlastP on this gene

EPS30826

hypothetical protein
  
Accession: EPS30827
  
Location: 3862599-3862895
  
 NCBI BlastP on this gene

EPS30827

hypothetical protein
  
Accession: EPS30828
  
Location: 3863540-3865591
  
  
**BlastP hit with Mycgr3G68458\_Mycgr3T**
  
Percentage identity: 64 %
  
BlastP bit score: 836
  
Sequence coverage: 90 %
  
E-value: 0.0
  
  
 NCBI BlastP on this gene

EPS30828

hypothetical protein
  
Accession: EPS30829
  
Location: 3865995-3867267
  
 NCBI BlastP on this gene

EPS30829

hypothetical protein
  
Accession: EPS30830
  
Location: 3868184-3870787
  
 NCBI BlastP on this gene

EPS30830

hypothetical protein
  
Accession: EPS30831
  
Location: 3873192-3875741
  
 NCBI BlastP on this gene

EPS30831

hypothetical protein
  
Accession: EPS30832
  
Location: 3876716-3877677
  
 NCBI BlastP on this gene

EPS30832

Query: Architecture Search FASTA input

CP003002 : Myceliophthora thermophila ATCC 42464 chromosome 1    Total score: 3.0     Cumulative Blast bit score: 1357

Hit cluster cross-links:

Mycgr3G90785 Mycgr3T
  
Location: 0-1047

Mycgr3G90785\_Mycgr3T

Mycgr3G103262 Mycgr3
  
Location: 1147-1390

Mycgr3G103262\_Mycgr3

Mycgr3G68458 Mycgr3T
  
Location: 1490-3602

Mycgr3G68458\_Mycgr3T

Mycgr3G99145 Mycgr3T
  
Location: 3702-4326

Mycgr3G99145\_Mycgr3T

Mycgr3G103274 Mycgr3
  
Location: 4426-4957

Mycgr3G103274\_Mycgr3

Mycgr3G103264 Mycgr3
  
Location: 5057-5390

Mycgr3G103264\_Mycgr3

Mycgr3G37570 Mycgr3T
  
Location: 5490-6006

Mycgr3G37570\_Mycgr3T

Mycgr3G108094 Mycgr3
  
Location: 6106-10555

Mycgr3G108094\_Mycgr3

Mycgr3G90786 Mycgr3T
  
Location: 10655-12080

Mycgr3G90786\_Mycgr3T

Mycgr3G68429 Mycgr3T
  
Location: 12180-13440

Mycgr3G68429\_Mycgr3T

Mycgr3G68421 Mycgr3T
  
Location: 13540-17086

Mycgr3G68421\_Mycgr3T

Mycgr3G90801 Mycgr3T
  
Location: 17186-18056

Mycgr3G90801\_Mycgr3T

Mycgr3G84646 Mycgr3T
  
Location: 18156-20235

Mycgr3G84646\_Mycgr3T

Mycgr3G68456 Mycgr3T
  
Location: 20335-21970

Mycgr3G68456\_Mycgr3T

Mycgr3G103270 Mycgr3
  
Location: 22070-22355

Mycgr3G103270\_Mycgr3

Mycgr3G90803 Mycgr3T
  
Location: 22455-23019

Mycgr3G90803\_Mycgr3T

Mycgr3G36941 Mycgr3T
  
Location: 23119-24064

Mycgr3G36941\_Mycgr3T

Mycgr3G25746 Mycgr3T
  
Location: 24164-25241

Mycgr3G25746\_Mycgr3T

Mycgr3G90788 Mycgr3T
  
Location: 25341-25803

Mycgr3G90788\_Mycgr3T

Mycgr3G103260 Mycgr3
  
Location: 25903-26635

Mycgr3G103260\_Mycgr3

Mycgr3G84644 Mycgr3T
  
Location: 26735-28457

Mycgr3G84644\_Mycgr3T

Mycgr3G29227 Mycgr3T
  
Location: 28557-28863

Mycgr3G29227\_Mycgr3T

Mycgr3G36271 Mycgr3T
  
Location: 28963-29854

Mycgr3G36271\_Mycgr3T

Mycgr3G68433 Mycgr3T
  
Location: 29954-33041

Mycgr3G68433\_Mycgr3T

Mycgr3G79452 Mycgr3T
  
Location: 33141-33399

Mycgr3G79452\_Mycgr3T

Mycgr3G55345 Mycgr3T
  
Location: 33499-34126

Mycgr3G55345\_Mycgr3T

Mycgr3G103278 Mycgr3
  
Location: 34226-35195

Mycgr3G103278\_Mycgr3

Mycgr3G84654 Mycgr3T
  
Location: 35295-36630

Mycgr3G84654\_Mycgr3T

Mycgr3G108090 Mycgr3
  
Location: 36730-37591

Mycgr3G108090\_Mycgr3

Mycgr3G21922 Mycgr3T
  
Location: 37691-39149

Mycgr3G21922\_Mycgr3T

Mycgr3G99148 Mycgr3T
  
Location: 39249-42819

Mycgr3G99148\_Mycgr3T

hypothetical protein
  
Accession: AEO55347
  
Location: 9069837-9070888
  
 NCBI BlastP on this gene

MYCTH\_2124283

hypothetical protein
  
Accession: AEO55346
  
Location: 9067117-9067923
  
 NCBI BlastP on this gene

MYCTH\_2142488

hypothetical protein
  
Accession: AEO55345
  
Location: 9065400-9066267
  
 NCBI BlastP on this gene

MYCTH\_2314212

hypothetical protein
  
Accession: AEO55344
  
Location: 9063982-9064429
  
 NCBI BlastP on this gene

MYCTH\_2299070

hypothetical protein
  
Accession: AEO55343
  
Location: 9062891-9063490
  
 NCBI BlastP on this gene

MYCTH\_2299068

hypothetical protein
  
Accession: AEO55342
  
Location: 9062117-9062576
  
 NCBI BlastP on this gene

MYCTH\_2299067

hypothetical protein
  
Accession: AEO55341
  
Location: 9060838-9061245
  
 NCBI BlastP on this gene

MYCTH\_2299066

ARP5-like protein
  
Accession: AEO55340
  
Location: 9056987-9059260
  
 NCBI BlastP on this gene

MYCTH\_2299064

hypothetical protein
  
Accession: AEO55339
  
Location: 9055360-9056020
  
 NCBI BlastP on this gene

MYCTH\_2299061

hypothetical protein
  
Accession: AEO55338
  
Location: 9051489-9051887
  
 NCBI BlastP on this gene

MYCTH\_2299056

methylsterol monooxygenase
  
Accession: AEO55337
  
Location: 9049575-9050674
  
  
**BlastP hit with Mycgr3G36271\_Mycgr3T**
  
Percentage identity: 72 %
  
BlastP bit score: 459
  
Sequence coverage: 95 %
  
E-value: 1e-159
  
  
 NCBI BlastP on this gene

MYCTH\_109638

hypothetical protein
  
Accession: AEO55336
  
Location: 9046163-9048350
  
  
**BlastP hit with Mycgr3G84646\_Mycgr3T**
  
Percentage identity: 44 %
  
BlastP bit score: 543
  
Sequence coverage: 103 %
  
E-value: 0.0
  
  
 NCBI BlastP on this gene

MYCTH\_97642

hypothetical protein
  
Accession: AEO55335
  
Location: 9044169-9044981
  
  
**BlastP hit with Mycgr3G99145\_Mycgr3T**
  
Percentage identity: 82 %
  
BlastP bit score: 355
  
Sequence coverage: 99 %
  
E-value: 1e-121
  
  
 NCBI BlastP on this gene

MYCTH\_2314205

hypothetical protein
  
Accession: AEO55334
  
Location: 9036757-9042828
  
 NCBI BlastP on this gene

MYCTH\_2299043

hypothetical protein
  
Accession: AEO55333
  
Location: 9035197-9035970
  
 NCBI BlastP on this gene

MYCTH\_2107979

hypothetical protein
  
Accession: AEO55332
  
Location: 9033211-9034473
  
 NCBI BlastP on this gene

MYCTH\_2299042

hypothetical protein
  
Accession: AEO55331
  
Location: 9032396-9032668
  
 NCBI BlastP on this gene

MYCTH\_2299041

hypothetical protein
  
Accession: AEO55330
  
Location: 9030985-9031910
  
 NCBI BlastP on this gene

MYCTH\_2299040

hypothetical protein
  
Accession: AEO55329
  
Location: 9028063-9030554
  
 NCBI BlastP on this gene

MYCTH\_2299039

hypothetical protein
  
Accession: AEO55328
  
Location: 9026402-9027154
  
 NCBI BlastP on this gene

MYCTH\_2299037

Query: Architecture Search FASTA input

KB707370 : Eutypa lata UCREL1 unplaced genomic scaffold EL1\_03\_scaffold\_2032    Total score: 3.0     Cumulative Blast bit score: 1326

Hit cluster cross-links:

Mycgr3G90785 Mycgr3T
  
Location: 0-1047

Mycgr3G90785\_Mycgr3T

Mycgr3G103262 Mycgr3
  
Location: 1147-1390

Mycgr3G103262\_Mycgr3

Mycgr3G68458 Mycgr3T
  
Location: 1490-3602

Mycgr3G68458\_Mycgr3T

Mycgr3G99145 Mycgr3T
  
Location: 3702-4326

Mycgr3G99145\_Mycgr3T

Mycgr3G103274 Mycgr3
  
Location: 4426-4957

Mycgr3G103274\_Mycgr3

Mycgr3G103264 Mycgr3
  
Location: 5057-5390

Mycgr3G103264\_Mycgr3

Mycgr3G37570 Mycgr3T
  
Location: 5490-6006

Mycgr3G37570\_Mycgr3T

Mycgr3G108094 Mycgr3
  
Location: 6106-10555

Mycgr3G108094\_Mycgr3

Mycgr3G90786 Mycgr3T
  
Location: 10655-12080

Mycgr3G90786\_Mycgr3T

Mycgr3G68429 Mycgr3T
  
Location: 12180-13440

Mycgr3G68429\_Mycgr3T

Mycgr3G68421 Mycgr3T
  
Location: 13540-17086

Mycgr3G68421\_Mycgr3T

Mycgr3G90801 Mycgr3T
  
Location: 17186-18056

Mycgr3G90801\_Mycgr3T

Mycgr3G84646 Mycgr3T
  
Location: 18156-20235

Mycgr3G84646\_Mycgr3T

Mycgr3G68456 Mycgr3T
  
Location: 20335-21970

Mycgr3G68456\_Mycgr3T

Mycgr3G103270 Mycgr3
  
Location: 22070-22355

Mycgr3G103270\_Mycgr3

Mycgr3G90803 Mycgr3T
  
Location: 22455-23019

Mycgr3G90803\_Mycgr3T

Mycgr3G36941 Mycgr3T
  
Location: 23119-24064

Mycgr3G36941\_Mycgr3T

Mycgr3G25746 Mycgr3T
  
Location: 24164-25241

Mycgr3G25746\_Mycgr3T

Mycgr3G90788 Mycgr3T
  
Location: 25341-25803

Mycgr3G90788\_Mycgr3T

Mycgr3G103260 Mycgr3
  
Location: 25903-26635

Mycgr3G103260\_Mycgr3

Mycgr3G84644 Mycgr3T
  
Location: 26735-28457

Mycgr3G84644\_Mycgr3T

Mycgr3G29227 Mycgr3T
  
Location: 28557-28863

Mycgr3G29227\_Mycgr3T

Mycgr3G36271 Mycgr3T
  
Location: 28963-29854

Mycgr3G36271\_Mycgr3T

Mycgr3G68433 Mycgr3T
  
Location: 29954-33041

Mycgr3G68433\_Mycgr3T

Mycgr3G79452 Mycgr3T
  
Location: 33141-33399

Mycgr3G79452\_Mycgr3T

Mycgr3G55345 Mycgr3T
  
Location: 33499-34126

Mycgr3G55345\_Mycgr3T

Mycgr3G103278 Mycgr3
  
Location: 34226-35195

Mycgr3G103278\_Mycgr3

Mycgr3G84654 Mycgr3T
  
Location: 35295-36630

Mycgr3G84654\_Mycgr3T

Mycgr3G108090 Mycgr3
  
Location: 36730-37591

Mycgr3G108090\_Mycgr3

Mycgr3G21922 Mycgr3T
  
Location: 37691-39149

Mycgr3G21922\_Mycgr3T

Mycgr3G99148 Mycgr3T
  
Location: 39249-42819

Mycgr3G99148\_Mycgr3T

putative pterin 4 alpha carbinolamine dehydratase protein
  
Accession: EMR62832
  
Location: 700-918
  
 NCBI BlastP on this gene

EMR62832

putative c-4 methylsterol variant protein
  
Accession: EMR62826
  
Location: 1515-2635
  
  
**BlastP hit with Mycgr3G36271\_Mycgr3T**
  
Percentage identity: 68 %
  
BlastP bit score: 416
  
Sequence coverage: 97 %
  
E-value: 3e-143
  
  
 NCBI BlastP on this gene

EMR62826

putative atp synthase regulation protein nca2 protein
  
Accession: EMR62821
  
Location: 4053-6124
  
  
**BlastP hit with Mycgr3G84646\_Mycgr3T**
  
Percentage identity: 44 %
  
BlastP bit score: 554
  
Sequence coverage: 101 %
  
E-value: 0.0
  
  
 NCBI BlastP on this gene

EMR62821

putative rab gtpase protein
  
Accession: EMR62820
  
Location: 7527-8553
  
  
**BlastP hit with Mycgr3G99145\_Mycgr3T**
  
Percentage identity: 84 %
  
BlastP bit score: 356
  
Sequence coverage: 99 %
  
E-value: 4e-122
  
  
 NCBI BlastP on this gene

EMR62820

hypothetical protein
  
Accession: EMR62831
  
Location: 9770-11721
  
 NCBI BlastP on this gene

EMR62831

putative aaa family atpase protein
  
Accession: EMR62827
  
Location: 12387-14737
  
 NCBI BlastP on this gene

EMR62827

hypothetical protein
  
Accession: EMR62824
  
Location: 16515-17322
  
 NCBI BlastP on this gene

EMR62824

hypothetical protein
  
Accession: EMR62833
  
Location: 18302-18942
  
 NCBI BlastP on this gene

EMR62833

putative aromatic-l-amino-acid decarboxylase protein
  
Accession: EMR62818
  
Location: 21629-23378
  
 NCBI BlastP on this gene

EMR62818

hypothetical protein
  
Accession: EMR62823
  
Location: 23728-24270
  
 NCBI BlastP on this gene

EMR62823

Query: Architecture Search FASTA input

KB446566 : Pseudocercospora fijiensis CIRAD86 unplaced genomic scaffold MYCFIscaffold\_12    Total score: 3.0     Cumulative Blast bit score: 1322

Hit cluster cross-links:

Mycgr3G90785 Mycgr3T
  
Location: 0-1047

Mycgr3G90785\_Mycgr3T

Mycgr3G103262 Mycgr3
  
Location: 1147-1390

Mycgr3G103262\_Mycgr3

Mycgr3G68458 Mycgr3T
  
Location: 1490-3602

Mycgr3G68458\_Mycgr3T

Mycgr3G99145 Mycgr3T
  
Location: 3702-4326

Mycgr3G99145\_Mycgr3T

Mycgr3G103274 Mycgr3
  
Location: 4426-4957

Mycgr3G103274\_Mycgr3

Mycgr3G103264 Mycgr3
  
Location: 5057-5390

Mycgr3G103264\_Mycgr3

Mycgr3G37570 Mycgr3T
  
Location: 5490-6006

Mycgr3G37570\_Mycgr3T

Mycgr3G108094 Mycgr3
  
Location: 6106-10555

Mycgr3G108094\_Mycgr3

Mycgr3G90786 Mycgr3T
  
Location: 10655-12080

Mycgr3G90786\_Mycgr3T

Mycgr3G68429 Mycgr3T
  
Location: 12180-13440

Mycgr3G68429\_Mycgr3T

Mycgr3G68421 Mycgr3T
  
Location: 13540-17086

Mycgr3G68421\_Mycgr3T

Mycgr3G90801 Mycgr3T
  
Location: 17186-18056

Mycgr3G90801\_Mycgr3T

Mycgr3G84646 Mycgr3T
  
Location: 18156-20235

Mycgr3G84646\_Mycgr3T

Mycgr3G68456 Mycgr3T
  
Location: 20335-21970

Mycgr3G68456\_Mycgr3T

Mycgr3G103270 Mycgr3
  
Location: 22070-22355

Mycgr3G103270\_Mycgr3

Mycgr3G90803 Mycgr3T
  
Location: 22455-23019

Mycgr3G90803\_Mycgr3T

Mycgr3G36941 Mycgr3T
  
Location: 23119-24064

Mycgr3G36941\_Mycgr3T

Mycgr3G25746 Mycgr3T
  
Location: 24164-25241

Mycgr3G25746\_Mycgr3T

Mycgr3G90788 Mycgr3T
  
Location: 25341-25803

Mycgr3G90788\_Mycgr3T

Mycgr3G103260 Mycgr3
  
Location: 25903-26635

Mycgr3G103260\_Mycgr3

Mycgr3G84644 Mycgr3T
  
Location: 26735-28457

Mycgr3G84644\_Mycgr3T

Mycgr3G29227 Mycgr3T
  
Location: 28557-28863

Mycgr3G29227\_Mycgr3T

Mycgr3G36271 Mycgr3T
  
Location: 28963-29854

Mycgr3G36271\_Mycgr3T

Mycgr3G68433 Mycgr3T
  
Location: 29954-33041

Mycgr3G68433\_Mycgr3T

Mycgr3G79452 Mycgr3T
  
Location: 33141-33399

Mycgr3G79452\_Mycgr3T

Mycgr3G55345 Mycgr3T
  
Location: 33499-34126

Mycgr3G55345\_Mycgr3T

Mycgr3G103278 Mycgr3
  
Location: 34226-35195

Mycgr3G103278\_Mycgr3

Mycgr3G84654 Mycgr3T
  
Location: 35295-36630

Mycgr3G84654\_Mycgr3T

Mycgr3G108090 Mycgr3
  
Location: 36730-37591

Mycgr3G108090\_Mycgr3

Mycgr3G21922 Mycgr3T
  
Location: 37691-39149

Mycgr3G21922\_Mycgr3T

Mycgr3G99148 Mycgr3T
  
Location: 39249-42819

Mycgr3G99148\_Mycgr3T

hypothetical protein
  
Accession: EME77366
  
Location: 920062-922370
  
 NCBI BlastP on this gene

EME77366

hypothetical protein
  
Accession: EME77367
  
Location: 922504-923817
  
 NCBI BlastP on this gene

EME77367

hypothetical protein
  
Accession: EME77368
  
Location: 925041-925934
  
 NCBI BlastP on this gene

EME77368

hypothetical protein
  
Accession: EME77369
  
Location: 932702-932893
  
 NCBI BlastP on this gene

EME77369

hypothetical protein
  
Accession: EME77370
  
Location: 934075-935963
  
 NCBI BlastP on this gene

EME77370

hypothetical protein
  
Accession: EME77371
  
Location: 936380-937201
  
 NCBI BlastP on this gene

EME77371

hypothetical protein
  
Accession: EME77372
  
Location: 938799-941387
  
  
**BlastP hit with Mycgr3G68433\_Mycgr3T**
  
Percentage identity: 59 %
  
BlastP bit score: 937
  
Sequence coverage: 83 %
  
E-value: 0.0
  
  
 NCBI BlastP on this gene

EME77372

hypothetical protein
  
Accession: EME77373
  
Location: 942550-945648
  
  
**BlastP hit with Mycgr3G90785\_Mycgr3T**
  
Percentage identity: 27 %
  
BlastP bit score: 68
  
Sequence coverage: 89 %
  
E-value: 3e-09
  
  
  
**BlastP hit with Mycgr3G90786\_Mycgr3T**
  
Percentage identity: 47 %
  
BlastP bit score: 317
  
Sequence coverage: 97 %
  
E-value: 1e-95
  
  
 NCBI BlastP on this gene

EME77373

hypothetical protein
  
Accession: EME77374
  
Location: 946401-947795
  
 NCBI BlastP on this gene

EME77374

hypothetical protein
  
Accession: EME77375
  
Location: 949865-951562
  
 NCBI BlastP on this gene

EME77375

hypothetical protein
  
Accession: EME77376
  
Location: 954705-954960
  
 NCBI BlastP on this gene

EME77376

hypothetical protein
  
Accession: EME77377
  
Location: 954984-955358
  
 NCBI BlastP on this gene

EME77377

phosphatidylinositol 3-kinase tor2
  
Accession: EME77378
  
Location: 955417-962775
  
 NCBI BlastP on this gene

EME77378

hypothetical protein
  
Accession: EME77379
  
Location: 963166-965354
  
 NCBI BlastP on this gene

EME77379

Query: Architecture Search FASTA input

FP929137 : Leptosphaeria maculans JN3 lm\_SuperContig\_10\_v2 genomic supercontig    Total score: 3.0     Cumulative Blast bit score: 1318

Hit cluster cross-links:

Mycgr3G90785 Mycgr3T
  
Location: 0-1047

Mycgr3G90785\_Mycgr3T

Mycgr3G103262 Mycgr3
  
Location: 1147-1390

Mycgr3G103262\_Mycgr3

Mycgr3G68458 Mycgr3T
  
Location: 1490-3602

Mycgr3G68458\_Mycgr3T

Mycgr3G99145 Mycgr3T
  
Location: 3702-4326

Mycgr3G99145\_Mycgr3T

Mycgr3G103274 Mycgr3
  
Location: 4426-4957

Mycgr3G103274\_Mycgr3

Mycgr3G103264 Mycgr3
  
Location: 5057-5390

Mycgr3G103264\_Mycgr3

Mycgr3G37570 Mycgr3T
  
Location: 5490-6006

Mycgr3G37570\_Mycgr3T

Mycgr3G108094 Mycgr3
  
Location: 6106-10555

Mycgr3G108094\_Mycgr3

Mycgr3G90786 Mycgr3T
  
Location: 10655-12080

Mycgr3G90786\_Mycgr3T

Mycgr3G68429 Mycgr3T
  
Location: 12180-13440

Mycgr3G68429\_Mycgr3T

Mycgr3G68421 Mycgr3T
  
Location: 13540-17086

Mycgr3G68421\_Mycgr3T

Mycgr3G90801 Mycgr3T
  
Location: 17186-18056

Mycgr3G90801\_Mycgr3T

Mycgr3G84646 Mycgr3T
  
Location: 18156-20235

Mycgr3G84646\_Mycgr3T

Mycgr3G68456 Mycgr3T
  
Location: 20335-21970

Mycgr3G68456\_Mycgr3T

Mycgr3G103270 Mycgr3
  
Location: 22070-22355

Mycgr3G103270\_Mycgr3

Mycgr3G90803 Mycgr3T
  
Location: 22455-23019

Mycgr3G90803\_Mycgr3T

Mycgr3G36941 Mycgr3T
  
Location: 23119-24064

Mycgr3G36941\_Mycgr3T

Mycgr3G25746 Mycgr3T
  
Location: 24164-25241

Mycgr3G25746\_Mycgr3T

Mycgr3G90788 Mycgr3T
  
Location: 25341-25803

Mycgr3G90788\_Mycgr3T

Mycgr3G103260 Mycgr3
  
Location: 25903-26635

Mycgr3G103260\_Mycgr3

Mycgr3G84644 Mycgr3T
  
Location: 26735-28457

Mycgr3G84644\_Mycgr3T

Mycgr3G29227 Mycgr3T
  
Location: 28557-28863

Mycgr3G29227\_Mycgr3T

Mycgr3G36271 Mycgr3T
  
Location: 28963-29854

Mycgr3G36271\_Mycgr3T

Mycgr3G68433 Mycgr3T
  
Location: 29954-33041

Mycgr3G68433\_Mycgr3T

Mycgr3G79452 Mycgr3T
  
Location: 33141-33399

Mycgr3G79452\_Mycgr3T

Mycgr3G55345 Mycgr3T
  
Location: 33499-34126

Mycgr3G55345\_Mycgr3T

Mycgr3G103278 Mycgr3
  
Location: 34226-35195

Mycgr3G103278\_Mycgr3

Mycgr3G84654 Mycgr3T
  
Location: 35295-36630

Mycgr3G84654\_Mycgr3T

Mycgr3G108090 Mycgr3
  
Location: 36730-37591

Mycgr3G108090\_Mycgr3

Mycgr3G21922 Mycgr3T
  
Location: 37691-39149

Mycgr3G21922\_Mycgr3T

Mycgr3G99148 Mycgr3T
  
Location: 39249-42819

Mycgr3G99148\_Mycgr3T

predicted protein
  
Accession: CBY00049
  
Location: 1509441-1509941
  
 NCBI BlastP on this gene

LEMA\_P076380.1

similar to Mn2+ homeostasis protein Per1
  
Accession: CBY00050
  
Location: 1512271-1513437
  
 NCBI BlastP on this gene

LEMA\_P076390.1

predicted protein
  
Accession: CBY00051
  
Location: 1514471-1515267
  
 NCBI BlastP on this gene

LEMA\_P076400.1

predicted protein
  
Accession: CBY00052
  
Location: 1515559-1516067
  
 NCBI BlastP on this gene

LEMA\_P076410.1

predicted protein
  
Accession: CBY00053
  
Location: 1516791-1517108
  
 NCBI BlastP on this gene

LEMA\_uP076420.1

hypothetical protein
  
Accession: CBY00054
  
Location: 1517506-1519427
  
 NCBI BlastP on this gene

LEMA\_P076430.1

similar to DNA-binding protein HGH1
  
Accession: CBY00055
  
Location: 1519502-1520818
  
  
**BlastP hit with Mycgr3G25746\_Mycgr3T**
  
Percentage identity: 55 %
  
BlastP bit score: 371
  
Sequence coverage: 102 %
  
E-value: 5e-123
  
  
 NCBI BlastP on this gene

LEMA\_P076440.1

hypothetical protein
  
Accession: CBY00056
  
Location: 1522175-1524427
  
 NCBI BlastP on this gene

LEMA\_P076450.1

similar to carboxylesterase family protein
  
Accession: CBY00057
  
Location: 1524896-1526691
  
 NCBI BlastP on this gene

LEMA\_P076460.1

predicted protein
  
Accession: CBY00058
  
Location: 1526987-1527352
  
 NCBI BlastP on this gene

LEMA\_P076470.1

hypothetical protein
  
Accession: CBY00059
  
Location: 1527671-1529952
  
 NCBI BlastP on this gene

LEMA\_P076480.1

similar to MFS multidrug transporter
  
Accession: CBY00060
  
Location: 1531604-1533489
  
 NCBI BlastP on this gene

LEMA\_P076490.1

hypothetical protein
  
Accession: CBY00061
  
Location: 1535769-1537635
  
 NCBI BlastP on this gene

LEMA\_P076500.1

hypothetical protein
  
Accession: CBY00062
  
Location: 1538211-1540873
  
  
**BlastP hit with Mycgr3G84646\_Mycgr3T**
  
Percentage identity: 47 %
  
BlastP bit score: 581
  
Sequence coverage: 100 %
  
E-value: 0.0
  
  
 NCBI BlastP on this gene

LEMA\_P076510.1

similar to GTP-binding protein
  
Accession: CBY00063
  
Location: 1541206-1542152
  
  
**BlastP hit with Mycgr3G99145\_Mycgr3T**
  
Percentage identity: 87 %
  
BlastP bit score: 366
  
Sequence coverage: 99 %
  
E-value: 3e-126
  
  
 NCBI BlastP on this gene

LEMA\_P076520.1

hypothetical protein
  
Accession: CBY00064
  
Location: 1543529-1545322
  
 NCBI BlastP on this gene

LEMA\_P076530.1

predicted protein
  
Accession: CBY00065
  
Location: 1545628-1546059
  
 NCBI BlastP on this gene

LEMA\_P076540.1

similar to calcium permease family membrane transporter
  
Accession: CBY00066
  
Location: 1546447-1549948
  
 NCBI BlastP on this gene

LEMA\_P076550.1

similar to 26S protease regulatory subunit 8
  
Accession: CBY00067
  
Location: 1551329-1552554
  
 NCBI BlastP on this gene

LEMA\_P076560.1

similar to sugar transporter
  
Accession: CBY00068
  
Location: 1552712-1555070
  
 NCBI BlastP on this gene

LEMA\_P076570.1

Query: Architecture Search FASTA input

GG749488 : Ajellomyces dermatitidis ATCC 18188 genomic scaffold supercont1.82    Total score: 3.0     Cumulative Blast bit score: 1317

Hit cluster cross-links:

Mycgr3G90785 Mycgr3T
  
Location: 0-1047

Mycgr3G90785\_Mycgr3T

Mycgr3G103262 Mycgr3
  
Location: 1147-1390

Mycgr3G103262\_Mycgr3

Mycgr3G68458 Mycgr3T
  
Location: 1490-3602

Mycgr3G68458\_Mycgr3T

Mycgr3G99145 Mycgr3T
  
Location: 3702-4326

Mycgr3G99145\_Mycgr3T

Mycgr3G103274 Mycgr3
  
Location: 4426-4957

Mycgr3G103274\_Mycgr3

Mycgr3G103264 Mycgr3
  
Location: 5057-5390

Mycgr3G103264\_Mycgr3

Mycgr3G37570 Mycgr3T
  
Location: 5490-6006

Mycgr3G37570\_Mycgr3T

Mycgr3G108094 Mycgr3
  
Location: 6106-10555

Mycgr3G108094\_Mycgr3

Mycgr3G90786 Mycgr3T
  
Location: 10655-12080

Mycgr3G90786\_Mycgr3T

Mycgr3G68429 Mycgr3T
  
Location: 12180-13440

Mycgr3G68429\_Mycgr3T

Mycgr3G68421 Mycgr3T
  
Location: 13540-17086

Mycgr3G68421\_Mycgr3T

Mycgr3G90801 Mycgr3T
  
Location: 17186-18056

Mycgr3G90801\_Mycgr3T

Mycgr3G84646 Mycgr3T
  
Location: 18156-20235

Mycgr3G84646\_Mycgr3T

Mycgr3G68456 Mycgr3T
  
Location: 20335-21970

Mycgr3G68456\_Mycgr3T

Mycgr3G103270 Mycgr3
  
Location: 22070-22355

Mycgr3G103270\_Mycgr3

Mycgr3G90803 Mycgr3T
  
Location: 22455-23019

Mycgr3G90803\_Mycgr3T

Mycgr3G36941 Mycgr3T
  
Location: 23119-24064

Mycgr3G36941\_Mycgr3T

Mycgr3G25746 Mycgr3T
  
Location: 24164-25241

Mycgr3G25746\_Mycgr3T

Mycgr3G90788 Mycgr3T
  
Location: 25341-25803

Mycgr3G90788\_Mycgr3T

Mycgr3G103260 Mycgr3
  
Location: 25903-26635

Mycgr3G103260\_Mycgr3

Mycgr3G84644 Mycgr3T
  
Location: 26735-28457

Mycgr3G84644\_Mycgr3T

Mycgr3G29227 Mycgr3T
  
Location: 28557-28863

Mycgr3G29227\_Mycgr3T

Mycgr3G36271 Mycgr3T
  
Location: 28963-29854

Mycgr3G36271\_Mycgr3T

Mycgr3G68433 Mycgr3T
  
Location: 29954-33041

Mycgr3G68433\_Mycgr3T

Mycgr3G79452 Mycgr3T
  
Location: 33141-33399

Mycgr3G79452\_Mycgr3T

Mycgr3G55345 Mycgr3T
  
Location: 33499-34126

Mycgr3G55345\_Mycgr3T

Mycgr3G103278 Mycgr3
  
Location: 34226-35195

Mycgr3G103278\_Mycgr3

Mycgr3G84654 Mycgr3T
  
Location: 35295-36630

Mycgr3G84654\_Mycgr3T

Mycgr3G108090 Mycgr3
  
Location: 36730-37591

Mycgr3G108090\_Mycgr3

Mycgr3G21922 Mycgr3T
  
Location: 37691-39149

Mycgr3G21922\_Mycgr3T

Mycgr3G99148 Mycgr3T
  
Location: 39249-42819

Mycgr3G99148\_Mycgr3T

SnoRNA binding protein
  
Accession: EGE85260
  
Location: 90412-94130
  
 NCBI BlastP on this gene

EGE85260

hypothetical protein
  
Accession: EGE85261
  
Location: 95288-96118
  
 NCBI BlastP on this gene

EGE85261

hypothetical protein
  
Accession: EGE85262
  
Location: 96480-98746
  
 NCBI BlastP on this gene

EGE85262

DUF652 domain-containing protein
  
Accession: EGE85263
  
Location: 99796-100802
  
 NCBI BlastP on this gene

EGE85263

hypothetical protein
  
Accession: EGE85264
  
Location: 101595-103181
  
  
**BlastP hit with Mycgr3G36271\_Mycgr3T**
  
Percentage identity: 75 %
  
BlastP bit score: 482
  
Sequence coverage: 99 %
  
E-value: 6e-168
  
  
 NCBI BlastP on this gene

EGE85264

ATP synthase regulation protein NCA2
  
Accession: EGE85265
  
Location: 103769-106041
  
  
**BlastP hit with Mycgr3G84646\_Mycgr3T**
  
Percentage identity: 41 %
  
BlastP bit score: 483
  
Sequence coverage: 99 %
  
E-value: 9e-158
  
  
 NCBI BlastP on this gene

EGE85265

hypothetical protein
  
Accession: EGE85266
  
Location: 106528-109978
  
 NCBI BlastP on this gene

EGE85266

hypothetical protein
  
Accession: EGE85267
  
Location: 110394-112017
  
 NCBI BlastP on this gene

EGE85267

hypothetical protein
  
Accession: EGE85268
  
Location: 113262-113847
  
 NCBI BlastP on this gene

EGE85268

hypothetical protein
  
Accession: EGE85269
  
Location: 115187-118039
  
 NCBI BlastP on this gene

EGE85269

hypothetical protein
  
Accession: EGE85270
  
Location: 118767-119298
  
 NCBI BlastP on this gene

EGE85270

creatine transporter
  
Accession: EGE85271
  
Location: 122315-124396
  
 NCBI BlastP on this gene

EGE85271

hypothetical protein
  
Accession: EGE85272
  
Location: 124924-125303
  
 NCBI BlastP on this gene

EGE85272

hypothetical protein
  
Accession: EGE85273
  
Location: 128052-129153
  
 NCBI BlastP on this gene

EGE85273

GTP-binding protein
  
Accession: EGE85274
  
Location: 130528-131352
  
  
**BlastP hit with Mycgr3G99145\_Mycgr3T**
  
Percentage identity: 84 %
  
BlastP bit score: 352
  
Sequence coverage: 99 %
  
E-value: 2e-120
  
  
 NCBI BlastP on this gene

EGE85274

hypothetical protein
  
Accession: EGE85275
  
Location: 131983-133221
  
 NCBI BlastP on this gene

EGE85275

cell division cycle protein
  
Accession: EGE85276
  
Location: 136882-139206
  
 NCBI BlastP on this gene

EGE85276

hypothetical protein
  
Accession: EGE85277
  
Location: 139790-140835
  
 NCBI BlastP on this gene

EGE85277

Query: Architecture Search FASTA input

GG657483 : Ajellomyces dermatitidis SLH14081 genomic scaffold supercont1.36    Total score: 3.0     Cumulative Blast bit score: 1317

Hit cluster cross-links:

Mycgr3G90785 Mycgr3T
  
Location: 0-1047

Mycgr3G90785\_Mycgr3T

Mycgr3G103262 Mycgr3
  
Location: 1147-1390

Mycgr3G103262\_Mycgr3

Mycgr3G68458 Mycgr3T
  
Location: 1490-3602

Mycgr3G68458\_Mycgr3T

Mycgr3G99145 Mycgr3T
  
Location: 3702-4326

Mycgr3G99145\_Mycgr3T

Mycgr3G103274 Mycgr3
  
Location: 4426-4957

Mycgr3G103274\_Mycgr3

Mycgr3G103264 Mycgr3
  
Location: 5057-5390

Mycgr3G103264\_Mycgr3

Mycgr3G37570 Mycgr3T
  
Location: 5490-6006

Mycgr3G37570\_Mycgr3T

Mycgr3G108094 Mycgr3
  
Location: 6106-10555

Mycgr3G108094\_Mycgr3

Mycgr3G90786 Mycgr3T
  
Location: 10655-12080

Mycgr3G90786\_Mycgr3T

Mycgr3G68429 Mycgr3T
  
Location: 12180-13440

Mycgr3G68429\_Mycgr3T

Mycgr3G68421 Mycgr3T
  
Location: 13540-17086

Mycgr3G68421\_Mycgr3T

Mycgr3G90801 Mycgr3T
  
Location: 17186-18056

Mycgr3G90801\_Mycgr3T

Mycgr3G84646 Mycgr3T
  
Location: 18156-20235

Mycgr3G84646\_Mycgr3T

Mycgr3G68456 Mycgr3T
  
Location: 20335-21970

Mycgr3G68456\_Mycgr3T

Mycgr3G103270 Mycgr3
  
Location: 22070-22355

Mycgr3G103270\_Mycgr3

Mycgr3G90803 Mycgr3T
  
Location: 22455-23019

Mycgr3G90803\_Mycgr3T

Mycgr3G36941 Mycgr3T
  
Location: 23119-24064

Mycgr3G36941\_Mycgr3T

Mycgr3G25746 Mycgr3T
  
Location: 24164-25241

Mycgr3G25746\_Mycgr3T

Mycgr3G90788 Mycgr3T
  
Location: 25341-25803

Mycgr3G90788\_Mycgr3T

Mycgr3G103260 Mycgr3
  
Location: 25903-26635

Mycgr3G103260\_Mycgr3

Mycgr3G84644 Mycgr3T
  
Location: 26735-28457

Mycgr3G84644\_Mycgr3T

Mycgr3G29227 Mycgr3T
  
Location: 28557-28863

Mycgr3G29227\_Mycgr3T

Mycgr3G36271 Mycgr3T
  
Location: 28963-29854

Mycgr3G36271\_Mycgr3T

Mycgr3G68433 Mycgr3T
  
Location: 29954-33041

Mycgr3G68433\_Mycgr3T

Mycgr3G79452 Mycgr3T
  
Location: 33141-33399

Mycgr3G79452\_Mycgr3T

Mycgr3G55345 Mycgr3T
  
Location: 33499-34126

Mycgr3G55345\_Mycgr3T

Mycgr3G103278 Mycgr3
  
Location: 34226-35195

Mycgr3G103278\_Mycgr3

Mycgr3G84654 Mycgr3T
  
Location: 35295-36630

Mycgr3G84654\_Mycgr3T

Mycgr3G108090 Mycgr3
  
Location: 36730-37591

Mycgr3G108090\_Mycgr3

Mycgr3G21922 Mycgr3T
  
Location: 37691-39149

Mycgr3G21922\_Mycgr3T

Mycgr3G99148 Mycgr3T
  
Location: 39249-42819

Mycgr3G99148\_Mycgr3T

snoRNA binding protein
  
Accession: EEQ75948
  
Location: 276621-280283
  
 NCBI BlastP on this gene

EEQ75948

conserved hypothetical protein
  
Accession: EEQ75947
  
Location: 274520-275350
  
 NCBI BlastP on this gene

EEQ75947

conserved hypothetical protein
  
Accession: EEQ75946
  
Location: 271892-274158
  
 NCBI BlastP on this gene

EEQ75946

FCF1 small subunit
  
Accession: EEQ75945
  
Location: 269835-270841
  
 NCBI BlastP on this gene

EEQ75945

C-4 methyl sterol oxidase Erg25
  
Accession: EEQ75944
  
Location: 267878-269039
  
  
**BlastP hit with Mycgr3G36271\_Mycgr3T**
  
Percentage identity: 75 %
  
BlastP bit score: 480
  
Sequence coverage: 99 %
  
E-value: 4e-168
  
  
 NCBI BlastP on this gene

EEQ75944

ATP synthase regulation protein NCA2
  
Accession: EEQ75943
  
Location: 264581-266853
  
  
**BlastP hit with Mycgr3G84646\_Mycgr3T**
  
Percentage identity: 41 %
  
BlastP bit score: 485
  
Sequence coverage: 99 %
  
E-value: 2e-158
  
  
 NCBI BlastP on this gene

EEQ75943

conserved hypothetical protein
  
Accession: EEQ75942
  
Location: 260558-264094
  
 NCBI BlastP on this gene

EEQ75942

conserved hypothetical protein
  
Accession: EEQ75941
  
Location: 258878-260221
  
 NCBI BlastP on this gene

EEQ75941

predicted protein
  
Accession: EEQ75940
  
Location: 256278-257390
  
 NCBI BlastP on this gene

EEQ75940

conserved hypothetical protein
  
Accession: EEQ75939
  
Location: 252643-255406
  
 NCBI BlastP on this gene

EEQ75939

hypothetical protein
  
Accession: EEQ75938
  
Location: 251386-251917
  
 NCBI BlastP on this gene

EEQ75938

creatine transporter
  
Accession: EEQ75937
  
Location: 246297-248378
  
 NCBI BlastP on this gene

EEQ75937

predicted protein
  
Accession: EEQ75936
  
Location: 244361-245718
  
 NCBI BlastP on this gene

EEQ75936

hypothetical protein
  
Accession: EEQ75935
  
Location: 242215-242637
  
 NCBI BlastP on this gene

EEQ75935

GTP-binding protein SAS1
  
Accession: EEQ75934
  
Location: 239369-240193
  
  
**BlastP hit with Mycgr3G99145\_Mycgr3T**
  
Percentage identity: 84 %
  
BlastP bit score: 352
  
Sequence coverage: 99 %
  
E-value: 2e-120
  
  
 NCBI BlastP on this gene

EEQ75934

conserved hypothetical protein
  
Accession: EEQ75933
  
Location: 237499-238737
  
 NCBI BlastP on this gene

EEQ75933

AAA family ATPase
  
Accession: EEQ75932
  
Location: 234518-236842
  
 NCBI BlastP on this gene

EEQ75932

conserved hypothetical protein
  
Accession: EEQ75931
  
Location: 232892-233938
  
 NCBI BlastP on this gene

EEQ75931

phosphatidylinositol 3- and 4-kinase
  
Accession: EEQ75930
  
Location: 223739-232614
  
 NCBI BlastP on this gene

EEQ75930

Query: Architecture Search FASTA input

EQ999987 : Ajellomyces dermatitidis ER-3 genomic scaffold supercont1.15    Total score: 3.0     Cumulative Blast bit score: 1316

Hit cluster cross-links:

Mycgr3G90785 Mycgr3T
  
Location: 0-1047

Mycgr3G90785\_Mycgr3T

Mycgr3G103262 Mycgr3
  
Location: 1147-1390

Mycgr3G103262\_Mycgr3

Mycgr3G68458 Mycgr3T
  
Location: 1490-3602

Mycgr3G68458\_Mycgr3T

Mycgr3G99145 Mycgr3T
  
Location: 3702-4326

Mycgr3G99145\_Mycgr3T

Mycgr3G103274 Mycgr3
  
Location: 4426-4957

Mycgr3G103274\_Mycgr3

Mycgr3G103264 Mycgr3
  
Location: 5057-5390

Mycgr3G103264\_Mycgr3

Mycgr3G37570 Mycgr3T
  
Location: 5490-6006

Mycgr3G37570\_Mycgr3T

Mycgr3G108094 Mycgr3
  
Location: 6106-10555

Mycgr3G108094\_Mycgr3

Mycgr3G90786 Mycgr3T
  
Location: 10655-12080

Mycgr3G90786\_Mycgr3T

Mycgr3G68429 Mycgr3T
  
Location: 12180-13440

Mycgr3G68429\_Mycgr3T

Mycgr3G68421 Mycgr3T
  
Location: 13540-17086

Mycgr3G68421\_Mycgr3T

Mycgr3G90801 Mycgr3T
  
Location: 17186-18056

Mycgr3G90801\_Mycgr3T

Mycgr3G84646 Mycgr3T
  
Location: 18156-20235

Mycgr3G84646\_Mycgr3T

Mycgr3G68456 Mycgr3T
  
Location: 20335-21970

Mycgr3G68456\_Mycgr3T

Mycgr3G103270 Mycgr3
  
Location: 22070-22355

Mycgr3G103270\_Mycgr3

Mycgr3G90803 Mycgr3T
  
Location: 22455-23019

Mycgr3G90803\_Mycgr3T

Mycgr3G36941 Mycgr3T
  
Location: 23119-24064

Mycgr3G36941\_Mycgr3T

Mycgr3G25746 Mycgr3T
  
Location: 24164-25241

Mycgr3G25746\_Mycgr3T

Mycgr3G90788 Mycgr3T
  
Location: 25341-25803

Mycgr3G90788\_Mycgr3T

Mycgr3G103260 Mycgr3
  
Location: 25903-26635

Mycgr3G103260\_Mycgr3

Mycgr3G84644 Mycgr3T
  
Location: 26735-28457

Mycgr3G84644\_Mycgr3T

Mycgr3G29227 Mycgr3T
  
Location: 28557-28863

Mycgr3G29227\_Mycgr3T

Mycgr3G36271 Mycgr3T
  
Location: 28963-29854

Mycgr3G36271\_Mycgr3T

Mycgr3G68433 Mycgr3T
  
Location: 29954-33041

Mycgr3G68433\_Mycgr3T

Mycgr3G79452 Mycgr3T
  
Location: 33141-33399

Mycgr3G79452\_Mycgr3T

Mycgr3G55345 Mycgr3T
  
Location: 33499-34126

Mycgr3G55345\_Mycgr3T

Mycgr3G103278 Mycgr3
  
Location: 34226-35195

Mycgr3G103278\_Mycgr3

Mycgr3G84654 Mycgr3T
  
Location: 35295-36630

Mycgr3G84654\_Mycgr3T

Mycgr3G108090 Mycgr3
  
Location: 36730-37591

Mycgr3G108090\_Mycgr3

Mycgr3G21922 Mycgr3T
  
Location: 37691-39149

Mycgr3G21922\_Mycgr3T

Mycgr3G99148 Mycgr3T
  
Location: 39249-42819

Mycgr3G99148\_Mycgr3T

snoRNA binding protein
  
Accession: EEQ86096
  
Location: 411828-415544
  
 NCBI BlastP on this gene

EEQ86096

conserved hypothetical protein
  
Accession: EEQ86095
  
Location: 409860-410687
  
 NCBI BlastP on this gene

EEQ86095

conserved hypothetical protein
  
Accession: EEQ86094
  
Location: 407241-409498
  
 NCBI BlastP on this gene

EEQ86094

DUF652 domain-containing protein
  
Accession: EEQ86093
  
Location: 405188-406191
  
 NCBI BlastP on this gene

EEQ86093

C-4 methyl sterol oxidase Erg25
  
Accession: EEQ86092
  
Location: 403277-404436
  
  
**BlastP hit with Mycgr3G36271\_Mycgr3T**
  
Percentage identity: 75 %
  
BlastP bit score: 480
  
Sequence coverage: 99 %
  
E-value: 4e-168
  
  
 NCBI BlastP on this gene

EEQ86092

conserved hypothetical protein
  
Accession: EEQ86091
  
Location: 399982-402254
  
  
**BlastP hit with Mycgr3G84646\_Mycgr3T**
  
Percentage identity: 41 %
  
BlastP bit score: 484
  
Sequence coverage: 99 %
  
E-value: 7e-158
  
  
 NCBI BlastP on this gene

EEQ86091

conserved hypothetical protein
  
Accession: EEQ86090
  
Location: 395962-399495
  
 NCBI BlastP on this gene

EEQ86090

conserved hypothetical protein
  
Accession: EEQ86089
  
Location: 394282-395625
  
 NCBI BlastP on this gene

EEQ86089

predicted protein
  
Accession: EEQ86088
  
Location: 391696-392677
  
 NCBI BlastP on this gene

EEQ86088

conserved hypothetical protein
  
Accession: EEQ86087
  
Location: 388030-390793
  
 NCBI BlastP on this gene

EEQ86087

hypothetical protein
  
Accession: EEQ86086
  
Location: 386768-387302
  
 NCBI BlastP on this gene

EEQ86086

creatine transporter
  
Accession: EEQ86085
  
Location: 381671-383752
  
 NCBI BlastP on this gene

EEQ86085

predicted protein
  
Accession: EEQ86084
  
Location: 379581-381100
  
 NCBI BlastP on this gene

EEQ86084

predicted protein
  
Accession: EEQ86083
  
Location: 376944-378056
  
 NCBI BlastP on this gene

EEQ86083

GTP-binding protein SAS1
  
Accession: EEQ86082
  
Location: 374745-375569
  
  
**BlastP hit with Mycgr3G99145\_Mycgr3T**
  
Percentage identity: 84 %
  
BlastP bit score: 352
  
Sequence coverage: 99 %
  
E-value: 2e-120
  
  
 NCBI BlastP on this gene

EEQ86082

conserved hypothetical protein
  
Accession: EEQ86081
  
Location: 372875-374113
  
 NCBI BlastP on this gene

EEQ86081

AAA family ATPase
  
Accession: EEQ86080
  
Location: 369881-372205
  
 NCBI BlastP on this gene

EEQ86080

conserved hypothetical protein
  
Accession: EEQ86079
  
Location: 368255-369301
  
 NCBI BlastP on this gene

EEQ86079

phosphatidylinositol 3- and 4-kinase
  
Accession: EEQ86078
  
Location: 359099-367975
  
 NCBI BlastP on this gene

EEQ86078

Query: Architecture Search FASTA input

JH794989 : Magnaporthe oryzae P131 unplaced genomic scaffold P131\_scaffold00957    Total score: 3.0     Cumulative Blast bit score: 1313

Hit cluster cross-links:

Mycgr3G90785 Mycgr3T
  
Location: 0-1047

Mycgr3G90785\_Mycgr3T

Mycgr3G103262 Mycgr3
  
Location: 1147-1390

Mycgr3G103262\_Mycgr3

Mycgr3G68458 Mycgr3T
  
Location: 1490-3602

Mycgr3G68458\_Mycgr3T

Mycgr3G99145 Mycgr3T
  
Location: 3702-4326

Mycgr3G99145\_Mycgr3T

Mycgr3G103274 Mycgr3
  
Location: 4426-4957

Mycgr3G103274\_Mycgr3

Mycgr3G103264 Mycgr3
  
Location: 5057-5390

Mycgr3G103264\_Mycgr3

Mycgr3G37570 Mycgr3T
  
Location: 5490-6006

Mycgr3G37570\_Mycgr3T

Mycgr3G108094 Mycgr3
  
Location: 6106-10555

Mycgr3G108094\_Mycgr3

Mycgr3G90786 Mycgr3T
  
Location: 10655-12080

Mycgr3G90786\_Mycgr3T

Mycgr3G68429 Mycgr3T
  
Location: 12180-13440

Mycgr3G68429\_Mycgr3T

Mycgr3G68421 Mycgr3T
  
Location: 13540-17086

Mycgr3G68421\_Mycgr3T

Mycgr3G90801 Mycgr3T
  
Location: 17186-18056

Mycgr3G90801\_Mycgr3T

Mycgr3G84646 Mycgr3T
  
Location: 18156-20235

Mycgr3G84646\_Mycgr3T

Mycgr3G68456 Mycgr3T
  
Location: 20335-21970

Mycgr3G68456\_Mycgr3T

Mycgr3G103270 Mycgr3
  
Location: 22070-22355

Mycgr3G103270\_Mycgr3

Mycgr3G90803 Mycgr3T
  
Location: 22455-23019

Mycgr3G90803\_Mycgr3T

Mycgr3G36941 Mycgr3T
  
Location: 23119-24064

Mycgr3G36941\_Mycgr3T

Mycgr3G25746 Mycgr3T
  
Location: 24164-25241

Mycgr3G25746\_Mycgr3T

Mycgr3G90788 Mycgr3T
  
Location: 25341-25803

Mycgr3G90788\_Mycgr3T

Mycgr3G103260 Mycgr3
  
Location: 25903-26635

Mycgr3G103260\_Mycgr3

Mycgr3G84644 Mycgr3T
  
Location: 26735-28457

Mycgr3G84644\_Mycgr3T

Mycgr3G29227 Mycgr3T
  
Location: 28557-28863

Mycgr3G29227\_Mycgr3T

Mycgr3G36271 Mycgr3T
  
Location: 28963-29854

Mycgr3G36271\_Mycgr3T

Mycgr3G68433 Mycgr3T
  
Location: 29954-33041

Mycgr3G68433\_Mycgr3T

Mycgr3G79452 Mycgr3T
  
Location: 33141-33399

Mycgr3G79452\_Mycgr3T

Mycgr3G55345 Mycgr3T
  
Location: 33499-34126

Mycgr3G55345\_Mycgr3T

Mycgr3G103278 Mycgr3
  
Location: 34226-35195

Mycgr3G103278\_Mycgr3

Mycgr3G84654 Mycgr3T
  
Location: 35295-36630

Mycgr3G84654\_Mycgr3T

Mycgr3G108090 Mycgr3
  
Location: 36730-37591

Mycgr3G108090\_Mycgr3

Mycgr3G21922 Mycgr3T
  
Location: 37691-39149

Mycgr3G21922\_Mycgr3T

Mycgr3G99148 Mycgr3T
  
Location: 39249-42819

Mycgr3G99148\_Mycgr3T

profilin
  
Accession: ELQ63654
  
Location: 59934-60674
  
 NCBI BlastP on this gene

ELQ63654

hypothetical protein
  
Accession: ELQ63653
  
Location: 58351-59301
  
 NCBI BlastP on this gene

ELQ63653

hypothetical protein
  
Accession: ELQ63652
  
Location: 56873-57460
  
 NCBI BlastP on this gene

ELQ63652

actin-related protein 5
  
Accession: ELQ63651
  
Location: 52845-55385
  
 NCBI BlastP on this gene

ELQ63651

hypothetical protein
  
Accession: ELQ63650
  
Location: 51654-52538
  
 NCBI BlastP on this gene

ELQ63650

hypothetical protein
  
Accession: ELQ63649
  
Location: 48462-49127
  
 NCBI BlastP on this gene

ELQ63649

C-4 methylsterol oxidase
  
Accession: ELQ63648
  
Location: 46968-48081
  
  
**BlastP hit with Mycgr3G36271\_Mycgr3T**
  
Percentage identity: 70 %
  
BlastP bit score: 432
  
Sequence coverage: 94 %
  
E-value: 5e-149
  
  
 NCBI BlastP on this gene

ELQ63648

nuclear control of ATPase protein 2
  
Accession: ELQ63647
  
Location: 43145-45298
  
  
**BlastP hit with Mycgr3G84646\_Mycgr3T**
  
Percentage identity: 42 %
  
BlastP bit score: 526
  
Sequence coverage: 101 %
  
E-value: 1e-174
  
  
 NCBI BlastP on this gene

ELQ63647

GTP-binding protein SAS1
  
Accession: ELQ63646
  
Location: 40988-41834
  
  
**BlastP hit with Mycgr3G99145\_Mycgr3T**
  
Percentage identity: 85 %
  
BlastP bit score: 355
  
Sequence coverage: 99 %
  
E-value: 1e-121
  
  
 NCBI BlastP on this gene

ELQ63646

hypothetical protein
  
Accession: ELQ63645
  
Location: 38944-40206
  
 NCBI BlastP on this gene

ELQ63645

ATPase, AFG2 type
  
Accession: ELQ63644
  
Location: 36010-38401
  
 NCBI BlastP on this gene

ELQ63644

dolichyl-phosphate beta-glucosyltransferase
  
Accession: ELQ63643
  
Location: 34295-35633
  
 NCBI BlastP on this gene

ELQ63643

hypothetical protein
  
Accession: ELQ63642
  
Location: 31622-33275
  
 NCBI BlastP on this gene

ELQ63642

DUF625 domain-containing protein
  
Accession: ELQ63641
  
Location: 24515-27581
  
 NCBI BlastP on this gene

ELQ63641

squalene monooxygenase
  
Accession: ELQ63640
  
Location: 21429-23033
  
 NCBI BlastP on this gene

ELQ63640

Query: Architecture Search FASTA input

JH793752 : Magnaporthe oryzae Y34 unplaced genomic scaffold Y34\_scaffold00460    Total score: 3.0     Cumulative Blast bit score: 1313

Hit cluster cross-links:

Mycgr3G90785 Mycgr3T
  
Location: 0-1047

Mycgr3G90785\_Mycgr3T

Mycgr3G103262 Mycgr3
  
Location: 1147-1390

Mycgr3G103262\_Mycgr3

Mycgr3G68458 Mycgr3T
  
Location: 1490-3602

Mycgr3G68458\_Mycgr3T

Mycgr3G99145 Mycgr3T
  
Location: 3702-4326

Mycgr3G99145\_Mycgr3T

Mycgr3G103274 Mycgr3
  
Location: 4426-4957

Mycgr3G103274\_Mycgr3

Mycgr3G103264 Mycgr3
  
Location: 5057-5390

Mycgr3G103264\_Mycgr3

Mycgr3G37570 Mycgr3T
  
Location: 5490-6006

Mycgr3G37570\_Mycgr3T

Mycgr3G108094 Mycgr3
  
Location: 6106-10555

Mycgr3G108094\_Mycgr3

Mycgr3G90786 Mycgr3T
  
Location: 10655-12080

Mycgr3G90786\_Mycgr3T

Mycgr3G68429 Mycgr3T
  
Location: 12180-13440

Mycgr3G68429\_Mycgr3T

Mycgr3G68421 Mycgr3T
  
Location: 13540-17086

Mycgr3G68421\_Mycgr3T

Mycgr3G90801 Mycgr3T
  
Location: 17186-18056

Mycgr3G90801\_Mycgr3T

Mycgr3G84646 Mycgr3T
  
Location: 18156-20235

Mycgr3G84646\_Mycgr3T

Mycgr3G68456 Mycgr3T
  
Location: 20335-21970

Mycgr3G68456\_Mycgr3T

Mycgr3G103270 Mycgr3
  
Location: 22070-22355

Mycgr3G103270\_Mycgr3

Mycgr3G90803 Mycgr3T
  
Location: 22455-23019

Mycgr3G90803\_Mycgr3T

Mycgr3G36941 Mycgr3T
  
Location: 23119-24064

Mycgr3G36941\_Mycgr3T

Mycgr3G25746 Mycgr3T
  
Location: 24164-25241

Mycgr3G25746\_Mycgr3T

Mycgr3G90788 Mycgr3T
  
Location: 25341-25803

Mycgr3G90788\_Mycgr3T

Mycgr3G103260 Mycgr3
  
Location: 25903-26635

Mycgr3G103260\_Mycgr3

Mycgr3G84644 Mycgr3T
  
Location: 26735-28457

Mycgr3G84644\_Mycgr3T

Mycgr3G29227 Mycgr3T
  
Location: 28557-28863

Mycgr3G29227\_Mycgr3T

Mycgr3G36271 Mycgr3T
  
Location: 28963-29854

Mycgr3G36271\_Mycgr3T

Mycgr3G68433 Mycgr3T
  
Location: 29954-33041

Mycgr3G68433\_Mycgr3T

Mycgr3G79452 Mycgr3T
  
Location: 33141-33399

Mycgr3G79452\_Mycgr3T

Mycgr3G55345 Mycgr3T
  
Location: 33499-34126

Mycgr3G55345\_Mycgr3T

Mycgr3G103278 Mycgr3
  
Location: 34226-35195

Mycgr3G103278\_Mycgr3

Mycgr3G84654 Mycgr3T
  
Location: 35295-36630

Mycgr3G84654\_Mycgr3T

Mycgr3G108090 Mycgr3
  
Location: 36730-37591

Mycgr3G108090\_Mycgr3

Mycgr3G21922 Mycgr3T
  
Location: 37691-39149

Mycgr3G21922\_Mycgr3T

Mycgr3G99148 Mycgr3T
  
Location: 39249-42819

Mycgr3G99148\_Mycgr3T

hypothetical protein
  
Accession: ELQ40148
  
Location: 342-2591
  
 NCBI BlastP on this gene

ELQ40148

profilin
  
Accession: ELQ40149
  
Location: 7232-7972
  
 NCBI BlastP on this gene

ELQ40149

hypothetical protein
  
Accession: ELQ40150
  
Location: 8551-9553
  
 NCBI BlastP on this gene

ELQ40150

hypothetical protein
  
Accession: ELQ40151
  
Location: 10444-11031
  
 NCBI BlastP on this gene

ELQ40151

actin-related protein 5
  
Accession: ELQ40152
  
Location: 12519-15059
  
 NCBI BlastP on this gene

ELQ40152

hypothetical protein
  
Accession: ELQ40153
  
Location: 15366-16250
  
 NCBI BlastP on this gene

ELQ40153

hypothetical protein
  
Accession: ELQ40154
  
Location: 18775-19440
  
 NCBI BlastP on this gene

ELQ40154

C-4 methylsterol oxidase
  
Accession: ELQ40155
  
Location: 19821-20934
  
  
**BlastP hit with Mycgr3G36271\_Mycgr3T**
  
Percentage identity: 70 %
  
BlastP bit score: 432
  
Sequence coverage: 94 %
  
E-value: 5e-149
  
  
 NCBI BlastP on this gene

ELQ40155

nuclear control of ATPase protein 2
  
Accession: ELQ40156
  
Location: 22605-24758
  
  
**BlastP hit with Mycgr3G84646\_Mycgr3T**
  
Percentage identity: 42 %
  
BlastP bit score: 526
  
Sequence coverage: 101 %
  
E-value: 1e-174
  
  
 NCBI BlastP on this gene

ELQ40156

GTP-binding protein SAS1
  
Accession: ELQ40157
  
Location: 26069-26915
  
  
**BlastP hit with Mycgr3G99145\_Mycgr3T**
  
Percentage identity: 85 %
  
BlastP bit score: 355
  
Sequence coverage: 99 %
  
E-value: 1e-121
  
  
 NCBI BlastP on this gene

ELQ40157

hypothetical protein
  
Accession: ELQ40158
  
Location: 27697-28959
  
 NCBI BlastP on this gene

ELQ40158

ATPase, AFG2 type
  
Accession: ELQ40159
  
Location: 29502-31894
  
 NCBI BlastP on this gene

ELQ40159

dolichyl-phosphate beta-glucosyltransferase
  
Accession: ELQ40160
  
Location: 32271-33609
  
 NCBI BlastP on this gene

ELQ40160

hypothetical protein
  
Accession: ELQ40161
  
Location: 34629-36282
  
 NCBI BlastP on this gene

ELQ40161

DUF625 domain-containing protein
  
Accession: ELQ40162
  
Location: 40320-43386
  
 NCBI BlastP on this gene

ELQ40162

squalene monooxygenase
  
Accession: ELQ40163
  
Location: 44864-46468
  
 NCBI BlastP on this gene

ELQ40163

Query: Architecture Search FASTA input

CM001233 : Magnaporthe oryzae 70-15 chromosome 3    Total score: 3.0     Cumulative Blast bit score: 1313

Hit cluster cross-links:

Mycgr3G90785 Mycgr3T
  
Location: 0-1047

Mycgr3G90785\_Mycgr3T

Mycgr3G103262 Mycgr3
  
Location: 1147-1390

Mycgr3G103262\_Mycgr3

Mycgr3G68458 Mycgr3T
  
Location: 1490-3602

Mycgr3G68458\_Mycgr3T

Mycgr3G99145 Mycgr3T
  
Location: 3702-4326

Mycgr3G99145\_Mycgr3T

Mycgr3G103274 Mycgr3
  
Location: 4426-4957

Mycgr3G103274\_Mycgr3

Mycgr3G103264 Mycgr3
  
Location: 5057-5390

Mycgr3G103264\_Mycgr3

Mycgr3G37570 Mycgr3T
  
Location: 5490-6006

Mycgr3G37570\_Mycgr3T

Mycgr3G108094 Mycgr3
  
Location: 6106-10555

Mycgr3G108094\_Mycgr3

Mycgr3G90786 Mycgr3T
  
Location: 10655-12080

Mycgr3G90786\_Mycgr3T

Mycgr3G68429 Mycgr3T
  
Location: 12180-13440

Mycgr3G68429\_Mycgr3T

Mycgr3G68421 Mycgr3T
  
Location: 13540-17086

Mycgr3G68421\_Mycgr3T

Mycgr3G90801 Mycgr3T
  
Location: 17186-18056

Mycgr3G90801\_Mycgr3T

Mycgr3G84646 Mycgr3T
  
Location: 18156-20235

Mycgr3G84646\_Mycgr3T

Mycgr3G68456 Mycgr3T
  
Location: 20335-21970

Mycgr3G68456\_Mycgr3T

Mycgr3G103270 Mycgr3
  
Location: 22070-22355

Mycgr3G103270\_Mycgr3

Mycgr3G90803 Mycgr3T
  
Location: 22455-23019

Mycgr3G90803\_Mycgr3T

Mycgr3G36941 Mycgr3T
  
Location: 23119-24064

Mycgr3G36941\_Mycgr3T

Mycgr3G25746 Mycgr3T
  
Location: 24164-25241

Mycgr3G25746\_Mycgr3T

Mycgr3G90788 Mycgr3T
  
Location: 25341-25803

Mycgr3G90788\_Mycgr3T

Mycgr3G103260 Mycgr3
  
Location: 25903-26635

Mycgr3G103260\_Mycgr3

Mycgr3G84644 Mycgr3T
  
Location: 26735-28457

Mycgr3G84644\_Mycgr3T

Mycgr3G29227 Mycgr3T
  
Location: 28557-28863

Mycgr3G29227\_Mycgr3T

Mycgr3G36271 Mycgr3T
  
Location: 28963-29854

Mycgr3G36271\_Mycgr3T

Mycgr3G68433 Mycgr3T
  
Location: 29954-33041

Mycgr3G68433\_Mycgr3T

Mycgr3G79452 Mycgr3T
  
Location: 33141-33399

Mycgr3G79452\_Mycgr3T

Mycgr3G55345 Mycgr3T
  
Location: 33499-34126

Mycgr3G55345\_Mycgr3T

Mycgr3G103278 Mycgr3
  
Location: 34226-35195

Mycgr3G103278\_Mycgr3

Mycgr3G84654 Mycgr3T
  
Location: 35295-36630

Mycgr3G84654\_Mycgr3T

Mycgr3G108090 Mycgr3
  
Location: 36730-37591

Mycgr3G108090\_Mycgr3

Mycgr3G21922 Mycgr3T
  
Location: 37691-39149

Mycgr3G21922\_Mycgr3T

Mycgr3G99148 Mycgr3T
  
Location: 39249-42819

Mycgr3G99148\_Mycgr3T

hypothetical protein
  
Accession: EHA52186
  
Location: 2659095-2661341
  
 NCBI BlastP on this gene

EHA52186

hypothetical protein
  
Accession: EHA52187
  
Location: 2662481-2664568
  
 NCBI BlastP on this gene

EHA52187

profilin
  
Accession: EHA52188
  
Location: 2665982-2666722
  
 NCBI BlastP on this gene

EHA52188

Got1 family protein
  
Accession: EHA52189
  
Location: 2667416-2668183
  
 NCBI BlastP on this gene

EHA52189

hypothetical protein
  
Accession: EHA52190
  
Location: 2669195-2669782
  
 NCBI BlastP on this gene

EHA52190

DASH complex subunit dad4
  
Accession: EHA52191
  
Location: 2670188-2670606
  
 NCBI BlastP on this gene

EHA52191

hypothetical protein
  
Accession: EHA52192
  
Location: 2671270-2673810
  
 NCBI BlastP on this gene

EHA52192

hypothetical protein, variant 2
  
Accession: EHA52193
  
Location: 2674117-2675004
  
 NCBI BlastP on this gene

EHA52193

hypothetical protein
  
Accession: EHA52196
  
Location: 2677527-2678192
  
 NCBI BlastP on this gene

EHA52196

C-4 methylsterol oxidase
  
Accession: EHA52197
  
Location: 2678573-2679686
  
  
**BlastP hit with Mycgr3G36271\_Mycgr3T**
  
Percentage identity: 70 %
  
BlastP bit score: 432
  
Sequence coverage: 94 %
  
E-value: 5e-149
  
  
 NCBI BlastP on this gene

EHA52197

hypothetical protein
  
Accession: EHA52198
  
Location: 2680625-2681076
  
 NCBI BlastP on this gene

EHA52198

hypothetical protein
  
Accession: EHA52199
  
Location: 2681356-2683509
  
  
**BlastP hit with Mycgr3G84646\_Mycgr3T**
  
Percentage identity: 42 %
  
BlastP bit score: 526
  
Sequence coverage: 101 %
  
E-value: 1e-174
  
  
 NCBI BlastP on this gene

EHA52199

GTP-binding protein ypt2
  
Accession: EHA52200
  
Location: 2684820-2685666
  
  
**BlastP hit with Mycgr3G99145\_Mycgr3T**
  
Percentage identity: 85 %
  
BlastP bit score: 355
  
Sequence coverage: 99 %
  
E-value: 1e-121
  
  
 NCBI BlastP on this gene

EHA52200

hypothetical protein
  
Accession: EHA52201
  
Location: 2686448-2688009
  
 NCBI BlastP on this gene

EHA52201

ATPase
  
Accession: EHA52202
  
Location: 2688253-2690655
  
 NCBI BlastP on this gene

EHA52202

dolichyl-phosphate beta-glucosyltransferase
  
Accession: EHA52203
  
Location: 2691032-2692370
  
 NCBI BlastP on this gene

EHA52203

hypothetical protein
  
Accession: EHA52204
  
Location: 2693390-2695043
  
 NCBI BlastP on this gene

EHA52204

hypothetical protein
  
Accession: EHA52205
  
Location: 2699082-2702148
  
 NCBI BlastP on this gene

EHA52205

squalene monooxygenase
  
Accession: EHA52206
  
Location: 2703629-2705233
  
 NCBI BlastP on this gene

EHA52206

Query: Architecture Search FASTA input

KB908844 : Setosphaeria turcica Et28A unplaced genomic scaffold SETTUscaffold\_6    Total score: 3.0     Cumulative Blast bit score: 1312

Hit cluster cross-links:

Mycgr3G90785 Mycgr3T
  
Location: 0-1047

Mycgr3G90785\_Mycgr3T

Mycgr3G103262 Mycgr3
  
Location: 1147-1390

Mycgr3G103262\_Mycgr3

Mycgr3G68458 Mycgr3T
  
Location: 1490-3602

Mycgr3G68458\_Mycgr3T

Mycgr3G99145 Mycgr3T
  
Location: 3702-4326

Mycgr3G99145\_Mycgr3T

Mycgr3G103274 Mycgr3
  
Location: 4426-4957

Mycgr3G103274\_Mycgr3

Mycgr3G103264 Mycgr3
  
Location: 5057-5390

Mycgr3G103264\_Mycgr3

Mycgr3G37570 Mycgr3T
  
Location: 5490-6006

Mycgr3G37570\_Mycgr3T

Mycgr3G108094 Mycgr3
  
Location: 6106-10555

Mycgr3G108094\_Mycgr3

Mycgr3G90786 Mycgr3T
  
Location: 10655-12080

Mycgr3G90786\_Mycgr3T

Mycgr3G68429 Mycgr3T
  
Location: 12180-13440

Mycgr3G68429\_Mycgr3T

Mycgr3G68421 Mycgr3T
  
Location: 13540-17086

Mycgr3G68421\_Mycgr3T

Mycgr3G90801 Mycgr3T
  
Location: 17186-18056

Mycgr3G90801\_Mycgr3T

Mycgr3G84646 Mycgr3T
  
Location: 18156-20235

Mycgr3G84646\_Mycgr3T

Mycgr3G68456 Mycgr3T
  
Location: 20335-21970

Mycgr3G68456\_Mycgr3T

Mycgr3G103270 Mycgr3
  
Location: 22070-22355

Mycgr3G103270\_Mycgr3

Mycgr3G90803 Mycgr3T
  
Location: 22455-23019

Mycgr3G90803\_Mycgr3T

Mycgr3G36941 Mycgr3T
  
Location: 23119-24064

Mycgr3G36941\_Mycgr3T

Mycgr3G25746 Mycgr3T
  
Location: 24164-25241

Mycgr3G25746\_Mycgr3T

Mycgr3G90788 Mycgr3T
  
Location: 25341-25803

Mycgr3G90788\_Mycgr3T

Mycgr3G103260 Mycgr3
  
Location: 25903-26635

Mycgr3G103260\_Mycgr3

Mycgr3G84644 Mycgr3T
  
Location: 26735-28457

Mycgr3G84644\_Mycgr3T

Mycgr3G29227 Mycgr3T
  
Location: 28557-28863

Mycgr3G29227\_Mycgr3T

Mycgr3G36271 Mycgr3T
  
Location: 28963-29854

Mycgr3G36271\_Mycgr3T

Mycgr3G68433 Mycgr3T
  
Location: 29954-33041

Mycgr3G68433\_Mycgr3T

Mycgr3G79452 Mycgr3T
  
Location: 33141-33399

Mycgr3G79452\_Mycgr3T

Mycgr3G55345 Mycgr3T
  
Location: 33499-34126

Mycgr3G55345\_Mycgr3T

Mycgr3G103278 Mycgr3
  
Location: 34226-35195

Mycgr3G103278\_Mycgr3

Mycgr3G84654 Mycgr3T
  
Location: 35295-36630

Mycgr3G84654\_Mycgr3T

Mycgr3G108090 Mycgr3
  
Location: 36730-37591

Mycgr3G108090\_Mycgr3

Mycgr3G21922 Mycgr3T
  
Location: 37691-39149

Mycgr3G21922\_Mycgr3T

Mycgr3G99148 Mycgr3T
  
Location: 39249-42819

Mycgr3G99148\_Mycgr3T

hypothetical protein
  
Accession: EOA82896
  
Location: 1692451-1695948
  
 NCBI BlastP on this gene

EOA82896

hypothetical protein
  
Accession: EOA82897
  
Location: 1696383-1697024
  
 NCBI BlastP on this gene

EOA82897

hypothetical protein
  
Accession: EOA82898
  
Location: 1697063-1698052
  
 NCBI BlastP on this gene

EOA82898

hypothetical protein
  
Accession: EOA82899
  
Location: 1698464-1700054
  
 NCBI BlastP on this gene

EOA82899

hypothetical protein
  
Accession: EOA82900
  
Location: 1701162-1702179
  
 NCBI BlastP on this gene

EOA82900

carbohydrate esterase family 9 protein
  
Accession: EOA82901
  
Location: 1703065-1704372
  
 NCBI BlastP on this gene

EOA82901

glycoside hydrolase family 3 protein
  
Accession: EOA82902
  
Location: 1705027-1708080
  
 NCBI BlastP on this gene

EOA82902

hypothetical protein
  
Accession: EOA82903
  
Location: 1708413-1710944
  
  
**BlastP hit with Mycgr3G84654\_Mycgr3T**
  
Percentage identity: 55 %
  
BlastP bit score: 388
  
Sequence coverage: 85 %
  
E-value: 4e-123
  
  
 NCBI BlastP on this gene

EOA82903

hypothetical protein
  
Accession: EOA82904
  
Location: 1715589-1717350
  
 NCBI BlastP on this gene

EOA82904

hypothetical protein
  
Accession: EOA82905
  
Location: 1719073-1720719
  
 NCBI BlastP on this gene

EOA82905

hypothetical protein
  
Accession: EOA82906
  
Location: 1722133-1723389
  
 NCBI BlastP on this gene

EOA82906

hypothetical protein
  
Accession: EOA82907
  
Location: 1724102-1727533
  
  
**BlastP hit with Mycgr3G99145\_Mycgr3T**
  
Percentage identity: 76 %
  
BlastP bit score: 317
  
Sequence coverage: 98 %
  
E-value: 2e-99
  
  
  
**BlastP hit with Mycgr3G84646\_Mycgr3T**
  
Percentage identity: 48 %
  
BlastP bit score: 607
  
Sequence coverage: 98 %
  
E-value: 0.0
  
  
 NCBI BlastP on this gene

EOA82907

hypothetical protein
  
Accession: EOA82908
  
Location: 1728084-1735952
  
 NCBI BlastP on this gene

EOA82908

hypothetical protein
  
Accession: EOA82909
  
Location: 1736521-1736926
  
 NCBI BlastP on this gene

EOA82909

hypothetical protein
  
Accession: EOA82910
  
Location: 1737827-1740242
  
 NCBI BlastP on this gene

EOA82910

Query: Architecture Search FASTA input

GG704913 : Coccidioides immitis RS genomic scaffold supercont3.3    Total score: 3.0     Cumulative Blast bit score: 1308

Hit cluster cross-links:

Mycgr3G90785 Mycgr3T
  
Location: 0-1047

Mycgr3G90785\_Mycgr3T

Mycgr3G103262 Mycgr3
  
Location: 1147-1390

Mycgr3G103262\_Mycgr3

Mycgr3G68458 Mycgr3T
  
Location: 1490-3602

Mycgr3G68458\_Mycgr3T

Mycgr3G99145 Mycgr3T
  
Location: 3702-4326

Mycgr3G99145\_Mycgr3T

Mycgr3G103274 Mycgr3
  
Location: 4426-4957

Mycgr3G103274\_Mycgr3

Mycgr3G103264 Mycgr3
  
Location: 5057-5390

Mycgr3G103264\_Mycgr3

Mycgr3G37570 Mycgr3T
  
Location: 5490-6006

Mycgr3G37570\_Mycgr3T

Mycgr3G108094 Mycgr3
  
Location: 6106-10555

Mycgr3G108094\_Mycgr3

Mycgr3G90786 Mycgr3T
  
Location: 10655-12080

Mycgr3G90786\_Mycgr3T

Mycgr3G68429 Mycgr3T
  
Location: 12180-13440

Mycgr3G68429\_Mycgr3T

Mycgr3G68421 Mycgr3T
  
Location: 13540-17086

Mycgr3G68421\_Mycgr3T

Mycgr3G90801 Mycgr3T
  
Location: 17186-18056

Mycgr3G90801\_Mycgr3T

Mycgr3G84646 Mycgr3T
  
Location: 18156-20235

Mycgr3G84646\_Mycgr3T

Mycgr3G68456 Mycgr3T
  
Location: 20335-21970

Mycgr3G68456\_Mycgr3T

Mycgr3G103270 Mycgr3
  
Location: 22070-22355

Mycgr3G103270\_Mycgr3

Mycgr3G90803 Mycgr3T
  
Location: 22455-23019

Mycgr3G90803\_Mycgr3T

Mycgr3G36941 Mycgr3T
  
Location: 23119-24064

Mycgr3G36941\_Mycgr3T

Mycgr3G25746 Mycgr3T
  
Location: 24164-25241

Mycgr3G25746\_Mycgr3T

Mycgr3G90788 Mycgr3T
  
Location: 25341-25803

Mycgr3G90788\_Mycgr3T

Mycgr3G103260 Mycgr3
  
Location: 25903-26635

Mycgr3G103260\_Mycgr3

Mycgr3G84644 Mycgr3T
  
Location: 26735-28457

Mycgr3G84644\_Mycgr3T

Mycgr3G29227 Mycgr3T
  
Location: 28557-28863

Mycgr3G29227\_Mycgr3T

Mycgr3G36271 Mycgr3T
  
Location: 28963-29854

Mycgr3G36271\_Mycgr3T

Mycgr3G68433 Mycgr3T
  
Location: 29954-33041

Mycgr3G68433\_Mycgr3T

Mycgr3G79452 Mycgr3T
  
Location: 33141-33399

Mycgr3G79452\_Mycgr3T

Mycgr3G55345 Mycgr3T
  
Location: 33499-34126

Mycgr3G55345\_Mycgr3T

Mycgr3G103278 Mycgr3
  
Location: 34226-35195

Mycgr3G103278\_Mycgr3

Mycgr3G84654 Mycgr3T
  
Location: 35295-36630

Mycgr3G84654\_Mycgr3T

Mycgr3G108090 Mycgr3
  
Location: 36730-37591

Mycgr3G108090\_Mycgr3

Mycgr3G21922 Mycgr3T
  
Location: 37691-39149

Mycgr3G21922\_Mycgr3T

Mycgr3G99148 Mycgr3T
  
Location: 39249-42819

Mycgr3G99148\_Mycgr3T

chromosome segregation in meiosis protein 3
  
Accession: EAS30096
  
Location: 1097031-1098202
  
 NCBI BlastP on this gene

EAS30096

NADH-ubiquinone oxidoreductase 21 kDa subunit
  
Accession: EAS30095
  
Location: 1098653-1099478
  
 NCBI BlastP on this gene

EAS30095

tRNA specific adenosine deaminase
  
Accession: EAS30094
  
Location: 1099644-1100610
  
 NCBI BlastP on this gene

EAS30094

UVSB PI-3 kinase
  
Accession: EJB11175
  
Location: 1100872-1109613
  
 NCBI BlastP on this gene

EJB11175

hypothetical protein
  
Accession: EAS30091
  
Location: 1109928-1111029
  
 NCBI BlastP on this gene

EAS30091

AAA family ATPase
  
Accession: EAS30090
  
Location: 1112389-1114696
  
 NCBI BlastP on this gene

EAS30090

hypothetical protein
  
Accession: EAS30089
  
Location: 1115444-1116138
  
 NCBI BlastP on this gene

EAS30089

C-4 methyl sterol oxidase Erg25
  
Accession: EAS30088
  
Location: 1116619-1117947
  
  
**BlastP hit with Mycgr3G36271\_Mycgr3T**
  
Percentage identity: 77 %
  
BlastP bit score: 478
  
Sequence coverage: 97 %
  
E-value: 3e-166
  
  
 NCBI BlastP on this gene

EAS30088

hypothetical protein
  
Accession: EAS30087
  
Location: 1118363-1120570
  
  
**BlastP hit with Mycgr3G84646\_Mycgr3T**
  
Percentage identity: 40 %
  
BlastP bit score: 480
  
Sequence coverage: 100 %
  
E-value: 7e-157
  
  
 NCBI BlastP on this gene

EAS30087

GTP-binding protein ypt2
  
Accession: EAS30086
  
Location: 1121658-1122428
  
  
**BlastP hit with Mycgr3G99145\_Mycgr3T**
  
Percentage identity: 82 %
  
BlastP bit score: 350
  
Sequence coverage: 99 %
  
E-value: 6e-120
  
  
 NCBI BlastP on this gene

EAS30086

hypothetical protein
  
Accession: EAS30085
  
Location: 1123983-1124928
  
 NCBI BlastP on this gene

EAS30085

hypothetical protein
  
Accession: EJB11176
  
Location: 1125417-1126656
  
 NCBI BlastP on this gene

EJB11176

hypothetical protein
  
Accession: EAS30082
  
Location: 1127161-1127859
  
 NCBI BlastP on this gene

EAS30082

hypothetical protein
  
Accession: EAS30081
  
Location: 1128197-1129540
  
 NCBI BlastP on this gene

EAS30081

hypothetical protein
  
Accession: EAS30080
  
Location: 1129864-1130172
  
 NCBI BlastP on this gene

EAS30080

hypothetical protein
  
Accession: EJB11177
  
Location: 1131774-1132392
  
 NCBI BlastP on this gene

EJB11177

hypothetical protein
  
Accession: EAS30078
  
Location: 1133396-1134215
  
 NCBI BlastP on this gene

EAS30078

hypothetical protein
  
Accession: EJB11178
  
Location: 1134729-1136678
  
 NCBI BlastP on this gene

EJB11178

hypothetical protein
  
Accession: EAS30075
  
Location: 1138553-1139059
  
 NCBI BlastP on this gene

EAS30075

hypothetical protein
  
Accession: EAS30074
  
Location: 1140178-1140393
  
 NCBI BlastP on this gene

EAS30074

hypothetical protein
  
Accession: EAS30073
  
Location: 1140898-1147836
  
 NCBI BlastP on this gene

EAS30073

Query: Architecture Search FASTA input

GL385398 : Gaeumannomyces graminis var. tritici R3-111a-1 unplaced genomic scaffold supercont2.4    Total score: 3.0     Cumulative Blast bit score: 1302

Hit cluster cross-links:

Mycgr3G90785 Mycgr3T
  
Location: 0-1047

Mycgr3G90785\_Mycgr3T

Mycgr3G103262 Mycgr3
  
Location: 1147-1390

Mycgr3G103262\_Mycgr3

Mycgr3G68458 Mycgr3T
  
Location: 1490-3602

Mycgr3G68458\_Mycgr3T

Mycgr3G99145 Mycgr3T
  
Location: 3702-4326

Mycgr3G99145\_Mycgr3T

Mycgr3G103274 Mycgr3
  
Location: 4426-4957

Mycgr3G103274\_Mycgr3

Mycgr3G103264 Mycgr3
  
Location: 5057-5390

Mycgr3G103264\_Mycgr3

Mycgr3G37570 Mycgr3T
  
Location: 5490-6006

Mycgr3G37570\_Mycgr3T

Mycgr3G108094 Mycgr3
  
Location: 6106-10555

Mycgr3G108094\_Mycgr3

Mycgr3G90786 Mycgr3T
  
Location: 10655-12080

Mycgr3G90786\_Mycgr3T

Mycgr3G68429 Mycgr3T
  
Location: 12180-13440

Mycgr3G68429\_Mycgr3T

Mycgr3G68421 Mycgr3T
  
Location: 13540-17086

Mycgr3G68421\_Mycgr3T

Mycgr3G90801 Mycgr3T
  
Location: 17186-18056

Mycgr3G90801\_Mycgr3T

Mycgr3G84646 Mycgr3T
  
Location: 18156-20235

Mycgr3G84646\_Mycgr3T

Mycgr3G68456 Mycgr3T
  
Location: 20335-21970

Mycgr3G68456\_Mycgr3T

Mycgr3G103270 Mycgr3
  
Location: 22070-22355

Mycgr3G103270\_Mycgr3

Mycgr3G90803 Mycgr3T
  
Location: 22455-23019

Mycgr3G90803\_Mycgr3T

Mycgr3G36941 Mycgr3T
  
Location: 23119-24064

Mycgr3G36941\_Mycgr3T

Mycgr3G25746 Mycgr3T
  
Location: 24164-25241

Mycgr3G25746\_Mycgr3T

Mycgr3G90788 Mycgr3T
  
Location: 25341-25803

Mycgr3G90788\_Mycgr3T

Mycgr3G103260 Mycgr3
  
Location: 25903-26635

Mycgr3G103260\_Mycgr3

Mycgr3G84644 Mycgr3T
  
Location: 26735-28457

Mycgr3G84644\_Mycgr3T

Mycgr3G29227 Mycgr3T
  
Location: 28557-28863

Mycgr3G29227\_Mycgr3T

Mycgr3G36271 Mycgr3T
  
Location: 28963-29854

Mycgr3G36271\_Mycgr3T

Mycgr3G68433 Mycgr3T
  
Location: 29954-33041

Mycgr3G68433\_Mycgr3T

Mycgr3G79452 Mycgr3T
  
Location: 33141-33399

Mycgr3G79452\_Mycgr3T

Mycgr3G55345 Mycgr3T
  
Location: 33499-34126

Mycgr3G55345\_Mycgr3T

Mycgr3G103278 Mycgr3
  
Location: 34226-35195

Mycgr3G103278\_Mycgr3

Mycgr3G84654 Mycgr3T
  
Location: 35295-36630

Mycgr3G84654\_Mycgr3T

Mycgr3G108090 Mycgr3
  
Location: 36730-37591

Mycgr3G108090\_Mycgr3

Mycgr3G21922 Mycgr3T
  
Location: 37691-39149

Mycgr3G21922\_Mycgr3T

Mycgr3G99148 Mycgr3T
  
Location: 39249-42819

Mycgr3G99148\_Mycgr3T

hypothetical protein
  
Accession: EJT74551
  
Location: 2959309-2960184
  
 NCBI BlastP on this gene

EJT74551

hypothetical protein
  
Accession: EJT74550
  
Location: 2958573-2958923
  
 NCBI BlastP on this gene

EJT74550

hypothetical protein
  
Accession: EJT74549
  
Location: 2957630-2958151
  
 NCBI BlastP on this gene

EJT74549

hypothetical protein
  
Accession: EJT74548
  
Location: 2954701-2957216
  
 NCBI BlastP on this gene

EJT74548

hypothetical protein
  
Accession: EJT74547
  
Location: 2952815-2953827
  
 NCBI BlastP on this gene

EJT74547

hypothetical protein
  
Accession: EJT74546
  
Location: 2950610-2951321
  
 NCBI BlastP on this gene

EJT74546

hypothetical protein
  
Accession: EJT74545
  
Location: 2948742-2949155
  
 NCBI BlastP on this gene

EJT74545

hypothetical protein
  
Accession: EJT74544
  
Location: 2945869-2946496
  
 NCBI BlastP on this gene

EJT74544

hypothetical protein
  
Accession: EJT74543
  
Location: 2943356-2944649
  
 NCBI BlastP on this gene

EJT74543

C-4 methylsterol oxidase
  
Accession: EJT74542
  
Location: 2941856-2942979
  
  
**BlastP hit with Mycgr3G36271\_Mycgr3T**
  
Percentage identity: 70 %
  
BlastP bit score: 434
  
Sequence coverage: 93 %
  
E-value: 7e-150
  
  
 NCBI BlastP on this gene

EJT74542

hypothetical protein
  
Accession: EJT74541
  
Location: 2938286-2940411
  
  
**BlastP hit with Mycgr3G84646\_Mycgr3T**
  
Percentage identity: 42 %
  
BlastP bit score: 515
  
Sequence coverage: 102 %
  
E-value: 5e-170
  
  
 NCBI BlastP on this gene

EJT74541

GTP-binding protein ypt2
  
Accession: EJT74540
  
Location: 2935676-2936683
  
  
**BlastP hit with Mycgr3G99145\_Mycgr3T**
  
Percentage identity: 82 %
  
BlastP bit score: 353
  
Sequence coverage: 99 %
  
E-value: 5e-121
  
  
 NCBI BlastP on this gene

EJT74540

hypothetical protein
  
Accession: EJT74539
  
Location: 2932795-2934528
  
 NCBI BlastP on this gene

EJT74539

ATPase
  
Accession: EJT74538
  
Location: 2929456-2931831
  
 NCBI BlastP on this gene

EJT74538

dolichyl-phosphate beta-glucosyltransferase
  
Accession: EJT74537
  
Location: 2927702-2929062
  
 NCBI BlastP on this gene

EJT74537

hypothetical protein
  
Accession: EJT74536
  
Location: 2923498-2925173
  
 NCBI BlastP on this gene

EJT74536

Query: Architecture Search FASTA input

GL636502 : Coccidioides posadasii str. Silveira unplaced genomic scaffold supercont2.17    Total score: 3.0     Cumulative Blast bit score: 1299

Hit cluster cross-links:

Mycgr3G90785 Mycgr3T
  
Location: 0-1047

Mycgr3G90785\_Mycgr3T

Mycgr3G103262 Mycgr3
  
Location: 1147-1390

Mycgr3G103262\_Mycgr3

Mycgr3G68458 Mycgr3T
  
Location: 1490-3602

Mycgr3G68458\_Mycgr3T

Mycgr3G99145 Mycgr3T
  
Location: 3702-4326

Mycgr3G99145\_Mycgr3T

Mycgr3G103274 Mycgr3
  
Location: 4426-4957

Mycgr3G103274\_Mycgr3

Mycgr3G103264 Mycgr3
  
Location: 5057-5390

Mycgr3G103264\_Mycgr3

Mycgr3G37570 Mycgr3T
  
Location: 5490-6006

Mycgr3G37570\_Mycgr3T

Mycgr3G108094 Mycgr3
  
Location: 6106-10555

Mycgr3G108094\_Mycgr3

Mycgr3G90786 Mycgr3T
  
Location: 10655-12080

Mycgr3G90786\_Mycgr3T

Mycgr3G68429 Mycgr3T
  
Location: 12180-13440

Mycgr3G68429\_Mycgr3T

Mycgr3G68421 Mycgr3T
  
Location: 13540-17086

Mycgr3G68421\_Mycgr3T

Mycgr3G90801 Mycgr3T
  
Location: 17186-18056

Mycgr3G90801\_Mycgr3T

Mycgr3G84646 Mycgr3T
  
Location: 18156-20235

Mycgr3G84646\_Mycgr3T

Mycgr3G68456 Mycgr3T
  
Location: 20335-21970

Mycgr3G68456\_Mycgr3T

Mycgr3G103270 Mycgr3
  
Location: 22070-22355

Mycgr3G103270\_Mycgr3

Mycgr3G90803 Mycgr3T
  
Location: 22455-23019

Mycgr3G90803\_Mycgr3T

Mycgr3G36941 Mycgr3T
  
Location: 23119-24064

Mycgr3G36941\_Mycgr3T

Mycgr3G25746 Mycgr3T
  
Location: 24164-25241

Mycgr3G25746\_Mycgr3T

Mycgr3G90788 Mycgr3T
  
Location: 25341-25803

Mycgr3G90788\_Mycgr3T

Mycgr3G103260 Mycgr3
  
Location: 25903-26635

Mycgr3G103260\_Mycgr3

Mycgr3G84644 Mycgr3T
  
Location: 26735-28457

Mycgr3G84644\_Mycgr3T

Mycgr3G29227 Mycgr3T
  
Location: 28557-28863

Mycgr3G29227\_Mycgr3T

Mycgr3G36271 Mycgr3T
  
Location: 28963-29854

Mycgr3G36271\_Mycgr3T

Mycgr3G68433 Mycgr3T
  
Location: 29954-33041

Mycgr3G68433\_Mycgr3T

Mycgr3G79452 Mycgr3T
  
Location: 33141-33399

Mycgr3G79452\_Mycgr3T

Mycgr3G55345 Mycgr3T
  
Location: 33499-34126

Mycgr3G55345\_Mycgr3T

Mycgr3G103278 Mycgr3
  
Location: 34226-35195

Mycgr3G103278\_Mycgr3

Mycgr3G84654 Mycgr3T
  
Location: 35295-36630

Mycgr3G84654\_Mycgr3T

Mycgr3G108090 Mycgr3
  
Location: 36730-37591

Mycgr3G108090\_Mycgr3

Mycgr3G21922 Mycgr3T
  
Location: 37691-39149

Mycgr3G21922\_Mycgr3T

Mycgr3G99148 Mycgr3T
  
Location: 39249-42819

Mycgr3G99148\_Mycgr3T

chromosome segregation in meiosis protein 3
  
Accession: EFW15000
  
Location: 15288-16449
  
 NCBI BlastP on this gene

EFW15000

NADH-ubiquinone oxidoreductase 21 kDa subunit
  
Accession: EFW15001
  
Location: 16900-17729
  
 NCBI BlastP on this gene

EFW15001

tRNA-specific adenosine deaminase subunit TAD2
  
Accession: EFW15002
  
Location: 17895-18815
  
 NCBI BlastP on this gene

EFW15002

conserved hypothetical protein
  
Accession: EFW15003
  
Location: 19144-21761
  
 NCBI BlastP on this gene

EFW15003

conserved hypothetical protein
  
Accession: EFW15004
  
Location: 22411-27887
  
 NCBI BlastP on this gene

EFW15004

hypothetical protein
  
Accession: EFW15005
  
Location: 28216-29313
  
 NCBI BlastP on this gene

EFW15005

AAA family ATPase
  
Accession: EFW15006
  
Location: 30675-32982
  
 NCBI BlastP on this gene

EFW15006

conserved hypothetical protein
  
Accession: EFW15007
  
Location: 33732-34423
  
 NCBI BlastP on this gene

EFW15007

C-4 methylsterol oxidase
  
Accession: EFW15008
  
Location: 34904-35990
  
  
**BlastP hit with Mycgr3G36271\_Mycgr3T**
  
Percentage identity: 77 %
  
BlastP bit score: 476
  
Sequence coverage: 97 %
  
E-value: 1e-166
  
  
 NCBI BlastP on this gene

EFW15008

conserved hypothetical protein
  
Accession: EFW15009
  
Location: 36652-38859
  
  
**BlastP hit with Mycgr3G84646\_Mycgr3T**
  
Percentage identity: 39 %
  
BlastP bit score: 473
  
Sequence coverage: 100 %
  
E-value: 3e-154
  
  
 NCBI BlastP on this gene

EFW15009

GTP-binding protein SAS1
  
Accession: EFW15010
  
Location: 39970-40740
  
  
**BlastP hit with Mycgr3G99145\_Mycgr3T**
  
Percentage identity: 82 %
  
BlastP bit score: 350
  
Sequence coverage: 99 %
  
E-value: 6e-120
  
  
 NCBI BlastP on this gene

EFW15010

hypothetical protein
  
Accession: EFW15011
  
Location: 43776-45015
  
 NCBI BlastP on this gene

EFW15011

predicted protein
  
Accession: EFW15012
  
Location: 45524-46225
  
 NCBI BlastP on this gene

EFW15012

conserved hypothetical protein
  
Accession: EFW15013
  
Location: 46558-47709
  
 NCBI BlastP on this gene

EFW15013

predicted protein
  
Accession: EFW15014
  
Location: 48198-48935
  
 NCBI BlastP on this gene

EFW15014

predicted protein
  
Accession: EFW15015
  
Location: 50423-50779
  
 NCBI BlastP on this gene

EFW15015

FCF1 small subunit
  
Accession: EFW15016
  
Location: 51932-52855
  
 NCBI BlastP on this gene

EFW15016

conserved hypothetical protein
  
Accession: EFW15017
  
Location: 53849-55471
  
 NCBI BlastP on this gene

EFW15017

conserved hypothetical protein
  
Accession: EFW15018
  
Location: 56781-57200
  
 NCBI BlastP on this gene

EFW15018

hypothetical protein
  
Accession: EFW15019
  
Location: 58985-66023
  
 NCBI BlastP on this gene

EFW15019

Query: Architecture Search FASTA input

ACFW01000015 : Coccidioides posadasii C735 delta SOWgp    Total score: 3.0     Cumulative Blast bit score: 1299

Hit cluster cross-links:

Mycgr3G90785 Mycgr3T
  
Location: 0-1047

Mycgr3G90785\_Mycgr3T

Mycgr3G103262 Mycgr3
  
Location: 1147-1390

Mycgr3G103262\_Mycgr3

Mycgr3G68458 Mycgr3T
  
Location: 1490-3602

Mycgr3G68458\_Mycgr3T

Mycgr3G99145 Mycgr3T
  
Location: 3702-4326

Mycgr3G99145\_Mycgr3T

Mycgr3G103274 Mycgr3
  
Location: 4426-4957

Mycgr3G103274\_Mycgr3

Mycgr3G103264 Mycgr3
  
Location: 5057-5390

Mycgr3G103264\_Mycgr3

Mycgr3G37570 Mycgr3T
  
Location: 5490-6006

Mycgr3G37570\_Mycgr3T

Mycgr3G108094 Mycgr3
  
Location: 6106-10555

Mycgr3G108094\_Mycgr3

Mycgr3G90786 Mycgr3T
  
Location: 10655-12080

Mycgr3G90786\_Mycgr3T

Mycgr3G68429 Mycgr3T
  
Location: 12180-13440

Mycgr3G68429\_Mycgr3T

Mycgr3G68421 Mycgr3T
  
Location: 13540-17086

Mycgr3G68421\_Mycgr3T

Mycgr3G90801 Mycgr3T
  
Location: 17186-18056

Mycgr3G90801\_Mycgr3T

Mycgr3G84646 Mycgr3T
  
Location: 18156-20235

Mycgr3G84646\_Mycgr3T

Mycgr3G68456 Mycgr3T
  
Location: 20335-21970

Mycgr3G68456\_Mycgr3T

Mycgr3G103270 Mycgr3
  
Location: 22070-22355

Mycgr3G103270\_Mycgr3

Mycgr3G90803 Mycgr3T
  
Location: 22455-23019

Mycgr3G90803\_Mycgr3T

Mycgr3G36941 Mycgr3T
  
Location: 23119-24064

Mycgr3G36941\_Mycgr3T

Mycgr3G25746 Mycgr3T
  
Location: 24164-25241

Mycgr3G25746\_Mycgr3T

Mycgr3G90788 Mycgr3T
  
Location: 25341-25803

Mycgr3G90788\_Mycgr3T

Mycgr3G103260 Mycgr3
  
Location: 25903-26635

Mycgr3G103260\_Mycgr3

Mycgr3G84644 Mycgr3T
  
Location: 26735-28457

Mycgr3G84644\_Mycgr3T

Mycgr3G29227 Mycgr3T
  
Location: 28557-28863

Mycgr3G29227\_Mycgr3T

Mycgr3G36271 Mycgr3T
  
Location: 28963-29854

Mycgr3G36271\_Mycgr3T

Mycgr3G68433 Mycgr3T
  
Location: 29954-33041

Mycgr3G68433\_Mycgr3T

Mycgr3G79452 Mycgr3T
  
Location: 33141-33399

Mycgr3G79452\_Mycgr3T

Mycgr3G55345 Mycgr3T
  
Location: 33499-34126

Mycgr3G55345\_Mycgr3T

Mycgr3G103278 Mycgr3
  
Location: 34226-35195

Mycgr3G103278\_Mycgr3

Mycgr3G84654 Mycgr3T
  
Location: 35295-36630

Mycgr3G84654\_Mycgr3T

Mycgr3G108090 Mycgr3
  
Location: 36730-37591

Mycgr3G108090\_Mycgr3

Mycgr3G21922 Mycgr3T
  
Location: 37691-39149

Mycgr3G21922\_Mycgr3T

Mycgr3G99148 Mycgr3T
  
Location: 39249-42819

Mycgr3G99148\_Mycgr3T

hypothetical protein
  
Accession: EER28304
  
Location: 362297-363458
  
 NCBI BlastP on this gene

EER28304

NADH-ubiquinone oxidoreductase 21 kDa subunit, putative
  
Accession: EER28303
  
Location: 361017-361846
  
 NCBI BlastP on this gene

EER28303

Cytidine and deoxycytidylate deaminase zinc-binding domain containing protein
  
Accession: EER28302
  
Location: 359934-360851
  
 NCBI BlastP on this gene

EER28302

DNA repair protein, putative
  
Accession: EER28301
  
Location: 350859-359602
  
 NCBI BlastP on this gene

EER28301

ATPase, AAA family protein
  
Accession: EER28300
  
Location: 345755-348062
  
 NCBI BlastP on this gene

EER28300

pterin-4-alpha-carbinolamine dehydratase family protein
  
Accession: EER28299
  
Location: 344314-345005
  
 NCBI BlastP on this gene

EER28299

C-4 methylsterol oxidase, putative
  
Accession: EER28298
  
Location: 342741-343833
  
  
**BlastP hit with Mycgr3G36271\_Mycgr3T**
  
Percentage identity: 77 %
  
BlastP bit score: 476
  
Sequence coverage: 97 %
  
E-value: 1e-166
  
  
 NCBI BlastP on this gene

EER28298

hypothetical protein
  
Accession: EER28297
  
Location: 339872-342079
  
  
**BlastP hit with Mycgr3G84646\_Mycgr3T**
  
Percentage identity: 39 %
  
BlastP bit score: 473
  
Sequence coverage: 100 %
  
E-value: 3e-154
  
  
 NCBI BlastP on this gene

EER28297

Ras family protein
  
Accession: EER28296
  
Location: 337980-338750
  
  
**BlastP hit with Mycgr3G99145\_Mycgr3T**
  
Percentage identity: 82 %
  
BlastP bit score: 350
  
Sequence coverage: 99 %
  
E-value: 6e-120
  
  
 NCBI BlastP on this gene

EER28296

copia-like retrotransposon family protein
  
Accession: EER28295
  
Location: 331882-336498
  
 NCBI BlastP on this gene

EER28295

hypothetical protein
  
Accession: EER28294
  
Location: 328387-329626
  
 NCBI BlastP on this gene

EER28294

hypothetical protein
  
Accession: EER28293
  
Location: 327177-327878
  
 NCBI BlastP on this gene

EER28293

hypothetical protein
  
Accession: EER28292
  
Location: 325672-326844
  
 NCBI BlastP on this gene

EER28292

hypothetical protein
  
Accession: EER28291
  
Location: 320549-321472
  
 NCBI BlastP on this gene

EER28291

Query: Architecture Search FASTA input

KB446566 : Pseudocercospora fijiensis CIRAD86 unplaced genomic scaffold MYCFIscaffold\_12    Total score: 3.0     Cumulative Blast bit score: 1294

Hit cluster cross-links:

Mycgr3G90785 Mycgr3T
  
Location: 0-1047

Mycgr3G90785\_Mycgr3T

Mycgr3G103262 Mycgr3
  
Location: 1147-1390

Mycgr3G103262\_Mycgr3

Mycgr3G68458 Mycgr3T
  
Location: 1490-3602

Mycgr3G68458\_Mycgr3T

Mycgr3G99145 Mycgr3T
  
Location: 3702-4326

Mycgr3G99145\_Mycgr3T

Mycgr3G103274 Mycgr3
  
Location: 4426-4957

Mycgr3G103274\_Mycgr3

Mycgr3G103264 Mycgr3
  
Location: 5057-5390

Mycgr3G103264\_Mycgr3

Mycgr3G37570 Mycgr3T
  
Location: 5490-6006

Mycgr3G37570\_Mycgr3T

Mycgr3G108094 Mycgr3
  
Location: 6106-10555

Mycgr3G108094\_Mycgr3

Mycgr3G90786 Mycgr3T
  
Location: 10655-12080

Mycgr3G90786\_Mycgr3T

Mycgr3G68429 Mycgr3T
  
Location: 12180-13440

Mycgr3G68429\_Mycgr3T

Mycgr3G68421 Mycgr3T
  
Location: 13540-17086

Mycgr3G68421\_Mycgr3T

Mycgr3G90801 Mycgr3T
  
Location: 17186-18056

Mycgr3G90801\_Mycgr3T

Mycgr3G84646 Mycgr3T
  
Location: 18156-20235

Mycgr3G84646\_Mycgr3T

Mycgr3G68456 Mycgr3T
  
Location: 20335-21970

Mycgr3G68456\_Mycgr3T

Mycgr3G103270 Mycgr3
  
Location: 22070-22355

Mycgr3G103270\_Mycgr3

Mycgr3G90803 Mycgr3T
  
Location: 22455-23019

Mycgr3G90803\_Mycgr3T

Mycgr3G36941 Mycgr3T
  
Location: 23119-24064

Mycgr3G36941\_Mycgr3T

Mycgr3G25746 Mycgr3T
  
Location: 24164-25241

Mycgr3G25746\_Mycgr3T

Mycgr3G90788 Mycgr3T
  
Location: 25341-25803

Mycgr3G90788\_Mycgr3T

Mycgr3G103260 Mycgr3
  
Location: 25903-26635

Mycgr3G103260\_Mycgr3

Mycgr3G84644 Mycgr3T
  
Location: 26735-28457

Mycgr3G84644\_Mycgr3T

Mycgr3G29227 Mycgr3T
  
Location: 28557-28863

Mycgr3G29227\_Mycgr3T

Mycgr3G36271 Mycgr3T
  
Location: 28963-29854

Mycgr3G36271\_Mycgr3T

Mycgr3G68433 Mycgr3T
  
Location: 29954-33041

Mycgr3G68433\_Mycgr3T

Mycgr3G79452 Mycgr3T
  
Location: 33141-33399

Mycgr3G79452\_Mycgr3T

Mycgr3G55345 Mycgr3T
  
Location: 33499-34126

Mycgr3G55345\_Mycgr3T

Mycgr3G103278 Mycgr3
  
Location: 34226-35195

Mycgr3G103278\_Mycgr3

Mycgr3G84654 Mycgr3T
  
Location: 35295-36630

Mycgr3G84654\_Mycgr3T

Mycgr3G108090 Mycgr3
  
Location: 36730-37591

Mycgr3G108090\_Mycgr3

Mycgr3G21922 Mycgr3T
  
Location: 37691-39149

Mycgr3G21922\_Mycgr3T

Mycgr3G99148 Mycgr3T
  
Location: 39249-42819

Mycgr3G99148\_Mycgr3T

glycoside hydrolase family 13 protein
  
Accession: EME77258
  
Location: 284353-285861
  
 NCBI BlastP on this gene

EME77258

hypothetical protein
  
Accession: EME77257
  
Location: 272383-276236
  
 NCBI BlastP on this gene

EME77257

serine/threonine protein kinase
  
Accession: EME77256
  
Location: 269951-271369
  
  
**BlastP hit with Mycgr3G103260\_Mycgr3**
  
Percentage identity: 74 %
  
BlastP bit score: 392
  
Sequence coverage: 100 %
  
E-value: 9e-132
  
  
 NCBI BlastP on this gene

EME77256

hypothetical protein
  
Accession: EME77255
  
Location: 268919-269143
  
 NCBI BlastP on this gene

EME77255

hypothetical protein
  
Accession: EME77254
  
Location: 259305-260315
  
  
**BlastP hit with Mycgr3G68429\_Mycgr3T**
  
Percentage identity: 83 %
  
BlastP bit score: 558
  
Sequence coverage: 75 %
  
E-value: 0.0
  
  
 NCBI BlastP on this gene

EME77254

hypothetical protein
  
Accession: EME77253
  
Location: 255336-255506
  
 NCBI BlastP on this gene

EME77253

hypothetical protein
  
Accession: EME77252
  
Location: 253586-254708
  
 NCBI BlastP on this gene

EME77252

hypothetical protein
  
Accession: EME77251
  
Location: 252128-253563
  
 NCBI BlastP on this gene

EME77251

hypothetical protein
  
Accession: EME77250
  
Location: 251044-251824
  
  
**BlastP hit with Mycgr3G55345\_Mycgr3T**
  
Percentage identity: 81 %
  
BlastP bit score: 344
  
Sequence coverage: 97 %
  
E-value: 2e-117
  
  
 NCBI BlastP on this gene

EME77250

hypothetical protein
  
Accession: EME77249
  
Location: 248686-249795
  
 NCBI BlastP on this gene

EME77249

hypothetical protein
  
Accession: EME77248
  
Location: 243650-245851
  
 NCBI BlastP on this gene

EME77248

hypothetical protein
  
Accession: EME77247
  
Location: 242456-243393
  
 NCBI BlastP on this gene

EME77247

hypothetical protein
  
Accession: EME77246
  
Location: 241602-242410
  
 NCBI BlastP on this gene

EME77246

hypothetical protein
  
Accession: EME77245
  
Location: 240045-241434
  
 NCBI BlastP on this gene

EME77245

Query: Architecture Search FASTA input

CABT02000002 : Sordaria macrospora k-hell    Total score: 3.0     Cumulative Blast bit score: 1287

Hit cluster cross-links:

Mycgr3G90785 Mycgr3T
  
Location: 0-1047

Mycgr3G90785\_Mycgr3T

Mycgr3G103262 Mycgr3
  
Location: 1147-1390

Mycgr3G103262\_Mycgr3

Mycgr3G68458 Mycgr3T
  
Location: 1490-3602

Mycgr3G68458\_Mycgr3T

Mycgr3G99145 Mycgr3T
  
Location: 3702-4326

Mycgr3G99145\_Mycgr3T

Mycgr3G103274 Mycgr3
  
Location: 4426-4957

Mycgr3G103274\_Mycgr3

Mycgr3G103264 Mycgr3
  
Location: 5057-5390

Mycgr3G103264\_Mycgr3

Mycgr3G37570 Mycgr3T
  
Location: 5490-6006

Mycgr3G37570\_Mycgr3T

Mycgr3G108094 Mycgr3
  
Location: 6106-10555

Mycgr3G108094\_Mycgr3

Mycgr3G90786 Mycgr3T
  
Location: 10655-12080

Mycgr3G90786\_Mycgr3T

Mycgr3G68429 Mycgr3T
  
Location: 12180-13440

Mycgr3G68429\_Mycgr3T

Mycgr3G68421 Mycgr3T
  
Location: 13540-17086

Mycgr3G68421\_Mycgr3T

Mycgr3G90801 Mycgr3T
  
Location: 17186-18056

Mycgr3G90801\_Mycgr3T

Mycgr3G84646 Mycgr3T
  
Location: 18156-20235

Mycgr3G84646\_Mycgr3T

Mycgr3G68456 Mycgr3T
  
Location: 20335-21970

Mycgr3G68456\_Mycgr3T

Mycgr3G103270 Mycgr3
  
Location: 22070-22355

Mycgr3G103270\_Mycgr3

Mycgr3G90803 Mycgr3T
  
Location: 22455-23019

Mycgr3G90803\_Mycgr3T

Mycgr3G36941 Mycgr3T
  
Location: 23119-24064

Mycgr3G36941\_Mycgr3T

Mycgr3G25746 Mycgr3T
  
Location: 24164-25241

Mycgr3G25746\_Mycgr3T

Mycgr3G90788 Mycgr3T
  
Location: 25341-25803

Mycgr3G90788\_Mycgr3T

Mycgr3G103260 Mycgr3
  
Location: 25903-26635

Mycgr3G103260\_Mycgr3

Mycgr3G84644 Mycgr3T
  
Location: 26735-28457

Mycgr3G84644\_Mycgr3T

Mycgr3G29227 Mycgr3T
  
Location: 28557-28863

Mycgr3G29227\_Mycgr3T

Mycgr3G36271 Mycgr3T
  
Location: 28963-29854

Mycgr3G36271\_Mycgr3T

Mycgr3G68433 Mycgr3T
  
Location: 29954-33041

Mycgr3G68433\_Mycgr3T

Mycgr3G79452 Mycgr3T
  
Location: 33141-33399

Mycgr3G79452\_Mycgr3T

Mycgr3G55345 Mycgr3T
  
Location: 33499-34126

Mycgr3G55345\_Mycgr3T

Mycgr3G103278 Mycgr3
  
Location: 34226-35195

Mycgr3G103278\_Mycgr3

Mycgr3G84654 Mycgr3T
  
Location: 35295-36630

Mycgr3G84654\_Mycgr3T

Mycgr3G108090 Mycgr3
  
Location: 36730-37591

Mycgr3G108090\_Mycgr3

Mycgr3G21922 Mycgr3T
  
Location: 37691-39149

Mycgr3G21922\_Mycgr3T

Mycgr3G99148 Mycgr3T
  
Location: 39249-42819

Mycgr3G99148\_Mycgr3T

not annotated
  
Accession: CCC06934
  
Location: 1270485-1271599
  
 NCBI BlastP on this gene

CCC06934

not annotated
  
Accession: CCC06933
  
Location: 1264410-1267328
  
 NCBI BlastP on this gene

CCC06933

not annotated
  
Accession: CCC06932
  
Location: 1261886-1262416
  
 NCBI BlastP on this gene

CCC06932

not annotated
  
Accession: CCC06931
  
Location: 1258586-1260083
  
 NCBI BlastP on this gene

CCC06931

not annotated
  
Accession: CCC06930
  
Location: 1254883-1255982
  
 NCBI BlastP on this gene

CCC06930

not annotated
  
Accession: CCC06929
  
Location: 1252856-1254081
  
 NCBI BlastP on this gene

CCC06929

not annotated
  
Accession: CCC06928
  
Location: 1251128-1252266
  
  
**BlastP hit with Mycgr3G36271\_Mycgr3T**
  
Percentage identity: 72 %
  
BlastP bit score: 437
  
Sequence coverage: 94 %
  
E-value: 5e-151
  
  
 NCBI BlastP on this gene

CCC06928

not annotated
  
Accession: CCC06927
  
Location: 1246865-1249055
  
  
**BlastP hit with Mycgr3G84646\_Mycgr3T**
  
Percentage identity: 41 %
  
BlastP bit score: 488
  
Sequence coverage: 101 %
  
E-value: 1e-159
  
  
 NCBI BlastP on this gene

CCC06927

not annotated
  
Accession: CCC06926
  
Location: 1243977-1244948
  
  
**BlastP hit with Mycgr3G99145\_Mycgr3T**
  
Percentage identity: 84 %
  
BlastP bit score: 362
  
Sequence coverage: 99 %
  
E-value: 2e-124
  
  
 NCBI BlastP on this gene

CCC06926

not annotated
  
Accession: CCC06925
  
Location: 1236346-1242626
  
 NCBI BlastP on this gene

CCC06925

not annotated
  
Accession: CCC06924
  
Location: 1234970-1236190
  
 NCBI BlastP on this gene

CCC06924

not annotated
  
Accession: CCC06923
  
Location: 1233359-1234319
  
 NCBI BlastP on this gene

CCC06923

not annotated
  
Accession: CCC06922
  
Location: 1227748-1232623
  
 NCBI BlastP on this gene

CCC06922

not annotated
  
Accession: CCC06921
  
Location: 1224908-1226006
  
 NCBI BlastP on this gene

CCC06921

Query: Architecture Search FASTA input

DS572750 : Paracoccidioides brasiliensis Pb18 supercont1.1 genomic scaffold    Total score: 3.0     Cumulative Blast bit score: 1279

Hit cluster cross-links:

Mycgr3G90785 Mycgr3T
  
Location: 0-1047

Mycgr3G90785\_Mycgr3T

Mycgr3G103262 Mycgr3
  
Location: 1147-1390

Mycgr3G103262\_Mycgr3

Mycgr3G68458 Mycgr3T
  
Location: 1490-3602

Mycgr3G68458\_Mycgr3T

Mycgr3G99145 Mycgr3T
  
Location: 3702-4326

Mycgr3G99145\_Mycgr3T

Mycgr3G103274 Mycgr3
  
Location: 4426-4957

Mycgr3G103274\_Mycgr3

Mycgr3G103264 Mycgr3
  
Location: 5057-5390

Mycgr3G103264\_Mycgr3

Mycgr3G37570 Mycgr3T
  
Location: 5490-6006

Mycgr3G37570\_Mycgr3T

Mycgr3G108094 Mycgr3
  
Location: 6106-10555

Mycgr3G108094\_Mycgr3

Mycgr3G90786 Mycgr3T
  
Location: 10655-12080

Mycgr3G90786\_Mycgr3T

Mycgr3G68429 Mycgr3T
  
Location: 12180-13440

Mycgr3G68429\_Mycgr3T

Mycgr3G68421 Mycgr3T
  
Location: 13540-17086

Mycgr3G68421\_Mycgr3T

Mycgr3G90801 Mycgr3T
  
Location: 17186-18056

Mycgr3G90801\_Mycgr3T

Mycgr3G84646 Mycgr3T
  
Location: 18156-20235

Mycgr3G84646\_Mycgr3T

Mycgr3G68456 Mycgr3T
  
Location: 20335-21970

Mycgr3G68456\_Mycgr3T

Mycgr3G103270 Mycgr3
  
Location: 22070-22355

Mycgr3G103270\_Mycgr3

Mycgr3G90803 Mycgr3T
  
Location: 22455-23019

Mycgr3G90803\_Mycgr3T

Mycgr3G36941 Mycgr3T
  
Location: 23119-24064

Mycgr3G36941\_Mycgr3T

Mycgr3G25746 Mycgr3T
  
Location: 24164-25241

Mycgr3G25746\_Mycgr3T

Mycgr3G90788 Mycgr3T
  
Location: 25341-25803

Mycgr3G90788\_Mycgr3T

Mycgr3G103260 Mycgr3
  
Location: 25903-26635

Mycgr3G103260\_Mycgr3

Mycgr3G84644 Mycgr3T
  
Location: 26735-28457

Mycgr3G84644\_Mycgr3T

Mycgr3G29227 Mycgr3T
  
Location: 28557-28863

Mycgr3G29227\_Mycgr3T

Mycgr3G36271 Mycgr3T
  
Location: 28963-29854

Mycgr3G36271\_Mycgr3T

Mycgr3G68433 Mycgr3T
  
Location: 29954-33041

Mycgr3G68433\_Mycgr3T

Mycgr3G79452 Mycgr3T
  
Location: 33141-33399

Mycgr3G79452\_Mycgr3T

Mycgr3G55345 Mycgr3T
  
Location: 33499-34126

Mycgr3G55345\_Mycgr3T

Mycgr3G103278 Mycgr3
  
Location: 34226-35195

Mycgr3G103278\_Mycgr3

Mycgr3G84654 Mycgr3T
  
Location: 35295-36630

Mycgr3G84654\_Mycgr3T

Mycgr3G108090 Mycgr3
  
Location: 36730-37591

Mycgr3G108090\_Mycgr3

Mycgr3G21922 Mycgr3T
  
Location: 37691-39149

Mycgr3G21922\_Mycgr3T

Mycgr3G99148 Mycgr3T
  
Location: 39249-42819

Mycgr3G99148\_Mycgr3T

conserved hypothetical protein
  
Accession: EEH43980
  
Location: 898018-901203
  
 NCBI BlastP on this gene

EEH43980

conserved hypothetical protein
  
Accession: EEH43981
  
Location: 902421-903206
  
 NCBI BlastP on this gene

EEH43981

conserved hypothetical protein
  
Accession: EEH43982
  
Location: 903558-905249
  
 NCBI BlastP on this gene

EEH43982

rRNA-processing protein FCF1
  
Accession: EEH43983
  
Location: 906944-907934
  
 NCBI BlastP on this gene

EEH43983

hypothetical protein
  
Accession: EEH43984
  
Location: 908618-909670
  
 NCBI BlastP on this gene

EEH43984

C-4 methylsterol oxidase
  
Accession: EEH43985
  
Location: 910552-912112
  
  
**BlastP hit with Mycgr3G36271\_Mycgr3T**
  
Percentage identity: 73 %
  
BlastP bit score: 457
  
Sequence coverage: 97 %
  
E-value: 5e-159
  
  
 NCBI BlastP on this gene

EEH43985

predicted protein
  
Accession: EEH43986
  
Location: 916982-917251
  
 NCBI BlastP on this gene

EEH43986

conserved hypothetical protein
  
Accession: EEH43987
  
Location: 918373-919149
  
 NCBI BlastP on this gene

EEH43987

conserved hypothetical protein
  
Accession: EEH43988
  
Location: 919452-921521
  
 NCBI BlastP on this gene

EEH43988

hypothetical protein
  
Accession: EEH43989
  
Location: 922806-926903
  
 NCBI BlastP on this gene

EEH43989

conserved hypothetical protein
  
Accession: EEH43990
  
Location: 927224-928561
  
 NCBI BlastP on this gene

EEH43990

conserved hypothetical protein
  
Accession: EEH43991
  
Location: 929126-931594
  
  
**BlastP hit with Mycgr3G84646\_Mycgr3T**
  
Percentage identity: 40 %
  
BlastP bit score: 476
  
Sequence coverage: 104 %
  
E-value: 8e-155
  
  
 NCBI BlastP on this gene

EEH43991

conserved hypothetical protein
  
Accession: EEH43992
  
Location: 934598-935938
  
 NCBI BlastP on this gene

EEH43992

GTP-binding protein SAS1
  
Accession: EEH43993
  
Location: 937234-938101
  
  
**BlastP hit with Mycgr3G99145\_Mycgr3T**
  
Percentage identity: 83 %
  
BlastP bit score: 346
  
Sequence coverage: 99 %
  
E-value: 2e-118
  
  
 NCBI BlastP on this gene

EEH43993

conserved hypothetical protein
  
Accession: EEH43994
  
Location: 939267-940299
  
 NCBI BlastP on this gene

EEH43994

AAA family ATPase
  
Accession: EEH43995
  
Location: 940941-943289
  
 NCBI BlastP on this gene

EEH43995

hypothetical protein
  
Accession: EEH43996
  
Location: 943683-944940
  
 NCBI BlastP on this gene

EEH43996

conserved hypothetical protein
  
Accession: EEH43997
  
Location: 945424-954064
  
 NCBI BlastP on this gene

EEH43997

Query: Architecture Search FASTA input

GL891303 : Neurospora tetrasperma FGSC 2508 unplaced genomic scaffold NEUTE1scaffold\_2    Total score: 3.0     Cumulative Blast bit score: 1277

Hit cluster cross-links:

Mycgr3G90785 Mycgr3T
  
Location: 0-1047

Mycgr3G90785\_Mycgr3T

Mycgr3G103262 Mycgr3
  
Location: 1147-1390

Mycgr3G103262\_Mycgr3

Mycgr3G68458 Mycgr3T
  
Location: 1490-3602

Mycgr3G68458\_Mycgr3T

Mycgr3G99145 Mycgr3T
  
Location: 3702-4326

Mycgr3G99145\_Mycgr3T

Mycgr3G103274 Mycgr3
  
Location: 4426-4957

Mycgr3G103274\_Mycgr3

Mycgr3G103264 Mycgr3
  
Location: 5057-5390

Mycgr3G103264\_Mycgr3

Mycgr3G37570 Mycgr3T
  
Location: 5490-6006

Mycgr3G37570\_Mycgr3T

Mycgr3G108094 Mycgr3
  
Location: 6106-10555

Mycgr3G108094\_Mycgr3

Mycgr3G90786 Mycgr3T
  
Location: 10655-12080

Mycgr3G90786\_Mycgr3T

Mycgr3G68429 Mycgr3T
  
Location: 12180-13440

Mycgr3G68429\_Mycgr3T

Mycgr3G68421 Mycgr3T
  
Location: 13540-17086

Mycgr3G68421\_Mycgr3T

Mycgr3G90801 Mycgr3T
  
Location: 17186-18056

Mycgr3G90801\_Mycgr3T

Mycgr3G84646 Mycgr3T
  
Location: 18156-20235

Mycgr3G84646\_Mycgr3T

Mycgr3G68456 Mycgr3T
  
Location: 20335-21970

Mycgr3G68456\_Mycgr3T

Mycgr3G103270 Mycgr3
  
Location: 22070-22355

Mycgr3G103270\_Mycgr3

Mycgr3G90803 Mycgr3T
  
Location: 22455-23019

Mycgr3G90803\_Mycgr3T

Mycgr3G36941 Mycgr3T
  
Location: 23119-24064

Mycgr3G36941\_Mycgr3T

Mycgr3G25746 Mycgr3T
  
Location: 24164-25241

Mycgr3G25746\_Mycgr3T

Mycgr3G90788 Mycgr3T
  
Location: 25341-25803

Mycgr3G90788\_Mycgr3T

Mycgr3G103260 Mycgr3
  
Location: 25903-26635

Mycgr3G103260\_Mycgr3

Mycgr3G84644 Mycgr3T
  
Location: 26735-28457

Mycgr3G84644\_Mycgr3T

Mycgr3G29227 Mycgr3T
  
Location: 28557-28863

Mycgr3G29227\_Mycgr3T

Mycgr3G36271 Mycgr3T
  
Location: 28963-29854

Mycgr3G36271\_Mycgr3T

Mycgr3G68433 Mycgr3T
  
Location: 29954-33041

Mycgr3G68433\_Mycgr3T

Mycgr3G79452 Mycgr3T
  
Location: 33141-33399

Mycgr3G79452\_Mycgr3T

Mycgr3G55345 Mycgr3T
  
Location: 33499-34126

Mycgr3G55345\_Mycgr3T

Mycgr3G103278 Mycgr3
  
Location: 34226-35195

Mycgr3G103278\_Mycgr3

Mycgr3G84654 Mycgr3T
  
Location: 35295-36630

Mycgr3G84654\_Mycgr3T

Mycgr3G108090 Mycgr3
  
Location: 36730-37591

Mycgr3G108090\_Mycgr3

Mycgr3G21922 Mycgr3T
  
Location: 37691-39149

Mycgr3G21922\_Mycgr3T

Mycgr3G99148 Mycgr3T
  
Location: 39249-42819

Mycgr3G99148\_Mycgr3T

hypothetical protein
  
Accession: EGO59159
  
Location: 2479089-2479721
  
 NCBI BlastP on this gene

EGO59159

profilin
  
Accession: EGO59158
  
Location: 2475174-2476263
  
 NCBI BlastP on this gene

EGO59158

hypothetical protein
  
Accession: EGO59157
  
Location: 2469696-2472251
  
 NCBI BlastP on this gene

EGO59157

hypothetical protein
  
Accession: EGO59156
  
Location: 2467032-2467942
  
 NCBI BlastP on this gene

EGO59156

hypothetical protein
  
Accession: EGO59155
  
Location: 2462720-2464177
  
 NCBI BlastP on this gene

EGO59155

C-4 methylsterol oxidase, variant
  
Accession: EGO59154
  
Location: 2458783-2459902
  
  
**BlastP hit with Mycgr3G36271\_Mycgr3T**
  
Percentage identity: 71 %
  
BlastP bit score: 435
  
Sequence coverage: 93 %
  
E-value: 2e-150
  
  
 NCBI BlastP on this gene

EGO59154

hypothetical protein
  
Accession: EGO59153
  
Location: 2454656-2456834
  
  
**BlastP hit with Mycgr3G84646\_Mycgr3T**
  
Percentage identity: 41 %
  
BlastP bit score: 487
  
Sequence coverage: 101 %
  
E-value: 2e-159
  
  
 NCBI BlastP on this gene

EGO59153

GTP-binding protein SAS1
  
Accession: EGO59152
  
Location: 2451741-2452697
  
  
**BlastP hit with Mycgr3G99145\_Mycgr3T**
  
Percentage identity: 88 %
  
BlastP bit score: 355
  
Sequence coverage: 92 %
  
E-value: 1e-121
  
  
 NCBI BlastP on this gene

EGO59152

activating signal cointegrator 1 complex subunit 3
  
Accession: EGO59151
  
Location: 2444132-2450399
  
 NCBI BlastP on this gene

EGO59151

pyridoxamine 5'-phosphate oxidase
  
Accession: EGO59150
  
Location: 2440958-2441826
  
 NCBI BlastP on this gene

EGO59150

hypothetical protein
  
Accession: EGO59149
  
Location: 2435390-2440255
  
 NCBI BlastP on this gene

EGO59149

hypothetical protein
  
Accession: EGO59148
  
Location: 2432505-2434137
  
 NCBI BlastP on this gene

EGO59148

Query: Architecture Search FASTA input

GL891217 : Neurospora tetrasperma FGSC 2509 unplaced genomic scaffold NEUTE2scaffold\_3    Total score: 3.0     Cumulative Blast bit score: 1277

Hit cluster cross-links:

Mycgr3G90785 Mycgr3T
  
Location: 0-1047

Mycgr3G90785\_Mycgr3T

Mycgr3G103262 Mycgr3
  
Location: 1147-1390

Mycgr3G103262\_Mycgr3

Mycgr3G68458 Mycgr3T
  
Location: 1490-3602

Mycgr3G68458\_Mycgr3T

Mycgr3G99145 Mycgr3T
  
Location: 3702-4326

Mycgr3G99145\_Mycgr3T

Mycgr3G103274 Mycgr3
  
Location: 4426-4957

Mycgr3G103274\_Mycgr3

Mycgr3G103264 Mycgr3
  
Location: 5057-5390

Mycgr3G103264\_Mycgr3

Mycgr3G37570 Mycgr3T
  
Location: 5490-6006

Mycgr3G37570\_Mycgr3T

Mycgr3G108094 Mycgr3
  
Location: 6106-10555

Mycgr3G108094\_Mycgr3

Mycgr3G90786 Mycgr3T
  
Location: 10655-12080

Mycgr3G90786\_Mycgr3T

Mycgr3G68429 Mycgr3T
  
Location: 12180-13440

Mycgr3G68429\_Mycgr3T

Mycgr3G68421 Mycgr3T
  
Location: 13540-17086

Mycgr3G68421\_Mycgr3T

Mycgr3G90801 Mycgr3T
  
Location: 17186-18056

Mycgr3G90801\_Mycgr3T

Mycgr3G84646 Mycgr3T
  
Location: 18156-20235

Mycgr3G84646\_Mycgr3T

Mycgr3G68456 Mycgr3T
  
Location: 20335-21970

Mycgr3G68456\_Mycgr3T

Mycgr3G103270 Mycgr3
  
Location: 22070-22355

Mycgr3G103270\_Mycgr3

Mycgr3G90803 Mycgr3T
  
Location: 22455-23019

Mycgr3G90803\_Mycgr3T

Mycgr3G36941 Mycgr3T
  
Location: 23119-24064

Mycgr3G36941\_Mycgr3T

Mycgr3G25746 Mycgr3T
  
Location: 24164-25241

Mycgr3G25746\_Mycgr3T

Mycgr3G90788 Mycgr3T
  
Location: 25341-25803

Mycgr3G90788\_Mycgr3T

Mycgr3G103260 Mycgr3
  
Location: 25903-26635

Mycgr3G103260\_Mycgr3

Mycgr3G84644 Mycgr3T
  
Location: 26735-28457

Mycgr3G84644\_Mycgr3T

Mycgr3G29227 Mycgr3T
  
Location: 28557-28863

Mycgr3G29227\_Mycgr3T

Mycgr3G36271 Mycgr3T
  
Location: 28963-29854

Mycgr3G36271\_Mycgr3T

Mycgr3G68433 Mycgr3T
  
Location: 29954-33041

Mycgr3G68433\_Mycgr3T

Mycgr3G79452 Mycgr3T
  
Location: 33141-33399

Mycgr3G79452\_Mycgr3T

Mycgr3G55345 Mycgr3T
  
Location: 33499-34126

Mycgr3G55345\_Mycgr3T

Mycgr3G103278 Mycgr3
  
Location: 34226-35195

Mycgr3G103278\_Mycgr3

Mycgr3G84654 Mycgr3T
  
Location: 35295-36630

Mycgr3G84654\_Mycgr3T

Mycgr3G108090 Mycgr3
  
Location: 36730-37591

Mycgr3G108090\_Mycgr3

Mycgr3G21922 Mycgr3T
  
Location: 37691-39149

Mycgr3G21922\_Mycgr3T

Mycgr3G99148 Mycgr3T
  
Location: 39249-42819

Mycgr3G99148\_Mycgr3T

hypothetical protein
  
Accession: EGZ73270
  
Location: 2481193-2481825
  
 NCBI BlastP on this gene

EGZ73270

profilin
  
Accession: EGZ73269
  
Location: 2477278-2478367
  
 NCBI BlastP on this gene

EGZ73269

hypothetical protein
  
Accession: EGZ73268
  
Location: 2471800-2474355
  
 NCBI BlastP on this gene

EGZ73268

hypothetical protein
  
Accession: EGZ73267
  
Location: 2469136-2470046
  
 NCBI BlastP on this gene

EGZ73267

hypothetical protein
  
Accession: EGZ73266
  
Location: 2464824-2466281
  
 NCBI BlastP on this gene

EGZ73266

C-4 methylsterol oxidase, variant
  
Accession: EGZ73265
  
Location: 2460887-2462006
  
  
**BlastP hit with Mycgr3G36271\_Mycgr3T**
  
Percentage identity: 71 %
  
BlastP bit score: 435
  
Sequence coverage: 93 %
  
E-value: 2e-150
  
  
 NCBI BlastP on this gene

EGZ73265

NCA2-domain-containing protein
  
Accession: EGZ73264
  
Location: 2456760-2458938
  
  
**BlastP hit with Mycgr3G84646\_Mycgr3T**
  
Percentage identity: 41 %
  
BlastP bit score: 487
  
Sequence coverage: 101 %
  
E-value: 2e-159
  
  
 NCBI BlastP on this gene

EGZ73264

GTP-binding protein SAS1
  
Accession: EGZ73263
  
Location: 2453845-2454801
  
  
**BlastP hit with Mycgr3G99145\_Mycgr3T**
  
Percentage identity: 88 %
  
BlastP bit score: 355
  
Sequence coverage: 92 %
  
E-value: 1e-121
  
  
 NCBI BlastP on this gene

EGZ73263

activating signal cointegrator 1 complex subunit 3
  
Accession: EGZ73262
  
Location: 2446236-2452503
  
 NCBI BlastP on this gene

EGZ73262

pyridoxamine 5'-phosphate oxidase
  
Accession: EGZ73261
  
Location: 2443095-2443930
  
 NCBI BlastP on this gene

EGZ73261

hypothetical protein
  
Accession: EGZ73260
  
Location: 2437493-2442358
  
 NCBI BlastP on this gene

EGZ73260

ras-domain-containing protein
  
Accession: EGZ73259
  
Location: 2434608-2436240
  
 NCBI BlastP on this gene

EGZ73259

Query: Architecture Search FASTA input

KE148177 : Ophiostoma piceae UAMH 11346 chromosome Unknown scf32    Total score: 3.0     Cumulative Blast bit score: 1269

Hit cluster cross-links:

Mycgr3G90785 Mycgr3T
  
Location: 0-1047

Mycgr3G90785\_Mycgr3T

Mycgr3G103262 Mycgr3
  
Location: 1147-1390

Mycgr3G103262\_Mycgr3

Mycgr3G68458 Mycgr3T
  
Location: 1490-3602

Mycgr3G68458\_Mycgr3T

Mycgr3G99145 Mycgr3T
  
Location: 3702-4326

Mycgr3G99145\_Mycgr3T

Mycgr3G103274 Mycgr3
  
Location: 4426-4957

Mycgr3G103274\_Mycgr3

Mycgr3G103264 Mycgr3
  
Location: 5057-5390

Mycgr3G103264\_Mycgr3

Mycgr3G37570 Mycgr3T
  
Location: 5490-6006

Mycgr3G37570\_Mycgr3T

Mycgr3G108094 Mycgr3
  
Location: 6106-10555

Mycgr3G108094\_Mycgr3

Mycgr3G90786 Mycgr3T
  
Location: 10655-12080

Mycgr3G90786\_Mycgr3T

Mycgr3G68429 Mycgr3T
  
Location: 12180-13440

Mycgr3G68429\_Mycgr3T

Mycgr3G68421 Mycgr3T
  
Location: 13540-17086

Mycgr3G68421\_Mycgr3T

Mycgr3G90801 Mycgr3T
  
Location: 17186-18056

Mycgr3G90801\_Mycgr3T

Mycgr3G84646 Mycgr3T
  
Location: 18156-20235

Mycgr3G84646\_Mycgr3T

Mycgr3G68456 Mycgr3T
  
Location: 20335-21970

Mycgr3G68456\_Mycgr3T

Mycgr3G103270 Mycgr3
  
Location: 22070-22355

Mycgr3G103270\_Mycgr3

Mycgr3G90803 Mycgr3T
  
Location: 22455-23019

Mycgr3G90803\_Mycgr3T

Mycgr3G36941 Mycgr3T
  
Location: 23119-24064

Mycgr3G36941\_Mycgr3T

Mycgr3G25746 Mycgr3T
  
Location: 24164-25241

Mycgr3G25746\_Mycgr3T

Mycgr3G90788 Mycgr3T
  
Location: 25341-25803

Mycgr3G90788\_Mycgr3T

Mycgr3G103260 Mycgr3
  
Location: 25903-26635

Mycgr3G103260\_Mycgr3

Mycgr3G84644 Mycgr3T
  
Location: 26735-28457

Mycgr3G84644\_Mycgr3T

Mycgr3G29227 Mycgr3T
  
Location: 28557-28863

Mycgr3G29227\_Mycgr3T

Mycgr3G36271 Mycgr3T
  
Location: 28963-29854

Mycgr3G36271\_Mycgr3T

Mycgr3G68433 Mycgr3T
  
Location: 29954-33041

Mycgr3G68433\_Mycgr3T

Mycgr3G79452 Mycgr3T
  
Location: 33141-33399

Mycgr3G79452\_Mycgr3T

Mycgr3G55345 Mycgr3T
  
Location: 33499-34126

Mycgr3G55345\_Mycgr3T

Mycgr3G103278 Mycgr3
  
Location: 34226-35195

Mycgr3G103278\_Mycgr3

Mycgr3G84654 Mycgr3T
  
Location: 35295-36630

Mycgr3G84654\_Mycgr3T

Mycgr3G108090 Mycgr3
  
Location: 36730-37591

Mycgr3G108090\_Mycgr3

Mycgr3G21922 Mycgr3T
  
Location: 37691-39149

Mycgr3G21922\_Mycgr3T

Mycgr3G99148 Mycgr3T
  
Location: 39249-42819

Mycgr3G99148\_Mycgr3T

got1 family protein
  
Accession: EPE02531
  
Location: 78146-78598
  
 NCBI BlastP on this gene

EPE02531

profilin
  
Accession: EPE02532
  
Location: 79461-79947
  
 NCBI BlastP on this gene

EPE02532

chromatin remodeling complex subunit
  
Accession: EPE02533
  
Location: 82320-84806
  
 NCBI BlastP on this gene

EPE02533

transmembrane protein
  
Accession: EPE02534
  
Location: 85582-86421
  
 NCBI BlastP on this gene

EPE02534

hypothetical protein
  
Accession: EPE02535
  
Location: 87981-88629
  
 NCBI BlastP on this gene

EPE02535

sphingomyelin phosphodiesterase
  
Accession: EPE02536
  
Location: 91466-93498
  
 NCBI BlastP on this gene

EPE02536

pterin 4 alpha carbinolamine dehydratase
  
Accession: EPE02537
  
Location: 94609-95553
  
 NCBI BlastP on this gene

EPE02537

c-4 methylsterol oxidase
  
Accession: EPE02538
  
Location: 96072-97164
  
  
**BlastP hit with Mycgr3G36271\_Mycgr3T**
  
Percentage identity: 72 %
  
BlastP bit score: 460
  
Sequence coverage: 95 %
  
E-value: 5e-160
  
  
 NCBI BlastP on this gene

EPE02538

atp synthase regulation protein nca2
  
Accession: EPE02539
  
Location: 99029-101436
  
  
**BlastP hit with Mycgr3G84646\_Mycgr3T**
  
Percentage identity: 42 %
  
BlastP bit score: 451
  
Sequence coverage: 84 %
  
E-value: 1e-144
  
  
 NCBI BlastP on this gene

EPE02539

rab gtpase
  
Accession: EPE02540
  
Location: 103269-104120
  
  
**BlastP hit with Mycgr3G99145\_Mycgr3T**
  
Percentage identity: 85 %
  
BlastP bit score: 358
  
Sequence coverage: 99 %
  
E-value: 4e-123
  
  
 NCBI BlastP on this gene

EPE02540

hypothetical protein
  
Accession: EPE02541
  
Location: 104996-107137
  
 NCBI BlastP on this gene

EPE02541

aaa family atpase
  
Accession: EPE02542
  
Location: 107978-110437
  
 NCBI BlastP on this gene

EPE02542

dolichyl-phosphate beta-glucosyltransferase
  
Accession: EPE02543
  
Location: 110681-111988
  
 NCBI BlastP on this gene

EPE02543

snrnp and snornp protein
  
Accession: EPE02544
  
Location: 113123-113506
  
 NCBI BlastP on this gene

EPE02544

hypothetical protein
  
Accession: EPE02545
  
Location: 118159-118817
  
 NCBI BlastP on this gene

EPE02545

Query: Architecture Search FASTA input

CH408032 : Chaetomium globosum CBS 148.51 scaffold\_4 genomic scaffold    Total score: 3.0     Cumulative Blast bit score: 1253

Hit cluster cross-links:

Mycgr3G90785 Mycgr3T
  
Location: 0-1047

Mycgr3G90785\_Mycgr3T

Mycgr3G103262 Mycgr3
  
Location: 1147-1390

Mycgr3G103262\_Mycgr3

Mycgr3G68458 Mycgr3T
  
Location: 1490-3602

Mycgr3G68458\_Mycgr3T

Mycgr3G99145 Mycgr3T
  
Location: 3702-4326

Mycgr3G99145\_Mycgr3T

Mycgr3G103274 Mycgr3
  
Location: 4426-4957

Mycgr3G103274\_Mycgr3

Mycgr3G103264 Mycgr3
  
Location: 5057-5390

Mycgr3G103264\_Mycgr3

Mycgr3G37570 Mycgr3T
  
Location: 5490-6006

Mycgr3G37570\_Mycgr3T

Mycgr3G108094 Mycgr3
  
Location: 6106-10555

Mycgr3G108094\_Mycgr3

Mycgr3G90786 Mycgr3T
  
Location: 10655-12080

Mycgr3G90786\_Mycgr3T

Mycgr3G68429 Mycgr3T
  
Location: 12180-13440

Mycgr3G68429\_Mycgr3T

Mycgr3G68421 Mycgr3T
  
Location: 13540-17086

Mycgr3G68421\_Mycgr3T

Mycgr3G90801 Mycgr3T
  
Location: 17186-18056

Mycgr3G90801\_Mycgr3T

Mycgr3G84646 Mycgr3T
  
Location: 18156-20235

Mycgr3G84646\_Mycgr3T

Mycgr3G68456 Mycgr3T
  
Location: 20335-21970

Mycgr3G68456\_Mycgr3T

Mycgr3G103270 Mycgr3
  
Location: 22070-22355

Mycgr3G103270\_Mycgr3

Mycgr3G90803 Mycgr3T
  
Location: 22455-23019

Mycgr3G90803\_Mycgr3T

Mycgr3G36941 Mycgr3T
  
Location: 23119-24064

Mycgr3G36941\_Mycgr3T

Mycgr3G25746 Mycgr3T
  
Location: 24164-25241

Mycgr3G25746\_Mycgr3T

Mycgr3G90788 Mycgr3T
  
Location: 25341-25803

Mycgr3G90788\_Mycgr3T

Mycgr3G103260 Mycgr3
  
Location: 25903-26635

Mycgr3G103260\_Mycgr3

Mycgr3G84644 Mycgr3T
  
Location: 26735-28457

Mycgr3G84644\_Mycgr3T

Mycgr3G29227 Mycgr3T
  
Location: 28557-28863

Mycgr3G29227\_Mycgr3T

Mycgr3G36271 Mycgr3T
  
Location: 28963-29854

Mycgr3G36271\_Mycgr3T

Mycgr3G68433 Mycgr3T
  
Location: 29954-33041

Mycgr3G68433\_Mycgr3T

Mycgr3G79452 Mycgr3T
  
Location: 33141-33399

Mycgr3G79452\_Mycgr3T

Mycgr3G55345 Mycgr3T
  
Location: 33499-34126

Mycgr3G55345\_Mycgr3T

Mycgr3G103278 Mycgr3
  
Location: 34226-35195

Mycgr3G103278\_Mycgr3

Mycgr3G84654 Mycgr3T
  
Location: 35295-36630

Mycgr3G84654\_Mycgr3T

Mycgr3G108090 Mycgr3
  
Location: 36730-37591

Mycgr3G108090\_Mycgr3

Mycgr3G21922 Mycgr3T
  
Location: 37691-39149

Mycgr3G21922\_Mycgr3T

Mycgr3G99148 Mycgr3T
  
Location: 39249-42819

Mycgr3G99148\_Mycgr3T

hypothetical protein
  
Accession: EAQ88133
  
Location: 3176603-3180263
  
 NCBI BlastP on this gene

EAQ88133

predicted protein
  
Accession: EAQ88132
  
Location: 3173200-3173968
  
 NCBI BlastP on this gene

EAQ88132

hypothetical protein
  
Accession: EAQ88131
  
Location: 3171570-3172284
  
 NCBI BlastP on this gene

EAQ88131

hypothetical protein
  
Accession: EAQ88130
  
Location: 3169209-3169811
  
 NCBI BlastP on this gene

EAQ88130

hypothetical protein
  
Accession: EAQ88129
  
Location: 3166875-3168334
  
 NCBI BlastP on this gene

EAQ88129

hypothetical protein
  
Accession: EAQ88128
  
Location: 3163862-3166355
  
 NCBI BlastP on this gene

EAQ88128

hypothetical protein
  
Accession: EAQ88127
  
Location: 3162488-3163119
  
 NCBI BlastP on this gene

EAQ88127

hypothetical protein
  
Accession: EAQ88126
  
Location: 3158297-3159091
  
 NCBI BlastP on this gene

EAQ88126

hypothetical protein
  
Accession: EAQ88125
  
Location: 3156246-3157343
  
  
**BlastP hit with Mycgr3G36271\_Mycgr3T**
  
Percentage identity: 65 %
  
BlastP bit score: 389
  
Sequence coverage: 95 %
  
E-value: 8e-133
  
  
 NCBI BlastP on this gene

EAQ88125

hypothetical protein
  
Accession: EAQ88124
  
Location: 3152860-3155032
  
  
**BlastP hit with Mycgr3G84646\_Mycgr3T**
  
Percentage identity: 42 %
  
BlastP bit score: 518
  
Sequence coverage: 103 %
  
E-value: 2e-171
  
  
 NCBI BlastP on this gene

EAQ88124

hypothetical protein
  
Accession: EAQ88123
  
Location: 3150792-3151552
  
  
**BlastP hit with Mycgr3G99145\_Mycgr3T**
  
Percentage identity: 82 %
  
BlastP bit score: 346
  
Sequence coverage: 99 %
  
E-value: 2e-118
  
  
 NCBI BlastP on this gene

EAQ88123

hypothetical protein
  
Accession: EAQ88122
  
Location: 3143418-3149459
  
 NCBI BlastP on this gene

EAQ88122

hypothetical protein
  
Accession: EAQ88121
  
Location: 3142002-3142730
  
 NCBI BlastP on this gene

EAQ88121

hypothetical protein
  
Accession: EAQ88120
  
Location: 3138985-3140993
  
 NCBI BlastP on this gene

EAQ88120

hypothetical protein
  
Accession: EAQ88119
  
Location: 3137794-3138597
  
 NCBI BlastP on this gene

EAQ88119

hypothetical protein
  
Accession: EAQ88118
  
Location: 3134963-3137399
  
 NCBI BlastP on this gene

EAQ88118

hypothetical protein
  
Accession: EAQ88117
  
Location: 3133464-3134210
  
 NCBI BlastP on this gene

EAQ88117

Query: Architecture Search FASTA input

GL629769 : Grosmannia clavigera kw1407 unplaced genomic scaffold GCSC\_144    Total score: 3.0     Cumulative Blast bit score: 1241

Hit cluster cross-links:

Mycgr3G90785 Mycgr3T
  
Location: 0-1047

Mycgr3G90785\_Mycgr3T

Mycgr3G103262 Mycgr3
  
Location: 1147-1390

Mycgr3G103262\_Mycgr3

Mycgr3G68458 Mycgr3T
  
Location: 1490-3602

Mycgr3G68458\_Mycgr3T

Mycgr3G99145 Mycgr3T
  
Location: 3702-4326

Mycgr3G99145\_Mycgr3T

Mycgr3G103274 Mycgr3
  
Location: 4426-4957

Mycgr3G103274\_Mycgr3

Mycgr3G103264 Mycgr3
  
Location: 5057-5390

Mycgr3G103264\_Mycgr3

Mycgr3G37570 Mycgr3T
  
Location: 5490-6006

Mycgr3G37570\_Mycgr3T

Mycgr3G108094 Mycgr3
  
Location: 6106-10555

Mycgr3G108094\_Mycgr3

Mycgr3G90786 Mycgr3T
  
Location: 10655-12080

Mycgr3G90786\_Mycgr3T

Mycgr3G68429 Mycgr3T
  
Location: 12180-13440

Mycgr3G68429\_Mycgr3T

Mycgr3G68421 Mycgr3T
  
Location: 13540-17086

Mycgr3G68421\_Mycgr3T

Mycgr3G90801 Mycgr3T
  
Location: 17186-18056

Mycgr3G90801\_Mycgr3T

Mycgr3G84646 Mycgr3T
  
Location: 18156-20235

Mycgr3G84646\_Mycgr3T

Mycgr3G68456 Mycgr3T
  
Location: 20335-21970

Mycgr3G68456\_Mycgr3T

Mycgr3G103270 Mycgr3
  
Location: 22070-22355

Mycgr3G103270\_Mycgr3

Mycgr3G90803 Mycgr3T
  
Location: 22455-23019

Mycgr3G90803\_Mycgr3T

Mycgr3G36941 Mycgr3T
  
Location: 23119-24064

Mycgr3G36941\_Mycgr3T

Mycgr3G25746 Mycgr3T
  
Location: 24164-25241

Mycgr3G25746\_Mycgr3T

Mycgr3G90788 Mycgr3T
  
Location: 25341-25803

Mycgr3G90788\_Mycgr3T

Mycgr3G103260 Mycgr3
  
Location: 25903-26635

Mycgr3G103260\_Mycgr3

Mycgr3G84644 Mycgr3T
  
Location: 26735-28457

Mycgr3G84644\_Mycgr3T

Mycgr3G29227 Mycgr3T
  
Location: 28557-28863

Mycgr3G29227\_Mycgr3T

Mycgr3G36271 Mycgr3T
  
Location: 28963-29854

Mycgr3G36271\_Mycgr3T

Mycgr3G68433 Mycgr3T
  
Location: 29954-33041

Mycgr3G68433\_Mycgr3T

Mycgr3G79452 Mycgr3T
  
Location: 33141-33399

Mycgr3G79452\_Mycgr3T

Mycgr3G55345 Mycgr3T
  
Location: 33499-34126

Mycgr3G55345\_Mycgr3T

Mycgr3G103278 Mycgr3
  
Location: 34226-35195

Mycgr3G103278\_Mycgr3

Mycgr3G84654 Mycgr3T
  
Location: 35295-36630

Mycgr3G84654\_Mycgr3T

Mycgr3G108090 Mycgr3
  
Location: 36730-37591

Mycgr3G108090\_Mycgr3

Mycgr3G21922 Mycgr3T
  
Location: 37691-39149

Mycgr3G21922\_Mycgr3T

Mycgr3G99148 Mycgr3T
  
Location: 39249-42819

Mycgr3G99148\_Mycgr3T

got1 family protein
  
Accession: EFX02879
  
Location: 368914-369690
  
 NCBI BlastP on this gene

EFX02879

profilin
  
Accession: EFX03070
  
Location: 370309-371088
  
 NCBI BlastP on this gene

EFX03070

chromatin remodeling complex subunit
  
Accession: EFX02970
  
Location: 372426-374960
  
 NCBI BlastP on this gene

EFX02970

bzip transcription factor
  
Accession: EFX02620
  
Location: 375530-377503
  
 NCBI BlastP on this gene

EFX02620

stress responsive a b barrel domain containing protein
  
Accession: EFX03226
  
Location: 380092-380552
  
 NCBI BlastP on this gene

EFX03226

pterin-4-alpha-carbinolamine dehydratase
  
Accession: EFX03320
  
Location: 382105-382865
  
 NCBI BlastP on this gene

EFX03320

hypothetical protein
  
Accession: EFX03157
  
Location: 382355-382837
  
 NCBI BlastP on this gene

EFX03157

c-4 methyl sterol oxidase
  
Accession: EFX03141
  
Location: 383367-384429
  
  
**BlastP hit with Mycgr3G36271\_Mycgr3T**
  
Percentage identity: 72 %
  
BlastP bit score: 434
  
Sequence coverage: 91 %
  
E-value: 6e-150
  
  
 NCBI BlastP on this gene

EFX03141

nuclear control of ATP synthase 2 protein
  
Accession: EFX02912
  
Location: 385369-387663
  
  
**BlastP hit with Mycgr3G84646\_Mycgr3T**
  
Percentage identity: 43 %
  
BlastP bit score: 450
  
Sequence coverage: 84 %
  
E-value: 1e-144
  
  
 NCBI BlastP on this gene

EFX02912

rab GTPase
  
Accession: EFX03003
  
Location: 388446-389194
  
  
**BlastP hit with Mycgr3G99145\_Mycgr3T**
  
Percentage identity: 85 %
  
BlastP bit score: 357
  
Sequence coverage: 99 %
  
E-value: 2e-122
  
  
 NCBI BlastP on this gene

EFX03003

hypothetical protein
  
Accession: EFX02964
  
Location: 389704-391725
  
 NCBI BlastP on this gene

EFX02964

duf625 domain containing protein
  
Accession: EFX03091
  
Location: 392652-395714
  
 NCBI BlastP on this gene

EFX03091

hypothetical protein
  
Accession: EFX02675
  
Location: 399590-400127
  
 NCBI BlastP on this gene

EFX02675

hypothetical protein
  
Accession: EFX02849
  
Location: 401133-402206
  
 NCBI BlastP on this gene

EFX02849

snrnp and snornp protein
  
Accession: EFX03270
  
Location: 403641-404176
  
 NCBI BlastP on this gene

EFX03270

dolichyl-phosphate beta-transferase
  
Accession: EFX02688
  
Location: 405072-406286
  
 NCBI BlastP on this gene

EFX02688

aaa family ATPase
  
Accession: EFX02828
  
Location: 406727-408985
  
 NCBI BlastP on this gene

EFX02828

Query: Architecture Search FASTA input

CH476615 : Uncinocarpus reesii 1704 scaffold\_1 genomic scaffold    Total score: 3.0     Cumulative Blast bit score: 1238

Hit cluster cross-links:

Mycgr3G90785 Mycgr3T
  
Location: 0-1047

Mycgr3G90785\_Mycgr3T

Mycgr3G103262 Mycgr3
  
Location: 1147-1390

Mycgr3G103262\_Mycgr3

Mycgr3G68458 Mycgr3T
  
Location: 1490-3602

Mycgr3G68458\_Mycgr3T

Mycgr3G99145 Mycgr3T
  
Location: 3702-4326

Mycgr3G99145\_Mycgr3T

Mycgr3G103274 Mycgr3
  
Location: 4426-4957

Mycgr3G103274\_Mycgr3

Mycgr3G103264 Mycgr3
  
Location: 5057-5390

Mycgr3G103264\_Mycgr3

Mycgr3G37570 Mycgr3T
  
Location: 5490-6006

Mycgr3G37570\_Mycgr3T

Mycgr3G108094 Mycgr3
  
Location: 6106-10555

Mycgr3G108094\_Mycgr3

Mycgr3G90786 Mycgr3T
  
Location: 10655-12080

Mycgr3G90786\_Mycgr3T

Mycgr3G68429 Mycgr3T
  
Location: 12180-13440

Mycgr3G68429\_Mycgr3T

Mycgr3G68421 Mycgr3T
  
Location: 13540-17086

Mycgr3G68421\_Mycgr3T

Mycgr3G90801 Mycgr3T
  
Location: 17186-18056

Mycgr3G90801\_Mycgr3T

Mycgr3G84646 Mycgr3T
  
Location: 18156-20235

Mycgr3G84646\_Mycgr3T

Mycgr3G68456 Mycgr3T
  
Location: 20335-21970

Mycgr3G68456\_Mycgr3T

Mycgr3G103270 Mycgr3
  
Location: 22070-22355

Mycgr3G103270\_Mycgr3

Mycgr3G90803 Mycgr3T
  
Location: 22455-23019

Mycgr3G90803\_Mycgr3T

Mycgr3G36941 Mycgr3T
  
Location: 23119-24064

Mycgr3G36941\_Mycgr3T

Mycgr3G25746 Mycgr3T
  
Location: 24164-25241

Mycgr3G25746\_Mycgr3T

Mycgr3G90788 Mycgr3T
  
Location: 25341-25803

Mycgr3G90788\_Mycgr3T

Mycgr3G103260 Mycgr3
  
Location: 25903-26635

Mycgr3G103260\_Mycgr3

Mycgr3G84644 Mycgr3T
  
Location: 26735-28457

Mycgr3G84644\_Mycgr3T

Mycgr3G29227 Mycgr3T
  
Location: 28557-28863

Mycgr3G29227\_Mycgr3T

Mycgr3G36271 Mycgr3T
  
Location: 28963-29854

Mycgr3G36271\_Mycgr3T

Mycgr3G68433 Mycgr3T
  
Location: 29954-33041

Mycgr3G68433\_Mycgr3T

Mycgr3G79452 Mycgr3T
  
Location: 33141-33399

Mycgr3G79452\_Mycgr3T

Mycgr3G55345 Mycgr3T
  
Location: 33499-34126

Mycgr3G55345\_Mycgr3T

Mycgr3G103278 Mycgr3
  
Location: 34226-35195

Mycgr3G103278\_Mycgr3

Mycgr3G84654 Mycgr3T
  
Location: 35295-36630

Mycgr3G84654\_Mycgr3T

Mycgr3G108090 Mycgr3
  
Location: 36730-37591

Mycgr3G108090\_Mycgr3

Mycgr3G21922 Mycgr3T
  
Location: 37691-39149

Mycgr3G21922\_Mycgr3T

Mycgr3G99148 Mycgr3T
  
Location: 39249-42819

Mycgr3G99148\_Mycgr3T

conserved hypothetical protein
  
Accession: EEP77732
  
Location: 6853553-6855345
  
 NCBI BlastP on this gene

EEP77732

predicted protein
  
Accession: EEP77731
  
Location: 6852077-6853007
  
 NCBI BlastP on this gene

EEP77731

conserved hypothetical protein
  
Accession: EEP77730
  
Location: 6850922-6851751
  
 NCBI BlastP on this gene

EEP77730

hypothetical protein
  
Accession: EEP77729
  
Location: 6840903-6849576
  
 NCBI BlastP on this gene

EEP77729

ATPase
  
Accession: EEP77728
  
Location: 6836861-6839170
  
 NCBI BlastP on this gene

EEP77728

predicted protein
  
Accession: EEP77727
  
Location: 6835582-6836656
  
 NCBI BlastP on this gene

EEP77727

C-4 methylsterol oxidase
  
Accession: EEP77726
  
Location: 6834301-6835161
  
  
**BlastP hit with Mycgr3G36271\_Mycgr3T**
  
Percentage identity: 81 %
  
BlastP bit score: 422
  
Sequence coverage: 80 %
  
E-value: 4e-146
  
  
 NCBI BlastP on this gene

EEP77726

predicted protein
  
Accession: EEP77725
  
Location: 6831336-6833511
  
  
**BlastP hit with Mycgr3G84646\_Mycgr3T**
  
Percentage identity: 39 %
  
BlastP bit score: 463
  
Sequence coverage: 100 %
  
E-value: 3e-150
  
  
 NCBI BlastP on this gene

EEP77725

GTP-binding protein SAS1
  
Accession: EEP77724
  
Location: 6829677-6830445
  
  
**BlastP hit with Mycgr3G99145\_Mycgr3T**
  
Percentage identity: 82 %
  
BlastP bit score: 353
  
Sequence coverage: 99 %
  
E-value: 3e-121
  
  
 NCBI BlastP on this gene

EEP77724

predicted protein
  
Accession: EEP77723
  
Location: 6827738-6828976
  
 NCBI BlastP on this gene

EEP77723

predicted protein
  
Accession: EEP77722
  
Location: 6826125-6827111
  
 NCBI BlastP on this gene

EEP77722

hypothetical protein
  
Accession: EEP77721
  
Location: 6824266-6825095
  
 NCBI BlastP on this gene

EEP77721

predicted protein
  
Accession: EEP77720
  
Location: 6821443-6823383
  
 NCBI BlastP on this gene

EEP77720

conserved hypothetical protein
  
Accession: EEP77719
  
Location: 6819536-6820461
  
 NCBI BlastP on this gene

EEP77719

predicted protein
  
Accession: EEP77718
  
Location: 6817253-6818824
  
 NCBI BlastP on this gene

EEP77718

predicted protein
  
Accession: EEP77717
  
Location: 6816382-6816979
  
 NCBI BlastP on this gene

EEP77717

conserved hypothetical protein
  
Accession: EEP77716
  
Location: 6812395-6815757
  
 NCBI BlastP on this gene

EEP77716

conserved hypothetical protein
  
Accession: EEP77715
  
Location: 6807300-6811550
  
 NCBI BlastP on this gene

EEP77715

Query: Architecture Search FASTA input

AM920428 : Penicillium chrysogenum Wisconsin 54-1255 complete genome, contig Pc00c13.    Total score: 3.0     Cumulative Blast bit score: 1235

Hit cluster cross-links:

Mycgr3G90785 Mycgr3T
  
Location: 0-1047

Mycgr3G90785\_Mycgr3T

Mycgr3G103262 Mycgr3
  
Location: 1147-1390

Mycgr3G103262\_Mycgr3

Mycgr3G68458 Mycgr3T
  
Location: 1490-3602

Mycgr3G68458\_Mycgr3T

Mycgr3G99145 Mycgr3T
  
Location: 3702-4326

Mycgr3G99145\_Mycgr3T

Mycgr3G103274 Mycgr3
  
Location: 4426-4957

Mycgr3G103274\_Mycgr3

Mycgr3G103264 Mycgr3
  
Location: 5057-5390

Mycgr3G103264\_Mycgr3

Mycgr3G37570 Mycgr3T
  
Location: 5490-6006

Mycgr3G37570\_Mycgr3T

Mycgr3G108094 Mycgr3
  
Location: 6106-10555

Mycgr3G108094\_Mycgr3

Mycgr3G90786 Mycgr3T
  
Location: 10655-12080

Mycgr3G90786\_Mycgr3T

Mycgr3G68429 Mycgr3T
  
Location: 12180-13440

Mycgr3G68429\_Mycgr3T

Mycgr3G68421 Mycgr3T
  
Location: 13540-17086

Mycgr3G68421\_Mycgr3T

Mycgr3G90801 Mycgr3T
  
Location: 17186-18056

Mycgr3G90801\_Mycgr3T

Mycgr3G84646 Mycgr3T
  
Location: 18156-20235

Mycgr3G84646\_Mycgr3T

Mycgr3G68456 Mycgr3T
  
Location: 20335-21970

Mycgr3G68456\_Mycgr3T

Mycgr3G103270 Mycgr3
  
Location: 22070-22355

Mycgr3G103270\_Mycgr3

Mycgr3G90803 Mycgr3T
  
Location: 22455-23019

Mycgr3G90803\_Mycgr3T

Mycgr3G36941 Mycgr3T
  
Location: 23119-24064

Mycgr3G36941\_Mycgr3T

Mycgr3G25746 Mycgr3T
  
Location: 24164-25241

Mycgr3G25746\_Mycgr3T

Mycgr3G90788 Mycgr3T
  
Location: 25341-25803

Mycgr3G90788\_Mycgr3T

Mycgr3G103260 Mycgr3
  
Location: 25903-26635

Mycgr3G103260\_Mycgr3

Mycgr3G84644 Mycgr3T
  
Location: 26735-28457

Mycgr3G84644\_Mycgr3T

Mycgr3G29227 Mycgr3T
  
Location: 28557-28863

Mycgr3G29227\_Mycgr3T

Mycgr3G36271 Mycgr3T
  
Location: 28963-29854

Mycgr3G36271\_Mycgr3T

Mycgr3G68433 Mycgr3T
  
Location: 29954-33041

Mycgr3G68433\_Mycgr3T

Mycgr3G79452 Mycgr3T
  
Location: 33141-33399

Mycgr3G79452\_Mycgr3T

Mycgr3G55345 Mycgr3T
  
Location: 33499-34126

Mycgr3G55345\_Mycgr3T

Mycgr3G103278 Mycgr3
  
Location: 34226-35195

Mycgr3G103278\_Mycgr3

Mycgr3G84654 Mycgr3T
  
Location: 35295-36630

Mycgr3G84654\_Mycgr3T

Mycgr3G108090 Mycgr3
  
Location: 36730-37591

Mycgr3G108090\_Mycgr3

Mycgr3G21922 Mycgr3T
  
Location: 37691-39149

Mycgr3G21922\_Mycgr3T

Mycgr3G99148 Mycgr3T
  
Location: 39249-42819

Mycgr3G99148\_Mycgr3T

not annotated
  
Accession: CAP91619
  
Location: 1361097-1364643
  
 NCBI BlastP on this gene

Pc13g05500

not annotated
  
Accession: CAP91620
  
Location: 1364765-1368949
  
 NCBI BlastP on this gene

Pc13g05510

unnamed
  
Accession: CAP91621
  
Location: 1369356-1371039
  
 NCBI BlastP on this gene

Pc13g05520

not annotated
  
Accession: CAP91622
  
Location: 1372110-1373410
  
 NCBI BlastP on this gene

Pc13g05530

not annotated
  
Accession: CAP91623
  
Location: 1373850-1374672
  
 NCBI BlastP on this gene

Pc13g05540

not annotated
  
Accession: CAP91624
  
Location: 1374976-1376468
  
 NCBI BlastP on this gene

Pc13g05550

not annotated
  
Accession: CAP91625
  
Location: 1376863-1378648
  
 NCBI BlastP on this gene

Pc13g05560

not annotated
  
Accession: CAP91626
  
Location: 1379129-1380628
  
  
**BlastP hit with Mycgr3G36271\_Mycgr3T**
  
Percentage identity: 76 %
  
BlastP bit score: 471
  
Sequence coverage: 98 %
  
E-value: 5e-164
  
  
 NCBI BlastP on this gene

Pc13g05570

not annotated
  
Accession: CAP91627
  
Location: 1380859-1382926
  
  
**BlastP hit with Mycgr3G84646\_Mycgr3T**
  
Percentage identity: 39 %
  
BlastP bit score: 421
  
Sequence coverage: 99 %
  
E-value: 2e-134
  
  
 NCBI BlastP on this gene

Pc13g05580

not annotated
  
Accession: CAP91628
  
Location: 1383694-1384452
  
  
**BlastP hit with Mycgr3G99145\_Mycgr3T**
  
Percentage identity: 86 %
  
BlastP bit score: 343
  
Sequence coverage: 93 %
  
E-value: 3e-117
  
  
 NCBI BlastP on this gene

Pc13g05590

not annotated
  
Accession: CAP91629
  
Location: 1385167-1387480
  
 NCBI BlastP on this gene

Pc13g05600

not annotated
  
Accession: CAP91630
  
Location: 1387853-1388873
  
 NCBI BlastP on this gene

Pc13g05610

not annotated
  
Accession: CAP91631
  
Location: 1389244-1398061
  
 NCBI BlastP on this gene

Pc13g05620

not annotated
  
Accession: CAP91632
  
Location: 1398708-1399484
  
 NCBI BlastP on this gene

Pc13g05630

not annotated
  
Accession: CAP91633
  
Location: 1399751-1400568
  
 NCBI BlastP on this gene

Pc13g05640

not annotated
  
Accession: CAP91634
  
Location: 1400909-1401886
  
 NCBI BlastP on this gene

Pc13g05650

not annotated
  
Accession: CAP91635
  
Location: 1402253-1404036
  
 NCBI BlastP on this gene

Pc13g05660

not annotated
  
Accession: CAP91636
  
Location: 1404374-1406197
  
 NCBI BlastP on this gene

Pc13g05670

Query: Architecture Search FASTA input

CH476663 : Ajellomyces capsulatus NAm1 scaffold\_9 genomic scaffold    Total score: 3.0     Cumulative Blast bit score: 1202

Hit cluster cross-links:

Mycgr3G90785 Mycgr3T
  
Location: 0-1047

Mycgr3G90785\_Mycgr3T

Mycgr3G103262 Mycgr3
  
Location: 1147-1390

Mycgr3G103262\_Mycgr3

Mycgr3G68458 Mycgr3T
  
Location: 1490-3602

Mycgr3G68458\_Mycgr3T

Mycgr3G99145 Mycgr3T
  
Location: 3702-4326

Mycgr3G99145\_Mycgr3T

Mycgr3G103274 Mycgr3
  
Location: 4426-4957

Mycgr3G103274\_Mycgr3

Mycgr3G103264 Mycgr3
  
Location: 5057-5390

Mycgr3G103264\_Mycgr3

Mycgr3G37570 Mycgr3T
  
Location: 5490-6006

Mycgr3G37570\_Mycgr3T

Mycgr3G108094 Mycgr3
  
Location: 6106-10555

Mycgr3G108094\_Mycgr3

Mycgr3G90786 Mycgr3T
  
Location: 10655-12080

Mycgr3G90786\_Mycgr3T

Mycgr3G68429 Mycgr3T
  
Location: 12180-13440

Mycgr3G68429\_Mycgr3T

Mycgr3G68421 Mycgr3T
  
Location: 13540-17086

Mycgr3G68421\_Mycgr3T

Mycgr3G90801 Mycgr3T
  
Location: 17186-18056

Mycgr3G90801\_Mycgr3T

Mycgr3G84646 Mycgr3T
  
Location: 18156-20235

Mycgr3G84646\_Mycgr3T

Mycgr3G68456 Mycgr3T
  
Location: 20335-21970

Mycgr3G68456\_Mycgr3T

Mycgr3G103270 Mycgr3
  
Location: 22070-22355

Mycgr3G103270\_Mycgr3

Mycgr3G90803 Mycgr3T
  
Location: 22455-23019

Mycgr3G90803\_Mycgr3T

Mycgr3G36941 Mycgr3T
  
Location: 23119-24064

Mycgr3G36941\_Mycgr3T

Mycgr3G25746 Mycgr3T
  
Location: 24164-25241

Mycgr3G25746\_Mycgr3T

Mycgr3G90788 Mycgr3T
  
Location: 25341-25803

Mycgr3G90788\_Mycgr3T

Mycgr3G103260 Mycgr3
  
Location: 25903-26635

Mycgr3G103260\_Mycgr3

Mycgr3G84644 Mycgr3T
  
Location: 26735-28457

Mycgr3G84644\_Mycgr3T

Mycgr3G29227 Mycgr3T
  
Location: 28557-28863

Mycgr3G29227\_Mycgr3T

Mycgr3G36271 Mycgr3T
  
Location: 28963-29854

Mycgr3G36271\_Mycgr3T

Mycgr3G68433 Mycgr3T
  
Location: 29954-33041

Mycgr3G68433\_Mycgr3T

Mycgr3G79452 Mycgr3T
  
Location: 33141-33399

Mycgr3G79452\_Mycgr3T

Mycgr3G55345 Mycgr3T
  
Location: 33499-34126

Mycgr3G55345\_Mycgr3T

Mycgr3G103278 Mycgr3
  
Location: 34226-35195

Mycgr3G103278\_Mycgr3

Mycgr3G84654 Mycgr3T
  
Location: 35295-36630

Mycgr3G84654\_Mycgr3T

Mycgr3G108090 Mycgr3
  
Location: 36730-37591

Mycgr3G108090\_Mycgr3

Mycgr3G21922 Mycgr3T
  
Location: 37691-39149

Mycgr3G21922\_Mycgr3T

Mycgr3G99148 Mycgr3T
  
Location: 39249-42819

Mycgr3G99148\_Mycgr3T

conserved hypothetical protein
  
Accession: EDN11193
  
Location: 577399-581249
  
 NCBI BlastP on this gene

EDN11193

predicted protein
  
Accession: EDN11194
  
Location: 582420-583244
  
 NCBI BlastP on this gene

EDN11194

predicted protein
  
Accession: EDN11195
  
Location: 583786-585591
  
 NCBI BlastP on this gene

EDN11195

hypothetical protein
  
Accession: EDN11196
  
Location: 587694-588675
  
 NCBI BlastP on this gene

EDN11196

C-4 methylsterol oxidase
  
Accession: EDN11197
  
Location: 589643-590494
  
  
**BlastP hit with Mycgr3G36271\_Mycgr3T**
  
Percentage identity: 80 %
  
BlastP bit score: 387
  
Sequence coverage: 75 %
  
E-value: 1e-132
  
  
 NCBI BlastP on this gene

EDN11197

predicted protein
  
Accession: EDN11198
  
Location: 590746-593927
  
  
**BlastP hit with Mycgr3G84646\_Mycgr3T**
  
Percentage identity: 39 %
  
BlastP bit score: 460
  
Sequence coverage: 100 %
  
E-value: 1e-147
  
  
 NCBI BlastP on this gene

EDN11198

predicted protein
  
Accession: EDN11199
  
Location: 596276-596795
  
 NCBI BlastP on this gene

EDN11199

predicted protein
  
Accession: EDN11200
  
Location: 598546-601485
  
 NCBI BlastP on this gene

EDN11200

predicted protein
  
Accession: EDN11201
  
Location: 602596-605701
  
 NCBI BlastP on this gene

EDN11201

predicted protein
  
Accession: EDN11202
  
Location: 605966-607741
  
 NCBI BlastP on this gene

EDN11202

predicted protein
  
Accession: EDN11203
  
Location: 609074-609425
  
 NCBI BlastP on this gene

EDN11203

conserved hypothetical protein
  
Accession: EDN11204
  
Location: 610654-612771
  
 NCBI BlastP on this gene

EDN11204

predicted protein
  
Accession: EDN11205
  
Location: 615876-616416
  
 NCBI BlastP on this gene

EDN11205

GTP-binding protein SAS1
  
Accession: EDN11206
  
Location: 618048-618957
  
  
**BlastP hit with Mycgr3G99145\_Mycgr3T**
  
Percentage identity: 84 %
  
BlastP bit score: 355
  
Sequence coverage: 99 %
  
E-value: 8e-122
  
  
 NCBI BlastP on this gene

EDN11206

predicted protein
  
Accession: EDN11207
  
Location: 619694-620808
  
 NCBI BlastP on this gene

EDN11207

conserved hypothetical protein
  
Accession: EDN11208
  
Location: 621560-623911
  
 NCBI BlastP on this gene

EDN11208

predicted protein
  
Accession: EDN11209
  
Location: 624832-625272
  
 NCBI BlastP on this gene

EDN11209

predicted protein
  
Accession: EDN11210
  
Location: 625530-626181
  
 NCBI BlastP on this gene

EDN11210

hypothetical protein
  
Accession: EDN11211
  
Location: 626551-635497
  
 NCBI BlastP on this gene

EDN11211

Query: Architecture Search FASTA input

KB933328 : Togninia minima UCRPA7 unplaced genomic scaffold PA7\_03\_scaffold\_560    Total score: 3.0     Cumulative Blast bit score: 1200

Hit cluster cross-links:

Mycgr3G90785 Mycgr3T
  
Location: 0-1047

Mycgr3G90785\_Mycgr3T

Mycgr3G103262 Mycgr3
  
Location: 1147-1390

Mycgr3G103262\_Mycgr3

Mycgr3G68458 Mycgr3T
  
Location: 1490-3602

Mycgr3G68458\_Mycgr3T

Mycgr3G99145 Mycgr3T
  
Location: 3702-4326

Mycgr3G99145\_Mycgr3T

Mycgr3G103274 Mycgr3
  
Location: 4426-4957

Mycgr3G103274\_Mycgr3

Mycgr3G103264 Mycgr3
  
Location: 5057-5390

Mycgr3G103264\_Mycgr3

Mycgr3G37570 Mycgr3T
  
Location: 5490-6006

Mycgr3G37570\_Mycgr3T

Mycgr3G108094 Mycgr3
  
Location: 6106-10555

Mycgr3G108094\_Mycgr3

Mycgr3G90786 Mycgr3T
  
Location: 10655-12080

Mycgr3G90786\_Mycgr3T

Mycgr3G68429 Mycgr3T
  
Location: 12180-13440

Mycgr3G68429\_Mycgr3T

Mycgr3G68421 Mycgr3T
  
Location: 13540-17086

Mycgr3G68421\_Mycgr3T

Mycgr3G90801 Mycgr3T
  
Location: 17186-18056

Mycgr3G90801\_Mycgr3T

Mycgr3G84646 Mycgr3T
  
Location: 18156-20235

Mycgr3G84646\_Mycgr3T

Mycgr3G68456 Mycgr3T
  
Location: 20335-21970

Mycgr3G68456\_Mycgr3T

Mycgr3G103270 Mycgr3
  
Location: 22070-22355

Mycgr3G103270\_Mycgr3

Mycgr3G90803 Mycgr3T
  
Location: 22455-23019

Mycgr3G90803\_Mycgr3T

Mycgr3G36941 Mycgr3T
  
Location: 23119-24064

Mycgr3G36941\_Mycgr3T

Mycgr3G25746 Mycgr3T
  
Location: 24164-25241

Mycgr3G25746\_Mycgr3T

Mycgr3G90788 Mycgr3T
  
Location: 25341-25803

Mycgr3G90788\_Mycgr3T

Mycgr3G103260 Mycgr3
  
Location: 25903-26635

Mycgr3G103260\_Mycgr3

Mycgr3G84644 Mycgr3T
  
Location: 26735-28457

Mycgr3G84644\_Mycgr3T

Mycgr3G29227 Mycgr3T
  
Location: 28557-28863

Mycgr3G29227\_Mycgr3T

Mycgr3G36271 Mycgr3T
  
Location: 28963-29854

Mycgr3G36271\_Mycgr3T

Mycgr3G68433 Mycgr3T
  
Location: 29954-33041

Mycgr3G68433\_Mycgr3T

Mycgr3G79452 Mycgr3T
  
Location: 33141-33399

Mycgr3G79452\_Mycgr3T

Mycgr3G55345 Mycgr3T
  
Location: 33499-34126

Mycgr3G55345\_Mycgr3T

Mycgr3G103278 Mycgr3
  
Location: 34226-35195

Mycgr3G103278\_Mycgr3

Mycgr3G84654 Mycgr3T
  
Location: 35295-36630

Mycgr3G84654\_Mycgr3T

Mycgr3G108090 Mycgr3
  
Location: 36730-37591

Mycgr3G108090\_Mycgr3

Mycgr3G21922 Mycgr3T
  
Location: 37691-39149

Mycgr3G21922\_Mycgr3T

Mycgr3G99148 Mycgr3T
  
Location: 39249-42819

Mycgr3G99148\_Mycgr3T

putative eukaryotic translation initiation factor 2 alpha subunit protein
  
Accession: EON96623
  
Location: 99389-100534
  
 NCBI BlastP on this gene

EON96623

putative chromatin remodeling complex subunit protein
  
Accession: EON96616
  
Location: 95822-98298
  
 NCBI BlastP on this gene

EON96616

putative amidohydrolase protein
  
Accession: EON96602
  
Location: 83602-85214
  
 NCBI BlastP on this gene

EON96602

putative c-4 sterol methyl oxidase protein
  
Accession: EON96620
  
Location: 80296-81411
  
  
**BlastP hit with Mycgr3G36271\_Mycgr3T**
  
Percentage identity: 72 %
  
BlastP bit score: 457
  
Sequence coverage: 95 %
  
E-value: 4e-159
  
  
 NCBI BlastP on this gene

EON96620

putative atp synthase regulation protein nca2 protein
  
Accession: EON96549
  
Location: 76384-77655
  
  
**BlastP hit with Mycgr3G84646\_Mycgr3T**
  
Percentage identity: 49 %
  
BlastP bit score: 405
  
Sequence coverage: 62 %
  
E-value: 4e-131
  
  
 NCBI BlastP on this gene

EON96549

putative gtp-binding protein sas1 protein
  
Accession: EON96550
  
Location: 74663-75993
  
  
**BlastP hit with Mycgr3G99145\_Mycgr3T**
  
Percentage identity: 86 %
  
BlastP bit score: 338
  
Sequence coverage: 92 %
  
E-value: 2e-115
  
  
 NCBI BlastP on this gene

EON96550

hypothetical protein
  
Accession: EON96560
  
Location: 73532-74161
  
 NCBI BlastP on this gene

EON96560

putative aaa family atpase protein
  
Accession: EON96578
  
Location: 69971-72223
  
 NCBI BlastP on this gene

EON96578

putative dash complex subunit dad4 protein
  
Accession: EON96551
  
Location: 69054-69509
  
 NCBI BlastP on this gene

EON96551

putative yl1 nuclear protein
  
Accession: EON96595
  
Location: 68141-68752
  
 NCBI BlastP on this gene

EON96595

putative got1 family protein
  
Accession: EON96604
  
Location: 66507-67202
  
 NCBI BlastP on this gene

EON96604

putative profilin protein
  
Accession: EON96599
  
Location: 65228-65960
  
 NCBI BlastP on this gene

EON96599

putative snare ykt6 protein
  
Accession: EON96605
  
Location: 57344-58522
  
 NCBI BlastP on this gene

EON96605

Query: Architecture Search FASTA input

EQ963487 : Aspergillus flavus NRRL3357 scf\_1106286419368 genomic scaffold    Total score: 3.0     Cumulative Blast bit score: 1188

Hit cluster cross-links:

Mycgr3G90785 Mycgr3T
  
Location: 0-1047

Mycgr3G90785\_Mycgr3T

Mycgr3G103262 Mycgr3
  
Location: 1147-1390

Mycgr3G103262\_Mycgr3

Mycgr3G68458 Mycgr3T
  
Location: 1490-3602

Mycgr3G68458\_Mycgr3T

Mycgr3G99145 Mycgr3T
  
Location: 3702-4326

Mycgr3G99145\_Mycgr3T

Mycgr3G103274 Mycgr3
  
Location: 4426-4957

Mycgr3G103274\_Mycgr3

Mycgr3G103264 Mycgr3
  
Location: 5057-5390

Mycgr3G103264\_Mycgr3

Mycgr3G37570 Mycgr3T
  
Location: 5490-6006

Mycgr3G37570\_Mycgr3T

Mycgr3G108094 Mycgr3
  
Location: 6106-10555

Mycgr3G108094\_Mycgr3

Mycgr3G90786 Mycgr3T
  
Location: 10655-12080

Mycgr3G90786\_Mycgr3T

Mycgr3G68429 Mycgr3T
  
Location: 12180-13440

Mycgr3G68429\_Mycgr3T

Mycgr3G68421 Mycgr3T
  
Location: 13540-17086

Mycgr3G68421\_Mycgr3T

Mycgr3G90801 Mycgr3T
  
Location: 17186-18056

Mycgr3G90801\_Mycgr3T

Mycgr3G84646 Mycgr3T
  
Location: 18156-20235

Mycgr3G84646\_Mycgr3T

Mycgr3G68456 Mycgr3T
  
Location: 20335-21970

Mycgr3G68456\_Mycgr3T

Mycgr3G103270 Mycgr3
  
Location: 22070-22355

Mycgr3G103270\_Mycgr3

Mycgr3G90803 Mycgr3T
  
Location: 22455-23019

Mycgr3G90803\_Mycgr3T

Mycgr3G36941 Mycgr3T
  
Location: 23119-24064

Mycgr3G36941\_Mycgr3T

Mycgr3G25746 Mycgr3T
  
Location: 24164-25241

Mycgr3G25746\_Mycgr3T

Mycgr3G90788 Mycgr3T
  
Location: 25341-25803

Mycgr3G90788\_Mycgr3T

Mycgr3G103260 Mycgr3
  
Location: 25903-26635

Mycgr3G103260\_Mycgr3

Mycgr3G84644 Mycgr3T
  
Location: 26735-28457

Mycgr3G84644\_Mycgr3T

Mycgr3G29227 Mycgr3T
  
Location: 28557-28863

Mycgr3G29227\_Mycgr3T

Mycgr3G36271 Mycgr3T
  
Location: 28963-29854

Mycgr3G36271\_Mycgr3T

Mycgr3G68433 Mycgr3T
  
Location: 29954-33041

Mycgr3G68433\_Mycgr3T

Mycgr3G79452 Mycgr3T
  
Location: 33141-33399

Mycgr3G79452\_Mycgr3T

Mycgr3G55345 Mycgr3T
  
Location: 33499-34126

Mycgr3G55345\_Mycgr3T

Mycgr3G103278 Mycgr3
  
Location: 34226-35195

Mycgr3G103278\_Mycgr3

Mycgr3G84654 Mycgr3T
  
Location: 35295-36630

Mycgr3G84654\_Mycgr3T

Mycgr3G108090 Mycgr3
  
Location: 36730-37591

Mycgr3G108090\_Mycgr3

Mycgr3G21922 Mycgr3T
  
Location: 37691-39149

Mycgr3G21922\_Mycgr3T

Mycgr3G99148 Mycgr3T
  
Location: 39249-42819

Mycgr3G99148\_Mycgr3T

F-box domain protein
  
Accession: EED44814
  
Location: 254459-255224
  
 NCBI BlastP on this gene

EED44814

flocculation suppression protein
  
Accession: EED44813
  
Location: 251235-253346
  
 NCBI BlastP on this gene

EED44813

conserved hypothetical protein
  
Accession: EED44812
  
Location: 249687-250516
  
 NCBI BlastP on this gene

EED44812

ATP-dependent RNA helicase, putative
  
Accession: EED44811
  
Location: 247703-249357
  
 NCBI BlastP on this gene

EED44811

conserved hypothetical protein
  
Accession: EED44810
  
Location: 244363-245151
  
 NCBI BlastP on this gene

EED44810

60S ribosomal protein L13
  
Accession: EED44809
  
Location: 240450-241556
  
 NCBI BlastP on this gene

EED44809

conserved hypothetical protein
  
Accession: EED44808
  
Location: 238079-239165
  
  
**BlastP hit with Mycgr3G90786\_Mycgr3T**
  
Percentage identity: 27 %
  
BlastP bit score: 69
  
Sequence coverage: 55 %
  
E-value: 1e-09
  
  
 NCBI BlastP on this gene

EED44808

conserved leucine-rich repeat protein
  
Accession: EED44807
  
Location: 232924-235413
  
  
**BlastP hit with Mycgr3G68433\_Mycgr3T**
  
Percentage identity: 40 %
  
BlastP bit score: 469
  
Sequence coverage: 78 %
  
E-value: 2e-146
  
  
 NCBI BlastP on this gene

EED44807

conserved hypothetical protein
  
Accession: EED44806
  
Location: 231519-232655
  
 NCBI BlastP on this gene

EED44806

actin family protein
  
Accession: EED44805
  
Location: 229107-230684
  
 NCBI BlastP on this gene

EED44805

hypothetical protein
  
Accession: EED44804
  
Location: 227603-227905
  
 NCBI BlastP on this gene

EED44804

IBR domain protein
  
Accession: EED44803
  
Location: 226701-227560
  
 NCBI BlastP on this gene

EED44803

3-hydroxybutyryl-CoA dehydrogenase, putative
  
Accession: EED44802
  
Location: 225418-226532
  
 NCBI BlastP on this gene

EED44802

DNA repair protein (Tof1), putative
  
Accession: EED44801
  
Location: 222525-224483
  
  
**BlastP hit with Mycgr3G68421\_Mycgr3T**
  
Percentage identity: 51 %
  
BlastP bit score: 650
  
Sequence coverage: 55 %
  
E-value: 0.0
  
  
 NCBI BlastP on this gene

EED44801

conserved hypothetical protein
  
Accession: EED44800
  
Location: 218319-219362
  
 NCBI BlastP on this gene

EED44800

TFIIH and nucleotide excision repair factor 3 complexes subunit
  
Accession: EED44799
  
Location: 216203-217772
  
 NCBI BlastP on this gene

EED44799

conserved hypothetical protein
  
Accession: EED44798
  
Location: 214771-215942
  
 NCBI BlastP on this gene

EED44798

DEAD helicases superfamily protein (Aquarius), putative
  
Accession: EED44797
  
Location: 209853-214306
  
 NCBI BlastP on this gene

EED44797

THO complex subunit Tho1, putative
  
Accession: EED44796
  
Location: 207249-209609
  
 NCBI BlastP on this gene

EED44796

Query: Architecture Search FASTA input

FN430379 : Tuber melanosporum whole genome shotgun sequence assembly, scaffold\_95, strain Mel28.    Total score: 3.0     Cumulative Blast bit score: 1179

Hit cluster cross-links:

Mycgr3G90785 Mycgr3T
  
Location: 0-1047

Mycgr3G90785\_Mycgr3T

Mycgr3G103262 Mycgr3
  
Location: 1147-1390

Mycgr3G103262\_Mycgr3

Mycgr3G68458 Mycgr3T
  
Location: 1490-3602

Mycgr3G68458\_Mycgr3T

Mycgr3G99145 Mycgr3T
  
Location: 3702-4326

Mycgr3G99145\_Mycgr3T

Mycgr3G103274 Mycgr3
  
Location: 4426-4957

Mycgr3G103274\_Mycgr3

Mycgr3G103264 Mycgr3
  
Location: 5057-5390

Mycgr3G103264\_Mycgr3

Mycgr3G37570 Mycgr3T
  
Location: 5490-6006

Mycgr3G37570\_Mycgr3T

Mycgr3G108094 Mycgr3
  
Location: 6106-10555

Mycgr3G108094\_Mycgr3

Mycgr3G90786 Mycgr3T
  
Location: 10655-12080

Mycgr3G90786\_Mycgr3T

Mycgr3G68429 Mycgr3T
  
Location: 12180-13440

Mycgr3G68429\_Mycgr3T

Mycgr3G68421 Mycgr3T
  
Location: 13540-17086

Mycgr3G68421\_Mycgr3T

Mycgr3G90801 Mycgr3T
  
Location: 17186-18056

Mycgr3G90801\_Mycgr3T

Mycgr3G84646 Mycgr3T
  
Location: 18156-20235

Mycgr3G84646\_Mycgr3T

Mycgr3G68456 Mycgr3T
  
Location: 20335-21970

Mycgr3G68456\_Mycgr3T

Mycgr3G103270 Mycgr3
  
Location: 22070-22355

Mycgr3G103270\_Mycgr3

Mycgr3G90803 Mycgr3T
  
Location: 22455-23019

Mycgr3G90803\_Mycgr3T

Mycgr3G36941 Mycgr3T
  
Location: 23119-24064

Mycgr3G36941\_Mycgr3T

Mycgr3G25746 Mycgr3T
  
Location: 24164-25241

Mycgr3G25746\_Mycgr3T

Mycgr3G90788 Mycgr3T
  
Location: 25341-25803

Mycgr3G90788\_Mycgr3T

Mycgr3G103260 Mycgr3
  
Location: 25903-26635

Mycgr3G103260\_Mycgr3

Mycgr3G84644 Mycgr3T
  
Location: 26735-28457

Mycgr3G84644\_Mycgr3T

Mycgr3G29227 Mycgr3T
  
Location: 28557-28863

Mycgr3G29227\_Mycgr3T

Mycgr3G36271 Mycgr3T
  
Location: 28963-29854

Mycgr3G36271\_Mycgr3T

Mycgr3G68433 Mycgr3T
  
Location: 29954-33041

Mycgr3G68433\_Mycgr3T

Mycgr3G79452 Mycgr3T
  
Location: 33141-33399

Mycgr3G79452\_Mycgr3T

Mycgr3G55345 Mycgr3T
  
Location: 33499-34126

Mycgr3G55345\_Mycgr3T

Mycgr3G103278 Mycgr3
  
Location: 34226-35195

Mycgr3G103278\_Mycgr3

Mycgr3G84654 Mycgr3T
  
Location: 35295-36630

Mycgr3G84654\_Mycgr3T

Mycgr3G108090 Mycgr3
  
Location: 36730-37591

Mycgr3G108090\_Mycgr3

Mycgr3G21922 Mycgr3T
  
Location: 37691-39149

Mycgr3G21922\_Mycgr3T

Mycgr3G99148 Mycgr3T
  
Location: 39249-42819

Mycgr3G99148\_Mycgr3T

not annotated
  
Accession: CAZ86551
  
Location: 151586-152517
  
 NCBI BlastP on this gene

CAZ86551

not annotated
  
Accession: CAZ86552
  
Location: 155103-158795
  
  
**BlastP hit with Mycgr3G108094\_Mycgr3**
  
Percentage identity: 35 %
  
BlastP bit score: 310
  
Sequence coverage: 45 %
  
E-value: 8e-84
  
  
 NCBI BlastP on this gene

CAZ86552

not annotated
  
Accession: CAZ86553
  
Location: 159919-162135
  
  
**BlastP hit with Mycgr3G84646\_Mycgr3T**
  
Percentage identity: 44 %
  
BlastP bit score: 510
  
Sequence coverage: 104 %
  
E-value: 3e-168
  
  
 NCBI BlastP on this gene

CAZ86553

not annotated
  
Accession: CAZ86554
  
Location: 163092-163997
  
  
**BlastP hit with Mycgr3G99145\_Mycgr3T**
  
Percentage identity: 84 %
  
BlastP bit score: 359
  
Sequence coverage: 101 %
  
E-value: 2e-123
  
  
 NCBI BlastP on this gene

CAZ86554

not annotated
  
Accession: CAZ86555
  
Location: 164888-165301
  
 NCBI BlastP on this gene

CAZ86555

not annotated
  
Accession: CAZ86556
  
Location: 167971-168573
  
 NCBI BlastP on this gene

CAZ86556

not annotated
  
Accession: CAZ86557
  
Location: 168579-168680
  
 NCBI BlastP on this gene

CAZ86557

not annotated
  
Accession: CAZ86558
  
Location: 177276-178037
  
 NCBI BlastP on this gene

CAZ86558

not annotated
  
Accession: CAZ86559
  
Location: 180119-182273
  
 NCBI BlastP on this gene

CAZ86559

not annotated
  
Accession: CAZ86560
  
Location: 183024-185233
  
 NCBI BlastP on this gene

CAZ86560

Query: Architecture Search FASTA input

AP007172 : Aspergillus oryzae RIB40 DNA, SC206.    Total score: 3.0     Cumulative Blast bit score: 1179

Hit cluster cross-links:

Mycgr3G90785 Mycgr3T
  
Location: 0-1047

Mycgr3G90785\_Mycgr3T

Mycgr3G103262 Mycgr3
  
Location: 1147-1390

Mycgr3G103262\_Mycgr3

Mycgr3G68458 Mycgr3T
  
Location: 1490-3602

Mycgr3G68458\_Mycgr3T

Mycgr3G99145 Mycgr3T
  
Location: 3702-4326

Mycgr3G99145\_Mycgr3T

Mycgr3G103274 Mycgr3
  
Location: 4426-4957

Mycgr3G103274\_Mycgr3

Mycgr3G103264 Mycgr3
  
Location: 5057-5390

Mycgr3G103264\_Mycgr3

Mycgr3G37570 Mycgr3T
  
Location: 5490-6006

Mycgr3G37570\_Mycgr3T

Mycgr3G108094 Mycgr3
  
Location: 6106-10555

Mycgr3G108094\_Mycgr3

Mycgr3G90786 Mycgr3T
  
Location: 10655-12080

Mycgr3G90786\_Mycgr3T

Mycgr3G68429 Mycgr3T
  
Location: 12180-13440

Mycgr3G68429\_Mycgr3T

Mycgr3G68421 Mycgr3T
  
Location: 13540-17086

Mycgr3G68421\_Mycgr3T

Mycgr3G90801 Mycgr3T
  
Location: 17186-18056

Mycgr3G90801\_Mycgr3T

Mycgr3G84646 Mycgr3T
  
Location: 18156-20235

Mycgr3G84646\_Mycgr3T

Mycgr3G68456 Mycgr3T
  
Location: 20335-21970

Mycgr3G68456\_Mycgr3T

Mycgr3G103270 Mycgr3
  
Location: 22070-22355

Mycgr3G103270\_Mycgr3

Mycgr3G90803 Mycgr3T
  
Location: 22455-23019

Mycgr3G90803\_Mycgr3T

Mycgr3G36941 Mycgr3T
  
Location: 23119-24064

Mycgr3G36941\_Mycgr3T

Mycgr3G25746 Mycgr3T
  
Location: 24164-25241

Mycgr3G25746\_Mycgr3T

Mycgr3G90788 Mycgr3T
  
Location: 25341-25803

Mycgr3G90788\_Mycgr3T

Mycgr3G103260 Mycgr3
  
Location: 25903-26635

Mycgr3G103260\_Mycgr3

Mycgr3G84644 Mycgr3T
  
Location: 26735-28457

Mycgr3G84644\_Mycgr3T

Mycgr3G29227 Mycgr3T
  
Location: 28557-28863

Mycgr3G29227\_Mycgr3T

Mycgr3G36271 Mycgr3T
  
Location: 28963-29854

Mycgr3G36271\_Mycgr3T

Mycgr3G68433 Mycgr3T
  
Location: 29954-33041

Mycgr3G68433\_Mycgr3T

Mycgr3G79452 Mycgr3T
  
Location: 33141-33399

Mycgr3G79452\_Mycgr3T

Mycgr3G55345 Mycgr3T
  
Location: 33499-34126

Mycgr3G55345\_Mycgr3T

Mycgr3G103278 Mycgr3
  
Location: 34226-35195

Mycgr3G103278\_Mycgr3

Mycgr3G84654 Mycgr3T
  
Location: 35295-36630

Mycgr3G84654\_Mycgr3T

Mycgr3G108090 Mycgr3
  
Location: 36730-37591

Mycgr3G108090\_Mycgr3

Mycgr3G21922 Mycgr3T
  
Location: 37691-39149

Mycgr3G21922\_Mycgr3T

Mycgr3G99148 Mycgr3T
  
Location: 39249-42819

Mycgr3G99148\_Mycgr3T

not annotated
  
Accession: BAE65381
  
Location: 1556-3373
  
  
**BlastP hit with Mycgr3G36271\_Mycgr3T**
  
Percentage identity: 74 %
  
BlastP bit score: 469
  
Sequence coverage: 99 %
  
E-value: 6e-164
  
  
 NCBI BlastP on this gene

AO090206000001

not annotated
  
Accession: BAE65382
  
Location: 3502-5607
  
  
**BlastP hit with Mycgr3G84646\_Mycgr3T**
  
Percentage identity: 37 %
  
BlastP bit score: 363
  
Sequence coverage: 82 %
  
E-value: 7e-112
  
  
 NCBI BlastP on this gene

AO090206000002

not annotated
  
Accession: BAE65383
  
Location: 6851-7590
  
  
**BlastP hit with Mycgr3G99145\_Mycgr3T**
  
Percentage identity: 82 %
  
BlastP bit score: 347
  
Sequence coverage: 100 %
  
E-value: 9e-119
  
  
 NCBI BlastP on this gene

AO090206000003

not annotated
  
Accession: BAE65384
  
Location: 9980-12289
  
 NCBI BlastP on this gene

AO090206000004

not annotated
  
Accession: BAE65385
  
Location: 12764-13859
  
 NCBI BlastP on this gene

AO090206000005

not annotated
  
Accession: BAE65386
  
Location: 14114-20724
  
 NCBI BlastP on this gene

AO090206000006

not annotated
  
Accession: BAE65387
  
Location: 23784-24408
  
 NCBI BlastP on this gene

AO090206000009

not annotated
  
Accession: BAE65388
  
Location: 24633-25427
  
 NCBI BlastP on this gene

AO090206000010

not annotated
  
Accession: BAE65389
  
Location: 27395-29014
  
 NCBI BlastP on this gene

AO090206000012

Query: Architecture Search FASTA input

AKHY01000145 : Aspergillus oryzae 3.042    Total score: 3.0     Cumulative Blast bit score: 1179

Hit cluster cross-links:

Mycgr3G90785 Mycgr3T
  
Location: 0-1047

Mycgr3G90785\_Mycgr3T

Mycgr3G103262 Mycgr3
  
Location: 1147-1390

Mycgr3G103262\_Mycgr3

Mycgr3G68458 Mycgr3T
  
Location: 1490-3602

Mycgr3G68458\_Mycgr3T

Mycgr3G99145 Mycgr3T
  
Location: 3702-4326

Mycgr3G99145\_Mycgr3T

Mycgr3G103274 Mycgr3
  
Location: 4426-4957

Mycgr3G103274\_Mycgr3

Mycgr3G103264 Mycgr3
  
Location: 5057-5390

Mycgr3G103264\_Mycgr3

Mycgr3G37570 Mycgr3T
  
Location: 5490-6006

Mycgr3G37570\_Mycgr3T

Mycgr3G108094 Mycgr3
  
Location: 6106-10555

Mycgr3G108094\_Mycgr3

Mycgr3G90786 Mycgr3T
  
Location: 10655-12080

Mycgr3G90786\_Mycgr3T

Mycgr3G68429 Mycgr3T
  
Location: 12180-13440

Mycgr3G68429\_Mycgr3T

Mycgr3G68421 Mycgr3T
  
Location: 13540-17086

Mycgr3G68421\_Mycgr3T

Mycgr3G90801 Mycgr3T
  
Location: 17186-18056

Mycgr3G90801\_Mycgr3T

Mycgr3G84646 Mycgr3T
  
Location: 18156-20235

Mycgr3G84646\_Mycgr3T

Mycgr3G68456 Mycgr3T
  
Location: 20335-21970

Mycgr3G68456\_Mycgr3T

Mycgr3G103270 Mycgr3
  
Location: 22070-22355

Mycgr3G103270\_Mycgr3

Mycgr3G90803 Mycgr3T
  
Location: 22455-23019

Mycgr3G90803\_Mycgr3T

Mycgr3G36941 Mycgr3T
  
Location: 23119-24064

Mycgr3G36941\_Mycgr3T

Mycgr3G25746 Mycgr3T
  
Location: 24164-25241

Mycgr3G25746\_Mycgr3T

Mycgr3G90788 Mycgr3T
  
Location: 25341-25803

Mycgr3G90788\_Mycgr3T

Mycgr3G103260 Mycgr3
  
Location: 25903-26635

Mycgr3G103260\_Mycgr3

Mycgr3G84644 Mycgr3T
  
Location: 26735-28457

Mycgr3G84644\_Mycgr3T

Mycgr3G29227 Mycgr3T
  
Location: 28557-28863

Mycgr3G29227\_Mycgr3T

Mycgr3G36271 Mycgr3T
  
Location: 28963-29854

Mycgr3G36271\_Mycgr3T

Mycgr3G68433 Mycgr3T
  
Location: 29954-33041

Mycgr3G68433\_Mycgr3T

Mycgr3G79452 Mycgr3T
  
Location: 33141-33399

Mycgr3G79452\_Mycgr3T

Mycgr3G55345 Mycgr3T
  
Location: 33499-34126

Mycgr3G55345\_Mycgr3T

Mycgr3G103278 Mycgr3
  
Location: 34226-35195

Mycgr3G103278\_Mycgr3

Mycgr3G84654 Mycgr3T
  
Location: 35295-36630

Mycgr3G84654\_Mycgr3T

Mycgr3G108090 Mycgr3
  
Location: 36730-37591

Mycgr3G108090\_Mycgr3

Mycgr3G21922 Mycgr3T
  
Location: 37691-39149

Mycgr3G21922\_Mycgr3T

Mycgr3G99148 Mycgr3T
  
Location: 39249-42819

Mycgr3G99148\_Mycgr3T

C-4 sterol methyl oxidase
  
Accession: EIT77737
  
Location: 577-1639
  
  
**BlastP hit with Mycgr3G36271\_Mycgr3T**
  
Percentage identity: 74 %
  
BlastP bit score: 469
  
Sequence coverage: 99 %
  
E-value: 6e-164
  
  
 NCBI BlastP on this gene

EIT77737

hypothetical protein
  
Accession: EIT77740
  
Location: 2522-4627
  
  
**BlastP hit with Mycgr3G84646\_Mycgr3T**
  
Percentage identity: 37 %
  
BlastP bit score: 363
  
Sequence coverage: 82 %
  
E-value: 7e-112
  
  
 NCBI BlastP on this gene

EIT77740

GTP-binding protein
  
Accession: EIT77742
  
Location: 5870-6609
  
  
**BlastP hit with Mycgr3G99145\_Mycgr3T**
  
Percentage identity: 82 %
  
BlastP bit score: 347
  
Sequence coverage: 100 %
  
E-value: 9e-119
  
  
 NCBI BlastP on this gene

EIT77742

AAA+-type ATPase
  
Accession: EIT77733
  
Location: 9000-11309
  
 NCBI BlastP on this gene

EIT77733

hypothetical protein
  
Accession: EIT77729
  
Location: 11784-12879
  
 NCBI BlastP on this gene

EIT77729

protein kinase of the PI-3 kinase family
  
Accession: EIT77736
  
Location: 13134-22007
  
 NCBI BlastP on this gene

EIT77736

adenosine deaminase
  
Accession: EIT77741
  
Location: 22804-23428
  
 NCBI BlastP on this gene

EIT77741

NADH-ubiquinone oxidoreductase subunit
  
Accession: EIT77746
  
Location: 23653-24447
  
 NCBI BlastP on this gene

EIT77746

RCC1 domain protein
  
Accession: EIT77728
  
Location: 26415-28034
  
 NCBI BlastP on this gene

EIT77728

Query: Architecture Search FASTA input

KB644412 : Penicillium oxalicum 114-2 unplaced genomic scaffold scaffold\_5    Total score: 3.0     Cumulative Blast bit score: 1154

Hit cluster cross-links:

Mycgr3G90785 Mycgr3T
  
Location: 0-1047

Mycgr3G90785\_Mycgr3T

Mycgr3G103262 Mycgr3
  
Location: 1147-1390

Mycgr3G103262\_Mycgr3

Mycgr3G68458 Mycgr3T
  
Location: 1490-3602

Mycgr3G68458\_Mycgr3T

Mycgr3G99145 Mycgr3T
  
Location: 3702-4326

Mycgr3G99145\_Mycgr3T

Mycgr3G103274 Mycgr3
  
Location: 4426-4957

Mycgr3G103274\_Mycgr3

Mycgr3G103264 Mycgr3
  
Location: 5057-5390

Mycgr3G103264\_Mycgr3

Mycgr3G37570 Mycgr3T
  
Location: 5490-6006

Mycgr3G37570\_Mycgr3T

Mycgr3G108094 Mycgr3
  
Location: 6106-10555

Mycgr3G108094\_Mycgr3

Mycgr3G90786 Mycgr3T
  
Location: 10655-12080

Mycgr3G90786\_Mycgr3T

Mycgr3G68429 Mycgr3T
  
Location: 12180-13440

Mycgr3G68429\_Mycgr3T

Mycgr3G68421 Mycgr3T
  
Location: 13540-17086

Mycgr3G68421\_Mycgr3T

Mycgr3G90801 Mycgr3T
  
Location: 17186-18056

Mycgr3G90801\_Mycgr3T

Mycgr3G84646 Mycgr3T
  
Location: 18156-20235

Mycgr3G84646\_Mycgr3T

Mycgr3G68456 Mycgr3T
  
Location: 20335-21970

Mycgr3G68456\_Mycgr3T

Mycgr3G103270 Mycgr3
  
Location: 22070-22355

Mycgr3G103270\_Mycgr3

Mycgr3G90803 Mycgr3T
  
Location: 22455-23019

Mycgr3G90803\_Mycgr3T

Mycgr3G36941 Mycgr3T
  
Location: 23119-24064

Mycgr3G36941\_Mycgr3T

Mycgr3G25746 Mycgr3T
  
Location: 24164-25241

Mycgr3G25746\_Mycgr3T

Mycgr3G90788 Mycgr3T
  
Location: 25341-25803

Mycgr3G90788\_Mycgr3T

Mycgr3G103260 Mycgr3
  
Location: 25903-26635

Mycgr3G103260\_Mycgr3

Mycgr3G84644 Mycgr3T
  
Location: 26735-28457

Mycgr3G84644\_Mycgr3T

Mycgr3G29227 Mycgr3T
  
Location: 28557-28863

Mycgr3G29227\_Mycgr3T

Mycgr3G36271 Mycgr3T
  
Location: 28963-29854

Mycgr3G36271\_Mycgr3T

Mycgr3G68433 Mycgr3T
  
Location: 29954-33041

Mycgr3G68433\_Mycgr3T

Mycgr3G79452 Mycgr3T
  
Location: 33141-33399

Mycgr3G79452\_Mycgr3T

Mycgr3G55345 Mycgr3T
  
Location: 33499-34126

Mycgr3G55345\_Mycgr3T

Mycgr3G103278 Mycgr3
  
Location: 34226-35195

Mycgr3G103278\_Mycgr3

Mycgr3G84654 Mycgr3T
  
Location: 35295-36630

Mycgr3G84654\_Mycgr3T

Mycgr3G108090 Mycgr3
  
Location: 36730-37591

Mycgr3G108090\_Mycgr3

Mycgr3G21922 Mycgr3T
  
Location: 37691-39149

Mycgr3G21922\_Mycgr3T

Mycgr3G99148 Mycgr3T
  
Location: 39249-42819

Mycgr3G99148\_Mycgr3T

hypothetical protein
  
Accession: EPS30860
  
Location: 3964233-3966734
  
 NCBI BlastP on this gene

EPS30860

hypothetical protein
  
Accession: EPS30859
  
Location: 3960559-3963864
  
 NCBI BlastP on this gene

EPS30859

hypothetical protein
  
Accession: EPS30858
  
Location: 3956391-3959698
  
 NCBI BlastP on this gene

EPS30858

hypothetical protein
  
Accession: EPS30857
  
Location: 3952055-3952258
  
 NCBI BlastP on this gene

EPS30857

hypothetical protein
  
Accession: EPS30856
  
Location: 3948553-3949469
  
  
**BlastP hit with Mycgr3G36271\_Mycgr3T**
  
Percentage identity: 76 %
  
BlastP bit score: 403
  
Sequence coverage: 82 %
  
E-value: 2e-138
  
  
 NCBI BlastP on this gene

EPS30856

hypothetical protein
  
Accession: EPS30855
  
Location: 3944607-3946756
  
  
**BlastP hit with Mycgr3G84646\_Mycgr3T**
  
Percentage identity: 40 %
  
BlastP bit score: 409
  
Sequence coverage: 85 %
  
E-value: 1e-129
  
  
 NCBI BlastP on this gene

EPS30855

hypothetical protein
  
Accession: EPS30854
  
Location: 3942875-3943679
  
  
**BlastP hit with Mycgr3G99145\_Mycgr3T**
  
Percentage identity: 83 %
  
BlastP bit score: 342
  
Sequence coverage: 99 %
  
E-value: 1e-116
  
  
 NCBI BlastP on this gene

EPS30854

hypothetical protein
  
Accession: EPS30853
  
Location: 3939210-3941569
  
 NCBI BlastP on this gene

EPS30853

hypothetical protein
  
Accession: EPS30852
  
Location: 3937775-3938780
  
 NCBI BlastP on this gene

EPS30852

hypothetical protein
  
Accession: EPS30851
  
Location: 3928481-3937364
  
 NCBI BlastP on this gene

EPS30851

hypothetical protein
  
Accession: EPS30850
  
Location: 3927027-3927966
  
 NCBI BlastP on this gene

EPS30850

hypothetical protein
  
Accession: EPS30849
  
Location: 3925933-3926774
  
 NCBI BlastP on this gene

EPS30849

hypothetical protein
  
Accession: EPS30848
  
Location: 3924612-3925674
  
 NCBI BlastP on this gene

EPS30848

Query: Architecture Search FASTA input

DS499603 : Aspergillus fumigatus A1163 scf\_000010 genomic scaffold    Total score: 3.0     Cumulative Blast bit score: 1129

Hit cluster cross-links:

Mycgr3G90785 Mycgr3T
  
Location: 0-1047

Mycgr3G90785\_Mycgr3T

Mycgr3G103262 Mycgr3
  
Location: 1147-1390

Mycgr3G103262\_Mycgr3

Mycgr3G68458 Mycgr3T
  
Location: 1490-3602

Mycgr3G68458\_Mycgr3T

Mycgr3G99145 Mycgr3T
  
Location: 3702-4326

Mycgr3G99145\_Mycgr3T

Mycgr3G103274 Mycgr3
  
Location: 4426-4957

Mycgr3G103274\_Mycgr3

Mycgr3G103264 Mycgr3
  
Location: 5057-5390

Mycgr3G103264\_Mycgr3

Mycgr3G37570 Mycgr3T
  
Location: 5490-6006

Mycgr3G37570\_Mycgr3T

Mycgr3G108094 Mycgr3
  
Location: 6106-10555

Mycgr3G108094\_Mycgr3

Mycgr3G90786 Mycgr3T
  
Location: 10655-12080

Mycgr3G90786\_Mycgr3T

Mycgr3G68429 Mycgr3T
  
Location: 12180-13440

Mycgr3G68429\_Mycgr3T

Mycgr3G68421 Mycgr3T
  
Location: 13540-17086

Mycgr3G68421\_Mycgr3T

Mycgr3G90801 Mycgr3T
  
Location: 17186-18056

Mycgr3G90801\_Mycgr3T

Mycgr3G84646 Mycgr3T
  
Location: 18156-20235

Mycgr3G84646\_Mycgr3T

Mycgr3G68456 Mycgr3T
  
Location: 20335-21970

Mycgr3G68456\_Mycgr3T

Mycgr3G103270 Mycgr3
  
Location: 22070-22355

Mycgr3G103270\_Mycgr3

Mycgr3G90803 Mycgr3T
  
Location: 22455-23019

Mycgr3G90803\_Mycgr3T

Mycgr3G36941 Mycgr3T
  
Location: 23119-24064

Mycgr3G36941\_Mycgr3T

Mycgr3G25746 Mycgr3T
  
Location: 24164-25241

Mycgr3G25746\_Mycgr3T

Mycgr3G90788 Mycgr3T
  
Location: 25341-25803

Mycgr3G90788\_Mycgr3T

Mycgr3G103260 Mycgr3
  
Location: 25903-26635

Mycgr3G103260\_Mycgr3

Mycgr3G84644 Mycgr3T
  
Location: 26735-28457

Mycgr3G84644\_Mycgr3T

Mycgr3G29227 Mycgr3T
  
Location: 28557-28863

Mycgr3G29227\_Mycgr3T

Mycgr3G36271 Mycgr3T
  
Location: 28963-29854

Mycgr3G36271\_Mycgr3T

Mycgr3G68433 Mycgr3T
  
Location: 29954-33041

Mycgr3G68433\_Mycgr3T

Mycgr3G79452 Mycgr3T
  
Location: 33141-33399

Mycgr3G79452\_Mycgr3T

Mycgr3G55345 Mycgr3T
  
Location: 33499-34126

Mycgr3G55345\_Mycgr3T

Mycgr3G103278 Mycgr3
  
Location: 34226-35195

Mycgr3G103278\_Mycgr3

Mycgr3G84654 Mycgr3T
  
Location: 35295-36630

Mycgr3G84654\_Mycgr3T

Mycgr3G108090 Mycgr3
  
Location: 36730-37591

Mycgr3G108090\_Mycgr3

Mycgr3G21922 Mycgr3T
  
Location: 37691-39149

Mycgr3G21922\_Mycgr3T

Mycgr3G99148 Mycgr3T
  
Location: 39249-42819

Mycgr3G99148\_Mycgr3T

C-4 methyl sterol oxidase (Erg25), putative
  
Accession: EDP47217
  
Location: 10520-11519
  
  
**BlastP hit with Mycgr3G36271\_Mycgr3T**
  
Percentage identity: 75 %
  
BlastP bit score: 469
  
Sequence coverage: 95 %
  
E-value: 9e-164
  
  
 NCBI BlastP on this gene

EDP47217

hypothetical protein
  
Accession: EDP47218
  
Location: 12807-14084
  
  
**BlastP hit with Mycgr3G84646\_Mycgr3T**
  
Percentage identity: 41 %
  
BlastP bit score: 320
  
Sequence coverage: 65 %
  
E-value: 4e-98
  
  
 NCBI BlastP on this gene

EDP47218

Rab GTPase SrgA, putative
  
Accession: EDP47219
  
Location: 15998-16742
  
  
**BlastP hit with Mycgr3G99145\_Mycgr3T**
  
Percentage identity: 89 %
  
BlastP bit score: 340
  
Sequence coverage: 87 %
  
E-value: 1e-115
  
  
 NCBI BlastP on this gene

EDP47219

AAA family ATPase, putative
  
Accession: EDP47220
  
Location: 17605-19932
  
 NCBI BlastP on this gene

EDP47220

conserved hypothetical protein
  
Accession: EDP47221
  
Location: 20115-20552
  
 NCBI BlastP on this gene

EDP47221

conserved hypothetical protein
  
Accession: EDP47222
  
Location: 20919-21290
  
 NCBI BlastP on this gene

EDP47222

inositol kinase kinase (UvsB), putative
  
Accession: EDP47223
  
Location: 21684-30524
  
 NCBI BlastP on this gene

EDP47223

NADH-ubiquinone oxidoreductase 21 kDa subunit, putative
  
Accession: EDP47224
  
Location: 32227-32783
  
 NCBI BlastP on this gene

EDP47224

Swi3-like protein
  
Accession: EDP47225
  
Location: 33723-34655
  
 NCBI BlastP on this gene

EDP47225

Ran exchange factor Prp20/Pim1, putative
  
Accession: EDP47226
  
Location: 35138-36878
  
 NCBI BlastP on this gene

EDP47226

Query: Architecture Search FASTA input

CH476596 : Aspergillus terreus NIH2624 scaffold\_3 genomic scaffold    Total score: 3.0     Cumulative Blast bit score: 1114

Hit cluster cross-links:

Mycgr3G90785 Mycgr3T
  
Location: 0-1047

Mycgr3G90785\_Mycgr3T

Mycgr3G103262 Mycgr3
  
Location: 1147-1390

Mycgr3G103262\_Mycgr3

Mycgr3G68458 Mycgr3T
  
Location: 1490-3602

Mycgr3G68458\_Mycgr3T

Mycgr3G99145 Mycgr3T
  
Location: 3702-4326

Mycgr3G99145\_Mycgr3T

Mycgr3G103274 Mycgr3
  
Location: 4426-4957

Mycgr3G103274\_Mycgr3

Mycgr3G103264 Mycgr3
  
Location: 5057-5390

Mycgr3G103264\_Mycgr3

Mycgr3G37570 Mycgr3T
  
Location: 5490-6006

Mycgr3G37570\_Mycgr3T

Mycgr3G108094 Mycgr3
  
Location: 6106-10555

Mycgr3G108094\_Mycgr3

Mycgr3G90786 Mycgr3T
  
Location: 10655-12080

Mycgr3G90786\_Mycgr3T

Mycgr3G68429 Mycgr3T
  
Location: 12180-13440

Mycgr3G68429\_Mycgr3T

Mycgr3G68421 Mycgr3T
  
Location: 13540-17086

Mycgr3G68421\_Mycgr3T

Mycgr3G90801 Mycgr3T
  
Location: 17186-18056

Mycgr3G90801\_Mycgr3T

Mycgr3G84646 Mycgr3T
  
Location: 18156-20235

Mycgr3G84646\_Mycgr3T

Mycgr3G68456 Mycgr3T
  
Location: 20335-21970

Mycgr3G68456\_Mycgr3T

Mycgr3G103270 Mycgr3
  
Location: 22070-22355

Mycgr3G103270\_Mycgr3

Mycgr3G90803 Mycgr3T
  
Location: 22455-23019

Mycgr3G90803\_Mycgr3T

Mycgr3G36941 Mycgr3T
  
Location: 23119-24064

Mycgr3G36941\_Mycgr3T

Mycgr3G25746 Mycgr3T
  
Location: 24164-25241

Mycgr3G25746\_Mycgr3T

Mycgr3G90788 Mycgr3T
  
Location: 25341-25803

Mycgr3G90788\_Mycgr3T

Mycgr3G103260 Mycgr3
  
Location: 25903-26635

Mycgr3G103260\_Mycgr3

Mycgr3G84644 Mycgr3T
  
Location: 26735-28457

Mycgr3G84644\_Mycgr3T

Mycgr3G29227 Mycgr3T
  
Location: 28557-28863

Mycgr3G29227\_Mycgr3T

Mycgr3G36271 Mycgr3T
  
Location: 28963-29854

Mycgr3G36271\_Mycgr3T

Mycgr3G68433 Mycgr3T
  
Location: 29954-33041

Mycgr3G68433\_Mycgr3T

Mycgr3G79452 Mycgr3T
  
Location: 33141-33399

Mycgr3G79452\_Mycgr3T

Mycgr3G55345 Mycgr3T
  
Location: 33499-34126

Mycgr3G55345\_Mycgr3T

Mycgr3G103278 Mycgr3
  
Location: 34226-35195

Mycgr3G103278\_Mycgr3

Mycgr3G84654 Mycgr3T
  
Location: 35295-36630

Mycgr3G84654\_Mycgr3T

Mycgr3G108090 Mycgr3
  
Location: 36730-37591

Mycgr3G108090\_Mycgr3

Mycgr3G21922 Mycgr3T
  
Location: 37691-39149

Mycgr3G21922\_Mycgr3T

Mycgr3G99148 Mycgr3T
  
Location: 39249-42819

Mycgr3G99148\_Mycgr3T

conserved hypothetical protein
  
Accession: EAU37738
  
Location: 2467530-2469104
  
 NCBI BlastP on this gene

EAU37738

C-4 methylsterol oxidase
  
Accession: EAU37737
  
Location: 2466097-2466962
  
  
**BlastP hit with Mycgr3G36271\_Mycgr3T**
  
Percentage identity: 81 %
  
BlastP bit score: 422
  
Sequence coverage: 80 %
  
E-value: 4e-146
  
  
 NCBI BlastP on this gene

EAU37737

predicted protein
  
Accession: EAU37736
  
Location: 2463031-2464936
  
  
**BlastP hit with Mycgr3G84646\_Mycgr3T**
  
Percentage identity: 41 %
  
BlastP bit score: 353
  
Sequence coverage: 72 %
  
E-value: 5e-109
  
  
 NCBI BlastP on this gene

EAU37736

GTP-binding protein SAS1
  
Accession: EAU37735
  
Location: 2460896-2461633
  
  
**BlastP hit with Mycgr3G99145\_Mycgr3T**
  
Percentage identity: 88 %
  
BlastP bit score: 339
  
Sequence coverage: 87 %
  
E-value: 2e-115
  
  
 NCBI BlastP on this gene

EAU37735

conserved hypothetical protein
  
Accession: EAU37734
  
Location: 2457631-2459940
  
 NCBI BlastP on this gene

EAU37734

hypothetical protein
  
Accession: EAU37733
  
Location: 2448879-2455967
  
 NCBI BlastP on this gene

EAU37733

hypothetical protein
  
Accession: EAU37732
  
Location: 2447365-2448015
  
 NCBI BlastP on this gene

EAU37732

NADH-ubiquinone oxidoreductase 21 kDa subunit
  
Accession: EAU37731
  
Location: 2445181-2445971
  
 NCBI BlastP on this gene

EAU37731

predicted protein
  
Accession: EAU37730
  
Location: 2443255-2444622
  
 NCBI BlastP on this gene

EAU37730

conserved hypothetical protein
  
Accession: EAU37729
  
Location: 2441389-2443038
  
 NCBI BlastP on this gene

EAU37729

Query: Architecture Search FASTA input

ACYE01000064 : Trichophyton verrucosum HKI 0517    Total score: 3.0     Cumulative Blast bit score: 1082

Hit cluster cross-links:

Mycgr3G90785 Mycgr3T
  
Location: 0-1047

Mycgr3G90785\_Mycgr3T

Mycgr3G103262 Mycgr3
  
Location: 1147-1390

Mycgr3G103262\_Mycgr3

Mycgr3G68458 Mycgr3T
  
Location: 1490-3602

Mycgr3G68458\_Mycgr3T

Mycgr3G99145 Mycgr3T
  
Location: 3702-4326

Mycgr3G99145\_Mycgr3T

Mycgr3G103274 Mycgr3
  
Location: 4426-4957

Mycgr3G103274\_Mycgr3

Mycgr3G103264 Mycgr3
  
Location: 5057-5390

Mycgr3G103264\_Mycgr3

Mycgr3G37570 Mycgr3T
  
Location: 5490-6006

Mycgr3G37570\_Mycgr3T

Mycgr3G108094 Mycgr3
  
Location: 6106-10555

Mycgr3G108094\_Mycgr3

Mycgr3G90786 Mycgr3T
  
Location: 10655-12080

Mycgr3G90786\_Mycgr3T

Mycgr3G68429 Mycgr3T
  
Location: 12180-13440

Mycgr3G68429\_Mycgr3T

Mycgr3G68421 Mycgr3T
  
Location: 13540-17086

Mycgr3G68421\_Mycgr3T

Mycgr3G90801 Mycgr3T
  
Location: 17186-18056

Mycgr3G90801\_Mycgr3T

Mycgr3G84646 Mycgr3T
  
Location: 18156-20235

Mycgr3G84646\_Mycgr3T

Mycgr3G68456 Mycgr3T
  
Location: 20335-21970

Mycgr3G68456\_Mycgr3T

Mycgr3G103270 Mycgr3
  
Location: 22070-22355

Mycgr3G103270\_Mycgr3

Mycgr3G90803 Mycgr3T
  
Location: 22455-23019

Mycgr3G90803\_Mycgr3T

Mycgr3G36941 Mycgr3T
  
Location: 23119-24064

Mycgr3G36941\_Mycgr3T

Mycgr3G25746 Mycgr3T
  
Location: 24164-25241

Mycgr3G25746\_Mycgr3T

Mycgr3G90788 Mycgr3T
  
Location: 25341-25803

Mycgr3G90788\_Mycgr3T

Mycgr3G103260 Mycgr3
  
Location: 25903-26635

Mycgr3G103260\_Mycgr3

Mycgr3G84644 Mycgr3T
  
Location: 26735-28457

Mycgr3G84644\_Mycgr3T

Mycgr3G29227 Mycgr3T
  
Location: 28557-28863

Mycgr3G29227\_Mycgr3T

Mycgr3G36271 Mycgr3T
  
Location: 28963-29854

Mycgr3G36271\_Mycgr3T

Mycgr3G68433 Mycgr3T
  
Location: 29954-33041

Mycgr3G68433\_Mycgr3T

Mycgr3G79452 Mycgr3T
  
Location: 33141-33399

Mycgr3G79452\_Mycgr3T

Mycgr3G55345 Mycgr3T
  
Location: 33499-34126

Mycgr3G55345\_Mycgr3T

Mycgr3G103278 Mycgr3
  
Location: 34226-35195

Mycgr3G103278\_Mycgr3

Mycgr3G84654 Mycgr3T
  
Location: 35295-36630

Mycgr3G84654\_Mycgr3T

Mycgr3G108090 Mycgr3
  
Location: 36730-37591

Mycgr3G108090\_Mycgr3

Mycgr3G21922 Mycgr3T
  
Location: 37691-39149

Mycgr3G21922\_Mycgr3T

Mycgr3G99148 Mycgr3T
  
Location: 39249-42819

Mycgr3G99148\_Mycgr3T

RING finger protein
  
Accession: EFE44038
  
Location: 328-1089
  
 NCBI BlastP on this gene

EFE44038

hypothetical protein
  
Accession: EFE44039
  
Location: 1323-3342
  
 NCBI BlastP on this gene

EFE44039

inositol monophosphatase QutG, putative
  
Accession: EFE44040
  
Location: 4332-5335
  
 NCBI BlastP on this gene

EFE44040

hypothetical protein
  
Accession: EFE44041
  
Location: 5553-10407
  
 NCBI BlastP on this gene

EFE44041

hypothetical protein
  
Accession: EFE44042
  
Location: 12291-13562
  
 NCBI BlastP on this gene

EFE44042

hypothetical protein
  
Accession: EFE44043
  
Location: 14269-18588
  
  
**BlastP hit with Mycgr3G99145\_Mycgr3T**
  
Percentage identity: 79 %
  
BlastP bit score: 328
  
Sequence coverage: 97 %
  
E-value: 2e-101
  
  
  
**BlastP hit with Mycgr3G84646\_Mycgr3T**
  
Percentage identity: 37 %
  
BlastP bit score: 437
  
Sequence coverage: 112 %
  
E-value: 5e-135
  
  
 NCBI BlastP on this gene

EFE44043

hypothetical protein
  
Accession: EFE44044
  
Location: 19035-19696
  
 NCBI BlastP on this gene

EFE44044

C-4 methyl sterol oxidase, putative
  
Accession: EFE44045
  
Location: 19800-20339
  
  
**BlastP hit with Mycgr3G36271\_Mycgr3T**
  
Percentage identity: 81 %
  
BlastP bit score: 317
  
Sequence coverage: 60 %
  
E-value: 1e-105
  
  
 NCBI BlastP on this gene

EFE44045

hypothetical protein
  
Accession: EFE44046
  
Location: 21443-23947
  
 NCBI BlastP on this gene

EFE44046

inositol kinase kinase (UvsB), putative
  
Accession: EFE44047
  
Location: 29514-36775
  
 NCBI BlastP on this gene

EFE44047

replication fork protection component Swi3, putative
  
Accession: EFE44048
  
Location: 39256-40377
  
 NCBI BlastP on this gene

EFE44048

Query: Architecture Search FASTA input

JH767573 : Coniosporium apollinis CBS 100218 chromosome Unknown supercont1.20    Total score: 3.0     Cumulative Blast bit score: 1052

Hit cluster cross-links:

Mycgr3G90785 Mycgr3T
  
Location: 0-1047

Mycgr3G90785\_Mycgr3T

Mycgr3G103262 Mycgr3
  
Location: 1147-1390

Mycgr3G103262\_Mycgr3

Mycgr3G68458 Mycgr3T
  
Location: 1490-3602

Mycgr3G68458\_Mycgr3T

Mycgr3G99145 Mycgr3T
  
Location: 3702-4326

Mycgr3G99145\_Mycgr3T

Mycgr3G103274 Mycgr3
  
Location: 4426-4957

Mycgr3G103274\_Mycgr3

Mycgr3G103264 Mycgr3
  
Location: 5057-5390

Mycgr3G103264\_Mycgr3

Mycgr3G37570 Mycgr3T
  
Location: 5490-6006

Mycgr3G37570\_Mycgr3T

Mycgr3G108094 Mycgr3
  
Location: 6106-10555

Mycgr3G108094\_Mycgr3

Mycgr3G90786 Mycgr3T
  
Location: 10655-12080

Mycgr3G90786\_Mycgr3T

Mycgr3G68429 Mycgr3T
  
Location: 12180-13440

Mycgr3G68429\_Mycgr3T

Mycgr3G68421 Mycgr3T
  
Location: 13540-17086

Mycgr3G68421\_Mycgr3T

Mycgr3G90801 Mycgr3T
  
Location: 17186-18056

Mycgr3G90801\_Mycgr3T

Mycgr3G84646 Mycgr3T
  
Location: 18156-20235

Mycgr3G84646\_Mycgr3T

Mycgr3G68456 Mycgr3T
  
Location: 20335-21970

Mycgr3G68456\_Mycgr3T

Mycgr3G103270 Mycgr3
  
Location: 22070-22355

Mycgr3G103270\_Mycgr3

Mycgr3G90803 Mycgr3T
  
Location: 22455-23019

Mycgr3G90803\_Mycgr3T

Mycgr3G36941 Mycgr3T
  
Location: 23119-24064

Mycgr3G36941\_Mycgr3T

Mycgr3G25746 Mycgr3T
  
Location: 24164-25241

Mycgr3G25746\_Mycgr3T

Mycgr3G90788 Mycgr3T
  
Location: 25341-25803

Mycgr3G90788\_Mycgr3T

Mycgr3G103260 Mycgr3
  
Location: 25903-26635

Mycgr3G103260\_Mycgr3

Mycgr3G84644 Mycgr3T
  
Location: 26735-28457

Mycgr3G84644\_Mycgr3T

Mycgr3G29227 Mycgr3T
  
Location: 28557-28863

Mycgr3G29227\_Mycgr3T

Mycgr3G36271 Mycgr3T
  
Location: 28963-29854

Mycgr3G36271\_Mycgr3T

Mycgr3G68433 Mycgr3T
  
Location: 29954-33041

Mycgr3G68433\_Mycgr3T

Mycgr3G79452 Mycgr3T
  
Location: 33141-33399

Mycgr3G79452\_Mycgr3T

Mycgr3G55345 Mycgr3T
  
Location: 33499-34126

Mycgr3G55345\_Mycgr3T

Mycgr3G103278 Mycgr3
  
Location: 34226-35195

Mycgr3G103278\_Mycgr3

Mycgr3G84654 Mycgr3T
  
Location: 35295-36630

Mycgr3G84654\_Mycgr3T

Mycgr3G108090 Mycgr3
  
Location: 36730-37591

Mycgr3G108090\_Mycgr3

Mycgr3G21922 Mycgr3T
  
Location: 37691-39149

Mycgr3G21922\_Mycgr3T

Mycgr3G99148 Mycgr3T
  
Location: 39249-42819

Mycgr3G99148\_Mycgr3T

pantothenate kinase
  
Accession: EON65344
  
Location: 284394-285964
  
 NCBI BlastP on this gene

EON65344

hypothetical protein
  
Accession: EON65345
  
Location: 286462-287683
  
 NCBI BlastP on this gene

EON65345

hypothetical protein
  
Accession: EON65346
  
Location: 289160-291049
  
 NCBI BlastP on this gene

EON65346

hypothetical protein
  
Accession: EON65347
  
Location: 291603-293328
  
 NCBI BlastP on this gene

EON65347

hypothetical protein
  
Accession: EON65348
  
Location: 294650-296903
  
 NCBI BlastP on this gene

EON65348

methylsterol monooxygenase
  
Accession: EON65349
  
Location: 297639-298854
  
  
**BlastP hit with Mycgr3G36271\_Mycgr3T**
  
Percentage identity: 84 %
  
BlastP bit score: 535
  
Sequence coverage: 98 %
  
E-value: 0.0
  
  
 NCBI BlastP on this gene

EON65349

hypothetical protein
  
Accession: EON65350
  
Location: 299508-300138
  
 NCBI BlastP on this gene

EON65350

hypothetical protein
  
Accession: EON65351
  
Location: 300541-302976
  
  
**BlastP hit with Mycgr3G68433\_Mycgr3T**
  
Percentage identity: 44 %
  
BlastP bit score: 459
  
Sequence coverage: 66 %
  
E-value: 3e-143
  
  
 NCBI BlastP on this gene

EON65351

hypothetical protein
  
Accession: EON65352
  
Location: 304091-305197
  
  
**BlastP hit with Mycgr3G90786\_Mycgr3T**
  
Percentage identity: 26 %
  
BlastP bit score: 58
  
Sequence coverage: 72 %
  
E-value: 2e-06
  
  
 NCBI BlastP on this gene

EON65352

FKBP12-rapamycin complex-associated protein
  
Accession: EON65353
  
Location: 306195-313744
  
 NCBI BlastP on this gene

EON65353

hypothetical protein
  
Accession: EON65354
  
Location: 314500-318013
  
 NCBI BlastP on this gene

EON65354

hypothetical protein
  
Accession: EON65355
  
Location: 318649-322746
  
 NCBI BlastP on this gene

EON65355

hypothetical protein
  
Accession: EON65356
  
Location: 323249-324208
  
 NCBI BlastP on this gene

EON65356

hypothetical protein
  
Accession: EON65357
  
Location: 324742-326947
  
 NCBI BlastP on this gene

EON65357

Query: Architecture Search FASTA input

AKCU01000109 : Penicillium digitatum Pd1    Total score: 3.0     Cumulative Blast bit score: 1033

Hit cluster cross-links:

Mycgr3G90785 Mycgr3T
  
Location: 0-1047

Mycgr3G90785\_Mycgr3T

Mycgr3G103262 Mycgr3
  
Location: 1147-1390

Mycgr3G103262\_Mycgr3

Mycgr3G68458 Mycgr3T
  
Location: 1490-3602

Mycgr3G68458\_Mycgr3T

Mycgr3G99145 Mycgr3T
  
Location: 3702-4326

Mycgr3G99145\_Mycgr3T

Mycgr3G103274 Mycgr3
  
Location: 4426-4957

Mycgr3G103274\_Mycgr3

Mycgr3G103264 Mycgr3
  
Location: 5057-5390

Mycgr3G103264\_Mycgr3

Mycgr3G37570 Mycgr3T
  
Location: 5490-6006

Mycgr3G37570\_Mycgr3T

Mycgr3G108094 Mycgr3
  
Location: 6106-10555

Mycgr3G108094\_Mycgr3

Mycgr3G90786 Mycgr3T
  
Location: 10655-12080

Mycgr3G90786\_Mycgr3T

Mycgr3G68429 Mycgr3T
  
Location: 12180-13440

Mycgr3G68429\_Mycgr3T

Mycgr3G68421 Mycgr3T
  
Location: 13540-17086

Mycgr3G68421\_Mycgr3T

Mycgr3G90801 Mycgr3T
  
Location: 17186-18056

Mycgr3G90801\_Mycgr3T

Mycgr3G84646 Mycgr3T
  
Location: 18156-20235

Mycgr3G84646\_Mycgr3T

Mycgr3G68456 Mycgr3T
  
Location: 20335-21970

Mycgr3G68456\_Mycgr3T

Mycgr3G103270 Mycgr3
  
Location: 22070-22355

Mycgr3G103270\_Mycgr3

Mycgr3G90803 Mycgr3T
  
Location: 22455-23019

Mycgr3G90803\_Mycgr3T

Mycgr3G36941 Mycgr3T
  
Location: 23119-24064

Mycgr3G36941\_Mycgr3T

Mycgr3G25746 Mycgr3T
  
Location: 24164-25241

Mycgr3G25746\_Mycgr3T

Mycgr3G90788 Mycgr3T
  
Location: 25341-25803

Mycgr3G90788\_Mycgr3T

Mycgr3G103260 Mycgr3
  
Location: 25903-26635

Mycgr3G103260\_Mycgr3

Mycgr3G84644 Mycgr3T
  
Location: 26735-28457

Mycgr3G84644\_Mycgr3T

Mycgr3G29227 Mycgr3T
  
Location: 28557-28863

Mycgr3G29227\_Mycgr3T

Mycgr3G36271 Mycgr3T
  
Location: 28963-29854

Mycgr3G36271\_Mycgr3T

Mycgr3G68433 Mycgr3T
  
Location: 29954-33041

Mycgr3G68433\_Mycgr3T

Mycgr3G79452 Mycgr3T
  
Location: 33141-33399

Mycgr3G79452\_Mycgr3T

Mycgr3G55345 Mycgr3T
  
Location: 33499-34126

Mycgr3G55345\_Mycgr3T

Mycgr3G103278 Mycgr3
  
Location: 34226-35195

Mycgr3G103278\_Mycgr3

Mycgr3G84654 Mycgr3T
  
Location: 35295-36630

Mycgr3G84654\_Mycgr3T

Mycgr3G108090 Mycgr3
  
Location: 36730-37591

Mycgr3G108090\_Mycgr3

Mycgr3G21922 Mycgr3T
  
Location: 37691-39149

Mycgr3G21922\_Mycgr3T

Mycgr3G99148 Mycgr3T
  
Location: 39249-42819

Mycgr3G99148\_Mycgr3T

C-4 methyl sterol oxidase Erg25, putative
  
Accession: EKV20600
  
Location: 917-1757
  
  
**BlastP hit with Mycgr3G36271\_Mycgr3T**
  
Percentage identity: 77 %
  
BlastP bit score: 404
  
Sequence coverage: 81 %
  
E-value: 6e-139
  
  
 NCBI BlastP on this gene

EKV20600

hypothetical protein
  
Accession: EKV20601
  
Location: 3779-4725
  
  
**BlastP hit with Mycgr3G84646\_Mycgr3T**
  
Percentage identity: 51 %
  
BlastP bit score: 286
  
Sequence coverage: 40 %
  
E-value: 2e-87
  
  
 NCBI BlastP on this gene

EKV20601

Rab GTPase SrgA, putative
  
Accession: EKV20602
  
Location: 5484-6241
  
  
**BlastP hit with Mycgr3G99145\_Mycgr3T**
  
Percentage identity: 86 %
  
BlastP bit score: 343
  
Sequence coverage: 93 %
  
E-value: 3e-117
  
  
 NCBI BlastP on this gene

EKV20602

AAA family ATPase, putative
  
Accession: EKV20603
  
Location: 6950-9272
  
 NCBI BlastP on this gene

EKV20603

Inositol kinase kinase (UvsB), putative
  
Accession: EKV20604
  
Location: 10336-17049
  
 NCBI BlastP on this gene

EKV20604

hypothetical protein
  
Accession: EKV20605
  
Location: 19043-19696
  
 NCBI BlastP on this gene

EKV20605

hypothetical protein
  
Accession: EKV20606
  
Location: 20319-21098
  
 NCBI BlastP on this gene

EKV20606

NADH-ubiquinone oxidoreductase 21 kDa subunit, putative
  
Accession: EKV20607
  
Location: 21354-22178
  
 NCBI BlastP on this gene

EKV20607

hypothetical protein
  
Accession: EKV20608
  
Location: 22520-23494
  
 NCBI BlastP on this gene

EKV20608

Ran exchange factor Prp20/Pim1, putative
  
Accession: EKV20609
  
Location: 23873-25564
  
 NCBI BlastP on this gene

EKV20609

Riboflavin-specific deaminase
  
Accession: EKV20610
  
Location: 25926-27752
  
 NCBI BlastP on this gene

EKV20610

Query: Architecture Search FASTA input

AKCT01000108 : Penicillium digitatum PHI26    Total score: 3.0     Cumulative Blast bit score: 1033

Hit cluster cross-links:

Mycgr3G90785 Mycgr3T
  
Location: 0-1047

Mycgr3G90785\_Mycgr3T

Mycgr3G103262 Mycgr3
  
Location: 1147-1390

Mycgr3G103262\_Mycgr3

Mycgr3G68458 Mycgr3T
  
Location: 1490-3602

Mycgr3G68458\_Mycgr3T

Mycgr3G99145 Mycgr3T
  
Location: 3702-4326

Mycgr3G99145\_Mycgr3T

Mycgr3G103274 Mycgr3
  
Location: 4426-4957

Mycgr3G103274\_Mycgr3

Mycgr3G103264 Mycgr3
  
Location: 5057-5390

Mycgr3G103264\_Mycgr3

Mycgr3G37570 Mycgr3T
  
Location: 5490-6006

Mycgr3G37570\_Mycgr3T

Mycgr3G108094 Mycgr3
  
Location: 6106-10555

Mycgr3G108094\_Mycgr3

Mycgr3G90786 Mycgr3T
  
Location: 10655-12080

Mycgr3G90786\_Mycgr3T

Mycgr3G68429 Mycgr3T
  
Location: 12180-13440

Mycgr3G68429\_Mycgr3T

Mycgr3G68421 Mycgr3T
  
Location: 13540-17086

Mycgr3G68421\_Mycgr3T

Mycgr3G90801 Mycgr3T
  
Location: 17186-18056

Mycgr3G90801\_Mycgr3T

Mycgr3G84646 Mycgr3T
  
Location: 18156-20235

Mycgr3G84646\_Mycgr3T

Mycgr3G68456 Mycgr3T
  
Location: 20335-21970

Mycgr3G68456\_Mycgr3T

Mycgr3G103270 Mycgr3
  
Location: 22070-22355

Mycgr3G103270\_Mycgr3

Mycgr3G90803 Mycgr3T
  
Location: 22455-23019

Mycgr3G90803\_Mycgr3T

Mycgr3G36941 Mycgr3T
  
Location: 23119-24064

Mycgr3G36941\_Mycgr3T

Mycgr3G25746 Mycgr3T
  
Location: 24164-25241

Mycgr3G25746\_Mycgr3T

Mycgr3G90788 Mycgr3T
  
Location: 25341-25803

Mycgr3G90788\_Mycgr3T

Mycgr3G103260 Mycgr3
  
Location: 25903-26635

Mycgr3G103260\_Mycgr3

Mycgr3G84644 Mycgr3T
  
Location: 26735-28457

Mycgr3G84644\_Mycgr3T

Mycgr3G29227 Mycgr3T
  
Location: 28557-28863

Mycgr3G29227\_Mycgr3T

Mycgr3G36271 Mycgr3T
  
Location: 28963-29854

Mycgr3G36271\_Mycgr3T

Mycgr3G68433 Mycgr3T
  
Location: 29954-33041

Mycgr3G68433\_Mycgr3T

Mycgr3G79452 Mycgr3T
  
Location: 33141-33399

Mycgr3G79452\_Mycgr3T

Mycgr3G55345 Mycgr3T
  
Location: 33499-34126

Mycgr3G55345\_Mycgr3T

Mycgr3G103278 Mycgr3
  
Location: 34226-35195

Mycgr3G103278\_Mycgr3

Mycgr3G84654 Mycgr3T
  
Location: 35295-36630

Mycgr3G84654\_Mycgr3T

Mycgr3G108090 Mycgr3
  
Location: 36730-37591

Mycgr3G108090\_Mycgr3

Mycgr3G21922 Mycgr3T
  
Location: 37691-39149

Mycgr3G21922\_Mycgr3T

Mycgr3G99148 Mycgr3T
  
Location: 39249-42819

Mycgr3G99148\_Mycgr3T

C-4 methyl sterol oxidase Erg25, putative
  
Accession: EKV15878
  
Location: 2639-3479
  
  
**BlastP hit with Mycgr3G36271\_Mycgr3T**
  
Percentage identity: 77 %
  
BlastP bit score: 404
  
Sequence coverage: 81 %
  
E-value: 6e-139
  
  
 NCBI BlastP on this gene

EKV15878

hypothetical protein
  
Accession: EKV15879
  
Location: 5504-6450
  
  
**BlastP hit with Mycgr3G84646\_Mycgr3T**
  
Percentage identity: 51 %
  
BlastP bit score: 286
  
Sequence coverage: 40 %
  
E-value: 2e-87
  
  
 NCBI BlastP on this gene

EKV15879

Rab GTPase SrgA, putative
  
Accession: EKV15880
  
Location: 7209-7966
  
  
**BlastP hit with Mycgr3G99145\_Mycgr3T**
  
Percentage identity: 86 %
  
BlastP bit score: 343
  
Sequence coverage: 93 %
  
E-value: 3e-117
  
  
 NCBI BlastP on this gene

EKV15880

AAA family ATPase, putative
  
Accession: EKV15881
  
Location: 8676-10998
  
 NCBI BlastP on this gene

EKV15881

Inositol kinase kinase (UvsB), putative
  
Accession: EKV15882
  
Location: 12062-18775
  
 NCBI BlastP on this gene

EKV15882

hypothetical protein
  
Accession: EKV15883
  
Location: 20769-21422
  
 NCBI BlastP on this gene

EKV15883

hypothetical protein
  
Accession: EKV15884
  
Location: 22045-22824
  
 NCBI BlastP on this gene

EKV15884

NADH-ubiquinone oxidoreductase 21 kDa subunit, putative
  
Accession: EKV15885
  
Location: 23080-23904
  
 NCBI BlastP on this gene

EKV15885

hypothetical protein
  
Accession: EKV15886
  
Location: 24246-25220
  
 NCBI BlastP on this gene

EKV15886

Ran exchange factor Prp20/Pim1, putative
  
Accession: EKV15887
  
Location: 25599-27290
  
 NCBI BlastP on this gene

EKV15887

Riboflavin-specific deaminase
  
Accession: EKV15888
  
Location: 27653-29479
  
 NCBI BlastP on this gene

EKV15888

Query: Architecture Search FASTA input

GL573178 : Geomyces destructans 20631-21 unplaced genomic scaffold supercont1.10    Total score: 3.0     Cumulative Blast bit score: 1030

Hit cluster cross-links:

Mycgr3G90785 Mycgr3T
  
Location: 0-1047

Mycgr3G90785\_Mycgr3T

Mycgr3G103262 Mycgr3
  
Location: 1147-1390

Mycgr3G103262\_Mycgr3

Mycgr3G68458 Mycgr3T
  
Location: 1490-3602

Mycgr3G68458\_Mycgr3T

Mycgr3G99145 Mycgr3T
  
Location: 3702-4326

Mycgr3G99145\_Mycgr3T

Mycgr3G103274 Mycgr3
  
Location: 4426-4957

Mycgr3G103274\_Mycgr3

Mycgr3G103264 Mycgr3
  
Location: 5057-5390

Mycgr3G103264\_Mycgr3

Mycgr3G37570 Mycgr3T
  
Location: 5490-6006

Mycgr3G37570\_Mycgr3T

Mycgr3G108094 Mycgr3
  
Location: 6106-10555

Mycgr3G108094\_Mycgr3

Mycgr3G90786 Mycgr3T
  
Location: 10655-12080

Mycgr3G90786\_Mycgr3T

Mycgr3G68429 Mycgr3T
  
Location: 12180-13440

Mycgr3G68429\_Mycgr3T

Mycgr3G68421 Mycgr3T
  
Location: 13540-17086

Mycgr3G68421\_Mycgr3T

Mycgr3G90801 Mycgr3T
  
Location: 17186-18056

Mycgr3G90801\_Mycgr3T

Mycgr3G84646 Mycgr3T
  
Location: 18156-20235

Mycgr3G84646\_Mycgr3T

Mycgr3G68456 Mycgr3T
  
Location: 20335-21970

Mycgr3G68456\_Mycgr3T

Mycgr3G103270 Mycgr3
  
Location: 22070-22355

Mycgr3G103270\_Mycgr3

Mycgr3G90803 Mycgr3T
  
Location: 22455-23019

Mycgr3G90803\_Mycgr3T

Mycgr3G36941 Mycgr3T
  
Location: 23119-24064

Mycgr3G36941\_Mycgr3T

Mycgr3G25746 Mycgr3T
  
Location: 24164-25241

Mycgr3G25746\_Mycgr3T

Mycgr3G90788 Mycgr3T
  
Location: 25341-25803

Mycgr3G90788\_Mycgr3T

Mycgr3G103260 Mycgr3
  
Location: 25903-26635

Mycgr3G103260\_Mycgr3

Mycgr3G84644 Mycgr3T
  
Location: 26735-28457

Mycgr3G84644\_Mycgr3T

Mycgr3G29227 Mycgr3T
  
Location: 28557-28863

Mycgr3G29227\_Mycgr3T

Mycgr3G36271 Mycgr3T
  
Location: 28963-29854

Mycgr3G36271\_Mycgr3T

Mycgr3G68433 Mycgr3T
  
Location: 29954-33041

Mycgr3G68433\_Mycgr3T

Mycgr3G79452 Mycgr3T
  
Location: 33141-33399

Mycgr3G79452\_Mycgr3T

Mycgr3G55345 Mycgr3T
  
Location: 33499-34126

Mycgr3G55345\_Mycgr3T

Mycgr3G103278 Mycgr3
  
Location: 34226-35195

Mycgr3G103278\_Mycgr3

Mycgr3G84654 Mycgr3T
  
Location: 35295-36630

Mycgr3G84654\_Mycgr3T

Mycgr3G108090 Mycgr3
  
Location: 36730-37591

Mycgr3G108090\_Mycgr3

Mycgr3G21922 Mycgr3T
  
Location: 37691-39149

Mycgr3G21922\_Mycgr3T

Mycgr3G99148 Mycgr3T
  
Location: 39249-42819

Mycgr3G99148\_Mycgr3T

hypothetical protein
  
Accession: ELR01837
  
Location: 215462-216889
  
 NCBI BlastP on this gene

ELR01837

hypothetical protein
  
Accession: ELR01836
  
Location: 212320-214186
  
 NCBI BlastP on this gene

ELR01836

hypothetical protein
  
Accession: ELR01835
  
Location: 209424-210270
  
 NCBI BlastP on this gene

ELR01835

hypothetical protein
  
Accession: ELR01834
  
Location: 201120-204999
  
  
**BlastP hit with Mycgr3G21922\_Mycgr3T**
  
Percentage identity: 31 %
  
BlastP bit score: 120
  
Sequence coverage: 47 %
  
E-value: 2e-25
  
  
 NCBI BlastP on this gene

ELR01834

hypothetical protein
  
Accession: ELR01833
  
Location: 198659-199912
  
 NCBI BlastP on this gene

ELR01833

hypothetical protein
  
Accession: ELR01832
  
Location: 195064-198266
  
  
**BlastP hit with Mycgr3G108094\_Mycgr3**
  
Percentage identity: 31 %
  
BlastP bit score: 140
  
Sequence coverage: 33 %
  
E-value: 2e-30
  
  
 NCBI BlastP on this gene

ELR01832

CMGC/SRPK protein kinase
  
Accession: ELR01831
  
Location: 182650-184938
  
  
**BlastP hit with Mycgr3G84644\_Mycgr3T**
  
Percentage identity: 66 %
  
BlastP bit score: 770
  
Sequence coverage: 104 %
  
E-value: 0.0
  
  
 NCBI BlastP on this gene

ELR01831

hypothetical protein
  
Accession: ELR01829
  
Location: 180590-181969
  
 NCBI BlastP on this gene

ELR01829

hypothetical protein
  
Accession: ELR01828
  
Location: 169521-173241
  
 NCBI BlastP on this gene

ELR01828

Query: Architecture Search FASTA input

DS027693 : Neosartorya fischeri NRRL 181 1099437636261 genomic scaffold    Total score: 3.0     Cumulative Blast bit score: 1025

Hit cluster cross-links:

Mycgr3G90785 Mycgr3T
  
Location: 0-1047

Mycgr3G90785\_Mycgr3T

Mycgr3G103262 Mycgr3
  
Location: 1147-1390

Mycgr3G103262\_Mycgr3

Mycgr3G68458 Mycgr3T
  
Location: 1490-3602

Mycgr3G68458\_Mycgr3T

Mycgr3G99145 Mycgr3T
  
Location: 3702-4326

Mycgr3G99145\_Mycgr3T

Mycgr3G103274 Mycgr3
  
Location: 4426-4957

Mycgr3G103274\_Mycgr3

Mycgr3G103264 Mycgr3
  
Location: 5057-5390

Mycgr3G103264\_Mycgr3

Mycgr3G37570 Mycgr3T
  
Location: 5490-6006

Mycgr3G37570\_Mycgr3T

Mycgr3G108094 Mycgr3
  
Location: 6106-10555

Mycgr3G108094\_Mycgr3

Mycgr3G90786 Mycgr3T
  
Location: 10655-12080

Mycgr3G90786\_Mycgr3T

Mycgr3G68429 Mycgr3T
  
Location: 12180-13440

Mycgr3G68429\_Mycgr3T

Mycgr3G68421 Mycgr3T
  
Location: 13540-17086

Mycgr3G68421\_Mycgr3T

Mycgr3G90801 Mycgr3T
  
Location: 17186-18056

Mycgr3G90801\_Mycgr3T

Mycgr3G84646 Mycgr3T
  
Location: 18156-20235

Mycgr3G84646\_Mycgr3T

Mycgr3G68456 Mycgr3T
  
Location: 20335-21970

Mycgr3G68456\_Mycgr3T

Mycgr3G103270 Mycgr3
  
Location: 22070-22355

Mycgr3G103270\_Mycgr3

Mycgr3G90803 Mycgr3T
  
Location: 22455-23019

Mycgr3G90803\_Mycgr3T

Mycgr3G36941 Mycgr3T
  
Location: 23119-24064

Mycgr3G36941\_Mycgr3T

Mycgr3G25746 Mycgr3T
  
Location: 24164-25241

Mycgr3G25746\_Mycgr3T

Mycgr3G90788 Mycgr3T
  
Location: 25341-25803

Mycgr3G90788\_Mycgr3T

Mycgr3G103260 Mycgr3
  
Location: 25903-26635

Mycgr3G103260\_Mycgr3

Mycgr3G84644 Mycgr3T
  
Location: 26735-28457

Mycgr3G84644\_Mycgr3T

Mycgr3G29227 Mycgr3T
  
Location: 28557-28863

Mycgr3G29227\_Mycgr3T

Mycgr3G36271 Mycgr3T
  
Location: 28963-29854

Mycgr3G36271\_Mycgr3T

Mycgr3G68433 Mycgr3T
  
Location: 29954-33041

Mycgr3G68433\_Mycgr3T

Mycgr3G79452 Mycgr3T
  
Location: 33141-33399

Mycgr3G79452\_Mycgr3T

Mycgr3G55345 Mycgr3T
  
Location: 33499-34126

Mycgr3G55345\_Mycgr3T

Mycgr3G103278 Mycgr3
  
Location: 34226-35195

Mycgr3G103278\_Mycgr3

Mycgr3G84654 Mycgr3T
  
Location: 35295-36630

Mycgr3G84654\_Mycgr3T

Mycgr3G108090 Mycgr3
  
Location: 36730-37591

Mycgr3G108090\_Mycgr3

Mycgr3G21922 Mycgr3T
  
Location: 37691-39149

Mycgr3G21922\_Mycgr3T

Mycgr3G99148 Mycgr3T
  
Location: 39249-42819

Mycgr3G99148\_Mycgr3T

C-4 methyl sterol oxidase (Erg25), putative
  
Accession: EAW20419
  
Location: 67962-68811
  
  
**BlastP hit with Mycgr3G36271\_Mycgr3T**
  
Percentage identity: 70 %
  
BlastP bit score: 354
  
Sequence coverage: 81 %
  
E-value: 7e-120
  
  
 NCBI BlastP on this gene

EAW20419

hypothetical protein
  
Accession: EAW20420
  
Location: 70303-71580
  
  
**BlastP hit with Mycgr3G84646\_Mycgr3T**
  
Percentage identity: 41 %
  
BlastP bit score: 331
  
Sequence coverage: 65 %
  
E-value: 2e-102
  
  
 NCBI BlastP on this gene

EAW20420

Rab GTPase SrgA, putative
  
Accession: EAW20421
  
Location: 72984-73733
  
  
**BlastP hit with Mycgr3G99145\_Mycgr3T**
  
Percentage identity: 89 %
  
BlastP bit score: 340
  
Sequence coverage: 87 %
  
E-value: 1e-115
  
  
 NCBI BlastP on this gene

EAW20421

AAA family ATPase, putative
  
Accession: EAW20422
  
Location: 74596-76911
  
 NCBI BlastP on this gene

EAW20422

hypothetical protein
  
Accession: EAW20423
  
Location: 78030-78527
  
 NCBI BlastP on this gene

EAW20423

phosphatidylinositol 3- and 4-kinase, putative
  
Accession: EAW20424
  
Location: 78666-86258
  
 NCBI BlastP on this gene

EAW20424

hypothetical protein
  
Accession: EAW20425
  
Location: 88641-89016
  
 NCBI BlastP on this gene

EAW20425

NADH-ubiquinone oxidoreductase 21 kDa subunit, putative
  
Accession: EAW20426
  
Location: 89290-89859
  
 NCBI BlastP on this gene

EAW20426

replication fork protection component Swi3, putative
  
Accession: EAW20427
  
Location: 90778-91712
  
 NCBI BlastP on this gene

EAW20427

Ran exchange factor Prp20/Pim1, putative
  
Accession: EAW20428
  
Location: 92243-93984
  
 NCBI BlastP on this gene

EAW20428

Query: Architecture Search FASTA input

FM992691 : Candida dubliniensis CD36 chromosome 4    Total score: 3.0     Cumulative Blast bit score: 967

Hit cluster cross-links:

Mycgr3G90785 Mycgr3T
  
Location: 0-1047

Mycgr3G90785\_Mycgr3T

Mycgr3G103262 Mycgr3
  
Location: 1147-1390

Mycgr3G103262\_Mycgr3

Mycgr3G68458 Mycgr3T
  
Location: 1490-3602

Mycgr3G68458\_Mycgr3T

Mycgr3G99145 Mycgr3T
  
Location: 3702-4326

Mycgr3G99145\_Mycgr3T

Mycgr3G103274 Mycgr3
  
Location: 4426-4957

Mycgr3G103274\_Mycgr3

Mycgr3G103264 Mycgr3
  
Location: 5057-5390

Mycgr3G103264\_Mycgr3

Mycgr3G37570 Mycgr3T
  
Location: 5490-6006

Mycgr3G37570\_Mycgr3T

Mycgr3G108094 Mycgr3
  
Location: 6106-10555

Mycgr3G108094\_Mycgr3

Mycgr3G90786 Mycgr3T
  
Location: 10655-12080

Mycgr3G90786\_Mycgr3T

Mycgr3G68429 Mycgr3T
  
Location: 12180-13440

Mycgr3G68429\_Mycgr3T

Mycgr3G68421 Mycgr3T
  
Location: 13540-17086

Mycgr3G68421\_Mycgr3T

Mycgr3G90801 Mycgr3T
  
Location: 17186-18056

Mycgr3G90801\_Mycgr3T

Mycgr3G84646 Mycgr3T
  
Location: 18156-20235

Mycgr3G84646\_Mycgr3T

Mycgr3G68456 Mycgr3T
  
Location: 20335-21970

Mycgr3G68456\_Mycgr3T

Mycgr3G103270 Mycgr3
  
Location: 22070-22355

Mycgr3G103270\_Mycgr3

Mycgr3G90803 Mycgr3T
  
Location: 22455-23019

Mycgr3G90803\_Mycgr3T

Mycgr3G36941 Mycgr3T
  
Location: 23119-24064

Mycgr3G36941\_Mycgr3T

Mycgr3G25746 Mycgr3T
  
Location: 24164-25241

Mycgr3G25746\_Mycgr3T

Mycgr3G90788 Mycgr3T
  
Location: 25341-25803

Mycgr3G90788\_Mycgr3T

Mycgr3G103260 Mycgr3
  
Location: 25903-26635

Mycgr3G103260\_Mycgr3

Mycgr3G84644 Mycgr3T
  
Location: 26735-28457

Mycgr3G84644\_Mycgr3T

Mycgr3G29227 Mycgr3T
  
Location: 28557-28863

Mycgr3G29227\_Mycgr3T

Mycgr3G36271 Mycgr3T
  
Location: 28963-29854

Mycgr3G36271\_Mycgr3T

Mycgr3G68433 Mycgr3T
  
Location: 29954-33041

Mycgr3G68433\_Mycgr3T

Mycgr3G79452 Mycgr3T
  
Location: 33141-33399

Mycgr3G79452\_Mycgr3T

Mycgr3G55345 Mycgr3T
  
Location: 33499-34126

Mycgr3G55345\_Mycgr3T

Mycgr3G103278 Mycgr3
  
Location: 34226-35195

Mycgr3G103278\_Mycgr3

Mycgr3G84654 Mycgr3T
  
Location: 35295-36630

Mycgr3G84654\_Mycgr3T

Mycgr3G108090 Mycgr3
  
Location: 36730-37591

Mycgr3G108090\_Mycgr3

Mycgr3G21922 Mycgr3T
  
Location: 37691-39149

Mycgr3G21922\_Mycgr3T

Mycgr3G99148 Mycgr3T
  
Location: 39249-42819

Mycgr3G99148\_Mycgr3T

not annotated
  
Accession: CAX42093
  
Location: 454686-455348
  
 NCBI BlastP on this gene

CD36\_42130

catechol 1,2-dioxygenase, putative
  
Accession: CAX42092
  
Location: 453006-453917
  
 NCBI BlastP on this gene

HQD2

not annotated
  
Accession: CAX42091
  
Location: 449987-452383
  
 NCBI BlastP on this gene

CD36\_42110

not annotated
  
Accession: CAX42090
  
Location: 449143-449937
  
 NCBI BlastP on this gene

CD36\_42100

not annotated
  
Accession: CAX42089
  
Location: 448402-448839
  
 NCBI BlastP on this gene

CD36\_42090

not annotated
  
Accession: CAX42088
  
Location: 446364-448010
  
 NCBI BlastP on this gene

CD36\_42080

not annotated
  
Accession: CAX42087
  
Location: 443255-445753
  
 NCBI BlastP on this gene

CD36\_42070

conserved hypothetical protein
  
Accession: CAX42086
  
Location: 441327-442976
  
 NCBI BlastP on this gene

CD36\_42060

ABC1 family protein, mitochondrial precursor, putative
  
Accession: CAX42085
  
Location: 438731-441057
  
  
**BlastP hit with Mycgr3G68458\_Mycgr3T**
  
Percentage identity: 50 %
  
BlastP bit score: 608
  
Sequence coverage: 90 %
  
E-value: 0.0
  
  
 NCBI BlastP on this gene

CD36\_42050

subunit of guanine nucleotide-binding protein, putative
  
Accession: CAX42084
  
Location: 434771-438100
  
 NCBI BlastP on this gene

CD36\_42040

mitochondrial import inner membrane translocase subunit, putative
  
Accession: CAX42083
  
Location: 434271-434546
  
 NCBI BlastP on this gene

CD36\_42030

cytochrome c1 heme lyase, putative
  
Accession: CAX42082
  
Location: 433331-434086
  
 NCBI BlastP on this gene

CD36\_42020

ER-derived vesicles protein, putative
  
Accession: CAX42081
  
Location: 432005-432931
  
 NCBI BlastP on this gene

CD36\_42010

not annotated
  
Accession: CAX42080
  
Location: 430400-430945
  
 NCBI BlastP on this gene

CD36\_42000

GPI mannosyltransferase, putative
  
Accession: CAX42079
  
Location: 428957-430165
  
 NCBI BlastP on this gene

CD36\_41990

3'-to-5' phosphorolytic exoribonuclease, putative
  
Accession: CAX42078
  
Location: 428058-428780
  
 NCBI BlastP on this gene

CD36\_41980

carnitine/acylcarnitine carrier protein, putative
  
Accession: CAX42077
  
Location: 427036-427965
  
 NCBI BlastP on this gene

CD36\_41970

acid phosphatase, putative
  
Accession: CAX42076
  
Location: 425320-426693
  
 NCBI BlastP on this gene

CD36\_41960

subunit of TFIIF (transcription factor II), putative
  
Accession: CAX42075
  
Location: 422583-424406
  
  
**BlastP hit with Mycgr3G103278\_Mycgr3**
  
Percentage identity: 40 %
  
BlastP bit score: 84
  
Sequence coverage: 26 %
  
E-value: 9e-15
  
  
 NCBI BlastP on this gene

CD36\_41950

HMG1/2-related protein, putative
  
Accession: CAX42074
  
Location: 421378-422466
  
  
**BlastP hit with Mycgr3G25746\_Mycgr3T**
  
Percentage identity: 42 %
  
BlastP bit score: 275
  
Sequence coverage: 100 %
  
E-value: 7e-86
  
  
 NCBI BlastP on this gene

CD36\_41940

acetylspermidine oxidase, putative
  
Accession: CAX42073
  
Location: 419794-421227
  
 NCBI BlastP on this gene

CD36\_41930

conserved hypothetical protein
  
Accession: CAX42072
  
Location: 415889-419230
  
 NCBI BlastP on this gene

CD36\_41920

carnitine O-acetyltransferase, mitochondrial precursor, putative
  
Accession: CAX42071
  
Location: 408726-410624
  
 NCBI BlastP on this gene

CAT2

Query: Architecture Search FASTA input

KB733455 : Bipolaris maydis ATCC 48331 unplaced genomic scaffold COCC4scaffold\_12    Total score: 3.0     Cumulative Blast bit score: 953

Hit cluster cross-links:

Mycgr3G90785 Mycgr3T
  
Location: 0-1047

Mycgr3G90785\_Mycgr3T

Mycgr3G103262 Mycgr3
  
Location: 1147-1390

Mycgr3G103262\_Mycgr3

Mycgr3G68458 Mycgr3T
  
Location: 1490-3602

Mycgr3G68458\_Mycgr3T

Mycgr3G99145 Mycgr3T
  
Location: 3702-4326

Mycgr3G99145\_Mycgr3T

Mycgr3G103274 Mycgr3
  
Location: 4426-4957

Mycgr3G103274\_Mycgr3

Mycgr3G103264 Mycgr3
  
Location: 5057-5390

Mycgr3G103264\_Mycgr3

Mycgr3G37570 Mycgr3T
  
Location: 5490-6006

Mycgr3G37570\_Mycgr3T

Mycgr3G108094 Mycgr3
  
Location: 6106-10555

Mycgr3G108094\_Mycgr3

Mycgr3G90786 Mycgr3T
  
Location: 10655-12080

Mycgr3G90786\_Mycgr3T

Mycgr3G68429 Mycgr3T
  
Location: 12180-13440

Mycgr3G68429\_Mycgr3T

Mycgr3G68421 Mycgr3T
  
Location: 13540-17086

Mycgr3G68421\_Mycgr3T

Mycgr3G90801 Mycgr3T
  
Location: 17186-18056

Mycgr3G90801\_Mycgr3T

Mycgr3G84646 Mycgr3T
  
Location: 18156-20235

Mycgr3G84646\_Mycgr3T

Mycgr3G68456 Mycgr3T
  
Location: 20335-21970

Mycgr3G68456\_Mycgr3T

Mycgr3G103270 Mycgr3
  
Location: 22070-22355

Mycgr3G103270\_Mycgr3

Mycgr3G90803 Mycgr3T
  
Location: 22455-23019

Mycgr3G90803\_Mycgr3T

Mycgr3G36941 Mycgr3T
  
Location: 23119-24064

Mycgr3G36941\_Mycgr3T

Mycgr3G25746 Mycgr3T
  
Location: 24164-25241

Mycgr3G25746\_Mycgr3T

Mycgr3G90788 Mycgr3T
  
Location: 25341-25803

Mycgr3G90788\_Mycgr3T

Mycgr3G103260 Mycgr3
  
Location: 25903-26635

Mycgr3G103260\_Mycgr3

Mycgr3G84644 Mycgr3T
  
Location: 26735-28457

Mycgr3G84644\_Mycgr3T

Mycgr3G29227 Mycgr3T
  
Location: 28557-28863

Mycgr3G29227\_Mycgr3T

Mycgr3G36271 Mycgr3T
  
Location: 28963-29854

Mycgr3G36271\_Mycgr3T

Mycgr3G68433 Mycgr3T
  
Location: 29954-33041

Mycgr3G68433\_Mycgr3T

Mycgr3G79452 Mycgr3T
  
Location: 33141-33399

Mycgr3G79452\_Mycgr3T

Mycgr3G55345 Mycgr3T
  
Location: 33499-34126

Mycgr3G55345\_Mycgr3T

Mycgr3G103278 Mycgr3
  
Location: 34226-35195

Mycgr3G103278\_Mycgr3

Mycgr3G84654 Mycgr3T
  
Location: 35295-36630

Mycgr3G84654\_Mycgr3T

Mycgr3G108090 Mycgr3
  
Location: 36730-37591

Mycgr3G108090\_Mycgr3

Mycgr3G21922 Mycgr3T
  
Location: 37691-39149

Mycgr3G21922\_Mycgr3T

Mycgr3G99148 Mycgr3T
  
Location: 39249-42819

Mycgr3G99148\_Mycgr3T

hypothetical protein
  
Accession: ENI05063
  
Location: 391576-392858
  
 NCBI BlastP on this gene

ENI05063

hypothetical protein
  
Accession: ENI05064
  
Location: 393416-393920
  
 NCBI BlastP on this gene

ENI05064

hypothetical protein
  
Accession: ENI05065
  
Location: 395875-396629
  
 NCBI BlastP on this gene

ENI05065

hypothetical protein
  
Accession: ENI05066
  
Location: 397130-397976
  
 NCBI BlastP on this gene

ENI05066

hypothetical protein
  
Accession: ENI05067
  
Location: 399288-399935
  
 NCBI BlastP on this gene

ENI05067

hypothetical protein
  
Accession: ENI05068
  
Location: 401608-402086
  
 NCBI BlastP on this gene

ENI05068

hypothetical protein
  
Accession: ENI05069
  
Location: 406076-407044
  
 NCBI BlastP on this gene

ENI05069

hypothetical protein
  
Accession: ENI05070
  
Location: 407506-409581
  
 NCBI BlastP on this gene

ENI05070

hypothetical protein
  
Accession: ENI05071
  
Location: 411081-412390
  
  
**BlastP hit with Mycgr3G25746\_Mycgr3T**
  
Percentage identity: 56 %
  
BlastP bit score: 380
  
Sequence coverage: 102 %
  
E-value: 8e-127
  
  
 NCBI BlastP on this gene

ENI05071

hypothetical protein
  
Accession: ENI05072
  
Location: 412739-414638
  
 NCBI BlastP on this gene

ENI05072

hypothetical protein
  
Accession: ENI05073
  
Location: 414865-416991
  
  
**BlastP hit with Mycgr3G103278\_Mycgr3**
  
Percentage identity: 33 %
  
BlastP bit score: 118
  
Sequence coverage: 94 %
  
E-value: 4e-26
  
  
 NCBI BlastP on this gene

ENI05073

hypothetical protein
  
Accession: ENI05074
  
Location: 417643-419898
  
  
**BlastP hit with Mycgr3G21922\_Mycgr3T**
  
Percentage identity: 47 %
  
BlastP bit score: 455
  
Sequence coverage: 105 %
  
E-value: 2e-149
  
  
 NCBI BlastP on this gene

ENI05074

hypothetical protein
  
Accession: ENI05075
  
Location: 422519-425282
  
 NCBI BlastP on this gene

ENI05075

hypothetical protein
  
Accession: ENI05076
  
Location: 431658-433192
  
 NCBI BlastP on this gene

ENI05076

hypothetical protein
  
Accession: ENI05077
  
Location: 433206-435244
  
 NCBI BlastP on this gene

ENI05077

hypothetical protein
  
Accession: ENI05078
  
Location: 437812-438321
  
 NCBI BlastP on this gene

ENI05078

Query: Architecture Search FASTA input

51. :  KB020429 Colletotrichum gloeosporioides Nara gc5 unplaced genomic scaffold scaffold120     Total score: 3.0     Cumulative Blast bit score: 1376

Mycgr3G90785 Mycgr3T
  
Location: 0-1047
  
 NCBI BlastP on this gene

Mycgr3G90785\_Mycgr3T

Mycgr3G103262 Mycgr3
  
Location: 1147-1390
  
 NCBI BlastP on this gene

Mycgr3G103262\_Mycgr3

Mycgr3G68458 Mycgr3T
  
Location: 1490-3602
  
 NCBI BlastP on this gene

Mycgr3G68458\_Mycgr3T

Mycgr3G99145 Mycgr3T
  
Location: 3702-4326
  
 NCBI BlastP on this gene

Mycgr3G99145\_Mycgr3T

Mycgr3G103274 Mycgr3
  
Location: 4426-4957
  
 NCBI BlastP on this gene

Mycgr3G103274\_Mycgr3

Mycgr3G103264 Mycgr3
  
Location: 5057-5390
  
 NCBI BlastP on this gene

Mycgr3G103264\_Mycgr3

Mycgr3G37570 Mycgr3T
  
Location: 5490-6006
  
 NCBI BlastP on this gene

Mycgr3G37570\_Mycgr3T

Mycgr3G108094 Mycgr3
  
Location: 6106-10555
  
 NCBI BlastP on this gene

Mycgr3G108094\_Mycgr3

Mycgr3G90786 Mycgr3T
  
Location: 10655-12080
  
 NCBI BlastP on this gene

Mycgr3G90786\_Mycgr3T

Mycgr3G68429 Mycgr3T
  
Location: 12180-13440
  
 NCBI BlastP on this gene

Mycgr3G68429\_Mycgr3T

Mycgr3G68421 Mycgr3T
  
Location: 13540-17086
  
 NCBI BlastP on this gene

Mycgr3G68421\_Mycgr3T

Mycgr3G90801 Mycgr3T
  
Location: 17186-18056
  
 NCBI BlastP on this gene

Mycgr3G90801\_Mycgr3T

Mycgr3G84646 Mycgr3T
  
Location: 18156-20235
  
 NCBI BlastP on this gene

Mycgr3G84646\_Mycgr3T

Mycgr3G68456 Mycgr3T
  
Location: 20335-21970
  
 NCBI BlastP on this gene

Mycgr3G68456\_Mycgr3T

Mycgr3G103270 Mycgr3
  
Location: 22070-22355
  
 NCBI BlastP on this gene

Mycgr3G103270\_Mycgr3

Mycgr3G90803 Mycgr3T
  
Location: 22455-23019
  
 NCBI BlastP on this gene

Mycgr3G90803\_Mycgr3T

Mycgr3G36941 Mycgr3T
  
Location: 23119-24064
  
 NCBI BlastP on this gene

Mycgr3G36941\_Mycgr3T

Mycgr3G25746 Mycgr3T
  
Location: 24164-25241
  
 NCBI BlastP on this gene

Mycgr3G25746\_Mycgr3T

Mycgr3G90788 Mycgr3T
  
Location: 25341-25803
  
 NCBI BlastP on this gene

Mycgr3G90788\_Mycgr3T

Mycgr3G103260 Mycgr3
  
Location: 25903-26635
  
 NCBI BlastP on this gene

Mycgr3G103260\_Mycgr3

Mycgr3G84644 Mycgr3T
  
Location: 26735-28457
  
 NCBI BlastP on this gene

Mycgr3G84644\_Mycgr3T

Mycgr3G29227 Mycgr3T
  
Location: 28557-28863
  
 NCBI BlastP on this gene

Mycgr3G29227\_Mycgr3T

Mycgr3G36271 Mycgr3T
  
Location: 28963-29854
  
 NCBI BlastP on this gene

Mycgr3G36271\_Mycgr3T

Mycgr3G68433 Mycgr3T
  
Location: 29954-33041
  
 NCBI BlastP on this gene

Mycgr3G68433\_Mycgr3T

Mycgr3G79452 Mycgr3T
  
Location: 33141-33399
  
 NCBI BlastP on this gene

Mycgr3G79452\_Mycgr3T

Mycgr3G55345 Mycgr3T
  
Location: 33499-34126
  
 NCBI BlastP on this gene

Mycgr3G55345\_Mycgr3T

Mycgr3G103278 Mycgr3
  
Location: 34226-35195
  
 NCBI BlastP on this gene

Mycgr3G103278\_Mycgr3

Mycgr3G84654 Mycgr3T
  
Location: 35295-36630
  
 NCBI BlastP on this gene

Mycgr3G84654\_Mycgr3T

Mycgr3G108090 Mycgr3
  
Location: 36730-37591
  
 NCBI BlastP on this gene

Mycgr3G108090\_Mycgr3

Mycgr3G21922 Mycgr3T
  
Location: 37691-39149
  
 NCBI BlastP on this gene

Mycgr3G21922\_Mycgr3T

Mycgr3G99148 Mycgr3T
  
Location: 39249-42819
  
 NCBI BlastP on this gene

Mycgr3G99148\_Mycgr3T

pterin-4-alpha-carbinolamine dehydratase family protein
  
Accession: ELA37624
  
Location: 339976-340622
  
 NCBI BlastP on this gene

ELA37624

c-4 sterol methyl oxidase
  
Accession: ELA37623
  
Location: 338544-339653
  
  
**BlastP hit with Mycgr3G36271\_Mycgr3T**
  
Percentage identity: 73 %
  
BlastP bit score: 459
  
Sequence coverage: 96 %
  
E-value: 2e-159
  
  
 NCBI BlastP on this gene

ELA37623

ATP synthase regulation protein nca2
  
Accession: ELA37622
  
Location: 334342-336458
  
  
**BlastP hit with Mycgr3G84646\_Mycgr3T**
  
Percentage identity: 44 %
  
BlastP bit score: 561
  
Sequence coverage: 100 %
  
E-value: 0.0
  
  
 NCBI BlastP on this gene

ELA37622

rab GTPase
  
Accession: ELA37621
  
Location: 332139-333057
  
  
**BlastP hit with Mycgr3G99145\_Mycgr3T**
  
Percentage identity: 84 %
  
BlastP bit score: 356
  
Sequence coverage: 99 %
  
E-value: 2e-122
  
  
 NCBI BlastP on this gene

ELA37621

hypothetical protein
  
Accession: ELA37620
  
Location: 330179-331558
  
 NCBI BlastP on this gene

ELA37620

AAA family ATPase
  
Accession: ELA37619
  
Location: 327476-329002
  
 NCBI BlastP on this gene

ELA37619

AAA family ATPase
  
Accession: ELA37618
  
Location: 326398-327024
  
 NCBI BlastP on this gene

ELA37618

squalene epoxidase
  
Accession: ELA37617
  
Location: 323383-324869
  
 NCBI BlastP on this gene

ELA37617

dolichyl-phosphate beta-glucosyltransferase
  
Accession: ELA37616
  
Location: 321339-322654
  
 NCBI BlastP on this gene

ELA37616

hypothetical protein
  
Accession: ELA37615
  
Location: 316724-318128
  
 NCBI BlastP on this gene

ELA37615

52. :  DS572751 Paracoccidioides brasiliensis Pb18 supercont1.2 genomic scaffold     Total score: 3.0     Cumulative Blast bit score: 1369

conserved hypothetical protein
  
Accession: EEH45067
  
Location: 103715-106192
  
 NCBI BlastP on this gene

EEH45067

conserved hypothetical protein
  
Accession: EEH45068
  
Location: 106837-107862
  
 NCBI BlastP on this gene

EEH45068

conserved hypothetical protein
  
Accession: EEH45069
  
Location: 108236-110470
  
 NCBI BlastP on this gene

EEH45069

conserved hypothetical protein
  
Accession: EEH45070
  
Location: 111937-113233
  
 NCBI BlastP on this gene

EEH45070

conserved hypothetical protein
  
Accession: EEH45071
  
Location: 114154-115518
  
  
**BlastP hit with Mycgr3G90786\_Mycgr3T**
  
Percentage identity: 28 %
  
BlastP bit score: 84
  
Sequence coverage: 103 %
  
E-value: 2e-14
  
  
 NCBI BlastP on this gene

EEH45071

leucine-rich repeat-containing protein
  
Accession: EEH45072
  
Location: 116921-119959
  
  
**BlastP hit with Mycgr3G68433\_Mycgr3T**
  
Percentage identity: 37 %
  
BlastP bit score: 479
  
Sequence coverage: 95 %
  
E-value: 5e-148
  
  
 NCBI BlastP on this gene

EEH45072

conserved hypothetical protein
  
Accession: EEH45073
  
Location: 120585-121826
  
 NCBI BlastP on this gene

EEH45073

predicted protein
  
Accession: EEH45074
  
Location: 122284-123125
  
 NCBI BlastP on this gene

EEH45074

actin family protein
  
Accession: EEH45075
  
Location: 124434-126084
  
 NCBI BlastP on this gene

EEH45075

IBR domain-containing protein
  
Accession: EEH45076
  
Location: 129354-131723
  
 NCBI BlastP on this gene

EEH45076

predicted protein
  
Accession: EEH45077
  
Location: 132662-134098
  
 NCBI BlastP on this gene

EEH45077

3-hydroxybutyryl-CoA dehydrogenase
  
Accession: EEH45078
  
Location: 134531-135690
  
 NCBI BlastP on this gene

EEH45078

topoisomerase 1-associated factor 1
  
Accession: EEH45079
  
Location: 136062-139873
  
  
**BlastP hit with Mycgr3G68421\_Mycgr3T**
  
Percentage identity: 41 %
  
BlastP bit score: 806
  
Sequence coverage: 103 %
  
E-value: 0.0
  
  
 NCBI BlastP on this gene

EEH45079

predicted protein
  
Accession: EEH45080
  
Location: 140910-141266
  
 NCBI BlastP on this gene

EEH45080

conserved hypothetical protein
  
Accession: EEH45081
  
Location: 141749-143525
  
 NCBI BlastP on this gene

EEH45081

TFIIH basal transcription factor complex p52 subunit
  
Accession: EEH45083
  
Location: 144111-145699
  
 NCBI BlastP on this gene

EEH45083

predicted protein
  
Accession: EEH45082
  
Location: 145711-147251
  
 NCBI BlastP on this gene

EEH45082

intron-binding protein aquarius
  
Accession: EEH45084
  
Location: 147936-152389
  
 NCBI BlastP on this gene

EEH45084

53. :  KB726077 Colletotrichum orbiculare MAFF 240422 unplaced genomic scaffold Scaffold\_499     Total score: 3.0     Cumulative Blast bit score: 1368

hypothetical protein
  
Accession: ENH77775
  
Location: 1043553-1044227
  
 NCBI BlastP on this gene

ENH77775

WD repeat protein
  
Accession: ENH77776
  
Location: 1051744-1056440
  
 NCBI BlastP on this gene

ENH77776

ubiquitin conjugating enzyme
  
Accession: ENH77777
  
Location: 1057406-1058048
  
 NCBI BlastP on this gene

ENH77777

hypothetical protein
  
Accession: ENH77778
  
Location: 1058980-1059363
  
 NCBI BlastP on this gene

ENH77778

amidohydrolase
  
Accession: ENH77779
  
Location: 1060502-1061884
  
 NCBI BlastP on this gene

ENH77779

pterin-4-alpha-carbinolamine dehydratase family protein
  
Accession: ENH77780
  
Location: 1061998-1062472
  
 NCBI BlastP on this gene

ENH77780

c-4 methyl sterol oxidase
  
Accession: ENH77781
  
Location: 1062926-1064064
  
  
**BlastP hit with Mycgr3G36271\_Mycgr3T**
  
Percentage identity: 74 %
  
BlastP bit score: 462
  
Sequence coverage: 96 %
  
E-value: 5e-161
  
  
 NCBI BlastP on this gene

ENH77781

ATP synthase regulation protein nca2
  
Accession: ENH77782
  
Location: 1066304-1068496
  
  
**BlastP hit with Mycgr3G84646\_Mycgr3T**
  
Percentage identity: 43 %
  
BlastP bit score: 551
  
Sequence coverage: 102 %
  
E-value: 0.0
  
  
 NCBI BlastP on this gene

ENH77782

RAB GTPase
  
Accession: ENH77783
  
Location: 1070215-1071103
  
  
**BlastP hit with Mycgr3G99145\_Mycgr3T**
  
Percentage identity: 83 %
  
BlastP bit score: 355
  
Sequence coverage: 99 %
  
E-value: 5e-122
  
  
 NCBI BlastP on this gene

ENH77783

hypothetical protein
  
Accession: ENH77784
  
Location: 1071880-1073508
  
 NCBI BlastP on this gene

ENH77784

AAA family ATPase
  
Accession: ENH77785
  
Location: 1074599-1076912
  
 NCBI BlastP on this gene

ENH77785

squalene epoxidase
  
Accession: ENH77786
  
Location: 1078768-1080267
  
 NCBI BlastP on this gene

ENH77786

C6 zinc finger domain protein
  
Accession: ENH77787
  
Location: 1081064-1082314
  
 NCBI BlastP on this gene

ENH77787

ABC multidrug transporter
  
Accession: ENH77788
  
Location: 1083794-1088145
  
 NCBI BlastP on this gene

ENH77788

hypothetical protein
  
Accession: ENH77789
  
Location: 1089088-1089954
  
 NCBI BlastP on this gene

ENH77789

nonribosomal peptide synthetase
  
Accession: ENH77790
  
Location: 1090911-1094250
  
 NCBI BlastP on this gene

ENH77790

54. :  KB733455 Bipolaris maydis ATCC 48331 unplaced genomic scaffold COCC4scaffold\_12     Total score: 3.0     Cumulative Blast bit score: 1366

hypothetical protein
  
Accession: ENI05253
  
Location: 879556-883208
  
 NCBI BlastP on this gene

ENI05253

hypothetical protein
  
Accession: ENI05252
  
Location: 878521-879144
  
 NCBI BlastP on this gene

ENI05252

hypothetical protein
  
Accession: ENI05251
  
Location: 877466-878471
  
 NCBI BlastP on this gene

ENI05251

hypothetical protein
  
Accession: ENI05250
  
Location: 875499-877086
  
 NCBI BlastP on this gene

ENI05250

hypothetical protein
  
Accession: ENI05249
  
Location: 873558-874579
  
 NCBI BlastP on this gene

ENI05249

hypothetical protein
  
Accession: ENI05248
  
Location: 873258-873428
  
 NCBI BlastP on this gene

ENI05248

carbohydrate esterase family 9 protein
  
Accession: ENI05247
  
Location: 871739-873152
  
 NCBI BlastP on this gene

ENI05247

glycoside hydrolase family 3 protein
  
Accession: ENI05246
  
Location: 868116-871171
  
 NCBI BlastP on this gene

ENI05246

hypothetical protein
  
Accession: ENI05245
  
Location: 865240-866902
  
  
**BlastP hit with Mycgr3G84654\_Mycgr3T**
  
Percentage identity: 58 %
  
BlastP bit score: 395
  
Sequence coverage: 81 %
  
E-value: 5e-129
  
  
 NCBI BlastP on this gene

ENI05245

hypothetical protein
  
Accession: ENI05244
  
Location: 861018-862793
  
 NCBI BlastP on this gene

ENI05244

hypothetical protein
  
Accession: ENI05243
  
Location: 852143-853762
  
 NCBI BlastP on this gene

ENI05243

hypothetical protein
  
Accession: ENI05242
  
Location: 849230-850723
  
 NCBI BlastP on this gene

ENI05242

hypothetical protein
  
Accession: ENI05241
  
Location: 846575-848596
  
  
**BlastP hit with Mycgr3G84646\_Mycgr3T**
  
Percentage identity: 47 %
  
BlastP bit score: 607
  
Sequence coverage: 99 %
  
E-value: 0.0
  
  
 NCBI BlastP on this gene

ENI05241

hypothetical protein
  
Accession: ENI05240
  
Location: 845189-846053
  
  
**BlastP hit with Mycgr3G99145\_Mycgr3T**
  
Percentage identity: 86 %
  
BlastP bit score: 364
  
Sequence coverage: 99 %
  
E-value: 2e-125
  
  
 NCBI BlastP on this gene

ENI05240

hypothetical protein
  
Accession: ENI05239
  
Location: 836794-844658
  
 NCBI BlastP on this gene

ENI05239

hypothetical protein
  
Accession: ENI05238
  
Location: 835855-836258
  
 NCBI BlastP on this gene

ENI05238

hypothetical protein
  
Accession: ENI05237
  
Location: 832412-834819
  
 NCBI BlastP on this gene

ENI05237

55. :  KB445579 Cochliobolus heterostrophus C5 unplaced genomic scaffold COCHEscaffold\_11     Total score: 3.0     Cumulative Blast bit score: 1366

hypothetical protein
  
Accession: EMD89026
  
Location: 121449-125101
  
 NCBI BlastP on this gene

EMD89026

hypothetical protein
  
Accession: EMD89027
  
Location: 125513-126136
  
 NCBI BlastP on this gene

EMD89027

hypothetical protein
  
Accession: EMD89028
  
Location: 126186-127191
  
 NCBI BlastP on this gene

EMD89028

hypothetical protein
  
Accession: EMD89029
  
Location: 127571-129158
  
 NCBI BlastP on this gene

EMD89029

hypothetical protein
  
Accession: EMD89030
  
Location: 130078-131099
  
 NCBI BlastP on this gene

EMD89030

hypothetical protein
  
Accession: EMD89031
  
Location: 131230-131400
  
 NCBI BlastP on this gene

EMD89031

carbohydrate esterase family 9 protein
  
Accession: EMD89032
  
Location: 131606-132919
  
 NCBI BlastP on this gene

EMD89032

glycoside hydrolase family 3 protein
  
Accession: EMD89033
  
Location: 133487-136542
  
 NCBI BlastP on this gene

EMD89033

hypothetical protein
  
Accession: EMD89034
  
Location: 137756-139418
  
  
**BlastP hit with Mycgr3G84654\_Mycgr3T**
  
Percentage identity: 58 %
  
BlastP bit score: 395
  
Sequence coverage: 81 %
  
E-value: 5e-129
  
  
 NCBI BlastP on this gene

EMD89034

hypothetical protein
  
Accession: EMD89035
  
Location: 141865-143640
  
 NCBI BlastP on this gene

EMD89035

hypothetical protein
  
Accession: EMD89036
  
Location: 149496-150482
  
 NCBI BlastP on this gene

EMD89036

hypothetical protein
  
Accession: EMD89037
  
Location: 151080-152699
  
 NCBI BlastP on this gene

EMD89037

hypothetical protein
  
Accession: EMD89038
  
Location: 154119-155612
  
 NCBI BlastP on this gene

EMD89038

hypothetical protein
  
Accession: EMD89039
  
Location: 156246-158267
  
  
**BlastP hit with Mycgr3G84646\_Mycgr3T**
  
Percentage identity: 47 %
  
BlastP bit score: 607
  
Sequence coverage: 99 %
  
E-value: 0.0
  
  
 NCBI BlastP on this gene

EMD89039

hypothetical protein
  
Accession: EMD89040
  
Location: 158789-159653
  
  
**BlastP hit with Mycgr3G99145\_Mycgr3T**
  
Percentage identity: 86 %
  
BlastP bit score: 364
  
Sequence coverage: 99 %
  
E-value: 2e-125
  
  
 NCBI BlastP on this gene

EMD89040

hypothetical protein
  
Accession: EMD89041
  
Location: 160184-168048
  
 NCBI BlastP on this gene

EMD89041

hypothetical protein
  
Accession: EMD89042
  
Location: 168584-168987
  
 NCBI BlastP on this gene

EMD89042

hypothetical protein
  
Accession: EMD89043
  
Location: 170023-172430
  
 NCBI BlastP on this gene

EMD89043

56. :  KB445649 Cochliobolus sativus ND90Pr unplaced genomic scaffold COCSAscaffold\_13     Total score: 3.0     Cumulative Blast bit score: 1364

hypothetical protein
  
Accession: EMD60800
  
Location: 187265-190746
  
 NCBI BlastP on this gene

EMD60800

hypothetical protein
  
Accession: EMD60801
  
Location: 191317-191940
  
 NCBI BlastP on this gene

EMD60801

hypothetical protein
  
Accession: EMD60802
  
Location: 191990-192994
  
 NCBI BlastP on this gene

EMD60802

hypothetical protein
  
Accession: EMD60803
  
Location: 193668-195253
  
 NCBI BlastP on this gene

EMD60803

hypothetical protein
  
Accession: EMD60804
  
Location: 196158-197179
  
 NCBI BlastP on this gene

EMD60804

carbohydrate esterase family 9 protein
  
Accession: EMD60805
  
Location: 197676-198971
  
 NCBI BlastP on this gene

EMD60805

glycoside hydrolase family 3 protein
  
Accession: EMD60806
  
Location: 199560-202617
  
 NCBI BlastP on this gene

EMD60806

hypothetical protein
  
Accession: EMD60807
  
Location: 203814-205476
  
  
**BlastP hit with Mycgr3G84654\_Mycgr3T**
  
Percentage identity: 58 %
  
BlastP bit score: 395
  
Sequence coverage: 81 %
  
E-value: 7e-129
  
  
 NCBI BlastP on this gene

EMD60807

hypothetical protein
  
Accession: EMD60808
  
Location: 208333-210108
  
 NCBI BlastP on this gene

EMD60808

hypothetical protein
  
Accession: EMD60809
  
Location: 211684-213303
  
 NCBI BlastP on this gene

EMD60809

hypothetical protein
  
Accession: EMD60810
  
Location: 214787-216136
  
 NCBI BlastP on this gene

EMD60810

hypothetical protein
  
Accession: EMD60811
  
Location: 216775-218796
  
  
**BlastP hit with Mycgr3G84646\_Mycgr3T**
  
Percentage identity: 47 %
  
BlastP bit score: 605
  
Sequence coverage: 99 %
  
E-value: 0.0
  
  
 NCBI BlastP on this gene

EMD60811

hypothetical protein
  
Accession: EMD60812
  
Location: 219317-220181
  
  
**BlastP hit with Mycgr3G99145\_Mycgr3T**
  
Percentage identity: 86 %
  
BlastP bit score: 364
  
Sequence coverage: 99 %
  
E-value: 2e-125
  
  
 NCBI BlastP on this gene

EMD60812

hypothetical protein
  
Accession: EMD60813
  
Location: 220698-228562
  
 NCBI BlastP on this gene

EMD60813

hypothetical protein
  
Accession: EMD60814
  
Location: 229102-229505
  
 NCBI BlastP on this gene

EMD60814

hypothetical protein
  
Accession: EMD60815
  
Location: 230541-232948
  
 NCBI BlastP on this gene

EMD60815

hypothetical protein
  
Accession: EMD60816
  
Location: 235133-236736
  
 NCBI BlastP on this gene

EMD60816

57. :  GG697338 Glomerella graminicola M1.001 genomic scaffold supercont1.8     Total score: 3.0     Cumulative Blast bit score: 1362

profilin
  
Accession: EFQ27668
  
Location: 477127-477885
  
 NCBI BlastP on this gene

EFQ27668

Got1-like family protein
  
Accession: EFQ27669
  
Location: 478408-479180
  
 NCBI BlastP on this gene

EFQ27669

hypothetical protein
  
Accession: EFQ27670
  
Location: 480482-481096
  
 NCBI BlastP on this gene

EFQ27670

DASH complex subunit Dad4
  
Accession: EFQ27671
  
Location: 481424-481796
  
 NCBI BlastP on this gene

EFQ27671

actin
  
Accession: EFQ27672
  
Location: 482379-484864
  
 NCBI BlastP on this gene

EFQ27672

bZIP transcription factor
  
Accession: EFQ27673
  
Location: 485480-486365
  
 NCBI BlastP on this gene

EFQ27673

amidohydrolase
  
Accession: EFQ27674
  
Location: 491008-492046
  
 NCBI BlastP on this gene

EFQ27674

pterin 4 alpha carbinolamine dehydratase
  
Accession: EFQ27675
  
Location: 493360-494119
  
 NCBI BlastP on this gene

EFQ27675

fatty acid hydroxylase superfamily protein
  
Accession: EFQ27676
  
Location: 494661-495796
  
  
**BlastP hit with Mycgr3G36271\_Mycgr3T**
  
Percentage identity: 74 %
  
BlastP bit score: 461
  
Sequence coverage: 96 %
  
E-value: 2e-160
  
  
 NCBI BlastP on this gene

EFQ27676

ATP synthase regulation protein NCA2
  
Accession: EFQ27677
  
Location: 498128-500231
  
  
**BlastP hit with Mycgr3G84646\_Mycgr3T**
  
Percentage identity: 43 %
  
BlastP bit score: 546
  
Sequence coverage: 101 %
  
E-value: 0.0
  
  
 NCBI BlastP on this gene

EFQ27677

Ras family protein
  
Accession: EFQ27678
  
Location: 501788-502706
  
  
**BlastP hit with Mycgr3G99145\_Mycgr3T**
  
Percentage identity: 84 %
  
BlastP bit score: 355
  
Sequence coverage: 99 %
  
E-value: 1e-121
  
  
 NCBI BlastP on this gene

EFQ27678

hypothetical protein
  
Accession: EFQ27679
  
Location: 503250-505030
  
 NCBI BlastP on this gene

EFQ27679

ATPase
  
Accession: EFQ27680
  
Location: 505936-508329
  
 NCBI BlastP on this gene

EFQ27680

squalene epoxidase
  
Accession: EFQ27681
  
Location: 510106-511611
  
 NCBI BlastP on this gene

EFQ27681

glycosyl transferase family 2
  
Accession: EFQ27682
  
Location: 513442-514798
  
 NCBI BlastP on this gene

EFQ27682

hypothetical protein
  
Accession: EFQ27683
  
Location: 518101-519495
  
 NCBI BlastP on this gene

EFQ27683

58. :  KB644412 Penicillium oxalicum 114-2 unplaced genomic scaffold scaffold\_5     Total score: 3.0     Cumulative Blast bit score: 1358

hypothetical protein
  
Accession: EPS30815
  
Location: 3829129-3829935
  
 NCBI BlastP on this gene

EPS30815

hypothetical protein
  
Accession: EPS30816
  
Location: 3830099-3832030
  
 NCBI BlastP on this gene

EPS30816

hypothetical protein
  
Accession: EPS30817
  
Location: 3835368-3837000
  
 NCBI BlastP on this gene

EPS30817

hypothetical protein
  
Accession: EPS30818
  
Location: 3837647-3838235
  
 NCBI BlastP on this gene

EPS30818

hypothetical protein
  
Accession: EPS30819
  
Location: 3840318-3841460
  
 NCBI BlastP on this gene

EPS30819

hypothetical protein
  
Accession: EPS30820
  
Location: 3841744-3844827
  
  
**BlastP hit with Mycgr3G68433\_Mycgr3T**
  
Percentage identity: 37 %
  
BlastP bit score: 459
  
Sequence coverage: 88 %
  
E-value: 2e-140
  
  
 NCBI BlastP on this gene

EPS30820

hypothetical protein
  
Accession: EPS30821
  
Location: 3847235-3848524
  
  
**BlastP hit with Mycgr3G90786\_Mycgr3T**
  
Percentage identity: 29 %
  
BlastP bit score: 63
  
Sequence coverage: 45 %
  
E-value: 7e-08
  
  
 NCBI BlastP on this gene

EPS30821

hypothetical protein
  
Accession: EPS30822
  
Location: 3849825-3851058
  
 NCBI BlastP on this gene

EPS30822

hypothetical protein
  
Accession: EPS30823
  
Location: 3854324-3854545
  
 NCBI BlastP on this gene

EPS30823

hypothetical protein
  
Accession: EPS30824
  
Location: 3856811-3857342
  
 NCBI BlastP on this gene

EPS30824

hypothetical protein
  
Accession: EPS30825
  
Location: 3858415-3859480
  
 NCBI BlastP on this gene

EPS30825

hypothetical protein
  
Accession: EPS30826
  
Location: 3861186-3862494
  
 NCBI BlastP on this gene

EPS30826

hypothetical protein
  
Accession: EPS30827
  
Location: 3862599-3862895
  
 NCBI BlastP on this gene

EPS30827

hypothetical protein
  
Accession: EPS30828
  
Location: 3863540-3865591
  
  
**BlastP hit with Mycgr3G68458\_Mycgr3T**
  
Percentage identity: 64 %
  
BlastP bit score: 836
  
Sequence coverage: 90 %
  
E-value: 0.0
  
  
 NCBI BlastP on this gene

EPS30828

hypothetical protein
  
Accession: EPS30829
  
Location: 3865995-3867267
  
 NCBI BlastP on this gene

EPS30829

hypothetical protein
  
Accession: EPS30830
  
Location: 3868184-3870787
  
 NCBI BlastP on this gene

EPS30830

hypothetical protein
  
Accession: EPS30831
  
Location: 3873192-3875741
  
 NCBI BlastP on this gene

EPS30831

hypothetical protein
  
Accession: EPS30832
  
Location: 3876716-3877677
  
 NCBI BlastP on this gene

EPS30832

59. :  CP003002 Myceliophthora thermophila ATCC 42464 chromosome 1     Total score: 3.0     Cumulative Blast bit score: 1357

hypothetical protein
  
Accession: AEO55347
  
Location: 9069837-9070888
  
 NCBI BlastP on this gene

MYCTH\_2124283

hypothetical protein
  
Accession: AEO55346
  
Location: 9067117-9067923
  
 NCBI BlastP on this gene

MYCTH\_2142488

hypothetical protein
  
Accession: AEO55345
  
Location: 9065400-9066267
  
 NCBI BlastP on this gene

MYCTH\_2314212

hypothetical protein
  
Accession: AEO55344
  
Location: 9063982-9064429
  
 NCBI BlastP on this gene

MYCTH\_2299070

hypothetical protein
  
Accession: AEO55343
  
Location: 9062891-9063490
  
 NCBI BlastP on this gene

MYCTH\_2299068

hypothetical protein
  
Accession: AEO55342
  
Location: 9062117-9062576
  
 NCBI BlastP on this gene

MYCTH\_2299067

hypothetical protein
  
Accession: AEO55341
  
Location: 9060838-9061245
  
 NCBI BlastP on this gene

MYCTH\_2299066

ARP5-like protein
  
Accession: AEO55340
  
Location: 9056987-9059260
  
 NCBI BlastP on this gene

MYCTH\_2299064

hypothetical protein
  
Accession: AEO55339
  
Location: 9055360-9056020
  
 NCBI BlastP on this gene

MYCTH\_2299061

hypothetical protein
  
Accession: AEO55338
  
Location: 9051489-9051887
  
 NCBI BlastP on this gene

MYCTH\_2299056

methylsterol monooxygenase
  
Accession: AEO55337
  
Location: 9049575-9050674
  
  
**BlastP hit with Mycgr3G36271\_Mycgr3T**
  
Percentage identity: 72 %
  
BlastP bit score: 459
  
Sequence coverage: 95 %
  
E-value: 1e-159
  
  
 NCBI BlastP on this gene

MYCTH\_109638

hypothetical protein
  
Accession: AEO55336
  
Location: 9046163-9048350
  
  
**BlastP hit with Mycgr3G84646\_Mycgr3T**
  
Percentage identity: 44 %
  
BlastP bit score: 543
  
Sequence coverage: 103 %
  
E-value: 0.0
  
  
 NCBI BlastP on this gene

MYCTH\_97642

hypothetical protein
  
Accession: AEO55335
  
Location: 9044169-9044981
  
  
**BlastP hit with Mycgr3G99145\_Mycgr3T**
  
Percentage identity: 82 %
  
BlastP bit score: 355
  
Sequence coverage: 99 %
  
E-value: 1e-121
  
  
 NCBI BlastP on this gene

MYCTH\_2314205

hypothetical protein
  
Accession: AEO55334
  
Location: 9036757-9042828
  
 NCBI BlastP on this gene

MYCTH\_2299043

hypothetical protein
  
Accession: AEO55333
  
Location: 9035197-9035970
  
 NCBI BlastP on this gene

MYCTH\_2107979

hypothetical protein
  
Accession: AEO55332
  
Location: 9033211-9034473
  
 NCBI BlastP on this gene

MYCTH\_2299042

hypothetical protein
  
Accession: AEO55331
  
Location: 9032396-9032668
  
 NCBI BlastP on this gene

MYCTH\_2299041

hypothetical protein
  
Accession: AEO55330
  
Location: 9030985-9031910
  
 NCBI BlastP on this gene

MYCTH\_2299040

hypothetical protein
  
Accession: AEO55329
  
Location: 9028063-9030554
  
 NCBI BlastP on this gene

MYCTH\_2299039

hypothetical protein
  
Accession: AEO55328
  
Location: 9026402-9027154
  
 NCBI BlastP on this gene

MYCTH\_2299037

60. :  KB707370 Eutypa lata UCREL1 unplaced genomic scaffold EL1\_03\_scaffold\_2032     Total score: 3.0     Cumulative Blast bit score: 1326

putative pterin 4 alpha carbinolamine dehydratase protein
  
Accession: EMR62832
  
Location: 700-918
  
 NCBI BlastP on this gene

EMR62832

putative c-4 methylsterol variant protein
  
Accession: EMR62826
  
Location: 1515-2635
  
  
**BlastP hit with Mycgr3G36271\_Mycgr3T**
  
Percentage identity: 68 %
  
BlastP bit score: 416
  
Sequence coverage: 97 %
  
E-value: 3e-143
  
  
 NCBI BlastP on this gene

EMR62826

putative atp synthase regulation protein nca2 protein
  
Accession: EMR62821
  
Location: 4053-6124
  
  
**BlastP hit with Mycgr3G84646\_Mycgr3T**
  
Percentage identity: 44 %
  
BlastP bit score: 554
  
Sequence coverage: 101 %
  
E-value: 0.0
  
  
 NCBI BlastP on this gene

EMR62821

putative rab gtpase protein
  
Accession: EMR62820
  
Location: 7527-8553
  
  
**BlastP hit with Mycgr3G99145\_Mycgr3T**
  
Percentage identity: 84 %
  
BlastP bit score: 356
  
Sequence coverage: 99 %
  
E-value: 4e-122
  
  
 NCBI BlastP on this gene

EMR62820

hypothetical protein
  
Accession: EMR62831
  
Location: 9770-11721
  
 NCBI BlastP on this gene

EMR62831

putative aaa family atpase protein
  
Accession: EMR62827
  
Location: 12387-14737
  
 NCBI BlastP on this gene

EMR62827

hypothetical protein
  
Accession: EMR62824
  
Location: 16515-17322
  
 NCBI BlastP on this gene

EMR62824

hypothetical protein
  
Accession: EMR62833
  
Location: 18302-18942
  
 NCBI BlastP on this gene

EMR62833

putative aromatic-l-amino-acid decarboxylase protein
  
Accession: EMR62818
  
Location: 21629-23378
  
 NCBI BlastP on this gene

EMR62818

hypothetical protein
  
Accession: EMR62823
  
Location: 23728-24270
  
 NCBI BlastP on this gene

EMR62823

61. :  KB446566 Pseudocercospora fijiensis CIRAD86 unplaced genomic scaffold MYCFIscaffold\_12     Total score: 3.0     Cumulative Blast bit score: 1322

hypothetical protein
  
Accession: EME77366
  
Location: 920062-922370
  
 NCBI BlastP on this gene

EME77366

hypothetical protein
  
Accession: EME77367
  
Location: 922504-923817
  
 NCBI BlastP on this gene

EME77367

hypothetical protein
  
Accession: EME77368
  
Location: 925041-925934
  
 NCBI BlastP on this gene

EME77368

hypothetical protein
  
Accession: EME77369
  
Location: 932702-932893
  
 NCBI BlastP on this gene

EME77369

hypothetical protein
  
Accession: EME77370
  
Location: 934075-935963
  
 NCBI BlastP on this gene

EME77370

hypothetical protein
  
Accession: EME77371
  
Location: 936380-937201
  
 NCBI BlastP on this gene

EME77371

hypothetical protein
  
Accession: EME77372
  
Location: 938799-941387
  
  
**BlastP hit with Mycgr3G68433\_Mycgr3T**
  
Percentage identity: 59 %
  
BlastP bit score: 937
  
Sequence coverage: 83 %
  
E-value: 0.0
  
  
 NCBI BlastP on this gene

EME77372

hypothetical protein
  
Accession: EME77373
  
Location: 942550-945648
  
  
**BlastP hit with Mycgr3G90785\_Mycgr3T**
  
Percentage identity: 27 %
  
BlastP bit score: 68
  
Sequence coverage: 89 %
  
E-value: 3e-09
  
  
  
**BlastP hit with Mycgr3G90786\_Mycgr3T**
  
Percentage identity: 47 %
  
BlastP bit score: 317
  
Sequence coverage: 97 %
  
E-value: 1e-95
  
  
 NCBI BlastP on this gene

EME77373

hypothetical protein
  
Accession: EME77374
  
Location: 946401-947795
  
 NCBI BlastP on this gene

EME77374

hypothetical protein
  
Accession: EME77375
  
Location: 949865-951562
  
 NCBI BlastP on this gene

EME77375

hypothetical protein
  
Accession: EME77376
  
Location: 954705-954960
  
 NCBI BlastP on this gene

EME77376

hypothetical protein
  
Accession: EME77377
  
Location: 954984-955358
  
 NCBI BlastP on this gene

EME77377

phosphatidylinositol 3-kinase tor2
  
Accession: EME77378
  
Location: 955417-962775
  
 NCBI BlastP on this gene

EME77378

hypothetical protein
  
Accession: EME77379
  
Location: 963166-965354
  
 NCBI BlastP on this gene

EME77379

62. :  FP929137 Leptosphaeria maculans JN3 lm\_SuperContig\_10\_v2 genomic supercontig     Total score: 3.0     Cumulative Blast bit score: 1318

predicted protein
  
Accession: CBY00049
  
Location: 1509441-1509941
  
 NCBI BlastP on this gene

LEMA\_P076380.1

similar to Mn2+ homeostasis protein Per1
  
Accession: CBY00050
  
Location: 1512271-1513437
  
 NCBI BlastP on this gene

LEMA\_P076390.1

predicted protein
  
Accession: CBY00051
  
Location: 1514471-1515267
  
 NCBI BlastP on this gene

LEMA\_P076400.1

predicted protein
  
Accession: CBY00052
  
Location: 1515559-1516067
  
 NCBI BlastP on this gene

LEMA\_P076410.1

predicted protein
  
Accession: CBY00053
  
Location: 1516791-1517108
  
 NCBI BlastP on this gene

LEMA\_uP076420.1

hypothetical protein
  
Accession: CBY00054
  
Location: 1517506-1519427
  
 NCBI BlastP on this gene

LEMA\_P076430.1

similar to DNA-binding protein HGH1
  
Accession: CBY00055
  
Location: 1519502-1520818
  
  
**BlastP hit with Mycgr3G25746\_Mycgr3T**
  
Percentage identity: 55 %
  
BlastP bit score: 371
  
Sequence coverage: 102 %
  
E-value: 5e-123
  
  
 NCBI BlastP on this gene

LEMA\_P076440.1

hypothetical protein
  
Accession: CBY00056
  
Location: 1522175-1524427
  
 NCBI BlastP on this gene

LEMA\_P076450.1

similar to carboxylesterase family protein
  
Accession: CBY00057
  
Location: 1524896-1526691
  
 NCBI BlastP on this gene

LEMA\_P076460.1

predicted protein
  
Accession: CBY00058
  
Location: 1526987-1527352
  
 NCBI BlastP on this gene

LEMA\_P076470.1

hypothetical protein
  
Accession: CBY00059
  
Location: 1527671-1529952
  
 NCBI BlastP on this gene

LEMA\_P076480.1

similar to MFS multidrug transporter
  
Accession: CBY00060
  
Location: 1531604-1533489
  
 NCBI BlastP on this gene

LEMA\_P076490.1

hypothetical protein
  
Accession: CBY00061
  
Location: 1535769-1537635
  
 NCBI BlastP on this gene

LEMA\_P076500.1

hypothetical protein
  
Accession: CBY00062
  
Location: 1538211-1540873
  
  
**BlastP hit with Mycgr3G84646\_Mycgr3T**
  
Percentage identity: 47 %
  
BlastP bit score: 581
  
Sequence coverage: 100 %
  
E-value: 0.0
  
  
 NCBI BlastP on this gene

LEMA\_P076510.1

similar to GTP-binding protein
  
Accession: CBY00063
  
Location: 1541206-1542152
  
  
**BlastP hit with Mycgr3G99145\_Mycgr3T**
  
Percentage identity: 87 %
  
BlastP bit score: 366
  
Sequence coverage: 99 %
  
E-value: 3e-126
  
  
 NCBI BlastP on this gene

LEMA\_P076520.1

hypothetical protein
  
Accession: CBY00064
  
Location: 1543529-1545322
  
 NCBI BlastP on this gene

LEMA\_P076530.1

predicted protein
  
Accession: CBY00065
  
Location: 1545628-1546059
  
 NCBI BlastP on this gene

LEMA\_P076540.1

similar to calcium permease family membrane transporter
  
Accession: CBY00066
  
Location: 1546447-1549948
  
 NCBI BlastP on this gene

LEMA\_P076550.1

similar to 26S protease regulatory subunit 8
  
Accession: CBY00067
  
Location: 1551329-1552554
  
 NCBI BlastP on this gene

LEMA\_P076560.1

similar to sugar transporter
  
Accession: CBY00068
  
Location: 1552712-1555070
  
 NCBI BlastP on this gene

LEMA\_P076570.1

63. :  GG749488 Ajellomyces dermatitidis ATCC 18188 genomic scaffold supercont1.82     Total score: 3.0     Cumulative Blast bit score: 1317

SnoRNA binding protein
  
Accession: EGE85260
  
Location: 90412-94130
  
 NCBI BlastP on this gene

EGE85260

hypothetical protein
  
Accession: EGE85261
  
Location: 95288-96118
  
 NCBI BlastP on this gene

EGE85261

hypothetical protein
  
Accession: EGE85262
  
Location: 96480-98746
  
 NCBI BlastP on this gene

EGE85262

DUF652 domain-containing protein
  
Accession: EGE85263
  
Location: 99796-100802
  
 NCBI BlastP on this gene

EGE85263

hypothetical protein
  
Accession: EGE85264
  
Location: 101595-103181
  
  
**BlastP hit with Mycgr3G36271\_Mycgr3T**
  
Percentage identity: 75 %
  
BlastP bit score: 482
  
Sequence coverage: 99 %
  
E-value: 6e-168
  
  
 NCBI BlastP on this gene

EGE85264

ATP synthase regulation protein NCA2
  
Accession: EGE85265
  
Location: 103769-106041
  
  
**BlastP hit with Mycgr3G84646\_Mycgr3T**
  
Percentage identity: 41 %
  
BlastP bit score: 483
  
Sequence coverage: 99 %
  
E-value: 9e-158
  
  
 NCBI BlastP on this gene

EGE85265

hypothetical protein
  
Accession: EGE85266
  
Location: 106528-109978
  
 NCBI BlastP on this gene

EGE85266

hypothetical protein
  
Accession: EGE85267
  
Location: 110394-112017
  
 NCBI BlastP on this gene

EGE85267

hypothetical protein
  
Accession: EGE85268
  
Location: 113262-113847
  
 NCBI BlastP on this gene

EGE85268

hypothetical protein
  
Accession: EGE85269
  
Location: 115187-118039
  
 NCBI BlastP on this gene

EGE85269

hypothetical protein
  
Accession: EGE85270
  
Location: 118767-119298
  
 NCBI BlastP on this gene

EGE85270

creatine transporter
  
Accession: EGE85271
  
Location: 122315-124396
  
 NCBI BlastP on this gene

EGE85271

hypothetical protein
  
Accession: EGE85272
  
Location: 124924-125303
  
 NCBI BlastP on this gene

EGE85272

hypothetical protein
  
Accession: EGE85273
  
Location: 128052-129153
  
 NCBI BlastP on this gene

EGE85273

GTP-binding protein
  
Accession: EGE85274
  
Location: 130528-131352
  
  
**BlastP hit with Mycgr3G99145\_Mycgr3T**
  
Percentage identity: 84 %
  
BlastP bit score: 352
  
Sequence coverage: 99 %
  
E-value: 2e-120
  
  
 NCBI BlastP on this gene

EGE85274

hypothetical protein
  
Accession: EGE85275
  
Location: 131983-133221
  
 NCBI BlastP on this gene

EGE85275

cell division cycle protein
  
Accession: EGE85276
  
Location: 136882-139206
  
 NCBI BlastP on this gene

EGE85276

hypothetical protein
  
Accession: EGE85277
  
Location: 139790-140835
  
 NCBI BlastP on this gene

EGE85277

64. :  GG657483 Ajellomyces dermatitidis SLH14081 genomic scaffold supercont1.36     Total score: 3.0     Cumulative Blast bit score: 1317

snoRNA binding protein
  
Accession: EEQ75948
  
Location: 276621-280283
  
 NCBI BlastP on this gene

EEQ75948

conserved hypothetical protein
  
Accession: EEQ75947
  
Location: 274520-275350
  
 NCBI BlastP on this gene

EEQ75947

conserved hypothetical protein
  
Accession: EEQ75946
  
Location: 271892-274158
  
 NCBI BlastP on this gene

EEQ75946

FCF1 small subunit
  
Accession: EEQ75945
  
Location: 269835-270841
  
 NCBI BlastP on this gene

EEQ75945

C-4 methyl sterol oxidase Erg25
  
Accession: EEQ75944
  
Location: 267878-269039
  
  
**BlastP hit with Mycgr3G36271\_Mycgr3T**
  
Percentage identity: 75 %
  
BlastP bit score: 480
  
Sequence coverage: 99 %
  
E-value: 4e-168
  
  
 NCBI BlastP on this gene

EEQ75944

ATP synthase regulation protein NCA2
  
Accession: EEQ75943
  
Location: 264581-266853
  
  
**BlastP hit with Mycgr3G84646\_Mycgr3T**
  
Percentage identity: 41 %
  
BlastP bit score: 485
  
Sequence coverage: 99 %
  
E-value: 2e-158
  
  
 NCBI BlastP on this gene

EEQ75943

conserved hypothetical protein
  
Accession: EEQ75942
  
Location: 260558-264094
  
 NCBI BlastP on this gene

EEQ75942

conserved hypothetical protein
  
Accession: EEQ75941
  
Location: 258878-260221
  
 NCBI BlastP on this gene

EEQ75941

predicted protein
  
Accession: EEQ75940
  
Location: 256278-257390
  
 NCBI BlastP on this gene

EEQ75940

conserved hypothetical protein
  
Accession: EEQ75939
  
Location: 252643-255406
  
 NCBI BlastP on this gene

EEQ75939

hypothetical protein
  
Accession: EEQ75938
  
Location: 251386-251917
  
 NCBI BlastP on this gene

EEQ75938

creatine transporter
  
Accession: EEQ75937
  
Location: 246297-248378
  
 NCBI BlastP on this gene

EEQ75937

predicted protein
  
Accession: EEQ75936
  
Location: 244361-245718
  
 NCBI BlastP on this gene

EEQ75936

hypothetical protein
  
Accession: EEQ75935
  
Location: 242215-242637
  
 NCBI BlastP on this gene

EEQ75935

GTP-binding protein SAS1
  
Accession: EEQ75934
  
Location: 239369-240193
  
  
**BlastP hit with Mycgr3G99145\_Mycgr3T**
  
Percentage identity: 84 %
  
BlastP bit score: 352
  
Sequence coverage: 99 %
  
E-value: 2e-120
  
  
 NCBI BlastP on this gene

EEQ75934

conserved hypothetical protein
  
Accession: EEQ75933
  
Location: 237499-238737
  
 NCBI BlastP on this gene

EEQ75933

AAA family ATPase
  
Accession: EEQ75932
  
Location: 234518-236842
  
 NCBI BlastP on this gene

EEQ75932

conserved hypothetical protein
  
Accession: EEQ75931
  
Location: 232892-233938
  
 NCBI BlastP on this gene

EEQ75931

phosphatidylinositol 3- and 4-kinase
  
Accession: EEQ75930
  
Location: 223739-232614
  
 NCBI BlastP on this gene

EEQ75930

65. :  EQ999987 Ajellomyces dermatitidis ER-3 genomic scaffold supercont1.15     Total score: 3.0     Cumulative Blast bit score: 1316

snoRNA binding protein
  
Accession: EEQ86096
  
Location: 411828-415544
  
 NCBI BlastP on this gene

EEQ86096

conserved hypothetical protein
  
Accession: EEQ86095
  
Location: 409860-410687
  
 NCBI BlastP on this gene

EEQ86095

conserved hypothetical protein
  
Accession: EEQ86094
  
Location: 407241-409498
  
 NCBI BlastP on this gene

EEQ86094

DUF652 domain-containing protein
  
Accession: EEQ86093
  
Location: 405188-406191
  
 NCBI BlastP on this gene

EEQ86093

C-4 methyl sterol oxidase Erg25
  
Accession: EEQ86092
  
Location: 403277-404436
  
  
**BlastP hit with Mycgr3G36271\_Mycgr3T**
  
Percentage identity: 75 %
  
BlastP bit score: 480
  
Sequence coverage: 99 %
  
E-value: 4e-168
  
  
 NCBI BlastP on this gene

EEQ86092

conserved hypothetical protein
  
Accession: EEQ86091
  
Location: 399982-402254
  
  
**BlastP hit with Mycgr3G84646\_Mycgr3T**
  
Percentage identity: 41 %
  
BlastP bit score: 484
  
Sequence coverage: 99 %
  
E-value: 7e-158
  
  
 NCBI BlastP on this gene

EEQ86091

conserved hypothetical protein
  
Accession: EEQ86090
  
Location: 395962-399495
  
 NCBI BlastP on this gene

EEQ86090

conserved hypothetical protein
  
Accession: EEQ86089
  
Location: 394282-395625
  
 NCBI BlastP on this gene

EEQ86089

predicted protein
  
Accession: EEQ86088
  
Location: 391696-392677
  
 NCBI BlastP on this gene

EEQ86088

conserved hypothetical protein
  
Accession: EEQ86087
  
Location: 388030-390793
  
 NCBI BlastP on this gene

EEQ86087

hypothetical protein
  
Accession: EEQ86086
  
Location: 386768-387302
  
 NCBI BlastP on this gene

EEQ86086

creatine transporter
  
Accession: EEQ86085
  
Location: 381671-383752
  
 NCBI BlastP on this gene

EEQ86085

predicted protein
  
Accession: EEQ86084
  
Location: 379581-381100
  
 NCBI BlastP on this gene

EEQ86084

predicted protein
  
Accession: EEQ86083
  
Location: 376944-378056
  
 NCBI BlastP on this gene

EEQ86083

GTP-binding protein SAS1
  
Accession: EEQ86082
  
Location: 374745-375569
  
  
**BlastP hit with Mycgr3G99145\_Mycgr3T**
  
Percentage identity: 84 %
  
BlastP bit score: 352
  
Sequence coverage: 99 %
  
E-value: 2e-120
  
  
 NCBI BlastP on this gene

EEQ86082

conserved hypothetical protein
  
Accession: EEQ86081
  
Location: 372875-374113
  
 NCBI BlastP on this gene

EEQ86081

AAA family ATPase
  
Accession: EEQ86080
  
Location: 369881-372205
  
 NCBI BlastP on this gene

EEQ86080

conserved hypothetical protein
  
Accession: EEQ86079
  
Location: 368255-369301
  
 NCBI BlastP on this gene

EEQ86079

phosphatidylinositol 3- and 4-kinase
  
Accession: EEQ86078
  
Location: 359099-367975
  
 NCBI BlastP on this gene

EEQ86078

66. :  JH794989 Magnaporthe oryzae P131 unplaced genomic scaffold P131\_scaffold00957     Total score: 3.0     Cumulative Blast bit score: 1313

profilin
  
Accession: ELQ63654
  
Location: 59934-60674
  
 NCBI BlastP on this gene

ELQ63654

hypothetical protein
  
Accession: ELQ63653
  
Location: 58351-59301
  
 NCBI BlastP on this gene

ELQ63653

hypothetical protein
  
Accession: ELQ63652
  
Location: 56873-57460
  
 NCBI BlastP on this gene

ELQ63652

actin-related protein 5
  
Accession: ELQ63651
  
Location: 52845-55385
  
 NCBI BlastP on this gene

ELQ63651

hypothetical protein
  
Accession: ELQ63650
  
Location: 51654-52538
  
 NCBI BlastP on this gene

ELQ63650

hypothetical protein
  
Accession: ELQ63649
  
Location: 48462-49127
  
 NCBI BlastP on this gene

ELQ63649

C-4 methylsterol oxidase
  
Accession: ELQ63648
  
Location: 46968-48081
  
  
**BlastP hit with Mycgr3G36271\_Mycgr3T**
  
Percentage identity: 70 %
  
BlastP bit score: 432
  
Sequence coverage: 94 %
  
E-value: 5e-149
  
  
 NCBI BlastP on this gene

ELQ63648

nuclear control of ATPase protein 2
  
Accession: ELQ63647
  
Location: 43145-45298
  
  
**BlastP hit with Mycgr3G84646\_Mycgr3T**
  
Percentage identity: 42 %
  
BlastP bit score: 526
  
Sequence coverage: 101 %
  
E-value: 1e-174
  
  
 NCBI BlastP on this gene

ELQ63647

GTP-binding protein SAS1
  
Accession: ELQ63646
  
Location: 40988-41834
  
  
**BlastP hit with Mycgr3G99145\_Mycgr3T**
  
Percentage identity: 85 %
  
BlastP bit score: 355
  
Sequence coverage: 99 %
  
E-value: 1e-121
  
  
 NCBI BlastP on this gene

ELQ63646

hypothetical protein
  
Accession: ELQ63645
  
Location: 38944-40206
  
 NCBI BlastP on this gene

ELQ63645

ATPase, AFG2 type
  
Accession: ELQ63644
  
Location: 36010-38401
  
 NCBI BlastP on this gene

ELQ63644

dolichyl-phosphate beta-glucosyltransferase
  
Accession: ELQ63643
  
Location: 34295-35633
  
 NCBI BlastP on this gene

ELQ63643

hypothetical protein
  
Accession: ELQ63642
  
Location: 31622-33275
  
 NCBI BlastP on this gene

ELQ63642

DUF625 domain-containing protein
  
Accession: ELQ63641
  
Location: 24515-27581
  
 NCBI BlastP on this gene

ELQ63641

squalene monooxygenase
  
Accession: ELQ63640
  
Location: 21429-23033
  
 NCBI BlastP on this gene

ELQ63640

67. :  JH793752 Magnaporthe oryzae Y34 unplaced genomic scaffold Y34\_scaffold00460     Total score: 3.0     Cumulative Blast bit score: 1313

hypothetical protein
  
Accession: ELQ40148
  
Location: 342-2591
  
 NCBI BlastP on this gene

ELQ40148

profilin
  
Accession: ELQ40149
  
Location: 7232-7972
  
 NCBI BlastP on this gene

ELQ40149

hypothetical protein
  
Accession: ELQ40150
  
Location: 8551-9553
  
 NCBI BlastP on this gene

ELQ40150

hypothetical protein
  
Accession: ELQ40151
  
Location: 10444-11031
  
 NCBI BlastP on this gene

ELQ40151

actin-related protein 5
  
Accession: ELQ40152
  
Location: 12519-15059
  
 NCBI BlastP on this gene

ELQ40152

hypothetical protein
  
Accession: ELQ40153
  
Location: 15366-16250
  
 NCBI BlastP on this gene

ELQ40153

hypothetical protein
  
Accession: ELQ40154
  
Location: 18775-19440
  
 NCBI BlastP on this gene

ELQ40154

C-4 methylsterol oxidase
  
Accession: ELQ40155
  
Location: 19821-20934
  
  
**BlastP hit with Mycgr3G36271\_Mycgr3T**
  
Percentage identity: 70 %
  
BlastP bit score: 432
  
Sequence coverage: 94 %
  
E-value: 5e-149
  
  
 NCBI BlastP on this gene

ELQ40155

nuclear control of ATPase protein 2
  
Accession: ELQ40156
  
Location: 22605-24758
  
  
**BlastP hit with Mycgr3G84646\_Mycgr3T**
  
Percentage identity: 42 %
  
BlastP bit score: 526
  
Sequence coverage: 101 %
  
E-value: 1e-174
  
  
 NCBI BlastP on this gene

ELQ40156

GTP-binding protein SAS1
  
Accession: ELQ40157
  
Location: 26069-26915
  
  
**BlastP hit with Mycgr3G99145\_Mycgr3T**
  
Percentage identity: 85 %
  
BlastP bit score: 355
  
Sequence coverage: 99 %
  
E-value: 1e-121
  
  
 NCBI BlastP on this gene

ELQ40157

hypothetical protein
  
Accession: ELQ40158
  
Location: 27697-28959
  
 NCBI BlastP on this gene

ELQ40158

ATPase, AFG2 type
  
Accession: ELQ40159
  
Location: 29502-31894
  
 NCBI BlastP on this gene

ELQ40159

dolichyl-phosphate beta-glucosyltransferase
  
Accession: ELQ40160
  
Location: 32271-33609
  
 NCBI BlastP on this gene

ELQ40160

hypothetical protein
  
Accession: ELQ40161
  
Location: 34629-36282
  
 NCBI BlastP on this gene

ELQ40161

DUF625 domain-containing protein
  
Accession: ELQ40162
  
Location: 40320-43386
  
 NCBI BlastP on this gene

ELQ40162

squalene monooxygenase
  
Accession: ELQ40163
  
Location: 44864-46468
  
 NCBI BlastP on this gene

ELQ40163

68. :  CM001233 Magnaporthe oryzae 70-15 chromosome 3     Total score: 3.0     Cumulative Blast bit score: 1313

hypothetical protein
  
Accession: EHA52186
  
Location: 2659095-2661341
  
 NCBI BlastP on this gene

EHA52186

hypothetical protein
  
Accession: EHA52187
  
Location: 2662481-2664568
  
 NCBI BlastP on this gene

EHA52187

profilin
  
Accession: EHA52188
  
Location: 2665982-2666722
  
 NCBI BlastP on this gene

EHA52188

Got1 family protein
  
Accession: EHA52189
  
Location: 2667416-2668183
  
 NCBI BlastP on this gene

EHA52189

hypothetical protein
  
Accession: EHA52190
  
Location: 2669195-2669782
  
 NCBI BlastP on this gene

EHA52190

DASH complex subunit dad4
  
Accession: EHA52191
  
Location: 2670188-2670606
  
 NCBI BlastP on this gene

EHA52191

hypothetical protein
  
Accession: EHA52192
  
Location: 2671270-2673810
  
 NCBI BlastP on this gene

EHA52192

hypothetical protein, variant 2
  
Accession: EHA52193
  
Location: 2674117-2675004
  
 NCBI BlastP on this gene

EHA52193

hypothetical protein
  
Accession: EHA52196
  
Location: 2677527-2678192
  
 NCBI BlastP on this gene

EHA52196

C-4 methylsterol oxidase
  
Accession: EHA52197
  
Location: 2678573-2679686
  
  
**BlastP hit with Mycgr3G36271\_Mycgr3T**
  
Percentage identity: 70 %
  
BlastP bit score: 432
  
Sequence coverage: 94 %
  
E-value: 5e-149
  
  
 NCBI BlastP on this gene

EHA52197

hypothetical protein
  
Accession: EHA52198
  
Location: 2680625-2681076
  
 NCBI BlastP on this gene

EHA52198

hypothetical protein
  
Accession: EHA52199
  
Location: 2681356-2683509
  
  
**BlastP hit with Mycgr3G84646\_Mycgr3T**
  
Percentage identity: 42 %
  
BlastP bit score: 526
  
Sequence coverage: 101 %
  
E-value: 1e-174
  
  
 NCBI BlastP on this gene

EHA52199

GTP-binding protein ypt2
  
Accession: EHA52200
  
Location: 2684820-2685666
  
  
**BlastP hit with Mycgr3G99145\_Mycgr3T**
  
Percentage identity: 85 %
  
BlastP bit score: 355
  
Sequence coverage: 99 %
  
E-value: 1e-121
  
  
 NCBI BlastP on this gene

EHA52200

hypothetical protein
  
Accession: EHA52201
  
Location: 2686448-2688009
  
 NCBI BlastP on this gene

EHA52201

ATPase
  
Accession: EHA52202
  
Location: 2688253-2690655
  
 NCBI BlastP on this gene

EHA52202

dolichyl-phosphate beta-glucosyltransferase
  
Accession: EHA52203
  
Location: 2691032-2692370
  
 NCBI BlastP on this gene

EHA52203

hypothetical protein
  
Accession: EHA52204
  
Location: 2693390-2695043
  
 NCBI BlastP on this gene

EHA52204

hypothetical protein
  
Accession: EHA52205
  
Location: 2699082-2702148
  
 NCBI BlastP on this gene

EHA52205

squalene monooxygenase
  
Accession: EHA52206
  
Location: 2703629-2705233
  
 NCBI BlastP on this gene

EHA52206

69. :  KB908844 Setosphaeria turcica Et28A unplaced genomic scaffold SETTUscaffold\_6     Total score: 3.0     Cumulative Blast bit score: 1312

hypothetical protein
  
Accession: EOA82896
  
Location: 1692451-1695948
  
 NCBI BlastP on this gene

EOA82896

hypothetical protein
  
Accession: EOA82897
  
Location: 1696383-1697024
  
 NCBI BlastP on this gene

EOA82897

hypothetical protein
  
Accession: EOA82898
  
Location: 1697063-1698052
  
 NCBI BlastP on this gene

EOA82898

hypothetical protein
  
Accession: EOA82899
  
Location: 1698464-1700054
  
 NCBI BlastP on this gene

EOA82899

hypothetical protein
  
Accession: EOA82900
  
Location: 1701162-1702179
  
 NCBI BlastP on this gene

EOA82900

carbohydrate esterase family 9 protein
  
Accession: EOA82901
  
Location: 1703065-1704372
  
 NCBI BlastP on this gene

EOA82901

glycoside hydrolase family 3 protein
  
Accession: EOA82902
  
Location: 1705027-1708080
  
 NCBI BlastP on this gene

EOA82902

hypothetical protein
  
Accession: EOA82903
  
Location: 1708413-1710944
  
  
**BlastP hit with Mycgr3G84654\_Mycgr3T**
  
Percentage identity: 55 %
  
BlastP bit score: 388
  
Sequence coverage: 85 %
  
E-value: 4e-123
  
  
 NCBI BlastP on this gene

EOA82903

hypothetical protein
  
Accession: EOA82904
  
Location: 1715589-1717350
  
 NCBI BlastP on this gene

EOA82904

hypothetical protein
  
Accession: EOA82905
  
Location: 1719073-1720719
  
 NCBI BlastP on this gene

EOA82905

hypothetical protein
  
Accession: EOA82906
  
Location: 1722133-1723389
  
 NCBI BlastP on this gene

EOA82906

hypothetical protein
  
Accession: EOA82907
  
Location: 1724102-1727533
  
  
**BlastP hit with Mycgr3G99145\_Mycgr3T**
  
Percentage identity: 76 %
  
BlastP bit score: 317
  
Sequence coverage: 98 %
  
E-value: 2e-99
  
  
  
**BlastP hit with Mycgr3G84646\_Mycgr3T**
  
Percentage identity: 48 %
  
BlastP bit score: 607
  
Sequence coverage: 98 %
  
E-value: 0.0
  
  
 NCBI BlastP on this gene

EOA82907

hypothetical protein
  
Accession: EOA82908
  
Location: 1728084-1735952
  
 NCBI BlastP on this gene

EOA82908

hypothetical protein
  
Accession: EOA82909
  
Location: 1736521-1736926
  
 NCBI BlastP on this gene

EOA82909

hypothetical protein
  
Accession: EOA82910
  
Location: 1737827-1740242
  
 NCBI BlastP on this gene

EOA82910

70. :  GG704913 Coccidioides immitis RS genomic scaffold supercont3.3     Total score: 3.0     Cumulative Blast bit score: 1308

chromosome segregation in meiosis protein 3
  
Accession: EAS30096
  
Location: 1097031-1098202
  
 NCBI BlastP on this gene

EAS30096

NADH-ubiquinone oxidoreductase 21 kDa subunit
  
Accession: EAS30095
  
Location: 1098653-1099478
  
 NCBI BlastP on this gene

EAS30095

tRNA specific adenosine deaminase
  
Accession: EAS30094
  
Location: 1099644-1100610
  
 NCBI BlastP on this gene

EAS30094

UVSB PI-3 kinase
  
Accession: EJB11175
  
Location: 1100872-1109613
  
 NCBI BlastP on this gene

EJB11175

hypothetical protein
  
Accession: EAS30091
  
Location: 1109928-1111029
  
 NCBI BlastP on this gene

EAS30091

AAA family ATPase
  
Accession: EAS30090
  
Location: 1112389-1114696
  
 NCBI BlastP on this gene

EAS30090

hypothetical protein
  
Accession: EAS30089
  
Location: 1115444-1116138
  
 NCBI BlastP on this gene

EAS30089

C-4 methyl sterol oxidase Erg25
  
Accession: EAS30088
  
Location: 1116619-1117947
  
  
**BlastP hit with Mycgr3G36271\_Mycgr3T**
  
Percentage identity: 77 %
  
BlastP bit score: 478
  
Sequence coverage: 97 %
  
E-value: 3e-166
  
  
 NCBI BlastP on this gene

EAS30088

hypothetical protein
  
Accession: EAS30087
  
Location: 1118363-1120570
  
  
**BlastP hit with Mycgr3G84646\_Mycgr3T**
  
Percentage identity: 40 %
  
BlastP bit score: 480
  
Sequence coverage: 100 %
  
E-value: 7e-157
  
  
 NCBI BlastP on this gene

EAS30087

GTP-binding protein ypt2
  
Accession: EAS30086
  
Location: 1121658-1122428
  
  
**BlastP hit with Mycgr3G99145\_Mycgr3T**
  
Percentage identity: 82 %
  
BlastP bit score: 350
  
Sequence coverage: 99 %
  
E-value: 6e-120
  
  
 NCBI BlastP on this gene

EAS30086

hypothetical protein
  
Accession: EAS30085
  
Location: 1123983-1124928
  
 NCBI BlastP on this gene

EAS30085

hypothetical protein
  
Accession: EJB11176
  
Location: 1125417-1126656
  
 NCBI BlastP on this gene

EJB11176

hypothetical protein
  
Accession: EAS30082
  
Location: 1127161-1127859
  
 NCBI BlastP on this gene

EAS30082

hypothetical protein
  
Accession: EAS30081
  
Location: 1128197-1129540
  
 NCBI BlastP on this gene

EAS30081

hypothetical protein
  
Accession: EAS30080
  
Location: 1129864-1130172
  
 NCBI BlastP on this gene

EAS30080

hypothetical protein
  
Accession: EJB11177
  
Location: 1131774-1132392
  
 NCBI BlastP on this gene

EJB11177

hypothetical protein
  
Accession: EAS30078
  
Location: 1133396-1134215
  
 NCBI BlastP on this gene

EAS30078

hypothetical protein
  
Accession: EJB11178
  
Location: 1134729-1136678
  
 NCBI BlastP on this gene

EJB11178

hypothetical protein
  
Accession: EAS30075
  
Location: 1138553-1139059
  
 NCBI BlastP on this gene

EAS30075

hypothetical protein
  
Accession: EAS30074
  
Location: 1140178-1140393
  
 NCBI BlastP on this gene

EAS30074

hypothetical protein
  
Accession: EAS30073
  
Location: 1140898-1147836
  
 NCBI BlastP on this gene

EAS30073

71. :  GL385398 Gaeumannomyces graminis var. tritici R3-111a-1 unplaced genomic scaffold supercont2.4     Total score: 3.0     Cumulative Blast bit score: 1302

hypothetical protein
  
Accession: EJT74551
  
Location: 2959309-2960184
  
 NCBI BlastP on this gene

EJT74551

hypothetical protein
  
Accession: EJT74550
  
Location: 2958573-2958923
  
 NCBI BlastP on this gene

EJT74550

hypothetical protein
  
Accession: EJT74549
  
Location: 2957630-2958151
  
 NCBI BlastP on this gene

EJT74549

hypothetical protein
  
Accession: EJT74548
  
Location: 2954701-2957216
  
 NCBI BlastP on this gene

EJT74548

hypothetical protein
  
Accession: EJT74547
  
Location: 2952815-2953827
  
 NCBI BlastP on this gene

EJT74547

hypothetical protein
  
Accession: EJT74546
  
Location: 2950610-2951321
  
 NCBI BlastP on this gene

EJT74546

hypothetical protein
  
Accession: EJT74545
  
Location: 2948742-2949155
  
 NCBI BlastP on this gene

EJT74545

hypothetical protein
  
Accession: EJT74544
  
Location: 2945869-2946496
  
 NCBI BlastP on this gene

EJT74544

hypothetical protein
  
Accession: EJT74543
  
Location: 2943356-2944649
  
 NCBI BlastP on this gene

EJT74543

C-4 methylsterol oxidase
  
Accession: EJT74542
  
Location: 2941856-2942979
  
  
**BlastP hit with Mycgr3G36271\_Mycgr3T**
  
Percentage identity: 70 %
  
BlastP bit score: 434
  
Sequence coverage: 93 %
  
E-value: 7e-150
  
  
 NCBI BlastP on this gene

EJT74542

hypothetical protein
  
Accession: EJT74541
  
Location: 2938286-2940411
  
  
**BlastP hit with Mycgr3G84646\_Mycgr3T**
  
Percentage identity: 42 %
  
BlastP bit score: 515
  
Sequence coverage: 102 %
  
E-value: 5e-170
  
  
 NCBI BlastP on this gene

EJT74541

GTP-binding protein ypt2
  
Accession: EJT74540
  
Location: 2935676-2936683
  
  
**BlastP hit with Mycgr3G99145\_Mycgr3T**
  
Percentage identity: 82 %
  
BlastP bit score: 353
  
Sequence coverage: 99 %
  
E-value: 5e-121
  
  
 NCBI BlastP on this gene

EJT74540

hypothetical protein
  
Accession: EJT74539
  
Location: 2932795-2934528
  
 NCBI BlastP on this gene

EJT74539

ATPase
  
Accession: EJT74538
  
Location: 2929456-2931831
  
 NCBI BlastP on this gene

EJT74538

dolichyl-phosphate beta-glucosyltransferase
  
Accession: EJT74537
  
Location: 2927702-2929062
  
 NCBI BlastP on this gene

EJT74537

hypothetical protein
  
Accession: EJT74536
  
Location: 2923498-2925173
  
 NCBI BlastP on this gene

EJT74536

72. :  GL636502 Coccidioides posadasii str. Silveira unplaced genomic scaffold supercont2.17     Total score: 3.0     Cumulative Blast bit score: 1299

chromosome segregation in meiosis protein 3
  
Accession: EFW15000
  
Location: 15288-16449
  
 NCBI BlastP on this gene

EFW15000

NADH-ubiquinone oxidoreductase 21 kDa subunit
  
Accession: EFW15001
  
Location: 16900-17729
  
 NCBI BlastP on this gene

EFW15001

tRNA-specific adenosine deaminase subunit TAD2
  
Accession: EFW15002
  
Location: 17895-18815
  
 NCBI BlastP on this gene

EFW15002

conserved hypothetical protein
  
Accession: EFW15003
  
Location: 19144-21761
  
 NCBI BlastP on this gene

EFW15003

conserved hypothetical protein
  
Accession: EFW15004
  
Location: 22411-27887
  
 NCBI BlastP on this gene

EFW15004

hypothetical protein
  
Accession: EFW15005
  
Location: 28216-29313
  
 NCBI BlastP on this gene

EFW15005

AAA family ATPase
  
Accession: EFW15006
  
Location: 30675-32982
  
 NCBI BlastP on this gene

EFW15006

conserved hypothetical protein
  
Accession: EFW15007
  
Location: 33732-34423
  
 NCBI BlastP on this gene

EFW15007

C-4 methylsterol oxidase
  
Accession: EFW15008
  
Location: 34904-35990
  
  
**BlastP hit with Mycgr3G36271\_Mycgr3T**
  
Percentage identity: 77 %
  
BlastP bit score: 476
  
Sequence coverage: 97 %
  
E-value: 1e-166
  
  
 NCBI BlastP on this gene

EFW15008

conserved hypothetical protein
  
Accession: EFW15009
  
Location: 36652-38859
  
  
**BlastP hit with Mycgr3G84646\_Mycgr3T**
  
Percentage identity: 39 %
  
BlastP bit score: 473
  
Sequence coverage: 100 %
  
E-value: 3e-154
  
  
 NCBI BlastP on this gene

EFW15009

GTP-binding protein SAS1
  
Accession: EFW15010
  
Location: 39970-40740
  
  
**BlastP hit with Mycgr3G99145\_Mycgr3T**
  
Percentage identity: 82 %
  
BlastP bit score: 350
  
Sequence coverage: 99 %
  
E-value: 6e-120
  
  
 NCBI BlastP on this gene

EFW15010

hypothetical protein
  
Accession: EFW15011
  
Location: 43776-45015
  
 NCBI BlastP on this gene

EFW15011

predicted protein
  
Accession: EFW15012
  
Location: 45524-46225
  
 NCBI BlastP on this gene

EFW15012

conserved hypothetical protein
  
Accession: EFW15013
  
Location: 46558-47709
  
 NCBI BlastP on this gene

EFW15013

predicted protein
  
Accession: EFW15014
  
Location: 48198-48935
  
 NCBI BlastP on this gene

EFW15014

predicted protein
  
Accession: EFW15015
  
Location: 50423-50779
  
 NCBI BlastP on this gene

EFW15015

FCF1 small subunit
  
Accession: EFW15016
  
Location: 51932-52855
  
 NCBI BlastP on this gene

EFW15016

conserved hypothetical protein
  
Accession: EFW15017
  
Location: 53849-55471
  
 NCBI BlastP on this gene

EFW15017

conserved hypothetical protein
  
Accession: EFW15018
  
Location: 56781-57200
  
 NCBI BlastP on this gene

EFW15018

hypothetical protein
  
Accession: EFW15019
  
Location: 58985-66023
  
 NCBI BlastP on this gene

EFW15019

73. :  ACFW01000015 Coccidioides posadasii C735 delta SOWgp     Total score: 3.0     Cumulative Blast bit score: 1299

hypothetical protein
  
Accession: EER28304
  
Location: 362297-363458
  
 NCBI BlastP on this gene

EER28304

NADH-ubiquinone oxidoreductase 21 kDa subunit, putative
  
Accession: EER28303
  
Location: 361017-361846
  
 NCBI BlastP on this gene

EER28303

Cytidine and deoxycytidylate deaminase zinc-binding domain containing protein
  
Accession: EER28302
  
Location: 359934-360851
  
 NCBI BlastP on this gene

EER28302

DNA repair protein, putative
  
Accession: EER28301
  
Location: 350859-359602
  
 NCBI BlastP on this gene

EER28301

ATPase, AAA family protein
  
Accession: EER28300
  
Location: 345755-348062
  
 NCBI BlastP on this gene

EER28300

pterin-4-alpha-carbinolamine dehydratase family protein
  
Accession: EER28299
  
Location: 344314-345005
  
 NCBI BlastP on this gene

EER28299

C-4 methylsterol oxidase, putative
  
Accession: EER28298
  
Location: 342741-343833
  
  
**BlastP hit with Mycgr3G36271\_Mycgr3T**
  
Percentage identity: 77 %
  
BlastP bit score: 476
  
Sequence coverage: 97 %
  
E-value: 1e-166
  
  
 NCBI BlastP on this gene

EER28298

hypothetical protein
  
Accession: EER28297
  
Location: 339872-342079
  
  
**BlastP hit with Mycgr3G84646\_Mycgr3T**
  
Percentage identity: 39 %
  
BlastP bit score: 473
  
Sequence coverage: 100 %
  
E-value: 3e-154
  
  
 NCBI BlastP on this gene

EER28297

Ras family protein
  
Accession: EER28296
  
Location: 337980-338750
  
  
**BlastP hit with Mycgr3G99145\_Mycgr3T**
  
Percentage identity: 82 %
  
BlastP bit score: 350
  
Sequence coverage: 99 %
  
E-value: 6e-120
  
  
 NCBI BlastP on this gene

EER28296

copia-like retrotransposon family protein
  
Accession: EER28295
  
Location: 331882-336498
  
 NCBI BlastP on this gene

EER28295

hypothetical protein
  
Accession: EER28294
  
Location: 328387-329626
  
 NCBI BlastP on this gene

EER28294

hypothetical protein
  
Accession: EER28293
  
Location: 327177-327878
  
 NCBI BlastP on this gene

EER28293

hypothetical protein
  
Accession: EER28292
  
Location: 325672-326844
  
 NCBI BlastP on this gene

EER28292

hypothetical protein
  
Accession: EER28291
  
Location: 320549-321472
  
 NCBI BlastP on this gene

EER28291

74. :  KB446566 Pseudocercospora fijiensis CIRAD86 unplaced genomic scaffold MYCFIscaffold\_12     Total score: 3.0     Cumulative Blast bit score: 1294

glycoside hydrolase family 13 protein
  
Accession: EME77258
  
Location: 284353-285861
  
 NCBI BlastP on this gene

EME77258

hypothetical protein
  
Accession: EME77257
  
Location: 272383-276236
  
 NCBI BlastP on this gene

EME77257

serine/threonine protein kinase
  
Accession: EME77256
  
Location: 269951-271369
  
  
**BlastP hit with Mycgr3G103260\_Mycgr3**
  
Percentage identity: 74 %
  
BlastP bit score: 392
  
Sequence coverage: 100 %
  
E-value: 9e-132
  
  
 NCBI BlastP on this gene

EME77256

hypothetical protein
  
Accession: EME77255
  
Location: 268919-269143
  
 NCBI BlastP on this gene

EME77255

hypothetical protein
  
Accession: EME77254
  
Location: 259305-260315
  
  
**BlastP hit with Mycgr3G68429\_Mycgr3T**
  
Percentage identity: 83 %
  
BlastP bit score: 558
  
Sequence coverage: 75 %
  
E-value: 0.0
  
  
 NCBI BlastP on this gene

EME77254

hypothetical protein
  
Accession: EME77253
  
Location: 255336-255506
  
 NCBI BlastP on this gene

EME77253

hypothetical protein
  
Accession: EME77252
  
Location: 253586-254708
  
 NCBI BlastP on this gene

EME77252

hypothetical protein
  
Accession: EME77251
  
Location: 252128-253563
  
 NCBI BlastP on this gene

EME77251

hypothetical protein
  
Accession: EME77250
  
Location: 251044-251824
  
  
**BlastP hit with Mycgr3G55345\_Mycgr3T**
  
Percentage identity: 81 %
  
BlastP bit score: 344
  
Sequence coverage: 97 %
  
E-value: 2e-117
  
  
 NCBI BlastP on this gene

EME77250

hypothetical protein
  
Accession: EME77249
  
Location: 248686-249795
  
 NCBI BlastP on this gene

EME77249

hypothetical protein
  
Accession: EME77248
  
Location: 243650-245851
  
 NCBI BlastP on this gene

EME77248

hypothetical protein
  
Accession: EME77247
  
Location: 242456-243393
  
 NCBI BlastP on this gene

EME77247

hypothetical protein
  
Accession: EME77246
  
Location: 241602-242410
  
 NCBI BlastP on this gene

EME77246

hypothetical protein
  
Accession: EME77245
  
Location: 240045-241434
  
 NCBI BlastP on this gene

EME77245

75. :  CABT02000002 Sordaria macrospora k-hell     Total score: 3.0     Cumulative Blast bit score: 1287

not annotated
  
Accession: CCC06934
  
Location: 1270485-1271599
  
 NCBI BlastP on this gene

CCC06934

not annotated
  
Accession: CCC06933
  
Location: 1264410-1267328
  
 NCBI BlastP on this gene

CCC06933

not annotated
  
Accession: CCC06932
  
Location: 1261886-1262416
  
 NCBI BlastP on this gene

CCC06932

not annotated
  
Accession: CCC06931
  
Location: 1258586-1260083
  
 NCBI BlastP on this gene

CCC06931

not annotated
  
Accession: CCC06930
  
Location: 1254883-1255982
  
 NCBI BlastP on this gene

CCC06930

not annotated
  
Accession: CCC06929
  
Location: 1252856-1254081
  
 NCBI BlastP on this gene

CCC06929

not annotated
  
Accession: CCC06928
  
Location: 1251128-1252266
  
  
**BlastP hit with Mycgr3G36271\_Mycgr3T**
  
Percentage identity: 72 %
  
BlastP bit score: 437
  
Sequence coverage: 94 %
  
E-value: 5e-151
  
  
 NCBI BlastP on this gene

CCC06928

not annotated
  
Accession: CCC06927
  
Location: 1246865-1249055
  
  
**BlastP hit with Mycgr3G84646\_Mycgr3T**
  
Percentage identity: 41 %
  
BlastP bit score: 488
  
Sequence coverage: 101 %
  
E-value: 1e-159
  
  
 NCBI BlastP on this gene

CCC06927

not annotated
  
Accession: CCC06926
  
Location: 1243977-1244948
  
  
**BlastP hit with Mycgr3G99145\_Mycgr3T**
  
Percentage identity: 84 %
  
BlastP bit score: 362
  
Sequence coverage: 99 %
  
E-value: 2e-124
  
  
 NCBI BlastP on this gene

CCC06926

not annotated
  
Accession: CCC06925
  
Location: 1236346-1242626
  
 NCBI BlastP on this gene

CCC06925

not annotated
  
Accession: CCC06924
  
Location: 1234970-1236190
  
 NCBI BlastP on this gene

CCC06924

not annotated
  
Accession: CCC06923
  
Location: 1233359-1234319
  
 NCBI BlastP on this gene

CCC06923

not annotated
  
Accession: CCC06922
  
Location: 1227748-1232623
  
 NCBI BlastP on this gene

CCC06922

not annotated
  
Accession: CCC06921
  
Location: 1224908-1226006
  
 NCBI BlastP on this gene

CCC06921

76. :  DS572750 Paracoccidioides brasiliensis Pb18 supercont1.1 genomic scaffold     Total score: 3.0     Cumulative Blast bit score: 1279

conserved hypothetical protein
  
Accession: EEH43980
  
Location: 898018-901203
  
 NCBI BlastP on this gene

EEH43980

conserved hypothetical protein
  
Accession: EEH43981
  
Location: 902421-903206
  
 NCBI BlastP on this gene

EEH43981

conserved hypothetical protein
  
Accession: EEH43982
  
Location: 903558-905249
  
 NCBI BlastP on this gene

EEH43982

rRNA-processing protein FCF1
  
Accession: EEH43983
  
Location: 906944-907934
  
 NCBI BlastP on this gene

EEH43983

hypothetical protein
  
Accession: EEH43984
  
Location: 908618-909670
  
 NCBI BlastP on this gene

EEH43984

C-4 methylsterol oxidase
  
Accession: EEH43985
  
Location: 910552-912112
  
  
**BlastP hit with Mycgr3G36271\_Mycgr3T**
  
Percentage identity: 73 %
  
BlastP bit score: 457
  
Sequence coverage: 97 %
  
E-value: 5e-159
  
  
 NCBI BlastP on this gene

EEH43985

predicted protein
  
Accession: EEH43986
  
Location: 916982-917251
  
 NCBI BlastP on this gene

EEH43986

conserved hypothetical protein
  
Accession: EEH43987
  
Location: 918373-919149
  
 NCBI BlastP on this gene

EEH43987

conserved hypothetical protein
  
Accession: EEH43988
  
Location: 919452-921521
  
 NCBI BlastP on this gene

EEH43988

hypothetical protein
  
Accession: EEH43989
  
Location: 922806-926903
  
 NCBI BlastP on this gene

EEH43989

conserved hypothetical protein
  
Accession: EEH43990
  
Location: 927224-928561
  
 NCBI BlastP on this gene

EEH43990

conserved hypothetical protein
  
Accession: EEH43991
  
Location: 929126-931594
  
  
**BlastP hit with Mycgr3G84646\_Mycgr3T**
  
Percentage identity: 40 %
  
BlastP bit score: 476
  
Sequence coverage: 104 %
  
E-value: 8e-155
  
  
 NCBI BlastP on this gene

EEH43991

conserved hypothetical protein
  
Accession: EEH43992
  
Location: 934598-935938
  
 NCBI BlastP on this gene

EEH43992

GTP-binding protein SAS1
  
Accession: EEH43993
  
Location: 937234-938101
  
  
**BlastP hit with Mycgr3G99145\_Mycgr3T**
  
Percentage identity: 83 %
  
BlastP bit score: 346
  
Sequence coverage: 99 %
  
E-value: 2e-118
  
  
 NCBI BlastP on this gene

EEH43993

conserved hypothetical protein
  
Accession: EEH43994
  
Location: 939267-940299
  
 NCBI BlastP on this gene

EEH43994

AAA family ATPase
  
Accession: EEH43995
  
Location: 940941-943289
  
 NCBI BlastP on this gene

EEH43995

hypothetical protein
  
Accession: EEH43996
  
Location: 943683-944940
  
 NCBI BlastP on this gene

EEH43996

conserved hypothetical protein
  
Accession: EEH43997
  
Location: 945424-954064
  
 NCBI BlastP on this gene

EEH43997

77. :  GL891303 Neurospora tetrasperma FGSC 2508 unplaced genomic scaffold NEUTE1scaffold\_2     Total score: 3.0     Cumulative Blast bit score: 1277

hypothetical protein
  
Accession: EGO59159
  
Location: 2479089-2479721
  
 NCBI BlastP on this gene

EGO59159

profilin
  
Accession: EGO59158
  
Location: 2475174-2476263
  
 NCBI BlastP on this gene

EGO59158

hypothetical protein
  
Accession: EGO59157
  
Location: 2469696-2472251
  
 NCBI BlastP on this gene

EGO59157

hypothetical protein
  
Accession: EGO59156
  
Location: 2467032-2467942
  
 NCBI BlastP on this gene

EGO59156

hypothetical protein
  
Accession: EGO59155
  
Location: 2462720-2464177
  
 NCBI BlastP on this gene

EGO59155

C-4 methylsterol oxidase, variant
  
Accession: EGO59154
  
Location: 2458783-2459902
  
  
**BlastP hit with Mycgr3G36271\_Mycgr3T**
  
Percentage identity: 71 %
  
BlastP bit score: 435
  
Sequence coverage: 93 %
  
E-value: 2e-150
  
  
 NCBI BlastP on this gene

EGO59154

hypothetical protein
  
Accession: EGO59153
  
Location: 2454656-2456834
  
  
**BlastP hit with Mycgr3G84646\_Mycgr3T**
  
Percentage identity: 41 %
  
BlastP bit score: 487
  
Sequence coverage: 101 %
  
E-value: 2e-159
  
  
 NCBI BlastP on this gene

EGO59153

GTP-binding protein SAS1
  
Accession: EGO59152
  
Location: 2451741-2452697
  
  
**BlastP hit with Mycgr3G99145\_Mycgr3T**
  
Percentage identity: 88 %
  
BlastP bit score: 355
  
Sequence coverage: 92 %
  
E-value: 1e-121
  
  
 NCBI BlastP on this gene

EGO59152

activating signal cointegrator 1 complex subunit 3
  
Accession: EGO59151
  
Location: 2444132-2450399
  
 NCBI BlastP on this gene

EGO59151

pyridoxamine 5'-phosphate oxidase
  
Accession: EGO59150
  
Location: 2440958-2441826
  
 NCBI BlastP on this gene

EGO59150

hypothetical protein
  
Accession: EGO59149
  
Location: 2435390-2440255
  
 NCBI BlastP on this gene

EGO59149

hypothetical protein
  
Accession: EGO59148
  
Location: 2432505-2434137
  
 NCBI BlastP on this gene

EGO59148

78. :  GL891217 Neurospora tetrasperma FGSC 2509 unplaced genomic scaffold NEUTE2scaffold\_3     Total score: 3.0     Cumulative Blast bit score: 1277

hypothetical protein
  
Accession: EGZ73270
  
Location: 2481193-2481825
  
 NCBI BlastP on this gene

EGZ73270

profilin
  
Accession: EGZ73269
  
Location: 2477278-2478367
  
 NCBI BlastP on this gene

EGZ73269

hypothetical protein
  
Accession: EGZ73268
  
Location: 2471800-2474355
  
 NCBI BlastP on this gene

EGZ73268

hypothetical protein
  
Accession: EGZ73267
  
Location: 2469136-2470046
  
 NCBI BlastP on this gene

EGZ73267

hypothetical protein
  
Accession: EGZ73266
  
Location: 2464824-2466281
  
 NCBI BlastP on this gene

EGZ73266

C-4 methylsterol oxidase, variant
  
Accession: EGZ73265
  
Location: 2460887-2462006
  
  
**BlastP hit with Mycgr3G36271\_Mycgr3T**
  
Percentage identity: 71 %
  
BlastP bit score: 435
  
Sequence coverage: 93 %
  
E-value: 2e-150
  
  
 NCBI BlastP on this gene

EGZ73265

NCA2-domain-containing protein
  
Accession: EGZ73264
  
Location: 2456760-2458938
  
  
**BlastP hit with Mycgr3G84646\_Mycgr3T**
  
Percentage identity: 41 %
  
BlastP bit score: 487
  
Sequence coverage: 101 %
  
E-value: 2e-159
  
  
 NCBI BlastP on this gene

EGZ73264

GTP-binding protein SAS1
  
Accession: EGZ73263
  
Location: 2453845-2454801
  
  
**BlastP hit with Mycgr3G99145\_Mycgr3T**
  
Percentage identity: 88 %
  
BlastP bit score: 355
  
Sequence coverage: 92 %
  
E-value: 1e-121
  
  
 NCBI BlastP on this gene

EGZ73263

activating signal cointegrator 1 complex subunit 3
  
Accession: EGZ73262
  
Location: 2446236-2452503
  
 NCBI BlastP on this gene

EGZ73262

pyridoxamine 5'-phosphate oxidase
  
Accession: EGZ73261
  
Location: 2443095-2443930
  
 NCBI BlastP on this gene

EGZ73261

hypothetical protein
  
Accession: EGZ73260
  
Location: 2437493-2442358
  
 NCBI BlastP on this gene

EGZ73260

ras-domain-containing protein
  
Accession: EGZ73259
  
Location: 2434608-2436240
  
 NCBI BlastP on this gene

EGZ73259

79. :  KE148177 Ophiostoma piceae UAMH 11346 chromosome Unknown scf32     Total score: 3.0     Cumulative Blast bit score: 1269

got1 family protein
  
Accession: EPE02531
  
Location: 78146-78598
  
 NCBI BlastP on this gene

EPE02531

profilin
  
Accession: EPE02532
  
Location: 79461-79947
  
 NCBI BlastP on this gene

EPE02532

chromatin remodeling complex subunit
  
Accession: EPE02533
  
Location: 82320-84806
  
 NCBI BlastP on this gene

EPE02533

transmembrane protein
  
Accession: EPE02534
  
Location: 85582-86421
  
 NCBI BlastP on this gene

EPE02534

hypothetical protein
  
Accession: EPE02535
  
Location: 87981-88629
  
 NCBI BlastP on this gene

EPE02535

sphingomyelin phosphodiesterase
  
Accession: EPE02536
  
Location: 91466-93498
  
 NCBI BlastP on this gene

EPE02536

pterin 4 alpha carbinolamine dehydratase
  
Accession: EPE02537
  
Location: 94609-95553
  
 NCBI BlastP on this gene

EPE02537

c-4 methylsterol oxidase
  
Accession: EPE02538
  
Location: 96072-97164
  
  
**BlastP hit with Mycgr3G36271\_Mycgr3T**
  
Percentage identity: 72 %
  
BlastP bit score: 460
  
Sequence coverage: 95 %
  
E-value: 5e-160
  
  
 NCBI BlastP on this gene

EPE02538

atp synthase regulation protein nca2
  
Accession: EPE02539
  
Location: 99029-101436
  
  
**BlastP hit with Mycgr3G84646\_Mycgr3T**
  
Percentage identity: 42 %
  
BlastP bit score: 451
  
Sequence coverage: 84 %
  
E-value: 1e-144
  
  
 NCBI BlastP on this gene

EPE02539

rab gtpase
  
Accession: EPE02540
  
Location: 103269-104120
  
  
**BlastP hit with Mycgr3G99145\_Mycgr3T**
  
Percentage identity: 85 %
  
BlastP bit score: 358
  
Sequence coverage: 99 %
  
E-value: 4e-123
  
  
 NCBI BlastP on this gene

EPE02540

hypothetical protein
  
Accession: EPE02541
  
Location: 104996-107137
  
 NCBI BlastP on this gene

EPE02541

aaa family atpase
  
Accession: EPE02542
  
Location: 107978-110437
  
 NCBI BlastP on this gene

EPE02542

dolichyl-phosphate beta-glucosyltransferase
  
Accession: EPE02543
  
Location: 110681-111988
  
 NCBI BlastP on this gene

EPE02543

snrnp and snornp protein
  
Accession: EPE02544
  
Location: 113123-113506
  
 NCBI BlastP on this gene

EPE02544

hypothetical protein
  
Accession: EPE02545
  
Location: 118159-118817
  
 NCBI BlastP on this gene

EPE02545

80. :  CH408032 Chaetomium globosum CBS 148.51 scaffold\_4 genomic scaffold     Total score: 3.0     Cumulative Blast bit score: 1253

hypothetical protein
  
Accession: EAQ88133
  
Location: 3176603-3180263
  
 NCBI BlastP on this gene

EAQ88133

predicted protein
  
Accession: EAQ88132
  
Location: 3173200-3173968
  
 NCBI BlastP on this gene

EAQ88132

hypothetical protein
  
Accession: EAQ88131
  
Location: 3171570-3172284
  
 NCBI BlastP on this gene

EAQ88131

hypothetical protein
  
Accession: EAQ88130
  
Location: 3169209-3169811
  
 NCBI BlastP on this gene

EAQ88130

hypothetical protein
  
Accession: EAQ88129
  
Location: 3166875-3168334
  
 NCBI BlastP on this gene

EAQ88129

hypothetical protein
  
Accession: EAQ88128
  
Location: 3163862-3166355
  
 NCBI BlastP on this gene

EAQ88128

hypothetical protein
  
Accession: EAQ88127
  
Location: 3162488-3163119
  
 NCBI BlastP on this gene

EAQ88127

hypothetical protein
  
Accession: EAQ88126
  
Location: 3158297-3159091
  
 NCBI BlastP on this gene

EAQ88126

hypothetical protein
  
Accession: EAQ88125
  
Location: 3156246-3157343
  
  
**BlastP hit with Mycgr3G36271\_Mycgr3T**
  
Percentage identity: 65 %
  
BlastP bit score: 389
  
Sequence coverage: 95 %
  
E-value: 8e-133
  
  
 NCBI BlastP on this gene

EAQ88125

hypothetical protein
  
Accession: EAQ88124
  
Location: 3152860-3155032
  
  
**BlastP hit with Mycgr3G84646\_Mycgr3T**
  
Percentage identity: 42 %
  
BlastP bit score: 518
  
Sequence coverage: 103 %
  
E-value: 2e-171
  
  
 NCBI BlastP on this gene

EAQ88124

hypothetical protein
  
Accession: EAQ88123
  
Location: 3150792-3151552
  
  
**BlastP hit with Mycgr3G99145\_Mycgr3T**
  
Percentage identity: 82 %
  
BlastP bit score: 346
  
Sequence coverage: 99 %
  
E-value: 2e-118
  
  
 NCBI BlastP on this gene

EAQ88123

hypothetical protein
  
Accession: EAQ88122
  
Location: 3143418-3149459
  
 NCBI BlastP on this gene

EAQ88122

hypothetical protein
  
Accession: EAQ88121
  
Location: 3142002-3142730
  
 NCBI BlastP on this gene

EAQ88121

hypothetical protein
  
Accession: EAQ88120
  
Location: 3138985-3140993
  
 NCBI BlastP on this gene

EAQ88120

hypothetical protein
  
Accession: EAQ88119
  
Location: 3137794-3138597
  
 NCBI BlastP on this gene

EAQ88119

hypothetical protein
  
Accession: EAQ88118
  
Location: 3134963-3137399
  
 NCBI BlastP on this gene

EAQ88118

hypothetical protein
  
Accession: EAQ88117
  
Location: 3133464-3134210
  
 NCBI BlastP on this gene

EAQ88117

81. :  GL629769 Grosmannia clavigera kw1407 unplaced genomic scaffold GCSC\_144     Total score: 3.0     Cumulative Blast bit score: 1241

got1 family protein
  
Accession: EFX02879
  
Location: 368914-369690
  
 NCBI BlastP on this gene

EFX02879

profilin
  
Accession: EFX03070
  
Location: 370309-371088
  
 NCBI BlastP on this gene

EFX03070

chromatin remodeling complex subunit
  
Accession: EFX02970
  
Location: 372426-374960
  
 NCBI BlastP on this gene

EFX02970

bzip transcription factor
  
Accession: EFX02620
  
Location: 375530-377503
  
 NCBI BlastP on this gene

EFX02620

stress responsive a b barrel domain containing protein
  
Accession: EFX03226
  
Location: 380092-380552
  
 NCBI BlastP on this gene

EFX03226

pterin-4-alpha-carbinolamine dehydratase
  
Accession: EFX03320
  
Location: 382105-382865
  
 NCBI BlastP on this gene

EFX03320

hypothetical protein
  
Accession: EFX03157
  
Location: 382355-382837
  
 NCBI BlastP on this gene

EFX03157

c-4 methyl sterol oxidase
  
Accession: EFX03141
  
Location: 383367-384429
  
  
**BlastP hit with Mycgr3G36271\_Mycgr3T**
  
Percentage identity: 72 %
  
BlastP bit score: 434
  
Sequence coverage: 91 %
  
E-value: 6e-150
  
  
 NCBI BlastP on this gene

EFX03141

nuclear control of ATP synthase 2 protein
  
Accession: EFX02912
  
Location: 385369-387663
  
  
**BlastP hit with Mycgr3G84646\_Mycgr3T**
  
Percentage identity: 43 %
  
BlastP bit score: 450
  
Sequence coverage: 84 %
  
E-value: 1e-144
  
  
 NCBI BlastP on this gene

EFX02912

rab GTPase
  
Accession: EFX03003
  
Location: 388446-389194
  
  
**BlastP hit with Mycgr3G99145\_Mycgr3T**
  
Percentage identity: 85 %
  
BlastP bit score: 357
  
Sequence coverage: 99 %
  
E-value: 2e-122
  
  
 NCBI BlastP on this gene

EFX03003

hypothetical protein
  
Accession: EFX02964
  
Location: 389704-391725
  
 NCBI BlastP on this gene

EFX02964

duf625 domain containing protein
  
Accession: EFX03091
  
Location: 392652-395714
  
 NCBI BlastP on this gene

EFX03091

hypothetical protein
  
Accession: EFX02675
  
Location: 399590-400127
  
 NCBI BlastP on this gene

EFX02675

hypothetical protein
  
Accession: EFX02849
  
Location: 401133-402206
  
 NCBI BlastP on this gene

EFX02849

snrnp and snornp protein
  
Accession: EFX03270
  
Location: 403641-404176
  
 NCBI BlastP on this gene

EFX03270

dolichyl-phosphate beta-transferase
  
Accession: EFX02688
  
Location: 405072-406286
  
 NCBI BlastP on this gene

EFX02688

aaa family ATPase
  
Accession: EFX02828
  
Location: 406727-408985
  
 NCBI BlastP on this gene

EFX02828

82. :  CH476615 Uncinocarpus reesii 1704 scaffold\_1 genomic scaffold     Total score: 3.0     Cumulative Blast bit score: 1238

conserved hypothetical protein
  
Accession: EEP77732
  
Location: 6853553-6855345
  
 NCBI BlastP on this gene

EEP77732

predicted protein
  
Accession: EEP77731
  
Location: 6852077-6853007
  
 NCBI BlastP on this gene

EEP77731

conserved hypothetical protein
  
Accession: EEP77730
  
Location: 6850922-6851751
  
 NCBI BlastP on this gene

EEP77730

hypothetical protein
  
Accession: EEP77729
  
Location: 6840903-6849576
  
 NCBI BlastP on this gene

EEP77729

ATPase
  
Accession: EEP77728
  
Location: 6836861-6839170
  
 NCBI BlastP on this gene

EEP77728

predicted protein
  
Accession: EEP77727
  
Location: 6835582-6836656
  
 NCBI BlastP on this gene

EEP77727

C-4 methylsterol oxidase
  
Accession: EEP77726
  
Location: 6834301-6835161
  
  
**BlastP hit with Mycgr3G36271\_Mycgr3T**
  
Percentage identity: 81 %
  
BlastP bit score: 422
  
Sequence coverage: 80 %
  
E-value: 4e-146
  
  
 NCBI BlastP on this gene

EEP77726

predicted protein
  
Accession: EEP77725
  
Location: 6831336-6833511
  
  
**BlastP hit with Mycgr3G84646\_Mycgr3T**
  
Percentage identity: 39 %
  
BlastP bit score: 463
  
Sequence coverage: 100 %
  
E-value: 3e-150
  
  
 NCBI BlastP on this gene

EEP77725

GTP-binding protein SAS1
  
Accession: EEP77724
  
Location: 6829677-6830445
  
  
**BlastP hit with Mycgr3G99145\_Mycgr3T**
  
Percentage identity: 82 %
  
BlastP bit score: 353
  
Sequence coverage: 99 %
  
E-value: 3e-121
  
  
 NCBI BlastP on this gene

EEP77724

predicted protein
  
Accession: EEP77723
  
Location: 6827738-6828976
  
 NCBI BlastP on this gene

EEP77723

predicted protein
  
Accession: EEP77722
  
Location: 6826125-6827111
  
 NCBI BlastP on this gene

EEP77722

hypothetical protein
  
Accession: EEP77721
  
Location: 6824266-6825095
  
 NCBI BlastP on this gene

EEP77721

predicted protein
  
Accession: EEP77720
  
Location: 6821443-6823383
  
 NCBI BlastP on this gene

EEP77720

conserved hypothetical protein
  
Accession: EEP77719
  
Location: 6819536-6820461
  
 NCBI BlastP on this gene

EEP77719

predicted protein
  
Accession: EEP77718
  
Location: 6817253-6818824
  
 NCBI BlastP on this gene

EEP77718

predicted protein
  
Accession: EEP77717
  
Location: 6816382-6816979
  
 NCBI BlastP on this gene

EEP77717

conserved hypothetical protein
  
Accession: EEP77716
  
Location: 6812395-6815757
  
 NCBI BlastP on this gene

EEP77716

conserved hypothetical protein
  
Accession: EEP77715
  
Location: 6807300-6811550
  
 NCBI BlastP on this gene

EEP77715

83. :  AM920428 Penicillium chrysogenum Wisconsin 54-1255 complete genome, contig Pc00c13.     Total score: 3.0     Cumulative Blast bit score: 1235

not annotated
  
Accession: CAP91619
  
Location: 1361097-1364643
  
 NCBI BlastP on this gene

Pc13g05500

not annotated
  
Accession: CAP91620
  
Location: 1364765-1368949
  
 NCBI BlastP on this gene

Pc13g05510

unnamed
  
Accession: CAP91621
  
Location: 1369356-1371039
  
 NCBI BlastP on this gene

Pc13g05520

not annotated
  
Accession: CAP91622
  
Location: 1372110-1373410
  
 NCBI BlastP on this gene

Pc13g05530

not annotated
  
Accession: CAP91623
  
Location: 1373850-1374672
  
 NCBI BlastP on this gene

Pc13g05540

not annotated
  
Accession: CAP91624
  
Location: 1374976-1376468
  
 NCBI BlastP on this gene

Pc13g05550

not annotated
  
Accession: CAP91625
  
Location: 1376863-1378648
  
 NCBI BlastP on this gene

Pc13g05560

not annotated
  
Accession: CAP91626
  
Location: 1379129-1380628
  
  
**BlastP hit with Mycgr3G36271\_Mycgr3T**
  
Percentage identity: 76 %
  
BlastP bit score: 471
  
Sequence coverage: 98 %
  
E-value: 5e-164
  
  
 NCBI BlastP on this gene

Pc13g05570

not annotated
  
Accession: CAP91627
  
Location: 1380859-1382926
  
  
**BlastP hit with Mycgr3G84646\_Mycgr3T**
  
Percentage identity: 39 %
  
BlastP bit score: 421
  
Sequence coverage: 99 %
  
E-value: 2e-134
  
  
 NCBI BlastP on this gene

Pc13g05580

not annotated
  
Accession: CAP91628
  
Location: 1383694-1384452
  
  
**BlastP hit with Mycgr3G99145\_Mycgr3T**
  
Percentage identity: 86 %
  
BlastP bit score: 343
  
Sequence coverage: 93 %
  
E-value: 3e-117
  
  
 NCBI BlastP on this gene

Pc13g05590

not annotated
  
Accession: CAP91629
  
Location: 1385167-1387480
  
 NCBI BlastP on this gene

Pc13g05600

not annotated
  
Accession: CAP91630
  
Location: 1387853-1388873
  
 NCBI BlastP on this gene

Pc13g05610

not annotated
  
Accession: CAP91631
  
Location: 1389244-1398061
  
 NCBI BlastP on this gene

Pc13g05620

not annotated
  
Accession: CAP91632
  
Location: 1398708-1399484
  
 NCBI BlastP on this gene

Pc13g05630

not annotated
  
Accession: CAP91633
  
Location: 1399751-1400568
  
 NCBI BlastP on this gene

Pc13g05640

not annotated
  
Accession: CAP91634
  
Location: 1400909-1401886
  
 NCBI BlastP on this gene

Pc13g05650

not annotated
  
Accession: CAP91635
  
Location: 1402253-1404036
  
 NCBI BlastP on this gene

Pc13g05660

not annotated
  
Accession: CAP91636
  
Location: 1404374-1406197
  
 NCBI BlastP on this gene

Pc13g05670

84. :  CH476663 Ajellomyces capsulatus NAm1 scaffold\_9 genomic scaffold     Total score: 3.0     Cumulative Blast bit score: 1202

conserved hypothetical protein
  
Accession: EDN11193
  
Location: 577399-581249
  
 NCBI BlastP on this gene

EDN11193

predicted protein
  
Accession: EDN11194
  
Location: 582420-583244
  
 NCBI BlastP on this gene

EDN11194

predicted protein
  
Accession: EDN11195
  
Location: 583786-585591
  
 NCBI BlastP on this gene

EDN11195

hypothetical protein
  
Accession: EDN11196
  
Location: 587694-588675
  
 NCBI BlastP on this gene

EDN11196

C-4 methylsterol oxidase
  
Accession: EDN11197
  
Location: 589643-590494
  
  
**BlastP hit with Mycgr3G36271\_Mycgr3T**
  
Percentage identity: 80 %
  
BlastP bit score: 387
  
Sequence coverage: 75 %
  
E-value: 1e-132
  
  
 NCBI BlastP on this gene

EDN11197

predicted protein
  
Accession: EDN11198
  
Location: 590746-593927
  
  
**BlastP hit with Mycgr3G84646\_Mycgr3T**
  
Percentage identity: 39 %
  
BlastP bit score: 460
  
Sequence coverage: 100 %
  
E-value: 1e-147
  
  
 NCBI BlastP on this gene

EDN11198

predicted protein
  
Accession: EDN11199
  
Location: 596276-596795
  
 NCBI BlastP on this gene

EDN11199

predicted protein
  
Accession: EDN11200
  
Location: 598546-601485
  
 NCBI BlastP on this gene

EDN11200

predicted protein
  
Accession: EDN11201
  
Location: 602596-605701
  
 NCBI BlastP on this gene

EDN11201

predicted protein
  
Accession: EDN11202
  
Location: 605966-607741
  
 NCBI BlastP on this gene

EDN11202

predicted protein
  
Accession: EDN11203
  
Location: 609074-609425
  
 NCBI BlastP on this gene

EDN11203

conserved hypothetical protein
  
Accession: EDN11204
  
Location: 610654-612771
  
 NCBI BlastP on this gene

EDN11204

predicted protein
  
Accession: EDN11205
  
Location: 615876-616416
  
 NCBI BlastP on this gene

EDN11205

GTP-binding protein SAS1
  
Accession: EDN11206
  
Location: 618048-618957
  
  
**BlastP hit with Mycgr3G99145\_Mycgr3T**
  
Percentage identity: 84 %
  
BlastP bit score: 355
  
Sequence coverage: 99 %
  
E-value: 8e-122
  
  
 NCBI BlastP on this gene

EDN11206

predicted protein
  
Accession: EDN11207
  
Location: 619694-620808
  
 NCBI BlastP on this gene

EDN11207

conserved hypothetical protein
  
Accession: EDN11208
  
Location: 621560-623911
  
 NCBI BlastP on this gene

EDN11208

predicted protein
  
Accession: EDN11209
  
Location: 624832-625272
  
 NCBI BlastP on this gene

EDN11209

predicted protein
  
Accession: EDN11210
  
Location: 625530-626181
  
 NCBI BlastP on this gene

EDN11210

hypothetical protein
  
Accession: EDN11211
  
Location: 626551-635497
  
 NCBI BlastP on this gene

EDN11211

85. :  KB933328 Togninia minima UCRPA7 unplaced genomic scaffold PA7\_03\_scaffold\_560     Total score: 3.0     Cumulative Blast bit score: 1200

putative eukaryotic translation initiation factor 2 alpha subunit protein
  
Accession: EON96623
  
Location: 99389-100534
  
 NCBI BlastP on this gene

EON96623

putative chromatin remodeling complex subunit protein
  
Accession: EON96616
  
Location: 95822-98298
  
 NCBI BlastP on this gene

EON96616

putative amidohydrolase protein
  
Accession: EON96602
  
Location: 83602-85214
  
 NCBI BlastP on this gene

EON96602

putative c-4 sterol methyl oxidase protein
  
Accession: EON96620
  
Location: 80296-81411
  
  
**BlastP hit with Mycgr3G36271\_Mycgr3T**
  
Percentage identity: 72 %
  
BlastP bit score: 457
  
Sequence coverage: 95 %
  
E-value: 4e-159
  
  
 NCBI BlastP on this gene

EON96620

putative atp synthase regulation protein nca2 protein
  
Accession: EON96549
  
Location: 76384-77655
  
  
**BlastP hit with Mycgr3G84646\_Mycgr3T**
  
Percentage identity: 49 %
  
BlastP bit score: 405
  
Sequence coverage: 62 %
  
E-value: 4e-131
  
  
 NCBI BlastP on this gene

EON96549

putative gtp-binding protein sas1 protein
  
Accession: EON96550
  
Location: 74663-75993
  
  
**BlastP hit with Mycgr3G99145\_Mycgr3T**
  
Percentage identity: 86 %
  
BlastP bit score: 338
  
Sequence coverage: 92 %
  
E-value: 2e-115
  
  
 NCBI BlastP on this gene

EON96550

hypothetical protein
  
Accession: EON96560
  
Location: 73532-74161
  
 NCBI BlastP on this gene

EON96560

putative aaa family atpase protein
  
Accession: EON96578
  
Location: 69971-72223
  
 NCBI BlastP on this gene

EON96578

putative dash complex subunit dad4 protein
  
Accession: EON96551
  
Location: 69054-69509
  
 NCBI BlastP on this gene

EON96551

putative yl1 nuclear protein
  
Accession: EON96595
  
Location: 68141-68752
  
 NCBI BlastP on this gene

EON96595

putative got1 family protein
  
Accession: EON96604
  
Location: 66507-67202
  
 NCBI BlastP on this gene

EON96604

putative profilin protein
  
Accession: EON96599
  
Location: 65228-65960
  
 NCBI BlastP on this gene

EON96599

putative snare ykt6 protein
  
Accession: EON96605
  
Location: 57344-58522
  
 NCBI BlastP on this gene

EON96605

86. :  EQ963487 Aspergillus flavus NRRL3357 scf\_1106286419368 genomic scaffold     Total score: 3.0     Cumulative Blast bit score: 1188

F-box domain protein
  
Accession: EED44814
  
Location: 254459-255224
  
 NCBI BlastP on this gene

EED44814

flocculation suppression protein
  
Accession: EED44813
  
Location: 251235-253346
  
 NCBI BlastP on this gene

EED44813

conserved hypothetical protein
  
Accession: EED44812
  
Location: 249687-250516
  
 NCBI BlastP on this gene

EED44812

ATP-dependent RNA helicase, putative
  
Accession: EED44811
  
Location: 247703-249357
  
 NCBI BlastP on this gene

EED44811

conserved hypothetical protein
  
Accession: EED44810
  
Location: 244363-245151
  
 NCBI BlastP on this gene

EED44810

60S ribosomal protein L13
  
Accession: EED44809
  
Location: 240450-241556
  
 NCBI BlastP on this gene

EED44809

conserved hypothetical protein
  
Accession: EED44808
  
Location: 238079-239165
  
  
**BlastP hit with Mycgr3G90786\_Mycgr3T**
  
Percentage identity: 27 %
  
BlastP bit score: 69
  
Sequence coverage: 55 %
  
E-value: 1e-09
  
  
 NCBI BlastP on this gene

EED44808

conserved leucine-rich repeat protein
  
Accession: EED44807
  
Location: 232924-235413
  
  
**BlastP hit with Mycgr3G68433\_Mycgr3T**
  
Percentage identity: 40 %
  
BlastP bit score: 469
  
Sequence coverage: 78 %
  
E-value: 2e-146
  
  
 NCBI BlastP on this gene

EED44807

conserved hypothetical protein
  
Accession: EED44806
  
Location: 231519-232655
  
 NCBI BlastP on this gene

EED44806

actin family protein
  
Accession: EED44805
  
Location: 229107-230684
  
 NCBI BlastP on this gene

EED44805

hypothetical protein
  
Accession: EED44804
  
Location: 227603-227905
  
 NCBI BlastP on this gene

EED44804

IBR domain protein
  
Accession: EED44803
  
Location: 226701-227560
  
 NCBI BlastP on this gene

EED44803

3-hydroxybutyryl-CoA dehydrogenase, putative
  
Accession: EED44802
  
Location: 225418-226532
  
 NCBI BlastP on this gene

EED44802

DNA repair protein (Tof1), putative
  
Accession: EED44801
  
Location: 222525-224483
  
  
**BlastP hit with Mycgr3G68421\_Mycgr3T**
  
Percentage identity: 51 %
  
BlastP bit score: 650
  
Sequence coverage: 55 %
  
E-value: 0.0
  
  
 NCBI BlastP on this gene

EED44801

conserved hypothetical protein
  
Accession: EED44800
  
Location: 218319-219362
  
 NCBI BlastP on this gene

EED44800

TFIIH and nucleotide excision repair factor 3 complexes subunit
  
Accession: EED44799
  
Location: 216203-217772
  
 NCBI BlastP on this gene

EED44799

conserved hypothetical protein
  
Accession: EED44798
  
Location: 214771-215942
  
 NCBI BlastP on this gene

EED44798

DEAD helicases superfamily protein (Aquarius), putative
  
Accession: EED44797
  
Location: 209853-214306
  
 NCBI BlastP on this gene

EED44797

THO complex subunit Tho1, putative
  
Accession: EED44796
  
Location: 207249-209609
  
 NCBI BlastP on this gene

EED44796

87. :  FN430379 Tuber melanosporum whole genome shotgun sequence assembly, scaffold\_95, strain Mel28.     Total score: 3.0     Cumulative Blast bit score: 1179

not annotated
  
Accession: CAZ86551
  
Location: 151586-152517
  
 NCBI BlastP on this gene

CAZ86551

not annotated
  
Accession: CAZ86552
  
Location: 155103-158795
  
  
**BlastP hit with Mycgr3G108094\_Mycgr3**
  
Percentage identity: 35 %
  
BlastP bit score: 310
  
Sequence coverage: 45 %
  
E-value: 8e-84
  
  
 NCBI BlastP on this gene

CAZ86552

not annotated
  
Accession: CAZ86553
  
Location: 159919-162135
  
  
**BlastP hit with Mycgr3G84646\_Mycgr3T**
  
Percentage identity: 44 %
  
BlastP bit score: 510
  
Sequence coverage: 104 %
  
E-value: 3e-168
  
  
 NCBI BlastP on this gene

CAZ86553

not annotated
  
Accession: CAZ86554
  
Location: 163092-163997
  
  
**BlastP hit with Mycgr3G99145\_Mycgr3T**
  
Percentage identity: 84 %
  
BlastP bit score: 359
  
Sequence coverage: 101 %
  
E-value: 2e-123
  
  
 NCBI BlastP on this gene

CAZ86554

not annotated
  
Accession: CAZ86555
  
Location: 164888-165301
  
 NCBI BlastP on this gene

CAZ86555

not annotated
  
Accession: CAZ86556
  
Location: 167971-168573
  
 NCBI BlastP on this gene

CAZ86556

not annotated
  
Accession: CAZ86557
  
Location: 168579-168680
  
 NCBI BlastP on this gene

CAZ86557

not annotated
  
Accession: CAZ86558
  
Location: 177276-178037
  
 NCBI BlastP on this gene

CAZ86558

not annotated
  
Accession: CAZ86559
  
Location: 180119-182273
  
 NCBI BlastP on this gene

CAZ86559

not annotated
  
Accession: CAZ86560
  
Location: 183024-185233
  
 NCBI BlastP on this gene

CAZ86560

88. :  AP007172 Aspergillus oryzae RIB40 DNA, SC206.     Total score: 3.0     Cumulative Blast bit score: 1179

not annotated
  
Accession: BAE65381
  
Location: 1556-3373
  
  
**BlastP hit with Mycgr3G36271\_Mycgr3T**
  
Percentage identity: 74 %
  
BlastP bit score: 469
  
Sequence coverage: 99 %
  
E-value: 6e-164
  
  
 NCBI BlastP on this gene

AO090206000001

not annotated
  
Accession: BAE65382
  
Location: 3502-5607
  
  
**BlastP hit with Mycgr3G84646\_Mycgr3T**
  
Percentage identity: 37 %
  
BlastP bit score: 363
  
Sequence coverage: 82 %
  
E-value: 7e-112
  
  
 NCBI BlastP on this gene

AO090206000002

not annotated
  
Accession: BAE65383
  
Location: 6851-7590
  
  
**BlastP hit with Mycgr3G99145\_Mycgr3T**
  
Percentage identity: 82 %
  
BlastP bit score: 347
  
Sequence coverage: 100 %
  
E-value: 9e-119
  
  
 NCBI BlastP on this gene

AO090206000003

not annotated
  
Accession: BAE65384
  
Location: 9980-12289
  
 NCBI BlastP on this gene

AO090206000004

not annotated
  
Accession: BAE65385
  
Location: 12764-13859
  
 NCBI BlastP on this gene

AO090206000005

not annotated
  
Accession: BAE65386
  
Location: 14114-20724
  
 NCBI BlastP on this gene

AO090206000006

not annotated
  
Accession: BAE65387
  
Location: 23784-24408
  
 NCBI BlastP on this gene

AO090206000009

not annotated
  
Accession: BAE65388
  
Location: 24633-25427
  
 NCBI BlastP on this gene

AO090206000010

not annotated
  
Accession: BAE65389
  
Location: 27395-29014
  
 NCBI BlastP on this gene

AO090206000012

89. :  AKHY01000145 Aspergillus oryzae 3.042     Total score: 3.0     Cumulative Blast bit score: 1179

C-4 sterol methyl oxidase
  
Accession: EIT77737
  
Location: 577-1639
  
  
**BlastP hit with Mycgr3G36271\_Mycgr3T**
  
Percentage identity: 74 %
  
BlastP bit score: 469
  
Sequence coverage: 99 %
  
E-value: 6e-164
  
  
 NCBI BlastP on this gene

EIT77737

hypothetical protein
  
Accession: EIT77740
  
Location: 2522-4627
  
  
**BlastP hit with Mycgr3G84646\_Mycgr3T**
  
Percentage identity: 37 %
  
BlastP bit score: 363
  
Sequence coverage: 82 %
  
E-value: 7e-112
  
  
 NCBI BlastP on this gene

EIT77740

GTP-binding protein
  
Accession: EIT77742
  
Location: 5870-6609
  
  
**BlastP hit with Mycgr3G99145\_Mycgr3T**
  
Percentage identity: 82 %
  
BlastP bit score: 347
  
Sequence coverage: 100 %
  
E-value: 9e-119
  
  
 NCBI BlastP on this gene

EIT77742

AAA+-type ATPase
  
Accession: EIT77733
  
Location: 9000-11309
  
 NCBI BlastP on this gene

EIT77733

hypothetical protein
  
Accession: EIT77729
  
Location: 11784-12879
  
 NCBI BlastP on this gene

EIT77729

protein kinase of the PI-3 kinase family
  
Accession: EIT77736
  
Location: 13134-22007
  
 NCBI BlastP on this gene

EIT77736

adenosine deaminase
  
Accession: EIT77741
  
Location: 22804-23428
  
 NCBI BlastP on this gene

EIT77741

NADH-ubiquinone oxidoreductase subunit
  
Accession: EIT77746
  
Location: 23653-24447
  
 NCBI BlastP on this gene

EIT77746

RCC1 domain protein
  
Accession: EIT77728
  
Location: 26415-28034
  
 NCBI BlastP on this gene

EIT77728

90. :  KB644412 Penicillium oxalicum 114-2 unplaced genomic scaffold scaffold\_5     Total score: 3.0     Cumulative Blast bit score: 1154

hypothetical protein
  
Accession: EPS30860
  
Location: 3964233-3966734
  
 NCBI BlastP on this gene

EPS30860

hypothetical protein
  
Accession: EPS30859
  
Location: 3960559-3963864
  
 NCBI BlastP on this gene

EPS30859

hypothetical protein
  
Accession: EPS30858
  
Location: 3956391-3959698
  
 NCBI BlastP on this gene

EPS30858

hypothetical protein
  
Accession: EPS30857
  
Location: 3952055-3952258
  
 NCBI BlastP on this gene

EPS30857

hypothetical protein
  
Accession: EPS30856
  
Location: 3948553-3949469
  
  
**BlastP hit with Mycgr3G36271\_Mycgr3T**
  
Percentage identity: 76 %
  
BlastP bit score: 403
  
Sequence coverage: 82 %
  
E-value: 2e-138
  
  
 NCBI BlastP on this gene

EPS30856

hypothetical protein
  
Accession: EPS30855
  
Location: 3944607-3946756
  
  
**BlastP hit with Mycgr3G84646\_Mycgr3T**
  
Percentage identity: 40 %
  
BlastP bit score: 409
  
Sequence coverage: 85 %
  
E-value: 1e-129
  
  
 NCBI BlastP on this gene

EPS30855

hypothetical protein
  
Accession: EPS30854
  
Location: 3942875-3943679
  
  
**BlastP hit with Mycgr3G99145\_Mycgr3T**
  
Percentage identity: 83 %
  
BlastP bit score: 342
  
Sequence coverage: 99 %
  
E-value: 1e-116
  
  
 NCBI BlastP on this gene

EPS30854

hypothetical protein
  
Accession: EPS30853
  
Location: 3939210-3941569
  
 NCBI BlastP on this gene

EPS30853

hypothetical protein
  
Accession: EPS30852
  
Location: 3937775-3938780
  
 NCBI BlastP on this gene

EPS30852

hypothetical protein
  
Accession: EPS30851
  
Location: 3928481-3937364
  
 NCBI BlastP on this gene

EPS30851

hypothetical protein
  
Accession: EPS30850
  
Location: 3927027-3927966
  
 NCBI BlastP on this gene

EPS30850

hypothetical protein
  
Accession: EPS30849
  
Location: 3925933-3926774
  
 NCBI BlastP on this gene

EPS30849

hypothetical protein
  
Accession: EPS30848
  
Location: 3924612-3925674
  
 NCBI BlastP on this gene

EPS30848

91. :  DS499603 Aspergillus fumigatus A1163 scf\_000010 genomic scaffold     Total score: 3.0     Cumulative Blast bit score: 1129

C-4 methyl sterol oxidase (Erg25), putative
  
Accession: EDP47217
  
Location: 10520-11519
  
  
**BlastP hit with Mycgr3G36271\_Mycgr3T**
  
Percentage identity: 75 %
  
BlastP bit score: 469
  
Sequence coverage: 95 %
  
E-value: 9e-164
  
  
 NCBI BlastP on this gene

EDP47217

hypothetical protein
  
Accession: EDP47218
  
Location: 12807-14084
  
  
**BlastP hit with Mycgr3G84646\_Mycgr3T**
  
Percentage identity: 41 %
  
BlastP bit score: 320
  
Sequence coverage: 65 %
  
E-value: 4e-98
  
  
 NCBI BlastP on this gene

EDP47218

Rab GTPase SrgA, putative
  
Accession: EDP47219
  
Location: 15998-16742
  
  
**BlastP hit with Mycgr3G99145\_Mycgr3T**
  
Percentage identity: 89 %
  
BlastP bit score: 340
  
Sequence coverage: 87 %
  
E-value: 1e-115
  
  
 NCBI BlastP on this gene

EDP47219

AAA family ATPase, putative
  
Accession: EDP47220
  
Location: 17605-19932
  
 NCBI BlastP on this gene

EDP47220

conserved hypothetical protein
  
Accession: EDP47221
  
Location: 20115-20552
  
 NCBI BlastP on this gene

EDP47221

conserved hypothetical protein
  
Accession: EDP47222
  
Location: 20919-21290
  
 NCBI BlastP on this gene

EDP47222

inositol kinase kinase (UvsB), putative
  
Accession: EDP47223
  
Location: 21684-30524
  
 NCBI BlastP on this gene

EDP47223

NADH-ubiquinone oxidoreductase 21 kDa subunit, putative
  
Accession: EDP47224
  
Location: 32227-32783
  
 NCBI BlastP on this gene

EDP47224

Swi3-like protein
  
Accession: EDP47225
  
Location: 33723-34655
  
 NCBI BlastP on this gene

EDP47225

Ran exchange factor Prp20/Pim1, putative
  
Accession: EDP47226
  
Location: 35138-36878
  
 NCBI BlastP on this gene

EDP47226

92. :  CH476596 Aspergillus terreus NIH2624 scaffold\_3 genomic scaffold     Total score: 3.0     Cumulative Blast bit score: 1114

conserved hypothetical protein
  
Accession: EAU37738
  
Location: 2467530-2469104
  
 NCBI BlastP on this gene

EAU37738

C-4 methylsterol oxidase
  
Accession: EAU37737
  
Location: 2466097-2466962
  
  
**BlastP hit with Mycgr3G36271\_Mycgr3T**
  
Percentage identity: 81 %
  
BlastP bit score: 422
  
Sequence coverage: 80 %
  
E-value: 4e-146
  
  
 NCBI BlastP on this gene

EAU37737

predicted protein
  
Accession: EAU37736
  
Location: 2463031-2464936
  
  
**BlastP hit with Mycgr3G84646\_Mycgr3T**
  
Percentage identity: 41 %
  
BlastP bit score: 353
  
Sequence coverage: 72 %
  
E-value: 5e-109
  
  
 NCBI BlastP on this gene

EAU37736

GTP-binding protein SAS1
  
Accession: EAU37735
  
Location: 2460896-2461633
  
  
**BlastP hit with Mycgr3G99145\_Mycgr3T**
  
Percentage identity: 88 %
  
BlastP bit score: 339
  
Sequence coverage: 87 %
  
E-value: 2e-115
  
  
 NCBI BlastP on this gene

EAU37735

conserved hypothetical protein
  
Accession: EAU37734
  
Location: 2457631-2459940
  
 NCBI BlastP on this gene

EAU37734

hypothetical protein
  
Accession: EAU37733
  
Location: 2448879-2455967
  
 NCBI BlastP on this gene

EAU37733

hypothetical protein
  
Accession: EAU37732
  
Location: 2447365-2448015
  
 NCBI BlastP on this gene

EAU37732

NADH-ubiquinone oxidoreductase 21 kDa subunit
  
Accession: EAU37731
  
Location: 2445181-2445971
  
 NCBI BlastP on this gene

EAU37731

predicted protein
  
Accession: EAU37730
  
Location: 2443255-2444622
  
 NCBI BlastP on this gene

EAU37730

conserved hypothetical protein
  
Accession: EAU37729
  
Location: 2441389-2443038
  
 NCBI BlastP on this gene

EAU37729

93. :  ACYE01000064 Trichophyton verrucosum HKI 0517     Total score: 3.0     Cumulative Blast bit score: 1082

RING finger protein
  
Accession: EFE44038
  
Location: 328-1089
  
 NCBI BlastP on this gene

EFE44038

hypothetical protein
  
Accession: EFE44039
  
Location: 1323-3342
  
 NCBI BlastP on this gene

EFE44039

inositol monophosphatase QutG, putative
  
Accession: EFE44040
  
Location: 4332-5335
  
 NCBI BlastP on this gene

EFE44040

hypothetical protein
  
Accession: EFE44041
  
Location: 5553-10407
  
 NCBI BlastP on this gene

EFE44041

hypothetical protein
  
Accession: EFE44042
  
Location: 12291-13562
  
 NCBI BlastP on this gene

EFE44042

hypothetical protein
  
Accession: EFE44043
  
Location: 14269-18588
  
  
**BlastP hit with Mycgr3G99145\_Mycgr3T**
  
Percentage identity: 79 %
  
BlastP bit score: 328
  
Sequence coverage: 97 %
  
E-value: 2e-101
  
  
  
**BlastP hit with Mycgr3G84646\_Mycgr3T**
  
Percentage identity: 37 %
  
BlastP bit score: 437
  
Sequence coverage: 112 %
  
E-value: 5e-135
  
  
 NCBI BlastP on this gene

EFE44043

hypothetical protein
  
Accession: EFE44044
  
Location: 19035-19696
  
 NCBI BlastP on this gene

EFE44044

C-4 methyl sterol oxidase, putative
  
Accession: EFE44045
  
Location: 19800-20339
  
  
**BlastP hit with Mycgr3G36271\_Mycgr3T**
  
Percentage identity: 81 %
  
BlastP bit score: 317
  
Sequence coverage: 60 %
  
E-value: 1e-105
  
  
 NCBI BlastP on this gene

EFE44045

hypothetical protein
  
Accession: EFE44046
  
Location: 21443-23947
  
 NCBI BlastP on this gene

EFE44046

inositol kinase kinase (UvsB), putative
  
Accession: EFE44047
  
Location: 29514-36775
  
 NCBI BlastP on this gene

EFE44047

replication fork protection component Swi3, putative
  
Accession: EFE44048
  
Location: 39256-40377
  
 NCBI BlastP on this gene

EFE44048

94. :  JH767573 Coniosporium apollinis CBS 100218 chromosome Unknown supercont1.20     Total score: 3.0     Cumulative Blast bit score: 1052

pantothenate kinase
  
Accession: EON65344
  
Location: 284394-285964
  
 NCBI BlastP on this gene

EON65344

hypothetical protein
  
Accession: EON65345
  
Location: 286462-287683
  
 NCBI BlastP on this gene

EON65345

hypothetical protein
  
Accession: EON65346
  
Location: 289160-291049
  
 NCBI BlastP on this gene

EON65346

hypothetical protein
  
Accession: EON65347
  
Location: 291603-293328
  
 NCBI BlastP on this gene

EON65347

hypothetical protein
  
Accession: EON65348
  
Location: 294650-296903
  
 NCBI BlastP on this gene

EON65348

methylsterol monooxygenase
  
Accession: EON65349
  
Location: 297639-298854
  
  
**BlastP hit with Mycgr3G36271\_Mycgr3T**
  
Percentage identity: 84 %
  
BlastP bit score: 535
  
Sequence coverage: 98 %
  
E-value: 0.0
  
  
 NCBI BlastP on this gene

EON65349

hypothetical protein
  
Accession: EON65350
  
Location: 299508-300138
  
 NCBI BlastP on this gene

EON65350

hypothetical protein
  
Accession: EON65351
  
Location: 300541-302976
  
  
**BlastP hit with Mycgr3G68433\_Mycgr3T**
  
Percentage identity: 44 %
  
BlastP bit score: 459
  
Sequence coverage: 66 %
  
E-value: 3e-143
  
  
 NCBI BlastP on this gene

EON65351

hypothetical protein
  
Accession: EON65352
  
Location: 304091-305197
  
  
**BlastP hit with Mycgr3G90786\_Mycgr3T**
  
Percentage identity: 26 %
  
BlastP bit score: 58
  
Sequence coverage: 72 %
  
E-value: 2e-06
  
  
 NCBI BlastP on this gene

EON65352

FKBP12-rapamycin complex-associated protein
  
Accession: EON65353
  
Location: 306195-313744
  
 NCBI BlastP on this gene

EON65353

hypothetical protein
  
Accession: EON65354
  
Location: 314500-318013
  
 NCBI BlastP on this gene

EON65354

hypothetical protein
  
Accession: EON65355
  
Location: 318649-322746
  
 NCBI BlastP on this gene

EON65355

hypothetical protein
  
Accession: EON65356
  
Location: 323249-324208
  
 NCBI BlastP on this gene

EON65356

hypothetical protein
  
Accession: EON65357
  
Location: 324742-326947
  
 NCBI BlastP on this gene

EON65357

95. :  AKCU01000109 Penicillium digitatum Pd1     Total score: 3.0     Cumulative Blast bit score: 1033

C-4 methyl sterol oxidase Erg25, putative
  
Accession: EKV20600
  
Location: 917-1757
  
  
**BlastP hit with Mycgr3G36271\_Mycgr3T**
  
Percentage identity: 77 %
  
BlastP bit score: 404
  
Sequence coverage: 81 %
  
E-value: 6e-139
  
  
 NCBI BlastP on this gene

EKV20600

hypothetical protein
  
Accession: EKV20601
  
Location: 3779-4725
  
  
**BlastP hit with Mycgr3G84646\_Mycgr3T**
  
Percentage identity: 51 %
  
BlastP bit score: 286
  
Sequence coverage: 40 %
  
E-value: 2e-87
  
  
 NCBI BlastP on this gene

EKV20601

Rab GTPase SrgA, putative
  
Accession: EKV20602
  
Location: 5484-6241
  
  
**BlastP hit with Mycgr3G99145\_Mycgr3T**
  
Percentage identity: 86 %
  
BlastP bit score: 343
  
Sequence coverage: 93 %
  
E-value: 3e-117
  
  
 NCBI BlastP on this gene

EKV20602

AAA family ATPase, putative
  
Accession: EKV20603
  
Location: 6950-9272
  
 NCBI BlastP on this gene

EKV20603

Inositol kinase kinase (UvsB), putative
  
Accession: EKV20604
  
Location: 10336-17049
  
 NCBI BlastP on this gene

EKV20604

hypothetical protein
  
Accession: EKV20605
  
Location: 19043-19696
  
 NCBI BlastP on this gene

EKV20605

hypothetical protein
  
Accession: EKV20606
  
Location: 20319-21098
  
 NCBI BlastP on this gene

EKV20606

NADH-ubiquinone oxidoreductase 21 kDa subunit, putative
  
Accession: EKV20607
  
Location: 21354-22178
  
 NCBI BlastP on this gene

EKV20607

hypothetical protein
  
Accession: EKV20608
  
Location: 22520-23494
  
 NCBI BlastP on this gene

EKV20608

Ran exchange factor Prp20/Pim1, putative
  
Accession: EKV20609
  
Location: 23873-25564
  
 NCBI BlastP on this gene

EKV20609

Riboflavin-specific deaminase
  
Accession: EKV20610
  
Location: 25926-27752
  
 NCBI BlastP on this gene

EKV20610

96. :  AKCT01000108 Penicillium digitatum PHI26     Total score: 3.0     Cumulative Blast bit score: 1033

C-4 methyl sterol oxidase Erg25, putative
  
Accession: EKV15878
  
Location: 2639-3479
  
  
**BlastP hit with Mycgr3G36271\_Mycgr3T**
  
Percentage identity: 77 %
  
BlastP bit score: 404
  
Sequence coverage: 81 %
  
E-value: 6e-139
  
  
 NCBI BlastP on this gene

EKV15878

hypothetical protein
  
Accession: EKV15879
  
Location: 5504-6450
  
  
**BlastP hit with Mycgr3G84646\_Mycgr3T**
  
Percentage identity: 51 %
  
BlastP bit score: 286
  
Sequence coverage: 40 %
  
E-value: 2e-87
  
  
 NCBI BlastP on this gene

EKV15879

Rab GTPase SrgA, putative
  
Accession: EKV15880
  
Location: 7209-7966
  
  
**BlastP hit with Mycgr3G99145\_Mycgr3T**
  
Percentage identity: 86 %
  
BlastP bit score: 343
  
Sequence coverage: 93 %
  
E-value: 3e-117
  
  
 NCBI BlastP on this gene

EKV15880

AAA family ATPase, putative
  
Accession: EKV15881
  
Location: 8676-10998
  
 NCBI BlastP on this gene

EKV15881

Inositol kinase kinase (UvsB), putative
  
Accession: EKV15882
  
Location: 12062-18775
  
 NCBI BlastP on this gene

EKV15882

hypothetical protein
  
Accession: EKV15883
  
Location: 20769-21422
  
 NCBI BlastP on this gene

EKV15883

hypothetical protein
  
Accession: EKV15884
  
Location: 22045-22824
  
 NCBI BlastP on this gene

EKV15884

NADH-ubiquinone oxidoreductase 21 kDa subunit, putative
  
Accession: EKV15885
  
Location: 23080-23904
  
 NCBI BlastP on this gene

EKV15885

hypothetical protein
  
Accession: EKV15886
  
Location: 24246-25220
  
 NCBI BlastP on this gene

EKV15886

Ran exchange factor Prp20/Pim1, putative
  
Accession: EKV15887
  
Location: 25599-27290
  
 NCBI BlastP on this gene

EKV15887

Riboflavin-specific deaminase
  
Accession: EKV15888
  
Location: 27653-29479
  
 NCBI BlastP on this gene

EKV15888

97. :  GL573178 Geomyces destructans 20631-21 unplaced genomic scaffold supercont1.10     Total score: 3.0     Cumulative Blast bit score: 1030

hypothetical protein
  
Accession: ELR01837
  
Location: 215462-216889
  
 NCBI BlastP on this gene

ELR01837

hypothetical protein
  
Accession: ELR01836
  
Location: 212320-214186
  
 NCBI BlastP on this gene

ELR01836

hypothetical protein
  
Accession: ELR01835
  
Location: 209424-210270
  
 NCBI BlastP on this gene

ELR01835

hypothetical protein
  
Accession: ELR01834
  
Location: 201120-204999
  
  
**BlastP hit with Mycgr3G21922\_Mycgr3T**
  
Percentage identity: 31 %
  
BlastP bit score: 120
  
Sequence coverage: 47 %
  
E-value: 2e-25
  
  
 NCBI BlastP on this gene

ELR01834

hypothetical protein
  
Accession: ELR01833
  
Location: 198659-199912
  
 NCBI BlastP on this gene

ELR01833

hypothetical protein
  
Accession: ELR01832
  
Location: 195064-198266
  
  
**BlastP hit with Mycgr3G108094\_Mycgr3**
  
Percentage identity: 31 %
  
BlastP bit score: 140
  
Sequence coverage: 33 %
  
E-value: 2e-30
  
  
 NCBI BlastP on this gene

ELR01832

CMGC/SRPK protein kinase
  
Accession: ELR01831
  
Location: 182650-184938
  
  
**BlastP hit with Mycgr3G84644\_Mycgr3T**
  
Percentage identity: 66 %
  
BlastP bit score: 770
  
Sequence coverage: 104 %
  
E-value: 0.0
  
  
 NCBI BlastP on this gene

ELR01831

hypothetical protein
  
Accession: ELR01829
  
Location: 180590-181969
  
 NCBI BlastP on this gene

ELR01829

hypothetical protein
  
Accession: ELR01828
  
Location: 169521-173241
  
 NCBI BlastP on this gene

ELR01828

98. :  DS027693 Neosartorya fischeri NRRL 181 1099437636261 genomic scaffold     Total score: 3.0     Cumulative Blast bit score: 1025

C-4 methyl sterol oxidase (Erg25), putative
  
Accession: EAW20419
  
Location: 67962-68811
  
  
**BlastP hit with Mycgr3G36271\_Mycgr3T**
  
Percentage identity: 70 %
  
BlastP bit score: 354
  
Sequence coverage: 81 %
  
E-value: 7e-120
  
  
 NCBI BlastP on this gene

EAW20419

hypothetical protein
  
Accession: EAW20420
  
Location: 70303-71580
  
  
**BlastP hit with Mycgr3G84646\_Mycgr3T**
  
Percentage identity: 41 %
  
BlastP bit score: 331
  
Sequence coverage: 65 %
  
E-value: 2e-102
  
  
 NCBI BlastP on this gene

EAW20420

Rab GTPase SrgA, putative
  
Accession: EAW20421
  
Location: 72984-73733
  
  
**BlastP hit with Mycgr3G99145\_Mycgr3T**
  
Percentage identity: 89 %
  
BlastP bit score: 340
  
Sequence coverage: 87 %
  
E-value: 1e-115
  
  
 NCBI BlastP on this gene

EAW20421

AAA family ATPase, putative
  
Accession: EAW20422
  
Location: 74596-76911
  
 NCBI BlastP on this gene

EAW20422

hypothetical protein
  
Accession: EAW20423
  
Location: 78030-78527
  
 NCBI BlastP on this gene

EAW20423

phosphatidylinositol 3- and 4-kinase, putative
  
Accession: EAW20424
  
Location: 78666-86258
  
 NCBI BlastP on this gene

EAW20424

hypothetical protein
  
Accession: EAW20425
  
Location: 88641-89016
  
 NCBI BlastP on this gene

EAW20425

NADH-ubiquinone oxidoreductase 21 kDa subunit, putative
  
Accession: EAW20426
  
Location: 89290-89859
  
 NCBI BlastP on this gene

EAW20426

replication fork protection component Swi3, putative
  
Accession: EAW20427
  
Location: 90778-91712
  
 NCBI BlastP on this gene

EAW20427

Ran exchange factor Prp20/Pim1, putative
  
Accession: EAW20428
  
Location: 92243-93984
  
 NCBI BlastP on this gene

EAW20428

99. :  FM992691 Candida dubliniensis CD36 chromosome 4     Total score: 3.0     Cumulative Blast bit score: 967

not annotated
  
Accession: CAX42093
  
Location: 454686-455348
  
 NCBI BlastP on this gene

CD36\_42130

catechol 1,2-dioxygenase, putative
  
Accession: CAX42092
  
Location: 453006-453917
  
 NCBI BlastP on this gene

HQD2

not annotated
  
Accession: CAX42091
  
Location: 449987-452383
  
 NCBI BlastP on this gene

CD36\_42110

not annotated
  
Accession: CAX42090
  
Location: 449143-449937
  
 NCBI BlastP on this gene

CD36\_42100

not annotated
  
Accession: CAX42089
  
Location: 448402-448839
  
 NCBI BlastP on this gene

CD36\_42090

not annotated
  
Accession: CAX42088
  
Location: 446364-448010
  
 NCBI BlastP on this gene

CD36\_42080

not annotated
  
Accession: CAX42087
  
Location: 443255-445753
  
 NCBI BlastP on this gene

CD36\_42070

conserved hypothetical protein
  
Accession: CAX42086
  
Location: 441327-442976
  
 NCBI BlastP on this gene

CD36\_42060

ABC1 family protein, mitochondrial precursor, putative
  
Accession: CAX42085
  
Location: 438731-441057
  
  
**BlastP hit with Mycgr3G68458\_Mycgr3T**
  
Percentage identity: 50 %
  
BlastP bit score: 608
  
Sequence coverage: 90 %
  
E-value: 0.0
  
  
 NCBI BlastP on this gene

CD36\_42050

subunit of guanine nucleotide-binding protein, putative
  
Accession: CAX42084
  
Location: 434771-438100
  
 NCBI BlastP on this gene

CD36\_42040

mitochondrial import inner membrane translocase subunit, putative
  
Accession: CAX42083
  
Location: 434271-434546
  
 NCBI BlastP on this gene

CD36\_42030

cytochrome c1 heme lyase, putative
  
Accession: CAX42082
  
Location: 433331-434086
  
 NCBI BlastP on this gene

CD36\_42020

ER-derived vesicles protein, putative
  
Accession: CAX42081
  
Location: 432005-432931
  
 NCBI BlastP on this gene

CD36\_42010

not annotated
  
Accession: CAX42080
  
Location: 430400-430945
  
 NCBI BlastP on this gene

CD36\_42000

GPI mannosyltransferase, putative
  
Accession: CAX42079
  
Location: 428957-430165
  
 NCBI BlastP on this gene

CD36\_41990

3'-to-5' phosphorolytic exoribonuclease, putative
  
Accession: CAX42078
  
Location: 428058-428780
  
 NCBI BlastP on this gene

CD36\_41980

carnitine/acylcarnitine carrier protein, putative
  
Accession: CAX42077
  
Location: 427036-427965
  
 NCBI BlastP on this gene

CD36\_41970

acid phosphatase, putative
  
Accession: CAX42076
  
Location: 425320-426693
  
 NCBI BlastP on this gene

CD36\_41960

subunit of TFIIF (transcription factor II), putative
  
Accession: CAX42075
  
Location: 422583-424406
  
  
**BlastP hit with Mycgr3G103278\_Mycgr3**
  
Percentage identity: 40 %
  
BlastP bit score: 84
  
Sequence coverage: 26 %
  
E-value: 9e-15
  
  
 NCBI BlastP on this gene

CD36\_41950

HMG1/2-related protein, putative
  
Accession: CAX42074
  
Location: 421378-422466
  
  
**BlastP hit with Mycgr3G25746\_Mycgr3T**
  
Percentage identity: 42 %
  
BlastP bit score: 275
  
Sequence coverage: 100 %
  
E-value: 7e-86
  
  
 NCBI BlastP on this gene

CD36\_41940

acetylspermidine oxidase, putative
  
Accession: CAX42073
  
Location: 419794-421227
  
 NCBI BlastP on this gene

CD36\_41930

conserved hypothetical protein
  
Accession: CAX42072
  
Location: 415889-419230
  
 NCBI BlastP on this gene

CD36\_41920

carnitine O-acetyltransferase, mitochondrial precursor, putative
  
Accession: CAX42071
  
Location: 408726-410624
  
 NCBI BlastP on this gene

CAT2

100. :  KB733455 Bipolaris maydis ATCC 48331 unplaced genomic scaffold COCC4scaffold\_12     Total score: 3.0     Cumulative Blast bit score: 953

hypothetical protein
  
Accession: ENI05063
  
Location: 391576-392858
  
 NCBI BlastP on this gene

ENI05063

hypothetical protein
  
Accession: ENI05064
  
Location: 393416-393920
  
 NCBI BlastP on this gene

ENI05064

hypothetical protein
  
Accession: ENI05065
  
Location: 395875-396629
  
 NCBI BlastP on this gene

ENI05065

hypothetical protein
  
Accession: ENI05066
  
Location: 397130-397976
  
 NCBI BlastP on this gene

ENI05066

hypothetical protein
  
Accession: ENI05067
  
Location: 399288-399935
  
 NCBI BlastP on this gene

ENI05067

hypothetical protein
  
Accession: ENI05068
  
Location: 401608-402086
  
 NCBI BlastP on this gene

ENI05068

hypothetical protein
  
Accession: ENI05069
  
Location: 406076-407044
  
 NCBI BlastP on this gene

ENI05069

hypothetical protein
  
Accession: ENI05070
  
Location: 407506-409581
  
 NCBI BlastP on this gene

ENI05070

hypothetical protein
  
Accession: ENI05071
  
Location: 411081-412390
  
  
**BlastP hit with Mycgr3G25746\_Mycgr3T**
  
Percentage identity: 56 %
  
BlastP bit score: 380
  
Sequence coverage: 102 %
  
E-value: 8e-127
  
  
 NCBI BlastP on this gene

ENI05071

hypothetical protein
  
Accession: ENI05072
  
Location: 412739-414638
  
 NCBI BlastP on this gene

ENI05072

hypothetical protein
  
Accession: ENI05073
  
Location: 414865-416991
  
  
**BlastP hit with Mycgr3G103278\_Mycgr3**
  
Percentage identity: 33 %
  
BlastP bit score: 118
  
Sequence coverage: 94 %
  
E-value: 4e-26
  
  
 NCBI BlastP on this gene

ENI05073

hypothetical protein
  
Accession: ENI05074
  
Location: 417643-419898
  
  
**BlastP hit with Mycgr3G21922\_Mycgr3T**
  
Percentage identity: 47 %
  
BlastP bit score: 455
  
Sequence coverage: 105 %
  
E-value: 2e-149
  
  
 NCBI BlastP on this gene

ENI05074

hypothetical protein
  
Accession: ENI05075
  
Location: 422519-425282
  
 NCBI BlastP on this gene

ENI05075

hypothetical protein
  
Accession: ENI05076
  
Location: 431658-433192
  
 NCBI BlastP on this gene

ENI05076

hypothetical protein
  
Accession: ENI05077
  
Location: 433206-435244
  
 NCBI BlastP on this gene

ENI05077

hypothetical protein
  
Accession: ENI05078
  
Location: 437812-438321
  
 NCBI BlastP on this gene

ENI05078

Detecting sequence homology at the gene cluster level with MultiGeneBlast.
  
Marnix H. Medema, Rainer Breitling & Eriko Takano (2013)
  
*Molecular Biology and Evolution* , 30: 1218-1223.
