# Supplementary material for: In silico prediction and characterization of secondary metabolite biosynthetic gene clusters in the wheat pathogen Zymoseptoria tritici
Source: BMC Genomics. 2017 Aug 17;18:631. doi: 10.1186/s12864-017-3969-y (PMC5561558; doi:10.1186/s12864-017-3969-y)
Supplement: Supplementary file 1 — MultiGeneBLAST analysis of putative secondary metabolite clusters. All encoded amino acid sequences from genes residing in clusters predicted by AntiSMASH are given as FASTA file format. All output data from MultiGeneBLASTs are also provided. (ZIP 42911 kb) [file 12864_2017_3969_MOESM1_ESM.zip › Cluster MultiGene BLAST/out/Clusters_1_34/Cluster_12/displaypage3.xhtml]

xml version="1.0" encoding="UTF-8"?


Search Results
  
  
 Results pages: 1, 2, 3, 4, 5

**MultiGeneBlast hits**

Select gene cluster alignment
101. KB445579\_3 Cochliobolus heterostrophus C5 unplaced genomic scaffold COCH...
102. KB445649\_3 Cochliobolus sativus ND90Pr unplaced genomic scaffold COCSAsc...
103. DS231636\_0 Pyrenophora tritici-repentis Pt-1C-BFP supercont1.22 genomic ...
104. KB908844\_6 Setosphaeria turcica Et28A unplaced genomic scaffold SETTUsca...
105. CR382139\_2 Debaryomyces hansenii CBS767 chromosome G complete sequence.
106. CH672349\_0 Candida albicans WO-1 chromosome 4 supercont1.4 genomic scaff...
107. AAVQ01000001\_2 Pichia stipitis CBS 6054 chromosome 1, whole genome shotg...
108. CH408081\_0 Clavispora lusitaniae ATCC 42720 scaffold\_6 genomic scaffold,...
109. CH408157\_0 Pichia guilliermondii ATCC 6260 scaffold\_3 genomic scaffold, ...
110. KE145361\_0 Glarea lozoyensis ATCC 20868 chromosome Unknown GLAREA18, who...
111. GL996524\_0 Candida tenuis ATCC 10573 unplaced genomic scaffold CANTEscaf...
112. GG698897\_0 Nectria haematococca mpVI 77-13-4 chromosome 3 genomic scaffo...
113. KB707952\_0 Botryotinia fuckeliana BcDW1 unplaced genomic scaffold Scaffo...
114. FQ790278\_1 Botryotinia fuckeliana T4 SuperContig\_34\_1 genomic supercontig.
115. JH767573\_4 Coniosporium apollinis CBS 100218 chromosome Unknown supercon...
116. GL385395\_1 Gaeumannomyces graminis var. tritici R3-111a-1 unplaced genom...
117. JH921428\_1 Marssonina brunnea f. sp. 'multigermtubi' MB\_m1 unplaced geno...
118. EQ963480\_0 Aspergillus flavus NRRL3357 scf\_1106286418846 genomic scaffol...
119. AP007165\_0 Aspergillus oryzae RIB40 DNA, SC124.
120. CH476625\_1 Sclerotinia sclerotiorum 1980 scaffold\_5 genomic scaffold, wh...
121. GL629801\_2 Grosmannia clavigera kw1407 unplaced genomic scaffold GCSC\_17...
122. GG704913\_6 Coccidioides immitis RS genomic scaffold supercont3.3, whole ...
123. AKHY01000107\_0 Aspergillus oryzae 3.042, whole genome shotgun sequencing...
124. GL636502\_1 Coccidioides posadasii str. Silveira unplaced genomic scaffol...
125. DS572813\_4 Paracoccidioides brasiliensis Pb01 supercont1.3 genomic scaff...
126. CH476599\_0 Aspergillus terreus NIH2624 scaffold\_6 genomic scaffold, whol...
127. ACFW01000015\_0 Coccidioides posadasii C735 delta SOWgp, whole genome sho...
128. CH476615\_0 Uncinocarpus reesii 1704 scaffold\_1 genomic scaffold, whole g...
129. ACYE01000028\_0 Trichophyton verrucosum HKI 0517, whole genome shotgun se...
130. KE148155\_0 Ophiostoma piceae UAMH 11346 chromosome Unknown scf10, whole ...
131. AGUE01000023\_0 Glarea lozoyensis 74030, whole genome shotgun sequencing ...
132. KB445561\_2 Baudoinia compniacensis UAMH 10762 unplaced genomic scaffold ...
133. KB446573\_1 Pseudocercospora fijiensis CIRAD86 unplaced genomic scaffold ...
134. AKCU01000112\_0 Penicillium digitatum Pd1, whole genome shotgun sequencin...
135. AKCT01000108\_2 Penicillium digitatum PHI26, whole genome shotgun sequenc...
136. KB446542\_3 Dothistroma septosporum NZE10 unplaced genomic scaffold DOTSE...
137. KB446555\_0 Pseudocercospora fijiensis CIRAD86 unplaced genomic scaffold ...
138. AM920428\_2 Penicillium chrysogenum Wisconsin 54-1255 complete genome, co...
139. KB916472\_1 Neofusicoccum parvum UCRNP2 chromosome Unknown NP2\_03\_scaffol...
140. AHHD01000092\_0 Macrophomina phaseolina MS6, whole genome shotgun sequenc...
141. DS231623\_7 Pyrenophora tritici-repentis Pt-1C-BFP supercont1.9 genomic s...
142. JH767653\_0 Coniosporium apollinis CBS 100218 chromosome Unknown supercon...
143. KB644412\_2 Penicillium oxalicum 114-2 unplaced genomic scaffold scaffold...
144. GL533200\_0 Pyrenophora teres f. teres 0-1 unplaced genomic scaffold scaf...
145. FP929065\_0 Leptosphaeria maculans JN3 lm\_SuperContig\_8\_v2 genomic superc...
146. EQ963487\_1 Aspergillus flavus NRRL3357 scf\_1106286419368 genomic scaffol...
147. DS572750\_3 Paracoccidioides brasiliensis Pb18 supercont1.1 genomic scaff...
148. DS544806\_0 Paracoccidioides brasiliensis Pb03 supercont1.4 genomic scaff...
149. CH476599\_4 Aspergillus terreus NIH2624 scaffold\_6 genomic scaffold, whol...
150. DS544807\_1 Paracoccidioides brasiliensis Pb03 supercont1.5 genomic scaff...

Query: Architecture Search FASTA input

KB445579 : Cochliobolus heterostrophus C5 unplaced genomic scaffold COCHEscaffold\_11    Total score: 3.0     Cumulative Blast bit score: 953

Hit cluster cross-links:

Mycgr3G90785 Mycgr3T
  
Location: 0-1047

Mycgr3G90785\_Mycgr3T

Mycgr3G103262 Mycgr3
  
Location: 1147-1390

Mycgr3G103262\_Mycgr3

Mycgr3G68458 Mycgr3T
  
Location: 1490-3602

Mycgr3G68458\_Mycgr3T

Mycgr3G99145 Mycgr3T
  
Location: 3702-4326

Mycgr3G99145\_Mycgr3T

Mycgr3G103274 Mycgr3
  
Location: 4426-4957

Mycgr3G103274\_Mycgr3

Mycgr3G103264 Mycgr3
  
Location: 5057-5390

Mycgr3G103264\_Mycgr3

Mycgr3G37570 Mycgr3T
  
Location: 5490-6006

Mycgr3G37570\_Mycgr3T

Mycgr3G108094 Mycgr3
  
Location: 6106-10555

Mycgr3G108094\_Mycgr3

Mycgr3G90786 Mycgr3T
  
Location: 10655-12080

Mycgr3G90786\_Mycgr3T

Mycgr3G68429 Mycgr3T
  
Location: 12180-13440

Mycgr3G68429\_Mycgr3T

Mycgr3G68421 Mycgr3T
  
Location: 13540-17086

Mycgr3G68421\_Mycgr3T

Mycgr3G90801 Mycgr3T
  
Location: 17186-18056

Mycgr3G90801\_Mycgr3T

Mycgr3G84646 Mycgr3T
  
Location: 18156-20235

Mycgr3G84646\_Mycgr3T

Mycgr3G68456 Mycgr3T
  
Location: 20335-21970

Mycgr3G68456\_Mycgr3T

Mycgr3G103270 Mycgr3
  
Location: 22070-22355

Mycgr3G103270\_Mycgr3

Mycgr3G90803 Mycgr3T
  
Location: 22455-23019

Mycgr3G90803\_Mycgr3T

Mycgr3G36941 Mycgr3T
  
Location: 23119-24064

Mycgr3G36941\_Mycgr3T

Mycgr3G25746 Mycgr3T
  
Location: 24164-25241

Mycgr3G25746\_Mycgr3T

Mycgr3G90788 Mycgr3T
  
Location: 25341-25803

Mycgr3G90788\_Mycgr3T

Mycgr3G103260 Mycgr3
  
Location: 25903-26635

Mycgr3G103260\_Mycgr3

Mycgr3G84644 Mycgr3T
  
Location: 26735-28457

Mycgr3G84644\_Mycgr3T

Mycgr3G29227 Mycgr3T
  
Location: 28557-28863

Mycgr3G29227\_Mycgr3T

Mycgr3G36271 Mycgr3T
  
Location: 28963-29854

Mycgr3G36271\_Mycgr3T

Mycgr3G68433 Mycgr3T
  
Location: 29954-33041

Mycgr3G68433\_Mycgr3T

Mycgr3G79452 Mycgr3T
  
Location: 33141-33399

Mycgr3G79452\_Mycgr3T

Mycgr3G55345 Mycgr3T
  
Location: 33499-34126

Mycgr3G55345\_Mycgr3T

Mycgr3G103278 Mycgr3
  
Location: 34226-35195

Mycgr3G103278\_Mycgr3

Mycgr3G84654 Mycgr3T
  
Location: 35295-36630

Mycgr3G84654\_Mycgr3T

Mycgr3G108090 Mycgr3
  
Location: 36730-37591

Mycgr3G108090\_Mycgr3

Mycgr3G21922 Mycgr3T
  
Location: 37691-39149

Mycgr3G21922\_Mycgr3T

Mycgr3G99148 Mycgr3T
  
Location: 39249-42819

Mycgr3G99148\_Mycgr3T

hypothetical protein
  
Accession: EMD89220
  
Location: 612232-613805
  
 NCBI BlastP on this gene

EMD89220

hypothetical protein
  
Accession: EMD89219
  
Location: 611170-611674
  
 NCBI BlastP on this gene

EMD89219

hypothetical protein
  
Accession: EMD89218
  
Location: 608461-609215
  
 NCBI BlastP on this gene

EMD89218

hypothetical protein
  
Accession: EMD89217
  
Location: 606885-607960
  
 NCBI BlastP on this gene

EMD89217

hypothetical protein
  
Accession: EMD89216
  
Location: 605155-605802
  
 NCBI BlastP on this gene

EMD89216

hypothetical protein
  
Accession: EMD89215
  
Location: 602985-603463
  
 NCBI BlastP on this gene

EMD89215

hypothetical protein
  
Accession: EMD89214
  
Location: 597922-598890
  
 NCBI BlastP on this gene

EMD89214

hypothetical protein
  
Accession: EMD89213
  
Location: 595385-597460
  
 NCBI BlastP on this gene

EMD89213

hypothetical protein
  
Accession: EMD89212
  
Location: 592576-593885
  
  
**BlastP hit with Mycgr3G25746\_Mycgr3T**
  
Percentage identity: 56 %
  
BlastP bit score: 380
  
Sequence coverage: 102 %
  
E-value: 8e-127
  
  
 NCBI BlastP on this gene

EMD89212

hypothetical protein
  
Accession: EMD89211
  
Location: 590328-591647
  
 NCBI BlastP on this gene

EMD89211

hypothetical protein
  
Accession: EMD89210
  
Location: 587975-590101
  
  
**BlastP hit with Mycgr3G103278\_Mycgr3**
  
Percentage identity: 33 %
  
BlastP bit score: 118
  
Sequence coverage: 94 %
  
E-value: 4e-26
  
  
 NCBI BlastP on this gene

EMD89210

hypothetical protein
  
Accession: EMD89209
  
Location: 585068-587323
  
  
**BlastP hit with Mycgr3G21922\_Mycgr3T**
  
Percentage identity: 47 %
  
BlastP bit score: 455
  
Sequence coverage: 105 %
  
E-value: 2e-149
  
  
 NCBI BlastP on this gene

EMD89209

hypothetical protein
  
Accession: EMD89208
  
Location: 579684-582447
  
 NCBI BlastP on this gene

EMD89208

hypothetical protein
  
Accession: EMD89207
  
Location: 574478-574804
  
 NCBI BlastP on this gene

EMD89207

hypothetical protein
  
Accession: EMD89206
  
Location: 571774-573308
  
 NCBI BlastP on this gene

EMD89206

hypothetical protein
  
Accession: EMD89205
  
Location: 569722-571658
  
 NCBI BlastP on this gene

EMD89205

hypothetical protein
  
Accession: EMD89204
  
Location: 566645-567154
  
 NCBI BlastP on this gene

EMD89204

Query: Architecture Search FASTA input

KB445649 : Cochliobolus sativus ND90Pr unplaced genomic scaffold COCSAscaffold\_13    Total score: 3.0     Cumulative Blast bit score: 950

Hit cluster cross-links:

Mycgr3G90785 Mycgr3T
  
Location: 0-1047

Mycgr3G90785\_Mycgr3T

Mycgr3G103262 Mycgr3
  
Location: 1147-1390

Mycgr3G103262\_Mycgr3

Mycgr3G68458 Mycgr3T
  
Location: 1490-3602

Mycgr3G68458\_Mycgr3T

Mycgr3G99145 Mycgr3T
  
Location: 3702-4326

Mycgr3G99145\_Mycgr3T

Mycgr3G103274 Mycgr3
  
Location: 4426-4957

Mycgr3G103274\_Mycgr3

Mycgr3G103264 Mycgr3
  
Location: 5057-5390

Mycgr3G103264\_Mycgr3

Mycgr3G37570 Mycgr3T
  
Location: 5490-6006

Mycgr3G37570\_Mycgr3T

Mycgr3G108094 Mycgr3
  
Location: 6106-10555

Mycgr3G108094\_Mycgr3

Mycgr3G90786 Mycgr3T
  
Location: 10655-12080

Mycgr3G90786\_Mycgr3T

Mycgr3G68429 Mycgr3T
  
Location: 12180-13440

Mycgr3G68429\_Mycgr3T

Mycgr3G68421 Mycgr3T
  
Location: 13540-17086

Mycgr3G68421\_Mycgr3T

Mycgr3G90801 Mycgr3T
  
Location: 17186-18056

Mycgr3G90801\_Mycgr3T

Mycgr3G84646 Mycgr3T
  
Location: 18156-20235

Mycgr3G84646\_Mycgr3T

Mycgr3G68456 Mycgr3T
  
Location: 20335-21970

Mycgr3G68456\_Mycgr3T

Mycgr3G103270 Mycgr3
  
Location: 22070-22355

Mycgr3G103270\_Mycgr3

Mycgr3G90803 Mycgr3T
  
Location: 22455-23019

Mycgr3G90803\_Mycgr3T

Mycgr3G36941 Mycgr3T
  
Location: 23119-24064

Mycgr3G36941\_Mycgr3T

Mycgr3G25746 Mycgr3T
  
Location: 24164-25241

Mycgr3G25746\_Mycgr3T

Mycgr3G90788 Mycgr3T
  
Location: 25341-25803

Mycgr3G90788\_Mycgr3T

Mycgr3G103260 Mycgr3
  
Location: 25903-26635

Mycgr3G103260\_Mycgr3

Mycgr3G84644 Mycgr3T
  
Location: 26735-28457

Mycgr3G84644\_Mycgr3T

Mycgr3G29227 Mycgr3T
  
Location: 28557-28863

Mycgr3G29227\_Mycgr3T

Mycgr3G36271 Mycgr3T
  
Location: 28963-29854

Mycgr3G36271\_Mycgr3T

Mycgr3G68433 Mycgr3T
  
Location: 29954-33041

Mycgr3G68433\_Mycgr3T

Mycgr3G79452 Mycgr3T
  
Location: 33141-33399

Mycgr3G79452\_Mycgr3T

Mycgr3G55345 Mycgr3T
  
Location: 33499-34126

Mycgr3G55345\_Mycgr3T

Mycgr3G103278 Mycgr3
  
Location: 34226-35195

Mycgr3G103278\_Mycgr3

Mycgr3G84654 Mycgr3T
  
Location: 35295-36630

Mycgr3G84654\_Mycgr3T

Mycgr3G108090 Mycgr3
  
Location: 36730-37591

Mycgr3G108090\_Mycgr3

Mycgr3G21922 Mycgr3T
  
Location: 37691-39149

Mycgr3G21922\_Mycgr3T

Mycgr3G99148 Mycgr3T
  
Location: 39249-42819

Mycgr3G99148\_Mycgr3T

hypothetical protein
  
Accession: EMD60987
  
Location: 670499-671818
  
 NCBI BlastP on this gene

EMD60987

hypothetical protein
  
Accession: EMD60986
  
Location: 667686-669631
  
 NCBI BlastP on this gene

EMD60986

hypothetical protein
  
Accession: EMD60985
  
Location: 663961-666133
  
 NCBI BlastP on this gene

EMD60985

hypothetical protein
  
Accession: EMD60984
  
Location: 663040-663558
  
 NCBI BlastP on this gene

EMD60984

hypothetical protein
  
Accession: EMD60983
  
Location: 660788-661541
  
 NCBI BlastP on this gene

EMD60983

hypothetical protein
  
Accession: EMD60982
  
Location: 659432-660278
  
 NCBI BlastP on this gene

EMD60982

hypothetical protein
  
Accession: EMD60981
  
Location: 657469-658126
  
 NCBI BlastP on this gene

EMD60981

hypothetical protein
  
Accession: EMD60980
  
Location: 655228-656187
  
 NCBI BlastP on this gene

EMD60980

hypothetical protein
  
Accession: EMD60979
  
Location: 652686-654764
  
 NCBI BlastP on this gene

EMD60979

hypothetical protein
  
Accession: EMD60978
  
Location: 649880-651189
  
  
**BlastP hit with Mycgr3G25746\_Mycgr3T**
  
Percentage identity: 56 %
  
BlastP bit score: 379
  
Sequence coverage: 102 %
  
E-value: 4e-126
  
  
 NCBI BlastP on this gene

EMD60978

hypothetical protein
  
Accession: EMD60977
  
Location: 645295-647433
  
  
**BlastP hit with Mycgr3G103278\_Mycgr3**
  
Percentage identity: 33 %
  
BlastP bit score: 117
  
Sequence coverage: 94 %
  
E-value: 1e-25
  
  
 NCBI BlastP on this gene

EMD60977

hypothetical protein
  
Accession: EMD60976
  
Location: 642385-644655
  
  
**BlastP hit with Mycgr3G21922\_Mycgr3T**
  
Percentage identity: 48 %
  
BlastP bit score: 455
  
Sequence coverage: 105 %
  
E-value: 2e-149
  
  
 NCBI BlastP on this gene

EMD60976

hypothetical protein
  
Accession: EMD60975
  
Location: 640860-641916
  
 NCBI BlastP on this gene

EMD60975

hypothetical protein
  
Accession: EMD60974
  
Location: 638342-640574
  
 NCBI BlastP on this gene

EMD60974

hypothetical protein
  
Accession: EMD60973
  
Location: 636733-637335
  
 NCBI BlastP on this gene

EMD60973

hypothetical protein
  
Accession: EMD60972
  
Location: 635259-635741
  
 NCBI BlastP on this gene

EMD60972

hypothetical protein
  
Accession: EMD60971
  
Location: 634581-635045
  
 NCBI BlastP on this gene

EMD60971

hypothetical protein
  
Accession: EMD60970
  
Location: 634073-634414
  
 NCBI BlastP on this gene

EMD60970

hypothetical protein
  
Accession: EMD60969
  
Location: 631275-632800
  
 NCBI BlastP on this gene

EMD60969

hypothetical protein
  
Accession: EMD60968
  
Location: 628174-628539
  
 NCBI BlastP on this gene

EMD60968

hypothetical protein
  
Accession: EMD60967
  
Location: 627342-627716
  
 NCBI BlastP on this gene

EMD60967

hypothetical protein
  
Accession: EMD60966
  
Location: 626021-626437
  
 NCBI BlastP on this gene

EMD60966

Query: Architecture Search FASTA input

DS231636 : Pyrenophora tritici-repentis Pt-1C-BFP supercont1.22 genomic scaffold    Total score: 3.0     Cumulative Blast bit score: 946

Hit cluster cross-links:

Mycgr3G90785 Mycgr3T
  
Location: 0-1047

Mycgr3G90785\_Mycgr3T

Mycgr3G103262 Mycgr3
  
Location: 1147-1390

Mycgr3G103262\_Mycgr3

Mycgr3G68458 Mycgr3T
  
Location: 1490-3602

Mycgr3G68458\_Mycgr3T

Mycgr3G99145 Mycgr3T
  
Location: 3702-4326

Mycgr3G99145\_Mycgr3T

Mycgr3G103274 Mycgr3
  
Location: 4426-4957

Mycgr3G103274\_Mycgr3

Mycgr3G103264 Mycgr3
  
Location: 5057-5390

Mycgr3G103264\_Mycgr3

Mycgr3G37570 Mycgr3T
  
Location: 5490-6006

Mycgr3G37570\_Mycgr3T

Mycgr3G108094 Mycgr3
  
Location: 6106-10555

Mycgr3G108094\_Mycgr3

Mycgr3G90786 Mycgr3T
  
Location: 10655-12080

Mycgr3G90786\_Mycgr3T

Mycgr3G68429 Mycgr3T
  
Location: 12180-13440

Mycgr3G68429\_Mycgr3T

Mycgr3G68421 Mycgr3T
  
Location: 13540-17086

Mycgr3G68421\_Mycgr3T

Mycgr3G90801 Mycgr3T
  
Location: 17186-18056

Mycgr3G90801\_Mycgr3T

Mycgr3G84646 Mycgr3T
  
Location: 18156-20235

Mycgr3G84646\_Mycgr3T

Mycgr3G68456 Mycgr3T
  
Location: 20335-21970

Mycgr3G68456\_Mycgr3T

Mycgr3G103270 Mycgr3
  
Location: 22070-22355

Mycgr3G103270\_Mycgr3

Mycgr3G90803 Mycgr3T
  
Location: 22455-23019

Mycgr3G90803\_Mycgr3T

Mycgr3G36941 Mycgr3T
  
Location: 23119-24064

Mycgr3G36941\_Mycgr3T

Mycgr3G25746 Mycgr3T
  
Location: 24164-25241

Mycgr3G25746\_Mycgr3T

Mycgr3G90788 Mycgr3T
  
Location: 25341-25803

Mycgr3G90788\_Mycgr3T

Mycgr3G103260 Mycgr3
  
Location: 25903-26635

Mycgr3G103260\_Mycgr3

Mycgr3G84644 Mycgr3T
  
Location: 26735-28457

Mycgr3G84644\_Mycgr3T

Mycgr3G29227 Mycgr3T
  
Location: 28557-28863

Mycgr3G29227\_Mycgr3T

Mycgr3G36271 Mycgr3T
  
Location: 28963-29854

Mycgr3G36271\_Mycgr3T

Mycgr3G68433 Mycgr3T
  
Location: 29954-33041

Mycgr3G68433\_Mycgr3T

Mycgr3G79452 Mycgr3T
  
Location: 33141-33399

Mycgr3G79452\_Mycgr3T

Mycgr3G55345 Mycgr3T
  
Location: 33499-34126

Mycgr3G55345\_Mycgr3T

Mycgr3G103278 Mycgr3
  
Location: 34226-35195

Mycgr3G103278\_Mycgr3

Mycgr3G84654 Mycgr3T
  
Location: 35295-36630

Mycgr3G84654\_Mycgr3T

Mycgr3G108090 Mycgr3
  
Location: 36730-37591

Mycgr3G108090\_Mycgr3

Mycgr3G21922 Mycgr3T
  
Location: 37691-39149

Mycgr3G21922\_Mycgr3T

Mycgr3G99148 Mycgr3T
  
Location: 39249-42819

Mycgr3G99148\_Mycgr3T

RING finger domain containing protein
  
Accession: EDU46037
  
Location: 89859-91377
  
 NCBI BlastP on this gene

EDU46037

conserved hypothetical protein
  
Accession: EDU46036
  
Location: 88480-89575
  
 NCBI BlastP on this gene

EDU46036

conserved hypothetical protein
  
Accession: EDU46035
  
Location: 86131-87576
  
 NCBI BlastP on this gene

EDU46035

nitrilase
  
Accession: EDU46034
  
Location: 84192-85309
  
 NCBI BlastP on this gene

EDU46034

aldehyde reductase 1
  
Accession: EDU46033
  
Location: 82071-83012
  
 NCBI BlastP on this gene

EDU46033

hypothetical protein
  
Accession: EDU46032
  
Location: 76181-76486
  
 NCBI BlastP on this gene

EDU46032

predicted protein
  
Accession: EDU46031
  
Location: 71937-74290
  
 NCBI BlastP on this gene

EDU46031

DNA-binding protein HGH1
  
Accession: EDU46030
  
Location: 69222-70694
  
  
**BlastP hit with Mycgr3G25746\_Mycgr3T**
  
Percentage identity: 53 %
  
BlastP bit score: 358
  
Sequence coverage: 101 %
  
E-value: 7e-118
  
  
 NCBI BlastP on this gene

EDU46030

major facilitator family transporter
  
Accession: EDU46029
  
Location: 66913-68878
  
 NCBI BlastP on this gene

EDU46029

transcription factor TFIIF complex alpha subunit Tfg1
  
Accession: EDU46028
  
Location: 64715-66820
  
  
**BlastP hit with Mycgr3G103278\_Mycgr3**
  
Percentage identity: 34 %
  
BlastP bit score: 126
  
Sequence coverage: 99 %
  
E-value: 9e-29
  
  
 NCBI BlastP on this gene

EDU46028

Poly(A) RNA polymerase cid13
  
Accession: EDU46027
  
Location: 62285-64195
  
  
**BlastP hit with Mycgr3G21922\_Mycgr3T**
  
Percentage identity: 50 %
  
BlastP bit score: 463
  
Sequence coverage: 101 %
  
E-value: 1e-153
  
  
 NCBI BlastP on this gene

EDU46027

predicted protein
  
Accession: EDU46026
  
Location: 61032-61474
  
 NCBI BlastP on this gene

EDU46026

conserved hypothetical protein
  
Accession: EDU46025
  
Location: 59313-60326
  
 NCBI BlastP on this gene

EDU46025

predicted protein
  
Accession: EDU46024
  
Location: 56447-58780
  
 NCBI BlastP on this gene

EDU46024

predicted protein
  
Accession: EDU46023
  
Location: 54485-55401
  
 NCBI BlastP on this gene

EDU46023

conserved hypothetical protein
  
Accession: EDU46022
  
Location: 50955-51437
  
 NCBI BlastP on this gene

EDU46022

predicted protein
  
Accession: EDU46021
  
Location: 42626-44384
  
 NCBI BlastP on this gene

EDU46021

Query: Architecture Search FASTA input

KB908844 : Setosphaeria turcica Et28A unplaced genomic scaffold SETTUscaffold\_6    Total score: 3.0     Cumulative Blast bit score: 933

Hit cluster cross-links:

Mycgr3G90785 Mycgr3T
  
Location: 0-1047

Mycgr3G90785\_Mycgr3T

Mycgr3G103262 Mycgr3
  
Location: 1147-1390

Mycgr3G103262\_Mycgr3

Mycgr3G68458 Mycgr3T
  
Location: 1490-3602

Mycgr3G68458\_Mycgr3T

Mycgr3G99145 Mycgr3T
  
Location: 3702-4326

Mycgr3G99145\_Mycgr3T

Mycgr3G103274 Mycgr3
  
Location: 4426-4957

Mycgr3G103274\_Mycgr3

Mycgr3G103264 Mycgr3
  
Location: 5057-5390

Mycgr3G103264\_Mycgr3

Mycgr3G37570 Mycgr3T
  
Location: 5490-6006

Mycgr3G37570\_Mycgr3T

Mycgr3G108094 Mycgr3
  
Location: 6106-10555

Mycgr3G108094\_Mycgr3

Mycgr3G90786 Mycgr3T
  
Location: 10655-12080

Mycgr3G90786\_Mycgr3T

Mycgr3G68429 Mycgr3T
  
Location: 12180-13440

Mycgr3G68429\_Mycgr3T

Mycgr3G68421 Mycgr3T
  
Location: 13540-17086

Mycgr3G68421\_Mycgr3T

Mycgr3G90801 Mycgr3T
  
Location: 17186-18056

Mycgr3G90801\_Mycgr3T

Mycgr3G84646 Mycgr3T
  
Location: 18156-20235

Mycgr3G84646\_Mycgr3T

Mycgr3G68456 Mycgr3T
  
Location: 20335-21970

Mycgr3G68456\_Mycgr3T

Mycgr3G103270 Mycgr3
  
Location: 22070-22355

Mycgr3G103270\_Mycgr3

Mycgr3G90803 Mycgr3T
  
Location: 22455-23019

Mycgr3G90803\_Mycgr3T

Mycgr3G36941 Mycgr3T
  
Location: 23119-24064

Mycgr3G36941\_Mycgr3T

Mycgr3G25746 Mycgr3T
  
Location: 24164-25241

Mycgr3G25746\_Mycgr3T

Mycgr3G90788 Mycgr3T
  
Location: 25341-25803

Mycgr3G90788\_Mycgr3T

Mycgr3G103260 Mycgr3
  
Location: 25903-26635

Mycgr3G103260\_Mycgr3

Mycgr3G84644 Mycgr3T
  
Location: 26735-28457

Mycgr3G84644\_Mycgr3T

Mycgr3G29227 Mycgr3T
  
Location: 28557-28863

Mycgr3G29227\_Mycgr3T

Mycgr3G36271 Mycgr3T
  
Location: 28963-29854

Mycgr3G36271\_Mycgr3T

Mycgr3G68433 Mycgr3T
  
Location: 29954-33041

Mycgr3G68433\_Mycgr3T

Mycgr3G79452 Mycgr3T
  
Location: 33141-33399

Mycgr3G79452\_Mycgr3T

Mycgr3G55345 Mycgr3T
  
Location: 33499-34126

Mycgr3G55345\_Mycgr3T

Mycgr3G103278 Mycgr3
  
Location: 34226-35195

Mycgr3G103278\_Mycgr3

Mycgr3G84654 Mycgr3T
  
Location: 35295-36630

Mycgr3G84654\_Mycgr3T

Mycgr3G108090 Mycgr3
  
Location: 36730-37591

Mycgr3G108090\_Mycgr3

Mycgr3G21922 Mycgr3T
  
Location: 37691-39149

Mycgr3G21922\_Mycgr3T

Mycgr3G99148 Mycgr3T
  
Location: 39249-42819

Mycgr3G99148\_Mycgr3T

hypothetical protein
  
Accession: EOA82789
  
Location: 1338652-1340595
  
 NCBI BlastP on this gene

EOA82789

hypothetical protein
  
Accession: EOA82790
  
Location: 1342133-1343894
  
 NCBI BlastP on this gene

EOA82790

hypothetical protein
  
Accession: EOA82791
  
Location: 1344453-1344647
  
 NCBI BlastP on this gene

EOA82791

hypothetical protein
  
Accession: EOA82792
  
Location: 1344860-1345625
  
 NCBI BlastP on this gene

EOA82792

hypothetical protein
  
Accession: EOA82793
  
Location: 1346347-1347431
  
 NCBI BlastP on this gene

EOA82793

hypothetical protein
  
Accession: EOA82794
  
Location: 1348578-1349748
  
 NCBI BlastP on this gene

EOA82794

hypothetical protein
  
Accession: EOA82795
  
Location: 1350312-1350969
  
 NCBI BlastP on this gene

EOA82795

hypothetical protein
  
Accession: EOA82796
  
Location: 1351960-1352910
  
 NCBI BlastP on this gene

EOA82796

hypothetical protein
  
Accession: EOA82797
  
Location: 1353378-1354436
  
 NCBI BlastP on this gene

EOA82797

hypothetical protein
  
Accession: EOA82798
  
Location: 1355086-1357095
  
 NCBI BlastP on this gene

EOA82798

hypothetical protein
  
Accession: EOA82799
  
Location: 1358579-1359893
  
  
**BlastP hit with Mycgr3G25746\_Mycgr3T**
  
Percentage identity: 56 %
  
BlastP bit score: 372
  
Sequence coverage: 100 %
  
E-value: 1e-123
  
  
 NCBI BlastP on this gene

EOA82799

hypothetical protein
  
Accession: EOA82800
  
Location: 1360820-1362192
  
 NCBI BlastP on this gene

EOA82800

hypothetical protein
  
Accession: EOA82801
  
Location: 1362354-1364474
  
  
**BlastP hit with Mycgr3G103278\_Mycgr3**
  
Percentage identity: 35 %
  
BlastP bit score: 120
  
Sequence coverage: 92 %
  
E-value: 9e-27
  
  
 NCBI BlastP on this gene

EOA82801

hypothetical protein
  
Accession: EOA82802
  
Location: 1365535-1367031
  
  
**BlastP hit with Mycgr3G21922\_Mycgr3T**
  
Percentage identity: 46 %
  
BlastP bit score: 442
  
Sequence coverage: 103 %
  
E-value: 4e-147
  
  
 NCBI BlastP on this gene

EOA82802

hypothetical protein
  
Accession: EOA82803
  
Location: 1367426-1368031
  
 NCBI BlastP on this gene

EOA82803

Query: Architecture Search FASTA input

CR382139 : Debaryomyces hansenii CBS767 chromosome G complete sequence.    Total score: 3.0     Cumulative Blast bit score: 920

Hit cluster cross-links:

Mycgr3G90785 Mycgr3T
  
Location: 0-1047

Mycgr3G90785\_Mycgr3T

Mycgr3G103262 Mycgr3
  
Location: 1147-1390

Mycgr3G103262\_Mycgr3

Mycgr3G68458 Mycgr3T
  
Location: 1490-3602

Mycgr3G68458\_Mycgr3T

Mycgr3G99145 Mycgr3T
  
Location: 3702-4326

Mycgr3G99145\_Mycgr3T

Mycgr3G103274 Mycgr3
  
Location: 4426-4957

Mycgr3G103274\_Mycgr3

Mycgr3G103264 Mycgr3
  
Location: 5057-5390

Mycgr3G103264\_Mycgr3

Mycgr3G37570 Mycgr3T
  
Location: 5490-6006

Mycgr3G37570\_Mycgr3T

Mycgr3G108094 Mycgr3
  
Location: 6106-10555

Mycgr3G108094\_Mycgr3

Mycgr3G90786 Mycgr3T
  
Location: 10655-12080

Mycgr3G90786\_Mycgr3T

Mycgr3G68429 Mycgr3T
  
Location: 12180-13440

Mycgr3G68429\_Mycgr3T

Mycgr3G68421 Mycgr3T
  
Location: 13540-17086

Mycgr3G68421\_Mycgr3T

Mycgr3G90801 Mycgr3T
  
Location: 17186-18056

Mycgr3G90801\_Mycgr3T

Mycgr3G84646 Mycgr3T
  
Location: 18156-20235

Mycgr3G84646\_Mycgr3T

Mycgr3G68456 Mycgr3T
  
Location: 20335-21970

Mycgr3G68456\_Mycgr3T

Mycgr3G103270 Mycgr3
  
Location: 22070-22355

Mycgr3G103270\_Mycgr3

Mycgr3G90803 Mycgr3T
  
Location: 22455-23019

Mycgr3G90803\_Mycgr3T

Mycgr3G36941 Mycgr3T
  
Location: 23119-24064

Mycgr3G36941\_Mycgr3T

Mycgr3G25746 Mycgr3T
  
Location: 24164-25241

Mycgr3G25746\_Mycgr3T

Mycgr3G90788 Mycgr3T
  
Location: 25341-25803

Mycgr3G90788\_Mycgr3T

Mycgr3G103260 Mycgr3
  
Location: 25903-26635

Mycgr3G103260\_Mycgr3

Mycgr3G84644 Mycgr3T
  
Location: 26735-28457

Mycgr3G84644\_Mycgr3T

Mycgr3G29227 Mycgr3T
  
Location: 28557-28863

Mycgr3G29227\_Mycgr3T

Mycgr3G36271 Mycgr3T
  
Location: 28963-29854

Mycgr3G36271\_Mycgr3T

Mycgr3G68433 Mycgr3T
  
Location: 29954-33041

Mycgr3G68433\_Mycgr3T

Mycgr3G79452 Mycgr3T
  
Location: 33141-33399

Mycgr3G79452\_Mycgr3T

Mycgr3G55345 Mycgr3T
  
Location: 33499-34126

Mycgr3G55345\_Mycgr3T

Mycgr3G103278 Mycgr3
  
Location: 34226-35195

Mycgr3G103278\_Mycgr3

Mycgr3G84654 Mycgr3T
  
Location: 35295-36630

Mycgr3G84654\_Mycgr3T

Mycgr3G108090 Mycgr3
  
Location: 36730-37591

Mycgr3G108090\_Mycgr3

Mycgr3G21922 Mycgr3T
  
Location: 37691-39149

Mycgr3G21922\_Mycgr3T

Mycgr3G99148 Mycgr3T
  
Location: 39249-42819

Mycgr3G99148\_Mycgr3T

DEHA2G17842p
  
Accession: CAG90821
  
Location: 1453597-1455762
  
 NCBI BlastP on this gene

DEHA2G17842g

DEHA2G17864p
  
Accession: CAG90822
  
Location: 1456140-1456661
  
 NCBI BlastP on this gene

DEHA2G17864g

DEHA2G17886p
  
Accession: CAG90823
  
Location: 1457089-1457799
  
 NCBI BlastP on this gene

DEHA2G17886g

DEHA2G17908p
  
Accession: CAG90824
  
Location: 1457913-1461941
  
 NCBI BlastP on this gene

DEHA2G17908g

DEHA2G17930p
  
Accession: CAG90825
  
Location: 1462868-1464097
  
 NCBI BlastP on this gene

DEHA2G17930g

DEHA2G17952p
  
Accession: CAG90826
  
Location: 1464555-1465556
  
 NCBI BlastP on this gene

DEHA2G17952g

DEHA2G17974p
  
Accession: CAG90827
  
Location: 1465676-1466314
  
 NCBI BlastP on this gene

DEHA2G17974g

DEHA2G17996p
  
Accession: CAG90828
  
Location: 1466393-1467121
  
 NCBI BlastP on this gene

DEHA2G17996g

DEHA2G18018p
  
Accession: CAG90829
  
Location: 1467780-1468076
  
 NCBI BlastP on this gene

DEHA2G18018g

DEHA2G18040p
  
Accession: CAG90830
  
Location: 1468794-1470464
  
 NCBI BlastP on this gene

DEHA2G18040g

DEHA2G18062p
  
Accession: CAG90831
  
Location: 1470627-1472639
  
  
**BlastP hit with Mycgr3G68458\_Mycgr3T**
  
Percentage identity: 49 %
  
BlastP bit score: 556
  
Sequence coverage: 85 %
  
E-value: 0.0
  
  
 NCBI BlastP on this gene

DEHA2G18062g

DEHA2G18084p
  
Accession: CAR65994
  
Location: 1473251-1476406
  
 NCBI BlastP on this gene

DEHA2G18084g

DEHA2G18106p
  
Accession: CAG90834
  
Location: 1476616-1476891
  
 NCBI BlastP on this gene

DEHA2G18106g

DEHA2G18128p
  
Accession: CAG90835
  
Location: 1477052-1477819
  
 NCBI BlastP on this gene

DEHA2G18128g

DEHA2G18150p
  
Accession: CAG90836
  
Location: 1478047-1478973
  
 NCBI BlastP on this gene

DEHA2G18150g

DEHA2G18194p
  
Accession: CAG90837
  
Location: 1479821-1480558
  
 NCBI BlastP on this gene

DEHA2G18194g

DEHA2G18216p
  
Accession: CAG90838
  
Location: 1480739-1481983
  
 NCBI BlastP on this gene

DEHA2G18216g

DEHA2G18238p
  
Accession: CAG90839
  
Location: 1482112-1482621
  
 NCBI BlastP on this gene

DEHA2G18238g

DEHA2G18260p
  
Accession: CAG90840
  
Location: 1482693-1483613
  
 NCBI BlastP on this gene

DEHA2G18260g

DEHA2G18282p
  
Accession: CAG90841
  
Location: 1484264-1486069
  
  
**BlastP hit with Mycgr3G103278\_Mycgr3**
  
Percentage identity: 38 %
  
BlastP bit score: 91
  
Sequence coverage: 37 %
  
E-value: 4e-17
  
  
 NCBI BlastP on this gene

DEHA2G18282g

DEHA2G18304p
  
Accession: CAG90842
  
Location: 1486170-1487273
  
  
**BlastP hit with Mycgr3G25746\_Mycgr3T**
  
Percentage identity: 43 %
  
BlastP bit score: 273
  
Sequence coverage: 101 %
  
E-value: 4e-85
  
  
 NCBI BlastP on this gene

DEHA2G18304g

DEHA2G18326p
  
Accession: CAG90843
  
Location: 1487428-1489206
  
 NCBI BlastP on this gene

DEHA2G18326g

DEHA2G18348p
  
Accession: CAG90844
  
Location: 1489476-1491245
  
 NCBI BlastP on this gene

DEHA2G18348g

DEHA2G18370p
  
Accession: CAG90845
  
Location: 1491466-1492848
  
 NCBI BlastP on this gene

DEHA2G18370g

DEHA2G18392p
  
Accession: CAG90846
  
Location: 1493018-1494487
  
 NCBI BlastP on this gene

DEHA2G18392g

DEHA2G18414p
  
Accession: CAG90847
  
Location: 1494533-1495858
  
 NCBI BlastP on this gene

DEHA2G18414g

DEHA2G18436p
  
Accession: CAG90848
  
Location: 1495968-1496525
  
 NCBI BlastP on this gene

DEHA2G18436g

DEHA2G18458p
  
Accession: CAG90849
  
Location: 1496927-1497541
  
 NCBI BlastP on this gene

DEHA2G18458g

DEHA2G18480p
  
Accession: CAG90850
  
Location: 1497648-1498943
  
 NCBI BlastP on this gene

DEHA2G18480g

DEHA2G18502p
  
Accession: CAG90851
  
Location: 1499224-1499988
  
 NCBI BlastP on this gene

DEHA2G18502g

DEHA2G18524p
  
Accession: CAG90852
  
Location: 1500371-1500998
  
 NCBI BlastP on this gene

DEHA2G18524g

DEHA2G18546p
  
Accession: CAG90853
  
Location: 1501187-1502389
  
 NCBI BlastP on this gene

DEHA2G18546g

DEHA2G18568p
  
Accession: CAG90854
  
Location: 1502597-1508041
  
 NCBI BlastP on this gene

DEHA2G18568g

Query: Architecture Search FASTA input

CH672349 : Candida albicans WO-1 chromosome 4 supercont1.4 genomic scaffold    Total score: 3.0     Cumulative Blast bit score: 919

Hit cluster cross-links:

Mycgr3G90785 Mycgr3T
  
Location: 0-1047

Mycgr3G90785\_Mycgr3T

Mycgr3G103262 Mycgr3
  
Location: 1147-1390

Mycgr3G103262\_Mycgr3

Mycgr3G68458 Mycgr3T
  
Location: 1490-3602

Mycgr3G68458\_Mycgr3T

Mycgr3G99145 Mycgr3T
  
Location: 3702-4326

Mycgr3G99145\_Mycgr3T

Mycgr3G103274 Mycgr3
  
Location: 4426-4957

Mycgr3G103274\_Mycgr3

Mycgr3G103264 Mycgr3
  
Location: 5057-5390

Mycgr3G103264\_Mycgr3

Mycgr3G37570 Mycgr3T
  
Location: 5490-6006

Mycgr3G37570\_Mycgr3T

Mycgr3G108094 Mycgr3
  
Location: 6106-10555

Mycgr3G108094\_Mycgr3

Mycgr3G90786 Mycgr3T
  
Location: 10655-12080

Mycgr3G90786\_Mycgr3T

Mycgr3G68429 Mycgr3T
  
Location: 12180-13440

Mycgr3G68429\_Mycgr3T

Mycgr3G68421 Mycgr3T
  
Location: 13540-17086

Mycgr3G68421\_Mycgr3T

Mycgr3G90801 Mycgr3T
  
Location: 17186-18056

Mycgr3G90801\_Mycgr3T

Mycgr3G84646 Mycgr3T
  
Location: 18156-20235

Mycgr3G84646\_Mycgr3T

Mycgr3G68456 Mycgr3T
  
Location: 20335-21970

Mycgr3G68456\_Mycgr3T

Mycgr3G103270 Mycgr3
  
Location: 22070-22355

Mycgr3G103270\_Mycgr3

Mycgr3G90803 Mycgr3T
  
Location: 22455-23019

Mycgr3G90803\_Mycgr3T

Mycgr3G36941 Mycgr3T
  
Location: 23119-24064

Mycgr3G36941\_Mycgr3T

Mycgr3G25746 Mycgr3T
  
Location: 24164-25241

Mycgr3G25746\_Mycgr3T

Mycgr3G90788 Mycgr3T
  
Location: 25341-25803

Mycgr3G90788\_Mycgr3T

Mycgr3G103260 Mycgr3
  
Location: 25903-26635

Mycgr3G103260\_Mycgr3

Mycgr3G84644 Mycgr3T
  
Location: 26735-28457

Mycgr3G84644\_Mycgr3T

Mycgr3G29227 Mycgr3T
  
Location: 28557-28863

Mycgr3G29227\_Mycgr3T

Mycgr3G36271 Mycgr3T
  
Location: 28963-29854

Mycgr3G36271\_Mycgr3T

Mycgr3G68433 Mycgr3T
  
Location: 29954-33041

Mycgr3G68433\_Mycgr3T

Mycgr3G79452 Mycgr3T
  
Location: 33141-33399

Mycgr3G79452\_Mycgr3T

Mycgr3G55345 Mycgr3T
  
Location: 33499-34126

Mycgr3G55345\_Mycgr3T

Mycgr3G103278 Mycgr3
  
Location: 34226-35195

Mycgr3G103278\_Mycgr3

Mycgr3G84654 Mycgr3T
  
Location: 35295-36630

Mycgr3G84654\_Mycgr3T

Mycgr3G108090 Mycgr3
  
Location: 36730-37591

Mycgr3G108090\_Mycgr3

Mycgr3G21922 Mycgr3T
  
Location: 37691-39149

Mycgr3G21922\_Mycgr3T

Mycgr3G99148 Mycgr3T
  
Location: 39249-42819

Mycgr3G99148\_Mycgr3T

conserved hypothetical protein
  
Accession: EEQ45254
  
Location: 1187802-1188383
  
 NCBI BlastP on this gene

EEQ45254

hypothetical protein
  
Accession: EEQ45255
  
Location: 1189146-1190057
  
 NCBI BlastP on this gene

EEQ45255

conserved hypothetical protein
  
Accession: EEQ45256
  
Location: 1190917-1193334
  
 NCBI BlastP on this gene

EEQ45256

conserved hypothetical protein
  
Accession: EEQ45257
  
Location: 1193391-1194185
  
 NCBI BlastP on this gene

EEQ45257

conserved hypothetical protein
  
Accession: EEQ45258
  
Location: 1194473-1194913
  
 NCBI BlastP on this gene

EEQ45258

hypothetical protein
  
Accession: EEQ45259
  
Location: 1195297-1196946
  
 NCBI BlastP on this gene

EEQ45259

conserved hypothetical protein
  
Accession: EEQ45260
  
Location: 1197509-1200001
  
 NCBI BlastP on this gene

EEQ45260

conserved hypothetical protein
  
Accession: EEQ45261
  
Location: 1200287-1201957
  
 NCBI BlastP on this gene

EEQ45261

ABC1 family protein
  
Accession: EEQ45262
  
Location: 1202180-1204171
  
  
**BlastP hit with Mycgr3G68458\_Mycgr3T**
  
Percentage identity: 51 %
  
BlastP bit score: 556
  
Sequence coverage: 82 %
  
E-value: 0.0
  
  
 NCBI BlastP on this gene

EEQ45262

conserved hypothetical protein
  
Accession: EEQ45263
  
Location: 1205075-1208386
  
 NCBI BlastP on this gene

EEQ45263

mitochondrial import inner membrane translocase subunit TIM10
  
Accession: EEQ45264
  
Location: 1208616-1208891
  
 NCBI BlastP on this gene

EEQ45264

cytochrome C1 heme lyase
  
Accession: EEQ45265
  
Location: 1209026-1209781
  
 NCBI BlastP on this gene

EEQ45265

conserved hypothetical protein
  
Accession: EEQ45266
  
Location: 1210114-1211040
  
 NCBI BlastP on this gene

EEQ45266

conserved hypothetical protein
  
Accession: EEQ45267
  
Location: 1212078-1212611
  
 NCBI BlastP on this gene

EEQ45267

conserved hypothetical protein
  
Accession: EEQ45268
  
Location: 1212819-1214015
  
 NCBI BlastP on this gene

EEQ45268

exosome complex exonuclease RRP41
  
Accession: EEQ45269
  
Location: 1214203-1214925
  
 NCBI BlastP on this gene

EEQ45269

hypothetical protein
  
Accession: EEQ45270
  
Location: 1215005-1215934
  
 NCBI BlastP on this gene

EEQ45270

hypothetical protein
  
Accession: EEQ45271
  
Location: 1216261-1217631
  
 NCBI BlastP on this gene

EEQ45271

conserved hypothetical protein
  
Accession: EEQ45272
  
Location: 1218505-1220331
  
  
**BlastP hit with Mycgr3G103278\_Mycgr3**
  
Percentage identity: 40 %
  
BlastP bit score: 84
  
Sequence coverage: 26 %
  
E-value: 8e-15
  
  
 NCBI BlastP on this gene

EEQ45272

protein HGH1
  
Accession: EEQ45273
  
Location: 1220446-1221534
  
  
**BlastP hit with Mycgr3G25746\_Mycgr3T**
  
Percentage identity: 43 %
  
BlastP bit score: 279
  
Sequence coverage: 100 %
  
E-value: 2e-87
  
  
 NCBI BlastP on this gene

EEQ45273

conserved hypothetical protein
  
Accession: EEQ45274
  
Location: 1221666-1223099
  
 NCBI BlastP on this gene

EEQ45274

carnitine O-acetyltransferase, mitochondrial precursor
  
Accession: EEQ45275
  
Location: 1232041-1233939
  
 NCBI BlastP on this gene

EEQ45275

hypothetical protein
  
Accession: EEQ45276
  
Location: 1235576-1237210
  
 NCBI BlastP on this gene

EEQ45276

Query: Architecture Search FASTA input

AAVQ01000001 : Pichia stipitis CBS 6054 chromosome 1    Total score: 3.0     Cumulative Blast bit score: 905

Hit cluster cross-links:

Mycgr3G90785 Mycgr3T
  
Location: 0-1047

Mycgr3G90785\_Mycgr3T

Mycgr3G103262 Mycgr3
  
Location: 1147-1390

Mycgr3G103262\_Mycgr3

Mycgr3G68458 Mycgr3T
  
Location: 1490-3602

Mycgr3G68458\_Mycgr3T

Mycgr3G99145 Mycgr3T
  
Location: 3702-4326

Mycgr3G99145\_Mycgr3T

Mycgr3G103274 Mycgr3
  
Location: 4426-4957

Mycgr3G103274\_Mycgr3

Mycgr3G103264 Mycgr3
  
Location: 5057-5390

Mycgr3G103264\_Mycgr3

Mycgr3G37570 Mycgr3T
  
Location: 5490-6006

Mycgr3G37570\_Mycgr3T

Mycgr3G108094 Mycgr3
  
Location: 6106-10555

Mycgr3G108094\_Mycgr3

Mycgr3G90786 Mycgr3T
  
Location: 10655-12080

Mycgr3G90786\_Mycgr3T

Mycgr3G68429 Mycgr3T
  
Location: 12180-13440

Mycgr3G68429\_Mycgr3T

Mycgr3G68421 Mycgr3T
  
Location: 13540-17086

Mycgr3G68421\_Mycgr3T

Mycgr3G90801 Mycgr3T
  
Location: 17186-18056

Mycgr3G90801\_Mycgr3T

Mycgr3G84646 Mycgr3T
  
Location: 18156-20235

Mycgr3G84646\_Mycgr3T

Mycgr3G68456 Mycgr3T
  
Location: 20335-21970

Mycgr3G68456\_Mycgr3T

Mycgr3G103270 Mycgr3
  
Location: 22070-22355

Mycgr3G103270\_Mycgr3

Mycgr3G90803 Mycgr3T
  
Location: 22455-23019

Mycgr3G90803\_Mycgr3T

Mycgr3G36941 Mycgr3T
  
Location: 23119-24064

Mycgr3G36941\_Mycgr3T

Mycgr3G25746 Mycgr3T
  
Location: 24164-25241

Mycgr3G25746\_Mycgr3T

Mycgr3G90788 Mycgr3T
  
Location: 25341-25803

Mycgr3G90788\_Mycgr3T

Mycgr3G103260 Mycgr3
  
Location: 25903-26635

Mycgr3G103260\_Mycgr3

Mycgr3G84644 Mycgr3T
  
Location: 26735-28457

Mycgr3G84644\_Mycgr3T

Mycgr3G29227 Mycgr3T
  
Location: 28557-28863

Mycgr3G29227\_Mycgr3T

Mycgr3G36271 Mycgr3T
  
Location: 28963-29854

Mycgr3G36271\_Mycgr3T

Mycgr3G68433 Mycgr3T
  
Location: 29954-33041

Mycgr3G68433\_Mycgr3T

Mycgr3G79452 Mycgr3T
  
Location: 33141-33399

Mycgr3G79452\_Mycgr3T

Mycgr3G55345 Mycgr3T
  
Location: 33499-34126

Mycgr3G55345\_Mycgr3T

Mycgr3G103278 Mycgr3
  
Location: 34226-35195

Mycgr3G103278\_Mycgr3

Mycgr3G84654 Mycgr3T
  
Location: 35295-36630

Mycgr3G84654\_Mycgr3T

Mycgr3G108090 Mycgr3
  
Location: 36730-37591

Mycgr3G108090\_Mycgr3

Mycgr3G21922 Mycgr3T
  
Location: 37691-39149

Mycgr3G21922\_Mycgr3T

Mycgr3G99148 Mycgr3T
  
Location: 39249-42819

Mycgr3G99148\_Mycgr3T

hyphal wall protein (putative)
  
Accession: EAZ63557
  
Location: 1807332-1808774
  
 NCBI BlastP on this gene

EAZ63557

predicted protein
  
Accession: EAZ63959
  
Location: 1805837-1806836
  
 NCBI BlastP on this gene

EAZ63959

predicted protein
  
Accession: EAZ63958
  
Location: 1804602-1805240
  
 NCBI BlastP on this gene

EAZ63958

predicted protein
  
Accession: EAZ63556
  
Location: 1803498-1804226
  
 NCBI BlastP on this gene

EAZ63556

predicted protein
  
Accession: EAZ63555
  
Location: 1802132-1802422
  
 NCBI BlastP on this gene

EAZ63555

predicted protein
  
Accession: EAZ63957
  
Location: 1799893-1801578
  
 NCBI BlastP on this gene

EAZ63957

predicted protein
  
Accession: EAZ63956
  
Location: 1797482-1799425
  
  
**BlastP hit with Mycgr3G68458\_Mycgr3T**
  
Percentage identity: 47 %
  
BlastP bit score: 543
  
Sequence coverage: 93 %
  
E-value: 0.0
  
  
 NCBI BlastP on this gene

EAZ63956

predicted protein
  
Accession: EAZ63955
  
Location: 1791373-1791648
  
 NCBI BlastP on this gene

EAZ63955

cytochrome c1 heme lyase
  
Accession: EAZ63554
  
Location: 1790112-1790915
  
 NCBI BlastP on this gene

EAZ63554

predicted protein
  
Accession: EAZ63553
  
Location: 1788618-1789538
  
 NCBI BlastP on this gene

EAZ63553

predicted protein
  
Accession: EAZ63552
  
Location: 1779134-1779859
  
 NCBI BlastP on this gene

EAZ63552

mannosyltransferase
  
Accession: EAZ63551
  
Location: 1777613-1778803
  
 NCBI BlastP on this gene

EAZ63551

predicted protein
  
Accession: EAZ63954
  
Location: 1776324-1776932
  
 NCBI BlastP on this gene

EAZ63954

mitochondrial carrier protein
  
Accession: EAZ63550
  
Location: 1775207-1776130
  
 NCBI BlastP on this gene

EAZ63550

predicted protein
  
Accession: EAZ63953
  
Location: 1772821-1774638
  
  
**BlastP hit with Mycgr3G103278\_Mycgr3**
  
Percentage identity: 32 %
  
BlastP bit score: 90
  
Sequence coverage: 90 %
  
E-value: 4e-17
  
  
 NCBI BlastP on this gene

EAZ63953

predicted protein
  
Accession: EAZ63549
  
Location: 1771522-1772607
  
  
**BlastP hit with Mycgr3G25746\_Mycgr3T**
  
Percentage identity: 42 %
  
BlastP bit score: 272
  
Sequence coverage: 100 %
  
E-value: 9e-85
  
  
 NCBI BlastP on this gene

EAZ63549

predicted protein
  
Accession: EAZ63548
  
Location: 1769322-1771124
  
 NCBI BlastP on this gene

EAZ63548

predicted protein
  
Accession: EAZ63547
  
Location: 1768206-1768919
  
 NCBI BlastP on this gene

EAZ63547

pyruvate decarboxylase
  
Accession: EAZ63546
  
Location: 1765968-1767758
  
 NCBI BlastP on this gene

EAZ63546

predicted protein
  
Accession: EAZ63545
  
Location: 1763148-1764149
  
 NCBI BlastP on this gene

EAZ63545

Phosphomevalonate kinase
  
Accession: EAZ63544
  
Location: 1760839-1762236
  
 NCBI BlastP on this gene

EAZ63544

Query: Architecture Search FASTA input

CH408081 : Clavispora lusitaniae ATCC 42720 scaffold\_6 genomic scaffold    Total score: 3.0     Cumulative Blast bit score: 889

Hit cluster cross-links:

Mycgr3G90785 Mycgr3T
  
Location: 0-1047

Mycgr3G90785\_Mycgr3T

Mycgr3G103262 Mycgr3
  
Location: 1147-1390

Mycgr3G103262\_Mycgr3

Mycgr3G68458 Mycgr3T
  
Location: 1490-3602

Mycgr3G68458\_Mycgr3T

Mycgr3G99145 Mycgr3T
  
Location: 3702-4326

Mycgr3G99145\_Mycgr3T

Mycgr3G103274 Mycgr3
  
Location: 4426-4957

Mycgr3G103274\_Mycgr3

Mycgr3G103264 Mycgr3
  
Location: 5057-5390

Mycgr3G103264\_Mycgr3

Mycgr3G37570 Mycgr3T
  
Location: 5490-6006

Mycgr3G37570\_Mycgr3T

Mycgr3G108094 Mycgr3
  
Location: 6106-10555

Mycgr3G108094\_Mycgr3

Mycgr3G90786 Mycgr3T
  
Location: 10655-12080

Mycgr3G90786\_Mycgr3T

Mycgr3G68429 Mycgr3T
  
Location: 12180-13440

Mycgr3G68429\_Mycgr3T

Mycgr3G68421 Mycgr3T
  
Location: 13540-17086

Mycgr3G68421\_Mycgr3T

Mycgr3G90801 Mycgr3T
  
Location: 17186-18056

Mycgr3G90801\_Mycgr3T

Mycgr3G84646 Mycgr3T
  
Location: 18156-20235

Mycgr3G84646\_Mycgr3T

Mycgr3G68456 Mycgr3T
  
Location: 20335-21970

Mycgr3G68456\_Mycgr3T

Mycgr3G103270 Mycgr3
  
Location: 22070-22355

Mycgr3G103270\_Mycgr3

Mycgr3G90803 Mycgr3T
  
Location: 22455-23019

Mycgr3G90803\_Mycgr3T

Mycgr3G36941 Mycgr3T
  
Location: 23119-24064

Mycgr3G36941\_Mycgr3T

Mycgr3G25746 Mycgr3T
  
Location: 24164-25241

Mycgr3G25746\_Mycgr3T

Mycgr3G90788 Mycgr3T
  
Location: 25341-25803

Mycgr3G90788\_Mycgr3T

Mycgr3G103260 Mycgr3
  
Location: 25903-26635

Mycgr3G103260\_Mycgr3

Mycgr3G84644 Mycgr3T
  
Location: 26735-28457

Mycgr3G84644\_Mycgr3T

Mycgr3G29227 Mycgr3T
  
Location: 28557-28863

Mycgr3G29227\_Mycgr3T

Mycgr3G36271 Mycgr3T
  
Location: 28963-29854

Mycgr3G36271\_Mycgr3T

Mycgr3G68433 Mycgr3T
  
Location: 29954-33041

Mycgr3G68433\_Mycgr3T

Mycgr3G79452 Mycgr3T
  
Location: 33141-33399

Mycgr3G79452\_Mycgr3T

Mycgr3G55345 Mycgr3T
  
Location: 33499-34126

Mycgr3G55345\_Mycgr3T

Mycgr3G103278 Mycgr3
  
Location: 34226-35195

Mycgr3G103278\_Mycgr3

Mycgr3G84654 Mycgr3T
  
Location: 35295-36630

Mycgr3G84654\_Mycgr3T

Mycgr3G108090 Mycgr3
  
Location: 36730-37591

Mycgr3G108090\_Mycgr3

Mycgr3G21922 Mycgr3T
  
Location: 37691-39149

Mycgr3G21922\_Mycgr3T

Mycgr3G99148 Mycgr3T
  
Location: 39249-42819

Mycgr3G99148\_Mycgr3T

predicted protein
  
Accession: EEQ40867
  
Location: 346796-348211
  
 NCBI BlastP on this gene

EEQ40867

predicted protein
  
Accession: EEQ40868
  
Location: 346850-348178
  
 NCBI BlastP on this gene

EEQ40868

hypothetical protein
  
Accession: EEQ40869
  
Location: 349167-350123
  
 NCBI BlastP on this gene

EEQ40869

hypothetical protein
  
Accession: EEQ40870
  
Location: 350909-351547
  
 NCBI BlastP on this gene

EEQ40870

hypothetical protein
  
Accession: EEQ40871
  
Location: 351053-351586
  
 NCBI BlastP on this gene

EEQ40871

conserved hypothetical protein
  
Accession: EEQ40872
  
Location: 352135-352860
  
 NCBI BlastP on this gene

EEQ40872

hypothetical protein
  
Accession: EEQ40873
  
Location: 353588-353863
  
 NCBI BlastP on this gene

EEQ40873

hypothetical protein
  
Accession: EEQ40874
  
Location: 355269-356939
  
 NCBI BlastP on this gene

EEQ40874

hypothetical protein
  
Accession: EEQ40875
  
Location: 357509-359584
  
  
**BlastP hit with Mycgr3G68458\_Mycgr3T**
  
Percentage identity: 48 %
  
BlastP bit score: 529
  
Sequence coverage: 83 %
  
E-value: 2e-175
  
  
 NCBI BlastP on this gene

EEQ40875

hypothetical protein
  
Accession: EEQ40876
  
Location: 361069-361779
  
 NCBI BlastP on this gene

EEQ40876

hypothetical protein
  
Accession: EEQ40877
  
Location: 363279-363998
  
 NCBI BlastP on this gene

EEQ40877

hypothetical protein
  
Accession: EEQ40878
  
Location: 365044-365811
  
 NCBI BlastP on this gene

EEQ40878

predicted protein
  
Accession: EEQ40879
  
Location: 366831-368375
  
 NCBI BlastP on this gene

EEQ40879

predicted protein
  
Accession: EEQ40880
  
Location: 369526-371013
  
 NCBI BlastP on this gene

EEQ40880

predicted protein
  
Accession: EEQ40881
  
Location: 372102-373535
  
 NCBI BlastP on this gene

EEQ40881

hypothetical protein
  
Accession: EEQ40882
  
Location: 374855-376708
  
 NCBI BlastP on this gene

EEQ40882

hypothetical protein
  
Accession: EEQ40883
  
Location: 377280-378374
  
  
**BlastP hit with Mycgr3G25746\_Mycgr3T**
  
Percentage identity: 42 %
  
BlastP bit score: 271
  
Sequence coverage: 101 %
  
E-value: 2e-84
  
  
 NCBI BlastP on this gene

EEQ40883

hypothetical protein
  
Accession: EEQ40884
  
Location: 379144-380805
  
  
**BlastP hit with Mycgr3G103278\_Mycgr3**
  
Percentage identity: 48 %
  
BlastP bit score: 89
  
Sequence coverage: 26 %
  
E-value: 1e-16
  
  
 NCBI BlastP on this gene

EEQ40884

hypothetical protein
  
Accession: EEQ40886
  
Location: 383182-384135
  
 NCBI BlastP on this gene

EEQ40886

hypothetical protein
  
Accession: EEQ40885
  
Location: 383224-384090
  
 NCBI BlastP on this gene

EEQ40885

hypothetical protein
  
Accession: EEQ40888
  
Location: 384506-385042
  
 NCBI BlastP on this gene

EEQ40888

hypothetical protein
  
Accession: EEQ40887
  
Location: 384740-385207
  
 NCBI BlastP on this gene

EEQ40887

hypothetical protein
  
Accession: EEQ40889
  
Location: 385315-386562
  
 NCBI BlastP on this gene

EEQ40889

predicted protein
  
Accession: EEQ40890
  
Location: 387070-387789
  
 NCBI BlastP on this gene

EEQ40890

hypothetical protein
  
Accession: EEQ40891
  
Location: 388932-389666
  
 NCBI BlastP on this gene

EEQ40891

hypothetical protein
  
Accession: EEQ40892
  
Location: 391518-392435
  
 NCBI BlastP on this gene

EEQ40892

hypothetical protein
  
Accession: EEQ40893
  
Location: 392998-393717
  
 NCBI BlastP on this gene

EEQ40893

Query: Architecture Search FASTA input

CH408157 : Pichia guilliermondii ATCC 6260 scaffold\_3 genomic scaffold    Total score: 3.0     Cumulative Blast bit score: 886

Hit cluster cross-links:

Mycgr3G90785 Mycgr3T
  
Location: 0-1047

Mycgr3G90785\_Mycgr3T

Mycgr3G103262 Mycgr3
  
Location: 1147-1390

Mycgr3G103262\_Mycgr3

Mycgr3G68458 Mycgr3T
  
Location: 1490-3602

Mycgr3G68458\_Mycgr3T

Mycgr3G99145 Mycgr3T
  
Location: 3702-4326

Mycgr3G99145\_Mycgr3T

Mycgr3G103274 Mycgr3
  
Location: 4426-4957

Mycgr3G103274\_Mycgr3

Mycgr3G103264 Mycgr3
  
Location: 5057-5390

Mycgr3G103264\_Mycgr3

Mycgr3G37570 Mycgr3T
  
Location: 5490-6006

Mycgr3G37570\_Mycgr3T

Mycgr3G108094 Mycgr3
  
Location: 6106-10555

Mycgr3G108094\_Mycgr3

Mycgr3G90786 Mycgr3T
  
Location: 10655-12080

Mycgr3G90786\_Mycgr3T

Mycgr3G68429 Mycgr3T
  
Location: 12180-13440

Mycgr3G68429\_Mycgr3T

Mycgr3G68421 Mycgr3T
  
Location: 13540-17086

Mycgr3G68421\_Mycgr3T

Mycgr3G90801 Mycgr3T
  
Location: 17186-18056

Mycgr3G90801\_Mycgr3T

Mycgr3G84646 Mycgr3T
  
Location: 18156-20235

Mycgr3G84646\_Mycgr3T

Mycgr3G68456 Mycgr3T
  
Location: 20335-21970

Mycgr3G68456\_Mycgr3T

Mycgr3G103270 Mycgr3
  
Location: 22070-22355

Mycgr3G103270\_Mycgr3

Mycgr3G90803 Mycgr3T
  
Location: 22455-23019

Mycgr3G90803\_Mycgr3T

Mycgr3G36941 Mycgr3T
  
Location: 23119-24064

Mycgr3G36941\_Mycgr3T

Mycgr3G25746 Mycgr3T
  
Location: 24164-25241

Mycgr3G25746\_Mycgr3T

Mycgr3G90788 Mycgr3T
  
Location: 25341-25803

Mycgr3G90788\_Mycgr3T

Mycgr3G103260 Mycgr3
  
Location: 25903-26635

Mycgr3G103260\_Mycgr3

Mycgr3G84644 Mycgr3T
  
Location: 26735-28457

Mycgr3G84644\_Mycgr3T

Mycgr3G29227 Mycgr3T
  
Location: 28557-28863

Mycgr3G29227\_Mycgr3T

Mycgr3G36271 Mycgr3T
  
Location: 28963-29854

Mycgr3G36271\_Mycgr3T

Mycgr3G68433 Mycgr3T
  
Location: 29954-33041

Mycgr3G68433\_Mycgr3T

Mycgr3G79452 Mycgr3T
  
Location: 33141-33399

Mycgr3G79452\_Mycgr3T

Mycgr3G55345 Mycgr3T
  
Location: 33499-34126

Mycgr3G55345\_Mycgr3T

Mycgr3G103278 Mycgr3
  
Location: 34226-35195

Mycgr3G103278\_Mycgr3

Mycgr3G84654 Mycgr3T
  
Location: 35295-36630

Mycgr3G84654\_Mycgr3T

Mycgr3G108090 Mycgr3
  
Location: 36730-37591

Mycgr3G108090\_Mycgr3

Mycgr3G21922 Mycgr3T
  
Location: 37691-39149

Mycgr3G21922\_Mycgr3T

Mycgr3G99148 Mycgr3T
  
Location: 39249-42819

Mycgr3G99148\_Mycgr3T

hypothetical protein
  
Accession: EDK38328
  
Location: 222910-224205
  
 NCBI BlastP on this gene

EDK38328

hypothetical protein
  
Accession: EDK38327
  
Location: 221087-222892
  
 NCBI BlastP on this gene

EDK38327

hypothetical protein
  
Accession: EDK38326
  
Location: 219436-221079
  
 NCBI BlastP on this gene

EDK38326

hypothetical protein
  
Accession: EDK38325
  
Location: 217998-219398
  
 NCBI BlastP on this gene

EDK38325

conserved hypothetical protein
  
Accession: EDK38324
  
Location: 216469-217512
  
 NCBI BlastP on this gene

EDK38324

conserved hypothetical protein
  
Accession: EDK38322
  
Location: 215234-215899
  
 NCBI BlastP on this gene

EDK38322

conserved hypothetical protein
  
Accession: EDK38323
  
Location: 215081-215890
  
 NCBI BlastP on this gene

EDK38323

conserved hypothetical protein
  
Accession: EDK38321
  
Location: 213836-214882
  
 NCBI BlastP on this gene

EDK38321

hypothetical protein
  
Accession: EDK38320
  
Location: 211150-213693
  
 NCBI BlastP on this gene

EDK38320

hypothetical protein
  
Accession: EDK38319
  
Location: 207545-209083
  
 NCBI BlastP on this gene

EDK38319

hypothetical protein
  
Accession: EDK38318
  
Location: 205287-207464
  
  
**BlastP hit with Mycgr3G68458\_Mycgr3T**
  
Percentage identity: 49 %
  
BlastP bit score: 555
  
Sequence coverage: 85 %
  
E-value: 0.0
  
  
 NCBI BlastP on this gene

EDK38318

hypothetical protein
  
Accession: EDK38317
  
Location: 201944-204889
  
 NCBI BlastP on this gene

EDK38317

mitochondrial import inner membrane translocase subunit TIM10
  
Accession: EDK38316
  
Location: 201560-201910
  
 NCBI BlastP on this gene

EDK38316

hypothetical protein
  
Accession: EDK38315
  
Location: 199728-200657
  
 NCBI BlastP on this gene

EDK38315

hypothetical protein
  
Accession: EDK38314
  
Location: 198448-199176
  
 NCBI BlastP on this gene

EDK38314

hypothetical protein
  
Accession: EDK38313
  
Location: 197122-198375
  
 NCBI BlastP on this gene

EDK38313

hypothetical protein
  
Accession: EDK38312
  
Location: 196553-197050
  
 NCBI BlastP on this gene

EDK38312

hypothetical protein
  
Accession: EDK38311
  
Location: 195619-196527
  
 NCBI BlastP on this gene

EDK38311

hypothetical protein
  
Accession: EDK38310
  
Location: 193515-195113
  
  
**BlastP hit with Mycgr3G103278\_Mycgr3**
  
Percentage identity: 41 %
  
BlastP bit score: 84
  
Sequence coverage: 26 %
  
E-value: 7e-15
  
  
 NCBI BlastP on this gene

EDK38310

hypothetical protein
  
Accession: EDK38309
  
Location: 192372-193313
  
  
**BlastP hit with Mycgr3G25746\_Mycgr3T**
  
Percentage identity: 42 %
  
BlastP bit score: 247
  
Sequence coverage: 90 %
  
E-value: 2e-75
  
  
 NCBI BlastP on this gene

EDK38309

hypothetical protein
  
Accession: EDK38308
  
Location: 190423-192225
  
 NCBI BlastP on this gene

EDK38308

hypothetical protein
  
Accession: EDK38307
  
Location: 189128-190294
  
 NCBI BlastP on this gene

EDK38307

hypothetical protein
  
Accession: EDK38305
  
Location: 188105-188938
  
 NCBI BlastP on this gene

EDK38305

hypothetical protein
  
Accession: EDK38306
  
Location: 187654-188148
  
 NCBI BlastP on this gene

EDK38306

hypothetical protein
  
Accession: EDK38304
  
Location: 187504-188247
  
 NCBI BlastP on this gene

EDK38304

hypothetical protein
  
Accession: EDK38302
  
Location: 186168-186455
  
 NCBI BlastP on this gene

EDK38302

hypothetical protein
  
Accession: EDK38303
  
Location: 186134-187450
  
 NCBI BlastP on this gene

EDK38303

hypothetical protein
  
Accession: EDK38301
  
Location: 185484-186041
  
 NCBI BlastP on this gene

EDK38301

hypothetical protein
  
Accession: EDK38300
  
Location: 184569-185174
  
 NCBI BlastP on this gene

EDK38300

hypothetical protein
  
Accession: EDK38299
  
Location: 183075-184514
  
 NCBI BlastP on this gene

EDK38299

hypothetical protein
  
Accession: EDK38298
  
Location: 182248-183009
  
 NCBI BlastP on this gene

EDK38298

hypothetical protein
  
Accession: EDK38297
  
Location: 180121-181335
  
 NCBI BlastP on this gene

EDK38297

hypothetical protein
  
Accession: EDK38296
  
Location: 177866-179890
  
 NCBI BlastP on this gene

EDK38296

hypothetical protein
  
Accession: EDK38295
  
Location: 174622-177816
  
 NCBI BlastP on this gene

EDK38295

Query: Architecture Search FASTA input

KE145361 : Glarea lozoyensis ATCC 20868 chromosome Unknown GLAREA18    Total score: 3.0     Cumulative Blast bit score: 868

Hit cluster cross-links:

Mycgr3G90785 Mycgr3T
  
Location: 0-1047

Mycgr3G90785\_Mycgr3T

Mycgr3G103262 Mycgr3
  
Location: 1147-1390

Mycgr3G103262\_Mycgr3

Mycgr3G68458 Mycgr3T
  
Location: 1490-3602

Mycgr3G68458\_Mycgr3T

Mycgr3G99145 Mycgr3T
  
Location: 3702-4326

Mycgr3G99145\_Mycgr3T

Mycgr3G103274 Mycgr3
  
Location: 4426-4957

Mycgr3G103274\_Mycgr3

Mycgr3G103264 Mycgr3
  
Location: 5057-5390

Mycgr3G103264\_Mycgr3

Mycgr3G37570 Mycgr3T
  
Location: 5490-6006

Mycgr3G37570\_Mycgr3T

Mycgr3G108094 Mycgr3
  
Location: 6106-10555

Mycgr3G108094\_Mycgr3

Mycgr3G90786 Mycgr3T
  
Location: 10655-12080

Mycgr3G90786\_Mycgr3T

Mycgr3G68429 Mycgr3T
  
Location: 12180-13440

Mycgr3G68429\_Mycgr3T

Mycgr3G68421 Mycgr3T
  
Location: 13540-17086

Mycgr3G68421\_Mycgr3T

Mycgr3G90801 Mycgr3T
  
Location: 17186-18056

Mycgr3G90801\_Mycgr3T

Mycgr3G84646 Mycgr3T
  
Location: 18156-20235

Mycgr3G84646\_Mycgr3T

Mycgr3G68456 Mycgr3T
  
Location: 20335-21970

Mycgr3G68456\_Mycgr3T

Mycgr3G103270 Mycgr3
  
Location: 22070-22355

Mycgr3G103270\_Mycgr3

Mycgr3G90803 Mycgr3T
  
Location: 22455-23019

Mycgr3G90803\_Mycgr3T

Mycgr3G36941 Mycgr3T
  
Location: 23119-24064

Mycgr3G36941\_Mycgr3T

Mycgr3G25746 Mycgr3T
  
Location: 24164-25241

Mycgr3G25746\_Mycgr3T

Mycgr3G90788 Mycgr3T
  
Location: 25341-25803

Mycgr3G90788\_Mycgr3T

Mycgr3G103260 Mycgr3
  
Location: 25903-26635

Mycgr3G103260\_Mycgr3

Mycgr3G84644 Mycgr3T
  
Location: 26735-28457

Mycgr3G84644\_Mycgr3T

Mycgr3G29227 Mycgr3T
  
Location: 28557-28863

Mycgr3G29227\_Mycgr3T

Mycgr3G36271 Mycgr3T
  
Location: 28963-29854

Mycgr3G36271\_Mycgr3T

Mycgr3G68433 Mycgr3T
  
Location: 29954-33041

Mycgr3G68433\_Mycgr3T

Mycgr3G79452 Mycgr3T
  
Location: 33141-33399

Mycgr3G79452\_Mycgr3T

Mycgr3G55345 Mycgr3T
  
Location: 33499-34126

Mycgr3G55345\_Mycgr3T

Mycgr3G103278 Mycgr3
  
Location: 34226-35195

Mycgr3G103278\_Mycgr3

Mycgr3G84654 Mycgr3T
  
Location: 35295-36630

Mycgr3G84654\_Mycgr3T

Mycgr3G108090 Mycgr3
  
Location: 36730-37591

Mycgr3G108090\_Mycgr3

Mycgr3G21922 Mycgr3T
  
Location: 37691-39149

Mycgr3G21922\_Mycgr3T

Mycgr3G99148 Mycgr3T
  
Location: 39249-42819

Mycgr3G99148\_Mycgr3T

hypothetical protein
  
Accession: EPE31621
  
Location: 431036-433040
  
 NCBI BlastP on this gene

EPE31621

hypothetical protein
  
Accession: EPE31622
  
Location: 434309-435860
  
 NCBI BlastP on this gene

EPE31622

Thioesterase/thiol ester dehydrase-isomerase
  
Accession: EPE31623
  
Location: 436130-437285
  
 NCBI BlastP on this gene

EPE31623

MFS general substrate transporter
  
Accession: EPE31624
  
Location: 438738-440689
  
 NCBI BlastP on this gene

EPE31624

hypothetical protein
  
Accession: EPE31625
  
Location: 442023-442718
  
 NCBI BlastP on this gene

EPE31625

hypothetical protein
  
Accession: EPE31626
  
Location: 443951-445071
  
 NCBI BlastP on this gene

EPE31626

ARM repeat-containing protein
  
Accession: EPE31627
  
Location: 445719-448231
  
 NCBI BlastP on this gene

EPE31627

hypothetical protein
  
Accession: EPE31628
  
Location: 449652-451163
  
  
**BlastP hit with Mycgr3G90786\_Mycgr3T**
  
Percentage identity: 29 %
  
BlastP bit score: 95
  
Sequence coverage: 110 %
  
E-value: 5e-18
  
  
 NCBI BlastP on this gene

EPE31628

L
  
Accession: EPE31629
  
Location: 452227-455505
  
  
**BlastP hit with Mycgr3G68433\_Mycgr3T**
  
Percentage identity: 35 %
  
BlastP bit score: 465
  
Sequence coverage: 107 %
  
E-value: 3e-142
  
  
 NCBI BlastP on this gene

EPE31629

hypothetical protein
  
Accession: EPE31630
  
Location: 455847-457618
  
 NCBI BlastP on this gene

EPE31630

Metallo-hydrolase/oxidoreductase
  
Accession: EPE31631
  
Location: 457953-459035
  
 NCBI BlastP on this gene

EPE31631

PRTase-like protein
  
Accession: EPE31632
  
Location: 459722-460505
  
  
**BlastP hit with Mycgr3G55345\_Mycgr3T**
  
Percentage identity: 73 %
  
BlastP bit score: 308
  
Sequence coverage: 100 %
  
E-value: 4e-103
  
  
 NCBI BlastP on this gene

EPE31632

NAD(P)-binding Rossmann-fold containing protein
  
Accession: EPE31633
  
Location: 460890-462232
  
 NCBI BlastP on this gene

EPE31633

vegetative cell wall protein gp1
  
Accession: EPE31634
  
Location: 472528-473697
  
 NCBI BlastP on this gene

EPE31634

S-adenosyl-L-methionine-dependent methyltransferase
  
Accession: EPE31635
  
Location: 476541-477654
  
 NCBI BlastP on this gene

EPE31635

EF-hand
  
Accession: EPE31636
  
Location: 478187-480929
  
 NCBI BlastP on this gene

EPE31636

Query: Architecture Search FASTA input

GL996524 : Candida tenuis ATCC 10573 unplaced genomic scaffold CANTEscaffold\_00018    Total score: 3.0     Cumulative Blast bit score: 839

Hit cluster cross-links:

Mycgr3G90785 Mycgr3T
  
Location: 0-1047

Mycgr3G90785\_Mycgr3T

Mycgr3G103262 Mycgr3
  
Location: 1147-1390

Mycgr3G103262\_Mycgr3

Mycgr3G68458 Mycgr3T
  
Location: 1490-3602

Mycgr3G68458\_Mycgr3T

Mycgr3G99145 Mycgr3T
  
Location: 3702-4326

Mycgr3G99145\_Mycgr3T

Mycgr3G103274 Mycgr3
  
Location: 4426-4957

Mycgr3G103274\_Mycgr3

Mycgr3G103264 Mycgr3
  
Location: 5057-5390

Mycgr3G103264\_Mycgr3

Mycgr3G37570 Mycgr3T
  
Location: 5490-6006

Mycgr3G37570\_Mycgr3T

Mycgr3G108094 Mycgr3
  
Location: 6106-10555

Mycgr3G108094\_Mycgr3

Mycgr3G90786 Mycgr3T
  
Location: 10655-12080

Mycgr3G90786\_Mycgr3T

Mycgr3G68429 Mycgr3T
  
Location: 12180-13440

Mycgr3G68429\_Mycgr3T

Mycgr3G68421 Mycgr3T
  
Location: 13540-17086

Mycgr3G68421\_Mycgr3T

Mycgr3G90801 Mycgr3T
  
Location: 17186-18056

Mycgr3G90801\_Mycgr3T

Mycgr3G84646 Mycgr3T
  
Location: 18156-20235

Mycgr3G84646\_Mycgr3T

Mycgr3G68456 Mycgr3T
  
Location: 20335-21970

Mycgr3G68456\_Mycgr3T

Mycgr3G103270 Mycgr3
  
Location: 22070-22355

Mycgr3G103270\_Mycgr3

Mycgr3G90803 Mycgr3T
  
Location: 22455-23019

Mycgr3G90803\_Mycgr3T

Mycgr3G36941 Mycgr3T
  
Location: 23119-24064

Mycgr3G36941\_Mycgr3T

Mycgr3G25746 Mycgr3T
  
Location: 24164-25241

Mycgr3G25746\_Mycgr3T

Mycgr3G90788 Mycgr3T
  
Location: 25341-25803

Mycgr3G90788\_Mycgr3T

Mycgr3G103260 Mycgr3
  
Location: 25903-26635

Mycgr3G103260\_Mycgr3

Mycgr3G84644 Mycgr3T
  
Location: 26735-28457

Mycgr3G84644\_Mycgr3T

Mycgr3G29227 Mycgr3T
  
Location: 28557-28863

Mycgr3G29227\_Mycgr3T

Mycgr3G36271 Mycgr3T
  
Location: 28963-29854

Mycgr3G36271\_Mycgr3T

Mycgr3G68433 Mycgr3T
  
Location: 29954-33041

Mycgr3G68433\_Mycgr3T

Mycgr3G79452 Mycgr3T
  
Location: 33141-33399

Mycgr3G79452\_Mycgr3T

Mycgr3G55345 Mycgr3T
  
Location: 33499-34126

Mycgr3G55345\_Mycgr3T

Mycgr3G103278 Mycgr3
  
Location: 34226-35195

Mycgr3G103278\_Mycgr3

Mycgr3G84654 Mycgr3T
  
Location: 35295-36630

Mycgr3G84654\_Mycgr3T

Mycgr3G108090 Mycgr3
  
Location: 36730-37591

Mycgr3G108090\_Mycgr3

Mycgr3G21922 Mycgr3T
  
Location: 37691-39149

Mycgr3G21922\_Mycgr3T

Mycgr3G99148 Mycgr3T
  
Location: 39249-42819

Mycgr3G99148\_Mycgr3T

hypothetical protein
  
Accession: EGV63419
  
Location: 945520-947079
  
 NCBI BlastP on this gene

EGV63419

kynurenine 3-monooxygenase mitochondrial precursor
  
Accession: EGV63661
  
Location: 947236-948600
  
 NCBI BlastP on this gene

EGV63661

Tim44-domain-containing protein
  
Accession: EGV63662
  
Location: 949312-950034
  
 NCBI BlastP on this gene

EGV63662

hypothetical protein
  
Accession: EGV63665
  
Location: 950158-951591
  
 NCBI BlastP on this gene

EGV63665

hypothetical protein
  
Accession: EGV63420
  
Location: 951858-952352
  
 NCBI BlastP on this gene

EGV63420

hypothetical protein
  
Accession: EGV63421
  
Location: 953971-958418
  
 NCBI BlastP on this gene

EGV63421

hypothetical protein
  
Accession: EGV63422
  
Location: 953971-955884
  
 NCBI BlastP on this gene

EGV63422

arabinose-proton symporter
  
Accession: EGV63423
  
Location: 956694-958418
  
 NCBI BlastP on this gene

EGV63423

hypothetical protein
  
Accession: EGV63424
  
Location: 958615-959313
  
 NCBI BlastP on this gene

EGV63424

hypothetical protein
  
Accession: EGV63426
  
Location: 959979-960207
  
 NCBI BlastP on this gene

EGV63426

hypothetical protein
  
Accession: EGV63427
  
Location: 960246-961439
  
 NCBI BlastP on this gene

EGV63427

ABC1-domain-containing protein
  
Accession: EGV63428
  
Location: 962509-964218
  
  
**BlastP hit with Mycgr3G68458\_Mycgr3T**
  
Percentage identity: 46 %
  
BlastP bit score: 483
  
Sequence coverage: 81 %
  
E-value: 6e-159
  
  
 NCBI BlastP on this gene

EGV63428

hypothetical protein
  
Accession: EGV63429
  
Location: 966144-967583
  
 NCBI BlastP on this gene

EGV63429

hypothetical protein
  
Accession: EGV63667
  
Location: 968038-968814
  
 NCBI BlastP on this gene

EGV63667

SURF4-domain-containing protein
  
Accession: EGV63431
  
Location: 968886-969764
  
 NCBI BlastP on this gene

EGV63431

ribosomal protein S5 domain 2-like protein
  
Accession: EGV63668
  
Location: 970024-970752
  
 NCBI BlastP on this gene

EGV63668

hypothetical protein
  
Accession: EGV63670
  
Location: 971211-972060
  
 NCBI BlastP on this gene

EGV63670

hypothetical protein
  
Accession: EGV63433
  
Location: 972166-972714
  
 NCBI BlastP on this gene

EGV63433

mitochondrial carrier
  
Accession: EGV63672
  
Location: 972749-973681
  
 NCBI BlastP on this gene

EGV63672

Rap30/74 interaction domain-containing protein
  
Accession: EGV63434
  
Location: 973980-975791
  
  
**BlastP hit with Mycgr3G103278\_Mycgr3**
  
Percentage identity: 45 %
  
BlastP bit score: 86
  
Sequence coverage: 26 %
  
E-value: 1e-15
  
  
 NCBI BlastP on this gene

EGV63434

hypothetical protein
  
Accession: EGV63675
  
Location: 975826-976929
  
  
**BlastP hit with Mycgr3G25746\_Mycgr3T**
  
Percentage identity: 42 %
  
BlastP bit score: 270
  
Sequence coverage: 101 %
  
E-value: 6e-84
  
  
 NCBI BlastP on this gene

EGV63675

hypothetical protein
  
Accession: EGV63435
  
Location: 976994-978730
  
 NCBI BlastP on this gene

EGV63435

dienelactone hydrolase
  
Accession: EGV63436
  
Location: 978864-979598
  
 NCBI BlastP on this gene

EGV63436

hypothetical protein
  
Accession: EGV63677
  
Location: 982754-983470
  
 NCBI BlastP on this gene

EGV63677

pyruvate decarboxylase
  
Accession: EGV63437
  
Location: 984030-985802
  
 NCBI BlastP on this gene

EGV63437

hypothetical protein
  
Accession: EGV63438
  
Location: 986489-987663
  
 NCBI BlastP on this gene

EGV63438

hypothetical protein
  
Accession: EGV63439
  
Location: 987808-989163
  
 NCBI BlastP on this gene

EGV63439

hypothetical protein
  
Accession: EGV63440
  
Location: 990632-991189
  
 NCBI BlastP on this gene

EGV63440

hypothetical protein
  
Accession: EGV63442
  
Location: 991425-992012
  
 NCBI BlastP on this gene

EGV63442

hypothetical protein
  
Accession: EGV63679
  
Location: 992100-993389
  
 NCBI BlastP on this gene

EGV63679

Shwachman-Bodian-diamond syndrome protein
  
Accession: EGV63443
  
Location: 993486-994247
  
 NCBI BlastP on this gene

EGV63443

Query: Architecture Search FASTA input

GG698897 : Nectria haematococca mpVI 77-13-4 chromosome 3 genomic scaffold NECHAsca\_2\_chr3\_3\_0    Total score: 3.0     Cumulative Blast bit score: 801

Hit cluster cross-links:

Mycgr3G90785 Mycgr3T
  
Location: 0-1047

Mycgr3G90785\_Mycgr3T

Mycgr3G103262 Mycgr3
  
Location: 1147-1390

Mycgr3G103262\_Mycgr3

Mycgr3G68458 Mycgr3T
  
Location: 1490-3602

Mycgr3G68458\_Mycgr3T

Mycgr3G99145 Mycgr3T
  
Location: 3702-4326

Mycgr3G99145\_Mycgr3T

Mycgr3G103274 Mycgr3
  
Location: 4426-4957

Mycgr3G103274\_Mycgr3

Mycgr3G103264 Mycgr3
  
Location: 5057-5390

Mycgr3G103264\_Mycgr3

Mycgr3G37570 Mycgr3T
  
Location: 5490-6006

Mycgr3G37570\_Mycgr3T

Mycgr3G108094 Mycgr3
  
Location: 6106-10555

Mycgr3G108094\_Mycgr3

Mycgr3G90786 Mycgr3T
  
Location: 10655-12080

Mycgr3G90786\_Mycgr3T

Mycgr3G68429 Mycgr3T
  
Location: 12180-13440

Mycgr3G68429\_Mycgr3T

Mycgr3G68421 Mycgr3T
  
Location: 13540-17086

Mycgr3G68421\_Mycgr3T

Mycgr3G90801 Mycgr3T
  
Location: 17186-18056

Mycgr3G90801\_Mycgr3T

Mycgr3G84646 Mycgr3T
  
Location: 18156-20235

Mycgr3G84646\_Mycgr3T

Mycgr3G68456 Mycgr3T
  
Location: 20335-21970

Mycgr3G68456\_Mycgr3T

Mycgr3G103270 Mycgr3
  
Location: 22070-22355

Mycgr3G103270\_Mycgr3

Mycgr3G90803 Mycgr3T
  
Location: 22455-23019

Mycgr3G90803\_Mycgr3T

Mycgr3G36941 Mycgr3T
  
Location: 23119-24064

Mycgr3G36941\_Mycgr3T

Mycgr3G25746 Mycgr3T
  
Location: 24164-25241

Mycgr3G25746\_Mycgr3T

Mycgr3G90788 Mycgr3T
  
Location: 25341-25803

Mycgr3G90788\_Mycgr3T

Mycgr3G103260 Mycgr3
  
Location: 25903-26635

Mycgr3G103260\_Mycgr3

Mycgr3G84644 Mycgr3T
  
Location: 26735-28457

Mycgr3G84644\_Mycgr3T

Mycgr3G29227 Mycgr3T
  
Location: 28557-28863

Mycgr3G29227\_Mycgr3T

Mycgr3G36271 Mycgr3T
  
Location: 28963-29854

Mycgr3G36271\_Mycgr3T

Mycgr3G68433 Mycgr3T
  
Location: 29954-33041

Mycgr3G68433\_Mycgr3T

Mycgr3G79452 Mycgr3T
  
Location: 33141-33399

Mycgr3G79452\_Mycgr3T

Mycgr3G55345 Mycgr3T
  
Location: 33499-34126

Mycgr3G55345\_Mycgr3T

Mycgr3G103278 Mycgr3
  
Location: 34226-35195

Mycgr3G103278\_Mycgr3

Mycgr3G84654 Mycgr3T
  
Location: 35295-36630

Mycgr3G84654\_Mycgr3T

Mycgr3G108090 Mycgr3
  
Location: 36730-37591

Mycgr3G108090\_Mycgr3

Mycgr3G21922 Mycgr3T
  
Location: 37691-39149

Mycgr3G21922\_Mycgr3T

Mycgr3G99148 Mycgr3T
  
Location: 39249-42819

Mycgr3G99148\_Mycgr3T

hypothetical protein
  
Accession: EEU47196
  
Location: 347397-349573
  
 NCBI BlastP on this gene

EEU47196

hypothetical protein
  
Accession: EEU47195
  
Location: 344200-345891
  
 NCBI BlastP on this gene

EEU47195

hypothetical protein
  
Accession: EEU46728
  
Location: 338382-344149
  
 NCBI BlastP on this gene

EEU46728

hypothetical protein
  
Accession: EEU46727
  
Location: 336500-337724
  
 NCBI BlastP on this gene

EEU46727

hypothetical protein
  
Accession: EEU47194
  
Location: 334896-336139
  
 NCBI BlastP on this gene

EEU47194

hypothetical protein
  
Accession: EEU47193
  
Location: 330055-333663
  
  
**BlastP hit with Mycgr3G68433\_Mycgr3T**
  
Percentage identity: 35 %
  
BlastP bit score: 426
  
Sequence coverage: 93 %
  
E-value: 1e-126
  
  
 NCBI BlastP on this gene

EEU47193

hypothetical protein
  
Accession: EEU47192
  
Location: 328806-329322
  
 NCBI BlastP on this gene

EEU47192

hypothetical protein
  
Accession: EEU47191
  
Location: 324389-328038
  
  
**BlastP hit with Mycgr3G90786\_Mycgr3T**
  
Percentage identity: 26 %
  
BlastP bit score: 74
  
Sequence coverage: 88 %
  
E-value: 7e-11
  
  
 NCBI BlastP on this gene

EEU47191

hypothetical protein
  
Accession: EEU46726
  
Location: 321583-323990
  
 NCBI BlastP on this gene

EEU46726

hypothetical protein
  
Accession: EEU47190
  
Location: 317504-318242
  
 NCBI BlastP on this gene

EEU47190

hypothetical protein
  
Accession: EEU47189
  
Location: 315991-316867
  
  
**BlastP hit with Mycgr3G55345\_Mycgr3T**
  
Percentage identity: 71 %
  
BlastP bit score: 301
  
Sequence coverage: 99 %
  
E-value: 2e-100
  
  
 NCBI BlastP on this gene

EEU47189

predicted protein
  
Accession: EEU46725
  
Location: 314400-315719
  
 NCBI BlastP on this gene

EEU46725

hypothetical protein
  
Accession: EEU46724
  
Location: 313501-314226
  
 NCBI BlastP on this gene

EEU46724

hypothetical protein
  
Accession: EEU47188
  
Location: 310450-312847
  
 NCBI BlastP on this gene

EEU47188

hypothetical protein
  
Accession: EEU47187
  
Location: 304130-308162
  
 NCBI BlastP on this gene

EEU47187

hypothetical protein
  
Accession: EEU47186
  
Location: 299576-303459
  
 NCBI BlastP on this gene

EEU47186

Query: Architecture Search FASTA input

KB707952 : Botryotinia fuckeliana BcDW1 unplaced genomic scaffold Scaffold\_280    Total score: 3.0     Cumulative Blast bit score: 795

Hit cluster cross-links:

Mycgr3G90785 Mycgr3T
  
Location: 0-1047

Mycgr3G90785\_Mycgr3T

Mycgr3G103262 Mycgr3
  
Location: 1147-1390

Mycgr3G103262\_Mycgr3

Mycgr3G68458 Mycgr3T
  
Location: 1490-3602

Mycgr3G68458\_Mycgr3T

Mycgr3G99145 Mycgr3T
  
Location: 3702-4326

Mycgr3G99145\_Mycgr3T

Mycgr3G103274 Mycgr3
  
Location: 4426-4957

Mycgr3G103274\_Mycgr3

Mycgr3G103264 Mycgr3
  
Location: 5057-5390

Mycgr3G103264\_Mycgr3

Mycgr3G37570 Mycgr3T
  
Location: 5490-6006

Mycgr3G37570\_Mycgr3T

Mycgr3G108094 Mycgr3
  
Location: 6106-10555

Mycgr3G108094\_Mycgr3

Mycgr3G90786 Mycgr3T
  
Location: 10655-12080

Mycgr3G90786\_Mycgr3T

Mycgr3G68429 Mycgr3T
  
Location: 12180-13440

Mycgr3G68429\_Mycgr3T

Mycgr3G68421 Mycgr3T
  
Location: 13540-17086

Mycgr3G68421\_Mycgr3T

Mycgr3G90801 Mycgr3T
  
Location: 17186-18056

Mycgr3G90801\_Mycgr3T

Mycgr3G84646 Mycgr3T
  
Location: 18156-20235

Mycgr3G84646\_Mycgr3T

Mycgr3G68456 Mycgr3T
  
Location: 20335-21970

Mycgr3G68456\_Mycgr3T

Mycgr3G103270 Mycgr3
  
Location: 22070-22355

Mycgr3G103270\_Mycgr3

Mycgr3G90803 Mycgr3T
  
Location: 22455-23019

Mycgr3G90803\_Mycgr3T

Mycgr3G36941 Mycgr3T
  
Location: 23119-24064

Mycgr3G36941\_Mycgr3T

Mycgr3G25746 Mycgr3T
  
Location: 24164-25241

Mycgr3G25746\_Mycgr3T

Mycgr3G90788 Mycgr3T
  
Location: 25341-25803

Mycgr3G90788\_Mycgr3T

Mycgr3G103260 Mycgr3
  
Location: 25903-26635

Mycgr3G103260\_Mycgr3

Mycgr3G84644 Mycgr3T
  
Location: 26735-28457

Mycgr3G84644\_Mycgr3T

Mycgr3G29227 Mycgr3T
  
Location: 28557-28863

Mycgr3G29227\_Mycgr3T

Mycgr3G36271 Mycgr3T
  
Location: 28963-29854

Mycgr3G36271\_Mycgr3T

Mycgr3G68433 Mycgr3T
  
Location: 29954-33041

Mycgr3G68433\_Mycgr3T

Mycgr3G79452 Mycgr3T
  
Location: 33141-33399

Mycgr3G79452\_Mycgr3T

Mycgr3G55345 Mycgr3T
  
Location: 33499-34126

Mycgr3G55345\_Mycgr3T

Mycgr3G103278 Mycgr3
  
Location: 34226-35195

Mycgr3G103278\_Mycgr3

Mycgr3G84654 Mycgr3T
  
Location: 35295-36630

Mycgr3G84654\_Mycgr3T

Mycgr3G108090 Mycgr3
  
Location: 36730-37591

Mycgr3G108090\_Mycgr3

Mycgr3G21922 Mycgr3T
  
Location: 37691-39149

Mycgr3G21922\_Mycgr3T

Mycgr3G99148 Mycgr3T
  
Location: 39249-42819

Mycgr3G99148\_Mycgr3T

putative endopolygalacturonase 5 protein
  
Accession: EMR84421
  
Location: 150867-152236
  
 NCBI BlastP on this gene

EMR84421

hypothetical protein
  
Accession: EMR84420
  
Location: 140528-145822
  
 NCBI BlastP on this gene

EMR84420

hypothetical protein
  
Accession: EMR84419
  
Location: 139323-139749
  
 NCBI BlastP on this gene

EMR84419

putative pre-mrna-splicing factor cwc26 protein
  
Accession: EMR84418
  
Location: 137969-138919
  
 NCBI BlastP on this gene

EMR84418

putative transcription initiation factor iif subunit alpha protein
  
Accession: EMR84417
  
Location: 135128-137409
  
  
**BlastP hit with Mycgr3G103278\_Mycgr3**
  
Percentage identity: 37 %
  
BlastP bit score: 128
  
Sequence coverage: 100 %
  
E-value: 2e-29
  
  
 NCBI BlastP on this gene

EMR84417

putative caffeine-induced death protein
  
Accession: EMR84416
  
Location: 130554-133955
  
  
**BlastP hit with Mycgr3G21922\_Mycgr3T**
  
Percentage identity: 47 %
  
BlastP bit score: 283
  
Sequence coverage: 63 %
  
E-value: 5e-81
  
  
 NCBI BlastP on this gene

EMR84416

putative sugar transporter protein
  
Accession: EMR84415
  
Location: 127737-129584
  
 NCBI BlastP on this gene

EMR84415

putative glycosyl hydrolase protein
  
Accession: EMR84414
  
Location: 125011-126883
  
 NCBI BlastP on this gene

EMR84414

putative polygalacturonase protein
  
Accession: EMR84413
  
Location: 120719-124207
  
  
**BlastP hit with Mycgr3G25746\_Mycgr3T**
  
Percentage identity: 53 %
  
BlastP bit score: 384
  
Sequence coverage: 100 %
  
E-value: 1e-122
  
  
 NCBI BlastP on this gene

EMR84413

putative neutral ceramidase protein
  
Accession: EMR84412
  
Location: 117930-120582
  
 NCBI BlastP on this gene

EMR84412

putative abc a-pheromone efflux pump protein
  
Accession: EMR84411
  
Location: 110968-115518
  
 NCBI BlastP on this gene

EMR84411

putative alpha mannosidase family protein
  
Accession: EMR84410
  
Location: 106482-109009
  
 NCBI BlastP on this gene

EMR84410

putative extracellular serine-rich protein
  
Accession: EMR84409
  
Location: 105053-105710
  
 NCBI BlastP on this gene

EMR84409

Query: Architecture Search FASTA input

FQ790278 : Botryotinia fuckeliana T4 SuperContig\_34\_1 genomic supercontig.    Total score: 3.0     Cumulative Blast bit score: 795

Hit cluster cross-links:

Mycgr3G90785 Mycgr3T
  
Location: 0-1047

Mycgr3G90785\_Mycgr3T

Mycgr3G103262 Mycgr3
  
Location: 1147-1390

Mycgr3G103262\_Mycgr3

Mycgr3G68458 Mycgr3T
  
Location: 1490-3602

Mycgr3G68458\_Mycgr3T

Mycgr3G99145 Mycgr3T
  
Location: 3702-4326

Mycgr3G99145\_Mycgr3T

Mycgr3G103274 Mycgr3
  
Location: 4426-4957

Mycgr3G103274\_Mycgr3

Mycgr3G103264 Mycgr3
  
Location: 5057-5390

Mycgr3G103264\_Mycgr3

Mycgr3G37570 Mycgr3T
  
Location: 5490-6006

Mycgr3G37570\_Mycgr3T

Mycgr3G108094 Mycgr3
  
Location: 6106-10555

Mycgr3G108094\_Mycgr3

Mycgr3G90786 Mycgr3T
  
Location: 10655-12080

Mycgr3G90786\_Mycgr3T

Mycgr3G68429 Mycgr3T
  
Location: 12180-13440

Mycgr3G68429\_Mycgr3T

Mycgr3G68421 Mycgr3T
  
Location: 13540-17086

Mycgr3G68421\_Mycgr3T

Mycgr3G90801 Mycgr3T
  
Location: 17186-18056

Mycgr3G90801\_Mycgr3T

Mycgr3G84646 Mycgr3T
  
Location: 18156-20235

Mycgr3G84646\_Mycgr3T

Mycgr3G68456 Mycgr3T
  
Location: 20335-21970

Mycgr3G68456\_Mycgr3T

Mycgr3G103270 Mycgr3
  
Location: 22070-22355

Mycgr3G103270\_Mycgr3

Mycgr3G90803 Mycgr3T
  
Location: 22455-23019

Mycgr3G90803\_Mycgr3T

Mycgr3G36941 Mycgr3T
  
Location: 23119-24064

Mycgr3G36941\_Mycgr3T

Mycgr3G25746 Mycgr3T
  
Location: 24164-25241

Mycgr3G25746\_Mycgr3T

Mycgr3G90788 Mycgr3T
  
Location: 25341-25803

Mycgr3G90788\_Mycgr3T

Mycgr3G103260 Mycgr3
  
Location: 25903-26635

Mycgr3G103260\_Mycgr3

Mycgr3G84644 Mycgr3T
  
Location: 26735-28457

Mycgr3G84644\_Mycgr3T

Mycgr3G29227 Mycgr3T
  
Location: 28557-28863

Mycgr3G29227\_Mycgr3T

Mycgr3G36271 Mycgr3T
  
Location: 28963-29854

Mycgr3G36271\_Mycgr3T

Mycgr3G68433 Mycgr3T
  
Location: 29954-33041

Mycgr3G68433\_Mycgr3T

Mycgr3G79452 Mycgr3T
  
Location: 33141-33399

Mycgr3G79452\_Mycgr3T

Mycgr3G55345 Mycgr3T
  
Location: 33499-34126

Mycgr3G55345\_Mycgr3T

Mycgr3G103278 Mycgr3
  
Location: 34226-35195

Mycgr3G103278\_Mycgr3

Mycgr3G84654 Mycgr3T
  
Location: 35295-36630

Mycgr3G84654\_Mycgr3T

Mycgr3G108090 Mycgr3
  
Location: 36730-37591

Mycgr3G108090\_Mycgr3

Mycgr3G21922 Mycgr3T
  
Location: 37691-39149

Mycgr3G21922\_Mycgr3T

Mycgr3G99148 Mycgr3T
  
Location: 39249-42819

Mycgr3G99148\_Mycgr3T

BcPG5, endopolygalacturonase 5
  
Accession: CCD45769
  
Location: 881374-882743
  
 NCBI BlastP on this gene

BofuT4P34000021001

predicted protein
  
Accession: CCD45768
  
Location: 880355-880662
  
 NCBI BlastP on this gene

BofuT4\_uP047860.1

predicted protein
  
Accession: CCD45767
  
Location: 879253-879454
  
 NCBI BlastP on this gene

BofuT4\_uP047850.1

predicted protein
  
Accession: CCD45766
  
Location: 876398-876592
  
 NCBI BlastP on this gene

BofuT4\_uP047840.1

CND7
  
Accession: CCD45765
  
Location: 874436-875493
  
 NCBI BlastP on this gene

BofuT4\_P047830.1

hypothetical protein
  
Accession: CCD45764
  
Location: 869636-871614
  
 NCBI BlastP on this gene

BofuT4\_P047820.1

hypothetical protein
  
Accession: CCD45763
  
Location: 868433-869215
  
 NCBI BlastP on this gene

BofuT4\_P047810.1

similar to pre-mRNA-splicing factor cwc26
  
Accession: CCD45762
  
Location: 867079-868029
  
 NCBI BlastP on this gene

BofuT4\_P047800.1

similar to transcription initiation factor IIF subunit alpha
  
Accession: CCD45761
  
Location: 864235-866519
  
  
**BlastP hit with Mycgr3G103278\_Mycgr3**
  
Percentage identity: 37 %
  
BlastP bit score: 128
  
Sequence coverage: 100 %
  
E-value: 2e-29
  
  
 NCBI BlastP on this gene

BofuT4\_P047790.1

similar to caffeine-induced death protein
  
Accession: CCD45760
  
Location: 859661-863062
  
  
**BlastP hit with Mycgr3G21922\_Mycgr3T**
  
Percentage identity: 47 %
  
BlastP bit score: 283
  
Sequence coverage: 63 %
  
E-value: 5e-81
  
  
 NCBI BlastP on this gene

BofuT4\_P047780.1

hypothetical protein
  
Accession: CCD45759
  
Location: 857537-858691
  
 NCBI BlastP on this gene

BofuT4\_P047770.1

hypothetical protein
  
Accession: CCD45758
  
Location: 856370-857237
  
 NCBI BlastP on this gene

BofuT4\_P047760.1

glycoside hydrolase family 43 protein
  
Accession: CCD45757
  
Location: 853640-855512
  
 NCBI BlastP on this gene

BofuT4P34000020001

glycoside hydrolase family 28 protein
  
Accession: CCD45756
  
Location: 851648-852836
  
 NCBI BlastP on this gene

BofuT4\_P047740.1

similar to DNA-binding protein HGH1
  
Accession: CCD45755
  
Location: 849353-850759
  
  
**BlastP hit with Mycgr3G25746\_Mycgr3T**
  
Percentage identity: 53 %
  
BlastP bit score: 384
  
Sequence coverage: 100 %
  
E-value: 6e-128
  
  
 NCBI BlastP on this gene

BofuT4\_P047730.1

similar to neutral/alkaline nonlysosomal ceramidase
  
Accession: CCD45754
  
Location: 846583-849235
  
 NCBI BlastP on this gene

BofuT4\_P047720.1

similar to ABC transporter
  
Accession: CCD45753
  
Location: 839623-844173
  
 NCBI BlastP on this gene

BofuT4\_P047710.1

glycoside hydrolase family 92 protein
  
Accession: CCD45752
  
Location: 835137-837664
  
 NCBI BlastP on this gene

BofuT4P34000018001

hypothetical protein
  
Accession: CCD45751
  
Location: 833708-834365
  
 NCBI BlastP on this gene

BofuT4\_P047690.1

Query: Architecture Search FASTA input

JH767573 : Coniosporium apollinis CBS 100218 chromosome Unknown supercont1.20    Total score: 3.0     Cumulative Blast bit score: 789

Hit cluster cross-links:

Mycgr3G90785 Mycgr3T
  
Location: 0-1047

Mycgr3G90785\_Mycgr3T

Mycgr3G103262 Mycgr3
  
Location: 1147-1390

Mycgr3G103262\_Mycgr3

Mycgr3G68458 Mycgr3T
  
Location: 1490-3602

Mycgr3G68458\_Mycgr3T

Mycgr3G99145 Mycgr3T
  
Location: 3702-4326

Mycgr3G99145\_Mycgr3T

Mycgr3G103274 Mycgr3
  
Location: 4426-4957

Mycgr3G103274\_Mycgr3

Mycgr3G103264 Mycgr3
  
Location: 5057-5390

Mycgr3G103264\_Mycgr3

Mycgr3G37570 Mycgr3T
  
Location: 5490-6006

Mycgr3G37570\_Mycgr3T

Mycgr3G108094 Mycgr3
  
Location: 6106-10555

Mycgr3G108094\_Mycgr3

Mycgr3G90786 Mycgr3T
  
Location: 10655-12080

Mycgr3G90786\_Mycgr3T

Mycgr3G68429 Mycgr3T
  
Location: 12180-13440

Mycgr3G68429\_Mycgr3T

Mycgr3G68421 Mycgr3T
  
Location: 13540-17086

Mycgr3G68421\_Mycgr3T

Mycgr3G90801 Mycgr3T
  
Location: 17186-18056

Mycgr3G90801\_Mycgr3T

Mycgr3G84646 Mycgr3T
  
Location: 18156-20235

Mycgr3G84646\_Mycgr3T

Mycgr3G68456 Mycgr3T
  
Location: 20335-21970

Mycgr3G68456\_Mycgr3T

Mycgr3G103270 Mycgr3
  
Location: 22070-22355

Mycgr3G103270\_Mycgr3

Mycgr3G90803 Mycgr3T
  
Location: 22455-23019

Mycgr3G90803\_Mycgr3T

Mycgr3G36941 Mycgr3T
  
Location: 23119-24064

Mycgr3G36941\_Mycgr3T

Mycgr3G25746 Mycgr3T
  
Location: 24164-25241

Mycgr3G25746\_Mycgr3T

Mycgr3G90788 Mycgr3T
  
Location: 25341-25803

Mycgr3G90788\_Mycgr3T

Mycgr3G103260 Mycgr3
  
Location: 25903-26635

Mycgr3G103260\_Mycgr3

Mycgr3G84644 Mycgr3T
  
Location: 26735-28457

Mycgr3G84644\_Mycgr3T

Mycgr3G29227 Mycgr3T
  
Location: 28557-28863

Mycgr3G29227\_Mycgr3T

Mycgr3G36271 Mycgr3T
  
Location: 28963-29854

Mycgr3G36271\_Mycgr3T

Mycgr3G68433 Mycgr3T
  
Location: 29954-33041

Mycgr3G68433\_Mycgr3T

Mycgr3G79452 Mycgr3T
  
Location: 33141-33399

Mycgr3G79452\_Mycgr3T

Mycgr3G55345 Mycgr3T
  
Location: 33499-34126

Mycgr3G55345\_Mycgr3T

Mycgr3G103278 Mycgr3
  
Location: 34226-35195

Mycgr3G103278\_Mycgr3

Mycgr3G84654 Mycgr3T
  
Location: 35295-36630

Mycgr3G84654\_Mycgr3T

Mycgr3G108090 Mycgr3
  
Location: 36730-37591

Mycgr3G108090\_Mycgr3

Mycgr3G21922 Mycgr3T
  
Location: 37691-39149

Mycgr3G21922\_Mycgr3T

Mycgr3G99148 Mycgr3T
  
Location: 39249-42819

Mycgr3G99148\_Mycgr3T

hypothetical protein
  
Accession: EON65393
  
Location: 417896-420000
  
 NCBI BlastP on this gene

EON65393

hypothetical protein
  
Accession: EON65394
  
Location: 432403-433044
  
 NCBI BlastP on this gene

EON65394

hypothetical protein
  
Accession: EON65395
  
Location: 433667-435126
  
 NCBI BlastP on this gene

EON65395

hypothetical protein
  
Accession: EON65396
  
Location: 435948-438515
  
  
**BlastP hit with Mycgr3G21922\_Mycgr3T**
  
Percentage identity: 49 %
  
BlastP bit score: 286
  
Sequence coverage: 63 %
  
E-value: 3e-83
  
  
 NCBI BlastP on this gene

EON65396

hypothetical protein
  
Accession: EON65397
  
Location: 439578-441487
  
  
**BlastP hit with Mycgr3G103278\_Mycgr3**
  
Percentage identity: 35 %
  
BlastP bit score: 115
  
Sequence coverage: 78 %
  
E-value: 1e-25
  
  
 NCBI BlastP on this gene

EON65397

hypothetical protein
  
Accession: EON65398
  
Location: 442225-444153
  
 NCBI BlastP on this gene

EON65398

hypothetical protein
  
Accession: EON65399
  
Location: 445165-446514
  
  
**BlastP hit with Mycgr3G25746\_Mycgr3T**
  
Percentage identity: 58 %
  
BlastP bit score: 388
  
Sequence coverage: 100 %
  
E-value: 5e-130
  
  
 NCBI BlastP on this gene

EON65399

hypothetical protein
  
Accession: EON65400
  
Location: 447004-448896
  
 NCBI BlastP on this gene

EON65400

hypothetical protein
  
Accession: EON65401
  
Location: 449502-450344
  
 NCBI BlastP on this gene

EON65401

hypothetical protein
  
Accession: EON65402
  
Location: 451104-451415
  
 NCBI BlastP on this gene

EON65402

hypothetical protein
  
Accession: EON65403
  
Location: 455275-457007
  
 NCBI BlastP on this gene

EON65403

ubiquitin-conjugating enzyme E2 J2
  
Accession: EON65404
  
Location: 457324-458175
  
 NCBI BlastP on this gene

EON65404

hypothetical protein
  
Accession: EON65405
  
Location: 458766-459394
  
 NCBI BlastP on this gene

EON65405

hypothetical protein
  
Accession: EON65406
  
Location: 461386-462254
  
 NCBI BlastP on this gene

EON65406

hypothetical protein
  
Accession: EON65407
  
Location: 463928-465017
  
 NCBI BlastP on this gene

EON65407

Query: Architecture Search FASTA input

GL385395 : Gaeumannomyces graminis var. tritici R3-111a-1 unplaced genomic scaffold supercont2.1    Total score: 3.0     Cumulative Blast bit score: 780

Hit cluster cross-links:

Mycgr3G90785 Mycgr3T
  
Location: 0-1047

Mycgr3G90785\_Mycgr3T

Mycgr3G103262 Mycgr3
  
Location: 1147-1390

Mycgr3G103262\_Mycgr3

Mycgr3G68458 Mycgr3T
  
Location: 1490-3602

Mycgr3G68458\_Mycgr3T

Mycgr3G99145 Mycgr3T
  
Location: 3702-4326

Mycgr3G99145\_Mycgr3T

Mycgr3G103274 Mycgr3
  
Location: 4426-4957

Mycgr3G103274\_Mycgr3

Mycgr3G103264 Mycgr3
  
Location: 5057-5390

Mycgr3G103264\_Mycgr3

Mycgr3G37570 Mycgr3T
  
Location: 5490-6006

Mycgr3G37570\_Mycgr3T

Mycgr3G108094 Mycgr3
  
Location: 6106-10555

Mycgr3G108094\_Mycgr3

Mycgr3G90786 Mycgr3T
  
Location: 10655-12080

Mycgr3G90786\_Mycgr3T

Mycgr3G68429 Mycgr3T
  
Location: 12180-13440

Mycgr3G68429\_Mycgr3T

Mycgr3G68421 Mycgr3T
  
Location: 13540-17086

Mycgr3G68421\_Mycgr3T

Mycgr3G90801 Mycgr3T
  
Location: 17186-18056

Mycgr3G90801\_Mycgr3T

Mycgr3G84646 Mycgr3T
  
Location: 18156-20235

Mycgr3G84646\_Mycgr3T

Mycgr3G68456 Mycgr3T
  
Location: 20335-21970

Mycgr3G68456\_Mycgr3T

Mycgr3G103270 Mycgr3
  
Location: 22070-22355

Mycgr3G103270\_Mycgr3

Mycgr3G90803 Mycgr3T
  
Location: 22455-23019

Mycgr3G90803\_Mycgr3T

Mycgr3G36941 Mycgr3T
  
Location: 23119-24064

Mycgr3G36941\_Mycgr3T

Mycgr3G25746 Mycgr3T
  
Location: 24164-25241

Mycgr3G25746\_Mycgr3T

Mycgr3G90788 Mycgr3T
  
Location: 25341-25803

Mycgr3G90788\_Mycgr3T

Mycgr3G103260 Mycgr3
  
Location: 25903-26635

Mycgr3G103260\_Mycgr3

Mycgr3G84644 Mycgr3T
  
Location: 26735-28457

Mycgr3G84644\_Mycgr3T

Mycgr3G29227 Mycgr3T
  
Location: 28557-28863

Mycgr3G29227\_Mycgr3T

Mycgr3G36271 Mycgr3T
  
Location: 28963-29854

Mycgr3G36271\_Mycgr3T

Mycgr3G68433 Mycgr3T
  
Location: 29954-33041

Mycgr3G68433\_Mycgr3T

Mycgr3G79452 Mycgr3T
  
Location: 33141-33399

Mycgr3G79452\_Mycgr3T

Mycgr3G55345 Mycgr3T
  
Location: 33499-34126

Mycgr3G55345\_Mycgr3T

Mycgr3G103278 Mycgr3
  
Location: 34226-35195

Mycgr3G103278\_Mycgr3

Mycgr3G84654 Mycgr3T
  
Location: 35295-36630

Mycgr3G84654\_Mycgr3T

Mycgr3G108090 Mycgr3
  
Location: 36730-37591

Mycgr3G108090\_Mycgr3

Mycgr3G21922 Mycgr3T
  
Location: 37691-39149

Mycgr3G21922\_Mycgr3T

Mycgr3G99148 Mycgr3T
  
Location: 39249-42819

Mycgr3G99148\_Mycgr3T

hypothetical protein
  
Accession: EJT82071
  
Location: 6544921-6547078
  
 NCBI BlastP on this gene

EJT82071

hypothetical protein
  
Accession: EJT82072
  
Location: 6547854-6548687
  
 NCBI BlastP on this gene

EJT82072

hypothetical protein
  
Accession: EJT82073
  
Location: 6549073-6550009
  
 NCBI BlastP on this gene

EJT82073

dolichyl-phosphate mannosyltransferase polypeptide 3
  
Accession: EJT82074
  
Location: 6552185-6552680
  
 NCBI BlastP on this gene

EJT82074

hypothetical protein
  
Accession: EJT82075
  
Location: 6553081-6555434
  
 NCBI BlastP on this gene

EJT82075

hypothetical protein
  
Accession: EJT82076
  
Location: 6556111-6557013
  
 NCBI BlastP on this gene

EJT82076

hypoxanthine guanine phosphoribosyltransferase
  
Accession: EJT82077
  
Location: 6557662-6559050
  
  
**BlastP hit with Mycgr3G55345\_Mycgr3T**
  
Percentage identity: 74 %
  
BlastP bit score: 323
  
Sequence coverage: 99 %
  
E-value: 3e-109
  
  
 NCBI BlastP on this gene

EJT82077

hypothetical protein
  
Accession: EJT82078
  
Location: 6559818-6560890
  
 NCBI BlastP on this gene

EJT82078

hypothetical protein
  
Accession: EJT82079
  
Location: 6561499-6562200
  
 NCBI BlastP on this gene

EJT82079

hypothetical protein
  
Accession: EJT82080
  
Location: 6563131-6565022
  
 NCBI BlastP on this gene

EJT82080

leucine-rich repeat-containing protein 40
  
Accession: EJT82081
  
Location: 6567962-6570874
  
  
**BlastP hit with Mycgr3G68433\_Mycgr3T**
  
Percentage identity: 35 %
  
BlastP bit score: 390
  
Sequence coverage: 73 %
  
E-value: 2e-115
  
  
 NCBI BlastP on this gene

EJT82081

hypothetical protein
  
Accession: EJT82082
  
Location: 6571948-6575652
  
 NCBI BlastP on this gene

EJT82082

hypothetical protein
  
Accession: EJT82083
  
Location: 6575962-6577226
  
 NCBI BlastP on this gene

EJT82083

hypothetical protein
  
Accession: EJT82084
  
Location: 6577589-6578903
  
 NCBI BlastP on this gene

EJT82084

trichothecene 3-O-acetyltransferase
  
Accession: EJT82085
  
Location: 6579609-6581060
  
 NCBI BlastP on this gene

EJT82085

hypothetical protein
  
Accession: EJT82086
  
Location: 6582121-6583878
  
  
**BlastP hit with Mycgr3G90786\_Mycgr3T**
  
Percentage identity: 27 %
  
BlastP bit score: 67
  
Sequence coverage: 79 %
  
E-value: 6e-09
  
  
 NCBI BlastP on this gene

EJT82086

hypothetical protein
  
Accession: EJT82087
  
Location: 6584292-6585554
  
 NCBI BlastP on this gene

EJT82087

hypothetical protein
  
Accession: EJT82088
  
Location: 6586708-6587319
  
 NCBI BlastP on this gene

EJT82088

hypothetical protein
  
Accession: EJT82089
  
Location: 6587706-6588628
  
 NCBI BlastP on this gene

EJT82089

hypothetical protein
  
Accession: EJT82090
  
Location: 6589157-6589657
  
 NCBI BlastP on this gene

EJT82090

hypothetical protein
  
Accession: EJT82091
  
Location: 6590058-6592598
  
 NCBI BlastP on this gene

EJT82091

50S ribosomal protein L13e
  
Accession: EJT82092
  
Location: 6592976-6594038
  
 NCBI BlastP on this gene

EJT82092

Query: Architecture Search FASTA input

JH921428 : Marssonina brunnea f. sp. 'multigermtubi' MB\_m1 unplaced genomic scaffold M6\_S00001    Total score: 3.0     Cumulative Blast bit score: 769

Hit cluster cross-links:

Mycgr3G90785 Mycgr3T
  
Location: 0-1047

Mycgr3G90785\_Mycgr3T

Mycgr3G103262 Mycgr3
  
Location: 1147-1390

Mycgr3G103262\_Mycgr3

Mycgr3G68458 Mycgr3T
  
Location: 1490-3602

Mycgr3G68458\_Mycgr3T

Mycgr3G99145 Mycgr3T
  
Location: 3702-4326

Mycgr3G99145\_Mycgr3T

Mycgr3G103274 Mycgr3
  
Location: 4426-4957

Mycgr3G103274\_Mycgr3

Mycgr3G103264 Mycgr3
  
Location: 5057-5390

Mycgr3G103264\_Mycgr3

Mycgr3G37570 Mycgr3T
  
Location: 5490-6006

Mycgr3G37570\_Mycgr3T

Mycgr3G108094 Mycgr3
  
Location: 6106-10555

Mycgr3G108094\_Mycgr3

Mycgr3G90786 Mycgr3T
  
Location: 10655-12080

Mycgr3G90786\_Mycgr3T

Mycgr3G68429 Mycgr3T
  
Location: 12180-13440

Mycgr3G68429\_Mycgr3T

Mycgr3G68421 Mycgr3T
  
Location: 13540-17086

Mycgr3G68421\_Mycgr3T

Mycgr3G90801 Mycgr3T
  
Location: 17186-18056

Mycgr3G90801\_Mycgr3T

Mycgr3G84646 Mycgr3T
  
Location: 18156-20235

Mycgr3G84646\_Mycgr3T

Mycgr3G68456 Mycgr3T
  
Location: 20335-21970

Mycgr3G68456\_Mycgr3T

Mycgr3G103270 Mycgr3
  
Location: 22070-22355

Mycgr3G103270\_Mycgr3

Mycgr3G90803 Mycgr3T
  
Location: 22455-23019

Mycgr3G90803\_Mycgr3T

Mycgr3G36941 Mycgr3T
  
Location: 23119-24064

Mycgr3G36941\_Mycgr3T

Mycgr3G25746 Mycgr3T
  
Location: 24164-25241

Mycgr3G25746\_Mycgr3T

Mycgr3G90788 Mycgr3T
  
Location: 25341-25803

Mycgr3G90788\_Mycgr3T

Mycgr3G103260 Mycgr3
  
Location: 25903-26635

Mycgr3G103260\_Mycgr3

Mycgr3G84644 Mycgr3T
  
Location: 26735-28457

Mycgr3G84644\_Mycgr3T

Mycgr3G29227 Mycgr3T
  
Location: 28557-28863

Mycgr3G29227\_Mycgr3T

Mycgr3G36271 Mycgr3T
  
Location: 28963-29854

Mycgr3G36271\_Mycgr3T

Mycgr3G68433 Mycgr3T
  
Location: 29954-33041

Mycgr3G68433\_Mycgr3T

Mycgr3G79452 Mycgr3T
  
Location: 33141-33399

Mycgr3G79452\_Mycgr3T

Mycgr3G55345 Mycgr3T
  
Location: 33499-34126

Mycgr3G55345\_Mycgr3T

Mycgr3G103278 Mycgr3
  
Location: 34226-35195

Mycgr3G103278\_Mycgr3

Mycgr3G84654 Mycgr3T
  
Location: 35295-36630

Mycgr3G84654\_Mycgr3T

Mycgr3G108090 Mycgr3
  
Location: 36730-37591

Mycgr3G108090\_Mycgr3

Mycgr3G21922 Mycgr3T
  
Location: 37691-39149

Mycgr3G21922\_Mycgr3T

Mycgr3G99148 Mycgr3T
  
Location: 39249-42819

Mycgr3G99148\_Mycgr3T

fatty acid synthase beta subunit dehydratase
  
Accession: EKD21446
  
Location: 3118465-3125509
  
 NCBI BlastP on this gene

EKD21446

synaptobrevin
  
Accession: EKD21447
  
Location: 3125837-3126849
  
 NCBI BlastP on this gene

EKD21447

Ras family protein
  
Accession: EKD21448
  
Location: 3127457-3128664
  
 NCBI BlastP on this gene

EKD21448

FMN-dependent dehydrogenase
  
Accession: EKD21449
  
Location: 3129654-3131396
  
 NCBI BlastP on this gene

EKD21449

hypothetical protein
  
Accession: EKD21450
  
Location: 3134610-3135168
  
 NCBI BlastP on this gene

EKD21450

DNA-binding protein HGH1
  
Accession: EKD21451
  
Location: 3136498-3137998
  
  
**BlastP hit with Mycgr3G25746\_Mycgr3T**
  
Percentage identity: 52 %
  
BlastP bit score: 372
  
Sequence coverage: 100 %
  
E-value: 1e-122
  
  
 NCBI BlastP on this gene

EKD21451

neutral/alkaline nonlysosomal ceramidase
  
Accession: EKD21452
  
Location: 3139004-3139479
  
 NCBI BlastP on this gene

EKD21452

RING-like domain-containing protein
  
Accession: EKD21453
  
Location: 3139570-3141358
  
 NCBI BlastP on this gene

EKD21453

cid13-like poly(A) RNA polymerase
  
Accession: EKD21454
  
Location: 3145135-3148443
  
  
**BlastP hit with Mycgr3G21922\_Mycgr3T**
  
Percentage identity: 49 %
  
BlastP bit score: 273
  
Sequence coverage: 61 %
  
E-value: 6e-78
  
  
 NCBI BlastP on this gene

EKD21454

transcription initiation factor iif subunit
  
Accession: EKD21455
  
Location: 3149611-3152039
  
  
**BlastP hit with Mycgr3G103278\_Mycgr3**
  
Percentage identity: 35 %
  
BlastP bit score: 124
  
Sequence coverage: 81 %
  
E-value: 4e-28
  
  
 NCBI BlastP on this gene

EKD21455

pre-mRNA-splicing factor cwc26
  
Accession: EKD21456
  
Location: 3152259-3153275
  
 NCBI BlastP on this gene

EKD21456

hypothetical protein
  
Accession: EKD21457
  
Location: 3155013-3155810
  
 NCBI BlastP on this gene

EKD21457

cutinase
  
Accession: EKD21458
  
Location: 3156788-3157892
  
 NCBI BlastP on this gene

EKD21458

cytochrome P450 4A10
  
Accession: EKD21459
  
Location: 3160869-3162403
  
 NCBI BlastP on this gene

EKD21459

bystin
  
Accession: EKD21460
  
Location: 3163576-3165072
  
 NCBI BlastP on this gene

EKD21460

3-dehydroshikimate dehydratase
  
Accession: EKD21461
  
Location: 3166374-3167533
  
 NCBI BlastP on this gene

EKD21461

Query: Architecture Search FASTA input

EQ963480 : Aspergillus flavus NRRL3357 scf\_1106286418846 genomic scaffold    Total score: 3.0     Cumulative Blast bit score: 745

Hit cluster cross-links:

Mycgr3G90785 Mycgr3T
  
Location: 0-1047

Mycgr3G90785\_Mycgr3T

Mycgr3G103262 Mycgr3
  
Location: 1147-1390

Mycgr3G103262\_Mycgr3

Mycgr3G68458 Mycgr3T
  
Location: 1490-3602

Mycgr3G68458\_Mycgr3T

Mycgr3G99145 Mycgr3T
  
Location: 3702-4326

Mycgr3G99145\_Mycgr3T

Mycgr3G103274 Mycgr3
  
Location: 4426-4957

Mycgr3G103274\_Mycgr3

Mycgr3G103264 Mycgr3
  
Location: 5057-5390

Mycgr3G103264\_Mycgr3

Mycgr3G37570 Mycgr3T
  
Location: 5490-6006

Mycgr3G37570\_Mycgr3T

Mycgr3G108094 Mycgr3
  
Location: 6106-10555

Mycgr3G108094\_Mycgr3

Mycgr3G90786 Mycgr3T
  
Location: 10655-12080

Mycgr3G90786\_Mycgr3T

Mycgr3G68429 Mycgr3T
  
Location: 12180-13440

Mycgr3G68429\_Mycgr3T

Mycgr3G68421 Mycgr3T
  
Location: 13540-17086

Mycgr3G68421\_Mycgr3T

Mycgr3G90801 Mycgr3T
  
Location: 17186-18056

Mycgr3G90801\_Mycgr3T

Mycgr3G84646 Mycgr3T
  
Location: 18156-20235

Mycgr3G84646\_Mycgr3T

Mycgr3G68456 Mycgr3T
  
Location: 20335-21970

Mycgr3G68456\_Mycgr3T

Mycgr3G103270 Mycgr3
  
Location: 22070-22355

Mycgr3G103270\_Mycgr3

Mycgr3G90803 Mycgr3T
  
Location: 22455-23019

Mycgr3G90803\_Mycgr3T

Mycgr3G36941 Mycgr3T
  
Location: 23119-24064

Mycgr3G36941\_Mycgr3T

Mycgr3G25746 Mycgr3T
  
Location: 24164-25241

Mycgr3G25746\_Mycgr3T

Mycgr3G90788 Mycgr3T
  
Location: 25341-25803

Mycgr3G90788\_Mycgr3T

Mycgr3G103260 Mycgr3
  
Location: 25903-26635

Mycgr3G103260\_Mycgr3

Mycgr3G84644 Mycgr3T
  
Location: 26735-28457

Mycgr3G84644\_Mycgr3T

Mycgr3G29227 Mycgr3T
  
Location: 28557-28863

Mycgr3G29227\_Mycgr3T

Mycgr3G36271 Mycgr3T
  
Location: 28963-29854

Mycgr3G36271\_Mycgr3T

Mycgr3G68433 Mycgr3T
  
Location: 29954-33041

Mycgr3G68433\_Mycgr3T

Mycgr3G79452 Mycgr3T
  
Location: 33141-33399

Mycgr3G79452\_Mycgr3T

Mycgr3G55345 Mycgr3T
  
Location: 33499-34126

Mycgr3G55345\_Mycgr3T

Mycgr3G103278 Mycgr3
  
Location: 34226-35195

Mycgr3G103278\_Mycgr3

Mycgr3G84654 Mycgr3T
  
Location: 35295-36630

Mycgr3G84654\_Mycgr3T

Mycgr3G108090 Mycgr3
  
Location: 36730-37591

Mycgr3G108090\_Mycgr3

Mycgr3G21922 Mycgr3T
  
Location: 37691-39149

Mycgr3G21922\_Mycgr3T

Mycgr3G99148 Mycgr3T
  
Location: 39249-42819

Mycgr3G99148\_Mycgr3T

conserved hypothetical protein
  
Accession: EED48827
  
Location: 1137-2970
  
 NCBI BlastP on this gene

EED48827

snoRNA binding protein, putative
  
Accession: EED48828
  
Location: 3102-5388
  
 NCBI BlastP on this gene

EED48828

actin cytoskeleton organization and biogenesis protein, putative
  
Accession: EED48829
  
Location: 7708-11511
  
 NCBI BlastP on this gene

EED48829

conserved hypothetical protein
  
Accession: EED48830
  
Location: 12759-13532
  
 NCBI BlastP on this gene

EED48830

lipase/serine esterase, putative
  
Accession: EED48831
  
Location: 14813-17818
  
 NCBI BlastP on this gene

EED48831

inositol monophosphatase, putative
  
Accession: EED48832
  
Location: 18235-19314
  
 NCBI BlastP on this gene

EED48832

DNA-binding protein HGH1, putative
  
Accession: EED48833
  
Location: 20165-21506
  
  
**BlastP hit with Mycgr3G25746\_Mycgr3T**
  
Percentage identity: 58 %
  
BlastP bit score: 387
  
Sequence coverage: 100 %
  
E-value: 1e-129
  
  
 NCBI BlastP on this gene

EED48833

zinc finger protein, cchc domain containing protein, putative
  
Accession: EED48834
  
Location: 21679-23739
  
  
**BlastP hit with Mycgr3G21922\_Mycgr3T**
  
Percentage identity: 48 %
  
BlastP bit score: 218
  
Sequence coverage: 47 %
  
E-value: 2e-59
  
  
 NCBI BlastP on this gene

EED48834

transcription initiation factor IIF subunit alpha, putative
  
Accession: EED48835
  
Location: 24404-26660
  
  
**BlastP hit with Mycgr3G103278\_Mycgr3**
  
Percentage identity: 38 %
  
BlastP bit score: 140
  
Sequence coverage: 67 %
  
E-value: 1e-33
  
  
 NCBI BlastP on this gene

EED48835

fatty acid synthase beta subunit, putative
  
Accession: EED48836
  
Location: 27030-33372
  
 NCBI BlastP on this gene

EED48836

fatty acid synthase alpha subunit FasA
  
Accession: EED48837
  
Location: 36079-41772
  
 NCBI BlastP on this gene

EED48837

N2,N2-dimethylguanosine tRNA methyltransferase
  
Accession: EED48838
  
Location: 42154-44253
  
 NCBI BlastP on this gene

EED48838

conserved hypothetical protein
  
Accession: EED48839
  
Location: 44786-45859
  
 NCBI BlastP on this gene

EED48839

C2H2 finger domain protein, putative
  
Accession: EED48840
  
Location: 46318-47101
  
 NCBI BlastP on this gene

EED48840

Query: Architecture Search FASTA input

AP007165 : Aspergillus oryzae RIB40 DNA, SC124.    Total score: 3.0     Cumulative Blast bit score: 729

Hit cluster cross-links:

Mycgr3G90785 Mycgr3T
  
Location: 0-1047

Mycgr3G90785\_Mycgr3T

Mycgr3G103262 Mycgr3
  
Location: 1147-1390

Mycgr3G103262\_Mycgr3

Mycgr3G68458 Mycgr3T
  
Location: 1490-3602

Mycgr3G68458\_Mycgr3T

Mycgr3G99145 Mycgr3T
  
Location: 3702-4326

Mycgr3G99145\_Mycgr3T

Mycgr3G103274 Mycgr3
  
Location: 4426-4957

Mycgr3G103274\_Mycgr3

Mycgr3G103264 Mycgr3
  
Location: 5057-5390

Mycgr3G103264\_Mycgr3

Mycgr3G37570 Mycgr3T
  
Location: 5490-6006

Mycgr3G37570\_Mycgr3T

Mycgr3G108094 Mycgr3
  
Location: 6106-10555

Mycgr3G108094\_Mycgr3

Mycgr3G90786 Mycgr3T
  
Location: 10655-12080

Mycgr3G90786\_Mycgr3T

Mycgr3G68429 Mycgr3T
  
Location: 12180-13440

Mycgr3G68429\_Mycgr3T

Mycgr3G68421 Mycgr3T
  
Location: 13540-17086

Mycgr3G68421\_Mycgr3T

Mycgr3G90801 Mycgr3T
  
Location: 17186-18056

Mycgr3G90801\_Mycgr3T

Mycgr3G84646 Mycgr3T
  
Location: 18156-20235

Mycgr3G84646\_Mycgr3T

Mycgr3G68456 Mycgr3T
  
Location: 20335-21970

Mycgr3G68456\_Mycgr3T

Mycgr3G103270 Mycgr3
  
Location: 22070-22355

Mycgr3G103270\_Mycgr3

Mycgr3G90803 Mycgr3T
  
Location: 22455-23019

Mycgr3G90803\_Mycgr3T

Mycgr3G36941 Mycgr3T
  
Location: 23119-24064

Mycgr3G36941\_Mycgr3T

Mycgr3G25746 Mycgr3T
  
Location: 24164-25241

Mycgr3G25746\_Mycgr3T

Mycgr3G90788 Mycgr3T
  
Location: 25341-25803

Mycgr3G90788\_Mycgr3T

Mycgr3G103260 Mycgr3
  
Location: 25903-26635

Mycgr3G103260\_Mycgr3

Mycgr3G84644 Mycgr3T
  
Location: 26735-28457

Mycgr3G84644\_Mycgr3T

Mycgr3G29227 Mycgr3T
  
Location: 28557-28863

Mycgr3G29227\_Mycgr3T

Mycgr3G36271 Mycgr3T
  
Location: 28963-29854

Mycgr3G36271\_Mycgr3T

Mycgr3G68433 Mycgr3T
  
Location: 29954-33041

Mycgr3G68433\_Mycgr3T

Mycgr3G79452 Mycgr3T
  
Location: 33141-33399

Mycgr3G79452\_Mycgr3T

Mycgr3G55345 Mycgr3T
  
Location: 33499-34126

Mycgr3G55345\_Mycgr3T

Mycgr3G103278 Mycgr3
  
Location: 34226-35195

Mycgr3G103278\_Mycgr3

Mycgr3G84654 Mycgr3T
  
Location: 35295-36630

Mycgr3G84654\_Mycgr3T

Mycgr3G108090 Mycgr3
  
Location: 36730-37591

Mycgr3G108090\_Mycgr3

Mycgr3G21922 Mycgr3T
  
Location: 37691-39149

Mycgr3G21922\_Mycgr3T

Mycgr3G99148 Mycgr3T
  
Location: 39249-42819

Mycgr3G99148\_Mycgr3T

not annotated
  
Accession: BAE62640
  
Location: 246156-247968
  
 NCBI BlastP on this gene

AO090124000092

not annotated
  
Accession: BAE62639
  
Location: 242691-246024
  
 NCBI BlastP on this gene

AO090124000091

not annotated
  
Accession: BAE62638
  
Location: 237521-241178
  
 NCBI BlastP on this gene

AO090124000090

not annotated
  
Accession: BAE62637
  
Location: 231311-236024
  
 NCBI BlastP on this gene

AO090124000089

not annotated
  
Accession: BAE62636
  
Location: 229815-230894
  
 NCBI BlastP on this gene

AO090124000088

not annotated
  
Accession: BAE62635
  
Location: 227621-228962
  
  
**BlastP hit with Mycgr3G25746\_Mycgr3T**
  
Percentage identity: 58 %
  
BlastP bit score: 387
  
Sequence coverage: 100 %
  
E-value: 1e-129
  
  
 NCBI BlastP on this gene

AO090124000087

not annotated
  
Accession: BAE62634
  
Location: 225667-226920
  
  
**BlastP hit with Mycgr3G21922\_Mycgr3T**
  
Percentage identity: 48 %
  
BlastP bit score: 203
  
Sequence coverage: 44 %
  
E-value: 3e-56
  
  
 NCBI BlastP on this gene

AO090124000086

not annotated
  
Accession: BAE62633
  
Location: 220519-222850
  
  
**BlastP hit with Mycgr3G103278\_Mycgr3**
  
Percentage identity: 38 %
  
BlastP bit score: 139
  
Sequence coverage: 67 %
  
E-value: 2e-33
  
  
 NCBI BlastP on this gene

AO090124000085

not annotated
  
Accession: BAE62632
  
Location: 213881-220223
  
 NCBI BlastP on this gene

AO090124000084

not annotated
  
Accession: BAE62631
  
Location: 205477-211170
  
 NCBI BlastP on this gene

AO090124000083

not annotated
  
Accession: BAE62630
  
Location: 202997-205096
  
 NCBI BlastP on this gene

AO090124000082

not annotated
  
Accession: BAE62629
  
Location: 201393-202466
  
 NCBI BlastP on this gene

AO090124000081

Query: Architecture Search FASTA input

CH476625 : Sclerotinia sclerotiorum 1980 scaffold\_5 genomic scaffold    Total score: 3.0     Cumulative Blast bit score: 723

Hit cluster cross-links:

Mycgr3G90785 Mycgr3T
  
Location: 0-1047

Mycgr3G90785\_Mycgr3T

Mycgr3G103262 Mycgr3
  
Location: 1147-1390

Mycgr3G103262\_Mycgr3

Mycgr3G68458 Mycgr3T
  
Location: 1490-3602

Mycgr3G68458\_Mycgr3T

Mycgr3G99145 Mycgr3T
  
Location: 3702-4326

Mycgr3G99145\_Mycgr3T

Mycgr3G103274 Mycgr3
  
Location: 4426-4957

Mycgr3G103274\_Mycgr3

Mycgr3G103264 Mycgr3
  
Location: 5057-5390

Mycgr3G103264\_Mycgr3

Mycgr3G37570 Mycgr3T
  
Location: 5490-6006

Mycgr3G37570\_Mycgr3T

Mycgr3G108094 Mycgr3
  
Location: 6106-10555

Mycgr3G108094\_Mycgr3

Mycgr3G90786 Mycgr3T
  
Location: 10655-12080

Mycgr3G90786\_Mycgr3T

Mycgr3G68429 Mycgr3T
  
Location: 12180-13440

Mycgr3G68429\_Mycgr3T

Mycgr3G68421 Mycgr3T
  
Location: 13540-17086

Mycgr3G68421\_Mycgr3T

Mycgr3G90801 Mycgr3T
  
Location: 17186-18056

Mycgr3G90801\_Mycgr3T

Mycgr3G84646 Mycgr3T
  
Location: 18156-20235

Mycgr3G84646\_Mycgr3T

Mycgr3G68456 Mycgr3T
  
Location: 20335-21970

Mycgr3G68456\_Mycgr3T

Mycgr3G103270 Mycgr3
  
Location: 22070-22355

Mycgr3G103270\_Mycgr3

Mycgr3G90803 Mycgr3T
  
Location: 22455-23019

Mycgr3G90803\_Mycgr3T

Mycgr3G36941 Mycgr3T
  
Location: 23119-24064

Mycgr3G36941\_Mycgr3T

Mycgr3G25746 Mycgr3T
  
Location: 24164-25241

Mycgr3G25746\_Mycgr3T

Mycgr3G90788 Mycgr3T
  
Location: 25341-25803

Mycgr3G90788\_Mycgr3T

Mycgr3G103260 Mycgr3
  
Location: 25903-26635

Mycgr3G103260\_Mycgr3

Mycgr3G84644 Mycgr3T
  
Location: 26735-28457

Mycgr3G84644\_Mycgr3T

Mycgr3G29227 Mycgr3T
  
Location: 28557-28863

Mycgr3G29227\_Mycgr3T

Mycgr3G36271 Mycgr3T
  
Location: 28963-29854

Mycgr3G36271\_Mycgr3T

Mycgr3G68433 Mycgr3T
  
Location: 29954-33041

Mycgr3G68433\_Mycgr3T

Mycgr3G79452 Mycgr3T
  
Location: 33141-33399

Mycgr3G79452\_Mycgr3T

Mycgr3G55345 Mycgr3T
  
Location: 33499-34126

Mycgr3G55345\_Mycgr3T

Mycgr3G103278 Mycgr3
  
Location: 34226-35195

Mycgr3G103278\_Mycgr3

Mycgr3G84654 Mycgr3T
  
Location: 35295-36630

Mycgr3G84654\_Mycgr3T

Mycgr3G108090 Mycgr3
  
Location: 36730-37591

Mycgr3G108090\_Mycgr3

Mycgr3G21922 Mycgr3T
  
Location: 37691-39149

Mycgr3G21922\_Mycgr3T

Mycgr3G99148 Mycgr3T
  
Location: 39249-42819

Mycgr3G99148\_Mycgr3T

hypothetical protein
  
Accession: EDO01741
  
Location: 608924-610688
  
 NCBI BlastP on this gene

EDO01741

hypothetical protein
  
Accession: EDO01740
  
Location: 606463-608448
  
 NCBI BlastP on this gene

EDO01740

hypothetical protein
  
Accession: EDO01739
  
Location: 603350-604824
  
 NCBI BlastP on this gene

EDO01739

hypothetical protein
  
Accession: EDO01738
  
Location: 597305-602693
  
 NCBI BlastP on this gene

EDO01738

hypothetical protein
  
Accession: EDO01737
  
Location: 594838-595809
  
 NCBI BlastP on this gene

EDO01737

hypothetical protein
  
Accession: EDO01736
  
Location: 591865-594178
  
  
**BlastP hit with Mycgr3G103278\_Mycgr3**
  
Percentage identity: 35 %
  
BlastP bit score: 124
  
Sequence coverage: 105 %
  
E-value: 5e-28
  
  
 NCBI BlastP on this gene

EDO01736

hypothetical protein
  
Accession: EDO01735
  
Location: 590169-590513
  
 NCBI BlastP on this gene

EDO01735

hypothetical protein
  
Accession: EDO01734
  
Location: 587145-589706
  
  
**BlastP hit with Mycgr3G21922\_Mycgr3T**
  
Percentage identity: 48 %
  
BlastP bit score: 275
  
Sequence coverage: 61 %
  
E-value: 2e-79
  
  
 NCBI BlastP on this gene

EDO01734

hypothetical protein
  
Accession: EDO01733
  
Location: 584292-586193
  
 NCBI BlastP on this gene

EDO01733

hypothetical protein
  
Accession: EDO01732
  
Location: 582026-583679
  
 NCBI BlastP on this gene

EDO01732

hypothetical protein
  
Accession: EDO01731
  
Location: 579355-581288
  
 NCBI BlastP on this gene

EDO01731

hypothetical protein
  
Accession: EDO01730
  
Location: 576824-578227
  
  
**BlastP hit with Mycgr3G25746\_Mycgr3T**
  
Percentage identity: 48 %
  
BlastP bit score: 324
  
Sequence coverage: 100 %
  
E-value: 4e-105
  
  
 NCBI BlastP on this gene

EDO01730

hypothetical protein
  
Accession: EDO01729
  
Location: 574006-576570
  
 NCBI BlastP on this gene

EDO01729

predicted protein
  
Accession: EDO01728
  
Location: 572246-572900
  
 NCBI BlastP on this gene

EDO01728

hypothetical protein
  
Accession: EDO01727
  
Location: 567140-571683
  
 NCBI BlastP on this gene

EDO01727

predicted protein
  
Accession: EDO01726
  
Location: 563846-563950
  
 NCBI BlastP on this gene

EDO01726

hypothetical protein
  
Accession: EDO01725
  
Location: 559972-562483
  
 NCBI BlastP on this gene

EDO01725

Query: Architecture Search FASTA input

GL629801 : Grosmannia clavigera kw1407 unplaced genomic scaffold GCSC\_173    Total score: 3.0     Cumulative Blast bit score: 709

Hit cluster cross-links:

Mycgr3G90785 Mycgr3T
  
Location: 0-1047

Mycgr3G90785\_Mycgr3T

Mycgr3G103262 Mycgr3
  
Location: 1147-1390

Mycgr3G103262\_Mycgr3

Mycgr3G68458 Mycgr3T
  
Location: 1490-3602

Mycgr3G68458\_Mycgr3T

Mycgr3G99145 Mycgr3T
  
Location: 3702-4326

Mycgr3G99145\_Mycgr3T

Mycgr3G103274 Mycgr3
  
Location: 4426-4957

Mycgr3G103274\_Mycgr3

Mycgr3G103264 Mycgr3
  
Location: 5057-5390

Mycgr3G103264\_Mycgr3

Mycgr3G37570 Mycgr3T
  
Location: 5490-6006

Mycgr3G37570\_Mycgr3T

Mycgr3G108094 Mycgr3
  
Location: 6106-10555

Mycgr3G108094\_Mycgr3

Mycgr3G90786 Mycgr3T
  
Location: 10655-12080

Mycgr3G90786\_Mycgr3T

Mycgr3G68429 Mycgr3T
  
Location: 12180-13440

Mycgr3G68429\_Mycgr3T

Mycgr3G68421 Mycgr3T
  
Location: 13540-17086

Mycgr3G68421\_Mycgr3T

Mycgr3G90801 Mycgr3T
  
Location: 17186-18056

Mycgr3G90801\_Mycgr3T

Mycgr3G84646 Mycgr3T
  
Location: 18156-20235

Mycgr3G84646\_Mycgr3T

Mycgr3G68456 Mycgr3T
  
Location: 20335-21970

Mycgr3G68456\_Mycgr3T

Mycgr3G103270 Mycgr3
  
Location: 22070-22355

Mycgr3G103270\_Mycgr3

Mycgr3G90803 Mycgr3T
  
Location: 22455-23019

Mycgr3G90803\_Mycgr3T

Mycgr3G36941 Mycgr3T
  
Location: 23119-24064

Mycgr3G36941\_Mycgr3T

Mycgr3G25746 Mycgr3T
  
Location: 24164-25241

Mycgr3G25746\_Mycgr3T

Mycgr3G90788 Mycgr3T
  
Location: 25341-25803

Mycgr3G90788\_Mycgr3T

Mycgr3G103260 Mycgr3
  
Location: 25903-26635

Mycgr3G103260\_Mycgr3

Mycgr3G84644 Mycgr3T
  
Location: 26735-28457

Mycgr3G84644\_Mycgr3T

Mycgr3G29227 Mycgr3T
  
Location: 28557-28863

Mycgr3G29227\_Mycgr3T

Mycgr3G36271 Mycgr3T
  
Location: 28963-29854

Mycgr3G36271\_Mycgr3T

Mycgr3G68433 Mycgr3T
  
Location: 29954-33041

Mycgr3G68433\_Mycgr3T

Mycgr3G79452 Mycgr3T
  
Location: 33141-33399

Mycgr3G79452\_Mycgr3T

Mycgr3G55345 Mycgr3T
  
Location: 33499-34126

Mycgr3G55345\_Mycgr3T

Mycgr3G103278 Mycgr3
  
Location: 34226-35195

Mycgr3G103278\_Mycgr3

Mycgr3G84654 Mycgr3T
  
Location: 35295-36630

Mycgr3G84654\_Mycgr3T

Mycgr3G108090 Mycgr3
  
Location: 36730-37591

Mycgr3G108090\_Mycgr3

Mycgr3G21922 Mycgr3T
  
Location: 37691-39149

Mycgr3G21922\_Mycgr3T

Mycgr3G99148 Mycgr3T
  
Location: 39249-42819

Mycgr3G99148\_Mycgr3T

glycoprotein
  
Accession: EFX00737
  
Location: 1948623-1949100
  
 NCBI BlastP on this gene

EFX00737

short chain dehydrogenase reductase
  
Accession: EFX00515
  
Location: 1949856-1950845
  
 NCBI BlastP on this gene

EFX00515

xanthine-guanine phosphoribosyl transferase
  
Accession: EFX00626
  
Location: 1952205-1953146
  
  
**BlastP hit with Mycgr3G55345\_Mycgr3T**
  
Percentage identity: 72 %
  
BlastP bit score: 301
  
Sequence coverage: 97 %
  
E-value: 2e-100
  
  
 NCBI BlastP on this gene

EFX00626

hypothetical protein
  
Accession: EFX00465
  
Location: 1953461-1954303
  
 NCBI BlastP on this gene

EFX00465

hypothetical protein
  
Accession: EFX00657
  
Location: 1954661-1956877
  
 NCBI BlastP on this gene

EFX00657

dolichyl-phosphate mannosyltransferase polypeptide 3
  
Accession: EFX00248
  
Location: 1957314-1957712
  
 NCBI BlastP on this gene

EFX00248

major facilitator superfamily transporter multidrug resistance
  
Accession: EFX00211
  
Location: 1960830-1962822
  
 NCBI BlastP on this gene

EFX00211

FAD-binding domain containing protein
  
Accession: EFX00315
  
Location: 1965495-1967240
  
 NCBI BlastP on this gene

EFX00315

hypothetical protein
  
Accession: EFX00122
  
Location: 1967994-1969140
  
 NCBI BlastP on this gene

EFX00122

hypothetical protein
  
Accession: EFX00230
  
Location: 1970482-1973998
  
 NCBI BlastP on this gene

EFX00230

hypothetical protein
  
Accession: EFX00061
  
Location: 1975243-1975935
  
 NCBI BlastP on this gene

EFX00061

FAD-dependent oxidoreductase-like enzyme
  
Accession: EFX00526
  
Location: 1976463-1978535
  
  
**BlastP hit with Mycgr3G90786\_Mycgr3T**
  
Percentage identity: 27 %
  
BlastP bit score: 75
  
Sequence coverage: 69 %
  
E-value: 2e-11
  
  
 NCBI BlastP on this gene

EFX00526

flavin-binding monooxygenase-like protein
  
Accession: EFX00495
  
Location: 1978936-1981633
  
 NCBI BlastP on this gene

EFX00495

3-ketoacyl-thiolase
  
Accession: EFX00072
  
Location: 1982405-1983724
  
 NCBI BlastP on this gene

EFX00072

hypothetical protein
  
Accession: EFX00545
  
Location: 1983987-1984667
  
 NCBI BlastP on this gene

EFX00545

conserved leucine-rich repeat protein
  
Accession: EFX00602
  
Location: 1985471-1989174
  
  
**BlastP hit with Mycgr3G68433\_Mycgr3T**
  
Percentage identity: 32 %
  
BlastP bit score: 333
  
Sequence coverage: 91 %
  
E-value: 4e-93
  
  
 NCBI BlastP on this gene

EFX00602

hypothetical protein
  
Accession: EFX00269
  
Location: 1992896-1993114
  
 NCBI BlastP on this gene

EFX00269

hypothetical protein
  
Accession: EFX00555
  
Location: 1994024-1994817
  
 NCBI BlastP on this gene

EFX00555

Query: Architecture Search FASTA input

GG704913 : Coccidioides immitis RS genomic scaffold supercont3.3    Total score: 3.0     Cumulative Blast bit score: 638

Hit cluster cross-links:

Mycgr3G90785 Mycgr3T
  
Location: 0-1047

Mycgr3G90785\_Mycgr3T

Mycgr3G103262 Mycgr3
  
Location: 1147-1390

Mycgr3G103262\_Mycgr3

Mycgr3G68458 Mycgr3T
  
Location: 1490-3602

Mycgr3G68458\_Mycgr3T

Mycgr3G99145 Mycgr3T
  
Location: 3702-4326

Mycgr3G99145\_Mycgr3T

Mycgr3G103274 Mycgr3
  
Location: 4426-4957

Mycgr3G103274\_Mycgr3

Mycgr3G103264 Mycgr3
  
Location: 5057-5390

Mycgr3G103264\_Mycgr3

Mycgr3G37570 Mycgr3T
  
Location: 5490-6006

Mycgr3G37570\_Mycgr3T

Mycgr3G108094 Mycgr3
  
Location: 6106-10555

Mycgr3G108094\_Mycgr3

Mycgr3G90786 Mycgr3T
  
Location: 10655-12080

Mycgr3G90786\_Mycgr3T

Mycgr3G68429 Mycgr3T
  
Location: 12180-13440

Mycgr3G68429\_Mycgr3T

Mycgr3G68421 Mycgr3T
  
Location: 13540-17086

Mycgr3G68421\_Mycgr3T

Mycgr3G90801 Mycgr3T
  
Location: 17186-18056

Mycgr3G90801\_Mycgr3T

Mycgr3G84646 Mycgr3T
  
Location: 18156-20235

Mycgr3G84646\_Mycgr3T

Mycgr3G68456 Mycgr3T
  
Location: 20335-21970

Mycgr3G68456\_Mycgr3T

Mycgr3G103270 Mycgr3
  
Location: 22070-22355

Mycgr3G103270\_Mycgr3

Mycgr3G90803 Mycgr3T
  
Location: 22455-23019

Mycgr3G90803\_Mycgr3T

Mycgr3G36941 Mycgr3T
  
Location: 23119-24064

Mycgr3G36941\_Mycgr3T

Mycgr3G25746 Mycgr3T
  
Location: 24164-25241

Mycgr3G25746\_Mycgr3T

Mycgr3G90788 Mycgr3T
  
Location: 25341-25803

Mycgr3G90788\_Mycgr3T

Mycgr3G103260 Mycgr3
  
Location: 25903-26635

Mycgr3G103260\_Mycgr3

Mycgr3G84644 Mycgr3T
  
Location: 26735-28457

Mycgr3G84644\_Mycgr3T

Mycgr3G29227 Mycgr3T
  
Location: 28557-28863

Mycgr3G29227\_Mycgr3T

Mycgr3G36271 Mycgr3T
  
Location: 28963-29854

Mycgr3G36271\_Mycgr3T

Mycgr3G68433 Mycgr3T
  
Location: 29954-33041

Mycgr3G68433\_Mycgr3T

Mycgr3G79452 Mycgr3T
  
Location: 33141-33399

Mycgr3G79452\_Mycgr3T

Mycgr3G55345 Mycgr3T
  
Location: 33499-34126

Mycgr3G55345\_Mycgr3T

Mycgr3G103278 Mycgr3
  
Location: 34226-35195

Mycgr3G103278\_Mycgr3

Mycgr3G84654 Mycgr3T
  
Location: 35295-36630

Mycgr3G84654\_Mycgr3T

Mycgr3G108090 Mycgr3
  
Location: 36730-37591

Mycgr3G108090\_Mycgr3

Mycgr3G21922 Mycgr3T
  
Location: 37691-39149

Mycgr3G21922\_Mycgr3T

Mycgr3G99148 Mycgr3T
  
Location: 39249-42819

Mycgr3G99148\_Mycgr3T

hypothetical protein
  
Accession: EAS30066
  
Location: 1165257-1166675
  
 NCBI BlastP on this gene

EAS30066

hypothetical protein
  
Accession: EAS30065
  
Location: 1167226-1167767
  
 NCBI BlastP on this gene

EAS30065

hypothetical protein
  
Accession: EAS30064
  
Location: 1168750-1169510
  
 NCBI BlastP on this gene

EAS30064

lipase/serine esterase
  
Accession: EAS30063
  
Location: 1170183-1173597
  
 NCBI BlastP on this gene

EAS30063

hypothetical protein
  
Accession: EAS30062
  
Location: 1174511-1176343
  
 NCBI BlastP on this gene

EAS30062

inositol monophosphatase
  
Accession: EAS30061
  
Location: 1177535-1178564
  
 NCBI BlastP on this gene

EAS30061

hypothetical protein
  
Accession: EAS30060
  
Location: 1179848-1181383
  
 NCBI BlastP on this gene

EAS30060

hypothetical protein
  
Accession: EAS30059
  
Location: 1181981-1182407
  
 NCBI BlastP on this gene

EAS30059

hypothetical protein
  
Accession: EAS30058
  
Location: 1183070-1184507
  
  
**BlastP hit with Mycgr3G25746\_Mycgr3T**
  
Percentage identity: 54 %
  
BlastP bit score: 362
  
Sequence coverage: 100 %
  
E-value: 8e-120
  
  
 NCBI BlastP on this gene

EAS30058

hypothetical protein
  
Accession: EJB11179
  
Location: 1184850-1186402
  
 NCBI BlastP on this gene

EJB11179

hypothetical protein
  
Accession: EAS30055
  
Location: 1187046-1188572
  
  
**BlastP hit with Mycgr3G21922\_Mycgr3T**
  
Percentage identity: 37 %
  
BlastP bit score: 152
  
Sequence coverage: 47 %
  
E-value: 3e-37
  
  
 NCBI BlastP on this gene

EAS30055

transcription initiation factor IIF subunit alpha
  
Accession: EAS30054
  
Location: 1190139-1192429
  
  
**BlastP hit with Mycgr3G103278\_Mycgr3**
  
Percentage identity: 40 %
  
BlastP bit score: 124
  
Sequence coverage: 54 %
  
E-value: 6e-28
  
  
 NCBI BlastP on this gene

EAS30054

fatty acid synthase beta subunit dehydratase, variant
  
Accession: EJB11181
  
Location: 1193653-1199493
  
 NCBI BlastP on this gene

EJB11181

hypothetical protein
  
Accession: EJB11182
  
Location: 1199896-1200734
  
 NCBI BlastP on this gene

EJB11182

fatty acid synthase subunit alpha
  
Accession: EAS30052
  
Location: 1201441-1207156
  
 NCBI BlastP on this gene

EAS30052

3-isopropylmalate dehydrogenase B
  
Accession: EAS30051
  
Location: 1208236-1209517
  
 NCBI BlastP on this gene

EAS30051

N2,N2-dimethylguanosine tRNA methyltransferase
  
Accession: EAS30050
  
Location: 1209772-1212093
  
 NCBI BlastP on this gene

EAS30050

Query: Architecture Search FASTA input

AKHY01000107 : Aspergillus oryzae 3.042    Total score: 3.0     Cumulative Blast bit score: 638

Hit cluster cross-links:

Mycgr3G90785 Mycgr3T
  
Location: 0-1047

Mycgr3G90785\_Mycgr3T

Mycgr3G103262 Mycgr3
  
Location: 1147-1390

Mycgr3G103262\_Mycgr3

Mycgr3G68458 Mycgr3T
  
Location: 1490-3602

Mycgr3G68458\_Mycgr3T

Mycgr3G99145 Mycgr3T
  
Location: 3702-4326

Mycgr3G99145\_Mycgr3T

Mycgr3G103274 Mycgr3
  
Location: 4426-4957

Mycgr3G103274\_Mycgr3

Mycgr3G103264 Mycgr3
  
Location: 5057-5390

Mycgr3G103264\_Mycgr3

Mycgr3G37570 Mycgr3T
  
Location: 5490-6006

Mycgr3G37570\_Mycgr3T

Mycgr3G108094 Mycgr3
  
Location: 6106-10555

Mycgr3G108094\_Mycgr3

Mycgr3G90786 Mycgr3T
  
Location: 10655-12080

Mycgr3G90786\_Mycgr3T

Mycgr3G68429 Mycgr3T
  
Location: 12180-13440

Mycgr3G68429\_Mycgr3T

Mycgr3G68421 Mycgr3T
  
Location: 13540-17086

Mycgr3G68421\_Mycgr3T

Mycgr3G90801 Mycgr3T
  
Location: 17186-18056

Mycgr3G90801\_Mycgr3T

Mycgr3G84646 Mycgr3T
  
Location: 18156-20235

Mycgr3G84646\_Mycgr3T

Mycgr3G68456 Mycgr3T
  
Location: 20335-21970

Mycgr3G68456\_Mycgr3T

Mycgr3G103270 Mycgr3
  
Location: 22070-22355

Mycgr3G103270\_Mycgr3

Mycgr3G90803 Mycgr3T
  
Location: 22455-23019

Mycgr3G90803\_Mycgr3T

Mycgr3G36941 Mycgr3T
  
Location: 23119-24064

Mycgr3G36941\_Mycgr3T

Mycgr3G25746 Mycgr3T
  
Location: 24164-25241

Mycgr3G25746\_Mycgr3T

Mycgr3G90788 Mycgr3T
  
Location: 25341-25803

Mycgr3G90788\_Mycgr3T

Mycgr3G103260 Mycgr3
  
Location: 25903-26635

Mycgr3G103260\_Mycgr3

Mycgr3G84644 Mycgr3T
  
Location: 26735-28457

Mycgr3G84644\_Mycgr3T

Mycgr3G29227 Mycgr3T
  
Location: 28557-28863

Mycgr3G29227\_Mycgr3T

Mycgr3G36271 Mycgr3T
  
Location: 28963-29854

Mycgr3G36271\_Mycgr3T

Mycgr3G68433 Mycgr3T
  
Location: 29954-33041

Mycgr3G68433\_Mycgr3T

Mycgr3G79452 Mycgr3T
  
Location: 33141-33399

Mycgr3G79452\_Mycgr3T

Mycgr3G55345 Mycgr3T
  
Location: 33499-34126

Mycgr3G55345\_Mycgr3T

Mycgr3G103278 Mycgr3
  
Location: 34226-35195

Mycgr3G103278\_Mycgr3

Mycgr3G84654 Mycgr3T
  
Location: 35295-36630

Mycgr3G84654\_Mycgr3T

Mycgr3G108090 Mycgr3
  
Location: 36730-37591

Mycgr3G108090\_Mycgr3

Mycgr3G21922 Mycgr3T
  
Location: 37691-39149

Mycgr3G21922\_Mycgr3T

Mycgr3G99148 Mycgr3T
  
Location: 39249-42819

Mycgr3G99148\_Mycgr3T

hypothetical protein
  
Accession: EIT81276
  
Location: 87637-89360
  
 NCBI BlastP on this gene

EIT81276

hypothetical protein
  
Accession: EIT81266
  
Location: 84172-87505
  
 NCBI BlastP on this gene

EIT81266

actin cytoskeleton organization and biogenesis protein, putative
  
Accession: EIT81272
  
Location: 79047-82701
  
 NCBI BlastP on this gene

EIT81272

hypothetical protein
  
Accession: EIT81288
  
Location: 72882-75890
  
 NCBI BlastP on this gene

EIT81288

inositol monophosphatase
  
Accession: EIT81284
  
Location: 71386-72465
  
 NCBI BlastP on this gene

EIT81284

DNA-binding protein
  
Accession: EIT81270
  
Location: 69177-70115
  
  
**BlastP hit with Mycgr3G25746\_Mycgr3T**
  
Percentage identity: 53 %
  
BlastP bit score: 282
  
Sequence coverage: 81 %
  
E-value: 1e-89
  
  
 NCBI BlastP on this gene

EIT81270

zinc finger protein, cchc domain containing protein, putative
  
Accession: EIT81282
  
Location: 67223-69004
  
  
**BlastP hit with Mycgr3G21922\_Mycgr3T**
  
Percentage identity: 48 %
  
BlastP bit score: 216
  
Sequence coverage: 47 %
  
E-value: 1e-59
  
  
 NCBI BlastP on this gene

EIT81282

hypothetical protein
  
Accession: EIT81290
  
Location: 63948-66279
  
  
**BlastP hit with Mycgr3G103278\_Mycgr3**
  
Percentage identity: 38 %
  
BlastP bit score: 140
  
Sequence coverage: 67 %
  
E-value: 1e-33
  
  
 NCBI BlastP on this gene

EIT81290

fatty acid synthase beta subunit, putative
  
Accession: EIT81271
  
Location: 57310-63652
  
 NCBI BlastP on this gene

EIT81271

3-oxoacyl-[acyl-carrier-protein] synthase
  
Accession: EIT81285
  
Location: 48928-54621
  
 NCBI BlastP on this gene

EIT81285

hypothetical protein
  
Accession: EIT81269
  
Location: 44841-45914
  
 NCBI BlastP on this gene

EIT81269

Query: Architecture Search FASTA input

GL636502 : Coccidioides posadasii str. Silveira unplaced genomic scaffold supercont2.17    Total score: 3.0     Cumulative Blast bit score: 636

Hit cluster cross-links:

Mycgr3G90785 Mycgr3T
  
Location: 0-1047

Mycgr3G90785\_Mycgr3T

Mycgr3G103262 Mycgr3
  
Location: 1147-1390

Mycgr3G103262\_Mycgr3

Mycgr3G68458 Mycgr3T
  
Location: 1490-3602

Mycgr3G68458\_Mycgr3T

Mycgr3G99145 Mycgr3T
  
Location: 3702-4326

Mycgr3G99145\_Mycgr3T

Mycgr3G103274 Mycgr3
  
Location: 4426-4957

Mycgr3G103274\_Mycgr3

Mycgr3G103264 Mycgr3
  
Location: 5057-5390

Mycgr3G103264\_Mycgr3

Mycgr3G37570 Mycgr3T
  
Location: 5490-6006

Mycgr3G37570\_Mycgr3T

Mycgr3G108094 Mycgr3
  
Location: 6106-10555

Mycgr3G108094\_Mycgr3

Mycgr3G90786 Mycgr3T
  
Location: 10655-12080

Mycgr3G90786\_Mycgr3T

Mycgr3G68429 Mycgr3T
  
Location: 12180-13440

Mycgr3G68429\_Mycgr3T

Mycgr3G68421 Mycgr3T
  
Location: 13540-17086

Mycgr3G68421\_Mycgr3T

Mycgr3G90801 Mycgr3T
  
Location: 17186-18056

Mycgr3G90801\_Mycgr3T

Mycgr3G84646 Mycgr3T
  
Location: 18156-20235

Mycgr3G84646\_Mycgr3T

Mycgr3G68456 Mycgr3T
  
Location: 20335-21970

Mycgr3G68456\_Mycgr3T

Mycgr3G103270 Mycgr3
  
Location: 22070-22355

Mycgr3G103270\_Mycgr3

Mycgr3G90803 Mycgr3T
  
Location: 22455-23019

Mycgr3G90803\_Mycgr3T

Mycgr3G36941 Mycgr3T
  
Location: 23119-24064

Mycgr3G36941\_Mycgr3T

Mycgr3G25746 Mycgr3T
  
Location: 24164-25241

Mycgr3G25746\_Mycgr3T

Mycgr3G90788 Mycgr3T
  
Location: 25341-25803

Mycgr3G90788\_Mycgr3T

Mycgr3G103260 Mycgr3
  
Location: 25903-26635

Mycgr3G103260\_Mycgr3

Mycgr3G84644 Mycgr3T
  
Location: 26735-28457

Mycgr3G84644\_Mycgr3T

Mycgr3G29227 Mycgr3T
  
Location: 28557-28863

Mycgr3G29227\_Mycgr3T

Mycgr3G36271 Mycgr3T
  
Location: 28963-29854

Mycgr3G36271\_Mycgr3T

Mycgr3G68433 Mycgr3T
  
Location: 29954-33041

Mycgr3G68433\_Mycgr3T

Mycgr3G79452 Mycgr3T
  
Location: 33141-33399

Mycgr3G79452\_Mycgr3T

Mycgr3G55345 Mycgr3T
  
Location: 33499-34126

Mycgr3G55345\_Mycgr3T

Mycgr3G103278 Mycgr3
  
Location: 34226-35195

Mycgr3G103278\_Mycgr3

Mycgr3G84654 Mycgr3T
  
Location: 35295-36630

Mycgr3G84654\_Mycgr3T

Mycgr3G108090 Mycgr3
  
Location: 36730-37591

Mycgr3G108090\_Mycgr3

Mycgr3G21922 Mycgr3T
  
Location: 37691-39149

Mycgr3G21922\_Mycgr3T

Mycgr3G99148 Mycgr3T
  
Location: 39249-42819

Mycgr3G99148\_Mycgr3T

conserved hypothetical protein
  
Accession: EFW15027
  
Location: 87984-88478
  
 NCBI BlastP on this gene

EFW15027

conserved hypothetical protein
  
Accession: EFW15028
  
Location: 88875-90052
  
 NCBI BlastP on this gene

EFW15028

lipase/serine esterase
  
Accession: EFW15029
  
Location: 90730-94144
  
 NCBI BlastP on this gene

EFW15029

conserved hypothetical protein
  
Accession: EFW15030
  
Location: 95058-96890
  
 NCBI BlastP on this gene

EFW15030

predicted protein
  
Accession: EFW15031
  
Location: 97104-97913
  
 NCBI BlastP on this gene

EFW15031

inositol monophosphatase
  
Accession: EFW15032
  
Location: 98077-99106
  
 NCBI BlastP on this gene

EFW15032

conserved hypothetical protein
  
Accession: EFW15033
  
Location: 100291-101931
  
 NCBI BlastP on this gene

EFW15033

predicted protein
  
Accession: EFW15034
  
Location: 102475-102993
  
 NCBI BlastP on this gene

EFW15034

DNA-binding protein HGH1
  
Accession: EFW15035
  
Location: 103718-105133
  
  
**BlastP hit with Mycgr3G25746\_Mycgr3T**
  
Percentage identity: 54 %
  
BlastP bit score: 364
  
Sequence coverage: 100 %
  
E-value: 1e-120
  
  
 NCBI BlastP on this gene

EFW15035

conserved hypothetical protein
  
Accession: EFW15036
  
Location: 105475-107027
  
 NCBI BlastP on this gene

EFW15036

conserved hypothetical protein
  
Accession: EFW15037
  
Location: 107638-109164
  
  
**BlastP hit with Mycgr3G21922\_Mycgr3T**
  
Percentage identity: 37 %
  
BlastP bit score: 148
  
Sequence coverage: 47 %
  
E-value: 7e-36
  
  
 NCBI BlastP on this gene

EFW15037

transcription initiation factor IIF subunit alpha
  
Accession: EFW15038
  
Location: 110754-113044
  
  
**BlastP hit with Mycgr3G103278\_Mycgr3**
  
Percentage identity: 40 %
  
BlastP bit score: 124
  
Sequence coverage: 54 %
  
E-value: 5e-28
  
  
 NCBI BlastP on this gene

EFW15038

fatty acid synthase beta subunit
  
Accession: EFW15039
  
Location: 113768-118090
  
 NCBI BlastP on this gene

EFW15039

fatty acid synthase beta subunit
  
Accession: EFW15040
  
Location: 118267-119469
  
 NCBI BlastP on this gene

EFW15040

predicted protein
  
Accession: EFW15041
  
Location: 121368-121708
  
 NCBI BlastP on this gene

EFW15041

fatty acid synthase subunit alpha reductase
  
Accession: EFW15042
  
Location: 122081-127798
  
 NCBI BlastP on this gene

EFW15042

3-isopropylmalate dehydrogenase B
  
Accession: EFW15043
  
Location: 128873-130175
  
 NCBI BlastP on this gene

EFW15043

N2,N2-dimethylguanosine tRNA methyltransferase
  
Accession: EFW15044
  
Location: 130431-132605
  
 NCBI BlastP on this gene

EFW15044

Query: Architecture Search FASTA input

DS572813 : Paracoccidioides brasiliensis Pb01 supercont1.3 genomic scaffold    Total score: 3.0     Cumulative Blast bit score: 635

Hit cluster cross-links:

Mycgr3G90785 Mycgr3T
  
Location: 0-1047

Mycgr3G90785\_Mycgr3T

Mycgr3G103262 Mycgr3
  
Location: 1147-1390

Mycgr3G103262\_Mycgr3

Mycgr3G68458 Mycgr3T
  
Location: 1490-3602

Mycgr3G68458\_Mycgr3T

Mycgr3G99145 Mycgr3T
  
Location: 3702-4326

Mycgr3G99145\_Mycgr3T

Mycgr3G103274 Mycgr3
  
Location: 4426-4957

Mycgr3G103274\_Mycgr3

Mycgr3G103264 Mycgr3
  
Location: 5057-5390

Mycgr3G103264\_Mycgr3

Mycgr3G37570 Mycgr3T
  
Location: 5490-6006

Mycgr3G37570\_Mycgr3T

Mycgr3G108094 Mycgr3
  
Location: 6106-10555

Mycgr3G108094\_Mycgr3

Mycgr3G90786 Mycgr3T
  
Location: 10655-12080

Mycgr3G90786\_Mycgr3T

Mycgr3G68429 Mycgr3T
  
Location: 12180-13440

Mycgr3G68429\_Mycgr3T

Mycgr3G68421 Mycgr3T
  
Location: 13540-17086

Mycgr3G68421\_Mycgr3T

Mycgr3G90801 Mycgr3T
  
Location: 17186-18056

Mycgr3G90801\_Mycgr3T

Mycgr3G84646 Mycgr3T
  
Location: 18156-20235

Mycgr3G84646\_Mycgr3T

Mycgr3G68456 Mycgr3T
  
Location: 20335-21970

Mycgr3G68456\_Mycgr3T

Mycgr3G103270 Mycgr3
  
Location: 22070-22355

Mycgr3G103270\_Mycgr3

Mycgr3G90803 Mycgr3T
  
Location: 22455-23019

Mycgr3G90803\_Mycgr3T

Mycgr3G36941 Mycgr3T
  
Location: 23119-24064

Mycgr3G36941\_Mycgr3T

Mycgr3G25746 Mycgr3T
  
Location: 24164-25241

Mycgr3G25746\_Mycgr3T

Mycgr3G90788 Mycgr3T
  
Location: 25341-25803

Mycgr3G90788\_Mycgr3T

Mycgr3G103260 Mycgr3
  
Location: 25903-26635

Mycgr3G103260\_Mycgr3

Mycgr3G84644 Mycgr3T
  
Location: 26735-28457

Mycgr3G84644\_Mycgr3T

Mycgr3G29227 Mycgr3T
  
Location: 28557-28863

Mycgr3G29227\_Mycgr3T

Mycgr3G36271 Mycgr3T
  
Location: 28963-29854

Mycgr3G36271\_Mycgr3T

Mycgr3G68433 Mycgr3T
  
Location: 29954-33041

Mycgr3G68433\_Mycgr3T

Mycgr3G79452 Mycgr3T
  
Location: 33141-33399

Mycgr3G79452\_Mycgr3T

Mycgr3G55345 Mycgr3T
  
Location: 33499-34126

Mycgr3G55345\_Mycgr3T

Mycgr3G103278 Mycgr3
  
Location: 34226-35195

Mycgr3G103278\_Mycgr3

Mycgr3G84654 Mycgr3T
  
Location: 35295-36630

Mycgr3G84654\_Mycgr3T

Mycgr3G108090 Mycgr3
  
Location: 36730-37591

Mycgr3G108090\_Mycgr3

Mycgr3G21922 Mycgr3T
  
Location: 37691-39149

Mycgr3G21922\_Mycgr3T

Mycgr3G99148 Mycgr3T
  
Location: 39249-42819

Mycgr3G99148\_Mycgr3T

conserved hypothetical protein
  
Accession: EEH39052
  
Location: 645058-646620
  
 NCBI BlastP on this gene

EEH39052

conserved hypothetical protein
  
Accession: EEH39053
  
Location: 647312-652101
  
 NCBI BlastP on this gene

EEH39053

rieske domain-containing protein
  
Accession: EEH39054
  
Location: 653983-655458
  
 NCBI BlastP on this gene

EEH39054

IBR domain-containing protein
  
Accession: EEH39055
  
Location: 656385-658199
  
 NCBI BlastP on this gene

EEH39055

predicted protein
  
Accession: EEH39056
  
Location: 659139-659379
  
 NCBI BlastP on this gene

EEH39056

inositol monophosphatase
  
Accession: EEH39057
  
Location: 660256-661354
  
 NCBI BlastP on this gene

EEH39057

DNA-binding protein HGH1
  
Accession: EEH39058
  
Location: 663585-665096
  
  
**BlastP hit with Mycgr3G25746\_Mycgr3T**
  
Percentage identity: 55 %
  
BlastP bit score: 359
  
Sequence coverage: 99 %
  
E-value: 2e-118
  
  
 NCBI BlastP on this gene

EEH39058

conserved hypothetical protein
  
Accession: EEH39059
  
Location: 665814-667351
  
 NCBI BlastP on this gene

EEH39059

hypothetical protein
  
Accession: EEH39060
  
Location: 668088-669397
  
  
**BlastP hit with Mycgr3G21922\_Mycgr3T**
  
Percentage identity: 37 %
  
BlastP bit score: 146
  
Sequence coverage: 48 %
  
E-value: 6e-36
  
  
 NCBI BlastP on this gene

EEH39060

conserved hypothetical protein
  
Accession: EEH39061
  
Location: 670216-672456
  
  
**BlastP hit with Mycgr3G103278\_Mycgr3**
  
Percentage identity: 34 %
  
BlastP bit score: 130
  
Sequence coverage: 94 %
  
E-value: 5e-30
  
  
 NCBI BlastP on this gene

EEH39061

fatty acid synthase subunit beta dehydratase
  
Accession: EEH39062
  
Location: 673616-679936
  
 NCBI BlastP on this gene

EEH39062

fatty acid synthase subunit alpha reductase
  
Accession: EEH39063
  
Location: 681712-687542
  
 NCBI BlastP on this gene

EEH39063

N2,N2-dimethylguanosine tRNA methyltransferase
  
Accession: EEH39064
  
Location: 688359-691045
  
 NCBI BlastP on this gene

EEH39064

3-isopropylmalate dehydrogenase
  
Accession: EEH39065
  
Location: 691638-694949
  
 NCBI BlastP on this gene

EEH39065

Query: Architecture Search FASTA input

CH476599 : Aspergillus terreus NIH2624 scaffold\_6 genomic scaffold    Total score: 3.0     Cumulative Blast bit score: 613

Hit cluster cross-links:

Mycgr3G90785 Mycgr3T
  
Location: 0-1047

Mycgr3G90785\_Mycgr3T

Mycgr3G103262 Mycgr3
  
Location: 1147-1390

Mycgr3G103262\_Mycgr3

Mycgr3G68458 Mycgr3T
  
Location: 1490-3602

Mycgr3G68458\_Mycgr3T

Mycgr3G99145 Mycgr3T
  
Location: 3702-4326

Mycgr3G99145\_Mycgr3T

Mycgr3G103274 Mycgr3
  
Location: 4426-4957

Mycgr3G103274\_Mycgr3

Mycgr3G103264 Mycgr3
  
Location: 5057-5390

Mycgr3G103264\_Mycgr3

Mycgr3G37570 Mycgr3T
  
Location: 5490-6006

Mycgr3G37570\_Mycgr3T

Mycgr3G108094 Mycgr3
  
Location: 6106-10555

Mycgr3G108094\_Mycgr3

Mycgr3G90786 Mycgr3T
  
Location: 10655-12080

Mycgr3G90786\_Mycgr3T

Mycgr3G68429 Mycgr3T
  
Location: 12180-13440

Mycgr3G68429\_Mycgr3T

Mycgr3G68421 Mycgr3T
  
Location: 13540-17086

Mycgr3G68421\_Mycgr3T

Mycgr3G90801 Mycgr3T
  
Location: 17186-18056

Mycgr3G90801\_Mycgr3T

Mycgr3G84646 Mycgr3T
  
Location: 18156-20235

Mycgr3G84646\_Mycgr3T

Mycgr3G68456 Mycgr3T
  
Location: 20335-21970

Mycgr3G68456\_Mycgr3T

Mycgr3G103270 Mycgr3
  
Location: 22070-22355

Mycgr3G103270\_Mycgr3

Mycgr3G90803 Mycgr3T
  
Location: 22455-23019

Mycgr3G90803\_Mycgr3T

Mycgr3G36941 Mycgr3T
  
Location: 23119-24064

Mycgr3G36941\_Mycgr3T

Mycgr3G25746 Mycgr3T
  
Location: 24164-25241

Mycgr3G25746\_Mycgr3T

Mycgr3G90788 Mycgr3T
  
Location: 25341-25803

Mycgr3G90788\_Mycgr3T

Mycgr3G103260 Mycgr3
  
Location: 25903-26635

Mycgr3G103260\_Mycgr3

Mycgr3G84644 Mycgr3T
  
Location: 26735-28457

Mycgr3G84644\_Mycgr3T

Mycgr3G29227 Mycgr3T
  
Location: 28557-28863

Mycgr3G29227\_Mycgr3T

Mycgr3G36271 Mycgr3T
  
Location: 28963-29854

Mycgr3G36271\_Mycgr3T

Mycgr3G68433 Mycgr3T
  
Location: 29954-33041

Mycgr3G68433\_Mycgr3T

Mycgr3G79452 Mycgr3T
  
Location: 33141-33399

Mycgr3G79452\_Mycgr3T

Mycgr3G55345 Mycgr3T
  
Location: 33499-34126

Mycgr3G55345\_Mycgr3T

Mycgr3G103278 Mycgr3
  
Location: 34226-35195

Mycgr3G103278\_Mycgr3

Mycgr3G84654 Mycgr3T
  
Location: 35295-36630

Mycgr3G84654\_Mycgr3T

Mycgr3G108090 Mycgr3
  
Location: 36730-37591

Mycgr3G108090\_Mycgr3

Mycgr3G21922 Mycgr3T
  
Location: 37691-39149

Mycgr3G21922\_Mycgr3T

Mycgr3G99148 Mycgr3T
  
Location: 39249-42819

Mycgr3G99148\_Mycgr3T

hypothetical protein
  
Accession: EAU34696
  
Location: 5189-5981
  
 NCBI BlastP on this gene

EAU34696

predicted protein
  
Accession: EAU34697
  
Location: 6930-8444
  
 NCBI BlastP on this gene

EAU34697

conserved hypothetical protein
  
Accession: EAU34698
  
Location: 8955-12312
  
 NCBI BlastP on this gene

EAU34698

conserved hypothetical protein
  
Accession: EAU34699
  
Location: 13404-17352
  
 NCBI BlastP on this gene

EAU34699

conserved hypothetical protein
  
Accession: EAU34700
  
Location: 17979-21852
  
 NCBI BlastP on this gene

EAU34700

hypothetical protein
  
Accession: EAU34701
  
Location: 22966-23991
  
 NCBI BlastP on this gene

EAU34701

conserved hypothetical protein
  
Accession: EAU34702
  
Location: 24657-25616
  
  
**BlastP hit with Mycgr3G25746\_Mycgr3T**
  
Percentage identity: 54 %
  
BlastP bit score: 277
  
Sequence coverage: 82 %
  
E-value: 2e-87
  
  
 NCBI BlastP on this gene

EAU34702

predicted protein
  
Accession: EAU34703
  
Location: 26177-27924
  
  
**BlastP hit with Mycgr3G21922\_Mycgr3T**
  
Percentage identity: 47 %
  
BlastP bit score: 209
  
Sequence coverage: 46 %
  
E-value: 3e-57
  
  
 NCBI BlastP on this gene

EAU34703

predicted protein
  
Accession: EAU34704
  
Location: 28495-30630
  
  
**BlastP hit with Mycgr3G103278\_Mycgr3**
  
Percentage identity: 40 %
  
BlastP bit score: 127
  
Sequence coverage: 61 %
  
E-value: 4e-29
  
  
 NCBI BlastP on this gene

EAU34704

fatty acid synthase beta subunit dehydratase
  
Accession: EAU34705
  
Location: 31180-37514
  
 NCBI BlastP on this gene

EAU34705

hypothetical protein
  
Accession: EAU34706
  
Location: 39270-44963
  
 NCBI BlastP on this gene

EAU34706

conserved hypothetical protein
  
Accession: EAU34707
  
Location: 45188-48799
  
 NCBI BlastP on this gene

EAU34707

conserved hypothetical protein
  
Accession: EAU34708
  
Location: 49300-50043
  
 NCBI BlastP on this gene

EAU34708

pyruvate dehydrogenase E1 component beta subunit, mitochondrial precursor
  
Accession: EAU34709
  
Location: 50511-51852
  
 NCBI BlastP on this gene

EAU34709

Query: Architecture Search FASTA input

ACFW01000015 : Coccidioides posadasii C735 delta SOWgp    Total score: 3.0     Cumulative Blast bit score: 589

Hit cluster cross-links:

Mycgr3G90785 Mycgr3T
  
Location: 0-1047

Mycgr3G90785\_Mycgr3T

Mycgr3G103262 Mycgr3
  
Location: 1147-1390

Mycgr3G103262\_Mycgr3

Mycgr3G68458 Mycgr3T
  
Location: 1490-3602

Mycgr3G68458\_Mycgr3T

Mycgr3G99145 Mycgr3T
  
Location: 3702-4326

Mycgr3G99145\_Mycgr3T

Mycgr3G103274 Mycgr3
  
Location: 4426-4957

Mycgr3G103274\_Mycgr3

Mycgr3G103264 Mycgr3
  
Location: 5057-5390

Mycgr3G103264\_Mycgr3

Mycgr3G37570 Mycgr3T
  
Location: 5490-6006

Mycgr3G37570\_Mycgr3T

Mycgr3G108094 Mycgr3
  
Location: 6106-10555

Mycgr3G108094\_Mycgr3

Mycgr3G90786 Mycgr3T
  
Location: 10655-12080

Mycgr3G90786\_Mycgr3T

Mycgr3G68429 Mycgr3T
  
Location: 12180-13440

Mycgr3G68429\_Mycgr3T

Mycgr3G68421 Mycgr3T
  
Location: 13540-17086

Mycgr3G68421\_Mycgr3T

Mycgr3G90801 Mycgr3T
  
Location: 17186-18056

Mycgr3G90801\_Mycgr3T

Mycgr3G84646 Mycgr3T
  
Location: 18156-20235

Mycgr3G84646\_Mycgr3T

Mycgr3G68456 Mycgr3T
  
Location: 20335-21970

Mycgr3G68456\_Mycgr3T

Mycgr3G103270 Mycgr3
  
Location: 22070-22355

Mycgr3G103270\_Mycgr3

Mycgr3G90803 Mycgr3T
  
Location: 22455-23019

Mycgr3G90803\_Mycgr3T

Mycgr3G36941 Mycgr3T
  
Location: 23119-24064

Mycgr3G36941\_Mycgr3T

Mycgr3G25746 Mycgr3T
  
Location: 24164-25241

Mycgr3G25746\_Mycgr3T

Mycgr3G90788 Mycgr3T
  
Location: 25341-25803

Mycgr3G90788\_Mycgr3T

Mycgr3G103260 Mycgr3
  
Location: 25903-26635

Mycgr3G103260\_Mycgr3

Mycgr3G84644 Mycgr3T
  
Location: 26735-28457

Mycgr3G84644\_Mycgr3T

Mycgr3G29227 Mycgr3T
  
Location: 28557-28863

Mycgr3G29227\_Mycgr3T

Mycgr3G36271 Mycgr3T
  
Location: 28963-29854

Mycgr3G36271\_Mycgr3T

Mycgr3G68433 Mycgr3T
  
Location: 29954-33041

Mycgr3G68433\_Mycgr3T

Mycgr3G79452 Mycgr3T
  
Location: 33141-33399

Mycgr3G79452\_Mycgr3T

Mycgr3G55345 Mycgr3T
  
Location: 33499-34126

Mycgr3G55345\_Mycgr3T

Mycgr3G103278 Mycgr3
  
Location: 34226-35195

Mycgr3G103278\_Mycgr3

Mycgr3G84654 Mycgr3T
  
Location: 35295-36630

Mycgr3G84654\_Mycgr3T

Mycgr3G108090 Mycgr3
  
Location: 36730-37591

Mycgr3G108090\_Mycgr3

Mycgr3G21922 Mycgr3T
  
Location: 37691-39149

Mycgr3G21922\_Mycgr3T

Mycgr3G99148 Mycgr3T
  
Location: 39249-42819

Mycgr3G99148\_Mycgr3T

cortical actin cytoskeleton protein VIP1, putative
  
Accession: EER28284
  
Location: 290432-294853
  
 NCBI BlastP on this gene

EER28284

hypothetical protein
  
Accession: EER28283
  
Location: 288528-289946
  
 NCBI BlastP on this gene

EER28283

hypothetical protein
  
Accession: EER28282
  
Location: 285692-286448
  
 NCBI BlastP on this gene

EER28282

Putative serine esterase family protein
  
Accession: EER28281
  
Location: 281596-285010
  
 NCBI BlastP on this gene

EER28281

zinc knuckle containing protein
  
Accession: EER28280
  
Location: 278851-280683
  
 NCBI BlastP on this gene

EER28280

Inositol monophosphatase, putative
  
Accession: EER28279
  
Location: 276634-277663
  
 NCBI BlastP on this gene

EER28279

methyltransferase, putative
  
Accession: EER28278
  
Location: 274037-275352
  
 NCBI BlastP on this gene

EER28278

hypothetical protein
  
Accession: EER28277
  
Location: 270607-271895
  
  
**BlastP hit with Mycgr3G25746\_Mycgr3T**
  
Percentage identity: 51 %
  
BlastP bit score: 316
  
Sequence coverage: 94 %
  
E-value: 1e-101
  
  
 NCBI BlastP on this gene

EER28277

hypothetical protein
  
Accession: EER28276
  
Location: 268713-270155
  
 NCBI BlastP on this gene

EER28276

PAP/25A associated domain containing protein
  
Accession: EER28275
  
Location: 266576-268102
  
  
**BlastP hit with Mycgr3G21922\_Mycgr3T**
  
Percentage identity: 37 %
  
BlastP bit score: 149
  
Sequence coverage: 47 %
  
E-value: 3e-36
  
  
 NCBI BlastP on this gene

EER28275

transcription initiation factor IIF subunit alpha, putative
  
Accession: EER28274
  
Location: 262691-264981
  
  
**BlastP hit with Mycgr3G103278\_Mycgr3**
  
Percentage identity: 40 %
  
BlastP bit score: 124
  
Sequence coverage: 54 %
  
E-value: 5e-28
  
  
 NCBI BlastP on this gene

EER28274

Fatty acid synthase subunit beta, putative
  
Accession: EER28273
  
Location: 255638-261959
  
 NCBI BlastP on this gene

EER28273

Fatty acid synthase subunit alpha, putative
  
Accession: EER28272
  
Location: 247977-253694
  
 NCBI BlastP on this gene

EER28272

3-isopropylmalate dehydrogenase B, putative
  
Accession: EER28271
  
Location: 245603-246902
  
 NCBI BlastP on this gene

EER28271

N2,N2-dimethylguanosine tRNA methyltransferase family protein
  
Accession: EER28270
  
Location: 243173-245347
  
 NCBI BlastP on this gene

EER28270

Query: Architecture Search FASTA input

CH476615 : Uncinocarpus reesii 1704 scaffold\_1 genomic scaffold    Total score: 3.0     Cumulative Blast bit score: 491

Hit cluster cross-links:

Mycgr3G90785 Mycgr3T
  
Location: 0-1047

Mycgr3G90785\_Mycgr3T

Mycgr3G103262 Mycgr3
  
Location: 1147-1390

Mycgr3G103262\_Mycgr3

Mycgr3G68458 Mycgr3T
  
Location: 1490-3602

Mycgr3G68458\_Mycgr3T

Mycgr3G99145 Mycgr3T
  
Location: 3702-4326

Mycgr3G99145\_Mycgr3T

Mycgr3G103274 Mycgr3
  
Location: 4426-4957

Mycgr3G103274\_Mycgr3

Mycgr3G103264 Mycgr3
  
Location: 5057-5390

Mycgr3G103264\_Mycgr3

Mycgr3G37570 Mycgr3T
  
Location: 5490-6006

Mycgr3G37570\_Mycgr3T

Mycgr3G108094 Mycgr3
  
Location: 6106-10555

Mycgr3G108094\_Mycgr3

Mycgr3G90786 Mycgr3T
  
Location: 10655-12080

Mycgr3G90786\_Mycgr3T

Mycgr3G68429 Mycgr3T
  
Location: 12180-13440

Mycgr3G68429\_Mycgr3T

Mycgr3G68421 Mycgr3T
  
Location: 13540-17086

Mycgr3G68421\_Mycgr3T

Mycgr3G90801 Mycgr3T
  
Location: 17186-18056

Mycgr3G90801\_Mycgr3T

Mycgr3G84646 Mycgr3T
  
Location: 18156-20235

Mycgr3G84646\_Mycgr3T

Mycgr3G68456 Mycgr3T
  
Location: 20335-21970

Mycgr3G68456\_Mycgr3T

Mycgr3G103270 Mycgr3
  
Location: 22070-22355

Mycgr3G103270\_Mycgr3

Mycgr3G90803 Mycgr3T
  
Location: 22455-23019

Mycgr3G90803\_Mycgr3T

Mycgr3G36941 Mycgr3T
  
Location: 23119-24064

Mycgr3G36941\_Mycgr3T

Mycgr3G25746 Mycgr3T
  
Location: 24164-25241

Mycgr3G25746\_Mycgr3T

Mycgr3G90788 Mycgr3T
  
Location: 25341-25803

Mycgr3G90788\_Mycgr3T

Mycgr3G103260 Mycgr3
  
Location: 25903-26635

Mycgr3G103260\_Mycgr3

Mycgr3G84644 Mycgr3T
  
Location: 26735-28457

Mycgr3G84644\_Mycgr3T

Mycgr3G29227 Mycgr3T
  
Location: 28557-28863

Mycgr3G29227\_Mycgr3T

Mycgr3G36271 Mycgr3T
  
Location: 28963-29854

Mycgr3G36271\_Mycgr3T

Mycgr3G68433 Mycgr3T
  
Location: 29954-33041

Mycgr3G68433\_Mycgr3T

Mycgr3G79452 Mycgr3T
  
Location: 33141-33399

Mycgr3G79452\_Mycgr3T

Mycgr3G55345 Mycgr3T
  
Location: 33499-34126

Mycgr3G55345\_Mycgr3T

Mycgr3G103278 Mycgr3
  
Location: 34226-35195

Mycgr3G103278\_Mycgr3

Mycgr3G84654 Mycgr3T
  
Location: 35295-36630

Mycgr3G84654\_Mycgr3T

Mycgr3G108090 Mycgr3
  
Location: 36730-37591

Mycgr3G108090\_Mycgr3

Mycgr3G21922 Mycgr3T
  
Location: 37691-39149

Mycgr3G21922\_Mycgr3T

Mycgr3G99148 Mycgr3T
  
Location: 39249-42819

Mycgr3G99148\_Mycgr3T

conserved hypothetical protein
  
Accession: EEP77715
  
Location: 6807300-6811550
  
 NCBI BlastP on this gene

EEP77715

conserved hypothetical protein
  
Accession: EEP77714
  
Location: 6801526-6806862
  
 NCBI BlastP on this gene

EEP77714

predicted protein
  
Accession: EEP77713
  
Location: 6799705-6800751
  
 NCBI BlastP on this gene

EEP77713

conserved hypothetical protein
  
Accession: EEP77712
  
Location: 6796875-6797920
  
 NCBI BlastP on this gene

EEP77712

conserved hypothetical protein
  
Accession: EEP77711
  
Location: 6794059-6795614
  
 NCBI BlastP on this gene

EEP77711

conserved hypothetical protein
  
Accession: EEP77710
  
Location: 6791870-6792649
  
  
**BlastP hit with Mycgr3G25746\_Mycgr3T**
  
Percentage identity: 52 %
  
BlastP bit score: 226
  
Sequence coverage: 69 %
  
E-value: 3e-68
  
  
 NCBI BlastP on this gene

EEP77710

predicted protein
  
Accession: EEP77709
  
Location: 6790166-6790753
  
 NCBI BlastP on this gene

EEP77709

predicted protein
  
Accession: EEP77708
  
Location: 6787424-6788983
  
 NCBI BlastP on this gene

EEP77708

predicted protein
  
Accession: EEP77707
  
Location: 6785259-6786824
  
  
**BlastP hit with Mycgr3G21922\_Mycgr3T**
  
Percentage identity: 36 %
  
BlastP bit score: 145
  
Sequence coverage: 45 %
  
E-value: 9e-35
  
  
 NCBI BlastP on this gene

EEP77707

predicted protein
  
Accession: EEP77706
  
Location: 6782031-6784083
  
  
**BlastP hit with Mycgr3G103278\_Mycgr3**
  
Percentage identity: 40 %
  
BlastP bit score: 120
  
Sequence coverage: 55 %
  
E-value: 6e-27
  
  
 NCBI BlastP on this gene

EEP77706

fatty acid synthase beta subunit dehydratase
  
Accession: EEP77705
  
Location: 6775162-6781479
  
 NCBI BlastP on this gene

EEP77705

fatty acid synthase alpha subunit
  
Accession: EEP77704
  
Location: 6768037-6773745
  
 NCBI BlastP on this gene

EEP77704

3-isopropylmalate dehydrogenase
  
Accession: EEP77703
  
Location: 6765871-6767119
  
 NCBI BlastP on this gene

EEP77703

conserved hypothetical protein
  
Accession: EEP77702
  
Location: 6763412-6765595
  
 NCBI BlastP on this gene

EEP77702

Query: Architecture Search FASTA input

ACYE01000028 : Trichophyton verrucosum HKI 0517    Total score: 3.0     Cumulative Blast bit score: 487

Hit cluster cross-links:

Mycgr3G90785 Mycgr3T
  
Location: 0-1047

Mycgr3G90785\_Mycgr3T

Mycgr3G103262 Mycgr3
  
Location: 1147-1390

Mycgr3G103262\_Mycgr3

Mycgr3G68458 Mycgr3T
  
Location: 1490-3602

Mycgr3G68458\_Mycgr3T

Mycgr3G99145 Mycgr3T
  
Location: 3702-4326

Mycgr3G99145\_Mycgr3T

Mycgr3G103274 Mycgr3
  
Location: 4426-4957

Mycgr3G103274\_Mycgr3

Mycgr3G103264 Mycgr3
  
Location: 5057-5390

Mycgr3G103264\_Mycgr3

Mycgr3G37570 Mycgr3T
  
Location: 5490-6006

Mycgr3G37570\_Mycgr3T

Mycgr3G108094 Mycgr3
  
Location: 6106-10555

Mycgr3G108094\_Mycgr3

Mycgr3G90786 Mycgr3T
  
Location: 10655-12080

Mycgr3G90786\_Mycgr3T

Mycgr3G68429 Mycgr3T
  
Location: 12180-13440

Mycgr3G68429\_Mycgr3T

Mycgr3G68421 Mycgr3T
  
Location: 13540-17086

Mycgr3G68421\_Mycgr3T

Mycgr3G90801 Mycgr3T
  
Location: 17186-18056

Mycgr3G90801\_Mycgr3T

Mycgr3G84646 Mycgr3T
  
Location: 18156-20235

Mycgr3G84646\_Mycgr3T

Mycgr3G68456 Mycgr3T
  
Location: 20335-21970

Mycgr3G68456\_Mycgr3T

Mycgr3G103270 Mycgr3
  
Location: 22070-22355

Mycgr3G103270\_Mycgr3

Mycgr3G90803 Mycgr3T
  
Location: 22455-23019

Mycgr3G90803\_Mycgr3T

Mycgr3G36941 Mycgr3T
  
Location: 23119-24064

Mycgr3G36941\_Mycgr3T

Mycgr3G25746 Mycgr3T
  
Location: 24164-25241

Mycgr3G25746\_Mycgr3T

Mycgr3G90788 Mycgr3T
  
Location: 25341-25803

Mycgr3G90788\_Mycgr3T

Mycgr3G103260 Mycgr3
  
Location: 25903-26635

Mycgr3G103260\_Mycgr3

Mycgr3G84644 Mycgr3T
  
Location: 26735-28457

Mycgr3G84644\_Mycgr3T

Mycgr3G29227 Mycgr3T
  
Location: 28557-28863

Mycgr3G29227\_Mycgr3T

Mycgr3G36271 Mycgr3T
  
Location: 28963-29854

Mycgr3G36271\_Mycgr3T

Mycgr3G68433 Mycgr3T
  
Location: 29954-33041

Mycgr3G68433\_Mycgr3T

Mycgr3G79452 Mycgr3T
  
Location: 33141-33399

Mycgr3G79452\_Mycgr3T

Mycgr3G55345 Mycgr3T
  
Location: 33499-34126

Mycgr3G55345\_Mycgr3T

Mycgr3G103278 Mycgr3
  
Location: 34226-35195

Mycgr3G103278\_Mycgr3

Mycgr3G84654 Mycgr3T
  
Location: 35295-36630

Mycgr3G84654\_Mycgr3T

Mycgr3G108090 Mycgr3
  
Location: 36730-37591

Mycgr3G108090\_Mycgr3

Mycgr3G21922 Mycgr3T
  
Location: 37691-39149

Mycgr3G21922\_Mycgr3T

Mycgr3G99148 Mycgr3T
  
Location: 39249-42819

Mycgr3G99148\_Mycgr3T

hypothetical protein
  
Accession: EFE44690
  
Location: 32-3580
  
 NCBI BlastP on this gene

EFE44690

hypothetical protein
  
Accession: EFE44691
  
Location: 4094-4765
  
 NCBI BlastP on this gene

EFE44691

DNA-binding protein HGH1, putative
  
Accession: EFE44692
  
Location: 6074-7473
  
  
**BlastP hit with Mycgr3G25746\_Mycgr3T**
  
Percentage identity: 56 %
  
BlastP bit score: 195
  
Sequence coverage: 46 %
  
E-value: 1e-55
  
  
 NCBI BlastP on this gene

EFE44692

hypothetical protein
  
Accession: EFE44693
  
Location: 7617-8927
  
 NCBI BlastP on this gene

EFE44693

hypothetical protein
  
Accession: EFE44694
  
Location: 9075-11296
  
  
**BlastP hit with Mycgr3G21922\_Mycgr3T**
  
Percentage identity: 41 %
  
BlastP bit score: 156
  
Sequence coverage: 45 %
  
E-value: 3e-38
  
  
 NCBI BlastP on this gene

EFE44694

conserved hypothetical protein
  
Accession: EFE44695
  
Location: 12043-14426
  
  
**BlastP hit with Mycgr3G103278\_Mycgr3**
  
Percentage identity: 34 %
  
BlastP bit score: 136
  
Sequence coverage: 95 %
  
E-value: 4e-32
  
  
 NCBI BlastP on this gene

EFE44695

Query: Architecture Search FASTA input

KE148155 : Ophiostoma piceae UAMH 11346 chromosome Unknown scf10    Total score: 3.0     Cumulative Blast bit score: 486

Hit cluster cross-links:

Mycgr3G90785 Mycgr3T
  
Location: 0-1047

Mycgr3G90785\_Mycgr3T

Mycgr3G103262 Mycgr3
  
Location: 1147-1390

Mycgr3G103262\_Mycgr3

Mycgr3G68458 Mycgr3T
  
Location: 1490-3602

Mycgr3G68458\_Mycgr3T

Mycgr3G99145 Mycgr3T
  
Location: 3702-4326

Mycgr3G99145\_Mycgr3T

Mycgr3G103274 Mycgr3
  
Location: 4426-4957

Mycgr3G103274\_Mycgr3

Mycgr3G103264 Mycgr3
  
Location: 5057-5390

Mycgr3G103264\_Mycgr3

Mycgr3G37570 Mycgr3T
  
Location: 5490-6006

Mycgr3G37570\_Mycgr3T

Mycgr3G108094 Mycgr3
  
Location: 6106-10555

Mycgr3G108094\_Mycgr3

Mycgr3G90786 Mycgr3T
  
Location: 10655-12080

Mycgr3G90786\_Mycgr3T

Mycgr3G68429 Mycgr3T
  
Location: 12180-13440

Mycgr3G68429\_Mycgr3T

Mycgr3G68421 Mycgr3T
  
Location: 13540-17086

Mycgr3G68421\_Mycgr3T

Mycgr3G90801 Mycgr3T
  
Location: 17186-18056

Mycgr3G90801\_Mycgr3T

Mycgr3G84646 Mycgr3T
  
Location: 18156-20235

Mycgr3G84646\_Mycgr3T

Mycgr3G68456 Mycgr3T
  
Location: 20335-21970

Mycgr3G68456\_Mycgr3T

Mycgr3G103270 Mycgr3
  
Location: 22070-22355

Mycgr3G103270\_Mycgr3

Mycgr3G90803 Mycgr3T
  
Location: 22455-23019

Mycgr3G90803\_Mycgr3T

Mycgr3G36941 Mycgr3T
  
Location: 23119-24064

Mycgr3G36941\_Mycgr3T

Mycgr3G25746 Mycgr3T
  
Location: 24164-25241

Mycgr3G25746\_Mycgr3T

Mycgr3G90788 Mycgr3T
  
Location: 25341-25803

Mycgr3G90788\_Mycgr3T

Mycgr3G103260 Mycgr3
  
Location: 25903-26635

Mycgr3G103260\_Mycgr3

Mycgr3G84644 Mycgr3T
  
Location: 26735-28457

Mycgr3G84644\_Mycgr3T

Mycgr3G29227 Mycgr3T
  
Location: 28557-28863

Mycgr3G29227\_Mycgr3T

Mycgr3G36271 Mycgr3T
  
Location: 28963-29854

Mycgr3G36271\_Mycgr3T

Mycgr3G68433 Mycgr3T
  
Location: 29954-33041

Mycgr3G68433\_Mycgr3T

Mycgr3G79452 Mycgr3T
  
Location: 33141-33399

Mycgr3G79452\_Mycgr3T

Mycgr3G55345 Mycgr3T
  
Location: 33499-34126

Mycgr3G55345\_Mycgr3T

Mycgr3G103278 Mycgr3
  
Location: 34226-35195

Mycgr3G103278\_Mycgr3

Mycgr3G84654 Mycgr3T
  
Location: 35295-36630

Mycgr3G84654\_Mycgr3T

Mycgr3G108090 Mycgr3
  
Location: 36730-37591

Mycgr3G108090\_Mycgr3

Mycgr3G21922 Mycgr3T
  
Location: 37691-39149

Mycgr3G21922\_Mycgr3T

Mycgr3G99148 Mycgr3T
  
Location: 39249-42819

Mycgr3G99148\_Mycgr3T

acetyl- carboxylase
  
Accession: EPE05929
  
Location: 1139478-1146323
  
 NCBI BlastP on this gene

EPE05929

integral membrane protein
  
Accession: EPE05928
  
Location: 1137326-1138062
  
 NCBI BlastP on this gene

EPE05928

hypoxanthine guanine phosphoribosyltransferase
  
Accession: EPE05927
  
Location: 1134075-1135014
  
  
**BlastP hit with Mycgr3G55345\_Mycgr3T**
  
Percentage identity: 72 %
  
BlastP bit score: 306
  
Sequence coverage: 96 %
  
E-value: 2e-102
  
  
 NCBI BlastP on this gene

EPE05927

oxidoreductase-like protein
  
Accession: EPE05926
  
Location: 1132740-1133774
  
 NCBI BlastP on this gene

EPE05926

hypothetical protein
  
Accession: EPE05925
  
Location: 1129600-1132095
  
 NCBI BlastP on this gene

EPE05925

dolichyl-phosphate mannosyltransferase polypeptide 3
  
Accession: EPE05924
  
Location: 1128722-1129233
  
 NCBI BlastP on this gene

EPE05924

multidrug resistant protein
  
Accession: EPE05923
  
Location: 1121135-1123063
  
 NCBI BlastP on this gene

EPE05923

hypothetical protein
  
Accession: EPE05922
  
Location: 1119994-1120485
  
 NCBI BlastP on this gene

EPE05922

leucine-rich repeat-containing protein 40
  
Accession: EPE05921
  
Location: 1113921-1117716
  
  
**BlastP hit with Mycgr3G68433\_Mycgr3T**
  
Percentage identity: 37 %
  
BlastP bit score: 114
  
Sequence coverage: 22 %
  
E-value: 2e-22
  
  
 NCBI BlastP on this gene

EPE05921

hypothetical protein
  
Accession: EPE05920
  
Location: 1111323-1112698
  
 NCBI BlastP on this gene

EPE05920

3-ketoacyl- thiolase
  
Accession: EPE05919
  
Location: 1109647-1110963
  
 NCBI BlastP on this gene

EPE05919

fad-dependent oxidoreductase-like enzyme
  
Accession: EPE05918
  
Location: 1106019-1108487
  
  
**BlastP hit with Mycgr3G90786\_Mycgr3T**
  
Percentage identity: 26 %
  
BlastP bit score: 66
  
Sequence coverage: 54 %
  
E-value: 2e-08
  
  
 NCBI BlastP on this gene

EPE05918

enolase
  
Accession: EPE05917
  
Location: 1103315-1104932
  
 NCBI BlastP on this gene

EPE05917

salicylate hydroxylase
  
Accession: EPE05916
  
Location: 1101001-1102323
  
 NCBI BlastP on this gene

EPE05916

allantoate permease
  
Accession: EPE05915
  
Location: 1097982-1099622
  
 NCBI BlastP on this gene

EPE05915

tat pathway signal sequence
  
Accession: EPE05914
  
Location: 1093507-1097298
  
 NCBI BlastP on this gene

EPE05914

Query: Architecture Search FASTA input

AGUE01000023 : Glarea lozoyensis 74030    Total score: 3.0     Cumulative Blast bit score: 478

Hit cluster cross-links:

Mycgr3G90785 Mycgr3T
  
Location: 0-1047

Mycgr3G90785\_Mycgr3T

Mycgr3G103262 Mycgr3
  
Location: 1147-1390

Mycgr3G103262\_Mycgr3

Mycgr3G68458 Mycgr3T
  
Location: 1490-3602

Mycgr3G68458\_Mycgr3T

Mycgr3G99145 Mycgr3T
  
Location: 3702-4326

Mycgr3G99145\_Mycgr3T

Mycgr3G103274 Mycgr3
  
Location: 4426-4957

Mycgr3G103274\_Mycgr3

Mycgr3G103264 Mycgr3
  
Location: 5057-5390

Mycgr3G103264\_Mycgr3

Mycgr3G37570 Mycgr3T
  
Location: 5490-6006

Mycgr3G37570\_Mycgr3T

Mycgr3G108094 Mycgr3
  
Location: 6106-10555

Mycgr3G108094\_Mycgr3

Mycgr3G90786 Mycgr3T
  
Location: 10655-12080

Mycgr3G90786\_Mycgr3T

Mycgr3G68429 Mycgr3T
  
Location: 12180-13440

Mycgr3G68429\_Mycgr3T

Mycgr3G68421 Mycgr3T
  
Location: 13540-17086

Mycgr3G68421\_Mycgr3T

Mycgr3G90801 Mycgr3T
  
Location: 17186-18056

Mycgr3G90801\_Mycgr3T

Mycgr3G84646 Mycgr3T
  
Location: 18156-20235

Mycgr3G84646\_Mycgr3T

Mycgr3G68456 Mycgr3T
  
Location: 20335-21970

Mycgr3G68456\_Mycgr3T

Mycgr3G103270 Mycgr3
  
Location: 22070-22355

Mycgr3G103270\_Mycgr3

Mycgr3G90803 Mycgr3T
  
Location: 22455-23019

Mycgr3G90803\_Mycgr3T

Mycgr3G36941 Mycgr3T
  
Location: 23119-24064

Mycgr3G36941\_Mycgr3T

Mycgr3G25746 Mycgr3T
  
Location: 24164-25241

Mycgr3G25746\_Mycgr3T

Mycgr3G90788 Mycgr3T
  
Location: 25341-25803

Mycgr3G90788\_Mycgr3T

Mycgr3G103260 Mycgr3
  
Location: 25903-26635

Mycgr3G103260\_Mycgr3

Mycgr3G84644 Mycgr3T
  
Location: 26735-28457

Mycgr3G84644\_Mycgr3T

Mycgr3G29227 Mycgr3T
  
Location: 28557-28863

Mycgr3G29227\_Mycgr3T

Mycgr3G36271 Mycgr3T
  
Location: 28963-29854

Mycgr3G36271\_Mycgr3T

Mycgr3G68433 Mycgr3T
  
Location: 29954-33041

Mycgr3G68433\_Mycgr3T

Mycgr3G79452 Mycgr3T
  
Location: 33141-33399

Mycgr3G79452\_Mycgr3T

Mycgr3G55345 Mycgr3T
  
Location: 33499-34126

Mycgr3G55345\_Mycgr3T

Mycgr3G103278 Mycgr3
  
Location: 34226-35195

Mycgr3G103278\_Mycgr3

Mycgr3G84654 Mycgr3T
  
Location: 35295-36630

Mycgr3G84654\_Mycgr3T

Mycgr3G108090 Mycgr3
  
Location: 36730-37591

Mycgr3G108090\_Mycgr3

Mycgr3G21922 Mycgr3T
  
Location: 37691-39149

Mycgr3G21922\_Mycgr3T

Mycgr3G99148 Mycgr3T
  
Location: 39249-42819

Mycgr3G99148\_Mycgr3T

hypothetical protein
  
Accession: EHL02457
  
Location: 422648-424518
  
 NCBI BlastP on this gene

EHL02457

hypothetical protein
  
Accession: EHL02458
  
Location: 424841-425421
  
 NCBI BlastP on this gene

EHL02458

putative RNA polymerase II transcription factor B subunit 2
  
Accession: EHL02459
  
Location: 425787-426437
  
 NCBI BlastP on this gene

EHL02459

putative High-affinity glucose transporter
  
Accession: EHL02460
  
Location: 430914-432167
  
 NCBI BlastP on this gene

EHL02460

hypothetical protein
  
Accession: EHL02461
  
Location: 435107-436549
  
 NCBI BlastP on this gene

EHL02461

hypothetical protein
  
Accession: EHL02462
  
Location: 437197-437820
  
 NCBI BlastP on this gene

EHL02462

hypothetical protein
  
Accession: EHL02463
  
Location: 441130-442641
  
  
**BlastP hit with Mycgr3G90786\_Mycgr3T**
  
Percentage identity: 28 %
  
BlastP bit score: 73
  
Sequence coverage: 90 %
  
E-value: 5e-11
  
  
 NCBI BlastP on this gene

EHL02463

putative Leucine-rich repeat-containing protein 40
  
Accession: EHL02464
  
Location: 444731-446235
  
  
**BlastP hit with Mycgr3G68433\_Mycgr3T**
  
Percentage identity: 47 %
  
BlastP bit score: 147
  
Sequence coverage: 17 %
  
E-value: 2e-35
  
  
 NCBI BlastP on this gene

EHL02464

hypothetical protein
  
Accession: EHL02465
  
Location: 447325-449096
  
 NCBI BlastP on this gene

EHL02465

putative N-acyl homoserine lactonase AttM
  
Accession: EHL02466
  
Location: 449165-450513
  
 NCBI BlastP on this gene

EHL02466

putative Xanthine phosphoribosyltransferase 1
  
Accession: EHL02467
  
Location: 451360-451983
  
  
**BlastP hit with Mycgr3G55345\_Mycgr3T**
  
Percentage identity: 73 %
  
BlastP bit score: 258
  
Sequence coverage: 85 %
  
E-value: 9e-84
  
  
 NCBI BlastP on this gene

EHL02467

putative D-3-phosphoglycerate dehydrogenase
  
Accession: EHL02468
  
Location: 452368-453934
  
 NCBI BlastP on this gene

EHL02468

hypothetical protein
  
Accession: EHL02469
  
Location: 464804-465175
  
 NCBI BlastP on this gene

EHL02469

putative UPF0665 family protein C23C4.06c
  
Accession: EHL02470
  
Location: 468391-472283
  
 NCBI BlastP on this gene

EHL02470

Query: Architecture Search FASTA input

KB445561 : Baudoinia compniacensis UAMH 10762 unplaced genomic scaffold BAUCOscaffold\_12    Total score: 2.0     Cumulative Blast bit score: 1570

Hit cluster cross-links:

Mycgr3G90785 Mycgr3T
  
Location: 0-1047

Mycgr3G90785\_Mycgr3T

Mycgr3G103262 Mycgr3
  
Location: 1147-1390

Mycgr3G103262\_Mycgr3

Mycgr3G68458 Mycgr3T
  
Location: 1490-3602

Mycgr3G68458\_Mycgr3T

Mycgr3G99145 Mycgr3T
  
Location: 3702-4326

Mycgr3G99145\_Mycgr3T

Mycgr3G103274 Mycgr3
  
Location: 4426-4957

Mycgr3G103274\_Mycgr3

Mycgr3G103264 Mycgr3
  
Location: 5057-5390

Mycgr3G103264\_Mycgr3

Mycgr3G37570 Mycgr3T
  
Location: 5490-6006

Mycgr3G37570\_Mycgr3T

Mycgr3G108094 Mycgr3
  
Location: 6106-10555

Mycgr3G108094\_Mycgr3

Mycgr3G90786 Mycgr3T
  
Location: 10655-12080

Mycgr3G90786\_Mycgr3T

Mycgr3G68429 Mycgr3T
  
Location: 12180-13440

Mycgr3G68429\_Mycgr3T

Mycgr3G68421 Mycgr3T
  
Location: 13540-17086

Mycgr3G68421\_Mycgr3T

Mycgr3G90801 Mycgr3T
  
Location: 17186-18056

Mycgr3G90801\_Mycgr3T

Mycgr3G84646 Mycgr3T
  
Location: 18156-20235

Mycgr3G84646\_Mycgr3T

Mycgr3G68456 Mycgr3T
  
Location: 20335-21970

Mycgr3G68456\_Mycgr3T

Mycgr3G103270 Mycgr3
  
Location: 22070-22355

Mycgr3G103270\_Mycgr3

Mycgr3G90803 Mycgr3T
  
Location: 22455-23019

Mycgr3G90803\_Mycgr3T

Mycgr3G36941 Mycgr3T
  
Location: 23119-24064

Mycgr3G36941\_Mycgr3T

Mycgr3G25746 Mycgr3T
  
Location: 24164-25241

Mycgr3G25746\_Mycgr3T

Mycgr3G90788 Mycgr3T
  
Location: 25341-25803

Mycgr3G90788\_Mycgr3T

Mycgr3G103260 Mycgr3
  
Location: 25903-26635

Mycgr3G103260\_Mycgr3

Mycgr3G84644 Mycgr3T
  
Location: 26735-28457

Mycgr3G84644\_Mycgr3T

Mycgr3G29227 Mycgr3T
  
Location: 28557-28863

Mycgr3G29227\_Mycgr3T

Mycgr3G36271 Mycgr3T
  
Location: 28963-29854

Mycgr3G36271\_Mycgr3T

Mycgr3G68433 Mycgr3T
  
Location: 29954-33041

Mycgr3G68433\_Mycgr3T

Mycgr3G79452 Mycgr3T
  
Location: 33141-33399

Mycgr3G79452\_Mycgr3T

Mycgr3G55345 Mycgr3T
  
Location: 33499-34126

Mycgr3G55345\_Mycgr3T

Mycgr3G103278 Mycgr3
  
Location: 34226-35195

Mycgr3G103278\_Mycgr3

Mycgr3G84654 Mycgr3T
  
Location: 35295-36630

Mycgr3G84654\_Mycgr3T

Mycgr3G108090 Mycgr3
  
Location: 36730-37591

Mycgr3G108090\_Mycgr3

Mycgr3G21922 Mycgr3T
  
Location: 37691-39149

Mycgr3G21922\_Mycgr3T

Mycgr3G99148 Mycgr3T
  
Location: 39249-42819

Mycgr3G99148\_Mycgr3T

hypothetical protein
  
Accession: EMC92701
  
Location: 325868-326641
  
 NCBI BlastP on this gene

EMC92701

hypothetical protein
  
Accession: EMC92702
  
Location: 327131-327544
  
 NCBI BlastP on this gene

EMC92702

hypothetical protein
  
Accession: EMC92703
  
Location: 328253-329119
  
 NCBI BlastP on this gene

EMC92703

hypothetical protein
  
Accession: EMC92704
  
Location: 329180-330739
  
 NCBI BlastP on this gene

EMC92704

hypothetical protein
  
Accession: EMC92705
  
Location: 331313-335497
  
 NCBI BlastP on this gene

EMC92705

hypothetical protein
  
Accession: EMC92706
  
Location: 335985-337720
  
 NCBI BlastP on this gene

EMC92706

hypothetical protein
  
Accession: EMC92707
  
Location: 338175-338828
  
 NCBI BlastP on this gene

EMC92707

carbohydrate esterase family 8 protein
  
Accession: EMC92708
  
Location: 339208-340488
  
 NCBI BlastP on this gene

EMC92708

hypothetical protein
  
Accession: EMC92709
  
Location: 341046-342137
  
 NCBI BlastP on this gene

EMC92709

hypothetical protein
  
Accession: EMC92710
  
Location: 342639-344320
  
  
**BlastP hit with Mycgr3G84654\_Mycgr3T**
  
Percentage identity: 74 %
  
BlastP bit score: 498
  
Sequence coverage: 74 %
  
E-value: 5e-169
  
  
 NCBI BlastP on this gene

EMC92710

glycoside hydrolase family 3 protein
  
Accession: EMC92711
  
Location: 344957-347878
  
 NCBI BlastP on this gene

EMC92711

carbohydrate esterase family 9 protein
  
Accession: EMC92712
  
Location: 348546-349859
  
 NCBI BlastP on this gene

EMC92712

hypothetical protein
  
Accession: EMC92713
  
Location: 350204-352345
  
  
**BlastP hit with Mycgr3G68458\_Mycgr3T**
  
Percentage identity: 74 %
  
BlastP bit score: 1073
  
Sequence coverage: 100 %
  
E-value: 0.0
  
  
 NCBI BlastP on this gene

EMC92713

hypothetical protein
  
Accession: EMC92714
  
Location: 352562-352891
  
 NCBI BlastP on this gene

EMC92714

hypothetical protein
  
Accession: EMC92715
  
Location: 353474-354937
  
 NCBI BlastP on this gene

EMC92715

hypothetical protein
  
Accession: EMC92716
  
Location: 355236-356741
  
 NCBI BlastP on this gene

EMC92716

hypothetical protein
  
Accession: EMC92717
  
Location: 357461-357655
  
 NCBI BlastP on this gene

EMC92717

hypothetical protein
  
Accession: EMC92718
  
Location: 358144-359587
  
 NCBI BlastP on this gene

EMC92718

hypothetical protein
  
Accession: EMC92719
  
Location: 360407-362740
  
 NCBI BlastP on this gene

EMC92719

hypothetical protein
  
Accession: EMC92720
  
Location: 363457-364234
  
 NCBI BlastP on this gene

EMC92720

hypothetical protein
  
Accession: EMC92721
  
Location: 364886-367775
  
 NCBI BlastP on this gene

EMC92721

hypothetical protein
  
Accession: EMC92722
  
Location: 368811-371612
  
 NCBI BlastP on this gene

EMC92722

Query: Architecture Search FASTA input

KB446573 : Pseudocercospora fijiensis CIRAD86 unplaced genomic scaffold MYCFIscaffold\_19    Total score: 2.0     Cumulative Blast bit score: 1450

Hit cluster cross-links:

Mycgr3G90785 Mycgr3T
  
Location: 0-1047

Mycgr3G90785\_Mycgr3T

Mycgr3G103262 Mycgr3
  
Location: 1147-1390

Mycgr3G103262\_Mycgr3

Mycgr3G68458 Mycgr3T
  
Location: 1490-3602

Mycgr3G68458\_Mycgr3T

Mycgr3G99145 Mycgr3T
  
Location: 3702-4326

Mycgr3G99145\_Mycgr3T

Mycgr3G103274 Mycgr3
  
Location: 4426-4957

Mycgr3G103274\_Mycgr3

Mycgr3G103264 Mycgr3
  
Location: 5057-5390

Mycgr3G103264\_Mycgr3

Mycgr3G37570 Mycgr3T
  
Location: 5490-6006

Mycgr3G37570\_Mycgr3T

Mycgr3G108094 Mycgr3
  
Location: 6106-10555

Mycgr3G108094\_Mycgr3

Mycgr3G90786 Mycgr3T
  
Location: 10655-12080

Mycgr3G90786\_Mycgr3T

Mycgr3G68429 Mycgr3T
  
Location: 12180-13440

Mycgr3G68429\_Mycgr3T

Mycgr3G68421 Mycgr3T
  
Location: 13540-17086

Mycgr3G68421\_Mycgr3T

Mycgr3G90801 Mycgr3T
  
Location: 17186-18056

Mycgr3G90801\_Mycgr3T

Mycgr3G84646 Mycgr3T
  
Location: 18156-20235

Mycgr3G84646\_Mycgr3T

Mycgr3G68456 Mycgr3T
  
Location: 20335-21970

Mycgr3G68456\_Mycgr3T

Mycgr3G103270 Mycgr3
  
Location: 22070-22355

Mycgr3G103270\_Mycgr3

Mycgr3G90803 Mycgr3T
  
Location: 22455-23019

Mycgr3G90803\_Mycgr3T

Mycgr3G36941 Mycgr3T
  
Location: 23119-24064

Mycgr3G36941\_Mycgr3T

Mycgr3G25746 Mycgr3T
  
Location: 24164-25241

Mycgr3G25746\_Mycgr3T

Mycgr3G90788 Mycgr3T
  
Location: 25341-25803

Mycgr3G90788\_Mycgr3T

Mycgr3G103260 Mycgr3
  
Location: 25903-26635

Mycgr3G103260\_Mycgr3

Mycgr3G84644 Mycgr3T
  
Location: 26735-28457

Mycgr3G84644\_Mycgr3T

Mycgr3G29227 Mycgr3T
  
Location: 28557-28863

Mycgr3G29227\_Mycgr3T

Mycgr3G36271 Mycgr3T
  
Location: 28963-29854

Mycgr3G36271\_Mycgr3T

Mycgr3G68433 Mycgr3T
  
Location: 29954-33041

Mycgr3G68433\_Mycgr3T

Mycgr3G79452 Mycgr3T
  
Location: 33141-33399

Mycgr3G79452\_Mycgr3T

Mycgr3G55345 Mycgr3T
  
Location: 33499-34126

Mycgr3G55345\_Mycgr3T

Mycgr3G103278 Mycgr3
  
Location: 34226-35195

Mycgr3G103278\_Mycgr3

Mycgr3G84654 Mycgr3T
  
Location: 35295-36630

Mycgr3G84654\_Mycgr3T

Mycgr3G108090 Mycgr3
  
Location: 36730-37591

Mycgr3G108090\_Mycgr3

Mycgr3G21922 Mycgr3T
  
Location: 37691-39149

Mycgr3G21922\_Mycgr3T

Mycgr3G99148 Mycgr3T
  
Location: 39249-42819

Mycgr3G99148\_Mycgr3T

hypothetical protein
  
Accession: EME76936
  
Location: 443548-444135
  
 NCBI BlastP on this gene

EME76936

hypothetical protein
  
Accession: EME76937
  
Location: 445109-445748
  
 NCBI BlastP on this gene

EME76937

hypothetical protein
  
Accession: EME76938
  
Location: 447028-448760
  
 NCBI BlastP on this gene

EME76938

hypothetical protein
  
Accession: EME76939
  
Location: 450124-451679
  
 NCBI BlastP on this gene

EME76939

hypothetical protein
  
Accession: EME76940
  
Location: 451889-452646
  
 NCBI BlastP on this gene

EME76940

hypothetical protein
  
Accession: EME76941
  
Location: 453293-454615
  
 NCBI BlastP on this gene

EME76941

hypothetical protein
  
Accession: EME76942
  
Location: 454928-455726
  
 NCBI BlastP on this gene

EME76942

hypothetical protein
  
Accession: EME76943
  
Location: 456819-458525
  
 NCBI BlastP on this gene

EME76943

hypothetical protein
  
Accession: EME76944
  
Location: 460319-462682
  
 NCBI BlastP on this gene

EME76944

hypothetical protein
  
Accession: EME76945
  
Location: 463066-464403
  
  
**BlastP hit with Mycgr3G25746\_Mycgr3T**
  
Percentage identity: 69 %
  
BlastP bit score: 491
  
Sequence coverage: 100 %
  
E-value: 6e-170
  
  
 NCBI BlastP on this gene

EME76945

hypothetical protein
  
Accession: EME76946
  
Location: 464689-469309
  
  
**BlastP hit with Mycgr3G108094\_Mycgr3**
  
Percentage identity: 42 %
  
BlastP bit score: 959
  
Sequence coverage: 106 %
  
E-value: 0.0
  
  
 NCBI BlastP on this gene

EME76946

hypothetical protein
  
Accession: EME76947
  
Location: 469383-471699
  
 NCBI BlastP on this gene

EME76947

Query: Architecture Search FASTA input

AKCU01000112 : Penicillium digitatum Pd1    Total score: 2.0     Cumulative Blast bit score: 1424

Hit cluster cross-links:

Mycgr3G90785 Mycgr3T
  
Location: 0-1047

Mycgr3G90785\_Mycgr3T

Mycgr3G103262 Mycgr3
  
Location: 1147-1390

Mycgr3G103262\_Mycgr3

Mycgr3G68458 Mycgr3T
  
Location: 1490-3602

Mycgr3G68458\_Mycgr3T

Mycgr3G99145 Mycgr3T
  
Location: 3702-4326

Mycgr3G99145\_Mycgr3T

Mycgr3G103274 Mycgr3
  
Location: 4426-4957

Mycgr3G103274\_Mycgr3

Mycgr3G103264 Mycgr3
  
Location: 5057-5390

Mycgr3G103264\_Mycgr3

Mycgr3G37570 Mycgr3T
  
Location: 5490-6006

Mycgr3G37570\_Mycgr3T

Mycgr3G108094 Mycgr3
  
Location: 6106-10555

Mycgr3G108094\_Mycgr3

Mycgr3G90786 Mycgr3T
  
Location: 10655-12080

Mycgr3G90786\_Mycgr3T

Mycgr3G68429 Mycgr3T
  
Location: 12180-13440

Mycgr3G68429\_Mycgr3T

Mycgr3G68421 Mycgr3T
  
Location: 13540-17086

Mycgr3G68421\_Mycgr3T

Mycgr3G90801 Mycgr3T
  
Location: 17186-18056

Mycgr3G90801\_Mycgr3T

Mycgr3G84646 Mycgr3T
  
Location: 18156-20235

Mycgr3G84646\_Mycgr3T

Mycgr3G68456 Mycgr3T
  
Location: 20335-21970

Mycgr3G68456\_Mycgr3T

Mycgr3G103270 Mycgr3
  
Location: 22070-22355

Mycgr3G103270\_Mycgr3

Mycgr3G90803 Mycgr3T
  
Location: 22455-23019

Mycgr3G90803\_Mycgr3T

Mycgr3G36941 Mycgr3T
  
Location: 23119-24064

Mycgr3G36941\_Mycgr3T

Mycgr3G25746 Mycgr3T
  
Location: 24164-25241

Mycgr3G25746\_Mycgr3T

Mycgr3G90788 Mycgr3T
  
Location: 25341-25803

Mycgr3G90788\_Mycgr3T

Mycgr3G103260 Mycgr3
  
Location: 25903-26635

Mycgr3G103260\_Mycgr3

Mycgr3G84644 Mycgr3T
  
Location: 26735-28457

Mycgr3G84644\_Mycgr3T

Mycgr3G29227 Mycgr3T
  
Location: 28557-28863

Mycgr3G29227\_Mycgr3T

Mycgr3G36271 Mycgr3T
  
Location: 28963-29854

Mycgr3G36271\_Mycgr3T

Mycgr3G68433 Mycgr3T
  
Location: 29954-33041

Mycgr3G68433\_Mycgr3T

Mycgr3G79452 Mycgr3T
  
Location: 33141-33399

Mycgr3G79452\_Mycgr3T

Mycgr3G55345 Mycgr3T
  
Location: 33499-34126

Mycgr3G55345\_Mycgr3T

Mycgr3G103278 Mycgr3
  
Location: 34226-35195

Mycgr3G103278\_Mycgr3

Mycgr3G84654 Mycgr3T
  
Location: 35295-36630

Mycgr3G84654\_Mycgr3T

Mycgr3G108090 Mycgr3
  
Location: 36730-37591

Mycgr3G108090\_Mycgr3

Mycgr3G21922 Mycgr3T
  
Location: 37691-39149

Mycgr3G21922\_Mycgr3T

Mycgr3G99148 Mycgr3T
  
Location: 39249-42819

Mycgr3G99148\_Mycgr3T

hypothetical protein
  
Accession: EKV20491
  
Location: 113201-113971
  
 NCBI BlastP on this gene

EKV20491

hypothetical protein
  
Accession: EKV20492
  
Location: 114606-116077
  
 NCBI BlastP on this gene

EKV20492

hypothetical protein
  
Accession: EKV20493
  
Location: 118411-118863
  
 NCBI BlastP on this gene

EKV20493

hypothetical protein
  
Accession: EKV20494
  
Location: 119780-121267
  
 NCBI BlastP on this gene

EKV20494

60S ribosomal protein L13
  
Accession: EKV20495
  
Location: 121879-122996
  
 NCBI BlastP on this gene

EKV20495

hypothetical protein
  
Accession: EKV20496
  
Location: 123682-124929
  
 NCBI BlastP on this gene

EKV20496

putative leucine-rich repeat protein
  
Accession: EKV20497
  
Location: 126623-129736
  
  
**BlastP hit with Mycgr3G68433\_Mycgr3T**
  
Percentage identity: 39 %
  
BlastP bit score: 505
  
Sequence coverage: 89 %
  
E-value: 7e-158
  
  
 NCBI BlastP on this gene

EKV20497

hypothetical protein
  
Accession: EKV20498
  
Location: 129884-130987
  
 NCBI BlastP on this gene

EKV20498

hypothetical protein
  
Accession: EKV20499
  
Location: 131729-132961
  
 NCBI BlastP on this gene

EKV20499

Alpha/beta hydrolase, putative
  
Accession: EKV20500
  
Location: 133560-134595
  
 NCBI BlastP on this gene

EKV20500

Actin family protein
  
Accession: EKV20501
  
Location: 135167-136748
  
 NCBI BlastP on this gene

EKV20501

hypothetical protein
  
Accession: EKV20502
  
Location: 137642-138112
  
 NCBI BlastP on this gene

EKV20502

3-hydroxybutyryl-CoA dehydrogenase, putative
  
Accession: EKV20503
  
Location: 138413-139487
  
 NCBI BlastP on this gene

EKV20503

DNA repair protein (Tof1), putative
  
Accession: EKV20504
  
Location: 142765-146391
  
  
**BlastP hit with Mycgr3G68421\_Mycgr3T**
  
Percentage identity: 43 %
  
BlastP bit score: 919
  
Sequence coverage: 102 %
  
E-value: 0.0
  
  
 NCBI BlastP on this gene

EKV20504

hypothetical protein
  
Accession: EKV20505
  
Location: 146552-146848
  
 NCBI BlastP on this gene

EKV20505

hypothetical protein
  
Accession: EKV20506
  
Location: 147447-147590
  
 NCBI BlastP on this gene

EKV20506

Phenazine biosynthesis-like protein, putative
  
Accession: EKV20507
  
Location: 147720-148664
  
 NCBI BlastP on this gene

EKV20507

hypothetical protein
  
Accession: EKV20508
  
Location: 149775-150260
  
 NCBI BlastP on this gene

EKV20508

hypothetical protein
  
Accession: EKV20509
  
Location: 155167-155610
  
 NCBI BlastP on this gene

EKV20509

hypothetical protein
  
Accession: EKV20510
  
Location: 157854-158042
  
 NCBI BlastP on this gene

EKV20510

hypothetical protein
  
Accession: EKV20511
  
Location: 158546-158797
  
 NCBI BlastP on this gene

EKV20511

Query: Architecture Search FASTA input

AKCT01000108 : Penicillium digitatum PHI26    Total score: 2.0     Cumulative Blast bit score: 1424

Hit cluster cross-links:

Mycgr3G90785 Mycgr3T
  
Location: 0-1047

Mycgr3G90785\_Mycgr3T

Mycgr3G103262 Mycgr3
  
Location: 1147-1390

Mycgr3G103262\_Mycgr3

Mycgr3G68458 Mycgr3T
  
Location: 1490-3602

Mycgr3G68458\_Mycgr3T

Mycgr3G99145 Mycgr3T
  
Location: 3702-4326

Mycgr3G99145\_Mycgr3T

Mycgr3G103274 Mycgr3
  
Location: 4426-4957

Mycgr3G103274\_Mycgr3

Mycgr3G103264 Mycgr3
  
Location: 5057-5390

Mycgr3G103264\_Mycgr3

Mycgr3G37570 Mycgr3T
  
Location: 5490-6006

Mycgr3G37570\_Mycgr3T

Mycgr3G108094 Mycgr3
  
Location: 6106-10555

Mycgr3G108094\_Mycgr3

Mycgr3G90786 Mycgr3T
  
Location: 10655-12080

Mycgr3G90786\_Mycgr3T

Mycgr3G68429 Mycgr3T
  
Location: 12180-13440

Mycgr3G68429\_Mycgr3T

Mycgr3G68421 Mycgr3T
  
Location: 13540-17086

Mycgr3G68421\_Mycgr3T

Mycgr3G90801 Mycgr3T
  
Location: 17186-18056

Mycgr3G90801\_Mycgr3T

Mycgr3G84646 Mycgr3T
  
Location: 18156-20235

Mycgr3G84646\_Mycgr3T

Mycgr3G68456 Mycgr3T
  
Location: 20335-21970

Mycgr3G68456\_Mycgr3T

Mycgr3G103270 Mycgr3
  
Location: 22070-22355

Mycgr3G103270\_Mycgr3

Mycgr3G90803 Mycgr3T
  
Location: 22455-23019

Mycgr3G90803\_Mycgr3T

Mycgr3G36941 Mycgr3T
  
Location: 23119-24064

Mycgr3G36941\_Mycgr3T

Mycgr3G25746 Mycgr3T
  
Location: 24164-25241

Mycgr3G25746\_Mycgr3T

Mycgr3G90788 Mycgr3T
  
Location: 25341-25803

Mycgr3G90788\_Mycgr3T

Mycgr3G103260 Mycgr3
  
Location: 25903-26635

Mycgr3G103260\_Mycgr3

Mycgr3G84644 Mycgr3T
  
Location: 26735-28457

Mycgr3G84644\_Mycgr3T

Mycgr3G29227 Mycgr3T
  
Location: 28557-28863

Mycgr3G29227\_Mycgr3T

Mycgr3G36271 Mycgr3T
  
Location: 28963-29854

Mycgr3G36271\_Mycgr3T

Mycgr3G68433 Mycgr3T
  
Location: 29954-33041

Mycgr3G68433\_Mycgr3T

Mycgr3G79452 Mycgr3T
  
Location: 33141-33399

Mycgr3G79452\_Mycgr3T

Mycgr3G55345 Mycgr3T
  
Location: 33499-34126

Mycgr3G55345\_Mycgr3T

Mycgr3G103278 Mycgr3
  
Location: 34226-35195

Mycgr3G103278\_Mycgr3

Mycgr3G84654 Mycgr3T
  
Location: 35295-36630

Mycgr3G84654\_Mycgr3T

Mycgr3G108090 Mycgr3
  
Location: 36730-37591

Mycgr3G108090\_Mycgr3

Mycgr3G21922 Mycgr3T
  
Location: 37691-39149

Mycgr3G21922\_Mycgr3T

Mycgr3G99148 Mycgr3T
  
Location: 39249-42819

Mycgr3G99148\_Mycgr3T

hypothetical protein
  
Accession: EKV15979
  
Location: 267228-267998
  
 NCBI BlastP on this gene

EKV15979

hypothetical protein
  
Accession: EKV15980
  
Location: 268635-270106
  
 NCBI BlastP on this gene

EKV15980

hypothetical protein
  
Accession: EKV15981
  
Location: 272442-272894
  
 NCBI BlastP on this gene

EKV15981

hypothetical protein
  
Accession: EKV15982
  
Location: 273811-275298
  
 NCBI BlastP on this gene

EKV15982

60S ribosomal protein L13
  
Accession: EKV15983
  
Location: 275910-277027
  
 NCBI BlastP on this gene

EKV15983

hypothetical protein
  
Accession: EKV15984
  
Location: 277714-278961
  
 NCBI BlastP on this gene

EKV15984

putative leucine-rich repeat protein
  
Accession: EKV15985
  
Location: 280655-283768
  
  
**BlastP hit with Mycgr3G68433\_Mycgr3T**
  
Percentage identity: 39 %
  
BlastP bit score: 505
  
Sequence coverage: 89 %
  
E-value: 7e-158
  
  
 NCBI BlastP on this gene

EKV15985

hypothetical protein
  
Accession: EKV15986
  
Location: 283916-285019
  
 NCBI BlastP on this gene

EKV15986

hypothetical protein
  
Accession: EKV15987
  
Location: 285761-286993
  
 NCBI BlastP on this gene

EKV15987

Alpha/beta hydrolase, putative
  
Accession: EKV15988
  
Location: 287592-288627
  
 NCBI BlastP on this gene

EKV15988

Actin family protein
  
Accession: EKV15989
  
Location: 289199-290780
  
 NCBI BlastP on this gene

EKV15989

hypothetical protein
  
Accession: EKV15990
  
Location: 291674-292144
  
 NCBI BlastP on this gene

EKV15990

3-hydroxybutyryl-CoA dehydrogenase, putative
  
Accession: EKV15991
  
Location: 292445-293519
  
 NCBI BlastP on this gene

EKV15991

DNA repair protein (Tof1), putative
  
Accession: EKV15992
  
Location: 296797-300423
  
  
**BlastP hit with Mycgr3G68421\_Mycgr3T**
  
Percentage identity: 43 %
  
BlastP bit score: 919
  
Sequence coverage: 102 %
  
E-value: 0.0
  
  
 NCBI BlastP on this gene

EKV15992

hypothetical protein
  
Accession: EKV15993
  
Location: 300584-300880
  
 NCBI BlastP on this gene

EKV15993

hypothetical protein
  
Accession: EKV15994
  
Location: 301479-301622
  
 NCBI BlastP on this gene

EKV15994

Phenazine biosynthesis-like protein, putative
  
Accession: EKV15995
  
Location: 301752-302696
  
 NCBI BlastP on this gene

EKV15995

hypothetical protein
  
Accession: EKV15996
  
Location: 303807-304292
  
 NCBI BlastP on this gene

EKV15996

hypothetical protein
  
Accession: EKV15997
  
Location: 309199-309642
  
 NCBI BlastP on this gene

EKV15997

hypothetical protein
  
Accession: EKV15998
  
Location: 311916-312104
  
 NCBI BlastP on this gene

EKV15998

hypothetical protein
  
Accession: EKV15999
  
Location: 312608-312859
  
 NCBI BlastP on this gene

EKV15999

Query: Architecture Search FASTA input

KB446542 : Dothistroma septosporum NZE10 unplaced genomic scaffold DOTSEscaffold\_8    Total score: 2.0     Cumulative Blast bit score: 1410

Hit cluster cross-links:

Mycgr3G90785 Mycgr3T
  
Location: 0-1047

Mycgr3G90785\_Mycgr3T

Mycgr3G103262 Mycgr3
  
Location: 1147-1390

Mycgr3G103262\_Mycgr3

Mycgr3G68458 Mycgr3T
  
Location: 1490-3602

Mycgr3G68458\_Mycgr3T

Mycgr3G99145 Mycgr3T
  
Location: 3702-4326

Mycgr3G99145\_Mycgr3T

Mycgr3G103274 Mycgr3
  
Location: 4426-4957

Mycgr3G103274\_Mycgr3

Mycgr3G103264 Mycgr3
  
Location: 5057-5390

Mycgr3G103264\_Mycgr3

Mycgr3G37570 Mycgr3T
  
Location: 5490-6006

Mycgr3G37570\_Mycgr3T

Mycgr3G108094 Mycgr3
  
Location: 6106-10555

Mycgr3G108094\_Mycgr3

Mycgr3G90786 Mycgr3T
  
Location: 10655-12080

Mycgr3G90786\_Mycgr3T

Mycgr3G68429 Mycgr3T
  
Location: 12180-13440

Mycgr3G68429\_Mycgr3T

Mycgr3G68421 Mycgr3T
  
Location: 13540-17086

Mycgr3G68421\_Mycgr3T

Mycgr3G90801 Mycgr3T
  
Location: 17186-18056

Mycgr3G90801\_Mycgr3T

Mycgr3G84646 Mycgr3T
  
Location: 18156-20235

Mycgr3G84646\_Mycgr3T

Mycgr3G68456 Mycgr3T
  
Location: 20335-21970

Mycgr3G68456\_Mycgr3T

Mycgr3G103270 Mycgr3
  
Location: 22070-22355

Mycgr3G103270\_Mycgr3

Mycgr3G90803 Mycgr3T
  
Location: 22455-23019

Mycgr3G90803\_Mycgr3T

Mycgr3G36941 Mycgr3T
  
Location: 23119-24064

Mycgr3G36941\_Mycgr3T

Mycgr3G25746 Mycgr3T
  
Location: 24164-25241

Mycgr3G25746\_Mycgr3T

Mycgr3G90788 Mycgr3T
  
Location: 25341-25803

Mycgr3G90788\_Mycgr3T

Mycgr3G103260 Mycgr3
  
Location: 25903-26635

Mycgr3G103260\_Mycgr3

Mycgr3G84644 Mycgr3T
  
Location: 26735-28457

Mycgr3G84644\_Mycgr3T

Mycgr3G29227 Mycgr3T
  
Location: 28557-28863

Mycgr3G29227\_Mycgr3T

Mycgr3G36271 Mycgr3T
  
Location: 28963-29854

Mycgr3G36271\_Mycgr3T

Mycgr3G68433 Mycgr3T
  
Location: 29954-33041

Mycgr3G68433\_Mycgr3T

Mycgr3G79452 Mycgr3T
  
Location: 33141-33399

Mycgr3G79452\_Mycgr3T

Mycgr3G55345 Mycgr3T
  
Location: 33499-34126

Mycgr3G55345\_Mycgr3T

Mycgr3G103278 Mycgr3
  
Location: 34226-35195

Mycgr3G103278\_Mycgr3

Mycgr3G84654 Mycgr3T
  
Location: 35295-36630

Mycgr3G84654\_Mycgr3T

Mycgr3G108090 Mycgr3
  
Location: 36730-37591

Mycgr3G108090\_Mycgr3

Mycgr3G21922 Mycgr3T
  
Location: 37691-39149

Mycgr3G21922\_Mycgr3T

Mycgr3G99148 Mycgr3T
  
Location: 39249-42819

Mycgr3G99148\_Mycgr3T

hypothetical protein
  
Accession: EME41440
  
Location: 743033-743892
  
 NCBI BlastP on this gene

EME41440

hypothetical protein
  
Accession: EME41441
  
Location: 744207-746390
  
 NCBI BlastP on this gene

EME41441

hypothetical protein
  
Accession: EME41442
  
Location: 749963-750870
  
 NCBI BlastP on this gene

EME41442

hypothetical protein
  
Accession: EME41443
  
Location: 751308-752027
  
 NCBI BlastP on this gene

EME41443

hypothetical protein
  
Accession: EME41444
  
Location: 752091-752501
  
 NCBI BlastP on this gene

EME41444

hypothetical protein
  
Accession: EME41445
  
Location: 753572-755300
  
  
**BlastP hit with Mycgr3G68456\_Mycgr3T**
  
Percentage identity: 79 %
  
BlastP bit score: 857
  
Sequence coverage: 99 %
  
E-value: 0.0
  
  
 NCBI BlastP on this gene

EME41445

hypothetical protein
  
Accession: EME41446
  
Location: 755773-757342
  
 NCBI BlastP on this gene

EME41446

hypothetical protein
  
Accession: EME41447
  
Location: 758422-760068
  
 NCBI BlastP on this gene

EME41447

hypothetical protein
  
Accession: EME41448
  
Location: 760784-761353
  
 NCBI BlastP on this gene

EME41448

hypothetical protein
  
Accession: EME41449
  
Location: 761959-763479
  
 NCBI BlastP on this gene

EME41449

hypothetical protein
  
Accession: EME41450
  
Location: 766200-767933
  
  
**BlastP hit with Mycgr3G84654\_Mycgr3T**
  
Percentage identity: 81 %
  
BlastP bit score: 553
  
Sequence coverage: 74 %
  
E-value: 0.0
  
  
 NCBI BlastP on this gene

EME41450

glycoside hydrolase family 3 protein
  
Accession: EME41452
  
Location: 768761-771803
  
 NCBI BlastP on this gene

EME41452

carbohydrate esterase family 9 protein
  
Accession: EME41453
  
Location: 772634-773938
  
 NCBI BlastP on this gene

EME41453

hypothetical protein
  
Accession: EME41454
  
Location: 774950-776122
  
 NCBI BlastP on this gene

EME41454

hypothetical protein
  
Accession: EME41455
  
Location: 776518-776840
  
 NCBI BlastP on this gene

EME41455

hypothetical protein
  
Accession: EME41456
  
Location: 778701-779054
  
 NCBI BlastP on this gene

EME41456

hypothetical protein
  
Accession: EME41457
  
Location: 780513-780782
  
 NCBI BlastP on this gene

EME41457

hypothetical protein
  
Accession: EME41458
  
Location: 781693-782618
  
 NCBI BlastP on this gene

EME41458

hypothetical protein
  
Accession: EME41459
  
Location: 783398-783862
  
 NCBI BlastP on this gene

EME41459

Query: Architecture Search FASTA input

KB446555 : Pseudocercospora fijiensis CIRAD86 unplaced genomic scaffold MYCFIscaffold\_1    Total score: 2.0     Cumulative Blast bit score: 1396

Hit cluster cross-links:

Mycgr3G90785 Mycgr3T
  
Location: 0-1047

Mycgr3G90785\_Mycgr3T

Mycgr3G103262 Mycgr3
  
Location: 1147-1390

Mycgr3G103262\_Mycgr3

Mycgr3G68458 Mycgr3T
  
Location: 1490-3602

Mycgr3G68458\_Mycgr3T

Mycgr3G99145 Mycgr3T
  
Location: 3702-4326

Mycgr3G99145\_Mycgr3T

Mycgr3G103274 Mycgr3
  
Location: 4426-4957

Mycgr3G103274\_Mycgr3

Mycgr3G103264 Mycgr3
  
Location: 5057-5390

Mycgr3G103264\_Mycgr3

Mycgr3G37570 Mycgr3T
  
Location: 5490-6006

Mycgr3G37570\_Mycgr3T

Mycgr3G108094 Mycgr3
  
Location: 6106-10555

Mycgr3G108094\_Mycgr3

Mycgr3G90786 Mycgr3T
  
Location: 10655-12080

Mycgr3G90786\_Mycgr3T

Mycgr3G68429 Mycgr3T
  
Location: 12180-13440

Mycgr3G68429\_Mycgr3T

Mycgr3G68421 Mycgr3T
  
Location: 13540-17086

Mycgr3G68421\_Mycgr3T

Mycgr3G90801 Mycgr3T
  
Location: 17186-18056

Mycgr3G90801\_Mycgr3T

Mycgr3G84646 Mycgr3T
  
Location: 18156-20235

Mycgr3G84646\_Mycgr3T

Mycgr3G68456 Mycgr3T
  
Location: 20335-21970

Mycgr3G68456\_Mycgr3T

Mycgr3G103270 Mycgr3
  
Location: 22070-22355

Mycgr3G103270\_Mycgr3

Mycgr3G90803 Mycgr3T
  
Location: 22455-23019

Mycgr3G90803\_Mycgr3T

Mycgr3G36941 Mycgr3T
  
Location: 23119-24064

Mycgr3G36941\_Mycgr3T

Mycgr3G25746 Mycgr3T
  
Location: 24164-25241

Mycgr3G25746\_Mycgr3T

Mycgr3G90788 Mycgr3T
  
Location: 25341-25803

Mycgr3G90788\_Mycgr3T

Mycgr3G103260 Mycgr3
  
Location: 25903-26635

Mycgr3G103260\_Mycgr3

Mycgr3G84644 Mycgr3T
  
Location: 26735-28457

Mycgr3G84644\_Mycgr3T

Mycgr3G29227 Mycgr3T
  
Location: 28557-28863

Mycgr3G29227\_Mycgr3T

Mycgr3G36271 Mycgr3T
  
Location: 28963-29854

Mycgr3G36271\_Mycgr3T

Mycgr3G68433 Mycgr3T
  
Location: 29954-33041

Mycgr3G68433\_Mycgr3T

Mycgr3G79452 Mycgr3T
  
Location: 33141-33399

Mycgr3G79452\_Mycgr3T

Mycgr3G55345 Mycgr3T
  
Location: 33499-34126

Mycgr3G55345\_Mycgr3T

Mycgr3G103278 Mycgr3
  
Location: 34226-35195

Mycgr3G103278\_Mycgr3

Mycgr3G84654 Mycgr3T
  
Location: 35295-36630

Mycgr3G84654\_Mycgr3T

Mycgr3G108090 Mycgr3
  
Location: 36730-37591

Mycgr3G108090\_Mycgr3

Mycgr3G21922 Mycgr3T
  
Location: 37691-39149

Mycgr3G21922\_Mycgr3T

Mycgr3G99148 Mycgr3T
  
Location: 39249-42819

Mycgr3G99148\_Mycgr3T

hypothetical protein
  
Accession: EME88473
  
Location: 5598661-5598825
  
 NCBI BlastP on this gene

EME88473

hypothetical protein
  
Accession: EME88472
  
Location: 5596804-5597541
  
 NCBI BlastP on this gene

EME88472

serine/threonine protein kinase, CMGC family
  
Accession: EME88471
  
Location: 5593047-5595415
  
  
**BlastP hit with Mycgr3G84644\_Mycgr3T**
  
Percentage identity: 74 %
  
BlastP bit score: 888
  
Sequence coverage: 108 %
  
E-value: 0.0
  
  
 NCBI BlastP on this gene

EME88471

hypothetical protein
  
Accession: EME88470
  
Location: 5590136-5591084
  
  
**BlastP hit with Mycgr3G36271\_Mycgr3T**
  
Percentage identity: 80 %
  
BlastP bit score: 508
  
Sequence coverage: 98 %
  
E-value: 2e-179
  
  
 NCBI BlastP on this gene

EME88470

hypothetical protein
  
Accession: EME88469
  
Location: 5587545-5588663
  
 NCBI BlastP on this gene

EME88469

hypothetical protein
  
Accession: EME88468
  
Location: 5586786-5587184
  
 NCBI BlastP on this gene

EME88468

Query: Architecture Search FASTA input

AM920428 : Penicillium chrysogenum Wisconsin 54-1255 complete genome, contig Pc00c13.    Total score: 2.0     Cumulative Blast bit score: 1376

Hit cluster cross-links:

Mycgr3G90785 Mycgr3T
  
Location: 0-1047

Mycgr3G90785\_Mycgr3T

Mycgr3G103262 Mycgr3
  
Location: 1147-1390

Mycgr3G103262\_Mycgr3

Mycgr3G68458 Mycgr3T
  
Location: 1490-3602

Mycgr3G68458\_Mycgr3T

Mycgr3G99145 Mycgr3T
  
Location: 3702-4326

Mycgr3G99145\_Mycgr3T

Mycgr3G103274 Mycgr3
  
Location: 4426-4957

Mycgr3G103274\_Mycgr3

Mycgr3G103264 Mycgr3
  
Location: 5057-5390

Mycgr3G103264\_Mycgr3

Mycgr3G37570 Mycgr3T
  
Location: 5490-6006

Mycgr3G37570\_Mycgr3T

Mycgr3G108094 Mycgr3
  
Location: 6106-10555

Mycgr3G108094\_Mycgr3

Mycgr3G90786 Mycgr3T
  
Location: 10655-12080

Mycgr3G90786\_Mycgr3T

Mycgr3G68429 Mycgr3T
  
Location: 12180-13440

Mycgr3G68429\_Mycgr3T

Mycgr3G68421 Mycgr3T
  
Location: 13540-17086

Mycgr3G68421\_Mycgr3T

Mycgr3G90801 Mycgr3T
  
Location: 17186-18056

Mycgr3G90801\_Mycgr3T

Mycgr3G84646 Mycgr3T
  
Location: 18156-20235

Mycgr3G84646\_Mycgr3T

Mycgr3G68456 Mycgr3T
  
Location: 20335-21970

Mycgr3G68456\_Mycgr3T

Mycgr3G103270 Mycgr3
  
Location: 22070-22355

Mycgr3G103270\_Mycgr3

Mycgr3G90803 Mycgr3T
  
Location: 22455-23019

Mycgr3G90803\_Mycgr3T

Mycgr3G36941 Mycgr3T
  
Location: 23119-24064

Mycgr3G36941\_Mycgr3T

Mycgr3G25746 Mycgr3T
  
Location: 24164-25241

Mycgr3G25746\_Mycgr3T

Mycgr3G90788 Mycgr3T
  
Location: 25341-25803

Mycgr3G90788\_Mycgr3T

Mycgr3G103260 Mycgr3
  
Location: 25903-26635

Mycgr3G103260\_Mycgr3

Mycgr3G84644 Mycgr3T
  
Location: 26735-28457

Mycgr3G84644\_Mycgr3T

Mycgr3G29227 Mycgr3T
  
Location: 28557-28863

Mycgr3G29227\_Mycgr3T

Mycgr3G36271 Mycgr3T
  
Location: 28963-29854

Mycgr3G36271\_Mycgr3T

Mycgr3G68433 Mycgr3T
  
Location: 29954-33041

Mycgr3G68433\_Mycgr3T

Mycgr3G79452 Mycgr3T
  
Location: 33141-33399

Mycgr3G79452\_Mycgr3T

Mycgr3G55345 Mycgr3T
  
Location: 33499-34126

Mycgr3G55345\_Mycgr3T

Mycgr3G103278 Mycgr3
  
Location: 34226-35195

Mycgr3G103278\_Mycgr3

Mycgr3G84654 Mycgr3T
  
Location: 35295-36630

Mycgr3G84654\_Mycgr3T

Mycgr3G108090 Mycgr3
  
Location: 36730-37591

Mycgr3G108090\_Mycgr3

Mycgr3G21922 Mycgr3T
  
Location: 37691-39149

Mycgr3G21922\_Mycgr3T

Mycgr3G99148 Mycgr3T
  
Location: 39249-42819

Mycgr3G99148\_Mycgr3T

not annotated
  
Accession: CAP91738
  
Location: 1650613-1652318
  
 NCBI BlastP on this gene

Pc13g06690

not annotated
  
Accession: CAP91739
  
Location: 1652884-1654358
  
 NCBI BlastP on this gene

Pc13g06700

not annotated
  
Accession: CAP91740
  
Location: 1654982-1658124
  
 NCBI BlastP on this gene

Pc13g06710

not annotated
  
Accession: CAP91741
  
Location: 1658958-1659773
  
 NCBI BlastP on this gene

Pc13g06720

not annotated
  
Accession: CAP91742
  
Location: 1659918-1661820
  
 NCBI BlastP on this gene

Pc13g06730

not annotated
  
Accession: CAP91743
  
Location: 1662428-1663550
  
 NCBI BlastP on this gene

Pc13g06740

not annotated
  
Accession: CAP91744
  
Location: 1664198-1665445
  
 NCBI BlastP on this gene

Pc13g06750

not annotated
  
Accession: CAP91745
  
Location: 1667181-1670294
  
  
**BlastP hit with Mycgr3G68433\_Mycgr3T**
  
Percentage identity: 37 %
  
BlastP bit score: 508
  
Sequence coverage: 102 %
  
E-value: 7e-159
  
  
 NCBI BlastP on this gene

Pc13g06760

not annotated
  
Accession: CAP91746
  
Location: 1670452-1671555
  
 NCBI BlastP on this gene

Pc13g06770

not annotated
  
Accession: CAP91747
  
Location: 1672279-1673430
  
 NCBI BlastP on this gene

Pc13g06780

not annotated
  
Accession: CAP91748
  
Location: 1674151-1675177
  
 NCBI BlastP on this gene

Pc13g06790

hypothetical protein
  
Accession: CAP91749
  
Location: 1675220-1675989
  
 NCBI BlastP on this gene

Pc13g06800

not annotated
  
Accession: CAP91750
  
Location: 1675991-1677581
  
 NCBI BlastP on this gene

Pc13g06810

not annotated
  
Accession: CAP91751
  
Location: 1678453-1678941
  
 NCBI BlastP on this gene

Pc13g06820

not annotated
  
Accession: CAP91752
  
Location: 1679220-1680295
  
 NCBI BlastP on this gene

Pc13g06830

not annotated
  
Accession: CAP91753
  
Location: 1681119-1684756
  
  
**BlastP hit with Mycgr3G68421\_Mycgr3T**
  
Percentage identity: 41 %
  
BlastP bit score: 868
  
Sequence coverage: 101 %
  
E-value: 0.0
  
  
 NCBI BlastP on this gene

Pc13g06840

not annotated
  
Accession: CAP91754
  
Location: 1685049-1686083
  
 NCBI BlastP on this gene

Pc13g06850

not annotated
  
Accession: CAP91755
  
Location: 1686296-1687243
  
 NCBI BlastP on this gene

Pc13g06860

hypothetical protein
  
Accession: CAP91756
  
Location: 1688019-1689234
  
 NCBI BlastP on this gene

Pc13g06870

not annotated
  
Accession: CAP91757
  
Location: 1689276-1689761
  
 NCBI BlastP on this gene

Pc13g06880

not annotated
  
Accession: CAP91758
  
Location: 1690852-1691318
  
 NCBI BlastP on this gene

Pc13g06890

not annotated
  
Accession: CAP91759
  
Location: 1692104-1692603
  
 NCBI BlastP on this gene

Pc13g06900

hypothetical protein
  
Accession: CAP91760
  
Location: 1692837-1694615
  
 NCBI BlastP on this gene

Pc13g06910

not annotated
  
Accession: CAP91761
  
Location: 1695235-1696185
  
 NCBI BlastP on this gene

Pc13g06920

unnamed
  
Accession: CAP91762
  
Location: 1697047-1697487
  
 NCBI BlastP on this gene

Pc13g06930

not annotated
  
Accession: Pc13g06940
  
Location: 1698779-1699354
  
 NCBI BlastP on this gene

Pc13g06940

not annotated
  
Accession: CAP91764
  
Location: 1699687-1700607
  
 NCBI BlastP on this gene

Pc13g06950

Query: Architecture Search FASTA input

KB916472 : Neofusicoccum parvum UCRNP2 chromosome Unknown NP2\_03\_scaffold\_834    Total score: 2.0     Cumulative Blast bit score: 1284

Hit cluster cross-links:

Mycgr3G90785 Mycgr3T
  
Location: 0-1047

Mycgr3G90785\_Mycgr3T

Mycgr3G103262 Mycgr3
  
Location: 1147-1390

Mycgr3G103262\_Mycgr3

Mycgr3G68458 Mycgr3T
  
Location: 1490-3602

Mycgr3G68458\_Mycgr3T

Mycgr3G99145 Mycgr3T
  
Location: 3702-4326

Mycgr3G99145\_Mycgr3T

Mycgr3G103274 Mycgr3
  
Location: 4426-4957

Mycgr3G103274\_Mycgr3

Mycgr3G103264 Mycgr3
  
Location: 5057-5390

Mycgr3G103264\_Mycgr3

Mycgr3G37570 Mycgr3T
  
Location: 5490-6006

Mycgr3G37570\_Mycgr3T

Mycgr3G108094 Mycgr3
  
Location: 6106-10555

Mycgr3G108094\_Mycgr3

Mycgr3G90786 Mycgr3T
  
Location: 10655-12080

Mycgr3G90786\_Mycgr3T

Mycgr3G68429 Mycgr3T
  
Location: 12180-13440

Mycgr3G68429\_Mycgr3T

Mycgr3G68421 Mycgr3T
  
Location: 13540-17086

Mycgr3G68421\_Mycgr3T

Mycgr3G90801 Mycgr3T
  
Location: 17186-18056

Mycgr3G90801\_Mycgr3T

Mycgr3G84646 Mycgr3T
  
Location: 18156-20235

Mycgr3G84646\_Mycgr3T

Mycgr3G68456 Mycgr3T
  
Location: 20335-21970

Mycgr3G68456\_Mycgr3T

Mycgr3G103270 Mycgr3
  
Location: 22070-22355

Mycgr3G103270\_Mycgr3

Mycgr3G90803 Mycgr3T
  
Location: 22455-23019

Mycgr3G90803\_Mycgr3T

Mycgr3G36941 Mycgr3T
  
Location: 23119-24064

Mycgr3G36941\_Mycgr3T

Mycgr3G25746 Mycgr3T
  
Location: 24164-25241

Mycgr3G25746\_Mycgr3T

Mycgr3G90788 Mycgr3T
  
Location: 25341-25803

Mycgr3G90788\_Mycgr3T

Mycgr3G103260 Mycgr3
  
Location: 25903-26635

Mycgr3G103260\_Mycgr3

Mycgr3G84644 Mycgr3T
  
Location: 26735-28457

Mycgr3G84644\_Mycgr3T

Mycgr3G29227 Mycgr3T
  
Location: 28557-28863

Mycgr3G29227\_Mycgr3T

Mycgr3G36271 Mycgr3T
  
Location: 28963-29854

Mycgr3G36271\_Mycgr3T

Mycgr3G68433 Mycgr3T
  
Location: 29954-33041

Mycgr3G68433\_Mycgr3T

Mycgr3G79452 Mycgr3T
  
Location: 33141-33399

Mycgr3G79452\_Mycgr3T

Mycgr3G55345 Mycgr3T
  
Location: 33499-34126

Mycgr3G55345\_Mycgr3T

Mycgr3G103278 Mycgr3
  
Location: 34226-35195

Mycgr3G103278\_Mycgr3

Mycgr3G84654 Mycgr3T
  
Location: 35295-36630

Mycgr3G84654\_Mycgr3T

Mycgr3G108090 Mycgr3
  
Location: 36730-37591

Mycgr3G108090\_Mycgr3

Mycgr3G21922 Mycgr3T
  
Location: 37691-39149

Mycgr3G21922\_Mycgr3T

Mycgr3G99148 Mycgr3T
  
Location: 39249-42819

Mycgr3G99148\_Mycgr3T

putative golgi membrane protein
  
Accession: EOD46287
  
Location: 64170-65275
  
 NCBI BlastP on this gene

EOD46287

putative sodium nucleoside cotransporter protein
  
Accession: EOD46265
  
Location: 62251-63445
  
 NCBI BlastP on this gene

EOD46265

putative nadh-ubiquinone oxidoreductase 21 kda protein
  
Accession: EOD46277
  
Location: 58834-59620
  
 NCBI BlastP on this gene

EOD46277

putative dead helicases superfamily protein
  
Accession: EOD46272
  
Location: 54150-58586
  
 NCBI BlastP on this gene

EOD46272

putative ferric reductase transmembrane component protein
  
Accession: EOD46274
  
Location: 51674-53722
  
 NCBI BlastP on this gene

EOD46274

putative universal stress protein family domain protein
  
Accession: EOD46269
  
Location: 49590-50688
  
 NCBI BlastP on this gene

EOD46269

hypothetical protein
  
Accession: EOD46281
  
Location: 46899-48292
  
 NCBI BlastP on this gene

EOD46281

putative viral a-type inclusion protein repeat protein
  
Accession: EOD46261
  
Location: 43049-46642
  
  
**BlastP hit with Mycgr3G108094\_Mycgr3**
  
Percentage identity: 44 %
  
BlastP bit score: 439
  
Sequence coverage: 40 %
  
E-value: 2e-128
  
  
 NCBI BlastP on this gene

EOD46261

putative serine protein kinase protein
  
Accession: EOD46268
  
Location: 39988-42058
  
  
**BlastP hit with Mycgr3G84644\_Mycgr3T**
  
Percentage identity: 72 %
  
BlastP bit score: 845
  
Sequence coverage: 105 %
  
E-value: 0.0
  
  
 NCBI BlastP on this gene

EOD46268

putative antigenic thaumatin domain-containing protein
  
Accession: EOD46276
  
Location: 37113-38145
  
 NCBI BlastP on this gene

EOD46276

putative fructose-bisphosphate class ii protein
  
Accession: EOD46290
  
Location: 29159-30342
  
 NCBI BlastP on this gene

EOD46290

putative ribosomal protein l9 rnase h1 protein
  
Accession: EOD46283
  
Location: 27128-28220
  
 NCBI BlastP on this gene

EOD46283

putative ubiquitin c-terminal protein
  
Accession: EOD46280
  
Location: 24970-26805
  
 NCBI BlastP on this gene

EOD46280

putative siderochrome-iron transporter sit1 protein
  
Accession: EOD46273
  
Location: 21669-23679
  
 NCBI BlastP on this gene

EOD46273

putative peroxisomal membrane anchor protein
  
Accession: EOD46262
  
Location: 19330-20672
  
 NCBI BlastP on this gene

EOD46262

Query: Architecture Search FASTA input

AHHD01000092 : Macrophomina phaseolina MS6    Total score: 2.0     Cumulative Blast bit score: 1266

Hit cluster cross-links:

Mycgr3G90785 Mycgr3T
  
Location: 0-1047

Mycgr3G90785\_Mycgr3T

Mycgr3G103262 Mycgr3
  
Location: 1147-1390

Mycgr3G103262\_Mycgr3

Mycgr3G68458 Mycgr3T
  
Location: 1490-3602

Mycgr3G68458\_Mycgr3T

Mycgr3G99145 Mycgr3T
  
Location: 3702-4326

Mycgr3G99145\_Mycgr3T

Mycgr3G103274 Mycgr3
  
Location: 4426-4957

Mycgr3G103274\_Mycgr3

Mycgr3G103264 Mycgr3
  
Location: 5057-5390

Mycgr3G103264\_Mycgr3

Mycgr3G37570 Mycgr3T
  
Location: 5490-6006

Mycgr3G37570\_Mycgr3T

Mycgr3G108094 Mycgr3
  
Location: 6106-10555

Mycgr3G108094\_Mycgr3

Mycgr3G90786 Mycgr3T
  
Location: 10655-12080

Mycgr3G90786\_Mycgr3T

Mycgr3G68429 Mycgr3T
  
Location: 12180-13440

Mycgr3G68429\_Mycgr3T

Mycgr3G68421 Mycgr3T
  
Location: 13540-17086

Mycgr3G68421\_Mycgr3T

Mycgr3G90801 Mycgr3T
  
Location: 17186-18056

Mycgr3G90801\_Mycgr3T

Mycgr3G84646 Mycgr3T
  
Location: 18156-20235

Mycgr3G84646\_Mycgr3T

Mycgr3G68456 Mycgr3T
  
Location: 20335-21970

Mycgr3G68456\_Mycgr3T

Mycgr3G103270 Mycgr3
  
Location: 22070-22355

Mycgr3G103270\_Mycgr3

Mycgr3G90803 Mycgr3T
  
Location: 22455-23019

Mycgr3G90803\_Mycgr3T

Mycgr3G36941 Mycgr3T
  
Location: 23119-24064

Mycgr3G36941\_Mycgr3T

Mycgr3G25746 Mycgr3T
  
Location: 24164-25241

Mycgr3G25746\_Mycgr3T

Mycgr3G90788 Mycgr3T
  
Location: 25341-25803

Mycgr3G90788\_Mycgr3T

Mycgr3G103260 Mycgr3
  
Location: 25903-26635

Mycgr3G103260\_Mycgr3

Mycgr3G84644 Mycgr3T
  
Location: 26735-28457

Mycgr3G84644\_Mycgr3T

Mycgr3G29227 Mycgr3T
  
Location: 28557-28863

Mycgr3G29227\_Mycgr3T

Mycgr3G36271 Mycgr3T
  
Location: 28963-29854

Mycgr3G36271\_Mycgr3T

Mycgr3G68433 Mycgr3T
  
Location: 29954-33041

Mycgr3G68433\_Mycgr3T

Mycgr3G79452 Mycgr3T
  
Location: 33141-33399

Mycgr3G79452\_Mycgr3T

Mycgr3G55345 Mycgr3T
  
Location: 33499-34126

Mycgr3G55345\_Mycgr3T

Mycgr3G103278 Mycgr3
  
Location: 34226-35195

Mycgr3G103278\_Mycgr3

Mycgr3G84654 Mycgr3T
  
Location: 35295-36630

Mycgr3G84654\_Mycgr3T

Mycgr3G108090 Mycgr3
  
Location: 36730-37591

Mycgr3G108090\_Mycgr3

Mycgr3G21922 Mycgr3T
  
Location: 37691-39149

Mycgr3G21922\_Mycgr3T

Mycgr3G99148 Mycgr3T
  
Location: 39249-42819

Mycgr3G99148\_Mycgr3T

Peroxisome membrane anchor protein Pex14p
  
Accession: EKG20208
  
Location: 18787-20112
  
 NCBI BlastP on this gene

EKG20208

General substrate transporter
  
Accession: EKG20207
  
Location: 15802-17771
  
 NCBI BlastP on this gene

EKG20207

FAD-binding 8
  
Accession: EKG20206
  
Location: 11798-13870
  
 NCBI BlastP on this gene

EKG20206

UspA
  
Accession: EKG20205
  
Location: 10149-11104
  
 NCBI BlastP on this gene

EKG20205

Heat shock protein DnaJ
  
Accession: EKG20204
  
Location: 7066-8455
  
 NCBI BlastP on this gene

EKG20204

GRIP domain-containing protein
  
Accession: EKG20203
  
Location: 3219-6809
  
  
**BlastP hit with Mycgr3G108094\_Mycgr3**
  
Percentage identity: 43 %
  
BlastP bit score: 434
  
Sequence coverage: 40 %
  
E-value: 2e-126
  
  
 NCBI BlastP on this gene

EKG20203

hypothetical protein
  
Accession: EKG20202
  
Location: 192-2252
  
  
**BlastP hit with Mycgr3G84644\_Mycgr3T**
  
Percentage identity: 70 %
  
BlastP bit score: 832
  
Sequence coverage: 109 %
  
E-value: 0.0
  
  
 NCBI BlastP on this gene

EKG20202

Query: Architecture Search FASTA input

DS231623 : Pyrenophora tritici-repentis Pt-1C-BFP supercont1.9 genomic scaffold    Total score: 2.0     Cumulative Blast bit score: 1237

Hit cluster cross-links:

Mycgr3G90785 Mycgr3T
  
Location: 0-1047

Mycgr3G90785\_Mycgr3T

Mycgr3G103262 Mycgr3
  
Location: 1147-1390

Mycgr3G103262\_Mycgr3

Mycgr3G68458 Mycgr3T
  
Location: 1490-3602

Mycgr3G68458\_Mycgr3T

Mycgr3G99145 Mycgr3T
  
Location: 3702-4326

Mycgr3G99145\_Mycgr3T

Mycgr3G103274 Mycgr3
  
Location: 4426-4957

Mycgr3G103274\_Mycgr3

Mycgr3G103264 Mycgr3
  
Location: 5057-5390

Mycgr3G103264\_Mycgr3

Mycgr3G37570 Mycgr3T
  
Location: 5490-6006

Mycgr3G37570\_Mycgr3T

Mycgr3G108094 Mycgr3
  
Location: 6106-10555

Mycgr3G108094\_Mycgr3

Mycgr3G90786 Mycgr3T
  
Location: 10655-12080

Mycgr3G90786\_Mycgr3T

Mycgr3G68429 Mycgr3T
  
Location: 12180-13440

Mycgr3G68429\_Mycgr3T

Mycgr3G68421 Mycgr3T
  
Location: 13540-17086

Mycgr3G68421\_Mycgr3T

Mycgr3G90801 Mycgr3T
  
Location: 17186-18056

Mycgr3G90801\_Mycgr3T

Mycgr3G84646 Mycgr3T
  
Location: 18156-20235

Mycgr3G84646\_Mycgr3T

Mycgr3G68456 Mycgr3T
  
Location: 20335-21970

Mycgr3G68456\_Mycgr3T

Mycgr3G103270 Mycgr3
  
Location: 22070-22355

Mycgr3G103270\_Mycgr3

Mycgr3G90803 Mycgr3T
  
Location: 22455-23019

Mycgr3G90803\_Mycgr3T

Mycgr3G36941 Mycgr3T
  
Location: 23119-24064

Mycgr3G36941\_Mycgr3T

Mycgr3G25746 Mycgr3T
  
Location: 24164-25241

Mycgr3G25746\_Mycgr3T

Mycgr3G90788 Mycgr3T
  
Location: 25341-25803

Mycgr3G90788\_Mycgr3T

Mycgr3G103260 Mycgr3
  
Location: 25903-26635

Mycgr3G103260\_Mycgr3

Mycgr3G84644 Mycgr3T
  
Location: 26735-28457

Mycgr3G84644\_Mycgr3T

Mycgr3G29227 Mycgr3T
  
Location: 28557-28863

Mycgr3G29227\_Mycgr3T

Mycgr3G36271 Mycgr3T
  
Location: 28963-29854

Mycgr3G36271\_Mycgr3T

Mycgr3G68433 Mycgr3T
  
Location: 29954-33041

Mycgr3G68433\_Mycgr3T

Mycgr3G79452 Mycgr3T
  
Location: 33141-33399

Mycgr3G79452\_Mycgr3T

Mycgr3G55345 Mycgr3T
  
Location: 33499-34126

Mycgr3G55345\_Mycgr3T

Mycgr3G103278 Mycgr3
  
Location: 34226-35195

Mycgr3G103278\_Mycgr3

Mycgr3G84654 Mycgr3T
  
Location: 35295-36630

Mycgr3G84654\_Mycgr3T

Mycgr3G108090 Mycgr3
  
Location: 36730-37591

Mycgr3G108090\_Mycgr3

Mycgr3G21922 Mycgr3T
  
Location: 37691-39149

Mycgr3G21922\_Mycgr3T

Mycgr3G99148 Mycgr3T
  
Location: 39249-42819

Mycgr3G99148\_Mycgr3T

choline dehydrogenase
  
Accession: EDU51576
  
Location: 1635595-1637283
  
 NCBI BlastP on this gene

EDU51576

predicted protein
  
Accession: EDU51575
  
Location: 1634398-1635356
  
 NCBI BlastP on this gene

EDU51575

predicted protein
  
Accession: EDU51574
  
Location: 1632243-1633283
  
 NCBI BlastP on this gene

EDU51574

predicted protein
  
Accession: EDU51573
  
Location: 1631307-1631769
  
 NCBI BlastP on this gene

EDU51573

conserved hypothetical protein
  
Accession: EDU51572
  
Location: 1628949-1630504
  
 NCBI BlastP on this gene

EDU51572

eukaryotic translation initiation factor 2C 2
  
Accession: EDU51571
  
Location: 1625285-1628560
  
 NCBI BlastP on this gene

EDU51571

hypothetical protein
  
Accession: EDU51570
  
Location: 1622073-1622285
  
 NCBI BlastP on this gene

EDU51570

conserved hypothetical protein
  
Accession: EDU51569
  
Location: 1619842-1621435
  
 NCBI BlastP on this gene

EDU51569

hypothetical protein
  
Accession: EDU51568
  
Location: 1618010-1619442
  
 NCBI BlastP on this gene

EDU51568

zuotin
  
Accession: EDU51567
  
Location: 1616036-1617478
  
 NCBI BlastP on this gene

EDU51567

leucine zipper protein 1
  
Accession: EDU51566
  
Location: 1612130-1615755
  
  
**BlastP hit with Mycgr3G108094\_Mycgr3**
  
Percentage identity: 40 %
  
BlastP bit score: 392
  
Sequence coverage: 44 %
  
E-value: 2e-111
  
  
 NCBI BlastP on this gene

EDU51566

serine/threonine-protein kinase SRPK2
  
Accession: EDU51565
  
Location: 1609059-1611176
  
  
**BlastP hit with Mycgr3G84644\_Mycgr3T**
  
Percentage identity: 70 %
  
BlastP bit score: 845
  
Sequence coverage: 108 %
  
E-value: 0.0
  
  
 NCBI BlastP on this gene

EDU51565

predicted protein
  
Accession: EDU51564
  
Location: 1607901-1608300
  
 NCBI BlastP on this gene

EDU51564

acetolactate synthase, mitochondrial precursor
  
Accession: EDU51563
  
Location: 1605111-1607306
  
 NCBI BlastP on this gene

EDU51563

conserved hypothetical protein
  
Accession: EDU51562
  
Location: 1603399-1604460
  
 NCBI BlastP on this gene

EDU51562

conserved hypothetical protein
  
Accession: EDU51561
  
Location: 1602167-1603338
  
 NCBI BlastP on this gene

EDU51561

conserved hypothetical protein
  
Accession: EDU51560
  
Location: 1599881-1601968
  
 NCBI BlastP on this gene

EDU51560

conserved hypothetical protein
  
Accession: EDU51559
  
Location: 1597530-1599308
  
 NCBI BlastP on this gene

EDU51559

haloacid dehalogenase
  
Accession: EDU51558
  
Location: 1596184-1597122
  
 NCBI BlastP on this gene

EDU51558

dolichol-phosphate mannosyltransferase
  
Accession: EDU51557
  
Location: 1595093-1595878
  
 NCBI BlastP on this gene

EDU51557

predicted protein
  
Accession: EDU51556
  
Location: 1590543-1590923
  
 NCBI BlastP on this gene

EDU51556

predicted protein
  
Accession: EDU51555
  
Location: 1589618-1589866
  
 NCBI BlastP on this gene

EDU51555

Query: Architecture Search FASTA input

JH767653 : Coniosporium apollinis CBS 100218 chromosome Unknown supercont1.100    Total score: 2.0     Cumulative Blast bit score: 1226

Hit cluster cross-links:

Mycgr3G90785 Mycgr3T
  
Location: 0-1047

Mycgr3G90785\_Mycgr3T

Mycgr3G103262 Mycgr3
  
Location: 1147-1390

Mycgr3G103262\_Mycgr3

Mycgr3G68458 Mycgr3T
  
Location: 1490-3602

Mycgr3G68458\_Mycgr3T

Mycgr3G99145 Mycgr3T
  
Location: 3702-4326

Mycgr3G99145\_Mycgr3T

Mycgr3G103274 Mycgr3
  
Location: 4426-4957

Mycgr3G103274\_Mycgr3

Mycgr3G103264 Mycgr3
  
Location: 5057-5390

Mycgr3G103264\_Mycgr3

Mycgr3G37570 Mycgr3T
  
Location: 5490-6006

Mycgr3G37570\_Mycgr3T

Mycgr3G108094 Mycgr3
  
Location: 6106-10555

Mycgr3G108094\_Mycgr3

Mycgr3G90786 Mycgr3T
  
Location: 10655-12080

Mycgr3G90786\_Mycgr3T

Mycgr3G68429 Mycgr3T
  
Location: 12180-13440

Mycgr3G68429\_Mycgr3T

Mycgr3G68421 Mycgr3T
  
Location: 13540-17086

Mycgr3G68421\_Mycgr3T

Mycgr3G90801 Mycgr3T
  
Location: 17186-18056

Mycgr3G90801\_Mycgr3T

Mycgr3G84646 Mycgr3T
  
Location: 18156-20235

Mycgr3G84646\_Mycgr3T

Mycgr3G68456 Mycgr3T
  
Location: 20335-21970

Mycgr3G68456\_Mycgr3T

Mycgr3G103270 Mycgr3
  
Location: 22070-22355

Mycgr3G103270\_Mycgr3

Mycgr3G90803 Mycgr3T
  
Location: 22455-23019

Mycgr3G90803\_Mycgr3T

Mycgr3G36941 Mycgr3T
  
Location: 23119-24064

Mycgr3G36941\_Mycgr3T

Mycgr3G25746 Mycgr3T
  
Location: 24164-25241

Mycgr3G25746\_Mycgr3T

Mycgr3G90788 Mycgr3T
  
Location: 25341-25803

Mycgr3G90788\_Mycgr3T

Mycgr3G103260 Mycgr3
  
Location: 25903-26635

Mycgr3G103260\_Mycgr3

Mycgr3G84644 Mycgr3T
  
Location: 26735-28457

Mycgr3G84644\_Mycgr3T

Mycgr3G29227 Mycgr3T
  
Location: 28557-28863

Mycgr3G29227\_Mycgr3T

Mycgr3G36271 Mycgr3T
  
Location: 28963-29854

Mycgr3G36271\_Mycgr3T

Mycgr3G68433 Mycgr3T
  
Location: 29954-33041

Mycgr3G68433\_Mycgr3T

Mycgr3G79452 Mycgr3T
  
Location: 33141-33399

Mycgr3G79452\_Mycgr3T

Mycgr3G55345 Mycgr3T
  
Location: 33499-34126

Mycgr3G55345\_Mycgr3T

Mycgr3G103278 Mycgr3
  
Location: 34226-35195

Mycgr3G103278\_Mycgr3

Mycgr3G84654 Mycgr3T
  
Location: 35295-36630

Mycgr3G84654\_Mycgr3T

Mycgr3G108090 Mycgr3
  
Location: 36730-37591

Mycgr3G108090\_Mycgr3

Mycgr3G21922 Mycgr3T
  
Location: 37691-39149

Mycgr3G21922\_Mycgr3T

Mycgr3G99148 Mycgr3T
  
Location: 39249-42819

Mycgr3G99148\_Mycgr3T

hypothetical protein
  
Accession: EON69997
  
Location: 4802-7261
  
 NCBI BlastP on this gene

EON69997

hypothetical protein
  
Accession: EON69998
  
Location: 7572-9365
  
 NCBI BlastP on this gene

EON69998

hypothetical protein
  
Accession: EON69999
  
Location: 9989-11356
  
 NCBI BlastP on this gene

EON69999

hypothetical protein
  
Accession: EON70000
  
Location: 12453-13901
  
 NCBI BlastP on this gene

EON70000

hypothetical protein
  
Accession: EON70001
  
Location: 14114-17852
  
  
**BlastP hit with Mycgr3G108094\_Mycgr3**
  
Percentage identity: 44 %
  
BlastP bit score: 384
  
Sequence coverage: 34 %
  
E-value: 8e-109
  
  
 NCBI BlastP on this gene

EON70001

CMGC/SRPK protein kinase
  
Accession: EON70002
  
Location: 19128-21202
  
  
**BlastP hit with Mycgr3G84644\_Mycgr3T**
  
Percentage identity: 72 %
  
BlastP bit score: 842
  
Sequence coverage: 105 %
  
E-value: 0.0
  
  
 NCBI BlastP on this gene

EON70002

Query: Architecture Search FASTA input

KB644412 : Penicillium oxalicum 114-2 unplaced genomic scaffold scaffold\_5    Total score: 2.0     Cumulative Blast bit score: 1211

Hit cluster cross-links:

Mycgr3G90785 Mycgr3T
  
Location: 0-1047

Mycgr3G90785\_Mycgr3T

Mycgr3G103262 Mycgr3
  
Location: 1147-1390

Mycgr3G103262\_Mycgr3

Mycgr3G68458 Mycgr3T
  
Location: 1490-3602

Mycgr3G68458\_Mycgr3T

Mycgr3G99145 Mycgr3T
  
Location: 3702-4326

Mycgr3G99145\_Mycgr3T

Mycgr3G103274 Mycgr3
  
Location: 4426-4957

Mycgr3G103274\_Mycgr3

Mycgr3G103264 Mycgr3
  
Location: 5057-5390

Mycgr3G103264\_Mycgr3

Mycgr3G37570 Mycgr3T
  
Location: 5490-6006

Mycgr3G37570\_Mycgr3T

Mycgr3G108094 Mycgr3
  
Location: 6106-10555

Mycgr3G108094\_Mycgr3

Mycgr3G90786 Mycgr3T
  
Location: 10655-12080

Mycgr3G90786\_Mycgr3T

Mycgr3G68429 Mycgr3T
  
Location: 12180-13440

Mycgr3G68429\_Mycgr3T

Mycgr3G68421 Mycgr3T
  
Location: 13540-17086

Mycgr3G68421\_Mycgr3T

Mycgr3G90801 Mycgr3T
  
Location: 17186-18056

Mycgr3G90801\_Mycgr3T

Mycgr3G84646 Mycgr3T
  
Location: 18156-20235

Mycgr3G84646\_Mycgr3T

Mycgr3G68456 Mycgr3T
  
Location: 20335-21970

Mycgr3G68456\_Mycgr3T

Mycgr3G103270 Mycgr3
  
Location: 22070-22355

Mycgr3G103270\_Mycgr3

Mycgr3G90803 Mycgr3T
  
Location: 22455-23019

Mycgr3G90803\_Mycgr3T

Mycgr3G36941 Mycgr3T
  
Location: 23119-24064

Mycgr3G36941\_Mycgr3T

Mycgr3G25746 Mycgr3T
  
Location: 24164-25241

Mycgr3G25746\_Mycgr3T

Mycgr3G90788 Mycgr3T
  
Location: 25341-25803

Mycgr3G90788\_Mycgr3T

Mycgr3G103260 Mycgr3
  
Location: 25903-26635

Mycgr3G103260\_Mycgr3

Mycgr3G84644 Mycgr3T
  
Location: 26735-28457

Mycgr3G84644\_Mycgr3T

Mycgr3G29227 Mycgr3T
  
Location: 28557-28863

Mycgr3G29227\_Mycgr3T

Mycgr3G36271 Mycgr3T
  
Location: 28963-29854

Mycgr3G36271\_Mycgr3T

Mycgr3G68433 Mycgr3T
  
Location: 29954-33041

Mycgr3G68433\_Mycgr3T

Mycgr3G79452 Mycgr3T
  
Location: 33141-33399

Mycgr3G79452\_Mycgr3T

Mycgr3G55345 Mycgr3T
  
Location: 33499-34126

Mycgr3G55345\_Mycgr3T

Mycgr3G103278 Mycgr3
  
Location: 34226-35195

Mycgr3G103278\_Mycgr3

Mycgr3G84654 Mycgr3T
  
Location: 35295-36630

Mycgr3G84654\_Mycgr3T

Mycgr3G108090 Mycgr3
  
Location: 36730-37591

Mycgr3G108090\_Mycgr3

Mycgr3G21922 Mycgr3T
  
Location: 37691-39149

Mycgr3G21922\_Mycgr3T

Mycgr3G99148 Mycgr3T
  
Location: 39249-42819

Mycgr3G99148\_Mycgr3T

hypothetical protein
  
Accession: EPS30771
  
Location: 3705358-3707085
  
 NCBI BlastP on this gene

EPS30771

hypothetical protein
  
Accession: EPS30772
  
Location: 3708707-3710293
  
 NCBI BlastP on this gene

EPS30772

hypothetical protein
  
Accession: EPS30773
  
Location: 3711016-3711318
  
 NCBI BlastP on this gene

EPS30773

hypothetical protein
  
Accession: EPS30774
  
Location: 3711791-3713232
  
 NCBI BlastP on this gene

EPS30774

hypothetical protein
  
Accession: EPS30775
  
Location: 3715090-3716451
  
 NCBI BlastP on this gene

EPS30775

hypothetical protein
  
Accession: EPS30776
  
Location: 3717255-3718367
  
 NCBI BlastP on this gene

EPS30776

hypothetical protein
  
Accession: EPS30777
  
Location: 3719904-3720474
  
 NCBI BlastP on this gene

EPS30777

hypothetical protein
  
Accession: EPS30778
  
Location: 3720875-3721839
  
  
**BlastP hit with Mycgr3G55345\_Mycgr3T**
  
Percentage identity: 73 %
  
BlastP bit score: 301
  
Sequence coverage: 96 %
  
E-value: 1e-100
  
  
 NCBI BlastP on this gene

EPS30778

hypothetical protein
  
Accession: EPS30779
  
Location: 3722406-3722867
  
 NCBI BlastP on this gene

EPS30779

hypothetical protein
  
Accession: EPS30780
  
Location: 3723698-3726117
  
 NCBI BlastP on this gene

EPS30780

hypothetical protein
  
Accession: EPS30781
  
Location: 3726950-3728082
  
 NCBI BlastP on this gene

EPS30781

hypothetical protein
  
Accession: EPS30782
  
Location: 3728332-3732066
  
  
**BlastP hit with Mycgr3G68421\_Mycgr3T**
  
Percentage identity: 43 %
  
BlastP bit score: 910
  
Sequence coverage: 102 %
  
E-value: 0.0
  
  
 NCBI BlastP on this gene

EPS30782

hypothetical protein
  
Accession: EPS30783
  
Location: 3732925-3733881
  
 NCBI BlastP on this gene

EPS30783

hypothetical protein
  
Accession: EPS30784
  
Location: 3734237-3734725
  
 NCBI BlastP on this gene

EPS30784

hypothetical protein
  
Accession: EPS30785
  
Location: 3738125-3738733
  
 NCBI BlastP on this gene

EPS30785

hypothetical protein
  
Accession: EPS30786
  
Location: 3741643-3742738
  
 NCBI BlastP on this gene

EPS30786

hypothetical protein
  
Accession: EPS30787
  
Location: 3744500-3745000
  
 NCBI BlastP on this gene

EPS30787

hypothetical protein
  
Accession: EPS30788
  
Location: 3746983-3749305
  
 NCBI BlastP on this gene

EPS30788

Query: Architecture Search FASTA input

GL533200 : Pyrenophora teres f. teres 0-1 unplaced genomic scaffold scaffold\_189433    Total score: 2.0     Cumulative Blast bit score: 1206

Hit cluster cross-links:

Mycgr3G90785 Mycgr3T
  
Location: 0-1047

Mycgr3G90785\_Mycgr3T

Mycgr3G103262 Mycgr3
  
Location: 1147-1390

Mycgr3G103262\_Mycgr3

Mycgr3G68458 Mycgr3T
  
Location: 1490-3602

Mycgr3G68458\_Mycgr3T

Mycgr3G99145 Mycgr3T
  
Location: 3702-4326

Mycgr3G99145\_Mycgr3T

Mycgr3G103274 Mycgr3
  
Location: 4426-4957

Mycgr3G103274\_Mycgr3

Mycgr3G103264 Mycgr3
  
Location: 5057-5390

Mycgr3G103264\_Mycgr3

Mycgr3G37570 Mycgr3T
  
Location: 5490-6006

Mycgr3G37570\_Mycgr3T

Mycgr3G108094 Mycgr3
  
Location: 6106-10555

Mycgr3G108094\_Mycgr3

Mycgr3G90786 Mycgr3T
  
Location: 10655-12080

Mycgr3G90786\_Mycgr3T

Mycgr3G68429 Mycgr3T
  
Location: 12180-13440

Mycgr3G68429\_Mycgr3T

Mycgr3G68421 Mycgr3T
  
Location: 13540-17086

Mycgr3G68421\_Mycgr3T

Mycgr3G90801 Mycgr3T
  
Location: 17186-18056

Mycgr3G90801\_Mycgr3T

Mycgr3G84646 Mycgr3T
  
Location: 18156-20235

Mycgr3G84646\_Mycgr3T

Mycgr3G68456 Mycgr3T
  
Location: 20335-21970

Mycgr3G68456\_Mycgr3T

Mycgr3G103270 Mycgr3
  
Location: 22070-22355

Mycgr3G103270\_Mycgr3

Mycgr3G90803 Mycgr3T
  
Location: 22455-23019

Mycgr3G90803\_Mycgr3T

Mycgr3G36941 Mycgr3T
  
Location: 23119-24064

Mycgr3G36941\_Mycgr3T

Mycgr3G25746 Mycgr3T
  
Location: 24164-25241

Mycgr3G25746\_Mycgr3T

Mycgr3G90788 Mycgr3T
  
Location: 25341-25803

Mycgr3G90788\_Mycgr3T

Mycgr3G103260 Mycgr3
  
Location: 25903-26635

Mycgr3G103260\_Mycgr3

Mycgr3G84644 Mycgr3T
  
Location: 26735-28457

Mycgr3G84644\_Mycgr3T

Mycgr3G29227 Mycgr3T
  
Location: 28557-28863

Mycgr3G29227\_Mycgr3T

Mycgr3G36271 Mycgr3T
  
Location: 28963-29854

Mycgr3G36271\_Mycgr3T

Mycgr3G68433 Mycgr3T
  
Location: 29954-33041

Mycgr3G68433\_Mycgr3T

Mycgr3G79452 Mycgr3T
  
Location: 33141-33399

Mycgr3G79452\_Mycgr3T

Mycgr3G55345 Mycgr3T
  
Location: 33499-34126

Mycgr3G55345\_Mycgr3T

Mycgr3G103278 Mycgr3
  
Location: 34226-35195

Mycgr3G103278\_Mycgr3

Mycgr3G84654 Mycgr3T
  
Location: 35295-36630

Mycgr3G84654\_Mycgr3T

Mycgr3G108090 Mycgr3
  
Location: 36730-37591

Mycgr3G108090\_Mycgr3

Mycgr3G21922 Mycgr3T
  
Location: 37691-39149

Mycgr3G21922\_Mycgr3T

Mycgr3G99148 Mycgr3T
  
Location: 39249-42819

Mycgr3G99148\_Mycgr3T

hypothetical protein
  
Accession: EFQ94646
  
Location: 32940-34389
  
 NCBI BlastP on this gene

EFQ94646

hypothetical protein
  
Accession: EFQ94645
  
Location: 30962-32407
  
 NCBI BlastP on this gene

EFQ94645

hypothetical protein
  
Accession: EFQ94644
  
Location: 27052-30695
  
  
**BlastP hit with Mycgr3G108094\_Mycgr3**
  
Percentage identity: 41 %
  
BlastP bit score: 393
  
Sequence coverage: 44 %
  
E-value: 6e-112
  
  
 NCBI BlastP on this gene

EFQ94644

hypothetical protein
  
Accession: EFQ94643
  
Location: 24073-26210
  
  
**BlastP hit with Mycgr3G84644\_Mycgr3T**
  
Percentage identity: 69 %
  
BlastP bit score: 813
  
Sequence coverage: 108 %
  
E-value: 0.0
  
  
 NCBI BlastP on this gene

EFQ94643

hypothetical protein
  
Accession: EFQ94642
  
Location: 19998-22196
  
 NCBI BlastP on this gene

EFQ94642

hypothetical protein
  
Accession: EFQ94641
  
Location: 18265-19659
  
 NCBI BlastP on this gene

EFQ94641

hypothetical protein
  
Accession: EFQ94640
  
Location: 16991-18192
  
 NCBI BlastP on this gene

EFQ94640

hypothetical protein
  
Accession: EFQ94639
  
Location: 14678-16771
  
 NCBI BlastP on this gene

EFQ94639

hypothetical protein
  
Accession: EFQ94638
  
Location: 12307-14093
  
 NCBI BlastP on this gene

EFQ94638

hypothetical protein
  
Accession: EFQ94637
  
Location: 10925-11863
  
 NCBI BlastP on this gene

EFQ94637

hypothetical protein
  
Accession: EFQ94636
  
Location: 9826-10616
  
 NCBI BlastP on this gene

EFQ94636

hypothetical protein
  
Accession: EFQ94635
  
Location: 8012-8605
  
 NCBI BlastP on this gene

EFQ94635

Query: Architecture Search FASTA input

FP929065 : Leptosphaeria maculans JN3 lm\_SuperContig\_8\_v2 genomic supercontig    Total score: 2.0     Cumulative Blast bit score: 1172

Hit cluster cross-links:

Mycgr3G90785 Mycgr3T
  
Location: 0-1047

Mycgr3G90785\_Mycgr3T

Mycgr3G103262 Mycgr3
  
Location: 1147-1390

Mycgr3G103262\_Mycgr3

Mycgr3G68458 Mycgr3T
  
Location: 1490-3602

Mycgr3G68458\_Mycgr3T

Mycgr3G99145 Mycgr3T
  
Location: 3702-4326

Mycgr3G99145\_Mycgr3T

Mycgr3G103274 Mycgr3
  
Location: 4426-4957

Mycgr3G103274\_Mycgr3

Mycgr3G103264 Mycgr3
  
Location: 5057-5390

Mycgr3G103264\_Mycgr3

Mycgr3G37570 Mycgr3T
  
Location: 5490-6006

Mycgr3G37570\_Mycgr3T

Mycgr3G108094 Mycgr3
  
Location: 6106-10555

Mycgr3G108094\_Mycgr3

Mycgr3G90786 Mycgr3T
  
Location: 10655-12080

Mycgr3G90786\_Mycgr3T

Mycgr3G68429 Mycgr3T
  
Location: 12180-13440

Mycgr3G68429\_Mycgr3T

Mycgr3G68421 Mycgr3T
  
Location: 13540-17086

Mycgr3G68421\_Mycgr3T

Mycgr3G90801 Mycgr3T
  
Location: 17186-18056

Mycgr3G90801\_Mycgr3T

Mycgr3G84646 Mycgr3T
  
Location: 18156-20235

Mycgr3G84646\_Mycgr3T

Mycgr3G68456 Mycgr3T
  
Location: 20335-21970

Mycgr3G68456\_Mycgr3T

Mycgr3G103270 Mycgr3
  
Location: 22070-22355

Mycgr3G103270\_Mycgr3

Mycgr3G90803 Mycgr3T
  
Location: 22455-23019

Mycgr3G90803\_Mycgr3T

Mycgr3G36941 Mycgr3T
  
Location: 23119-24064

Mycgr3G36941\_Mycgr3T

Mycgr3G25746 Mycgr3T
  
Location: 24164-25241

Mycgr3G25746\_Mycgr3T

Mycgr3G90788 Mycgr3T
  
Location: 25341-25803

Mycgr3G90788\_Mycgr3T

Mycgr3G103260 Mycgr3
  
Location: 25903-26635

Mycgr3G103260\_Mycgr3

Mycgr3G84644 Mycgr3T
  
Location: 26735-28457

Mycgr3G84644\_Mycgr3T

Mycgr3G29227 Mycgr3T
  
Location: 28557-28863

Mycgr3G29227\_Mycgr3T

Mycgr3G36271 Mycgr3T
  
Location: 28963-29854

Mycgr3G36271\_Mycgr3T

Mycgr3G68433 Mycgr3T
  
Location: 29954-33041

Mycgr3G68433\_Mycgr3T

Mycgr3G79452 Mycgr3T
  
Location: 33141-33399

Mycgr3G79452\_Mycgr3T

Mycgr3G55345 Mycgr3T
  
Location: 33499-34126

Mycgr3G55345\_Mycgr3T

Mycgr3G103278 Mycgr3
  
Location: 34226-35195

Mycgr3G103278\_Mycgr3

Mycgr3G84654 Mycgr3T
  
Location: 35295-36630

Mycgr3G84654\_Mycgr3T

Mycgr3G108090 Mycgr3
  
Location: 36730-37591

Mycgr3G108090\_Mycgr3

Mycgr3G21922 Mycgr3T
  
Location: 37691-39149

Mycgr3G21922\_Mycgr3T

Mycgr3G99148 Mycgr3T
  
Location: 39249-42819

Mycgr3G99148\_Mycgr3T

hypothetical protein
  
Accession: CBX90987
  
Location: 1228694-1232129
  
 NCBI BlastP on this gene

LEMA\_P060210.1

similar to ascus development protein
  
Accession: CBX90988
  
Location: 1232363-1233112
  
 NCBI BlastP on this gene

LEMA\_P060220.1

hypothetical protein
  
Accession: CBX90989
  
Location: 1233164-1234295
  
 NCBI BlastP on this gene

LEMA\_P060230.1

hypothetical protein
  
Accession: CBX90990
  
Location: 1236195-1237865
  
 NCBI BlastP on this gene

LEMA\_P060240.1

hypothetical protein
  
Accession: CBX90991
  
Location: 1238840-1239055
  
 NCBI BlastP on this gene

LEMA\_P060250.1

hypothetical protein
  
Accession: CBX90992
  
Location: 1239457-1242326
  
 NCBI BlastP on this gene

LEMA\_P060260.1

predicted protein
  
Accession: CBX90993
  
Location: 1242684-1242961
  
 NCBI BlastP on this gene

LEMA\_P060270.1

hypothetical protein
  
Accession: CBX90994
  
Location: 1243435-1244089
  
 NCBI BlastP on this gene

LEMA\_P060280.1

similar to viral A-type inclusion protein repeat protein
  
Accession: CBX90995
  
Location: 1244465-1248154
  
  
**BlastP hit with Mycgr3G108094\_Mycgr3**
  
Percentage identity: 39 %
  
BlastP bit score: 399
  
Sequence coverage: 46 %
  
E-value: 7e-114
  
  
 NCBI BlastP on this gene

LEMA\_P060290.1

similar to serine protein kinase Sky1
  
Accession: CBX90996
  
Location: 1250201-1252003
  
  
**BlastP hit with Mycgr3G84644\_Mycgr3T**
  
Percentage identity: 71 %
  
BlastP bit score: 773
  
Sequence coverage: 98 %
  
E-value: 0.0
  
  
 NCBI BlastP on this gene

LEMA\_P060300.1

predicted protein
  
Accession: CBX90997
  
Location: 1252952-1253378
  
 NCBI BlastP on this gene

LEMA\_uP060310.1

hypothetical protein
  
Accession: CBX90998
  
Location: 1253680-1254209
  
 NCBI BlastP on this gene

LEMA\_P060320.1

hypothetical protein
  
Accession: CBX90999
  
Location: 1254448-1255091
  
 NCBI BlastP on this gene

LEMA\_P060330.1

hypothetical protein
  
Accession: CBX91000
  
Location: 1255311-1256465
  
 NCBI BlastP on this gene

LEMA\_P060340.1

predicted protein
  
Accession: CBX91001
  
Location: 1257044-1258091
  
 NCBI BlastP on this gene

LEMA\_P060350.1

hypothetical protein
  
Accession: CBX91002
  
Location: 1258440-1259180
  
 NCBI BlastP on this gene

LEMA\_P060360.1

hypothetical protein
  
Accession: CBX91003
  
Location: 1259868-1261115
  
 NCBI BlastP on this gene

LEMA\_P060370.1

predicted protein
  
Accession: CBX91004
  
Location: 1262044-1263695
  
 NCBI BlastP on this gene

LEMA\_P060380.1

similar to C6 zinc finger domain protein
  
Accession: CBX91005
  
Location: 1264070-1265588
  
 NCBI BlastP on this gene

LEMA\_P060390.1

Query: Architecture Search FASTA input

EQ963487 : Aspergillus flavus NRRL3357 scf\_1106286419368 genomic scaffold    Total score: 2.0     Cumulative Blast bit score: 1172

Hit cluster cross-links:

Mycgr3G90785 Mycgr3T
  
Location: 0-1047

Mycgr3G90785\_Mycgr3T

Mycgr3G103262 Mycgr3
  
Location: 1147-1390

Mycgr3G103262\_Mycgr3

Mycgr3G68458 Mycgr3T
  
Location: 1490-3602

Mycgr3G68458\_Mycgr3T

Mycgr3G99145 Mycgr3T
  
Location: 3702-4326

Mycgr3G99145\_Mycgr3T

Mycgr3G103274 Mycgr3
  
Location: 4426-4957

Mycgr3G103274\_Mycgr3

Mycgr3G103264 Mycgr3
  
Location: 5057-5390

Mycgr3G103264\_Mycgr3

Mycgr3G37570 Mycgr3T
  
Location: 5490-6006

Mycgr3G37570\_Mycgr3T

Mycgr3G108094 Mycgr3
  
Location: 6106-10555

Mycgr3G108094\_Mycgr3

Mycgr3G90786 Mycgr3T
  
Location: 10655-12080

Mycgr3G90786\_Mycgr3T

Mycgr3G68429 Mycgr3T
  
Location: 12180-13440

Mycgr3G68429\_Mycgr3T

Mycgr3G68421 Mycgr3T
  
Location: 13540-17086

Mycgr3G68421\_Mycgr3T

Mycgr3G90801 Mycgr3T
  
Location: 17186-18056

Mycgr3G90801\_Mycgr3T

Mycgr3G84646 Mycgr3T
  
Location: 18156-20235

Mycgr3G84646\_Mycgr3T

Mycgr3G68456 Mycgr3T
  
Location: 20335-21970

Mycgr3G68456\_Mycgr3T

Mycgr3G103270 Mycgr3
  
Location: 22070-22355

Mycgr3G103270\_Mycgr3

Mycgr3G90803 Mycgr3T
  
Location: 22455-23019

Mycgr3G90803\_Mycgr3T

Mycgr3G36941 Mycgr3T
  
Location: 23119-24064

Mycgr3G36941\_Mycgr3T

Mycgr3G25746 Mycgr3T
  
Location: 24164-25241

Mycgr3G25746\_Mycgr3T

Mycgr3G90788 Mycgr3T
  
Location: 25341-25803

Mycgr3G90788\_Mycgr3T

Mycgr3G103260 Mycgr3
  
Location: 25903-26635

Mycgr3G103260\_Mycgr3

Mycgr3G84644 Mycgr3T
  
Location: 26735-28457

Mycgr3G84644\_Mycgr3T

Mycgr3G29227 Mycgr3T
  
Location: 28557-28863

Mycgr3G29227\_Mycgr3T

Mycgr3G36271 Mycgr3T
  
Location: 28963-29854

Mycgr3G36271\_Mycgr3T

Mycgr3G68433 Mycgr3T
  
Location: 29954-33041

Mycgr3G68433\_Mycgr3T

Mycgr3G79452 Mycgr3T
  
Location: 33141-33399

Mycgr3G79452\_Mycgr3T

Mycgr3G55345 Mycgr3T
  
Location: 33499-34126

Mycgr3G55345\_Mycgr3T

Mycgr3G103278 Mycgr3
  
Location: 34226-35195

Mycgr3G103278\_Mycgr3

Mycgr3G84654 Mycgr3T
  
Location: 35295-36630

Mycgr3G84654\_Mycgr3T

Mycgr3G108090 Mycgr3
  
Location: 36730-37591

Mycgr3G108090\_Mycgr3

Mycgr3G21922 Mycgr3T
  
Location: 37691-39149

Mycgr3G21922\_Mycgr3T

Mycgr3G99148 Mycgr3T
  
Location: 39249-42819

Mycgr3G99148\_Mycgr3T

ATP-dependent RNA helicase, putative
  
Accession: EED44811
  
Location: 247703-249357
  
 NCBI BlastP on this gene

EED44811

conserved hypothetical protein
  
Accession: EED44812
  
Location: 249687-250516
  
 NCBI BlastP on this gene

EED44812

flocculation suppression protein
  
Accession: EED44813
  
Location: 251235-253346
  
 NCBI BlastP on this gene

EED44813

F-box domain protein
  
Accession: EED44814
  
Location: 254459-255224
  
 NCBI BlastP on this gene

EED44814

succinyl-CoA synthetase beta subunit, putative
  
Accession: EED44815
  
Location: 256722-258530
  
 NCBI BlastP on this gene

EED44815

hypothetical protein
  
Accession: EED44816
  
Location: 259231-260447
  
 NCBI BlastP on this gene

EED44816

C4-dicarboxylate transporter/malic acid transport protein, putative
  
Accession: EED44817
  
Location: 263278-264858
  
 NCBI BlastP on this gene

EED44817

xanthine-guanine phosphoribosyl transferase Xpt1, putative
  
Accession: EED44818
  
Location: 265516-266536
  
  
**BlastP hit with Mycgr3G55345\_Mycgr3T**
  
Percentage identity: 73 %
  
BlastP bit score: 303
  
Sequence coverage: 96 %
  
E-value: 3e-101
  
  
 NCBI BlastP on this gene

EED44818

conserved hypothetical protein
  
Accession: EED44819
  
Location: 267094-267512
  
 NCBI BlastP on this gene

EED44819

hypothetical protein
  
Accession: EED44820
  
Location: 267601-267978
  
 NCBI BlastP on this gene

EED44820

conserved hypothetical protein
  
Accession: EED44821
  
Location: 268617-269181
  
 NCBI BlastP on this gene

EED44821

GTP binding protein, putative
  
Accession: EED44822
  
Location: 269589-270730
  
 NCBI BlastP on this gene

EED44822

conserved hypothetical protein
  
Accession: EED44823
  
Location: 270639-272236
  
 NCBI BlastP on this gene

EED44823

ubiquinone biosynthesis protein, putative
  
Accession: EED44824
  
Location: 272795-275038
  
  
**BlastP hit with Mycgr3G68458\_Mycgr3T**
  
Percentage identity: 61 %
  
BlastP bit score: 869
  
Sequence coverage: 100 %
  
E-value: 0.0
  
  
 NCBI BlastP on this gene

EED44824

cytochrome c heme lyase, putative
  
Accession: EED44825
  
Location: 275949-277045
  
 NCBI BlastP on this gene

EED44825

serine/threonine protein kinase, putative
  
Accession: EED44826
  
Location: 277909-278792
  
 NCBI BlastP on this gene

EED44826

ubiquitin-conjugating enzyme Ubc6, putative
  
Accession: EED44827
  
Location: 282661-283608
  
 NCBI BlastP on this gene

EED44827

mitochondrial outer membrane protein (Sam50), putative
  
Accession: EED44828
  
Location: 283904-285709
  
 NCBI BlastP on this gene

EED44828

Golgi membrane protein, putative
  
Accession: EED44829
  
Location: 286064-287069
  
 NCBI BlastP on this gene

EED44829

ubiquitin fusion degradation protein (Ufd1), putative
  
Accession: EED44830
  
Location: 287460-289856
  
 NCBI BlastP on this gene

EED44830

hypothetical protein
  
Accession: EED44831
  
Location: 292009-293088
  
 NCBI BlastP on this gene

EED44831

Query: Architecture Search FASTA input

DS572750 : Paracoccidioides brasiliensis Pb18 supercont1.1 genomic scaffold    Total score: 2.0     Cumulative Blast bit score: 1162

Hit cluster cross-links:

Mycgr3G90785 Mycgr3T
  
Location: 0-1047

Mycgr3G90785\_Mycgr3T

Mycgr3G103262 Mycgr3
  
Location: 1147-1390

Mycgr3G103262\_Mycgr3

Mycgr3G68458 Mycgr3T
  
Location: 1490-3602

Mycgr3G68458\_Mycgr3T

Mycgr3G99145 Mycgr3T
  
Location: 3702-4326

Mycgr3G99145\_Mycgr3T

Mycgr3G103274 Mycgr3
  
Location: 4426-4957

Mycgr3G103274\_Mycgr3

Mycgr3G103264 Mycgr3
  
Location: 5057-5390

Mycgr3G103264\_Mycgr3

Mycgr3G37570 Mycgr3T
  
Location: 5490-6006

Mycgr3G37570\_Mycgr3T

Mycgr3G108094 Mycgr3
  
Location: 6106-10555

Mycgr3G108094\_Mycgr3

Mycgr3G90786 Mycgr3T
  
Location: 10655-12080

Mycgr3G90786\_Mycgr3T

Mycgr3G68429 Mycgr3T
  
Location: 12180-13440

Mycgr3G68429\_Mycgr3T

Mycgr3G68421 Mycgr3T
  
Location: 13540-17086

Mycgr3G68421\_Mycgr3T

Mycgr3G90801 Mycgr3T
  
Location: 17186-18056

Mycgr3G90801\_Mycgr3T

Mycgr3G84646 Mycgr3T
  
Location: 18156-20235

Mycgr3G84646\_Mycgr3T

Mycgr3G68456 Mycgr3T
  
Location: 20335-21970

Mycgr3G68456\_Mycgr3T

Mycgr3G103270 Mycgr3
  
Location: 22070-22355

Mycgr3G103270\_Mycgr3

Mycgr3G90803 Mycgr3T
  
Location: 22455-23019

Mycgr3G90803\_Mycgr3T

Mycgr3G36941 Mycgr3T
  
Location: 23119-24064

Mycgr3G36941\_Mycgr3T

Mycgr3G25746 Mycgr3T
  
Location: 24164-25241

Mycgr3G25746\_Mycgr3T

Mycgr3G90788 Mycgr3T
  
Location: 25341-25803

Mycgr3G90788\_Mycgr3T

Mycgr3G103260 Mycgr3
  
Location: 25903-26635

Mycgr3G103260\_Mycgr3

Mycgr3G84644 Mycgr3T
  
Location: 26735-28457

Mycgr3G84644\_Mycgr3T

Mycgr3G29227 Mycgr3T
  
Location: 28557-28863

Mycgr3G29227\_Mycgr3T

Mycgr3G36271 Mycgr3T
  
Location: 28963-29854

Mycgr3G36271\_Mycgr3T

Mycgr3G68433 Mycgr3T
  
Location: 29954-33041

Mycgr3G68433\_Mycgr3T

Mycgr3G79452 Mycgr3T
  
Location: 33141-33399

Mycgr3G79452\_Mycgr3T

Mycgr3G55345 Mycgr3T
  
Location: 33499-34126

Mycgr3G55345\_Mycgr3T

Mycgr3G103278 Mycgr3
  
Location: 34226-35195

Mycgr3G103278\_Mycgr3

Mycgr3G84654 Mycgr3T
  
Location: 35295-36630

Mycgr3G84654\_Mycgr3T

Mycgr3G108090 Mycgr3
  
Location: 36730-37591

Mycgr3G108090\_Mycgr3

Mycgr3G21922 Mycgr3T
  
Location: 37691-39149

Mycgr3G21922\_Mycgr3T

Mycgr3G99148 Mycgr3T
  
Location: 39249-42819

Mycgr3G99148\_Mycgr3T

tachykinin family protein
  
Accession: EEH44022
  
Location: 1024941-1027145
  
 NCBI BlastP on this gene

EEH44022

cytochrome c heme lyase
  
Accession: EEH44023
  
Location: 1028443-1029657
  
 NCBI BlastP on this gene

EEH44023

conserved hypothetical protein
  
Accession: EEH44024
  
Location: 1030447-1032403
  
 NCBI BlastP on this gene

EEH44024

ABC1 family protein
  
Accession: EEH44025
  
Location: 1034244-1036623
  
  
**BlastP hit with Mycgr3G68458\_Mycgr3T**
  
Percentage identity: 64 %
  
BlastP bit score: 848
  
Sequence coverage: 92 %
  
E-value: 0.0
  
  
 NCBI BlastP on this gene

EEH44025

carbonic anhydrase
  
Accession: EEH44026
  
Location: 1039641-1040250
  
 NCBI BlastP on this gene

EEH44026

hypothetical protein
  
Accession: EEH44027
  
Location: 1042156-1043026
  
 NCBI BlastP on this gene

EEH44027

succinyl-CoA ligase subunit beta
  
Accession: EEH44028
  
Location: 1043711-1045646
  
 NCBI BlastP on this gene

EEH44028

conserved hypothetical protein
  
Accession: EEH44029
  
Location: 1046118-1046854
  
 NCBI BlastP on this gene

EEH44029

hypothetical protein
  
Accession: EEH44030
  
Location: 1049812-1051342
  
 NCBI BlastP on this gene

EEH44030

hypothetical protein
  
Accession: EEH44031
  
Location: 1052002-1055896
  
 NCBI BlastP on this gene

EEH44031

HSF-type DNA-binding domain-containing protein
  
Accession: EEH44032
  
Location: 1055937-1058400
  
 NCBI BlastP on this gene

EEH44032

hypothetical protein
  
Accession: EEH44033
  
Location: 1061509-1063250
  
  
**BlastP hit with Mycgr3G55345\_Mycgr3T**
  
Percentage identity: 75 %
  
BlastP bit score: 314
  
Sequence coverage: 97 %
  
E-value: 9e-106
  
  
 NCBI BlastP on this gene

EEH44033

conserved hypothetical protein
  
Accession: EEH44034
  
Location: 1063943-1064563
  
 NCBI BlastP on this gene

EEH44034

peroxisomal catalase
  
Accession: EEH44035
  
Location: 1068931-1070836
  
 NCBI BlastP on this gene

EEH44035

Query: Architecture Search FASTA input

DS544806 : Paracoccidioides brasiliensis Pb03 supercont1.4 genomic scaffold    Total score: 2.0     Cumulative Blast bit score: 1142

Hit cluster cross-links:

Mycgr3G90785 Mycgr3T
  
Location: 0-1047

Mycgr3G90785\_Mycgr3T

Mycgr3G103262 Mycgr3
  
Location: 1147-1390

Mycgr3G103262\_Mycgr3

Mycgr3G68458 Mycgr3T
  
Location: 1490-3602

Mycgr3G68458\_Mycgr3T

Mycgr3G99145 Mycgr3T
  
Location: 3702-4326

Mycgr3G99145\_Mycgr3T

Mycgr3G103274 Mycgr3
  
Location: 4426-4957

Mycgr3G103274\_Mycgr3

Mycgr3G103264 Mycgr3
  
Location: 5057-5390

Mycgr3G103264\_Mycgr3

Mycgr3G37570 Mycgr3T
  
Location: 5490-6006

Mycgr3G37570\_Mycgr3T

Mycgr3G108094 Mycgr3
  
Location: 6106-10555

Mycgr3G108094\_Mycgr3

Mycgr3G90786 Mycgr3T
  
Location: 10655-12080

Mycgr3G90786\_Mycgr3T

Mycgr3G68429 Mycgr3T
  
Location: 12180-13440

Mycgr3G68429\_Mycgr3T

Mycgr3G68421 Mycgr3T
  
Location: 13540-17086

Mycgr3G68421\_Mycgr3T

Mycgr3G90801 Mycgr3T
  
Location: 17186-18056

Mycgr3G90801\_Mycgr3T

Mycgr3G84646 Mycgr3T
  
Location: 18156-20235

Mycgr3G84646\_Mycgr3T

Mycgr3G68456 Mycgr3T
  
Location: 20335-21970

Mycgr3G68456\_Mycgr3T

Mycgr3G103270 Mycgr3
  
Location: 22070-22355

Mycgr3G103270\_Mycgr3

Mycgr3G90803 Mycgr3T
  
Location: 22455-23019

Mycgr3G90803\_Mycgr3T

Mycgr3G36941 Mycgr3T
  
Location: 23119-24064

Mycgr3G36941\_Mycgr3T

Mycgr3G25746 Mycgr3T
  
Location: 24164-25241

Mycgr3G25746\_Mycgr3T

Mycgr3G90788 Mycgr3T
  
Location: 25341-25803

Mycgr3G90788\_Mycgr3T

Mycgr3G103260 Mycgr3
  
Location: 25903-26635

Mycgr3G103260\_Mycgr3

Mycgr3G84644 Mycgr3T
  
Location: 26735-28457

Mycgr3G84644\_Mycgr3T

Mycgr3G29227 Mycgr3T
  
Location: 28557-28863

Mycgr3G29227\_Mycgr3T

Mycgr3G36271 Mycgr3T
  
Location: 28963-29854

Mycgr3G36271\_Mycgr3T

Mycgr3G68433 Mycgr3T
  
Location: 29954-33041

Mycgr3G68433\_Mycgr3T

Mycgr3G79452 Mycgr3T
  
Location: 33141-33399

Mycgr3G79452\_Mycgr3T

Mycgr3G55345 Mycgr3T
  
Location: 33499-34126

Mycgr3G55345\_Mycgr3T

Mycgr3G103278 Mycgr3
  
Location: 34226-35195

Mycgr3G103278\_Mycgr3

Mycgr3G84654 Mycgr3T
  
Location: 35295-36630

Mycgr3G84654\_Mycgr3T

Mycgr3G108090 Mycgr3
  
Location: 36730-37591

Mycgr3G108090\_Mycgr3

Mycgr3G21922 Mycgr3T
  
Location: 37691-39149

Mycgr3G21922\_Mycgr3T

Mycgr3G99148 Mycgr3T
  
Location: 39249-42819

Mycgr3G99148\_Mycgr3T

60S ribosomal protein L13
  
Accession: EEH20500
  
Location: 1474-2763
  
 NCBI BlastP on this gene

EEH20500

leucine-rich repeat-containing protein
  
Accession: EEH20501
  
Location: 7187-9497
  
  
**BlastP hit with Mycgr3G68433\_Mycgr3T**
  
Percentage identity: 35 %
  
BlastP bit score: 228
  
Sequence coverage: 51 %
  
E-value: 3e-60
  
  
 NCBI BlastP on this gene

EEH20501

conserved hypothetical protein
  
Accession: EEH20502
  
Location: 10124-11365
  
 NCBI BlastP on this gene

EEH20502

predicted protein
  
Accession: EEH20503
  
Location: 11886-12663
  
 NCBI BlastP on this gene

EEH20503

conserved hypothetical protein
  
Accession: EEH20504
  
Location: 13984-15628
  
 NCBI BlastP on this gene

EEH20504

IBR domain-containing protein
  
Accession: EEH20505
  
Location: 18297-21263
  
 NCBI BlastP on this gene

EEH20505

predicted protein
  
Accession: EEH20506
  
Location: 22198-23634
  
 NCBI BlastP on this gene

EEH20506

3-hydroxybutyryl-CoA dehydrogenase
  
Accession: EEH20507
  
Location: 24067-25228
  
 NCBI BlastP on this gene

EEH20507

mating-type switching protein
  
Accession: EEH20508
  
Location: 25600-29413
  
  
**BlastP hit with Mycgr3G68421\_Mycgr3T**
  
Percentage identity: 44 %
  
BlastP bit score: 914
  
Sequence coverage: 103 %
  
E-value: 0.0
  
  
 NCBI BlastP on this gene

EEH20508

predicted protein
  
Accession: EEH20509
  
Location: 30444-33059
  
 NCBI BlastP on this gene

EEH20509

RNA polymerase II transcription factor B subunit 2
  
Accession: EEH20511
  
Location: 33652-35240
  
 NCBI BlastP on this gene

EEH20511

predicted protein
  
Accession: EEH20510
  
Location: 35252-36788
  
 NCBI BlastP on this gene

EEH20510

conserved hypothetical protein
  
Accession: EEH20512
  
Location: 37473-41925
  
 NCBI BlastP on this gene

EEH20512

Query: Architecture Search FASTA input

CH476599 : Aspergillus terreus NIH2624 scaffold\_6 genomic scaffold    Total score: 2.0     Cumulative Blast bit score: 1133

Hit cluster cross-links:

Mycgr3G90785 Mycgr3T
  
Location: 0-1047

Mycgr3G90785\_Mycgr3T

Mycgr3G103262 Mycgr3
  
Location: 1147-1390

Mycgr3G103262\_Mycgr3

Mycgr3G68458 Mycgr3T
  
Location: 1490-3602

Mycgr3G68458\_Mycgr3T

Mycgr3G99145 Mycgr3T
  
Location: 3702-4326

Mycgr3G99145\_Mycgr3T

Mycgr3G103274 Mycgr3
  
Location: 4426-4957

Mycgr3G103274\_Mycgr3

Mycgr3G103264 Mycgr3
  
Location: 5057-5390

Mycgr3G103264\_Mycgr3

Mycgr3G37570 Mycgr3T
  
Location: 5490-6006

Mycgr3G37570\_Mycgr3T

Mycgr3G108094 Mycgr3
  
Location: 6106-10555

Mycgr3G108094\_Mycgr3

Mycgr3G90786 Mycgr3T
  
Location: 10655-12080

Mycgr3G90786\_Mycgr3T

Mycgr3G68429 Mycgr3T
  
Location: 12180-13440

Mycgr3G68429\_Mycgr3T

Mycgr3G68421 Mycgr3T
  
Location: 13540-17086

Mycgr3G68421\_Mycgr3T

Mycgr3G90801 Mycgr3T
  
Location: 17186-18056

Mycgr3G90801\_Mycgr3T

Mycgr3G84646 Mycgr3T
  
Location: 18156-20235

Mycgr3G84646\_Mycgr3T

Mycgr3G68456 Mycgr3T
  
Location: 20335-21970

Mycgr3G68456\_Mycgr3T

Mycgr3G103270 Mycgr3
  
Location: 22070-22355

Mycgr3G103270\_Mycgr3

Mycgr3G90803 Mycgr3T
  
Location: 22455-23019

Mycgr3G90803\_Mycgr3T

Mycgr3G36941 Mycgr3T
  
Location: 23119-24064

Mycgr3G36941\_Mycgr3T

Mycgr3G25746 Mycgr3T
  
Location: 24164-25241

Mycgr3G25746\_Mycgr3T

Mycgr3G90788 Mycgr3T
  
Location: 25341-25803

Mycgr3G90788\_Mycgr3T

Mycgr3G103260 Mycgr3
  
Location: 25903-26635

Mycgr3G103260\_Mycgr3

Mycgr3G84644 Mycgr3T
  
Location: 26735-28457

Mycgr3G84644\_Mycgr3T

Mycgr3G29227 Mycgr3T
  
Location: 28557-28863

Mycgr3G29227\_Mycgr3T

Mycgr3G36271 Mycgr3T
  
Location: 28963-29854

Mycgr3G36271\_Mycgr3T

Mycgr3G68433 Mycgr3T
  
Location: 29954-33041

Mycgr3G68433\_Mycgr3T

Mycgr3G79452 Mycgr3T
  
Location: 33141-33399

Mycgr3G79452\_Mycgr3T

Mycgr3G55345 Mycgr3T
  
Location: 33499-34126

Mycgr3G55345\_Mycgr3T

Mycgr3G103278 Mycgr3
  
Location: 34226-35195

Mycgr3G103278\_Mycgr3

Mycgr3G84654 Mycgr3T
  
Location: 35295-36630

Mycgr3G84654\_Mycgr3T

Mycgr3G108090 Mycgr3
  
Location: 36730-37591

Mycgr3G108090\_Mycgr3

Mycgr3G21922 Mycgr3T
  
Location: 37691-39149

Mycgr3G21922\_Mycgr3T

Mycgr3G99148 Mycgr3T
  
Location: 39249-42819

Mycgr3G99148\_Mycgr3T

conserved hypothetical protein
  
Accession: EAU35256
  
Location: 1600210-1602698
  
 NCBI BlastP on this gene

EAU35256

conserved hypothetical protein
  
Accession: EAU35255
  
Location: 1598113-1598796
  
 NCBI BlastP on this gene

EAU35255

hypothetical protein
  
Accession: EAU35254
  
Location: 1593041-1597683
  
 NCBI BlastP on this gene

EAU35254

conserved hypothetical protein
  
Accession: EAU35253
  
Location: 1588452-1592271
  
  
**BlastP hit with Mycgr3G108094\_Mycgr3**
  
Percentage identity: 37 %
  
BlastP bit score: 370
  
Sequence coverage: 50 %
  
E-value: 3e-104
  
  
 NCBI BlastP on this gene

EAU35253

conserved hypothetical protein
  
Accession: EAU35252
  
Location: 1587649-1588247
  
 NCBI BlastP on this gene

EAU35252

predicted protein
  
Accession: EAU35251
  
Location: 1585458-1587008
  
 NCBI BlastP on this gene

EAU35251

conserved hypothetical protein
  
Accession: EAU35250
  
Location: 1584013-1585092
  
 NCBI BlastP on this gene

EAU35250

predicted protein
  
Accession: EAU35249
  
Location: 1581846-1583103
  
 NCBI BlastP on this gene

EAU35249

hypothetical protein
  
Accession: EAU35248
  
Location: 1579932-1580935
  
 NCBI BlastP on this gene

EAU35248

hypothetical protein
  
Accession: EAU35247
  
Location: 1577506-1579258
  
 NCBI BlastP on this gene

EAU35247

cytochrome b2, mitochondrial precursor
  
Accession: EAU35246
  
Location: 1572609-1574216
  
 NCBI BlastP on this gene

EAU35246

conserved hypothetical protein
  
Accession: EAU35245
  
Location: 1571474-1572073
  
 NCBI BlastP on this gene

EAU35245

protein kinase dsk1
  
Accession: EAU35244
  
Location: 1567805-1569680
  
  
**BlastP hit with Mycgr3G84644\_Mycgr3T**
  
Percentage identity: 68 %
  
BlastP bit score: 763
  
Sequence coverage: 101 %
  
E-value: 0.0
  
  
 NCBI BlastP on this gene

EAU35244

conserved hypothetical protein
  
Accession: EAU35243
  
Location: 1565657-1566667
  
 NCBI BlastP on this gene

EAU35243

predicted protein
  
Accession: EAU35242
  
Location: 1563835-1564458
  
 NCBI BlastP on this gene

EAU35242

conserved hypothetical protein
  
Accession: EAU35241
  
Location: 1561761-1563238
  
 NCBI BlastP on this gene

EAU35241

alpha,alpha-trehalose-phosphate synthase 1
  
Accession: EAU35240
  
Location: 1559555-1561227
  
 NCBI BlastP on this gene

EAU35240

conserved hypothetical protein
  
Accession: EAU35239
  
Location: 1557300-1558821
  
 NCBI BlastP on this gene

EAU35239

cutinase precursor
  
Accession: EAU35238
  
Location: 1555654-1556459
  
 NCBI BlastP on this gene

EAU35238

Query: Architecture Search FASTA input

DS544807 : Paracoccidioides brasiliensis Pb03 supercont1.5 genomic scaffold    Total score: 2.0     Cumulative Blast bit score: 1132

Hit cluster cross-links:

Mycgr3G90785 Mycgr3T
  
Location: 0-1047

Mycgr3G90785\_Mycgr3T

Mycgr3G103262 Mycgr3
  
Location: 1147-1390

Mycgr3G103262\_Mycgr3

Mycgr3G68458 Mycgr3T
  
Location: 1490-3602

Mycgr3G68458\_Mycgr3T

Mycgr3G99145 Mycgr3T
  
Location: 3702-4326

Mycgr3G99145\_Mycgr3T

Mycgr3G103274 Mycgr3
  
Location: 4426-4957

Mycgr3G103274\_Mycgr3

Mycgr3G103264 Mycgr3
  
Location: 5057-5390

Mycgr3G103264\_Mycgr3

Mycgr3G37570 Mycgr3T
  
Location: 5490-6006

Mycgr3G37570\_Mycgr3T

Mycgr3G108094 Mycgr3
  
Location: 6106-10555

Mycgr3G108094\_Mycgr3

Mycgr3G90786 Mycgr3T
  
Location: 10655-12080

Mycgr3G90786\_Mycgr3T

Mycgr3G68429 Mycgr3T
  
Location: 12180-13440

Mycgr3G68429\_Mycgr3T

Mycgr3G68421 Mycgr3T
  
Location: 13540-17086

Mycgr3G68421\_Mycgr3T

Mycgr3G90801 Mycgr3T
  
Location: 17186-18056

Mycgr3G90801\_Mycgr3T

Mycgr3G84646 Mycgr3T
  
Location: 18156-20235

Mycgr3G84646\_Mycgr3T

Mycgr3G68456 Mycgr3T
  
Location: 20335-21970

Mycgr3G68456\_Mycgr3T

Mycgr3G103270 Mycgr3
  
Location: 22070-22355

Mycgr3G103270\_Mycgr3

Mycgr3G90803 Mycgr3T
  
Location: 22455-23019

Mycgr3G90803\_Mycgr3T

Mycgr3G36941 Mycgr3T
  
Location: 23119-24064

Mycgr3G36941\_Mycgr3T

Mycgr3G25746 Mycgr3T
  
Location: 24164-25241

Mycgr3G25746\_Mycgr3T

Mycgr3G90788 Mycgr3T
  
Location: 25341-25803

Mycgr3G90788\_Mycgr3T

Mycgr3G103260 Mycgr3
  
Location: 25903-26635

Mycgr3G103260\_Mycgr3

Mycgr3G84644 Mycgr3T
  
Location: 26735-28457

Mycgr3G84644\_Mycgr3T

Mycgr3G29227 Mycgr3T
  
Location: 28557-28863

Mycgr3G29227\_Mycgr3T

Mycgr3G36271 Mycgr3T
  
Location: 28963-29854

Mycgr3G36271\_Mycgr3T

Mycgr3G68433 Mycgr3T
  
Location: 29954-33041

Mycgr3G68433\_Mycgr3T

Mycgr3G79452 Mycgr3T
  
Location: 33141-33399

Mycgr3G79452\_Mycgr3T

Mycgr3G55345 Mycgr3T
  
Location: 33499-34126

Mycgr3G55345\_Mycgr3T

Mycgr3G103278 Mycgr3
  
Location: 34226-35195

Mycgr3G103278\_Mycgr3

Mycgr3G84654 Mycgr3T
  
Location: 35295-36630

Mycgr3G84654\_Mycgr3T

Mycgr3G108090 Mycgr3
  
Location: 36730-37591

Mycgr3G108090\_Mycgr3

Mycgr3G21922 Mycgr3T
  
Location: 37691-39149

Mycgr3G21922\_Mycgr3T

Mycgr3G99148 Mycgr3T
  
Location: 39249-42819

Mycgr3G99148\_Mycgr3T

sarcoma antigen NY-SAR-16
  
Accession: EEH21437
  
Location: 334224-336832
  
 NCBI BlastP on this gene

EEH21437

predicted protein
  
Accession: EEH21438
  
Location: 338853-339273
  
 NCBI BlastP on this gene

EEH21438

conserved hypothetical protein
  
Accession: EEH21439
  
Location: 342409-344974
  
 NCBI BlastP on this gene

EEH21439

conserved hypothetical protein
  
Accession: EEH21440
  
Location: 345768-351940
  
  
**BlastP hit with Mycgr3G108094\_Mycgr3**
  
Percentage identity: 40 %
  
BlastP bit score: 362
  
Sequence coverage: 40 %
  
E-value: 4e-100
  
  
 NCBI BlastP on this gene

EEH21440

predicted protein
  
Accession: EEH21441
  
Location: 352332-353463
  
 NCBI BlastP on this gene

EEH21441

conserved hypothetical protein
  
Accession: EEH21442
  
Location: 355955-357793
  
 NCBI BlastP on this gene

EEH21442

L-lactate dehydrogenase
  
Accession: EEH21443
  
Location: 361370-363084
  
 NCBI BlastP on this gene

EEH21443

ser/Thr protein phosphatase family protein
  
Accession: EEH21444
  
Location: 363343-364500
  
 NCBI BlastP on this gene

EEH21444

hypothetical protein
  
Accession: EEH21445
  
Location: 365221-365800
  
 NCBI BlastP on this gene

EEH21445

predicted protein
  
Accession: EEH21446
  
Location: 366742-367147
  
 NCBI BlastP on this gene

EEH21446

serine/threonine-protein kinase SRPK1
  
Accession: EEH21447
  
Location: 367883-370439
  
  
**BlastP hit with Mycgr3G84644\_Mycgr3T**
  
Percentage identity: 67 %
  
BlastP bit score: 770
  
Sequence coverage: 108 %
  
E-value: 0.0
  
  
 NCBI BlastP on this gene

EEH21447

pre-mRNA-splicing factor cwc26
  
Accession: EEH21448
  
Location: 372249-373337
  
 NCBI BlastP on this gene

EEH21448

MGMT family protein
  
Accession: EEH21449
  
Location: 373608-374420
  
 NCBI BlastP on this gene

EEH21449

predicted protein
  
Accession: EEH21450
  
Location: 378970-379577
  
 NCBI BlastP on this gene

EEH21450

Query: Architecture Search FASTA input

101. :  KB445579 Cochliobolus heterostrophus C5 unplaced genomic scaffold COCHEscaffold\_11     Total score: 3.0     Cumulative Blast bit score: 953

Mycgr3G90785 Mycgr3T
  
Location: 0-1047
  
 NCBI BlastP on this gene

Mycgr3G90785\_Mycgr3T

Mycgr3G103262 Mycgr3
  
Location: 1147-1390
  
 NCBI BlastP on this gene

Mycgr3G103262\_Mycgr3

Mycgr3G68458 Mycgr3T
  
Location: 1490-3602
  
 NCBI BlastP on this gene

Mycgr3G68458\_Mycgr3T

Mycgr3G99145 Mycgr3T
  
Location: 3702-4326
  
 NCBI BlastP on this gene

Mycgr3G99145\_Mycgr3T

Mycgr3G103274 Mycgr3
  
Location: 4426-4957
  
 NCBI BlastP on this gene

Mycgr3G103274\_Mycgr3

Mycgr3G103264 Mycgr3
  
Location: 5057-5390
  
 NCBI BlastP on this gene

Mycgr3G103264\_Mycgr3

Mycgr3G37570 Mycgr3T
  
Location: 5490-6006
  
 NCBI BlastP on this gene

Mycgr3G37570\_Mycgr3T

Mycgr3G108094 Mycgr3
  
Location: 6106-10555
  
 NCBI BlastP on this gene

Mycgr3G108094\_Mycgr3

Mycgr3G90786 Mycgr3T
  
Location: 10655-12080
  
 NCBI BlastP on this gene

Mycgr3G90786\_Mycgr3T

Mycgr3G68429 Mycgr3T
  
Location: 12180-13440
  
 NCBI BlastP on this gene

Mycgr3G68429\_Mycgr3T

Mycgr3G68421 Mycgr3T
  
Location: 13540-17086
  
 NCBI BlastP on this gene

Mycgr3G68421\_Mycgr3T

Mycgr3G90801 Mycgr3T
  
Location: 17186-18056
  
 NCBI BlastP on this gene

Mycgr3G90801\_Mycgr3T

Mycgr3G84646 Mycgr3T
  
Location: 18156-20235
  
 NCBI BlastP on this gene

Mycgr3G84646\_Mycgr3T

Mycgr3G68456 Mycgr3T
  
Location: 20335-21970
  
 NCBI BlastP on this gene

Mycgr3G68456\_Mycgr3T

Mycgr3G103270 Mycgr3
  
Location: 22070-22355
  
 NCBI BlastP on this gene

Mycgr3G103270\_Mycgr3

Mycgr3G90803 Mycgr3T
  
Location: 22455-23019
  
 NCBI BlastP on this gene

Mycgr3G90803\_Mycgr3T

Mycgr3G36941 Mycgr3T
  
Location: 23119-24064
  
 NCBI BlastP on this gene

Mycgr3G36941\_Mycgr3T

Mycgr3G25746 Mycgr3T
  
Location: 24164-25241
  
 NCBI BlastP on this gene

Mycgr3G25746\_Mycgr3T

Mycgr3G90788 Mycgr3T
  
Location: 25341-25803
  
 NCBI BlastP on this gene

Mycgr3G90788\_Mycgr3T

Mycgr3G103260 Mycgr3
  
Location: 25903-26635
  
 NCBI BlastP on this gene

Mycgr3G103260\_Mycgr3

Mycgr3G84644 Mycgr3T
  
Location: 26735-28457
  
 NCBI BlastP on this gene

Mycgr3G84644\_Mycgr3T

Mycgr3G29227 Mycgr3T
  
Location: 28557-28863
  
 NCBI BlastP on this gene

Mycgr3G29227\_Mycgr3T

Mycgr3G36271 Mycgr3T
  
Location: 28963-29854
  
 NCBI BlastP on this gene

Mycgr3G36271\_Mycgr3T

Mycgr3G68433 Mycgr3T
  
Location: 29954-33041
  
 NCBI BlastP on this gene

Mycgr3G68433\_Mycgr3T

Mycgr3G79452 Mycgr3T
  
Location: 33141-33399
  
 NCBI BlastP on this gene

Mycgr3G79452\_Mycgr3T

Mycgr3G55345 Mycgr3T
  
Location: 33499-34126
  
 NCBI BlastP on this gene

Mycgr3G55345\_Mycgr3T

Mycgr3G103278 Mycgr3
  
Location: 34226-35195
  
 NCBI BlastP on this gene

Mycgr3G103278\_Mycgr3

Mycgr3G84654 Mycgr3T
  
Location: 35295-36630
  
 NCBI BlastP on this gene

Mycgr3G84654\_Mycgr3T

Mycgr3G108090 Mycgr3
  
Location: 36730-37591
  
 NCBI BlastP on this gene

Mycgr3G108090\_Mycgr3

Mycgr3G21922 Mycgr3T
  
Location: 37691-39149
  
 NCBI BlastP on this gene

Mycgr3G21922\_Mycgr3T

Mycgr3G99148 Mycgr3T
  
Location: 39249-42819
  
 NCBI BlastP on this gene

Mycgr3G99148\_Mycgr3T

hypothetical protein
  
Accession: EMD89220
  
Location: 612232-613805
  
 NCBI BlastP on this gene

EMD89220

hypothetical protein
  
Accession: EMD89219
  
Location: 611170-611674
  
 NCBI BlastP on this gene

EMD89219

hypothetical protein
  
Accession: EMD89218
  
Location: 608461-609215
  
 NCBI BlastP on this gene

EMD89218

hypothetical protein
  
Accession: EMD89217
  
Location: 606885-607960
  
 NCBI BlastP on this gene

EMD89217

hypothetical protein
  
Accession: EMD89216
  
Location: 605155-605802
  
 NCBI BlastP on this gene

EMD89216

hypothetical protein
  
Accession: EMD89215
  
Location: 602985-603463
  
 NCBI BlastP on this gene

EMD89215

hypothetical protein
  
Accession: EMD89214
  
Location: 597922-598890
  
 NCBI BlastP on this gene

EMD89214

hypothetical protein
  
Accession: EMD89213
  
Location: 595385-597460
  
 NCBI BlastP on this gene

EMD89213

hypothetical protein
  
Accession: EMD89212
  
Location: 592576-593885
  
  
**BlastP hit with Mycgr3G25746\_Mycgr3T**
  
Percentage identity: 56 %
  
BlastP bit score: 380
  
Sequence coverage: 102 %
  
E-value: 8e-127
  
  
 NCBI BlastP on this gene

EMD89212

hypothetical protein
  
Accession: EMD89211
  
Location: 590328-591647
  
 NCBI BlastP on this gene

EMD89211

hypothetical protein
  
Accession: EMD89210
  
Location: 587975-590101
  
  
**BlastP hit with Mycgr3G103278\_Mycgr3**
  
Percentage identity: 33 %
  
BlastP bit score: 118
  
Sequence coverage: 94 %
  
E-value: 4e-26
  
  
 NCBI BlastP on this gene

EMD89210

hypothetical protein
  
Accession: EMD89209
  
Location: 585068-587323
  
  
**BlastP hit with Mycgr3G21922\_Mycgr3T**
  
Percentage identity: 47 %
  
BlastP bit score: 455
  
Sequence coverage: 105 %
  
E-value: 2e-149
  
  
 NCBI BlastP on this gene

EMD89209

hypothetical protein
  
Accession: EMD89208
  
Location: 579684-582447
  
 NCBI BlastP on this gene

EMD89208

hypothetical protein
  
Accession: EMD89207
  
Location: 574478-574804
  
 NCBI BlastP on this gene

EMD89207

hypothetical protein
  
Accession: EMD89206
  
Location: 571774-573308
  
 NCBI BlastP on this gene

EMD89206

hypothetical protein
  
Accession: EMD89205
  
Location: 569722-571658
  
 NCBI BlastP on this gene

EMD89205

hypothetical protein
  
Accession: EMD89204
  
Location: 566645-567154
  
 NCBI BlastP on this gene

EMD89204

102. :  KB445649 Cochliobolus sativus ND90Pr unplaced genomic scaffold COCSAscaffold\_13     Total score: 3.0     Cumulative Blast bit score: 950

hypothetical protein
  
Accession: EMD60987
  
Location: 670499-671818
  
 NCBI BlastP on this gene

EMD60987

hypothetical protein
  
Accession: EMD60986
  
Location: 667686-669631
  
 NCBI BlastP on this gene

EMD60986

hypothetical protein
  
Accession: EMD60985
  
Location: 663961-666133
  
 NCBI BlastP on this gene

EMD60985

hypothetical protein
  
Accession: EMD60984
  
Location: 663040-663558
  
 NCBI BlastP on this gene

EMD60984

hypothetical protein
  
Accession: EMD60983
  
Location: 660788-661541
  
 NCBI BlastP on this gene

EMD60983

hypothetical protein
  
Accession: EMD60982
  
Location: 659432-660278
  
 NCBI BlastP on this gene

EMD60982

hypothetical protein
  
Accession: EMD60981
  
Location: 657469-658126
  
 NCBI BlastP on this gene

EMD60981

hypothetical protein
  
Accession: EMD60980
  
Location: 655228-656187
  
 NCBI BlastP on this gene

EMD60980

hypothetical protein
  
Accession: EMD60979
  
Location: 652686-654764
  
 NCBI BlastP on this gene

EMD60979

hypothetical protein
  
Accession: EMD60978
  
Location: 649880-651189
  
  
**BlastP hit with Mycgr3G25746\_Mycgr3T**
  
Percentage identity: 56 %
  
BlastP bit score: 379
  
Sequence coverage: 102 %
  
E-value: 4e-126
  
  
 NCBI BlastP on this gene

EMD60978

hypothetical protein
  
Accession: EMD60977
  
Location: 645295-647433
  
  
**BlastP hit with Mycgr3G103278\_Mycgr3**
  
Percentage identity: 33 %
  
BlastP bit score: 117
  
Sequence coverage: 94 %
  
E-value: 1e-25
  
  
 NCBI BlastP on this gene

EMD60977

hypothetical protein
  
Accession: EMD60976
  
Location: 642385-644655
  
  
**BlastP hit with Mycgr3G21922\_Mycgr3T**
  
Percentage identity: 48 %
  
BlastP bit score: 455
  
Sequence coverage: 105 %
  
E-value: 2e-149
  
  
 NCBI BlastP on this gene

EMD60976

hypothetical protein
  
Accession: EMD60975
  
Location: 640860-641916
  
 NCBI BlastP on this gene

EMD60975

hypothetical protein
  
Accession: EMD60974
  
Location: 638342-640574
  
 NCBI BlastP on this gene

EMD60974

hypothetical protein
  
Accession: EMD60973
  
Location: 636733-637335
  
 NCBI BlastP on this gene

EMD60973

hypothetical protein
  
Accession: EMD60972
  
Location: 635259-635741
  
 NCBI BlastP on this gene

EMD60972

hypothetical protein
  
Accession: EMD60971
  
Location: 634581-635045
  
 NCBI BlastP on this gene

EMD60971

hypothetical protein
  
Accession: EMD60970
  
Location: 634073-634414
  
 NCBI BlastP on this gene

EMD60970

hypothetical protein
  
Accession: EMD60969
  
Location: 631275-632800
  
 NCBI BlastP on this gene

EMD60969

hypothetical protein
  
Accession: EMD60968
  
Location: 628174-628539
  
 NCBI BlastP on this gene

EMD60968

hypothetical protein
  
Accession: EMD60967
  
Location: 627342-627716
  
 NCBI BlastP on this gene

EMD60967

hypothetical protein
  
Accession: EMD60966
  
Location: 626021-626437
  
 NCBI BlastP on this gene

EMD60966

103. :  DS231636 Pyrenophora tritici-repentis Pt-1C-BFP supercont1.22 genomic scaffold     Total score: 3.0     Cumulative Blast bit score: 946

RING finger domain containing protein
  
Accession: EDU46037
  
Location: 89859-91377
  
 NCBI BlastP on this gene

EDU46037

conserved hypothetical protein
  
Accession: EDU46036
  
Location: 88480-89575
  
 NCBI BlastP on this gene

EDU46036

conserved hypothetical protein
  
Accession: EDU46035
  
Location: 86131-87576
  
 NCBI BlastP on this gene

EDU46035

nitrilase
  
Accession: EDU46034
  
Location: 84192-85309
  
 NCBI BlastP on this gene

EDU46034

aldehyde reductase 1
  
Accession: EDU46033
  
Location: 82071-83012
  
 NCBI BlastP on this gene

EDU46033

hypothetical protein
  
Accession: EDU46032
  
Location: 76181-76486
  
 NCBI BlastP on this gene

EDU46032

predicted protein
  
Accession: EDU46031
  
Location: 71937-74290
  
 NCBI BlastP on this gene

EDU46031

DNA-binding protein HGH1
  
Accession: EDU46030
  
Location: 69222-70694
  
  
**BlastP hit with Mycgr3G25746\_Mycgr3T**
  
Percentage identity: 53 %
  
BlastP bit score: 358
  
Sequence coverage: 101 %
  
E-value: 7e-118
  
  
 NCBI BlastP on this gene

EDU46030

major facilitator family transporter
  
Accession: EDU46029
  
Location: 66913-68878
  
 NCBI BlastP on this gene

EDU46029

transcription factor TFIIF complex alpha subunit Tfg1
  
Accession: EDU46028
  
Location: 64715-66820
  
  
**BlastP hit with Mycgr3G103278\_Mycgr3**
  
Percentage identity: 34 %
  
BlastP bit score: 126
  
Sequence coverage: 99 %
  
E-value: 9e-29
  
  
 NCBI BlastP on this gene

EDU46028

Poly(A) RNA polymerase cid13
  
Accession: EDU46027
  
Location: 62285-64195
  
  
**BlastP hit with Mycgr3G21922\_Mycgr3T**
  
Percentage identity: 50 %
  
BlastP bit score: 463
  
Sequence coverage: 101 %
  
E-value: 1e-153
  
  
 NCBI BlastP on this gene

EDU46027

predicted protein
  
Accession: EDU46026
  
Location: 61032-61474
  
 NCBI BlastP on this gene

EDU46026

conserved hypothetical protein
  
Accession: EDU46025
  
Location: 59313-60326
  
 NCBI BlastP on this gene

EDU46025

predicted protein
  
Accession: EDU46024
  
Location: 56447-58780
  
 NCBI BlastP on this gene

EDU46024

predicted protein
  
Accession: EDU46023
  
Location: 54485-55401
  
 NCBI BlastP on this gene

EDU46023

conserved hypothetical protein
  
Accession: EDU46022
  
Location: 50955-51437
  
 NCBI BlastP on this gene

EDU46022

predicted protein
  
Accession: EDU46021
  
Location: 42626-44384
  
 NCBI BlastP on this gene

EDU46021

104. :  KB908844 Setosphaeria turcica Et28A unplaced genomic scaffold SETTUscaffold\_6     Total score: 3.0     Cumulative Blast bit score: 933

hypothetical protein
  
Accession: EOA82789
  
Location: 1338652-1340595
  
 NCBI BlastP on this gene

EOA82789

hypothetical protein
  
Accession: EOA82790
  
Location: 1342133-1343894
  
 NCBI BlastP on this gene

EOA82790

hypothetical protein
  
Accession: EOA82791
  
Location: 1344453-1344647
  
 NCBI BlastP on this gene

EOA82791

hypothetical protein
  
Accession: EOA82792
  
Location: 1344860-1345625
  
 NCBI BlastP on this gene

EOA82792

hypothetical protein
  
Accession: EOA82793
  
Location: 1346347-1347431
  
 NCBI BlastP on this gene

EOA82793

hypothetical protein
  
Accession: EOA82794
  
Location: 1348578-1349748
  
 NCBI BlastP on this gene

EOA82794

hypothetical protein
  
Accession: EOA82795
  
Location: 1350312-1350969
  
 NCBI BlastP on this gene

EOA82795

hypothetical protein
  
Accession: EOA82796
  
Location: 1351960-1352910
  
 NCBI BlastP on this gene

EOA82796

hypothetical protein
  
Accession: EOA82797
  
Location: 1353378-1354436
  
 NCBI BlastP on this gene

EOA82797

hypothetical protein
  
Accession: EOA82798
  
Location: 1355086-1357095
  
 NCBI BlastP on this gene

EOA82798

hypothetical protein
  
Accession: EOA82799
  
Location: 1358579-1359893
  
  
**BlastP hit with Mycgr3G25746\_Mycgr3T**
  
Percentage identity: 56 %
  
BlastP bit score: 372
  
Sequence coverage: 100 %
  
E-value: 1e-123
  
  
 NCBI BlastP on this gene

EOA82799

hypothetical protein
  
Accession: EOA82800
  
Location: 1360820-1362192
  
 NCBI BlastP on this gene

EOA82800

hypothetical protein
  
Accession: EOA82801
  
Location: 1362354-1364474
  
  
**BlastP hit with Mycgr3G103278\_Mycgr3**
  
Percentage identity: 35 %
  
BlastP bit score: 120
  
Sequence coverage: 92 %
  
E-value: 9e-27
  
  
 NCBI BlastP on this gene

EOA82801

hypothetical protein
  
Accession: EOA82802
  
Location: 1365535-1367031
  
  
**BlastP hit with Mycgr3G21922\_Mycgr3T**
  
Percentage identity: 46 %
  
BlastP bit score: 442
  
Sequence coverage: 103 %
  
E-value: 4e-147
  
  
 NCBI BlastP on this gene

EOA82802

hypothetical protein
  
Accession: EOA82803
  
Location: 1367426-1368031
  
 NCBI BlastP on this gene

EOA82803

105. :  CR382139 Debaryomyces hansenii CBS767 chromosome G complete sequence.     Total score: 3.0     Cumulative Blast bit score: 920

DEHA2G17842p
  
Accession: CAG90821
  
Location: 1453597-1455762
  
 NCBI BlastP on this gene

DEHA2G17842g

DEHA2G17864p
  
Accession: CAG90822
  
Location: 1456140-1456661
  
 NCBI BlastP on this gene

DEHA2G17864g

DEHA2G17886p
  
Accession: CAG90823
  
Location: 1457089-1457799
  
 NCBI BlastP on this gene

DEHA2G17886g

DEHA2G17908p
  
Accession: CAG90824
  
Location: 1457913-1461941
  
 NCBI BlastP on this gene

DEHA2G17908g

DEHA2G17930p
  
Accession: CAG90825
  
Location: 1462868-1464097
  
 NCBI BlastP on this gene

DEHA2G17930g

DEHA2G17952p
  
Accession: CAG90826
  
Location: 1464555-1465556
  
 NCBI BlastP on this gene

DEHA2G17952g

DEHA2G17974p
  
Accession: CAG90827
  
Location: 1465676-1466314
  
 NCBI BlastP on this gene

DEHA2G17974g

DEHA2G17996p
  
Accession: CAG90828
  
Location: 1466393-1467121
  
 NCBI BlastP on this gene

DEHA2G17996g

DEHA2G18018p
  
Accession: CAG90829
  
Location: 1467780-1468076
  
 NCBI BlastP on this gene

DEHA2G18018g

DEHA2G18040p
  
Accession: CAG90830
  
Location: 1468794-1470464
  
 NCBI BlastP on this gene

DEHA2G18040g

DEHA2G18062p
  
Accession: CAG90831
  
Location: 1470627-1472639
  
  
**BlastP hit with Mycgr3G68458\_Mycgr3T**
  
Percentage identity: 49 %
  
BlastP bit score: 556
  
Sequence coverage: 85 %
  
E-value: 0.0
  
  
 NCBI BlastP on this gene

DEHA2G18062g

DEHA2G18084p
  
Accession: CAR65994
  
Location: 1473251-1476406
  
 NCBI BlastP on this gene

DEHA2G18084g

DEHA2G18106p
  
Accession: CAG90834
  
Location: 1476616-1476891
  
 NCBI BlastP on this gene

DEHA2G18106g

DEHA2G18128p
  
Accession: CAG90835
  
Location: 1477052-1477819
  
 NCBI BlastP on this gene

DEHA2G18128g

DEHA2G18150p
  
Accession: CAG90836
  
Location: 1478047-1478973
  
 NCBI BlastP on this gene

DEHA2G18150g

DEHA2G18194p
  
Accession: CAG90837
  
Location: 1479821-1480558
  
 NCBI BlastP on this gene

DEHA2G18194g

DEHA2G18216p
  
Accession: CAG90838
  
Location: 1480739-1481983
  
 NCBI BlastP on this gene

DEHA2G18216g

DEHA2G18238p
  
Accession: CAG90839
  
Location: 1482112-1482621
  
 NCBI BlastP on this gene

DEHA2G18238g

DEHA2G18260p
  
Accession: CAG90840
  
Location: 1482693-1483613
  
 NCBI BlastP on this gene

DEHA2G18260g

DEHA2G18282p
  
Accession: CAG90841
  
Location: 1484264-1486069
  
  
**BlastP hit with Mycgr3G103278\_Mycgr3**
  
Percentage identity: 38 %
  
BlastP bit score: 91
  
Sequence coverage: 37 %
  
E-value: 4e-17
  
  
 NCBI BlastP on this gene

DEHA2G18282g

DEHA2G18304p
  
Accession: CAG90842
  
Location: 1486170-1487273
  
  
**BlastP hit with Mycgr3G25746\_Mycgr3T**
  
Percentage identity: 43 %
  
BlastP bit score: 273
  
Sequence coverage: 101 %
  
E-value: 4e-85
  
  
 NCBI BlastP on this gene

DEHA2G18304g

DEHA2G18326p
  
Accession: CAG90843
  
Location: 1487428-1489206
  
 NCBI BlastP on this gene

DEHA2G18326g

DEHA2G18348p
  
Accession: CAG90844
  
Location: 1489476-1491245
  
 NCBI BlastP on this gene

DEHA2G18348g

DEHA2G18370p
  
Accession: CAG90845
  
Location: 1491466-1492848
  
 NCBI BlastP on this gene

DEHA2G18370g

DEHA2G18392p
  
Accession: CAG90846
  
Location: 1493018-1494487
  
 NCBI BlastP on this gene

DEHA2G18392g

DEHA2G18414p
  
Accession: CAG90847
  
Location: 1494533-1495858
  
 NCBI BlastP on this gene

DEHA2G18414g

DEHA2G18436p
  
Accession: CAG90848
  
Location: 1495968-1496525
  
 NCBI BlastP on this gene

DEHA2G18436g

DEHA2G18458p
  
Accession: CAG90849
  
Location: 1496927-1497541
  
 NCBI BlastP on this gene

DEHA2G18458g

DEHA2G18480p
  
Accession: CAG90850
  
Location: 1497648-1498943
  
 NCBI BlastP on this gene

DEHA2G18480g

DEHA2G18502p
  
Accession: CAG90851
  
Location: 1499224-1499988
  
 NCBI BlastP on this gene

DEHA2G18502g

DEHA2G18524p
  
Accession: CAG90852
  
Location: 1500371-1500998
  
 NCBI BlastP on this gene

DEHA2G18524g

DEHA2G18546p
  
Accession: CAG90853
  
Location: 1501187-1502389
  
 NCBI BlastP on this gene

DEHA2G18546g

DEHA2G18568p
  
Accession: CAG90854
  
Location: 1502597-1508041
  
 NCBI BlastP on this gene

DEHA2G18568g

106. :  CH672349 Candida albicans WO-1 chromosome 4 supercont1.4 genomic scaffold     Total score: 3.0     Cumulative Blast bit score: 919

conserved hypothetical protein
  
Accession: EEQ45254
  
Location: 1187802-1188383
  
 NCBI BlastP on this gene

EEQ45254

hypothetical protein
  
Accession: EEQ45255
  
Location: 1189146-1190057
  
 NCBI BlastP on this gene

EEQ45255

conserved hypothetical protein
  
Accession: EEQ45256
  
Location: 1190917-1193334
  
 NCBI BlastP on this gene

EEQ45256

conserved hypothetical protein
  
Accession: EEQ45257
  
Location: 1193391-1194185
  
 NCBI BlastP on this gene

EEQ45257

conserved hypothetical protein
  
Accession: EEQ45258
  
Location: 1194473-1194913
  
 NCBI BlastP on this gene

EEQ45258

hypothetical protein
  
Accession: EEQ45259
  
Location: 1195297-1196946
  
 NCBI BlastP on this gene

EEQ45259

conserved hypothetical protein
  
Accession: EEQ45260
  
Location: 1197509-1200001
  
 NCBI BlastP on this gene

EEQ45260

conserved hypothetical protein
  
Accession: EEQ45261
  
Location: 1200287-1201957
  
 NCBI BlastP on this gene

EEQ45261

ABC1 family protein
  
Accession: EEQ45262
  
Location: 1202180-1204171
  
  
**BlastP hit with Mycgr3G68458\_Mycgr3T**
  
Percentage identity: 51 %
  
BlastP bit score: 556
  
Sequence coverage: 82 %
  
E-value: 0.0
  
  
 NCBI BlastP on this gene

EEQ45262

conserved hypothetical protein
  
Accession: EEQ45263
  
Location: 1205075-1208386
  
 NCBI BlastP on this gene

EEQ45263

mitochondrial import inner membrane translocase subunit TIM10
  
Accession: EEQ45264
  
Location: 1208616-1208891
  
 NCBI BlastP on this gene

EEQ45264

cytochrome C1 heme lyase
  
Accession: EEQ45265
  
Location: 1209026-1209781
  
 NCBI BlastP on this gene

EEQ45265

conserved hypothetical protein
  
Accession: EEQ45266
  
Location: 1210114-1211040
  
 NCBI BlastP on this gene

EEQ45266

conserved hypothetical protein
  
Accession: EEQ45267
  
Location: 1212078-1212611
  
 NCBI BlastP on this gene

EEQ45267

conserved hypothetical protein
  
Accession: EEQ45268
  
Location: 1212819-1214015
  
 NCBI BlastP on this gene

EEQ45268

exosome complex exonuclease RRP41
  
Accession: EEQ45269
  
Location: 1214203-1214925
  
 NCBI BlastP on this gene

EEQ45269

hypothetical protein
  
Accession: EEQ45270
  
Location: 1215005-1215934
  
 NCBI BlastP on this gene

EEQ45270

hypothetical protein
  
Accession: EEQ45271
  
Location: 1216261-1217631
  
 NCBI BlastP on this gene

EEQ45271

conserved hypothetical protein
  
Accession: EEQ45272
  
Location: 1218505-1220331
  
  
**BlastP hit with Mycgr3G103278\_Mycgr3**
  
Percentage identity: 40 %
  
BlastP bit score: 84
  
Sequence coverage: 26 %
  
E-value: 8e-15
  
  
 NCBI BlastP on this gene

EEQ45272

protein HGH1
  
Accession: EEQ45273
  
Location: 1220446-1221534
  
  
**BlastP hit with Mycgr3G25746\_Mycgr3T**
  
Percentage identity: 43 %
  
BlastP bit score: 279
  
Sequence coverage: 100 %
  
E-value: 2e-87
  
  
 NCBI BlastP on this gene

EEQ45273

conserved hypothetical protein
  
Accession: EEQ45274
  
Location: 1221666-1223099
  
 NCBI BlastP on this gene

EEQ45274

carnitine O-acetyltransferase, mitochondrial precursor
  
Accession: EEQ45275
  
Location: 1232041-1233939
  
 NCBI BlastP on this gene

EEQ45275

hypothetical protein
  
Accession: EEQ45276
  
Location: 1235576-1237210
  
 NCBI BlastP on this gene

EEQ45276

107. :  AAVQ01000001 Pichia stipitis CBS 6054 chromosome 1     Total score: 3.0     Cumulative Blast bit score: 905

hyphal wall protein (putative)
  
Accession: EAZ63557
  
Location: 1807332-1808774
  
 NCBI BlastP on this gene

EAZ63557

predicted protein
  
Accession: EAZ63959
  
Location: 1805837-1806836
  
 NCBI BlastP on this gene

EAZ63959

predicted protein
  
Accession: EAZ63958
  
Location: 1804602-1805240
  
 NCBI BlastP on this gene

EAZ63958

predicted protein
  
Accession: EAZ63556
  
Location: 1803498-1804226
  
 NCBI BlastP on this gene

EAZ63556

predicted protein
  
Accession: EAZ63555
  
Location: 1802132-1802422
  
 NCBI BlastP on this gene

EAZ63555

predicted protein
  
Accession: EAZ63957
  
Location: 1799893-1801578
  
 NCBI BlastP on this gene

EAZ63957

predicted protein
  
Accession: EAZ63956
  
Location: 1797482-1799425
  
  
**BlastP hit with Mycgr3G68458\_Mycgr3T**
  
Percentage identity: 47 %
  
BlastP bit score: 543
  
Sequence coverage: 93 %
  
E-value: 0.0
  
  
 NCBI BlastP on this gene

EAZ63956

predicted protein
  
Accession: EAZ63955
  
Location: 1791373-1791648
  
 NCBI BlastP on this gene

EAZ63955

cytochrome c1 heme lyase
  
Accession: EAZ63554
  
Location: 1790112-1790915
  
 NCBI BlastP on this gene

EAZ63554

predicted protein
  
Accession: EAZ63553
  
Location: 1788618-1789538
  
 NCBI BlastP on this gene

EAZ63553

predicted protein
  
Accession: EAZ63552
  
Location: 1779134-1779859
  
 NCBI BlastP on this gene

EAZ63552

mannosyltransferase
  
Accession: EAZ63551
  
Location: 1777613-1778803
  
 NCBI BlastP on this gene

EAZ63551

predicted protein
  
Accession: EAZ63954
  
Location: 1776324-1776932
  
 NCBI BlastP on this gene

EAZ63954

mitochondrial carrier protein
  
Accession: EAZ63550
  
Location: 1775207-1776130
  
 NCBI BlastP on this gene

EAZ63550

predicted protein
  
Accession: EAZ63953
  
Location: 1772821-1774638
  
  
**BlastP hit with Mycgr3G103278\_Mycgr3**
  
Percentage identity: 32 %
  
BlastP bit score: 90
  
Sequence coverage: 90 %
  
E-value: 4e-17
  
  
 NCBI BlastP on this gene

EAZ63953

predicted protein
  
Accession: EAZ63549
  
Location: 1771522-1772607
  
  
**BlastP hit with Mycgr3G25746\_Mycgr3T**
  
Percentage identity: 42 %
  
BlastP bit score: 272
  
Sequence coverage: 100 %
  
E-value: 9e-85
  
  
 NCBI BlastP on this gene

EAZ63549

predicted protein
  
Accession: EAZ63548
  
Location: 1769322-1771124
  
 NCBI BlastP on this gene

EAZ63548

predicted protein
  
Accession: EAZ63547
  
Location: 1768206-1768919
  
 NCBI BlastP on this gene

EAZ63547

pyruvate decarboxylase
  
Accession: EAZ63546
  
Location: 1765968-1767758
  
 NCBI BlastP on this gene

EAZ63546

predicted protein
  
Accession: EAZ63545
  
Location: 1763148-1764149
  
 NCBI BlastP on this gene

EAZ63545

Phosphomevalonate kinase
  
Accession: EAZ63544
  
Location: 1760839-1762236
  
 NCBI BlastP on this gene

EAZ63544

108. :  CH408081 Clavispora lusitaniae ATCC 42720 scaffold\_6 genomic scaffold     Total score: 3.0     Cumulative Blast bit score: 889

predicted protein
  
Accession: EEQ40867
  
Location: 346796-348211
  
 NCBI BlastP on this gene

EEQ40867

predicted protein
  
Accession: EEQ40868
  
Location: 346850-348178
  
 NCBI BlastP on this gene

EEQ40868

hypothetical protein
  
Accession: EEQ40869
  
Location: 349167-350123
  
 NCBI BlastP on this gene

EEQ40869

hypothetical protein
  
Accession: EEQ40870
  
Location: 350909-351547
  
 NCBI BlastP on this gene

EEQ40870

hypothetical protein
  
Accession: EEQ40871
  
Location: 351053-351586
  
 NCBI BlastP on this gene

EEQ40871

conserved hypothetical protein
  
Accession: EEQ40872
  
Location: 352135-352860
  
 NCBI BlastP on this gene

EEQ40872

hypothetical protein
  
Accession: EEQ40873
  
Location: 353588-353863
  
 NCBI BlastP on this gene

EEQ40873

hypothetical protein
  
Accession: EEQ40874
  
Location: 355269-356939
  
 NCBI BlastP on this gene

EEQ40874

hypothetical protein
  
Accession: EEQ40875
  
Location: 357509-359584
  
  
**BlastP hit with Mycgr3G68458\_Mycgr3T**
  
Percentage identity: 48 %
  
BlastP bit score: 529
  
Sequence coverage: 83 %
  
E-value: 2e-175
  
  
 NCBI BlastP on this gene

EEQ40875

hypothetical protein
  
Accession: EEQ40876
  
Location: 361069-361779
  
 NCBI BlastP on this gene

EEQ40876

hypothetical protein
  
Accession: EEQ40877
  
Location: 363279-363998
  
 NCBI BlastP on this gene

EEQ40877

hypothetical protein
  
Accession: EEQ40878
  
Location: 365044-365811
  
 NCBI BlastP on this gene

EEQ40878

predicted protein
  
Accession: EEQ40879
  
Location: 366831-368375
  
 NCBI BlastP on this gene

EEQ40879

predicted protein
  
Accession: EEQ40880
  
Location: 369526-371013
  
 NCBI BlastP on this gene

EEQ40880

predicted protein
  
Accession: EEQ40881
  
Location: 372102-373535
  
 NCBI BlastP on this gene

EEQ40881

hypothetical protein
  
Accession: EEQ40882
  
Location: 374855-376708
  
 NCBI BlastP on this gene

EEQ40882

hypothetical protein
  
Accession: EEQ40883
  
Location: 377280-378374
  
  
**BlastP hit with Mycgr3G25746\_Mycgr3T**
  
Percentage identity: 42 %
  
BlastP bit score: 271
  
Sequence coverage: 101 %
  
E-value: 2e-84
  
  
 NCBI BlastP on this gene

EEQ40883

hypothetical protein
  
Accession: EEQ40884
  
Location: 379144-380805
  
  
**BlastP hit with Mycgr3G103278\_Mycgr3**
  
Percentage identity: 48 %
  
BlastP bit score: 89
  
Sequence coverage: 26 %
  
E-value: 1e-16
  
  
 NCBI BlastP on this gene

EEQ40884

hypothetical protein
  
Accession: EEQ40886
  
Location: 383182-384135
  
 NCBI BlastP on this gene

EEQ40886

hypothetical protein
  
Accession: EEQ40885
  
Location: 383224-384090
  
 NCBI BlastP on this gene

EEQ40885

hypothetical protein
  
Accession: EEQ40888
  
Location: 384506-385042
  
 NCBI BlastP on this gene

EEQ40888

hypothetical protein
  
Accession: EEQ40887
  
Location: 384740-385207
  
 NCBI BlastP on this gene

EEQ40887

hypothetical protein
  
Accession: EEQ40889
  
Location: 385315-386562
  
 NCBI BlastP on this gene

EEQ40889

predicted protein
  
Accession: EEQ40890
  
Location: 387070-387789
  
 NCBI BlastP on this gene

EEQ40890

hypothetical protein
  
Accession: EEQ40891
  
Location: 388932-389666
  
 NCBI BlastP on this gene

EEQ40891

hypothetical protein
  
Accession: EEQ40892
  
Location: 391518-392435
  
 NCBI BlastP on this gene

EEQ40892

hypothetical protein
  
Accession: EEQ40893
  
Location: 392998-393717
  
 NCBI BlastP on this gene

EEQ40893

109. :  CH408157 Pichia guilliermondii ATCC 6260 scaffold\_3 genomic scaffold     Total score: 3.0     Cumulative Blast bit score: 886

hypothetical protein
  
Accession: EDK38328
  
Location: 222910-224205
  
 NCBI BlastP on this gene

EDK38328

hypothetical protein
  
Accession: EDK38327
  
Location: 221087-222892
  
 NCBI BlastP on this gene

EDK38327

hypothetical protein
  
Accession: EDK38326
  
Location: 219436-221079
  
 NCBI BlastP on this gene

EDK38326

hypothetical protein
  
Accession: EDK38325
  
Location: 217998-219398
  
 NCBI BlastP on this gene

EDK38325

conserved hypothetical protein
  
Accession: EDK38324
  
Location: 216469-217512
  
 NCBI BlastP on this gene

EDK38324

conserved hypothetical protein
  
Accession: EDK38322
  
Location: 215234-215899
  
 NCBI BlastP on this gene

EDK38322

conserved hypothetical protein
  
Accession: EDK38323
  
Location: 215081-215890
  
 NCBI BlastP on this gene

EDK38323

conserved hypothetical protein
  
Accession: EDK38321
  
Location: 213836-214882
  
 NCBI BlastP on this gene

EDK38321

hypothetical protein
  
Accession: EDK38320
  
Location: 211150-213693
  
 NCBI BlastP on this gene

EDK38320

hypothetical protein
  
Accession: EDK38319
  
Location: 207545-209083
  
 NCBI BlastP on this gene

EDK38319

hypothetical protein
  
Accession: EDK38318
  
Location: 205287-207464
  
  
**BlastP hit with Mycgr3G68458\_Mycgr3T**
  
Percentage identity: 49 %
  
BlastP bit score: 555
  
Sequence coverage: 85 %
  
E-value: 0.0
  
  
 NCBI BlastP on this gene

EDK38318

hypothetical protein
  
Accession: EDK38317
  
Location: 201944-204889
  
 NCBI BlastP on this gene

EDK38317

mitochondrial import inner membrane translocase subunit TIM10
  
Accession: EDK38316
  
Location: 201560-201910
  
 NCBI BlastP on this gene

EDK38316

hypothetical protein
  
Accession: EDK38315
  
Location: 199728-200657
  
 NCBI BlastP on this gene

EDK38315

hypothetical protein
  
Accession: EDK38314
  
Location: 198448-199176
  
 NCBI BlastP on this gene

EDK38314

hypothetical protein
  
Accession: EDK38313
  
Location: 197122-198375
  
 NCBI BlastP on this gene

EDK38313

hypothetical protein
  
Accession: EDK38312
  
Location: 196553-197050
  
 NCBI BlastP on this gene

EDK38312

hypothetical protein
  
Accession: EDK38311
  
Location: 195619-196527
  
 NCBI BlastP on this gene

EDK38311

hypothetical protein
  
Accession: EDK38310
  
Location: 193515-195113
  
  
**BlastP hit with Mycgr3G103278\_Mycgr3**
  
Percentage identity: 41 %
  
BlastP bit score: 84
  
Sequence coverage: 26 %
  
E-value: 7e-15
  
  
 NCBI BlastP on this gene

EDK38310

hypothetical protein
  
Accession: EDK38309
  
Location: 192372-193313
  
  
**BlastP hit with Mycgr3G25746\_Mycgr3T**
  
Percentage identity: 42 %
  
BlastP bit score: 247
  
Sequence coverage: 90 %
  
E-value: 2e-75
  
  
 NCBI BlastP on this gene

EDK38309

hypothetical protein
  
Accession: EDK38308
  
Location: 190423-192225
  
 NCBI BlastP on this gene

EDK38308

hypothetical protein
  
Accession: EDK38307
  
Location: 189128-190294
  
 NCBI BlastP on this gene

EDK38307

hypothetical protein
  
Accession: EDK38305
  
Location: 188105-188938
  
 NCBI BlastP on this gene

EDK38305

hypothetical protein
  
Accession: EDK38306
  
Location: 187654-188148
  
 NCBI BlastP on this gene

EDK38306

hypothetical protein
  
Accession: EDK38304
  
Location: 187504-188247
  
 NCBI BlastP on this gene

EDK38304

hypothetical protein
  
Accession: EDK38302
  
Location: 186168-186455
  
 NCBI BlastP on this gene

EDK38302

hypothetical protein
  
Accession: EDK38303
  
Location: 186134-187450
  
 NCBI BlastP on this gene

EDK38303

hypothetical protein
  
Accession: EDK38301
  
Location: 185484-186041
  
 NCBI BlastP on this gene

EDK38301

hypothetical protein
  
Accession: EDK38300
  
Location: 184569-185174
  
 NCBI BlastP on this gene

EDK38300

hypothetical protein
  
Accession: EDK38299
  
Location: 183075-184514
  
 NCBI BlastP on this gene

EDK38299

hypothetical protein
  
Accession: EDK38298
  
Location: 182248-183009
  
 NCBI BlastP on this gene

EDK38298

hypothetical protein
  
Accession: EDK38297
  
Location: 180121-181335
  
 NCBI BlastP on this gene

EDK38297

hypothetical protein
  
Accession: EDK38296
  
Location: 177866-179890
  
 NCBI BlastP on this gene

EDK38296

hypothetical protein
  
Accession: EDK38295
  
Location: 174622-177816
  
 NCBI BlastP on this gene

EDK38295

110. :  KE145361 Glarea lozoyensis ATCC 20868 chromosome Unknown GLAREA18     Total score: 3.0     Cumulative Blast bit score: 868

hypothetical protein
  
Accession: EPE31621
  
Location: 431036-433040
  
 NCBI BlastP on this gene

EPE31621

hypothetical protein
  
Accession: EPE31622
  
Location: 434309-435860
  
 NCBI BlastP on this gene

EPE31622

Thioesterase/thiol ester dehydrase-isomerase
  
Accession: EPE31623
  
Location: 436130-437285
  
 NCBI BlastP on this gene

EPE31623

MFS general substrate transporter
  
Accession: EPE31624
  
Location: 438738-440689
  
 NCBI BlastP on this gene

EPE31624

hypothetical protein
  
Accession: EPE31625
  
Location: 442023-442718
  
 NCBI BlastP on this gene

EPE31625

hypothetical protein
  
Accession: EPE31626
  
Location: 443951-445071
  
 NCBI BlastP on this gene

EPE31626

ARM repeat-containing protein
  
Accession: EPE31627
  
Location: 445719-448231
  
 NCBI BlastP on this gene

EPE31627

hypothetical protein
  
Accession: EPE31628
  
Location: 449652-451163
  
  
**BlastP hit with Mycgr3G90786\_Mycgr3T**
  
Percentage identity: 29 %
  
BlastP bit score: 95
  
Sequence coverage: 110 %
  
E-value: 5e-18
  
  
 NCBI BlastP on this gene

EPE31628

L
  
Accession: EPE31629
  
Location: 452227-455505
  
  
**BlastP hit with Mycgr3G68433\_Mycgr3T**
  
Percentage identity: 35 %
  
BlastP bit score: 465
  
Sequence coverage: 107 %
  
E-value: 3e-142
  
  
 NCBI BlastP on this gene

EPE31629

hypothetical protein
  
Accession: EPE31630
  
Location: 455847-457618
  
 NCBI BlastP on this gene

EPE31630

Metallo-hydrolase/oxidoreductase
  
Accession: EPE31631
  
Location: 457953-459035
  
 NCBI BlastP on this gene

EPE31631

PRTase-like protein
  
Accession: EPE31632
  
Location: 459722-460505
  
  
**BlastP hit with Mycgr3G55345\_Mycgr3T**
  
Percentage identity: 73 %
  
BlastP bit score: 308
  
Sequence coverage: 100 %
  
E-value: 4e-103
  
  
 NCBI BlastP on this gene

EPE31632

NAD(P)-binding Rossmann-fold containing protein
  
Accession: EPE31633
  
Location: 460890-462232
  
 NCBI BlastP on this gene

EPE31633

vegetative cell wall protein gp1
  
Accession: EPE31634
  
Location: 472528-473697
  
 NCBI BlastP on this gene

EPE31634

S-adenosyl-L-methionine-dependent methyltransferase
  
Accession: EPE31635
  
Location: 476541-477654
  
 NCBI BlastP on this gene

EPE31635

EF-hand
  
Accession: EPE31636
  
Location: 478187-480929
  
 NCBI BlastP on this gene

EPE31636

111. :  GL996524 Candida tenuis ATCC 10573 unplaced genomic scaffold CANTEscaffold\_00018     Total score: 3.0     Cumulative Blast bit score: 839

hypothetical protein
  
Accession: EGV63419
  
Location: 945520-947079
  
 NCBI BlastP on this gene

EGV63419

kynurenine 3-monooxygenase mitochondrial precursor
  
Accession: EGV63661
  
Location: 947236-948600
  
 NCBI BlastP on this gene

EGV63661

Tim44-domain-containing protein
  
Accession: EGV63662
  
Location: 949312-950034
  
 NCBI BlastP on this gene

EGV63662

hypothetical protein
  
Accession: EGV63665
  
Location: 950158-951591
  
 NCBI BlastP on this gene

EGV63665

hypothetical protein
  
Accession: EGV63420
  
Location: 951858-952352
  
 NCBI BlastP on this gene

EGV63420

hypothetical protein
  
Accession: EGV63421
  
Location: 953971-958418
  
 NCBI BlastP on this gene

EGV63421

hypothetical protein
  
Accession: EGV63422
  
Location: 953971-955884
  
 NCBI BlastP on this gene

EGV63422

arabinose-proton symporter
  
Accession: EGV63423
  
Location: 956694-958418
  
 NCBI BlastP on this gene

EGV63423

hypothetical protein
  
Accession: EGV63424
  
Location: 958615-959313
  
 NCBI BlastP on this gene

EGV63424

hypothetical protein
  
Accession: EGV63426
  
Location: 959979-960207
  
 NCBI BlastP on this gene

EGV63426

hypothetical protein
  
Accession: EGV63427
  
Location: 960246-961439
  
 NCBI BlastP on this gene

EGV63427

ABC1-domain-containing protein
  
Accession: EGV63428
  
Location: 962509-964218
  
  
**BlastP hit with Mycgr3G68458\_Mycgr3T**
  
Percentage identity: 46 %
  
BlastP bit score: 483
  
Sequence coverage: 81 %
  
E-value: 6e-159
  
  
 NCBI BlastP on this gene

EGV63428

hypothetical protein
  
Accession: EGV63429
  
Location: 966144-967583
  
 NCBI BlastP on this gene

EGV63429

hypothetical protein
  
Accession: EGV63667
  
Location: 968038-968814
  
 NCBI BlastP on this gene

EGV63667

SURF4-domain-containing protein
  
Accession: EGV63431
  
Location: 968886-969764
  
 NCBI BlastP on this gene

EGV63431

ribosomal protein S5 domain 2-like protein
  
Accession: EGV63668
  
Location: 970024-970752
  
 NCBI BlastP on this gene

EGV63668

hypothetical protein
  
Accession: EGV63670
  
Location: 971211-972060
  
 NCBI BlastP on this gene

EGV63670

hypothetical protein
  
Accession: EGV63433
  
Location: 972166-972714
  
 NCBI BlastP on this gene

EGV63433

mitochondrial carrier
  
Accession: EGV63672
  
Location: 972749-973681
  
 NCBI BlastP on this gene

EGV63672

Rap30/74 interaction domain-containing protein
  
Accession: EGV63434
  
Location: 973980-975791
  
  
**BlastP hit with Mycgr3G103278\_Mycgr3**
  
Percentage identity: 45 %
  
BlastP bit score: 86
  
Sequence coverage: 26 %
  
E-value: 1e-15
  
  
 NCBI BlastP on this gene

EGV63434

hypothetical protein
  
Accession: EGV63675
  
Location: 975826-976929
  
  
**BlastP hit with Mycgr3G25746\_Mycgr3T**
  
Percentage identity: 42 %
  
BlastP bit score: 270
  
Sequence coverage: 101 %
  
E-value: 6e-84
  
  
 NCBI BlastP on this gene

EGV63675

hypothetical protein
  
Accession: EGV63435
  
Location: 976994-978730
  
 NCBI BlastP on this gene

EGV63435

dienelactone hydrolase
  
Accession: EGV63436
  
Location: 978864-979598
  
 NCBI BlastP on this gene

EGV63436

hypothetical protein
  
Accession: EGV63677
  
Location: 982754-983470
  
 NCBI BlastP on this gene

EGV63677

pyruvate decarboxylase
  
Accession: EGV63437
  
Location: 984030-985802
  
 NCBI BlastP on this gene

EGV63437

hypothetical protein
  
Accession: EGV63438
  
Location: 986489-987663
  
 NCBI BlastP on this gene

EGV63438

hypothetical protein
  
Accession: EGV63439
  
Location: 987808-989163
  
 NCBI BlastP on this gene

EGV63439

hypothetical protein
  
Accession: EGV63440
  
Location: 990632-991189
  
 NCBI BlastP on this gene

EGV63440

hypothetical protein
  
Accession: EGV63442
  
Location: 991425-992012
  
 NCBI BlastP on this gene

EGV63442

hypothetical protein
  
Accession: EGV63679
  
Location: 992100-993389
  
 NCBI BlastP on this gene

EGV63679

Shwachman-Bodian-diamond syndrome protein
  
Accession: EGV63443
  
Location: 993486-994247
  
 NCBI BlastP on this gene

EGV63443

112. :  GG698897 Nectria haematococca mpVI 77-13-4 chromosome 3 genomic scaffold NECHAsca\_2\_chr3\_3\_0     Total score: 3.0     Cumulative Blast bit score: 801

hypothetical protein
  
Accession: EEU47196
  
Location: 347397-349573
  
 NCBI BlastP on this gene

EEU47196

hypothetical protein
  
Accession: EEU47195
  
Location: 344200-345891
  
 NCBI BlastP on this gene

EEU47195

hypothetical protein
  
Accession: EEU46728
  
Location: 338382-344149
  
 NCBI BlastP on this gene

EEU46728

hypothetical protein
  
Accession: EEU46727
  
Location: 336500-337724
  
 NCBI BlastP on this gene

EEU46727

hypothetical protein
  
Accession: EEU47194
  
Location: 334896-336139
  
 NCBI BlastP on this gene

EEU47194

hypothetical protein
  
Accession: EEU47193
  
Location: 330055-333663
  
  
**BlastP hit with Mycgr3G68433\_Mycgr3T**
  
Percentage identity: 35 %
  
BlastP bit score: 426
  
Sequence coverage: 93 %
  
E-value: 1e-126
  
  
 NCBI BlastP on this gene

EEU47193

hypothetical protein
  
Accession: EEU47192
  
Location: 328806-329322
  
 NCBI BlastP on this gene

EEU47192

hypothetical protein
  
Accession: EEU47191
  
Location: 324389-328038
  
  
**BlastP hit with Mycgr3G90786\_Mycgr3T**
  
Percentage identity: 26 %
  
BlastP bit score: 74
  
Sequence coverage: 88 %
  
E-value: 7e-11
  
  
 NCBI BlastP on this gene

EEU47191

hypothetical protein
  
Accession: EEU46726
  
Location: 321583-323990
  
 NCBI BlastP on this gene

EEU46726

hypothetical protein
  
Accession: EEU47190
  
Location: 317504-318242
  
 NCBI BlastP on this gene

EEU47190

hypothetical protein
  
Accession: EEU47189
  
Location: 315991-316867
  
  
**BlastP hit with Mycgr3G55345\_Mycgr3T**
  
Percentage identity: 71 %
  
BlastP bit score: 301
  
Sequence coverage: 99 %
  
E-value: 2e-100
  
  
 NCBI BlastP on this gene

EEU47189

predicted protein
  
Accession: EEU46725
  
Location: 314400-315719
  
 NCBI BlastP on this gene

EEU46725

hypothetical protein
  
Accession: EEU46724
  
Location: 313501-314226
  
 NCBI BlastP on this gene

EEU46724

hypothetical protein
  
Accession: EEU47188
  
Location: 310450-312847
  
 NCBI BlastP on this gene

EEU47188

hypothetical protein
  
Accession: EEU47187
  
Location: 304130-308162
  
 NCBI BlastP on this gene

EEU47187

hypothetical protein
  
Accession: EEU47186
  
Location: 299576-303459
  
 NCBI BlastP on this gene

EEU47186

113. :  KB707952 Botryotinia fuckeliana BcDW1 unplaced genomic scaffold Scaffold\_280     Total score: 3.0     Cumulative Blast bit score: 795

putative endopolygalacturonase 5 protein
  
Accession: EMR84421
  
Location: 150867-152236
  
 NCBI BlastP on this gene

EMR84421

hypothetical protein
  
Accession: EMR84420
  
Location: 140528-145822
  
 NCBI BlastP on this gene

EMR84420

hypothetical protein
  
Accession: EMR84419
  
Location: 139323-139749
  
 NCBI BlastP on this gene

EMR84419

putative pre-mrna-splicing factor cwc26 protein
  
Accession: EMR84418
  
Location: 137969-138919
  
 NCBI BlastP on this gene

EMR84418

putative transcription initiation factor iif subunit alpha protein
  
Accession: EMR84417
  
Location: 135128-137409
  
  
**BlastP hit with Mycgr3G103278\_Mycgr3**
  
Percentage identity: 37 %
  
BlastP bit score: 128
  
Sequence coverage: 100 %
  
E-value: 2e-29
  
  
 NCBI BlastP on this gene

EMR84417

putative caffeine-induced death protein
  
Accession: EMR84416
  
Location: 130554-133955
  
  
**BlastP hit with Mycgr3G21922\_Mycgr3T**
  
Percentage identity: 47 %
  
BlastP bit score: 283
  
Sequence coverage: 63 %
  
E-value: 5e-81
  
  
 NCBI BlastP on this gene

EMR84416

putative sugar transporter protein
  
Accession: EMR84415
  
Location: 127737-129584
  
 NCBI BlastP on this gene

EMR84415

putative glycosyl hydrolase protein
  
Accession: EMR84414
  
Location: 125011-126883
  
 NCBI BlastP on this gene

EMR84414

putative polygalacturonase protein
  
Accession: EMR84413
  
Location: 120719-124207
  
  
**BlastP hit with Mycgr3G25746\_Mycgr3T**
  
Percentage identity: 53 %
  
BlastP bit score: 384
  
Sequence coverage: 100 %
  
E-value: 1e-122
  
  
 NCBI BlastP on this gene

EMR84413

putative neutral ceramidase protein
  
Accession: EMR84412
  
Location: 117930-120582
  
 NCBI BlastP on this gene

EMR84412

putative abc a-pheromone efflux pump protein
  
Accession: EMR84411
  
Location: 110968-115518
  
 NCBI BlastP on this gene

EMR84411

putative alpha mannosidase family protein
  
Accession: EMR84410
  
Location: 106482-109009
  
 NCBI BlastP on this gene

EMR84410

putative extracellular serine-rich protein
  
Accession: EMR84409
  
Location: 105053-105710
  
 NCBI BlastP on this gene

EMR84409

114. :  FQ790278 Botryotinia fuckeliana T4 SuperContig\_34\_1 genomic supercontig.     Total score: 3.0     Cumulative Blast bit score: 795

BcPG5, endopolygalacturonase 5
  
Accession: CCD45769
  
Location: 881374-882743
  
 NCBI BlastP on this gene

BofuT4P34000021001

predicted protein
  
Accession: CCD45768
  
Location: 880355-880662
  
 NCBI BlastP on this gene

BofuT4\_uP047860.1

predicted protein
  
Accession: CCD45767
  
Location: 879253-879454
  
 NCBI BlastP on this gene

BofuT4\_uP047850.1

predicted protein
  
Accession: CCD45766
  
Location: 876398-876592
  
 NCBI BlastP on this gene

BofuT4\_uP047840.1

CND7
  
Accession: CCD45765
  
Location: 874436-875493
  
 NCBI BlastP on this gene

BofuT4\_P047830.1

hypothetical protein
  
Accession: CCD45764
  
Location: 869636-871614
  
 NCBI BlastP on this gene

BofuT4\_P047820.1

hypothetical protein
  
Accession: CCD45763
  
Location: 868433-869215
  
 NCBI BlastP on this gene

BofuT4\_P047810.1

similar to pre-mRNA-splicing factor cwc26
  
Accession: CCD45762
  
Location: 867079-868029
  
 NCBI BlastP on this gene

BofuT4\_P047800.1

similar to transcription initiation factor IIF subunit alpha
  
Accession: CCD45761
  
Location: 864235-866519
  
  
**BlastP hit with Mycgr3G103278\_Mycgr3**
  
Percentage identity: 37 %
  
BlastP bit score: 128
  
Sequence coverage: 100 %
  
E-value: 2e-29
  
  
 NCBI BlastP on this gene

BofuT4\_P047790.1

similar to caffeine-induced death protein
  
Accession: CCD45760
  
Location: 859661-863062
  
  
**BlastP hit with Mycgr3G21922\_Mycgr3T**
  
Percentage identity: 47 %
  
BlastP bit score: 283
  
Sequence coverage: 63 %
  
E-value: 5e-81
  
  
 NCBI BlastP on this gene

BofuT4\_P047780.1

hypothetical protein
  
Accession: CCD45759
  
Location: 857537-858691
  
 NCBI BlastP on this gene

BofuT4\_P047770.1

hypothetical protein
  
Accession: CCD45758
  
Location: 856370-857237
  
 NCBI BlastP on this gene

BofuT4\_P047760.1

glycoside hydrolase family 43 protein
  
Accession: CCD45757
  
Location: 853640-855512
  
 NCBI BlastP on this gene

BofuT4P34000020001

glycoside hydrolase family 28 protein
  
Accession: CCD45756
  
Location: 851648-852836
  
 NCBI BlastP on this gene

BofuT4\_P047740.1

similar to DNA-binding protein HGH1
  
Accession: CCD45755
  
Location: 849353-850759
  
  
**BlastP hit with Mycgr3G25746\_Mycgr3T**
  
Percentage identity: 53 %
  
BlastP bit score: 384
  
Sequence coverage: 100 %
  
E-value: 6e-128
  
  
 NCBI BlastP on this gene

BofuT4\_P047730.1

similar to neutral/alkaline nonlysosomal ceramidase
  
Accession: CCD45754
  
Location: 846583-849235
  
 NCBI BlastP on this gene

BofuT4\_P047720.1

similar to ABC transporter
  
Accession: CCD45753
  
Location: 839623-844173
  
 NCBI BlastP on this gene

BofuT4\_P047710.1

glycoside hydrolase family 92 protein
  
Accession: CCD45752
  
Location: 835137-837664
  
 NCBI BlastP on this gene

BofuT4P34000018001

hypothetical protein
  
Accession: CCD45751
  
Location: 833708-834365
  
 NCBI BlastP on this gene

BofuT4\_P047690.1

115. :  JH767573 Coniosporium apollinis CBS 100218 chromosome Unknown supercont1.20     Total score: 3.0     Cumulative Blast bit score: 789

hypothetical protein
  
Accession: EON65393
  
Location: 417896-420000
  
 NCBI BlastP on this gene

EON65393

hypothetical protein
  
Accession: EON65394
  
Location: 432403-433044
  
 NCBI BlastP on this gene

EON65394

hypothetical protein
  
Accession: EON65395
  
Location: 433667-435126
  
 NCBI BlastP on this gene

EON65395

hypothetical protein
  
Accession: EON65396
  
Location: 435948-438515
  
  
**BlastP hit with Mycgr3G21922\_Mycgr3T**
  
Percentage identity: 49 %
  
BlastP bit score: 286
  
Sequence coverage: 63 %
  
E-value: 3e-83
  
  
 NCBI BlastP on this gene

EON65396

hypothetical protein
  
Accession: EON65397
  
Location: 439578-441487
  
  
**BlastP hit with Mycgr3G103278\_Mycgr3**
  
Percentage identity: 35 %
  
BlastP bit score: 115
  
Sequence coverage: 78 %
  
E-value: 1e-25
  
  
 NCBI BlastP on this gene

EON65397

hypothetical protein
  
Accession: EON65398
  
Location: 442225-444153
  
 NCBI BlastP on this gene

EON65398

hypothetical protein
  
Accession: EON65399
  
Location: 445165-446514
  
  
**BlastP hit with Mycgr3G25746\_Mycgr3T**
  
Percentage identity: 58 %
  
BlastP bit score: 388
  
Sequence coverage: 100 %
  
E-value: 5e-130
  
  
 NCBI BlastP on this gene

EON65399

hypothetical protein
  
Accession: EON65400
  
Location: 447004-448896
  
 NCBI BlastP on this gene

EON65400

hypothetical protein
  
Accession: EON65401
  
Location: 449502-450344
  
 NCBI BlastP on this gene

EON65401

hypothetical protein
  
Accession: EON65402
  
Location: 451104-451415
  
 NCBI BlastP on this gene

EON65402

hypothetical protein
  
Accession: EON65403
  
Location: 455275-457007
  
 NCBI BlastP on this gene

EON65403

ubiquitin-conjugating enzyme E2 J2
  
Accession: EON65404
  
Location: 457324-458175
  
 NCBI BlastP on this gene

EON65404

hypothetical protein
  
Accession: EON65405
  
Location: 458766-459394
  
 NCBI BlastP on this gene

EON65405

hypothetical protein
  
Accession: EON65406
  
Location: 461386-462254
  
 NCBI BlastP on this gene

EON65406

hypothetical protein
  
Accession: EON65407
  
Location: 463928-465017
  
 NCBI BlastP on this gene

EON65407

116. :  GL385395 Gaeumannomyces graminis var. tritici R3-111a-1 unplaced genomic scaffold supercont2.1     Total score: 3.0     Cumulative Blast bit score: 780

hypothetical protein
  
Accession: EJT82071
  
Location: 6544921-6547078
  
 NCBI BlastP on this gene

EJT82071

hypothetical protein
  
Accession: EJT82072
  
Location: 6547854-6548687
  
 NCBI BlastP on this gene

EJT82072

hypothetical protein
  
Accession: EJT82073
  
Location: 6549073-6550009
  
 NCBI BlastP on this gene

EJT82073

dolichyl-phosphate mannosyltransferase polypeptide 3
  
Accession: EJT82074
  
Location: 6552185-6552680
  
 NCBI BlastP on this gene

EJT82074

hypothetical protein
  
Accession: EJT82075
  
Location: 6553081-6555434
  
 NCBI BlastP on this gene

EJT82075

hypothetical protein
  
Accession: EJT82076
  
Location: 6556111-6557013
  
 NCBI BlastP on this gene

EJT82076

hypoxanthine guanine phosphoribosyltransferase
  
Accession: EJT82077
  
Location: 6557662-6559050
  
  
**BlastP hit with Mycgr3G55345\_Mycgr3T**
  
Percentage identity: 74 %
  
BlastP bit score: 323
  
Sequence coverage: 99 %
  
E-value: 3e-109
  
  
 NCBI BlastP on this gene

EJT82077

hypothetical protein
  
Accession: EJT82078
  
Location: 6559818-6560890
  
 NCBI BlastP on this gene

EJT82078

hypothetical protein
  
Accession: EJT82079
  
Location: 6561499-6562200
  
 NCBI BlastP on this gene

EJT82079

hypothetical protein
  
Accession: EJT82080
  
Location: 6563131-6565022
  
 NCBI BlastP on this gene

EJT82080

leucine-rich repeat-containing protein 40
  
Accession: EJT82081
  
Location: 6567962-6570874
  
  
**BlastP hit with Mycgr3G68433\_Mycgr3T**
  
Percentage identity: 35 %
  
BlastP bit score: 390
  
Sequence coverage: 73 %
  
E-value: 2e-115
  
  
 NCBI BlastP on this gene

EJT82081

hypothetical protein
  
Accession: EJT82082
  
Location: 6571948-6575652
  
 NCBI BlastP on this gene

EJT82082

hypothetical protein
  
Accession: EJT82083
  
Location: 6575962-6577226
  
 NCBI BlastP on this gene

EJT82083

hypothetical protein
  
Accession: EJT82084
  
Location: 6577589-6578903
  
 NCBI BlastP on this gene

EJT82084

trichothecene 3-O-acetyltransferase
  
Accession: EJT82085
  
Location: 6579609-6581060
  
 NCBI BlastP on this gene

EJT82085

hypothetical protein
  
Accession: EJT82086
  
Location: 6582121-6583878
  
  
**BlastP hit with Mycgr3G90786\_Mycgr3T**
  
Percentage identity: 27 %
  
BlastP bit score: 67
  
Sequence coverage: 79 %
  
E-value: 6e-09
  
  
 NCBI BlastP on this gene

EJT82086

hypothetical protein
  
Accession: EJT82087
  
Location: 6584292-6585554
  
 NCBI BlastP on this gene

EJT82087

hypothetical protein
  
Accession: EJT82088
  
Location: 6586708-6587319
  
 NCBI BlastP on this gene

EJT82088

hypothetical protein
  
Accession: EJT82089
  
Location: 6587706-6588628
  
 NCBI BlastP on this gene

EJT82089

hypothetical protein
  
Accession: EJT82090
  
Location: 6589157-6589657
  
 NCBI BlastP on this gene

EJT82090

hypothetical protein
  
Accession: EJT82091
  
Location: 6590058-6592598
  
 NCBI BlastP on this gene

EJT82091

50S ribosomal protein L13e
  
Accession: EJT82092
  
Location: 6592976-6594038
  
 NCBI BlastP on this gene

EJT82092

117. :  JH921428 Marssonina brunnea f. sp. 'multigermtubi' MB\_m1 unplaced genomic scaffold M6\_S00001     Total score: 3.0     Cumulative Blast bit score: 769

fatty acid synthase beta subunit dehydratase
  
Accession: EKD21446
  
Location: 3118465-3125509
  
 NCBI BlastP on this gene

EKD21446

synaptobrevin
  
Accession: EKD21447
  
Location: 3125837-3126849
  
 NCBI BlastP on this gene

EKD21447

Ras family protein
  
Accession: EKD21448
  
Location: 3127457-3128664
  
 NCBI BlastP on this gene

EKD21448

FMN-dependent dehydrogenase
  
Accession: EKD21449
  
Location: 3129654-3131396
  
 NCBI BlastP on this gene

EKD21449

hypothetical protein
  
Accession: EKD21450
  
Location: 3134610-3135168
  
 NCBI BlastP on this gene

EKD21450

DNA-binding protein HGH1
  
Accession: EKD21451
  
Location: 3136498-3137998
  
  
**BlastP hit with Mycgr3G25746\_Mycgr3T**
  
Percentage identity: 52 %
  
BlastP bit score: 372
  
Sequence coverage: 100 %
  
E-value: 1e-122
  
  
 NCBI BlastP on this gene

EKD21451

neutral/alkaline nonlysosomal ceramidase
  
Accession: EKD21452
  
Location: 3139004-3139479
  
 NCBI BlastP on this gene

EKD21452

RING-like domain-containing protein
  
Accession: EKD21453
  
Location: 3139570-3141358
  
 NCBI BlastP on this gene

EKD21453

cid13-like poly(A) RNA polymerase
  
Accession: EKD21454
  
Location: 3145135-3148443
  
  
**BlastP hit with Mycgr3G21922\_Mycgr3T**
  
Percentage identity: 49 %
  
BlastP bit score: 273
  
Sequence coverage: 61 %
  
E-value: 6e-78
  
  
 NCBI BlastP on this gene

EKD21454

transcription initiation factor iif subunit
  
Accession: EKD21455
  
Location: 3149611-3152039
  
  
**BlastP hit with Mycgr3G103278\_Mycgr3**
  
Percentage identity: 35 %
  
BlastP bit score: 124
  
Sequence coverage: 81 %
  
E-value: 4e-28
  
  
 NCBI BlastP on this gene

EKD21455

pre-mRNA-splicing factor cwc26
  
Accession: EKD21456
  
Location: 3152259-3153275
  
 NCBI BlastP on this gene

EKD21456

hypothetical protein
  
Accession: EKD21457
  
Location: 3155013-3155810
  
 NCBI BlastP on this gene

EKD21457

cutinase
  
Accession: EKD21458
  
Location: 3156788-3157892
  
 NCBI BlastP on this gene

EKD21458

cytochrome P450 4A10
  
Accession: EKD21459
  
Location: 3160869-3162403
  
 NCBI BlastP on this gene

EKD21459

bystin
  
Accession: EKD21460
  
Location: 3163576-3165072
  
 NCBI BlastP on this gene

EKD21460

3-dehydroshikimate dehydratase
  
Accession: EKD21461
  
Location: 3166374-3167533
  
 NCBI BlastP on this gene

EKD21461

118. :  EQ963480 Aspergillus flavus NRRL3357 scf\_1106286418846 genomic scaffold     Total score: 3.0     Cumulative Blast bit score: 745

conserved hypothetical protein
  
Accession: EED48827
  
Location: 1137-2970
  
 NCBI BlastP on this gene

EED48827

snoRNA binding protein, putative
  
Accession: EED48828
  
Location: 3102-5388
  
 NCBI BlastP on this gene

EED48828

actin cytoskeleton organization and biogenesis protein, putative
  
Accession: EED48829
  
Location: 7708-11511
  
 NCBI BlastP on this gene

EED48829

conserved hypothetical protein
  
Accession: EED48830
  
Location: 12759-13532
  
 NCBI BlastP on this gene

EED48830

lipase/serine esterase, putative
  
Accession: EED48831
  
Location: 14813-17818
  
 NCBI BlastP on this gene

EED48831

inositol monophosphatase, putative
  
Accession: EED48832
  
Location: 18235-19314
  
 NCBI BlastP on this gene

EED48832

DNA-binding protein HGH1, putative
  
Accession: EED48833
  
Location: 20165-21506
  
  
**BlastP hit with Mycgr3G25746\_Mycgr3T**
  
Percentage identity: 58 %
  
BlastP bit score: 387
  
Sequence coverage: 100 %
  
E-value: 1e-129
  
  
 NCBI BlastP on this gene

EED48833

zinc finger protein, cchc domain containing protein, putative
  
Accession: EED48834
  
Location: 21679-23739
  
  
**BlastP hit with Mycgr3G21922\_Mycgr3T**
  
Percentage identity: 48 %
  
BlastP bit score: 218
  
Sequence coverage: 47 %
  
E-value: 2e-59
  
  
 NCBI BlastP on this gene

EED48834

transcription initiation factor IIF subunit alpha, putative
  
Accession: EED48835
  
Location: 24404-26660
  
  
**BlastP hit with Mycgr3G103278\_Mycgr3**
  
Percentage identity: 38 %
  
BlastP bit score: 140
  
Sequence coverage: 67 %
  
E-value: 1e-33
  
  
 NCBI BlastP on this gene

EED48835

fatty acid synthase beta subunit, putative
  
Accession: EED48836
  
Location: 27030-33372
  
 NCBI BlastP on this gene

EED48836

fatty acid synthase alpha subunit FasA
  
Accession: EED48837
  
Location: 36079-41772
  
 NCBI BlastP on this gene

EED48837

N2,N2-dimethylguanosine tRNA methyltransferase
  
Accession: EED48838
  
Location: 42154-44253
  
 NCBI BlastP on this gene

EED48838

conserved hypothetical protein
  
Accession: EED48839
  
Location: 44786-45859
  
 NCBI BlastP on this gene

EED48839

C2H2 finger domain protein, putative
  
Accession: EED48840
  
Location: 46318-47101
  
 NCBI BlastP on this gene

EED48840

119. :  AP007165 Aspergillus oryzae RIB40 DNA, SC124.     Total score: 3.0     Cumulative Blast bit score: 729

not annotated
  
Accession: BAE62640
  
Location: 246156-247968
  
 NCBI BlastP on this gene

AO090124000092

not annotated
  
Accession: BAE62639
  
Location: 242691-246024
  
 NCBI BlastP on this gene

AO090124000091

not annotated
  
Accession: BAE62638
  
Location: 237521-241178
  
 NCBI BlastP on this gene

AO090124000090

not annotated
  
Accession: BAE62637
  
Location: 231311-236024
  
 NCBI BlastP on this gene

AO090124000089

not annotated
  
Accession: BAE62636
  
Location: 229815-230894
  
 NCBI BlastP on this gene

AO090124000088

not annotated
  
Accession: BAE62635
  
Location: 227621-228962
  
  
**BlastP hit with Mycgr3G25746\_Mycgr3T**
  
Percentage identity: 58 %
  
BlastP bit score: 387
  
Sequence coverage: 100 %
  
E-value: 1e-129
  
  
 NCBI BlastP on this gene

AO090124000087

not annotated
  
Accession: BAE62634
  
Location: 225667-226920
  
  
**BlastP hit with Mycgr3G21922\_Mycgr3T**
  
Percentage identity: 48 %
  
BlastP bit score: 203
  
Sequence coverage: 44 %
  
E-value: 3e-56
  
  
 NCBI BlastP on this gene

AO090124000086

not annotated
  
Accession: BAE62633
  
Location: 220519-222850
  
  
**BlastP hit with Mycgr3G103278\_Mycgr3**
  
Percentage identity: 38 %
  
BlastP bit score: 139
  
Sequence coverage: 67 %
  
E-value: 2e-33
  
  
 NCBI BlastP on this gene

AO090124000085

not annotated
  
Accession: BAE62632
  
Location: 213881-220223
  
 NCBI BlastP on this gene

AO090124000084

not annotated
  
Accession: BAE62631
  
Location: 205477-211170
  
 NCBI BlastP on this gene

AO090124000083

not annotated
  
Accession: BAE62630
  
Location: 202997-205096
  
 NCBI BlastP on this gene

AO090124000082

not annotated
  
Accession: BAE62629
  
Location: 201393-202466
  
 NCBI BlastP on this gene

AO090124000081

120. :  CH476625 Sclerotinia sclerotiorum 1980 scaffold\_5 genomic scaffold     Total score: 3.0     Cumulative Blast bit score: 723

hypothetical protein
  
Accession: EDO01741
  
Location: 608924-610688
  
 NCBI BlastP on this gene

EDO01741

hypothetical protein
  
Accession: EDO01740
  
Location: 606463-608448
  
 NCBI BlastP on this gene

EDO01740

hypothetical protein
  
Accession: EDO01739
  
Location: 603350-604824
  
 NCBI BlastP on this gene

EDO01739

hypothetical protein
  
Accession: EDO01738
  
Location: 597305-602693
  
 NCBI BlastP on this gene

EDO01738

hypothetical protein
  
Accession: EDO01737
  
Location: 594838-595809
  
 NCBI BlastP on this gene

EDO01737

hypothetical protein
  
Accession: EDO01736
  
Location: 591865-594178
  
  
**BlastP hit with Mycgr3G103278\_Mycgr3**
  
Percentage identity: 35 %
  
BlastP bit score: 124
  
Sequence coverage: 105 %
  
E-value: 5e-28
  
  
 NCBI BlastP on this gene

EDO01736

hypothetical protein
  
Accession: EDO01735
  
Location: 590169-590513
  
 NCBI BlastP on this gene

EDO01735

hypothetical protein
  
Accession: EDO01734
  
Location: 587145-589706
  
  
**BlastP hit with Mycgr3G21922\_Mycgr3T**
  
Percentage identity: 48 %
  
BlastP bit score: 275
  
Sequence coverage: 61 %
  
E-value: 2e-79
  
  
 NCBI BlastP on this gene

EDO01734

hypothetical protein
  
Accession: EDO01733
  
Location: 584292-586193
  
 NCBI BlastP on this gene

EDO01733

hypothetical protein
  
Accession: EDO01732
  
Location: 582026-583679
  
 NCBI BlastP on this gene

EDO01732

hypothetical protein
  
Accession: EDO01731
  
Location: 579355-581288
  
 NCBI BlastP on this gene

EDO01731

hypothetical protein
  
Accession: EDO01730
  
Location: 576824-578227
  
  
**BlastP hit with Mycgr3G25746\_Mycgr3T**
  
Percentage identity: 48 %
  
BlastP bit score: 324
  
Sequence coverage: 100 %
  
E-value: 4e-105
  
  
 NCBI BlastP on this gene

EDO01730

hypothetical protein
  
Accession: EDO01729
  
Location: 574006-576570
  
 NCBI BlastP on this gene

EDO01729

predicted protein
  
Accession: EDO01728
  
Location: 572246-572900
  
 NCBI BlastP on this gene

EDO01728

hypothetical protein
  
Accession: EDO01727
  
Location: 567140-571683
  
 NCBI BlastP on this gene

EDO01727

predicted protein
  
Accession: EDO01726
  
Location: 563846-563950
  
 NCBI BlastP on this gene

EDO01726

hypothetical protein
  
Accession: EDO01725
  
Location: 559972-562483
  
 NCBI BlastP on this gene

EDO01725

121. :  GL629801 Grosmannia clavigera kw1407 unplaced genomic scaffold GCSC\_173     Total score: 3.0     Cumulative Blast bit score: 709

glycoprotein
  
Accession: EFX00737
  
Location: 1948623-1949100
  
 NCBI BlastP on this gene

EFX00737

short chain dehydrogenase reductase
  
Accession: EFX00515
  
Location: 1949856-1950845
  
 NCBI BlastP on this gene

EFX00515

xanthine-guanine phosphoribosyl transferase
  
Accession: EFX00626
  
Location: 1952205-1953146
  
  
**BlastP hit with Mycgr3G55345\_Mycgr3T**
  
Percentage identity: 72 %
  
BlastP bit score: 301
  
Sequence coverage: 97 %
  
E-value: 2e-100
  
  
 NCBI BlastP on this gene

EFX00626

hypothetical protein
  
Accession: EFX00465
  
Location: 1953461-1954303
  
 NCBI BlastP on this gene

EFX00465

hypothetical protein
  
Accession: EFX00657
  
Location: 1954661-1956877
  
 NCBI BlastP on this gene

EFX00657

dolichyl-phosphate mannosyltransferase polypeptide 3
  
Accession: EFX00248
  
Location: 1957314-1957712
  
 NCBI BlastP on this gene

EFX00248

major facilitator superfamily transporter multidrug resistance
  
Accession: EFX00211
  
Location: 1960830-1962822
  
 NCBI BlastP on this gene

EFX00211

FAD-binding domain containing protein
  
Accession: EFX00315
  
Location: 1965495-1967240
  
 NCBI BlastP on this gene

EFX00315

hypothetical protein
  
Accession: EFX00122
  
Location: 1967994-1969140
  
 NCBI BlastP on this gene

EFX00122

hypothetical protein
  
Accession: EFX00230
  
Location: 1970482-1973998
  
 NCBI BlastP on this gene

EFX00230

hypothetical protein
  
Accession: EFX00061
  
Location: 1975243-1975935
  
 NCBI BlastP on this gene

EFX00061

FAD-dependent oxidoreductase-like enzyme
  
Accession: EFX00526
  
Location: 1976463-1978535
  
  
**BlastP hit with Mycgr3G90786\_Mycgr3T**
  
Percentage identity: 27 %
  
BlastP bit score: 75
  
Sequence coverage: 69 %
  
E-value: 2e-11
  
  
 NCBI BlastP on this gene

EFX00526

flavin-binding monooxygenase-like protein
  
Accession: EFX00495
  
Location: 1978936-1981633
  
 NCBI BlastP on this gene

EFX00495

3-ketoacyl-thiolase
  
Accession: EFX00072
  
Location: 1982405-1983724
  
 NCBI BlastP on this gene

EFX00072

hypothetical protein
  
Accession: EFX00545
  
Location: 1983987-1984667
  
 NCBI BlastP on this gene

EFX00545

conserved leucine-rich repeat protein
  
Accession: EFX00602
  
Location: 1985471-1989174
  
  
**BlastP hit with Mycgr3G68433\_Mycgr3T**
  
Percentage identity: 32 %
  
BlastP bit score: 333
  
Sequence coverage: 91 %
  
E-value: 4e-93
  
  
 NCBI BlastP on this gene

EFX00602

hypothetical protein
  
Accession: EFX00269
  
Location: 1992896-1993114
  
 NCBI BlastP on this gene

EFX00269

hypothetical protein
  
Accession: EFX00555
  
Location: 1994024-1994817
  
 NCBI BlastP on this gene

EFX00555

122. :  GG704913 Coccidioides immitis RS genomic scaffold supercont3.3     Total score: 3.0     Cumulative Blast bit score: 638

hypothetical protein
  
Accession: EAS30066
  
Location: 1165257-1166675
  
 NCBI BlastP on this gene

EAS30066

hypothetical protein
  
Accession: EAS30065
  
Location: 1167226-1167767
  
 NCBI BlastP on this gene

EAS30065

hypothetical protein
  
Accession: EAS30064
  
Location: 1168750-1169510
  
 NCBI BlastP on this gene

EAS30064

lipase/serine esterase
  
Accession: EAS30063
  
Location: 1170183-1173597
  
 NCBI BlastP on this gene

EAS30063

hypothetical protein
  
Accession: EAS30062
  
Location: 1174511-1176343
  
 NCBI BlastP on this gene

EAS30062

inositol monophosphatase
  
Accession: EAS30061
  
Location: 1177535-1178564
  
 NCBI BlastP on this gene

EAS30061

hypothetical protein
  
Accession: EAS30060
  
Location: 1179848-1181383
  
 NCBI BlastP on this gene

EAS30060

hypothetical protein
  
Accession: EAS30059
  
Location: 1181981-1182407
  
 NCBI BlastP on this gene

EAS30059

hypothetical protein
  
Accession: EAS30058
  
Location: 1183070-1184507
  
  
**BlastP hit with Mycgr3G25746\_Mycgr3T**
  
Percentage identity: 54 %
  
BlastP bit score: 362
  
Sequence coverage: 100 %
  
E-value: 8e-120
  
  
 NCBI BlastP on this gene

EAS30058

hypothetical protein
  
Accession: EJB11179
  
Location: 1184850-1186402
  
 NCBI BlastP on this gene

EJB11179

hypothetical protein
  
Accession: EAS30055
  
Location: 1187046-1188572
  
  
**BlastP hit with Mycgr3G21922\_Mycgr3T**
  
Percentage identity: 37 %
  
BlastP bit score: 152
  
Sequence coverage: 47 %
  
E-value: 3e-37
  
  
 NCBI BlastP on this gene

EAS30055

transcription initiation factor IIF subunit alpha
  
Accession: EAS30054
  
Location: 1190139-1192429
  
  
**BlastP hit with Mycgr3G103278\_Mycgr3**
  
Percentage identity: 40 %
  
BlastP bit score: 124
  
Sequence coverage: 54 %
  
E-value: 6e-28
  
  
 NCBI BlastP on this gene

EAS30054

fatty acid synthase beta subunit dehydratase, variant
  
Accession: EJB11181
  
Location: 1193653-1199493
  
 NCBI BlastP on this gene

EJB11181

hypothetical protein
  
Accession: EJB11182
  
Location: 1199896-1200734
  
 NCBI BlastP on this gene

EJB11182

fatty acid synthase subunit alpha
  
Accession: EAS30052
  
Location: 1201441-1207156
  
 NCBI BlastP on this gene

EAS30052

3-isopropylmalate dehydrogenase B
  
Accession: EAS30051
  
Location: 1208236-1209517
  
 NCBI BlastP on this gene

EAS30051

N2,N2-dimethylguanosine tRNA methyltransferase
  
Accession: EAS30050
  
Location: 1209772-1212093
  
 NCBI BlastP on this gene

EAS30050

123. :  AKHY01000107 Aspergillus oryzae 3.042     Total score: 3.0     Cumulative Blast bit score: 638

hypothetical protein
  
Accession: EIT81276
  
Location: 87637-89360
  
 NCBI BlastP on this gene

EIT81276

hypothetical protein
  
Accession: EIT81266
  
Location: 84172-87505
  
 NCBI BlastP on this gene

EIT81266

actin cytoskeleton organization and biogenesis protein, putative
  
Accession: EIT81272
  
Location: 79047-82701
  
 NCBI BlastP on this gene

EIT81272

hypothetical protein
  
Accession: EIT81288
  
Location: 72882-75890
  
 NCBI BlastP on this gene

EIT81288

inositol monophosphatase
  
Accession: EIT81284
  
Location: 71386-72465
  
 NCBI BlastP on this gene

EIT81284

DNA-binding protein
  
Accession: EIT81270
  
Location: 69177-70115
  
  
**BlastP hit with Mycgr3G25746\_Mycgr3T**
  
Percentage identity: 53 %
  
BlastP bit score: 282
  
Sequence coverage: 81 %
  
E-value: 1e-89
  
  
 NCBI BlastP on this gene

EIT81270

zinc finger protein, cchc domain containing protein, putative
  
Accession: EIT81282
  
Location: 67223-69004
  
  
**BlastP hit with Mycgr3G21922\_Mycgr3T**
  
Percentage identity: 48 %
  
BlastP bit score: 216
  
Sequence coverage: 47 %
  
E-value: 1e-59
  
  
 NCBI BlastP on this gene

EIT81282

hypothetical protein
  
Accession: EIT81290
  
Location: 63948-66279
  
  
**BlastP hit with Mycgr3G103278\_Mycgr3**
  
Percentage identity: 38 %
  
BlastP bit score: 140
  
Sequence coverage: 67 %
  
E-value: 1e-33
  
  
 NCBI BlastP on this gene

EIT81290

fatty acid synthase beta subunit, putative
  
Accession: EIT81271
  
Location: 57310-63652
  
 NCBI BlastP on this gene

EIT81271

3-oxoacyl-[acyl-carrier-protein] synthase
  
Accession: EIT81285
  
Location: 48928-54621
  
 NCBI BlastP on this gene

EIT81285

hypothetical protein
  
Accession: EIT81269
  
Location: 44841-45914
  
 NCBI BlastP on this gene

EIT81269

124. :  GL636502 Coccidioides posadasii str. Silveira unplaced genomic scaffold supercont2.17     Total score: 3.0     Cumulative Blast bit score: 636

conserved hypothetical protein
  
Accession: EFW15027
  
Location: 87984-88478
  
 NCBI BlastP on this gene

EFW15027

conserved hypothetical protein
  
Accession: EFW15028
  
Location: 88875-90052
  
 NCBI BlastP on this gene

EFW15028

lipase/serine esterase
  
Accession: EFW15029
  
Location: 90730-94144
  
 NCBI BlastP on this gene

EFW15029

conserved hypothetical protein
  
Accession: EFW15030
  
Location: 95058-96890
  
 NCBI BlastP on this gene

EFW15030

predicted protein
  
Accession: EFW15031
  
Location: 97104-97913
  
 NCBI BlastP on this gene

EFW15031

inositol monophosphatase
  
Accession: EFW15032
  
Location: 98077-99106
  
 NCBI BlastP on this gene

EFW15032

conserved hypothetical protein
  
Accession: EFW15033
  
Location: 100291-101931
  
 NCBI BlastP on this gene

EFW15033

predicted protein
  
Accession: EFW15034
  
Location: 102475-102993
  
 NCBI BlastP on this gene

EFW15034

DNA-binding protein HGH1
  
Accession: EFW15035
  
Location: 103718-105133
  
  
**BlastP hit with Mycgr3G25746\_Mycgr3T**
  
Percentage identity: 54 %
  
BlastP bit score: 364
  
Sequence coverage: 100 %
  
E-value: 1e-120
  
  
 NCBI BlastP on this gene

EFW15035

conserved hypothetical protein
  
Accession: EFW15036
  
Location: 105475-107027
  
 NCBI BlastP on this gene

EFW15036

conserved hypothetical protein
  
Accession: EFW15037
  
Location: 107638-109164
  
  
**BlastP hit with Mycgr3G21922\_Mycgr3T**
  
Percentage identity: 37 %
  
BlastP bit score: 148
  
Sequence coverage: 47 %
  
E-value: 7e-36
  
  
 NCBI BlastP on this gene

EFW15037

transcription initiation factor IIF subunit alpha
  
Accession: EFW15038
  
Location: 110754-113044
  
  
**BlastP hit with Mycgr3G103278\_Mycgr3**
  
Percentage identity: 40 %
  
BlastP bit score: 124
  
Sequence coverage: 54 %
  
E-value: 5e-28
  
  
 NCBI BlastP on this gene

EFW15038

fatty acid synthase beta subunit
  
Accession: EFW15039
  
Location: 113768-118090
  
 NCBI BlastP on this gene

EFW15039

fatty acid synthase beta subunit
  
Accession: EFW15040
  
Location: 118267-119469
  
 NCBI BlastP on this gene

EFW15040

predicted protein
  
Accession: EFW15041
  
Location: 121368-121708
  
 NCBI BlastP on this gene

EFW15041

fatty acid synthase subunit alpha reductase
  
Accession: EFW15042
  
Location: 122081-127798
  
 NCBI BlastP on this gene

EFW15042

3-isopropylmalate dehydrogenase B
  
Accession: EFW15043
  
Location: 128873-130175
  
 NCBI BlastP on this gene

EFW15043

N2,N2-dimethylguanosine tRNA methyltransferase
  
Accession: EFW15044
  
Location: 130431-132605
  
 NCBI BlastP on this gene

EFW15044

125. :  DS572813 Paracoccidioides brasiliensis Pb01 supercont1.3 genomic scaffold     Total score: 3.0     Cumulative Blast bit score: 635

conserved hypothetical protein
  
Accession: EEH39052
  
Location: 645058-646620
  
 NCBI BlastP on this gene

EEH39052

conserved hypothetical protein
  
Accession: EEH39053
  
Location: 647312-652101
  
 NCBI BlastP on this gene

EEH39053

rieske domain-containing protein
  
Accession: EEH39054
  
Location: 653983-655458
  
 NCBI BlastP on this gene

EEH39054

IBR domain-containing protein
  
Accession: EEH39055
  
Location: 656385-658199
  
 NCBI BlastP on this gene

EEH39055

predicted protein
  
Accession: EEH39056
  
Location: 659139-659379
  
 NCBI BlastP on this gene

EEH39056

inositol monophosphatase
  
Accession: EEH39057
  
Location: 660256-661354
  
 NCBI BlastP on this gene

EEH39057

DNA-binding protein HGH1
  
Accession: EEH39058
  
Location: 663585-665096
  
  
**BlastP hit with Mycgr3G25746\_Mycgr3T**
  
Percentage identity: 55 %
  
BlastP bit score: 359
  
Sequence coverage: 99 %
  
E-value: 2e-118
  
  
 NCBI BlastP on this gene

EEH39058

conserved hypothetical protein
  
Accession: EEH39059
  
Location: 665814-667351
  
 NCBI BlastP on this gene

EEH39059

hypothetical protein
  
Accession: EEH39060
  
Location: 668088-669397
  
  
**BlastP hit with Mycgr3G21922\_Mycgr3T**
  
Percentage identity: 37 %
  
BlastP bit score: 146
  
Sequence coverage: 48 %
  
E-value: 6e-36
  
  
 NCBI BlastP on this gene

EEH39060

conserved hypothetical protein
  
Accession: EEH39061
  
Location: 670216-672456
  
  
**BlastP hit with Mycgr3G103278\_Mycgr3**
  
Percentage identity: 34 %
  
BlastP bit score: 130
  
Sequence coverage: 94 %
  
E-value: 5e-30
  
  
 NCBI BlastP on this gene

EEH39061

fatty acid synthase subunit beta dehydratase
  
Accession: EEH39062
  
Location: 673616-679936
  
 NCBI BlastP on this gene

EEH39062

fatty acid synthase subunit alpha reductase
  
Accession: EEH39063
  
Location: 681712-687542
  
 NCBI BlastP on this gene

EEH39063

N2,N2-dimethylguanosine tRNA methyltransferase
  
Accession: EEH39064
  
Location: 688359-691045
  
 NCBI BlastP on this gene

EEH39064

3-isopropylmalate dehydrogenase
  
Accession: EEH39065
  
Location: 691638-694949
  
 NCBI BlastP on this gene

EEH39065

126. :  CH476599 Aspergillus terreus NIH2624 scaffold\_6 genomic scaffold     Total score: 3.0     Cumulative Blast bit score: 613

hypothetical protein
  
Accession: EAU34696
  
Location: 5189-5981
  
 NCBI BlastP on this gene

EAU34696

predicted protein
  
Accession: EAU34697
  
Location: 6930-8444
  
 NCBI BlastP on this gene

EAU34697

conserved hypothetical protein
  
Accession: EAU34698
  
Location: 8955-12312
  
 NCBI BlastP on this gene

EAU34698

conserved hypothetical protein
  
Accession: EAU34699
  
Location: 13404-17352
  
 NCBI BlastP on this gene

EAU34699

conserved hypothetical protein
  
Accession: EAU34700
  
Location: 17979-21852
  
 NCBI BlastP on this gene

EAU34700

hypothetical protein
  
Accession: EAU34701
  
Location: 22966-23991
  
 NCBI BlastP on this gene

EAU34701

conserved hypothetical protein
  
Accession: EAU34702
  
Location: 24657-25616
  
  
**BlastP hit with Mycgr3G25746\_Mycgr3T**
  
Percentage identity: 54 %
  
BlastP bit score: 277
  
Sequence coverage: 82 %
  
E-value: 2e-87
  
  
 NCBI BlastP on this gene

EAU34702

predicted protein
  
Accession: EAU34703
  
Location: 26177-27924
  
  
**BlastP hit with Mycgr3G21922\_Mycgr3T**
  
Percentage identity: 47 %
  
BlastP bit score: 209
  
Sequence coverage: 46 %
  
E-value: 3e-57
  
  
 NCBI BlastP on this gene

EAU34703

predicted protein
  
Accession: EAU34704
  
Location: 28495-30630
  
  
**BlastP hit with Mycgr3G103278\_Mycgr3**
  
Percentage identity: 40 %
  
BlastP bit score: 127
  
Sequence coverage: 61 %
  
E-value: 4e-29
  
  
 NCBI BlastP on this gene

EAU34704

fatty acid synthase beta subunit dehydratase
  
Accession: EAU34705
  
Location: 31180-37514
  
 NCBI BlastP on this gene

EAU34705

hypothetical protein
  
Accession: EAU34706
  
Location: 39270-44963
  
 NCBI BlastP on this gene

EAU34706

conserved hypothetical protein
  
Accession: EAU34707
  
Location: 45188-48799
  
 NCBI BlastP on this gene

EAU34707

conserved hypothetical protein
  
Accession: EAU34708
  
Location: 49300-50043
  
 NCBI BlastP on this gene

EAU34708

pyruvate dehydrogenase E1 component beta subunit, mitochondrial precursor
  
Accession: EAU34709
  
Location: 50511-51852
  
 NCBI BlastP on this gene

EAU34709

127. :  ACFW01000015 Coccidioides posadasii C735 delta SOWgp     Total score: 3.0     Cumulative Blast bit score: 589

cortical actin cytoskeleton protein VIP1, putative
  
Accession: EER28284
  
Location: 290432-294853
  
 NCBI BlastP on this gene

EER28284

hypothetical protein
  
Accession: EER28283
  
Location: 288528-289946
  
 NCBI BlastP on this gene

EER28283

hypothetical protein
  
Accession: EER28282
  
Location: 285692-286448
  
 NCBI BlastP on this gene

EER28282

Putative serine esterase family protein
  
Accession: EER28281
  
Location: 281596-285010
  
 NCBI BlastP on this gene

EER28281

zinc knuckle containing protein
  
Accession: EER28280
  
Location: 278851-280683
  
 NCBI BlastP on this gene

EER28280

Inositol monophosphatase, putative
  
Accession: EER28279
  
Location: 276634-277663
  
 NCBI BlastP on this gene

EER28279

methyltransferase, putative
  
Accession: EER28278
  
Location: 274037-275352
  
 NCBI BlastP on this gene

EER28278

hypothetical protein
  
Accession: EER28277
  
Location: 270607-271895
  
  
**BlastP hit with Mycgr3G25746\_Mycgr3T**
  
Percentage identity: 51 %
  
BlastP bit score: 316
  
Sequence coverage: 94 %
  
E-value: 1e-101
  
  
 NCBI BlastP on this gene

EER28277

hypothetical protein
  
Accession: EER28276
  
Location: 268713-270155
  
 NCBI BlastP on this gene

EER28276

PAP/25A associated domain containing protein
  
Accession: EER28275
  
Location: 266576-268102
  
  
**BlastP hit with Mycgr3G21922\_Mycgr3T**
  
Percentage identity: 37 %
  
BlastP bit score: 149
  
Sequence coverage: 47 %
  
E-value: 3e-36
  
  
 NCBI BlastP on this gene

EER28275

transcription initiation factor IIF subunit alpha, putative
  
Accession: EER28274
  
Location: 262691-264981
  
  
**BlastP hit with Mycgr3G103278\_Mycgr3**
  
Percentage identity: 40 %
  
BlastP bit score: 124
  
Sequence coverage: 54 %
  
E-value: 5e-28
  
  
 NCBI BlastP on this gene

EER28274

Fatty acid synthase subunit beta, putative
  
Accession: EER28273
  
Location: 255638-261959
  
 NCBI BlastP on this gene

EER28273

Fatty acid synthase subunit alpha, putative
  
Accession: EER28272
  
Location: 247977-253694
  
 NCBI BlastP on this gene

EER28272

3-isopropylmalate dehydrogenase B, putative
  
Accession: EER28271
  
Location: 245603-246902
  
 NCBI BlastP on this gene

EER28271

N2,N2-dimethylguanosine tRNA methyltransferase family protein
  
Accession: EER28270
  
Location: 243173-245347
  
 NCBI BlastP on this gene

EER28270

128. :  CH476615 Uncinocarpus reesii 1704 scaffold\_1 genomic scaffold     Total score: 3.0     Cumulative Blast bit score: 491

conserved hypothetical protein
  
Accession: EEP77715
  
Location: 6807300-6811550
  
 NCBI BlastP on this gene

EEP77715

conserved hypothetical protein
  
Accession: EEP77714
  
Location: 6801526-6806862
  
 NCBI BlastP on this gene

EEP77714

predicted protein
  
Accession: EEP77713
  
Location: 6799705-6800751
  
 NCBI BlastP on this gene

EEP77713

conserved hypothetical protein
  
Accession: EEP77712
  
Location: 6796875-6797920
  
 NCBI BlastP on this gene

EEP77712

conserved hypothetical protein
  
Accession: EEP77711
  
Location: 6794059-6795614
  
 NCBI BlastP on this gene

EEP77711

conserved hypothetical protein
  
Accession: EEP77710
  
Location: 6791870-6792649
  
  
**BlastP hit with Mycgr3G25746\_Mycgr3T**
  
Percentage identity: 52 %
  
BlastP bit score: 226
  
Sequence coverage: 69 %
  
E-value: 3e-68
  
  
 NCBI BlastP on this gene

EEP77710

predicted protein
  
Accession: EEP77709
  
Location: 6790166-6790753
  
 NCBI BlastP on this gene

EEP77709

predicted protein
  
Accession: EEP77708
  
Location: 6787424-6788983
  
 NCBI BlastP on this gene

EEP77708

predicted protein
  
Accession: EEP77707
  
Location: 6785259-6786824
  
  
**BlastP hit with Mycgr3G21922\_Mycgr3T**
  
Percentage identity: 36 %
  
BlastP bit score: 145
  
Sequence coverage: 45 %
  
E-value: 9e-35
  
  
 NCBI BlastP on this gene

EEP77707

predicted protein
  
Accession: EEP77706
  
Location: 6782031-6784083
  
  
**BlastP hit with Mycgr3G103278\_Mycgr3**
  
Percentage identity: 40 %
  
BlastP bit score: 120
  
Sequence coverage: 55 %
  
E-value: 6e-27
  
  
 NCBI BlastP on this gene

EEP77706

fatty acid synthase beta subunit dehydratase
  
Accession: EEP77705
  
Location: 6775162-6781479
  
 NCBI BlastP on this gene

EEP77705

fatty acid synthase alpha subunit
  
Accession: EEP77704
  
Location: 6768037-6773745
  
 NCBI BlastP on this gene

EEP77704

3-isopropylmalate dehydrogenase
  
Accession: EEP77703
  
Location: 6765871-6767119
  
 NCBI BlastP on this gene

EEP77703

conserved hypothetical protein
  
Accession: EEP77702
  
Location: 6763412-6765595
  
 NCBI BlastP on this gene

EEP77702

129. :  ACYE01000028 Trichophyton verrucosum HKI 0517     Total score: 3.0     Cumulative Blast bit score: 487

hypothetical protein
  
Accession: EFE44690
  
Location: 32-3580
  
 NCBI BlastP on this gene

EFE44690

hypothetical protein
  
Accession: EFE44691
  
Location: 4094-4765
  
 NCBI BlastP on this gene

EFE44691

DNA-binding protein HGH1, putative
  
Accession: EFE44692
  
Location: 6074-7473
  
  
**BlastP hit with Mycgr3G25746\_Mycgr3T**
  
Percentage identity: 56 %
  
BlastP bit score: 195
  
Sequence coverage: 46 %
  
E-value: 1e-55
  
  
 NCBI BlastP on this gene

EFE44692

hypothetical protein
  
Accession: EFE44693
  
Location: 7617-8927
  
 NCBI BlastP on this gene

EFE44693

hypothetical protein
  
Accession: EFE44694
  
Location: 9075-11296
  
  
**BlastP hit with Mycgr3G21922\_Mycgr3T**
  
Percentage identity: 41 %
  
BlastP bit score: 156
  
Sequence coverage: 45 %
  
E-value: 3e-38
  
  
 NCBI BlastP on this gene

EFE44694

conserved hypothetical protein
  
Accession: EFE44695
  
Location: 12043-14426
  
  
**BlastP hit with Mycgr3G103278\_Mycgr3**
  
Percentage identity: 34 %
  
BlastP bit score: 136
  
Sequence coverage: 95 %
  
E-value: 4e-32
  
  
 NCBI BlastP on this gene

EFE44695

130. :  KE148155 Ophiostoma piceae UAMH 11346 chromosome Unknown scf10     Total score: 3.0     Cumulative Blast bit score: 486

acetyl- carboxylase
  
Accession: EPE05929
  
Location: 1139478-1146323
  
 NCBI BlastP on this gene

EPE05929

integral membrane protein
  
Accession: EPE05928
  
Location: 1137326-1138062
  
 NCBI BlastP on this gene

EPE05928

hypoxanthine guanine phosphoribosyltransferase
  
Accession: EPE05927
  
Location: 1134075-1135014
  
  
**BlastP hit with Mycgr3G55345\_Mycgr3T**
  
Percentage identity: 72 %
  
BlastP bit score: 306
  
Sequence coverage: 96 %
  
E-value: 2e-102
  
  
 NCBI BlastP on this gene

EPE05927

oxidoreductase-like protein
  
Accession: EPE05926
  
Location: 1132740-1133774
  
 NCBI BlastP on this gene

EPE05926

hypothetical protein
  
Accession: EPE05925
  
Location: 1129600-1132095
  
 NCBI BlastP on this gene

EPE05925

dolichyl-phosphate mannosyltransferase polypeptide 3
  
Accession: EPE05924
  
Location: 1128722-1129233
  
 NCBI BlastP on this gene

EPE05924

multidrug resistant protein
  
Accession: EPE05923
  
Location: 1121135-1123063
  
 NCBI BlastP on this gene

EPE05923

hypothetical protein
  
Accession: EPE05922
  
Location: 1119994-1120485
  
 NCBI BlastP on this gene

EPE05922

leucine-rich repeat-containing protein 40
  
Accession: EPE05921
  
Location: 1113921-1117716
  
  
**BlastP hit with Mycgr3G68433\_Mycgr3T**
  
Percentage identity: 37 %
  
BlastP bit score: 114
  
Sequence coverage: 22 %
  
E-value: 2e-22
  
  
 NCBI BlastP on this gene

EPE05921

hypothetical protein
  
Accession: EPE05920
  
Location: 1111323-1112698
  
 NCBI BlastP on this gene

EPE05920

3-ketoacyl- thiolase
  
Accession: EPE05919
  
Location: 1109647-1110963
  
 NCBI BlastP on this gene

EPE05919

fad-dependent oxidoreductase-like enzyme
  
Accession: EPE05918
  
Location: 1106019-1108487
  
  
**BlastP hit with Mycgr3G90786\_Mycgr3T**
  
Percentage identity: 26 %
  
BlastP bit score: 66
  
Sequence coverage: 54 %
  
E-value: 2e-08
  
  
 NCBI BlastP on this gene

EPE05918

enolase
  
Accession: EPE05917
  
Location: 1103315-1104932
  
 NCBI BlastP on this gene

EPE05917

salicylate hydroxylase
  
Accession: EPE05916
  
Location: 1101001-1102323
  
 NCBI BlastP on this gene

EPE05916

allantoate permease
  
Accession: EPE05915
  
Location: 1097982-1099622
  
 NCBI BlastP on this gene

EPE05915

tat pathway signal sequence
  
Accession: EPE05914
  
Location: 1093507-1097298
  
 NCBI BlastP on this gene

EPE05914

131. :  AGUE01000023 Glarea lozoyensis 74030     Total score: 3.0     Cumulative Blast bit score: 478

hypothetical protein
  
Accession: EHL02457
  
Location: 422648-424518
  
 NCBI BlastP on this gene

EHL02457

hypothetical protein
  
Accession: EHL02458
  
Location: 424841-425421
  
 NCBI BlastP on this gene

EHL02458

putative RNA polymerase II transcription factor B subunit 2
  
Accession: EHL02459
  
Location: 425787-426437
  
 NCBI BlastP on this gene

EHL02459

putative High-affinity glucose transporter
  
Accession: EHL02460
  
Location: 430914-432167
  
 NCBI BlastP on this gene

EHL02460

hypothetical protein
  
Accession: EHL02461
  
Location: 435107-436549
  
 NCBI BlastP on this gene

EHL02461

hypothetical protein
  
Accession: EHL02462
  
Location: 437197-437820
  
 NCBI BlastP on this gene

EHL02462

hypothetical protein
  
Accession: EHL02463
  
Location: 441130-442641
  
  
**BlastP hit with Mycgr3G90786\_Mycgr3T**
  
Percentage identity: 28 %
  
BlastP bit score: 73
  
Sequence coverage: 90 %
  
E-value: 5e-11
  
  
 NCBI BlastP on this gene

EHL02463

putative Leucine-rich repeat-containing protein 40
  
Accession: EHL02464
  
Location: 444731-446235
  
  
**BlastP hit with Mycgr3G68433\_Mycgr3T**
  
Percentage identity: 47 %
  
BlastP bit score: 147
  
Sequence coverage: 17 %
  
E-value: 2e-35
  
  
 NCBI BlastP on this gene

EHL02464

hypothetical protein
  
Accession: EHL02465
  
Location: 447325-449096
  
 NCBI BlastP on this gene

EHL02465

putative N-acyl homoserine lactonase AttM
  
Accession: EHL02466
  
Location: 449165-450513
  
 NCBI BlastP on this gene

EHL02466

putative Xanthine phosphoribosyltransferase 1
  
Accession: EHL02467
  
Location: 451360-451983
  
  
**BlastP hit with Mycgr3G55345\_Mycgr3T**
  
Percentage identity: 73 %
  
BlastP bit score: 258
  
Sequence coverage: 85 %
  
E-value: 9e-84
  
  
 NCBI BlastP on this gene

EHL02467

putative D-3-phosphoglycerate dehydrogenase
  
Accession: EHL02468
  
Location: 452368-453934
  
 NCBI BlastP on this gene

EHL02468

hypothetical protein
  
Accession: EHL02469
  
Location: 464804-465175
  
 NCBI BlastP on this gene

EHL02469

putative UPF0665 family protein C23C4.06c
  
Accession: EHL02470
  
Location: 468391-472283
  
 NCBI BlastP on this gene

EHL02470

132. :  KB445561 Baudoinia compniacensis UAMH 10762 unplaced genomic scaffold BAUCOscaffold\_12     Total score: 2.0     Cumulative Blast bit score: 1570

hypothetical protein
  
Accession: EMC92701
  
Location: 325868-326641
  
 NCBI BlastP on this gene

EMC92701

hypothetical protein
  
Accession: EMC92702
  
Location: 327131-327544
  
 NCBI BlastP on this gene

EMC92702

hypothetical protein
  
Accession: EMC92703
  
Location: 328253-329119
  
 NCBI BlastP on this gene

EMC92703

hypothetical protein
  
Accession: EMC92704
  
Location: 329180-330739
  
 NCBI BlastP on this gene

EMC92704

hypothetical protein
  
Accession: EMC92705
  
Location: 331313-335497
  
 NCBI BlastP on this gene

EMC92705

hypothetical protein
  
Accession: EMC92706
  
Location: 335985-337720
  
 NCBI BlastP on this gene

EMC92706

hypothetical protein
  
Accession: EMC92707
  
Location: 338175-338828
  
 NCBI BlastP on this gene

EMC92707

carbohydrate esterase family 8 protein
  
Accession: EMC92708
  
Location: 339208-340488
  
 NCBI BlastP on this gene

EMC92708

hypothetical protein
  
Accession: EMC92709
  
Location: 341046-342137
  
 NCBI BlastP on this gene

EMC92709

hypothetical protein
  
Accession: EMC92710
  
Location: 342639-344320
  
  
**BlastP hit with Mycgr3G84654\_Mycgr3T**
  
Percentage identity: 74 %
  
BlastP bit score: 498
  
Sequence coverage: 74 %
  
E-value: 5e-169
  
  
 NCBI BlastP on this gene

EMC92710

glycoside hydrolase family 3 protein
  
Accession: EMC92711
  
Location: 344957-347878
  
 NCBI BlastP on this gene

EMC92711

carbohydrate esterase family 9 protein
  
Accession: EMC92712
  
Location: 348546-349859
  
 NCBI BlastP on this gene

EMC92712

hypothetical protein
  
Accession: EMC92713
  
Location: 350204-352345
  
  
**BlastP hit with Mycgr3G68458\_Mycgr3T**
  
Percentage identity: 74 %
  
BlastP bit score: 1073
  
Sequence coverage: 100 %
  
E-value: 0.0
  
  
 NCBI BlastP on this gene

EMC92713

hypothetical protein
  
Accession: EMC92714
  
Location: 352562-352891
  
 NCBI BlastP on this gene

EMC92714

hypothetical protein
  
Accession: EMC92715
  
Location: 353474-354937
  
 NCBI BlastP on this gene

EMC92715

hypothetical protein
  
Accession: EMC92716
  
Location: 355236-356741
  
 NCBI BlastP on this gene

EMC92716

hypothetical protein
  
Accession: EMC92717
  
Location: 357461-357655
  
 NCBI BlastP on this gene

EMC92717

hypothetical protein
  
Accession: EMC92718
  
Location: 358144-359587
  
 NCBI BlastP on this gene

EMC92718

hypothetical protein
  
Accession: EMC92719
  
Location: 360407-362740
  
 NCBI BlastP on this gene

EMC92719

hypothetical protein
  
Accession: EMC92720
  
Location: 363457-364234
  
 NCBI BlastP on this gene

EMC92720

hypothetical protein
  
Accession: EMC92721
  
Location: 364886-367775
  
 NCBI BlastP on this gene

EMC92721

hypothetical protein
  
Accession: EMC92722
  
Location: 368811-371612
  
 NCBI BlastP on this gene

EMC92722

133. :  KB446573 Pseudocercospora fijiensis CIRAD86 unplaced genomic scaffold MYCFIscaffold\_19     Total score: 2.0     Cumulative Blast bit score: 1450

hypothetical protein
  
Accession: EME76936
  
Location: 443548-444135
  
 NCBI BlastP on this gene

EME76936

hypothetical protein
  
Accession: EME76937
  
Location: 445109-445748
  
 NCBI BlastP on this gene

EME76937

hypothetical protein
  
Accession: EME76938
  
Location: 447028-448760
  
 NCBI BlastP on this gene

EME76938

hypothetical protein
  
Accession: EME76939
  
Location: 450124-451679
  
 NCBI BlastP on this gene

EME76939

hypothetical protein
  
Accession: EME76940
  
Location: 451889-452646
  
 NCBI BlastP on this gene

EME76940

hypothetical protein
  
Accession: EME76941
  
Location: 453293-454615
  
 NCBI BlastP on this gene

EME76941

hypothetical protein
  
Accession: EME76942
  
Location: 454928-455726
  
 NCBI BlastP on this gene

EME76942

hypothetical protein
  
Accession: EME76943
  
Location: 456819-458525
  
 NCBI BlastP on this gene

EME76943

hypothetical protein
  
Accession: EME76944
  
Location: 460319-462682
  
 NCBI BlastP on this gene

EME76944

hypothetical protein
  
Accession: EME76945
  
Location: 463066-464403
  
  
**BlastP hit with Mycgr3G25746\_Mycgr3T**
  
Percentage identity: 69 %
  
BlastP bit score: 491
  
Sequence coverage: 100 %
  
E-value: 6e-170
  
  
 NCBI BlastP on this gene

EME76945

hypothetical protein
  
Accession: EME76946
  
Location: 464689-469309
  
  
**BlastP hit with Mycgr3G108094\_Mycgr3**
  
Percentage identity: 42 %
  
BlastP bit score: 959
  
Sequence coverage: 106 %
  
E-value: 0.0
  
  
 NCBI BlastP on this gene

EME76946

hypothetical protein
  
Accession: EME76947
  
Location: 469383-471699
  
 NCBI BlastP on this gene

EME76947

134. :  AKCU01000112 Penicillium digitatum Pd1     Total score: 2.0     Cumulative Blast bit score: 1424

hypothetical protein
  
Accession: EKV20491
  
Location: 113201-113971
  
 NCBI BlastP on this gene

EKV20491

hypothetical protein
  
Accession: EKV20492
  
Location: 114606-116077
  
 NCBI BlastP on this gene

EKV20492

hypothetical protein
  
Accession: EKV20493
  
Location: 118411-118863
  
 NCBI BlastP on this gene

EKV20493

hypothetical protein
  
Accession: EKV20494
  
Location: 119780-121267
  
 NCBI BlastP on this gene

EKV20494

60S ribosomal protein L13
  
Accession: EKV20495
  
Location: 121879-122996
  
 NCBI BlastP on this gene

EKV20495

hypothetical protein
  
Accession: EKV20496
  
Location: 123682-124929
  
 NCBI BlastP on this gene

EKV20496

putative leucine-rich repeat protein
  
Accession: EKV20497
  
Location: 126623-129736
  
  
**BlastP hit with Mycgr3G68433\_Mycgr3T**
  
Percentage identity: 39 %
  
BlastP bit score: 505
  
Sequence coverage: 89 %
  
E-value: 7e-158
  
  
 NCBI BlastP on this gene

EKV20497

hypothetical protein
  
Accession: EKV20498
  
Location: 129884-130987
  
 NCBI BlastP on this gene

EKV20498

hypothetical protein
  
Accession: EKV20499
  
Location: 131729-132961
  
 NCBI BlastP on this gene

EKV20499

Alpha/beta hydrolase, putative
  
Accession: EKV20500
  
Location: 133560-134595
  
 NCBI BlastP on this gene

EKV20500

Actin family protein
  
Accession: EKV20501
  
Location: 135167-136748
  
 NCBI BlastP on this gene

EKV20501

hypothetical protein
  
Accession: EKV20502
  
Location: 137642-138112
  
 NCBI BlastP on this gene

EKV20502

3-hydroxybutyryl-CoA dehydrogenase, putative
  
Accession: EKV20503
  
Location: 138413-139487
  
 NCBI BlastP on this gene

EKV20503

DNA repair protein (Tof1), putative
  
Accession: EKV20504
  
Location: 142765-146391
  
  
**BlastP hit with Mycgr3G68421\_Mycgr3T**
  
Percentage identity: 43 %
  
BlastP bit score: 919
  
Sequence coverage: 102 %
  
E-value: 0.0
  
  
 NCBI BlastP on this gene

EKV20504

hypothetical protein
  
Accession: EKV20505
  
Location: 146552-146848
  
 NCBI BlastP on this gene

EKV20505

hypothetical protein
  
Accession: EKV20506
  
Location: 147447-147590
  
 NCBI BlastP on this gene

EKV20506

Phenazine biosynthesis-like protein, putative
  
Accession: EKV20507
  
Location: 147720-148664
  
 NCBI BlastP on this gene

EKV20507

hypothetical protein
  
Accession: EKV20508
  
Location: 149775-150260
  
 NCBI BlastP on this gene

EKV20508

hypothetical protein
  
Accession: EKV20509
  
Location: 155167-155610
  
 NCBI BlastP on this gene

EKV20509

hypothetical protein
  
Accession: EKV20510
  
Location: 157854-158042
  
 NCBI BlastP on this gene

EKV20510

hypothetical protein
  
Accession: EKV20511
  
Location: 158546-158797
  
 NCBI BlastP on this gene

EKV20511

135. :  AKCT01000108 Penicillium digitatum PHI26     Total score: 2.0     Cumulative Blast bit score: 1424

hypothetical protein
  
Accession: EKV15979
  
Location: 267228-267998
  
 NCBI BlastP on this gene

EKV15979

hypothetical protein
  
Accession: EKV15980
  
Location: 268635-270106
  
 NCBI BlastP on this gene

EKV15980

hypothetical protein
  
Accession: EKV15981
  
Location: 272442-272894
  
 NCBI BlastP on this gene

EKV15981

hypothetical protein
  
Accession: EKV15982
  
Location: 273811-275298
  
 NCBI BlastP on this gene

EKV15982

60S ribosomal protein L13
  
Accession: EKV15983
  
Location: 275910-277027
  
 NCBI BlastP on this gene

EKV15983

hypothetical protein
  
Accession: EKV15984
  
Location: 277714-278961
  
 NCBI BlastP on this gene

EKV15984

putative leucine-rich repeat protein
  
Accession: EKV15985
  
Location: 280655-283768
  
  
**BlastP hit with Mycgr3G68433\_Mycgr3T**
  
Percentage identity: 39 %
  
BlastP bit score: 505
  
Sequence coverage: 89 %
  
E-value: 7e-158
  
  
 NCBI BlastP on this gene

EKV15985

hypothetical protein
  
Accession: EKV15986
  
Location: 283916-285019
  
 NCBI BlastP on this gene

EKV15986

hypothetical protein
  
Accession: EKV15987
  
Location: 285761-286993
  
 NCBI BlastP on this gene

EKV15987

Alpha/beta hydrolase, putative
  
Accession: EKV15988
  
Location: 287592-288627
  
 NCBI BlastP on this gene

EKV15988

Actin family protein
  
Accession: EKV15989
  
Location: 289199-290780
  
 NCBI BlastP on this gene

EKV15989

hypothetical protein
  
Accession: EKV15990
  
Location: 291674-292144
  
 NCBI BlastP on this gene

EKV15990

3-hydroxybutyryl-CoA dehydrogenase, putative
  
Accession: EKV15991
  
Location: 292445-293519
  
 NCBI BlastP on this gene

EKV15991

DNA repair protein (Tof1), putative
  
Accession: EKV15992
  
Location: 296797-300423
  
  
**BlastP hit with Mycgr3G68421\_Mycgr3T**
  
Percentage identity: 43 %
  
BlastP bit score: 919
  
Sequence coverage: 102 %
  
E-value: 0.0
  
  
 NCBI BlastP on this gene

EKV15992

hypothetical protein
  
Accession: EKV15993
  
Location: 300584-300880
  
 NCBI BlastP on this gene

EKV15993

hypothetical protein
  
Accession: EKV15994
  
Location: 301479-301622
  
 NCBI BlastP on this gene

EKV15994

Phenazine biosynthesis-like protein, putative
  
Accession: EKV15995
  
Location: 301752-302696
  
 NCBI BlastP on this gene

EKV15995

hypothetical protein
  
Accession: EKV15996
  
Location: 303807-304292
  
 NCBI BlastP on this gene

EKV15996

hypothetical protein
  
Accession: EKV15997
  
Location: 309199-309642
  
 NCBI BlastP on this gene

EKV15997

hypothetical protein
  
Accession: EKV15998
  
Location: 311916-312104
  
 NCBI BlastP on this gene

EKV15998

hypothetical protein
  
Accession: EKV15999
  
Location: 312608-312859
  
 NCBI BlastP on this gene

EKV15999

136. :  KB446542 Dothistroma septosporum NZE10 unplaced genomic scaffold DOTSEscaffold\_8     Total score: 2.0     Cumulative Blast bit score: 1410

hypothetical protein
  
Accession: EME41440
  
Location: 743033-743892
  
 NCBI BlastP on this gene

EME41440

hypothetical protein
  
Accession: EME41441
  
Location: 744207-746390
  
 NCBI BlastP on this gene

EME41441

hypothetical protein
  
Accession: EME41442
  
Location: 749963-750870
  
 NCBI BlastP on this gene

EME41442

hypothetical protein
  
Accession: EME41443
  
Location: 751308-752027
  
 NCBI BlastP on this gene

EME41443

hypothetical protein
  
Accession: EME41444
  
Location: 752091-752501
  
 NCBI BlastP on this gene

EME41444

hypothetical protein
  
Accession: EME41445
  
Location: 753572-755300
  
  
**BlastP hit with Mycgr3G68456\_Mycgr3T**
  
Percentage identity: 79 %
  
BlastP bit score: 857
  
Sequence coverage: 99 %
  
E-value: 0.0
  
  
 NCBI BlastP on this gene

EME41445

hypothetical protein
  
Accession: EME41446
  
Location: 755773-757342
  
 NCBI BlastP on this gene

EME41446

hypothetical protein
  
Accession: EME41447
  
Location: 758422-760068
  
 NCBI BlastP on this gene

EME41447

hypothetical protein
  
Accession: EME41448
  
Location: 760784-761353
  
 NCBI BlastP on this gene

EME41448

hypothetical protein
  
Accession: EME41449
  
Location: 761959-763479
  
 NCBI BlastP on this gene

EME41449

hypothetical protein
  
Accession: EME41450
  
Location: 766200-767933
  
  
**BlastP hit with Mycgr3G84654\_Mycgr3T**
  
Percentage identity: 81 %
  
BlastP bit score: 553
  
Sequence coverage: 74 %
  
E-value: 0.0
  
  
 NCBI BlastP on this gene

EME41450

glycoside hydrolase family 3 protein
  
Accession: EME41452
  
Location: 768761-771803
  
 NCBI BlastP on this gene

EME41452

carbohydrate esterase family 9 protein
  
Accession: EME41453
  
Location: 772634-773938
  
 NCBI BlastP on this gene

EME41453

hypothetical protein
  
Accession: EME41454
  
Location: 774950-776122
  
 NCBI BlastP on this gene

EME41454

hypothetical protein
  
Accession: EME41455
  
Location: 776518-776840
  
 NCBI BlastP on this gene

EME41455

hypothetical protein
  
Accession: EME41456
  
Location: 778701-779054
  
 NCBI BlastP on this gene

EME41456

hypothetical protein
  
Accession: EME41457
  
Location: 780513-780782
  
 NCBI BlastP on this gene

EME41457

hypothetical protein
  
Accession: EME41458
  
Location: 781693-782618
  
 NCBI BlastP on this gene

EME41458

hypothetical protein
  
Accession: EME41459
  
Location: 783398-783862
  
 NCBI BlastP on this gene

EME41459

137. :  KB446555 Pseudocercospora fijiensis CIRAD86 unplaced genomic scaffold MYCFIscaffold\_1     Total score: 2.0     Cumulative Blast bit score: 1396

hypothetical protein
  
Accession: EME88473
  
Location: 5598661-5598825
  
 NCBI BlastP on this gene

EME88473

hypothetical protein
  
Accession: EME88472
  
Location: 5596804-5597541
  
 NCBI BlastP on this gene

EME88472

serine/threonine protein kinase, CMGC family
  
Accession: EME88471
  
Location: 5593047-5595415
  
  
**BlastP hit with Mycgr3G84644\_Mycgr3T**
  
Percentage identity: 74 %
  
BlastP bit score: 888
  
Sequence coverage: 108 %
  
E-value: 0.0
  
  
 NCBI BlastP on this gene

EME88471

hypothetical protein
  
Accession: EME88470
  
Location: 5590136-5591084
  
  
**BlastP hit with Mycgr3G36271\_Mycgr3T**
  
Percentage identity: 80 %
  
BlastP bit score: 508
  
Sequence coverage: 98 %
  
E-value: 2e-179
  
  
 NCBI BlastP on this gene

EME88470

hypothetical protein
  
Accession: EME88469
  
Location: 5587545-5588663
  
 NCBI BlastP on this gene

EME88469

hypothetical protein
  
Accession: EME88468
  
Location: 5586786-5587184
  
 NCBI BlastP on this gene

EME88468

138. :  AM920428 Penicillium chrysogenum Wisconsin 54-1255 complete genome, contig Pc00c13.     Total score: 2.0     Cumulative Blast bit score: 1376

not annotated
  
Accession: CAP91738
  
Location: 1650613-1652318
  
 NCBI BlastP on this gene

Pc13g06690

not annotated
  
Accession: CAP91739
  
Location: 1652884-1654358
  
 NCBI BlastP on this gene

Pc13g06700

not annotated
  
Accession: CAP91740
  
Location: 1654982-1658124
  
 NCBI BlastP on this gene

Pc13g06710

not annotated
  
Accession: CAP91741
  
Location: 1658958-1659773
  
 NCBI BlastP on this gene

Pc13g06720

not annotated
  
Accession: CAP91742
  
Location: 1659918-1661820
  
 NCBI BlastP on this gene

Pc13g06730

not annotated
  
Accession: CAP91743
  
Location: 1662428-1663550
  
 NCBI BlastP on this gene

Pc13g06740

not annotated
  
Accession: CAP91744
  
Location: 1664198-1665445
  
 NCBI BlastP on this gene

Pc13g06750

not annotated
  
Accession: CAP91745
  
Location: 1667181-1670294
  
  
**BlastP hit with Mycgr3G68433\_Mycgr3T**
  
Percentage identity: 37 %
  
BlastP bit score: 508
  
Sequence coverage: 102 %
  
E-value: 7e-159
  
  
 NCBI BlastP on this gene

Pc13g06760

not annotated
  
Accession: CAP91746
  
Location: 1670452-1671555
  
 NCBI BlastP on this gene

Pc13g06770

not annotated
  
Accession: CAP91747
  
Location: 1672279-1673430
  
 NCBI BlastP on this gene

Pc13g06780

not annotated
  
Accession: CAP91748
  
Location: 1674151-1675177
  
 NCBI BlastP on this gene

Pc13g06790

hypothetical protein
  
Accession: CAP91749
  
Location: 1675220-1675989
  
 NCBI BlastP on this gene

Pc13g06800

not annotated
  
Accession: CAP91750
  
Location: 1675991-1677581
  
 NCBI BlastP on this gene

Pc13g06810

not annotated
  
Accession: CAP91751
  
Location: 1678453-1678941
  
 NCBI BlastP on this gene

Pc13g06820

not annotated
  
Accession: CAP91752
  
Location: 1679220-1680295
  
 NCBI BlastP on this gene

Pc13g06830

not annotated
  
Accession: CAP91753
  
Location: 1681119-1684756
  
  
**BlastP hit with Mycgr3G68421\_Mycgr3T**
  
Percentage identity: 41 %
  
BlastP bit score: 868
  
Sequence coverage: 101 %
  
E-value: 0.0
  
  
 NCBI BlastP on this gene

Pc13g06840

not annotated
  
Accession: CAP91754
  
Location: 1685049-1686083
  
 NCBI BlastP on this gene

Pc13g06850

not annotated
  
Accession: CAP91755
  
Location: 1686296-1687243
  
 NCBI BlastP on this gene

Pc13g06860

hypothetical protein
  
Accession: CAP91756
  
Location: 1688019-1689234
  
 NCBI BlastP on this gene

Pc13g06870

not annotated
  
Accession: CAP91757
  
Location: 1689276-1689761
  
 NCBI BlastP on this gene

Pc13g06880

not annotated
  
Accession: CAP91758
  
Location: 1690852-1691318
  
 NCBI BlastP on this gene

Pc13g06890

not annotated
  
Accession: CAP91759
  
Location: 1692104-1692603
  
 NCBI BlastP on this gene

Pc13g06900

hypothetical protein
  
Accession: CAP91760
  
Location: 1692837-1694615
  
 NCBI BlastP on this gene

Pc13g06910

not annotated
  
Accession: CAP91761
  
Location: 1695235-1696185
  
 NCBI BlastP on this gene

Pc13g06920

unnamed
  
Accession: CAP91762
  
Location: 1697047-1697487
  
 NCBI BlastP on this gene

Pc13g06930

not annotated
  
Accession: Pc13g06940
  
Location: 1698779-1699354
  
 NCBI BlastP on this gene

Pc13g06940

not annotated
  
Accession: CAP91764
  
Location: 1699687-1700607
  
 NCBI BlastP on this gene

Pc13g06950

139. :  KB916472 Neofusicoccum parvum UCRNP2 chromosome Unknown NP2\_03\_scaffold\_834     Total score: 2.0     Cumulative Blast bit score: 1284

putative golgi membrane protein
  
Accession: EOD46287
  
Location: 64170-65275
  
 NCBI BlastP on this gene

EOD46287

putative sodium nucleoside cotransporter protein
  
Accession: EOD46265
  
Location: 62251-63445
  
 NCBI BlastP on this gene

EOD46265

putative nadh-ubiquinone oxidoreductase 21 kda protein
  
Accession: EOD46277
  
Location: 58834-59620
  
 NCBI BlastP on this gene

EOD46277

putative dead helicases superfamily protein
  
Accession: EOD46272
  
Location: 54150-58586
  
 NCBI BlastP on this gene

EOD46272

putative ferric reductase transmembrane component protein
  
Accession: EOD46274
  
Location: 51674-53722
  
 NCBI BlastP on this gene

EOD46274

putative universal stress protein family domain protein
  
Accession: EOD46269
  
Location: 49590-50688
  
 NCBI BlastP on this gene

EOD46269

hypothetical protein
  
Accession: EOD46281
  
Location: 46899-48292
  
 NCBI BlastP on this gene

EOD46281

putative viral a-type inclusion protein repeat protein
  
Accession: EOD46261
  
Location: 43049-46642
  
  
**BlastP hit with Mycgr3G108094\_Mycgr3**
  
Percentage identity: 44 %
  
BlastP bit score: 439
  
Sequence coverage: 40 %
  
E-value: 2e-128
  
  
 NCBI BlastP on this gene

EOD46261

putative serine protein kinase protein
  
Accession: EOD46268
  
Location: 39988-42058
  
  
**BlastP hit with Mycgr3G84644\_Mycgr3T**
  
Percentage identity: 72 %
  
BlastP bit score: 845
  
Sequence coverage: 105 %
  
E-value: 0.0
  
  
 NCBI BlastP on this gene

EOD46268

putative antigenic thaumatin domain-containing protein
  
Accession: EOD46276
  
Location: 37113-38145
  
 NCBI BlastP on this gene

EOD46276

putative fructose-bisphosphate class ii protein
  
Accession: EOD46290
  
Location: 29159-30342
  
 NCBI BlastP on this gene

EOD46290

putative ribosomal protein l9 rnase h1 protein
  
Accession: EOD46283
  
Location: 27128-28220
  
 NCBI BlastP on this gene

EOD46283

putative ubiquitin c-terminal protein
  
Accession: EOD46280
  
Location: 24970-26805
  
 NCBI BlastP on this gene

EOD46280

putative siderochrome-iron transporter sit1 protein
  
Accession: EOD46273
  
Location: 21669-23679
  
 NCBI BlastP on this gene

EOD46273

putative peroxisomal membrane anchor protein
  
Accession: EOD46262
  
Location: 19330-20672
  
 NCBI BlastP on this gene

EOD46262

140. :  AHHD01000092 Macrophomina phaseolina MS6     Total score: 2.0     Cumulative Blast bit score: 1266

Peroxisome membrane anchor protein Pex14p
  
Accession: EKG20208
  
Location: 18787-20112
  
 NCBI BlastP on this gene

EKG20208

General substrate transporter
  
Accession: EKG20207
  
Location: 15802-17771
  
 NCBI BlastP on this gene

EKG20207

FAD-binding 8
  
Accession: EKG20206
  
Location: 11798-13870
  
 NCBI BlastP on this gene

EKG20206

UspA
  
Accession: EKG20205
  
Location: 10149-11104
  
 NCBI BlastP on this gene

EKG20205

Heat shock protein DnaJ
  
Accession: EKG20204
  
Location: 7066-8455
  
 NCBI BlastP on this gene

EKG20204

GRIP domain-containing protein
  
Accession: EKG20203
  
Location: 3219-6809
  
  
**BlastP hit with Mycgr3G108094\_Mycgr3**
  
Percentage identity: 43 %
  
BlastP bit score: 434
  
Sequence coverage: 40 %
  
E-value: 2e-126
  
  
 NCBI BlastP on this gene

EKG20203

hypothetical protein
  
Accession: EKG20202
  
Location: 192-2252
  
  
**BlastP hit with Mycgr3G84644\_Mycgr3T**
  
Percentage identity: 70 %
  
BlastP bit score: 832
  
Sequence coverage: 109 %
  
E-value: 0.0
  
  
 NCBI BlastP on this gene

EKG20202

141. :  DS231623 Pyrenophora tritici-repentis Pt-1C-BFP supercont1.9 genomic scaffold     Total score: 2.0     Cumulative Blast bit score: 1237

choline dehydrogenase
  
Accession: EDU51576
  
Location: 1635595-1637283
  
 NCBI BlastP on this gene

EDU51576

predicted protein
  
Accession: EDU51575
  
Location: 1634398-1635356
  
 NCBI BlastP on this gene

EDU51575

predicted protein
  
Accession: EDU51574
  
Location: 1632243-1633283
  
 NCBI BlastP on this gene

EDU51574

predicted protein
  
Accession: EDU51573
  
Location: 1631307-1631769
  
 NCBI BlastP on this gene

EDU51573

conserved hypothetical protein
  
Accession: EDU51572
  
Location: 1628949-1630504
  
 NCBI BlastP on this gene

EDU51572

eukaryotic translation initiation factor 2C 2
  
Accession: EDU51571
  
Location: 1625285-1628560
  
 NCBI BlastP on this gene

EDU51571

hypothetical protein
  
Accession: EDU51570
  
Location: 1622073-1622285
  
 NCBI BlastP on this gene

EDU51570

conserved hypothetical protein
  
Accession: EDU51569
  
Location: 1619842-1621435
  
 NCBI BlastP on this gene

EDU51569

hypothetical protein
  
Accession: EDU51568
  
Location: 1618010-1619442
  
 NCBI BlastP on this gene

EDU51568

zuotin
  
Accession: EDU51567
  
Location: 1616036-1617478
  
 NCBI BlastP on this gene

EDU51567

leucine zipper protein 1
  
Accession: EDU51566
  
Location: 1612130-1615755
  
  
**BlastP hit with Mycgr3G108094\_Mycgr3**
  
Percentage identity: 40 %
  
BlastP bit score: 392
  
Sequence coverage: 44 %
  
E-value: 2e-111
  
  
 NCBI BlastP on this gene

EDU51566

serine/threonine-protein kinase SRPK2
  
Accession: EDU51565
  
Location: 1609059-1611176
  
  
**BlastP hit with Mycgr3G84644\_Mycgr3T**
  
Percentage identity: 70 %
  
BlastP bit score: 845
  
Sequence coverage: 108 %
  
E-value: 0.0
  
  
 NCBI BlastP on this gene

EDU51565

predicted protein
  
Accession: EDU51564
  
Location: 1607901-1608300
  
 NCBI BlastP on this gene

EDU51564

acetolactate synthase, mitochondrial precursor
  
Accession: EDU51563
  
Location: 1605111-1607306
  
 NCBI BlastP on this gene

EDU51563

conserved hypothetical protein
  
Accession: EDU51562
  
Location: 1603399-1604460
  
 NCBI BlastP on this gene

EDU51562

conserved hypothetical protein
  
Accession: EDU51561
  
Location: 1602167-1603338
  
 NCBI BlastP on this gene

EDU51561

conserved hypothetical protein
  
Accession: EDU51560
  
Location: 1599881-1601968
  
 NCBI BlastP on this gene

EDU51560

conserved hypothetical protein
  
Accession: EDU51559
  
Location: 1597530-1599308
  
 NCBI BlastP on this gene

EDU51559

haloacid dehalogenase
  
Accession: EDU51558
  
Location: 1596184-1597122
  
 NCBI BlastP on this gene

EDU51558

dolichol-phosphate mannosyltransferase
  
Accession: EDU51557
  
Location: 1595093-1595878
  
 NCBI BlastP on this gene

EDU51557

predicted protein
  
Accession: EDU51556
  
Location: 1590543-1590923
  
 NCBI BlastP on this gene

EDU51556

predicted protein
  
Accession: EDU51555
  
Location: 1589618-1589866
  
 NCBI BlastP on this gene

EDU51555

142. :  JH767653 Coniosporium apollinis CBS 100218 chromosome Unknown supercont1.100     Total score: 2.0     Cumulative Blast bit score: 1226

hypothetical protein
  
Accession: EON69997
  
Location: 4802-7261
  
 NCBI BlastP on this gene

EON69997

hypothetical protein
  
Accession: EON69998
  
Location: 7572-9365
  
 NCBI BlastP on this gene

EON69998

hypothetical protein
  
Accession: EON69999
  
Location: 9989-11356
  
 NCBI BlastP on this gene

EON69999

hypothetical protein
  
Accession: EON70000
  
Location: 12453-13901
  
 NCBI BlastP on this gene

EON70000

hypothetical protein
  
Accession: EON70001
  
Location: 14114-17852
  
  
**BlastP hit with Mycgr3G108094\_Mycgr3**
  
Percentage identity: 44 %
  
BlastP bit score: 384
  
Sequence coverage: 34 %
  
E-value: 8e-109
  
  
 NCBI BlastP on this gene

EON70001

CMGC/SRPK protein kinase
  
Accession: EON70002
  
Location: 19128-21202
  
  
**BlastP hit with Mycgr3G84644\_Mycgr3T**
  
Percentage identity: 72 %
  
BlastP bit score: 842
  
Sequence coverage: 105 %
  
E-value: 0.0
  
  
 NCBI BlastP on this gene

EON70002

143. :  KB644412 Penicillium oxalicum 114-2 unplaced genomic scaffold scaffold\_5     Total score: 2.0     Cumulative Blast bit score: 1211

hypothetical protein
  
Accession: EPS30771
  
Location: 3705358-3707085
  
 NCBI BlastP on this gene

EPS30771

hypothetical protein
  
Accession: EPS30772
  
Location: 3708707-3710293
  
 NCBI BlastP on this gene

EPS30772

hypothetical protein
  
Accession: EPS30773
  
Location: 3711016-3711318
  
 NCBI BlastP on this gene

EPS30773

hypothetical protein
  
Accession: EPS30774
  
Location: 3711791-3713232
  
 NCBI BlastP on this gene

EPS30774

hypothetical protein
  
Accession: EPS30775
  
Location: 3715090-3716451
  
 NCBI BlastP on this gene

EPS30775

hypothetical protein
  
Accession: EPS30776
  
Location: 3717255-3718367
  
 NCBI BlastP on this gene

EPS30776

hypothetical protein
  
Accession: EPS30777
  
Location: 3719904-3720474
  
 NCBI BlastP on this gene

EPS30777

hypothetical protein
  
Accession: EPS30778
  
Location: 3720875-3721839
  
  
**BlastP hit with Mycgr3G55345\_Mycgr3T**
  
Percentage identity: 73 %
  
BlastP bit score: 301
  
Sequence coverage: 96 %
  
E-value: 1e-100
  
  
 NCBI BlastP on this gene

EPS30778

hypothetical protein
  
Accession: EPS30779
  
Location: 3722406-3722867
  
 NCBI BlastP on this gene

EPS30779

hypothetical protein
  
Accession: EPS30780
  
Location: 3723698-3726117
  
 NCBI BlastP on this gene

EPS30780

hypothetical protein
  
Accession: EPS30781
  
Location: 3726950-3728082
  
 NCBI BlastP on this gene

EPS30781

hypothetical protein
  
Accession: EPS30782
  
Location: 3728332-3732066
  
  
**BlastP hit with Mycgr3G68421\_Mycgr3T**
  
Percentage identity: 43 %
  
BlastP bit score: 910
  
Sequence coverage: 102 %
  
E-value: 0.0
  
  
 NCBI BlastP on this gene

EPS30782

hypothetical protein
  
Accession: EPS30783
  
Location: 3732925-3733881
  
 NCBI BlastP on this gene

EPS30783

hypothetical protein
  
Accession: EPS30784
  
Location: 3734237-3734725
  
 NCBI BlastP on this gene

EPS30784

hypothetical protein
  
Accession: EPS30785
  
Location: 3738125-3738733
  
 NCBI BlastP on this gene

EPS30785

hypothetical protein
  
Accession: EPS30786
  
Location: 3741643-3742738
  
 NCBI BlastP on this gene

EPS30786

hypothetical protein
  
Accession: EPS30787
  
Location: 3744500-3745000
  
 NCBI BlastP on this gene

EPS30787

hypothetical protein
  
Accession: EPS30788
  
Location: 3746983-3749305
  
 NCBI BlastP on this gene

EPS30788

144. :  GL533200 Pyrenophora teres f. teres 0-1 unplaced genomic scaffold scaffold\_189433     Total score: 2.0     Cumulative Blast bit score: 1206

hypothetical protein
  
Accession: EFQ94646
  
Location: 32940-34389
  
 NCBI BlastP on this gene

EFQ94646

hypothetical protein
  
Accession: EFQ94645
  
Location: 30962-32407
  
 NCBI BlastP on this gene

EFQ94645

hypothetical protein
  
Accession: EFQ94644
  
Location: 27052-30695
  
  
**BlastP hit with Mycgr3G108094\_Mycgr3**
  
Percentage identity: 41 %
  
BlastP bit score: 393
  
Sequence coverage: 44 %
  
E-value: 6e-112
  
  
 NCBI BlastP on this gene

EFQ94644

hypothetical protein
  
Accession: EFQ94643
  
Location: 24073-26210
  
  
**BlastP hit with Mycgr3G84644\_Mycgr3T**
  
Percentage identity: 69 %
  
BlastP bit score: 813
  
Sequence coverage: 108 %
  
E-value: 0.0
  
  
 NCBI BlastP on this gene

EFQ94643

hypothetical protein
  
Accession: EFQ94642
  
Location: 19998-22196
  
 NCBI BlastP on this gene

EFQ94642

hypothetical protein
  
Accession: EFQ94641
  
Location: 18265-19659
  
 NCBI BlastP on this gene

EFQ94641

hypothetical protein
  
Accession: EFQ94640
  
Location: 16991-18192
  
 NCBI BlastP on this gene

EFQ94640

hypothetical protein
  
Accession: EFQ94639
  
Location: 14678-16771
  
 NCBI BlastP on this gene

EFQ94639

hypothetical protein
  
Accession: EFQ94638
  
Location: 12307-14093
  
 NCBI BlastP on this gene

EFQ94638

hypothetical protein
  
Accession: EFQ94637
  
Location: 10925-11863
  
 NCBI BlastP on this gene

EFQ94637

hypothetical protein
  
Accession: EFQ94636
  
Location: 9826-10616
  
 NCBI BlastP on this gene

EFQ94636

hypothetical protein
  
Accession: EFQ94635
  
Location: 8012-8605
  
 NCBI BlastP on this gene

EFQ94635

145. :  FP929065 Leptosphaeria maculans JN3 lm\_SuperContig\_8\_v2 genomic supercontig     Total score: 2.0     Cumulative Blast bit score: 1172

hypothetical protein
  
Accession: CBX90987
  
Location: 1228694-1232129
  
 NCBI BlastP on this gene

LEMA\_P060210.1

similar to ascus development protein
  
Accession: CBX90988
  
Location: 1232363-1233112
  
 NCBI BlastP on this gene

LEMA\_P060220.1

hypothetical protein
  
Accession: CBX90989
  
Location: 1233164-1234295
  
 NCBI BlastP on this gene

LEMA\_P060230.1

hypothetical protein
  
Accession: CBX90990
  
Location: 1236195-1237865
  
 NCBI BlastP on this gene

LEMA\_P060240.1

hypothetical protein
  
Accession: CBX90991
  
Location: 1238840-1239055
  
 NCBI BlastP on this gene

LEMA\_P060250.1

hypothetical protein
  
Accession: CBX90992
  
Location: 1239457-1242326
  
 NCBI BlastP on this gene

LEMA\_P060260.1

predicted protein
  
Accession: CBX90993
  
Location: 1242684-1242961
  
 NCBI BlastP on this gene

LEMA\_P060270.1

hypothetical protein
  
Accession: CBX90994
  
Location: 1243435-1244089
  
 NCBI BlastP on this gene

LEMA\_P060280.1

similar to viral A-type inclusion protein repeat protein
  
Accession: CBX90995
  
Location: 1244465-1248154
  
  
**BlastP hit with Mycgr3G108094\_Mycgr3**
  
Percentage identity: 39 %
  
BlastP bit score: 399
  
Sequence coverage: 46 %
  
E-value: 7e-114
  
  
 NCBI BlastP on this gene

LEMA\_P060290.1

similar to serine protein kinase Sky1
  
Accession: CBX90996
  
Location: 1250201-1252003
  
  
**BlastP hit with Mycgr3G84644\_Mycgr3T**
  
Percentage identity: 71 %
  
BlastP bit score: 773
  
Sequence coverage: 98 %
  
E-value: 0.0
  
  
 NCBI BlastP on this gene

LEMA\_P060300.1

predicted protein
  
Accession: CBX90997
  
Location: 1252952-1253378
  
 NCBI BlastP on this gene

LEMA\_uP060310.1

hypothetical protein
  
Accession: CBX90998
  
Location: 1253680-1254209
  
 NCBI BlastP on this gene

LEMA\_P060320.1

hypothetical protein
  
Accession: CBX90999
  
Location: 1254448-1255091
  
 NCBI BlastP on this gene

LEMA\_P060330.1

hypothetical protein
  
Accession: CBX91000
  
Location: 1255311-1256465
  
 NCBI BlastP on this gene

LEMA\_P060340.1

predicted protein
  
Accession: CBX91001
  
Location: 1257044-1258091
  
 NCBI BlastP on this gene

LEMA\_P060350.1

hypothetical protein
  
Accession: CBX91002
  
Location: 1258440-1259180
  
 NCBI BlastP on this gene

LEMA\_P060360.1

hypothetical protein
  
Accession: CBX91003
  
Location: 1259868-1261115
  
 NCBI BlastP on this gene

LEMA\_P060370.1

predicted protein
  
Accession: CBX91004
  
Location: 1262044-1263695
  
 NCBI BlastP on this gene

LEMA\_P060380.1

similar to C6 zinc finger domain protein
  
Accession: CBX91005
  
Location: 1264070-1265588
  
 NCBI BlastP on this gene

LEMA\_P060390.1

146. :  EQ963487 Aspergillus flavus NRRL3357 scf\_1106286419368 genomic scaffold     Total score: 2.0     Cumulative Blast bit score: 1172

ATP-dependent RNA helicase, putative
  
Accession: EED44811
  
Location: 247703-249357
  
 NCBI BlastP on this gene

EED44811

conserved hypothetical protein
  
Accession: EED44812
  
Location: 249687-250516
  
 NCBI BlastP on this gene

EED44812

flocculation suppression protein
  
Accession: EED44813
  
Location: 251235-253346
  
 NCBI BlastP on this gene

EED44813

F-box domain protein
  
Accession: EED44814
  
Location: 254459-255224
  
 NCBI BlastP on this gene

EED44814

succinyl-CoA synthetase beta subunit, putative
  
Accession: EED44815
  
Location: 256722-258530
  
 NCBI BlastP on this gene

EED44815

hypothetical protein
  
Accession: EED44816
  
Location: 259231-260447
  
 NCBI BlastP on this gene

EED44816

C4-dicarboxylate transporter/malic acid transport protein, putative
  
Accession: EED44817
  
Location: 263278-264858
  
 NCBI BlastP on this gene

EED44817

xanthine-guanine phosphoribosyl transferase Xpt1, putative
  
Accession: EED44818
  
Location: 265516-266536
  
  
**BlastP hit with Mycgr3G55345\_Mycgr3T**
  
Percentage identity: 73 %
  
BlastP bit score: 303
  
Sequence coverage: 96 %
  
E-value: 3e-101
  
  
 NCBI BlastP on this gene

EED44818

conserved hypothetical protein
  
Accession: EED44819
  
Location: 267094-267512
  
 NCBI BlastP on this gene

EED44819

hypothetical protein
  
Accession: EED44820
  
Location: 267601-267978
  
 NCBI BlastP on this gene

EED44820

conserved hypothetical protein
  
Accession: EED44821
  
Location: 268617-269181
  
 NCBI BlastP on this gene

EED44821

GTP binding protein, putative
  
Accession: EED44822
  
Location: 269589-270730
  
 NCBI BlastP on this gene

EED44822

conserved hypothetical protein
  
Accession: EED44823
  
Location: 270639-272236
  
 NCBI BlastP on this gene

EED44823

ubiquinone biosynthesis protein, putative
  
Accession: EED44824
  
Location: 272795-275038
  
  
**BlastP hit with Mycgr3G68458\_Mycgr3T**
  
Percentage identity: 61 %
  
BlastP bit score: 869
  
Sequence coverage: 100 %
  
E-value: 0.0
  
  
 NCBI BlastP on this gene

EED44824

cytochrome c heme lyase, putative
  
Accession: EED44825
  
Location: 275949-277045
  
 NCBI BlastP on this gene

EED44825

serine/threonine protein kinase, putative
  
Accession: EED44826
  
Location: 277909-278792
  
 NCBI BlastP on this gene

EED44826

ubiquitin-conjugating enzyme Ubc6, putative
  
Accession: EED44827
  
Location: 282661-283608
  
 NCBI BlastP on this gene

EED44827

mitochondrial outer membrane protein (Sam50), putative
  
Accession: EED44828
  
Location: 283904-285709
  
 NCBI BlastP on this gene

EED44828

Golgi membrane protein, putative
  
Accession: EED44829
  
Location: 286064-287069
  
 NCBI BlastP on this gene

EED44829

ubiquitin fusion degradation protein (Ufd1), putative
  
Accession: EED44830
  
Location: 287460-289856
  
 NCBI BlastP on this gene

EED44830

hypothetical protein
  
Accession: EED44831
  
Location: 292009-293088
  
 NCBI BlastP on this gene

EED44831

147. :  DS572750 Paracoccidioides brasiliensis Pb18 supercont1.1 genomic scaffold     Total score: 2.0     Cumulative Blast bit score: 1162

tachykinin family protein
  
Accession: EEH44022
  
Location: 1024941-1027145
  
 NCBI BlastP on this gene

EEH44022

cytochrome c heme lyase
  
Accession: EEH44023
  
Location: 1028443-1029657
  
 NCBI BlastP on this gene

EEH44023

conserved hypothetical protein
  
Accession: EEH44024
  
Location: 1030447-1032403
  
 NCBI BlastP on this gene

EEH44024

ABC1 family protein
  
Accession: EEH44025
  
Location: 1034244-1036623
  
  
**BlastP hit with Mycgr3G68458\_Mycgr3T**
  
Percentage identity: 64 %
  
BlastP bit score: 848
  
Sequence coverage: 92 %
  
E-value: 0.0
  
  
 NCBI BlastP on this gene

EEH44025

carbonic anhydrase
  
Accession: EEH44026
  
Location: 1039641-1040250
  
 NCBI BlastP on this gene

EEH44026

hypothetical protein
  
Accession: EEH44027
  
Location: 1042156-1043026
  
 NCBI BlastP on this gene

EEH44027

succinyl-CoA ligase subunit beta
  
Accession: EEH44028
  
Location: 1043711-1045646
  
 NCBI BlastP on this gene

EEH44028

conserved hypothetical protein
  
Accession: EEH44029
  
Location: 1046118-1046854
  
 NCBI BlastP on this gene

EEH44029

hypothetical protein
  
Accession: EEH44030
  
Location: 1049812-1051342
  
 NCBI BlastP on this gene

EEH44030

hypothetical protein
  
Accession: EEH44031
  
Location: 1052002-1055896
  
 NCBI BlastP on this gene

EEH44031

HSF-type DNA-binding domain-containing protein
  
Accession: EEH44032
  
Location: 1055937-1058400
  
 NCBI BlastP on this gene

EEH44032

hypothetical protein
  
Accession: EEH44033
  
Location: 1061509-1063250
  
  
**BlastP hit with Mycgr3G55345\_Mycgr3T**
  
Percentage identity: 75 %
  
BlastP bit score: 314
  
Sequence coverage: 97 %
  
E-value: 9e-106
  
  
 NCBI BlastP on this gene

EEH44033

conserved hypothetical protein
  
Accession: EEH44034
  
Location: 1063943-1064563
  
 NCBI BlastP on this gene

EEH44034

peroxisomal catalase
  
Accession: EEH44035
  
Location: 1068931-1070836
  
 NCBI BlastP on this gene

EEH44035

148. :  DS544806 Paracoccidioides brasiliensis Pb03 supercont1.4 genomic scaffold     Total score: 2.0     Cumulative Blast bit score: 1142

60S ribosomal protein L13
  
Accession: EEH20500
  
Location: 1474-2763
  
 NCBI BlastP on this gene

EEH20500

leucine-rich repeat-containing protein
  
Accession: EEH20501
  
Location: 7187-9497
  
  
**BlastP hit with Mycgr3G68433\_Mycgr3T**
  
Percentage identity: 35 %
  
BlastP bit score: 228
  
Sequence coverage: 51 %
  
E-value: 3e-60
  
  
 NCBI BlastP on this gene

EEH20501

conserved hypothetical protein
  
Accession: EEH20502
  
Location: 10124-11365
  
 NCBI BlastP on this gene

EEH20502

predicted protein
  
Accession: EEH20503
  
Location: 11886-12663
  
 NCBI BlastP on this gene

EEH20503

conserved hypothetical protein
  
Accession: EEH20504
  
Location: 13984-15628
  
 NCBI BlastP on this gene

EEH20504

IBR domain-containing protein
  
Accession: EEH20505
  
Location: 18297-21263
  
 NCBI BlastP on this gene

EEH20505

predicted protein
  
Accession: EEH20506
  
Location: 22198-23634
  
 NCBI BlastP on this gene

EEH20506

3-hydroxybutyryl-CoA dehydrogenase
  
Accession: EEH20507
  
Location: 24067-25228
  
 NCBI BlastP on this gene

EEH20507

mating-type switching protein
  
Accession: EEH20508
  
Location: 25600-29413
  
  
**BlastP hit with Mycgr3G68421\_Mycgr3T**
  
Percentage identity: 44 %
  
BlastP bit score: 914
  
Sequence coverage: 103 %
  
E-value: 0.0
  
  
 NCBI BlastP on this gene

EEH20508

predicted protein
  
Accession: EEH20509
  
Location: 30444-33059
  
 NCBI BlastP on this gene

EEH20509

RNA polymerase II transcription factor B subunit 2
  
Accession: EEH20511
  
Location: 33652-35240
  
 NCBI BlastP on this gene

EEH20511

predicted protein
  
Accession: EEH20510
  
Location: 35252-36788
  
 NCBI BlastP on this gene

EEH20510

conserved hypothetical protein
  
Accession: EEH20512
  
Location: 37473-41925
  
 NCBI BlastP on this gene

EEH20512

149. :  CH476599 Aspergillus terreus NIH2624 scaffold\_6 genomic scaffold     Total score: 2.0     Cumulative Blast bit score: 1133

conserved hypothetical protein
  
Accession: EAU35256
  
Location: 1600210-1602698
  
 NCBI BlastP on this gene

EAU35256

conserved hypothetical protein
  
Accession: EAU35255
  
Location: 1598113-1598796
  
 NCBI BlastP on this gene

EAU35255

hypothetical protein
  
Accession: EAU35254
  
Location: 1593041-1597683
  
 NCBI BlastP on this gene

EAU35254

conserved hypothetical protein
  
Accession: EAU35253
  
Location: 1588452-1592271
  
  
**BlastP hit with Mycgr3G108094\_Mycgr3**
  
Percentage identity: 37 %
  
BlastP bit score: 370
  
Sequence coverage: 50 %
  
E-value: 3e-104
  
  
 NCBI BlastP on this gene

EAU35253

conserved hypothetical protein
  
Accession: EAU35252
  
Location: 1587649-1588247
  
 NCBI BlastP on this gene

EAU35252

predicted protein
  
Accession: EAU35251
  
Location: 1585458-1587008
  
 NCBI BlastP on this gene

EAU35251

conserved hypothetical protein
  
Accession: EAU35250
  
Location: 1584013-1585092
  
 NCBI BlastP on this gene

EAU35250

predicted protein
  
Accession: EAU35249
  
Location: 1581846-1583103
  
 NCBI BlastP on this gene

EAU35249

hypothetical protein
  
Accession: EAU35248
  
Location: 1579932-1580935
  
 NCBI BlastP on this gene

EAU35248

hypothetical protein
  
Accession: EAU35247
  
Location: 1577506-1579258
  
 NCBI BlastP on this gene

EAU35247

cytochrome b2, mitochondrial precursor
  
Accession: EAU35246
  
Location: 1572609-1574216
  
 NCBI BlastP on this gene

EAU35246

conserved hypothetical protein
  
Accession: EAU35245
  
Location: 1571474-1572073
  
 NCBI BlastP on this gene

EAU35245

protein kinase dsk1
  
Accession: EAU35244
  
Location: 1567805-1569680
  
  
**BlastP hit with Mycgr3G84644\_Mycgr3T**
  
Percentage identity: 68 %
  
BlastP bit score: 763
  
Sequence coverage: 101 %
  
E-value: 0.0
  
  
 NCBI BlastP on this gene

EAU35244

conserved hypothetical protein
  
Accession: EAU35243
  
Location: 1565657-1566667
  
 NCBI BlastP on this gene

EAU35243

predicted protein
  
Accession: EAU35242
  
Location: 1563835-1564458
  
 NCBI BlastP on this gene

EAU35242

conserved hypothetical protein
  
Accession: EAU35241
  
Location: 1561761-1563238
  
 NCBI BlastP on this gene

EAU35241

alpha,alpha-trehalose-phosphate synthase 1
  
Accession: EAU35240
  
Location: 1559555-1561227
  
 NCBI BlastP on this gene

EAU35240

conserved hypothetical protein
  
Accession: EAU35239
  
Location: 1557300-1558821
  
 NCBI BlastP on this gene

EAU35239

cutinase precursor
  
Accession: EAU35238
  
Location: 1555654-1556459
  
 NCBI BlastP on this gene

EAU35238

150. :  DS544807 Paracoccidioides brasiliensis Pb03 supercont1.5 genomic scaffold     Total score: 2.0     Cumulative Blast bit score: 1132

sarcoma antigen NY-SAR-16
  
Accession: EEH21437
  
Location: 334224-336832
  
 NCBI BlastP on this gene

EEH21437

predicted protein
  
Accession: EEH21438
  
Location: 338853-339273
  
 NCBI BlastP on this gene

EEH21438

conserved hypothetical protein
  
Accession: EEH21439
  
Location: 342409-344974
  
 NCBI BlastP on this gene

EEH21439

conserved hypothetical protein
  
Accession: EEH21440
  
Location: 345768-351940
  
  
**BlastP hit with Mycgr3G108094\_Mycgr3**
  
Percentage identity: 40 %
  
BlastP bit score: 362
  
Sequence coverage: 40 %
  
E-value: 4e-100
  
  
 NCBI BlastP on this gene

EEH21440

predicted protein
  
Accession: EEH21441
  
Location: 352332-353463
  
 NCBI BlastP on this gene

EEH21441

conserved hypothetical protein
  
Accession: EEH21442
  
Location: 355955-357793
  
 NCBI BlastP on this gene

EEH21442

L-lactate dehydrogenase
  
Accession: EEH21443
  
Location: 361370-363084
  
 NCBI BlastP on this gene

EEH21443

ser/Thr protein phosphatase family protein
  
Accession: EEH21444
  
Location: 363343-364500
  
 NCBI BlastP on this gene

EEH21444

hypothetical protein
  
Accession: EEH21445
  
Location: 365221-365800
  
 NCBI BlastP on this gene

EEH21445

predicted protein
  
Accession: EEH21446
  
Location: 366742-367147
  
 NCBI BlastP on this gene

EEH21446

serine/threonine-protein kinase SRPK1
  
Accession: EEH21447
  
Location: 367883-370439
  
  
**BlastP hit with Mycgr3G84644\_Mycgr3T**
  
Percentage identity: 67 %
  
BlastP bit score: 770
  
Sequence coverage: 108 %
  
E-value: 0.0
  
  
 NCBI BlastP on this gene

EEH21447

pre-mRNA-splicing factor cwc26
  
Accession: EEH21448
  
Location: 372249-373337
  
 NCBI BlastP on this gene

EEH21448

MGMT family protein
  
Accession: EEH21449
  
Location: 373608-374420
  
 NCBI BlastP on this gene

EEH21449

predicted protein
  
Accession: EEH21450
  
Location: 378970-379577
  
 NCBI BlastP on this gene

EEH21450

Detecting sequence homology at the gene cluster level with MultiGeneBlast.
  
Marnix H. Medema, Rainer Breitling & Eriko Takano (2013)
  
*Molecular Biology and Evolution* , 30: 1218-1223.
