# Supplementary material for: In silico prediction and characterization of secondary metabolite biosynthetic gene clusters in the wheat pathogen Zymoseptoria tritici
Source: BMC Genomics. 2017 Aug 17;18:631. doi: 10.1186/s12864-017-3969-y (PMC5561558; doi:10.1186/s12864-017-3969-y)
Supplement: Supplementary file 1 — MultiGeneBLAST analysis of putative secondary metabolite clusters. All encoded amino acid sequences from genes residing in clusters predicted by AntiSMASH are given as FASTA file format. All output data from MultiGeneBLASTs are also provided. (ZIP 42911 kb) [file 12864_2017_3969_MOESM1_ESM.zip › Cluster MultiGene BLAST/out/Clusters_1_34/Cluster_12/displaypage4.xhtml]

xml version="1.0" encoding="UTF-8"?


Search Results
  
  
 Results pages: 1, 2, 3, 4, 5

**MultiGeneBlast hits**

Select gene cluster alignment
151. DS572750\_0 Paracoccidioides brasiliensis Pb18 supercont1.1 genomic scaff...
152. AKHY01000145\_1 Aspergillus oryzae 3.042, whole genome shotgun sequencing...
153. DS572813\_5 Paracoccidioides brasiliensis Pb01 supercont1.3 genomic scaff...
154. AKCU01000110\_0 Penicillium digitatum Pd1, whole genome shotgun sequencin...
155. AKCT01000108\_1 Penicillium digitatum PHI26, whole genome shotgun sequenc...
156. CH476624\_0 Sclerotinia sclerotiorum 1980 scaffold\_4 genomic scaffold, wh...
157. CH445336\_2 Phaeosphaeria nodorum SN15 scaffold\_12, whole genome shotgun ...
158. KB445579\_8 Cochliobolus heterostrophus C5 unplaced genomic scaffold COCH...
159. AP007172\_1 Aspergillus oryzae RIB40 DNA, SC206.
160. CP002686\_6 Arabidopsis thaliana chromosome 3, complete sequence.
161. KE145352\_0 Glarea lozoyensis ATCC 20868 chromosome Unknown GLAREA1, whol...
162. DS995737\_0 Trichophyton equinum CBS 127.97 supercont1.20 genomic scaffol...
163. GG698482\_1 Trichophyton tonsurans CBS 112818 genomic scaffold supercont1...
164. JH921446\_0 Marssonina brunnea f. sp. 'multigermtubi' MB\_m1 unplaced geno...
165. AM920428\_1 Penicillium chrysogenum Wisconsin 54-1255 complete genome, co...
166. KB726203\_0 Fusarium oxysporum f. sp. cubense race 4 unplaced genomic sca...
167. KB730140\_0 Fusarium oxysporum f. sp. cubense race 1 unplaced genomic sca...
168. AFNW01000113\_0 Fusarium pseudograminearum CS3096, whole genome shotgun s...
169. CAGA01000024\_0 Claviceps purpurea 20.1, whole genome shotgun sequencing ...
170. AFQF01000897\_0 Fusarium oxysporum Fo5176, whole genome shotgun sequencin...
171. CAGA01000038\_0 Claviceps purpurea 20.1, whole genome shotgun sequencing ...
172. AMYD01003902\_0 Colletotrichum gloeosporioides Cg-14, whole genome shotgu...
173. GG749476\_0 Ajellomyces dermatitidis ATCC 18188 genomic scaffold supercon...
174. GG657474\_0 Ajellomyces dermatitidis SLH14081 genomic scaffold supercont1...
175. EQ999987\_3 Ajellomyces dermatitidis ER-3 genomic scaffold supercont1.15,...
176. GL385396\_5 Gaeumannomyces graminis var. tritici R3-111a-1 unplaced genom...
177. KB446555\_3 Pseudocercospora fijiensis CIRAD86 unplaced genomic scaffold ...
178. GL698568\_0 Metarhizium acridum CQMa 102 unplaced genomic scaffold Scf\_09...
179. GL698734\_0 Metarhizium anisopliae ARSEF 23 unplaced genomic scaffold Scf...
180. FQ790349\_0 Botryotinia fuckeliana T4 SupSuperContig\_62\_54\_1 genomic supe...
181. GG698942\_0 Nectria haematococca mpVI 77-13-4 chromosome 3 genomic scaffo...
182. GL698717\_0 Metarhizium anisopliae ARSEF 23 unplaced genomic scaffold Scf...
183. GL985057\_1 Trichoderma reesei QM6a unplaced genomic scaffold TRIREscaffo...
184. AHHD01000090\_0 Macrophomina phaseolina MS6, whole genome shotgun sequenc...
185. ABDG02000026\_0 Trichoderma atroviride IMI 206040, whole genome shotgun s...
186. KB445649\_8 Cochliobolus sativus ND90Pr unplaced genomic scaffold COCSAsc...
187. HF679028\_0 Fusarium fujikuroi IMI 58289 draft genome, chromosome FFUJ\_ch...
188. ABDF02000006\_1 Trichoderma virens Gv29-8, whole genome shotgun sequencin...
189. KB456266\_1 Mycosphaerella populorum SO2202 unplaced genomic scaffold SEP...
190. KB730484\_0 Fusarium oxysporum f. sp. cubense race 1 unplaced genomic sca...
191. KB726997\_0 Fusarium oxysporum f. sp. cubense race 4 unplaced genomic sca...
192. AFQF01000058\_0 Fusarium oxysporum Fo5176, whole genome shotgun sequencin...
193. CP003010\_3 Thielavia terrestris NRRL 8126 chromosome 2, complete sequence.
194. ABDF02000072\_0 Trichoderma virens Gv29-8, whole genome shotgun sequencin...
195. ABDG02000026\_2 Trichoderma atroviride IMI 206040, whole genome shotgun s...
196. AFNW01000339\_0 Fusarium pseudograminearum CS3096, whole genome shotgun s...
197. JH126401\_0 Cordyceps militaris CM01 unplaced genomic scaffold CCM\_S00003...
198. GL698525\_0 Metarhizium acridum CQMa 102 unplaced genomic scaffold Scf\_05...
199. GG698910\_3 Nectria haematococca mpVI 77-13-4 chromosome 2 genomic scaffo...
200. GL985061\_0 Trichoderma reesei QM6a unplaced genomic scaffold TRIREscaffo...

Query: Architecture Search FASTA input

DS572750 : Paracoccidioides brasiliensis Pb18 supercont1.1 genomic scaffold    Total score: 2.0     Cumulative Blast bit score: 1131

Hit cluster cross-links:

Mycgr3G90785 Mycgr3T
  
Location: 0-1047

Mycgr3G90785\_Mycgr3T

Mycgr3G103262 Mycgr3
  
Location: 1147-1390

Mycgr3G103262\_Mycgr3

Mycgr3G68458 Mycgr3T
  
Location: 1490-3602

Mycgr3G68458\_Mycgr3T

Mycgr3G99145 Mycgr3T
  
Location: 3702-4326

Mycgr3G99145\_Mycgr3T

Mycgr3G103274 Mycgr3
  
Location: 4426-4957

Mycgr3G103274\_Mycgr3

Mycgr3G103264 Mycgr3
  
Location: 5057-5390

Mycgr3G103264\_Mycgr3

Mycgr3G37570 Mycgr3T
  
Location: 5490-6006

Mycgr3G37570\_Mycgr3T

Mycgr3G108094 Mycgr3
  
Location: 6106-10555

Mycgr3G108094\_Mycgr3

Mycgr3G90786 Mycgr3T
  
Location: 10655-12080

Mycgr3G90786\_Mycgr3T

Mycgr3G68429 Mycgr3T
  
Location: 12180-13440

Mycgr3G68429\_Mycgr3T

Mycgr3G68421 Mycgr3T
  
Location: 13540-17086

Mycgr3G68421\_Mycgr3T

Mycgr3G90801 Mycgr3T
  
Location: 17186-18056

Mycgr3G90801\_Mycgr3T

Mycgr3G84646 Mycgr3T
  
Location: 18156-20235

Mycgr3G84646\_Mycgr3T

Mycgr3G68456 Mycgr3T
  
Location: 20335-21970

Mycgr3G68456\_Mycgr3T

Mycgr3G103270 Mycgr3
  
Location: 22070-22355

Mycgr3G103270\_Mycgr3

Mycgr3G90803 Mycgr3T
  
Location: 22455-23019

Mycgr3G90803\_Mycgr3T

Mycgr3G36941 Mycgr3T
  
Location: 23119-24064

Mycgr3G36941\_Mycgr3T

Mycgr3G25746 Mycgr3T
  
Location: 24164-25241

Mycgr3G25746\_Mycgr3T

Mycgr3G90788 Mycgr3T
  
Location: 25341-25803

Mycgr3G90788\_Mycgr3T

Mycgr3G103260 Mycgr3
  
Location: 25903-26635

Mycgr3G103260\_Mycgr3

Mycgr3G84644 Mycgr3T
  
Location: 26735-28457

Mycgr3G84644\_Mycgr3T

Mycgr3G29227 Mycgr3T
  
Location: 28557-28863

Mycgr3G29227\_Mycgr3T

Mycgr3G36271 Mycgr3T
  
Location: 28963-29854

Mycgr3G36271\_Mycgr3T

Mycgr3G68433 Mycgr3T
  
Location: 29954-33041

Mycgr3G68433\_Mycgr3T

Mycgr3G79452 Mycgr3T
  
Location: 33141-33399

Mycgr3G79452\_Mycgr3T

Mycgr3G55345 Mycgr3T
  
Location: 33499-34126

Mycgr3G55345\_Mycgr3T

Mycgr3G103278 Mycgr3
  
Location: 34226-35195

Mycgr3G103278\_Mycgr3

Mycgr3G84654 Mycgr3T
  
Location: 35295-36630

Mycgr3G84654\_Mycgr3T

Mycgr3G108090 Mycgr3
  
Location: 36730-37591

Mycgr3G108090\_Mycgr3

Mycgr3G21922 Mycgr3T
  
Location: 37691-39149

Mycgr3G21922\_Mycgr3T

Mycgr3G99148 Mycgr3T
  
Location: 39249-42819

Mycgr3G99148\_Mycgr3T

conserved hypothetical protein
  
Accession: EEH43876
  
Location: 550840-551439
  
 NCBI BlastP on this gene

EEH43876

MGMT family protein
  
Accession: EEH43877
  
Location: 558876-559688
  
 NCBI BlastP on this gene

EEH43877

pre-mRNA-splicing factor cwc26
  
Accession: EEH43878
  
Location: 559959-561047
  
 NCBI BlastP on this gene

EEH43878

serine/threonine-protein kinase SKY1
  
Accession: EEH43879
  
Location: 562856-564956
  
  
**BlastP hit with Mycgr3G84644\_Mycgr3T**
  
Percentage identity: 67 %
  
BlastP bit score: 753
  
Sequence coverage: 104 %
  
E-value: 0.0
  
  
 NCBI BlastP on this gene

EEH43879

hypothetical protein
  
Accession: EEH43880
  
Location: 567534-568113
  
 NCBI BlastP on this gene

EEH43880

conserved hypothetical protein
  
Accession: EEH43881
  
Location: 568840-570003
  
 NCBI BlastP on this gene

EEH43881

cytochrome b2
  
Accession: EEH43882
  
Location: 570306-572020
  
 NCBI BlastP on this gene

EEH43882

ras-like GTP-binding protein
  
Accession: EEH43883
  
Location: 575249-576242
  
 NCBI BlastP on this gene

EEH43883

conserved hypothetical protein
  
Accession: EEH43884
  
Location: 579948-586324
  
  
**BlastP hit with Mycgr3G108094\_Mycgr3**
  
Percentage identity: 40 %
  
BlastP bit score: 378
  
Sequence coverage: 40 %
  
E-value: 6e-105
  
  
 NCBI BlastP on this gene

EEH43884

N-acetyltransferase ats1
  
Accession: EEH43885
  
Location: 586954-587642
  
 NCBI BlastP on this gene

EEH43885

conserved hypothetical protein
  
Accession: EEH43886
  
Location: 588436-591002
  
 NCBI BlastP on this gene

EEH43886

predicted protein
  
Accession: EEH43887
  
Location: 592590-593372
  
 NCBI BlastP on this gene

EEH43887

chromatin remodeling complex subunit (Arp5)
  
Accession: EEH43888
  
Location: 596969-599594
  
 NCBI BlastP on this gene

EEH43888

Query: Architecture Search FASTA input

AKHY01000145 : Aspergillus oryzae 3.042    Total score: 2.0     Cumulative Blast bit score: 1124

Hit cluster cross-links:

Mycgr3G90785 Mycgr3T
  
Location: 0-1047

Mycgr3G90785\_Mycgr3T

Mycgr3G103262 Mycgr3
  
Location: 1147-1390

Mycgr3G103262\_Mycgr3

Mycgr3G68458 Mycgr3T
  
Location: 1490-3602

Mycgr3G68458\_Mycgr3T

Mycgr3G99145 Mycgr3T
  
Location: 3702-4326

Mycgr3G99145\_Mycgr3T

Mycgr3G103274 Mycgr3
  
Location: 4426-4957

Mycgr3G103274\_Mycgr3

Mycgr3G103264 Mycgr3
  
Location: 5057-5390

Mycgr3G103264\_Mycgr3

Mycgr3G37570 Mycgr3T
  
Location: 5490-6006

Mycgr3G37570\_Mycgr3T

Mycgr3G108094 Mycgr3
  
Location: 6106-10555

Mycgr3G108094\_Mycgr3

Mycgr3G90786 Mycgr3T
  
Location: 10655-12080

Mycgr3G90786\_Mycgr3T

Mycgr3G68429 Mycgr3T
  
Location: 12180-13440

Mycgr3G68429\_Mycgr3T

Mycgr3G68421 Mycgr3T
  
Location: 13540-17086

Mycgr3G68421\_Mycgr3T

Mycgr3G90801 Mycgr3T
  
Location: 17186-18056

Mycgr3G90801\_Mycgr3T

Mycgr3G84646 Mycgr3T
  
Location: 18156-20235

Mycgr3G84646\_Mycgr3T

Mycgr3G68456 Mycgr3T
  
Location: 20335-21970

Mycgr3G68456\_Mycgr3T

Mycgr3G103270 Mycgr3
  
Location: 22070-22355

Mycgr3G103270\_Mycgr3

Mycgr3G90803 Mycgr3T
  
Location: 22455-23019

Mycgr3G90803\_Mycgr3T

Mycgr3G36941 Mycgr3T
  
Location: 23119-24064

Mycgr3G36941\_Mycgr3T

Mycgr3G25746 Mycgr3T
  
Location: 24164-25241

Mycgr3G25746\_Mycgr3T

Mycgr3G90788 Mycgr3T
  
Location: 25341-25803

Mycgr3G90788\_Mycgr3T

Mycgr3G103260 Mycgr3
  
Location: 25903-26635

Mycgr3G103260\_Mycgr3

Mycgr3G84644 Mycgr3T
  
Location: 26735-28457

Mycgr3G84644\_Mycgr3T

Mycgr3G29227 Mycgr3T
  
Location: 28557-28863

Mycgr3G29227\_Mycgr3T

Mycgr3G36271 Mycgr3T
  
Location: 28963-29854

Mycgr3G36271\_Mycgr3T

Mycgr3G68433 Mycgr3T
  
Location: 29954-33041

Mycgr3G68433\_Mycgr3T

Mycgr3G79452 Mycgr3T
  
Location: 33141-33399

Mycgr3G79452\_Mycgr3T

Mycgr3G55345 Mycgr3T
  
Location: 33499-34126

Mycgr3G55345\_Mycgr3T

Mycgr3G103278 Mycgr3
  
Location: 34226-35195

Mycgr3G103278\_Mycgr3

Mycgr3G84654 Mycgr3T
  
Location: 35295-36630

Mycgr3G84654\_Mycgr3T

Mycgr3G108090 Mycgr3
  
Location: 36730-37591

Mycgr3G108090\_Mycgr3

Mycgr3G21922 Mycgr3T
  
Location: 37691-39149

Mycgr3G21922\_Mycgr3T

Mycgr3G99148 Mycgr3T
  
Location: 39249-42819

Mycgr3G99148\_Mycgr3T

ATP-dependent RNA helicase A
  
Accession: EIT77726
  
Location: 102212-104760
  
 NCBI BlastP on this gene

EIT77726

hypothetical protein
  
Accession: EIT77713
  
Location: 100849-101678
  
 NCBI BlastP on this gene

EIT77713

hypothetical protein
  
Accession: EIT77735
  
Location: 98019-100130
  
 NCBI BlastP on this gene

EIT77735

F-box domain protein
  
Accession: EIT77725
  
Location: 95211-96906
  
 NCBI BlastP on this gene

EIT77725

succinyl-CoA synthetase, beta subunit
  
Accession: EIT77717
  
Location: 92834-94643
  
 NCBI BlastP on this gene

EIT77717

C4-dicarboxylate/malic acid transporter
  
Accession: EIT77722
  
Location: 86505-88122
  
 NCBI BlastP on this gene

EIT77722

putative phosphoribosyltransferase
  
Accession: EIT77719
  
Location: 84829-85536
  
  
**BlastP hit with Mycgr3G55345\_Mycgr3T**
  
Percentage identity: 74 %
  
BlastP bit score: 255
  
Sequence coverage: 82 %
  
E-value: 8e-83
  
  
 NCBI BlastP on this gene

EIT77719

hypothetical protein
  
Accession: EIT77714
  
Location: 82184-82982
  
 NCBI BlastP on this gene

EIT77714

hypothetical protein
  
Accession: EIT77747
  
Location: 80635-81797
  
 NCBI BlastP on this gene

EIT77747

hypothetical protein
  
Accession: EIT77748
  
Location: 79129-80534
  
 NCBI BlastP on this gene

EIT77748

putative unusual protein kinase
  
Accession: EIT77756
  
Location: 76327-78570
  
  
**BlastP hit with Mycgr3G68458\_Mycgr3T**
  
Percentage identity: 61 %
  
BlastP bit score: 869
  
Sequence coverage: 100 %
  
E-value: 0.0
  
  
 NCBI BlastP on this gene

EIT77756

holocytochrome c synthase/heme-lyase
  
Accession: EIT77731
  
Location: 74320-75416
  
 NCBI BlastP on this gene

EIT77731

hypothetical protein
  
Accession: EIT77718
  
Location: 71597-73457
  
 NCBI BlastP on this gene

EIT77718

ubiquitin-protein ligase
  
Accession: EIT77753
  
Location: 67845-68792
  
 NCBI BlastP on this gene

EIT77753

Rab GTPase interacting factor, Golgi family membrane protein
  
Accession: EIT77723
  
Location: 64384-65389
  
 NCBI BlastP on this gene

EIT77723

hypothetical protein
  
Accession: EIT77724
  
Location: 61597-63993
  
 NCBI BlastP on this gene

EIT77724

hypothetical protein
  
Accession: EIT77752
  
Location: 57442-59445
  
 NCBI BlastP on this gene

EIT77752

Query: Architecture Search FASTA input

DS572813 : Paracoccidioides brasiliensis Pb01 supercont1.3 genomic scaffold    Total score: 2.0     Cumulative Blast bit score: 1110

Hit cluster cross-links:

Mycgr3G90785 Mycgr3T
  
Location: 0-1047

Mycgr3G90785\_Mycgr3T

Mycgr3G103262 Mycgr3
  
Location: 1147-1390

Mycgr3G103262\_Mycgr3

Mycgr3G68458 Mycgr3T
  
Location: 1490-3602

Mycgr3G68458\_Mycgr3T

Mycgr3G99145 Mycgr3T
  
Location: 3702-4326

Mycgr3G99145\_Mycgr3T

Mycgr3G103274 Mycgr3
  
Location: 4426-4957

Mycgr3G103274\_Mycgr3

Mycgr3G103264 Mycgr3
  
Location: 5057-5390

Mycgr3G103264\_Mycgr3

Mycgr3G37570 Mycgr3T
  
Location: 5490-6006

Mycgr3G37570\_Mycgr3T

Mycgr3G108094 Mycgr3
  
Location: 6106-10555

Mycgr3G108094\_Mycgr3

Mycgr3G90786 Mycgr3T
  
Location: 10655-12080

Mycgr3G90786\_Mycgr3T

Mycgr3G68429 Mycgr3T
  
Location: 12180-13440

Mycgr3G68429\_Mycgr3T

Mycgr3G68421 Mycgr3T
  
Location: 13540-17086

Mycgr3G68421\_Mycgr3T

Mycgr3G90801 Mycgr3T
  
Location: 17186-18056

Mycgr3G90801\_Mycgr3T

Mycgr3G84646 Mycgr3T
  
Location: 18156-20235

Mycgr3G84646\_Mycgr3T

Mycgr3G68456 Mycgr3T
  
Location: 20335-21970

Mycgr3G68456\_Mycgr3T

Mycgr3G103270 Mycgr3
  
Location: 22070-22355

Mycgr3G103270\_Mycgr3

Mycgr3G90803 Mycgr3T
  
Location: 22455-23019

Mycgr3G90803\_Mycgr3T

Mycgr3G36941 Mycgr3T
  
Location: 23119-24064

Mycgr3G36941\_Mycgr3T

Mycgr3G25746 Mycgr3T
  
Location: 24164-25241

Mycgr3G25746\_Mycgr3T

Mycgr3G90788 Mycgr3T
  
Location: 25341-25803

Mycgr3G90788\_Mycgr3T

Mycgr3G103260 Mycgr3
  
Location: 25903-26635

Mycgr3G103260\_Mycgr3

Mycgr3G84644 Mycgr3T
  
Location: 26735-28457

Mycgr3G84644\_Mycgr3T

Mycgr3G29227 Mycgr3T
  
Location: 28557-28863

Mycgr3G29227\_Mycgr3T

Mycgr3G36271 Mycgr3T
  
Location: 28963-29854

Mycgr3G36271\_Mycgr3T

Mycgr3G68433 Mycgr3T
  
Location: 29954-33041

Mycgr3G68433\_Mycgr3T

Mycgr3G79452 Mycgr3T
  
Location: 33141-33399

Mycgr3G79452\_Mycgr3T

Mycgr3G55345 Mycgr3T
  
Location: 33499-34126

Mycgr3G55345\_Mycgr3T

Mycgr3G103278 Mycgr3
  
Location: 34226-35195

Mycgr3G103278\_Mycgr3

Mycgr3G84654 Mycgr3T
  
Location: 35295-36630

Mycgr3G84654\_Mycgr3T

Mycgr3G108090 Mycgr3
  
Location: 36730-37591

Mycgr3G108090\_Mycgr3

Mycgr3G21922 Mycgr3T
  
Location: 37691-39149

Mycgr3G21922\_Mycgr3T

Mycgr3G99148 Mycgr3T
  
Location: 39249-42819

Mycgr3G99148\_Mycgr3T

predicted protein
  
Accession: EEH39135
  
Location: 914273-915848
  
 NCBI BlastP on this gene

EEH39135

conserved hypothetical protein
  
Accession: EEH39136
  
Location: 917780-918497
  
 NCBI BlastP on this gene

EEH39136

conserved hypothetical protein
  
Accession: EEH39137
  
Location: 920355-922918
  
 NCBI BlastP on this gene

EEH39137

N-acetyltransferase ats1
  
Accession: EEH39138
  
Location: 923704-924009
  
 NCBI BlastP on this gene

EEH39138

conserved hypothetical protein
  
Accession: EEH39139
  
Location: 925024-931450
  
  
**BlastP hit with Mycgr3G108094\_Mycgr3**
  
Percentage identity: 36 %
  
BlastP bit score: 358
  
Sequence coverage: 47 %
  
E-value: 6e-98
  
  
 NCBI BlastP on this gene

EEH39139

ras-like GTP-binding protein
  
Accession: EEH39140
  
Location: 935352-936345
  
 NCBI BlastP on this gene

EEH39140

cytochrome b2
  
Accession: EEH39141
  
Location: 939557-941272
  
 NCBI BlastP on this gene

EEH39141

conserved hypothetical protein
  
Accession: EEH39142
  
Location: 941517-942677
  
 NCBI BlastP on this gene

EEH39142

protein kinase dsk1
  
Accession: EEH39143
  
Location: 946495-948589
  
  
**BlastP hit with Mycgr3G84644\_Mycgr3T**
  
Percentage identity: 67 %
  
BlastP bit score: 752
  
Sequence coverage: 104 %
  
E-value: 0.0
  
  
 NCBI BlastP on this gene

EEH39143

pre-mRNA-splicing factor cwc26
  
Accession: EEH39144
  
Location: 950426-951520
  
 NCBI BlastP on this gene

EEH39144

MGMT family protein
  
Accession: EEH39145
  
Location: 951785-952595
  
 NCBI BlastP on this gene

EEH39145

Query: Architecture Search FASTA input

AKCU01000110 : Penicillium digitatum Pd1    Total score: 2.0     Cumulative Blast bit score: 1106

Hit cluster cross-links:

Mycgr3G90785 Mycgr3T
  
Location: 0-1047

Mycgr3G90785\_Mycgr3T

Mycgr3G103262 Mycgr3
  
Location: 1147-1390

Mycgr3G103262\_Mycgr3

Mycgr3G68458 Mycgr3T
  
Location: 1490-3602

Mycgr3G68458\_Mycgr3T

Mycgr3G99145 Mycgr3T
  
Location: 3702-4326

Mycgr3G99145\_Mycgr3T

Mycgr3G103274 Mycgr3
  
Location: 4426-4957

Mycgr3G103274\_Mycgr3

Mycgr3G103264 Mycgr3
  
Location: 5057-5390

Mycgr3G103264\_Mycgr3

Mycgr3G37570 Mycgr3T
  
Location: 5490-6006

Mycgr3G37570\_Mycgr3T

Mycgr3G108094 Mycgr3
  
Location: 6106-10555

Mycgr3G108094\_Mycgr3

Mycgr3G90786 Mycgr3T
  
Location: 10655-12080

Mycgr3G90786\_Mycgr3T

Mycgr3G68429 Mycgr3T
  
Location: 12180-13440

Mycgr3G68429\_Mycgr3T

Mycgr3G68421 Mycgr3T
  
Location: 13540-17086

Mycgr3G68421\_Mycgr3T

Mycgr3G90801 Mycgr3T
  
Location: 17186-18056

Mycgr3G90801\_Mycgr3T

Mycgr3G84646 Mycgr3T
  
Location: 18156-20235

Mycgr3G84646\_Mycgr3T

Mycgr3G68456 Mycgr3T
  
Location: 20335-21970

Mycgr3G68456\_Mycgr3T

Mycgr3G103270 Mycgr3
  
Location: 22070-22355

Mycgr3G103270\_Mycgr3

Mycgr3G90803 Mycgr3T
  
Location: 22455-23019

Mycgr3G90803\_Mycgr3T

Mycgr3G36941 Mycgr3T
  
Location: 23119-24064

Mycgr3G36941\_Mycgr3T

Mycgr3G25746 Mycgr3T
  
Location: 24164-25241

Mycgr3G25746\_Mycgr3T

Mycgr3G90788 Mycgr3T
  
Location: 25341-25803

Mycgr3G90788\_Mycgr3T

Mycgr3G103260 Mycgr3
  
Location: 25903-26635

Mycgr3G103260\_Mycgr3

Mycgr3G84644 Mycgr3T
  
Location: 26735-28457

Mycgr3G84644\_Mycgr3T

Mycgr3G29227 Mycgr3T
  
Location: 28557-28863

Mycgr3G29227\_Mycgr3T

Mycgr3G36271 Mycgr3T
  
Location: 28963-29854

Mycgr3G36271\_Mycgr3T

Mycgr3G68433 Mycgr3T
  
Location: 29954-33041

Mycgr3G68433\_Mycgr3T

Mycgr3G79452 Mycgr3T
  
Location: 33141-33399

Mycgr3G79452\_Mycgr3T

Mycgr3G55345 Mycgr3T
  
Location: 33499-34126

Mycgr3G55345\_Mycgr3T

Mycgr3G103278 Mycgr3
  
Location: 34226-35195

Mycgr3G103278\_Mycgr3

Mycgr3G84654 Mycgr3T
  
Location: 35295-36630

Mycgr3G84654\_Mycgr3T

Mycgr3G108090 Mycgr3
  
Location: 36730-37591

Mycgr3G108090\_Mycgr3

Mycgr3G21922 Mycgr3T
  
Location: 37691-39149

Mycgr3G21922\_Mycgr3T

Mycgr3G99148 Mycgr3T
  
Location: 39249-42819

Mycgr3G99148\_Mycgr3T

Pyoverdine/dityrosine biosynthesis protein, putative
  
Accession: EKV20588
  
Location: 60576-61625
  
 NCBI BlastP on this gene

EKV20588

hypothetical protein
  
Accession: EKV20587
  
Location: 58660-59397
  
 NCBI BlastP on this gene

EKV20587

RING finger protein (Zin), putative
  
Accession: EKV20586
  
Location: 55314-57656
  
 NCBI BlastP on this gene

EKV20586

Peroxisomal multifunctional beta-oxidation protein (MFP), putative
  
Accession: EKV20585
  
Location: 51581-54459
  
 NCBI BlastP on this gene

EKV20585

hypothetical protein
  
Accession: EKV20584
  
Location: 49674-50904
  
 NCBI BlastP on this gene

EKV20584

60S ribosomal protein L7
  
Accession: EKV20583
  
Location: 47129-48718
  
 NCBI BlastP on this gene

EKV20583

hypothetical protein
  
Accession: EKV20582
  
Location: 45967-46821
  
 NCBI BlastP on this gene

EKV20582

Thermophilic desulfurizing enzyme family protein
  
Accession: EKV20581
  
Location: 43792-45212
  
 NCBI BlastP on this gene

EKV20581

Xanthine-guanine phosphoribosyl transferase Xpt1, putative
  
Accession: EKV20580
  
Location: 42374-43453
  
  
**BlastP hit with Mycgr3G55345\_Mycgr3T**
  
Percentage identity: 67 %
  
BlastP bit score: 264
  
Sequence coverage: 97 %
  
E-value: 8e-86
  
  
 NCBI BlastP on this gene

EKV20580

hypothetical protein
  
Accession: EKV20579
  
Location: 41324-41757
  
 NCBI BlastP on this gene

EKV20579

NADPH-dependent 1-acyl dihydroxyacetone phosphate reductase, putative
  
Accession: EKV20578
  
Location: 39867-40710
  
 NCBI BlastP on this gene

EKV20578

GTP binding protein, putative
  
Accession: EKV20577
  
Location: 35453-37618
  
 NCBI BlastP on this gene

EKV20577

hypothetical protein
  
Accession: EKV20576
  
Location: 33941-35252
  
 NCBI BlastP on this gene

EKV20576

Ubiquinone biosynthesis protein, putative
  
Accession: EKV20575
  
Location: 31196-33208
  
  
**BlastP hit with Mycgr3G68458\_Mycgr3T**
  
Percentage identity: 63 %
  
BlastP bit score: 842
  
Sequence coverage: 90 %
  
E-value: 0.0
  
  
 NCBI BlastP on this gene

EKV20575

Cytochrome c heme lyase, putative
  
Accession: EKV20574
  
Location: 29744-30787
  
 NCBI BlastP on this gene

EKV20574

hypothetical protein
  
Accession: EKV20573
  
Location: 28751-29350
  
 NCBI BlastP on this gene

EKV20573

hypothetical protein
  
Accession: EKV20572
  
Location: 28021-28113
  
 NCBI BlastP on this gene

EKV20572

Ubiquitin-conjugating enzyme Ubc6, putative
  
Accession: EKV20571
  
Location: 25751-26708
  
 NCBI BlastP on this gene

EKV20571

Mitochondrial outer membrane protein (Sam50), putative
  
Accession: EKV20570
  
Location: 23728-25497
  
 NCBI BlastP on this gene

EKV20570

Golgi membrane protein, putative
  
Accession: EKV20569
  
Location: 22364-23412
  
 NCBI BlastP on this gene

EKV20569

Ubiquitin fusion degradation protein (Ufd1), putative
  
Accession: EKV20568
  
Location: 19790-22084
  
 NCBI BlastP on this gene

EKV20568

hypothetical protein
  
Accession: EKV20567
  
Location: 17397-18881
  
 NCBI BlastP on this gene

EKV20567

Proteasome regulatory particle subunit Rpt6, putative
  
Accession: EKV20566
  
Location: 14398-15757
  
 NCBI BlastP on this gene

EKV20566

Query: Architecture Search FASTA input

AKCT01000108 : Penicillium digitatum PHI26    Total score: 2.0     Cumulative Blast bit score: 1106

Hit cluster cross-links:

Mycgr3G90785 Mycgr3T
  
Location: 0-1047

Mycgr3G90785\_Mycgr3T

Mycgr3G103262 Mycgr3
  
Location: 1147-1390

Mycgr3G103262\_Mycgr3

Mycgr3G68458 Mycgr3T
  
Location: 1490-3602

Mycgr3G68458\_Mycgr3T

Mycgr3G99145 Mycgr3T
  
Location: 3702-4326

Mycgr3G99145\_Mycgr3T

Mycgr3G103274 Mycgr3
  
Location: 4426-4957

Mycgr3G103274\_Mycgr3

Mycgr3G103264 Mycgr3
  
Location: 5057-5390

Mycgr3G103264\_Mycgr3

Mycgr3G37570 Mycgr3T
  
Location: 5490-6006

Mycgr3G37570\_Mycgr3T

Mycgr3G108094 Mycgr3
  
Location: 6106-10555

Mycgr3G108094\_Mycgr3

Mycgr3G90786 Mycgr3T
  
Location: 10655-12080

Mycgr3G90786\_Mycgr3T

Mycgr3G68429 Mycgr3T
  
Location: 12180-13440

Mycgr3G68429\_Mycgr3T

Mycgr3G68421 Mycgr3T
  
Location: 13540-17086

Mycgr3G68421\_Mycgr3T

Mycgr3G90801 Mycgr3T
  
Location: 17186-18056

Mycgr3G90801\_Mycgr3T

Mycgr3G84646 Mycgr3T
  
Location: 18156-20235

Mycgr3G84646\_Mycgr3T

Mycgr3G68456 Mycgr3T
  
Location: 20335-21970

Mycgr3G68456\_Mycgr3T

Mycgr3G103270 Mycgr3
  
Location: 22070-22355

Mycgr3G103270\_Mycgr3

Mycgr3G90803 Mycgr3T
  
Location: 22455-23019

Mycgr3G90803\_Mycgr3T

Mycgr3G36941 Mycgr3T
  
Location: 23119-24064

Mycgr3G36941\_Mycgr3T

Mycgr3G25746 Mycgr3T
  
Location: 24164-25241

Mycgr3G25746\_Mycgr3T

Mycgr3G90788 Mycgr3T
  
Location: 25341-25803

Mycgr3G90788\_Mycgr3T

Mycgr3G103260 Mycgr3
  
Location: 25903-26635

Mycgr3G103260\_Mycgr3

Mycgr3G84644 Mycgr3T
  
Location: 26735-28457

Mycgr3G84644\_Mycgr3T

Mycgr3G29227 Mycgr3T
  
Location: 28557-28863

Mycgr3G29227\_Mycgr3T

Mycgr3G36271 Mycgr3T
  
Location: 28963-29854

Mycgr3G36271\_Mycgr3T

Mycgr3G68433 Mycgr3T
  
Location: 29954-33041

Mycgr3G68433\_Mycgr3T

Mycgr3G79452 Mycgr3T
  
Location: 33141-33399

Mycgr3G79452\_Mycgr3T

Mycgr3G55345 Mycgr3T
  
Location: 33499-34126

Mycgr3G55345\_Mycgr3T

Mycgr3G103278 Mycgr3
  
Location: 34226-35195

Mycgr3G103278\_Mycgr3

Mycgr3G84654 Mycgr3T
  
Location: 35295-36630

Mycgr3G84654\_Mycgr3T

Mycgr3G108090 Mycgr3
  
Location: 36730-37591

Mycgr3G108090\_Mycgr3

Mycgr3G21922 Mycgr3T
  
Location: 37691-39149

Mycgr3G21922\_Mycgr3T

Mycgr3G99148 Mycgr3T
  
Location: 39249-42819

Mycgr3G99148\_Mycgr3T

Pyoverdine/dityrosine biosynthesis protein, putative
  
Accession: EKV15917
  
Location: 94927-95976
  
 NCBI BlastP on this gene

EKV15917

hypothetical protein
  
Accession: EKV15916
  
Location: 93011-93748
  
 NCBI BlastP on this gene

EKV15916

RING finger protein (Zin), putative
  
Accession: EKV15915
  
Location: 89665-92007
  
 NCBI BlastP on this gene

EKV15915

Peroxisomal multifunctional beta-oxidation protein (MFP), putative
  
Accession: EKV15914
  
Location: 85932-88810
  
 NCBI BlastP on this gene

EKV15914

hypothetical protein
  
Accession: EKV15913
  
Location: 84029-85259
  
 NCBI BlastP on this gene

EKV15913

60S ribosomal protein L7
  
Accession: EKV15912
  
Location: 81482-83071
  
 NCBI BlastP on this gene

EKV15912

hypothetical protein
  
Accession: EKV15911
  
Location: 80320-81174
  
 NCBI BlastP on this gene

EKV15911

Thermophilic desulfurizing enzyme family protein
  
Accession: EKV15910
  
Location: 78145-79565
  
 NCBI BlastP on this gene

EKV15910

Xanthine-guanine phosphoribosyl transferase Xpt1, putative
  
Accession: EKV15909
  
Location: 76721-77801
  
  
**BlastP hit with Mycgr3G55345\_Mycgr3T**
  
Percentage identity: 67 %
  
BlastP bit score: 264
  
Sequence coverage: 97 %
  
E-value: 8e-86
  
  
 NCBI BlastP on this gene

EKV15909

hypothetical protein
  
Accession: EKV15908
  
Location: 75671-76104
  
 NCBI BlastP on this gene

EKV15908

NADPH-dependent 1-acyl dihydroxyacetone phosphate reductase, putative
  
Accession: EKV15907
  
Location: 74214-75057
  
 NCBI BlastP on this gene

EKV15907

GTP binding protein, putative
  
Accession: EKV15906
  
Location: 69800-71965
  
 NCBI BlastP on this gene

EKV15906

hypothetical protein
  
Accession: EKV15905
  
Location: 68288-69599
  
 NCBI BlastP on this gene

EKV15905

Ubiquinone biosynthesis protein, putative
  
Accession: EKV15904
  
Location: 65543-67555
  
  
**BlastP hit with Mycgr3G68458\_Mycgr3T**
  
Percentage identity: 63 %
  
BlastP bit score: 842
  
Sequence coverage: 90 %
  
E-value: 0.0
  
  
 NCBI BlastP on this gene

EKV15904

Cytochrome c heme lyase, putative
  
Accession: EKV15903
  
Location: 64091-65134
  
 NCBI BlastP on this gene

EKV15903

hypothetical protein
  
Accession: EKV15902
  
Location: 62367-63697
  
 NCBI BlastP on this gene

EKV15902

Ubiquitin-conjugating enzyme Ubc6, putative
  
Accession: EKV15901
  
Location: 60117-61074
  
 NCBI BlastP on this gene

EKV15901

Mitochondrial outer membrane protein (Sam50), putative
  
Accession: EKV15900
  
Location: 58094-59863
  
 NCBI BlastP on this gene

EKV15900

Golgi membrane protein, putative
  
Accession: EKV15899
  
Location: 56730-57778
  
 NCBI BlastP on this gene

EKV15899

Ubiquitin fusion degradation protein (Ufd1), putative
  
Accession: EKV15898
  
Location: 54156-56450
  
 NCBI BlastP on this gene

EKV15898

hypothetical protein
  
Accession: EKV15897
  
Location: 51763-53247
  
 NCBI BlastP on this gene

EKV15897

Proteasome regulatory particle subunit Rpt6, putative
  
Accession: EKV15896
  
Location: 48764-50123
  
 NCBI BlastP on this gene

EKV15896

Query: Architecture Search FASTA input

CH476624 : Sclerotinia sclerotiorum 1980 scaffold\_4 genomic scaffold    Total score: 2.0     Cumulative Blast bit score: 1105

Hit cluster cross-links:

Mycgr3G90785 Mycgr3T
  
Location: 0-1047

Mycgr3G90785\_Mycgr3T

Mycgr3G103262 Mycgr3
  
Location: 1147-1390

Mycgr3G103262\_Mycgr3

Mycgr3G68458 Mycgr3T
  
Location: 1490-3602

Mycgr3G68458\_Mycgr3T

Mycgr3G99145 Mycgr3T
  
Location: 3702-4326

Mycgr3G99145\_Mycgr3T

Mycgr3G103274 Mycgr3
  
Location: 4426-4957

Mycgr3G103274\_Mycgr3

Mycgr3G103264 Mycgr3
  
Location: 5057-5390

Mycgr3G103264\_Mycgr3

Mycgr3G37570 Mycgr3T
  
Location: 5490-6006

Mycgr3G37570\_Mycgr3T

Mycgr3G108094 Mycgr3
  
Location: 6106-10555

Mycgr3G108094\_Mycgr3

Mycgr3G90786 Mycgr3T
  
Location: 10655-12080

Mycgr3G90786\_Mycgr3T

Mycgr3G68429 Mycgr3T
  
Location: 12180-13440

Mycgr3G68429\_Mycgr3T

Mycgr3G68421 Mycgr3T
  
Location: 13540-17086

Mycgr3G68421\_Mycgr3T

Mycgr3G90801 Mycgr3T
  
Location: 17186-18056

Mycgr3G90801\_Mycgr3T

Mycgr3G84646 Mycgr3T
  
Location: 18156-20235

Mycgr3G84646\_Mycgr3T

Mycgr3G68456 Mycgr3T
  
Location: 20335-21970

Mycgr3G68456\_Mycgr3T

Mycgr3G103270 Mycgr3
  
Location: 22070-22355

Mycgr3G103270\_Mycgr3

Mycgr3G90803 Mycgr3T
  
Location: 22455-23019

Mycgr3G90803\_Mycgr3T

Mycgr3G36941 Mycgr3T
  
Location: 23119-24064

Mycgr3G36941\_Mycgr3T

Mycgr3G25746 Mycgr3T
  
Location: 24164-25241

Mycgr3G25746\_Mycgr3T

Mycgr3G90788 Mycgr3T
  
Location: 25341-25803

Mycgr3G90788\_Mycgr3T

Mycgr3G103260 Mycgr3
  
Location: 25903-26635

Mycgr3G103260\_Mycgr3

Mycgr3G84644 Mycgr3T
  
Location: 26735-28457

Mycgr3G84644\_Mycgr3T

Mycgr3G29227 Mycgr3T
  
Location: 28557-28863

Mycgr3G29227\_Mycgr3T

Mycgr3G36271 Mycgr3T
  
Location: 28963-29854

Mycgr3G36271\_Mycgr3T

Mycgr3G68433 Mycgr3T
  
Location: 29954-33041

Mycgr3G68433\_Mycgr3T

Mycgr3G79452 Mycgr3T
  
Location: 33141-33399

Mycgr3G79452\_Mycgr3T

Mycgr3G55345 Mycgr3T
  
Location: 33499-34126

Mycgr3G55345\_Mycgr3T

Mycgr3G103278 Mycgr3
  
Location: 34226-35195

Mycgr3G103278\_Mycgr3

Mycgr3G84654 Mycgr3T
  
Location: 35295-36630

Mycgr3G84654\_Mycgr3T

Mycgr3G108090 Mycgr3
  
Location: 36730-37591

Mycgr3G108090\_Mycgr3

Mycgr3G21922 Mycgr3T
  
Location: 37691-39149

Mycgr3G21922\_Mycgr3T

Mycgr3G99148 Mycgr3T
  
Location: 39249-42819

Mycgr3G99148\_Mycgr3T

hypothetical protein
  
Accession: EDO00795
  
Location: 438662-439279
  
 NCBI BlastP on this gene

EDO00795

vacuolar protein sorting protein
  
Accession: EDO00796
  
Location: 439612-440441
  
 NCBI BlastP on this gene

EDO00796

predicted protein
  
Accession: EDO00797
  
Location: 441976-442754
  
 NCBI BlastP on this gene

EDO00797

predicted protein
  
Accession: EDO00798
  
Location: 444281-444682
  
 NCBI BlastP on this gene

EDO00798

predicted protein
  
Accession: EDO00799
  
Location: 446628-447275
  
 NCBI BlastP on this gene

EDO00799

hypothetical protein
  
Accession: EDO00800
  
Location: 447590-448873
  
 NCBI BlastP on this gene

EDO00800

hypothetical protein
  
Accession: EDO00801
  
Location: 449847-453983
  
  
**BlastP hit with Mycgr3G108094\_Mycgr3**
  
Percentage identity: 33 %
  
BlastP bit score: 305
  
Sequence coverage: 47 %
  
E-value: 9e-82
  
  
 NCBI BlastP on this gene

EDO00801

hypothetical protein
  
Accession: EDO00802
  
Location: 455717-456481
  
 NCBI BlastP on this gene

EDO00802

hypothetical protein
  
Accession: EDO00803
  
Location: 460903-462976
  
  
**BlastP hit with Mycgr3G84644\_Mycgr3T**
  
Percentage identity: 71 %
  
BlastP bit score: 800
  
Sequence coverage: 100 %
  
E-value: 0.0
  
  
 NCBI BlastP on this gene

EDO00803

hypothetical protein
  
Accession: EDO00804
  
Location: 464344-465852
  
 NCBI BlastP on this gene

EDO00804

hypothetical protein
  
Accession: EDO00805
  
Location: 466248-467722
  
 NCBI BlastP on this gene

EDO00805

hypothetical protein
  
Accession: EDO00806
  
Location: 469795-471332
  
 NCBI BlastP on this gene

EDO00806

Query: Architecture Search FASTA input

CH445336 : Phaeosphaeria nodorum SN15 scaffold\_12    Total score: 2.0     Cumulative Blast bit score: 1101

Hit cluster cross-links:

Mycgr3G90785 Mycgr3T
  
Location: 0-1047

Mycgr3G90785\_Mycgr3T

Mycgr3G103262 Mycgr3
  
Location: 1147-1390

Mycgr3G103262\_Mycgr3

Mycgr3G68458 Mycgr3T
  
Location: 1490-3602

Mycgr3G68458\_Mycgr3T

Mycgr3G99145 Mycgr3T
  
Location: 3702-4326

Mycgr3G99145\_Mycgr3T

Mycgr3G103274 Mycgr3
  
Location: 4426-4957

Mycgr3G103274\_Mycgr3

Mycgr3G103264 Mycgr3
  
Location: 5057-5390

Mycgr3G103264\_Mycgr3

Mycgr3G37570 Mycgr3T
  
Location: 5490-6006

Mycgr3G37570\_Mycgr3T

Mycgr3G108094 Mycgr3
  
Location: 6106-10555

Mycgr3G108094\_Mycgr3

Mycgr3G90786 Mycgr3T
  
Location: 10655-12080

Mycgr3G90786\_Mycgr3T

Mycgr3G68429 Mycgr3T
  
Location: 12180-13440

Mycgr3G68429\_Mycgr3T

Mycgr3G68421 Mycgr3T
  
Location: 13540-17086

Mycgr3G68421\_Mycgr3T

Mycgr3G90801 Mycgr3T
  
Location: 17186-18056

Mycgr3G90801\_Mycgr3T

Mycgr3G84646 Mycgr3T
  
Location: 18156-20235

Mycgr3G84646\_Mycgr3T

Mycgr3G68456 Mycgr3T
  
Location: 20335-21970

Mycgr3G68456\_Mycgr3T

Mycgr3G103270 Mycgr3
  
Location: 22070-22355

Mycgr3G103270\_Mycgr3

Mycgr3G90803 Mycgr3T
  
Location: 22455-23019

Mycgr3G90803\_Mycgr3T

Mycgr3G36941 Mycgr3T
  
Location: 23119-24064

Mycgr3G36941\_Mycgr3T

Mycgr3G25746 Mycgr3T
  
Location: 24164-25241

Mycgr3G25746\_Mycgr3T

Mycgr3G90788 Mycgr3T
  
Location: 25341-25803

Mycgr3G90788\_Mycgr3T

Mycgr3G103260 Mycgr3
  
Location: 25903-26635

Mycgr3G103260\_Mycgr3

Mycgr3G84644 Mycgr3T
  
Location: 26735-28457

Mycgr3G84644\_Mycgr3T

Mycgr3G29227 Mycgr3T
  
Location: 28557-28863

Mycgr3G29227\_Mycgr3T

Mycgr3G36271 Mycgr3T
  
Location: 28963-29854

Mycgr3G36271\_Mycgr3T

Mycgr3G68433 Mycgr3T
  
Location: 29954-33041

Mycgr3G68433\_Mycgr3T

Mycgr3G79452 Mycgr3T
  
Location: 33141-33399

Mycgr3G79452\_Mycgr3T

Mycgr3G55345 Mycgr3T
  
Location: 33499-34126

Mycgr3G55345\_Mycgr3T

Mycgr3G103278 Mycgr3
  
Location: 34226-35195

Mycgr3G103278\_Mycgr3

Mycgr3G84654 Mycgr3T
  
Location: 35295-36630

Mycgr3G84654\_Mycgr3T

Mycgr3G108090 Mycgr3
  
Location: 36730-37591

Mycgr3G108090\_Mycgr3

Mycgr3G21922 Mycgr3T
  
Location: 37691-39149

Mycgr3G21922\_Mycgr3T

Mycgr3G99148 Mycgr3T
  
Location: 39249-42819

Mycgr3G99148\_Mycgr3T

hypothetical protein
  
Accession: EAT84361
  
Location: 417739-418575
  
 NCBI BlastP on this gene

EAT84361

hypothetical protein
  
Accession: EAT84360
  
Location: 416194-417251
  
 NCBI BlastP on this gene

EAT84360

hypothetical protein
  
Accession: EAT84359
  
Location: 413450-414969
  
 NCBI BlastP on this gene

EAT84359

hypothetical protein
  
Accession: EAT84358
  
Location: 412307-412770
  
 NCBI BlastP on this gene

EAT84358

hypothetical protein
  
Accession: EAT84357
  
Location: 409263-410377
  
 NCBI BlastP on this gene

EAT84357

hypothetical protein
  
Accession: EAT84356
  
Location: 407638-408019
  
 NCBI BlastP on this gene

EAT84356

hypothetical protein
  
Accession: EAT84355
  
Location: 405658-407311
  
 NCBI BlastP on this gene

EAT84355

hypothetical protein
  
Accession: EAT84354
  
Location: 403301-405201
  
 NCBI BlastP on this gene

EAT84354

hypothetical protein
  
Accession: EAT84353
  
Location: 402962-403131
  
 NCBI BlastP on this gene

EAT84353

hypothetical protein
  
Accession: EAT84352
  
Location: 401427-402866
  
 NCBI BlastP on this gene

EAT84352

hypothetical protein
  
Accession: EAT84351
  
Location: 397565-401273
  
  
**BlastP hit with Mycgr3G108094\_Mycgr3**
  
Percentage identity: 40 %
  
BlastP bit score: 403
  
Sequence coverage: 44 %
  
E-value: 8e-116
  
  
 NCBI BlastP on this gene

EAT84351

hypothetical protein
  
Accession: EAT84350
  
Location: 394277-396410
  
  
**BlastP hit with Mycgr3G84644\_Mycgr3T**
  
Percentage identity: 68 %
  
BlastP bit score: 698
  
Sequence coverage: 96 %
  
E-value: 0.0
  
  
 NCBI BlastP on this gene

EAT84350

hypothetical protein
  
Accession: EAT84349
  
Location: 391778-393680
  
 NCBI BlastP on this gene

EAT84349

hypothetical protein
  
Accession: EAT84348
  
Location: 389919-390751
  
 NCBI BlastP on this gene

EAT84348

hypothetical protein
  
Accession: EAT84347
  
Location: 386501-388900
  
 NCBI BlastP on this gene

EAT84347

hypothetical protein
  
Accession: EAT84346
  
Location: 386285-386499
  
 NCBI BlastP on this gene

EAT84346

hypothetical protein
  
Accession: EDP89783
  
Location: 385869-386030
  
 NCBI BlastP on this gene

EDP89783

hypothetical protein
  
Accession: EAT84345
  
Location: 385010-385698
  
 NCBI BlastP on this gene

EAT84345

hypothetical protein
  
Accession: EAT84344
  
Location: 383410-384550
  
 NCBI BlastP on this gene

EAT84344

hypothetical protein
  
Accession: EAT84343
  
Location: 378879-382416
  
 NCBI BlastP on this gene

EAT84343

hypothetical protein
  
Accession: EAT84342
  
Location: 375960-378077
  
 NCBI BlastP on this gene

EAT84342

hypothetical protein
  
Accession: EAT84340
  
Location: 374297-375393
  
 NCBI BlastP on this gene

EAT84340

Query: Architecture Search FASTA input

KB445579 : Cochliobolus heterostrophus C5 unplaced genomic scaffold COCHEscaffold\_11    Total score: 2.0     Cumulative Blast bit score: 1091

Hit cluster cross-links:

Mycgr3G90785 Mycgr3T
  
Location: 0-1047

Mycgr3G90785\_Mycgr3T

Mycgr3G103262 Mycgr3
  
Location: 1147-1390

Mycgr3G103262\_Mycgr3

Mycgr3G68458 Mycgr3T
  
Location: 1490-3602

Mycgr3G68458\_Mycgr3T

Mycgr3G99145 Mycgr3T
  
Location: 3702-4326

Mycgr3G99145\_Mycgr3T

Mycgr3G103274 Mycgr3
  
Location: 4426-4957

Mycgr3G103274\_Mycgr3

Mycgr3G103264 Mycgr3
  
Location: 5057-5390

Mycgr3G103264\_Mycgr3

Mycgr3G37570 Mycgr3T
  
Location: 5490-6006

Mycgr3G37570\_Mycgr3T

Mycgr3G108094 Mycgr3
  
Location: 6106-10555

Mycgr3G108094\_Mycgr3

Mycgr3G90786 Mycgr3T
  
Location: 10655-12080

Mycgr3G90786\_Mycgr3T

Mycgr3G68429 Mycgr3T
  
Location: 12180-13440

Mycgr3G68429\_Mycgr3T

Mycgr3G68421 Mycgr3T
  
Location: 13540-17086

Mycgr3G68421\_Mycgr3T

Mycgr3G90801 Mycgr3T
  
Location: 17186-18056

Mycgr3G90801\_Mycgr3T

Mycgr3G84646 Mycgr3T
  
Location: 18156-20235

Mycgr3G84646\_Mycgr3T

Mycgr3G68456 Mycgr3T
  
Location: 20335-21970

Mycgr3G68456\_Mycgr3T

Mycgr3G103270 Mycgr3
  
Location: 22070-22355

Mycgr3G103270\_Mycgr3

Mycgr3G90803 Mycgr3T
  
Location: 22455-23019

Mycgr3G90803\_Mycgr3T

Mycgr3G36941 Mycgr3T
  
Location: 23119-24064

Mycgr3G36941\_Mycgr3T

Mycgr3G25746 Mycgr3T
  
Location: 24164-25241

Mycgr3G25746\_Mycgr3T

Mycgr3G90788 Mycgr3T
  
Location: 25341-25803

Mycgr3G90788\_Mycgr3T

Mycgr3G103260 Mycgr3
  
Location: 25903-26635

Mycgr3G103260\_Mycgr3

Mycgr3G84644 Mycgr3T
  
Location: 26735-28457

Mycgr3G84644\_Mycgr3T

Mycgr3G29227 Mycgr3T
  
Location: 28557-28863

Mycgr3G29227\_Mycgr3T

Mycgr3G36271 Mycgr3T
  
Location: 28963-29854

Mycgr3G36271\_Mycgr3T

Mycgr3G68433 Mycgr3T
  
Location: 29954-33041

Mycgr3G68433\_Mycgr3T

Mycgr3G79452 Mycgr3T
  
Location: 33141-33399

Mycgr3G79452\_Mycgr3T

Mycgr3G55345 Mycgr3T
  
Location: 33499-34126

Mycgr3G55345\_Mycgr3T

Mycgr3G103278 Mycgr3
  
Location: 34226-35195

Mycgr3G103278\_Mycgr3

Mycgr3G84654 Mycgr3T
  
Location: 35295-36630

Mycgr3G84654\_Mycgr3T

Mycgr3G108090 Mycgr3
  
Location: 36730-37591

Mycgr3G108090\_Mycgr3

Mycgr3G21922 Mycgr3T
  
Location: 37691-39149

Mycgr3G21922\_Mycgr3T

Mycgr3G99148 Mycgr3T
  
Location: 39249-42819

Mycgr3G99148\_Mycgr3T

hypothetical protein
  
Accession: EMD89514
  
Location: 1368503-1369972
  
 NCBI BlastP on this gene

EMD89514

hypothetical protein
  
Accession: EMD89513
  
Location: 1367701-1367988
  
 NCBI BlastP on this gene

EMD89513

hypothetical protein
  
Accession: EMD89512
  
Location: 1366055-1367145
  
 NCBI BlastP on this gene

EMD89512

hypothetical protein
  
Accession: EMD89511
  
Location: 1365185-1365835
  
 NCBI BlastP on this gene

EMD89511

hypothetical protein
  
Accession: EMD89510
  
Location: 1363444-1364453
  
 NCBI BlastP on this gene

EMD89510

hypothetical protein
  
Accession: EMD89509
  
Location: 1362713-1362985
  
 NCBI BlastP on this gene

EMD89509

hypothetical protein
  
Accession: EMD89508
  
Location: 1359913-1362565
  
 NCBI BlastP on this gene

EMD89508

hypothetical protein
  
Accession: EMD89507
  
Location: 1357496-1357708
  
 NCBI BlastP on this gene

EMD89507

hypothetical protein
  
Accession: EMD89506
  
Location: 1355047-1356710
  
 NCBI BlastP on this gene

EMD89506

hypothetical protein
  
Accession: EMD89505
  
Location: 1353031-1354624
  
 NCBI BlastP on this gene

EMD89505

hypothetical protein
  
Accession: EMD89504
  
Location: 1350981-1352422
  
 NCBI BlastP on this gene

EMD89504

hypothetical protein
  
Accession: EMD89503
  
Location: 1346864-1350714
  
  
**BlastP hit with Mycgr3G108094\_Mycgr3**
  
Percentage identity: 49 %
  
BlastP bit score: 246
  
Sequence coverage: 20 %
  
E-value: 3e-64
  
  
 NCBI BlastP on this gene

EMD89503

hypothetical protein
  
Accession: EMD89502
  
Location: 1343319-1345514
  
  
**BlastP hit with Mycgr3G84644\_Mycgr3T**
  
Percentage identity: 70 %
  
BlastP bit score: 845
  
Sequence coverage: 109 %
  
E-value: 0.0
  
  
 NCBI BlastP on this gene

EMD89502

glycoside hydrolase family 16 protein
  
Accession: EMD89501
  
Location: 1340877-1342093
  
 NCBI BlastP on this gene

EMD89501

hypothetical protein
  
Accession: EMD89500
  
Location: 1337047-1339228
  
 NCBI BlastP on this gene

EMD89500

hypothetical protein
  
Accession: EMD89499
  
Location: 1335252-1336611
  
 NCBI BlastP on this gene

EMD89499

hypothetical protein
  
Accession: EMD89498
  
Location: 1333930-1335178
  
 NCBI BlastP on this gene

EMD89498

hypothetical protein
  
Accession: EMD89497
  
Location: 1331594-1333705
  
 NCBI BlastP on this gene

EMD89497

hypothetical protein
  
Accession: EMD89496
  
Location: 1329148-1330940
  
 NCBI BlastP on this gene

EMD89496

hypothetical protein
  
Accession: EMD89495
  
Location: 1327717-1328634
  
 NCBI BlastP on this gene

EMD89495

glycosyltransferase family 2 protein
  
Accession: EMD89494
  
Location: 1326668-1327463
  
 NCBI BlastP on this gene

EMD89494

hypothetical protein
  
Accession: EMD89493
  
Location: 1325942-1326349
  
 NCBI BlastP on this gene

EMD89493

Query: Architecture Search FASTA input

AP007172 : Aspergillus oryzae RIB40 DNA, SC206.    Total score: 2.0     Cumulative Blast bit score: 1078

Hit cluster cross-links:

Mycgr3G90785 Mycgr3T
  
Location: 0-1047

Mycgr3G90785\_Mycgr3T

Mycgr3G103262 Mycgr3
  
Location: 1147-1390

Mycgr3G103262\_Mycgr3

Mycgr3G68458 Mycgr3T
  
Location: 1490-3602

Mycgr3G68458\_Mycgr3T

Mycgr3G99145 Mycgr3T
  
Location: 3702-4326

Mycgr3G99145\_Mycgr3T

Mycgr3G103274 Mycgr3
  
Location: 4426-4957

Mycgr3G103274\_Mycgr3

Mycgr3G103264 Mycgr3
  
Location: 5057-5390

Mycgr3G103264\_Mycgr3

Mycgr3G37570 Mycgr3T
  
Location: 5490-6006

Mycgr3G37570\_Mycgr3T

Mycgr3G108094 Mycgr3
  
Location: 6106-10555

Mycgr3G108094\_Mycgr3

Mycgr3G90786 Mycgr3T
  
Location: 10655-12080

Mycgr3G90786\_Mycgr3T

Mycgr3G68429 Mycgr3T
  
Location: 12180-13440

Mycgr3G68429\_Mycgr3T

Mycgr3G68421 Mycgr3T
  
Location: 13540-17086

Mycgr3G68421\_Mycgr3T

Mycgr3G90801 Mycgr3T
  
Location: 17186-18056

Mycgr3G90801\_Mycgr3T

Mycgr3G84646 Mycgr3T
  
Location: 18156-20235

Mycgr3G84646\_Mycgr3T

Mycgr3G68456 Mycgr3T
  
Location: 20335-21970

Mycgr3G68456\_Mycgr3T

Mycgr3G103270 Mycgr3
  
Location: 22070-22355

Mycgr3G103270\_Mycgr3

Mycgr3G90803 Mycgr3T
  
Location: 22455-23019

Mycgr3G90803\_Mycgr3T

Mycgr3G36941 Mycgr3T
  
Location: 23119-24064

Mycgr3G36941\_Mycgr3T

Mycgr3G25746 Mycgr3T
  
Location: 24164-25241

Mycgr3G25746\_Mycgr3T

Mycgr3G90788 Mycgr3T
  
Location: 25341-25803

Mycgr3G90788\_Mycgr3T

Mycgr3G103260 Mycgr3
  
Location: 25903-26635

Mycgr3G103260\_Mycgr3

Mycgr3G84644 Mycgr3T
  
Location: 26735-28457

Mycgr3G84644\_Mycgr3T

Mycgr3G29227 Mycgr3T
  
Location: 28557-28863

Mycgr3G29227\_Mycgr3T

Mycgr3G36271 Mycgr3T
  
Location: 28963-29854

Mycgr3G36271\_Mycgr3T

Mycgr3G68433 Mycgr3T
  
Location: 29954-33041

Mycgr3G68433\_Mycgr3T

Mycgr3G79452 Mycgr3T
  
Location: 33141-33399

Mycgr3G79452\_Mycgr3T

Mycgr3G55345 Mycgr3T
  
Location: 33499-34126

Mycgr3G55345\_Mycgr3T

Mycgr3G103278 Mycgr3
  
Location: 34226-35195

Mycgr3G103278\_Mycgr3

Mycgr3G84654 Mycgr3T
  
Location: 35295-36630

Mycgr3G84654\_Mycgr3T

Mycgr3G108090 Mycgr3
  
Location: 36730-37591

Mycgr3G108090\_Mycgr3

Mycgr3G21922 Mycgr3T
  
Location: 37691-39149

Mycgr3G21922\_Mycgr3T

Mycgr3G99148 Mycgr3T
  
Location: 39249-42819

Mycgr3G99148\_Mycgr3T

not annotated
  
Accession: BAE65417
  
Location: 103211-105760
  
 NCBI BlastP on this gene

AO090206000045

not annotated
  
Accession: BAE65416
  
Location: 101848-102677
  
 NCBI BlastP on this gene

AO090206000044

not annotated
  
Accession: BAE65415
  
Location: 100010-101129
  
 NCBI BlastP on this gene

AO090206000043

not annotated
  
Accession: BAE65414
  
Location: 99018-99840
  
 NCBI BlastP on this gene

AO090206000042

not annotated
  
Accession: BAE65413
  
Location: 97141-97906
  
 NCBI BlastP on this gene

AO090206000041

not annotated
  
Accession: BAE65412
  
Location: 93835-95643
  
 NCBI BlastP on this gene

AO090206000040

not annotated
  
Accession: BAE65411
  
Location: 87506-89087
  
 NCBI BlastP on this gene

AO090206000038

not annotated
  
Accession: BAE65410
  
Location: 85830-86850
  
  
**BlastP hit with Mycgr3G55345\_Mycgr3T**
  
Percentage identity: 73 %
  
BlastP bit score: 303
  
Sequence coverage: 96 %
  
E-value: 3e-101
  
  
 NCBI BlastP on this gene

AO090206000037

not annotated
  
Accession: BAE65409
  
Location: 83185-83749
  
 NCBI BlastP on this gene

AO090206000034

not annotated
  
Accession: BAE65408
  
Location: 81680-82777
  
 NCBI BlastP on this gene

AO090206000033

not annotated
  
Accession: BAE65407
  
Location: 80130-81535
  
 NCBI BlastP on this gene

AO090206000032

not annotated
  
Accession: BAE65406
  
Location: 76525-79328
  
  
**BlastP hit with Mycgr3G68458\_Mycgr3T**
  
Percentage identity: 67 %
  
BlastP bit score: 775
  
Sequence coverage: 78 %
  
E-value: 0.0
  
  
 NCBI BlastP on this gene

AO090206000031

not annotated
  
Accession: BAE65405
  
Location: 75321-76417
  
 NCBI BlastP on this gene

AO090206000030

not annotated
  
Accession: BAE65404
  
Location: 72561-74456
  
 NCBI BlastP on this gene

AO090206000029

not annotated
  
Accession: BAE65403
  
Location: 68846-69793
  
 NCBI BlastP on this gene

AO090206000028

not annotated
  
Accession: BAE65402
  
Location: 66745-68550
  
 NCBI BlastP on this gene

AO090206000027

not annotated
  
Accession: BAE65401
  
Location: 65385-66390
  
 NCBI BlastP on this gene

AO090206000026

not annotated
  
Accession: BAE65400
  
Location: 62598-64994
  
 NCBI BlastP on this gene

AO090206000025

not annotated
  
Accession: BAE65399
  
Location: 58506-60107
  
 NCBI BlastP on this gene

AO090206000024

Query: Architecture Search FASTA input

CP002686 : Arabidopsis thaliana chromosome 3    Total score: 2.0     Cumulative Blast bit score: 1076

Hit cluster cross-links:

Mycgr3G90785 Mycgr3T
  
Location: 0-1047

Mycgr3G90785\_Mycgr3T

Mycgr3G103262 Mycgr3
  
Location: 1147-1390

Mycgr3G103262\_Mycgr3

Mycgr3G68458 Mycgr3T
  
Location: 1490-3602

Mycgr3G68458\_Mycgr3T

Mycgr3G99145 Mycgr3T
  
Location: 3702-4326

Mycgr3G99145\_Mycgr3T

Mycgr3G103274 Mycgr3
  
Location: 4426-4957

Mycgr3G103274\_Mycgr3

Mycgr3G103264 Mycgr3
  
Location: 5057-5390

Mycgr3G103264\_Mycgr3

Mycgr3G37570 Mycgr3T
  
Location: 5490-6006

Mycgr3G37570\_Mycgr3T

Mycgr3G108094 Mycgr3
  
Location: 6106-10555

Mycgr3G108094\_Mycgr3

Mycgr3G90786 Mycgr3T
  
Location: 10655-12080

Mycgr3G90786\_Mycgr3T

Mycgr3G68429 Mycgr3T
  
Location: 12180-13440

Mycgr3G68429\_Mycgr3T

Mycgr3G68421 Mycgr3T
  
Location: 13540-17086

Mycgr3G68421\_Mycgr3T

Mycgr3G90801 Mycgr3T
  
Location: 17186-18056

Mycgr3G90801\_Mycgr3T

Mycgr3G84646 Mycgr3T
  
Location: 18156-20235

Mycgr3G84646\_Mycgr3T

Mycgr3G68456 Mycgr3T
  
Location: 20335-21970

Mycgr3G68456\_Mycgr3T

Mycgr3G103270 Mycgr3
  
Location: 22070-22355

Mycgr3G103270\_Mycgr3

Mycgr3G90803 Mycgr3T
  
Location: 22455-23019

Mycgr3G90803\_Mycgr3T

Mycgr3G36941 Mycgr3T
  
Location: 23119-24064

Mycgr3G36941\_Mycgr3T

Mycgr3G25746 Mycgr3T
  
Location: 24164-25241

Mycgr3G25746\_Mycgr3T

Mycgr3G90788 Mycgr3T
  
Location: 25341-25803

Mycgr3G90788\_Mycgr3T

Mycgr3G103260 Mycgr3
  
Location: 25903-26635

Mycgr3G103260\_Mycgr3

Mycgr3G84644 Mycgr3T
  
Location: 26735-28457

Mycgr3G84644\_Mycgr3T

Mycgr3G29227 Mycgr3T
  
Location: 28557-28863

Mycgr3G29227\_Mycgr3T

Mycgr3G36271 Mycgr3T
  
Location: 28963-29854

Mycgr3G36271\_Mycgr3T

Mycgr3G68433 Mycgr3T
  
Location: 29954-33041

Mycgr3G68433\_Mycgr3T

Mycgr3G79452 Mycgr3T
  
Location: 33141-33399

Mycgr3G79452\_Mycgr3T

Mycgr3G55345 Mycgr3T
  
Location: 33499-34126

Mycgr3G55345\_Mycgr3T

Mycgr3G103278 Mycgr3
  
Location: 34226-35195

Mycgr3G103278\_Mycgr3

Mycgr3G84654 Mycgr3T
  
Location: 35295-36630

Mycgr3G84654\_Mycgr3T

Mycgr3G108090 Mycgr3
  
Location: 36730-37591

Mycgr3G108090\_Mycgr3

Mycgr3G21922 Mycgr3T
  
Location: 37691-39149

Mycgr3G21922\_Mycgr3T

Mycgr3G99148 Mycgr3T
  
Location: 39249-42819

Mycgr3G99148\_Mycgr3T

cation/H(+) antiporter 20
  
Accession: AEE79132
  
Location: 19905826-19910027
  
 NCBI BlastP on this gene

CHX20

putative ADP-ribosylation factor GTPase-activating protein AGD6
  
Accession: AEE79131
  
Location: 19903730-19905419
  
 NCBI BlastP on this gene

AGD6

putative ADP-ribosylation factor GTPase-activating protein AGD6
  
Accession: AEE79130
  
Location: 19903730-19905419
  
 NCBI BlastP on this gene

AGD6

pentatricopeptide repeat-containing protein
  
Accession: AEE79129
  
Location: 19900303-19902567
  
 NCBI BlastP on this gene

MEE40

RING/U-box superfamily protein
  
Accession: AEE79128
  
Location: 19898997-19900044
  
 NCBI BlastP on this gene

AT3G53690

Acyl-CoA N-acyltransferase with
  
Accession: AEE79127
  
Location: 19892863-19897412
  
 NCBI BlastP on this gene

AT3G53680

uncharacterized protein
  
Accession: AEE79125
  
Location: 19891104-19892214
  
 NCBI BlastP on this gene

AT3G53670

uncharacterized protein
  
Accession: AEE79124
  
Location: 19891104-19892214
  
 NCBI BlastP on this gene

AT3G53670

conserved peptide upstream open reading frame 51
  
Accession: AEE79126
  
Location: 19890708-19890833
  
 NCBI BlastP on this gene

CPuORF51

histone H2B
  
Accession: AEE79123
  
Location: 19889358-19889774
  
 NCBI BlastP on this gene

AT3G53650

protein kinase family protein
  
Accession: AEE79122
  
Location: 19887007-19888935
  
  
**BlastP hit with Mycgr3G103260\_Mycgr3**
  
Percentage identity: 41 %
  
BlastP bit score: 203
  
Sequence coverage: 96 %
  
E-value: 3e-57
  
  
 NCBI BlastP on this gene

AT3G53640

uncharacterized protein
  
Accession: AEE79121
  
Location: 19883986-19885568
  
 NCBI BlastP on this gene

AT3G53630

pyrophosphorylase 4
  
Accession: AEE79120
  
Location: 19881491-19883308
  
 NCBI BlastP on this gene

PPa4

uncharacterized protein
  
Accession: AEE79119
  
Location: 19878542-19878694
  
 NCBI BlastP on this gene

AT3G53611

RAB GTPase-8
  
Accession: AEE79118
  
Location: 19876531-19878264
  
  
**BlastP hit with Mycgr3G99145\_Mycgr3T**
  
Percentage identity: 70 %
  
BlastP bit score: 291
  
Sequence coverage: 97 %
  
E-value: 1e-96
  
  
 NCBI BlastP on this gene

RAB8

RAB GTPase-8
  
Accession: AEE79117
  
Location: 19876531-19878264
  
  
**BlastP hit with Mycgr3G99145\_Mycgr3T**
  
Percentage identity: 70 %
  
BlastP bit score: 291
  
Sequence coverage: 97 %
  
E-value: 1e-96
  
  
 NCBI BlastP on this gene

RAB8

RAB GTPase-8
  
Accession: AEE79116
  
Location: 19876531-19878264
  
  
**BlastP hit with Mycgr3G99145\_Mycgr3T**
  
Percentage identity: 70 %
  
BlastP bit score: 291
  
Sequence coverage: 97 %
  
E-value: 1e-96
  
  
 NCBI BlastP on this gene

RAB8

C2H2-type zinc finger protein
  
Accession: AEE79115
  
Location: 19875532-19876059
  
 NCBI BlastP on this gene

AT3G53600

putative leucine-rich repeat receptor-like
  
Accession: AEE79114
  
Location: 19867379-19871651
  
 NCBI BlastP on this gene

AT3G53590

diaminopimelate epimerase
  
Accession: AEE79113
  
Location: 19864784-19866907
  
 NCBI BlastP on this gene

AT3G53580

serine/threonine-protein kinase AFC1
  
Accession: AEE79112
  
Location: 19861449-19864125
  
 NCBI BlastP on this gene

FC1

serine/threonine-protein kinase AFC1
  
Accession: AEE79111
  
Location: 19861449-19864125
  
 NCBI BlastP on this gene

FC1

serine/threonine-protein kinase AFC1
  
Accession: AEE79110
  
Location: 19861449-19864125
  
 NCBI BlastP on this gene

FC1

serine/threonine-protein kinase AFC1
  
Accession: AEE79109
  
Location: 19861449-19864083
  
 NCBI BlastP on this gene

FC1

tetratricopeptide repeat domain-containing protein
  
Accession: AEE79108
  
Location: 19859954-19860976
  
 NCBI BlastP on this gene

AT3G53560

Query: Architecture Search FASTA input

KE145352 : Glarea lozoyensis ATCC 20868 chromosome Unknown GLAREA1    Total score: 2.0     Cumulative Blast bit score: 1073

Hit cluster cross-links:

Mycgr3G90785 Mycgr3T
  
Location: 0-1047

Mycgr3G90785\_Mycgr3T

Mycgr3G103262 Mycgr3
  
Location: 1147-1390

Mycgr3G103262\_Mycgr3

Mycgr3G68458 Mycgr3T
  
Location: 1490-3602

Mycgr3G68458\_Mycgr3T

Mycgr3G99145 Mycgr3T
  
Location: 3702-4326

Mycgr3G99145\_Mycgr3T

Mycgr3G103274 Mycgr3
  
Location: 4426-4957

Mycgr3G103274\_Mycgr3

Mycgr3G103264 Mycgr3
  
Location: 5057-5390

Mycgr3G103264\_Mycgr3

Mycgr3G37570 Mycgr3T
  
Location: 5490-6006

Mycgr3G37570\_Mycgr3T

Mycgr3G108094 Mycgr3
  
Location: 6106-10555

Mycgr3G108094\_Mycgr3

Mycgr3G90786 Mycgr3T
  
Location: 10655-12080

Mycgr3G90786\_Mycgr3T

Mycgr3G68429 Mycgr3T
  
Location: 12180-13440

Mycgr3G68429\_Mycgr3T

Mycgr3G68421 Mycgr3T
  
Location: 13540-17086

Mycgr3G68421\_Mycgr3T

Mycgr3G90801 Mycgr3T
  
Location: 17186-18056

Mycgr3G90801\_Mycgr3T

Mycgr3G84646 Mycgr3T
  
Location: 18156-20235

Mycgr3G84646\_Mycgr3T

Mycgr3G68456 Mycgr3T
  
Location: 20335-21970

Mycgr3G68456\_Mycgr3T

Mycgr3G103270 Mycgr3
  
Location: 22070-22355

Mycgr3G103270\_Mycgr3

Mycgr3G90803 Mycgr3T
  
Location: 22455-23019

Mycgr3G90803\_Mycgr3T

Mycgr3G36941 Mycgr3T
  
Location: 23119-24064

Mycgr3G36941\_Mycgr3T

Mycgr3G25746 Mycgr3T
  
Location: 24164-25241

Mycgr3G25746\_Mycgr3T

Mycgr3G90788 Mycgr3T
  
Location: 25341-25803

Mycgr3G90788\_Mycgr3T

Mycgr3G103260 Mycgr3
  
Location: 25903-26635

Mycgr3G103260\_Mycgr3

Mycgr3G84644 Mycgr3T
  
Location: 26735-28457

Mycgr3G84644\_Mycgr3T

Mycgr3G29227 Mycgr3T
  
Location: 28557-28863

Mycgr3G29227\_Mycgr3T

Mycgr3G36271 Mycgr3T
  
Location: 28963-29854

Mycgr3G36271\_Mycgr3T

Mycgr3G68433 Mycgr3T
  
Location: 29954-33041

Mycgr3G68433\_Mycgr3T

Mycgr3G79452 Mycgr3T
  
Location: 33141-33399

Mycgr3G79452\_Mycgr3T

Mycgr3G55345 Mycgr3T
  
Location: 33499-34126

Mycgr3G55345\_Mycgr3T

Mycgr3G103278 Mycgr3
  
Location: 34226-35195

Mycgr3G103278\_Mycgr3

Mycgr3G84654 Mycgr3T
  
Location: 35295-36630

Mycgr3G84654\_Mycgr3T

Mycgr3G108090 Mycgr3
  
Location: 36730-37591

Mycgr3G108090\_Mycgr3

Mycgr3G21922 Mycgr3T
  
Location: 37691-39149

Mycgr3G21922\_Mycgr3T

Mycgr3G99148 Mycgr3T
  
Location: 39249-42819

Mycgr3G99148\_Mycgr3T

FAD/NAD(P)-binding protein
  
Accession: EPE37030
  
Location: 1648002-1649477
  
 NCBI BlastP on this gene

EPE37030

S-adenosyl-L-methionine-dependent methyltransferase
  
Accession: EPE37031
  
Location: 1650883-1652341
  
 NCBI BlastP on this gene

EPE37031

hypothetical protein
  
Accession: EPE37032
  
Location: 1652598-1654256
  
 NCBI BlastP on this gene

EPE37032

hypothetical protein
  
Accession: EPE37033
  
Location: 1656460-1657691
  
 NCBI BlastP on this gene

EPE37033

Prefoldin
  
Accession: EPE37034
  
Location: 1658120-1661586
  
  
**BlastP hit with Mycgr3G108094\_Mycgr3**
  
Percentage identity: 36 %
  
BlastP bit score: 295
  
Sequence coverage: 44 %
  
E-value: 3e-79
  
  
 NCBI BlastP on this gene

EPE37034

Protein kinase-like (PK-like)
  
Accession: EPE37035
  
Location: 1663145-1665302
  
  
**BlastP hit with Mycgr3G84644\_Mycgr3T**
  
Percentage identity: 70 %
  
BlastP bit score: 778
  
Sequence coverage: 101 %
  
E-value: 0.0
  
  
 NCBI BlastP on this gene

EPE37035

hypothetical protein
  
Accession: EPE37036
  
Location: 1666341-1667707
  
 NCBI BlastP on this gene

EPE37036

hypothetical protein
  
Accession: EPE37037
  
Location: 1668170-1669599
  
 NCBI BlastP on this gene

EPE37037

HLH, helix-loop-helix DNA-binding protein
  
Accession: EPE37038
  
Location: 1670550-1672004
  
 NCBI BlastP on this gene

EPE37038

Zn2/Cys6 DNA-binding protein
  
Accession: EPE37039
  
Location: 1674031-1676578
  
 NCBI BlastP on this gene

EPE37039

Cupredoxin
  
Accession: EPE37040
  
Location: 1677904-1679991
  
 NCBI BlastP on this gene

EPE37040

non-hemolytic phospholipase C precursor, putative
  
Accession: EPE37041
  
Location: 1680129-1683300
  
 NCBI BlastP on this gene

EPE37041

hypothetical protein
  
Accession: EPE37042
  
Location: 1684175-1685135
  
 NCBI BlastP on this gene

EPE37042

Query: Architecture Search FASTA input

DS995737 : Trichophyton equinum CBS 127.97 supercont1.20 genomic scaffold    Total score: 2.0     Cumulative Blast bit score: 1073

Hit cluster cross-links:

Mycgr3G90785 Mycgr3T
  
Location: 0-1047

Mycgr3G90785\_Mycgr3T

Mycgr3G103262 Mycgr3
  
Location: 1147-1390

Mycgr3G103262\_Mycgr3

Mycgr3G68458 Mycgr3T
  
Location: 1490-3602

Mycgr3G68458\_Mycgr3T

Mycgr3G99145 Mycgr3T
  
Location: 3702-4326

Mycgr3G99145\_Mycgr3T

Mycgr3G103274 Mycgr3
  
Location: 4426-4957

Mycgr3G103274\_Mycgr3

Mycgr3G103264 Mycgr3
  
Location: 5057-5390

Mycgr3G103264\_Mycgr3

Mycgr3G37570 Mycgr3T
  
Location: 5490-6006

Mycgr3G37570\_Mycgr3T

Mycgr3G108094 Mycgr3
  
Location: 6106-10555

Mycgr3G108094\_Mycgr3

Mycgr3G90786 Mycgr3T
  
Location: 10655-12080

Mycgr3G90786\_Mycgr3T

Mycgr3G68429 Mycgr3T
  
Location: 12180-13440

Mycgr3G68429\_Mycgr3T

Mycgr3G68421 Mycgr3T
  
Location: 13540-17086

Mycgr3G68421\_Mycgr3T

Mycgr3G90801 Mycgr3T
  
Location: 17186-18056

Mycgr3G90801\_Mycgr3T

Mycgr3G84646 Mycgr3T
  
Location: 18156-20235

Mycgr3G84646\_Mycgr3T

Mycgr3G68456 Mycgr3T
  
Location: 20335-21970

Mycgr3G68456\_Mycgr3T

Mycgr3G103270 Mycgr3
  
Location: 22070-22355

Mycgr3G103270\_Mycgr3

Mycgr3G90803 Mycgr3T
  
Location: 22455-23019

Mycgr3G90803\_Mycgr3T

Mycgr3G36941 Mycgr3T
  
Location: 23119-24064

Mycgr3G36941\_Mycgr3T

Mycgr3G25746 Mycgr3T
  
Location: 24164-25241

Mycgr3G25746\_Mycgr3T

Mycgr3G90788 Mycgr3T
  
Location: 25341-25803

Mycgr3G90788\_Mycgr3T

Mycgr3G103260 Mycgr3
  
Location: 25903-26635

Mycgr3G103260\_Mycgr3

Mycgr3G84644 Mycgr3T
  
Location: 26735-28457

Mycgr3G84644\_Mycgr3T

Mycgr3G29227 Mycgr3T
  
Location: 28557-28863

Mycgr3G29227\_Mycgr3T

Mycgr3G36271 Mycgr3T
  
Location: 28963-29854

Mycgr3G36271\_Mycgr3T

Mycgr3G68433 Mycgr3T
  
Location: 29954-33041

Mycgr3G68433\_Mycgr3T

Mycgr3G79452 Mycgr3T
  
Location: 33141-33399

Mycgr3G79452\_Mycgr3T

Mycgr3G55345 Mycgr3T
  
Location: 33499-34126

Mycgr3G55345\_Mycgr3T

Mycgr3G103278 Mycgr3
  
Location: 34226-35195

Mycgr3G103278\_Mycgr3

Mycgr3G84654 Mycgr3T
  
Location: 35295-36630

Mycgr3G84654\_Mycgr3T

Mycgr3G108090 Mycgr3
  
Location: 36730-37591

Mycgr3G108090\_Mycgr3

Mycgr3G21922 Mycgr3T
  
Location: 37691-39149

Mycgr3G21922\_Mycgr3T

Mycgr3G99148 Mycgr3T
  
Location: 39249-42819

Mycgr3G99148\_Mycgr3T

hypothetical protein
  
Accession: EGE05061
  
Location: 96646-97962
  
 NCBI BlastP on this gene

EGE05061

ras-like GTP-binding protein
  
Accession: EGE05062
  
Location: 99169-99751
  
 NCBI BlastP on this gene

EGE05062

cytochrome b2
  
Accession: EGE05063
  
Location: 100538-102177
  
 NCBI BlastP on this gene

EGE05063

phosphoric ester hydrolase
  
Accession: EGE05064
  
Location: 102342-103395
  
 NCBI BlastP on this gene

EGE05064

CMGC/SRPK protein kinase
  
Accession: EGE05065
  
Location: 104246-106272
  
  
**BlastP hit with Mycgr3G84644\_Mycgr3T**
  
Percentage identity: 68 %
  
BlastP bit score: 735
  
Sequence coverage: 98 %
  
E-value: 0.0
  
  
 NCBI BlastP on this gene

EGE05065

pre-mRNA-splicing factor cwc-26
  
Accession: EGE05066
  
Location: 107189-108167
  
 NCBI BlastP on this gene

EGE05066

hypothetical protein
  
Accession: EGE05067
  
Location: 108966-109898
  
 NCBI BlastP on this gene

EGE05067

MGMT family protein
  
Accession: EGE05068
  
Location: 110501-111095
  
 NCBI BlastP on this gene

EGE05068

hypothetical protein
  
Accession: EGE05069
  
Location: 111740-112313
  
 NCBI BlastP on this gene

EGE05069

hypothetical protein
  
Accession: EGE05070
  
Location: 113280-113895
  
 NCBI BlastP on this gene

EGE05070

hypothetical protein
  
Accession: EGE05071
  
Location: 115621-117775
  
 NCBI BlastP on this gene

EGE05071

nuclear envelope protein Brr6
  
Accession: EGE05072
  
Location: 118700-120188
  
 NCBI BlastP on this gene

EGE05072

amino acid permease
  
Accession: EGE05073
  
Location: 120561-122623
  
 NCBI BlastP on this gene

EGE05073

GABA-specific permease
  
Accession: EGE05074
  
Location: 123441-125538
  
 NCBI BlastP on this gene

EGE05074

integral membrane protein
  
Accession: EGE05075
  
Location: 127169-128504
  
 NCBI BlastP on this gene

EGE05075

hypothetical protein
  
Accession: EGE05076
  
Location: 130085-130679
  
 NCBI BlastP on this gene

EGE05076

hypothetical protein
  
Accession: EGE05077
  
Location: 131142-134753
  
  
**BlastP hit with Mycgr3G108094\_Mycgr3**
  
Percentage identity: 40 %
  
BlastP bit score: 338
  
Sequence coverage: 38 %
  
E-value: 2e-93
  
  
 NCBI BlastP on this gene

EGE05077

hypothetical protein
  
Accession: EGE05078
  
Location: 135277-136658
  
 NCBI BlastP on this gene

EGE05078

patatin-like phospholipase
  
Accession: EGE05079
  
Location: 137029-141580
  
 NCBI BlastP on this gene

EGE05079

sarcosine oxidase
  
Accession: EGE05080
  
Location: 143045-144500
  
 NCBI BlastP on this gene

EGE05080

Query: Architecture Search FASTA input

GG698482 : Trichophyton tonsurans CBS 112818 genomic scaffold supercont1.6    Total score: 2.0     Cumulative Blast bit score: 1069

Hit cluster cross-links:

Mycgr3G90785 Mycgr3T
  
Location: 0-1047

Mycgr3G90785\_Mycgr3T

Mycgr3G103262 Mycgr3
  
Location: 1147-1390

Mycgr3G103262\_Mycgr3

Mycgr3G68458 Mycgr3T
  
Location: 1490-3602

Mycgr3G68458\_Mycgr3T

Mycgr3G99145 Mycgr3T
  
Location: 3702-4326

Mycgr3G99145\_Mycgr3T

Mycgr3G103274 Mycgr3
  
Location: 4426-4957

Mycgr3G103274\_Mycgr3

Mycgr3G103264 Mycgr3
  
Location: 5057-5390

Mycgr3G103264\_Mycgr3

Mycgr3G37570 Mycgr3T
  
Location: 5490-6006

Mycgr3G37570\_Mycgr3T

Mycgr3G108094 Mycgr3
  
Location: 6106-10555

Mycgr3G108094\_Mycgr3

Mycgr3G90786 Mycgr3T
  
Location: 10655-12080

Mycgr3G90786\_Mycgr3T

Mycgr3G68429 Mycgr3T
  
Location: 12180-13440

Mycgr3G68429\_Mycgr3T

Mycgr3G68421 Mycgr3T
  
Location: 13540-17086

Mycgr3G68421\_Mycgr3T

Mycgr3G90801 Mycgr3T
  
Location: 17186-18056

Mycgr3G90801\_Mycgr3T

Mycgr3G84646 Mycgr3T
  
Location: 18156-20235

Mycgr3G84646\_Mycgr3T

Mycgr3G68456 Mycgr3T
  
Location: 20335-21970

Mycgr3G68456\_Mycgr3T

Mycgr3G103270 Mycgr3
  
Location: 22070-22355

Mycgr3G103270\_Mycgr3

Mycgr3G90803 Mycgr3T
  
Location: 22455-23019

Mycgr3G90803\_Mycgr3T

Mycgr3G36941 Mycgr3T
  
Location: 23119-24064

Mycgr3G36941\_Mycgr3T

Mycgr3G25746 Mycgr3T
  
Location: 24164-25241

Mycgr3G25746\_Mycgr3T

Mycgr3G90788 Mycgr3T
  
Location: 25341-25803

Mycgr3G90788\_Mycgr3T

Mycgr3G103260 Mycgr3
  
Location: 25903-26635

Mycgr3G103260\_Mycgr3

Mycgr3G84644 Mycgr3T
  
Location: 26735-28457

Mycgr3G84644\_Mycgr3T

Mycgr3G29227 Mycgr3T
  
Location: 28557-28863

Mycgr3G29227\_Mycgr3T

Mycgr3G36271 Mycgr3T
  
Location: 28963-29854

Mycgr3G36271\_Mycgr3T

Mycgr3G68433 Mycgr3T
  
Location: 29954-33041

Mycgr3G68433\_Mycgr3T

Mycgr3G79452 Mycgr3T
  
Location: 33141-33399

Mycgr3G79452\_Mycgr3T

Mycgr3G55345 Mycgr3T
  
Location: 33499-34126

Mycgr3G55345\_Mycgr3T

Mycgr3G103278 Mycgr3
  
Location: 34226-35195

Mycgr3G103278\_Mycgr3

Mycgr3G84654 Mycgr3T
  
Location: 35295-36630

Mycgr3G84654\_Mycgr3T

Mycgr3G108090 Mycgr3
  
Location: 36730-37591

Mycgr3G108090\_Mycgr3

Mycgr3G21922 Mycgr3T
  
Location: 37691-39149

Mycgr3G21922\_Mycgr3T

Mycgr3G99148 Mycgr3T
  
Location: 39249-42819

Mycgr3G99148\_Mycgr3T

CMGC/CLK protein kinase
  
Accession: EGD94221
  
Location: 377119-378425
  
 NCBI BlastP on this gene

EGD94221

MFS transporter
  
Accession: EGD94222
  
Location: 379113-380684
  
 NCBI BlastP on this gene

EGD94222

GTP-binding protein yptV5
  
Accession: EGD94223
  
Location: 381047-382118
  
 NCBI BlastP on this gene

EGD94223

mitochondrial cytochrome b2
  
Accession: EGD94224
  
Location: 382880-384519
  
 NCBI BlastP on this gene

EGD94224

hypothetical protein
  
Accession: EGD94225
  
Location: 384684-385736
  
 NCBI BlastP on this gene

EGD94225

CMGC/SRPK protein kinase
  
Accession: EGD94226
  
Location: 386544-388570
  
  
**BlastP hit with Mycgr3G84644\_Mycgr3T**
  
Percentage identity: 68 %
  
BlastP bit score: 732
  
Sequence coverage: 98 %
  
E-value: 0.0
  
  
 NCBI BlastP on this gene

EGD94226

hypothetical protein
  
Accession: EGD94227
  
Location: 389406-390482
  
 NCBI BlastP on this gene

EGD94227

hypothetical protein
  
Accession: EGD94228
  
Location: 391281-392213
  
 NCBI BlastP on this gene

EGD94228

MGMT family protein
  
Accession: EGD94229
  
Location: 392799-393393
  
 NCBI BlastP on this gene

EGD94229

hypothetical protein
  
Accession: EGD94230
  
Location: 394036-394446
  
 NCBI BlastP on this gene

EGD94230

hypothetical protein
  
Accession: EGD94231
  
Location: 395576-396191
  
 NCBI BlastP on this gene

EGD94231

hypothetical protein
  
Accession: EGD94232
  
Location: 397491-397929
  
 NCBI BlastP on this gene

EGD94232

hypothetical protein
  
Accession: EGD94233
  
Location: 397867-400021
  
 NCBI BlastP on this gene

EGD94233

hypothetical protein
  
Accession: EGD94234
  
Location: 400950-402438
  
 NCBI BlastP on this gene

EGD94234

amino acid permease
  
Accession: EGD94235
  
Location: 402840-404877
  
 NCBI BlastP on this gene

EGD94235

GABA permease
  
Accession: EGD94236
  
Location: 405697-407794
  
 NCBI BlastP on this gene

EGD94236

hypothetical protein
  
Accession: EGD94237
  
Location: 409450-410785
  
 NCBI BlastP on this gene

EGD94237

acetyltransferase
  
Accession: EGD94238
  
Location: 412383-412977
  
 NCBI BlastP on this gene

EGD94238

viral A-type inclusion protein repeat protein
  
Accession: EGD94239
  
Location: 413440-417051
  
  
**BlastP hit with Mycgr3G108094\_Mycgr3**
  
Percentage identity: 41 %
  
BlastP bit score: 337
  
Sequence coverage: 38 %
  
E-value: 4e-93
  
  
 NCBI BlastP on this gene

EGD94239

hypothetical protein
  
Accession: EGD94240
  
Location: 417575-418956
  
 NCBI BlastP on this gene

EGD94240

hypothetical protein
  
Accession: EGD94241
  
Location: 419325-423873
  
 NCBI BlastP on this gene

EGD94241

hypothetical protein
  
Accession: EGD94242
  
Location: 425337-426792
  
 NCBI BlastP on this gene

EGD94242

Query: Architecture Search FASTA input

JH921446 : Marssonina brunnea f. sp. 'multigermtubi' MB\_m1 unplaced genomic scaffold M6\_S00019    Total score: 2.0     Cumulative Blast bit score: 1047

Hit cluster cross-links:

Mycgr3G90785 Mycgr3T
  
Location: 0-1047

Mycgr3G90785\_Mycgr3T

Mycgr3G103262 Mycgr3
  
Location: 1147-1390

Mycgr3G103262\_Mycgr3

Mycgr3G68458 Mycgr3T
  
Location: 1490-3602

Mycgr3G68458\_Mycgr3T

Mycgr3G99145 Mycgr3T
  
Location: 3702-4326

Mycgr3G99145\_Mycgr3T

Mycgr3G103274 Mycgr3
  
Location: 4426-4957

Mycgr3G103274\_Mycgr3

Mycgr3G103264 Mycgr3
  
Location: 5057-5390

Mycgr3G103264\_Mycgr3

Mycgr3G37570 Mycgr3T
  
Location: 5490-6006

Mycgr3G37570\_Mycgr3T

Mycgr3G108094 Mycgr3
  
Location: 6106-10555

Mycgr3G108094\_Mycgr3

Mycgr3G90786 Mycgr3T
  
Location: 10655-12080

Mycgr3G90786\_Mycgr3T

Mycgr3G68429 Mycgr3T
  
Location: 12180-13440

Mycgr3G68429\_Mycgr3T

Mycgr3G68421 Mycgr3T
  
Location: 13540-17086

Mycgr3G68421\_Mycgr3T

Mycgr3G90801 Mycgr3T
  
Location: 17186-18056

Mycgr3G90801\_Mycgr3T

Mycgr3G84646 Mycgr3T
  
Location: 18156-20235

Mycgr3G84646\_Mycgr3T

Mycgr3G68456 Mycgr3T
  
Location: 20335-21970

Mycgr3G68456\_Mycgr3T

Mycgr3G103270 Mycgr3
  
Location: 22070-22355

Mycgr3G103270\_Mycgr3

Mycgr3G90803 Mycgr3T
  
Location: 22455-23019

Mycgr3G90803\_Mycgr3T

Mycgr3G36941 Mycgr3T
  
Location: 23119-24064

Mycgr3G36941\_Mycgr3T

Mycgr3G25746 Mycgr3T
  
Location: 24164-25241

Mycgr3G25746\_Mycgr3T

Mycgr3G90788 Mycgr3T
  
Location: 25341-25803

Mycgr3G90788\_Mycgr3T

Mycgr3G103260 Mycgr3
  
Location: 25903-26635

Mycgr3G103260\_Mycgr3

Mycgr3G84644 Mycgr3T
  
Location: 26735-28457

Mycgr3G84644\_Mycgr3T

Mycgr3G29227 Mycgr3T
  
Location: 28557-28863

Mycgr3G29227\_Mycgr3T

Mycgr3G36271 Mycgr3T
  
Location: 28963-29854

Mycgr3G36271\_Mycgr3T

Mycgr3G68433 Mycgr3T
  
Location: 29954-33041

Mycgr3G68433\_Mycgr3T

Mycgr3G79452 Mycgr3T
  
Location: 33141-33399

Mycgr3G79452\_Mycgr3T

Mycgr3G55345 Mycgr3T
  
Location: 33499-34126

Mycgr3G55345\_Mycgr3T

Mycgr3G103278 Mycgr3
  
Location: 34226-35195

Mycgr3G103278\_Mycgr3

Mycgr3G84654 Mycgr3T
  
Location: 35295-36630

Mycgr3G84654\_Mycgr3T

Mycgr3G108090 Mycgr3
  
Location: 36730-37591

Mycgr3G108090\_Mycgr3

Mycgr3G21922 Mycgr3T
  
Location: 37691-39149

Mycgr3G21922\_Mycgr3T

Mycgr3G99148 Mycgr3T
  
Location: 39249-42819

Mycgr3G99148\_Mycgr3T

hypothetical protein
  
Accession: EKD14353
  
Location: 821932-822588
  
 NCBI BlastP on this gene

EKD14353

hypothetical protein
  
Accession: EKD14352
  
Location: 819204-820400
  
 NCBI BlastP on this gene

EKD14352

hypothetical protein
  
Accession: EKD14351
  
Location: 814859-816402
  
 NCBI BlastP on this gene

EKD14351

putative Protein lunapark-B
  
Accession: EKD14350
  
Location: 813552-814826
  
 NCBI BlastP on this gene

EKD14350

viral A-type inclusion protein repeat protein
  
Accession: EKD14349
  
Location: 809723-813150
  
  
**BlastP hit with Mycgr3G108094\_Mycgr3**
  
Percentage identity: 34 %
  
BlastP bit score: 281
  
Sequence coverage: 44 %
  
E-value: 8e-75
  
  
 NCBI BlastP on this gene

EKD14349

isoprenylcysteine carboxyl methyltransferase
  
Accession: EKD14348
  
Location: 804611-805300
  
 NCBI BlastP on this gene

EKD14348

DSHCT domain-containing protein
  
Accession: EKD14347
  
Location: 800432-804373
  
 NCBI BlastP on this gene

EKD14347

replication fork protection component Swi3
  
Accession: EKD14346
  
Location: 798929-800124
  
 NCBI BlastP on this gene

EKD14346

putative NADH-ubiquinone oxidoreductase 21 kDa subunit
  
Accession: EKD14345
  
Location: 797923-798651
  
 NCBI BlastP on this gene

EKD14345

tRNA-specific adenosine deaminase subunit TAD2
  
Accession: EKD14344
  
Location: 796762-797685
  
 NCBI BlastP on this gene

EKD14344

hypothetical protein
  
Accession: EKD14343
  
Location: 793402-795946
  
 NCBI BlastP on this gene

EKD14343

hypothetical protein
  
Accession: EKD14342
  
Location: 791469-792236
  
 NCBI BlastP on this gene

EKD14342

serine/threonine-protein kinase SRPK2
  
Accession: EKD14341
  
Location: 786637-788953
  
  
**BlastP hit with Mycgr3G84644\_Mycgr3T**
  
Percentage identity: 67 %
  
BlastP bit score: 766
  
Sequence coverage: 101 %
  
E-value: 0.0
  
  
 NCBI BlastP on this gene

EKD14341

nuclear membrane protein
  
Accession: EKD14340
  
Location: 783687-785155
  
 NCBI BlastP on this gene

EKD14340

transcription factor IWS1
  
Accession: EKD14339
  
Location: 781974-783376
  
 NCBI BlastP on this gene

EKD14339

hypothetical protein
  
Accession: EKD14338
  
Location: 779884-780875
  
 NCBI BlastP on this gene

EKD14338

homoserine O-acetyltransferase
  
Accession: EKD14337
  
Location: 777091-778659
  
 NCBI BlastP on this gene

EKD14337

hypothetical protein
  
Accession: EKD14336
  
Location: 775536-776717
  
 NCBI BlastP on this gene

EKD14336

Query: Architecture Search FASTA input

AM920428 : Penicillium chrysogenum Wisconsin 54-1255 complete genome, contig Pc00c13.    Total score: 2.0     Cumulative Blast bit score: 1047

Hit cluster cross-links:

Mycgr3G90785 Mycgr3T
  
Location: 0-1047

Mycgr3G90785\_Mycgr3T

Mycgr3G103262 Mycgr3
  
Location: 1147-1390

Mycgr3G103262\_Mycgr3

Mycgr3G68458 Mycgr3T
  
Location: 1490-3602

Mycgr3G68458\_Mycgr3T

Mycgr3G99145 Mycgr3T
  
Location: 3702-4326

Mycgr3G99145\_Mycgr3T

Mycgr3G103274 Mycgr3
  
Location: 4426-4957

Mycgr3G103274\_Mycgr3

Mycgr3G103264 Mycgr3
  
Location: 5057-5390

Mycgr3G103264\_Mycgr3

Mycgr3G37570 Mycgr3T
  
Location: 5490-6006

Mycgr3G37570\_Mycgr3T

Mycgr3G108094 Mycgr3
  
Location: 6106-10555

Mycgr3G108094\_Mycgr3

Mycgr3G90786 Mycgr3T
  
Location: 10655-12080

Mycgr3G90786\_Mycgr3T

Mycgr3G68429 Mycgr3T
  
Location: 12180-13440

Mycgr3G68429\_Mycgr3T

Mycgr3G68421 Mycgr3T
  
Location: 13540-17086

Mycgr3G68421\_Mycgr3T

Mycgr3G90801 Mycgr3T
  
Location: 17186-18056

Mycgr3G90801\_Mycgr3T

Mycgr3G84646 Mycgr3T
  
Location: 18156-20235

Mycgr3G84646\_Mycgr3T

Mycgr3G68456 Mycgr3T
  
Location: 20335-21970

Mycgr3G68456\_Mycgr3T

Mycgr3G103270 Mycgr3
  
Location: 22070-22355

Mycgr3G103270\_Mycgr3

Mycgr3G90803 Mycgr3T
  
Location: 22455-23019

Mycgr3G90803\_Mycgr3T

Mycgr3G36941 Mycgr3T
  
Location: 23119-24064

Mycgr3G36941\_Mycgr3T

Mycgr3G25746 Mycgr3T
  
Location: 24164-25241

Mycgr3G25746\_Mycgr3T

Mycgr3G90788 Mycgr3T
  
Location: 25341-25803

Mycgr3G90788\_Mycgr3T

Mycgr3G103260 Mycgr3
  
Location: 25903-26635

Mycgr3G103260\_Mycgr3

Mycgr3G84644 Mycgr3T
  
Location: 26735-28457

Mycgr3G84644\_Mycgr3T

Mycgr3G29227 Mycgr3T
  
Location: 28557-28863

Mycgr3G29227\_Mycgr3T

Mycgr3G36271 Mycgr3T
  
Location: 28963-29854

Mycgr3G36271\_Mycgr3T

Mycgr3G68433 Mycgr3T
  
Location: 29954-33041

Mycgr3G68433\_Mycgr3T

Mycgr3G79452 Mycgr3T
  
Location: 33141-33399

Mycgr3G79452\_Mycgr3T

Mycgr3G55345 Mycgr3T
  
Location: 33499-34126

Mycgr3G55345\_Mycgr3T

Mycgr3G103278 Mycgr3
  
Location: 34226-35195

Mycgr3G103278\_Mycgr3

Mycgr3G84654 Mycgr3T
  
Location: 35295-36630

Mycgr3G84654\_Mycgr3T

Mycgr3G108090 Mycgr3
  
Location: 36730-37591

Mycgr3G108090\_Mycgr3

Mycgr3G21922 Mycgr3T
  
Location: 37691-39149

Mycgr3G21922\_Mycgr3T

Mycgr3G99148 Mycgr3T
  
Location: 39249-42819

Mycgr3G99148\_Mycgr3T

hypothetical protein
  
Accession: CAP91665
  
Location: 1469663-1473034
  
 NCBI BlastP on this gene

Pc13g05960

not annotated
  
Accession: CAP91664
  
Location: 1466895-1469234
  
 NCBI BlastP on this gene

Pc13g05950

not annotated
  
Accession: CAP91663
  
Location: 1463159-1466033
  
 NCBI BlastP on this gene

Pc13g05940

not annotated
  
Accession: CAP91662
  
Location: 1461276-1462506
  
 NCBI BlastP on this gene

Pc13g05930

not annotated
  
Accession: CAP91661
  
Location: 1459016-1460466
  
 NCBI BlastP on this gene

Pc13g05920

unnamed
  
Accession: CAP91660
  
Location: 1457734-1458588
  
 NCBI BlastP on this gene

Pc13g05910

not annotated
  
Accession: CAP91659
  
Location: 1455274-1456697
  
 NCBI BlastP on this gene

Pc13g05900

not annotated
  
Accession: CAP91658
  
Location: 1453816-1454914
  
  
**BlastP hit with Mycgr3G55345\_Mycgr3T**
  
Percentage identity: 64 %
  
BlastP bit score: 221
  
Sequence coverage: 98 %
  
E-value: 8e-69
  
  
 NCBI BlastP on this gene

Pc13g05890

unnamed
  
Accession: CAP91657
  
Location: 1452801-1453235
  
 NCBI BlastP on this gene

Pc13g05880

not annotated
  
Accession: CAP91656
  
Location: 1451285-1452324
  
 NCBI BlastP on this gene

Pc13g05870

not annotated
  
Accession: CAP91655
  
Location: 1447167-1449507
  
 NCBI BlastP on this gene

Pc13g05860

not annotated
  
Accession: CAP91654
  
Location: 1445549-1446835
  
 NCBI BlastP on this gene

Pc13g05850

not annotated
  
Accession: CAP91653
  
Location: 1442694-1444710
  
  
**BlastP hit with Mycgr3G68458\_Mycgr3T**
  
Percentage identity: 63 %
  
BlastP bit score: 826
  
Sequence coverage: 91 %
  
E-value: 0.0
  
  
 NCBI BlastP on this gene

Pc13g05840

not annotated
  
Accession: CAP91652
  
Location: 1441195-1442297
  
 NCBI BlastP on this gene

Pc13g05830

unnamed
  
Accession: CAP91651
  
Location: 1439071-1440895
  
 NCBI BlastP on this gene

Pc13g05820

not annotated
  
Accession: CAP91650
  
Location: 1437289-1438253
  
 NCBI BlastP on this gene

Pc13g05810

not annotated
  
Accession: CAP91649
  
Location: 1435276-1437034
  
 NCBI BlastP on this gene

Pc13g05800

not annotated
  
Accession: CAP91648
  
Location: 1433843-1434942
  
 NCBI BlastP on this gene

Pc13g05790

not annotated
  
Accession: CAP91647
  
Location: 1431274-1433571
  
 NCBI BlastP on this gene

Pc13g05780

not annotated
  
Accession: CAP91646
  
Location: 1428901-1430744
  
 NCBI BlastP on this gene

Pc13g05770

not annotated
  
Accession: CAP91645
  
Location: 1425937-1427288
  
 NCBI BlastP on this gene

Pc13g05760

Query: Architecture Search FASTA input

KB726203 : Fusarium oxysporum f. sp. cubense race 4 unplaced genomic scaffold scaffold2    Total score: 2.0     Cumulative Blast bit score: 1035

Hit cluster cross-links:

Mycgr3G90785 Mycgr3T
  
Location: 0-1047

Mycgr3G90785\_Mycgr3T

Mycgr3G103262 Mycgr3
  
Location: 1147-1390

Mycgr3G103262\_Mycgr3

Mycgr3G68458 Mycgr3T
  
Location: 1490-3602

Mycgr3G68458\_Mycgr3T

Mycgr3G99145 Mycgr3T
  
Location: 3702-4326

Mycgr3G99145\_Mycgr3T

Mycgr3G103274 Mycgr3
  
Location: 4426-4957

Mycgr3G103274\_Mycgr3

Mycgr3G103264 Mycgr3
  
Location: 5057-5390

Mycgr3G103264\_Mycgr3

Mycgr3G37570 Mycgr3T
  
Location: 5490-6006

Mycgr3G37570\_Mycgr3T

Mycgr3G108094 Mycgr3
  
Location: 6106-10555

Mycgr3G108094\_Mycgr3

Mycgr3G90786 Mycgr3T
  
Location: 10655-12080

Mycgr3G90786\_Mycgr3T

Mycgr3G68429 Mycgr3T
  
Location: 12180-13440

Mycgr3G68429\_Mycgr3T

Mycgr3G68421 Mycgr3T
  
Location: 13540-17086

Mycgr3G68421\_Mycgr3T

Mycgr3G90801 Mycgr3T
  
Location: 17186-18056

Mycgr3G90801\_Mycgr3T

Mycgr3G84646 Mycgr3T
  
Location: 18156-20235

Mycgr3G84646\_Mycgr3T

Mycgr3G68456 Mycgr3T
  
Location: 20335-21970

Mycgr3G68456\_Mycgr3T

Mycgr3G103270 Mycgr3
  
Location: 22070-22355

Mycgr3G103270\_Mycgr3

Mycgr3G90803 Mycgr3T
  
Location: 22455-23019

Mycgr3G90803\_Mycgr3T

Mycgr3G36941 Mycgr3T
  
Location: 23119-24064

Mycgr3G36941\_Mycgr3T

Mycgr3G25746 Mycgr3T
  
Location: 24164-25241

Mycgr3G25746\_Mycgr3T

Mycgr3G90788 Mycgr3T
  
Location: 25341-25803

Mycgr3G90788\_Mycgr3T

Mycgr3G103260 Mycgr3
  
Location: 25903-26635

Mycgr3G103260\_Mycgr3

Mycgr3G84644 Mycgr3T
  
Location: 26735-28457

Mycgr3G84644\_Mycgr3T

Mycgr3G29227 Mycgr3T
  
Location: 28557-28863

Mycgr3G29227\_Mycgr3T

Mycgr3G36271 Mycgr3T
  
Location: 28963-29854

Mycgr3G36271\_Mycgr3T

Mycgr3G68433 Mycgr3T
  
Location: 29954-33041

Mycgr3G68433\_Mycgr3T

Mycgr3G79452 Mycgr3T
  
Location: 33141-33399

Mycgr3G79452\_Mycgr3T

Mycgr3G55345 Mycgr3T
  
Location: 33499-34126

Mycgr3G55345\_Mycgr3T

Mycgr3G103278 Mycgr3
  
Location: 34226-35195

Mycgr3G103278\_Mycgr3

Mycgr3G84654 Mycgr3T
  
Location: 35295-36630

Mycgr3G84654\_Mycgr3T

Mycgr3G108090 Mycgr3
  
Location: 36730-37591

Mycgr3G108090\_Mycgr3

Mycgr3G21922 Mycgr3T
  
Location: 37691-39149

Mycgr3G21922\_Mycgr3T

Mycgr3G99148 Mycgr3T
  
Location: 39249-42819

Mycgr3G99148\_Mycgr3T

Nitrogen assimilation transcription factor nit-4
  
Accession: EMT74522
  
Location: 87210-89985
  
 NCBI BlastP on this gene

EMT74522

Nucleus export protein BRL1
  
Accession: EMT74523
  
Location: 92765-94323
  
 NCBI BlastP on this gene

EMT74523

hypothetical protein
  
Accession: EMT74524
  
Location: 100531-100958
  
 NCBI BlastP on this gene

EMT74524

Protein kinase dsk1
  
Accession: EMT74525
  
Location: 102582-104300
  
  
**BlastP hit with Mycgr3G84644\_Mycgr3T**
  
Percentage identity: 75 %
  
BlastP bit score: 759
  
Sequence coverage: 89 %
  
E-value: 0.0
  
  
 NCBI BlastP on this gene

EMT74525

Protein lunapark
  
Accession: EMT74526
  
Location: 105391-106620
  
 NCBI BlastP on this gene

EMT74526

Golgin IMH1
  
Accession: EMT74527
  
Location: 107677-111234
  
  
**BlastP hit with Mycgr3G108094\_Mycgr3**
  
Percentage identity: 35 %
  
BlastP bit score: 276
  
Sequence coverage: 44 %
  
E-value: 3e-73
  
  
 NCBI BlastP on this gene

EMT74527

Rhamnolipids biosynthesis 3-oxoacyl-[acyl-carrier-protein] reductase
  
Accession: EMT74528
  
Location: 112080-113031
  
 NCBI BlastP on this gene

EMT74528

Rhamnolipids biosynthesis 3-oxoacyl-[acyl-carrier-protein] reductase
  
Accession: EMT74529
  
Location: 114212-115163
  
 NCBI BlastP on this gene

EMT74529

hypothetical protein
  
Accession: EMT74530
  
Location: 117752-118861
  
 NCBI BlastP on this gene

EMT74530

hypothetical protein
  
Accession: EMT74531
  
Location: 126546-128075
  
 NCBI BlastP on this gene

EMT74531

hypothetical protein
  
Accession: EMT74532
  
Location: 130736-132424
  
 NCBI BlastP on this gene

EMT74532

Query: Architecture Search FASTA input

KB730140 : Fusarium oxysporum f. sp. cubense race 1 unplaced genomic scaffold scaffold224    Total score: 2.0     Cumulative Blast bit score: 1034

Hit cluster cross-links:

Mycgr3G90785 Mycgr3T
  
Location: 0-1047

Mycgr3G90785\_Mycgr3T

Mycgr3G103262 Mycgr3
  
Location: 1147-1390

Mycgr3G103262\_Mycgr3

Mycgr3G68458 Mycgr3T
  
Location: 1490-3602

Mycgr3G68458\_Mycgr3T

Mycgr3G99145 Mycgr3T
  
Location: 3702-4326

Mycgr3G99145\_Mycgr3T

Mycgr3G103274 Mycgr3
  
Location: 4426-4957

Mycgr3G103274\_Mycgr3

Mycgr3G103264 Mycgr3
  
Location: 5057-5390

Mycgr3G103264\_Mycgr3

Mycgr3G37570 Mycgr3T
  
Location: 5490-6006

Mycgr3G37570\_Mycgr3T

Mycgr3G108094 Mycgr3
  
Location: 6106-10555

Mycgr3G108094\_Mycgr3

Mycgr3G90786 Mycgr3T
  
Location: 10655-12080

Mycgr3G90786\_Mycgr3T

Mycgr3G68429 Mycgr3T
  
Location: 12180-13440

Mycgr3G68429\_Mycgr3T

Mycgr3G68421 Mycgr3T
  
Location: 13540-17086

Mycgr3G68421\_Mycgr3T

Mycgr3G90801 Mycgr3T
  
Location: 17186-18056

Mycgr3G90801\_Mycgr3T

Mycgr3G84646 Mycgr3T
  
Location: 18156-20235

Mycgr3G84646\_Mycgr3T

Mycgr3G68456 Mycgr3T
  
Location: 20335-21970

Mycgr3G68456\_Mycgr3T

Mycgr3G103270 Mycgr3
  
Location: 22070-22355

Mycgr3G103270\_Mycgr3

Mycgr3G90803 Mycgr3T
  
Location: 22455-23019

Mycgr3G90803\_Mycgr3T

Mycgr3G36941 Mycgr3T
  
Location: 23119-24064

Mycgr3G36941\_Mycgr3T

Mycgr3G25746 Mycgr3T
  
Location: 24164-25241

Mycgr3G25746\_Mycgr3T

Mycgr3G90788 Mycgr3T
  
Location: 25341-25803

Mycgr3G90788\_Mycgr3T

Mycgr3G103260 Mycgr3
  
Location: 25903-26635

Mycgr3G103260\_Mycgr3

Mycgr3G84644 Mycgr3T
  
Location: 26735-28457

Mycgr3G84644\_Mycgr3T

Mycgr3G29227 Mycgr3T
  
Location: 28557-28863

Mycgr3G29227\_Mycgr3T

Mycgr3G36271 Mycgr3T
  
Location: 28963-29854

Mycgr3G36271\_Mycgr3T

Mycgr3G68433 Mycgr3T
  
Location: 29954-33041

Mycgr3G68433\_Mycgr3T

Mycgr3G79452 Mycgr3T
  
Location: 33141-33399

Mycgr3G79452\_Mycgr3T

Mycgr3G55345 Mycgr3T
  
Location: 33499-34126

Mycgr3G55345\_Mycgr3T

Mycgr3G103278 Mycgr3
  
Location: 34226-35195

Mycgr3G103278\_Mycgr3

Mycgr3G84654 Mycgr3T
  
Location: 35295-36630

Mycgr3G84654\_Mycgr3T

Mycgr3G108090 Mycgr3
  
Location: 36730-37591

Mycgr3G108090\_Mycgr3

Mycgr3G21922 Mycgr3T
  
Location: 37691-39149

Mycgr3G21922\_Mycgr3T

Mycgr3G99148 Mycgr3T
  
Location: 39249-42819

Mycgr3G99148\_Mycgr3T

hypothetical protein
  
Accession: ENH71669
  
Location: 92032-92448
  
 NCBI BlastP on this gene

ENH71669

Helicase required for RNAi-mediated heterochromatin assembly 1
  
Accession: ENH71668
  
Location: 88599-91477
  
 NCBI BlastP on this gene

ENH71668

Nitrogen assimilation transcription factor nit-4
  
Accession: ENH71667
  
Location: 83594-86369
  
 NCBI BlastP on this gene

ENH71667

Nucleus export protein BRL1
  
Accession: ENH71666
  
Location: 79239-80797
  
 NCBI BlastP on this gene

ENH71666

Transcription factor IWS1
  
Accession: ENH71665
  
Location: 77429-78788
  
 NCBI BlastP on this gene

ENH71665

hypothetical protein
  
Accession: ENH71664
  
Location: 74734-75161
  
 NCBI BlastP on this gene

ENH71664

Protein kinase dsk1
  
Accession: ENH71663
  
Location: 71389-73107
  
  
**BlastP hit with Mycgr3G84644\_Mycgr3T**
  
Percentage identity: 75 %
  
BlastP bit score: 759
  
Sequence coverage: 89 %
  
E-value: 0.0
  
  
 NCBI BlastP on this gene

ENH71663

Protein lunapark
  
Accession: ENH71662
  
Location: 69068-70298
  
 NCBI BlastP on this gene

ENH71662

Golgin IMH1
  
Accession: ENH71661
  
Location: 64454-68011
  
  
**BlastP hit with Mycgr3G108094\_Mycgr3**
  
Percentage identity: 35 %
  
BlastP bit score: 275
  
Sequence coverage: 44 %
  
E-value: 1e-72
  
  
 NCBI BlastP on this gene

ENH71661

Rhamnolipids biosynthesis 3-oxoacyl-[acyl-carrier-protein] reductase
  
Accession: ENH71660
  
Location: 62656-63607
  
 NCBI BlastP on this gene

ENH71660

SWIRM domain-containing protein FUN19
  
Accession: ENH71659
  
Location: 58931-60040
  
 NCBI BlastP on this gene

ENH71659

hypothetical protein
  
Accession: ENH71658
  
Location: 51843-53373
  
 NCBI BlastP on this gene

ENH71658

hypothetical protein
  
Accession: ENH71657
  
Location: 47499-49187
  
 NCBI BlastP on this gene

ENH71657

hypothetical protein
  
Accession: ENH71656
  
Location: 45794-46921
  
 NCBI BlastP on this gene

ENH71656

G patch domain-containing protein 8
  
Accession: ENH71655
  
Location: 44767-45319
  
 NCBI BlastP on this gene

ENH71655

Query: Architecture Search FASTA input

AFNW01000113 : Fusarium pseudograminearum CS3096    Total score: 2.0     Cumulative Blast bit score: 1028

Hit cluster cross-links:

Mycgr3G90785 Mycgr3T
  
Location: 0-1047

Mycgr3G90785\_Mycgr3T

Mycgr3G103262 Mycgr3
  
Location: 1147-1390

Mycgr3G103262\_Mycgr3

Mycgr3G68458 Mycgr3T
  
Location: 1490-3602

Mycgr3G68458\_Mycgr3T

Mycgr3G99145 Mycgr3T
  
Location: 3702-4326

Mycgr3G99145\_Mycgr3T

Mycgr3G103274 Mycgr3
  
Location: 4426-4957

Mycgr3G103274\_Mycgr3

Mycgr3G103264 Mycgr3
  
Location: 5057-5390

Mycgr3G103264\_Mycgr3

Mycgr3G37570 Mycgr3T
  
Location: 5490-6006

Mycgr3G37570\_Mycgr3T

Mycgr3G108094 Mycgr3
  
Location: 6106-10555

Mycgr3G108094\_Mycgr3

Mycgr3G90786 Mycgr3T
  
Location: 10655-12080

Mycgr3G90786\_Mycgr3T

Mycgr3G68429 Mycgr3T
  
Location: 12180-13440

Mycgr3G68429\_Mycgr3T

Mycgr3G68421 Mycgr3T
  
Location: 13540-17086

Mycgr3G68421\_Mycgr3T

Mycgr3G90801 Mycgr3T
  
Location: 17186-18056

Mycgr3G90801\_Mycgr3T

Mycgr3G84646 Mycgr3T
  
Location: 18156-20235

Mycgr3G84646\_Mycgr3T

Mycgr3G68456 Mycgr3T
  
Location: 20335-21970

Mycgr3G68456\_Mycgr3T

Mycgr3G103270 Mycgr3
  
Location: 22070-22355

Mycgr3G103270\_Mycgr3

Mycgr3G90803 Mycgr3T
  
Location: 22455-23019

Mycgr3G90803\_Mycgr3T

Mycgr3G36941 Mycgr3T
  
Location: 23119-24064

Mycgr3G36941\_Mycgr3T

Mycgr3G25746 Mycgr3T
  
Location: 24164-25241

Mycgr3G25746\_Mycgr3T

Mycgr3G90788 Mycgr3T
  
Location: 25341-25803

Mycgr3G90788\_Mycgr3T

Mycgr3G103260 Mycgr3
  
Location: 25903-26635

Mycgr3G103260\_Mycgr3

Mycgr3G84644 Mycgr3T
  
Location: 26735-28457

Mycgr3G84644\_Mycgr3T

Mycgr3G29227 Mycgr3T
  
Location: 28557-28863

Mycgr3G29227\_Mycgr3T

Mycgr3G36271 Mycgr3T
  
Location: 28963-29854

Mycgr3G36271\_Mycgr3T

Mycgr3G68433 Mycgr3T
  
Location: 29954-33041

Mycgr3G68433\_Mycgr3T

Mycgr3G79452 Mycgr3T
  
Location: 33141-33399

Mycgr3G79452\_Mycgr3T

Mycgr3G55345 Mycgr3T
  
Location: 33499-34126

Mycgr3G55345\_Mycgr3T

Mycgr3G103278 Mycgr3
  
Location: 34226-35195

Mycgr3G103278\_Mycgr3

Mycgr3G84654 Mycgr3T
  
Location: 35295-36630

Mycgr3G84654\_Mycgr3T

Mycgr3G108090 Mycgr3
  
Location: 36730-37591

Mycgr3G108090\_Mycgr3

Mycgr3G21922 Mycgr3T
  
Location: 37691-39149

Mycgr3G21922\_Mycgr3T

Mycgr3G99148 Mycgr3T
  
Location: 39249-42819

Mycgr3G99148\_Mycgr3T

hypothetical protein
  
Accession: EKJ74295
  
Location: 255465-259430
  
 NCBI BlastP on this gene

EKJ74295

hypothetical protein
  
Accession: EKJ74296
  
Location: 261087-263844
  
 NCBI BlastP on this gene

EKJ74296

hypothetical protein
  
Accession: EKJ74297
  
Location: 266790-268338
  
 NCBI BlastP on this gene

EKJ74297

hypothetical protein
  
Accession: EKJ74298
  
Location: 268790-270180
  
 NCBI BlastP on this gene

EKJ74298

hypothetical protein
  
Accession: EKJ74299
  
Location: 272593-273460
  
 NCBI BlastP on this gene

EKJ74299

hypothetical protein
  
Accession: EKJ74300
  
Location: 274654-276378
  
  
**BlastP hit with Mycgr3G84644\_Mycgr3T**
  
Percentage identity: 74 %
  
BlastP bit score: 753
  
Sequence coverage: 89 %
  
E-value: 0.0
  
  
 NCBI BlastP on this gene

EKJ74300

hypothetical protein
  
Accession: EKJ74301
  
Location: 276983-278200
  
 NCBI BlastP on this gene

EKJ74301

hypothetical protein
  
Accession: EKJ74302
  
Location: 279541-283147
  
  
**BlastP hit with Mycgr3G108094\_Mycgr3**
  
Percentage identity: 33 %
  
BlastP bit score: 275
  
Sequence coverage: 45 %
  
E-value: 2e-72
  
  
 NCBI BlastP on this gene

EKJ74302

hypothetical protein
  
Accession: EKJ74303
  
Location: 283914-285040
  
 NCBI BlastP on this gene

EKJ74303

hypothetical protein
  
Accession: EKJ74304
  
Location: 287321-288463
  
 NCBI BlastP on this gene

EKJ74304

hypothetical protein
  
Accession: EKJ74305
  
Location: 297868-299586
  
 NCBI BlastP on this gene

EKJ74305

hypothetical protein
  
Accession: EKJ74306
  
Location: 300196-301331
  
 NCBI BlastP on this gene

EKJ74306

hypothetical protein
  
Accession: EKJ74307
  
Location: 301815-302390
  
 NCBI BlastP on this gene

EKJ74307

hypothetical protein
  
Accession: EKJ74308
  
Location: 302491-305148
  
 NCBI BlastP on this gene

EKJ74308

Query: Architecture Search FASTA input

CAGA01000024 : Claviceps purpurea 20.1    Total score: 2.0     Cumulative Blast bit score: 1024

Hit cluster cross-links:

Mycgr3G90785 Mycgr3T
  
Location: 0-1047

Mycgr3G90785\_Mycgr3T

Mycgr3G103262 Mycgr3
  
Location: 1147-1390

Mycgr3G103262\_Mycgr3

Mycgr3G68458 Mycgr3T
  
Location: 1490-3602

Mycgr3G68458\_Mycgr3T

Mycgr3G99145 Mycgr3T
  
Location: 3702-4326

Mycgr3G99145\_Mycgr3T

Mycgr3G103274 Mycgr3
  
Location: 4426-4957

Mycgr3G103274\_Mycgr3

Mycgr3G103264 Mycgr3
  
Location: 5057-5390

Mycgr3G103264\_Mycgr3

Mycgr3G37570 Mycgr3T
  
Location: 5490-6006

Mycgr3G37570\_Mycgr3T

Mycgr3G108094 Mycgr3
  
Location: 6106-10555

Mycgr3G108094\_Mycgr3

Mycgr3G90786 Mycgr3T
  
Location: 10655-12080

Mycgr3G90786\_Mycgr3T

Mycgr3G68429 Mycgr3T
  
Location: 12180-13440

Mycgr3G68429\_Mycgr3T

Mycgr3G68421 Mycgr3T
  
Location: 13540-17086

Mycgr3G68421\_Mycgr3T

Mycgr3G90801 Mycgr3T
  
Location: 17186-18056

Mycgr3G90801\_Mycgr3T

Mycgr3G84646 Mycgr3T
  
Location: 18156-20235

Mycgr3G84646\_Mycgr3T

Mycgr3G68456 Mycgr3T
  
Location: 20335-21970

Mycgr3G68456\_Mycgr3T

Mycgr3G103270 Mycgr3
  
Location: 22070-22355

Mycgr3G103270\_Mycgr3

Mycgr3G90803 Mycgr3T
  
Location: 22455-23019

Mycgr3G90803\_Mycgr3T

Mycgr3G36941 Mycgr3T
  
Location: 23119-24064

Mycgr3G36941\_Mycgr3T

Mycgr3G25746 Mycgr3T
  
Location: 24164-25241

Mycgr3G25746\_Mycgr3T

Mycgr3G90788 Mycgr3T
  
Location: 25341-25803

Mycgr3G90788\_Mycgr3T

Mycgr3G103260 Mycgr3
  
Location: 25903-26635

Mycgr3G103260\_Mycgr3

Mycgr3G84644 Mycgr3T
  
Location: 26735-28457

Mycgr3G84644\_Mycgr3T

Mycgr3G29227 Mycgr3T
  
Location: 28557-28863

Mycgr3G29227\_Mycgr3T

Mycgr3G36271 Mycgr3T
  
Location: 28963-29854

Mycgr3G36271\_Mycgr3T

Mycgr3G68433 Mycgr3T
  
Location: 29954-33041

Mycgr3G68433\_Mycgr3T

Mycgr3G79452 Mycgr3T
  
Location: 33141-33399

Mycgr3G79452\_Mycgr3T

Mycgr3G55345 Mycgr3T
  
Location: 33499-34126

Mycgr3G55345\_Mycgr3T

Mycgr3G103278 Mycgr3
  
Location: 34226-35195

Mycgr3G103278\_Mycgr3

Mycgr3G84654 Mycgr3T
  
Location: 35295-36630

Mycgr3G84654\_Mycgr3T

Mycgr3G108090 Mycgr3
  
Location: 36730-37591

Mycgr3G108090\_Mycgr3

Mycgr3G21922 Mycgr3T
  
Location: 37691-39149

Mycgr3G21922\_Mycgr3T

Mycgr3G99148 Mycgr3T
  
Location: 39249-42819

Mycgr3G99148\_Mycgr3T

related to NAM7-nonsense-mediated mRNA decay protein
  
Accession: CCE30747
  
Location: 347041-349386
  
 NCBI BlastP on this gene

CCE30747

uncharacterized protein
  
Accession: CCE30746
  
Location: 346421-346790
  
 NCBI BlastP on this gene

CCE30746

related to transcription factor SPT8
  
Accession: CCE30745
  
Location: 343827-346308
  
 NCBI BlastP on this gene

CCE30745

related to nitrate assimilation regulatory protein nirA
  
Accession: CCE30744
  
Location: 340111-342844
  
 NCBI BlastP on this gene

CCE30744

uncharacterized protein
  
Accession: CCE30743
  
Location: 336381-337929
  
 NCBI BlastP on this gene

CCE30743

uncharacterized protein
  
Accession: CCE30742
  
Location: 334319-335777
  
 NCBI BlastP on this gene

CCE30742

probable dis1-suppressing protein kinase dsk1
  
Accession: CCE30741
  
Location: 325326-329296
  
  
**BlastP hit with Mycgr3G84644\_Mycgr3T**
  
Percentage identity: 68 %
  
BlastP bit score: 771
  
Sequence coverage: 98 %
  
E-value: 0.0
  
  
 NCBI BlastP on this gene

CCE30741

uncharacterized protein
  
Accession: CCE30740
  
Location: 322603-324204
  
 NCBI BlastP on this gene

CCE30740

related to vesicular transport protein
  
Accession: CCE30739
  
Location: 318695-322478
  
  
**BlastP hit with Mycgr3G108094\_Mycgr3**
  
Percentage identity: 36 %
  
BlastP bit score: 253
  
Sequence coverage: 36 %
  
E-value: 1e-65
  
  
 NCBI BlastP on this gene

CCE30739

uncharacterized protein
  
Accession: CCE30738
  
Location: 315615-317003
  
 NCBI BlastP on this gene

CCE30738

uncharacterized protein
  
Accession: CCE30737
  
Location: 303929-305995
  
 NCBI BlastP on this gene

CCE30737

related to glycinamide ribonucleotide transformylase
  
Accession: CCE30736
  
Location: 302594-303431
  
 NCBI BlastP on this gene

CCE30736

uncharacterized protein
  
Accession: CCE30735
  
Location: 300985-302193
  
 NCBI BlastP on this gene

CCE30735

uncharacterized protein
  
Accession: CCE30734
  
Location: 299933-300501
  
 NCBI BlastP on this gene

CCE30734

Query: Architecture Search FASTA input

AFQF01000897 : Fusarium oxysporum Fo5176    Total score: 2.0     Cumulative Blast bit score: 1021

Hit cluster cross-links:

Mycgr3G90785 Mycgr3T
  
Location: 0-1047

Mycgr3G90785\_Mycgr3T

Mycgr3G103262 Mycgr3
  
Location: 1147-1390

Mycgr3G103262\_Mycgr3

Mycgr3G68458 Mycgr3T
  
Location: 1490-3602

Mycgr3G68458\_Mycgr3T

Mycgr3G99145 Mycgr3T
  
Location: 3702-4326

Mycgr3G99145\_Mycgr3T

Mycgr3G103274 Mycgr3
  
Location: 4426-4957

Mycgr3G103274\_Mycgr3

Mycgr3G103264 Mycgr3
  
Location: 5057-5390

Mycgr3G103264\_Mycgr3

Mycgr3G37570 Mycgr3T
  
Location: 5490-6006

Mycgr3G37570\_Mycgr3T

Mycgr3G108094 Mycgr3
  
Location: 6106-10555

Mycgr3G108094\_Mycgr3

Mycgr3G90786 Mycgr3T
  
Location: 10655-12080

Mycgr3G90786\_Mycgr3T

Mycgr3G68429 Mycgr3T
  
Location: 12180-13440

Mycgr3G68429\_Mycgr3T

Mycgr3G68421 Mycgr3T
  
Location: 13540-17086

Mycgr3G68421\_Mycgr3T

Mycgr3G90801 Mycgr3T
  
Location: 17186-18056

Mycgr3G90801\_Mycgr3T

Mycgr3G84646 Mycgr3T
  
Location: 18156-20235

Mycgr3G84646\_Mycgr3T

Mycgr3G68456 Mycgr3T
  
Location: 20335-21970

Mycgr3G68456\_Mycgr3T

Mycgr3G103270 Mycgr3
  
Location: 22070-22355

Mycgr3G103270\_Mycgr3

Mycgr3G90803 Mycgr3T
  
Location: 22455-23019

Mycgr3G90803\_Mycgr3T

Mycgr3G36941 Mycgr3T
  
Location: 23119-24064

Mycgr3G36941\_Mycgr3T

Mycgr3G25746 Mycgr3T
  
Location: 24164-25241

Mycgr3G25746\_Mycgr3T

Mycgr3G90788 Mycgr3T
  
Location: 25341-25803

Mycgr3G90788\_Mycgr3T

Mycgr3G103260 Mycgr3
  
Location: 25903-26635

Mycgr3G103260\_Mycgr3

Mycgr3G84644 Mycgr3T
  
Location: 26735-28457

Mycgr3G84644\_Mycgr3T

Mycgr3G29227 Mycgr3T
  
Location: 28557-28863

Mycgr3G29227\_Mycgr3T

Mycgr3G36271 Mycgr3T
  
Location: 28963-29854

Mycgr3G36271\_Mycgr3T

Mycgr3G68433 Mycgr3T
  
Location: 29954-33041

Mycgr3G68433\_Mycgr3T

Mycgr3G79452 Mycgr3T
  
Location: 33141-33399

Mycgr3G79452\_Mycgr3T

Mycgr3G55345 Mycgr3T
  
Location: 33499-34126

Mycgr3G55345\_Mycgr3T

Mycgr3G103278 Mycgr3
  
Location: 34226-35195

Mycgr3G103278\_Mycgr3

Mycgr3G84654 Mycgr3T
  
Location: 35295-36630

Mycgr3G84654\_Mycgr3T

Mycgr3G108090 Mycgr3
  
Location: 36730-37591

Mycgr3G108090\_Mycgr3

Mycgr3G21922 Mycgr3T
  
Location: 37691-39149

Mycgr3G21922\_Mycgr3T

Mycgr3G99148 Mycgr3T
  
Location: 39249-42819

Mycgr3G99148\_Mycgr3T

hypothetical protein
  
Accession: EGU86743
  
Location: 89232-93081
  
 NCBI BlastP on this gene

EGU86743

hypothetical protein
  
Accession: EGU86742
  
Location: 84193-86968
  
 NCBI BlastP on this gene

EGU86742

hypothetical protein
  
Accession: EGU86741
  
Location: 79838-81396
  
 NCBI BlastP on this gene

EGU86741

hypothetical protein
  
Accession: EGU86740
  
Location: 77996-79387
  
 NCBI BlastP on this gene

EGU86740

hypothetical protein
  
Accession: EGU86739
  
Location: 71956-73674
  
  
**BlastP hit with Mycgr3G84644\_Mycgr3T**
  
Percentage identity: 75 %
  
BlastP bit score: 759
  
Sequence coverage: 89 %
  
E-value: 0.0
  
  
 NCBI BlastP on this gene

EGU86739

hypothetical protein
  
Accession: EGU86738
  
Location: 69635-70865
  
 NCBI BlastP on this gene

EGU86738

hypothetical protein
  
Accession: EGU86737
  
Location: 63483-68837
  
  
**BlastP hit with Mycgr3G108094\_Mycgr3**
  
Percentage identity: 36 %
  
BlastP bit score: 262
  
Sequence coverage: 44 %
  
E-value: 5e-68
  
  
 NCBI BlastP on this gene

EGU86737

hypothetical protein
  
Accession: EGU86736
  
Location: 59661-60917
  
 NCBI BlastP on this gene

EGU86736

hypothetical protein
  
Accession: EGU86735
  
Location: 48374-50062
  
 NCBI BlastP on this gene

EGU86735

hypothetical protein
  
Accession: EGU86734
  
Location: 46669-47796
  
 NCBI BlastP on this gene

EGU86734

hypothetical protein
  
Accession: EGU86733
  
Location: 45642-46194
  
 NCBI BlastP on this gene

EGU86733

Query: Architecture Search FASTA input

CAGA01000038 : Claviceps purpurea 20.1    Total score: 2.0     Cumulative Blast bit score: 1018

Hit cluster cross-links:

Mycgr3G90785 Mycgr3T
  
Location: 0-1047

Mycgr3G90785\_Mycgr3T

Mycgr3G103262 Mycgr3
  
Location: 1147-1390

Mycgr3G103262\_Mycgr3

Mycgr3G68458 Mycgr3T
  
Location: 1490-3602

Mycgr3G68458\_Mycgr3T

Mycgr3G99145 Mycgr3T
  
Location: 3702-4326

Mycgr3G99145\_Mycgr3T

Mycgr3G103274 Mycgr3
  
Location: 4426-4957

Mycgr3G103274\_Mycgr3

Mycgr3G103264 Mycgr3
  
Location: 5057-5390

Mycgr3G103264\_Mycgr3

Mycgr3G37570 Mycgr3T
  
Location: 5490-6006

Mycgr3G37570\_Mycgr3T

Mycgr3G108094 Mycgr3
  
Location: 6106-10555

Mycgr3G108094\_Mycgr3

Mycgr3G90786 Mycgr3T
  
Location: 10655-12080

Mycgr3G90786\_Mycgr3T

Mycgr3G68429 Mycgr3T
  
Location: 12180-13440

Mycgr3G68429\_Mycgr3T

Mycgr3G68421 Mycgr3T
  
Location: 13540-17086

Mycgr3G68421\_Mycgr3T

Mycgr3G90801 Mycgr3T
  
Location: 17186-18056

Mycgr3G90801\_Mycgr3T

Mycgr3G84646 Mycgr3T
  
Location: 18156-20235

Mycgr3G84646\_Mycgr3T

Mycgr3G68456 Mycgr3T
  
Location: 20335-21970

Mycgr3G68456\_Mycgr3T

Mycgr3G103270 Mycgr3
  
Location: 22070-22355

Mycgr3G103270\_Mycgr3

Mycgr3G90803 Mycgr3T
  
Location: 22455-23019

Mycgr3G90803\_Mycgr3T

Mycgr3G36941 Mycgr3T
  
Location: 23119-24064

Mycgr3G36941\_Mycgr3T

Mycgr3G25746 Mycgr3T
  
Location: 24164-25241

Mycgr3G25746\_Mycgr3T

Mycgr3G90788 Mycgr3T
  
Location: 25341-25803

Mycgr3G90788\_Mycgr3T

Mycgr3G103260 Mycgr3
  
Location: 25903-26635

Mycgr3G103260\_Mycgr3

Mycgr3G84644 Mycgr3T
  
Location: 26735-28457

Mycgr3G84644\_Mycgr3T

Mycgr3G29227 Mycgr3T
  
Location: 28557-28863

Mycgr3G29227\_Mycgr3T

Mycgr3G36271 Mycgr3T
  
Location: 28963-29854

Mycgr3G36271\_Mycgr3T

Mycgr3G68433 Mycgr3T
  
Location: 29954-33041

Mycgr3G68433\_Mycgr3T

Mycgr3G79452 Mycgr3T
  
Location: 33141-33399

Mycgr3G79452\_Mycgr3T

Mycgr3G55345 Mycgr3T
  
Location: 33499-34126

Mycgr3G55345\_Mycgr3T

Mycgr3G103278 Mycgr3
  
Location: 34226-35195

Mycgr3G103278\_Mycgr3

Mycgr3G84654 Mycgr3T
  
Location: 35295-36630

Mycgr3G84654\_Mycgr3T

Mycgr3G108090 Mycgr3
  
Location: 36730-37591

Mycgr3G108090\_Mycgr3

Mycgr3G21922 Mycgr3T
  
Location: 37691-39149

Mycgr3G21922\_Mycgr3T

Mycgr3G99148 Mycgr3T
  
Location: 39249-42819

Mycgr3G99148\_Mycgr3T

uncharacterized protein
  
Accession: CCE32191
  
Location: 262552-263466
  
 NCBI BlastP on this gene

CCE32191

uncharacterized protein
  
Accession: CCE32192
  
Location: 264141-265455
  
 NCBI BlastP on this gene

CCE32192

uncharacterized protein
  
Accession: CCE32193
  
Location: 267452-268595
  
 NCBI BlastP on this gene

CCE32193

uncharacterized protein
  
Accession: CCE32194
  
Location: 268688-270280
  
 NCBI BlastP on this gene

CCE32194

uncharacterized protein
  
Accession: CCE32195
  
Location: 271412-272190
  
 NCBI BlastP on this gene

CCE32195

probable ARP5-Actin-related protein
  
Accession: CCE32196
  
Location: 273521-276156
  
 NCBI BlastP on this gene

CCE32196

uncharacterized protein
  
Accession: CCE32197
  
Location: 277265-278533
  
 NCBI BlastP on this gene

CCE32197

related to linoleate diol synthase
  
Accession: CCE32198
  
Location: 279403-280740
  
 NCBI BlastP on this gene

CCE32198

related to pterin-4-alpha-carbinolamine dehydratase
  
Accession: CCE32199
  
Location: 281194-281601
  
 NCBI BlastP on this gene

CCE32199

probable C-4 methyl sterol oxidase
  
Accession: CCE32200
  
Location: 282534-283615
  
  
**BlastP hit with Mycgr3G36271\_Mycgr3T**
  
Percentage identity: 70 %
  
BlastP bit score: 453
  
Sequence coverage: 98 %
  
E-value: 2e-157
  
  
 NCBI BlastP on this gene

CCE32200

related to NCA2 protein
  
Accession: CCE32201
  
Location: 284878-286954
  
  
**BlastP hit with Mycgr3G84646\_Mycgr3T**
  
Percentage identity: 45 %
  
BlastP bit score: 566
  
Sequence coverage: 101 %
  
E-value: 0.0
  
  
 NCBI BlastP on this gene

CCE32201

related to malic acid transport protein
  
Accession: CCE32202
  
Location: 287395-289172
  
 NCBI BlastP on this gene

CCE32202

related to cysteine dioxygenase type I
  
Accession: CCE32203
  
Location: 291463-292161
  
 NCBI BlastP on this gene

CCE32203

related to helicase-like transcription factor
  
Accession: CCE32204
  
Location: 293035-296199
  
 NCBI BlastP on this gene

CCE32204

uncharacterized protein
  
Accession: CCE32205
  
Location: 298454-298834
  
 NCBI BlastP on this gene

CCE32205

uncharacterized protein
  
Accession: CCE32206
  
Location: 300539-306384
  
 NCBI BlastP on this gene

CCE32206

Query: Architecture Search FASTA input

AMYD01003902 : Colletotrichum gloeosporioides Cg-14    Total score: 2.0     Cumulative Blast bit score: 1018

Hit cluster cross-links:

Mycgr3G90785 Mycgr3T
  
Location: 0-1047

Mycgr3G90785\_Mycgr3T

Mycgr3G103262 Mycgr3
  
Location: 1147-1390

Mycgr3G103262\_Mycgr3

Mycgr3G68458 Mycgr3T
  
Location: 1490-3602

Mycgr3G68458\_Mycgr3T

Mycgr3G99145 Mycgr3T
  
Location: 3702-4326

Mycgr3G99145\_Mycgr3T

Mycgr3G103274 Mycgr3
  
Location: 4426-4957

Mycgr3G103274\_Mycgr3

Mycgr3G103264 Mycgr3
  
Location: 5057-5390

Mycgr3G103264\_Mycgr3

Mycgr3G37570 Mycgr3T
  
Location: 5490-6006

Mycgr3G37570\_Mycgr3T

Mycgr3G108094 Mycgr3
  
Location: 6106-10555

Mycgr3G108094\_Mycgr3

Mycgr3G90786 Mycgr3T
  
Location: 10655-12080

Mycgr3G90786\_Mycgr3T

Mycgr3G68429 Mycgr3T
  
Location: 12180-13440

Mycgr3G68429\_Mycgr3T

Mycgr3G68421 Mycgr3T
  
Location: 13540-17086

Mycgr3G68421\_Mycgr3T

Mycgr3G90801 Mycgr3T
  
Location: 17186-18056

Mycgr3G90801\_Mycgr3T

Mycgr3G84646 Mycgr3T
  
Location: 18156-20235

Mycgr3G84646\_Mycgr3T

Mycgr3G68456 Mycgr3T
  
Location: 20335-21970

Mycgr3G68456\_Mycgr3T

Mycgr3G103270 Mycgr3
  
Location: 22070-22355

Mycgr3G103270\_Mycgr3

Mycgr3G90803 Mycgr3T
  
Location: 22455-23019

Mycgr3G90803\_Mycgr3T

Mycgr3G36941 Mycgr3T
  
Location: 23119-24064

Mycgr3G36941\_Mycgr3T

Mycgr3G25746 Mycgr3T
  
Location: 24164-25241

Mycgr3G25746\_Mycgr3T

Mycgr3G90788 Mycgr3T
  
Location: 25341-25803

Mycgr3G90788\_Mycgr3T

Mycgr3G103260 Mycgr3
  
Location: 25903-26635

Mycgr3G103260\_Mycgr3

Mycgr3G84644 Mycgr3T
  
Location: 26735-28457

Mycgr3G84644\_Mycgr3T

Mycgr3G29227 Mycgr3T
  
Location: 28557-28863

Mycgr3G29227\_Mycgr3T

Mycgr3G36271 Mycgr3T
  
Location: 28963-29854

Mycgr3G36271\_Mycgr3T

Mycgr3G68433 Mycgr3T
  
Location: 29954-33041

Mycgr3G68433\_Mycgr3T

Mycgr3G79452 Mycgr3T
  
Location: 33141-33399

Mycgr3G79452\_Mycgr3T

Mycgr3G55345 Mycgr3T
  
Location: 33499-34126

Mycgr3G55345\_Mycgr3T

Mycgr3G103278 Mycgr3
  
Location: 34226-35195

Mycgr3G103278\_Mycgr3

Mycgr3G84654 Mycgr3T
  
Location: 35295-36630

Mycgr3G84654\_Mycgr3T

Mycgr3G108090 Mycgr3
  
Location: 36730-37591

Mycgr3G108090\_Mycgr3

Mycgr3G21922 Mycgr3T
  
Location: 37691-39149

Mycgr3G21922\_Mycgr3T

Mycgr3G99148 Mycgr3T
  
Location: 39249-42819

Mycgr3G99148\_Mycgr3T

ATP synthase regulation protein NCA2
  
Accession: EQB44753
  
Location: 1083-3199
  
  
**BlastP hit with Mycgr3G84646\_Mycgr3T**
  
Percentage identity: 44 %
  
BlastP bit score: 560
  
Sequence coverage: 100 %
  
E-value: 0.0
  
  
 NCBI BlastP on this gene

EQB44753

hypothetical protein
  
Accession: EQB44754
  
Location: 5296-6405
  
  
**BlastP hit with Mycgr3G36271\_Mycgr3T**
  
Percentage identity: 73 %
  
BlastP bit score: 459
  
Sequence coverage: 96 %
  
E-value: 2e-159
  
  
 NCBI BlastP on this gene

EQB44754

Query: Architecture Search FASTA input

GG749476 : Ajellomyces dermatitidis ATCC 18188 genomic scaffold supercont1.70    Total score: 2.0     Cumulative Blast bit score: 1018

Hit cluster cross-links:

Mycgr3G90785 Mycgr3T
  
Location: 0-1047

Mycgr3G90785\_Mycgr3T

Mycgr3G103262 Mycgr3
  
Location: 1147-1390

Mycgr3G103262\_Mycgr3

Mycgr3G68458 Mycgr3T
  
Location: 1490-3602

Mycgr3G68458\_Mycgr3T

Mycgr3G99145 Mycgr3T
  
Location: 3702-4326

Mycgr3G99145\_Mycgr3T

Mycgr3G103274 Mycgr3
  
Location: 4426-4957

Mycgr3G103274\_Mycgr3

Mycgr3G103264 Mycgr3
  
Location: 5057-5390

Mycgr3G103264\_Mycgr3

Mycgr3G37570 Mycgr3T
  
Location: 5490-6006

Mycgr3G37570\_Mycgr3T

Mycgr3G108094 Mycgr3
  
Location: 6106-10555

Mycgr3G108094\_Mycgr3

Mycgr3G90786 Mycgr3T
  
Location: 10655-12080

Mycgr3G90786\_Mycgr3T

Mycgr3G68429 Mycgr3T
  
Location: 12180-13440

Mycgr3G68429\_Mycgr3T

Mycgr3G68421 Mycgr3T
  
Location: 13540-17086

Mycgr3G68421\_Mycgr3T

Mycgr3G90801 Mycgr3T
  
Location: 17186-18056

Mycgr3G90801\_Mycgr3T

Mycgr3G84646 Mycgr3T
  
Location: 18156-20235

Mycgr3G84646\_Mycgr3T

Mycgr3G68456 Mycgr3T
  
Location: 20335-21970

Mycgr3G68456\_Mycgr3T

Mycgr3G103270 Mycgr3
  
Location: 22070-22355

Mycgr3G103270\_Mycgr3

Mycgr3G90803 Mycgr3T
  
Location: 22455-23019

Mycgr3G90803\_Mycgr3T

Mycgr3G36941 Mycgr3T
  
Location: 23119-24064

Mycgr3G36941\_Mycgr3T

Mycgr3G25746 Mycgr3T
  
Location: 24164-25241

Mycgr3G25746\_Mycgr3T

Mycgr3G90788 Mycgr3T
  
Location: 25341-25803

Mycgr3G90788\_Mycgr3T

Mycgr3G103260 Mycgr3
  
Location: 25903-26635

Mycgr3G103260\_Mycgr3

Mycgr3G84644 Mycgr3T
  
Location: 26735-28457

Mycgr3G84644\_Mycgr3T

Mycgr3G29227 Mycgr3T
  
Location: 28557-28863

Mycgr3G29227\_Mycgr3T

Mycgr3G36271 Mycgr3T
  
Location: 28963-29854

Mycgr3G36271\_Mycgr3T

Mycgr3G68433 Mycgr3T
  
Location: 29954-33041

Mycgr3G68433\_Mycgr3T

Mycgr3G79452 Mycgr3T
  
Location: 33141-33399

Mycgr3G79452\_Mycgr3T

Mycgr3G55345 Mycgr3T
  
Location: 33499-34126

Mycgr3G55345\_Mycgr3T

Mycgr3G103278 Mycgr3
  
Location: 34226-35195

Mycgr3G103278\_Mycgr3

Mycgr3G84654 Mycgr3T
  
Location: 35295-36630

Mycgr3G84654\_Mycgr3T

Mycgr3G108090 Mycgr3
  
Location: 36730-37591

Mycgr3G108090\_Mycgr3

Mycgr3G21922 Mycgr3T
  
Location: 37691-39149

Mycgr3G21922\_Mycgr3T

Mycgr3G99148 Mycgr3T
  
Location: 39249-42819

Mycgr3G99148\_Mycgr3T

viral A-type inclusion protein repeat protein
  
Accession: EGE84735
  
Location: 59839-63661
  
  
**BlastP hit with Mycgr3G108094\_Mycgr3**
  
Percentage identity: 37 %
  
BlastP bit score: 384
  
Sequence coverage: 45 %
  
E-value: 2e-108
  
  
 NCBI BlastP on this gene

EGE84735

hypothetical protein
  
Accession: EGE84736
  
Location: 64283-66262
  
 NCBI BlastP on this gene

EGE84736

Ras family protein
  
Accession: EGE84737
  
Location: 69261-70243
  
 NCBI BlastP on this gene

EGE84737

cytochrome b2
  
Accession: EGE84738
  
Location: 71917-73612
  
 NCBI BlastP on this gene

EGE84738

phosphoric ester hydrolase
  
Accession: EGE84739
  
Location: 73946-75079
  
 NCBI BlastP on this gene

EGE84739

hypothetical protein
  
Accession: EGE84740
  
Location: 75742-76350
  
 NCBI BlastP on this gene

EGE84740

hypothetical protein
  
Accession: EGE84741
  
Location: 78032-79028
  
 NCBI BlastP on this gene

EGE84741

protein kinase dsk1
  
Accession: EGE84742
  
Location: 79329-81448
  
  
**BlastP hit with Mycgr3G84644\_Mycgr3T**
  
Percentage identity: 66 %
  
BlastP bit score: 634
  
Sequence coverage: 88 %
  
E-value: 0.0
  
  
 NCBI BlastP on this gene

EGE84742

Query: Architecture Search FASTA input

GG657474 : Ajellomyces dermatitidis SLH14081 genomic scaffold supercont1.27    Total score: 2.0     Cumulative Blast bit score: 1018

Hit cluster cross-links:

Mycgr3G90785 Mycgr3T
  
Location: 0-1047

Mycgr3G90785\_Mycgr3T

Mycgr3G103262 Mycgr3
  
Location: 1147-1390

Mycgr3G103262\_Mycgr3

Mycgr3G68458 Mycgr3T
  
Location: 1490-3602

Mycgr3G68458\_Mycgr3T

Mycgr3G99145 Mycgr3T
  
Location: 3702-4326

Mycgr3G99145\_Mycgr3T

Mycgr3G103274 Mycgr3
  
Location: 4426-4957

Mycgr3G103274\_Mycgr3

Mycgr3G103264 Mycgr3
  
Location: 5057-5390

Mycgr3G103264\_Mycgr3

Mycgr3G37570 Mycgr3T
  
Location: 5490-6006

Mycgr3G37570\_Mycgr3T

Mycgr3G108094 Mycgr3
  
Location: 6106-10555

Mycgr3G108094\_Mycgr3

Mycgr3G90786 Mycgr3T
  
Location: 10655-12080

Mycgr3G90786\_Mycgr3T

Mycgr3G68429 Mycgr3T
  
Location: 12180-13440

Mycgr3G68429\_Mycgr3T

Mycgr3G68421 Mycgr3T
  
Location: 13540-17086

Mycgr3G68421\_Mycgr3T

Mycgr3G90801 Mycgr3T
  
Location: 17186-18056

Mycgr3G90801\_Mycgr3T

Mycgr3G84646 Mycgr3T
  
Location: 18156-20235

Mycgr3G84646\_Mycgr3T

Mycgr3G68456 Mycgr3T
  
Location: 20335-21970

Mycgr3G68456\_Mycgr3T

Mycgr3G103270 Mycgr3
  
Location: 22070-22355

Mycgr3G103270\_Mycgr3

Mycgr3G90803 Mycgr3T
  
Location: 22455-23019

Mycgr3G90803\_Mycgr3T

Mycgr3G36941 Mycgr3T
  
Location: 23119-24064

Mycgr3G36941\_Mycgr3T

Mycgr3G25746 Mycgr3T
  
Location: 24164-25241

Mycgr3G25746\_Mycgr3T

Mycgr3G90788 Mycgr3T
  
Location: 25341-25803

Mycgr3G90788\_Mycgr3T

Mycgr3G103260 Mycgr3
  
Location: 25903-26635

Mycgr3G103260\_Mycgr3

Mycgr3G84644 Mycgr3T
  
Location: 26735-28457

Mycgr3G84644\_Mycgr3T

Mycgr3G29227 Mycgr3T
  
Location: 28557-28863

Mycgr3G29227\_Mycgr3T

Mycgr3G36271 Mycgr3T
  
Location: 28963-29854

Mycgr3G36271\_Mycgr3T

Mycgr3G68433 Mycgr3T
  
Location: 29954-33041

Mycgr3G68433\_Mycgr3T

Mycgr3G79452 Mycgr3T
  
Location: 33141-33399

Mycgr3G79452\_Mycgr3T

Mycgr3G55345 Mycgr3T
  
Location: 33499-34126

Mycgr3G55345\_Mycgr3T

Mycgr3G103278 Mycgr3
  
Location: 34226-35195

Mycgr3G103278\_Mycgr3

Mycgr3G84654 Mycgr3T
  
Location: 35295-36630

Mycgr3G84654\_Mycgr3T

Mycgr3G108090 Mycgr3
  
Location: 36730-37591

Mycgr3G108090\_Mycgr3

Mycgr3G21922 Mycgr3T
  
Location: 37691-39149

Mycgr3G21922\_Mycgr3T

Mycgr3G99148 Mycgr3T
  
Location: 39249-42819

Mycgr3G99148\_Mycgr3T

C6 finger domain-containing protein
  
Accession: EEQ74517
  
Location: 389150-391608
  
 NCBI BlastP on this gene

EEQ74517

methionine permease
  
Accession: EEQ74518
  
Location: 392166-393645
  
 NCBI BlastP on this gene

EEQ74518

C6 finger domain-containing protein
  
Accession: EEQ74519
  
Location: 394452-396318
  
 NCBI BlastP on this gene

EEQ74519

conserved hypothetical protein
  
Accession: EEQ74520
  
Location: 397919-398569
  
 NCBI BlastP on this gene

EEQ74520

MGMT family protein
  
Accession: EEQ74521
  
Location: 400376-401093
  
 NCBI BlastP on this gene

EEQ74521

pre-mRNA-splicing factor cwc26
  
Accession: EEQ74522
  
Location: 401318-402415
  
 NCBI BlastP on this gene

EEQ74522

protein kinase dsk1
  
Accession: EEQ74523
  
Location: 404206-406325
  
  
**BlastP hit with Mycgr3G84644\_Mycgr3T**
  
Percentage identity: 66 %
  
BlastP bit score: 634
  
Sequence coverage: 88 %
  
E-value: 0.0
  
  
 NCBI BlastP on this gene

EEQ74523

predicted protein
  
Accession: EEQ74524
  
Location: 406647-407646
  
 NCBI BlastP on this gene

EEQ74524

predicted protein
  
Accession: EEQ74525
  
Location: 409199-410043
  
 NCBI BlastP on this gene

EEQ74525

phosphoric ester hydrolase
  
Accession: EEQ74526
  
Location: 410471-411601
  
 NCBI BlastP on this gene

EEQ74526

cytochrome b2
  
Accession: EEQ74527
  
Location: 411946-413641
  
 NCBI BlastP on this gene

EEQ74527

ras-like GTP-binding protein RYL2
  
Accession: EEQ74528
  
Location: 415356-416338
  
 NCBI BlastP on this gene

EEQ74528

conserved hypothetical protein
  
Accession: EEQ74529
  
Location: 419346-421325
  
 NCBI BlastP on this gene

EEQ74529

viral A-type inclusion protein repeat protein
  
Accession: EEQ74530
  
Location: 421991-425813
  
  
**BlastP hit with Mycgr3G108094\_Mycgr3**
  
Percentage identity: 37 %
  
BlastP bit score: 384
  
Sequence coverage: 45 %
  
E-value: 9e-109
  
  
 NCBI BlastP on this gene

EEQ74530

Query: Architecture Search FASTA input

EQ999987 : Ajellomyces dermatitidis ER-3 genomic scaffold supercont1.15    Total score: 2.0     Cumulative Blast bit score: 1018

Hit cluster cross-links:

Mycgr3G90785 Mycgr3T
  
Location: 0-1047

Mycgr3G90785\_Mycgr3T

Mycgr3G103262 Mycgr3
  
Location: 1147-1390

Mycgr3G103262\_Mycgr3

Mycgr3G68458 Mycgr3T
  
Location: 1490-3602

Mycgr3G68458\_Mycgr3T

Mycgr3G99145 Mycgr3T
  
Location: 3702-4326

Mycgr3G99145\_Mycgr3T

Mycgr3G103274 Mycgr3
  
Location: 4426-4957

Mycgr3G103274\_Mycgr3

Mycgr3G103264 Mycgr3
  
Location: 5057-5390

Mycgr3G103264\_Mycgr3

Mycgr3G37570 Mycgr3T
  
Location: 5490-6006

Mycgr3G37570\_Mycgr3T

Mycgr3G108094 Mycgr3
  
Location: 6106-10555

Mycgr3G108094\_Mycgr3

Mycgr3G90786 Mycgr3T
  
Location: 10655-12080

Mycgr3G90786\_Mycgr3T

Mycgr3G68429 Mycgr3T
  
Location: 12180-13440

Mycgr3G68429\_Mycgr3T

Mycgr3G68421 Mycgr3T
  
Location: 13540-17086

Mycgr3G68421\_Mycgr3T

Mycgr3G90801 Mycgr3T
  
Location: 17186-18056

Mycgr3G90801\_Mycgr3T

Mycgr3G84646 Mycgr3T
  
Location: 18156-20235

Mycgr3G84646\_Mycgr3T

Mycgr3G68456 Mycgr3T
  
Location: 20335-21970

Mycgr3G68456\_Mycgr3T

Mycgr3G103270 Mycgr3
  
Location: 22070-22355

Mycgr3G103270\_Mycgr3

Mycgr3G90803 Mycgr3T
  
Location: 22455-23019

Mycgr3G90803\_Mycgr3T

Mycgr3G36941 Mycgr3T
  
Location: 23119-24064

Mycgr3G36941\_Mycgr3T

Mycgr3G25746 Mycgr3T
  
Location: 24164-25241

Mycgr3G25746\_Mycgr3T

Mycgr3G90788 Mycgr3T
  
Location: 25341-25803

Mycgr3G90788\_Mycgr3T

Mycgr3G103260 Mycgr3
  
Location: 25903-26635

Mycgr3G103260\_Mycgr3

Mycgr3G84644 Mycgr3T
  
Location: 26735-28457

Mycgr3G84644\_Mycgr3T

Mycgr3G29227 Mycgr3T
  
Location: 28557-28863

Mycgr3G29227\_Mycgr3T

Mycgr3G36271 Mycgr3T
  
Location: 28963-29854

Mycgr3G36271\_Mycgr3T

Mycgr3G68433 Mycgr3T
  
Location: 29954-33041

Mycgr3G68433\_Mycgr3T

Mycgr3G79452 Mycgr3T
  
Location: 33141-33399

Mycgr3G79452\_Mycgr3T

Mycgr3G55345 Mycgr3T
  
Location: 33499-34126

Mycgr3G55345\_Mycgr3T

Mycgr3G103278 Mycgr3
  
Location: 34226-35195

Mycgr3G103278\_Mycgr3

Mycgr3G84654 Mycgr3T
  
Location: 35295-36630

Mycgr3G84654\_Mycgr3T

Mycgr3G108090 Mycgr3
  
Location: 36730-37591

Mycgr3G108090\_Mycgr3

Mycgr3G21922 Mycgr3T
  
Location: 37691-39149

Mycgr3G21922\_Mycgr3T

Mycgr3G99148 Mycgr3T
  
Location: 39249-42819

Mycgr3G99148\_Mycgr3T

C6 finger domain-containing protein
  
Accession: EEQ86197
  
Location: 974815-977273
  
 NCBI BlastP on this gene

EEQ86197

methionine permease
  
Accession: EEQ86198
  
Location: 977832-979311
  
 NCBI BlastP on this gene

EEQ86198

C6 finger domain-containing protein
  
Accession: EEQ86199
  
Location: 980261-982681
  
 NCBI BlastP on this gene

EEQ86199

conserved hypothetical protein
  
Accession: EEQ86200
  
Location: 983590-984240
  
 NCBI BlastP on this gene

EEQ86200

MGMT family protein
  
Accession: EEQ86201
  
Location: 986028-986745
  
 NCBI BlastP on this gene

EEQ86201

pre-mRNA-splicing factor cwc26
  
Accession: EEQ86202
  
Location: 986971-988068
  
 NCBI BlastP on this gene

EEQ86202

protein kinase dsk1
  
Accession: EEQ86203
  
Location: 989869-991988
  
  
**BlastP hit with Mycgr3G84644\_Mycgr3T**
  
Percentage identity: 66 %
  
BlastP bit score: 634
  
Sequence coverage: 88 %
  
E-value: 0.0
  
  
 NCBI BlastP on this gene

EEQ86203

predicted protein
  
Accession: EEQ86204
  
Location: 992288-993285
  
 NCBI BlastP on this gene

EEQ86204

predicted protein
  
Accession: EEQ86205
  
Location: 994968-995576
  
 NCBI BlastP on this gene

EEQ86205

phosphoric ester hydrolase
  
Accession: EEQ86206
  
Location: 996243-997376
  
 NCBI BlastP on this gene

EEQ86206

cytochrome b2
  
Accession: EEQ86207
  
Location: 997720-999417
  
 NCBI BlastP on this gene

EEQ86207

ras-like GTP-binding protein RYL2
  
Accession: EEQ86208
  
Location: 1001055-1002037
  
 NCBI BlastP on this gene

EEQ86208

conserved hypothetical protein
  
Accession: EEQ86209
  
Location: 1005043-1007022
  
 NCBI BlastP on this gene

EEQ86209

viral A-type inclusion protein repeat protein
  
Accession: EEQ86210
  
Location: 1007644-1011466
  
  
**BlastP hit with Mycgr3G108094\_Mycgr3**
  
Percentage identity: 37 %
  
BlastP bit score: 384
  
Sequence coverage: 45 %
  
E-value: 9e-109
  
  
 NCBI BlastP on this gene

EEQ86210

conserved hypothetical protein
  
Accession: EEQ86211
  
Location: 1013201-1013858
  
 NCBI BlastP on this gene

EEQ86211

Query: Architecture Search FASTA input

GL385396 : Gaeumannomyces graminis var. tritici R3-111a-1 unplaced genomic scaffold supercont2.2    Total score: 2.0     Cumulative Blast bit score: 1016

Hit cluster cross-links:

Mycgr3G90785 Mycgr3T
  
Location: 0-1047

Mycgr3G90785\_Mycgr3T

Mycgr3G103262 Mycgr3
  
Location: 1147-1390

Mycgr3G103262\_Mycgr3

Mycgr3G68458 Mycgr3T
  
Location: 1490-3602

Mycgr3G68458\_Mycgr3T

Mycgr3G99145 Mycgr3T
  
Location: 3702-4326

Mycgr3G99145\_Mycgr3T

Mycgr3G103274 Mycgr3
  
Location: 4426-4957

Mycgr3G103274\_Mycgr3

Mycgr3G103264 Mycgr3
  
Location: 5057-5390

Mycgr3G103264\_Mycgr3

Mycgr3G37570 Mycgr3T
  
Location: 5490-6006

Mycgr3G37570\_Mycgr3T

Mycgr3G108094 Mycgr3
  
Location: 6106-10555

Mycgr3G108094\_Mycgr3

Mycgr3G90786 Mycgr3T
  
Location: 10655-12080

Mycgr3G90786\_Mycgr3T

Mycgr3G68429 Mycgr3T
  
Location: 12180-13440

Mycgr3G68429\_Mycgr3T

Mycgr3G68421 Mycgr3T
  
Location: 13540-17086

Mycgr3G68421\_Mycgr3T

Mycgr3G90801 Mycgr3T
  
Location: 17186-18056

Mycgr3G90801\_Mycgr3T

Mycgr3G84646 Mycgr3T
  
Location: 18156-20235

Mycgr3G84646\_Mycgr3T

Mycgr3G68456 Mycgr3T
  
Location: 20335-21970

Mycgr3G68456\_Mycgr3T

Mycgr3G103270 Mycgr3
  
Location: 22070-22355

Mycgr3G103270\_Mycgr3

Mycgr3G90803 Mycgr3T
  
Location: 22455-23019

Mycgr3G90803\_Mycgr3T

Mycgr3G36941 Mycgr3T
  
Location: 23119-24064

Mycgr3G36941\_Mycgr3T

Mycgr3G25746 Mycgr3T
  
Location: 24164-25241

Mycgr3G25746\_Mycgr3T

Mycgr3G90788 Mycgr3T
  
Location: 25341-25803

Mycgr3G90788\_Mycgr3T

Mycgr3G103260 Mycgr3
  
Location: 25903-26635

Mycgr3G103260\_Mycgr3

Mycgr3G84644 Mycgr3T
  
Location: 26735-28457

Mycgr3G84644\_Mycgr3T

Mycgr3G29227 Mycgr3T
  
Location: 28557-28863

Mycgr3G29227\_Mycgr3T

Mycgr3G36271 Mycgr3T
  
Location: 28963-29854

Mycgr3G36271\_Mycgr3T

Mycgr3G68433 Mycgr3T
  
Location: 29954-33041

Mycgr3G68433\_Mycgr3T

Mycgr3G79452 Mycgr3T
  
Location: 33141-33399

Mycgr3G79452\_Mycgr3T

Mycgr3G55345 Mycgr3T
  
Location: 33499-34126

Mycgr3G55345\_Mycgr3T

Mycgr3G103278 Mycgr3
  
Location: 34226-35195

Mycgr3G103278\_Mycgr3

Mycgr3G84654 Mycgr3T
  
Location: 35295-36630

Mycgr3G84654\_Mycgr3T

Mycgr3G108090 Mycgr3
  
Location: 36730-37591

Mycgr3G108090\_Mycgr3

Mycgr3G21922 Mycgr3T
  
Location: 37691-39149

Mycgr3G21922\_Mycgr3T

Mycgr3G99148 Mycgr3T
  
Location: 39249-42819

Mycgr3G99148\_Mycgr3T

hypothetical protein
  
Accession: EJT79900
  
Location: 7299176-7300264
  
 NCBI BlastP on this gene

EJT79900

hypothetical protein
  
Accession: EJT79901
  
Location: 7300314-7300565
  
 NCBI BlastP on this gene

EJT79901

hypothetical protein
  
Accession: EJT79902
  
Location: 7301145-7301369
  
 NCBI BlastP on this gene

EJT79902

hypothetical protein
  
Accession: EJT79903
  
Location: 7302141-7303289
  
 NCBI BlastP on this gene

EJT79903

hypothetical protein
  
Accession: EJT79904
  
Location: 7303926-7304521
  
 NCBI BlastP on this gene

EJT79904

hypothetical protein
  
Accession: EJT79905
  
Location: 7304876-7306237
  
 NCBI BlastP on this gene

EJT79905

hypothetical protein
  
Accession: EJT79906
  
Location: 7307443-7311293
  
  
**BlastP hit with Mycgr3G108094\_Mycgr3**
  
Percentage identity: 33 %
  
BlastP bit score: 270
  
Sequence coverage: 44 %
  
E-value: 5e-71
  
  
 NCBI BlastP on this gene

EJT79906

hypothetical protein
  
Accession: EJT79907
  
Location: 7311820-7312748
  
 NCBI BlastP on this gene

EJT79907

hypothetical protein
  
Accession: EJT79908
  
Location: 7312973-7313548
  
 NCBI BlastP on this gene

EJT79908

hypothetical protein
  
Accession: EJT79909
  
Location: 7313908-7315017
  
 NCBI BlastP on this gene

EJT79909

hypothetical protein
  
Accession: EJT79910
  
Location: 7316476-7316906
  
 NCBI BlastP on this gene

EJT79910

alcohol dehydrogenase
  
Accession: EJT79911
  
Location: 7317299-7319179
  
 NCBI BlastP on this gene

EJT79911

Znf1
  
Accession: EJT79912
  
Location: 7319244-7320404
  
 NCBI BlastP on this gene

EJT79912

hypothetical protein
  
Accession: EJT79913
  
Location: 7322156-7324422
  
 NCBI BlastP on this gene

EJT79913

hypothetical protein
  
Accession: EJT79914
  
Location: 7324979-7328381
  
 NCBI BlastP on this gene

EJT79914

hypothetical protein
  
Accession: EJT79915
  
Location: 7329821-7331384
  
 NCBI BlastP on this gene

EJT79915

transcription factor IWS1
  
Accession: EJT79916
  
Location: 7332146-7333611
  
 NCBI BlastP on this gene

EJT79916

CMGC/SRPK protein kinase
  
Accession: EJT79917
  
Location: 7335008-7336923
  
  
**BlastP hit with Mycgr3G84644\_Mycgr3T**
  
Percentage identity: 73 %
  
BlastP bit score: 746
  
Sequence coverage: 88 %
  
E-value: 0.0
  
  
 NCBI BlastP on this gene

EJT79917

hypothetical protein
  
Accession: EJT79918
  
Location: 7338081-7339061
  
 NCBI BlastP on this gene

EJT79918

hypothetical protein
  
Accession: EJT79919
  
Location: 7339452-7340505
  
 NCBI BlastP on this gene

EJT79919

hypothetical protein
  
Accession: EJT79920
  
Location: 7342367-7344405
  
 NCBI BlastP on this gene

EJT79920

hypothetical protein
  
Accession: EJT79921
  
Location: 7345301-7345654
  
 NCBI BlastP on this gene

EJT79921

Query: Architecture Search FASTA input

KB446555 : Pseudocercospora fijiensis CIRAD86 unplaced genomic scaffold MYCFIscaffold\_1    Total score: 2.0     Cumulative Blast bit score: 1014

Hit cluster cross-links:

Mycgr3G90785 Mycgr3T
  
Location: 0-1047

Mycgr3G90785\_Mycgr3T

Mycgr3G103262 Mycgr3
  
Location: 1147-1390

Mycgr3G103262\_Mycgr3

Mycgr3G68458 Mycgr3T
  
Location: 1490-3602

Mycgr3G68458\_Mycgr3T

Mycgr3G99145 Mycgr3T
  
Location: 3702-4326

Mycgr3G99145\_Mycgr3T

Mycgr3G103274 Mycgr3
  
Location: 4426-4957

Mycgr3G103274\_Mycgr3

Mycgr3G103264 Mycgr3
  
Location: 5057-5390

Mycgr3G103264\_Mycgr3

Mycgr3G37570 Mycgr3T
  
Location: 5490-6006

Mycgr3G37570\_Mycgr3T

Mycgr3G108094 Mycgr3
  
Location: 6106-10555

Mycgr3G108094\_Mycgr3

Mycgr3G90786 Mycgr3T
  
Location: 10655-12080

Mycgr3G90786\_Mycgr3T

Mycgr3G68429 Mycgr3T
  
Location: 12180-13440

Mycgr3G68429\_Mycgr3T

Mycgr3G68421 Mycgr3T
  
Location: 13540-17086

Mycgr3G68421\_Mycgr3T

Mycgr3G90801 Mycgr3T
  
Location: 17186-18056

Mycgr3G90801\_Mycgr3T

Mycgr3G84646 Mycgr3T
  
Location: 18156-20235

Mycgr3G84646\_Mycgr3T

Mycgr3G68456 Mycgr3T
  
Location: 20335-21970

Mycgr3G68456\_Mycgr3T

Mycgr3G103270 Mycgr3
  
Location: 22070-22355

Mycgr3G103270\_Mycgr3

Mycgr3G90803 Mycgr3T
  
Location: 22455-23019

Mycgr3G90803\_Mycgr3T

Mycgr3G36941 Mycgr3T
  
Location: 23119-24064

Mycgr3G36941\_Mycgr3T

Mycgr3G25746 Mycgr3T
  
Location: 24164-25241

Mycgr3G25746\_Mycgr3T

Mycgr3G90788 Mycgr3T
  
Location: 25341-25803

Mycgr3G90788\_Mycgr3T

Mycgr3G103260 Mycgr3
  
Location: 25903-26635

Mycgr3G103260\_Mycgr3

Mycgr3G84644 Mycgr3T
  
Location: 26735-28457

Mycgr3G84644\_Mycgr3T

Mycgr3G29227 Mycgr3T
  
Location: 28557-28863

Mycgr3G29227\_Mycgr3T

Mycgr3G36271 Mycgr3T
  
Location: 28963-29854

Mycgr3G36271\_Mycgr3T

Mycgr3G68433 Mycgr3T
  
Location: 29954-33041

Mycgr3G68433\_Mycgr3T

Mycgr3G79452 Mycgr3T
  
Location: 33141-33399

Mycgr3G79452\_Mycgr3T

Mycgr3G55345 Mycgr3T
  
Location: 33499-34126

Mycgr3G55345\_Mycgr3T

Mycgr3G103278 Mycgr3
  
Location: 34226-35195

Mycgr3G103278\_Mycgr3

Mycgr3G84654 Mycgr3T
  
Location: 35295-36630

Mycgr3G84654\_Mycgr3T

Mycgr3G108090 Mycgr3
  
Location: 36730-37591

Mycgr3G108090\_Mycgr3

Mycgr3G21922 Mycgr3T
  
Location: 37691-39149

Mycgr3G21922\_Mycgr3T

Mycgr3G99148 Mycgr3T
  
Location: 39249-42819

Mycgr3G99148\_Mycgr3T

ABC transporter, ABC-F family, GCN-EF3 type
  
Accession: EME88709
  
Location: 6964072-6966303
  
 NCBI BlastP on this gene

EME88709

hypothetical protein
  
Accession: EME88710
  
Location: 6966556-6967286
  
 NCBI BlastP on this gene

EME88710

hypothetical protein
  
Accession: EME88711
  
Location: 6968642-6971565
  
 NCBI BlastP on this gene

EME88711

hypothetical protein
  
Accession: EME88712
  
Location: 6973492-6974058
  
 NCBI BlastP on this gene

EME88712

hypothetical protein
  
Accession: EME88713
  
Location: 6974362-6975093
  
 NCBI BlastP on this gene

EME88713

hypothetical protein
  
Accession: EME88714
  
Location: 6977058-6978629
  
 NCBI BlastP on this gene

EME88714

hypothetical protein
  
Accession: EME88715
  
Location: 6978891-6979213
  
 NCBI BlastP on this gene

EME88715

hypothetical protein
  
Accession: EME88716
  
Location: 6979301-6980263
  
 NCBI BlastP on this gene

EME88716

hypothetical protein
  
Accession: EME88717
  
Location: 6981449-6983995
  
  
**BlastP hit with Mycgr3G21922\_Mycgr3T**
  
Percentage identity: 67 %
  
BlastP bit score: 671
  
Sequence coverage: 101 %
  
E-value: 0.0
  
  
 NCBI BlastP on this gene

EME88717

hypothetical protein
  
Accession: EME88718
  
Location: 6984539-6986612
  
  
**BlastP hit with Mycgr3G103278\_Mycgr3**
  
Percentage identity: 62 %
  
BlastP bit score: 344
  
Sequence coverage: 95 %
  
E-value: 2e-109
  
  
 NCBI BlastP on this gene

EME88718

hypothetical protein
  
Accession: EME88719
  
Location: 6987251-6988958
  
 NCBI BlastP on this gene

EME88719

hypothetical protein
  
Accession: EME88720
  
Location: 6989778-6990779
  
 NCBI BlastP on this gene

EME88720

Query: Architecture Search FASTA input

GL698568 : Metarhizium acridum CQMa 102 unplaced genomic scaffold Scf\_099    Total score: 2.0     Cumulative Blast bit score: 1010

Hit cluster cross-links:

Mycgr3G90785 Mycgr3T
  
Location: 0-1047

Mycgr3G90785\_Mycgr3T

Mycgr3G103262 Mycgr3
  
Location: 1147-1390

Mycgr3G103262\_Mycgr3

Mycgr3G68458 Mycgr3T
  
Location: 1490-3602

Mycgr3G68458\_Mycgr3T

Mycgr3G99145 Mycgr3T
  
Location: 3702-4326

Mycgr3G99145\_Mycgr3T

Mycgr3G103274 Mycgr3
  
Location: 4426-4957

Mycgr3G103274\_Mycgr3

Mycgr3G103264 Mycgr3
  
Location: 5057-5390

Mycgr3G103264\_Mycgr3

Mycgr3G37570 Mycgr3T
  
Location: 5490-6006

Mycgr3G37570\_Mycgr3T

Mycgr3G108094 Mycgr3
  
Location: 6106-10555

Mycgr3G108094\_Mycgr3

Mycgr3G90786 Mycgr3T
  
Location: 10655-12080

Mycgr3G90786\_Mycgr3T

Mycgr3G68429 Mycgr3T
  
Location: 12180-13440

Mycgr3G68429\_Mycgr3T

Mycgr3G68421 Mycgr3T
  
Location: 13540-17086

Mycgr3G68421\_Mycgr3T

Mycgr3G90801 Mycgr3T
  
Location: 17186-18056

Mycgr3G90801\_Mycgr3T

Mycgr3G84646 Mycgr3T
  
Location: 18156-20235

Mycgr3G84646\_Mycgr3T

Mycgr3G68456 Mycgr3T
  
Location: 20335-21970

Mycgr3G68456\_Mycgr3T

Mycgr3G103270 Mycgr3
  
Location: 22070-22355

Mycgr3G103270\_Mycgr3

Mycgr3G90803 Mycgr3T
  
Location: 22455-23019

Mycgr3G90803\_Mycgr3T

Mycgr3G36941 Mycgr3T
  
Location: 23119-24064

Mycgr3G36941\_Mycgr3T

Mycgr3G25746 Mycgr3T
  
Location: 24164-25241

Mycgr3G25746\_Mycgr3T

Mycgr3G90788 Mycgr3T
  
Location: 25341-25803

Mycgr3G90788\_Mycgr3T

Mycgr3G103260 Mycgr3
  
Location: 25903-26635

Mycgr3G103260\_Mycgr3

Mycgr3G84644 Mycgr3T
  
Location: 26735-28457

Mycgr3G84644\_Mycgr3T

Mycgr3G29227 Mycgr3T
  
Location: 28557-28863

Mycgr3G29227\_Mycgr3T

Mycgr3G36271 Mycgr3T
  
Location: 28963-29854

Mycgr3G36271\_Mycgr3T

Mycgr3G68433 Mycgr3T
  
Location: 29954-33041

Mycgr3G68433\_Mycgr3T

Mycgr3G79452 Mycgr3T
  
Location: 33141-33399

Mycgr3G79452\_Mycgr3T

Mycgr3G55345 Mycgr3T
  
Location: 33499-34126

Mycgr3G55345\_Mycgr3T

Mycgr3G103278 Mycgr3
  
Location: 34226-35195

Mycgr3G103278\_Mycgr3

Mycgr3G84654 Mycgr3T
  
Location: 35295-36630

Mycgr3G84654\_Mycgr3T

Mycgr3G108090 Mycgr3
  
Location: 36730-37591

Mycgr3G108090\_Mycgr3

Mycgr3G21922 Mycgr3T
  
Location: 37691-39149

Mycgr3G21922\_Mycgr3T

Mycgr3G99148 Mycgr3T
  
Location: 39249-42819

Mycgr3G99148\_Mycgr3T

putative nitrate assimilation regulatory protein nirA
  
Accession: EFY85718
  
Location: 108464-113002
  
 NCBI BlastP on this gene

EFY85718

hypothetical protein
  
Accession: EFY85717
  
Location: 105033-106561
  
 NCBI BlastP on this gene

EFY85717

transcription factor IWS1
  
Accession: EFY85716
  
Location: 103131-104548
  
 NCBI BlastP on this gene

EFY85716

serine/threonine-protein kinase SRPK2
  
Accession: EFY85715
  
Location: 98215-101450
  
  
**BlastP hit with Mycgr3G84644\_Mycgr3T**
  
Percentage identity: 70 %
  
BlastP bit score: 770
  
Sequence coverage: 98 %
  
E-value: 0.0
  
  
 NCBI BlastP on this gene

EFY85715

hypothetical protein
  
Accession: EFY85714
  
Location: 96263-97413
  
 NCBI BlastP on this gene

EFY85714

putative vesicular transport protein
  
Accession: EFY85713
  
Location: 91801-95386
  
  
**BlastP hit with Mycgr3G108094\_Mycgr3**
  
Percentage identity: 33 %
  
BlastP bit score: 241
  
Sequence coverage: 44 %
  
E-value: 9e-62
  
  
 NCBI BlastP on this gene

EFY85713

SWIRM domain-containing protein
  
Accession: EFY85712
  
Location: 86783-88279
  
 NCBI BlastP on this gene

EFY85712

F-box domain containing protein
  
Accession: EFY85711
  
Location: 77475-79295
  
 NCBI BlastP on this gene

EFY85711

N2,N2-dimethylguanosine tRNA methyltransferase
  
Accession: EFY85710
  
Location: 74494-75600
  
 NCBI BlastP on this gene

EFY85710

C2H2 type zinc finger containing protein
  
Accession: EFY85709
  
Location: 73638-74170
  
 NCBI BlastP on this gene

EFY85709

Query: Architecture Search FASTA input

GL698734 : Metarhizium anisopliae ARSEF 23 unplaced genomic scaffold Scf\_024    Total score: 2.0     Cumulative Blast bit score: 1009

Hit cluster cross-links:

Mycgr3G90785 Mycgr3T
  
Location: 0-1047

Mycgr3G90785\_Mycgr3T

Mycgr3G103262 Mycgr3
  
Location: 1147-1390

Mycgr3G103262\_Mycgr3

Mycgr3G68458 Mycgr3T
  
Location: 1490-3602

Mycgr3G68458\_Mycgr3T

Mycgr3G99145 Mycgr3T
  
Location: 3702-4326

Mycgr3G99145\_Mycgr3T

Mycgr3G103274 Mycgr3
  
Location: 4426-4957

Mycgr3G103274\_Mycgr3

Mycgr3G103264 Mycgr3
  
Location: 5057-5390

Mycgr3G103264\_Mycgr3

Mycgr3G37570 Mycgr3T
  
Location: 5490-6006

Mycgr3G37570\_Mycgr3T

Mycgr3G108094 Mycgr3
  
Location: 6106-10555

Mycgr3G108094\_Mycgr3

Mycgr3G90786 Mycgr3T
  
Location: 10655-12080

Mycgr3G90786\_Mycgr3T

Mycgr3G68429 Mycgr3T
  
Location: 12180-13440

Mycgr3G68429\_Mycgr3T

Mycgr3G68421 Mycgr3T
  
Location: 13540-17086

Mycgr3G68421\_Mycgr3T

Mycgr3G90801 Mycgr3T
  
Location: 17186-18056

Mycgr3G90801\_Mycgr3T

Mycgr3G84646 Mycgr3T
  
Location: 18156-20235

Mycgr3G84646\_Mycgr3T

Mycgr3G68456 Mycgr3T
  
Location: 20335-21970

Mycgr3G68456\_Mycgr3T

Mycgr3G103270 Mycgr3
  
Location: 22070-22355

Mycgr3G103270\_Mycgr3

Mycgr3G90803 Mycgr3T
  
Location: 22455-23019

Mycgr3G90803\_Mycgr3T

Mycgr3G36941 Mycgr3T
  
Location: 23119-24064

Mycgr3G36941\_Mycgr3T

Mycgr3G25746 Mycgr3T
  
Location: 24164-25241

Mycgr3G25746\_Mycgr3T

Mycgr3G90788 Mycgr3T
  
Location: 25341-25803

Mycgr3G90788\_Mycgr3T

Mycgr3G103260 Mycgr3
  
Location: 25903-26635

Mycgr3G103260\_Mycgr3

Mycgr3G84644 Mycgr3T
  
Location: 26735-28457

Mycgr3G84644\_Mycgr3T

Mycgr3G29227 Mycgr3T
  
Location: 28557-28863

Mycgr3G29227\_Mycgr3T

Mycgr3G36271 Mycgr3T
  
Location: 28963-29854

Mycgr3G36271\_Mycgr3T

Mycgr3G68433 Mycgr3T
  
Location: 29954-33041

Mycgr3G68433\_Mycgr3T

Mycgr3G79452 Mycgr3T
  
Location: 33141-33399

Mycgr3G79452\_Mycgr3T

Mycgr3G55345 Mycgr3T
  
Location: 33499-34126

Mycgr3G55345\_Mycgr3T

Mycgr3G103278 Mycgr3
  
Location: 34226-35195

Mycgr3G103278\_Mycgr3

Mycgr3G84654 Mycgr3T
  
Location: 35295-36630

Mycgr3G84654\_Mycgr3T

Mycgr3G108090 Mycgr3
  
Location: 36730-37591

Mycgr3G108090\_Mycgr3

Mycgr3G21922 Mycgr3T
  
Location: 37691-39149

Mycgr3G21922\_Mycgr3T

Mycgr3G99148 Mycgr3T
  
Location: 39249-42819

Mycgr3G99148\_Mycgr3T

peroxisomal membrane protein pex16
  
Accession: EFY95993
  
Location: 54208-55470
  
 NCBI BlastP on this gene

EFY95993

putative nitrate assimilation regulatory protein nirA
  
Accession: EFY95992
  
Location: 52806-53510
  
 NCBI BlastP on this gene

EFY95992

nuclear envelope protein Brr6, putative
  
Accession: EFY95991
  
Location: 47394-48918
  
 NCBI BlastP on this gene

EFY95991

transcription factor IWS1
  
Accession: EFY95990
  
Location: 45512-46930
  
 NCBI BlastP on this gene

EFY95990

serine/threonine-protein kinase SRPK2
  
Accession: EFY95989
  
Location: 40314-44917
  
  
**BlastP hit with Mycgr3G84644\_Mycgr3T**
  
Percentage identity: 70 %
  
BlastP bit score: 768
  
Sequence coverage: 97 %
  
E-value: 0.0
  
  
 NCBI BlastP on this gene

EFY95989

hypothetical protein
  
Accession: EFY95988
  
Location: 38326-39512
  
 NCBI BlastP on this gene

EFY95988

putative vesicular transport protein
  
Accession: EFY95987
  
Location: 34112-37434
  
  
**BlastP hit with Mycgr3G108094\_Mycgr3**
  
Percentage identity: 37 %
  
BlastP bit score: 241
  
Sequence coverage: 39 %
  
E-value: 6e-62
  
  
 NCBI BlastP on this gene

EFY95987

SWIRM domain protein Fun19, putative
  
Accession: EFY95986
  
Location: 29550-30869
  
 NCBI BlastP on this gene

EFY95986

F-box domain containing protein
  
Accession: EFY95985
  
Location: 20129-21949
  
 NCBI BlastP on this gene

EFY95985

N2,N2-dimethylguanosine tRNA methyltransferase
  
Accession: EFY95984
  
Location: 16961-18052
  
 NCBI BlastP on this gene

EFY95984

C2H2 type zinc finger containing protein
  
Accession: EFY95983
  
Location: 16036-16571
  
 NCBI BlastP on this gene

EFY95983

Query: Architecture Search FASTA input

FQ790349 : Botryotinia fuckeliana T4 SupSuperContig\_62\_54\_1 genomic supercontig.    Total score: 2.0     Cumulative Blast bit score: 1009

Hit cluster cross-links:

Mycgr3G90785 Mycgr3T
  
Location: 0-1047

Mycgr3G90785\_Mycgr3T

Mycgr3G103262 Mycgr3
  
Location: 1147-1390

Mycgr3G103262\_Mycgr3

Mycgr3G68458 Mycgr3T
  
Location: 1490-3602

Mycgr3G68458\_Mycgr3T

Mycgr3G99145 Mycgr3T
  
Location: 3702-4326

Mycgr3G99145\_Mycgr3T

Mycgr3G103274 Mycgr3
  
Location: 4426-4957

Mycgr3G103274\_Mycgr3

Mycgr3G103264 Mycgr3
  
Location: 5057-5390

Mycgr3G103264\_Mycgr3

Mycgr3G37570 Mycgr3T
  
Location: 5490-6006

Mycgr3G37570\_Mycgr3T

Mycgr3G108094 Mycgr3
  
Location: 6106-10555

Mycgr3G108094\_Mycgr3

Mycgr3G90786 Mycgr3T
  
Location: 10655-12080

Mycgr3G90786\_Mycgr3T

Mycgr3G68429 Mycgr3T
  
Location: 12180-13440

Mycgr3G68429\_Mycgr3T

Mycgr3G68421 Mycgr3T
  
Location: 13540-17086

Mycgr3G68421\_Mycgr3T

Mycgr3G90801 Mycgr3T
  
Location: 17186-18056

Mycgr3G90801\_Mycgr3T

Mycgr3G84646 Mycgr3T
  
Location: 18156-20235

Mycgr3G84646\_Mycgr3T

Mycgr3G68456 Mycgr3T
  
Location: 20335-21970

Mycgr3G68456\_Mycgr3T

Mycgr3G103270 Mycgr3
  
Location: 22070-22355

Mycgr3G103270\_Mycgr3

Mycgr3G90803 Mycgr3T
  
Location: 22455-23019

Mycgr3G90803\_Mycgr3T

Mycgr3G36941 Mycgr3T
  
Location: 23119-24064

Mycgr3G36941\_Mycgr3T

Mycgr3G25746 Mycgr3T
  
Location: 24164-25241

Mycgr3G25746\_Mycgr3T

Mycgr3G90788 Mycgr3T
  
Location: 25341-25803

Mycgr3G90788\_Mycgr3T

Mycgr3G103260 Mycgr3
  
Location: 25903-26635

Mycgr3G103260\_Mycgr3

Mycgr3G84644 Mycgr3T
  
Location: 26735-28457

Mycgr3G84644\_Mycgr3T

Mycgr3G29227 Mycgr3T
  
Location: 28557-28863

Mycgr3G29227\_Mycgr3T

Mycgr3G36271 Mycgr3T
  
Location: 28963-29854

Mycgr3G36271\_Mycgr3T

Mycgr3G68433 Mycgr3T
  
Location: 29954-33041

Mycgr3G68433\_Mycgr3T

Mycgr3G79452 Mycgr3T
  
Location: 33141-33399

Mycgr3G79452\_Mycgr3T

Mycgr3G55345 Mycgr3T
  
Location: 33499-34126

Mycgr3G55345\_Mycgr3T

Mycgr3G103278 Mycgr3
  
Location: 34226-35195

Mycgr3G103278\_Mycgr3

Mycgr3G84654 Mycgr3T
  
Location: 35295-36630

Mycgr3G84654\_Mycgr3T

Mycgr3G108090 Mycgr3
  
Location: 36730-37591

Mycgr3G108090\_Mycgr3

Mycgr3G21922 Mycgr3T
  
Location: 37691-39149

Mycgr3G21922\_Mycgr3T

Mycgr3G99148 Mycgr3T
  
Location: 39249-42819

Mycgr3G99148\_Mycgr3T

similar to subtilisin-like serine protease (secreted protein)
  
Accession: CCD53977
  
Location: 285126-286942
  
 NCBI BlastP on this gene

BofuT4\_P131570.1

hypothetical protein
  
Accession: CCD53978
  
Location: 287522-291091
  
 NCBI BlastP on this gene

BofuT4\_P131580.1

predicted protein
  
Accession: CCD53979
  
Location: 293879-294193
  
 NCBI BlastP on this gene

BofuT4\_uP131590.1

predicted protein
  
Accession: CCD53980
  
Location: 295746-295921
  
 NCBI BlastP on this gene

BofuT4\_uP131600.1

predicted protein
  
Accession: CCD53981
  
Location: 296168-296775
  
 NCBI BlastP on this gene

BofuT4\_P131610.1

hypothetical protein
  
Accession: CCD53982
  
Location: 297694-298020
  
 NCBI BlastP on this gene

BofuT4\_P131620.1

hypothetical protein
  
Accession: CCD53983
  
Location: 299203-299355
  
 NCBI BlastP on this gene

BofuT4\_uP131630.1

hypothetical protein
  
Accession: CCD53984
  
Location: 299654-300721
  
 NCBI BlastP on this gene

BofuT4\_P131640.1

hypothetical protein
  
Accession: CCD53985
  
Location: 300921-302201
  
 NCBI BlastP on this gene

BofuT4\_P131650.1

hypothetical protein
  
Accession: CCD53986
  
Location: 302649-307255
  
  
**BlastP hit with Mycgr3G108094\_Mycgr3**
  
Percentage identity: 31 %
  
BlastP bit score: 236
  
Sequence coverage: 47 %
  
E-value: 9e-60
  
  
 NCBI BlastP on this gene

BofuT4\_P131660.1

hypothetical protein
  
Accession: CCD53987
  
Location: 307731-308954
  
 NCBI BlastP on this gene

BofuT4\_P131670.1

hypothetical protein
  
Accession: CCD53988
  
Location: 309284-309557
  
 NCBI BlastP on this gene

BofuT4\_uP131680.1

hypothetical protein
  
Accession: CCD53989
  
Location: 311757-312023
  
 NCBI BlastP on this gene

BofuT4\_uP131690.1

similar to serine protein kinase Sky1
  
Accession: CCD53990
  
Location: 312730-314667
  
  
**BlastP hit with Mycgr3G84644\_Mycgr3T**
  
Percentage identity: 76 %
  
BlastP bit score: 773
  
Sequence coverage: 89 %
  
E-value: 0.0
  
  
 NCBI BlastP on this gene

BofuT4\_P131700.1

hypothetical protein
  
Accession: CCD53991
  
Location: 315740-317244
  
 NCBI BlastP on this gene

BofuT4\_P131710.1

similar to transcriptional elongation factor Iws1
  
Accession: CCD53992
  
Location: 317648-319131
  
 NCBI BlastP on this gene

BofuT4\_P131720.1

similar to transcription factor bHLH
  
Accession: CCD53993
  
Location: 321040-322569
  
 NCBI BlastP on this gene

BofuT4\_P131730.1

predicted protein
  
Accession: CCD53994
  
Location: 325438-325593
  
 NCBI BlastP on this gene

BofuT4\_uP131740.1

predicted protein
  
Accession: CCD53995
  
Location: 329368-330061
  
 NCBI BlastP on this gene

BofuT4\_uP131750.1

predicted protein
  
Accession: CCD53996
  
Location: 330336-330821
  
 NCBI BlastP on this gene

BofuT4\_uP131760.1

similar to charged multivesicular body protein 1b
  
Accession: CCD53997
  
Location: 331814-332666
  
 NCBI BlastP on this gene

BofuT4\_P131770.1

Query: Architecture Search FASTA input

GG698942 : Nectria haematococca mpVI 77-13-4 chromosome 3 genomic scaffold NECHAsca\_54\_chr3\_4\_0    Total score: 2.0     Cumulative Blast bit score: 1005

Hit cluster cross-links:

Mycgr3G90785 Mycgr3T
  
Location: 0-1047

Mycgr3G90785\_Mycgr3T

Mycgr3G103262 Mycgr3
  
Location: 1147-1390

Mycgr3G103262\_Mycgr3

Mycgr3G68458 Mycgr3T
  
Location: 1490-3602

Mycgr3G68458\_Mycgr3T

Mycgr3G99145 Mycgr3T
  
Location: 3702-4326

Mycgr3G99145\_Mycgr3T

Mycgr3G103274 Mycgr3
  
Location: 4426-4957

Mycgr3G103274\_Mycgr3

Mycgr3G103264 Mycgr3
  
Location: 5057-5390

Mycgr3G103264\_Mycgr3

Mycgr3G37570 Mycgr3T
  
Location: 5490-6006

Mycgr3G37570\_Mycgr3T

Mycgr3G108094 Mycgr3
  
Location: 6106-10555

Mycgr3G108094\_Mycgr3

Mycgr3G90786 Mycgr3T
  
Location: 10655-12080

Mycgr3G90786\_Mycgr3T

Mycgr3G68429 Mycgr3T
  
Location: 12180-13440

Mycgr3G68429\_Mycgr3T

Mycgr3G68421 Mycgr3T
  
Location: 13540-17086

Mycgr3G68421\_Mycgr3T

Mycgr3G90801 Mycgr3T
  
Location: 17186-18056

Mycgr3G90801\_Mycgr3T

Mycgr3G84646 Mycgr3T
  
Location: 18156-20235

Mycgr3G84646\_Mycgr3T

Mycgr3G68456 Mycgr3T
  
Location: 20335-21970

Mycgr3G68456\_Mycgr3T

Mycgr3G103270 Mycgr3
  
Location: 22070-22355

Mycgr3G103270\_Mycgr3

Mycgr3G90803 Mycgr3T
  
Location: 22455-23019

Mycgr3G90803\_Mycgr3T

Mycgr3G36941 Mycgr3T
  
Location: 23119-24064

Mycgr3G36941\_Mycgr3T

Mycgr3G25746 Mycgr3T
  
Location: 24164-25241

Mycgr3G25746\_Mycgr3T

Mycgr3G90788 Mycgr3T
  
Location: 25341-25803

Mycgr3G90788\_Mycgr3T

Mycgr3G103260 Mycgr3
  
Location: 25903-26635

Mycgr3G103260\_Mycgr3

Mycgr3G84644 Mycgr3T
  
Location: 26735-28457

Mycgr3G84644\_Mycgr3T

Mycgr3G29227 Mycgr3T
  
Location: 28557-28863

Mycgr3G29227\_Mycgr3T

Mycgr3G36271 Mycgr3T
  
Location: 28963-29854

Mycgr3G36271\_Mycgr3T

Mycgr3G68433 Mycgr3T
  
Location: 29954-33041

Mycgr3G68433\_Mycgr3T

Mycgr3G79452 Mycgr3T
  
Location: 33141-33399

Mycgr3G79452\_Mycgr3T

Mycgr3G55345 Mycgr3T
  
Location: 33499-34126

Mycgr3G55345\_Mycgr3T

Mycgr3G103278 Mycgr3
  
Location: 34226-35195

Mycgr3G103278\_Mycgr3

Mycgr3G84654 Mycgr3T
  
Location: 35295-36630

Mycgr3G84654\_Mycgr3T

Mycgr3G108090 Mycgr3
  
Location: 36730-37591

Mycgr3G108090\_Mycgr3

Mycgr3G21922 Mycgr3T
  
Location: 37691-39149

Mycgr3G21922\_Mycgr3T

Mycgr3G99148 Mycgr3T
  
Location: 39249-42819

Mycgr3G99148\_Mycgr3T

hypothetical protein
  
Accession: EEU35286
  
Location: 78801-79937
  
 NCBI BlastP on this gene

EEU35286

predicted protein
  
Accession: EEU35321
  
Location: 82006-82956
  
 NCBI BlastP on this gene

EEU35321

hypothetical protein
  
Accession: EEU35322
  
Location: 83616-87218
  
  
**BlastP hit with Mycgr3G108094\_Mycgr3**
  
Percentage identity: 34 %
  
BlastP bit score: 247
  
Sequence coverage: 44 %
  
E-value: 8e-64
  
  
 NCBI BlastP on this gene

EEU35322

hypothetical protein
  
Accession: EEU35287
  
Location: 89090-90286
  
 NCBI BlastP on this gene

EEU35287

predicted protein
  
Accession: EEU35323
  
Location: 91019-93702
  
 NCBI BlastP on this gene

EEU35323

hypothetical protein
  
Accession: EEU35324
  
Location: 96414-97988
  
 NCBI BlastP on this gene

EEU35324

predicted protein
  
Accession: EEU35288
  
Location: 98429-99824
  
 NCBI BlastP on this gene

EEU35288

predicted protein
  
Accession: EEU35289
  
Location: 102327-103446
  
 NCBI BlastP on this gene

EEU35289

predicted protein
  
Accession: EEU35290
  
Location: 104522-106263
  
  
**BlastP hit with Mycgr3G84644\_Mycgr3T**
  
Percentage identity: 74 %
  
BlastP bit score: 758
  
Sequence coverage: 89 %
  
E-value: 0.0
  
  
 NCBI BlastP on this gene

EEU35290

hypothetical protein
  
Accession: EEU35325
  
Location: 106970-108637
  
 NCBI BlastP on this gene

EEU35325

hypothetical protein
  
Accession: EEU35291
  
Location: 109262-110827
  
 NCBI BlastP on this gene

EEU35291

hypothetical protein
  
Accession: EEU35292
  
Location: 111710-112860
  
 NCBI BlastP on this gene

EEU35292

hypothetical protein
  
Accession: EEU35293
  
Location: 118565-120421
  
 NCBI BlastP on this gene

EEU35293

Query: Architecture Search FASTA input

GL698717 : Metarhizium anisopliae ARSEF 23 unplaced genomic scaffold Scf\_007    Total score: 2.0     Cumulative Blast bit score: 1004

Hit cluster cross-links:

Mycgr3G90785 Mycgr3T
  
Location: 0-1047

Mycgr3G90785\_Mycgr3T

Mycgr3G103262 Mycgr3
  
Location: 1147-1390

Mycgr3G103262\_Mycgr3

Mycgr3G68458 Mycgr3T
  
Location: 1490-3602

Mycgr3G68458\_Mycgr3T

Mycgr3G99145 Mycgr3T
  
Location: 3702-4326

Mycgr3G99145\_Mycgr3T

Mycgr3G103274 Mycgr3
  
Location: 4426-4957

Mycgr3G103274\_Mycgr3

Mycgr3G103264 Mycgr3
  
Location: 5057-5390

Mycgr3G103264\_Mycgr3

Mycgr3G37570 Mycgr3T
  
Location: 5490-6006

Mycgr3G37570\_Mycgr3T

Mycgr3G108094 Mycgr3
  
Location: 6106-10555

Mycgr3G108094\_Mycgr3

Mycgr3G90786 Mycgr3T
  
Location: 10655-12080

Mycgr3G90786\_Mycgr3T

Mycgr3G68429 Mycgr3T
  
Location: 12180-13440

Mycgr3G68429\_Mycgr3T

Mycgr3G68421 Mycgr3T
  
Location: 13540-17086

Mycgr3G68421\_Mycgr3T

Mycgr3G90801 Mycgr3T
  
Location: 17186-18056

Mycgr3G90801\_Mycgr3T

Mycgr3G84646 Mycgr3T
  
Location: 18156-20235

Mycgr3G84646\_Mycgr3T

Mycgr3G68456 Mycgr3T
  
Location: 20335-21970

Mycgr3G68456\_Mycgr3T

Mycgr3G103270 Mycgr3
  
Location: 22070-22355

Mycgr3G103270\_Mycgr3

Mycgr3G90803 Mycgr3T
  
Location: 22455-23019

Mycgr3G90803\_Mycgr3T

Mycgr3G36941 Mycgr3T
  
Location: 23119-24064

Mycgr3G36941\_Mycgr3T

Mycgr3G25746 Mycgr3T
  
Location: 24164-25241

Mycgr3G25746\_Mycgr3T

Mycgr3G90788 Mycgr3T
  
Location: 25341-25803

Mycgr3G90788\_Mycgr3T

Mycgr3G103260 Mycgr3
  
Location: 25903-26635

Mycgr3G103260\_Mycgr3

Mycgr3G84644 Mycgr3T
  
Location: 26735-28457

Mycgr3G84644\_Mycgr3T

Mycgr3G29227 Mycgr3T
  
Location: 28557-28863

Mycgr3G29227\_Mycgr3T

Mycgr3G36271 Mycgr3T
  
Location: 28963-29854

Mycgr3G36271\_Mycgr3T

Mycgr3G68433 Mycgr3T
  
Location: 29954-33041

Mycgr3G68433\_Mycgr3T

Mycgr3G79452 Mycgr3T
  
Location: 33141-33399

Mycgr3G79452\_Mycgr3T

Mycgr3G55345 Mycgr3T
  
Location: 33499-34126

Mycgr3G55345\_Mycgr3T

Mycgr3G103278 Mycgr3
  
Location: 34226-35195

Mycgr3G103278\_Mycgr3

Mycgr3G84654 Mycgr3T
  
Location: 35295-36630

Mycgr3G84654\_Mycgr3T

Mycgr3G108090 Mycgr3
  
Location: 36730-37591

Mycgr3G108090\_Mycgr3

Mycgr3G21922 Mycgr3T
  
Location: 37691-39149

Mycgr3G21922\_Mycgr3T

Mycgr3G99148 Mycgr3T
  
Location: 39249-42819

Mycgr3G99148\_Mycgr3T

serine/threonine protein phosphatase PP2A catalytic subunit
  
Accession: EFY99004
  
Location: 114455-115965
  
 NCBI BlastP on this gene

EFY99004

Exocyst complex component EXO84
  
Accession: EFY99005
  
Location: 116866-119031
  
 NCBI BlastP on this gene

EFY99005

mitochondrial import receptor subunit tom-20
  
Accession: EFY99006
  
Location: 119696-120485
  
 NCBI BlastP on this gene

EFY99006

flavodoxin and radical SAM domain protein
  
Accession: EFY99007
  
Location: 120969-123403
  
 NCBI BlastP on this gene

EFY99007

DNA repair and recombination protein RAD5B
  
Accession: EFY99008
  
Location: 124050-127199
  
 NCBI BlastP on this gene

EFY99008

cysteine dioxygenase
  
Accession: EFY99009
  
Location: 127977-128770
  
 NCBI BlastP on this gene

EFY99009

C4-dicarboxylate transporter, putative
  
Accession: EFY99010
  
Location: 131258-132187
  
 NCBI BlastP on this gene

EFY99010

ATP synthase regulation protein NCA2
  
Accession: EFY99011
  
Location: 132727-134812
  
  
**BlastP hit with Mycgr3G84646\_Mycgr3T**
  
Percentage identity: 42 %
  
BlastP bit score: 541
  
Sequence coverage: 100 %
  
E-value: 2e-180
  
  
 NCBI BlastP on this gene

EFY99011

C-4 sterol methyl oxidase
  
Accession: EFY99012
  
Location: 136082-137187
  
  
**BlastP hit with Mycgr3G36271\_Mycgr3T**
  
Percentage identity: 72 %
  
BlastP bit score: 463
  
Sequence coverage: 96 %
  
E-value: 2e-161
  
  
 NCBI BlastP on this gene

EFY99012

pterin-4-alpha-carbinolamine dehydratase family protein
  
Accession: EFY99013
  
Location: 137725-138438
  
 NCBI BlastP on this gene

EFY99013

amidohydrolase
  
Accession: EFY99014
  
Location: 138864-140213
  
 NCBI BlastP on this gene

EFY99014

transmembrane protein
  
Accession: EFY99015
  
Location: 141903-143376
  
 NCBI BlastP on this gene

EFY99015

chromatin remodeling complex subunit (Arp5), putative
  
Accession: EFY99016
  
Location: 143972-146445
  
 NCBI BlastP on this gene

EFY99016

hypothetical protein
  
Accession: EFY99017
  
Location: 147687-148265
  
 NCBI BlastP on this gene

EFY99017

Got1 family protein
  
Accession: EFY99018
  
Location: 149294-150049
  
 NCBI BlastP on this gene

EFY99018

profilin
  
Accession: EFY99019
  
Location: 150709-151400
  
 NCBI BlastP on this gene

EFY99019

hypothetical protein
  
Accession: EFY99020
  
Location: 156037-157689
  
 NCBI BlastP on this gene

EFY99020

Query: Architecture Search FASTA input

GL985057 : Trichoderma reesei QM6a unplaced genomic scaffold TRIREscaffold\_2    Total score: 2.0     Cumulative Blast bit score: 997

Hit cluster cross-links:

Mycgr3G90785 Mycgr3T
  
Location: 0-1047

Mycgr3G90785\_Mycgr3T

Mycgr3G103262 Mycgr3
  
Location: 1147-1390

Mycgr3G103262\_Mycgr3

Mycgr3G68458 Mycgr3T
  
Location: 1490-3602

Mycgr3G68458\_Mycgr3T

Mycgr3G99145 Mycgr3T
  
Location: 3702-4326

Mycgr3G99145\_Mycgr3T

Mycgr3G103274 Mycgr3
  
Location: 4426-4957

Mycgr3G103274\_Mycgr3

Mycgr3G103264 Mycgr3
  
Location: 5057-5390

Mycgr3G103264\_Mycgr3

Mycgr3G37570 Mycgr3T
  
Location: 5490-6006

Mycgr3G37570\_Mycgr3T

Mycgr3G108094 Mycgr3
  
Location: 6106-10555

Mycgr3G108094\_Mycgr3

Mycgr3G90786 Mycgr3T
  
Location: 10655-12080

Mycgr3G90786\_Mycgr3T

Mycgr3G68429 Mycgr3T
  
Location: 12180-13440

Mycgr3G68429\_Mycgr3T

Mycgr3G68421 Mycgr3T
  
Location: 13540-17086

Mycgr3G68421\_Mycgr3T

Mycgr3G90801 Mycgr3T
  
Location: 17186-18056

Mycgr3G90801\_Mycgr3T

Mycgr3G84646 Mycgr3T
  
Location: 18156-20235

Mycgr3G84646\_Mycgr3T

Mycgr3G68456 Mycgr3T
  
Location: 20335-21970

Mycgr3G68456\_Mycgr3T

Mycgr3G103270 Mycgr3
  
Location: 22070-22355

Mycgr3G103270\_Mycgr3

Mycgr3G90803 Mycgr3T
  
Location: 22455-23019

Mycgr3G90803\_Mycgr3T

Mycgr3G36941 Mycgr3T
  
Location: 23119-24064

Mycgr3G36941\_Mycgr3T

Mycgr3G25746 Mycgr3T
  
Location: 24164-25241

Mycgr3G25746\_Mycgr3T

Mycgr3G90788 Mycgr3T
  
Location: 25341-25803

Mycgr3G90788\_Mycgr3T

Mycgr3G103260 Mycgr3
  
Location: 25903-26635

Mycgr3G103260\_Mycgr3

Mycgr3G84644 Mycgr3T
  
Location: 26735-28457

Mycgr3G84644\_Mycgr3T

Mycgr3G29227 Mycgr3T
  
Location: 28557-28863

Mycgr3G29227\_Mycgr3T

Mycgr3G36271 Mycgr3T
  
Location: 28963-29854

Mycgr3G36271\_Mycgr3T

Mycgr3G68433 Mycgr3T
  
Location: 29954-33041

Mycgr3G68433\_Mycgr3T

Mycgr3G79452 Mycgr3T
  
Location: 33141-33399

Mycgr3G79452\_Mycgr3T

Mycgr3G55345 Mycgr3T
  
Location: 33499-34126

Mycgr3G55345\_Mycgr3T

Mycgr3G103278 Mycgr3
  
Location: 34226-35195

Mycgr3G103278\_Mycgr3

Mycgr3G84654 Mycgr3T
  
Location: 35295-36630

Mycgr3G84654\_Mycgr3T

Mycgr3G108090 Mycgr3
  
Location: 36730-37591

Mycgr3G108090\_Mycgr3

Mycgr3G21922 Mycgr3T
  
Location: 37691-39149

Mycgr3G21922\_Mycgr3T

Mycgr3G99148 Mycgr3T
  
Location: 39249-42819

Mycgr3G99148\_Mycgr3T

predicted protein
  
Accession: EGR52017
  
Location: 1242446-1243432
  
 NCBI BlastP on this gene

EGR52017

predicted protein
  
Accession: EGR52018
  
Location: 1251041-1254197
  
 NCBI BlastP on this gene

EGR52018

hypothetical protein
  
Accession: EGR52019
  
Location: 1255166-1255952
  
 NCBI BlastP on this gene

EGR52019

predicted protein
  
Accession: EGR51742
  
Location: 1259267-1260836
  
 NCBI BlastP on this gene

EGR51742

predicted protein
  
Accession: EGR52020
  
Location: 1261252-1263389
  
  
**BlastP hit with Mycgr3G84646\_Mycgr3T**
  
Percentage identity: 45 %
  
BlastP bit score: 550
  
Sequence coverage: 102 %
  
E-value: 0.0
  
  
 NCBI BlastP on this gene

EGR52020

sterol desaturase-like protein
  
Accession: EGR51743
  
Location: 1265105-1266157
  
  
**BlastP hit with Mycgr3G36271\_Mycgr3T**
  
Percentage identity: 71 %
  
BlastP bit score: 448
  
Sequence coverage: 95 %
  
E-value: 2e-155
  
  
 NCBI BlastP on this gene

EGR51743

predicted protein
  
Accession: EGR52021
  
Location: 1267002-1267948
  
 NCBI BlastP on this gene

EGR52021

predicted protein
  
Accession: EGR51744
  
Location: 1268313-1268718
  
 NCBI BlastP on this gene

EGR51744

actin-like protein
  
Accession: EGR51745
  
Location: 1269290-1271778
  
 NCBI BlastP on this gene

EGR51745

DNA binding domain-containing/Basic-Leucine zipper transcription factor domain-containing protein
  
Accession: EGR52022
  
Location: 1272638-1273746
  
 NCBI BlastP on this gene

EGR52022

predicted protein
  
Accession: EGR51746
  
Location: 1273896-1274360
  
 NCBI BlastP on this gene

EGR51746

predicted protein
  
Accession: EGR52023
  
Location: 1276821-1279084
  
 NCBI BlastP on this gene

EGR52023

predicted protein
  
Accession: EGR51747
  
Location: 1280095-1280683
  
 NCBI BlastP on this gene

EGR51747

predicted protein
  
Accession: EGR52024
  
Location: 1284346-1285628
  
 NCBI BlastP on this gene

EGR52024

Query: Architecture Search FASTA input

AHHD01000090 : Macrophomina phaseolina MS6    Total score: 2.0     Cumulative Blast bit score: 995

Hit cluster cross-links:

Mycgr3G90785 Mycgr3T
  
Location: 0-1047

Mycgr3G90785\_Mycgr3T

Mycgr3G103262 Mycgr3
  
Location: 1147-1390

Mycgr3G103262\_Mycgr3

Mycgr3G68458 Mycgr3T
  
Location: 1490-3602

Mycgr3G68458\_Mycgr3T

Mycgr3G99145 Mycgr3T
  
Location: 3702-4326

Mycgr3G99145\_Mycgr3T

Mycgr3G103274 Mycgr3
  
Location: 4426-4957

Mycgr3G103274\_Mycgr3

Mycgr3G103264 Mycgr3
  
Location: 5057-5390

Mycgr3G103264\_Mycgr3

Mycgr3G37570 Mycgr3T
  
Location: 5490-6006

Mycgr3G37570\_Mycgr3T

Mycgr3G108094 Mycgr3
  
Location: 6106-10555

Mycgr3G108094\_Mycgr3

Mycgr3G90786 Mycgr3T
  
Location: 10655-12080

Mycgr3G90786\_Mycgr3T

Mycgr3G68429 Mycgr3T
  
Location: 12180-13440

Mycgr3G68429\_Mycgr3T

Mycgr3G68421 Mycgr3T
  
Location: 13540-17086

Mycgr3G68421\_Mycgr3T

Mycgr3G90801 Mycgr3T
  
Location: 17186-18056

Mycgr3G90801\_Mycgr3T

Mycgr3G84646 Mycgr3T
  
Location: 18156-20235

Mycgr3G84646\_Mycgr3T

Mycgr3G68456 Mycgr3T
  
Location: 20335-21970

Mycgr3G68456\_Mycgr3T

Mycgr3G103270 Mycgr3
  
Location: 22070-22355

Mycgr3G103270\_Mycgr3

Mycgr3G90803 Mycgr3T
  
Location: 22455-23019

Mycgr3G90803\_Mycgr3T

Mycgr3G36941 Mycgr3T
  
Location: 23119-24064

Mycgr3G36941\_Mycgr3T

Mycgr3G25746 Mycgr3T
  
Location: 24164-25241

Mycgr3G25746\_Mycgr3T

Mycgr3G90788 Mycgr3T
  
Location: 25341-25803

Mycgr3G90788\_Mycgr3T

Mycgr3G103260 Mycgr3
  
Location: 25903-26635

Mycgr3G103260\_Mycgr3

Mycgr3G84644 Mycgr3T
  
Location: 26735-28457

Mycgr3G84644\_Mycgr3T

Mycgr3G29227 Mycgr3T
  
Location: 28557-28863

Mycgr3G29227\_Mycgr3T

Mycgr3G36271 Mycgr3T
  
Location: 28963-29854

Mycgr3G36271\_Mycgr3T

Mycgr3G68433 Mycgr3T
  
Location: 29954-33041

Mycgr3G68433\_Mycgr3T

Mycgr3G79452 Mycgr3T
  
Location: 33141-33399

Mycgr3G79452\_Mycgr3T

Mycgr3G55345 Mycgr3T
  
Location: 33499-34126

Mycgr3G55345\_Mycgr3T

Mycgr3G103278 Mycgr3
  
Location: 34226-35195

Mycgr3G103278\_Mycgr3

Mycgr3G84654 Mycgr3T
  
Location: 35295-36630

Mycgr3G84654\_Mycgr3T

Mycgr3G108090 Mycgr3
  
Location: 36730-37591

Mycgr3G108090\_Mycgr3

Mycgr3G21922 Mycgr3T
  
Location: 37691-39149

Mycgr3G21922\_Mycgr3T

Mycgr3G99148 Mycgr3T
  
Location: 39249-42819

Mycgr3G99148\_Mycgr3T

MaoC-like dehydratase
  
Accession: EKG20274
  
Location: 74332-75779
  
 NCBI BlastP on this gene

EKG20274

hypothetical protein
  
Accession: EKG20275
  
Location: 77426-79390
  
 NCBI BlastP on this gene

EKG20275

Profilin/allergen
  
Accession: EKG20276
  
Location: 79944-80705
  
 NCBI BlastP on this gene

EKG20276

Ubiquitin-conjugating enzyme E2
  
Accession: EKG20277
  
Location: 83266-83734
  
 NCBI BlastP on this gene

EKG20277

hypothetical protein
  
Accession: EKG20278
  
Location: 85715-88300
  
 NCBI BlastP on this gene

EKG20278

Nuclear control of ATP synthase 2
  
Accession: EKG20279
  
Location: 90662-92958
  
  
**BlastP hit with Mycgr3G84646\_Mycgr3T**
  
Percentage identity: 45 %
  
BlastP bit score: 623
  
Sequence coverage: 104 %
  
E-value: 0.0
  
  
 NCBI BlastP on this gene

EKG20279

Ras GTPase
  
Accession: EKG20280
  
Location: 93780-94902
  
  
**BlastP hit with Mycgr3G99145\_Mycgr3T**
  
Percentage identity: 88 %
  
BlastP bit score: 372
  
Sequence coverage: 100 %
  
E-value: 2e-128
  
  
 NCBI BlastP on this gene

EKG20280

hypothetical protein
  
Accession: EKG20281
  
Location: 98726-100219
  
 NCBI BlastP on this gene

EKG20281

Major facilitator superfamily
  
Accession: EKG20282
  
Location: 102489-107077
  
 NCBI BlastP on this gene

EKG20282

Query: Architecture Search FASTA input

ABDG02000026 : Trichoderma atroviride IMI 206040    Total score: 2.0     Cumulative Blast bit score: 993

Hit cluster cross-links:

Mycgr3G90785 Mycgr3T
  
Location: 0-1047

Mycgr3G90785\_Mycgr3T

Mycgr3G103262 Mycgr3
  
Location: 1147-1390

Mycgr3G103262\_Mycgr3

Mycgr3G68458 Mycgr3T
  
Location: 1490-3602

Mycgr3G68458\_Mycgr3T

Mycgr3G99145 Mycgr3T
  
Location: 3702-4326

Mycgr3G99145\_Mycgr3T

Mycgr3G103274 Mycgr3
  
Location: 4426-4957

Mycgr3G103274\_Mycgr3

Mycgr3G103264 Mycgr3
  
Location: 5057-5390

Mycgr3G103264\_Mycgr3

Mycgr3G37570 Mycgr3T
  
Location: 5490-6006

Mycgr3G37570\_Mycgr3T

Mycgr3G108094 Mycgr3
  
Location: 6106-10555

Mycgr3G108094\_Mycgr3

Mycgr3G90786 Mycgr3T
  
Location: 10655-12080

Mycgr3G90786\_Mycgr3T

Mycgr3G68429 Mycgr3T
  
Location: 12180-13440

Mycgr3G68429\_Mycgr3T

Mycgr3G68421 Mycgr3T
  
Location: 13540-17086

Mycgr3G68421\_Mycgr3T

Mycgr3G90801 Mycgr3T
  
Location: 17186-18056

Mycgr3G90801\_Mycgr3T

Mycgr3G84646 Mycgr3T
  
Location: 18156-20235

Mycgr3G84646\_Mycgr3T

Mycgr3G68456 Mycgr3T
  
Location: 20335-21970

Mycgr3G68456\_Mycgr3T

Mycgr3G103270 Mycgr3
  
Location: 22070-22355

Mycgr3G103270\_Mycgr3

Mycgr3G90803 Mycgr3T
  
Location: 22455-23019

Mycgr3G90803\_Mycgr3T

Mycgr3G36941 Mycgr3T
  
Location: 23119-24064

Mycgr3G36941\_Mycgr3T

Mycgr3G25746 Mycgr3T
  
Location: 24164-25241

Mycgr3G25746\_Mycgr3T

Mycgr3G90788 Mycgr3T
  
Location: 25341-25803

Mycgr3G90788\_Mycgr3T

Mycgr3G103260 Mycgr3
  
Location: 25903-26635

Mycgr3G103260\_Mycgr3

Mycgr3G84644 Mycgr3T
  
Location: 26735-28457

Mycgr3G84644\_Mycgr3T

Mycgr3G29227 Mycgr3T
  
Location: 28557-28863

Mycgr3G29227\_Mycgr3T

Mycgr3G36271 Mycgr3T
  
Location: 28963-29854

Mycgr3G36271\_Mycgr3T

Mycgr3G68433 Mycgr3T
  
Location: 29954-33041

Mycgr3G68433\_Mycgr3T

Mycgr3G79452 Mycgr3T
  
Location: 33141-33399

Mycgr3G79452\_Mycgr3T

Mycgr3G55345 Mycgr3T
  
Location: 33499-34126

Mycgr3G55345\_Mycgr3T

Mycgr3G103278 Mycgr3
  
Location: 34226-35195

Mycgr3G103278\_Mycgr3

Mycgr3G84654 Mycgr3T
  
Location: 35295-36630

Mycgr3G84654\_Mycgr3T

Mycgr3G108090 Mycgr3
  
Location: 36730-37591

Mycgr3G108090\_Mycgr3

Mycgr3G21922 Mycgr3T
  
Location: 37691-39149

Mycgr3G21922\_Mycgr3T

Mycgr3G99148 Mycgr3T
  
Location: 39249-42819

Mycgr3G99148\_Mycgr3T

hypothetical protein
  
Accession: EHK42683
  
Location: 1015647-1017674
  
 NCBI BlastP on this gene

EHK42683

hypothetical protein
  
Accession: EHK42684
  
Location: 1018983-1020733
  
 NCBI BlastP on this gene

EHK42684

hypothetical protein
  
Accession: EHK42685
  
Location: 1020809-1021470
  
 NCBI BlastP on this gene

EHK42685

hypothetical protein
  
Accession: EHK42686
  
Location: 1024484-1025833
  
 NCBI BlastP on this gene

EHK42686

serine/threonine protein kinase, CMGC group
  
Accession: EHK42687
  
Location: 1031960-1033723
  
  
**BlastP hit with Mycgr3G84644\_Mycgr3T**
  
Percentage identity: 72 %
  
BlastP bit score: 734
  
Sequence coverage: 86 %
  
E-value: 0.0
  
  
 NCBI BlastP on this gene

EHK42687

hypothetical protein
  
Accession: EHK42688
  
Location: 1034648-1036192
  
 NCBI BlastP on this gene

EHK42688

hypothetical protein
  
Accession: EHK42689
  
Location: 1038333-1039382
  
 NCBI BlastP on this gene

EHK42689

hypothetical protein
  
Accession: EHK42690
  
Location: 1045891-1049857
  
  
**BlastP hit with Mycgr3G108094\_Mycgr3**
  
Percentage identity: 32 %
  
BlastP bit score: 259
  
Sequence coverage: 44 %
  
E-value: 1e-67
  
  
 NCBI BlastP on this gene

EHK42690

hypothetical protein
  
Accession: EHK42691
  
Location: 1051014-1051232
  
 NCBI BlastP on this gene

EHK42691

hypothetical protein
  
Accession: EHK42692
  
Location: 1052204-1053478
  
 NCBI BlastP on this gene

EHK42692

glycosyltransferase family 1 protein
  
Accession: EHK43614
  
Location: 1063433-1065084
  
 NCBI BlastP on this gene

EHK43614

Query: Architecture Search FASTA input

KB445649 : Cochliobolus sativus ND90Pr unplaced genomic scaffold COCSAscaffold\_13    Total score: 2.0     Cumulative Blast bit score: 991

Hit cluster cross-links:

Mycgr3G90785 Mycgr3T
  
Location: 0-1047

Mycgr3G90785\_Mycgr3T

Mycgr3G103262 Mycgr3
  
Location: 1147-1390

Mycgr3G103262\_Mycgr3

Mycgr3G68458 Mycgr3T
  
Location: 1490-3602

Mycgr3G68458\_Mycgr3T

Mycgr3G99145 Mycgr3T
  
Location: 3702-4326

Mycgr3G99145\_Mycgr3T

Mycgr3G103274 Mycgr3
  
Location: 4426-4957

Mycgr3G103274\_Mycgr3

Mycgr3G103264 Mycgr3
  
Location: 5057-5390

Mycgr3G103264\_Mycgr3

Mycgr3G37570 Mycgr3T
  
Location: 5490-6006

Mycgr3G37570\_Mycgr3T

Mycgr3G108094 Mycgr3
  
Location: 6106-10555

Mycgr3G108094\_Mycgr3

Mycgr3G90786 Mycgr3T
  
Location: 10655-12080

Mycgr3G90786\_Mycgr3T

Mycgr3G68429 Mycgr3T
  
Location: 12180-13440

Mycgr3G68429\_Mycgr3T

Mycgr3G68421 Mycgr3T
  
Location: 13540-17086

Mycgr3G68421\_Mycgr3T

Mycgr3G90801 Mycgr3T
  
Location: 17186-18056

Mycgr3G90801\_Mycgr3T

Mycgr3G84646 Mycgr3T
  
Location: 18156-20235

Mycgr3G84646\_Mycgr3T

Mycgr3G68456 Mycgr3T
  
Location: 20335-21970

Mycgr3G68456\_Mycgr3T

Mycgr3G103270 Mycgr3
  
Location: 22070-22355

Mycgr3G103270\_Mycgr3

Mycgr3G90803 Mycgr3T
  
Location: 22455-23019

Mycgr3G90803\_Mycgr3T

Mycgr3G36941 Mycgr3T
  
Location: 23119-24064

Mycgr3G36941\_Mycgr3T

Mycgr3G25746 Mycgr3T
  
Location: 24164-25241

Mycgr3G25746\_Mycgr3T

Mycgr3G90788 Mycgr3T
  
Location: 25341-25803

Mycgr3G90788\_Mycgr3T

Mycgr3G103260 Mycgr3
  
Location: 25903-26635

Mycgr3G103260\_Mycgr3

Mycgr3G84644 Mycgr3T
  
Location: 26735-28457

Mycgr3G84644\_Mycgr3T

Mycgr3G29227 Mycgr3T
  
Location: 28557-28863

Mycgr3G29227\_Mycgr3T

Mycgr3G36271 Mycgr3T
  
Location: 28963-29854

Mycgr3G36271\_Mycgr3T

Mycgr3G68433 Mycgr3T
  
Location: 29954-33041

Mycgr3G68433\_Mycgr3T

Mycgr3G79452 Mycgr3T
  
Location: 33141-33399

Mycgr3G79452\_Mycgr3T

Mycgr3G55345 Mycgr3T
  
Location: 33499-34126

Mycgr3G55345\_Mycgr3T

Mycgr3G103278 Mycgr3
  
Location: 34226-35195

Mycgr3G103278\_Mycgr3

Mycgr3G84654 Mycgr3T
  
Location: 35295-36630

Mycgr3G84654\_Mycgr3T

Mycgr3G108090 Mycgr3
  
Location: 36730-37591

Mycgr3G108090\_Mycgr3

Mycgr3G21922 Mycgr3T
  
Location: 37691-39149

Mycgr3G21922\_Mycgr3T

Mycgr3G99148 Mycgr3T
  
Location: 39249-42819

Mycgr3G99148\_Mycgr3T

hypothetical protein
  
Accession: EMD61270
  
Location: 1370424-1370760
  
 NCBI BlastP on this gene

EMD61270

hypothetical protein
  
Accession: EMD61269
  
Location: 1368649-1368918
  
 NCBI BlastP on this gene

EMD61269

hypothetical protein
  
Accession: EMD61268
  
Location: 1367191-1368282
  
 NCBI BlastP on this gene

EMD61268

hypothetical protein
  
Accession: EMD61267
  
Location: 1366347-1366997
  
 NCBI BlastP on this gene

EMD61267

hypothetical protein
  
Accession: EMD61266
  
Location: 1364592-1365601
  
 NCBI BlastP on this gene

EMD61266

hypothetical protein
  
Accession: EMD61265
  
Location: 1363796-1364257
  
 NCBI BlastP on this gene

EMD61265

hypothetical protein
  
Accession: EMD61264
  
Location: 1361003-1362652
  
 NCBI BlastP on this gene

EMD61264

hypothetical protein
  
Accession: EMD61263
  
Location: 1358538-1358750
  
 NCBI BlastP on this gene

EMD61263

hypothetical protein
  
Accession: EMD61262
  
Location: 1356085-1357748
  
 NCBI BlastP on this gene

EMD61262

hypothetical protein
  
Accession: EMD61261
  
Location: 1354100-1355693
  
 NCBI BlastP on this gene

EMD61261

hypothetical protein
  
Accession: EMD61260
  
Location: 1352049-1353490
  
 NCBI BlastP on this gene

EMD61260

hypothetical protein
  
Accession: EMD61259
  
Location: 1349441-1351781
  
  
**BlastP hit with Mycgr3G108094\_Mycgr3**
  
Percentage identity: 35 %
  
BlastP bit score: 146
  
Sequence coverage: 22 %
  
E-value: 7e-33
  
  
 NCBI BlastP on this gene

EMD61259

hypothetical protein
  
Accession: EMD61258
  
Location: 1347778-1348056
  
 NCBI BlastP on this gene

EMD61258

hypothetical protein
  
Accession: EMD61257
  
Location: 1344408-1346602
  
  
**BlastP hit with Mycgr3G84644\_Mycgr3T**
  
Percentage identity: 70 %
  
BlastP bit score: 845
  
Sequence coverage: 109 %
  
E-value: 0.0
  
  
 NCBI BlastP on this gene

EMD61257

glycoside hydrolase family 16 protein
  
Accession: EMD61256
  
Location: 1341986-1343184
  
 NCBI BlastP on this gene

EMD61256

hypothetical protein
  
Accession: EMD61255
  
Location: 1338191-1340371
  
 NCBI BlastP on this gene

EMD61255

hypothetical protein
  
Accession: EMD61254
  
Location: 1336380-1337444
  
 NCBI BlastP on this gene

EMD61254

hypothetical protein
  
Accession: EMD61253
  
Location: 1335057-1336284
  
 NCBI BlastP on this gene

EMD61253

hypothetical protein
  
Accession: EMD61252
  
Location: 1332721-1334832
  
 NCBI BlastP on this gene

EMD61252

hypothetical protein
  
Accession: EMD61251
  
Location: 1330275-1332066
  
 NCBI BlastP on this gene

EMD61251

hypothetical protein
  
Accession: EMD61250
  
Location: 1328847-1329773
  
 NCBI BlastP on this gene

EMD61250

glycosyltransferase family 2 protein
  
Accession: EMD61249
  
Location: 1327795-1328589
  
 NCBI BlastP on this gene

EMD61249

Query: Architecture Search FASTA input

HF679028 : Fusarium fujikuroi IMI 58289 draft genome, chromosome FFUJ\_chr06.    Total score: 2.0     Cumulative Blast bit score: 988

Hit cluster cross-links:

Mycgr3G90785 Mycgr3T
  
Location: 0-1047

Mycgr3G90785\_Mycgr3T

Mycgr3G103262 Mycgr3
  
Location: 1147-1390

Mycgr3G103262\_Mycgr3

Mycgr3G68458 Mycgr3T
  
Location: 1490-3602

Mycgr3G68458\_Mycgr3T

Mycgr3G99145 Mycgr3T
  
Location: 3702-4326

Mycgr3G99145\_Mycgr3T

Mycgr3G103274 Mycgr3
  
Location: 4426-4957

Mycgr3G103274\_Mycgr3

Mycgr3G103264 Mycgr3
  
Location: 5057-5390

Mycgr3G103264\_Mycgr3

Mycgr3G37570 Mycgr3T
  
Location: 5490-6006

Mycgr3G37570\_Mycgr3T

Mycgr3G108094 Mycgr3
  
Location: 6106-10555

Mycgr3G108094\_Mycgr3

Mycgr3G90786 Mycgr3T
  
Location: 10655-12080

Mycgr3G90786\_Mycgr3T

Mycgr3G68429 Mycgr3T
  
Location: 12180-13440

Mycgr3G68429\_Mycgr3T

Mycgr3G68421 Mycgr3T
  
Location: 13540-17086

Mycgr3G68421\_Mycgr3T

Mycgr3G90801 Mycgr3T
  
Location: 17186-18056

Mycgr3G90801\_Mycgr3T

Mycgr3G84646 Mycgr3T
  
Location: 18156-20235

Mycgr3G84646\_Mycgr3T

Mycgr3G68456 Mycgr3T
  
Location: 20335-21970

Mycgr3G68456\_Mycgr3T

Mycgr3G103270 Mycgr3
  
Location: 22070-22355

Mycgr3G103270\_Mycgr3

Mycgr3G90803 Mycgr3T
  
Location: 22455-23019

Mycgr3G90803\_Mycgr3T

Mycgr3G36941 Mycgr3T
  
Location: 23119-24064

Mycgr3G36941\_Mycgr3T

Mycgr3G25746 Mycgr3T
  
Location: 24164-25241

Mycgr3G25746\_Mycgr3T

Mycgr3G90788 Mycgr3T
  
Location: 25341-25803

Mycgr3G90788\_Mycgr3T

Mycgr3G103260 Mycgr3
  
Location: 25903-26635

Mycgr3G103260\_Mycgr3

Mycgr3G84644 Mycgr3T
  
Location: 26735-28457

Mycgr3G84644\_Mycgr3T

Mycgr3G29227 Mycgr3T
  
Location: 28557-28863

Mycgr3G29227\_Mycgr3T

Mycgr3G36271 Mycgr3T
  
Location: 28963-29854

Mycgr3G36271\_Mycgr3T

Mycgr3G68433 Mycgr3T
  
Location: 29954-33041

Mycgr3G68433\_Mycgr3T

Mycgr3G79452 Mycgr3T
  
Location: 33141-33399

Mycgr3G79452\_Mycgr3T

Mycgr3G55345 Mycgr3T
  
Location: 33499-34126

Mycgr3G55345\_Mycgr3T

Mycgr3G103278 Mycgr3
  
Location: 34226-35195

Mycgr3G103278\_Mycgr3

Mycgr3G84654 Mycgr3T
  
Location: 35295-36630

Mycgr3G84654\_Mycgr3T

Mycgr3G108090 Mycgr3
  
Location: 36730-37591

Mycgr3G108090\_Mycgr3

Mycgr3G21922 Mycgr3T
  
Location: 37691-39149

Mycgr3G21922\_Mycgr3T

Mycgr3G99148 Mycgr3T
  
Location: 39249-42819

Mycgr3G99148\_Mycgr3T

related to nitrate assimilation regulatory protein nirA
  
Accession: CCT69673
  
Location: 673788-676563
  
 NCBI BlastP on this gene

FFUJ\_05577

uncharacterized protein
  
Accession: CCT69674
  
Location: 679409-680964
  
 NCBI BlastP on this gene

FFUJ\_05578

Transcription factor IWS1
  
Accession: CCT69675
  
Location: 681413-682803
  
 NCBI BlastP on this gene

FFUJ\_05579

uncharacterized protein
  
Accession: CCT69676
  
Location: 684604-685125
  
 NCBI BlastP on this gene

FFUJ\_05580

uncharacterized protein
  
Accession: CCT69677
  
Location: 685587-686504
  
 NCBI BlastP on this gene

FFUJ\_05581

probable dis1-suppressing protein kinase dsk1
  
Accession: CCT69678
  
Location: 690303-692490
  
  
**BlastP hit with Mycgr3G84644\_Mycgr3T**
  
Percentage identity: 72 %
  
BlastP bit score: 723
  
Sequence coverage: 89 %
  
E-value: 0.0
  
  
 NCBI BlastP on this gene

FFUJ\_05582

uncharacterized protein
  
Accession: CCT69679
  
Location: 693879-695048
  
 NCBI BlastP on this gene

FFUJ\_05583

related to vesicular transport protein
  
Accession: CCT70708
  
Location: 696163-699670
  
  
**BlastP hit with Mycgr3G108094\_Mycgr3**
  
Percentage identity: 35 %
  
BlastP bit score: 265
  
Sequence coverage: 44 %
  
E-value: 1e-69
  
  
 NCBI BlastP on this gene

FFUJ\_05584

related to NAD(P)H-dependent oxidoreductase
  
Accession: CCT69680
  
Location: 700556-701510
  
 NCBI BlastP on this gene

FFUJ\_05585

related to protein involved in sporulation and meiosis
  
Accession: CCT69681
  
Location: 703959-705077
  
 NCBI BlastP on this gene

FFUJ\_05586

uncharacterized protein
  
Accession: CCT69682
  
Location: 714950-716638
  
 NCBI BlastP on this gene

FFUJ\_05587

uncharacterized protein
  
Accession: CCT69683
  
Location: 717195-718322
  
 NCBI BlastP on this gene

FFUJ\_05588

uncharacterized protein
  
Accession: CCT70732
  
Location: 718799-719348
  
 NCBI BlastP on this gene

FFUJ\_05589

Query: Architecture Search FASTA input

ABDF02000006 : Trichoderma virens Gv29-8    Total score: 2.0     Cumulative Blast bit score: 988

Hit cluster cross-links:

Mycgr3G90785 Mycgr3T
  
Location: 0-1047

Mycgr3G90785\_Mycgr3T

Mycgr3G103262 Mycgr3
  
Location: 1147-1390

Mycgr3G103262\_Mycgr3

Mycgr3G68458 Mycgr3T
  
Location: 1490-3602

Mycgr3G68458\_Mycgr3T

Mycgr3G99145 Mycgr3T
  
Location: 3702-4326

Mycgr3G99145\_Mycgr3T

Mycgr3G103274 Mycgr3
  
Location: 4426-4957

Mycgr3G103274\_Mycgr3

Mycgr3G103264 Mycgr3
  
Location: 5057-5390

Mycgr3G103264\_Mycgr3

Mycgr3G37570 Mycgr3T
  
Location: 5490-6006

Mycgr3G37570\_Mycgr3T

Mycgr3G108094 Mycgr3
  
Location: 6106-10555

Mycgr3G108094\_Mycgr3

Mycgr3G90786 Mycgr3T
  
Location: 10655-12080

Mycgr3G90786\_Mycgr3T

Mycgr3G68429 Mycgr3T
  
Location: 12180-13440

Mycgr3G68429\_Mycgr3T

Mycgr3G68421 Mycgr3T
  
Location: 13540-17086

Mycgr3G68421\_Mycgr3T

Mycgr3G90801 Mycgr3T
  
Location: 17186-18056

Mycgr3G90801\_Mycgr3T

Mycgr3G84646 Mycgr3T
  
Location: 18156-20235

Mycgr3G84646\_Mycgr3T

Mycgr3G68456 Mycgr3T
  
Location: 20335-21970

Mycgr3G68456\_Mycgr3T

Mycgr3G103270 Mycgr3
  
Location: 22070-22355

Mycgr3G103270\_Mycgr3

Mycgr3G90803 Mycgr3T
  
Location: 22455-23019

Mycgr3G90803\_Mycgr3T

Mycgr3G36941 Mycgr3T
  
Location: 23119-24064

Mycgr3G36941\_Mycgr3T

Mycgr3G25746 Mycgr3T
  
Location: 24164-25241

Mycgr3G25746\_Mycgr3T

Mycgr3G90788 Mycgr3T
  
Location: 25341-25803

Mycgr3G90788\_Mycgr3T

Mycgr3G103260 Mycgr3
  
Location: 25903-26635

Mycgr3G103260\_Mycgr3

Mycgr3G84644 Mycgr3T
  
Location: 26735-28457

Mycgr3G84644\_Mycgr3T

Mycgr3G29227 Mycgr3T
  
Location: 28557-28863

Mycgr3G29227\_Mycgr3T

Mycgr3G36271 Mycgr3T
  
Location: 28963-29854

Mycgr3G36271\_Mycgr3T

Mycgr3G68433 Mycgr3T
  
Location: 29954-33041

Mycgr3G68433\_Mycgr3T

Mycgr3G79452 Mycgr3T
  
Location: 33141-33399

Mycgr3G79452\_Mycgr3T

Mycgr3G55345 Mycgr3T
  
Location: 33499-34126

Mycgr3G55345\_Mycgr3T

Mycgr3G103278 Mycgr3
  
Location: 34226-35195

Mycgr3G103278\_Mycgr3

Mycgr3G84654 Mycgr3T
  
Location: 35295-36630

Mycgr3G84654\_Mycgr3T

Mycgr3G108090 Mycgr3
  
Location: 36730-37591

Mycgr3G108090\_Mycgr3

Mycgr3G21922 Mycgr3T
  
Location: 37691-39149

Mycgr3G21922\_Mycgr3T

Mycgr3G99148 Mycgr3T
  
Location: 39249-42819

Mycgr3G99148\_Mycgr3T

hypothetical protein
  
Accession: EHK22808
  
Location: 2346077-2348083
  
 NCBI BlastP on this gene

EHK22808

hypothetical protein
  
Accession: EHK22807
  
Location: 2341744-2344253
  
 NCBI BlastP on this gene

EHK22807

hypothetical protein
  
Accession: EHK22806
  
Location: 2338632-2339825
  
 NCBI BlastP on this gene

EHK22806

hypothetical protein
  
Accession: EHK22805
  
Location: 2336725-2338044
  
 NCBI BlastP on this gene

EHK22805

hypothetical protein
  
Accession: EHK22804
  
Location: 2335010-2335324
  
 NCBI BlastP on this gene

EHK22804

serine/threonine protein kinase, CMGC group
  
Accession: EHK22803
  
Location: 2328629-2332959
  
  
**BlastP hit with Mycgr3G84644\_Mycgr3T**
  
Percentage identity: 69 %
  
BlastP bit score: 763
  
Sequence coverage: 99 %
  
E-value: 0.0
  
  
 NCBI BlastP on this gene

EHK22803

hypothetical protein
  
Accession: EHK22802
  
Location: 2326051-2327449
  
 NCBI BlastP on this gene

EHK22802

NACHT and ankyrin domain protein
  
Accession: EHK22801
  
Location: 2323548-2324321
  
 NCBI BlastP on this gene

EHK22801

hypothetical protein
  
Accession: EHK22800
  
Location: 2319459-2320552
  
 NCBI BlastP on this gene

EHK22800

hypothetical protein
  
Accession: EHK22799
  
Location: 2318022-2318414
  
 NCBI BlastP on this gene

EHK22799

hypothetical protein
  
Accession: EHK22798
  
Location: 2315645-2316675
  
 NCBI BlastP on this gene

EHK22798

hypothetical protein
  
Accession: EHK22797
  
Location: 2311666-2315106
  
  
**BlastP hit with Mycgr3G108094\_Mycgr3**
  
Percentage identity: 31 %
  
BlastP bit score: 225
  
Sequence coverage: 44 %
  
E-value: 9e-57
  
  
 NCBI BlastP on this gene

EHK22797

hypothetical protein
  
Accession: EHK22796
  
Location: 2307969-2309129
  
 NCBI BlastP on this gene

EHK22796

glycosyltransferase family 1 protein
  
Accession: EHK22795
  
Location: 2297637-2299297
  
 NCBI BlastP on this gene

EHK22795

Query: Architecture Search FASTA input

KB456266 : Mycosphaerella populorum SO2202 unplaced genomic scaffold SEPMUscaffold\_7    Total score: 2.0     Cumulative Blast bit score: 982

Hit cluster cross-links:

Mycgr3G90785 Mycgr3T
  
Location: 0-1047

Mycgr3G90785\_Mycgr3T

Mycgr3G103262 Mycgr3
  
Location: 1147-1390

Mycgr3G103262\_Mycgr3

Mycgr3G68458 Mycgr3T
  
Location: 1490-3602

Mycgr3G68458\_Mycgr3T

Mycgr3G99145 Mycgr3T
  
Location: 3702-4326

Mycgr3G99145\_Mycgr3T

Mycgr3G103274 Mycgr3
  
Location: 4426-4957

Mycgr3G103274\_Mycgr3

Mycgr3G103264 Mycgr3
  
Location: 5057-5390

Mycgr3G103264\_Mycgr3

Mycgr3G37570 Mycgr3T
  
Location: 5490-6006

Mycgr3G37570\_Mycgr3T

Mycgr3G108094 Mycgr3
  
Location: 6106-10555

Mycgr3G108094\_Mycgr3

Mycgr3G90786 Mycgr3T
  
Location: 10655-12080

Mycgr3G90786\_Mycgr3T

Mycgr3G68429 Mycgr3T
  
Location: 12180-13440

Mycgr3G68429\_Mycgr3T

Mycgr3G68421 Mycgr3T
  
Location: 13540-17086

Mycgr3G68421\_Mycgr3T

Mycgr3G90801 Mycgr3T
  
Location: 17186-18056

Mycgr3G90801\_Mycgr3T

Mycgr3G84646 Mycgr3T
  
Location: 18156-20235

Mycgr3G84646\_Mycgr3T

Mycgr3G68456 Mycgr3T
  
Location: 20335-21970

Mycgr3G68456\_Mycgr3T

Mycgr3G103270 Mycgr3
  
Location: 22070-22355

Mycgr3G103270\_Mycgr3

Mycgr3G90803 Mycgr3T
  
Location: 22455-23019

Mycgr3G90803\_Mycgr3T

Mycgr3G36941 Mycgr3T
  
Location: 23119-24064

Mycgr3G36941\_Mycgr3T

Mycgr3G25746 Mycgr3T
  
Location: 24164-25241

Mycgr3G25746\_Mycgr3T

Mycgr3G90788 Mycgr3T
  
Location: 25341-25803

Mycgr3G90788\_Mycgr3T

Mycgr3G103260 Mycgr3
  
Location: 25903-26635

Mycgr3G103260\_Mycgr3

Mycgr3G84644 Mycgr3T
  
Location: 26735-28457

Mycgr3G84644\_Mycgr3T

Mycgr3G29227 Mycgr3T
  
Location: 28557-28863

Mycgr3G29227\_Mycgr3T

Mycgr3G36271 Mycgr3T
  
Location: 28963-29854

Mycgr3G36271\_Mycgr3T

Mycgr3G68433 Mycgr3T
  
Location: 29954-33041

Mycgr3G68433\_Mycgr3T

Mycgr3G79452 Mycgr3T
  
Location: 33141-33399

Mycgr3G79452\_Mycgr3T

Mycgr3G55345 Mycgr3T
  
Location: 33499-34126

Mycgr3G55345\_Mycgr3T

Mycgr3G103278 Mycgr3
  
Location: 34226-35195

Mycgr3G103278\_Mycgr3

Mycgr3G84654 Mycgr3T
  
Location: 35295-36630

Mycgr3G84654\_Mycgr3T

Mycgr3G108090 Mycgr3
  
Location: 36730-37591

Mycgr3G108090\_Mycgr3

Mycgr3G21922 Mycgr3T
  
Location: 37691-39149

Mycgr3G21922\_Mycgr3T

Mycgr3G99148 Mycgr3T
  
Location: 39249-42819

Mycgr3G99148\_Mycgr3T

phosphatidylinositol 3 and 4-kinase
  
Accession: EMF11057
  
Location: 265588-271300
  
 NCBI BlastP on this gene

EMF11057

FAD/NAD(P)-binding domain-containing protein
  
Accession: EMF11058
  
Location: 274306-276243
  
 NCBI BlastP on this gene

EMF11058

cochaperone Pam16
  
Accession: EMF11060
  
Location: 279114-279677
  
 NCBI BlastP on this gene

EMF11060

hypothetical protein
  
Accession: EMF11061
  
Location: 280382-281200
  
 NCBI BlastP on this gene

EMF11061

hypothetical protein
  
Accession: EMF11062
  
Location: 283420-284268
  
 NCBI BlastP on this gene

EMF11062

kinase-like protein
  
Accession: EMF11063
  
Location: 285484-287853
  
  
**BlastP hit with Mycgr3G103260\_Mycgr3**
  
Percentage identity: 72 %
  
BlastP bit score: 383
  
Sequence coverage: 96 %
  
E-value: 1e-124
  
  
 NCBI BlastP on this gene

EMF11063

kinase-like protein
  
Accession: EMF11064
  
Location: 290267-291587
  
  
**BlastP hit with Mycgr3G68429\_Mycgr3T**
  
Percentage identity: 70 %
  
BlastP bit score: 599
  
Sequence coverage: 98 %
  
E-value: 0.0
  
  
 NCBI BlastP on this gene

EMF11064

hypothetical protein
  
Accession: EMF11065
  
Location: 301161-301358
  
 NCBI BlastP on this gene

EMF11065

FAD dependent oxidoreductase
  
Accession: EMF11066
  
Location: 302247-303467
  
 NCBI BlastP on this gene

EMF11066

carbohydrate-binding module family 14 protein
  
Accession: EMF11067
  
Location: 305564-305773
  
 NCBI BlastP on this gene

EMF11067

PTR2-domain-containing protein
  
Accession: EMF11068
  
Location: 307070-309210
  
 NCBI BlastP on this gene

EMF11068

NAD(P)-binding protein
  
Accession: EMF11069
  
Location: 310832-311593
  
 NCBI BlastP on this gene

EMF11069

Query: Architecture Search FASTA input

KB730484 : Fusarium oxysporum f. sp. cubense race 1 unplaced genomic scaffold scaffold489    Total score: 2.0     Cumulative Blast bit score: 980

Hit cluster cross-links:

Mycgr3G90785 Mycgr3T
  
Location: 0-1047

Mycgr3G90785\_Mycgr3T

Mycgr3G103262 Mycgr3
  
Location: 1147-1390

Mycgr3G103262\_Mycgr3

Mycgr3G68458 Mycgr3T
  
Location: 1490-3602

Mycgr3G68458\_Mycgr3T

Mycgr3G99145 Mycgr3T
  
Location: 3702-4326

Mycgr3G99145\_Mycgr3T

Mycgr3G103274 Mycgr3
  
Location: 4426-4957

Mycgr3G103274\_Mycgr3

Mycgr3G103264 Mycgr3
  
Location: 5057-5390

Mycgr3G103264\_Mycgr3

Mycgr3G37570 Mycgr3T
  
Location: 5490-6006

Mycgr3G37570\_Mycgr3T

Mycgr3G108094 Mycgr3
  
Location: 6106-10555

Mycgr3G108094\_Mycgr3

Mycgr3G90786 Mycgr3T
  
Location: 10655-12080

Mycgr3G90786\_Mycgr3T

Mycgr3G68429 Mycgr3T
  
Location: 12180-13440

Mycgr3G68429\_Mycgr3T

Mycgr3G68421 Mycgr3T
  
Location: 13540-17086

Mycgr3G68421\_Mycgr3T

Mycgr3G90801 Mycgr3T
  
Location: 17186-18056

Mycgr3G90801\_Mycgr3T

Mycgr3G84646 Mycgr3T
  
Location: 18156-20235

Mycgr3G84646\_Mycgr3T

Mycgr3G68456 Mycgr3T
  
Location: 20335-21970

Mycgr3G68456\_Mycgr3T

Mycgr3G103270 Mycgr3
  
Location: 22070-22355

Mycgr3G103270\_Mycgr3

Mycgr3G90803 Mycgr3T
  
Location: 22455-23019

Mycgr3G90803\_Mycgr3T

Mycgr3G36941 Mycgr3T
  
Location: 23119-24064

Mycgr3G36941\_Mycgr3T

Mycgr3G25746 Mycgr3T
  
Location: 24164-25241

Mycgr3G25746\_Mycgr3T

Mycgr3G90788 Mycgr3T
  
Location: 25341-25803

Mycgr3G90788\_Mycgr3T

Mycgr3G103260 Mycgr3
  
Location: 25903-26635

Mycgr3G103260\_Mycgr3

Mycgr3G84644 Mycgr3T
  
Location: 26735-28457

Mycgr3G84644\_Mycgr3T

Mycgr3G29227 Mycgr3T
  
Location: 28557-28863

Mycgr3G29227\_Mycgr3T

Mycgr3G36271 Mycgr3T
  
Location: 28963-29854

Mycgr3G36271\_Mycgr3T

Mycgr3G68433 Mycgr3T
  
Location: 29954-33041

Mycgr3G68433\_Mycgr3T

Mycgr3G79452 Mycgr3T
  
Location: 33141-33399

Mycgr3G79452\_Mycgr3T

Mycgr3G55345 Mycgr3T
  
Location: 33499-34126

Mycgr3G55345\_Mycgr3T

Mycgr3G103278 Mycgr3
  
Location: 34226-35195

Mycgr3G103278\_Mycgr3

Mycgr3G84654 Mycgr3T
  
Location: 35295-36630

Mycgr3G84654\_Mycgr3T

Mycgr3G108090 Mycgr3
  
Location: 36730-37591

Mycgr3G108090\_Mycgr3

Mycgr3G21922 Mycgr3T
  
Location: 37691-39149

Mycgr3G21922\_Mycgr3T

Mycgr3G99148 Mycgr3T
  
Location: 39249-42819

Mycgr3G99148\_Mycgr3T

hypothetical protein
  
Accession: ENH65641
  
Location: 212141-213022
  
 NCBI BlastP on this gene

ENH65641

Lipase 4
  
Accession: ENH65642
  
Location: 214376-215884
  
 NCBI BlastP on this gene

ENH65642

hypothetical protein
  
Accession: ENH65643
  
Location: 216884-217969
  
 NCBI BlastP on this gene

ENH65643

hypothetical protein
  
Accession: ENH65644
  
Location: 219640-220004
  
 NCBI BlastP on this gene

ENH65644

hypothetical protein
  
Accession: ENH65645
  
Location: 223678-225008
  
 NCBI BlastP on this gene

ENH65645

hypothetical protein
  
Accession: ENH65646
  
Location: 226507-227067
  
 NCBI BlastP on this gene

ENH65646

Putative SWI/SNF-related matrix-associated
  
Accession: ENH65647
  
Location: 228320-231454
  
 NCBI BlastP on this gene

ENH65647

Nuclear control of ATPase protein 2
  
Accession: ENH65648
  
Location: 231902-233993
  
  
**BlastP hit with Mycgr3G84646\_Mycgr3T**
  
Percentage identity: 43 %
  
BlastP bit score: 547
  
Sequence coverage: 101 %
  
E-value: 0.0
  
  
 NCBI BlastP on this gene

ENH65648

C-4 methylsterol oxidase
  
Accession: ENH65649
  
Location: 236706-237756
  
  
**BlastP hit with Mycgr3G36271\_Mycgr3T**
  
Percentage identity: 67 %
  
BlastP bit score: 434
  
Sequence coverage: 97 %
  
E-value: 1e-149
  
  
 NCBI BlastP on this gene

ENH65649

Putative pterin-4-alpha-carbinolamine dehydratase
  
Accession: ENH65650
  
Location: 238263-238865
  
 NCBI BlastP on this gene

ENH65650

Putative transcription factor kapC
  
Accession: ENH65651
  
Location: 241126-242510
  
 NCBI BlastP on this gene

ENH65651

Actin-like protein arp5
  
Accession: ENH65652
  
Location: 242987-245387
  
 NCBI BlastP on this gene

ENH65652

DASH complex subunit dad4
  
Accession: ENH65653
  
Location: 245985-246419
  
 NCBI BlastP on this gene

ENH65653

Chromatin-remodeling complex subunit ies6
  
Accession: ENH65654
  
Location: 246732-247295
  
 NCBI BlastP on this gene

ENH65654

Protein transport protein GOT1
  
Accession: ENH65655
  
Location: 248188-248903
  
 NCBI BlastP on this gene

ENH65655

Profilin
  
Accession: ENH65656
  
Location: 249455-250261
  
 NCBI BlastP on this gene

ENH65656

hypothetical protein
  
Accession: ENH65657
  
Location: 253767-255719
  
 NCBI BlastP on this gene

ENH65657

Synaptobrevin like protein ykt6
  
Accession: ENH65658
  
Location: 256433-257292
  
 NCBI BlastP on this gene

ENH65658

Query: Architecture Search FASTA input

KB726997 : Fusarium oxysporum f. sp. cubense race 4 unplaced genomic scaffold scaffold71    Total score: 2.0     Cumulative Blast bit score: 980

Hit cluster cross-links:

Mycgr3G90785 Mycgr3T
  
Location: 0-1047

Mycgr3G90785\_Mycgr3T

Mycgr3G103262 Mycgr3
  
Location: 1147-1390

Mycgr3G103262\_Mycgr3

Mycgr3G68458 Mycgr3T
  
Location: 1490-3602

Mycgr3G68458\_Mycgr3T

Mycgr3G99145 Mycgr3T
  
Location: 3702-4326

Mycgr3G99145\_Mycgr3T

Mycgr3G103274 Mycgr3
  
Location: 4426-4957

Mycgr3G103274\_Mycgr3

Mycgr3G103264 Mycgr3
  
Location: 5057-5390

Mycgr3G103264\_Mycgr3

Mycgr3G37570 Mycgr3T
  
Location: 5490-6006

Mycgr3G37570\_Mycgr3T

Mycgr3G108094 Mycgr3
  
Location: 6106-10555

Mycgr3G108094\_Mycgr3

Mycgr3G90786 Mycgr3T
  
Location: 10655-12080

Mycgr3G90786\_Mycgr3T

Mycgr3G68429 Mycgr3T
  
Location: 12180-13440

Mycgr3G68429\_Mycgr3T

Mycgr3G68421 Mycgr3T
  
Location: 13540-17086

Mycgr3G68421\_Mycgr3T

Mycgr3G90801 Mycgr3T
  
Location: 17186-18056

Mycgr3G90801\_Mycgr3T

Mycgr3G84646 Mycgr3T
  
Location: 18156-20235

Mycgr3G84646\_Mycgr3T

Mycgr3G68456 Mycgr3T
  
Location: 20335-21970

Mycgr3G68456\_Mycgr3T

Mycgr3G103270 Mycgr3
  
Location: 22070-22355

Mycgr3G103270\_Mycgr3

Mycgr3G90803 Mycgr3T
  
Location: 22455-23019

Mycgr3G90803\_Mycgr3T

Mycgr3G36941 Mycgr3T
  
Location: 23119-24064

Mycgr3G36941\_Mycgr3T

Mycgr3G25746 Mycgr3T
  
Location: 24164-25241

Mycgr3G25746\_Mycgr3T

Mycgr3G90788 Mycgr3T
  
Location: 25341-25803

Mycgr3G90788\_Mycgr3T

Mycgr3G103260 Mycgr3
  
Location: 25903-26635

Mycgr3G103260\_Mycgr3

Mycgr3G84644 Mycgr3T
  
Location: 26735-28457

Mycgr3G84644\_Mycgr3T

Mycgr3G29227 Mycgr3T
  
Location: 28557-28863

Mycgr3G29227\_Mycgr3T

Mycgr3G36271 Mycgr3T
  
Location: 28963-29854

Mycgr3G36271\_Mycgr3T

Mycgr3G68433 Mycgr3T
  
Location: 29954-33041

Mycgr3G68433\_Mycgr3T

Mycgr3G79452 Mycgr3T
  
Location: 33141-33399

Mycgr3G79452\_Mycgr3T

Mycgr3G55345 Mycgr3T
  
Location: 33499-34126

Mycgr3G55345\_Mycgr3T

Mycgr3G103278 Mycgr3
  
Location: 34226-35195

Mycgr3G103278\_Mycgr3

Mycgr3G84654 Mycgr3T
  
Location: 35295-36630

Mycgr3G84654\_Mycgr3T

Mycgr3G108090 Mycgr3
  
Location: 36730-37591

Mycgr3G108090\_Mycgr3

Mycgr3G21922 Mycgr3T
  
Location: 37691-39149

Mycgr3G21922\_Mycgr3T

Mycgr3G99148 Mycgr3T
  
Location: 39249-42819

Mycgr3G99148\_Mycgr3T

Profilin
  
Accession: EMT60295
  
Location: 146801-147607
  
 NCBI BlastP on this gene

EMT60295

Protein transport protein GOT1
  
Accession: EMT60296
  
Location: 148171-148888
  
 NCBI BlastP on this gene

EMT60296

Chromatin-remodeling complex subunit ies6
  
Accession: EMT60297
  
Location: 149788-150351
  
 NCBI BlastP on this gene

EMT60297

DASH complex subunit dad4
  
Accession: EMT60298
  
Location: 150665-151099
  
 NCBI BlastP on this gene

EMT60298

Actin-like protein arp5
  
Accession: EMT60299
  
Location: 151696-156195
  
 NCBI BlastP on this gene

EMT60299

Putative transcription factor kapC
  
Accession: EMT60300
  
Location: 156669-158053
  
 NCBI BlastP on this gene

EMT60300

Putative pterin-4-alpha-carbinolamine dehydratase
  
Accession: EMT60301
  
Location: 160312-160914
  
 NCBI BlastP on this gene

EMT60301

C-4 methylsterol oxidase
  
Accession: EMT60302
  
Location: 161421-162471
  
  
**BlastP hit with Mycgr3G36271\_Mycgr3T**
  
Percentage identity: 67 %
  
BlastP bit score: 434
  
Sequence coverage: 97 %
  
E-value: 1e-149
  
  
 NCBI BlastP on this gene

EMT60302

Nuclear control of ATPase protein 2
  
Accession: EMT60303
  
Location: 165181-167272
  
  
**BlastP hit with Mycgr3G84646\_Mycgr3T**
  
Percentage identity: 43 %
  
BlastP bit score: 546
  
Sequence coverage: 101 %
  
E-value: 0.0
  
  
 NCBI BlastP on this gene

EMT60303

hypothetical protein
  
Accession: EMT60304
  
Location: 167720-170854
  
 NCBI BlastP on this gene

EMT60304

hypothetical protein
  
Accession: EMT60305
  
Location: 172107-172667
  
 NCBI BlastP on this gene

EMT60305

hypothetical protein
  
Accession: EMT60306
  
Location: 174167-175497
  
 NCBI BlastP on this gene

EMT60306

hypothetical protein
  
Accession: EMT60307
  
Location: 179290-181297
  
 NCBI BlastP on this gene

EMT60307

hypothetical protein
  
Accession: EMT60308
  
Location: 183190-184275
  
 NCBI BlastP on this gene

EMT60308

hypothetical protein
  
Accession: EMT60309
  
Location: 185096-186181
  
 NCBI BlastP on this gene

EMT60309

Lipase 4
  
Accession: EMT60310
  
Location: 186943-188685
  
 NCBI BlastP on this gene

EMT60310

Query: Architecture Search FASTA input

AFQF01000058 : Fusarium oxysporum Fo5176    Total score: 2.0     Cumulative Blast bit score: 976

Hit cluster cross-links:

Mycgr3G90785 Mycgr3T
  
Location: 0-1047

Mycgr3G90785\_Mycgr3T

Mycgr3G103262 Mycgr3
  
Location: 1147-1390

Mycgr3G103262\_Mycgr3

Mycgr3G68458 Mycgr3T
  
Location: 1490-3602

Mycgr3G68458\_Mycgr3T

Mycgr3G99145 Mycgr3T
  
Location: 3702-4326

Mycgr3G99145\_Mycgr3T

Mycgr3G103274 Mycgr3
  
Location: 4426-4957

Mycgr3G103274\_Mycgr3

Mycgr3G103264 Mycgr3
  
Location: 5057-5390

Mycgr3G103264\_Mycgr3

Mycgr3G37570 Mycgr3T
  
Location: 5490-6006

Mycgr3G37570\_Mycgr3T

Mycgr3G108094 Mycgr3
  
Location: 6106-10555

Mycgr3G108094\_Mycgr3

Mycgr3G90786 Mycgr3T
  
Location: 10655-12080

Mycgr3G90786\_Mycgr3T

Mycgr3G68429 Mycgr3T
  
Location: 12180-13440

Mycgr3G68429\_Mycgr3T

Mycgr3G68421 Mycgr3T
  
Location: 13540-17086

Mycgr3G68421\_Mycgr3T

Mycgr3G90801 Mycgr3T
  
Location: 17186-18056

Mycgr3G90801\_Mycgr3T

Mycgr3G84646 Mycgr3T
  
Location: 18156-20235

Mycgr3G84646\_Mycgr3T

Mycgr3G68456 Mycgr3T
  
Location: 20335-21970

Mycgr3G68456\_Mycgr3T

Mycgr3G103270 Mycgr3
  
Location: 22070-22355

Mycgr3G103270\_Mycgr3

Mycgr3G90803 Mycgr3T
  
Location: 22455-23019

Mycgr3G90803\_Mycgr3T

Mycgr3G36941 Mycgr3T
  
Location: 23119-24064

Mycgr3G36941\_Mycgr3T

Mycgr3G25746 Mycgr3T
  
Location: 24164-25241

Mycgr3G25746\_Mycgr3T

Mycgr3G90788 Mycgr3T
  
Location: 25341-25803

Mycgr3G90788\_Mycgr3T

Mycgr3G103260 Mycgr3
  
Location: 25903-26635

Mycgr3G103260\_Mycgr3

Mycgr3G84644 Mycgr3T
  
Location: 26735-28457

Mycgr3G84644\_Mycgr3T

Mycgr3G29227 Mycgr3T
  
Location: 28557-28863

Mycgr3G29227\_Mycgr3T

Mycgr3G36271 Mycgr3T
  
Location: 28963-29854

Mycgr3G36271\_Mycgr3T

Mycgr3G68433 Mycgr3T
  
Location: 29954-33041

Mycgr3G68433\_Mycgr3T

Mycgr3G79452 Mycgr3T
  
Location: 33141-33399

Mycgr3G79452\_Mycgr3T

Mycgr3G55345 Mycgr3T
  
Location: 33499-34126

Mycgr3G55345\_Mycgr3T

Mycgr3G103278 Mycgr3
  
Location: 34226-35195

Mycgr3G103278\_Mycgr3

Mycgr3G84654 Mycgr3T
  
Location: 35295-36630

Mycgr3G84654\_Mycgr3T

Mycgr3G108090 Mycgr3
  
Location: 36730-37591

Mycgr3G108090\_Mycgr3

Mycgr3G21922 Mycgr3T
  
Location: 37691-39149

Mycgr3G21922\_Mycgr3T

Mycgr3G99148 Mycgr3T
  
Location: 39249-42819

Mycgr3G99148\_Mycgr3T

hypothetical protein
  
Accession: EGU89375
  
Location: 7801-8808
  
 NCBI BlastP on this gene

EGU89375

hypothetical protein
  
Accession: EGU89376
  
Location: 9368-11362
  
 NCBI BlastP on this gene

EGU89376

hypothetical protein
  
Accession: EGU89377
  
Location: 14866-15673
  
 NCBI BlastP on this gene

EGU89377

hypothetical protein
  
Accession: EGU89378
  
Location: 16237-16948
  
 NCBI BlastP on this gene

EGU89378

hypothetical protein
  
Accession: EGU89379
  
Location: 17847-18410
  
 NCBI BlastP on this gene

EGU89379

hypothetical protein
  
Accession: EGU89380
  
Location: 18721-19155
  
 NCBI BlastP on this gene

EGU89380

hypothetical protein
  
Accession: EGU89381
  
Location: 19753-22153
  
 NCBI BlastP on this gene

EGU89381

hypothetical protein
  
Accession: EGU89382
  
Location: 22626-24006
  
 NCBI BlastP on this gene

EGU89382

hypothetical protein
  
Accession: EGU89383
  
Location: 26268-26870
  
 NCBI BlastP on this gene

EGU89383

hypothetical protein
  
Accession: EGU89384
  
Location: 27377-28427
  
  
**BlastP hit with Mycgr3G36271\_Mycgr3T**
  
Percentage identity: 67 %
  
BlastP bit score: 434
  
Sequence coverage: 97 %
  
E-value: 1e-149
  
  
 NCBI BlastP on this gene

EGU89384

hypothetical protein
  
Accession: EGU89385
  
Location: 31139-33230
  
  
**BlastP hit with Mycgr3G84646\_Mycgr3T**
  
Percentage identity: 46 %
  
BlastP bit score: 543
  
Sequence coverage: 86 %
  
E-value: 0.0
  
  
 NCBI BlastP on this gene

EGU89385

hypothetical protein
  
Accession: EGU89386
  
Location: 33678-36812
  
 NCBI BlastP on this gene

EGU89386

hypothetical protein
  
Accession: EGU89387
  
Location: 38066-38626
  
 NCBI BlastP on this gene

EGU89387

hypothetical protein
  
Accession: EGU89388
  
Location: 39821-41455
  
 NCBI BlastP on this gene

EGU89388

hypothetical protein
  
Accession: EGU89389
  
Location: 44376-44583
  
 NCBI BlastP on this gene

EGU89389

hypothetical protein
  
Accession: EGU89390
  
Location: 45128-45542
  
 NCBI BlastP on this gene

EGU89390

hypothetical protein
  
Accession: EGU89391
  
Location: 47166-48251
  
 NCBI BlastP on this gene

EGU89391

hypothetical protein
  
Accession: EGU89392
  
Location: 49005-50756
  
 NCBI BlastP on this gene

EGU89392

hypothetical protein
  
Accession: EGU89393
  
Location: 51666-52992
  
 NCBI BlastP on this gene

EGU89393

Query: Architecture Search FASTA input

CP003010 : Thielavia terrestris NRRL 8126 chromosome 2    Total score: 2.0     Cumulative Blast bit score: 974

Hit cluster cross-links:

Mycgr3G90785 Mycgr3T
  
Location: 0-1047

Mycgr3G90785\_Mycgr3T

Mycgr3G103262 Mycgr3
  
Location: 1147-1390

Mycgr3G103262\_Mycgr3

Mycgr3G68458 Mycgr3T
  
Location: 1490-3602

Mycgr3G68458\_Mycgr3T

Mycgr3G99145 Mycgr3T
  
Location: 3702-4326

Mycgr3G99145\_Mycgr3T

Mycgr3G103274 Mycgr3
  
Location: 4426-4957

Mycgr3G103274\_Mycgr3

Mycgr3G103264 Mycgr3
  
Location: 5057-5390

Mycgr3G103264\_Mycgr3

Mycgr3G37570 Mycgr3T
  
Location: 5490-6006

Mycgr3G37570\_Mycgr3T

Mycgr3G108094 Mycgr3
  
Location: 6106-10555

Mycgr3G108094\_Mycgr3

Mycgr3G90786 Mycgr3T
  
Location: 10655-12080

Mycgr3G90786\_Mycgr3T

Mycgr3G68429 Mycgr3T
  
Location: 12180-13440

Mycgr3G68429\_Mycgr3T

Mycgr3G68421 Mycgr3T
  
Location: 13540-17086

Mycgr3G68421\_Mycgr3T

Mycgr3G90801 Mycgr3T
  
Location: 17186-18056

Mycgr3G90801\_Mycgr3T

Mycgr3G84646 Mycgr3T
  
Location: 18156-20235

Mycgr3G84646\_Mycgr3T

Mycgr3G68456 Mycgr3T
  
Location: 20335-21970

Mycgr3G68456\_Mycgr3T

Mycgr3G103270 Mycgr3
  
Location: 22070-22355

Mycgr3G103270\_Mycgr3

Mycgr3G90803 Mycgr3T
  
Location: 22455-23019

Mycgr3G90803\_Mycgr3T

Mycgr3G36941 Mycgr3T
  
Location: 23119-24064

Mycgr3G36941\_Mycgr3T

Mycgr3G25746 Mycgr3T
  
Location: 24164-25241

Mycgr3G25746\_Mycgr3T

Mycgr3G90788 Mycgr3T
  
Location: 25341-25803

Mycgr3G90788\_Mycgr3T

Mycgr3G103260 Mycgr3
  
Location: 25903-26635

Mycgr3G103260\_Mycgr3

Mycgr3G84644 Mycgr3T
  
Location: 26735-28457

Mycgr3G84644\_Mycgr3T

Mycgr3G29227 Mycgr3T
  
Location: 28557-28863

Mycgr3G29227\_Mycgr3T

Mycgr3G36271 Mycgr3T
  
Location: 28963-29854

Mycgr3G36271\_Mycgr3T

Mycgr3G68433 Mycgr3T
  
Location: 29954-33041

Mycgr3G68433\_Mycgr3T

Mycgr3G79452 Mycgr3T
  
Location: 33141-33399

Mycgr3G79452\_Mycgr3T

Mycgr3G55345 Mycgr3T
  
Location: 33499-34126

Mycgr3G55345\_Mycgr3T

Mycgr3G103278 Mycgr3
  
Location: 34226-35195

Mycgr3G103278\_Mycgr3

Mycgr3G84654 Mycgr3T
  
Location: 35295-36630

Mycgr3G84654\_Mycgr3T

Mycgr3G108090 Mycgr3
  
Location: 36730-37591

Mycgr3G108090\_Mycgr3

Mycgr3G21922 Mycgr3T
  
Location: 37691-39149

Mycgr3G21922\_Mycgr3T

Mycgr3G99148 Mycgr3T
  
Location: 39249-42819

Mycgr3G99148\_Mycgr3T

hypothetical protein
  
Accession: AEO66920
  
Location: 7475788-7477507
  
 NCBI BlastP on this gene

THITE\_2045895

hypothetical protein
  
Accession: AEO66921
  
Location: 7478671-7480848
  
 NCBI BlastP on this gene

THITE\_2115480

hypothetical protein
  
Accession: AEO66922
  
Location: 7481542-7482375
  
 NCBI BlastP on this gene

THITE\_2115482

hypothetical protein
  
Accession: AEO66923
  
Location: 7482745-7484890
  
 NCBI BlastP on this gene

THITE\_2154729

hypothetical protein
  
Accession: AEO66924
  
Location: 7485335-7486021
  
 NCBI BlastP on this gene

THITE\_31960

hypothetical protein
  
Accession: AEO66925
  
Location: 7486785-7492979
  
 NCBI BlastP on this gene

THITE\_2115485

hypothetical protein
  
Accession: AEO66926
  
Location: 7495019-7498357
  
  
**BlastP hit with Mycgr3G84646\_Mycgr3T**
  
Percentage identity: 42 %
  
BlastP bit score: 517
  
Sequence coverage: 104 %
  
E-value: 8e-170
  
  
 NCBI BlastP on this gene

THITE\_2150534

methylsterol monooxygenase
  
Accession: AEO66927
  
Location: 7499573-7500681
  
  
**BlastP hit with Mycgr3G36271\_Mycgr3T**
  
Percentage identity: 72 %
  
BlastP bit score: 457
  
Sequence coverage: 95 %
  
E-value: 4e-159
  
  
 NCBI BlastP on this gene

THITE\_125790

hypothetical protein
  
Accession: AEO66928
  
Location: 7501250-7502257
  
 NCBI BlastP on this gene

THITE\_2115493

hypothetical protein
  
Accession: AEO66929
  
Location: 7505843-7506514
  
 NCBI BlastP on this gene

THITE\_2115495

ARP5-like protein
  
Accession: AEO66930
  
Location: 7507460-7510046
  
 NCBI BlastP on this gene

THITE\_2115499

hypothetical protein
  
Accession: AEO66931
  
Location: 7510511-7510981
  
 NCBI BlastP on this gene

THITE\_2115503

hypothetical protein
  
Accession: AEO66932
  
Location: 7511300-7511911
  
 NCBI BlastP on this gene

THITE\_2115508

hypothetical protein
  
Accession: AEO66933
  
Location: 7512378-7512578
  
 NCBI BlastP on this gene

THITE\_2115510

hypothetical protein
  
Accession: AEO66934
  
Location: 7513893-7514684
  
 NCBI BlastP on this gene

THITE\_37801

hypothetical protein
  
Accession: AEO66935
  
Location: 7515426-7516189
  
 NCBI BlastP on this gene

THITE\_2115513

hypothetical protein
  
Accession: AEO66936
  
Location: 7520673-7522808
  
 NCBI BlastP on this gene

THITE\_2115520

Query: Architecture Search FASTA input

ABDF02000072 : Trichoderma virens Gv29-8    Total score: 2.0     Cumulative Blast bit score: 972

Hit cluster cross-links:

Mycgr3G90785 Mycgr3T
  
Location: 0-1047

Mycgr3G90785\_Mycgr3T

Mycgr3G103262 Mycgr3
  
Location: 1147-1390

Mycgr3G103262\_Mycgr3

Mycgr3G68458 Mycgr3T
  
Location: 1490-3602

Mycgr3G68458\_Mycgr3T

Mycgr3G99145 Mycgr3T
  
Location: 3702-4326

Mycgr3G99145\_Mycgr3T

Mycgr3G103274 Mycgr3
  
Location: 4426-4957

Mycgr3G103274\_Mycgr3

Mycgr3G103264 Mycgr3
  
Location: 5057-5390

Mycgr3G103264\_Mycgr3

Mycgr3G37570 Mycgr3T
  
Location: 5490-6006

Mycgr3G37570\_Mycgr3T

Mycgr3G108094 Mycgr3
  
Location: 6106-10555

Mycgr3G108094\_Mycgr3

Mycgr3G90786 Mycgr3T
  
Location: 10655-12080

Mycgr3G90786\_Mycgr3T

Mycgr3G68429 Mycgr3T
  
Location: 12180-13440

Mycgr3G68429\_Mycgr3T

Mycgr3G68421 Mycgr3T
  
Location: 13540-17086

Mycgr3G68421\_Mycgr3T

Mycgr3G90801 Mycgr3T
  
Location: 17186-18056

Mycgr3G90801\_Mycgr3T

Mycgr3G84646 Mycgr3T
  
Location: 18156-20235

Mycgr3G84646\_Mycgr3T

Mycgr3G68456 Mycgr3T
  
Location: 20335-21970

Mycgr3G68456\_Mycgr3T

Mycgr3G103270 Mycgr3
  
Location: 22070-22355

Mycgr3G103270\_Mycgr3

Mycgr3G90803 Mycgr3T
  
Location: 22455-23019

Mycgr3G90803\_Mycgr3T

Mycgr3G36941 Mycgr3T
  
Location: 23119-24064

Mycgr3G36941\_Mycgr3T

Mycgr3G25746 Mycgr3T
  
Location: 24164-25241

Mycgr3G25746\_Mycgr3T

Mycgr3G90788 Mycgr3T
  
Location: 25341-25803

Mycgr3G90788\_Mycgr3T

Mycgr3G103260 Mycgr3
  
Location: 25903-26635

Mycgr3G103260\_Mycgr3

Mycgr3G84644 Mycgr3T
  
Location: 26735-28457

Mycgr3G84644\_Mycgr3T

Mycgr3G29227 Mycgr3T
  
Location: 28557-28863

Mycgr3G29227\_Mycgr3T

Mycgr3G36271 Mycgr3T
  
Location: 28963-29854

Mycgr3G36271\_Mycgr3T

Mycgr3G68433 Mycgr3T
  
Location: 29954-33041

Mycgr3G68433\_Mycgr3T

Mycgr3G79452 Mycgr3T
  
Location: 33141-33399

Mycgr3G79452\_Mycgr3T

Mycgr3G55345 Mycgr3T
  
Location: 33499-34126

Mycgr3G55345\_Mycgr3T

Mycgr3G103278 Mycgr3
  
Location: 34226-35195

Mycgr3G103278\_Mycgr3

Mycgr3G84654 Mycgr3T
  
Location: 35295-36630

Mycgr3G84654\_Mycgr3T

Mycgr3G108090 Mycgr3
  
Location: 36730-37591

Mycgr3G108090\_Mycgr3

Mycgr3G21922 Mycgr3T
  
Location: 37691-39149

Mycgr3G21922\_Mycgr3T

Mycgr3G99148 Mycgr3T
  
Location: 39249-42819

Mycgr3G99148\_Mycgr3T

hypothetical protein
  
Accession: EHK21299
  
Location: 13923-14282
  
 NCBI BlastP on this gene

EHK21299

hypothetical protein
  
Accession: EHK21300
  
Location: 16197-17756
  
 NCBI BlastP on this gene

EHK21300

hypothetical protein
  
Accession: EHK21301
  
Location: 19532-21121
  
 NCBI BlastP on this gene

EHK21301

hypothetical protein
  
Accession: EHK21302
  
Location: 21537-23631
  
  
**BlastP hit with Mycgr3G84646\_Mycgr3T**
  
Percentage identity: 45 %
  
BlastP bit score: 540
  
Sequence coverage: 101 %
  
E-value: 3e-180
  
  
 NCBI BlastP on this gene

EHK21302

hypothetical protein
  
Accession: EHK21303
  
Location: 25365-26415
  
  
**BlastP hit with Mycgr3G36271\_Mycgr3T**
  
Percentage identity: 70 %
  
BlastP bit score: 432
  
Sequence coverage: 92 %
  
E-value: 3e-149
  
  
 NCBI BlastP on this gene

EHK21303

hypothetical protein
  
Accession: EHK21304
  
Location: 27569-28156
  
 NCBI BlastP on this gene

EHK21304

hypothetical protein
  
Accession: EHK21305
  
Location: 28533-28938
  
 NCBI BlastP on this gene

EHK21305

hypothetical protein
  
Accession: EHK21306
  
Location: 29494-31967
  
 NCBI BlastP on this gene

EHK21306

hypothetical protein
  
Accession: EHK21307
  
Location: 32830-34740
  
 NCBI BlastP on this gene

EHK21307

hypothetical protein
  
Accession: EHK21308
  
Location: 37768-39111
  
 NCBI BlastP on this gene

EHK21308

hypothetical protein
  
Accession: EHK21309
  
Location: 39896-40422
  
 NCBI BlastP on this gene

EHK21309

hypothetical protein
  
Accession: EHK21310
  
Location: 40815-41066
  
 NCBI BlastP on this gene

EHK21310

hypothetical protein
  
Accession: EHK21311
  
Location: 43782-44249
  
 NCBI BlastP on this gene

EHK21311

Query: Architecture Search FASTA input

ABDG02000026 : Trichoderma atroviride IMI 206040    Total score: 2.0     Cumulative Blast bit score: 967

Hit cluster cross-links:

Mycgr3G90785 Mycgr3T
  
Location: 0-1047

Mycgr3G90785\_Mycgr3T

Mycgr3G103262 Mycgr3
  
Location: 1147-1390

Mycgr3G103262\_Mycgr3

Mycgr3G68458 Mycgr3T
  
Location: 1490-3602

Mycgr3G68458\_Mycgr3T

Mycgr3G99145 Mycgr3T
  
Location: 3702-4326

Mycgr3G99145\_Mycgr3T

Mycgr3G103274 Mycgr3
  
Location: 4426-4957

Mycgr3G103274\_Mycgr3

Mycgr3G103264 Mycgr3
  
Location: 5057-5390

Mycgr3G103264\_Mycgr3

Mycgr3G37570 Mycgr3T
  
Location: 5490-6006

Mycgr3G37570\_Mycgr3T

Mycgr3G108094 Mycgr3
  
Location: 6106-10555

Mycgr3G108094\_Mycgr3

Mycgr3G90786 Mycgr3T
  
Location: 10655-12080

Mycgr3G90786\_Mycgr3T

Mycgr3G68429 Mycgr3T
  
Location: 12180-13440

Mycgr3G68429\_Mycgr3T

Mycgr3G68421 Mycgr3T
  
Location: 13540-17086

Mycgr3G68421\_Mycgr3T

Mycgr3G90801 Mycgr3T
  
Location: 17186-18056

Mycgr3G90801\_Mycgr3T

Mycgr3G84646 Mycgr3T
  
Location: 18156-20235

Mycgr3G84646\_Mycgr3T

Mycgr3G68456 Mycgr3T
  
Location: 20335-21970

Mycgr3G68456\_Mycgr3T

Mycgr3G103270 Mycgr3
  
Location: 22070-22355

Mycgr3G103270\_Mycgr3

Mycgr3G90803 Mycgr3T
  
Location: 22455-23019

Mycgr3G90803\_Mycgr3T

Mycgr3G36941 Mycgr3T
  
Location: 23119-24064

Mycgr3G36941\_Mycgr3T

Mycgr3G25746 Mycgr3T
  
Location: 24164-25241

Mycgr3G25746\_Mycgr3T

Mycgr3G90788 Mycgr3T
  
Location: 25341-25803

Mycgr3G90788\_Mycgr3T

Mycgr3G103260 Mycgr3
  
Location: 25903-26635

Mycgr3G103260\_Mycgr3

Mycgr3G84644 Mycgr3T
  
Location: 26735-28457

Mycgr3G84644\_Mycgr3T

Mycgr3G29227 Mycgr3T
  
Location: 28557-28863

Mycgr3G29227\_Mycgr3T

Mycgr3G36271 Mycgr3T
  
Location: 28963-29854

Mycgr3G36271\_Mycgr3T

Mycgr3G68433 Mycgr3T
  
Location: 29954-33041

Mycgr3G68433\_Mycgr3T

Mycgr3G79452 Mycgr3T
  
Location: 33141-33399

Mycgr3G79452\_Mycgr3T

Mycgr3G55345 Mycgr3T
  
Location: 33499-34126

Mycgr3G55345\_Mycgr3T

Mycgr3G103278 Mycgr3
  
Location: 34226-35195

Mycgr3G103278\_Mycgr3

Mycgr3G84654 Mycgr3T
  
Location: 35295-36630

Mycgr3G84654\_Mycgr3T

Mycgr3G108090 Mycgr3
  
Location: 36730-37591

Mycgr3G108090\_Mycgr3

Mycgr3G21922 Mycgr3T
  
Location: 37691-39149

Mycgr3G21922\_Mycgr3T

Mycgr3G99148 Mycgr3T
  
Location: 39249-42819

Mycgr3G99148\_Mycgr3T

hypothetical protein
  
Accession: EHK43358
  
Location: 3345242-3346480
  
 NCBI BlastP on this gene

EHK43358

hypothetical protein
  
Accession: EHK43359
  
Location: 3347359-3352500
  
 NCBI BlastP on this gene

EHK43359

hypothetical protein
  
Accession: EHK43360
  
Location: 3352826-3353667
  
 NCBI BlastP on this gene

EHK43360

hypothetical protein
  
Accession: EHK43361
  
Location: 3355017-3358168
  
 NCBI BlastP on this gene

EHK43361

hypothetical protein
  
Accession: EHK43362
  
Location: 3359014-3359716
  
 NCBI BlastP on this gene

EHK43362

hypothetical protein
  
Accession: EHK43363
  
Location: 3362660-3363881
  
 NCBI BlastP on this gene

EHK43363

hypothetical protein
  
Accession: EHK43364
  
Location: 3364329-3366459
  
  
**BlastP hit with Mycgr3G84646\_Mycgr3T**
  
Percentage identity: 44 %
  
BlastP bit score: 536
  
Sequence coverage: 100 %
  
E-value: 2e-178
  
  
 NCBI BlastP on this gene

EHK43364

C-4 sterol methyl oxidase
  
Accession: EHK43365
  
Location: 3368164-3369223
  
  
**BlastP hit with Mycgr3G36271\_Mycgr3T**
  
Percentage identity: 70 %
  
BlastP bit score: 432
  
Sequence coverage: 93 %
  
E-value: 3e-149
  
  
 NCBI BlastP on this gene

EHK43365

hypothetical protein
  
Accession: EHK43366
  
Location: 3370402-3370989
  
 NCBI BlastP on this gene

EHK43366

hypothetical protein
  
Accession: EHK43367
  
Location: 3371167-3371716
  
 NCBI BlastP on this gene

EHK43367

hypothetical protein
  
Accession: EHK43368
  
Location: 3372283-3374729
  
 NCBI BlastP on this gene

EHK43368

hypothetical protein
  
Accession: EHK43369
  
Location: 3375753-3376907
  
 NCBI BlastP on this gene

EHK43369

hypothetical protein
  
Accession: EHK43370
  
Location: 3380834-3382192
  
 NCBI BlastP on this gene

EHK43370

hypothetical protein
  
Accession: EHK43371
  
Location: 3382930-3383452
  
 NCBI BlastP on this gene

EHK43371

hypothetical protein
  
Accession: EHK43372
  
Location: 3387168-3387872
  
 NCBI BlastP on this gene

EHK43372

hypothetical protein
  
Accession: EHK43373
  
Location: 3388481-3389241
  
 NCBI BlastP on this gene

EHK43373

Query: Architecture Search FASTA input

AFNW01000339 : Fusarium pseudograminearum CS3096    Total score: 2.0     Cumulative Blast bit score: 963

Hit cluster cross-links:

Mycgr3G90785 Mycgr3T
  
Location: 0-1047

Mycgr3G90785\_Mycgr3T

Mycgr3G103262 Mycgr3
  
Location: 1147-1390

Mycgr3G103262\_Mycgr3

Mycgr3G68458 Mycgr3T
  
Location: 1490-3602

Mycgr3G68458\_Mycgr3T

Mycgr3G99145 Mycgr3T
  
Location: 3702-4326

Mycgr3G99145\_Mycgr3T

Mycgr3G103274 Mycgr3
  
Location: 4426-4957

Mycgr3G103274\_Mycgr3

Mycgr3G103264 Mycgr3
  
Location: 5057-5390

Mycgr3G103264\_Mycgr3

Mycgr3G37570 Mycgr3T
  
Location: 5490-6006

Mycgr3G37570\_Mycgr3T

Mycgr3G108094 Mycgr3
  
Location: 6106-10555

Mycgr3G108094\_Mycgr3

Mycgr3G90786 Mycgr3T
  
Location: 10655-12080

Mycgr3G90786\_Mycgr3T

Mycgr3G68429 Mycgr3T
  
Location: 12180-13440

Mycgr3G68429\_Mycgr3T

Mycgr3G68421 Mycgr3T
  
Location: 13540-17086

Mycgr3G68421\_Mycgr3T

Mycgr3G90801 Mycgr3T
  
Location: 17186-18056

Mycgr3G90801\_Mycgr3T

Mycgr3G84646 Mycgr3T
  
Location: 18156-20235

Mycgr3G84646\_Mycgr3T

Mycgr3G68456 Mycgr3T
  
Location: 20335-21970

Mycgr3G68456\_Mycgr3T

Mycgr3G103270 Mycgr3
  
Location: 22070-22355

Mycgr3G103270\_Mycgr3

Mycgr3G90803 Mycgr3T
  
Location: 22455-23019

Mycgr3G90803\_Mycgr3T

Mycgr3G36941 Mycgr3T
  
Location: 23119-24064

Mycgr3G36941\_Mycgr3T

Mycgr3G25746 Mycgr3T
  
Location: 24164-25241

Mycgr3G25746\_Mycgr3T

Mycgr3G90788 Mycgr3T
  
Location: 25341-25803

Mycgr3G90788\_Mycgr3T

Mycgr3G103260 Mycgr3
  
Location: 25903-26635

Mycgr3G103260\_Mycgr3

Mycgr3G84644 Mycgr3T
  
Location: 26735-28457

Mycgr3G84644\_Mycgr3T

Mycgr3G29227 Mycgr3T
  
Location: 28557-28863

Mycgr3G29227\_Mycgr3T

Mycgr3G36271 Mycgr3T
  
Location: 28963-29854

Mycgr3G36271\_Mycgr3T

Mycgr3G68433 Mycgr3T
  
Location: 29954-33041

Mycgr3G68433\_Mycgr3T

Mycgr3G79452 Mycgr3T
  
Location: 33141-33399

Mycgr3G79452\_Mycgr3T

Mycgr3G55345 Mycgr3T
  
Location: 33499-34126

Mycgr3G55345\_Mycgr3T

Mycgr3G103278 Mycgr3
  
Location: 34226-35195

Mycgr3G103278\_Mycgr3

Mycgr3G84654 Mycgr3T
  
Location: 35295-36630

Mycgr3G84654\_Mycgr3T

Mycgr3G108090 Mycgr3
  
Location: 36730-37591

Mycgr3G108090\_Mycgr3

Mycgr3G21922 Mycgr3T
  
Location: 37691-39149

Mycgr3G21922\_Mycgr3T

Mycgr3G99148 Mycgr3T
  
Location: 39249-42819

Mycgr3G99148\_Mycgr3T

hypothetical protein
  
Accession: EKJ69464
  
Location: 54-2944
  
 NCBI BlastP on this gene

EKJ69464

hypothetical protein
  
Accession: EKJ69465
  
Location: 3382-6724
  
  
**BlastP hit with Mycgr3G84646\_Mycgr3T**
  
Percentage identity: 47 %
  
BlastP bit score: 533
  
Sequence coverage: 84 %
  
E-value: 2e-175
  
  
 NCBI BlastP on this gene

EKJ69465

hypothetical protein
  
Accession: EKJ69466
  
Location: 8285-9338
  
  
**BlastP hit with Mycgr3G36271\_Mycgr3T**
  
Percentage identity: 67 %
  
BlastP bit score: 430
  
Sequence coverage: 97 %
  
E-value: 3e-148
  
  
 NCBI BlastP on this gene

EKJ69466

hypothetical protein
  
Accession: EKJ69467
  
Location: 9846-10518
  
 NCBI BlastP on this gene

EKJ69467

hypothetical protein
  
Accession: EKJ69468
  
Location: 12542-13846
  
 NCBI BlastP on this gene

EKJ69468

hypothetical protein
  
Accession: EKJ69469
  
Location: 16583-17198
  
 NCBI BlastP on this gene

EKJ69469

hypothetical protein
  
Accession: EKJ69470
  
Location: 20058-21894
  
 NCBI BlastP on this gene

EKJ69470

hypothetical protein
  
Accession: EKJ69471
  
Location: 22531-23827
  
 NCBI BlastP on this gene

EKJ69471

hypothetical protein
  
Accession: EKJ69472
  
Location: 26955-28580
  
 NCBI BlastP on this gene

EKJ69472

hypothetical protein
  
Accession: EKJ69473
  
Location: 28769-30732
  
 NCBI BlastP on this gene

EKJ69473

Query: Architecture Search FASTA input

JH126401 : Cordyceps militaris CM01 unplaced genomic scaffold CCM\_S00003    Total score: 2.0     Cumulative Blast bit score: 959

Hit cluster cross-links:

Mycgr3G90785 Mycgr3T
  
Location: 0-1047

Mycgr3G90785\_Mycgr3T

Mycgr3G103262 Mycgr3
  
Location: 1147-1390

Mycgr3G103262\_Mycgr3

Mycgr3G68458 Mycgr3T
  
Location: 1490-3602

Mycgr3G68458\_Mycgr3T

Mycgr3G99145 Mycgr3T
  
Location: 3702-4326

Mycgr3G99145\_Mycgr3T

Mycgr3G103274 Mycgr3
  
Location: 4426-4957

Mycgr3G103274\_Mycgr3

Mycgr3G103264 Mycgr3
  
Location: 5057-5390

Mycgr3G103264\_Mycgr3

Mycgr3G37570 Mycgr3T
  
Location: 5490-6006

Mycgr3G37570\_Mycgr3T

Mycgr3G108094 Mycgr3
  
Location: 6106-10555

Mycgr3G108094\_Mycgr3

Mycgr3G90786 Mycgr3T
  
Location: 10655-12080

Mycgr3G90786\_Mycgr3T

Mycgr3G68429 Mycgr3T
  
Location: 12180-13440

Mycgr3G68429\_Mycgr3T

Mycgr3G68421 Mycgr3T
  
Location: 13540-17086

Mycgr3G68421\_Mycgr3T

Mycgr3G90801 Mycgr3T
  
Location: 17186-18056

Mycgr3G90801\_Mycgr3T

Mycgr3G84646 Mycgr3T
  
Location: 18156-20235

Mycgr3G84646\_Mycgr3T

Mycgr3G68456 Mycgr3T
  
Location: 20335-21970

Mycgr3G68456\_Mycgr3T

Mycgr3G103270 Mycgr3
  
Location: 22070-22355

Mycgr3G103270\_Mycgr3

Mycgr3G90803 Mycgr3T
  
Location: 22455-23019

Mycgr3G90803\_Mycgr3T

Mycgr3G36941 Mycgr3T
  
Location: 23119-24064

Mycgr3G36941\_Mycgr3T

Mycgr3G25746 Mycgr3T
  
Location: 24164-25241

Mycgr3G25746\_Mycgr3T

Mycgr3G90788 Mycgr3T
  
Location: 25341-25803

Mycgr3G90788\_Mycgr3T

Mycgr3G103260 Mycgr3
  
Location: 25903-26635

Mycgr3G103260\_Mycgr3

Mycgr3G84644 Mycgr3T
  
Location: 26735-28457

Mycgr3G84644\_Mycgr3T

Mycgr3G29227 Mycgr3T
  
Location: 28557-28863

Mycgr3G29227\_Mycgr3T

Mycgr3G36271 Mycgr3T
  
Location: 28963-29854

Mycgr3G36271\_Mycgr3T

Mycgr3G68433 Mycgr3T
  
Location: 29954-33041

Mycgr3G68433\_Mycgr3T

Mycgr3G79452 Mycgr3T
  
Location: 33141-33399

Mycgr3G79452\_Mycgr3T

Mycgr3G55345 Mycgr3T
  
Location: 33499-34126

Mycgr3G55345\_Mycgr3T

Mycgr3G103278 Mycgr3
  
Location: 34226-35195

Mycgr3G103278\_Mycgr3

Mycgr3G84654 Mycgr3T
  
Location: 35295-36630

Mycgr3G84654\_Mycgr3T

Mycgr3G108090 Mycgr3
  
Location: 36730-37591

Mycgr3G108090\_Mycgr3

Mycgr3G21922 Mycgr3T
  
Location: 37691-39149

Mycgr3G21922\_Mycgr3T

Mycgr3G99148 Mycgr3T
  
Location: 39249-42819

Mycgr3G99148\_Mycgr3T

profilin
  
Accession: EGX92884
  
Location: 2065025-2065598
  
 NCBI BlastP on this gene

EGX92884

Got1 family protein
  
Accession: EGX92885
  
Location: 2066345-2067122
  
 NCBI BlastP on this gene

EGX92885

hypothetical protein
  
Accession: EGX92886
  
Location: 2068444-2068996
  
 NCBI BlastP on this gene

EGX92886

Protein kinase-like domain
  
Accession: EGX92887
  
Location: 2070117-2072285
  
 NCBI BlastP on this gene

EGX92887

YL1 nuclear
  
Accession: EGX92888
  
Location: 2072707-2073315
  
 NCBI BlastP on this gene

EGX92888

DASH complex subunit DAD4
  
Accession: EGX92889
  
Location: 2073599-2074003
  
 NCBI BlastP on this gene

EGX92889

chromatin remodeling complex subunit (Arp5), putative
  
Accession: EGX92890
  
Location: 2074418-2076889
  
 NCBI BlastP on this gene

EGX92890

bZIP transcription factor
  
Accession: EGX92891
  
Location: 2077577-2078936
  
 NCBI BlastP on this gene

EGX92891

pterin-4-alpha-carbinolamine dehydratase, putative
  
Accession: EGX92892
  
Location: 2081051-2081685
  
 NCBI BlastP on this gene

EGX92892

C-4 methylsterol oxidase, variant
  
Accession: EGX92893
  
Location: 2082091-2083149
  
  
**BlastP hit with Mycgr3G36271\_Mycgr3T**
  
Percentage identity: 69 %
  
BlastP bit score: 433
  
Sequence coverage: 96 %
  
E-value: 1e-149
  
  
 NCBI BlastP on this gene

EGX92893

ATP synthase regulation protein NCA2
  
Accession: EGX92894
  
Location: 2084555-2086612
  
  
**BlastP hit with Mycgr3G84646\_Mycgr3T**
  
Percentage identity: 41 %
  
BlastP bit score: 527
  
Sequence coverage: 101 %
  
E-value: 6e-175
  
  
 NCBI BlastP on this gene

EGX92894

C4-dicarboxylate transporter/malic acid transport protein, putative
  
Accession: EGX92895
  
Location: 2086967-2088458
  
 NCBI BlastP on this gene

EGX92895

cysteine dioxygenase
  
Accession: EGX92896
  
Location: 2090463-2091281
  
 NCBI BlastP on this gene

EGX92896

flavodoxin and radical SAM domain protein
  
Accession: EGX92897
  
Location: 2092171-2094544
  
 NCBI BlastP on this gene

EGX92897

mitochondrial import receptor subunit tom-20
  
Accession: EGX92898
  
Location: 2095010-2095789
  
 NCBI BlastP on this gene

EGX92898

exocyst complex component EXO84
  
Accession: EGX92899
  
Location: 2096363-2098496
  
 NCBI BlastP on this gene

EGX92899

serine/threonine-protein phosphatase PP2A catalytic subunit
  
Accession: EGX92900
  
Location: 2099420-2100821
  
 NCBI BlastP on this gene

EGX92900

hypothetical protein
  
Accession: EGX92901
  
Location: 2101337-2102495
  
 NCBI BlastP on this gene

EGX92901

Query: Architecture Search FASTA input

GL698525 : Metarhizium acridum CQMa 102 unplaced genomic scaffold Scf\_056    Total score: 2.0     Cumulative Blast bit score: 959

Hit cluster cross-links:

Mycgr3G90785 Mycgr3T
  
Location: 0-1047

Mycgr3G90785\_Mycgr3T

Mycgr3G103262 Mycgr3
  
Location: 1147-1390

Mycgr3G103262\_Mycgr3

Mycgr3G68458 Mycgr3T
  
Location: 1490-3602

Mycgr3G68458\_Mycgr3T

Mycgr3G99145 Mycgr3T
  
Location: 3702-4326

Mycgr3G99145\_Mycgr3T

Mycgr3G103274 Mycgr3
  
Location: 4426-4957

Mycgr3G103274\_Mycgr3

Mycgr3G103264 Mycgr3
  
Location: 5057-5390

Mycgr3G103264\_Mycgr3

Mycgr3G37570 Mycgr3T
  
Location: 5490-6006

Mycgr3G37570\_Mycgr3T

Mycgr3G108094 Mycgr3
  
Location: 6106-10555

Mycgr3G108094\_Mycgr3

Mycgr3G90786 Mycgr3T
  
Location: 10655-12080

Mycgr3G90786\_Mycgr3T

Mycgr3G68429 Mycgr3T
  
Location: 12180-13440

Mycgr3G68429\_Mycgr3T

Mycgr3G68421 Mycgr3T
  
Location: 13540-17086

Mycgr3G68421\_Mycgr3T

Mycgr3G90801 Mycgr3T
  
Location: 17186-18056

Mycgr3G90801\_Mycgr3T

Mycgr3G84646 Mycgr3T
  
Location: 18156-20235

Mycgr3G84646\_Mycgr3T

Mycgr3G68456 Mycgr3T
  
Location: 20335-21970

Mycgr3G68456\_Mycgr3T

Mycgr3G103270 Mycgr3
  
Location: 22070-22355

Mycgr3G103270\_Mycgr3

Mycgr3G90803 Mycgr3T
  
Location: 22455-23019

Mycgr3G90803\_Mycgr3T

Mycgr3G36941 Mycgr3T
  
Location: 23119-24064

Mycgr3G36941\_Mycgr3T

Mycgr3G25746 Mycgr3T
  
Location: 24164-25241

Mycgr3G25746\_Mycgr3T

Mycgr3G90788 Mycgr3T
  
Location: 25341-25803

Mycgr3G90788\_Mycgr3T

Mycgr3G103260 Mycgr3
  
Location: 25903-26635

Mycgr3G103260\_Mycgr3

Mycgr3G84644 Mycgr3T
  
Location: 26735-28457

Mycgr3G84644\_Mycgr3T

Mycgr3G29227 Mycgr3T
  
Location: 28557-28863

Mycgr3G29227\_Mycgr3T

Mycgr3G36271 Mycgr3T
  
Location: 28963-29854

Mycgr3G36271\_Mycgr3T

Mycgr3G68433 Mycgr3T
  
Location: 29954-33041

Mycgr3G68433\_Mycgr3T

Mycgr3G79452 Mycgr3T
  
Location: 33141-33399

Mycgr3G79452\_Mycgr3T

Mycgr3G55345 Mycgr3T
  
Location: 33499-34126

Mycgr3G55345\_Mycgr3T

Mycgr3G103278 Mycgr3
  
Location: 34226-35195

Mycgr3G103278\_Mycgr3

Mycgr3G84654 Mycgr3T
  
Location: 35295-36630

Mycgr3G84654\_Mycgr3T

Mycgr3G108090 Mycgr3
  
Location: 36730-37591

Mycgr3G108090\_Mycgr3

Mycgr3G21922 Mycgr3T
  
Location: 37691-39149

Mycgr3G21922\_Mycgr3T

Mycgr3G99148 Mycgr3T
  
Location: 39249-42819

Mycgr3G99148\_Mycgr3T

profilin
  
Accession: EFY87554
  
Location: 112544-113547
  
 NCBI BlastP on this gene

EFY87554

Got1 family protein
  
Accession: EFY87555
  
Location: 114213-114962
  
 NCBI BlastP on this gene

EFY87555

hypothetical protein
  
Accession: EFY87556
  
Location: 115984-116562
  
 NCBI BlastP on this gene

EFY87556

chromatin remodeling complex subunit (Arp5), putative
  
Accession: EFY87557
  
Location: 117807-120266
  
 NCBI BlastP on this gene

EFY87557

transmembrane protein
  
Accession: EFY87558
  
Location: 120852-122296
  
 NCBI BlastP on this gene

EFY87558

amidohydrolase
  
Accession: EFY87559
  
Location: 124303-125415
  
 NCBI BlastP on this gene

EFY87559

pterin-4-alpha-carbinolamine dehydratase family protein
  
Accession: EFY87560
  
Location: 126064-126690
  
 NCBI BlastP on this gene

EFY87560

C-4 sterol methyl oxidase
  
Accession: EFY87561
  
Location: 127230-128015
  
  
**BlastP hit with Mycgr3G36271\_Mycgr3T**
  
Percentage identity: 74 %
  
BlastP bit score: 422
  
Sequence coverage: 86 %
  
E-value: 6e-146
  
  
 NCBI BlastP on this gene

EFY87561

ATP synthase regulation protein NCA2
  
Accession: EFY87562
  
Location: 129596-131682
  
  
**BlastP hit with Mycgr3G84646\_Mycgr3T**
  
Percentage identity: 43 %
  
BlastP bit score: 538
  
Sequence coverage: 100 %
  
E-value: 2e-179
  
  
 NCBI BlastP on this gene

EFY87562

C4-dicarboxylate transporter, putative
  
Accession: EFY87563
  
Location: 132205-133507
  
 NCBI BlastP on this gene

EFY87563

DNA repair and recombination protein RAD5B
  
Accession: EFY87564
  
Location: 135538-140246
  
 NCBI BlastP on this gene

EFY87564

flavodoxin and radical SAM domain protein
  
Accession: EFY87565
  
Location: 140699-143131
  
 NCBI BlastP on this gene

EFY87565

mitochondrial import receptor subunit tom-20
  
Accession: EFY87566
  
Location: 143615-144409
  
 NCBI BlastP on this gene

EFY87566

Exocyst complex component EXO84
  
Accession: EFY87567
  
Location: 145488-147654
  
 NCBI BlastP on this gene

EFY87567

serine/threonine protein phosphatase PP2A catalytic subunit
  
Accession: EFY87568
  
Location: 148491-149997
  
 NCBI BlastP on this gene

EFY87568

hypothetical protein
  
Accession: EFY87569
  
Location: 150668-151726
  
 NCBI BlastP on this gene

EFY87569

Query: Architecture Search FASTA input

GG698910 : Nectria haematococca mpVI 77-13-4 chromosome 2 genomic scaffold NECHAsca\_18\_chr2\_1\_0    Total score: 2.0     Cumulative Blast bit score: 958

Hit cluster cross-links:

Mycgr3G90785 Mycgr3T
  
Location: 0-1047

Mycgr3G90785\_Mycgr3T

Mycgr3G103262 Mycgr3
  
Location: 1147-1390

Mycgr3G103262\_Mycgr3

Mycgr3G68458 Mycgr3T
  
Location: 1490-3602

Mycgr3G68458\_Mycgr3T

Mycgr3G99145 Mycgr3T
  
Location: 3702-4326

Mycgr3G99145\_Mycgr3T

Mycgr3G103274 Mycgr3
  
Location: 4426-4957

Mycgr3G103274\_Mycgr3

Mycgr3G103264 Mycgr3
  
Location: 5057-5390

Mycgr3G103264\_Mycgr3

Mycgr3G37570 Mycgr3T
  
Location: 5490-6006

Mycgr3G37570\_Mycgr3T

Mycgr3G108094 Mycgr3
  
Location: 6106-10555

Mycgr3G108094\_Mycgr3

Mycgr3G90786 Mycgr3T
  
Location: 10655-12080

Mycgr3G90786\_Mycgr3T

Mycgr3G68429 Mycgr3T
  
Location: 12180-13440

Mycgr3G68429\_Mycgr3T

Mycgr3G68421 Mycgr3T
  
Location: 13540-17086

Mycgr3G68421\_Mycgr3T

Mycgr3G90801 Mycgr3T
  
Location: 17186-18056

Mycgr3G90801\_Mycgr3T

Mycgr3G84646 Mycgr3T
  
Location: 18156-20235

Mycgr3G84646\_Mycgr3T

Mycgr3G68456 Mycgr3T
  
Location: 20335-21970

Mycgr3G68456\_Mycgr3T

Mycgr3G103270 Mycgr3
  
Location: 22070-22355

Mycgr3G103270\_Mycgr3

Mycgr3G90803 Mycgr3T
  
Location: 22455-23019

Mycgr3G90803\_Mycgr3T

Mycgr3G36941 Mycgr3T
  
Location: 23119-24064

Mycgr3G36941\_Mycgr3T

Mycgr3G25746 Mycgr3T
  
Location: 24164-25241

Mycgr3G25746\_Mycgr3T

Mycgr3G90788 Mycgr3T
  
Location: 25341-25803

Mycgr3G90788\_Mycgr3T

Mycgr3G103260 Mycgr3
  
Location: 25903-26635

Mycgr3G103260\_Mycgr3

Mycgr3G84644 Mycgr3T
  
Location: 26735-28457

Mycgr3G84644\_Mycgr3T

Mycgr3G29227 Mycgr3T
  
Location: 28557-28863

Mycgr3G29227\_Mycgr3T

Mycgr3G36271 Mycgr3T
  
Location: 28963-29854

Mycgr3G36271\_Mycgr3T

Mycgr3G68433 Mycgr3T
  
Location: 29954-33041

Mycgr3G68433\_Mycgr3T

Mycgr3G79452 Mycgr3T
  
Location: 33141-33399

Mycgr3G79452\_Mycgr3T

Mycgr3G55345 Mycgr3T
  
Location: 33499-34126

Mycgr3G55345\_Mycgr3T

Mycgr3G103278 Mycgr3
  
Location: 34226-35195

Mycgr3G103278\_Mycgr3

Mycgr3G84654 Mycgr3T
  
Location: 35295-36630

Mycgr3G84654\_Mycgr3T

Mycgr3G108090 Mycgr3
  
Location: 36730-37591

Mycgr3G108090\_Mycgr3

Mycgr3G21922 Mycgr3T
  
Location: 37691-39149

Mycgr3G21922\_Mycgr3T

Mycgr3G99148 Mycgr3T
  
Location: 39249-42819

Mycgr3G99148\_Mycgr3T

hypothetical protein
  
Accession: EEU40349
  
Location: 2301402-2302753
  
 NCBI BlastP on this gene

EEU40349

predicted protein
  
Accession: EEU40926
  
Location: 2304724-2305671
  
 NCBI BlastP on this gene

EEU40926

hypothetical protein
  
Accession: EEU40927
  
Location: 2306561-2307942
  
 NCBI BlastP on this gene

EEU40927

hypothetical protein
  
Accession: EEU40350
  
Location: 2310078-2310723
  
 NCBI BlastP on this gene

EEU40350

hypothetical protein
  
Accession: EEU40351
  
Location: 2313782-2317019
  
 NCBI BlastP on this gene

EEU40351

hypothetical protein
  
Accession: EEU40928
  
Location: 2317387-2319364
  
  
**BlastP hit with Mycgr3G84646\_Mycgr3T**
  
Percentage identity: 42 %
  
BlastP bit score: 514
  
Sequence coverage: 100 %
  
E-value: 3e-170
  
  
 NCBI BlastP on this gene

EEU40928

predicted protein
  
Accession: EEU40352
  
Location: 2322408-2325131
  
 NCBI BlastP on this gene

EEU40352

hypothetical protein
  
Accession: EEU40929
  
Location: 2325220-2327089
  
 NCBI BlastP on this gene

EEU40929

hypothetical protein
  
Accession: EEU40353
  
Location: 2327667-2329510
  
 NCBI BlastP on this gene

EEU40353

predicted protein
  
Accession: EEU40354
  
Location: 2331904-2332963
  
  
**BlastP hit with Mycgr3G36271\_Mycgr3T**
  
Percentage identity: 69 %
  
BlastP bit score: 444
  
Sequence coverage: 97 %
  
E-value: 8e-154
  
  
 NCBI BlastP on this gene

EEU40354

hypothetical protein
  
Accession: EEU40930
  
Location: 2333444-2334056
  
 NCBI BlastP on this gene

EEU40930

predicted protein
  
Accession: EEU40931
  
Location: 2334552-2335185
  
 NCBI BlastP on this gene

EEU40931

hypothetical protein
  
Accession: EEU40355
  
Location: 2336268-2337573
  
 NCBI BlastP on this gene

EEU40355

Actin-related protein, ARP5 class
  
Accession: EEU40932
  
Location: 2338075-2340481
  
 NCBI BlastP on this gene

EEU40932

hypothetical protein
  
Accession: EEU40356
  
Location: 2341863-2342429
  
 NCBI BlastP on this gene

EEU40356

predicted protein
  
Accession: EEU40357
  
Location: 2343438-2344198
  
 NCBI BlastP on this gene

EEU40357

predicted protein
  
Accession: EEU40933
  
Location: 2344760-2345569
  
 NCBI BlastP on this gene

EEU40933

predicted protein
  
Accession: EEU40934
  
Location: 2346472-2347530
  
 NCBI BlastP on this gene

EEU40934

Query: Architecture Search FASTA input

GL985061 : Trichoderma reesei QM6a unplaced genomic scaffold TRIREscaffold\_6    Total score: 2.0     Cumulative Blast bit score: 948

Hit cluster cross-links:

Mycgr3G90785 Mycgr3T
  
Location: 0-1047

Mycgr3G90785\_Mycgr3T

Mycgr3G103262 Mycgr3
  
Location: 1147-1390

Mycgr3G103262\_Mycgr3

Mycgr3G68458 Mycgr3T
  
Location: 1490-3602

Mycgr3G68458\_Mycgr3T

Mycgr3G99145 Mycgr3T
  
Location: 3702-4326

Mycgr3G99145\_Mycgr3T

Mycgr3G103274 Mycgr3
  
Location: 4426-4957

Mycgr3G103274\_Mycgr3

Mycgr3G103264 Mycgr3
  
Location: 5057-5390

Mycgr3G103264\_Mycgr3

Mycgr3G37570 Mycgr3T
  
Location: 5490-6006

Mycgr3G37570\_Mycgr3T

Mycgr3G108094 Mycgr3
  
Location: 6106-10555

Mycgr3G108094\_Mycgr3

Mycgr3G90786 Mycgr3T
  
Location: 10655-12080

Mycgr3G90786\_Mycgr3T

Mycgr3G68429 Mycgr3T
  
Location: 12180-13440

Mycgr3G68429\_Mycgr3T

Mycgr3G68421 Mycgr3T
  
Location: 13540-17086

Mycgr3G68421\_Mycgr3T

Mycgr3G90801 Mycgr3T
  
Location: 17186-18056

Mycgr3G90801\_Mycgr3T

Mycgr3G84646 Mycgr3T
  
Location: 18156-20235

Mycgr3G84646\_Mycgr3T

Mycgr3G68456 Mycgr3T
  
Location: 20335-21970

Mycgr3G68456\_Mycgr3T

Mycgr3G103270 Mycgr3
  
Location: 22070-22355

Mycgr3G103270\_Mycgr3

Mycgr3G90803 Mycgr3T
  
Location: 22455-23019

Mycgr3G90803\_Mycgr3T

Mycgr3G36941 Mycgr3T
  
Location: 23119-24064

Mycgr3G36941\_Mycgr3T

Mycgr3G25746 Mycgr3T
  
Location: 24164-25241

Mycgr3G25746\_Mycgr3T

Mycgr3G90788 Mycgr3T
  
Location: 25341-25803

Mycgr3G90788\_Mycgr3T

Mycgr3G103260 Mycgr3
  
Location: 25903-26635

Mycgr3G103260\_Mycgr3

Mycgr3G84644 Mycgr3T
  
Location: 26735-28457

Mycgr3G84644\_Mycgr3T

Mycgr3G29227 Mycgr3T
  
Location: 28557-28863

Mycgr3G29227\_Mycgr3T

Mycgr3G36271 Mycgr3T
  
Location: 28963-29854

Mycgr3G36271\_Mycgr3T

Mycgr3G68433 Mycgr3T
  
Location: 29954-33041

Mycgr3G68433\_Mycgr3T

Mycgr3G79452 Mycgr3T
  
Location: 33141-33399

Mycgr3G79452\_Mycgr3T

Mycgr3G55345 Mycgr3T
  
Location: 33499-34126

Mycgr3G55345\_Mycgr3T

Mycgr3G103278 Mycgr3
  
Location: 34226-35195

Mycgr3G103278\_Mycgr3

Mycgr3G84654 Mycgr3T
  
Location: 35295-36630

Mycgr3G84654\_Mycgr3T

Mycgr3G108090 Mycgr3
  
Location: 36730-37591

Mycgr3G108090\_Mycgr3

Mycgr3G21922 Mycgr3T
  
Location: 37691-39149

Mycgr3G21922\_Mycgr3T

Mycgr3G99148 Mycgr3T
  
Location: 39249-42819

Mycgr3G99148\_Mycgr3T

predicted protein
  
Accession: EGR49937
  
Location: 437138-440483
  
 NCBI BlastP on this gene

EGR49937

flavohemoglobin
  
Accession: EGR49938
  
Location: 442244-443563
  
 NCBI BlastP on this gene

EGR49938

serine/threonine protein kinase
  
Accession: EGR49749
  
Location: 453024-454811
  
  
**BlastP hit with Mycgr3G84644\_Mycgr3T**
  
Percentage identity: 73 %
  
BlastP bit score: 735
  
Sequence coverage: 86 %
  
E-value: 0.0
  
  
 NCBI BlastP on this gene

EGR49749

predicted protein
  
Accession: EGR49939
  
Location: 456186-457641
  
 NCBI BlastP on this gene

EGR49939

predicted protein
  
Accession: EGR49940
  
Location: 459709-460191
  
 NCBI BlastP on this gene

EGR49940

vesicular transport protein
  
Accession: EGR49750
  
Location: 467138-470596
  
  
**BlastP hit with Mycgr3G108094\_Mycgr3**
  
Percentage identity: 31 %
  
BlastP bit score: 213
  
Sequence coverage: 44 %
  
E-value: 4e-53
  
  
 NCBI BlastP on this gene

EGR49750

predicted protein
  
Accession: EGR49941
  
Location: 472904-474067
  
 NCBI BlastP on this gene

EGR49941

glycosyltransferase family 1
  
Accession: EGR49942
  
Location: 484124-485739
  
 NCBI BlastP on this gene

EGR49942

Query: Architecture Search FASTA input

151. :  DS572750 Paracoccidioides brasiliensis Pb18 supercont1.1 genomic scaffold     Total score: 2.0     Cumulative Blast bit score: 1131

Mycgr3G90785 Mycgr3T
  
Location: 0-1047
  
 NCBI BlastP on this gene

Mycgr3G90785\_Mycgr3T

Mycgr3G103262 Mycgr3
  
Location: 1147-1390
  
 NCBI BlastP on this gene

Mycgr3G103262\_Mycgr3

Mycgr3G68458 Mycgr3T
  
Location: 1490-3602
  
 NCBI BlastP on this gene

Mycgr3G68458\_Mycgr3T

Mycgr3G99145 Mycgr3T
  
Location: 3702-4326
  
 NCBI BlastP on this gene

Mycgr3G99145\_Mycgr3T

Mycgr3G103274 Mycgr3
  
Location: 4426-4957
  
 NCBI BlastP on this gene

Mycgr3G103274\_Mycgr3

Mycgr3G103264 Mycgr3
  
Location: 5057-5390
  
 NCBI BlastP on this gene

Mycgr3G103264\_Mycgr3

Mycgr3G37570 Mycgr3T
  
Location: 5490-6006
  
 NCBI BlastP on this gene

Mycgr3G37570\_Mycgr3T

Mycgr3G108094 Mycgr3
  
Location: 6106-10555
  
 NCBI BlastP on this gene

Mycgr3G108094\_Mycgr3

Mycgr3G90786 Mycgr3T
  
Location: 10655-12080
  
 NCBI BlastP on this gene

Mycgr3G90786\_Mycgr3T

Mycgr3G68429 Mycgr3T
  
Location: 12180-13440
  
 NCBI BlastP on this gene

Mycgr3G68429\_Mycgr3T

Mycgr3G68421 Mycgr3T
  
Location: 13540-17086
  
 NCBI BlastP on this gene

Mycgr3G68421\_Mycgr3T

Mycgr3G90801 Mycgr3T
  
Location: 17186-18056
  
 NCBI BlastP on this gene

Mycgr3G90801\_Mycgr3T

Mycgr3G84646 Mycgr3T
  
Location: 18156-20235
  
 NCBI BlastP on this gene

Mycgr3G84646\_Mycgr3T

Mycgr3G68456 Mycgr3T
  
Location: 20335-21970
  
 NCBI BlastP on this gene

Mycgr3G68456\_Mycgr3T

Mycgr3G103270 Mycgr3
  
Location: 22070-22355
  
 NCBI BlastP on this gene

Mycgr3G103270\_Mycgr3

Mycgr3G90803 Mycgr3T
  
Location: 22455-23019
  
 NCBI BlastP on this gene

Mycgr3G90803\_Mycgr3T

Mycgr3G36941 Mycgr3T
  
Location: 23119-24064
  
 NCBI BlastP on this gene

Mycgr3G36941\_Mycgr3T

Mycgr3G25746 Mycgr3T
  
Location: 24164-25241
  
 NCBI BlastP on this gene

Mycgr3G25746\_Mycgr3T

Mycgr3G90788 Mycgr3T
  
Location: 25341-25803
  
 NCBI BlastP on this gene

Mycgr3G90788\_Mycgr3T

Mycgr3G103260 Mycgr3
  
Location: 25903-26635
  
 NCBI BlastP on this gene

Mycgr3G103260\_Mycgr3

Mycgr3G84644 Mycgr3T
  
Location: 26735-28457
  
 NCBI BlastP on this gene

Mycgr3G84644\_Mycgr3T

Mycgr3G29227 Mycgr3T
  
Location: 28557-28863
  
 NCBI BlastP on this gene

Mycgr3G29227\_Mycgr3T

Mycgr3G36271 Mycgr3T
  
Location: 28963-29854
  
 NCBI BlastP on this gene

Mycgr3G36271\_Mycgr3T

Mycgr3G68433 Mycgr3T
  
Location: 29954-33041
  
 NCBI BlastP on this gene

Mycgr3G68433\_Mycgr3T

Mycgr3G79452 Mycgr3T
  
Location: 33141-33399
  
 NCBI BlastP on this gene

Mycgr3G79452\_Mycgr3T

Mycgr3G55345 Mycgr3T
  
Location: 33499-34126
  
 NCBI BlastP on this gene

Mycgr3G55345\_Mycgr3T

Mycgr3G103278 Mycgr3
  
Location: 34226-35195
  
 NCBI BlastP on this gene

Mycgr3G103278\_Mycgr3

Mycgr3G84654 Mycgr3T
  
Location: 35295-36630
  
 NCBI BlastP on this gene

Mycgr3G84654\_Mycgr3T

Mycgr3G108090 Mycgr3
  
Location: 36730-37591
  
 NCBI BlastP on this gene

Mycgr3G108090\_Mycgr3

Mycgr3G21922 Mycgr3T
  
Location: 37691-39149
  
 NCBI BlastP on this gene

Mycgr3G21922\_Mycgr3T

Mycgr3G99148 Mycgr3T
  
Location: 39249-42819
  
 NCBI BlastP on this gene

Mycgr3G99148\_Mycgr3T

conserved hypothetical protein
  
Accession: EEH43876
  
Location: 550840-551439
  
 NCBI BlastP on this gene

EEH43876

MGMT family protein
  
Accession: EEH43877
  
Location: 558876-559688
  
 NCBI BlastP on this gene

EEH43877

pre-mRNA-splicing factor cwc26
  
Accession: EEH43878
  
Location: 559959-561047
  
 NCBI BlastP on this gene

EEH43878

serine/threonine-protein kinase SKY1
  
Accession: EEH43879
  
Location: 562856-564956
  
  
**BlastP hit with Mycgr3G84644\_Mycgr3T**
  
Percentage identity: 67 %
  
BlastP bit score: 753
  
Sequence coverage: 104 %
  
E-value: 0.0
  
  
 NCBI BlastP on this gene

EEH43879

hypothetical protein
  
Accession: EEH43880
  
Location: 567534-568113
  
 NCBI BlastP on this gene

EEH43880

conserved hypothetical protein
  
Accession: EEH43881
  
Location: 568840-570003
  
 NCBI BlastP on this gene

EEH43881

cytochrome b2
  
Accession: EEH43882
  
Location: 570306-572020
  
 NCBI BlastP on this gene

EEH43882

ras-like GTP-binding protein
  
Accession: EEH43883
  
Location: 575249-576242
  
 NCBI BlastP on this gene

EEH43883

conserved hypothetical protein
  
Accession: EEH43884
  
Location: 579948-586324
  
  
**BlastP hit with Mycgr3G108094\_Mycgr3**
  
Percentage identity: 40 %
  
BlastP bit score: 378
  
Sequence coverage: 40 %
  
E-value: 6e-105
  
  
 NCBI BlastP on this gene

EEH43884

N-acetyltransferase ats1
  
Accession: EEH43885
  
Location: 586954-587642
  
 NCBI BlastP on this gene

EEH43885

conserved hypothetical protein
  
Accession: EEH43886
  
Location: 588436-591002
  
 NCBI BlastP on this gene

EEH43886

predicted protein
  
Accession: EEH43887
  
Location: 592590-593372
  
 NCBI BlastP on this gene

EEH43887

chromatin remodeling complex subunit (Arp5)
  
Accession: EEH43888
  
Location: 596969-599594
  
 NCBI BlastP on this gene

EEH43888

152. :  AKHY01000145 Aspergillus oryzae 3.042     Total score: 2.0     Cumulative Blast bit score: 1124

ATP-dependent RNA helicase A
  
Accession: EIT77726
  
Location: 102212-104760
  
 NCBI BlastP on this gene

EIT77726

hypothetical protein
  
Accession: EIT77713
  
Location: 100849-101678
  
 NCBI BlastP on this gene

EIT77713

hypothetical protein
  
Accession: EIT77735
  
Location: 98019-100130
  
 NCBI BlastP on this gene

EIT77735

F-box domain protein
  
Accession: EIT77725
  
Location: 95211-96906
  
 NCBI BlastP on this gene

EIT77725

succinyl-CoA synthetase, beta subunit
  
Accession: EIT77717
  
Location: 92834-94643
  
 NCBI BlastP on this gene

EIT77717

C4-dicarboxylate/malic acid transporter
  
Accession: EIT77722
  
Location: 86505-88122
  
 NCBI BlastP on this gene

EIT77722

putative phosphoribosyltransferase
  
Accession: EIT77719
  
Location: 84829-85536
  
  
**BlastP hit with Mycgr3G55345\_Mycgr3T**
  
Percentage identity: 74 %
  
BlastP bit score: 255
  
Sequence coverage: 82 %
  
E-value: 8e-83
  
  
 NCBI BlastP on this gene

EIT77719

hypothetical protein
  
Accession: EIT77714
  
Location: 82184-82982
  
 NCBI BlastP on this gene

EIT77714

hypothetical protein
  
Accession: EIT77747
  
Location: 80635-81797
  
 NCBI BlastP on this gene

EIT77747

hypothetical protein
  
Accession: EIT77748
  
Location: 79129-80534
  
 NCBI BlastP on this gene

EIT77748

putative unusual protein kinase
  
Accession: EIT77756
  
Location: 76327-78570
  
  
**BlastP hit with Mycgr3G68458\_Mycgr3T**
  
Percentage identity: 61 %
  
BlastP bit score: 869
  
Sequence coverage: 100 %
  
E-value: 0.0
  
  
 NCBI BlastP on this gene

EIT77756

holocytochrome c synthase/heme-lyase
  
Accession: EIT77731
  
Location: 74320-75416
  
 NCBI BlastP on this gene

EIT77731

hypothetical protein
  
Accession: EIT77718
  
Location: 71597-73457
  
 NCBI BlastP on this gene

EIT77718

ubiquitin-protein ligase
  
Accession: EIT77753
  
Location: 67845-68792
  
 NCBI BlastP on this gene

EIT77753

Rab GTPase interacting factor, Golgi family membrane protein
  
Accession: EIT77723
  
Location: 64384-65389
  
 NCBI BlastP on this gene

EIT77723

hypothetical protein
  
Accession: EIT77724
  
Location: 61597-63993
  
 NCBI BlastP on this gene

EIT77724

hypothetical protein
  
Accession: EIT77752
  
Location: 57442-59445
  
 NCBI BlastP on this gene

EIT77752

153. :  DS572813 Paracoccidioides brasiliensis Pb01 supercont1.3 genomic scaffold     Total score: 2.0     Cumulative Blast bit score: 1110

predicted protein
  
Accession: EEH39135
  
Location: 914273-915848
  
 NCBI BlastP on this gene

EEH39135

conserved hypothetical protein
  
Accession: EEH39136
  
Location: 917780-918497
  
 NCBI BlastP on this gene

EEH39136

conserved hypothetical protein
  
Accession: EEH39137
  
Location: 920355-922918
  
 NCBI BlastP on this gene

EEH39137

N-acetyltransferase ats1
  
Accession: EEH39138
  
Location: 923704-924009
  
 NCBI BlastP on this gene

EEH39138

conserved hypothetical protein
  
Accession: EEH39139
  
Location: 925024-931450
  
  
**BlastP hit with Mycgr3G108094\_Mycgr3**
  
Percentage identity: 36 %
  
BlastP bit score: 358
  
Sequence coverage: 47 %
  
E-value: 6e-98
  
  
 NCBI BlastP on this gene

EEH39139

ras-like GTP-binding protein
  
Accession: EEH39140
  
Location: 935352-936345
  
 NCBI BlastP on this gene

EEH39140

cytochrome b2
  
Accession: EEH39141
  
Location: 939557-941272
  
 NCBI BlastP on this gene

EEH39141

conserved hypothetical protein
  
Accession: EEH39142
  
Location: 941517-942677
  
 NCBI BlastP on this gene

EEH39142

protein kinase dsk1
  
Accession: EEH39143
  
Location: 946495-948589
  
  
**BlastP hit with Mycgr3G84644\_Mycgr3T**
  
Percentage identity: 67 %
  
BlastP bit score: 752
  
Sequence coverage: 104 %
  
E-value: 0.0
  
  
 NCBI BlastP on this gene

EEH39143

pre-mRNA-splicing factor cwc26
  
Accession: EEH39144
  
Location: 950426-951520
  
 NCBI BlastP on this gene

EEH39144

MGMT family protein
  
Accession: EEH39145
  
Location: 951785-952595
  
 NCBI BlastP on this gene

EEH39145

154. :  AKCU01000110 Penicillium digitatum Pd1     Total score: 2.0     Cumulative Blast bit score: 1106

Pyoverdine/dityrosine biosynthesis protein, putative
  
Accession: EKV20588
  
Location: 60576-61625
  
 NCBI BlastP on this gene

EKV20588

hypothetical protein
  
Accession: EKV20587
  
Location: 58660-59397
  
 NCBI BlastP on this gene

EKV20587

RING finger protein (Zin), putative
  
Accession: EKV20586
  
Location: 55314-57656
  
 NCBI BlastP on this gene

EKV20586

Peroxisomal multifunctional beta-oxidation protein (MFP), putative
  
Accession: EKV20585
  
Location: 51581-54459
  
 NCBI BlastP on this gene

EKV20585

hypothetical protein
  
Accession: EKV20584
  
Location: 49674-50904
  
 NCBI BlastP on this gene

EKV20584

60S ribosomal protein L7
  
Accession: EKV20583
  
Location: 47129-48718
  
 NCBI BlastP on this gene

EKV20583

hypothetical protein
  
Accession: EKV20582
  
Location: 45967-46821
  
 NCBI BlastP on this gene

EKV20582

Thermophilic desulfurizing enzyme family protein
  
Accession: EKV20581
  
Location: 43792-45212
  
 NCBI BlastP on this gene

EKV20581

Xanthine-guanine phosphoribosyl transferase Xpt1, putative
  
Accession: EKV20580
  
Location: 42374-43453
  
  
**BlastP hit with Mycgr3G55345\_Mycgr3T**
  
Percentage identity: 67 %
  
BlastP bit score: 264
  
Sequence coverage: 97 %
  
E-value: 8e-86
  
  
 NCBI BlastP on this gene

EKV20580

hypothetical protein
  
Accession: EKV20579
  
Location: 41324-41757
  
 NCBI BlastP on this gene

EKV20579

NADPH-dependent 1-acyl dihydroxyacetone phosphate reductase, putative
  
Accession: EKV20578
  
Location: 39867-40710
  
 NCBI BlastP on this gene

EKV20578

GTP binding protein, putative
  
Accession: EKV20577
  
Location: 35453-37618
  
 NCBI BlastP on this gene

EKV20577

hypothetical protein
  
Accession: EKV20576
  
Location: 33941-35252
  
 NCBI BlastP on this gene

EKV20576

Ubiquinone biosynthesis protein, putative
  
Accession: EKV20575
  
Location: 31196-33208
  
  
**BlastP hit with Mycgr3G68458\_Mycgr3T**
  
Percentage identity: 63 %
  
BlastP bit score: 842
  
Sequence coverage: 90 %
  
E-value: 0.0
  
  
 NCBI BlastP on this gene

EKV20575

Cytochrome c heme lyase, putative
  
Accession: EKV20574
  
Location: 29744-30787
  
 NCBI BlastP on this gene

EKV20574

hypothetical protein
  
Accession: EKV20573
  
Location: 28751-29350
  
 NCBI BlastP on this gene

EKV20573

hypothetical protein
  
Accession: EKV20572
  
Location: 28021-28113
  
 NCBI BlastP on this gene

EKV20572

Ubiquitin-conjugating enzyme Ubc6, putative
  
Accession: EKV20571
  
Location: 25751-26708
  
 NCBI BlastP on this gene

EKV20571

Mitochondrial outer membrane protein (Sam50), putative
  
Accession: EKV20570
  
Location: 23728-25497
  
 NCBI BlastP on this gene

EKV20570

Golgi membrane protein, putative
  
Accession: EKV20569
  
Location: 22364-23412
  
 NCBI BlastP on this gene

EKV20569

Ubiquitin fusion degradation protein (Ufd1), putative
  
Accession: EKV20568
  
Location: 19790-22084
  
 NCBI BlastP on this gene

EKV20568

hypothetical protein
  
Accession: EKV20567
  
Location: 17397-18881
  
 NCBI BlastP on this gene

EKV20567

Proteasome regulatory particle subunit Rpt6, putative
  
Accession: EKV20566
  
Location: 14398-15757
  
 NCBI BlastP on this gene

EKV20566

155. :  AKCT01000108 Penicillium digitatum PHI26     Total score: 2.0     Cumulative Blast bit score: 1106

Pyoverdine/dityrosine biosynthesis protein, putative
  
Accession: EKV15917
  
Location: 94927-95976
  
 NCBI BlastP on this gene

EKV15917

hypothetical protein
  
Accession: EKV15916
  
Location: 93011-93748
  
 NCBI BlastP on this gene

EKV15916

RING finger protein (Zin), putative
  
Accession: EKV15915
  
Location: 89665-92007
  
 NCBI BlastP on this gene

EKV15915

Peroxisomal multifunctional beta-oxidation protein (MFP), putative
  
Accession: EKV15914
  
Location: 85932-88810
  
 NCBI BlastP on this gene

EKV15914

hypothetical protein
  
Accession: EKV15913
  
Location: 84029-85259
  
 NCBI BlastP on this gene

EKV15913

60S ribosomal protein L7
  
Accession: EKV15912
  
Location: 81482-83071
  
 NCBI BlastP on this gene

EKV15912

hypothetical protein
  
Accession: EKV15911
  
Location: 80320-81174
  
 NCBI BlastP on this gene

EKV15911

Thermophilic desulfurizing enzyme family protein
  
Accession: EKV15910
  
Location: 78145-79565
  
 NCBI BlastP on this gene

EKV15910

Xanthine-guanine phosphoribosyl transferase Xpt1, putative
  
Accession: EKV15909
  
Location: 76721-77801
  
  
**BlastP hit with Mycgr3G55345\_Mycgr3T**
  
Percentage identity: 67 %
  
BlastP bit score: 264
  
Sequence coverage: 97 %
  
E-value: 8e-86
  
  
 NCBI BlastP on this gene

EKV15909

hypothetical protein
  
Accession: EKV15908
  
Location: 75671-76104
  
 NCBI BlastP on this gene

EKV15908

NADPH-dependent 1-acyl dihydroxyacetone phosphate reductase, putative
  
Accession: EKV15907
  
Location: 74214-75057
  
 NCBI BlastP on this gene

EKV15907

GTP binding protein, putative
  
Accession: EKV15906
  
Location: 69800-71965
  
 NCBI BlastP on this gene

EKV15906

hypothetical protein
  
Accession: EKV15905
  
Location: 68288-69599
  
 NCBI BlastP on this gene

EKV15905

Ubiquinone biosynthesis protein, putative
  
Accession: EKV15904
  
Location: 65543-67555
  
  
**BlastP hit with Mycgr3G68458\_Mycgr3T**
  
Percentage identity: 63 %
  
BlastP bit score: 842
  
Sequence coverage: 90 %
  
E-value: 0.0
  
  
 NCBI BlastP on this gene

EKV15904

Cytochrome c heme lyase, putative
  
Accession: EKV15903
  
Location: 64091-65134
  
 NCBI BlastP on this gene

EKV15903

hypothetical protein
  
Accession: EKV15902
  
Location: 62367-63697
  
 NCBI BlastP on this gene

EKV15902

Ubiquitin-conjugating enzyme Ubc6, putative
  
Accession: EKV15901
  
Location: 60117-61074
  
 NCBI BlastP on this gene

EKV15901

Mitochondrial outer membrane protein (Sam50), putative
  
Accession: EKV15900
  
Location: 58094-59863
  
 NCBI BlastP on this gene

EKV15900

Golgi membrane protein, putative
  
Accession: EKV15899
  
Location: 56730-57778
  
 NCBI BlastP on this gene

EKV15899

Ubiquitin fusion degradation protein (Ufd1), putative
  
Accession: EKV15898
  
Location: 54156-56450
  
 NCBI BlastP on this gene

EKV15898

hypothetical protein
  
Accession: EKV15897
  
Location: 51763-53247
  
 NCBI BlastP on this gene

EKV15897

Proteasome regulatory particle subunit Rpt6, putative
  
Accession: EKV15896
  
Location: 48764-50123
  
 NCBI BlastP on this gene

EKV15896

156. :  CH476624 Sclerotinia sclerotiorum 1980 scaffold\_4 genomic scaffold     Total score: 2.0     Cumulative Blast bit score: 1105

hypothetical protein
  
Accession: EDO00795
  
Location: 438662-439279
  
 NCBI BlastP on this gene

EDO00795

vacuolar protein sorting protein
  
Accession: EDO00796
  
Location: 439612-440441
  
 NCBI BlastP on this gene

EDO00796

predicted protein
  
Accession: EDO00797
  
Location: 441976-442754
  
 NCBI BlastP on this gene

EDO00797

predicted protein
  
Accession: EDO00798
  
Location: 444281-444682
  
 NCBI BlastP on this gene

EDO00798

predicted protein
  
Accession: EDO00799
  
Location: 446628-447275
  
 NCBI BlastP on this gene

EDO00799

hypothetical protein
  
Accession: EDO00800
  
Location: 447590-448873
  
 NCBI BlastP on this gene

EDO00800

hypothetical protein
  
Accession: EDO00801
  
Location: 449847-453983
  
  
**BlastP hit with Mycgr3G108094\_Mycgr3**
  
Percentage identity: 33 %
  
BlastP bit score: 305
  
Sequence coverage: 47 %
  
E-value: 9e-82
  
  
 NCBI BlastP on this gene

EDO00801

hypothetical protein
  
Accession: EDO00802
  
Location: 455717-456481
  
 NCBI BlastP on this gene

EDO00802

hypothetical protein
  
Accession: EDO00803
  
Location: 460903-462976
  
  
**BlastP hit with Mycgr3G84644\_Mycgr3T**
  
Percentage identity: 71 %
  
BlastP bit score: 800
  
Sequence coverage: 100 %
  
E-value: 0.0
  
  
 NCBI BlastP on this gene

EDO00803

hypothetical protein
  
Accession: EDO00804
  
Location: 464344-465852
  
 NCBI BlastP on this gene

EDO00804

hypothetical protein
  
Accession: EDO00805
  
Location: 466248-467722
  
 NCBI BlastP on this gene

EDO00805

hypothetical protein
  
Accession: EDO00806
  
Location: 469795-471332
  
 NCBI BlastP on this gene

EDO00806

157. :  CH445336 Phaeosphaeria nodorum SN15 scaffold\_12     Total score: 2.0     Cumulative Blast bit score: 1101

hypothetical protein
  
Accession: EAT84361
  
Location: 417739-418575
  
 NCBI BlastP on this gene

EAT84361

hypothetical protein
  
Accession: EAT84360
  
Location: 416194-417251
  
 NCBI BlastP on this gene

EAT84360

hypothetical protein
  
Accession: EAT84359
  
Location: 413450-414969
  
 NCBI BlastP on this gene

EAT84359

hypothetical protein
  
Accession: EAT84358
  
Location: 412307-412770
  
 NCBI BlastP on this gene

EAT84358

hypothetical protein
  
Accession: EAT84357
  
Location: 409263-410377
  
 NCBI BlastP on this gene

EAT84357

hypothetical protein
  
Accession: EAT84356
  
Location: 407638-408019
  
 NCBI BlastP on this gene

EAT84356

hypothetical protein
  
Accession: EAT84355
  
Location: 405658-407311
  
 NCBI BlastP on this gene

EAT84355

hypothetical protein
  
Accession: EAT84354
  
Location: 403301-405201
  
 NCBI BlastP on this gene

EAT84354

hypothetical protein
  
Accession: EAT84353
  
Location: 402962-403131
  
 NCBI BlastP on this gene

EAT84353

hypothetical protein
  
Accession: EAT84352
  
Location: 401427-402866
  
 NCBI BlastP on this gene

EAT84352

hypothetical protein
  
Accession: EAT84351
  
Location: 397565-401273
  
  
**BlastP hit with Mycgr3G108094\_Mycgr3**
  
Percentage identity: 40 %
  
BlastP bit score: 403
  
Sequence coverage: 44 %
  
E-value: 8e-116
  
  
 NCBI BlastP on this gene

EAT84351

hypothetical protein
  
Accession: EAT84350
  
Location: 394277-396410
  
  
**BlastP hit with Mycgr3G84644\_Mycgr3T**
  
Percentage identity: 68 %
  
BlastP bit score: 698
  
Sequence coverage: 96 %
  
E-value: 0.0
  
  
 NCBI BlastP on this gene

EAT84350

hypothetical protein
  
Accession: EAT84349
  
Location: 391778-393680
  
 NCBI BlastP on this gene

EAT84349

hypothetical protein
  
Accession: EAT84348
  
Location: 389919-390751
  
 NCBI BlastP on this gene

EAT84348

hypothetical protein
  
Accession: EAT84347
  
Location: 386501-388900
  
 NCBI BlastP on this gene

EAT84347

hypothetical protein
  
Accession: EAT84346
  
Location: 386285-386499
  
 NCBI BlastP on this gene

EAT84346

hypothetical protein
  
Accession: EDP89783
  
Location: 385869-386030
  
 NCBI BlastP on this gene

EDP89783

hypothetical protein
  
Accession: EAT84345
  
Location: 385010-385698
  
 NCBI BlastP on this gene

EAT84345

hypothetical protein
  
Accession: EAT84344
  
Location: 383410-384550
  
 NCBI BlastP on this gene

EAT84344

hypothetical protein
  
Accession: EAT84343
  
Location: 378879-382416
  
 NCBI BlastP on this gene

EAT84343

hypothetical protein
  
Accession: EAT84342
  
Location: 375960-378077
  
 NCBI BlastP on this gene

EAT84342

hypothetical protein
  
Accession: EAT84340
  
Location: 374297-375393
  
 NCBI BlastP on this gene

EAT84340

158. :  KB445579 Cochliobolus heterostrophus C5 unplaced genomic scaffold COCHEscaffold\_11     Total score: 2.0     Cumulative Blast bit score: 1091

hypothetical protein
  
Accession: EMD89514
  
Location: 1368503-1369972
  
 NCBI BlastP on this gene

EMD89514

hypothetical protein
  
Accession: EMD89513
  
Location: 1367701-1367988
  
 NCBI BlastP on this gene

EMD89513

hypothetical protein
  
Accession: EMD89512
  
Location: 1366055-1367145
  
 NCBI BlastP on this gene

EMD89512

hypothetical protein
  
Accession: EMD89511
  
Location: 1365185-1365835
  
 NCBI BlastP on this gene

EMD89511

hypothetical protein
  
Accession: EMD89510
  
Location: 1363444-1364453
  
 NCBI BlastP on this gene

EMD89510

hypothetical protein
  
Accession: EMD89509
  
Location: 1362713-1362985
  
 NCBI BlastP on this gene

EMD89509

hypothetical protein
  
Accession: EMD89508
  
Location: 1359913-1362565
  
 NCBI BlastP on this gene

EMD89508

hypothetical protein
  
Accession: EMD89507
  
Location: 1357496-1357708
  
 NCBI BlastP on this gene

EMD89507

hypothetical protein
  
Accession: EMD89506
  
Location: 1355047-1356710
  
 NCBI BlastP on this gene

EMD89506

hypothetical protein
  
Accession: EMD89505
  
Location: 1353031-1354624
  
 NCBI BlastP on this gene

EMD89505

hypothetical protein
  
Accession: EMD89504
  
Location: 1350981-1352422
  
 NCBI BlastP on this gene

EMD89504

hypothetical protein
  
Accession: EMD89503
  
Location: 1346864-1350714
  
  
**BlastP hit with Mycgr3G108094\_Mycgr3**
  
Percentage identity: 49 %
  
BlastP bit score: 246
  
Sequence coverage: 20 %
  
E-value: 3e-64
  
  
 NCBI BlastP on this gene

EMD89503

hypothetical protein
  
Accession: EMD89502
  
Location: 1343319-1345514
  
  
**BlastP hit with Mycgr3G84644\_Mycgr3T**
  
Percentage identity: 70 %
  
BlastP bit score: 845
  
Sequence coverage: 109 %
  
E-value: 0.0
  
  
 NCBI BlastP on this gene

EMD89502

glycoside hydrolase family 16 protein
  
Accession: EMD89501
  
Location: 1340877-1342093
  
 NCBI BlastP on this gene

EMD89501

hypothetical protein
  
Accession: EMD89500
  
Location: 1337047-1339228
  
 NCBI BlastP on this gene

EMD89500

hypothetical protein
  
Accession: EMD89499
  
Location: 1335252-1336611
  
 NCBI BlastP on this gene

EMD89499

hypothetical protein
  
Accession: EMD89498
  
Location: 1333930-1335178
  
 NCBI BlastP on this gene

EMD89498

hypothetical protein
  
Accession: EMD89497
  
Location: 1331594-1333705
  
 NCBI BlastP on this gene

EMD89497

hypothetical protein
  
Accession: EMD89496
  
Location: 1329148-1330940
  
 NCBI BlastP on this gene

EMD89496

hypothetical protein
  
Accession: EMD89495
  
Location: 1327717-1328634
  
 NCBI BlastP on this gene

EMD89495

glycosyltransferase family 2 protein
  
Accession: EMD89494
  
Location: 1326668-1327463
  
 NCBI BlastP on this gene

EMD89494

hypothetical protein
  
Accession: EMD89493
  
Location: 1325942-1326349
  
 NCBI BlastP on this gene

EMD89493

159. :  AP007172 Aspergillus oryzae RIB40 DNA, SC206.     Total score: 2.0     Cumulative Blast bit score: 1078

not annotated
  
Accession: BAE65417
  
Location: 103211-105760
  
 NCBI BlastP on this gene

AO090206000045

not annotated
  
Accession: BAE65416
  
Location: 101848-102677
  
 NCBI BlastP on this gene

AO090206000044

not annotated
  
Accession: BAE65415
  
Location: 100010-101129
  
 NCBI BlastP on this gene

AO090206000043

not annotated
  
Accession: BAE65414
  
Location: 99018-99840
  
 NCBI BlastP on this gene

AO090206000042

not annotated
  
Accession: BAE65413
  
Location: 97141-97906
  
 NCBI BlastP on this gene

AO090206000041

not annotated
  
Accession: BAE65412
  
Location: 93835-95643
  
 NCBI BlastP on this gene

AO090206000040

not annotated
  
Accession: BAE65411
  
Location: 87506-89087
  
 NCBI BlastP on this gene

AO090206000038

not annotated
  
Accession: BAE65410
  
Location: 85830-86850
  
  
**BlastP hit with Mycgr3G55345\_Mycgr3T**
  
Percentage identity: 73 %
  
BlastP bit score: 303
  
Sequence coverage: 96 %
  
E-value: 3e-101
  
  
 NCBI BlastP on this gene

AO090206000037

not annotated
  
Accession: BAE65409
  
Location: 83185-83749
  
 NCBI BlastP on this gene

AO090206000034

not annotated
  
Accession: BAE65408
  
Location: 81680-82777
  
 NCBI BlastP on this gene

AO090206000033

not annotated
  
Accession: BAE65407
  
Location: 80130-81535
  
 NCBI BlastP on this gene

AO090206000032

not annotated
  
Accession: BAE65406
  
Location: 76525-79328
  
  
**BlastP hit with Mycgr3G68458\_Mycgr3T**
  
Percentage identity: 67 %
  
BlastP bit score: 775
  
Sequence coverage: 78 %
  
E-value: 0.0
  
  
 NCBI BlastP on this gene

AO090206000031

not annotated
  
Accession: BAE65405
  
Location: 75321-76417
  
 NCBI BlastP on this gene

AO090206000030

not annotated
  
Accession: BAE65404
  
Location: 72561-74456
  
 NCBI BlastP on this gene

AO090206000029

not annotated
  
Accession: BAE65403
  
Location: 68846-69793
  
 NCBI BlastP on this gene

AO090206000028

not annotated
  
Accession: BAE65402
  
Location: 66745-68550
  
 NCBI BlastP on this gene

AO090206000027

not annotated
  
Accession: BAE65401
  
Location: 65385-66390
  
 NCBI BlastP on this gene

AO090206000026

not annotated
  
Accession: BAE65400
  
Location: 62598-64994
  
 NCBI BlastP on this gene

AO090206000025

not annotated
  
Accession: BAE65399
  
Location: 58506-60107
  
 NCBI BlastP on this gene

AO090206000024

160. :  CP002686 Arabidopsis thaliana chromosome 3     Total score: 2.0     Cumulative Blast bit score: 1076

cation/H(+) antiporter 20
  
Accession: AEE79132
  
Location: 19905826-19910027
  
 NCBI BlastP on this gene

CHX20

putative ADP-ribosylation factor GTPase-activating protein AGD6
  
Accession: AEE79131
  
Location: 19903730-19905419
  
 NCBI BlastP on this gene

AGD6

putative ADP-ribosylation factor GTPase-activating protein AGD6
  
Accession: AEE79130
  
Location: 19903730-19905419
  
 NCBI BlastP on this gene

AGD6

pentatricopeptide repeat-containing protein
  
Accession: AEE79129
  
Location: 19900303-19902567
  
 NCBI BlastP on this gene

MEE40

RING/U-box superfamily protein
  
Accession: AEE79128
  
Location: 19898997-19900044
  
 NCBI BlastP on this gene

AT3G53690

Acyl-CoA N-acyltransferase with
  
Accession: AEE79127
  
Location: 19892863-19897412
  
 NCBI BlastP on this gene

AT3G53680

uncharacterized protein
  
Accession: AEE79125
  
Location: 19891104-19892214
  
 NCBI BlastP on this gene

AT3G53670

uncharacterized protein
  
Accession: AEE79124
  
Location: 19891104-19892214
  
 NCBI BlastP on this gene

AT3G53670

conserved peptide upstream open reading frame 51
  
Accession: AEE79126
  
Location: 19890708-19890833
  
 NCBI BlastP on this gene

CPuORF51

histone H2B
  
Accession: AEE79123
  
Location: 19889358-19889774
  
 NCBI BlastP on this gene

AT3G53650

protein kinase family protein
  
Accession: AEE79122
  
Location: 19887007-19888935
  
  
**BlastP hit with Mycgr3G103260\_Mycgr3**
  
Percentage identity: 41 %
  
BlastP bit score: 203
  
Sequence coverage: 96 %
  
E-value: 3e-57
  
  
 NCBI BlastP on this gene

AT3G53640

uncharacterized protein
  
Accession: AEE79121
  
Location: 19883986-19885568
  
 NCBI BlastP on this gene

AT3G53630

pyrophosphorylase 4
  
Accession: AEE79120
  
Location: 19881491-19883308
  
 NCBI BlastP on this gene

PPa4

uncharacterized protein
  
Accession: AEE79119
  
Location: 19878542-19878694
  
 NCBI BlastP on this gene

AT3G53611

RAB GTPase-8
  
Accession: AEE79118
  
Location: 19876531-19878264
  
  
**BlastP hit with Mycgr3G99145\_Mycgr3T**
  
Percentage identity: 70 %
  
BlastP bit score: 291
  
Sequence coverage: 97 %
  
E-value: 1e-96
  
  
 NCBI BlastP on this gene

RAB8

RAB GTPase-8
  
Accession: AEE79117
  
Location: 19876531-19878264
  
  
**BlastP hit with Mycgr3G99145\_Mycgr3T**
  
Percentage identity: 70 %
  
BlastP bit score: 291
  
Sequence coverage: 97 %
  
E-value: 1e-96
  
  
 NCBI BlastP on this gene

RAB8

RAB GTPase-8
  
Accession: AEE79116
  
Location: 19876531-19878264
  
  
**BlastP hit with Mycgr3G99145\_Mycgr3T**
  
Percentage identity: 70 %
  
BlastP bit score: 291
  
Sequence coverage: 97 %
  
E-value: 1e-96
  
  
 NCBI BlastP on this gene

RAB8

C2H2-type zinc finger protein
  
Accession: AEE79115
  
Location: 19875532-19876059
  
 NCBI BlastP on this gene

AT3G53600

putative leucine-rich repeat receptor-like
  
Accession: AEE79114
  
Location: 19867379-19871651
  
 NCBI BlastP on this gene

AT3G53590

diaminopimelate epimerase
  
Accession: AEE79113
  
Location: 19864784-19866907
  
 NCBI BlastP on this gene

AT3G53580

serine/threonine-protein kinase AFC1
  
Accession: AEE79112
  
Location: 19861449-19864125
  
 NCBI BlastP on this gene

FC1

serine/threonine-protein kinase AFC1
  
Accession: AEE79111
  
Location: 19861449-19864125
  
 NCBI BlastP on this gene

FC1

serine/threonine-protein kinase AFC1
  
Accession: AEE79110
  
Location: 19861449-19864125
  
 NCBI BlastP on this gene

FC1

serine/threonine-protein kinase AFC1
  
Accession: AEE79109
  
Location: 19861449-19864083
  
 NCBI BlastP on this gene

FC1

tetratricopeptide repeat domain-containing protein
  
Accession: AEE79108
  
Location: 19859954-19860976
  
 NCBI BlastP on this gene

AT3G53560

161. :  KE145352 Glarea lozoyensis ATCC 20868 chromosome Unknown GLAREA1     Total score: 2.0     Cumulative Blast bit score: 1073

FAD/NAD(P)-binding protein
  
Accession: EPE37030
  
Location: 1648002-1649477
  
 NCBI BlastP on this gene

EPE37030

S-adenosyl-L-methionine-dependent methyltransferase
  
Accession: EPE37031
  
Location: 1650883-1652341
  
 NCBI BlastP on this gene

EPE37031

hypothetical protein
  
Accession: EPE37032
  
Location: 1652598-1654256
  
 NCBI BlastP on this gene

EPE37032

hypothetical protein
  
Accession: EPE37033
  
Location: 1656460-1657691
  
 NCBI BlastP on this gene

EPE37033

Prefoldin
  
Accession: EPE37034
  
Location: 1658120-1661586
  
  
**BlastP hit with Mycgr3G108094\_Mycgr3**
  
Percentage identity: 36 %
  
BlastP bit score: 295
  
Sequence coverage: 44 %
  
E-value: 3e-79
  
  
 NCBI BlastP on this gene

EPE37034

Protein kinase-like (PK-like)
  
Accession: EPE37035
  
Location: 1663145-1665302
  
  
**BlastP hit with Mycgr3G84644\_Mycgr3T**
  
Percentage identity: 70 %
  
BlastP bit score: 778
  
Sequence coverage: 101 %
  
E-value: 0.0
  
  
 NCBI BlastP on this gene

EPE37035

hypothetical protein
  
Accession: EPE37036
  
Location: 1666341-1667707
  
 NCBI BlastP on this gene

EPE37036

hypothetical protein
  
Accession: EPE37037
  
Location: 1668170-1669599
  
 NCBI BlastP on this gene

EPE37037

HLH, helix-loop-helix DNA-binding protein
  
Accession: EPE37038
  
Location: 1670550-1672004
  
 NCBI BlastP on this gene

EPE37038

Zn2/Cys6 DNA-binding protein
  
Accession: EPE37039
  
Location: 1674031-1676578
  
 NCBI BlastP on this gene

EPE37039

Cupredoxin
  
Accession: EPE37040
  
Location: 1677904-1679991
  
 NCBI BlastP on this gene

EPE37040

non-hemolytic phospholipase C precursor, putative
  
Accession: EPE37041
  
Location: 1680129-1683300
  
 NCBI BlastP on this gene

EPE37041

hypothetical protein
  
Accession: EPE37042
  
Location: 1684175-1685135
  
 NCBI BlastP on this gene

EPE37042

162. :  DS995737 Trichophyton equinum CBS 127.97 supercont1.20 genomic scaffold     Total score: 2.0     Cumulative Blast bit score: 1073

hypothetical protein
  
Accession: EGE05061
  
Location: 96646-97962
  
 NCBI BlastP on this gene

EGE05061

ras-like GTP-binding protein
  
Accession: EGE05062
  
Location: 99169-99751
  
 NCBI BlastP on this gene

EGE05062

cytochrome b2
  
Accession: EGE05063
  
Location: 100538-102177
  
 NCBI BlastP on this gene

EGE05063

phosphoric ester hydrolase
  
Accession: EGE05064
  
Location: 102342-103395
  
 NCBI BlastP on this gene

EGE05064

CMGC/SRPK protein kinase
  
Accession: EGE05065
  
Location: 104246-106272
  
  
**BlastP hit with Mycgr3G84644\_Mycgr3T**
  
Percentage identity: 68 %
  
BlastP bit score: 735
  
Sequence coverage: 98 %
  
E-value: 0.0
  
  
 NCBI BlastP on this gene

EGE05065

pre-mRNA-splicing factor cwc-26
  
Accession: EGE05066
  
Location: 107189-108167
  
 NCBI BlastP on this gene

EGE05066

hypothetical protein
  
Accession: EGE05067
  
Location: 108966-109898
  
 NCBI BlastP on this gene

EGE05067

MGMT family protein
  
Accession: EGE05068
  
Location: 110501-111095
  
 NCBI BlastP on this gene

EGE05068

hypothetical protein
  
Accession: EGE05069
  
Location: 111740-112313
  
 NCBI BlastP on this gene

EGE05069

hypothetical protein
  
Accession: EGE05070
  
Location: 113280-113895
  
 NCBI BlastP on this gene

EGE05070

hypothetical protein
  
Accession: EGE05071
  
Location: 115621-117775
  
 NCBI BlastP on this gene

EGE05071

nuclear envelope protein Brr6
  
Accession: EGE05072
  
Location: 118700-120188
  
 NCBI BlastP on this gene

EGE05072

amino acid permease
  
Accession: EGE05073
  
Location: 120561-122623
  
 NCBI BlastP on this gene

EGE05073

GABA-specific permease
  
Accession: EGE05074
  
Location: 123441-125538
  
 NCBI BlastP on this gene

EGE05074

integral membrane protein
  
Accession: EGE05075
  
Location: 127169-128504
  
 NCBI BlastP on this gene

EGE05075

hypothetical protein
  
Accession: EGE05076
  
Location: 130085-130679
  
 NCBI BlastP on this gene

EGE05076

hypothetical protein
  
Accession: EGE05077
  
Location: 131142-134753
  
  
**BlastP hit with Mycgr3G108094\_Mycgr3**
  
Percentage identity: 40 %
  
BlastP bit score: 338
  
Sequence coverage: 38 %
  
E-value: 2e-93
  
  
 NCBI BlastP on this gene

EGE05077

hypothetical protein
  
Accession: EGE05078
  
Location: 135277-136658
  
 NCBI BlastP on this gene

EGE05078

patatin-like phospholipase
  
Accession: EGE05079
  
Location: 137029-141580
  
 NCBI BlastP on this gene

EGE05079

sarcosine oxidase
  
Accession: EGE05080
  
Location: 143045-144500
  
 NCBI BlastP on this gene

EGE05080

163. :  GG698482 Trichophyton tonsurans CBS 112818 genomic scaffold supercont1.6     Total score: 2.0     Cumulative Blast bit score: 1069

CMGC/CLK protein kinase
  
Accession: EGD94221
  
Location: 377119-378425
  
 NCBI BlastP on this gene

EGD94221

MFS transporter
  
Accession: EGD94222
  
Location: 379113-380684
  
 NCBI BlastP on this gene

EGD94222

GTP-binding protein yptV5
  
Accession: EGD94223
  
Location: 381047-382118
  
 NCBI BlastP on this gene

EGD94223

mitochondrial cytochrome b2
  
Accession: EGD94224
  
Location: 382880-384519
  
 NCBI BlastP on this gene

EGD94224

hypothetical protein
  
Accession: EGD94225
  
Location: 384684-385736
  
 NCBI BlastP on this gene

EGD94225

CMGC/SRPK protein kinase
  
Accession: EGD94226
  
Location: 386544-388570
  
  
**BlastP hit with Mycgr3G84644\_Mycgr3T**
  
Percentage identity: 68 %
  
BlastP bit score: 732
  
Sequence coverage: 98 %
  
E-value: 0.0
  
  
 NCBI BlastP on this gene

EGD94226

hypothetical protein
  
Accession: EGD94227
  
Location: 389406-390482
  
 NCBI BlastP on this gene

EGD94227

hypothetical protein
  
Accession: EGD94228
  
Location: 391281-392213
  
 NCBI BlastP on this gene

EGD94228

MGMT family protein
  
Accession: EGD94229
  
Location: 392799-393393
  
 NCBI BlastP on this gene

EGD94229

hypothetical protein
  
Accession: EGD94230
  
Location: 394036-394446
  
 NCBI BlastP on this gene

EGD94230

hypothetical protein
  
Accession: EGD94231
  
Location: 395576-396191
  
 NCBI BlastP on this gene

EGD94231

hypothetical protein
  
Accession: EGD94232
  
Location: 397491-397929
  
 NCBI BlastP on this gene

EGD94232

hypothetical protein
  
Accession: EGD94233
  
Location: 397867-400021
  
 NCBI BlastP on this gene

EGD94233

hypothetical protein
  
Accession: EGD94234
  
Location: 400950-402438
  
 NCBI BlastP on this gene

EGD94234

amino acid permease
  
Accession: EGD94235
  
Location: 402840-404877
  
 NCBI BlastP on this gene

EGD94235

GABA permease
  
Accession: EGD94236
  
Location: 405697-407794
  
 NCBI BlastP on this gene

EGD94236

hypothetical protein
  
Accession: EGD94237
  
Location: 409450-410785
  
 NCBI BlastP on this gene

EGD94237

acetyltransferase
  
Accession: EGD94238
  
Location: 412383-412977
  
 NCBI BlastP on this gene

EGD94238

viral A-type inclusion protein repeat protein
  
Accession: EGD94239
  
Location: 413440-417051
  
  
**BlastP hit with Mycgr3G108094\_Mycgr3**
  
Percentage identity: 41 %
  
BlastP bit score: 337
  
Sequence coverage: 38 %
  
E-value: 4e-93
  
  
 NCBI BlastP on this gene

EGD94239

hypothetical protein
  
Accession: EGD94240
  
Location: 417575-418956
  
 NCBI BlastP on this gene

EGD94240

hypothetical protein
  
Accession: EGD94241
  
Location: 419325-423873
  
 NCBI BlastP on this gene

EGD94241

hypothetical protein
  
Accession: EGD94242
  
Location: 425337-426792
  
 NCBI BlastP on this gene

EGD94242

164. :  JH921446 Marssonina brunnea f. sp. 'multigermtubi' MB\_m1 unplaced genomic scaffold M6\_S00019     Total score: 2.0     Cumulative Blast bit score: 1047

hypothetical protein
  
Accession: EKD14353
  
Location: 821932-822588
  
 NCBI BlastP on this gene

EKD14353

hypothetical protein
  
Accession: EKD14352
  
Location: 819204-820400
  
 NCBI BlastP on this gene

EKD14352

hypothetical protein
  
Accession: EKD14351
  
Location: 814859-816402
  
 NCBI BlastP on this gene

EKD14351

putative Protein lunapark-B
  
Accession: EKD14350
  
Location: 813552-814826
  
 NCBI BlastP on this gene

EKD14350

viral A-type inclusion protein repeat protein
  
Accession: EKD14349
  
Location: 809723-813150
  
  
**BlastP hit with Mycgr3G108094\_Mycgr3**
  
Percentage identity: 34 %
  
BlastP bit score: 281
  
Sequence coverage: 44 %
  
E-value: 8e-75
  
  
 NCBI BlastP on this gene

EKD14349

isoprenylcysteine carboxyl methyltransferase
  
Accession: EKD14348
  
Location: 804611-805300
  
 NCBI BlastP on this gene

EKD14348

DSHCT domain-containing protein
  
Accession: EKD14347
  
Location: 800432-804373
  
 NCBI BlastP on this gene

EKD14347

replication fork protection component Swi3
  
Accession: EKD14346
  
Location: 798929-800124
  
 NCBI BlastP on this gene

EKD14346

putative NADH-ubiquinone oxidoreductase 21 kDa subunit
  
Accession: EKD14345
  
Location: 797923-798651
  
 NCBI BlastP on this gene

EKD14345

tRNA-specific adenosine deaminase subunit TAD2
  
Accession: EKD14344
  
Location: 796762-797685
  
 NCBI BlastP on this gene

EKD14344

hypothetical protein
  
Accession: EKD14343
  
Location: 793402-795946
  
 NCBI BlastP on this gene

EKD14343

hypothetical protein
  
Accession: EKD14342
  
Location: 791469-792236
  
 NCBI BlastP on this gene

EKD14342

serine/threonine-protein kinase SRPK2
  
Accession: EKD14341
  
Location: 786637-788953
  
  
**BlastP hit with Mycgr3G84644\_Mycgr3T**
  
Percentage identity: 67 %
  
BlastP bit score: 766
  
Sequence coverage: 101 %
  
E-value: 0.0
  
  
 NCBI BlastP on this gene

EKD14341

nuclear membrane protein
  
Accession: EKD14340
  
Location: 783687-785155
  
 NCBI BlastP on this gene

EKD14340

transcription factor IWS1
  
Accession: EKD14339
  
Location: 781974-783376
  
 NCBI BlastP on this gene

EKD14339

hypothetical protein
  
Accession: EKD14338
  
Location: 779884-780875
  
 NCBI BlastP on this gene

EKD14338

homoserine O-acetyltransferase
  
Accession: EKD14337
  
Location: 777091-778659
  
 NCBI BlastP on this gene

EKD14337

hypothetical protein
  
Accession: EKD14336
  
Location: 775536-776717
  
 NCBI BlastP on this gene

EKD14336

165. :  AM920428 Penicillium chrysogenum Wisconsin 54-1255 complete genome, contig Pc00c13.     Total score: 2.0     Cumulative Blast bit score: 1047

hypothetical protein
  
Accession: CAP91665
  
Location: 1469663-1473034
  
 NCBI BlastP on this gene

Pc13g05960

not annotated
  
Accession: CAP91664
  
Location: 1466895-1469234
  
 NCBI BlastP on this gene

Pc13g05950

not annotated
  
Accession: CAP91663
  
Location: 1463159-1466033
  
 NCBI BlastP on this gene

Pc13g05940

not annotated
  
Accession: CAP91662
  
Location: 1461276-1462506
  
 NCBI BlastP on this gene

Pc13g05930

not annotated
  
Accession: CAP91661
  
Location: 1459016-1460466
  
 NCBI BlastP on this gene

Pc13g05920

unnamed
  
Accession: CAP91660
  
Location: 1457734-1458588
  
 NCBI BlastP on this gene

Pc13g05910

not annotated
  
Accession: CAP91659
  
Location: 1455274-1456697
  
 NCBI BlastP on this gene

Pc13g05900

not annotated
  
Accession: CAP91658
  
Location: 1453816-1454914
  
  
**BlastP hit with Mycgr3G55345\_Mycgr3T**
  
Percentage identity: 64 %
  
BlastP bit score: 221
  
Sequence coverage: 98 %
  
E-value: 8e-69
  
  
 NCBI BlastP on this gene

Pc13g05890

unnamed
  
Accession: CAP91657
  
Location: 1452801-1453235
  
 NCBI BlastP on this gene

Pc13g05880

not annotated
  
Accession: CAP91656
  
Location: 1451285-1452324
  
 NCBI BlastP on this gene

Pc13g05870

not annotated
  
Accession: CAP91655
  
Location: 1447167-1449507
  
 NCBI BlastP on this gene

Pc13g05860

not annotated
  
Accession: CAP91654
  
Location: 1445549-1446835
  
 NCBI BlastP on this gene

Pc13g05850

not annotated
  
Accession: CAP91653
  
Location: 1442694-1444710
  
  
**BlastP hit with Mycgr3G68458\_Mycgr3T**
  
Percentage identity: 63 %
  
BlastP bit score: 826
  
Sequence coverage: 91 %
  
E-value: 0.0
  
  
 NCBI BlastP on this gene

Pc13g05840

not annotated
  
Accession: CAP91652
  
Location: 1441195-1442297
  
 NCBI BlastP on this gene

Pc13g05830

unnamed
  
Accession: CAP91651
  
Location: 1439071-1440895
  
 NCBI BlastP on this gene

Pc13g05820

not annotated
  
Accession: CAP91650
  
Location: 1437289-1438253
  
 NCBI BlastP on this gene

Pc13g05810

not annotated
  
Accession: CAP91649
  
Location: 1435276-1437034
  
 NCBI BlastP on this gene

Pc13g05800

not annotated
  
Accession: CAP91648
  
Location: 1433843-1434942
  
 NCBI BlastP on this gene

Pc13g05790

not annotated
  
Accession: CAP91647
  
Location: 1431274-1433571
  
 NCBI BlastP on this gene

Pc13g05780

not annotated
  
Accession: CAP91646
  
Location: 1428901-1430744
  
 NCBI BlastP on this gene

Pc13g05770

not annotated
  
Accession: CAP91645
  
Location: 1425937-1427288
  
 NCBI BlastP on this gene

Pc13g05760

166. :  KB726203 Fusarium oxysporum f. sp. cubense race 4 unplaced genomic scaffold scaffold2     Total score: 2.0     Cumulative Blast bit score: 1035

Nitrogen assimilation transcription factor nit-4
  
Accession: EMT74522
  
Location: 87210-89985
  
 NCBI BlastP on this gene

EMT74522

Nucleus export protein BRL1
  
Accession: EMT74523
  
Location: 92765-94323
  
 NCBI BlastP on this gene

EMT74523

hypothetical protein
  
Accession: EMT74524
  
Location: 100531-100958
  
 NCBI BlastP on this gene

EMT74524

Protein kinase dsk1
  
Accession: EMT74525
  
Location: 102582-104300
  
  
**BlastP hit with Mycgr3G84644\_Mycgr3T**
  
Percentage identity: 75 %
  
BlastP bit score: 759
  
Sequence coverage: 89 %
  
E-value: 0.0
  
  
 NCBI BlastP on this gene

EMT74525

Protein lunapark
  
Accession: EMT74526
  
Location: 105391-106620
  
 NCBI BlastP on this gene

EMT74526

Golgin IMH1
  
Accession: EMT74527
  
Location: 107677-111234
  
  
**BlastP hit with Mycgr3G108094\_Mycgr3**
  
Percentage identity: 35 %
  
BlastP bit score: 276
  
Sequence coverage: 44 %
  
E-value: 3e-73
  
  
 NCBI BlastP on this gene

EMT74527

Rhamnolipids biosynthesis 3-oxoacyl-[acyl-carrier-protein] reductase
  
Accession: EMT74528
  
Location: 112080-113031
  
 NCBI BlastP on this gene

EMT74528

Rhamnolipids biosynthesis 3-oxoacyl-[acyl-carrier-protein] reductase
  
Accession: EMT74529
  
Location: 114212-115163
  
 NCBI BlastP on this gene

EMT74529

hypothetical protein
  
Accession: EMT74530
  
Location: 117752-118861
  
 NCBI BlastP on this gene

EMT74530

hypothetical protein
  
Accession: EMT74531
  
Location: 126546-128075
  
 NCBI BlastP on this gene

EMT74531

hypothetical protein
  
Accession: EMT74532
  
Location: 130736-132424
  
 NCBI BlastP on this gene

EMT74532

167. :  KB730140 Fusarium oxysporum f. sp. cubense race 1 unplaced genomic scaffold scaffold224     Total score: 2.0     Cumulative Blast bit score: 1034

hypothetical protein
  
Accession: ENH71669
  
Location: 92032-92448
  
 NCBI BlastP on this gene

ENH71669

Helicase required for RNAi-mediated heterochromatin assembly 1
  
Accession: ENH71668
  
Location: 88599-91477
  
 NCBI BlastP on this gene

ENH71668

Nitrogen assimilation transcription factor nit-4
  
Accession: ENH71667
  
Location: 83594-86369
  
 NCBI BlastP on this gene

ENH71667

Nucleus export protein BRL1
  
Accession: ENH71666
  
Location: 79239-80797
  
 NCBI BlastP on this gene

ENH71666

Transcription factor IWS1
  
Accession: ENH71665
  
Location: 77429-78788
  
 NCBI BlastP on this gene

ENH71665

hypothetical protein
  
Accession: ENH71664
  
Location: 74734-75161
  
 NCBI BlastP on this gene

ENH71664

Protein kinase dsk1
  
Accession: ENH71663
  
Location: 71389-73107
  
  
**BlastP hit with Mycgr3G84644\_Mycgr3T**
  
Percentage identity: 75 %
  
BlastP bit score: 759
  
Sequence coverage: 89 %
  
E-value: 0.0
  
  
 NCBI BlastP on this gene

ENH71663

Protein lunapark
  
Accession: ENH71662
  
Location: 69068-70298
  
 NCBI BlastP on this gene

ENH71662

Golgin IMH1
  
Accession: ENH71661
  
Location: 64454-68011
  
  
**BlastP hit with Mycgr3G108094\_Mycgr3**
  
Percentage identity: 35 %
  
BlastP bit score: 275
  
Sequence coverage: 44 %
  
E-value: 1e-72
  
  
 NCBI BlastP on this gene

ENH71661

Rhamnolipids biosynthesis 3-oxoacyl-[acyl-carrier-protein] reductase
  
Accession: ENH71660
  
Location: 62656-63607
  
 NCBI BlastP on this gene

ENH71660

SWIRM domain-containing protein FUN19
  
Accession: ENH71659
  
Location: 58931-60040
  
 NCBI BlastP on this gene

ENH71659

hypothetical protein
  
Accession: ENH71658
  
Location: 51843-53373
  
 NCBI BlastP on this gene

ENH71658

hypothetical protein
  
Accession: ENH71657
  
Location: 47499-49187
  
 NCBI BlastP on this gene

ENH71657

hypothetical protein
  
Accession: ENH71656
  
Location: 45794-46921
  
 NCBI BlastP on this gene

ENH71656

G patch domain-containing protein 8
  
Accession: ENH71655
  
Location: 44767-45319
  
 NCBI BlastP on this gene

ENH71655

168. :  AFNW01000113 Fusarium pseudograminearum CS3096     Total score: 2.0     Cumulative Blast bit score: 1028

hypothetical protein
  
Accession: EKJ74295
  
Location: 255465-259430
  
 NCBI BlastP on this gene

EKJ74295

hypothetical protein
  
Accession: EKJ74296
  
Location: 261087-263844
  
 NCBI BlastP on this gene

EKJ74296

hypothetical protein
  
Accession: EKJ74297
  
Location: 266790-268338
  
 NCBI BlastP on this gene

EKJ74297

hypothetical protein
  
Accession: EKJ74298
  
Location: 268790-270180
  
 NCBI BlastP on this gene

EKJ74298

hypothetical protein
  
Accession: EKJ74299
  
Location: 272593-273460
  
 NCBI BlastP on this gene

EKJ74299

hypothetical protein
  
Accession: EKJ74300
  
Location: 274654-276378
  
  
**BlastP hit with Mycgr3G84644\_Mycgr3T**
  
Percentage identity: 74 %
  
BlastP bit score: 753
  
Sequence coverage: 89 %
  
E-value: 0.0
  
  
 NCBI BlastP on this gene

EKJ74300

hypothetical protein
  
Accession: EKJ74301
  
Location: 276983-278200
  
 NCBI BlastP on this gene

EKJ74301

hypothetical protein
  
Accession: EKJ74302
  
Location: 279541-283147
  
  
**BlastP hit with Mycgr3G108094\_Mycgr3**
  
Percentage identity: 33 %
  
BlastP bit score: 275
  
Sequence coverage: 45 %
  
E-value: 2e-72
  
  
 NCBI BlastP on this gene

EKJ74302

hypothetical protein
  
Accession: EKJ74303
  
Location: 283914-285040
  
 NCBI BlastP on this gene

EKJ74303

hypothetical protein
  
Accession: EKJ74304
  
Location: 287321-288463
  
 NCBI BlastP on this gene

EKJ74304

hypothetical protein
  
Accession: EKJ74305
  
Location: 297868-299586
  
 NCBI BlastP on this gene

EKJ74305

hypothetical protein
  
Accession: EKJ74306
  
Location: 300196-301331
  
 NCBI BlastP on this gene

EKJ74306

hypothetical protein
  
Accession: EKJ74307
  
Location: 301815-302390
  
 NCBI BlastP on this gene

EKJ74307

hypothetical protein
  
Accession: EKJ74308
  
Location: 302491-305148
  
 NCBI BlastP on this gene

EKJ74308

169. :  CAGA01000024 Claviceps purpurea 20.1     Total score: 2.0     Cumulative Blast bit score: 1024

related to NAM7-nonsense-mediated mRNA decay protein
  
Accession: CCE30747
  
Location: 347041-349386
  
 NCBI BlastP on this gene

CCE30747

uncharacterized protein
  
Accession: CCE30746
  
Location: 346421-346790
  
 NCBI BlastP on this gene

CCE30746

related to transcription factor SPT8
  
Accession: CCE30745
  
Location: 343827-346308
  
 NCBI BlastP on this gene

CCE30745

related to nitrate assimilation regulatory protein nirA
  
Accession: CCE30744
  
Location: 340111-342844
  
 NCBI BlastP on this gene

CCE30744

uncharacterized protein
  
Accession: CCE30743
  
Location: 336381-337929
  
 NCBI BlastP on this gene

CCE30743

uncharacterized protein
  
Accession: CCE30742
  
Location: 334319-335777
  
 NCBI BlastP on this gene

CCE30742

probable dis1-suppressing protein kinase dsk1
  
Accession: CCE30741
  
Location: 325326-329296
  
  
**BlastP hit with Mycgr3G84644\_Mycgr3T**
  
Percentage identity: 68 %
  
BlastP bit score: 771
  
Sequence coverage: 98 %
  
E-value: 0.0
  
  
 NCBI BlastP on this gene

CCE30741

uncharacterized protein
  
Accession: CCE30740
  
Location: 322603-324204
  
 NCBI BlastP on this gene

CCE30740

related to vesicular transport protein
  
Accession: CCE30739
  
Location: 318695-322478
  
  
**BlastP hit with Mycgr3G108094\_Mycgr3**
  
Percentage identity: 36 %
  
BlastP bit score: 253
  
Sequence coverage: 36 %
  
E-value: 1e-65
  
  
 NCBI BlastP on this gene

CCE30739

uncharacterized protein
  
Accession: CCE30738
  
Location: 315615-317003
  
 NCBI BlastP on this gene

CCE30738

uncharacterized protein
  
Accession: CCE30737
  
Location: 303929-305995
  
 NCBI BlastP on this gene

CCE30737

related to glycinamide ribonucleotide transformylase
  
Accession: CCE30736
  
Location: 302594-303431
  
 NCBI BlastP on this gene

CCE30736

uncharacterized protein
  
Accession: CCE30735
  
Location: 300985-302193
  
 NCBI BlastP on this gene

CCE30735

uncharacterized protein
  
Accession: CCE30734
  
Location: 299933-300501
  
 NCBI BlastP on this gene

CCE30734

170. :  AFQF01000897 Fusarium oxysporum Fo5176     Total score: 2.0     Cumulative Blast bit score: 1021

hypothetical protein
  
Accession: EGU86743
  
Location: 89232-93081
  
 NCBI BlastP on this gene

EGU86743

hypothetical protein
  
Accession: EGU86742
  
Location: 84193-86968
  
 NCBI BlastP on this gene

EGU86742

hypothetical protein
  
Accession: EGU86741
  
Location: 79838-81396
  
 NCBI BlastP on this gene

EGU86741

hypothetical protein
  
Accession: EGU86740
  
Location: 77996-79387
  
 NCBI BlastP on this gene

EGU86740

hypothetical protein
  
Accession: EGU86739
  
Location: 71956-73674
  
  
**BlastP hit with Mycgr3G84644\_Mycgr3T**
  
Percentage identity: 75 %
  
BlastP bit score: 759
  
Sequence coverage: 89 %
  
E-value: 0.0
  
  
 NCBI BlastP on this gene

EGU86739

hypothetical protein
  
Accession: EGU86738
  
Location: 69635-70865
  
 NCBI BlastP on this gene

EGU86738

hypothetical protein
  
Accession: EGU86737
  
Location: 63483-68837
  
  
**BlastP hit with Mycgr3G108094\_Mycgr3**
  
Percentage identity: 36 %
  
BlastP bit score: 262
  
Sequence coverage: 44 %
  
E-value: 5e-68
  
  
 NCBI BlastP on this gene

EGU86737

hypothetical protein
  
Accession: EGU86736
  
Location: 59661-60917
  
 NCBI BlastP on this gene

EGU86736

hypothetical protein
  
Accession: EGU86735
  
Location: 48374-50062
  
 NCBI BlastP on this gene

EGU86735

hypothetical protein
  
Accession: EGU86734
  
Location: 46669-47796
  
 NCBI BlastP on this gene

EGU86734

hypothetical protein
  
Accession: EGU86733
  
Location: 45642-46194
  
 NCBI BlastP on this gene

EGU86733

171. :  CAGA01000038 Claviceps purpurea 20.1     Total score: 2.0     Cumulative Blast bit score: 1018

uncharacterized protein
  
Accession: CCE32191
  
Location: 262552-263466
  
 NCBI BlastP on this gene

CCE32191

uncharacterized protein
  
Accession: CCE32192
  
Location: 264141-265455
  
 NCBI BlastP on this gene

CCE32192

uncharacterized protein
  
Accession: CCE32193
  
Location: 267452-268595
  
 NCBI BlastP on this gene

CCE32193

uncharacterized protein
  
Accession: CCE32194
  
Location: 268688-270280
  
 NCBI BlastP on this gene

CCE32194

uncharacterized protein
  
Accession: CCE32195
  
Location: 271412-272190
  
 NCBI BlastP on this gene

CCE32195

probable ARP5-Actin-related protein
  
Accession: CCE32196
  
Location: 273521-276156
  
 NCBI BlastP on this gene

CCE32196

uncharacterized protein
  
Accession: CCE32197
  
Location: 277265-278533
  
 NCBI BlastP on this gene

CCE32197

related to linoleate diol synthase
  
Accession: CCE32198
  
Location: 279403-280740
  
 NCBI BlastP on this gene

CCE32198

related to pterin-4-alpha-carbinolamine dehydratase
  
Accession: CCE32199
  
Location: 281194-281601
  
 NCBI BlastP on this gene

CCE32199

probable C-4 methyl sterol oxidase
  
Accession: CCE32200
  
Location: 282534-283615
  
  
**BlastP hit with Mycgr3G36271\_Mycgr3T**
  
Percentage identity: 70 %
  
BlastP bit score: 453
  
Sequence coverage: 98 %
  
E-value: 2e-157
  
  
 NCBI BlastP on this gene

CCE32200

related to NCA2 protein
  
Accession: CCE32201
  
Location: 284878-286954
  
  
**BlastP hit with Mycgr3G84646\_Mycgr3T**
  
Percentage identity: 45 %
  
BlastP bit score: 566
  
Sequence coverage: 101 %
  
E-value: 0.0
  
  
 NCBI BlastP on this gene

CCE32201

related to malic acid transport protein
  
Accession: CCE32202
  
Location: 287395-289172
  
 NCBI BlastP on this gene

CCE32202

related to cysteine dioxygenase type I
  
Accession: CCE32203
  
Location: 291463-292161
  
 NCBI BlastP on this gene

CCE32203

related to helicase-like transcription factor
  
Accession: CCE32204
  
Location: 293035-296199
  
 NCBI BlastP on this gene

CCE32204

uncharacterized protein
  
Accession: CCE32205
  
Location: 298454-298834
  
 NCBI BlastP on this gene

CCE32205

uncharacterized protein
  
Accession: CCE32206
  
Location: 300539-306384
  
 NCBI BlastP on this gene

CCE32206

172. :  AMYD01003902 Colletotrichum gloeosporioides Cg-14     Total score: 2.0     Cumulative Blast bit score: 1018

ATP synthase regulation protein NCA2
  
Accession: EQB44753
  
Location: 1083-3199
  
  
**BlastP hit with Mycgr3G84646\_Mycgr3T**
  
Percentage identity: 44 %
  
BlastP bit score: 560
  
Sequence coverage: 100 %
  
E-value: 0.0
  
  
 NCBI BlastP on this gene

EQB44753

hypothetical protein
  
Accession: EQB44754
  
Location: 5296-6405
  
  
**BlastP hit with Mycgr3G36271\_Mycgr3T**
  
Percentage identity: 73 %
  
BlastP bit score: 459
  
Sequence coverage: 96 %
  
E-value: 2e-159
  
  
 NCBI BlastP on this gene

EQB44754

173. :  GG749476 Ajellomyces dermatitidis ATCC 18188 genomic scaffold supercont1.70     Total score: 2.0     Cumulative Blast bit score: 1018

viral A-type inclusion protein repeat protein
  
Accession: EGE84735
  
Location: 59839-63661
  
  
**BlastP hit with Mycgr3G108094\_Mycgr3**
  
Percentage identity: 37 %
  
BlastP bit score: 384
  
Sequence coverage: 45 %
  
E-value: 2e-108
  
  
 NCBI BlastP on this gene

EGE84735

hypothetical protein
  
Accession: EGE84736
  
Location: 64283-66262
  
 NCBI BlastP on this gene

EGE84736

Ras family protein
  
Accession: EGE84737
  
Location: 69261-70243
  
 NCBI BlastP on this gene

EGE84737

cytochrome b2
  
Accession: EGE84738
  
Location: 71917-73612
  
 NCBI BlastP on this gene

EGE84738

phosphoric ester hydrolase
  
Accession: EGE84739
  
Location: 73946-75079
  
 NCBI BlastP on this gene

EGE84739

hypothetical protein
  
Accession: EGE84740
  
Location: 75742-76350
  
 NCBI BlastP on this gene

EGE84740

hypothetical protein
  
Accession: EGE84741
  
Location: 78032-79028
  
 NCBI BlastP on this gene

EGE84741

protein kinase dsk1
  
Accession: EGE84742
  
Location: 79329-81448
  
  
**BlastP hit with Mycgr3G84644\_Mycgr3T**
  
Percentage identity: 66 %
  
BlastP bit score: 634
  
Sequence coverage: 88 %
  
E-value: 0.0
  
  
 NCBI BlastP on this gene

EGE84742

174. :  GG657474 Ajellomyces dermatitidis SLH14081 genomic scaffold supercont1.27     Total score: 2.0     Cumulative Blast bit score: 1018

C6 finger domain-containing protein
  
Accession: EEQ74517
  
Location: 389150-391608
  
 NCBI BlastP on this gene

EEQ74517

methionine permease
  
Accession: EEQ74518
  
Location: 392166-393645
  
 NCBI BlastP on this gene

EEQ74518

C6 finger domain-containing protein
  
Accession: EEQ74519
  
Location: 394452-396318
  
 NCBI BlastP on this gene

EEQ74519

conserved hypothetical protein
  
Accession: EEQ74520
  
Location: 397919-398569
  
 NCBI BlastP on this gene

EEQ74520

MGMT family protein
  
Accession: EEQ74521
  
Location: 400376-401093
  
 NCBI BlastP on this gene

EEQ74521

pre-mRNA-splicing factor cwc26
  
Accession: EEQ74522
  
Location: 401318-402415
  
 NCBI BlastP on this gene

EEQ74522

protein kinase dsk1
  
Accession: EEQ74523
  
Location: 404206-406325
  
  
**BlastP hit with Mycgr3G84644\_Mycgr3T**
  
Percentage identity: 66 %
  
BlastP bit score: 634
  
Sequence coverage: 88 %
  
E-value: 0.0
  
  
 NCBI BlastP on this gene

EEQ74523

predicted protein
  
Accession: EEQ74524
  
Location: 406647-407646
  
 NCBI BlastP on this gene

EEQ74524

predicted protein
  
Accession: EEQ74525
  
Location: 409199-410043
  
 NCBI BlastP on this gene

EEQ74525

phosphoric ester hydrolase
  
Accession: EEQ74526
  
Location: 410471-411601
  
 NCBI BlastP on this gene

EEQ74526

cytochrome b2
  
Accession: EEQ74527
  
Location: 411946-413641
  
 NCBI BlastP on this gene

EEQ74527

ras-like GTP-binding protein RYL2
  
Accession: EEQ74528
  
Location: 415356-416338
  
 NCBI BlastP on this gene

EEQ74528

conserved hypothetical protein
  
Accession: EEQ74529
  
Location: 419346-421325
  
 NCBI BlastP on this gene

EEQ74529

viral A-type inclusion protein repeat protein
  
Accession: EEQ74530
  
Location: 421991-425813
  
  
**BlastP hit with Mycgr3G108094\_Mycgr3**
  
Percentage identity: 37 %
  
BlastP bit score: 384
  
Sequence coverage: 45 %
  
E-value: 9e-109
  
  
 NCBI BlastP on this gene

EEQ74530

175. :  EQ999987 Ajellomyces dermatitidis ER-3 genomic scaffold supercont1.15     Total score: 2.0     Cumulative Blast bit score: 1018

C6 finger domain-containing protein
  
Accession: EEQ86197
  
Location: 974815-977273
  
 NCBI BlastP on this gene

EEQ86197

methionine permease
  
Accession: EEQ86198
  
Location: 977832-979311
  
 NCBI BlastP on this gene

EEQ86198

C6 finger domain-containing protein
  
Accession: EEQ86199
  
Location: 980261-982681
  
 NCBI BlastP on this gene

EEQ86199

conserved hypothetical protein
  
Accession: EEQ86200
  
Location: 983590-984240
  
 NCBI BlastP on this gene

EEQ86200

MGMT family protein
  
Accession: EEQ86201
  
Location: 986028-986745
  
 NCBI BlastP on this gene

EEQ86201

pre-mRNA-splicing factor cwc26
  
Accession: EEQ86202
  
Location: 986971-988068
  
 NCBI BlastP on this gene

EEQ86202

protein kinase dsk1
  
Accession: EEQ86203
  
Location: 989869-991988
  
  
**BlastP hit with Mycgr3G84644\_Mycgr3T**
  
Percentage identity: 66 %
  
BlastP bit score: 634
  
Sequence coverage: 88 %
  
E-value: 0.0
  
  
 NCBI BlastP on this gene

EEQ86203

predicted protein
  
Accession: EEQ86204
  
Location: 992288-993285
  
 NCBI BlastP on this gene

EEQ86204

predicted protein
  
Accession: EEQ86205
  
Location: 994968-995576
  
 NCBI BlastP on this gene

EEQ86205

phosphoric ester hydrolase
  
Accession: EEQ86206
  
Location: 996243-997376
  
 NCBI BlastP on this gene

EEQ86206

cytochrome b2
  
Accession: EEQ86207
  
Location: 997720-999417
  
 NCBI BlastP on this gene

EEQ86207

ras-like GTP-binding protein RYL2
  
Accession: EEQ86208
  
Location: 1001055-1002037
  
 NCBI BlastP on this gene

EEQ86208

conserved hypothetical protein
  
Accession: EEQ86209
  
Location: 1005043-1007022
  
 NCBI BlastP on this gene

EEQ86209

viral A-type inclusion protein repeat protein
  
Accession: EEQ86210
  
Location: 1007644-1011466
  
  
**BlastP hit with Mycgr3G108094\_Mycgr3**
  
Percentage identity: 37 %
  
BlastP bit score: 384
  
Sequence coverage: 45 %
  
E-value: 9e-109
  
  
 NCBI BlastP on this gene

EEQ86210

conserved hypothetical protein
  
Accession: EEQ86211
  
Location: 1013201-1013858
  
 NCBI BlastP on this gene

EEQ86211

176. :  GL385396 Gaeumannomyces graminis var. tritici R3-111a-1 unplaced genomic scaffold supercont2.2     Total score: 2.0     Cumulative Blast bit score: 1016

hypothetical protein
  
Accession: EJT79900
  
Location: 7299176-7300264
  
 NCBI BlastP on this gene

EJT79900

hypothetical protein
  
Accession: EJT79901
  
Location: 7300314-7300565
  
 NCBI BlastP on this gene

EJT79901

hypothetical protein
  
Accession: EJT79902
  
Location: 7301145-7301369
  
 NCBI BlastP on this gene

EJT79902

hypothetical protein
  
Accession: EJT79903
  
Location: 7302141-7303289
  
 NCBI BlastP on this gene

EJT79903

hypothetical protein
  
Accession: EJT79904
  
Location: 7303926-7304521
  
 NCBI BlastP on this gene

EJT79904

hypothetical protein
  
Accession: EJT79905
  
Location: 7304876-7306237
  
 NCBI BlastP on this gene

EJT79905

hypothetical protein
  
Accession: EJT79906
  
Location: 7307443-7311293
  
  
**BlastP hit with Mycgr3G108094\_Mycgr3**
  
Percentage identity: 33 %
  
BlastP bit score: 270
  
Sequence coverage: 44 %
  
E-value: 5e-71
  
  
 NCBI BlastP on this gene

EJT79906

hypothetical protein
  
Accession: EJT79907
  
Location: 7311820-7312748
  
 NCBI BlastP on this gene

EJT79907

hypothetical protein
  
Accession: EJT79908
  
Location: 7312973-7313548
  
 NCBI BlastP on this gene

EJT79908

hypothetical protein
  
Accession: EJT79909
  
Location: 7313908-7315017
  
 NCBI BlastP on this gene

EJT79909

hypothetical protein
  
Accession: EJT79910
  
Location: 7316476-7316906
  
 NCBI BlastP on this gene

EJT79910

alcohol dehydrogenase
  
Accession: EJT79911
  
Location: 7317299-7319179
  
 NCBI BlastP on this gene

EJT79911

Znf1
  
Accession: EJT79912
  
Location: 7319244-7320404
  
 NCBI BlastP on this gene

EJT79912

hypothetical protein
  
Accession: EJT79913
  
Location: 7322156-7324422
  
 NCBI BlastP on this gene

EJT79913

hypothetical protein
  
Accession: EJT79914
  
Location: 7324979-7328381
  
 NCBI BlastP on this gene

EJT79914

hypothetical protein
  
Accession: EJT79915
  
Location: 7329821-7331384
  
 NCBI BlastP on this gene

EJT79915

transcription factor IWS1
  
Accession: EJT79916
  
Location: 7332146-7333611
  
 NCBI BlastP on this gene

EJT79916

CMGC/SRPK protein kinase
  
Accession: EJT79917
  
Location: 7335008-7336923
  
  
**BlastP hit with Mycgr3G84644\_Mycgr3T**
  
Percentage identity: 73 %
  
BlastP bit score: 746
  
Sequence coverage: 88 %
  
E-value: 0.0
  
  
 NCBI BlastP on this gene

EJT79917

hypothetical protein
  
Accession: EJT79918
  
Location: 7338081-7339061
  
 NCBI BlastP on this gene

EJT79918

hypothetical protein
  
Accession: EJT79919
  
Location: 7339452-7340505
  
 NCBI BlastP on this gene

EJT79919

hypothetical protein
  
Accession: EJT79920
  
Location: 7342367-7344405
  
 NCBI BlastP on this gene

EJT79920

hypothetical protein
  
Accession: EJT79921
  
Location: 7345301-7345654
  
 NCBI BlastP on this gene

EJT79921

177. :  KB446555 Pseudocercospora fijiensis CIRAD86 unplaced genomic scaffold MYCFIscaffold\_1     Total score: 2.0     Cumulative Blast bit score: 1014

ABC transporter, ABC-F family, GCN-EF3 type
  
Accession: EME88709
  
Location: 6964072-6966303
  
 NCBI BlastP on this gene

EME88709

hypothetical protein
  
Accession: EME88710
  
Location: 6966556-6967286
  
 NCBI BlastP on this gene

EME88710

hypothetical protein
  
Accession: EME88711
  
Location: 6968642-6971565
  
 NCBI BlastP on this gene

EME88711

hypothetical protein
  
Accession: EME88712
  
Location: 6973492-6974058
  
 NCBI BlastP on this gene

EME88712

hypothetical protein
  
Accession: EME88713
  
Location: 6974362-6975093
  
 NCBI BlastP on this gene

EME88713

hypothetical protein
  
Accession: EME88714
  
Location: 6977058-6978629
  
 NCBI BlastP on this gene

EME88714

hypothetical protein
  
Accession: EME88715
  
Location: 6978891-6979213
  
 NCBI BlastP on this gene

EME88715

hypothetical protein
  
Accession: EME88716
  
Location: 6979301-6980263
  
 NCBI BlastP on this gene

EME88716

hypothetical protein
  
Accession: EME88717
  
Location: 6981449-6983995
  
  
**BlastP hit with Mycgr3G21922\_Mycgr3T**
  
Percentage identity: 67 %
  
BlastP bit score: 671
  
Sequence coverage: 101 %
  
E-value: 0.0
  
  
 NCBI BlastP on this gene

EME88717

hypothetical protein
  
Accession: EME88718
  
Location: 6984539-6986612
  
  
**BlastP hit with Mycgr3G103278\_Mycgr3**
  
Percentage identity: 62 %
  
BlastP bit score: 344
  
Sequence coverage: 95 %
  
E-value: 2e-109
  
  
 NCBI BlastP on this gene

EME88718

hypothetical protein
  
Accession: EME88719
  
Location: 6987251-6988958
  
 NCBI BlastP on this gene

EME88719

hypothetical protein
  
Accession: EME88720
  
Location: 6989778-6990779
  
 NCBI BlastP on this gene

EME88720

178. :  GL698568 Metarhizium acridum CQMa 102 unplaced genomic scaffold Scf\_099     Total score: 2.0     Cumulative Blast bit score: 1010

putative nitrate assimilation regulatory protein nirA
  
Accession: EFY85718
  
Location: 108464-113002
  
 NCBI BlastP on this gene

EFY85718

hypothetical protein
  
Accession: EFY85717
  
Location: 105033-106561
  
 NCBI BlastP on this gene

EFY85717

transcription factor IWS1
  
Accession: EFY85716
  
Location: 103131-104548
  
 NCBI BlastP on this gene

EFY85716

serine/threonine-protein kinase SRPK2
  
Accession: EFY85715
  
Location: 98215-101450
  
  
**BlastP hit with Mycgr3G84644\_Mycgr3T**
  
Percentage identity: 70 %
  
BlastP bit score: 770
  
Sequence coverage: 98 %
  
E-value: 0.0
  
  
 NCBI BlastP on this gene

EFY85715

hypothetical protein
  
Accession: EFY85714
  
Location: 96263-97413
  
 NCBI BlastP on this gene

EFY85714

putative vesicular transport protein
  
Accession: EFY85713
  
Location: 91801-95386
  
  
**BlastP hit with Mycgr3G108094\_Mycgr3**
  
Percentage identity: 33 %
  
BlastP bit score: 241
  
Sequence coverage: 44 %
  
E-value: 9e-62
  
  
 NCBI BlastP on this gene

EFY85713

SWIRM domain-containing protein
  
Accession: EFY85712
  
Location: 86783-88279
  
 NCBI BlastP on this gene

EFY85712

F-box domain containing protein
  
Accession: EFY85711
  
Location: 77475-79295
  
 NCBI BlastP on this gene

EFY85711

N2,N2-dimethylguanosine tRNA methyltransferase
  
Accession: EFY85710
  
Location: 74494-75600
  
 NCBI BlastP on this gene

EFY85710

C2H2 type zinc finger containing protein
  
Accession: EFY85709
  
Location: 73638-74170
  
 NCBI BlastP on this gene

EFY85709

179. :  GL698734 Metarhizium anisopliae ARSEF 23 unplaced genomic scaffold Scf\_024     Total score: 2.0     Cumulative Blast bit score: 1009

peroxisomal membrane protein pex16
  
Accession: EFY95993
  
Location: 54208-55470
  
 NCBI BlastP on this gene

EFY95993

putative nitrate assimilation regulatory protein nirA
  
Accession: EFY95992
  
Location: 52806-53510
  
 NCBI BlastP on this gene

EFY95992

nuclear envelope protein Brr6, putative
  
Accession: EFY95991
  
Location: 47394-48918
  
 NCBI BlastP on this gene

EFY95991

transcription factor IWS1
  
Accession: EFY95990
  
Location: 45512-46930
  
 NCBI BlastP on this gene

EFY95990

serine/threonine-protein kinase SRPK2
  
Accession: EFY95989
  
Location: 40314-44917
  
  
**BlastP hit with Mycgr3G84644\_Mycgr3T**
  
Percentage identity: 70 %
  
BlastP bit score: 768
  
Sequence coverage: 97 %
  
E-value: 0.0
  
  
 NCBI BlastP on this gene

EFY95989

hypothetical protein
  
Accession: EFY95988
  
Location: 38326-39512
  
 NCBI BlastP on this gene

EFY95988

putative vesicular transport protein
  
Accession: EFY95987
  
Location: 34112-37434
  
  
**BlastP hit with Mycgr3G108094\_Mycgr3**
  
Percentage identity: 37 %
  
BlastP bit score: 241
  
Sequence coverage: 39 %
  
E-value: 6e-62
  
  
 NCBI BlastP on this gene

EFY95987

SWIRM domain protein Fun19, putative
  
Accession: EFY95986
  
Location: 29550-30869
  
 NCBI BlastP on this gene

EFY95986

F-box domain containing protein
  
Accession: EFY95985
  
Location: 20129-21949
  
 NCBI BlastP on this gene

EFY95985

N2,N2-dimethylguanosine tRNA methyltransferase
  
Accession: EFY95984
  
Location: 16961-18052
  
 NCBI BlastP on this gene

EFY95984

C2H2 type zinc finger containing protein
  
Accession: EFY95983
  
Location: 16036-16571
  
 NCBI BlastP on this gene

EFY95983

180. :  FQ790349 Botryotinia fuckeliana T4 SupSuperContig\_62\_54\_1 genomic supercontig.     Total score: 2.0     Cumulative Blast bit score: 1009

similar to subtilisin-like serine protease (secreted protein)
  
Accession: CCD53977
  
Location: 285126-286942
  
 NCBI BlastP on this gene

BofuT4\_P131570.1

hypothetical protein
  
Accession: CCD53978
  
Location: 287522-291091
  
 NCBI BlastP on this gene

BofuT4\_P131580.1

predicted protein
  
Accession: CCD53979
  
Location: 293879-294193
  
 NCBI BlastP on this gene

BofuT4\_uP131590.1

predicted protein
  
Accession: CCD53980
  
Location: 295746-295921
  
 NCBI BlastP on this gene

BofuT4\_uP131600.1

predicted protein
  
Accession: CCD53981
  
Location: 296168-296775
  
 NCBI BlastP on this gene

BofuT4\_P131610.1

hypothetical protein
  
Accession: CCD53982
  
Location: 297694-298020
  
 NCBI BlastP on this gene

BofuT4\_P131620.1

hypothetical protein
  
Accession: CCD53983
  
Location: 299203-299355
  
 NCBI BlastP on this gene

BofuT4\_uP131630.1

hypothetical protein
  
Accession: CCD53984
  
Location: 299654-300721
  
 NCBI BlastP on this gene

BofuT4\_P131640.1

hypothetical protein
  
Accession: CCD53985
  
Location: 300921-302201
  
 NCBI BlastP on this gene

BofuT4\_P131650.1

hypothetical protein
  
Accession: CCD53986
  
Location: 302649-307255
  
  
**BlastP hit with Mycgr3G108094\_Mycgr3**
  
Percentage identity: 31 %
  
BlastP bit score: 236
  
Sequence coverage: 47 %
  
E-value: 9e-60
  
  
 NCBI BlastP on this gene

BofuT4\_P131660.1

hypothetical protein
  
Accession: CCD53987
  
Location: 307731-308954
  
 NCBI BlastP on this gene

BofuT4\_P131670.1

hypothetical protein
  
Accession: CCD53988
  
Location: 309284-309557
  
 NCBI BlastP on this gene

BofuT4\_uP131680.1

hypothetical protein
  
Accession: CCD53989
  
Location: 311757-312023
  
 NCBI BlastP on this gene

BofuT4\_uP131690.1

similar to serine protein kinase Sky1
  
Accession: CCD53990
  
Location: 312730-314667
  
  
**BlastP hit with Mycgr3G84644\_Mycgr3T**
  
Percentage identity: 76 %
  
BlastP bit score: 773
  
Sequence coverage: 89 %
  
E-value: 0.0
  
  
 NCBI BlastP on this gene

BofuT4\_P131700.1

hypothetical protein
  
Accession: CCD53991
  
Location: 315740-317244
  
 NCBI BlastP on this gene

BofuT4\_P131710.1

similar to transcriptional elongation factor Iws1
  
Accession: CCD53992
  
Location: 317648-319131
  
 NCBI BlastP on this gene

BofuT4\_P131720.1

similar to transcription factor bHLH
  
Accession: CCD53993
  
Location: 321040-322569
  
 NCBI BlastP on this gene

BofuT4\_P131730.1

predicted protein
  
Accession: CCD53994
  
Location: 325438-325593
  
 NCBI BlastP on this gene

BofuT4\_uP131740.1

predicted protein
  
Accession: CCD53995
  
Location: 329368-330061
  
 NCBI BlastP on this gene

BofuT4\_uP131750.1

predicted protein
  
Accession: CCD53996
  
Location: 330336-330821
  
 NCBI BlastP on this gene

BofuT4\_uP131760.1

similar to charged multivesicular body protein 1b
  
Accession: CCD53997
  
Location: 331814-332666
  
 NCBI BlastP on this gene

BofuT4\_P131770.1

181. :  GG698942 Nectria haematococca mpVI 77-13-4 chromosome 3 genomic scaffold NECHAsca\_54\_chr3\_4\_0     Total score: 2.0     Cumulative Blast bit score: 1005

hypothetical protein
  
Accession: EEU35286
  
Location: 78801-79937
  
 NCBI BlastP on this gene

EEU35286

predicted protein
  
Accession: EEU35321
  
Location: 82006-82956
  
 NCBI BlastP on this gene

EEU35321

hypothetical protein
  
Accession: EEU35322
  
Location: 83616-87218
  
  
**BlastP hit with Mycgr3G108094\_Mycgr3**
  
Percentage identity: 34 %
  
BlastP bit score: 247
  
Sequence coverage: 44 %
  
E-value: 8e-64
  
  
 NCBI BlastP on this gene

EEU35322

hypothetical protein
  
Accession: EEU35287
  
Location: 89090-90286
  
 NCBI BlastP on this gene

EEU35287

predicted protein
  
Accession: EEU35323
  
Location: 91019-93702
  
 NCBI BlastP on this gene

EEU35323

hypothetical protein
  
Accession: EEU35324
  
Location: 96414-97988
  
 NCBI BlastP on this gene

EEU35324

predicted protein
  
Accession: EEU35288
  
Location: 98429-99824
  
 NCBI BlastP on this gene

EEU35288

predicted protein
  
Accession: EEU35289
  
Location: 102327-103446
  
 NCBI BlastP on this gene

EEU35289

predicted protein
  
Accession: EEU35290
  
Location: 104522-106263
  
  
**BlastP hit with Mycgr3G84644\_Mycgr3T**
  
Percentage identity: 74 %
  
BlastP bit score: 758
  
Sequence coverage: 89 %
  
E-value: 0.0
  
  
 NCBI BlastP on this gene

EEU35290

hypothetical protein
  
Accession: EEU35325
  
Location: 106970-108637
  
 NCBI BlastP on this gene

EEU35325

hypothetical protein
  
Accession: EEU35291
  
Location: 109262-110827
  
 NCBI BlastP on this gene

EEU35291

hypothetical protein
  
Accession: EEU35292
  
Location: 111710-112860
  
 NCBI BlastP on this gene

EEU35292

hypothetical protein
  
Accession: EEU35293
  
Location: 118565-120421
  
 NCBI BlastP on this gene

EEU35293

182. :  GL698717 Metarhizium anisopliae ARSEF 23 unplaced genomic scaffold Scf\_007     Total score: 2.0     Cumulative Blast bit score: 1004

serine/threonine protein phosphatase PP2A catalytic subunit
  
Accession: EFY99004
  
Location: 114455-115965
  
 NCBI BlastP on this gene

EFY99004

Exocyst complex component EXO84
  
Accession: EFY99005
  
Location: 116866-119031
  
 NCBI BlastP on this gene

EFY99005

mitochondrial import receptor subunit tom-20
  
Accession: EFY99006
  
Location: 119696-120485
  
 NCBI BlastP on this gene

EFY99006

flavodoxin and radical SAM domain protein
  
Accession: EFY99007
  
Location: 120969-123403
  
 NCBI BlastP on this gene

EFY99007

DNA repair and recombination protein RAD5B
  
Accession: EFY99008
  
Location: 124050-127199
  
 NCBI BlastP on this gene

EFY99008

cysteine dioxygenase
  
Accession: EFY99009
  
Location: 127977-128770
  
 NCBI BlastP on this gene

EFY99009

C4-dicarboxylate transporter, putative
  
Accession: EFY99010
  
Location: 131258-132187
  
 NCBI BlastP on this gene

EFY99010

ATP synthase regulation protein NCA2
  
Accession: EFY99011
  
Location: 132727-134812
  
  
**BlastP hit with Mycgr3G84646\_Mycgr3T**
  
Percentage identity: 42 %
  
BlastP bit score: 541
  
Sequence coverage: 100 %
  
E-value: 2e-180
  
  
 NCBI BlastP on this gene

EFY99011

C-4 sterol methyl oxidase
  
Accession: EFY99012
  
Location: 136082-137187
  
  
**BlastP hit with Mycgr3G36271\_Mycgr3T**
  
Percentage identity: 72 %
  
BlastP bit score: 463
  
Sequence coverage: 96 %
  
E-value: 2e-161
  
  
 NCBI BlastP on this gene

EFY99012

pterin-4-alpha-carbinolamine dehydratase family protein
  
Accession: EFY99013
  
Location: 137725-138438
  
 NCBI BlastP on this gene

EFY99013

amidohydrolase
  
Accession: EFY99014
  
Location: 138864-140213
  
 NCBI BlastP on this gene

EFY99014

transmembrane protein
  
Accession: EFY99015
  
Location: 141903-143376
  
 NCBI BlastP on this gene

EFY99015

chromatin remodeling complex subunit (Arp5), putative
  
Accession: EFY99016
  
Location: 143972-146445
  
 NCBI BlastP on this gene

EFY99016

hypothetical protein
  
Accession: EFY99017
  
Location: 147687-148265
  
 NCBI BlastP on this gene

EFY99017

Got1 family protein
  
Accession: EFY99018
  
Location: 149294-150049
  
 NCBI BlastP on this gene

EFY99018

profilin
  
Accession: EFY99019
  
Location: 150709-151400
  
 NCBI BlastP on this gene

EFY99019

hypothetical protein
  
Accession: EFY99020
  
Location: 156037-157689
  
 NCBI BlastP on this gene

EFY99020

183. :  GL985057 Trichoderma reesei QM6a unplaced genomic scaffold TRIREscaffold\_2     Total score: 2.0     Cumulative Blast bit score: 997

predicted protein
  
Accession: EGR52017
  
Location: 1242446-1243432
  
 NCBI BlastP on this gene

EGR52017

predicted protein
  
Accession: EGR52018
  
Location: 1251041-1254197
  
 NCBI BlastP on this gene

EGR52018

hypothetical protein
  
Accession: EGR52019
  
Location: 1255166-1255952
  
 NCBI BlastP on this gene

EGR52019

predicted protein
  
Accession: EGR51742
  
Location: 1259267-1260836
  
 NCBI BlastP on this gene

EGR51742

predicted protein
  
Accession: EGR52020
  
Location: 1261252-1263389
  
  
**BlastP hit with Mycgr3G84646\_Mycgr3T**
  
Percentage identity: 45 %
  
BlastP bit score: 550
  
Sequence coverage: 102 %
  
E-value: 0.0
  
  
 NCBI BlastP on this gene

EGR52020

sterol desaturase-like protein
  
Accession: EGR51743
  
Location: 1265105-1266157
  
  
**BlastP hit with Mycgr3G36271\_Mycgr3T**
  
Percentage identity: 71 %
  
BlastP bit score: 448
  
Sequence coverage: 95 %
  
E-value: 2e-155
  
  
 NCBI BlastP on this gene

EGR51743

predicted protein
  
Accession: EGR52021
  
Location: 1267002-1267948
  
 NCBI BlastP on this gene

EGR52021

predicted protein
  
Accession: EGR51744
  
Location: 1268313-1268718
  
 NCBI BlastP on this gene

EGR51744

actin-like protein
  
Accession: EGR51745
  
Location: 1269290-1271778
  
 NCBI BlastP on this gene

EGR51745

DNA binding domain-containing/Basic-Leucine zipper transcription factor domain-containing protein
  
Accession: EGR52022
  
Location: 1272638-1273746
  
 NCBI BlastP on this gene

EGR52022

predicted protein
  
Accession: EGR51746
  
Location: 1273896-1274360
  
 NCBI BlastP on this gene

EGR51746

predicted protein
  
Accession: EGR52023
  
Location: 1276821-1279084
  
 NCBI BlastP on this gene

EGR52023

predicted protein
  
Accession: EGR51747
  
Location: 1280095-1280683
  
 NCBI BlastP on this gene

EGR51747

predicted protein
  
Accession: EGR52024
  
Location: 1284346-1285628
  
 NCBI BlastP on this gene

EGR52024

184. :  AHHD01000090 Macrophomina phaseolina MS6     Total score: 2.0     Cumulative Blast bit score: 995

MaoC-like dehydratase
  
Accession: EKG20274
  
Location: 74332-75779
  
 NCBI BlastP on this gene

EKG20274

hypothetical protein
  
Accession: EKG20275
  
Location: 77426-79390
  
 NCBI BlastP on this gene

EKG20275

Profilin/allergen
  
Accession: EKG20276
  
Location: 79944-80705
  
 NCBI BlastP on this gene

EKG20276

Ubiquitin-conjugating enzyme E2
  
Accession: EKG20277
  
Location: 83266-83734
  
 NCBI BlastP on this gene

EKG20277

hypothetical protein
  
Accession: EKG20278
  
Location: 85715-88300
  
 NCBI BlastP on this gene

EKG20278

Nuclear control of ATP synthase 2
  
Accession: EKG20279
  
Location: 90662-92958
  
  
**BlastP hit with Mycgr3G84646\_Mycgr3T**
  
Percentage identity: 45 %
  
BlastP bit score: 623
  
Sequence coverage: 104 %
  
E-value: 0.0
  
  
 NCBI BlastP on this gene

EKG20279

Ras GTPase
  
Accession: EKG20280
  
Location: 93780-94902
  
  
**BlastP hit with Mycgr3G99145\_Mycgr3T**
  
Percentage identity: 88 %
  
BlastP bit score: 372
  
Sequence coverage: 100 %
  
E-value: 2e-128
  
  
 NCBI BlastP on this gene

EKG20280

hypothetical protein
  
Accession: EKG20281
  
Location: 98726-100219
  
 NCBI BlastP on this gene

EKG20281

Major facilitator superfamily
  
Accession: EKG20282
  
Location: 102489-107077
  
 NCBI BlastP on this gene

EKG20282

185. :  ABDG02000026 Trichoderma atroviride IMI 206040     Total score: 2.0     Cumulative Blast bit score: 993

hypothetical protein
  
Accession: EHK42683
  
Location: 1015647-1017674
  
 NCBI BlastP on this gene

EHK42683

hypothetical protein
  
Accession: EHK42684
  
Location: 1018983-1020733
  
 NCBI BlastP on this gene

EHK42684

hypothetical protein
  
Accession: EHK42685
  
Location: 1020809-1021470
  
 NCBI BlastP on this gene

EHK42685

hypothetical protein
  
Accession: EHK42686
  
Location: 1024484-1025833
  
 NCBI BlastP on this gene

EHK42686

serine/threonine protein kinase, CMGC group
  
Accession: EHK42687
  
Location: 1031960-1033723
  
  
**BlastP hit with Mycgr3G84644\_Mycgr3T**
  
Percentage identity: 72 %
  
BlastP bit score: 734
  
Sequence coverage: 86 %
  
E-value: 0.0
  
  
 NCBI BlastP on this gene

EHK42687

hypothetical protein
  
Accession: EHK42688
  
Location: 1034648-1036192
  
 NCBI BlastP on this gene

EHK42688

hypothetical protein
  
Accession: EHK42689
  
Location: 1038333-1039382
  
 NCBI BlastP on this gene

EHK42689

hypothetical protein
  
Accession: EHK42690
  
Location: 1045891-1049857
  
  
**BlastP hit with Mycgr3G108094\_Mycgr3**
  
Percentage identity: 32 %
  
BlastP bit score: 259
  
Sequence coverage: 44 %
  
E-value: 1e-67
  
  
 NCBI BlastP on this gene

EHK42690

hypothetical protein
  
Accession: EHK42691
  
Location: 1051014-1051232
  
 NCBI BlastP on this gene

EHK42691

hypothetical protein
  
Accession: EHK42692
  
Location: 1052204-1053478
  
 NCBI BlastP on this gene

EHK42692

glycosyltransferase family 1 protein
  
Accession: EHK43614
  
Location: 1063433-1065084
  
 NCBI BlastP on this gene

EHK43614

186. :  KB445649 Cochliobolus sativus ND90Pr unplaced genomic scaffold COCSAscaffold\_13     Total score: 2.0     Cumulative Blast bit score: 991

hypothetical protein
  
Accession: EMD61270
  
Location: 1370424-1370760
  
 NCBI BlastP on this gene

EMD61270

hypothetical protein
  
Accession: EMD61269
  
Location: 1368649-1368918
  
 NCBI BlastP on this gene

EMD61269

hypothetical protein
  
Accession: EMD61268
  
Location: 1367191-1368282
  
 NCBI BlastP on this gene

EMD61268

hypothetical protein
  
Accession: EMD61267
  
Location: 1366347-1366997
  
 NCBI BlastP on this gene

EMD61267

hypothetical protein
  
Accession: EMD61266
  
Location: 1364592-1365601
  
 NCBI BlastP on this gene

EMD61266

hypothetical protein
  
Accession: EMD61265
  
Location: 1363796-1364257
  
 NCBI BlastP on this gene

EMD61265

hypothetical protein
  
Accession: EMD61264
  
Location: 1361003-1362652
  
 NCBI BlastP on this gene

EMD61264

hypothetical protein
  
Accession: EMD61263
  
Location: 1358538-1358750
  
 NCBI BlastP on this gene

EMD61263

hypothetical protein
  
Accession: EMD61262
  
Location: 1356085-1357748
  
 NCBI BlastP on this gene

EMD61262

hypothetical protein
  
Accession: EMD61261
  
Location: 1354100-1355693
  
 NCBI BlastP on this gene

EMD61261

hypothetical protein
  
Accession: EMD61260
  
Location: 1352049-1353490
  
 NCBI BlastP on this gene

EMD61260

hypothetical protein
  
Accession: EMD61259
  
Location: 1349441-1351781
  
  
**BlastP hit with Mycgr3G108094\_Mycgr3**
  
Percentage identity: 35 %
  
BlastP bit score: 146
  
Sequence coverage: 22 %
  
E-value: 7e-33
  
  
 NCBI BlastP on this gene

EMD61259

hypothetical protein
  
Accession: EMD61258
  
Location: 1347778-1348056
  
 NCBI BlastP on this gene

EMD61258

hypothetical protein
  
Accession: EMD61257
  
Location: 1344408-1346602
  
  
**BlastP hit with Mycgr3G84644\_Mycgr3T**
  
Percentage identity: 70 %
  
BlastP bit score: 845
  
Sequence coverage: 109 %
  
E-value: 0.0
  
  
 NCBI BlastP on this gene

EMD61257

glycoside hydrolase family 16 protein
  
Accession: EMD61256
  
Location: 1341986-1343184
  
 NCBI BlastP on this gene

EMD61256

hypothetical protein
  
Accession: EMD61255
  
Location: 1338191-1340371
  
 NCBI BlastP on this gene

EMD61255

hypothetical protein
  
Accession: EMD61254
  
Location: 1336380-1337444
  
 NCBI BlastP on this gene

EMD61254

hypothetical protein
  
Accession: EMD61253
  
Location: 1335057-1336284
  
 NCBI BlastP on this gene

EMD61253

hypothetical protein
  
Accession: EMD61252
  
Location: 1332721-1334832
  
 NCBI BlastP on this gene

EMD61252

hypothetical protein
  
Accession: EMD61251
  
Location: 1330275-1332066
  
 NCBI BlastP on this gene

EMD61251

hypothetical protein
  
Accession: EMD61250
  
Location: 1328847-1329773
  
 NCBI BlastP on this gene

EMD61250

glycosyltransferase family 2 protein
  
Accession: EMD61249
  
Location: 1327795-1328589
  
 NCBI BlastP on this gene

EMD61249

187. :  HF679028 Fusarium fujikuroi IMI 58289 draft genome, chromosome FFUJ\_chr06.     Total score: 2.0     Cumulative Blast bit score: 988

related to nitrate assimilation regulatory protein nirA
  
Accession: CCT69673
  
Location: 673788-676563
  
 NCBI BlastP on this gene

FFUJ\_05577

uncharacterized protein
  
Accession: CCT69674
  
Location: 679409-680964
  
 NCBI BlastP on this gene

FFUJ\_05578

Transcription factor IWS1
  
Accession: CCT69675
  
Location: 681413-682803
  
 NCBI BlastP on this gene

FFUJ\_05579

uncharacterized protein
  
Accession: CCT69676
  
Location: 684604-685125
  
 NCBI BlastP on this gene

FFUJ\_05580

uncharacterized protein
  
Accession: CCT69677
  
Location: 685587-686504
  
 NCBI BlastP on this gene

FFUJ\_05581

probable dis1-suppressing protein kinase dsk1
  
Accession: CCT69678
  
Location: 690303-692490
  
  
**BlastP hit with Mycgr3G84644\_Mycgr3T**
  
Percentage identity: 72 %
  
BlastP bit score: 723
  
Sequence coverage: 89 %
  
E-value: 0.0
  
  
 NCBI BlastP on this gene

FFUJ\_05582

uncharacterized protein
  
Accession: CCT69679
  
Location: 693879-695048
  
 NCBI BlastP on this gene

FFUJ\_05583

related to vesicular transport protein
  
Accession: CCT70708
  
Location: 696163-699670
  
  
**BlastP hit with Mycgr3G108094\_Mycgr3**
  
Percentage identity: 35 %
  
BlastP bit score: 265
  
Sequence coverage: 44 %
  
E-value: 1e-69
  
  
 NCBI BlastP on this gene

FFUJ\_05584

related to NAD(P)H-dependent oxidoreductase
  
Accession: CCT69680
  
Location: 700556-701510
  
 NCBI BlastP on this gene

FFUJ\_05585

related to protein involved in sporulation and meiosis
  
Accession: CCT69681
  
Location: 703959-705077
  
 NCBI BlastP on this gene

FFUJ\_05586

uncharacterized protein
  
Accession: CCT69682
  
Location: 714950-716638
  
 NCBI BlastP on this gene

FFUJ\_05587

uncharacterized protein
  
Accession: CCT69683
  
Location: 717195-718322
  
 NCBI BlastP on this gene

FFUJ\_05588

uncharacterized protein
  
Accession: CCT70732
  
Location: 718799-719348
  
 NCBI BlastP on this gene

FFUJ\_05589

188. :  ABDF02000006 Trichoderma virens Gv29-8     Total score: 2.0     Cumulative Blast bit score: 988

hypothetical protein
  
Accession: EHK22808
  
Location: 2346077-2348083
  
 NCBI BlastP on this gene

EHK22808

hypothetical protein
  
Accession: EHK22807
  
Location: 2341744-2344253
  
 NCBI BlastP on this gene

EHK22807

hypothetical protein
  
Accession: EHK22806
  
Location: 2338632-2339825
  
 NCBI BlastP on this gene

EHK22806

hypothetical protein
  
Accession: EHK22805
  
Location: 2336725-2338044
  
 NCBI BlastP on this gene

EHK22805

hypothetical protein
  
Accession: EHK22804
  
Location: 2335010-2335324
  
 NCBI BlastP on this gene

EHK22804

serine/threonine protein kinase, CMGC group
  
Accession: EHK22803
  
Location: 2328629-2332959
  
  
**BlastP hit with Mycgr3G84644\_Mycgr3T**
  
Percentage identity: 69 %
  
BlastP bit score: 763
  
Sequence coverage: 99 %
  
E-value: 0.0
  
  
 NCBI BlastP on this gene

EHK22803

hypothetical protein
  
Accession: EHK22802
  
Location: 2326051-2327449
  
 NCBI BlastP on this gene

EHK22802

NACHT and ankyrin domain protein
  
Accession: EHK22801
  
Location: 2323548-2324321
  
 NCBI BlastP on this gene

EHK22801

hypothetical protein
  
Accession: EHK22800
  
Location: 2319459-2320552
  
 NCBI BlastP on this gene

EHK22800

hypothetical protein
  
Accession: EHK22799
  
Location: 2318022-2318414
  
 NCBI BlastP on this gene

EHK22799

hypothetical protein
  
Accession: EHK22798
  
Location: 2315645-2316675
  
 NCBI BlastP on this gene

EHK22798

hypothetical protein
  
Accession: EHK22797
  
Location: 2311666-2315106
  
  
**BlastP hit with Mycgr3G108094\_Mycgr3**
  
Percentage identity: 31 %
  
BlastP bit score: 225
  
Sequence coverage: 44 %
  
E-value: 9e-57
  
  
 NCBI BlastP on this gene

EHK22797

hypothetical protein
  
Accession: EHK22796
  
Location: 2307969-2309129
  
 NCBI BlastP on this gene

EHK22796

glycosyltransferase family 1 protein
  
Accession: EHK22795
  
Location: 2297637-2299297
  
 NCBI BlastP on this gene

EHK22795

189. :  KB456266 Mycosphaerella populorum SO2202 unplaced genomic scaffold SEPMUscaffold\_7     Total score: 2.0     Cumulative Blast bit score: 982

phosphatidylinositol 3 and 4-kinase
  
Accession: EMF11057
  
Location: 265588-271300
  
 NCBI BlastP on this gene

EMF11057

FAD/NAD(P)-binding domain-containing protein
  
Accession: EMF11058
  
Location: 274306-276243
  
 NCBI BlastP on this gene

EMF11058

cochaperone Pam16
  
Accession: EMF11060
  
Location: 279114-279677
  
 NCBI BlastP on this gene

EMF11060

hypothetical protein
  
Accession: EMF11061
  
Location: 280382-281200
  
 NCBI BlastP on this gene

EMF11061

hypothetical protein
  
Accession: EMF11062
  
Location: 283420-284268
  
 NCBI BlastP on this gene

EMF11062

kinase-like protein
  
Accession: EMF11063
  
Location: 285484-287853
  
  
**BlastP hit with Mycgr3G103260\_Mycgr3**
  
Percentage identity: 72 %
  
BlastP bit score: 383
  
Sequence coverage: 96 %
  
E-value: 1e-124
  
  
 NCBI BlastP on this gene

EMF11063

kinase-like protein
  
Accession: EMF11064
  
Location: 290267-291587
  
  
**BlastP hit with Mycgr3G68429\_Mycgr3T**
  
Percentage identity: 70 %
  
BlastP bit score: 599
  
Sequence coverage: 98 %
  
E-value: 0.0
  
  
 NCBI BlastP on this gene

EMF11064

hypothetical protein
  
Accession: EMF11065
  
Location: 301161-301358
  
 NCBI BlastP on this gene

EMF11065

FAD dependent oxidoreductase
  
Accession: EMF11066
  
Location: 302247-303467
  
 NCBI BlastP on this gene

EMF11066

carbohydrate-binding module family 14 protein
  
Accession: EMF11067
  
Location: 305564-305773
  
 NCBI BlastP on this gene

EMF11067

PTR2-domain-containing protein
  
Accession: EMF11068
  
Location: 307070-309210
  
 NCBI BlastP on this gene

EMF11068

NAD(P)-binding protein
  
Accession: EMF11069
  
Location: 310832-311593
  
 NCBI BlastP on this gene

EMF11069

190. :  KB730484 Fusarium oxysporum f. sp. cubense race 1 unplaced genomic scaffold scaffold489     Total score: 2.0     Cumulative Blast bit score: 980

hypothetical protein
  
Accession: ENH65641
  
Location: 212141-213022
  
 NCBI BlastP on this gene

ENH65641

Lipase 4
  
Accession: ENH65642
  
Location: 214376-215884
  
 NCBI BlastP on this gene

ENH65642

hypothetical protein
  
Accession: ENH65643
  
Location: 216884-217969
  
 NCBI BlastP on this gene

ENH65643

hypothetical protein
  
Accession: ENH65644
  
Location: 219640-220004
  
 NCBI BlastP on this gene

ENH65644

hypothetical protein
  
Accession: ENH65645
  
Location: 223678-225008
  
 NCBI BlastP on this gene

ENH65645

hypothetical protein
  
Accession: ENH65646
  
Location: 226507-227067
  
 NCBI BlastP on this gene

ENH65646

Putative SWI/SNF-related matrix-associated
  
Accession: ENH65647
  
Location: 228320-231454
  
 NCBI BlastP on this gene

ENH65647

Nuclear control of ATPase protein 2
  
Accession: ENH65648
  
Location: 231902-233993
  
  
**BlastP hit with Mycgr3G84646\_Mycgr3T**
  
Percentage identity: 43 %
  
BlastP bit score: 547
  
Sequence coverage: 101 %
  
E-value: 0.0
  
  
 NCBI BlastP on this gene

ENH65648

C-4 methylsterol oxidase
  
Accession: ENH65649
  
Location: 236706-237756
  
  
**BlastP hit with Mycgr3G36271\_Mycgr3T**
  
Percentage identity: 67 %
  
BlastP bit score: 434
  
Sequence coverage: 97 %
  
E-value: 1e-149
  
  
 NCBI BlastP on this gene

ENH65649

Putative pterin-4-alpha-carbinolamine dehydratase
  
Accession: ENH65650
  
Location: 238263-238865
  
 NCBI BlastP on this gene

ENH65650

Putative transcription factor kapC
  
Accession: ENH65651
  
Location: 241126-242510
  
 NCBI BlastP on this gene

ENH65651

Actin-like protein arp5
  
Accession: ENH65652
  
Location: 242987-245387
  
 NCBI BlastP on this gene

ENH65652

DASH complex subunit dad4
  
Accession: ENH65653
  
Location: 245985-246419
  
 NCBI BlastP on this gene

ENH65653

Chromatin-remodeling complex subunit ies6
  
Accession: ENH65654
  
Location: 246732-247295
  
 NCBI BlastP on this gene

ENH65654

Protein transport protein GOT1
  
Accession: ENH65655
  
Location: 248188-248903
  
 NCBI BlastP on this gene

ENH65655

Profilin
  
Accession: ENH65656
  
Location: 249455-250261
  
 NCBI BlastP on this gene

ENH65656

hypothetical protein
  
Accession: ENH65657
  
Location: 253767-255719
  
 NCBI BlastP on this gene

ENH65657

Synaptobrevin like protein ykt6
  
Accession: ENH65658
  
Location: 256433-257292
  
 NCBI BlastP on this gene

ENH65658

191. :  KB726997 Fusarium oxysporum f. sp. cubense race 4 unplaced genomic scaffold scaffold71     Total score: 2.0     Cumulative Blast bit score: 980

Profilin
  
Accession: EMT60295
  
Location: 146801-147607
  
 NCBI BlastP on this gene

EMT60295

Protein transport protein GOT1
  
Accession: EMT60296
  
Location: 148171-148888
  
 NCBI BlastP on this gene

EMT60296

Chromatin-remodeling complex subunit ies6
  
Accession: EMT60297
  
Location: 149788-150351
  
 NCBI BlastP on this gene

EMT60297

DASH complex subunit dad4
  
Accession: EMT60298
  
Location: 150665-151099
  
 NCBI BlastP on this gene

EMT60298

Actin-like protein arp5
  
Accession: EMT60299
  
Location: 151696-156195
  
 NCBI BlastP on this gene

EMT60299

Putative transcription factor kapC
  
Accession: EMT60300
  
Location: 156669-158053
  
 NCBI BlastP on this gene

EMT60300

Putative pterin-4-alpha-carbinolamine dehydratase
  
Accession: EMT60301
  
Location: 160312-160914
  
 NCBI BlastP on this gene

EMT60301

C-4 methylsterol oxidase
  
Accession: EMT60302
  
Location: 161421-162471
  
  
**BlastP hit with Mycgr3G36271\_Mycgr3T**
  
Percentage identity: 67 %
  
BlastP bit score: 434
  
Sequence coverage: 97 %
  
E-value: 1e-149
  
  
 NCBI BlastP on this gene

EMT60302

Nuclear control of ATPase protein 2
  
Accession: EMT60303
  
Location: 165181-167272
  
  
**BlastP hit with Mycgr3G84646\_Mycgr3T**
  
Percentage identity: 43 %
  
BlastP bit score: 546
  
Sequence coverage: 101 %
  
E-value: 0.0
  
  
 NCBI BlastP on this gene

EMT60303

hypothetical protein
  
Accession: EMT60304
  
Location: 167720-170854
  
 NCBI BlastP on this gene

EMT60304

hypothetical protein
  
Accession: EMT60305
  
Location: 172107-172667
  
 NCBI BlastP on this gene

EMT60305

hypothetical protein
  
Accession: EMT60306
  
Location: 174167-175497
  
 NCBI BlastP on this gene

EMT60306

hypothetical protein
  
Accession: EMT60307
  
Location: 179290-181297
  
 NCBI BlastP on this gene

EMT60307

hypothetical protein
  
Accession: EMT60308
  
Location: 183190-184275
  
 NCBI BlastP on this gene

EMT60308

hypothetical protein
  
Accession: EMT60309
  
Location: 185096-186181
  
 NCBI BlastP on this gene

EMT60309

Lipase 4
  
Accession: EMT60310
  
Location: 186943-188685
  
 NCBI BlastP on this gene

EMT60310

192. :  AFQF01000058 Fusarium oxysporum Fo5176     Total score: 2.0     Cumulative Blast bit score: 976

hypothetical protein
  
Accession: EGU89375
  
Location: 7801-8808
  
 NCBI BlastP on this gene

EGU89375

hypothetical protein
  
Accession: EGU89376
  
Location: 9368-11362
  
 NCBI BlastP on this gene

EGU89376

hypothetical protein
  
Accession: EGU89377
  
Location: 14866-15673
  
 NCBI BlastP on this gene

EGU89377

hypothetical protein
  
Accession: EGU89378
  
Location: 16237-16948
  
 NCBI BlastP on this gene

EGU89378

hypothetical protein
  
Accession: EGU89379
  
Location: 17847-18410
  
 NCBI BlastP on this gene

EGU89379

hypothetical protein
  
Accession: EGU89380
  
Location: 18721-19155
  
 NCBI BlastP on this gene

EGU89380

hypothetical protein
  
Accession: EGU89381
  
Location: 19753-22153
  
 NCBI BlastP on this gene

EGU89381

hypothetical protein
  
Accession: EGU89382
  
Location: 22626-24006
  
 NCBI BlastP on this gene

EGU89382

hypothetical protein
  
Accession: EGU89383
  
Location: 26268-26870
  
 NCBI BlastP on this gene

EGU89383

hypothetical protein
  
Accession: EGU89384
  
Location: 27377-28427
  
  
**BlastP hit with Mycgr3G36271\_Mycgr3T**
  
Percentage identity: 67 %
  
BlastP bit score: 434
  
Sequence coverage: 97 %
  
E-value: 1e-149
  
  
 NCBI BlastP on this gene

EGU89384

hypothetical protein
  
Accession: EGU89385
  
Location: 31139-33230
  
  
**BlastP hit with Mycgr3G84646\_Mycgr3T**
  
Percentage identity: 46 %
  
BlastP bit score: 543
  
Sequence coverage: 86 %
  
E-value: 0.0
  
  
 NCBI BlastP on this gene

EGU89385

hypothetical protein
  
Accession: EGU89386
  
Location: 33678-36812
  
 NCBI BlastP on this gene

EGU89386

hypothetical protein
  
Accession: EGU89387
  
Location: 38066-38626
  
 NCBI BlastP on this gene

EGU89387

hypothetical protein
  
Accession: EGU89388
  
Location: 39821-41455
  
 NCBI BlastP on this gene

EGU89388

hypothetical protein
  
Accession: EGU89389
  
Location: 44376-44583
  
 NCBI BlastP on this gene

EGU89389

hypothetical protein
  
Accession: EGU89390
  
Location: 45128-45542
  
 NCBI BlastP on this gene

EGU89390

hypothetical protein
  
Accession: EGU89391
  
Location: 47166-48251
  
 NCBI BlastP on this gene

EGU89391

hypothetical protein
  
Accession: EGU89392
  
Location: 49005-50756
  
 NCBI BlastP on this gene

EGU89392

hypothetical protein
  
Accession: EGU89393
  
Location: 51666-52992
  
 NCBI BlastP on this gene

EGU89393

193. :  CP003010 Thielavia terrestris NRRL 8126 chromosome 2     Total score: 2.0     Cumulative Blast bit score: 974

hypothetical protein
  
Accession: AEO66920
  
Location: 7475788-7477507
  
 NCBI BlastP on this gene

THITE\_2045895

hypothetical protein
  
Accession: AEO66921
  
Location: 7478671-7480848
  
 NCBI BlastP on this gene

THITE\_2115480

hypothetical protein
  
Accession: AEO66922
  
Location: 7481542-7482375
  
 NCBI BlastP on this gene

THITE\_2115482

hypothetical protein
  
Accession: AEO66923
  
Location: 7482745-7484890
  
 NCBI BlastP on this gene

THITE\_2154729

hypothetical protein
  
Accession: AEO66924
  
Location: 7485335-7486021
  
 NCBI BlastP on this gene

THITE\_31960

hypothetical protein
  
Accession: AEO66925
  
Location: 7486785-7492979
  
 NCBI BlastP on this gene

THITE\_2115485

hypothetical protein
  
Accession: AEO66926
  
Location: 7495019-7498357
  
  
**BlastP hit with Mycgr3G84646\_Mycgr3T**
  
Percentage identity: 42 %
  
BlastP bit score: 517
  
Sequence coverage: 104 %
  
E-value: 8e-170
  
  
 NCBI BlastP on this gene

THITE\_2150534

methylsterol monooxygenase
  
Accession: AEO66927
  
Location: 7499573-7500681
  
  
**BlastP hit with Mycgr3G36271\_Mycgr3T**
  
Percentage identity: 72 %
  
BlastP bit score: 457
  
Sequence coverage: 95 %
  
E-value: 4e-159
  
  
 NCBI BlastP on this gene

THITE\_125790

hypothetical protein
  
Accession: AEO66928
  
Location: 7501250-7502257
  
 NCBI BlastP on this gene

THITE\_2115493

hypothetical protein
  
Accession: AEO66929
  
Location: 7505843-7506514
  
 NCBI BlastP on this gene

THITE\_2115495

ARP5-like protein
  
Accession: AEO66930
  
Location: 7507460-7510046
  
 NCBI BlastP on this gene

THITE\_2115499

hypothetical protein
  
Accession: AEO66931
  
Location: 7510511-7510981
  
 NCBI BlastP on this gene

THITE\_2115503

hypothetical protein
  
Accession: AEO66932
  
Location: 7511300-7511911
  
 NCBI BlastP on this gene

THITE\_2115508

hypothetical protein
  
Accession: AEO66933
  
Location: 7512378-7512578
  
 NCBI BlastP on this gene

THITE\_2115510

hypothetical protein
  
Accession: AEO66934
  
Location: 7513893-7514684
  
 NCBI BlastP on this gene

THITE\_37801

hypothetical protein
  
Accession: AEO66935
  
Location: 7515426-7516189
  
 NCBI BlastP on this gene

THITE\_2115513

hypothetical protein
  
Accession: AEO66936
  
Location: 7520673-7522808
  
 NCBI BlastP on this gene

THITE\_2115520

194. :  ABDF02000072 Trichoderma virens Gv29-8     Total score: 2.0     Cumulative Blast bit score: 972

hypothetical protein
  
Accession: EHK21299
  
Location: 13923-14282
  
 NCBI BlastP on this gene

EHK21299

hypothetical protein
  
Accession: EHK21300
  
Location: 16197-17756
  
 NCBI BlastP on this gene

EHK21300

hypothetical protein
  
Accession: EHK21301
  
Location: 19532-21121
  
 NCBI BlastP on this gene

EHK21301

hypothetical protein
  
Accession: EHK21302
  
Location: 21537-23631
  
  
**BlastP hit with Mycgr3G84646\_Mycgr3T**
  
Percentage identity: 45 %
  
BlastP bit score: 540
  
Sequence coverage: 101 %
  
E-value: 3e-180
  
  
 NCBI BlastP on this gene

EHK21302

hypothetical protein
  
Accession: EHK21303
  
Location: 25365-26415
  
  
**BlastP hit with Mycgr3G36271\_Mycgr3T**
  
Percentage identity: 70 %
  
BlastP bit score: 432
  
Sequence coverage: 92 %
  
E-value: 3e-149
  
  
 NCBI BlastP on this gene

EHK21303

hypothetical protein
  
Accession: EHK21304
  
Location: 27569-28156
  
 NCBI BlastP on this gene

EHK21304

hypothetical protein
  
Accession: EHK21305
  
Location: 28533-28938
  
 NCBI BlastP on this gene

EHK21305

hypothetical protein
  
Accession: EHK21306
  
Location: 29494-31967
  
 NCBI BlastP on this gene

EHK21306

hypothetical protein
  
Accession: EHK21307
  
Location: 32830-34740
  
 NCBI BlastP on this gene

EHK21307

hypothetical protein
  
Accession: EHK21308
  
Location: 37768-39111
  
 NCBI BlastP on this gene

EHK21308

hypothetical protein
  
Accession: EHK21309
  
Location: 39896-40422
  
 NCBI BlastP on this gene

EHK21309

hypothetical protein
  
Accession: EHK21310
  
Location: 40815-41066
  
 NCBI BlastP on this gene

EHK21310

hypothetical protein
  
Accession: EHK21311
  
Location: 43782-44249
  
 NCBI BlastP on this gene

EHK21311

195. :  ABDG02000026 Trichoderma atroviride IMI 206040     Total score: 2.0     Cumulative Blast bit score: 967

hypothetical protein
  
Accession: EHK43358
  
Location: 3345242-3346480
  
 NCBI BlastP on this gene

EHK43358

hypothetical protein
  
Accession: EHK43359
  
Location: 3347359-3352500
  
 NCBI BlastP on this gene

EHK43359

hypothetical protein
  
Accession: EHK43360
  
Location: 3352826-3353667
  
 NCBI BlastP on this gene

EHK43360

hypothetical protein
  
Accession: EHK43361
  
Location: 3355017-3358168
  
 NCBI BlastP on this gene

EHK43361

hypothetical protein
  
Accession: EHK43362
  
Location: 3359014-3359716
  
 NCBI BlastP on this gene

EHK43362

hypothetical protein
  
Accession: EHK43363
  
Location: 3362660-3363881
  
 NCBI BlastP on this gene

EHK43363

hypothetical protein
  
Accession: EHK43364
  
Location: 3364329-3366459
  
  
**BlastP hit with Mycgr3G84646\_Mycgr3T**
  
Percentage identity: 44 %
  
BlastP bit score: 536
  
Sequence coverage: 100 %
  
E-value: 2e-178
  
  
 NCBI BlastP on this gene

EHK43364

C-4 sterol methyl oxidase
  
Accession: EHK43365
  
Location: 3368164-3369223
  
  
**BlastP hit with Mycgr3G36271\_Mycgr3T**
  
Percentage identity: 70 %
  
BlastP bit score: 432
  
Sequence coverage: 93 %
  
E-value: 3e-149
  
  
 NCBI BlastP on this gene

EHK43365

hypothetical protein
  
Accession: EHK43366
  
Location: 3370402-3370989
  
 NCBI BlastP on this gene

EHK43366

hypothetical protein
  
Accession: EHK43367
  
Location: 3371167-3371716
  
 NCBI BlastP on this gene

EHK43367

hypothetical protein
  
Accession: EHK43368
  
Location: 3372283-3374729
  
 NCBI BlastP on this gene

EHK43368

hypothetical protein
  
Accession: EHK43369
  
Location: 3375753-3376907
  
 NCBI BlastP on this gene

EHK43369

hypothetical protein
  
Accession: EHK43370
  
Location: 3380834-3382192
  
 NCBI BlastP on this gene

EHK43370

hypothetical protein
  
Accession: EHK43371
  
Location: 3382930-3383452
  
 NCBI BlastP on this gene

EHK43371

hypothetical protein
  
Accession: EHK43372
  
Location: 3387168-3387872
  
 NCBI BlastP on this gene

EHK43372

hypothetical protein
  
Accession: EHK43373
  
Location: 3388481-3389241
  
 NCBI BlastP on this gene

EHK43373

196. :  AFNW01000339 Fusarium pseudograminearum CS3096     Total score: 2.0     Cumulative Blast bit score: 963

hypothetical protein
  
Accession: EKJ69464
  
Location: 54-2944
  
 NCBI BlastP on this gene

EKJ69464

hypothetical protein
  
Accession: EKJ69465
  
Location: 3382-6724
  
  
**BlastP hit with Mycgr3G84646\_Mycgr3T**
  
Percentage identity: 47 %
  
BlastP bit score: 533
  
Sequence coverage: 84 %
  
E-value: 2e-175
  
  
 NCBI BlastP on this gene

EKJ69465

hypothetical protein
  
Accession: EKJ69466
  
Location: 8285-9338
  
  
**BlastP hit with Mycgr3G36271\_Mycgr3T**
  
Percentage identity: 67 %
  
BlastP bit score: 430
  
Sequence coverage: 97 %
  
E-value: 3e-148
  
  
 NCBI BlastP on this gene

EKJ69466

hypothetical protein
  
Accession: EKJ69467
  
Location: 9846-10518
  
 NCBI BlastP on this gene

EKJ69467

hypothetical protein
  
Accession: EKJ69468
  
Location: 12542-13846
  
 NCBI BlastP on this gene

EKJ69468

hypothetical protein
  
Accession: EKJ69469
  
Location: 16583-17198
  
 NCBI BlastP on this gene

EKJ69469

hypothetical protein
  
Accession: EKJ69470
  
Location: 20058-21894
  
 NCBI BlastP on this gene

EKJ69470

hypothetical protein
  
Accession: EKJ69471
  
Location: 22531-23827
  
 NCBI BlastP on this gene

EKJ69471

hypothetical protein
  
Accession: EKJ69472
  
Location: 26955-28580
  
 NCBI BlastP on this gene

EKJ69472

hypothetical protein
  
Accession: EKJ69473
  
Location: 28769-30732
  
 NCBI BlastP on this gene

EKJ69473

197. :  JH126401 Cordyceps militaris CM01 unplaced genomic scaffold CCM\_S00003     Total score: 2.0     Cumulative Blast bit score: 959

profilin
  
Accession: EGX92884
  
Location: 2065025-2065598
  
 NCBI BlastP on this gene

EGX92884

Got1 family protein
  
Accession: EGX92885
  
Location: 2066345-2067122
  
 NCBI BlastP on this gene

EGX92885

hypothetical protein
  
Accession: EGX92886
  
Location: 2068444-2068996
  
 NCBI BlastP on this gene

EGX92886

Protein kinase-like domain
  
Accession: EGX92887
  
Location: 2070117-2072285
  
 NCBI BlastP on this gene

EGX92887

YL1 nuclear
  
Accession: EGX92888
  
Location: 2072707-2073315
  
 NCBI BlastP on this gene

EGX92888

DASH complex subunit DAD4
  
Accession: EGX92889
  
Location: 2073599-2074003
  
 NCBI BlastP on this gene

EGX92889

chromatin remodeling complex subunit (Arp5), putative
  
Accession: EGX92890
  
Location: 2074418-2076889
  
 NCBI BlastP on this gene

EGX92890

bZIP transcription factor
  
Accession: EGX92891
  
Location: 2077577-2078936
  
 NCBI BlastP on this gene

EGX92891

pterin-4-alpha-carbinolamine dehydratase, putative
  
Accession: EGX92892
  
Location: 2081051-2081685
  
 NCBI BlastP on this gene

EGX92892

C-4 methylsterol oxidase, variant
  
Accession: EGX92893
  
Location: 2082091-2083149
  
  
**BlastP hit with Mycgr3G36271\_Mycgr3T**
  
Percentage identity: 69 %
  
BlastP bit score: 433
  
Sequence coverage: 96 %
  
E-value: 1e-149
  
  
 NCBI BlastP on this gene

EGX92893

ATP synthase regulation protein NCA2
  
Accession: EGX92894
  
Location: 2084555-2086612
  
  
**BlastP hit with Mycgr3G84646\_Mycgr3T**
  
Percentage identity: 41 %
  
BlastP bit score: 527
  
Sequence coverage: 101 %
  
E-value: 6e-175
  
  
 NCBI BlastP on this gene

EGX92894

C4-dicarboxylate transporter/malic acid transport protein, putative
  
Accession: EGX92895
  
Location: 2086967-2088458
  
 NCBI BlastP on this gene

EGX92895

cysteine dioxygenase
  
Accession: EGX92896
  
Location: 2090463-2091281
  
 NCBI BlastP on this gene

EGX92896

flavodoxin and radical SAM domain protein
  
Accession: EGX92897
  
Location: 2092171-2094544
  
 NCBI BlastP on this gene

EGX92897

mitochondrial import receptor subunit tom-20
  
Accession: EGX92898
  
Location: 2095010-2095789
  
 NCBI BlastP on this gene

EGX92898

exocyst complex component EXO84
  
Accession: EGX92899
  
Location: 2096363-2098496
  
 NCBI BlastP on this gene

EGX92899

serine/threonine-protein phosphatase PP2A catalytic subunit
  
Accession: EGX92900
  
Location: 2099420-2100821
  
 NCBI BlastP on this gene

EGX92900

hypothetical protein
  
Accession: EGX92901
  
Location: 2101337-2102495
  
 NCBI BlastP on this gene

EGX92901

198. :  GL698525 Metarhizium acridum CQMa 102 unplaced genomic scaffold Scf\_056     Total score: 2.0     Cumulative Blast bit score: 959

profilin
  
Accession: EFY87554
  
Location: 112544-113547
  
 NCBI BlastP on this gene

EFY87554

Got1 family protein
  
Accession: EFY87555
  
Location: 114213-114962
  
 NCBI BlastP on this gene

EFY87555

hypothetical protein
  
Accession: EFY87556
  
Location: 115984-116562
  
 NCBI BlastP on this gene

EFY87556

chromatin remodeling complex subunit (Arp5), putative
  
Accession: EFY87557
  
Location: 117807-120266
  
 NCBI BlastP on this gene

EFY87557

transmembrane protein
  
Accession: EFY87558
  
Location: 120852-122296
  
 NCBI BlastP on this gene

EFY87558

amidohydrolase
  
Accession: EFY87559
  
Location: 124303-125415
  
 NCBI BlastP on this gene

EFY87559

pterin-4-alpha-carbinolamine dehydratase family protein
  
Accession: EFY87560
  
Location: 126064-126690
  
 NCBI BlastP on this gene

EFY87560

C-4 sterol methyl oxidase
  
Accession: EFY87561
  
Location: 127230-128015
  
  
**BlastP hit with Mycgr3G36271\_Mycgr3T**
  
Percentage identity: 74 %
  
BlastP bit score: 422
  
Sequence coverage: 86 %
  
E-value: 6e-146
  
  
 NCBI BlastP on this gene

EFY87561

ATP synthase regulation protein NCA2
  
Accession: EFY87562
  
Location: 129596-131682
  
  
**BlastP hit with Mycgr3G84646\_Mycgr3T**
  
Percentage identity: 43 %
  
BlastP bit score: 538
  
Sequence coverage: 100 %
  
E-value: 2e-179
  
  
 NCBI BlastP on this gene

EFY87562

C4-dicarboxylate transporter, putative
  
Accession: EFY87563
  
Location: 132205-133507
  
 NCBI BlastP on this gene

EFY87563

DNA repair and recombination protein RAD5B
  
Accession: EFY87564
  
Location: 135538-140246
  
 NCBI BlastP on this gene

EFY87564

flavodoxin and radical SAM domain protein
  
Accession: EFY87565
  
Location: 140699-143131
  
 NCBI BlastP on this gene

EFY87565

mitochondrial import receptor subunit tom-20
  
Accession: EFY87566
  
Location: 143615-144409
  
 NCBI BlastP on this gene

EFY87566

Exocyst complex component EXO84
  
Accession: EFY87567
  
Location: 145488-147654
  
 NCBI BlastP on this gene

EFY87567

serine/threonine protein phosphatase PP2A catalytic subunit
  
Accession: EFY87568
  
Location: 148491-149997
  
 NCBI BlastP on this gene

EFY87568

hypothetical protein
  
Accession: EFY87569
  
Location: 150668-151726
  
 NCBI BlastP on this gene

EFY87569

199. :  GG698910 Nectria haematococca mpVI 77-13-4 chromosome 2 genomic scaffold NECHAsca\_18\_chr2\_1\_0     Total score: 2.0     Cumulative Blast bit score: 958

hypothetical protein
  
Accession: EEU40349
  
Location: 2301402-2302753
  
 NCBI BlastP on this gene

EEU40349

predicted protein
  
Accession: EEU40926
  
Location: 2304724-2305671
  
 NCBI BlastP on this gene

EEU40926

hypothetical protein
  
Accession: EEU40927
  
Location: 2306561-2307942
  
 NCBI BlastP on this gene

EEU40927

hypothetical protein
  
Accession: EEU40350
  
Location: 2310078-2310723
  
 NCBI BlastP on this gene

EEU40350

hypothetical protein
  
Accession: EEU40351
  
Location: 2313782-2317019
  
 NCBI BlastP on this gene

EEU40351

hypothetical protein
  
Accession: EEU40928
  
Location: 2317387-2319364
  
  
**BlastP hit with Mycgr3G84646\_Mycgr3T**
  
Percentage identity: 42 %
  
BlastP bit score: 514
  
Sequence coverage: 100 %
  
E-value: 3e-170
  
  
 NCBI BlastP on this gene

EEU40928

predicted protein
  
Accession: EEU40352
  
Location: 2322408-2325131
  
 NCBI BlastP on this gene

EEU40352

hypothetical protein
  
Accession: EEU40929
  
Location: 2325220-2327089
  
 NCBI BlastP on this gene

EEU40929

hypothetical protein
  
Accession: EEU40353
  
Location: 2327667-2329510
  
 NCBI BlastP on this gene

EEU40353

predicted protein
  
Accession: EEU40354
  
Location: 2331904-2332963
  
  
**BlastP hit with Mycgr3G36271\_Mycgr3T**
  
Percentage identity: 69 %
  
BlastP bit score: 444
  
Sequence coverage: 97 %
  
E-value: 8e-154
  
  
 NCBI BlastP on this gene

EEU40354

hypothetical protein
  
Accession: EEU40930
  
Location: 2333444-2334056
  
 NCBI BlastP on this gene

EEU40930

predicted protein
  
Accession: EEU40931
  
Location: 2334552-2335185
  
 NCBI BlastP on this gene

EEU40931

hypothetical protein
  
Accession: EEU40355
  
Location: 2336268-2337573
  
 NCBI BlastP on this gene

EEU40355

Actin-related protein, ARP5 class
  
Accession: EEU40932
  
Location: 2338075-2340481
  
 NCBI BlastP on this gene

EEU40932

hypothetical protein
  
Accession: EEU40356
  
Location: 2341863-2342429
  
 NCBI BlastP on this gene

EEU40356

predicted protein
  
Accession: EEU40357
  
Location: 2343438-2344198
  
 NCBI BlastP on this gene

EEU40357

predicted protein
  
Accession: EEU40933
  
Location: 2344760-2345569
  
 NCBI BlastP on this gene

EEU40933

predicted protein
  
Accession: EEU40934
  
Location: 2346472-2347530
  
 NCBI BlastP on this gene

EEU40934

200. :  GL985061 Trichoderma reesei QM6a unplaced genomic scaffold TRIREscaffold\_6     Total score: 2.0     Cumulative Blast bit score: 948

predicted protein
  
Accession: EGR49937
  
Location: 437138-440483
  
 NCBI BlastP on this gene

EGR49937

flavohemoglobin
  
Accession: EGR49938
  
Location: 442244-443563
  
 NCBI BlastP on this gene

EGR49938

serine/threonine protein kinase
  
Accession: EGR49749
  
Location: 453024-454811
  
  
**BlastP hit with Mycgr3G84644\_Mycgr3T**
  
Percentage identity: 73 %
  
BlastP bit score: 735
  
Sequence coverage: 86 %
  
E-value: 0.0
  
  
 NCBI BlastP on this gene

EGR49749

predicted protein
  
Accession: EGR49939
  
Location: 456186-457641
  
 NCBI BlastP on this gene

EGR49939

predicted protein
  
Accession: EGR49940
  
Location: 459709-460191
  
 NCBI BlastP on this gene

EGR49940

vesicular transport protein
  
Accession: EGR49750
  
Location: 467138-470596
  
  
**BlastP hit with Mycgr3G108094\_Mycgr3**
  
Percentage identity: 31 %
  
BlastP bit score: 213
  
Sequence coverage: 44 %
  
E-value: 4e-53
  
  
 NCBI BlastP on this gene

EGR49750

predicted protein
  
Accession: EGR49941
  
Location: 472904-474067
  
 NCBI BlastP on this gene

EGR49941

glycosyltransferase family 1
  
Accession: EGR49942
  
Location: 484124-485739
  
 NCBI BlastP on this gene

EGR49942

Detecting sequence homology at the gene cluster level with MultiGeneBlast.
  
Marnix H. Medema, Rainer Breitling & Eriko Takano (2013)
  
*Molecular Biology and Evolution* , 30: 1218-1223.
