# Supplementary material for: In silico prediction and characterization of secondary metabolite biosynthetic gene clusters in the wheat pathogen Zymoseptoria tritici
Source: BMC Genomics. 2017 Aug 17;18:631. doi: 10.1186/s12864-017-3969-y (PMC5561558; doi:10.1186/s12864-017-3969-y)
Supplement: Supplementary file 1 — MultiGeneBLAST analysis of putative secondary metabolite clusters. All encoded amino acid sequences from genes residing in clusters predicted by AntiSMASH are given as FASTA file format. All output data from MultiGeneBLASTs are also provided. (ZIP 42911 kb) [file 12864_2017_3969_MOESM1_ESM.zip › Cluster MultiGene BLAST/out/Clusters_1_34/Cluster_12/displaypage5.xhtml]

xml version="1.0" encoding="UTF-8"?


Search Results
  
  
 Results pages: 1, 2, 3, 4, 5

**MultiGeneBlast hits**

Select gene cluster alignment
201. AGUE01000255\_0 Glarea lozoyensis 74030, whole genome shotgun sequencing ...
202. JH725150\_0 Beauveria bassiana ARSEF 2860 unplaced genomic scaffold BBA\_S...
203. DS985219\_0 Verticillium albo-atrum VaMs.102 supercont1.6 genomic scaffol...
204. KE145373\_2 Glarea lozoyensis ATCC 20868 chromosome Unknown GLAREA9, whol...
205. DS572718\_0 Verticillium dahliae VdLs.17 supercont1.24 genomic scaffold, ...
206. CH476643\_1 Sclerotinia sclerotiorum 1980 scaffold\_23 genomic scaffold, w...
207. KB707720\_0 Botryotinia fuckeliana BcDW1 unplaced genomic scaffold Scaffo...
208. FQ790356\_1 Botryotinia fuckeliana T4 SupSuperContig\_182\_154r\_1 genomic s...
209. JH921428\_2 Marssonina brunnea f. sp. 'multigermtubi' MB\_m1 unplaced geno...
210. GL988041\_4 Chaetomium thermophilum var. thermophilum DSM 1495 unplaced g...
211. CACQ02000613\_0 Colletotrichum higginsianum strain IMI 349063, whole geno...
212. CU638744\_0 Podospora anserina S mat+ genomic DNA chromosome 6, supercont...
213. JH226136\_2 Exophiala dermatitidis NIH/UT8656 unplaced genomic scaffold s...
214. GG663377\_2 Ajellomyces capsulatus G186AR genomic scaffold supercont2.15,...
215. DS990639\_3 Ajellomyces capsulatus H88 supercont1.4 genomic scaffold, who...
216. CAUH01003861\_0 Blumeria graminis f. sp. hordei DH14, whole genome shotgu...
217. DS544805\_0 Paracoccidioides brasiliensis Pb03 supercont1.3 genomic scaff...
218. DS027045\_0 Aspergillus clavatus NRRL 1 1099423829791 genomic scaffold, w...
219. KE374991\_0 Blumeria graminis f. sp. tritici 96224 unplaced genomic scaff...
220. FP929137\_4 Leptosphaeria maculans JN3 lm\_SuperContig\_10\_v2 genomic super...
221. AAHF01000016\_3 Aspergillus fumigatus Af293, whole genome shotgun sequenc...
222. CH408034\_2 Chaetomium globosum CBS 148.51 scaffold\_6 genomic scaffold, w...
223. AM920430\_0 Penicillium chrysogenum Wisconsin 54-1255 complete genome, co...
224. ABDF02000085\_1 Trichoderma virens Gv29-8, whole genome shotgun sequencin...
225. EQ962655\_2 Talaromyces stipitatus ATCC 10500 scf\_1105507295555 genomic s...
226. CP003005\_1 Myceliophthora thermophila ATCC 42464 chromosome 4, complete ...
227. DS995901\_2 Penicillium marneffei ATCC 18224 scf\_1105668340960 genomic sc...
228. DF126458\_3 Aspergillus kawachii IFO 4308 DNA, contig: scaffold00012, who...
229. GL988043\_0 Chaetomium thermophilum var. thermophilum DSM 1495 unplaced g...
230. DS572813\_3 Paracoccidioides brasiliensis Pb01 supercont1.3 genomic scaff...
231. ACJE01000002\_0 Aspergillus niger ATCC 1015, whole genome shotgun sequenc...
232. DS027690\_2 Neosartorya fischeri NRRL 181 1099437636252 genomic scaffold,...
233. DS989822\_0 Arthroderma gypseum CBS 118893 supercont1.1 genomic scaffold,...
234. DS499596\_1 Aspergillus fumigatus A1163 scf\_000003 genomic scaffold, whol...
235. AAHF01000002\_1 Aspergillus fumigatus Af293, whole genome shotgun sequenc...
236. DS027056\_0 Aspergillus clavatus NRRL 1 1099423829802 genomic scaffold, w...
237. GL636488\_0 Coccidioides posadasii str. Silveira unplaced genomic scaffol...
238. GG698485\_0 Trichophyton tonsurans CBS 112818 genomic scaffold supercont1...
239. DS995701\_0 Microsporum canis CBS 113480 supercont1.1 genomic scaffold, w...
240. ACFW01000030\_0 Coccidioides posadasii C735 delta SOWgp, whole genome sho...
241. KB644412\_0 Penicillium oxalicum 114-2 unplaced genomic scaffold scaffold...
242. GG700648\_0 Trichophyton rubrum CBS 118892 genomic scaffold supercont2.1,...
243. AACD01000084\_0 Aspergillus nidulans FGSC A4, whole genome shotgun sequen...
244. DF126452\_0 Aspergillus kawachii IFO 4308 DNA, contig: scaffold00006, who...
245. ABSU01000003\_0 Arthroderma benhamiae CBS 112371, whole genome shotgun se...
246. AM270368\_0 Aspergillus niger contig An16c0160, genomic contig.
247. ACJE01000013\_0 Aspergillus niger ATCC 1015, whole genome shotgun sequenc...
248. GG749418\_0 Ajellomyces dermatitidis ATCC 18188 genomic scaffold supercon...
249. KB445649\_7 Cochliobolus sativus ND90Pr unplaced genomic scaffold COCSAsc...
250. EQ999973\_0 Ajellomyces dermatitidis ER-3 genomic scaffold supercont1.1, ...

Query: Architecture Search FASTA input

AGUE01000255 : Glarea lozoyensis 74030    Total score: 2.0     Cumulative Blast bit score: 944

Hit cluster cross-links:

Mycgr3G90785 Mycgr3T
  
Location: 0-1047

Mycgr3G90785\_Mycgr3T

Mycgr3G103262 Mycgr3
  
Location: 1147-1390

Mycgr3G103262\_Mycgr3

Mycgr3G68458 Mycgr3T
  
Location: 1490-3602

Mycgr3G68458\_Mycgr3T

Mycgr3G99145 Mycgr3T
  
Location: 3702-4326

Mycgr3G99145\_Mycgr3T

Mycgr3G103274 Mycgr3
  
Location: 4426-4957

Mycgr3G103274\_Mycgr3

Mycgr3G103264 Mycgr3
  
Location: 5057-5390

Mycgr3G103264\_Mycgr3

Mycgr3G37570 Mycgr3T
  
Location: 5490-6006

Mycgr3G37570\_Mycgr3T

Mycgr3G108094 Mycgr3
  
Location: 6106-10555

Mycgr3G108094\_Mycgr3

Mycgr3G90786 Mycgr3T
  
Location: 10655-12080

Mycgr3G90786\_Mycgr3T

Mycgr3G68429 Mycgr3T
  
Location: 12180-13440

Mycgr3G68429\_Mycgr3T

Mycgr3G68421 Mycgr3T
  
Location: 13540-17086

Mycgr3G68421\_Mycgr3T

Mycgr3G90801 Mycgr3T
  
Location: 17186-18056

Mycgr3G90801\_Mycgr3T

Mycgr3G84646 Mycgr3T
  
Location: 18156-20235

Mycgr3G84646\_Mycgr3T

Mycgr3G68456 Mycgr3T
  
Location: 20335-21970

Mycgr3G68456\_Mycgr3T

Mycgr3G103270 Mycgr3
  
Location: 22070-22355

Mycgr3G103270\_Mycgr3

Mycgr3G90803 Mycgr3T
  
Location: 22455-23019

Mycgr3G90803\_Mycgr3T

Mycgr3G36941 Mycgr3T
  
Location: 23119-24064

Mycgr3G36941\_Mycgr3T

Mycgr3G25746 Mycgr3T
  
Location: 24164-25241

Mycgr3G25746\_Mycgr3T

Mycgr3G90788 Mycgr3T
  
Location: 25341-25803

Mycgr3G90788\_Mycgr3T

Mycgr3G103260 Mycgr3
  
Location: 25903-26635

Mycgr3G103260\_Mycgr3

Mycgr3G84644 Mycgr3T
  
Location: 26735-28457

Mycgr3G84644\_Mycgr3T

Mycgr3G29227 Mycgr3T
  
Location: 28557-28863

Mycgr3G29227\_Mycgr3T

Mycgr3G36271 Mycgr3T
  
Location: 28963-29854

Mycgr3G36271\_Mycgr3T

Mycgr3G68433 Mycgr3T
  
Location: 29954-33041

Mycgr3G68433\_Mycgr3T

Mycgr3G79452 Mycgr3T
  
Location: 33141-33399

Mycgr3G79452\_Mycgr3T

Mycgr3G55345 Mycgr3T
  
Location: 33499-34126

Mycgr3G55345\_Mycgr3T

Mycgr3G103278 Mycgr3
  
Location: 34226-35195

Mycgr3G103278\_Mycgr3

Mycgr3G84654 Mycgr3T
  
Location: 35295-36630

Mycgr3G84654\_Mycgr3T

Mycgr3G108090 Mycgr3
  
Location: 36730-37591

Mycgr3G108090\_Mycgr3

Mycgr3G21922 Mycgr3T
  
Location: 37691-39149

Mycgr3G21922\_Mycgr3T

Mycgr3G99148 Mycgr3T
  
Location: 39249-42819

Mycgr3G99148\_Mycgr3T

putative Tetracycline resistance protein from transposon/Tn4400
  
Accession: EHK96368
  
Location: 78675-79424
  
 NCBI BlastP on this gene

EHK96368

hypothetical protein
  
Accession: EHK96367
  
Location: 77427-77869
  
 NCBI BlastP on this gene

EHK96367

hypothetical protein
  
Accession: EHK96366
  
Location: 74730-76000
  
 NCBI BlastP on this gene

EHK96366

hypothetical protein
  
Accession: EHK96365
  
Location: 72631-73400
  
 NCBI BlastP on this gene

EHK96365

putative protein lunapark like protein
  
Accession: EHK96364
  
Location: 69800-70719
  
 NCBI BlastP on this gene

EHK96364

putative Golgin IMH1
  
Accession: EHK96363
  
Location: 65358-69143
  
  
**BlastP hit with Mycgr3G108094\_Mycgr3**
  
Percentage identity: 35 %
  
BlastP bit score: 260
  
Sequence coverage: 40 %
  
E-value: 6e-68
  
  
 NCBI BlastP on this gene

EHK96363

putative protein kinase dsk1
  
Accession: EHK96362
  
Location: 61877-64034
  
  
**BlastP hit with Mycgr3G84644\_Mycgr3T**
  
Percentage identity: 64 %
  
BlastP bit score: 684
  
Sequence coverage: 99 %
  
E-value: 0.0
  
  
 NCBI BlastP on this gene

EHK96362

hypothetical protein
  
Accession: EHK96361
  
Location: 59479-59757
  
 NCBI BlastP on this gene

EHK96361

putative Transcription factor IWS1
  
Accession: EHK96360
  
Location: 57580-59009
  
 NCBI BlastP on this gene

EHK96360

hypothetical protein
  
Accession: EHK96359
  
Location: 54121-54525
  
 NCBI BlastP on this gene

EHK96359

hypothetical protein
  
Accession: EHK96358
  
Location: 52663-53930
  
 NCBI BlastP on this gene

EHK96358

putative Laccase-2
  
Accession: EHK96357
  
Location: 47677-49275
  
 NCBI BlastP on this gene

EHK96357

Query: Architecture Search FASTA input

JH725150 : Beauveria bassiana ARSEF 2860 unplaced genomic scaffold BBA\_S00001    Total score: 2.0     Cumulative Blast bit score: 942

Hit cluster cross-links:

Mycgr3G90785 Mycgr3T
  
Location: 0-1047

Mycgr3G90785\_Mycgr3T

Mycgr3G103262 Mycgr3
  
Location: 1147-1390

Mycgr3G103262\_Mycgr3

Mycgr3G68458 Mycgr3T
  
Location: 1490-3602

Mycgr3G68458\_Mycgr3T

Mycgr3G99145 Mycgr3T
  
Location: 3702-4326

Mycgr3G99145\_Mycgr3T

Mycgr3G103274 Mycgr3
  
Location: 4426-4957

Mycgr3G103274\_Mycgr3

Mycgr3G103264 Mycgr3
  
Location: 5057-5390

Mycgr3G103264\_Mycgr3

Mycgr3G37570 Mycgr3T
  
Location: 5490-6006

Mycgr3G37570\_Mycgr3T

Mycgr3G108094 Mycgr3
  
Location: 6106-10555

Mycgr3G108094\_Mycgr3

Mycgr3G90786 Mycgr3T
  
Location: 10655-12080

Mycgr3G90786\_Mycgr3T

Mycgr3G68429 Mycgr3T
  
Location: 12180-13440

Mycgr3G68429\_Mycgr3T

Mycgr3G68421 Mycgr3T
  
Location: 13540-17086

Mycgr3G68421\_Mycgr3T

Mycgr3G90801 Mycgr3T
  
Location: 17186-18056

Mycgr3G90801\_Mycgr3T

Mycgr3G84646 Mycgr3T
  
Location: 18156-20235

Mycgr3G84646\_Mycgr3T

Mycgr3G68456 Mycgr3T
  
Location: 20335-21970

Mycgr3G68456\_Mycgr3T

Mycgr3G103270 Mycgr3
  
Location: 22070-22355

Mycgr3G103270\_Mycgr3

Mycgr3G90803 Mycgr3T
  
Location: 22455-23019

Mycgr3G90803\_Mycgr3T

Mycgr3G36941 Mycgr3T
  
Location: 23119-24064

Mycgr3G36941\_Mycgr3T

Mycgr3G25746 Mycgr3T
  
Location: 24164-25241

Mycgr3G25746\_Mycgr3T

Mycgr3G90788 Mycgr3T
  
Location: 25341-25803

Mycgr3G90788\_Mycgr3T

Mycgr3G103260 Mycgr3
  
Location: 25903-26635

Mycgr3G103260\_Mycgr3

Mycgr3G84644 Mycgr3T
  
Location: 26735-28457

Mycgr3G84644\_Mycgr3T

Mycgr3G29227 Mycgr3T
  
Location: 28557-28863

Mycgr3G29227\_Mycgr3T

Mycgr3G36271 Mycgr3T
  
Location: 28963-29854

Mycgr3G36271\_Mycgr3T

Mycgr3G68433 Mycgr3T
  
Location: 29954-33041

Mycgr3G68433\_Mycgr3T

Mycgr3G79452 Mycgr3T
  
Location: 33141-33399

Mycgr3G79452\_Mycgr3T

Mycgr3G55345 Mycgr3T
  
Location: 33499-34126

Mycgr3G55345\_Mycgr3T

Mycgr3G103278 Mycgr3
  
Location: 34226-35195

Mycgr3G103278\_Mycgr3

Mycgr3G84654 Mycgr3T
  
Location: 35295-36630

Mycgr3G84654\_Mycgr3T

Mycgr3G108090 Mycgr3
  
Location: 36730-37591

Mycgr3G108090\_Mycgr3

Mycgr3G21922 Mycgr3T
  
Location: 37691-39149

Mycgr3G21922\_Mycgr3T

Mycgr3G99148 Mycgr3T
  
Location: 39249-42819

Mycgr3G99148\_Mycgr3T

hypothetical protein
  
Accession: EJP70847
  
Location: 1452114-1453324
  
 NCBI BlastP on this gene

EJP70847

calcineurin-like phosphoesterase
  
Accession: EJP70848
  
Location: 1453856-1455256
  
 NCBI BlastP on this gene

EJP70848

exocyst complex component EXO84
  
Accession: EJP70849
  
Location: 1456377-1458506
  
 NCBI BlastP on this gene

EJP70849

hypothetical protein
  
Accession: EJP70850
  
Location: 1460053-1460914
  
 NCBI BlastP on this gene

EJP70850

MAS20 protein import receptor
  
Accession: EJP70851
  
Location: 1461919-1462718
  
 NCBI BlastP on this gene

EJP70851

wyosine base formation
  
Accession: EJP70852
  
Location: 1463189-1465576
  
 NCBI BlastP on this gene

EJP70852

cysteine dioxygenase
  
Accession: EJP70853
  
Location: 1466438-1467256
  
 NCBI BlastP on this gene

EJP70853

sulfite transporter Ssu2
  
Accession: EJP70854
  
Location: 1469300-1470938
  
 NCBI BlastP on this gene

EJP70854

ATP synthase regulation protein NCA2
  
Accession: EJP70855
  
Location: 1471287-1473354
  
  
**BlastP hit with Mycgr3G84646\_Mycgr3T**
  
Percentage identity: 40 %
  
BlastP bit score: 504
  
Sequence coverage: 102 %
  
E-value: 3e-166
  
  
 NCBI BlastP on this gene

EJP70855

fatty acid hydroxylase superfamily protein
  
Accession: EJP70856
  
Location: 1474564-1475617
  
  
**BlastP hit with Mycgr3G36271\_Mycgr3T**
  
Percentage identity: 70 %
  
BlastP bit score: 439
  
Sequence coverage: 95 %
  
E-value: 5e-152
  
  
 NCBI BlastP on this gene

EJP70856

pterin 4 alpha carbinolamine dehydratase
  
Accession: EJP70857
  
Location: 1476030-1476743
  
 NCBI BlastP on this gene

EJP70857

bZIP transcription factor
  
Accession: EJP70858
  
Location: 1478299-1479656
  
 NCBI BlastP on this gene

EJP70858

Actin-like protein, ARP5 class
  
Accession: EJP70859
  
Location: 1480301-1482774
  
 NCBI BlastP on this gene

EJP70859

DASH complex subunit Dad4
  
Accession: EJP70860
  
Location: 1483195-1483705
  
 NCBI BlastP on this gene

EJP70860

chromatin-remodeling complex subunit ies6
  
Accession: EJP70861
  
Location: 1483875-1484468
  
 NCBI BlastP on this gene

EJP70861

Got1 family protein
  
Accession: EJP70862
  
Location: 1485493-1486254
  
 NCBI BlastP on this gene

EJP70862

profilin-like protein
  
Accession: EJP70863
  
Location: 1486986-1487792
  
 NCBI BlastP on this gene

EJP70863

hypothetical protein
  
Accession: EJP70864
  
Location: 1491044-1492639
  
 NCBI BlastP on this gene

EJP70864

SNARE protein, putative
  
Accession: EJP70865
  
Location: 1493479-1494462
  
 NCBI BlastP on this gene

EJP70865

Query: Architecture Search FASTA input

DS985219 : Verticillium albo-atrum VaMs.102 supercont1.6 genomic scaffold    Total score: 2.0     Cumulative Blast bit score: 942

Hit cluster cross-links:

Mycgr3G90785 Mycgr3T
  
Location: 0-1047

Mycgr3G90785\_Mycgr3T

Mycgr3G103262 Mycgr3
  
Location: 1147-1390

Mycgr3G103262\_Mycgr3

Mycgr3G68458 Mycgr3T
  
Location: 1490-3602

Mycgr3G68458\_Mycgr3T

Mycgr3G99145 Mycgr3T
  
Location: 3702-4326

Mycgr3G99145\_Mycgr3T

Mycgr3G103274 Mycgr3
  
Location: 4426-4957

Mycgr3G103274\_Mycgr3

Mycgr3G103264 Mycgr3
  
Location: 5057-5390

Mycgr3G103264\_Mycgr3

Mycgr3G37570 Mycgr3T
  
Location: 5490-6006

Mycgr3G37570\_Mycgr3T

Mycgr3G108094 Mycgr3
  
Location: 6106-10555

Mycgr3G108094\_Mycgr3

Mycgr3G90786 Mycgr3T
  
Location: 10655-12080

Mycgr3G90786\_Mycgr3T

Mycgr3G68429 Mycgr3T
  
Location: 12180-13440

Mycgr3G68429\_Mycgr3T

Mycgr3G68421 Mycgr3T
  
Location: 13540-17086

Mycgr3G68421\_Mycgr3T

Mycgr3G90801 Mycgr3T
  
Location: 17186-18056

Mycgr3G90801\_Mycgr3T

Mycgr3G84646 Mycgr3T
  
Location: 18156-20235

Mycgr3G84646\_Mycgr3T

Mycgr3G68456 Mycgr3T
  
Location: 20335-21970

Mycgr3G68456\_Mycgr3T

Mycgr3G103270 Mycgr3
  
Location: 22070-22355

Mycgr3G103270\_Mycgr3

Mycgr3G90803 Mycgr3T
  
Location: 22455-23019

Mycgr3G90803\_Mycgr3T

Mycgr3G36941 Mycgr3T
  
Location: 23119-24064

Mycgr3G36941\_Mycgr3T

Mycgr3G25746 Mycgr3T
  
Location: 24164-25241

Mycgr3G25746\_Mycgr3T

Mycgr3G90788 Mycgr3T
  
Location: 25341-25803

Mycgr3G90788\_Mycgr3T

Mycgr3G103260 Mycgr3
  
Location: 25903-26635

Mycgr3G103260\_Mycgr3

Mycgr3G84644 Mycgr3T
  
Location: 26735-28457

Mycgr3G84644\_Mycgr3T

Mycgr3G29227 Mycgr3T
  
Location: 28557-28863

Mycgr3G29227\_Mycgr3T

Mycgr3G36271 Mycgr3T
  
Location: 28963-29854

Mycgr3G36271\_Mycgr3T

Mycgr3G68433 Mycgr3T
  
Location: 29954-33041

Mycgr3G68433\_Mycgr3T

Mycgr3G79452 Mycgr3T
  
Location: 33141-33399

Mycgr3G79452\_Mycgr3T

Mycgr3G55345 Mycgr3T
  
Location: 33499-34126

Mycgr3G55345\_Mycgr3T

Mycgr3G103278 Mycgr3
  
Location: 34226-35195

Mycgr3G103278\_Mycgr3

Mycgr3G84654 Mycgr3T
  
Location: 35295-36630

Mycgr3G84654\_Mycgr3T

Mycgr3G108090 Mycgr3
  
Location: 36730-37591

Mycgr3G108090\_Mycgr3

Mycgr3G21922 Mycgr3T
  
Location: 37691-39149

Mycgr3G21922\_Mycgr3T

Mycgr3G99148 Mycgr3T
  
Location: 39249-42819

Mycgr3G99148\_Mycgr3T

conserved hypothetical protein
  
Accession: EEY19063
  
Location: 281540-281804
  
 NCBI BlastP on this gene

EEY19063

Got1 family protein
  
Accession: EEY19064
  
Location: 282416-283116
  
 NCBI BlastP on this gene

EEY19064

conserved hypothetical protein
  
Accession: EEY19065
  
Location: 284341-284937
  
 NCBI BlastP on this gene

EEY19065

hypothetical protein
  
Accession: EEY19066
  
Location: 285203-285639
  
 NCBI BlastP on this gene

EEY19066

conserved hypothetical protein
  
Accession: EEY19067
  
Location: 287110-289517
  
 NCBI BlastP on this gene

EEY19067

hypothetical protein
  
Accession: EEY19068
  
Location: 290900-291365
  
 NCBI BlastP on this gene

EEY19068

conserved hypothetical protein
  
Accession: EEY19069
  
Location: 296215-296768
  
 NCBI BlastP on this gene

EEY19069

C-4 methylsterol oxidase
  
Accession: EEY19070
  
Location: 297455-298540
  
  
**BlastP hit with Mycgr3G36271\_Mycgr3T**
  
Percentage identity: 71 %
  
BlastP bit score: 456
  
Sequence coverage: 97 %
  
E-value: 2e-158
  
  
 NCBI BlastP on this gene

EEY19070

nuclear control of ATPase protein
  
Accession: EEY19071
  
Location: 300277-302523
  
  
**BlastP hit with Mycgr3G84646\_Mycgr3T**
  
Percentage identity: 42 %
  
BlastP bit score: 486
  
Sequence coverage: 87 %
  
E-value: 4e-159
  
  
 NCBI BlastP on this gene

EEY19071

NADP-dependent mannitol dehydrogenase
  
Accession: EEY19072
  
Location: 303225-304651
  
 NCBI BlastP on this gene

EEY19072

40S ribosomal protein S10-B
  
Accession: EEY19073
  
Location: 308481-309318
  
 NCBI BlastP on this gene

EEY19073

conserved hypothetical protein
  
Accession: EEY19074
  
Location: 309913-310779
  
 NCBI BlastP on this gene

EEY19074

cytochrome c oxidase polypeptide vib
  
Accession: EEY19075
  
Location: 311070-311627
  
 NCBI BlastP on this gene

EEY19075

hypothetical protein
  
Accession: EEY19076
  
Location: 311951-312468
  
 NCBI BlastP on this gene

EEY19076

peptidyl-prolyl cis-trans isomerase E
  
Accession: EEY19077
  
Location: 312732-313246
  
 NCBI BlastP on this gene

EEY19077

transport protein SEC31
  
Accession: EEY19078
  
Location: 313819-317712
  
 NCBI BlastP on this gene

EEY19078

conserved hypothetical protein
  
Accession: EEY19079
  
Location: 318159-319291
  
 NCBI BlastP on this gene

EEY19079

conserved hypothetical protein
  
Accession: EEY19080
  
Location: 320875-321851
  
 NCBI BlastP on this gene

EEY19080

Query: Architecture Search FASTA input

KE145373 : Glarea lozoyensis ATCC 20868 chromosome Unknown GLAREA9    Total score: 2.0     Cumulative Blast bit score: 936

Hit cluster cross-links:

Mycgr3G90785 Mycgr3T
  
Location: 0-1047

Mycgr3G90785\_Mycgr3T

Mycgr3G103262 Mycgr3
  
Location: 1147-1390

Mycgr3G103262\_Mycgr3

Mycgr3G68458 Mycgr3T
  
Location: 1490-3602

Mycgr3G68458\_Mycgr3T

Mycgr3G99145 Mycgr3T
  
Location: 3702-4326

Mycgr3G99145\_Mycgr3T

Mycgr3G103274 Mycgr3
  
Location: 4426-4957

Mycgr3G103274\_Mycgr3

Mycgr3G103264 Mycgr3
  
Location: 5057-5390

Mycgr3G103264\_Mycgr3

Mycgr3G37570 Mycgr3T
  
Location: 5490-6006

Mycgr3G37570\_Mycgr3T

Mycgr3G108094 Mycgr3
  
Location: 6106-10555

Mycgr3G108094\_Mycgr3

Mycgr3G90786 Mycgr3T
  
Location: 10655-12080

Mycgr3G90786\_Mycgr3T

Mycgr3G68429 Mycgr3T
  
Location: 12180-13440

Mycgr3G68429\_Mycgr3T

Mycgr3G68421 Mycgr3T
  
Location: 13540-17086

Mycgr3G68421\_Mycgr3T

Mycgr3G90801 Mycgr3T
  
Location: 17186-18056

Mycgr3G90801\_Mycgr3T

Mycgr3G84646 Mycgr3T
  
Location: 18156-20235

Mycgr3G84646\_Mycgr3T

Mycgr3G68456 Mycgr3T
  
Location: 20335-21970

Mycgr3G68456\_Mycgr3T

Mycgr3G103270 Mycgr3
  
Location: 22070-22355

Mycgr3G103270\_Mycgr3

Mycgr3G90803 Mycgr3T
  
Location: 22455-23019

Mycgr3G90803\_Mycgr3T

Mycgr3G36941 Mycgr3T
  
Location: 23119-24064

Mycgr3G36941\_Mycgr3T

Mycgr3G25746 Mycgr3T
  
Location: 24164-25241

Mycgr3G25746\_Mycgr3T

Mycgr3G90788 Mycgr3T
  
Location: 25341-25803

Mycgr3G90788\_Mycgr3T

Mycgr3G103260 Mycgr3
  
Location: 25903-26635

Mycgr3G103260\_Mycgr3

Mycgr3G84644 Mycgr3T
  
Location: 26735-28457

Mycgr3G84644\_Mycgr3T

Mycgr3G29227 Mycgr3T
  
Location: 28557-28863

Mycgr3G29227\_Mycgr3T

Mycgr3G36271 Mycgr3T
  
Location: 28963-29854

Mycgr3G36271\_Mycgr3T

Mycgr3G68433 Mycgr3T
  
Location: 29954-33041

Mycgr3G68433\_Mycgr3T

Mycgr3G79452 Mycgr3T
  
Location: 33141-33399

Mycgr3G79452\_Mycgr3T

Mycgr3G55345 Mycgr3T
  
Location: 33499-34126

Mycgr3G55345\_Mycgr3T

Mycgr3G103278 Mycgr3
  
Location: 34226-35195

Mycgr3G103278\_Mycgr3

Mycgr3G84654 Mycgr3T
  
Location: 35295-36630

Mycgr3G84654\_Mycgr3T

Mycgr3G108090 Mycgr3
  
Location: 36730-37591

Mycgr3G108090\_Mycgr3

Mycgr3G21922 Mycgr3T
  
Location: 37691-39149

Mycgr3G21922\_Mycgr3T

Mycgr3G99148 Mycgr3T
  
Location: 39249-42819

Mycgr3G99148\_Mycgr3T

MFS general substrate transporter
  
Accession: EPE24482
  
Location: 1188115-1190598
  
 NCBI BlastP on this gene

EPE24482

hypothetical protein
  
Accession: EPE24483
  
Location: 1191291-1192791
  
 NCBI BlastP on this gene

EPE24483

NAD(P)-binding Rossmann-fold containing protein
  
Accession: EPE24484
  
Location: 1193263-1194428
  
 NCBI BlastP on this gene

EPE24484

Metallo-dependent hydrolase
  
Accession: EPE24485
  
Location: 1194909-1196381
  
 NCBI BlastP on this gene

EPE24485

FabD/lysophospholipase-like protein
  
Accession: EPE24486
  
Location: 1197038-1201805
  
 NCBI BlastP on this gene

EPE24486

eIF-2-alpha, C-terminal
  
Accession: EPE24487
  
Location: 1202337-1203419
  
 NCBI BlastP on this gene

EPE24487

LexA/Signal peptidase
  
Accession: EPE24488
  
Location: 1203743-1204383
  
 NCBI BlastP on this gene

EPE24488

Mitochondrial carrier
  
Accession: EPE24489
  
Location: 1205000-1206321
  
 NCBI BlastP on this gene

EPE24489

hypothetical protein
  
Accession: EPE24490
  
Location: 1206887-1209952
  
 NCBI BlastP on this gene

EPE24490

hypothetical protein
  
Accession: EPE24491
  
Location: 1210439-1212494
  
  
**BlastP hit with Mycgr3G84646\_Mycgr3T**
  
Percentage identity: 45 %
  
BlastP bit score: 581
  
Sequence coverage: 101 %
  
E-value: 0.0
  
  
 NCBI BlastP on this gene

EPE24491

P-loop containing nucleoside triphosphate hydrolase
  
Accession: EPE24492
  
Location: 1213161-1213892
  
  
**BlastP hit with Mycgr3G99145\_Mycgr3T**
  
Percentage identity: 90 %
  
BlastP bit score: 355
  
Sequence coverage: 92 %
  
E-value: 8e-122
  
  
 NCBI BlastP on this gene

EPE24492

hypothetical protein
  
Accession: EPE24493
  
Location: 1214139-1216048
  
 NCBI BlastP on this gene

EPE24493

P-loop containing nucleoside triphosphate hydrolase
  
Accession: EPE24494
  
Location: 1216276-1218847
  
 NCBI BlastP on this gene

EPE24494

Nucleotide-diphospho-sugar transferase
  
Accession: EPE24495
  
Location: 1219360-1220620
  
 NCBI BlastP on this gene

EPE24495

ARM repeat-containing protein
  
Accession: EPE24496
  
Location: 1221547-1224507
  
 NCBI BlastP on this gene

EPE24496

hypothetical protein
  
Accession: EPE24497
  
Location: 1228687-1229595
  
 NCBI BlastP on this gene

EPE24497

Query: Architecture Search FASTA input

DS572718 : Verticillium dahliae VdLs.17 supercont1.24 genomic scaffold    Total score: 2.0     Cumulative Blast bit score: 936

Hit cluster cross-links:

Mycgr3G90785 Mycgr3T
  
Location: 0-1047

Mycgr3G90785\_Mycgr3T

Mycgr3G103262 Mycgr3
  
Location: 1147-1390

Mycgr3G103262\_Mycgr3

Mycgr3G68458 Mycgr3T
  
Location: 1490-3602

Mycgr3G68458\_Mycgr3T

Mycgr3G99145 Mycgr3T
  
Location: 3702-4326

Mycgr3G99145\_Mycgr3T

Mycgr3G103274 Mycgr3
  
Location: 4426-4957

Mycgr3G103274\_Mycgr3

Mycgr3G103264 Mycgr3
  
Location: 5057-5390

Mycgr3G103264\_Mycgr3

Mycgr3G37570 Mycgr3T
  
Location: 5490-6006

Mycgr3G37570\_Mycgr3T

Mycgr3G108094 Mycgr3
  
Location: 6106-10555

Mycgr3G108094\_Mycgr3

Mycgr3G90786 Mycgr3T
  
Location: 10655-12080

Mycgr3G90786\_Mycgr3T

Mycgr3G68429 Mycgr3T
  
Location: 12180-13440

Mycgr3G68429\_Mycgr3T

Mycgr3G68421 Mycgr3T
  
Location: 13540-17086

Mycgr3G68421\_Mycgr3T

Mycgr3G90801 Mycgr3T
  
Location: 17186-18056

Mycgr3G90801\_Mycgr3T

Mycgr3G84646 Mycgr3T
  
Location: 18156-20235

Mycgr3G84646\_Mycgr3T

Mycgr3G68456 Mycgr3T
  
Location: 20335-21970

Mycgr3G68456\_Mycgr3T

Mycgr3G103270 Mycgr3
  
Location: 22070-22355

Mycgr3G103270\_Mycgr3

Mycgr3G90803 Mycgr3T
  
Location: 22455-23019

Mycgr3G90803\_Mycgr3T

Mycgr3G36941 Mycgr3T
  
Location: 23119-24064

Mycgr3G36941\_Mycgr3T

Mycgr3G25746 Mycgr3T
  
Location: 24164-25241

Mycgr3G25746\_Mycgr3T

Mycgr3G90788 Mycgr3T
  
Location: 25341-25803

Mycgr3G90788\_Mycgr3T

Mycgr3G103260 Mycgr3
  
Location: 25903-26635

Mycgr3G103260\_Mycgr3

Mycgr3G84644 Mycgr3T
  
Location: 26735-28457

Mycgr3G84644\_Mycgr3T

Mycgr3G29227 Mycgr3T
  
Location: 28557-28863

Mycgr3G29227\_Mycgr3T

Mycgr3G36271 Mycgr3T
  
Location: 28963-29854

Mycgr3G36271\_Mycgr3T

Mycgr3G68433 Mycgr3T
  
Location: 29954-33041

Mycgr3G68433\_Mycgr3T

Mycgr3G79452 Mycgr3T
  
Location: 33141-33399

Mycgr3G79452\_Mycgr3T

Mycgr3G55345 Mycgr3T
  
Location: 33499-34126

Mycgr3G55345\_Mycgr3T

Mycgr3G103278 Mycgr3
  
Location: 34226-35195

Mycgr3G103278\_Mycgr3

Mycgr3G84654 Mycgr3T
  
Location: 35295-36630

Mycgr3G84654\_Mycgr3T

Mycgr3G108090 Mycgr3
  
Location: 36730-37591

Mycgr3G108090\_Mycgr3

Mycgr3G21922 Mycgr3T
  
Location: 37691-39149

Mycgr3G21922\_Mycgr3T

Mycgr3G99148 Mycgr3T
  
Location: 39249-42819

Mycgr3G99148\_Mycgr3T

hypothetical protein
  
Accession: EGY18760
  
Location: 166788-168601
  
 NCBI BlastP on this gene

EGY18760

hypothetical protein
  
Accession: EGY18761
  
Location: 169495-170564
  
 NCBI BlastP on this gene

EGY18761

transport protein SEC31
  
Accession: EGY18762
  
Location: 171007-171715
  
 NCBI BlastP on this gene

EGY18762

transport protein SEC31
  
Accession: EGY18763
  
Location: 174103-174893
  
 NCBI BlastP on this gene

EGY18763

peptidyl-prolyl cis-trans isomerase E
  
Accession: EGY18764
  
Location: 175487-176001
  
 NCBI BlastP on this gene

EGY18764

hypothetical protein
  
Accession: EGY18765
  
Location: 176263-176781
  
 NCBI BlastP on this gene

EGY18765

cytochrome c oxidase polypeptide VIb
  
Accession: EGY18766
  
Location: 177107-177658
  
 NCBI BlastP on this gene

EGY18766

hypothetical protein
  
Accession: EGY18767
  
Location: 177950-178816
  
 NCBI BlastP on this gene

EGY18767

40S ribosomal protein S10-A
  
Accession: EGY18768
  
Location: 179411-180187
  
 NCBI BlastP on this gene

EGY18768

L-threonine 3-dehydrogenase
  
Accession: EGY18769
  
Location: 183567-184879
  
 NCBI BlastP on this gene

EGY18769

nuclear control of ATPase protein
  
Accession: EGY18770
  
Location: 185519-187739
  
  
**BlastP hit with Mycgr3G84646\_Mycgr3T**
  
Percentage identity: 42 %
  
BlastP bit score: 479
  
Sequence coverage: 88 %
  
E-value: 3e-156
  
  
 NCBI BlastP on this gene

EGY18770

C-4 methylsterol oxidase
  
Accession: EGY18771
  
Location: 189418-190500
  
  
**BlastP hit with Mycgr3G36271\_Mycgr3T**
  
Percentage identity: 71 %
  
BlastP bit score: 457
  
Sequence coverage: 97 %
  
E-value: 7e-159
  
  
 NCBI BlastP on this gene

EGY18771

hypothetical protein
  
Accession: EGY18772
  
Location: 190957-191593
  
 NCBI BlastP on this gene

EGY18772

hypothetical protein
  
Accession: EGY18773
  
Location: 197610-198532
  
 NCBI BlastP on this gene

EGY18773

hypothetical protein
  
Accession: EGY18774
  
Location: 198935-199474
  
 NCBI BlastP on this gene

EGY18774

hypothetical protein
  
Accession: EGY18775
  
Location: 201511-203903
  
 NCBI BlastP on this gene

EGY18775

hypothetical protein
  
Accession: EGY18776
  
Location: 205194-205631
  
 NCBI BlastP on this gene

EGY18776

hypothetical protein
  
Accession: EGY18777
  
Location: 205889-206485
  
 NCBI BlastP on this gene

EGY18777

Got1 family protein
  
Accession: EGY18778
  
Location: 207739-208449
  
 NCBI BlastP on this gene

EGY18778

profilin
  
Accession: EGY18779
  
Location: 209059-210466
  
 NCBI BlastP on this gene

EGY18779

Query: Architecture Search FASTA input

CH476643 : Sclerotinia sclerotiorum 1980 scaffold\_23 genomic scaffold    Total score: 2.0     Cumulative Blast bit score: 931

Hit cluster cross-links:

Mycgr3G90785 Mycgr3T
  
Location: 0-1047

Mycgr3G90785\_Mycgr3T

Mycgr3G103262 Mycgr3
  
Location: 1147-1390

Mycgr3G103262\_Mycgr3

Mycgr3G68458 Mycgr3T
  
Location: 1490-3602

Mycgr3G68458\_Mycgr3T

Mycgr3G99145 Mycgr3T
  
Location: 3702-4326

Mycgr3G99145\_Mycgr3T

Mycgr3G103274 Mycgr3
  
Location: 4426-4957

Mycgr3G103274\_Mycgr3

Mycgr3G103264 Mycgr3
  
Location: 5057-5390

Mycgr3G103264\_Mycgr3

Mycgr3G37570 Mycgr3T
  
Location: 5490-6006

Mycgr3G37570\_Mycgr3T

Mycgr3G108094 Mycgr3
  
Location: 6106-10555

Mycgr3G108094\_Mycgr3

Mycgr3G90786 Mycgr3T
  
Location: 10655-12080

Mycgr3G90786\_Mycgr3T

Mycgr3G68429 Mycgr3T
  
Location: 12180-13440

Mycgr3G68429\_Mycgr3T

Mycgr3G68421 Mycgr3T
  
Location: 13540-17086

Mycgr3G68421\_Mycgr3T

Mycgr3G90801 Mycgr3T
  
Location: 17186-18056

Mycgr3G90801\_Mycgr3T

Mycgr3G84646 Mycgr3T
  
Location: 18156-20235

Mycgr3G84646\_Mycgr3T

Mycgr3G68456 Mycgr3T
  
Location: 20335-21970

Mycgr3G68456\_Mycgr3T

Mycgr3G103270 Mycgr3
  
Location: 22070-22355

Mycgr3G103270\_Mycgr3

Mycgr3G90803 Mycgr3T
  
Location: 22455-23019

Mycgr3G90803\_Mycgr3T

Mycgr3G36941 Mycgr3T
  
Location: 23119-24064

Mycgr3G36941\_Mycgr3T

Mycgr3G25746 Mycgr3T
  
Location: 24164-25241

Mycgr3G25746\_Mycgr3T

Mycgr3G90788 Mycgr3T
  
Location: 25341-25803

Mycgr3G90788\_Mycgr3T

Mycgr3G103260 Mycgr3
  
Location: 25903-26635

Mycgr3G103260\_Mycgr3

Mycgr3G84644 Mycgr3T
  
Location: 26735-28457

Mycgr3G84644\_Mycgr3T

Mycgr3G29227 Mycgr3T
  
Location: 28557-28863

Mycgr3G29227\_Mycgr3T

Mycgr3G36271 Mycgr3T
  
Location: 28963-29854

Mycgr3G36271\_Mycgr3T

Mycgr3G68433 Mycgr3T
  
Location: 29954-33041

Mycgr3G68433\_Mycgr3T

Mycgr3G79452 Mycgr3T
  
Location: 33141-33399

Mycgr3G79452\_Mycgr3T

Mycgr3G55345 Mycgr3T
  
Location: 33499-34126

Mycgr3G55345\_Mycgr3T

Mycgr3G103278 Mycgr3
  
Location: 34226-35195

Mycgr3G103278\_Mycgr3

Mycgr3G84654 Mycgr3T
  
Location: 35295-36630

Mycgr3G84654\_Mycgr3T

Mycgr3G108090 Mycgr3
  
Location: 36730-37591

Mycgr3G108090\_Mycgr3

Mycgr3G21922 Mycgr3T
  
Location: 37691-39149

Mycgr3G21922\_Mycgr3T

Mycgr3G99148 Mycgr3T
  
Location: 39249-42819

Mycgr3G99148\_Mycgr3T

hypothetical protein
  
Accession: EDN98214
  
Location: 341846-347770
  
 NCBI BlastP on this gene

EDN98214

hypothetical protein
  
Accession: EDN98213
  
Location: 340713-341526
  
 NCBI BlastP on this gene

EDN98213

hypothetical protein
  
Accession: EDN98212
  
Location: 340028-340276
  
 NCBI BlastP on this gene

EDN98212

predicted protein
  
Accession: EDN98211
  
Location: 339553-339926
  
 NCBI BlastP on this gene

EDN98211

hypothetical protein
  
Accession: EDN98210
  
Location: 338046-338615
  
 NCBI BlastP on this gene

EDN98210

hypothetical protein
  
Accession: EDN98209
  
Location: 334483-337049
  
 NCBI BlastP on this gene

EDN98209

hypothetical protein
  
Accession: EDN98208
  
Location: 332590-333413
  
 NCBI BlastP on this gene

EDN98208

predicted protein
  
Accession: EDN98207
  
Location: 331390-331780
  
 NCBI BlastP on this gene

EDN98207

predicted protein
  
Accession: EDN98206
  
Location: 330893-331105
  
 NCBI BlastP on this gene

EDN98206

predicted protein
  
Accession: EDN98205
  
Location: 329051-330370
  
 NCBI BlastP on this gene

EDN98205

hypothetical protein
  
Accession: EDN98204
  
Location: 324128-326983
  
 NCBI BlastP on this gene

EDN98204

hypothetical protein
  
Accession: EDN98203
  
Location: 321257-323585
  
  
**BlastP hit with Mycgr3G84646\_Mycgr3T**
  
Percentage identity: 44 %
  
BlastP bit score: 580
  
Sequence coverage: 102 %
  
E-value: 0.0
  
  
 NCBI BlastP on this gene

EDN98203

GTP-binding protein SAS1
  
Accession: EDN98202
  
Location: 319222-320215
  
  
**BlastP hit with Mycgr3G99145\_Mycgr3T**
  
Percentage identity: 94 %
  
BlastP bit score: 351
  
Sequence coverage: 85 %
  
E-value: 2e-120
  
  
 NCBI BlastP on this gene

EDN98202

predicted protein
  
Accession: EDN98201
  
Location: 316128-317499
  
 NCBI BlastP on this gene

EDN98201

hypothetical protein
  
Accession: EDN98200
  
Location: 313417-315779
  
 NCBI BlastP on this gene

EDN98200

hypothetical protein
  
Accession: EDN98199
  
Location: 310839-311924
  
 NCBI BlastP on this gene

EDN98199

predicted protein
  
Accession: EDN98198
  
Location: 309010-310415
  
 NCBI BlastP on this gene

EDN98198

predicted protein
  
Accession: EDN98197
  
Location: 307206-307520
  
 NCBI BlastP on this gene

EDN98197

predicted protein
  
Accession: EDN98196
  
Location: 306001-306699
  
 NCBI BlastP on this gene

EDN98196

hypothetical protein
  
Accession: EDN98195
  
Location: 303221-305072
  
 NCBI BlastP on this gene

EDN98195

hypothetical protein
  
Accession: EDN98194
  
Location: 300735-302790
  
 NCBI BlastP on this gene

EDN98194

Query: Architecture Search FASTA input

KB707720 : Botryotinia fuckeliana BcDW1 unplaced genomic scaffold Scaffold\_48    Total score: 2.0     Cumulative Blast bit score: 925

Hit cluster cross-links:

Mycgr3G90785 Mycgr3T
  
Location: 0-1047

Mycgr3G90785\_Mycgr3T

Mycgr3G103262 Mycgr3
  
Location: 1147-1390

Mycgr3G103262\_Mycgr3

Mycgr3G68458 Mycgr3T
  
Location: 1490-3602

Mycgr3G68458\_Mycgr3T

Mycgr3G99145 Mycgr3T
  
Location: 3702-4326

Mycgr3G99145\_Mycgr3T

Mycgr3G103274 Mycgr3
  
Location: 4426-4957

Mycgr3G103274\_Mycgr3

Mycgr3G103264 Mycgr3
  
Location: 5057-5390

Mycgr3G103264\_Mycgr3

Mycgr3G37570 Mycgr3T
  
Location: 5490-6006

Mycgr3G37570\_Mycgr3T

Mycgr3G108094 Mycgr3
  
Location: 6106-10555

Mycgr3G108094\_Mycgr3

Mycgr3G90786 Mycgr3T
  
Location: 10655-12080

Mycgr3G90786\_Mycgr3T

Mycgr3G68429 Mycgr3T
  
Location: 12180-13440

Mycgr3G68429\_Mycgr3T

Mycgr3G68421 Mycgr3T
  
Location: 13540-17086

Mycgr3G68421\_Mycgr3T

Mycgr3G90801 Mycgr3T
  
Location: 17186-18056

Mycgr3G90801\_Mycgr3T

Mycgr3G84646 Mycgr3T
  
Location: 18156-20235

Mycgr3G84646\_Mycgr3T

Mycgr3G68456 Mycgr3T
  
Location: 20335-21970

Mycgr3G68456\_Mycgr3T

Mycgr3G103270 Mycgr3
  
Location: 22070-22355

Mycgr3G103270\_Mycgr3

Mycgr3G90803 Mycgr3T
  
Location: 22455-23019

Mycgr3G90803\_Mycgr3T

Mycgr3G36941 Mycgr3T
  
Location: 23119-24064

Mycgr3G36941\_Mycgr3T

Mycgr3G25746 Mycgr3T
  
Location: 24164-25241

Mycgr3G25746\_Mycgr3T

Mycgr3G90788 Mycgr3T
  
Location: 25341-25803

Mycgr3G90788\_Mycgr3T

Mycgr3G103260 Mycgr3
  
Location: 25903-26635

Mycgr3G103260\_Mycgr3

Mycgr3G84644 Mycgr3T
  
Location: 26735-28457

Mycgr3G84644\_Mycgr3T

Mycgr3G29227 Mycgr3T
  
Location: 28557-28863

Mycgr3G29227\_Mycgr3T

Mycgr3G36271 Mycgr3T
  
Location: 28963-29854

Mycgr3G36271\_Mycgr3T

Mycgr3G68433 Mycgr3T
  
Location: 29954-33041

Mycgr3G68433\_Mycgr3T

Mycgr3G79452 Mycgr3T
  
Location: 33141-33399

Mycgr3G79452\_Mycgr3T

Mycgr3G55345 Mycgr3T
  
Location: 33499-34126

Mycgr3G55345\_Mycgr3T

Mycgr3G103278 Mycgr3
  
Location: 34226-35195

Mycgr3G103278\_Mycgr3

Mycgr3G84654 Mycgr3T
  
Location: 35295-36630

Mycgr3G84654\_Mycgr3T

Mycgr3G108090 Mycgr3
  
Location: 36730-37591

Mycgr3G108090\_Mycgr3

Mycgr3G21922 Mycgr3T
  
Location: 37691-39149

Mycgr3G21922\_Mycgr3T

Mycgr3G99148 Mycgr3T
  
Location: 39249-42819

Mycgr3G99148\_Mycgr3T

putative activating signal cointegrator 1 complex subunit 3 protein
  
Accession: EMR89991
  
Location: 558000-564056
  
 NCBI BlastP on this gene

EMR89991

putative snare ykt6 protein
  
Accession: EMR89990
  
Location: 556889-557703
  
 NCBI BlastP on this gene

EMR89990

putative chromatin-remodeling complex subunit ies6 protein
  
Accession: EMR89989
  
Location: 554219-554788
  
 NCBI BlastP on this gene

EMR89989

putative dash complex subunit dad4 protein
  
Accession: EMR89988
  
Location: 553563-553984
  
 NCBI BlastP on this gene

EMR89988

putative chromatin remodeling complex subunit protein
  
Accession: EMR89987
  
Location: 550606-553199
  
 NCBI BlastP on this gene

EMR89987

putative transmembrane protein
  
Accession: EMR89986
  
Location: 548102-549581
  
 NCBI BlastP on this gene

EMR89986

hypothetical protein
  
Accession: EMR89985
  
Location: 540203-543064
  
 NCBI BlastP on this gene

EMR89985

putative atp synthase regulation protein nca2 protein
  
Accession: EMR89984
  
Location: 537203-539657
  
  
**BlastP hit with Mycgr3G84646\_Mycgr3T**
  
Percentage identity: 44 %
  
BlastP bit score: 570
  
Sequence coverage: 102 %
  
E-value: 0.0
  
  
 NCBI BlastP on this gene

EMR89984

putative rab gtpase protein
  
Accession: EMR89983
  
Location: 534779-535767
  
  
**BlastP hit with Mycgr3G99145\_Mycgr3T**
  
Percentage identity: 91 %
  
BlastP bit score: 355
  
Sequence coverage: 90 %
  
E-value: 8e-122
  
  
 NCBI BlastP on this gene

EMR89983

hypothetical protein
  
Accession: EMR89982
  
Location: 531974-533373
  
 NCBI BlastP on this gene

EMR89982

putative aaa family atpase protein
  
Accession: EMR89981
  
Location: 529202-531683
  
 NCBI BlastP on this gene

EMR89981

putative caleosin domain-containing protein
  
Accession: EMR89980
  
Location: 526996-528257
  
 NCBI BlastP on this gene

EMR89980

putative pterin-4-alpha-carbinolamine dehydratase family protein
  
Accession: EMR89979
  
Location: 525531-526568
  
 NCBI BlastP on this gene

EMR89979

putative aaa family atpase protein
  
Accession: EMR89978
  
Location: 520001-523417
  
 NCBI BlastP on this gene

EMR89978

hypothetical protein
  
Accession: EMR89977
  
Location: 517739-519507
  
 NCBI BlastP on this gene

EMR89977

putative solute carrier family 25 member 38 protein
  
Accession: EMR89976
  
Location: 515779-517130
  
 NCBI BlastP on this gene

EMR89976

Query: Architecture Search FASTA input

FQ790356 : Botryotinia fuckeliana T4 SupSuperContig\_182\_154r\_1 genomic supercontig.    Total score: 2.0     Cumulative Blast bit score: 925

Hit cluster cross-links:

Mycgr3G90785 Mycgr3T
  
Location: 0-1047

Mycgr3G90785\_Mycgr3T

Mycgr3G103262 Mycgr3
  
Location: 1147-1390

Mycgr3G103262\_Mycgr3

Mycgr3G68458 Mycgr3T
  
Location: 1490-3602

Mycgr3G68458\_Mycgr3T

Mycgr3G99145 Mycgr3T
  
Location: 3702-4326

Mycgr3G99145\_Mycgr3T

Mycgr3G103274 Mycgr3
  
Location: 4426-4957

Mycgr3G103274\_Mycgr3

Mycgr3G103264 Mycgr3
  
Location: 5057-5390

Mycgr3G103264\_Mycgr3

Mycgr3G37570 Mycgr3T
  
Location: 5490-6006

Mycgr3G37570\_Mycgr3T

Mycgr3G108094 Mycgr3
  
Location: 6106-10555

Mycgr3G108094\_Mycgr3

Mycgr3G90786 Mycgr3T
  
Location: 10655-12080

Mycgr3G90786\_Mycgr3T

Mycgr3G68429 Mycgr3T
  
Location: 12180-13440

Mycgr3G68429\_Mycgr3T

Mycgr3G68421 Mycgr3T
  
Location: 13540-17086

Mycgr3G68421\_Mycgr3T

Mycgr3G90801 Mycgr3T
  
Location: 17186-18056

Mycgr3G90801\_Mycgr3T

Mycgr3G84646 Mycgr3T
  
Location: 18156-20235

Mycgr3G84646\_Mycgr3T

Mycgr3G68456 Mycgr3T
  
Location: 20335-21970

Mycgr3G68456\_Mycgr3T

Mycgr3G103270 Mycgr3
  
Location: 22070-22355

Mycgr3G103270\_Mycgr3

Mycgr3G90803 Mycgr3T
  
Location: 22455-23019

Mycgr3G90803\_Mycgr3T

Mycgr3G36941 Mycgr3T
  
Location: 23119-24064

Mycgr3G36941\_Mycgr3T

Mycgr3G25746 Mycgr3T
  
Location: 24164-25241

Mycgr3G25746\_Mycgr3T

Mycgr3G90788 Mycgr3T
  
Location: 25341-25803

Mycgr3G90788\_Mycgr3T

Mycgr3G103260 Mycgr3
  
Location: 25903-26635

Mycgr3G103260\_Mycgr3

Mycgr3G84644 Mycgr3T
  
Location: 26735-28457

Mycgr3G84644\_Mycgr3T

Mycgr3G29227 Mycgr3T
  
Location: 28557-28863

Mycgr3G29227\_Mycgr3T

Mycgr3G36271 Mycgr3T
  
Location: 28963-29854

Mycgr3G36271\_Mycgr3T

Mycgr3G68433 Mycgr3T
  
Location: 29954-33041

Mycgr3G68433\_Mycgr3T

Mycgr3G79452 Mycgr3T
  
Location: 33141-33399

Mycgr3G79452\_Mycgr3T

Mycgr3G55345 Mycgr3T
  
Location: 33499-34126

Mycgr3G55345\_Mycgr3T

Mycgr3G103278 Mycgr3
  
Location: 34226-35195

Mycgr3G103278\_Mycgr3

Mycgr3G84654 Mycgr3T
  
Location: 35295-36630

Mycgr3G84654\_Mycgr3T

Mycgr3G108090 Mycgr3
  
Location: 36730-37591

Mycgr3G108090\_Mycgr3

Mycgr3G21922 Mycgr3T
  
Location: 37691-39149

Mycgr3G21922\_Mycgr3T

Mycgr3G99148 Mycgr3T
  
Location: 39249-42819

Mycgr3G99148\_Mycgr3T

similar to snare protein ykt6
  
Accession: CCD55628
  
Location: 84189-85003
  
 NCBI BlastP on this gene

BofuT4\_P154400.1

similar to Golgi transport protein
  
Accession: CCD55629
  
Location: 85457-86169
  
 NCBI BlastP on this gene

BofuT4\_P154410.1

similar to chromatin-remodeling complex subunit ies6
  
Accession: CCD55630
  
Location: 87336-87905
  
 NCBI BlastP on this gene

BofuT4\_P154420.1

hypothetical protein
  
Accession: CCD55631
  
Location: 88322-88561
  
 NCBI BlastP on this gene

BofuT4\_P154430.1

similar to chromatin remodeling complex subunit Arp5
  
Accession: CCD55632
  
Location: 88925-91519
  
 NCBI BlastP on this gene

BofuT4\_P154440.1

similar to transcription factor bZIP
  
Accession: CCD55633
  
Location: 92561-93397
  
 NCBI BlastP on this gene

BofuT4\_P154450.1

hypothetical protein
  
Accession: CCD55634
  
Location: 94770-95521
  
 NCBI BlastP on this gene

BofuT4\_P154460.1

hypothetical protein
  
Accession: CCD55635
  
Location: 96114-96327
  
 NCBI BlastP on this gene

BofuT4\_uP154470.1

hypothetical protein
  
Accession: CCD55636
  
Location: 99433-102294
  
 NCBI BlastP on this gene

BofuT4\_P154480.1

similar to nuclear control of ATPase protein
  
Accession: CCD55637
  
Location: 102829-105289
  
  
**BlastP hit with Mycgr3G84646\_Mycgr3T**
  
Percentage identity: 44 %
  
BlastP bit score: 570
  
Sequence coverage: 102 %
  
E-value: 0.0
  
  
 NCBI BlastP on this gene

BofuT4\_P154490.1

similar to GTP-binding protein
  
Accession: CCD55638
  
Location: 106735-107723
  
  
**BlastP hit with Mycgr3G99145\_Mycgr3T**
  
Percentage identity: 91 %
  
BlastP bit score: 355
  
Sequence coverage: 90 %
  
E-value: 8e-122
  
  
 NCBI BlastP on this gene

BofuT4\_P154500.1

hypothetical protein
  
Accession: CCD55639
  
Location: 118274-119434
  
 NCBI BlastP on this gene

BofuT4\_P154510.1

similar to AAA family ATPase
  
Accession: CCD55640
  
Location: 119725-122206
  
 NCBI BlastP on this gene

BofuT4\_P154520.1

hypothetical protein
  
Accession: CCD55641
  
Location: 123151-124412
  
 NCBI BlastP on this gene

BofuT4\_P154530.1

hypothetical protein
  
Accession: CCD55642
  
Location: 124840-125877
  
 NCBI BlastP on this gene

BofuT4\_P154540.1

hypothetical protein
  
Accession: CCD55643
  
Location: 127142-127882
  
 NCBI BlastP on this gene

BofuT4\_P154550.1

Query: Architecture Search FASTA input

JH921428 : Marssonina brunnea f. sp. 'multigermtubi' MB\_m1 unplaced genomic scaffold M6\_S00001    Total score: 2.0     Cumulative Blast bit score: 916

Hit cluster cross-links:

Mycgr3G90785 Mycgr3T
  
Location: 0-1047

Mycgr3G90785\_Mycgr3T

Mycgr3G103262 Mycgr3
  
Location: 1147-1390

Mycgr3G103262\_Mycgr3

Mycgr3G68458 Mycgr3T
  
Location: 1490-3602

Mycgr3G68458\_Mycgr3T

Mycgr3G99145 Mycgr3T
  
Location: 3702-4326

Mycgr3G99145\_Mycgr3T

Mycgr3G103274 Mycgr3
  
Location: 4426-4957

Mycgr3G103274\_Mycgr3

Mycgr3G103264 Mycgr3
  
Location: 5057-5390

Mycgr3G103264\_Mycgr3

Mycgr3G37570 Mycgr3T
  
Location: 5490-6006

Mycgr3G37570\_Mycgr3T

Mycgr3G108094 Mycgr3
  
Location: 6106-10555

Mycgr3G108094\_Mycgr3

Mycgr3G90786 Mycgr3T
  
Location: 10655-12080

Mycgr3G90786\_Mycgr3T

Mycgr3G68429 Mycgr3T
  
Location: 12180-13440

Mycgr3G68429\_Mycgr3T

Mycgr3G68421 Mycgr3T
  
Location: 13540-17086

Mycgr3G68421\_Mycgr3T

Mycgr3G90801 Mycgr3T
  
Location: 17186-18056

Mycgr3G90801\_Mycgr3T

Mycgr3G84646 Mycgr3T
  
Location: 18156-20235

Mycgr3G84646\_Mycgr3T

Mycgr3G68456 Mycgr3T
  
Location: 20335-21970

Mycgr3G68456\_Mycgr3T

Mycgr3G103270 Mycgr3
  
Location: 22070-22355

Mycgr3G103270\_Mycgr3

Mycgr3G90803 Mycgr3T
  
Location: 22455-23019

Mycgr3G90803\_Mycgr3T

Mycgr3G36941 Mycgr3T
  
Location: 23119-24064

Mycgr3G36941\_Mycgr3T

Mycgr3G25746 Mycgr3T
  
Location: 24164-25241

Mycgr3G25746\_Mycgr3T

Mycgr3G90788 Mycgr3T
  
Location: 25341-25803

Mycgr3G90788\_Mycgr3T

Mycgr3G103260 Mycgr3
  
Location: 25903-26635

Mycgr3G103260\_Mycgr3

Mycgr3G84644 Mycgr3T
  
Location: 26735-28457

Mycgr3G84644\_Mycgr3T

Mycgr3G29227 Mycgr3T
  
Location: 28557-28863

Mycgr3G29227\_Mycgr3T

Mycgr3G36271 Mycgr3T
  
Location: 28963-29854

Mycgr3G36271\_Mycgr3T

Mycgr3G68433 Mycgr3T
  
Location: 29954-33041

Mycgr3G68433\_Mycgr3T

Mycgr3G79452 Mycgr3T
  
Location: 33141-33399

Mycgr3G79452\_Mycgr3T

Mycgr3G55345 Mycgr3T
  
Location: 33499-34126

Mycgr3G55345\_Mycgr3T

Mycgr3G103278 Mycgr3
  
Location: 34226-35195

Mycgr3G103278\_Mycgr3

Mycgr3G84654 Mycgr3T
  
Location: 35295-36630

Mycgr3G84654\_Mycgr3T

Mycgr3G108090 Mycgr3
  
Location: 36730-37591

Mycgr3G108090\_Mycgr3

Mycgr3G21922 Mycgr3T
  
Location: 37691-39149

Mycgr3G21922\_Mycgr3T

Mycgr3G99148 Mycgr3T
  
Location: 39249-42819

Mycgr3G99148\_Mycgr3T

60S ribosomal protein L37
  
Accession: EKD21497
  
Location: 3270392-3270988
  
 NCBI BlastP on this gene

EKD21497

hypothetical protein
  
Accession: EKD21498
  
Location: 3271295-3272281
  
 NCBI BlastP on this gene

EKD21498

FAD binding domain protein
  
Accession: EKD21499
  
Location: 3272649-3274169
  
 NCBI BlastP on this gene

EKD21499

guanine deaminase
  
Accession: EKD21500
  
Location: 3274735-3276168
  
 NCBI BlastP on this gene

EKD21500

putative Lysophospholipase NTE1
  
Accession: EKD21501
  
Location: 3276623-3281467
  
 NCBI BlastP on this gene

EKD21501

eukaryotic translation initiation factor 2 alpha subunit
  
Accession: EKD21502
  
Location: 3281925-3283022
  
 NCBI BlastP on this gene

EKD21502

hypothetical protein
  
Accession: EKD21503
  
Location: 3283348-3283990
  
 NCBI BlastP on this gene

EKD21503

hypothetical protein
  
Accession: EKD21504
  
Location: 3284651-3286002
  
 NCBI BlastP on this gene

EKD21504

hypothetical protein
  
Accession: EKD21505
  
Location: 3286559-3289546
  
 NCBI BlastP on this gene

EKD21505

ATP synthase regulation protein NCA2
  
Accession: EKD21506
  
Location: 3289990-3292421
  
  
**BlastP hit with Mycgr3G84646\_Mycgr3T**
  
Percentage identity: 43 %
  
BlastP bit score: 558
  
Sequence coverage: 100 %
  
E-value: 0.0
  
  
 NCBI BlastP on this gene

EKD21506

secretion related Rab/GTPase
  
Accession: EKD21507
  
Location: 3293067-3293818
  
  
**BlastP hit with Mycgr3G99145\_Mycgr3T**
  
Percentage identity: 86 %
  
BlastP bit score: 359
  
Sequence coverage: 99 %
  
E-value: 1e-123
  
  
 NCBI BlastP on this gene

EKD21507

hypothetical protein
  
Accession: EKD21508
  
Location: 3294045-3295494
  
 NCBI BlastP on this gene

EKD21508

ATPase
  
Accession: EKD21509
  
Location: 3296181-3298559
  
 NCBI BlastP on this gene

EKD21509

caleosin domain containing protein
  
Accession: EKD21510
  
Location: 3298966-3300070
  
 NCBI BlastP on this gene

EKD21510

glycosyl transferase family 2
  
Accession: EKD21511
  
Location: 3301724-3303029
  
 NCBI BlastP on this gene

EKD21511

suppressor of Mek1
  
Accession: EKD21512
  
Location: 3303713-3306747
  
 NCBI BlastP on this gene

EKD21512

hypothetical protein
  
Accession: EKD21513
  
Location: 3310446-3312294
  
 NCBI BlastP on this gene

EKD21513

hypothetical protein
  
Accession: EKD21514
  
Location: 3312394-3313297
  
 NCBI BlastP on this gene

EKD21514

Query: Architecture Search FASTA input

GL988041 : Chaetomium thermophilum var. thermophilum DSM 1495 unplaced genomic scaffold scf7180000...    Total score: 2.0     Cumulative Blast bit score: 915

Hit cluster cross-links:

Mycgr3G90785 Mycgr3T
  
Location: 0-1047

Mycgr3G90785\_Mycgr3T

Mycgr3G103262 Mycgr3
  
Location: 1147-1390

Mycgr3G103262\_Mycgr3

Mycgr3G68458 Mycgr3T
  
Location: 1490-3602

Mycgr3G68458\_Mycgr3T

Mycgr3G99145 Mycgr3T
  
Location: 3702-4326

Mycgr3G99145\_Mycgr3T

Mycgr3G103274 Mycgr3
  
Location: 4426-4957

Mycgr3G103274\_Mycgr3

Mycgr3G103264 Mycgr3
  
Location: 5057-5390

Mycgr3G103264\_Mycgr3

Mycgr3G37570 Mycgr3T
  
Location: 5490-6006

Mycgr3G37570\_Mycgr3T

Mycgr3G108094 Mycgr3
  
Location: 6106-10555

Mycgr3G108094\_Mycgr3

Mycgr3G90786 Mycgr3T
  
Location: 10655-12080

Mycgr3G90786\_Mycgr3T

Mycgr3G68429 Mycgr3T
  
Location: 12180-13440

Mycgr3G68429\_Mycgr3T

Mycgr3G68421 Mycgr3T
  
Location: 13540-17086

Mycgr3G68421\_Mycgr3T

Mycgr3G90801 Mycgr3T
  
Location: 17186-18056

Mycgr3G90801\_Mycgr3T

Mycgr3G84646 Mycgr3T
  
Location: 18156-20235

Mycgr3G84646\_Mycgr3T

Mycgr3G68456 Mycgr3T
  
Location: 20335-21970

Mycgr3G68456\_Mycgr3T

Mycgr3G103270 Mycgr3
  
Location: 22070-22355

Mycgr3G103270\_Mycgr3

Mycgr3G90803 Mycgr3T
  
Location: 22455-23019

Mycgr3G90803\_Mycgr3T

Mycgr3G36941 Mycgr3T
  
Location: 23119-24064

Mycgr3G36941\_Mycgr3T

Mycgr3G25746 Mycgr3T
  
Location: 24164-25241

Mycgr3G25746\_Mycgr3T

Mycgr3G90788 Mycgr3T
  
Location: 25341-25803

Mycgr3G90788\_Mycgr3T

Mycgr3G103260 Mycgr3
  
Location: 25903-26635

Mycgr3G103260\_Mycgr3

Mycgr3G84644 Mycgr3T
  
Location: 26735-28457

Mycgr3G84644\_Mycgr3T

Mycgr3G29227 Mycgr3T
  
Location: 28557-28863

Mycgr3G29227\_Mycgr3T

Mycgr3G36271 Mycgr3T
  
Location: 28963-29854

Mycgr3G36271\_Mycgr3T

Mycgr3G68433 Mycgr3T
  
Location: 29954-33041

Mycgr3G68433\_Mycgr3T

Mycgr3G79452 Mycgr3T
  
Location: 33141-33399

Mycgr3G79452\_Mycgr3T

Mycgr3G55345 Mycgr3T
  
Location: 33499-34126

Mycgr3G55345\_Mycgr3T

Mycgr3G103278 Mycgr3
  
Location: 34226-35195

Mycgr3G103278\_Mycgr3

Mycgr3G84654 Mycgr3T
  
Location: 35295-36630

Mycgr3G84654\_Mycgr3T

Mycgr3G108090 Mycgr3
  
Location: 36730-37591

Mycgr3G108090\_Mycgr3

Mycgr3G21922 Mycgr3T
  
Location: 37691-39149

Mycgr3G21922\_Mycgr3T

Mycgr3G99148 Mycgr3T
  
Location: 39249-42819

Mycgr3G99148\_Mycgr3T

ion channel-like protein
  
Accession: EGS21401
  
Location: 4300558-4302395
  
 NCBI BlastP on this gene

EGS21401

hypothetical protein
  
Accession: EGS21402
  
Location: 4306797-4313738
  
 NCBI BlastP on this gene

EGS21402

hypothetical protein
  
Accession: EGS21403
  
Location: 4315506-4316352
  
 NCBI BlastP on this gene

EGS21403

hypothetical protein
  
Accession: EGS21404
  
Location: 4318402-4320749
  
  
**BlastP hit with Mycgr3G84646\_Mycgr3T**
  
Percentage identity: 42 %
  
BlastP bit score: 493
  
Sequence coverage: 90 %
  
E-value: 4e-161
  
  
 NCBI BlastP on this gene

EGS21404

hypothetical protein
  
Accession: EGS21405
  
Location: 4322359-4323490
  
  
**BlastP hit with Mycgr3G36271\_Mycgr3T**
  
Percentage identity: 68 %
  
BlastP bit score: 422
  
Sequence coverage: 97 %
  
E-value: 1e-144
  
  
 NCBI BlastP on this gene

EGS21405

hypothetical protein
  
Accession: EGS21406
  
Location: 4323958-4324879
  
 NCBI BlastP on this gene

EGS21406

putative sequence-specific DNA binding protein
  
Accession: EGS21407
  
Location: 4326400-4329440
  
 NCBI BlastP on this gene

EGS21407

hypothetical protein
  
Accession: EGS21408
  
Location: 4330182-4333803
  
 NCBI BlastP on this gene

EGS21408

hypothetical protein
  
Accession: EGS21409
  
Location: 4334121-4334780
  
 NCBI BlastP on this gene

EGS21409

hypothetical protein
  
Accession: EGS21410
  
Location: 4335138-4335667
  
 NCBI BlastP on this gene

EGS21410

putative golgi transport protein
  
Accession: EGS21411
  
Location: 4336769-4337495
  
 NCBI BlastP on this gene

EGS21411

hypothetical protein
  
Accession: EGS21412
  
Location: 4338813-4339735
  
 NCBI BlastP on this gene

EGS21412

hypothetical protein
  
Accession: EGS21413
  
Location: 4343130-4346489
  
 NCBI BlastP on this gene

EGS21413

Query: Architecture Search FASTA input

CACQ02000613 : Colletotrichum higginsianum strain IMI 349063    Total score: 2.0     Cumulative Blast bit score: 912

Hit cluster cross-links:

Mycgr3G90785 Mycgr3T
  
Location: 0-1047

Mycgr3G90785\_Mycgr3T

Mycgr3G103262 Mycgr3
  
Location: 1147-1390

Mycgr3G103262\_Mycgr3

Mycgr3G68458 Mycgr3T
  
Location: 1490-3602

Mycgr3G68458\_Mycgr3T

Mycgr3G99145 Mycgr3T
  
Location: 3702-4326

Mycgr3G99145\_Mycgr3T

Mycgr3G103274 Mycgr3
  
Location: 4426-4957

Mycgr3G103274\_Mycgr3

Mycgr3G103264 Mycgr3
  
Location: 5057-5390

Mycgr3G103264\_Mycgr3

Mycgr3G37570 Mycgr3T
  
Location: 5490-6006

Mycgr3G37570\_Mycgr3T

Mycgr3G108094 Mycgr3
  
Location: 6106-10555

Mycgr3G108094\_Mycgr3

Mycgr3G90786 Mycgr3T
  
Location: 10655-12080

Mycgr3G90786\_Mycgr3T

Mycgr3G68429 Mycgr3T
  
Location: 12180-13440

Mycgr3G68429\_Mycgr3T

Mycgr3G68421 Mycgr3T
  
Location: 13540-17086

Mycgr3G68421\_Mycgr3T

Mycgr3G90801 Mycgr3T
  
Location: 17186-18056

Mycgr3G90801\_Mycgr3T

Mycgr3G84646 Mycgr3T
  
Location: 18156-20235

Mycgr3G84646\_Mycgr3T

Mycgr3G68456 Mycgr3T
  
Location: 20335-21970

Mycgr3G68456\_Mycgr3T

Mycgr3G103270 Mycgr3
  
Location: 22070-22355

Mycgr3G103270\_Mycgr3

Mycgr3G90803 Mycgr3T
  
Location: 22455-23019

Mycgr3G90803\_Mycgr3T

Mycgr3G36941 Mycgr3T
  
Location: 23119-24064

Mycgr3G36941\_Mycgr3T

Mycgr3G25746 Mycgr3T
  
Location: 24164-25241

Mycgr3G25746\_Mycgr3T

Mycgr3G90788 Mycgr3T
  
Location: 25341-25803

Mycgr3G90788\_Mycgr3T

Mycgr3G103260 Mycgr3
  
Location: 25903-26635

Mycgr3G103260\_Mycgr3

Mycgr3G84644 Mycgr3T
  
Location: 26735-28457

Mycgr3G84644\_Mycgr3T

Mycgr3G29227 Mycgr3T
  
Location: 28557-28863

Mycgr3G29227\_Mycgr3T

Mycgr3G36271 Mycgr3T
  
Location: 28963-29854

Mycgr3G36271\_Mycgr3T

Mycgr3G68433 Mycgr3T
  
Location: 29954-33041

Mycgr3G68433\_Mycgr3T

Mycgr3G79452 Mycgr3T
  
Location: 33141-33399

Mycgr3G79452\_Mycgr3T

Mycgr3G55345 Mycgr3T
  
Location: 33499-34126

Mycgr3G55345\_Mycgr3T

Mycgr3G103278 Mycgr3
  
Location: 34226-35195

Mycgr3G103278\_Mycgr3

Mycgr3G84654 Mycgr3T
  
Location: 35295-36630

Mycgr3G84654\_Mycgr3T

Mycgr3G108090 Mycgr3
  
Location: 36730-37591

Mycgr3G108090\_Mycgr3

Mycgr3G21922 Mycgr3T
  
Location: 37691-39149

Mycgr3G21922\_Mycgr3T

Mycgr3G99148 Mycgr3T
  
Location: 39249-42819

Mycgr3G99148\_Mycgr3T

ATP synthase regulation protein NCA2
  
Accession: CCF33019
  
Location: 2067-4171
  
  
**BlastP hit with Mycgr3G84646\_Mycgr3T**
  
Percentage identity: 44 %
  
BlastP bit score: 557
  
Sequence coverage: 101 %
  
E-value: 0.0
  
  
 NCBI BlastP on this gene

CCF33019

Ras-like protein Rab-8A
  
Accession: CCF33020
  
Location: 5662-6556
  
  
**BlastP hit with Mycgr3G99145\_Mycgr3T**
  
Percentage identity: 84 %
  
BlastP bit score: 356
  
Sequence coverage: 99 %
  
E-value: 3e-122
  
  
 NCBI BlastP on this gene

CCF33020

hypothetical protein
  
Accession: CCF33021
  
Location: 7296-8918
  
 NCBI BlastP on this gene

CCF33021

ATPase
  
Accession: CCF33022
  
Location: 9790-12217
  
 NCBI BlastP on this gene

CCF33022

squalene epoxidase
  
Accession: CCF33023
  
Location: 14119-15624
  
 NCBI BlastP on this gene

CCF33023

Query: Architecture Search FASTA input

CU638744 : Podospora anserina S mat+ genomic DNA chromosome 6, supercontig 2.    Total score: 2.0     Cumulative Blast bit score: 903

Hit cluster cross-links:

Mycgr3G90785 Mycgr3T
  
Location: 0-1047

Mycgr3G90785\_Mycgr3T

Mycgr3G103262 Mycgr3
  
Location: 1147-1390

Mycgr3G103262\_Mycgr3

Mycgr3G68458 Mycgr3T
  
Location: 1490-3602

Mycgr3G68458\_Mycgr3T

Mycgr3G99145 Mycgr3T
  
Location: 3702-4326

Mycgr3G99145\_Mycgr3T

Mycgr3G103274 Mycgr3
  
Location: 4426-4957

Mycgr3G103274\_Mycgr3

Mycgr3G103264 Mycgr3
  
Location: 5057-5390

Mycgr3G103264\_Mycgr3

Mycgr3G37570 Mycgr3T
  
Location: 5490-6006

Mycgr3G37570\_Mycgr3T

Mycgr3G108094 Mycgr3
  
Location: 6106-10555

Mycgr3G108094\_Mycgr3

Mycgr3G90786 Mycgr3T
  
Location: 10655-12080

Mycgr3G90786\_Mycgr3T

Mycgr3G68429 Mycgr3T
  
Location: 12180-13440

Mycgr3G68429\_Mycgr3T

Mycgr3G68421 Mycgr3T
  
Location: 13540-17086

Mycgr3G68421\_Mycgr3T

Mycgr3G90801 Mycgr3T
  
Location: 17186-18056

Mycgr3G90801\_Mycgr3T

Mycgr3G84646 Mycgr3T
  
Location: 18156-20235

Mycgr3G84646\_Mycgr3T

Mycgr3G68456 Mycgr3T
  
Location: 20335-21970

Mycgr3G68456\_Mycgr3T

Mycgr3G103270 Mycgr3
  
Location: 22070-22355

Mycgr3G103270\_Mycgr3

Mycgr3G90803 Mycgr3T
  
Location: 22455-23019

Mycgr3G90803\_Mycgr3T

Mycgr3G36941 Mycgr3T
  
Location: 23119-24064

Mycgr3G36941\_Mycgr3T

Mycgr3G25746 Mycgr3T
  
Location: 24164-25241

Mycgr3G25746\_Mycgr3T

Mycgr3G90788 Mycgr3T
  
Location: 25341-25803

Mycgr3G90788\_Mycgr3T

Mycgr3G103260 Mycgr3
  
Location: 25903-26635

Mycgr3G103260\_Mycgr3

Mycgr3G84644 Mycgr3T
  
Location: 26735-28457

Mycgr3G84644\_Mycgr3T

Mycgr3G29227 Mycgr3T
  
Location: 28557-28863

Mycgr3G29227\_Mycgr3T

Mycgr3G36271 Mycgr3T
  
Location: 28963-29854

Mycgr3G36271\_Mycgr3T

Mycgr3G68433 Mycgr3T
  
Location: 29954-33041

Mycgr3G68433\_Mycgr3T

Mycgr3G79452 Mycgr3T
  
Location: 33141-33399

Mycgr3G79452\_Mycgr3T

Mycgr3G55345 Mycgr3T
  
Location: 33499-34126

Mycgr3G55345\_Mycgr3T

Mycgr3G103278 Mycgr3
  
Location: 34226-35195

Mycgr3G103278\_Mycgr3

Mycgr3G84654 Mycgr3T
  
Location: 35295-36630

Mycgr3G84654\_Mycgr3T

Mycgr3G108090 Mycgr3
  
Location: 36730-37591

Mycgr3G108090\_Mycgr3

Mycgr3G21922 Mycgr3T
  
Location: 37691-39149

Mycgr3G21922\_Mycgr3T

Mycgr3G99148 Mycgr3T
  
Location: 39249-42819

Mycgr3G99148\_Mycgr3T

not annotated
  
Accession: CAP71229
  
Location: 903275-904577
  
 NCBI BlastP on this gene

CAP71229

not annotated
  
Accession: CAP71230
  
Location: 905327-905941
  
 NCBI BlastP on this gene

CAP71230

not annotated
  
Accession: CAP71231
  
Location: 906488-907586
  
 NCBI BlastP on this gene

CAP71231

not annotated
  
Accession: CAP71232
  
Location: 908423-912982
  
 NCBI BlastP on this gene

CAP71232

not annotated
  
Accession: CAP71233
  
Location: 913476-914753
  
 NCBI BlastP on this gene

CAP71233

not annotated
  
Accession: CAP71234
  
Location: 915059-917374
  
 NCBI BlastP on this gene

CAP71234

not annotated
  
Accession: CAP71235
  
Location: 918669-919811
  
 NCBI BlastP on this gene

CAP71235

not annotated
  
Accession: CAP71236
  
Location: 920895-921999
  
  
**BlastP hit with Mycgr3G36271\_Mycgr3T**
  
Percentage identity: 69 %
  
BlastP bit score: 447
  
Sequence coverage: 98 %
  
E-value: 3e-155
  
  
 NCBI BlastP on this gene

CAP71236

not annotated
  
Accession: CAP71237
  
Location: 923198-925464
  
  
**BlastP hit with Mycgr3G84646\_Mycgr3T**
  
Percentage identity: 39 %
  
BlastP bit score: 456
  
Sequence coverage: 108 %
  
E-value: 8e-147
  
  
 NCBI BlastP on this gene

CAP71237

tRNA-Ala
  
Accession: CAP71238
  
Location: 926368-927182
  
 NCBI BlastP on this gene

CAP71238

not annotated
  
Accession: CAP71239
  
Location: 930372-931421
  
 NCBI BlastP on this gene

CAP71239

not annotated
  
Accession: CAP71240
  
Location: 933731-934204
  
 NCBI BlastP on this gene

CAP71240

not annotated
  
Accession: CAP71241
  
Location: 939483-940483
  
 NCBI BlastP on this gene

CAP71241

tRNA-Arg
  
Accession: CAP71242
  
Location: 942643-946596
  
 NCBI BlastP on this gene

CAP71242

Query: Architecture Search FASTA input

JH226136 : Exophiala dermatitidis NIH/UT8656 unplaced genomic scaffold supercont1.7    Total score: 2.0     Cumulative Blast bit score: 884

Hit cluster cross-links:

Mycgr3G90785 Mycgr3T
  
Location: 0-1047

Mycgr3G90785\_Mycgr3T

Mycgr3G103262 Mycgr3
  
Location: 1147-1390

Mycgr3G103262\_Mycgr3

Mycgr3G68458 Mycgr3T
  
Location: 1490-3602

Mycgr3G68458\_Mycgr3T

Mycgr3G99145 Mycgr3T
  
Location: 3702-4326

Mycgr3G99145\_Mycgr3T

Mycgr3G103274 Mycgr3
  
Location: 4426-4957

Mycgr3G103274\_Mycgr3

Mycgr3G103264 Mycgr3
  
Location: 5057-5390

Mycgr3G103264\_Mycgr3

Mycgr3G37570 Mycgr3T
  
Location: 5490-6006

Mycgr3G37570\_Mycgr3T

Mycgr3G108094 Mycgr3
  
Location: 6106-10555

Mycgr3G108094\_Mycgr3

Mycgr3G90786 Mycgr3T
  
Location: 10655-12080

Mycgr3G90786\_Mycgr3T

Mycgr3G68429 Mycgr3T
  
Location: 12180-13440

Mycgr3G68429\_Mycgr3T

Mycgr3G68421 Mycgr3T
  
Location: 13540-17086

Mycgr3G68421\_Mycgr3T

Mycgr3G90801 Mycgr3T
  
Location: 17186-18056

Mycgr3G90801\_Mycgr3T

Mycgr3G84646 Mycgr3T
  
Location: 18156-20235

Mycgr3G84646\_Mycgr3T

Mycgr3G68456 Mycgr3T
  
Location: 20335-21970

Mycgr3G68456\_Mycgr3T

Mycgr3G103270 Mycgr3
  
Location: 22070-22355

Mycgr3G103270\_Mycgr3

Mycgr3G90803 Mycgr3T
  
Location: 22455-23019

Mycgr3G90803\_Mycgr3T

Mycgr3G36941 Mycgr3T
  
Location: 23119-24064

Mycgr3G36941\_Mycgr3T

Mycgr3G25746 Mycgr3T
  
Location: 24164-25241

Mycgr3G25746\_Mycgr3T

Mycgr3G90788 Mycgr3T
  
Location: 25341-25803

Mycgr3G90788\_Mycgr3T

Mycgr3G103260 Mycgr3
  
Location: 25903-26635

Mycgr3G103260\_Mycgr3

Mycgr3G84644 Mycgr3T
  
Location: 26735-28457

Mycgr3G84644\_Mycgr3T

Mycgr3G29227 Mycgr3T
  
Location: 28557-28863

Mycgr3G29227\_Mycgr3T

Mycgr3G36271 Mycgr3T
  
Location: 28963-29854

Mycgr3G36271\_Mycgr3T

Mycgr3G68433 Mycgr3T
  
Location: 29954-33041

Mycgr3G68433\_Mycgr3T

Mycgr3G79452 Mycgr3T
  
Location: 33141-33399

Mycgr3G79452\_Mycgr3T

Mycgr3G55345 Mycgr3T
  
Location: 33499-34126

Mycgr3G55345\_Mycgr3T

Mycgr3G103278 Mycgr3
  
Location: 34226-35195

Mycgr3G103278\_Mycgr3

Mycgr3G84654 Mycgr3T
  
Location: 35295-36630

Mycgr3G84654\_Mycgr3T

Mycgr3G108090 Mycgr3
  
Location: 36730-37591

Mycgr3G108090\_Mycgr3

Mycgr3G21922 Mycgr3T
  
Location: 37691-39149

Mycgr3G21922\_Mycgr3T

Mycgr3G99148 Mycgr3T
  
Location: 39249-42819

Mycgr3G99148\_Mycgr3T

hypothetical protein
  
Accession: EHY60550
  
Location: 1953958-1955725
  
 NCBI BlastP on this gene

EHY60550

hypothetical protein
  
Accession: EHY60551
  
Location: 1957271-1957708
  
 NCBI BlastP on this gene

EHY60551

hypothetical protein
  
Accession: EHY60552
  
Location: 1958956-1960023
  
 NCBI BlastP on this gene

EHY60552

hypothetical protein
  
Accession: EHY60553
  
Location: 1961023-1961700
  
 NCBI BlastP on this gene

EHY60553

hypothetical protein
  
Accession: EHY60554
  
Location: 1961875-1962518
  
 NCBI BlastP on this gene

EHY60554

hypothetical protein
  
Accession: EHY60555
  
Location: 1964768-1967450
  
 NCBI BlastP on this gene

EHY60555

hypothetical protein
  
Accession: EHY60556
  
Location: 1968311-1971445
  
 NCBI BlastP on this gene

EHY60556

hypothetical protein
  
Accession: EHY60557
  
Location: 1973332-1975362
  
  
**BlastP hit with Mycgr3G84646\_Mycgr3T**
  
Percentage identity: 41 %
  
BlastP bit score: 516
  
Sequence coverage: 100 %
  
E-value: 2e-171
  
  
 NCBI BlastP on this gene

EHY60557

GTP-binding protein ypt2
  
Accession: EHY60558
  
Location: 1976922-1977744
  
  
**BlastP hit with Mycgr3G99145\_Mycgr3T**
  
Percentage identity: 84 %
  
BlastP bit score: 368
  
Sequence coverage: 101 %
  
E-value: 9e-127
  
  
 NCBI BlastP on this gene

EHY60558

profilin
  
Accession: EHY60559
  
Location: 1978587-1979345
  
 NCBI BlastP on this gene

EHY60559

hypothetical protein
  
Accession: EHY60560
  
Location: 1980774-1981773
  
 NCBI BlastP on this gene

EHY60560

hypothetical protein
  
Accession: EHY60561
  
Location: 1983354-1985286
  
 NCBI BlastP on this gene

EHY60561

hypothetical protein
  
Accession: EHY60562
  
Location: 1986139-1989289
  
 NCBI BlastP on this gene

EHY60562

hypothetical protein
  
Accession: EHY60563
  
Location: 1989855-1994509
  
 NCBI BlastP on this gene

EHY60563

hypothetical protein
  
Accession: EHY60564
  
Location: 1996491-1996712
  
 NCBI BlastP on this gene

EHY60564

Query: Architecture Search FASTA input

GG663377 : Ajellomyces capsulatus G186AR genomic scaffold supercont2.15    Total score: 2.0     Cumulative Blast bit score: 865

Hit cluster cross-links:

Mycgr3G90785 Mycgr3T
  
Location: 0-1047

Mycgr3G90785\_Mycgr3T

Mycgr3G103262 Mycgr3
  
Location: 1147-1390

Mycgr3G103262\_Mycgr3

Mycgr3G68458 Mycgr3T
  
Location: 1490-3602

Mycgr3G68458\_Mycgr3T

Mycgr3G99145 Mycgr3T
  
Location: 3702-4326

Mycgr3G99145\_Mycgr3T

Mycgr3G103274 Mycgr3
  
Location: 4426-4957

Mycgr3G103274\_Mycgr3

Mycgr3G103264 Mycgr3
  
Location: 5057-5390

Mycgr3G103264\_Mycgr3

Mycgr3G37570 Mycgr3T
  
Location: 5490-6006

Mycgr3G37570\_Mycgr3T

Mycgr3G108094 Mycgr3
  
Location: 6106-10555

Mycgr3G108094\_Mycgr3

Mycgr3G90786 Mycgr3T
  
Location: 10655-12080

Mycgr3G90786\_Mycgr3T

Mycgr3G68429 Mycgr3T
  
Location: 12180-13440

Mycgr3G68429\_Mycgr3T

Mycgr3G68421 Mycgr3T
  
Location: 13540-17086

Mycgr3G68421\_Mycgr3T

Mycgr3G90801 Mycgr3T
  
Location: 17186-18056

Mycgr3G90801\_Mycgr3T

Mycgr3G84646 Mycgr3T
  
Location: 18156-20235

Mycgr3G84646\_Mycgr3T

Mycgr3G68456 Mycgr3T
  
Location: 20335-21970

Mycgr3G68456\_Mycgr3T

Mycgr3G103270 Mycgr3
  
Location: 22070-22355

Mycgr3G103270\_Mycgr3

Mycgr3G90803 Mycgr3T
  
Location: 22455-23019

Mycgr3G90803\_Mycgr3T

Mycgr3G36941 Mycgr3T
  
Location: 23119-24064

Mycgr3G36941\_Mycgr3T

Mycgr3G25746 Mycgr3T
  
Location: 24164-25241

Mycgr3G25746\_Mycgr3T

Mycgr3G90788 Mycgr3T
  
Location: 25341-25803

Mycgr3G90788\_Mycgr3T

Mycgr3G103260 Mycgr3
  
Location: 25903-26635

Mycgr3G103260\_Mycgr3

Mycgr3G84644 Mycgr3T
  
Location: 26735-28457

Mycgr3G84644\_Mycgr3T

Mycgr3G29227 Mycgr3T
  
Location: 28557-28863

Mycgr3G29227\_Mycgr3T

Mycgr3G36271 Mycgr3T
  
Location: 28963-29854

Mycgr3G36271\_Mycgr3T

Mycgr3G68433 Mycgr3T
  
Location: 29954-33041

Mycgr3G68433\_Mycgr3T

Mycgr3G79452 Mycgr3T
  
Location: 33141-33399

Mycgr3G79452\_Mycgr3T

Mycgr3G55345 Mycgr3T
  
Location: 33499-34126

Mycgr3G55345\_Mycgr3T

Mycgr3G103278 Mycgr3
  
Location: 34226-35195

Mycgr3G103278\_Mycgr3

Mycgr3G84654 Mycgr3T
  
Location: 35295-36630

Mycgr3G84654\_Mycgr3T

Mycgr3G108090 Mycgr3
  
Location: 36730-37591

Mycgr3G108090\_Mycgr3

Mycgr3G21922 Mycgr3T
  
Location: 37691-39149

Mycgr3G21922\_Mycgr3T

Mycgr3G99148 Mycgr3T
  
Location: 39249-42819

Mycgr3G99148\_Mycgr3T

conserved hypothetical protein
  
Accession: EEH03402
  
Location: 333687-336407
  
 NCBI BlastP on this gene

EEH03402

protein kinase
  
Accession: EEH03403
  
Location: 338652-340181
  
 NCBI BlastP on this gene

EEH03403

conserved hypothetical protein
  
Accession: EEH03404
  
Location: 341631-343823
  
  
**BlastP hit with Mycgr3G84646\_Mycgr3T**
  
Percentage identity: 38 %
  
BlastP bit score: 436
  
Sequence coverage: 102 %
  
E-value: 8e-140
  
  
 NCBI BlastP on this gene

EEH03404

predicted protein
  
Accession: EEH03405
  
Location: 344338-344706
  
 NCBI BlastP on this gene

EEH03405

C-4 methylsterol oxidase
  
Accession: EEH03406
  
Location: 344964-345899
  
  
**BlastP hit with Mycgr3G36271\_Mycgr3T**
  
Percentage identity: 80 %
  
BlastP bit score: 429
  
Sequence coverage: 84 %
  
E-value: 1e-148
  
  
 NCBI BlastP on this gene

EEH03406

DUF652 domain-containing protein
  
Accession: EEH03407
  
Location: 346868-347884
  
 NCBI BlastP on this gene

EEH03407

conserved hypothetical protein
  
Accession: EEH03408
  
Location: 348977-351373
  
 NCBI BlastP on this gene

EEH03408

conserved hypothetical protein
  
Accession: EEH03409
  
Location: 351923-352762
  
 NCBI BlastP on this gene

EEH03409

U3 small nucleolar RNA-associated protein
  
Accession: EEH03410
  
Location: 353868-357685
  
 NCBI BlastP on this gene

EEH03410

inositol pyrophosphate synthase
  
Accession: EEH03411
  
Location: 358484-363532
  
 NCBI BlastP on this gene

EEH03411

conserved hypothetical protein
  
Accession: EEH03412
  
Location: 364076-365647
  
 NCBI BlastP on this gene

EEH03412

conserved hypothetical protein
  
Accession: EEH03413
  
Location: 365863-369933
  
 NCBI BlastP on this gene

EEH03413

Query: Architecture Search FASTA input

DS990639 : Ajellomyces capsulatus H88 supercont1.4 genomic scaffold    Total score: 2.0     Cumulative Blast bit score: 850

Hit cluster cross-links:

Mycgr3G90785 Mycgr3T
  
Location: 0-1047

Mycgr3G90785\_Mycgr3T

Mycgr3G103262 Mycgr3
  
Location: 1147-1390

Mycgr3G103262\_Mycgr3

Mycgr3G68458 Mycgr3T
  
Location: 1490-3602

Mycgr3G68458\_Mycgr3T

Mycgr3G99145 Mycgr3T
  
Location: 3702-4326

Mycgr3G99145\_Mycgr3T

Mycgr3G103274 Mycgr3
  
Location: 4426-4957

Mycgr3G103274\_Mycgr3

Mycgr3G103264 Mycgr3
  
Location: 5057-5390

Mycgr3G103264\_Mycgr3

Mycgr3G37570 Mycgr3T
  
Location: 5490-6006

Mycgr3G37570\_Mycgr3T

Mycgr3G108094 Mycgr3
  
Location: 6106-10555

Mycgr3G108094\_Mycgr3

Mycgr3G90786 Mycgr3T
  
Location: 10655-12080

Mycgr3G90786\_Mycgr3T

Mycgr3G68429 Mycgr3T
  
Location: 12180-13440

Mycgr3G68429\_Mycgr3T

Mycgr3G68421 Mycgr3T
  
Location: 13540-17086

Mycgr3G68421\_Mycgr3T

Mycgr3G90801 Mycgr3T
  
Location: 17186-18056

Mycgr3G90801\_Mycgr3T

Mycgr3G84646 Mycgr3T
  
Location: 18156-20235

Mycgr3G84646\_Mycgr3T

Mycgr3G68456 Mycgr3T
  
Location: 20335-21970

Mycgr3G68456\_Mycgr3T

Mycgr3G103270 Mycgr3
  
Location: 22070-22355

Mycgr3G103270\_Mycgr3

Mycgr3G90803 Mycgr3T
  
Location: 22455-23019

Mycgr3G90803\_Mycgr3T

Mycgr3G36941 Mycgr3T
  
Location: 23119-24064

Mycgr3G36941\_Mycgr3T

Mycgr3G25746 Mycgr3T
  
Location: 24164-25241

Mycgr3G25746\_Mycgr3T

Mycgr3G90788 Mycgr3T
  
Location: 25341-25803

Mycgr3G90788\_Mycgr3T

Mycgr3G103260 Mycgr3
  
Location: 25903-26635

Mycgr3G103260\_Mycgr3

Mycgr3G84644 Mycgr3T
  
Location: 26735-28457

Mycgr3G84644\_Mycgr3T

Mycgr3G29227 Mycgr3T
  
Location: 28557-28863

Mycgr3G29227\_Mycgr3T

Mycgr3G36271 Mycgr3T
  
Location: 28963-29854

Mycgr3G36271\_Mycgr3T

Mycgr3G68433 Mycgr3T
  
Location: 29954-33041

Mycgr3G68433\_Mycgr3T

Mycgr3G79452 Mycgr3T
  
Location: 33141-33399

Mycgr3G79452\_Mycgr3T

Mycgr3G55345 Mycgr3T
  
Location: 33499-34126

Mycgr3G55345\_Mycgr3T

Mycgr3G103278 Mycgr3
  
Location: 34226-35195

Mycgr3G103278\_Mycgr3

Mycgr3G84654 Mycgr3T
  
Location: 35295-36630

Mycgr3G84654\_Mycgr3T

Mycgr3G108090 Mycgr3
  
Location: 36730-37591

Mycgr3G108090\_Mycgr3

Mycgr3G21922 Mycgr3T
  
Location: 37691-39149

Mycgr3G21922\_Mycgr3T

Mycgr3G99148 Mycgr3T
  
Location: 39249-42819

Mycgr3G99148\_Mycgr3T

cortical actin cytoskeleton protein asp1
  
Accession: EGC45488
  
Location: 756516-761566
  
 NCBI BlastP on this gene

EGC45488

beta transducin
  
Accession: EGC45489
  
Location: 762380-766208
  
 NCBI BlastP on this gene

EGC45489

serine protein kinase
  
Accession: EGC45490
  
Location: 768870-770207
  
 NCBI BlastP on this gene

EGC45490

conserved hypothetical protein
  
Accession: EGC45491
  
Location: 771072-773703
  
 NCBI BlastP on this gene

EGC45491

conserved hypothetical protein
  
Accession: EGC45492
  
Location: 774493-775338
  
 NCBI BlastP on this gene

EGC45492

conserved hypothetical protein
  
Accession: EGC45493
  
Location: 775874-778278
  
 NCBI BlastP on this gene

EGC45493

hypothetical protein
  
Accession: EGC45494
  
Location: 779371-780379
  
 NCBI BlastP on this gene

EGC45494

C4-methylsterol oxidase
  
Accession: EGC45495
  
Location: 781335-782266
  
  
**BlastP hit with Mycgr3G36271\_Mycgr3T**
  
Percentage identity: 80 %
  
BlastP bit score: 414
  
Sequence coverage: 81 %
  
E-value: 1e-142
  
  
 NCBI BlastP on this gene

EGC45495

conserved hypothetical protein
  
Accession: EGC45496
  
Location: 782538-785599
  
  
**BlastP hit with Mycgr3G84646\_Mycgr3T**
  
Percentage identity: 38 %
  
BlastP bit score: 436
  
Sequence coverage: 100 %
  
E-value: 9e-139
  
  
 NCBI BlastP on this gene

EGC45496

conserved hypothetical protein
  
Accession: EGC45497
  
Location: 788893-791085
  
 NCBI BlastP on this gene

EGC45497

protein kinase
  
Accession: EGC45498
  
Location: 792720-794115
  
 NCBI BlastP on this gene

EGC45498

C2H2 finger domain-containing protein
  
Accession: EGC45499
  
Location: 795973-798708
  
 NCBI BlastP on this gene

EGC45499

conserved hypothetical protein
  
Accession: EGC45500
  
Location: 800605-803091
  
 NCBI BlastP on this gene

EGC45500

conserved hypothetical protein
  
Accession: EGC45501
  
Location: 803160-804065
  
 NCBI BlastP on this gene

EGC45501

predicted protein
  
Accession: EGC45502
  
Location: 804347-805908
  
 NCBI BlastP on this gene

EGC45502

Query: Architecture Search FASTA input

CAUH01003861 : Blumeria graminis f. sp. hordei DH14    Total score: 2.0     Cumulative Blast bit score: 841

Hit cluster cross-links:

Mycgr3G90785 Mycgr3T
  
Location: 0-1047

Mycgr3G90785\_Mycgr3T

Mycgr3G103262 Mycgr3
  
Location: 1147-1390

Mycgr3G103262\_Mycgr3

Mycgr3G68458 Mycgr3T
  
Location: 1490-3602

Mycgr3G68458\_Mycgr3T

Mycgr3G99145 Mycgr3T
  
Location: 3702-4326

Mycgr3G99145\_Mycgr3T

Mycgr3G103274 Mycgr3
  
Location: 4426-4957

Mycgr3G103274\_Mycgr3

Mycgr3G103264 Mycgr3
  
Location: 5057-5390

Mycgr3G103264\_Mycgr3

Mycgr3G37570 Mycgr3T
  
Location: 5490-6006

Mycgr3G37570\_Mycgr3T

Mycgr3G108094 Mycgr3
  
Location: 6106-10555

Mycgr3G108094\_Mycgr3

Mycgr3G90786 Mycgr3T
  
Location: 10655-12080

Mycgr3G90786\_Mycgr3T

Mycgr3G68429 Mycgr3T
  
Location: 12180-13440

Mycgr3G68429\_Mycgr3T

Mycgr3G68421 Mycgr3T
  
Location: 13540-17086

Mycgr3G68421\_Mycgr3T

Mycgr3G90801 Mycgr3T
  
Location: 17186-18056

Mycgr3G90801\_Mycgr3T

Mycgr3G84646 Mycgr3T
  
Location: 18156-20235

Mycgr3G84646\_Mycgr3T

Mycgr3G68456 Mycgr3T
  
Location: 20335-21970

Mycgr3G68456\_Mycgr3T

Mycgr3G103270 Mycgr3
  
Location: 22070-22355

Mycgr3G103270\_Mycgr3

Mycgr3G90803 Mycgr3T
  
Location: 22455-23019

Mycgr3G90803\_Mycgr3T

Mycgr3G36941 Mycgr3T
  
Location: 23119-24064

Mycgr3G36941\_Mycgr3T

Mycgr3G25746 Mycgr3T
  
Location: 24164-25241

Mycgr3G25746\_Mycgr3T

Mycgr3G90788 Mycgr3T
  
Location: 25341-25803

Mycgr3G90788\_Mycgr3T

Mycgr3G103260 Mycgr3
  
Location: 25903-26635

Mycgr3G103260\_Mycgr3

Mycgr3G84644 Mycgr3T
  
Location: 26735-28457

Mycgr3G84644\_Mycgr3T

Mycgr3G29227 Mycgr3T
  
Location: 28557-28863

Mycgr3G29227\_Mycgr3T

Mycgr3G36271 Mycgr3T
  
Location: 28963-29854

Mycgr3G36271\_Mycgr3T

Mycgr3G68433 Mycgr3T
  
Location: 29954-33041

Mycgr3G68433\_Mycgr3T

Mycgr3G79452 Mycgr3T
  
Location: 33141-33399

Mycgr3G79452\_Mycgr3T

Mycgr3G55345 Mycgr3T
  
Location: 33499-34126

Mycgr3G55345\_Mycgr3T

Mycgr3G103278 Mycgr3
  
Location: 34226-35195

Mycgr3G103278\_Mycgr3

Mycgr3G84654 Mycgr3T
  
Location: 35295-36630

Mycgr3G84654\_Mycgr3T

Mycgr3G108090 Mycgr3
  
Location: 36730-37591

Mycgr3G108090\_Mycgr3

Mycgr3G21922 Mycgr3T
  
Location: 37691-39149

Mycgr3G21922\_Mycgr3T

Mycgr3G99148 Mycgr3T
  
Location: 39249-42819

Mycgr3G99148\_Mycgr3T

eIF-2-alpha/eukaryotic translation initiation factor 2 alpha subunit
  
Accession: CCU77668
  
Location: 43118-44228
  
 NCBI BlastP on this gene

CCU77668

mitochondrial inner membrane protease subunit 2
  
Accession: CCU77667
  
Location: 42197-42848
  
 NCBI BlastP on this gene

CCU77667

mitochondrial carrier protein
  
Accession: CCU77666
  
Location: 40610-41906
  
 NCBI BlastP on this gene

CCU77666

hypothetical protein
  
Accession: CCU77665
  
Location: 24304-27111
  
 NCBI BlastP on this gene

CCU77665

NCA2/nuclear control of ATPase
  
Accession: CCU77663
  
Location: 21842-23877
  
  
**BlastP hit with Mycgr3G84646\_Mycgr3T**
  
Percentage identity: 39 %
  
BlastP bit score: 488
  
Sequence coverage: 100 %
  
E-value: 4e-160
  
  
 NCBI BlastP on this gene

CCU77663

GTP-binding protein SAS1
  
Accession: CCU77662
  
Location: 20519-21256
  
  
**BlastP hit with Mycgr3G99145\_Mycgr3T**
  
Percentage identity: 83 %
  
BlastP bit score: 353
  
Sequence coverage: 99 %
  
E-value: 4e-121
  
  
 NCBI BlastP on this gene

CCU77662

hypothetical protein
  
Accession: CCU77661
  
Location: 18567-20356
  
 NCBI BlastP on this gene

CCU77661

Query: Architecture Search FASTA input

DS544805 : Paracoccidioides brasiliensis Pb03 supercont1.3 genomic scaffold    Total score: 2.0     Cumulative Blast bit score: 834

Hit cluster cross-links:

Mycgr3G90785 Mycgr3T
  
Location: 0-1047

Mycgr3G90785\_Mycgr3T

Mycgr3G103262 Mycgr3
  
Location: 1147-1390

Mycgr3G103262\_Mycgr3

Mycgr3G68458 Mycgr3T
  
Location: 1490-3602

Mycgr3G68458\_Mycgr3T

Mycgr3G99145 Mycgr3T
  
Location: 3702-4326

Mycgr3G99145\_Mycgr3T

Mycgr3G103274 Mycgr3
  
Location: 4426-4957

Mycgr3G103274\_Mycgr3

Mycgr3G103264 Mycgr3
  
Location: 5057-5390

Mycgr3G103264\_Mycgr3

Mycgr3G37570 Mycgr3T
  
Location: 5490-6006

Mycgr3G37570\_Mycgr3T

Mycgr3G108094 Mycgr3
  
Location: 6106-10555

Mycgr3G108094\_Mycgr3

Mycgr3G90786 Mycgr3T
  
Location: 10655-12080

Mycgr3G90786\_Mycgr3T

Mycgr3G68429 Mycgr3T
  
Location: 12180-13440

Mycgr3G68429\_Mycgr3T

Mycgr3G68421 Mycgr3T
  
Location: 13540-17086

Mycgr3G68421\_Mycgr3T

Mycgr3G90801 Mycgr3T
  
Location: 17186-18056

Mycgr3G90801\_Mycgr3T

Mycgr3G84646 Mycgr3T
  
Location: 18156-20235

Mycgr3G84646\_Mycgr3T

Mycgr3G68456 Mycgr3T
  
Location: 20335-21970

Mycgr3G68456\_Mycgr3T

Mycgr3G103270 Mycgr3
  
Location: 22070-22355

Mycgr3G103270\_Mycgr3

Mycgr3G90803 Mycgr3T
  
Location: 22455-23019

Mycgr3G90803\_Mycgr3T

Mycgr3G36941 Mycgr3T
  
Location: 23119-24064

Mycgr3G36941\_Mycgr3T

Mycgr3G25746 Mycgr3T
  
Location: 24164-25241

Mycgr3G25746\_Mycgr3T

Mycgr3G90788 Mycgr3T
  
Location: 25341-25803

Mycgr3G90788\_Mycgr3T

Mycgr3G103260 Mycgr3
  
Location: 25903-26635

Mycgr3G103260\_Mycgr3

Mycgr3G84644 Mycgr3T
  
Location: 26735-28457

Mycgr3G84644\_Mycgr3T

Mycgr3G29227 Mycgr3T
  
Location: 28557-28863

Mycgr3G29227\_Mycgr3T

Mycgr3G36271 Mycgr3T
  
Location: 28963-29854

Mycgr3G36271\_Mycgr3T

Mycgr3G68433 Mycgr3T
  
Location: 29954-33041

Mycgr3G68433\_Mycgr3T

Mycgr3G79452 Mycgr3T
  
Location: 33141-33399

Mycgr3G79452\_Mycgr3T

Mycgr3G55345 Mycgr3T
  
Location: 33499-34126

Mycgr3G55345\_Mycgr3T

Mycgr3G103278 Mycgr3
  
Location: 34226-35195

Mycgr3G103278\_Mycgr3

Mycgr3G84654 Mycgr3T
  
Location: 35295-36630

Mycgr3G84654\_Mycgr3T

Mycgr3G108090 Mycgr3
  
Location: 36730-37591

Mycgr3G108090\_Mycgr3

Mycgr3G21922 Mycgr3T
  
Location: 37691-39149

Mycgr3G21922\_Mycgr3T

Mycgr3G99148 Mycgr3T
  
Location: 39249-42819

Mycgr3G99148\_Mycgr3T

predicted protein
  
Accession: EEH19631
  
Location: 6104-6775
  
 NCBI BlastP on this gene

EEH19631

predicted protein
  
Accession: EEH19632
  
Location: 7191-9169
  
 NCBI BlastP on this gene

EEH19632

hypothetical protein
  
Accession: EEH19633
  
Location: 12848-14711
  
 NCBI BlastP on this gene

EEH19633

conserved hypothetical protein
  
Accession: EEH19634
  
Location: 16936-19371
  
  
**BlastP hit with Mycgr3G84646\_Mycgr3T**
  
Percentage identity: 40 %
  
BlastP bit score: 488
  
Sequence coverage: 103 %
  
E-value: 2e-159
  
  
 NCBI BlastP on this gene

EEH19634

predicted protein
  
Accession: EEH19635
  
Location: 19659-21169
  
 NCBI BlastP on this gene

EEH19635

conserved hypothetical protein
  
Accession: EEH19636
  
Location: 22377-23717
  
 NCBI BlastP on this gene

EEH19636

GTP-binding protein SAS1
  
Accession: EEH19637
  
Location: 25012-25877
  
  
**BlastP hit with Mycgr3G99145\_Mycgr3T**
  
Percentage identity: 83 %
  
BlastP bit score: 346
  
Sequence coverage: 99 %
  
E-value: 2e-118
  
  
 NCBI BlastP on this gene

EEH19637

predicted protein
  
Accession: EEH19638
  
Location: 26789-28064
  
 NCBI BlastP on this gene

EEH19638

cell division cycle protein
  
Accession: EEH19639
  
Location: 28704-31052
  
 NCBI BlastP on this gene

EEH19639

hypothetical protein
  
Accession: EEH19640
  
Location: 31446-32644
  
 NCBI BlastP on this gene

EEH19640

protein kinase rad3
  
Accession: EEH19641
  
Location: 32949-41818
  
 NCBI BlastP on this gene

EEH19641

tRNA-specific adenosine deaminase subunit TAD2
  
Accession: EEH19642
  
Location: 42153-43018
  
 NCBI BlastP on this gene

EEH19642

conserved hypothetical protein
  
Accession: EEH19643
  
Location: 43250-44099
  
 NCBI BlastP on this gene

EEH19643

predicted protein
  
Accession: EEH19644
  
Location: 44707-45804
  
 NCBI BlastP on this gene

EEH19644

Query: Architecture Search FASTA input

DS027045 : Aspergillus clavatus NRRL 1 1099423829791 genomic scaffold    Total score: 2.0     Cumulative Blast bit score: 834

Hit cluster cross-links:

Mycgr3G90785 Mycgr3T
  
Location: 0-1047

Mycgr3G90785\_Mycgr3T

Mycgr3G103262 Mycgr3
  
Location: 1147-1390

Mycgr3G103262\_Mycgr3

Mycgr3G68458 Mycgr3T
  
Location: 1490-3602

Mycgr3G68458\_Mycgr3T

Mycgr3G99145 Mycgr3T
  
Location: 3702-4326

Mycgr3G99145\_Mycgr3T

Mycgr3G103274 Mycgr3
  
Location: 4426-4957

Mycgr3G103274\_Mycgr3

Mycgr3G103264 Mycgr3
  
Location: 5057-5390

Mycgr3G103264\_Mycgr3

Mycgr3G37570 Mycgr3T
  
Location: 5490-6006

Mycgr3G37570\_Mycgr3T

Mycgr3G108094 Mycgr3
  
Location: 6106-10555

Mycgr3G108094\_Mycgr3

Mycgr3G90786 Mycgr3T
  
Location: 10655-12080

Mycgr3G90786\_Mycgr3T

Mycgr3G68429 Mycgr3T
  
Location: 12180-13440

Mycgr3G68429\_Mycgr3T

Mycgr3G68421 Mycgr3T
  
Location: 13540-17086

Mycgr3G68421\_Mycgr3T

Mycgr3G90801 Mycgr3T
  
Location: 17186-18056

Mycgr3G90801\_Mycgr3T

Mycgr3G84646 Mycgr3T
  
Location: 18156-20235

Mycgr3G84646\_Mycgr3T

Mycgr3G68456 Mycgr3T
  
Location: 20335-21970

Mycgr3G68456\_Mycgr3T

Mycgr3G103270 Mycgr3
  
Location: 22070-22355

Mycgr3G103270\_Mycgr3

Mycgr3G90803 Mycgr3T
  
Location: 22455-23019

Mycgr3G90803\_Mycgr3T

Mycgr3G36941 Mycgr3T
  
Location: 23119-24064

Mycgr3G36941\_Mycgr3T

Mycgr3G25746 Mycgr3T
  
Location: 24164-25241

Mycgr3G25746\_Mycgr3T

Mycgr3G90788 Mycgr3T
  
Location: 25341-25803

Mycgr3G90788\_Mycgr3T

Mycgr3G103260 Mycgr3
  
Location: 25903-26635

Mycgr3G103260\_Mycgr3

Mycgr3G84644 Mycgr3T
  
Location: 26735-28457

Mycgr3G84644\_Mycgr3T

Mycgr3G29227 Mycgr3T
  
Location: 28557-28863

Mycgr3G29227\_Mycgr3T

Mycgr3G36271 Mycgr3T
  
Location: 28963-29854

Mycgr3G36271\_Mycgr3T

Mycgr3G68433 Mycgr3T
  
Location: 29954-33041

Mycgr3G68433\_Mycgr3T

Mycgr3G79452 Mycgr3T
  
Location: 33141-33399

Mycgr3G79452\_Mycgr3T

Mycgr3G55345 Mycgr3T
  
Location: 33499-34126

Mycgr3G55345\_Mycgr3T

Mycgr3G103278 Mycgr3
  
Location: 34226-35195

Mycgr3G103278\_Mycgr3

Mycgr3G84654 Mycgr3T
  
Location: 35295-36630

Mycgr3G84654\_Mycgr3T

Mycgr3G108090 Mycgr3
  
Location: 36730-37591

Mycgr3G108090\_Mycgr3

Mycgr3G21922 Mycgr3T
  
Location: 37691-39149

Mycgr3G21922\_Mycgr3T

Mycgr3G99148 Mycgr3T
  
Location: 39249-42819

Mycgr3G99148\_Mycgr3T

C-4 methyl sterol oxidase (Erg25), putative
  
Accession: EAW13770
  
Location: 62225-63082
  
  
**BlastP hit with Mycgr3G36271\_Mycgr3T**
  
Percentage identity: 80 %
  
BlastP bit score: 417
  
Sequence coverage: 80 %
  
E-value: 3e-144
  
  
 NCBI BlastP on this gene

EAW13770

conserved hypothetical protein
  
Accession: EAW13771
  
Location: 64637-66332
  
  
**BlastP hit with Mycgr3G84646\_Mycgr3T**
  
Percentage identity: 40 %
  
BlastP bit score: 417
  
Sequence coverage: 79 %
  
E-value: 3e-134
  
  
 NCBI BlastP on this gene

EAW13771

ubiquitin C-terminal hydrolase, putative
  
Accession: EAW13772
  
Location: 66542-69177
  
 NCBI BlastP on this gene

EAW13772

replication factor A 1, rfa1
  
Accession: EAW13773
  
Location: 70194-72332
  
 NCBI BlastP on this gene

EAW13773

dead box ATP-dependent rna helicase
  
Accession: EAW13774
  
Location: 73527-75166
  
 NCBI BlastP on this gene

EAW13774

Rft domain protein
  
Accession: EAW13775
  
Location: 75169-76650
  
 NCBI BlastP on this gene

EAW13775

oligosaccharyl transferase subunit (gamma), putative
  
Accession: EAW13776
  
Location: 77613-78780
  
 NCBI BlastP on this gene

EAW13776

hypothetical protein
  
Accession: EAW13777
  
Location: 79489-81063
  
 NCBI BlastP on this gene

EAW13777

cell division protein ftsj
  
Accession: EAW13778
  
Location: 82264-83467
  
 NCBI BlastP on this gene

EAW13778

autophagy ubiquitin-activating enzyme ApgG, putative
  
Accession: EAW13779
  
Location: 83586-86151
  
 NCBI BlastP on this gene

EAW13779

Query: Architecture Search FASTA input

KE374991 : Blumeria graminis f. sp. tritici 96224 unplaced genomic scaffold Scaffold-128    Total score: 2.0     Cumulative Blast bit score: 830

Hit cluster cross-links:

Mycgr3G90785 Mycgr3T
  
Location: 0-1047

Mycgr3G90785\_Mycgr3T

Mycgr3G103262 Mycgr3
  
Location: 1147-1390

Mycgr3G103262\_Mycgr3

Mycgr3G68458 Mycgr3T
  
Location: 1490-3602

Mycgr3G68458\_Mycgr3T

Mycgr3G99145 Mycgr3T
  
Location: 3702-4326

Mycgr3G99145\_Mycgr3T

Mycgr3G103274 Mycgr3
  
Location: 4426-4957

Mycgr3G103274\_Mycgr3

Mycgr3G103264 Mycgr3
  
Location: 5057-5390

Mycgr3G103264\_Mycgr3

Mycgr3G37570 Mycgr3T
  
Location: 5490-6006

Mycgr3G37570\_Mycgr3T

Mycgr3G108094 Mycgr3
  
Location: 6106-10555

Mycgr3G108094\_Mycgr3

Mycgr3G90786 Mycgr3T
  
Location: 10655-12080

Mycgr3G90786\_Mycgr3T

Mycgr3G68429 Mycgr3T
  
Location: 12180-13440

Mycgr3G68429\_Mycgr3T

Mycgr3G68421 Mycgr3T
  
Location: 13540-17086

Mycgr3G68421\_Mycgr3T

Mycgr3G90801 Mycgr3T
  
Location: 17186-18056

Mycgr3G90801\_Mycgr3T

Mycgr3G84646 Mycgr3T
  
Location: 18156-20235

Mycgr3G84646\_Mycgr3T

Mycgr3G68456 Mycgr3T
  
Location: 20335-21970

Mycgr3G68456\_Mycgr3T

Mycgr3G103270 Mycgr3
  
Location: 22070-22355

Mycgr3G103270\_Mycgr3

Mycgr3G90803 Mycgr3T
  
Location: 22455-23019

Mycgr3G90803\_Mycgr3T

Mycgr3G36941 Mycgr3T
  
Location: 23119-24064

Mycgr3G36941\_Mycgr3T

Mycgr3G25746 Mycgr3T
  
Location: 24164-25241

Mycgr3G25746\_Mycgr3T

Mycgr3G90788 Mycgr3T
  
Location: 25341-25803

Mycgr3G90788\_Mycgr3T

Mycgr3G103260 Mycgr3
  
Location: 25903-26635

Mycgr3G103260\_Mycgr3

Mycgr3G84644 Mycgr3T
  
Location: 26735-28457

Mycgr3G84644\_Mycgr3T

Mycgr3G29227 Mycgr3T
  
Location: 28557-28863

Mycgr3G29227\_Mycgr3T

Mycgr3G36271 Mycgr3T
  
Location: 28963-29854

Mycgr3G36271\_Mycgr3T

Mycgr3G68433 Mycgr3T
  
Location: 29954-33041

Mycgr3G68433\_Mycgr3T

Mycgr3G79452 Mycgr3T
  
Location: 33141-33399

Mycgr3G79452\_Mycgr3T

Mycgr3G55345 Mycgr3T
  
Location: 33499-34126

Mycgr3G55345\_Mycgr3T

Mycgr3G103278 Mycgr3
  
Location: 34226-35195

Mycgr3G103278\_Mycgr3

Mycgr3G84654 Mycgr3T
  
Location: 35295-36630

Mycgr3G84654\_Mycgr3T

Mycgr3G108090 Mycgr3
  
Location: 36730-37591

Mycgr3G108090\_Mycgr3

Mycgr3G21922 Mycgr3T
  
Location: 37691-39149

Mycgr3G21922\_Mycgr3T

Mycgr3G99148 Mycgr3T
  
Location: 39249-42819

Mycgr3G99148\_Mycgr3T

Serine esterase
  
Accession: EPQ66681
  
Location: 131024-135445
  
 NCBI BlastP on this gene

EPQ66681

Alpha subunit of the translation initiation factor eIF2
  
Accession: EPQ66682
  
Location: 136099-136429
  
 NCBI BlastP on this gene

EPQ66682

hypothetical protein
  
Accession: EPQ66683
  
Location: 136535-137209
  
 NCBI BlastP on this gene

EPQ66683

Catalytic subunit of the mitochondrial inner membrane peptidase complex
  
Accession: EPQ66684
  
Location: 137480-138132
  
 NCBI BlastP on this gene

EPQ66684

transporter of the mitochondrial inner membrane
  
Accession: EPQ66685
  
Location: 138426-139722
  
 NCBI BlastP on this gene

EPQ66685

hypothetical protein
  
Accession: EPQ66686
  
Location: 146089-148896
  
 NCBI BlastP on this gene

EPQ66686

hypothetical protein
  
Accession: EPQ66687
  
Location: 149325-151360
  
  
**BlastP hit with Mycgr3G84646\_Mycgr3T**
  
Percentage identity: 38 %
  
BlastP bit score: 475
  
Sequence coverage: 100 %
  
E-value: 9e-155
  
  
 NCBI BlastP on this gene

EPQ66687

Secretory vesicle-associated Rab GTPase
  
Accession: EPQ66688
  
Location: 151948-152685
  
  
**BlastP hit with Mycgr3G99145\_Mycgr3T**
  
Percentage identity: 84 %
  
BlastP bit score: 355
  
Sequence coverage: 99 %
  
E-value: 7e-122
  
  
 NCBI BlastP on this gene

EPQ66688

hypothetical protein
  
Accession: EPQ66689
  
Location: 152848-154636
  
 NCBI BlastP on this gene

EPQ66689

Query: Architecture Search FASTA input

FP929137 : Leptosphaeria maculans JN3 lm\_SuperContig\_10\_v2 genomic supercontig    Total score: 2.0     Cumulative Blast bit score: 810

Hit cluster cross-links:

Mycgr3G90785 Mycgr3T
  
Location: 0-1047

Mycgr3G90785\_Mycgr3T

Mycgr3G103262 Mycgr3
  
Location: 1147-1390

Mycgr3G103262\_Mycgr3

Mycgr3G68458 Mycgr3T
  
Location: 1490-3602

Mycgr3G68458\_Mycgr3T

Mycgr3G99145 Mycgr3T
  
Location: 3702-4326

Mycgr3G99145\_Mycgr3T

Mycgr3G103274 Mycgr3
  
Location: 4426-4957

Mycgr3G103274\_Mycgr3

Mycgr3G103264 Mycgr3
  
Location: 5057-5390

Mycgr3G103264\_Mycgr3

Mycgr3G37570 Mycgr3T
  
Location: 5490-6006

Mycgr3G37570\_Mycgr3T

Mycgr3G108094 Mycgr3
  
Location: 6106-10555

Mycgr3G108094\_Mycgr3

Mycgr3G90786 Mycgr3T
  
Location: 10655-12080

Mycgr3G90786\_Mycgr3T

Mycgr3G68429 Mycgr3T
  
Location: 12180-13440

Mycgr3G68429\_Mycgr3T

Mycgr3G68421 Mycgr3T
  
Location: 13540-17086

Mycgr3G68421\_Mycgr3T

Mycgr3G90801 Mycgr3T
  
Location: 17186-18056

Mycgr3G90801\_Mycgr3T

Mycgr3G84646 Mycgr3T
  
Location: 18156-20235

Mycgr3G84646\_Mycgr3T

Mycgr3G68456 Mycgr3T
  
Location: 20335-21970

Mycgr3G68456\_Mycgr3T

Mycgr3G103270 Mycgr3
  
Location: 22070-22355

Mycgr3G103270\_Mycgr3

Mycgr3G90803 Mycgr3T
  
Location: 22455-23019

Mycgr3G90803\_Mycgr3T

Mycgr3G36941 Mycgr3T
  
Location: 23119-24064

Mycgr3G36941\_Mycgr3T

Mycgr3G25746 Mycgr3T
  
Location: 24164-25241

Mycgr3G25746\_Mycgr3T

Mycgr3G90788 Mycgr3T
  
Location: 25341-25803

Mycgr3G90788\_Mycgr3T

Mycgr3G103260 Mycgr3
  
Location: 25903-26635

Mycgr3G103260\_Mycgr3

Mycgr3G84644 Mycgr3T
  
Location: 26735-28457

Mycgr3G84644\_Mycgr3T

Mycgr3G29227 Mycgr3T
  
Location: 28557-28863

Mycgr3G29227\_Mycgr3T

Mycgr3G36271 Mycgr3T
  
Location: 28963-29854

Mycgr3G36271\_Mycgr3T

Mycgr3G68433 Mycgr3T
  
Location: 29954-33041

Mycgr3G68433\_Mycgr3T

Mycgr3G79452 Mycgr3T
  
Location: 33141-33399

Mycgr3G79452\_Mycgr3T

Mycgr3G55345 Mycgr3T
  
Location: 33499-34126

Mycgr3G55345\_Mycgr3T

Mycgr3G103278 Mycgr3
  
Location: 34226-35195

Mycgr3G103278\_Mycgr3

Mycgr3G84654 Mycgr3T
  
Location: 35295-36630

Mycgr3G84654\_Mycgr3T

Mycgr3G108090 Mycgr3
  
Location: 36730-37591

Mycgr3G108090\_Mycgr3

Mycgr3G21922 Mycgr3T
  
Location: 37691-39149

Mycgr3G21922\_Mycgr3T

Mycgr3G99148 Mycgr3T
  
Location: 39249-42819

Mycgr3G99148\_Mycgr3T

predicted protein
  
Accession: CBY00095
  
Location: 1616833-1617024
  
 NCBI BlastP on this gene

LEMA\_uP076840.1

similar to DNA repair protein Rhp26/Rad26
  
Accession: CBY00094
  
Location: 1612589-1616263
  
 NCBI BlastP on this gene

LEMA\_P076830.1

predicted protein
  
Accession: CBY00093
  
Location: 1611614-1611859
  
 NCBI BlastP on this gene

LEMA\_P076820.1

hypothetical protein
  
Accession: CBY00092
  
Location: 1604091-1605375
  
  
**BlastP hit with Mycgr3G36271\_Mycgr3T**
  
Percentage identity: 78 %
  
BlastP bit score: 475
  
Sequence coverage: 97 %
  
E-value: 3e-165
  
  
 NCBI BlastP on this gene

LEMA\_P076810.1

hypothetical protein
  
Accession: CBY00091
  
Location: 1602045-1603501
  
 NCBI BlastP on this gene

LEMA\_P076800.1

hypothetical protein
  
Accession: CBY00090
  
Location: 1600793-1601514
  
 NCBI BlastP on this gene

LEMA\_P076790.1

hypothetical protein
  
Accession: CBY00089
  
Location: 1596937-1600039
  
 NCBI BlastP on this gene

LEMA\_P076780.1

hypothetical protein
  
Accession: CBY00088
  
Location: 1594876-1595501
  
 NCBI BlastP on this gene

LEMA\_P076770.1

similar to ubiquitin-conjugating enzyme
  
Accession: CBY00087
  
Location: 1593095-1593922
  
 NCBI BlastP on this gene

LEMA\_P076760.1

similar to mitochondrial outer membrane protein (Sam50)
  
Accession: CBY00086
  
Location: 1591028-1592817
  
 NCBI BlastP on this gene

LEMA\_P076750.1

hypothetical protein
  
Accession: CBY00085
  
Location: 1587412-1590210
  
 NCBI BlastP on this gene

LEMA\_P076740.1

hypothetical protein
  
Accession: CBY00084
  
Location: 1583920-1585846
  
 NCBI BlastP on this gene

LEMA\_P076730.1

similar to xanthine phosphoribosyltransferase
  
Accession: CBY00083
  
Location: 1582161-1583053
  
  
**BlastP hit with Mycgr3G55345\_Mycgr3T**
  
Percentage identity: 80 %
  
BlastP bit score: 335
  
Sequence coverage: 95 %
  
E-value: 1e-113
  
  
 NCBI BlastP on this gene

LEMA\_P076720.1

predicted protein
  
Accession: CBY00082
  
Location: 1581098-1581253
  
 NCBI BlastP on this gene

LEMA\_P076710.1

hypothetical protein
  
Accession: CBY00081
  
Location: 1578259-1580625
  
 NCBI BlastP on this gene

LEMA\_P076700.1

predicted protein
  
Accession: CBY00080
  
Location: 1576541-1577198
  
 NCBI BlastP on this gene

LEMA\_P076690.1

predicted protein
  
Accession: CBY00079
  
Location: 1575621-1576371
  
 NCBI BlastP on this gene

LEMA\_P076680.1

predicted protein
  
Accession: CBY00078
  
Location: 1574997-1575290
  
 NCBI BlastP on this gene

LEMA\_P076670.1

similar to ubiquitin-conjugating enzyme E2
  
Accession: CBY00077
  
Location: 1574059-1574665
  
 NCBI BlastP on this gene

LEMA\_P076660.1

predicted protein
  
Accession: CBY00076
  
Location: 1573044-1573382
  
 NCBI BlastP on this gene

LEMA\_P076650.1

hypothetical protein
  
Accession: CBY00075
  
Location: 1569971-1572972
  
 NCBI BlastP on this gene

LEMA\_P076640.1

Query: Architecture Search FASTA input

AAHF01000016 : Aspergillus fumigatus Af293    Total score: 2.0     Cumulative Blast bit score: 809

Hit cluster cross-links:

Mycgr3G90785 Mycgr3T
  
Location: 0-1047

Mycgr3G90785\_Mycgr3T

Mycgr3G103262 Mycgr3
  
Location: 1147-1390

Mycgr3G103262\_Mycgr3

Mycgr3G68458 Mycgr3T
  
Location: 1490-3602

Mycgr3G68458\_Mycgr3T

Mycgr3G99145 Mycgr3T
  
Location: 3702-4326

Mycgr3G99145\_Mycgr3T

Mycgr3G103274 Mycgr3
  
Location: 4426-4957

Mycgr3G103274\_Mycgr3

Mycgr3G103264 Mycgr3
  
Location: 5057-5390

Mycgr3G103264\_Mycgr3

Mycgr3G37570 Mycgr3T
  
Location: 5490-6006

Mycgr3G37570\_Mycgr3T

Mycgr3G108094 Mycgr3
  
Location: 6106-10555

Mycgr3G108094\_Mycgr3

Mycgr3G90786 Mycgr3T
  
Location: 10655-12080

Mycgr3G90786\_Mycgr3T

Mycgr3G68429 Mycgr3T
  
Location: 12180-13440

Mycgr3G68429\_Mycgr3T

Mycgr3G68421 Mycgr3T
  
Location: 13540-17086

Mycgr3G68421\_Mycgr3T

Mycgr3G90801 Mycgr3T
  
Location: 17186-18056

Mycgr3G90801\_Mycgr3T

Mycgr3G84646 Mycgr3T
  
Location: 18156-20235

Mycgr3G84646\_Mycgr3T

Mycgr3G68456 Mycgr3T
  
Location: 20335-21970

Mycgr3G68456\_Mycgr3T

Mycgr3G103270 Mycgr3
  
Location: 22070-22355

Mycgr3G103270\_Mycgr3

Mycgr3G90803 Mycgr3T
  
Location: 22455-23019

Mycgr3G90803\_Mycgr3T

Mycgr3G36941 Mycgr3T
  
Location: 23119-24064

Mycgr3G36941\_Mycgr3T

Mycgr3G25746 Mycgr3T
  
Location: 24164-25241

Mycgr3G25746\_Mycgr3T

Mycgr3G90788 Mycgr3T
  
Location: 25341-25803

Mycgr3G90788\_Mycgr3T

Mycgr3G103260 Mycgr3
  
Location: 25903-26635

Mycgr3G103260\_Mycgr3

Mycgr3G84644 Mycgr3T
  
Location: 26735-28457

Mycgr3G84644\_Mycgr3T

Mycgr3G29227 Mycgr3T
  
Location: 28557-28863

Mycgr3G29227\_Mycgr3T

Mycgr3G36271 Mycgr3T
  
Location: 28963-29854

Mycgr3G36271\_Mycgr3T

Mycgr3G68433 Mycgr3T
  
Location: 29954-33041

Mycgr3G68433\_Mycgr3T

Mycgr3G79452 Mycgr3T
  
Location: 33141-33399

Mycgr3G79452\_Mycgr3T

Mycgr3G55345 Mycgr3T
  
Location: 33499-34126

Mycgr3G55345\_Mycgr3T

Mycgr3G103278 Mycgr3
  
Location: 34226-35195

Mycgr3G103278\_Mycgr3

Mycgr3G84654 Mycgr3T
  
Location: 35295-36630

Mycgr3G84654\_Mycgr3T

Mycgr3G108090 Mycgr3
  
Location: 36730-37591

Mycgr3G108090\_Mycgr3

Mycgr3G21922 Mycgr3T
  
Location: 37691-39149

Mycgr3G21922\_Mycgr3T

Mycgr3G99148 Mycgr3T
  
Location: 39249-42819

Mycgr3G99148\_Mycgr3T

replication fork protection component Swi3, putative
  
Accession: EBA27200
  
Location: 629994-630926
  
 NCBI BlastP on this gene

EBA27200

NADH-ubiquinone oxidoreductase 21 kDa subunit, putative
  
Accession: EAL84623
  
Location: 631866-632422
  
 NCBI BlastP on this gene

EAL84623

inositol kinase kinase (UvsB), putative
  
Accession: EAL84624
  
Location: 634125-642965
  
 NCBI BlastP on this gene

EAL84624

AAA family ATPase, putative
  
Accession: EAL84628
  
Location: 644717-647044
  
 NCBI BlastP on this gene

EAL84628

Rab GTPase SrgA, putative
  
Accession: EAL84629
  
Location: 647907-648651
  
  
**BlastP hit with Mycgr3G99145\_Mycgr3T**
  
Percentage identity: 89 %
  
BlastP bit score: 340
  
Sequence coverage: 87 %
  
E-value: 1e-115
  
  
 NCBI BlastP on this gene

EAL84629

C-4 methyl sterol oxidase Erg25, putative
  
Accession: EAL84630
  
Location: 653072-654129
  
  
**BlastP hit with Mycgr3G36271\_Mycgr3T**
  
Percentage identity: 75 %
  
BlastP bit score: 469
  
Sequence coverage: 95 %
  
E-value: 7e-164
  
  
 NCBI BlastP on this gene

EAL84630

Query: Architecture Search FASTA input

CH408034 : Chaetomium globosum CBS 148.51 scaffold\_6 genomic scaffold    Total score: 2.0     Cumulative Blast bit score: 793

Hit cluster cross-links:

Mycgr3G90785 Mycgr3T
  
Location: 0-1047

Mycgr3G90785\_Mycgr3T

Mycgr3G103262 Mycgr3
  
Location: 1147-1390

Mycgr3G103262\_Mycgr3

Mycgr3G68458 Mycgr3T
  
Location: 1490-3602

Mycgr3G68458\_Mycgr3T

Mycgr3G99145 Mycgr3T
  
Location: 3702-4326

Mycgr3G99145\_Mycgr3T

Mycgr3G103274 Mycgr3
  
Location: 4426-4957

Mycgr3G103274\_Mycgr3

Mycgr3G103264 Mycgr3
  
Location: 5057-5390

Mycgr3G103264\_Mycgr3

Mycgr3G37570 Mycgr3T
  
Location: 5490-6006

Mycgr3G37570\_Mycgr3T

Mycgr3G108094 Mycgr3
  
Location: 6106-10555

Mycgr3G108094\_Mycgr3

Mycgr3G90786 Mycgr3T
  
Location: 10655-12080

Mycgr3G90786\_Mycgr3T

Mycgr3G68429 Mycgr3T
  
Location: 12180-13440

Mycgr3G68429\_Mycgr3T

Mycgr3G68421 Mycgr3T
  
Location: 13540-17086

Mycgr3G68421\_Mycgr3T

Mycgr3G90801 Mycgr3T
  
Location: 17186-18056

Mycgr3G90801\_Mycgr3T

Mycgr3G84646 Mycgr3T
  
Location: 18156-20235

Mycgr3G84646\_Mycgr3T

Mycgr3G68456 Mycgr3T
  
Location: 20335-21970

Mycgr3G68456\_Mycgr3T

Mycgr3G103270 Mycgr3
  
Location: 22070-22355

Mycgr3G103270\_Mycgr3

Mycgr3G90803 Mycgr3T
  
Location: 22455-23019

Mycgr3G90803\_Mycgr3T

Mycgr3G36941 Mycgr3T
  
Location: 23119-24064

Mycgr3G36941\_Mycgr3T

Mycgr3G25746 Mycgr3T
  
Location: 24164-25241

Mycgr3G25746\_Mycgr3T

Mycgr3G90788 Mycgr3T
  
Location: 25341-25803

Mycgr3G90788\_Mycgr3T

Mycgr3G103260 Mycgr3
  
Location: 25903-26635

Mycgr3G103260\_Mycgr3

Mycgr3G84644 Mycgr3T
  
Location: 26735-28457

Mycgr3G84644\_Mycgr3T

Mycgr3G29227 Mycgr3T
  
Location: 28557-28863

Mycgr3G29227\_Mycgr3T

Mycgr3G36271 Mycgr3T
  
Location: 28963-29854

Mycgr3G36271\_Mycgr3T

Mycgr3G68433 Mycgr3T
  
Location: 29954-33041

Mycgr3G68433\_Mycgr3T

Mycgr3G79452 Mycgr3T
  
Location: 33141-33399

Mycgr3G79452\_Mycgr3T

Mycgr3G55345 Mycgr3T
  
Location: 33499-34126

Mycgr3G55345\_Mycgr3T

Mycgr3G103278 Mycgr3
  
Location: 34226-35195

Mycgr3G103278\_Mycgr3

Mycgr3G84654 Mycgr3T
  
Location: 35295-36630

Mycgr3G84654\_Mycgr3T

Mycgr3G108090 Mycgr3
  
Location: 36730-37591

Mycgr3G108090\_Mycgr3

Mycgr3G21922 Mycgr3T
  
Location: 37691-39149

Mycgr3G21922\_Mycgr3T

Mycgr3G99148 Mycgr3T
  
Location: 39249-42819

Mycgr3G99148\_Mycgr3T

hypothetical protein
  
Accession: EAQ84787
  
Location: 1431377-1432347
  
 NCBI BlastP on this gene

EAQ84787

conserved hypothetical protein
  
Accession: EAQ84788
  
Location: 1437231-1438556
  
 NCBI BlastP on this gene

EAQ84788

predicted protein
  
Accession: EAQ84789
  
Location: 1439143-1439926
  
 NCBI BlastP on this gene

EAQ84789

hypothetical protein
  
Accession: EAQ84790
  
Location: 1440788-1442685
  
 NCBI BlastP on this gene

EAQ84790

hypothetical protein
  
Accession: EAQ84791
  
Location: 1443880-1445941
  
 NCBI BlastP on this gene

EAQ84791

predicted protein
  
Accession: EAQ84792
  
Location: 1446677-1448003
  
 NCBI BlastP on this gene

EAQ84792

hypothetical protein
  
Accession: EAQ84793
  
Location: 1448878-1452371
  
  
**BlastP hit with Mycgr3G68433\_Mycgr3T**
  
Percentage identity: 37 %
  
BlastP bit score: 484
  
Sequence coverage: 96 %
  
E-value: 1e-148
  
  
 NCBI BlastP on this gene

EAQ84793

hypothetical protein
  
Accession: EAQ84794
  
Location: 1453755-1454221
  
 NCBI BlastP on this gene

EAQ84794

hypothetical protein
  
Accession: EAQ84795
  
Location: 1455641-1456847
  
 NCBI BlastP on this gene

EAQ84795

predicted protein
  
Accession: EAQ84796
  
Location: 1457200-1458630
  
 NCBI BlastP on this gene

EAQ84796

conserved hypothetical protein
  
Accession: EAQ84797
  
Location: 1459088-1459867
  
  
**BlastP hit with Mycgr3G55345\_Mycgr3T**
  
Percentage identity: 74 %
  
BlastP bit score: 309
  
Sequence coverage: 99 %
  
E-value: 1e-103
  
  
 NCBI BlastP on this gene

EAQ84797

hypothetical protein
  
Accession: EAQ84798
  
Location: 1461272-1462414
  
 NCBI BlastP on this gene

EAQ84798

hypothetical protein
  
Accession: EAQ84799
  
Location: 1463789-1464340
  
 NCBI BlastP on this gene

EAQ84799

hypothetical protein
  
Accession: EAQ84800
  
Location: 1466459-1467697
  
 NCBI BlastP on this gene

EAQ84800

predicted protein
  
Accession: EAQ84801
  
Location: 1469841-1471274
  
 NCBI BlastP on this gene

EAQ84801

hypothetical protein
  
Accession: EAQ84802
  
Location: 1472181-1474545
  
 NCBI BlastP on this gene

EAQ84802

predicted protein
  
Accession: EAQ84803
  
Location: 1474922-1475612
  
 NCBI BlastP on this gene

EAQ84803

predicted protein
  
Accession: EAQ84804
  
Location: 1476811-1477149
  
 NCBI BlastP on this gene

EAQ84804

Query: Architecture Search FASTA input

AM920430 : Penicillium chrysogenum Wisconsin 54-1255 complete genome, contig Pc00c15.    Total score: 2.0     Cumulative Blast bit score: 788

Hit cluster cross-links:

Mycgr3G90785 Mycgr3T
  
Location: 0-1047

Mycgr3G90785\_Mycgr3T

Mycgr3G103262 Mycgr3
  
Location: 1147-1390

Mycgr3G103262\_Mycgr3

Mycgr3G68458 Mycgr3T
  
Location: 1490-3602

Mycgr3G68458\_Mycgr3T

Mycgr3G99145 Mycgr3T
  
Location: 3702-4326

Mycgr3G99145\_Mycgr3T

Mycgr3G103274 Mycgr3
  
Location: 4426-4957

Mycgr3G103274\_Mycgr3

Mycgr3G103264 Mycgr3
  
Location: 5057-5390

Mycgr3G103264\_Mycgr3

Mycgr3G37570 Mycgr3T
  
Location: 5490-6006

Mycgr3G37570\_Mycgr3T

Mycgr3G108094 Mycgr3
  
Location: 6106-10555

Mycgr3G108094\_Mycgr3

Mycgr3G90786 Mycgr3T
  
Location: 10655-12080

Mycgr3G90786\_Mycgr3T

Mycgr3G68429 Mycgr3T
  
Location: 12180-13440

Mycgr3G68429\_Mycgr3T

Mycgr3G68421 Mycgr3T
  
Location: 13540-17086

Mycgr3G68421\_Mycgr3T

Mycgr3G90801 Mycgr3T
  
Location: 17186-18056

Mycgr3G90801\_Mycgr3T

Mycgr3G84646 Mycgr3T
  
Location: 18156-20235

Mycgr3G84646\_Mycgr3T

Mycgr3G68456 Mycgr3T
  
Location: 20335-21970

Mycgr3G68456\_Mycgr3T

Mycgr3G103270 Mycgr3
  
Location: 22070-22355

Mycgr3G103270\_Mycgr3

Mycgr3G90803 Mycgr3T
  
Location: 22455-23019

Mycgr3G90803\_Mycgr3T

Mycgr3G36941 Mycgr3T
  
Location: 23119-24064

Mycgr3G36941\_Mycgr3T

Mycgr3G25746 Mycgr3T
  
Location: 24164-25241

Mycgr3G25746\_Mycgr3T

Mycgr3G90788 Mycgr3T
  
Location: 25341-25803

Mycgr3G90788\_Mycgr3T

Mycgr3G103260 Mycgr3
  
Location: 25903-26635

Mycgr3G103260\_Mycgr3

Mycgr3G84644 Mycgr3T
  
Location: 26735-28457

Mycgr3G84644\_Mycgr3T

Mycgr3G29227 Mycgr3T
  
Location: 28557-28863

Mycgr3G29227\_Mycgr3T

Mycgr3G36271 Mycgr3T
  
Location: 28963-29854

Mycgr3G36271\_Mycgr3T

Mycgr3G68433 Mycgr3T
  
Location: 29954-33041

Mycgr3G68433\_Mycgr3T

Mycgr3G79452 Mycgr3T
  
Location: 33141-33399

Mycgr3G79452\_Mycgr3T

Mycgr3G55345 Mycgr3T
  
Location: 33499-34126

Mycgr3G55345\_Mycgr3T

Mycgr3G103278 Mycgr3
  
Location: 34226-35195

Mycgr3G103278\_Mycgr3

Mycgr3G84654 Mycgr3T
  
Location: 35295-36630

Mycgr3G84654\_Mycgr3T

Mycgr3G108090 Mycgr3
  
Location: 36730-37591

Mycgr3G108090\_Mycgr3

Mycgr3G21922 Mycgr3T
  
Location: 37691-39149

Mycgr3G21922\_Mycgr3T

Mycgr3G99148 Mycgr3T
  
Location: 39249-42819

Mycgr3G99148\_Mycgr3T

not annotated
  
Accession: CAP82896
  
Location: 22858-23857
  
 NCBI BlastP on this gene

Pc15g00100

unnamed
  
Accession: CAP82897
  
Location: 24264-25199
  
 NCBI BlastP on this gene

Pc15g00110

not annotated
  
Accession: CAP82898
  
Location: 25458-27296
  
 NCBI BlastP on this gene

Pc15g00120

not annotated
  
Accession: CAP82899
  
Location: 27929-28997
  
 NCBI BlastP on this gene

Pc15g00130

not annotated
  
Accession: CAP82900
  
Location: 29714-30998
  
 NCBI BlastP on this gene

Pc15g00140

unnamed
  
Accession: CAP82901
  
Location: 31201-31680
  
 NCBI BlastP on this gene

Pc15g00150

not annotated
  
Accession: CAP82902
  
Location: 31867-32969
  
 NCBI BlastP on this gene

Pc15g00160

not annotated
  
Accession: CAP82903
  
Location: 33323-34048
  
 NCBI BlastP on this gene

Pc15g00170

not annotated
  
Accession: CAP82904
  
Location: 35931-36762
  
 NCBI BlastP on this gene

Pc15g00180

not annotated
  
Accession: CAP82905
  
Location: 38020-38999
  
  
**BlastP hit with Mycgr3G29227\_Mycgr3T**
  
Percentage identity: 38 %
  
BlastP bit score: 87
  
Sequence coverage: 100 %
  
E-value: 1e-18
  
  
 NCBI BlastP on this gene

Pc15g00190

not annotated
  
Accession: CAP82906
  
Location: 39583-41199
  
 NCBI BlastP on this gene

Pc15g00200

unnamed
  
Accession: CAP82907
  
Location: 42876-44360
  
 NCBI BlastP on this gene

Pc15g00210

not annotated
  
Accession: CAP82908
  
Location: 45100-46122
  
 NCBI BlastP on this gene

Pc15g00220

not annotated
  
Accession: Pc15g00230
  
Location: 47726-49288
  
 NCBI BlastP on this gene

Pc15g00230

unnamed
  
Accession: CAP82910
  
Location: 50742-51370
  
 NCBI BlastP on this gene

Pc15g00240

not annotated
  
Accession: CAP82911
  
Location: 51860-53308
  
 NCBI BlastP on this gene

Pc15g00250

not annotated
  
Accession: CAP82912
  
Location: 53667-55543
  
  
**BlastP hit with Mycgr3G68456\_Mycgr3T**
  
Percentage identity: 69 %
  
BlastP bit score: 701
  
Sequence coverage: 91 %
  
E-value: 0.0
  
  
 NCBI BlastP on this gene

Pc15g00260

not annotated
  
Accession: CAP82913
  
Location: 56105-57285
  
 NCBI BlastP on this gene

Pc15g00270

not annotated
  
Accession: CAP82914
  
Location: 59443-60839
  
 NCBI BlastP on this gene

Pc15g00280

hypothetical protein
  
Accession: CAP82915
  
Location: 61551-62374
  
 NCBI BlastP on this gene

Pc15g00290

not annotated
  
Accession: CAP82916
  
Location: 63685-64632
  
 NCBI BlastP on this gene

Pc15g00300

not annotated
  
Accession: CAP82917
  
Location: 65299-68102
  
 NCBI BlastP on this gene

Pc15g00310

not annotated
  
Accession: CAP82918
  
Location: 68443-69499
  
 NCBI BlastP on this gene

Pc15g00320

not annotated
  
Accession: CAP82919
  
Location: 69723-71264
  
 NCBI BlastP on this gene

Pc15g00330

Query: Architecture Search FASTA input

ABDF02000085 : Trichoderma virens Gv29-8    Total score: 2.0     Cumulative Blast bit score: 784

Hit cluster cross-links:

Mycgr3G90785 Mycgr3T
  
Location: 0-1047

Mycgr3G90785\_Mycgr3T

Mycgr3G103262 Mycgr3
  
Location: 1147-1390

Mycgr3G103262\_Mycgr3

Mycgr3G68458 Mycgr3T
  
Location: 1490-3602

Mycgr3G68458\_Mycgr3T

Mycgr3G99145 Mycgr3T
  
Location: 3702-4326

Mycgr3G99145\_Mycgr3T

Mycgr3G103274 Mycgr3
  
Location: 4426-4957

Mycgr3G103274\_Mycgr3

Mycgr3G103264 Mycgr3
  
Location: 5057-5390

Mycgr3G103264\_Mycgr3

Mycgr3G37570 Mycgr3T
  
Location: 5490-6006

Mycgr3G37570\_Mycgr3T

Mycgr3G108094 Mycgr3
  
Location: 6106-10555

Mycgr3G108094\_Mycgr3

Mycgr3G90786 Mycgr3T
  
Location: 10655-12080

Mycgr3G90786\_Mycgr3T

Mycgr3G68429 Mycgr3T
  
Location: 12180-13440

Mycgr3G68429\_Mycgr3T

Mycgr3G68421 Mycgr3T
  
Location: 13540-17086

Mycgr3G68421\_Mycgr3T

Mycgr3G90801 Mycgr3T
  
Location: 17186-18056

Mycgr3G90801\_Mycgr3T

Mycgr3G84646 Mycgr3T
  
Location: 18156-20235

Mycgr3G84646\_Mycgr3T

Mycgr3G68456 Mycgr3T
  
Location: 20335-21970

Mycgr3G68456\_Mycgr3T

Mycgr3G103270 Mycgr3
  
Location: 22070-22355

Mycgr3G103270\_Mycgr3

Mycgr3G90803 Mycgr3T
  
Location: 22455-23019

Mycgr3G90803\_Mycgr3T

Mycgr3G36941 Mycgr3T
  
Location: 23119-24064

Mycgr3G36941\_Mycgr3T

Mycgr3G25746 Mycgr3T
  
Location: 24164-25241

Mycgr3G25746\_Mycgr3T

Mycgr3G90788 Mycgr3T
  
Location: 25341-25803

Mycgr3G90788\_Mycgr3T

Mycgr3G103260 Mycgr3
  
Location: 25903-26635

Mycgr3G103260\_Mycgr3

Mycgr3G84644 Mycgr3T
  
Location: 26735-28457

Mycgr3G84644\_Mycgr3T

Mycgr3G29227 Mycgr3T
  
Location: 28557-28863

Mycgr3G29227\_Mycgr3T

Mycgr3G36271 Mycgr3T
  
Location: 28963-29854

Mycgr3G36271\_Mycgr3T

Mycgr3G68433 Mycgr3T
  
Location: 29954-33041

Mycgr3G68433\_Mycgr3T

Mycgr3G79452 Mycgr3T
  
Location: 33141-33399

Mycgr3G79452\_Mycgr3T

Mycgr3G55345 Mycgr3T
  
Location: 33499-34126

Mycgr3G55345\_Mycgr3T

Mycgr3G103278 Mycgr3
  
Location: 34226-35195

Mycgr3G103278\_Mycgr3

Mycgr3G84654 Mycgr3T
  
Location: 35295-36630

Mycgr3G84654\_Mycgr3T

Mycgr3G108090 Mycgr3
  
Location: 36730-37591

Mycgr3G108090\_Mycgr3

Mycgr3G21922 Mycgr3T
  
Location: 37691-39149

Mycgr3G21922\_Mycgr3T

Mycgr3G99148 Mycgr3T
  
Location: 39249-42819

Mycgr3G99148\_Mycgr3T

glycosyltransferase family 32 protein
  
Accession: EHK18966
  
Location: 1084261-1085449
  
 NCBI BlastP on this gene

EHK18966

hypothetical protein
  
Accession: EHK18967
  
Location: 1088580-1090301
  
 NCBI BlastP on this gene

EHK18967

hypothetical protein
  
Accession: EHK18968
  
Location: 1090788-1093164
  
 NCBI BlastP on this gene

EHK18968

hypothetical protein
  
Accession: EHK18969
  
Location: 1093421-1094365
  
 NCBI BlastP on this gene

EHK18969

glycosyltransferase family 8 protein
  
Accession: EHK19031
  
Location: 1094963-1096131
  
 NCBI BlastP on this gene

EHK19031

hypothetical protein
  
Accession: EHK18970
  
Location: 1097172-1098890
  
 NCBI BlastP on this gene

EHK18970

hypothetical protein
  
Accession: EHK18971
  
Location: 1099282-1100949
  
  
**BlastP hit with Mycgr3G68456\_Mycgr3T**
  
Percentage identity: 61 %
  
BlastP bit score: 645
  
Sequence coverage: 97 %
  
E-value: 0.0
  
  
 NCBI BlastP on this gene

EHK18971

hypothetical protein
  
Accession: EHK18972
  
Location: 1106489-1107190
  
 NCBI BlastP on this gene

EHK18972

hypothetical protein
  
Accession: EHK18973
  
Location: 1107359-1108267
  
 NCBI BlastP on this gene

EHK18973

glycoside hydrolase family 18 protein
  
Accession: EHK19035
  
Location: 1109120-1113383
  
 NCBI BlastP on this gene

EHK19035

hypothetical protein
  
Accession: EHK18974
  
Location: 1114868-1116901
  
 NCBI BlastP on this gene

EHK18974

hypothetical protein
  
Accession: EHK18975
  
Location: 1118038-1118910
  
  
**BlastP hit with Mycgr3G108090\_Mycgr3**
  
Percentage identity: 35 %
  
BlastP bit score: 140
  
Sequence coverage: 83 %
  
E-value: 7e-36
  
  
 NCBI BlastP on this gene

EHK18975

hypothetical protein
  
Accession: EHK18976
  
Location: 1118941-1119804
  
 NCBI BlastP on this gene

EHK18976

hypothetical protein
  
Accession: EHK18977
  
Location: 1120335-1121677
  
 NCBI BlastP on this gene

EHK18977

hypothetical protein
  
Accession: EHK18978
  
Location: 1122745-1124465
  
 NCBI BlastP on this gene

EHK18978

hypothetical protein
  
Accession: EHK18979
  
Location: 1124553-1125989
  
 NCBI BlastP on this gene

EHK18979

hypothetical protein
  
Accession: EHK18980
  
Location: 1127889-1128587
  
 NCBI BlastP on this gene

EHK18980

hypothetical protein
  
Accession: EHK18981
  
Location: 1129535-1130227
  
 NCBI BlastP on this gene

EHK18981

hypothetical protein
  
Accession: EHK18982
  
Location: 1130561-1131325
  
 NCBI BlastP on this gene

EHK18982

hypothetical protein
  
Accession: EHK19036
  
Location: 1131794-1133378
  
 NCBI BlastP on this gene

EHK19036

Query: Architecture Search FASTA input

EQ962655 : Talaromyces stipitatus ATCC 10500 scf\_1105507295555 genomic scaffold    Total score: 2.0     Cumulative Blast bit score: 776

Hit cluster cross-links:

Mycgr3G90785 Mycgr3T
  
Location: 0-1047

Mycgr3G90785\_Mycgr3T

Mycgr3G103262 Mycgr3
  
Location: 1147-1390

Mycgr3G103262\_Mycgr3

Mycgr3G68458 Mycgr3T
  
Location: 1490-3602

Mycgr3G68458\_Mycgr3T

Mycgr3G99145 Mycgr3T
  
Location: 3702-4326

Mycgr3G99145\_Mycgr3T

Mycgr3G103274 Mycgr3
  
Location: 4426-4957

Mycgr3G103274\_Mycgr3

Mycgr3G103264 Mycgr3
  
Location: 5057-5390

Mycgr3G103264\_Mycgr3

Mycgr3G37570 Mycgr3T
  
Location: 5490-6006

Mycgr3G37570\_Mycgr3T

Mycgr3G108094 Mycgr3
  
Location: 6106-10555

Mycgr3G108094\_Mycgr3

Mycgr3G90786 Mycgr3T
  
Location: 10655-12080

Mycgr3G90786\_Mycgr3T

Mycgr3G68429 Mycgr3T
  
Location: 12180-13440

Mycgr3G68429\_Mycgr3T

Mycgr3G68421 Mycgr3T
  
Location: 13540-17086

Mycgr3G68421\_Mycgr3T

Mycgr3G90801 Mycgr3T
  
Location: 17186-18056

Mycgr3G90801\_Mycgr3T

Mycgr3G84646 Mycgr3T
  
Location: 18156-20235

Mycgr3G84646\_Mycgr3T

Mycgr3G68456 Mycgr3T
  
Location: 20335-21970

Mycgr3G68456\_Mycgr3T

Mycgr3G103270 Mycgr3
  
Location: 22070-22355

Mycgr3G103270\_Mycgr3

Mycgr3G90803 Mycgr3T
  
Location: 22455-23019

Mycgr3G90803\_Mycgr3T

Mycgr3G36941 Mycgr3T
  
Location: 23119-24064

Mycgr3G36941\_Mycgr3T

Mycgr3G25746 Mycgr3T
  
Location: 24164-25241

Mycgr3G25746\_Mycgr3T

Mycgr3G90788 Mycgr3T
  
Location: 25341-25803

Mycgr3G90788\_Mycgr3T

Mycgr3G103260 Mycgr3
  
Location: 25903-26635

Mycgr3G103260\_Mycgr3

Mycgr3G84644 Mycgr3T
  
Location: 26735-28457

Mycgr3G84644\_Mycgr3T

Mycgr3G29227 Mycgr3T
  
Location: 28557-28863

Mycgr3G29227\_Mycgr3T

Mycgr3G36271 Mycgr3T
  
Location: 28963-29854

Mycgr3G36271\_Mycgr3T

Mycgr3G68433 Mycgr3T
  
Location: 29954-33041

Mycgr3G68433\_Mycgr3T

Mycgr3G79452 Mycgr3T
  
Location: 33141-33399

Mycgr3G79452\_Mycgr3T

Mycgr3G55345 Mycgr3T
  
Location: 33499-34126

Mycgr3G55345\_Mycgr3T

Mycgr3G103278 Mycgr3
  
Location: 34226-35195

Mycgr3G103278\_Mycgr3

Mycgr3G84654 Mycgr3T
  
Location: 35295-36630

Mycgr3G84654\_Mycgr3T

Mycgr3G108090 Mycgr3
  
Location: 36730-37591

Mycgr3G108090\_Mycgr3

Mycgr3G21922 Mycgr3T
  
Location: 37691-39149

Mycgr3G21922\_Mycgr3T

Mycgr3G99148 Mycgr3T
  
Location: 39249-42819

Mycgr3G99148\_Mycgr3T

reverse transcriptase, putative
  
Accession: EED18393
  
Location: 2800302-2802185
  
 NCBI BlastP on this gene

EED18393

reverse transcriptase, putative
  
Accession: EED18392
  
Location: 2795868-2799940
  
 NCBI BlastP on this gene

EED18392

retrotransposon polyprotein, putative
  
Accession: EED18391
  
Location: 2788334-2793746
  
 NCBI BlastP on this gene

EED18391

hypothetical protein
  
Accession: EED18390
  
Location: 2784739-2785777
  
 NCBI BlastP on this gene

EED18390

conserved hypothetical protein
  
Accession: EED18389
  
Location: 2780380-2782510
  
  
**BlastP hit with Mycgr3G84646\_Mycgr3T**
  
Percentage identity: 37 %
  
BlastP bit score: 424
  
Sequence coverage: 100 %
  
E-value: 2e-135
  
  
 NCBI BlastP on this gene

EED18389

Rab GTPase SrgA, putative
  
Accession: EED18388
  
Location: 2779102-2779833
  
  
**BlastP hit with Mycgr3G99145\_Mycgr3T**
  
Percentage identity: 83 %
  
BlastP bit score: 352
  
Sequence coverage: 100 %
  
E-value: 8e-121
  
  
 NCBI BlastP on this gene

EED18388

AAA family ATPase, putative
  
Accession: EED18387
  
Location: 2776092-2778372
  
 NCBI BlastP on this gene

EED18387

hypothetical protein
  
Accession: EED18386
  
Location: 2774305-2774989
  
 NCBI BlastP on this gene

EED18386

inositol kinase kinase (UvsB), putative
  
Accession: EED18385
  
Location: 2765341-2774207
  
 NCBI BlastP on this gene

EED18385

cytosine deaminase, putative
  
Accession: EED18384
  
Location: 2764077-2764896
  
 NCBI BlastP on this gene

EED18384

NADH-ubiquinone oxidoreductase 21 kDa subunit, putative
  
Accession: EED18383
  
Location: 2763061-2763888
  
 NCBI BlastP on this gene

EED18383

replication fork protection component Swi3, putative
  
Accession: EED18382
  
Location: 2761793-2762500
  
 NCBI BlastP on this gene

EED18382

FGGY-family carbohydrate kinase, putative
  
Accession: EED18380
  
Location: 2759479-2761463
  
 NCBI BlastP on this gene

EED18380

Query: Architecture Search FASTA input

CP003005 : Myceliophthora thermophila ATCC 42464 chromosome 4    Total score: 2.0     Cumulative Blast bit score: 772

Hit cluster cross-links:

Mycgr3G90785 Mycgr3T
  
Location: 0-1047

Mycgr3G90785\_Mycgr3T

Mycgr3G103262 Mycgr3
  
Location: 1147-1390

Mycgr3G103262\_Mycgr3

Mycgr3G68458 Mycgr3T
  
Location: 1490-3602

Mycgr3G68458\_Mycgr3T

Mycgr3G99145 Mycgr3T
  
Location: 3702-4326

Mycgr3G99145\_Mycgr3T

Mycgr3G103274 Mycgr3
  
Location: 4426-4957

Mycgr3G103274\_Mycgr3

Mycgr3G103264 Mycgr3
  
Location: 5057-5390

Mycgr3G103264\_Mycgr3

Mycgr3G37570 Mycgr3T
  
Location: 5490-6006

Mycgr3G37570\_Mycgr3T

Mycgr3G108094 Mycgr3
  
Location: 6106-10555

Mycgr3G108094\_Mycgr3

Mycgr3G90786 Mycgr3T
  
Location: 10655-12080

Mycgr3G90786\_Mycgr3T

Mycgr3G68429 Mycgr3T
  
Location: 12180-13440

Mycgr3G68429\_Mycgr3T

Mycgr3G68421 Mycgr3T
  
Location: 13540-17086

Mycgr3G68421\_Mycgr3T

Mycgr3G90801 Mycgr3T
  
Location: 17186-18056

Mycgr3G90801\_Mycgr3T

Mycgr3G84646 Mycgr3T
  
Location: 18156-20235

Mycgr3G84646\_Mycgr3T

Mycgr3G68456 Mycgr3T
  
Location: 20335-21970

Mycgr3G68456\_Mycgr3T

Mycgr3G103270 Mycgr3
  
Location: 22070-22355

Mycgr3G103270\_Mycgr3

Mycgr3G90803 Mycgr3T
  
Location: 22455-23019

Mycgr3G90803\_Mycgr3T

Mycgr3G36941 Mycgr3T
  
Location: 23119-24064

Mycgr3G36941\_Mycgr3T

Mycgr3G25746 Mycgr3T
  
Location: 24164-25241

Mycgr3G25746\_Mycgr3T

Mycgr3G90788 Mycgr3T
  
Location: 25341-25803

Mycgr3G90788\_Mycgr3T

Mycgr3G103260 Mycgr3
  
Location: 25903-26635

Mycgr3G103260\_Mycgr3

Mycgr3G84644 Mycgr3T
  
Location: 26735-28457

Mycgr3G84644\_Mycgr3T

Mycgr3G29227 Mycgr3T
  
Location: 28557-28863

Mycgr3G29227\_Mycgr3T

Mycgr3G36271 Mycgr3T
  
Location: 28963-29854

Mycgr3G36271\_Mycgr3T

Mycgr3G68433 Mycgr3T
  
Location: 29954-33041

Mycgr3G68433\_Mycgr3T

Mycgr3G79452 Mycgr3T
  
Location: 33141-33399

Mycgr3G79452\_Mycgr3T

Mycgr3G55345 Mycgr3T
  
Location: 33499-34126

Mycgr3G55345\_Mycgr3T

Mycgr3G103278 Mycgr3
  
Location: 34226-35195

Mycgr3G103278\_Mycgr3

Mycgr3G84654 Mycgr3T
  
Location: 35295-36630

Mycgr3G84654\_Mycgr3T

Mycgr3G108090 Mycgr3
  
Location: 36730-37591

Mycgr3G108090\_Mycgr3

Mycgr3G21922 Mycgr3T
  
Location: 37691-39149

Mycgr3G21922\_Mycgr3T

Mycgr3G99148 Mycgr3T
  
Location: 39249-42819

Mycgr3G99148\_Mycgr3T

hypothetical protein
  
Accession: AEO59137
  
Location: 3131585-3133817
  
 NCBI BlastP on this gene

MYCTH\_2307148

hypothetical protein
  
Accession: AEO59138
  
Location: 3136159-3136812
  
 NCBI BlastP on this gene

MYCTH\_116261

hypothetical protein
  
Accession: AEO59139
  
Location: 3138876-3139892
  
 NCBI BlastP on this gene

MYCTH\_52498

hypothetical protein
  
Accession: AEO59140
  
Location: 3142636-3143848
  
 NCBI BlastP on this gene

MYCTH\_2307154

hypothetical protein
  
Accession: AEO59141
  
Location: 3143995-3145158
  
 NCBI BlastP on this gene

MYCTH\_2307157

hypothetical protein
  
Accession: AEO59142
  
Location: 3145883-3146559
  
 NCBI BlastP on this gene

MYCTH\_52789

hypothetical protein
  
Accession: AEO59143
  
Location: 3148958-3149737
  
  
**BlastP hit with Mycgr3G55345\_Mycgr3T**
  
Percentage identity: 74 %
  
BlastP bit score: 318
  
Sequence coverage: 99 %
  
E-value: 2e-107
  
  
 NCBI BlastP on this gene

MYCTH\_2134806

hypothetical protein
  
Accession: AEO59144
  
Location: 3150248-3151444
  
 NCBI BlastP on this gene

MYCTH\_2307162

hypothetical protein
  
Accession: AEO59145
  
Location: 3152384-3154284
  
 NCBI BlastP on this gene

MYCTH\_2307164

hypothetical protein
  
Accession: AEO59146
  
Location: 3154654-3155166
  
 NCBI BlastP on this gene

MYCTH\_2307165

hypothetical protein
  
Accession: AEO59147
  
Location: 3155921-3159427
  
  
**BlastP hit with Mycgr3G68433\_Mycgr3T**
  
Percentage identity: 36 %
  
BlastP bit score: 454
  
Sequence coverage: 95 %
  
E-value: 2e-137
  
  
 NCBI BlastP on this gene

MYCTH\_2307166

hypothetical protein
  
Accession: AEO59148
  
Location: 3160872-3162192
  
 NCBI BlastP on this gene

MYCTH\_108061

hypothetical protein
  
Accession: AEO59149
  
Location: 3162624-3164529
  
 NCBI BlastP on this gene

MYCTH\_2307170

hypothetical protein
  
Accession: AEO59150
  
Location: 3166349-3166931
  
 NCBI BlastP on this gene

MYCTH\_2019371

hypothetical protein
  
Accession: AEO59151
  
Location: 3167490-3168828
  
 NCBI BlastP on this gene

MYCTH\_2307175

hypothetical protein
  
Accession: AEO59152
  
Location: 3170165-3171361
  
 NCBI BlastP on this gene

MYCTH\_2307177

hypothetical protein
  
Accession: AEO59153
  
Location: 3172326-3173560
  
 NCBI BlastP on this gene

MYCTH\_2307178

hypothetical protein
  
Accession: AEO59154
  
Location: 3174015-3178483
  
 NCBI BlastP on this gene

MYCTH\_2119434

Query: Architecture Search FASTA input

DS995901 : Penicillium marneffei ATCC 18224 scf\_1105668340960 genomic scaffold    Total score: 2.0     Cumulative Blast bit score: 765

Hit cluster cross-links:

Mycgr3G90785 Mycgr3T
  
Location: 0-1047

Mycgr3G90785\_Mycgr3T

Mycgr3G103262 Mycgr3
  
Location: 1147-1390

Mycgr3G103262\_Mycgr3

Mycgr3G68458 Mycgr3T
  
Location: 1490-3602

Mycgr3G68458\_Mycgr3T

Mycgr3G99145 Mycgr3T
  
Location: 3702-4326

Mycgr3G99145\_Mycgr3T

Mycgr3G103274 Mycgr3
  
Location: 4426-4957

Mycgr3G103274\_Mycgr3

Mycgr3G103264 Mycgr3
  
Location: 5057-5390

Mycgr3G103264\_Mycgr3

Mycgr3G37570 Mycgr3T
  
Location: 5490-6006

Mycgr3G37570\_Mycgr3T

Mycgr3G108094 Mycgr3
  
Location: 6106-10555

Mycgr3G108094\_Mycgr3

Mycgr3G90786 Mycgr3T
  
Location: 10655-12080

Mycgr3G90786\_Mycgr3T

Mycgr3G68429 Mycgr3T
  
Location: 12180-13440

Mycgr3G68429\_Mycgr3T

Mycgr3G68421 Mycgr3T
  
Location: 13540-17086

Mycgr3G68421\_Mycgr3T

Mycgr3G90801 Mycgr3T
  
Location: 17186-18056

Mycgr3G90801\_Mycgr3T

Mycgr3G84646 Mycgr3T
  
Location: 18156-20235

Mycgr3G84646\_Mycgr3T

Mycgr3G68456 Mycgr3T
  
Location: 20335-21970

Mycgr3G68456\_Mycgr3T

Mycgr3G103270 Mycgr3
  
Location: 22070-22355

Mycgr3G103270\_Mycgr3

Mycgr3G90803 Mycgr3T
  
Location: 22455-23019

Mycgr3G90803\_Mycgr3T

Mycgr3G36941 Mycgr3T
  
Location: 23119-24064

Mycgr3G36941\_Mycgr3T

Mycgr3G25746 Mycgr3T
  
Location: 24164-25241

Mycgr3G25746\_Mycgr3T

Mycgr3G90788 Mycgr3T
  
Location: 25341-25803

Mycgr3G90788\_Mycgr3T

Mycgr3G103260 Mycgr3
  
Location: 25903-26635

Mycgr3G103260\_Mycgr3

Mycgr3G84644 Mycgr3T
  
Location: 26735-28457

Mycgr3G84644\_Mycgr3T

Mycgr3G29227 Mycgr3T
  
Location: 28557-28863

Mycgr3G29227\_Mycgr3T

Mycgr3G36271 Mycgr3T
  
Location: 28963-29854

Mycgr3G36271\_Mycgr3T

Mycgr3G68433 Mycgr3T
  
Location: 29954-33041

Mycgr3G68433\_Mycgr3T

Mycgr3G79452 Mycgr3T
  
Location: 33141-33399

Mycgr3G79452\_Mycgr3T

Mycgr3G55345 Mycgr3T
  
Location: 33499-34126

Mycgr3G55345\_Mycgr3T

Mycgr3G103278 Mycgr3
  
Location: 34226-35195

Mycgr3G103278\_Mycgr3

Mycgr3G84654 Mycgr3T
  
Location: 35295-36630

Mycgr3G84654\_Mycgr3T

Mycgr3G108090 Mycgr3
  
Location: 36730-37591

Mycgr3G108090\_Mycgr3

Mycgr3G21922 Mycgr3T
  
Location: 37691-39149

Mycgr3G21922\_Mycgr3T

Mycgr3G99148 Mycgr3T
  
Location: 39249-42819

Mycgr3G99148\_Mycgr3T

transposon, putative
  
Accession: EEA24637
  
Location: 2625325-2626953
  
 NCBI BlastP on this gene

EEA24637

transposable element tc1 transposase, putative
  
Accession: EEA24636
  
Location: 2616774-2617871
  
 NCBI BlastP on this gene

EEA24636

hypothetical protein
  
Accession: EEA24635
  
Location: 2614101-2614860
  
 NCBI BlastP on this gene

EEA24635

conserved hypothetical protein
  
Accession: EEA24634
  
Location: 2613104-2613988
  
 NCBI BlastP on this gene

EEA24634

conserved hypothetical protein
  
Accession: EEA24632
  
Location: 2611512-2612213
  
 NCBI BlastP on this gene

EEA24632

conserved hypothetical protein
  
Accession: EEA24631
  
Location: 2606606-2608729
  
  
**BlastP hit with Mycgr3G84646\_Mycgr3T**
  
Percentage identity: 40 %
  
BlastP bit score: 412
  
Sequence coverage: 84 %
  
E-value: 9e-131
  
  
 NCBI BlastP on this gene

EEA24631

Rab GTPase SrgA, putative
  
Accession: EEA24630
  
Location: 2605188-2605934
  
  
**BlastP hit with Mycgr3G99145\_Mycgr3T**
  
Percentage identity: 84 %
  
BlastP bit score: 353
  
Sequence coverage: 100 %
  
E-value: 6e-121
  
  
 NCBI BlastP on this gene

EEA24630

AAA family ATPase, putative
  
Accession: EEA24629
  
Location: 2602161-2604439
  
 NCBI BlastP on this gene

EEA24629

conserved hypothetical protein
  
Accession: EEA24628
  
Location: 2600425-2601480
  
 NCBI BlastP on this gene

EEA24628

inositol kinase kinase (UvsB), putative
  
Accession: EEA24627
  
Location: 2591336-2599959
  
 NCBI BlastP on this gene

EEA24627

tRNA-specific adenosine deaminase subunit TAD2, putative
  
Accession: EEA24626
  
Location: 2590091-2590891
  
 NCBI BlastP on this gene

EEA24626

NADH-ubiquinone oxidoreductase 21 kDa subunit, putative
  
Accession: EEA24625
  
Location: 2589079-2589900
  
 NCBI BlastP on this gene

EEA24625

replication fork protection component Swi3, putative
  
Accession: EEA24624
  
Location: 2587726-2588819
  
 NCBI BlastP on this gene

EEA24624

FGGY-family carbohydrate kinase, putative
  
Accession: EEA24623
  
Location: 2585338-2587347
  
 NCBI BlastP on this gene

EEA24623

Query: Architecture Search FASTA input

DF126458 : Aspergillus kawachii IFO 4308 DNA, contig: scaffold00012    Total score: 2.0     Cumulative Blast bit score: 748

Hit cluster cross-links:

Mycgr3G90785 Mycgr3T
  
Location: 0-1047

Mycgr3G90785\_Mycgr3T

Mycgr3G103262 Mycgr3
  
Location: 1147-1390

Mycgr3G103262\_Mycgr3

Mycgr3G68458 Mycgr3T
  
Location: 1490-3602

Mycgr3G68458\_Mycgr3T

Mycgr3G99145 Mycgr3T
  
Location: 3702-4326

Mycgr3G99145\_Mycgr3T

Mycgr3G103274 Mycgr3
  
Location: 4426-4957

Mycgr3G103274\_Mycgr3

Mycgr3G103264 Mycgr3
  
Location: 5057-5390

Mycgr3G103264\_Mycgr3

Mycgr3G37570 Mycgr3T
  
Location: 5490-6006

Mycgr3G37570\_Mycgr3T

Mycgr3G108094 Mycgr3
  
Location: 6106-10555

Mycgr3G108094\_Mycgr3

Mycgr3G90786 Mycgr3T
  
Location: 10655-12080

Mycgr3G90786\_Mycgr3T

Mycgr3G68429 Mycgr3T
  
Location: 12180-13440

Mycgr3G68429\_Mycgr3T

Mycgr3G68421 Mycgr3T
  
Location: 13540-17086

Mycgr3G68421\_Mycgr3T

Mycgr3G90801 Mycgr3T
  
Location: 17186-18056

Mycgr3G90801\_Mycgr3T

Mycgr3G84646 Mycgr3T
  
Location: 18156-20235

Mycgr3G84646\_Mycgr3T

Mycgr3G68456 Mycgr3T
  
Location: 20335-21970

Mycgr3G68456\_Mycgr3T

Mycgr3G103270 Mycgr3
  
Location: 22070-22355

Mycgr3G103270\_Mycgr3

Mycgr3G90803 Mycgr3T
  
Location: 22455-23019

Mycgr3G90803\_Mycgr3T

Mycgr3G36941 Mycgr3T
  
Location: 23119-24064

Mycgr3G36941\_Mycgr3T

Mycgr3G25746 Mycgr3T
  
Location: 24164-25241

Mycgr3G25746\_Mycgr3T

Mycgr3G90788 Mycgr3T
  
Location: 25341-25803

Mycgr3G90788\_Mycgr3T

Mycgr3G103260 Mycgr3
  
Location: 25903-26635

Mycgr3G103260\_Mycgr3

Mycgr3G84644 Mycgr3T
  
Location: 26735-28457

Mycgr3G84644\_Mycgr3T

Mycgr3G29227 Mycgr3T
  
Location: 28557-28863

Mycgr3G29227\_Mycgr3T

Mycgr3G36271 Mycgr3T
  
Location: 28963-29854

Mycgr3G36271\_Mycgr3T

Mycgr3G68433 Mycgr3T
  
Location: 29954-33041

Mycgr3G68433\_Mycgr3T

Mycgr3G79452 Mycgr3T
  
Location: 33141-33399

Mycgr3G79452\_Mycgr3T

Mycgr3G55345 Mycgr3T
  
Location: 33499-34126

Mycgr3G55345\_Mycgr3T

Mycgr3G103278 Mycgr3
  
Location: 34226-35195

Mycgr3G103278\_Mycgr3

Mycgr3G84654 Mycgr3T
  
Location: 35295-36630

Mycgr3G84654\_Mycgr3T

Mycgr3G108090 Mycgr3
  
Location: 36730-37591

Mycgr3G108090\_Mycgr3

Mycgr3G21922 Mycgr3T
  
Location: 37691-39149

Mycgr3G21922\_Mycgr3T

Mycgr3G99148 Mycgr3T
  
Location: 39249-42819

Mycgr3G99148\_Mycgr3T

ATP synthase regulation protein NCA2
  
Accession: GAA87272
  
Location: 933912-936038
  
  
**BlastP hit with Mycgr3G84646\_Mycgr3T**
  
Percentage identity: 39 %
  
BlastP bit score: 400
  
Sequence coverage: 83 %
  
E-value: 5e-126
  
  
 NCBI BlastP on this gene

GAA87272

secretion related GTPase (SrgA)
  
Accession: GAA87271
  
Location: 932089-932838
  
  
**BlastP hit with Mycgr3G99145\_Mycgr3T**
  
Percentage identity: 83 %
  
BlastP bit score: 348
  
Sequence coverage: 99 %
  
E-value: 5e-119
  
  
 NCBI BlastP on this gene

GAA87271

AAA family ATPase
  
Accession: GAA87270
  
Location: 928467-930216
  
 NCBI BlastP on this gene

GAA87270

UVSB
  
Accession: GAA87269
  
Location: 917764-926608
  
 NCBI BlastP on this gene

GAA87269

NADH-ubiquinone oxidoreductase 21 kDa subunit
  
Accession: GAA87268
  
Location: 915156-915987
  
 NCBI BlastP on this gene

GAA87268

chromosome segregation in meiosis protein 3
  
Accession: GAA87267
  
Location: 913776-914661
  
 NCBI BlastP on this gene

GAA87267

Query: Architecture Search FASTA input

GL988043 : Chaetomium thermophilum var. thermophilum DSM 1495 unplaced genomic scaffold scf7180000...    Total score: 2.0     Cumulative Blast bit score: 738

Hit cluster cross-links:

Mycgr3G90785 Mycgr3T
  
Location: 0-1047

Mycgr3G90785\_Mycgr3T

Mycgr3G103262 Mycgr3
  
Location: 1147-1390

Mycgr3G103262\_Mycgr3

Mycgr3G68458 Mycgr3T
  
Location: 1490-3602

Mycgr3G68458\_Mycgr3T

Mycgr3G99145 Mycgr3T
  
Location: 3702-4326

Mycgr3G99145\_Mycgr3T

Mycgr3G103274 Mycgr3
  
Location: 4426-4957

Mycgr3G103274\_Mycgr3

Mycgr3G103264 Mycgr3
  
Location: 5057-5390

Mycgr3G103264\_Mycgr3

Mycgr3G37570 Mycgr3T
  
Location: 5490-6006

Mycgr3G37570\_Mycgr3T

Mycgr3G108094 Mycgr3
  
Location: 6106-10555

Mycgr3G108094\_Mycgr3

Mycgr3G90786 Mycgr3T
  
Location: 10655-12080

Mycgr3G90786\_Mycgr3T

Mycgr3G68429 Mycgr3T
  
Location: 12180-13440

Mycgr3G68429\_Mycgr3T

Mycgr3G68421 Mycgr3T
  
Location: 13540-17086

Mycgr3G68421\_Mycgr3T

Mycgr3G90801 Mycgr3T
  
Location: 17186-18056

Mycgr3G90801\_Mycgr3T

Mycgr3G84646 Mycgr3T
  
Location: 18156-20235

Mycgr3G84646\_Mycgr3T

Mycgr3G68456 Mycgr3T
  
Location: 20335-21970

Mycgr3G68456\_Mycgr3T

Mycgr3G103270 Mycgr3
  
Location: 22070-22355

Mycgr3G103270\_Mycgr3

Mycgr3G90803 Mycgr3T
  
Location: 22455-23019

Mycgr3G90803\_Mycgr3T

Mycgr3G36941 Mycgr3T
  
Location: 23119-24064

Mycgr3G36941\_Mycgr3T

Mycgr3G25746 Mycgr3T
  
Location: 24164-25241

Mycgr3G25746\_Mycgr3T

Mycgr3G90788 Mycgr3T
  
Location: 25341-25803

Mycgr3G90788\_Mycgr3T

Mycgr3G103260 Mycgr3
  
Location: 25903-26635

Mycgr3G103260\_Mycgr3

Mycgr3G84644 Mycgr3T
  
Location: 26735-28457

Mycgr3G84644\_Mycgr3T

Mycgr3G29227 Mycgr3T
  
Location: 28557-28863

Mycgr3G29227\_Mycgr3T

Mycgr3G36271 Mycgr3T
  
Location: 28963-29854

Mycgr3G36271\_Mycgr3T

Mycgr3G68433 Mycgr3T
  
Location: 29954-33041

Mycgr3G68433\_Mycgr3T

Mycgr3G79452 Mycgr3T
  
Location: 33141-33399

Mycgr3G79452\_Mycgr3T

Mycgr3G55345 Mycgr3T
  
Location: 33499-34126

Mycgr3G55345\_Mycgr3T

Mycgr3G103278 Mycgr3
  
Location: 34226-35195

Mycgr3G103278\_Mycgr3

Mycgr3G84654 Mycgr3T
  
Location: 35295-36630

Mycgr3G84654\_Mycgr3T

Mycgr3G108090 Mycgr3
  
Location: 36730-37591

Mycgr3G108090\_Mycgr3

Mycgr3G21922 Mycgr3T
  
Location: 37691-39149

Mycgr3G21922\_Mycgr3T

Mycgr3G99148 Mycgr3T
  
Location: 39249-42819

Mycgr3G99148\_Mycgr3T

hypothetical protein
  
Accession: EGS20001
  
Location: 1820192-1821367
  
 NCBI BlastP on this gene

EGS20001

hypothetical protein
  
Accession: EGS20000
  
Location: 1817802-1819013
  
 NCBI BlastP on this gene

EGS20000

hypothetical protein
  
Accession: EGS19999
  
Location: 1813250-1814579
  
 NCBI BlastP on this gene

EGS19999

hypothetical protein
  
Accession: EGS19998
  
Location: 1812032-1812754
  
 NCBI BlastP on this gene

EGS19998

hypothetical protein
  
Accession: EGS19997
  
Location: 1808606-1810673
  
 NCBI BlastP on this gene

EGS19997

hypothetical protein
  
Accession: EGS19996
  
Location: 1803091-1804774
  
 NCBI BlastP on this gene

EGS19996

putative leucine-rich protein
  
Accession: EGS19995
  
Location: 1796704-1800216
  
  
**BlastP hit with Mycgr3G68433\_Mycgr3T**
  
Percentage identity: 35 %
  
BlastP bit score: 430
  
Sequence coverage: 97 %
  
E-value: 2e-128
  
  
 NCBI BlastP on this gene

EGS19995

xanthine phosphoribosyltransferase-like protein
  
Accession: EGS19994
  
Location: 1794472-1795208
  
  
**BlastP hit with Mycgr3G55345\_Mycgr3T**
  
Percentage identity: 72 %
  
BlastP bit score: 308
  
Sequence coverage: 99 %
  
E-value: 2e-103
  
  
 NCBI BlastP on this gene

EGS19994

hypothetical protein
  
Accession: EGS19993
  
Location: 1792499-1793786
  
 NCBI BlastP on this gene

EGS19993

hypothetical protein
  
Accession: EGS19992
  
Location: 1789581-1791783
  
 NCBI BlastP on this gene

EGS19992

hypothetical protein
  
Accession: EGS19991
  
Location: 1788653-1789085
  
 NCBI BlastP on this gene

EGS19991

hypothetical protein
  
Accession: EGS19990
  
Location: 1783459-1785379
  
 NCBI BlastP on this gene

EGS19990

Query: Architecture Search FASTA input

DS572813 : Paracoccidioides brasiliensis Pb01 supercont1.3 genomic scaffold    Total score: 2.0     Cumulative Blast bit score: 730

Hit cluster cross-links:

Mycgr3G90785 Mycgr3T
  
Location: 0-1047

Mycgr3G90785\_Mycgr3T

Mycgr3G103262 Mycgr3
  
Location: 1147-1390

Mycgr3G103262\_Mycgr3

Mycgr3G68458 Mycgr3T
  
Location: 1490-3602

Mycgr3G68458\_Mycgr3T

Mycgr3G99145 Mycgr3T
  
Location: 3702-4326

Mycgr3G99145\_Mycgr3T

Mycgr3G103274 Mycgr3
  
Location: 4426-4957

Mycgr3G103274\_Mycgr3

Mycgr3G103264 Mycgr3
  
Location: 5057-5390

Mycgr3G103264\_Mycgr3

Mycgr3G37570 Mycgr3T
  
Location: 5490-6006

Mycgr3G37570\_Mycgr3T

Mycgr3G108094 Mycgr3
  
Location: 6106-10555

Mycgr3G108094\_Mycgr3

Mycgr3G90786 Mycgr3T
  
Location: 10655-12080

Mycgr3G90786\_Mycgr3T

Mycgr3G68429 Mycgr3T
  
Location: 12180-13440

Mycgr3G68429\_Mycgr3T

Mycgr3G68421 Mycgr3T
  
Location: 13540-17086

Mycgr3G68421\_Mycgr3T

Mycgr3G90801 Mycgr3T
  
Location: 17186-18056

Mycgr3G90801\_Mycgr3T

Mycgr3G84646 Mycgr3T
  
Location: 18156-20235

Mycgr3G84646\_Mycgr3T

Mycgr3G68456 Mycgr3T
  
Location: 20335-21970

Mycgr3G68456\_Mycgr3T

Mycgr3G103270 Mycgr3
  
Location: 22070-22355

Mycgr3G103270\_Mycgr3

Mycgr3G90803 Mycgr3T
  
Location: 22455-23019

Mycgr3G90803\_Mycgr3T

Mycgr3G36941 Mycgr3T
  
Location: 23119-24064

Mycgr3G36941\_Mycgr3T

Mycgr3G25746 Mycgr3T
  
Location: 24164-25241

Mycgr3G25746\_Mycgr3T

Mycgr3G90788 Mycgr3T
  
Location: 25341-25803

Mycgr3G90788\_Mycgr3T

Mycgr3G103260 Mycgr3
  
Location: 25903-26635

Mycgr3G103260\_Mycgr3

Mycgr3G84644 Mycgr3T
  
Location: 26735-28457

Mycgr3G84644\_Mycgr3T

Mycgr3G29227 Mycgr3T
  
Location: 28557-28863

Mycgr3G29227\_Mycgr3T

Mycgr3G36271 Mycgr3T
  
Location: 28963-29854

Mycgr3G36271\_Mycgr3T

Mycgr3G68433 Mycgr3T
  
Location: 29954-33041

Mycgr3G68433\_Mycgr3T

Mycgr3G79452 Mycgr3T
  
Location: 33141-33399

Mycgr3G79452\_Mycgr3T

Mycgr3G55345 Mycgr3T
  
Location: 33499-34126

Mycgr3G55345\_Mycgr3T

Mycgr3G103278 Mycgr3
  
Location: 34226-35195

Mycgr3G103278\_Mycgr3

Mycgr3G84654 Mycgr3T
  
Location: 35295-36630

Mycgr3G84654\_Mycgr3T

Mycgr3G108090 Mycgr3
  
Location: 36730-37591

Mycgr3G108090\_Mycgr3

Mycgr3G21922 Mycgr3T
  
Location: 37691-39149

Mycgr3G21922\_Mycgr3T

Mycgr3G99148 Mycgr3T
  
Location: 39249-42819

Mycgr3G99148\_Mycgr3T

conserved hypothetical protein
  
Accession: EEH39046
  
Location: 617868-619211
  
 NCBI BlastP on this gene

EEH39046

predicted protein
  
Accession: EEH39045
  
Location: 615225-616314
  
 NCBI BlastP on this gene

EEH39045

C-4 methylsterol oxidase
  
Accession: EEH39044
  
Location: 613839-614390
  
 NCBI BlastP on this gene

EEH39044

conserved hypothetical protein
  
Accession: EEH39043
  
Location: 605548-607731
  
 NCBI BlastP on this gene

EEH39043

conserved hypothetical protein
  
Accession: EEH39042
  
Location: 604102-605053
  
 NCBI BlastP on this gene

EEH39042

conserved hypothetical protein
  
Accession: EEH39041
  
Location: 599261-602711
  
 NCBI BlastP on this gene

EEH39041

conserved hypothetical protein
  
Accession: EEH39040
  
Location: 594902-599119
  
  
**BlastP hit with Mycgr3G84646\_Mycgr3T**
  
Percentage identity: 39 %
  
BlastP bit score: 384
  
Sequence coverage: 93 %
  
E-value: 2e-119
  
  
 NCBI BlastP on this gene

EEH39040

conserved hypothetical protein
  
Accession: EEH39039
  
Location: 591077-592417
  
 NCBI BlastP on this gene

EEH39039

GTP-binding protein SAS1
  
Accession: EEH39038
  
Location: 588940-589798
  
  
**BlastP hit with Mycgr3G99145\_Mycgr3T**
  
Percentage identity: 83 %
  
BlastP bit score: 346
  
Sequence coverage: 99 %
  
E-value: 2e-118
  
  
 NCBI BlastP on this gene

EEH39038

conserved hypothetical protein
  
Accession: EEH39037
  
Location: 586757-588075
  
 NCBI BlastP on this gene

EEH39037

AAA family ATPase
  
Accession: EEH39036
  
Location: 583765-586113
  
 NCBI BlastP on this gene

EEH39036

hypothetical protein
  
Accession: EEH39035
  
Location: 582138-583382
  
 NCBI BlastP on this gene

EEH39035

UVSB PI-3 kinase
  
Accession: EEH39034
  
Location: 573004-581454
  
 NCBI BlastP on this gene

EEH39034

tRNA-specific adenosine deaminase
  
Accession: EEH39033
  
Location: 571824-572676
  
 NCBI BlastP on this gene

EEH39033

NADH-ubiquinone oxidoreductase 21 kDa subunit
  
Accession: EEH39032
  
Location: 570749-571594
  
 NCBI BlastP on this gene

EEH39032

Query: Architecture Search FASTA input

ACJE01000002 : Aspergillus niger ATCC 1015    Total score: 2.0     Cumulative Blast bit score: 724

Hit cluster cross-links:

Mycgr3G90785 Mycgr3T
  
Location: 0-1047

Mycgr3G90785\_Mycgr3T

Mycgr3G103262 Mycgr3
  
Location: 1147-1390

Mycgr3G103262\_Mycgr3

Mycgr3G68458 Mycgr3T
  
Location: 1490-3602

Mycgr3G68458\_Mycgr3T

Mycgr3G99145 Mycgr3T
  
Location: 3702-4326

Mycgr3G99145\_Mycgr3T

Mycgr3G103274 Mycgr3
  
Location: 4426-4957

Mycgr3G103274\_Mycgr3

Mycgr3G103264 Mycgr3
  
Location: 5057-5390

Mycgr3G103264\_Mycgr3

Mycgr3G37570 Mycgr3T
  
Location: 5490-6006

Mycgr3G37570\_Mycgr3T

Mycgr3G108094 Mycgr3
  
Location: 6106-10555

Mycgr3G108094\_Mycgr3

Mycgr3G90786 Mycgr3T
  
Location: 10655-12080

Mycgr3G90786\_Mycgr3T

Mycgr3G68429 Mycgr3T
  
Location: 12180-13440

Mycgr3G68429\_Mycgr3T

Mycgr3G68421 Mycgr3T
  
Location: 13540-17086

Mycgr3G68421\_Mycgr3T

Mycgr3G90801 Mycgr3T
  
Location: 17186-18056

Mycgr3G90801\_Mycgr3T

Mycgr3G84646 Mycgr3T
  
Location: 18156-20235

Mycgr3G84646\_Mycgr3T

Mycgr3G68456 Mycgr3T
  
Location: 20335-21970

Mycgr3G68456\_Mycgr3T

Mycgr3G103270 Mycgr3
  
Location: 22070-22355

Mycgr3G103270\_Mycgr3

Mycgr3G90803 Mycgr3T
  
Location: 22455-23019

Mycgr3G90803\_Mycgr3T

Mycgr3G36941 Mycgr3T
  
Location: 23119-24064

Mycgr3G36941\_Mycgr3T

Mycgr3G25746 Mycgr3T
  
Location: 24164-25241

Mycgr3G25746\_Mycgr3T

Mycgr3G90788 Mycgr3T
  
Location: 25341-25803

Mycgr3G90788\_Mycgr3T

Mycgr3G103260 Mycgr3
  
Location: 25903-26635

Mycgr3G103260\_Mycgr3

Mycgr3G84644 Mycgr3T
  
Location: 26735-28457

Mycgr3G84644\_Mycgr3T

Mycgr3G29227 Mycgr3T
  
Location: 28557-28863

Mycgr3G29227\_Mycgr3T

Mycgr3G36271 Mycgr3T
  
Location: 28963-29854

Mycgr3G36271\_Mycgr3T

Mycgr3G68433 Mycgr3T
  
Location: 29954-33041

Mycgr3G68433\_Mycgr3T

Mycgr3G79452 Mycgr3T
  
Location: 33141-33399

Mycgr3G79452\_Mycgr3T

Mycgr3G55345 Mycgr3T
  
Location: 33499-34126

Mycgr3G55345\_Mycgr3T

Mycgr3G103278 Mycgr3
  
Location: 34226-35195

Mycgr3G103278\_Mycgr3

Mycgr3G84654 Mycgr3T
  
Location: 35295-36630

Mycgr3G84654\_Mycgr3T

Mycgr3G108090 Mycgr3
  
Location: 36730-37591

Mycgr3G108090\_Mycgr3

Mycgr3G21922 Mycgr3T
  
Location: 37691-39149

Mycgr3G21922\_Mycgr3T

Mycgr3G99148 Mycgr3T
  
Location: 39249-42819

Mycgr3G99148\_Mycgr3T

hypothetical protein
  
Accession: EHA27517
  
Location: 4053-5906
  
  
**BlastP hit with Mycgr3G84646\_Mycgr3T**
  
Percentage identity: 38 %
  
BlastP bit score: 376
  
Sequence coverage: 80 %
  
E-value: 1e-117
  
  
 NCBI BlastP on this gene

EHA27517

hypothetical protein
  
Accession: EHA27518
  
Location: 7060-7804
  
  
**BlastP hit with Mycgr3G99145\_Mycgr3T**
  
Percentage identity: 83 %
  
BlastP bit score: 348
  
Sequence coverage: 99 %
  
E-value: 4e-119
  
  
 NCBI BlastP on this gene

EHA27518

hypothetical protein
  
Accession: EHA27519
  
Location: 9104-11408
  
 NCBI BlastP on this gene

EHA27519

hypothetical protein
  
Accession: EHA27520
  
Location: 12080-12926
  
 NCBI BlastP on this gene

EHA27520

hypothetical protein
  
Accession: EHA27521
  
Location: 13238-22108
  
 NCBI BlastP on this gene

EHA27521

hypothetical protein
  
Accession: EHA27522
  
Location: 23934-24757
  
 NCBI BlastP on this gene

EHA27522

hypothetical protein
  
Accession: EHA27523
  
Location: 25211-26141
  
 NCBI BlastP on this gene

EHA27523

hypothetical protein
  
Accession: EHA27524
  
Location: 26604-28384
  
 NCBI BlastP on this gene

EHA27524

Query: Architecture Search FASTA input

DS027690 : Neosartorya fischeri NRRL 181 1099437636252 genomic scaffold    Total score: 2.0     Cumulative Blast bit score: 720

Hit cluster cross-links:

Mycgr3G90785 Mycgr3T
  
Location: 0-1047

Mycgr3G90785\_Mycgr3T

Mycgr3G103262 Mycgr3
  
Location: 1147-1390

Mycgr3G103262\_Mycgr3

Mycgr3G68458 Mycgr3T
  
Location: 1490-3602

Mycgr3G68458\_Mycgr3T

Mycgr3G99145 Mycgr3T
  
Location: 3702-4326

Mycgr3G99145\_Mycgr3T

Mycgr3G103274 Mycgr3
  
Location: 4426-4957

Mycgr3G103274\_Mycgr3

Mycgr3G103264 Mycgr3
  
Location: 5057-5390

Mycgr3G103264\_Mycgr3

Mycgr3G37570 Mycgr3T
  
Location: 5490-6006

Mycgr3G37570\_Mycgr3T

Mycgr3G108094 Mycgr3
  
Location: 6106-10555

Mycgr3G108094\_Mycgr3

Mycgr3G90786 Mycgr3T
  
Location: 10655-12080

Mycgr3G90786\_Mycgr3T

Mycgr3G68429 Mycgr3T
  
Location: 12180-13440

Mycgr3G68429\_Mycgr3T

Mycgr3G68421 Mycgr3T
  
Location: 13540-17086

Mycgr3G68421\_Mycgr3T

Mycgr3G90801 Mycgr3T
  
Location: 17186-18056

Mycgr3G90801\_Mycgr3T

Mycgr3G84646 Mycgr3T
  
Location: 18156-20235

Mycgr3G84646\_Mycgr3T

Mycgr3G68456 Mycgr3T
  
Location: 20335-21970

Mycgr3G68456\_Mycgr3T

Mycgr3G103270 Mycgr3
  
Location: 22070-22355

Mycgr3G103270\_Mycgr3

Mycgr3G90803 Mycgr3T
  
Location: 22455-23019

Mycgr3G90803\_Mycgr3T

Mycgr3G36941 Mycgr3T
  
Location: 23119-24064

Mycgr3G36941\_Mycgr3T

Mycgr3G25746 Mycgr3T
  
Location: 24164-25241

Mycgr3G25746\_Mycgr3T

Mycgr3G90788 Mycgr3T
  
Location: 25341-25803

Mycgr3G90788\_Mycgr3T

Mycgr3G103260 Mycgr3
  
Location: 25903-26635

Mycgr3G103260\_Mycgr3

Mycgr3G84644 Mycgr3T
  
Location: 26735-28457

Mycgr3G84644\_Mycgr3T

Mycgr3G29227 Mycgr3T
  
Location: 28557-28863

Mycgr3G29227\_Mycgr3T

Mycgr3G36271 Mycgr3T
  
Location: 28963-29854

Mycgr3G36271\_Mycgr3T

Mycgr3G68433 Mycgr3T
  
Location: 29954-33041

Mycgr3G68433\_Mycgr3T

Mycgr3G79452 Mycgr3T
  
Location: 33141-33399

Mycgr3G79452\_Mycgr3T

Mycgr3G55345 Mycgr3T
  
Location: 33499-34126

Mycgr3G55345\_Mycgr3T

Mycgr3G103278 Mycgr3
  
Location: 34226-35195

Mycgr3G103278\_Mycgr3

Mycgr3G84654 Mycgr3T
  
Location: 35295-36630

Mycgr3G84654\_Mycgr3T

Mycgr3G108090 Mycgr3
  
Location: 36730-37591

Mycgr3G108090\_Mycgr3

Mycgr3G21922 Mycgr3T
  
Location: 37691-39149

Mycgr3G21922\_Mycgr3T

Mycgr3G99148 Mycgr3T
  
Location: 39249-42819

Mycgr3G99148\_Mycgr3T

conserved hypothetical protein
  
Accession: EAW21518
  
Location: 1503691-1505820
  
 NCBI BlastP on this gene

EAW21518

conserved hypothetical protein
  
Accession: EAW21517
  
Location: 1499856-1501408
  
 NCBI BlastP on this gene

EAW21517

conserved hypothetical protein
  
Accession: EAW21516
  
Location: 1497193-1499643
  
 NCBI BlastP on this gene

EAW21516

nuclear transport factor NTF-2, putative
  
Accession: EAW21515
  
Location: 1496059-1496890
  
 NCBI BlastP on this gene

EAW21515

PQ loop repeat protein
  
Accession: EAW21514
  
Location: 1494612-1495633
  
 NCBI BlastP on this gene

EAW21514

stress response protein (Ish1), putative
  
Accession: EAW21513
  
Location: 1491841-1493639
  
 NCBI BlastP on this gene

EAW21513

DNA damage response protein (Dap1), putative
  
Accession: EAW21512
  
Location: 1490832-1491364
  
 NCBI BlastP on this gene

EAW21512

conserved hypothetical protein
  
Accession: EAW21511
  
Location: 1487756-1489507
  
 NCBI BlastP on this gene

EAW21511

conserved hypothetical protein
  
Accession: EAW21510
  
Location: 1486843-1487183
  
 NCBI BlastP on this gene

EAW21510

serine/threonine protein kinase (Prp4), putative
  
Accession: EAW21509
  
Location: 1483748-1486294
  
  
**BlastP hit with Mycgr3G103260\_Mycgr3**
  
Percentage identity: 53 %
  
BlastP bit score: 281
  
Sequence coverage: 98 %
  
E-value: 1e-85
  
  
 NCBI BlastP on this gene

EAW21509

protein kinase, putative
  
Accession: EAW21508
  
Location: 1477534-1478836
  
  
**BlastP hit with Mycgr3G68429\_Mycgr3T**
  
Percentage identity: 68 %
  
BlastP bit score: 439
  
Sequence coverage: 75 %
  
E-value: 4e-148
  
  
 NCBI BlastP on this gene

EAW21508

3-oxoacyl-(acyl-carrier-protein) reductase, putative
  
Accession: EAW21507
  
Location: 1475462-1476511
  
 NCBI BlastP on this gene

EAW21507

C6 zinc finger domain protein
  
Accession: EAW21506
  
Location: 1474064-1475223
  
 NCBI BlastP on this gene

EAW21506

haemolysin-III channel protein Izh2, putative
  
Accession: EAW21505
  
Location: 1472038-1473003
  
 NCBI BlastP on this gene

EAW21505

conserved hypothetical protein
  
Accession: EAW21504
  
Location: 1470563-1471606
  
 NCBI BlastP on this gene

EAW21504

conserved hypothetical protein
  
Accession: EAW21503
  
Location: 1469120-1470315
  
 NCBI BlastP on this gene

EAW21503

clathrin-coated vesicle protein, putative
  
Accession: EAW21502
  
Location: 1467560-1468396
  
 NCBI BlastP on this gene

EAW21502

conserved hypothetical protein
  
Accession: EAW21501
  
Location: 1460857-1463688
  
 NCBI BlastP on this gene

EAW21501

transcription initiation protein
  
Accession: EAW21500
  
Location: 1458994-1460046
  
 NCBI BlastP on this gene

EAW21500

mitochondrial F1F0 ATP synthase subunit Atp14, putative
  
Accession: EAW21499
  
Location: 1458166-1458717
  
 NCBI BlastP on this gene

EAW21499

Query: Architecture Search FASTA input

DS989822 : Arthroderma gypseum CBS 118893 supercont1.1 genomic scaffold    Total score: 2.0     Cumulative Blast bit score: 719

Hit cluster cross-links:

Mycgr3G90785 Mycgr3T
  
Location: 0-1047

Mycgr3G90785\_Mycgr3T

Mycgr3G103262 Mycgr3
  
Location: 1147-1390

Mycgr3G103262\_Mycgr3

Mycgr3G68458 Mycgr3T
  
Location: 1490-3602

Mycgr3G68458\_Mycgr3T

Mycgr3G99145 Mycgr3T
  
Location: 3702-4326

Mycgr3G99145\_Mycgr3T

Mycgr3G103274 Mycgr3
  
Location: 4426-4957

Mycgr3G103274\_Mycgr3

Mycgr3G103264 Mycgr3
  
Location: 5057-5390

Mycgr3G103264\_Mycgr3

Mycgr3G37570 Mycgr3T
  
Location: 5490-6006

Mycgr3G37570\_Mycgr3T

Mycgr3G108094 Mycgr3
  
Location: 6106-10555

Mycgr3G108094\_Mycgr3

Mycgr3G90786 Mycgr3T
  
Location: 10655-12080

Mycgr3G90786\_Mycgr3T

Mycgr3G68429 Mycgr3T
  
Location: 12180-13440

Mycgr3G68429\_Mycgr3T

Mycgr3G68421 Mycgr3T
  
Location: 13540-17086

Mycgr3G68421\_Mycgr3T

Mycgr3G90801 Mycgr3T
  
Location: 17186-18056

Mycgr3G90801\_Mycgr3T

Mycgr3G84646 Mycgr3T
  
Location: 18156-20235

Mycgr3G84646\_Mycgr3T

Mycgr3G68456 Mycgr3T
  
Location: 20335-21970

Mycgr3G68456\_Mycgr3T

Mycgr3G103270 Mycgr3
  
Location: 22070-22355

Mycgr3G103270\_Mycgr3

Mycgr3G90803 Mycgr3T
  
Location: 22455-23019

Mycgr3G90803\_Mycgr3T

Mycgr3G36941 Mycgr3T
  
Location: 23119-24064

Mycgr3G36941\_Mycgr3T

Mycgr3G25746 Mycgr3T
  
Location: 24164-25241

Mycgr3G25746\_Mycgr3T

Mycgr3G90788 Mycgr3T
  
Location: 25341-25803

Mycgr3G90788\_Mycgr3T

Mycgr3G103260 Mycgr3
  
Location: 25903-26635

Mycgr3G103260\_Mycgr3

Mycgr3G84644 Mycgr3T
  
Location: 26735-28457

Mycgr3G84644\_Mycgr3T

Mycgr3G29227 Mycgr3T
  
Location: 28557-28863

Mycgr3G29227\_Mycgr3T

Mycgr3G36271 Mycgr3T
  
Location: 28963-29854

Mycgr3G36271\_Mycgr3T

Mycgr3G68433 Mycgr3T
  
Location: 29954-33041

Mycgr3G68433\_Mycgr3T

Mycgr3G79452 Mycgr3T
  
Location: 33141-33399

Mycgr3G79452\_Mycgr3T

Mycgr3G55345 Mycgr3T
  
Location: 33499-34126

Mycgr3G55345\_Mycgr3T

Mycgr3G103278 Mycgr3
  
Location: 34226-35195

Mycgr3G103278\_Mycgr3

Mycgr3G84654 Mycgr3T
  
Location: 35295-36630

Mycgr3G84654\_Mycgr3T

Mycgr3G108090 Mycgr3
  
Location: 36730-37591

Mycgr3G108090\_Mycgr3

Mycgr3G21922 Mycgr3T
  
Location: 37691-39149

Mycgr3G21922\_Mycgr3T

Mycgr3G99148 Mycgr3T
  
Location: 39249-42819

Mycgr3G99148\_Mycgr3T

hypothetical protein
  
Accession: EFQ98155
  
Location: 3256082-3258505
  
 NCBI BlastP on this gene

EFQ98155

hypothetical protein
  
Accession: EFQ98156
  
Location: 3259510-3259919
  
 NCBI BlastP on this gene

EFQ98156

PQ loop repeat protein
  
Accession: EFQ98157
  
Location: 3261639-3262682
  
 NCBI BlastP on this gene

EFQ98157

hypothetical protein
  
Accession: EFQ98158
  
Location: 3263685-3265502
  
 NCBI BlastP on this gene

EFQ98158

hypothetical protein
  
Accession: EFQ98159
  
Location: 3265872-3266577
  
 NCBI BlastP on this gene

EFQ98159

hypothetical protein
  
Accession: EFQ98160
  
Location: 3267436-3269100
  
 NCBI BlastP on this gene

EFQ98160

hypothetical protein
  
Accession: EFQ98161
  
Location: 3269448-3269798
  
 NCBI BlastP on this gene

EFQ98161

CMGC/DYRK/PRP4 protein kinase
  
Accession: EFQ98162
  
Location: 3270390-3272900
  
  
**BlastP hit with Mycgr3G103260\_Mycgr3**
  
Percentage identity: 54 %
  
BlastP bit score: 283
  
Sequence coverage: 96 %
  
E-value: 2e-86
  
  
 NCBI BlastP on this gene

EFQ98162

hypothetical protein
  
Accession: EFQ98163
  
Location: 3277146-3277592
  
 NCBI BlastP on this gene

EFQ98163

hypothetical protein
  
Accession: EFQ98164
  
Location: 3281065-3282049
  
 NCBI BlastP on this gene

EFQ98164

hypothetical protein
  
Accession: EFQ98165
  
Location: 3282776-3283214
  
 NCBI BlastP on this gene

EFQ98165

hypothetical protein
  
Accession: EFQ98166
  
Location: 3284702-3284965
  
 NCBI BlastP on this gene

EFQ98166

serine/threonine protein kinase
  
Accession: EFQ98167
  
Location: 3285769-3287073
  
  
**BlastP hit with Mycgr3G68429\_Mycgr3T**
  
Percentage identity: 64 %
  
BlastP bit score: 436
  
Sequence coverage: 82 %
  
E-value: 7e-147
  
  
 NCBI BlastP on this gene

EFQ98167

3-oxoacyl-[acyl-carrier-protein] reductase
  
Accession: EFQ98168
  
Location: 3290451-3291638
  
 NCBI BlastP on this gene

EFQ98168

adiponectin receptor protein 1
  
Accession: EFQ98169
  
Location: 3292121-3293086
  
 NCBI BlastP on this gene

EFQ98169

hypothetical protein
  
Accession: EFQ98170
  
Location: 3293473-3294537
  
 NCBI BlastP on this gene

EFQ98170

hypothetical protein
  
Accession: EFQ98171
  
Location: 3294829-3296390
  
 NCBI BlastP on this gene

EFQ98171

dolichol-phosphate mannosyltransferase
  
Accession: EFQ98172
  
Location: 3296773-3297715
  
 NCBI BlastP on this gene

EFQ98172

dolichol-phosphate mannosyltransferase
  
Accession: EFQ98173
  
Location: 3297985-3299097
  
 NCBI BlastP on this gene

EFQ98173

hypothetical protein
  
Accession: EFQ98174
  
Location: 3299507-3301781
  
 NCBI BlastP on this gene

EFQ98174

hypothetical protein
  
Accession: EFQ98175
  
Location: 3302333-3302965
  
 NCBI BlastP on this gene

EFQ98175

Query: Architecture Search FASTA input

DS499596 : Aspergillus fumigatus A1163 scf\_000003 genomic scaffold    Total score: 2.0     Cumulative Blast bit score: 719

Hit cluster cross-links:

Mycgr3G90785 Mycgr3T
  
Location: 0-1047

Mycgr3G90785\_Mycgr3T

Mycgr3G103262 Mycgr3
  
Location: 1147-1390

Mycgr3G103262\_Mycgr3

Mycgr3G68458 Mycgr3T
  
Location: 1490-3602

Mycgr3G68458\_Mycgr3T

Mycgr3G99145 Mycgr3T
  
Location: 3702-4326

Mycgr3G99145\_Mycgr3T

Mycgr3G103274 Mycgr3
  
Location: 4426-4957

Mycgr3G103274\_Mycgr3

Mycgr3G103264 Mycgr3
  
Location: 5057-5390

Mycgr3G103264\_Mycgr3

Mycgr3G37570 Mycgr3T
  
Location: 5490-6006

Mycgr3G37570\_Mycgr3T

Mycgr3G108094 Mycgr3
  
Location: 6106-10555

Mycgr3G108094\_Mycgr3

Mycgr3G90786 Mycgr3T
  
Location: 10655-12080

Mycgr3G90786\_Mycgr3T

Mycgr3G68429 Mycgr3T
  
Location: 12180-13440

Mycgr3G68429\_Mycgr3T

Mycgr3G68421 Mycgr3T
  
Location: 13540-17086

Mycgr3G68421\_Mycgr3T

Mycgr3G90801 Mycgr3T
  
Location: 17186-18056

Mycgr3G90801\_Mycgr3T

Mycgr3G84646 Mycgr3T
  
Location: 18156-20235

Mycgr3G84646\_Mycgr3T

Mycgr3G68456 Mycgr3T
  
Location: 20335-21970

Mycgr3G68456\_Mycgr3T

Mycgr3G103270 Mycgr3
  
Location: 22070-22355

Mycgr3G103270\_Mycgr3

Mycgr3G90803 Mycgr3T
  
Location: 22455-23019

Mycgr3G90803\_Mycgr3T

Mycgr3G36941 Mycgr3T
  
Location: 23119-24064

Mycgr3G36941\_Mycgr3T

Mycgr3G25746 Mycgr3T
  
Location: 24164-25241

Mycgr3G25746\_Mycgr3T

Mycgr3G90788 Mycgr3T
  
Location: 25341-25803

Mycgr3G90788\_Mycgr3T

Mycgr3G103260 Mycgr3
  
Location: 25903-26635

Mycgr3G103260\_Mycgr3

Mycgr3G84644 Mycgr3T
  
Location: 26735-28457

Mycgr3G84644\_Mycgr3T

Mycgr3G29227 Mycgr3T
  
Location: 28557-28863

Mycgr3G29227\_Mycgr3T

Mycgr3G36271 Mycgr3T
  
Location: 28963-29854

Mycgr3G36271\_Mycgr3T

Mycgr3G68433 Mycgr3T
  
Location: 29954-33041

Mycgr3G68433\_Mycgr3T

Mycgr3G79452 Mycgr3T
  
Location: 33141-33399

Mycgr3G79452\_Mycgr3T

Mycgr3G55345 Mycgr3T
  
Location: 33499-34126

Mycgr3G55345\_Mycgr3T

Mycgr3G103278 Mycgr3
  
Location: 34226-35195

Mycgr3G103278\_Mycgr3

Mycgr3G84654 Mycgr3T
  
Location: 35295-36630

Mycgr3G84654\_Mycgr3T

Mycgr3G108090 Mycgr3
  
Location: 36730-37591

Mycgr3G108090\_Mycgr3

Mycgr3G21922 Mycgr3T
  
Location: 37691-39149

Mycgr3G21922\_Mycgr3T

Mycgr3G99148 Mycgr3T
  
Location: 39249-42819

Mycgr3G99148\_Mycgr3T

conserved hypothetical protein
  
Accession: EDP52704
  
Location: 1368580-1370709
  
 NCBI BlastP on this gene

EDP52704

conserved hypothetical protein
  
Accession: EDP52703
  
Location: 1365344-1366075
  
 NCBI BlastP on this gene

EDP52703

conserved hypothetical protein
  
Accession: EDP52702
  
Location: 1362205-1364655
  
 NCBI BlastP on this gene

EDP52702

nuclear transport factor NTF-2, putative
  
Accession: EDP52701
  
Location: 1361067-1361900
  
 NCBI BlastP on this gene

EDP52701

PQ loop repeat protein
  
Accession: EDP52700
  
Location: 1359621-1360850
  
 NCBI BlastP on this gene

EDP52700

stress response protein (Ish1), putative
  
Accession: EDP52699
  
Location: 1356836-1358631
  
 NCBI BlastP on this gene

EDP52699

DNA damage response protein (Dap1), putative
  
Accession: EDP52698
  
Location: 1355831-1356363
  
 NCBI BlastP on this gene

EDP52698

conserved hypothetical protein
  
Accession: EDP52697
  
Location: 1352784-1354112
  
 NCBI BlastP on this gene

EDP52697

conserved hypothetical protein
  
Accession: EDP52696
  
Location: 1351880-1352215
  
 NCBI BlastP on this gene

EDP52696

serine/threonine protein kinase (Prp4), putative
  
Accession: EDP52695
  
Location: 1348747-1351298
  
  
**BlastP hit with Mycgr3G103260\_Mycgr3**
  
Percentage identity: 53 %
  
BlastP bit score: 281
  
Sequence coverage: 98 %
  
E-value: 1e-85
  
  
 NCBI BlastP on this gene

EDP52695

protein serine/threonine kinase (Ran1), putative
  
Accession: EDP52694
  
Location: 1342528-1343855
  
  
**BlastP hit with Mycgr3G68429\_Mycgr3T**
  
Percentage identity: 67 %
  
BlastP bit score: 438
  
Sequence coverage: 76 %
  
E-value: 7e-148
  
  
 NCBI BlastP on this gene

EDP52694

3-oxoacyl-(acyl-carrier-protein) reductase, putative
  
Accession: EDP52693
  
Location: 1340396-1341463
  
 NCBI BlastP on this gene

EDP52693

conserved hypothetical protein
  
Accession: EDP52692
  
Location: 1339511-1340158
  
 NCBI BlastP on this gene

EDP52692

C6 finger domain protein, putative
  
Accession: EDP52691
  
Location: 1339000-1339362
  
 NCBI BlastP on this gene

EDP52691

haemolysin-III channel protein Izh2, putative
  
Accession: EDP52690
  
Location: 1336985-1337950
  
 NCBI BlastP on this gene

EDP52690

conserved hypothetical protein
  
Accession: EDP52689
  
Location: 1335516-1336559
  
 NCBI BlastP on this gene

EDP52689

conserved hypothetical protein
  
Accession: EDP52688
  
Location: 1334791-1335269
  
 NCBI BlastP on this gene

EDP52688

clathrin-coated vesicle protein, putative
  
Accession: EDP52687
  
Location: 1332516-1333350
  
 NCBI BlastP on this gene

EDP52687

conserved hypothetical protein
  
Accession: EDP52686
  
Location: 1325808-1328624
  
 NCBI BlastP on this gene

EDP52686

transcription initiation protein
  
Accession: EDP52685
  
Location: 1323728-1324996
  
 NCBI BlastP on this gene

EDP52685

mitochondrial F1F0 ATP synthase subunit Atp14, putative
  
Accession: EDP52684
  
Location: 1323118-1323665
  
 NCBI BlastP on this gene

EDP52684

Query: Architecture Search FASTA input

AAHF01000002 : Aspergillus fumigatus Af293    Total score: 2.0     Cumulative Blast bit score: 719

Hit cluster cross-links:

Mycgr3G90785 Mycgr3T
  
Location: 0-1047

Mycgr3G90785\_Mycgr3T

Mycgr3G103262 Mycgr3
  
Location: 1147-1390

Mycgr3G103262\_Mycgr3

Mycgr3G68458 Mycgr3T
  
Location: 1490-3602

Mycgr3G68458\_Mycgr3T

Mycgr3G99145 Mycgr3T
  
Location: 3702-4326

Mycgr3G99145\_Mycgr3T

Mycgr3G103274 Mycgr3
  
Location: 4426-4957

Mycgr3G103274\_Mycgr3

Mycgr3G103264 Mycgr3
  
Location: 5057-5390

Mycgr3G103264\_Mycgr3

Mycgr3G37570 Mycgr3T
  
Location: 5490-6006

Mycgr3G37570\_Mycgr3T

Mycgr3G108094 Mycgr3
  
Location: 6106-10555

Mycgr3G108094\_Mycgr3

Mycgr3G90786 Mycgr3T
  
Location: 10655-12080

Mycgr3G90786\_Mycgr3T

Mycgr3G68429 Mycgr3T
  
Location: 12180-13440

Mycgr3G68429\_Mycgr3T

Mycgr3G68421 Mycgr3T
  
Location: 13540-17086

Mycgr3G68421\_Mycgr3T

Mycgr3G90801 Mycgr3T
  
Location: 17186-18056

Mycgr3G90801\_Mycgr3T

Mycgr3G84646 Mycgr3T
  
Location: 18156-20235

Mycgr3G84646\_Mycgr3T

Mycgr3G68456 Mycgr3T
  
Location: 20335-21970

Mycgr3G68456\_Mycgr3T

Mycgr3G103270 Mycgr3
  
Location: 22070-22355

Mycgr3G103270\_Mycgr3

Mycgr3G90803 Mycgr3T
  
Location: 22455-23019

Mycgr3G90803\_Mycgr3T

Mycgr3G36941 Mycgr3T
  
Location: 23119-24064

Mycgr3G36941\_Mycgr3T

Mycgr3G25746 Mycgr3T
  
Location: 24164-25241

Mycgr3G25746\_Mycgr3T

Mycgr3G90788 Mycgr3T
  
Location: 25341-25803

Mycgr3G90788\_Mycgr3T

Mycgr3G103260 Mycgr3
  
Location: 25903-26635

Mycgr3G103260\_Mycgr3

Mycgr3G84644 Mycgr3T
  
Location: 26735-28457

Mycgr3G84644\_Mycgr3T

Mycgr3G29227 Mycgr3T
  
Location: 28557-28863

Mycgr3G29227\_Mycgr3T

Mycgr3G36271 Mycgr3T
  
Location: 28963-29854

Mycgr3G36271\_Mycgr3T

Mycgr3G68433 Mycgr3T
  
Location: 29954-33041

Mycgr3G68433\_Mycgr3T

Mycgr3G79452 Mycgr3T
  
Location: 33141-33399

Mycgr3G79452\_Mycgr3T

Mycgr3G55345 Mycgr3T
  
Location: 33499-34126

Mycgr3G55345\_Mycgr3T

Mycgr3G103278 Mycgr3
  
Location: 34226-35195

Mycgr3G103278\_Mycgr3

Mycgr3G84654 Mycgr3T
  
Location: 35295-36630

Mycgr3G84654\_Mycgr3T

Mycgr3G108090 Mycgr3
  
Location: 36730-37591

Mycgr3G108090\_Mycgr3

Mycgr3G21922 Mycgr3T
  
Location: 37691-39149

Mycgr3G21922\_Mycgr3T

Mycgr3G99148 Mycgr3T
  
Location: 39249-42819

Mycgr3G99148\_Mycgr3T

conserved hypothetical protein
  
Accession: EAL92539
  
Location: 1378885-1381014
  
 NCBI BlastP on this gene

EAL92539

conserved hypothetical protein
  
Accession: EAL92537
  
Location: 1373765-1374496
  
 NCBI BlastP on this gene

EAL92537

conserved hypothetical protein
  
Accession: EAL92536
  
Location: 1370626-1373076
  
 NCBI BlastP on this gene

EAL92536

nuclear transport factor NTF-2, putative
  
Accession: EAL92535
  
Location: 1369488-1370321
  
 NCBI BlastP on this gene

EAL92535

PQ loop repeat protein
  
Accession: EAL92534
  
Location: 1368042-1369271
  
 NCBI BlastP on this gene

EAL92534

meiotic sister chromatid recombination protein Ish1/Msc1, putative
  
Accession: EAL92533
  
Location: 1365257-1367052
  
 NCBI BlastP on this gene

EAL92533

DNA damage response protein (Dap1), putative
  
Accession: EAL92532
  
Location: 1364251-1364783
  
 NCBI BlastP on this gene

EAL92532

conserved hypothetical protein
  
Accession: EAL92531
  
Location: 1361204-1362532
  
 NCBI BlastP on this gene

EAL92531

conserved hypothetical protein
  
Accession: EAL92530
  
Location: 1360300-1360635
  
 NCBI BlastP on this gene

EAL92530

serine/threonine protein kinase (Prp4), putative
  
Accession: EAL92529
  
Location: 1357168-1359719
  
  
**BlastP hit with Mycgr3G103260\_Mycgr3**
  
Percentage identity: 53 %
  
BlastP bit score: 281
  
Sequence coverage: 98 %
  
E-value: 1e-85
  
  
 NCBI BlastP on this gene

EAL92529

protein serine/threonine kinase (Ran1), putative
  
Accession: EAL92528
  
Location: 1350952-1352279
  
  
**BlastP hit with Mycgr3G68429\_Mycgr3T**
  
Percentage identity: 67 %
  
BlastP bit score: 438
  
Sequence coverage: 76 %
  
E-value: 8e-148
  
  
 NCBI BlastP on this gene

EAL92528

3-oxoacyl-(acyl-carrier-protein) reductase, putative
  
Accession: EAL92527
  
Location: 1348810-1349877
  
 NCBI BlastP on this gene

EAL92527

conserved hypothetical protein
  
Accession: EAL92526
  
Location: 1347925-1348572
  
 NCBI BlastP on this gene

EAL92526

C6 finger domain protein, putative
  
Accession: EAL92525
  
Location: 1347414-1347776
  
 NCBI BlastP on this gene

EAL92525

haemolysin-III channel protein Izh2, putative
  
Accession: EAL92524
  
Location: 1345399-1346364
  
 NCBI BlastP on this gene

EAL92524

conserved hypothetical protein
  
Accession: EAL92523
  
Location: 1343930-1344973
  
 NCBI BlastP on this gene

EAL92523

conserved hypothetical protein
  
Accession: EAL92522
  
Location: 1343205-1343683
  
 NCBI BlastP on this gene

EAL92522

clathrin-coated vesicle protein, putative
  
Accession: EAL92521
  
Location: 1340930-1341764
  
 NCBI BlastP on this gene

EAL92521

conserved hypothetical protein
  
Accession: EAL92520
  
Location: 1334228-1337044
  
 NCBI BlastP on this gene

EAL92520

transcription initiation protein
  
Accession: EAL92519
  
Location: 1332148-1333416
  
 NCBI BlastP on this gene

EAL92519

mitochondrial F1F0 ATP synthase subunit Atp14, putative
  
Accession: EAL92518
  
Location: 1331538-1332085
  
 NCBI BlastP on this gene

EAL92518

Query: Architecture Search FASTA input

DS027056 : Aspergillus clavatus NRRL 1 1099423829802 genomic scaffold    Total score: 2.0     Cumulative Blast bit score: 716

Hit cluster cross-links:

Mycgr3G90785 Mycgr3T
  
Location: 0-1047

Mycgr3G90785\_Mycgr3T

Mycgr3G103262 Mycgr3
  
Location: 1147-1390

Mycgr3G103262\_Mycgr3

Mycgr3G68458 Mycgr3T
  
Location: 1490-3602

Mycgr3G68458\_Mycgr3T

Mycgr3G99145 Mycgr3T
  
Location: 3702-4326

Mycgr3G99145\_Mycgr3T

Mycgr3G103274 Mycgr3
  
Location: 4426-4957

Mycgr3G103274\_Mycgr3

Mycgr3G103264 Mycgr3
  
Location: 5057-5390

Mycgr3G103264\_Mycgr3

Mycgr3G37570 Mycgr3T
  
Location: 5490-6006

Mycgr3G37570\_Mycgr3T

Mycgr3G108094 Mycgr3
  
Location: 6106-10555

Mycgr3G108094\_Mycgr3

Mycgr3G90786 Mycgr3T
  
Location: 10655-12080

Mycgr3G90786\_Mycgr3T

Mycgr3G68429 Mycgr3T
  
Location: 12180-13440

Mycgr3G68429\_Mycgr3T

Mycgr3G68421 Mycgr3T
  
Location: 13540-17086

Mycgr3G68421\_Mycgr3T

Mycgr3G90801 Mycgr3T
  
Location: 17186-18056

Mycgr3G90801\_Mycgr3T

Mycgr3G84646 Mycgr3T
  
Location: 18156-20235

Mycgr3G84646\_Mycgr3T

Mycgr3G68456 Mycgr3T
  
Location: 20335-21970

Mycgr3G68456\_Mycgr3T

Mycgr3G103270 Mycgr3
  
Location: 22070-22355

Mycgr3G103270\_Mycgr3

Mycgr3G90803 Mycgr3T
  
Location: 22455-23019

Mycgr3G90803\_Mycgr3T

Mycgr3G36941 Mycgr3T
  
Location: 23119-24064

Mycgr3G36941\_Mycgr3T

Mycgr3G25746 Mycgr3T
  
Location: 24164-25241

Mycgr3G25746\_Mycgr3T

Mycgr3G90788 Mycgr3T
  
Location: 25341-25803

Mycgr3G90788\_Mycgr3T

Mycgr3G103260 Mycgr3
  
Location: 25903-26635

Mycgr3G103260\_Mycgr3

Mycgr3G84644 Mycgr3T
  
Location: 26735-28457

Mycgr3G84644\_Mycgr3T

Mycgr3G29227 Mycgr3T
  
Location: 28557-28863

Mycgr3G29227\_Mycgr3T

Mycgr3G36271 Mycgr3T
  
Location: 28963-29854

Mycgr3G36271\_Mycgr3T

Mycgr3G68433 Mycgr3T
  
Location: 29954-33041

Mycgr3G68433\_Mycgr3T

Mycgr3G79452 Mycgr3T
  
Location: 33141-33399

Mycgr3G79452\_Mycgr3T

Mycgr3G55345 Mycgr3T
  
Location: 33499-34126

Mycgr3G55345\_Mycgr3T

Mycgr3G103278 Mycgr3
  
Location: 34226-35195

Mycgr3G103278\_Mycgr3

Mycgr3G84654 Mycgr3T
  
Location: 35295-36630

Mycgr3G84654\_Mycgr3T

Mycgr3G108090 Mycgr3
  
Location: 36730-37591

Mycgr3G108090\_Mycgr3

Mycgr3G21922 Mycgr3T
  
Location: 37691-39149

Mycgr3G21922\_Mycgr3T

Mycgr3G99148 Mycgr3T
  
Location: 39249-42819

Mycgr3G99148\_Mycgr3T

hypothetical protein
  
Accession: EAW09622
  
Location: 1458058-1459116
  
 NCBI BlastP on this gene

EAW09622

conserved hypothetical protein
  
Accession: EAW09623
  
Location: 1459828-1461022
  
 NCBI BlastP on this gene

EAW09623

conserved hypothetical protein
  
Accession: EAW09624
  
Location: 1461245-1463701
  
 NCBI BlastP on this gene

EAW09624

nuclear transport factor NTF-2, putative
  
Accession: EAW09625
  
Location: 1464053-1464995
  
 NCBI BlastP on this gene

EAW09625

PQ loop repeat protein
  
Accession: EAW09626
  
Location: 1465410-1466437
  
 NCBI BlastP on this gene

EAW09626

stress response protein (Ish1), putative
  
Accession: EAW09627
  
Location: 1467470-1469270
  
 NCBI BlastP on this gene

EAW09627

DNA damage response protein (Dap1), putative
  
Accession: EAW09628
  
Location: 1469770-1470303
  
 NCBI BlastP on this gene

EAW09628

conserved hypothetical protein
  
Accession: EAW09629
  
Location: 1471475-1473236
  
 NCBI BlastP on this gene

EAW09629

conserved hypothetical protein
  
Accession: EAW09630
  
Location: 1473821-1474165
  
 NCBI BlastP on this gene

EAW09630

serine/threonine protein kinase (Prp4), putative
  
Accession: EAW09631
  
Location: 1474813-1477340
  
  
**BlastP hit with Mycgr3G103260\_Mycgr3**
  
Percentage identity: 52 %
  
BlastP bit score: 279
  
Sequence coverage: 98 %
  
E-value: 9e-85
  
  
 NCBI BlastP on this gene

EAW09631

protein serine/threonine kinase (Ran1), putative
  
Accession: EAW09632
  
Location: 1481962-1483293
  
  
**BlastP hit with Mycgr3G68429\_Mycgr3T**
  
Percentage identity: 68 %
  
BlastP bit score: 437
  
Sequence coverage: 75 %
  
E-value: 2e-147
  
  
 NCBI BlastP on this gene

EAW09632

3-oxoacyl-(acyl-carrier-protein) reductase, putative
  
Accession: EAW09633
  
Location: 1484549-1485898
  
 NCBI BlastP on this gene

EAW09633

C6 zinc finger domain protein
  
Accession: EAW09634
  
Location: 1486198-1487357
  
 NCBI BlastP on this gene

EAW09634

haemolysin-III channel protein Izh2, putative
  
Accession: EAW09635
  
Location: 1488406-1489371
  
 NCBI BlastP on this gene

EAW09635

conserved hypothetical protein
  
Accession: EAW09636
  
Location: 1489807-1490837
  
 NCBI BlastP on this gene

EAW09636

conserved hypothetical protein
  
Accession: EAW09637
  
Location: 1491085-1492605
  
 NCBI BlastP on this gene

EAW09637

clathrin-coated vesicle protein, putative
  
Accession: EAW09638
  
Location: 1492978-1493835
  
 NCBI BlastP on this gene

EAW09638

conserved hypothetical protein
  
Accession: EAW09639
  
Location: 1497385-1500274
  
 NCBI BlastP on this gene

EAW09639

transcription initiation protein
  
Accession: EAW09640
  
Location: 1501230-1502282
  
 NCBI BlastP on this gene

EAW09640

mitochondrial F1F0 ATP synthase subunit Atp14, putative
  
Accession: EAW09641
  
Location: 1502639-1503191
  
 NCBI BlastP on this gene

EAW09641

Query: Architecture Search FASTA input

GL636488 : Coccidioides posadasii str. Silveira unplaced genomic scaffold supercont2.3    Total score: 2.0     Cumulative Blast bit score: 715

Hit cluster cross-links:

Mycgr3G90785 Mycgr3T
  
Location: 0-1047

Mycgr3G90785\_Mycgr3T

Mycgr3G103262 Mycgr3
  
Location: 1147-1390

Mycgr3G103262\_Mycgr3

Mycgr3G68458 Mycgr3T
  
Location: 1490-3602

Mycgr3G68458\_Mycgr3T

Mycgr3G99145 Mycgr3T
  
Location: 3702-4326

Mycgr3G99145\_Mycgr3T

Mycgr3G103274 Mycgr3
  
Location: 4426-4957

Mycgr3G103274\_Mycgr3

Mycgr3G103264 Mycgr3
  
Location: 5057-5390

Mycgr3G103264\_Mycgr3

Mycgr3G37570 Mycgr3T
  
Location: 5490-6006

Mycgr3G37570\_Mycgr3T

Mycgr3G108094 Mycgr3
  
Location: 6106-10555

Mycgr3G108094\_Mycgr3

Mycgr3G90786 Mycgr3T
  
Location: 10655-12080

Mycgr3G90786\_Mycgr3T

Mycgr3G68429 Mycgr3T
  
Location: 12180-13440

Mycgr3G68429\_Mycgr3T

Mycgr3G68421 Mycgr3T
  
Location: 13540-17086

Mycgr3G68421\_Mycgr3T

Mycgr3G90801 Mycgr3T
  
Location: 17186-18056

Mycgr3G90801\_Mycgr3T

Mycgr3G84646 Mycgr3T
  
Location: 18156-20235

Mycgr3G84646\_Mycgr3T

Mycgr3G68456 Mycgr3T
  
Location: 20335-21970

Mycgr3G68456\_Mycgr3T

Mycgr3G103270 Mycgr3
  
Location: 22070-22355

Mycgr3G103270\_Mycgr3

Mycgr3G90803 Mycgr3T
  
Location: 22455-23019

Mycgr3G90803\_Mycgr3T

Mycgr3G36941 Mycgr3T
  
Location: 23119-24064

Mycgr3G36941\_Mycgr3T

Mycgr3G25746 Mycgr3T
  
Location: 24164-25241

Mycgr3G25746\_Mycgr3T

Mycgr3G90788 Mycgr3T
  
Location: 25341-25803

Mycgr3G90788\_Mycgr3T

Mycgr3G103260 Mycgr3
  
Location: 25903-26635

Mycgr3G103260\_Mycgr3

Mycgr3G84644 Mycgr3T
  
Location: 26735-28457

Mycgr3G84644\_Mycgr3T

Mycgr3G29227 Mycgr3T
  
Location: 28557-28863

Mycgr3G29227\_Mycgr3T

Mycgr3G36271 Mycgr3T
  
Location: 28963-29854

Mycgr3G36271\_Mycgr3T

Mycgr3G68433 Mycgr3T
  
Location: 29954-33041

Mycgr3G68433\_Mycgr3T

Mycgr3G79452 Mycgr3T
  
Location: 33141-33399

Mycgr3G79452\_Mycgr3T

Mycgr3G55345 Mycgr3T
  
Location: 33499-34126

Mycgr3G55345\_Mycgr3T

Mycgr3G103278 Mycgr3
  
Location: 34226-35195

Mycgr3G103278\_Mycgr3

Mycgr3G84654 Mycgr3T
  
Location: 35295-36630

Mycgr3G84654\_Mycgr3T

Mycgr3G108090 Mycgr3
  
Location: 36730-37591

Mycgr3G108090\_Mycgr3

Mycgr3G21922 Mycgr3T
  
Location: 37691-39149

Mycgr3G21922\_Mycgr3T

Mycgr3G99148 Mycgr3T
  
Location: 39249-42819

Mycgr3G99148\_Mycgr3T

conserved hypothetical protein
  
Accession: EFW20497
  
Location: 194254-196032
  
 NCBI BlastP on this gene

EFW20497

PQ loop repeat protein
  
Accession: EFW20498
  
Location: 196587-197679
  
 NCBI BlastP on this gene

EFW20498

conserved hypothetical protein
  
Accession: EFW20499
  
Location: 200048-200665
  
 NCBI BlastP on this gene

EFW20499

conserved hypothetical protein
  
Accession: EFW20500
  
Location: 201216-204260
  
 NCBI BlastP on this gene

EFW20500

conserved hypothetical protein
  
Accession: EFW20501
  
Location: 204920-205235
  
 NCBI BlastP on this gene

EFW20501

DNA damage response protein
  
Accession: EFW20502
  
Location: 207171-207622
  
 NCBI BlastP on this gene

EFW20502

conserved hypothetical protein
  
Accession: EFW20503
  
Location: 208471-210248
  
 NCBI BlastP on this gene

EFW20503

hypothetical protein
  
Accession: EFW20504
  
Location: 210774-211150
  
 NCBI BlastP on this gene

EFW20504

serine/threonine-protein kinase prp4
  
Accession: EFW20505
  
Location: 212326-214178
  
  
**BlastP hit with Mycgr3G103260\_Mycgr3**
  
Percentage identity: 52 %
  
BlastP bit score: 280
  
Sequence coverage: 96 %
  
E-value: 9e-87
  
  
 NCBI BlastP on this gene

EFW20505

predicted protein
  
Accession: EFW20506
  
Location: 215706-216181
  
 NCBI BlastP on this gene

EFW20506

hypothetical protein
  
Accession: EFW20507
  
Location: 216539-216730
  
 NCBI BlastP on this gene

EFW20507

predicted protein
  
Accession: EFW20508
  
Location: 217585-219467
  
 NCBI BlastP on this gene

EFW20508

protein serine/threonine kinase
  
Accession: EFW20509
  
Location: 221596-222885
  
  
**BlastP hit with Mycgr3G68429\_Mycgr3T**
  
Percentage identity: 67 %
  
BlastP bit score: 435
  
Sequence coverage: 74 %
  
E-value: 1e-146
  
  
 NCBI BlastP on this gene

EFW20509

conserved hypothetical protein
  
Accession: EFW20511
  
Location: 224002-224561
  
 NCBI BlastP on this gene

EFW20511

hypothetical protein
  
Accession: EFW20510
  
Location: 224974-225534
  
 NCBI BlastP on this gene

EFW20510

3-oxoacyl-(acyl-carrier-protein) reductase
  
Accession: EFW20512
  
Location: 227049-228179
  
 NCBI BlastP on this gene

EFW20512

hemolysin-III channel protein Izh2
  
Accession: EFW20513
  
Location: 228699-229661
  
 NCBI BlastP on this gene

EFW20513

conserved hypothetical protein
  
Accession: EFW20514
  
Location: 229979-231026
  
 NCBI BlastP on this gene

EFW20514

conserved hypothetical protein
  
Accession: EFW20515
  
Location: 231324-232695
  
 NCBI BlastP on this gene

EFW20515

glycosyltransferase
  
Accession: EFW20516
  
Location: 233051-233965
  
 NCBI BlastP on this gene

EFW20516

conserved hypothetical protein
  
Accession: EFW20517
  
Location: 234215-235319
  
 NCBI BlastP on this gene

EFW20517

conserved hypothetical protein
  
Accession: EFW20518
  
Location: 235725-238014
  
 NCBI BlastP on this gene

EFW20518

hypothetical protein
  
Accession: EFW20519
  
Location: 238580-239140
  
 NCBI BlastP on this gene

EFW20519

vacuolar sorting protein 35
  
Accession: EFW20520
  
Location: 241221-244226
  
 NCBI BlastP on this gene

EFW20520

Query: Architecture Search FASTA input

GG698485 : Trichophyton tonsurans CBS 112818 genomic scaffold supercont1.9    Total score: 2.0     Cumulative Blast bit score: 715

Hit cluster cross-links:

Mycgr3G90785 Mycgr3T
  
Location: 0-1047

Mycgr3G90785\_Mycgr3T

Mycgr3G103262 Mycgr3
  
Location: 1147-1390

Mycgr3G103262\_Mycgr3

Mycgr3G68458 Mycgr3T
  
Location: 1490-3602

Mycgr3G68458\_Mycgr3T

Mycgr3G99145 Mycgr3T
  
Location: 3702-4326

Mycgr3G99145\_Mycgr3T

Mycgr3G103274 Mycgr3
  
Location: 4426-4957

Mycgr3G103274\_Mycgr3

Mycgr3G103264 Mycgr3
  
Location: 5057-5390

Mycgr3G103264\_Mycgr3

Mycgr3G37570 Mycgr3T
  
Location: 5490-6006

Mycgr3G37570\_Mycgr3T

Mycgr3G108094 Mycgr3
  
Location: 6106-10555

Mycgr3G108094\_Mycgr3

Mycgr3G90786 Mycgr3T
  
Location: 10655-12080

Mycgr3G90786\_Mycgr3T

Mycgr3G68429 Mycgr3T
  
Location: 12180-13440

Mycgr3G68429\_Mycgr3T

Mycgr3G68421 Mycgr3T
  
Location: 13540-17086

Mycgr3G68421\_Mycgr3T

Mycgr3G90801 Mycgr3T
  
Location: 17186-18056

Mycgr3G90801\_Mycgr3T

Mycgr3G84646 Mycgr3T
  
Location: 18156-20235

Mycgr3G84646\_Mycgr3T

Mycgr3G68456 Mycgr3T
  
Location: 20335-21970

Mycgr3G68456\_Mycgr3T

Mycgr3G103270 Mycgr3
  
Location: 22070-22355

Mycgr3G103270\_Mycgr3

Mycgr3G90803 Mycgr3T
  
Location: 22455-23019

Mycgr3G90803\_Mycgr3T

Mycgr3G36941 Mycgr3T
  
Location: 23119-24064

Mycgr3G36941\_Mycgr3T

Mycgr3G25746 Mycgr3T
  
Location: 24164-25241

Mycgr3G25746\_Mycgr3T

Mycgr3G90788 Mycgr3T
  
Location: 25341-25803

Mycgr3G90788\_Mycgr3T

Mycgr3G103260 Mycgr3
  
Location: 25903-26635

Mycgr3G103260\_Mycgr3

Mycgr3G84644 Mycgr3T
  
Location: 26735-28457

Mycgr3G84644\_Mycgr3T

Mycgr3G29227 Mycgr3T
  
Location: 28557-28863

Mycgr3G29227\_Mycgr3T

Mycgr3G36271 Mycgr3T
  
Location: 28963-29854

Mycgr3G36271\_Mycgr3T

Mycgr3G68433 Mycgr3T
  
Location: 29954-33041

Mycgr3G68433\_Mycgr3T

Mycgr3G79452 Mycgr3T
  
Location: 33141-33399

Mycgr3G79452\_Mycgr3T

Mycgr3G55345 Mycgr3T
  
Location: 33499-34126

Mycgr3G55345\_Mycgr3T

Mycgr3G103278 Mycgr3
  
Location: 34226-35195

Mycgr3G103278\_Mycgr3

Mycgr3G84654 Mycgr3T
  
Location: 35295-36630

Mycgr3G84654\_Mycgr3T

Mycgr3G108090 Mycgr3
  
Location: 36730-37591

Mycgr3G108090\_Mycgr3

Mycgr3G21922 Mycgr3T
  
Location: 37691-39149

Mycgr3G21922\_Mycgr3T

Mycgr3G99148 Mycgr3T
  
Location: 39249-42819

Mycgr3G99148\_Mycgr3T

hypothetical protein
  
Accession: EGD94986
  
Location: 434415-436807
  
 NCBI BlastP on this gene

EGD94986

hypothetical protein
  
Accession: EGD94987
  
Location: 438167-438706
  
 NCBI BlastP on this gene

EGD94987

PQ loop repeat protein
  
Accession: EGD94988
  
Location: 440194-441255
  
 NCBI BlastP on this gene

EGD94988

hypothetical protein
  
Accession: EGD94989
  
Location: 442277-444095
  
 NCBI BlastP on this gene

EGD94989

DNA damage response protein
  
Accession: EGD94990
  
Location: 444458-445152
  
 NCBI BlastP on this gene

EGD94990

hypothetical protein
  
Accession: EGD94991
  
Location: 446008-447673
  
 NCBI BlastP on this gene

EGD94991

hypothetical protein
  
Accession: EGD94992
  
Location: 448031-448379
  
 NCBI BlastP on this gene

EGD94992

CMGC/DYRK/PRP4 protein kinase
  
Accession: EGD94993
  
Location: 448961-451483
  
  
**BlastP hit with Mycgr3G103260\_Mycgr3**
  
Percentage identity: 54 %
  
BlastP bit score: 284
  
Sequence coverage: 96 %
  
E-value: 2e-86
  
  
 NCBI BlastP on this gene

EGD94993

hypothetical protein
  
Accession: EGD94994
  
Location: 452159-452617
  
 NCBI BlastP on this gene

EGD94994

hypothetical protein
  
Accession: EGD94995
  
Location: 453076-454096
  
 NCBI BlastP on this gene

EGD94995

hypothetical protein
  
Accession: EGD94996
  
Location: 456030-456572
  
 NCBI BlastP on this gene

EGD94996

hypothetical protein
  
Accession: EGD94997
  
Location: 458114-458665
  
 NCBI BlastP on this gene

EGD94997

hypothetical protein
  
Accession: EGD94998
  
Location: 459430-460318
  
 NCBI BlastP on this gene

EGD94998

serine/threonine protein kinase
  
Accession: EGD94999
  
Location: 464346-465661
  
  
**BlastP hit with Mycgr3G68429\_Mycgr3T**
  
Percentage identity: 62 %
  
BlastP bit score: 431
  
Sequence coverage: 82 %
  
E-value: 6e-145
  
  
 NCBI BlastP on this gene

EGD94999

3-oxoacyl-(acyl-carrier-protein) reductase
  
Accession: EGD95000
  
Location: 469097-470282
  
 NCBI BlastP on this gene

EGD95000

hemolysin-III channel protein Izh2
  
Accession: EGD95001
  
Location: 470782-471768
  
 NCBI BlastP on this gene

EGD95001

hypothetical protein
  
Accession: EGD95002
  
Location: 472158-473249
  
 NCBI BlastP on this gene

EGD95002

hypothetical protein
  
Accession: EGD95003
  
Location: 473535-475153
  
 NCBI BlastP on this gene

EGD95003

dolichol-phosphate mannosyltransferase
  
Accession: EGD95004
  
Location: 475569-476528
  
 NCBI BlastP on this gene

EGD95004

hypothetical protein
  
Accession: EGD95005
  
Location: 476787-477885
  
 NCBI BlastP on this gene

EGD95005

hypothetical protein
  
Accession: EGD95006
  
Location: 478346-480609
  
 NCBI BlastP on this gene

EGD95006

hypothetical protein
  
Accession: EGD95007
  
Location: 481132-481770
  
 NCBI BlastP on this gene

EGD95007

Query: Architecture Search FASTA input

DS995701 : Microsporum canis CBS 113480 supercont1.1 genomic scaffold    Total score: 2.0     Cumulative Blast bit score: 715

Hit cluster cross-links:

Mycgr3G90785 Mycgr3T
  
Location: 0-1047

Mycgr3G90785\_Mycgr3T

Mycgr3G103262 Mycgr3
  
Location: 1147-1390

Mycgr3G103262\_Mycgr3

Mycgr3G68458 Mycgr3T
  
Location: 1490-3602

Mycgr3G68458\_Mycgr3T

Mycgr3G99145 Mycgr3T
  
Location: 3702-4326

Mycgr3G99145\_Mycgr3T

Mycgr3G103274 Mycgr3
  
Location: 4426-4957

Mycgr3G103274\_Mycgr3

Mycgr3G103264 Mycgr3
  
Location: 5057-5390

Mycgr3G103264\_Mycgr3

Mycgr3G37570 Mycgr3T
  
Location: 5490-6006

Mycgr3G37570\_Mycgr3T

Mycgr3G108094 Mycgr3
  
Location: 6106-10555

Mycgr3G108094\_Mycgr3

Mycgr3G90786 Mycgr3T
  
Location: 10655-12080

Mycgr3G90786\_Mycgr3T

Mycgr3G68429 Mycgr3T
  
Location: 12180-13440

Mycgr3G68429\_Mycgr3T

Mycgr3G68421 Mycgr3T
  
Location: 13540-17086

Mycgr3G68421\_Mycgr3T

Mycgr3G90801 Mycgr3T
  
Location: 17186-18056

Mycgr3G90801\_Mycgr3T

Mycgr3G84646 Mycgr3T
  
Location: 18156-20235

Mycgr3G84646\_Mycgr3T

Mycgr3G68456 Mycgr3T
  
Location: 20335-21970

Mycgr3G68456\_Mycgr3T

Mycgr3G103270 Mycgr3
  
Location: 22070-22355

Mycgr3G103270\_Mycgr3

Mycgr3G90803 Mycgr3T
  
Location: 22455-23019

Mycgr3G90803\_Mycgr3T

Mycgr3G36941 Mycgr3T
  
Location: 23119-24064

Mycgr3G36941\_Mycgr3T

Mycgr3G25746 Mycgr3T
  
Location: 24164-25241

Mycgr3G25746\_Mycgr3T

Mycgr3G90788 Mycgr3T
  
Location: 25341-25803

Mycgr3G90788\_Mycgr3T

Mycgr3G103260 Mycgr3
  
Location: 25903-26635

Mycgr3G103260\_Mycgr3

Mycgr3G84644 Mycgr3T
  
Location: 26735-28457

Mycgr3G84644\_Mycgr3T

Mycgr3G29227 Mycgr3T
  
Location: 28557-28863

Mycgr3G29227\_Mycgr3T

Mycgr3G36271 Mycgr3T
  
Location: 28963-29854

Mycgr3G36271\_Mycgr3T

Mycgr3G68433 Mycgr3T
  
Location: 29954-33041

Mycgr3G68433\_Mycgr3T

Mycgr3G79452 Mycgr3T
  
Location: 33141-33399

Mycgr3G79452\_Mycgr3T

Mycgr3G55345 Mycgr3T
  
Location: 33499-34126

Mycgr3G55345\_Mycgr3T

Mycgr3G103278 Mycgr3
  
Location: 34226-35195

Mycgr3G103278\_Mycgr3

Mycgr3G84654 Mycgr3T
  
Location: 35295-36630

Mycgr3G84654\_Mycgr3T

Mycgr3G108090 Mycgr3
  
Location: 36730-37591

Mycgr3G108090\_Mycgr3

Mycgr3G21922 Mycgr3T
  
Location: 37691-39149

Mycgr3G21922\_Mycgr3T

Mycgr3G99148 Mycgr3T
  
Location: 39249-42819

Mycgr3G99148\_Mycgr3T

conserved hypothetical protein
  
Accession: EEQ27880
  
Location: 2077916-2080178
  
 NCBI BlastP on this gene

EEQ27880

conserved hypothetical protein
  
Accession: EEQ27879
  
Location: 2074401-2076061
  
 NCBI BlastP on this gene

EEQ27879

conserved hypothetical protein
  
Accession: EEQ27878
  
Location: 2073704-2074046
  
 NCBI BlastP on this gene

EEQ27878

serine/threonine-protein kinase prp4
  
Accession: EEQ27877
  
Location: 2070654-2073180
  
  
**BlastP hit with Mycgr3G103260\_Mycgr3**
  
Percentage identity: 52 %
  
BlastP bit score: 279
  
Sequence coverage: 96 %
  
E-value: 9e-85
  
  
 NCBI BlastP on this gene

EEQ27877

predicted protein
  
Accession: EEQ27876
  
Location: 2068171-2069374
  
 NCBI BlastP on this gene

EEQ27876

predicted protein
  
Accession: EEQ27875
  
Location: 2066082-2066638
  
 NCBI BlastP on this gene

EEQ27875

negative regulator of sexual conjugation and meiosis
  
Accession: EEQ27874
  
Location: 2057852-2059163
  
  
**BlastP hit with Mycgr3G68429\_Mycgr3T**
  
Percentage identity: 63 %
  
BlastP bit score: 436
  
Sequence coverage: 82 %
  
E-value: 7e-147
  
  
 NCBI BlastP on this gene

EEQ27874

predicted protein
  
Accession: EEQ27873
  
Location: 2055556-2056376
  
 NCBI BlastP on this gene

EEQ27873

short-chain dehydrogenase/reductase SDR
  
Accession: EEQ27872
  
Location: 2053702-2054819
  
 NCBI BlastP on this gene

EEQ27872

adiponectin receptor protein 1
  
Accession: EEQ27871
  
Location: 2052454-2053401
  
 NCBI BlastP on this gene

EEQ27871

conserved hypothetical protein
  
Accession: EEQ27870
  
Location: 2051047-2052117
  
 NCBI BlastP on this gene

EEQ27870

conserved hypothetical protein
  
Accession: EEQ27869
  
Location: 2049237-2050812
  
 NCBI BlastP on this gene

EEQ27869

dolichol-phosphate mannosyltransferase
  
Accession: EEQ27868
  
Location: 2047931-2048786
  
 NCBI BlastP on this gene

EEQ27868

dolichol-phosphate mannosyltransferase
  
Accession: EEQ27867
  
Location: 2046494-2047607
  
 NCBI BlastP on this gene

EEQ27867

transcription factor tfiiic complex subunit sfc6
  
Accession: EEQ27866
  
Location: 2043808-2046070
  
 NCBI BlastP on this gene

EEQ27866

A-agglutinin anchorage subunit
  
Accession: EEQ27865
  
Location: 2041834-2043323
  
 NCBI BlastP on this gene

EEQ27865

vacuolar sorting protein 35
  
Accession: EEQ27864
  
Location: 2039732-2041759
  
 NCBI BlastP on this gene

EEQ27864

Query: Architecture Search FASTA input

ACFW01000030 : Coccidioides posadasii C735 delta SOWgp    Total score: 2.0     Cumulative Blast bit score: 714

Hit cluster cross-links:

Mycgr3G90785 Mycgr3T
  
Location: 0-1047

Mycgr3G90785\_Mycgr3T

Mycgr3G103262 Mycgr3
  
Location: 1147-1390

Mycgr3G103262\_Mycgr3

Mycgr3G68458 Mycgr3T
  
Location: 1490-3602

Mycgr3G68458\_Mycgr3T

Mycgr3G99145 Mycgr3T
  
Location: 3702-4326

Mycgr3G99145\_Mycgr3T

Mycgr3G103274 Mycgr3
  
Location: 4426-4957

Mycgr3G103274\_Mycgr3

Mycgr3G103264 Mycgr3
  
Location: 5057-5390

Mycgr3G103264\_Mycgr3

Mycgr3G37570 Mycgr3T
  
Location: 5490-6006

Mycgr3G37570\_Mycgr3T

Mycgr3G108094 Mycgr3
  
Location: 6106-10555

Mycgr3G108094\_Mycgr3

Mycgr3G90786 Mycgr3T
  
Location: 10655-12080

Mycgr3G90786\_Mycgr3T

Mycgr3G68429 Mycgr3T
  
Location: 12180-13440

Mycgr3G68429\_Mycgr3T

Mycgr3G68421 Mycgr3T
  
Location: 13540-17086

Mycgr3G68421\_Mycgr3T

Mycgr3G90801 Mycgr3T
  
Location: 17186-18056

Mycgr3G90801\_Mycgr3T

Mycgr3G84646 Mycgr3T
  
Location: 18156-20235

Mycgr3G84646\_Mycgr3T

Mycgr3G68456 Mycgr3T
  
Location: 20335-21970

Mycgr3G68456\_Mycgr3T

Mycgr3G103270 Mycgr3
  
Location: 22070-22355

Mycgr3G103270\_Mycgr3

Mycgr3G90803 Mycgr3T
  
Location: 22455-23019

Mycgr3G90803\_Mycgr3T

Mycgr3G36941 Mycgr3T
  
Location: 23119-24064

Mycgr3G36941\_Mycgr3T

Mycgr3G25746 Mycgr3T
  
Location: 24164-25241

Mycgr3G25746\_Mycgr3T

Mycgr3G90788 Mycgr3T
  
Location: 25341-25803

Mycgr3G90788\_Mycgr3T

Mycgr3G103260 Mycgr3
  
Location: 25903-26635

Mycgr3G103260\_Mycgr3

Mycgr3G84644 Mycgr3T
  
Location: 26735-28457

Mycgr3G84644\_Mycgr3T

Mycgr3G29227 Mycgr3T
  
Location: 28557-28863

Mycgr3G29227\_Mycgr3T

Mycgr3G36271 Mycgr3T
  
Location: 28963-29854

Mycgr3G36271\_Mycgr3T

Mycgr3G68433 Mycgr3T
  
Location: 29954-33041

Mycgr3G68433\_Mycgr3T

Mycgr3G79452 Mycgr3T
  
Location: 33141-33399

Mycgr3G79452\_Mycgr3T

Mycgr3G55345 Mycgr3T
  
Location: 33499-34126

Mycgr3G55345\_Mycgr3T

Mycgr3G103278 Mycgr3
  
Location: 34226-35195

Mycgr3G103278\_Mycgr3

Mycgr3G84654 Mycgr3T
  
Location: 35295-36630

Mycgr3G84654\_Mycgr3T

Mycgr3G108090 Mycgr3
  
Location: 36730-37591

Mycgr3G108090\_Mycgr3

Mycgr3G21922 Mycgr3T
  
Location: 37691-39149

Mycgr3G21922\_Mycgr3T

Mycgr3G99148 Mycgr3T
  
Location: 39249-42819

Mycgr3G99148\_Mycgr3T

hypothetical protein
  
Accession: EER26484
  
Location: 1637807-1639585
  
 NCBI BlastP on this gene

EER26484

hypothetical protein
  
Accession: EER26485
  
Location: 1640140-1641232
  
 NCBI BlastP on this gene

EER26485

hypothetical protein
  
Accession: EER26486
  
Location: 1643600-1644218
  
 NCBI BlastP on this gene

EER26486

hypothetical protein
  
Accession: EER26487
  
Location: 1645209-1647812
  
 NCBI BlastP on this gene

EER26487

Cytochrome b5-like Heme/Steroid binding domain containing protein
  
Accession: EER26488
  
Location: 1650453-1651167
  
 NCBI BlastP on this gene

EER26488

hypothetical protein
  
Accession: EER26489
  
Location: 1652015-1653792
  
 NCBI BlastP on this gene

EER26489

hypothetical protein
  
Accession: EER26490
  
Location: 1654318-1654694
  
 NCBI BlastP on this gene

EER26490

serine/threonine-protein kinase, putative
  
Accession: EER26491
  
Location: 1655225-1657722
  
  
**BlastP hit with Mycgr3G103260\_Mycgr3**
  
Percentage identity: 52 %
  
BlastP bit score: 280
  
Sequence coverage: 96 %
  
E-value: 6e-85
  
  
 NCBI BlastP on this gene

EER26491

Ran1-like protein kinase, putative
  
Accession: EER26492
  
Location: 1664833-1666515
  
  
**BlastP hit with Mycgr3G68429\_Mycgr3T**
  
Percentage identity: 67 %
  
BlastP bit score: 434
  
Sequence coverage: 74 %
  
E-value: 1e-144
  
  
 NCBI BlastP on this gene

EER26492

oxidoreductase,short chain dehydrogenase, putative
  
Accession: EER26493
  
Location: 1670663-1671793
  
 NCBI BlastP on this gene

EER26493

Hemolysin-III related family protein
  
Accession: EER26494
  
Location: 1672313-1673275
  
 NCBI BlastP on this gene

EER26494

hypothetical protein
  
Accession: EER26495
  
Location: 1673593-1674640
  
 NCBI BlastP on this gene

EER26495

hypothetical protein
  
Accession: EER26496
  
Location: 1674799-1676135
  
 NCBI BlastP on this gene

EER26496

dolichol-phosphate mannosyltransferase, putative
  
Accession: EER26497
  
Location: 1676665-1677579
  
 NCBI BlastP on this gene

EER26497

hypothetical protein
  
Accession: EER26498
  
Location: 1677829-1678933
  
 NCBI BlastP on this gene

EER26498

hypothetical protein
  
Accession: EER26499
  
Location: 1679339-1681628
  
 NCBI BlastP on this gene

EER26499

hypothetical protein
  
Accession: EER26500
  
Location: 1682008-1682789
  
 NCBI BlastP on this gene

EER26500

Query: Architecture Search FASTA input

KB644412 : Penicillium oxalicum 114-2 unplaced genomic scaffold scaffold\_5    Total score: 2.0     Cumulative Blast bit score: 712

Hit cluster cross-links:

Mycgr3G90785 Mycgr3T
  
Location: 0-1047

Mycgr3G90785\_Mycgr3T

Mycgr3G103262 Mycgr3
  
Location: 1147-1390

Mycgr3G103262\_Mycgr3

Mycgr3G68458 Mycgr3T
  
Location: 1490-3602

Mycgr3G68458\_Mycgr3T

Mycgr3G99145 Mycgr3T
  
Location: 3702-4326

Mycgr3G99145\_Mycgr3T

Mycgr3G103274 Mycgr3
  
Location: 4426-4957

Mycgr3G103274\_Mycgr3

Mycgr3G103264 Mycgr3
  
Location: 5057-5390

Mycgr3G103264\_Mycgr3

Mycgr3G37570 Mycgr3T
  
Location: 5490-6006

Mycgr3G37570\_Mycgr3T

Mycgr3G108094 Mycgr3
  
Location: 6106-10555

Mycgr3G108094\_Mycgr3

Mycgr3G90786 Mycgr3T
  
Location: 10655-12080

Mycgr3G90786\_Mycgr3T

Mycgr3G68429 Mycgr3T
  
Location: 12180-13440

Mycgr3G68429\_Mycgr3T

Mycgr3G68421 Mycgr3T
  
Location: 13540-17086

Mycgr3G68421\_Mycgr3T

Mycgr3G90801 Mycgr3T
  
Location: 17186-18056

Mycgr3G90801\_Mycgr3T

Mycgr3G84646 Mycgr3T
  
Location: 18156-20235

Mycgr3G84646\_Mycgr3T

Mycgr3G68456 Mycgr3T
  
Location: 20335-21970

Mycgr3G68456\_Mycgr3T

Mycgr3G103270 Mycgr3
  
Location: 22070-22355

Mycgr3G103270\_Mycgr3

Mycgr3G90803 Mycgr3T
  
Location: 22455-23019

Mycgr3G90803\_Mycgr3T

Mycgr3G36941 Mycgr3T
  
Location: 23119-24064

Mycgr3G36941\_Mycgr3T

Mycgr3G25746 Mycgr3T
  
Location: 24164-25241

Mycgr3G25746\_Mycgr3T

Mycgr3G90788 Mycgr3T
  
Location: 25341-25803

Mycgr3G90788\_Mycgr3T

Mycgr3G103260 Mycgr3
  
Location: 25903-26635

Mycgr3G103260\_Mycgr3

Mycgr3G84644 Mycgr3T
  
Location: 26735-28457

Mycgr3G84644\_Mycgr3T

Mycgr3G29227 Mycgr3T
  
Location: 28557-28863

Mycgr3G29227\_Mycgr3T

Mycgr3G36271 Mycgr3T
  
Location: 28963-29854

Mycgr3G36271\_Mycgr3T

Mycgr3G68433 Mycgr3T
  
Location: 29954-33041

Mycgr3G68433\_Mycgr3T

Mycgr3G79452 Mycgr3T
  
Location: 33141-33399

Mycgr3G79452\_Mycgr3T

Mycgr3G55345 Mycgr3T
  
Location: 33499-34126

Mycgr3G55345\_Mycgr3T

Mycgr3G103278 Mycgr3
  
Location: 34226-35195

Mycgr3G103278\_Mycgr3

Mycgr3G84654 Mycgr3T
  
Location: 35295-36630

Mycgr3G84654\_Mycgr3T

Mycgr3G108090 Mycgr3
  
Location: 36730-37591

Mycgr3G108090\_Mycgr3

Mycgr3G21922 Mycgr3T
  
Location: 37691-39149

Mycgr3G21922\_Mycgr3T

Mycgr3G99148 Mycgr3T
  
Location: 39249-42819

Mycgr3G99148\_Mycgr3T

hypothetical protein
  
Accession: EPS29769
  
Location: 807471-809933
  
 NCBI BlastP on this gene

EPS29769

hypothetical protein
  
Accession: EPS29770
  
Location: 810416-810767
  
 NCBI BlastP on this gene

EPS29770

hypothetical protein
  
Accession: EPS29771
  
Location: 811615-812643
  
 NCBI BlastP on this gene

EPS29771

hypothetical protein
  
Accession: EPS29772
  
Location: 813112-814250
  
 NCBI BlastP on this gene

EPS29772

hypothetical protein
  
Accession: EPS29773
  
Location: 817431-819259
  
 NCBI BlastP on this gene

EPS29773

hypothetical protein
  
Accession: EPS29774
  
Location: 819797-820566
  
 NCBI BlastP on this gene

EPS29774

hypothetical protein
  
Accession: EPS29775
  
Location: 821891-823478
  
 NCBI BlastP on this gene

EPS29775

hypothetical protein
  
Accession: EPS29776
  
Location: 824271-826821
  
  
**BlastP hit with Mycgr3G103260\_Mycgr3**
  
Percentage identity: 54 %
  
BlastP bit score: 276
  
Sequence coverage: 96 %
  
E-value: 2e-83
  
  
 NCBI BlastP on this gene

EPS29776

hypothetical protein
  
Accession: EPS29777
  
Location: 827224-827555
  
 NCBI BlastP on this gene

EPS29777

hypothetical protein
  
Accession: EPS29778
  
Location: 831072-831347
  
 NCBI BlastP on this gene

EPS29778

hypothetical protein
  
Accession: EPS29779
  
Location: 834984-835291
  
 NCBI BlastP on this gene

EPS29779

hypothetical protein
  
Accession: EPS29780
  
Location: 836364-836597
  
 NCBI BlastP on this gene

EPS29780

hypothetical protein
  
Accession: EPS29781
  
Location: 837856-839489
  
  
**BlastP hit with Mycgr3G68429\_Mycgr3T**
  
Percentage identity: 63 %
  
BlastP bit score: 436
  
Sequence coverage: 82 %
  
E-value: 5e-147
  
  
 NCBI BlastP on this gene

EPS29781

hypothetical protein
  
Accession: EPS29782
  
Location: 841066-842414
  
 NCBI BlastP on this gene

EPS29782

hypothetical protein
  
Accession: EPS29783
  
Location: 843063-844431
  
 NCBI BlastP on this gene

EPS29783

hypothetical protein
  
Accession: EPS29784
  
Location: 845955-846914
  
 NCBI BlastP on this gene

EPS29784

hypothetical protein
  
Accession: EPS29785
  
Location: 847517-848542
  
 NCBI BlastP on this gene

EPS29785

hypothetical protein
  
Accession: EPS29786
  
Location: 848788-850483
  
 NCBI BlastP on this gene

EPS29786

putative dolichyl-phosphate beta-D-mannosyltransferase
  
Accession: EPS29787
  
Location: 850620-851421
  
 NCBI BlastP on this gene

EPS29787

hypothetical protein
  
Accession: EPS29788
  
Location: 851701-852801
  
 NCBI BlastP on this gene

EPS29788

hypothetical protein
  
Accession: EPS29789
  
Location: 854436-856738
  
 NCBI BlastP on this gene

EPS29789

Query: Architecture Search FASTA input

GG700648 : Trichophyton rubrum CBS 118892 genomic scaffold supercont2.1    Total score: 2.0     Cumulative Blast bit score: 712

Hit cluster cross-links:

Mycgr3G90785 Mycgr3T
  
Location: 0-1047

Mycgr3G90785\_Mycgr3T

Mycgr3G103262 Mycgr3
  
Location: 1147-1390

Mycgr3G103262\_Mycgr3

Mycgr3G68458 Mycgr3T
  
Location: 1490-3602

Mycgr3G68458\_Mycgr3T

Mycgr3G99145 Mycgr3T
  
Location: 3702-4326

Mycgr3G99145\_Mycgr3T

Mycgr3G103274 Mycgr3
  
Location: 4426-4957

Mycgr3G103274\_Mycgr3

Mycgr3G103264 Mycgr3
  
Location: 5057-5390

Mycgr3G103264\_Mycgr3

Mycgr3G37570 Mycgr3T
  
Location: 5490-6006

Mycgr3G37570\_Mycgr3T

Mycgr3G108094 Mycgr3
  
Location: 6106-10555

Mycgr3G108094\_Mycgr3

Mycgr3G90786 Mycgr3T
  
Location: 10655-12080

Mycgr3G90786\_Mycgr3T

Mycgr3G68429 Mycgr3T
  
Location: 12180-13440

Mycgr3G68429\_Mycgr3T

Mycgr3G68421 Mycgr3T
  
Location: 13540-17086

Mycgr3G68421\_Mycgr3T

Mycgr3G90801 Mycgr3T
  
Location: 17186-18056

Mycgr3G90801\_Mycgr3T

Mycgr3G84646 Mycgr3T
  
Location: 18156-20235

Mycgr3G84646\_Mycgr3T

Mycgr3G68456 Mycgr3T
  
Location: 20335-21970

Mycgr3G68456\_Mycgr3T

Mycgr3G103270 Mycgr3
  
Location: 22070-22355

Mycgr3G103270\_Mycgr3

Mycgr3G90803 Mycgr3T
  
Location: 22455-23019

Mycgr3G90803\_Mycgr3T

Mycgr3G36941 Mycgr3T
  
Location: 23119-24064

Mycgr3G36941\_Mycgr3T

Mycgr3G25746 Mycgr3T
  
Location: 24164-25241

Mycgr3G25746\_Mycgr3T

Mycgr3G90788 Mycgr3T
  
Location: 25341-25803

Mycgr3G90788\_Mycgr3T

Mycgr3G103260 Mycgr3
  
Location: 25903-26635

Mycgr3G103260\_Mycgr3

Mycgr3G84644 Mycgr3T
  
Location: 26735-28457

Mycgr3G84644\_Mycgr3T

Mycgr3G29227 Mycgr3T
  
Location: 28557-28863

Mycgr3G29227\_Mycgr3T

Mycgr3G36271 Mycgr3T
  
Location: 28963-29854

Mycgr3G36271\_Mycgr3T

Mycgr3G68433 Mycgr3T
  
Location: 29954-33041

Mycgr3G68433\_Mycgr3T

Mycgr3G79452 Mycgr3T
  
Location: 33141-33399

Mycgr3G79452\_Mycgr3T

Mycgr3G55345 Mycgr3T
  
Location: 33499-34126

Mycgr3G55345\_Mycgr3T

Mycgr3G103278 Mycgr3
  
Location: 34226-35195

Mycgr3G103278\_Mycgr3

Mycgr3G84654 Mycgr3T
  
Location: 35295-36630

Mycgr3G84654\_Mycgr3T

Mycgr3G108090 Mycgr3
  
Location: 36730-37591

Mycgr3G108090\_Mycgr3

Mycgr3G21922 Mycgr3T
  
Location: 37691-39149

Mycgr3G21922\_Mycgr3T

Mycgr3G99148 Mycgr3T
  
Location: 39249-42819

Mycgr3G99148\_Mycgr3T

hypothetical protein
  
Accession: EGD84046
  
Location: 830935-833327
  
 NCBI BlastP on this gene

EGD84046

hypothetical protein
  
Accession: EGD84045
  
Location: 829137-829749
  
 NCBI BlastP on this gene

EGD84045

PQ loop repeat protein
  
Accession: EGD84044
  
Location: 826608-827656
  
 NCBI BlastP on this gene

EGD84044

hypothetical protein
  
Accession: EGD84043
  
Location: 823801-825596
  
 NCBI BlastP on this gene

EGD84043

DNA damage response protein
  
Accession: EGD84042
  
Location: 822756-823450
  
 NCBI BlastP on this gene

EGD84042

hypothetical protein
  
Accession: EGD84041
  
Location: 820221-821783
  
 NCBI BlastP on this gene

EGD84041

hypothetical protein
  
Accession: EGD84040
  
Location: 819516-819864
  
 NCBI BlastP on this gene

EGD84040

CMGC/DYRK/PRP4 protein kinase
  
Accession: EGD84039
  
Location: 816424-818946
  
  
**BlastP hit with Mycgr3G103260\_Mycgr3**
  
Percentage identity: 54 %
  
BlastP bit score: 283
  
Sequence coverage: 96 %
  
E-value: 2e-86
  
  
 NCBI BlastP on this gene

EGD84039

hypothetical protein
  
Accession: EGD84038
  
Location: 814483-814907
  
 NCBI BlastP on this gene

EGD84038

hypothetical protein
  
Accession: EGD84037
  
Location: 810024-810461
  
 NCBI BlastP on this gene

EGD84037

hypothetical protein
  
Accession: EGD84036
  
Location: 807486-807903
  
 NCBI BlastP on this gene

EGD84036

hypothetical protein
  
Accession: EGD84035
  
Location: 805655-806349
  
 NCBI BlastP on this gene

EGD84035

serine/threonine protein kinase
  
Accession: EGD84034
  
Location: 802280-803594
  
  
**BlastP hit with Mycgr3G68429\_Mycgr3T**
  
Percentage identity: 63 %
  
BlastP bit score: 429
  
Sequence coverage: 79 %
  
E-value: 4e-144
  
  
 NCBI BlastP on this gene

EGD84034

3-oxoacyl-(acyl-carrier-protein) reductase
  
Accession: EGD84033
  
Location: 797657-798844
  
 NCBI BlastP on this gene

EGD84033

hemolysin-III channel protein Izh2
  
Accession: EGD84032
  
Location: 796174-797157
  
 NCBI BlastP on this gene

EGD84032

hypothetical protein
  
Accession: EGD84031
  
Location: 794695-795777
  
 NCBI BlastP on this gene

EGD84031

hypothetical protein
  
Accession: EGD84030
  
Location: 792825-794395
  
 NCBI BlastP on this gene

EGD84030

dolichol-phosphate mannosyltransferase
  
Accession: EGD84029
  
Location: 791473-792433
  
 NCBI BlastP on this gene

EGD84029

hypothetical protein
  
Accession: EGD84028
  
Location: 790117-791203
  
 NCBI BlastP on this gene

EGD84028

hypothetical protein
  
Accession: EGD84027
  
Location: 787395-789656
  
 NCBI BlastP on this gene

EGD84027

hypothetical protein
  
Accession: EGD84026
  
Location: 786249-786890
  
 NCBI BlastP on this gene

EGD84026

Query: Architecture Search FASTA input

AACD01000084 : Aspergillus nidulans FGSC A4    Total score: 2.0     Cumulative Blast bit score: 710

Hit cluster cross-links:

Mycgr3G90785 Mycgr3T
  
Location: 0-1047

Mycgr3G90785\_Mycgr3T

Mycgr3G103262 Mycgr3
  
Location: 1147-1390

Mycgr3G103262\_Mycgr3

Mycgr3G68458 Mycgr3T
  
Location: 1490-3602

Mycgr3G68458\_Mycgr3T

Mycgr3G99145 Mycgr3T
  
Location: 3702-4326

Mycgr3G99145\_Mycgr3T

Mycgr3G103274 Mycgr3
  
Location: 4426-4957

Mycgr3G103274\_Mycgr3

Mycgr3G103264 Mycgr3
  
Location: 5057-5390

Mycgr3G103264\_Mycgr3

Mycgr3G37570 Mycgr3T
  
Location: 5490-6006

Mycgr3G37570\_Mycgr3T

Mycgr3G108094 Mycgr3
  
Location: 6106-10555

Mycgr3G108094\_Mycgr3

Mycgr3G90786 Mycgr3T
  
Location: 10655-12080

Mycgr3G90786\_Mycgr3T

Mycgr3G68429 Mycgr3T
  
Location: 12180-13440

Mycgr3G68429\_Mycgr3T

Mycgr3G68421 Mycgr3T
  
Location: 13540-17086

Mycgr3G68421\_Mycgr3T

Mycgr3G90801 Mycgr3T
  
Location: 17186-18056

Mycgr3G90801\_Mycgr3T

Mycgr3G84646 Mycgr3T
  
Location: 18156-20235

Mycgr3G84646\_Mycgr3T

Mycgr3G68456 Mycgr3T
  
Location: 20335-21970

Mycgr3G68456\_Mycgr3T

Mycgr3G103270 Mycgr3
  
Location: 22070-22355

Mycgr3G103270\_Mycgr3

Mycgr3G90803 Mycgr3T
  
Location: 22455-23019

Mycgr3G90803\_Mycgr3T

Mycgr3G36941 Mycgr3T
  
Location: 23119-24064

Mycgr3G36941\_Mycgr3T

Mycgr3G25746 Mycgr3T
  
Location: 24164-25241

Mycgr3G25746\_Mycgr3T

Mycgr3G90788 Mycgr3T
  
Location: 25341-25803

Mycgr3G90788\_Mycgr3T

Mycgr3G103260 Mycgr3
  
Location: 25903-26635

Mycgr3G103260\_Mycgr3

Mycgr3G84644 Mycgr3T
  
Location: 26735-28457

Mycgr3G84644\_Mycgr3T

Mycgr3G29227 Mycgr3T
  
Location: 28557-28863

Mycgr3G29227\_Mycgr3T

Mycgr3G36271 Mycgr3T
  
Location: 28963-29854

Mycgr3G36271\_Mycgr3T

Mycgr3G68433 Mycgr3T
  
Location: 29954-33041

Mycgr3G68433\_Mycgr3T

Mycgr3G79452 Mycgr3T
  
Location: 33141-33399

Mycgr3G79452\_Mycgr3T

Mycgr3G55345 Mycgr3T
  
Location: 33499-34126

Mycgr3G55345\_Mycgr3T

Mycgr3G103278 Mycgr3
  
Location: 34226-35195

Mycgr3G103278\_Mycgr3

Mycgr3G84654 Mycgr3T
  
Location: 35295-36630

Mycgr3G84654\_Mycgr3T

Mycgr3G108090 Mycgr3
  
Location: 36730-37591

Mycgr3G108090\_Mycgr3

Mycgr3G21922 Mycgr3T
  
Location: 37691-39149

Mycgr3G21922\_Mycgr3T

Mycgr3G99148 Mycgr3T
  
Location: 39249-42819

Mycgr3G99148\_Mycgr3T

hypothetical protein
  
Accession: EAA61023
  
Location: 286718-288859
  
 NCBI BlastP on this gene

EAA61023

hypothetical protein
  
Accession: EAA61022
  
Location: 283802-285041
  
 NCBI BlastP on this gene

EAA61022

hypothetical protein
  
Accession: EAA61021
  
Location: 280871-283294
  
 NCBI BlastP on this gene

EAA61021

hypothetical protein
  
Accession: EAA61020
  
Location: 279683-280552
  
 NCBI BlastP on this gene

EAA61020

hypothetical protein
  
Accession: EAA61019
  
Location: 278273-279267
  
 NCBI BlastP on this gene

EAA61019

hypothetical protein
  
Accession: EAA61018
  
Location: 275740-277490
  
 NCBI BlastP on this gene

EAA61018

hypothetical protein
  
Accession: EAA61017
  
Location: 274571-275273
  
 NCBI BlastP on this gene

EAA61017

hypothetical protein
  
Accession: EAA61016
  
Location: 270143-271860
  
 NCBI BlastP on this gene

EAA61016

hypothetical protein
  
Accession: EAA61015
  
Location: 269374-269710
  
 NCBI BlastP on this gene

EAA61015

hypothetical protein
  
Accession: EAA61014
  
Location: 266473-268912
  
  
**BlastP hit with Mycgr3G103260\_Mycgr3**
  
Percentage identity: 55 %
  
BlastP bit score: 286
  
Sequence coverage: 98 %
  
E-value: 2e-87
  
  
 NCBI BlastP on this gene

EAA61014

hypothetical protein
  
Accession: EAA61013
  
Location: 261811-263149
  
  
**BlastP hit with Mycgr3G68429\_Mycgr3T**
  
Percentage identity: 71 %
  
BlastP bit score: 424
  
Sequence coverage: 66 %
  
E-value: 5e-142
  
  
 NCBI BlastP on this gene

EAA61013

hypothetical protein
  
Accession: EAA61012
  
Location: 259737-260839
  
 NCBI BlastP on this gene

EAA61012

hypothetical protein
  
Accession: EAA61011
  
Location: 258354-259480
  
 NCBI BlastP on this gene

EAA61011

hypothetical protein
  
Accession: EAA61010
  
Location: 256438-257394
  
 NCBI BlastP on this gene

EAA61010

hypothetical protein
  
Accession: EAA61009
  
Location: 254960-255996
  
 NCBI BlastP on this gene

EAA61009

hypothetical protein
  
Accession: EAA61008
  
Location: 253493-254831
  
 NCBI BlastP on this gene

EAA61008

hypothetical protein
  
Accession: EAA61007
  
Location: 244449-247347
  
 NCBI BlastP on this gene

EAA61007

hypothetical protein
  
Accession: EAA61006
  
Location: 242652-243707
  
 NCBI BlastP on this gene

EAA61006

Query: Architecture Search FASTA input

DF126452 : Aspergillus kawachii IFO 4308 DNA, contig: scaffold00006    Total score: 2.0     Cumulative Blast bit score: 708

Hit cluster cross-links:

Mycgr3G90785 Mycgr3T
  
Location: 0-1047

Mycgr3G90785\_Mycgr3T

Mycgr3G103262 Mycgr3
  
Location: 1147-1390

Mycgr3G103262\_Mycgr3

Mycgr3G68458 Mycgr3T
  
Location: 1490-3602

Mycgr3G68458\_Mycgr3T

Mycgr3G99145 Mycgr3T
  
Location: 3702-4326

Mycgr3G99145\_Mycgr3T

Mycgr3G103274 Mycgr3
  
Location: 4426-4957

Mycgr3G103274\_Mycgr3

Mycgr3G103264 Mycgr3
  
Location: 5057-5390

Mycgr3G103264\_Mycgr3

Mycgr3G37570 Mycgr3T
  
Location: 5490-6006

Mycgr3G37570\_Mycgr3T

Mycgr3G108094 Mycgr3
  
Location: 6106-10555

Mycgr3G108094\_Mycgr3

Mycgr3G90786 Mycgr3T
  
Location: 10655-12080

Mycgr3G90786\_Mycgr3T

Mycgr3G68429 Mycgr3T
  
Location: 12180-13440

Mycgr3G68429\_Mycgr3T

Mycgr3G68421 Mycgr3T
  
Location: 13540-17086

Mycgr3G68421\_Mycgr3T

Mycgr3G90801 Mycgr3T
  
Location: 17186-18056

Mycgr3G90801\_Mycgr3T

Mycgr3G84646 Mycgr3T
  
Location: 18156-20235

Mycgr3G84646\_Mycgr3T

Mycgr3G68456 Mycgr3T
  
Location: 20335-21970

Mycgr3G68456\_Mycgr3T

Mycgr3G103270 Mycgr3
  
Location: 22070-22355

Mycgr3G103270\_Mycgr3

Mycgr3G90803 Mycgr3T
  
Location: 22455-23019

Mycgr3G90803\_Mycgr3T

Mycgr3G36941 Mycgr3T
  
Location: 23119-24064

Mycgr3G36941\_Mycgr3T

Mycgr3G25746 Mycgr3T
  
Location: 24164-25241

Mycgr3G25746\_Mycgr3T

Mycgr3G90788 Mycgr3T
  
Location: 25341-25803

Mycgr3G90788\_Mycgr3T

Mycgr3G103260 Mycgr3
  
Location: 25903-26635

Mycgr3G103260\_Mycgr3

Mycgr3G84644 Mycgr3T
  
Location: 26735-28457

Mycgr3G84644\_Mycgr3T

Mycgr3G29227 Mycgr3T
  
Location: 28557-28863

Mycgr3G29227\_Mycgr3T

Mycgr3G36271 Mycgr3T
  
Location: 28963-29854

Mycgr3G36271\_Mycgr3T

Mycgr3G68433 Mycgr3T
  
Location: 29954-33041

Mycgr3G68433\_Mycgr3T

Mycgr3G79452 Mycgr3T
  
Location: 33141-33399

Mycgr3G79452\_Mycgr3T

Mycgr3G55345 Mycgr3T
  
Location: 33499-34126

Mycgr3G55345\_Mycgr3T

Mycgr3G103278 Mycgr3
  
Location: 34226-35195

Mycgr3G103278\_Mycgr3

Mycgr3G84654 Mycgr3T
  
Location: 35295-36630

Mycgr3G84654\_Mycgr3T

Mycgr3G108090 Mycgr3
  
Location: 36730-37591

Mycgr3G108090\_Mycgr3

Mycgr3G21922 Mycgr3T
  
Location: 37691-39149

Mycgr3G21922\_Mycgr3T

Mycgr3G99148 Mycgr3T
  
Location: 39249-42819

Mycgr3G99148\_Mycgr3T

similar to An16g04370
  
Accession: GAA85019
  
Location: 990261-991520
  
 NCBI BlastP on this gene

GAA85019

coatamer subunit protein
  
Accession: GAA85020
  
Location: 995278-997707
  
 NCBI BlastP on this gene

GAA85020

nuclear transport factor 2
  
Accession: GAA85021
  
Location: 998095-999079
  
 NCBI BlastP on this gene

GAA85021

PQ loop repeat protein
  
Accession: GAA85022
  
Location: 999570-1000596
  
 NCBI BlastP on this gene

GAA85022

stress response protein
  
Accession: GAA85023
  
Location: 1001582-1003396
  
 NCBI BlastP on this gene

GAA85023

DNA damage response protein
  
Accession: GAA85024
  
Location: 1003919-1004481
  
 NCBI BlastP on this gene

GAA85024

similar to An16g04440
  
Accession: GAA85025
  
Location: 1006026-1007822
  
 NCBI BlastP on this gene

GAA85025

coiled-coil domain-containing protein
  
Accession: GAA85026
  
Location: 1008318-1008656
  
 NCBI BlastP on this gene

GAA85026

serine/threonine protein kinase
  
Accession: GAA85027
  
Location: 1009237-1011764
  
  
**BlastP hit with Mycgr3G103260\_Mycgr3**
  
Percentage identity: 53 %
  
BlastP bit score: 275
  
Sequence coverage: 98 %
  
E-value: 3e-83
  
  
 NCBI BlastP on this gene

GAA85027

hypothetical protein
  
Accession: GAA85028
  
Location: 1018827-1020174
  
  
**BlastP hit with Mycgr3G68429\_Mycgr3T**
  
Percentage identity: 68 %
  
BlastP bit score: 433
  
Sequence coverage: 73 %
  
E-value: 7e-146
  
  
 NCBI BlastP on this gene

GAA85028

3-oxoacyl-(acyl-carrier-protein) reductase
  
Accession: GAA85029
  
Location: 1021724-1024366
  
 NCBI BlastP on this gene

GAA85029

haemolysin-III channel protein Izh2
  
Accession: GAA85030
  
Location: 1025464-1026429
  
 NCBI BlastP on this gene

GAA85030

similar to An16g04550
  
Accession: GAA85031
  
Location: 1026913-1027948
  
 NCBI BlastP on this gene

GAA85031

hypothetical protein
  
Accession: GAA85032
  
Location: 1028147-1029660
  
 NCBI BlastP on this gene

GAA85032

clathrin-coated vesicle protein
  
Accession: GAA85033
  
Location: 1030082-1030922
  
 NCBI BlastP on this gene

GAA85033

eukaryotic translation initiation factor 3 subunit 6-interacting protein
  
Accession: GAA85034
  
Location: 1031780-1033362
  
 NCBI BlastP on this gene

GAA85034

iron-sulfur cluster assembly accessory protein Isa2
  
Accession: GAA85035
  
Location: 1034160-1035318
  
 NCBI BlastP on this gene

GAA85035

kynureninase
  
Accession: GAA85036
  
Location: 1035693-1037324
  
 NCBI BlastP on this gene

GAA85036

ER membrane DUF1077 domain protein
  
Accession: GAA85037
  
Location: 1037661-1038394
  
 NCBI BlastP on this gene

GAA85037

Query: Architecture Search FASTA input

ABSU01000003 : Arthroderma benhamiae CBS 112371    Total score: 2.0     Cumulative Blast bit score: 708

Hit cluster cross-links:

Mycgr3G90785 Mycgr3T
  
Location: 0-1047

Mycgr3G90785\_Mycgr3T

Mycgr3G103262 Mycgr3
  
Location: 1147-1390

Mycgr3G103262\_Mycgr3

Mycgr3G68458 Mycgr3T
  
Location: 1490-3602

Mycgr3G68458\_Mycgr3T

Mycgr3G99145 Mycgr3T
  
Location: 3702-4326

Mycgr3G99145\_Mycgr3T

Mycgr3G103274 Mycgr3
  
Location: 4426-4957

Mycgr3G103274\_Mycgr3

Mycgr3G103264 Mycgr3
  
Location: 5057-5390

Mycgr3G103264\_Mycgr3

Mycgr3G37570 Mycgr3T
  
Location: 5490-6006

Mycgr3G37570\_Mycgr3T

Mycgr3G108094 Mycgr3
  
Location: 6106-10555

Mycgr3G108094\_Mycgr3

Mycgr3G90786 Mycgr3T
  
Location: 10655-12080

Mycgr3G90786\_Mycgr3T

Mycgr3G68429 Mycgr3T
  
Location: 12180-13440

Mycgr3G68429\_Mycgr3T

Mycgr3G68421 Mycgr3T
  
Location: 13540-17086

Mycgr3G68421\_Mycgr3T

Mycgr3G90801 Mycgr3T
  
Location: 17186-18056

Mycgr3G90801\_Mycgr3T

Mycgr3G84646 Mycgr3T
  
Location: 18156-20235

Mycgr3G84646\_Mycgr3T

Mycgr3G68456 Mycgr3T
  
Location: 20335-21970

Mycgr3G68456\_Mycgr3T

Mycgr3G103270 Mycgr3
  
Location: 22070-22355

Mycgr3G103270\_Mycgr3

Mycgr3G90803 Mycgr3T
  
Location: 22455-23019

Mycgr3G90803\_Mycgr3T

Mycgr3G36941 Mycgr3T
  
Location: 23119-24064

Mycgr3G36941\_Mycgr3T

Mycgr3G25746 Mycgr3T
  
Location: 24164-25241

Mycgr3G25746\_Mycgr3T

Mycgr3G90788 Mycgr3T
  
Location: 25341-25803

Mycgr3G90788\_Mycgr3T

Mycgr3G103260 Mycgr3
  
Location: 25903-26635

Mycgr3G103260\_Mycgr3

Mycgr3G84644 Mycgr3T
  
Location: 26735-28457

Mycgr3G84644\_Mycgr3T

Mycgr3G29227 Mycgr3T
  
Location: 28557-28863

Mycgr3G29227\_Mycgr3T

Mycgr3G36271 Mycgr3T
  
Location: 28963-29854

Mycgr3G36271\_Mycgr3T

Mycgr3G68433 Mycgr3T
  
Location: 29954-33041

Mycgr3G68433\_Mycgr3T

Mycgr3G79452 Mycgr3T
  
Location: 33141-33399

Mycgr3G79452\_Mycgr3T

Mycgr3G55345 Mycgr3T
  
Location: 33499-34126

Mycgr3G55345\_Mycgr3T

Mycgr3G103278 Mycgr3
  
Location: 34226-35195

Mycgr3G103278\_Mycgr3

Mycgr3G84654 Mycgr3T
  
Location: 35295-36630

Mycgr3G84654\_Mycgr3T

Mycgr3G108090 Mycgr3
  
Location: 36730-37591

Mycgr3G108090\_Mycgr3

Mycgr3G21922 Mycgr3T
  
Location: 37691-39149

Mycgr3G21922\_Mycgr3T

Mycgr3G99148 Mycgr3T
  
Location: 39249-42819

Mycgr3G99148\_Mycgr3T

hypothetical protein
  
Accession: EFE35458
  
Location: 346001-346626
  
 NCBI BlastP on this gene

EFE35458

conserved hypothetical protein
  
Accession: EFE35457
  
Location: 343364-345740
  
 NCBI BlastP on this gene

EFE35457

conserved serine-rich protein
  
Accession: EFE35456
  
Location: 341542-341844
  
 NCBI BlastP on this gene

EFE35456

hypothetical protein
  
Accession: EFE35455
  
Location: 338975-340024
  
 NCBI BlastP on this gene

EFE35455

hypothetical protein
  
Accession: EFE35454
  
Location: 336145-337836
  
 NCBI BlastP on this gene

EFE35454

hypothetical protein
  
Accession: EFE35453
  
Location: 335093-335787
  
 NCBI BlastP on this gene

EFE35453

hypothetical protein
  
Accession: EFE35452
  
Location: 332556-333923
  
 NCBI BlastP on this gene

EFE35452

hypothetical protein
  
Accession: EFE35451
  
Location: 331852-332198
  
 NCBI BlastP on this gene

EFE35451

hypothetical protein
  
Accession: EFE35450
  
Location: 328764-331283
  
  
**BlastP hit with Mycgr3G103260\_Mycgr3**
  
Percentage identity: 54 %
  
BlastP bit score: 283
  
Sequence coverage: 96 %
  
E-value: 2e-86
  
  
 NCBI BlastP on this gene

EFE35450

hypothetical protein
  
Accession: EFE35449
  
Location: 321609-322166
  
 NCBI BlastP on this gene

EFE35449

hypothetical protein
  
Accession: EFE35448
  
Location: 317830-318141
  
 NCBI BlastP on this gene

EFE35448

hypothetical protein
  
Accession: EFE35447
  
Location: 314436-315746
  
  
**BlastP hit with Mycgr3G68429\_Mycgr3T**
  
Percentage identity: 62 %
  
BlastP bit score: 425
  
Sequence coverage: 83 %
  
E-value: 1e-142
  
  
 NCBI BlastP on this gene

EFE35447

hypothetical protein
  
Accession: EFE35446
  
Location: 312125-313063
  
 NCBI BlastP on this gene

EFE35446

hypothetical protein
  
Accession: EFE35445
  
Location: 309819-311005
  
 NCBI BlastP on this gene

EFE35445

hypothetical protein
  
Accession: EFE35444
  
Location: 308354-309334
  
 NCBI BlastP on this gene

EFE35444

conserved hypothetical protein
  
Accession: EFE35443
  
Location: 306848-307678
  
 NCBI BlastP on this gene

EFE35443

hypothetical protein
  
Accession: EFE35442
  
Location: 304745-306547
  
 NCBI BlastP on this gene

EFE35442

hypothetical protein
  
Accession: EFE35441
  
Location: 303589-304449
  
 NCBI BlastP on this gene

EFE35441

esterase, putative
  
Accession: EFE35440
  
Location: 302244-303317
  
 NCBI BlastP on this gene

EFE35440

transcription factor TFIIIC complex subunit Tfc6, putative
  
Accession: EFE35439
  
Location: 299491-301752
  
 NCBI BlastP on this gene

EFE35439

Query: Architecture Search FASTA input

AM270368 : Aspergillus niger contig An16c0160, genomic contig.    Total score: 2.0     Cumulative Blast bit score: 707

Hit cluster cross-links:

Mycgr3G90785 Mycgr3T
  
Location: 0-1047

Mycgr3G90785\_Mycgr3T

Mycgr3G103262 Mycgr3
  
Location: 1147-1390

Mycgr3G103262\_Mycgr3

Mycgr3G68458 Mycgr3T
  
Location: 1490-3602

Mycgr3G68458\_Mycgr3T

Mycgr3G99145 Mycgr3T
  
Location: 3702-4326

Mycgr3G99145\_Mycgr3T

Mycgr3G103274 Mycgr3
  
Location: 4426-4957

Mycgr3G103274\_Mycgr3

Mycgr3G103264 Mycgr3
  
Location: 5057-5390

Mycgr3G103264\_Mycgr3

Mycgr3G37570 Mycgr3T
  
Location: 5490-6006

Mycgr3G37570\_Mycgr3T

Mycgr3G108094 Mycgr3
  
Location: 6106-10555

Mycgr3G108094\_Mycgr3

Mycgr3G90786 Mycgr3T
  
Location: 10655-12080

Mycgr3G90786\_Mycgr3T

Mycgr3G68429 Mycgr3T
  
Location: 12180-13440

Mycgr3G68429\_Mycgr3T

Mycgr3G68421 Mycgr3T
  
Location: 13540-17086

Mycgr3G68421\_Mycgr3T

Mycgr3G90801 Mycgr3T
  
Location: 17186-18056

Mycgr3G90801\_Mycgr3T

Mycgr3G84646 Mycgr3T
  
Location: 18156-20235

Mycgr3G84646\_Mycgr3T

Mycgr3G68456 Mycgr3T
  
Location: 20335-21970

Mycgr3G68456\_Mycgr3T

Mycgr3G103270 Mycgr3
  
Location: 22070-22355

Mycgr3G103270\_Mycgr3

Mycgr3G90803 Mycgr3T
  
Location: 22455-23019

Mycgr3G90803\_Mycgr3T

Mycgr3G36941 Mycgr3T
  
Location: 23119-24064

Mycgr3G36941\_Mycgr3T

Mycgr3G25746 Mycgr3T
  
Location: 24164-25241

Mycgr3G25746\_Mycgr3T

Mycgr3G90788 Mycgr3T
  
Location: 25341-25803

Mycgr3G90788\_Mycgr3T

Mycgr3G103260 Mycgr3
  
Location: 25903-26635

Mycgr3G103260\_Mycgr3

Mycgr3G84644 Mycgr3T
  
Location: 26735-28457

Mycgr3G84644\_Mycgr3T

Mycgr3G29227 Mycgr3T
  
Location: 28557-28863

Mycgr3G29227\_Mycgr3T

Mycgr3G36271 Mycgr3T
  
Location: 28963-29854

Mycgr3G36271\_Mycgr3T

Mycgr3G68433 Mycgr3T
  
Location: 29954-33041

Mycgr3G68433\_Mycgr3T

Mycgr3G79452 Mycgr3T
  
Location: 33141-33399

Mycgr3G79452\_Mycgr3T

Mycgr3G55345 Mycgr3T
  
Location: 33499-34126

Mycgr3G55345\_Mycgr3T

Mycgr3G103278 Mycgr3
  
Location: 34226-35195

Mycgr3G103278\_Mycgr3

Mycgr3G84654 Mycgr3T
  
Location: 35295-36630

Mycgr3G84654\_Mycgr3T

Mycgr3G108090 Mycgr3
  
Location: 36730-37591

Mycgr3G108090\_Mycgr3

Mycgr3G21922 Mycgr3T
  
Location: 37691-39149

Mycgr3G21922\_Mycgr3T

Mycgr3G99148 Mycgr3T
  
Location: 39249-42819

Mycgr3G99148\_Mycgr3T

not annotated
  
Accession: CAK46854
  
Location: 60474-61727
  
 NCBI BlastP on this gene

An16g04370

not annotated
  
Accession: CAK46855
  
Location: 62222-63822
  
 NCBI BlastP on this gene

An16g04380

not annotated
  
Accession: CAK46856
  
Location: 64887-67316
  
 NCBI BlastP on this gene

An16g04390

not annotated
  
Accession: CAK46857
  
Location: 67710-68670
  
 NCBI BlastP on this gene

An16g04400

not annotated
  
Accession: CAK46858
  
Location: 68899-70201
  
 NCBI BlastP on this gene

An16g04410

unnamed
  
Accession: CAK46859
  
Location: 71177-72995
  
 NCBI BlastP on this gene

An16g04420

not annotated
  
Accession: CAK46860
  
Location: 73522-74272
  
 NCBI BlastP on this gene

An16g04430

hypothetical protein
  
Accession: CAK46861
  
Location: 75519-77317
  
 NCBI BlastP on this gene

An16g04440

not annotated
  
Accession: CAK46862
  
Location: 77817-78159
  
 NCBI BlastP on this gene

An16g04450

not annotated
  
Accession: CAK46863
  
Location: 78749-81300
  
  
**BlastP hit with Mycgr3G103260\_Mycgr3**
  
Percentage identity: 53 %
  
BlastP bit score: 275
  
Sequence coverage: 98 %
  
E-value: 3e-83
  
  
 NCBI BlastP on this gene

An16g04460

not annotated
  
Accession: CAK46864
  
Location: 82403-82969
  
 NCBI BlastP on this gene

An16g04470

hypothetical protein
  
Accession: CAK46865
  
Location: 84832-85167
  
 NCBI BlastP on this gene

An16g04480

not annotated
  
Accession: CAK46866
  
Location: 86913-87457
  
 NCBI BlastP on this gene

An16g04490

not annotated
  
Accession: CAK46867
  
Location: 88233-89533
  
  
**BlastP hit with Mycgr3G68429\_Mycgr3T**
  
Percentage identity: 68 %
  
BlastP bit score: 432
  
Sequence coverage: 73 %
  
E-value: 7e-146
  
  
 NCBI BlastP on this gene

An16g04500

not annotated
  
Accession: CAK46868
  
Location: 90262-90777
  
 NCBI BlastP on this gene

An16g04510

not annotated
  
Accession: CAK46869
  
Location: 91025-92268
  
 NCBI BlastP on this gene

An16g04520

not annotated
  
Accession: CAK46870
  
Location: 92528-93671
  
 NCBI BlastP on this gene

An16g04530

Query: Architecture Search FASTA input

ACJE01000013 : Aspergillus niger ATCC 1015    Total score: 2.0     Cumulative Blast bit score: 707

Hit cluster cross-links:

Mycgr3G90785 Mycgr3T
  
Location: 0-1047

Mycgr3G90785\_Mycgr3T

Mycgr3G103262 Mycgr3
  
Location: 1147-1390

Mycgr3G103262\_Mycgr3

Mycgr3G68458 Mycgr3T
  
Location: 1490-3602

Mycgr3G68458\_Mycgr3T

Mycgr3G99145 Mycgr3T
  
Location: 3702-4326

Mycgr3G99145\_Mycgr3T

Mycgr3G103274 Mycgr3
  
Location: 4426-4957

Mycgr3G103274\_Mycgr3

Mycgr3G103264 Mycgr3
  
Location: 5057-5390

Mycgr3G103264\_Mycgr3

Mycgr3G37570 Mycgr3T
  
Location: 5490-6006

Mycgr3G37570\_Mycgr3T

Mycgr3G108094 Mycgr3
  
Location: 6106-10555

Mycgr3G108094\_Mycgr3

Mycgr3G90786 Mycgr3T
  
Location: 10655-12080

Mycgr3G90786\_Mycgr3T

Mycgr3G68429 Mycgr3T
  
Location: 12180-13440

Mycgr3G68429\_Mycgr3T

Mycgr3G68421 Mycgr3T
  
Location: 13540-17086

Mycgr3G68421\_Mycgr3T

Mycgr3G90801 Mycgr3T
  
Location: 17186-18056

Mycgr3G90801\_Mycgr3T

Mycgr3G84646 Mycgr3T
  
Location: 18156-20235

Mycgr3G84646\_Mycgr3T

Mycgr3G68456 Mycgr3T
  
Location: 20335-21970

Mycgr3G68456\_Mycgr3T

Mycgr3G103270 Mycgr3
  
Location: 22070-22355

Mycgr3G103270\_Mycgr3

Mycgr3G90803 Mycgr3T
  
Location: 22455-23019

Mycgr3G90803\_Mycgr3T

Mycgr3G36941 Mycgr3T
  
Location: 23119-24064

Mycgr3G36941\_Mycgr3T

Mycgr3G25746 Mycgr3T
  
Location: 24164-25241

Mycgr3G25746\_Mycgr3T

Mycgr3G90788 Mycgr3T
  
Location: 25341-25803

Mycgr3G90788\_Mycgr3T

Mycgr3G103260 Mycgr3
  
Location: 25903-26635

Mycgr3G103260\_Mycgr3

Mycgr3G84644 Mycgr3T
  
Location: 26735-28457

Mycgr3G84644\_Mycgr3T

Mycgr3G29227 Mycgr3T
  
Location: 28557-28863

Mycgr3G29227\_Mycgr3T

Mycgr3G36271 Mycgr3T
  
Location: 28963-29854

Mycgr3G36271\_Mycgr3T

Mycgr3G68433 Mycgr3T
  
Location: 29954-33041

Mycgr3G68433\_Mycgr3T

Mycgr3G79452 Mycgr3T
  
Location: 33141-33399

Mycgr3G79452\_Mycgr3T

Mycgr3G55345 Mycgr3T
  
Location: 33499-34126

Mycgr3G55345\_Mycgr3T

Mycgr3G103278 Mycgr3
  
Location: 34226-35195

Mycgr3G103278\_Mycgr3

Mycgr3G84654 Mycgr3T
  
Location: 35295-36630

Mycgr3G84654\_Mycgr3T

Mycgr3G108090 Mycgr3
  
Location: 36730-37591

Mycgr3G108090\_Mycgr3

Mycgr3G21922 Mycgr3T
  
Location: 37691-39149

Mycgr3G21922\_Mycgr3T

Mycgr3G99148 Mycgr3T
  
Location: 39249-42819

Mycgr3G99148\_Mycgr3T

hypothetical protein
  
Accession: EHA22031
  
Location: 1315775-1317034
  
 NCBI BlastP on this gene

EHA22031

hypothetical protein
  
Accession: EHA22032
  
Location: 1320196-1322625
  
 NCBI BlastP on this gene

EHA22032

hypothetical protein
  
Accession: EHA22033
  
Location: 1323019-1323979
  
 NCBI BlastP on this gene

EHA22033

hypothetical protein
  
Accession: EHA22034
  
Location: 1324489-1325510
  
 NCBI BlastP on this gene

EHA22034

hypothetical protein
  
Accession: EHA22035
  
Location: 1326482-1328300
  
 NCBI BlastP on this gene

EHA22035

hypothetical protein
  
Accession: EHA22036
  
Location: 1328827-1329416
  
 NCBI BlastP on this gene

EHA22036

hypothetical protein
  
Accession: EHA22037
  
Location: 1331265-1332623
  
 NCBI BlastP on this gene

EHA22037

hypothetical protein
  
Accession: EHA22038
  
Location: 1334055-1336605
  
  
**BlastP hit with Mycgr3G103260\_Mycgr3**
  
Percentage identity: 53 %
  
BlastP bit score: 275
  
Sequence coverage: 98 %
  
E-value: 2e-83
  
  
 NCBI BlastP on this gene

EHA22038

hypothetical protein
  
Accession: EHA22039
  
Location: 1343514-1344816
  
  
**BlastP hit with Mycgr3G68429\_Mycgr3T**
  
Percentage identity: 68 %
  
BlastP bit score: 432
  
Sequence coverage: 73 %
  
E-value: 7e-146
  
  
 NCBI BlastP on this gene

EHA22039

hypothetical protein
  
Accession: EHA22040
  
Location: 1346365-1347557
  
 NCBI BlastP on this gene

EHA22040

hypothetical protein
  
Accession: EHA22041
  
Location: 1347831-1348968
  
 NCBI BlastP on this gene

EHA22041

hypothetical protein
  
Accession: EHA22042
  
Location: 1350049-1351014
  
 NCBI BlastP on this gene

EHA22042

hypothetical protein
  
Accession: EHA22043
  
Location: 1351496-1352531
  
 NCBI BlastP on this gene

EHA22043

hypothetical protein
  
Accession: EHA22044
  
Location: 1352736-1354050
  
 NCBI BlastP on this gene

EHA22044

hypothetical protein
  
Accession: EHA22045
  
Location: 1354670-1355503
  
 NCBI BlastP on this gene

EHA22045

hypothetical protein
  
Accession: EHA22046
  
Location: 1356357-1357939
  
 NCBI BlastP on this gene

EHA22046

hypothetical protein
  
Accession: EHA22047
  
Location: 1358752-1359854
  
 NCBI BlastP on this gene

EHA22047

hypothetical protein
  
Accession: EHA22048
  
Location: 1360225-1361856
  
 NCBI BlastP on this gene

EHA22048

hypothetical protein
  
Accession: EHA22049
  
Location: 1362222-1362950
  
 NCBI BlastP on this gene

EHA22049

Query: Architecture Search FASTA input

GG749418 : Ajellomyces dermatitidis ATCC 18188 genomic scaffold supercont1.12    Total score: 2.0     Cumulative Blast bit score: 706

Hit cluster cross-links:

Mycgr3G90785 Mycgr3T
  
Location: 0-1047

Mycgr3G90785\_Mycgr3T

Mycgr3G103262 Mycgr3
  
Location: 1147-1390

Mycgr3G103262\_Mycgr3

Mycgr3G68458 Mycgr3T
  
Location: 1490-3602

Mycgr3G68458\_Mycgr3T

Mycgr3G99145 Mycgr3T
  
Location: 3702-4326

Mycgr3G99145\_Mycgr3T

Mycgr3G103274 Mycgr3
  
Location: 4426-4957

Mycgr3G103274\_Mycgr3

Mycgr3G103264 Mycgr3
  
Location: 5057-5390

Mycgr3G103264\_Mycgr3

Mycgr3G37570 Mycgr3T
  
Location: 5490-6006

Mycgr3G37570\_Mycgr3T

Mycgr3G108094 Mycgr3
  
Location: 6106-10555

Mycgr3G108094\_Mycgr3

Mycgr3G90786 Mycgr3T
  
Location: 10655-12080

Mycgr3G90786\_Mycgr3T

Mycgr3G68429 Mycgr3T
  
Location: 12180-13440

Mycgr3G68429\_Mycgr3T

Mycgr3G68421 Mycgr3T
  
Location: 13540-17086

Mycgr3G68421\_Mycgr3T

Mycgr3G90801 Mycgr3T
  
Location: 17186-18056

Mycgr3G90801\_Mycgr3T

Mycgr3G84646 Mycgr3T
  
Location: 18156-20235

Mycgr3G84646\_Mycgr3T

Mycgr3G68456 Mycgr3T
  
Location: 20335-21970

Mycgr3G68456\_Mycgr3T

Mycgr3G103270 Mycgr3
  
Location: 22070-22355

Mycgr3G103270\_Mycgr3

Mycgr3G90803 Mycgr3T
  
Location: 22455-23019

Mycgr3G90803\_Mycgr3T

Mycgr3G36941 Mycgr3T
  
Location: 23119-24064

Mycgr3G36941\_Mycgr3T

Mycgr3G25746 Mycgr3T
  
Location: 24164-25241

Mycgr3G25746\_Mycgr3T

Mycgr3G90788 Mycgr3T
  
Location: 25341-25803

Mycgr3G90788\_Mycgr3T

Mycgr3G103260 Mycgr3
  
Location: 25903-26635

Mycgr3G103260\_Mycgr3

Mycgr3G84644 Mycgr3T
  
Location: 26735-28457

Mycgr3G84644\_Mycgr3T

Mycgr3G29227 Mycgr3T
  
Location: 28557-28863

Mycgr3G29227\_Mycgr3T

Mycgr3G36271 Mycgr3T
  
Location: 28963-29854

Mycgr3G36271\_Mycgr3T

Mycgr3G68433 Mycgr3T
  
Location: 29954-33041

Mycgr3G68433\_Mycgr3T

Mycgr3G79452 Mycgr3T
  
Location: 33141-33399

Mycgr3G79452\_Mycgr3T

Mycgr3G55345 Mycgr3T
  
Location: 33499-34126

Mycgr3G55345\_Mycgr3T

Mycgr3G103278 Mycgr3
  
Location: 34226-35195

Mycgr3G103278\_Mycgr3

Mycgr3G84654 Mycgr3T
  
Location: 35295-36630

Mycgr3G84654\_Mycgr3T

Mycgr3G108090 Mycgr3
  
Location: 36730-37591

Mycgr3G108090\_Mycgr3

Mycgr3G21922 Mycgr3T
  
Location: 37691-39149

Mycgr3G21922\_Mycgr3T

Mycgr3G99148 Mycgr3T
  
Location: 39249-42819

Mycgr3G99148\_Mycgr3T

hypothetical protein
  
Accession: EGE80176
  
Location: 598558-600808
  
 NCBI BlastP on this gene

EGE80176

hypothetical protein
  
Accession: EGE80177
  
Location: 604471-605108
  
 NCBI BlastP on this gene

EGE80177

PQ loop repeat protein
  
Accession: EGE80178
  
Location: 605564-606635
  
 NCBI BlastP on this gene

EGE80178

stress response protein ish1
  
Accession: EGE80179
  
Location: 607431-609278
  
 NCBI BlastP on this gene

EGE80179

membrane-associated progesterone receptor component 1
  
Accession: EGE80180
  
Location: 610207-611127
  
 NCBI BlastP on this gene

EGE80180

hypothetical protein
  
Accession: EGE80181
  
Location: 611819-613773
  
 NCBI BlastP on this gene

EGE80181

hypothetical protein
  
Accession: EGE80182
  
Location: 614736-615120
  
 NCBI BlastP on this gene

EGE80182

serine/threonine-protein kinase prp4
  
Accession: EGE80183
  
Location: 615728-618220
  
  
**BlastP hit with Mycgr3G103260\_Mycgr3**
  
Percentage identity: 54 %
  
BlastP bit score: 280
  
Sequence coverage: 97 %
  
E-value: 6e-85
  
  
 NCBI BlastP on this gene

EGE80183

integral membrane protein
  
Accession: EGE80184
  
Location: 618662-620173
  
 NCBI BlastP on this gene

EGE80184

hypothetical protein
  
Accession: EGE80185
  
Location: 623503-626728
  
 NCBI BlastP on this gene

EGE80185

hypothetical protein
  
Accession: EGE80186
  
Location: 627100-628985
  
 NCBI BlastP on this gene

EGE80186

protein serine/threonine kinase
  
Accession: EGE80187
  
Location: 630472-631830
  
  
**BlastP hit with Mycgr3G68429\_Mycgr3T**
  
Percentage identity: 68 %
  
BlastP bit score: 426
  
Sequence coverage: 71 %
  
E-value: 3e-143
  
  
 NCBI BlastP on this gene

EGE80187

short-chain dehydrogenase/reductase SDR
  
Accession: EGE80188
  
Location: 633398-634597
  
 NCBI BlastP on this gene

EGE80188

hemolysin-III channel protein Izh2
  
Accession: EGE80189
  
Location: 635189-636220
  
 NCBI BlastP on this gene

EGE80189

hypothetical protein
  
Accession: EGE80190
  
Location: 636418-637534
  
 NCBI BlastP on this gene

EGE80190

hypothetical protein
  
Accession: EGE80191
  
Location: 637748-639317
  
 NCBI BlastP on this gene

EGE80191

dolichol-phosphate mannosyltransferase
  
Accession: EGE80192
  
Location: 639739-640651
  
 NCBI BlastP on this gene

EGE80192

esterase
  
Accession: EGE80193
  
Location: 641122-642249
  
 NCBI BlastP on this gene

EGE80193

transcription factor tfiiic complex subunit tfc6
  
Accession: EGE80194
  
Location: 642818-645215
  
 NCBI BlastP on this gene

EGE80194

hypothetical protein
  
Accession: EGE80195
  
Location: 645972-646538
  
 NCBI BlastP on this gene

EGE80195

hypothetical protein
  
Accession: EGE80196
  
Location: 647147-648403
  
 NCBI BlastP on this gene

EGE80196

Query: Architecture Search FASTA input

KB445649 : Cochliobolus sativus ND90Pr unplaced genomic scaffold COCSAscaffold\_13    Total score: 2.0     Cumulative Blast bit score: 704

Hit cluster cross-links:

Mycgr3G90785 Mycgr3T
  
Location: 0-1047

Mycgr3G90785\_Mycgr3T

Mycgr3G103262 Mycgr3
  
Location: 1147-1390

Mycgr3G103262\_Mycgr3

Mycgr3G68458 Mycgr3T
  
Location: 1490-3602

Mycgr3G68458\_Mycgr3T

Mycgr3G99145 Mycgr3T
  
Location: 3702-4326

Mycgr3G99145\_Mycgr3T

Mycgr3G103274 Mycgr3
  
Location: 4426-4957

Mycgr3G103274\_Mycgr3

Mycgr3G103264 Mycgr3
  
Location: 5057-5390

Mycgr3G103264\_Mycgr3

Mycgr3G37570 Mycgr3T
  
Location: 5490-6006

Mycgr3G37570\_Mycgr3T

Mycgr3G108094 Mycgr3
  
Location: 6106-10555

Mycgr3G108094\_Mycgr3

Mycgr3G90786 Mycgr3T
  
Location: 10655-12080

Mycgr3G90786\_Mycgr3T

Mycgr3G68429 Mycgr3T
  
Location: 12180-13440

Mycgr3G68429\_Mycgr3T

Mycgr3G68421 Mycgr3T
  
Location: 13540-17086

Mycgr3G68421\_Mycgr3T

Mycgr3G90801 Mycgr3T
  
Location: 17186-18056

Mycgr3G90801\_Mycgr3T

Mycgr3G84646 Mycgr3T
  
Location: 18156-20235

Mycgr3G84646\_Mycgr3T

Mycgr3G68456 Mycgr3T
  
Location: 20335-21970

Mycgr3G68456\_Mycgr3T

Mycgr3G103270 Mycgr3
  
Location: 22070-22355

Mycgr3G103270\_Mycgr3

Mycgr3G90803 Mycgr3T
  
Location: 22455-23019

Mycgr3G90803\_Mycgr3T

Mycgr3G36941 Mycgr3T
  
Location: 23119-24064

Mycgr3G36941\_Mycgr3T

Mycgr3G25746 Mycgr3T
  
Location: 24164-25241

Mycgr3G25746\_Mycgr3T

Mycgr3G90788 Mycgr3T
  
Location: 25341-25803

Mycgr3G90788\_Mycgr3T

Mycgr3G103260 Mycgr3
  
Location: 25903-26635

Mycgr3G103260\_Mycgr3

Mycgr3G84644 Mycgr3T
  
Location: 26735-28457

Mycgr3G84644\_Mycgr3T

Mycgr3G29227 Mycgr3T
  
Location: 28557-28863

Mycgr3G29227\_Mycgr3T

Mycgr3G36271 Mycgr3T
  
Location: 28963-29854

Mycgr3G36271\_Mycgr3T

Mycgr3G68433 Mycgr3T
  
Location: 29954-33041

Mycgr3G68433\_Mycgr3T

Mycgr3G79452 Mycgr3T
  
Location: 33141-33399

Mycgr3G79452\_Mycgr3T

Mycgr3G55345 Mycgr3T
  
Location: 33499-34126

Mycgr3G55345\_Mycgr3T

Mycgr3G103278 Mycgr3
  
Location: 34226-35195

Mycgr3G103278\_Mycgr3

Mycgr3G84654 Mycgr3T
  
Location: 35295-36630

Mycgr3G84654\_Mycgr3T

Mycgr3G108090 Mycgr3
  
Location: 36730-37591

Mycgr3G108090\_Mycgr3

Mycgr3G21922 Mycgr3T
  
Location: 37691-39149

Mycgr3G21922\_Mycgr3T

Mycgr3G99148 Mycgr3T
  
Location: 39249-42819

Mycgr3G99148\_Mycgr3T

hypothetical protein
  
Accession: EMD61193
  
Location: 1182865-1184103
  
 NCBI BlastP on this gene

EMD61193

hypothetical protein
  
Accession: EMD61194
  
Location: 1184804-1186119
  
 NCBI BlastP on this gene

EMD61194

hypothetical protein
  
Accession: EMD61195
  
Location: 1186635-1187406
  
 NCBI BlastP on this gene

EMD61195

hypothetical protein
  
Accession: EMD61196
  
Location: 1187981-1189630
  
 NCBI BlastP on this gene

EMD61196

hypothetical protein
  
Accession: EMD61197
  
Location: 1190061-1190693
  
 NCBI BlastP on this gene

EMD61197

hypothetical protein
  
Accession: EMD61198
  
Location: 1195220-1198205
  
 NCBI BlastP on this gene

EMD61198

hypothetical protein
  
Accession: EMD61199
  
Location: 1198819-1199687
  
 NCBI BlastP on this gene

EMD61199

hypothetical protein
  
Accession: EMD61200
  
Location: 1200407-1203758
  
  
**BlastP hit with Mycgr3G68433\_Mycgr3T**
  
Percentage identity: 42 %
  
BlastP bit score: 577
  
Sequence coverage: 86 %
  
E-value: 0.0
  
  
 NCBI BlastP on this gene

EMD61200

hypothetical protein
  
Accession: EMD61201
  
Location: 1204376-1205740
  
  
**BlastP hit with Mycgr3G90786\_Mycgr3T**
  
Percentage identity: 30 %
  
BlastP bit score: 127
  
Sequence coverage: 92 %
  
E-value: 5e-29
  
  
 NCBI BlastP on this gene

EMD61201

hypothetical protein
  
Accession: EMD61202
  
Location: 1206541-1207901
  
 NCBI BlastP on this gene

EMD61202

hypothetical protein
  
Accession: EMD61203
  
Location: 1208201-1209321
  
 NCBI BlastP on this gene

EMD61203

hypothetical protein
  
Accession: EMD61204
  
Location: 1209806-1211101
  
 NCBI BlastP on this gene

EMD61204

hypothetical protein
  
Accession: EMD61205
  
Location: 1211333-1211767
  
 NCBI BlastP on this gene

EMD61205

hypothetical protein
  
Accession: EMD61206
  
Location: 1211862-1213013
  
 NCBI BlastP on this gene

EMD61206

hypothetical protein
  
Accession: EMD61207
  
Location: 1213473-1214712
  
 NCBI BlastP on this gene

EMD61207

hypothetical protein
  
Accession: EMD61208
  
Location: 1215338-1216756
  
 NCBI BlastP on this gene

EMD61208

hypothetical protein
  
Accession: EMD61209
  
Location: 1219347-1219835
  
 NCBI BlastP on this gene

EMD61209

hypothetical protein
  
Accession: EMD61210
  
Location: 1222789-1224415
  
 NCBI BlastP on this gene

EMD61210

hypothetical protein
  
Accession: EMD61211
  
Location: 1225655-1227043
  
 NCBI BlastP on this gene

EMD61211

Query: Architecture Search FASTA input

EQ999973 : Ajellomyces dermatitidis ER-3 genomic scaffold supercont1.1    Total score: 2.0     Cumulative Blast bit score: 704

Hit cluster cross-links:

Mycgr3G90785 Mycgr3T
  
Location: 0-1047

Mycgr3G90785\_Mycgr3T

Mycgr3G103262 Mycgr3
  
Location: 1147-1390

Mycgr3G103262\_Mycgr3

Mycgr3G68458 Mycgr3T
  
Location: 1490-3602

Mycgr3G68458\_Mycgr3T

Mycgr3G99145 Mycgr3T
  
Location: 3702-4326

Mycgr3G99145\_Mycgr3T

Mycgr3G103274 Mycgr3
  
Location: 4426-4957

Mycgr3G103274\_Mycgr3

Mycgr3G103264 Mycgr3
  
Location: 5057-5390

Mycgr3G103264\_Mycgr3

Mycgr3G37570 Mycgr3T
  
Location: 5490-6006

Mycgr3G37570\_Mycgr3T

Mycgr3G108094 Mycgr3
  
Location: 6106-10555

Mycgr3G108094\_Mycgr3

Mycgr3G90786 Mycgr3T
  
Location: 10655-12080

Mycgr3G90786\_Mycgr3T

Mycgr3G68429 Mycgr3T
  
Location: 12180-13440

Mycgr3G68429\_Mycgr3T

Mycgr3G68421 Mycgr3T
  
Location: 13540-17086

Mycgr3G68421\_Mycgr3T

Mycgr3G90801 Mycgr3T
  
Location: 17186-18056

Mycgr3G90801\_Mycgr3T

Mycgr3G84646 Mycgr3T
  
Location: 18156-20235

Mycgr3G84646\_Mycgr3T

Mycgr3G68456 Mycgr3T
  
Location: 20335-21970

Mycgr3G68456\_Mycgr3T

Mycgr3G103270 Mycgr3
  
Location: 22070-22355

Mycgr3G103270\_Mycgr3

Mycgr3G90803 Mycgr3T
  
Location: 22455-23019

Mycgr3G90803\_Mycgr3T

Mycgr3G36941 Mycgr3T
  
Location: 23119-24064

Mycgr3G36941\_Mycgr3T

Mycgr3G25746 Mycgr3T
  
Location: 24164-25241

Mycgr3G25746\_Mycgr3T

Mycgr3G90788 Mycgr3T
  
Location: 25341-25803

Mycgr3G90788\_Mycgr3T

Mycgr3G103260 Mycgr3
  
Location: 25903-26635

Mycgr3G103260\_Mycgr3

Mycgr3G84644 Mycgr3T
  
Location: 26735-28457

Mycgr3G84644\_Mycgr3T

Mycgr3G29227 Mycgr3T
  
Location: 28557-28863

Mycgr3G29227\_Mycgr3T

Mycgr3G36271 Mycgr3T
  
Location: 28963-29854

Mycgr3G36271\_Mycgr3T

Mycgr3G68433 Mycgr3T
  
Location: 29954-33041

Mycgr3G68433\_Mycgr3T

Mycgr3G79452 Mycgr3T
  
Location: 33141-33399

Mycgr3G79452\_Mycgr3T

Mycgr3G55345 Mycgr3T
  
Location: 33499-34126

Mycgr3G55345\_Mycgr3T

Mycgr3G103278 Mycgr3
  
Location: 34226-35195

Mycgr3G103278\_Mycgr3

Mycgr3G84654 Mycgr3T
  
Location: 35295-36630

Mycgr3G84654\_Mycgr3T

Mycgr3G108090 Mycgr3
  
Location: 36730-37591

Mycgr3G108090\_Mycgr3

Mycgr3G21922 Mycgr3T
  
Location: 37691-39149

Mycgr3G21922\_Mycgr3T

Mycgr3G99148 Mycgr3T
  
Location: 39249-42819

Mycgr3G99148\_Mycgr3T

conserved hypothetical protein
  
Accession: EEQ83842
  
Location: 5063516-5065766
  
 NCBI BlastP on this gene

EEQ83842

predicted protein
  
Accession: EEQ83843
  
Location: 5068129-5068890
  
 NCBI BlastP on this gene

EEQ83843

conserved hypothetical protein
  
Accession: EEQ83844
  
Location: 5069518-5070155
  
 NCBI BlastP on this gene

EEQ83844

PQ loop repeat protein
  
Accession: EEQ83845
  
Location: 5070611-5071682
  
 NCBI BlastP on this gene

EEQ83845

conserved hypothetical protein
  
Accession: EEQ83846
  
Location: 5072476-5074323
  
 NCBI BlastP on this gene

EEQ83846

DNA damage response protein
  
Accession: EEQ83847
  
Location: 5075290-5076100
  
 NCBI BlastP on this gene

EEQ83847

conserved hypothetical protein
  
Accession: EEQ83848
  
Location: 5076890-5078844
  
 NCBI BlastP on this gene

EEQ83848

hypothetical protein
  
Accession: EEQ83849
  
Location: 5079813-5080197
  
 NCBI BlastP on this gene

EEQ83849

serine/threonine-protein kinase prp4
  
Accession: EEQ83850
  
Location: 5080804-5083296
  
  
**BlastP hit with Mycgr3G103260\_Mycgr3**
  
Percentage identity: 54 %
  
BlastP bit score: 278
  
Sequence coverage: 96 %
  
E-value: 3e-84
  
  
 NCBI BlastP on this gene

EEQ83850

integral membrane protein
  
Accession: EEQ83851
  
Location: 5083738-5085249
  
 NCBI BlastP on this gene

EEQ83851

conserved hypothetical protein
  
Accession: EEQ83852
  
Location: 5087537-5091831
  
 NCBI BlastP on this gene

EEQ83852

predicted protein
  
Accession: EEQ83853
  
Location: 5092207-5094081
  
 NCBI BlastP on this gene

EEQ83853

protein serine/threonine kinase
  
Accession: EEQ83854
  
Location: 5094929-5096275
  
  
**BlastP hit with Mycgr3G68429\_Mycgr3T**
  
Percentage identity: 68 %
  
BlastP bit score: 426
  
Sequence coverage: 71 %
  
E-value: 3e-143
  
  
 NCBI BlastP on this gene

EEQ83854

3-oxoacyl-(acyl-carrier-protein) reductase
  
Accession: EEQ83855
  
Location: 5097842-5099043
  
 NCBI BlastP on this gene

EEQ83855

hemolysin-III channel protein Izh2
  
Accession: EEQ83856
  
Location: 5099634-5100611
  
 NCBI BlastP on this gene

EEQ83856

conserved hypothetical protein
  
Accession: EEQ83857
  
Location: 5100863-5101979
  
 NCBI BlastP on this gene

EEQ83857

conserved hypothetical protein
  
Accession: EEQ83858
  
Location: 5102265-5103764
  
 NCBI BlastP on this gene

EEQ83858

dolichol-phosphate mannosyltransferase
  
Accession: EEQ83859
  
Location: 5104290-5105097
  
 NCBI BlastP on this gene

EEQ83859

esterase
  
Accession: EEQ83860
  
Location: 5105568-5106695
  
 NCBI BlastP on this gene

EEQ83860

transcription factor tfiiic complex subunit tfc6
  
Accession: EEQ83861
  
Location: 5107263-5109660
  
 NCBI BlastP on this gene

EEQ83861

hypothetical protein
  
Accession: EEQ83862
  
Location: 5110413-5110931
  
 NCBI BlastP on this gene

EEQ83862

hypothetical protein
  
Accession: EEQ83863
  
Location: 5111545-5112801
  
 NCBI BlastP on this gene

EEQ83863

Query: Architecture Search FASTA input

201. :  AGUE01000255 Glarea lozoyensis 74030     Total score: 2.0     Cumulative Blast bit score: 944

Mycgr3G90785 Mycgr3T
  
Location: 0-1047
  
 NCBI BlastP on this gene

Mycgr3G90785\_Mycgr3T

Mycgr3G103262 Mycgr3
  
Location: 1147-1390
  
 NCBI BlastP on this gene

Mycgr3G103262\_Mycgr3

Mycgr3G68458 Mycgr3T
  
Location: 1490-3602
  
 NCBI BlastP on this gene

Mycgr3G68458\_Mycgr3T

Mycgr3G99145 Mycgr3T
  
Location: 3702-4326
  
 NCBI BlastP on this gene

Mycgr3G99145\_Mycgr3T

Mycgr3G103274 Mycgr3
  
Location: 4426-4957
  
 NCBI BlastP on this gene

Mycgr3G103274\_Mycgr3

Mycgr3G103264 Mycgr3
  
Location: 5057-5390
  
 NCBI BlastP on this gene

Mycgr3G103264\_Mycgr3

Mycgr3G37570 Mycgr3T
  
Location: 5490-6006
  
 NCBI BlastP on this gene

Mycgr3G37570\_Mycgr3T

Mycgr3G108094 Mycgr3
  
Location: 6106-10555
  
 NCBI BlastP on this gene

Mycgr3G108094\_Mycgr3

Mycgr3G90786 Mycgr3T
  
Location: 10655-12080
  
 NCBI BlastP on this gene

Mycgr3G90786\_Mycgr3T

Mycgr3G68429 Mycgr3T
  
Location: 12180-13440
  
 NCBI BlastP on this gene

Mycgr3G68429\_Mycgr3T

Mycgr3G68421 Mycgr3T
  
Location: 13540-17086
  
 NCBI BlastP on this gene

Mycgr3G68421\_Mycgr3T

Mycgr3G90801 Mycgr3T
  
Location: 17186-18056
  
 NCBI BlastP on this gene

Mycgr3G90801\_Mycgr3T

Mycgr3G84646 Mycgr3T
  
Location: 18156-20235
  
 NCBI BlastP on this gene

Mycgr3G84646\_Mycgr3T

Mycgr3G68456 Mycgr3T
  
Location: 20335-21970
  
 NCBI BlastP on this gene

Mycgr3G68456\_Mycgr3T

Mycgr3G103270 Mycgr3
  
Location: 22070-22355
  
 NCBI BlastP on this gene

Mycgr3G103270\_Mycgr3

Mycgr3G90803 Mycgr3T
  
Location: 22455-23019
  
 NCBI BlastP on this gene

Mycgr3G90803\_Mycgr3T

Mycgr3G36941 Mycgr3T
  
Location: 23119-24064
  
 NCBI BlastP on this gene

Mycgr3G36941\_Mycgr3T

Mycgr3G25746 Mycgr3T
  
Location: 24164-25241
  
 NCBI BlastP on this gene

Mycgr3G25746\_Mycgr3T

Mycgr3G90788 Mycgr3T
  
Location: 25341-25803
  
 NCBI BlastP on this gene

Mycgr3G90788\_Mycgr3T

Mycgr3G103260 Mycgr3
  
Location: 25903-26635
  
 NCBI BlastP on this gene

Mycgr3G103260\_Mycgr3

Mycgr3G84644 Mycgr3T
  
Location: 26735-28457
  
 NCBI BlastP on this gene

Mycgr3G84644\_Mycgr3T

Mycgr3G29227 Mycgr3T
  
Location: 28557-28863
  
 NCBI BlastP on this gene

Mycgr3G29227\_Mycgr3T

Mycgr3G36271 Mycgr3T
  
Location: 28963-29854
  
 NCBI BlastP on this gene

Mycgr3G36271\_Mycgr3T

Mycgr3G68433 Mycgr3T
  
Location: 29954-33041
  
 NCBI BlastP on this gene

Mycgr3G68433\_Mycgr3T

Mycgr3G79452 Mycgr3T
  
Location: 33141-33399
  
 NCBI BlastP on this gene

Mycgr3G79452\_Mycgr3T

Mycgr3G55345 Mycgr3T
  
Location: 33499-34126
  
 NCBI BlastP on this gene

Mycgr3G55345\_Mycgr3T

Mycgr3G103278 Mycgr3
  
Location: 34226-35195
  
 NCBI BlastP on this gene

Mycgr3G103278\_Mycgr3

Mycgr3G84654 Mycgr3T
  
Location: 35295-36630
  
 NCBI BlastP on this gene

Mycgr3G84654\_Mycgr3T

Mycgr3G108090 Mycgr3
  
Location: 36730-37591
  
 NCBI BlastP on this gene

Mycgr3G108090\_Mycgr3

Mycgr3G21922 Mycgr3T
  
Location: 37691-39149
  
 NCBI BlastP on this gene

Mycgr3G21922\_Mycgr3T

Mycgr3G99148 Mycgr3T
  
Location: 39249-42819
  
 NCBI BlastP on this gene

Mycgr3G99148\_Mycgr3T

putative Tetracycline resistance protein from transposon/Tn4400
  
Accession: EHK96368
  
Location: 78675-79424
  
 NCBI BlastP on this gene

EHK96368

hypothetical protein
  
Accession: EHK96367
  
Location: 77427-77869
  
 NCBI BlastP on this gene

EHK96367

hypothetical protein
  
Accession: EHK96366
  
Location: 74730-76000
  
 NCBI BlastP on this gene

EHK96366

hypothetical protein
  
Accession: EHK96365
  
Location: 72631-73400
  
 NCBI BlastP on this gene

EHK96365

putative protein lunapark like protein
  
Accession: EHK96364
  
Location: 69800-70719
  
 NCBI BlastP on this gene

EHK96364

putative Golgin IMH1
  
Accession: EHK96363
  
Location: 65358-69143
  
  
**BlastP hit with Mycgr3G108094\_Mycgr3**
  
Percentage identity: 35 %
  
BlastP bit score: 260
  
Sequence coverage: 40 %
  
E-value: 6e-68
  
  
 NCBI BlastP on this gene

EHK96363

putative protein kinase dsk1
  
Accession: EHK96362
  
Location: 61877-64034
  
  
**BlastP hit with Mycgr3G84644\_Mycgr3T**
  
Percentage identity: 64 %
  
BlastP bit score: 684
  
Sequence coverage: 99 %
  
E-value: 0.0
  
  
 NCBI BlastP on this gene

EHK96362

hypothetical protein
  
Accession: EHK96361
  
Location: 59479-59757
  
 NCBI BlastP on this gene

EHK96361

putative Transcription factor IWS1
  
Accession: EHK96360
  
Location: 57580-59009
  
 NCBI BlastP on this gene

EHK96360

hypothetical protein
  
Accession: EHK96359
  
Location: 54121-54525
  
 NCBI BlastP on this gene

EHK96359

hypothetical protein
  
Accession: EHK96358
  
Location: 52663-53930
  
 NCBI BlastP on this gene

EHK96358

putative Laccase-2
  
Accession: EHK96357
  
Location: 47677-49275
  
 NCBI BlastP on this gene

EHK96357

202. :  JH725150 Beauveria bassiana ARSEF 2860 unplaced genomic scaffold BBA\_S00001     Total score: 2.0     Cumulative Blast bit score: 942

hypothetical protein
  
Accession: EJP70847
  
Location: 1452114-1453324
  
 NCBI BlastP on this gene

EJP70847

calcineurin-like phosphoesterase
  
Accession: EJP70848
  
Location: 1453856-1455256
  
 NCBI BlastP on this gene

EJP70848

exocyst complex component EXO84
  
Accession: EJP70849
  
Location: 1456377-1458506
  
 NCBI BlastP on this gene

EJP70849

hypothetical protein
  
Accession: EJP70850
  
Location: 1460053-1460914
  
 NCBI BlastP on this gene

EJP70850

MAS20 protein import receptor
  
Accession: EJP70851
  
Location: 1461919-1462718
  
 NCBI BlastP on this gene

EJP70851

wyosine base formation
  
Accession: EJP70852
  
Location: 1463189-1465576
  
 NCBI BlastP on this gene

EJP70852

cysteine dioxygenase
  
Accession: EJP70853
  
Location: 1466438-1467256
  
 NCBI BlastP on this gene

EJP70853

sulfite transporter Ssu2
  
Accession: EJP70854
  
Location: 1469300-1470938
  
 NCBI BlastP on this gene

EJP70854

ATP synthase regulation protein NCA2
  
Accession: EJP70855
  
Location: 1471287-1473354
  
  
**BlastP hit with Mycgr3G84646\_Mycgr3T**
  
Percentage identity: 40 %
  
BlastP bit score: 504
  
Sequence coverage: 102 %
  
E-value: 3e-166
  
  
 NCBI BlastP on this gene

EJP70855

fatty acid hydroxylase superfamily protein
  
Accession: EJP70856
  
Location: 1474564-1475617
  
  
**BlastP hit with Mycgr3G36271\_Mycgr3T**
  
Percentage identity: 70 %
  
BlastP bit score: 439
  
Sequence coverage: 95 %
  
E-value: 5e-152
  
  
 NCBI BlastP on this gene

EJP70856

pterin 4 alpha carbinolamine dehydratase
  
Accession: EJP70857
  
Location: 1476030-1476743
  
 NCBI BlastP on this gene

EJP70857

bZIP transcription factor
  
Accession: EJP70858
  
Location: 1478299-1479656
  
 NCBI BlastP on this gene

EJP70858

Actin-like protein, ARP5 class
  
Accession: EJP70859
  
Location: 1480301-1482774
  
 NCBI BlastP on this gene

EJP70859

DASH complex subunit Dad4
  
Accession: EJP70860
  
Location: 1483195-1483705
  
 NCBI BlastP on this gene

EJP70860

chromatin-remodeling complex subunit ies6
  
Accession: EJP70861
  
Location: 1483875-1484468
  
 NCBI BlastP on this gene

EJP70861

Got1 family protein
  
Accession: EJP70862
  
Location: 1485493-1486254
  
 NCBI BlastP on this gene

EJP70862

profilin-like protein
  
Accession: EJP70863
  
Location: 1486986-1487792
  
 NCBI BlastP on this gene

EJP70863

hypothetical protein
  
Accession: EJP70864
  
Location: 1491044-1492639
  
 NCBI BlastP on this gene

EJP70864

SNARE protein, putative
  
Accession: EJP70865
  
Location: 1493479-1494462
  
 NCBI BlastP on this gene

EJP70865

203. :  DS985219 Verticillium albo-atrum VaMs.102 supercont1.6 genomic scaffold     Total score: 2.0     Cumulative Blast bit score: 942

conserved hypothetical protein
  
Accession: EEY19063
  
Location: 281540-281804
  
 NCBI BlastP on this gene

EEY19063

Got1 family protein
  
Accession: EEY19064
  
Location: 282416-283116
  
 NCBI BlastP on this gene

EEY19064

conserved hypothetical protein
  
Accession: EEY19065
  
Location: 284341-284937
  
 NCBI BlastP on this gene

EEY19065

hypothetical protein
  
Accession: EEY19066
  
Location: 285203-285639
  
 NCBI BlastP on this gene

EEY19066

conserved hypothetical protein
  
Accession: EEY19067
  
Location: 287110-289517
  
 NCBI BlastP on this gene

EEY19067

hypothetical protein
  
Accession: EEY19068
  
Location: 290900-291365
  
 NCBI BlastP on this gene

EEY19068

conserved hypothetical protein
  
Accession: EEY19069
  
Location: 296215-296768
  
 NCBI BlastP on this gene

EEY19069

C-4 methylsterol oxidase
  
Accession: EEY19070
  
Location: 297455-298540
  
  
**BlastP hit with Mycgr3G36271\_Mycgr3T**
  
Percentage identity: 71 %
  
BlastP bit score: 456
  
Sequence coverage: 97 %
  
E-value: 2e-158
  
  
 NCBI BlastP on this gene

EEY19070

nuclear control of ATPase protein
  
Accession: EEY19071
  
Location: 300277-302523
  
  
**BlastP hit with Mycgr3G84646\_Mycgr3T**
  
Percentage identity: 42 %
  
BlastP bit score: 486
  
Sequence coverage: 87 %
  
E-value: 4e-159
  
  
 NCBI BlastP on this gene

EEY19071

NADP-dependent mannitol dehydrogenase
  
Accession: EEY19072
  
Location: 303225-304651
  
 NCBI BlastP on this gene

EEY19072

40S ribosomal protein S10-B
  
Accession: EEY19073
  
Location: 308481-309318
  
 NCBI BlastP on this gene

EEY19073

conserved hypothetical protein
  
Accession: EEY19074
  
Location: 309913-310779
  
 NCBI BlastP on this gene

EEY19074

cytochrome c oxidase polypeptide vib
  
Accession: EEY19075
  
Location: 311070-311627
  
 NCBI BlastP on this gene

EEY19075

hypothetical protein
  
Accession: EEY19076
  
Location: 311951-312468
  
 NCBI BlastP on this gene

EEY19076

peptidyl-prolyl cis-trans isomerase E
  
Accession: EEY19077
  
Location: 312732-313246
  
 NCBI BlastP on this gene

EEY19077

transport protein SEC31
  
Accession: EEY19078
  
Location: 313819-317712
  
 NCBI BlastP on this gene

EEY19078

conserved hypothetical protein
  
Accession: EEY19079
  
Location: 318159-319291
  
 NCBI BlastP on this gene

EEY19079

conserved hypothetical protein
  
Accession: EEY19080
  
Location: 320875-321851
  
 NCBI BlastP on this gene

EEY19080

204. :  KE145373 Glarea lozoyensis ATCC 20868 chromosome Unknown GLAREA9     Total score: 2.0     Cumulative Blast bit score: 936

MFS general substrate transporter
  
Accession: EPE24482
  
Location: 1188115-1190598
  
 NCBI BlastP on this gene

EPE24482

hypothetical protein
  
Accession: EPE24483
  
Location: 1191291-1192791
  
 NCBI BlastP on this gene

EPE24483

NAD(P)-binding Rossmann-fold containing protein
  
Accession: EPE24484
  
Location: 1193263-1194428
  
 NCBI BlastP on this gene

EPE24484

Metallo-dependent hydrolase
  
Accession: EPE24485
  
Location: 1194909-1196381
  
 NCBI BlastP on this gene

EPE24485

FabD/lysophospholipase-like protein
  
Accession: EPE24486
  
Location: 1197038-1201805
  
 NCBI BlastP on this gene

EPE24486

eIF-2-alpha, C-terminal
  
Accession: EPE24487
  
Location: 1202337-1203419
  
 NCBI BlastP on this gene

EPE24487

LexA/Signal peptidase
  
Accession: EPE24488
  
Location: 1203743-1204383
  
 NCBI BlastP on this gene

EPE24488

Mitochondrial carrier
  
Accession: EPE24489
  
Location: 1205000-1206321
  
 NCBI BlastP on this gene

EPE24489

hypothetical protein
  
Accession: EPE24490
  
Location: 1206887-1209952
  
 NCBI BlastP on this gene

EPE24490

hypothetical protein
  
Accession: EPE24491
  
Location: 1210439-1212494
  
  
**BlastP hit with Mycgr3G84646\_Mycgr3T**
  
Percentage identity: 45 %
  
BlastP bit score: 581
  
Sequence coverage: 101 %
  
E-value: 0.0
  
  
 NCBI BlastP on this gene

EPE24491

P-loop containing nucleoside triphosphate hydrolase
  
Accession: EPE24492
  
Location: 1213161-1213892
  
  
**BlastP hit with Mycgr3G99145\_Mycgr3T**
  
Percentage identity: 90 %
  
BlastP bit score: 355
  
Sequence coverage: 92 %
  
E-value: 8e-122
  
  
 NCBI BlastP on this gene

EPE24492

hypothetical protein
  
Accession: EPE24493
  
Location: 1214139-1216048
  
 NCBI BlastP on this gene

EPE24493

P-loop containing nucleoside triphosphate hydrolase
  
Accession: EPE24494
  
Location: 1216276-1218847
  
 NCBI BlastP on this gene

EPE24494

Nucleotide-diphospho-sugar transferase
  
Accession: EPE24495
  
Location: 1219360-1220620
  
 NCBI BlastP on this gene

EPE24495

ARM repeat-containing protein
  
Accession: EPE24496
  
Location: 1221547-1224507
  
 NCBI BlastP on this gene

EPE24496

hypothetical protein
  
Accession: EPE24497
  
Location: 1228687-1229595
  
 NCBI BlastP on this gene

EPE24497

205. :  DS572718 Verticillium dahliae VdLs.17 supercont1.24 genomic scaffold     Total score: 2.0     Cumulative Blast bit score: 936

hypothetical protein
  
Accession: EGY18760
  
Location: 166788-168601
  
 NCBI BlastP on this gene

EGY18760

hypothetical protein
  
Accession: EGY18761
  
Location: 169495-170564
  
 NCBI BlastP on this gene

EGY18761

transport protein SEC31
  
Accession: EGY18762
  
Location: 171007-171715
  
 NCBI BlastP on this gene

EGY18762

transport protein SEC31
  
Accession: EGY18763
  
Location: 174103-174893
  
 NCBI BlastP on this gene

EGY18763

peptidyl-prolyl cis-trans isomerase E
  
Accession: EGY18764
  
Location: 175487-176001
  
 NCBI BlastP on this gene

EGY18764

hypothetical protein
  
Accession: EGY18765
  
Location: 176263-176781
  
 NCBI BlastP on this gene

EGY18765

cytochrome c oxidase polypeptide VIb
  
Accession: EGY18766
  
Location: 177107-177658
  
 NCBI BlastP on this gene

EGY18766

hypothetical protein
  
Accession: EGY18767
  
Location: 177950-178816
  
 NCBI BlastP on this gene

EGY18767

40S ribosomal protein S10-A
  
Accession: EGY18768
  
Location: 179411-180187
  
 NCBI BlastP on this gene

EGY18768

L-threonine 3-dehydrogenase
  
Accession: EGY18769
  
Location: 183567-184879
  
 NCBI BlastP on this gene

EGY18769

nuclear control of ATPase protein
  
Accession: EGY18770
  
Location: 185519-187739
  
  
**BlastP hit with Mycgr3G84646\_Mycgr3T**
  
Percentage identity: 42 %
  
BlastP bit score: 479
  
Sequence coverage: 88 %
  
E-value: 3e-156
  
  
 NCBI BlastP on this gene

EGY18770

C-4 methylsterol oxidase
  
Accession: EGY18771
  
Location: 189418-190500
  
  
**BlastP hit with Mycgr3G36271\_Mycgr3T**
  
Percentage identity: 71 %
  
BlastP bit score: 457
  
Sequence coverage: 97 %
  
E-value: 7e-159
  
  
 NCBI BlastP on this gene

EGY18771

hypothetical protein
  
Accession: EGY18772
  
Location: 190957-191593
  
 NCBI BlastP on this gene

EGY18772

hypothetical protein
  
Accession: EGY18773
  
Location: 197610-198532
  
 NCBI BlastP on this gene

EGY18773

hypothetical protein
  
Accession: EGY18774
  
Location: 198935-199474
  
 NCBI BlastP on this gene

EGY18774

hypothetical protein
  
Accession: EGY18775
  
Location: 201511-203903
  
 NCBI BlastP on this gene

EGY18775

hypothetical protein
  
Accession: EGY18776
  
Location: 205194-205631
  
 NCBI BlastP on this gene

EGY18776

hypothetical protein
  
Accession: EGY18777
  
Location: 205889-206485
  
 NCBI BlastP on this gene

EGY18777

Got1 family protein
  
Accession: EGY18778
  
Location: 207739-208449
  
 NCBI BlastP on this gene

EGY18778

profilin
  
Accession: EGY18779
  
Location: 209059-210466
  
 NCBI BlastP on this gene

EGY18779

206. :  CH476643 Sclerotinia sclerotiorum 1980 scaffold\_23 genomic scaffold     Total score: 2.0     Cumulative Blast bit score: 931

hypothetical protein
  
Accession: EDN98214
  
Location: 341846-347770
  
 NCBI BlastP on this gene

EDN98214

hypothetical protein
  
Accession: EDN98213
  
Location: 340713-341526
  
 NCBI BlastP on this gene

EDN98213

hypothetical protein
  
Accession: EDN98212
  
Location: 340028-340276
  
 NCBI BlastP on this gene

EDN98212

predicted protein
  
Accession: EDN98211
  
Location: 339553-339926
  
 NCBI BlastP on this gene

EDN98211

hypothetical protein
  
Accession: EDN98210
  
Location: 338046-338615
  
 NCBI BlastP on this gene

EDN98210

hypothetical protein
  
Accession: EDN98209
  
Location: 334483-337049
  
 NCBI BlastP on this gene

EDN98209

hypothetical protein
  
Accession: EDN98208
  
Location: 332590-333413
  
 NCBI BlastP on this gene

EDN98208

predicted protein
  
Accession: EDN98207
  
Location: 331390-331780
  
 NCBI BlastP on this gene

EDN98207

predicted protein
  
Accession: EDN98206
  
Location: 330893-331105
  
 NCBI BlastP on this gene

EDN98206

predicted protein
  
Accession: EDN98205
  
Location: 329051-330370
  
 NCBI BlastP on this gene

EDN98205

hypothetical protein
  
Accession: EDN98204
  
Location: 324128-326983
  
 NCBI BlastP on this gene

EDN98204

hypothetical protein
  
Accession: EDN98203
  
Location: 321257-323585
  
  
**BlastP hit with Mycgr3G84646\_Mycgr3T**
  
Percentage identity: 44 %
  
BlastP bit score: 580
  
Sequence coverage: 102 %
  
E-value: 0.0
  
  
 NCBI BlastP on this gene

EDN98203

GTP-binding protein SAS1
  
Accession: EDN98202
  
Location: 319222-320215
  
  
**BlastP hit with Mycgr3G99145\_Mycgr3T**
  
Percentage identity: 94 %
  
BlastP bit score: 351
  
Sequence coverage: 85 %
  
E-value: 2e-120
  
  
 NCBI BlastP on this gene

EDN98202

predicted protein
  
Accession: EDN98201
  
Location: 316128-317499
  
 NCBI BlastP on this gene

EDN98201

hypothetical protein
  
Accession: EDN98200
  
Location: 313417-315779
  
 NCBI BlastP on this gene

EDN98200

hypothetical protein
  
Accession: EDN98199
  
Location: 310839-311924
  
 NCBI BlastP on this gene

EDN98199

predicted protein
  
Accession: EDN98198
  
Location: 309010-310415
  
 NCBI BlastP on this gene

EDN98198

predicted protein
  
Accession: EDN98197
  
Location: 307206-307520
  
 NCBI BlastP on this gene

EDN98197

predicted protein
  
Accession: EDN98196
  
Location: 306001-306699
  
 NCBI BlastP on this gene

EDN98196

hypothetical protein
  
Accession: EDN98195
  
Location: 303221-305072
  
 NCBI BlastP on this gene

EDN98195

hypothetical protein
  
Accession: EDN98194
  
Location: 300735-302790
  
 NCBI BlastP on this gene

EDN98194

207. :  KB707720 Botryotinia fuckeliana BcDW1 unplaced genomic scaffold Scaffold\_48     Total score: 2.0     Cumulative Blast bit score: 925

putative activating signal cointegrator 1 complex subunit 3 protein
  
Accession: EMR89991
  
Location: 558000-564056
  
 NCBI BlastP on this gene

EMR89991

putative snare ykt6 protein
  
Accession: EMR89990
  
Location: 556889-557703
  
 NCBI BlastP on this gene

EMR89990

putative chromatin-remodeling complex subunit ies6 protein
  
Accession: EMR89989
  
Location: 554219-554788
  
 NCBI BlastP on this gene

EMR89989

putative dash complex subunit dad4 protein
  
Accession: EMR89988
  
Location: 553563-553984
  
 NCBI BlastP on this gene

EMR89988

putative chromatin remodeling complex subunit protein
  
Accession: EMR89987
  
Location: 550606-553199
  
 NCBI BlastP on this gene

EMR89987

putative transmembrane protein
  
Accession: EMR89986
  
Location: 548102-549581
  
 NCBI BlastP on this gene

EMR89986

hypothetical protein
  
Accession: EMR89985
  
Location: 540203-543064
  
 NCBI BlastP on this gene

EMR89985

putative atp synthase regulation protein nca2 protein
  
Accession: EMR89984
  
Location: 537203-539657
  
  
**BlastP hit with Mycgr3G84646\_Mycgr3T**
  
Percentage identity: 44 %
  
BlastP bit score: 570
  
Sequence coverage: 102 %
  
E-value: 0.0
  
  
 NCBI BlastP on this gene

EMR89984

putative rab gtpase protein
  
Accession: EMR89983
  
Location: 534779-535767
  
  
**BlastP hit with Mycgr3G99145\_Mycgr3T**
  
Percentage identity: 91 %
  
BlastP bit score: 355
  
Sequence coverage: 90 %
  
E-value: 8e-122
  
  
 NCBI BlastP on this gene

EMR89983

hypothetical protein
  
Accession: EMR89982
  
Location: 531974-533373
  
 NCBI BlastP on this gene

EMR89982

putative aaa family atpase protein
  
Accession: EMR89981
  
Location: 529202-531683
  
 NCBI BlastP on this gene

EMR89981

putative caleosin domain-containing protein
  
Accession: EMR89980
  
Location: 526996-528257
  
 NCBI BlastP on this gene

EMR89980

putative pterin-4-alpha-carbinolamine dehydratase family protein
  
Accession: EMR89979
  
Location: 525531-526568
  
 NCBI BlastP on this gene

EMR89979

putative aaa family atpase protein
  
Accession: EMR89978
  
Location: 520001-523417
  
 NCBI BlastP on this gene

EMR89978

hypothetical protein
  
Accession: EMR89977
  
Location: 517739-519507
  
 NCBI BlastP on this gene

EMR89977

putative solute carrier family 25 member 38 protein
  
Accession: EMR89976
  
Location: 515779-517130
  
 NCBI BlastP on this gene

EMR89976

208. :  FQ790356 Botryotinia fuckeliana T4 SupSuperContig\_182\_154r\_1 genomic supercontig.     Total score: 2.0     Cumulative Blast bit score: 925

similar to snare protein ykt6
  
Accession: CCD55628
  
Location: 84189-85003
  
 NCBI BlastP on this gene

BofuT4\_P154400.1

similar to Golgi transport protein
  
Accession: CCD55629
  
Location: 85457-86169
  
 NCBI BlastP on this gene

BofuT4\_P154410.1

similar to chromatin-remodeling complex subunit ies6
  
Accession: CCD55630
  
Location: 87336-87905
  
 NCBI BlastP on this gene

BofuT4\_P154420.1

hypothetical protein
  
Accession: CCD55631
  
Location: 88322-88561
  
 NCBI BlastP on this gene

BofuT4\_P154430.1

similar to chromatin remodeling complex subunit Arp5
  
Accession: CCD55632
  
Location: 88925-91519
  
 NCBI BlastP on this gene

BofuT4\_P154440.1

similar to transcription factor bZIP
  
Accession: CCD55633
  
Location: 92561-93397
  
 NCBI BlastP on this gene

BofuT4\_P154450.1

hypothetical protein
  
Accession: CCD55634
  
Location: 94770-95521
  
 NCBI BlastP on this gene

BofuT4\_P154460.1

hypothetical protein
  
Accession: CCD55635
  
Location: 96114-96327
  
 NCBI BlastP on this gene

BofuT4\_uP154470.1

hypothetical protein
  
Accession: CCD55636
  
Location: 99433-102294
  
 NCBI BlastP on this gene

BofuT4\_P154480.1

similar to nuclear control of ATPase protein
  
Accession: CCD55637
  
Location: 102829-105289
  
  
**BlastP hit with Mycgr3G84646\_Mycgr3T**
  
Percentage identity: 44 %
  
BlastP bit score: 570
  
Sequence coverage: 102 %
  
E-value: 0.0
  
  
 NCBI BlastP on this gene

BofuT4\_P154490.1

similar to GTP-binding protein
  
Accession: CCD55638
  
Location: 106735-107723
  
  
**BlastP hit with Mycgr3G99145\_Mycgr3T**
  
Percentage identity: 91 %
  
BlastP bit score: 355
  
Sequence coverage: 90 %
  
E-value: 8e-122
  
  
 NCBI BlastP on this gene

BofuT4\_P154500.1

hypothetical protein
  
Accession: CCD55639
  
Location: 118274-119434
  
 NCBI BlastP on this gene

BofuT4\_P154510.1

similar to AAA family ATPase
  
Accession: CCD55640
  
Location: 119725-122206
  
 NCBI BlastP on this gene

BofuT4\_P154520.1

hypothetical protein
  
Accession: CCD55641
  
Location: 123151-124412
  
 NCBI BlastP on this gene

BofuT4\_P154530.1

hypothetical protein
  
Accession: CCD55642
  
Location: 124840-125877
  
 NCBI BlastP on this gene

BofuT4\_P154540.1

hypothetical protein
  
Accession: CCD55643
  
Location: 127142-127882
  
 NCBI BlastP on this gene

BofuT4\_P154550.1

209. :  JH921428 Marssonina brunnea f. sp. 'multigermtubi' MB\_m1 unplaced genomic scaffold M6\_S00001     Total score: 2.0     Cumulative Blast bit score: 916

60S ribosomal protein L37
  
Accession: EKD21497
  
Location: 3270392-3270988
  
 NCBI BlastP on this gene

EKD21497

hypothetical protein
  
Accession: EKD21498
  
Location: 3271295-3272281
  
 NCBI BlastP on this gene

EKD21498

FAD binding domain protein
  
Accession: EKD21499
  
Location: 3272649-3274169
  
 NCBI BlastP on this gene

EKD21499

guanine deaminase
  
Accession: EKD21500
  
Location: 3274735-3276168
  
 NCBI BlastP on this gene

EKD21500

putative Lysophospholipase NTE1
  
Accession: EKD21501
  
Location: 3276623-3281467
  
 NCBI BlastP on this gene

EKD21501

eukaryotic translation initiation factor 2 alpha subunit
  
Accession: EKD21502
  
Location: 3281925-3283022
  
 NCBI BlastP on this gene

EKD21502

hypothetical protein
  
Accession: EKD21503
  
Location: 3283348-3283990
  
 NCBI BlastP on this gene

EKD21503

hypothetical protein
  
Accession: EKD21504
  
Location: 3284651-3286002
  
 NCBI BlastP on this gene

EKD21504

hypothetical protein
  
Accession: EKD21505
  
Location: 3286559-3289546
  
 NCBI BlastP on this gene

EKD21505

ATP synthase regulation protein NCA2
  
Accession: EKD21506
  
Location: 3289990-3292421
  
  
**BlastP hit with Mycgr3G84646\_Mycgr3T**
  
Percentage identity: 43 %
  
BlastP bit score: 558
  
Sequence coverage: 100 %
  
E-value: 0.0
  
  
 NCBI BlastP on this gene

EKD21506

secretion related Rab/GTPase
  
Accession: EKD21507
  
Location: 3293067-3293818
  
  
**BlastP hit with Mycgr3G99145\_Mycgr3T**
  
Percentage identity: 86 %
  
BlastP bit score: 359
  
Sequence coverage: 99 %
  
E-value: 1e-123
  
  
 NCBI BlastP on this gene

EKD21507

hypothetical protein
  
Accession: EKD21508
  
Location: 3294045-3295494
  
 NCBI BlastP on this gene

EKD21508

ATPase
  
Accession: EKD21509
  
Location: 3296181-3298559
  
 NCBI BlastP on this gene

EKD21509

caleosin domain containing protein
  
Accession: EKD21510
  
Location: 3298966-3300070
  
 NCBI BlastP on this gene

EKD21510

glycosyl transferase family 2
  
Accession: EKD21511
  
Location: 3301724-3303029
  
 NCBI BlastP on this gene

EKD21511

suppressor of Mek1
  
Accession: EKD21512
  
Location: 3303713-3306747
  
 NCBI BlastP on this gene

EKD21512

hypothetical protein
  
Accession: EKD21513
  
Location: 3310446-3312294
  
 NCBI BlastP on this gene

EKD21513

hypothetical protein
  
Accession: EKD21514
  
Location: 3312394-3313297
  
 NCBI BlastP on this gene

EKD21514

210. :  GL988041 Chaetomium thermophilum var. thermophilum DSM 1495 unplaced genomic scaffold scf7180000...     Total score: 2.0     Cumulative Blast bit score: 915

ion channel-like protein
  
Accession: EGS21401
  
Location: 4300558-4302395
  
 NCBI BlastP on this gene

EGS21401

hypothetical protein
  
Accession: EGS21402
  
Location: 4306797-4313738
  
 NCBI BlastP on this gene

EGS21402

hypothetical protein
  
Accession: EGS21403
  
Location: 4315506-4316352
  
 NCBI BlastP on this gene

EGS21403

hypothetical protein
  
Accession: EGS21404
  
Location: 4318402-4320749
  
  
**BlastP hit with Mycgr3G84646\_Mycgr3T**
  
Percentage identity: 42 %
  
BlastP bit score: 493
  
Sequence coverage: 90 %
  
E-value: 4e-161
  
  
 NCBI BlastP on this gene

EGS21404

hypothetical protein
  
Accession: EGS21405
  
Location: 4322359-4323490
  
  
**BlastP hit with Mycgr3G36271\_Mycgr3T**
  
Percentage identity: 68 %
  
BlastP bit score: 422
  
Sequence coverage: 97 %
  
E-value: 1e-144
  
  
 NCBI BlastP on this gene

EGS21405

hypothetical protein
  
Accession: EGS21406
  
Location: 4323958-4324879
  
 NCBI BlastP on this gene

EGS21406

putative sequence-specific DNA binding protein
  
Accession: EGS21407
  
Location: 4326400-4329440
  
 NCBI BlastP on this gene

EGS21407

hypothetical protein
  
Accession: EGS21408
  
Location: 4330182-4333803
  
 NCBI BlastP on this gene

EGS21408

hypothetical protein
  
Accession: EGS21409
  
Location: 4334121-4334780
  
 NCBI BlastP on this gene

EGS21409

hypothetical protein
  
Accession: EGS21410
  
Location: 4335138-4335667
  
 NCBI BlastP on this gene

EGS21410

putative golgi transport protein
  
Accession: EGS21411
  
Location: 4336769-4337495
  
 NCBI BlastP on this gene

EGS21411

hypothetical protein
  
Accession: EGS21412
  
Location: 4338813-4339735
  
 NCBI BlastP on this gene

EGS21412

hypothetical protein
  
Accession: EGS21413
  
Location: 4343130-4346489
  
 NCBI BlastP on this gene

EGS21413

211. :  CACQ02000613 Colletotrichum higginsianum strain IMI 349063     Total score: 2.0     Cumulative Blast bit score: 912

ATP synthase regulation protein NCA2
  
Accession: CCF33019
  
Location: 2067-4171
  
  
**BlastP hit with Mycgr3G84646\_Mycgr3T**
  
Percentage identity: 44 %
  
BlastP bit score: 557
  
Sequence coverage: 101 %
  
E-value: 0.0
  
  
 NCBI BlastP on this gene

CCF33019

Ras-like protein Rab-8A
  
Accession: CCF33020
  
Location: 5662-6556
  
  
**BlastP hit with Mycgr3G99145\_Mycgr3T**
  
Percentage identity: 84 %
  
BlastP bit score: 356
  
Sequence coverage: 99 %
  
E-value: 3e-122
  
  
 NCBI BlastP on this gene

CCF33020

hypothetical protein
  
Accession: CCF33021
  
Location: 7296-8918
  
 NCBI BlastP on this gene

CCF33021

ATPase
  
Accession: CCF33022
  
Location: 9790-12217
  
 NCBI BlastP on this gene

CCF33022

squalene epoxidase
  
Accession: CCF33023
  
Location: 14119-15624
  
 NCBI BlastP on this gene

CCF33023

212. :  CU638744 Podospora anserina S mat+ genomic DNA chromosome 6, supercontig 2.     Total score: 2.0     Cumulative Blast bit score: 903

not annotated
  
Accession: CAP71229
  
Location: 903275-904577
  
 NCBI BlastP on this gene

CAP71229

not annotated
  
Accession: CAP71230
  
Location: 905327-905941
  
 NCBI BlastP on this gene

CAP71230

not annotated
  
Accession: CAP71231
  
Location: 906488-907586
  
 NCBI BlastP on this gene

CAP71231

not annotated
  
Accession: CAP71232
  
Location: 908423-912982
  
 NCBI BlastP on this gene

CAP71232

not annotated
  
Accession: CAP71233
  
Location: 913476-914753
  
 NCBI BlastP on this gene

CAP71233

not annotated
  
Accession: CAP71234
  
Location: 915059-917374
  
 NCBI BlastP on this gene

CAP71234

not annotated
  
Accession: CAP71235
  
Location: 918669-919811
  
 NCBI BlastP on this gene

CAP71235

not annotated
  
Accession: CAP71236
  
Location: 920895-921999
  
  
**BlastP hit with Mycgr3G36271\_Mycgr3T**
  
Percentage identity: 69 %
  
BlastP bit score: 447
  
Sequence coverage: 98 %
  
E-value: 3e-155
  
  
 NCBI BlastP on this gene

CAP71236

not annotated
  
Accession: CAP71237
  
Location: 923198-925464
  
  
**BlastP hit with Mycgr3G84646\_Mycgr3T**
  
Percentage identity: 39 %
  
BlastP bit score: 456
  
Sequence coverage: 108 %
  
E-value: 8e-147
  
  
 NCBI BlastP on this gene

CAP71237

tRNA-Ala
  
Accession: CAP71238
  
Location: 926368-927182
  
 NCBI BlastP on this gene

CAP71238

not annotated
  
Accession: CAP71239
  
Location: 930372-931421
  
 NCBI BlastP on this gene

CAP71239

not annotated
  
Accession: CAP71240
  
Location: 933731-934204
  
 NCBI BlastP on this gene

CAP71240

not annotated
  
Accession: CAP71241
  
Location: 939483-940483
  
 NCBI BlastP on this gene

CAP71241

tRNA-Arg
  
Accession: CAP71242
  
Location: 942643-946596
  
 NCBI BlastP on this gene

CAP71242

213. :  JH226136 Exophiala dermatitidis NIH/UT8656 unplaced genomic scaffold supercont1.7     Total score: 2.0     Cumulative Blast bit score: 884

hypothetical protein
  
Accession: EHY60550
  
Location: 1953958-1955725
  
 NCBI BlastP on this gene

EHY60550

hypothetical protein
  
Accession: EHY60551
  
Location: 1957271-1957708
  
 NCBI BlastP on this gene

EHY60551

hypothetical protein
  
Accession: EHY60552
  
Location: 1958956-1960023
  
 NCBI BlastP on this gene

EHY60552

hypothetical protein
  
Accession: EHY60553
  
Location: 1961023-1961700
  
 NCBI BlastP on this gene

EHY60553

hypothetical protein
  
Accession: EHY60554
  
Location: 1961875-1962518
  
 NCBI BlastP on this gene

EHY60554

hypothetical protein
  
Accession: EHY60555
  
Location: 1964768-1967450
  
 NCBI BlastP on this gene

EHY60555

hypothetical protein
  
Accession: EHY60556
  
Location: 1968311-1971445
  
 NCBI BlastP on this gene

EHY60556

hypothetical protein
  
Accession: EHY60557
  
Location: 1973332-1975362
  
  
**BlastP hit with Mycgr3G84646\_Mycgr3T**
  
Percentage identity: 41 %
  
BlastP bit score: 516
  
Sequence coverage: 100 %
  
E-value: 2e-171
  
  
 NCBI BlastP on this gene

EHY60557

GTP-binding protein ypt2
  
Accession: EHY60558
  
Location: 1976922-1977744
  
  
**BlastP hit with Mycgr3G99145\_Mycgr3T**
  
Percentage identity: 84 %
  
BlastP bit score: 368
  
Sequence coverage: 101 %
  
E-value: 9e-127
  
  
 NCBI BlastP on this gene

EHY60558

profilin
  
Accession: EHY60559
  
Location: 1978587-1979345
  
 NCBI BlastP on this gene

EHY60559

hypothetical protein
  
Accession: EHY60560
  
Location: 1980774-1981773
  
 NCBI BlastP on this gene

EHY60560

hypothetical protein
  
Accession: EHY60561
  
Location: 1983354-1985286
  
 NCBI BlastP on this gene

EHY60561

hypothetical protein
  
Accession: EHY60562
  
Location: 1986139-1989289
  
 NCBI BlastP on this gene

EHY60562

hypothetical protein
  
Accession: EHY60563
  
Location: 1989855-1994509
  
 NCBI BlastP on this gene

EHY60563

hypothetical protein
  
Accession: EHY60564
  
Location: 1996491-1996712
  
 NCBI BlastP on this gene

EHY60564

214. :  GG663377 Ajellomyces capsulatus G186AR genomic scaffold supercont2.15     Total score: 2.0     Cumulative Blast bit score: 865

conserved hypothetical protein
  
Accession: EEH03402
  
Location: 333687-336407
  
 NCBI BlastP on this gene

EEH03402

protein kinase
  
Accession: EEH03403
  
Location: 338652-340181
  
 NCBI BlastP on this gene

EEH03403

conserved hypothetical protein
  
Accession: EEH03404
  
Location: 341631-343823
  
  
**BlastP hit with Mycgr3G84646\_Mycgr3T**
  
Percentage identity: 38 %
  
BlastP bit score: 436
  
Sequence coverage: 102 %
  
E-value: 8e-140
  
  
 NCBI BlastP on this gene

EEH03404

predicted protein
  
Accession: EEH03405
  
Location: 344338-344706
  
 NCBI BlastP on this gene

EEH03405

C-4 methylsterol oxidase
  
Accession: EEH03406
  
Location: 344964-345899
  
  
**BlastP hit with Mycgr3G36271\_Mycgr3T**
  
Percentage identity: 80 %
  
BlastP bit score: 429
  
Sequence coverage: 84 %
  
E-value: 1e-148
  
  
 NCBI BlastP on this gene

EEH03406

DUF652 domain-containing protein
  
Accession: EEH03407
  
Location: 346868-347884
  
 NCBI BlastP on this gene

EEH03407

conserved hypothetical protein
  
Accession: EEH03408
  
Location: 348977-351373
  
 NCBI BlastP on this gene

EEH03408

conserved hypothetical protein
  
Accession: EEH03409
  
Location: 351923-352762
  
 NCBI BlastP on this gene

EEH03409

U3 small nucleolar RNA-associated protein
  
Accession: EEH03410
  
Location: 353868-357685
  
 NCBI BlastP on this gene

EEH03410

inositol pyrophosphate synthase
  
Accession: EEH03411
  
Location: 358484-363532
  
 NCBI BlastP on this gene

EEH03411

conserved hypothetical protein
  
Accession: EEH03412
  
Location: 364076-365647
  
 NCBI BlastP on this gene

EEH03412

conserved hypothetical protein
  
Accession: EEH03413
  
Location: 365863-369933
  
 NCBI BlastP on this gene

EEH03413

215. :  DS990639 Ajellomyces capsulatus H88 supercont1.4 genomic scaffold     Total score: 2.0     Cumulative Blast bit score: 850

cortical actin cytoskeleton protein asp1
  
Accession: EGC45488
  
Location: 756516-761566
  
 NCBI BlastP on this gene

EGC45488

beta transducin
  
Accession: EGC45489
  
Location: 762380-766208
  
 NCBI BlastP on this gene

EGC45489

serine protein kinase
  
Accession: EGC45490
  
Location: 768870-770207
  
 NCBI BlastP on this gene

EGC45490

conserved hypothetical protein
  
Accession: EGC45491
  
Location: 771072-773703
  
 NCBI BlastP on this gene

EGC45491

conserved hypothetical protein
  
Accession: EGC45492
  
Location: 774493-775338
  
 NCBI BlastP on this gene

EGC45492

conserved hypothetical protein
  
Accession: EGC45493
  
Location: 775874-778278
  
 NCBI BlastP on this gene

EGC45493

hypothetical protein
  
Accession: EGC45494
  
Location: 779371-780379
  
 NCBI BlastP on this gene

EGC45494

C4-methylsterol oxidase
  
Accession: EGC45495
  
Location: 781335-782266
  
  
**BlastP hit with Mycgr3G36271\_Mycgr3T**
  
Percentage identity: 80 %
  
BlastP bit score: 414
  
Sequence coverage: 81 %
  
E-value: 1e-142
  
  
 NCBI BlastP on this gene

EGC45495

conserved hypothetical protein
  
Accession: EGC45496
  
Location: 782538-785599
  
  
**BlastP hit with Mycgr3G84646\_Mycgr3T**
  
Percentage identity: 38 %
  
BlastP bit score: 436
  
Sequence coverage: 100 %
  
E-value: 9e-139
  
  
 NCBI BlastP on this gene

EGC45496

conserved hypothetical protein
  
Accession: EGC45497
  
Location: 788893-791085
  
 NCBI BlastP on this gene

EGC45497

protein kinase
  
Accession: EGC45498
  
Location: 792720-794115
  
 NCBI BlastP on this gene

EGC45498

C2H2 finger domain-containing protein
  
Accession: EGC45499
  
Location: 795973-798708
  
 NCBI BlastP on this gene

EGC45499

conserved hypothetical protein
  
Accession: EGC45500
  
Location: 800605-803091
  
 NCBI BlastP on this gene

EGC45500

conserved hypothetical protein
  
Accession: EGC45501
  
Location: 803160-804065
  
 NCBI BlastP on this gene

EGC45501

predicted protein
  
Accession: EGC45502
  
Location: 804347-805908
  
 NCBI BlastP on this gene

EGC45502

216. :  CAUH01003861 Blumeria graminis f. sp. hordei DH14     Total score: 2.0     Cumulative Blast bit score: 841

eIF-2-alpha/eukaryotic translation initiation factor 2 alpha subunit
  
Accession: CCU77668
  
Location: 43118-44228
  
 NCBI BlastP on this gene

CCU77668

mitochondrial inner membrane protease subunit 2
  
Accession: CCU77667
  
Location: 42197-42848
  
 NCBI BlastP on this gene

CCU77667

mitochondrial carrier protein
  
Accession: CCU77666
  
Location: 40610-41906
  
 NCBI BlastP on this gene

CCU77666

hypothetical protein
  
Accession: CCU77665
  
Location: 24304-27111
  
 NCBI BlastP on this gene

CCU77665

NCA2/nuclear control of ATPase
  
Accession: CCU77663
  
Location: 21842-23877
  
  
**BlastP hit with Mycgr3G84646\_Mycgr3T**
  
Percentage identity: 39 %
  
BlastP bit score: 488
  
Sequence coverage: 100 %
  
E-value: 4e-160
  
  
 NCBI BlastP on this gene

CCU77663

GTP-binding protein SAS1
  
Accession: CCU77662
  
Location: 20519-21256
  
  
**BlastP hit with Mycgr3G99145\_Mycgr3T**
  
Percentage identity: 83 %
  
BlastP bit score: 353
  
Sequence coverage: 99 %
  
E-value: 4e-121
  
  
 NCBI BlastP on this gene

CCU77662

hypothetical protein
  
Accession: CCU77661
  
Location: 18567-20356
  
 NCBI BlastP on this gene

CCU77661

217. :  DS544805 Paracoccidioides brasiliensis Pb03 supercont1.3 genomic scaffold     Total score: 2.0     Cumulative Blast bit score: 834

predicted protein
  
Accession: EEH19631
  
Location: 6104-6775
  
 NCBI BlastP on this gene

EEH19631

predicted protein
  
Accession: EEH19632
  
Location: 7191-9169
  
 NCBI BlastP on this gene

EEH19632

hypothetical protein
  
Accession: EEH19633
  
Location: 12848-14711
  
 NCBI BlastP on this gene

EEH19633

conserved hypothetical protein
  
Accession: EEH19634
  
Location: 16936-19371
  
  
**BlastP hit with Mycgr3G84646\_Mycgr3T**
  
Percentage identity: 40 %
  
BlastP bit score: 488
  
Sequence coverage: 103 %
  
E-value: 2e-159
  
  
 NCBI BlastP on this gene

EEH19634

predicted protein
  
Accession: EEH19635
  
Location: 19659-21169
  
 NCBI BlastP on this gene

EEH19635

conserved hypothetical protein
  
Accession: EEH19636
  
Location: 22377-23717
  
 NCBI BlastP on this gene

EEH19636

GTP-binding protein SAS1
  
Accession: EEH19637
  
Location: 25012-25877
  
  
**BlastP hit with Mycgr3G99145\_Mycgr3T**
  
Percentage identity: 83 %
  
BlastP bit score: 346
  
Sequence coverage: 99 %
  
E-value: 2e-118
  
  
 NCBI BlastP on this gene

EEH19637

predicted protein
  
Accession: EEH19638
  
Location: 26789-28064
  
 NCBI BlastP on this gene

EEH19638

cell division cycle protein
  
Accession: EEH19639
  
Location: 28704-31052
  
 NCBI BlastP on this gene

EEH19639

hypothetical protein
  
Accession: EEH19640
  
Location: 31446-32644
  
 NCBI BlastP on this gene

EEH19640

protein kinase rad3
  
Accession: EEH19641
  
Location: 32949-41818
  
 NCBI BlastP on this gene

EEH19641

tRNA-specific adenosine deaminase subunit TAD2
  
Accession: EEH19642
  
Location: 42153-43018
  
 NCBI BlastP on this gene

EEH19642

conserved hypothetical protein
  
Accession: EEH19643
  
Location: 43250-44099
  
 NCBI BlastP on this gene

EEH19643

predicted protein
  
Accession: EEH19644
  
Location: 44707-45804
  
 NCBI BlastP on this gene

EEH19644

218. :  DS027045 Aspergillus clavatus NRRL 1 1099423829791 genomic scaffold     Total score: 2.0     Cumulative Blast bit score: 834

C-4 methyl sterol oxidase (Erg25), putative
  
Accession: EAW13770
  
Location: 62225-63082
  
  
**BlastP hit with Mycgr3G36271\_Mycgr3T**
  
Percentage identity: 80 %
  
BlastP bit score: 417
  
Sequence coverage: 80 %
  
E-value: 3e-144
  
  
 NCBI BlastP on this gene

EAW13770

conserved hypothetical protein
  
Accession: EAW13771
  
Location: 64637-66332
  
  
**BlastP hit with Mycgr3G84646\_Mycgr3T**
  
Percentage identity: 40 %
  
BlastP bit score: 417
  
Sequence coverage: 79 %
  
E-value: 3e-134
  
  
 NCBI BlastP on this gene

EAW13771

ubiquitin C-terminal hydrolase, putative
  
Accession: EAW13772
  
Location: 66542-69177
  
 NCBI BlastP on this gene

EAW13772

replication factor A 1, rfa1
  
Accession: EAW13773
  
Location: 70194-72332
  
 NCBI BlastP on this gene

EAW13773

dead box ATP-dependent rna helicase
  
Accession: EAW13774
  
Location: 73527-75166
  
 NCBI BlastP on this gene

EAW13774

Rft domain protein
  
Accession: EAW13775
  
Location: 75169-76650
  
 NCBI BlastP on this gene

EAW13775

oligosaccharyl transferase subunit (gamma), putative
  
Accession: EAW13776
  
Location: 77613-78780
  
 NCBI BlastP on this gene

EAW13776

hypothetical protein
  
Accession: EAW13777
  
Location: 79489-81063
  
 NCBI BlastP on this gene

EAW13777

cell division protein ftsj
  
Accession: EAW13778
  
Location: 82264-83467
  
 NCBI BlastP on this gene

EAW13778

autophagy ubiquitin-activating enzyme ApgG, putative
  
Accession: EAW13779
  
Location: 83586-86151
  
 NCBI BlastP on this gene

EAW13779

219. :  KE374991 Blumeria graminis f. sp. tritici 96224 unplaced genomic scaffold Scaffold-128     Total score: 2.0     Cumulative Blast bit score: 830

Serine esterase
  
Accession: EPQ66681
  
Location: 131024-135445
  
 NCBI BlastP on this gene

EPQ66681

Alpha subunit of the translation initiation factor eIF2
  
Accession: EPQ66682
  
Location: 136099-136429
  
 NCBI BlastP on this gene

EPQ66682

hypothetical protein
  
Accession: EPQ66683
  
Location: 136535-137209
  
 NCBI BlastP on this gene

EPQ66683

Catalytic subunit of the mitochondrial inner membrane peptidase complex
  
Accession: EPQ66684
  
Location: 137480-138132
  
 NCBI BlastP on this gene

EPQ66684

transporter of the mitochondrial inner membrane
  
Accession: EPQ66685
  
Location: 138426-139722
  
 NCBI BlastP on this gene

EPQ66685

hypothetical protein
  
Accession: EPQ66686
  
Location: 146089-148896
  
 NCBI BlastP on this gene

EPQ66686

hypothetical protein
  
Accession: EPQ66687
  
Location: 149325-151360
  
  
**BlastP hit with Mycgr3G84646\_Mycgr3T**
  
Percentage identity: 38 %
  
BlastP bit score: 475
  
Sequence coverage: 100 %
  
E-value: 9e-155
  
  
 NCBI BlastP on this gene

EPQ66687

Secretory vesicle-associated Rab GTPase
  
Accession: EPQ66688
  
Location: 151948-152685
  
  
**BlastP hit with Mycgr3G99145\_Mycgr3T**
  
Percentage identity: 84 %
  
BlastP bit score: 355
  
Sequence coverage: 99 %
  
E-value: 7e-122
  
  
 NCBI BlastP on this gene

EPQ66688

hypothetical protein
  
Accession: EPQ66689
  
Location: 152848-154636
  
 NCBI BlastP on this gene

EPQ66689

220. :  FP929137 Leptosphaeria maculans JN3 lm\_SuperContig\_10\_v2 genomic supercontig     Total score: 2.0     Cumulative Blast bit score: 810

predicted protein
  
Accession: CBY00095
  
Location: 1616833-1617024
  
 NCBI BlastP on this gene

LEMA\_uP076840.1

similar to DNA repair protein Rhp26/Rad26
  
Accession: CBY00094
  
Location: 1612589-1616263
  
 NCBI BlastP on this gene

LEMA\_P076830.1

predicted protein
  
Accession: CBY00093
  
Location: 1611614-1611859
  
 NCBI BlastP on this gene

LEMA\_P076820.1

hypothetical protein
  
Accession: CBY00092
  
Location: 1604091-1605375
  
  
**BlastP hit with Mycgr3G36271\_Mycgr3T**
  
Percentage identity: 78 %
  
BlastP bit score: 475
  
Sequence coverage: 97 %
  
E-value: 3e-165
  
  
 NCBI BlastP on this gene

LEMA\_P076810.1

hypothetical protein
  
Accession: CBY00091
  
Location: 1602045-1603501
  
 NCBI BlastP on this gene

LEMA\_P076800.1

hypothetical protein
  
Accession: CBY00090
  
Location: 1600793-1601514
  
 NCBI BlastP on this gene

LEMA\_P076790.1

hypothetical protein
  
Accession: CBY00089
  
Location: 1596937-1600039
  
 NCBI BlastP on this gene

LEMA\_P076780.1

hypothetical protein
  
Accession: CBY00088
  
Location: 1594876-1595501
  
 NCBI BlastP on this gene

LEMA\_P076770.1

similar to ubiquitin-conjugating enzyme
  
Accession: CBY00087
  
Location: 1593095-1593922
  
 NCBI BlastP on this gene

LEMA\_P076760.1

similar to mitochondrial outer membrane protein (Sam50)
  
Accession: CBY00086
  
Location: 1591028-1592817
  
 NCBI BlastP on this gene

LEMA\_P076750.1

hypothetical protein
  
Accession: CBY00085
  
Location: 1587412-1590210
  
 NCBI BlastP on this gene

LEMA\_P076740.1

hypothetical protein
  
Accession: CBY00084
  
Location: 1583920-1585846
  
 NCBI BlastP on this gene

LEMA\_P076730.1

similar to xanthine phosphoribosyltransferase
  
Accession: CBY00083
  
Location: 1582161-1583053
  
  
**BlastP hit with Mycgr3G55345\_Mycgr3T**
  
Percentage identity: 80 %
  
BlastP bit score: 335
  
Sequence coverage: 95 %
  
E-value: 1e-113
  
  
 NCBI BlastP on this gene

LEMA\_P076720.1

predicted protein
  
Accession: CBY00082
  
Location: 1581098-1581253
  
 NCBI BlastP on this gene

LEMA\_P076710.1

hypothetical protein
  
Accession: CBY00081
  
Location: 1578259-1580625
  
 NCBI BlastP on this gene

LEMA\_P076700.1

predicted protein
  
Accession: CBY00080
  
Location: 1576541-1577198
  
 NCBI BlastP on this gene

LEMA\_P076690.1

predicted protein
  
Accession: CBY00079
  
Location: 1575621-1576371
  
 NCBI BlastP on this gene

LEMA\_P076680.1

predicted protein
  
Accession: CBY00078
  
Location: 1574997-1575290
  
 NCBI BlastP on this gene

LEMA\_P076670.1

similar to ubiquitin-conjugating enzyme E2
  
Accession: CBY00077
  
Location: 1574059-1574665
  
 NCBI BlastP on this gene

LEMA\_P076660.1

predicted protein
  
Accession: CBY00076
  
Location: 1573044-1573382
  
 NCBI BlastP on this gene

LEMA\_P076650.1

hypothetical protein
  
Accession: CBY00075
  
Location: 1569971-1572972
  
 NCBI BlastP on this gene

LEMA\_P076640.1

221. :  AAHF01000016 Aspergillus fumigatus Af293     Total score: 2.0     Cumulative Blast bit score: 809

replication fork protection component Swi3, putative
  
Accession: EBA27200
  
Location: 629994-630926
  
 NCBI BlastP on this gene

EBA27200

NADH-ubiquinone oxidoreductase 21 kDa subunit, putative
  
Accession: EAL84623
  
Location: 631866-632422
  
 NCBI BlastP on this gene

EAL84623

inositol kinase kinase (UvsB), putative
  
Accession: EAL84624
  
Location: 634125-642965
  
 NCBI BlastP on this gene

EAL84624

AAA family ATPase, putative
  
Accession: EAL84628
  
Location: 644717-647044
  
 NCBI BlastP on this gene

EAL84628

Rab GTPase SrgA, putative
  
Accession: EAL84629
  
Location: 647907-648651
  
  
**BlastP hit with Mycgr3G99145\_Mycgr3T**
  
Percentage identity: 89 %
  
BlastP bit score: 340
  
Sequence coverage: 87 %
  
E-value: 1e-115
  
  
 NCBI BlastP on this gene

EAL84629

C-4 methyl sterol oxidase Erg25, putative
  
Accession: EAL84630
  
Location: 653072-654129
  
  
**BlastP hit with Mycgr3G36271\_Mycgr3T**
  
Percentage identity: 75 %
  
BlastP bit score: 469
  
Sequence coverage: 95 %
  
E-value: 7e-164
  
  
 NCBI BlastP on this gene

EAL84630

222. :  CH408034 Chaetomium globosum CBS 148.51 scaffold\_6 genomic scaffold     Total score: 2.0     Cumulative Blast bit score: 793

hypothetical protein
  
Accession: EAQ84787
  
Location: 1431377-1432347
  
 NCBI BlastP on this gene

EAQ84787

conserved hypothetical protein
  
Accession: EAQ84788
  
Location: 1437231-1438556
  
 NCBI BlastP on this gene

EAQ84788

predicted protein
  
Accession: EAQ84789
  
Location: 1439143-1439926
  
 NCBI BlastP on this gene

EAQ84789

hypothetical protein
  
Accession: EAQ84790
  
Location: 1440788-1442685
  
 NCBI BlastP on this gene

EAQ84790

hypothetical protein
  
Accession: EAQ84791
  
Location: 1443880-1445941
  
 NCBI BlastP on this gene

EAQ84791

predicted protein
  
Accession: EAQ84792
  
Location: 1446677-1448003
  
 NCBI BlastP on this gene

EAQ84792

hypothetical protein
  
Accession: EAQ84793
  
Location: 1448878-1452371
  
  
**BlastP hit with Mycgr3G68433\_Mycgr3T**
  
Percentage identity: 37 %
  
BlastP bit score: 484
  
Sequence coverage: 96 %
  
E-value: 1e-148
  
  
 NCBI BlastP on this gene

EAQ84793

hypothetical protein
  
Accession: EAQ84794
  
Location: 1453755-1454221
  
 NCBI BlastP on this gene

EAQ84794

hypothetical protein
  
Accession: EAQ84795
  
Location: 1455641-1456847
  
 NCBI BlastP on this gene

EAQ84795

predicted protein
  
Accession: EAQ84796
  
Location: 1457200-1458630
  
 NCBI BlastP on this gene

EAQ84796

conserved hypothetical protein
  
Accession: EAQ84797
  
Location: 1459088-1459867
  
  
**BlastP hit with Mycgr3G55345\_Mycgr3T**
  
Percentage identity: 74 %
  
BlastP bit score: 309
  
Sequence coverage: 99 %
  
E-value: 1e-103
  
  
 NCBI BlastP on this gene

EAQ84797

hypothetical protein
  
Accession: EAQ84798
  
Location: 1461272-1462414
  
 NCBI BlastP on this gene

EAQ84798

hypothetical protein
  
Accession: EAQ84799
  
Location: 1463789-1464340
  
 NCBI BlastP on this gene

EAQ84799

hypothetical protein
  
Accession: EAQ84800
  
Location: 1466459-1467697
  
 NCBI BlastP on this gene

EAQ84800

predicted protein
  
Accession: EAQ84801
  
Location: 1469841-1471274
  
 NCBI BlastP on this gene

EAQ84801

hypothetical protein
  
Accession: EAQ84802
  
Location: 1472181-1474545
  
 NCBI BlastP on this gene

EAQ84802

predicted protein
  
Accession: EAQ84803
  
Location: 1474922-1475612
  
 NCBI BlastP on this gene

EAQ84803

predicted protein
  
Accession: EAQ84804
  
Location: 1476811-1477149
  
 NCBI BlastP on this gene

EAQ84804

223. :  AM920430 Penicillium chrysogenum Wisconsin 54-1255 complete genome, contig Pc00c15.     Total score: 2.0     Cumulative Blast bit score: 788

not annotated
  
Accession: CAP82896
  
Location: 22858-23857
  
 NCBI BlastP on this gene

Pc15g00100

unnamed
  
Accession: CAP82897
  
Location: 24264-25199
  
 NCBI BlastP on this gene

Pc15g00110

not annotated
  
Accession: CAP82898
  
Location: 25458-27296
  
 NCBI BlastP on this gene

Pc15g00120

not annotated
  
Accession: CAP82899
  
Location: 27929-28997
  
 NCBI BlastP on this gene

Pc15g00130

not annotated
  
Accession: CAP82900
  
Location: 29714-30998
  
 NCBI BlastP on this gene

Pc15g00140

unnamed
  
Accession: CAP82901
  
Location: 31201-31680
  
 NCBI BlastP on this gene

Pc15g00150

not annotated
  
Accession: CAP82902
  
Location: 31867-32969
  
 NCBI BlastP on this gene

Pc15g00160

not annotated
  
Accession: CAP82903
  
Location: 33323-34048
  
 NCBI BlastP on this gene

Pc15g00170

not annotated
  
Accession: CAP82904
  
Location: 35931-36762
  
 NCBI BlastP on this gene

Pc15g00180

not annotated
  
Accession: CAP82905
  
Location: 38020-38999
  
  
**BlastP hit with Mycgr3G29227\_Mycgr3T**
  
Percentage identity: 38 %
  
BlastP bit score: 87
  
Sequence coverage: 100 %
  
E-value: 1e-18
  
  
 NCBI BlastP on this gene

Pc15g00190

not annotated
  
Accession: CAP82906
  
Location: 39583-41199
  
 NCBI BlastP on this gene

Pc15g00200

unnamed
  
Accession: CAP82907
  
Location: 42876-44360
  
 NCBI BlastP on this gene

Pc15g00210

not annotated
  
Accession: CAP82908
  
Location: 45100-46122
  
 NCBI BlastP on this gene

Pc15g00220

not annotated
  
Accession: Pc15g00230
  
Location: 47726-49288
  
 NCBI BlastP on this gene

Pc15g00230

unnamed
  
Accession: CAP82910
  
Location: 50742-51370
  
 NCBI BlastP on this gene

Pc15g00240

not annotated
  
Accession: CAP82911
  
Location: 51860-53308
  
 NCBI BlastP on this gene

Pc15g00250

not annotated
  
Accession: CAP82912
  
Location: 53667-55543
  
  
**BlastP hit with Mycgr3G68456\_Mycgr3T**
  
Percentage identity: 69 %
  
BlastP bit score: 701
  
Sequence coverage: 91 %
  
E-value: 0.0
  
  
 NCBI BlastP on this gene

Pc15g00260

not annotated
  
Accession: CAP82913
  
Location: 56105-57285
  
 NCBI BlastP on this gene

Pc15g00270

not annotated
  
Accession: CAP82914
  
Location: 59443-60839
  
 NCBI BlastP on this gene

Pc15g00280

hypothetical protein
  
Accession: CAP82915
  
Location: 61551-62374
  
 NCBI BlastP on this gene

Pc15g00290

not annotated
  
Accession: CAP82916
  
Location: 63685-64632
  
 NCBI BlastP on this gene

Pc15g00300

not annotated
  
Accession: CAP82917
  
Location: 65299-68102
  
 NCBI BlastP on this gene

Pc15g00310

not annotated
  
Accession: CAP82918
  
Location: 68443-69499
  
 NCBI BlastP on this gene

Pc15g00320

not annotated
  
Accession: CAP82919
  
Location: 69723-71264
  
 NCBI BlastP on this gene

Pc15g00330

224. :  ABDF02000085 Trichoderma virens Gv29-8     Total score: 2.0     Cumulative Blast bit score: 784

glycosyltransferase family 32 protein
  
Accession: EHK18966
  
Location: 1084261-1085449
  
 NCBI BlastP on this gene

EHK18966

hypothetical protein
  
Accession: EHK18967
  
Location: 1088580-1090301
  
 NCBI BlastP on this gene

EHK18967

hypothetical protein
  
Accession: EHK18968
  
Location: 1090788-1093164
  
 NCBI BlastP on this gene

EHK18968

hypothetical protein
  
Accession: EHK18969
  
Location: 1093421-1094365
  
 NCBI BlastP on this gene

EHK18969

glycosyltransferase family 8 protein
  
Accession: EHK19031
  
Location: 1094963-1096131
  
 NCBI BlastP on this gene

EHK19031

hypothetical protein
  
Accession: EHK18970
  
Location: 1097172-1098890
  
 NCBI BlastP on this gene

EHK18970

hypothetical protein
  
Accession: EHK18971
  
Location: 1099282-1100949
  
  
**BlastP hit with Mycgr3G68456\_Mycgr3T**
  
Percentage identity: 61 %
  
BlastP bit score: 645
  
Sequence coverage: 97 %
  
E-value: 0.0
  
  
 NCBI BlastP on this gene

EHK18971

hypothetical protein
  
Accession: EHK18972
  
Location: 1106489-1107190
  
 NCBI BlastP on this gene

EHK18972

hypothetical protein
  
Accession: EHK18973
  
Location: 1107359-1108267
  
 NCBI BlastP on this gene

EHK18973

glycoside hydrolase family 18 protein
  
Accession: EHK19035
  
Location: 1109120-1113383
  
 NCBI BlastP on this gene

EHK19035

hypothetical protein
  
Accession: EHK18974
  
Location: 1114868-1116901
  
 NCBI BlastP on this gene

EHK18974

hypothetical protein
  
Accession: EHK18975
  
Location: 1118038-1118910
  
  
**BlastP hit with Mycgr3G108090\_Mycgr3**
  
Percentage identity: 35 %
  
BlastP bit score: 140
  
Sequence coverage: 83 %
  
E-value: 7e-36
  
  
 NCBI BlastP on this gene

EHK18975

hypothetical protein
  
Accession: EHK18976
  
Location: 1118941-1119804
  
 NCBI BlastP on this gene

EHK18976

hypothetical protein
  
Accession: EHK18977
  
Location: 1120335-1121677
  
 NCBI BlastP on this gene

EHK18977

hypothetical protein
  
Accession: EHK18978
  
Location: 1122745-1124465
  
 NCBI BlastP on this gene

EHK18978

hypothetical protein
  
Accession: EHK18979
  
Location: 1124553-1125989
  
 NCBI BlastP on this gene

EHK18979

hypothetical protein
  
Accession: EHK18980
  
Location: 1127889-1128587
  
 NCBI BlastP on this gene

EHK18980

hypothetical protein
  
Accession: EHK18981
  
Location: 1129535-1130227
  
 NCBI BlastP on this gene

EHK18981

hypothetical protein
  
Accession: EHK18982
  
Location: 1130561-1131325
  
 NCBI BlastP on this gene

EHK18982

hypothetical protein
  
Accession: EHK19036
  
Location: 1131794-1133378
  
 NCBI BlastP on this gene

EHK19036

225. :  EQ962655 Talaromyces stipitatus ATCC 10500 scf\_1105507295555 genomic scaffold     Total score: 2.0     Cumulative Blast bit score: 776

reverse transcriptase, putative
  
Accession: EED18393
  
Location: 2800302-2802185
  
 NCBI BlastP on this gene

EED18393

reverse transcriptase, putative
  
Accession: EED18392
  
Location: 2795868-2799940
  
 NCBI BlastP on this gene

EED18392

retrotransposon polyprotein, putative
  
Accession: EED18391
  
Location: 2788334-2793746
  
 NCBI BlastP on this gene

EED18391

hypothetical protein
  
Accession: EED18390
  
Location: 2784739-2785777
  
 NCBI BlastP on this gene

EED18390

conserved hypothetical protein
  
Accession: EED18389
  
Location: 2780380-2782510
  
  
**BlastP hit with Mycgr3G84646\_Mycgr3T**
  
Percentage identity: 37 %
  
BlastP bit score: 424
  
Sequence coverage: 100 %
  
E-value: 2e-135
  
  
 NCBI BlastP on this gene

EED18389

Rab GTPase SrgA, putative
  
Accession: EED18388
  
Location: 2779102-2779833
  
  
**BlastP hit with Mycgr3G99145\_Mycgr3T**
  
Percentage identity: 83 %
  
BlastP bit score: 352
  
Sequence coverage: 100 %
  
E-value: 8e-121
  
  
 NCBI BlastP on this gene

EED18388

AAA family ATPase, putative
  
Accession: EED18387
  
Location: 2776092-2778372
  
 NCBI BlastP on this gene

EED18387

hypothetical protein
  
Accession: EED18386
  
Location: 2774305-2774989
  
 NCBI BlastP on this gene

EED18386

inositol kinase kinase (UvsB), putative
  
Accession: EED18385
  
Location: 2765341-2774207
  
 NCBI BlastP on this gene

EED18385

cytosine deaminase, putative
  
Accession: EED18384
  
Location: 2764077-2764896
  
 NCBI BlastP on this gene

EED18384

NADH-ubiquinone oxidoreductase 21 kDa subunit, putative
  
Accession: EED18383
  
Location: 2763061-2763888
  
 NCBI BlastP on this gene

EED18383

replication fork protection component Swi3, putative
  
Accession: EED18382
  
Location: 2761793-2762500
  
 NCBI BlastP on this gene

EED18382

FGGY-family carbohydrate kinase, putative
  
Accession: EED18380
  
Location: 2759479-2761463
  
 NCBI BlastP on this gene

EED18380

226. :  CP003005 Myceliophthora thermophila ATCC 42464 chromosome 4     Total score: 2.0     Cumulative Blast bit score: 772

hypothetical protein
  
Accession: AEO59137
  
Location: 3131585-3133817
  
 NCBI BlastP on this gene

MYCTH\_2307148

hypothetical protein
  
Accession: AEO59138
  
Location: 3136159-3136812
  
 NCBI BlastP on this gene

MYCTH\_116261

hypothetical protein
  
Accession: AEO59139
  
Location: 3138876-3139892
  
 NCBI BlastP on this gene

MYCTH\_52498

hypothetical protein
  
Accession: AEO59140
  
Location: 3142636-3143848
  
 NCBI BlastP on this gene

MYCTH\_2307154

hypothetical protein
  
Accession: AEO59141
  
Location: 3143995-3145158
  
 NCBI BlastP on this gene

MYCTH\_2307157

hypothetical protein
  
Accession: AEO59142
  
Location: 3145883-3146559
  
 NCBI BlastP on this gene

MYCTH\_52789

hypothetical protein
  
Accession: AEO59143
  
Location: 3148958-3149737
  
  
**BlastP hit with Mycgr3G55345\_Mycgr3T**
  
Percentage identity: 74 %
  
BlastP bit score: 318
  
Sequence coverage: 99 %
  
E-value: 2e-107
  
  
 NCBI BlastP on this gene

MYCTH\_2134806

hypothetical protein
  
Accession: AEO59144
  
Location: 3150248-3151444
  
 NCBI BlastP on this gene

MYCTH\_2307162

hypothetical protein
  
Accession: AEO59145
  
Location: 3152384-3154284
  
 NCBI BlastP on this gene

MYCTH\_2307164

hypothetical protein
  
Accession: AEO59146
  
Location: 3154654-3155166
  
 NCBI BlastP on this gene

MYCTH\_2307165

hypothetical protein
  
Accession: AEO59147
  
Location: 3155921-3159427
  
  
**BlastP hit with Mycgr3G68433\_Mycgr3T**
  
Percentage identity: 36 %
  
BlastP bit score: 454
  
Sequence coverage: 95 %
  
E-value: 2e-137
  
  
 NCBI BlastP on this gene

MYCTH\_2307166

hypothetical protein
  
Accession: AEO59148
  
Location: 3160872-3162192
  
 NCBI BlastP on this gene

MYCTH\_108061

hypothetical protein
  
Accession: AEO59149
  
Location: 3162624-3164529
  
 NCBI BlastP on this gene

MYCTH\_2307170

hypothetical protein
  
Accession: AEO59150
  
Location: 3166349-3166931
  
 NCBI BlastP on this gene

MYCTH\_2019371

hypothetical protein
  
Accession: AEO59151
  
Location: 3167490-3168828
  
 NCBI BlastP on this gene

MYCTH\_2307175

hypothetical protein
  
Accession: AEO59152
  
Location: 3170165-3171361
  
 NCBI BlastP on this gene

MYCTH\_2307177

hypothetical protein
  
Accession: AEO59153
  
Location: 3172326-3173560
  
 NCBI BlastP on this gene

MYCTH\_2307178

hypothetical protein
  
Accession: AEO59154
  
Location: 3174015-3178483
  
 NCBI BlastP on this gene

MYCTH\_2119434

227. :  DS995901 Penicillium marneffei ATCC 18224 scf\_1105668340960 genomic scaffold     Total score: 2.0     Cumulative Blast bit score: 765

transposon, putative
  
Accession: EEA24637
  
Location: 2625325-2626953
  
 NCBI BlastP on this gene

EEA24637

transposable element tc1 transposase, putative
  
Accession: EEA24636
  
Location: 2616774-2617871
  
 NCBI BlastP on this gene

EEA24636

hypothetical protein
  
Accession: EEA24635
  
Location: 2614101-2614860
  
 NCBI BlastP on this gene

EEA24635

conserved hypothetical protein
  
Accession: EEA24634
  
Location: 2613104-2613988
  
 NCBI BlastP on this gene

EEA24634

conserved hypothetical protein
  
Accession: EEA24632
  
Location: 2611512-2612213
  
 NCBI BlastP on this gene

EEA24632

conserved hypothetical protein
  
Accession: EEA24631
  
Location: 2606606-2608729
  
  
**BlastP hit with Mycgr3G84646\_Mycgr3T**
  
Percentage identity: 40 %
  
BlastP bit score: 412
  
Sequence coverage: 84 %
  
E-value: 9e-131
  
  
 NCBI BlastP on this gene

EEA24631

Rab GTPase SrgA, putative
  
Accession: EEA24630
  
Location: 2605188-2605934
  
  
**BlastP hit with Mycgr3G99145\_Mycgr3T**
  
Percentage identity: 84 %
  
BlastP bit score: 353
  
Sequence coverage: 100 %
  
E-value: 6e-121
  
  
 NCBI BlastP on this gene

EEA24630

AAA family ATPase, putative
  
Accession: EEA24629
  
Location: 2602161-2604439
  
 NCBI BlastP on this gene

EEA24629

conserved hypothetical protein
  
Accession: EEA24628
  
Location: 2600425-2601480
  
 NCBI BlastP on this gene

EEA24628

inositol kinase kinase (UvsB), putative
  
Accession: EEA24627
  
Location: 2591336-2599959
  
 NCBI BlastP on this gene

EEA24627

tRNA-specific adenosine deaminase subunit TAD2, putative
  
Accession: EEA24626
  
Location: 2590091-2590891
  
 NCBI BlastP on this gene

EEA24626

NADH-ubiquinone oxidoreductase 21 kDa subunit, putative
  
Accession: EEA24625
  
Location: 2589079-2589900
  
 NCBI BlastP on this gene

EEA24625

replication fork protection component Swi3, putative
  
Accession: EEA24624
  
Location: 2587726-2588819
  
 NCBI BlastP on this gene

EEA24624

FGGY-family carbohydrate kinase, putative
  
Accession: EEA24623
  
Location: 2585338-2587347
  
 NCBI BlastP on this gene

EEA24623

228. :  DF126458 Aspergillus kawachii IFO 4308 DNA, contig: scaffold00012     Total score: 2.0     Cumulative Blast bit score: 748

ATP synthase regulation protein NCA2
  
Accession: GAA87272
  
Location: 933912-936038
  
  
**BlastP hit with Mycgr3G84646\_Mycgr3T**
  
Percentage identity: 39 %
  
BlastP bit score: 400
  
Sequence coverage: 83 %
  
E-value: 5e-126
  
  
 NCBI BlastP on this gene

GAA87272

secretion related GTPase (SrgA)
  
Accession: GAA87271
  
Location: 932089-932838
  
  
**BlastP hit with Mycgr3G99145\_Mycgr3T**
  
Percentage identity: 83 %
  
BlastP bit score: 348
  
Sequence coverage: 99 %
  
E-value: 5e-119
  
  
 NCBI BlastP on this gene

GAA87271

AAA family ATPase
  
Accession: GAA87270
  
Location: 928467-930216
  
 NCBI BlastP on this gene

GAA87270

UVSB
  
Accession: GAA87269
  
Location: 917764-926608
  
 NCBI BlastP on this gene

GAA87269

NADH-ubiquinone oxidoreductase 21 kDa subunit
  
Accession: GAA87268
  
Location: 915156-915987
  
 NCBI BlastP on this gene

GAA87268

chromosome segregation in meiosis protein 3
  
Accession: GAA87267
  
Location: 913776-914661
  
 NCBI BlastP on this gene

GAA87267

229. :  GL988043 Chaetomium thermophilum var. thermophilum DSM 1495 unplaced genomic scaffold scf7180000...     Total score: 2.0     Cumulative Blast bit score: 738

hypothetical protein
  
Accession: EGS20001
  
Location: 1820192-1821367
  
 NCBI BlastP on this gene

EGS20001

hypothetical protein
  
Accession: EGS20000
  
Location: 1817802-1819013
  
 NCBI BlastP on this gene

EGS20000

hypothetical protein
  
Accession: EGS19999
  
Location: 1813250-1814579
  
 NCBI BlastP on this gene

EGS19999

hypothetical protein
  
Accession: EGS19998
  
Location: 1812032-1812754
  
 NCBI BlastP on this gene

EGS19998

hypothetical protein
  
Accession: EGS19997
  
Location: 1808606-1810673
  
 NCBI BlastP on this gene

EGS19997

hypothetical protein
  
Accession: EGS19996
  
Location: 1803091-1804774
  
 NCBI BlastP on this gene

EGS19996

putative leucine-rich protein
  
Accession: EGS19995
  
Location: 1796704-1800216
  
  
**BlastP hit with Mycgr3G68433\_Mycgr3T**
  
Percentage identity: 35 %
  
BlastP bit score: 430
  
Sequence coverage: 97 %
  
E-value: 2e-128
  
  
 NCBI BlastP on this gene

EGS19995

xanthine phosphoribosyltransferase-like protein
  
Accession: EGS19994
  
Location: 1794472-1795208
  
  
**BlastP hit with Mycgr3G55345\_Mycgr3T**
  
Percentage identity: 72 %
  
BlastP bit score: 308
  
Sequence coverage: 99 %
  
E-value: 2e-103
  
  
 NCBI BlastP on this gene

EGS19994

hypothetical protein
  
Accession: EGS19993
  
Location: 1792499-1793786
  
 NCBI BlastP on this gene

EGS19993

hypothetical protein
  
Accession: EGS19992
  
Location: 1789581-1791783
  
 NCBI BlastP on this gene

EGS19992

hypothetical protein
  
Accession: EGS19991
  
Location: 1788653-1789085
  
 NCBI BlastP on this gene

EGS19991

hypothetical protein
  
Accession: EGS19990
  
Location: 1783459-1785379
  
 NCBI BlastP on this gene

EGS19990

230. :  DS572813 Paracoccidioides brasiliensis Pb01 supercont1.3 genomic scaffold     Total score: 2.0     Cumulative Blast bit score: 730

conserved hypothetical protein
  
Accession: EEH39046
  
Location: 617868-619211
  
 NCBI BlastP on this gene

EEH39046

predicted protein
  
Accession: EEH39045
  
Location: 615225-616314
  
 NCBI BlastP on this gene

EEH39045

C-4 methylsterol oxidase
  
Accession: EEH39044
  
Location: 613839-614390
  
 NCBI BlastP on this gene

EEH39044

conserved hypothetical protein
  
Accession: EEH39043
  
Location: 605548-607731
  
 NCBI BlastP on this gene

EEH39043

conserved hypothetical protein
  
Accession: EEH39042
  
Location: 604102-605053
  
 NCBI BlastP on this gene

EEH39042

conserved hypothetical protein
  
Accession: EEH39041
  
Location: 599261-602711
  
 NCBI BlastP on this gene

EEH39041

conserved hypothetical protein
  
Accession: EEH39040
  
Location: 594902-599119
  
  
**BlastP hit with Mycgr3G84646\_Mycgr3T**
  
Percentage identity: 39 %
  
BlastP bit score: 384
  
Sequence coverage: 93 %
  
E-value: 2e-119
  
  
 NCBI BlastP on this gene

EEH39040

conserved hypothetical protein
  
Accession: EEH39039
  
Location: 591077-592417
  
 NCBI BlastP on this gene

EEH39039

GTP-binding protein SAS1
  
Accession: EEH39038
  
Location: 588940-589798
  
  
**BlastP hit with Mycgr3G99145\_Mycgr3T**
  
Percentage identity: 83 %
  
BlastP bit score: 346
  
Sequence coverage: 99 %
  
E-value: 2e-118
  
  
 NCBI BlastP on this gene

EEH39038

conserved hypothetical protein
  
Accession: EEH39037
  
Location: 586757-588075
  
 NCBI BlastP on this gene

EEH39037

AAA family ATPase
  
Accession: EEH39036
  
Location: 583765-586113
  
 NCBI BlastP on this gene

EEH39036

hypothetical protein
  
Accession: EEH39035
  
Location: 582138-583382
  
 NCBI BlastP on this gene

EEH39035

UVSB PI-3 kinase
  
Accession: EEH39034
  
Location: 573004-581454
  
 NCBI BlastP on this gene

EEH39034

tRNA-specific adenosine deaminase
  
Accession: EEH39033
  
Location: 571824-572676
  
 NCBI BlastP on this gene

EEH39033

NADH-ubiquinone oxidoreductase 21 kDa subunit
  
Accession: EEH39032
  
Location: 570749-571594
  
 NCBI BlastP on this gene

EEH39032

231. :  ACJE01000002 Aspergillus niger ATCC 1015     Total score: 2.0     Cumulative Blast bit score: 724

hypothetical protein
  
Accession: EHA27517
  
Location: 4053-5906
  
  
**BlastP hit with Mycgr3G84646\_Mycgr3T**
  
Percentage identity: 38 %
  
BlastP bit score: 376
  
Sequence coverage: 80 %
  
E-value: 1e-117
  
  
 NCBI BlastP on this gene

EHA27517

hypothetical protein
  
Accession: EHA27518
  
Location: 7060-7804
  
  
**BlastP hit with Mycgr3G99145\_Mycgr3T**
  
Percentage identity: 83 %
  
BlastP bit score: 348
  
Sequence coverage: 99 %
  
E-value: 4e-119
  
  
 NCBI BlastP on this gene

EHA27518

hypothetical protein
  
Accession: EHA27519
  
Location: 9104-11408
  
 NCBI BlastP on this gene

EHA27519

hypothetical protein
  
Accession: EHA27520
  
Location: 12080-12926
  
 NCBI BlastP on this gene

EHA27520

hypothetical protein
  
Accession: EHA27521
  
Location: 13238-22108
  
 NCBI BlastP on this gene

EHA27521

hypothetical protein
  
Accession: EHA27522
  
Location: 23934-24757
  
 NCBI BlastP on this gene

EHA27522

hypothetical protein
  
Accession: EHA27523
  
Location: 25211-26141
  
 NCBI BlastP on this gene

EHA27523

hypothetical protein
  
Accession: EHA27524
  
Location: 26604-28384
  
 NCBI BlastP on this gene

EHA27524

232. :  DS027690 Neosartorya fischeri NRRL 181 1099437636252 genomic scaffold     Total score: 2.0     Cumulative Blast bit score: 720

conserved hypothetical protein
  
Accession: EAW21518
  
Location: 1503691-1505820
  
 NCBI BlastP on this gene

EAW21518

conserved hypothetical protein
  
Accession: EAW21517
  
Location: 1499856-1501408
  
 NCBI BlastP on this gene

EAW21517

conserved hypothetical protein
  
Accession: EAW21516
  
Location: 1497193-1499643
  
 NCBI BlastP on this gene

EAW21516

nuclear transport factor NTF-2, putative
  
Accession: EAW21515
  
Location: 1496059-1496890
  
 NCBI BlastP on this gene

EAW21515

PQ loop repeat protein
  
Accession: EAW21514
  
Location: 1494612-1495633
  
 NCBI BlastP on this gene

EAW21514

stress response protein (Ish1), putative
  
Accession: EAW21513
  
Location: 1491841-1493639
  
 NCBI BlastP on this gene

EAW21513

DNA damage response protein (Dap1), putative
  
Accession: EAW21512
  
Location: 1490832-1491364
  
 NCBI BlastP on this gene

EAW21512

conserved hypothetical protein
  
Accession: EAW21511
  
Location: 1487756-1489507
  
 NCBI BlastP on this gene

EAW21511

conserved hypothetical protein
  
Accession: EAW21510
  
Location: 1486843-1487183
  
 NCBI BlastP on this gene

EAW21510

serine/threonine protein kinase (Prp4), putative
  
Accession: EAW21509
  
Location: 1483748-1486294
  
  
**BlastP hit with Mycgr3G103260\_Mycgr3**
  
Percentage identity: 53 %
  
BlastP bit score: 281
  
Sequence coverage: 98 %
  
E-value: 1e-85
  
  
 NCBI BlastP on this gene

EAW21509

protein kinase, putative
  
Accession: EAW21508
  
Location: 1477534-1478836
  
  
**BlastP hit with Mycgr3G68429\_Mycgr3T**
  
Percentage identity: 68 %
  
BlastP bit score: 439
  
Sequence coverage: 75 %
  
E-value: 4e-148
  
  
 NCBI BlastP on this gene

EAW21508

3-oxoacyl-(acyl-carrier-protein) reductase, putative
  
Accession: EAW21507
  
Location: 1475462-1476511
  
 NCBI BlastP on this gene

EAW21507

C6 zinc finger domain protein
  
Accession: EAW21506
  
Location: 1474064-1475223
  
 NCBI BlastP on this gene

EAW21506

haemolysin-III channel protein Izh2, putative
  
Accession: EAW21505
  
Location: 1472038-1473003
  
 NCBI BlastP on this gene

EAW21505

conserved hypothetical protein
  
Accession: EAW21504
  
Location: 1470563-1471606
  
 NCBI BlastP on this gene

EAW21504

conserved hypothetical protein
  
Accession: EAW21503
  
Location: 1469120-1470315
  
 NCBI BlastP on this gene

EAW21503

clathrin-coated vesicle protein, putative
  
Accession: EAW21502
  
Location: 1467560-1468396
  
 NCBI BlastP on this gene

EAW21502

conserved hypothetical protein
  
Accession: EAW21501
  
Location: 1460857-1463688
  
 NCBI BlastP on this gene

EAW21501

transcription initiation protein
  
Accession: EAW21500
  
Location: 1458994-1460046
  
 NCBI BlastP on this gene

EAW21500

mitochondrial F1F0 ATP synthase subunit Atp14, putative
  
Accession: EAW21499
  
Location: 1458166-1458717
  
 NCBI BlastP on this gene

EAW21499

233. :  DS989822 Arthroderma gypseum CBS 118893 supercont1.1 genomic scaffold     Total score: 2.0     Cumulative Blast bit score: 719

hypothetical protein
  
Accession: EFQ98155
  
Location: 3256082-3258505
  
 NCBI BlastP on this gene

EFQ98155

hypothetical protein
  
Accession: EFQ98156
  
Location: 3259510-3259919
  
 NCBI BlastP on this gene

EFQ98156

PQ loop repeat protein
  
Accession: EFQ98157
  
Location: 3261639-3262682
  
 NCBI BlastP on this gene

EFQ98157

hypothetical protein
  
Accession: EFQ98158
  
Location: 3263685-3265502
  
 NCBI BlastP on this gene

EFQ98158

hypothetical protein
  
Accession: EFQ98159
  
Location: 3265872-3266577
  
 NCBI BlastP on this gene

EFQ98159

hypothetical protein
  
Accession: EFQ98160
  
Location: 3267436-3269100
  
 NCBI BlastP on this gene

EFQ98160

hypothetical protein
  
Accession: EFQ98161
  
Location: 3269448-3269798
  
 NCBI BlastP on this gene

EFQ98161

CMGC/DYRK/PRP4 protein kinase
  
Accession: EFQ98162
  
Location: 3270390-3272900
  
  
**BlastP hit with Mycgr3G103260\_Mycgr3**
  
Percentage identity: 54 %
  
BlastP bit score: 283
  
Sequence coverage: 96 %
  
E-value: 2e-86
  
  
 NCBI BlastP on this gene

EFQ98162

hypothetical protein
  
Accession: EFQ98163
  
Location: 3277146-3277592
  
 NCBI BlastP on this gene

EFQ98163

hypothetical protein
  
Accession: EFQ98164
  
Location: 3281065-3282049
  
 NCBI BlastP on this gene

EFQ98164

hypothetical protein
  
Accession: EFQ98165
  
Location: 3282776-3283214
  
 NCBI BlastP on this gene

EFQ98165

hypothetical protein
  
Accession: EFQ98166
  
Location: 3284702-3284965
  
 NCBI BlastP on this gene

EFQ98166

serine/threonine protein kinase
  
Accession: EFQ98167
  
Location: 3285769-3287073
  
  
**BlastP hit with Mycgr3G68429\_Mycgr3T**
  
Percentage identity: 64 %
  
BlastP bit score: 436
  
Sequence coverage: 82 %
  
E-value: 7e-147
  
  
 NCBI BlastP on this gene

EFQ98167

3-oxoacyl-[acyl-carrier-protein] reductase
  
Accession: EFQ98168
  
Location: 3290451-3291638
  
 NCBI BlastP on this gene

EFQ98168

adiponectin receptor protein 1
  
Accession: EFQ98169
  
Location: 3292121-3293086
  
 NCBI BlastP on this gene

EFQ98169

hypothetical protein
  
Accession: EFQ98170
  
Location: 3293473-3294537
  
 NCBI BlastP on this gene

EFQ98170

hypothetical protein
  
Accession: EFQ98171
  
Location: 3294829-3296390
  
 NCBI BlastP on this gene

EFQ98171

dolichol-phosphate mannosyltransferase
  
Accession: EFQ98172
  
Location: 3296773-3297715
  
 NCBI BlastP on this gene

EFQ98172

dolichol-phosphate mannosyltransferase
  
Accession: EFQ98173
  
Location: 3297985-3299097
  
 NCBI BlastP on this gene

EFQ98173

hypothetical protein
  
Accession: EFQ98174
  
Location: 3299507-3301781
  
 NCBI BlastP on this gene

EFQ98174

hypothetical protein
  
Accession: EFQ98175
  
Location: 3302333-3302965
  
 NCBI BlastP on this gene

EFQ98175

234. :  DS499596 Aspergillus fumigatus A1163 scf\_000003 genomic scaffold     Total score: 2.0     Cumulative Blast bit score: 719

conserved hypothetical protein
  
Accession: EDP52704
  
Location: 1368580-1370709
  
 NCBI BlastP on this gene

EDP52704

conserved hypothetical protein
  
Accession: EDP52703
  
Location: 1365344-1366075
  
 NCBI BlastP on this gene

EDP52703

conserved hypothetical protein
  
Accession: EDP52702
  
Location: 1362205-1364655
  
 NCBI BlastP on this gene

EDP52702

nuclear transport factor NTF-2, putative
  
Accession: EDP52701
  
Location: 1361067-1361900
  
 NCBI BlastP on this gene

EDP52701

PQ loop repeat protein
  
Accession: EDP52700
  
Location: 1359621-1360850
  
 NCBI BlastP on this gene

EDP52700

stress response protein (Ish1), putative
  
Accession: EDP52699
  
Location: 1356836-1358631
  
 NCBI BlastP on this gene

EDP52699

DNA damage response protein (Dap1), putative
  
Accession: EDP52698
  
Location: 1355831-1356363
  
 NCBI BlastP on this gene

EDP52698

conserved hypothetical protein
  
Accession: EDP52697
  
Location: 1352784-1354112
  
 NCBI BlastP on this gene

EDP52697

conserved hypothetical protein
  
Accession: EDP52696
  
Location: 1351880-1352215
  
 NCBI BlastP on this gene

EDP52696

serine/threonine protein kinase (Prp4), putative
  
Accession: EDP52695
  
Location: 1348747-1351298
  
  
**BlastP hit with Mycgr3G103260\_Mycgr3**
  
Percentage identity: 53 %
  
BlastP bit score: 281
  
Sequence coverage: 98 %
  
E-value: 1e-85
  
  
 NCBI BlastP on this gene

EDP52695

protein serine/threonine kinase (Ran1), putative
  
Accession: EDP52694
  
Location: 1342528-1343855
  
  
**BlastP hit with Mycgr3G68429\_Mycgr3T**
  
Percentage identity: 67 %
  
BlastP bit score: 438
  
Sequence coverage: 76 %
  
E-value: 7e-148
  
  
 NCBI BlastP on this gene

EDP52694

3-oxoacyl-(acyl-carrier-protein) reductase, putative
  
Accession: EDP52693
  
Location: 1340396-1341463
  
 NCBI BlastP on this gene

EDP52693

conserved hypothetical protein
  
Accession: EDP52692
  
Location: 1339511-1340158
  
 NCBI BlastP on this gene

EDP52692

C6 finger domain protein, putative
  
Accession: EDP52691
  
Location: 1339000-1339362
  
 NCBI BlastP on this gene

EDP52691

haemolysin-III channel protein Izh2, putative
  
Accession: EDP52690
  
Location: 1336985-1337950
  
 NCBI BlastP on this gene

EDP52690

conserved hypothetical protein
  
Accession: EDP52689
  
Location: 1335516-1336559
  
 NCBI BlastP on this gene

EDP52689

conserved hypothetical protein
  
Accession: EDP52688
  
Location: 1334791-1335269
  
 NCBI BlastP on this gene

EDP52688

clathrin-coated vesicle protein, putative
  
Accession: EDP52687
  
Location: 1332516-1333350
  
 NCBI BlastP on this gene

EDP52687

conserved hypothetical protein
  
Accession: EDP52686
  
Location: 1325808-1328624
  
 NCBI BlastP on this gene

EDP52686

transcription initiation protein
  
Accession: EDP52685
  
Location: 1323728-1324996
  
 NCBI BlastP on this gene

EDP52685

mitochondrial F1F0 ATP synthase subunit Atp14, putative
  
Accession: EDP52684
  
Location: 1323118-1323665
  
 NCBI BlastP on this gene

EDP52684

235. :  AAHF01000002 Aspergillus fumigatus Af293     Total score: 2.0     Cumulative Blast bit score: 719

conserved hypothetical protein
  
Accession: EAL92539
  
Location: 1378885-1381014
  
 NCBI BlastP on this gene

EAL92539

conserved hypothetical protein
  
Accession: EAL92537
  
Location: 1373765-1374496
  
 NCBI BlastP on this gene

EAL92537

conserved hypothetical protein
  
Accession: EAL92536
  
Location: 1370626-1373076
  
 NCBI BlastP on this gene

EAL92536

nuclear transport factor NTF-2, putative
  
Accession: EAL92535
  
Location: 1369488-1370321
  
 NCBI BlastP on this gene

EAL92535

PQ loop repeat protein
  
Accession: EAL92534
  
Location: 1368042-1369271
  
 NCBI BlastP on this gene

EAL92534

meiotic sister chromatid recombination protein Ish1/Msc1, putative
  
Accession: EAL92533
  
Location: 1365257-1367052
  
 NCBI BlastP on this gene

EAL92533

DNA damage response protein (Dap1), putative
  
Accession: EAL92532
  
Location: 1364251-1364783
  
 NCBI BlastP on this gene

EAL92532

conserved hypothetical protein
  
Accession: EAL92531
  
Location: 1361204-1362532
  
 NCBI BlastP on this gene

EAL92531

conserved hypothetical protein
  
Accession: EAL92530
  
Location: 1360300-1360635
  
 NCBI BlastP on this gene

EAL92530

serine/threonine protein kinase (Prp4), putative
  
Accession: EAL92529
  
Location: 1357168-1359719
  
  
**BlastP hit with Mycgr3G103260\_Mycgr3**
  
Percentage identity: 53 %
  
BlastP bit score: 281
  
Sequence coverage: 98 %
  
E-value: 1e-85
  
  
 NCBI BlastP on this gene

EAL92529

protein serine/threonine kinase (Ran1), putative
  
Accession: EAL92528
  
Location: 1350952-1352279
  
  
**BlastP hit with Mycgr3G68429\_Mycgr3T**
  
Percentage identity: 67 %
  
BlastP bit score: 438
  
Sequence coverage: 76 %
  
E-value: 8e-148
  
  
 NCBI BlastP on this gene

EAL92528

3-oxoacyl-(acyl-carrier-protein) reductase, putative
  
Accession: EAL92527
  
Location: 1348810-1349877
  
 NCBI BlastP on this gene

EAL92527

conserved hypothetical protein
  
Accession: EAL92526
  
Location: 1347925-1348572
  
 NCBI BlastP on this gene

EAL92526

C6 finger domain protein, putative
  
Accession: EAL92525
  
Location: 1347414-1347776
  
 NCBI BlastP on this gene

EAL92525

haemolysin-III channel protein Izh2, putative
  
Accession: EAL92524
  
Location: 1345399-1346364
  
 NCBI BlastP on this gene

EAL92524

conserved hypothetical protein
  
Accession: EAL92523
  
Location: 1343930-1344973
  
 NCBI BlastP on this gene

EAL92523

conserved hypothetical protein
  
Accession: EAL92522
  
Location: 1343205-1343683
  
 NCBI BlastP on this gene

EAL92522

clathrin-coated vesicle protein, putative
  
Accession: EAL92521
  
Location: 1340930-1341764
  
 NCBI BlastP on this gene

EAL92521

conserved hypothetical protein
  
Accession: EAL92520
  
Location: 1334228-1337044
  
 NCBI BlastP on this gene

EAL92520

transcription initiation protein
  
Accession: EAL92519
  
Location: 1332148-1333416
  
 NCBI BlastP on this gene

EAL92519

mitochondrial F1F0 ATP synthase subunit Atp14, putative
  
Accession: EAL92518
  
Location: 1331538-1332085
  
 NCBI BlastP on this gene

EAL92518

236. :  DS027056 Aspergillus clavatus NRRL 1 1099423829802 genomic scaffold     Total score: 2.0     Cumulative Blast bit score: 716

hypothetical protein
  
Accession: EAW09622
  
Location: 1458058-1459116
  
 NCBI BlastP on this gene

EAW09622

conserved hypothetical protein
  
Accession: EAW09623
  
Location: 1459828-1461022
  
 NCBI BlastP on this gene

EAW09623

conserved hypothetical protein
  
Accession: EAW09624
  
Location: 1461245-1463701
  
 NCBI BlastP on this gene

EAW09624

nuclear transport factor NTF-2, putative
  
Accession: EAW09625
  
Location: 1464053-1464995
  
 NCBI BlastP on this gene

EAW09625

PQ loop repeat protein
  
Accession: EAW09626
  
Location: 1465410-1466437
  
 NCBI BlastP on this gene

EAW09626

stress response protein (Ish1), putative
  
Accession: EAW09627
  
Location: 1467470-1469270
  
 NCBI BlastP on this gene

EAW09627

DNA damage response protein (Dap1), putative
  
Accession: EAW09628
  
Location: 1469770-1470303
  
 NCBI BlastP on this gene

EAW09628

conserved hypothetical protein
  
Accession: EAW09629
  
Location: 1471475-1473236
  
 NCBI BlastP on this gene

EAW09629

conserved hypothetical protein
  
Accession: EAW09630
  
Location: 1473821-1474165
  
 NCBI BlastP on this gene

EAW09630

serine/threonine protein kinase (Prp4), putative
  
Accession: EAW09631
  
Location: 1474813-1477340
  
  
**BlastP hit with Mycgr3G103260\_Mycgr3**
  
Percentage identity: 52 %
  
BlastP bit score: 279
  
Sequence coverage: 98 %
  
E-value: 9e-85
  
  
 NCBI BlastP on this gene

EAW09631

protein serine/threonine kinase (Ran1), putative
  
Accession: EAW09632
  
Location: 1481962-1483293
  
  
**BlastP hit with Mycgr3G68429\_Mycgr3T**
  
Percentage identity: 68 %
  
BlastP bit score: 437
  
Sequence coverage: 75 %
  
E-value: 2e-147
  
  
 NCBI BlastP on this gene

EAW09632

3-oxoacyl-(acyl-carrier-protein) reductase, putative
  
Accession: EAW09633
  
Location: 1484549-1485898
  
 NCBI BlastP on this gene

EAW09633

C6 zinc finger domain protein
  
Accession: EAW09634
  
Location: 1486198-1487357
  
 NCBI BlastP on this gene

EAW09634

haemolysin-III channel protein Izh2, putative
  
Accession: EAW09635
  
Location: 1488406-1489371
  
 NCBI BlastP on this gene

EAW09635

conserved hypothetical protein
  
Accession: EAW09636
  
Location: 1489807-1490837
  
 NCBI BlastP on this gene

EAW09636

conserved hypothetical protein
  
Accession: EAW09637
  
Location: 1491085-1492605
  
 NCBI BlastP on this gene

EAW09637

clathrin-coated vesicle protein, putative
  
Accession: EAW09638
  
Location: 1492978-1493835
  
 NCBI BlastP on this gene

EAW09638

conserved hypothetical protein
  
Accession: EAW09639
  
Location: 1497385-1500274
  
 NCBI BlastP on this gene

EAW09639

transcription initiation protein
  
Accession: EAW09640
  
Location: 1501230-1502282
  
 NCBI BlastP on this gene

EAW09640

mitochondrial F1F0 ATP synthase subunit Atp14, putative
  
Accession: EAW09641
  
Location: 1502639-1503191
  
 NCBI BlastP on this gene

EAW09641

237. :  GL636488 Coccidioides posadasii str. Silveira unplaced genomic scaffold supercont2.3     Total score: 2.0     Cumulative Blast bit score: 715

conserved hypothetical protein
  
Accession: EFW20497
  
Location: 194254-196032
  
 NCBI BlastP on this gene

EFW20497

PQ loop repeat protein
  
Accession: EFW20498
  
Location: 196587-197679
  
 NCBI BlastP on this gene

EFW20498

conserved hypothetical protein
  
Accession: EFW20499
  
Location: 200048-200665
  
 NCBI BlastP on this gene

EFW20499

conserved hypothetical protein
  
Accession: EFW20500
  
Location: 201216-204260
  
 NCBI BlastP on this gene

EFW20500

conserved hypothetical protein
  
Accession: EFW20501
  
Location: 204920-205235
  
 NCBI BlastP on this gene

EFW20501

DNA damage response protein
  
Accession: EFW20502
  
Location: 207171-207622
  
 NCBI BlastP on this gene

EFW20502

conserved hypothetical protein
  
Accession: EFW20503
  
Location: 208471-210248
  
 NCBI BlastP on this gene

EFW20503

hypothetical protein
  
Accession: EFW20504
  
Location: 210774-211150
  
 NCBI BlastP on this gene

EFW20504

serine/threonine-protein kinase prp4
  
Accession: EFW20505
  
Location: 212326-214178
  
  
**BlastP hit with Mycgr3G103260\_Mycgr3**
  
Percentage identity: 52 %
  
BlastP bit score: 280
  
Sequence coverage: 96 %
  
E-value: 9e-87
  
  
 NCBI BlastP on this gene

EFW20505

predicted protein
  
Accession: EFW20506
  
Location: 215706-216181
  
 NCBI BlastP on this gene

EFW20506

hypothetical protein
  
Accession: EFW20507
  
Location: 216539-216730
  
 NCBI BlastP on this gene

EFW20507

predicted protein
  
Accession: EFW20508
  
Location: 217585-219467
  
 NCBI BlastP on this gene

EFW20508

protein serine/threonine kinase
  
Accession: EFW20509
  
Location: 221596-222885
  
  
**BlastP hit with Mycgr3G68429\_Mycgr3T**
  
Percentage identity: 67 %
  
BlastP bit score: 435
  
Sequence coverage: 74 %
  
E-value: 1e-146
  
  
 NCBI BlastP on this gene

EFW20509

conserved hypothetical protein
  
Accession: EFW20511
  
Location: 224002-224561
  
 NCBI BlastP on this gene

EFW20511

hypothetical protein
  
Accession: EFW20510
  
Location: 224974-225534
  
 NCBI BlastP on this gene

EFW20510

3-oxoacyl-(acyl-carrier-protein) reductase
  
Accession: EFW20512
  
Location: 227049-228179
  
 NCBI BlastP on this gene

EFW20512

hemolysin-III channel protein Izh2
  
Accession: EFW20513
  
Location: 228699-229661
  
 NCBI BlastP on this gene

EFW20513

conserved hypothetical protein
  
Accession: EFW20514
  
Location: 229979-231026
  
 NCBI BlastP on this gene

EFW20514

conserved hypothetical protein
  
Accession: EFW20515
  
Location: 231324-232695
  
 NCBI BlastP on this gene

EFW20515

glycosyltransferase
  
Accession: EFW20516
  
Location: 233051-233965
  
 NCBI BlastP on this gene

EFW20516

conserved hypothetical protein
  
Accession: EFW20517
  
Location: 234215-235319
  
 NCBI BlastP on this gene

EFW20517

conserved hypothetical protein
  
Accession: EFW20518
  
Location: 235725-238014
  
 NCBI BlastP on this gene

EFW20518

hypothetical protein
  
Accession: EFW20519
  
Location: 238580-239140
  
 NCBI BlastP on this gene

EFW20519

vacuolar sorting protein 35
  
Accession: EFW20520
  
Location: 241221-244226
  
 NCBI BlastP on this gene

EFW20520

238. :  GG698485 Trichophyton tonsurans CBS 112818 genomic scaffold supercont1.9     Total score: 2.0     Cumulative Blast bit score: 715

hypothetical protein
  
Accession: EGD94986
  
Location: 434415-436807
  
 NCBI BlastP on this gene

EGD94986

hypothetical protein
  
Accession: EGD94987
  
Location: 438167-438706
  
 NCBI BlastP on this gene

EGD94987

PQ loop repeat protein
  
Accession: EGD94988
  
Location: 440194-441255
  
 NCBI BlastP on this gene

EGD94988

hypothetical protein
  
Accession: EGD94989
  
Location: 442277-444095
  
 NCBI BlastP on this gene

EGD94989

DNA damage response protein
  
Accession: EGD94990
  
Location: 444458-445152
  
 NCBI BlastP on this gene

EGD94990

hypothetical protein
  
Accession: EGD94991
  
Location: 446008-447673
  
 NCBI BlastP on this gene

EGD94991

hypothetical protein
  
Accession: EGD94992
  
Location: 448031-448379
  
 NCBI BlastP on this gene

EGD94992

CMGC/DYRK/PRP4 protein kinase
  
Accession: EGD94993
  
Location: 448961-451483
  
  
**BlastP hit with Mycgr3G103260\_Mycgr3**
  
Percentage identity: 54 %
  
BlastP bit score: 284
  
Sequence coverage: 96 %
  
E-value: 2e-86
  
  
 NCBI BlastP on this gene

EGD94993

hypothetical protein
  
Accession: EGD94994
  
Location: 452159-452617
  
 NCBI BlastP on this gene

EGD94994

hypothetical protein
  
Accession: EGD94995
  
Location: 453076-454096
  
 NCBI BlastP on this gene

EGD94995

hypothetical protein
  
Accession: EGD94996
  
Location: 456030-456572
  
 NCBI BlastP on this gene

EGD94996

hypothetical protein
  
Accession: EGD94997
  
Location: 458114-458665
  
 NCBI BlastP on this gene

EGD94997

hypothetical protein
  
Accession: EGD94998
  
Location: 459430-460318
  
 NCBI BlastP on this gene

EGD94998

serine/threonine protein kinase
  
Accession: EGD94999
  
Location: 464346-465661
  
  
**BlastP hit with Mycgr3G68429\_Mycgr3T**
  
Percentage identity: 62 %
  
BlastP bit score: 431
  
Sequence coverage: 82 %
  
E-value: 6e-145
  
  
 NCBI BlastP on this gene

EGD94999

3-oxoacyl-(acyl-carrier-protein) reductase
  
Accession: EGD95000
  
Location: 469097-470282
  
 NCBI BlastP on this gene

EGD95000

hemolysin-III channel protein Izh2
  
Accession: EGD95001
  
Location: 470782-471768
  
 NCBI BlastP on this gene

EGD95001

hypothetical protein
  
Accession: EGD95002
  
Location: 472158-473249
  
 NCBI BlastP on this gene

EGD95002

hypothetical protein
  
Accession: EGD95003
  
Location: 473535-475153
  
 NCBI BlastP on this gene

EGD95003

dolichol-phosphate mannosyltransferase
  
Accession: EGD95004
  
Location: 475569-476528
  
 NCBI BlastP on this gene

EGD95004

hypothetical protein
  
Accession: EGD95005
  
Location: 476787-477885
  
 NCBI BlastP on this gene

EGD95005

hypothetical protein
  
Accession: EGD95006
  
Location: 478346-480609
  
 NCBI BlastP on this gene

EGD95006

hypothetical protein
  
Accession: EGD95007
  
Location: 481132-481770
  
 NCBI BlastP on this gene

EGD95007

239. :  DS995701 Microsporum canis CBS 113480 supercont1.1 genomic scaffold     Total score: 2.0     Cumulative Blast bit score: 715

conserved hypothetical protein
  
Accession: EEQ27880
  
Location: 2077916-2080178
  
 NCBI BlastP on this gene

EEQ27880

conserved hypothetical protein
  
Accession: EEQ27879
  
Location: 2074401-2076061
  
 NCBI BlastP on this gene

EEQ27879

conserved hypothetical protein
  
Accession: EEQ27878
  
Location: 2073704-2074046
  
 NCBI BlastP on this gene

EEQ27878

serine/threonine-protein kinase prp4
  
Accession: EEQ27877
  
Location: 2070654-2073180
  
  
**BlastP hit with Mycgr3G103260\_Mycgr3**
  
Percentage identity: 52 %
  
BlastP bit score: 279
  
Sequence coverage: 96 %
  
E-value: 9e-85
  
  
 NCBI BlastP on this gene

EEQ27877

predicted protein
  
Accession: EEQ27876
  
Location: 2068171-2069374
  
 NCBI BlastP on this gene

EEQ27876

predicted protein
  
Accession: EEQ27875
  
Location: 2066082-2066638
  
 NCBI BlastP on this gene

EEQ27875

negative regulator of sexual conjugation and meiosis
  
Accession: EEQ27874
  
Location: 2057852-2059163
  
  
**BlastP hit with Mycgr3G68429\_Mycgr3T**
  
Percentage identity: 63 %
  
BlastP bit score: 436
  
Sequence coverage: 82 %
  
E-value: 7e-147
  
  
 NCBI BlastP on this gene

EEQ27874

predicted protein
  
Accession: EEQ27873
  
Location: 2055556-2056376
  
 NCBI BlastP on this gene

EEQ27873

short-chain dehydrogenase/reductase SDR
  
Accession: EEQ27872
  
Location: 2053702-2054819
  
 NCBI BlastP on this gene

EEQ27872

adiponectin receptor protein 1
  
Accession: EEQ27871
  
Location: 2052454-2053401
  
 NCBI BlastP on this gene

EEQ27871

conserved hypothetical protein
  
Accession: EEQ27870
  
Location: 2051047-2052117
  
 NCBI BlastP on this gene

EEQ27870

conserved hypothetical protein
  
Accession: EEQ27869
  
Location: 2049237-2050812
  
 NCBI BlastP on this gene

EEQ27869

dolichol-phosphate mannosyltransferase
  
Accession: EEQ27868
  
Location: 2047931-2048786
  
 NCBI BlastP on this gene

EEQ27868

dolichol-phosphate mannosyltransferase
  
Accession: EEQ27867
  
Location: 2046494-2047607
  
 NCBI BlastP on this gene

EEQ27867

transcription factor tfiiic complex subunit sfc6
  
Accession: EEQ27866
  
Location: 2043808-2046070
  
 NCBI BlastP on this gene

EEQ27866

A-agglutinin anchorage subunit
  
Accession: EEQ27865
  
Location: 2041834-2043323
  
 NCBI BlastP on this gene

EEQ27865

vacuolar sorting protein 35
  
Accession: EEQ27864
  
Location: 2039732-2041759
  
 NCBI BlastP on this gene

EEQ27864

240. :  ACFW01000030 Coccidioides posadasii C735 delta SOWgp     Total score: 2.0     Cumulative Blast bit score: 714

hypothetical protein
  
Accession: EER26484
  
Location: 1637807-1639585
  
 NCBI BlastP on this gene

EER26484

hypothetical protein
  
Accession: EER26485
  
Location: 1640140-1641232
  
 NCBI BlastP on this gene

EER26485

hypothetical protein
  
Accession: EER26486
  
Location: 1643600-1644218
  
 NCBI BlastP on this gene

EER26486

hypothetical protein
  
Accession: EER26487
  
Location: 1645209-1647812
  
 NCBI BlastP on this gene

EER26487

Cytochrome b5-like Heme/Steroid binding domain containing protein
  
Accession: EER26488
  
Location: 1650453-1651167
  
 NCBI BlastP on this gene

EER26488

hypothetical protein
  
Accession: EER26489
  
Location: 1652015-1653792
  
 NCBI BlastP on this gene

EER26489

hypothetical protein
  
Accession: EER26490
  
Location: 1654318-1654694
  
 NCBI BlastP on this gene

EER26490

serine/threonine-protein kinase, putative
  
Accession: EER26491
  
Location: 1655225-1657722
  
  
**BlastP hit with Mycgr3G103260\_Mycgr3**
  
Percentage identity: 52 %
  
BlastP bit score: 280
  
Sequence coverage: 96 %
  
E-value: 6e-85
  
  
 NCBI BlastP on this gene

EER26491

Ran1-like protein kinase, putative
  
Accession: EER26492
  
Location: 1664833-1666515
  
  
**BlastP hit with Mycgr3G68429\_Mycgr3T**
  
Percentage identity: 67 %
  
BlastP bit score: 434
  
Sequence coverage: 74 %
  
E-value: 1e-144
  
  
 NCBI BlastP on this gene

EER26492

oxidoreductase,short chain dehydrogenase, putative
  
Accession: EER26493
  
Location: 1670663-1671793
  
 NCBI BlastP on this gene

EER26493

Hemolysin-III related family protein
  
Accession: EER26494
  
Location: 1672313-1673275
  
 NCBI BlastP on this gene

EER26494

hypothetical protein
  
Accession: EER26495
  
Location: 1673593-1674640
  
 NCBI BlastP on this gene

EER26495

hypothetical protein
  
Accession: EER26496
  
Location: 1674799-1676135
  
 NCBI BlastP on this gene

EER26496

dolichol-phosphate mannosyltransferase, putative
  
Accession: EER26497
  
Location: 1676665-1677579
  
 NCBI BlastP on this gene

EER26497

hypothetical protein
  
Accession: EER26498
  
Location: 1677829-1678933
  
 NCBI BlastP on this gene

EER26498

hypothetical protein
  
Accession: EER26499
  
Location: 1679339-1681628
  
 NCBI BlastP on this gene

EER26499

hypothetical protein
  
Accession: EER26500
  
Location: 1682008-1682789
  
 NCBI BlastP on this gene

EER26500

241. :  KB644412 Penicillium oxalicum 114-2 unplaced genomic scaffold scaffold\_5     Total score: 2.0     Cumulative Blast bit score: 712

hypothetical protein
  
Accession: EPS29769
  
Location: 807471-809933
  
 NCBI BlastP on this gene

EPS29769

hypothetical protein
  
Accession: EPS29770
  
Location: 810416-810767
  
 NCBI BlastP on this gene

EPS29770

hypothetical protein
  
Accession: EPS29771
  
Location: 811615-812643
  
 NCBI BlastP on this gene

EPS29771

hypothetical protein
  
Accession: EPS29772
  
Location: 813112-814250
  
 NCBI BlastP on this gene

EPS29772

hypothetical protein
  
Accession: EPS29773
  
Location: 817431-819259
  
 NCBI BlastP on this gene

EPS29773

hypothetical protein
  
Accession: EPS29774
  
Location: 819797-820566
  
 NCBI BlastP on this gene

EPS29774

hypothetical protein
  
Accession: EPS29775
  
Location: 821891-823478
  
 NCBI BlastP on this gene

EPS29775

hypothetical protein
  
Accession: EPS29776
  
Location: 824271-826821
  
  
**BlastP hit with Mycgr3G103260\_Mycgr3**
  
Percentage identity: 54 %
  
BlastP bit score: 276
  
Sequence coverage: 96 %
  
E-value: 2e-83
  
  
 NCBI BlastP on this gene

EPS29776

hypothetical protein
  
Accession: EPS29777
  
Location: 827224-827555
  
 NCBI BlastP on this gene

EPS29777

hypothetical protein
  
Accession: EPS29778
  
Location: 831072-831347
  
 NCBI BlastP on this gene

EPS29778

hypothetical protein
  
Accession: EPS29779
  
Location: 834984-835291
  
 NCBI BlastP on this gene

EPS29779

hypothetical protein
  
Accession: EPS29780
  
Location: 836364-836597
  
 NCBI BlastP on this gene

EPS29780

hypothetical protein
  
Accession: EPS29781
  
Location: 837856-839489
  
  
**BlastP hit with Mycgr3G68429\_Mycgr3T**
  
Percentage identity: 63 %
  
BlastP bit score: 436
  
Sequence coverage: 82 %
  
E-value: 5e-147
  
  
 NCBI BlastP on this gene

EPS29781

hypothetical protein
  
Accession: EPS29782
  
Location: 841066-842414
  
 NCBI BlastP on this gene

EPS29782

hypothetical protein
  
Accession: EPS29783
  
Location: 843063-844431
  
 NCBI BlastP on this gene

EPS29783

hypothetical protein
  
Accession: EPS29784
  
Location: 845955-846914
  
 NCBI BlastP on this gene

EPS29784

hypothetical protein
  
Accession: EPS29785
  
Location: 847517-848542
  
 NCBI BlastP on this gene

EPS29785

hypothetical protein
  
Accession: EPS29786
  
Location: 848788-850483
  
 NCBI BlastP on this gene

EPS29786

putative dolichyl-phosphate beta-D-mannosyltransferase
  
Accession: EPS29787
  
Location: 850620-851421
  
 NCBI BlastP on this gene

EPS29787

hypothetical protein
  
Accession: EPS29788
  
Location: 851701-852801
  
 NCBI BlastP on this gene

EPS29788

hypothetical protein
  
Accession: EPS29789
  
Location: 854436-856738
  
 NCBI BlastP on this gene

EPS29789

242. :  GG700648 Trichophyton rubrum CBS 118892 genomic scaffold supercont2.1     Total score: 2.0     Cumulative Blast bit score: 712

hypothetical protein
  
Accession: EGD84046
  
Location: 830935-833327
  
 NCBI BlastP on this gene

EGD84046

hypothetical protein
  
Accession: EGD84045
  
Location: 829137-829749
  
 NCBI BlastP on this gene

EGD84045

PQ loop repeat protein
  
Accession: EGD84044
  
Location: 826608-827656
  
 NCBI BlastP on this gene

EGD84044

hypothetical protein
  
Accession: EGD84043
  
Location: 823801-825596
  
 NCBI BlastP on this gene

EGD84043

DNA damage response protein
  
Accession: EGD84042
  
Location: 822756-823450
  
 NCBI BlastP on this gene

EGD84042

hypothetical protein
  
Accession: EGD84041
  
Location: 820221-821783
  
 NCBI BlastP on this gene

EGD84041

hypothetical protein
  
Accession: EGD84040
  
Location: 819516-819864
  
 NCBI BlastP on this gene

EGD84040

CMGC/DYRK/PRP4 protein kinase
  
Accession: EGD84039
  
Location: 816424-818946
  
  
**BlastP hit with Mycgr3G103260\_Mycgr3**
  
Percentage identity: 54 %
  
BlastP bit score: 283
  
Sequence coverage: 96 %
  
E-value: 2e-86
  
  
 NCBI BlastP on this gene

EGD84039

hypothetical protein
  
Accession: EGD84038
  
Location: 814483-814907
  
 NCBI BlastP on this gene

EGD84038

hypothetical protein
  
Accession: EGD84037
  
Location: 810024-810461
  
 NCBI BlastP on this gene

EGD84037

hypothetical protein
  
Accession: EGD84036
  
Location: 807486-807903
  
 NCBI BlastP on this gene

EGD84036

hypothetical protein
  
Accession: EGD84035
  
Location: 805655-806349
  
 NCBI BlastP on this gene

EGD84035

serine/threonine protein kinase
  
Accession: EGD84034
  
Location: 802280-803594
  
  
**BlastP hit with Mycgr3G68429\_Mycgr3T**
  
Percentage identity: 63 %
  
BlastP bit score: 429
  
Sequence coverage: 79 %
  
E-value: 4e-144
  
  
 NCBI BlastP on this gene

EGD84034

3-oxoacyl-(acyl-carrier-protein) reductase
  
Accession: EGD84033
  
Location: 797657-798844
  
 NCBI BlastP on this gene

EGD84033

hemolysin-III channel protein Izh2
  
Accession: EGD84032
  
Location: 796174-797157
  
 NCBI BlastP on this gene

EGD84032

hypothetical protein
  
Accession: EGD84031
  
Location: 794695-795777
  
 NCBI BlastP on this gene

EGD84031

hypothetical protein
  
Accession: EGD84030
  
Location: 792825-794395
  
 NCBI BlastP on this gene

EGD84030

dolichol-phosphate mannosyltransferase
  
Accession: EGD84029
  
Location: 791473-792433
  
 NCBI BlastP on this gene

EGD84029

hypothetical protein
  
Accession: EGD84028
  
Location: 790117-791203
  
 NCBI BlastP on this gene

EGD84028

hypothetical protein
  
Accession: EGD84027
  
Location: 787395-789656
  
 NCBI BlastP on this gene

EGD84027

hypothetical protein
  
Accession: EGD84026
  
Location: 786249-786890
  
 NCBI BlastP on this gene

EGD84026

243. :  AACD01000084 Aspergillus nidulans FGSC A4     Total score: 2.0     Cumulative Blast bit score: 710

hypothetical protein
  
Accession: EAA61023
  
Location: 286718-288859
  
 NCBI BlastP on this gene

EAA61023

hypothetical protein
  
Accession: EAA61022
  
Location: 283802-285041
  
 NCBI BlastP on this gene

EAA61022

hypothetical protein
  
Accession: EAA61021
  
Location: 280871-283294
  
 NCBI BlastP on this gene

EAA61021

hypothetical protein
  
Accession: EAA61020
  
Location: 279683-280552
  
 NCBI BlastP on this gene

EAA61020

hypothetical protein
  
Accession: EAA61019
  
Location: 278273-279267
  
 NCBI BlastP on this gene

EAA61019

hypothetical protein
  
Accession: EAA61018
  
Location: 275740-277490
  
 NCBI BlastP on this gene

EAA61018

hypothetical protein
  
Accession: EAA61017
  
Location: 274571-275273
  
 NCBI BlastP on this gene

EAA61017

hypothetical protein
  
Accession: EAA61016
  
Location: 270143-271860
  
 NCBI BlastP on this gene

EAA61016

hypothetical protein
  
Accession: EAA61015
  
Location: 269374-269710
  
 NCBI BlastP on this gene

EAA61015

hypothetical protein
  
Accession: EAA61014
  
Location: 266473-268912
  
  
**BlastP hit with Mycgr3G103260\_Mycgr3**
  
Percentage identity: 55 %
  
BlastP bit score: 286
  
Sequence coverage: 98 %
  
E-value: 2e-87
  
  
 NCBI BlastP on this gene

EAA61014

hypothetical protein
  
Accession: EAA61013
  
Location: 261811-263149
  
  
**BlastP hit with Mycgr3G68429\_Mycgr3T**
  
Percentage identity: 71 %
  
BlastP bit score: 424
  
Sequence coverage: 66 %
  
E-value: 5e-142
  
  
 NCBI BlastP on this gene

EAA61013

hypothetical protein
  
Accession: EAA61012
  
Location: 259737-260839
  
 NCBI BlastP on this gene

EAA61012

hypothetical protein
  
Accession: EAA61011
  
Location: 258354-259480
  
 NCBI BlastP on this gene

EAA61011

hypothetical protein
  
Accession: EAA61010
  
Location: 256438-257394
  
 NCBI BlastP on this gene

EAA61010

hypothetical protein
  
Accession: EAA61009
  
Location: 254960-255996
  
 NCBI BlastP on this gene

EAA61009

hypothetical protein
  
Accession: EAA61008
  
Location: 253493-254831
  
 NCBI BlastP on this gene

EAA61008

hypothetical protein
  
Accession: EAA61007
  
Location: 244449-247347
  
 NCBI BlastP on this gene

EAA61007

hypothetical protein
  
Accession: EAA61006
  
Location: 242652-243707
  
 NCBI BlastP on this gene

EAA61006

244. :  DF126452 Aspergillus kawachii IFO 4308 DNA, contig: scaffold00006     Total score: 2.0     Cumulative Blast bit score: 708

similar to An16g04370
  
Accession: GAA85019
  
Location: 990261-991520
  
 NCBI BlastP on this gene

GAA85019

coatamer subunit protein
  
Accession: GAA85020
  
Location: 995278-997707
  
 NCBI BlastP on this gene

GAA85020

nuclear transport factor 2
  
Accession: GAA85021
  
Location: 998095-999079
  
 NCBI BlastP on this gene

GAA85021

PQ loop repeat protein
  
Accession: GAA85022
  
Location: 999570-1000596
  
 NCBI BlastP on this gene

GAA85022

stress response protein
  
Accession: GAA85023
  
Location: 1001582-1003396
  
 NCBI BlastP on this gene

GAA85023

DNA damage response protein
  
Accession: GAA85024
  
Location: 1003919-1004481
  
 NCBI BlastP on this gene

GAA85024

similar to An16g04440
  
Accession: GAA85025
  
Location: 1006026-1007822
  
 NCBI BlastP on this gene

GAA85025

coiled-coil domain-containing protein
  
Accession: GAA85026
  
Location: 1008318-1008656
  
 NCBI BlastP on this gene

GAA85026

serine/threonine protein kinase
  
Accession: GAA85027
  
Location: 1009237-1011764
  
  
**BlastP hit with Mycgr3G103260\_Mycgr3**
  
Percentage identity: 53 %
  
BlastP bit score: 275
  
Sequence coverage: 98 %
  
E-value: 3e-83
  
  
 NCBI BlastP on this gene

GAA85027

hypothetical protein
  
Accession: GAA85028
  
Location: 1018827-1020174
  
  
**BlastP hit with Mycgr3G68429\_Mycgr3T**
  
Percentage identity: 68 %
  
BlastP bit score: 433
  
Sequence coverage: 73 %
  
E-value: 7e-146
  
  
 NCBI BlastP on this gene

GAA85028

3-oxoacyl-(acyl-carrier-protein) reductase
  
Accession: GAA85029
  
Location: 1021724-1024366
  
 NCBI BlastP on this gene

GAA85029

haemolysin-III channel protein Izh2
  
Accession: GAA85030
  
Location: 1025464-1026429
  
 NCBI BlastP on this gene

GAA85030

similar to An16g04550
  
Accession: GAA85031
  
Location: 1026913-1027948
  
 NCBI BlastP on this gene

GAA85031

hypothetical protein
  
Accession: GAA85032
  
Location: 1028147-1029660
  
 NCBI BlastP on this gene

GAA85032

clathrin-coated vesicle protein
  
Accession: GAA85033
  
Location: 1030082-1030922
  
 NCBI BlastP on this gene

GAA85033

eukaryotic translation initiation factor 3 subunit 6-interacting protein
  
Accession: GAA85034
  
Location: 1031780-1033362
  
 NCBI BlastP on this gene

GAA85034

iron-sulfur cluster assembly accessory protein Isa2
  
Accession: GAA85035
  
Location: 1034160-1035318
  
 NCBI BlastP on this gene

GAA85035

kynureninase
  
Accession: GAA85036
  
Location: 1035693-1037324
  
 NCBI BlastP on this gene

GAA85036

ER membrane DUF1077 domain protein
  
Accession: GAA85037
  
Location: 1037661-1038394
  
 NCBI BlastP on this gene

GAA85037

245. :  ABSU01000003 Arthroderma benhamiae CBS 112371     Total score: 2.0     Cumulative Blast bit score: 708

hypothetical protein
  
Accession: EFE35458
  
Location: 346001-346626
  
 NCBI BlastP on this gene

EFE35458

conserved hypothetical protein
  
Accession: EFE35457
  
Location: 343364-345740
  
 NCBI BlastP on this gene

EFE35457

conserved serine-rich protein
  
Accession: EFE35456
  
Location: 341542-341844
  
 NCBI BlastP on this gene

EFE35456

hypothetical protein
  
Accession: EFE35455
  
Location: 338975-340024
  
 NCBI BlastP on this gene

EFE35455

hypothetical protein
  
Accession: EFE35454
  
Location: 336145-337836
  
 NCBI BlastP on this gene

EFE35454

hypothetical protein
  
Accession: EFE35453
  
Location: 335093-335787
  
 NCBI BlastP on this gene

EFE35453

hypothetical protein
  
Accession: EFE35452
  
Location: 332556-333923
  
 NCBI BlastP on this gene

EFE35452

hypothetical protein
  
Accession: EFE35451
  
Location: 331852-332198
  
 NCBI BlastP on this gene

EFE35451

hypothetical protein
  
Accession: EFE35450
  
Location: 328764-331283
  
  
**BlastP hit with Mycgr3G103260\_Mycgr3**
  
Percentage identity: 54 %
  
BlastP bit score: 283
  
Sequence coverage: 96 %
  
E-value: 2e-86
  
  
 NCBI BlastP on this gene

EFE35450

hypothetical protein
  
Accession: EFE35449
  
Location: 321609-322166
  
 NCBI BlastP on this gene

EFE35449

hypothetical protein
  
Accession: EFE35448
  
Location: 317830-318141
  
 NCBI BlastP on this gene

EFE35448

hypothetical protein
  
Accession: EFE35447
  
Location: 314436-315746
  
  
**BlastP hit with Mycgr3G68429\_Mycgr3T**
  
Percentage identity: 62 %
  
BlastP bit score: 425
  
Sequence coverage: 83 %
  
E-value: 1e-142
  
  
 NCBI BlastP on this gene

EFE35447

hypothetical protein
  
Accession: EFE35446
  
Location: 312125-313063
  
 NCBI BlastP on this gene

EFE35446

hypothetical protein
  
Accession: EFE35445
  
Location: 309819-311005
  
 NCBI BlastP on this gene

EFE35445

hypothetical protein
  
Accession: EFE35444
  
Location: 308354-309334
  
 NCBI BlastP on this gene

EFE35444

conserved hypothetical protein
  
Accession: EFE35443
  
Location: 306848-307678
  
 NCBI BlastP on this gene

EFE35443

hypothetical protein
  
Accession: EFE35442
  
Location: 304745-306547
  
 NCBI BlastP on this gene

EFE35442

hypothetical protein
  
Accession: EFE35441
  
Location: 303589-304449
  
 NCBI BlastP on this gene

EFE35441

esterase, putative
  
Accession: EFE35440
  
Location: 302244-303317
  
 NCBI BlastP on this gene

EFE35440

transcription factor TFIIIC complex subunit Tfc6, putative
  
Accession: EFE35439
  
Location: 299491-301752
  
 NCBI BlastP on this gene

EFE35439

246. :  AM270368 Aspergillus niger contig An16c0160, genomic contig.     Total score: 2.0     Cumulative Blast bit score: 707

not annotated
  
Accession: CAK46854
  
Location: 60474-61727
  
 NCBI BlastP on this gene

An16g04370

not annotated
  
Accession: CAK46855
  
Location: 62222-63822
  
 NCBI BlastP on this gene

An16g04380

not annotated
  
Accession: CAK46856
  
Location: 64887-67316
  
 NCBI BlastP on this gene

An16g04390

not annotated
  
Accession: CAK46857
  
Location: 67710-68670
  
 NCBI BlastP on this gene

An16g04400

not annotated
  
Accession: CAK46858
  
Location: 68899-70201
  
 NCBI BlastP on this gene

An16g04410

unnamed
  
Accession: CAK46859
  
Location: 71177-72995
  
 NCBI BlastP on this gene

An16g04420

not annotated
  
Accession: CAK46860
  
Location: 73522-74272
  
 NCBI BlastP on this gene

An16g04430

hypothetical protein
  
Accession: CAK46861
  
Location: 75519-77317
  
 NCBI BlastP on this gene

An16g04440

not annotated
  
Accession: CAK46862
  
Location: 77817-78159
  
 NCBI BlastP on this gene

An16g04450

not annotated
  
Accession: CAK46863
  
Location: 78749-81300
  
  
**BlastP hit with Mycgr3G103260\_Mycgr3**
  
Percentage identity: 53 %
  
BlastP bit score: 275
  
Sequence coverage: 98 %
  
E-value: 3e-83
  
  
 NCBI BlastP on this gene

An16g04460

not annotated
  
Accession: CAK46864
  
Location: 82403-82969
  
 NCBI BlastP on this gene

An16g04470

hypothetical protein
  
Accession: CAK46865
  
Location: 84832-85167
  
 NCBI BlastP on this gene

An16g04480

not annotated
  
Accession: CAK46866
  
Location: 86913-87457
  
 NCBI BlastP on this gene

An16g04490

not annotated
  
Accession: CAK46867
  
Location: 88233-89533
  
  
**BlastP hit with Mycgr3G68429\_Mycgr3T**
  
Percentage identity: 68 %
  
BlastP bit score: 432
  
Sequence coverage: 73 %
  
E-value: 7e-146
  
  
 NCBI BlastP on this gene

An16g04500

not annotated
  
Accession: CAK46868
  
Location: 90262-90777
  
 NCBI BlastP on this gene

An16g04510

not annotated
  
Accession: CAK46869
  
Location: 91025-92268
  
 NCBI BlastP on this gene

An16g04520

not annotated
  
Accession: CAK46870
  
Location: 92528-93671
  
 NCBI BlastP on this gene

An16g04530

247. :  ACJE01000013 Aspergillus niger ATCC 1015     Total score: 2.0     Cumulative Blast bit score: 707

hypothetical protein
  
Accession: EHA22031
  
Location: 1315775-1317034
  
 NCBI BlastP on this gene

EHA22031

hypothetical protein
  
Accession: EHA22032
  
Location: 1320196-1322625
  
 NCBI BlastP on this gene

EHA22032

hypothetical protein
  
Accession: EHA22033
  
Location: 1323019-1323979
  
 NCBI BlastP on this gene

EHA22033

hypothetical protein
  
Accession: EHA22034
  
Location: 1324489-1325510
  
 NCBI BlastP on this gene

EHA22034

hypothetical protein
  
Accession: EHA22035
  
Location: 1326482-1328300
  
 NCBI BlastP on this gene

EHA22035

hypothetical protein
  
Accession: EHA22036
  
Location: 1328827-1329416
  
 NCBI BlastP on this gene

EHA22036

hypothetical protein
  
Accession: EHA22037
  
Location: 1331265-1332623
  
 NCBI BlastP on this gene

EHA22037

hypothetical protein
  
Accession: EHA22038
  
Location: 1334055-1336605
  
  
**BlastP hit with Mycgr3G103260\_Mycgr3**
  
Percentage identity: 53 %
  
BlastP bit score: 275
  
Sequence coverage: 98 %
  
E-value: 2e-83
  
  
 NCBI BlastP on this gene

EHA22038

hypothetical protein
  
Accession: EHA22039
  
Location: 1343514-1344816
  
  
**BlastP hit with Mycgr3G68429\_Mycgr3T**
  
Percentage identity: 68 %
  
BlastP bit score: 432
  
Sequence coverage: 73 %
  
E-value: 7e-146
  
  
 NCBI BlastP on this gene

EHA22039

hypothetical protein
  
Accession: EHA22040
  
Location: 1346365-1347557
  
 NCBI BlastP on this gene

EHA22040

hypothetical protein
  
Accession: EHA22041
  
Location: 1347831-1348968
  
 NCBI BlastP on this gene

EHA22041

hypothetical protein
  
Accession: EHA22042
  
Location: 1350049-1351014
  
 NCBI BlastP on this gene

EHA22042

hypothetical protein
  
Accession: EHA22043
  
Location: 1351496-1352531
  
 NCBI BlastP on this gene

EHA22043

hypothetical protein
  
Accession: EHA22044
  
Location: 1352736-1354050
  
 NCBI BlastP on this gene

EHA22044

hypothetical protein
  
Accession: EHA22045
  
Location: 1354670-1355503
  
 NCBI BlastP on this gene

EHA22045

hypothetical protein
  
Accession: EHA22046
  
Location: 1356357-1357939
  
 NCBI BlastP on this gene

EHA22046

hypothetical protein
  
Accession: EHA22047
  
Location: 1358752-1359854
  
 NCBI BlastP on this gene

EHA22047

hypothetical protein
  
Accession: EHA22048
  
Location: 1360225-1361856
  
 NCBI BlastP on this gene

EHA22048

hypothetical protein
  
Accession: EHA22049
  
Location: 1362222-1362950
  
 NCBI BlastP on this gene

EHA22049

248. :  GG749418 Ajellomyces dermatitidis ATCC 18188 genomic scaffold supercont1.12     Total score: 2.0     Cumulative Blast bit score: 706

hypothetical protein
  
Accession: EGE80176
  
Location: 598558-600808
  
 NCBI BlastP on this gene

EGE80176

hypothetical protein
  
Accession: EGE80177
  
Location: 604471-605108
  
 NCBI BlastP on this gene

EGE80177

PQ loop repeat protein
  
Accession: EGE80178
  
Location: 605564-606635
  
 NCBI BlastP on this gene

EGE80178

stress response protein ish1
  
Accession: EGE80179
  
Location: 607431-609278
  
 NCBI BlastP on this gene

EGE80179

membrane-associated progesterone receptor component 1
  
Accession: EGE80180
  
Location: 610207-611127
  
 NCBI BlastP on this gene

EGE80180

hypothetical protein
  
Accession: EGE80181
  
Location: 611819-613773
  
 NCBI BlastP on this gene

EGE80181

hypothetical protein
  
Accession: EGE80182
  
Location: 614736-615120
  
 NCBI BlastP on this gene

EGE80182

serine/threonine-protein kinase prp4
  
Accession: EGE80183
  
Location: 615728-618220
  
  
**BlastP hit with Mycgr3G103260\_Mycgr3**
  
Percentage identity: 54 %
  
BlastP bit score: 280
  
Sequence coverage: 97 %
  
E-value: 6e-85
  
  
 NCBI BlastP on this gene

EGE80183

integral membrane protein
  
Accession: EGE80184
  
Location: 618662-620173
  
 NCBI BlastP on this gene

EGE80184

hypothetical protein
  
Accession: EGE80185
  
Location: 623503-626728
  
 NCBI BlastP on this gene

EGE80185

hypothetical protein
  
Accession: EGE80186
  
Location: 627100-628985
  
 NCBI BlastP on this gene

EGE80186

protein serine/threonine kinase
  
Accession: EGE80187
  
Location: 630472-631830
  
  
**BlastP hit with Mycgr3G68429\_Mycgr3T**
  
Percentage identity: 68 %
  
BlastP bit score: 426
  
Sequence coverage: 71 %
  
E-value: 3e-143
  
  
 NCBI BlastP on this gene

EGE80187

short-chain dehydrogenase/reductase SDR
  
Accession: EGE80188
  
Location: 633398-634597
  
 NCBI BlastP on this gene

EGE80188

hemolysin-III channel protein Izh2
  
Accession: EGE80189
  
Location: 635189-636220
  
 NCBI BlastP on this gene

EGE80189

hypothetical protein
  
Accession: EGE80190
  
Location: 636418-637534
  
 NCBI BlastP on this gene

EGE80190

hypothetical protein
  
Accession: EGE80191
  
Location: 637748-639317
  
 NCBI BlastP on this gene

EGE80191

dolichol-phosphate mannosyltransferase
  
Accession: EGE80192
  
Location: 639739-640651
  
 NCBI BlastP on this gene

EGE80192

esterase
  
Accession: EGE80193
  
Location: 641122-642249
  
 NCBI BlastP on this gene

EGE80193

transcription factor tfiiic complex subunit tfc6
  
Accession: EGE80194
  
Location: 642818-645215
  
 NCBI BlastP on this gene

EGE80194

hypothetical protein
  
Accession: EGE80195
  
Location: 645972-646538
  
 NCBI BlastP on this gene

EGE80195

hypothetical protein
  
Accession: EGE80196
  
Location: 647147-648403
  
 NCBI BlastP on this gene

EGE80196

249. :  KB445649 Cochliobolus sativus ND90Pr unplaced genomic scaffold COCSAscaffold\_13     Total score: 2.0     Cumulative Blast bit score: 704

hypothetical protein
  
Accession: EMD61193
  
Location: 1182865-1184103
  
 NCBI BlastP on this gene

EMD61193

hypothetical protein
  
Accession: EMD61194
  
Location: 1184804-1186119
  
 NCBI BlastP on this gene

EMD61194

hypothetical protein
  
Accession: EMD61195
  
Location: 1186635-1187406
  
 NCBI BlastP on this gene

EMD61195

hypothetical protein
  
Accession: EMD61196
  
Location: 1187981-1189630
  
 NCBI BlastP on this gene

EMD61196

hypothetical protein
  
Accession: EMD61197
  
Location: 1190061-1190693
  
 NCBI BlastP on this gene

EMD61197

hypothetical protein
  
Accession: EMD61198
  
Location: 1195220-1198205
  
 NCBI BlastP on this gene

EMD61198

hypothetical protein
  
Accession: EMD61199
  
Location: 1198819-1199687
  
 NCBI BlastP on this gene

EMD61199

hypothetical protein
  
Accession: EMD61200
  
Location: 1200407-1203758
  
  
**BlastP hit with Mycgr3G68433\_Mycgr3T**
  
Percentage identity: 42 %
  
BlastP bit score: 577
  
Sequence coverage: 86 %
  
E-value: 0.0
  
  
 NCBI BlastP on this gene

EMD61200

hypothetical protein
  
Accession: EMD61201
  
Location: 1204376-1205740
  
  
**BlastP hit with Mycgr3G90786\_Mycgr3T**
  
Percentage identity: 30 %
  
BlastP bit score: 127
  
Sequence coverage: 92 %
  
E-value: 5e-29
  
  
 NCBI BlastP on this gene

EMD61201

hypothetical protein
  
Accession: EMD61202
  
Location: 1206541-1207901
  
 NCBI BlastP on this gene

EMD61202

hypothetical protein
  
Accession: EMD61203
  
Location: 1208201-1209321
  
 NCBI BlastP on this gene

EMD61203

hypothetical protein
  
Accession: EMD61204
  
Location: 1209806-1211101
  
 NCBI BlastP on this gene

EMD61204

hypothetical protein
  
Accession: EMD61205
  
Location: 1211333-1211767
  
 NCBI BlastP on this gene

EMD61205

hypothetical protein
  
Accession: EMD61206
  
Location: 1211862-1213013
  
 NCBI BlastP on this gene

EMD61206

hypothetical protein
  
Accession: EMD61207
  
Location: 1213473-1214712
  
 NCBI BlastP on this gene

EMD61207

hypothetical protein
  
Accession: EMD61208
  
Location: 1215338-1216756
  
 NCBI BlastP on this gene

EMD61208

hypothetical protein
  
Accession: EMD61209
  
Location: 1219347-1219835
  
 NCBI BlastP on this gene

EMD61209

hypothetical protein
  
Accession: EMD61210
  
Location: 1222789-1224415
  
 NCBI BlastP on this gene

EMD61210

hypothetical protein
  
Accession: EMD61211
  
Location: 1225655-1227043
  
 NCBI BlastP on this gene

EMD61211

250. :  EQ999973 Ajellomyces dermatitidis ER-3 genomic scaffold supercont1.1     Total score: 2.0     Cumulative Blast bit score: 704

conserved hypothetical protein
  
Accession: EEQ83842
  
Location: 5063516-5065766
  
 NCBI BlastP on this gene

EEQ83842

predicted protein
  
Accession: EEQ83843
  
Location: 5068129-5068890
  
 NCBI BlastP on this gene

EEQ83843

conserved hypothetical protein
  
Accession: EEQ83844
  
Location: 5069518-5070155
  
 NCBI BlastP on this gene

EEQ83844

PQ loop repeat protein
  
Accession: EEQ83845
  
Location: 5070611-5071682
  
 NCBI BlastP on this gene

EEQ83845

conserved hypothetical protein
  
Accession: EEQ83846
  
Location: 5072476-5074323
  
 NCBI BlastP on this gene

EEQ83846

DNA damage response protein
  
Accession: EEQ83847
  
Location: 5075290-5076100
  
 NCBI BlastP on this gene

EEQ83847

conserved hypothetical protein
  
Accession: EEQ83848
  
Location: 5076890-5078844
  
 NCBI BlastP on this gene

EEQ83848

hypothetical protein
  
Accession: EEQ83849
  
Location: 5079813-5080197
  
 NCBI BlastP on this gene

EEQ83849

serine/threonine-protein kinase prp4
  
Accession: EEQ83850
  
Location: 5080804-5083296
  
  
**BlastP hit with Mycgr3G103260\_Mycgr3**
  
Percentage identity: 54 %
  
BlastP bit score: 278
  
Sequence coverage: 96 %
  
E-value: 3e-84
  
  
 NCBI BlastP on this gene

EEQ83850

integral membrane protein
  
Accession: EEQ83851
  
Location: 5083738-5085249
  
 NCBI BlastP on this gene

EEQ83851

conserved hypothetical protein
  
Accession: EEQ83852
  
Location: 5087537-5091831
  
 NCBI BlastP on this gene

EEQ83852

predicted protein
  
Accession: EEQ83853
  
Location: 5092207-5094081
  
 NCBI BlastP on this gene

EEQ83853

protein serine/threonine kinase
  
Accession: EEQ83854
  
Location: 5094929-5096275
  
  
**BlastP hit with Mycgr3G68429\_Mycgr3T**
  
Percentage identity: 68 %
  
BlastP bit score: 426
  
Sequence coverage: 71 %
  
E-value: 3e-143
  
  
 NCBI BlastP on this gene

EEQ83854

3-oxoacyl-(acyl-carrier-protein) reductase
  
Accession: EEQ83855
  
Location: 5097842-5099043
  
 NCBI BlastP on this gene

EEQ83855

hemolysin-III channel protein Izh2
  
Accession: EEQ83856
  
Location: 5099634-5100611
  
 NCBI BlastP on this gene

EEQ83856

conserved hypothetical protein
  
Accession: EEQ83857
  
Location: 5100863-5101979
  
 NCBI BlastP on this gene

EEQ83857

conserved hypothetical protein
  
Accession: EEQ83858
  
Location: 5102265-5103764
  
 NCBI BlastP on this gene

EEQ83858

dolichol-phosphate mannosyltransferase
  
Accession: EEQ83859
  
Location: 5104290-5105097
  
 NCBI BlastP on this gene

EEQ83859

esterase
  
Accession: EEQ83860
  
Location: 5105568-5106695
  
 NCBI BlastP on this gene

EEQ83860

transcription factor tfiiic complex subunit tfc6
  
Accession: EEQ83861
  
Location: 5107263-5109660
  
 NCBI BlastP on this gene

EEQ83861

hypothetical protein
  
Accession: EEQ83862
  
Location: 5110413-5110931
  
 NCBI BlastP on this gene

EEQ83862

hypothetical protein
  
Accession: EEQ83863
  
Location: 5111545-5112801
  
 NCBI BlastP on this gene

EEQ83863

Detecting sequence homology at the gene cluster level with MultiGeneBlast.
  
Marnix H. Medema, Rainer Breitling & Eriko Takano (2013)
  
*Molecular Biology and Evolution* , 30: 1218-1223.
