# Supplementary material for: In silico prediction and characterization of secondary metabolite biosynthetic gene clusters in the wheat pathogen Zymoseptoria tritici
Source: BMC Genomics. 2017 Aug 17;18:631. doi: 10.1186/s12864-017-3969-y (PMC5561558; doi:10.1186/s12864-017-3969-y)
Supplement: Supplementary file 1 — MultiGeneBLAST analysis of putative secondary metabolite clusters. All encoded amino acid sequences from genes residing in clusters predicted by AntiSMASH are given as FASTA file format. All output data from MultiGeneBLASTs are also provided. (ZIP 42911 kb) [file 12864_2017_3969_MOESM1_ESM.zip › Cluster MultiGene BLAST/out/Clusters_1_34/Cluster_13/displaypage1.xhtml]

xml version="1.0" encoding="UTF-8"?


Search Results
  
  
 Results pages: 1, 2, 3, 4, 5

**MultiGeneBlast hits**

Select gene cluster alignment
1. CM001198\_0 Mycosphaerella graminicola IPO323 chromosome 3, whole genome sh...
2. JH767568\_0 Coniosporium apollinis CBS 100218 chromosome Unknown supercont1...
3. AHHD01000457\_0 Macrophomina phaseolina MS6, whole genome shotgun sequencin...
4. KB446557\_1 Pseudocercospora fijiensis CIRAD86 unplaced genomic scaffold MY...
5. KB456267\_1 Mycosphaerella populorum SO2202 unplaced genomic scaffold SEPMU...
6. KB916303\_0 Neofusicoccum parvum UCRNP2 chromosome Unknown NP2\_03\_scaffold\_...
7. KB446538\_1 Dothistroma septosporum NZE10 unplaced genomic scaffold DOTSEsc...
8. KB733456\_1 Bipolaris maydis ATCC 48331 unplaced genomic scaffold COCC4scaf...
9. KB445574\_1 Cochliobolus heterostrophus C5 unplaced genomic scaffold COCHEs...
10. KB445644\_1 Cochliobolus sativus ND90Pr unplaced genomic scaffold COCSAsca...
11. FP929131\_1 Leptosphaeria maculans JN3 lm\_SuperContig\_16\_v2 genomic superc...
12. DS231629\_0 Pyrenophora tritici-repentis Pt-1C-BFP supercont1.15 genomic s...
13. KB908833\_1 Setosphaeria turcica Et28A unplaced genomic scaffold SETTUscaf...
14. CH445352\_0 Phaeosphaeria nodorum SN15 scaffold\_28, whole genome shotgun s...
15. KB446557\_3 Pseudocercospora fijiensis CIRAD86 unplaced genomic scaffold M...
16. KB445552\_2 Baudoinia compniacensis UAMH 10762 unplaced genomic scaffold B...
17. KB908833\_2 Setosphaeria turcica Et28A unplaced genomic scaffold SETTUscaf...
18. FP929131\_0 Leptosphaeria maculans JN3 lm\_SuperContig\_16\_v2 genomic superc...
19. KB445559\_0 Baudoinia compniacensis UAMH 10762 unplaced genomic scaffold B...
20. KB445644\_0 Cochliobolus sativus ND90Pr unplaced genomic scaffold COCSAsca...
21. KB733456\_2 Bipolaris maydis ATCC 48331 unplaced genomic scaffold COCC4sca...
22. KB445587\_0 Cochliobolus heterostrophus C5 unplaced genomic scaffold COCHE...
23. KB445574\_0 Cochliobolus heterostrophus C5 unplaced genomic scaffold COCHE...
24. GL532905\_0 Pyrenophora teres f. teres 0-1 unplaced genomic scaffold scaff...
25. DS231625\_0 Pyrenophora tritici-repentis Pt-1C-BFP supercont1.11 genomic s...
26. CH476632\_0 Sclerotinia sclerotiorum 1980 scaffold\_12 genomic scaffold, wh...
27. KB456267\_0 Mycosphaerella populorum SO2202 unplaced genomic scaffold SEPM...
28. KB446538\_4 Dothistroma septosporum NZE10 unplaced genomic scaffold DOTSEs...
29. KB445552\_1 Baudoinia compniacensis UAMH 10762 unplaced genomic scaffold B...
30. GL534066\_0 Pyrenophora teres f. teres 0-1 unplaced genomic scaffold scaff...
31. GG697355\_0 Glomerella graminicola M1.001 genomic scaffold supercont1.25, ...
32. KB021237\_0 Colletotrichum gloeosporioides Nara gc5 unplaced genomic scaff...
33. HF679031\_0 Fusarium fujikuroi IMI 58289 draft genome, chromosome FFUJ\_chr09.
34. AMYD01001766\_0 Colletotrichum gloeosporioides Cg-14, whole genome shotgun...
35. KB725935\_0 Colletotrichum orbiculare MAFF 240422 unplaced genomic scaffol...
36. KB730180\_0 Fusarium oxysporum f. sp. cubense race 1 unplaced genomic scaf...
37. KB726995\_0 Fusarium oxysporum f. sp. cubense race 4 unplaced genomic scaf...
38. AFQF01003174\_0 Fusarium oxysporum Fo5176, whole genome shotgun sequencing...
39. AFNW01000285\_0 Fusarium pseudograminearum CS3096, whole genome shotgun se...
40. CP003003\_1 Myceliophthora thermophila ATCC 42464 chromosome 2, complete s...
41. DS572698\_0 Verticillium dahliae VdLs.17 supercont1.4 genomic scaffold, wh...
42. GG698904\_0 Nectria haematococca mpVI 77-13-4 chromosome 8 genomic scaffol...
43. CABT02000038\_0 Sordaria macrospora k-hell, whole genome shotgun sequencin...
44. JH921437\_0 Marssonina brunnea f. sp. 'multigermtubi' MB\_m1 unplaced genom...
45. DS985215\_0 Verticillium albo-atrum VaMs.102 supercont1.2 genomic scaffold...
46. CACQ02001341\_0 Colletotrichum higginsianum strain IMI 349063, whole genom...
47. GL891302\_0 Neurospora tetrasperma FGSC 2508 unplaced genomic scaffold NEU...
48. GL891107\_1 Neurospora tetrasperma FGSC 2509 unplaced genomic scaffold NEU...
49. BX294027\_0 Neurospora crassa DNA linkage group V BAC contig B8G12.
50. FQ790270\_0 Botryotinia fuckeliana T4 SuperContig\_51\_1 genomic supercontig.

Query: Architecture Search FASTA input

CM001198 : Mycosphaerella graminicola IPO323 chromosome 3    Total score: 13.0     Cumulative Blast bit score: 8325

Hit cluster cross-links:

Mycgr3G70471
  
Location: 0-405

Mycgr3G70471

Mycgr3G39149
  
Location: 505-1798

Mycgr3G39149

Mycgr3G92130
  
Location: 1898-2396

Mycgr3G92130

Mycgr3G38483
  
Location: 2496-3576

Mycgr3G38483

Mycgr3G108869
  
Location: 3676-5056

Mycgr3G108869

Mycgr3G103943
  
Location: 5156-5762

Mycgr3G103943

Mycgr3G57362
  
Location: 5862-7296

Mycgr3G57362

Mycgr3G39086
  
Location: 7396-8368

Mycgr3G39086

Mycgr3G103942
  
Location: 8468-8714

Mycgr3G103942

Mycgr3G108865
  
Location: 8814-10239

Mycgr3G108865

Mycgr3G70475
  
Location: 10339-11821

Mycgr3G70475

Mycgr3G108866
  
Location: 11921-13010

Mycgr3G108866

Mycgr3G92136
  
Location: 13110-13593

Mycgr3G92136

hypothetical protein
  
Accession: EGP89173
  
Location: 3175573-3179202
  
 NCBI BlastP on this gene

EGP89173

hypothetical protein
  
Accession: EGP89172
  
Location: 3179594-3181026
  
 NCBI BlastP on this gene

EGP89172

hypothetical protein
  
Accession: EGP89070
  
Location: 3181485-3182969
  
  
**BlastP hit with Mycgr3G57362**
  
Percentage identity: 100 %
  
BlastP bit score: 986
  
Sequence coverage: 99 %
  
E-value: 0.0
  
  
 NCBI BlastP on this gene

EGP89070

hypothetical protein
  
Accession: EGP89071
  
Location: 3184562-3185266
  
  
**BlastP hit with Mycgr3G92130**
  
Percentage identity: 100 %
  
BlastP bit score: 347
  
Sequence coverage: 99 %
  
E-value: 5e-120
  
  
 NCBI BlastP on this gene

EGP89071

hypothetical protein
  
Accession: EGP89171
  
Location: 3187686-3188893
  
  
**BlastP hit with Mycgr3G39086**
  
Percentage identity: 100 %
  
BlastP bit score: 667
  
Sequence coverage: 99 %
  
E-value: 0.0
  
  
 NCBI BlastP on this gene

EGP89171

ketoacyl synthase domain-containing protein
  
Accession: EGP89072
  
Location: 3190853-3192248
  
  
**BlastP hit with Mycgr3G39149**
  
Percentage identity: 100 %
  
BlastP bit score: 882
  
Sequence coverage: 99 %
  
E-value: 0.0
  
  
 NCBI BlastP on this gene

EGP89072

hypothetical protein
  
Accession: EGP89073
  
Location: 3192609-3194129
  
  
**BlastP hit with Mycgr3G108865**
  
Percentage identity: 100 %
  
BlastP bit score: 951
  
Sequence coverage: 99 %
  
E-value: 0.0
  
  
 NCBI BlastP on this gene

EGP89073

hypothetical protein
  
Accession: EGP89170
  
Location: 3194727-3195964
  
  
**BlastP hit with Mycgr3G108866**
  
Percentage identity: 100 %
  
BlastP bit score: 733
  
Sequence coverage: 99 %
  
E-value: 0.0
  
  
 NCBI BlastP on this gene

EGP89170

hypothetical protein
  
Accession: EGP89169
  
Location: 3199537-3200221
  
  
**BlastP hit with Mycgr3G70471**
  
Percentage identity: 100 %
  
BlastP bit score: 271
  
Sequence coverage: 99 %
  
E-value: 4e-91
  
  
 NCBI BlastP on this gene

EGP89169

hypothetical protein
  
Accession: EGP89074
  
Location: 3200446-3201663
  
  
**BlastP hit with Mycgr3G38483**
  
Percentage identity: 100 %
  
BlastP bit score: 740
  
Sequence coverage: 99 %
  
E-value: 0.0
  
  
 NCBI BlastP on this gene

EGP89074

hypothetical protein
  
Accession: EGP89168
  
Location: 3203515-3203999
  
  
**BlastP hit with Mycgr3G92136**
  
Percentage identity: 100 %
  
BlastP bit score: 245
  
Sequence coverage: 72 %
  
E-value: 1e-80
  
  
 NCBI BlastP on this gene

EGP89168

hypothetical protein
  
Accession: EGP89075
  
Location: 3204785-3206344
  
  
**BlastP hit with Mycgr3G70475**
  
Percentage identity: 100 %
  
BlastP bit score: 998
  
Sequence coverage: 99 %
  
E-value: 0.0
  
  
 NCBI BlastP on this gene

EGP89075

hypothetical protein
  
Accession: EGP89167
  
Location: 3206700-3206945
  
  
**BlastP hit with Mycgr3G103942**
  
Percentage identity: 100 %
  
BlastP bit score: 166
  
Sequence coverage: 98 %
  
E-value: 2e-51
  
  
 NCBI BlastP on this gene

EGP89167

hypothetical protein
  
Accession: EGP89076
  
Location: 3208316-3208992
  
  
**BlastP hit with Mycgr3G103943**
  
Percentage identity: 100 %
  
BlastP bit score: 421
  
Sequence coverage: 100 %
  
E-value: 7e-148
  
  
 NCBI BlastP on this gene

EGP89076

hypothetical protein
  
Accession: EGP89077
  
Location: 3211185-3213111
  
  
**BlastP hit with Mycgr3G108869**
  
Percentage identity: 100 %
  
BlastP bit score: 918
  
Sequence coverage: 99 %
  
E-value: 0.0
  
  
 NCBI BlastP on this gene

EGP89077

hypothetical protein
  
Accession: EGP89166
  
Location: 3213380-3214434
  
 NCBI BlastP on this gene

EGP89166

Query: Architecture Search FASTA input

JH767568 : Coniosporium apollinis CBS 100218 chromosome Unknown supercont1.15    Total score: 5.0     Cumulative Blast bit score: 1651

Hit cluster cross-links:

Mycgr3G70471
  
Location: 0-405

Mycgr3G70471

Mycgr3G39149
  
Location: 505-1798

Mycgr3G39149

Mycgr3G92130
  
Location: 1898-2396

Mycgr3G92130

Mycgr3G38483
  
Location: 2496-3576

Mycgr3G38483

Mycgr3G108869
  
Location: 3676-5056

Mycgr3G108869

Mycgr3G103943
  
Location: 5156-5762

Mycgr3G103943

Mycgr3G57362
  
Location: 5862-7296

Mycgr3G57362

Mycgr3G39086
  
Location: 7396-8368

Mycgr3G39086

Mycgr3G103942
  
Location: 8468-8714

Mycgr3G103942

Mycgr3G108865
  
Location: 8814-10239

Mycgr3G108865

Mycgr3G70475
  
Location: 10339-11821

Mycgr3G70475

Mycgr3G108866
  
Location: 11921-13010

Mycgr3G108866

Mycgr3G92136
  
Location: 13110-13593

Mycgr3G92136

hypothetical protein
  
Accession: EON64373
  
Location: 107130-109350
  
 NCBI BlastP on this gene

EON64373

small subunit ribosomal protein S24e
  
Accession: EON64372
  
Location: 104982-105589
  
  
**BlastP hit with Mycgr3G70471**
  
Percentage identity: 85 %
  
BlastP bit score: 234
  
Sequence coverage: 99 %
  
E-value: 2e-76
  
  
 NCBI BlastP on this gene

EON64372

hypothetical protein
  
Accession: EON64371
  
Location: 103016-104236
  
 NCBI BlastP on this gene

EON64371

hypothetical protein
  
Accession: EON64370
  
Location: 100139-101270
  
 NCBI BlastP on this gene

EON64370

hypothetical protein
  
Accession: EON64369
  
Location: 98332-99346
  
  
**BlastP hit with Mycgr3G38483**
  
Percentage identity: 46 %
  
BlastP bit score: 234
  
Sequence coverage: 76 %
  
E-value: 6e-71
  
  
 NCBI BlastP on this gene

EON64369

hypothetical protein
  
Accession: EON64368
  
Location: 95381-96589
  
  
**BlastP hit with Mycgr3G108866**
  
Percentage identity: 35 %
  
BlastP bit score: 120
  
Sequence coverage: 95 %
  
E-value: 1e-27
  
  
 NCBI BlastP on this gene

EON64368

hypothetical protein
  
Accession: EON64367
  
Location: 92710-94510
  
  
**BlastP hit with Mycgr3G108865**
  
Percentage identity: 54 %
  
BlastP bit score: 425
  
Sequence coverage: 95 %
  
E-value: 4e-141
  
  
 NCBI BlastP on this gene

EON64367

3-oxoacyl-[acyl-carrier-protein] synthase II
  
Accession: EON64366
  
Location: 90692-92337
  
  
**BlastP hit with Mycgr3G39149**
  
Percentage identity: 73 %
  
BlastP bit score: 638
  
Sequence coverage: 99 %
  
E-value: 0.0
  
  
 NCBI BlastP on this gene

EON64366

hypothetical protein
  
Accession: EON64365
  
Location: 88557-90042
  
 NCBI BlastP on this gene

EON64365

Query: Architecture Search FASTA input

AHHD01000457 : Macrophomina phaseolina MS6    Total score: 5.0     Cumulative Blast bit score: 1561

Hit cluster cross-links:

Mycgr3G70471
  
Location: 0-405

Mycgr3G70471

Mycgr3G39149
  
Location: 505-1798

Mycgr3G39149

Mycgr3G92130
  
Location: 1898-2396

Mycgr3G92130

Mycgr3G38483
  
Location: 2496-3576

Mycgr3G38483

Mycgr3G108869
  
Location: 3676-5056

Mycgr3G108869

Mycgr3G103943
  
Location: 5156-5762

Mycgr3G103943

Mycgr3G57362
  
Location: 5862-7296

Mycgr3G57362

Mycgr3G39086
  
Location: 7396-8368

Mycgr3G39086

Mycgr3G103942
  
Location: 8468-8714

Mycgr3G103942

Mycgr3G108865
  
Location: 8814-10239

Mycgr3G108865

Mycgr3G70475
  
Location: 10339-11821

Mycgr3G70475

Mycgr3G108866
  
Location: 11921-13010

Mycgr3G108866

Mycgr3G92136
  
Location: 13110-13593

Mycgr3G92136

Pyridine nucleotide-disulfide oxidoreductase
  
Accession: EKG12156
  
Location: 22950-24069
  
 NCBI BlastP on this gene

EKG12156

Beta-ketoacyl synthase
  
Accession: EKG12157
  
Location: 24995-26597
  
  
**BlastP hit with Mycgr3G39149**
  
Percentage identity: 72 %
  
BlastP bit score: 629
  
Sequence coverage: 99 %
  
E-value: 0.0
  
  
 NCBI BlastP on this gene

EKG12157

Nickel/cobalt transporter high-affinity
  
Accession: EKG12158
  
Location: 27220-28663
  
  
**BlastP hit with Mycgr3G108865**
  
Percentage identity: 53 %
  
BlastP bit score: 315
  
Sequence coverage: 70 %
  
E-value: 8e-100
  
  
 NCBI BlastP on this gene

EKG12158

hypothetical protein
  
Accession: EKG12159
  
Location: 28853-31024
  
 NCBI BlastP on this gene

EKG12159

Ribosomal protein S24e
  
Accession: EKG12160
  
Location: 32279-32820
  
  
**BlastP hit with Mycgr3G70471**
  
Percentage identity: 87 %
  
BlastP bit score: 225
  
Sequence coverage: 91 %
  
E-value: 5e-73
  
  
 NCBI BlastP on this gene

EKG12160

Nicotinamide N-methyltransferase putative
  
Accession: EKG12161
  
Location: 33259-34732
  
  
**BlastP hit with Mycgr3G38483**
  
Percentage identity: 48 %
  
BlastP bit score: 316
  
Sequence coverage: 93 %
  
E-value: 3e-102
  
  
 NCBI BlastP on this gene

EKG12161

hypothetical protein
  
Accession: EKG12162
  
Location: 36612-37506
  
  
**BlastP hit with Mycgr3G108866**
  
Percentage identity: 32 %
  
BlastP bit score: 76
  
Sequence coverage: 77 %
  
E-value: 8e-13
  
  
 NCBI BlastP on this gene

EKG12162

hypothetical protein
  
Accession: EKG12163
  
Location: 40038-42366
  
 NCBI BlastP on this gene

EKG12163

Query: Architecture Search FASTA input

KB446557 : Pseudocercospora fijiensis CIRAD86 unplaced genomic scaffold MYCFIscaffold\_3    Total score: 4.0     Cumulative Blast bit score: 1994

Hit cluster cross-links:

Mycgr3G70471
  
Location: 0-405

Mycgr3G70471

Mycgr3G39149
  
Location: 505-1798

Mycgr3G39149

Mycgr3G92130
  
Location: 1898-2396

Mycgr3G92130

Mycgr3G38483
  
Location: 2496-3576

Mycgr3G38483

Mycgr3G108869
  
Location: 3676-5056

Mycgr3G108869

Mycgr3G103943
  
Location: 5156-5762

Mycgr3G103943

Mycgr3G57362
  
Location: 5862-7296

Mycgr3G57362

Mycgr3G39086
  
Location: 7396-8368

Mycgr3G39086

Mycgr3G103942
  
Location: 8468-8714

Mycgr3G103942

Mycgr3G108865
  
Location: 8814-10239

Mycgr3G108865

Mycgr3G70475
  
Location: 10339-11821

Mycgr3G70475

Mycgr3G108866
  
Location: 11921-13010

Mycgr3G108866

Mycgr3G92136
  
Location: 13110-13593

Mycgr3G92136

hypothetical protein
  
Accession: EME84130
  
Location: 2033126-2033710
  
 NCBI BlastP on this gene

EME84130

hypothetical protein
  
Accession: EME84131
  
Location: 2036472-2037647
  
  
**BlastP hit with Mycgr3G108866**
  
Percentage identity: 42 %
  
BlastP bit score: 209
  
Sequence coverage: 97 %
  
E-value: 2e-60
  
  
 NCBI BlastP on this gene

EME84131

hypothetical protein
  
Accession: EME84132
  
Location: 2038009-2039283
  
  
**BlastP hit with Mycgr3G108865**
  
Percentage identity: 69 %
  
BlastP bit score: 555
  
Sequence coverage: 83 %
  
E-value: 0.0
  
  
 NCBI BlastP on this gene

EME84132

hypothetical protein
  
Accession: EME84133
  
Location: 2039530-2040929
  
  
**BlastP hit with Mycgr3G39149**
  
Percentage identity: 86 %
  
BlastP bit score: 768
  
Sequence coverage: 99 %
  
E-value: 0.0
  
  
 NCBI BlastP on this gene

EME84133

hypothetical protein
  
Accession: EME84134
  
Location: 2042100-2043965
  
  
**BlastP hit with Mycgr3G39086**
  
Percentage identity: 69 %
  
BlastP bit score: 462
  
Sequence coverage: 99 %
  
E-value: 3e-157
  
  
 NCBI BlastP on this gene

EME84134

hypothetical protein
  
Accession: EME84135
  
Location: 2044682-2045722
  
 NCBI BlastP on this gene

EME84135

hypothetical protein
  
Accession: EME84136
  
Location: 2046462-2047517
  
 NCBI BlastP on this gene

EME84136

hypothetical protein
  
Accession: EME84137
  
Location: 2048436-2050220
  
 NCBI BlastP on this gene

EME84137

Query: Architecture Search FASTA input

KB456267 : Mycosphaerella populorum SO2202 unplaced genomic scaffold SEPMUscaffold\_8    Total score: 4.0     Cumulative Blast bit score: 1833

Hit cluster cross-links:

Mycgr3G70471
  
Location: 0-405

Mycgr3G70471

Mycgr3G39149
  
Location: 505-1798

Mycgr3G39149

Mycgr3G92130
  
Location: 1898-2396

Mycgr3G92130

Mycgr3G38483
  
Location: 2496-3576

Mycgr3G38483

Mycgr3G108869
  
Location: 3676-5056

Mycgr3G108869

Mycgr3G103943
  
Location: 5156-5762

Mycgr3G103943

Mycgr3G57362
  
Location: 5862-7296

Mycgr3G57362

Mycgr3G39086
  
Location: 7396-8368

Mycgr3G39086

Mycgr3G103942
  
Location: 8468-8714

Mycgr3G103942

Mycgr3G108865
  
Location: 8814-10239

Mycgr3G108865

Mycgr3G70475
  
Location: 10339-11821

Mycgr3G70475

Mycgr3G108866
  
Location: 11921-13010

Mycgr3G108866

Mycgr3G92136
  
Location: 13110-13593

Mycgr3G92136

hypothetical protein
  
Accession: EMF10549
  
Location: 684861-687588
  
 NCBI BlastP on this gene

EMF10549

hypothetical protein
  
Accession: EMF10550
  
Location: 692543-693722
  
  
**BlastP hit with Mycgr3G108866**
  
Percentage identity: 38 %
  
BlastP bit score: 132
  
Sequence coverage: 99 %
  
E-value: 1e-31
  
  
 NCBI BlastP on this gene

EMF10550

NicO-domain-containing protein
  
Accession: EMF10551
  
Location: 694737-696056
  
  
**BlastP hit with Mycgr3G108865**
  
Percentage identity: 70 %
  
BlastP bit score: 484
  
Sequence coverage: 76 %
  
E-value: 8e-165
  
  
 NCBI BlastP on this gene

EMF10551

beta-ketoacyl synthase
  
Accession: EMF10552
  
Location: 696401-697821
  
  
**BlastP hit with Mycgr3G39149**
  
Percentage identity: 85 %
  
BlastP bit score: 758
  
Sequence coverage: 99 %
  
E-value: 0.0
  
  
 NCBI BlastP on this gene

EMF10552

hypothetical protein
  
Accession: EMF10553
  
Location: 699441-700898
  
  
**BlastP hit with Mycgr3G39086**
  
Percentage identity: 66 %
  
BlastP bit score: 459
  
Sequence coverage: 99 %
  
E-value: 2e-157
  
  
 NCBI BlastP on this gene

EMF10553

hypothetical protein
  
Accession: EMF10554
  
Location: 701245-705834
  
 NCBI BlastP on this gene

EMF10554

Query: Architecture Search FASTA input

KB916303 : Neofusicoccum parvum UCRNP2 chromosome Unknown NP2\_03\_scaffold\_665    Total score: 4.0     Cumulative Blast bit score: 955

Hit cluster cross-links:

Mycgr3G70471
  
Location: 0-405

Mycgr3G70471

Mycgr3G39149
  
Location: 505-1798

Mycgr3G39149

Mycgr3G92130
  
Location: 1898-2396

Mycgr3G92130

Mycgr3G38483
  
Location: 2496-3576

Mycgr3G38483

Mycgr3G108869
  
Location: 3676-5056

Mycgr3G108869

Mycgr3G103943
  
Location: 5156-5762

Mycgr3G103943

Mycgr3G57362
  
Location: 5862-7296

Mycgr3G57362

Mycgr3G39086
  
Location: 7396-8368

Mycgr3G39086

Mycgr3G103942
  
Location: 8468-8714

Mycgr3G103942

Mycgr3G108865
  
Location: 8814-10239

Mycgr3G108865

Mycgr3G70475
  
Location: 10339-11821

Mycgr3G70475

Mycgr3G108866
  
Location: 11921-13010

Mycgr3G108866

Mycgr3G92136
  
Location: 13110-13593

Mycgr3G92136

putative high affinity nickel transport protein nic1 protein
  
Accession: EOD47792
  
Location: 235058-236897
  
  
**BlastP hit with Mycgr3G108865**
  
Percentage identity: 52 %
  
BlastP bit score: 395
  
Sequence coverage: 88 %
  
E-value: 5e-130
  
  
 NCBI BlastP on this gene

EOD47792

putative sorting nexin-41 protein
  
Accession: EOD47803
  
Location: 237021-239189
  
 NCBI BlastP on this gene

EOD47803

putative 40s ribosomal protein s24 protein
  
Accession: EOD47785
  
Location: 240535-241084
  
  
**BlastP hit with Mycgr3G70471**
  
Percentage identity: 87 %
  
BlastP bit score: 226
  
Sequence coverage: 91 %
  
E-value: 2e-73
  
  
 NCBI BlastP on this gene

EOD47785

putative nicotinamide n-methyltransferase protein
  
Accession: EOD47808
  
Location: 241437-242857
  
  
**BlastP hit with Mycgr3G38483**
  
Percentage identity: 47 %
  
BlastP bit score: 259
  
Sequence coverage: 83 %
  
E-value: 1e-80
  
  
 NCBI BlastP on this gene

EOD47808

hypothetical protein
  
Accession: EOD47760
  
Location: 244791-245860
  
  
**BlastP hit with Mycgr3G108866**
  
Percentage identity: 38 %
  
BlastP bit score: 75
  
Sequence coverage: 36 %
  
E-value: 1e-12
  
  
 NCBI BlastP on this gene

EOD47760

putative nucleoside-diphosphate-sugar epimerase protein
  
Accession: EOD47817
  
Location: 248391-249267
  
 NCBI BlastP on this gene

EOD47817

Query: Architecture Search FASTA input

KB446538 : Dothistroma septosporum NZE10 unplaced genomic scaffold DOTSEscaffold\_4    Total score: 3.0     Cumulative Blast bit score: 1515

Hit cluster cross-links:

Mycgr3G70471
  
Location: 0-405

Mycgr3G70471

Mycgr3G39149
  
Location: 505-1798

Mycgr3G39149

Mycgr3G92130
  
Location: 1898-2396

Mycgr3G92130

Mycgr3G38483
  
Location: 2496-3576

Mycgr3G38483

Mycgr3G108869
  
Location: 3676-5056

Mycgr3G108869

Mycgr3G103943
  
Location: 5156-5762

Mycgr3G103943

Mycgr3G57362
  
Location: 5862-7296

Mycgr3G57362

Mycgr3G39086
  
Location: 7396-8368

Mycgr3G39086

Mycgr3G103942
  
Location: 8468-8714

Mycgr3G103942

Mycgr3G108865
  
Location: 8814-10239

Mycgr3G108865

Mycgr3G70475
  
Location: 10339-11821

Mycgr3G70475

Mycgr3G108866
  
Location: 11921-13010

Mycgr3G108866

Mycgr3G92136
  
Location: 13110-13593

Mycgr3G92136

hypothetical protein
  
Accession: EME45124
  
Location: 707252-707680
  
 NCBI BlastP on this gene

EME45124

hypothetical protein
  
Accession: EME45123
  
Location: 702226-706509
  
 NCBI BlastP on this gene

EME45123

ketoacyl synthase domain-containing protein
  
Accession: EME45122
  
Location: 699669-701075
  
  
**BlastP hit with Mycgr3G39149**
  
Percentage identity: 83 %
  
BlastP bit score: 757
  
Sequence coverage: 99 %
  
E-value: 0.0
  
  
 NCBI BlastP on this gene

EME45122

hypothetical protein
  
Accession: EME45121
  
Location: 698053-699307
  
  
**BlastP hit with Mycgr3G108865**
  
Percentage identity: 71 %
  
BlastP bit score: 540
  
Sequence coverage: 81 %
  
E-value: 0.0
  
  
 NCBI BlastP on this gene

EME45121

hypothetical protein
  
Accession: EME45120
  
Location: 696549-697690
  
  
**BlastP hit with Mycgr3G108866**
  
Percentage identity: 45 %
  
BlastP bit score: 218
  
Sequence coverage: 98 %
  
E-value: 3e-64
  
  
 NCBI BlastP on this gene

EME45120

hypothetical protein
  
Accession: EME45119
  
Location: 690646-693824
  
 NCBI BlastP on this gene

EME45119

Query: Architecture Search FASTA input

KB733456 : Bipolaris maydis ATCC 48331 unplaced genomic scaffold COCC4scaffold\_13    Total score: 3.0     Cumulative Blast bit score: 626

Hit cluster cross-links:

Mycgr3G70471
  
Location: 0-405

Mycgr3G70471

Mycgr3G39149
  
Location: 505-1798

Mycgr3G39149

Mycgr3G92130
  
Location: 1898-2396

Mycgr3G92130

Mycgr3G38483
  
Location: 2496-3576

Mycgr3G38483

Mycgr3G108869
  
Location: 3676-5056

Mycgr3G108869

Mycgr3G103943
  
Location: 5156-5762

Mycgr3G103943

Mycgr3G57362
  
Location: 5862-7296

Mycgr3G57362

Mycgr3G39086
  
Location: 7396-8368

Mycgr3G39086

Mycgr3G103942
  
Location: 8468-8714

Mycgr3G103942

Mycgr3G108865
  
Location: 8814-10239

Mycgr3G108865

Mycgr3G70475
  
Location: 10339-11821

Mycgr3G70475

Mycgr3G108866
  
Location: 11921-13010

Mycgr3G108866

Mycgr3G92136
  
Location: 13110-13593

Mycgr3G92136

hypothetical protein
  
Accession: ENI04809
  
Location: 721305-724710
  
 NCBI BlastP on this gene

ENI04809

hypothetical protein
  
Accession: ENI04810
  
Location: 724861-725304
  
 NCBI BlastP on this gene

ENI04810

hypothetical protein
  
Accession: ENI04811
  
Location: 726999-729182
  
 NCBI BlastP on this gene

ENI04811

hypothetical protein
  
Accession: ENI04812
  
Location: 729703-730328
  
  
**BlastP hit with Mycgr3G70471**
  
Percentage identity: 83 %
  
BlastP bit score: 223
  
Sequence coverage: 96 %
  
E-value: 4e-72
  
  
 NCBI BlastP on this gene

ENI04812

hypothetical protein
  
Accession: ENI04813
  
Location: 730696-732035
  
  
**BlastP hit with Mycgr3G38483**
  
Percentage identity: 48 %
  
BlastP bit score: 306
  
Sequence coverage: 100 %
  
E-value: 4e-98
  
  
 NCBI BlastP on this gene

ENI04813

hypothetical protein
  
Accession: ENI04814
  
Location: 732379-732690
  
 NCBI BlastP on this gene

ENI04814

hypothetical protein
  
Accession: ENI04815
  
Location: 733719-734772
  
  
**BlastP hit with Mycgr3G108866**
  
Percentage identity: 33 %
  
BlastP bit score: 97
  
Sequence coverage: 96 %
  
E-value: 7e-20
  
  
 NCBI BlastP on this gene

ENI04815

hypothetical protein
  
Accession: ENI04816
  
Location: 737043-737510
  
 NCBI BlastP on this gene

ENI04816

hypothetical protein
  
Accession: ENI04817
  
Location: 738110-738389
  
 NCBI BlastP on this gene

ENI04817

hypothetical protein
  
Accession: ENI04818
  
Location: 739442-740140
  
 NCBI BlastP on this gene

ENI04818

Query: Architecture Search FASTA input

KB445574 : Cochliobolus heterostrophus C5 unplaced genomic scaffold COCHEscaffold\_6    Total score: 3.0     Cumulative Blast bit score: 626

Hit cluster cross-links:

Mycgr3G70471
  
Location: 0-405

Mycgr3G70471

Mycgr3G39149
  
Location: 505-1798

Mycgr3G39149

Mycgr3G92130
  
Location: 1898-2396

Mycgr3G92130

Mycgr3G38483
  
Location: 2496-3576

Mycgr3G38483

Mycgr3G108869
  
Location: 3676-5056

Mycgr3G108869

Mycgr3G103943
  
Location: 5156-5762

Mycgr3G103943

Mycgr3G57362
  
Location: 5862-7296

Mycgr3G57362

Mycgr3G39086
  
Location: 7396-8368

Mycgr3G39086

Mycgr3G103942
  
Location: 8468-8714

Mycgr3G103942

Mycgr3G108865
  
Location: 8814-10239

Mycgr3G108865

Mycgr3G70475
  
Location: 10339-11821

Mycgr3G70475

Mycgr3G108866
  
Location: 11921-13010

Mycgr3G108866

Mycgr3G92136
  
Location: 13110-13593

Mycgr3G92136

hypothetical protein
  
Accession: EMD92802
  
Location: 255509-258914
  
 NCBI BlastP on this gene

EMD92802

hypothetical protein
  
Accession: EMD92801
  
Location: 251037-253220
  
 NCBI BlastP on this gene

EMD92801

hypothetical protein
  
Accession: EMD92800
  
Location: 249891-250516
  
  
**BlastP hit with Mycgr3G70471**
  
Percentage identity: 83 %
  
BlastP bit score: 223
  
Sequence coverage: 96 %
  
E-value: 4e-72
  
  
 NCBI BlastP on this gene

EMD92800

hypothetical protein
  
Accession: EMD92799
  
Location: 248184-249523
  
  
**BlastP hit with Mycgr3G38483**
  
Percentage identity: 48 %
  
BlastP bit score: 306
  
Sequence coverage: 100 %
  
E-value: 4e-98
  
  
 NCBI BlastP on this gene

EMD92799

hypothetical protein
  
Accession: EMD92798
  
Location: 247529-247840
  
 NCBI BlastP on this gene

EMD92798

hypothetical protein
  
Accession: EMD92797
  
Location: 245447-246500
  
  
**BlastP hit with Mycgr3G108866**
  
Percentage identity: 33 %
  
BlastP bit score: 97
  
Sequence coverage: 96 %
  
E-value: 7e-20
  
  
 NCBI BlastP on this gene

EMD92797

hypothetical protein
  
Accession: EMD92796
  
Location: 242709-243176
  
 NCBI BlastP on this gene

EMD92796

hypothetical protein
  
Accession: EMD92795
  
Location: 241830-242108
  
 NCBI BlastP on this gene

EMD92795

hypothetical protein
  
Accession: EMD92794
  
Location: 240079-240777
  
 NCBI BlastP on this gene

EMD92794

Query: Architecture Search FASTA input

KB445644 : Cochliobolus sativus ND90Pr unplaced genomic scaffold COCSAscaffold\_8    Total score: 3.0     Cumulative Blast bit score: 618

Hit cluster cross-links:

Mycgr3G70471
  
Location: 0-405

Mycgr3G70471

Mycgr3G39149
  
Location: 505-1798

Mycgr3G39149

Mycgr3G92130
  
Location: 1898-2396

Mycgr3G92130

Mycgr3G38483
  
Location: 2496-3576

Mycgr3G38483

Mycgr3G108869
  
Location: 3676-5056

Mycgr3G108869

Mycgr3G103943
  
Location: 5156-5762

Mycgr3G103943

Mycgr3G57362
  
Location: 5862-7296

Mycgr3G57362

Mycgr3G39086
  
Location: 7396-8368

Mycgr3G39086

Mycgr3G103942
  
Location: 8468-8714

Mycgr3G103942

Mycgr3G108865
  
Location: 8814-10239

Mycgr3G108865

Mycgr3G70475
  
Location: 10339-11821

Mycgr3G70475

Mycgr3G108866
  
Location: 11921-13010

Mycgr3G108866

Mycgr3G92136
  
Location: 13110-13593

Mycgr3G92136

hypothetical protein
  
Accession: EMD63620
  
Location: 678112-681517
  
 NCBI BlastP on this gene

EMD63620

hypothetical protein
  
Accession: EMD63619
  
Location: 677526-677927
  
 NCBI BlastP on this gene

EMD63619

hypothetical protein
  
Accession: EMD63618
  
Location: 673690-675966
  
 NCBI BlastP on this gene

EMD63618

hypothetical protein
  
Accession: EMD63617
  
Location: 672564-673191
  
  
**BlastP hit with Mycgr3G70471**
  
Percentage identity: 85 %
  
BlastP bit score: 212
  
Sequence coverage: 88 %
  
E-value: 9e-68
  
  
 NCBI BlastP on this gene

EMD63617

hypothetical protein
  
Accession: EMD63616
  
Location: 670858-672198
  
  
**BlastP hit with Mycgr3G38483**
  
Percentage identity: 48 %
  
BlastP bit score: 311
  
Sequence coverage: 100 %
  
E-value: 7e-100
  
  
 NCBI BlastP on this gene

EMD63616

hypothetical protein
  
Accession: EMD63615
  
Location: 669502-670508
  
 NCBI BlastP on this gene

EMD63615

hypothetical protein
  
Accession: EMD63614
  
Location: 668094-669143
  
  
**BlastP hit with Mycgr3G108866**
  
Percentage identity: 33 %
  
BlastP bit score: 95
  
Sequence coverage: 96 %
  
E-value: 2e-19
  
  
 NCBI BlastP on this gene

EMD63614

hypothetical protein
  
Accession: EMD63613
  
Location: 667665-667880
  
 NCBI BlastP on this gene

EMD63613

hypothetical protein
  
Accession: EMD63612
  
Location: 665483-665950
  
 NCBI BlastP on this gene

EMD63612

hypothetical protein
  
Accession: EMD63611
  
Location: 662896-663569
  
 NCBI BlastP on this gene

EMD63611

Query: Architecture Search FASTA input

FP929131 : Leptosphaeria maculans JN3 lm\_SuperContig\_16\_v2 genomic supercontig    Total score: 3.0     Cumulative Blast bit score: 610

Hit cluster cross-links:

Mycgr3G70471
  
Location: 0-405

Mycgr3G70471

Mycgr3G39149
  
Location: 505-1798

Mycgr3G39149

Mycgr3G92130
  
Location: 1898-2396

Mycgr3G92130

Mycgr3G38483
  
Location: 2496-3576

Mycgr3G38483

Mycgr3G108869
  
Location: 3676-5056

Mycgr3G108869

Mycgr3G103943
  
Location: 5156-5762

Mycgr3G103943

Mycgr3G57362
  
Location: 5862-7296

Mycgr3G57362

Mycgr3G39086
  
Location: 7396-8368

Mycgr3G39086

Mycgr3G103942
  
Location: 8468-8714

Mycgr3G103942

Mycgr3G108865
  
Location: 8814-10239

Mycgr3G108865

Mycgr3G70475
  
Location: 10339-11821

Mycgr3G70475

Mycgr3G108866
  
Location: 11921-13010

Mycgr3G108866

Mycgr3G92136
  
Location: 13110-13593

Mycgr3G92136

similar to RNA binding protein Jsn1
  
Accession: CBX97373
  
Location: 968198-971628
  
 NCBI BlastP on this gene

LEMA\_P105040.1

hypothetical protein
  
Accession: CBX97374
  
Location: 973909-976203
  
 NCBI BlastP on this gene

LEMA\_P105050.1

hypothetical protein
  
Accession: CBX97375
  
Location: 976823-977634
  
  
**BlastP hit with Mycgr3G70471**
  
Percentage identity: 81 %
  
BlastP bit score: 222
  
Sequence coverage: 99 %
  
E-value: 2e-71
  
  
 NCBI BlastP on this gene

LEMA\_P105060.1

hypothetical protein
  
Accession: CBX97376
  
Location: 977755-979062
  
  
**BlastP hit with Mycgr3G38483**
  
Percentage identity: 42 %
  
BlastP bit score: 286
  
Sequence coverage: 106 %
  
E-value: 3e-90
  
  
 NCBI BlastP on this gene

LEMA\_P105070.1

predicted protein
  
Accession: CBX97377
  
Location: 979452-979718
  
 NCBI BlastP on this gene

LEMA\_uP105080.1

hypothetical protein
  
Accession: CBX97378
  
Location: 980984-982016
  
  
**BlastP hit with Mycgr3G108866**
  
Percentage identity: 37 %
  
BlastP bit score: 102
  
Sequence coverage: 63 %
  
E-value: 9e-22
  
  
 NCBI BlastP on this gene

LEMA\_P105090.1

hypothetical protein
  
Accession: CBX97379
  
Location: 983003-983685
  
 NCBI BlastP on this gene

LEMA\_P105100.1

predicted protein
  
Accession: CBX97380
  
Location: 984841-985311
  
 NCBI BlastP on this gene

LEMA\_P105110.1

hypothetical protein
  
Accession: CBX97381
  
Location: 985688-986357
  
 NCBI BlastP on this gene

LEMA\_P105120.1

similar to oxidoreductase
  
Accession: CBX97382
  
Location: 987431-988558
  
 NCBI BlastP on this gene

LEMA\_P105130.1

Query: Architecture Search FASTA input

DS231629 : Pyrenophora tritici-repentis Pt-1C-BFP supercont1.15 genomic scaffold    Total score: 3.0     Cumulative Blast bit score: 608

Hit cluster cross-links:

Mycgr3G70471
  
Location: 0-405

Mycgr3G70471

Mycgr3G39149
  
Location: 505-1798

Mycgr3G39149

Mycgr3G92130
  
Location: 1898-2396

Mycgr3G92130

Mycgr3G38483
  
Location: 2496-3576

Mycgr3G38483

Mycgr3G108869
  
Location: 3676-5056

Mycgr3G108869

Mycgr3G103943
  
Location: 5156-5762

Mycgr3G103943

Mycgr3G57362
  
Location: 5862-7296

Mycgr3G57362

Mycgr3G39086
  
Location: 7396-8368

Mycgr3G39086

Mycgr3G103942
  
Location: 8468-8714

Mycgr3G103942

Mycgr3G108865
  
Location: 8814-10239

Mycgr3G108865

Mycgr3G70475
  
Location: 10339-11821

Mycgr3G70475

Mycgr3G108866
  
Location: 11921-13010

Mycgr3G108866

Mycgr3G92136
  
Location: 13110-13593

Mycgr3G92136

RNA binding protein
  
Accession: EDU43844
  
Location: 95501-98925
  
 NCBI BlastP on this gene

EDU43844

conserved hypothetical protein
  
Accession: EDU43843
  
Location: 91478-93700
  
 NCBI BlastP on this gene

EDU43843

40S ribosomal protein S24
  
Accession: EDU43842
  
Location: 90259-90818
  
  
**BlastP hit with Mycgr3G70471**
  
Percentage identity: 82 %
  
BlastP bit score: 226
  
Sequence coverage: 99 %
  
E-value: 2e-73
  
  
 NCBI BlastP on this gene

EDU43842

conserved hypothetical protein
  
Accession: EDU43841
  
Location: 88576-89893
  
  
**BlastP hit with Mycgr3G38483**
  
Percentage identity: 45 %
  
BlastP bit score: 295
  
Sequence coverage: 100 %
  
E-value: 1e-93
  
  
 NCBI BlastP on this gene

EDU43841

predicted protein
  
Accession: EDU43840
  
Location: 86035-87061
  
  
**BlastP hit with Mycgr3G108866**
  
Percentage identity: 34 %
  
BlastP bit score: 87
  
Sequence coverage: 78 %
  
E-value: 1e-16
  
  
 NCBI BlastP on this gene

EDU43840

heat-stable 19 kDa antigen precursor
  
Accession: EDU43839
  
Location: 80115-80525
  
 NCBI BlastP on this gene

EDU43839

Query: Architecture Search FASTA input

KB908833 : Setosphaeria turcica Et28A unplaced genomic scaffold SETTUscaffold\_5    Total score: 3.0     Cumulative Blast bit score: 603

Hit cluster cross-links:

Mycgr3G70471
  
Location: 0-405

Mycgr3G70471

Mycgr3G39149
  
Location: 505-1798

Mycgr3G39149

Mycgr3G92130
  
Location: 1898-2396

Mycgr3G92130

Mycgr3G38483
  
Location: 2496-3576

Mycgr3G38483

Mycgr3G108869
  
Location: 3676-5056

Mycgr3G108869

Mycgr3G103943
  
Location: 5156-5762

Mycgr3G103943

Mycgr3G57362
  
Location: 5862-7296

Mycgr3G57362

Mycgr3G39086
  
Location: 7396-8368

Mycgr3G39086

Mycgr3G103942
  
Location: 8468-8714

Mycgr3G103942

Mycgr3G108865
  
Location: 8814-10239

Mycgr3G108865

Mycgr3G70475
  
Location: 10339-11821

Mycgr3G70475

Mycgr3G108866
  
Location: 11921-13010

Mycgr3G108866

Mycgr3G92136
  
Location: 13110-13593

Mycgr3G92136

hypothetical protein
  
Accession: EOA83427
  
Location: 1612227-1615636
  
 NCBI BlastP on this gene

EOA83427

hypothetical protein
  
Accession: EOA83428
  
Location: 1617481-1619349
  
 NCBI BlastP on this gene

EOA83428

hypothetical protein
  
Accession: EOA83429
  
Location: 1620065-1620697
  
  
**BlastP hit with Mycgr3G70471**
  
Percentage identity: 85 %
  
BlastP bit score: 212
  
Sequence coverage: 88 %
  
E-value: 8e-68
  
  
 NCBI BlastP on this gene

EOA83429

hypothetical protein
  
Accession: EOA83430
  
Location: 1621083-1622430
  
  
**BlastP hit with Mycgr3G38483**
  
Percentage identity: 46 %
  
BlastP bit score: 298
  
Sequence coverage: 100 %
  
E-value: 1e-94
  
  
 NCBI BlastP on this gene

EOA83430

hypothetical protein
  
Accession: EOA83431
  
Location: 1624221-1625277
  
  
**BlastP hit with Mycgr3G108866**
  
Percentage identity: 33 %
  
BlastP bit score: 93
  
Sequence coverage: 79 %
  
E-value: 1e-18
  
  
 NCBI BlastP on this gene

EOA83431

hypothetical protein
  
Accession: EOA83432
  
Location: 1627076-1627527
  
 NCBI BlastP on this gene

EOA83432

hypothetical protein
  
Accession: EOA83433
  
Location: 1629598-1630279
  
 NCBI BlastP on this gene

EOA83433

hypothetical protein
  
Accession: EOA83434
  
Location: 1631031-1632390
  
 NCBI BlastP on this gene

EOA83434

Query: Architecture Search FASTA input

CH445352 : Phaeosphaeria nodorum SN15 scaffold\_28    Total score: 3.0     Cumulative Blast bit score: 591

Hit cluster cross-links:

Mycgr3G70471
  
Location: 0-405

Mycgr3G70471

Mycgr3G39149
  
Location: 505-1798

Mycgr3G39149

Mycgr3G92130
  
Location: 1898-2396

Mycgr3G92130

Mycgr3G38483
  
Location: 2496-3576

Mycgr3G38483

Mycgr3G108869
  
Location: 3676-5056

Mycgr3G108869

Mycgr3G103943
  
Location: 5156-5762

Mycgr3G103943

Mycgr3G57362
  
Location: 5862-7296

Mycgr3G57362

Mycgr3G39086
  
Location: 7396-8368

Mycgr3G39086

Mycgr3G103942
  
Location: 8468-8714

Mycgr3G103942

Mycgr3G108865
  
Location: 8814-10239

Mycgr3G108865

Mycgr3G70475
  
Location: 10339-11821

Mycgr3G70475

Mycgr3G108866
  
Location: 11921-13010

Mycgr3G108866

Mycgr3G92136
  
Location: 13110-13593

Mycgr3G92136

hypothetical protein
  
Accession: EAT78761
  
Location: 95141-98567
  
 NCBI BlastP on this gene

EAT78761

hypothetical protein
  
Accession: EAT78760
  
Location: 91150-93321
  
 NCBI BlastP on this gene

EAT78760

hypothetical protein
  
Accession: EAT78759
  
Location: 90103-90661
  
  
**BlastP hit with Mycgr3G70471**
  
Percentage identity: 84 %
  
BlastP bit score: 230
  
Sequence coverage: 99 %
  
E-value: 7e-75
  
  
 NCBI BlastP on this gene

EAT78759

hypothetical protein
  
Accession: EAT78758
  
Location: 88541-89715
  
  
**BlastP hit with Mycgr3G38483**
  
Percentage identity: 51 %
  
BlastP bit score: 303
  
Sequence coverage: 85 %
  
E-value: 3e-97
  
  
 NCBI BlastP on this gene

EAT78758

hypothetical protein
  
Accession: EAT78757
  
Location: 87366-88193
  
 NCBI BlastP on this gene

EAT78757

hypothetical protein
  
Accession: EAT78756
  
Location: 86071-86860
  
  
**BlastP hit with Mycgr3G108866**
  
Percentage identity: 34 %
  
BlastP bit score: 58
  
Sequence coverage: 52 %
  
E-value: 5e-07
  
  
 NCBI BlastP on this gene

EAT78756

hypothetical protein
  
Accession: EAT78755
  
Location: 84342-85158
  
 NCBI BlastP on this gene

EAT78755

hypothetical protein
  
Accession: EAT78754
  
Location: 82552-84177
  
 NCBI BlastP on this gene

EAT78754

hypothetical protein
  
Accession: EAT78753
  
Location: 81227-81755
  
 NCBI BlastP on this gene

EAT78753

hypothetical protein
  
Accession: EAT78752
  
Location: 79366-80427
  
 NCBI BlastP on this gene

EAT78752

Query: Architecture Search FASTA input

KB446557 : Pseudocercospora fijiensis CIRAD86 unplaced genomic scaffold MYCFIscaffold\_3    Total score: 2.0     Cumulative Blast bit score: 1202

Hit cluster cross-links:

Mycgr3G70471
  
Location: 0-405

Mycgr3G70471

Mycgr3G39149
  
Location: 505-1798

Mycgr3G39149

Mycgr3G92130
  
Location: 1898-2396

Mycgr3G92130

Mycgr3G38483
  
Location: 2496-3576

Mycgr3G38483

Mycgr3G108869
  
Location: 3676-5056

Mycgr3G108869

Mycgr3G103943
  
Location: 5156-5762

Mycgr3G103943

Mycgr3G57362
  
Location: 5862-7296

Mycgr3G57362

Mycgr3G39086
  
Location: 7396-8368

Mycgr3G39086

Mycgr3G103942
  
Location: 8468-8714

Mycgr3G103942

Mycgr3G108865
  
Location: 8814-10239

Mycgr3G108865

Mycgr3G70475
  
Location: 10339-11821

Mycgr3G70475

Mycgr3G108866
  
Location: 11921-13010

Mycgr3G108866

Mycgr3G92136
  
Location: 13110-13593

Mycgr3G92136

hypothetical protein
  
Accession: EME84836
  
Location: 4978306-4978808
  
 NCBI BlastP on this gene

EME84836

glycoside hydrolase family 43 carbohydrate-binding module family 35 protein
  
Accession: EME84835
  
Location: 4975841-4977347
  
 NCBI BlastP on this gene

EME84835

hypothetical protein
  
Accession: EME84834
  
Location: 4973389-4974885
  
  
**BlastP hit with Mycgr3G70475**
  
Percentage identity: 71 %
  
BlastP bit score: 650
  
Sequence coverage: 101 %
  
E-value: 0.0
  
  
 NCBI BlastP on this gene

EME84834

hypothetical protein
  
Accession: EME84833
  
Location: 4970491-4973301
  
 NCBI BlastP on this gene

EME84833

hypothetical protein
  
Accession: EME84832
  
Location: 4968740-4969938
  
 NCBI BlastP on this gene

EME84832

hypothetical protein
  
Accession: EME84831
  
Location: 4966382-4967851
  
  
**BlastP hit with Mycgr3G57362**
  
Percentage identity: 58 %
  
BlastP bit score: 552
  
Sequence coverage: 100 %
  
E-value: 0.0
  
  
 NCBI BlastP on this gene

EME84831

hypothetical protein
  
Accession: EME84830
  
Location: 4964030-4966173
  
 NCBI BlastP on this gene

EME84830

hypothetical protein
  
Accession: EME84829
  
Location: 4961729-4963731
  
 NCBI BlastP on this gene

EME84829

hypothetical protein
  
Accession: EME84828
  
Location: 4960054-4961412
  
 NCBI BlastP on this gene

EME84828

Query: Architecture Search FASTA input

KB445552 : Baudoinia compniacensis UAMH 10762 unplaced genomic scaffold BAUCOscaffold\_3    Total score: 2.0     Cumulative Blast bit score: 1159

Hit cluster cross-links:

Mycgr3G70471
  
Location: 0-405

Mycgr3G70471

Mycgr3G39149
  
Location: 505-1798

Mycgr3G39149

Mycgr3G92130
  
Location: 1898-2396

Mycgr3G92130

Mycgr3G38483
  
Location: 2496-3576

Mycgr3G38483

Mycgr3G108869
  
Location: 3676-5056

Mycgr3G108869

Mycgr3G103943
  
Location: 5156-5762

Mycgr3G103943

Mycgr3G57362
  
Location: 5862-7296

Mycgr3G57362

Mycgr3G39086
  
Location: 7396-8368

Mycgr3G39086

Mycgr3G103942
  
Location: 8468-8714

Mycgr3G103942

Mycgr3G108865
  
Location: 8814-10239

Mycgr3G108865

Mycgr3G70475
  
Location: 10339-11821

Mycgr3G70475

Mycgr3G108866
  
Location: 11921-13010

Mycgr3G108866

Mycgr3G92136
  
Location: 13110-13593

Mycgr3G92136

hypothetical protein
  
Accession: EMC98845
  
Location: 583821-585170
  
 NCBI BlastP on this gene

EMC98845

hypothetical protein
  
Accession: EMC98846
  
Location: 585583-586122
  
 NCBI BlastP on this gene

EMC98846

hypothetical protein
  
Accession: EMC98847
  
Location: 587114-591421
  
 NCBI BlastP on this gene

EMC98847

hypothetical protein
  
Accession: EMC98848
  
Location: 591780-593122
  
  
**BlastP hit with Mycgr3G39149**
  
Percentage identity: 82 %
  
BlastP bit score: 708
  
Sequence coverage: 99 %
  
E-value: 0.0
  
  
 NCBI BlastP on this gene

EMC98848

hypothetical protein
  
Accession: EMC98849
  
Location: 593343-594413
  
  
**BlastP hit with Mycgr3G108865**
  
Percentage identity: 66 %
  
BlastP bit score: 451
  
Sequence coverage: 75 %
  
E-value: 8e-153
  
  
 NCBI BlastP on this gene

EMC98849

hypothetical protein
  
Accession: EMC98850
  
Location: 595688-596067
  
 NCBI BlastP on this gene

EMC98850

hypothetical protein
  
Accession: EMC98851
  
Location: 599050-599262
  
 NCBI BlastP on this gene

EMC98851

hypothetical protein
  
Accession: EMC98852
  
Location: 600132-600293
  
 NCBI BlastP on this gene

EMC98852

hypothetical protein
  
Accession: EMC98853
  
Location: 600432-600620
  
 NCBI BlastP on this gene

EMC98853

hypothetical protein
  
Accession: EMC98854
  
Location: 601611-601934
  
 NCBI BlastP on this gene

EMC98854

Query: Architecture Search FASTA input

KB908833 : Setosphaeria turcica Et28A unplaced genomic scaffold SETTUscaffold\_5    Total score: 2.0     Cumulative Blast bit score: 1092

Hit cluster cross-links:

Mycgr3G70471
  
Location: 0-405

Mycgr3G70471

Mycgr3G39149
  
Location: 505-1798

Mycgr3G39149

Mycgr3G92130
  
Location: 1898-2396

Mycgr3G92130

Mycgr3G38483
  
Location: 2496-3576

Mycgr3G38483

Mycgr3G108869
  
Location: 3676-5056

Mycgr3G108869

Mycgr3G103943
  
Location: 5156-5762

Mycgr3G103943

Mycgr3G57362
  
Location: 5862-7296

Mycgr3G57362

Mycgr3G39086
  
Location: 7396-8368

Mycgr3G39086

Mycgr3G103942
  
Location: 8468-8714

Mycgr3G103942

Mycgr3G108865
  
Location: 8814-10239

Mycgr3G108865

Mycgr3G70475
  
Location: 10339-11821

Mycgr3G70475

Mycgr3G108866
  
Location: 11921-13010

Mycgr3G108866

Mycgr3G92136
  
Location: 13110-13593

Mycgr3G92136

hypothetical protein
  
Accession: EOA83504
  
Location: 1842249-1843535
  
 NCBI BlastP on this gene

EOA83504

hypothetical protein
  
Accession: EOA83503
  
Location: 1839721-1841530
  
 NCBI BlastP on this gene

EOA83503

hypothetical protein
  
Accession: EOA83502
  
Location: 1838235-1838918
  
 NCBI BlastP on this gene

EOA83502

hypothetical protein
  
Accession: EOA83501
  
Location: 1836403-1838032
  
  
**BlastP hit with Mycgr3G39149**
  
Percentage identity: 73 %
  
BlastP bit score: 642
  
Sequence coverage: 99 %
  
E-value: 0.0
  
  
 NCBI BlastP on this gene

EOA83501

hypothetical protein
  
Accession: EOA83500
  
Location: 1834695-1836086
  
  
**BlastP hit with Mycgr3G108865**
  
Percentage identity: 58 %
  
BlastP bit score: 450
  
Sequence coverage: 82 %
  
E-value: 1e-151
  
  
 NCBI BlastP on this gene

EOA83500

hypothetical protein
  
Accession: EOA83499
  
Location: 1834072-1834385
  
 NCBI BlastP on this gene

EOA83499

hypothetical protein
  
Accession: EOA83498
  
Location: 1831837-1832153
  
 NCBI BlastP on this gene

EOA83498

hypothetical protein
  
Accession: EOA83497
  
Location: 1828088-1830017
  
 NCBI BlastP on this gene

EOA83497

hypothetical protein
  
Accession: EOA83496
  
Location: 1826128-1827530
  
 NCBI BlastP on this gene

EOA83496

Query: Architecture Search FASTA input

FP929131 : Leptosphaeria maculans JN3 lm\_SuperContig\_16\_v2 genomic supercontig    Total score: 2.0     Cumulative Blast bit score: 1091

Hit cluster cross-links:

Mycgr3G70471
  
Location: 0-405

Mycgr3G70471

Mycgr3G39149
  
Location: 505-1798

Mycgr3G39149

Mycgr3G92130
  
Location: 1898-2396

Mycgr3G92130

Mycgr3G38483
  
Location: 2496-3576

Mycgr3G38483

Mycgr3G108869
  
Location: 3676-5056

Mycgr3G108869

Mycgr3G103943
  
Location: 5156-5762

Mycgr3G103943

Mycgr3G57362
  
Location: 5862-7296

Mycgr3G57362

Mycgr3G39086
  
Location: 7396-8368

Mycgr3G39086

Mycgr3G103942
  
Location: 8468-8714

Mycgr3G103942

Mycgr3G108865
  
Location: 8814-10239

Mycgr3G108865

Mycgr3G70475
  
Location: 10339-11821

Mycgr3G70475

Mycgr3G108866
  
Location: 11921-13010

Mycgr3G108866

Mycgr3G92136
  
Location: 13110-13593

Mycgr3G92136

hypothetical protein
  
Accession: CBX97251
  
Location: 399960-403793
  
 NCBI BlastP on this gene

LEMA\_P103820.1

similar to Arylacetamide deacetylase
  
Accession: CBX97250
  
Location: 397738-398981
  
 NCBI BlastP on this gene

LEMA\_P103810.1

similar to homoserine o-acetyltransferase
  
Accession: CBX97249
  
Location: 395624-397438
  
 NCBI BlastP on this gene

LEMA\_P103800.1

predicted protein
  
Accession: CBX97248
  
Location: 394243-394892
  
 NCBI BlastP on this gene

LEMA\_P103790.1

similar to 3-oxoacyl-(acyl-carrier-protein) synthase
  
Accession: CBX97247
  
Location: 392481-394120
  
  
**BlastP hit with Mycgr3G39149**
  
Percentage identity: 69 %
  
BlastP bit score: 619
  
Sequence coverage: 103 %
  
E-value: 0.0
  
  
 NCBI BlastP on this gene

LEMA\_P103780.1

similar to high-affinity nickel transport protein
  
Accession: CBX97246
  
Location: 390825-392171
  
  
**BlastP hit with Mycgr3G108865**
  
Percentage identity: 58 %
  
BlastP bit score: 472
  
Sequence coverage: 88 %
  
E-value: 5e-160
  
  
 NCBI BlastP on this gene

LEMA\_P103770.1

similar to mitochondrial GTPase
  
Accession: CBX97245
  
Location: 389505-390587
  
 NCBI BlastP on this gene

LEMA\_P103760.1

hypothetical protein
  
Accession: CBX97244
  
Location: 387486-389281
  
 NCBI BlastP on this gene

LEMA\_P103750.1

hypothetical protein
  
Accession: CBX97243
  
Location: 381717-387202
  
 NCBI BlastP on this gene

LEMA\_P103740.1

Query: Architecture Search FASTA input

KB445559 : Baudoinia compniacensis UAMH 10762 unplaced genomic scaffold BAUCOscaffold\_10    Total score: 2.0     Cumulative Blast bit score: 1086

Hit cluster cross-links:

Mycgr3G70471
  
Location: 0-405

Mycgr3G70471

Mycgr3G39149
  
Location: 505-1798

Mycgr3G39149

Mycgr3G92130
  
Location: 1898-2396

Mycgr3G92130

Mycgr3G38483
  
Location: 2496-3576

Mycgr3G38483

Mycgr3G108869
  
Location: 3676-5056

Mycgr3G108869

Mycgr3G103943
  
Location: 5156-5762

Mycgr3G103943

Mycgr3G57362
  
Location: 5862-7296

Mycgr3G57362

Mycgr3G39086
  
Location: 7396-8368

Mycgr3G39086

Mycgr3G103942
  
Location: 8468-8714

Mycgr3G103942

Mycgr3G108865
  
Location: 8814-10239

Mycgr3G108865

Mycgr3G70475
  
Location: 10339-11821

Mycgr3G70475

Mycgr3G108866
  
Location: 11921-13010

Mycgr3G108866

Mycgr3G92136
  
Location: 13110-13593

Mycgr3G92136

hypothetical protein
  
Accession: EMC94018
  
Location: 650864-651973
  
 NCBI BlastP on this gene

EMC94018

hypothetical protein
  
Accession: EMC94019
  
Location: 652126-653972
  
 NCBI BlastP on this gene

EMC94019

hypothetical protein
  
Accession: EMC94020
  
Location: 654670-655739
  
 NCBI BlastP on this gene

EMC94020

hypothetical protein
  
Accession: EMC94021
  
Location: 656080-657543
  
 NCBI BlastP on this gene

EMC94021

hypothetical protein
  
Accession: EMC94022
  
Location: 658145-659698
  
  
**BlastP hit with Mycgr3G57362**
  
Percentage identity: 56 %
  
BlastP bit score: 530
  
Sequence coverage: 102 %
  
E-value: 0.0
  
  
 NCBI BlastP on this gene

EMC94022

hypothetical protein
  
Accession: EMC94023
  
Location: 659869-661506
  
  
**BlastP hit with Mycgr3G70475**
  
Percentage identity: 59 %
  
BlastP bit score: 556
  
Sequence coverage: 111 %
  
E-value: 0.0
  
  
 NCBI BlastP on this gene

EMC94023

hypothetical protein
  
Accession: EMC94024
  
Location: 661864-662598
  
 NCBI BlastP on this gene

EMC94024

hypothetical protein
  
Accession: EMC94025
  
Location: 663805-664513
  
 NCBI BlastP on this gene

EMC94025

hypothetical protein
  
Accession: EMC94026
  
Location: 665120-665359
  
 NCBI BlastP on this gene

EMC94026

hypothetical protein
  
Accession: EMC94027
  
Location: 666088-667537
  
 NCBI BlastP on this gene

EMC94027

hypothetical protein
  
Accession: EMC94028
  
Location: 668909-669394
  
 NCBI BlastP on this gene

EMC94028

Query: Architecture Search FASTA input

KB445644 : Cochliobolus sativus ND90Pr unplaced genomic scaffold COCSAscaffold\_8    Total score: 2.0     Cumulative Blast bit score: 1081

Hit cluster cross-links:

Mycgr3G70471
  
Location: 0-405

Mycgr3G70471

Mycgr3G39149
  
Location: 505-1798

Mycgr3G39149

Mycgr3G92130
  
Location: 1898-2396

Mycgr3G92130

Mycgr3G38483
  
Location: 2496-3576

Mycgr3G38483

Mycgr3G108869
  
Location: 3676-5056

Mycgr3G108869

Mycgr3G103943
  
Location: 5156-5762

Mycgr3G103943

Mycgr3G57362
  
Location: 5862-7296

Mycgr3G57362

Mycgr3G39086
  
Location: 7396-8368

Mycgr3G39086

Mycgr3G103942
  
Location: 8468-8714

Mycgr3G103942

Mycgr3G108865
  
Location: 8814-10239

Mycgr3G108865

Mycgr3G70475
  
Location: 10339-11821

Mycgr3G70475

Mycgr3G108866
  
Location: 11921-13010

Mycgr3G108866

Mycgr3G92136
  
Location: 13110-13593

Mycgr3G92136

hypothetical protein
  
Accession: EMD63534
  
Location: 450495-451622
  
 NCBI BlastP on this gene

EMD63534

hypothetical protein
  
Accession: EMD63535
  
Location: 452440-454252
  
 NCBI BlastP on this gene

EMD63535

hypothetical protein
  
Accession: EMD63536
  
Location: 455168-456798
  
  
**BlastP hit with Mycgr3G39149**
  
Percentage identity: 71 %
  
BlastP bit score: 623
  
Sequence coverage: 99 %
  
E-value: 0.0
  
  
 NCBI BlastP on this gene

EMD63536

hypothetical protein
  
Accession: EMD63537
  
Location: 457130-458540
  
  
**BlastP hit with Mycgr3G108865**
  
Percentage identity: 61 %
  
BlastP bit score: 458
  
Sequence coverage: 78 %
  
E-value: 1e-154
  
  
 NCBI BlastP on this gene

EMD63537

hypothetical protein
  
Accession: EMD63538
  
Location: 461075-462993
  
 NCBI BlastP on this gene

EMD63538

hypothetical protein
  
Accession: EMD63539
  
Location: 463542-464894
  
 NCBI BlastP on this gene

EMD63539

hypothetical protein
  
Accession: EMD63540
  
Location: 465137-466060
  
 NCBI BlastP on this gene

EMD63540

Query: Architecture Search FASTA input

KB733456 : Bipolaris maydis ATCC 48331 unplaced genomic scaffold COCC4scaffold\_13    Total score: 2.0     Cumulative Blast bit score: 1070

Hit cluster cross-links:

Mycgr3G70471
  
Location: 0-405

Mycgr3G70471

Mycgr3G39149
  
Location: 505-1798

Mycgr3G39149

Mycgr3G92130
  
Location: 1898-2396

Mycgr3G92130

Mycgr3G38483
  
Location: 2496-3576

Mycgr3G38483

Mycgr3G108869
  
Location: 3676-5056

Mycgr3G108869

Mycgr3G103943
  
Location: 5156-5762

Mycgr3G103943

Mycgr3G57362
  
Location: 5862-7296

Mycgr3G57362

Mycgr3G39086
  
Location: 7396-8368

Mycgr3G39086

Mycgr3G103942
  
Location: 8468-8714

Mycgr3G103942

Mycgr3G108865
  
Location: 8814-10239

Mycgr3G108865

Mycgr3G70475
  
Location: 10339-11821

Mycgr3G70475

Mycgr3G108866
  
Location: 11921-13010

Mycgr3G108866

Mycgr3G92136
  
Location: 13110-13593

Mycgr3G92136

hypothetical protein
  
Accession: ENI04890
  
Location: 926685-927816
  
 NCBI BlastP on this gene

ENI04890

hypothetical protein
  
Accession: ENI04889
  
Location: 923698-925513
  
 NCBI BlastP on this gene

ENI04889

hypothetical protein
  
Accession: ENI04888
  
Location: 921171-922799
  
  
**BlastP hit with Mycgr3G39149**
  
Percentage identity: 70 %
  
BlastP bit score: 610
  
Sequence coverage: 99 %
  
E-value: 0.0
  
  
 NCBI BlastP on this gene

ENI04888

hypothetical protein
  
Accession: ENI04887
  
Location: 919447-920829
  
  
**BlastP hit with Mycgr3G108865**
  
Percentage identity: 61 %
  
BlastP bit score: 460
  
Sequence coverage: 78 %
  
E-value: 1e-155
  
  
 NCBI BlastP on this gene

ENI04887

hypothetical protein
  
Accession: ENI04886
  
Location: 918868-919208
  
 NCBI BlastP on this gene

ENI04886

hypothetical protein
  
Accession: ENI04885
  
Location: 914796-916714
  
 NCBI BlastP on this gene

ENI04885

hypothetical protein
  
Accession: ENI04884
  
Location: 912896-914248
  
 NCBI BlastP on this gene

ENI04884

hypothetical protein
  
Accession: ENI04883
  
Location: 911728-912650
  
 NCBI BlastP on this gene

ENI04883

Query: Architecture Search FASTA input

KB445587 : Cochliobolus heterostrophus C5 unplaced genomic scaffold COCHEscaffold\_19    Total score: 2.0     Cumulative Blast bit score: 1070

Hit cluster cross-links:

Mycgr3G70471
  
Location: 0-405

Mycgr3G70471

Mycgr3G39149
  
Location: 505-1798

Mycgr3G39149

Mycgr3G92130
  
Location: 1898-2396

Mycgr3G92130

Mycgr3G38483
  
Location: 2496-3576

Mycgr3G38483

Mycgr3G108869
  
Location: 3676-5056

Mycgr3G108869

Mycgr3G103943
  
Location: 5156-5762

Mycgr3G103943

Mycgr3G57362
  
Location: 5862-7296

Mycgr3G57362

Mycgr3G39086
  
Location: 7396-8368

Mycgr3G39086

Mycgr3G103942
  
Location: 8468-8714

Mycgr3G103942

Mycgr3G108865
  
Location: 8814-10239

Mycgr3G108865

Mycgr3G70475
  
Location: 10339-11821

Mycgr3G70475

Mycgr3G108866
  
Location: 11921-13010

Mycgr3G108866

Mycgr3G92136
  
Location: 13110-13593

Mycgr3G92136

hypothetical protein
  
Accession: EMD85850
  
Location: 702254-703385
  
 NCBI BlastP on this gene

EMD85850

hypothetical protein
  
Accession: EMD85851
  
Location: 704557-706372
  
 NCBI BlastP on this gene

EMD85851

hypothetical protein
  
Accession: EMD85852
  
Location: 707271-708899
  
  
**BlastP hit with Mycgr3G39149**
  
Percentage identity: 70 %
  
BlastP bit score: 610
  
Sequence coverage: 99 %
  
E-value: 0.0
  
  
 NCBI BlastP on this gene

EMD85852

hypothetical protein
  
Accession: EMD85853
  
Location: 709241-710623
  
  
**BlastP hit with Mycgr3G108865**
  
Percentage identity: 61 %
  
BlastP bit score: 460
  
Sequence coverage: 78 %
  
E-value: 1e-155
  
  
 NCBI BlastP on this gene

EMD85853

hypothetical protein
  
Accession: EMD85854
  
Location: 710862-711202
  
 NCBI BlastP on this gene

EMD85854

hypothetical protein
  
Accession: EMD85855
  
Location: 713356-715274
  
 NCBI BlastP on this gene

EMD85855

hypothetical protein
  
Accession: EMD85856
  
Location: 715822-717174
  
 NCBI BlastP on this gene

EMD85856

hypothetical protein
  
Accession: EMD85857
  
Location: 717420-718342
  
 NCBI BlastP on this gene

EMD85857

Query: Architecture Search FASTA input

KB445574 : Cochliobolus heterostrophus C5 unplaced genomic scaffold COCHEscaffold\_6    Total score: 2.0     Cumulative Blast bit score: 1070

Hit cluster cross-links:

Mycgr3G70471
  
Location: 0-405

Mycgr3G70471

Mycgr3G39149
  
Location: 505-1798

Mycgr3G39149

Mycgr3G92130
  
Location: 1898-2396

Mycgr3G92130

Mycgr3G38483
  
Location: 2496-3576

Mycgr3G38483

Mycgr3G108869
  
Location: 3676-5056

Mycgr3G108869

Mycgr3G103943
  
Location: 5156-5762

Mycgr3G103943

Mycgr3G57362
  
Location: 5862-7296

Mycgr3G57362

Mycgr3G39086
  
Location: 7396-8368

Mycgr3G39086

Mycgr3G103942
  
Location: 8468-8714

Mycgr3G103942

Mycgr3G108865
  
Location: 8814-10239

Mycgr3G108865

Mycgr3G70475
  
Location: 10339-11821

Mycgr3G70475

Mycgr3G108866
  
Location: 11921-13010

Mycgr3G108866

Mycgr3G92136
  
Location: 13110-13593

Mycgr3G92136

hypothetical protein
  
Accession: EMD92720
  
Location: 52025-53501
  
 NCBI BlastP on this gene

EMD92720

hypothetical protein
  
Accession: EMD92721
  
Location: 54328-56143
  
 NCBI BlastP on this gene

EMD92721

hypothetical protein
  
Accession: EMD92722
  
Location: 57042-58670
  
  
**BlastP hit with Mycgr3G39149**
  
Percentage identity: 70 %
  
BlastP bit score: 610
  
Sequence coverage: 99 %
  
E-value: 0.0
  
  
 NCBI BlastP on this gene

EMD92722

hypothetical protein
  
Accession: EMD92723
  
Location: 59012-60394
  
  
**BlastP hit with Mycgr3G108865**
  
Percentage identity: 61 %
  
BlastP bit score: 460
  
Sequence coverage: 78 %
  
E-value: 1e-155
  
  
 NCBI BlastP on this gene

EMD92723

hypothetical protein
  
Accession: EMD92724
  
Location: 60633-60973
  
 NCBI BlastP on this gene

EMD92724

hypothetical protein
  
Accession: EMD92725
  
Location: 63127-65045
  
 NCBI BlastP on this gene

EMD92725

hypothetical protein
  
Accession: EMD92726
  
Location: 65593-66945
  
 NCBI BlastP on this gene

EMD92726

hypothetical protein
  
Accession: EMD92727
  
Location: 67191-68113
  
 NCBI BlastP on this gene

EMD92727

Query: Architecture Search FASTA input

GL532905 : Pyrenophora teres f. teres 0-1 unplaced genomic scaffold scaffold\_189131    Total score: 2.0     Cumulative Blast bit score: 1053

Hit cluster cross-links:

Mycgr3G70471
  
Location: 0-405

Mycgr3G70471

Mycgr3G39149
  
Location: 505-1798

Mycgr3G39149

Mycgr3G92130
  
Location: 1898-2396

Mycgr3G92130

Mycgr3G38483
  
Location: 2496-3576

Mycgr3G38483

Mycgr3G108869
  
Location: 3676-5056

Mycgr3G108869

Mycgr3G103943
  
Location: 5156-5762

Mycgr3G103943

Mycgr3G57362
  
Location: 5862-7296

Mycgr3G57362

Mycgr3G39086
  
Location: 7396-8368

Mycgr3G39086

Mycgr3G103942
  
Location: 8468-8714

Mycgr3G103942

Mycgr3G108865
  
Location: 8814-10239

Mycgr3G108865

Mycgr3G70475
  
Location: 10339-11821

Mycgr3G70475

Mycgr3G108866
  
Location: 11921-13010

Mycgr3G108866

Mycgr3G92136
  
Location: 13110-13593

Mycgr3G92136

hypothetical protein
  
Accession: EFQ95179
  
Location: 26602-27299
  
 NCBI BlastP on this gene

EFQ95179

hypothetical protein
  
Accession: EFQ95180
  
Location: 30234-32053
  
 NCBI BlastP on this gene

EFQ95180

hypothetical protein
  
Accession: EFQ95181
  
Location: 33077-35525
  
  
**BlastP hit with Mycgr3G39149**
  
Percentage identity: 71 %
  
BlastP bit score: 598
  
Sequence coverage: 95 %
  
E-value: 0.0
  
  
 NCBI BlastP on this gene

EFQ95181

hypothetical protein
  
Accession: EFQ95182
  
Location: 35809-37210
  
  
**BlastP hit with Mycgr3G108865**
  
Percentage identity: 57 %
  
BlastP bit score: 455
  
Sequence coverage: 83 %
  
E-value: 1e-153
  
  
 NCBI BlastP on this gene

EFQ95182

hypothetical protein
  
Accession: EFQ95183
  
Location: 41182-41636
  
 NCBI BlastP on this gene

EFQ95183

hypothetical protein
  
Accession: EFQ95184
  
Location: 42424-44279
  
 NCBI BlastP on this gene

EFQ95184

Query: Architecture Search FASTA input

DS231625 : Pyrenophora tritici-repentis Pt-1C-BFP supercont1.11 genomic scaffold    Total score: 2.0     Cumulative Blast bit score: 903

Hit cluster cross-links:

Mycgr3G70471
  
Location: 0-405

Mycgr3G70471

Mycgr3G39149
  
Location: 505-1798

Mycgr3G39149

Mycgr3G92130
  
Location: 1898-2396

Mycgr3G92130

Mycgr3G38483
  
Location: 2496-3576

Mycgr3G38483

Mycgr3G108869
  
Location: 3676-5056

Mycgr3G108869

Mycgr3G103943
  
Location: 5156-5762

Mycgr3G103943

Mycgr3G57362
  
Location: 5862-7296

Mycgr3G57362

Mycgr3G39086
  
Location: 7396-8368

Mycgr3G39086

Mycgr3G103942
  
Location: 8468-8714

Mycgr3G103942

Mycgr3G108865
  
Location: 8814-10239

Mycgr3G108865

Mycgr3G70475
  
Location: 10339-11821

Mycgr3G70475

Mycgr3G108866
  
Location: 11921-13010

Mycgr3G108866

Mycgr3G92136
  
Location: 13110-13593

Mycgr3G92136

homoserine O-acetyltransferase
  
Accession: EDU42763
  
Location: 1532287-1534106
  
 NCBI BlastP on this gene

EDU42763

3-oxoacyl-(acyl-carrier-protein) synthase, mitochondrial precursor
  
Accession: EDU42762
  
Location: 1523280-1524592
  
  
**BlastP hit with Mycgr3G39149**
  
Percentage identity: 65 %
  
BlastP bit score: 460
  
Sequence coverage: 85 %
  
E-value: 2e-157
  
  
 NCBI BlastP on this gene

EDU42762

high affinity nickel transport protein nic1
  
Accession: EDU42761
  
Location: 1521616-1523014
  
  
**BlastP hit with Mycgr3G108865**
  
Percentage identity: 54 %
  
BlastP bit score: 444
  
Sequence coverage: 90 %
  
E-value: 4e-149
  
  
 NCBI BlastP on this gene

EDU42761

L-ascorbate oxidase
  
Accession: EDU42760
  
Location: 1514126-1515697
  
 NCBI BlastP on this gene

EDU42760

Query: Architecture Search FASTA input

CH476632 : Sclerotinia sclerotiorum 1980 scaffold\_12 genomic scaffold    Total score: 2.0     Cumulative Blast bit score: 865

Hit cluster cross-links:

Mycgr3G70471
  
Location: 0-405

Mycgr3G70471

Mycgr3G39149
  
Location: 505-1798

Mycgr3G39149

Mycgr3G92130
  
Location: 1898-2396

Mycgr3G92130

Mycgr3G38483
  
Location: 2496-3576

Mycgr3G38483

Mycgr3G108869
  
Location: 3676-5056

Mycgr3G108869

Mycgr3G103943
  
Location: 5156-5762

Mycgr3G103943

Mycgr3G57362
  
Location: 5862-7296

Mycgr3G57362

Mycgr3G39086
  
Location: 7396-8368

Mycgr3G39086

Mycgr3G103942
  
Location: 8468-8714

Mycgr3G103942

Mycgr3G108865
  
Location: 8814-10239

Mycgr3G108865

Mycgr3G70475
  
Location: 10339-11821

Mycgr3G70475

Mycgr3G108866
  
Location: 11921-13010

Mycgr3G108866

Mycgr3G92136
  
Location: 13110-13593

Mycgr3G92136

predicted protein
  
Accession: EDN92925
  
Location: 360742-361477
  
 NCBI BlastP on this gene

EDN92925

predicted protein
  
Accession: EDN92926
  
Location: 362048-362771
  
 NCBI BlastP on this gene

EDN92926

hypothetical protein
  
Accession: EDN92927
  
Location: 363910-365114
  
  
**BlastP hit with Mycgr3G39149**
  
Percentage identity: 68 %
  
BlastP bit score: 524
  
Sequence coverage: 87 %
  
E-value: 0.0
  
  
 NCBI BlastP on this gene

EDN92927

predicted protein
  
Accession: EDN92928
  
Location: 366572-366898
  
 NCBI BlastP on this gene

EDN92928

hypothetical protein
  
Accession: EDN92929
  
Location: 368882-370800
  
 NCBI BlastP on this gene

EDN92929

hypothetical protein
  
Accession: EDN92930
  
Location: 372638-373999
  
 NCBI BlastP on this gene

EDN92930

hypothetical protein
  
Accession: EDN92931
  
Location: 374251-375352
  
 NCBI BlastP on this gene

EDN92931

predicted protein
  
Accession: EDN92932
  
Location: 375868-376760
  
 NCBI BlastP on this gene

EDN92932

hypothetical protein
  
Accession: EDN92933
  
Location: 377269-379281
  
 NCBI BlastP on this gene

EDN92933

hypothetical protein
  
Accession: EDN92934
  
Location: 379922-380772
  
 NCBI BlastP on this gene

EDN92934

hypothetical protein
  
Accession: EDN92935
  
Location: 382826-383888
  
  
**BlastP hit with Mycgr3G108865**
  
Percentage identity: 56 %
  
BlastP bit score: 342
  
Sequence coverage: 70 %
  
E-value: 1e-110
  
  
 NCBI BlastP on this gene

EDN92935

hypothetical protein
  
Accession: EDN92936
  
Location: 384548-386462
  
 NCBI BlastP on this gene

EDN92936

Query: Architecture Search FASTA input

KB456267 : Mycosphaerella populorum SO2202 unplaced genomic scaffold SEPMUscaffold\_8    Total score: 2.0     Cumulative Blast bit score: 786

Hit cluster cross-links:

Mycgr3G70471
  
Location: 0-405

Mycgr3G70471

Mycgr3G39149
  
Location: 505-1798

Mycgr3G39149

Mycgr3G92130
  
Location: 1898-2396

Mycgr3G92130

Mycgr3G38483
  
Location: 2496-3576

Mycgr3G38483

Mycgr3G108869
  
Location: 3676-5056

Mycgr3G108869

Mycgr3G103943
  
Location: 5156-5762

Mycgr3G103943

Mycgr3G57362
  
Location: 5862-7296

Mycgr3G57362

Mycgr3G39086
  
Location: 7396-8368

Mycgr3G39086

Mycgr3G103942
  
Location: 8468-8714

Mycgr3G103942

Mycgr3G108865
  
Location: 8814-10239

Mycgr3G108865

Mycgr3G70475
  
Location: 10339-11821

Mycgr3G70475

Mycgr3G108866
  
Location: 11921-13010

Mycgr3G108866

Mycgr3G92136
  
Location: 13110-13593

Mycgr3G92136

hypothetical protein
  
Accession: EMF10399
  
Location: 274002-274334
  
 NCBI BlastP on this gene

EMF10399

glycerol kinase
  
Accession: EMF10400
  
Location: 275712-277457
  
 NCBI BlastP on this gene

EMF10400

hypothetical protein
  
Accession: EMF10401
  
Location: 279441-280705
  
  
**BlastP hit with Mycgr3G38483**
  
Percentage identity: 71 %
  
BlastP bit score: 536
  
Sequence coverage: 99 %
  
E-value: 0.0
  
  
 NCBI BlastP on this gene

EMF10401

Ribosomal S24e-domain-containing protein
  
Accession: EMF10402
  
Location: 280973-281564
  
  
**BlastP hit with Mycgr3G70471**
  
Percentage identity: 92 %
  
BlastP bit score: 250
  
Sequence coverage: 99 %
  
E-value: 1e-82
  
  
 NCBI BlastP on this gene

EMF10402

hypothetical protein
  
Accession: EMF10403
  
Location: 282518-283054
  
 NCBI BlastP on this gene

EMF10403

protein serine/threonine phosphatase 2C
  
Accession: EMF10404
  
Location: 283833-285536
  
 NCBI BlastP on this gene

EMF10404

hypothetical protein
  
Accession: EMF10405
  
Location: 286097-289279
  
 NCBI BlastP on this gene

EMF10405

Query: Architecture Search FASTA input

KB446538 : Dothistroma septosporum NZE10 unplaced genomic scaffold DOTSEscaffold\_4    Total score: 2.0     Cumulative Blast bit score: 780

Hit cluster cross-links:

Mycgr3G70471
  
Location: 0-405

Mycgr3G70471

Mycgr3G39149
  
Location: 505-1798

Mycgr3G39149

Mycgr3G92130
  
Location: 1898-2396

Mycgr3G92130

Mycgr3G38483
  
Location: 2496-3576

Mycgr3G38483

Mycgr3G108869
  
Location: 3676-5056

Mycgr3G108869

Mycgr3G103943
  
Location: 5156-5762

Mycgr3G103943

Mycgr3G57362
  
Location: 5862-7296

Mycgr3G57362

Mycgr3G39086
  
Location: 7396-8368

Mycgr3G39086

Mycgr3G103942
  
Location: 8468-8714

Mycgr3G103942

Mycgr3G108865
  
Location: 8814-10239

Mycgr3G108865

Mycgr3G70475
  
Location: 10339-11821

Mycgr3G70475

Mycgr3G108866
  
Location: 11921-13010

Mycgr3G108866

Mycgr3G92136
  
Location: 13110-13593

Mycgr3G92136

hypothetical protein
  
Accession: EME45792
  
Location: 2399626-2401407
  
 NCBI BlastP on this gene

EME45792

hypothetical protein
  
Accession: EME45793
  
Location: 2402472-2402918
  
 NCBI BlastP on this gene

EME45793

hypothetical protein
  
Accession: EME45795
  
Location: 2405022-2407075
  
 NCBI BlastP on this gene

EME45795

hypothetical protein
  
Accession: EME45797
  
Location: 2407522-2408746
  
  
**BlastP hit with Mycgr3G38483**
  
Percentage identity: 70 %
  
BlastP bit score: 530
  
Sequence coverage: 99 %
  
E-value: 0.0
  
  
 NCBI BlastP on this gene

EME45797

hypothetical protein
  
Accession: EME45798
  
Location: 2409173-2409772
  
  
**BlastP hit with Mycgr3G70471**
  
Percentage identity: 91 %
  
BlastP bit score: 250
  
Sequence coverage: 99 %
  
E-value: 7e-83
  
  
 NCBI BlastP on this gene

EME45798

hypothetical protein
  
Accession: EME45799
  
Location: 2411049-2412458
  
 NCBI BlastP on this gene

EME45799

hypothetical protein
  
Accession: EME45800
  
Location: 2413509-2413814
  
 NCBI BlastP on this gene

EME45800

hypothetical protein
  
Accession: EME45801
  
Location: 2415523-2415867
  
 NCBI BlastP on this gene

EME45801

hypothetical protein
  
Accession: EME45802
  
Location: 2416943-2418808
  
 NCBI BlastP on this gene

EME45802

Query: Architecture Search FASTA input

KB445552 : Baudoinia compniacensis UAMH 10762 unplaced genomic scaffold BAUCOscaffold\_3    Total score: 2.0     Cumulative Blast bit score: 724

Hit cluster cross-links:

Mycgr3G70471
  
Location: 0-405

Mycgr3G70471

Mycgr3G39149
  
Location: 505-1798

Mycgr3G39149

Mycgr3G92130
  
Location: 1898-2396

Mycgr3G92130

Mycgr3G38483
  
Location: 2496-3576

Mycgr3G38483

Mycgr3G108869
  
Location: 3676-5056

Mycgr3G108869

Mycgr3G103943
  
Location: 5156-5762

Mycgr3G103943

Mycgr3G57362
  
Location: 5862-7296

Mycgr3G57362

Mycgr3G39086
  
Location: 7396-8368

Mycgr3G39086

Mycgr3G103942
  
Location: 8468-8714

Mycgr3G103942

Mycgr3G108865
  
Location: 8814-10239

Mycgr3G108865

Mycgr3G70475
  
Location: 10339-11821

Mycgr3G70475

Mycgr3G108866
  
Location: 11921-13010

Mycgr3G108866

Mycgr3G92136
  
Location: 13110-13593

Mycgr3G92136

hypothetical protein
  
Accession: EMC98771
  
Location: 435470-437253
  
 NCBI BlastP on this gene

EMC98771

hypothetical protein
  
Accession: EMC98772
  
Location: 437795-439849
  
 NCBI BlastP on this gene

EMC98772

hypothetical protein
  
Accession: EMC98773
  
Location: 440950-444547
  
 NCBI BlastP on this gene

EMC98773

hypothetical protein
  
Accession: EMC98774
  
Location: 445372-446613
  
  
**BlastP hit with Mycgr3G38483**
  
Percentage identity: 65 %
  
BlastP bit score: 498
  
Sequence coverage: 99 %
  
E-value: 2e-173
  
  
 NCBI BlastP on this gene

EMC98774

hypothetical protein
  
Accession: EMC98775
  
Location: 446897-447468
  
  
**BlastP hit with Mycgr3G70471**
  
Percentage identity: 88 %
  
BlastP bit score: 226
  
Sequence coverage: 91 %
  
E-value: 3e-73
  
  
 NCBI BlastP on this gene

EMC98775

hypothetical protein
  
Accession: EMC98776
  
Location: 448485-448811
  
 NCBI BlastP on this gene

EMC98776

hypothetical protein
  
Accession: EMC98777
  
Location: 448960-449136
  
 NCBI BlastP on this gene

EMC98777

hypothetical protein
  
Accession: EMC98778
  
Location: 453142-454063
  
 NCBI BlastP on this gene

EMC98778

hypothetical protein
  
Accession: EMC98779
  
Location: 454697-456865
  
 NCBI BlastP on this gene

EMC98779

Query: Architecture Search FASTA input

GL534066 : Pyrenophora teres f. teres 0-1 unplaced genomic scaffold scaffold\_190324    Total score: 2.0     Cumulative Blast bit score: 522

Hit cluster cross-links:

Mycgr3G70471
  
Location: 0-405

Mycgr3G70471

Mycgr3G39149
  
Location: 505-1798

Mycgr3G39149

Mycgr3G92130
  
Location: 1898-2396

Mycgr3G92130

Mycgr3G38483
  
Location: 2496-3576

Mycgr3G38483

Mycgr3G108869
  
Location: 3676-5056

Mycgr3G108869

Mycgr3G103943
  
Location: 5156-5762

Mycgr3G103943

Mycgr3G57362
  
Location: 5862-7296

Mycgr3G57362

Mycgr3G39086
  
Location: 7396-8368

Mycgr3G39086

Mycgr3G103942
  
Location: 8468-8714

Mycgr3G103942

Mycgr3G108865
  
Location: 8814-10239

Mycgr3G108865

Mycgr3G70475
  
Location: 10339-11821

Mycgr3G70475

Mycgr3G108866
  
Location: 11921-13010

Mycgr3G108866

Mycgr3G92136
  
Location: 13110-13593

Mycgr3G92136

hypothetical protein
  
Accession: EFQ92977
  
Location: 743-2062
  
  
**BlastP hit with Mycgr3G38483**
  
Percentage identity: 45 %
  
BlastP bit score: 296
  
Sequence coverage: 100 %
  
E-value: 3e-94
  
  
 NCBI BlastP on this gene

EFQ92977

hypothetical protein
  
Accession: EFQ92978
  
Location: 2408-2966
  
  
**BlastP hit with Mycgr3G70471**
  
Percentage identity: 82 %
  
BlastP bit score: 226
  
Sequence coverage: 99 %
  
E-value: 2e-73
  
  
 NCBI BlastP on this gene

EFQ92978

hypothetical protein
  
Accession: EFQ92979
  
Location: 3611-5921
  
 NCBI BlastP on this gene

EFQ92979

Query: Architecture Search FASTA input

GG697355 : Glomerella graminicola M1.001 genomic scaffold supercont1.25    Total score: 2.0     Cumulative Blast bit score: 475

Hit cluster cross-links:

Mycgr3G70471
  
Location: 0-405

Mycgr3G70471

Mycgr3G39149
  
Location: 505-1798

Mycgr3G39149

Mycgr3G92130
  
Location: 1898-2396

Mycgr3G92130

Mycgr3G38483
  
Location: 2496-3576

Mycgr3G38483

Mycgr3G108869
  
Location: 3676-5056

Mycgr3G108869

Mycgr3G103943
  
Location: 5156-5762

Mycgr3G103943

Mycgr3G57362
  
Location: 5862-7296

Mycgr3G57362

Mycgr3G39086
  
Location: 7396-8368

Mycgr3G39086

Mycgr3G103942
  
Location: 8468-8714

Mycgr3G103942

Mycgr3G108865
  
Location: 8814-10239

Mycgr3G108865

Mycgr3G70475
  
Location: 10339-11821

Mycgr3G70475

Mycgr3G108866
  
Location: 11921-13010

Mycgr3G108866

Mycgr3G92136
  
Location: 13110-13593

Mycgr3G92136

RNA recognition domain-containing protein
  
Accession: EFQ31470
  
Location: 448628-450239
  
 NCBI BlastP on this gene

EFQ31470

hypothetical protein
  
Accession: EFQ31471
  
Location: 451651-452163
  
 NCBI BlastP on this gene

EFQ31471

hypothetical protein
  
Accession: EFQ31472
  
Location: 458042-459202
  
  
**BlastP hit with Mycgr3G38483**
  
Percentage identity: 43 %
  
BlastP bit score: 260
  
Sequence coverage: 98 %
  
E-value: 5e-80
  
  
 NCBI BlastP on this gene

EFQ31472

ribosomal protein S24e
  
Accession: EFQ31473
  
Location: 459913-460765
  
  
**BlastP hit with Mycgr3G70471**
  
Percentage identity: 84 %
  
BlastP bit score: 215
  
Sequence coverage: 88 %
  
E-value: 6e-69
  
  
 NCBI BlastP on this gene

EFQ31473

hypothetical protein
  
Accession: EFQ31474
  
Location: 462165-463592
  
 NCBI BlastP on this gene

EFQ31474

actin
  
Accession: EFQ31475
  
Location: 466706-468113
  
 NCBI BlastP on this gene

EFQ31475

hydantoin racemase
  
Accession: EFQ31476
  
Location: 468640-469541
  
 NCBI BlastP on this gene

EFQ31476

Query: Architecture Search FASTA input

KB021237 : Colletotrichum gloeosporioides Nara gc5 unplaced genomic scaffold scaffold810    Total score: 2.0     Cumulative Blast bit score: 473

Hit cluster cross-links:

Mycgr3G70471
  
Location: 0-405

Mycgr3G70471

Mycgr3G39149
  
Location: 505-1798

Mycgr3G39149

Mycgr3G92130
  
Location: 1898-2396

Mycgr3G92130

Mycgr3G38483
  
Location: 2496-3576

Mycgr3G38483

Mycgr3G108869
  
Location: 3676-5056

Mycgr3G108869

Mycgr3G103943
  
Location: 5156-5762

Mycgr3G103943

Mycgr3G57362
  
Location: 5862-7296

Mycgr3G57362

Mycgr3G39086
  
Location: 7396-8368

Mycgr3G39086

Mycgr3G103942
  
Location: 8468-8714

Mycgr3G103942

Mycgr3G108865
  
Location: 8814-10239

Mycgr3G108865

Mycgr3G70475
  
Location: 10339-11821

Mycgr3G70475

Mycgr3G108866
  
Location: 11921-13010

Mycgr3G108866

Mycgr3G92136
  
Location: 13110-13593

Mycgr3G92136

RNA binding protein
  
Accession: ELA24798
  
Location: 7462-9127
  
 NCBI BlastP on this gene

ELA24798

hypothetical protein
  
Accession: ELA24799
  
Location: 10533-11023
  
 NCBI BlastP on this gene

ELA24799

hypothetical protein
  
Accession: ELA24800
  
Location: 16827-17959
  
  
**BlastP hit with Mycgr3G38483**
  
Percentage identity: 44 %
  
BlastP bit score: 262
  
Sequence coverage: 100 %
  
E-value: 5e-81
  
  
 NCBI BlastP on this gene

ELA24800

40s ribosomal protein s24
  
Accession: ELA24801
  
Location: 18699-19557
  
  
**BlastP hit with Mycgr3G70471**
  
Percentage identity: 83 %
  
BlastP bit score: 211
  
Sequence coverage: 88 %
  
E-value: 3e-67
  
  
 NCBI BlastP on this gene

ELA24801

Query: Architecture Search FASTA input

HF679031 : Fusarium fujikuroi IMI 58289 draft genome, chromosome FFUJ\_chr09.    Total score: 2.0     Cumulative Blast bit score: 472

Hit cluster cross-links:

Mycgr3G70471
  
Location: 0-405

Mycgr3G70471

Mycgr3G39149
  
Location: 505-1798

Mycgr3G39149

Mycgr3G92130
  
Location: 1898-2396

Mycgr3G92130

Mycgr3G38483
  
Location: 2496-3576

Mycgr3G38483

Mycgr3G108869
  
Location: 3676-5056

Mycgr3G108869

Mycgr3G103943
  
Location: 5156-5762

Mycgr3G103943

Mycgr3G57362
  
Location: 5862-7296

Mycgr3G57362

Mycgr3G39086
  
Location: 7396-8368

Mycgr3G39086

Mycgr3G103942
  
Location: 8468-8714

Mycgr3G103942

Mycgr3G108865
  
Location: 8814-10239

Mycgr3G108865

Mycgr3G70475
  
Location: 10339-11821

Mycgr3G70475

Mycgr3G108866
  
Location: 11921-13010

Mycgr3G108866

Mycgr3G92136
  
Location: 13110-13593

Mycgr3G92136

related to peptidylprolyl isomerase (cyclophilin)-like protein
  
Accession: CCT73349
  
Location: 639645-641158
  
 NCBI BlastP on this gene

FFUJ\_09965

uncharacterized protein
  
Accession: CCT73350
  
Location: 643741-644235
  
 NCBI BlastP on this gene

FFUJ\_09964

uncharacterized protein
  
Accession: CCT73351
  
Location: 649115-650204
  
  
**BlastP hit with Mycgr3G38483**
  
Percentage identity: 45 %
  
BlastP bit score: 265
  
Sequence coverage: 98 %
  
E-value: 5e-82
  
  
 NCBI BlastP on this gene

FFUJ\_09963

probable 40S RIBOSOMAL PROTEIN S24
  
Accession: CCT73352
  
Location: 650665-651435
  
  
**BlastP hit with Mycgr3G70471**
  
Percentage identity: 81 %
  
BlastP bit score: 207
  
Sequence coverage: 89 %
  
E-value: 4e-66
  
  
 NCBI BlastP on this gene

FFUJ\_09962

uncharacterized protein
  
Accession: CCT73998
  
Location: 651903-652331
  
 NCBI BlastP on this gene

FFUJ\_09961

related to SRP40-suppressor of mutant AC40 of RNA polymerase I and III
  
Accession: CCT73353
  
Location: 653205-657770
  
 NCBI BlastP on this gene

FFUJ\_09960

probable GTP cyclohydrolase II
  
Accession: CCT73354
  
Location: 658559-659784
  
 NCBI BlastP on this gene

FFUJ\_09959

Query: Architecture Search FASTA input

AMYD01001766 : Colletotrichum gloeosporioides Cg-14    Total score: 2.0     Cumulative Blast bit score: 472

Hit cluster cross-links:

Mycgr3G70471
  
Location: 0-405

Mycgr3G70471

Mycgr3G39149
  
Location: 505-1798

Mycgr3G39149

Mycgr3G92130
  
Location: 1898-2396

Mycgr3G92130

Mycgr3G38483
  
Location: 2496-3576

Mycgr3G38483

Mycgr3G108869
  
Location: 3676-5056

Mycgr3G108869

Mycgr3G103943
  
Location: 5156-5762

Mycgr3G103943

Mycgr3G57362
  
Location: 5862-7296

Mycgr3G57362

Mycgr3G39086
  
Location: 7396-8368

Mycgr3G39086

Mycgr3G103942
  
Location: 8468-8714

Mycgr3G103942

Mycgr3G108865
  
Location: 8814-10239

Mycgr3G108865

Mycgr3G70475
  
Location: 10339-11821

Mycgr3G70475

Mycgr3G108866
  
Location: 11921-13010

Mycgr3G108866

Mycgr3G92136
  
Location: 13110-13593

Mycgr3G92136

ribosomal protein S24e
  
Accession: EQB51666
  
Location: 5677-6534
  
  
**BlastP hit with Mycgr3G70471**
  
Percentage identity: 83 %
  
BlastP bit score: 211
  
Sequence coverage: 88 %
  
E-value: 3e-67
  
  
 NCBI BlastP on this gene

EQB51666

hypothetical protein
  
Accession: EQB51667
  
Location: 7299-8614
  
  
**BlastP hit with Mycgr3G38483**
  
Percentage identity: 44 %
  
BlastP bit score: 261
  
Sequence coverage: 98 %
  
E-value: 2e-80
  
  
 NCBI BlastP on this gene

EQB51667

hypothetical protein
  
Accession: EQB51668
  
Location: 14249-14746
  
 NCBI BlastP on this gene

EQB51668

Query: Architecture Search FASTA input

KB725935 : Colletotrichum orbiculare MAFF 240422 unplaced genomic scaffold Scaffold\_370    Total score: 2.0     Cumulative Blast bit score: 471

Hit cluster cross-links:

Mycgr3G70471
  
Location: 0-405

Mycgr3G70471

Mycgr3G39149
  
Location: 505-1798

Mycgr3G39149

Mycgr3G92130
  
Location: 1898-2396

Mycgr3G92130

Mycgr3G38483
  
Location: 2496-3576

Mycgr3G38483

Mycgr3G108869
  
Location: 3676-5056

Mycgr3G108869

Mycgr3G103943
  
Location: 5156-5762

Mycgr3G103943

Mycgr3G57362
  
Location: 5862-7296

Mycgr3G57362

Mycgr3G39086
  
Location: 7396-8368

Mycgr3G39086

Mycgr3G103942
  
Location: 8468-8714

Mycgr3G103942

Mycgr3G108865
  
Location: 8814-10239

Mycgr3G108865

Mycgr3G70475
  
Location: 10339-11821

Mycgr3G70475

Mycgr3G108866
  
Location: 11921-13010

Mycgr3G108866

Mycgr3G92136
  
Location: 13110-13593

Mycgr3G92136

serine threonine-protein kinase ssn3
  
Accession: ENH82179
  
Location: 103223-104664
  
 NCBI BlastP on this gene

ENH82179

40s ribosomal protein s24
  
Accession: ENH82180
  
Location: 108826-109641
  
  
**BlastP hit with Mycgr3G70471**
  
Percentage identity: 82 %
  
BlastP bit score: 211
  
Sequence coverage: 88 %
  
E-value: 3e-67
  
  
 NCBI BlastP on this gene

ENH82180

hypothetical protein
  
Accession: ENH82181
  
Location: 110335-111453
  
  
**BlastP hit with Mycgr3G38483**
  
Percentage identity: 45 %
  
BlastP bit score: 260
  
Sequence coverage: 99 %
  
E-value: 2e-80
  
  
 NCBI BlastP on this gene

ENH82181

hypothetical protein
  
Accession: ENH82182
  
Location: 117292-117795
  
 NCBI BlastP on this gene

ENH82182

RNA-binding protein
  
Accession: ENH82183
  
Location: 119254-120811
  
 NCBI BlastP on this gene

ENH82183

Query: Architecture Search FASTA input

KB730180 : Fusarium oxysporum f. sp. cubense race 1 unplaced genomic scaffold scaffold243    Total score: 2.0     Cumulative Blast bit score: 470

Hit cluster cross-links:

Mycgr3G70471
  
Location: 0-405

Mycgr3G70471

Mycgr3G39149
  
Location: 505-1798

Mycgr3G39149

Mycgr3G92130
  
Location: 1898-2396

Mycgr3G92130

Mycgr3G38483
  
Location: 2496-3576

Mycgr3G38483

Mycgr3G108869
  
Location: 3676-5056

Mycgr3G108869

Mycgr3G103943
  
Location: 5156-5762

Mycgr3G103943

Mycgr3G57362
  
Location: 5862-7296

Mycgr3G57362

Mycgr3G39086
  
Location: 7396-8368

Mycgr3G39086

Mycgr3G103942
  
Location: 8468-8714

Mycgr3G103942

Mycgr3G108865
  
Location: 8814-10239

Mycgr3G108865

Mycgr3G70475
  
Location: 10339-11821

Mycgr3G70475

Mycgr3G108866
  
Location: 11921-13010

Mycgr3G108866

Mycgr3G92136
  
Location: 13110-13593

Mycgr3G92136

Peptidyl-prolyl cis-trans isomerase-like 4
  
Accession: ENH70759
  
Location: 218766-220277
  
 NCBI BlastP on this gene

ENH70759

hypothetical protein
  
Accession: ENH70760
  
Location: 222903-223349
  
 NCBI BlastP on this gene

ENH70760

Protein FAM86A
  
Accession: ENH70761
  
Location: 228202-229288
  
  
**BlastP hit with Mycgr3G38483**
  
Percentage identity: 45 %
  
BlastP bit score: 263
  
Sequence coverage: 98 %
  
E-value: 1e-81
  
  
 NCBI BlastP on this gene

ENH70761

40S ribosomal protein S24-B
  
Accession: ENH70762
  
Location: 229755-230529
  
  
**BlastP hit with Mycgr3G70471**
  
Percentage identity: 81 %
  
BlastP bit score: 207
  
Sequence coverage: 89 %
  
E-value: 4e-66
  
  
 NCBI BlastP on this gene

ENH70762

hypothetical protein
  
Accession: ENH70763
  
Location: 231002-231430
  
 NCBI BlastP on this gene

ENH70763

hypothetical protein
  
Accession: ENH70764
  
Location: 232307-236827
  
 NCBI BlastP on this gene

ENH70764

Putative GTP cyclohydrolase-2
  
Accession: ENH70765
  
Location: 237645-238870
  
 NCBI BlastP on this gene

ENH70765

Query: Architecture Search FASTA input

KB726995 : Fusarium oxysporum f. sp. cubense race 4 unplaced genomic scaffold scaffold85    Total score: 2.0     Cumulative Blast bit score: 470

Hit cluster cross-links:

Mycgr3G70471
  
Location: 0-405

Mycgr3G70471

Mycgr3G39149
  
Location: 505-1798

Mycgr3G39149

Mycgr3G92130
  
Location: 1898-2396

Mycgr3G92130

Mycgr3G38483
  
Location: 2496-3576

Mycgr3G38483

Mycgr3G108869
  
Location: 3676-5056

Mycgr3G108869

Mycgr3G103943
  
Location: 5156-5762

Mycgr3G103943

Mycgr3G57362
  
Location: 5862-7296

Mycgr3G57362

Mycgr3G39086
  
Location: 7396-8368

Mycgr3G39086

Mycgr3G103942
  
Location: 8468-8714

Mycgr3G103942

Mycgr3G108865
  
Location: 8814-10239

Mycgr3G108865

Mycgr3G70475
  
Location: 10339-11821

Mycgr3G70475

Mycgr3G108866
  
Location: 11921-13010

Mycgr3G108866

Mycgr3G92136
  
Location: 13110-13593

Mycgr3G92136

hypothetical protein
  
Accession: EMT62598
  
Location: 866954-873623
  
 NCBI BlastP on this gene

EMT62598

hypothetical protein
  
Accession: EMT62599
  
Location: 874493-874921
  
 NCBI BlastP on this gene

EMT62599

40S ribosomal protein S24-B
  
Accession: EMT62600
  
Location: 875399-876174
  
  
**BlastP hit with Mycgr3G70471**
  
Percentage identity: 81 %
  
BlastP bit score: 207
  
Sequence coverage: 89 %
  
E-value: 4e-66
  
  
 NCBI BlastP on this gene

EMT62600

Protein FAM86A
  
Accession: EMT62601
  
Location: 876641-877727
  
  
**BlastP hit with Mycgr3G38483**
  
Percentage identity: 45 %
  
BlastP bit score: 263
  
Sequence coverage: 98 %
  
E-value: 1e-81
  
  
 NCBI BlastP on this gene

EMT62601

hypothetical protein
  
Accession: EMT62602
  
Location: 882538-883032
  
 NCBI BlastP on this gene

EMT62602

Peptidyl-prolyl cis-trans isomerase-like 4
  
Accession: EMT62603
  
Location: 885659-887170
  
 NCBI BlastP on this gene

EMT62603

Query: Architecture Search FASTA input

AFQF01003174 : Fusarium oxysporum Fo5176    Total score: 2.0     Cumulative Blast bit score: 470

Hit cluster cross-links:

Mycgr3G70471
  
Location: 0-405

Mycgr3G70471

Mycgr3G39149
  
Location: 505-1798

Mycgr3G39149

Mycgr3G92130
  
Location: 1898-2396

Mycgr3G92130

Mycgr3G38483
  
Location: 2496-3576

Mycgr3G38483

Mycgr3G108869
  
Location: 3676-5056

Mycgr3G108869

Mycgr3G103943
  
Location: 5156-5762

Mycgr3G103943

Mycgr3G57362
  
Location: 5862-7296

Mycgr3G57362

Mycgr3G39086
  
Location: 7396-8368

Mycgr3G39086

Mycgr3G103942
  
Location: 8468-8714

Mycgr3G103942

Mycgr3G108865
  
Location: 8814-10239

Mycgr3G108865

Mycgr3G70475
  
Location: 10339-11821

Mycgr3G70475

Mycgr3G108866
  
Location: 11921-13010

Mycgr3G108866

Mycgr3G92136
  
Location: 13110-13593

Mycgr3G92136

hypothetical protein
  
Accession: EGU76793
  
Location: 68397-72095
  
 NCBI BlastP on this gene

EGU76793

hypothetical protein
  
Accession: EGU76794
  
Location: 73464-73955
  
 NCBI BlastP on this gene

EGU76794

hypothetical protein
  
Accession: EGU76795
  
Location: 78766-79852
  
  
**BlastP hit with Mycgr3G38483**
  
Percentage identity: 45 %
  
BlastP bit score: 263
  
Sequence coverage: 98 %
  
E-value: 2e-81
  
  
 NCBI BlastP on this gene

EGU76795

hypothetical protein
  
Accession: EGU76796
  
Location: 80319-81093
  
  
**BlastP hit with Mycgr3G70471**
  
Percentage identity: 81 %
  
BlastP bit score: 207
  
Sequence coverage: 89 %
  
E-value: 4e-66
  
  
 NCBI BlastP on this gene

EGU76796

hypothetical protein
  
Accession: EGU76797
  
Location: 81567-81995
  
 NCBI BlastP on this gene

EGU76797

hypothetical protein
  
Accession: EGU76798
  
Location: 82873-87418
  
 NCBI BlastP on this gene

EGU76798

hypothetical protein
  
Accession: EGU76799
  
Location: 88236-89461
  
 NCBI BlastP on this gene

EGU76799

Query: Architecture Search FASTA input

AFNW01000285 : Fusarium pseudograminearum CS3096    Total score: 2.0     Cumulative Blast bit score: 464

Hit cluster cross-links:

Mycgr3G70471
  
Location: 0-405

Mycgr3G70471

Mycgr3G39149
  
Location: 505-1798

Mycgr3G39149

Mycgr3G92130
  
Location: 1898-2396

Mycgr3G92130

Mycgr3G38483
  
Location: 2496-3576

Mycgr3G38483

Mycgr3G108869
  
Location: 3676-5056

Mycgr3G108869

Mycgr3G103943
  
Location: 5156-5762

Mycgr3G103943

Mycgr3G57362
  
Location: 5862-7296

Mycgr3G57362

Mycgr3G39086
  
Location: 7396-8368

Mycgr3G39086

Mycgr3G103942
  
Location: 8468-8714

Mycgr3G103942

Mycgr3G108865
  
Location: 8814-10239

Mycgr3G108865

Mycgr3G70475
  
Location: 10339-11821

Mycgr3G70475

Mycgr3G108866
  
Location: 11921-13010

Mycgr3G108866

Mycgr3G92136
  
Location: 13110-13593

Mycgr3G92136

hypothetical protein
  
Accession: EKJ71544
  
Location: 50956-51450
  
 NCBI BlastP on this gene

EKJ71544

hypothetical protein
  
Accession: EKJ71545
  
Location: 56485-57582
  
  
**BlastP hit with Mycgr3G38483**
  
Percentage identity: 49 %
  
BlastP bit score: 258
  
Sequence coverage: 84 %
  
E-value: 2e-79
  
  
 NCBI BlastP on this gene

EKJ71545

hypothetical protein
  
Accession: EKJ71546
  
Location: 58087-58852
  
  
**BlastP hit with Mycgr3G70471**
  
Percentage identity: 81 %
  
BlastP bit score: 207
  
Sequence coverage: 89 %
  
E-value: 4e-66
  
  
 NCBI BlastP on this gene

EKJ71546

hypothetical protein
  
Accession: EKJ71547
  
Location: 59370-59786
  
 NCBI BlastP on this gene

EKJ71547

hypothetical protein
  
Accession: EKJ71548
  
Location: 60741-65321
  
 NCBI BlastP on this gene

EKJ71548

hypothetical protein
  
Accession: EKJ71549
  
Location: 66066-67302
  
 NCBI BlastP on this gene

EKJ71549

Query: Architecture Search FASTA input

CP003003 : Myceliophthora thermophila ATCC 42464 chromosome 2    Total score: 2.0     Cumulative Blast bit score: 464

Hit cluster cross-links:

Mycgr3G70471
  
Location: 0-405

Mycgr3G70471

Mycgr3G39149
  
Location: 505-1798

Mycgr3G39149

Mycgr3G92130
  
Location: 1898-2396

Mycgr3G92130

Mycgr3G38483
  
Location: 2496-3576

Mycgr3G38483

Mycgr3G108869
  
Location: 3676-5056

Mycgr3G108869

Mycgr3G103943
  
Location: 5156-5762

Mycgr3G103943

Mycgr3G57362
  
Location: 5862-7296

Mycgr3G57362

Mycgr3G39086
  
Location: 7396-8368

Mycgr3G39086

Mycgr3G103942
  
Location: 8468-8714

Mycgr3G103942

Mycgr3G108865
  
Location: 8814-10239

Mycgr3G108865

Mycgr3G70475
  
Location: 10339-11821

Mycgr3G70475

Mycgr3G108866
  
Location: 11921-13010

Mycgr3G108866

Mycgr3G92136
  
Location: 13110-13593

Mycgr3G92136

hypothetical protein
  
Accession: AEO57185
  
Location: 5375072-5376650
  
 NCBI BlastP on this gene

MYCTH\_47428

hypothetical protein
  
Accession: AEO57186
  
Location: 5378323-5379448
  
  
**BlastP hit with Mycgr3G38483**
  
Percentage identity: 44 %
  
BlastP bit score: 246
  
Sequence coverage: 100 %
  
E-value: 9e-75
  
  
 NCBI BlastP on this gene

MYCTH\_47934

hypothetical protein
  
Accession: AEO57187
  
Location: 5379805-5380573
  
  
**BlastP hit with Mycgr3G70471**
  
Percentage identity: 78 %
  
BlastP bit score: 218
  
Sequence coverage: 98 %
  
E-value: 7e-70
  
  
 NCBI BlastP on this gene

MYCTH\_2303040

hypothetical protein
  
Accession: AEO57188
  
Location: 5381892-5383712
  
 NCBI BlastP on this gene

MYCTH\_2056315

hypothetical protein
  
Accession: AEO57189
  
Location: 5387021-5388317
  
 NCBI BlastP on this gene

MYCTH\_2303043

Query: Architecture Search FASTA input

DS572698 : Verticillium dahliae VdLs.17 supercont1.4 genomic scaffold    Total score: 2.0     Cumulative Blast bit score: 462

Hit cluster cross-links:

Mycgr3G70471
  
Location: 0-405

Mycgr3G70471

Mycgr3G39149
  
Location: 505-1798

Mycgr3G39149

Mycgr3G92130
  
Location: 1898-2396

Mycgr3G92130

Mycgr3G38483
  
Location: 2496-3576

Mycgr3G38483

Mycgr3G108869
  
Location: 3676-5056

Mycgr3G108869

Mycgr3G103943
  
Location: 5156-5762

Mycgr3G103943

Mycgr3G57362
  
Location: 5862-7296

Mycgr3G57362

Mycgr3G39086
  
Location: 7396-8368

Mycgr3G39086

Mycgr3G103942
  
Location: 8468-8714

Mycgr3G103942

Mycgr3G108865
  
Location: 8814-10239

Mycgr3G108865

Mycgr3G70475
  
Location: 10339-11821

Mycgr3G70475

Mycgr3G108866
  
Location: 11921-13010

Mycgr3G108866

Mycgr3G92136
  
Location: 13110-13593

Mycgr3G92136

hypothetical protein
  
Accession: EGY21075
  
Location: 793835-794690
  
 NCBI BlastP on this gene

EGY21075

actin
  
Accession: EGY21076
  
Location: 795240-796551
  
 NCBI BlastP on this gene

EGY21076

meiotic mRNA stability protein kinase SSN3
  
Accession: EGY21077
  
Location: 798886-800268
  
 NCBI BlastP on this gene

EGY21077

40S ribosomal protein S24
  
Accession: EGY21078
  
Location: 800939-801816
  
  
**BlastP hit with Mycgr3G70471**
  
Percentage identity: 81 %
  
BlastP bit score: 209
  
Sequence coverage: 89 %
  
E-value: 1e-66
  
  
 NCBI BlastP on this gene

EGY21078

FAM86A protein
  
Accession: EGY21079
  
Location: 802369-803460
  
  
**BlastP hit with Mycgr3G38483**
  
Percentage identity: 42 %
  
BlastP bit score: 254
  
Sequence coverage: 98 %
  
E-value: 8e-78
  
  
 NCBI BlastP on this gene

EGY21079

hypothetical protein
  
Accession: EGY21080
  
Location: 805312-806636
  
 NCBI BlastP on this gene

EGY21080

hypothetical protein
  
Accession: EGY21081
  
Location: 808667-809236
  
 NCBI BlastP on this gene

EGY21081

peptidyl-prolyl cis-trans isomerase cyp6
  
Accession: EGY21082
  
Location: 809870-811459
  
 NCBI BlastP on this gene

EGY21082

Query: Architecture Search FASTA input

GG698904 : Nectria haematococca mpVI 77-13-4 chromosome 8 genomic scaffold NECHAsca\_11\_chr8\_3\_0    Total score: 2.0     Cumulative Blast bit score: 462

Hit cluster cross-links:

Mycgr3G70471
  
Location: 0-405

Mycgr3G70471

Mycgr3G39149
  
Location: 505-1798

Mycgr3G39149

Mycgr3G92130
  
Location: 1898-2396

Mycgr3G92130

Mycgr3G38483
  
Location: 2496-3576

Mycgr3G38483

Mycgr3G108869
  
Location: 3676-5056

Mycgr3G108869

Mycgr3G103943
  
Location: 5156-5762

Mycgr3G103943

Mycgr3G57362
  
Location: 5862-7296

Mycgr3G57362

Mycgr3G39086
  
Location: 7396-8368

Mycgr3G39086

Mycgr3G103942
  
Location: 8468-8714

Mycgr3G103942

Mycgr3G108865
  
Location: 8814-10239

Mycgr3G108865

Mycgr3G70475
  
Location: 10339-11821

Mycgr3G70475

Mycgr3G108866
  
Location: 11921-13010

Mycgr3G108866

Mycgr3G92136
  
Location: 13110-13593

Mycgr3G92136

hypothetical protein
  
Accession: EEU42681
  
Location: 814195-815414
  
 NCBI BlastP on this gene

EEU42681

hypothetical protein
  
Accession: EEU42682
  
Location: 816258-820855
  
 NCBI BlastP on this gene

EEU42682

predicted protein
  
Accession: EEU42683
  
Location: 821775-822179
  
 NCBI BlastP on this gene

EEU42683

predicted protein
  
Accession: EEU42869
  
Location: 822635-823338
  
  
**BlastP hit with Mycgr3G70471**
  
Percentage identity: 81 %
  
BlastP bit score: 207
  
Sequence coverage: 89 %
  
E-value: 8e-66
  
  
 NCBI BlastP on this gene

EEU42869

hypothetical protein
  
Accession: EEU42684
  
Location: 823840-824937
  
  
**BlastP hit with Mycgr3G38483**
  
Percentage identity: 42 %
  
BlastP bit score: 255
  
Sequence coverage: 100 %
  
E-value: 3e-78
  
  
 NCBI BlastP on this gene

EEU42684

hypothetical protein
  
Accession: EEU42685
  
Location: 829837-830331
  
 NCBI BlastP on this gene

EEU42685

Query: Architecture Search FASTA input

CABT02000038 : Sordaria macrospora k-hell    Total score: 2.0     Cumulative Blast bit score: 462

Hit cluster cross-links:

Mycgr3G70471
  
Location: 0-405

Mycgr3G70471

Mycgr3G39149
  
Location: 505-1798

Mycgr3G39149

Mycgr3G92130
  
Location: 1898-2396

Mycgr3G92130

Mycgr3G38483
  
Location: 2496-3576

Mycgr3G38483

Mycgr3G108869
  
Location: 3676-5056

Mycgr3G108869

Mycgr3G103943
  
Location: 5156-5762

Mycgr3G103943

Mycgr3G57362
  
Location: 5862-7296

Mycgr3G57362

Mycgr3G39086
  
Location: 7396-8368

Mycgr3G39086

Mycgr3G103942
  
Location: 8468-8714

Mycgr3G103942

Mycgr3G108865
  
Location: 8814-10239

Mycgr3G108865

Mycgr3G70475
  
Location: 10339-11821

Mycgr3G70475

Mycgr3G108866
  
Location: 11921-13010

Mycgr3G108866

Mycgr3G92136
  
Location: 13110-13593

Mycgr3G92136

not annotated
  
Accession: CCC13415
  
Location: 159343-160871
  
 NCBI BlastP on this gene

CCC13415

not annotated
  
Accession: CCC13414
  
Location: 153078-154329
  
  
**BlastP hit with Mycgr3G38483**
  
Percentage identity: 44 %
  
BlastP bit score: 241
  
Sequence coverage: 102 %
  
E-value: 9e-73
  
  
 NCBI BlastP on this gene

CCC13414

not annotated
  
Accession: CCC13413
  
Location: 151354-152414
  
  
**BlastP hit with Mycgr3G70471**
  
Percentage identity: 80 %
  
BlastP bit score: 221
  
Sequence coverage: 98 %
  
E-value: 4e-71
  
  
 NCBI BlastP on this gene

CCC13413

not annotated
  
Accession: CCC13412
  
Location: 149566-150888
  
 NCBI BlastP on this gene

CCC13412

not annotated
  
Accession: CCC13411
  
Location: 147403-148710
  
 NCBI BlastP on this gene

CCC13411

not annotated
  
Accession: CCC13410
  
Location: 142589-144193
  
 NCBI BlastP on this gene

CCC13410

Query: Architecture Search FASTA input

JH921437 : Marssonina brunnea f. sp. 'multigermtubi' MB\_m1 unplaced genomic scaffold M6\_S00010    Total score: 2.0     Cumulative Blast bit score: 461

Hit cluster cross-links:

Mycgr3G70471
  
Location: 0-405

Mycgr3G70471

Mycgr3G39149
  
Location: 505-1798

Mycgr3G39149

Mycgr3G92130
  
Location: 1898-2396

Mycgr3G92130

Mycgr3G38483
  
Location: 2496-3576

Mycgr3G38483

Mycgr3G108869
  
Location: 3676-5056

Mycgr3G108869

Mycgr3G103943
  
Location: 5156-5762

Mycgr3G103943

Mycgr3G57362
  
Location: 5862-7296

Mycgr3G57362

Mycgr3G39086
  
Location: 7396-8368

Mycgr3G39086

Mycgr3G103942
  
Location: 8468-8714

Mycgr3G103942

Mycgr3G108865
  
Location: 8814-10239

Mycgr3G108865

Mycgr3G70475
  
Location: 10339-11821

Mycgr3G70475

Mycgr3G108866
  
Location: 11921-13010

Mycgr3G108866

Mycgr3G92136
  
Location: 13110-13593

Mycgr3G92136

sugar transporter
  
Accession: EKD17080
  
Location: 905914-907905
  
 NCBI BlastP on this gene

EKD17080

arrestin
  
Accession: EKD17081
  
Location: 908753-910296
  
 NCBI BlastP on this gene

EKD17081

beta-glucosidase D
  
Accession: EKD17082
  
Location: 910701-913983
  
 NCBI BlastP on this gene

EKD17082

40S ribosomal protein S24
  
Accession: EKD17083
  
Location: 915618-916301
  
  
**BlastP hit with Mycgr3G70471**
  
Percentage identity: 76 %
  
BlastP bit score: 211
  
Sequence coverage: 100 %
  
E-value: 2e-67
  
  
 NCBI BlastP on this gene

EKD17083

putative Protein FAM86A
  
Accession: EKD17084
  
Location: 916620-917718
  
  
**BlastP hit with Mycgr3G38483**
  
Percentage identity: 41 %
  
BlastP bit score: 250
  
Sequence coverage: 98 %
  
E-value: 1e-76
  
  
 NCBI BlastP on this gene

EKD17084

hypothetical protein
  
Accession: EKD17085
  
Location: 918073-918835
  
 NCBI BlastP on this gene

EKD17085

hypothetical protein
  
Accession: EKD17086
  
Location: 920014-925753
  
 NCBI BlastP on this gene

EKD17086

Query: Architecture Search FASTA input

DS985215 : Verticillium albo-atrum VaMs.102 supercont1.2 genomic scaffold    Total score: 2.0     Cumulative Blast bit score: 460

Hit cluster cross-links:

Mycgr3G70471
  
Location: 0-405

Mycgr3G70471

Mycgr3G39149
  
Location: 505-1798

Mycgr3G39149

Mycgr3G92130
  
Location: 1898-2396

Mycgr3G92130

Mycgr3G38483
  
Location: 2496-3576

Mycgr3G38483

Mycgr3G108869
  
Location: 3676-5056

Mycgr3G108869

Mycgr3G103943
  
Location: 5156-5762

Mycgr3G103943

Mycgr3G57362
  
Location: 5862-7296

Mycgr3G57362

Mycgr3G39086
  
Location: 7396-8368

Mycgr3G39086

Mycgr3G103942
  
Location: 8468-8714

Mycgr3G103942

Mycgr3G108865
  
Location: 8814-10239

Mycgr3G108865

Mycgr3G70475
  
Location: 10339-11821

Mycgr3G70475

Mycgr3G108866
  
Location: 11921-13010

Mycgr3G108866

Mycgr3G92136
  
Location: 13110-13593

Mycgr3G92136

conserved hypothetical protein
  
Accession: EEY16023
  
Location: 2180195-2181050
  
 NCBI BlastP on this gene

EEY16023

actin
  
Accession: EEY16024
  
Location: 2181688-2182992
  
 NCBI BlastP on this gene

EEY16024

meiotic mRNA stability protein kinase SSN3
  
Accession: EEY16025
  
Location: 2185288-2186670
  
 NCBI BlastP on this gene

EEY16025

40S ribosomal protein S24
  
Accession: EEY16026
  
Location: 2187340-2188215
  
  
**BlastP hit with Mycgr3G70471**
  
Percentage identity: 81 %
  
BlastP bit score: 209
  
Sequence coverage: 89 %
  
E-value: 1e-66
  
  
 NCBI BlastP on this gene

EEY16026

FAM86A
  
Accession: EEY16027
  
Location: 2188794-2189885
  
  
**BlastP hit with Mycgr3G38483**
  
Percentage identity: 42 %
  
BlastP bit score: 251
  
Sequence coverage: 98 %
  
E-value: 9e-77
  
  
 NCBI BlastP on this gene

EEY16027

hypothetical protein
  
Accession: EEY16028
  
Location: 2194957-2195526
  
 NCBI BlastP on this gene

EEY16028

peptidyl-prolyl cis-trans isomerase cyp6
  
Accession: EEY16029
  
Location: 2196162-2197715
  
 NCBI BlastP on this gene

EEY16029

Query: Architecture Search FASTA input

CACQ02001341 : Colletotrichum higginsianum strain IMI 349063    Total score: 2.0     Cumulative Blast bit score: 459

Hit cluster cross-links:

Mycgr3G70471
  
Location: 0-405

Mycgr3G70471

Mycgr3G39149
  
Location: 505-1798

Mycgr3G39149

Mycgr3G92130
  
Location: 1898-2396

Mycgr3G92130

Mycgr3G38483
  
Location: 2496-3576

Mycgr3G38483

Mycgr3G108869
  
Location: 3676-5056

Mycgr3G108869

Mycgr3G103943
  
Location: 5156-5762

Mycgr3G103943

Mycgr3G57362
  
Location: 5862-7296

Mycgr3G57362

Mycgr3G39086
  
Location: 7396-8368

Mycgr3G39086

Mycgr3G103942
  
Location: 8468-8714

Mycgr3G103942

Mycgr3G108865
  
Location: 8814-10239

Mycgr3G108865

Mycgr3G70475
  
Location: 10339-11821

Mycgr3G70475

Mycgr3G108866
  
Location: 11921-13010

Mycgr3G108866

Mycgr3G92136
  
Location: 13110-13593

Mycgr3G92136

ribosomal protein S24e
  
Accession: CCF34943
  
Location: 1104-1935
  
  
**BlastP hit with Mycgr3G70471**
  
Percentage identity: 83 %
  
BlastP bit score: 213
  
Sequence coverage: 89 %
  
E-value: 5e-68
  
  
 NCBI BlastP on this gene

CCF34943

hypothetical protein
  
Accession: CCF34944
  
Location: 2606-3817
  
  
**BlastP hit with Mycgr3G38483**
  
Percentage identity: 48 %
  
BlastP bit score: 246
  
Sequence coverage: 83 %
  
E-value: 6e-75
  
  
 NCBI BlastP on this gene

CCF34944

hypothetical protein
  
Accession: CCF34945
  
Location: 6200-6772
  
 NCBI BlastP on this gene

CCF34945

Query: Architecture Search FASTA input

GL891302 : Neurospora tetrasperma FGSC 2508 unplaced genomic scaffold NEUTE1scaffold\_1    Total score: 2.0     Cumulative Blast bit score: 453

Hit cluster cross-links:

Mycgr3G70471
  
Location: 0-405

Mycgr3G70471

Mycgr3G39149
  
Location: 505-1798

Mycgr3G39149

Mycgr3G92130
  
Location: 1898-2396

Mycgr3G92130

Mycgr3G38483
  
Location: 2496-3576

Mycgr3G38483

Mycgr3G108869
  
Location: 3676-5056

Mycgr3G108869

Mycgr3G103943
  
Location: 5156-5762

Mycgr3G103943

Mycgr3G57362
  
Location: 5862-7296

Mycgr3G57362

Mycgr3G39086
  
Location: 7396-8368

Mycgr3G39086

Mycgr3G103942
  
Location: 8468-8714

Mycgr3G103942

Mycgr3G108865
  
Location: 8814-10239

Mycgr3G108865

Mycgr3G70475
  
Location: 10339-11821

Mycgr3G70475

Mycgr3G108866
  
Location: 11921-13010

Mycgr3G108866

Mycgr3G92136
  
Location: 13110-13593

Mycgr3G92136

hypothetical protein
  
Accession: EGO60064
  
Location: 106035-107801
  
 NCBI BlastP on this gene

EGO60064

hypothetical protein
  
Accession: EGO60065
  
Location: 111785-113049
  
  
**BlastP hit with Mycgr3G38483**
  
Percentage identity: 42 %
  
BlastP bit score: 232
  
Sequence coverage: 105 %
  
E-value: 3e-69
  
  
 NCBI BlastP on this gene

EGO60065

hypothetical protein
  
Accession: EGO60066
  
Location: 113653-114706
  
  
**BlastP hit with Mycgr3G70471**
  
Percentage identity: 80 %
  
BlastP bit score: 221
  
Sequence coverage: 98 %
  
E-value: 4e-71
  
  
 NCBI BlastP on this gene

EGO60066

hypothetical protein
  
Accession: EGO60067
  
Location: 115000-115964
  
 NCBI BlastP on this gene

EGO60067

hypothetical protein
  
Accession: EGO60068
  
Location: 116836-117500
  
 NCBI BlastP on this gene

EGO60068

hypothetical protein
  
Accession: EGO60069
  
Location: 117816-119126
  
 NCBI BlastP on this gene

EGO60069

hypothetical protein
  
Accession: EGO60070
  
Location: 120181-121425
  
 NCBI BlastP on this gene

EGO60070

hypothetical protein
  
Accession: EGO60071
  
Location: 122519-127395
  
 NCBI BlastP on this gene

EGO60071

Query: Architecture Search FASTA input

GL891107 : Neurospora tetrasperma FGSC 2509 unplaced genomic scaffold NEUTE2scaffold\_2    Total score: 2.0     Cumulative Blast bit score: 453

Hit cluster cross-links:

Mycgr3G70471
  
Location: 0-405

Mycgr3G70471

Mycgr3G39149
  
Location: 505-1798

Mycgr3G39149

Mycgr3G92130
  
Location: 1898-2396

Mycgr3G92130

Mycgr3G38483
  
Location: 2496-3576

Mycgr3G38483

Mycgr3G108869
  
Location: 3676-5056

Mycgr3G108869

Mycgr3G103943
  
Location: 5156-5762

Mycgr3G103943

Mycgr3G57362
  
Location: 5862-7296

Mycgr3G57362

Mycgr3G39086
  
Location: 7396-8368

Mycgr3G39086

Mycgr3G103942
  
Location: 8468-8714

Mycgr3G103942

Mycgr3G108865
  
Location: 8814-10239

Mycgr3G108865

Mycgr3G70475
  
Location: 10339-11821

Mycgr3G70475

Mycgr3G108866
  
Location: 11921-13010

Mycgr3G108866

Mycgr3G92136
  
Location: 13110-13593

Mycgr3G92136

hypothetical protein
  
Accession: EGZ75985
  
Location: 6080991-6082757
  
 NCBI BlastP on this gene

EGZ75985

hypothetical protein
  
Accession: EGZ75984
  
Location: 6075743-6077007
  
  
**BlastP hit with Mycgr3G38483**
  
Percentage identity: 42 %
  
BlastP bit score: 232
  
Sequence coverage: 105 %
  
E-value: 3e-69
  
  
 NCBI BlastP on this gene

EGZ75984

putative 40S ribosomal protein S24
  
Accession: EGZ75983
  
Location: 6074087-6075139
  
  
**BlastP hit with Mycgr3G70471**
  
Percentage identity: 80 %
  
BlastP bit score: 221
  
Sequence coverage: 98 %
  
E-value: 4e-71
  
  
 NCBI BlastP on this gene

EGZ75983

hypothetical protein
  
Accession: EGZ75982
  
Location: 6072829-6073793
  
 NCBI BlastP on this gene

EGZ75982

hypothetical protein
  
Accession: EGZ75981
  
Location: 6071293-6071957
  
 NCBI BlastP on this gene

EGZ75981

hypothetical protein
  
Accession: EGZ75980
  
Location: 6069667-6070977
  
 NCBI BlastP on this gene

EGZ75980

hypothetical protein
  
Accession: EGZ75979
  
Location: 6067569-6068375
  
 NCBI BlastP on this gene

EGZ75979

hypothetical protein
  
Accession: EGZ75978
  
Location: 6061398-6066274
  
 NCBI BlastP on this gene

EGZ75978

Query: Architecture Search FASTA input

BX294027 : Neurospora crassa DNA linkage group V BAC contig B8G12.    Total score: 2.0     Cumulative Blast bit score: 451

Hit cluster cross-links:

Mycgr3G70471
  
Location: 0-405

Mycgr3G70471

Mycgr3G39149
  
Location: 505-1798

Mycgr3G39149

Mycgr3G92130
  
Location: 1898-2396

Mycgr3G92130

Mycgr3G38483
  
Location: 2496-3576

Mycgr3G38483

Mycgr3G108869
  
Location: 3676-5056

Mycgr3G108869

Mycgr3G103943
  
Location: 5156-5762

Mycgr3G103943

Mycgr3G57362
  
Location: 5862-7296

Mycgr3G57362

Mycgr3G39086
  
Location: 7396-8368

Mycgr3G39086

Mycgr3G103942
  
Location: 8468-8714

Mycgr3G103942

Mycgr3G108865
  
Location: 8814-10239

Mycgr3G108865

Mycgr3G70475
  
Location: 10339-11821

Mycgr3G70475

Mycgr3G108866
  
Location: 11921-13010

Mycgr3G108866

Mycgr3G92136
  
Location: 13110-13593

Mycgr3G92136

conserved hypothetical protein
  
Accession: CAD71102
  
Location: 125734-127499
  
 NCBI BlastP on this gene

B8G12.430

conserved hypothetical protein
  
Accession: CAD71101
  
Location: 120250-121514
  
  
**BlastP hit with Mycgr3G38483**
  
Percentage identity: 43 %
  
BlastP bit score: 231
  
Sequence coverage: 105 %
  
E-value: 1e-68
  
  
 NCBI BlastP on this gene

B8G12.410

probable 40S RIBOSOMAL PROTEIN S24
  
Accession: CAD71100
  
Location: 118582-119638
  
  
**BlastP hit with Mycgr3G70471**
  
Percentage identity: 80 %
  
BlastP bit score: 221
  
Sequence coverage: 98 %
  
E-value: 4e-71
  
  
 NCBI BlastP on this gene

B8G12.400

hypothetical protein
  
Accession: CAD71099
  
Location: 117334-118238
  
 NCBI BlastP on this gene

B8G12.390

hypothetical protein
  
Accession: CAD71098
  
Location: 116117-116805
  
 NCBI BlastP on this gene

B8G12.380

hypothetical protein
  
Accession: CAD71097
  
Location: 114426-115762
  
 NCBI BlastP on this gene

B8G12.370

hypothetical protein
  
Accession: CAD71096
  
Location: 112141-113397
  
 NCBI BlastP on this gene

B8G12.360

hypothetical protein
  
Accession: CAD71095
  
Location: 105921-110788
  
 NCBI BlastP on this gene

B8G12.350

Query: Architecture Search FASTA input

FQ790270 : Botryotinia fuckeliana T4 SuperContig\_51\_1 genomic supercontig.    Total score: 2.0     Cumulative Blast bit score: 451

Hit cluster cross-links:

Mycgr3G70471
  
Location: 0-405

Mycgr3G70471

Mycgr3G39149
  
Location: 505-1798

Mycgr3G39149

Mycgr3G92130
  
Location: 1898-2396

Mycgr3G92130

Mycgr3G38483
  
Location: 2496-3576

Mycgr3G38483

Mycgr3G108869
  
Location: 3676-5056

Mycgr3G108869

Mycgr3G103943
  
Location: 5156-5762

Mycgr3G103943

Mycgr3G57362
  
Location: 5862-7296

Mycgr3G57362

Mycgr3G39086
  
Location: 7396-8368

Mycgr3G39086

Mycgr3G103942
  
Location: 8468-8714

Mycgr3G103942

Mycgr3G108865
  
Location: 8814-10239

Mycgr3G108865

Mycgr3G70475
  
Location: 10339-11821

Mycgr3G70475

Mycgr3G108866
  
Location: 11921-13010

Mycgr3G108866

Mycgr3G92136
  
Location: 13110-13593

Mycgr3G92136

similar to cysteine desulfurase
  
Accession: CCD44207
  
Location: 184833-186617
  
 NCBI BlastP on this gene

BofuT4\_P057870.1

similar to beta-catenin-like protein 1
  
Accession: CCD44208
  
Location: 188300-190177
  
 NCBI BlastP on this gene

BofuT4\_P057880.1

hypothetical protein
  
Accession: CCD44209
  
Location: 190700-191541
  
 NCBI BlastP on this gene

BofuT4\_P057890.1

hypothetical protein
  
Accession: CCD44210
  
Location: 192543-193822
  
  
**BlastP hit with Mycgr3G38483**
  
Percentage identity: 42 %
  
BlastP bit score: 243
  
Sequence coverage: 92 %
  
E-value: 2e-73
  
  
 NCBI BlastP on this gene

BofuT4\_P057900.1

similar to 40S ribosomal protein S24
  
Accession: CCD44211
  
Location: 194252-194897
  
  
**BlastP hit with Mycgr3G70471**
  
Percentage identity: 81 %
  
BlastP bit score: 208
  
Sequence coverage: 89 %
  
E-value: 3e-66
  
  
 NCBI BlastP on this gene

BofuT4\_P057910.1

similar to transcription factor Cys6
  
Accession: CCD44212
  
Location: 198219-199833
  
 NCBI BlastP on this gene

BofuT4\_P057920.1

hypothetical protein
  
Accession: CCD44213
  
Location: 200643-201038
  
 NCBI BlastP on this gene

BofuT4\_P057930.1

predicted protein
  
Accession: CCD44214
  
Location: 201138-201296
  
 NCBI BlastP on this gene

BofuT4\_uP057940.1

predicted protein
  
Accession: CCD44215
  
Location: 201962-202291
  
 NCBI BlastP on this gene

BofuT4\_P057950.1

hypothetical protein
  
Accession: CCD44216
  
Location: 202830-204345
  
 NCBI BlastP on this gene

BofuT4\_P057960.1

Query: Architecture Search FASTA input

1. :  CM001198 Mycosphaerella graminicola IPO323 chromosome 3     Total score: 13.0     Cumulative Blast bit score: 8325

Mycgr3G70471
  
Location: 0-405
  
 NCBI BlastP on this gene

Mycgr3G70471

Mycgr3G39149
  
Location: 505-1798
  
 NCBI BlastP on this gene

Mycgr3G39149

Mycgr3G92130
  
Location: 1898-2396
  
 NCBI BlastP on this gene

Mycgr3G92130

Mycgr3G38483
  
Location: 2496-3576
  
 NCBI BlastP on this gene

Mycgr3G38483

Mycgr3G108869
  
Location: 3676-5056
  
 NCBI BlastP on this gene

Mycgr3G108869

Mycgr3G103943
  
Location: 5156-5762
  
 NCBI BlastP on this gene

Mycgr3G103943

Mycgr3G57362
  
Location: 5862-7296
  
 NCBI BlastP on this gene

Mycgr3G57362

Mycgr3G39086
  
Location: 7396-8368
  
 NCBI BlastP on this gene

Mycgr3G39086

Mycgr3G103942
  
Location: 8468-8714
  
 NCBI BlastP on this gene

Mycgr3G103942

Mycgr3G108865
  
Location: 8814-10239
  
 NCBI BlastP on this gene

Mycgr3G108865

Mycgr3G70475
  
Location: 10339-11821
  
 NCBI BlastP on this gene

Mycgr3G70475

Mycgr3G108866
  
Location: 11921-13010
  
 NCBI BlastP on this gene

Mycgr3G108866

Mycgr3G92136
  
Location: 13110-13593
  
 NCBI BlastP on this gene

Mycgr3G92136

hypothetical protein
  
Accession: EGP89173
  
Location: 3175573-3179202
  
 NCBI BlastP on this gene

EGP89173

hypothetical protein
  
Accession: EGP89172
  
Location: 3179594-3181026
  
 NCBI BlastP on this gene

EGP89172

hypothetical protein
  
Accession: EGP89070
  
Location: 3181485-3182969
  
  
**BlastP hit with Mycgr3G57362**
  
Percentage identity: 100 %
  
BlastP bit score: 986
  
Sequence coverage: 99 %
  
E-value: 0.0
  
  
 NCBI BlastP on this gene

EGP89070

hypothetical protein
  
Accession: EGP89071
  
Location: 3184562-3185266
  
  
**BlastP hit with Mycgr3G92130**
  
Percentage identity: 100 %
  
BlastP bit score: 347
  
Sequence coverage: 99 %
  
E-value: 5e-120
  
  
 NCBI BlastP on this gene

EGP89071

hypothetical protein
  
Accession: EGP89171
  
Location: 3187686-3188893
  
  
**BlastP hit with Mycgr3G39086**
  
Percentage identity: 100 %
  
BlastP bit score: 667
  
Sequence coverage: 99 %
  
E-value: 0.0
  
  
 NCBI BlastP on this gene

EGP89171

ketoacyl synthase domain-containing protein
  
Accession: EGP89072
  
Location: 3190853-3192248
  
  
**BlastP hit with Mycgr3G39149**
  
Percentage identity: 100 %
  
BlastP bit score: 882
  
Sequence coverage: 99 %
  
E-value: 0.0
  
  
 NCBI BlastP on this gene

EGP89072

hypothetical protein
  
Accession: EGP89073
  
Location: 3192609-3194129
  
  
**BlastP hit with Mycgr3G108865**
  
Percentage identity: 100 %
  
BlastP bit score: 951
  
Sequence coverage: 99 %
  
E-value: 0.0
  
  
 NCBI BlastP on this gene

EGP89073

hypothetical protein
  
Accession: EGP89170
  
Location: 3194727-3195964
  
  
**BlastP hit with Mycgr3G108866**
  
Percentage identity: 100 %
  
BlastP bit score: 733
  
Sequence coverage: 99 %
  
E-value: 0.0
  
  
 NCBI BlastP on this gene

EGP89170

hypothetical protein
  
Accession: EGP89169
  
Location: 3199537-3200221
  
  
**BlastP hit with Mycgr3G70471**
  
Percentage identity: 100 %
  
BlastP bit score: 271
  
Sequence coverage: 99 %
  
E-value: 4e-91
  
  
 NCBI BlastP on this gene

EGP89169

hypothetical protein
  
Accession: EGP89074
  
Location: 3200446-3201663
  
  
**BlastP hit with Mycgr3G38483**
  
Percentage identity: 100 %
  
BlastP bit score: 740
  
Sequence coverage: 99 %
  
E-value: 0.0
  
  
 NCBI BlastP on this gene

EGP89074

hypothetical protein
  
Accession: EGP89168
  
Location: 3203515-3203999
  
  
**BlastP hit with Mycgr3G92136**
  
Percentage identity: 100 %
  
BlastP bit score: 245
  
Sequence coverage: 72 %
  
E-value: 1e-80
  
  
 NCBI BlastP on this gene

EGP89168

hypothetical protein
  
Accession: EGP89075
  
Location: 3204785-3206344
  
  
**BlastP hit with Mycgr3G70475**
  
Percentage identity: 100 %
  
BlastP bit score: 998
  
Sequence coverage: 99 %
  
E-value: 0.0
  
  
 NCBI BlastP on this gene

EGP89075

hypothetical protein
  
Accession: EGP89167
  
Location: 3206700-3206945
  
  
**BlastP hit with Mycgr3G103942**
  
Percentage identity: 100 %
  
BlastP bit score: 166
  
Sequence coverage: 98 %
  
E-value: 2e-51
  
  
 NCBI BlastP on this gene

EGP89167

hypothetical protein
  
Accession: EGP89076
  
Location: 3208316-3208992
  
  
**BlastP hit with Mycgr3G103943**
  
Percentage identity: 100 %
  
BlastP bit score: 421
  
Sequence coverage: 100 %
  
E-value: 7e-148
  
  
 NCBI BlastP on this gene

EGP89076

hypothetical protein
  
Accession: EGP89077
  
Location: 3211185-3213111
  
  
**BlastP hit with Mycgr3G108869**
  
Percentage identity: 100 %
  
BlastP bit score: 918
  
Sequence coverage: 99 %
  
E-value: 0.0
  
  
 NCBI BlastP on this gene

EGP89077

hypothetical protein
  
Accession: EGP89166
  
Location: 3213380-3214434
  
 NCBI BlastP on this gene

EGP89166

2. :  JH767568 Coniosporium apollinis CBS 100218 chromosome Unknown supercont1.15     Total score: 5.0     Cumulative Blast bit score: 1651

hypothetical protein
  
Accession: EON64374
  
Location: 110705-125613
  
 NCBI BlastP on this gene

EON64374

hypothetical protein
  
Accession: EON64373
  
Location: 107130-109350
  
 NCBI BlastP on this gene

EON64373

small subunit ribosomal protein S24e
  
Accession: EON64372
  
Location: 104982-105589
  
  
**BlastP hit with Mycgr3G70471**
  
Percentage identity: 85 %
  
BlastP bit score: 234
  
Sequence coverage: 99 %
  
E-value: 2e-76
  
  
 NCBI BlastP on this gene

EON64372

hypothetical protein
  
Accession: EON64371
  
Location: 103016-104236
  
 NCBI BlastP on this gene

EON64371

hypothetical protein
  
Accession: EON64370
  
Location: 100139-101270
  
 NCBI BlastP on this gene

EON64370

hypothetical protein
  
Accession: EON64369
  
Location: 98332-99346
  
  
**BlastP hit with Mycgr3G38483**
  
Percentage identity: 46 %
  
BlastP bit score: 234
  
Sequence coverage: 76 %
  
E-value: 6e-71
  
  
 NCBI BlastP on this gene

EON64369

hypothetical protein
  
Accession: EON64368
  
Location: 95381-96589
  
  
**BlastP hit with Mycgr3G108866**
  
Percentage identity: 35 %
  
BlastP bit score: 120
  
Sequence coverage: 95 %
  
E-value: 1e-27
  
  
 NCBI BlastP on this gene

EON64368

hypothetical protein
  
Accession: EON64367
  
Location: 92710-94510
  
  
**BlastP hit with Mycgr3G108865**
  
Percentage identity: 54 %
  
BlastP bit score: 425
  
Sequence coverage: 95 %
  
E-value: 4e-141
  
  
 NCBI BlastP on this gene

EON64367

3-oxoacyl-[acyl-carrier-protein] synthase II
  
Accession: EON64366
  
Location: 90692-92337
  
  
**BlastP hit with Mycgr3G39149**
  
Percentage identity: 73 %
  
BlastP bit score: 638
  
Sequence coverage: 99 %
  
E-value: 0.0
  
  
 NCBI BlastP on this gene

EON64366

hypothetical protein
  
Accession: EON64365
  
Location: 88557-90042
  
 NCBI BlastP on this gene

EON64365

hypothetical protein
  
Accession: EON64364
  
Location: 86951-88067
  
 NCBI BlastP on this gene

EON64364

hypothetical protein
  
Accession: EON64363
  
Location: 81880-86503
  
 NCBI BlastP on this gene

EON64363

hypothetical protein
  
Accession: EON64362
  
Location: 78170-81098
  
 NCBI BlastP on this gene

EON64362

3. :  AHHD01000457 Macrophomina phaseolina MS6     Total score: 5.0     Cumulative Blast bit score: 1561

hypothetical protein
  
Accession: EKG12152
  
Location: 13573-16092
  
 NCBI BlastP on this gene

EKG12152

hypothetical protein
  
Accession: EKG12153
  
Location: 16281-16607
  
 NCBI BlastP on this gene

EKG12153

Aldehyde dehydrogenase NAD(P)-dependent
  
Accession: EKG12154
  
Location: 18555-20346
  
 NCBI BlastP on this gene

EKG12154

Ubiquinol-cytochrome c chaperone CBP3
  
Accession: EKG12155
  
Location: 20913-21389
  
 NCBI BlastP on this gene

EKG12155

Pyridine nucleotide-disulfide oxidoreductase
  
Accession: EKG12156
  
Location: 22950-24069
  
 NCBI BlastP on this gene

EKG12156

Beta-ketoacyl synthase
  
Accession: EKG12157
  
Location: 24995-26597
  
  
**BlastP hit with Mycgr3G39149**
  
Percentage identity: 72 %
  
BlastP bit score: 629
  
Sequence coverage: 99 %
  
E-value: 0.0
  
  
 NCBI BlastP on this gene

EKG12157

Nickel/cobalt transporter high-affinity
  
Accession: EKG12158
  
Location: 27220-28663
  
  
**BlastP hit with Mycgr3G108865**
  
Percentage identity: 53 %
  
BlastP bit score: 315
  
Sequence coverage: 70 %
  
E-value: 8e-100
  
  
 NCBI BlastP on this gene

EKG12158

hypothetical protein
  
Accession: EKG12159
  
Location: 28853-31024
  
 NCBI BlastP on this gene

EKG12159

Ribosomal protein S24e
  
Accession: EKG12160
  
Location: 32279-32820
  
  
**BlastP hit with Mycgr3G70471**
  
Percentage identity: 87 %
  
BlastP bit score: 225
  
Sequence coverage: 91 %
  
E-value: 5e-73
  
  
 NCBI BlastP on this gene

EKG12160

Nicotinamide N-methyltransferase putative
  
Accession: EKG12161
  
Location: 33259-34732
  
  
**BlastP hit with Mycgr3G38483**
  
Percentage identity: 48 %
  
BlastP bit score: 316
  
Sequence coverage: 93 %
  
E-value: 3e-102
  
  
 NCBI BlastP on this gene

EKG12161

hypothetical protein
  
Accession: EKG12162
  
Location: 36612-37506
  
  
**BlastP hit with Mycgr3G108866**
  
Percentage identity: 32 %
  
BlastP bit score: 76
  
Sequence coverage: 77 %
  
E-value: 8e-13
  
  
 NCBI BlastP on this gene

EKG12162

hypothetical protein
  
Accession: EKG12163
  
Location: 40038-42366
  
 NCBI BlastP on this gene

EKG12163

hypothetical protein
  
Accession: EKG12164
  
Location: 43032-43555
  
 NCBI BlastP on this gene

EKG12164

Proline racemase
  
Accession: EKG12165
  
Location: 45173-46230
  
 NCBI BlastP on this gene

EKG12165

hypothetical protein
  
Accession: EKG12166
  
Location: 46336-49296
  
 NCBI BlastP on this gene

EKG12166

4. :  KB446557 Pseudocercospora fijiensis CIRAD86 unplaced genomic scaffold MYCFIscaffold\_3     Total score: 4.0     Cumulative Blast bit score: 1994

hypothetical protein
  
Accession: EME84125
  
Location: 2020600-2023150
  
 NCBI BlastP on this gene

EME84125

hypothetical protein
  
Accession: EME84126
  
Location: 2023376-2024066
  
 NCBI BlastP on this gene

EME84126

hypothetical protein
  
Accession: EME84127
  
Location: 2025963-2028110
  
 NCBI BlastP on this gene

EME84127

hypothetical protein
  
Accession: EME84128
  
Location: 2028662-2029157
  
 NCBI BlastP on this gene

EME84128

hypothetical protein
  
Accession: EME84129
  
Location: 2029209-2030674
  
 NCBI BlastP on this gene

EME84129

hypothetical protein
  
Accession: EME84130
  
Location: 2033126-2033710
  
 NCBI BlastP on this gene

EME84130

hypothetical protein
  
Accession: EME84131
  
Location: 2036472-2037647
  
  
**BlastP hit with Mycgr3G108866**
  
Percentage identity: 42 %
  
BlastP bit score: 209
  
Sequence coverage: 97 %
  
E-value: 2e-60
  
  
 NCBI BlastP on this gene

EME84131

hypothetical protein
  
Accession: EME84132
  
Location: 2038009-2039283
  
  
**BlastP hit with Mycgr3G108865**
  
Percentage identity: 69 %
  
BlastP bit score: 555
  
Sequence coverage: 83 %
  
E-value: 0.0
  
  
 NCBI BlastP on this gene

EME84132

hypothetical protein
  
Accession: EME84133
  
Location: 2039530-2040929
  
  
**BlastP hit with Mycgr3G39149**
  
Percentage identity: 86 %
  
BlastP bit score: 768
  
Sequence coverage: 99 %
  
E-value: 0.0
  
  
 NCBI BlastP on this gene

EME84133

hypothetical protein
  
Accession: EME84134
  
Location: 2042100-2043965
  
  
**BlastP hit with Mycgr3G39086**
  
Percentage identity: 69 %
  
BlastP bit score: 462
  
Sequence coverage: 99 %
  
E-value: 3e-157
  
  
 NCBI BlastP on this gene

EME84134

hypothetical protein
  
Accession: EME84135
  
Location: 2044682-2045722
  
 NCBI BlastP on this gene

EME84135

hypothetical protein
  
Accession: EME84136
  
Location: 2046462-2047517
  
 NCBI BlastP on this gene

EME84136

hypothetical protein
  
Accession: EME84137
  
Location: 2048436-2050220
  
 NCBI BlastP on this gene

EME84137

hypothetical protein
  
Accession: EME84138
  
Location: 2051382-2051810
  
 NCBI BlastP on this gene

EME84138

hypothetical protein
  
Accession: EME84139
  
Location: 2052755-2053647
  
 NCBI BlastP on this gene

EME84139

hypothetical protein
  
Accession: EME84140
  
Location: 2055175-2056196
  
 NCBI BlastP on this gene

EME84140

5. :  KB456267 Mycosphaerella populorum SO2202 unplaced genomic scaffold SEPMUscaffold\_8     Total score: 4.0     Cumulative Blast bit score: 1833

hypothetical protein
  
Accession: EMF10547
  
Location: 678409-680098
  
 NCBI BlastP on this gene

EMF10547

hypothetical protein
  
Accession: EMF10548
  
Location: 682213-683490
  
 NCBI BlastP on this gene

EMF10548

hypothetical protein
  
Accession: EMF10549
  
Location: 684861-687588
  
 NCBI BlastP on this gene

EMF10549

hypothetical protein
  
Accession: EMF10550
  
Location: 692543-693722
  
  
**BlastP hit with Mycgr3G108866**
  
Percentage identity: 38 %
  
BlastP bit score: 132
  
Sequence coverage: 99 %
  
E-value: 1e-31
  
  
 NCBI BlastP on this gene

EMF10550

NicO-domain-containing protein
  
Accession: EMF10551
  
Location: 694737-696056
  
  
**BlastP hit with Mycgr3G108865**
  
Percentage identity: 70 %
  
BlastP bit score: 484
  
Sequence coverage: 76 %
  
E-value: 8e-165
  
  
 NCBI BlastP on this gene

EMF10551

beta-ketoacyl synthase
  
Accession: EMF10552
  
Location: 696401-697821
  
  
**BlastP hit with Mycgr3G39149**
  
Percentage identity: 85 %
  
BlastP bit score: 758
  
Sequence coverage: 99 %
  
E-value: 0.0
  
  
 NCBI BlastP on this gene

EMF10552

hypothetical protein
  
Accession: EMF10553
  
Location: 699441-700898
  
  
**BlastP hit with Mycgr3G39086**
  
Percentage identity: 66 %
  
BlastP bit score: 459
  
Sequence coverage: 99 %
  
E-value: 2e-157
  
  
 NCBI BlastP on this gene

EMF10553

hypothetical protein
  
Accession: EMF10554
  
Location: 701245-705834
  
 NCBI BlastP on this gene

EMF10554

zf-Tim10 DDP-domain-containing protein
  
Accession: EMF10555
  
Location: 706644-707022
  
 NCBI BlastP on this gene

EMF10555

hypothetical protein
  
Accession: EMF10556
  
Location: 707620-708426
  
 NCBI BlastP on this gene

EMF10556

t-complex protein 1 theta subunit
  
Accession: EMF10557
  
Location: 708679-710471
  
 NCBI BlastP on this gene

EMF10557

hypothetical protein
  
Accession: EMF10558
  
Location: 714276-714656
  
 NCBI BlastP on this gene

EMF10558

6. :  KB916303 Neofusicoccum parvum UCRNP2 chromosome Unknown NP2\_03\_scaffold\_665     Total score: 4.0     Cumulative Blast bit score: 955

putative carbohydrate-binding module family 48 protein
  
Accession: EOD47794
  
Location: 221426-223846
  
 NCBI BlastP on this gene

EOD47794

putative aldehyde dehydrogenase protein
  
Accession: EOD47815
  
Location: 226590-228476
  
 NCBI BlastP on this gene

EOD47815

putative ubiquinol-cytochrome c protein
  
Accession: EOD47798
  
Location: 229097-229884
  
 NCBI BlastP on this gene

EOD47798

putative high affinity nickel transport protein nic1 protein
  
Accession: EOD47792
  
Location: 235058-236897
  
  
**BlastP hit with Mycgr3G108865**
  
Percentage identity: 52 %
  
BlastP bit score: 395
  
Sequence coverage: 88 %
  
E-value: 5e-130
  
  
 NCBI BlastP on this gene

EOD47792

putative sorting nexin-41 protein
  
Accession: EOD47803
  
Location: 237021-239189
  
 NCBI BlastP on this gene

EOD47803

putative 40s ribosomal protein s24 protein
  
Accession: EOD47785
  
Location: 240535-241084
  
  
**BlastP hit with Mycgr3G70471**
  
Percentage identity: 87 %
  
BlastP bit score: 226
  
Sequence coverage: 91 %
  
E-value: 2e-73
  
  
 NCBI BlastP on this gene

EOD47785

putative nicotinamide n-methyltransferase protein
  
Accession: EOD47808
  
Location: 241437-242857
  
  
**BlastP hit with Mycgr3G38483**
  
Percentage identity: 47 %
  
BlastP bit score: 259
  
Sequence coverage: 83 %
  
E-value: 1e-80
  
  
 NCBI BlastP on this gene

EOD47808

hypothetical protein
  
Accession: EOD47760
  
Location: 244791-245860
  
  
**BlastP hit with Mycgr3G108866**
  
Percentage identity: 38 %
  
BlastP bit score: 75
  
Sequence coverage: 36 %
  
E-value: 1e-12
  
  
 NCBI BlastP on this gene

EOD47760

putative nucleoside-diphosphate-sugar epimerase protein
  
Accession: EOD47817
  
Location: 248391-249267
  
 NCBI BlastP on this gene

EOD47817

7. :  KB446538 Dothistroma septosporum NZE10 unplaced genomic scaffold DOTSEscaffold\_4     Total score: 3.0     Cumulative Blast bit score: 1515

hypothetical protein
  
Accession: EME45127
  
Location: 714357-716259
  
 NCBI BlastP on this gene

EME45127

hypothetical protein
  
Accession: EME45126
  
Location: 711024-713810
  
 NCBI BlastP on this gene

EME45126

hypothetical protein
  
Accession: EME45125
  
Location: 709691-710462
  
 NCBI BlastP on this gene

EME45125

hypothetical protein
  
Accession: EME45124
  
Location: 707252-707680
  
 NCBI BlastP on this gene

EME45124

hypothetical protein
  
Accession: EME45123
  
Location: 702226-706509
  
 NCBI BlastP on this gene

EME45123

ketoacyl synthase domain-containing protein
  
Accession: EME45122
  
Location: 699669-701075
  
  
**BlastP hit with Mycgr3G39149**
  
Percentage identity: 83 %
  
BlastP bit score: 757
  
Sequence coverage: 99 %
  
E-value: 0.0
  
  
 NCBI BlastP on this gene

EME45122

hypothetical protein
  
Accession: EME45121
  
Location: 698053-699307
  
  
**BlastP hit with Mycgr3G108865**
  
Percentage identity: 71 %
  
BlastP bit score: 540
  
Sequence coverage: 81 %
  
E-value: 0.0
  
  
 NCBI BlastP on this gene

EME45121

hypothetical protein
  
Accession: EME45120
  
Location: 696549-697690
  
  
**BlastP hit with Mycgr3G108866**
  
Percentage identity: 45 %
  
BlastP bit score: 218
  
Sequence coverage: 98 %
  
E-value: 3e-64
  
  
 NCBI BlastP on this gene

EME45120

hypothetical protein
  
Accession: EME45119
  
Location: 690646-693824
  
 NCBI BlastP on this gene

EME45119

hypothetical protein
  
Accession: EME45118
  
Location: 688047-689255
  
 NCBI BlastP on this gene

EME45118

hypothetical protein
  
Accession: EME45117
  
Location: 685068-686813
  
 NCBI BlastP on this gene

EME45117

hypothetical protein
  
Accession: EME45116
  
Location: 683436-684155
  
 NCBI BlastP on this gene

EME45116

hypothetical protein
  
Accession: EME45115
  
Location: 682485-683294
  
 NCBI BlastP on this gene

EME45115

hypothetical protein
  
Accession: EME45114
  
Location: 680830-681342
  
 NCBI BlastP on this gene

EME45114

8. :  KB733456 Bipolaris maydis ATCC 48331 unplaced genomic scaffold COCC4scaffold\_13     Total score: 3.0     Cumulative Blast bit score: 626

hypothetical protein
  
Accession: ENI04807
  
Location: 713882-714420
  
 NCBI BlastP on this gene

ENI04807

hypothetical protein
  
Accession: ENI04808
  
Location: 714650-715809
  
 NCBI BlastP on this gene

ENI04808

hypothetical protein
  
Accession: ENI04809
  
Location: 721305-724710
  
 NCBI BlastP on this gene

ENI04809

hypothetical protein
  
Accession: ENI04810
  
Location: 724861-725304
  
 NCBI BlastP on this gene

ENI04810

hypothetical protein
  
Accession: ENI04811
  
Location: 726999-729182
  
 NCBI BlastP on this gene

ENI04811

hypothetical protein
  
Accession: ENI04812
  
Location: 729703-730328
  
  
**BlastP hit with Mycgr3G70471**
  
Percentage identity: 83 %
  
BlastP bit score: 223
  
Sequence coverage: 96 %
  
E-value: 4e-72
  
  
 NCBI BlastP on this gene

ENI04812

hypothetical protein
  
Accession: ENI04813
  
Location: 730696-732035
  
  
**BlastP hit with Mycgr3G38483**
  
Percentage identity: 48 %
  
BlastP bit score: 306
  
Sequence coverage: 100 %
  
E-value: 4e-98
  
  
 NCBI BlastP on this gene

ENI04813

hypothetical protein
  
Accession: ENI04814
  
Location: 732379-732690
  
 NCBI BlastP on this gene

ENI04814

hypothetical protein
  
Accession: ENI04815
  
Location: 733719-734772
  
  
**BlastP hit with Mycgr3G108866**
  
Percentage identity: 33 %
  
BlastP bit score: 97
  
Sequence coverage: 96 %
  
E-value: 7e-20
  
  
 NCBI BlastP on this gene

ENI04815

hypothetical protein
  
Accession: ENI04816
  
Location: 737043-737510
  
 NCBI BlastP on this gene

ENI04816

hypothetical protein
  
Accession: ENI04817
  
Location: 738110-738389
  
 NCBI BlastP on this gene

ENI04817

hypothetical protein
  
Accession: ENI04818
  
Location: 739442-740140
  
 NCBI BlastP on this gene

ENI04818

hypothetical protein
  
Accession: ENI04819
  
Location: 742301-743041
  
 NCBI BlastP on this gene

ENI04819

hypothetical protein
  
Accession: ENI04820
  
Location: 743118-743981
  
 NCBI BlastP on this gene

ENI04820

hypothetical protein
  
Accession: ENI04821
  
Location: 744342-744755
  
 NCBI BlastP on this gene

ENI04821

hypothetical protein
  
Accession: ENI04822
  
Location: 746131-747402
  
 NCBI BlastP on this gene

ENI04822

hypothetical protein
  
Accession: ENI04823
  
Location: 747822-748688
  
 NCBI BlastP on this gene

ENI04823

9. :  KB445574 Cochliobolus heterostrophus C5 unplaced genomic scaffold COCHEscaffold\_6     Total score: 3.0     Cumulative Blast bit score: 626

hypothetical protein
  
Accession: EMD92804
  
Location: 265876-266414
  
 NCBI BlastP on this gene

EMD92804

hypothetical protein
  
Accession: EMD92803
  
Location: 264487-265646
  
 NCBI BlastP on this gene

EMD92803

hypothetical protein
  
Accession: EMD92802
  
Location: 255509-258914
  
 NCBI BlastP on this gene

EMD92802

hypothetical protein
  
Accession: EMD92801
  
Location: 251037-253220
  
 NCBI BlastP on this gene

EMD92801

hypothetical protein
  
Accession: EMD92800
  
Location: 249891-250516
  
  
**BlastP hit with Mycgr3G70471**
  
Percentage identity: 83 %
  
BlastP bit score: 223
  
Sequence coverage: 96 %
  
E-value: 4e-72
  
  
 NCBI BlastP on this gene

EMD92800

hypothetical protein
  
Accession: EMD92799
  
Location: 248184-249523
  
  
**BlastP hit with Mycgr3G38483**
  
Percentage identity: 48 %
  
BlastP bit score: 306
  
Sequence coverage: 100 %
  
E-value: 4e-98
  
  
 NCBI BlastP on this gene

EMD92799

hypothetical protein
  
Accession: EMD92798
  
Location: 247529-247840
  
 NCBI BlastP on this gene

EMD92798

hypothetical protein
  
Accession: EMD92797
  
Location: 245447-246500
  
  
**BlastP hit with Mycgr3G108866**
  
Percentage identity: 33 %
  
BlastP bit score: 97
  
Sequence coverage: 96 %
  
E-value: 7e-20
  
  
 NCBI BlastP on this gene

EMD92797

hypothetical protein
  
Accession: EMD92796
  
Location: 242709-243176
  
 NCBI BlastP on this gene

EMD92796

hypothetical protein
  
Accession: EMD92795
  
Location: 241830-242108
  
 NCBI BlastP on this gene

EMD92795

hypothetical protein
  
Accession: EMD92794
  
Location: 240079-240777
  
 NCBI BlastP on this gene

EMD92794

hypothetical protein
  
Accession: EMD92793
  
Location: 237178-237918
  
 NCBI BlastP on this gene

EMD92793

hypothetical protein
  
Accession: EMD92792
  
Location: 236238-237101
  
 NCBI BlastP on this gene

EMD92792

hypothetical protein
  
Accession: EMD92791
  
Location: 235464-235877
  
 NCBI BlastP on this gene

EMD92791

hypothetical protein
  
Accession: EMD92790
  
Location: 232817-234088
  
 NCBI BlastP on this gene

EMD92790

hypothetical protein
  
Accession: EMD92789
  
Location: 231531-232397
  
 NCBI BlastP on this gene

EMD92789

10. :  KB445644 Cochliobolus sativus ND90Pr unplaced genomic scaffold COCSAscaffold\_8     Total score: 3.0     Cumulative Blast bit score: 618

hypothetical protein
  
Accession: EMD63623
  
Location: 687704-689824
  
 NCBI BlastP on this gene

EMD63623

hypothetical protein
  
Accession: EMD63622
  
Location: 685956-686494
  
 NCBI BlastP on this gene

EMD63622

hypothetical protein
  
Accession: EMD63621
  
Location: 684566-685725
  
 NCBI BlastP on this gene

EMD63621

hypothetical protein
  
Accession: EMD63620
  
Location: 678112-681517
  
 NCBI BlastP on this gene

EMD63620

hypothetical protein
  
Accession: EMD63619
  
Location: 677526-677927
  
 NCBI BlastP on this gene

EMD63619

hypothetical protein
  
Accession: EMD63618
  
Location: 673690-675966
  
 NCBI BlastP on this gene

EMD63618

hypothetical protein
  
Accession: EMD63617
  
Location: 672564-673191
  
  
**BlastP hit with Mycgr3G70471**
  
Percentage identity: 85 %
  
BlastP bit score: 212
  
Sequence coverage: 88 %
  
E-value: 9e-68
  
  
 NCBI BlastP on this gene

EMD63617

hypothetical protein
  
Accession: EMD63616
  
Location: 670858-672198
  
  
**BlastP hit with Mycgr3G38483**
  
Percentage identity: 48 %
  
BlastP bit score: 311
  
Sequence coverage: 100 %
  
E-value: 7e-100
  
  
 NCBI BlastP on this gene

EMD63616

hypothetical protein
  
Accession: EMD63615
  
Location: 669502-670508
  
 NCBI BlastP on this gene

EMD63615

hypothetical protein
  
Accession: EMD63614
  
Location: 668094-669143
  
  
**BlastP hit with Mycgr3G108866**
  
Percentage identity: 33 %
  
BlastP bit score: 95
  
Sequence coverage: 96 %
  
E-value: 2e-19
  
  
 NCBI BlastP on this gene

EMD63614

hypothetical protein
  
Accession: EMD63613
  
Location: 667665-667880
  
 NCBI BlastP on this gene

EMD63613

hypothetical protein
  
Accession: EMD63612
  
Location: 665483-665950
  
 NCBI BlastP on this gene

EMD63612

hypothetical protein
  
Accession: EMD63611
  
Location: 662896-663569
  
 NCBI BlastP on this gene

EMD63611

hypothetical protein
  
Accession: EMD63610
  
Location: 660005-660744
  
 NCBI BlastP on this gene

EMD63610

hypothetical protein
  
Accession: EMD63609
  
Location: 659065-659928
  
 NCBI BlastP on this gene

EMD63609

hypothetical protein
  
Accession: EMD63608
  
Location: 658303-658521
  
 NCBI BlastP on this gene

EMD63608

hypothetical protein
  
Accession: EMD63607
  
Location: 655343-656612
  
 NCBI BlastP on this gene

EMD63607

hypothetical protein
  
Accession: EMD63606
  
Location: 654022-654888
  
 NCBI BlastP on this gene

EMD63606

11. :  FP929131 Leptosphaeria maculans JN3 lm\_SuperContig\_16\_v2 genomic supercontig     Total score: 3.0     Cumulative Blast bit score: 610

hypothetical protein
  
Accession: CBX97370
  
Location: 961808-963289
  
 NCBI BlastP on this gene

LEMA\_P105010.1

hypothetical protein
  
Accession: CBX97371
  
Location: 963617-965232
  
 NCBI BlastP on this gene

LEMA\_P105020.1

hypothetical protein
  
Accession: CBX97372
  
Location: 965425-966938
  
 NCBI BlastP on this gene

LEMA\_P105030.1

similar to RNA binding protein Jsn1
  
Accession: CBX97373
  
Location: 968198-971628
  
 NCBI BlastP on this gene

LEMA\_P105040.1

hypothetical protein
  
Accession: CBX97374
  
Location: 973909-976203
  
 NCBI BlastP on this gene

LEMA\_P105050.1

hypothetical protein
  
Accession: CBX97375
  
Location: 976823-977634
  
  
**BlastP hit with Mycgr3G70471**
  
Percentage identity: 81 %
  
BlastP bit score: 222
  
Sequence coverage: 99 %
  
E-value: 2e-71
  
  
 NCBI BlastP on this gene

LEMA\_P105060.1

hypothetical protein
  
Accession: CBX97376
  
Location: 977755-979062
  
  
**BlastP hit with Mycgr3G38483**
  
Percentage identity: 42 %
  
BlastP bit score: 286
  
Sequence coverage: 106 %
  
E-value: 3e-90
  
  
 NCBI BlastP on this gene

LEMA\_P105070.1

predicted protein
  
Accession: CBX97377
  
Location: 979452-979718
  
 NCBI BlastP on this gene

LEMA\_uP105080.1

hypothetical protein
  
Accession: CBX97378
  
Location: 980984-982016
  
  
**BlastP hit with Mycgr3G108866**
  
Percentage identity: 37 %
  
BlastP bit score: 102
  
Sequence coverage: 63 %
  
E-value: 9e-22
  
  
 NCBI BlastP on this gene

LEMA\_P105090.1

hypothetical protein
  
Accession: CBX97379
  
Location: 983003-983685
  
 NCBI BlastP on this gene

LEMA\_P105100.1

predicted protein
  
Accession: CBX97380
  
Location: 984841-985311
  
 NCBI BlastP on this gene

LEMA\_P105110.1

hypothetical protein
  
Accession: CBX97381
  
Location: 985688-986357
  
 NCBI BlastP on this gene

LEMA\_P105120.1

similar to oxidoreductase
  
Accession: CBX97382
  
Location: 987431-988558
  
 NCBI BlastP on this gene

LEMA\_P105130.1

hypothetical protein
  
Accession: CBX97383
  
Location: 989644-992000
  
 NCBI BlastP on this gene

LEMA\_P105140.1

predicted protein
  
Accession: CBX97384
  
Location: 992072-992755
  
 NCBI BlastP on this gene

LEMA\_uP105150.1

similar to 50S ribosomal subunit L30
  
Accession: CBX97385
  
Location: 993457-994586
  
 NCBI BlastP on this gene

LEMA\_P105160.1

similar to t-complex protein 1
  
Accession: CBX97386
  
Location: 995384-997167
  
 NCBI BlastP on this gene

LEMA\_P105170.1

12. :  DS231629 Pyrenophora tritici-repentis Pt-1C-BFP supercont1.15 genomic scaffold     Total score: 3.0     Cumulative Blast bit score: 608

conserved hypothetical protein
  
Accession: EDU43848
  
Location: 104067-107916
  
 NCBI BlastP on this gene

EDU43848

conserved hypothetical protein
  
Accession: EDU43847
  
Location: 102576-103347
  
 NCBI BlastP on this gene

EDU43847

conserved hypothetical protein
  
Accession: EDU43846
  
Location: 101624-102478
  
 NCBI BlastP on this gene

EDU43846

conserved hypothetical protein
  
Accession: EDU43845
  
Location: 100802-101250
  
 NCBI BlastP on this gene

EDU43845

RNA binding protein
  
Accession: EDU43844
  
Location: 95501-98925
  
 NCBI BlastP on this gene

EDU43844

conserved hypothetical protein
  
Accession: EDU43843
  
Location: 91478-93700
  
 NCBI BlastP on this gene

EDU43843

40S ribosomal protein S24
  
Accession: EDU43842
  
Location: 90259-90818
  
  
**BlastP hit with Mycgr3G70471**
  
Percentage identity: 82 %
  
BlastP bit score: 226
  
Sequence coverage: 99 %
  
E-value: 2e-73
  
  
 NCBI BlastP on this gene

EDU43842

conserved hypothetical protein
  
Accession: EDU43841
  
Location: 88576-89893
  
  
**BlastP hit with Mycgr3G38483**
  
Percentage identity: 45 %
  
BlastP bit score: 295
  
Sequence coverage: 100 %
  
E-value: 1e-93
  
  
 NCBI BlastP on this gene

EDU43841

predicted protein
  
Accession: EDU43840
  
Location: 86035-87061
  
  
**BlastP hit with Mycgr3G108866**
  
Percentage identity: 34 %
  
BlastP bit score: 87
  
Sequence coverage: 78 %
  
E-value: 1e-16
  
  
 NCBI BlastP on this gene

EDU43840

heat-stable 19 kDa antigen precursor
  
Accession: EDU43839
  
Location: 80115-80525
  
 NCBI BlastP on this gene

EDU43839

hypothetical protein
  
Accession: EDU43838
  
Location: 77806-78196
  
 NCBI BlastP on this gene

EDU43838

conserved hypothetical protein
  
Accession: EDU43837
  
Location: 76111-76752
  
 NCBI BlastP on this gene

EDU43837

zinc-binding oxidoreductase CipB
  
Accession: EDU43836
  
Location: 74180-75299
  
 NCBI BlastP on this gene

EDU43836

mRNA-capping enzyme subunit alpha
  
Accession: EDU43835
  
Location: 72123-73394
  
 NCBI BlastP on this gene

EDU43835

ubiquinol cytochrome-c reductase assembly protein Cbp3
  
Accession: EDU43834
  
Location: 70940-71696
  
 NCBI BlastP on this gene

EDU43834

hypothetical protein
  
Accession: EDU43833
  
Location: 68768-70201
  
 NCBI BlastP on this gene

EDU43833

13. :  KB908833 Setosphaeria turcica Et28A unplaced genomic scaffold SETTUscaffold\_5     Total score: 3.0     Cumulative Blast bit score: 603

hypothetical protein
  
Accession: EOA83424
  
Location: 1602945-1604987
  
 NCBI BlastP on this gene

EOA83424

hypothetical protein
  
Accession: EOA83425
  
Location: 1606297-1606851
  
 NCBI BlastP on this gene

EOA83425

hypothetical protein
  
Accession: EOA83426
  
Location: 1607078-1608252
  
 NCBI BlastP on this gene

EOA83426

hypothetical protein
  
Accession: EOA83427
  
Location: 1612227-1615636
  
 NCBI BlastP on this gene

EOA83427

hypothetical protein
  
Accession: EOA83428
  
Location: 1617481-1619349
  
 NCBI BlastP on this gene

EOA83428

hypothetical protein
  
Accession: EOA83429
  
Location: 1620065-1620697
  
  
**BlastP hit with Mycgr3G70471**
  
Percentage identity: 85 %
  
BlastP bit score: 212
  
Sequence coverage: 88 %
  
E-value: 8e-68
  
  
 NCBI BlastP on this gene

EOA83429

hypothetical protein
  
Accession: EOA83430
  
Location: 1621083-1622430
  
  
**BlastP hit with Mycgr3G38483**
  
Percentage identity: 46 %
  
BlastP bit score: 298
  
Sequence coverage: 100 %
  
E-value: 1e-94
  
  
 NCBI BlastP on this gene

EOA83430

hypothetical protein
  
Accession: EOA83431
  
Location: 1624221-1625277
  
  
**BlastP hit with Mycgr3G108866**
  
Percentage identity: 33 %
  
BlastP bit score: 93
  
Sequence coverage: 79 %
  
E-value: 1e-18
  
  
 NCBI BlastP on this gene

EOA83431

hypothetical protein
  
Accession: EOA83432
  
Location: 1627076-1627527
  
 NCBI BlastP on this gene

EOA83432

hypothetical protein
  
Accession: EOA83433
  
Location: 1629598-1630279
  
 NCBI BlastP on this gene

EOA83433

hypothetical protein
  
Accession: EOA83434
  
Location: 1631031-1632390
  
 NCBI BlastP on this gene

EOA83434

hypothetical protein
  
Accession: EOA83435
  
Location: 1634121-1634866
  
 NCBI BlastP on this gene

EOA83435

hypothetical protein
  
Accession: EOA83436
  
Location: 1634966-1635820
  
 NCBI BlastP on this gene

EOA83436

hypothetical protein
  
Accession: EOA83437
  
Location: 1636159-1636546
  
 NCBI BlastP on this gene

EOA83437

hypothetical protein
  
Accession: EOA83438
  
Location: 1639801-1641068
  
 NCBI BlastP on this gene

EOA83438

14. :  CH445352 Phaeosphaeria nodorum SN15 scaffold\_28     Total score: 3.0     Cumulative Blast bit score: 591

hypothetical protein
  
Accession: EAT78766
  
Location: 105338-107437
  
 NCBI BlastP on this gene

EAT78766

hypothetical protein
  
Accession: EAT78765
  
Location: 103241-104935
  
 NCBI BlastP on this gene

EAT78765

hypothetical protein
  
Accession: EAT78764
  
Location: 101423-102849
  
 NCBI BlastP on this gene

EAT78764

hypothetical protein
  
Accession: EAT78763
  
Location: 100244-101118
  
 NCBI BlastP on this gene

EAT78763

hypothetical protein
  
Accession: EAT78762
  
Location: 99285-99922
  
 NCBI BlastP on this gene

EAT78762

hypothetical protein
  
Accession: EAT78761
  
Location: 95141-98567
  
 NCBI BlastP on this gene

EAT78761

hypothetical protein
  
Accession: EAT78760
  
Location: 91150-93321
  
 NCBI BlastP on this gene

EAT78760

hypothetical protein
  
Accession: EAT78759
  
Location: 90103-90661
  
  
**BlastP hit with Mycgr3G70471**
  
Percentage identity: 84 %
  
BlastP bit score: 230
  
Sequence coverage: 99 %
  
E-value: 7e-75
  
  
 NCBI BlastP on this gene

EAT78759

hypothetical protein
  
Accession: EAT78758
  
Location: 88541-89715
  
  
**BlastP hit with Mycgr3G38483**
  
Percentage identity: 51 %
  
BlastP bit score: 303
  
Sequence coverage: 85 %
  
E-value: 3e-97
  
  
 NCBI BlastP on this gene

EAT78758

hypothetical protein
  
Accession: EAT78757
  
Location: 87366-88193
  
 NCBI BlastP on this gene

EAT78757

hypothetical protein
  
Accession: EAT78756
  
Location: 86071-86860
  
  
**BlastP hit with Mycgr3G108866**
  
Percentage identity: 34 %
  
BlastP bit score: 58
  
Sequence coverage: 52 %
  
E-value: 5e-07
  
  
 NCBI BlastP on this gene

EAT78756

hypothetical protein
  
Accession: EAT78755
  
Location: 84342-85158
  
 NCBI BlastP on this gene

EAT78755

hypothetical protein
  
Accession: EAT78754
  
Location: 82552-84177
  
 NCBI BlastP on this gene

EAT78754

hypothetical protein
  
Accession: EAT78753
  
Location: 81227-81755
  
 NCBI BlastP on this gene

EAT78753

hypothetical protein
  
Accession: EAT78752
  
Location: 79366-80427
  
 NCBI BlastP on this gene

EAT78752

hypothetical protein
  
Accession: EAT78751
  
Location: 77161-78468
  
 NCBI BlastP on this gene

EAT78751

hypothetical protein
  
Accession: EAT78750
  
Location: 75160-75627
  
 NCBI BlastP on this gene

EAT78750

hypothetical protein
  
Accession: EAT78749
  
Location: 73284-73835
  
 NCBI BlastP on this gene

EAT78749

hypothetical protein
  
Accession: EAT78748
  
Location: 72247-72715
  
 NCBI BlastP on this gene

EAT78748

hypothetical protein
  
Accession: EAT78747
  
Location: 71383-71985
  
 NCBI BlastP on this gene

EAT78747

15. :  KB446557 Pseudocercospora fijiensis CIRAD86 unplaced genomic scaffold MYCFIscaffold\_3     Total score: 2.0     Cumulative Blast bit score: 1202

hypothetical protein
  
Accession: EME84841
  
Location: 4987442-4989060
  
 NCBI BlastP on this gene

EME84841

hypothetical protein
  
Accession: EME84840
  
Location: 4985439-4987039
  
 NCBI BlastP on this gene

EME84840

hypothetical protein
  
Accession: EME84839
  
Location: 4983466-4984883
  
 NCBI BlastP on this gene

EME84839

hypothetical protein
  
Accession: EME84838
  
Location: 4981404-4982865
  
 NCBI BlastP on this gene

EME84838

hypothetical protein
  
Accession: EME84837
  
Location: 4980799-4981160
  
 NCBI BlastP on this gene

EME84837

hypothetical protein
  
Accession: EME84836
  
Location: 4978306-4978808
  
 NCBI BlastP on this gene

EME84836

glycoside hydrolase family 43 carbohydrate-binding module family 35 protein
  
Accession: EME84835
  
Location: 4975841-4977347
  
 NCBI BlastP on this gene

EME84835

hypothetical protein
  
Accession: EME84834
  
Location: 4973389-4974885
  
  
**BlastP hit with Mycgr3G70475**
  
Percentage identity: 71 %
  
BlastP bit score: 650
  
Sequence coverage: 101 %
  
E-value: 0.0
  
  
 NCBI BlastP on this gene

EME84834

hypothetical protein
  
Accession: EME84833
  
Location: 4970491-4973301
  
 NCBI BlastP on this gene

EME84833

hypothetical protein
  
Accession: EME84832
  
Location: 4968740-4969938
  
 NCBI BlastP on this gene

EME84832

hypothetical protein
  
Accession: EME84831
  
Location: 4966382-4967851
  
  
**BlastP hit with Mycgr3G57362**
  
Percentage identity: 58 %
  
BlastP bit score: 552
  
Sequence coverage: 100 %
  
E-value: 0.0
  
  
 NCBI BlastP on this gene

EME84831

hypothetical protein
  
Accession: EME84830
  
Location: 4964030-4966173
  
 NCBI BlastP on this gene

EME84830

hypothetical protein
  
Accession: EME84829
  
Location: 4961729-4963731
  
 NCBI BlastP on this gene

EME84829

hypothetical protein
  
Accession: EME84828
  
Location: 4960054-4961412
  
 NCBI BlastP on this gene

EME84828

hypothetical protein
  
Accession: EME84827
  
Location: 4957852-4959585
  
 NCBI BlastP on this gene

EME84827

hypothetical protein
  
Accession: EME84826
  
Location: 4956093-4957036
  
 NCBI BlastP on this gene

EME84826

16. :  KB445552 Baudoinia compniacensis UAMH 10762 unplaced genomic scaffold BAUCOscaffold\_3     Total score: 2.0     Cumulative Blast bit score: 1159

hypothetical protein
  
Accession: EMC98839
  
Location: 573279-575468
  
 NCBI BlastP on this gene

EMC98839

hypothetical protein
  
Accession: EMC98840
  
Location: 575850-576011
  
 NCBI BlastP on this gene

EMC98840

hypothetical protein
  
Accession: EMC98841
  
Location: 576551-576871
  
 NCBI BlastP on this gene

EMC98841

hypothetical protein
  
Accession: EMC98842
  
Location: 579213-579604
  
 NCBI BlastP on this gene

EMC98842

hypothetical protein
  
Accession: EMC98843
  
Location: 580067-580828
  
 NCBI BlastP on this gene

EMC98843

hypothetical protein
  
Accession: EMC98844
  
Location: 582676-583650
  
 NCBI BlastP on this gene

EMC98844

hypothetical protein
  
Accession: EMC98845
  
Location: 583821-585170
  
 NCBI BlastP on this gene

EMC98845

hypothetical protein
  
Accession: EMC98846
  
Location: 585583-586122
  
 NCBI BlastP on this gene

EMC98846

hypothetical protein
  
Accession: EMC98847
  
Location: 587114-591421
  
 NCBI BlastP on this gene

EMC98847

hypothetical protein
  
Accession: EMC98848
  
Location: 591780-593122
  
  
**BlastP hit with Mycgr3G39149**
  
Percentage identity: 82 %
  
BlastP bit score: 708
  
Sequence coverage: 99 %
  
E-value: 0.0
  
  
 NCBI BlastP on this gene

EMC98848

hypothetical protein
  
Accession: EMC98849
  
Location: 593343-594413
  
  
**BlastP hit with Mycgr3G108865**
  
Percentage identity: 66 %
  
BlastP bit score: 451
  
Sequence coverage: 75 %
  
E-value: 8e-153
  
  
 NCBI BlastP on this gene

EMC98849

hypothetical protein
  
Accession: EMC98850
  
Location: 595688-596067
  
 NCBI BlastP on this gene

EMC98850

hypothetical protein
  
Accession: EMC98851
  
Location: 599050-599262
  
 NCBI BlastP on this gene

EMC98851

hypothetical protein
  
Accession: EMC98852
  
Location: 600132-600293
  
 NCBI BlastP on this gene

EMC98852

hypothetical protein
  
Accession: EMC98853
  
Location: 600432-600620
  
 NCBI BlastP on this gene

EMC98853

hypothetical protein
  
Accession: EMC98854
  
Location: 601611-601934
  
 NCBI BlastP on this gene

EMC98854

hypothetical protein
  
Accession: EMC98855
  
Location: 603239-608521
  
 NCBI BlastP on this gene

EMC98855

hypothetical protein
  
Accession: EMC98856
  
Location: 608530-609314
  
 NCBI BlastP on this gene

EMC98856

hypothetical protein
  
Accession: EMC98857
  
Location: 609684-610557
  
 NCBI BlastP on this gene

EMC98857

hypothetical protein
  
Accession: EMC98858
  
Location: 611060-612512
  
 NCBI BlastP on this gene

EMC98858

17. :  KB908833 Setosphaeria turcica Et28A unplaced genomic scaffold SETTUscaffold\_5     Total score: 2.0     Cumulative Blast bit score: 1092

hypothetical protein
  
Accession: EOA83505
  
Location: 1846259-1847074
  
 NCBI BlastP on this gene

EOA83505

hypothetical protein
  
Accession: EOA83504
  
Location: 1842249-1843535
  
 NCBI BlastP on this gene

EOA83504

hypothetical protein
  
Accession: EOA83503
  
Location: 1839721-1841530
  
 NCBI BlastP on this gene

EOA83503

hypothetical protein
  
Accession: EOA83502
  
Location: 1838235-1838918
  
 NCBI BlastP on this gene

EOA83502

hypothetical protein
  
Accession: EOA83501
  
Location: 1836403-1838032
  
  
**BlastP hit with Mycgr3G39149**
  
Percentage identity: 73 %
  
BlastP bit score: 642
  
Sequence coverage: 99 %
  
E-value: 0.0
  
  
 NCBI BlastP on this gene

EOA83501

hypothetical protein
  
Accession: EOA83500
  
Location: 1834695-1836086
  
  
**BlastP hit with Mycgr3G108865**
  
Percentage identity: 58 %
  
BlastP bit score: 450
  
Sequence coverage: 82 %
  
E-value: 1e-151
  
  
 NCBI BlastP on this gene

EOA83500

hypothetical protein
  
Accession: EOA83499
  
Location: 1834072-1834385
  
 NCBI BlastP on this gene

EOA83499

hypothetical protein
  
Accession: EOA83498
  
Location: 1831837-1832153
  
 NCBI BlastP on this gene

EOA83498

hypothetical protein
  
Accession: EOA83497
  
Location: 1828088-1830017
  
 NCBI BlastP on this gene

EOA83497

hypothetical protein
  
Accession: EOA83496
  
Location: 1826128-1827530
  
 NCBI BlastP on this gene

EOA83496

hypothetical protein
  
Accession: EOA83495
  
Location: 1824708-1825583
  
 NCBI BlastP on this gene

EOA83495

hypothetical protein
  
Accession: EOA83494
  
Location: 1822728-1823738
  
 NCBI BlastP on this gene

EOA83494

hypothetical protein
  
Accession: EOA83493
  
Location: 1818511-1820250
  
 NCBI BlastP on this gene

EOA83493

hypothetical protein
  
Accession: EOA83492
  
Location: 1817391-1818371
  
 NCBI BlastP on this gene

EOA83492

18. :  FP929131 Leptosphaeria maculans JN3 lm\_SuperContig\_16\_v2 genomic supercontig     Total score: 2.0     Cumulative Blast bit score: 1091

hypothetical protein
  
Accession: CBX97255
  
Location: 407619-422270
  
 NCBI BlastP on this gene

LEMA\_P103860.1

similar to ribosomal protein L12
  
Accession: CBX97254
  
Location: 406541-407223
  
 NCBI BlastP on this gene

LEMA\_P103850.1

hypothetical protein
  
Accession: CBX97253
  
Location: 405517-406212
  
 NCBI BlastP on this gene

LEMA\_P103840.1

similar to pyrroline-5-carboxylate reductase
  
Accession: CBX97252
  
Location: 404123-405149
  
 NCBI BlastP on this gene

LEMA\_P103830.1

hypothetical protein
  
Accession: CBX97251
  
Location: 399960-403793
  
 NCBI BlastP on this gene

LEMA\_P103820.1

similar to Arylacetamide deacetylase
  
Accession: CBX97250
  
Location: 397738-398981
  
 NCBI BlastP on this gene

LEMA\_P103810.1

similar to homoserine o-acetyltransferase
  
Accession: CBX97249
  
Location: 395624-397438
  
 NCBI BlastP on this gene

LEMA\_P103800.1

predicted protein
  
Accession: CBX97248
  
Location: 394243-394892
  
 NCBI BlastP on this gene

LEMA\_P103790.1

similar to 3-oxoacyl-(acyl-carrier-protein) synthase
  
Accession: CBX97247
  
Location: 392481-394120
  
  
**BlastP hit with Mycgr3G39149**
  
Percentage identity: 69 %
  
BlastP bit score: 619
  
Sequence coverage: 103 %
  
E-value: 0.0
  
  
 NCBI BlastP on this gene

LEMA\_P103780.1

similar to high-affinity nickel transport protein
  
Accession: CBX97246
  
Location: 390825-392171
  
  
**BlastP hit with Mycgr3G108865**
  
Percentage identity: 58 %
  
BlastP bit score: 472
  
Sequence coverage: 88 %
  
E-value: 5e-160
  
  
 NCBI BlastP on this gene

LEMA\_P103770.1

similar to mitochondrial GTPase
  
Accession: CBX97245
  
Location: 389505-390587
  
 NCBI BlastP on this gene

LEMA\_P103760.1

hypothetical protein
  
Accession: CBX97244
  
Location: 387486-389281
  
 NCBI BlastP on this gene

LEMA\_P103750.1

hypothetical protein
  
Accession: CBX97243
  
Location: 381717-387202
  
 NCBI BlastP on this gene

LEMA\_P103740.1

hypothetical protein
  
Accession: CBX97242
  
Location: 379049-379666
  
 NCBI BlastP on this gene

LEMA\_P103730.1

predicted protein
  
Accession: CBX97241
  
Location: 376075-378757
  
 NCBI BlastP on this gene

LEMA\_P103720.1

hypothetical protein
  
Accession: CBX97240
  
Location: 374020-375576
  
 NCBI BlastP on this gene

LEMA\_P103710.1

19. :  KB445559 Baudoinia compniacensis UAMH 10762 unplaced genomic scaffold BAUCOscaffold\_10     Total score: 2.0     Cumulative Blast bit score: 1086

hypothetical protein
  
Accession: EMC94015
  
Location: 642599-643522
  
 NCBI BlastP on this gene

EMC94015

hypothetical protein
  
Accession: EMC94016
  
Location: 646239-649041
  
 NCBI BlastP on this gene

EMC94016

hypothetical protein
  
Accession: EMC94017
  
Location: 649218-650206
  
 NCBI BlastP on this gene

EMC94017

hypothetical protein
  
Accession: EMC94018
  
Location: 650864-651973
  
 NCBI BlastP on this gene

EMC94018

hypothetical protein
  
Accession: EMC94019
  
Location: 652126-653972
  
 NCBI BlastP on this gene

EMC94019

hypothetical protein
  
Accession: EMC94020
  
Location: 654670-655739
  
 NCBI BlastP on this gene

EMC94020

hypothetical protein
  
Accession: EMC94021
  
Location: 656080-657543
  
 NCBI BlastP on this gene

EMC94021

hypothetical protein
  
Accession: EMC94022
  
Location: 658145-659698
  
  
**BlastP hit with Mycgr3G57362**
  
Percentage identity: 56 %
  
BlastP bit score: 530
  
Sequence coverage: 102 %
  
E-value: 0.0
  
  
 NCBI BlastP on this gene

EMC94022

hypothetical protein
  
Accession: EMC94023
  
Location: 659869-661506
  
  
**BlastP hit with Mycgr3G70475**
  
Percentage identity: 59 %
  
BlastP bit score: 556
  
Sequence coverage: 111 %
  
E-value: 0.0
  
  
 NCBI BlastP on this gene

EMC94023

hypothetical protein
  
Accession: EMC94024
  
Location: 661864-662598
  
 NCBI BlastP on this gene

EMC94024

hypothetical protein
  
Accession: EMC94025
  
Location: 663805-664513
  
 NCBI BlastP on this gene

EMC94025

hypothetical protein
  
Accession: EMC94026
  
Location: 665120-665359
  
 NCBI BlastP on this gene

EMC94026

hypothetical protein
  
Accession: EMC94027
  
Location: 666088-667537
  
 NCBI BlastP on this gene

EMC94027

hypothetical protein
  
Accession: EMC94028
  
Location: 668909-669394
  
 NCBI BlastP on this gene

EMC94028

hypothetical protein
  
Accession: EMC94029
  
Location: 671670-671944
  
 NCBI BlastP on this gene

EMC94029

hypothetical protein
  
Accession: EMC94030
  
Location: 672249-672626
  
 NCBI BlastP on this gene

EMC94030

hypothetical protein
  
Accession: EMC94031
  
Location: 673820-674105
  
 NCBI BlastP on this gene

EMC94031

hypothetical protein
  
Accession: EMC94032
  
Location: 674858-677450
  
 NCBI BlastP on this gene

EMC94032

hypothetical protein
  
Accession: EMC94033
  
Location: 677822-678457
  
 NCBI BlastP on this gene

EMC94033

20. :  KB445644 Cochliobolus sativus ND90Pr unplaced genomic scaffold COCSAscaffold\_8     Total score: 2.0     Cumulative Blast bit score: 1081

hypothetical protein
  
Accession: EMD63533
  
Location: 447143-447503
  
 NCBI BlastP on this gene

EMD63533

hypothetical protein
  
Accession: EMD63534
  
Location: 450495-451622
  
 NCBI BlastP on this gene

EMD63534

hypothetical protein
  
Accession: EMD63535
  
Location: 452440-454252
  
 NCBI BlastP on this gene

EMD63535

hypothetical protein
  
Accession: EMD63536
  
Location: 455168-456798
  
  
**BlastP hit with Mycgr3G39149**
  
Percentage identity: 71 %
  
BlastP bit score: 623
  
Sequence coverage: 99 %
  
E-value: 0.0
  
  
 NCBI BlastP on this gene

EMD63536

hypothetical protein
  
Accession: EMD63537
  
Location: 457130-458540
  
  
**BlastP hit with Mycgr3G108865**
  
Percentage identity: 61 %
  
BlastP bit score: 458
  
Sequence coverage: 78 %
  
E-value: 1e-154
  
  
 NCBI BlastP on this gene

EMD63537

hypothetical protein
  
Accession: EMD63538
  
Location: 461075-462993
  
 NCBI BlastP on this gene

EMD63538

hypothetical protein
  
Accession: EMD63539
  
Location: 463542-464894
  
 NCBI BlastP on this gene

EMD63539

hypothetical protein
  
Accession: EMD63540
  
Location: 465137-466060
  
 NCBI BlastP on this gene

EMD63540

hypothetical protein
  
Accession: EMD63541
  
Location: 467103-467822
  
 NCBI BlastP on this gene

EMD63541

hypothetical protein
  
Accession: EMD63542
  
Location: 468722-470452
  
 NCBI BlastP on this gene

EMD63542

hypothetical protein
  
Accession: EMD63543
  
Location: 470629-471537
  
 NCBI BlastP on this gene

EMD63543

hypothetical protein
  
Accession: EMD63544
  
Location: 472721-473719
  
 NCBI BlastP on this gene

EMD63544

hypothetical protein
  
Accession: EMD63545
  
Location: 473909-476890
  
 NCBI BlastP on this gene

EMD63545

21. :  KB733456 Bipolaris maydis ATCC 48331 unplaced genomic scaffold COCC4scaffold\_13     Total score: 2.0     Cumulative Blast bit score: 1070

hypothetical protein
  
Accession: ENI04892
  
Location: 934217-934428
  
 NCBI BlastP on this gene

ENI04892

hypothetical protein
  
Accession: ENI04891
  
Location: 930902-931253
  
 NCBI BlastP on this gene

ENI04891

hypothetical protein
  
Accession: ENI04890
  
Location: 926685-927816
  
 NCBI BlastP on this gene

ENI04890

hypothetical protein
  
Accession: ENI04889
  
Location: 923698-925513
  
 NCBI BlastP on this gene

ENI04889

hypothetical protein
  
Accession: ENI04888
  
Location: 921171-922799
  
  
**BlastP hit with Mycgr3G39149**
  
Percentage identity: 70 %
  
BlastP bit score: 610
  
Sequence coverage: 99 %
  
E-value: 0.0
  
  
 NCBI BlastP on this gene

ENI04888

hypothetical protein
  
Accession: ENI04887
  
Location: 919447-920829
  
  
**BlastP hit with Mycgr3G108865**
  
Percentage identity: 61 %
  
BlastP bit score: 460
  
Sequence coverage: 78 %
  
E-value: 1e-155
  
  
 NCBI BlastP on this gene

ENI04887

hypothetical protein
  
Accession: ENI04886
  
Location: 918868-919208
  
 NCBI BlastP on this gene

ENI04886

hypothetical protein
  
Accession: ENI04885
  
Location: 914796-916714
  
 NCBI BlastP on this gene

ENI04885

hypothetical protein
  
Accession: ENI04884
  
Location: 912896-914248
  
 NCBI BlastP on this gene

ENI04884

hypothetical protein
  
Accession: ENI04883
  
Location: 911728-912650
  
 NCBI BlastP on this gene

ENI04883

hypothetical protein
  
Accession: ENI04882
  
Location: 910010-910990
  
 NCBI BlastP on this gene

ENI04882

hypothetical protein
  
Accession: ENI04881
  
Location: 907371-909101
  
 NCBI BlastP on this gene

ENI04881

hypothetical protein
  
Accession: ENI04880
  
Location: 906316-907191
  
 NCBI BlastP on this gene

ENI04880

hypothetical protein
  
Accession: ENI04879
  
Location: 905736-906167
  
 NCBI BlastP on this gene

ENI04879

hypothetical protein
  
Accession: ENI04878
  
Location: 904154-905149
  
 NCBI BlastP on this gene

ENI04878

hypothetical protein
  
Accession: ENI04877
  
Location: 900924-903923
  
 NCBI BlastP on this gene

ENI04877

22. :  KB445587 Cochliobolus heterostrophus C5 unplaced genomic scaffold COCHEscaffold\_19     Total score: 2.0     Cumulative Blast bit score: 1070

hypothetical protein
  
Accession: EMD85848
  
Location: 695642-695853
  
 NCBI BlastP on this gene

EMD85848

hypothetical protein
  
Accession: EMD85849
  
Location: 698817-699168
  
 NCBI BlastP on this gene

EMD85849

hypothetical protein
  
Accession: EMD85850
  
Location: 702254-703385
  
 NCBI BlastP on this gene

EMD85850

hypothetical protein
  
Accession: EMD85851
  
Location: 704557-706372
  
 NCBI BlastP on this gene

EMD85851

hypothetical protein
  
Accession: EMD85852
  
Location: 707271-708899
  
  
**BlastP hit with Mycgr3G39149**
  
Percentage identity: 70 %
  
BlastP bit score: 610
  
Sequence coverage: 99 %
  
E-value: 0.0
  
  
 NCBI BlastP on this gene

EMD85852

hypothetical protein
  
Accession: EMD85853
  
Location: 709241-710623
  
  
**BlastP hit with Mycgr3G108865**
  
Percentage identity: 61 %
  
BlastP bit score: 460
  
Sequence coverage: 78 %
  
E-value: 1e-155
  
  
 NCBI BlastP on this gene

EMD85853

hypothetical protein
  
Accession: EMD85854
  
Location: 710862-711202
  
 NCBI BlastP on this gene

EMD85854

hypothetical protein
  
Accession: EMD85855
  
Location: 713356-715274
  
 NCBI BlastP on this gene

EMD85855

hypothetical protein
  
Accession: EMD85856
  
Location: 715822-717174
  
 NCBI BlastP on this gene

EMD85856

hypothetical protein
  
Accession: EMD85857
  
Location: 717420-718342
  
 NCBI BlastP on this gene

EMD85857

hypothetical protein
  
Accession: EMD85858
  
Location: 719080-720060
  
 NCBI BlastP on this gene

EMD85858

hypothetical protein
  
Accession: EMD85859
  
Location: 720969-722699
  
 NCBI BlastP on this gene

EMD85859

23. :  KB445574 Cochliobolus heterostrophus C5 unplaced genomic scaffold COCHEscaffold\_6     Total score: 2.0     Cumulative Blast bit score: 1070

hypothetical protein
  
Accession: EMD92718
  
Location: 45413-45624
  
 NCBI BlastP on this gene

EMD92718

hypothetical protein
  
Accession: EMD92719
  
Location: 48588-48939
  
 NCBI BlastP on this gene

EMD92719

hypothetical protein
  
Accession: EMD92720
  
Location: 52025-53501
  
 NCBI BlastP on this gene

EMD92720

hypothetical protein
  
Accession: EMD92721
  
Location: 54328-56143
  
 NCBI BlastP on this gene

EMD92721

hypothetical protein
  
Accession: EMD92722
  
Location: 57042-58670
  
  
**BlastP hit with Mycgr3G39149**
  
Percentage identity: 70 %
  
BlastP bit score: 610
  
Sequence coverage: 99 %
  
E-value: 0.0
  
  
 NCBI BlastP on this gene

EMD92722

hypothetical protein
  
Accession: EMD92723
  
Location: 59012-60394
  
  
**BlastP hit with Mycgr3G108865**
  
Percentage identity: 61 %
  
BlastP bit score: 460
  
Sequence coverage: 78 %
  
E-value: 1e-155
  
  
 NCBI BlastP on this gene

EMD92723

hypothetical protein
  
Accession: EMD92724
  
Location: 60633-60973
  
 NCBI BlastP on this gene

EMD92724

hypothetical protein
  
Accession: EMD92725
  
Location: 63127-65045
  
 NCBI BlastP on this gene

EMD92725

hypothetical protein
  
Accession: EMD92726
  
Location: 65593-66945
  
 NCBI BlastP on this gene

EMD92726

hypothetical protein
  
Accession: EMD92727
  
Location: 67191-68113
  
 NCBI BlastP on this gene

EMD92727

hypothetical protein
  
Accession: EMD92728
  
Location: 68851-69819
  
 NCBI BlastP on this gene

EMD92728

hypothetical protein
  
Accession: EMD92729
  
Location: 70740-72470
  
 NCBI BlastP on this gene

EMD92729

hypothetical protein
  
Accession: EMD92730
  
Location: 72650-73525
  
 NCBI BlastP on this gene

EMD92730

hypothetical protein
  
Accession: EMD92731
  
Location: 73674-74105
  
 NCBI BlastP on this gene

EMD92731

hypothetical protein
  
Accession: EMD92732
  
Location: 74692-75687
  
 NCBI BlastP on this gene

EMD92732

hypothetical protein
  
Accession: EMD92733
  
Location: 75918-78917
  
 NCBI BlastP on this gene

EMD92733

24. :  GL532905 Pyrenophora teres f. teres 0-1 unplaced genomic scaffold scaffold\_189131     Total score: 2.0     Cumulative Blast bit score: 1053

hypothetical protein
  
Accession: EFQ95174
  
Location: 16309-17205
  
 NCBI BlastP on this gene

EFQ95174

hypothetical protein
  
Accession: EFQ95175
  
Location: 17461-18177
  
 NCBI BlastP on this gene

EFQ95175

hypothetical protein
  
Accession: EFQ95176
  
Location: 18456-19468
  
 NCBI BlastP on this gene

EFQ95176

hypothetical protein
  
Accession: EFQ95177
  
Location: 19850-23664
  
 NCBI BlastP on this gene

EFQ95177

hypothetical protein
  
Accession: EFQ95178
  
Location: 24219-25445
  
 NCBI BlastP on this gene

EFQ95178

hypothetical protein
  
Accession: EFQ95179
  
Location: 26602-27299
  
 NCBI BlastP on this gene

EFQ95179

hypothetical protein
  
Accession: EFQ95180
  
Location: 30234-32053
  
 NCBI BlastP on this gene

EFQ95180

hypothetical protein
  
Accession: EFQ95181
  
Location: 33077-35525
  
  
**BlastP hit with Mycgr3G39149**
  
Percentage identity: 71 %
  
BlastP bit score: 598
  
Sequence coverage: 95 %
  
E-value: 0.0
  
  
 NCBI BlastP on this gene

EFQ95181

hypothetical protein
  
Accession: EFQ95182
  
Location: 35809-37210
  
  
**BlastP hit with Mycgr3G108865**
  
Percentage identity: 57 %
  
BlastP bit score: 455
  
Sequence coverage: 83 %
  
E-value: 1e-153
  
  
 NCBI BlastP on this gene

EFQ95182

hypothetical protein
  
Accession: EFQ95183
  
Location: 41182-41636
  
 NCBI BlastP on this gene

EFQ95183

hypothetical protein
  
Accession: EFQ95184
  
Location: 42424-44279
  
 NCBI BlastP on this gene

EFQ95184

hypothetical protein
  
Accession: EFQ95185
  
Location: 45024-45552
  
 NCBI BlastP on this gene

EFQ95185

hypothetical protein
  
Accession: EFQ95186
  
Location: 45721-47667
  
 NCBI BlastP on this gene

EFQ95186

25. :  DS231625 Pyrenophora tritici-repentis Pt-1C-BFP supercont1.11 genomic scaffold     Total score: 2.0     Cumulative Blast bit score: 903

predicted protein
  
Accession: EDU42764
  
Location: 1540793-1541163
  
 NCBI BlastP on this gene

EDU42764

homoserine O-acetyltransferase
  
Accession: EDU42763
  
Location: 1532287-1534106
  
 NCBI BlastP on this gene

EDU42763

3-oxoacyl-(acyl-carrier-protein) synthase, mitochondrial precursor
  
Accession: EDU42762
  
Location: 1523280-1524592
  
  
**BlastP hit with Mycgr3G39149**
  
Percentage identity: 65 %
  
BlastP bit score: 460
  
Sequence coverage: 85 %
  
E-value: 2e-157
  
  
 NCBI BlastP on this gene

EDU42762

high affinity nickel transport protein nic1
  
Accession: EDU42761
  
Location: 1521616-1523014
  
  
**BlastP hit with Mycgr3G108865**
  
Percentage identity: 54 %
  
BlastP bit score: 444
  
Sequence coverage: 90 %
  
E-value: 4e-149
  
  
 NCBI BlastP on this gene

EDU42761

L-ascorbate oxidase
  
Accession: EDU42760
  
Location: 1514126-1515697
  
 NCBI BlastP on this gene

EDU42760

conserved hypothetical protein
  
Accession: EDU42759
  
Location: 1512540-1513072
  
 NCBI BlastP on this gene

EDU42759

conserved hypothetical protein
  
Accession: EDU42758
  
Location: 1510434-1512384
  
 NCBI BlastP on this gene

EDU42758

conserved hypothetical protein
  
Accession: EDU42757
  
Location: 1508594-1510083
  
 NCBI BlastP on this gene

EDU42757

26. :  CH476632 Sclerotinia sclerotiorum 1980 scaffold\_12 genomic scaffold     Total score: 2.0     Cumulative Blast bit score: 865

predicted protein
  
Accession: EDN92924
  
Location: 356773-356972
  
 NCBI BlastP on this gene

EDN92924

predicted protein
  
Accession: EDN92925
  
Location: 360742-361477
  
 NCBI BlastP on this gene

EDN92925

predicted protein
  
Accession: EDN92926
  
Location: 362048-362771
  
 NCBI BlastP on this gene

EDN92926

hypothetical protein
  
Accession: EDN92927
  
Location: 363910-365114
  
  
**BlastP hit with Mycgr3G39149**
  
Percentage identity: 68 %
  
BlastP bit score: 524
  
Sequence coverage: 87 %
  
E-value: 0.0
  
  
 NCBI BlastP on this gene

EDN92927

predicted protein
  
Accession: EDN92928
  
Location: 366572-366898
  
 NCBI BlastP on this gene

EDN92928

hypothetical protein
  
Accession: EDN92929
  
Location: 368882-370800
  
 NCBI BlastP on this gene

EDN92929

hypothetical protein
  
Accession: EDN92930
  
Location: 372638-373999
  
 NCBI BlastP on this gene

EDN92930

hypothetical protein
  
Accession: EDN92931
  
Location: 374251-375352
  
 NCBI BlastP on this gene

EDN92931

predicted protein
  
Accession: EDN92932
  
Location: 375868-376760
  
 NCBI BlastP on this gene

EDN92932

hypothetical protein
  
Accession: EDN92933
  
Location: 377269-379281
  
 NCBI BlastP on this gene

EDN92933

hypothetical protein
  
Accession: EDN92934
  
Location: 379922-380772
  
 NCBI BlastP on this gene

EDN92934

hypothetical protein
  
Accession: EDN92935
  
Location: 382826-383888
  
  
**BlastP hit with Mycgr3G108865**
  
Percentage identity: 56 %
  
BlastP bit score: 342
  
Sequence coverage: 70 %
  
E-value: 1e-110
  
  
 NCBI BlastP on this gene

EDN92935

hypothetical protein
  
Accession: EDN92936
  
Location: 384548-386462
  
 NCBI BlastP on this gene

EDN92936

predicted protein
  
Accession: EDN92937
  
Location: 388318-389856
  
 NCBI BlastP on this gene

EDN92937

hypothetical protein
  
Accession: EDN92938
  
Location: 391048-391983
  
 NCBI BlastP on this gene

EDN92938

27. :  KB456267 Mycosphaerella populorum SO2202 unplaced genomic scaffold SEPMUscaffold\_8     Total score: 2.0     Cumulative Blast bit score: 786

hypothetical protein
  
Accession: EMF10393
  
Location: 262906-264051
  
 NCBI BlastP on this gene

EMF10393

hypothetical protein
  
Accession: EMF10394
  
Location: 264091-264454
  
 NCBI BlastP on this gene

EMF10394

hypothetical protein
  
Accession: EMF10395
  
Location: 265947-268601
  
 NCBI BlastP on this gene

EMF10395

hypothetical protein
  
Accession: EMF10396
  
Location: 270361-270837
  
 NCBI BlastP on this gene

EMF10396

hypothetical protein
  
Accession: EMF10397
  
Location: 270901-271077
  
 NCBI BlastP on this gene

EMF10397

hypothetical protein
  
Accession: EMF10399
  
Location: 274002-274334
  
 NCBI BlastP on this gene

EMF10399

glycerol kinase
  
Accession: EMF10400
  
Location: 275712-277457
  
 NCBI BlastP on this gene

EMF10400

hypothetical protein
  
Accession: EMF10401
  
Location: 279441-280705
  
  
**BlastP hit with Mycgr3G38483**
  
Percentage identity: 71 %
  
BlastP bit score: 536
  
Sequence coverage: 99 %
  
E-value: 0.0
  
  
 NCBI BlastP on this gene

EMF10401

Ribosomal S24e-domain-containing protein
  
Accession: EMF10402
  
Location: 280973-281564
  
  
**BlastP hit with Mycgr3G70471**
  
Percentage identity: 92 %
  
BlastP bit score: 250
  
Sequence coverage: 99 %
  
E-value: 1e-82
  
  
 NCBI BlastP on this gene

EMF10402

hypothetical protein
  
Accession: EMF10403
  
Location: 282518-283054
  
 NCBI BlastP on this gene

EMF10403

protein serine/threonine phosphatase 2C
  
Accession: EMF10404
  
Location: 283833-285536
  
 NCBI BlastP on this gene

EMF10404

hypothetical protein
  
Accession: EMF10405
  
Location: 286097-289279
  
 NCBI BlastP on this gene

EMF10405

hypothetical protein
  
Accession: EMF10406
  
Location: 289939-290346
  
 NCBI BlastP on this gene

EMF10406

NIF-domain-containing protein
  
Accession: EMF10407
  
Location: 291980-293626
  
 NCBI BlastP on this gene

EMF10407

FAD/NAD(P)-binding domain-containing protein
  
Accession: EMF10408
  
Location: 295425-297942
  
 NCBI BlastP on this gene

EMF10408

28. :  KB446538 Dothistroma septosporum NZE10 unplaced genomic scaffold DOTSEscaffold\_4     Total score: 2.0     Cumulative Blast bit score: 780

hypothetical protein
  
Accession: EME45787
  
Location: 2388800-2390531
  
 NCBI BlastP on this gene

EME45787

hypothetical protein
  
Accession: EME45788
  
Location: 2390776-2392100
  
 NCBI BlastP on this gene

EME45788

hypothetical protein
  
Accession: EME45789
  
Location: 2392796-2393773
  
 NCBI BlastP on this gene

EME45789

hypothetical protein
  
Accession: EME45790
  
Location: 2394026-2394484
  
 NCBI BlastP on this gene

EME45790

hypothetical protein
  
Accession: EME45791
  
Location: 2398623-2399349
  
 NCBI BlastP on this gene

EME45791

hypothetical protein
  
Accession: EME45792
  
Location: 2399626-2401407
  
 NCBI BlastP on this gene

EME45792

hypothetical protein
  
Accession: EME45793
  
Location: 2402472-2402918
  
 NCBI BlastP on this gene

EME45793

hypothetical protein
  
Accession: EME45795
  
Location: 2405022-2407075
  
 NCBI BlastP on this gene

EME45795

hypothetical protein
  
Accession: EME45797
  
Location: 2407522-2408746
  
  
**BlastP hit with Mycgr3G38483**
  
Percentage identity: 70 %
  
BlastP bit score: 530
  
Sequence coverage: 99 %
  
E-value: 0.0
  
  
 NCBI BlastP on this gene

EME45797

hypothetical protein
  
Accession: EME45798
  
Location: 2409173-2409772
  
  
**BlastP hit with Mycgr3G70471**
  
Percentage identity: 91 %
  
BlastP bit score: 250
  
Sequence coverage: 99 %
  
E-value: 7e-83
  
  
 NCBI BlastP on this gene

EME45798

hypothetical protein
  
Accession: EME45799
  
Location: 2411049-2412458
  
 NCBI BlastP on this gene

EME45799

hypothetical protein
  
Accession: EME45800
  
Location: 2413509-2413814
  
 NCBI BlastP on this gene

EME45800

hypothetical protein
  
Accession: EME45801
  
Location: 2415523-2415867
  
 NCBI BlastP on this gene

EME45801

hypothetical protein
  
Accession: EME45802
  
Location: 2416943-2418808
  
 NCBI BlastP on this gene

EME45802

hypothetical protein
  
Accession: EME45803
  
Location: 2418962-2419429
  
 NCBI BlastP on this gene

EME45803

hypothetical protein
  
Accession: EME45804
  
Location: 2420128-2421180
  
 NCBI BlastP on this gene

EME45804

hypothetical protein
  
Accession: EME45805
  
Location: 2422548-2423732
  
 NCBI BlastP on this gene

EME45805

hypothetical protein
  
Accession: EME45806
  
Location: 2425459-2426791
  
 NCBI BlastP on this gene

EME45806

29. :  KB445552 Baudoinia compniacensis UAMH 10762 unplaced genomic scaffold BAUCOscaffold\_3     Total score: 2.0     Cumulative Blast bit score: 724

hypothetical protein
  
Accession: EMC98767
  
Location: 427025-428719
  
 NCBI BlastP on this gene

EMC98767

hypothetical protein
  
Accession: EMC98768
  
Location: 430476-431479
  
 NCBI BlastP on this gene

EMC98768

hypothetical protein
  
Accession: EMC98769
  
Location: 431829-433718
  
 NCBI BlastP on this gene

EMC98769

hypothetical protein
  
Accession: EMC98770
  
Location: 434080-434520
  
 NCBI BlastP on this gene

EMC98770

hypothetical protein
  
Accession: EMC98771
  
Location: 435470-437253
  
 NCBI BlastP on this gene

EMC98771

hypothetical protein
  
Accession: EMC98772
  
Location: 437795-439849
  
 NCBI BlastP on this gene

EMC98772

hypothetical protein
  
Accession: EMC98773
  
Location: 440950-444547
  
 NCBI BlastP on this gene

EMC98773

hypothetical protein
  
Accession: EMC98774
  
Location: 445372-446613
  
  
**BlastP hit with Mycgr3G38483**
  
Percentage identity: 65 %
  
BlastP bit score: 498
  
Sequence coverage: 99 %
  
E-value: 2e-173
  
  
 NCBI BlastP on this gene

EMC98774

hypothetical protein
  
Accession: EMC98775
  
Location: 446897-447468
  
  
**BlastP hit with Mycgr3G70471**
  
Percentage identity: 88 %
  
BlastP bit score: 226
  
Sequence coverage: 91 %
  
E-value: 3e-73
  
  
 NCBI BlastP on this gene

EMC98775

hypothetical protein
  
Accession: EMC98776
  
Location: 448485-448811
  
 NCBI BlastP on this gene

EMC98776

hypothetical protein
  
Accession: EMC98777
  
Location: 448960-449136
  
 NCBI BlastP on this gene

EMC98777

hypothetical protein
  
Accession: EMC98778
  
Location: 453142-454063
  
 NCBI BlastP on this gene

EMC98778

hypothetical protein
  
Accession: EMC98779
  
Location: 454697-456865
  
 NCBI BlastP on this gene

EMC98779

hypothetical protein
  
Accession: EMC98780
  
Location: 457041-458147
  
 NCBI BlastP on this gene

EMC98780

hypothetical protein
  
Accession: EMC98781
  
Location: 458437-459324
  
 NCBI BlastP on this gene

EMC98781

hypothetical protein
  
Accession: EMC98782
  
Location: 459337-461436
  
 NCBI BlastP on this gene

EMC98782

hypothetical protein
  
Accession: EMC98783
  
Location: 461633-462414
  
 NCBI BlastP on this gene

EMC98783

hypothetical protein
  
Accession: EMC98784
  
Location: 463120-463461
  
 NCBI BlastP on this gene

EMC98784

30. :  GL534066 Pyrenophora teres f. teres 0-1 unplaced genomic scaffold scaffold\_190324     Total score: 2.0     Cumulative Blast bit score: 522

hypothetical protein
  
Accession: EFQ92977
  
Location: 743-2062
  
  
**BlastP hit with Mycgr3G38483**
  
Percentage identity: 45 %
  
BlastP bit score: 296
  
Sequence coverage: 100 %
  
E-value: 3e-94
  
  
 NCBI BlastP on this gene

EFQ92977

hypothetical protein
  
Accession: EFQ92978
  
Location: 2408-2966
  
  
**BlastP hit with Mycgr3G70471**
  
Percentage identity: 82 %
  
BlastP bit score: 226
  
Sequence coverage: 99 %
  
E-value: 2e-73
  
  
 NCBI BlastP on this gene

EFQ92978

hypothetical protein
  
Accession: EFQ92979
  
Location: 3611-5921
  
 NCBI BlastP on this gene

EFQ92979

31. :  GG697355 Glomerella graminicola M1.001 genomic scaffold supercont1.25     Total score: 2.0     Cumulative Blast bit score: 475

RNA recognition domain-containing protein
  
Accession: EFQ31470
  
Location: 448628-450239
  
 NCBI BlastP on this gene

EFQ31470

hypothetical protein
  
Accession: EFQ31471
  
Location: 451651-452163
  
 NCBI BlastP on this gene

EFQ31471

hypothetical protein
  
Accession: EFQ31472
  
Location: 458042-459202
  
  
**BlastP hit with Mycgr3G38483**
  
Percentage identity: 43 %
  
BlastP bit score: 260
  
Sequence coverage: 98 %
  
E-value: 5e-80
  
  
 NCBI BlastP on this gene

EFQ31472

ribosomal protein S24e
  
Accession: EFQ31473
  
Location: 459913-460765
  
  
**BlastP hit with Mycgr3G70471**
  
Percentage identity: 84 %
  
BlastP bit score: 215
  
Sequence coverage: 88 %
  
E-value: 6e-69
  
  
 NCBI BlastP on this gene

EFQ31473

hypothetical protein
  
Accession: EFQ31474
  
Location: 462165-463592
  
 NCBI BlastP on this gene

EFQ31474

actin
  
Accession: EFQ31475
  
Location: 466706-468113
  
 NCBI BlastP on this gene

EFQ31475

hydantoin racemase
  
Accession: EFQ31476
  
Location: 468640-469541
  
 NCBI BlastP on this gene

EFQ31476

hypothetical protein
  
Accession: EFQ31477
  
Location: 469665-471291
  
 NCBI BlastP on this gene

EFQ31477

NmrA-like family protein
  
Accession: EFQ31478
  
Location: 471827-472900
  
 NCBI BlastP on this gene

EFQ31478

RNA polymerase Rpb7-like domain-containing protein
  
Accession: EFQ31479
  
Location: 473795-475303
  
 NCBI BlastP on this gene

EFQ31479

hypothetical protein
  
Accession: EFQ31480
  
Location: 475841-476306
  
 NCBI BlastP on this gene

EFQ31480

32. :  KB021237 Colletotrichum gloeosporioides Nara gc5 unplaced genomic scaffold scaffold810     Total score: 2.0     Cumulative Blast bit score: 473

hypothetical protein
  
Accession: ELA24797
  
Location: 217-993
  
 NCBI BlastP on this gene

ELA24797

RNA binding protein
  
Accession: ELA24798
  
Location: 7462-9127
  
 NCBI BlastP on this gene

ELA24798

hypothetical protein
  
Accession: ELA24799
  
Location: 10533-11023
  
 NCBI BlastP on this gene

ELA24799

hypothetical protein
  
Accession: ELA24800
  
Location: 16827-17959
  
  
**BlastP hit with Mycgr3G38483**
  
Percentage identity: 44 %
  
BlastP bit score: 262
  
Sequence coverage: 100 %
  
E-value: 5e-81
  
  
 NCBI BlastP on this gene

ELA24800

40s ribosomal protein s24
  
Accession: ELA24801
  
Location: 18699-19557
  
  
**BlastP hit with Mycgr3G70471**
  
Percentage identity: 83 %
  
BlastP bit score: 211
  
Sequence coverage: 88 %
  
E-value: 3e-67
  
  
 NCBI BlastP on this gene

ELA24801

essential component of the arp2 3 complex
  
Accession: ELA24802
  
Location: 29371-30744
  
 NCBI BlastP on this gene

ELA24802

dcg1 protein
  
Accession: ELA24803
  
Location: 31213-32085
  
 NCBI BlastP on this gene

ELA24803

MFS glucose
  
Accession: ELA24804
  
Location: 32182-33786
  
 NCBI BlastP on this gene

ELA24804

isoflavone reductase family protein
  
Accession: ELA24805
  
Location: 34501-35499
  
 NCBI BlastP on this gene

ELA24805

33. :  HF679031 Fusarium fujikuroi IMI 58289 draft genome, chromosome FFUJ\_chr09.     Total score: 2.0     Cumulative Blast bit score: 472

uncharacterized protein
  
Accession: CCT73348
  
Location: 633120-634579
  
 NCBI BlastP on this gene

FFUJ\_09966

related to peptidylprolyl isomerase (cyclophilin)-like protein
  
Accession: CCT73349
  
Location: 639645-641158
  
 NCBI BlastP on this gene

FFUJ\_09965

uncharacterized protein
  
Accession: CCT73350
  
Location: 643741-644235
  
 NCBI BlastP on this gene

FFUJ\_09964

uncharacterized protein
  
Accession: CCT73351
  
Location: 649115-650204
  
  
**BlastP hit with Mycgr3G38483**
  
Percentage identity: 45 %
  
BlastP bit score: 265
  
Sequence coverage: 98 %
  
E-value: 5e-82
  
  
 NCBI BlastP on this gene

FFUJ\_09963

probable 40S RIBOSOMAL PROTEIN S24
  
Accession: CCT73352
  
Location: 650665-651435
  
  
**BlastP hit with Mycgr3G70471**
  
Percentage identity: 81 %
  
BlastP bit score: 207
  
Sequence coverage: 89 %
  
E-value: 4e-66
  
  
 NCBI BlastP on this gene

FFUJ\_09962

uncharacterized protein
  
Accession: CCT73998
  
Location: 651903-652331
  
 NCBI BlastP on this gene

FFUJ\_09961

related to SRP40-suppressor of mutant AC40 of RNA polymerase I and III
  
Accession: CCT73353
  
Location: 653205-657770
  
 NCBI BlastP on this gene

FFUJ\_09960

probable GTP cyclohydrolase II
  
Accession: CCT73354
  
Location: 658559-659784
  
 NCBI BlastP on this gene

FFUJ\_09959

uncharacterized protein
  
Accession: CCT73355
  
Location: 662248-662910
  
 NCBI BlastP on this gene

FFUJ\_09958

related to dna-dependent rna polymerase I subunit a43 (rpa43)
  
Accession: CCT73356
  
Location: 667260-668651
  
 NCBI BlastP on this gene

FFUJ\_09957

34. :  AMYD01001766 Colletotrichum gloeosporioides Cg-14     Total score: 2.0     Cumulative Blast bit score: 472

ribosomal protein S24e
  
Accession: EQB51666
  
Location: 5677-6534
  
  
**BlastP hit with Mycgr3G70471**
  
Percentage identity: 83 %
  
BlastP bit score: 211
  
Sequence coverage: 88 %
  
E-value: 3e-67
  
  
 NCBI BlastP on this gene

EQB51666

hypothetical protein
  
Accession: EQB51667
  
Location: 7299-8614
  
  
**BlastP hit with Mycgr3G38483**
  
Percentage identity: 44 %
  
BlastP bit score: 261
  
Sequence coverage: 98 %
  
E-value: 2e-80
  
  
 NCBI BlastP on this gene

EQB51667

hypothetical protein
  
Accession: EQB51668
  
Location: 14249-14746
  
 NCBI BlastP on this gene

EQB51668

35. :  KB725935 Colletotrichum orbiculare MAFF 240422 unplaced genomic scaffold Scaffold\_370     Total score: 2.0     Cumulative Blast bit score: 471

DNA-dependent RNA polymerase i subunit a43
  
Accession: ENH82174
  
Location: 90729-92255
  
 NCBI BlastP on this gene

ENH82174

isoflavone reductase family protein
  
Accession: ENH82175
  
Location: 93072-94068
  
 NCBI BlastP on this gene

ENH82175

MFS glucose
  
Accession: ENH82176
  
Location: 94712-96325
  
 NCBI BlastP on this gene

ENH82176

dcg1 protein
  
Accession: ENH82177
  
Location: 96416-97293
  
 NCBI BlastP on this gene

ENH82177

actin
  
Accession: ENH82178
  
Location: 98105-99451
  
 NCBI BlastP on this gene

ENH82178

serine threonine-protein kinase ssn3
  
Accession: ENH82179
  
Location: 103223-104664
  
 NCBI BlastP on this gene

ENH82179

40s ribosomal protein s24
  
Accession: ENH82180
  
Location: 108826-109641
  
  
**BlastP hit with Mycgr3G70471**
  
Percentage identity: 82 %
  
BlastP bit score: 211
  
Sequence coverage: 88 %
  
E-value: 3e-67
  
  
 NCBI BlastP on this gene

ENH82180

hypothetical protein
  
Accession: ENH82181
  
Location: 110335-111453
  
  
**BlastP hit with Mycgr3G38483**
  
Percentage identity: 45 %
  
BlastP bit score: 260
  
Sequence coverage: 99 %
  
E-value: 2e-80
  
  
 NCBI BlastP on this gene

ENH82181

hypothetical protein
  
Accession: ENH82182
  
Location: 117292-117795
  
 NCBI BlastP on this gene

ENH82182

RNA-binding protein
  
Accession: ENH82183
  
Location: 119254-120811
  
 NCBI BlastP on this gene

ENH82183

hypothetical protein
  
Accession: ENH82184
  
Location: 122608-123505
  
 NCBI BlastP on this gene

ENH82184

36. :  KB730180 Fusarium oxysporum f. sp. cubense race 1 unplaced genomic scaffold scaffold243     Total score: 2.0     Cumulative Blast bit score: 470

Putative transcriptional regulatory protein C2H10.01
  
Accession: ENH70757
  
Location: 212271-213731
  
 NCBI BlastP on this gene

ENH70757

hypothetical protein
  
Accession: ENH70758
  
Location: 217277-217726
  
 NCBI BlastP on this gene

ENH70758

Peptidyl-prolyl cis-trans isomerase-like 4
  
Accession: ENH70759
  
Location: 218766-220277
  
 NCBI BlastP on this gene

ENH70759

hypothetical protein
  
Accession: ENH70760
  
Location: 222903-223349
  
 NCBI BlastP on this gene

ENH70760

Protein FAM86A
  
Accession: ENH70761
  
Location: 228202-229288
  
  
**BlastP hit with Mycgr3G38483**
  
Percentage identity: 45 %
  
BlastP bit score: 263
  
Sequence coverage: 98 %
  
E-value: 1e-81
  
  
 NCBI BlastP on this gene

ENH70761

40S ribosomal protein S24-B
  
Accession: ENH70762
  
Location: 229755-230529
  
  
**BlastP hit with Mycgr3G70471**
  
Percentage identity: 81 %
  
BlastP bit score: 207
  
Sequence coverage: 89 %
  
E-value: 4e-66
  
  
 NCBI BlastP on this gene

ENH70762

hypothetical protein
  
Accession: ENH70763
  
Location: 231002-231430
  
 NCBI BlastP on this gene

ENH70763

hypothetical protein
  
Accession: ENH70764
  
Location: 232307-236827
  
 NCBI BlastP on this gene

ENH70764

Putative GTP cyclohydrolase-2
  
Accession: ENH70765
  
Location: 237645-238870
  
 NCBI BlastP on this gene

ENH70765

hypothetical protein
  
Accession: ENH70766
  
Location: 241332-241994
  
 NCBI BlastP on this gene

ENH70766

DNA-directed RNA polymerase I subunit RPA43
  
Accession: ENH70767
  
Location: 246235-247623
  
 NCBI BlastP on this gene

ENH70767

37. :  KB726995 Fusarium oxysporum f. sp. cubense race 4 unplaced genomic scaffold scaffold85     Total score: 2.0     Cumulative Blast bit score: 470

hypothetical protein
  
Accession: EMT62595
  
Location: 858166-860133
  
 NCBI BlastP on this gene

EMT62595

hypothetical protein
  
Accession: EMT62596
  
Location: 861785-862450
  
 NCBI BlastP on this gene

EMT62596

Putative GTP cyclohydrolase-2
  
Accession: EMT62597
  
Location: 864915-866140
  
 NCBI BlastP on this gene

EMT62597

hypothetical protein
  
Accession: EMT62598
  
Location: 866954-873623
  
 NCBI BlastP on this gene

EMT62598

hypothetical protein
  
Accession: EMT62599
  
Location: 874493-874921
  
 NCBI BlastP on this gene

EMT62599

40S ribosomal protein S24-B
  
Accession: EMT62600
  
Location: 875399-876174
  
  
**BlastP hit with Mycgr3G70471**
  
Percentage identity: 81 %
  
BlastP bit score: 207
  
Sequence coverage: 89 %
  
E-value: 4e-66
  
  
 NCBI BlastP on this gene

EMT62600

Protein FAM86A
  
Accession: EMT62601
  
Location: 876641-877727
  
  
**BlastP hit with Mycgr3G38483**
  
Percentage identity: 45 %
  
BlastP bit score: 263
  
Sequence coverage: 98 %
  
E-value: 1e-81
  
  
 NCBI BlastP on this gene

EMT62601

hypothetical protein
  
Accession: EMT62602
  
Location: 882538-883032
  
 NCBI BlastP on this gene

EMT62602

Peptidyl-prolyl cis-trans isomerase-like 4
  
Accession: EMT62603
  
Location: 885659-887170
  
 NCBI BlastP on this gene

EMT62603

hypothetical protein
  
Accession: EMT62604
  
Location: 888206-891491
  
 NCBI BlastP on this gene

EMT62604

Putative transcriptional regulatory protein C2H10.01
  
Accession: EMT62605
  
Location: 892201-893661
  
 NCBI BlastP on this gene

EMT62605

38. :  AFQF01003174 Fusarium oxysporum Fo5176     Total score: 2.0     Cumulative Blast bit score: 470

hypothetical protein
  
Accession: EGU76792
  
Location: 62839-64299
  
 NCBI BlastP on this gene

EGU76792

hypothetical protein
  
Accession: EGU76793
  
Location: 68397-72095
  
 NCBI BlastP on this gene

EGU76793

hypothetical protein
  
Accession: EGU76794
  
Location: 73464-73955
  
 NCBI BlastP on this gene

EGU76794

hypothetical protein
  
Accession: EGU76795
  
Location: 78766-79852
  
  
**BlastP hit with Mycgr3G38483**
  
Percentage identity: 45 %
  
BlastP bit score: 263
  
Sequence coverage: 98 %
  
E-value: 2e-81
  
  
 NCBI BlastP on this gene

EGU76795

hypothetical protein
  
Accession: EGU76796
  
Location: 80319-81093
  
  
**BlastP hit with Mycgr3G70471**
  
Percentage identity: 81 %
  
BlastP bit score: 207
  
Sequence coverage: 89 %
  
E-value: 4e-66
  
  
 NCBI BlastP on this gene

EGU76796

hypothetical protein
  
Accession: EGU76797
  
Location: 81567-81995
  
 NCBI BlastP on this gene

EGU76797

hypothetical protein
  
Accession: EGU76798
  
Location: 82873-87418
  
 NCBI BlastP on this gene

EGU76798

hypothetical protein
  
Accession: EGU76799
  
Location: 88236-89461
  
 NCBI BlastP on this gene

EGU76799

hypothetical protein
  
Accession: EGU76800
  
Location: 91924-92586
  
 NCBI BlastP on this gene

EGU76800

hypothetical protein
  
Accession: EGU76801
  
Location: 96842-98230
  
 NCBI BlastP on this gene

EGU76801

39. :  AFNW01000285 Fusarium pseudograminearum CS3096     Total score: 2.0     Cumulative Blast bit score: 464

hypothetical protein
  
Accession: EKJ71542
  
Location: 39658-41112
  
 NCBI BlastP on this gene

EKJ71542

hypothetical protein
  
Accession: EKJ71543
  
Location: 46103-47623
  
 NCBI BlastP on this gene

EKJ71543

hypothetical protein
  
Accession: EKJ71544
  
Location: 50956-51450
  
 NCBI BlastP on this gene

EKJ71544

hypothetical protein
  
Accession: EKJ71545
  
Location: 56485-57582
  
  
**BlastP hit with Mycgr3G38483**
  
Percentage identity: 49 %
  
BlastP bit score: 258
  
Sequence coverage: 84 %
  
E-value: 2e-79
  
  
 NCBI BlastP on this gene

EKJ71545

hypothetical protein
  
Accession: EKJ71546
  
Location: 58087-58852
  
  
**BlastP hit with Mycgr3G70471**
  
Percentage identity: 81 %
  
BlastP bit score: 207
  
Sequence coverage: 89 %
  
E-value: 4e-66
  
  
 NCBI BlastP on this gene

EKJ71546

hypothetical protein
  
Accession: EKJ71547
  
Location: 59370-59786
  
 NCBI BlastP on this gene

EKJ71547

hypothetical protein
  
Accession: EKJ71548
  
Location: 60741-65321
  
 NCBI BlastP on this gene

EKJ71548

hypothetical protein
  
Accession: EKJ71549
  
Location: 66066-67302
  
 NCBI BlastP on this gene

EKJ71549

hypothetical protein
  
Accession: EKJ71550
  
Location: 69755-70417
  
 NCBI BlastP on this gene

EKJ71550

hypothetical protein
  
Accession: EKJ71551
  
Location: 75052-76407
  
 NCBI BlastP on this gene

EKJ71551

40. :  CP003003 Myceliophthora thermophila ATCC 42464 chromosome 2     Total score: 2.0     Cumulative Blast bit score: 464

hypothetical protein
  
Accession: AEO57181
  
Location: 5359812-5361314
  
 NCBI BlastP on this gene

MYCTH\_2303031

hypothetical protein
  
Accession: AEO57182
  
Location: 5362620-5363417
  
 NCBI BlastP on this gene

MYCTH\_2303033

hypothetical protein
  
Accession: AEO57183
  
Location: 5365555-5366154
  
 NCBI BlastP on this gene

MYCTH\_2303034

hypothetical protein
  
Accession: AEO57184
  
Location: 5367031-5368695
  
 NCBI BlastP on this gene

MYCTH\_2303035

hypothetical protein
  
Accession: AEO57185
  
Location: 5375072-5376650
  
 NCBI BlastP on this gene

MYCTH\_47428

hypothetical protein
  
Accession: AEO57186
  
Location: 5378323-5379448
  
  
**BlastP hit with Mycgr3G38483**
  
Percentage identity: 44 %
  
BlastP bit score: 246
  
Sequence coverage: 100 %
  
E-value: 9e-75
  
  
 NCBI BlastP on this gene

MYCTH\_47934

hypothetical protein
  
Accession: AEO57187
  
Location: 5379805-5380573
  
  
**BlastP hit with Mycgr3G70471**
  
Percentage identity: 78 %
  
BlastP bit score: 218
  
Sequence coverage: 98 %
  
E-value: 7e-70
  
  
 NCBI BlastP on this gene

MYCTH\_2303040

hypothetical protein
  
Accession: AEO57188
  
Location: 5381892-5383712
  
 NCBI BlastP on this gene

MYCTH\_2056315

hypothetical protein
  
Accession: AEO57189
  
Location: 5387021-5388317
  
 NCBI BlastP on this gene

MYCTH\_2303043

glycoside hydrolase family 6 protein
  
Accession: AEO57190
  
Location: 5389825-5391047
  
 NCBI BlastP on this gene

MYCTH\_2303045

hypothetical protein
  
Accession: AEO57191
  
Location: 5391655-5393017
  
 NCBI BlastP on this gene

MYCTH\_2303046

hypothetical protein
  
Accession: AEO57192
  
Location: 5393414-5394058
  
 NCBI BlastP on this gene

MYCTH\_2029812

hypothetical protein
  
Accession: AEO57193
  
Location: 5395613-5396953
  
 NCBI BlastP on this gene

MYCTH\_2303048

hypothetical protein
  
Accession: AEO57194
  
Location: 5397603-5398177
  
 NCBI BlastP on this gene

MYCTH\_2133773

41. :  DS572698 Verticillium dahliae VdLs.17 supercont1.4 genomic scaffold     Total score: 2.0     Cumulative Blast bit score: 462

hypothetical protein
  
Accession: EGY21070
  
Location: 783084-784269
  
 NCBI BlastP on this gene

EGY21070

DNA-dependent RNA polymerase I subunit A43
  
Accession: EGY21071
  
Location: 785052-786512
  
 NCBI BlastP on this gene

EGY21071

isoflavone reductase family protein
  
Accession: EGY21072
  
Location: 787000-788053
  
 NCBI BlastP on this gene

EGY21072

hypothetical protein
  
Accession: EGY21073
  
Location: 789353-789964
  
 NCBI BlastP on this gene

EGY21073

MFS hexose transporter
  
Accession: EGY21074
  
Location: 790955-792820
  
 NCBI BlastP on this gene

EGY21074

hypothetical protein
  
Accession: EGY21075
  
Location: 793835-794690
  
 NCBI BlastP on this gene

EGY21075

actin
  
Accession: EGY21076
  
Location: 795240-796551
  
 NCBI BlastP on this gene

EGY21076

meiotic mRNA stability protein kinase SSN3
  
Accession: EGY21077
  
Location: 798886-800268
  
 NCBI BlastP on this gene

EGY21077

40S ribosomal protein S24
  
Accession: EGY21078
  
Location: 800939-801816
  
  
**BlastP hit with Mycgr3G70471**
  
Percentage identity: 81 %
  
BlastP bit score: 209
  
Sequence coverage: 89 %
  
E-value: 1e-66
  
  
 NCBI BlastP on this gene

EGY21078

FAM86A protein
  
Accession: EGY21079
  
Location: 802369-803460
  
  
**BlastP hit with Mycgr3G38483**
  
Percentage identity: 42 %
  
BlastP bit score: 254
  
Sequence coverage: 98 %
  
E-value: 8e-78
  
  
 NCBI BlastP on this gene

EGY21079

hypothetical protein
  
Accession: EGY21080
  
Location: 805312-806636
  
 NCBI BlastP on this gene

EGY21080

hypothetical protein
  
Accession: EGY21081
  
Location: 808667-809236
  
 NCBI BlastP on this gene

EGY21081

peptidyl-prolyl cis-trans isomerase cyp6
  
Accession: EGY21082
  
Location: 809870-811459
  
 NCBI BlastP on this gene

EGY21082

hypothetical protein
  
Accession: EGY21083
  
Location: 817126-818625
  
 NCBI BlastP on this gene

EGY21083

42. :  GG698904 Nectria haematococca mpVI 77-13-4 chromosome 8 genomic scaffold NECHAsca\_11\_chr8\_3\_0     Total score: 2.0     Cumulative Blast bit score: 462

hypothetical protein
  
Accession: EEU42868
  
Location: 805055-806428
  
 NCBI BlastP on this gene

EEU42868

predicted protein
  
Accession: EEU42680
  
Location: 810976-811641
  
 NCBI BlastP on this gene

EEU42680

hypothetical protein
  
Accession: EEU42681
  
Location: 814195-815414
  
 NCBI BlastP on this gene

EEU42681

hypothetical protein
  
Accession: EEU42682
  
Location: 816258-820855
  
 NCBI BlastP on this gene

EEU42682

predicted protein
  
Accession: EEU42683
  
Location: 821775-822179
  
 NCBI BlastP on this gene

EEU42683

predicted protein
  
Accession: EEU42869
  
Location: 822635-823338
  
  
**BlastP hit with Mycgr3G70471**
  
Percentage identity: 81 %
  
BlastP bit score: 207
  
Sequence coverage: 89 %
  
E-value: 8e-66
  
  
 NCBI BlastP on this gene

EEU42869

hypothetical protein
  
Accession: EEU42684
  
Location: 823840-824937
  
  
**BlastP hit with Mycgr3G38483**
  
Percentage identity: 42 %
  
BlastP bit score: 255
  
Sequence coverage: 100 %
  
E-value: 3e-78
  
  
 NCBI BlastP on this gene

EEU42684

hypothetical protein
  
Accession: EEU42685
  
Location: 829837-830331
  
 NCBI BlastP on this gene

EEU42685

hypothetical protein
  
Accession: EEU42686
  
Location: 833239-834746
  
 NCBI BlastP on this gene

EEU42686

hypothetical protein
  
Accession: EEU42687
  
Location: 839970-841415
  
 NCBI BlastP on this gene

EEU42687

43. :  CABT02000038 Sordaria macrospora k-hell     Total score: 2.0     Cumulative Blast bit score: 462

not annotated
  
Accession: CCC13416
  
Location: 170112-171711
  
 NCBI BlastP on this gene

CCC13416

not annotated
  
Accession: CCC13415
  
Location: 159343-160871
  
 NCBI BlastP on this gene

CCC13415

not annotated
  
Accession: CCC13414
  
Location: 153078-154329
  
  
**BlastP hit with Mycgr3G38483**
  
Percentage identity: 44 %
  
BlastP bit score: 241
  
Sequence coverage: 102 %
  
E-value: 9e-73
  
  
 NCBI BlastP on this gene

CCC13414

not annotated
  
Accession: CCC13413
  
Location: 151354-152414
  
  
**BlastP hit with Mycgr3G70471**
  
Percentage identity: 80 %
  
BlastP bit score: 221
  
Sequence coverage: 98 %
  
E-value: 4e-71
  
  
 NCBI BlastP on this gene

CCC13413

not annotated
  
Accession: CCC13412
  
Location: 149566-150888
  
 NCBI BlastP on this gene

CCC13412

not annotated
  
Accession: CCC13411
  
Location: 147403-148710
  
 NCBI BlastP on this gene

CCC13411

not annotated
  
Accession: CCC13410
  
Location: 142589-144193
  
 NCBI BlastP on this gene

CCC13410

not annotated
  
Accession: CCC13409
  
Location: 139423-140732
  
 NCBI BlastP on this gene

CCC13409

not annotated
  
Accession: CCC13408
  
Location: 135554-136964
  
 NCBI BlastP on this gene

CCC13408

not annotated
  
Accession: CCC13407
  
Location: 133206-134537
  
 NCBI BlastP on this gene

CCC13407

44. :  JH921437 Marssonina brunnea f. sp. 'multigermtubi' MB\_m1 unplaced genomic scaffold M6\_S00010     Total score: 2.0     Cumulative Blast bit score: 461

hypothetical protein
  
Accession: EKD17078
  
Location: 898355-902560
  
 NCBI BlastP on this gene

EKD17078

hypothetical protein
  
Accession: EKD17079
  
Location: 902942-903892
  
 NCBI BlastP on this gene

EKD17079

sugar transporter
  
Accession: EKD17080
  
Location: 905914-907905
  
 NCBI BlastP on this gene

EKD17080

arrestin
  
Accession: EKD17081
  
Location: 908753-910296
  
 NCBI BlastP on this gene

EKD17081

beta-glucosidase D
  
Accession: EKD17082
  
Location: 910701-913983
  
 NCBI BlastP on this gene

EKD17082

40S ribosomal protein S24
  
Accession: EKD17083
  
Location: 915618-916301
  
  
**BlastP hit with Mycgr3G70471**
  
Percentage identity: 76 %
  
BlastP bit score: 211
  
Sequence coverage: 100 %
  
E-value: 2e-67
  
  
 NCBI BlastP on this gene

EKD17083

putative Protein FAM86A
  
Accession: EKD17084
  
Location: 916620-917718
  
  
**BlastP hit with Mycgr3G38483**
  
Percentage identity: 41 %
  
BlastP bit score: 250
  
Sequence coverage: 98 %
  
E-value: 1e-76
  
  
 NCBI BlastP on this gene

EKD17084

hypothetical protein
  
Accession: EKD17085
  
Location: 918073-918835
  
 NCBI BlastP on this gene

EKD17085

hypothetical protein
  
Accession: EKD17086
  
Location: 920014-925753
  
 NCBI BlastP on this gene

EKD17086

hypothetical protein
  
Accession: EKD17087
  
Location: 926587-927341
  
 NCBI BlastP on this gene

EKD17087

meiotic recombination protein DMC1
  
Accession: EKD17088
  
Location: 931031-931951
  
 NCBI BlastP on this gene

EKD17088

meiotic recombination protein dmc1
  
Accession: EKD17089
  
Location: 932255-933825
  
 NCBI BlastP on this gene

EKD17089

45. :  DS985215 Verticillium albo-atrum VaMs.102 supercont1.2 genomic scaffold     Total score: 2.0     Cumulative Blast bit score: 460

DNA-dependent RNA polymerase I subunit A43
  
Accession: EEY16019
  
Location: 2170981-2172217
  
 NCBI BlastP on this gene

EEY16019

isoflavone reductase family protein
  
Accession: EEY16020
  
Location: 2172914-2173972
  
 NCBI BlastP on this gene

EEY16020

conserved hypothetical protein
  
Accession: EEY16021
  
Location: 2175276-2175884
  
 NCBI BlastP on this gene

EEY16021

sugar transport protein
  
Accession: EEY16022
  
Location: 2177249-2178697
  
 NCBI BlastP on this gene

EEY16022

conserved hypothetical protein
  
Accession: EEY16023
  
Location: 2180195-2181050
  
 NCBI BlastP on this gene

EEY16023

actin
  
Accession: EEY16024
  
Location: 2181688-2182992
  
 NCBI BlastP on this gene

EEY16024

meiotic mRNA stability protein kinase SSN3
  
Accession: EEY16025
  
Location: 2185288-2186670
  
 NCBI BlastP on this gene

EEY16025

40S ribosomal protein S24
  
Accession: EEY16026
  
Location: 2187340-2188215
  
  
**BlastP hit with Mycgr3G70471**
  
Percentage identity: 81 %
  
BlastP bit score: 209
  
Sequence coverage: 89 %
  
E-value: 1e-66
  
  
 NCBI BlastP on this gene

EEY16026

FAM86A
  
Accession: EEY16027
  
Location: 2188794-2189885
  
  
**BlastP hit with Mycgr3G38483**
  
Percentage identity: 42 %
  
BlastP bit score: 251
  
Sequence coverage: 98 %
  
E-value: 9e-77
  
  
 NCBI BlastP on this gene

EEY16027

hypothetical protein
  
Accession: EEY16028
  
Location: 2194957-2195526
  
 NCBI BlastP on this gene

EEY16028

peptidyl-prolyl cis-trans isomerase cyp6
  
Accession: EEY16029
  
Location: 2196162-2197715
  
 NCBI BlastP on this gene

EEY16029

conserved hypothetical protein
  
Accession: EEY16030
  
Location: 2203279-2204455
  
 NCBI BlastP on this gene

EEY16030

46. :  CACQ02001341 Colletotrichum higginsianum strain IMI 349063     Total score: 2.0     Cumulative Blast bit score: 459

ribosomal protein S24e
  
Accession: CCF34943
  
Location: 1104-1935
  
  
**BlastP hit with Mycgr3G70471**
  
Percentage identity: 83 %
  
BlastP bit score: 213
  
Sequence coverage: 89 %
  
E-value: 5e-68
  
  
 NCBI BlastP on this gene

CCF34943

hypothetical protein
  
Accession: CCF34944
  
Location: 2606-3817
  
  
**BlastP hit with Mycgr3G38483**
  
Percentage identity: 48 %
  
BlastP bit score: 246
  
Sequence coverage: 83 %
  
E-value: 6e-75
  
  
 NCBI BlastP on this gene

CCF34944

hypothetical protein
  
Accession: CCF34945
  
Location: 6200-6772
  
 NCBI BlastP on this gene

CCF34945

47. :  GL891302 Neurospora tetrasperma FGSC 2508 unplaced genomic scaffold NEUTE1scaffold\_1     Total score: 2.0     Cumulative Blast bit score: 453

hypothetical protein
  
Accession: EGO60062
  
Location: 95879-97194
  
 NCBI BlastP on this gene

EGO60062

hypothetical protein
  
Accession: EGO60063
  
Location: 101185-101370
  
 NCBI BlastP on this gene

EGO60063

hypothetical protein
  
Accession: EGO60064
  
Location: 106035-107801
  
 NCBI BlastP on this gene

EGO60064

hypothetical protein
  
Accession: EGO60065
  
Location: 111785-113049
  
  
**BlastP hit with Mycgr3G38483**
  
Percentage identity: 42 %
  
BlastP bit score: 232
  
Sequence coverage: 105 %
  
E-value: 3e-69
  
  
 NCBI BlastP on this gene

EGO60065

hypothetical protein
  
Accession: EGO60066
  
Location: 113653-114706
  
  
**BlastP hit with Mycgr3G70471**
  
Percentage identity: 80 %
  
BlastP bit score: 221
  
Sequence coverage: 98 %
  
E-value: 4e-71
  
  
 NCBI BlastP on this gene

EGO60066

hypothetical protein
  
Accession: EGO60067
  
Location: 115000-115964
  
 NCBI BlastP on this gene

EGO60067

hypothetical protein
  
Accession: EGO60068
  
Location: 116836-117500
  
 NCBI BlastP on this gene

EGO60068

hypothetical protein
  
Accession: EGO60069
  
Location: 117816-119126
  
 NCBI BlastP on this gene

EGO60069

hypothetical protein
  
Accession: EGO60070
  
Location: 120181-121425
  
 NCBI BlastP on this gene

EGO60070

hypothetical protein
  
Accession: EGO60071
  
Location: 122519-127395
  
 NCBI BlastP on this gene

EGO60071

hypothetical protein
  
Accession: EGO60072
  
Location: 128505-129817
  
 NCBI BlastP on this gene

EGO60072

hypothetical protein
  
Accession: EGO60073
  
Location: 131265-131745
  
 NCBI BlastP on this gene

EGO60073

48. :  GL891107 Neurospora tetrasperma FGSC 2509 unplaced genomic scaffold NEUTE2scaffold\_2     Total score: 2.0     Cumulative Blast bit score: 453

cyclophilin-like protein
  
Accession: EGZ75988
  
Location: 6091595-6092910
  
 NCBI BlastP on this gene

EGZ75988

hypothetical protein
  
Accession: EGZ75987
  
Location: 6089578-6091056
  
 NCBI BlastP on this gene

EGZ75987

hypothetical protein
  
Accession: EGZ75986
  
Location: 6087422-6087607
  
 NCBI BlastP on this gene

EGZ75986

hypothetical protein
  
Accession: EGZ75985
  
Location: 6080991-6082757
  
 NCBI BlastP on this gene

EGZ75985

hypothetical protein
  
Accession: EGZ75984
  
Location: 6075743-6077007
  
  
**BlastP hit with Mycgr3G38483**
  
Percentage identity: 42 %
  
BlastP bit score: 232
  
Sequence coverage: 105 %
  
E-value: 3e-69
  
  
 NCBI BlastP on this gene

EGZ75984

putative 40S ribosomal protein S24
  
Accession: EGZ75983
  
Location: 6074087-6075139
  
  
**BlastP hit with Mycgr3G70471**
  
Percentage identity: 80 %
  
BlastP bit score: 221
  
Sequence coverage: 98 %
  
E-value: 4e-71
  
  
 NCBI BlastP on this gene

EGZ75983

hypothetical protein
  
Accession: EGZ75982
  
Location: 6072829-6073793
  
 NCBI BlastP on this gene

EGZ75982

hypothetical protein
  
Accession: EGZ75981
  
Location: 6071293-6071957
  
 NCBI BlastP on this gene

EGZ75981

hypothetical protein
  
Accession: EGZ75980
  
Location: 6069667-6070977
  
 NCBI BlastP on this gene

EGZ75980

hypothetical protein
  
Accession: EGZ75979
  
Location: 6067569-6068375
  
 NCBI BlastP on this gene

EGZ75979

hypothetical protein
  
Accession: EGZ75978
  
Location: 6061398-6066274
  
 NCBI BlastP on this gene

EGZ75978

putative GTP cyclohydrolase II
  
Accession: EGZ75977
  
Location: 6058976-6060288
  
 NCBI BlastP on this gene

EGZ75977

hypothetical protein
  
Accession: EGZ75976
  
Location: 6057048-6057528
  
 NCBI BlastP on this gene

EGZ75976

49. :  BX294027 Neurospora crassa DNA linkage group V BAC contig B8G12.     Total score: 2.0     Cumulative Blast bit score: 451

conserved hypothetical protein
  
Accession: CAD71104
  
Location: 136139-137682
  
 NCBI BlastP on this gene

B8G12.450

hypothetical protein
  
Accession: CAD71103
  
Location: 134351-135769
  
 NCBI BlastP on this gene

B8G12.440

conserved hypothetical protein
  
Accession: CAD71102
  
Location: 125734-127499
  
 NCBI BlastP on this gene

B8G12.430

conserved hypothetical protein
  
Accession: CAD71101
  
Location: 120250-121514
  
  
**BlastP hit with Mycgr3G38483**
  
Percentage identity: 43 %
  
BlastP bit score: 231
  
Sequence coverage: 105 %
  
E-value: 1e-68
  
  
 NCBI BlastP on this gene

B8G12.410

probable 40S RIBOSOMAL PROTEIN S24
  
Accession: CAD71100
  
Location: 118582-119638
  
  
**BlastP hit with Mycgr3G70471**
  
Percentage identity: 80 %
  
BlastP bit score: 221
  
Sequence coverage: 98 %
  
E-value: 4e-71
  
  
 NCBI BlastP on this gene

B8G12.400

hypothetical protein
  
Accession: CAD71099
  
Location: 117334-118238
  
 NCBI BlastP on this gene

B8G12.390

hypothetical protein
  
Accession: CAD71098
  
Location: 116117-116805
  
 NCBI BlastP on this gene

B8G12.380

hypothetical protein
  
Accession: CAD71097
  
Location: 114426-115762
  
 NCBI BlastP on this gene

B8G12.370

hypothetical protein
  
Accession: CAD71096
  
Location: 112141-113397
  
 NCBI BlastP on this gene

B8G12.360

hypothetical protein
  
Accession: CAD71095
  
Location: 105921-110788
  
 NCBI BlastP on this gene

B8G12.350

probable GTP cyclohydrolase II
  
Accession: CAD71094
  
Location: 103463-104787
  
 NCBI BlastP on this gene

B8G12.340

hypothetical protein
  
Accession: CAD71093
  
Location: 101557-102037
  
 NCBI BlastP on this gene

B8G12.330

50. :  FQ790270 Botryotinia fuckeliana T4 SuperContig\_51\_1 genomic supercontig.     Total score: 2.0     Cumulative Blast bit score: 451

similar to MFS transporter
  
Accession: CCD44202
  
Location: 174404-175972
  
 NCBI BlastP on this gene

BofuT4\_P057820.1

hypothetical protein
  
Accession: CCD44203
  
Location: 176676-176954
  
 NCBI BlastP on this gene

BofuT4\_uP057830.1

hypothetical protein
  
Accession: CCD44204
  
Location: 178012-180335
  
 NCBI BlastP on this gene

BofuT4\_P057840.1

hypothetical protein
  
Accession: CCD44205
  
Location: 180950-181371
  
 NCBI BlastP on this gene

BofuT4\_uP057850.1

similar to reduced viability upon starvation protein
  
Accession: CCD44206
  
Location: 182411-183849
  
 NCBI BlastP on this gene

BofuT4\_P057860.1

similar to cysteine desulfurase
  
Accession: CCD44207
  
Location: 184833-186617
  
 NCBI BlastP on this gene

BofuT4\_P057870.1

similar to beta-catenin-like protein 1
  
Accession: CCD44208
  
Location: 188300-190177
  
 NCBI BlastP on this gene

BofuT4\_P057880.1

hypothetical protein
  
Accession: CCD44209
  
Location: 190700-191541
  
 NCBI BlastP on this gene

BofuT4\_P057890.1

hypothetical protein
  
Accession: CCD44210
  
Location: 192543-193822
  
  
**BlastP hit with Mycgr3G38483**
  
Percentage identity: 42 %
  
BlastP bit score: 243
  
Sequence coverage: 92 %
  
E-value: 2e-73
  
  
 NCBI BlastP on this gene

BofuT4\_P057900.1

similar to 40S ribosomal protein S24
  
Accession: CCD44211
  
Location: 194252-194897
  
  
**BlastP hit with Mycgr3G70471**
  
Percentage identity: 81 %
  
BlastP bit score: 208
  
Sequence coverage: 89 %
  
E-value: 3e-66
  
  
 NCBI BlastP on this gene

BofuT4\_P057910.1

similar to transcription factor Cys6
  
Accession: CCD44212
  
Location: 198219-199833
  
 NCBI BlastP on this gene

BofuT4\_P057920.1

hypothetical protein
  
Accession: CCD44213
  
Location: 200643-201038
  
 NCBI BlastP on this gene

BofuT4\_P057930.1

predicted protein
  
Accession: CCD44214
  
Location: 201138-201296
  
 NCBI BlastP on this gene

BofuT4\_uP057940.1

predicted protein
  
Accession: CCD44215
  
Location: 201962-202291
  
 NCBI BlastP on this gene

BofuT4\_P057950.1

hypothetical protein
  
Accession: CCD44216
  
Location: 202830-204345
  
 NCBI BlastP on this gene

BofuT4\_P057960.1

hypothetical protein
  
Accession: CCD44217
  
Location: 205490-207018
  
 NCBI BlastP on this gene

BofuT4\_P057970.1

similar to sugar transporter
  
Accession: CCD44218
  
Location: 207834-209836
  
 NCBI BlastP on this gene

BofuT4\_P057980.1

Detecting sequence homology at the gene cluster level with MultiGeneBlast.
  
Marnix H. Medema, Rainer Breitling & Eriko Takano (2013)
  
*Molecular Biology and Evolution* , 30: 1218-1223.
