# Supplementary material for: In silico prediction and characterization of secondary metabolite biosynthetic gene clusters in the wheat pathogen Zymoseptoria tritici
Source: BMC Genomics. 2017 Aug 17;18:631. doi: 10.1186/s12864-017-3969-y (PMC5561558; doi:10.1186/s12864-017-3969-y)
Supplement: Supplementary file 1 — MultiGeneBLAST analysis of putative secondary metabolite clusters. All encoded amino acid sequences from genes residing in clusters predicted by AntiSMASH are given as FASTA file format. All output data from MultiGeneBLASTs are also provided. (ZIP 42911 kb) [file 12864_2017_3969_MOESM1_ESM.zip › Cluster MultiGene BLAST/out/Clusters_1_34/Cluster_13/displaypage2.xhtml]

xml version="1.0" encoding="UTF-8"?


Search Results
  
  
 Results pages: 1, 2, 3, 4, 5

**MultiGeneBlast hits**

Select gene cluster alignment
51. DS499601\_0 Aspergillus fumigatus A1163 scf\_000008 genomic scaffold, whole...
52. AAHF01000015\_0 Aspergillus fumigatus Af293, whole genome shotgun sequenci...
53. DS027692\_0 Neosartorya fischeri NRRL 181 1099437636259 genomic scaffold, ...
54. AM920436\_0 Penicillium chrysogenum Wisconsin 54-1255 complete genome, con...
55. CP003009\_1 Thielavia terrestris NRRL 8126 chromosome 1, complete sequence.
56. CAGA01000037\_1 Claviceps purpurea 20.1, whole genome shotgun sequencing p...
57. DS027053\_0 Aspergillus clavatus NRRL 1 1099423829799 genomic scaffold, wh...
58. CU633447\_0 Podospora anserina S mat+ genomic DNA chromosome 2, superconti...
59. GL698484\_0 Metarhizium acridum CQMa 102 unplaced genomic scaffold Scf\_015...
60. DS572753\_0 Paracoccidioides brasiliensis Pb18 supercont1.4 genomic scaffo...
61. KB644412\_1 Penicillium oxalicum 114-2 unplaced genomic scaffold scaffold\_...
62. DS544804\_0 Paracoccidioides brasiliensis Pb03 supercont1.2 genomic scaffo...
63. AKCU01000381\_0 Penicillium digitatum Pd1, whole genome shotgun sequencing...
64. AKCT01000084\_0 Penicillium digitatum PHI26, whole genome shotgun sequenci...
65. KB705480\_0 Eutypa lata UCREL1 unplaced genomic scaffold EL1\_03\_scaffold\_1...
66. KE145356\_0 Glarea lozoyensis ATCC 20868 chromosome Unknown GLAREA13, whol...
67. JH725156\_0 Beauveria bassiana ARSEF 2860 unplaced genomic scaffold BBA\_S0...
68. DS572830\_0 Paracoccidioides brasiliensis Pb01 supercont1.20 genomic scaff...
69. GL573339\_0 Geomyces destructans 20631-21 unplaced genomic scaffold superc...
70. JH795346\_0 Magnaporthe oryzae P131 unplaced genomic scaffold P131\_scaffol...
71. JH793790\_0 Magnaporthe oryzae Y34 unplaced genomic scaffold Y34\_scaffold0...
72. CM001234\_0 Magnaporthe oryzae 70-15 chromosome 4, whole genome shotgun se...
73. GL985082\_0 Trichoderma reesei QM6a unplaced genomic scaffold TRIREscaffol...
74. KE148175\_0 Ophiostoma piceae UAMH 11346 chromosome Unknown scf30, whole g...
75. GG749437\_0 Ajellomyces dermatitidis ATCC 18188 genomic scaffold supercont...
76. EQ999974\_0 Ajellomyces dermatitidis ER-3 genomic scaffold supercont1.2, w...
77. GG657453\_0 Ajellomyces dermatitidis SLH14081 genomic scaffold supercont1....
78. DS990639\_0 Ajellomyces capsulatus H88 supercont1.4 genomic scaffold, whol...
79. DS989826\_0 Arthroderma gypseum CBS 118893 supercont1.5 genomic scaffold, ...
80. GG704913\_0 Coccidioides immitis RS genomic scaffold supercont3.3, whole g...
81. GG663363\_0 Ajellomyces capsulatus G186AR genomic scaffold supercont2.1, w...
82. ABDF02000003\_1 Trichoderma virens Gv29-8, whole genome shotgun sequencing...
83. ABDG02000029\_0 Trichoderma atroviride IMI 206040, whole genome shotgun se...
84. GL636509\_0 Coccidioides posadasii str. Silveira unplaced genomic scaffold...
85. AABX02000037\_0 Neurospora crassa OR74A, whole genome shotgun sequencing p...
86. JH126408\_0 Cordyceps militaris CM01 unplaced genomic scaffold CCM\_S00010,...
87. CH476621\_0 Sclerotinia sclerotiorum 1980 scaffold\_1 genomic scaffold, who...
88. GG698544\_0 Trichophyton tonsurans CBS 112818 genomic scaffold supercont1....
89. CH476615\_1 Uncinocarpus reesii 1704 scaffold\_1 genomic scaffold, whole ge...
90. DS995906\_0 Penicillium marneffei ATCC 18224 scf\_1105668340770 genomic sca...
91. DS995705\_0 Microsporum canis CBS 113480 supercont1.5 genomic scaffold, wh...
92. AM270259\_0 Aspergillus niger contig An12c0020, genomic contig.
93. JH226133\_2 Exophiala dermatitidis NIH/UT8656 unplaced genomic scaffold su...
94. ACJE01000005\_0 Aspergillus niger ATCC 1015, whole genome shotgun sequenci...
95. GL698729\_0 Metarhizium anisopliae ARSEF 23 unplaced genomic scaffold Scf\_...
96. GG700654\_0 Trichophyton rubrum CBS 118892 genomic scaffold supercont2.7, ...
97. EQ963479\_0 Aspergillus flavus NRRL3357 scf\_1106286418500 genomic scaffold...
98. DF126478\_0 Aspergillus kawachii IFO 4308 DNA, contig: scaffold00032, whol...
99. AP007169\_0 Aspergillus oryzae RIB40 DNA, SC038.
100. ACYE01000161\_0 Trichophyton verrucosum HKI 0517, whole genome shotgun se...

Query: Architecture Search FASTA input

DS499601 : Aspergillus fumigatus A1163 scf\_000008 genomic scaffold    Total score: 2.0     Cumulative Blast bit score: 444

Hit cluster cross-links:

Mycgr3G70471
  
Location: 0-405

Mycgr3G70471

Mycgr3G39149
  
Location: 505-1798

Mycgr3G39149

Mycgr3G92130
  
Location: 1898-2396

Mycgr3G92130

Mycgr3G38483
  
Location: 2496-3576

Mycgr3G38483

Mycgr3G108869
  
Location: 3676-5056

Mycgr3G108869

Mycgr3G103943
  
Location: 5156-5762

Mycgr3G103943

Mycgr3G57362
  
Location: 5862-7296

Mycgr3G57362

Mycgr3G39086
  
Location: 7396-8368

Mycgr3G39086

Mycgr3G103942
  
Location: 8468-8714

Mycgr3G103942

Mycgr3G108865
  
Location: 8814-10239

Mycgr3G108865

Mycgr3G70475
  
Location: 10339-11821

Mycgr3G70475

Mycgr3G108866
  
Location: 11921-13010

Mycgr3G108866

Mycgr3G92136
  
Location: 13110-13593

Mycgr3G92136

Ran-binding protein (RanBPM), putative
  
Accession: EDP48157
  
Location: 564946-567299
  
 NCBI BlastP on this gene

EDP48157

conserved hypothetical protein
  
Accession: EDP48158
  
Location: 568968-571196
  
 NCBI BlastP on this gene

EDP48158

conserved hypothetical protein
  
Accession: EDP48159
  
Location: 571683-573136
  
  
**BlastP hit with Mycgr3G38483**
  
Percentage identity: 35 %
  
BlastP bit score: 217
  
Sequence coverage: 100 %
  
E-value: 3e-63
  
  
 NCBI BlastP on this gene

EDP48159

37S ribosomal protein S24
  
Accession: EDP48160
  
Location: 573451-574295
  
  
**BlastP hit with Mycgr3G70471**
  
Percentage identity: 82 %
  
BlastP bit score: 227
  
Sequence coverage: 97 %
  
E-value: 1e-73
  
  
 NCBI BlastP on this gene

EDP48160

streptococcal hemagglutinin protein, putative
  
Accession: EDP48161
  
Location: 575479-576612
  
 NCBI BlastP on this gene

EDP48161

RNA binding protein, putative
  
Accession: EDP48162
  
Location: 580308-581919
  
 NCBI BlastP on this gene

EDP48162

Query: Architecture Search FASTA input

AAHF01000015 : Aspergillus fumigatus Af293    Total score: 2.0     Cumulative Blast bit score: 444

Hit cluster cross-links:

Mycgr3G70471
  
Location: 0-405

Mycgr3G70471

Mycgr3G39149
  
Location: 505-1798

Mycgr3G39149

Mycgr3G92130
  
Location: 1898-2396

Mycgr3G92130

Mycgr3G38483
  
Location: 2496-3576

Mycgr3G38483

Mycgr3G108869
  
Location: 3676-5056

Mycgr3G108869

Mycgr3G103943
  
Location: 5156-5762

Mycgr3G103943

Mycgr3G57362
  
Location: 5862-7296

Mycgr3G57362

Mycgr3G39086
  
Location: 7396-8368

Mycgr3G39086

Mycgr3G103942
  
Location: 8468-8714

Mycgr3G103942

Mycgr3G108865
  
Location: 8814-10239

Mycgr3G108865

Mycgr3G70475
  
Location: 10339-11821

Mycgr3G70475

Mycgr3G108866
  
Location: 11921-13010

Mycgr3G108866

Mycgr3G92136
  
Location: 13110-13593

Mycgr3G92136

cyclophilin-type peptidyl-prolyl cis-trans isomerase, putative
  
Accession: EAL84677
  
Location: 146935-148546
  
 NCBI BlastP on this gene

EAL84677

streptococcal hemagglutinin protein, putative
  
Accession: EAL84679
  
Location: 154058-155191
  
 NCBI BlastP on this gene

EAL84679

37S ribosomal protein S24
  
Accession: EAL84680
  
Location: 156377-157221
  
  
**BlastP hit with Mycgr3G70471**
  
Percentage identity: 82 %
  
BlastP bit score: 227
  
Sequence coverage: 97 %
  
E-value: 1e-73
  
  
 NCBI BlastP on this gene

EAL84680

conserved hypothetical protein
  
Accession: EAL84681
  
Location: 157536-158989
  
  
**BlastP hit with Mycgr3G38483**
  
Percentage identity: 35 %
  
BlastP bit score: 217
  
Sequence coverage: 100 %
  
E-value: 3e-63
  
  
 NCBI BlastP on this gene

EAL84681

conserved hypothetical protein
  
Accession: EAL84682
  
Location: 159476-161701
  
 NCBI BlastP on this gene

EAL84682

Ran-binding protein (RanBP10), putative
  
Accession: EAL84683
  
Location: 163370-165723
  
 NCBI BlastP on this gene

EAL84683

Query: Architecture Search FASTA input

DS027692 : Neosartorya fischeri NRRL 181 1099437636259 genomic scaffold    Total score: 2.0     Cumulative Blast bit score: 443

Hit cluster cross-links:

Mycgr3G70471
  
Location: 0-405

Mycgr3G70471

Mycgr3G39149
  
Location: 505-1798

Mycgr3G39149

Mycgr3G92130
  
Location: 1898-2396

Mycgr3G92130

Mycgr3G38483
  
Location: 2496-3576

Mycgr3G38483

Mycgr3G108869
  
Location: 3676-5056

Mycgr3G108869

Mycgr3G103943
  
Location: 5156-5762

Mycgr3G103943

Mycgr3G57362
  
Location: 5862-7296

Mycgr3G57362

Mycgr3G39086
  
Location: 7396-8368

Mycgr3G39086

Mycgr3G103942
  
Location: 8468-8714

Mycgr3G103942

Mycgr3G108865
  
Location: 8814-10239

Mycgr3G108865

Mycgr3G70475
  
Location: 10339-11821

Mycgr3G70475

Mycgr3G108866
  
Location: 11921-13010

Mycgr3G108866

Mycgr3G92136
  
Location: 13110-13593

Mycgr3G92136

Ran-binding protein (RanBPM), putative
  
Accession: EAW20951
  
Location: 817712-820064
  
 NCBI BlastP on this gene

EAW20951

conserved hypothetical protein
  
Accession: EAW20952
  
Location: 821619-823844
  
 NCBI BlastP on this gene

EAW20952

conserved hypothetical protein
  
Accession: EAW20953
  
Location: 824322-825775
  
  
**BlastP hit with Mycgr3G38483**
  
Percentage identity: 36 %
  
BlastP bit score: 216
  
Sequence coverage: 100 %
  
E-value: 4e-63
  
  
 NCBI BlastP on this gene

EAW20953

37S ribosomal protein S24
  
Accession: EAW20954
  
Location: 826091-826927
  
  
**BlastP hit with Mycgr3G70471**
  
Percentage identity: 83 %
  
BlastP bit score: 227
  
Sequence coverage: 97 %
  
E-value: 8e-74
  
  
 NCBI BlastP on this gene

EAW20954

streptococcal hemagglutinin protein, putative
  
Accession: EAW20955
  
Location: 828164-829258
  
 NCBI BlastP on this gene

EAW20955

RNA binding protein, putative
  
Accession: EAW20956
  
Location: 832963-834582
  
 NCBI BlastP on this gene

EAW20956

Query: Architecture Search FASTA input

AM920436 : Penicillium chrysogenum Wisconsin 54-1255 complete genome, contig Pc00c21.    Total score: 2.0     Cumulative Blast bit score: 442

Hit cluster cross-links:

Mycgr3G70471
  
Location: 0-405

Mycgr3G70471

Mycgr3G39149
  
Location: 505-1798

Mycgr3G39149

Mycgr3G92130
  
Location: 1898-2396

Mycgr3G92130

Mycgr3G38483
  
Location: 2496-3576

Mycgr3G38483

Mycgr3G108869
  
Location: 3676-5056

Mycgr3G108869

Mycgr3G103943
  
Location: 5156-5762

Mycgr3G103943

Mycgr3G57362
  
Location: 5862-7296

Mycgr3G57362

Mycgr3G39086
  
Location: 7396-8368

Mycgr3G39086

Mycgr3G103942
  
Location: 8468-8714

Mycgr3G103942

Mycgr3G108865
  
Location: 8814-10239

Mycgr3G108865

Mycgr3G70475
  
Location: 10339-11821

Mycgr3G70475

Mycgr3G108866
  
Location: 11921-13010

Mycgr3G108866

Mycgr3G92136
  
Location: 13110-13593

Mycgr3G92136

not annotated
  
Accession: CAP96093
  
Location: 2840978-2842495
  
 NCBI BlastP on this gene

Pc21g11960

hypothetical protein
  
Accession: CAP96094
  
Location: 2842794-2843588
  
 NCBI BlastP on this gene

Pc21g11970

not annotated
  
Accession: CAP96095
  
Location: 2845075-2846573
  
 NCBI BlastP on this gene

Pc21g11980

hypothetical protein
  
Accession: CAP96096
  
Location: 2847105-2847745
  
 NCBI BlastP on this gene

Pc21g11990

hypothetical protein
  
Accession: CAP96097
  
Location: 2848539-2849367
  
 NCBI BlastP on this gene

Pc21g12000

not annotated
  
Accession: CAP96098
  
Location: 2849757-2850458
  
  
**BlastP hit with Mycgr3G70471**
  
Percentage identity: 80 %
  
BlastP bit score: 221
  
Sequence coverage: 97 %
  
E-value: 3e-71
  
  
 NCBI BlastP on this gene

Pc21g12010

not annotated
  
Accession: CAP96099
  
Location: 2850733-2852061
  
  
**BlastP hit with Mycgr3G38483**
  
Percentage identity: 38 %
  
BlastP bit score: 221
  
Sequence coverage: 99 %
  
E-value: 3e-65
  
  
 NCBI BlastP on this gene

Pc21g12020

not annotated
  
Accession: CAP96100
  
Location: 2852522-2854711
  
 NCBI BlastP on this gene

Pc21g12030

not annotated
  
Accession: CAP96101
  
Location: 2855767-2858093
  
 NCBI BlastP on this gene

Pc21g12040

not annotated
  
Accession: CAP96102
  
Location: 2859150-2860021
  
 NCBI BlastP on this gene

Pc21g12050

Query: Architecture Search FASTA input

CP003009 : Thielavia terrestris NRRL 8126 chromosome 1    Total score: 2.0     Cumulative Blast bit score: 441

Hit cluster cross-links:

Mycgr3G70471
  
Location: 0-405

Mycgr3G70471

Mycgr3G39149
  
Location: 505-1798

Mycgr3G39149

Mycgr3G92130
  
Location: 1898-2396

Mycgr3G92130

Mycgr3G38483
  
Location: 2496-3576

Mycgr3G38483

Mycgr3G108869
  
Location: 3676-5056

Mycgr3G108869

Mycgr3G103943
  
Location: 5156-5762

Mycgr3G103943

Mycgr3G57362
  
Location: 5862-7296

Mycgr3G57362

Mycgr3G39086
  
Location: 7396-8368

Mycgr3G39086

Mycgr3G103942
  
Location: 8468-8714

Mycgr3G103942

Mycgr3G108865
  
Location: 8814-10239

Mycgr3G108865

Mycgr3G70475
  
Location: 10339-11821

Mycgr3G70475

Mycgr3G108866
  
Location: 11921-13010

Mycgr3G108866

Mycgr3G92136
  
Location: 13110-13593

Mycgr3G92136

hypothetical protein
  
Accession: AEO63713
  
Location: 6001616-6003207
  
 NCBI BlastP on this gene

THITE\_39797

hypothetical protein
  
Accession: AEO63714
  
Location: 6004213-6005288
  
  
**BlastP hit with Mycgr3G38483**
  
Percentage identity: 41 %
  
BlastP bit score: 223
  
Sequence coverage: 100 %
  
E-value: 4e-66
  
  
 NCBI BlastP on this gene

THITE\_2109257

hypothetical protein
  
Accession: AEO63715
  
Location: 6005597-6006325
  
  
**BlastP hit with Mycgr3G70471**
  
Percentage identity: 78 %
  
BlastP bit score: 218
  
Sequence coverage: 98 %
  
E-value: 5e-70
  
  
 NCBI BlastP on this gene

THITE\_2109261

hypothetical protein
  
Accession: AEO63716
  
Location: 6008167-6012675
  
 NCBI BlastP on this gene

THITE\_2109263

hypothetical protein
  
Accession: AEO63717
  
Location: 6013102-6014363
  
 NCBI BlastP on this gene

THITE\_2169538

Query: Architecture Search FASTA input

CAGA01000037 : Claviceps purpurea 20.1    Total score: 2.0     Cumulative Blast bit score: 439

Hit cluster cross-links:

Mycgr3G70471
  
Location: 0-405

Mycgr3G70471

Mycgr3G39149
  
Location: 505-1798

Mycgr3G39149

Mycgr3G92130
  
Location: 1898-2396

Mycgr3G92130

Mycgr3G38483
  
Location: 2496-3576

Mycgr3G38483

Mycgr3G108869
  
Location: 3676-5056

Mycgr3G108869

Mycgr3G103943
  
Location: 5156-5762

Mycgr3G103943

Mycgr3G57362
  
Location: 5862-7296

Mycgr3G57362

Mycgr3G39086
  
Location: 7396-8368

Mycgr3G39086

Mycgr3G103942
  
Location: 8468-8714

Mycgr3G103942

Mycgr3G108865
  
Location: 8814-10239

Mycgr3G108865

Mycgr3G70475
  
Location: 10339-11821

Mycgr3G70475

Mycgr3G108866
  
Location: 11921-13010

Mycgr3G108866

Mycgr3G92136
  
Location: 13110-13593

Mycgr3G92136

uncharacterized protein
  
Accession: CCE32103
  
Location: 290726-291208
  
 NCBI BlastP on this gene

CCE32103

uncharacterized protein
  
Accession: CCE32104
  
Location: 296580-297681
  
  
**BlastP hit with Mycgr3G38483**
  
Percentage identity: 40 %
  
BlastP bit score: 231
  
Sequence coverage: 98 %
  
E-value: 5e-69
  
  
 NCBI BlastP on this gene

CCE32104

probable 40S RIBOSOMAL PROTEIN S24
  
Accession: CCE32105
  
Location: 298092-299045
  
  
**BlastP hit with Mycgr3G70471**
  
Percentage identity: 82 %
  
BlastP bit score: 208
  
Sequence coverage: 89 %
  
E-value: 3e-66
  
  
 NCBI BlastP on this gene

CCE32105

uncharacterized protein
  
Accession: CCE32106
  
Location: 300849-303723
  
 NCBI BlastP on this gene

CCE32106

probable GTP cyclohydrolase II
  
Accession: CCE32107
  
Location: 304335-305601
  
 NCBI BlastP on this gene

CCE32107

uncharacterized protein
  
Accession: CCE32108
  
Location: 306479-307135
  
 NCBI BlastP on this gene

CCE32108

Query: Architecture Search FASTA input

DS027053 : Aspergillus clavatus NRRL 1 1099423829799 genomic scaffold    Total score: 2.0     Cumulative Blast bit score: 438

Hit cluster cross-links:

Mycgr3G70471
  
Location: 0-405

Mycgr3G70471

Mycgr3G39149
  
Location: 505-1798

Mycgr3G39149

Mycgr3G92130
  
Location: 1898-2396

Mycgr3G92130

Mycgr3G38483
  
Location: 2496-3576

Mycgr3G38483

Mycgr3G108869
  
Location: 3676-5056

Mycgr3G108869

Mycgr3G103943
  
Location: 5156-5762

Mycgr3G103943

Mycgr3G57362
  
Location: 5862-7296

Mycgr3G57362

Mycgr3G39086
  
Location: 7396-8368

Mycgr3G39086

Mycgr3G103942
  
Location: 8468-8714

Mycgr3G103942

Mycgr3G108865
  
Location: 8814-10239

Mycgr3G108865

Mycgr3G70475
  
Location: 10339-11821

Mycgr3G70475

Mycgr3G108866
  
Location: 11921-13010

Mycgr3G108866

Mycgr3G92136
  
Location: 13110-13593

Mycgr3G92136

RNA binding protein, putative
  
Accession: EAW10902
  
Location: 184548-186192
  
 NCBI BlastP on this gene

EAW10902

streptococcal hemagglutinin protein, putative
  
Accession: EAW10903
  
Location: 190114-191181
  
 NCBI BlastP on this gene

EAW10903

37S ribosomal protein S24
  
Accession: EAW10904
  
Location: 192493-193365
  
  
**BlastP hit with Mycgr3G70471**
  
Percentage identity: 82 %
  
BlastP bit score: 224
  
Sequence coverage: 97 %
  
E-value: 9e-73
  
  
 NCBI BlastP on this gene

EAW10904

conserved hypothetical protein
  
Accession: EAW10905
  
Location: 193698-195092
  
  
**BlastP hit with Mycgr3G38483**
  
Percentage identity: 35 %
  
BlastP bit score: 214
  
Sequence coverage: 99 %
  
E-value: 2e-62
  
  
 NCBI BlastP on this gene

EAW10905

conserved hypothetical protein
  
Accession: EAW10906
  
Location: 195978-198206
  
 NCBI BlastP on this gene

EAW10906

Ran-binding protein (RanBPM), putative
  
Accession: EAW10907
  
Location: 200430-202830
  
 NCBI BlastP on this gene

EAW10907

Query: Architecture Search FASTA input

CU633447 : Podospora anserina S mat+ genomic DNA chromosome 2, supercontig 3.    Total score: 2.0     Cumulative Blast bit score: 436

Hit cluster cross-links:

Mycgr3G70471
  
Location: 0-405

Mycgr3G70471

Mycgr3G39149
  
Location: 505-1798

Mycgr3G39149

Mycgr3G92130
  
Location: 1898-2396

Mycgr3G92130

Mycgr3G38483
  
Location: 2496-3576

Mycgr3G38483

Mycgr3G108869
  
Location: 3676-5056

Mycgr3G108869

Mycgr3G103943
  
Location: 5156-5762

Mycgr3G103943

Mycgr3G57362
  
Location: 5862-7296

Mycgr3G57362

Mycgr3G39086
  
Location: 7396-8368

Mycgr3G39086

Mycgr3G103942
  
Location: 8468-8714

Mycgr3G103942

Mycgr3G108865
  
Location: 8814-10239

Mycgr3G108865

Mycgr3G70475
  
Location: 10339-11821

Mycgr3G70475

Mycgr3G108866
  
Location: 11921-13010

Mycgr3G108866

Mycgr3G92136
  
Location: 13110-13593

Mycgr3G92136

not annotated
  
Accession: CAP60986
  
Location: 113242-114995
  
 NCBI BlastP on this gene

CAP60986

not annotated
  
Accession: CAP60987
  
Location: 115411-116533
  
  
**BlastP hit with Mycgr3G38483**
  
Percentage identity: 42 %
  
BlastP bit score: 228
  
Sequence coverage: 98 %
  
E-value: 6e-68
  
  
 NCBI BlastP on this gene

CAP60987

not annotated
  
Accession: CAP60988
  
Location: 116882-117554
  
  
**BlastP hit with Mycgr3G70471**
  
Percentage identity: 81 %
  
BlastP bit score: 208
  
Sequence coverage: 90 %
  
E-value: 2e-66
  
  
 NCBI BlastP on this gene

CAP60988

not annotated
  
Accession: CAP60989
  
Location: 118869-119663
  
 NCBI BlastP on this gene

CAP60989

not annotated
  
Accession: CAP60990
  
Location: 121609-123078
  
 NCBI BlastP on this gene

CAP60990

not annotated
  
Accession: CAP60991
  
Location: 123358-124379
  
 NCBI BlastP on this gene

CAP60991

not annotated
  
Accession: CAP60992
  
Location: 125425-127029
  
 NCBI BlastP on this gene

CAP60992

Query: Architecture Search FASTA input

GL698484 : Metarhizium acridum CQMa 102 unplaced genomic scaffold Scf\_015    Total score: 2.0     Cumulative Blast bit score: 434

Hit cluster cross-links:

Mycgr3G70471
  
Location: 0-405

Mycgr3G70471

Mycgr3G39149
  
Location: 505-1798

Mycgr3G39149

Mycgr3G92130
  
Location: 1898-2396

Mycgr3G92130

Mycgr3G38483
  
Location: 2496-3576

Mycgr3G38483

Mycgr3G108869
  
Location: 3676-5056

Mycgr3G108869

Mycgr3G103943
  
Location: 5156-5762

Mycgr3G103943

Mycgr3G57362
  
Location: 5862-7296

Mycgr3G57362

Mycgr3G39086
  
Location: 7396-8368

Mycgr3G39086

Mycgr3G103942
  
Location: 8468-8714

Mycgr3G103942

Mycgr3G108865
  
Location: 8814-10239

Mycgr3G108865

Mycgr3G70475
  
Location: 10339-11821

Mycgr3G70475

Mycgr3G108866
  
Location: 11921-13010

Mycgr3G108866

Mycgr3G92136
  
Location: 13110-13593

Mycgr3G92136

hypothetical protein
  
Accession: EFY91162
  
Location: 352226-352807
  
 NCBI BlastP on this gene

EFY91162

40S ribosomal protein S24
  
Accession: EFY91163
  
Location: 353488-354416
  
  
**BlastP hit with Mycgr3G70471**
  
Percentage identity: 80 %
  
BlastP bit score: 202
  
Sequence coverage: 89 %
  
E-value: 6e-64
  
  
 NCBI BlastP on this gene

EFY91163

hypothetical protein
  
Accession: EFY91164
  
Location: 354842-356245
  
  
**BlastP hit with Mycgr3G38483**
  
Percentage identity: 44 %
  
BlastP bit score: 233
  
Sequence coverage: 85 %
  
E-value: 8e-69
  
  
 NCBI BlastP on this gene

EFY91164

hypothetical protein
  
Accession: EFY91165
  
Location: 359454-360415
  
 NCBI BlastP on this gene

EFY91165

RNA binding protein
  
Accession: EFY91166
  
Location: 361204-362731
  
 NCBI BlastP on this gene

EFY91166

Query: Architecture Search FASTA input

DS572753 : Paracoccidioides brasiliensis Pb18 supercont1.4 genomic scaffold    Total score: 2.0     Cumulative Blast bit score: 434

Hit cluster cross-links:

Mycgr3G70471
  
Location: 0-405

Mycgr3G70471

Mycgr3G39149
  
Location: 505-1798

Mycgr3G39149

Mycgr3G92130
  
Location: 1898-2396

Mycgr3G92130

Mycgr3G38483
  
Location: 2496-3576

Mycgr3G38483

Mycgr3G108869
  
Location: 3676-5056

Mycgr3G108869

Mycgr3G103943
  
Location: 5156-5762

Mycgr3G103943

Mycgr3G57362
  
Location: 5862-7296

Mycgr3G57362

Mycgr3G39086
  
Location: 7396-8368

Mycgr3G39086

Mycgr3G103942
  
Location: 8468-8714

Mycgr3G103942

Mycgr3G108865
  
Location: 8814-10239

Mycgr3G108865

Mycgr3G70475
  
Location: 10339-11821

Mycgr3G70475

Mycgr3G108866
  
Location: 11921-13010

Mycgr3G108866

Mycgr3G92136
  
Location: 13110-13593

Mycgr3G92136

RNA binding protein
  
Accession: EEH48229
  
Location: 2542296-2543917
  
 NCBI BlastP on this gene

EEH48229

predicted protein
  
Accession: EEH48230
  
Location: 2547070-2548678
  
 NCBI BlastP on this gene

EEH48230

40S ribosomal protein S24
  
Accession: EEH48231
  
Location: 2549780-2550477
  
  
**BlastP hit with Mycgr3G70471**
  
Percentage identity: 74 %
  
BlastP bit score: 210
  
Sequence coverage: 99 %
  
E-value: 6e-67
  
  
 NCBI BlastP on this gene

EEH48231

conserved hypothetical protein
  
Accession: EEH48232
  
Location: 2550839-2552272
  
  
**BlastP hit with Mycgr3G38483**
  
Percentage identity: 35 %
  
BlastP bit score: 224
  
Sequence coverage: 97 %
  
E-value: 3e-66
  
  
 NCBI BlastP on this gene

EEH48232

conserved hypothetical protein
  
Accession: EEH48233
  
Location: 2554685-2557234
  
 NCBI BlastP on this gene

EEH48233

conserved hypothetical protein
  
Accession: EEH48234
  
Location: 2558469-2558864
  
 NCBI BlastP on this gene

EEH48234

conserved hypothetical protein
  
Accession: EEH48235
  
Location: 2559144-2562880
  
 NCBI BlastP on this gene

EEH48235

Query: Architecture Search FASTA input

KB644412 : Penicillium oxalicum 114-2 unplaced genomic scaffold scaffold\_5    Total score: 2.0     Cumulative Blast bit score: 432

Hit cluster cross-links:

Mycgr3G70471
  
Location: 0-405

Mycgr3G70471

Mycgr3G39149
  
Location: 505-1798

Mycgr3G39149

Mycgr3G92130
  
Location: 1898-2396

Mycgr3G92130

Mycgr3G38483
  
Location: 2496-3576

Mycgr3G38483

Mycgr3G108869
  
Location: 3676-5056

Mycgr3G108869

Mycgr3G103943
  
Location: 5156-5762

Mycgr3G103943

Mycgr3G57362
  
Location: 5862-7296

Mycgr3G57362

Mycgr3G39086
  
Location: 7396-8368

Mycgr3G39086

Mycgr3G103942
  
Location: 8468-8714

Mycgr3G103942

Mycgr3G108865
  
Location: 8814-10239

Mycgr3G108865

Mycgr3G70475
  
Location: 10339-11821

Mycgr3G70475

Mycgr3G108866
  
Location: 11921-13010

Mycgr3G108866

Mycgr3G92136
  
Location: 13110-13593

Mycgr3G92136

hypothetical protein
  
Accession: EPS30916
  
Location: 4235061-4235285
  
 NCBI BlastP on this gene

EPS30916

hypothetical protein
  
Accession: EPS30917
  
Location: 4237816-4239420
  
 NCBI BlastP on this gene

EPS30917

hypothetical protein
  
Accession: EPS30918
  
Location: 4242978-4243830
  
  
**BlastP hit with Mycgr3G70471**
  
Percentage identity: 81 %
  
BlastP bit score: 223
  
Sequence coverage: 97 %
  
E-value: 6e-72
  
  
 NCBI BlastP on this gene

EPS30918

hypothetical protein
  
Accession: EPS30919
  
Location: 4244088-4245327
  
  
**BlastP hit with Mycgr3G38483**
  
Percentage identity: 36 %
  
BlastP bit score: 210
  
Sequence coverage: 98 %
  
E-value: 6e-61
  
  
 NCBI BlastP on this gene

EPS30919

hypothetical protein
  
Accession: EPS30920
  
Location: 4245763-4247992
  
 NCBI BlastP on this gene

EPS30920

Query: Architecture Search FASTA input

DS544804 : Paracoccidioides brasiliensis Pb03 supercont1.2 genomic scaffold    Total score: 2.0     Cumulative Blast bit score: 432

Hit cluster cross-links:

Mycgr3G70471
  
Location: 0-405

Mycgr3G70471

Mycgr3G39149
  
Location: 505-1798

Mycgr3G39149

Mycgr3G92130
  
Location: 1898-2396

Mycgr3G92130

Mycgr3G38483
  
Location: 2496-3576

Mycgr3G38483

Mycgr3G108869
  
Location: 3676-5056

Mycgr3G108869

Mycgr3G103943
  
Location: 5156-5762

Mycgr3G103943

Mycgr3G57362
  
Location: 5862-7296

Mycgr3G57362

Mycgr3G39086
  
Location: 7396-8368

Mycgr3G39086

Mycgr3G103942
  
Location: 8468-8714

Mycgr3G103942

Mycgr3G108865
  
Location: 8814-10239

Mycgr3G108865

Mycgr3G70475
  
Location: 10339-11821

Mycgr3G70475

Mycgr3G108866
  
Location: 11921-13010

Mycgr3G108866

Mycgr3G92136
  
Location: 13110-13593

Mycgr3G92136

conserved hypothetical protein
  
Accession: EEH18832
  
Location: 465128-469197
  
 NCBI BlastP on this gene

EEH18832

conserved hypothetical protein
  
Accession: EEH18833
  
Location: 469715-473307
  
 NCBI BlastP on this gene

EEH18833

conserved hypothetical protein
  
Accession: EEH18834
  
Location: 475707-477139
  
  
**BlastP hit with Mycgr3G38483**
  
Percentage identity: 36 %
  
BlastP bit score: 223
  
Sequence coverage: 96 %
  
E-value: 5e-66
  
  
 NCBI BlastP on this gene

EEH18834

40S ribosomal protein S24
  
Accession: EEH18835
  
Location: 477501-478207
  
  
**BlastP hit with Mycgr3G70471**
  
Percentage identity: 74 %
  
BlastP bit score: 210
  
Sequence coverage: 99 %
  
E-value: 6e-67
  
  
 NCBI BlastP on this gene

EEH18835

predicted protein
  
Accession: EEH18836
  
Location: 479310-480915
  
 NCBI BlastP on this gene

EEH18836

RNA binding protein
  
Accession: EEH18837
  
Location: 483258-485771
  
 NCBI BlastP on this gene

EEH18837

Query: Architecture Search FASTA input

AKCU01000381 : Penicillium digitatum Pd1    Total score: 2.0     Cumulative Blast bit score: 432

Hit cluster cross-links:

Mycgr3G70471
  
Location: 0-405

Mycgr3G70471

Mycgr3G39149
  
Location: 505-1798

Mycgr3G39149

Mycgr3G92130
  
Location: 1898-2396

Mycgr3G92130

Mycgr3G38483
  
Location: 2496-3576

Mycgr3G38483

Mycgr3G108869
  
Location: 3676-5056

Mycgr3G108869

Mycgr3G103943
  
Location: 5156-5762

Mycgr3G103943

Mycgr3G57362
  
Location: 5862-7296

Mycgr3G57362

Mycgr3G39086
  
Location: 7396-8368

Mycgr3G39086

Mycgr3G103942
  
Location: 8468-8714

Mycgr3G103942

Mycgr3G108865
  
Location: 8814-10239

Mycgr3G108865

Mycgr3G70475
  
Location: 10339-11821

Mycgr3G70475

Mycgr3G108866
  
Location: 11921-13010

Mycgr3G108866

Mycgr3G92136
  
Location: 13110-13593

Mycgr3G92136

hypothetical protein
  
Accession: EKV11440
  
Location: 15761-16650
  
 NCBI BlastP on this gene

EKV11440

Ran-binding protein (RanBP10), putative
  
Accession: EKV11441
  
Location: 17797-20135
  
 NCBI BlastP on this gene

EKV11441

hypothetical protein
  
Accession: EKV11442
  
Location: 21282-23384
  
 NCBI BlastP on this gene

EKV11442

hypothetical protein
  
Accession: EKV11443
  
Location: 23861-25199
  
  
**BlastP hit with Mycgr3G38483**
  
Percentage identity: 37 %
  
BlastP bit score: 211
  
Sequence coverage: 99 %
  
E-value: 2e-61
  
  
 NCBI BlastP on this gene

EKV11443

40S ribosomal protein S24
  
Accession: EKV11444
  
Location: 25480-26189
  
  
**BlastP hit with Mycgr3G70471**
  
Percentage identity: 81 %
  
BlastP bit score: 222
  
Sequence coverage: 97 %
  
E-value: 9e-72
  
  
 NCBI BlastP on this gene

EKV11444

hypothetical protein
  
Accession: EKV11445
  
Location: 26590-26781
  
 NCBI BlastP on this gene

EKV11445

hemagglutinin protein, putative
  
Accession: EKV11446
  
Location: 29353-30420
  
 NCBI BlastP on this gene

EKV11446

hypothetical protein
  
Accession: EKV11447
  
Location: 32340-32612
  
 NCBI BlastP on this gene

EKV11447

Peptidyl-prolyl cis-trans isomerase-like 4
  
Accession: EKV11448
  
Location: 33513-35062
  
 NCBI BlastP on this gene

EKV11448

Query: Architecture Search FASTA input

AKCT01000084 : Penicillium digitatum PHI26    Total score: 2.0     Cumulative Blast bit score: 432

Hit cluster cross-links:

Mycgr3G70471
  
Location: 0-405

Mycgr3G70471

Mycgr3G39149
  
Location: 505-1798

Mycgr3G39149

Mycgr3G92130
  
Location: 1898-2396

Mycgr3G92130

Mycgr3G38483
  
Location: 2496-3576

Mycgr3G38483

Mycgr3G108869
  
Location: 3676-5056

Mycgr3G108869

Mycgr3G103943
  
Location: 5156-5762

Mycgr3G103943

Mycgr3G57362
  
Location: 5862-7296

Mycgr3G57362

Mycgr3G39086
  
Location: 7396-8368

Mycgr3G39086

Mycgr3G103942
  
Location: 8468-8714

Mycgr3G103942

Mycgr3G108865
  
Location: 8814-10239

Mycgr3G108865

Mycgr3G70475
  
Location: 10339-11821

Mycgr3G70475

Mycgr3G108866
  
Location: 11921-13010

Mycgr3G108866

Mycgr3G92136
  
Location: 13110-13593

Mycgr3G92136

hypothetical protein
  
Accession: EKV16927
  
Location: 194344-195233
  
 NCBI BlastP on this gene

EKV16927

Ran-binding protein (RanBP10), putative
  
Accession: EKV16928
  
Location: 196380-198718
  
 NCBI BlastP on this gene

EKV16928

hypothetical protein
  
Accession: EKV16929
  
Location: 199865-201967
  
 NCBI BlastP on this gene

EKV16929

hypothetical protein
  
Accession: EKV16930
  
Location: 202445-203783
  
  
**BlastP hit with Mycgr3G38483**
  
Percentage identity: 37 %
  
BlastP bit score: 211
  
Sequence coverage: 99 %
  
E-value: 2e-61
  
  
 NCBI BlastP on this gene

EKV16930

40S ribosomal protein S24
  
Accession: EKV16931
  
Location: 204064-204773
  
  
**BlastP hit with Mycgr3G70471**
  
Percentage identity: 81 %
  
BlastP bit score: 222
  
Sequence coverage: 97 %
  
E-value: 9e-72
  
  
 NCBI BlastP on this gene

EKV16931

hypothetical protein
  
Accession: EKV16932
  
Location: 205174-205365
  
 NCBI BlastP on this gene

EKV16932

hemagglutinin protein, putative
  
Accession: EKV16933
  
Location: 207940-209007
  
 NCBI BlastP on this gene

EKV16933

hypothetical protein
  
Accession: EKV16934
  
Location: 210970-211212
  
 NCBI BlastP on this gene

EKV16934

Peptidyl-prolyl cis-trans isomerase-like 4
  
Accession: EKV16935
  
Location: 212117-213666
  
 NCBI BlastP on this gene

EKV16935

Query: Architecture Search FASTA input

KB705480 : Eutypa lata UCREL1 unplaced genomic scaffold EL1\_03\_scaffold\_142    Total score: 2.0     Cumulative Blast bit score: 432

Hit cluster cross-links:

Mycgr3G70471
  
Location: 0-405

Mycgr3G70471

Mycgr3G39149
  
Location: 505-1798

Mycgr3G39149

Mycgr3G92130
  
Location: 1898-2396

Mycgr3G92130

Mycgr3G38483
  
Location: 2496-3576

Mycgr3G38483

Mycgr3G108869
  
Location: 3676-5056

Mycgr3G108869

Mycgr3G103943
  
Location: 5156-5762

Mycgr3G103943

Mycgr3G57362
  
Location: 5862-7296

Mycgr3G57362

Mycgr3G39086
  
Location: 7396-8368

Mycgr3G39086

Mycgr3G103942
  
Location: 8468-8714

Mycgr3G103942

Mycgr3G108865
  
Location: 8814-10239

Mycgr3G108865

Mycgr3G70475
  
Location: 10339-11821

Mycgr3G70475

Mycgr3G108866
  
Location: 11921-13010

Mycgr3G108866

Mycgr3G92136
  
Location: 13110-13593

Mycgr3G92136

hypothetical protein
  
Accession: EMR72255
  
Location: 42202-42980
  
 NCBI BlastP on this gene

EMR72255

putative gdsl esterase lipase protein
  
Accession: EMR72241
  
Location: 45308-46144
  
 NCBI BlastP on this gene

EMR72241

hypothetical protein
  
Accession: EMR72219
  
Location: 48230-48922
  
 NCBI BlastP on this gene

EMR72219

putative 40s ribosomal protein s24 protein
  
Accession: EMR72230
  
Location: 49613-50394
  
  
**BlastP hit with Mycgr3G70471**
  
Percentage identity: 76 %
  
BlastP bit score: 173
  
Sequence coverage: 89 %
  
E-value: 2e-52
  
  
 NCBI BlastP on this gene

EMR72230

putative fam86a protein
  
Accession: EMR72248
  
Location: 50865-52029
  
  
**BlastP hit with Mycgr3G38483**
  
Percentage identity: 44 %
  
BlastP bit score: 259
  
Sequence coverage: 97 %
  
E-value: 5e-80
  
  
 NCBI BlastP on this gene

EMR72248

hypothetical protein
  
Accession: EMR72242
  
Location: 52913-53527
  
 NCBI BlastP on this gene

EMR72242

hypothetical protein
  
Accession: EMR72268
  
Location: 57582-58253
  
 NCBI BlastP on this gene

EMR72268

putative alpha beta hydrolase domain-containing protein
  
Accession: EMR72224
  
Location: 59023-60147
  
 NCBI BlastP on this gene

EMR72224

Query: Architecture Search FASTA input

KE145356 : Glarea lozoyensis ATCC 20868 chromosome Unknown GLAREA13    Total score: 2.0     Cumulative Blast bit score: 427

Hit cluster cross-links:

Mycgr3G70471
  
Location: 0-405

Mycgr3G70471

Mycgr3G39149
  
Location: 505-1798

Mycgr3G39149

Mycgr3G92130
  
Location: 1898-2396

Mycgr3G92130

Mycgr3G38483
  
Location: 2496-3576

Mycgr3G38483

Mycgr3G108869
  
Location: 3676-5056

Mycgr3G108869

Mycgr3G103943
  
Location: 5156-5762

Mycgr3G103943

Mycgr3G57362
  
Location: 5862-7296

Mycgr3G57362

Mycgr3G39086
  
Location: 7396-8368

Mycgr3G39086

Mycgr3G103942
  
Location: 8468-8714

Mycgr3G103942

Mycgr3G108865
  
Location: 8814-10239

Mycgr3G108865

Mycgr3G70475
  
Location: 10339-11821

Mycgr3G70475

Mycgr3G108866
  
Location: 11921-13010

Mycgr3G108866

Mycgr3G92136
  
Location: 13110-13593

Mycgr3G92136

Oxidoreductase molybdopterin-binding protein
  
Accession: EPE34677
  
Location: 1266321-1268647
  
 NCBI BlastP on this gene

EPE34677

hypothetical protein
  
Accession: EPE34678
  
Location: 1270396-1270794
  
 NCBI BlastP on this gene

EPE34678

hypothetical protein
  
Accession: EPE34679
  
Location: 1271905-1273470
  
 NCBI BlastP on this gene

EPE34679

hypothetical protein
  
Accession: EPE34680
  
Location: 1274308-1275273
  
 NCBI BlastP on this gene

EPE34680

S-adenosyl-L-methionine-dependent methyltransferase
  
Accession: EPE34681
  
Location: 1276387-1277480
  
  
**BlastP hit with Mycgr3G38483**
  
Percentage identity: 40 %
  
BlastP bit score: 221
  
Sequence coverage: 97 %
  
E-value: 4e-65
  
  
 NCBI BlastP on this gene

EPE34681

Ribosomal proteins S24e, L23 and L15e
  
Accession: EPE34682
  
Location: 1277894-1278472
  
  
**BlastP hit with Mycgr3G70471**
  
Percentage identity: 79 %
  
BlastP bit score: 206
  
Sequence coverage: 91 %
  
E-value: 3e-65
  
  
 NCBI BlastP on this gene

EPE34682

hypothetical protein
  
Accession: EPE34683
  
Location: 1280819-1283134
  
 NCBI BlastP on this gene

EPE34683

Cyclophilin-like protein
  
Accession: EPE34684
  
Location: 1283493-1284963
  
 NCBI BlastP on this gene

EPE34684

Query: Architecture Search FASTA input

JH725156 : Beauveria bassiana ARSEF 2860 unplaced genomic scaffold BBA\_S00007    Total score: 2.0     Cumulative Blast bit score: 424

Hit cluster cross-links:

Mycgr3G70471
  
Location: 0-405

Mycgr3G70471

Mycgr3G39149
  
Location: 505-1798

Mycgr3G39149

Mycgr3G92130
  
Location: 1898-2396

Mycgr3G92130

Mycgr3G38483
  
Location: 2496-3576

Mycgr3G38483

Mycgr3G108869
  
Location: 3676-5056

Mycgr3G108869

Mycgr3G103943
  
Location: 5156-5762

Mycgr3G103943

Mycgr3G57362
  
Location: 5862-7296

Mycgr3G57362

Mycgr3G39086
  
Location: 7396-8368

Mycgr3G39086

Mycgr3G103942
  
Location: 8468-8714

Mycgr3G103942

Mycgr3G108865
  
Location: 8814-10239

Mycgr3G108865

Mycgr3G70475
  
Location: 10339-11821

Mycgr3G70475

Mycgr3G108866
  
Location: 11921-13010

Mycgr3G108866

Mycgr3G92136
  
Location: 13110-13593

Mycgr3G92136

peptidyl-prolyl cis-trans isomerase cyp6
  
Accession: EJP67562
  
Location: 382431-384066
  
 NCBI BlastP on this gene

EJP67562

hypothetical protein
  
Accession: EJP67563
  
Location: 384899-385399
  
 NCBI BlastP on this gene

EJP67563

Nicotinamide N-methyltransferase, putative
  
Accession: EJP67564
  
Location: 390346-391476
  
  
**BlastP hit with Mycgr3G38483**
  
Percentage identity: 42 %
  
BlastP bit score: 221
  
Sequence coverage: 98 %
  
E-value: 4e-65
  
  
 NCBI BlastP on this gene

EJP67564

40S ribosomal protein S24
  
Accession: EJP67565
  
Location: 391842-392763
  
  
**BlastP hit with Mycgr3G70471**
  
Percentage identity: 72 %
  
BlastP bit score: 203
  
Sequence coverage: 98 %
  
E-value: 4e-64
  
  
 NCBI BlastP on this gene

EJP67565

hypothetical protein
  
Accession: EJP67566
  
Location: 393800-394300
  
 NCBI BlastP on this gene

EJP67566

clumping factor B
  
Accession: EJP67567
  
Location: 396371-400101
  
 NCBI BlastP on this gene

EJP67567

GTP cyclohydrolase II
  
Accession: EJP67568
  
Location: 400542-401733
  
 NCBI BlastP on this gene

EJP67568

Query: Architecture Search FASTA input

DS572830 : Paracoccidioides brasiliensis Pb01 supercont1.20 genomic scaffold    Total score: 2.0     Cumulative Blast bit score: 422

Hit cluster cross-links:

Mycgr3G70471
  
Location: 0-405

Mycgr3G70471

Mycgr3G39149
  
Location: 505-1798

Mycgr3G39149

Mycgr3G92130
  
Location: 1898-2396

Mycgr3G92130

Mycgr3G38483
  
Location: 2496-3576

Mycgr3G38483

Mycgr3G108869
  
Location: 3676-5056

Mycgr3G108869

Mycgr3G103943
  
Location: 5156-5762

Mycgr3G103943

Mycgr3G57362
  
Location: 5862-7296

Mycgr3G57362

Mycgr3G39086
  
Location: 7396-8368

Mycgr3G39086

Mycgr3G103942
  
Location: 8468-8714

Mycgr3G103942

Mycgr3G108865
  
Location: 8814-10239

Mycgr3G108865

Mycgr3G70475
  
Location: 10339-11821

Mycgr3G70475

Mycgr3G108866
  
Location: 11921-13010

Mycgr3G108866

Mycgr3G92136
  
Location: 13110-13593

Mycgr3G92136

hypothetical protein
  
Accession: EEH36460
  
Location: 528533-529395
  
 NCBI BlastP on this gene

EEH36460

predicted protein
  
Accession: EEH36461
  
Location: 529773-530179
  
 NCBI BlastP on this gene

EEH36461

conserved hypothetical protein
  
Accession: EEH36462
  
Location: 531759-533930
  
 NCBI BlastP on this gene

EEH36462

conserved hypothetical protein
  
Accession: EEH36463
  
Location: 536350-537780
  
  
**BlastP hit with Mycgr3G38483**
  
Percentage identity: 36 %
  
BlastP bit score: 222
  
Sequence coverage: 98 %
  
E-value: 2e-65
  
  
 NCBI BlastP on this gene

EEH36463

40S ribosomal protein S24
  
Accession: EEH36464
  
Location: 538132-538831
  
  
**BlastP hit with Mycgr3G70471**
  
Percentage identity: 76 %
  
BlastP bit score: 200
  
Sequence coverage: 90 %
  
E-value: 5e-63
  
  
 NCBI BlastP on this gene

EEH36464

predicted protein
  
Accession: EEH36465
  
Location: 539951-542208
  
 NCBI BlastP on this gene

EEH36465

RNA binding protein
  
Accession: EEH36466
  
Location: 544625-546243
  
 NCBI BlastP on this gene

EEH36466

UDP-N-acetylglucosamine pyrophosphorylase
  
Accession: EEH36467
  
Location: 546821-548547
  
 NCBI BlastP on this gene

EEH36467

Query: Architecture Search FASTA input

GL573339 : Geomyces destructans 20631-21 unplaced genomic scaffold supercont1.171    Total score: 2.0     Cumulative Blast bit score: 417

Hit cluster cross-links:

Mycgr3G70471
  
Location: 0-405

Mycgr3G70471

Mycgr3G39149
  
Location: 505-1798

Mycgr3G39149

Mycgr3G92130
  
Location: 1898-2396

Mycgr3G92130

Mycgr3G38483
  
Location: 2496-3576

Mycgr3G38483

Mycgr3G108869
  
Location: 3676-5056

Mycgr3G108869

Mycgr3G103943
  
Location: 5156-5762

Mycgr3G103943

Mycgr3G57362
  
Location: 5862-7296

Mycgr3G57362

Mycgr3G39086
  
Location: 7396-8368

Mycgr3G39086

Mycgr3G103942
  
Location: 8468-8714

Mycgr3G103942

Mycgr3G108865
  
Location: 8814-10239

Mycgr3G108865

Mycgr3G70475
  
Location: 10339-11821

Mycgr3G70475

Mycgr3G108866
  
Location: 11921-13010

Mycgr3G108866

Mycgr3G92136
  
Location: 13110-13593

Mycgr3G92136

hypothetical protein
  
Accession: ELR04191
  
Location: 31140-34239
  
 NCBI BlastP on this gene

ELR04191

hypothetical protein
  
Accession: ELR04192
  
Location: 35308-38627
  
 NCBI BlastP on this gene

ELR04192

hypothetical protein
  
Accession: ELR04193
  
Location: 39394-40637
  
  
**BlastP hit with Mycgr3G38483**
  
Percentage identity: 40 %
  
BlastP bit score: 223
  
Sequence coverage: 101 %
  
E-value: 1e-65
  
  
 NCBI BlastP on this gene

ELR04193

small subunit ribosomal protein S24e
  
Accession: ELR04194
  
Location: 41011-41640
  
  
**BlastP hit with Mycgr3G70471**
  
Percentage identity: 74 %
  
BlastP bit score: 195
  
Sequence coverage: 92 %
  
E-value: 5e-61
  
  
 NCBI BlastP on this gene

ELR04194

hypothetical protein
  
Accession: ELR04195
  
Location: 43156-44583
  
 NCBI BlastP on this gene

ELR04195

hypothetical protein
  
Accession: ELR04196
  
Location: 45875-46183
  
 NCBI BlastP on this gene

ELR04196

hypothetical protein
  
Accession: ELR04197
  
Location: 47537-49186
  
 NCBI BlastP on this gene

ELR04197

hypothetical protein
  
Accession: ELR04198
  
Location: 49720-50342
  
 NCBI BlastP on this gene

ELR04198

Query: Architecture Search FASTA input

JH795346 : Magnaporthe oryzae P131 unplaced genomic scaffold P131\_scaffold00065    Total score: 2.0     Cumulative Blast bit score: 417

Hit cluster cross-links:

Mycgr3G70471
  
Location: 0-405

Mycgr3G70471

Mycgr3G39149
  
Location: 505-1798

Mycgr3G39149

Mycgr3G92130
  
Location: 1898-2396

Mycgr3G92130

Mycgr3G38483
  
Location: 2496-3576

Mycgr3G38483

Mycgr3G108869
  
Location: 3676-5056

Mycgr3G108869

Mycgr3G103943
  
Location: 5156-5762

Mycgr3G103943

Mycgr3G57362
  
Location: 5862-7296

Mycgr3G57362

Mycgr3G39086
  
Location: 7396-8368

Mycgr3G39086

Mycgr3G103942
  
Location: 8468-8714

Mycgr3G103942

Mycgr3G108865
  
Location: 8814-10239

Mycgr3G108865

Mycgr3G70475
  
Location: 10339-11821

Mycgr3G70475

Mycgr3G108866
  
Location: 11921-13010

Mycgr3G108866

Mycgr3G92136
  
Location: 13110-13593

Mycgr3G92136

hypothetical protein
  
Accession: ELQ70235
  
Location: 25896-26732
  
 NCBI BlastP on this gene

ELQ70235

hypothetical protein
  
Accession: ELQ70236
  
Location: 27915-29142
  
  
**BlastP hit with Mycgr3G38483**
  
Percentage identity: 44 %
  
BlastP bit score: 211
  
Sequence coverage: 83 %
  
E-value: 2e-61
  
  
 NCBI BlastP on this gene

ELQ70236

hypothetical protein
  
Accession: ELQ70237
  
Location: 30559-33139
  
 NCBI BlastP on this gene

ELQ70237

hypothetical protein
  
Accession: ELQ70238
  
Location: 40247-43000
  
 NCBI BlastP on this gene

ELQ70238

peptidyl-prolyl cis-trans isomerase cyp6
  
Accession: ELQ70239
  
Location: 45048-46565
  
 NCBI BlastP on this gene

ELQ70239

40S ribosomal protein S24
  
Accession: ELQ70240
  
Location: 47145-47889
  
  
**BlastP hit with Mycgr3G70471**
  
Percentage identity: 80 %
  
BlastP bit score: 206
  
Sequence coverage: 88 %
  
E-value: 3e-65
  
  
 NCBI BlastP on this gene

ELQ70240

hypothetical protein
  
Accession: ELQ70241
  
Location: 48551-49027
  
 NCBI BlastP on this gene

ELQ70241

Query: Architecture Search FASTA input

JH793790 : Magnaporthe oryzae Y34 unplaced genomic scaffold Y34\_scaffold00456    Total score: 2.0     Cumulative Blast bit score: 417

Hit cluster cross-links:

Mycgr3G70471
  
Location: 0-405

Mycgr3G70471

Mycgr3G39149
  
Location: 505-1798

Mycgr3G39149

Mycgr3G92130
  
Location: 1898-2396

Mycgr3G92130

Mycgr3G38483
  
Location: 2496-3576

Mycgr3G38483

Mycgr3G108869
  
Location: 3676-5056

Mycgr3G108869

Mycgr3G103943
  
Location: 5156-5762

Mycgr3G103943

Mycgr3G57362
  
Location: 5862-7296

Mycgr3G57362

Mycgr3G39086
  
Location: 7396-8368

Mycgr3G39086

Mycgr3G103942
  
Location: 8468-8714

Mycgr3G103942

Mycgr3G108865
  
Location: 8814-10239

Mycgr3G108865

Mycgr3G70475
  
Location: 10339-11821

Mycgr3G70475

Mycgr3G108866
  
Location: 11921-13010

Mycgr3G108866

Mycgr3G92136
  
Location: 13110-13593

Mycgr3G92136

hypothetical protein
  
Accession: ELQ40237
  
Location: 25817-26653
  
 NCBI BlastP on this gene

ELQ40237

hypothetical protein
  
Accession: ELQ40238
  
Location: 27835-29062
  
  
**BlastP hit with Mycgr3G38483**
  
Percentage identity: 44 %
  
BlastP bit score: 211
  
Sequence coverage: 83 %
  
E-value: 2e-61
  
  
 NCBI BlastP on this gene

ELQ40238

hypothetical protein
  
Accession: ELQ40239
  
Location: 30481-33062
  
 NCBI BlastP on this gene

ELQ40239

hypothetical protein
  
Accession: ELQ40240
  
Location: 40156-42909
  
 NCBI BlastP on this gene

ELQ40240

peptidyl-prolyl cis-trans isomerase cyp6
  
Accession: ELQ40241
  
Location: 44955-46472
  
 NCBI BlastP on this gene

ELQ40241

40S ribosomal protein S24
  
Accession: ELQ40242
  
Location: 47052-47796
  
  
**BlastP hit with Mycgr3G70471**
  
Percentage identity: 80 %
  
BlastP bit score: 206
  
Sequence coverage: 88 %
  
E-value: 3e-65
  
  
 NCBI BlastP on this gene

ELQ40242

hypothetical protein
  
Accession: ELQ40243
  
Location: 48458-48934
  
 NCBI BlastP on this gene

ELQ40243

Query: Architecture Search FASTA input

CM001234 : Magnaporthe oryzae 70-15 chromosome 4    Total score: 2.0     Cumulative Blast bit score: 417

Hit cluster cross-links:

Mycgr3G70471
  
Location: 0-405

Mycgr3G70471

Mycgr3G39149
  
Location: 505-1798

Mycgr3G39149

Mycgr3G92130
  
Location: 1898-2396

Mycgr3G92130

Mycgr3G38483
  
Location: 2496-3576

Mycgr3G38483

Mycgr3G108869
  
Location: 3676-5056

Mycgr3G108869

Mycgr3G103943
  
Location: 5156-5762

Mycgr3G103943

Mycgr3G57362
  
Location: 5862-7296

Mycgr3G57362

Mycgr3G39086
  
Location: 7396-8368

Mycgr3G39086

Mycgr3G103942
  
Location: 8468-8714

Mycgr3G103942

Mycgr3G108865
  
Location: 8814-10239

Mycgr3G108865

Mycgr3G70475
  
Location: 10339-11821

Mycgr3G70475

Mycgr3G108866
  
Location: 11921-13010

Mycgr3G108866

Mycgr3G92136
  
Location: 13110-13593

Mycgr3G92136

hypothetical protein
  
Accession: EHA49574
  
Location: 53623-54459
  
 NCBI BlastP on this gene

EHA49574

hypothetical protein
  
Accession: EHA49575
  
Location: 55646-56870
  
  
**BlastP hit with Mycgr3G38483**
  
Percentage identity: 45 %
  
BlastP bit score: 211
  
Sequence coverage: 83 %
  
E-value: 2e-61
  
  
 NCBI BlastP on this gene

EHA49575

hypothetical protein
  
Accession: EHA49576
  
Location: 58267-59887
  
 NCBI BlastP on this gene

EHA49576

hypothetical protein
  
Accession: EHA49577
  
Location: 67968-70722
  
 NCBI BlastP on this gene

EHA49577

peptidyl-prolyl cis-trans isomerase-like 4
  
Accession: EHA49578
  
Location: 72770-74287
  
 NCBI BlastP on this gene

EHA49578

40S ribosomal protein S24
  
Accession: EHA49579
  
Location: 74867-75611
  
  
**BlastP hit with Mycgr3G70471**
  
Percentage identity: 80 %
  
BlastP bit score: 206
  
Sequence coverage: 88 %
  
E-value: 3e-65
  
  
 NCBI BlastP on this gene

EHA49579

hypothetical protein
  
Accession: EHA49580
  
Location: 76273-76749
  
 NCBI BlastP on this gene

EHA49580

Query: Architecture Search FASTA input

GL985082 : Trichoderma reesei QM6a unplaced genomic scaffold TRIREscaffold\_27    Total score: 2.0     Cumulative Blast bit score: 415

Hit cluster cross-links:

Mycgr3G70471
  
Location: 0-405

Mycgr3G70471

Mycgr3G39149
  
Location: 505-1798

Mycgr3G39149

Mycgr3G92130
  
Location: 1898-2396

Mycgr3G92130

Mycgr3G38483
  
Location: 2496-3576

Mycgr3G38483

Mycgr3G108869
  
Location: 3676-5056

Mycgr3G108869

Mycgr3G103943
  
Location: 5156-5762

Mycgr3G103943

Mycgr3G57362
  
Location: 5862-7296

Mycgr3G57362

Mycgr3G39086
  
Location: 7396-8368

Mycgr3G39086

Mycgr3G103942
  
Location: 8468-8714

Mycgr3G103942

Mycgr3G108865
  
Location: 8814-10239

Mycgr3G108865

Mycgr3G70475
  
Location: 10339-11821

Mycgr3G70475

Mycgr3G108866
  
Location: 11921-13010

Mycgr3G108866

Mycgr3G92136
  
Location: 13110-13593

Mycgr3G92136

hypothetical protein
  
Accession: EGR45101
  
Location: 66209-67550
  
 NCBI BlastP on this gene

EGR45101

predicted protein
  
Accession: EGR45102
  
Location: 67980-69419
  
 NCBI BlastP on this gene

EGR45102

predicted protein
  
Accession: EGR45020
  
Location: 71282-71974
  
 NCBI BlastP on this gene

EGR45020

predicted protein
  
Accession: EGR45021
  
Location: 72824-74476
  
 NCBI BlastP on this gene

EGR45021

hypothetical protein
  
Accession: EGR45103
  
Location: 75119-76002
  
  
**BlastP hit with Mycgr3G70471**
  
Percentage identity: 76 %
  
BlastP bit score: 195
  
Sequence coverage: 89 %
  
E-value: 5e-61
  
  
 NCBI BlastP on this gene

EGR45103

predicted protein
  
Accession: EGR45022
  
Location: 76505-77584
  
  
**BlastP hit with Mycgr3G38483**
  
Percentage identity: 40 %
  
BlastP bit score: 221
  
Sequence coverage: 99 %
  
E-value: 2e-65
  
  
 NCBI BlastP on this gene

EGR45022

predicted protein
  
Accession: EGR45023
  
Location: 83280-83753
  
 NCBI BlastP on this gene

EGR45023

predicted protein
  
Accession: EGR45024
  
Location: 85482-86951
  
 NCBI BlastP on this gene

EGR45024

Query: Architecture Search FASTA input

KE148175 : Ophiostoma piceae UAMH 11346 chromosome Unknown scf30    Total score: 2.0     Cumulative Blast bit score: 415

Hit cluster cross-links:

Mycgr3G70471
  
Location: 0-405

Mycgr3G70471

Mycgr3G39149
  
Location: 505-1798

Mycgr3G39149

Mycgr3G92130
  
Location: 1898-2396

Mycgr3G92130

Mycgr3G38483
  
Location: 2496-3576

Mycgr3G38483

Mycgr3G108869
  
Location: 3676-5056

Mycgr3G108869

Mycgr3G103943
  
Location: 5156-5762

Mycgr3G103943

Mycgr3G57362
  
Location: 5862-7296

Mycgr3G57362

Mycgr3G39086
  
Location: 7396-8368

Mycgr3G39086

Mycgr3G103942
  
Location: 8468-8714

Mycgr3G103942

Mycgr3G108865
  
Location: 8814-10239

Mycgr3G108865

Mycgr3G70475
  
Location: 10339-11821

Mycgr3G70475

Mycgr3G108866
  
Location: 11921-13010

Mycgr3G108866

Mycgr3G92136
  
Location: 13110-13593

Mycgr3G92136

n-terminal fungal transcription factor-containing protein
  
Accession: EPE02643
  
Location: 50472-52637
  
 NCBI BlastP on this gene

EPE02643

mannose-6-phosphate isomerase
  
Accession: EPE02644
  
Location: 53807-55156
  
 NCBI BlastP on this gene

EPE02644

fam86a protein
  
Accession: EPE02645
  
Location: 55576-56616
  
  
**BlastP hit with Mycgr3G38483**
  
Percentage identity: 41 %
  
BlastP bit score: 214
  
Sequence coverage: 99 %
  
E-value: 2e-62
  
  
 NCBI BlastP on this gene

EPE02645

40s ribosomal protein s24
  
Accession: EPE02646
  
Location: 57153-58130
  
  
**BlastP hit with Mycgr3G70471**
  
Percentage identity: 78 %
  
BlastP bit score: 201
  
Sequence coverage: 88 %
  
E-value: 2e-63
  
  
 NCBI BlastP on this gene

EPE02646

hypothetical protein
  
Accession: EPE02647
  
Location: 58757-59440
  
 NCBI BlastP on this gene

EPE02647

gtp cyclohydrolase-2
  
Accession: EPE02648
  
Location: 60763-64375
  
 NCBI BlastP on this gene

EPE02648

zinc c6 finger domain protein
  
Accession: EPE02649
  
Location: 65182-66450
  
 NCBI BlastP on this gene

EPE02649

Query: Architecture Search FASTA input

GG749437 : Ajellomyces dermatitidis ATCC 18188 genomic scaffold supercont1.31    Total score: 2.0     Cumulative Blast bit score: 415

Hit cluster cross-links:

Mycgr3G70471
  
Location: 0-405

Mycgr3G70471

Mycgr3G39149
  
Location: 505-1798

Mycgr3G39149

Mycgr3G92130
  
Location: 1898-2396

Mycgr3G92130

Mycgr3G38483
  
Location: 2496-3576

Mycgr3G38483

Mycgr3G108869
  
Location: 3676-5056

Mycgr3G108869

Mycgr3G103943
  
Location: 5156-5762

Mycgr3G103943

Mycgr3G57362
  
Location: 5862-7296

Mycgr3G57362

Mycgr3G39086
  
Location: 7396-8368

Mycgr3G39086

Mycgr3G103942
  
Location: 8468-8714

Mycgr3G103942

Mycgr3G108865
  
Location: 8814-10239

Mycgr3G108865

Mycgr3G70475
  
Location: 10339-11821

Mycgr3G70475

Mycgr3G108866
  
Location: 11921-13010

Mycgr3G108866

Mycgr3G92136
  
Location: 13110-13593

Mycgr3G92136

hypothetical protein
  
Accession: EGE82680
  
Location: 43224-44427
  
 NCBI BlastP on this gene

EGE82680

hypothetical protein
  
Accession: EGE82681
  
Location: 44789-46015
  
  
**BlastP hit with Mycgr3G38483**
  
Percentage identity: 39 %
  
BlastP bit score: 211
  
Sequence coverage: 83 %
  
E-value: 1e-61
  
  
 NCBI BlastP on this gene

EGE82681

37S ribosomal protein S24
  
Accession: EGE82682
  
Location: 46336-47002
  
  
**BlastP hit with Mycgr3G70471**
  
Percentage identity: 78 %
  
BlastP bit score: 204
  
Sequence coverage: 91 %
  
E-value: 1e-64
  
  
 NCBI BlastP on this gene

EGE82682

hypothetical protein
  
Accession: EGE82683
  
Location: 48021-49616
  
 NCBI BlastP on this gene

EGE82683

hypothetical protein
  
Accession: EGE82684
  
Location: 51152-52415
  
 NCBI BlastP on this gene

EGE82684

RNA binding protein
  
Accession: EGE82685
  
Location: 52542-54136
  
 NCBI BlastP on this gene

EGE82685

UDP-N-acetylglucosamine pyrophosphorylase
  
Accession: EGE82686
  
Location: 54731-56469
  
 NCBI BlastP on this gene

EGE82686

Query: Architecture Search FASTA input

EQ999974 : Ajellomyces dermatitidis ER-3 genomic scaffold supercont1.2    Total score: 2.0     Cumulative Blast bit score: 415

Hit cluster cross-links:

Mycgr3G70471
  
Location: 0-405

Mycgr3G70471

Mycgr3G39149
  
Location: 505-1798

Mycgr3G39149

Mycgr3G92130
  
Location: 1898-2396

Mycgr3G92130

Mycgr3G38483
  
Location: 2496-3576

Mycgr3G38483

Mycgr3G108869
  
Location: 3676-5056

Mycgr3G108869

Mycgr3G103943
  
Location: 5156-5762

Mycgr3G103943

Mycgr3G57362
  
Location: 5862-7296

Mycgr3G57362

Mycgr3G39086
  
Location: 7396-8368

Mycgr3G39086

Mycgr3G103942
  
Location: 8468-8714

Mycgr3G103942

Mycgr3G108865
  
Location: 8814-10239

Mycgr3G108865

Mycgr3G70475
  
Location: 10339-11821

Mycgr3G70475

Mycgr3G108866
  
Location: 11921-13010

Mycgr3G108866

Mycgr3G92136
  
Location: 13110-13593

Mycgr3G92136

UDP-N-acetylglucosamine pyrophosphorylase
  
Accession: EEQ86774
  
Location: 4622530-4624268
  
 NCBI BlastP on this gene

EEQ86774

peptidyl-prolyl cis-trans isomerase-like 4
  
Accession: EEQ86775
  
Location: 4624863-4626282
  
 NCBI BlastP on this gene

EEQ86775

predicted protein
  
Accession: EEQ86776
  
Location: 4626619-4627831
  
 NCBI BlastP on this gene

EEQ86776

predicted protein
  
Accession: EEQ86777
  
Location: 4629473-4630962
  
 NCBI BlastP on this gene

EEQ86777

37S ribosomal protein S24
  
Accession: EEQ86778
  
Location: 4631977-4632643
  
  
**BlastP hit with Mycgr3G70471**
  
Percentage identity: 78 %
  
BlastP bit score: 204
  
Sequence coverage: 91 %
  
E-value: 1e-64
  
  
 NCBI BlastP on this gene

EEQ86778

conserved hypothetical protein
  
Accession: EEQ86779
  
Location: 4632966-4634378
  
  
**BlastP hit with Mycgr3G38483**
  
Percentage identity: 39 %
  
BlastP bit score: 211
  
Sequence coverage: 83 %
  
E-value: 1e-61
  
  
 NCBI BlastP on this gene

EEQ86779

Query: Architecture Search FASTA input

GG657453 : Ajellomyces dermatitidis SLH14081 genomic scaffold supercont1.6    Total score: 2.0     Cumulative Blast bit score: 413

Hit cluster cross-links:

Mycgr3G70471
  
Location: 0-405

Mycgr3G70471

Mycgr3G39149
  
Location: 505-1798

Mycgr3G39149

Mycgr3G92130
  
Location: 1898-2396

Mycgr3G92130

Mycgr3G38483
  
Location: 2496-3576

Mycgr3G38483

Mycgr3G108869
  
Location: 3676-5056

Mycgr3G108869

Mycgr3G103943
  
Location: 5156-5762

Mycgr3G103943

Mycgr3G57362
  
Location: 5862-7296

Mycgr3G57362

Mycgr3G39086
  
Location: 7396-8368

Mycgr3G39086

Mycgr3G103942
  
Location: 8468-8714

Mycgr3G103942

Mycgr3G108865
  
Location: 8814-10239

Mycgr3G108865

Mycgr3G70475
  
Location: 10339-11821

Mycgr3G70475

Mycgr3G108866
  
Location: 11921-13010

Mycgr3G108866

Mycgr3G92136
  
Location: 13110-13593

Mycgr3G92136

conserved hypothetical protein
  
Accession: EEQ77680
  
Location: 2539526-2540940
  
  
**BlastP hit with Mycgr3G38483**
  
Percentage identity: 39 %
  
BlastP bit score: 209
  
Sequence coverage: 83 %
  
E-value: 6e-61
  
  
 NCBI BlastP on this gene

EEQ77680

37S ribosomal protein S24
  
Accession: EEQ77681
  
Location: 2541270-2541936
  
  
**BlastP hit with Mycgr3G70471**
  
Percentage identity: 78 %
  
BlastP bit score: 204
  
Sequence coverage: 91 %
  
E-value: 1e-64
  
  
 NCBI BlastP on this gene

EEQ77681

predicted protein
  
Accession: EEQ77682
  
Location: 2542951-2544440
  
 NCBI BlastP on this gene

EEQ77682

peptidyl-prolyl cis-trans isomerase-like 4
  
Accession: EEQ77683
  
Location: 2547463-2549057
  
 NCBI BlastP on this gene

EEQ77683

UDP-N-acetylglucosamine pyrophosphorylase
  
Accession: EEQ77684
  
Location: 2549652-2551390
  
 NCBI BlastP on this gene

EEQ77684

Query: Architecture Search FASTA input

DS990639 : Ajellomyces capsulatus H88 supercont1.4 genomic scaffold    Total score: 2.0     Cumulative Blast bit score: 413

Hit cluster cross-links:

Mycgr3G70471
  
Location: 0-405

Mycgr3G70471

Mycgr3G39149
  
Location: 505-1798

Mycgr3G39149

Mycgr3G92130
  
Location: 1898-2396

Mycgr3G92130

Mycgr3G38483
  
Location: 2496-3576

Mycgr3G38483

Mycgr3G108869
  
Location: 3676-5056

Mycgr3G108869

Mycgr3G103943
  
Location: 5156-5762

Mycgr3G103943

Mycgr3G57362
  
Location: 5862-7296

Mycgr3G57362

Mycgr3G39086
  
Location: 7396-8368

Mycgr3G39086

Mycgr3G103942
  
Location: 8468-8714

Mycgr3G103942

Mycgr3G108865
  
Location: 8814-10239

Mycgr3G108865

Mycgr3G70475
  
Location: 10339-11821

Mycgr3G70475

Mycgr3G108866
  
Location: 11921-13010

Mycgr3G108866

Mycgr3G92136
  
Location: 13110-13593

Mycgr3G92136

UDP-N-acetylglucosamine pyrophosphorylase
  
Accession: EGC45837
  
Location: 2163181-2164934
  
 NCBI BlastP on this gene

EGC45837

peptidyl-prolyl cis-trans isomerase
  
Accession: EGC45838
  
Location: 2165507-2167072
  
 NCBI BlastP on this gene

EGC45838

predicted protein
  
Accession: EGC45839
  
Location: 2169853-2171431
  
 NCBI BlastP on this gene

EGC45839

40S ribosomal protein S24
  
Accession: EGC45840
  
Location: 2172375-2173020
  
  
**BlastP hit with Mycgr3G70471**
  
Percentage identity: 78 %
  
BlastP bit score: 202
  
Sequence coverage: 90 %
  
E-value: 4e-64
  
  
 NCBI BlastP on this gene

EGC45840

conserved hypothetical protein
  
Accession: EGC45841
  
Location: 2173343-2174656
  
  
**BlastP hit with Mycgr3G38483**
  
Percentage identity: 35 %
  
BlastP bit score: 211
  
Sequence coverage: 98 %
  
E-value: 2e-61
  
  
 NCBI BlastP on this gene

EGC45841

conserved hypothetical protein
  
Accession: EGC45842
  
Location: 2177163-2179682
  
 NCBI BlastP on this gene

EGC45842

cytochrome c oxidase assembly protein
  
Accession: EGC45843
  
Location: 2180938-2181298
  
 NCBI BlastP on this gene

EGC45843

conserved hypothetical protein
  
Accession: EGC45844
  
Location: 2181600-2182484
  
 NCBI BlastP on this gene

EGC45844

Query: Architecture Search FASTA input

DS989826 : Arthroderma gypseum CBS 118893 supercont1.5 genomic scaffold    Total score: 2.0     Cumulative Blast bit score: 413

Hit cluster cross-links:

Mycgr3G70471
  
Location: 0-405

Mycgr3G70471

Mycgr3G39149
  
Location: 505-1798

Mycgr3G39149

Mycgr3G92130
  
Location: 1898-2396

Mycgr3G92130

Mycgr3G38483
  
Location: 2496-3576

Mycgr3G38483

Mycgr3G108869
  
Location: 3676-5056

Mycgr3G108869

Mycgr3G103943
  
Location: 5156-5762

Mycgr3G103943

Mycgr3G57362
  
Location: 5862-7296

Mycgr3G57362

Mycgr3G39086
  
Location: 7396-8368

Mycgr3G39086

Mycgr3G103942
  
Location: 8468-8714

Mycgr3G103942

Mycgr3G108865
  
Location: 8814-10239

Mycgr3G108865

Mycgr3G70475
  
Location: 10339-11821

Mycgr3G70475

Mycgr3G108866
  
Location: 11921-13010

Mycgr3G108866

Mycgr3G92136
  
Location: 13110-13593

Mycgr3G92136

26S proteasome non-ATPase regulatory subunit 8
  
Accession: EFR02878
  
Location: 87104-88038
  
 NCBI BlastP on this gene

EFR02878

hypothetical protein
  
Accession: EFR02879
  
Location: 89213-90208
  
 NCBI BlastP on this gene

EFR02879

tripeptidyl-peptidase 1
  
Accession: EFR02880
  
Location: 90533-92402
  
 NCBI BlastP on this gene

EFR02880

aflatoxin biosynthesis ketoreductase nor-1
  
Accession: EFR02881
  
Location: 93566-94342
  
 NCBI BlastP on this gene

EFR02881

hypothetical protein
  
Accession: EFR02882
  
Location: 95217-96848
  
  
**BlastP hit with Mycgr3G38483**
  
Percentage identity: 38 %
  
BlastP bit score: 215
  
Sequence coverage: 97 %
  
E-value: 6e-63
  
  
 NCBI BlastP on this gene

EFR02882

40S ribosomal protein S24
  
Accession: EFR02883
  
Location: 97216-97974
  
  
**BlastP hit with Mycgr3G70471**
  
Percentage identity: 75 %
  
BlastP bit score: 198
  
Sequence coverage: 90 %
  
E-value: 2e-62
  
  
 NCBI BlastP on this gene

EFR02883

hypothetical protein
  
Accession: EFR02884
  
Location: 98901-100389
  
 NCBI BlastP on this gene

EFR02884

peptidyl-prolyl cis-trans isomerase cyp6
  
Accession: EFR02885
  
Location: 103072-104654
  
 NCBI BlastP on this gene

EFR02885

UDP-N-acetylglucosamine pyrophosphorylase
  
Accession: EFR02886
  
Location: 105264-106949
  
 NCBI BlastP on this gene

EFR02886

Query: Architecture Search FASTA input

GG704913 : Coccidioides immitis RS genomic scaffold supercont3.3    Total score: 2.0     Cumulative Blast bit score: 412

Hit cluster cross-links:

Mycgr3G70471
  
Location: 0-405

Mycgr3G70471

Mycgr3G39149
  
Location: 505-1798

Mycgr3G39149

Mycgr3G92130
  
Location: 1898-2396

Mycgr3G92130

Mycgr3G38483
  
Location: 2496-3576

Mycgr3G38483

Mycgr3G108869
  
Location: 3676-5056

Mycgr3G108869

Mycgr3G103943
  
Location: 5156-5762

Mycgr3G103943

Mycgr3G57362
  
Location: 5862-7296

Mycgr3G57362

Mycgr3G39086
  
Location: 7396-8368

Mycgr3G39086

Mycgr3G103942
  
Location: 8468-8714

Mycgr3G103942

Mycgr3G108865
  
Location: 8814-10239

Mycgr3G108865

Mycgr3G70475
  
Location: 10339-11821

Mycgr3G70475

Mycgr3G108866
  
Location: 11921-13010

Mycgr3G108866

Mycgr3G92136
  
Location: 13110-13593

Mycgr3G92136

arrestin domain-containing protein
  
Accession: EJB11329
  
Location: 3144066-3146232
  
 NCBI BlastP on this gene

EJB11329

V-type ATPase, G subunit
  
Accession: EAS29343
  
Location: 3147571-3148278
  
 NCBI BlastP on this gene

EAS29343

cytochrome c oxidase assembly protein
  
Accession: EAS29342
  
Location: 3148585-3148927
  
 NCBI BlastP on this gene

EAS29342

hypothetical protein
  
Accession: EAS29341
  
Location: 3150053-3152365
  
 NCBI BlastP on this gene

EAS29341

hypothetical protein
  
Accession: EAS29340
  
Location: 3153806-3155135
  
  
**BlastP hit with Mycgr3G38483**
  
Percentage identity: 36 %
  
BlastP bit score: 209
  
Sequence coverage: 98 %
  
E-value: 1e-60
  
  
 NCBI BlastP on this gene

EAS29340

40S ribosomal protein S24
  
Accession: EJB11330
  
Location: 3155465-3156118
  
  
**BlastP hit with Mycgr3G70471**
  
Percentage identity: 77 %
  
BlastP bit score: 203
  
Sequence coverage: 91 %
  
E-value: 3e-64
  
  
 NCBI BlastP on this gene

EJB11330

hypothetical protein
  
Accession: EAS29337
  
Location: 3156928-3158393
  
 NCBI BlastP on this gene

EAS29337

peptidyl-prolyl cis-trans isomerase-like 4
  
Accession: EAS29335
  
Location: 3161371-3162891
  
 NCBI BlastP on this gene

EAS29335

UDP-N-acetylglucosamine pyrophosphorylase
  
Accession: EAS29334
  
Location: 3163507-3165167
  
 NCBI BlastP on this gene

EAS29334

Query: Architecture Search FASTA input

GG663363 : Ajellomyces capsulatus G186AR genomic scaffold supercont2.1    Total score: 2.0     Cumulative Blast bit score: 412

Hit cluster cross-links:

Mycgr3G70471
  
Location: 0-405

Mycgr3G70471

Mycgr3G39149
  
Location: 505-1798

Mycgr3G39149

Mycgr3G92130
  
Location: 1898-2396

Mycgr3G92130

Mycgr3G38483
  
Location: 2496-3576

Mycgr3G38483

Mycgr3G108869
  
Location: 3676-5056

Mycgr3G108869

Mycgr3G103943
  
Location: 5156-5762

Mycgr3G103943

Mycgr3G57362
  
Location: 5862-7296

Mycgr3G57362

Mycgr3G39086
  
Location: 7396-8368

Mycgr3G39086

Mycgr3G103942
  
Location: 8468-8714

Mycgr3G103942

Mycgr3G108865
  
Location: 8814-10239

Mycgr3G108865

Mycgr3G70475
  
Location: 10339-11821

Mycgr3G70475

Mycgr3G108866
  
Location: 11921-13010

Mycgr3G108866

Mycgr3G92136
  
Location: 13110-13593

Mycgr3G92136

UDP-N-acetylglucosamine pyrophosphorylase
  
Accession: EEH10871
  
Location: 1062872-1064635
  
 NCBI BlastP on this gene

EEH10871

peptidyl-prolyl cis-trans isomerase-like protein 4
  
Accession: EEH10872
  
Location: 1065208-1066773
  
 NCBI BlastP on this gene

EEH10872

predicted protein
  
Accession: EEH10873
  
Location: 1069539-1071117
  
 NCBI BlastP on this gene

EEH10873

40S ribosomal protein S24
  
Accession: EEH10874
  
Location: 1072060-1072713
  
  
**BlastP hit with Mycgr3G70471**
  
Percentage identity: 78 %
  
BlastP bit score: 202
  
Sequence coverage: 90 %
  
E-value: 4e-64
  
  
 NCBI BlastP on this gene

EEH10874

conserved hypothetical protein
  
Accession: EEH10875
  
Location: 1073005-1074344
  
  
**BlastP hit with Mycgr3G38483**
  
Percentage identity: 34 %
  
BlastP bit score: 210
  
Sequence coverage: 100 %
  
E-value: 5e-61
  
  
 NCBI BlastP on this gene

EEH10875

conserved hypothetical protein
  
Accession: EEH10876
  
Location: 1076855-1079374
  
 NCBI BlastP on this gene

EEH10876

conserved hypothetical protein
  
Accession: EEH10877
  
Location: 1080631-1080991
  
 NCBI BlastP on this gene

EEH10877

conserved hypothetical protein
  
Accession: EEH10878
  
Location: 1081289-1082174
  
 NCBI BlastP on this gene

EEH10878

Query: Architecture Search FASTA input

ABDF02000003 : Trichoderma virens Gv29-8    Total score: 2.0     Cumulative Blast bit score: 412

Hit cluster cross-links:

Mycgr3G70471
  
Location: 0-405

Mycgr3G70471

Mycgr3G39149
  
Location: 505-1798

Mycgr3G39149

Mycgr3G92130
  
Location: 1898-2396

Mycgr3G92130

Mycgr3G38483
  
Location: 2496-3576

Mycgr3G38483

Mycgr3G108869
  
Location: 3676-5056

Mycgr3G108869

Mycgr3G103943
  
Location: 5156-5762

Mycgr3G103943

Mycgr3G57362
  
Location: 5862-7296

Mycgr3G57362

Mycgr3G39086
  
Location: 7396-8368

Mycgr3G39086

Mycgr3G103942
  
Location: 8468-8714

Mycgr3G103942

Mycgr3G108865
  
Location: 8814-10239

Mycgr3G108865

Mycgr3G70475
  
Location: 10339-11821

Mycgr3G70475

Mycgr3G108866
  
Location: 11921-13010

Mycgr3G108866

Mycgr3G92136
  
Location: 13110-13593

Mycgr3G92136

hypothetical protein
  
Accession: EHK25803
  
Location: 2316322-2317849
  
 NCBI BlastP on this gene

EHK25803

hypothetical protein
  
Accession: EHK25804
  
Location: 2319541-2319984
  
 NCBI BlastP on this gene

EHK25804

hypothetical protein
  
Accession: EHK25805
  
Location: 2320045-2320671
  
 NCBI BlastP on this gene

EHK25805

hypothetical protein
  
Accession: EHK25806
  
Location: 2322024-2323320
  
 NCBI BlastP on this gene

EHK25806

hypothetical protein
  
Accession: EHK25807
  
Location: 2324822-2325889
  
  
**BlastP hit with Mycgr3G38483**
  
Percentage identity: 45 %
  
BlastP bit score: 219
  
Sequence coverage: 83 %
  
E-value: 9e-65
  
  
 NCBI BlastP on this gene

EHK25807

hypothetical protein
  
Accession: EHK25808
  
Location: 2326459-2327300
  
  
**BlastP hit with Mycgr3G70471**
  
Percentage identity: 76 %
  
BlastP bit score: 193
  
Sequence coverage: 89 %
  
E-value: 3e-60
  
  
 NCBI BlastP on this gene

EHK25808

hypothetical protein
  
Accession: EHK25809
  
Location: 2327993-2329644
  
 NCBI BlastP on this gene

EHK25809

hypothetical protein
  
Accession: EHK25810
  
Location: 2330551-2331273
  
 NCBI BlastP on this gene

EHK25810

hypothetical protein
  
Accession: EHK25811
  
Location: 2333335-2334672
  
 NCBI BlastP on this gene

EHK25811

hypothetical protein
  
Accession: EHK25812
  
Location: 2335119-2336459
  
 NCBI BlastP on this gene

EHK25812

Query: Architecture Search FASTA input

ABDG02000029 : Trichoderma atroviride IMI 206040    Total score: 2.0     Cumulative Blast bit score: 411

Hit cluster cross-links:

Mycgr3G70471
  
Location: 0-405

Mycgr3G70471

Mycgr3G39149
  
Location: 505-1798

Mycgr3G39149

Mycgr3G92130
  
Location: 1898-2396

Mycgr3G92130

Mycgr3G38483
  
Location: 2496-3576

Mycgr3G38483

Mycgr3G108869
  
Location: 3676-5056

Mycgr3G108869

Mycgr3G103943
  
Location: 5156-5762

Mycgr3G103943

Mycgr3G57362
  
Location: 5862-7296

Mycgr3G57362

Mycgr3G39086
  
Location: 7396-8368

Mycgr3G39086

Mycgr3G103942
  
Location: 8468-8714

Mycgr3G103942

Mycgr3G108865
  
Location: 8814-10239

Mycgr3G108865

Mycgr3G70475
  
Location: 10339-11821

Mycgr3G70475

Mycgr3G108866
  
Location: 11921-13010

Mycgr3G108866

Mycgr3G92136
  
Location: 13110-13593

Mycgr3G92136

hypothetical protein
  
Accession: EHK39412
  
Location: 235064-236401
  
 NCBI BlastP on this gene

EHK39412

hypothetical protein
  
Accession: EHK39413
  
Location: 236821-238165
  
 NCBI BlastP on this gene

EHK39413

hypothetical protein
  
Accession: EHK39414
  
Location: 239802-240524
  
 NCBI BlastP on this gene

EHK39414

hypothetical protein
  
Accession: EHK39415
  
Location: 241334-242959
  
 NCBI BlastP on this gene

EHK39415

hypothetical protein
  
Accession: EHK39416
  
Location: 243535-244364
  
  
**BlastP hit with Mycgr3G70471**
  
Percentage identity: 76 %
  
BlastP bit score: 195
  
Sequence coverage: 89 %
  
E-value: 5e-61
  
  
 NCBI BlastP on this gene

EHK39416

hypothetical protein
  
Accession: EHK39417
  
Location: 244840-245913
  
  
**BlastP hit with Mycgr3G38483**
  
Percentage identity: 39 %
  
BlastP bit score: 216
  
Sequence coverage: 99 %
  
E-value: 2e-63
  
  
 NCBI BlastP on this gene

EHK39417

hypothetical protein
  
Accession: EHK39418
  
Location: 250870-251358
  
 NCBI BlastP on this gene

EHK39418

hypothetical protein
  
Accession: EHK39419
  
Location: 252955-254214
  
 NCBI BlastP on this gene

EHK39419

Query: Architecture Search FASTA input

GL636509 : Coccidioides posadasii str. Silveira unplaced genomic scaffold supercont2.24    Total score: 2.0     Cumulative Blast bit score: 409

Hit cluster cross-links:

Mycgr3G70471
  
Location: 0-405

Mycgr3G70471

Mycgr3G39149
  
Location: 505-1798

Mycgr3G39149

Mycgr3G92130
  
Location: 1898-2396

Mycgr3G92130

Mycgr3G38483
  
Location: 2496-3576

Mycgr3G38483

Mycgr3G108869
  
Location: 3676-5056

Mycgr3G108869

Mycgr3G103943
  
Location: 5156-5762

Mycgr3G103943

Mycgr3G57362
  
Location: 5862-7296

Mycgr3G57362

Mycgr3G39086
  
Location: 7396-8368

Mycgr3G39086

Mycgr3G103942
  
Location: 8468-8714

Mycgr3G103942

Mycgr3G108865
  
Location: 8814-10239

Mycgr3G108865

Mycgr3G70475
  
Location: 10339-11821

Mycgr3G70475

Mycgr3G108866
  
Location: 11921-13010

Mycgr3G108866

Mycgr3G92136
  
Location: 13110-13593

Mycgr3G92136

hypothetical protein
  
Accession: EFW13958
  
Location: 65653-67814
  
 NCBI BlastP on this gene

EFW13958

predicted protein
  
Accession: EFW13959
  
Location: 68145-69069
  
 NCBI BlastP on this gene

EFW13959

vacuolar ATPase
  
Accession: EFW13960
  
Location: 69169-69877
  
 NCBI BlastP on this gene

EFW13960

cytochrome c oxidase assembly protein
  
Accession: EFW13961
  
Location: 70184-70526
  
 NCBI BlastP on this gene

EFW13961

conserved hypothetical protein
  
Accession: EFW13962
  
Location: 71649-73964
  
 NCBI BlastP on this gene

EFW13962

conserved hypothetical protein
  
Accession: EFW13963
  
Location: 75389-76718
  
  
**BlastP hit with Mycgr3G38483**
  
Percentage identity: 35 %
  
BlastP bit score: 206
  
Sequence coverage: 98 %
  
E-value: 1e-59
  
  
 NCBI BlastP on this gene

EFW13963

40S ribosomal protein S24-A
  
Accession: EFW13964
  
Location: 77048-77697
  
  
**BlastP hit with Mycgr3G70471**
  
Percentage identity: 77 %
  
BlastP bit score: 203
  
Sequence coverage: 91 %
  
E-value: 3e-64
  
  
 NCBI BlastP on this gene

EFW13964

conserved hypothetical protein
  
Accession: EFW13965
  
Location: 78507-79972
  
 NCBI BlastP on this gene

EFW13965

peptidyl-prolyl cis-trans isomerase
  
Accession: EFW13966
  
Location: 82984-84504
  
 NCBI BlastP on this gene

EFW13966

UDP-N-acetylglucosamine pyrophosphorylase
  
Accession: EFW13967
  
Location: 85089-86749
  
 NCBI BlastP on this gene

EFW13967

Query: Architecture Search FASTA input

AABX02000037 : Neurospora crassa OR74A    Total score: 2.0     Cumulative Blast bit score: 407

Hit cluster cross-links:

Mycgr3G70471
  
Location: 0-405

Mycgr3G70471

Mycgr3G39149
  
Location: 505-1798

Mycgr3G39149

Mycgr3G92130
  
Location: 1898-2396

Mycgr3G92130

Mycgr3G38483
  
Location: 2496-3576

Mycgr3G38483

Mycgr3G108869
  
Location: 3676-5056

Mycgr3G108869

Mycgr3G103943
  
Location: 5156-5762

Mycgr3G103943

Mycgr3G57362
  
Location: 5862-7296

Mycgr3G57362

Mycgr3G39086
  
Location: 7396-8368

Mycgr3G39086

Mycgr3G103942
  
Location: 8468-8714

Mycgr3G103942

Mycgr3G108865
  
Location: 8814-10239

Mycgr3G108865

Mycgr3G70475
  
Location: 10339-11821

Mycgr3G70475

Mycgr3G108866
  
Location: 11921-13010

Mycgr3G108866

Mycgr3G92136
  
Location: 13110-13593

Mycgr3G92136

predicted protein
  
Accession: EAA28169
  
Location: 232781-234547
  
 NCBI BlastP on this gene

EAA28169

conserved hypothetical protein
  
Accession: EAA28170
  
Location: 227120-228561
  
  
**BlastP hit with Mycgr3G38483**
  
Percentage identity: 38 %
  
BlastP bit score: 186
  
Sequence coverage: 102 %
  
E-value: 8e-52
  
  
 NCBI BlastP on this gene

EAA28170

40S ribosomal protein S24
  
Accession: EAA28171
  
Location: 225629-226685
  
  
**BlastP hit with Mycgr3G70471**
  
Percentage identity: 80 %
  
BlastP bit score: 221
  
Sequence coverage: 98 %
  
E-value: 4e-71
  
  
 NCBI BlastP on this gene

EAA28171

predicted protein
  
Accession: EAA28172
  
Location: 224381-225285
  
 NCBI BlastP on this gene

EAA28172

predicted protein
  
Accession: EAA28173
  
Location: 223164-223852
  
 NCBI BlastP on this gene

EAA28173

predicted protein
  
Accession: EAA28174
  
Location: 221473-222809
  
 NCBI BlastP on this gene

EAA28174

predicted protein
  
Accession: EAA28175
  
Location: 219188-220444
  
 NCBI BlastP on this gene

EAA28175

predicted protein
  
Accession: EAA28176
  
Location: 212968-217835
  
 NCBI BlastP on this gene

EAA28176

Query: Architecture Search FASTA input

JH126408 : Cordyceps militaris CM01 unplaced genomic scaffold CCM\_S00010    Total score: 2.0     Cumulative Blast bit score: 403

Hit cluster cross-links:

Mycgr3G70471
  
Location: 0-405

Mycgr3G70471

Mycgr3G39149
  
Location: 505-1798

Mycgr3G39149

Mycgr3G92130
  
Location: 1898-2396

Mycgr3G92130

Mycgr3G38483
  
Location: 2496-3576

Mycgr3G38483

Mycgr3G108869
  
Location: 3676-5056

Mycgr3G108869

Mycgr3G103943
  
Location: 5156-5762

Mycgr3G103943

Mycgr3G57362
  
Location: 5862-7296

Mycgr3G57362

Mycgr3G39086
  
Location: 7396-8368

Mycgr3G39086

Mycgr3G103942
  
Location: 8468-8714

Mycgr3G103942

Mycgr3G108865
  
Location: 8814-10239

Mycgr3G108865

Mycgr3G70475
  
Location: 10339-11821

Mycgr3G70475

Mycgr3G108866
  
Location: 11921-13010

Mycgr3G108866

Mycgr3G92136
  
Location: 13110-13593

Mycgr3G92136

cyclophilin-type peptidyl-prolyl cis-trans isomerase
  
Accession: EGX87684
  
Location: 404866-406436
  
 NCBI BlastP on this gene

EGX87684

hypothetical protein
  
Accession: EGX87685
  
Location: 407061-410641
  
 NCBI BlastP on this gene

EGX87685

Methyltransferase-16, putative
  
Accession: EGX87686
  
Location: 411214-412340
  
  
**BlastP hit with Mycgr3G38483**
  
Percentage identity: 42 %
  
BlastP bit score: 230
  
Sequence coverage: 98 %
  
E-value: 9e-69
  
  
 NCBI BlastP on this gene

EGX87686

40S ribosomal protein S24
  
Accession: EGX87687
  
Location: 412669-413585
  
  
**BlastP hit with Mycgr3G70471**
  
Percentage identity: 55 %
  
BlastP bit score: 173
  
Sequence coverage: 122 %
  
E-value: 8e-52
  
  
 NCBI BlastP on this gene

EGX87687

hypothetical protein
  
Accession: EGX87688
  
Location: 414562-414984
  
 NCBI BlastP on this gene

EGX87688

hypothetical protein
  
Accession: EGX87689
  
Location: 415888-416340
  
 NCBI BlastP on this gene

EGX87689

hypothetical protein
  
Accession: EGX87690
  
Location: 416611-420349
  
 NCBI BlastP on this gene

EGX87690

GTP cyclohydrolase-2
  
Accession: EGX87691
  
Location: 420812-421999
  
 NCBI BlastP on this gene

EGX87691

Query: Architecture Search FASTA input

CH476621 : Sclerotinia sclerotiorum 1980 scaffold\_1 genomic scaffold    Total score: 2.0     Cumulative Blast bit score: 403

Hit cluster cross-links:

Mycgr3G70471
  
Location: 0-405

Mycgr3G70471

Mycgr3G39149
  
Location: 505-1798

Mycgr3G39149

Mycgr3G92130
  
Location: 1898-2396

Mycgr3G92130

Mycgr3G38483
  
Location: 2496-3576

Mycgr3G38483

Mycgr3G108869
  
Location: 3676-5056

Mycgr3G108869

Mycgr3G103943
  
Location: 5156-5762

Mycgr3G103943

Mycgr3G57362
  
Location: 5862-7296

Mycgr3G57362

Mycgr3G39086
  
Location: 7396-8368

Mycgr3G39086

Mycgr3G103942
  
Location: 8468-8714

Mycgr3G103942

Mycgr3G108865
  
Location: 8814-10239

Mycgr3G108865

Mycgr3G70475
  
Location: 10339-11821

Mycgr3G70475

Mycgr3G108866
  
Location: 11921-13010

Mycgr3G108866

Mycgr3G92136
  
Location: 13110-13593

Mycgr3G92136

hypothetical protein
  
Accession: EDN91543
  
Location: 2436377-2437615
  
 NCBI BlastP on this gene

EDN91543

hypothetical protein
  
Accession: EDN91544
  
Location: 2438063-2439955
  
 NCBI BlastP on this gene

EDN91544

hypothetical protein
  
Accession: EDN91545
  
Location: 2440484-2441369
  
 NCBI BlastP on this gene

EDN91545

predicted protein
  
Accession: EDN91546
  
Location: 2441673-2441807
  
 NCBI BlastP on this gene

EDN91546

hypothetical protein
  
Accession: EDN91547
  
Location: 2442389-2443833
  
  
**BlastP hit with Mycgr3G38483**
  
Percentage identity: 41 %
  
BlastP bit score: 196
  
Sequence coverage: 92 %
  
E-value: 6e-55
  
  
 NCBI BlastP on this gene

EDN91547

40S ribosomal protein S24
  
Accession: EDN91548
  
Location: 2444103-2444744
  
  
**BlastP hit with Mycgr3G70471**
  
Percentage identity: 80 %
  
BlastP bit score: 207
  
Sequence coverage: 89 %
  
E-value: 9e-66
  
  
 NCBI BlastP on this gene

EDN91548

predicted protein
  
Accession: EDN91549
  
Location: 2446178-2446542
  
 NCBI BlastP on this gene

EDN91549

predicted protein
  
Accession: EDN91550
  
Location: 2448080-2450124
  
 NCBI BlastP on this gene

EDN91550

predicted protein
  
Accession: EDN91551
  
Location: 2450609-2451017
  
 NCBI BlastP on this gene

EDN91551

predicted protein
  
Accession: EDN91552
  
Location: 2451683-2451978
  
 NCBI BlastP on this gene

EDN91552

hypothetical protein
  
Accession: EDN91553
  
Location: 2452354-2453865
  
 NCBI BlastP on this gene

EDN91553

Query: Architecture Search FASTA input

GG698544 : Trichophyton tonsurans CBS 112818 genomic scaffold supercont1.68    Total score: 2.0     Cumulative Blast bit score: 396

Hit cluster cross-links:

Mycgr3G70471
  
Location: 0-405

Mycgr3G70471

Mycgr3G39149
  
Location: 505-1798

Mycgr3G39149

Mycgr3G92130
  
Location: 1898-2396

Mycgr3G92130

Mycgr3G38483
  
Location: 2496-3576

Mycgr3G38483

Mycgr3G108869
  
Location: 3676-5056

Mycgr3G108869

Mycgr3G103943
  
Location: 5156-5762

Mycgr3G103943

Mycgr3G57362
  
Location: 5862-7296

Mycgr3G57362

Mycgr3G39086
  
Location: 7396-8368

Mycgr3G39086

Mycgr3G103942
  
Location: 8468-8714

Mycgr3G103942

Mycgr3G108865
  
Location: 8814-10239

Mycgr3G108865

Mycgr3G70475
  
Location: 10339-11821

Mycgr3G70475

Mycgr3G108866
  
Location: 11921-13010

Mycgr3G108866

Mycgr3G92136
  
Location: 13110-13593

Mycgr3G92136

hypothetical protein
  
Accession: EGE00592
  
Location: 55055-55989
  
 NCBI BlastP on this gene

EGE00592

hypothetical protein
  
Accession: EGE00593
  
Location: 57156-58151
  
 NCBI BlastP on this gene

EGE00593

tripeptidyl peptidase SED3
  
Accession: EGE00594
  
Location: 58507-60232
  
 NCBI BlastP on this gene

EGE00594

hypothetical protein
  
Accession: EGE00595
  
Location: 61488-62264
  
 NCBI BlastP on this gene

EGE00595

hypothetical protein
  
Accession: EGE00596
  
Location: 63367-64786
  
  
**BlastP hit with Mycgr3G38483**
  
Percentage identity: 34 %
  
BlastP bit score: 202
  
Sequence coverage: 98 %
  
E-value: 9e-58
  
  
 NCBI BlastP on this gene

EGE00596

ribosomal protein S24
  
Accession: EGE00597
  
Location: 65146-65896
  
  
**BlastP hit with Mycgr3G70471**
  
Percentage identity: 72 %
  
BlastP bit score: 194
  
Sequence coverage: 90 %
  
E-value: 1e-60
  
  
 NCBI BlastP on this gene

EGE00597

hypothetical protein
  
Accession: EGE00598
  
Location: 66802-68369
  
 NCBI BlastP on this gene

EGE00598

peptidyl-prolyl cis-trans isomerase
  
Accession: EGE00599
  
Location: 71419-72431
  
 NCBI BlastP on this gene

EGE00599

UDP-N-acetylglucosamine pyrophosphorylase
  
Accession: EGE00600
  
Location: 73683-75342
  
 NCBI BlastP on this gene

EGE00600

Query: Architecture Search FASTA input

CH476615 : Uncinocarpus reesii 1704 scaffold\_1 genomic scaffold    Total score: 2.0     Cumulative Blast bit score: 396

Hit cluster cross-links:

Mycgr3G70471
  
Location: 0-405

Mycgr3G70471

Mycgr3G39149
  
Location: 505-1798

Mycgr3G39149

Mycgr3G92130
  
Location: 1898-2396

Mycgr3G92130

Mycgr3G38483
  
Location: 2496-3576

Mycgr3G38483

Mycgr3G108869
  
Location: 3676-5056

Mycgr3G108869

Mycgr3G103943
  
Location: 5156-5762

Mycgr3G103943

Mycgr3G57362
  
Location: 5862-7296

Mycgr3G57362

Mycgr3G39086
  
Location: 7396-8368

Mycgr3G39086

Mycgr3G103942
  
Location: 8468-8714

Mycgr3G103942

Mycgr3G108865
  
Location: 8814-10239

Mycgr3G108865

Mycgr3G70475
  
Location: 10339-11821

Mycgr3G70475

Mycgr3G108866
  
Location: 11921-13010

Mycgr3G108866

Mycgr3G92136
  
Location: 13110-13593

Mycgr3G92136

hypothetical protein
  
Accession: EEP77263
  
Location: 5536631-5538238
  
 NCBI BlastP on this gene

EEP77263

hypothetical protein
  
Accession: EEP77264
  
Location: 5538811-5540331
  
 NCBI BlastP on this gene

EEP77264

predicted protein
  
Accession: EEP77265
  
Location: 5543194-5544669
  
 NCBI BlastP on this gene

EEP77265

40S ribosomal protein S24-A
  
Accession: EEP77266
  
Location: 5545436-5546094
  
  
**BlastP hit with Mycgr3G70471**
  
Percentage identity: 78 %
  
BlastP bit score: 206
  
Sequence coverage: 91 %
  
E-value: 2e-65
  
  
 NCBI BlastP on this gene

EEP77266

conserved hypothetical protein
  
Accession: EEP77267
  
Location: 5546553-5547723
  
  
**BlastP hit with Mycgr3G38483**
  
Percentage identity: 38 %
  
BlastP bit score: 190
  
Sequence coverage: 88 %
  
E-value: 2e-53
  
  
 NCBI BlastP on this gene

EEP77267

predicted protein
  
Accession: EEP77268
  
Location: 5549149-5551478
  
 NCBI BlastP on this gene

EEP77268

V-type ATPase, G subunit
  
Accession: EEP77269
  
Location: 5553228-5553903
  
 NCBI BlastP on this gene

EEP77269

conserved hypothetical protein
  
Accession: EEP77270
  
Location: 5555120-5557289
  
 NCBI BlastP on this gene

EEP77270

Query: Architecture Search FASTA input

DS995906 : Penicillium marneffei ATCC 18224 scf\_1105668340770 genomic scaffold    Total score: 2.0     Cumulative Blast bit score: 395

Hit cluster cross-links:

Mycgr3G70471
  
Location: 0-405

Mycgr3G70471

Mycgr3G39149
  
Location: 505-1798

Mycgr3G39149

Mycgr3G92130
  
Location: 1898-2396

Mycgr3G92130

Mycgr3G38483
  
Location: 2496-3576

Mycgr3G38483

Mycgr3G108869
  
Location: 3676-5056

Mycgr3G108869

Mycgr3G103943
  
Location: 5156-5762

Mycgr3G103943

Mycgr3G57362
  
Location: 5862-7296

Mycgr3G57362

Mycgr3G39086
  
Location: 7396-8368

Mycgr3G39086

Mycgr3G103942
  
Location: 8468-8714

Mycgr3G103942

Mycgr3G108865
  
Location: 8814-10239

Mycgr3G108865

Mycgr3G70475
  
Location: 10339-11821

Mycgr3G70475

Mycgr3G108866
  
Location: 11921-13010

Mycgr3G108866

Mycgr3G92136
  
Location: 13110-13593

Mycgr3G92136

alcohol dehydrogenase, putative
  
Accession: EEA18773
  
Location: 574741-576008
  
 NCBI BlastP on this gene

EEA18773

conserved hypothetical protein
  
Accession: EEA18774
  
Location: 576805-577662
  
 NCBI BlastP on this gene

EEA18774

membrane associated DnaJ chaperone, putative
  
Accession: EEA18775
  
Location: 579202-580404
  
 NCBI BlastP on this gene

EEA18775

RNA polymerase I subunit Rpa43, putative
  
Accession: EEA18776
  
Location: 580723-582048
  
 NCBI BlastP on this gene

EEA18776

conserved hypothetical protein
  
Accession: EEA18777
  
Location: 582679-584095
  
  
**BlastP hit with Mycgr3G38483**
  
Percentage identity: 34 %
  
BlastP bit score: 175
  
Sequence coverage: 101 %
  
E-value: 1e-47
  
  
 NCBI BlastP on this gene

EEA18777

37S ribosomal protein S24
  
Accession: EEA18778
  
Location: 584446-585337
  
  
**BlastP hit with Mycgr3G70471**
  
Percentage identity: 81 %
  
BlastP bit score: 220
  
Sequence coverage: 97 %
  
E-value: 6e-71
  
  
 NCBI BlastP on this gene

EEA18778

conserved hypothetical protein
  
Accession: EEA18780
  
Location: 586518-589583
  
 NCBI BlastP on this gene

EEA18780

2,3-dihydroxybenzoic acid decarboxylase, putative
  
Accession: EEA18781
  
Location: 590076-591035
  
 NCBI BlastP on this gene

EEA18781

conserved hypothetical protein
  
Accession: EEA18782
  
Location: 591562-592831
  
 NCBI BlastP on this gene

EEA18782

Query: Architecture Search FASTA input

DS995705 : Microsporum canis CBS 113480 supercont1.5 genomic scaffold    Total score: 2.0     Cumulative Blast bit score: 395

Hit cluster cross-links:

Mycgr3G70471
  
Location: 0-405

Mycgr3G70471

Mycgr3G39149
  
Location: 505-1798

Mycgr3G39149

Mycgr3G92130
  
Location: 1898-2396

Mycgr3G92130

Mycgr3G38483
  
Location: 2496-3576

Mycgr3G38483

Mycgr3G108869
  
Location: 3676-5056

Mycgr3G108869

Mycgr3G103943
  
Location: 5156-5762

Mycgr3G103943

Mycgr3G57362
  
Location: 5862-7296

Mycgr3G57362

Mycgr3G39086
  
Location: 7396-8368

Mycgr3G39086

Mycgr3G103942
  
Location: 8468-8714

Mycgr3G103942

Mycgr3G108865
  
Location: 8814-10239

Mycgr3G108865

Mycgr3G70475
  
Location: 10339-11821

Mycgr3G70475

Mycgr3G108866
  
Location: 11921-13010

Mycgr3G108866

Mycgr3G92136
  
Location: 13110-13593

Mycgr3G92136

UDP-N-acetylglucosamine pyrophosphorylase
  
Accession: EEQ33253
  
Location: 2663548-2665225
  
 NCBI BlastP on this gene

EEQ33253

peptidyl-prolyl cis-trans isomerase-like 4
  
Accession: EEQ33254
  
Location: 2665838-2667414
  
 NCBI BlastP on this gene

EEQ33254

predicted protein
  
Accession: EEQ33255
  
Location: 2669920-2671458
  
 NCBI BlastP on this gene

EEQ33255

40S ribosomal protein S24
  
Accession: EEQ33256
  
Location: 2672351-2673093
  
  
**BlastP hit with Mycgr3G70471**
  
Percentage identity: 72 %
  
BlastP bit score: 191
  
Sequence coverage: 90 %
  
E-value: 1e-59
  
  
 NCBI BlastP on this gene

EEQ33256

conserved hypothetical protein
  
Accession: EEQ33257
  
Location: 2673419-2674655
  
  
**BlastP hit with Mycgr3G38483**
  
Percentage identity: 38 %
  
BlastP bit score: 204
  
Sequence coverage: 89 %
  
E-value: 8e-59
  
  
 NCBI BlastP on this gene

EEQ33257

tripeptidyl peptidase SED3
  
Accession: EEQ33258
  
Location: 2677505-2679414
  
 NCBI BlastP on this gene

EEQ33258

conserved hypothetical protein
  
Accession: EEQ33259
  
Location: 2679743-2680738
  
 NCBI BlastP on this gene

EEQ33259

predicted protein
  
Accession: EEQ33260
  
Location: 2680904-2681560
  
 NCBI BlastP on this gene

EEQ33260

26S proteasome non-ATPase regulatory subunit 8
  
Accession: EEQ33261
  
Location: 2681888-2682841
  
 NCBI BlastP on this gene

EEQ33261

Query: Architecture Search FASTA input

AM270259 : Aspergillus niger contig An12c0020, genomic contig.    Total score: 2.0     Cumulative Blast bit score: 394

Hit cluster cross-links:

Mycgr3G70471
  
Location: 0-405

Mycgr3G70471

Mycgr3G39149
  
Location: 505-1798

Mycgr3G39149

Mycgr3G92130
  
Location: 1898-2396

Mycgr3G92130

Mycgr3G38483
  
Location: 2496-3576

Mycgr3G38483

Mycgr3G108869
  
Location: 3676-5056

Mycgr3G108869

Mycgr3G103943
  
Location: 5156-5762

Mycgr3G103943

Mycgr3G57362
  
Location: 5862-7296

Mycgr3G57362

Mycgr3G39086
  
Location: 7396-8368

Mycgr3G39086

Mycgr3G103942
  
Location: 8468-8714

Mycgr3G103942

Mycgr3G108865
  
Location: 8814-10239

Mycgr3G108865

Mycgr3G70475
  
Location: 10339-11821

Mycgr3G70475

Mycgr3G108866
  
Location: 11921-13010

Mycgr3G108866

Mycgr3G92136
  
Location: 13110-13593

Mycgr3G92136

not annotated
  
Accession: CAK40978
  
Location: 51174-52792
  
 NCBI BlastP on this gene

An12g00490

not annotated
  
Accession: CAK40979
  
Location: 56452-58039
  
 NCBI BlastP on this gene

An12g00500

not annotated
  
Accession: CAK40980
  
Location: 60277-61028
  
  
**BlastP hit with Mycgr3G70471**
  
Percentage identity: 79 %
  
BlastP bit score: 217
  
Sequence coverage: 97 %
  
E-value: 9e-70
  
  
 NCBI BlastP on this gene

An12g00510

not annotated
  
Accession: CAK40981
  
Location: 61430-63087
  
  
**BlastP hit with Mycgr3G38483**
  
Percentage identity: 31 %
  
BlastP bit score: 177
  
Sequence coverage: 103 %
  
E-value: 4e-48
  
  
 NCBI BlastP on this gene

An12g00520

not annotated
  
Accession: CAK40982
  
Location: 63485-65563
  
 NCBI BlastP on this gene

An12g00530

hypothetical protein
  
Accession: CAK40983
  
Location: 65710-66171
  
 NCBI BlastP on this gene

An12g00540

unnamed
  
Accession: CAK40984
  
Location: 66712-70157
  
 NCBI BlastP on this gene

An12g00550

Query: Architecture Search FASTA input

JH226133 : Exophiala dermatitidis NIH/UT8656 unplaced genomic scaffold supercont1.4    Total score: 2.0     Cumulative Blast bit score: 393

Hit cluster cross-links:

Mycgr3G70471
  
Location: 0-405

Mycgr3G70471

Mycgr3G39149
  
Location: 505-1798

Mycgr3G39149

Mycgr3G92130
  
Location: 1898-2396

Mycgr3G92130

Mycgr3G38483
  
Location: 2496-3576

Mycgr3G38483

Mycgr3G108869
  
Location: 3676-5056

Mycgr3G108869

Mycgr3G103943
  
Location: 5156-5762

Mycgr3G103943

Mycgr3G57362
  
Location: 5862-7296

Mycgr3G57362

Mycgr3G39086
  
Location: 7396-8368

Mycgr3G39086

Mycgr3G103942
  
Location: 8468-8714

Mycgr3G103942

Mycgr3G108865
  
Location: 8814-10239

Mycgr3G108865

Mycgr3G70475
  
Location: 10339-11821

Mycgr3G70475

Mycgr3G108866
  
Location: 11921-13010

Mycgr3G108866

Mycgr3G92136
  
Location: 13110-13593

Mycgr3G92136

retrograde regulation protein 2
  
Accession: EHY57274
  
Location: 2784352-2786004
  
 NCBI BlastP on this gene

EHY57274

gibberellin 2-oxidase
  
Accession: EHY57275
  
Location: 2787125-2788285
  
 NCBI BlastP on this gene

EHY57275

hypothetical protein
  
Accession: EHY57276
  
Location: 2788969-2790113
  
 NCBI BlastP on this gene

EHY57276

hypothetical protein
  
Accession: EHY57277
  
Location: 2790695-2793641
  
 NCBI BlastP on this gene

EHY57277

hypothetical protein
  
Accession: EHY57278
  
Location: 2794187-2795428
  
  
**BlastP hit with Mycgr3G38483**
  
Percentage identity: 34 %
  
BlastP bit score: 179
  
Sequence coverage: 101 %
  
E-value: 4e-49
  
  
 NCBI BlastP on this gene

EHY57278

30S ribosomal protein S24e
  
Accession: EHY57279
  
Location: 2795782-2796380
  
  
**BlastP hit with Mycgr3G70471**
  
Percentage identity: 77 %
  
BlastP bit score: 214
  
Sequence coverage: 100 %
  
E-value: 2e-68
  
  
 NCBI BlastP on this gene

EHY57279

hypothetical protein, variant
  
Accession: EHY57280
  
Location: 2798525-2800217
  
 NCBI BlastP on this gene

EHY57280

peptidylprolyl isomerase
  
Accession: EHY57282
  
Location: 2803105-2804610
  
 NCBI BlastP on this gene

EHY57282

Query: Architecture Search FASTA input

ACJE01000005 : Aspergillus niger ATCC 1015    Total score: 2.0     Cumulative Blast bit score: 393

Hit cluster cross-links:

Mycgr3G70471
  
Location: 0-405

Mycgr3G70471

Mycgr3G39149
  
Location: 505-1798

Mycgr3G39149

Mycgr3G92130
  
Location: 1898-2396

Mycgr3G92130

Mycgr3G38483
  
Location: 2496-3576

Mycgr3G38483

Mycgr3G108869
  
Location: 3676-5056

Mycgr3G108869

Mycgr3G103943
  
Location: 5156-5762

Mycgr3G103943

Mycgr3G57362
  
Location: 5862-7296

Mycgr3G57362

Mycgr3G39086
  
Location: 7396-8368

Mycgr3G39086

Mycgr3G103942
  
Location: 8468-8714

Mycgr3G103942

Mycgr3G108865
  
Location: 8814-10239

Mycgr3G108865

Mycgr3G70475
  
Location: 10339-11821

Mycgr3G70475

Mycgr3G108866
  
Location: 11921-13010

Mycgr3G108866

Mycgr3G92136
  
Location: 13110-13593

Mycgr3G92136

hypothetical protein
  
Accession: EHA25968
  
Location: 1084395-1091067
  
 NCBI BlastP on this gene

EHA25968

hypothetical protein
  
Accession: EHA25969
  
Location: 1091465-1093130
  
  
**BlastP hit with Mycgr3G38483**
  
Percentage identity: 31 %
  
BlastP bit score: 176
  
Sequence coverage: 103 %
  
E-value: 8e-48
  
  
 NCBI BlastP on this gene

EHA25969

hypothetical protein
  
Accession: EHA25970
  
Location: 1093532-1094283
  
  
**BlastP hit with Mycgr3G70471**
  
Percentage identity: 79 %
  
BlastP bit score: 217
  
Sequence coverage: 97 %
  
E-value: 9e-70
  
  
 NCBI BlastP on this gene

EHA25970

hypothetical protein
  
Accession: EHA25971
  
Location: 1096521-1098108
  
 NCBI BlastP on this gene

EHA25971

hypothetical protein
  
Accession: EHA25972
  
Location: 1101768-1103386
  
 NCBI BlastP on this gene

EHA25972

Query: Architecture Search FASTA input

GL698729 : Metarhizium anisopliae ARSEF 23 unplaced genomic scaffold Scf\_019    Total score: 2.0     Cumulative Blast bit score: 391

Hit cluster cross-links:

Mycgr3G70471
  
Location: 0-405

Mycgr3G70471

Mycgr3G39149
  
Location: 505-1798

Mycgr3G39149

Mycgr3G92130
  
Location: 1898-2396

Mycgr3G92130

Mycgr3G38483
  
Location: 2496-3576

Mycgr3G38483

Mycgr3G108869
  
Location: 3676-5056

Mycgr3G108869

Mycgr3G103943
  
Location: 5156-5762

Mycgr3G103943

Mycgr3G57362
  
Location: 5862-7296

Mycgr3G57362

Mycgr3G39086
  
Location: 7396-8368

Mycgr3G39086

Mycgr3G103942
  
Location: 8468-8714

Mycgr3G103942

Mycgr3G108865
  
Location: 8814-10239

Mycgr3G108865

Mycgr3G70475
  
Location: 10339-11821

Mycgr3G70475

Mycgr3G108866
  
Location: 11921-13010

Mycgr3G108866

Mycgr3G92136
  
Location: 13110-13593

Mycgr3G92136

hypothetical protein
  
Accession: EFY96537
  
Location: 258201-258854
  
 NCBI BlastP on this gene

EFY96537

GTP cyclohydrolase-2
  
Accession: EFY96538
  
Location: 259852-261079
  
 NCBI BlastP on this gene

EFY96538

hypothetical protein
  
Accession: EFY96539
  
Location: 265425-266009
  
 NCBI BlastP on this gene

EFY96539

40S ribosomal protein S24
  
Accession: EFY96540
  
Location: 266646-267549
  
  
**BlastP hit with Mycgr3G70471**
  
Percentage identity: 78 %
  
BlastP bit score: 200
  
Sequence coverage: 89 %
  
E-value: 5e-63
  
  
 NCBI BlastP on this gene

EFY96540

hypothetical protein
  
Accession: EFY96541
  
Location: 268266-269084
  
  
**BlastP hit with Mycgr3G38483**
  
Percentage identity: 47 %
  
BlastP bit score: 191
  
Sequence coverage: 62 %
  
E-value: 2e-54
  
  
 NCBI BlastP on this gene

EFY96541

hypothetical protein
  
Accession: EFY96542
  
Location: 272267-272743
  
 NCBI BlastP on this gene

EFY96542

RNA binding protein
  
Accession: EFY96543
  
Location: 274134-275661
  
 NCBI BlastP on this gene

EFY96543

Query: Architecture Search FASTA input

GG700654 : Trichophyton rubrum CBS 118892 genomic scaffold supercont2.7    Total score: 2.0     Cumulative Blast bit score: 388

Hit cluster cross-links:

Mycgr3G70471
  
Location: 0-405

Mycgr3G70471

Mycgr3G39149
  
Location: 505-1798

Mycgr3G39149

Mycgr3G92130
  
Location: 1898-2396

Mycgr3G92130

Mycgr3G38483
  
Location: 2496-3576

Mycgr3G38483

Mycgr3G108869
  
Location: 3676-5056

Mycgr3G108869

Mycgr3G103943
  
Location: 5156-5762

Mycgr3G103943

Mycgr3G57362
  
Location: 5862-7296

Mycgr3G57362

Mycgr3G39086
  
Location: 7396-8368

Mycgr3G39086

Mycgr3G103942
  
Location: 8468-8714

Mycgr3G103942

Mycgr3G108865
  
Location: 8814-10239

Mycgr3G108865

Mycgr3G70475
  
Location: 10339-11821

Mycgr3G70475

Mycgr3G108866
  
Location: 11921-13010

Mycgr3G108866

Mycgr3G92136
  
Location: 13110-13593

Mycgr3G92136

hypothetical protein
  
Accession: EGD89783
  
Location: 81883-82816
  
 NCBI BlastP on this gene

EGD89783

hypothetical protein
  
Accession: EGD89784
  
Location: 83907-84902
  
 NCBI BlastP on this gene

EGD89784

tripeptidyl peptidase SED3
  
Accession: EGD89785
  
Location: 85232-87115
  
 NCBI BlastP on this gene

EGD89785

hypothetical protein
  
Accession: EGD89786
  
Location: 88269-89045
  
 NCBI BlastP on this gene

EGD89786

hypothetical protein
  
Accession: EGD89787
  
Location: 90141-91633
  
  
**BlastP hit with Mycgr3G38483**
  
Percentage identity: 34 %
  
BlastP bit score: 194
  
Sequence coverage: 97 %
  
E-value: 2e-54
  
  
 NCBI BlastP on this gene

EGD89787

40S ribosomal protein S24
  
Accession: EGD89788
  
Location: 91916-92663
  
  
**BlastP hit with Mycgr3G70471**
  
Percentage identity: 72 %
  
BlastP bit score: 194
  
Sequence coverage: 90 %
  
E-value: 1e-60
  
  
 NCBI BlastP on this gene

EGD89788

hypothetical protein
  
Accession: EGD89789
  
Location: 93549-94703
  
 NCBI BlastP on this gene

EGD89789

peptidyl-prolyl cis-trans isomerase
  
Accession: EGD89790
  
Location: 98412-99955
  
 NCBI BlastP on this gene

EGD89790

Query: Architecture Search FASTA input

EQ963479 : Aspergillus flavus NRRL3357 scf\_1106286418500 genomic scaffold    Total score: 2.0     Cumulative Blast bit score: 388

Hit cluster cross-links:

Mycgr3G70471
  
Location: 0-405

Mycgr3G70471

Mycgr3G39149
  
Location: 505-1798

Mycgr3G39149

Mycgr3G92130
  
Location: 1898-2396

Mycgr3G92130

Mycgr3G38483
  
Location: 2496-3576

Mycgr3G38483

Mycgr3G108869
  
Location: 3676-5056

Mycgr3G108869

Mycgr3G103943
  
Location: 5156-5762

Mycgr3G103943

Mycgr3G57362
  
Location: 5862-7296

Mycgr3G57362

Mycgr3G39086
  
Location: 7396-8368

Mycgr3G39086

Mycgr3G103942
  
Location: 8468-8714

Mycgr3G103942

Mycgr3G108865
  
Location: 8814-10239

Mycgr3G108865

Mycgr3G70475
  
Location: 10339-11821

Mycgr3G70475

Mycgr3G108866
  
Location: 11921-13010

Mycgr3G108866

Mycgr3G92136
  
Location: 13110-13593

Mycgr3G92136

hypothetical protein
  
Accession: EED50306
  
Location: 1945597-1945957
  
 NCBI BlastP on this gene

EED50306

Ran-binding protein (RanBP10), putative
  
Accession: EED50307
  
Location: 1946552-1948930
  
 NCBI BlastP on this gene

EED50307

conserved hypothetical protein
  
Accession: EED50308
  
Location: 1950601-1952770
  
 NCBI BlastP on this gene

EED50308

conserved hypothetical protein
  
Accession: EED50309
  
Location: 1953141-1954452
  
  
**BlastP hit with Mycgr3G38483**
  
Percentage identity: 32 %
  
BlastP bit score: 170
  
Sequence coverage: 106 %
  
E-value: 2e-45
  
  
 NCBI BlastP on this gene

EED50309

37S ribosomal protein S24
  
Accession: EED50310
  
Location: 1954744-1955516
  
  
**BlastP hit with Mycgr3G70471**
  
Percentage identity: 81 %
  
BlastP bit score: 218
  
Sequence coverage: 97 %
  
E-value: 3e-70
  
  
 NCBI BlastP on this gene

EED50310

hemagglutinin protein, putative
  
Accession: EED50311
  
Location: 1957574-1958722
  
 NCBI BlastP on this gene

EED50311

cyclophilin-type peptidyl-prolyl cis-trans isomerase, putative
  
Accession: EED50312
  
Location: 1962011-1963624
  
 NCBI BlastP on this gene

EED50312

Query: Architecture Search FASTA input

DF126478 : Aspergillus kawachii IFO 4308 DNA, contig: scaffold00032    Total score: 2.0     Cumulative Blast bit score: 388

Hit cluster cross-links:

Mycgr3G70471
  
Location: 0-405

Mycgr3G70471

Mycgr3G39149
  
Location: 505-1798

Mycgr3G39149

Mycgr3G92130
  
Location: 1898-2396

Mycgr3G92130

Mycgr3G38483
  
Location: 2496-3576

Mycgr3G38483

Mycgr3G108869
  
Location: 3676-5056

Mycgr3G108869

Mycgr3G103943
  
Location: 5156-5762

Mycgr3G103943

Mycgr3G57362
  
Location: 5862-7296

Mycgr3G57362

Mycgr3G39086
  
Location: 7396-8368

Mycgr3G39086

Mycgr3G103942
  
Location: 8468-8714

Mycgr3G103942

Mycgr3G108865
  
Location: 8814-10239

Mycgr3G108865

Mycgr3G70475
  
Location: 10339-11821

Mycgr3G70475

Mycgr3G108866
  
Location: 11921-13010

Mycgr3G108866

Mycgr3G92136
  
Location: 13110-13593

Mycgr3G92136

cyclophilin-type peptidyl-prolyl cis-trans isomerase
  
Accession: GAA91128
  
Location: 134748-136366
  
 NCBI BlastP on this gene

GAA91128

streptococcal hemagglutinin protein
  
Accession: GAA91129
  
Location: 140624-141604
  
 NCBI BlastP on this gene

GAA91129

37S ribosomal protein S24
  
Accession: GAA91130
  
Location: 144000-144764
  
  
**BlastP hit with Mycgr3G70471**
  
Percentage identity: 79 %
  
BlastP bit score: 217
  
Sequence coverage: 97 %
  
E-value: 9e-70
  
  
 NCBI BlastP on this gene

GAA91130

hypothetical protein
  
Accession: GAA91131
  
Location: 145186-146613
  
  
**BlastP hit with Mycgr3G38483**
  
Percentage identity: 35 %
  
BlastP bit score: 171
  
Sequence coverage: 91 %
  
E-value: 2e-46
  
  
 NCBI BlastP on this gene

GAA91131

similar to An12g00530
  
Accession: GAA91132
  
Location: 147245-149422
  
 NCBI BlastP on this gene

GAA91132

hypothetical protein
  
Accession: GAA91133
  
Location: 149467-150234
  
 NCBI BlastP on this gene

GAA91133

Ran-binding protein (RanBPM)
  
Accession: GAA91134
  
Location: 151471-153932
  
 NCBI BlastP on this gene

GAA91134

Query: Architecture Search FASTA input

AP007169 : Aspergillus oryzae RIB40 DNA, SC038.    Total score: 2.0     Cumulative Blast bit score: 385

Hit cluster cross-links:

Mycgr3G70471
  
Location: 0-405

Mycgr3G70471

Mycgr3G39149
  
Location: 505-1798

Mycgr3G39149

Mycgr3G92130
  
Location: 1898-2396

Mycgr3G92130

Mycgr3G38483
  
Location: 2496-3576

Mycgr3G38483

Mycgr3G108869
  
Location: 3676-5056

Mycgr3G108869

Mycgr3G103943
  
Location: 5156-5762

Mycgr3G103943

Mycgr3G57362
  
Location: 5862-7296

Mycgr3G57362

Mycgr3G39086
  
Location: 7396-8368

Mycgr3G39086

Mycgr3G103942
  
Location: 8468-8714

Mycgr3G103942

Mycgr3G108865
  
Location: 8814-10239

Mycgr3G108865

Mycgr3G70475
  
Location: 10339-11821

Mycgr3G70475

Mycgr3G108866
  
Location: 11921-13010

Mycgr3G108866

Mycgr3G92136
  
Location: 13110-13593

Mycgr3G92136

not annotated
  
Accession: BAE64354
  
Location: 1594776-1597154
  
 NCBI BlastP on this gene

AO090038000589

not annotated
  
Accession: BAE64355
  
Location: 1598882-1600993
  
 NCBI BlastP on this gene

AO090038000590

not annotated
  
Accession: BAE64356
  
Location: 1601491-1602675
  
  
**BlastP hit with Mycgr3G38483**
  
Percentage identity: 39 %
  
BlastP bit score: 167
  
Sequence coverage: 76 %
  
E-value: 7e-45
  
  
 NCBI BlastP on this gene

AO090038000591

not annotated
  
Accession: BAE64357
  
Location: 1602965-1603737
  
  
**BlastP hit with Mycgr3G70471**
  
Percentage identity: 81 %
  
BlastP bit score: 218
  
Sequence coverage: 97 %
  
E-value: 3e-70
  
  
 NCBI BlastP on this gene

AO090038000592

not annotated
  
Accession: BAE64358
  
Location: 1605796-1606944
  
 NCBI BlastP on this gene

AO090038000593

not annotated
  
Accession: BAE64359
  
Location: 1610200-1611813
  
 NCBI BlastP on this gene

AO090038000594

Query: Architecture Search FASTA input

ACYE01000161 : Trichophyton verrucosum HKI 0517    Total score: 2.0     Cumulative Blast bit score: 384

Hit cluster cross-links:

Mycgr3G70471
  
Location: 0-405

Mycgr3G70471

Mycgr3G39149
  
Location: 505-1798

Mycgr3G39149

Mycgr3G92130
  
Location: 1898-2396

Mycgr3G92130

Mycgr3G38483
  
Location: 2496-3576

Mycgr3G38483

Mycgr3G108869
  
Location: 3676-5056

Mycgr3G108869

Mycgr3G103943
  
Location: 5156-5762

Mycgr3G103943

Mycgr3G57362
  
Location: 5862-7296

Mycgr3G57362

Mycgr3G39086
  
Location: 7396-8368

Mycgr3G39086

Mycgr3G103942
  
Location: 8468-8714

Mycgr3G103942

Mycgr3G108865
  
Location: 8814-10239

Mycgr3G108865

Mycgr3G70475
  
Location: 10339-11821

Mycgr3G70475

Mycgr3G108866
  
Location: 11921-13010

Mycgr3G108866

Mycgr3G92136
  
Location: 13110-13593

Mycgr3G92136

hypothetical protein
  
Accession: EFE42114
  
Location: 6994-8681
  
 NCBI BlastP on this gene

EFE42114

hypothetical protein
  
Accession: EFE42115
  
Location: 9404-11641
  
 NCBI BlastP on this gene

EFE42115

putative hemagglutinin protein
  
Accession: EFE42116
  
Location: 14284-15441
  
 NCBI BlastP on this gene

EFE42116

hypothetical protein
  
Accession: EFE42117
  
Location: 16358-17093
  
  
**BlastP hit with Mycgr3G70471**
  
Percentage identity: 64 %
  
BlastP bit score: 186
  
Sequence coverage: 103 %
  
E-value: 4e-57
  
  
 NCBI BlastP on this gene

EFE42117

hypothetical protein
  
Accession: EFE42118
  
Location: 17465-18663
  
  
**BlastP hit with Mycgr3G38483**
  
Percentage identity: 39 %
  
BlastP bit score: 198
  
Sequence coverage: 83 %
  
E-value: 8e-57
  
  
 NCBI BlastP on this gene

EFE42118

toxin biosynthesis ketoreductase, putative
  
Accession: EFE42119
  
Location: 19858-20634
  
 NCBI BlastP on this gene

EFE42119

hypothetical protein
  
Accession: EFE42120
  
Location: 21894-23381
  
 NCBI BlastP on this gene

EFE42120

hypothetical protein
  
Accession: EFE42121
  
Location: 24048-25043
  
 NCBI BlastP on this gene

EFE42121

hypothetical protein
  
Accession: EFE42122
  
Location: 26212-27146
  
 NCBI BlastP on this gene

EFE42122

Query: Architecture Search FASTA input

51. :  DS499601 Aspergillus fumigatus A1163 scf\_000008 genomic scaffold     Total score: 2.0     Cumulative Blast bit score: 444

Mycgr3G70471
  
Location: 0-405
  
 NCBI BlastP on this gene

Mycgr3G70471

Mycgr3G39149
  
Location: 505-1798
  
 NCBI BlastP on this gene

Mycgr3G39149

Mycgr3G92130
  
Location: 1898-2396
  
 NCBI BlastP on this gene

Mycgr3G92130

Mycgr3G38483
  
Location: 2496-3576
  
 NCBI BlastP on this gene

Mycgr3G38483

Mycgr3G108869
  
Location: 3676-5056
  
 NCBI BlastP on this gene

Mycgr3G108869

Mycgr3G103943
  
Location: 5156-5762
  
 NCBI BlastP on this gene

Mycgr3G103943

Mycgr3G57362
  
Location: 5862-7296
  
 NCBI BlastP on this gene

Mycgr3G57362

Mycgr3G39086
  
Location: 7396-8368
  
 NCBI BlastP on this gene

Mycgr3G39086

Mycgr3G103942
  
Location: 8468-8714
  
 NCBI BlastP on this gene

Mycgr3G103942

Mycgr3G108865
  
Location: 8814-10239
  
 NCBI BlastP on this gene

Mycgr3G108865

Mycgr3G70475
  
Location: 10339-11821
  
 NCBI BlastP on this gene

Mycgr3G70475

Mycgr3G108866
  
Location: 11921-13010
  
 NCBI BlastP on this gene

Mycgr3G108866

Mycgr3G92136
  
Location: 13110-13593
  
 NCBI BlastP on this gene

Mycgr3G92136

conserved hypothetical protein
  
Accession: EDP48156
  
Location: 562349-563202
  
 NCBI BlastP on this gene

EDP48156

Ran-binding protein (RanBPM), putative
  
Accession: EDP48157
  
Location: 564946-567299
  
 NCBI BlastP on this gene

EDP48157

conserved hypothetical protein
  
Accession: EDP48158
  
Location: 568968-571196
  
 NCBI BlastP on this gene

EDP48158

conserved hypothetical protein
  
Accession: EDP48159
  
Location: 571683-573136
  
  
**BlastP hit with Mycgr3G38483**
  
Percentage identity: 35 %
  
BlastP bit score: 217
  
Sequence coverage: 100 %
  
E-value: 3e-63
  
  
 NCBI BlastP on this gene

EDP48159

37S ribosomal protein S24
  
Accession: EDP48160
  
Location: 573451-574295
  
  
**BlastP hit with Mycgr3G70471**
  
Percentage identity: 82 %
  
BlastP bit score: 227
  
Sequence coverage: 97 %
  
E-value: 1e-73
  
  
 NCBI BlastP on this gene

EDP48160

streptococcal hemagglutinin protein, putative
  
Accession: EDP48161
  
Location: 575479-576612
  
 NCBI BlastP on this gene

EDP48161

RNA binding protein, putative
  
Accession: EDP48162
  
Location: 580308-581919
  
 NCBI BlastP on this gene

EDP48162

UDP-N-acetylglucosamine pyrophosphorylase
  
Accession: EDP48163
  
Location: 582601-584267
  
 NCBI BlastP on this gene

EDP48163

52. :  AAHF01000015 Aspergillus fumigatus Af293     Total score: 2.0     Cumulative Blast bit score: 444

UDP-N-acetylglucosamine pyrophosphorylase
  
Accession: EAL84676
  
Location: 144587-146253
  
 NCBI BlastP on this gene

EAL84676

cyclophilin-type peptidyl-prolyl cis-trans isomerase, putative
  
Accession: EAL84677
  
Location: 146935-148546
  
 NCBI BlastP on this gene

EAL84677

streptococcal hemagglutinin protein, putative
  
Accession: EAL84679
  
Location: 154058-155191
  
 NCBI BlastP on this gene

EAL84679

37S ribosomal protein S24
  
Accession: EAL84680
  
Location: 156377-157221
  
  
**BlastP hit with Mycgr3G70471**
  
Percentage identity: 82 %
  
BlastP bit score: 227
  
Sequence coverage: 97 %
  
E-value: 1e-73
  
  
 NCBI BlastP on this gene

EAL84680

conserved hypothetical protein
  
Accession: EAL84681
  
Location: 157536-158989
  
  
**BlastP hit with Mycgr3G38483**
  
Percentage identity: 35 %
  
BlastP bit score: 217
  
Sequence coverage: 100 %
  
E-value: 3e-63
  
  
 NCBI BlastP on this gene

EAL84681

conserved hypothetical protein
  
Accession: EAL84682
  
Location: 159476-161701
  
 NCBI BlastP on this gene

EAL84682

Ran-binding protein (RanBP10), putative
  
Accession: EAL84683
  
Location: 163370-165723
  
 NCBI BlastP on this gene

EAL84683

conserved hypothetical protein
  
Accession: EAL84684
  
Location: 167467-168320
  
 NCBI BlastP on this gene

EAL84684

53. :  DS027692 Neosartorya fischeri NRRL 181 1099437636259 genomic scaffold     Total score: 2.0     Cumulative Blast bit score: 443

conserved hypothetical protein
  
Accession: EAW20950
  
Location: 815119-815972
  
 NCBI BlastP on this gene

EAW20950

Ran-binding protein (RanBPM), putative
  
Accession: EAW20951
  
Location: 817712-820064
  
 NCBI BlastP on this gene

EAW20951

conserved hypothetical protein
  
Accession: EAW20952
  
Location: 821619-823844
  
 NCBI BlastP on this gene

EAW20952

conserved hypothetical protein
  
Accession: EAW20953
  
Location: 824322-825775
  
  
**BlastP hit with Mycgr3G38483**
  
Percentage identity: 36 %
  
BlastP bit score: 216
  
Sequence coverage: 100 %
  
E-value: 4e-63
  
  
 NCBI BlastP on this gene

EAW20953

37S ribosomal protein S24
  
Accession: EAW20954
  
Location: 826091-826927
  
  
**BlastP hit with Mycgr3G70471**
  
Percentage identity: 83 %
  
BlastP bit score: 227
  
Sequence coverage: 97 %
  
E-value: 8e-74
  
  
 NCBI BlastP on this gene

EAW20954

streptococcal hemagglutinin protein, putative
  
Accession: EAW20955
  
Location: 828164-829258
  
 NCBI BlastP on this gene

EAW20955

RNA binding protein, putative
  
Accession: EAW20956
  
Location: 832963-834582
  
 NCBI BlastP on this gene

EAW20956

UDP-N-acetylglucosamine pyrophosphorylase
  
Accession: EAW20957
  
Location: 835256-836926
  
 NCBI BlastP on this gene

EAW20957

54. :  AM920436 Penicillium chrysogenum Wisconsin 54-1255 complete genome, contig Pc00c21.     Total score: 2.0     Cumulative Blast bit score: 442

not annotated
  
Accession: CAP96092
  
Location: 2838955-2840586
  
 NCBI BlastP on this gene

Pc21g11950

not annotated
  
Accession: CAP96093
  
Location: 2840978-2842495
  
 NCBI BlastP on this gene

Pc21g11960

hypothetical protein
  
Accession: CAP96094
  
Location: 2842794-2843588
  
 NCBI BlastP on this gene

Pc21g11970

not annotated
  
Accession: CAP96095
  
Location: 2845075-2846573
  
 NCBI BlastP on this gene

Pc21g11980

hypothetical protein
  
Accession: CAP96096
  
Location: 2847105-2847745
  
 NCBI BlastP on this gene

Pc21g11990

hypothetical protein
  
Accession: CAP96097
  
Location: 2848539-2849367
  
 NCBI BlastP on this gene

Pc21g12000

not annotated
  
Accession: CAP96098
  
Location: 2849757-2850458
  
  
**BlastP hit with Mycgr3G70471**
  
Percentage identity: 80 %
  
BlastP bit score: 221
  
Sequence coverage: 97 %
  
E-value: 3e-71
  
  
 NCBI BlastP on this gene

Pc21g12010

not annotated
  
Accession: CAP96099
  
Location: 2850733-2852061
  
  
**BlastP hit with Mycgr3G38483**
  
Percentage identity: 38 %
  
BlastP bit score: 221
  
Sequence coverage: 99 %
  
E-value: 3e-65
  
  
 NCBI BlastP on this gene

Pc21g12020

not annotated
  
Accession: CAP96100
  
Location: 2852522-2854711
  
 NCBI BlastP on this gene

Pc21g12030

not annotated
  
Accession: CAP96101
  
Location: 2855767-2858093
  
 NCBI BlastP on this gene

Pc21g12040

not annotated
  
Accession: CAP96102
  
Location: 2859150-2860021
  
 NCBI BlastP on this gene

Pc21g12050

unnamed
  
Accession: CAP96103
  
Location: 2861041-2862233
  
 NCBI BlastP on this gene

Pc21g12060

not annotated
  
Accession: CAP96104
  
Location: 2862538-2863740
  
 NCBI BlastP on this gene

Pc21g12070

55. :  CP003009 Thielavia terrestris NRRL 8126 chromosome 1     Total score: 2.0     Cumulative Blast bit score: 441

hypothetical protein
  
Accession: AEO63711
  
Location: 5992972-5993499
  
 NCBI BlastP on this gene

THITE\_2109251

hypothetical protein
  
Accession: AEO63712
  
Location: 5994117-5995707
  
 NCBI BlastP on this gene

THITE\_2109253

hypothetical protein
  
Accession: AEO63713
  
Location: 6001616-6003207
  
 NCBI BlastP on this gene

THITE\_39797

hypothetical protein
  
Accession: AEO63714
  
Location: 6004213-6005288
  
  
**BlastP hit with Mycgr3G38483**
  
Percentage identity: 41 %
  
BlastP bit score: 223
  
Sequence coverage: 100 %
  
E-value: 4e-66
  
  
 NCBI BlastP on this gene

THITE\_2109257

hypothetical protein
  
Accession: AEO63715
  
Location: 6005597-6006325
  
  
**BlastP hit with Mycgr3G70471**
  
Percentage identity: 78 %
  
BlastP bit score: 218
  
Sequence coverage: 98 %
  
E-value: 5e-70
  
  
 NCBI BlastP on this gene

THITE\_2109261

hypothetical protein
  
Accession: AEO63716
  
Location: 6008167-6012675
  
 NCBI BlastP on this gene

THITE\_2109263

hypothetical protein
  
Accession: AEO63717
  
Location: 6013102-6014363
  
 NCBI BlastP on this gene

THITE\_2169538

hypothetical protein
  
Accession: AEO63718
  
Location: 6016896-6019160
  
 NCBI BlastP on this gene

THITE\_2109268

56. :  CAGA01000037 Claviceps purpurea 20.1     Total score: 2.0     Cumulative Blast bit score: 439

uncharacterized protein
  
Accession: CCE32103
  
Location: 290726-291208
  
 NCBI BlastP on this gene

CCE32103

uncharacterized protein
  
Accession: CCE32104
  
Location: 296580-297681
  
  
**BlastP hit with Mycgr3G38483**
  
Percentage identity: 40 %
  
BlastP bit score: 231
  
Sequence coverage: 98 %
  
E-value: 5e-69
  
  
 NCBI BlastP on this gene

CCE32104

probable 40S RIBOSOMAL PROTEIN S24
  
Accession: CCE32105
  
Location: 298092-299045
  
  
**BlastP hit with Mycgr3G70471**
  
Percentage identity: 82 %
  
BlastP bit score: 208
  
Sequence coverage: 89 %
  
E-value: 3e-66
  
  
 NCBI BlastP on this gene

CCE32105

uncharacterized protein
  
Accession: CCE32106
  
Location: 300849-303723
  
 NCBI BlastP on this gene

CCE32106

probable GTP cyclohydrolase II
  
Accession: CCE32107
  
Location: 304335-305601
  
 NCBI BlastP on this gene

CCE32107

uncharacterized protein
  
Accession: CCE32108
  
Location: 306479-307135
  
 NCBI BlastP on this gene

CCE32108

related to dna-dependent rna polymerase I subunit a43 (rpa43)
  
Accession: CCE32109
  
Location: 308741-310162
  
 NCBI BlastP on this gene

CCE32109

57. :  DS027053 Aspergillus clavatus NRRL 1 1099423829799 genomic scaffold     Total score: 2.0     Cumulative Blast bit score: 438

UDP-N-acetylglucosamine pyrophosphorylase
  
Accession: EAW10901
  
Location: 182142-183815
  
 NCBI BlastP on this gene

EAW10901

RNA binding protein, putative
  
Accession: EAW10902
  
Location: 184548-186192
  
 NCBI BlastP on this gene

EAW10902

streptococcal hemagglutinin protein, putative
  
Accession: EAW10903
  
Location: 190114-191181
  
 NCBI BlastP on this gene

EAW10903

37S ribosomal protein S24
  
Accession: EAW10904
  
Location: 192493-193365
  
  
**BlastP hit with Mycgr3G70471**
  
Percentage identity: 82 %
  
BlastP bit score: 224
  
Sequence coverage: 97 %
  
E-value: 9e-73
  
  
 NCBI BlastP on this gene

EAW10904

conserved hypothetical protein
  
Accession: EAW10905
  
Location: 193698-195092
  
  
**BlastP hit with Mycgr3G38483**
  
Percentage identity: 35 %
  
BlastP bit score: 214
  
Sequence coverage: 99 %
  
E-value: 2e-62
  
  
 NCBI BlastP on this gene

EAW10905

conserved hypothetical protein
  
Accession: EAW10906
  
Location: 195978-198206
  
 NCBI BlastP on this gene

EAW10906

Ran-binding protein (RanBPM), putative
  
Accession: EAW10907
  
Location: 200430-202830
  
 NCBI BlastP on this gene

EAW10907

DUF1711 domain protein
  
Accession: EAW10908
  
Location: 204636-205514
  
 NCBI BlastP on this gene

EAW10908

58. :  CU633447 Podospora anserina S mat+ genomic DNA chromosome 2, supercontig 3.     Total score: 2.0     Cumulative Blast bit score: 436

not annotated
  
Accession: CAP60985
  
Location: 104844-106545
  
 NCBI BlastP on this gene

CAP60985

not annotated
  
Accession: CAP60986
  
Location: 113242-114995
  
 NCBI BlastP on this gene

CAP60986

not annotated
  
Accession: CAP60987
  
Location: 115411-116533
  
  
**BlastP hit with Mycgr3G38483**
  
Percentage identity: 42 %
  
BlastP bit score: 228
  
Sequence coverage: 98 %
  
E-value: 6e-68
  
  
 NCBI BlastP on this gene

CAP60987

not annotated
  
Accession: CAP60988
  
Location: 116882-117554
  
  
**BlastP hit with Mycgr3G70471**
  
Percentage identity: 81 %
  
BlastP bit score: 208
  
Sequence coverage: 90 %
  
E-value: 2e-66
  
  
 NCBI BlastP on this gene

CAP60988

not annotated
  
Accession: CAP60989
  
Location: 118869-119663
  
 NCBI BlastP on this gene

CAP60989

not annotated
  
Accession: CAP60990
  
Location: 121609-123078
  
 NCBI BlastP on this gene

CAP60990

not annotated
  
Accession: CAP60991
  
Location: 123358-124379
  
 NCBI BlastP on this gene

CAP60991

not annotated
  
Accession: CAP60992
  
Location: 125425-127029
  
 NCBI BlastP on this gene

CAP60992

not annotated
  
Accession: CAP60993
  
Location: 127166-128122
  
 NCBI BlastP on this gene

CAP60993

not annotated
  
Accession: CAP60994
  
Location: 128565-129941
  
 NCBI BlastP on this gene

CAP60994

59. :  GL698484 Metarhizium acridum CQMa 102 unplaced genomic scaffold Scf\_015     Total score: 2.0     Cumulative Blast bit score: 434

hypothetical protein
  
Accession: EFY91161
  
Location: 344633-345383
  
 NCBI BlastP on this gene

EFY91161

hypothetical protein
  
Accession: EFY91162
  
Location: 352226-352807
  
 NCBI BlastP on this gene

EFY91162

40S ribosomal protein S24
  
Accession: EFY91163
  
Location: 353488-354416
  
  
**BlastP hit with Mycgr3G70471**
  
Percentage identity: 80 %
  
BlastP bit score: 202
  
Sequence coverage: 89 %
  
E-value: 6e-64
  
  
 NCBI BlastP on this gene

EFY91163

hypothetical protein
  
Accession: EFY91164
  
Location: 354842-356245
  
  
**BlastP hit with Mycgr3G38483**
  
Percentage identity: 44 %
  
BlastP bit score: 233
  
Sequence coverage: 85 %
  
E-value: 8e-69
  
  
 NCBI BlastP on this gene

EFY91164

hypothetical protein
  
Accession: EFY91165
  
Location: 359454-360415
  
 NCBI BlastP on this gene

EFY91165

RNA binding protein
  
Accession: EFY91166
  
Location: 361204-362731
  
 NCBI BlastP on this gene

EFY91166

60. :  DS572753 Paracoccidioides brasiliensis Pb18 supercont1.4 genomic scaffold     Total score: 2.0     Cumulative Blast bit score: 434

conserved hypothetical protein
  
Accession: EEH48227
  
Location: 2537796-2538865
  
 NCBI BlastP on this gene

EEH48227

UDP-N-acetylglucosamine pyrophosphorylase
  
Accession: EEH48228
  
Location: 2540014-2541726
  
 NCBI BlastP on this gene

EEH48228

RNA binding protein
  
Accession: EEH48229
  
Location: 2542296-2543917
  
 NCBI BlastP on this gene

EEH48229

predicted protein
  
Accession: EEH48230
  
Location: 2547070-2548678
  
 NCBI BlastP on this gene

EEH48230

40S ribosomal protein S24
  
Accession: EEH48231
  
Location: 2549780-2550477
  
  
**BlastP hit with Mycgr3G70471**
  
Percentage identity: 74 %
  
BlastP bit score: 210
  
Sequence coverage: 99 %
  
E-value: 6e-67
  
  
 NCBI BlastP on this gene

EEH48231

conserved hypothetical protein
  
Accession: EEH48232
  
Location: 2550839-2552272
  
  
**BlastP hit with Mycgr3G38483**
  
Percentage identity: 35 %
  
BlastP bit score: 224
  
Sequence coverage: 97 %
  
E-value: 3e-66
  
  
 NCBI BlastP on this gene

EEH48232

conserved hypothetical protein
  
Accession: EEH48233
  
Location: 2554685-2557234
  
 NCBI BlastP on this gene

EEH48233

conserved hypothetical protein
  
Accession: EEH48234
  
Location: 2558469-2558864
  
 NCBI BlastP on this gene

EEH48234

conserved hypothetical protein
  
Accession: EEH48235
  
Location: 2559144-2562880
  
 NCBI BlastP on this gene

EEH48235

61. :  KB644412 Penicillium oxalicum 114-2 unplaced genomic scaffold scaffold\_5     Total score: 2.0     Cumulative Blast bit score: 432

hypothetical protein
  
Accession: EPS30915
  
Location: 4231813-4233541
  
 NCBI BlastP on this gene

EPS30915

hypothetical protein
  
Accession: EPS30916
  
Location: 4235061-4235285
  
 NCBI BlastP on this gene

EPS30916

hypothetical protein
  
Accession: EPS30917
  
Location: 4237816-4239420
  
 NCBI BlastP on this gene

EPS30917

hypothetical protein
  
Accession: EPS30918
  
Location: 4242978-4243830
  
  
**BlastP hit with Mycgr3G70471**
  
Percentage identity: 81 %
  
BlastP bit score: 223
  
Sequence coverage: 97 %
  
E-value: 6e-72
  
  
 NCBI BlastP on this gene

EPS30918

hypothetical protein
  
Accession: EPS30919
  
Location: 4244088-4245327
  
  
**BlastP hit with Mycgr3G38483**
  
Percentage identity: 36 %
  
BlastP bit score: 210
  
Sequence coverage: 98 %
  
E-value: 6e-61
  
  
 NCBI BlastP on this gene

EPS30919

hypothetical protein
  
Accession: EPS30920
  
Location: 4245763-4247992
  
 NCBI BlastP on this gene

EPS30920

hypothetical protein
  
Accession: EPS30921
  
Location: 4256118-4256929
  
 NCBI BlastP on this gene

EPS30921

62. :  DS544804 Paracoccidioides brasiliensis Pb03 supercont1.2 genomic scaffold     Total score: 2.0     Cumulative Blast bit score: 432

conserved hypothetical protein
  
Accession: EEH18832
  
Location: 465128-469197
  
 NCBI BlastP on this gene

EEH18832

conserved hypothetical protein
  
Accession: EEH18833
  
Location: 469715-473307
  
 NCBI BlastP on this gene

EEH18833

conserved hypothetical protein
  
Accession: EEH18834
  
Location: 475707-477139
  
  
**BlastP hit with Mycgr3G38483**
  
Percentage identity: 36 %
  
BlastP bit score: 223
  
Sequence coverage: 96 %
  
E-value: 5e-66
  
  
 NCBI BlastP on this gene

EEH18834

40S ribosomal protein S24
  
Accession: EEH18835
  
Location: 477501-478207
  
  
**BlastP hit with Mycgr3G70471**
  
Percentage identity: 74 %
  
BlastP bit score: 210
  
Sequence coverage: 99 %
  
E-value: 6e-67
  
  
 NCBI BlastP on this gene

EEH18835

predicted protein
  
Accession: EEH18836
  
Location: 479310-480915
  
 NCBI BlastP on this gene

EEH18836

RNA binding protein
  
Accession: EEH18837
  
Location: 483258-485771
  
 NCBI BlastP on this gene

EEH18837

UDP-N-acetylglucosamine pyrophosphorylase
  
Accession: EEH18838
  
Location: 486341-488053
  
 NCBI BlastP on this gene

EEH18838

conserved hypothetical protein
  
Accession: EEH18839
  
Location: 489202-490270
  
 NCBI BlastP on this gene

EEH18839

63. :  AKCU01000381 Penicillium digitatum Pd1     Total score: 2.0     Cumulative Blast bit score: 432

RNA polymerase I subunit Rpa43, putative
  
Accession: EKV11438
  
Location: 12010-13206
  
 NCBI BlastP on this gene

EKV11438

Membrane associated DnaJ chaperone, putative
  
Accession: EKV11439
  
Location: 13521-14724
  
 NCBI BlastP on this gene

EKV11439

hypothetical protein
  
Accession: EKV11440
  
Location: 15761-16650
  
 NCBI BlastP on this gene

EKV11440

Ran-binding protein (RanBP10), putative
  
Accession: EKV11441
  
Location: 17797-20135
  
 NCBI BlastP on this gene

EKV11441

hypothetical protein
  
Accession: EKV11442
  
Location: 21282-23384
  
 NCBI BlastP on this gene

EKV11442

hypothetical protein
  
Accession: EKV11443
  
Location: 23861-25199
  
  
**BlastP hit with Mycgr3G38483**
  
Percentage identity: 37 %
  
BlastP bit score: 211
  
Sequence coverage: 99 %
  
E-value: 2e-61
  
  
 NCBI BlastP on this gene

EKV11443

40S ribosomal protein S24
  
Accession: EKV11444
  
Location: 25480-26189
  
  
**BlastP hit with Mycgr3G70471**
  
Percentage identity: 81 %
  
BlastP bit score: 222
  
Sequence coverage: 97 %
  
E-value: 9e-72
  
  
 NCBI BlastP on this gene

EKV11444

hypothetical protein
  
Accession: EKV11445
  
Location: 26590-26781
  
 NCBI BlastP on this gene

EKV11445

hemagglutinin protein, putative
  
Accession: EKV11446
  
Location: 29353-30420
  
 NCBI BlastP on this gene

EKV11446

hypothetical protein
  
Accession: EKV11447
  
Location: 32340-32612
  
 NCBI BlastP on this gene

EKV11447

Peptidyl-prolyl cis-trans isomerase-like 4
  
Accession: EKV11448
  
Location: 33513-35062
  
 NCBI BlastP on this gene

EKV11448

UDP-N-acetylglucosamine pyrophosphorylase
  
Accession: EKV11449
  
Location: 35437-37073
  
 NCBI BlastP on this gene

EKV11449

64. :  AKCT01000084 Penicillium digitatum PHI26     Total score: 2.0     Cumulative Blast bit score: 432

RNA polymerase I subunit Rpa43, putative
  
Accession: EKV16925
  
Location: 190591-191787
  
 NCBI BlastP on this gene

EKV16925

Membrane associated DnaJ chaperone, putative
  
Accession: EKV16926
  
Location: 192103-193306
  
 NCBI BlastP on this gene

EKV16926

hypothetical protein
  
Accession: EKV16927
  
Location: 194344-195233
  
 NCBI BlastP on this gene

EKV16927

Ran-binding protein (RanBP10), putative
  
Accession: EKV16928
  
Location: 196380-198718
  
 NCBI BlastP on this gene

EKV16928

hypothetical protein
  
Accession: EKV16929
  
Location: 199865-201967
  
 NCBI BlastP on this gene

EKV16929

hypothetical protein
  
Accession: EKV16930
  
Location: 202445-203783
  
  
**BlastP hit with Mycgr3G38483**
  
Percentage identity: 37 %
  
BlastP bit score: 211
  
Sequence coverage: 99 %
  
E-value: 2e-61
  
  
 NCBI BlastP on this gene

EKV16930

40S ribosomal protein S24
  
Accession: EKV16931
  
Location: 204064-204773
  
  
**BlastP hit with Mycgr3G70471**
  
Percentage identity: 81 %
  
BlastP bit score: 222
  
Sequence coverage: 97 %
  
E-value: 9e-72
  
  
 NCBI BlastP on this gene

EKV16931

hypothetical protein
  
Accession: EKV16932
  
Location: 205174-205365
  
 NCBI BlastP on this gene

EKV16932

hemagglutinin protein, putative
  
Accession: EKV16933
  
Location: 207940-209007
  
 NCBI BlastP on this gene

EKV16933

hypothetical protein
  
Accession: EKV16934
  
Location: 210970-211212
  
 NCBI BlastP on this gene

EKV16934

Peptidyl-prolyl cis-trans isomerase-like 4
  
Accession: EKV16935
  
Location: 212117-213666
  
 NCBI BlastP on this gene

EKV16935

UDP-N-acetylglucosamine pyrophosphorylase
  
Accession: EKV16936
  
Location: 214041-215677
  
 NCBI BlastP on this gene

EKV16936

65. :  KB705480 Eutypa lata UCREL1 unplaced genomic scaffold EL1\_03\_scaffold\_142     Total score: 2.0     Cumulative Blast bit score: 432

hypothetical protein
  
Accession: EMR72232
  
Location: 38685-39569
  
 NCBI BlastP on this gene

EMR72232

hypothetical protein
  
Accession: EMR72255
  
Location: 42202-42980
  
 NCBI BlastP on this gene

EMR72255

putative gdsl esterase lipase protein
  
Accession: EMR72241
  
Location: 45308-46144
  
 NCBI BlastP on this gene

EMR72241

hypothetical protein
  
Accession: EMR72219
  
Location: 48230-48922
  
 NCBI BlastP on this gene

EMR72219

putative 40s ribosomal protein s24 protein
  
Accession: EMR72230
  
Location: 49613-50394
  
  
**BlastP hit with Mycgr3G70471**
  
Percentage identity: 76 %
  
BlastP bit score: 173
  
Sequence coverage: 89 %
  
E-value: 2e-52
  
  
 NCBI BlastP on this gene

EMR72230

putative fam86a protein
  
Accession: EMR72248
  
Location: 50865-52029
  
  
**BlastP hit with Mycgr3G38483**
  
Percentage identity: 44 %
  
BlastP bit score: 259
  
Sequence coverage: 97 %
  
E-value: 5e-80
  
  
 NCBI BlastP on this gene

EMR72248

hypothetical protein
  
Accession: EMR72242
  
Location: 52913-53527
  
 NCBI BlastP on this gene

EMR72242

hypothetical protein
  
Accession: EMR72268
  
Location: 57582-58253
  
 NCBI BlastP on this gene

EMR72268

putative alpha beta hydrolase domain-containing protein
  
Accession: EMR72224
  
Location: 59023-60147
  
 NCBI BlastP on this gene

EMR72224

66. :  KE145356 Glarea lozoyensis ATCC 20868 chromosome Unknown GLAREA13     Total score: 2.0     Cumulative Blast bit score: 427

hypothetical protein
  
Accession: EPE34676
  
Location: 1263634-1265347
  
 NCBI BlastP on this gene

EPE34676

Oxidoreductase molybdopterin-binding protein
  
Accession: EPE34677
  
Location: 1266321-1268647
  
 NCBI BlastP on this gene

EPE34677

hypothetical protein
  
Accession: EPE34678
  
Location: 1270396-1270794
  
 NCBI BlastP on this gene

EPE34678

hypothetical protein
  
Accession: EPE34679
  
Location: 1271905-1273470
  
 NCBI BlastP on this gene

EPE34679

hypothetical protein
  
Accession: EPE34680
  
Location: 1274308-1275273
  
 NCBI BlastP on this gene

EPE34680

S-adenosyl-L-methionine-dependent methyltransferase
  
Accession: EPE34681
  
Location: 1276387-1277480
  
  
**BlastP hit with Mycgr3G38483**
  
Percentage identity: 40 %
  
BlastP bit score: 221
  
Sequence coverage: 97 %
  
E-value: 4e-65
  
  
 NCBI BlastP on this gene

EPE34681

Ribosomal proteins S24e, L23 and L15e
  
Accession: EPE34682
  
Location: 1277894-1278472
  
  
**BlastP hit with Mycgr3G70471**
  
Percentage identity: 79 %
  
BlastP bit score: 206
  
Sequence coverage: 91 %
  
E-value: 3e-65
  
  
 NCBI BlastP on this gene

EPE34682

hypothetical protein
  
Accession: EPE34683
  
Location: 1280819-1283134
  
 NCBI BlastP on this gene

EPE34683

Cyclophilin-like protein
  
Accession: EPE34684
  
Location: 1283493-1284963
  
 NCBI BlastP on this gene

EPE34684

Zn2/Cys6 DNA-binding protein
  
Accession: EPE34685
  
Location: 1288271-1289756
  
 NCBI BlastP on this gene

EPE34685

67. :  JH725156 Beauveria bassiana ARSEF 2860 unplaced genomic scaffold BBA\_S00007     Total score: 2.0     Cumulative Blast bit score: 424

peptidyl-prolyl cis-trans isomerase cyp6
  
Accession: EJP67562
  
Location: 382431-384066
  
 NCBI BlastP on this gene

EJP67562

hypothetical protein
  
Accession: EJP67563
  
Location: 384899-385399
  
 NCBI BlastP on this gene

EJP67563

Nicotinamide N-methyltransferase, putative
  
Accession: EJP67564
  
Location: 390346-391476
  
  
**BlastP hit with Mycgr3G38483**
  
Percentage identity: 42 %
  
BlastP bit score: 221
  
Sequence coverage: 98 %
  
E-value: 4e-65
  
  
 NCBI BlastP on this gene

EJP67564

40S ribosomal protein S24
  
Accession: EJP67565
  
Location: 391842-392763
  
  
**BlastP hit with Mycgr3G70471**
  
Percentage identity: 72 %
  
BlastP bit score: 203
  
Sequence coverage: 98 %
  
E-value: 4e-64
  
  
 NCBI BlastP on this gene

EJP67565

hypothetical protein
  
Accession: EJP67566
  
Location: 393800-394300
  
 NCBI BlastP on this gene

EJP67566

clumping factor B
  
Accession: EJP67567
  
Location: 396371-400101
  
 NCBI BlastP on this gene

EJP67567

GTP cyclohydrolase II
  
Accession: EJP67568
  
Location: 400542-401733
  
 NCBI BlastP on this gene

EJP67568

RNA polymerase Rpb7-like domain-containing protein
  
Accession: EJP67569
  
Location: 403088-404458
  
 NCBI BlastP on this gene

EJP67569

68. :  DS572830 Paracoccidioides brasiliensis Pb01 supercont1.20 genomic scaffold     Total score: 2.0     Cumulative Blast bit score: 422

conserved hypothetical protein
  
Accession: EEH36459
  
Location: 525896-527475
  
 NCBI BlastP on this gene

EEH36459

hypothetical protein
  
Accession: EEH36460
  
Location: 528533-529395
  
 NCBI BlastP on this gene

EEH36460

predicted protein
  
Accession: EEH36461
  
Location: 529773-530179
  
 NCBI BlastP on this gene

EEH36461

conserved hypothetical protein
  
Accession: EEH36462
  
Location: 531759-533930
  
 NCBI BlastP on this gene

EEH36462

conserved hypothetical protein
  
Accession: EEH36463
  
Location: 536350-537780
  
  
**BlastP hit with Mycgr3G38483**
  
Percentage identity: 36 %
  
BlastP bit score: 222
  
Sequence coverage: 98 %
  
E-value: 2e-65
  
  
 NCBI BlastP on this gene

EEH36463

40S ribosomal protein S24
  
Accession: EEH36464
  
Location: 538132-538831
  
  
**BlastP hit with Mycgr3G70471**
  
Percentage identity: 76 %
  
BlastP bit score: 200
  
Sequence coverage: 90 %
  
E-value: 5e-63
  
  
 NCBI BlastP on this gene

EEH36464

predicted protein
  
Accession: EEH36465
  
Location: 539951-542208
  
 NCBI BlastP on this gene

EEH36465

RNA binding protein
  
Accession: EEH36466
  
Location: 544625-546243
  
 NCBI BlastP on this gene

EEH36466

UDP-N-acetylglucosamine pyrophosphorylase
  
Accession: EEH36467
  
Location: 546821-548547
  
 NCBI BlastP on this gene

EEH36467

zinc finger protein GIS2
  
Accession: EEH36468
  
Location: 549629-550764
  
 NCBI BlastP on this gene

EEH36468

69. :  GL573339 Geomyces destructans 20631-21 unplaced genomic scaffold supercont1.171     Total score: 2.0     Cumulative Blast bit score: 417

hypothetical protein
  
Accession: ELR04190
  
Location: 27913-29953
  
 NCBI BlastP on this gene

ELR04190

hypothetical protein
  
Accession: ELR04191
  
Location: 31140-34239
  
 NCBI BlastP on this gene

ELR04191

hypothetical protein
  
Accession: ELR04192
  
Location: 35308-38627
  
 NCBI BlastP on this gene

ELR04192

hypothetical protein
  
Accession: ELR04193
  
Location: 39394-40637
  
  
**BlastP hit with Mycgr3G38483**
  
Percentage identity: 40 %
  
BlastP bit score: 223
  
Sequence coverage: 101 %
  
E-value: 1e-65
  
  
 NCBI BlastP on this gene

ELR04193

small subunit ribosomal protein S24e
  
Accession: ELR04194
  
Location: 41011-41640
  
  
**BlastP hit with Mycgr3G70471**
  
Percentage identity: 74 %
  
BlastP bit score: 195
  
Sequence coverage: 92 %
  
E-value: 5e-61
  
  
 NCBI BlastP on this gene

ELR04194

hypothetical protein
  
Accession: ELR04195
  
Location: 43156-44583
  
 NCBI BlastP on this gene

ELR04195

hypothetical protein
  
Accession: ELR04196
  
Location: 45875-46183
  
 NCBI BlastP on this gene

ELR04196

hypothetical protein
  
Accession: ELR04197
  
Location: 47537-49186
  
 NCBI BlastP on this gene

ELR04197

hypothetical protein
  
Accession: ELR04198
  
Location: 49720-50342
  
 NCBI BlastP on this gene

ELR04198

70. :  JH795346 Magnaporthe oryzae P131 unplaced genomic scaffold P131\_scaffold00065     Total score: 2.0     Cumulative Blast bit score: 417

hypothetical protein
  
Accession: ELQ70235
  
Location: 25896-26732
  
 NCBI BlastP on this gene

ELQ70235

hypothetical protein
  
Accession: ELQ70236
  
Location: 27915-29142
  
  
**BlastP hit with Mycgr3G38483**
  
Percentage identity: 44 %
  
BlastP bit score: 211
  
Sequence coverage: 83 %
  
E-value: 2e-61
  
  
 NCBI BlastP on this gene

ELQ70236

hypothetical protein
  
Accession: ELQ70237
  
Location: 30559-33139
  
 NCBI BlastP on this gene

ELQ70237

hypothetical protein
  
Accession: ELQ70238
  
Location: 40247-43000
  
 NCBI BlastP on this gene

ELQ70238

peptidyl-prolyl cis-trans isomerase cyp6
  
Accession: ELQ70239
  
Location: 45048-46565
  
 NCBI BlastP on this gene

ELQ70239

40S ribosomal protein S24
  
Accession: ELQ70240
  
Location: 47145-47889
  
  
**BlastP hit with Mycgr3G70471**
  
Percentage identity: 80 %
  
BlastP bit score: 206
  
Sequence coverage: 88 %
  
E-value: 3e-65
  
  
 NCBI BlastP on this gene

ELQ70240

hypothetical protein
  
Accession: ELQ70241
  
Location: 48551-49027
  
 NCBI BlastP on this gene

ELQ70241

71. :  JH793790 Magnaporthe oryzae Y34 unplaced genomic scaffold Y34\_scaffold00456     Total score: 2.0     Cumulative Blast bit score: 417

hypothetical protein
  
Accession: ELQ40237
  
Location: 25817-26653
  
 NCBI BlastP on this gene

ELQ40237

hypothetical protein
  
Accession: ELQ40238
  
Location: 27835-29062
  
  
**BlastP hit with Mycgr3G38483**
  
Percentage identity: 44 %
  
BlastP bit score: 211
  
Sequence coverage: 83 %
  
E-value: 2e-61
  
  
 NCBI BlastP on this gene

ELQ40238

hypothetical protein
  
Accession: ELQ40239
  
Location: 30481-33062
  
 NCBI BlastP on this gene

ELQ40239

hypothetical protein
  
Accession: ELQ40240
  
Location: 40156-42909
  
 NCBI BlastP on this gene

ELQ40240

peptidyl-prolyl cis-trans isomerase cyp6
  
Accession: ELQ40241
  
Location: 44955-46472
  
 NCBI BlastP on this gene

ELQ40241

40S ribosomal protein S24
  
Accession: ELQ40242
  
Location: 47052-47796
  
  
**BlastP hit with Mycgr3G70471**
  
Percentage identity: 80 %
  
BlastP bit score: 206
  
Sequence coverage: 88 %
  
E-value: 3e-65
  
  
 NCBI BlastP on this gene

ELQ40242

hypothetical protein
  
Accession: ELQ40243
  
Location: 48458-48934
  
 NCBI BlastP on this gene

ELQ40243

72. :  CM001234 Magnaporthe oryzae 70-15 chromosome 4     Total score: 2.0     Cumulative Blast bit score: 417

hypothetical protein
  
Accession: EHA49574
  
Location: 53623-54459
  
 NCBI BlastP on this gene

EHA49574

hypothetical protein
  
Accession: EHA49575
  
Location: 55646-56870
  
  
**BlastP hit with Mycgr3G38483**
  
Percentage identity: 45 %
  
BlastP bit score: 211
  
Sequence coverage: 83 %
  
E-value: 2e-61
  
  
 NCBI BlastP on this gene

EHA49575

hypothetical protein
  
Accession: EHA49576
  
Location: 58267-59887
  
 NCBI BlastP on this gene

EHA49576

hypothetical protein
  
Accession: EHA49577
  
Location: 67968-70722
  
 NCBI BlastP on this gene

EHA49577

peptidyl-prolyl cis-trans isomerase-like 4
  
Accession: EHA49578
  
Location: 72770-74287
  
 NCBI BlastP on this gene

EHA49578

40S ribosomal protein S24
  
Accession: EHA49579
  
Location: 74867-75611
  
  
**BlastP hit with Mycgr3G70471**
  
Percentage identity: 80 %
  
BlastP bit score: 206
  
Sequence coverage: 88 %
  
E-value: 3e-65
  
  
 NCBI BlastP on this gene

EHA49579

hypothetical protein
  
Accession: EHA49580
  
Location: 76273-76749
  
 NCBI BlastP on this gene

EHA49580

73. :  GL985082 Trichoderma reesei QM6a unplaced genomic scaffold TRIREscaffold\_27     Total score: 2.0     Cumulative Blast bit score: 415

predicted protein
  
Accession: EGR45019
  
Location: 64779-65730
  
 NCBI BlastP on this gene

EGR45019

hypothetical protein
  
Accession: EGR45101
  
Location: 66209-67550
  
 NCBI BlastP on this gene

EGR45101

predicted protein
  
Accession: EGR45102
  
Location: 67980-69419
  
 NCBI BlastP on this gene

EGR45102

predicted protein
  
Accession: EGR45020
  
Location: 71282-71974
  
 NCBI BlastP on this gene

EGR45020

predicted protein
  
Accession: EGR45021
  
Location: 72824-74476
  
 NCBI BlastP on this gene

EGR45021

hypothetical protein
  
Accession: EGR45103
  
Location: 75119-76002
  
  
**BlastP hit with Mycgr3G70471**
  
Percentage identity: 76 %
  
BlastP bit score: 195
  
Sequence coverage: 89 %
  
E-value: 5e-61
  
  
 NCBI BlastP on this gene

EGR45103

predicted protein
  
Accession: EGR45022
  
Location: 76505-77584
  
  
**BlastP hit with Mycgr3G38483**
  
Percentage identity: 40 %
  
BlastP bit score: 221
  
Sequence coverage: 99 %
  
E-value: 2e-65
  
  
 NCBI BlastP on this gene

EGR45022

predicted protein
  
Accession: EGR45023
  
Location: 83280-83753
  
 NCBI BlastP on this gene

EGR45023

predicted protein
  
Accession: EGR45024
  
Location: 85482-86951
  
 NCBI BlastP on this gene

EGR45024

74. :  KE148175 Ophiostoma piceae UAMH 11346 chromosome Unknown scf30     Total score: 2.0     Cumulative Blast bit score: 415

peptidyl-prolyl cis-trans isomerase cyp6
  
Accession: EPE02642
  
Location: 44486-46208
  
 NCBI BlastP on this gene

EPE02642

n-terminal fungal transcription factor-containing protein
  
Accession: EPE02643
  
Location: 50472-52637
  
 NCBI BlastP on this gene

EPE02643

mannose-6-phosphate isomerase
  
Accession: EPE02644
  
Location: 53807-55156
  
 NCBI BlastP on this gene

EPE02644

fam86a protein
  
Accession: EPE02645
  
Location: 55576-56616
  
  
**BlastP hit with Mycgr3G38483**
  
Percentage identity: 41 %
  
BlastP bit score: 214
  
Sequence coverage: 99 %
  
E-value: 2e-62
  
  
 NCBI BlastP on this gene

EPE02645

40s ribosomal protein s24
  
Accession: EPE02646
  
Location: 57153-58130
  
  
**BlastP hit with Mycgr3G70471**
  
Percentage identity: 78 %
  
BlastP bit score: 201
  
Sequence coverage: 88 %
  
E-value: 2e-63
  
  
 NCBI BlastP on this gene

EPE02646

hypothetical protein
  
Accession: EPE02647
  
Location: 58757-59440
  
 NCBI BlastP on this gene

EPE02647

gtp cyclohydrolase-2
  
Accession: EPE02648
  
Location: 60763-64375
  
 NCBI BlastP on this gene

EPE02648

zinc c6 finger domain protein
  
Accession: EPE02649
  
Location: 65182-66450
  
 NCBI BlastP on this gene

EPE02649

nad dependent epimerase
  
Accession: EPE02650
  
Location: 66875-67831
  
 NCBI BlastP on this gene

EPE02650

aldehyde reductase ii
  
Accession: EPE02651
  
Location: 68139-69281
  
 NCBI BlastP on this gene

EPE02651

75. :  GG749437 Ajellomyces dermatitidis ATCC 18188 genomic scaffold supercont1.31     Total score: 2.0     Cumulative Blast bit score: 415

hypothetical protein
  
Accession: EGE82680
  
Location: 43224-44427
  
 NCBI BlastP on this gene

EGE82680

hypothetical protein
  
Accession: EGE82681
  
Location: 44789-46015
  
  
**BlastP hit with Mycgr3G38483**
  
Percentage identity: 39 %
  
BlastP bit score: 211
  
Sequence coverage: 83 %
  
E-value: 1e-61
  
  
 NCBI BlastP on this gene

EGE82681

37S ribosomal protein S24
  
Accession: EGE82682
  
Location: 46336-47002
  
  
**BlastP hit with Mycgr3G70471**
  
Percentage identity: 78 %
  
BlastP bit score: 204
  
Sequence coverage: 91 %
  
E-value: 1e-64
  
  
 NCBI BlastP on this gene

EGE82682

hypothetical protein
  
Accession: EGE82683
  
Location: 48021-49616
  
 NCBI BlastP on this gene

EGE82683

hypothetical protein
  
Accession: EGE82684
  
Location: 51152-52415
  
 NCBI BlastP on this gene

EGE82684

RNA binding protein
  
Accession: EGE82685
  
Location: 52542-54136
  
 NCBI BlastP on this gene

EGE82685

UDP-N-acetylglucosamine pyrophosphorylase
  
Accession: EGE82686
  
Location: 54731-56469
  
 NCBI BlastP on this gene

EGE82686

zinc knuckle domain-containing protein
  
Accession: EGE82687
  
Location: 57568-58727
  
 NCBI BlastP on this gene

EGE82687

76. :  EQ999974 Ajellomyces dermatitidis ER-3 genomic scaffold supercont1.2     Total score: 2.0     Cumulative Blast bit score: 415

zinc knuckle domain-containing protein
  
Accession: EEQ86773
  
Location: 4620280-4621439
  
 NCBI BlastP on this gene

EEQ86773

UDP-N-acetylglucosamine pyrophosphorylase
  
Accession: EEQ86774
  
Location: 4622530-4624268
  
 NCBI BlastP on this gene

EEQ86774

peptidyl-prolyl cis-trans isomerase-like 4
  
Accession: EEQ86775
  
Location: 4624863-4626282
  
 NCBI BlastP on this gene

EEQ86775

predicted protein
  
Accession: EEQ86776
  
Location: 4626619-4627831
  
 NCBI BlastP on this gene

EEQ86776

predicted protein
  
Accession: EEQ86777
  
Location: 4629473-4630962
  
 NCBI BlastP on this gene

EEQ86777

37S ribosomal protein S24
  
Accession: EEQ86778
  
Location: 4631977-4632643
  
  
**BlastP hit with Mycgr3G70471**
  
Percentage identity: 78 %
  
BlastP bit score: 204
  
Sequence coverage: 91 %
  
E-value: 1e-64
  
  
 NCBI BlastP on this gene

EEQ86778

conserved hypothetical protein
  
Accession: EEQ86779
  
Location: 4632966-4634378
  
  
**BlastP hit with Mycgr3G38483**
  
Percentage identity: 39 %
  
BlastP bit score: 211
  
Sequence coverage: 83 %
  
E-value: 1e-61
  
  
 NCBI BlastP on this gene

EEQ86779

77. :  GG657453 Ajellomyces dermatitidis SLH14081 genomic scaffold supercont1.6     Total score: 2.0     Cumulative Blast bit score: 413

conserved hypothetical protein
  
Accession: EEQ77680
  
Location: 2539526-2540940
  
  
**BlastP hit with Mycgr3G38483**
  
Percentage identity: 39 %
  
BlastP bit score: 209
  
Sequence coverage: 83 %
  
E-value: 6e-61
  
  
 NCBI BlastP on this gene

EEQ77680

37S ribosomal protein S24
  
Accession: EEQ77681
  
Location: 2541270-2541936
  
  
**BlastP hit with Mycgr3G70471**
  
Percentage identity: 78 %
  
BlastP bit score: 204
  
Sequence coverage: 91 %
  
E-value: 1e-64
  
  
 NCBI BlastP on this gene

EEQ77681

predicted protein
  
Accession: EEQ77682
  
Location: 2542951-2544440
  
 NCBI BlastP on this gene

EEQ77682

peptidyl-prolyl cis-trans isomerase-like 4
  
Accession: EEQ77683
  
Location: 2547463-2549057
  
 NCBI BlastP on this gene

EEQ77683

UDP-N-acetylglucosamine pyrophosphorylase
  
Accession: EEQ77684
  
Location: 2549652-2551390
  
 NCBI BlastP on this gene

EEQ77684

zinc knuckle domain-containing protein
  
Accession: EEQ77685
  
Location: 2552490-2553649
  
 NCBI BlastP on this gene

EEQ77685

78. :  DS990639 Ajellomyces capsulatus H88 supercont1.4 genomic scaffold     Total score: 2.0     Cumulative Blast bit score: 413

zinc-finger protein GIS2
  
Accession: EGC45836
  
Location: 2160800-2161948
  
 NCBI BlastP on this gene

EGC45836

UDP-N-acetylglucosamine pyrophosphorylase
  
Accession: EGC45837
  
Location: 2163181-2164934
  
 NCBI BlastP on this gene

EGC45837

peptidyl-prolyl cis-trans isomerase
  
Accession: EGC45838
  
Location: 2165507-2167072
  
 NCBI BlastP on this gene

EGC45838

predicted protein
  
Accession: EGC45839
  
Location: 2169853-2171431
  
 NCBI BlastP on this gene

EGC45839

40S ribosomal protein S24
  
Accession: EGC45840
  
Location: 2172375-2173020
  
  
**BlastP hit with Mycgr3G70471**
  
Percentage identity: 78 %
  
BlastP bit score: 202
  
Sequence coverage: 90 %
  
E-value: 4e-64
  
  
 NCBI BlastP on this gene

EGC45840

conserved hypothetical protein
  
Accession: EGC45841
  
Location: 2173343-2174656
  
  
**BlastP hit with Mycgr3G38483**
  
Percentage identity: 35 %
  
BlastP bit score: 211
  
Sequence coverage: 98 %
  
E-value: 2e-61
  
  
 NCBI BlastP on this gene

EGC45841

conserved hypothetical protein
  
Accession: EGC45842
  
Location: 2177163-2179682
  
 NCBI BlastP on this gene

EGC45842

cytochrome c oxidase assembly protein
  
Accession: EGC45843
  
Location: 2180938-2181298
  
 NCBI BlastP on this gene

EGC45843

conserved hypothetical protein
  
Accession: EGC45844
  
Location: 2181600-2182484
  
 NCBI BlastP on this gene

EGC45844

triacylglycerol lipase
  
Accession: EGC45845
  
Location: 2183015-2184734
  
 NCBI BlastP on this gene

EGC45845

arrestin
  
Accession: EGC45846
  
Location: 2185706-2187912
  
 NCBI BlastP on this gene

EGC45846

79. :  DS989826 Arthroderma gypseum CBS 118893 supercont1.5 genomic scaffold     Total score: 2.0     Cumulative Blast bit score: 413

hypothetical protein
  
Accession: EFR02876
  
Location: 84029-84862
  
 NCBI BlastP on this gene

EFR02876

hypothetical protein
  
Accession: EFR02877
  
Location: 85676-86962
  
 NCBI BlastP on this gene

EFR02877

26S proteasome non-ATPase regulatory subunit 8
  
Accession: EFR02878
  
Location: 87104-88038
  
 NCBI BlastP on this gene

EFR02878

hypothetical protein
  
Accession: EFR02879
  
Location: 89213-90208
  
 NCBI BlastP on this gene

EFR02879

tripeptidyl-peptidase 1
  
Accession: EFR02880
  
Location: 90533-92402
  
 NCBI BlastP on this gene

EFR02880

aflatoxin biosynthesis ketoreductase nor-1
  
Accession: EFR02881
  
Location: 93566-94342
  
 NCBI BlastP on this gene

EFR02881

hypothetical protein
  
Accession: EFR02882
  
Location: 95217-96848
  
  
**BlastP hit with Mycgr3G38483**
  
Percentage identity: 38 %
  
BlastP bit score: 215
  
Sequence coverage: 97 %
  
E-value: 6e-63
  
  
 NCBI BlastP on this gene

EFR02882

40S ribosomal protein S24
  
Accession: EFR02883
  
Location: 97216-97974
  
  
**BlastP hit with Mycgr3G70471**
  
Percentage identity: 75 %
  
BlastP bit score: 198
  
Sequence coverage: 90 %
  
E-value: 2e-62
  
  
 NCBI BlastP on this gene

EFR02883

hypothetical protein
  
Accession: EFR02884
  
Location: 98901-100389
  
 NCBI BlastP on this gene

EFR02884

peptidyl-prolyl cis-trans isomerase cyp6
  
Accession: EFR02885
  
Location: 103072-104654
  
 NCBI BlastP on this gene

EFR02885

UDP-N-acetylglucosamine pyrophosphorylase
  
Accession: EFR02886
  
Location: 105264-106949
  
 NCBI BlastP on this gene

EFR02886

DNA-binding protein HEXBP
  
Accession: EFR02887
  
Location: 108222-109064
  
 NCBI BlastP on this gene

EFR02887

80. :  GG704913 Coccidioides immitis RS genomic scaffold supercont3.3     Total score: 2.0     Cumulative Blast bit score: 412

hypothetical protein
  
Accession: EAS29346
  
Location: 3139359-3143297
  
 NCBI BlastP on this gene

EAS29346

arrestin domain-containing protein
  
Accession: EJB11329
  
Location: 3144066-3146232
  
 NCBI BlastP on this gene

EJB11329

V-type ATPase, G subunit
  
Accession: EAS29343
  
Location: 3147571-3148278
  
 NCBI BlastP on this gene

EAS29343

cytochrome c oxidase assembly protein
  
Accession: EAS29342
  
Location: 3148585-3148927
  
 NCBI BlastP on this gene

EAS29342

hypothetical protein
  
Accession: EAS29341
  
Location: 3150053-3152365
  
 NCBI BlastP on this gene

EAS29341

hypothetical protein
  
Accession: EAS29340
  
Location: 3153806-3155135
  
  
**BlastP hit with Mycgr3G38483**
  
Percentage identity: 36 %
  
BlastP bit score: 209
  
Sequence coverage: 98 %
  
E-value: 1e-60
  
  
 NCBI BlastP on this gene

EAS29340

40S ribosomal protein S24
  
Accession: EJB11330
  
Location: 3155465-3156118
  
  
**BlastP hit with Mycgr3G70471**
  
Percentage identity: 77 %
  
BlastP bit score: 203
  
Sequence coverage: 91 %
  
E-value: 3e-64
  
  
 NCBI BlastP on this gene

EJB11330

hypothetical protein
  
Accession: EAS29337
  
Location: 3156928-3158393
  
 NCBI BlastP on this gene

EAS29337

peptidyl-prolyl cis-trans isomerase-like 4
  
Accession: EAS29335
  
Location: 3161371-3162891
  
 NCBI BlastP on this gene

EAS29335

UDP-N-acetylglucosamine pyrophosphorylase
  
Accession: EAS29334
  
Location: 3163507-3165167
  
 NCBI BlastP on this gene

EAS29334

zinc knuckle nucleic acid binding protein
  
Accession: EAS29333
  
Location: 3166022-3167068
  
 NCBI BlastP on this gene

EAS29333

81. :  GG663363 Ajellomyces capsulatus G186AR genomic scaffold supercont2.1     Total score: 2.0     Cumulative Blast bit score: 412

zinc knuckle domain-containing protein
  
Accession: EEH10870
  
Location: 1060491-1061639
  
 NCBI BlastP on this gene

EEH10870

UDP-N-acetylglucosamine pyrophosphorylase
  
Accession: EEH10871
  
Location: 1062872-1064635
  
 NCBI BlastP on this gene

EEH10871

peptidyl-prolyl cis-trans isomerase-like protein 4
  
Accession: EEH10872
  
Location: 1065208-1066773
  
 NCBI BlastP on this gene

EEH10872

predicted protein
  
Accession: EEH10873
  
Location: 1069539-1071117
  
 NCBI BlastP on this gene

EEH10873

40S ribosomal protein S24
  
Accession: EEH10874
  
Location: 1072060-1072713
  
  
**BlastP hit with Mycgr3G70471**
  
Percentage identity: 78 %
  
BlastP bit score: 202
  
Sequence coverage: 90 %
  
E-value: 4e-64
  
  
 NCBI BlastP on this gene

EEH10874

conserved hypothetical protein
  
Accession: EEH10875
  
Location: 1073005-1074344
  
  
**BlastP hit with Mycgr3G38483**
  
Percentage identity: 34 %
  
BlastP bit score: 210
  
Sequence coverage: 100 %
  
E-value: 5e-61
  
  
 NCBI BlastP on this gene

EEH10875

conserved hypothetical protein
  
Accession: EEH10876
  
Location: 1076855-1079374
  
 NCBI BlastP on this gene

EEH10876

conserved hypothetical protein
  
Accession: EEH10877
  
Location: 1080631-1080991
  
 NCBI BlastP on this gene

EEH10877

conserved hypothetical protein
  
Accession: EEH10878
  
Location: 1081289-1082174
  
 NCBI BlastP on this gene

EEH10878

triacylglycerol lipase
  
Accession: EEH10879
  
Location: 1082705-1084374
  
 NCBI BlastP on this gene

EEH10879

arrestin
  
Accession: EEH10880
  
Location: 1085398-1087595
  
 NCBI BlastP on this gene

EEH10880

82. :  ABDF02000003 Trichoderma virens Gv29-8     Total score: 2.0     Cumulative Blast bit score: 412

hypothetical protein
  
Accession: EHK25802
  
Location: 2315207-2316219
  
 NCBI BlastP on this gene

EHK25802

hypothetical protein
  
Accession: EHK25803
  
Location: 2316322-2317849
  
 NCBI BlastP on this gene

EHK25803

hypothetical protein
  
Accession: EHK25804
  
Location: 2319541-2319984
  
 NCBI BlastP on this gene

EHK25804

hypothetical protein
  
Accession: EHK25805
  
Location: 2320045-2320671
  
 NCBI BlastP on this gene

EHK25805

hypothetical protein
  
Accession: EHK25806
  
Location: 2322024-2323320
  
 NCBI BlastP on this gene

EHK25806

hypothetical protein
  
Accession: EHK25807
  
Location: 2324822-2325889
  
  
**BlastP hit with Mycgr3G38483**
  
Percentage identity: 45 %
  
BlastP bit score: 219
  
Sequence coverage: 83 %
  
E-value: 9e-65
  
  
 NCBI BlastP on this gene

EHK25807

hypothetical protein
  
Accession: EHK25808
  
Location: 2326459-2327300
  
  
**BlastP hit with Mycgr3G70471**
  
Percentage identity: 76 %
  
BlastP bit score: 193
  
Sequence coverage: 89 %
  
E-value: 3e-60
  
  
 NCBI BlastP on this gene

EHK25808

hypothetical protein
  
Accession: EHK25809
  
Location: 2327993-2329644
  
 NCBI BlastP on this gene

EHK25809

hypothetical protein
  
Accession: EHK25810
  
Location: 2330551-2331273
  
 NCBI BlastP on this gene

EHK25810

hypothetical protein
  
Accession: EHK25811
  
Location: 2333335-2334672
  
 NCBI BlastP on this gene

EHK25811

hypothetical protein
  
Accession: EHK25812
  
Location: 2335119-2336459
  
 NCBI BlastP on this gene

EHK25812

hypothetical protein
  
Accession: EHK25813
  
Location: 2336966-2337872
  
 NCBI BlastP on this gene

EHK25813

83. :  ABDG02000029 Trichoderma atroviride IMI 206040     Total score: 2.0     Cumulative Blast bit score: 411

hypothetical protein
  
Accession: EHK39410
  
Location: 230694-232401
  
 NCBI BlastP on this gene

EHK39410

hypothetical protein
  
Accession: EHK39411
  
Location: 233660-234574
  
 NCBI BlastP on this gene

EHK39411

hypothetical protein
  
Accession: EHK39412
  
Location: 235064-236401
  
 NCBI BlastP on this gene

EHK39412

hypothetical protein
  
Accession: EHK39413
  
Location: 236821-238165
  
 NCBI BlastP on this gene

EHK39413

hypothetical protein
  
Accession: EHK39414
  
Location: 239802-240524
  
 NCBI BlastP on this gene

EHK39414

hypothetical protein
  
Accession: EHK39415
  
Location: 241334-242959
  
 NCBI BlastP on this gene

EHK39415

hypothetical protein
  
Accession: EHK39416
  
Location: 243535-244364
  
  
**BlastP hit with Mycgr3G70471**
  
Percentage identity: 76 %
  
BlastP bit score: 195
  
Sequence coverage: 89 %
  
E-value: 5e-61
  
  
 NCBI BlastP on this gene

EHK39416

hypothetical protein
  
Accession: EHK39417
  
Location: 244840-245913
  
  
**BlastP hit with Mycgr3G38483**
  
Percentage identity: 39 %
  
BlastP bit score: 216
  
Sequence coverage: 99 %
  
E-value: 2e-63
  
  
 NCBI BlastP on this gene

EHK39417

hypothetical protein
  
Accession: EHK39418
  
Location: 250870-251358
  
 NCBI BlastP on this gene

EHK39418

hypothetical protein
  
Accession: EHK39419
  
Location: 252955-254214
  
 NCBI BlastP on this gene

EHK39419

phosphatidyltransferase
  
Accession: EHK39420
  
Location: 254562-255651
  
 NCBI BlastP on this gene

EHK39420

84. :  GL636509 Coccidioides posadasii str. Silveira unplaced genomic scaffold supercont2.24     Total score: 2.0     Cumulative Blast bit score: 409

conserved hypothetical protein
  
Accession: EFW13957
  
Location: 60941-64879
  
 NCBI BlastP on this gene

EFW13957

hypothetical protein
  
Accession: EFW13958
  
Location: 65653-67814
  
 NCBI BlastP on this gene

EFW13958

predicted protein
  
Accession: EFW13959
  
Location: 68145-69069
  
 NCBI BlastP on this gene

EFW13959

vacuolar ATPase
  
Accession: EFW13960
  
Location: 69169-69877
  
 NCBI BlastP on this gene

EFW13960

cytochrome c oxidase assembly protein
  
Accession: EFW13961
  
Location: 70184-70526
  
 NCBI BlastP on this gene

EFW13961

conserved hypothetical protein
  
Accession: EFW13962
  
Location: 71649-73964
  
 NCBI BlastP on this gene

EFW13962

conserved hypothetical protein
  
Accession: EFW13963
  
Location: 75389-76718
  
  
**BlastP hit with Mycgr3G38483**
  
Percentage identity: 35 %
  
BlastP bit score: 206
  
Sequence coverage: 98 %
  
E-value: 1e-59
  
  
 NCBI BlastP on this gene

EFW13963

40S ribosomal protein S24-A
  
Accession: EFW13964
  
Location: 77048-77697
  
  
**BlastP hit with Mycgr3G70471**
  
Percentage identity: 77 %
  
BlastP bit score: 203
  
Sequence coverage: 91 %
  
E-value: 3e-64
  
  
 NCBI BlastP on this gene

EFW13964

conserved hypothetical protein
  
Accession: EFW13965
  
Location: 78507-79972
  
 NCBI BlastP on this gene

EFW13965

peptidyl-prolyl cis-trans isomerase
  
Accession: EFW13966
  
Location: 82984-84504
  
 NCBI BlastP on this gene

EFW13966

UDP-N-acetylglucosamine pyrophosphorylase
  
Accession: EFW13967
  
Location: 85089-86749
  
 NCBI BlastP on this gene

EFW13967

zinc knuckle nucleic acid binding protein
  
Accession: EFW13968
  
Location: 87602-88649
  
 NCBI BlastP on this gene

EFW13968

85. :  AABX02000037 Neurospora crassa OR74A     Total score: 2.0     Cumulative Blast bit score: 407

predicted protein
  
Accession: EAA28169
  
Location: 232781-234547
  
 NCBI BlastP on this gene

EAA28169

conserved hypothetical protein
  
Accession: EAA28170
  
Location: 227120-228561
  
  
**BlastP hit with Mycgr3G38483**
  
Percentage identity: 38 %
  
BlastP bit score: 186
  
Sequence coverage: 102 %
  
E-value: 8e-52
  
  
 NCBI BlastP on this gene

EAA28170

40S ribosomal protein S24
  
Accession: EAA28171
  
Location: 225629-226685
  
  
**BlastP hit with Mycgr3G70471**
  
Percentage identity: 80 %
  
BlastP bit score: 221
  
Sequence coverage: 98 %
  
E-value: 4e-71
  
  
 NCBI BlastP on this gene

EAA28171

predicted protein
  
Accession: EAA28172
  
Location: 224381-225285
  
 NCBI BlastP on this gene

EAA28172

predicted protein
  
Accession: EAA28173
  
Location: 223164-223852
  
 NCBI BlastP on this gene

EAA28173

predicted protein
  
Accession: EAA28174
  
Location: 221473-222809
  
 NCBI BlastP on this gene

EAA28174

predicted protein
  
Accession: EAA28175
  
Location: 219188-220444
  
 NCBI BlastP on this gene

EAA28175

predicted protein
  
Accession: EAA28176
  
Location: 212968-217835
  
 NCBI BlastP on this gene

EAA28176

86. :  JH126408 Cordyceps militaris CM01 unplaced genomic scaffold CCM\_S00010     Total score: 2.0     Cumulative Blast bit score: 403

cyclophilin-type peptidyl-prolyl cis-trans isomerase
  
Accession: EGX87684
  
Location: 404866-406436
  
 NCBI BlastP on this gene

EGX87684

hypothetical protein
  
Accession: EGX87685
  
Location: 407061-410641
  
 NCBI BlastP on this gene

EGX87685

Methyltransferase-16, putative
  
Accession: EGX87686
  
Location: 411214-412340
  
  
**BlastP hit with Mycgr3G38483**
  
Percentage identity: 42 %
  
BlastP bit score: 230
  
Sequence coverage: 98 %
  
E-value: 9e-69
  
  
 NCBI BlastP on this gene

EGX87686

40S ribosomal protein S24
  
Accession: EGX87687
  
Location: 412669-413585
  
  
**BlastP hit with Mycgr3G70471**
  
Percentage identity: 55 %
  
BlastP bit score: 173
  
Sequence coverage: 122 %
  
E-value: 8e-52
  
  
 NCBI BlastP on this gene

EGX87687

hypothetical protein
  
Accession: EGX87688
  
Location: 414562-414984
  
 NCBI BlastP on this gene

EGX87688

hypothetical protein
  
Accession: EGX87689
  
Location: 415888-416340
  
 NCBI BlastP on this gene

EGX87689

hypothetical protein
  
Accession: EGX87690
  
Location: 416611-420349
  
 NCBI BlastP on this gene

EGX87690

GTP cyclohydrolase-2
  
Accession: EGX87691
  
Location: 420812-421999
  
 NCBI BlastP on this gene

EGX87691

RNA polymerase I subunit Rpa43, putative
  
Accession: EGX87692
  
Location: 423367-424746
  
 NCBI BlastP on this gene

EGX87692

87. :  CH476621 Sclerotinia sclerotiorum 1980 scaffold\_1 genomic scaffold     Total score: 2.0     Cumulative Blast bit score: 403

hypothetical protein
  
Accession: EDN91542
  
Location: 2431695-2434219
  
 NCBI BlastP on this gene

EDN91542

hypothetical protein
  
Accession: EDN91543
  
Location: 2436377-2437615
  
 NCBI BlastP on this gene

EDN91543

hypothetical protein
  
Accession: EDN91544
  
Location: 2438063-2439955
  
 NCBI BlastP on this gene

EDN91544

hypothetical protein
  
Accession: EDN91545
  
Location: 2440484-2441369
  
 NCBI BlastP on this gene

EDN91545

predicted protein
  
Accession: EDN91546
  
Location: 2441673-2441807
  
 NCBI BlastP on this gene

EDN91546

hypothetical protein
  
Accession: EDN91547
  
Location: 2442389-2443833
  
  
**BlastP hit with Mycgr3G38483**
  
Percentage identity: 41 %
  
BlastP bit score: 196
  
Sequence coverage: 92 %
  
E-value: 6e-55
  
  
 NCBI BlastP on this gene

EDN91547

40S ribosomal protein S24
  
Accession: EDN91548
  
Location: 2444103-2444744
  
  
**BlastP hit with Mycgr3G70471**
  
Percentage identity: 80 %
  
BlastP bit score: 207
  
Sequence coverage: 89 %
  
E-value: 9e-66
  
  
 NCBI BlastP on this gene

EDN91548

predicted protein
  
Accession: EDN91549
  
Location: 2446178-2446542
  
 NCBI BlastP on this gene

EDN91549

predicted protein
  
Accession: EDN91550
  
Location: 2448080-2450124
  
 NCBI BlastP on this gene

EDN91550

predicted protein
  
Accession: EDN91551
  
Location: 2450609-2451017
  
 NCBI BlastP on this gene

EDN91551

predicted protein
  
Accession: EDN91552
  
Location: 2451683-2451978
  
 NCBI BlastP on this gene

EDN91552

hypothetical protein
  
Accession: EDN91553
  
Location: 2452354-2453865
  
 NCBI BlastP on this gene

EDN91553

hypothetical protein
  
Accession: EDN91554
  
Location: 2454764-2456289
  
 NCBI BlastP on this gene

EDN91554

88. :  GG698544 Trichophyton tonsurans CBS 112818 genomic scaffold supercont1.68     Total score: 2.0     Cumulative Blast bit score: 396

hypothetical protein
  
Accession: EGE00590
  
Location: 52130-53198
  
 NCBI BlastP on this gene

EGE00590

hypothetical protein
  
Accession: EGE00591
  
Location: 53608-54938
  
 NCBI BlastP on this gene

EGE00591

hypothetical protein
  
Accession: EGE00592
  
Location: 55055-55989
  
 NCBI BlastP on this gene

EGE00592

hypothetical protein
  
Accession: EGE00593
  
Location: 57156-58151
  
 NCBI BlastP on this gene

EGE00593

tripeptidyl peptidase SED3
  
Accession: EGE00594
  
Location: 58507-60232
  
 NCBI BlastP on this gene

EGE00594

hypothetical protein
  
Accession: EGE00595
  
Location: 61488-62264
  
 NCBI BlastP on this gene

EGE00595

hypothetical protein
  
Accession: EGE00596
  
Location: 63367-64786
  
  
**BlastP hit with Mycgr3G38483**
  
Percentage identity: 34 %
  
BlastP bit score: 202
  
Sequence coverage: 98 %
  
E-value: 9e-58
  
  
 NCBI BlastP on this gene

EGE00596

ribosomal protein S24
  
Accession: EGE00597
  
Location: 65146-65896
  
  
**BlastP hit with Mycgr3G70471**
  
Percentage identity: 72 %
  
BlastP bit score: 194
  
Sequence coverage: 90 %
  
E-value: 1e-60
  
  
 NCBI BlastP on this gene

EGE00597

hypothetical protein
  
Accession: EGE00598
  
Location: 66802-68369
  
 NCBI BlastP on this gene

EGE00598

peptidyl-prolyl cis-trans isomerase
  
Accession: EGE00599
  
Location: 71419-72431
  
 NCBI BlastP on this gene

EGE00599

UDP-N-acetylglucosamine pyrophosphorylase
  
Accession: EGE00600
  
Location: 73683-75342
  
 NCBI BlastP on this gene

EGE00600

zinc knuckle domain-containing protein
  
Accession: EGE00601
  
Location: 76666-77507
  
 NCBI BlastP on this gene

EGE00601

89. :  CH476615 Uncinocarpus reesii 1704 scaffold\_1 genomic scaffold     Total score: 2.0     Cumulative Blast bit score: 396

hypothetical protein
  
Accession: EEP77263
  
Location: 5536631-5538238
  
 NCBI BlastP on this gene

EEP77263

hypothetical protein
  
Accession: EEP77264
  
Location: 5538811-5540331
  
 NCBI BlastP on this gene

EEP77264

predicted protein
  
Accession: EEP77265
  
Location: 5543194-5544669
  
 NCBI BlastP on this gene

EEP77265

40S ribosomal protein S24-A
  
Accession: EEP77266
  
Location: 5545436-5546094
  
  
**BlastP hit with Mycgr3G70471**
  
Percentage identity: 78 %
  
BlastP bit score: 206
  
Sequence coverage: 91 %
  
E-value: 2e-65
  
  
 NCBI BlastP on this gene

EEP77266

conserved hypothetical protein
  
Accession: EEP77267
  
Location: 5546553-5547723
  
  
**BlastP hit with Mycgr3G38483**
  
Percentage identity: 38 %
  
BlastP bit score: 190
  
Sequence coverage: 88 %
  
E-value: 2e-53
  
  
 NCBI BlastP on this gene

EEP77267

predicted protein
  
Accession: EEP77268
  
Location: 5549149-5551478
  
 NCBI BlastP on this gene

EEP77268

V-type ATPase, G subunit
  
Accession: EEP77269
  
Location: 5553228-5553903
  
 NCBI BlastP on this gene

EEP77269

conserved hypothetical protein
  
Accession: EEP77270
  
Location: 5555120-5557289
  
 NCBI BlastP on this gene

EEP77270

predicted protein
  
Accession: EEP77271
  
Location: 5557879-5558538
  
 NCBI BlastP on this gene

EEP77271

90. :  DS995906 Penicillium marneffei ATCC 18224 scf\_1105668340770 genomic scaffold     Total score: 2.0     Cumulative Blast bit score: 395

Ran-binding protein (RanBP10), putative
  
Accession: EEA18772
  
Location: 571838-574209
  
 NCBI BlastP on this gene

EEA18772

alcohol dehydrogenase, putative
  
Accession: EEA18773
  
Location: 574741-576008
  
 NCBI BlastP on this gene

EEA18773

conserved hypothetical protein
  
Accession: EEA18774
  
Location: 576805-577662
  
 NCBI BlastP on this gene

EEA18774

membrane associated DnaJ chaperone, putative
  
Accession: EEA18775
  
Location: 579202-580404
  
 NCBI BlastP on this gene

EEA18775

RNA polymerase I subunit Rpa43, putative
  
Accession: EEA18776
  
Location: 580723-582048
  
 NCBI BlastP on this gene

EEA18776

conserved hypothetical protein
  
Accession: EEA18777
  
Location: 582679-584095
  
  
**BlastP hit with Mycgr3G38483**
  
Percentage identity: 34 %
  
BlastP bit score: 175
  
Sequence coverage: 101 %
  
E-value: 1e-47
  
  
 NCBI BlastP on this gene

EEA18777

37S ribosomal protein S24
  
Accession: EEA18778
  
Location: 584446-585337
  
  
**BlastP hit with Mycgr3G70471**
  
Percentage identity: 81 %
  
BlastP bit score: 220
  
Sequence coverage: 97 %
  
E-value: 6e-71
  
  
 NCBI BlastP on this gene

EEA18778

conserved hypothetical protein
  
Accession: EEA18780
  
Location: 586518-589583
  
 NCBI BlastP on this gene

EEA18780

2,3-dihydroxybenzoic acid decarboxylase, putative
  
Accession: EEA18781
  
Location: 590076-591035
  
 NCBI BlastP on this gene

EEA18781

conserved hypothetical protein
  
Accession: EEA18782
  
Location: 591562-592831
  
 NCBI BlastP on this gene

EEA18782

U5 snRNP complex subunit, putative
  
Accession: EEA18783
  
Location: 594221-595526
  
 NCBI BlastP on this gene

EEA18783

TRAPP complex component Bet3, putative
  
Accession: EEA18784
  
Location: 595879-596735
  
 NCBI BlastP on this gene

EEA18784

91. :  DS995705 Microsporum canis CBS 113480 supercont1.5 genomic scaffold     Total score: 2.0     Cumulative Blast bit score: 395

zinc knuckle domain-containing protein
  
Accession: EEQ33252
  
Location: 2661510-2662336
  
 NCBI BlastP on this gene

EEQ33252

UDP-N-acetylglucosamine pyrophosphorylase
  
Accession: EEQ33253
  
Location: 2663548-2665225
  
 NCBI BlastP on this gene

EEQ33253

peptidyl-prolyl cis-trans isomerase-like 4
  
Accession: EEQ33254
  
Location: 2665838-2667414
  
 NCBI BlastP on this gene

EEQ33254

predicted protein
  
Accession: EEQ33255
  
Location: 2669920-2671458
  
 NCBI BlastP on this gene

EEQ33255

40S ribosomal protein S24
  
Accession: EEQ33256
  
Location: 2672351-2673093
  
  
**BlastP hit with Mycgr3G70471**
  
Percentage identity: 72 %
  
BlastP bit score: 191
  
Sequence coverage: 90 %
  
E-value: 1e-59
  
  
 NCBI BlastP on this gene

EEQ33256

conserved hypothetical protein
  
Accession: EEQ33257
  
Location: 2673419-2674655
  
  
**BlastP hit with Mycgr3G38483**
  
Percentage identity: 38 %
  
BlastP bit score: 204
  
Sequence coverage: 89 %
  
E-value: 8e-59
  
  
 NCBI BlastP on this gene

EEQ33257

tripeptidyl peptidase SED3
  
Accession: EEQ33258
  
Location: 2677505-2679414
  
 NCBI BlastP on this gene

EEQ33258

conserved hypothetical protein
  
Accession: EEQ33259
  
Location: 2679743-2680738
  
 NCBI BlastP on this gene

EEQ33259

predicted protein
  
Accession: EEQ33260
  
Location: 2680904-2681560
  
 NCBI BlastP on this gene

EEQ33260

26S proteasome non-ATPase regulatory subunit 8
  
Accession: EEQ33261
  
Location: 2681888-2682841
  
 NCBI BlastP on this gene

EEQ33261

conserved hypothetical protein
  
Accession: EEQ33262
  
Location: 2683121-2684492
  
 NCBI BlastP on this gene

EEQ33262

conserved hypothetical protein
  
Accession: EEQ33263
  
Location: 2685271-2686148
  
 NCBI BlastP on this gene

EEQ33263

92. :  AM270259 Aspergillus niger contig An12c0020, genomic contig.     Total score: 2.0     Cumulative Blast bit score: 394

not annotated
  
Accession: CAK40977
  
Location: 48742-50405
  
 NCBI BlastP on this gene

An12g00480

not annotated
  
Accession: CAK40978
  
Location: 51174-52792
  
 NCBI BlastP on this gene

An12g00490

not annotated
  
Accession: CAK40979
  
Location: 56452-58039
  
 NCBI BlastP on this gene

An12g00500

not annotated
  
Accession: CAK40980
  
Location: 60277-61028
  
  
**BlastP hit with Mycgr3G70471**
  
Percentage identity: 79 %
  
BlastP bit score: 217
  
Sequence coverage: 97 %
  
E-value: 9e-70
  
  
 NCBI BlastP on this gene

An12g00510

not annotated
  
Accession: CAK40981
  
Location: 61430-63087
  
  
**BlastP hit with Mycgr3G38483**
  
Percentage identity: 31 %
  
BlastP bit score: 177
  
Sequence coverage: 103 %
  
E-value: 4e-48
  
  
 NCBI BlastP on this gene

An12g00520

not annotated
  
Accession: CAK40982
  
Location: 63485-65563
  
 NCBI BlastP on this gene

An12g00530

hypothetical protein
  
Accession: CAK40983
  
Location: 65710-66171
  
 NCBI BlastP on this gene

An12g00540

unnamed
  
Accession: CAK40984
  
Location: 66712-70157
  
 NCBI BlastP on this gene

An12g00550

hypothetical protein
  
Accession: CAK40985
  
Location: 71196-71905
  
 NCBI BlastP on this gene

An12g00560

not annotated
  
Accession: CAK40986
  
Location: 72211-73073
  
 NCBI BlastP on this gene

An12g00570

93. :  JH226133 Exophiala dermatitidis NIH/UT8656 unplaced genomic scaffold supercont1.4     Total score: 2.0     Cumulative Blast bit score: 393

hypothetical protein
  
Accession: EHY57273
  
Location: 2783170-2783607
  
 NCBI BlastP on this gene

EHY57273

retrograde regulation protein 2
  
Accession: EHY57274
  
Location: 2784352-2786004
  
 NCBI BlastP on this gene

EHY57274

gibberellin 2-oxidase
  
Accession: EHY57275
  
Location: 2787125-2788285
  
 NCBI BlastP on this gene

EHY57275

hypothetical protein
  
Accession: EHY57276
  
Location: 2788969-2790113
  
 NCBI BlastP on this gene

EHY57276

hypothetical protein
  
Accession: EHY57277
  
Location: 2790695-2793641
  
 NCBI BlastP on this gene

EHY57277

hypothetical protein
  
Accession: EHY57278
  
Location: 2794187-2795428
  
  
**BlastP hit with Mycgr3G38483**
  
Percentage identity: 34 %
  
BlastP bit score: 179
  
Sequence coverage: 101 %
  
E-value: 4e-49
  
  
 NCBI BlastP on this gene

EHY57278

30S ribosomal protein S24e
  
Accession: EHY57279
  
Location: 2795782-2796380
  
  
**BlastP hit with Mycgr3G70471**
  
Percentage identity: 77 %
  
BlastP bit score: 214
  
Sequence coverage: 100 %
  
E-value: 2e-68
  
  
 NCBI BlastP on this gene

EHY57279

hypothetical protein, variant
  
Accession: EHY57280
  
Location: 2798525-2800217
  
 NCBI BlastP on this gene

EHY57280

peptidylprolyl isomerase
  
Accession: EHY57282
  
Location: 2803105-2804610
  
 NCBI BlastP on this gene

EHY57282

UDP-N-acetylglucosamine pyrophosphorylase
  
Accession: EHY57283
  
Location: 2805081-2806672
  
 NCBI BlastP on this gene

EHY57283

cellular nucleic acid-binding protein
  
Accession: EHY57284
  
Location: 2807491-2808305
  
 NCBI BlastP on this gene

EHY57284

94. :  ACJE01000005 Aspergillus niger ATCC 1015     Total score: 2.0     Cumulative Blast bit score: 393

hypothetical protein
  
Accession: EHA25965
  
Location: 1080681-1081327
  
 NCBI BlastP on this gene

EHA25965

hypothetical protein
  
Accession: EHA25966
  
Location: 1081481-1082343
  
 NCBI BlastP on this gene

EHA25966

hypothetical protein
  
Accession: EHA25967
  
Location: 1082649-1083290
  
 NCBI BlastP on this gene

EHA25967

hypothetical protein
  
Accession: EHA25968
  
Location: 1084395-1091067
  
 NCBI BlastP on this gene

EHA25968

hypothetical protein
  
Accession: EHA25969
  
Location: 1091465-1093130
  
  
**BlastP hit with Mycgr3G38483**
  
Percentage identity: 31 %
  
BlastP bit score: 176
  
Sequence coverage: 103 %
  
E-value: 8e-48
  
  
 NCBI BlastP on this gene

EHA25969

hypothetical protein
  
Accession: EHA25970
  
Location: 1093532-1094283
  
  
**BlastP hit with Mycgr3G70471**
  
Percentage identity: 79 %
  
BlastP bit score: 217
  
Sequence coverage: 97 %
  
E-value: 9e-70
  
  
 NCBI BlastP on this gene

EHA25970

hypothetical protein
  
Accession: EHA25971
  
Location: 1096521-1098108
  
 NCBI BlastP on this gene

EHA25971

hypothetical protein
  
Accession: EHA25972
  
Location: 1101768-1103386
  
 NCBI BlastP on this gene

EHA25972

hypothetical protein
  
Accession: EHA25973
  
Location: 1104155-1105818
  
 NCBI BlastP on this gene

EHA25973

95. :  GL698729 Metarhizium anisopliae ARSEF 23 unplaced genomic scaffold Scf\_019     Total score: 2.0     Cumulative Blast bit score: 391

hypothetical protein
  
Accession: EFY96537
  
Location: 258201-258854
  
 NCBI BlastP on this gene

EFY96537

GTP cyclohydrolase-2
  
Accession: EFY96538
  
Location: 259852-261079
  
 NCBI BlastP on this gene

EFY96538

hypothetical protein
  
Accession: EFY96539
  
Location: 265425-266009
  
 NCBI BlastP on this gene

EFY96539

40S ribosomal protein S24
  
Accession: EFY96540
  
Location: 266646-267549
  
  
**BlastP hit with Mycgr3G70471**
  
Percentage identity: 78 %
  
BlastP bit score: 200
  
Sequence coverage: 89 %
  
E-value: 5e-63
  
  
 NCBI BlastP on this gene

EFY96540

hypothetical protein
  
Accession: EFY96541
  
Location: 268266-269084
  
  
**BlastP hit with Mycgr3G38483**
  
Percentage identity: 47 %
  
BlastP bit score: 191
  
Sequence coverage: 62 %
  
E-value: 2e-54
  
  
 NCBI BlastP on this gene

EFY96541

hypothetical protein
  
Accession: EFY96542
  
Location: 272267-272743
  
 NCBI BlastP on this gene

EFY96542

RNA binding protein
  
Accession: EFY96543
  
Location: 274134-275661
  
 NCBI BlastP on this gene

EFY96543

hypothetical protein
  
Accession: EFY96544
  
Location: 280038-281224
  
 NCBI BlastP on this gene

EFY96544

96. :  GG700654 Trichophyton rubrum CBS 118892 genomic scaffold supercont2.7     Total score: 2.0     Cumulative Blast bit score: 388

hypothetical protein
  
Accession: EGD89781
  
Location: 78946-79779
  
 NCBI BlastP on this gene

EGD89781

hypothetical protein
  
Accession: EGD89782
  
Location: 80448-81756
  
 NCBI BlastP on this gene

EGD89782

hypothetical protein
  
Accession: EGD89783
  
Location: 81883-82816
  
 NCBI BlastP on this gene

EGD89783

hypothetical protein
  
Accession: EGD89784
  
Location: 83907-84902
  
 NCBI BlastP on this gene

EGD89784

tripeptidyl peptidase SED3
  
Accession: EGD89785
  
Location: 85232-87115
  
 NCBI BlastP on this gene

EGD89785

hypothetical protein
  
Accession: EGD89786
  
Location: 88269-89045
  
 NCBI BlastP on this gene

EGD89786

hypothetical protein
  
Accession: EGD89787
  
Location: 90141-91633
  
  
**BlastP hit with Mycgr3G38483**
  
Percentage identity: 34 %
  
BlastP bit score: 194
  
Sequence coverage: 97 %
  
E-value: 2e-54
  
  
 NCBI BlastP on this gene

EGD89787

40S ribosomal protein S24
  
Accession: EGD89788
  
Location: 91916-92663
  
  
**BlastP hit with Mycgr3G70471**
  
Percentage identity: 72 %
  
BlastP bit score: 194
  
Sequence coverage: 90 %
  
E-value: 1e-60
  
  
 NCBI BlastP on this gene

EGD89788

hypothetical protein
  
Accession: EGD89789
  
Location: 93549-94703
  
 NCBI BlastP on this gene

EGD89789

peptidyl-prolyl cis-trans isomerase
  
Accession: EGD89790
  
Location: 98412-99955
  
 NCBI BlastP on this gene

EGD89790

UDP-N-acetylglucosamine pyrophosphorylase
  
Accession: EGD89791
  
Location: 100701-102388
  
 NCBI BlastP on this gene

EGD89791

zinc knuckle nucleic acid binding protein
  
Accession: EGD89792
  
Location: 103707-104544
  
 NCBI BlastP on this gene

EGD89792

97. :  EQ963479 Aspergillus flavus NRRL3357 scf\_1106286418500 genomic scaffold     Total score: 2.0     Cumulative Blast bit score: 388

membrane associated DnaJ chaperone, putative
  
Accession: EED50303
  
Location: 1940772-1941993
  
 NCBI BlastP on this gene

EED50303

hypothetical protein
  
Accession: EED50304
  
Location: 1943415-1943992
  
 NCBI BlastP on this gene

EED50304

conserved hypothetical protein
  
Accession: EED50305
  
Location: 1944048-1944915
  
 NCBI BlastP on this gene

EED50305

hypothetical protein
  
Accession: EED50306
  
Location: 1945597-1945957
  
 NCBI BlastP on this gene

EED50306

Ran-binding protein (RanBP10), putative
  
Accession: EED50307
  
Location: 1946552-1948930
  
 NCBI BlastP on this gene

EED50307

conserved hypothetical protein
  
Accession: EED50308
  
Location: 1950601-1952770
  
 NCBI BlastP on this gene

EED50308

conserved hypothetical protein
  
Accession: EED50309
  
Location: 1953141-1954452
  
  
**BlastP hit with Mycgr3G38483**
  
Percentage identity: 32 %
  
BlastP bit score: 170
  
Sequence coverage: 106 %
  
E-value: 2e-45
  
  
 NCBI BlastP on this gene

EED50309

37S ribosomal protein S24
  
Accession: EED50310
  
Location: 1954744-1955516
  
  
**BlastP hit with Mycgr3G70471**
  
Percentage identity: 81 %
  
BlastP bit score: 218
  
Sequence coverage: 97 %
  
E-value: 3e-70
  
  
 NCBI BlastP on this gene

EED50310

hemagglutinin protein, putative
  
Accession: EED50311
  
Location: 1957574-1958722
  
 NCBI BlastP on this gene

EED50311

cyclophilin-type peptidyl-prolyl cis-trans isomerase, putative
  
Accession: EED50312
  
Location: 1962011-1963624
  
 NCBI BlastP on this gene

EED50312

UDP-N-acetylglucosamine pyrophosphorylase
  
Accession: EED50313
  
Location: 1964301-1965959
  
 NCBI BlastP on this gene

EED50313

98. :  DF126478 Aspergillus kawachii IFO 4308 DNA, contig: scaffold00032     Total score: 2.0     Cumulative Blast bit score: 388

UDP-N-acetylglucosamine pyrophosphorylase
  
Accession: GAA91127
  
Location: 132338-134002
  
 NCBI BlastP on this gene

GAA91127

cyclophilin-type peptidyl-prolyl cis-trans isomerase
  
Accession: GAA91128
  
Location: 134748-136366
  
 NCBI BlastP on this gene

GAA91128

streptococcal hemagglutinin protein
  
Accession: GAA91129
  
Location: 140624-141604
  
 NCBI BlastP on this gene

GAA91129

37S ribosomal protein S24
  
Accession: GAA91130
  
Location: 144000-144764
  
  
**BlastP hit with Mycgr3G70471**
  
Percentage identity: 79 %
  
BlastP bit score: 217
  
Sequence coverage: 97 %
  
E-value: 9e-70
  
  
 NCBI BlastP on this gene

GAA91130

hypothetical protein
  
Accession: GAA91131
  
Location: 145186-146613
  
  
**BlastP hit with Mycgr3G38483**
  
Percentage identity: 35 %
  
BlastP bit score: 171
  
Sequence coverage: 91 %
  
E-value: 2e-46
  
  
 NCBI BlastP on this gene

GAA91131

similar to An12g00530
  
Accession: GAA91132
  
Location: 147245-149422
  
 NCBI BlastP on this gene

GAA91132

hypothetical protein
  
Accession: GAA91133
  
Location: 149467-150234
  
 NCBI BlastP on this gene

GAA91133

Ran-binding protein (RanBPM)
  
Accession: GAA91134
  
Location: 151471-153932
  
 NCBI BlastP on this gene

GAA91134

DUF1711 domain protein
  
Accession: GAA91135
  
Location: 156055-156916
  
 NCBI BlastP on this gene

GAA91135

hypothetical protein
  
Accession: GAA91136
  
Location: 157140-157484
  
 NCBI BlastP on this gene

GAA91136

99. :  AP007169 Aspergillus oryzae RIB40 DNA, SC038.     Total score: 2.0     Cumulative Blast bit score: 385

not annotated
  
Accession: BAE64352
  
Location: 1589103-1590218
  
 NCBI BlastP on this gene

AO090038000586

not annotated
  
Accession: BAE64353
  
Location: 1592272-1593139
  
 NCBI BlastP on this gene

AO090038000587

not annotated
  
Accession: BAE64354
  
Location: 1594776-1597154
  
 NCBI BlastP on this gene

AO090038000589

not annotated
  
Accession: BAE64355
  
Location: 1598882-1600993
  
 NCBI BlastP on this gene

AO090038000590

not annotated
  
Accession: BAE64356
  
Location: 1601491-1602675
  
  
**BlastP hit with Mycgr3G38483**
  
Percentage identity: 39 %
  
BlastP bit score: 167
  
Sequence coverage: 76 %
  
E-value: 7e-45
  
  
 NCBI BlastP on this gene

AO090038000591

not annotated
  
Accession: BAE64357
  
Location: 1602965-1603737
  
  
**BlastP hit with Mycgr3G70471**
  
Percentage identity: 81 %
  
BlastP bit score: 218
  
Sequence coverage: 97 %
  
E-value: 3e-70
  
  
 NCBI BlastP on this gene

AO090038000592

not annotated
  
Accession: BAE64358
  
Location: 1605796-1606944
  
 NCBI BlastP on this gene

AO090038000593

not annotated
  
Accession: BAE64359
  
Location: 1610200-1611813
  
 NCBI BlastP on this gene

AO090038000594

not annotated
  
Accession: BAE64360
  
Location: 1612490-1614148
  
 NCBI BlastP on this gene

AO090038000595

100. :  ACYE01000161 Trichophyton verrucosum HKI 0517     Total score: 2.0     Cumulative Blast bit score: 384

hypothetical protein
  
Accession: EFE42114
  
Location: 6994-8681
  
 NCBI BlastP on this gene

EFE42114

hypothetical protein
  
Accession: EFE42115
  
Location: 9404-11641
  
 NCBI BlastP on this gene

EFE42115

putative hemagglutinin protein
  
Accession: EFE42116
  
Location: 14284-15441
  
 NCBI BlastP on this gene

EFE42116

hypothetical protein
  
Accession: EFE42117
  
Location: 16358-17093
  
  
**BlastP hit with Mycgr3G70471**
  
Percentage identity: 64 %
  
BlastP bit score: 186
  
Sequence coverage: 103 %
  
E-value: 4e-57
  
  
 NCBI BlastP on this gene

EFE42117

hypothetical protein
  
Accession: EFE42118
  
Location: 17465-18663
  
  
**BlastP hit with Mycgr3G38483**
  
Percentage identity: 39 %
  
BlastP bit score: 198
  
Sequence coverage: 83 %
  
E-value: 8e-57
  
  
 NCBI BlastP on this gene

EFE42118

toxin biosynthesis ketoreductase, putative
  
Accession: EFE42119
  
Location: 19858-20634
  
 NCBI BlastP on this gene

EFE42119

hypothetical protein
  
Accession: EFE42120
  
Location: 21894-23381
  
 NCBI BlastP on this gene

EFE42120

hypothetical protein
  
Accession: EFE42121
  
Location: 24048-25043
  
 NCBI BlastP on this gene

EFE42121

hypothetical protein
  
Accession: EFE42122
  
Location: 26212-27146
  
 NCBI BlastP on this gene

EFE42122

conserved hypothetical protein
  
Accession: EFE42123
  
Location: 27261-28594
  
 NCBI BlastP on this gene

EFE42123

hypothetical protein
  
Accession: EFE42124
  
Location: 29254-30087
  
 NCBI BlastP on this gene

EFE42124

Detecting sequence homology at the gene cluster level with MultiGeneBlast.
  
Marnix H. Medema, Rainer Breitling & Eriko Takano (2013)
  
*Molecular Biology and Evolution* , 30: 1218-1223.
