# Supplementary material for: In silico prediction and characterization of secondary metabolite biosynthetic gene clusters in the wheat pathogen Zymoseptoria tritici
Source: BMC Genomics. 2017 Aug 17;18:631. doi: 10.1186/s12864-017-3969-y (PMC5561558; doi:10.1186/s12864-017-3969-y)
Supplement: Supplementary file 1 — MultiGeneBLAST analysis of putative secondary metabolite clusters. All encoded amino acid sequences from genes residing in clusters predicted by AntiSMASH are given as FASTA file format. All output data from MultiGeneBLASTs are also provided. (ZIP 42911 kb) [file 12864_2017_3969_MOESM1_ESM.zip › Cluster MultiGene BLAST/out/Clusters_1_34/Cluster_13/displaypage3.xhtml]

xml version="1.0" encoding="UTF-8"?


Search Results
  
  
 Results pages: 1, 2, 3, 4, 5

**MultiGeneBlast hits**

Select gene cluster alignment
101. ACFW01000035\_0 Coccidioides posadasii C735 delta SOWgp, whole genome sho...
102. ABSU01000001\_1 Arthroderma benhamiae CBS 112371, whole genome shotgun se...
103. EQ962660\_0 Talaromyces stipitatus ATCC 10500 scf\_1105507295517 genomic s...
104. GL385404\_0 Gaeumannomyces graminis var. tritici R3-111a-1 unplaced genom...
105. GG692419\_0 Ajellomyces capsulatus H143 genomic scaffold supercont2.1, wh...
106. KE375214\_0 Blumeria graminis f. sp. tritici 96224 unplaced genomic scaff...
107. CAUH01002404\_0 Blumeria graminis f. sp. hordei DH14, whole genome shotgu...
108. AACD01000169\_0 Aspergillus nidulans FGSC A4, whole genome shotgun sequen...
109. CH476658\_0 Ajellomyces capsulatus NAm1 scaffold\_4 genomic scaffold, whol...
110. DS995769\_0 Trichophyton equinum CBS 127.97 supercont1.52 genomic scaffol...
111. CH476595\_1 Aspergillus terreus NIH2624 scaffold\_2 genomic scaffold, whol...
112. AQGS01000244\_0 Dactylellina haptotyla CBS 200.50, whole genome shotgun s...
113. ADOT01000287\_0 Arthrobotrys oligospora ATCC 24927, whole genome shotgun ...
114. GG704911\_0 Coccidioides immitis RS genomic scaffold supercont3.1, whole ...
115. KB446538\_3 Dothistroma septosporum NZE10 unplaced genomic scaffold DOTSE...
116. AM270040\_1 Aspergillus niger contig An02c0460, genomic contig.
117. JH921428\_1 Marssonina brunnea f. sp. 'multigermtubi' MB\_m1 unplaced geno...
118. DS027696\_2 Neosartorya fischeri NRRL 181 1099437636264 genomic scaffold,...
119. AM920437\_1 Penicillium chrysogenum Wisconsin 54-1255 complete genome, co...
120. DS572755\_1 Paracoccidioides brasiliensis Pb18 supercont1.6 genomic scaff...
121. GG749429\_1 Ajellomyces dermatitidis ATCC 18188 genomic scaffold supercon...
122. GG657469\_1 Ajellomyces dermatitidis SLH14081 genomic scaffold supercont1...
123. EQ999973\_1 Ajellomyces dermatitidis ER-3 genomic scaffold supercont1.1, ...
124. GL636500\_0 Coccidioides posadasii str. Silveira unplaced genomic scaffol...
125. GG704911\_1 Coccidioides immitis RS genomic scaffold supercont3.1, whole ...
126. DS499595\_1 Aspergillus fumigatus A1163 scf\_000002 genomic scaffold, whol...
127. DS544809\_1 Paracoccidioides brasiliensis Pb03 supercont1.7 genomic scaff...
128. DF126451\_0 Aspergillus kawachii IFO 4308 DNA, contig: scaffold00005, who...
129. GL891304\_0 Neurospora tetrasperma FGSC 2508 unplaced genomic scaffold NE...
130. GL891236\_1 Neurospora tetrasperma FGSC 2509 unplaced genomic scaffold NE...
131. ACJE01000010\_2 Aspergillus niger ATCC 1015, whole genome shotgun sequenc...
132. DS995900\_1 Penicillium marneffei ATCC 18224 scf\_1105668340758 genomic sc...
133. AAHF01000008\_1 Aspergillus fumigatus Af293, whole genome shotgun sequenc...
134. GG663367\_0 Ajellomyces capsulatus G186AR genomic scaffold supercont2.5, ...
135. DS572815\_1 Paracoccidioides brasiliensis Pb01 supercont1.5 genomic scaff...
136. AQGS01000575\_0 Dactylellina haptotyla CBS 200.50, whole genome shotgun s...
137. DS989823\_1 Arthroderma gypseum CBS 118893 supercont1.2 genomic scaffold,...
138. JH226136\_1 Exophiala dermatitidis NIH/UT8656 unplaced genomic scaffold s...
139. KB446557\_0 Pseudocercospora fijiensis CIRAD86 unplaced genomic scaffold ...
140. HF679025\_0 Fusarium fujikuroi IMI 58289 draft genome, chromosome FFUJ\_ch...
141. KB446538\_0 Dothistroma septosporum NZE10 unplaced genomic scaffold DOTSE...
142. EQ962653\_1 Talaromyces stipitatus ATCC 10500 scf\_1105507295527 genomic s...
143. ABDG02000027\_0 Trichoderma atroviride IMI 206040, whole genome shotgun s...
144. CU633900\_1 Podospora anserina S mat+ genomic DNA chromosome 7, supercont...
145. ACFW01000049\_1 Coccidioides posadasii C735 delta SOWgp, whole genome sho...
146. ADOT01000059\_1 Arthrobotrys oligospora ATCC 24927, whole genome shotgun ...
147. AACD01000129\_1 Aspergillus nidulans FGSC A4, whole genome shotgun sequen...
148. GL988041\_1 Chaetomium thermophilum var. thermophilum DSM 1495 unplaced g...
149. KB644415\_0 Penicillium oxalicum 114-2 unplaced genomic scaffold scaffold...
150. AM920437\_2 Penicillium chrysogenum Wisconsin 54-1255 complete genome, co...

Query: Architecture Search FASTA input

ACFW01000035 : Coccidioides posadasii C735 delta SOWgp    Total score: 2.0     Cumulative Blast bit score: 384

Hit cluster cross-links:

Mycgr3G70471
  
Location: 0-405

Mycgr3G70471

Mycgr3G39149
  
Location: 505-1798

Mycgr3G39149

Mycgr3G92130
  
Location: 1898-2396

Mycgr3G92130

Mycgr3G38483
  
Location: 2496-3576

Mycgr3G38483

Mycgr3G108869
  
Location: 3676-5056

Mycgr3G108869

Mycgr3G103943
  
Location: 5156-5762

Mycgr3G103943

Mycgr3G57362
  
Location: 5862-7296

Mycgr3G57362

Mycgr3G39086
  
Location: 7396-8368

Mycgr3G39086

Mycgr3G103942
  
Location: 8468-8714

Mycgr3G103942

Mycgr3G108865
  
Location: 8814-10239

Mycgr3G108865

Mycgr3G70475
  
Location: 10339-11821

Mycgr3G70475

Mycgr3G108866
  
Location: 11921-13010

Mycgr3G108866

Mycgr3G92136
  
Location: 13110-13593

Mycgr3G92136

hypothetical protein
  
Accession: EER25763
  
Location: 340048-342209
  
 NCBI BlastP on this gene

EER25763

vacuolar ATP synthase subunit G, putative
  
Accession: EER25764
  
Location: 343564-344272
  
 NCBI BlastP on this gene

EER25764

hypothetical protein
  
Accession: EER25765
  
Location: 344579-344921
  
 NCBI BlastP on this gene

EER25765

hypothetical protein
  
Accession: EER25766
  
Location: 346045-348359
  
 NCBI BlastP on this gene

EER25766

hypothetical protein
  
Accession: EER25767
  
Location: 349925-351113
  
  
**BlastP hit with Mycgr3G38483**
  
Percentage identity: 35 %
  
BlastP bit score: 181
  
Sequence coverage: 91 %
  
E-value: 7e-50
  
  
 NCBI BlastP on this gene

EER25767

40S ribosomal protein S24, putative
  
Accession: EER25768
  
Location: 351443-352096
  
  
**BlastP hit with Mycgr3G70471**
  
Percentage identity: 77 %
  
BlastP bit score: 203
  
Sequence coverage: 91 %
  
E-value: 3e-64
  
  
 NCBI BlastP on this gene

EER25768

hypothetical protein
  
Accession: EER25769
  
Location: 357381-358920
  
 NCBI BlastP on this gene

EER25769

UDP-N-acetylglucosamine pyrophosphorylase, putative
  
Accession: EER25770
  
Location: 359500-361160
  
 NCBI BlastP on this gene

EER25770

Query: Architecture Search FASTA input

ABSU01000001 : Arthroderma benhamiae CBS 112371    Total score: 2.0     Cumulative Blast bit score: 384

Hit cluster cross-links:

Mycgr3G70471
  
Location: 0-405

Mycgr3G70471

Mycgr3G39149
  
Location: 505-1798

Mycgr3G39149

Mycgr3G92130
  
Location: 1898-2396

Mycgr3G92130

Mycgr3G38483
  
Location: 2496-3576

Mycgr3G38483

Mycgr3G108869
  
Location: 3676-5056

Mycgr3G108869

Mycgr3G103943
  
Location: 5156-5762

Mycgr3G103943

Mycgr3G57362
  
Location: 5862-7296

Mycgr3G57362

Mycgr3G39086
  
Location: 7396-8368

Mycgr3G39086

Mycgr3G103942
  
Location: 8468-8714

Mycgr3G103942

Mycgr3G108865
  
Location: 8814-10239

Mycgr3G108865

Mycgr3G70475
  
Location: 10339-11821

Mycgr3G70475

Mycgr3G108866
  
Location: 11921-13010

Mycgr3G108866

Mycgr3G92136
  
Location: 13110-13593

Mycgr3G92136

hypothetical protein
  
Accession: EFE37142
  
Location: 2010914-2012599
  
 NCBI BlastP on this gene

EFE37142

hypothetical protein
  
Accession: EFE37143
  
Location: 2013341-2015033
  
 NCBI BlastP on this gene

EFE37143

hemagglutinin protein, putative
  
Accession: EFE37144
  
Location: 2018214-2019371
  
 NCBI BlastP on this gene

EFE37144

hypothetical protein
  
Accession: EFE37145
  
Location: 2020282-2021017
  
  
**BlastP hit with Mycgr3G70471**
  
Percentage identity: 64 %
  
BlastP bit score: 185
  
Sequence coverage: 103 %
  
E-value: 4e-57
  
  
 NCBI BlastP on this gene

EFE37145

hypothetical protein
  
Accession: EFE37146
  
Location: 2021389-2022586
  
  
**BlastP hit with Mycgr3G38483**
  
Percentage identity: 39 %
  
BlastP bit score: 199
  
Sequence coverage: 83 %
  
E-value: 2e-57
  
  
 NCBI BlastP on this gene

EFE37146

toxin biosynthesis ketoreductase, putative
  
Accession: EFE37147
  
Location: 2023773-2024549
  
 NCBI BlastP on this gene

EFE37147

hypothetical protein
  
Accession: EFE37148
  
Location: 2025755-2026210
  
 NCBI BlastP on this gene

EFE37148

hypothetical protein
  
Accession: EFE37149
  
Location: 2026261-2027283
  
 NCBI BlastP on this gene

EFE37149

hypothetical protein
  
Accession: EFE37150
  
Location: 2027949-2028944
  
 NCBI BlastP on this gene

EFE37150

hypothetical protein
  
Accession: EFE37151
  
Location: 2030116-2031050
  
 NCBI BlastP on this gene

EFE37151

Query: Architecture Search FASTA input

EQ962660 : Talaromyces stipitatus ATCC 10500 scf\_1105507295517 genomic scaffold    Total score: 2.0     Cumulative Blast bit score: 379

Hit cluster cross-links:

Mycgr3G70471
  
Location: 0-405

Mycgr3G70471

Mycgr3G39149
  
Location: 505-1798

Mycgr3G39149

Mycgr3G92130
  
Location: 1898-2396

Mycgr3G92130

Mycgr3G38483
  
Location: 2496-3576

Mycgr3G38483

Mycgr3G108869
  
Location: 3676-5056

Mycgr3G108869

Mycgr3G103943
  
Location: 5156-5762

Mycgr3G103943

Mycgr3G57362
  
Location: 5862-7296

Mycgr3G57362

Mycgr3G39086
  
Location: 7396-8368

Mycgr3G39086

Mycgr3G103942
  
Location: 8468-8714

Mycgr3G103942

Mycgr3G108865
  
Location: 8814-10239

Mycgr3G108865

Mycgr3G70475
  
Location: 10339-11821

Mycgr3G70475

Mycgr3G108866
  
Location: 11921-13010

Mycgr3G108866

Mycgr3G92136
  
Location: 13110-13593

Mycgr3G92136

conserved hypothetical protein
  
Accession: EED12635
  
Location: 1705478-1706706
  
 NCBI BlastP on this gene

EED12635

2-amino-3-carboxymuconate-6-semialdehyde decarboxylase, putative
  
Accession: EED12636
  
Location: 1707367-1708323
  
 NCBI BlastP on this gene

EED12636

conserved hypothetical protein
  
Accession: EED12637
  
Location: 1708804-1711767
  
 NCBI BlastP on this gene

EED12637

37S ribosomal protein S24
  
Accession: EED12638
  
Location: 1712844-1713747
  
  
**BlastP hit with Mycgr3G70471**
  
Percentage identity: 82 %
  
BlastP bit score: 224
  
Sequence coverage: 97 %
  
E-value: 9e-73
  
  
 NCBI BlastP on this gene

EED12638

conserved hypothetical protein
  
Accession: EED12639
  
Location: 1714107-1715468
  
  
**BlastP hit with Mycgr3G38483**
  
Percentage identity: 35 %
  
BlastP bit score: 155
  
Sequence coverage: 85 %
  
E-value: 5e-40
  
  
 NCBI BlastP on this gene

EED12639

RNA polymerase I subunit Rpa43, putative
  
Accession: EED12640
  
Location: 1716664-1717983
  
 NCBI BlastP on this gene

EED12640

membrane associated DnaJ chaperone, putative
  
Accession: EED12641
  
Location: 1718274-1719473
  
 NCBI BlastP on this gene

EED12641

conserved hypothetical protein
  
Accession: EED12642
  
Location: 1720622-1721470
  
 NCBI BlastP on this gene

EED12642

alcohol dehydrogenase, putative
  
Accession: EED12644
  
Location: 1722133-1722972
  
 NCBI BlastP on this gene

EED12644

Query: Architecture Search FASTA input

GL385404 : Gaeumannomyces graminis var. tritici R3-111a-1 unplaced genomic scaffold supercont2.10    Total score: 2.0     Cumulative Blast bit score: 377

Hit cluster cross-links:

Mycgr3G70471
  
Location: 0-405

Mycgr3G70471

Mycgr3G39149
  
Location: 505-1798

Mycgr3G39149

Mycgr3G92130
  
Location: 1898-2396

Mycgr3G92130

Mycgr3G38483
  
Location: 2496-3576

Mycgr3G38483

Mycgr3G108869
  
Location: 3676-5056

Mycgr3G108869

Mycgr3G103943
  
Location: 5156-5762

Mycgr3G103943

Mycgr3G57362
  
Location: 5862-7296

Mycgr3G57362

Mycgr3G39086
  
Location: 7396-8368

Mycgr3G39086

Mycgr3G103942
  
Location: 8468-8714

Mycgr3G103942

Mycgr3G108865
  
Location: 8814-10239

Mycgr3G108865

Mycgr3G70475
  
Location: 10339-11821

Mycgr3G70475

Mycgr3G108866
  
Location: 11921-13010

Mycgr3G108866

Mycgr3G92136
  
Location: 13110-13593

Mycgr3G92136

hypothetical protein
  
Accession: EJT69585
  
Location: 987635-989037
  
  
**BlastP hit with Mycgr3G38483**
  
Percentage identity: 36 %
  
BlastP bit score: 172
  
Sequence coverage: 101 %
  
E-value: 1e-46
  
  
 NCBI BlastP on this gene

EJT69585

40S ribosomal protein S24
  
Accession: EJT69586
  
Location: 989505-990379
  
  
**BlastP hit with Mycgr3G70471**
  
Percentage identity: 79 %
  
BlastP bit score: 205
  
Sequence coverage: 89 %
  
E-value: 5e-65
  
  
 NCBI BlastP on this gene

EJT69586

hypothetical protein
  
Accession: EJT69587
  
Location: 991092-991635
  
 NCBI BlastP on this gene

EJT69587

hypothetical protein
  
Accession: EJT69588
  
Location: 993106-994761
  
 NCBI BlastP on this gene

EJT69588

mannose-6-phosphate isomerase
  
Accession: EJT69589
  
Location: 995862-997302
  
 NCBI BlastP on this gene

EJT69589

Query: Architecture Search FASTA input

GG692419 : Ajellomyces capsulatus H143 genomic scaffold supercont2.1    Total score: 2.0     Cumulative Blast bit score: 377

Hit cluster cross-links:

Mycgr3G70471
  
Location: 0-405

Mycgr3G70471

Mycgr3G39149
  
Location: 505-1798

Mycgr3G39149

Mycgr3G92130
  
Location: 1898-2396

Mycgr3G92130

Mycgr3G38483
  
Location: 2496-3576

Mycgr3G38483

Mycgr3G108869
  
Location: 3676-5056

Mycgr3G108869

Mycgr3G103943
  
Location: 5156-5762

Mycgr3G103943

Mycgr3G57362
  
Location: 5862-7296

Mycgr3G57362

Mycgr3G39086
  
Location: 7396-8368

Mycgr3G39086

Mycgr3G103942
  
Location: 8468-8714

Mycgr3G103942

Mycgr3G108865
  
Location: 8814-10239

Mycgr3G108865

Mycgr3G70475
  
Location: 10339-11821

Mycgr3G70475

Mycgr3G108866
  
Location: 11921-13010

Mycgr3G108866

Mycgr3G92136
  
Location: 13110-13593

Mycgr3G92136

conserved hypothetical protein
  
Accession: EER44478
  
Location: 300988-301872
  
 NCBI BlastP on this gene

EER44478

cytochrome c oxidase assembly protein
  
Accession: EER44479
  
Location: 302174-302534
  
 NCBI BlastP on this gene

EER44479

conserved hypothetical protein
  
Accession: EER44480
  
Location: 303790-306309
  
 NCBI BlastP on this gene

EER44480

conserved hypothetical protein
  
Accession: EER44481
  
Location: 309189-310157
  
  
**BlastP hit with Mycgr3G38483**
  
Percentage identity: 36 %
  
BlastP bit score: 175
  
Sequence coverage: 76 %
  
E-value: 3e-48
  
  
 NCBI BlastP on this gene

EER44481

40S ribosomal protein S24
  
Accession: EER44482
  
Location: 310453-311106
  
  
**BlastP hit with Mycgr3G70471**
  
Percentage identity: 78 %
  
BlastP bit score: 202
  
Sequence coverage: 90 %
  
E-value: 4e-64
  
  
 NCBI BlastP on this gene

EER44482

predicted protein
  
Accession: EER44483
  
Location: 312049-313628
  
 NCBI BlastP on this gene

EER44483

UDP-N-acetylglucosamine pyrophosphorylase
  
Accession: EER44484
  
Location: 316548-317518
  
 NCBI BlastP on this gene

EER44484

Query: Architecture Search FASTA input

KE375214 : Blumeria graminis f. sp. tritici 96224 unplaced genomic scaffold Scaffold-88    Total score: 2.0     Cumulative Blast bit score: 356

Hit cluster cross-links:

Mycgr3G70471
  
Location: 0-405

Mycgr3G70471

Mycgr3G39149
  
Location: 505-1798

Mycgr3G39149

Mycgr3G92130
  
Location: 1898-2396

Mycgr3G92130

Mycgr3G38483
  
Location: 2496-3576

Mycgr3G38483

Mycgr3G108869
  
Location: 3676-5056

Mycgr3G108869

Mycgr3G103943
  
Location: 5156-5762

Mycgr3G103943

Mycgr3G57362
  
Location: 5862-7296

Mycgr3G57362

Mycgr3G39086
  
Location: 7396-8368

Mycgr3G39086

Mycgr3G103942
  
Location: 8468-8714

Mycgr3G103942

Mycgr3G108865
  
Location: 8814-10239

Mycgr3G108865

Mycgr3G70475
  
Location: 10339-11821

Mycgr3G70475

Mycgr3G108866
  
Location: 11921-13010

Mycgr3G108866

Mycgr3G92136
  
Location: 13110-13593

Mycgr3G92136

hypothetical protein
  
Accession: EPQ61759
  
Location: 367697-369130
  
  
**BlastP hit with Mycgr3G38483**
  
Percentage identity: 34 %
  
BlastP bit score: 160
  
Sequence coverage: 89 %
  
E-value: 7e-42
  
  
 NCBI BlastP on this gene

EPQ61759

Protein component of the small (40S) ribosomal subunit
  
Accession: EPQ61760
  
Location: 369529-370161
  
  
**BlastP hit with Mycgr3G70471**
  
Percentage identity: 77 %
  
BlastP bit score: 196
  
Sequence coverage: 88 %
  
E-value: 2e-61
  
  
 NCBI BlastP on this gene

EPQ61760

Query: Architecture Search FASTA input

CAUH01002404 : Blumeria graminis f. sp. hordei DH14    Total score: 2.0     Cumulative Blast bit score: 355

Hit cluster cross-links:

Mycgr3G70471
  
Location: 0-405

Mycgr3G70471

Mycgr3G39149
  
Location: 505-1798

Mycgr3G39149

Mycgr3G92130
  
Location: 1898-2396

Mycgr3G92130

Mycgr3G38483
  
Location: 2496-3576

Mycgr3G38483

Mycgr3G108869
  
Location: 3676-5056

Mycgr3G108869

Mycgr3G103943
  
Location: 5156-5762

Mycgr3G103943

Mycgr3G57362
  
Location: 5862-7296

Mycgr3G57362

Mycgr3G39086
  
Location: 7396-8368

Mycgr3G39086

Mycgr3G103942
  
Location: 8468-8714

Mycgr3G103942

Mycgr3G108865
  
Location: 8814-10239

Mycgr3G108865

Mycgr3G70475
  
Location: 10339-11821

Mycgr3G70475

Mycgr3G108866
  
Location: 11921-13010

Mycgr3G108866

Mycgr3G92136
  
Location: 13110-13593

Mycgr3G92136

Putative methyltransferase
  
Accession: CCU76365
  
Location: 55927-57360
  
  
**BlastP hit with Mycgr3G38483**
  
Percentage identity: 34 %
  
BlastP bit score: 159
  
Sequence coverage: 91 %
  
E-value: 2e-41
  
  
 NCBI BlastP on this gene

CCU76365

40S ribosomal protein S24
  
Accession: CCU76366
  
Location: 57758-58392
  
  
**BlastP hit with Mycgr3G70471**
  
Percentage identity: 77 %
  
BlastP bit score: 196
  
Sequence coverage: 88 %
  
E-value: 2e-61
  
  
 NCBI BlastP on this gene

CCU76366

CSEP0353 putative effector protein
  
Accession: CCU76367
  
Location: 62665-63168
  
 NCBI BlastP on this gene

CCU76367

ATP-dependent helicase NAM7
  
Accession: CCU76368
  
Location: 64429-67905
  
 NCBI BlastP on this gene

CCU76368

Query: Architecture Search FASTA input

AACD01000169 : Aspergillus nidulans FGSC A4    Total score: 2.0     Cumulative Blast bit score: 353

Hit cluster cross-links:

Mycgr3G70471
  
Location: 0-405

Mycgr3G70471

Mycgr3G39149
  
Location: 505-1798

Mycgr3G39149

Mycgr3G92130
  
Location: 1898-2396

Mycgr3G92130

Mycgr3G38483
  
Location: 2496-3576

Mycgr3G38483

Mycgr3G108869
  
Location: 3676-5056

Mycgr3G108869

Mycgr3G103943
  
Location: 5156-5762

Mycgr3G103943

Mycgr3G57362
  
Location: 5862-7296

Mycgr3G57362

Mycgr3G39086
  
Location: 7396-8368

Mycgr3G39086

Mycgr3G103942
  
Location: 8468-8714

Mycgr3G103942

Mycgr3G108865
  
Location: 8814-10239

Mycgr3G108865

Mycgr3G70475
  
Location: 10339-11821

Mycgr3G70475

Mycgr3G108866
  
Location: 11921-13010

Mycgr3G108866

Mycgr3G92136
  
Location: 13110-13593

Mycgr3G92136

hypothetical protein
  
Accession: EAA61928
  
Location: 122158-123763
  
 NCBI BlastP on this gene

EAA61928

predicted protein
  
Accession: EAA61929
  
Location: 127023-128628
  
 NCBI BlastP on this gene

EAA61929

hypothetical protein
  
Accession: EAA61930
  
Location: 131040-131675
  
  
**BlastP hit with Mycgr3G70471**
  
Percentage identity: 78 %
  
BlastP bit score: 216
  
Sequence coverage: 97 %
  
E-value: 3e-69
  
  
 NCBI BlastP on this gene

EAA61930

hypothetical protein
  
Accession: EAA61931
  
Location: 132338-133610
  
  
**BlastP hit with Mycgr3G38483**
  
Percentage identity: 35 %
  
BlastP bit score: 137
  
Sequence coverage: 71 %
  
E-value: 2e-34
  
  
 NCBI BlastP on this gene

EAA61931

hypothetical protein
  
Accession: EAA61932
  
Location: 133840-139572
  
 NCBI BlastP on this gene

EAA61932

hypothetical protein
  
Accession: EAA61933
  
Location: 141098-141980
  
 NCBI BlastP on this gene

EAA61933

Query: Architecture Search FASTA input

CH476658 : Ajellomyces capsulatus NAm1 scaffold\_4 genomic scaffold    Total score: 2.0     Cumulative Blast bit score: 349

Hit cluster cross-links:

Mycgr3G70471
  
Location: 0-405

Mycgr3G70471

Mycgr3G39149
  
Location: 505-1798

Mycgr3G39149

Mycgr3G92130
  
Location: 1898-2396

Mycgr3G92130

Mycgr3G38483
  
Location: 2496-3576

Mycgr3G38483

Mycgr3G108869
  
Location: 3676-5056

Mycgr3G108869

Mycgr3G103943
  
Location: 5156-5762

Mycgr3G103943

Mycgr3G57362
  
Location: 5862-7296

Mycgr3G57362

Mycgr3G39086
  
Location: 7396-8368

Mycgr3G39086

Mycgr3G103942
  
Location: 8468-8714

Mycgr3G103942

Mycgr3G108865
  
Location: 8814-10239

Mycgr3G108865

Mycgr3G70475
  
Location: 10339-11821

Mycgr3G70475

Mycgr3G108866
  
Location: 11921-13010

Mycgr3G108866

Mycgr3G92136
  
Location: 13110-13593

Mycgr3G92136

UDP-N-acetylglucosamine pyrophosphorylase
  
Accession: EDN07906
  
Location: 1356245-1358009
  
 NCBI BlastP on this gene

EDN07906

hypothetical protein
  
Accession: EDN07907
  
Location: 1358914-1360154
  
 NCBI BlastP on this gene

EDN07907

40S ribosomal protein S24
  
Accession: EDN07908
  
Location: 1365457-1366103
  
  
**BlastP hit with Mycgr3G70471**
  
Percentage identity: 78 %
  
BlastP bit score: 202
  
Sequence coverage: 90 %
  
E-value: 4e-64
  
  
 NCBI BlastP on this gene

EDN07908

conserved hypothetical protein
  
Accession: EDN07909
  
Location: 1366521-1367351
  
  
**BlastP hit with Mycgr3G38483**
  
Percentage identity: 38 %
  
BlastP bit score: 147
  
Sequence coverage: 65 %
  
E-value: 3e-38
  
  
 NCBI BlastP on this gene

EDN07909

predicted protein
  
Accession: EDN07910
  
Location: 1370274-1372564
  
 NCBI BlastP on this gene

EDN07910

conserved hypothetical protein
  
Accession: EDN07911
  
Location: 1373995-1374355
  
 NCBI BlastP on this gene

EDN07911

predicted protein
  
Accession: EDN07912
  
Location: 1374669-1375553
  
 NCBI BlastP on this gene

EDN07912

Query: Architecture Search FASTA input

DS995769 : Trichophyton equinum CBS 127.97 supercont1.52 genomic scaffold    Total score: 2.0     Cumulative Blast bit score: 346

Hit cluster cross-links:

Mycgr3G70471
  
Location: 0-405

Mycgr3G70471

Mycgr3G39149
  
Location: 505-1798

Mycgr3G39149

Mycgr3G92130
  
Location: 1898-2396

Mycgr3G92130

Mycgr3G38483
  
Location: 2496-3576

Mycgr3G38483

Mycgr3G108869
  
Location: 3676-5056

Mycgr3G108869

Mycgr3G103943
  
Location: 5156-5762

Mycgr3G103943

Mycgr3G57362
  
Location: 5862-7296

Mycgr3G57362

Mycgr3G39086
  
Location: 7396-8368

Mycgr3G39086

Mycgr3G103942
  
Location: 8468-8714

Mycgr3G103942

Mycgr3G108865
  
Location: 8814-10239

Mycgr3G108865

Mycgr3G70475
  
Location: 10339-11821

Mycgr3G70475

Mycgr3G108866
  
Location: 11921-13010

Mycgr3G108866

Mycgr3G92136
  
Location: 13110-13593

Mycgr3G92136

UDP-N-acetylglucosamine pyrophosphorylase
  
Accession: EGE07945
  
Location: 8232-9919
  
 NCBI BlastP on this gene

EGE07945

peptidyl-prolyl cis-trans isomerase-like 4
  
Accession: EGE07946
  
Location: 10709-12248
  
 NCBI BlastP on this gene

EGE07946

hypothetical protein
  
Accession: EGE07947
  
Location: 15283-16850
  
 NCBI BlastP on this gene

EGE07947

40S ribosomal protein S24
  
Accession: EGE07948
  
Location: 17756-18506
  
  
**BlastP hit with Mycgr3G70471**
  
Percentage identity: 72 %
  
BlastP bit score: 194
  
Sequence coverage: 90 %
  
E-value: 1e-60
  
  
 NCBI BlastP on this gene

EGE07948

hypothetical protein
  
Accession: EGE07949
  
Location: 18987-20278
  
  
**BlastP hit with Mycgr3G38483**
  
Percentage identity: 31 %
  
BlastP bit score: 152
  
Sequence coverage: 88 %
  
E-value: 9e-40
  
  
 NCBI BlastP on this gene

EGE07949

toxin biosynthesis ketoreductase
  
Accession: EGE07950
  
Location: 21381-22157
  
 NCBI BlastP on this gene

EGE07950

tripeptidyl peptidase SED3
  
Accession: EGE07951
  
Location: 23310-25176
  
 NCBI BlastP on this gene

EGE07951

hypothetical protein
  
Accession: EGE07952
  
Location: 25499-26494
  
 NCBI BlastP on this gene

EGE07952

proteasome regulatory particle subunit
  
Accession: EGE07953
  
Location: 27663-28595
  
 NCBI BlastP on this gene

EGE07953

Query: Architecture Search FASTA input

CH476595 : Aspergillus terreus NIH2624 scaffold\_2 genomic scaffold    Total score: 2.0     Cumulative Blast bit score: 323

Hit cluster cross-links:

Mycgr3G70471
  
Location: 0-405

Mycgr3G70471

Mycgr3G39149
  
Location: 505-1798

Mycgr3G39149

Mycgr3G92130
  
Location: 1898-2396

Mycgr3G92130

Mycgr3G38483
  
Location: 2496-3576

Mycgr3G38483

Mycgr3G108869
  
Location: 3676-5056

Mycgr3G108869

Mycgr3G103943
  
Location: 5156-5762

Mycgr3G103943

Mycgr3G57362
  
Location: 5862-7296

Mycgr3G57362

Mycgr3G39086
  
Location: 7396-8368

Mycgr3G39086

Mycgr3G103942
  
Location: 8468-8714

Mycgr3G103942

Mycgr3G108865
  
Location: 8814-10239

Mycgr3G108865

Mycgr3G70475
  
Location: 10339-11821

Mycgr3G70475

Mycgr3G108866
  
Location: 11921-13010

Mycgr3G108866

Mycgr3G92136
  
Location: 13110-13593

Mycgr3G92136

predicted protein
  
Accession: EAU38601
  
Location: 2411969-2412850
  
 NCBI BlastP on this gene

EAU38601

conserved hypothetical protein
  
Accession: EAU38602
  
Location: 2414235-2416580
  
 NCBI BlastP on this gene

EAU38602

conserved hypothetical protein
  
Accession: EAU38603
  
Location: 2417729-2419873
  
 NCBI BlastP on this gene

EAU38603

conserved hypothetical protein
  
Accession: EAU38604
  
Location: 2420447-2421161
  
  
**BlastP hit with Mycgr3G38483**
  
Percentage identity: 35 %
  
BlastP bit score: 102
  
Sequence coverage: 53 %
  
E-value: 4e-22
  
  
 NCBI BlastP on this gene

EAU38604

40S ribosomal protein S24
  
Accession: EAU38605
  
Location: 2421678-2422328
  
  
**BlastP hit with Mycgr3G70471**
  
Percentage identity: 81 %
  
BlastP bit score: 221
  
Sequence coverage: 97 %
  
E-value: 1e-71
  
  
 NCBI BlastP on this gene

EAU38605

predicted protein
  
Accession: EAU38606
  
Location: 2424127-2425683
  
 NCBI BlastP on this gene

EAU38606

hypothetical protein
  
Accession: EAU38607
  
Location: 2428237-2430179
  
 NCBI BlastP on this gene

EAU38607

Query: Architecture Search FASTA input

AQGS01000244 : Dactylellina haptotyla CBS 200.50    Total score: 2.0     Cumulative Blast bit score: 316

Hit cluster cross-links:

Mycgr3G70471
  
Location: 0-405

Mycgr3G70471

Mycgr3G39149
  
Location: 505-1798

Mycgr3G39149

Mycgr3G92130
  
Location: 1898-2396

Mycgr3G92130

Mycgr3G38483
  
Location: 2496-3576

Mycgr3G38483

Mycgr3G108869
  
Location: 3676-5056

Mycgr3G108869

Mycgr3G103943
  
Location: 5156-5762

Mycgr3G103943

Mycgr3G57362
  
Location: 5862-7296

Mycgr3G57362

Mycgr3G39086
  
Location: 7396-8368

Mycgr3G39086

Mycgr3G103942
  
Location: 8468-8714

Mycgr3G103942

Mycgr3G108865
  
Location: 8814-10239

Mycgr3G108865

Mycgr3G70475
  
Location: 10339-11821

Mycgr3G70475

Mycgr3G108866
  
Location: 11921-13010

Mycgr3G108866

Mycgr3G92136
  
Location: 13110-13593

Mycgr3G92136

hypothetical protein
  
Accession: EPS41414
  
Location: 54282-55410
  
 NCBI BlastP on this gene

EPS41414

hypothetical protein
  
Accession: EPS41400
  
Location: 56884-57393
  
 NCBI BlastP on this gene

EPS41400

hypothetical protein
  
Accession: EPS41410
  
Location: 58075-60776
  
 NCBI BlastP on this gene

EPS41410

hypothetical protein
  
Accession: EPS41399
  
Location: 61436-62713
  
 NCBI BlastP on this gene

EPS41399

hypothetical protein
  
Accession: EPS41402
  
Location: 63187-64195
  
  
**BlastP hit with Mycgr3G70471**
  
Percentage identity: 77 %
  
BlastP bit score: 198
  
Sequence coverage: 90 %
  
E-value: 2e-62
  
  
 NCBI BlastP on this gene

EPS41402

hypothetical protein
  
Accession: EPS41406
  
Location: 64704-65832
  
  
**BlastP hit with Mycgr3G38483**
  
Percentage identity: 29 %
  
BlastP bit score: 118
  
Sequence coverage: 101 %
  
E-value: 5e-27
  
  
 NCBI BlastP on this gene

EPS41406

Query: Architecture Search FASTA input

ADOT01000287 : Arthrobotrys oligospora ATCC 24927    Total score: 2.0     Cumulative Blast bit score: 311

Hit cluster cross-links:

Mycgr3G70471
  
Location: 0-405

Mycgr3G70471

Mycgr3G39149
  
Location: 505-1798

Mycgr3G39149

Mycgr3G92130
  
Location: 1898-2396

Mycgr3G92130

Mycgr3G38483
  
Location: 2496-3576

Mycgr3G38483

Mycgr3G108869
  
Location: 3676-5056

Mycgr3G108869

Mycgr3G103943
  
Location: 5156-5762

Mycgr3G103943

Mycgr3G57362
  
Location: 5862-7296

Mycgr3G57362

Mycgr3G39086
  
Location: 7396-8368

Mycgr3G39086

Mycgr3G103942
  
Location: 8468-8714

Mycgr3G103942

Mycgr3G108865
  
Location: 8814-10239

Mycgr3G108865

Mycgr3G70475
  
Location: 10339-11821

Mycgr3G70475

Mycgr3G108866
  
Location: 11921-13010

Mycgr3G108866

Mycgr3G92136
  
Location: 13110-13593

Mycgr3G92136

hypothetical protein
  
Accession: EGX44326
  
Location: 170584-172544
  
 NCBI BlastP on this gene

EGX44326

hypothetical protein
  
Accession: EGX44327
  
Location: 173010-173998
  
 NCBI BlastP on this gene

EGX44327

hypothetical protein
  
Accession: EGX44328
  
Location: 174339-174581
  
 NCBI BlastP on this gene

EGX44328

hypothetical protein
  
Accession: EGX44329
  
Location: 175159-179622
  
 NCBI BlastP on this gene

EGX44329

hypothetical protein
  
Accession: EGX44330
  
Location: 180049-181179
  
  
**BlastP hit with Mycgr3G38483**
  
Percentage identity: 33 %
  
BlastP bit score: 119
  
Sequence coverage: 77 %
  
E-value: 3e-27
  
  
 NCBI BlastP on this gene

EGX44330

hypothetical protein
  
Accession: EGX44331
  
Location: 181677-182520
  
  
**BlastP hit with Mycgr3G70471**
  
Percentage identity: 76 %
  
BlastP bit score: 192
  
Sequence coverage: 90 %
  
E-value: 7e-60
  
  
 NCBI BlastP on this gene

EGX44331

hypothetical protein
  
Accession: EGX44332
  
Location: 182962-184230
  
 NCBI BlastP on this gene

EGX44332

hypothetical protein
  
Accession: EGX44333
  
Location: 184862-187481
  
 NCBI BlastP on this gene

EGX44333

hypothetical protein
  
Accession: EGX44334
  
Location: 187909-188526
  
 NCBI BlastP on this gene

EGX44334

Query: Architecture Search FASTA input

GG704911 : Coccidioides immitis RS genomic scaffold supercont3.1    Total score: 1.0     Cumulative Blast bit score: 703

Hit cluster cross-links:

Mycgr3G70471
  
Location: 0-405

Mycgr3G70471

Mycgr3G39149
  
Location: 505-1798

Mycgr3G39149

Mycgr3G92130
  
Location: 1898-2396

Mycgr3G92130

Mycgr3G38483
  
Location: 2496-3576

Mycgr3G38483

Mycgr3G108869
  
Location: 3676-5056

Mycgr3G108869

Mycgr3G103943
  
Location: 5156-5762

Mycgr3G103943

Mycgr3G57362
  
Location: 5862-7296

Mycgr3G57362

Mycgr3G39086
  
Location: 7396-8368

Mycgr3G39086

Mycgr3G103942
  
Location: 8468-8714

Mycgr3G103942

Mycgr3G108865
  
Location: 8814-10239

Mycgr3G108865

Mycgr3G70475
  
Location: 10339-11821

Mycgr3G70475

Mycgr3G108866
  
Location: 11921-13010

Mycgr3G108866

Mycgr3G92136
  
Location: 13110-13593

Mycgr3G92136

guanyl-nucleotide exchange factor
  
Accession: EAS35511
  
Location: 6364855-6369178
  
 NCBI BlastP on this gene

EAS35511

zinc carboxypeptidase
  
Accession: EAS35510
  
Location: 6370655-6372470
  
 NCBI BlastP on this gene

EAS35510

hypothetical protein
  
Accession: EAS35509
  
Location: 6372995-6373393
  
 NCBI BlastP on this gene

EAS35509

Ni2+-Co2+ transporter (NiCoT) family transition metal uptake transporter, variant
  
Accession: EJB10688
  
Location: 6374389-6376431
  
  
**BlastP hit with Mycgr3G108865**
  
Percentage identity: 55 %
  
BlastP bit score: 399
  
Sequence coverage: 86 %
  
E-value: 1e-131
  
  
 NCBI BlastP on this gene

EJB10688

Ni2+-Co2+ transporter (NiCoT) family transition metal uptake transporter
  
Accession: EJB10689
  
Location: 6375203-6376431
  
  
**BlastP hit with Mycgr3G108865**
  
Percentage identity: 54 %
  
BlastP bit score: 304
  
Sequence coverage: 69 %
  
E-value: 6e-96
  
  
 NCBI BlastP on this gene

EJB10689

hypothetical protein
  
Accession: EAS35506
  
Location: 6377009-6379591
  
 NCBI BlastP on this gene

EAS35506

hypothetical protein
  
Accession: EJB10690
  
Location: 6381865-6382892
  
 NCBI BlastP on this gene

EJB10690

Query: Architecture Search FASTA input

KB446538 : Dothistroma septosporum NZE10 unplaced genomic scaffold DOTSEscaffold\_4    Total score: 1.0     Cumulative Blast bit score: 677

Hit cluster cross-links:

Mycgr3G70471
  
Location: 0-405

Mycgr3G70471

Mycgr3G39149
  
Location: 505-1798

Mycgr3G39149

Mycgr3G92130
  
Location: 1898-2396

Mycgr3G92130

Mycgr3G38483
  
Location: 2496-3576

Mycgr3G38483

Mycgr3G108869
  
Location: 3676-5056

Mycgr3G108869

Mycgr3G103943
  
Location: 5156-5762

Mycgr3G103943

Mycgr3G57362
  
Location: 5862-7296

Mycgr3G57362

Mycgr3G39086
  
Location: 7396-8368

Mycgr3G39086

Mycgr3G103942
  
Location: 8468-8714

Mycgr3G103942

Mycgr3G108865
  
Location: 8814-10239

Mycgr3G108865

Mycgr3G70475
  
Location: 10339-11821

Mycgr3G70475

Mycgr3G108866
  
Location: 11921-13010

Mycgr3G108866

Mycgr3G92136
  
Location: 13110-13593

Mycgr3G92136

hypothetical protein
  
Accession: EME45622
  
Location: 1933381-1935369
  
 NCBI BlastP on this gene

EME45622

hypothetical protein
  
Accession: EME45621
  
Location: 1930154-1931875
  
 NCBI BlastP on this gene

EME45621

hypothetical protein
  
Accession: EME45620
  
Location: 1925925-1928112
  
  
**BlastP hit with Mycgr3G70475**
  
Percentage identity: 66 %
  
BlastP bit score: 677
  
Sequence coverage: 103 %
  
E-value: 0.0
  
  
 NCBI BlastP on this gene

EME45620

hypothetical protein
  
Accession: EME45618
  
Location: 1923210-1925046
  
 NCBI BlastP on this gene

EME45618

hypothetical protein
  
Accession: EME45617
  
Location: 1920929-1921210
  
 NCBI BlastP on this gene

EME45617

hypothetical protein
  
Accession: EME45616
  
Location: 1920102-1920635
  
 NCBI BlastP on this gene

EME45616

hypothetical protein
  
Accession: EME45615
  
Location: 1916856-1917959
  
 NCBI BlastP on this gene

EME45615

Query: Architecture Search FASTA input

AM270040 : Aspergillus niger contig An02c0460, genomic contig.    Total score: 1.0     Cumulative Blast bit score: 607

Hit cluster cross-links:

Mycgr3G70471
  
Location: 0-405

Mycgr3G70471

Mycgr3G39149
  
Location: 505-1798

Mycgr3G39149

Mycgr3G92130
  
Location: 1898-2396

Mycgr3G92130

Mycgr3G38483
  
Location: 2496-3576

Mycgr3G38483

Mycgr3G108869
  
Location: 3676-5056

Mycgr3G108869

Mycgr3G103943
  
Location: 5156-5762

Mycgr3G103943

Mycgr3G57362
  
Location: 5862-7296

Mycgr3G57362

Mycgr3G39086
  
Location: 7396-8368

Mycgr3G39086

Mycgr3G103942
  
Location: 8468-8714

Mycgr3G103942

Mycgr3G108865
  
Location: 8814-10239

Mycgr3G108865

Mycgr3G70475
  
Location: 10339-11821

Mycgr3G70475

Mycgr3G108866
  
Location: 11921-13010

Mycgr3G108866

Mycgr3G92136
  
Location: 13110-13593

Mycgr3G92136

not annotated
  
Accession: CAK48850
  
Location: 71963-74020
  
 NCBI BlastP on this gene

An02g14190

not annotated
  
Accession: CAK48851
  
Location: 76446-77460
  
 NCBI BlastP on this gene

An02g14200

unnamed
  
Accession: CAK48852
  
Location: 78504-79887
  
 NCBI BlastP on this gene

An02g14210

unnamed
  
Accession: CAK48853
  
Location: 80462-82249
  
  
**BlastP hit with Mycgr3G39149**
  
Percentage identity: 66 %
  
BlastP bit score: 607
  
Sequence coverage: 99 %
  
E-value: 0.0
  
  
 NCBI BlastP on this gene

An02g14220

Query: Architecture Search FASTA input

JH921428 : Marssonina brunnea f. sp. 'multigermtubi' MB\_m1 unplaced genomic scaffold M6\_S00001    Total score: 1.0     Cumulative Blast bit score: 605

Hit cluster cross-links:

Mycgr3G70471
  
Location: 0-405

Mycgr3G70471

Mycgr3G39149
  
Location: 505-1798

Mycgr3G39149

Mycgr3G92130
  
Location: 1898-2396

Mycgr3G92130

Mycgr3G38483
  
Location: 2496-3576

Mycgr3G38483

Mycgr3G108869
  
Location: 3676-5056

Mycgr3G108869

Mycgr3G103943
  
Location: 5156-5762

Mycgr3G103943

Mycgr3G57362
  
Location: 5862-7296

Mycgr3G57362

Mycgr3G39086
  
Location: 7396-8368

Mycgr3G39086

Mycgr3G103942
  
Location: 8468-8714

Mycgr3G103942

Mycgr3G108865
  
Location: 8814-10239

Mycgr3G108865

Mycgr3G70475
  
Location: 10339-11821

Mycgr3G70475

Mycgr3G108866
  
Location: 11921-13010

Mycgr3G108866

Mycgr3G92136
  
Location: 13110-13593

Mycgr3G92136

UPF0047 domain protein
  
Accession: EKD21284
  
Location: 2547541-2548170
  
 NCBI BlastP on this gene

EKD21284

hypothetical protein
  
Accession: EKD21285
  
Location: 2549660-2550654
  
 NCBI BlastP on this gene

EKD21285

OPT family small oligopeptide transporter
  
Accession: EKD21286
  
Location: 2552003-2555024
  
 NCBI BlastP on this gene

EKD21286

3-oxoacyl-[acyl-carrier-protein]-synthase
  
Accession: EKD21287
  
Location: 2556121-2557521
  
  
**BlastP hit with Mycgr3G39149**
  
Percentage identity: 67 %
  
BlastP bit score: 605
  
Sequence coverage: 100 %
  
E-value: 0.0
  
  
 NCBI BlastP on this gene

EKD21287

Query: Architecture Search FASTA input

DS027696 : Neosartorya fischeri NRRL 181 1099437636264 genomic scaffold    Total score: 1.0     Cumulative Blast bit score: 602

Hit cluster cross-links:

Mycgr3G70471
  
Location: 0-405

Mycgr3G70471

Mycgr3G39149
  
Location: 505-1798

Mycgr3G39149

Mycgr3G92130
  
Location: 1898-2396

Mycgr3G92130

Mycgr3G38483
  
Location: 2496-3576

Mycgr3G38483

Mycgr3G108869
  
Location: 3676-5056

Mycgr3G108869

Mycgr3G103943
  
Location: 5156-5762

Mycgr3G103943

Mycgr3G57362
  
Location: 5862-7296

Mycgr3G57362

Mycgr3G39086
  
Location: 7396-8368

Mycgr3G39086

Mycgr3G103942
  
Location: 8468-8714

Mycgr3G103942

Mycgr3G108865
  
Location: 8814-10239

Mycgr3G108865

Mycgr3G70475
  
Location: 10339-11821

Mycgr3G70475

Mycgr3G108866
  
Location: 11921-13010

Mycgr3G108866

Mycgr3G92136
  
Location: 13110-13593

Mycgr3G92136

siderochrome-iron transporter MirC
  
Accession: EAW18298
  
Location: 2930508-2932571
  
 NCBI BlastP on this gene

EAW18298

Rho GTPase ModA, putative
  
Accession: EAW18299
  
Location: 2935873-2936852
  
 NCBI BlastP on this gene

EAW18299

agmatinase, putative
  
Accession: EAW18300
  
Location: 2937930-2939294
  
 NCBI BlastP on this gene

EAW18300

beta-ketoacyl synthase (Cem1), putative
  
Accession: EAW18301
  
Location: 2939827-2941753
  
  
**BlastP hit with Mycgr3G39149**
  
Percentage identity: 65 %
  
BlastP bit score: 602
  
Sequence coverage: 99 %
  
E-value: 0.0
  
  
 NCBI BlastP on this gene

EAW18301

SAGA complex component (Sgf73), putative
  
Accession: EAW18302
  
Location: 2942146-2943567
  
 NCBI BlastP on this gene

EAW18302

mRNA capping enzyme alpha subunit, putative
  
Accession: EAW18303
  
Location: 2944261-2945508
  
 NCBI BlastP on this gene

EAW18303

oligosaccharyl transferase subunit (alpha), putative
  
Accession: EAW18304
  
Location: 2945918-2947621
  
 NCBI BlastP on this gene

EAW18304

IdgA domain protein
  
Accession: EAW18305
  
Location: 2948052-2950776
  
 NCBI BlastP on this gene

EAW18305

Query: Architecture Search FASTA input

AM920437 : Penicillium chrysogenum Wisconsin 54-1255 complete genome, contig Pc00c22.    Total score: 1.0     Cumulative Blast bit score: 593

Hit cluster cross-links:

Mycgr3G70471
  
Location: 0-405

Mycgr3G70471

Mycgr3G39149
  
Location: 505-1798

Mycgr3G39149

Mycgr3G92130
  
Location: 1898-2396

Mycgr3G92130

Mycgr3G38483
  
Location: 2496-3576

Mycgr3G38483

Mycgr3G108869
  
Location: 3676-5056

Mycgr3G108869

Mycgr3G103943
  
Location: 5156-5762

Mycgr3G103943

Mycgr3G57362
  
Location: 5862-7296

Mycgr3G57362

Mycgr3G39086
  
Location: 7396-8368

Mycgr3G39086

Mycgr3G103942
  
Location: 8468-8714

Mycgr3G103942

Mycgr3G108865
  
Location: 8814-10239

Mycgr3G108865

Mycgr3G70475
  
Location: 10339-11821

Mycgr3G70475

Mycgr3G108866
  
Location: 11921-13010

Mycgr3G108866

Mycgr3G92136
  
Location: 13110-13593

Mycgr3G92136

not annotated
  
Accession: CAP98187
  
Location: 2131087-2133146
  
 NCBI BlastP on this gene

Pc22g08990

hypothetical protein
  
Accession: CAP98188
  
Location: 2133380-2135046
  
 NCBI BlastP on this gene

Pc22g09000

not annotated
  
Accession: CAP98189
  
Location: 2135333-2136160
  
 NCBI BlastP on this gene

Pc22g09010

unnamed
  
Accession: CAP98190
  
Location: 2137414-2138774
  
 NCBI BlastP on this gene

Pc22g09020

unnamed
  
Accession: CAP98191
  
Location: 2139003-2141024
  
  
**BlastP hit with Mycgr3G39149**
  
Percentage identity: 65 %
  
BlastP bit score: 593
  
Sequence coverage: 99 %
  
E-value: 0.0
  
  
 NCBI BlastP on this gene

Pc22g09030

not annotated
  
Accession: CAP98192
  
Location: 2141461-2142830
  
 NCBI BlastP on this gene

Pc22g09040

not annotated
  
Accession: CAP98193
  
Location: 2143543-2144787
  
 NCBI BlastP on this gene

Pc22g09050

not annotated
  
Accession: CAP98194
  
Location: 2145221-2145304
  
 NCBI BlastP on this gene

Pc22g09060

not annotated
  
Accession: CAP98195
  
Location: 2145490-2147267
  
 NCBI BlastP on this gene

Pc22g09070

hypothetical protein
  
Accession: CAP98196
  
Location: 2147341-2148854
  
 NCBI BlastP on this gene

Pc22g09080

not annotated
  
Accession: CAP98197
  
Location: 2149190-2153376
  
 NCBI BlastP on this gene

Pc22g09090

Query: Architecture Search FASTA input

DS572755 : Paracoccidioides brasiliensis Pb18 supercont1.6 genomic scaffold    Total score: 1.0     Cumulative Blast bit score: 588

Hit cluster cross-links:

Mycgr3G70471
  
Location: 0-405

Mycgr3G70471

Mycgr3G39149
  
Location: 505-1798

Mycgr3G39149

Mycgr3G92130
  
Location: 1898-2396

Mycgr3G92130

Mycgr3G38483
  
Location: 2496-3576

Mycgr3G38483

Mycgr3G108869
  
Location: 3676-5056

Mycgr3G108869

Mycgr3G103943
  
Location: 5156-5762

Mycgr3G103943

Mycgr3G57362
  
Location: 5862-7296

Mycgr3G57362

Mycgr3G39086
  
Location: 7396-8368

Mycgr3G39086

Mycgr3G103942
  
Location: 8468-8714

Mycgr3G103942

Mycgr3G108865
  
Location: 8814-10239

Mycgr3G108865

Mycgr3G70475
  
Location: 10339-11821

Mycgr3G70475

Mycgr3G108866
  
Location: 11921-13010

Mycgr3G108866

Mycgr3G92136
  
Location: 13110-13593

Mycgr3G92136

3-oxoacyl-[acyl-carrier-protein] synthase
  
Accession: EEH49288
  
Location: 944577-946417
  
  
**BlastP hit with Mycgr3G39149**
  
Percentage identity: 65 %
  
BlastP bit score: 588
  
Sequence coverage: 99 %
  
E-value: 0.0
  
  
 NCBI BlastP on this gene

EEH49288

conserved hypothetical protein
  
Accession: EEH49287
  
Location: 942193-944030
  
 NCBI BlastP on this gene

EEH49287

mRNA-capping enzyme subunit alpha
  
Accession: EEH49286
  
Location: 940212-941687
  
 NCBI BlastP on this gene

EEH49286

DNA ligase
  
Accession: EEH49285
  
Location: 936470-939659
  
 NCBI BlastP on this gene

EEH49285

Query: Architecture Search FASTA input

GG749429 : Ajellomyces dermatitidis ATCC 18188 genomic scaffold supercont1.23    Total score: 1.0     Cumulative Blast bit score: 582

Hit cluster cross-links:

Mycgr3G70471
  
Location: 0-405

Mycgr3G70471

Mycgr3G39149
  
Location: 505-1798

Mycgr3G39149

Mycgr3G92130
  
Location: 1898-2396

Mycgr3G92130

Mycgr3G38483
  
Location: 2496-3576

Mycgr3G38483

Mycgr3G108869
  
Location: 3676-5056

Mycgr3G108869

Mycgr3G103943
  
Location: 5156-5762

Mycgr3G103943

Mycgr3G57362
  
Location: 5862-7296

Mycgr3G57362

Mycgr3G39086
  
Location: 7396-8368

Mycgr3G39086

Mycgr3G103942
  
Location: 8468-8714

Mycgr3G103942

Mycgr3G108865
  
Location: 8814-10239

Mycgr3G108865

Mycgr3G70475
  
Location: 10339-11821

Mycgr3G70475

Mycgr3G108866
  
Location: 11921-13010

Mycgr3G108866

Mycgr3G92136
  
Location: 13110-13593

Mycgr3G92136

beta-ketoacyl synthase
  
Accession: EGE81978
  
Location: 616867-618774
  
  
**BlastP hit with Mycgr3G39149**
  
Percentage identity: 64 %
  
BlastP bit score: 582
  
Sequence coverage: 99 %
  
E-value: 0.0
  
  
 NCBI BlastP on this gene

EGE81978

SAGA complex component
  
Accession: EGE81977
  
Location: 614753-616525
  
 NCBI BlastP on this gene

EGE81977

mRNA capping enzyme alpha subunit
  
Accession: EGE81976
  
Location: 612725-614029
  
 NCBI BlastP on this gene

EGE81976

DNA ligase
  
Accession: EGE81975
  
Location: 608703-612199
  
 NCBI BlastP on this gene

EGE81975

Query: Architecture Search FASTA input

GG657469 : Ajellomyces dermatitidis SLH14081 genomic scaffold supercont1.22    Total score: 1.0     Cumulative Blast bit score: 582

Hit cluster cross-links:

Mycgr3G70471
  
Location: 0-405

Mycgr3G70471

Mycgr3G39149
  
Location: 505-1798

Mycgr3G39149

Mycgr3G92130
  
Location: 1898-2396

Mycgr3G92130

Mycgr3G38483
  
Location: 2496-3576

Mycgr3G38483

Mycgr3G108869
  
Location: 3676-5056

Mycgr3G108869

Mycgr3G103943
  
Location: 5156-5762

Mycgr3G103943

Mycgr3G57362
  
Location: 5862-7296

Mycgr3G57362

Mycgr3G39086
  
Location: 7396-8368

Mycgr3G39086

Mycgr3G103942
  
Location: 8468-8714

Mycgr3G103942

Mycgr3G108865
  
Location: 8814-10239

Mycgr3G108865

Mycgr3G70475
  
Location: 10339-11821

Mycgr3G70475

Mycgr3G108866
  
Location: 11921-13010

Mycgr3G108866

Mycgr3G92136
  
Location: 13110-13593

Mycgr3G92136

small GTPase
  
Accession: EEQ73823
  
Location: 71727-72697
  
 NCBI BlastP on this gene

EEQ73823

agmatinase
  
Accession: EEQ73824
  
Location: 75214-76525
  
 NCBI BlastP on this gene

EEQ73824

conserved hypothetical protein
  
Accession: EEQ73825
  
Location: 77970-78485
  
 NCBI BlastP on this gene

EEQ73825

beta-ketoacyl synthase
  
Accession: EEQ73826
  
Location: 79141-81052
  
  
**BlastP hit with Mycgr3G39149**
  
Percentage identity: 64 %
  
BlastP bit score: 582
  
Sequence coverage: 99 %
  
E-value: 0.0
  
  
 NCBI BlastP on this gene

EEQ73826

SAGA complex component
  
Accession: EEQ73827
  
Location: 81394-83166
  
 NCBI BlastP on this gene

EEQ73827

mRNA capping enzyme alpha subunit
  
Accession: EEQ73828
  
Location: 83897-85201
  
 NCBI BlastP on this gene

EEQ73828

DNA ligase Cdc9
  
Accession: EEQ73829
  
Location: 85727-89223
  
 NCBI BlastP on this gene

EEQ73829

Query: Architecture Search FASTA input

EQ999973 : Ajellomyces dermatitidis ER-3 genomic scaffold supercont1.1    Total score: 1.0     Cumulative Blast bit score: 582

Hit cluster cross-links:

Mycgr3G70471
  
Location: 0-405

Mycgr3G70471

Mycgr3G39149
  
Location: 505-1798

Mycgr3G39149

Mycgr3G92130
  
Location: 1898-2396

Mycgr3G92130

Mycgr3G38483
  
Location: 2496-3576

Mycgr3G38483

Mycgr3G108869
  
Location: 3676-5056

Mycgr3G108869

Mycgr3G103943
  
Location: 5156-5762

Mycgr3G103943

Mycgr3G57362
  
Location: 5862-7296

Mycgr3G57362

Mycgr3G39086
  
Location: 7396-8368

Mycgr3G39086

Mycgr3G103942
  
Location: 8468-8714

Mycgr3G103942

Mycgr3G108865
  
Location: 8814-10239

Mycgr3G108865

Mycgr3G70475
  
Location: 10339-11821

Mycgr3G70475

Mycgr3G108866
  
Location: 11921-13010

Mycgr3G108866

Mycgr3G92136
  
Location: 13110-13593

Mycgr3G92136

predicted protein
  
Accession: EEQ83290
  
Location: 911297-911884
  
 NCBI BlastP on this gene

EEQ83290

small GTPase
  
Accession: EEQ83291
  
Location: 912328-913298
  
 NCBI BlastP on this gene

EEQ83291

agmatinase
  
Accession: EEQ83292
  
Location: 915784-917095
  
 NCBI BlastP on this gene

EEQ83292

conserved hypothetical protein
  
Accession: EEQ83293
  
Location: 918557-919072
  
 NCBI BlastP on this gene

EEQ83293

beta-ketoacyl synthase
  
Accession: EEQ83294
  
Location: 919716-921620
  
  
**BlastP hit with Mycgr3G39149**
  
Percentage identity: 64 %
  
BlastP bit score: 582
  
Sequence coverage: 99 %
  
E-value: 0.0
  
  
 NCBI BlastP on this gene

EEQ83294

SAGA complex component
  
Accession: EEQ83295
  
Location: 921962-923734
  
 NCBI BlastP on this gene

EEQ83295

mRNA capping enzyme alpha subunit
  
Accession: EEQ83296
  
Location: 924458-925762
  
 NCBI BlastP on this gene

EEQ83296

DNA ligase Cdc9
  
Accession: EEQ83297
  
Location: 926288-929775
  
 NCBI BlastP on this gene

EEQ83297

Query: Architecture Search FASTA input

GL636500 : Coccidioides posadasii str. Silveira unplaced genomic scaffold supercont2.15    Total score: 1.0     Cumulative Blast bit score: 579

Hit cluster cross-links:

Mycgr3G70471
  
Location: 0-405

Mycgr3G70471

Mycgr3G39149
  
Location: 505-1798

Mycgr3G39149

Mycgr3G92130
  
Location: 1898-2396

Mycgr3G92130

Mycgr3G38483
  
Location: 2496-3576

Mycgr3G38483

Mycgr3G108869
  
Location: 3676-5056

Mycgr3G108869

Mycgr3G103943
  
Location: 5156-5762

Mycgr3G103943

Mycgr3G57362
  
Location: 5862-7296

Mycgr3G57362

Mycgr3G39086
  
Location: 7396-8368

Mycgr3G39086

Mycgr3G103942
  
Location: 8468-8714

Mycgr3G103942

Mycgr3G108865
  
Location: 8814-10239

Mycgr3G108865

Mycgr3G70475
  
Location: 10339-11821

Mycgr3G70475

Mycgr3G108866
  
Location: 11921-13010

Mycgr3G108866

Mycgr3G92136
  
Location: 13110-13593

Mycgr3G92136

conserved hypothetical protein
  
Accession: EFW15561
  
Location: 187321-188754
  
 NCBI BlastP on this gene

EFW15561

conserved hypothetical protein
  
Accession: EFW15562
  
Location: 190162-190830
  
 NCBI BlastP on this gene

EFW15562

cell division control protein 42
  
Accession: EFW15563
  
Location: 193574-194479
  
 NCBI BlastP on this gene

EFW15563

agmatinase
  
Accession: EFW15564
  
Location: 195323-196644
  
 NCBI BlastP on this gene

EFW15564

beta-ketoacyl synthase
  
Accession: EFW15565
  
Location: 197143-198914
  
  
**BlastP hit with Mycgr3G39149**
  
Percentage identity: 65 %
  
BlastP bit score: 579
  
Sequence coverage: 100 %
  
E-value: 0.0
  
  
 NCBI BlastP on this gene

EFW15565

phosphoribosylaminoimidazole carboxylase
  
Accession: EFW15566
  
Location: 199115-201002
  
 NCBI BlastP on this gene

EFW15566

predicted protein
  
Accession: EFW15567
  
Location: 201947-202347
  
 NCBI BlastP on this gene

EFW15567

SAGA complex component
  
Accession: EFW15568
  
Location: 202451-203952
  
 NCBI BlastP on this gene

EFW15568

mRNA capping enzyme alpha subunit
  
Accession: EFW15569
  
Location: 204559-205788
  
 NCBI BlastP on this gene

EFW15569

DNA ligase
  
Accession: EFW15570
  
Location: 206443-209673
  
 NCBI BlastP on this gene

EFW15570

Query: Architecture Search FASTA input

GG704911 : Coccidioides immitis RS genomic scaffold supercont3.1    Total score: 1.0     Cumulative Blast bit score: 579

Hit cluster cross-links:

Mycgr3G70471
  
Location: 0-405

Mycgr3G70471

Mycgr3G39149
  
Location: 505-1798

Mycgr3G39149

Mycgr3G92130
  
Location: 1898-2396

Mycgr3G92130

Mycgr3G38483
  
Location: 2496-3576

Mycgr3G38483

Mycgr3G108869
  
Location: 3676-5056

Mycgr3G108869

Mycgr3G103943
  
Location: 5156-5762

Mycgr3G103943

Mycgr3G57362
  
Location: 5862-7296

Mycgr3G57362

Mycgr3G39086
  
Location: 7396-8368

Mycgr3G39086

Mycgr3G103942
  
Location: 8468-8714

Mycgr3G103942

Mycgr3G108865
  
Location: 8814-10239

Mycgr3G108865

Mycgr3G70475
  
Location: 10339-11821

Mycgr3G70475

Mycgr3G108866
  
Location: 11921-13010

Mycgr3G108866

Mycgr3G92136
  
Location: 13110-13593

Mycgr3G92136

beta-ketoacyl-acyl-carrier-protein synthase II
  
Accession: EAS35483
  
Location: 6441972-6443742
  
  
**BlastP hit with Mycgr3G39149**
  
Percentage identity: 65 %
  
BlastP bit score: 579
  
Sequence coverage: 100 %
  
E-value: 0.0
  
  
 NCBI BlastP on this gene

EAS35483

phosphoribosylaminoimidazole carboxylase, ATPase subunit
  
Accession: EAS35484
  
Location: 6439877-6441764
  
 NCBI BlastP on this gene

EAS35484

SAGA complex component
  
Accession: EAS35485
  
Location: 6436929-6438429
  
 NCBI BlastP on this gene

EAS35485

mRNA capping enzyme alpha subunit
  
Accession: EAS35486
  
Location: 6435094-6436323
  
 NCBI BlastP on this gene

EAS35486

DNA ligase I, ATP-dependent (dnl1)
  
Accession: EAS35487
  
Location: 6431201-6434441
  
 NCBI BlastP on this gene

EAS35487

Query: Architecture Search FASTA input

DS499595 : Aspergillus fumigatus A1163 scf\_000002 genomic scaffold    Total score: 1.0     Cumulative Blast bit score: 578

Hit cluster cross-links:

Mycgr3G70471
  
Location: 0-405

Mycgr3G70471

Mycgr3G39149
  
Location: 505-1798

Mycgr3G39149

Mycgr3G92130
  
Location: 1898-2396

Mycgr3G92130

Mycgr3G38483
  
Location: 2496-3576

Mycgr3G38483

Mycgr3G108869
  
Location: 3676-5056

Mycgr3G108869

Mycgr3G103943
  
Location: 5156-5762

Mycgr3G103943

Mycgr3G57362
  
Location: 5862-7296

Mycgr3G57362

Mycgr3G39086
  
Location: 7396-8368

Mycgr3G39086

Mycgr3G103942
  
Location: 8468-8714

Mycgr3G103942

Mycgr3G108865
  
Location: 8814-10239

Mycgr3G108865

Mycgr3G70475
  
Location: 10339-11821

Mycgr3G70475

Mycgr3G108866
  
Location: 11921-13010

Mycgr3G108866

Mycgr3G92136
  
Location: 13110-13593

Mycgr3G92136

siderochrome-iron transporter MirC
  
Accession: EDP54224
  
Location: 1599437-1601504
  
 NCBI BlastP on this gene

EDP54224

Rho GTPase ModA, putative
  
Accession: EDP54225
  
Location: 1604613-1605565
  
 NCBI BlastP on this gene

EDP54225

agmatinase, putative
  
Accession: EDP54226
  
Location: 1606644-1608008
  
 NCBI BlastP on this gene

EDP54226

beta-ketoacyl synthase (Cem1), putative
  
Accession: EDP54227
  
Location: 1608526-1610453
  
  
**BlastP hit with Mycgr3G39149**
  
Percentage identity: 60 %
  
BlastP bit score: 578
  
Sequence coverage: 108 %
  
E-value: 0.0
  
  
 NCBI BlastP on this gene

EDP54227

SAGA complex component (Sgf73), putative
  
Accession: EDP54228
  
Location: 1610835-1612254
  
 NCBI BlastP on this gene

EDP54228

mRNA capping enzyme alpha subunit, putative
  
Accession: EDP54229
  
Location: 1612955-1614202
  
 NCBI BlastP on this gene

EDP54229

oligosaccharyl transferase subunit (alpha), putative
  
Accession: EDP54230
  
Location: 1614621-1616296
  
 NCBI BlastP on this gene

EDP54230

IdgA domain protein
  
Accession: EDP54231
  
Location: 1616729-1619459
  
 NCBI BlastP on this gene

EDP54231

Query: Architecture Search FASTA input

DS544809 : Paracoccidioides brasiliensis Pb03 supercont1.7 genomic scaffold    Total score: 1.0     Cumulative Blast bit score: 576

Hit cluster cross-links:

Mycgr3G70471
  
Location: 0-405

Mycgr3G70471

Mycgr3G39149
  
Location: 505-1798

Mycgr3G39149

Mycgr3G92130
  
Location: 1898-2396

Mycgr3G92130

Mycgr3G38483
  
Location: 2496-3576

Mycgr3G38483

Mycgr3G108869
  
Location: 3676-5056

Mycgr3G108869

Mycgr3G103943
  
Location: 5156-5762

Mycgr3G103943

Mycgr3G57362
  
Location: 5862-7296

Mycgr3G57362

Mycgr3G39086
  
Location: 7396-8368

Mycgr3G39086

Mycgr3G103942
  
Location: 8468-8714

Mycgr3G103942

Mycgr3G108865
  
Location: 8814-10239

Mycgr3G108865

Mycgr3G70475
  
Location: 10339-11821

Mycgr3G70475

Mycgr3G108866
  
Location: 11921-13010

Mycgr3G108866

Mycgr3G92136
  
Location: 13110-13593

Mycgr3G92136

predicted protein
  
Accession: EEH22534
  
Location: 479094-480268
  
 NCBI BlastP on this gene

EEH22534

cell division control protein
  
Accession: EEH22533
  
Location: 474533-475609
  
 NCBI BlastP on this gene

EEH22533

arginase family protein
  
Accession: EEH22532
  
Location: 471407-472918
  
 NCBI BlastP on this gene

EEH22532

3-oxoacyl-(acyl-carrier-protein) synthase
  
Accession: EEH22531
  
Location: 469333-471170
  
  
**BlastP hit with Mycgr3G39149**
  
Percentage identity: 62 %
  
BlastP bit score: 576
  
Sequence coverage: 104 %
  
E-value: 0.0
  
  
 NCBI BlastP on this gene

EEH22531

conserved hypothetical protein
  
Accession: EEH22530
  
Location: 466967-468789
  
 NCBI BlastP on this gene

EEH22530

mRNA-capping enzyme subunit alpha
  
Accession: EEH22529
  
Location: 464990-466204
  
 NCBI BlastP on this gene

EEH22529

DNA ligase
  
Accession: EEH22528
  
Location: 461210-464232
  
 NCBI BlastP on this gene

EEH22528

Query: Architecture Search FASTA input

DF126451 : Aspergillus kawachii IFO 4308 DNA, contig: scaffold00005    Total score: 1.0     Cumulative Blast bit score: 574

Hit cluster cross-links:

Mycgr3G70471
  
Location: 0-405

Mycgr3G70471

Mycgr3G39149
  
Location: 505-1798

Mycgr3G39149

Mycgr3G92130
  
Location: 1898-2396

Mycgr3G92130

Mycgr3G38483
  
Location: 2496-3576

Mycgr3G38483

Mycgr3G108869
  
Location: 3676-5056

Mycgr3G108869

Mycgr3G103943
  
Location: 5156-5762

Mycgr3G103943

Mycgr3G57362
  
Location: 5862-7296

Mycgr3G57362

Mycgr3G39086
  
Location: 7396-8368

Mycgr3G39086

Mycgr3G103942
  
Location: 8468-8714

Mycgr3G103942

Mycgr3G108865
  
Location: 8814-10239

Mycgr3G108865

Mycgr3G70475
  
Location: 10339-11821

Mycgr3G70475

Mycgr3G108866
  
Location: 11921-13010

Mycgr3G108866

Mycgr3G92136
  
Location: 13110-13593

Mycgr3G92136

siderochrome-iron transporter
  
Accession: GAA84239
  
Location: 187421-189478
  
 NCBI BlastP on this gene

GAA84239

cell division control protein 42
  
Accession: GAA84238
  
Location: 183875-184888
  
 NCBI BlastP on this gene

GAA84238

agmatinase
  
Accession: GAA84237
  
Location: 181358-182748
  
 NCBI BlastP on this gene

GAA84237

beta-ketoacyl synthase
  
Accession: GAA84236
  
Location: 179001-180703
  
  
**BlastP hit with Mycgr3G39149**
  
Percentage identity: 64 %
  
BlastP bit score: 574
  
Sequence coverage: 95 %
  
E-value: 0.0
  
  
 NCBI BlastP on this gene

GAA84236

SAGA complex component
  
Accession: GAA84235
  
Location: 176749-178273
  
 NCBI BlastP on this gene

GAA84235

mRNA capping enzyme alpha subunit
  
Accession: GAA84234
  
Location: 174866-176095
  
 NCBI BlastP on this gene

GAA84234

Query: Architecture Search FASTA input

GL891304 : Neurospora tetrasperma FGSC 2508 unplaced genomic scaffold NEUTE1scaffold\_3    Total score: 1.0     Cumulative Blast bit score: 573

Hit cluster cross-links:

Mycgr3G70471
  
Location: 0-405

Mycgr3G70471

Mycgr3G39149
  
Location: 505-1798

Mycgr3G39149

Mycgr3G92130
  
Location: 1898-2396

Mycgr3G92130

Mycgr3G38483
  
Location: 2496-3576

Mycgr3G38483

Mycgr3G108869
  
Location: 3676-5056

Mycgr3G108869

Mycgr3G103943
  
Location: 5156-5762

Mycgr3G103943

Mycgr3G57362
  
Location: 5862-7296

Mycgr3G57362

Mycgr3G39086
  
Location: 7396-8368

Mycgr3G39086

Mycgr3G103942
  
Location: 8468-8714

Mycgr3G103942

Mycgr3G108865
  
Location: 8814-10239

Mycgr3G108865

Mycgr3G70475
  
Location: 10339-11821

Mycgr3G70475

Mycgr3G108866
  
Location: 11921-13010

Mycgr3G108866

Mycgr3G92136
  
Location: 13110-13593

Mycgr3G92136

hypothetical protein
  
Accession: EGO57744
  
Location: 2116110-2118036
  
 NCBI BlastP on this gene

EGO57744

hypothetical protein
  
Accession: EGO57745
  
Location: 2118506-2119498
  
 NCBI BlastP on this gene

EGO57745

hypothetical protein
  
Accession: EGO57746
  
Location: 2121429-2122282
  
 NCBI BlastP on this gene

EGO57746

hypothetical protein
  
Accession: EGO57747
  
Location: 2122474-2125394
  
 NCBI BlastP on this gene

EGO57747

3-oxoacyl--synthase
  
Accession: EGO57748
  
Location: 2126386-2127830
  
  
**BlastP hit with Mycgr3G39149**
  
Percentage identity: 63 %
  
BlastP bit score: 573
  
Sequence coverage: 101 %
  
E-value: 0.0
  
  
 NCBI BlastP on this gene

EGO57748

hypothetical protein
  
Accession: EGO57749
  
Location: 2128617-2130778
  
 NCBI BlastP on this gene

EGO57749

hypothetical protein
  
Accession: EGO57750
  
Location: 2131025-2136234
  
 NCBI BlastP on this gene

EGO57750

Query: Architecture Search FASTA input

GL891236 : Neurospora tetrasperma FGSC 2509 unplaced genomic scaffold NEUTE2scaffold\_4    Total score: 1.0     Cumulative Blast bit score: 573

Hit cluster cross-links:

Mycgr3G70471
  
Location: 0-405

Mycgr3G70471

Mycgr3G39149
  
Location: 505-1798

Mycgr3G39149

Mycgr3G92130
  
Location: 1898-2396

Mycgr3G92130

Mycgr3G38483
  
Location: 2496-3576

Mycgr3G38483

Mycgr3G108869
  
Location: 3676-5056

Mycgr3G108869

Mycgr3G103943
  
Location: 5156-5762

Mycgr3G103943

Mycgr3G57362
  
Location: 5862-7296

Mycgr3G57362

Mycgr3G39086
  
Location: 7396-8368

Mycgr3G39086

Mycgr3G103942
  
Location: 8468-8714

Mycgr3G103942

Mycgr3G108865
  
Location: 8814-10239

Mycgr3G108865

Mycgr3G70475
  
Location: 10339-11821

Mycgr3G70475

Mycgr3G108866
  
Location: 11921-13010

Mycgr3G108866

Mycgr3G92136
  
Location: 13110-13593

Mycgr3G92136

3-oxoacyl--synthase
  
Accession: EGZ71980
  
Location: 2872111-2873555
  
  
**BlastP hit with Mycgr3G39149**
  
Percentage identity: 63 %
  
BlastP bit score: 573
  
Sequence coverage: 101 %
  
E-value: 0.0
  
  
 NCBI BlastP on this gene

EGZ71980

hypothetical protein
  
Accession: EGZ71979
  
Location: 2870091-2871324
  
 NCBI BlastP on this gene

EGZ71979

hypothetical protein
  
Accession: EGZ71978
  
Location: 2864639-2869844
  
 NCBI BlastP on this gene

EGZ71978

hypothetical protein
  
Accession: EGZ71977
  
Location: 2861201-2863751
  
 NCBI BlastP on this gene

EGZ71977

Query: Architecture Search FASTA input

ACJE01000010 : Aspergillus niger ATCC 1015    Total score: 1.0     Cumulative Blast bit score: 573

Hit cluster cross-links:

Mycgr3G70471
  
Location: 0-405

Mycgr3G70471

Mycgr3G39149
  
Location: 505-1798

Mycgr3G39149

Mycgr3G92130
  
Location: 1898-2396

Mycgr3G92130

Mycgr3G38483
  
Location: 2496-3576

Mycgr3G38483

Mycgr3G108869
  
Location: 3676-5056

Mycgr3G108869

Mycgr3G103943
  
Location: 5156-5762

Mycgr3G103943

Mycgr3G57362
  
Location: 5862-7296

Mycgr3G57362

Mycgr3G39086
  
Location: 7396-8368

Mycgr3G39086

Mycgr3G103942
  
Location: 8468-8714

Mycgr3G103942

Mycgr3G108865
  
Location: 8814-10239

Mycgr3G108865

Mycgr3G70475
  
Location: 10339-11821

Mycgr3G70475

Mycgr3G108866
  
Location: 11921-13010

Mycgr3G108866

Mycgr3G92136
  
Location: 13110-13593

Mycgr3G92136

hypothetical protein
  
Accession: EHA23590
  
Location: 3567181-3569236
  
 NCBI BlastP on this gene

EHA23590

Rho type ras-related small GTPase
  
Accession: EHA23591
  
Location: 3571664-3572777
  
 NCBI BlastP on this gene

EHA23591

hypothetical protein
  
Accession: EHA23592
  
Location: 3573721-3575104
  
 NCBI BlastP on this gene

EHA23592

3-oxoacyl--synthase
  
Accession: EHA23593
  
Location: 3575679-3577466
  
  
**BlastP hit with Mycgr3G39149**
  
Percentage identity: 63 %
  
BlastP bit score: 573
  
Sequence coverage: 99 %
  
E-value: 0.0
  
  
 NCBI BlastP on this gene

EHA23593

Query: Architecture Search FASTA input

DS995900 : Penicillium marneffei ATCC 18224 scf\_1105668340758 genomic scaffold    Total score: 1.0     Cumulative Blast bit score: 571

Hit cluster cross-links:

Mycgr3G70471
  
Location: 0-405

Mycgr3G70471

Mycgr3G39149
  
Location: 505-1798

Mycgr3G39149

Mycgr3G92130
  
Location: 1898-2396

Mycgr3G92130

Mycgr3G38483
  
Location: 2496-3576

Mycgr3G38483

Mycgr3G108869
  
Location: 3676-5056

Mycgr3G108869

Mycgr3G103943
  
Location: 5156-5762

Mycgr3G103943

Mycgr3G57362
  
Location: 5862-7296

Mycgr3G57362

Mycgr3G39086
  
Location: 7396-8368

Mycgr3G39086

Mycgr3G103942
  
Location: 8468-8714

Mycgr3G103942

Mycgr3G108865
  
Location: 8814-10239

Mycgr3G108865

Mycgr3G70475
  
Location: 10339-11821

Mycgr3G70475

Mycgr3G108866
  
Location: 11921-13010

Mycgr3G108866

Mycgr3G92136
  
Location: 13110-13593

Mycgr3G92136

siderochrome-iron transporter MirC
  
Accession: EEA26054
  
Location: 2661544-2663572
  
 NCBI BlastP on this gene

EEA26054

Rho GTPase ModA, putative
  
Accession: EEA26052
  
Location: 2659563-2660278
  
 NCBI BlastP on this gene

EEA26052

agmatinase, putative
  
Accession: EEA26050
  
Location: 2656455-2657821
  
 NCBI BlastP on this gene

EEA26050

beta-ketoacyl synthase (Cem1), putative
  
Accession: EEA26049
  
Location: 2653965-2655649
  
  
**BlastP hit with Mycgr3G39149**
  
Percentage identity: 64 %
  
BlastP bit score: 571
  
Sequence coverage: 100 %
  
E-value: 0.0
  
  
 NCBI BlastP on this gene

EEA26049

SAGA complex component (Sgf73), putative
  
Accession: EEA26048
  
Location: 2652182-2653633
  
 NCBI BlastP on this gene

EEA26048

mRNA capping enzyme alpha subunit, putative
  
Accession: EEA26046
  
Location: 2650299-2651567
  
 NCBI BlastP on this gene

EEA26046

DNA ligase Cdc9, putative
  
Accession: EEA26045
  
Location: 2646897-2649672
  
 NCBI BlastP on this gene

EEA26045

conserved hypothetical protein
  
Accession: EEA26044
  
Location: 2642558-2646134
  
 NCBI BlastP on this gene

EEA26044

Query: Architecture Search FASTA input

AAHF01000008 : Aspergillus fumigatus Af293    Total score: 1.0     Cumulative Blast bit score: 571

Hit cluster cross-links:

Mycgr3G70471
  
Location: 0-405

Mycgr3G70471

Mycgr3G39149
  
Location: 505-1798

Mycgr3G39149

Mycgr3G92130
  
Location: 1898-2396

Mycgr3G92130

Mycgr3G38483
  
Location: 2496-3576

Mycgr3G38483

Mycgr3G108869
  
Location: 3676-5056

Mycgr3G108869

Mycgr3G103943
  
Location: 5156-5762

Mycgr3G103943

Mycgr3G57362
  
Location: 5862-7296

Mycgr3G57362

Mycgr3G39086
  
Location: 7396-8368

Mycgr3G39086

Mycgr3G103942
  
Location: 8468-8714

Mycgr3G103942

Mycgr3G108865
  
Location: 8814-10239

Mycgr3G108865

Mycgr3G70475
  
Location: 10339-11821

Mycgr3G70475

Mycgr3G108866
  
Location: 11921-13010

Mycgr3G108866

Mycgr3G92136
  
Location: 13110-13593

Mycgr3G92136

siderochrome-iron transporter MirC
  
Accession: EAL87664
  
Location: 1612624-1614691
  
 NCBI BlastP on this gene

EAL87664

Rho GTPase ModA, putative
  
Accession: EAL87665
  
Location: 1617787-1618739
  
 NCBI BlastP on this gene

EAL87665

agmatinase, putative
  
Accession: EAL87666
  
Location: 1619818-1621182
  
 NCBI BlastP on this gene

EAL87666

beta-ketoacyl synthase (Cem1), putative
  
Accession: EAL87667
  
Location: 1621987-1623626
  
  
**BlastP hit with Mycgr3G39149**
  
Percentage identity: 64 %
  
BlastP bit score: 571
  
Sequence coverage: 97 %
  
E-value: 0.0
  
  
 NCBI BlastP on this gene

EAL87667

Query: Architecture Search FASTA input

GG663367 : Ajellomyces capsulatus G186AR genomic scaffold supercont2.5    Total score: 1.0     Cumulative Blast bit score: 564

Hit cluster cross-links:

Mycgr3G70471
  
Location: 0-405

Mycgr3G70471

Mycgr3G39149
  
Location: 505-1798

Mycgr3G39149

Mycgr3G92130
  
Location: 1898-2396

Mycgr3G92130

Mycgr3G38483
  
Location: 2496-3576

Mycgr3G38483

Mycgr3G108869
  
Location: 3676-5056

Mycgr3G108869

Mycgr3G103943
  
Location: 5156-5762

Mycgr3G103943

Mycgr3G57362
  
Location: 5862-7296

Mycgr3G57362

Mycgr3G39086
  
Location: 7396-8368

Mycgr3G39086

Mycgr3G103942
  
Location: 8468-8714

Mycgr3G103942

Mycgr3G108865
  
Location: 8814-10239

Mycgr3G108865

Mycgr3G70475
  
Location: 10339-11821

Mycgr3G70475

Mycgr3G108866
  
Location: 11921-13010

Mycgr3G108866

Mycgr3G92136
  
Location: 13110-13593

Mycgr3G92136

predicted protein
  
Accession: EEH07630
  
Location: 1635701-1636742
  
 NCBI BlastP on this gene

EEH07630

conserved hypothetical protein
  
Accession: EEH07631
  
Location: 1637618-1638581
  
 NCBI BlastP on this gene

EEH07631

predicted protein
  
Accession: EEH07632
  
Location: 1639868-1640233
  
 NCBI BlastP on this gene

EEH07632

agmatine ureohydrolase
  
Accession: EEH07633
  
Location: 1640711-1642461
  
 NCBI BlastP on this gene

EEH07633

conserved hypothetical protein
  
Accession: EEH07634
  
Location: 1643233-1643745
  
 NCBI BlastP on this gene

EEH07634

3-oxoacyl-[acyl-carrier-protein]-synthase
  
Accession: EEH07635
  
Location: 1644317-1646202
  
  
**BlastP hit with Mycgr3G39149**
  
Percentage identity: 63 %
  
BlastP bit score: 564
  
Sequence coverage: 101 %
  
E-value: 0.0
  
  
 NCBI BlastP on this gene

EEH07635

conserved hypothetical protein
  
Accession: EEH07636
  
Location: 1646494-1648239
  
 NCBI BlastP on this gene

EEH07636

mRNA-capping enzyme subunit alpha
  
Accession: EEH07637
  
Location: 1648972-1650264
  
 NCBI BlastP on this gene

EEH07637

DNA ligase
  
Accession: EEH07638
  
Location: 1650599-1654065
  
 NCBI BlastP on this gene

EEH07638

Query: Architecture Search FASTA input

DS572815 : Paracoccidioides brasiliensis Pb01 supercont1.5 genomic scaffold    Total score: 1.0     Cumulative Blast bit score: 559

Hit cluster cross-links:

Mycgr3G70471
  
Location: 0-405

Mycgr3G70471

Mycgr3G39149
  
Location: 505-1798

Mycgr3G39149

Mycgr3G92130
  
Location: 1898-2396

Mycgr3G92130

Mycgr3G38483
  
Location: 2496-3576

Mycgr3G38483

Mycgr3G108869
  
Location: 3676-5056

Mycgr3G108869

Mycgr3G103943
  
Location: 5156-5762

Mycgr3G103943

Mycgr3G57362
  
Location: 5862-7296

Mycgr3G57362

Mycgr3G39086
  
Location: 7396-8368

Mycgr3G39086

Mycgr3G103942
  
Location: 8468-8714

Mycgr3G103942

Mycgr3G108865
  
Location: 8814-10239

Mycgr3G108865

Mycgr3G70475
  
Location: 10339-11821

Mycgr3G70475

Mycgr3G108866
  
Location: 11921-13010

Mycgr3G108866

Mycgr3G92136
  
Location: 13110-13593

Mycgr3G92136

cell division control protein
  
Accession: EEH40175
  
Location: 179805-180886
  
 NCBI BlastP on this gene

EEH40175

guanidinobutyrase
  
Accession: EEH40174
  
Location: 177078-178275
  
 NCBI BlastP on this gene

EEH40174

3-oxoacyl-[acyl-carrier-protein] synthase
  
Accession: EEH40173
  
Location: 172388-176587
  
  
**BlastP hit with Mycgr3G39149**
  
Percentage identity: 65 %
  
BlastP bit score: 559
  
Sequence coverage: 94 %
  
E-value: 0.0
  
  
 NCBI BlastP on this gene

EEH40173

mRNA-capping enzyme subunit alpha
  
Accession: EEH40172
  
Location: 170375-172351
  
 NCBI BlastP on this gene

EEH40172

DNA ligase
  
Accession: EEH40171
  
Location: 166667-169834
  
 NCBI BlastP on this gene

EEH40171

Query: Architecture Search FASTA input

AQGS01000575 : Dactylellina haptotyla CBS 200.50    Total score: 1.0     Cumulative Blast bit score: 555

Hit cluster cross-links:

Mycgr3G70471
  
Location: 0-405

Mycgr3G70471

Mycgr3G39149
  
Location: 505-1798

Mycgr3G39149

Mycgr3G92130
  
Location: 1898-2396

Mycgr3G92130

Mycgr3G38483
  
Location: 2496-3576

Mycgr3G38483

Mycgr3G108869
  
Location: 3676-5056

Mycgr3G108869

Mycgr3G103943
  
Location: 5156-5762

Mycgr3G103943

Mycgr3G57362
  
Location: 5862-7296

Mycgr3G57362

Mycgr3G39086
  
Location: 7396-8368

Mycgr3G39086

Mycgr3G103942
  
Location: 8468-8714

Mycgr3G103942

Mycgr3G108865
  
Location: 8814-10239

Mycgr3G108865

Mycgr3G70475
  
Location: 10339-11821

Mycgr3G70475

Mycgr3G108866
  
Location: 11921-13010

Mycgr3G108866

Mycgr3G92136
  
Location: 13110-13593

Mycgr3G92136

hypothetical protein
  
Accession: EPS38362
  
Location: 248074-249602
  
 NCBI BlastP on this gene

EPS38362

hypothetical protein
  
Accession: EPS38282
  
Location: 250777-252349
  
 NCBI BlastP on this gene

EPS38282

hypothetical protein
  
Accession: EPS38163
  
Location: 253526-255842
  
 NCBI BlastP on this gene

EPS38163

hypothetical protein
  
Accession: EPS38330
  
Location: 256605-260102
  
  
**BlastP hit with Mycgr3G39149**
  
Percentage identity: 62 %
  
BlastP bit score: 555
  
Sequence coverage: 100 %
  
E-value: 0.0
  
  
 NCBI BlastP on this gene

EPS38330

hypothetical protein
  
Accession: EPS38216
  
Location: 260632-262739
  
 NCBI BlastP on this gene

EPS38216

hypothetical protein
  
Accession: EPS38355
  
Location: 267138-267845
  
 NCBI BlastP on this gene

EPS38355

Query: Architecture Search FASTA input

DS989823 : Arthroderma gypseum CBS 118893 supercont1.2 genomic scaffold    Total score: 1.0     Cumulative Blast bit score: 551

Hit cluster cross-links:

Mycgr3G70471
  
Location: 0-405

Mycgr3G70471

Mycgr3G39149
  
Location: 505-1798

Mycgr3G39149

Mycgr3G92130
  
Location: 1898-2396

Mycgr3G92130

Mycgr3G38483
  
Location: 2496-3576

Mycgr3G38483

Mycgr3G108869
  
Location: 3676-5056

Mycgr3G108869

Mycgr3G103943
  
Location: 5156-5762

Mycgr3G103943

Mycgr3G57362
  
Location: 5862-7296

Mycgr3G57362

Mycgr3G39086
  
Location: 7396-8368

Mycgr3G39086

Mycgr3G103942
  
Location: 8468-8714

Mycgr3G103942

Mycgr3G108865
  
Location: 8814-10239

Mycgr3G108865

Mycgr3G70475
  
Location: 10339-11821

Mycgr3G70475

Mycgr3G108866
  
Location: 11921-13010

Mycgr3G108866

Mycgr3G92136
  
Location: 13110-13593

Mycgr3G92136

3-oxoacyl-[acyl-carrier-protein] synthase 2
  
Accession: EFR00118
  
Location: 2943070-2944793
  
  
**BlastP hit with Mycgr3G39149**
  
Percentage identity: 65 %
  
BlastP bit score: 551
  
Sequence coverage: 99 %
  
E-value: 0.0
  
  
 NCBI BlastP on this gene

EFR00118

hypothetical protein
  
Accession: EFR00117
  
Location: 2941422-2942876
  
 NCBI BlastP on this gene

EFR00117

hypothetical protein
  
Accession: EFR00116
  
Location: 2938709-2940157
  
 NCBI BlastP on this gene

EFR00116

hypothetical protein
  
Accession: EFR00115
  
Location: 2937062-2937509
  
 NCBI BlastP on this gene

EFR00115

hypothetical protein
  
Accession: EFR00114
  
Location: 2934888-2936346
  
 NCBI BlastP on this gene

EFR00114

Query: Architecture Search FASTA input

JH226136 : Exophiala dermatitidis NIH/UT8656 unplaced genomic scaffold supercont1.7    Total score: 1.0     Cumulative Blast bit score: 549

Hit cluster cross-links:

Mycgr3G70471
  
Location: 0-405

Mycgr3G70471

Mycgr3G39149
  
Location: 505-1798

Mycgr3G39149

Mycgr3G92130
  
Location: 1898-2396

Mycgr3G92130

Mycgr3G38483
  
Location: 2496-3576

Mycgr3G38483

Mycgr3G108869
  
Location: 3676-5056

Mycgr3G108869

Mycgr3G103943
  
Location: 5156-5762

Mycgr3G103943

Mycgr3G57362
  
Location: 5862-7296

Mycgr3G57362

Mycgr3G39086
  
Location: 7396-8368

Mycgr3G39086

Mycgr3G103942
  
Location: 8468-8714

Mycgr3G103942

Mycgr3G108865
  
Location: 8814-10239

Mycgr3G108865

Mycgr3G70475
  
Location: 10339-11821

Mycgr3G70475

Mycgr3G108866
  
Location: 11921-13010

Mycgr3G108866

Mycgr3G92136
  
Location: 13110-13593

Mycgr3G92136

dolichyl pyrophosphate Glc1Man9GlcNAc2 alpha-1,3-glucosyltransferase
  
Accession: EHY60116
  
Location: 735916-737625
  
 NCBI BlastP on this gene

EHY60116

hypothetical protein
  
Accession: EHY60117
  
Location: 738912-742097
  
 NCBI BlastP on this gene

EHY60117

agmatinase
  
Accession: EHY60118
  
Location: 742819-744512
  
 NCBI BlastP on this gene

EHY60118

3-oxoacyl-[acyl-carrier-protein] synthase II
  
Accession: EHY60119
  
Location: 745527-747394
  
  
**BlastP hit with Mycgr3G39149**
  
Percentage identity: 63 %
  
BlastP bit score: 549
  
Sequence coverage: 105 %
  
E-value: 0.0
  
  
 NCBI BlastP on this gene

EHY60119

SAGA-associated factor 73
  
Accession: EHY60120
  
Location: 747554-748890
  
 NCBI BlastP on this gene

EHY60120

mRNA guanylyltransferase
  
Accession: EHY60121
  
Location: 749547-750869
  
 NCBI BlastP on this gene

EHY60121

hypothetical protein
  
Accession: EHY60122
  
Location: 751545-754415
  
 NCBI BlastP on this gene

EHY60122

hypothetical protein
  
Accession: EHY60123
  
Location: 755435-758675
  
 NCBI BlastP on this gene

EHY60123

Query: Architecture Search FASTA input

KB446557 : Pseudocercospora fijiensis CIRAD86 unplaced genomic scaffold MYCFIscaffold\_3    Total score: 1.0     Cumulative Blast bit score: 544

Hit cluster cross-links:

Mycgr3G70471
  
Location: 0-405

Mycgr3G70471

Mycgr3G39149
  
Location: 505-1798

Mycgr3G39149

Mycgr3G92130
  
Location: 1898-2396

Mycgr3G92130

Mycgr3G38483
  
Location: 2496-3576

Mycgr3G38483

Mycgr3G108869
  
Location: 3676-5056

Mycgr3G108869

Mycgr3G103943
  
Location: 5156-5762

Mycgr3G103943

Mycgr3G57362
  
Location: 5862-7296

Mycgr3G57362

Mycgr3G39086
  
Location: 7396-8368

Mycgr3G39086

Mycgr3G103942
  
Location: 8468-8714

Mycgr3G103942

Mycgr3G108865
  
Location: 8814-10239

Mycgr3G108865

Mycgr3G70475
  
Location: 10339-11821

Mycgr3G70475

Mycgr3G108866
  
Location: 11921-13010

Mycgr3G108866

Mycgr3G92136
  
Location: 13110-13593

Mycgr3G92136

hypothetical protein
  
Accession: EME84082
  
Location: 1913273-1914889
  
 NCBI BlastP on this gene

EME84082

hypothetical protein
  
Accession: EME84083
  
Location: 1914961-1916909
  
 NCBI BlastP on this gene

EME84083

hypothetical protein
  
Accession: EME84084
  
Location: 1918175-1919675
  
 NCBI BlastP on this gene

EME84084

glycosyltransferase family 8 protein
  
Accession: EME84085
  
Location: 1920888-1922165
  
 NCBI BlastP on this gene

EME84085

hypothetical protein
  
Accession: EME84086
  
Location: 1922777-1924025
  
  
**BlastP hit with Mycgr3G38483**
  
Percentage identity: 72 %
  
BlastP bit score: 544
  
Sequence coverage: 100 %
  
E-value: 0.0
  
  
 NCBI BlastP on this gene

EME84086

hypothetical protein
  
Accession: EME84087
  
Location: 1925170-1926203
  
 NCBI BlastP on this gene

EME84087

hypothetical protein
  
Accession: EME84088
  
Location: 1926285-1927577
  
 NCBI BlastP on this gene

EME84088

Query: Architecture Search FASTA input

HF679025 : Fusarium fujikuroi IMI 58289 draft genome, chromosome FFUJ\_chr03.    Total score: 1.0     Cumulative Blast bit score: 541

Hit cluster cross-links:

Mycgr3G70471
  
Location: 0-405

Mycgr3G70471

Mycgr3G39149
  
Location: 505-1798

Mycgr3G39149

Mycgr3G92130
  
Location: 1898-2396

Mycgr3G92130

Mycgr3G38483
  
Location: 2496-3576

Mycgr3G38483

Mycgr3G108869
  
Location: 3676-5056

Mycgr3G108869

Mycgr3G103943
  
Location: 5156-5762

Mycgr3G103943

Mycgr3G57362
  
Location: 5862-7296

Mycgr3G57362

Mycgr3G39086
  
Location: 7396-8368

Mycgr3G39086

Mycgr3G103942
  
Location: 8468-8714

Mycgr3G103942

Mycgr3G108865
  
Location: 8814-10239

Mycgr3G108865

Mycgr3G70475
  
Location: 10339-11821

Mycgr3G70475

Mycgr3G108866
  
Location: 11921-13010

Mycgr3G108866

Mycgr3G92136
  
Location: 13110-13593

Mycgr3G92136

uncharacterized protein
  
Accession: CCT66223
  
Location: 3695062-3696552
  
 NCBI BlastP on this gene

FFUJ\_03233

probable isp4 protein
  
Accession: CCT66224
  
Location: 3698507-3701309
  
 NCBI BlastP on this gene

FFUJ\_03234

probable CEM1-beta-keto-acyl-ACP synthase, mitochondrial
  
Accession: CCT66225
  
Location: 3701896-3703280
  
  
**BlastP hit with Mycgr3G39149**
  
Percentage identity: 62 %
  
BlastP bit score: 541
  
Sequence coverage: 100 %
  
E-value: 0.0
  
  
 NCBI BlastP on this gene

FFUJ\_03235

related to CBP3-required for assembly of cytochrome bc1 complex
  
Accession: CCT66226
  
Location: 3703539-3704512
  
 NCBI BlastP on this gene

FFUJ\_03236

related to DFR1-dihydrofolate reductase
  
Accession: CCT66227
  
Location: 3704688-3705392
  
 NCBI BlastP on this gene

FFUJ\_03237

related to glutathione S-transferase
  
Accession: CCT66228
  
Location: 3705751-3706579
  
 NCBI BlastP on this gene

FFUJ\_03238

probable reductases with broad range of substrate specificities
  
Accession: CCT66229
  
Location: 3706878-3707969
  
 NCBI BlastP on this gene

FFUJ\_03239

related to 26S proteasome subunit YTA7
  
Accession: CCT66230
  
Location: 3708245-3713148
  
 NCBI BlastP on this gene

FFUJ\_03240

Query: Architecture Search FASTA input

KB446538 : Dothistroma septosporum NZE10 unplaced genomic scaffold DOTSEscaffold\_4    Total score: 1.0     Cumulative Blast bit score: 537

Hit cluster cross-links:

Mycgr3G70471
  
Location: 0-405

Mycgr3G70471

Mycgr3G39149
  
Location: 505-1798

Mycgr3G39149

Mycgr3G92130
  
Location: 1898-2396

Mycgr3G92130

Mycgr3G38483
  
Location: 2496-3576

Mycgr3G38483

Mycgr3G108869
  
Location: 3676-5056

Mycgr3G108869

Mycgr3G103943
  
Location: 5156-5762

Mycgr3G103943

Mycgr3G57362
  
Location: 5862-7296

Mycgr3G57362

Mycgr3G39086
  
Location: 7396-8368

Mycgr3G39086

Mycgr3G103942
  
Location: 8468-8714

Mycgr3G103942

Mycgr3G108865
  
Location: 8814-10239

Mycgr3G108865

Mycgr3G70475
  
Location: 10339-11821

Mycgr3G70475

Mycgr3G108866
  
Location: 11921-13010

Mycgr3G108866

Mycgr3G92136
  
Location: 13110-13593

Mycgr3G92136

hypothetical protein
  
Accession: EME44904
  
Location: 188161-191376
  
 NCBI BlastP on this gene

EME44904

hypothetical protein
  
Accession: EME44903
  
Location: 185248-185664
  
 NCBI BlastP on this gene

EME44903

hypothetical protein
  
Accession: EME44902
  
Location: 183856-184412
  
 NCBI BlastP on this gene

EME44902

hypothetical protein
  
Accession: EME44901
  
Location: 182368-183532
  
 NCBI BlastP on this gene

EME44901

hypothetical protein
  
Accession: EME44900
  
Location: 179851-181490
  
  
**BlastP hit with Mycgr3G57362**
  
Percentage identity: 55 %
  
BlastP bit score: 537
  
Sequence coverage: 102 %
  
E-value: 0.0
  
  
 NCBI BlastP on this gene

EME44900

hypothetical protein
  
Accession: EME44899
  
Location: 179148-179591
  
 NCBI BlastP on this gene

EME44899

hypothetical protein
  
Accession: EME44898
  
Location: 177734-179019
  
 NCBI BlastP on this gene

EME44898

hypothetical protein
  
Accession: EME44897
  
Location: 176564-177443
  
 NCBI BlastP on this gene

EME44897

hypothetical protein
  
Accession: EME44896
  
Location: 174017-176333
  
 NCBI BlastP on this gene

EME44896

hypothetical protein
  
Accession: EME44895
  
Location: 172066-172224
  
 NCBI BlastP on this gene

EME44895

Query: Architecture Search FASTA input

EQ962653 : Talaromyces stipitatus ATCC 10500 scf\_1105507295527 genomic scaffold    Total score: 1.0     Cumulative Blast bit score: 536

Hit cluster cross-links:

Mycgr3G70471
  
Location: 0-405

Mycgr3G70471

Mycgr3G39149
  
Location: 505-1798

Mycgr3G39149

Mycgr3G92130
  
Location: 1898-2396

Mycgr3G92130

Mycgr3G38483
  
Location: 2496-3576

Mycgr3G38483

Mycgr3G108869
  
Location: 3676-5056

Mycgr3G108869

Mycgr3G103943
  
Location: 5156-5762

Mycgr3G103943

Mycgr3G57362
  
Location: 5862-7296

Mycgr3G57362

Mycgr3G39086
  
Location: 7396-8368

Mycgr3G39086

Mycgr3G103942
  
Location: 8468-8714

Mycgr3G103942

Mycgr3G108865
  
Location: 8814-10239

Mycgr3G108865

Mycgr3G70475
  
Location: 10339-11821

Mycgr3G70475

Mycgr3G108866
  
Location: 11921-13010

Mycgr3G108866

Mycgr3G92136
  
Location: 13110-13593

Mycgr3G92136

conserved hypothetical protein
  
Accession: EED21945
  
Location: 3135534-3136130
  
 NCBI BlastP on this gene

EED21945

siderochrome-iron transporter MirC
  
Accession: EED21944
  
Location: 3132720-3134730
  
 NCBI BlastP on this gene

EED21944

Rho GTPase ModA, putative
  
Accession: EED21943
  
Location: 3130808-3131632
  
 NCBI BlastP on this gene

EED21943

agmatinase, putative
  
Accession: EED21942
  
Location: 3128636-3130000
  
 NCBI BlastP on this gene

EED21942

beta-ketoacyl synthase (Cem1), putative
  
Accession: EED21941
  
Location: 3124630-3128063
  
  
**BlastP hit with Mycgr3G39149**
  
Percentage identity: 63 %
  
BlastP bit score: 536
  
Sequence coverage: 94 %
  
E-value: 2e-180
  
  
 NCBI BlastP on this gene

EED21941

mRNA capping enzyme alpha subunit, putative
  
Accession: EED21940
  
Location: 3122803-3124074
  
 NCBI BlastP on this gene

EED21940

conserved hypothetical protein
  
Accession: EED21939
  
Location: 3120518-3122277
  
 NCBI BlastP on this gene

EED21939

DNA ligase Cdc9, putative
  
Accession: EED21938
  
Location: 3117323-3120072
  
 NCBI BlastP on this gene

EED21938

Query: Architecture Search FASTA input

ABDG02000027 : Trichoderma atroviride IMI 206040    Total score: 1.0     Cumulative Blast bit score: 532

Hit cluster cross-links:

Mycgr3G70471
  
Location: 0-405

Mycgr3G70471

Mycgr3G39149
  
Location: 505-1798

Mycgr3G39149

Mycgr3G92130
  
Location: 1898-2396

Mycgr3G92130

Mycgr3G38483
  
Location: 2496-3576

Mycgr3G38483

Mycgr3G108869
  
Location: 3676-5056

Mycgr3G108869

Mycgr3G103943
  
Location: 5156-5762

Mycgr3G103943

Mycgr3G57362
  
Location: 5862-7296

Mycgr3G57362

Mycgr3G39086
  
Location: 7396-8368

Mycgr3G39086

Mycgr3G103942
  
Location: 8468-8714

Mycgr3G103942

Mycgr3G108865
  
Location: 8814-10239

Mycgr3G108865

Mycgr3G70475
  
Location: 10339-11821

Mycgr3G70475

Mycgr3G108866
  
Location: 11921-13010

Mycgr3G108866

Mycgr3G92136
  
Location: 13110-13593

Mycgr3G92136

hypothetical protein
  
Accession: EHK41235
  
Location: 2369651-2373075
  
 NCBI BlastP on this gene

EHK41235

hypothetical protein
  
Accession: EHK41236
  
Location: 2373677-2376538
  
 NCBI BlastP on this gene

EHK41236

hypothetical protein
  
Accession: EHK41237
  
Location: 2377394-2378852
  
  
**BlastP hit with Mycgr3G39149**
  
Percentage identity: 62 %
  
BlastP bit score: 532
  
Sequence coverage: 100 %
  
E-value: 0.0
  
  
 NCBI BlastP on this gene

EHK41237

hypothetical protein
  
Accession: EHK41238
  
Location: 2379342-2380342
  
 NCBI BlastP on this gene

EHK41238

hypothetical protein
  
Accession: EHK41239
  
Location: 2380681-2381448
  
 NCBI BlastP on this gene

EHK41239

hypothetical protein
  
Accession: EHK41240
  
Location: 2381720-2382797
  
 NCBI BlastP on this gene

EHK41240

hypothetical protein
  
Accession: EHK41241
  
Location: 2383746-2384952
  
 NCBI BlastP on this gene

EHK41241

hypothetical protein
  
Accession: EHK41242
  
Location: 2385832-2390897
  
 NCBI BlastP on this gene

EHK41242

Query: Architecture Search FASTA input

CU633900 : Podospora anserina S mat+ genomic DNA chromosome 7, supercontig 1.    Total score: 1.0     Cumulative Blast bit score: 528

Hit cluster cross-links:

Mycgr3G70471
  
Location: 0-405

Mycgr3G70471

Mycgr3G39149
  
Location: 505-1798

Mycgr3G39149

Mycgr3G92130
  
Location: 1898-2396

Mycgr3G92130

Mycgr3G38483
  
Location: 2496-3576

Mycgr3G38483

Mycgr3G108869
  
Location: 3676-5056

Mycgr3G108869

Mycgr3G103943
  
Location: 5156-5762

Mycgr3G103943

Mycgr3G57362
  
Location: 5862-7296

Mycgr3G57362

Mycgr3G39086
  
Location: 7396-8368

Mycgr3G39086

Mycgr3G103942
  
Location: 8468-8714

Mycgr3G103942

Mycgr3G108865
  
Location: 8814-10239

Mycgr3G108865

Mycgr3G70475
  
Location: 10339-11821

Mycgr3G70475

Mycgr3G108866
  
Location: 11921-13010

Mycgr3G108866

Mycgr3G92136
  
Location: 13110-13593

Mycgr3G92136

not annotated
  
Accession: CAP68987
  
Location: 2689446-2692267
  
 NCBI BlastP on this gene

CAP68987

not annotated
  
Accession: CAP68988
  
Location: 2692962-2693996
  
 NCBI BlastP on this gene

CAP68988

not annotated
  
Accession: CAP68989
  
Location: 2697105-2699936
  
 NCBI BlastP on this gene

CAP68989

tRNA-Val
  
Accession: CAP68990
  
Location: 2700555-2701977
  
  
**BlastP hit with Mycgr3G39149**
  
Percentage identity: 61 %
  
BlastP bit score: 528
  
Sequence coverage: 102 %
  
E-value: 0.0
  
  
 NCBI BlastP on this gene

CAP68990

Query: Architecture Search FASTA input

ACFW01000049 : Coccidioides posadasii C735 delta SOWgp    Total score: 1.0     Cumulative Blast bit score: 526

Hit cluster cross-links:

Mycgr3G70471
  
Location: 0-405

Mycgr3G70471

Mycgr3G39149
  
Location: 505-1798

Mycgr3G39149

Mycgr3G92130
  
Location: 1898-2396

Mycgr3G92130

Mycgr3G38483
  
Location: 2496-3576

Mycgr3G38483

Mycgr3G108869
  
Location: 3676-5056

Mycgr3G108869

Mycgr3G103943
  
Location: 5156-5762

Mycgr3G103943

Mycgr3G57362
  
Location: 5862-7296

Mycgr3G57362

Mycgr3G39086
  
Location: 7396-8368

Mycgr3G39086

Mycgr3G103942
  
Location: 8468-8714

Mycgr3G103942

Mycgr3G108865
  
Location: 8814-10239

Mycgr3G108865

Mycgr3G70475
  
Location: 10339-11821

Mycgr3G70475

Mycgr3G108866
  
Location: 11921-13010

Mycgr3G108866

Mycgr3G92136
  
Location: 13110-13593

Mycgr3G92136

Beta-ketoacyl synthase, C-terminal domain containing protein
  
Accession: EER24144
  
Location: 3470753-3472524
  
  
**BlastP hit with Mycgr3G39149**
  
Percentage identity: 62 %
  
BlastP bit score: 526
  
Sequence coverage: 99 %
  
E-value: 0.0
  
  
 NCBI BlastP on this gene

EER24144

phosphoribosylaminoimidazole carboxylase, putative
  
Accession: EER24143
  
Location: 3468668-3470555
  
 NCBI BlastP on this gene

EER24143

hypothetical protein
  
Accession: EER24142
  
Location: 3465717-3466763
  
 NCBI BlastP on this gene

EER24142

mRNA capping enzyme alpha subunit, putative
  
Accession: EER24141
  
Location: 3463881-3465110
  
 NCBI BlastP on this gene

EER24141

ATP-dependent DNA ligase, putative
  
Accession: EER24140
  
Location: 3459996-3463226
  
 NCBI BlastP on this gene

EER24140

Query: Architecture Search FASTA input

ADOT01000059 : Arthrobotrys oligospora ATCC 24927    Total score: 1.0     Cumulative Blast bit score: 524

Hit cluster cross-links:

Mycgr3G70471
  
Location: 0-405

Mycgr3G70471

Mycgr3G39149
  
Location: 505-1798

Mycgr3G39149

Mycgr3G92130
  
Location: 1898-2396

Mycgr3G92130

Mycgr3G38483
  
Location: 2496-3576

Mycgr3G38483

Mycgr3G108869
  
Location: 3676-5056

Mycgr3G108869

Mycgr3G103943
  
Location: 5156-5762

Mycgr3G103943

Mycgr3G57362
  
Location: 5862-7296

Mycgr3G57362

Mycgr3G39086
  
Location: 7396-8368

Mycgr3G39086

Mycgr3G103942
  
Location: 8468-8714

Mycgr3G103942

Mycgr3G108865
  
Location: 8814-10239

Mycgr3G108865

Mycgr3G70475
  
Location: 10339-11821

Mycgr3G70475

Mycgr3G108866
  
Location: 11921-13010

Mycgr3G108866

Mycgr3G92136
  
Location: 13110-13593

Mycgr3G92136

hypothetical protein
  
Accession: EGX52125
  
Location: 422183-423811
  
  
**BlastP hit with Mycgr3G39149**
  
Percentage identity: 58 %
  
BlastP bit score: 524
  
Sequence coverage: 100 %
  
E-value: 0.0
  
  
 NCBI BlastP on this gene

EGX52125

hypothetical protein
  
Accession: EGX52124
  
Location: 419408-421261
  
 NCBI BlastP on this gene

EGX52124

hypothetical protein
  
Accession: EGX52123
  
Location: 416199-416879
  
 NCBI BlastP on this gene

EGX52123

hypothetical protein
  
Accession: EGX52122
  
Location: 412824-414530
  
 NCBI BlastP on this gene

EGX52122

Query: Architecture Search FASTA input

AACD01000129 : Aspergillus nidulans FGSC A4    Total score: 1.0     Cumulative Blast bit score: 521

Hit cluster cross-links:

Mycgr3G70471
  
Location: 0-405

Mycgr3G70471

Mycgr3G39149
  
Location: 505-1798

Mycgr3G39149

Mycgr3G92130
  
Location: 1898-2396

Mycgr3G92130

Mycgr3G38483
  
Location: 2496-3576

Mycgr3G38483

Mycgr3G108869
  
Location: 3676-5056

Mycgr3G108869

Mycgr3G103943
  
Location: 5156-5762

Mycgr3G103943

Mycgr3G57362
  
Location: 5862-7296

Mycgr3G57362

Mycgr3G39086
  
Location: 7396-8368

Mycgr3G39086

Mycgr3G103942
  
Location: 8468-8714

Mycgr3G103942

Mycgr3G108865
  
Location: 8814-10239

Mycgr3G108865

Mycgr3G70475
  
Location: 10339-11821

Mycgr3G70475

Mycgr3G108866
  
Location: 11921-13010

Mycgr3G108866

Mycgr3G92136
  
Location: 13110-13593

Mycgr3G92136

hypothetical protein
  
Accession: EAA62065
  
Location: 200559-202616
  
 NCBI BlastP on this gene

EAA62065

predicted protein
  
Accession: EAA62066
  
Location: 202920-203174
  
 NCBI BlastP on this gene

EAA62066

CD42 CHICK Cell division control protein 42 homolog (G25K GTP-binding protein)
  
Accession: EAA62067
  
Location: 204520-205477
  
 NCBI BlastP on this gene

EAA62067

hypothetical protein
  
Accession: EAA62068
  
Location: 206328-207725
  
 NCBI BlastP on this gene

EAA62068

hypothetical protein
  
Accession: EAA62069
  
Location: 208323-209965
  
  
**BlastP hit with Mycgr3G39149**
  
Percentage identity: 63 %
  
BlastP bit score: 521
  
Sequence coverage: 95 %
  
E-value: 5e-180
  
  
 NCBI BlastP on this gene

EAA62069

Query: Architecture Search FASTA input

GL988041 : Chaetomium thermophilum var. thermophilum DSM 1495 unplaced genomic scaffold scf7180000...    Total score: 1.0     Cumulative Blast bit score: 516

Hit cluster cross-links:

Mycgr3G70471
  
Location: 0-405

Mycgr3G70471

Mycgr3G39149
  
Location: 505-1798

Mycgr3G39149

Mycgr3G92130
  
Location: 1898-2396

Mycgr3G92130

Mycgr3G38483
  
Location: 2496-3576

Mycgr3G38483

Mycgr3G108869
  
Location: 3676-5056

Mycgr3G108869

Mycgr3G103943
  
Location: 5156-5762

Mycgr3G103943

Mycgr3G57362
  
Location: 5862-7296

Mycgr3G57362

Mycgr3G39086
  
Location: 7396-8368

Mycgr3G39086

Mycgr3G103942
  
Location: 8468-8714

Mycgr3G103942

Mycgr3G108865
  
Location: 8814-10239

Mycgr3G108865

Mycgr3G70475
  
Location: 10339-11821

Mycgr3G70475

Mycgr3G108866
  
Location: 11921-13010

Mycgr3G108866

Mycgr3G92136
  
Location: 13110-13593

Mycgr3G92136

oligopeptide transporter-like protein
  
Accession: EGS20673
  
Location: 1547903-1550817
  
 NCBI BlastP on this gene

EGS20673

hypothetical protein
  
Accession: EGS20674
  
Location: 1552984-1554434
  
 NCBI BlastP on this gene

EGS20674

3-oxoacyl-[acyl-carrier-protein] synthase-like protein
  
Accession: EGS20675
  
Location: 1556547-1558010
  
  
**BlastP hit with Mycgr3G39149**
  
Percentage identity: 60 %
  
BlastP bit score: 516
  
Sequence coverage: 102 %
  
E-value: 8e-178
  
  
 NCBI BlastP on this gene

EGS20675

Query: Architecture Search FASTA input

KB644415 : Penicillium oxalicum 114-2 unplaced genomic scaffold scaffold\_8    Total score: 1.0     Cumulative Blast bit score: 515

Hit cluster cross-links:

Mycgr3G70471
  
Location: 0-405

Mycgr3G70471

Mycgr3G39149
  
Location: 505-1798

Mycgr3G39149

Mycgr3G92130
  
Location: 1898-2396

Mycgr3G92130

Mycgr3G38483
  
Location: 2496-3576

Mycgr3G38483

Mycgr3G108869
  
Location: 3676-5056

Mycgr3G108869

Mycgr3G103943
  
Location: 5156-5762

Mycgr3G103943

Mycgr3G57362
  
Location: 5862-7296

Mycgr3G57362

Mycgr3G39086
  
Location: 7396-8368

Mycgr3G39086

Mycgr3G103942
  
Location: 8468-8714

Mycgr3G103942

Mycgr3G108865
  
Location: 8814-10239

Mycgr3G108865

Mycgr3G70475
  
Location: 10339-11821

Mycgr3G70475

Mycgr3G108866
  
Location: 11921-13010

Mycgr3G108866

Mycgr3G92136
  
Location: 13110-13593

Mycgr3G92136

hypothetical protein
  
Accession: EPS34722
  
Location: 5032782-5035352
  
 NCBI BlastP on this gene

EPS34722

hypothetical protein
  
Accession: EPS34723
  
Location: 5036790-5038878
  
 NCBI BlastP on this gene

EPS34723

hypothetical protein
  
Accession: EPS34724
  
Location: 5039498-5040613
  
 NCBI BlastP on this gene

EPS34724

hypothetical protein
  
Accession: EPS34725
  
Location: 5041043-5042575
  
  
**BlastP hit with Mycgr3G70475**
  
Percentage identity: 55 %
  
BlastP bit score: 515
  
Sequence coverage: 102 %
  
E-value: 2e-175
  
  
 NCBI BlastP on this gene

EPS34725

hypothetical protein
  
Accession: EPS34726
  
Location: 5042830-5043051
  
 NCBI BlastP on this gene

EPS34726

hypothetical protein
  
Accession: EPS34727
  
Location: 5043102-5043423
  
 NCBI BlastP on this gene

EPS34727

hypothetical protein
  
Accession: EPS34728
  
Location: 5043647-5045146
  
 NCBI BlastP on this gene

EPS34728

hypothetical protein
  
Accession: EPS34729
  
Location: 5046330-5048624
  
 NCBI BlastP on this gene

EPS34729

hypothetical protein
  
Accession: EPS34730
  
Location: 5049324-5049962
  
 NCBI BlastP on this gene

EPS34730

hypothetical protein
  
Accession: EPS34731
  
Location: 5050761-5051861
  
 NCBI BlastP on this gene

EPS34731

Query: Architecture Search FASTA input

AM920437 : Penicillium chrysogenum Wisconsin 54-1255 complete genome, contig Pc00c22.    Total score: 1.0     Cumulative Blast bit score: 511

Hit cluster cross-links:

Mycgr3G70471
  
Location: 0-405

Mycgr3G70471

Mycgr3G39149
  
Location: 505-1798

Mycgr3G39149

Mycgr3G92130
  
Location: 1898-2396

Mycgr3G92130

Mycgr3G38483
  
Location: 2496-3576

Mycgr3G38483

Mycgr3G108869
  
Location: 3676-5056

Mycgr3G108869

Mycgr3G103943
  
Location: 5156-5762

Mycgr3G103943

Mycgr3G57362
  
Location: 5862-7296

Mycgr3G57362

Mycgr3G39086
  
Location: 7396-8368

Mycgr3G39086

Mycgr3G103942
  
Location: 8468-8714

Mycgr3G103942

Mycgr3G108865
  
Location: 8814-10239

Mycgr3G108865

Mycgr3G70475
  
Location: 10339-11821

Mycgr3G70475

Mycgr3G108866
  
Location: 11921-13010

Mycgr3G108866

Mycgr3G92136
  
Location: 13110-13593

Mycgr3G92136

not annotated
  
Accession: CAP98782
  
Location: 3505578-3508403
  
 NCBI BlastP on this gene

Pc22g14940

not annotated
  
Accession: Pc22g14930
  
Location: 3504697-3504990
  
 NCBI BlastP on this gene

Pc22g14930

not annotated
  
Accession: Pc22g14920
  
Location: 3503994-3504653
  
 NCBI BlastP on this gene

Pc22g14920

hypothetical protein
  
Accession: CAP98779
  
Location: 3502346-3503485
  
 NCBI BlastP on this gene

Pc22g14910

phenylacetyl-CoA ligase pclA-Penicillium chrysogenum
  
Accession: CAP98778
  
Location: 3500136-3502160
  
 NCBI BlastP on this gene

pclA

not annotated
  
Accession: CAP98777
  
Location: 3497681-3499228
  
  
**BlastP hit with Mycgr3G70475**
  
Percentage identity: 52 %
  
BlastP bit score: 511
  
Sequence coverage: 104 %
  
E-value: 7e-174
  
  
 NCBI BlastP on this gene

Pc22g14890

not annotated
  
Accession: CAP98776
  
Location: 3496913-3497239
  
 NCBI BlastP on this gene

Pc22g14880

not annotated
  
Accession: CAP98775
  
Location: 3494479-3496664
  
 NCBI BlastP on this gene

Pc22g14870

not annotated
  
Accession: CAP98774
  
Location: 3492106-3494274
  
 NCBI BlastP on this gene

Pc22g14860

not annotated
  
Accession: CAP98773
  
Location: 3490955-3491599
  
 NCBI BlastP on this gene

Pc22g14850

not annotated
  
Accession: CAP98772
  
Location: 3489038-3490169
  
 NCBI BlastP on this gene

Pc22g14840

Query: Architecture Search FASTA input

101. :  ACFW01000035 Coccidioides posadasii C735 delta SOWgp     Total score: 2.0     Cumulative Blast bit score: 384

Mycgr3G70471
  
Location: 0-405
  
 NCBI BlastP on this gene

Mycgr3G70471

Mycgr3G39149
  
Location: 505-1798
  
 NCBI BlastP on this gene

Mycgr3G39149

Mycgr3G92130
  
Location: 1898-2396
  
 NCBI BlastP on this gene

Mycgr3G92130

Mycgr3G38483
  
Location: 2496-3576
  
 NCBI BlastP on this gene

Mycgr3G38483

Mycgr3G108869
  
Location: 3676-5056
  
 NCBI BlastP on this gene

Mycgr3G108869

Mycgr3G103943
  
Location: 5156-5762
  
 NCBI BlastP on this gene

Mycgr3G103943

Mycgr3G57362
  
Location: 5862-7296
  
 NCBI BlastP on this gene

Mycgr3G57362

Mycgr3G39086
  
Location: 7396-8368
  
 NCBI BlastP on this gene

Mycgr3G39086

Mycgr3G103942
  
Location: 8468-8714
  
 NCBI BlastP on this gene

Mycgr3G103942

Mycgr3G108865
  
Location: 8814-10239
  
 NCBI BlastP on this gene

Mycgr3G108865

Mycgr3G70475
  
Location: 10339-11821
  
 NCBI BlastP on this gene

Mycgr3G70475

Mycgr3G108866
  
Location: 11921-13010
  
 NCBI BlastP on this gene

Mycgr3G108866

Mycgr3G92136
  
Location: 13110-13593
  
 NCBI BlastP on this gene

Mycgr3G92136

hypothetical protein
  
Accession: EER25763
  
Location: 340048-342209
  
 NCBI BlastP on this gene

EER25763

vacuolar ATP synthase subunit G, putative
  
Accession: EER25764
  
Location: 343564-344272
  
 NCBI BlastP on this gene

EER25764

hypothetical protein
  
Accession: EER25765
  
Location: 344579-344921
  
 NCBI BlastP on this gene

EER25765

hypothetical protein
  
Accession: EER25766
  
Location: 346045-348359
  
 NCBI BlastP on this gene

EER25766

hypothetical protein
  
Accession: EER25767
  
Location: 349925-351113
  
  
**BlastP hit with Mycgr3G38483**
  
Percentage identity: 35 %
  
BlastP bit score: 181
  
Sequence coverage: 91 %
  
E-value: 7e-50
  
  
 NCBI BlastP on this gene

EER25767

40S ribosomal protein S24, putative
  
Accession: EER25768
  
Location: 351443-352096
  
  
**BlastP hit with Mycgr3G70471**
  
Percentage identity: 77 %
  
BlastP bit score: 203
  
Sequence coverage: 91 %
  
E-value: 3e-64
  
  
 NCBI BlastP on this gene

EER25768

hypothetical protein
  
Accession: EER25769
  
Location: 357381-358920
  
 NCBI BlastP on this gene

EER25769

UDP-N-acetylglucosamine pyrophosphorylase, putative
  
Accession: EER25770
  
Location: 359500-361160
  
 NCBI BlastP on this gene

EER25770

102. :  ABSU01000001 Arthroderma benhamiae CBS 112371     Total score: 2.0     Cumulative Blast bit score: 384

hypothetical protein
  
Accession: EFE37142
  
Location: 2010914-2012599
  
 NCBI BlastP on this gene

EFE37142

hypothetical protein
  
Accession: EFE37143
  
Location: 2013341-2015033
  
 NCBI BlastP on this gene

EFE37143

hemagglutinin protein, putative
  
Accession: EFE37144
  
Location: 2018214-2019371
  
 NCBI BlastP on this gene

EFE37144

hypothetical protein
  
Accession: EFE37145
  
Location: 2020282-2021017
  
  
**BlastP hit with Mycgr3G70471**
  
Percentage identity: 64 %
  
BlastP bit score: 185
  
Sequence coverage: 103 %
  
E-value: 4e-57
  
  
 NCBI BlastP on this gene

EFE37145

hypothetical protein
  
Accession: EFE37146
  
Location: 2021389-2022586
  
  
**BlastP hit with Mycgr3G38483**
  
Percentage identity: 39 %
  
BlastP bit score: 199
  
Sequence coverage: 83 %
  
E-value: 2e-57
  
  
 NCBI BlastP on this gene

EFE37146

toxin biosynthesis ketoreductase, putative
  
Accession: EFE37147
  
Location: 2023773-2024549
  
 NCBI BlastP on this gene

EFE37147

hypothetical protein
  
Accession: EFE37148
  
Location: 2025755-2026210
  
 NCBI BlastP on this gene

EFE37148

hypothetical protein
  
Accession: EFE37149
  
Location: 2026261-2027283
  
 NCBI BlastP on this gene

EFE37149

hypothetical protein
  
Accession: EFE37150
  
Location: 2027949-2028944
  
 NCBI BlastP on this gene

EFE37150

hypothetical protein
  
Accession: EFE37151
  
Location: 2030116-2031050
  
 NCBI BlastP on this gene

EFE37151

103. :  EQ962660 Talaromyces stipitatus ATCC 10500 scf\_1105507295517 genomic scaffold     Total score: 2.0     Cumulative Blast bit score: 379

conserved hypothetical protein
  
Accession: EED12635
  
Location: 1705478-1706706
  
 NCBI BlastP on this gene

EED12635

2-amino-3-carboxymuconate-6-semialdehyde decarboxylase, putative
  
Accession: EED12636
  
Location: 1707367-1708323
  
 NCBI BlastP on this gene

EED12636

conserved hypothetical protein
  
Accession: EED12637
  
Location: 1708804-1711767
  
 NCBI BlastP on this gene

EED12637

37S ribosomal protein S24
  
Accession: EED12638
  
Location: 1712844-1713747
  
  
**BlastP hit with Mycgr3G70471**
  
Percentage identity: 82 %
  
BlastP bit score: 224
  
Sequence coverage: 97 %
  
E-value: 9e-73
  
  
 NCBI BlastP on this gene

EED12638

conserved hypothetical protein
  
Accession: EED12639
  
Location: 1714107-1715468
  
  
**BlastP hit with Mycgr3G38483**
  
Percentage identity: 35 %
  
BlastP bit score: 155
  
Sequence coverage: 85 %
  
E-value: 5e-40
  
  
 NCBI BlastP on this gene

EED12639

RNA polymerase I subunit Rpa43, putative
  
Accession: EED12640
  
Location: 1716664-1717983
  
 NCBI BlastP on this gene

EED12640

membrane associated DnaJ chaperone, putative
  
Accession: EED12641
  
Location: 1718274-1719473
  
 NCBI BlastP on this gene

EED12641

conserved hypothetical protein
  
Accession: EED12642
  
Location: 1720622-1721470
  
 NCBI BlastP on this gene

EED12642

alcohol dehydrogenase, putative
  
Accession: EED12644
  
Location: 1722133-1722972
  
 NCBI BlastP on this gene

EED12644

104. :  GL385404 Gaeumannomyces graminis var. tritici R3-111a-1 unplaced genomic scaffold supercont2.10     Total score: 2.0     Cumulative Blast bit score: 377

hypothetical protein
  
Accession: EJT69585
  
Location: 987635-989037
  
  
**BlastP hit with Mycgr3G38483**
  
Percentage identity: 36 %
  
BlastP bit score: 172
  
Sequence coverage: 101 %
  
E-value: 1e-46
  
  
 NCBI BlastP on this gene

EJT69585

40S ribosomal protein S24
  
Accession: EJT69586
  
Location: 989505-990379
  
  
**BlastP hit with Mycgr3G70471**
  
Percentage identity: 79 %
  
BlastP bit score: 205
  
Sequence coverage: 89 %
  
E-value: 5e-65
  
  
 NCBI BlastP on this gene

EJT69586

hypothetical protein
  
Accession: EJT69587
  
Location: 991092-991635
  
 NCBI BlastP on this gene

EJT69587

hypothetical protein
  
Accession: EJT69588
  
Location: 993106-994761
  
 NCBI BlastP on this gene

EJT69588

mannose-6-phosphate isomerase
  
Accession: EJT69589
  
Location: 995862-997302
  
 NCBI BlastP on this gene

EJT69589

105. :  GG692419 Ajellomyces capsulatus H143 genomic scaffold supercont2.1     Total score: 2.0     Cumulative Blast bit score: 377

conserved hypothetical protein
  
Accession: EER44478
  
Location: 300988-301872
  
 NCBI BlastP on this gene

EER44478

cytochrome c oxidase assembly protein
  
Accession: EER44479
  
Location: 302174-302534
  
 NCBI BlastP on this gene

EER44479

conserved hypothetical protein
  
Accession: EER44480
  
Location: 303790-306309
  
 NCBI BlastP on this gene

EER44480

conserved hypothetical protein
  
Accession: EER44481
  
Location: 309189-310157
  
  
**BlastP hit with Mycgr3G38483**
  
Percentage identity: 36 %
  
BlastP bit score: 175
  
Sequence coverage: 76 %
  
E-value: 3e-48
  
  
 NCBI BlastP on this gene

EER44481

40S ribosomal protein S24
  
Accession: EER44482
  
Location: 310453-311106
  
  
**BlastP hit with Mycgr3G70471**
  
Percentage identity: 78 %
  
BlastP bit score: 202
  
Sequence coverage: 90 %
  
E-value: 4e-64
  
  
 NCBI BlastP on this gene

EER44482

predicted protein
  
Accession: EER44483
  
Location: 312049-313628
  
 NCBI BlastP on this gene

EER44483

UDP-N-acetylglucosamine pyrophosphorylase
  
Accession: EER44484
  
Location: 316548-317518
  
 NCBI BlastP on this gene

EER44484

106. :  KE375214 Blumeria graminis f. sp. tritici 96224 unplaced genomic scaffold Scaffold-88     Total score: 2.0     Cumulative Blast bit score: 356

hypothetical protein
  
Accession: EPQ61759
  
Location: 367697-369130
  
  
**BlastP hit with Mycgr3G38483**
  
Percentage identity: 34 %
  
BlastP bit score: 160
  
Sequence coverage: 89 %
  
E-value: 7e-42
  
  
 NCBI BlastP on this gene

EPQ61759

Protein component of the small (40S) ribosomal subunit
  
Accession: EPQ61760
  
Location: 369529-370161
  
  
**BlastP hit with Mycgr3G70471**
  
Percentage identity: 77 %
  
BlastP bit score: 196
  
Sequence coverage: 88 %
  
E-value: 2e-61
  
  
 NCBI BlastP on this gene

EPQ61760

107. :  CAUH01002404 Blumeria graminis f. sp. hordei DH14     Total score: 2.0     Cumulative Blast bit score: 355

Putative methyltransferase
  
Accession: CCU76365
  
Location: 55927-57360
  
  
**BlastP hit with Mycgr3G38483**
  
Percentage identity: 34 %
  
BlastP bit score: 159
  
Sequence coverage: 91 %
  
E-value: 2e-41
  
  
 NCBI BlastP on this gene

CCU76365

40S ribosomal protein S24
  
Accession: CCU76366
  
Location: 57758-58392
  
  
**BlastP hit with Mycgr3G70471**
  
Percentage identity: 77 %
  
BlastP bit score: 196
  
Sequence coverage: 88 %
  
E-value: 2e-61
  
  
 NCBI BlastP on this gene

CCU76366

CSEP0353 putative effector protein
  
Accession: CCU76367
  
Location: 62665-63168
  
 NCBI BlastP on this gene

CCU76367

ATP-dependent helicase NAM7
  
Accession: CCU76368
  
Location: 64429-67905
  
 NCBI BlastP on this gene

CCU76368

108. :  AACD01000169 Aspergillus nidulans FGSC A4     Total score: 2.0     Cumulative Blast bit score: 353

hypothetical protein
  
Accession: EAA61928
  
Location: 122158-123763
  
 NCBI BlastP on this gene

EAA61928

predicted protein
  
Accession: EAA61929
  
Location: 127023-128628
  
 NCBI BlastP on this gene

EAA61929

hypothetical protein
  
Accession: EAA61930
  
Location: 131040-131675
  
  
**BlastP hit with Mycgr3G70471**
  
Percentage identity: 78 %
  
BlastP bit score: 216
  
Sequence coverage: 97 %
  
E-value: 3e-69
  
  
 NCBI BlastP on this gene

EAA61930

hypothetical protein
  
Accession: EAA61931
  
Location: 132338-133610
  
  
**BlastP hit with Mycgr3G38483**
  
Percentage identity: 35 %
  
BlastP bit score: 137
  
Sequence coverage: 71 %
  
E-value: 2e-34
  
  
 NCBI BlastP on this gene

EAA61931

hypothetical protein
  
Accession: EAA61932
  
Location: 133840-139572
  
 NCBI BlastP on this gene

EAA61932

hypothetical protein
  
Accession: EAA61933
  
Location: 141098-141980
  
 NCBI BlastP on this gene

EAA61933

109. :  CH476658 Ajellomyces capsulatus NAm1 scaffold\_4 genomic scaffold     Total score: 2.0     Cumulative Blast bit score: 349

UDP-N-acetylglucosamine pyrophosphorylase
  
Accession: EDN07906
  
Location: 1356245-1358009
  
 NCBI BlastP on this gene

EDN07906

hypothetical protein
  
Accession: EDN07907
  
Location: 1358914-1360154
  
 NCBI BlastP on this gene

EDN07907

40S ribosomal protein S24
  
Accession: EDN07908
  
Location: 1365457-1366103
  
  
**BlastP hit with Mycgr3G70471**
  
Percentage identity: 78 %
  
BlastP bit score: 202
  
Sequence coverage: 90 %
  
E-value: 4e-64
  
  
 NCBI BlastP on this gene

EDN07908

conserved hypothetical protein
  
Accession: EDN07909
  
Location: 1366521-1367351
  
  
**BlastP hit with Mycgr3G38483**
  
Percentage identity: 38 %
  
BlastP bit score: 147
  
Sequence coverage: 65 %
  
E-value: 3e-38
  
  
 NCBI BlastP on this gene

EDN07909

predicted protein
  
Accession: EDN07910
  
Location: 1370274-1372564
  
 NCBI BlastP on this gene

EDN07910

conserved hypothetical protein
  
Accession: EDN07911
  
Location: 1373995-1374355
  
 NCBI BlastP on this gene

EDN07911

predicted protein
  
Accession: EDN07912
  
Location: 1374669-1375553
  
 NCBI BlastP on this gene

EDN07912

110. :  DS995769 Trichophyton equinum CBS 127.97 supercont1.52 genomic scaffold     Total score: 2.0     Cumulative Blast bit score: 346

UDP-N-acetylglucosamine pyrophosphorylase
  
Accession: EGE07945
  
Location: 8232-9919
  
 NCBI BlastP on this gene

EGE07945

peptidyl-prolyl cis-trans isomerase-like 4
  
Accession: EGE07946
  
Location: 10709-12248
  
 NCBI BlastP on this gene

EGE07946

hypothetical protein
  
Accession: EGE07947
  
Location: 15283-16850
  
 NCBI BlastP on this gene

EGE07947

40S ribosomal protein S24
  
Accession: EGE07948
  
Location: 17756-18506
  
  
**BlastP hit with Mycgr3G70471**
  
Percentage identity: 72 %
  
BlastP bit score: 194
  
Sequence coverage: 90 %
  
E-value: 1e-60
  
  
 NCBI BlastP on this gene

EGE07948

hypothetical protein
  
Accession: EGE07949
  
Location: 18987-20278
  
  
**BlastP hit with Mycgr3G38483**
  
Percentage identity: 31 %
  
BlastP bit score: 152
  
Sequence coverage: 88 %
  
E-value: 9e-40
  
  
 NCBI BlastP on this gene

EGE07949

toxin biosynthesis ketoreductase
  
Accession: EGE07950
  
Location: 21381-22157
  
 NCBI BlastP on this gene

EGE07950

tripeptidyl peptidase SED3
  
Accession: EGE07951
  
Location: 23310-25176
  
 NCBI BlastP on this gene

EGE07951

hypothetical protein
  
Accession: EGE07952
  
Location: 25499-26494
  
 NCBI BlastP on this gene

EGE07952

proteasome regulatory particle subunit
  
Accession: EGE07953
  
Location: 27663-28595
  
 NCBI BlastP on this gene

EGE07953

111. :  CH476595 Aspergillus terreus NIH2624 scaffold\_2 genomic scaffold     Total score: 2.0     Cumulative Blast bit score: 323

predicted protein
  
Accession: EAU38601
  
Location: 2411969-2412850
  
 NCBI BlastP on this gene

EAU38601

conserved hypothetical protein
  
Accession: EAU38602
  
Location: 2414235-2416580
  
 NCBI BlastP on this gene

EAU38602

conserved hypothetical protein
  
Accession: EAU38603
  
Location: 2417729-2419873
  
 NCBI BlastP on this gene

EAU38603

conserved hypothetical protein
  
Accession: EAU38604
  
Location: 2420447-2421161
  
  
**BlastP hit with Mycgr3G38483**
  
Percentage identity: 35 %
  
BlastP bit score: 102
  
Sequence coverage: 53 %
  
E-value: 4e-22
  
  
 NCBI BlastP on this gene

EAU38604

40S ribosomal protein S24
  
Accession: EAU38605
  
Location: 2421678-2422328
  
  
**BlastP hit with Mycgr3G70471**
  
Percentage identity: 81 %
  
BlastP bit score: 221
  
Sequence coverage: 97 %
  
E-value: 1e-71
  
  
 NCBI BlastP on this gene

EAU38605

predicted protein
  
Accession: EAU38606
  
Location: 2424127-2425683
  
 NCBI BlastP on this gene

EAU38606

hypothetical protein
  
Accession: EAU38607
  
Location: 2428237-2430179
  
 NCBI BlastP on this gene

EAU38607

112. :  AQGS01000244 Dactylellina haptotyla CBS 200.50     Total score: 2.0     Cumulative Blast bit score: 316

hypothetical protein
  
Accession: EPS41414
  
Location: 54282-55410
  
 NCBI BlastP on this gene

EPS41414

hypothetical protein
  
Accession: EPS41400
  
Location: 56884-57393
  
 NCBI BlastP on this gene

EPS41400

hypothetical protein
  
Accession: EPS41410
  
Location: 58075-60776
  
 NCBI BlastP on this gene

EPS41410

hypothetical protein
  
Accession: EPS41399
  
Location: 61436-62713
  
 NCBI BlastP on this gene

EPS41399

hypothetical protein
  
Accession: EPS41402
  
Location: 63187-64195
  
  
**BlastP hit with Mycgr3G70471**
  
Percentage identity: 77 %
  
BlastP bit score: 198
  
Sequence coverage: 90 %
  
E-value: 2e-62
  
  
 NCBI BlastP on this gene

EPS41402

hypothetical protein
  
Accession: EPS41406
  
Location: 64704-65832
  
  
**BlastP hit with Mycgr3G38483**
  
Percentage identity: 29 %
  
BlastP bit score: 118
  
Sequence coverage: 101 %
  
E-value: 5e-27
  
  
 NCBI BlastP on this gene

EPS41406

113. :  ADOT01000287 Arthrobotrys oligospora ATCC 24927     Total score: 2.0     Cumulative Blast bit score: 311

hypothetical protein
  
Accession: EGX44326
  
Location: 170584-172544
  
 NCBI BlastP on this gene

EGX44326

hypothetical protein
  
Accession: EGX44327
  
Location: 173010-173998
  
 NCBI BlastP on this gene

EGX44327

hypothetical protein
  
Accession: EGX44328
  
Location: 174339-174581
  
 NCBI BlastP on this gene

EGX44328

hypothetical protein
  
Accession: EGX44329
  
Location: 175159-179622
  
 NCBI BlastP on this gene

EGX44329

hypothetical protein
  
Accession: EGX44330
  
Location: 180049-181179
  
  
**BlastP hit with Mycgr3G38483**
  
Percentage identity: 33 %
  
BlastP bit score: 119
  
Sequence coverage: 77 %
  
E-value: 3e-27
  
  
 NCBI BlastP on this gene

EGX44330

hypothetical protein
  
Accession: EGX44331
  
Location: 181677-182520
  
  
**BlastP hit with Mycgr3G70471**
  
Percentage identity: 76 %
  
BlastP bit score: 192
  
Sequence coverage: 90 %
  
E-value: 7e-60
  
  
 NCBI BlastP on this gene

EGX44331

hypothetical protein
  
Accession: EGX44332
  
Location: 182962-184230
  
 NCBI BlastP on this gene

EGX44332

hypothetical protein
  
Accession: EGX44333
  
Location: 184862-187481
  
 NCBI BlastP on this gene

EGX44333

hypothetical protein
  
Accession: EGX44334
  
Location: 187909-188526
  
 NCBI BlastP on this gene

EGX44334

114. :  GG704911 Coccidioides immitis RS genomic scaffold supercont3.1     Total score: 1.0     Cumulative Blast bit score: 703

guanyl-nucleotide exchange factor
  
Accession: EAS35511
  
Location: 6364855-6369178
  
 NCBI BlastP on this gene

EAS35511

zinc carboxypeptidase
  
Accession: EAS35510
  
Location: 6370655-6372470
  
 NCBI BlastP on this gene

EAS35510

hypothetical protein
  
Accession: EAS35509
  
Location: 6372995-6373393
  
 NCBI BlastP on this gene

EAS35509

Ni2+-Co2+ transporter (NiCoT) family transition metal uptake transporter, variant
  
Accession: EJB10688
  
Location: 6374389-6376431
  
  
**BlastP hit with Mycgr3G108865**
  
Percentage identity: 55 %
  
BlastP bit score: 399
  
Sequence coverage: 86 %
  
E-value: 1e-131
  
  
 NCBI BlastP on this gene

EJB10688

Ni2+-Co2+ transporter (NiCoT) family transition metal uptake transporter
  
Accession: EJB10689
  
Location: 6375203-6376431
  
  
**BlastP hit with Mycgr3G108865**
  
Percentage identity: 54 %
  
BlastP bit score: 304
  
Sequence coverage: 69 %
  
E-value: 6e-96
  
  
 NCBI BlastP on this gene

EJB10689

hypothetical protein
  
Accession: EAS35506
  
Location: 6377009-6379591
  
 NCBI BlastP on this gene

EAS35506

hypothetical protein
  
Accession: EJB10690
  
Location: 6381865-6382892
  
 NCBI BlastP on this gene

EJB10690

115. :  KB446538 Dothistroma septosporum NZE10 unplaced genomic scaffold DOTSEscaffold\_4     Total score: 1.0     Cumulative Blast bit score: 677

hypothetical protein
  
Accession: EME45622
  
Location: 1933381-1935369
  
 NCBI BlastP on this gene

EME45622

hypothetical protein
  
Accession: EME45621
  
Location: 1930154-1931875
  
 NCBI BlastP on this gene

EME45621

hypothetical protein
  
Accession: EME45620
  
Location: 1925925-1928112
  
  
**BlastP hit with Mycgr3G70475**
  
Percentage identity: 66 %
  
BlastP bit score: 677
  
Sequence coverage: 103 %
  
E-value: 0.0
  
  
 NCBI BlastP on this gene

EME45620

hypothetical protein
  
Accession: EME45618
  
Location: 1923210-1925046
  
 NCBI BlastP on this gene

EME45618

hypothetical protein
  
Accession: EME45617
  
Location: 1920929-1921210
  
 NCBI BlastP on this gene

EME45617

hypothetical protein
  
Accession: EME45616
  
Location: 1920102-1920635
  
 NCBI BlastP on this gene

EME45616

hypothetical protein
  
Accession: EME45615
  
Location: 1916856-1917959
  
 NCBI BlastP on this gene

EME45615

116. :  AM270040 Aspergillus niger contig An02c0460, genomic contig.     Total score: 1.0     Cumulative Blast bit score: 607

not annotated
  
Accession: CAK48850
  
Location: 71963-74020
  
 NCBI BlastP on this gene

An02g14190

not annotated
  
Accession: CAK48851
  
Location: 76446-77460
  
 NCBI BlastP on this gene

An02g14200

unnamed
  
Accession: CAK48852
  
Location: 78504-79887
  
 NCBI BlastP on this gene

An02g14210

unnamed
  
Accession: CAK48853
  
Location: 80462-82249
  
  
**BlastP hit with Mycgr3G39149**
  
Percentage identity: 66 %
  
BlastP bit score: 607
  
Sequence coverage: 99 %
  
E-value: 0.0
  
  
 NCBI BlastP on this gene

An02g14220

117. :  JH921428 Marssonina brunnea f. sp. 'multigermtubi' MB\_m1 unplaced genomic scaffold M6\_S00001     Total score: 1.0     Cumulative Blast bit score: 605

UPF0047 domain protein
  
Accession: EKD21284
  
Location: 2547541-2548170
  
 NCBI BlastP on this gene

EKD21284

hypothetical protein
  
Accession: EKD21285
  
Location: 2549660-2550654
  
 NCBI BlastP on this gene

EKD21285

OPT family small oligopeptide transporter
  
Accession: EKD21286
  
Location: 2552003-2555024
  
 NCBI BlastP on this gene

EKD21286

3-oxoacyl-[acyl-carrier-protein]-synthase
  
Accession: EKD21287
  
Location: 2556121-2557521
  
  
**BlastP hit with Mycgr3G39149**
  
Percentage identity: 67 %
  
BlastP bit score: 605
  
Sequence coverage: 100 %
  
E-value: 0.0
  
  
 NCBI BlastP on this gene

EKD21287

118. :  DS027696 Neosartorya fischeri NRRL 181 1099437636264 genomic scaffold     Total score: 1.0     Cumulative Blast bit score: 602

siderochrome-iron transporter MirC
  
Accession: EAW18298
  
Location: 2930508-2932571
  
 NCBI BlastP on this gene

EAW18298

Rho GTPase ModA, putative
  
Accession: EAW18299
  
Location: 2935873-2936852
  
 NCBI BlastP on this gene

EAW18299

agmatinase, putative
  
Accession: EAW18300
  
Location: 2937930-2939294
  
 NCBI BlastP on this gene

EAW18300

beta-ketoacyl synthase (Cem1), putative
  
Accession: EAW18301
  
Location: 2939827-2941753
  
  
**BlastP hit with Mycgr3G39149**
  
Percentage identity: 65 %
  
BlastP bit score: 602
  
Sequence coverage: 99 %
  
E-value: 0.0
  
  
 NCBI BlastP on this gene

EAW18301

SAGA complex component (Sgf73), putative
  
Accession: EAW18302
  
Location: 2942146-2943567
  
 NCBI BlastP on this gene

EAW18302

mRNA capping enzyme alpha subunit, putative
  
Accession: EAW18303
  
Location: 2944261-2945508
  
 NCBI BlastP on this gene

EAW18303

oligosaccharyl transferase subunit (alpha), putative
  
Accession: EAW18304
  
Location: 2945918-2947621
  
 NCBI BlastP on this gene

EAW18304

IdgA domain protein
  
Accession: EAW18305
  
Location: 2948052-2950776
  
 NCBI BlastP on this gene

EAW18305

119. :  AM920437 Penicillium chrysogenum Wisconsin 54-1255 complete genome, contig Pc00c22.     Total score: 1.0     Cumulative Blast bit score: 593

not annotated
  
Accession: CAP98187
  
Location: 2131087-2133146
  
 NCBI BlastP on this gene

Pc22g08990

hypothetical protein
  
Accession: CAP98188
  
Location: 2133380-2135046
  
 NCBI BlastP on this gene

Pc22g09000

not annotated
  
Accession: CAP98189
  
Location: 2135333-2136160
  
 NCBI BlastP on this gene

Pc22g09010

unnamed
  
Accession: CAP98190
  
Location: 2137414-2138774
  
 NCBI BlastP on this gene

Pc22g09020

unnamed
  
Accession: CAP98191
  
Location: 2139003-2141024
  
  
**BlastP hit with Mycgr3G39149**
  
Percentage identity: 65 %
  
BlastP bit score: 593
  
Sequence coverage: 99 %
  
E-value: 0.0
  
  
 NCBI BlastP on this gene

Pc22g09030

not annotated
  
Accession: CAP98192
  
Location: 2141461-2142830
  
 NCBI BlastP on this gene

Pc22g09040

not annotated
  
Accession: CAP98193
  
Location: 2143543-2144787
  
 NCBI BlastP on this gene

Pc22g09050

not annotated
  
Accession: CAP98194
  
Location: 2145221-2145304
  
 NCBI BlastP on this gene

Pc22g09060

not annotated
  
Accession: CAP98195
  
Location: 2145490-2147267
  
 NCBI BlastP on this gene

Pc22g09070

hypothetical protein
  
Accession: CAP98196
  
Location: 2147341-2148854
  
 NCBI BlastP on this gene

Pc22g09080

not annotated
  
Accession: CAP98197
  
Location: 2149190-2153376
  
 NCBI BlastP on this gene

Pc22g09090

120. :  DS572755 Paracoccidioides brasiliensis Pb18 supercont1.6 genomic scaffold     Total score: 1.0     Cumulative Blast bit score: 588

3-oxoacyl-[acyl-carrier-protein] synthase
  
Accession: EEH49288
  
Location: 944577-946417
  
  
**BlastP hit with Mycgr3G39149**
  
Percentage identity: 65 %
  
BlastP bit score: 588
  
Sequence coverage: 99 %
  
E-value: 0.0
  
  
 NCBI BlastP on this gene

EEH49288

conserved hypothetical protein
  
Accession: EEH49287
  
Location: 942193-944030
  
 NCBI BlastP on this gene

EEH49287

mRNA-capping enzyme subunit alpha
  
Accession: EEH49286
  
Location: 940212-941687
  
 NCBI BlastP on this gene

EEH49286

DNA ligase
  
Accession: EEH49285
  
Location: 936470-939659
  
 NCBI BlastP on this gene

EEH49285

121. :  GG749429 Ajellomyces dermatitidis ATCC 18188 genomic scaffold supercont1.23     Total score: 1.0     Cumulative Blast bit score: 582

beta-ketoacyl synthase
  
Accession: EGE81978
  
Location: 616867-618774
  
  
**BlastP hit with Mycgr3G39149**
  
Percentage identity: 64 %
  
BlastP bit score: 582
  
Sequence coverage: 99 %
  
E-value: 0.0
  
  
 NCBI BlastP on this gene

EGE81978

SAGA complex component
  
Accession: EGE81977
  
Location: 614753-616525
  
 NCBI BlastP on this gene

EGE81977

mRNA capping enzyme alpha subunit
  
Accession: EGE81976
  
Location: 612725-614029
  
 NCBI BlastP on this gene

EGE81976

DNA ligase
  
Accession: EGE81975
  
Location: 608703-612199
  
 NCBI BlastP on this gene

EGE81975

122. :  GG657469 Ajellomyces dermatitidis SLH14081 genomic scaffold supercont1.22     Total score: 1.0     Cumulative Blast bit score: 582

small GTPase
  
Accession: EEQ73823
  
Location: 71727-72697
  
 NCBI BlastP on this gene

EEQ73823

agmatinase
  
Accession: EEQ73824
  
Location: 75214-76525
  
 NCBI BlastP on this gene

EEQ73824

conserved hypothetical protein
  
Accession: EEQ73825
  
Location: 77970-78485
  
 NCBI BlastP on this gene

EEQ73825

beta-ketoacyl synthase
  
Accession: EEQ73826
  
Location: 79141-81052
  
  
**BlastP hit with Mycgr3G39149**
  
Percentage identity: 64 %
  
BlastP bit score: 582
  
Sequence coverage: 99 %
  
E-value: 0.0
  
  
 NCBI BlastP on this gene

EEQ73826

SAGA complex component
  
Accession: EEQ73827
  
Location: 81394-83166
  
 NCBI BlastP on this gene

EEQ73827

mRNA capping enzyme alpha subunit
  
Accession: EEQ73828
  
Location: 83897-85201
  
 NCBI BlastP on this gene

EEQ73828

DNA ligase Cdc9
  
Accession: EEQ73829
  
Location: 85727-89223
  
 NCBI BlastP on this gene

EEQ73829

123. :  EQ999973 Ajellomyces dermatitidis ER-3 genomic scaffold supercont1.1     Total score: 1.0     Cumulative Blast bit score: 582

predicted protein
  
Accession: EEQ83290
  
Location: 911297-911884
  
 NCBI BlastP on this gene

EEQ83290

small GTPase
  
Accession: EEQ83291
  
Location: 912328-913298
  
 NCBI BlastP on this gene

EEQ83291

agmatinase
  
Accession: EEQ83292
  
Location: 915784-917095
  
 NCBI BlastP on this gene

EEQ83292

conserved hypothetical protein
  
Accession: EEQ83293
  
Location: 918557-919072
  
 NCBI BlastP on this gene

EEQ83293

beta-ketoacyl synthase
  
Accession: EEQ83294
  
Location: 919716-921620
  
  
**BlastP hit with Mycgr3G39149**
  
Percentage identity: 64 %
  
BlastP bit score: 582
  
Sequence coverage: 99 %
  
E-value: 0.0
  
  
 NCBI BlastP on this gene

EEQ83294

SAGA complex component
  
Accession: EEQ83295
  
Location: 921962-923734
  
 NCBI BlastP on this gene

EEQ83295

mRNA capping enzyme alpha subunit
  
Accession: EEQ83296
  
Location: 924458-925762
  
 NCBI BlastP on this gene

EEQ83296

DNA ligase Cdc9
  
Accession: EEQ83297
  
Location: 926288-929775
  
 NCBI BlastP on this gene

EEQ83297

124. :  GL636500 Coccidioides posadasii str. Silveira unplaced genomic scaffold supercont2.15     Total score: 1.0     Cumulative Blast bit score: 579

conserved hypothetical protein
  
Accession: EFW15561
  
Location: 187321-188754
  
 NCBI BlastP on this gene

EFW15561

conserved hypothetical protein
  
Accession: EFW15562
  
Location: 190162-190830
  
 NCBI BlastP on this gene

EFW15562

cell division control protein 42
  
Accession: EFW15563
  
Location: 193574-194479
  
 NCBI BlastP on this gene

EFW15563

agmatinase
  
Accession: EFW15564
  
Location: 195323-196644
  
 NCBI BlastP on this gene

EFW15564

beta-ketoacyl synthase
  
Accession: EFW15565
  
Location: 197143-198914
  
  
**BlastP hit with Mycgr3G39149**
  
Percentage identity: 65 %
  
BlastP bit score: 579
  
Sequence coverage: 100 %
  
E-value: 0.0
  
  
 NCBI BlastP on this gene

EFW15565

phosphoribosylaminoimidazole carboxylase
  
Accession: EFW15566
  
Location: 199115-201002
  
 NCBI BlastP on this gene

EFW15566

predicted protein
  
Accession: EFW15567
  
Location: 201947-202347
  
 NCBI BlastP on this gene

EFW15567

SAGA complex component
  
Accession: EFW15568
  
Location: 202451-203952
  
 NCBI BlastP on this gene

EFW15568

mRNA capping enzyme alpha subunit
  
Accession: EFW15569
  
Location: 204559-205788
  
 NCBI BlastP on this gene

EFW15569

DNA ligase
  
Accession: EFW15570
  
Location: 206443-209673
  
 NCBI BlastP on this gene

EFW15570

125. :  GG704911 Coccidioides immitis RS genomic scaffold supercont3.1     Total score: 1.0     Cumulative Blast bit score: 579

beta-ketoacyl-acyl-carrier-protein synthase II
  
Accession: EAS35483
  
Location: 6441972-6443742
  
  
**BlastP hit with Mycgr3G39149**
  
Percentage identity: 65 %
  
BlastP bit score: 579
  
Sequence coverage: 100 %
  
E-value: 0.0
  
  
 NCBI BlastP on this gene

EAS35483

phosphoribosylaminoimidazole carboxylase, ATPase subunit
  
Accession: EAS35484
  
Location: 6439877-6441764
  
 NCBI BlastP on this gene

EAS35484

SAGA complex component
  
Accession: EAS35485
  
Location: 6436929-6438429
  
 NCBI BlastP on this gene

EAS35485

mRNA capping enzyme alpha subunit
  
Accession: EAS35486
  
Location: 6435094-6436323
  
 NCBI BlastP on this gene

EAS35486

DNA ligase I, ATP-dependent (dnl1)
  
Accession: EAS35487
  
Location: 6431201-6434441
  
 NCBI BlastP on this gene

EAS35487

126. :  DS499595 Aspergillus fumigatus A1163 scf\_000002 genomic scaffold     Total score: 1.0     Cumulative Blast bit score: 578

siderochrome-iron transporter MirC
  
Accession: EDP54224
  
Location: 1599437-1601504
  
 NCBI BlastP on this gene

EDP54224

Rho GTPase ModA, putative
  
Accession: EDP54225
  
Location: 1604613-1605565
  
 NCBI BlastP on this gene

EDP54225

agmatinase, putative
  
Accession: EDP54226
  
Location: 1606644-1608008
  
 NCBI BlastP on this gene

EDP54226

beta-ketoacyl synthase (Cem1), putative
  
Accession: EDP54227
  
Location: 1608526-1610453
  
  
**BlastP hit with Mycgr3G39149**
  
Percentage identity: 60 %
  
BlastP bit score: 578
  
Sequence coverage: 108 %
  
E-value: 0.0
  
  
 NCBI BlastP on this gene

EDP54227

SAGA complex component (Sgf73), putative
  
Accession: EDP54228
  
Location: 1610835-1612254
  
 NCBI BlastP on this gene

EDP54228

mRNA capping enzyme alpha subunit, putative
  
Accession: EDP54229
  
Location: 1612955-1614202
  
 NCBI BlastP on this gene

EDP54229

oligosaccharyl transferase subunit (alpha), putative
  
Accession: EDP54230
  
Location: 1614621-1616296
  
 NCBI BlastP on this gene

EDP54230

IdgA domain protein
  
Accession: EDP54231
  
Location: 1616729-1619459
  
 NCBI BlastP on this gene

EDP54231

127. :  DS544809 Paracoccidioides brasiliensis Pb03 supercont1.7 genomic scaffold     Total score: 1.0     Cumulative Blast bit score: 576

predicted protein
  
Accession: EEH22534
  
Location: 479094-480268
  
 NCBI BlastP on this gene

EEH22534

cell division control protein
  
Accession: EEH22533
  
Location: 474533-475609
  
 NCBI BlastP on this gene

EEH22533

arginase family protein
  
Accession: EEH22532
  
Location: 471407-472918
  
 NCBI BlastP on this gene

EEH22532

3-oxoacyl-(acyl-carrier-protein) synthase
  
Accession: EEH22531
  
Location: 469333-471170
  
  
**BlastP hit with Mycgr3G39149**
  
Percentage identity: 62 %
  
BlastP bit score: 576
  
Sequence coverage: 104 %
  
E-value: 0.0
  
  
 NCBI BlastP on this gene

EEH22531

conserved hypothetical protein
  
Accession: EEH22530
  
Location: 466967-468789
  
 NCBI BlastP on this gene

EEH22530

mRNA-capping enzyme subunit alpha
  
Accession: EEH22529
  
Location: 464990-466204
  
 NCBI BlastP on this gene

EEH22529

DNA ligase
  
Accession: EEH22528
  
Location: 461210-464232
  
 NCBI BlastP on this gene

EEH22528

128. :  DF126451 Aspergillus kawachii IFO 4308 DNA, contig: scaffold00005     Total score: 1.0     Cumulative Blast bit score: 574

siderochrome-iron transporter
  
Accession: GAA84239
  
Location: 187421-189478
  
 NCBI BlastP on this gene

GAA84239

cell division control protein 42
  
Accession: GAA84238
  
Location: 183875-184888
  
 NCBI BlastP on this gene

GAA84238

agmatinase
  
Accession: GAA84237
  
Location: 181358-182748
  
 NCBI BlastP on this gene

GAA84237

beta-ketoacyl synthase
  
Accession: GAA84236
  
Location: 179001-180703
  
  
**BlastP hit with Mycgr3G39149**
  
Percentage identity: 64 %
  
BlastP bit score: 574
  
Sequence coverage: 95 %
  
E-value: 0.0
  
  
 NCBI BlastP on this gene

GAA84236

SAGA complex component
  
Accession: GAA84235
  
Location: 176749-178273
  
 NCBI BlastP on this gene

GAA84235

mRNA capping enzyme alpha subunit
  
Accession: GAA84234
  
Location: 174866-176095
  
 NCBI BlastP on this gene

GAA84234

129. :  GL891304 Neurospora tetrasperma FGSC 2508 unplaced genomic scaffold NEUTE1scaffold\_3     Total score: 1.0     Cumulative Blast bit score: 573

hypothetical protein
  
Accession: EGO57744
  
Location: 2116110-2118036
  
 NCBI BlastP on this gene

EGO57744

hypothetical protein
  
Accession: EGO57745
  
Location: 2118506-2119498
  
 NCBI BlastP on this gene

EGO57745

hypothetical protein
  
Accession: EGO57746
  
Location: 2121429-2122282
  
 NCBI BlastP on this gene

EGO57746

hypothetical protein
  
Accession: EGO57747
  
Location: 2122474-2125394
  
 NCBI BlastP on this gene

EGO57747

3-oxoacyl--synthase
  
Accession: EGO57748
  
Location: 2126386-2127830
  
  
**BlastP hit with Mycgr3G39149**
  
Percentage identity: 63 %
  
BlastP bit score: 573
  
Sequence coverage: 101 %
  
E-value: 0.0
  
  
 NCBI BlastP on this gene

EGO57748

hypothetical protein
  
Accession: EGO57749
  
Location: 2128617-2130778
  
 NCBI BlastP on this gene

EGO57749

hypothetical protein
  
Accession: EGO57750
  
Location: 2131025-2136234
  
 NCBI BlastP on this gene

EGO57750

130. :  GL891236 Neurospora tetrasperma FGSC 2509 unplaced genomic scaffold NEUTE2scaffold\_4     Total score: 1.0     Cumulative Blast bit score: 573

3-oxoacyl--synthase
  
Accession: EGZ71980
  
Location: 2872111-2873555
  
  
**BlastP hit with Mycgr3G39149**
  
Percentage identity: 63 %
  
BlastP bit score: 573
  
Sequence coverage: 101 %
  
E-value: 0.0
  
  
 NCBI BlastP on this gene

EGZ71980

hypothetical protein
  
Accession: EGZ71979
  
Location: 2870091-2871324
  
 NCBI BlastP on this gene

EGZ71979

hypothetical protein
  
Accession: EGZ71978
  
Location: 2864639-2869844
  
 NCBI BlastP on this gene

EGZ71978

hypothetical protein
  
Accession: EGZ71977
  
Location: 2861201-2863751
  
 NCBI BlastP on this gene

EGZ71977

131. :  ACJE01000010 Aspergillus niger ATCC 1015     Total score: 1.0     Cumulative Blast bit score: 573

hypothetical protein
  
Accession: EHA23590
  
Location: 3567181-3569236
  
 NCBI BlastP on this gene

EHA23590

Rho type ras-related small GTPase
  
Accession: EHA23591
  
Location: 3571664-3572777
  
 NCBI BlastP on this gene

EHA23591

hypothetical protein
  
Accession: EHA23592
  
Location: 3573721-3575104
  
 NCBI BlastP on this gene

EHA23592

3-oxoacyl--synthase
  
Accession: EHA23593
  
Location: 3575679-3577466
  
  
**BlastP hit with Mycgr3G39149**
  
Percentage identity: 63 %
  
BlastP bit score: 573
  
Sequence coverage: 99 %
  
E-value: 0.0
  
  
 NCBI BlastP on this gene

EHA23593

132. :  DS995900 Penicillium marneffei ATCC 18224 scf\_1105668340758 genomic scaffold     Total score: 1.0     Cumulative Blast bit score: 571

siderochrome-iron transporter MirC
  
Accession: EEA26054
  
Location: 2661544-2663572
  
 NCBI BlastP on this gene

EEA26054

Rho GTPase ModA, putative
  
Accession: EEA26052
  
Location: 2659563-2660278
  
 NCBI BlastP on this gene

EEA26052

agmatinase, putative
  
Accession: EEA26050
  
Location: 2656455-2657821
  
 NCBI BlastP on this gene

EEA26050

beta-ketoacyl synthase (Cem1), putative
  
Accession: EEA26049
  
Location: 2653965-2655649
  
  
**BlastP hit with Mycgr3G39149**
  
Percentage identity: 64 %
  
BlastP bit score: 571
  
Sequence coverage: 100 %
  
E-value: 0.0
  
  
 NCBI BlastP on this gene

EEA26049

SAGA complex component (Sgf73), putative
  
Accession: EEA26048
  
Location: 2652182-2653633
  
 NCBI BlastP on this gene

EEA26048

mRNA capping enzyme alpha subunit, putative
  
Accession: EEA26046
  
Location: 2650299-2651567
  
 NCBI BlastP on this gene

EEA26046

DNA ligase Cdc9, putative
  
Accession: EEA26045
  
Location: 2646897-2649672
  
 NCBI BlastP on this gene

EEA26045

conserved hypothetical protein
  
Accession: EEA26044
  
Location: 2642558-2646134
  
 NCBI BlastP on this gene

EEA26044

133. :  AAHF01000008 Aspergillus fumigatus Af293     Total score: 1.0     Cumulative Blast bit score: 571

siderochrome-iron transporter MirC
  
Accession: EAL87664
  
Location: 1612624-1614691
  
 NCBI BlastP on this gene

EAL87664

Rho GTPase ModA, putative
  
Accession: EAL87665
  
Location: 1617787-1618739
  
 NCBI BlastP on this gene

EAL87665

agmatinase, putative
  
Accession: EAL87666
  
Location: 1619818-1621182
  
 NCBI BlastP on this gene

EAL87666

beta-ketoacyl synthase (Cem1), putative
  
Accession: EAL87667
  
Location: 1621987-1623626
  
  
**BlastP hit with Mycgr3G39149**
  
Percentage identity: 64 %
  
BlastP bit score: 571
  
Sequence coverage: 97 %
  
E-value: 0.0
  
  
 NCBI BlastP on this gene

EAL87667

134. :  GG663367 Ajellomyces capsulatus G186AR genomic scaffold supercont2.5     Total score: 1.0     Cumulative Blast bit score: 564

predicted protein
  
Accession: EEH07630
  
Location: 1635701-1636742
  
 NCBI BlastP on this gene

EEH07630

conserved hypothetical protein
  
Accession: EEH07631
  
Location: 1637618-1638581
  
 NCBI BlastP on this gene

EEH07631

predicted protein
  
Accession: EEH07632
  
Location: 1639868-1640233
  
 NCBI BlastP on this gene

EEH07632

agmatine ureohydrolase
  
Accession: EEH07633
  
Location: 1640711-1642461
  
 NCBI BlastP on this gene

EEH07633

conserved hypothetical protein
  
Accession: EEH07634
  
Location: 1643233-1643745
  
 NCBI BlastP on this gene

EEH07634

3-oxoacyl-[acyl-carrier-protein]-synthase
  
Accession: EEH07635
  
Location: 1644317-1646202
  
  
**BlastP hit with Mycgr3G39149**
  
Percentage identity: 63 %
  
BlastP bit score: 564
  
Sequence coverage: 101 %
  
E-value: 0.0
  
  
 NCBI BlastP on this gene

EEH07635

conserved hypothetical protein
  
Accession: EEH07636
  
Location: 1646494-1648239
  
 NCBI BlastP on this gene

EEH07636

mRNA-capping enzyme subunit alpha
  
Accession: EEH07637
  
Location: 1648972-1650264
  
 NCBI BlastP on this gene

EEH07637

DNA ligase
  
Accession: EEH07638
  
Location: 1650599-1654065
  
 NCBI BlastP on this gene

EEH07638

135. :  DS572815 Paracoccidioides brasiliensis Pb01 supercont1.5 genomic scaffold     Total score: 1.0     Cumulative Blast bit score: 559

cell division control protein
  
Accession: EEH40175
  
Location: 179805-180886
  
 NCBI BlastP on this gene

EEH40175

guanidinobutyrase
  
Accession: EEH40174
  
Location: 177078-178275
  
 NCBI BlastP on this gene

EEH40174

3-oxoacyl-[acyl-carrier-protein] synthase
  
Accession: EEH40173
  
Location: 172388-176587
  
  
**BlastP hit with Mycgr3G39149**
  
Percentage identity: 65 %
  
BlastP bit score: 559
  
Sequence coverage: 94 %
  
E-value: 0.0
  
  
 NCBI BlastP on this gene

EEH40173

mRNA-capping enzyme subunit alpha
  
Accession: EEH40172
  
Location: 170375-172351
  
 NCBI BlastP on this gene

EEH40172

DNA ligase
  
Accession: EEH40171
  
Location: 166667-169834
  
 NCBI BlastP on this gene

EEH40171

136. :  AQGS01000575 Dactylellina haptotyla CBS 200.50     Total score: 1.0     Cumulative Blast bit score: 555

hypothetical protein
  
Accession: EPS38362
  
Location: 248074-249602
  
 NCBI BlastP on this gene

EPS38362

hypothetical protein
  
Accession: EPS38282
  
Location: 250777-252349
  
 NCBI BlastP on this gene

EPS38282

hypothetical protein
  
Accession: EPS38163
  
Location: 253526-255842
  
 NCBI BlastP on this gene

EPS38163

hypothetical protein
  
Accession: EPS38330
  
Location: 256605-260102
  
  
**BlastP hit with Mycgr3G39149**
  
Percentage identity: 62 %
  
BlastP bit score: 555
  
Sequence coverage: 100 %
  
E-value: 0.0
  
  
 NCBI BlastP on this gene

EPS38330

hypothetical protein
  
Accession: EPS38216
  
Location: 260632-262739
  
 NCBI BlastP on this gene

EPS38216

hypothetical protein
  
Accession: EPS38355
  
Location: 267138-267845
  
 NCBI BlastP on this gene

EPS38355

137. :  DS989823 Arthroderma gypseum CBS 118893 supercont1.2 genomic scaffold     Total score: 1.0     Cumulative Blast bit score: 551

3-oxoacyl-[acyl-carrier-protein] synthase 2
  
Accession: EFR00118
  
Location: 2943070-2944793
  
  
**BlastP hit with Mycgr3G39149**
  
Percentage identity: 65 %
  
BlastP bit score: 551
  
Sequence coverage: 99 %
  
E-value: 0.0
  
  
 NCBI BlastP on this gene

EFR00118

hypothetical protein
  
Accession: EFR00117
  
Location: 2941422-2942876
  
 NCBI BlastP on this gene

EFR00117

hypothetical protein
  
Accession: EFR00116
  
Location: 2938709-2940157
  
 NCBI BlastP on this gene

EFR00116

hypothetical protein
  
Accession: EFR00115
  
Location: 2937062-2937509
  
 NCBI BlastP on this gene

EFR00115

hypothetical protein
  
Accession: EFR00114
  
Location: 2934888-2936346
  
 NCBI BlastP on this gene

EFR00114

138. :  JH226136 Exophiala dermatitidis NIH/UT8656 unplaced genomic scaffold supercont1.7     Total score: 1.0     Cumulative Blast bit score: 549

dolichyl pyrophosphate Glc1Man9GlcNAc2 alpha-1,3-glucosyltransferase
  
Accession: EHY60116
  
Location: 735916-737625
  
 NCBI BlastP on this gene

EHY60116

hypothetical protein
  
Accession: EHY60117
  
Location: 738912-742097
  
 NCBI BlastP on this gene

EHY60117

agmatinase
  
Accession: EHY60118
  
Location: 742819-744512
  
 NCBI BlastP on this gene

EHY60118

3-oxoacyl-[acyl-carrier-protein] synthase II
  
Accession: EHY60119
  
Location: 745527-747394
  
  
**BlastP hit with Mycgr3G39149**
  
Percentage identity: 63 %
  
BlastP bit score: 549
  
Sequence coverage: 105 %
  
E-value: 0.0
  
  
 NCBI BlastP on this gene

EHY60119

SAGA-associated factor 73
  
Accession: EHY60120
  
Location: 747554-748890
  
 NCBI BlastP on this gene

EHY60120

mRNA guanylyltransferase
  
Accession: EHY60121
  
Location: 749547-750869
  
 NCBI BlastP on this gene

EHY60121

hypothetical protein
  
Accession: EHY60122
  
Location: 751545-754415
  
 NCBI BlastP on this gene

EHY60122

hypothetical protein
  
Accession: EHY60123
  
Location: 755435-758675
  
 NCBI BlastP on this gene

EHY60123

139. :  KB446557 Pseudocercospora fijiensis CIRAD86 unplaced genomic scaffold MYCFIscaffold\_3     Total score: 1.0     Cumulative Blast bit score: 544

hypothetical protein
  
Accession: EME84082
  
Location: 1913273-1914889
  
 NCBI BlastP on this gene

EME84082

hypothetical protein
  
Accession: EME84083
  
Location: 1914961-1916909
  
 NCBI BlastP on this gene

EME84083

hypothetical protein
  
Accession: EME84084
  
Location: 1918175-1919675
  
 NCBI BlastP on this gene

EME84084

glycosyltransferase family 8 protein
  
Accession: EME84085
  
Location: 1920888-1922165
  
 NCBI BlastP on this gene

EME84085

hypothetical protein
  
Accession: EME84086
  
Location: 1922777-1924025
  
  
**BlastP hit with Mycgr3G38483**
  
Percentage identity: 72 %
  
BlastP bit score: 544
  
Sequence coverage: 100 %
  
E-value: 0.0
  
  
 NCBI BlastP on this gene

EME84086

hypothetical protein
  
Accession: EME84087
  
Location: 1925170-1926203
  
 NCBI BlastP on this gene

EME84087

hypothetical protein
  
Accession: EME84088
  
Location: 1926285-1927577
  
 NCBI BlastP on this gene

EME84088

140. :  HF679025 Fusarium fujikuroi IMI 58289 draft genome, chromosome FFUJ\_chr03.     Total score: 1.0     Cumulative Blast bit score: 541

uncharacterized protein
  
Accession: CCT66223
  
Location: 3695062-3696552
  
 NCBI BlastP on this gene

FFUJ\_03233

probable isp4 protein
  
Accession: CCT66224
  
Location: 3698507-3701309
  
 NCBI BlastP on this gene

FFUJ\_03234

probable CEM1-beta-keto-acyl-ACP synthase, mitochondrial
  
Accession: CCT66225
  
Location: 3701896-3703280
  
  
**BlastP hit with Mycgr3G39149**
  
Percentage identity: 62 %
  
BlastP bit score: 541
  
Sequence coverage: 100 %
  
E-value: 0.0
  
  
 NCBI BlastP on this gene

FFUJ\_03235

related to CBP3-required for assembly of cytochrome bc1 complex
  
Accession: CCT66226
  
Location: 3703539-3704512
  
 NCBI BlastP on this gene

FFUJ\_03236

related to DFR1-dihydrofolate reductase
  
Accession: CCT66227
  
Location: 3704688-3705392
  
 NCBI BlastP on this gene

FFUJ\_03237

related to glutathione S-transferase
  
Accession: CCT66228
  
Location: 3705751-3706579
  
 NCBI BlastP on this gene

FFUJ\_03238

probable reductases with broad range of substrate specificities
  
Accession: CCT66229
  
Location: 3706878-3707969
  
 NCBI BlastP on this gene

FFUJ\_03239

related to 26S proteasome subunit YTA7
  
Accession: CCT66230
  
Location: 3708245-3713148
  
 NCBI BlastP on this gene

FFUJ\_03240

141. :  KB446538 Dothistroma septosporum NZE10 unplaced genomic scaffold DOTSEscaffold\_4     Total score: 1.0     Cumulative Blast bit score: 537

hypothetical protein
  
Accession: EME44904
  
Location: 188161-191376
  
 NCBI BlastP on this gene

EME44904

hypothetical protein
  
Accession: EME44903
  
Location: 185248-185664
  
 NCBI BlastP on this gene

EME44903

hypothetical protein
  
Accession: EME44902
  
Location: 183856-184412
  
 NCBI BlastP on this gene

EME44902

hypothetical protein
  
Accession: EME44901
  
Location: 182368-183532
  
 NCBI BlastP on this gene

EME44901

hypothetical protein
  
Accession: EME44900
  
Location: 179851-181490
  
  
**BlastP hit with Mycgr3G57362**
  
Percentage identity: 55 %
  
BlastP bit score: 537
  
Sequence coverage: 102 %
  
E-value: 0.0
  
  
 NCBI BlastP on this gene

EME44900

hypothetical protein
  
Accession: EME44899
  
Location: 179148-179591
  
 NCBI BlastP on this gene

EME44899

hypothetical protein
  
Accession: EME44898
  
Location: 177734-179019
  
 NCBI BlastP on this gene

EME44898

hypothetical protein
  
Accession: EME44897
  
Location: 176564-177443
  
 NCBI BlastP on this gene

EME44897

hypothetical protein
  
Accession: EME44896
  
Location: 174017-176333
  
 NCBI BlastP on this gene

EME44896

hypothetical protein
  
Accession: EME44895
  
Location: 172066-172224
  
 NCBI BlastP on this gene

EME44895

142. :  EQ962653 Talaromyces stipitatus ATCC 10500 scf\_1105507295527 genomic scaffold     Total score: 1.0     Cumulative Blast bit score: 536

conserved hypothetical protein
  
Accession: EED21945
  
Location: 3135534-3136130
  
 NCBI BlastP on this gene

EED21945

siderochrome-iron transporter MirC
  
Accession: EED21944
  
Location: 3132720-3134730
  
 NCBI BlastP on this gene

EED21944

Rho GTPase ModA, putative
  
Accession: EED21943
  
Location: 3130808-3131632
  
 NCBI BlastP on this gene

EED21943

agmatinase, putative
  
Accession: EED21942
  
Location: 3128636-3130000
  
 NCBI BlastP on this gene

EED21942

beta-ketoacyl synthase (Cem1), putative
  
Accession: EED21941
  
Location: 3124630-3128063
  
  
**BlastP hit with Mycgr3G39149**
  
Percentage identity: 63 %
  
BlastP bit score: 536
  
Sequence coverage: 94 %
  
E-value: 2e-180
  
  
 NCBI BlastP on this gene

EED21941

mRNA capping enzyme alpha subunit, putative
  
Accession: EED21940
  
Location: 3122803-3124074
  
 NCBI BlastP on this gene

EED21940

conserved hypothetical protein
  
Accession: EED21939
  
Location: 3120518-3122277
  
 NCBI BlastP on this gene

EED21939

DNA ligase Cdc9, putative
  
Accession: EED21938
  
Location: 3117323-3120072
  
 NCBI BlastP on this gene

EED21938

143. :  ABDG02000027 Trichoderma atroviride IMI 206040     Total score: 1.0     Cumulative Blast bit score: 532

hypothetical protein
  
Accession: EHK41235
  
Location: 2369651-2373075
  
 NCBI BlastP on this gene

EHK41235

hypothetical protein
  
Accession: EHK41236
  
Location: 2373677-2376538
  
 NCBI BlastP on this gene

EHK41236

hypothetical protein
  
Accession: EHK41237
  
Location: 2377394-2378852
  
  
**BlastP hit with Mycgr3G39149**
  
Percentage identity: 62 %
  
BlastP bit score: 532
  
Sequence coverage: 100 %
  
E-value: 0.0
  
  
 NCBI BlastP on this gene

EHK41237

hypothetical protein
  
Accession: EHK41238
  
Location: 2379342-2380342
  
 NCBI BlastP on this gene

EHK41238

hypothetical protein
  
Accession: EHK41239
  
Location: 2380681-2381448
  
 NCBI BlastP on this gene

EHK41239

hypothetical protein
  
Accession: EHK41240
  
Location: 2381720-2382797
  
 NCBI BlastP on this gene

EHK41240

hypothetical protein
  
Accession: EHK41241
  
Location: 2383746-2384952
  
 NCBI BlastP on this gene

EHK41241

hypothetical protein
  
Accession: EHK41242
  
Location: 2385832-2390897
  
 NCBI BlastP on this gene

EHK41242

144. :  CU633900 Podospora anserina S mat+ genomic DNA chromosome 7, supercontig 1.     Total score: 1.0     Cumulative Blast bit score: 528

not annotated
  
Accession: CAP68987
  
Location: 2689446-2692267
  
 NCBI BlastP on this gene

CAP68987

not annotated
  
Accession: CAP68988
  
Location: 2692962-2693996
  
 NCBI BlastP on this gene

CAP68988

not annotated
  
Accession: CAP68989
  
Location: 2697105-2699936
  
 NCBI BlastP on this gene

CAP68989

tRNA-Val
  
Accession: CAP68990
  
Location: 2700555-2701977
  
  
**BlastP hit with Mycgr3G39149**
  
Percentage identity: 61 %
  
BlastP bit score: 528
  
Sequence coverage: 102 %
  
E-value: 0.0
  
  
 NCBI BlastP on this gene

CAP68990

145. :  ACFW01000049 Coccidioides posadasii C735 delta SOWgp     Total score: 1.0     Cumulative Blast bit score: 526

Beta-ketoacyl synthase, C-terminal domain containing protein
  
Accession: EER24144
  
Location: 3470753-3472524
  
  
**BlastP hit with Mycgr3G39149**
  
Percentage identity: 62 %
  
BlastP bit score: 526
  
Sequence coverage: 99 %
  
E-value: 0.0
  
  
 NCBI BlastP on this gene

EER24144

phosphoribosylaminoimidazole carboxylase, putative
  
Accession: EER24143
  
Location: 3468668-3470555
  
 NCBI BlastP on this gene

EER24143

hypothetical protein
  
Accession: EER24142
  
Location: 3465717-3466763
  
 NCBI BlastP on this gene

EER24142

mRNA capping enzyme alpha subunit, putative
  
Accession: EER24141
  
Location: 3463881-3465110
  
 NCBI BlastP on this gene

EER24141

ATP-dependent DNA ligase, putative
  
Accession: EER24140
  
Location: 3459996-3463226
  
 NCBI BlastP on this gene

EER24140

146. :  ADOT01000059 Arthrobotrys oligospora ATCC 24927     Total score: 1.0     Cumulative Blast bit score: 524

hypothetical protein
  
Accession: EGX52125
  
Location: 422183-423811
  
  
**BlastP hit with Mycgr3G39149**
  
Percentage identity: 58 %
  
BlastP bit score: 524
  
Sequence coverage: 100 %
  
E-value: 0.0
  
  
 NCBI BlastP on this gene

EGX52125

hypothetical protein
  
Accession: EGX52124
  
Location: 419408-421261
  
 NCBI BlastP on this gene

EGX52124

hypothetical protein
  
Accession: EGX52123
  
Location: 416199-416879
  
 NCBI BlastP on this gene

EGX52123

hypothetical protein
  
Accession: EGX52122
  
Location: 412824-414530
  
 NCBI BlastP on this gene

EGX52122

147. :  AACD01000129 Aspergillus nidulans FGSC A4     Total score: 1.0     Cumulative Blast bit score: 521

hypothetical protein
  
Accession: EAA62065
  
Location: 200559-202616
  
 NCBI BlastP on this gene

EAA62065

predicted protein
  
Accession: EAA62066
  
Location: 202920-203174
  
 NCBI BlastP on this gene

EAA62066

CD42 CHICK Cell division control protein 42 homolog (G25K GTP-binding protein)
  
Accession: EAA62067
  
Location: 204520-205477
  
 NCBI BlastP on this gene

EAA62067

hypothetical protein
  
Accession: EAA62068
  
Location: 206328-207725
  
 NCBI BlastP on this gene

EAA62068

hypothetical protein
  
Accession: EAA62069
  
Location: 208323-209965
  
  
**BlastP hit with Mycgr3G39149**
  
Percentage identity: 63 %
  
BlastP bit score: 521
  
Sequence coverage: 95 %
  
E-value: 5e-180
  
  
 NCBI BlastP on this gene

EAA62069

148. :  GL988041 Chaetomium thermophilum var. thermophilum DSM 1495 unplaced genomic scaffold scf7180000...     Total score: 1.0     Cumulative Blast bit score: 516

oligopeptide transporter-like protein
  
Accession: EGS20673
  
Location: 1547903-1550817
  
 NCBI BlastP on this gene

EGS20673

hypothetical protein
  
Accession: EGS20674
  
Location: 1552984-1554434
  
 NCBI BlastP on this gene

EGS20674

3-oxoacyl-[acyl-carrier-protein] synthase-like protein
  
Accession: EGS20675
  
Location: 1556547-1558010
  
  
**BlastP hit with Mycgr3G39149**
  
Percentage identity: 60 %
  
BlastP bit score: 516
  
Sequence coverage: 102 %
  
E-value: 8e-178
  
  
 NCBI BlastP on this gene

EGS20675

149. :  KB644415 Penicillium oxalicum 114-2 unplaced genomic scaffold scaffold\_8     Total score: 1.0     Cumulative Blast bit score: 515

hypothetical protein
  
Accession: EPS34722
  
Location: 5032782-5035352
  
 NCBI BlastP on this gene

EPS34722

hypothetical protein
  
Accession: EPS34723
  
Location: 5036790-5038878
  
 NCBI BlastP on this gene

EPS34723

hypothetical protein
  
Accession: EPS34724
  
Location: 5039498-5040613
  
 NCBI BlastP on this gene

EPS34724

hypothetical protein
  
Accession: EPS34725
  
Location: 5041043-5042575
  
  
**BlastP hit with Mycgr3G70475**
  
Percentage identity: 55 %
  
BlastP bit score: 515
  
Sequence coverage: 102 %
  
E-value: 2e-175
  
  
 NCBI BlastP on this gene

EPS34725

hypothetical protein
  
Accession: EPS34726
  
Location: 5042830-5043051
  
 NCBI BlastP on this gene

EPS34726

hypothetical protein
  
Accession: EPS34727
  
Location: 5043102-5043423
  
 NCBI BlastP on this gene

EPS34727

hypothetical protein
  
Accession: EPS34728
  
Location: 5043647-5045146
  
 NCBI BlastP on this gene

EPS34728

hypothetical protein
  
Accession: EPS34729
  
Location: 5046330-5048624
  
 NCBI BlastP on this gene

EPS34729

hypothetical protein
  
Accession: EPS34730
  
Location: 5049324-5049962
  
 NCBI BlastP on this gene

EPS34730

hypothetical protein
  
Accession: EPS34731
  
Location: 5050761-5051861
  
 NCBI BlastP on this gene

EPS34731

150. :  AM920437 Penicillium chrysogenum Wisconsin 54-1255 complete genome, contig Pc00c22.     Total score: 1.0     Cumulative Blast bit score: 511

not annotated
  
Accession: CAP98782
  
Location: 3505578-3508403
  
 NCBI BlastP on this gene

Pc22g14940

not annotated
  
Accession: Pc22g14930
  
Location: 3504697-3504990
  
 NCBI BlastP on this gene

Pc22g14930

not annotated
  
Accession: Pc22g14920
  
Location: 3503994-3504653
  
 NCBI BlastP on this gene

Pc22g14920

hypothetical protein
  
Accession: CAP98779
  
Location: 3502346-3503485
  
 NCBI BlastP on this gene

Pc22g14910

phenylacetyl-CoA ligase pclA-Penicillium chrysogenum
  
Accession: CAP98778
  
Location: 3500136-3502160
  
 NCBI BlastP on this gene

pclA

not annotated
  
Accession: CAP98777
  
Location: 3497681-3499228
  
  
**BlastP hit with Mycgr3G70475**
  
Percentage identity: 52 %
  
BlastP bit score: 511
  
Sequence coverage: 104 %
  
E-value: 7e-174
  
  
 NCBI BlastP on this gene

Pc22g14890

not annotated
  
Accession: CAP98776
  
Location: 3496913-3497239
  
 NCBI BlastP on this gene

Pc22g14880

not annotated
  
Accession: CAP98775
  
Location: 3494479-3496664
  
 NCBI BlastP on this gene

Pc22g14870

not annotated
  
Accession: CAP98774
  
Location: 3492106-3494274
  
 NCBI BlastP on this gene

Pc22g14860

not annotated
  
Accession: CAP98773
  
Location: 3490955-3491599
  
 NCBI BlastP on this gene

Pc22g14850

not annotated
  
Accession: CAP98772
  
Location: 3489038-3490169
  
 NCBI BlastP on this gene

Pc22g14840

Detecting sequence homology at the gene cluster level with MultiGeneBlast.
  
Marnix H. Medema, Rainer Breitling & Eriko Takano (2013)
  
*Molecular Biology and Evolution* , 30: 1218-1223.
