# Supplementary material for: In silico prediction and characterization of secondary metabolite biosynthetic gene clusters in the wheat pathogen Zymoseptoria tritici
Source: BMC Genomics. 2017 Aug 17;18:631. doi: 10.1186/s12864-017-3969-y (PMC5561558; doi:10.1186/s12864-017-3969-y)
Supplement: Supplementary file 1 — MultiGeneBLAST analysis of putative secondary metabolite clusters. All encoded amino acid sequences from genes residing in clusters predicted by AntiSMASH are given as FASTA file format. All output data from MultiGeneBLASTs are also provided. (ZIP 42911 kb) [file 12864_2017_3969_MOESM1_ESM.zip › Cluster MultiGene BLAST/out/Clusters_1_34/Cluster_13/displaypage4.xhtml]

xml version="1.0" encoding="UTF-8"?


Search Results
  
  
 Results pages: 1, 2, 3, 4, 5

**MultiGeneBlast hits**

Select gene cluster alignment
151. CP003004\_0 Myceliophthora thermophila ATCC 42464 chromosome 3, complete ...
152. CM001232\_0 Magnaporthe oryzae 70-15 chromosome 2, whole genome shotgun s...
153. DS499597\_1 Aspergillus fumigatus A1163 scf\_000004 genomic scaffold, whol...
154. EQ963481\_1 Aspergillus flavus NRRL3357 scf\_1106286419476 genomic scaffol...
155. KE145367\_1 Glarea lozoyensis ATCC 20868 chromosome Unknown GLAREA3, whol...
156. CP003012\_1 Thielavia terrestris NRRL 8126 chromosome 4, complete sequence.
157. CH476616\_1 Uncinocarpus reesii 1704 scaffold\_2 genomic scaffold, whole g...
158. EQ963477\_1 Aspergillus flavus NRRL3357 scf\_1106286418280 genomic scaffol...
159. JH126399\_1 Cordyceps militaris CM01 unplaced genomic scaffold CCM\_S00001...
160. DS027696\_0 Neosartorya fischeri NRRL 181 1099437636264 genomic scaffold,...
161. ABSU01000001\_0 Arthroderma benhamiae CBS 112371, whole genome shotgun se...
162. DS990636\_0 Ajellomyces capsulatus H88 supercont1.1 genomic scaffold, who...
163. AEOI01000012\_0 Ogataea parapolymorpha DL-1, whole genome shotgun sequenc...
164. CR382134\_1 Debaryomyces hansenii CBS767 chromosome B complete sequence.
165. JH226133\_1 Exophiala dermatitidis NIH/UT8656 unplaced genomic scaffold s...
166. CU329671\_1 Schizosaccharomyces pombe chromosome II, complete sequence.
167. GG704912\_0 Coccidioides immitis RS genomic scaffold supercont3.2, whole ...
168. ACJE01000012\_0 Aspergillus niger ATCC 1015, whole genome shotgun sequenc...
169. ACFW01000025\_0 Coccidioides posadasii C735 delta SOWgp, whole genome sho...
170. FR839631\_0 Pichia pastoris CBS 7435 chromosome 4, complete replicon sequ...
171. FN392322\_1 Pichia pastoris GS115 chromosome 4, complete sequence.
172. FO082056\_0 Pichia sorbitophila strain CBS 7064 chromosome D complete seq...
173. FO082057\_0 Pichia sorbitophila strain CBS 7064 chromosome C complete seq...
174. DS231615\_1 Pyrenophora tritici-repentis Pt-1C-BFP supercont1.1 genomic s...
175. GL629801\_0 Grosmannia clavigera kw1407 unplaced genomic scaffold GCSC\_17...
176. CH408156\_0 Pichia guilliermondii ATCC 6260 scaffold\_2 genomic scaffold, ...
177. DS572752\_0 Paracoccidioides brasiliensis Pb18 supercont1.3 genomic scaff...
178. DS022225\_1 Schizosaccharomyces japonicus yFS275 supercont1.2 genomic sca...
179. DS544803\_0 Paracoccidioides brasiliensis Pb03 supercont1.1 genomic scaff...
180. FM992690\_1 Candida dubliniensis CD36 chromosome 3, complete sequence.
181. CP000494\_1 Bradyrhizobium sp. BTAi1, complete genome.
182. DS572698\_1 Verticillium dahliae VdLs.17 supercont1.4 genomic scaffold, w...
183. EQ962652\_0 Talaromyces stipitatus ATCC 10500 scf\_1105507295523 genomic s...
184. JH687379\_1 Stereum hirsutum FP-91666 SS1 unplaced genomic scaffold STEHI...
185. KE145368\_1 Glarea lozoyensis ATCC 20868 chromosome Unknown GLAREA4, whol...
186. HF679025\_1 Fusarium fujikuroi IMI 58289 draft genome, chromosome FFUJ\_ch...
187. GL636500\_1 Coccidioides posadasii str. Silveira unplaced genomic scaffol...
188. AQGS01000575\_1 Dactylellina haptotyla CBS 200.50, whole genome shotgun s...
189. GL891304\_1 Neurospora tetrasperma FGSC 2508 unplaced genomic scaffold NE...
190. GL891236\_0 Neurospora tetrasperma FGSC 2509 unplaced genomic scaffold NE...
191. DS231619\_0 Pyrenophora tritici-repentis Pt-1C-BFP supercont1.5 genomic s...
192. CH445339\_1 Phaeosphaeria nodorum SN15 scaffold\_15, whole genome shotgun ...
193. GG749429\_0 Ajellomyces dermatitidis ATCC 18188 genomic scaffold supercon...
194. EQ999973\_2 Ajellomyces dermatitidis ER-3 genomic scaffold supercont1.1, ...
195. GG657469\_2 Ajellomyces dermatitidis SLH14081 genomic scaffold supercont1...
196. DS027696\_3 Neosartorya fischeri NRRL 181 1099437636264 genomic scaffold,...
197. DS985215\_1 Verticillium albo-atrum VaMs.102 supercont1.2 genomic scaffol...
198. DS499595\_2 Aspergillus fumigatus A1163 scf\_000002 genomic scaffold, whol...
199. DS231615\_0 Pyrenophora tritici-repentis Pt-1C-BFP supercont1.1 genomic s...
200. CU633900\_0 Podospora anserina S mat+ genomic DNA chromosome 7, supercont...

Query: Architecture Search FASTA input

CP003004 : Myceliophthora thermophila ATCC 42464 chromosome 3    Total score: 1.0     Cumulative Blast bit score: 509

Hit cluster cross-links:

Mycgr3G70471
  
Location: 0-405

Mycgr3G70471

Mycgr3G39149
  
Location: 505-1798

Mycgr3G39149

Mycgr3G92130
  
Location: 1898-2396

Mycgr3G92130

Mycgr3G38483
  
Location: 2496-3576

Mycgr3G38483

Mycgr3G108869
  
Location: 3676-5056

Mycgr3G108869

Mycgr3G103943
  
Location: 5156-5762

Mycgr3G103943

Mycgr3G57362
  
Location: 5862-7296

Mycgr3G57362

Mycgr3G39086
  
Location: 7396-8368

Mycgr3G39086

Mycgr3G103942
  
Location: 8468-8714

Mycgr3G103942

Mycgr3G108865
  
Location: 8814-10239

Mycgr3G108865

Mycgr3G70475
  
Location: 10339-11821

Mycgr3G70475

Mycgr3G108866
  
Location: 11921-13010

Mycgr3G108866

Mycgr3G92136
  
Location: 13110-13593

Mycgr3G92136

hypothetical protein
  
Accession: AEO57592
  
Location: 1555372-1556061
  
 NCBI BlastP on this gene

MYCTH\_2303921

hypothetical protein
  
Accession: AEO57593
  
Location: 1556754-1557795
  
 NCBI BlastP on this gene

MYCTH\_2126595

hypothetical protein
  
Accession: AEO57594
  
Location: 1559983-1563171
  
 NCBI BlastP on this gene

MYCTH\_2303924

hypothetical protein
  
Accession: AEO57595
  
Location: 1564217-1565851
  
  
**BlastP hit with Mycgr3G39149**
  
Percentage identity: 61 %
  
BlastP bit score: 509
  
Sequence coverage: 105 %
  
E-value: 4e-175
  
  
 NCBI BlastP on this gene

MYCTH\_2303929

hypothetical protein
  
Accession: AEO57596
  
Location: 1566645-1567350
  
 NCBI BlastP on this gene

MYCTH\_2034929

hypothetical protein
  
Accession: AEO57597
  
Location: 1567665-1568522
  
 NCBI BlastP on this gene

MYCTH\_2303933

hypothetical protein
  
Accession: AEO57598
  
Location: 1569102-1573108
  
 NCBI BlastP on this gene

MYCTH\_2303934

hypothetical protein
  
Accession: AEO57599
  
Location: 1573758-1576284
  
 NCBI BlastP on this gene

MYCTH\_2303936

Query: Architecture Search FASTA input

CM001232 : Magnaporthe oryzae 70-15 chromosome 2    Total score: 1.0     Cumulative Blast bit score: 509

Hit cluster cross-links:

Mycgr3G70471
  
Location: 0-405

Mycgr3G70471

Mycgr3G39149
  
Location: 505-1798

Mycgr3G39149

Mycgr3G92130
  
Location: 1898-2396

Mycgr3G92130

Mycgr3G38483
  
Location: 2496-3576

Mycgr3G38483

Mycgr3G108869
  
Location: 3676-5056

Mycgr3G108869

Mycgr3G103943
  
Location: 5156-5762

Mycgr3G103943

Mycgr3G57362
  
Location: 5862-7296

Mycgr3G57362

Mycgr3G39086
  
Location: 7396-8368

Mycgr3G39086

Mycgr3G103942
  
Location: 8468-8714

Mycgr3G103942

Mycgr3G108865
  
Location: 8814-10239

Mycgr3G108865

Mycgr3G70475
  
Location: 10339-11821

Mycgr3G70475

Mycgr3G108866
  
Location: 11921-13010

Mycgr3G108866

Mycgr3G92136
  
Location: 13110-13593

Mycgr3G92136

leucine Rich Repeat domain-containing protein
  
Accession: EHA54023
  
Location: 1744274-1746946
  
 NCBI BlastP on this gene

EHA54023

OPT family small oligopeptide transporter
  
Accession: EHA54024
  
Location: 1748146-1751096
  
 NCBI BlastP on this gene

EHA54024

hypothetical protein
  
Accession: EHA54025
  
Location: 1751833-1752348
  
 NCBI BlastP on this gene

EHA54025

3-oxoacyl-[acyl-carrier-protein] synthase 2
  
Accession: EHA54026
  
Location: 1753628-1755203
  
  
**BlastP hit with Mycgr3G39149**
  
Percentage identity: 61 %
  
BlastP bit score: 509
  
Sequence coverage: 105 %
  
E-value: 4e-175
  
  
 NCBI BlastP on this gene

EHA54026

hypothetical protein
  
Accession: EHA54027
  
Location: 1755506-1756526
  
 NCBI BlastP on this gene

EHA54027

dihydrofolate reductase
  
Accession: EHA54028
  
Location: 1756906-1757732
  
 NCBI BlastP on this gene

EHA54028

hypothetical protein
  
Accession: EHA54029
  
Location: 1759300-1763183
  
 NCBI BlastP on this gene

EHA54029

Query: Architecture Search FASTA input

DS499597 : Aspergillus fumigatus A1163 scf\_000004 genomic scaffold    Total score: 1.0     Cumulative Blast bit score: 508

Hit cluster cross-links:

Mycgr3G70471
  
Location: 0-405

Mycgr3G70471

Mycgr3G39149
  
Location: 505-1798

Mycgr3G39149

Mycgr3G92130
  
Location: 1898-2396

Mycgr3G92130

Mycgr3G38483
  
Location: 2496-3576

Mycgr3G38483

Mycgr3G108869
  
Location: 3676-5056

Mycgr3G108869

Mycgr3G103943
  
Location: 5156-5762

Mycgr3G103943

Mycgr3G57362
  
Location: 5862-7296

Mycgr3G57362

Mycgr3G39086
  
Location: 7396-8368

Mycgr3G39086

Mycgr3G103942
  
Location: 8468-8714

Mycgr3G103942

Mycgr3G108865
  
Location: 8814-10239

Mycgr3G108865

Mycgr3G70475
  
Location: 10339-11821

Mycgr3G70475

Mycgr3G108866
  
Location: 11921-13010

Mycgr3G108866

Mycgr3G92136
  
Location: 13110-13593

Mycgr3G92136

60S ribosome biogenesis protein Sqt1, putative
  
Accession: EDP51486
  
Location: 1744906-1746453
  
  
**BlastP hit with Mycgr3G70475**
  
Percentage identity: 55 %
  
BlastP bit score: 508
  
Sequence coverage: 104 %
  
E-value: 2e-172
  
  
 NCBI BlastP on this gene

EDP51486

NADH-ubiquinone oxidoreductase B12 subunit, putative
  
Accession: EDP51485
  
Location: 1744110-1744414
  
 NCBI BlastP on this gene

EDP51485

CCR4-NOT core complex subunit Caf1, putative
  
Accession: EDP51484
  
Location: 1742179-1743749
  
 NCBI BlastP on this gene

EDP51484

peroxisomal copper amine oxidase
  
Accession: EDP51483
  
Location: 1738916-1741077
  
 NCBI BlastP on this gene

EDP51483

Query: Architecture Search FASTA input

EQ963481 : Aspergillus flavus NRRL3357 scf\_1106286419476 genomic scaffold    Total score: 1.0     Cumulative Blast bit score: 507

Hit cluster cross-links:

Mycgr3G70471
  
Location: 0-405

Mycgr3G70471

Mycgr3G39149
  
Location: 505-1798

Mycgr3G39149

Mycgr3G92130
  
Location: 1898-2396

Mycgr3G92130

Mycgr3G38483
  
Location: 2496-3576

Mycgr3G38483

Mycgr3G108869
  
Location: 3676-5056

Mycgr3G108869

Mycgr3G103943
  
Location: 5156-5762

Mycgr3G103943

Mycgr3G57362
  
Location: 5862-7296

Mycgr3G57362

Mycgr3G39086
  
Location: 7396-8368

Mycgr3G39086

Mycgr3G103942
  
Location: 8468-8714

Mycgr3G103942

Mycgr3G108865
  
Location: 8814-10239

Mycgr3G108865

Mycgr3G70475
  
Location: 10339-11821

Mycgr3G70475

Mycgr3G108866
  
Location: 11921-13010

Mycgr3G108866

Mycgr3G92136
  
Location: 13110-13593

Mycgr3G92136

siderochrome-iron transporter MirC
  
Accession: EED48767
  
Location: 1845774-1847810
  
 NCBI BlastP on this gene

EED48767

Rho GTPase ModA, putative
  
Accession: EED48768
  
Location: 1849649-1850619
  
 NCBI BlastP on this gene

EED48768

agmatinase, putative
  
Accession: EED48769
  
Location: 1852018-1853373
  
 NCBI BlastP on this gene

EED48769

3-oxoacyl carrier protein synthase, putative
  
Accession: EED48770
  
Location: 1853766-1855503
  
  
**BlastP hit with Mycgr3G39149**
  
Percentage identity: 66 %
  
BlastP bit score: 507
  
Sequence coverage: 86 %
  
E-value: 4e-175
  
  
 NCBI BlastP on this gene

EED48770

Query: Architecture Search FASTA input

KE145367 : Glarea lozoyensis ATCC 20868 chromosome Unknown GLAREA3    Total score: 1.0     Cumulative Blast bit score: 505

Hit cluster cross-links:

Mycgr3G70471
  
Location: 0-405

Mycgr3G70471

Mycgr3G39149
  
Location: 505-1798

Mycgr3G39149

Mycgr3G92130
  
Location: 1898-2396

Mycgr3G92130

Mycgr3G38483
  
Location: 2496-3576

Mycgr3G38483

Mycgr3G108869
  
Location: 3676-5056

Mycgr3G108869

Mycgr3G103943
  
Location: 5156-5762

Mycgr3G103943

Mycgr3G57362
  
Location: 5862-7296

Mycgr3G57362

Mycgr3G39086
  
Location: 7396-8368

Mycgr3G39086

Mycgr3G103942
  
Location: 8468-8714

Mycgr3G103942

Mycgr3G108865
  
Location: 8814-10239

Mycgr3G108865

Mycgr3G70475
  
Location: 10339-11821

Mycgr3G70475

Mycgr3G108866
  
Location: 11921-13010

Mycgr3G108866

Mycgr3G92136
  
Location: 13110-13593

Mycgr3G92136

RNA-binding, RBD
  
Accession: EPE29410
  
Location: 1773483-1776778
  
 NCBI BlastP on this gene

EPE29410

alpha/beta-Hydrolase
  
Accession: EPE29411
  
Location: 1777941-1779846
  
 NCBI BlastP on this gene

EPE29411

Subtilisin-like protein
  
Accession: EPE29412
  
Location: 1781080-1783277
  
 NCBI BlastP on this gene

EPE29412

WD40 repeat-like protein
  
Accession: EPE29413
  
Location: 1783770-1785281
  
  
**BlastP hit with Mycgr3G70475**
  
Percentage identity: 57 %
  
BlastP bit score: 505
  
Sequence coverage: 102 %
  
E-value: 7e-172
  
  
 NCBI BlastP on this gene

EPE29413

Query: Architecture Search FASTA input

CP003012 : Thielavia terrestris NRRL 8126 chromosome 4    Total score: 1.0     Cumulative Blast bit score: 505

Hit cluster cross-links:

Mycgr3G70471
  
Location: 0-405

Mycgr3G70471

Mycgr3G39149
  
Location: 505-1798

Mycgr3G39149

Mycgr3G92130
  
Location: 1898-2396

Mycgr3G92130

Mycgr3G38483
  
Location: 2496-3576

Mycgr3G38483

Mycgr3G108869
  
Location: 3676-5056

Mycgr3G108869

Mycgr3G103943
  
Location: 5156-5762

Mycgr3G103943

Mycgr3G57362
  
Location: 5862-7296

Mycgr3G57362

Mycgr3G39086
  
Location: 7396-8368

Mycgr3G39086

Mycgr3G103942
  
Location: 8468-8714

Mycgr3G103942

Mycgr3G108865
  
Location: 8814-10239

Mycgr3G108865

Mycgr3G70475
  
Location: 10339-11821

Mycgr3G70475

Mycgr3G108866
  
Location: 11921-13010

Mycgr3G108866

Mycgr3G92136
  
Location: 13110-13593

Mycgr3G92136

hypothetical protein
  
Accession: AEO69409
  
Location: 3105016-3106517
  
  
**BlastP hit with Mycgr3G39149**
  
Percentage identity: 60 %
  
BlastP bit score: 505
  
Sequence coverage: 104 %
  
E-value: 2e-173
  
  
 NCBI BlastP on this gene

THITE\_2119781

hypothetical protein
  
Accession: AEO69408
  
Location: 3103521-3104585
  
 NCBI BlastP on this gene

THITE\_2119778

hypothetical protein
  
Accession: AEO69407
  
Location: 3102512-3103432
  
 NCBI BlastP on this gene

THITE\_2080461

hypothetical protein
  
Accession: AEO69406
  
Location: 3098095-3102056
  
 NCBI BlastP on this gene

THITE\_2080459

hypothetical protein
  
Accession: AEO69405
  
Location: 3095045-3097572
  
 NCBI BlastP on this gene

THITE\_71850

Query: Architecture Search FASTA input

CH476616 : Uncinocarpus reesii 1704 scaffold\_2 genomic scaffold    Total score: 1.0     Cumulative Blast bit score: 504

Hit cluster cross-links:

Mycgr3G70471
  
Location: 0-405

Mycgr3G70471

Mycgr3G39149
  
Location: 505-1798

Mycgr3G39149

Mycgr3G92130
  
Location: 1898-2396

Mycgr3G92130

Mycgr3G38483
  
Location: 2496-3576

Mycgr3G38483

Mycgr3G108869
  
Location: 3676-5056

Mycgr3G108869

Mycgr3G103943
  
Location: 5156-5762

Mycgr3G103943

Mycgr3G57362
  
Location: 5862-7296

Mycgr3G57362

Mycgr3G39086
  
Location: 7396-8368

Mycgr3G39086

Mycgr3G103942
  
Location: 8468-8714

Mycgr3G103942

Mycgr3G108865
  
Location: 8814-10239

Mycgr3G108865

Mycgr3G70475
  
Location: 10339-11821

Mycgr3G70475

Mycgr3G108866
  
Location: 11921-13010

Mycgr3G108866

Mycgr3G92136
  
Location: 13110-13593

Mycgr3G92136

conserved hypothetical protein
  
Accession: EEP79412
  
Location: 3757493-3759043
  
  
**BlastP hit with Mycgr3G70475**
  
Percentage identity: 53 %
  
BlastP bit score: 504
  
Sequence coverage: 106 %
  
E-value: 6e-171
  
  
 NCBI BlastP on this gene

EEP79412

CCR4-NOT transcription complex subunit 7
  
Accession: EEP79411
  
Location: 3754601-3756227
  
 NCBI BlastP on this gene

EEP79411

peroxisomal copper amine oxidase
  
Accession: EEP79410
  
Location: 3750912-3753086
  
 NCBI BlastP on this gene

EEP79410

predicted protein
  
Accession: EEP79409
  
Location: 3747084-3749011
  
 NCBI BlastP on this gene

EEP79409

Query: Architecture Search FASTA input

EQ963477 : Aspergillus flavus NRRL3357 scf\_1106286418280 genomic scaffold    Total score: 1.0     Cumulative Blast bit score: 502

Hit cluster cross-links:

Mycgr3G70471
  
Location: 0-405

Mycgr3G70471

Mycgr3G39149
  
Location: 505-1798

Mycgr3G39149

Mycgr3G92130
  
Location: 1898-2396

Mycgr3G92130

Mycgr3G38483
  
Location: 2496-3576

Mycgr3G38483

Mycgr3G108869
  
Location: 3676-5056

Mycgr3G108869

Mycgr3G103943
  
Location: 5156-5762

Mycgr3G103943

Mycgr3G57362
  
Location: 5862-7296

Mycgr3G57362

Mycgr3G39086
  
Location: 7396-8368

Mycgr3G39086

Mycgr3G103942
  
Location: 8468-8714

Mycgr3G103942

Mycgr3G108865
  
Location: 8814-10239

Mycgr3G108865

Mycgr3G70475
  
Location: 10339-11821

Mycgr3G70475

Mycgr3G108866
  
Location: 11921-13010

Mycgr3G108866

Mycgr3G92136
  
Location: 13110-13593

Mycgr3G92136

60S ribosome biogenesis protein Sqt1, putative
  
Accession: EED51501
  
Location: 817701-819254
  
  
**BlastP hit with Mycgr3G70475**
  
Percentage identity: 53 %
  
BlastP bit score: 502
  
Sequence coverage: 105 %
  
E-value: 2e-170
  
  
 NCBI BlastP on this gene

EED51501

NADH-ubiquinone oxidoreductase B12 subunit, putative
  
Accession: EED51500
  
Location: 816912-817218
  
 NCBI BlastP on this gene

EED51500

CCR4-NOT core complex subunit Caf1, putative
  
Accession: EED51499
  
Location: 814979-816547
  
 NCBI BlastP on this gene

EED51499

peroxisomal copper amine oxidase
  
Accession: EED51498
  
Location: 811674-813847
  
 NCBI BlastP on this gene

EED51498

heat shock protein, putative
  
Accession: EED51497
  
Location: 809808-810827
  
 NCBI BlastP on this gene

EED51497

Query: Architecture Search FASTA input

JH126399 : Cordyceps militaris CM01 unplaced genomic scaffold CCM\_S00001    Total score: 1.0     Cumulative Blast bit score: 500

Hit cluster cross-links:

Mycgr3G70471
  
Location: 0-405

Mycgr3G70471

Mycgr3G39149
  
Location: 505-1798

Mycgr3G39149

Mycgr3G92130
  
Location: 1898-2396

Mycgr3G92130

Mycgr3G38483
  
Location: 2496-3576

Mycgr3G38483

Mycgr3G108869
  
Location: 3676-5056

Mycgr3G108869

Mycgr3G103943
  
Location: 5156-5762

Mycgr3G103943

Mycgr3G57362
  
Location: 5862-7296

Mycgr3G57362

Mycgr3G39086
  
Location: 7396-8368

Mycgr3G39086

Mycgr3G103942
  
Location: 8468-8714

Mycgr3G103942

Mycgr3G108865
  
Location: 8814-10239

Mycgr3G108865

Mycgr3G70475
  
Location: 10339-11821

Mycgr3G70475

Mycgr3G108866
  
Location: 11921-13010

Mycgr3G108866

Mycgr3G92136
  
Location: 13110-13593

Mycgr3G92136

Fungal transcriptional regulatory protein
  
Accession: EGX97150
  
Location: 5853828-5856408
  
 NCBI BlastP on this gene

EGX97150

small oligopeptide transporter, OPT family
  
Accession: EGX97151
  
Location: 5856679-5859583
  
 NCBI BlastP on this gene

EGX97151

3-oxoacyl-(acyl-carrier-protein) synthase 2
  
Accession: EGX97152
  
Location: 5860234-5861584
  
  
**BlastP hit with Mycgr3G39149**
  
Percentage identity: 60 %
  
BlastP bit score: 501
  
Sequence coverage: 99 %
  
E-value: 3e-172
  
  
 NCBI BlastP on this gene

EGX97152

Query: Architecture Search FASTA input

DS027696 : Neosartorya fischeri NRRL 181 1099437636264 genomic scaffold    Total score: 1.0     Cumulative Blast bit score: 500

Hit cluster cross-links:

Mycgr3G70471
  
Location: 0-405

Mycgr3G70471

Mycgr3G39149
  
Location: 505-1798

Mycgr3G39149

Mycgr3G92130
  
Location: 1898-2396

Mycgr3G92130

Mycgr3G38483
  
Location: 2496-3576

Mycgr3G38483

Mycgr3G108869
  
Location: 3676-5056

Mycgr3G108869

Mycgr3G103943
  
Location: 5156-5762

Mycgr3G103943

Mycgr3G57362
  
Location: 5862-7296

Mycgr3G57362

Mycgr3G39086
  
Location: 7396-8368

Mycgr3G39086

Mycgr3G103942
  
Location: 8468-8714

Mycgr3G103942

Mycgr3G108865
  
Location: 8814-10239

Mycgr3G108865

Mycgr3G70475
  
Location: 10339-11821

Mycgr3G70475

Mycgr3G108866
  
Location: 11921-13010

Mycgr3G108866

Mycgr3G92136
  
Location: 13110-13593

Mycgr3G92136

CNH domain protein
  
Accession: EAW18042
  
Location: 2107109-2112702
  
 NCBI BlastP on this gene

EAW18042

conserved hypothetical protein
  
Accession: EAW18043
  
Location: 2114196-2115825
  
 NCBI BlastP on this gene

EAW18043

AMP dependent CoA ligase
  
Accession: EAW18044
  
Location: 2118442-2120510
  
 NCBI BlastP on this gene

EAW18044

60S ribosome biogenesis protein Sqt1, putative
  
Accession: EAW18045
  
Location: 2120924-2122471
  
  
**BlastP hit with Mycgr3G70475**
  
Percentage identity: 55 %
  
BlastP bit score: 501
  
Sequence coverage: 104 %
  
E-value: 6e-170
  
  
 NCBI BlastP on this gene

EAW18045

NADH-ubiquinone oxidoreductase B12 subunit, putative
  
Accession: EAW18046
  
Location: 2122983-2123286
  
 NCBI BlastP on this gene

EAW18046

CCR4-NOT core complex subunit Caf1, putative
  
Accession: EAW18047
  
Location: 2123638-2125207
  
 NCBI BlastP on this gene

EAW18047

peroxisomal copper amine oxidase
  
Accession: EAW18048
  
Location: 2126306-2128467
  
 NCBI BlastP on this gene

EAW18048

Query: Architecture Search FASTA input

ABSU01000001 : Arthroderma benhamiae CBS 112371    Total score: 1.0     Cumulative Blast bit score: 493

Hit cluster cross-links:

Mycgr3G70471
  
Location: 0-405

Mycgr3G70471

Mycgr3G39149
  
Location: 505-1798

Mycgr3G39149

Mycgr3G92130
  
Location: 1898-2396

Mycgr3G92130

Mycgr3G38483
  
Location: 2496-3576

Mycgr3G38483

Mycgr3G108869
  
Location: 3676-5056

Mycgr3G108869

Mycgr3G103943
  
Location: 5156-5762

Mycgr3G103943

Mycgr3G57362
  
Location: 5862-7296

Mycgr3G57362

Mycgr3G39086
  
Location: 7396-8368

Mycgr3G39086

Mycgr3G103942
  
Location: 8468-8714

Mycgr3G103942

Mycgr3G108865
  
Location: 8814-10239

Mycgr3G108865

Mycgr3G70475
  
Location: 10339-11821

Mycgr3G70475

Mycgr3G108866
  
Location: 11921-13010

Mycgr3G108866

Mycgr3G92136
  
Location: 13110-13593

Mycgr3G92136

hypothetical protein
  
Accession: EFE36506
  
Location: 217368-218076
  
 NCBI BlastP on this gene

EFE36506

hypothetical protein
  
Accession: EFE36505
  
Location: 212805-215816
  
 NCBI BlastP on this gene

EFE36505

hypothetical protein
  
Accession: EFE36504
  
Location: 212241-212507
  
 NCBI BlastP on this gene

EFE36504

hypothetical protein
  
Accession: EFE36503
  
Location: 209314-211264
  
 NCBI BlastP on this gene

EFE36503

hypothetical protein
  
Accession: EFE36502
  
Location: 207460-208992
  
  
**BlastP hit with Mycgr3G70475**
  
Percentage identity: 54 %
  
BlastP bit score: 493
  
Sequence coverage: 105 %
  
E-value: 1e-166
  
  
 NCBI BlastP on this gene

EFE36502

hypothetical protein
  
Accession: EFE36501
  
Location: 206762-207099
  
 NCBI BlastP on this gene

EFE36501

hypothetical protein
  
Accession: EFE36500
  
Location: 205596-206132
  
 NCBI BlastP on this gene

EFE36500

hypothetical protein
  
Accession: EFE36499
  
Location: 204550-205512
  
 NCBI BlastP on this gene

EFE36499

peroxisomal copper amine oxidase, putative
  
Accession: EFE36498
  
Location: 201298-203493
  
 NCBI BlastP on this gene

EFE36498

hypothetical protein
  
Accession: EFE36497
  
Location: 199722-201108
  
 NCBI BlastP on this gene

EFE36497

Query: Architecture Search FASTA input

DS990636 : Ajellomyces capsulatus H88 supercont1.1 genomic scaffold    Total score: 1.0     Cumulative Blast bit score: 489

Hit cluster cross-links:

Mycgr3G70471
  
Location: 0-405

Mycgr3G70471

Mycgr3G39149
  
Location: 505-1798

Mycgr3G39149

Mycgr3G92130
  
Location: 1898-2396

Mycgr3G92130

Mycgr3G38483
  
Location: 2496-3576

Mycgr3G38483

Mycgr3G108869
  
Location: 3676-5056

Mycgr3G108869

Mycgr3G103943
  
Location: 5156-5762

Mycgr3G103943

Mycgr3G57362
  
Location: 5862-7296

Mycgr3G57362

Mycgr3G39086
  
Location: 7396-8368

Mycgr3G39086

Mycgr3G103942
  
Location: 8468-8714

Mycgr3G103942

Mycgr3G108865
  
Location: 8814-10239

Mycgr3G108865

Mycgr3G70475
  
Location: 10339-11821

Mycgr3G70475

Mycgr3G108866
  
Location: 11921-13010

Mycgr3G108866

Mycgr3G92136
  
Location: 13110-13593

Mycgr3G92136

predicted protein
  
Accession: EGC41729
  
Location: 4193461-4194391
  
 NCBI BlastP on this gene

EGC41729

small GTPase
  
Accession: EGC41730
  
Location: 4195267-4196230
  
 NCBI BlastP on this gene

EGC41730

agmatine ureohydrolase
  
Accession: EGC41731
  
Location: 4198350-4199882
  
 NCBI BlastP on this gene

EGC41731

3-oxoacyl-acyl-carrier-protein synthase
  
Accession: EGC41732
  
Location: 4200856-4203715
  
  
**BlastP hit with Mycgr3G39149**
  
Percentage identity: 58 %
  
BlastP bit score: 489
  
Sequence coverage: 99 %
  
E-value: 2e-165
  
  
 NCBI BlastP on this gene

EGC41732

SAGA complex component
  
Accession: EGC41733
  
Location: 4204089-4205826
  
 NCBI BlastP on this gene

EGC41733

mRNA capping enzyme alpha subunit
  
Accession: EGC41734
  
Location: 4206525-4207839
  
 NCBI BlastP on this gene

EGC41734

DNA ligase
  
Accession: EGC41735
  
Location: 4208460-4212396
  
 NCBI BlastP on this gene

EGC41735

Query: Architecture Search FASTA input

AEOI01000012 : Ogataea parapolymorpha DL-1    Total score: 1.0     Cumulative Blast bit score: 487

Hit cluster cross-links:

Mycgr3G70471
  
Location: 0-405

Mycgr3G70471

Mycgr3G39149
  
Location: 505-1798

Mycgr3G39149

Mycgr3G92130
  
Location: 1898-2396

Mycgr3G92130

Mycgr3G38483
  
Location: 2496-3576

Mycgr3G38483

Mycgr3G108869
  
Location: 3676-5056

Mycgr3G108869

Mycgr3G103943
  
Location: 5156-5762

Mycgr3G103943

Mycgr3G57362
  
Location: 5862-7296

Mycgr3G57362

Mycgr3G39086
  
Location: 7396-8368

Mycgr3G39086

Mycgr3G103942
  
Location: 8468-8714

Mycgr3G103942

Mycgr3G108865
  
Location: 8814-10239

Mycgr3G108865

Mycgr3G70475
  
Location: 10339-11821

Mycgr3G70475

Mycgr3G108866
  
Location: 11921-13010

Mycgr3G108866

Mycgr3G92136
  
Location: 13110-13593

Mycgr3G92136

hypothetical protein
  
Accession: EFW95289
  
Location: 1338863-1339523
  
 NCBI BlastP on this gene

EFW95289

hypothetical protein
  
Accession: EFW95290
  
Location: 1340264-1340773
  
 NCBI BlastP on this gene

EFW95290

hypothetical protein
  
Accession: EFW95291
  
Location: 1340901-1341980
  
 NCBI BlastP on this gene

EFW95291

Autophagy-related protein 9
  
Accession: EFW95292
  
Location: 1342098-1344767
  
 NCBI BlastP on this gene

EFW95292

hypothetical protein
  
Accession: EFW95293
  
Location: 1344778-1347162
  
 NCBI BlastP on this gene

EFW95293

Mitochondrial beta-keto-acyl synthase
  
Accession: EFW95294
  
Location: 1347281-1348579
  
  
**BlastP hit with Mycgr3G39149**
  
Percentage identity: 55 %
  
BlastP bit score: 487
  
Sequence coverage: 100 %
  
E-value: 1e-166
  
  
 NCBI BlastP on this gene

EFW95294

hypothetical protein
  
Accession: EFW95295
  
Location: 1349604-1351504
  
 NCBI BlastP on this gene

EFW95295

ATP synthase regulation protein NCA2
  
Accession: EFW95296
  
Location: 1351551-1353473
  
 NCBI BlastP on this gene

EFW95296

TBP-associated factor, putative
  
Accession: EFW95297
  
Location: 1353546-1354988
  
 NCBI BlastP on this gene

EFW95297

RNA Pol II CTD phosphatase component, putative
  
Accession: EFW95298
  
Location: 1355057-1357288
  
 NCBI BlastP on this gene

EFW95298

Query: Architecture Search FASTA input

CR382134 : Debaryomyces hansenii CBS767 chromosome B complete sequence.    Total score: 1.0     Cumulative Blast bit score: 483

Hit cluster cross-links:

Mycgr3G70471
  
Location: 0-405

Mycgr3G70471

Mycgr3G39149
  
Location: 505-1798

Mycgr3G39149

Mycgr3G92130
  
Location: 1898-2396

Mycgr3G92130

Mycgr3G38483
  
Location: 2496-3576

Mycgr3G38483

Mycgr3G108869
  
Location: 3676-5056

Mycgr3G108869

Mycgr3G103943
  
Location: 5156-5762

Mycgr3G103943

Mycgr3G57362
  
Location: 5862-7296

Mycgr3G57362

Mycgr3G39086
  
Location: 7396-8368

Mycgr3G39086

Mycgr3G103942
  
Location: 8468-8714

Mycgr3G103942

Mycgr3G108865
  
Location: 8814-10239

Mycgr3G108865

Mycgr3G70475
  
Location: 10339-11821

Mycgr3G70475

Mycgr3G108866
  
Location: 11921-13010

Mycgr3G108866

Mycgr3G92136
  
Location: 13110-13593

Mycgr3G92136

DEHA2B13772p
  
Accession: CAR65492
  
Location: 1081701-1082819
  
 NCBI BlastP on this gene

DEHA2B13772g

DEHA2B13794p
  
Accession: CAG85556
  
Location: 1082814-1084232
  
 NCBI BlastP on this gene

DEHA2B13794g

DEHA2B13816p
  
Accession: CAG85557
  
Location: 1084455-1084550
  
 NCBI BlastP on this gene

DEHA2B13816g

DEHA2B13838p
  
Accession: CAG85558
  
Location: 1084742-1085215
  
 NCBI BlastP on this gene

DEHA2B13838g

DEHA2B13860p
  
Accession: CAG85559
  
Location: 1085435-1086187
  
 NCBI BlastP on this gene

DEHA2B13860g

DEHA2B13882p
  
Accession: CAG85560
  
Location: 1086433-1088304
  
 NCBI BlastP on this gene

DEHA2B13882g

DEHA2B13904p
  
Accession: CAG85561
  
Location: 1088962-1089468
  
 NCBI BlastP on this gene

DEHA2B13904g

DEHA2B13926p
  
Accession: CAG85562
  
Location: 1089634-1091181
  
 NCBI BlastP on this gene

DEHA2B13926g

DEHA2B13948p
  
Accession: CAG85563
  
Location: 1091282-1092595
  
  
**BlastP hit with Mycgr3G39149**
  
Percentage identity: 51 %
  
BlastP bit score: 484
  
Sequence coverage: 102 %
  
E-value: 2e-165
  
  
 NCBI BlastP on this gene

DEHA2B13948g

Query: Architecture Search FASTA input

JH226133 : Exophiala dermatitidis NIH/UT8656 unplaced genomic scaffold supercont1.4    Total score: 1.0     Cumulative Blast bit score: 483

Hit cluster cross-links:

Mycgr3G70471
  
Location: 0-405

Mycgr3G70471

Mycgr3G39149
  
Location: 505-1798

Mycgr3G39149

Mycgr3G92130
  
Location: 1898-2396

Mycgr3G92130

Mycgr3G38483
  
Location: 2496-3576

Mycgr3G38483

Mycgr3G108869
  
Location: 3676-5056

Mycgr3G108869

Mycgr3G103943
  
Location: 5156-5762

Mycgr3G103943

Mycgr3G57362
  
Location: 5862-7296

Mycgr3G57362

Mycgr3G39086
  
Location: 7396-8368

Mycgr3G39086

Mycgr3G103942
  
Location: 8468-8714

Mycgr3G103942

Mycgr3G108865
  
Location: 8814-10239

Mycgr3G108865

Mycgr3G70475
  
Location: 10339-11821

Mycgr3G70475

Mycgr3G108866
  
Location: 11921-13010

Mycgr3G108866

Mycgr3G92136
  
Location: 13110-13593

Mycgr3G92136

ATP-dependent DNA helicase 2 subunit 1
  
Accession: EHY57067
  
Location: 2225101-2227201
  
 NCBI BlastP on this gene

EHY57067

ferrochelatase
  
Accession: EHY57066
  
Location: 2223194-2224507
  
 NCBI BlastP on this gene

EHY57066

protein-serine/threonine kinase
  
Accession: EHY57065
  
Location: 2219081-2221908
  
 NCBI BlastP on this gene

EHY57065

hypothetical protein
  
Accession: EHY57064
  
Location: 2215703-2217094
  
  
**BlastP hit with Mycgr3G70475**
  
Percentage identity: 57 %
  
BlastP bit score: 483
  
Sequence coverage: 98 %
  
E-value: 9e-164
  
  
 NCBI BlastP on this gene

EHY57064

3-oxoacyl-[acyl-carrier protein] reductase
  
Accession: EHY57063
  
Location: 2214086-2214868
  
 NCBI BlastP on this gene

EHY57063

hypothetical protein
  
Accession: EHY57062
  
Location: 2212398-2213264
  
 NCBI BlastP on this gene

EHY57062

hypothetical protein
  
Accession: EHY57061
  
Location: 2208054-2211671
  
 NCBI BlastP on this gene

EHY57061

Query: Architecture Search FASTA input

CU329671 : Schizosaccharomyces pombe chromosome II    Total score: 1.0     Cumulative Blast bit score: 481

Hit cluster cross-links:

Mycgr3G70471
  
Location: 0-405

Mycgr3G70471

Mycgr3G39149
  
Location: 505-1798

Mycgr3G39149

Mycgr3G92130
  
Location: 1898-2396

Mycgr3G92130

Mycgr3G38483
  
Location: 2496-3576

Mycgr3G38483

Mycgr3G108869
  
Location: 3676-5056

Mycgr3G108869

Mycgr3G103943
  
Location: 5156-5762

Mycgr3G103943

Mycgr3G57362
  
Location: 5862-7296

Mycgr3G57362

Mycgr3G39086
  
Location: 7396-8368

Mycgr3G39086

Mycgr3G103942
  
Location: 8468-8714

Mycgr3G103942

Mycgr3G108865
  
Location: 8814-10239

Mycgr3G108865

Mycgr3G70475
  
Location: 10339-11821

Mycgr3G70475

Mycgr3G108866
  
Location: 11921-13010

Mycgr3G108866

Mycgr3G92136
  
Location: 13110-13593

Mycgr3G92136

3-oxoacyl-[acyl-carrier-protein]-synthase condensing enzyme (predicted)
  
Accession: CAA21898
  
Location: 3565824-3567187
  
  
**BlastP hit with Mycgr3G39149**
  
Percentage identity: 55 %
  
BlastP bit score: 482
  
Sequence coverage: 99 %
  
E-value: 7e-165
  
  
 NCBI BlastP on this gene

SPBC887.13c

P-type ATPase (predicted)
  
Accession: CAA21897
  
Location: 3561887-3565663
  
 NCBI BlastP on this gene

SPBC887.12

tRNA pseudouridine synthase Pus2 (predicted)
  
Accession: CAA21896
  
Location: 3559396-3560788
  
 NCBI BlastP on this gene

pus2

response regulator Mcs4
  
Accession: CAA21895
  
Location: 3557274-3558842
  
 NCBI BlastP on this gene

mcs4

Query: Architecture Search FASTA input

GG704912 : Coccidioides immitis RS genomic scaffold supercont3.2    Total score: 1.0     Cumulative Blast bit score: 481

Hit cluster cross-links:

Mycgr3G70471
  
Location: 0-405

Mycgr3G70471

Mycgr3G39149
  
Location: 505-1798

Mycgr3G39149

Mycgr3G92130
  
Location: 1898-2396

Mycgr3G92130

Mycgr3G38483
  
Location: 2496-3576

Mycgr3G38483

Mycgr3G108869
  
Location: 3676-5056

Mycgr3G108869

Mycgr3G103943
  
Location: 5156-5762

Mycgr3G103943

Mycgr3G57362
  
Location: 5862-7296

Mycgr3G57362

Mycgr3G39086
  
Location: 7396-8368

Mycgr3G39086

Mycgr3G103942
  
Location: 8468-8714

Mycgr3G103942

Mycgr3G108865
  
Location: 8814-10239

Mycgr3G108865

Mycgr3G70475
  
Location: 10339-11821

Mycgr3G70475

Mycgr3G108866
  
Location: 11921-13010

Mycgr3G108866

Mycgr3G92136
  
Location: 13110-13593

Mycgr3G92136

hypothetical protein
  
Accession: EAS30864
  
Location: 1716541-1718999
  
 NCBI BlastP on this gene

EAS30864

phenylacetyl-CoA ligase
  
Accession: EAS30862
  
Location: 1711849-1713894
  
 NCBI BlastP on this gene

EAS30862

acetyltransferase
  
Accession: EAS30861
  
Location: 1710481-1711137
  
 NCBI BlastP on this gene

EAS30861

60S ribosome biogenesis protein Sqt1
  
Accession: EAS30860
  
Location: 1708065-1709621
  
  
**BlastP hit with Mycgr3G70475**
  
Percentage identity: 52 %
  
BlastP bit score: 481
  
Sequence coverage: 106 %
  
E-value: 4e-162
  
  
 NCBI BlastP on this gene

EAS30860

NADH-ubiquinone oxidoreductase B12 subunit
  
Accession: EAS30859
  
Location: 1707323-1707669
  
 NCBI BlastP on this gene

EAS30859

CCR4-NOT transcription complex subunit 7
  
Accession: EAS30858
  
Location: 1705160-1706794
  
 NCBI BlastP on this gene

EAS30858

peroxisomal copper amine oxidase
  
Accession: EAS30857
  
Location: 1701305-1703479
  
 NCBI BlastP on this gene

EAS30857

hypothetical protein
  
Accession: EAS30856
  
Location: 1699847-1700869
  
 NCBI BlastP on this gene

EAS30856

Query: Architecture Search FASTA input

ACJE01000012 : Aspergillus niger ATCC 1015    Total score: 1.0     Cumulative Blast bit score: 478

Hit cluster cross-links:

Mycgr3G70471
  
Location: 0-405

Mycgr3G70471

Mycgr3G39149
  
Location: 505-1798

Mycgr3G39149

Mycgr3G92130
  
Location: 1898-2396

Mycgr3G92130

Mycgr3G38483
  
Location: 2496-3576

Mycgr3G38483

Mycgr3G108869
  
Location: 3676-5056

Mycgr3G108869

Mycgr3G103943
  
Location: 5156-5762

Mycgr3G103943

Mycgr3G57362
  
Location: 5862-7296

Mycgr3G57362

Mycgr3G39086
  
Location: 7396-8368

Mycgr3G39086

Mycgr3G103942
  
Location: 8468-8714

Mycgr3G103942

Mycgr3G108865
  
Location: 8814-10239

Mycgr3G108865

Mycgr3G70475
  
Location: 10339-11821

Mycgr3G70475

Mycgr3G108866
  
Location: 11921-13010

Mycgr3G108866

Mycgr3G92136
  
Location: 13110-13593

Mycgr3G92136

hypothetical protein
  
Accession: EHA22286
  
Location: 308317-309267
  
 NCBI BlastP on this gene

EHA22286

hypothetical protein
  
Accession: EHA22287
  
Location: 310436-311153
  
 NCBI BlastP on this gene

EHA22287

hypothetical protein
  
Accession: EHA22288
  
Location: 311405-312263
  
 NCBI BlastP on this gene

EHA22288

hypothetical protein
  
Accession: EHA22289
  
Location: 314825-316872
  
 NCBI BlastP on this gene

EHA22289

hypothetical protein
  
Accession: EHA22290
  
Location: 317314-318873
  
  
**BlastP hit with Mycgr3G70475**
  
Percentage identity: 53 %
  
BlastP bit score: 478
  
Sequence coverage: 105 %
  
E-value: 4e-161
  
  
 NCBI BlastP on this gene

EHA22290

hypothetical protein
  
Accession: EHA22291
  
Location: 319378-319690
  
 NCBI BlastP on this gene

EHA22291

hypothetical protein
  
Accession: EHA22292
  
Location: 320088-321639
  
 NCBI BlastP on this gene

EHA22292

hypothetical protein
  
Accession: EHA22293
  
Location: 322896-325062
  
 NCBI BlastP on this gene

EHA22293

Query: Architecture Search FASTA input

ACFW01000025 : Coccidioides posadasii C735 delta SOWgp    Total score: 1.0     Cumulative Blast bit score: 477

Hit cluster cross-links:

Mycgr3G70471
  
Location: 0-405

Mycgr3G70471

Mycgr3G39149
  
Location: 505-1798

Mycgr3G39149

Mycgr3G92130
  
Location: 1898-2396

Mycgr3G92130

Mycgr3G38483
  
Location: 2496-3576

Mycgr3G38483

Mycgr3G108869
  
Location: 3676-5056

Mycgr3G108869

Mycgr3G103943
  
Location: 5156-5762

Mycgr3G103943

Mycgr3G57362
  
Location: 5862-7296

Mycgr3G57362

Mycgr3G39086
  
Location: 7396-8368

Mycgr3G39086

Mycgr3G103942
  
Location: 8468-8714

Mycgr3G103942

Mycgr3G108865
  
Location: 8814-10239

Mycgr3G108865

Mycgr3G70475
  
Location: 10339-11821

Mycgr3G70475

Mycgr3G108866
  
Location: 11921-13010

Mycgr3G108866

Mycgr3G92136
  
Location: 13110-13593

Mycgr3G92136

hypothetical protein
  
Accession: EER27339
  
Location: 1439655-1442131
  
 NCBI BlastP on this gene

EER27339

AMP-binding enzyme, putative
  
Accession: EER27338
  
Location: 1434935-1436979
  
 NCBI BlastP on this gene

EER27338

GNAT family acetyltransferase, putative
  
Accession: EER27337
  
Location: 1433561-1434217
  
 NCBI BlastP on this gene

EER27337

WD domain, G-beta repeat containing protein
  
Accession: EER27336
  
Location: 1431143-1432699
  
  
**BlastP hit with Mycgr3G70475**
  
Percentage identity: 52 %
  
BlastP bit score: 477
  
Sequence coverage: 106 %
  
E-value: 2e-160
  
  
 NCBI BlastP on this gene

EER27336

NADH-ubiquinone oxidoreductase B12 subunit family protein
  
Accession: EER27335
  
Location: 1430401-1430747
  
 NCBI BlastP on this gene

EER27335

CAF1 family ribonuclease containing protein
  
Accession: EER27334
  
Location: 1428242-1429873
  
 NCBI BlastP on this gene

EER27334

peroxisomal copper amine oxidase, putative
  
Accession: EER27333
  
Location: 1424386-1426560
  
 NCBI BlastP on this gene

EER27333

short-chain dehydrogenase, putative
  
Accession: EER27332
  
Location: 1422919-1423941
  
 NCBI BlastP on this gene

EER27332

Query: Architecture Search FASTA input

FR839631 : Pichia pastoris CBS 7435 chromosome 4    Total score: 1.0     Cumulative Blast bit score: 476

Hit cluster cross-links:

Mycgr3G70471
  
Location: 0-405

Mycgr3G70471

Mycgr3G39149
  
Location: 505-1798

Mycgr3G39149

Mycgr3G92130
  
Location: 1898-2396

Mycgr3G92130

Mycgr3G38483
  
Location: 2496-3576

Mycgr3G38483

Mycgr3G108869
  
Location: 3676-5056

Mycgr3G108869

Mycgr3G103943
  
Location: 5156-5762

Mycgr3G103943

Mycgr3G57362
  
Location: 5862-7296

Mycgr3G57362

Mycgr3G39086
  
Location: 7396-8368

Mycgr3G39086

Mycgr3G103942
  
Location: 8468-8714

Mycgr3G103942

Mycgr3G108865
  
Location: 8814-10239

Mycgr3G108865

Mycgr3G70475
  
Location: 10339-11821

Mycgr3G70475

Mycgr3G108866
  
Location: 11921-13010

Mycgr3G108866

Mycgr3G92136
  
Location: 13110-13593

Mycgr3G92136

hypothetical protein
  
Accession: CCA40628
  
Location: 779270-780543
  
 NCBI BlastP on this gene

PP7435\_Chr4-0462

Purine-cytosine permease FCY2
  
Accession: CCA40629
  
Location: 780666-782225
  
 NCBI BlastP on this gene

PP7435\_Chr4-0463

hypothetical protein
  
Accession: CCA40630
  
Location: 782502-784481
  
 NCBI BlastP on this gene

PP7435\_Chr4-0464

T-complex protein 1 subunit zeta
  
Accession: CCA40631
  
Location: 784630-786243
  
 NCBI BlastP on this gene

CCT6

Probable nucleolar complex protein 14
  
Accession: CCA40632
  
Location: 786269-788731
  
 NCBI BlastP on this gene

PP7435\_Chr4-0466

3-oxoacyl-
  
Accession: CCA40633
  
Location: 788879-790195
  
  
**BlastP hit with Mycgr3G39149**
  
Percentage identity: 54 %
  
BlastP bit score: 476
  
Sequence coverage: 102 %
  
E-value: 5e-162
  
  
 NCBI BlastP on this gene

CEM1

Autophagy-related protein 9
  
Accession: CCA40634
  
Location: 790203-792860
  
 NCBI BlastP on this gene

PP7435\_Chr4-0468

Beta-glucan synthesis-associated protein KRE6
  
Accession: CCA40635
  
Location: 793847-795796
  
 NCBI BlastP on this gene

KRE7

Transcription elongation regulator 1
  
Accession: CCA40636
  
Location: 795851-797170
  
 NCBI BlastP on this gene

PP7435\_Chr4-0470

20S proteasome subunit alpha 3
  
Accession: CCA40637
  
Location: 797187-798053
  
 NCBI BlastP on this gene

PP7435\_Chr4-0471

Prohibitin
  
Accession: CCA40638
  
Location: 798201-799111
  
 NCBI BlastP on this gene

PHB

Query: Architecture Search FASTA input

FN392322 : Pichia pastoris GS115 chromosome 4    Total score: 1.0     Cumulative Blast bit score: 476

Hit cluster cross-links:

Mycgr3G70471
  
Location: 0-405

Mycgr3G70471

Mycgr3G39149
  
Location: 505-1798

Mycgr3G39149

Mycgr3G92130
  
Location: 1898-2396

Mycgr3G92130

Mycgr3G38483
  
Location: 2496-3576

Mycgr3G38483

Mycgr3G108869
  
Location: 3676-5056

Mycgr3G108869

Mycgr3G103943
  
Location: 5156-5762

Mycgr3G103943

Mycgr3G57362
  
Location: 5862-7296

Mycgr3G57362

Mycgr3G39086
  
Location: 7396-8368

Mycgr3G39086

Mycgr3G103942
  
Location: 8468-8714

Mycgr3G103942

Mycgr3G108865
  
Location: 8814-10239

Mycgr3G108865

Mycgr3G70475
  
Location: 10339-11821

Mycgr3G70475

Mycgr3G108866
  
Location: 11921-13010

Mycgr3G108866

Mycgr3G92136
  
Location: 13110-13593

Mycgr3G92136

Mitochondrial beta-keto-acyl synthase with possible role in fatty acid synthesis
  
Accession: CAY71766
  
Location: 998649-999965
  
  
**BlastP hit with Mycgr3G39149**
  
Percentage identity: 54 %
  
BlastP bit score: 476
  
Sequence coverage: 102 %
  
E-value: 5e-162
  
  
 NCBI BlastP on this gene

PAS\_chr4\_0511

Transmembrane protein involved in formation of Cvt and autophagic vesicles
  
Accession: CAY71765
  
Location: 995984-998641
  
 NCBI BlastP on this gene

PAS\_chr4\_0510

Protein required for beta-1,6 glucan biosynthesis
  
Accession: CAY71764
  
Location: 993048-994997
  
 NCBI BlastP on this gene

PAS\_chr4\_0508

Hypothetical protein
  
Accession: CAY71763
  
Location: 991650-992993
  
 NCBI BlastP on this gene

PAS\_chr4\_0507

Alpha 3 subunit of the 20S proteasome, the only nonessential 20S subunit
  
Accession: CAY71762
  
Location: 990791-991549
  
 NCBI BlastP on this gene

PAS\_chr4\_0506

Subunit of the prohibitin complex (Phb1p-Phb2p)
  
Accession: CAY71761
  
Location: 989732-990643
  
 NCBI BlastP on this gene

PAS\_chr4\_0505

Query: Architecture Search FASTA input

FO082056 : Pichia sorbitophila strain CBS 7064 chromosome D complete sequence.    Total score: 1.0     Cumulative Blast bit score: 469

Hit cluster cross-links:

Mycgr3G70471
  
Location: 0-405

Mycgr3G70471

Mycgr3G39149
  
Location: 505-1798

Mycgr3G39149

Mycgr3G92130
  
Location: 1898-2396

Mycgr3G92130

Mycgr3G38483
  
Location: 2496-3576

Mycgr3G38483

Mycgr3G108869
  
Location: 3676-5056

Mycgr3G108869

Mycgr3G103943
  
Location: 5156-5762

Mycgr3G103943

Mycgr3G57362
  
Location: 5862-7296

Mycgr3G57362

Mycgr3G39086
  
Location: 7396-8368

Mycgr3G39086

Mycgr3G103942
  
Location: 8468-8714

Mycgr3G103942

Mycgr3G108865
  
Location: 8814-10239

Mycgr3G108865

Mycgr3G70475
  
Location: 10339-11821

Mycgr3G70475

Mycgr3G108866
  
Location: 11921-13010

Mycgr3G108866

Mycgr3G92136
  
Location: 13110-13593

Mycgr3G92136

not annotated
  
Accession: CCE78667
  
Location: 177131-177883
  
 NCBI BlastP on this gene

Piso0\_000693

not annotated
  
Accession: CCE78666
  
Location: 174714-176702
  
 NCBI BlastP on this gene

Piso0\_000692

not annotated
  
Accession: CCE78665
  
Location: 174160-174519
  
 NCBI BlastP on this gene

Piso0\_000691

not annotated
  
Accession: CCE78664
  
Location: 171460-172080
  
 NCBI BlastP on this gene

Piso0\_000690

not annotated
  
Accession: CCE78663
  
Location: 168926-170455
  
 NCBI BlastP on this gene

Piso0\_000689

not annotated
  
Accession: CCE78662
  
Location: 167325-168641
  
  
**BlastP hit with Mycgr3G39149**
  
Percentage identity: 51 %
  
BlastP bit score: 469
  
Sequence coverage: 102 %
  
E-value: 1e-159
  
  
 NCBI BlastP on this gene

Piso0\_000688

not annotated
  
Accession: CCE78661
  
Location: 164817-166895
  
 NCBI BlastP on this gene

Piso0\_000687

not annotated
  
Accession: CCE78660
  
Location: 161856-163979
  
 NCBI BlastP on this gene

Piso0\_000686

not annotated
  
Accession: CCE78659
  
Location: 156562-159348
  
 NCBI BlastP on this gene

Piso0\_000685

Query: Architecture Search FASTA input

FO082057 : Pichia sorbitophila strain CBS 7064 chromosome C complete sequence.    Total score: 1.0     Cumulative Blast bit score: 464

Hit cluster cross-links:

Mycgr3G70471
  
Location: 0-405

Mycgr3G70471

Mycgr3G39149
  
Location: 505-1798

Mycgr3G39149

Mycgr3G92130
  
Location: 1898-2396

Mycgr3G92130

Mycgr3G38483
  
Location: 2496-3576

Mycgr3G38483

Mycgr3G108869
  
Location: 3676-5056

Mycgr3G108869

Mycgr3G103943
  
Location: 5156-5762

Mycgr3G103943

Mycgr3G57362
  
Location: 5862-7296

Mycgr3G57362

Mycgr3G39086
  
Location: 7396-8368

Mycgr3G39086

Mycgr3G103942
  
Location: 8468-8714

Mycgr3G103942

Mycgr3G108865
  
Location: 8814-10239

Mycgr3G108865

Mycgr3G70475
  
Location: 10339-11821

Mycgr3G70475

Mycgr3G108866
  
Location: 11921-13010

Mycgr3G108866

Mycgr3G92136
  
Location: 13110-13593

Mycgr3G92136

not annotated
  
Accession: CCE78080
  
Location: 169982-170734
  
 NCBI BlastP on this gene

Piso0\_000693

not annotated
  
Accession: CCE78079
  
Location: 167570-169558
  
 NCBI BlastP on this gene

Piso0\_000692

not annotated
  
Accession: CCE78078
  
Location: 166943-167296
  
 NCBI BlastP on this gene

Piso0\_000691

not annotated
  
Accession: CCE78077
  
Location: 164284-164904
  
 NCBI BlastP on this gene

Piso0\_000690

not annotated
  
Accession: CCE78076
  
Location: 161753-163279
  
 NCBI BlastP on this gene

Piso0\_000689

not annotated
  
Accession: CCE78075
  
Location: 160147-161472
  
  
**BlastP hit with Mycgr3G39149**
  
Percentage identity: 51 %
  
BlastP bit score: 465
  
Sequence coverage: 102 %
  
E-value: 8e-158
  
  
 NCBI BlastP on this gene

Piso0\_000688

not annotated
  
Accession: CCE78074
  
Location: 157646-159724
  
 NCBI BlastP on this gene

Piso0\_000687

not annotated
  
Accession: CCE78073
  
Location: 154714-156828
  
 NCBI BlastP on this gene

Piso0\_000686

not annotated
  
Accession: CCE78072
  
Location: 149498-152284
  
 NCBI BlastP on this gene

Piso0\_000685

Query: Architecture Search FASTA input

DS231615 : Pyrenophora tritici-repentis Pt-1C-BFP supercont1.1 genomic scaffold    Total score: 1.0     Cumulative Blast bit score: 462

Hit cluster cross-links:

Mycgr3G70471
  
Location: 0-405

Mycgr3G70471

Mycgr3G39149
  
Location: 505-1798

Mycgr3G39149

Mycgr3G92130
  
Location: 1898-2396

Mycgr3G92130

Mycgr3G38483
  
Location: 2496-3576

Mycgr3G38483

Mycgr3G108869
  
Location: 3676-5056

Mycgr3G108869

Mycgr3G103943
  
Location: 5156-5762

Mycgr3G103943

Mycgr3G57362
  
Location: 5862-7296

Mycgr3G57362

Mycgr3G39086
  
Location: 7396-8368

Mycgr3G39086

Mycgr3G103942
  
Location: 8468-8714

Mycgr3G103942

Mycgr3G108865
  
Location: 8814-10239

Mycgr3G108865

Mycgr3G70475
  
Location: 10339-11821

Mycgr3G70475

Mycgr3G108866
  
Location: 11921-13010

Mycgr3G108866

Mycgr3G92136
  
Location: 13110-13593

Mycgr3G92136

ribosome biogenesis protein Sqt1
  
Accession: EDU41433
  
Location: 5737553-5739091
  
  
**BlastP hit with Mycgr3G70475**
  
Percentage identity: 52 %
  
BlastP bit score: 462
  
Sequence coverage: 102 %
  
E-value: 1e-154
  
  
 NCBI BlastP on this gene

EDU41433

conserved hypothetical protein
  
Accession: EDU41432
  
Location: 5733327-5734313
  
 NCBI BlastP on this gene

EDU41432

structural maintenance of chromosomes protein 3
  
Accession: EDU41431
  
Location: 5726661-5730357
  
 NCBI BlastP on this gene

EDU41431

Query: Architecture Search FASTA input

GL629801 : Grosmannia clavigera kw1407 unplaced genomic scaffold GCSC\_173    Total score: 1.0     Cumulative Blast bit score: 461

Hit cluster cross-links:

Mycgr3G70471
  
Location: 0-405

Mycgr3G70471

Mycgr3G39149
  
Location: 505-1798

Mycgr3G39149

Mycgr3G92130
  
Location: 1898-2396

Mycgr3G92130

Mycgr3G38483
  
Location: 2496-3576

Mycgr3G38483

Mycgr3G108869
  
Location: 3676-5056

Mycgr3G108869

Mycgr3G103943
  
Location: 5156-5762

Mycgr3G103943

Mycgr3G57362
  
Location: 5862-7296

Mycgr3G57362

Mycgr3G39086
  
Location: 7396-8368

Mycgr3G39086

Mycgr3G103942
  
Location: 8468-8714

Mycgr3G103942

Mycgr3G108865
  
Location: 8814-10239

Mycgr3G108865

Mycgr3G70475
  
Location: 10339-11821

Mycgr3G70475

Mycgr3G108866
  
Location: 11921-13010

Mycgr3G108866

Mycgr3G92136
  
Location: 13110-13593

Mycgr3G92136

hypothetical protein
  
Accession: EFX00389
  
Location: 1220252-1223010
  
 NCBI BlastP on this gene

EFX00389

small oligopeptide transporter
  
Accession: EFX00268
  
Location: 1216970-1219636
  
 NCBI BlastP on this gene

EFX00268

beta-ketoacyl synthase
  
Accession: EFX00048
  
Location: 1213828-1216469
  
  
**BlastP hit with Mycgr3G39149**
  
Percentage identity: 57 %
  
BlastP bit score: 461
  
Sequence coverage: 100 %
  
E-value: 4e-152
  
  
 NCBI BlastP on this gene

EFX00048

dihydrofolate reductase
  
Accession: EFX00528
  
Location: 1213101-1213767
  
 NCBI BlastP on this gene

EFX00528

hypothetical protein
  
Accession: EFX00161
  
Location: 1211468-1212601
  
 NCBI BlastP on this gene

EFX00161

Query: Architecture Search FASTA input

CH408156 : Pichia guilliermondii ATCC 6260 scaffold\_2 genomic scaffold    Total score: 1.0     Cumulative Blast bit score: 457

Hit cluster cross-links:

Mycgr3G70471
  
Location: 0-405

Mycgr3G70471

Mycgr3G39149
  
Location: 505-1798

Mycgr3G39149

Mycgr3G92130
  
Location: 1898-2396

Mycgr3G92130

Mycgr3G38483
  
Location: 2496-3576

Mycgr3G38483

Mycgr3G108869
  
Location: 3676-5056

Mycgr3G108869

Mycgr3G103943
  
Location: 5156-5762

Mycgr3G103943

Mycgr3G57362
  
Location: 5862-7296

Mycgr3G57362

Mycgr3G39086
  
Location: 7396-8368

Mycgr3G39086

Mycgr3G103942
  
Location: 8468-8714

Mycgr3G103942

Mycgr3G108865
  
Location: 8814-10239

Mycgr3G108865

Mycgr3G70475
  
Location: 10339-11821

Mycgr3G70475

Mycgr3G108866
  
Location: 11921-13010

Mycgr3G108866

Mycgr3G92136
  
Location: 13110-13593

Mycgr3G92136

hypothetical protein
  
Accession: EDK37719
  
Location: 1103945-1104736
  
 NCBI BlastP on this gene

EDK37719

hypothetical protein
  
Accession: EDK37720
  
Location: 1104733-1106079
  
 NCBI BlastP on this gene

EDK37720

hypothetical protein
  
Accession: EDK37721
  
Location: 1106184-1106651
  
 NCBI BlastP on this gene

EDK37721

hypothetical protein
  
Accession: EDK37722
  
Location: 1107037-1107840
  
 NCBI BlastP on this gene

EDK37722

hypothetical protein
  
Accession: EDK37723
  
Location: 1108028-1109812
  
 NCBI BlastP on this gene

EDK37723

hypothetical protein
  
Accession: EDK37724
  
Location: 1110341-1111162
  
 NCBI BlastP on this gene

EDK37724

hypothetical protein
  
Accession: EDK37725
  
Location: 1111335-1112549
  
 NCBI BlastP on this gene

EDK37725

hypothetical protein
  
Accession: EDK37726
  
Location: 1112629-1113927
  
  
**BlastP hit with Mycgr3G39149**
  
Percentage identity: 52 %
  
BlastP bit score: 457
  
Sequence coverage: 102 %
  
E-value: 9e-155
  
  
 NCBI BlastP on this gene

EDK37726

hypothetical protein
  
Accession: EDK37727
  
Location: 1113945-1115912
  
 NCBI BlastP on this gene

EDK37727

hypothetical protein
  
Accession: EDK37728
  
Location: 1117279-1119705
  
 NCBI BlastP on this gene

EDK37728

hypothetical protein
  
Accession: EDK37729
  
Location: 1119788-1122115
  
 NCBI BlastP on this gene

EDK37729

hypothetical protein
  
Accession: EDK37730
  
Location: 1122197-1122709
  
 NCBI BlastP on this gene

EDK37730

Query: Architecture Search FASTA input

DS572752 : Paracoccidioides brasiliensis Pb18 supercont1.3 genomic scaffold    Total score: 1.0     Cumulative Blast bit score: 454

Hit cluster cross-links:

Mycgr3G70471
  
Location: 0-405

Mycgr3G70471

Mycgr3G39149
  
Location: 505-1798

Mycgr3G39149

Mycgr3G92130
  
Location: 1898-2396

Mycgr3G92130

Mycgr3G38483
  
Location: 2496-3576

Mycgr3G38483

Mycgr3G108869
  
Location: 3676-5056

Mycgr3G108869

Mycgr3G103943
  
Location: 5156-5762

Mycgr3G103943

Mycgr3G57362
  
Location: 5862-7296

Mycgr3G57362

Mycgr3G39086
  
Location: 7396-8368

Mycgr3G39086

Mycgr3G103942
  
Location: 8468-8714

Mycgr3G103942

Mycgr3G108865
  
Location: 8814-10239

Mycgr3G108865

Mycgr3G70475
  
Location: 10339-11821

Mycgr3G70475

Mycgr3G108866
  
Location: 11921-13010

Mycgr3G108866

Mycgr3G92136
  
Location: 13110-13593

Mycgr3G92136

4-coumarate-CoA ligase
  
Accession: EEH46527
  
Location: 742808-744941
  
 NCBI BlastP on this gene

EEH46527

ribosome assembly protein SQT1
  
Accession: EEH46526
  
Location: 734050-735585
  
  
**BlastP hit with Mycgr3G70475**
  
Percentage identity: 52 %
  
BlastP bit score: 454
  
Sequence coverage: 103 %
  
E-value: 1e-151
  
  
 NCBI BlastP on this gene

EEH46526

conserved hypothetical protein
  
Accession: EEH46525
  
Location: 733086-733467
  
 NCBI BlastP on this gene

EEH46525

CCR4-NOT transcription complex subunit 7
  
Accession: EEH46524
  
Location: 729966-732566
  
 NCBI BlastP on this gene

EEH46524

peroxisomal copper amine oxidase
  
Accession: EEH46523
  
Location: 727419-729704
  
 NCBI BlastP on this gene

EEH46523

predicted protein
  
Accession: EEH46522
  
Location: 725739-726515
  
 NCBI BlastP on this gene

EEH46522

Query: Architecture Search FASTA input

DS022225 : Schizosaccharomyces japonicus yFS275 supercont1.2 genomic scaffold    Total score: 1.0     Cumulative Blast bit score: 451

Hit cluster cross-links:

Mycgr3G70471
  
Location: 0-405

Mycgr3G70471

Mycgr3G39149
  
Location: 505-1798

Mycgr3G39149

Mycgr3G92130
  
Location: 1898-2396

Mycgr3G92130

Mycgr3G38483
  
Location: 2496-3576

Mycgr3G38483

Mycgr3G108869
  
Location: 3676-5056

Mycgr3G108869

Mycgr3G103943
  
Location: 5156-5762

Mycgr3G103943

Mycgr3G57362
  
Location: 5862-7296

Mycgr3G57362

Mycgr3G39086
  
Location: 7396-8368

Mycgr3G39086

Mycgr3G103942
  
Location: 8468-8714

Mycgr3G103942

Mycgr3G108865
  
Location: 8814-10239

Mycgr3G108865

Mycgr3G70475
  
Location: 10339-11821

Mycgr3G70475

Mycgr3G108866
  
Location: 11921-13010

Mycgr3G108866

Mycgr3G92136
  
Location: 13110-13593

Mycgr3G92136

3-oxoacyl-[acyl-carrier-protein] synthase
  
Accession: EEB06885
  
Location: 1739365-1740758
  
  
**BlastP hit with Mycgr3G39149**
  
Percentage identity: 54 %
  
BlastP bit score: 451
  
Sequence coverage: 99 %
  
E-value: 1e-152
  
  
 NCBI BlastP on this gene

EEB06885

phospholipid-transporting ATPase
  
Accession: EEB06884
  
Location: 1735530-1739330
  
 NCBI BlastP on this gene

EEB06884

CCR4-Not complex subunit Caf16
  
Accession: EEB06883
  
Location: 1733252-1734234
  
 NCBI BlastP on this gene

EEB06883

predicted protein
  
Accession: EEB06882
  
Location: 1729168-1733220
  
 NCBI BlastP on this gene

EEB06882

Query: Architecture Search FASTA input

DS544803 : Paracoccidioides brasiliensis Pb03 supercont1.1 genomic scaffold    Total score: 1.0     Cumulative Blast bit score: 449

Hit cluster cross-links:

Mycgr3G70471
  
Location: 0-405

Mycgr3G70471

Mycgr3G39149
  
Location: 505-1798

Mycgr3G39149

Mycgr3G92130
  
Location: 1898-2396

Mycgr3G92130

Mycgr3G38483
  
Location: 2496-3576

Mycgr3G38483

Mycgr3G108869
  
Location: 3676-5056

Mycgr3G108869

Mycgr3G103943
  
Location: 5156-5762

Mycgr3G103943

Mycgr3G57362
  
Location: 5862-7296

Mycgr3G57362

Mycgr3G39086
  
Location: 7396-8368

Mycgr3G39086

Mycgr3G103942
  
Location: 8468-8714

Mycgr3G103942

Mycgr3G108865
  
Location: 8814-10239

Mycgr3G108865

Mycgr3G70475
  
Location: 10339-11821

Mycgr3G70475

Mycgr3G108866
  
Location: 11921-13010

Mycgr3G108866

Mycgr3G92136
  
Location: 13110-13593

Mycgr3G92136

conserved hypothetical protein
  
Accession: EEH17663
  
Location: 774377-777108
  
 NCBI BlastP on this gene

EEH17663

4-coumarate-CoA ligase
  
Accession: EEH17662
  
Location: 769027-771160
  
 NCBI BlastP on this gene

EEH17662

conserved hypothetical protein
  
Accession: EEH17661
  
Location: 766152-767687
  
  
**BlastP hit with Mycgr3G70475**
  
Percentage identity: 51 %
  
BlastP bit score: 450
  
Sequence coverage: 103 %
  
E-value: 4e-150
  
  
 NCBI BlastP on this gene

EEH17661

predicted protein
  
Accession: EEH17660
  
Location: 765188-765569
  
 NCBI BlastP on this gene

EEH17660

CCR4-NOT transcription complex subunit 7
  
Accession: EEH17659
  
Location: 762068-764668
  
 NCBI BlastP on this gene

EEH17659

peroxisomal copper amine oxidase
  
Accession: EEH17658
  
Location: 759522-760728
  
 NCBI BlastP on this gene

EEH17658

predicted protein
  
Accession: EEH17657
  
Location: 757832-758608
  
 NCBI BlastP on this gene

EEH17657

Query: Architecture Search FASTA input

FM992690 : Candida dubliniensis CD36 chromosome 3    Total score: 1.0     Cumulative Blast bit score: 442

Hit cluster cross-links:

Mycgr3G70471
  
Location: 0-405

Mycgr3G70471

Mycgr3G39149
  
Location: 505-1798

Mycgr3G39149

Mycgr3G92130
  
Location: 1898-2396

Mycgr3G92130

Mycgr3G38483
  
Location: 2496-3576

Mycgr3G38483

Mycgr3G108869
  
Location: 3676-5056

Mycgr3G108869

Mycgr3G103943
  
Location: 5156-5762

Mycgr3G103943

Mycgr3G57362
  
Location: 5862-7296

Mycgr3G57362

Mycgr3G39086
  
Location: 7396-8368

Mycgr3G39086

Mycgr3G103942
  
Location: 8468-8714

Mycgr3G103942

Mycgr3G108865
  
Location: 8814-10239

Mycgr3G108865

Mycgr3G70475
  
Location: 10339-11821

Mycgr3G70475

Mycgr3G108866
  
Location: 11921-13010

Mycgr3G108866

Mycgr3G92136
  
Location: 13110-13593

Mycgr3G92136

autophagy-related protein, putative
  
Accession: CAX43004
  
Location: 1129718-1132582
  
 NCBI BlastP on this gene

CD36\_85010

zinc-finger transcription factor, putative
  
Accession: CAX43005
  
Location: 1134990-1135871
  
 NCBI BlastP on this gene

CD36\_85020

conserved hypothetical protein
  
Accession: CAX43006
  
Location: 1137268-1138599
  
 NCBI BlastP on this gene

CD36\_85030

3-oxoacyl-[acyl-carrier-protein] synthase, putative
  
Accession: CAX43007
  
Location: 1138655-1139977
  
  
**BlastP hit with Mycgr3G39149**
  
Percentage identity: 50 %
  
BlastP bit score: 442
  
Sequence coverage: 103 %
  
E-value: 6e-149
  
  
 NCBI BlastP on this gene

CD36\_85040

Query: Architecture Search FASTA input

CP000494 : Bradyrhizobium sp. BTAi1    Total score: 1.0     Cumulative Blast bit score: 439

Hit cluster cross-links:

Mycgr3G70471
  
Location: 0-405

Mycgr3G70471

Mycgr3G39149
  
Location: 505-1798

Mycgr3G39149

Mycgr3G92130
  
Location: 1898-2396

Mycgr3G92130

Mycgr3G38483
  
Location: 2496-3576

Mycgr3G38483

Mycgr3G108869
  
Location: 3676-5056

Mycgr3G108869

Mycgr3G103943
  
Location: 5156-5762

Mycgr3G103943

Mycgr3G57362
  
Location: 5862-7296

Mycgr3G57362

Mycgr3G39086
  
Location: 7396-8368

Mycgr3G39086

Mycgr3G103942
  
Location: 8468-8714

Mycgr3G103942

Mycgr3G108865
  
Location: 8814-10239

Mycgr3G108865

Mycgr3G70475
  
Location: 10339-11821

Mycgr3G70475

Mycgr3G108866
  
Location: 11921-13010

Mycgr3G108866

Mycgr3G92136
  
Location: 13110-13593

Mycgr3G92136

primary replicative DNA helicase
  
Accession: ABQ35885
  
Location: 3991390-3992898
  
 NCBI BlastP on this gene

BBta\_3808

hypothetical protein
  
Accession: ABQ35886
  
Location: 3993178-3993276
  
 NCBI BlastP on this gene

BBta\_3809

cyclopropane-fatty-acyl-phospholipid synthase
  
Accession: ABQ35887
  
Location: 3993523-3994623
  
 NCBI BlastP on this gene

BBta\_3810

putative exported protein of unknown function
  
Accession: ABQ35888
  
Location: 3995193-3996005
  
 NCBI BlastP on this gene

BBta\_3811

LSU ribosomal protein L9P
  
Accession: ABQ35889
  
Location: 3996072-3996665
  
 NCBI BlastP on this gene

BBta\_3812

putative membrane protein of unknown function
  
Accession: ABQ35890
  
Location: 3996715-3997656
  
 NCBI BlastP on this gene

BBta\_3813

SSU ribosomal protein S18P
  
Accession: ABQ35891
  
Location: 3997798-3998037
  
 NCBI BlastP on this gene

rpsR

SSU ribosomal protein S6P
  
Accession: ABQ35892
  
Location: 3998043-3998504
  
 NCBI BlastP on this gene

rps6

[Acyl-carrier-protein] S-malonyltransferase
  
Accession: ABQ35893
  
Location: 3998878-3999837
  
 NCBI BlastP on this gene

fabD

3-oxoacyl-[acyl-carrier-protein] reductase
  
Accession: ABQ35894
  
Location: 3999872-4000609
  
 NCBI BlastP on this gene

fabG

Acyl carrier protein (ACP)
  
Accession: ABQ35895
  
Location: 4001048-4001287
  
 NCBI BlastP on this gene

acpP

3-oxoacyl-[acyl-carrier-protein] synthase II
  
Accession: ABQ35896
  
Location: 4001508-4002773
  
  
**BlastP hit with Mycgr3G39149**
  
Percentage identity: 53 %
  
BlastP bit score: 439
  
Sequence coverage: 99 %
  
E-value: 3e-148
  
  
 NCBI BlastP on this gene

fabF

Query: Architecture Search FASTA input

DS572698 : Verticillium dahliae VdLs.17 supercont1.4 genomic scaffold    Total score: 1.0     Cumulative Blast bit score: 426

Hit cluster cross-links:

Mycgr3G70471
  
Location: 0-405

Mycgr3G70471

Mycgr3G39149
  
Location: 505-1798

Mycgr3G39149

Mycgr3G92130
  
Location: 1898-2396

Mycgr3G92130

Mycgr3G38483
  
Location: 2496-3576

Mycgr3G38483

Mycgr3G108869
  
Location: 3676-5056

Mycgr3G108869

Mycgr3G103943
  
Location: 5156-5762

Mycgr3G103943

Mycgr3G57362
  
Location: 5862-7296

Mycgr3G57362

Mycgr3G39086
  
Location: 7396-8368

Mycgr3G39086

Mycgr3G103942
  
Location: 8468-8714

Mycgr3G103942

Mycgr3G108865
  
Location: 8814-10239

Mycgr3G108865

Mycgr3G70475
  
Location: 10339-11821

Mycgr3G70475

Mycgr3G108866
  
Location: 11921-13010

Mycgr3G108866

Mycgr3G92136
  
Location: 13110-13593

Mycgr3G92136

ribosome biogenesis protein Sqt1
  
Accession: EGY21202
  
Location: 1207186-1208688
  
  
**BlastP hit with Mycgr3G70475**
  
Percentage identity: 52 %
  
BlastP bit score: 426
  
Sequence coverage: 104 %
  
E-value: 7e-141
  
  
 NCBI BlastP on this gene

EGY21202

hypothetical protein
  
Accession: EGY21201
  
Location: 1205551-1206597
  
 NCBI BlastP on this gene

EGY21201

hypothetical protein
  
Accession: EGY21200
  
Location: 1201399-1202629
  
 NCBI BlastP on this gene

EGY21200

hypothetical protein
  
Accession: EGY21199
  
Location: 1198637-1199743
  
 NCBI BlastP on this gene

EGY21199

Query: Architecture Search FASTA input

EQ962652 : Talaromyces stipitatus ATCC 10500 scf\_1105507295523 genomic scaffold    Total score: 1.0     Cumulative Blast bit score: 417

Hit cluster cross-links:

Mycgr3G70471
  
Location: 0-405

Mycgr3G70471

Mycgr3G39149
  
Location: 505-1798

Mycgr3G39149

Mycgr3G92130
  
Location: 1898-2396

Mycgr3G92130

Mycgr3G38483
  
Location: 2496-3576

Mycgr3G38483

Mycgr3G108869
  
Location: 3676-5056

Mycgr3G108869

Mycgr3G103943
  
Location: 5156-5762

Mycgr3G103943

Mycgr3G57362
  
Location: 5862-7296

Mycgr3G57362

Mycgr3G39086
  
Location: 7396-8368

Mycgr3G39086

Mycgr3G103942
  
Location: 8468-8714

Mycgr3G103942

Mycgr3G108865
  
Location: 8814-10239

Mycgr3G108865

Mycgr3G70475
  
Location: 10339-11821

Mycgr3G70475

Mycgr3G108866
  
Location: 11921-13010

Mycgr3G108866

Mycgr3G92136
  
Location: 13110-13593

Mycgr3G92136

fungal specific transcription factor, putative
  
Accession: EED23989
  
Location: 3898465-3901254
  
 NCBI BlastP on this gene

EED23989

conserved hypothetical protein
  
Accession: EED23990
  
Location: 3903280-3904849
  
  
**BlastP hit with Mycgr3G57362**
  
Percentage identity: 32 %
  
BlastP bit score: 205
  
Sequence coverage: 98 %
  
E-value: 1e-56
  
  
 NCBI BlastP on this gene

EED23990

amino acid transporter, putative
  
Accession: EED23991
  
Location: 3905710-3907693
  
 NCBI BlastP on this gene

EED23991

conserved hypothetical protein
  
Accession: EED23992
  
Location: 3908221-3908943
  
 NCBI BlastP on this gene

EED23992

FAD dependent oxidoreductase superfamily
  
Accession: EED23993
  
Location: 3909870-3914043
  
  
**BlastP hit with Mycgr3G57362**
  
Percentage identity: 31 %
  
BlastP bit score: 213
  
Sequence coverage: 98 %
  
E-value: 3e-56
  
  
 NCBI BlastP on this gene

EED23993

amino acid permease, putative
  
Accession: EED23994
  
Location: 3914817-3916612
  
 NCBI BlastP on this gene

EED23994

2-haloalkanoic acid dehalogenase, putative
  
Accession: EED23995
  
Location: 3917414-3918239
  
 NCBI BlastP on this gene

EED23995

Query: Architecture Search FASTA input

JH687379 : Stereum hirsutum FP-91666 SS1 unplaced genomic scaffold STEHIscaffold\_1    Total score: 1.0     Cumulative Blast bit score: 417

Hit cluster cross-links:

Mycgr3G70471
  
Location: 0-405

Mycgr3G70471

Mycgr3G39149
  
Location: 505-1798

Mycgr3G39149

Mycgr3G92130
  
Location: 1898-2396

Mycgr3G92130

Mycgr3G38483
  
Location: 2496-3576

Mycgr3G38483

Mycgr3G108869
  
Location: 3676-5056

Mycgr3G108869

Mycgr3G103943
  
Location: 5156-5762

Mycgr3G103943

Mycgr3G57362
  
Location: 5862-7296

Mycgr3G57362

Mycgr3G39086
  
Location: 7396-8368

Mycgr3G39086

Mycgr3G103942
  
Location: 8468-8714

Mycgr3G103942

Mycgr3G108865
  
Location: 8814-10239

Mycgr3G108865

Mycgr3G70475
  
Location: 10339-11821

Mycgr3G70475

Mycgr3G108866
  
Location: 11921-13010

Mycgr3G108866

Mycgr3G92136
  
Location: 13110-13593

Mycgr3G92136

high affinity nickel transport protein nic1
  
Accession: EIM92684
  
Location: 2972008-2973547
  
  
**BlastP hit with Mycgr3G108865**
  
Percentage identity: 57 %
  
BlastP bit score: 417
  
Sequence coverage: 81 %
  
E-value: 5e-139
  
  
 NCBI BlastP on this gene

EIM92684

hypothetical protein
  
Accession: EIM92683
  
Location: 2969934-2970239
  
 NCBI BlastP on this gene

EIM92683

hypothetical protein
  
Accession: EIM92682
  
Location: 2968084-2968470
  
 NCBI BlastP on this gene

EIM92682

hypothetical protein
  
Accession: EIM92681
  
Location: 2965276-2966244
  
 NCBI BlastP on this gene

EIM92681

40S ribosomal protein S27
  
Accession: EIM92680
  
Location: 2963325-2963788
  
 NCBI BlastP on this gene

EIM92680

Query: Architecture Search FASTA input

KE145368 : Glarea lozoyensis ATCC 20868 chromosome Unknown GLAREA4    Total score: 1.0     Cumulative Blast bit score: 406

Hit cluster cross-links:

Mycgr3G70471
  
Location: 0-405

Mycgr3G70471

Mycgr3G39149
  
Location: 505-1798

Mycgr3G39149

Mycgr3G92130
  
Location: 1898-2396

Mycgr3G92130

Mycgr3G38483
  
Location: 2496-3576

Mycgr3G38483

Mycgr3G108869
  
Location: 3676-5056

Mycgr3G108869

Mycgr3G103943
  
Location: 5156-5762

Mycgr3G103943

Mycgr3G57362
  
Location: 5862-7296

Mycgr3G57362

Mycgr3G39086
  
Location: 7396-8368

Mycgr3G39086

Mycgr3G103942
  
Location: 8468-8714

Mycgr3G103942

Mycgr3G108865
  
Location: 8814-10239

Mycgr3G108865

Mycgr3G70475
  
Location: 10339-11821

Mycgr3G70475

Mycgr3G108866
  
Location: 11921-13010

Mycgr3G108866

Mycgr3G92136
  
Location: 13110-13593

Mycgr3G92136

hypothetical protein
  
Accession: EPE28767
  
Location: 1614790-1615686
  
 NCBI BlastP on this gene

EPE28767

hypothetical protein
  
Accession: EPE28768
  
Location: 1617066-1618346
  
 NCBI BlastP on this gene

EPE28768

Actin-like ATPase
  
Accession: EPE28769
  
Location: 1618829-1620319
  
 NCBI BlastP on this gene

EPE28769

hypothetical protein
  
Accession: EPE28770
  
Location: 1620345-1621154
  
 NCBI BlastP on this gene

EPE28770

hypothetical protein
  
Accession: EPE28771
  
Location: 1622620-1623911
  
  
**BlastP hit with Mycgr3G108865**
  
Percentage identity: 53 %
  
BlastP bit score: 406
  
Sequence coverage: 86 %
  
E-value: 1e-134
  
  
 NCBI BlastP on this gene

EPE28771

Query: Architecture Search FASTA input

HF679025 : Fusarium fujikuroi IMI 58289 draft genome, chromosome FFUJ\_chr03.    Total score: 1.0     Cumulative Blast bit score: 405

Hit cluster cross-links:

Mycgr3G70471
  
Location: 0-405

Mycgr3G70471

Mycgr3G39149
  
Location: 505-1798

Mycgr3G39149

Mycgr3G92130
  
Location: 1898-2396

Mycgr3G92130

Mycgr3G38483
  
Location: 2496-3576

Mycgr3G38483

Mycgr3G108869
  
Location: 3676-5056

Mycgr3G108869

Mycgr3G103943
  
Location: 5156-5762

Mycgr3G103943

Mycgr3G57362
  
Location: 5862-7296

Mycgr3G57362

Mycgr3G39086
  
Location: 7396-8368

Mycgr3G39086

Mycgr3G103942
  
Location: 8468-8714

Mycgr3G103942

Mycgr3G108865
  
Location: 8814-10239

Mycgr3G108865

Mycgr3G70475
  
Location: 10339-11821

Mycgr3G70475

Mycgr3G108866
  
Location: 11921-13010

Mycgr3G108866

Mycgr3G92136
  
Location: 13110-13593

Mycgr3G92136

related to thioesterase superfamily member 2
  
Accession: CCT66461
  
Location: 4510646-4511152
  
 NCBI BlastP on this gene

FFUJ\_03493

related to malate dehydrogenase
  
Accession: CCT66462
  
Location: 4511362-4512432
  
 NCBI BlastP on this gene

FFUJ\_03494

related to purine utilization positive regulator
  
Accession: CCT66780
  
Location: 4512709-4515045
  
 NCBI BlastP on this gene

FFUJ\_03495

related to dihydroxy-acid dehydratase
  
Accession: CCT66745
  
Location: 4515107-4517031
  
 NCBI BlastP on this gene

FFUJ\_03496

related to short-chain alcohol dehydrogenase
  
Accession: CCT66463
  
Location: 4517336-4518136
  
 NCBI BlastP on this gene

FFUJ\_03497

uncharacterized protein
  
Accession: CCT66464
  
Location: 4518916-4519385
  
 NCBI BlastP on this gene

FFUJ\_03498

probable high-affinity nickel transport protein nic1
  
Accession: CCT66465
  
Location: 4519752-4521012
  
  
**BlastP hit with Mycgr3G108865**
  
Percentage identity: 54 %
  
BlastP bit score: 405
  
Sequence coverage: 79 %
  
E-value: 2e-134
  
  
 NCBI BlastP on this gene

FFUJ\_03499

Query: Architecture Search FASTA input

GL636500 : Coccidioides posadasii str. Silveira unplaced genomic scaffold supercont2.15    Total score: 1.0     Cumulative Blast bit score: 398

Hit cluster cross-links:

Mycgr3G70471
  
Location: 0-405

Mycgr3G70471

Mycgr3G39149
  
Location: 505-1798

Mycgr3G39149

Mycgr3G92130
  
Location: 1898-2396

Mycgr3G92130

Mycgr3G38483
  
Location: 2496-3576

Mycgr3G38483

Mycgr3G108869
  
Location: 3676-5056

Mycgr3G108869

Mycgr3G103943
  
Location: 5156-5762

Mycgr3G103943

Mycgr3G57362
  
Location: 5862-7296

Mycgr3G57362

Mycgr3G39086
  
Location: 7396-8368

Mycgr3G39086

Mycgr3G103942
  
Location: 8468-8714

Mycgr3G103942

Mycgr3G108865
  
Location: 8814-10239

Mycgr3G108865

Mycgr3G70475
  
Location: 10339-11821

Mycgr3G70475

Mycgr3G108866
  
Location: 11921-13010

Mycgr3G108866

Mycgr3G92136
  
Location: 13110-13593

Mycgr3G92136

nickel transporter
  
Accession: EFW15593
  
Location: 258575-260596
  
  
**BlastP hit with Mycgr3G108865**
  
Percentage identity: 55 %
  
BlastP bit score: 398
  
Sequence coverage: 86 %
  
E-value: 4e-131
  
  
 NCBI BlastP on this gene

EFW15593

conserved hypothetical protein
  
Accession: EFW15592
  
Location: 255542-258137
  
 NCBI BlastP on this gene

EFW15592

hypothetical protein
  
Accession: EFW15591
  
Location: 254248-255521
  
 NCBI BlastP on this gene

EFW15591

conserved hypothetical protein
  
Accession: EFW15590
  
Location: 249561-251757
  
 NCBI BlastP on this gene

EFW15590

Query: Architecture Search FASTA input

AQGS01000575 : Dactylellina haptotyla CBS 200.50    Total score: 1.0     Cumulative Blast bit score: 396

Hit cluster cross-links:

Mycgr3G70471
  
Location: 0-405

Mycgr3G70471

Mycgr3G39149
  
Location: 505-1798

Mycgr3G39149

Mycgr3G92130
  
Location: 1898-2396

Mycgr3G92130

Mycgr3G38483
  
Location: 2496-3576

Mycgr3G38483

Mycgr3G108869
  
Location: 3676-5056

Mycgr3G108869

Mycgr3G103943
  
Location: 5156-5762

Mycgr3G103943

Mycgr3G57362
  
Location: 5862-7296

Mycgr3G57362

Mycgr3G39086
  
Location: 7396-8368

Mycgr3G39086

Mycgr3G103942
  
Location: 8468-8714

Mycgr3G103942

Mycgr3G108865
  
Location: 8814-10239

Mycgr3G108865

Mycgr3G70475
  
Location: 10339-11821

Mycgr3G70475

Mycgr3G108866
  
Location: 11921-13010

Mycgr3G108866

Mycgr3G92136
  
Location: 13110-13593

Mycgr3G92136

hypothetical protein
  
Accession: EPS38297
  
Location: 346908-349134
  
 NCBI BlastP on this gene

EPS38297

hypothetical protein
  
Accession: EPS38341
  
Location: 349408-350592
  
 NCBI BlastP on this gene

EPS38341

hypothetical protein
  
Accession: EPS38209
  
Location: 351528-353015
  
 NCBI BlastP on this gene

EPS38209

hypothetical protein
  
Accession: EPS38251
  
Location: 354237-358793
  
  
**BlastP hit with Mycgr3G108865**
  
Percentage identity: 59 %
  
BlastP bit score: 397
  
Sequence coverage: 79 %
  
E-value: 2e-125
  
  
 NCBI BlastP on this gene

EPS38251

Query: Architecture Search FASTA input

GL891304 : Neurospora tetrasperma FGSC 2508 unplaced genomic scaffold NEUTE1scaffold\_3    Total score: 1.0     Cumulative Blast bit score: 392

Hit cluster cross-links:

Mycgr3G70471
  
Location: 0-405

Mycgr3G70471

Mycgr3G39149
  
Location: 505-1798

Mycgr3G39149

Mycgr3G92130
  
Location: 1898-2396

Mycgr3G92130

Mycgr3G38483
  
Location: 2496-3576

Mycgr3G38483

Mycgr3G108869
  
Location: 3676-5056

Mycgr3G108869

Mycgr3G103943
  
Location: 5156-5762

Mycgr3G103943

Mycgr3G57362
  
Location: 5862-7296

Mycgr3G57362

Mycgr3G39086
  
Location: 7396-8368

Mycgr3G39086

Mycgr3G103942
  
Location: 8468-8714

Mycgr3G103942

Mycgr3G108865
  
Location: 8814-10239

Mycgr3G108865

Mycgr3G70475
  
Location: 10339-11821

Mycgr3G70475

Mycgr3G108866
  
Location: 11921-13010

Mycgr3G108866

Mycgr3G92136
  
Location: 13110-13593

Mycgr3G92136

hypothetical protein
  
Accession: EGO58357
  
Location: 4423195-4424918
  
 NCBI BlastP on this gene

EGO58357

hypothetical protein
  
Accession: EGO58358
  
Location: 4425651-4426843
  
 NCBI BlastP on this gene

EGO58358

hypothetical protein
  
Accession: EGO58359
  
Location: 4429040-4429420
  
 NCBI BlastP on this gene

EGO58359

hypothetical protein
  
Accession: EGO58360
  
Location: 4430490-4431789
  
  
**BlastP hit with Mycgr3G108865**
  
Percentage identity: 58 %
  
BlastP bit score: 392
  
Sequence coverage: 74 %
  
E-value: 3e-129
  
  
 NCBI BlastP on this gene

EGO58360

Query: Architecture Search FASTA input

GL891236 : Neurospora tetrasperma FGSC 2509 unplaced genomic scaffold NEUTE2scaffold\_4    Total score: 1.0     Cumulative Blast bit score: 392

Hit cluster cross-links:

Mycgr3G70471
  
Location: 0-405

Mycgr3G70471

Mycgr3G39149
  
Location: 505-1798

Mycgr3G39149

Mycgr3G92130
  
Location: 1898-2396

Mycgr3G92130

Mycgr3G38483
  
Location: 2496-3576

Mycgr3G38483

Mycgr3G108869
  
Location: 3676-5056

Mycgr3G108869

Mycgr3G103943
  
Location: 5156-5762

Mycgr3G103943

Mycgr3G57362
  
Location: 5862-7296

Mycgr3G57362

Mycgr3G39086
  
Location: 7396-8368

Mycgr3G39086

Mycgr3G103942
  
Location: 8468-8714

Mycgr3G103942

Mycgr3G108865
  
Location: 8814-10239

Mycgr3G108865

Mycgr3G70475
  
Location: 10339-11821

Mycgr3G70475

Mycgr3G108866
  
Location: 11921-13010

Mycgr3G108866

Mycgr3G92136
  
Location: 13110-13593

Mycgr3G92136

hypothetical protein
  
Accession: EGZ71317
  
Location: 568075-568275
  
 NCBI BlastP on this gene

EGZ71317

hypothetical protein
  
Accession: EGZ71316
  
Location: 566267-567990
  
 NCBI BlastP on this gene

EGZ71316

hypothetical protein
  
Accession: EGZ71315
  
Location: 564342-565534
  
 NCBI BlastP on this gene

EGZ71315

NicO-domain-containing protein
  
Accession: EGZ71314
  
Location: 559397-562145
  
  
**BlastP hit with Mycgr3G108865**
  
Percentage identity: 58 %
  
BlastP bit score: 392
  
Sequence coverage: 74 %
  
E-value: 5e-128
  
  
 NCBI BlastP on this gene

EGZ71314

nucleotide-diphospho-sugar transferase
  
Accession: EGZ71313
  
Location: 553066-555512
  
 NCBI BlastP on this gene

EGZ71313

concanavalin A-like lectin/glucanase
  
Accession: EGZ71312
  
Location: 550908-552178
  
 NCBI BlastP on this gene

EGZ71312

Query: Architecture Search FASTA input

DS231619 : Pyrenophora tritici-repentis Pt-1C-BFP supercont1.5 genomic scaffold    Total score: 1.0     Cumulative Blast bit score: 392

Hit cluster cross-links:

Mycgr3G70471
  
Location: 0-405

Mycgr3G70471

Mycgr3G39149
  
Location: 505-1798

Mycgr3G39149

Mycgr3G92130
  
Location: 1898-2396

Mycgr3G92130

Mycgr3G38483
  
Location: 2496-3576

Mycgr3G38483

Mycgr3G108869
  
Location: 3676-5056

Mycgr3G108869

Mycgr3G103943
  
Location: 5156-5762

Mycgr3G103943

Mycgr3G57362
  
Location: 5862-7296

Mycgr3G57362

Mycgr3G39086
  
Location: 7396-8368

Mycgr3G39086

Mycgr3G103942
  
Location: 8468-8714

Mycgr3G103942

Mycgr3G108865
  
Location: 8814-10239

Mycgr3G108865

Mycgr3G70475
  
Location: 10339-11821

Mycgr3G70475

Mycgr3G108866
  
Location: 11921-13010

Mycgr3G108866

Mycgr3G92136
  
Location: 13110-13593

Mycgr3G92136

choline dehydrogenase
  
Accession: EDU48816
  
Location: 1780781-1781371
  
 NCBI BlastP on this gene

EDU48816

hypothetical protein
  
Accession: EDU48815
  
Location: 1777890-1779639
  
 NCBI BlastP on this gene

EDU48815

FAD dependent oxidoreductase
  
Accession: EDU48814
  
Location: 1775664-1777263
  
  
**BlastP hit with Mycgr3G57362**
  
Percentage identity: 33 %
  
BlastP bit score: 209
  
Sequence coverage: 97 %
  
E-value: 7e-58
  
  
 NCBI BlastP on this gene

EDU48814

PrnA protein
  
Accession: EDU48813
  
Location: 1772868-1775519
  
 NCBI BlastP on this gene

EDU48813

succinate-semialdehyde dehydrogenase, mitochondrial precursor
  
Accession: EDU48812
  
Location: 1770799-1772526
  
 NCBI BlastP on this gene

EDU48812

haloacid dehalogenase
  
Accession: EDU48811
  
Location: 1769933-1770658
  
 NCBI BlastP on this gene

EDU48811

FAD dependent oxidoreductase
  
Accession: EDU48810
  
Location: 1767870-1769564
  
  
**BlastP hit with Mycgr3G57362**
  
Percentage identity: 32 %
  
BlastP bit score: 183
  
Sequence coverage: 99 %
  
E-value: 4e-48
  
  
 NCBI BlastP on this gene

EDU48810

conserved hypothetical protein
  
Accession: EDU48809
  
Location: 1766541-1767341
  
 NCBI BlastP on this gene

EDU48809

conserved hypothetical protein
  
Accession: EDU48808
  
Location: 1764967-1765820
  
 NCBI BlastP on this gene

EDU48808

Query: Architecture Search FASTA input

CH445339 : Phaeosphaeria nodorum SN15 scaffold\_15    Total score: 1.0     Cumulative Blast bit score: 392

Hit cluster cross-links:

Mycgr3G70471
  
Location: 0-405

Mycgr3G70471

Mycgr3G39149
  
Location: 505-1798

Mycgr3G39149

Mycgr3G92130
  
Location: 1898-2396

Mycgr3G92130

Mycgr3G38483
  
Location: 2496-3576

Mycgr3G38483

Mycgr3G108869
  
Location: 3676-5056

Mycgr3G108869

Mycgr3G103943
  
Location: 5156-5762

Mycgr3G103943

Mycgr3G57362
  
Location: 5862-7296

Mycgr3G57362

Mycgr3G39086
  
Location: 7396-8368

Mycgr3G39086

Mycgr3G103942
  
Location: 8468-8714

Mycgr3G103942

Mycgr3G108865
  
Location: 8814-10239

Mycgr3G108865

Mycgr3G70475
  
Location: 10339-11821

Mycgr3G70475

Mycgr3G108866
  
Location: 11921-13010

Mycgr3G108866

Mycgr3G92136
  
Location: 13110-13593

Mycgr3G92136

hypothetical protein
  
Accession: EAT83172
  
Location: 992184-992883
  
 NCBI BlastP on this gene

EAT83172

hypothetical protein
  
Accession: EAT83171
  
Location: 991103-991596
  
 NCBI BlastP on this gene

EAT83171

hypothetical protein
  
Accession: EAT83170
  
Location: 988461-989982
  
  
**BlastP hit with Mycgr3G57362**
  
Percentage identity: 33 %
  
BlastP bit score: 210
  
Sequence coverage: 99 %
  
E-value: 2e-58
  
  
 NCBI BlastP on this gene

EAT83170

hypothetical protein
  
Accession: EAT83169
  
Location: 985685-988315
  
 NCBI BlastP on this gene

EAT83169

hypothetical protein
  
Accession: EAT83168
  
Location: 984554-985279
  
 NCBI BlastP on this gene

EAT83168

hypothetical protein
  
Accession: EAT83167
  
Location: 982368-984181
  
  
**BlastP hit with Mycgr3G57362**
  
Percentage identity: 30 %
  
BlastP bit score: 182
  
Sequence coverage: 100 %
  
E-value: 6e-48
  
  
 NCBI BlastP on this gene

EAT83167

hypothetical protein
  
Accession: EAT83166
  
Location: 981076-981678
  
 NCBI BlastP on this gene

EAT83166

hypothetical protein
  
Accession: EAT83165
  
Location: 980151-980878
  
 NCBI BlastP on this gene

EAT83165

hypothetical protein
  
Accession: EAT83164
  
Location: 978211-979862
  
 NCBI BlastP on this gene

EAT83164

Query: Architecture Search FASTA input

GG749429 : Ajellomyces dermatitidis ATCC 18188 genomic scaffold supercont1.23    Total score: 1.0     Cumulative Blast bit score: 391

Hit cluster cross-links:

Mycgr3G70471
  
Location: 0-405

Mycgr3G70471

Mycgr3G39149
  
Location: 505-1798

Mycgr3G39149

Mycgr3G92130
  
Location: 1898-2396

Mycgr3G92130

Mycgr3G38483
  
Location: 2496-3576

Mycgr3G38483

Mycgr3G108869
  
Location: 3676-5056

Mycgr3G108869

Mycgr3G103943
  
Location: 5156-5762

Mycgr3G103943

Mycgr3G57362
  
Location: 5862-7296

Mycgr3G57362

Mycgr3G39086
  
Location: 7396-8368

Mycgr3G39086

Mycgr3G103942
  
Location: 8468-8714

Mycgr3G103942

Mycgr3G108865
  
Location: 8814-10239

Mycgr3G108865

Mycgr3G70475
  
Location: 10339-11821

Mycgr3G70475

Mycgr3G108866
  
Location: 11921-13010

Mycgr3G108866

Mycgr3G92136
  
Location: 13110-13593

Mycgr3G92136

hypothetical protein
  
Accession: EGE81952
  
Location: 542677-545817
  
 NCBI BlastP on this gene

EGE81952

zinc carboxypeptidase
  
Accession: EGE81953
  
Location: 546539-548402
  
 NCBI BlastP on this gene

EGE81953

hypothetical protein
  
Accession: EGE81954
  
Location: 549961-550937
  
 NCBI BlastP on this gene

EGE81954

hypothetical protein
  
Accession: EGE81955
  
Location: 551233-552204
  
 NCBI BlastP on this gene

EGE81955

nickel transporter
  
Accession: EGE81956
  
Location: 553228-555307
  
  
**BlastP hit with Mycgr3G108865**
  
Percentage identity: 53 %
  
BlastP bit score: 391
  
Sequence coverage: 89 %
  
E-value: 2e-127
  
  
 NCBI BlastP on this gene

EGE81956

terminal deoxynucleotidyl transferase
  
Accession: EGE81957
  
Location: 555898-558069
  
 NCBI BlastP on this gene

EGE81957

phospholipid-transporting ATPase
  
Accession: EGE81958
  
Location: 559074-563287
  
 NCBI BlastP on this gene

EGE81958

Query: Architecture Search FASTA input

EQ999973 : Ajellomyces dermatitidis ER-3 genomic scaffold supercont1.1    Total score: 1.0     Cumulative Blast bit score: 391

Hit cluster cross-links:

Mycgr3G70471
  
Location: 0-405

Mycgr3G70471

Mycgr3G39149
  
Location: 505-1798

Mycgr3G39149

Mycgr3G92130
  
Location: 1898-2396

Mycgr3G92130

Mycgr3G38483
  
Location: 2496-3576

Mycgr3G38483

Mycgr3G108869
  
Location: 3676-5056

Mycgr3G108869

Mycgr3G103943
  
Location: 5156-5762

Mycgr3G103943

Mycgr3G57362
  
Location: 5862-7296

Mycgr3G57362

Mycgr3G39086
  
Location: 7396-8368

Mycgr3G39086

Mycgr3G103942
  
Location: 8468-8714

Mycgr3G103942

Mycgr3G108865
  
Location: 8814-10239

Mycgr3G108865

Mycgr3G70475
  
Location: 10339-11821

Mycgr3G70475

Mycgr3G108866
  
Location: 11921-13010

Mycgr3G108866

Mycgr3G92136
  
Location: 13110-13593

Mycgr3G92136

nickel transporter
  
Accession: EEQ83315
  
Location: 987636-989714
  
  
**BlastP hit with Mycgr3G108865**
  
Percentage identity: 53 %
  
BlastP bit score: 391
  
Sequence coverage: 89 %
  
E-value: 2e-127
  
  
 NCBI BlastP on this gene

EEQ83315

terminal deoxynucleotidyl transferase
  
Accession: EEQ83314
  
Location: 984601-986772
  
 NCBI BlastP on this gene

EEQ83314

phospholipid-transporting ATPase
  
Accession: EEQ83313
  
Location: 979382-983595
  
 NCBI BlastP on this gene

EEQ83313

Query: Architecture Search FASTA input

GG657469 : Ajellomyces dermatitidis SLH14081 genomic scaffold supercont1.22    Total score: 1.0     Cumulative Blast bit score: 389

Hit cluster cross-links:

Mycgr3G70471
  
Location: 0-405

Mycgr3G70471

Mycgr3G39149
  
Location: 505-1798

Mycgr3G39149

Mycgr3G92130
  
Location: 1898-2396

Mycgr3G92130

Mycgr3G38483
  
Location: 2496-3576

Mycgr3G38483

Mycgr3G108869
  
Location: 3676-5056

Mycgr3G108869

Mycgr3G103943
  
Location: 5156-5762

Mycgr3G103943

Mycgr3G57362
  
Location: 5862-7296

Mycgr3G57362

Mycgr3G39086
  
Location: 7396-8368

Mycgr3G39086

Mycgr3G103942
  
Location: 8468-8714

Mycgr3G103942

Mycgr3G108865
  
Location: 8814-10239

Mycgr3G108865

Mycgr3G70475
  
Location: 10339-11821

Mycgr3G70475

Mycgr3G108866
  
Location: 11921-13010

Mycgr3G108866

Mycgr3G92136
  
Location: 13110-13593

Mycgr3G92136

nickel transporter
  
Accession: EEQ73846
  
Location: 142369-144448
  
  
**BlastP hit with Mycgr3G108865**
  
Percentage identity: 52 %
  
BlastP bit score: 389
  
Sequence coverage: 88 %
  
E-value: 2e-126
  
  
 NCBI BlastP on this gene

EEQ73846

terminal deoxynucleotidyl transferase
  
Accession: EEQ73845
  
Location: 139591-141687
  
 NCBI BlastP on this gene

EEQ73845

phospholipid-transporting ATPase
  
Accession: EEQ73844
  
Location: 134374-138587
  
 NCBI BlastP on this gene

EEQ73844

Query: Architecture Search FASTA input

DS027696 : Neosartorya fischeri NRRL 181 1099437636264 genomic scaffold    Total score: 1.0     Cumulative Blast bit score: 388

Hit cluster cross-links:

Mycgr3G70471
  
Location: 0-405

Mycgr3G70471

Mycgr3G39149
  
Location: 505-1798

Mycgr3G39149

Mycgr3G92130
  
Location: 1898-2396

Mycgr3G92130

Mycgr3G38483
  
Location: 2496-3576

Mycgr3G38483

Mycgr3G108869
  
Location: 3676-5056

Mycgr3G108869

Mycgr3G103943
  
Location: 5156-5762

Mycgr3G103943

Mycgr3G57362
  
Location: 5862-7296

Mycgr3G57362

Mycgr3G39086
  
Location: 7396-8368

Mycgr3G39086

Mycgr3G103942
  
Location: 8468-8714

Mycgr3G103942

Mycgr3G108865
  
Location: 8814-10239

Mycgr3G108865

Mycgr3G70475
  
Location: 10339-11821

Mycgr3G70475

Mycgr3G108866
  
Location: 11921-13010

Mycgr3G108866

Mycgr3G92136
  
Location: 13110-13593

Mycgr3G92136

zinc carboxypeptidase, putative
  
Accession: EAW18486
  
Location: 3616789-3618618
  
 NCBI BlastP on this gene

EAW18486

amino acid permease (Dip5), putative
  
Accession: EAW18487
  
Location: 3618923-3620847
  
 NCBI BlastP on this gene

EAW18487

conserved hypothetical protein
  
Accession: EAW18488
  
Location: 3624185-3625138
  
 NCBI BlastP on this gene

EAW18488

nickel transport protein, putative
  
Accession: EAW18489
  
Location: 3626150-3627855
  
  
**BlastP hit with Mycgr3G108865**
  
Percentage identity: 51 %
  
BlastP bit score: 388
  
Sequence coverage: 93 %
  
E-value: 6e-127
  
  
 NCBI BlastP on this gene

EAW18489

Query: Architecture Search FASTA input

DS985215 : Verticillium albo-atrum VaMs.102 supercont1.2 genomic scaffold    Total score: 1.0     Cumulative Blast bit score: 387

Hit cluster cross-links:

Mycgr3G70471
  
Location: 0-405

Mycgr3G70471

Mycgr3G39149
  
Location: 505-1798

Mycgr3G39149

Mycgr3G92130
  
Location: 1898-2396

Mycgr3G92130

Mycgr3G38483
  
Location: 2496-3576

Mycgr3G38483

Mycgr3G108869
  
Location: 3676-5056

Mycgr3G108869

Mycgr3G103943
  
Location: 5156-5762

Mycgr3G103943

Mycgr3G57362
  
Location: 5862-7296

Mycgr3G57362

Mycgr3G39086
  
Location: 7396-8368

Mycgr3G39086

Mycgr3G103942
  
Location: 8468-8714

Mycgr3G103942

Mycgr3G108865
  
Location: 8814-10239

Mycgr3G108865

Mycgr3G70475
  
Location: 10339-11821

Mycgr3G70475

Mycgr3G108866
  
Location: 11921-13010

Mycgr3G108866

Mycgr3G92136
  
Location: 13110-13593

Mycgr3G92136

conserved hypothetical protein
  
Accession: EEY16146
  
Location: 2581014-2582540
  
  
**BlastP hit with Mycgr3G70475**
  
Percentage identity: 50 %
  
BlastP bit score: 387
  
Sequence coverage: 100 %
  
E-value: 4e-126
  
  
 NCBI BlastP on this gene

EEY16146

conserved hypothetical protein
  
Accession: EEY16145
  
Location: 2579383-2580431
  
 NCBI BlastP on this gene

EEY16145

conserved hypothetical protein
  
Accession: EEY16144
  
Location: 2575322-2576690
  
 NCBI BlastP on this gene

EEY16144

Query: Architecture Search FASTA input

DS499595 : Aspergillus fumigatus A1163 scf\_000002 genomic scaffold    Total score: 1.0     Cumulative Blast bit score: 386

Hit cluster cross-links:

Mycgr3G70471
  
Location: 0-405

Mycgr3G70471

Mycgr3G39149
  
Location: 505-1798

Mycgr3G39149

Mycgr3G92130
  
Location: 1898-2396

Mycgr3G92130

Mycgr3G38483
  
Location: 2496-3576

Mycgr3G38483

Mycgr3G108869
  
Location: 3676-5056

Mycgr3G108869

Mycgr3G103943
  
Location: 5156-5762

Mycgr3G103943

Mycgr3G57362
  
Location: 5862-7296

Mycgr3G57362

Mycgr3G39086
  
Location: 7396-8368

Mycgr3G39086

Mycgr3G103942
  
Location: 8468-8714

Mycgr3G103942

Mycgr3G108865
  
Location: 8814-10239

Mycgr3G108865

Mycgr3G70475
  
Location: 10339-11821

Mycgr3G70475

Mycgr3G108866
  
Location: 11921-13010

Mycgr3G108866

Mycgr3G92136
  
Location: 13110-13593

Mycgr3G92136

zinc carboxypeptidase, putative
  
Accession: EDP54414
  
Location: 2244741-2246568
  
 NCBI BlastP on this gene

EDP54414

amino acid permease (Dip5), putative
  
Accession: EDP54415
  
Location: 2246900-2248827
  
 NCBI BlastP on this gene

EDP54415

conserved hypothetical protein
  
Accession: EDP54416
  
Location: 2248872-2249057
  
 NCBI BlastP on this gene

EDP54416

conserved hypothetical protein
  
Accession: EDP54417
  
Location: 2252292-2253245
  
 NCBI BlastP on this gene

EDP54417

nickel transport protein, putative
  
Accession: EDP54418
  
Location: 2254316-2256047
  
  
**BlastP hit with Mycgr3G108865**
  
Percentage identity: 51 %
  
BlastP bit score: 386
  
Sequence coverage: 88 %
  
E-value: 5e-126
  
  
 NCBI BlastP on this gene

EDP54418

Query: Architecture Search FASTA input

DS231615 : Pyrenophora tritici-repentis Pt-1C-BFP supercont1.1 genomic scaffold    Total score: 1.0     Cumulative Blast bit score: 385

Hit cluster cross-links:

Mycgr3G70471
  
Location: 0-405

Mycgr3G70471

Mycgr3G39149
  
Location: 505-1798

Mycgr3G39149

Mycgr3G92130
  
Location: 1898-2396

Mycgr3G92130

Mycgr3G38483
  
Location: 2496-3576

Mycgr3G38483

Mycgr3G108869
  
Location: 3676-5056

Mycgr3G108869

Mycgr3G103943
  
Location: 5156-5762

Mycgr3G103943

Mycgr3G57362
  
Location: 5862-7296

Mycgr3G57362

Mycgr3G39086
  
Location: 7396-8368

Mycgr3G39086

Mycgr3G103942
  
Location: 8468-8714

Mycgr3G103942

Mycgr3G108865
  
Location: 8814-10239

Mycgr3G108865

Mycgr3G70475
  
Location: 10339-11821

Mycgr3G70475

Mycgr3G108866
  
Location: 11921-13010

Mycgr3G108866

Mycgr3G92136
  
Location: 13110-13593

Mycgr3G92136

predicted protein
  
Accession: EDU39825
  
Location: 1032530-1033676
  
 NCBI BlastP on this gene

EDU39825

citrinin biosynthesis oxydoreductase CtnB
  
Accession: EDU39826
  
Location: 1033840-1034670
  
 NCBI BlastP on this gene

EDU39826

trichothecene 3-O-acetyltransferase
  
Accession: EDU39827
  
Location: 1036095-1037465
  
 NCBI BlastP on this gene

EDU39827

vacuolar basic amino acid transporter 3
  
Accession: EDU39828
  
Location: 1038717-1041239
  
 NCBI BlastP on this gene

EDU39828

FAD dependent oxidoreductase
  
Accession: EDU39829
  
Location: 1041833-1043310
  
  
**BlastP hit with Mycgr3G57362**
  
Percentage identity: 46 %
  
BlastP bit score: 385
  
Sequence coverage: 95 %
  
E-value: 2e-125
  
  
 NCBI BlastP on this gene

EDU39829

DJ-1/PfpI family protein
  
Accession: EDU39830
  
Location: 1044856-1045647
  
 NCBI BlastP on this gene

EDU39830

zinc-binding oxidoreductase CipB
  
Accession: EDU39831
  
Location: 1046169-1047287
  
 NCBI BlastP on this gene

EDU39831

serine/threonine-protein kinase YPK2/YKR2
  
Accession: EDU39832
  
Location: 1048342-1049826
  
 NCBI BlastP on this gene

EDU39832

vacuolar ATP synthase 16 kDa proteolipid subunit 2
  
Accession: EDU39833
  
Location: 1050511-1051143
  
 NCBI BlastP on this gene

EDU39833

Query: Architecture Search FASTA input

CU633900 : Podospora anserina S mat+ genomic DNA chromosome 7, supercontig 1.    Total score: 1.0     Cumulative Blast bit score: 383

Hit cluster cross-links:

Mycgr3G70471
  
Location: 0-405

Mycgr3G70471

Mycgr3G39149
  
Location: 505-1798

Mycgr3G39149

Mycgr3G92130
  
Location: 1898-2396

Mycgr3G92130

Mycgr3G38483
  
Location: 2496-3576

Mycgr3G38483

Mycgr3G108869
  
Location: 3676-5056

Mycgr3G108869

Mycgr3G103943
  
Location: 5156-5762

Mycgr3G103943

Mycgr3G57362
  
Location: 5862-7296

Mycgr3G57362

Mycgr3G39086
  
Location: 7396-8368

Mycgr3G39086

Mycgr3G103942
  
Location: 8468-8714

Mycgr3G103942

Mycgr3G108865
  
Location: 8814-10239

Mycgr3G108865

Mycgr3G70475
  
Location: 10339-11821

Mycgr3G70475

Mycgr3G108866
  
Location: 11921-13010

Mycgr3G108866

Mycgr3G92136
  
Location: 13110-13593

Mycgr3G92136

not annotated
  
Accession: CAP68345
  
Location: 524076-525195
  
 NCBI BlastP on this gene

CAP68345

not annotated
  
Accession: CAP68344
  
Location: 522203-523297
  
 NCBI BlastP on this gene

CAP68344

not annotated
  
Accession: CAP68343
  
Location: 519554-521446
  
 NCBI BlastP on this gene

CAP68343

not annotated
  
Accession: CAP68342
  
Location: 516116-517018
  
 NCBI BlastP on this gene

CAP68342

not annotated
  
Accession: CAP68341
  
Location: 514211-515513
  
  
**BlastP hit with Mycgr3G108865**
  
Percentage identity: 54 %
  
BlastP bit score: 383
  
Sequence coverage: 74 %
  
E-value: 2e-125
  
  
 NCBI BlastP on this gene

CAP68341

not annotated
  
Accession: CAP68340
  
Location: 511292-514105
  
 NCBI BlastP on this gene

CAP68340

not annotated
  
Accession: CAP68339
  
Location: 510079-510639
  
 NCBI BlastP on this gene

CAP68339

not annotated
  
Accession: CAP68338
  
Location: 509197-509637
  
 NCBI BlastP on this gene

CAP68338

not annotated
  
Accession: CAP68337
  
Location: 506857-508231
  
 NCBI BlastP on this gene

CAP68337

not annotated
  
Accession: CAP68336
  
Location: 505396-506295
  
 NCBI BlastP on this gene

CAP68336

Query: Architecture Search FASTA input

151. :  CP003004 Myceliophthora thermophila ATCC 42464 chromosome 3     Total score: 1.0     Cumulative Blast bit score: 509

Mycgr3G70471
  
Location: 0-405
  
 NCBI BlastP on this gene

Mycgr3G70471

Mycgr3G39149
  
Location: 505-1798
  
 NCBI BlastP on this gene

Mycgr3G39149

Mycgr3G92130
  
Location: 1898-2396
  
 NCBI BlastP on this gene

Mycgr3G92130

Mycgr3G38483
  
Location: 2496-3576
  
 NCBI BlastP on this gene

Mycgr3G38483

Mycgr3G108869
  
Location: 3676-5056
  
 NCBI BlastP on this gene

Mycgr3G108869

Mycgr3G103943
  
Location: 5156-5762
  
 NCBI BlastP on this gene

Mycgr3G103943

Mycgr3G57362
  
Location: 5862-7296
  
 NCBI BlastP on this gene

Mycgr3G57362

Mycgr3G39086
  
Location: 7396-8368
  
 NCBI BlastP on this gene

Mycgr3G39086

Mycgr3G103942
  
Location: 8468-8714
  
 NCBI BlastP on this gene

Mycgr3G103942

Mycgr3G108865
  
Location: 8814-10239
  
 NCBI BlastP on this gene

Mycgr3G108865

Mycgr3G70475
  
Location: 10339-11821
  
 NCBI BlastP on this gene

Mycgr3G70475

Mycgr3G108866
  
Location: 11921-13010
  
 NCBI BlastP on this gene

Mycgr3G108866

Mycgr3G92136
  
Location: 13110-13593
  
 NCBI BlastP on this gene

Mycgr3G92136

hypothetical protein
  
Accession: AEO57592
  
Location: 1555372-1556061
  
 NCBI BlastP on this gene

MYCTH\_2303921

hypothetical protein
  
Accession: AEO57593
  
Location: 1556754-1557795
  
 NCBI BlastP on this gene

MYCTH\_2126595

hypothetical protein
  
Accession: AEO57594
  
Location: 1559983-1563171
  
 NCBI BlastP on this gene

MYCTH\_2303924

hypothetical protein
  
Accession: AEO57595
  
Location: 1564217-1565851
  
  
**BlastP hit with Mycgr3G39149**
  
Percentage identity: 61 %
  
BlastP bit score: 509
  
Sequence coverage: 105 %
  
E-value: 4e-175
  
  
 NCBI BlastP on this gene

MYCTH\_2303929

hypothetical protein
  
Accession: AEO57596
  
Location: 1566645-1567350
  
 NCBI BlastP on this gene

MYCTH\_2034929

hypothetical protein
  
Accession: AEO57597
  
Location: 1567665-1568522
  
 NCBI BlastP on this gene

MYCTH\_2303933

hypothetical protein
  
Accession: AEO57598
  
Location: 1569102-1573108
  
 NCBI BlastP on this gene

MYCTH\_2303934

hypothetical protein
  
Accession: AEO57599
  
Location: 1573758-1576284
  
 NCBI BlastP on this gene

MYCTH\_2303936

152. :  CM001232 Magnaporthe oryzae 70-15 chromosome 2     Total score: 1.0     Cumulative Blast bit score: 509

leucine Rich Repeat domain-containing protein
  
Accession: EHA54023
  
Location: 1744274-1746946
  
 NCBI BlastP on this gene

EHA54023

OPT family small oligopeptide transporter
  
Accession: EHA54024
  
Location: 1748146-1751096
  
 NCBI BlastP on this gene

EHA54024

hypothetical protein
  
Accession: EHA54025
  
Location: 1751833-1752348
  
 NCBI BlastP on this gene

EHA54025

3-oxoacyl-[acyl-carrier-protein] synthase 2
  
Accession: EHA54026
  
Location: 1753628-1755203
  
  
**BlastP hit with Mycgr3G39149**
  
Percentage identity: 61 %
  
BlastP bit score: 509
  
Sequence coverage: 105 %
  
E-value: 4e-175
  
  
 NCBI BlastP on this gene

EHA54026

hypothetical protein
  
Accession: EHA54027
  
Location: 1755506-1756526
  
 NCBI BlastP on this gene

EHA54027

dihydrofolate reductase
  
Accession: EHA54028
  
Location: 1756906-1757732
  
 NCBI BlastP on this gene

EHA54028

hypothetical protein
  
Accession: EHA54029
  
Location: 1759300-1763183
  
 NCBI BlastP on this gene

EHA54029

153. :  DS499597 Aspergillus fumigatus A1163 scf\_000004 genomic scaffold     Total score: 1.0     Cumulative Blast bit score: 508

60S ribosome biogenesis protein Sqt1, putative
  
Accession: EDP51486
  
Location: 1744906-1746453
  
  
**BlastP hit with Mycgr3G70475**
  
Percentage identity: 55 %
  
BlastP bit score: 508
  
Sequence coverage: 104 %
  
E-value: 2e-172
  
  
 NCBI BlastP on this gene

EDP51486

NADH-ubiquinone oxidoreductase B12 subunit, putative
  
Accession: EDP51485
  
Location: 1744110-1744414
  
 NCBI BlastP on this gene

EDP51485

CCR4-NOT core complex subunit Caf1, putative
  
Accession: EDP51484
  
Location: 1742179-1743749
  
 NCBI BlastP on this gene

EDP51484

peroxisomal copper amine oxidase
  
Accession: EDP51483
  
Location: 1738916-1741077
  
 NCBI BlastP on this gene

EDP51483

154. :  EQ963481 Aspergillus flavus NRRL3357 scf\_1106286419476 genomic scaffold     Total score: 1.0     Cumulative Blast bit score: 507

siderochrome-iron transporter MirC
  
Accession: EED48767
  
Location: 1845774-1847810
  
 NCBI BlastP on this gene

EED48767

Rho GTPase ModA, putative
  
Accession: EED48768
  
Location: 1849649-1850619
  
 NCBI BlastP on this gene

EED48768

agmatinase, putative
  
Accession: EED48769
  
Location: 1852018-1853373
  
 NCBI BlastP on this gene

EED48769

3-oxoacyl carrier protein synthase, putative
  
Accession: EED48770
  
Location: 1853766-1855503
  
  
**BlastP hit with Mycgr3G39149**
  
Percentage identity: 66 %
  
BlastP bit score: 507
  
Sequence coverage: 86 %
  
E-value: 4e-175
  
  
 NCBI BlastP on this gene

EED48770

155. :  KE145367 Glarea lozoyensis ATCC 20868 chromosome Unknown GLAREA3     Total score: 1.0     Cumulative Blast bit score: 505

RNA-binding, RBD
  
Accession: EPE29410
  
Location: 1773483-1776778
  
 NCBI BlastP on this gene

EPE29410

alpha/beta-Hydrolase
  
Accession: EPE29411
  
Location: 1777941-1779846
  
 NCBI BlastP on this gene

EPE29411

Subtilisin-like protein
  
Accession: EPE29412
  
Location: 1781080-1783277
  
 NCBI BlastP on this gene

EPE29412

WD40 repeat-like protein
  
Accession: EPE29413
  
Location: 1783770-1785281
  
  
**BlastP hit with Mycgr3G70475**
  
Percentage identity: 57 %
  
BlastP bit score: 505
  
Sequence coverage: 102 %
  
E-value: 7e-172
  
  
 NCBI BlastP on this gene

EPE29413

156. :  CP003012 Thielavia terrestris NRRL 8126 chromosome 4     Total score: 1.0     Cumulative Blast bit score: 505

hypothetical protein
  
Accession: AEO69409
  
Location: 3105016-3106517
  
  
**BlastP hit with Mycgr3G39149**
  
Percentage identity: 60 %
  
BlastP bit score: 505
  
Sequence coverage: 104 %
  
E-value: 2e-173
  
  
 NCBI BlastP on this gene

THITE\_2119781

hypothetical protein
  
Accession: AEO69408
  
Location: 3103521-3104585
  
 NCBI BlastP on this gene

THITE\_2119778

hypothetical protein
  
Accession: AEO69407
  
Location: 3102512-3103432
  
 NCBI BlastP on this gene

THITE\_2080461

hypothetical protein
  
Accession: AEO69406
  
Location: 3098095-3102056
  
 NCBI BlastP on this gene

THITE\_2080459

hypothetical protein
  
Accession: AEO69405
  
Location: 3095045-3097572
  
 NCBI BlastP on this gene

THITE\_71850

157. :  CH476616 Uncinocarpus reesii 1704 scaffold\_2 genomic scaffold     Total score: 1.0     Cumulative Blast bit score: 504

conserved hypothetical protein
  
Accession: EEP79412
  
Location: 3757493-3759043
  
  
**BlastP hit with Mycgr3G70475**
  
Percentage identity: 53 %
  
BlastP bit score: 504
  
Sequence coverage: 106 %
  
E-value: 6e-171
  
  
 NCBI BlastP on this gene

EEP79412

CCR4-NOT transcription complex subunit 7
  
Accession: EEP79411
  
Location: 3754601-3756227
  
 NCBI BlastP on this gene

EEP79411

peroxisomal copper amine oxidase
  
Accession: EEP79410
  
Location: 3750912-3753086
  
 NCBI BlastP on this gene

EEP79410

predicted protein
  
Accession: EEP79409
  
Location: 3747084-3749011
  
 NCBI BlastP on this gene

EEP79409

158. :  EQ963477 Aspergillus flavus NRRL3357 scf\_1106286418280 genomic scaffold     Total score: 1.0     Cumulative Blast bit score: 502

60S ribosome biogenesis protein Sqt1, putative
  
Accession: EED51501
  
Location: 817701-819254
  
  
**BlastP hit with Mycgr3G70475**
  
Percentage identity: 53 %
  
BlastP bit score: 502
  
Sequence coverage: 105 %
  
E-value: 2e-170
  
  
 NCBI BlastP on this gene

EED51501

NADH-ubiquinone oxidoreductase B12 subunit, putative
  
Accession: EED51500
  
Location: 816912-817218
  
 NCBI BlastP on this gene

EED51500

CCR4-NOT core complex subunit Caf1, putative
  
Accession: EED51499
  
Location: 814979-816547
  
 NCBI BlastP on this gene

EED51499

peroxisomal copper amine oxidase
  
Accession: EED51498
  
Location: 811674-813847
  
 NCBI BlastP on this gene

EED51498

heat shock protein, putative
  
Accession: EED51497
  
Location: 809808-810827
  
 NCBI BlastP on this gene

EED51497

159. :  JH126399 Cordyceps militaris CM01 unplaced genomic scaffold CCM\_S00001     Total score: 1.0     Cumulative Blast bit score: 500

Fungal transcriptional regulatory protein
  
Accession: EGX97150
  
Location: 5853828-5856408
  
 NCBI BlastP on this gene

EGX97150

small oligopeptide transporter, OPT family
  
Accession: EGX97151
  
Location: 5856679-5859583
  
 NCBI BlastP on this gene

EGX97151

3-oxoacyl-(acyl-carrier-protein) synthase 2
  
Accession: EGX97152
  
Location: 5860234-5861584
  
  
**BlastP hit with Mycgr3G39149**
  
Percentage identity: 60 %
  
BlastP bit score: 501
  
Sequence coverage: 99 %
  
E-value: 3e-172
  
  
 NCBI BlastP on this gene

EGX97152

160. :  DS027696 Neosartorya fischeri NRRL 181 1099437636264 genomic scaffold     Total score: 1.0     Cumulative Blast bit score: 500

CNH domain protein
  
Accession: EAW18042
  
Location: 2107109-2112702
  
 NCBI BlastP on this gene

EAW18042

conserved hypothetical protein
  
Accession: EAW18043
  
Location: 2114196-2115825
  
 NCBI BlastP on this gene

EAW18043

AMP dependent CoA ligase
  
Accession: EAW18044
  
Location: 2118442-2120510
  
 NCBI BlastP on this gene

EAW18044

60S ribosome biogenesis protein Sqt1, putative
  
Accession: EAW18045
  
Location: 2120924-2122471
  
  
**BlastP hit with Mycgr3G70475**
  
Percentage identity: 55 %
  
BlastP bit score: 501
  
Sequence coverage: 104 %
  
E-value: 6e-170
  
  
 NCBI BlastP on this gene

EAW18045

NADH-ubiquinone oxidoreductase B12 subunit, putative
  
Accession: EAW18046
  
Location: 2122983-2123286
  
 NCBI BlastP on this gene

EAW18046

CCR4-NOT core complex subunit Caf1, putative
  
Accession: EAW18047
  
Location: 2123638-2125207
  
 NCBI BlastP on this gene

EAW18047

peroxisomal copper amine oxidase
  
Accession: EAW18048
  
Location: 2126306-2128467
  
 NCBI BlastP on this gene

EAW18048

161. :  ABSU01000001 Arthroderma benhamiae CBS 112371     Total score: 1.0     Cumulative Blast bit score: 493

hypothetical protein
  
Accession: EFE36506
  
Location: 217368-218076
  
 NCBI BlastP on this gene

EFE36506

hypothetical protein
  
Accession: EFE36505
  
Location: 212805-215816
  
 NCBI BlastP on this gene

EFE36505

hypothetical protein
  
Accession: EFE36504
  
Location: 212241-212507
  
 NCBI BlastP on this gene

EFE36504

hypothetical protein
  
Accession: EFE36503
  
Location: 209314-211264
  
 NCBI BlastP on this gene

EFE36503

hypothetical protein
  
Accession: EFE36502
  
Location: 207460-208992
  
  
**BlastP hit with Mycgr3G70475**
  
Percentage identity: 54 %
  
BlastP bit score: 493
  
Sequence coverage: 105 %
  
E-value: 1e-166
  
  
 NCBI BlastP on this gene

EFE36502

hypothetical protein
  
Accession: EFE36501
  
Location: 206762-207099
  
 NCBI BlastP on this gene

EFE36501

hypothetical protein
  
Accession: EFE36500
  
Location: 205596-206132
  
 NCBI BlastP on this gene

EFE36500

hypothetical protein
  
Accession: EFE36499
  
Location: 204550-205512
  
 NCBI BlastP on this gene

EFE36499

peroxisomal copper amine oxidase, putative
  
Accession: EFE36498
  
Location: 201298-203493
  
 NCBI BlastP on this gene

EFE36498

hypothetical protein
  
Accession: EFE36497
  
Location: 199722-201108
  
 NCBI BlastP on this gene

EFE36497

162. :  DS990636 Ajellomyces capsulatus H88 supercont1.1 genomic scaffold     Total score: 1.0     Cumulative Blast bit score: 489

predicted protein
  
Accession: EGC41729
  
Location: 4193461-4194391
  
 NCBI BlastP on this gene

EGC41729

small GTPase
  
Accession: EGC41730
  
Location: 4195267-4196230
  
 NCBI BlastP on this gene

EGC41730

agmatine ureohydrolase
  
Accession: EGC41731
  
Location: 4198350-4199882
  
 NCBI BlastP on this gene

EGC41731

3-oxoacyl-acyl-carrier-protein synthase
  
Accession: EGC41732
  
Location: 4200856-4203715
  
  
**BlastP hit with Mycgr3G39149**
  
Percentage identity: 58 %
  
BlastP bit score: 489
  
Sequence coverage: 99 %
  
E-value: 2e-165
  
  
 NCBI BlastP on this gene

EGC41732

SAGA complex component
  
Accession: EGC41733
  
Location: 4204089-4205826
  
 NCBI BlastP on this gene

EGC41733

mRNA capping enzyme alpha subunit
  
Accession: EGC41734
  
Location: 4206525-4207839
  
 NCBI BlastP on this gene

EGC41734

DNA ligase
  
Accession: EGC41735
  
Location: 4208460-4212396
  
 NCBI BlastP on this gene

EGC41735

163. :  AEOI01000012 Ogataea parapolymorpha DL-1     Total score: 1.0     Cumulative Blast bit score: 487

hypothetical protein
  
Accession: EFW95289
  
Location: 1338863-1339523
  
 NCBI BlastP on this gene

EFW95289

hypothetical protein
  
Accession: EFW95290
  
Location: 1340264-1340773
  
 NCBI BlastP on this gene

EFW95290

hypothetical protein
  
Accession: EFW95291
  
Location: 1340901-1341980
  
 NCBI BlastP on this gene

EFW95291

Autophagy-related protein 9
  
Accession: EFW95292
  
Location: 1342098-1344767
  
 NCBI BlastP on this gene

EFW95292

hypothetical protein
  
Accession: EFW95293
  
Location: 1344778-1347162
  
 NCBI BlastP on this gene

EFW95293

Mitochondrial beta-keto-acyl synthase
  
Accession: EFW95294
  
Location: 1347281-1348579
  
  
**BlastP hit with Mycgr3G39149**
  
Percentage identity: 55 %
  
BlastP bit score: 487
  
Sequence coverage: 100 %
  
E-value: 1e-166
  
  
 NCBI BlastP on this gene

EFW95294

hypothetical protein
  
Accession: EFW95295
  
Location: 1349604-1351504
  
 NCBI BlastP on this gene

EFW95295

ATP synthase regulation protein NCA2
  
Accession: EFW95296
  
Location: 1351551-1353473
  
 NCBI BlastP on this gene

EFW95296

TBP-associated factor, putative
  
Accession: EFW95297
  
Location: 1353546-1354988
  
 NCBI BlastP on this gene

EFW95297

RNA Pol II CTD phosphatase component, putative
  
Accession: EFW95298
  
Location: 1355057-1357288
  
 NCBI BlastP on this gene

EFW95298

164. :  CR382134 Debaryomyces hansenii CBS767 chromosome B complete sequence.     Total score: 1.0     Cumulative Blast bit score: 483

DEHA2B13772p
  
Accession: CAR65492
  
Location: 1081701-1082819
  
 NCBI BlastP on this gene

DEHA2B13772g

DEHA2B13794p
  
Accession: CAG85556
  
Location: 1082814-1084232
  
 NCBI BlastP on this gene

DEHA2B13794g

DEHA2B13816p
  
Accession: CAG85557
  
Location: 1084455-1084550
  
 NCBI BlastP on this gene

DEHA2B13816g

DEHA2B13838p
  
Accession: CAG85558
  
Location: 1084742-1085215
  
 NCBI BlastP on this gene

DEHA2B13838g

DEHA2B13860p
  
Accession: CAG85559
  
Location: 1085435-1086187
  
 NCBI BlastP on this gene

DEHA2B13860g

DEHA2B13882p
  
Accession: CAG85560
  
Location: 1086433-1088304
  
 NCBI BlastP on this gene

DEHA2B13882g

DEHA2B13904p
  
Accession: CAG85561
  
Location: 1088962-1089468
  
 NCBI BlastP on this gene

DEHA2B13904g

DEHA2B13926p
  
Accession: CAG85562
  
Location: 1089634-1091181
  
 NCBI BlastP on this gene

DEHA2B13926g

DEHA2B13948p
  
Accession: CAG85563
  
Location: 1091282-1092595
  
  
**BlastP hit with Mycgr3G39149**
  
Percentage identity: 51 %
  
BlastP bit score: 484
  
Sequence coverage: 102 %
  
E-value: 2e-165
  
  
 NCBI BlastP on this gene

DEHA2B13948g

165. :  JH226133 Exophiala dermatitidis NIH/UT8656 unplaced genomic scaffold supercont1.4     Total score: 1.0     Cumulative Blast bit score: 483

ATP-dependent DNA helicase 2 subunit 1
  
Accession: EHY57067
  
Location: 2225101-2227201
  
 NCBI BlastP on this gene

EHY57067

ferrochelatase
  
Accession: EHY57066
  
Location: 2223194-2224507
  
 NCBI BlastP on this gene

EHY57066

protein-serine/threonine kinase
  
Accession: EHY57065
  
Location: 2219081-2221908
  
 NCBI BlastP on this gene

EHY57065

hypothetical protein
  
Accession: EHY57064
  
Location: 2215703-2217094
  
  
**BlastP hit with Mycgr3G70475**
  
Percentage identity: 57 %
  
BlastP bit score: 483
  
Sequence coverage: 98 %
  
E-value: 9e-164
  
  
 NCBI BlastP on this gene

EHY57064

3-oxoacyl-[acyl-carrier protein] reductase
  
Accession: EHY57063
  
Location: 2214086-2214868
  
 NCBI BlastP on this gene

EHY57063

hypothetical protein
  
Accession: EHY57062
  
Location: 2212398-2213264
  
 NCBI BlastP on this gene

EHY57062

hypothetical protein
  
Accession: EHY57061
  
Location: 2208054-2211671
  
 NCBI BlastP on this gene

EHY57061

166. :  CU329671 Schizosaccharomyces pombe chromosome II     Total score: 1.0     Cumulative Blast bit score: 481

3-oxoacyl-[acyl-carrier-protein]-synthase condensing enzyme (predicted)
  
Accession: CAA21898
  
Location: 3565824-3567187
  
  
**BlastP hit with Mycgr3G39149**
  
Percentage identity: 55 %
  
BlastP bit score: 482
  
Sequence coverage: 99 %
  
E-value: 7e-165
  
  
 NCBI BlastP on this gene

SPBC887.13c

P-type ATPase (predicted)
  
Accession: CAA21897
  
Location: 3561887-3565663
  
 NCBI BlastP on this gene

SPBC887.12

tRNA pseudouridine synthase Pus2 (predicted)
  
Accession: CAA21896
  
Location: 3559396-3560788
  
 NCBI BlastP on this gene

pus2

response regulator Mcs4
  
Accession: CAA21895
  
Location: 3557274-3558842
  
 NCBI BlastP on this gene

mcs4

167. :  GG704912 Coccidioides immitis RS genomic scaffold supercont3.2     Total score: 1.0     Cumulative Blast bit score: 481

hypothetical protein
  
Accession: EAS30864
  
Location: 1716541-1718999
  
 NCBI BlastP on this gene

EAS30864

phenylacetyl-CoA ligase
  
Accession: EAS30862
  
Location: 1711849-1713894
  
 NCBI BlastP on this gene

EAS30862

acetyltransferase
  
Accession: EAS30861
  
Location: 1710481-1711137
  
 NCBI BlastP on this gene

EAS30861

60S ribosome biogenesis protein Sqt1
  
Accession: EAS30860
  
Location: 1708065-1709621
  
  
**BlastP hit with Mycgr3G70475**
  
Percentage identity: 52 %
  
BlastP bit score: 481
  
Sequence coverage: 106 %
  
E-value: 4e-162
  
  
 NCBI BlastP on this gene

EAS30860

NADH-ubiquinone oxidoreductase B12 subunit
  
Accession: EAS30859
  
Location: 1707323-1707669
  
 NCBI BlastP on this gene

EAS30859

CCR4-NOT transcription complex subunit 7
  
Accession: EAS30858
  
Location: 1705160-1706794
  
 NCBI BlastP on this gene

EAS30858

peroxisomal copper amine oxidase
  
Accession: EAS30857
  
Location: 1701305-1703479
  
 NCBI BlastP on this gene

EAS30857

hypothetical protein
  
Accession: EAS30856
  
Location: 1699847-1700869
  
 NCBI BlastP on this gene

EAS30856

168. :  ACJE01000012 Aspergillus niger ATCC 1015     Total score: 1.0     Cumulative Blast bit score: 478

hypothetical protein
  
Accession: EHA22286
  
Location: 308317-309267
  
 NCBI BlastP on this gene

EHA22286

hypothetical protein
  
Accession: EHA22287
  
Location: 310436-311153
  
 NCBI BlastP on this gene

EHA22287

hypothetical protein
  
Accession: EHA22288
  
Location: 311405-312263
  
 NCBI BlastP on this gene

EHA22288

hypothetical protein
  
Accession: EHA22289
  
Location: 314825-316872
  
 NCBI BlastP on this gene

EHA22289

hypothetical protein
  
Accession: EHA22290
  
Location: 317314-318873
  
  
**BlastP hit with Mycgr3G70475**
  
Percentage identity: 53 %
  
BlastP bit score: 478
  
Sequence coverage: 105 %
  
E-value: 4e-161
  
  
 NCBI BlastP on this gene

EHA22290

hypothetical protein
  
Accession: EHA22291
  
Location: 319378-319690
  
 NCBI BlastP on this gene

EHA22291

hypothetical protein
  
Accession: EHA22292
  
Location: 320088-321639
  
 NCBI BlastP on this gene

EHA22292

hypothetical protein
  
Accession: EHA22293
  
Location: 322896-325062
  
 NCBI BlastP on this gene

EHA22293

169. :  ACFW01000025 Coccidioides posadasii C735 delta SOWgp     Total score: 1.0     Cumulative Blast bit score: 477

hypothetical protein
  
Accession: EER27339
  
Location: 1439655-1442131
  
 NCBI BlastP on this gene

EER27339

AMP-binding enzyme, putative
  
Accession: EER27338
  
Location: 1434935-1436979
  
 NCBI BlastP on this gene

EER27338

GNAT family acetyltransferase, putative
  
Accession: EER27337
  
Location: 1433561-1434217
  
 NCBI BlastP on this gene

EER27337

WD domain, G-beta repeat containing protein
  
Accession: EER27336
  
Location: 1431143-1432699
  
  
**BlastP hit with Mycgr3G70475**
  
Percentage identity: 52 %
  
BlastP bit score: 477
  
Sequence coverage: 106 %
  
E-value: 2e-160
  
  
 NCBI BlastP on this gene

EER27336

NADH-ubiquinone oxidoreductase B12 subunit family protein
  
Accession: EER27335
  
Location: 1430401-1430747
  
 NCBI BlastP on this gene

EER27335

CAF1 family ribonuclease containing protein
  
Accession: EER27334
  
Location: 1428242-1429873
  
 NCBI BlastP on this gene

EER27334

peroxisomal copper amine oxidase, putative
  
Accession: EER27333
  
Location: 1424386-1426560
  
 NCBI BlastP on this gene

EER27333

short-chain dehydrogenase, putative
  
Accession: EER27332
  
Location: 1422919-1423941
  
 NCBI BlastP on this gene

EER27332

170. :  FR839631 Pichia pastoris CBS 7435 chromosome 4     Total score: 1.0     Cumulative Blast bit score: 476

hypothetical protein
  
Accession: CCA40628
  
Location: 779270-780543
  
 NCBI BlastP on this gene

PP7435\_Chr4-0462

Purine-cytosine permease FCY2
  
Accession: CCA40629
  
Location: 780666-782225
  
 NCBI BlastP on this gene

PP7435\_Chr4-0463

hypothetical protein
  
Accession: CCA40630
  
Location: 782502-784481
  
 NCBI BlastP on this gene

PP7435\_Chr4-0464

T-complex protein 1 subunit zeta
  
Accession: CCA40631
  
Location: 784630-786243
  
 NCBI BlastP on this gene

CCT6

Probable nucleolar complex protein 14
  
Accession: CCA40632
  
Location: 786269-788731
  
 NCBI BlastP on this gene

PP7435\_Chr4-0466

3-oxoacyl-
  
Accession: CCA40633
  
Location: 788879-790195
  
  
**BlastP hit with Mycgr3G39149**
  
Percentage identity: 54 %
  
BlastP bit score: 476
  
Sequence coverage: 102 %
  
E-value: 5e-162
  
  
 NCBI BlastP on this gene

CEM1

Autophagy-related protein 9
  
Accession: CCA40634
  
Location: 790203-792860
  
 NCBI BlastP on this gene

PP7435\_Chr4-0468

Beta-glucan synthesis-associated protein KRE6
  
Accession: CCA40635
  
Location: 793847-795796
  
 NCBI BlastP on this gene

KRE7

Transcription elongation regulator 1
  
Accession: CCA40636
  
Location: 795851-797170
  
 NCBI BlastP on this gene

PP7435\_Chr4-0470

20S proteasome subunit alpha 3
  
Accession: CCA40637
  
Location: 797187-798053
  
 NCBI BlastP on this gene

PP7435\_Chr4-0471

Prohibitin
  
Accession: CCA40638
  
Location: 798201-799111
  
 NCBI BlastP on this gene

PHB

171. :  FN392322 Pichia pastoris GS115 chromosome 4     Total score: 1.0     Cumulative Blast bit score: 476

Mitochondrial beta-keto-acyl synthase with possible role in fatty acid synthesis
  
Accession: CAY71766
  
Location: 998649-999965
  
  
**BlastP hit with Mycgr3G39149**
  
Percentage identity: 54 %
  
BlastP bit score: 476
  
Sequence coverage: 102 %
  
E-value: 5e-162
  
  
 NCBI BlastP on this gene

PAS\_chr4\_0511

Transmembrane protein involved in formation of Cvt and autophagic vesicles
  
Accession: CAY71765
  
Location: 995984-998641
  
 NCBI BlastP on this gene

PAS\_chr4\_0510

Protein required for beta-1,6 glucan biosynthesis
  
Accession: CAY71764
  
Location: 993048-994997
  
 NCBI BlastP on this gene

PAS\_chr4\_0508

Hypothetical protein
  
Accession: CAY71763
  
Location: 991650-992993
  
 NCBI BlastP on this gene

PAS\_chr4\_0507

Alpha 3 subunit of the 20S proteasome, the only nonessential 20S subunit
  
Accession: CAY71762
  
Location: 990791-991549
  
 NCBI BlastP on this gene

PAS\_chr4\_0506

Subunit of the prohibitin complex (Phb1p-Phb2p)
  
Accession: CAY71761
  
Location: 989732-990643
  
 NCBI BlastP on this gene

PAS\_chr4\_0505

172. :  FO082056 Pichia sorbitophila strain CBS 7064 chromosome D complete sequence.     Total score: 1.0     Cumulative Blast bit score: 469

not annotated
  
Accession: CCE78667
  
Location: 177131-177883
  
 NCBI BlastP on this gene

Piso0\_000693

not annotated
  
Accession: CCE78666
  
Location: 174714-176702
  
 NCBI BlastP on this gene

Piso0\_000692

not annotated
  
Accession: CCE78665
  
Location: 174160-174519
  
 NCBI BlastP on this gene

Piso0\_000691

not annotated
  
Accession: CCE78664
  
Location: 171460-172080
  
 NCBI BlastP on this gene

Piso0\_000690

not annotated
  
Accession: CCE78663
  
Location: 168926-170455
  
 NCBI BlastP on this gene

Piso0\_000689

not annotated
  
Accession: CCE78662
  
Location: 167325-168641
  
  
**BlastP hit with Mycgr3G39149**
  
Percentage identity: 51 %
  
BlastP bit score: 469
  
Sequence coverage: 102 %
  
E-value: 1e-159
  
  
 NCBI BlastP on this gene

Piso0\_000688

not annotated
  
Accession: CCE78661
  
Location: 164817-166895
  
 NCBI BlastP on this gene

Piso0\_000687

not annotated
  
Accession: CCE78660
  
Location: 161856-163979
  
 NCBI BlastP on this gene

Piso0\_000686

not annotated
  
Accession: CCE78659
  
Location: 156562-159348
  
 NCBI BlastP on this gene

Piso0\_000685

173. :  FO082057 Pichia sorbitophila strain CBS 7064 chromosome C complete sequence.     Total score: 1.0     Cumulative Blast bit score: 464

not annotated
  
Accession: CCE78080
  
Location: 169982-170734
  
 NCBI BlastP on this gene

Piso0\_000693

not annotated
  
Accession: CCE78079
  
Location: 167570-169558
  
 NCBI BlastP on this gene

Piso0\_000692

not annotated
  
Accession: CCE78078
  
Location: 166943-167296
  
 NCBI BlastP on this gene

Piso0\_000691

not annotated
  
Accession: CCE78077
  
Location: 164284-164904
  
 NCBI BlastP on this gene

Piso0\_000690

not annotated
  
Accession: CCE78076
  
Location: 161753-163279
  
 NCBI BlastP on this gene

Piso0\_000689

not annotated
  
Accession: CCE78075
  
Location: 160147-161472
  
  
**BlastP hit with Mycgr3G39149**
  
Percentage identity: 51 %
  
BlastP bit score: 465
  
Sequence coverage: 102 %
  
E-value: 8e-158
  
  
 NCBI BlastP on this gene

Piso0\_000688

not annotated
  
Accession: CCE78074
  
Location: 157646-159724
  
 NCBI BlastP on this gene

Piso0\_000687

not annotated
  
Accession: CCE78073
  
Location: 154714-156828
  
 NCBI BlastP on this gene

Piso0\_000686

not annotated
  
Accession: CCE78072
  
Location: 149498-152284
  
 NCBI BlastP on this gene

Piso0\_000685

174. :  DS231615 Pyrenophora tritici-repentis Pt-1C-BFP supercont1.1 genomic scaffold     Total score: 1.0     Cumulative Blast bit score: 462

ribosome biogenesis protein Sqt1
  
Accession: EDU41433
  
Location: 5737553-5739091
  
  
**BlastP hit with Mycgr3G70475**
  
Percentage identity: 52 %
  
BlastP bit score: 462
  
Sequence coverage: 102 %
  
E-value: 1e-154
  
  
 NCBI BlastP on this gene

EDU41433

conserved hypothetical protein
  
Accession: EDU41432
  
Location: 5733327-5734313
  
 NCBI BlastP on this gene

EDU41432

structural maintenance of chromosomes protein 3
  
Accession: EDU41431
  
Location: 5726661-5730357
  
 NCBI BlastP on this gene

EDU41431

175. :  GL629801 Grosmannia clavigera kw1407 unplaced genomic scaffold GCSC\_173     Total score: 1.0     Cumulative Blast bit score: 461

hypothetical protein
  
Accession: EFX00389
  
Location: 1220252-1223010
  
 NCBI BlastP on this gene

EFX00389

small oligopeptide transporter
  
Accession: EFX00268
  
Location: 1216970-1219636
  
 NCBI BlastP on this gene

EFX00268

beta-ketoacyl synthase
  
Accession: EFX00048
  
Location: 1213828-1216469
  
  
**BlastP hit with Mycgr3G39149**
  
Percentage identity: 57 %
  
BlastP bit score: 461
  
Sequence coverage: 100 %
  
E-value: 4e-152
  
  
 NCBI BlastP on this gene

EFX00048

dihydrofolate reductase
  
Accession: EFX00528
  
Location: 1213101-1213767
  
 NCBI BlastP on this gene

EFX00528

hypothetical protein
  
Accession: EFX00161
  
Location: 1211468-1212601
  
 NCBI BlastP on this gene

EFX00161

176. :  CH408156 Pichia guilliermondii ATCC 6260 scaffold\_2 genomic scaffold     Total score: 1.0     Cumulative Blast bit score: 457

hypothetical protein
  
Accession: EDK37719
  
Location: 1103945-1104736
  
 NCBI BlastP on this gene

EDK37719

hypothetical protein
  
Accession: EDK37720
  
Location: 1104733-1106079
  
 NCBI BlastP on this gene

EDK37720

hypothetical protein
  
Accession: EDK37721
  
Location: 1106184-1106651
  
 NCBI BlastP on this gene

EDK37721

hypothetical protein
  
Accession: EDK37722
  
Location: 1107037-1107840
  
 NCBI BlastP on this gene

EDK37722

hypothetical protein
  
Accession: EDK37723
  
Location: 1108028-1109812
  
 NCBI BlastP on this gene

EDK37723

hypothetical protein
  
Accession: EDK37724
  
Location: 1110341-1111162
  
 NCBI BlastP on this gene

EDK37724

hypothetical protein
  
Accession: EDK37725
  
Location: 1111335-1112549
  
 NCBI BlastP on this gene

EDK37725

hypothetical protein
  
Accession: EDK37726
  
Location: 1112629-1113927
  
  
**BlastP hit with Mycgr3G39149**
  
Percentage identity: 52 %
  
BlastP bit score: 457
  
Sequence coverage: 102 %
  
E-value: 9e-155
  
  
 NCBI BlastP on this gene

EDK37726

hypothetical protein
  
Accession: EDK37727
  
Location: 1113945-1115912
  
 NCBI BlastP on this gene

EDK37727

hypothetical protein
  
Accession: EDK37728
  
Location: 1117279-1119705
  
 NCBI BlastP on this gene

EDK37728

hypothetical protein
  
Accession: EDK37729
  
Location: 1119788-1122115
  
 NCBI BlastP on this gene

EDK37729

hypothetical protein
  
Accession: EDK37730
  
Location: 1122197-1122709
  
 NCBI BlastP on this gene

EDK37730

177. :  DS572752 Paracoccidioides brasiliensis Pb18 supercont1.3 genomic scaffold     Total score: 1.0     Cumulative Blast bit score: 454

4-coumarate-CoA ligase
  
Accession: EEH46527
  
Location: 742808-744941
  
 NCBI BlastP on this gene

EEH46527

ribosome assembly protein SQT1
  
Accession: EEH46526
  
Location: 734050-735585
  
  
**BlastP hit with Mycgr3G70475**
  
Percentage identity: 52 %
  
BlastP bit score: 454
  
Sequence coverage: 103 %
  
E-value: 1e-151
  
  
 NCBI BlastP on this gene

EEH46526

conserved hypothetical protein
  
Accession: EEH46525
  
Location: 733086-733467
  
 NCBI BlastP on this gene

EEH46525

CCR4-NOT transcription complex subunit 7
  
Accession: EEH46524
  
Location: 729966-732566
  
 NCBI BlastP on this gene

EEH46524

peroxisomal copper amine oxidase
  
Accession: EEH46523
  
Location: 727419-729704
  
 NCBI BlastP on this gene

EEH46523

predicted protein
  
Accession: EEH46522
  
Location: 725739-726515
  
 NCBI BlastP on this gene

EEH46522

178. :  DS022225 Schizosaccharomyces japonicus yFS275 supercont1.2 genomic scaffold     Total score: 1.0     Cumulative Blast bit score: 451

3-oxoacyl-[acyl-carrier-protein] synthase
  
Accession: EEB06885
  
Location: 1739365-1740758
  
  
**BlastP hit with Mycgr3G39149**
  
Percentage identity: 54 %
  
BlastP bit score: 451
  
Sequence coverage: 99 %
  
E-value: 1e-152
  
  
 NCBI BlastP on this gene

EEB06885

phospholipid-transporting ATPase
  
Accession: EEB06884
  
Location: 1735530-1739330
  
 NCBI BlastP on this gene

EEB06884

CCR4-Not complex subunit Caf16
  
Accession: EEB06883
  
Location: 1733252-1734234
  
 NCBI BlastP on this gene

EEB06883

predicted protein
  
Accession: EEB06882
  
Location: 1729168-1733220
  
 NCBI BlastP on this gene

EEB06882

179. :  DS544803 Paracoccidioides brasiliensis Pb03 supercont1.1 genomic scaffold     Total score: 1.0     Cumulative Blast bit score: 449

conserved hypothetical protein
  
Accession: EEH17663
  
Location: 774377-777108
  
 NCBI BlastP on this gene

EEH17663

4-coumarate-CoA ligase
  
Accession: EEH17662
  
Location: 769027-771160
  
 NCBI BlastP on this gene

EEH17662

conserved hypothetical protein
  
Accession: EEH17661
  
Location: 766152-767687
  
  
**BlastP hit with Mycgr3G70475**
  
Percentage identity: 51 %
  
BlastP bit score: 450
  
Sequence coverage: 103 %
  
E-value: 4e-150
  
  
 NCBI BlastP on this gene

EEH17661

predicted protein
  
Accession: EEH17660
  
Location: 765188-765569
  
 NCBI BlastP on this gene

EEH17660

CCR4-NOT transcription complex subunit 7
  
Accession: EEH17659
  
Location: 762068-764668
  
 NCBI BlastP on this gene

EEH17659

peroxisomal copper amine oxidase
  
Accession: EEH17658
  
Location: 759522-760728
  
 NCBI BlastP on this gene

EEH17658

predicted protein
  
Accession: EEH17657
  
Location: 757832-758608
  
 NCBI BlastP on this gene

EEH17657

180. :  FM992690 Candida dubliniensis CD36 chromosome 3     Total score: 1.0     Cumulative Blast bit score: 442

autophagy-related protein, putative
  
Accession: CAX43004
  
Location: 1129718-1132582
  
 NCBI BlastP on this gene

CD36\_85010

zinc-finger transcription factor, putative
  
Accession: CAX43005
  
Location: 1134990-1135871
  
 NCBI BlastP on this gene

CD36\_85020

conserved hypothetical protein
  
Accession: CAX43006
  
Location: 1137268-1138599
  
 NCBI BlastP on this gene

CD36\_85030

3-oxoacyl-[acyl-carrier-protein] synthase, putative
  
Accession: CAX43007
  
Location: 1138655-1139977
  
  
**BlastP hit with Mycgr3G39149**
  
Percentage identity: 50 %
  
BlastP bit score: 442
  
Sequence coverage: 103 %
  
E-value: 6e-149
  
  
 NCBI BlastP on this gene

CD36\_85040

181. :  CP000494 Bradyrhizobium sp. BTAi1     Total score: 1.0     Cumulative Blast bit score: 439

primary replicative DNA helicase
  
Accession: ABQ35885
  
Location: 3991390-3992898
  
 NCBI BlastP on this gene

BBta\_3808

hypothetical protein
  
Accession: ABQ35886
  
Location: 3993178-3993276
  
 NCBI BlastP on this gene

BBta\_3809

cyclopropane-fatty-acyl-phospholipid synthase
  
Accession: ABQ35887
  
Location: 3993523-3994623
  
 NCBI BlastP on this gene

BBta\_3810

putative exported protein of unknown function
  
Accession: ABQ35888
  
Location: 3995193-3996005
  
 NCBI BlastP on this gene

BBta\_3811

LSU ribosomal protein L9P
  
Accession: ABQ35889
  
Location: 3996072-3996665
  
 NCBI BlastP on this gene

BBta\_3812

putative membrane protein of unknown function
  
Accession: ABQ35890
  
Location: 3996715-3997656
  
 NCBI BlastP on this gene

BBta\_3813

SSU ribosomal protein S18P
  
Accession: ABQ35891
  
Location: 3997798-3998037
  
 NCBI BlastP on this gene

rpsR

SSU ribosomal protein S6P
  
Accession: ABQ35892
  
Location: 3998043-3998504
  
 NCBI BlastP on this gene

rps6

[Acyl-carrier-protein] S-malonyltransferase
  
Accession: ABQ35893
  
Location: 3998878-3999837
  
 NCBI BlastP on this gene

fabD

3-oxoacyl-[acyl-carrier-protein] reductase
  
Accession: ABQ35894
  
Location: 3999872-4000609
  
 NCBI BlastP on this gene

fabG

Acyl carrier protein (ACP)
  
Accession: ABQ35895
  
Location: 4001048-4001287
  
 NCBI BlastP on this gene

acpP

3-oxoacyl-[acyl-carrier-protein] synthase II
  
Accession: ABQ35896
  
Location: 4001508-4002773
  
  
**BlastP hit with Mycgr3G39149**
  
Percentage identity: 53 %
  
BlastP bit score: 439
  
Sequence coverage: 99 %
  
E-value: 3e-148
  
  
 NCBI BlastP on this gene

fabF

182. :  DS572698 Verticillium dahliae VdLs.17 supercont1.4 genomic scaffold     Total score: 1.0     Cumulative Blast bit score: 426

ribosome biogenesis protein Sqt1
  
Accession: EGY21202
  
Location: 1207186-1208688
  
  
**BlastP hit with Mycgr3G70475**
  
Percentage identity: 52 %
  
BlastP bit score: 426
  
Sequence coverage: 104 %
  
E-value: 7e-141
  
  
 NCBI BlastP on this gene

EGY21202

hypothetical protein
  
Accession: EGY21201
  
Location: 1205551-1206597
  
 NCBI BlastP on this gene

EGY21201

hypothetical protein
  
Accession: EGY21200
  
Location: 1201399-1202629
  
 NCBI BlastP on this gene

EGY21200

hypothetical protein
  
Accession: EGY21199
  
Location: 1198637-1199743
  
 NCBI BlastP on this gene

EGY21199

183. :  EQ962652 Talaromyces stipitatus ATCC 10500 scf\_1105507295523 genomic scaffold     Total score: 1.0     Cumulative Blast bit score: 417

fungal specific transcription factor, putative
  
Accession: EED23989
  
Location: 3898465-3901254
  
 NCBI BlastP on this gene

EED23989

conserved hypothetical protein
  
Accession: EED23990
  
Location: 3903280-3904849
  
  
**BlastP hit with Mycgr3G57362**
  
Percentage identity: 32 %
  
BlastP bit score: 205
  
Sequence coverage: 98 %
  
E-value: 1e-56
  
  
 NCBI BlastP on this gene

EED23990

amino acid transporter, putative
  
Accession: EED23991
  
Location: 3905710-3907693
  
 NCBI BlastP on this gene

EED23991

conserved hypothetical protein
  
Accession: EED23992
  
Location: 3908221-3908943
  
 NCBI BlastP on this gene

EED23992

FAD dependent oxidoreductase superfamily
  
Accession: EED23993
  
Location: 3909870-3914043
  
  
**BlastP hit with Mycgr3G57362**
  
Percentage identity: 31 %
  
BlastP bit score: 213
  
Sequence coverage: 98 %
  
E-value: 3e-56
  
  
 NCBI BlastP on this gene

EED23993

amino acid permease, putative
  
Accession: EED23994
  
Location: 3914817-3916612
  
 NCBI BlastP on this gene

EED23994

2-haloalkanoic acid dehalogenase, putative
  
Accession: EED23995
  
Location: 3917414-3918239
  
 NCBI BlastP on this gene

EED23995

184. :  JH687379 Stereum hirsutum FP-91666 SS1 unplaced genomic scaffold STEHIscaffold\_1     Total score: 1.0     Cumulative Blast bit score: 417

high affinity nickel transport protein nic1
  
Accession: EIM92684
  
Location: 2972008-2973547
  
  
**BlastP hit with Mycgr3G108865**
  
Percentage identity: 57 %
  
BlastP bit score: 417
  
Sequence coverage: 81 %
  
E-value: 5e-139
  
  
 NCBI BlastP on this gene

EIM92684

hypothetical protein
  
Accession: EIM92683
  
Location: 2969934-2970239
  
 NCBI BlastP on this gene

EIM92683

hypothetical protein
  
Accession: EIM92682
  
Location: 2968084-2968470
  
 NCBI BlastP on this gene

EIM92682

hypothetical protein
  
Accession: EIM92681
  
Location: 2965276-2966244
  
 NCBI BlastP on this gene

EIM92681

40S ribosomal protein S27
  
Accession: EIM92680
  
Location: 2963325-2963788
  
 NCBI BlastP on this gene

EIM92680

185. :  KE145368 Glarea lozoyensis ATCC 20868 chromosome Unknown GLAREA4     Total score: 1.0     Cumulative Blast bit score: 406

hypothetical protein
  
Accession: EPE28767
  
Location: 1614790-1615686
  
 NCBI BlastP on this gene

EPE28767

hypothetical protein
  
Accession: EPE28768
  
Location: 1617066-1618346
  
 NCBI BlastP on this gene

EPE28768

Actin-like ATPase
  
Accession: EPE28769
  
Location: 1618829-1620319
  
 NCBI BlastP on this gene

EPE28769

hypothetical protein
  
Accession: EPE28770
  
Location: 1620345-1621154
  
 NCBI BlastP on this gene

EPE28770

hypothetical protein
  
Accession: EPE28771
  
Location: 1622620-1623911
  
  
**BlastP hit with Mycgr3G108865**
  
Percentage identity: 53 %
  
BlastP bit score: 406
  
Sequence coverage: 86 %
  
E-value: 1e-134
  
  
 NCBI BlastP on this gene

EPE28771

186. :  HF679025 Fusarium fujikuroi IMI 58289 draft genome, chromosome FFUJ\_chr03.     Total score: 1.0     Cumulative Blast bit score: 405

related to thioesterase superfamily member 2
  
Accession: CCT66461
  
Location: 4510646-4511152
  
 NCBI BlastP on this gene

FFUJ\_03493

related to malate dehydrogenase
  
Accession: CCT66462
  
Location: 4511362-4512432
  
 NCBI BlastP on this gene

FFUJ\_03494

related to purine utilization positive regulator
  
Accession: CCT66780
  
Location: 4512709-4515045
  
 NCBI BlastP on this gene

FFUJ\_03495

related to dihydroxy-acid dehydratase
  
Accession: CCT66745
  
Location: 4515107-4517031
  
 NCBI BlastP on this gene

FFUJ\_03496

related to short-chain alcohol dehydrogenase
  
Accession: CCT66463
  
Location: 4517336-4518136
  
 NCBI BlastP on this gene

FFUJ\_03497

uncharacterized protein
  
Accession: CCT66464
  
Location: 4518916-4519385
  
 NCBI BlastP on this gene

FFUJ\_03498

probable high-affinity nickel transport protein nic1
  
Accession: CCT66465
  
Location: 4519752-4521012
  
  
**BlastP hit with Mycgr3G108865**
  
Percentage identity: 54 %
  
BlastP bit score: 405
  
Sequence coverage: 79 %
  
E-value: 2e-134
  
  
 NCBI BlastP on this gene

FFUJ\_03499

187. :  GL636500 Coccidioides posadasii str. Silveira unplaced genomic scaffold supercont2.15     Total score: 1.0     Cumulative Blast bit score: 398

nickel transporter
  
Accession: EFW15593
  
Location: 258575-260596
  
  
**BlastP hit with Mycgr3G108865**
  
Percentage identity: 55 %
  
BlastP bit score: 398
  
Sequence coverage: 86 %
  
E-value: 4e-131
  
  
 NCBI BlastP on this gene

EFW15593

conserved hypothetical protein
  
Accession: EFW15592
  
Location: 255542-258137
  
 NCBI BlastP on this gene

EFW15592

hypothetical protein
  
Accession: EFW15591
  
Location: 254248-255521
  
 NCBI BlastP on this gene

EFW15591

conserved hypothetical protein
  
Accession: EFW15590
  
Location: 249561-251757
  
 NCBI BlastP on this gene

EFW15590

188. :  AQGS01000575 Dactylellina haptotyla CBS 200.50     Total score: 1.0     Cumulative Blast bit score: 396

hypothetical protein
  
Accession: EPS38297
  
Location: 346908-349134
  
 NCBI BlastP on this gene

EPS38297

hypothetical protein
  
Accession: EPS38341
  
Location: 349408-350592
  
 NCBI BlastP on this gene

EPS38341

hypothetical protein
  
Accession: EPS38209
  
Location: 351528-353015
  
 NCBI BlastP on this gene

EPS38209

hypothetical protein
  
Accession: EPS38251
  
Location: 354237-358793
  
  
**BlastP hit with Mycgr3G108865**
  
Percentage identity: 59 %
  
BlastP bit score: 397
  
Sequence coverage: 79 %
  
E-value: 2e-125
  
  
 NCBI BlastP on this gene

EPS38251

189. :  GL891304 Neurospora tetrasperma FGSC 2508 unplaced genomic scaffold NEUTE1scaffold\_3     Total score: 1.0     Cumulative Blast bit score: 392

hypothetical protein
  
Accession: EGO58357
  
Location: 4423195-4424918
  
 NCBI BlastP on this gene

EGO58357

hypothetical protein
  
Accession: EGO58358
  
Location: 4425651-4426843
  
 NCBI BlastP on this gene

EGO58358

hypothetical protein
  
Accession: EGO58359
  
Location: 4429040-4429420
  
 NCBI BlastP on this gene

EGO58359

hypothetical protein
  
Accession: EGO58360
  
Location: 4430490-4431789
  
  
**BlastP hit with Mycgr3G108865**
  
Percentage identity: 58 %
  
BlastP bit score: 392
  
Sequence coverage: 74 %
  
E-value: 3e-129
  
  
 NCBI BlastP on this gene

EGO58360

190. :  GL891236 Neurospora tetrasperma FGSC 2509 unplaced genomic scaffold NEUTE2scaffold\_4     Total score: 1.0     Cumulative Blast bit score: 392

hypothetical protein
  
Accession: EGZ71317
  
Location: 568075-568275
  
 NCBI BlastP on this gene

EGZ71317

hypothetical protein
  
Accession: EGZ71316
  
Location: 566267-567990
  
 NCBI BlastP on this gene

EGZ71316

hypothetical protein
  
Accession: EGZ71315
  
Location: 564342-565534
  
 NCBI BlastP on this gene

EGZ71315

NicO-domain-containing protein
  
Accession: EGZ71314
  
Location: 559397-562145
  
  
**BlastP hit with Mycgr3G108865**
  
Percentage identity: 58 %
  
BlastP bit score: 392
  
Sequence coverage: 74 %
  
E-value: 5e-128
  
  
 NCBI BlastP on this gene

EGZ71314

nucleotide-diphospho-sugar transferase
  
Accession: EGZ71313
  
Location: 553066-555512
  
 NCBI BlastP on this gene

EGZ71313

concanavalin A-like lectin/glucanase
  
Accession: EGZ71312
  
Location: 550908-552178
  
 NCBI BlastP on this gene

EGZ71312

191. :  DS231619 Pyrenophora tritici-repentis Pt-1C-BFP supercont1.5 genomic scaffold     Total score: 1.0     Cumulative Blast bit score: 392

choline dehydrogenase
  
Accession: EDU48816
  
Location: 1780781-1781371
  
 NCBI BlastP on this gene

EDU48816

hypothetical protein
  
Accession: EDU48815
  
Location: 1777890-1779639
  
 NCBI BlastP on this gene

EDU48815

FAD dependent oxidoreductase
  
Accession: EDU48814
  
Location: 1775664-1777263
  
  
**BlastP hit with Mycgr3G57362**
  
Percentage identity: 33 %
  
BlastP bit score: 209
  
Sequence coverage: 97 %
  
E-value: 7e-58
  
  
 NCBI BlastP on this gene

EDU48814

PrnA protein
  
Accession: EDU48813
  
Location: 1772868-1775519
  
 NCBI BlastP on this gene

EDU48813

succinate-semialdehyde dehydrogenase, mitochondrial precursor
  
Accession: EDU48812
  
Location: 1770799-1772526
  
 NCBI BlastP on this gene

EDU48812

haloacid dehalogenase
  
Accession: EDU48811
  
Location: 1769933-1770658
  
 NCBI BlastP on this gene

EDU48811

FAD dependent oxidoreductase
  
Accession: EDU48810
  
Location: 1767870-1769564
  
  
**BlastP hit with Mycgr3G57362**
  
Percentage identity: 32 %
  
BlastP bit score: 183
  
Sequence coverage: 99 %
  
E-value: 4e-48
  
  
 NCBI BlastP on this gene

EDU48810

conserved hypothetical protein
  
Accession: EDU48809
  
Location: 1766541-1767341
  
 NCBI BlastP on this gene

EDU48809

conserved hypothetical protein
  
Accession: EDU48808
  
Location: 1764967-1765820
  
 NCBI BlastP on this gene

EDU48808

192. :  CH445339 Phaeosphaeria nodorum SN15 scaffold\_15     Total score: 1.0     Cumulative Blast bit score: 392

hypothetical protein
  
Accession: EAT83172
  
Location: 992184-992883
  
 NCBI BlastP on this gene

EAT83172

hypothetical protein
  
Accession: EAT83171
  
Location: 991103-991596
  
 NCBI BlastP on this gene

EAT83171

hypothetical protein
  
Accession: EAT83170
  
Location: 988461-989982
  
  
**BlastP hit with Mycgr3G57362**
  
Percentage identity: 33 %
  
BlastP bit score: 210
  
Sequence coverage: 99 %
  
E-value: 2e-58
  
  
 NCBI BlastP on this gene

EAT83170

hypothetical protein
  
Accession: EAT83169
  
Location: 985685-988315
  
 NCBI BlastP on this gene

EAT83169

hypothetical protein
  
Accession: EAT83168
  
Location: 984554-985279
  
 NCBI BlastP on this gene

EAT83168

hypothetical protein
  
Accession: EAT83167
  
Location: 982368-984181
  
  
**BlastP hit with Mycgr3G57362**
  
Percentage identity: 30 %
  
BlastP bit score: 182
  
Sequence coverage: 100 %
  
E-value: 6e-48
  
  
 NCBI BlastP on this gene

EAT83167

hypothetical protein
  
Accession: EAT83166
  
Location: 981076-981678
  
 NCBI BlastP on this gene

EAT83166

hypothetical protein
  
Accession: EAT83165
  
Location: 980151-980878
  
 NCBI BlastP on this gene

EAT83165

hypothetical protein
  
Accession: EAT83164
  
Location: 978211-979862
  
 NCBI BlastP on this gene

EAT83164

193. :  GG749429 Ajellomyces dermatitidis ATCC 18188 genomic scaffold supercont1.23     Total score: 1.0     Cumulative Blast bit score: 391

hypothetical protein
  
Accession: EGE81952
  
Location: 542677-545817
  
 NCBI BlastP on this gene

EGE81952

zinc carboxypeptidase
  
Accession: EGE81953
  
Location: 546539-548402
  
 NCBI BlastP on this gene

EGE81953

hypothetical protein
  
Accession: EGE81954
  
Location: 549961-550937
  
 NCBI BlastP on this gene

EGE81954

hypothetical protein
  
Accession: EGE81955
  
Location: 551233-552204
  
 NCBI BlastP on this gene

EGE81955

nickel transporter
  
Accession: EGE81956
  
Location: 553228-555307
  
  
**BlastP hit with Mycgr3G108865**
  
Percentage identity: 53 %
  
BlastP bit score: 391
  
Sequence coverage: 89 %
  
E-value: 2e-127
  
  
 NCBI BlastP on this gene

EGE81956

terminal deoxynucleotidyl transferase
  
Accession: EGE81957
  
Location: 555898-558069
  
 NCBI BlastP on this gene

EGE81957

phospholipid-transporting ATPase
  
Accession: EGE81958
  
Location: 559074-563287
  
 NCBI BlastP on this gene

EGE81958

194. :  EQ999973 Ajellomyces dermatitidis ER-3 genomic scaffold supercont1.1     Total score: 1.0     Cumulative Blast bit score: 391

nickel transporter
  
Accession: EEQ83315
  
Location: 987636-989714
  
  
**BlastP hit with Mycgr3G108865**
  
Percentage identity: 53 %
  
BlastP bit score: 391
  
Sequence coverage: 89 %
  
E-value: 2e-127
  
  
 NCBI BlastP on this gene

EEQ83315

terminal deoxynucleotidyl transferase
  
Accession: EEQ83314
  
Location: 984601-986772
  
 NCBI BlastP on this gene

EEQ83314

phospholipid-transporting ATPase
  
Accession: EEQ83313
  
Location: 979382-983595
  
 NCBI BlastP on this gene

EEQ83313

195. :  GG657469 Ajellomyces dermatitidis SLH14081 genomic scaffold supercont1.22     Total score: 1.0     Cumulative Blast bit score: 389

nickel transporter
  
Accession: EEQ73846
  
Location: 142369-144448
  
  
**BlastP hit with Mycgr3G108865**
  
Percentage identity: 52 %
  
BlastP bit score: 389
  
Sequence coverage: 88 %
  
E-value: 2e-126
  
  
 NCBI BlastP on this gene

EEQ73846

terminal deoxynucleotidyl transferase
  
Accession: EEQ73845
  
Location: 139591-141687
  
 NCBI BlastP on this gene

EEQ73845

phospholipid-transporting ATPase
  
Accession: EEQ73844
  
Location: 134374-138587
  
 NCBI BlastP on this gene

EEQ73844

196. :  DS027696 Neosartorya fischeri NRRL 181 1099437636264 genomic scaffold     Total score: 1.0     Cumulative Blast bit score: 388

zinc carboxypeptidase, putative
  
Accession: EAW18486
  
Location: 3616789-3618618
  
 NCBI BlastP on this gene

EAW18486

amino acid permease (Dip5), putative
  
Accession: EAW18487
  
Location: 3618923-3620847
  
 NCBI BlastP on this gene

EAW18487

conserved hypothetical protein
  
Accession: EAW18488
  
Location: 3624185-3625138
  
 NCBI BlastP on this gene

EAW18488

nickel transport protein, putative
  
Accession: EAW18489
  
Location: 3626150-3627855
  
  
**BlastP hit with Mycgr3G108865**
  
Percentage identity: 51 %
  
BlastP bit score: 388
  
Sequence coverage: 93 %
  
E-value: 6e-127
  
  
 NCBI BlastP on this gene

EAW18489

197. :  DS985215 Verticillium albo-atrum VaMs.102 supercont1.2 genomic scaffold     Total score: 1.0     Cumulative Blast bit score: 387

conserved hypothetical protein
  
Accession: EEY16146
  
Location: 2581014-2582540
  
  
**BlastP hit with Mycgr3G70475**
  
Percentage identity: 50 %
  
BlastP bit score: 387
  
Sequence coverage: 100 %
  
E-value: 4e-126
  
  
 NCBI BlastP on this gene

EEY16146

conserved hypothetical protein
  
Accession: EEY16145
  
Location: 2579383-2580431
  
 NCBI BlastP on this gene

EEY16145

conserved hypothetical protein
  
Accession: EEY16144
  
Location: 2575322-2576690
  
 NCBI BlastP on this gene

EEY16144

198. :  DS499595 Aspergillus fumigatus A1163 scf\_000002 genomic scaffold     Total score: 1.0     Cumulative Blast bit score: 386

zinc carboxypeptidase, putative
  
Accession: EDP54414
  
Location: 2244741-2246568
  
 NCBI BlastP on this gene

EDP54414

amino acid permease (Dip5), putative
  
Accession: EDP54415
  
Location: 2246900-2248827
  
 NCBI BlastP on this gene

EDP54415

conserved hypothetical protein
  
Accession: EDP54416
  
Location: 2248872-2249057
  
 NCBI BlastP on this gene

EDP54416

conserved hypothetical protein
  
Accession: EDP54417
  
Location: 2252292-2253245
  
 NCBI BlastP on this gene

EDP54417

nickel transport protein, putative
  
Accession: EDP54418
  
Location: 2254316-2256047
  
  
**BlastP hit with Mycgr3G108865**
  
Percentage identity: 51 %
  
BlastP bit score: 386
  
Sequence coverage: 88 %
  
E-value: 5e-126
  
  
 NCBI BlastP on this gene

EDP54418

199. :  DS231615 Pyrenophora tritici-repentis Pt-1C-BFP supercont1.1 genomic scaffold     Total score: 1.0     Cumulative Blast bit score: 385

predicted protein
  
Accession: EDU39825
  
Location: 1032530-1033676
  
 NCBI BlastP on this gene

EDU39825

citrinin biosynthesis oxydoreductase CtnB
  
Accession: EDU39826
  
Location: 1033840-1034670
  
 NCBI BlastP on this gene

EDU39826

trichothecene 3-O-acetyltransferase
  
Accession: EDU39827
  
Location: 1036095-1037465
  
 NCBI BlastP on this gene

EDU39827

vacuolar basic amino acid transporter 3
  
Accession: EDU39828
  
Location: 1038717-1041239
  
 NCBI BlastP on this gene

EDU39828

FAD dependent oxidoreductase
  
Accession: EDU39829
  
Location: 1041833-1043310
  
  
**BlastP hit with Mycgr3G57362**
  
Percentage identity: 46 %
  
BlastP bit score: 385
  
Sequence coverage: 95 %
  
E-value: 2e-125
  
  
 NCBI BlastP on this gene

EDU39829

DJ-1/PfpI family protein
  
Accession: EDU39830
  
Location: 1044856-1045647
  
 NCBI BlastP on this gene

EDU39830

zinc-binding oxidoreductase CipB
  
Accession: EDU39831
  
Location: 1046169-1047287
  
 NCBI BlastP on this gene

EDU39831

serine/threonine-protein kinase YPK2/YKR2
  
Accession: EDU39832
  
Location: 1048342-1049826
  
 NCBI BlastP on this gene

EDU39832

vacuolar ATP synthase 16 kDa proteolipid subunit 2
  
Accession: EDU39833
  
Location: 1050511-1051143
  
 NCBI BlastP on this gene

EDU39833

200. :  CU633900 Podospora anserina S mat+ genomic DNA chromosome 7, supercontig 1.     Total score: 1.0     Cumulative Blast bit score: 383

not annotated
  
Accession: CAP68345
  
Location: 524076-525195
  
 NCBI BlastP on this gene

CAP68345

not annotated
  
Accession: CAP68344
  
Location: 522203-523297
  
 NCBI BlastP on this gene

CAP68344

not annotated
  
Accession: CAP68343
  
Location: 519554-521446
  
 NCBI BlastP on this gene

CAP68343

not annotated
  
Accession: CAP68342
  
Location: 516116-517018
  
 NCBI BlastP on this gene

CAP68342

not annotated
  
Accession: CAP68341
  
Location: 514211-515513
  
  
**BlastP hit with Mycgr3G108865**
  
Percentage identity: 54 %
  
BlastP bit score: 383
  
Sequence coverage: 74 %
  
E-value: 2e-125
  
  
 NCBI BlastP on this gene

CAP68341

not annotated
  
Accession: CAP68340
  
Location: 511292-514105
  
 NCBI BlastP on this gene

CAP68340

not annotated
  
Accession: CAP68339
  
Location: 510079-510639
  
 NCBI BlastP on this gene

CAP68339

not annotated
  
Accession: CAP68338
  
Location: 509197-509637
  
 NCBI BlastP on this gene

CAP68338

not annotated
  
Accession: CAP68337
  
Location: 506857-508231
  
 NCBI BlastP on this gene

CAP68337

not annotated
  
Accession: CAP68336
  
Location: 505396-506295
  
 NCBI BlastP on this gene

CAP68336

Detecting sequence homology at the gene cluster level with MultiGeneBlast.
  
Marnix H. Medema, Rainer Breitling & Eriko Takano (2013)
  
*Molecular Biology and Evolution* , 30: 1218-1223.
