# Supplementary material for: In silico prediction and characterization of secondary metabolite biosynthetic gene clusters in the wheat pathogen Zymoseptoria tritici
Source: BMC Genomics. 2017 Aug 17;18:631. doi: 10.1186/s12864-017-3969-y (PMC5561558; doi:10.1186/s12864-017-3969-y)
Supplement: Supplementary file 1 — MultiGeneBLAST analysis of putative secondary metabolite clusters. All encoded amino acid sequences from genes residing in clusters predicted by AntiSMASH are given as FASTA file format. All output data from MultiGeneBLASTs are also provided. (ZIP 42911 kb) [file 12864_2017_3969_MOESM1_ESM.zip › Cluster MultiGene BLAST/out/Clusters_1_34/Cluster_13/displaypage5.xhtml]

xml version="1.0" encoding="UTF-8"?


Search Results
  
  
 Results pages: 1, 2, 3, 4, 5

**MultiGeneBlast hits**

Select gene cluster alignment
201. GG663367\_1 Ajellomyces capsulatus G186AR genomic scaffold supercont2.5, ...
202. DS995900\_0 Penicillium marneffei ATCC 18224 scf\_1105668340758 genomic sc...
203. KB445644\_2 Cochliobolus sativus ND90Pr unplaced genomic scaffold COCSAsc...
204. GG692419\_1 Ajellomyces capsulatus H143 genomic scaffold supercont2.1, wh...
205. EQ962653\_0 Talaromyces stipitatus ATCC 10500 scf\_1105507295527 genomic s...
206. DS990636\_1 Ajellomyces capsulatus H88 supercont1.1 genomic scaffold, who...
207. KB908833\_0 Setosphaeria turcica Et28A unplaced genomic scaffold SETTUsca...
208. KB733456\_0 Bipolaris maydis ATCC 48331 unplaced genomic scaffold COCC4sc...
209. KB445574\_2 Cochliobolus heterostrophus C5 unplaced genomic scaffold COCH...
210. ABDG02000027\_1 Trichoderma atroviride IMI 206040, whole genome shotgun s...
211. CH445339\_0 Phaeosphaeria nodorum SN15 scaffold\_15, whole genome shotgun ...
212. CP003004\_1 Myceliophthora thermophila ATCC 42464 chromosome 3, complete ...
213. GL988041\_0 Chaetomium thermophilum var. thermophilum DSM 1495 unplaced g...
214. DS572815\_0 Paracoccidioides brasiliensis Pb01 supercont1.5 genomic scaff...
215. DS544809\_0 Paracoccidioides brasiliensis Pb03 supercont1.7 genomic scaff...
216. JH921428\_0 Marssonina brunnea f. sp. 'multigermtubi' MB\_m1 unplaced geno...
217. CP003012\_0 Thielavia terrestris NRRL 8126 chromosome 4, complete sequence.
218. KB707715\_0 Botryotinia fuckeliana BcDW1 unplaced genomic scaffold Scaffo...
219. CP003003\_0 Myceliophthora thermophila ATCC 42464 chromosome 2, complete ...
220. ACFW01000049\_0 Coccidioides posadasii C735 delta SOWgp, whole genome sho...
221. KE145369\_1 Glarea lozoyensis ATCC 20868 chromosome Unknown GLAREA5, whol...
222. DS989823\_0 Arthroderma gypseum CBS 118893 supercont1.2 genomic scaffold,...
223. CAGA01000037\_0 Claviceps purpurea 20.1, whole genome shotgun sequencing ...
224. ABDF02000003\_0 Trichoderma virens Gv29-8, whole genome shotgun sequencin...
225. KB730323\_1 Fusarium oxysporum f. sp. cubense race 1 unplaced genomic sca...
226. KB726993\_1 Fusarium oxysporum f. sp. cubense race 4 unplaced genomic sca...
227. HF679027\_1 Fusarium fujikuroi IMI 58289 draft genome, chromosome FFUJ\_ch...
228. JH226136\_2 Exophiala dermatitidis NIH/UT8656 unplaced genomic scaffold s...
229. JH126399\_0 Cordyceps militaris CM01 unplaced genomic scaffold CCM\_S00001...
230. CP003009\_0 Thielavia terrestris NRRL 8126 chromosome 1, complete sequence.
231. ABDG02000029\_1 Trichoderma atroviride IMI 206040, whole genome shotgun s...
232. DS572755\_0 Paracoccidioides brasiliensis Pb18 supercont1.6 genomic scaff...
233. GL891302\_1 Neurospora tetrasperma FGSC 2508 unplaced genomic scaffold NE...
234. GL891107\_0 Neurospora tetrasperma FGSC 2509 unplaced genomic scaffold NE...
235. ADOT01000059\_0 Arthrobotrys oligospora ATCC 24927, whole genome shotgun ...
236. KB446557\_4 Pseudocercospora fijiensis CIRAD86 unplaced genomic scaffold ...
237. CP000494\_0 Bradyrhizobium sp. BTAi1, complete genome.
238. ACJE01000001\_1 Aspergillus niger ATCC 1015, whole genome shotgun sequenc...
239. AM920437\_3 Penicillium chrysogenum Wisconsin 54-1255 complete genome, co...
240. JH226131\_1 Exophiala dermatitidis NIH/UT8656 unplaced genomic scaffold s...
241. ACJE01000012\_1 Aspergillus niger ATCC 1015, whole genome shotgun sequenc...
242. KB726307\_1 Fusarium oxysporum f. sp. cubense race 4 unplaced genomic sca...
243. KB644412\_0 Penicillium oxalicum 114-2 unplaced genomic scaffold scaffold...
244. CP003009\_2 Thielavia terrestris NRRL 8126 chromosome 1, complete sequence.
245. HF679032\_1 Fusarium fujikuroi IMI 58289 draft genome, chromosome FFUJ\_ch...
246. GG704912\_1 Coccidioides immitis RS genomic scaffold supercont3.2, whole ...
247. ACFW01000025\_1 Coccidioides posadasii C735 delta SOWgp, whole genome sho...
248. KE145352\_0 Glarea lozoyensis ATCC 20868 chromosome Unknown GLAREA1, whol...
249. AEOI01000007\_0 Ogataea parapolymorpha DL-1, whole genome shotgun sequenc...
250. KE148151\_1 Ophiostoma piceae UAMH 11346 chromosome Unknown scf06, whole ...

Query: Architecture Search FASTA input

GG663367 : Ajellomyces capsulatus G186AR genomic scaffold supercont2.5    Total score: 1.0     Cumulative Blast bit score: 380

Hit cluster cross-links:

Mycgr3G70471
  
Location: 0-405

Mycgr3G70471

Mycgr3G39149
  
Location: 505-1798

Mycgr3G39149

Mycgr3G92130
  
Location: 1898-2396

Mycgr3G92130

Mycgr3G38483
  
Location: 2496-3576

Mycgr3G38483

Mycgr3G108869
  
Location: 3676-5056

Mycgr3G108869

Mycgr3G103943
  
Location: 5156-5762

Mycgr3G103943

Mycgr3G57362
  
Location: 5862-7296

Mycgr3G57362

Mycgr3G39086
  
Location: 7396-8368

Mycgr3G39086

Mycgr3G103942
  
Location: 8468-8714

Mycgr3G103942

Mycgr3G108865
  
Location: 8814-10239

Mycgr3G108865

Mycgr3G70475
  
Location: 10339-11821

Mycgr3G70475

Mycgr3G108866
  
Location: 11921-13010

Mycgr3G108866

Mycgr3G92136
  
Location: 13110-13593

Mycgr3G92136

high-affinity nickel transporter
  
Accession: EEH07657
  
Location: 1704060-1706324
  
  
**BlastP hit with Mycgr3G108865**
  
Percentage identity: 51 %
  
BlastP bit score: 380
  
Sequence coverage: 85 %
  
E-value: 4e-123
  
  
 NCBI BlastP on this gene

EEH07657

high-affinity nickel transporter
  
Accession: EEH07656
  
Location: 1701350-1703434
  
 NCBI BlastP on this gene

EEH07656

P-type ATPase
  
Accession: EEH07655
  
Location: 1696040-1700249
  
 NCBI BlastP on this gene

EEH07655

Query: Architecture Search FASTA input

DS995900 : Penicillium marneffei ATCC 18224 scf\_1105668340758 genomic scaffold    Total score: 1.0     Cumulative Blast bit score: 378

Hit cluster cross-links:

Mycgr3G70471
  
Location: 0-405

Mycgr3G70471

Mycgr3G39149
  
Location: 505-1798

Mycgr3G39149

Mycgr3G92130
  
Location: 1898-2396

Mycgr3G92130

Mycgr3G38483
  
Location: 2496-3576

Mycgr3G38483

Mycgr3G108869
  
Location: 3676-5056

Mycgr3G108869

Mycgr3G103943
  
Location: 5156-5762

Mycgr3G103943

Mycgr3G57362
  
Location: 5862-7296

Mycgr3G57362

Mycgr3G39086
  
Location: 7396-8368

Mycgr3G39086

Mycgr3G103942
  
Location: 8468-8714

Mycgr3G103942

Mycgr3G108865
  
Location: 8814-10239

Mycgr3G108865

Mycgr3G70475
  
Location: 10339-11821

Mycgr3G70475

Mycgr3G108866
  
Location: 11921-13010

Mycgr3G108866

Mycgr3G92136
  
Location: 13110-13593

Mycgr3G92136

BTB/POZ domain protein
  
Accession: EEA26027
  
Location: 2602332-2603560
  
 NCBI BlastP on this gene

EEA26027

conserved hypothetical protein
  
Accession: EEA26028
  
Location: 2606760-2607987
  
 NCBI BlastP on this gene

EEA26028

conserved hypothetical protein
  
Accession: EEA26029
  
Location: 2608992-2610488
  
 NCBI BlastP on this gene

EEA26029

nickel transport protein, putative
  
Accession: EEA26030
  
Location: 2610982-2612796
  
  
**BlastP hit with Mycgr3G108865**
  
Percentage identity: 49 %
  
BlastP bit score: 378
  
Sequence coverage: 91 %
  
E-value: 2e-122
  
  
 NCBI BlastP on this gene

EEA26030

terminal deoxynucleotidyl transferase, putative
  
Accession: EEA26031
  
Location: 2612852-2614984
  
 NCBI BlastP on this gene

EEA26031

phospholipid-transporting ATPase, putative
  
Accession: EEA26032
  
Location: 2615646-2619796
  
 NCBI BlastP on this gene

EEA26032

exosome complex endonuclease 2/ribosomal RNA processing protein, putative
  
Accession: EEA26033
  
Location: 2620746-2621737
  
 NCBI BlastP on this gene

EEA26033

Query: Architecture Search FASTA input

KB445644 : Cochliobolus sativus ND90Pr unplaced genomic scaffold COCSAscaffold\_8    Total score: 1.0     Cumulative Blast bit score: 375

Hit cluster cross-links:

Mycgr3G70471
  
Location: 0-405

Mycgr3G70471

Mycgr3G39149
  
Location: 505-1798

Mycgr3G39149

Mycgr3G92130
  
Location: 1898-2396

Mycgr3G92130

Mycgr3G38483
  
Location: 2496-3576

Mycgr3G38483

Mycgr3G108869
  
Location: 3676-5056

Mycgr3G108869

Mycgr3G103943
  
Location: 5156-5762

Mycgr3G103943

Mycgr3G57362
  
Location: 5862-7296

Mycgr3G57362

Mycgr3G39086
  
Location: 7396-8368

Mycgr3G39086

Mycgr3G103942
  
Location: 8468-8714

Mycgr3G103942

Mycgr3G108865
  
Location: 8814-10239

Mycgr3G108865

Mycgr3G70475
  
Location: 10339-11821

Mycgr3G70475

Mycgr3G108866
  
Location: 11921-13010

Mycgr3G108866

Mycgr3G92136
  
Location: 13110-13593

Mycgr3G92136

hypothetical protein
  
Accession: EMD63852
  
Location: 1309585-1311114
  
  
**BlastP hit with Mycgr3G57362**
  
Percentage identity: 45 %
  
BlastP bit score: 375
  
Sequence coverage: 96 %
  
E-value: 1e-121
  
  
 NCBI BlastP on this gene

EMD63852

hypothetical protein
  
Accession: EMD63851
  
Location: 1308420-1309197
  
 NCBI BlastP on this gene

EMD63851

glycoside hydrolase family 17 protein
  
Accession: EMD63850
  
Location: 1306233-1307762
  
 NCBI BlastP on this gene

EMD63850

hypothetical protein
  
Accession: EMD63849
  
Location: 1304085-1305942
  
 NCBI BlastP on this gene

EMD63849

hypothetical protein
  
Accession: EMD63848
  
Location: 1300856-1302070
  
 NCBI BlastP on this gene

EMD63848

Query: Architecture Search FASTA input

GG692419 : Ajellomyces capsulatus H143 genomic scaffold supercont2.1    Total score: 1.0     Cumulative Blast bit score: 375

Hit cluster cross-links:

Mycgr3G70471
  
Location: 0-405

Mycgr3G70471

Mycgr3G39149
  
Location: 505-1798

Mycgr3G39149

Mycgr3G92130
  
Location: 1898-2396

Mycgr3G92130

Mycgr3G38483
  
Location: 2496-3576

Mycgr3G38483

Mycgr3G108869
  
Location: 3676-5056

Mycgr3G108869

Mycgr3G103943
  
Location: 5156-5762

Mycgr3G103943

Mycgr3G57362
  
Location: 5862-7296

Mycgr3G57362

Mycgr3G39086
  
Location: 7396-8368

Mycgr3G39086

Mycgr3G103942
  
Location: 8468-8714

Mycgr3G103942

Mycgr3G108865
  
Location: 8814-10239

Mycgr3G108865

Mycgr3G70475
  
Location: 10339-11821

Mycgr3G70475

Mycgr3G108866
  
Location: 11921-13010

Mycgr3G108866

Mycgr3G92136
  
Location: 13110-13593

Mycgr3G92136

high-affinity nickel transporter
  
Accession: EER45808
  
Location: 5846199-5848447
  
  
**BlastP hit with Mycgr3G108865**
  
Percentage identity: 51 %
  
BlastP bit score: 375
  
Sequence coverage: 85 %
  
E-value: 4e-121
  
  
 NCBI BlastP on this gene

EER45808

high-affinity nickel transporter
  
Accession: EER45807
  
Location: 5843493-5845577
  
 NCBI BlastP on this gene

EER45807

phospholipid-transporting ATPase
  
Accession: EER45806
  
Location: 5838192-5842399
  
 NCBI BlastP on this gene

EER45806

Query: Architecture Search FASTA input

EQ962653 : Talaromyces stipitatus ATCC 10500 scf\_1105507295527 genomic scaffold    Total score: 1.0     Cumulative Blast bit score: 375

Hit cluster cross-links:

Mycgr3G70471
  
Location: 0-405

Mycgr3G70471

Mycgr3G39149
  
Location: 505-1798

Mycgr3G39149

Mycgr3G92130
  
Location: 1898-2396

Mycgr3G92130

Mycgr3G38483
  
Location: 2496-3576

Mycgr3G38483

Mycgr3G108869
  
Location: 3676-5056

Mycgr3G108869

Mycgr3G103943
  
Location: 5156-5762

Mycgr3G103943

Mycgr3G57362
  
Location: 5862-7296

Mycgr3G57362

Mycgr3G39086
  
Location: 7396-8368

Mycgr3G39086

Mycgr3G103942
  
Location: 8468-8714

Mycgr3G103942

Mycgr3G108865
  
Location: 8814-10239

Mycgr3G108865

Mycgr3G70475
  
Location: 10339-11821

Mycgr3G70475

Mycgr3G108866
  
Location: 11921-13010

Mycgr3G108866

Mycgr3G92136
  
Location: 13110-13593

Mycgr3G92136

arylsulfatase A, putative
  
Accession: EED21920
  
Location: 3072215-3074411
  
 NCBI BlastP on this gene

EED21920

hypothetical protein
  
Accession: EED21921
  
Location: 3074976-3075582
  
 NCBI BlastP on this gene

EED21921

conserved hypothetical protein
  
Accession: EED21922
  
Location: 3077326-3078592
  
 NCBI BlastP on this gene

EED21922

conserved hypothetical protein
  
Accession: EED21923
  
Location: 3079481-3080810
  
 NCBI BlastP on this gene

EED21923

nickel transport protein, putative
  
Accession: EED21924
  
Location: 3081230-3083070
  
  
**BlastP hit with Mycgr3G108865**
  
Percentage identity: 50 %
  
BlastP bit score: 375
  
Sequence coverage: 91 %
  
E-value: 5e-121
  
  
 NCBI BlastP on this gene

EED21924

terminal deoxynucleotidyl transferase, putative
  
Accession: EED21925
  
Location: 3083136-3085259
  
 NCBI BlastP on this gene

EED21925

phospholipid-transporting ATPase, putative
  
Accession: EED21926
  
Location: 3085908-3090048
  
 NCBI BlastP on this gene

EED21926

exosome complex endonuclease 2/ribosomal RNA processing protein, putative
  
Accession: EED21927
  
Location: 3090933-3091745
  
 NCBI BlastP on this gene

EED21927

Query: Architecture Search FASTA input

DS990636 : Ajellomyces capsulatus H88 supercont1.1 genomic scaffold    Total score: 1.0     Cumulative Blast bit score: 375

Hit cluster cross-links:

Mycgr3G70471
  
Location: 0-405

Mycgr3G70471

Mycgr3G39149
  
Location: 505-1798

Mycgr3G39149

Mycgr3G92130
  
Location: 1898-2396

Mycgr3G92130

Mycgr3G38483
  
Location: 2496-3576

Mycgr3G38483

Mycgr3G108869
  
Location: 3676-5056

Mycgr3G108869

Mycgr3G103943
  
Location: 5156-5762

Mycgr3G103943

Mycgr3G57362
  
Location: 5862-7296

Mycgr3G57362

Mycgr3G39086
  
Location: 7396-8368

Mycgr3G39086

Mycgr3G103942
  
Location: 8468-8714

Mycgr3G103942

Mycgr3G108865
  
Location: 8814-10239

Mycgr3G108865

Mycgr3G70475
  
Location: 10339-11821

Mycgr3G70475

Mycgr3G108866
  
Location: 11921-13010

Mycgr3G108866

Mycgr3G92136
  
Location: 13110-13593

Mycgr3G92136

high-affinity nickel transporter
  
Accession: EGC41754
  
Location: 4262493-4264741
  
  
**BlastP hit with Mycgr3G108865**
  
Percentage identity: 51 %
  
BlastP bit score: 375
  
Sequence coverage: 85 %
  
E-value: 4e-121
  
  
 NCBI BlastP on this gene

EGC41754

high-affinity nickel transporter
  
Accession: EGC41753
  
Location: 4259787-4261871
  
 NCBI BlastP on this gene

EGC41753

phospholipid-transporting ATPase
  
Accession: EGC41752
  
Location: 4254480-4259029
  
 NCBI BlastP on this gene

EGC41752

Query: Architecture Search FASTA input

KB908833 : Setosphaeria turcica Et28A unplaced genomic scaffold SETTUscaffold\_5    Total score: 1.0     Cumulative Blast bit score: 373

Hit cluster cross-links:

Mycgr3G70471
  
Location: 0-405

Mycgr3G70471

Mycgr3G39149
  
Location: 505-1798

Mycgr3G39149

Mycgr3G92130
  
Location: 1898-2396

Mycgr3G92130

Mycgr3G38483
  
Location: 2496-3576

Mycgr3G38483

Mycgr3G108869
  
Location: 3676-5056

Mycgr3G108869

Mycgr3G103943
  
Location: 5156-5762

Mycgr3G103943

Mycgr3G57362
  
Location: 5862-7296

Mycgr3G57362

Mycgr3G39086
  
Location: 7396-8368

Mycgr3G39086

Mycgr3G103942
  
Location: 8468-8714

Mycgr3G103942

Mycgr3G108865
  
Location: 8814-10239

Mycgr3G108865

Mycgr3G70475
  
Location: 10339-11821

Mycgr3G70475

Mycgr3G108866
  
Location: 11921-13010

Mycgr3G108866

Mycgr3G92136
  
Location: 13110-13593

Mycgr3G92136

hypothetical protein
  
Accession: EOA83289
  
Location: 1097926-1099094
  
 NCBI BlastP on this gene

EOA83289

hypothetical protein
  
Accession: EOA83290
  
Location: 1100562-1100996
  
 NCBI BlastP on this gene

EOA83290

hypothetical protein
  
Accession: EOA83291
  
Location: 1101860-1103377
  
  
**BlastP hit with Mycgr3G57362**
  
Percentage identity: 45 %
  
BlastP bit score: 373
  
Sequence coverage: 98 %
  
E-value: 8e-121
  
  
 NCBI BlastP on this gene

EOA83291

hypothetical protein
  
Accession: EOA83292
  
Location: 1103724-1104663
  
 NCBI BlastP on this gene

EOA83292

hypothetical protein
  
Accession: EOA83293
  
Location: 1105138-1106047
  
 NCBI BlastP on this gene

EOA83293

hypothetical protein
  
Accession: EOA83294
  
Location: 1107636-1111510
  
 NCBI BlastP on this gene

EOA83294

Query: Architecture Search FASTA input

KB733456 : Bipolaris maydis ATCC 48331 unplaced genomic scaffold COCC4scaffold\_13    Total score: 1.0     Cumulative Blast bit score: 373

Hit cluster cross-links:

Mycgr3G70471
  
Location: 0-405

Mycgr3G70471

Mycgr3G39149
  
Location: 505-1798

Mycgr3G39149

Mycgr3G92130
  
Location: 1898-2396

Mycgr3G92130

Mycgr3G38483
  
Location: 2496-3576

Mycgr3G38483

Mycgr3G108869
  
Location: 3676-5056

Mycgr3G108869

Mycgr3G103943
  
Location: 5156-5762

Mycgr3G103943

Mycgr3G57362
  
Location: 5862-7296

Mycgr3G57362

Mycgr3G39086
  
Location: 7396-8368

Mycgr3G39086

Mycgr3G103942
  
Location: 8468-8714

Mycgr3G103942

Mycgr3G108865
  
Location: 8814-10239

Mycgr3G108865

Mycgr3G70475
  
Location: 10339-11821

Mycgr3G70475

Mycgr3G108866
  
Location: 11921-13010

Mycgr3G108866

Mycgr3G92136
  
Location: 13110-13593

Mycgr3G92136

hypothetical protein
  
Accession: ENI04559
  
Location: 86956-89594
  
 NCBI BlastP on this gene

ENI04559

hypothetical protein
  
Accession: ENI04560
  
Location: 89859-91118
  
 NCBI BlastP on this gene

ENI04560

hypothetical protein
  
Accession: ENI04561
  
Location: 92971-93438
  
 NCBI BlastP on this gene

ENI04561

hypothetical protein
  
Accession: ENI04562
  
Location: 95127-96656
  
  
**BlastP hit with Mycgr3G57362**
  
Percentage identity: 45 %
  
BlastP bit score: 373
  
Sequence coverage: 96 %
  
E-value: 8e-121
  
  
 NCBI BlastP on this gene

ENI04562

hypothetical protein
  
Accession: ENI04563
  
Location: 97040-97819
  
 NCBI BlastP on this gene

ENI04563

hypothetical protein
  
Accession: ENI04564
  
Location: 98055-98417
  
 NCBI BlastP on this gene

ENI04564

glycoside hydrolase family 17 protein
  
Accession: ENI04565
  
Location: 98728-100023
  
 NCBI BlastP on this gene

ENI04565

hypothetical protein
  
Accession: ENI04566
  
Location: 100313-102170
  
 NCBI BlastP on this gene

ENI04566

hypothetical protein
  
Accession: ENI04567
  
Location: 104206-104745
  
 NCBI BlastP on this gene

ENI04567

Query: Architecture Search FASTA input

KB445574 : Cochliobolus heterostrophus C5 unplaced genomic scaffold COCHEscaffold\_6    Total score: 1.0     Cumulative Blast bit score: 373

Hit cluster cross-links:

Mycgr3G70471
  
Location: 0-405

Mycgr3G70471

Mycgr3G39149
  
Location: 505-1798

Mycgr3G39149

Mycgr3G92130
  
Location: 1898-2396

Mycgr3G92130

Mycgr3G38483
  
Location: 2496-3576

Mycgr3G38483

Mycgr3G108869
  
Location: 3676-5056

Mycgr3G108869

Mycgr3G103943
  
Location: 5156-5762

Mycgr3G103943

Mycgr3G57362
  
Location: 5862-7296

Mycgr3G57362

Mycgr3G39086
  
Location: 7396-8368

Mycgr3G39086

Mycgr3G103942
  
Location: 8468-8714

Mycgr3G103942

Mycgr3G108865
  
Location: 8814-10239

Mycgr3G108865

Mycgr3G70475
  
Location: 10339-11821

Mycgr3G70475

Mycgr3G108866
  
Location: 11921-13010

Mycgr3G108866

Mycgr3G92136
  
Location: 13110-13593

Mycgr3G92136

hypothetical protein
  
Accession: EMD93049
  
Location: 883889-885418
  
  
**BlastP hit with Mycgr3G57362**
  
Percentage identity: 45 %
  
BlastP bit score: 373
  
Sequence coverage: 96 %
  
E-value: 8e-121
  
  
 NCBI BlastP on this gene

EMD93049

hypothetical protein
  
Accession: EMD93048
  
Location: 882726-883505
  
 NCBI BlastP on this gene

EMD93048

hypothetical protein
  
Accession: EMD93047
  
Location: 882122-882490
  
 NCBI BlastP on this gene

EMD93047

glycoside hydrolase family 17 protein
  
Accession: EMD93046
  
Location: 880690-881667
  
 NCBI BlastP on this gene

EMD93046

hypothetical protein
  
Accession: EMD93045
  
Location: 878375-880232
  
 NCBI BlastP on this gene

EMD93045

hypothetical protein
  
Accession: EMD93044
  
Location: 875239-876339
  
 NCBI BlastP on this gene

EMD93044

Query: Architecture Search FASTA input

ABDG02000027 : Trichoderma atroviride IMI 206040    Total score: 1.0     Cumulative Blast bit score: 371

Hit cluster cross-links:

Mycgr3G70471
  
Location: 0-405

Mycgr3G70471

Mycgr3G39149
  
Location: 505-1798

Mycgr3G39149

Mycgr3G92130
  
Location: 1898-2396

Mycgr3G92130

Mycgr3G38483
  
Location: 2496-3576

Mycgr3G38483

Mycgr3G108869
  
Location: 3676-5056

Mycgr3G108869

Mycgr3G103943
  
Location: 5156-5762

Mycgr3G103943

Mycgr3G57362
  
Location: 5862-7296

Mycgr3G57362

Mycgr3G39086
  
Location: 7396-8368

Mycgr3G39086

Mycgr3G103942
  
Location: 8468-8714

Mycgr3G103942

Mycgr3G108865
  
Location: 8814-10239

Mycgr3G108865

Mycgr3G70475
  
Location: 10339-11821

Mycgr3G70475

Mycgr3G108866
  
Location: 11921-13010

Mycgr3G108866

Mycgr3G92136
  
Location: 13110-13593

Mycgr3G92136

high-affinity nickel transport protein
  
Accession: EHK41551
  
Location: 3381317-3382686
  
  
**BlastP hit with Mycgr3G108865**
  
Percentage identity: 52 %
  
BlastP bit score: 371
  
Sequence coverage: 83 %
  
E-value: 1e-120
  
  
 NCBI BlastP on this gene

EHK41551

hypothetical protein
  
Accession: EHK41550
  
Location: 3375780-3378519
  
 NCBI BlastP on this gene

EHK41550

hypothetical protein
  
Accession: EHK41549
  
Location: 3373354-3374853
  
 NCBI BlastP on this gene

EHK41549

hypothetical protein
  
Accession: EHK41548
  
Location: 3371554-3372778
  
 NCBI BlastP on this gene

EHK41548

Query: Architecture Search FASTA input

CH445339 : Phaeosphaeria nodorum SN15 scaffold\_15    Total score: 1.0     Cumulative Blast bit score: 369

Hit cluster cross-links:

Mycgr3G70471
  
Location: 0-405

Mycgr3G70471

Mycgr3G39149
  
Location: 505-1798

Mycgr3G39149

Mycgr3G92130
  
Location: 1898-2396

Mycgr3G92130

Mycgr3G38483
  
Location: 2496-3576

Mycgr3G38483

Mycgr3G108869
  
Location: 3676-5056

Mycgr3G108869

Mycgr3G103943
  
Location: 5156-5762

Mycgr3G103943

Mycgr3G57362
  
Location: 5862-7296

Mycgr3G57362

Mycgr3G39086
  
Location: 7396-8368

Mycgr3G39086

Mycgr3G103942
  
Location: 8468-8714

Mycgr3G103942

Mycgr3G108865
  
Location: 8814-10239

Mycgr3G108865

Mycgr3G70475
  
Location: 10339-11821

Mycgr3G70475

Mycgr3G108866
  
Location: 11921-13010

Mycgr3G108866

Mycgr3G92136
  
Location: 13110-13593

Mycgr3G92136

hypothetical protein
  
Accession: EAT82811
  
Location: 228887-231809
  
 NCBI BlastP on this gene

EAT82811

hypothetical protein
  
Accession: EAT82810
  
Location: 227501-228363
  
 NCBI BlastP on this gene

EAT82810

hypothetical protein
  
Accession: EAT82809
  
Location: 225258-226256
  
 NCBI BlastP on this gene

EAT82809

hypothetical protein
  
Accession: EAT82808
  
Location: 222829-224357
  
  
**BlastP hit with Mycgr3G57362**
  
Percentage identity: 44 %
  
BlastP bit score: 369
  
Sequence coverage: 96 %
  
E-value: 3e-119
  
  
 NCBI BlastP on this gene

EAT82808

hypothetical protein
  
Accession: EAT82807
  
Location: 221777-222490
  
 NCBI BlastP on this gene

EAT82807

hypothetical protein
  
Accession: EAT82806
  
Location: 220968-221780
  
 NCBI BlastP on this gene

EAT82806

hypothetical protein
  
Accession: EAT82805
  
Location: 219468-220310
  
 NCBI BlastP on this gene

EAT82805

hypothetical protein
  
Accession: EAT82804
  
Location: 217087-218019
  
 NCBI BlastP on this gene

EAT82804

hypothetical protein
  
Accession: EAT82803
  
Location: 213856-216044
  
 NCBI BlastP on this gene

EAT82803

Query: Architecture Search FASTA input

CP003004 : Myceliophthora thermophila ATCC 42464 chromosome 3    Total score: 1.0     Cumulative Blast bit score: 363

Hit cluster cross-links:

Mycgr3G70471
  
Location: 0-405

Mycgr3G70471

Mycgr3G39149
  
Location: 505-1798

Mycgr3G39149

Mycgr3G92130
  
Location: 1898-2396

Mycgr3G92130

Mycgr3G38483
  
Location: 2496-3576

Mycgr3G38483

Mycgr3G108869
  
Location: 3676-5056

Mycgr3G108869

Mycgr3G103943
  
Location: 5156-5762

Mycgr3G103943

Mycgr3G57362
  
Location: 5862-7296

Mycgr3G57362

Mycgr3G39086
  
Location: 7396-8368

Mycgr3G39086

Mycgr3G103942
  
Location: 8468-8714

Mycgr3G103942

Mycgr3G108865
  
Location: 8814-10239

Mycgr3G108865

Mycgr3G70475
  
Location: 10339-11821

Mycgr3G70475

Mycgr3G108866
  
Location: 11921-13010

Mycgr3G108866

Mycgr3G92136
  
Location: 13110-13593

Mycgr3G92136

hypothetical protein
  
Accession: AEO58193
  
Location: 4129163-4131114
  
  
**BlastP hit with Mycgr3G108865**
  
Percentage identity: 54 %
  
BlastP bit score: 363
  
Sequence coverage: 78 %
  
E-value: 1e-117
  
  
 NCBI BlastP on this gene

MYCTH\_2093133

hypothetical protein
  
Accession: AEO58192
  
Location: 4128260-4128778
  
 NCBI BlastP on this gene

MYCTH\_2110543

hypothetical protein
  
Accession: AEO58191
  
Location: 4126947-4127898
  
 NCBI BlastP on this gene

MYCTH\_2110542

hypothetical protein
  
Accession: AEO58190
  
Location: 4122089-4123429
  
 NCBI BlastP on this gene

MYCTH\_2060238

Query: Architecture Search FASTA input

GL988041 : Chaetomium thermophilum var. thermophilum DSM 1495 unplaced genomic scaffold scf7180000...    Total score: 1.0     Cumulative Blast bit score: 355

Hit cluster cross-links:

Mycgr3G70471
  
Location: 0-405

Mycgr3G70471

Mycgr3G39149
  
Location: 505-1798

Mycgr3G39149

Mycgr3G92130
  
Location: 1898-2396

Mycgr3G92130

Mycgr3G38483
  
Location: 2496-3576

Mycgr3G38483

Mycgr3G108869
  
Location: 3676-5056

Mycgr3G108869

Mycgr3G103943
  
Location: 5156-5762

Mycgr3G103943

Mycgr3G57362
  
Location: 5862-7296

Mycgr3G57362

Mycgr3G39086
  
Location: 7396-8368

Mycgr3G39086

Mycgr3G103942
  
Location: 8468-8714

Mycgr3G103942

Mycgr3G108865
  
Location: 8814-10239

Mycgr3G108865

Mycgr3G70475
  
Location: 10339-11821

Mycgr3G70475

Mycgr3G108866
  
Location: 11921-13010

Mycgr3G108866

Mycgr3G92136
  
Location: 13110-13593

Mycgr3G92136

hypothetical protein
  
Accession: EGS20367
  
Location: 331967-332986
  
 NCBI BlastP on this gene

EGS20367

hypothetical protein
  
Accession: EGS20368
  
Location: 334320-336175
  
 NCBI BlastP on this gene

EGS20368

hypothetical protein
  
Accession: EGS20369
  
Location: 340000-341305
  
  
**BlastP hit with Mycgr3G108865**
  
Percentage identity: 54 %
  
BlastP bit score: 355
  
Sequence coverage: 72 %
  
E-value: 9e-115
  
  
 NCBI BlastP on this gene

EGS20369

hypothetical protein
  
Accession: EGS20370
  
Location: 341877-345689
  
 NCBI BlastP on this gene

EGS20370

putative UDP-glucose protein
  
Accession: EGS20371
  
Location: 347532-349033
  
 NCBI BlastP on this gene

EGS20371

Query: Architecture Search FASTA input

DS572815 : Paracoccidioides brasiliensis Pb01 supercont1.5 genomic scaffold    Total score: 1.0     Cumulative Blast bit score: 350

Hit cluster cross-links:

Mycgr3G70471
  
Location: 0-405

Mycgr3G70471

Mycgr3G39149
  
Location: 505-1798

Mycgr3G39149

Mycgr3G92130
  
Location: 1898-2396

Mycgr3G92130

Mycgr3G38483
  
Location: 2496-3576

Mycgr3G38483

Mycgr3G108869
  
Location: 3676-5056

Mycgr3G108869

Mycgr3G103943
  
Location: 5156-5762

Mycgr3G103943

Mycgr3G57362
  
Location: 5862-7296

Mycgr3G57362

Mycgr3G39086
  
Location: 7396-8368

Mycgr3G39086

Mycgr3G103942
  
Location: 8468-8714

Mycgr3G103942

Mycgr3G108865
  
Location: 8814-10239

Mycgr3G108865

Mycgr3G70475
  
Location: 10339-11821

Mycgr3G70475

Mycgr3G108866
  
Location: 11921-13010

Mycgr3G108866

Mycgr3G92136
  
Location: 13110-13593

Mycgr3G92136

predicted protein
  
Accession: EEH40152
  
Location: 61345-62338
  
 NCBI BlastP on this gene

EEH40152

conserved hypothetical protein
  
Accession: EEH40153
  
Location: 62449-64994
  
  
**BlastP hit with Mycgr3G108865**
  
Percentage identity: 48 %
  
BlastP bit score: 350
  
Sequence coverage: 97 %
  
E-value: 2e-111
  
  
 NCBI BlastP on this gene

EEH40153

Query: Architecture Search FASTA input

DS544809 : Paracoccidioides brasiliensis Pb03 supercont1.7 genomic scaffold    Total score: 1.0     Cumulative Blast bit score: 342

Hit cluster cross-links:

Mycgr3G70471
  
Location: 0-405

Mycgr3G70471

Mycgr3G39149
  
Location: 505-1798

Mycgr3G39149

Mycgr3G92130
  
Location: 1898-2396

Mycgr3G92130

Mycgr3G38483
  
Location: 2496-3576

Mycgr3G38483

Mycgr3G108869
  
Location: 3676-5056

Mycgr3G108869

Mycgr3G103943
  
Location: 5156-5762

Mycgr3G103943

Mycgr3G57362
  
Location: 5862-7296

Mycgr3G57362

Mycgr3G39086
  
Location: 7396-8368

Mycgr3G39086

Mycgr3G103942
  
Location: 8468-8714

Mycgr3G103942

Mycgr3G108865
  
Location: 8814-10239

Mycgr3G108865

Mycgr3G70475
  
Location: 10339-11821

Mycgr3G70475

Mycgr3G108866
  
Location: 11921-13010

Mycgr3G108866

Mycgr3G92136
  
Location: 13110-13593

Mycgr3G92136

DDHD domain-containing protein
  
Accession: EEH22506
  
Location: 387006-390000
  
 NCBI BlastP on this gene

EEH22506

conserved hypothetical protein
  
Accession: EEH22507
  
Location: 390619-391294
  
 NCBI BlastP on this gene

EEH22507

NADH dehydrogenase 29/21K chain
  
Accession: EEH22508
  
Location: 392436-393607
  
 NCBI BlastP on this gene

EEH22508

conserved hypothetical protein
  
Accession: EEH22509
  
Location: 394343-395152
  
 NCBI BlastP on this gene

EEH22509

high-affinity nickel permease
  
Accession: EEH22510
  
Location: 396131-399088
  
  
**BlastP hit with Mycgr3G108865**
  
Percentage identity: 48 %
  
BlastP bit score: 342
  
Sequence coverage: 94 %
  
E-value: 3e-107
  
  
 NCBI BlastP on this gene

EEH22510

DNA polymerase beta
  
Accession: EEH22511
  
Location: 399312-401563
  
 NCBI BlastP on this gene

EEH22511

ATPase
  
Accession: EEH22512
  
Location: 402086-406325
  
 NCBI BlastP on this gene

EEH22512

Query: Architecture Search FASTA input

JH921428 : Marssonina brunnea f. sp. 'multigermtubi' MB\_m1 unplaced genomic scaffold M6\_S00001    Total score: 1.0     Cumulative Blast bit score: 338

Hit cluster cross-links:

Mycgr3G70471
  
Location: 0-405

Mycgr3G70471

Mycgr3G39149
  
Location: 505-1798

Mycgr3G39149

Mycgr3G92130
  
Location: 1898-2396

Mycgr3G92130

Mycgr3G38483
  
Location: 2496-3576

Mycgr3G38483

Mycgr3G108869
  
Location: 3676-5056

Mycgr3G108869

Mycgr3G103943
  
Location: 5156-5762

Mycgr3G103943

Mycgr3G57362
  
Location: 5862-7296

Mycgr3G57362

Mycgr3G39086
  
Location: 7396-8368

Mycgr3G39086

Mycgr3G103942
  
Location: 8468-8714

Mycgr3G103942

Mycgr3G108865
  
Location: 8814-10239

Mycgr3G108865

Mycgr3G70475
  
Location: 10339-11821

Mycgr3G70475

Mycgr3G108866
  
Location: 11921-13010

Mycgr3G108866

Mycgr3G92136
  
Location: 13110-13593

Mycgr3G92136

integral membrane protein (Pth11)
  
Accession: EKD20892
  
Location: 519567-520980
  
 NCBI BlastP on this gene

EKD20892

hypothetical protein
  
Accession: EKD20893
  
Location: 522615-523930
  
 NCBI BlastP on this gene

EKD20893

allantoate permease
  
Accession: EKD20894
  
Location: 524075-525749
  
 NCBI BlastP on this gene

EKD20894

FAD dependent oxidoreductase
  
Accession: EKD20895
  
Location: 528471-529955
  
  
**BlastP hit with Mycgr3G57362**
  
Percentage identity: 44 %
  
BlastP bit score: 338
  
Sequence coverage: 100 %
  
E-value: 2e-107
  
  
 NCBI BlastP on this gene

EKD20895

amidase family protein
  
Accession: EKD20896
  
Location: 532575-534704
  
 NCBI BlastP on this gene

EKD20896

integral membrane protein DUF92
  
Accession: EKD20897
  
Location: 535679-536937
  
 NCBI BlastP on this gene

EKD20897

hypothetical protein
  
Accession: EKD20898
  
Location: 537208-541135
  
 NCBI BlastP on this gene

EKD20898

Query: Architecture Search FASTA input

CP003012 : Thielavia terrestris NRRL 8126 chromosome 4    Total score: 1.0     Cumulative Blast bit score: 338

Hit cluster cross-links:

Mycgr3G70471
  
Location: 0-405

Mycgr3G70471

Mycgr3G39149
  
Location: 505-1798

Mycgr3G39149

Mycgr3G92130
  
Location: 1898-2396

Mycgr3G92130

Mycgr3G38483
  
Location: 2496-3576

Mycgr3G38483

Mycgr3G108869
  
Location: 3676-5056

Mycgr3G108869

Mycgr3G103943
  
Location: 5156-5762

Mycgr3G103943

Mycgr3G57362
  
Location: 5862-7296

Mycgr3G57362

Mycgr3G39086
  
Location: 7396-8368

Mycgr3G39086

Mycgr3G103942
  
Location: 8468-8714

Mycgr3G103942

Mycgr3G108865
  
Location: 8814-10239

Mycgr3G108865

Mycgr3G70475
  
Location: 10339-11821

Mycgr3G70475

Mycgr3G108866
  
Location: 11921-13010

Mycgr3G108866

Mycgr3G92136
  
Location: 13110-13593

Mycgr3G92136

hypothetical protein
  
Accession: AEO69085
  
Location: 1954685-1955371
  
 NCBI BlastP on this gene

THITE\_2119095

hypothetical protein
  
Accession: AEO69084
  
Location: 1951707-1953598
  
 NCBI BlastP on this gene

THITE\_43879

hypothetical protein
  
Accession: AEO69083
  
Location: 1947927-1950879
  
 NCBI BlastP on this gene

THITE\_113789

hypothetical protein
  
Accession: AEO69082
  
Location: 1945306-1946824
  
  
**BlastP hit with Mycgr3G108865**
  
Percentage identity: 50 %
  
BlastP bit score: 338
  
Sequence coverage: 78 %
  
E-value: 9e-108
  
  
 NCBI BlastP on this gene

THITE\_2119090

hypothetical protein
  
Accession: AEO69081
  
Location: 1942046-1943150
  
 NCBI BlastP on this gene

THITE\_2119087

hypothetical protein
  
Accession: AEO69080
  
Location: 1941471-1941680
  
 NCBI BlastP on this gene

THITE\_2119083

glycosyltransferase family 2 protein
  
Accession: AEO69079
  
Location: 1934469-1938733
  
 NCBI BlastP on this gene

THITE\_121780

Query: Architecture Search FASTA input

KB707715 : Botryotinia fuckeliana BcDW1 unplaced genomic scaffold Scaffold\_43    Total score: 1.0     Cumulative Blast bit score: 336

Hit cluster cross-links:

Mycgr3G70471
  
Location: 0-405

Mycgr3G70471

Mycgr3G39149
  
Location: 505-1798

Mycgr3G39149

Mycgr3G92130
  
Location: 1898-2396

Mycgr3G92130

Mycgr3G38483
  
Location: 2496-3576

Mycgr3G38483

Mycgr3G108869
  
Location: 3676-5056

Mycgr3G108869

Mycgr3G103943
  
Location: 5156-5762

Mycgr3G103943

Mycgr3G57362
  
Location: 5862-7296

Mycgr3G57362

Mycgr3G39086
  
Location: 7396-8368

Mycgr3G39086

Mycgr3G103942
  
Location: 8468-8714

Mycgr3G103942

Mycgr3G108865
  
Location: 8814-10239

Mycgr3G108865

Mycgr3G70475
  
Location: 10339-11821

Mycgr3G70475

Mycgr3G108866
  
Location: 11921-13010

Mycgr3G108866

Mycgr3G92136
  
Location: 13110-13593

Mycgr3G92136

putative mediator of rna polymerase ii transcription subunit 22 protein
  
Accession: EMR90153
  
Location: 128927-130664
  
 NCBI BlastP on this gene

EMR90153

putative atp-dependent rna helicase mss116 protein
  
Accession: EMR90152
  
Location: 126633-128477
  
 NCBI BlastP on this gene

EMR90152

putative mannitol dehydrogenase protein
  
Accession: EMR90151
  
Location: 124441-125305
  
 NCBI BlastP on this gene

EMR90151

putative high affinity nickel transport protein nic1 protein
  
Accession: EMR90150
  
Location: 120979-122037
  
  
**BlastP hit with Mycgr3G108865**
  
Percentage identity: 60 %
  
BlastP bit score: 336
  
Sequence coverage: 62 %
  
E-value: 1e-108
  
  
 NCBI BlastP on this gene

EMR90150

putative duf1295 domain protein
  
Accession: EMR90149
  
Location: 119118-120407
  
 NCBI BlastP on this gene

EMR90149

putative btb poz domain containing protein
  
Accession: EMR90148
  
Location: 116272-117693
  
 NCBI BlastP on this gene

EMR90148

putative dihydrofolate reductase protein
  
Accession: EMR90147
  
Location: 113446-114417
  
 NCBI BlastP on this gene

EMR90147

Query: Architecture Search FASTA input

CP003003 : Myceliophthora thermophila ATCC 42464 chromosome 2    Total score: 1.0     Cumulative Blast bit score: 324

Hit cluster cross-links:

Mycgr3G70471
  
Location: 0-405

Mycgr3G70471

Mycgr3G39149
  
Location: 505-1798

Mycgr3G39149

Mycgr3G92130
  
Location: 1898-2396

Mycgr3G92130

Mycgr3G38483
  
Location: 2496-3576

Mycgr3G38483

Mycgr3G108869
  
Location: 3676-5056

Mycgr3G108869

Mycgr3G103943
  
Location: 5156-5762

Mycgr3G103943

Mycgr3G57362
  
Location: 5862-7296

Mycgr3G57362

Mycgr3G39086
  
Location: 7396-8368

Mycgr3G39086

Mycgr3G103942
  
Location: 8468-8714

Mycgr3G103942

Mycgr3G108865
  
Location: 8814-10239

Mycgr3G108865

Mycgr3G70475
  
Location: 10339-11821

Mycgr3G70475

Mycgr3G108866
  
Location: 11921-13010

Mycgr3G108866

Mycgr3G92136
  
Location: 13110-13593

Mycgr3G92136

hypothetical protein
  
Accession: AEO55901
  
Location: 603300-605691
  
 NCBI BlastP on this gene

MYCTH\_2300199

hypothetical protein
  
Accession: AEO55900
  
Location: 601162-601671
  
 NCBI BlastP on this gene

MYCTH\_2314426

hypothetical protein
  
Accession: AEO55899
  
Location: 600167-600583
  
 NCBI BlastP on this gene

MYCTH\_2058204

hypothetical protein
  
Accession: AEO55898
  
Location: 598108-599518
  
 NCBI BlastP on this gene

MYCTH\_2300192

hypothetical protein
  
Accession: AEO55897
  
Location: 595421-597323
  
  
**BlastP hit with Mycgr3G57362**
  
Percentage identity: 41 %
  
BlastP bit score: 324
  
Sequence coverage: 105 %
  
E-value: 8e-101
  
  
 NCBI BlastP on this gene

MYCTH\_2116566

hypothetical protein
  
Accession: AEO55896
  
Location: 591961-593724
  
 NCBI BlastP on this gene

MYCTH\_93330

hypothetical protein
  
Accession: AEO55895
  
Location: 590091-590683
  
 NCBI BlastP on this gene

MYCTH\_2124857

hypothetical protein
  
Accession: AEO55894
  
Location: 587818-589643
  
 NCBI BlastP on this gene

MYCTH\_2300189

Query: Architecture Search FASTA input

ACFW01000049 : Coccidioides posadasii C735 delta SOWgp    Total score: 1.0     Cumulative Blast bit score: 324

Hit cluster cross-links:

Mycgr3G70471
  
Location: 0-405

Mycgr3G70471

Mycgr3G39149
  
Location: 505-1798

Mycgr3G39149

Mycgr3G92130
  
Location: 1898-2396

Mycgr3G92130

Mycgr3G38483
  
Location: 2496-3576

Mycgr3G38483

Mycgr3G108869
  
Location: 3676-5056

Mycgr3G108869

Mycgr3G103943
  
Location: 5156-5762

Mycgr3G103943

Mycgr3G57362
  
Location: 5862-7296

Mycgr3G57362

Mycgr3G39086
  
Location: 7396-8368

Mycgr3G39086

Mycgr3G103942
  
Location: 8468-8714

Mycgr3G103942

Mycgr3G108865
  
Location: 8814-10239

Mycgr3G108865

Mycgr3G70475
  
Location: 10339-11821

Mycgr3G70475

Mycgr3G108866
  
Location: 11921-13010

Mycgr3G108866

Mycgr3G92136
  
Location: 13110-13593

Mycgr3G92136

carboxypeptidase A4 precursor, putative
  
Accession: EER24122
  
Location: 3400736-3402551
  
 NCBI BlastP on this gene

EER24122

High-affinity nickel transport protein nic1, putative
  
Accession: EER24123
  
Location: 3409572-3410947
  
  
**BlastP hit with Mycgr3G108865**
  
Percentage identity: 55 %
  
BlastP bit score: 324
  
Sequence coverage: 70 %
  
E-value: 3e-103
  
  
 NCBI BlastP on this gene

EER24123

hypothetical protein
  
Accession: EER24124
  
Location: 3411385-3415345
  
 NCBI BlastP on this gene

EER24124

hypothetical protein
  
Accession: EER24125
  
Location: 3417838-3420033
  
 NCBI BlastP on this gene

EER24125

Query: Architecture Search FASTA input

KE145369 : Glarea lozoyensis ATCC 20868 chromosome Unknown GLAREA5    Total score: 1.0     Cumulative Blast bit score: 319

Hit cluster cross-links:

Mycgr3G70471
  
Location: 0-405

Mycgr3G70471

Mycgr3G39149
  
Location: 505-1798

Mycgr3G39149

Mycgr3G92130
  
Location: 1898-2396

Mycgr3G92130

Mycgr3G38483
  
Location: 2496-3576

Mycgr3G38483

Mycgr3G108869
  
Location: 3676-5056

Mycgr3G108869

Mycgr3G103943
  
Location: 5156-5762

Mycgr3G103943

Mycgr3G57362
  
Location: 5862-7296

Mycgr3G57362

Mycgr3G39086
  
Location: 7396-8368

Mycgr3G39086

Mycgr3G103942
  
Location: 8468-8714

Mycgr3G103942

Mycgr3G108865
  
Location: 8814-10239

Mycgr3G108865

Mycgr3G70475
  
Location: 10339-11821

Mycgr3G70475

Mycgr3G108866
  
Location: 11921-13010

Mycgr3G108866

Mycgr3G92136
  
Location: 13110-13593

Mycgr3G92136

FAD/NAD(P)-binding protein
  
Accession: EPE27543
  
Location: 411420-412924
  
  
**BlastP hit with Mycgr3G57362**
  
Percentage identity: 43 %
  
BlastP bit score: 319
  
Sequence coverage: 100 %
  
E-value: 8e-100
  
  
 NCBI BlastP on this gene

EPE27543

(Trans)glycosidase
  
Accession: EPE27542
  
Location: 410085-410984
  
 NCBI BlastP on this gene

EPE27542

Di-copper centre-containing
  
Accession: EPE27541
  
Location: 405404-408559
  
 NCBI BlastP on this gene

EPE27541

Cytochrome P450
  
Accession: EPE27540
  
Location: 400310-403844
  
 NCBI BlastP on this gene

EPE27540

Query: Architecture Search FASTA input

DS989823 : Arthroderma gypseum CBS 118893 supercont1.2 genomic scaffold    Total score: 1.0     Cumulative Blast bit score: 318

Hit cluster cross-links:

Mycgr3G70471
  
Location: 0-405

Mycgr3G70471

Mycgr3G39149
  
Location: 505-1798

Mycgr3G39149

Mycgr3G92130
  
Location: 1898-2396

Mycgr3G92130

Mycgr3G38483
  
Location: 2496-3576

Mycgr3G38483

Mycgr3G108869
  
Location: 3676-5056

Mycgr3G108869

Mycgr3G103943
  
Location: 5156-5762

Mycgr3G103943

Mycgr3G57362
  
Location: 5862-7296

Mycgr3G57362

Mycgr3G39086
  
Location: 7396-8368

Mycgr3G39086

Mycgr3G103942
  
Location: 8468-8714

Mycgr3G103942

Mycgr3G108865
  
Location: 8814-10239

Mycgr3G108865

Mycgr3G70475
  
Location: 10339-11821

Mycgr3G70475

Mycgr3G108866
  
Location: 11921-13010

Mycgr3G108866

Mycgr3G92136
  
Location: 13110-13593

Mycgr3G92136

cytohesin-2
  
Accession: EFQ99133
  
Location: 400681-404941
  
 NCBI BlastP on this gene

EFQ99133

hypothetical protein
  
Accession: EFQ99132
  
Location: 398497-399716
  
 NCBI BlastP on this gene

EFQ99132

hypothetical protein
  
Accession: EFQ99131
  
Location: 396411-397472
  
 NCBI BlastP on this gene

EFQ99131

ECM14
  
Accession: EFQ99130
  
Location: 394251-396100
  
 NCBI BlastP on this gene

EFQ99130

high-affinity nickel transporter nic1
  
Accession: EFQ99129
  
Location: 391564-393317
  
  
**BlastP hit with Mycgr3G108865**
  
Percentage identity: 48 %
  
BlastP bit score: 318
  
Sequence coverage: 95 %
  
E-value: 1e-99
  
  
 NCBI BlastP on this gene

EFQ99129

alkaline proteinase
  
Accession: EFQ99128
  
Location: 389526-391097
  
 NCBI BlastP on this gene

EFQ99128

hypothetical protein
  
Accession: EFQ99127
  
Location: 386917-387285
  
 NCBI BlastP on this gene

EFQ99127

DNA polymerase lambda
  
Accession: EFQ99126
  
Location: 383667-385709
  
 NCBI BlastP on this gene

EFQ99126

Query: Architecture Search FASTA input

CAGA01000037 : Claviceps purpurea 20.1    Total score: 1.0     Cumulative Blast bit score: 317

Hit cluster cross-links:

Mycgr3G70471
  
Location: 0-405

Mycgr3G70471

Mycgr3G39149
  
Location: 505-1798

Mycgr3G39149

Mycgr3G92130
  
Location: 1898-2396

Mycgr3G92130

Mycgr3G38483
  
Location: 2496-3576

Mycgr3G38483

Mycgr3G108869
  
Location: 3676-5056

Mycgr3G108869

Mycgr3G103943
  
Location: 5156-5762

Mycgr3G103943

Mycgr3G57362
  
Location: 5862-7296

Mycgr3G57362

Mycgr3G39086
  
Location: 7396-8368

Mycgr3G39086

Mycgr3G103942
  
Location: 8468-8714

Mycgr3G103942

Mycgr3G108865
  
Location: 8814-10239

Mycgr3G108865

Mycgr3G70475
  
Location: 10339-11821

Mycgr3G70475

Mycgr3G108866
  
Location: 11921-13010

Mycgr3G108866

Mycgr3G92136
  
Location: 13110-13593

Mycgr3G92136

related to protein kinase SWE1
  
Accession: CCE32052
  
Location: 119662-122403
  
 NCBI BlastP on this gene

CCE32052

uncharacterized protein
  
Accession: CCE32051
  
Location: 114669-117877
  
  
**BlastP hit with Mycgr3G57362**
  
Percentage identity: 41 %
  
BlastP bit score: 317
  
Sequence coverage: 98 %
  
E-value: 1e-95
  
  
 NCBI BlastP on this gene

CCE32051

uncharacterized protein
  
Accession: CCE32050
  
Location: 112256-114061
  
 NCBI BlastP on this gene

CCE32050

probable ribosomal protein L30, cytosolic
  
Accession: CCE32049
  
Location: 110008-110677
  
 NCBI BlastP on this gene

CCE32049

uncharacterized protein
  
Accession: CCE32048
  
Location: 107340-107728
  
 NCBI BlastP on this gene

CCE32048

Query: Architecture Search FASTA input

ABDF02000003 : Trichoderma virens Gv29-8    Total score: 1.0     Cumulative Blast bit score: 317

Hit cluster cross-links:

Mycgr3G70471
  
Location: 0-405

Mycgr3G70471

Mycgr3G39149
  
Location: 505-1798

Mycgr3G39149

Mycgr3G92130
  
Location: 1898-2396

Mycgr3G92130

Mycgr3G38483
  
Location: 2496-3576

Mycgr3G38483

Mycgr3G108869
  
Location: 3676-5056

Mycgr3G108869

Mycgr3G103943
  
Location: 5156-5762

Mycgr3G103943

Mycgr3G57362
  
Location: 5862-7296

Mycgr3G57362

Mycgr3G39086
  
Location: 7396-8368

Mycgr3G39086

Mycgr3G103942
  
Location: 8468-8714

Mycgr3G103942

Mycgr3G108865
  
Location: 8814-10239

Mycgr3G108865

Mycgr3G70475
  
Location: 10339-11821

Mycgr3G70475

Mycgr3G108866
  
Location: 11921-13010

Mycgr3G108866

Mycgr3G92136
  
Location: 13110-13593

Mycgr3G92136

hypothetical protein
  
Accession: EHK25543
  
Location: 1569554-1569880
  
 NCBI BlastP on this gene

EHK25543

hypothetical protein
  
Accession: EHK25544
  
Location: 1571649-1575228
  
 NCBI BlastP on this gene

EHK25544

hypothetical protein
  
Accession: EHK25545
  
Location: 1576509-1577990
  
  
**BlastP hit with Mycgr3G57362**
  
Percentage identity: 43 %
  
BlastP bit score: 317
  
Sequence coverage: 100 %
  
E-value: 5e-99
  
  
 NCBI BlastP on this gene

EHK25545

glycoside hydrolase family 76 protein
  
Accession: EHK25546
  
Location: 1579003-1580470
  
 NCBI BlastP on this gene

EHK25546

hypothetical protein
  
Accession: EHK25547
  
Location: 1581408-1583454
  
 NCBI BlastP on this gene

EHK25547

hypothetical protein
  
Accession: EHK25548
  
Location: 1584006-1586778
  
 NCBI BlastP on this gene

EHK25548

Query: Architecture Search FASTA input

KB730323 : Fusarium oxysporum f. sp. cubense race 1 unplaced genomic scaffold scaffold253    Total score: 1.0     Cumulative Blast bit score: 316

Hit cluster cross-links:

Mycgr3G70471
  
Location: 0-405

Mycgr3G70471

Mycgr3G39149
  
Location: 505-1798

Mycgr3G39149

Mycgr3G92130
  
Location: 1898-2396

Mycgr3G92130

Mycgr3G38483
  
Location: 2496-3576

Mycgr3G38483

Mycgr3G108869
  
Location: 3676-5056

Mycgr3G108869

Mycgr3G103943
  
Location: 5156-5762

Mycgr3G103943

Mycgr3G57362
  
Location: 5862-7296

Mycgr3G57362

Mycgr3G39086
  
Location: 7396-8368

Mycgr3G39086

Mycgr3G103942
  
Location: 8468-8714

Mycgr3G103942

Mycgr3G108865
  
Location: 8814-10239

Mycgr3G108865

Mycgr3G70475
  
Location: 10339-11821

Mycgr3G70475

Mycgr3G108866
  
Location: 11921-13010

Mycgr3G108866

Mycgr3G92136
  
Location: 13110-13593

Mycgr3G92136

hypothetical protein
  
Accession: ENH67701
  
Location: 843752-844221
  
 NCBI BlastP on this gene

ENH67701

Mitosis inhibitor protein kinase wee1
  
Accession: ENH67702
  
Location: 846912-850413
  
 NCBI BlastP on this gene

ENH67702

Gamma-glutamylputrescine oxidoreductase
  
Accession: ENH67703
  
Location: 851590-853024
  
  
**BlastP hit with Mycgr3G57362**
  
Percentage identity: 41 %
  
BlastP bit score: 316
  
Sequence coverage: 96 %
  
E-value: 9e-99
  
  
 NCBI BlastP on this gene

ENH67703

Query: Architecture Search FASTA input

KB726993 : Fusarium oxysporum f. sp. cubense race 4 unplaced genomic scaffold scaffold83    Total score: 1.0     Cumulative Blast bit score: 316

Hit cluster cross-links:

Mycgr3G70471
  
Location: 0-405

Mycgr3G70471

Mycgr3G39149
  
Location: 505-1798

Mycgr3G39149

Mycgr3G92130
  
Location: 1898-2396

Mycgr3G92130

Mycgr3G38483
  
Location: 2496-3576

Mycgr3G38483

Mycgr3G108869
  
Location: 3676-5056

Mycgr3G108869

Mycgr3G103943
  
Location: 5156-5762

Mycgr3G103943

Mycgr3G57362
  
Location: 5862-7296

Mycgr3G57362

Mycgr3G39086
  
Location: 7396-8368

Mycgr3G39086

Mycgr3G103942
  
Location: 8468-8714

Mycgr3G103942

Mycgr3G108865
  
Location: 8814-10239

Mycgr3G108865

Mycgr3G70475
  
Location: 10339-11821

Mycgr3G70475

Mycgr3G108866
  
Location: 11921-13010

Mycgr3G108866

Mycgr3G92136
  
Location: 13110-13593

Mycgr3G92136

hypothetical protein
  
Accession: EMT63343
  
Location: 1172229-1172698
  
 NCBI BlastP on this gene

EMT63343

Mitosis inhibitor protein kinase wee1
  
Accession: EMT63344
  
Location: 1175400-1178901
  
 NCBI BlastP on this gene

EMT63344

Gamma-glutamylputrescine oxidoreductase
  
Accession: EMT63345
  
Location: 1180077-1181511
  
  
**BlastP hit with Mycgr3G57362**
  
Percentage identity: 41 %
  
BlastP bit score: 316
  
Sequence coverage: 96 %
  
E-value: 9e-99
  
  
 NCBI BlastP on this gene

EMT63345

Query: Architecture Search FASTA input

HF679027 : Fusarium fujikuroi IMI 58289 draft genome, chromosome FFUJ\_chr05.    Total score: 1.0     Cumulative Blast bit score: 315

Hit cluster cross-links:

Mycgr3G70471
  
Location: 0-405

Mycgr3G70471

Mycgr3G39149
  
Location: 505-1798

Mycgr3G39149

Mycgr3G92130
  
Location: 1898-2396

Mycgr3G92130

Mycgr3G38483
  
Location: 2496-3576

Mycgr3G38483

Mycgr3G108869
  
Location: 3676-5056

Mycgr3G108869

Mycgr3G103943
  
Location: 5156-5762

Mycgr3G103943

Mycgr3G57362
  
Location: 5862-7296

Mycgr3G57362

Mycgr3G39086
  
Location: 7396-8368

Mycgr3G39086

Mycgr3G103942
  
Location: 8468-8714

Mycgr3G103942

Mycgr3G108865
  
Location: 8814-10239

Mycgr3G108865

Mycgr3G70475
  
Location: 10339-11821

Mycgr3G70475

Mycgr3G108866
  
Location: 11921-13010

Mycgr3G108866

Mycgr3G92136
  
Location: 13110-13593

Mycgr3G92136

related to protein kinase SWE1
  
Accession: CCT68406
  
Location: 1318145-1321640
  
 NCBI BlastP on this gene

FFUJ\_07186

uncharacterized protein
  
Accession: CCT68407
  
Location: 1322784-1324218
  
  
**BlastP hit with Mycgr3G57362**
  
Percentage identity: 41 %
  
BlastP bit score: 315
  
Sequence coverage: 96 %
  
E-value: 1e-98
  
  
 NCBI BlastP on this gene

FFUJ\_07187

Query: Architecture Search FASTA input

JH226136 : Exophiala dermatitidis NIH/UT8656 unplaced genomic scaffold supercont1.7    Total score: 1.0     Cumulative Blast bit score: 306

Hit cluster cross-links:

Mycgr3G70471
  
Location: 0-405

Mycgr3G70471

Mycgr3G39149
  
Location: 505-1798

Mycgr3G39149

Mycgr3G92130
  
Location: 1898-2396

Mycgr3G92130

Mycgr3G38483
  
Location: 2496-3576

Mycgr3G38483

Mycgr3G108869
  
Location: 3676-5056

Mycgr3G108869

Mycgr3G103943
  
Location: 5156-5762

Mycgr3G103943

Mycgr3G57362
  
Location: 5862-7296

Mycgr3G57362

Mycgr3G39086
  
Location: 7396-8368

Mycgr3G39086

Mycgr3G103942
  
Location: 8468-8714

Mycgr3G103942

Mycgr3G108865
  
Location: 8814-10239

Mycgr3G108865

Mycgr3G70475
  
Location: 10339-11821

Mycgr3G70475

Mycgr3G108866
  
Location: 11921-13010

Mycgr3G108866

Mycgr3G92136
  
Location: 13110-13593

Mycgr3G92136

high-affinity nickel-transporter
  
Accession: EHY60299
  
Location: 1246501-1248153
  
  
**BlastP hit with Mycgr3G108865**
  
Percentage identity: 59 %
  
BlastP bit score: 306
  
Sequence coverage: 60 %
  
E-value: 6e-94
  
  
 NCBI BlastP on this gene

EHY60299

CTP synthase
  
Accession: EHY60298
  
Location: 1244059-1245977
  
 NCBI BlastP on this gene

EHY60298

alkanesulfonate monooxygenase
  
Accession: EHY60297
  
Location: 1241592-1242920
  
 NCBI BlastP on this gene

EHY60297

hypothetical protein
  
Accession: EHY60296
  
Location: 1237198-1241089
  
 NCBI BlastP on this gene

EHY60296

Query: Architecture Search FASTA input

JH126399 : Cordyceps militaris CM01 unplaced genomic scaffold CCM\_S00001    Total score: 1.0     Cumulative Blast bit score: 301

Hit cluster cross-links:

Mycgr3G70471
  
Location: 0-405

Mycgr3G70471

Mycgr3G39149
  
Location: 505-1798

Mycgr3G39149

Mycgr3G92130
  
Location: 1898-2396

Mycgr3G92130

Mycgr3G38483
  
Location: 2496-3576

Mycgr3G38483

Mycgr3G108869
  
Location: 3676-5056

Mycgr3G108869

Mycgr3G103943
  
Location: 5156-5762

Mycgr3G103943

Mycgr3G57362
  
Location: 5862-7296

Mycgr3G57362

Mycgr3G39086
  
Location: 7396-8368

Mycgr3G39086

Mycgr3G103942
  
Location: 8468-8714

Mycgr3G103942

Mycgr3G108865
  
Location: 8814-10239

Mycgr3G108865

Mycgr3G70475
  
Location: 10339-11821

Mycgr3G70475

Mycgr3G108866
  
Location: 11921-13010

Mycgr3G108866

Mycgr3G92136
  
Location: 13110-13593

Mycgr3G92136

Protein kinase-like domain
  
Accession: EGX95626
  
Location: 1008510-1011746
  
 NCBI BlastP on this gene

EGX95626

FAD dependent oxidoreductase
  
Accession: EGX95627
  
Location: 1012814-1014300
  
  
**BlastP hit with Mycgr3G57362**
  
Percentage identity: 40 %
  
BlastP bit score: 301
  
Sequence coverage: 99 %
  
E-value: 9e-93
  
  
 NCBI BlastP on this gene

EGX95627

hypothetical protein
  
Accession: EGX95628
  
Location: 1014377-1014746
  
 NCBI BlastP on this gene

EGX95628

hypothetical protein
  
Accession: EGX95629
  
Location: 1015516-1018185
  
 NCBI BlastP on this gene

EGX95629

Protein kinase-like domain
  
Accession: EGX95630
  
Location: 1020658-1021823
  
 NCBI BlastP on this gene

EGX95630

NADH-ubiquinone oxidoreductase 24 kDa subunit
  
Accession: EGX95631
  
Location: 1022030-1023171
  
 NCBI BlastP on this gene

EGX95631

Query: Architecture Search FASTA input

CP003009 : Thielavia terrestris NRRL 8126 chromosome 1    Total score: 1.0     Cumulative Blast bit score: 296

Hit cluster cross-links:

Mycgr3G70471
  
Location: 0-405

Mycgr3G70471

Mycgr3G39149
  
Location: 505-1798

Mycgr3G39149

Mycgr3G92130
  
Location: 1898-2396

Mycgr3G92130

Mycgr3G38483
  
Location: 2496-3576

Mycgr3G38483

Mycgr3G108869
  
Location: 3676-5056

Mycgr3G108869

Mycgr3G103943
  
Location: 5156-5762

Mycgr3G103943

Mycgr3G57362
  
Location: 5862-7296

Mycgr3G57362

Mycgr3G39086
  
Location: 7396-8368

Mycgr3G39086

Mycgr3G103942
  
Location: 8468-8714

Mycgr3G103942

Mycgr3G108865
  
Location: 8814-10239

Mycgr3G108865

Mycgr3G70475
  
Location: 10339-11821

Mycgr3G70475

Mycgr3G108866
  
Location: 11921-13010

Mycgr3G108866

Mycgr3G92136
  
Location: 13110-13593

Mycgr3G92136

hypothetical protein
  
Accession: AEO62298
  
Location: 646127-648480
  
 NCBI BlastP on this gene

THITE\_2106325

hypothetical protein
  
Accession: AEO62297
  
Location: 643674-644183
  
 NCBI BlastP on this gene

THITE\_2106324

hypothetical protein
  
Accession: AEO62296
  
Location: 642590-643055
  
 NCBI BlastP on this gene

THITE\_2106323

hypothetical protein
  
Accession: AEO62295
  
Location: 640564-641969
  
 NCBI BlastP on this gene

THITE\_153800

hypothetical protein
  
Accession: AEO62294
  
Location: 637784-639803
  
  
**BlastP hit with Mycgr3G57362**
  
Percentage identity: 40 %
  
BlastP bit score: 296
  
Sequence coverage: 104 %
  
E-value: 2e-90
  
  
 NCBI BlastP on this gene

THITE\_2039081

hypothetical protein
  
Accession: AEO62293
  
Location: 633929-634489
  
 NCBI BlastP on this gene

THITE\_2106312

hypothetical protein
  
Accession: AEO62292
  
Location: 629216-630304
  
 NCBI BlastP on this gene

THITE\_2124977

Query: Architecture Search FASTA input

ABDG02000029 : Trichoderma atroviride IMI 206040    Total score: 1.0     Cumulative Blast bit score: 291

Hit cluster cross-links:

Mycgr3G70471
  
Location: 0-405

Mycgr3G70471

Mycgr3G39149
  
Location: 505-1798

Mycgr3G39149

Mycgr3G92130
  
Location: 1898-2396

Mycgr3G92130

Mycgr3G38483
  
Location: 2496-3576

Mycgr3G38483

Mycgr3G108869
  
Location: 3676-5056

Mycgr3G108869

Mycgr3G103943
  
Location: 5156-5762

Mycgr3G103943

Mycgr3G57362
  
Location: 5862-7296

Mycgr3G57362

Mycgr3G39086
  
Location: 7396-8368

Mycgr3G39086

Mycgr3G103942
  
Location: 8468-8714

Mycgr3G103942

Mycgr3G108865
  
Location: 8814-10239

Mycgr3G108865

Mycgr3G70475
  
Location: 10339-11821

Mycgr3G70475

Mycgr3G108866
  
Location: 11921-13010

Mycgr3G108866

Mycgr3G92136
  
Location: 13110-13593

Mycgr3G92136

hypothetical protein
  
Accession: EHK39641
  
Location: 885633-885987
  
 NCBI BlastP on this gene

EHK39641

hypothetical protein
  
Accession: EHK39642
  
Location: 887671-891218
  
 NCBI BlastP on this gene

EHK39642

hypothetical protein
  
Accession: EHK39643
  
Location: 892507-893914
  
  
**BlastP hit with Mycgr3G57362**
  
Percentage identity: 41 %
  
BlastP bit score: 291
  
Sequence coverage: 95 %
  
E-value: 2e-89
  
  
 NCBI BlastP on this gene

EHK39643

Query: Architecture Search FASTA input

DS572755 : Paracoccidioides brasiliensis Pb18 supercont1.6 genomic scaffold    Total score: 1.0     Cumulative Blast bit score: 271

Hit cluster cross-links:

Mycgr3G70471
  
Location: 0-405

Mycgr3G70471

Mycgr3G39149
  
Location: 505-1798

Mycgr3G39149

Mycgr3G92130
  
Location: 1898-2396

Mycgr3G92130

Mycgr3G38483
  
Location: 2496-3576

Mycgr3G38483

Mycgr3G108869
  
Location: 3676-5056

Mycgr3G108869

Mycgr3G103943
  
Location: 5156-5762

Mycgr3G103943

Mycgr3G57362
  
Location: 5862-7296

Mycgr3G57362

Mycgr3G39086
  
Location: 7396-8368

Mycgr3G39086

Mycgr3G103942
  
Location: 8468-8714

Mycgr3G103942

Mycgr3G108865
  
Location: 8814-10239

Mycgr3G108865

Mycgr3G70475
  
Location: 10339-11821

Mycgr3G70475

Mycgr3G108866
  
Location: 11921-13010

Mycgr3G108866

Mycgr3G92136
  
Location: 13110-13593

Mycgr3G92136

DDHD domain-containing protein
  
Accession: EEH49263
  
Location: 862141-866666
  
 NCBI BlastP on this gene

EEH49263

NADH-ubiquinone oxidoreductase 21.3 kDa subunit
  
Accession: EEH49264
  
Location: 867809-868516
  
 NCBI BlastP on this gene

EEH49264

conserved hypothetical protein
  
Accession: EEH49265
  
Location: 869716-870525
  
 NCBI BlastP on this gene

EEH49265

high-affinity nickel transport protein
  
Accession: EEH49266
  
Location: 871516-873006
  
  
**BlastP hit with Mycgr3G108865**
  
Percentage identity: 59 %
  
BlastP bit score: 271
  
Sequence coverage: 53 %
  
E-value: 1e-83
  
  
 NCBI BlastP on this gene

EEH49266

DNA polymerase beta
  
Accession: EEH49267
  
Location: 874673-876736
  
 NCBI BlastP on this gene

EEH49267

phospholipid-transporting ATPase
  
Accession: EEH49268
  
Location: 877449-881688
  
 NCBI BlastP on this gene

EEH49268

Query: Architecture Search FASTA input

GL891302 : Neurospora tetrasperma FGSC 2508 unplaced genomic scaffold NEUTE1scaffold\_1    Total score: 1.0     Cumulative Blast bit score: 264

Hit cluster cross-links:

Mycgr3G70471
  
Location: 0-405

Mycgr3G70471

Mycgr3G39149
  
Location: 505-1798

Mycgr3G39149

Mycgr3G92130
  
Location: 1898-2396

Mycgr3G92130

Mycgr3G38483
  
Location: 2496-3576

Mycgr3G38483

Mycgr3G108869
  
Location: 3676-5056

Mycgr3G108869

Mycgr3G103943
  
Location: 5156-5762

Mycgr3G103943

Mycgr3G57362
  
Location: 5862-7296

Mycgr3G57362

Mycgr3G39086
  
Location: 7396-8368

Mycgr3G39086

Mycgr3G103942
  
Location: 8468-8714

Mycgr3G103942

Mycgr3G108865
  
Location: 8814-10239

Mycgr3G108865

Mycgr3G70475
  
Location: 10339-11821

Mycgr3G70475

Mycgr3G108866
  
Location: 11921-13010

Mycgr3G108866

Mycgr3G92136
  
Location: 13110-13593

Mycgr3G92136

hypothetical protein
  
Accession: EGO61428
  
Location: 4937978-4939924
  
 NCBI BlastP on this gene

EGO61428

hypothetical protein
  
Accession: EGO61429
  
Location: 4942890-4943402
  
 NCBI BlastP on this gene

EGO61429

hypothetical protein
  
Accession: EGO61430
  
Location: 4944023-4944476
  
 NCBI BlastP on this gene

EGO61430

hypothetical protein
  
Accession: EGO61431
  
Location: 4946148-4947236
  
 NCBI BlastP on this gene

EGO61431

hypothetical protein
  
Accession: EGO61432
  
Location: 4948412-4949926
  
  
**BlastP hit with Mycgr3G57362**
  
Percentage identity: 38 %
  
BlastP bit score: 264
  
Sequence coverage: 107 %
  
E-value: 3e-78
  
  
 NCBI BlastP on this gene

EGO61432

Query: Architecture Search FASTA input

GL891107 : Neurospora tetrasperma FGSC 2509 unplaced genomic scaffold NEUTE2scaffold\_2    Total score: 1.0     Cumulative Blast bit score: 264

Hit cluster cross-links:

Mycgr3G70471
  
Location: 0-405

Mycgr3G70471

Mycgr3G39149
  
Location: 505-1798

Mycgr3G39149

Mycgr3G92130
  
Location: 1898-2396

Mycgr3G92130

Mycgr3G38483
  
Location: 2496-3576

Mycgr3G38483

Mycgr3G108869
  
Location: 3676-5056

Mycgr3G108869

Mycgr3G103943
  
Location: 5156-5762

Mycgr3G103943

Mycgr3G57362
  
Location: 5862-7296

Mycgr3G57362

Mycgr3G39086
  
Location: 7396-8368

Mycgr3G39086

Mycgr3G103942
  
Location: 8468-8714

Mycgr3G103942

Mycgr3G108865
  
Location: 8814-10239

Mycgr3G108865

Mycgr3G70475
  
Location: 10339-11821

Mycgr3G70475

Mycgr3G108866
  
Location: 11921-13010

Mycgr3G108866

Mycgr3G92136
  
Location: 13110-13593

Mycgr3G92136

hypothetical protein
  
Accession: EGZ74544
  
Location: 1217070-1219016
  
 NCBI BlastP on this gene

EGZ74544

hypothetical protein
  
Accession: EGZ74543
  
Location: 1213595-1214107
  
 NCBI BlastP on this gene

EGZ74543

hypothetical protein
  
Accession: EGZ74542
  
Location: 1212521-1212974
  
 NCBI BlastP on this gene

EGZ74542

eukaryotic translation initiation factor 2 beta subunit
  
Accession: EGZ74541
  
Location: 1209761-1210849
  
 NCBI BlastP on this gene

EGZ74541

FAD dependent oxidoreductase
  
Accession: EGZ74540
  
Location: 1207071-1208585
  
  
**BlastP hit with Mycgr3G57362**
  
Percentage identity: 38 %
  
BlastP bit score: 264
  
Sequence coverage: 107 %
  
E-value: 3e-78
  
  
 NCBI BlastP on this gene

EGZ74540

Metallo-dependent hydrolase
  
Accession: EGZ74539
  
Location: 1204129-1206029
  
 NCBI BlastP on this gene

EGZ74539

hypothetical protein
  
Accession: EGZ74538
  
Location: 1200862-1203487
  
 NCBI BlastP on this gene

EGZ74538

hypothetical protein
  
Accession: EGZ74537
  
Location: 1199321-1200245
  
 NCBI BlastP on this gene

EGZ74537

Query: Architecture Search FASTA input

ADOT01000059 : Arthrobotrys oligospora ATCC 24927    Total score: 1.0     Cumulative Blast bit score: 259

Hit cluster cross-links:

Mycgr3G70471
  
Location: 0-405

Mycgr3G70471

Mycgr3G39149
  
Location: 505-1798

Mycgr3G39149

Mycgr3G92130
  
Location: 1898-2396

Mycgr3G92130

Mycgr3G38483
  
Location: 2496-3576

Mycgr3G38483

Mycgr3G108869
  
Location: 3676-5056

Mycgr3G108869

Mycgr3G103943
  
Location: 5156-5762

Mycgr3G103943

Mycgr3G57362
  
Location: 5862-7296

Mycgr3G57362

Mycgr3G39086
  
Location: 7396-8368

Mycgr3G39086

Mycgr3G103942
  
Location: 8468-8714

Mycgr3G103942

Mycgr3G108865
  
Location: 8814-10239

Mycgr3G108865

Mycgr3G70475
  
Location: 10339-11821

Mycgr3G70475

Mycgr3G108866
  
Location: 11921-13010

Mycgr3G108866

Mycgr3G92136
  
Location: 13110-13593

Mycgr3G92136

hypothetical protein
  
Accession: EGX52096
  
Location: 335880-339502
  
 NCBI BlastP on this gene

EGX52096

hypothetical protein
  
Accession: EGX52097
  
Location: 342478-343694
  
  
**BlastP hit with Mycgr3G108865**
  
Percentage identity: 53 %
  
BlastP bit score: 259
  
Sequence coverage: 58 %
  
E-value: 3e-78
  
  
 NCBI BlastP on this gene

EGX52097

hypothetical protein
  
Accession: EGX52098
  
Location: 344180-345822
  
 NCBI BlastP on this gene

EGX52098

hypothetical protein
  
Accession: EGX52099
  
Location: 347860-349523
  
 NCBI BlastP on this gene

EGX52099

hypothetical protein
  
Accession: EGX52100
  
Location: 350340-351539
  
 NCBI BlastP on this gene

EGX52100

hypothetical protein
  
Accession: EGX52101
  
Location: 351954-354206
  
 NCBI BlastP on this gene

EGX52101

Query: Architecture Search FASTA input

KB446557 : Pseudocercospora fijiensis CIRAD86 unplaced genomic scaffold MYCFIscaffold\_3    Total score: 1.0     Cumulative Blast bit score: 245

Hit cluster cross-links:

Mycgr3G70471
  
Location: 0-405

Mycgr3G70471

Mycgr3G39149
  
Location: 505-1798

Mycgr3G39149

Mycgr3G92130
  
Location: 1898-2396

Mycgr3G92130

Mycgr3G38483
  
Location: 2496-3576

Mycgr3G38483

Mycgr3G108869
  
Location: 3676-5056

Mycgr3G108869

Mycgr3G103943
  
Location: 5156-5762

Mycgr3G103943

Mycgr3G57362
  
Location: 5862-7296

Mycgr3G57362

Mycgr3G39086
  
Location: 7396-8368

Mycgr3G39086

Mycgr3G103942
  
Location: 8468-8714

Mycgr3G103942

Mycgr3G108865
  
Location: 8814-10239

Mycgr3G108865

Mycgr3G70475
  
Location: 10339-11821

Mycgr3G70475

Mycgr3G108866
  
Location: 11921-13010

Mycgr3G108866

Mycgr3G92136
  
Location: 13110-13593

Mycgr3G92136

hypothetical protein
  
Accession: EME85143
  
Location: 5903096-5903698
  
  
**BlastP hit with Mycgr3G70471**
  
Percentage identity: 90 %
  
BlastP bit score: 245
  
Sequence coverage: 99 %
  
E-value: 8e-81
  
  
 NCBI BlastP on this gene

EME85143

hypothetical protein
  
Accession: EME85142
  
Location: 5901122-5901519
  
 NCBI BlastP on this gene

EME85142

hypothetical protein
  
Accession: EME85141
  
Location: 5898926-5901089
  
 NCBI BlastP on this gene

EME85141

hypothetical protein
  
Accession: EME85140
  
Location: 5896537-5897541
  
 NCBI BlastP on this gene

EME85140

hypothetical protein
  
Accession: EME85139
  
Location: 5894470-5895874
  
 NCBI BlastP on this gene

EME85139

Query: Architecture Search FASTA input

CP000494 : Bradyrhizobium sp. BTAi1    Total score: 1.0     Cumulative Blast bit score: 239

Hit cluster cross-links:

Mycgr3G70471
  
Location: 0-405

Mycgr3G70471

Mycgr3G39149
  
Location: 505-1798

Mycgr3G39149

Mycgr3G92130
  
Location: 1898-2396

Mycgr3G92130

Mycgr3G38483
  
Location: 2496-3576

Mycgr3G38483

Mycgr3G108869
  
Location: 3676-5056

Mycgr3G108869

Mycgr3G103943
  
Location: 5156-5762

Mycgr3G103943

Mycgr3G57362
  
Location: 5862-7296

Mycgr3G57362

Mycgr3G39086
  
Location: 7396-8368

Mycgr3G39086

Mycgr3G103942
  
Location: 8468-8714

Mycgr3G103942

Mycgr3G108865
  
Location: 8814-10239

Mycgr3G108865

Mycgr3G70475
  
Location: 10339-11821

Mycgr3G70475

Mycgr3G108866
  
Location: 11921-13010

Mycgr3G108866

Mycgr3G92136
  
Location: 13110-13593

Mycgr3G92136

conjugal transfer protein trbE
  
Accession: ABQ35466
  
Location: 3523046-3525490
  
 NCBI BlastP on this gene

trbE

Conjugal transfer protein trbD
  
Accession: ABQ35465
  
Location: 3522754-3523035
  
 NCBI BlastP on this gene

trbD

conjugal transfer protein trbC
  
Accession: ABQ35464
  
Location: 3522425-3522754
  
 NCBI BlastP on this gene

trbC

conjugal transfer protein trbB
  
Accession: ABQ35463
  
Location: 3521448-3522428
  
 NCBI BlastP on this gene

trbB

Putative tonB-dependent receptor family (Outer membrane siderophore receptor) precursor
  
Accession: ABQ35462
  
Location: 3518690-3521131
  
 NCBI BlastP on this gene

BBta\_3362

putative ABC transporter (ATP-binding protein)
  
Accession: ABQ35461
  
Location: 3517807-3518526
  
 NCBI BlastP on this gene

BBta\_3361

putative membrane protein of unknown function
  
Accession: ABQ35460
  
Location: 3516408-3517805
  
 NCBI BlastP on this gene

BBta\_3360

putative membrane protein of unknown function
  
Accession: ABQ35459
  
Location: 3514917-3516392
  
 NCBI BlastP on this gene

BBta\_3359

high-affinity nickel-transport protein
  
Accession: ABQ35458
  
Location: 3513490-3514584
  
  
**BlastP hit with Mycgr3G108865**
  
Percentage identity: 38 %
  
BlastP bit score: 240
  
Sequence coverage: 69 %
  
E-value: 6e-71
  
  
 NCBI BlastP on this gene

nxiA

hypothetical protein
  
Accession: ABQ35457
  
Location: 3513056-3513493
  
 NCBI BlastP on this gene

BBta\_3357

Putative Conjugal transfer protein traG
  
Accession: ABQ35456
  
Location: 3511046-3513046
  
 NCBI BlastP on this gene

BBta\_3356

putative membrane protein of unknown function
  
Accession: ABQ35455
  
Location: 3509626-3510888
  
 NCBI BlastP on this gene

BBta\_3355

hypothetical protein
  
Accession: ABQ35454
  
Location: 3509038-3509433
  
 NCBI BlastP on this gene

BBta\_3353

putative alpha/beta-Hydrolases superfamily
  
Accession: ABQ35453
  
Location: 3508055-3508999
  
 NCBI BlastP on this gene

BBta\_3352

hypothetical protein
  
Accession: ABQ35452
  
Location: 3506148-3507899
  
 NCBI BlastP on this gene

BBta\_3351

putative Lytic transglycosylase
  
Accession: ABQ35451
  
Location: 3505071-3505901
  
 NCBI BlastP on this gene

BBta\_3350

conjugation peptidase TraF, Serine peptidase, MEROPS family S26C
  
Accession: ABQ35450
  
Location: 3504553-3505068
  
 NCBI BlastP on this gene

BBta\_3349

Query: Architecture Search FASTA input

ACJE01000001 : Aspergillus niger ATCC 1015    Total score: 1.0     Cumulative Blast bit score: 237

Hit cluster cross-links:

Mycgr3G70471
  
Location: 0-405

Mycgr3G70471

Mycgr3G39149
  
Location: 505-1798

Mycgr3G39149

Mycgr3G92130
  
Location: 1898-2396

Mycgr3G92130

Mycgr3G38483
  
Location: 2496-3576

Mycgr3G38483

Mycgr3G108869
  
Location: 3676-5056

Mycgr3G108869

Mycgr3G103943
  
Location: 5156-5762

Mycgr3G103943

Mycgr3G57362
  
Location: 5862-7296

Mycgr3G57362

Mycgr3G39086
  
Location: 7396-8368

Mycgr3G39086

Mycgr3G103942
  
Location: 8468-8714

Mycgr3G103942

Mycgr3G108865
  
Location: 8814-10239

Mycgr3G108865

Mycgr3G70475
  
Location: 10339-11821

Mycgr3G70475

Mycgr3G108866
  
Location: 11921-13010

Mycgr3G108866

Mycgr3G92136
  
Location: 13110-13593

Mycgr3G92136

hypothetical protein
  
Accession: EHA28122
  
Location: 118177-119620
  
  
**BlastP hit with Mycgr3G57362**
  
Percentage identity: 33 %
  
BlastP bit score: 237
  
Sequence coverage: 96 %
  
E-value: 1e-68
  
  
 NCBI BlastP on this gene

EHA28122

hypothetical protein
  
Accession: EHA28121
  
Location: 113639-114925
  
 NCBI BlastP on this gene

EHA28121

hypothetical protein
  
Accession: EHA28120
  
Location: 112757-113329
  
 NCBI BlastP on this gene

EHA28120

hypothetical protein
  
Accession: EHA28119
  
Location: 109829-111562
  
 NCBI BlastP on this gene

EHA28119

Query: Architecture Search FASTA input

AM920437 : Penicillium chrysogenum Wisconsin 54-1255 complete genome, contig Pc00c22.    Total score: 1.0     Cumulative Blast bit score: 228

Hit cluster cross-links:

Mycgr3G70471
  
Location: 0-405

Mycgr3G70471

Mycgr3G39149
  
Location: 505-1798

Mycgr3G39149

Mycgr3G92130
  
Location: 1898-2396

Mycgr3G92130

Mycgr3G38483
  
Location: 2496-3576

Mycgr3G38483

Mycgr3G108869
  
Location: 3676-5056

Mycgr3G108869

Mycgr3G103943
  
Location: 5156-5762

Mycgr3G103943

Mycgr3G57362
  
Location: 5862-7296

Mycgr3G57362

Mycgr3G39086
  
Location: 7396-8368

Mycgr3G39086

Mycgr3G103942
  
Location: 8468-8714

Mycgr3G103942

Mycgr3G108865
  
Location: 8814-10239

Mycgr3G108865

Mycgr3G70475
  
Location: 10339-11821

Mycgr3G70475

Mycgr3G108866
  
Location: 11921-13010

Mycgr3G108866

Mycgr3G92136
  
Location: 13110-13593

Mycgr3G92136

not annotated
  
Accession: CAP98998
  
Location: 4033866-4036870
  
 NCBI BlastP on this gene

Pc22g17100

not annotated
  
Accession: CAP98999
  
Location: 4037383-4038717
  
 NCBI BlastP on this gene

Pc22g17110

not annotated
  
Accession: CAP99000
  
Location: 4040204-4042665
  
 NCBI BlastP on this gene

Pc22g17120

not annotated
  
Accession: CAP99001
  
Location: 4043053-4044438
  
  
**BlastP hit with Mycgr3G57362**
  
Percentage identity: 33 %
  
BlastP bit score: 228
  
Sequence coverage: 98 %
  
E-value: 3e-65
  
  
 NCBI BlastP on this gene

Pc22g17130

Query: Architecture Search FASTA input

JH226131 : Exophiala dermatitidis NIH/UT8656 unplaced genomic scaffold supercont1.2    Total score: 1.0     Cumulative Blast bit score: 223

Hit cluster cross-links:

Mycgr3G70471
  
Location: 0-405

Mycgr3G70471

Mycgr3G39149
  
Location: 505-1798

Mycgr3G39149

Mycgr3G92130
  
Location: 1898-2396

Mycgr3G92130

Mycgr3G38483
  
Location: 2496-3576

Mycgr3G38483

Mycgr3G108869
  
Location: 3676-5056

Mycgr3G108869

Mycgr3G103943
  
Location: 5156-5762

Mycgr3G103943

Mycgr3G57362
  
Location: 5862-7296

Mycgr3G57362

Mycgr3G39086
  
Location: 7396-8368

Mycgr3G39086

Mycgr3G103942
  
Location: 8468-8714

Mycgr3G103942

Mycgr3G108865
  
Location: 8814-10239

Mycgr3G108865

Mycgr3G70475
  
Location: 10339-11821

Mycgr3G70475

Mycgr3G108866
  
Location: 11921-13010

Mycgr3G108866

Mycgr3G92136
  
Location: 13110-13593

Mycgr3G92136

hypothetical protein
  
Accession: EHY54734
  
Location: 4002560-4002946
  
 NCBI BlastP on this gene

EHY54734

hypothetical protein
  
Accession: EHY54735
  
Location: 4005396-4006475
  
 NCBI BlastP on this gene

EHY54735

hypothetical protein
  
Accession: EHY54736
  
Location: 4007956-4010238
  
 NCBI BlastP on this gene

EHY54736

hypothetical protein
  
Accession: EHY54737
  
Location: 4011316-4012737
  
  
**BlastP hit with Mycgr3G57362**
  
Percentage identity: 33 %
  
BlastP bit score: 224
  
Sequence coverage: 101 %
  
E-value: 1e-63
  
  
 NCBI BlastP on this gene

EHY54737

Query: Architecture Search FASTA input

ACJE01000012 : Aspergillus niger ATCC 1015    Total score: 1.0     Cumulative Blast bit score: 223

Hit cluster cross-links:

Mycgr3G70471
  
Location: 0-405

Mycgr3G70471

Mycgr3G39149
  
Location: 505-1798

Mycgr3G39149

Mycgr3G92130
  
Location: 1898-2396

Mycgr3G92130

Mycgr3G38483
  
Location: 2496-3576

Mycgr3G38483

Mycgr3G108869
  
Location: 3676-5056

Mycgr3G108869

Mycgr3G103943
  
Location: 5156-5762

Mycgr3G103943

Mycgr3G57362
  
Location: 5862-7296

Mycgr3G57362

Mycgr3G39086
  
Location: 7396-8368

Mycgr3G39086

Mycgr3G103942
  
Location: 8468-8714

Mycgr3G103942

Mycgr3G108865
  
Location: 8814-10239

Mycgr3G108865

Mycgr3G70475
  
Location: 10339-11821

Mycgr3G70475

Mycgr3G108866
  
Location: 11921-13010

Mycgr3G108866

Mycgr3G92136
  
Location: 13110-13593

Mycgr3G92136

hypothetical protein
  
Accession: EHA22338
  
Location: 476001-477620
  
  
**BlastP hit with Mycgr3G57362**
  
Percentage identity: 32 %
  
BlastP bit score: 224
  
Sequence coverage: 98 %
  
E-value: 1e-63
  
  
 NCBI BlastP on this gene

EHA22338

hypothetical protein
  
Accession: EHA22337
  
Location: 473629-475591
  
 NCBI BlastP on this gene

EHA22337

hypothetical protein
  
Accession: EHA22336
  
Location: 472270-473304
  
 NCBI BlastP on this gene

EHA22336

hypothetical protein
  
Accession: EHA22335
  
Location: 469787-471367
  
 NCBI BlastP on this gene

EHA22335

Query: Architecture Search FASTA input

KB726307 : Fusarium oxysporum f. sp. cubense race 4 unplaced genomic scaffold scaffold26    Total score: 1.0     Cumulative Blast bit score: 223

Hit cluster cross-links:

Mycgr3G70471
  
Location: 0-405

Mycgr3G70471

Mycgr3G39149
  
Location: 505-1798

Mycgr3G39149

Mycgr3G92130
  
Location: 1898-2396

Mycgr3G92130

Mycgr3G38483
  
Location: 2496-3576

Mycgr3G38483

Mycgr3G108869
  
Location: 3676-5056

Mycgr3G108869

Mycgr3G103943
  
Location: 5156-5762

Mycgr3G103943

Mycgr3G57362
  
Location: 5862-7296

Mycgr3G57362

Mycgr3G39086
  
Location: 7396-8368

Mycgr3G39086

Mycgr3G103942
  
Location: 8468-8714

Mycgr3G103942

Mycgr3G108865
  
Location: 8814-10239

Mycgr3G108865

Mycgr3G70475
  
Location: 10339-11821

Mycgr3G70475

Mycgr3G108866
  
Location: 11921-13010

Mycgr3G108866

Mycgr3G92136
  
Location: 13110-13593

Mycgr3G92136

Purine-cytosine permease FCY21
  
Accession: EMT71627
  
Location: 1729203-1730871
  
 NCBI BlastP on this gene

EMT71627

hypothetical protein
  
Accession: EMT71628
  
Location: 1732899-1733326
  
 NCBI BlastP on this gene

EMT71628

3-oxoadipate enol-lactonase 2
  
Accession: EMT71629
  
Location: 1733856-1734677
  
 NCBI BlastP on this gene

EMT71629

hypothetical protein
  
Accession: EMT71630
  
Location: 1736117-1736455
  
 NCBI BlastP on this gene

EMT71630

hypothetical protein
  
Accession: EMT71631
  
Location: 1737778-1738350
  
 NCBI BlastP on this gene

EMT71631

Gamma-glutamylputrescine oxidoreductase
  
Accession: EMT71632
  
Location: 1739000-1740556
  
  
**BlastP hit with Mycgr3G57362**
  
Percentage identity: 32 %
  
BlastP bit score: 223
  
Sequence coverage: 98 %
  
E-value: 2e-63
  
  
 NCBI BlastP on this gene

EMT71632

Query: Architecture Search FASTA input

KB644412 : Penicillium oxalicum 114-2 unplaced genomic scaffold scaffold\_5    Total score: 1.0     Cumulative Blast bit score: 220

Hit cluster cross-links:

Mycgr3G70471
  
Location: 0-405

Mycgr3G70471

Mycgr3G39149
  
Location: 505-1798

Mycgr3G39149

Mycgr3G92130
  
Location: 1898-2396

Mycgr3G92130

Mycgr3G38483
  
Location: 2496-3576

Mycgr3G38483

Mycgr3G108869
  
Location: 3676-5056

Mycgr3G108869

Mycgr3G103943
  
Location: 5156-5762

Mycgr3G103943

Mycgr3G57362
  
Location: 5862-7296

Mycgr3G57362

Mycgr3G39086
  
Location: 7396-8368

Mycgr3G39086

Mycgr3G103942
  
Location: 8468-8714

Mycgr3G103942

Mycgr3G108865
  
Location: 8814-10239

Mycgr3G108865

Mycgr3G70475
  
Location: 10339-11821

Mycgr3G70475

Mycgr3G108866
  
Location: 11921-13010

Mycgr3G108866

Mycgr3G92136
  
Location: 13110-13593

Mycgr3G92136

hypothetical protein
  
Accession: EPS30040
  
Location: 1593446-1595954
  
 NCBI BlastP on this gene

EPS30040

hypothetical protein
  
Accession: EPS30041
  
Location: 1596035-1596757
  
 NCBI BlastP on this gene

EPS30041

hypothetical protein
  
Accession: EPS30042
  
Location: 1597849-1599498
  
 NCBI BlastP on this gene

EPS30042

hypothetical protein
  
Accession: EPS30043
  
Location: 1600204-1601751
  
  
**BlastP hit with Mycgr3G57362**
  
Percentage identity: 33 %
  
BlastP bit score: 220
  
Sequence coverage: 96 %
  
E-value: 8e-62
  
  
 NCBI BlastP on this gene

EPS30043

hypothetical protein
  
Accession: EPS30044
  
Location: 1602120-1605245
  
 NCBI BlastP on this gene

EPS30044

hypothetical protein
  
Accession: EPS30045
  
Location: 1606656-1607780
  
 NCBI BlastP on this gene

EPS30045

hypothetical protein
  
Accession: EPS30046
  
Location: 1609063-1612406
  
 NCBI BlastP on this gene

EPS30046

Query: Architecture Search FASTA input

CP003009 : Thielavia terrestris NRRL 8126 chromosome 1    Total score: 1.0     Cumulative Blast bit score: 220

Hit cluster cross-links:

Mycgr3G70471
  
Location: 0-405

Mycgr3G70471

Mycgr3G39149
  
Location: 505-1798

Mycgr3G39149

Mycgr3G92130
  
Location: 1898-2396

Mycgr3G92130

Mycgr3G38483
  
Location: 2496-3576

Mycgr3G38483

Mycgr3G108869
  
Location: 3676-5056

Mycgr3G108869

Mycgr3G103943
  
Location: 5156-5762

Mycgr3G103943

Mycgr3G57362
  
Location: 5862-7296

Mycgr3G57362

Mycgr3G39086
  
Location: 7396-8368

Mycgr3G39086

Mycgr3G103942
  
Location: 8468-8714

Mycgr3G103942

Mycgr3G108865
  
Location: 8814-10239

Mycgr3G108865

Mycgr3G70475
  
Location: 10339-11821

Mycgr3G70475

Mycgr3G108866
  
Location: 11921-13010

Mycgr3G108866

Mycgr3G92136
  
Location: 13110-13593

Mycgr3G92136

hypothetical protein
  
Accession: AEO64380
  
Location: 8446410-8448225
  
  
**BlastP hit with Mycgr3G57362**
  
Percentage identity: 34 %
  
BlastP bit score: 220
  
Sequence coverage: 100 %
  
E-value: 6e-62
  
  
 NCBI BlastP on this gene

THITE\_2042085

hypothetical protein
  
Accession: AEO64379
  
Location: 8443397-8445086
  
 NCBI BlastP on this gene

THITE\_2041902

hypothetical protein
  
Accession: AEO64378
  
Location: 8441120-8442611
  
 NCBI BlastP on this gene

THITE\_2110483

hypothetical protein
  
Accession: AEO64377
  
Location: 8439218-8439933
  
 NCBI BlastP on this gene

THITE\_2110481

Query: Architecture Search FASTA input

HF679032 : Fusarium fujikuroi IMI 58289 draft genome, chromosome FFUJ\_chr10.    Total score: 1.0     Cumulative Blast bit score: 217

Hit cluster cross-links:

Mycgr3G70471
  
Location: 0-405

Mycgr3G70471

Mycgr3G39149
  
Location: 505-1798

Mycgr3G39149

Mycgr3G92130
  
Location: 1898-2396

Mycgr3G92130

Mycgr3G38483
  
Location: 2496-3576

Mycgr3G38483

Mycgr3G108869
  
Location: 3676-5056

Mycgr3G108869

Mycgr3G103943
  
Location: 5156-5762

Mycgr3G103943

Mycgr3G57362
  
Location: 5862-7296

Mycgr3G57362

Mycgr3G39086
  
Location: 7396-8368

Mycgr3G39086

Mycgr3G103942
  
Location: 8468-8714

Mycgr3G103942

Mycgr3G108865
  
Location: 8814-10239

Mycgr3G108865

Mycgr3G70475
  
Location: 10339-11821

Mycgr3G70475

Mycgr3G108866
  
Location: 11921-13010

Mycgr3G108866

Mycgr3G92136
  
Location: 13110-13593

Mycgr3G92136

related to oxidoreductase
  
Accession: CCT75015
  
Location: 2273854-2275409
  
  
**BlastP hit with Mycgr3G57362**
  
Percentage identity: 33 %
  
BlastP bit score: 217
  
Sequence coverage: 100 %
  
E-value: 4e-61
  
  
 NCBI BlastP on this gene

FFUJ\_11090

uncharacterized protein
  
Accession: CCT75014
  
Location: 2273137-2273625
  
 NCBI BlastP on this gene

FFUJ\_11089

uncharacterized protein
  
Accession: CCT75013
  
Location: 2271411-2272550
  
 NCBI BlastP on this gene

FFUJ\_11088

Query: Architecture Search FASTA input

GG704912 : Coccidioides immitis RS genomic scaffold supercont3.2    Total score: 1.0     Cumulative Blast bit score: 217

Hit cluster cross-links:

Mycgr3G70471
  
Location: 0-405

Mycgr3G70471

Mycgr3G39149
  
Location: 505-1798

Mycgr3G39149

Mycgr3G92130
  
Location: 1898-2396

Mycgr3G92130

Mycgr3G38483
  
Location: 2496-3576

Mycgr3G38483

Mycgr3G108869
  
Location: 3676-5056

Mycgr3G108869

Mycgr3G103943
  
Location: 5156-5762

Mycgr3G103943

Mycgr3G57362
  
Location: 5862-7296

Mycgr3G57362

Mycgr3G39086
  
Location: 7396-8368

Mycgr3G39086

Mycgr3G103942
  
Location: 8468-8714

Mycgr3G103942

Mycgr3G108865
  
Location: 8814-10239

Mycgr3G108865

Mycgr3G70475
  
Location: 10339-11821

Mycgr3G70475

Mycgr3G108866
  
Location: 11921-13010

Mycgr3G108866

Mycgr3G92136
  
Location: 13110-13593

Mycgr3G92136

hypothetical protein
  
Accession: EAS31716
  
Location: 3697547-3699137
  
  
**BlastP hit with Mycgr3G57362**
  
Percentage identity: 33 %
  
BlastP bit score: 217
  
Sequence coverage: 98 %
  
E-value: 4e-61
  
  
 NCBI BlastP on this gene

EAS31716

RNA-binding La domain-containing protein
  
Accession: EAS31715
  
Location: 3695690-3697158
  
 NCBI BlastP on this gene

EAS31715

chitin synthase class VI
  
Accession: EAS31713
  
Location: 3690677-3693254
  
 NCBI BlastP on this gene

EAS31713

hsp88-like protein
  
Accession: EAS31711
  
Location: 3686899-3689522
  
 NCBI BlastP on this gene

EAS31711

Query: Architecture Search FASTA input

ACFW01000025 : Coccidioides posadasii C735 delta SOWgp    Total score: 1.0     Cumulative Blast bit score: 217

Hit cluster cross-links:

Mycgr3G70471
  
Location: 0-405

Mycgr3G70471

Mycgr3G39149
  
Location: 505-1798

Mycgr3G39149

Mycgr3G92130
  
Location: 1898-2396

Mycgr3G92130

Mycgr3G38483
  
Location: 2496-3576

Mycgr3G38483

Mycgr3G108869
  
Location: 3676-5056

Mycgr3G108869

Mycgr3G103943
  
Location: 5156-5762

Mycgr3G103943

Mycgr3G57362
  
Location: 5862-7296

Mycgr3G57362

Mycgr3G39086
  
Location: 7396-8368

Mycgr3G39086

Mycgr3G103942
  
Location: 8468-8714

Mycgr3G103942

Mycgr3G108865
  
Location: 8814-10239

Mycgr3G108865

Mycgr3G70475
  
Location: 10339-11821

Mycgr3G70475

Mycgr3G108866
  
Location: 11921-13010

Mycgr3G108866

Mycgr3G92136
  
Location: 13110-13593

Mycgr3G92136

FAD dependent oxidoreductase family protein
  
Accession: EER27931
  
Location: 3370382-3371971
  
  
**BlastP hit with Mycgr3G57362**
  
Percentage identity: 33 %
  
BlastP bit score: 217
  
Sequence coverage: 98 %
  
E-value: 4e-61
  
  
 NCBI BlastP on this gene

EER27931

La domain containing protein
  
Accession: EER27930
  
Location: 3368520-3369994
  
 NCBI BlastP on this gene

EER27930

Chitin synthase D , putative
  
Accession: EER27929
  
Location: 3363499-3366079
  
 NCBI BlastP on this gene

EER27929

Heat shock protein, putative
  
Accession: EER27928
  
Location: 3359683-3362341
  
 NCBI BlastP on this gene

EER27928

Query: Architecture Search FASTA input

KE145352 : Glarea lozoyensis ATCC 20868 chromosome Unknown GLAREA1    Total score: 1.0     Cumulative Blast bit score: 216

Hit cluster cross-links:

Mycgr3G70471
  
Location: 0-405

Mycgr3G70471

Mycgr3G39149
  
Location: 505-1798

Mycgr3G39149

Mycgr3G92130
  
Location: 1898-2396

Mycgr3G92130

Mycgr3G38483
  
Location: 2496-3576

Mycgr3G38483

Mycgr3G108869
  
Location: 3676-5056

Mycgr3G108869

Mycgr3G103943
  
Location: 5156-5762

Mycgr3G103943

Mycgr3G57362
  
Location: 5862-7296

Mycgr3G57362

Mycgr3G39086
  
Location: 7396-8368

Mycgr3G39086

Mycgr3G103942
  
Location: 8468-8714

Mycgr3G103942

Mycgr3G108865
  
Location: 8814-10239

Mycgr3G108865

Mycgr3G70475
  
Location: 10339-11821

Mycgr3G70475

Mycgr3G108866
  
Location: 11921-13010

Mycgr3G108866

Mycgr3G92136
  
Location: 13110-13593

Mycgr3G92136

hypothetical protein
  
Accession: EPE36636
  
Location: 433483-435552
  
 NCBI BlastP on this gene

EPE36636

hypothetical protein
  
Accession: EPE36635
  
Location: 430655-432306
  
 NCBI BlastP on this gene

EPE36635

Zn-dependent exopeptidase
  
Accession: EPE36634
  
Location: 428147-429758
  
 NCBI BlastP on this gene

EPE36634

FAD/NAD(P)-binding protein
  
Accession: EPE36633
  
Location: 424122-425658
  
  
**BlastP hit with Mycgr3G57362**
  
Percentage identity: 32 %
  
BlastP bit score: 216
  
Sequence coverage: 100 %
  
E-value: 7e-61
  
  
 NCBI BlastP on this gene

EPE36633

hypothetical protein
  
Accession: EPE36632
  
Location: 423093-423671
  
 NCBI BlastP on this gene

EPE36632

hypothetical protein
  
Accession: EPE36631
  
Location: 420934-422010
  
 NCBI BlastP on this gene

EPE36631

ATP synthase subunit 4, mitochondrial precursor
  
Accession: EPE36630
  
Location: 419331-420229
  
 NCBI BlastP on this gene

EPE36630

hypothetical protein
  
Accession: EPE36629
  
Location: 417386-419122
  
 NCBI BlastP on this gene

EPE36629

HCP-like protein
  
Accession: EPE36628
  
Location: 415396-416949
  
 NCBI BlastP on this gene

EPE36628

Query: Architecture Search FASTA input

AEOI01000007 : Ogataea parapolymorpha DL-1    Total score: 1.0     Cumulative Blast bit score: 216

Hit cluster cross-links:

Mycgr3G70471
  
Location: 0-405

Mycgr3G70471

Mycgr3G39149
  
Location: 505-1798

Mycgr3G39149

Mycgr3G92130
  
Location: 1898-2396

Mycgr3G92130

Mycgr3G38483
  
Location: 2496-3576

Mycgr3G38483

Mycgr3G108869
  
Location: 3676-5056

Mycgr3G108869

Mycgr3G103943
  
Location: 5156-5762

Mycgr3G103943

Mycgr3G57362
  
Location: 5862-7296

Mycgr3G57362

Mycgr3G39086
  
Location: 7396-8368

Mycgr3G39086

Mycgr3G103942
  
Location: 8468-8714

Mycgr3G103942

Mycgr3G108865
  
Location: 8814-10239

Mycgr3G108865

Mycgr3G70475
  
Location: 10339-11821

Mycgr3G70475

Mycgr3G108866
  
Location: 11921-13010

Mycgr3G108866

Mycgr3G92136
  
Location: 13110-13593

Mycgr3G92136

Na+/Pi cotransporter, active in early growth phase
  
Accession: EFW96479
  
Location: 777-2483
  
 NCBI BlastP on this gene

EFW96479

oxidoreductase, aldo/keto reductase family protein
  
Accession: EFW96480
  
Location: 2882-3814
  
 NCBI BlastP on this gene

EFW96480

pantothenate transporter, putative
  
Accession: EFW96481
  
Location: 3941-5506
  
 NCBI BlastP on this gene

EFW96481

FAD dependent oxidoreductase superfamily
  
Accession: EFW96482
  
Location: 5685-7019
  
  
**BlastP hit with Mycgr3G57362**
  
Percentage identity: 34 %
  
BlastP bit score: 216
  
Sequence coverage: 98 %
  
E-value: 5e-61
  
  
 NCBI BlastP on this gene

EFW96482

2-haloalkanoic acid dehalogenase, putative
  
Accession: EFW96483
  
Location: 7160-7882
  
 NCBI BlastP on this gene

EFW96483

transcriptional activator protein, putative
  
Accession: EFW96484
  
Location: 7898-8977
  
 NCBI BlastP on this gene

EFW96484

aryl-alcohol dehydrogenase (AAD4)
  
Accession: EFW96485
  
Location: 10032-11108
  
 NCBI BlastP on this gene

EFW96485

Histidine acid phosphatase family protein
  
Accession: EFW96486
  
Location: 11490-12704
  
 NCBI BlastP on this gene

EFW96486

Gly-X carboxypeptidase
  
Accession: EFW96487
  
Location: 12750-14408
  
 NCBI BlastP on this gene

EFW96487

siderophore-iron transporter Str3
  
Accession: EFW96488
  
Location: 15219-18052
  
 NCBI BlastP on this gene

EFW96488

Query: Architecture Search FASTA input

KE148151 : Ophiostoma piceae UAMH 11346 chromosome Unknown scf06    Total score: 1.0     Cumulative Blast bit score: 214

Hit cluster cross-links:

Mycgr3G70471
  
Location: 0-405

Mycgr3G70471

Mycgr3G39149
  
Location: 505-1798

Mycgr3G39149

Mycgr3G92130
  
Location: 1898-2396

Mycgr3G92130

Mycgr3G38483
  
Location: 2496-3576

Mycgr3G38483

Mycgr3G108869
  
Location: 3676-5056

Mycgr3G108869

Mycgr3G103943
  
Location: 5156-5762

Mycgr3G103943

Mycgr3G57362
  
Location: 5862-7296

Mycgr3G57362

Mycgr3G39086
  
Location: 7396-8368

Mycgr3G39086

Mycgr3G103942
  
Location: 8468-8714

Mycgr3G103942

Mycgr3G108865
  
Location: 8814-10239

Mycgr3G108865

Mycgr3G70475
  
Location: 10339-11821

Mycgr3G70475

Mycgr3G108866
  
Location: 11921-13010

Mycgr3G108866

Mycgr3G92136
  
Location: 13110-13593

Mycgr3G92136

amidohydrolase domain containing protein
  
Accession: EPE07448
  
Location: 1405314-1406662
  
 NCBI BlastP on this gene

EPE07448

cytochrome p450
  
Accession: EPE07449
  
Location: 1406927-1408746
  
 NCBI BlastP on this gene

EPE07449

short-chain dehydrogenase
  
Accession: EPE07450
  
Location: 1409217-1410231
  
 NCBI BlastP on this gene

EPE07450

mfs general substrate transporter
  
Accession: EPE07451
  
Location: 1410571-1412603
  
 NCBI BlastP on this gene

EPE07451

fad dependent oxidoreductase
  
Accession: EPE07452
  
Location: 1413198-1414742
  
  
**BlastP hit with Mycgr3G57362**
  
Percentage identity: 35 %
  
BlastP bit score: 214
  
Sequence coverage: 99 %
  
E-value: 8e-60
  
  
 NCBI BlastP on this gene

EPE07452

Query: Architecture Search FASTA input

201. :  GG663367 Ajellomyces capsulatus G186AR genomic scaffold supercont2.5     Total score: 1.0     Cumulative Blast bit score: 380

Mycgr3G70471
  
Location: 0-405
  
 NCBI BlastP on this gene

Mycgr3G70471

Mycgr3G39149
  
Location: 505-1798
  
 NCBI BlastP on this gene

Mycgr3G39149

Mycgr3G92130
  
Location: 1898-2396
  
 NCBI BlastP on this gene

Mycgr3G92130

Mycgr3G38483
  
Location: 2496-3576
  
 NCBI BlastP on this gene

Mycgr3G38483

Mycgr3G108869
  
Location: 3676-5056
  
 NCBI BlastP on this gene

Mycgr3G108869

Mycgr3G103943
  
Location: 5156-5762
  
 NCBI BlastP on this gene

Mycgr3G103943

Mycgr3G57362
  
Location: 5862-7296
  
 NCBI BlastP on this gene

Mycgr3G57362

Mycgr3G39086
  
Location: 7396-8368
  
 NCBI BlastP on this gene

Mycgr3G39086

Mycgr3G103942
  
Location: 8468-8714
  
 NCBI BlastP on this gene

Mycgr3G103942

Mycgr3G108865
  
Location: 8814-10239
  
 NCBI BlastP on this gene

Mycgr3G108865

Mycgr3G70475
  
Location: 10339-11821
  
 NCBI BlastP on this gene

Mycgr3G70475

Mycgr3G108866
  
Location: 11921-13010
  
 NCBI BlastP on this gene

Mycgr3G108866

Mycgr3G92136
  
Location: 13110-13593
  
 NCBI BlastP on this gene

Mycgr3G92136

high-affinity nickel transporter
  
Accession: EEH07657
  
Location: 1704060-1706324
  
  
**BlastP hit with Mycgr3G108865**
  
Percentage identity: 51 %
  
BlastP bit score: 380
  
Sequence coverage: 85 %
  
E-value: 4e-123
  
  
 NCBI BlastP on this gene

EEH07657

high-affinity nickel transporter
  
Accession: EEH07656
  
Location: 1701350-1703434
  
 NCBI BlastP on this gene

EEH07656

P-type ATPase
  
Accession: EEH07655
  
Location: 1696040-1700249
  
 NCBI BlastP on this gene

EEH07655

202. :  DS995900 Penicillium marneffei ATCC 18224 scf\_1105668340758 genomic scaffold     Total score: 1.0     Cumulative Blast bit score: 378

BTB/POZ domain protein
  
Accession: EEA26027
  
Location: 2602332-2603560
  
 NCBI BlastP on this gene

EEA26027

conserved hypothetical protein
  
Accession: EEA26028
  
Location: 2606760-2607987
  
 NCBI BlastP on this gene

EEA26028

conserved hypothetical protein
  
Accession: EEA26029
  
Location: 2608992-2610488
  
 NCBI BlastP on this gene

EEA26029

nickel transport protein, putative
  
Accession: EEA26030
  
Location: 2610982-2612796
  
  
**BlastP hit with Mycgr3G108865**
  
Percentage identity: 49 %
  
BlastP bit score: 378
  
Sequence coverage: 91 %
  
E-value: 2e-122
  
  
 NCBI BlastP on this gene

EEA26030

terminal deoxynucleotidyl transferase, putative
  
Accession: EEA26031
  
Location: 2612852-2614984
  
 NCBI BlastP on this gene

EEA26031

phospholipid-transporting ATPase, putative
  
Accession: EEA26032
  
Location: 2615646-2619796
  
 NCBI BlastP on this gene

EEA26032

exosome complex endonuclease 2/ribosomal RNA processing protein, putative
  
Accession: EEA26033
  
Location: 2620746-2621737
  
 NCBI BlastP on this gene

EEA26033

203. :  KB445644 Cochliobolus sativus ND90Pr unplaced genomic scaffold COCSAscaffold\_8     Total score: 1.0     Cumulative Blast bit score: 375

hypothetical protein
  
Accession: EMD63852
  
Location: 1309585-1311114
  
  
**BlastP hit with Mycgr3G57362**
  
Percentage identity: 45 %
  
BlastP bit score: 375
  
Sequence coverage: 96 %
  
E-value: 1e-121
  
  
 NCBI BlastP on this gene

EMD63852

hypothetical protein
  
Accession: EMD63851
  
Location: 1308420-1309197
  
 NCBI BlastP on this gene

EMD63851

glycoside hydrolase family 17 protein
  
Accession: EMD63850
  
Location: 1306233-1307762
  
 NCBI BlastP on this gene

EMD63850

hypothetical protein
  
Accession: EMD63849
  
Location: 1304085-1305942
  
 NCBI BlastP on this gene

EMD63849

hypothetical protein
  
Accession: EMD63848
  
Location: 1300856-1302070
  
 NCBI BlastP on this gene

EMD63848

204. :  GG692419 Ajellomyces capsulatus H143 genomic scaffold supercont2.1     Total score: 1.0     Cumulative Blast bit score: 375

high-affinity nickel transporter
  
Accession: EER45808
  
Location: 5846199-5848447
  
  
**BlastP hit with Mycgr3G108865**
  
Percentage identity: 51 %
  
BlastP bit score: 375
  
Sequence coverage: 85 %
  
E-value: 4e-121
  
  
 NCBI BlastP on this gene

EER45808

high-affinity nickel transporter
  
Accession: EER45807
  
Location: 5843493-5845577
  
 NCBI BlastP on this gene

EER45807

phospholipid-transporting ATPase
  
Accession: EER45806
  
Location: 5838192-5842399
  
 NCBI BlastP on this gene

EER45806

205. :  EQ962653 Talaromyces stipitatus ATCC 10500 scf\_1105507295527 genomic scaffold     Total score: 1.0     Cumulative Blast bit score: 375

arylsulfatase A, putative
  
Accession: EED21920
  
Location: 3072215-3074411
  
 NCBI BlastP on this gene

EED21920

hypothetical protein
  
Accession: EED21921
  
Location: 3074976-3075582
  
 NCBI BlastP on this gene

EED21921

conserved hypothetical protein
  
Accession: EED21922
  
Location: 3077326-3078592
  
 NCBI BlastP on this gene

EED21922

conserved hypothetical protein
  
Accession: EED21923
  
Location: 3079481-3080810
  
 NCBI BlastP on this gene

EED21923

nickel transport protein, putative
  
Accession: EED21924
  
Location: 3081230-3083070
  
  
**BlastP hit with Mycgr3G108865**
  
Percentage identity: 50 %
  
BlastP bit score: 375
  
Sequence coverage: 91 %
  
E-value: 5e-121
  
  
 NCBI BlastP on this gene

EED21924

terminal deoxynucleotidyl transferase, putative
  
Accession: EED21925
  
Location: 3083136-3085259
  
 NCBI BlastP on this gene

EED21925

phospholipid-transporting ATPase, putative
  
Accession: EED21926
  
Location: 3085908-3090048
  
 NCBI BlastP on this gene

EED21926

exosome complex endonuclease 2/ribosomal RNA processing protein, putative
  
Accession: EED21927
  
Location: 3090933-3091745
  
 NCBI BlastP on this gene

EED21927

206. :  DS990636 Ajellomyces capsulatus H88 supercont1.1 genomic scaffold     Total score: 1.0     Cumulative Blast bit score: 375

high-affinity nickel transporter
  
Accession: EGC41754
  
Location: 4262493-4264741
  
  
**BlastP hit with Mycgr3G108865**
  
Percentage identity: 51 %
  
BlastP bit score: 375
  
Sequence coverage: 85 %
  
E-value: 4e-121
  
  
 NCBI BlastP on this gene

EGC41754

high-affinity nickel transporter
  
Accession: EGC41753
  
Location: 4259787-4261871
  
 NCBI BlastP on this gene

EGC41753

phospholipid-transporting ATPase
  
Accession: EGC41752
  
Location: 4254480-4259029
  
 NCBI BlastP on this gene

EGC41752

207. :  KB908833 Setosphaeria turcica Et28A unplaced genomic scaffold SETTUscaffold\_5     Total score: 1.0     Cumulative Blast bit score: 373

hypothetical protein
  
Accession: EOA83289
  
Location: 1097926-1099094
  
 NCBI BlastP on this gene

EOA83289

hypothetical protein
  
Accession: EOA83290
  
Location: 1100562-1100996
  
 NCBI BlastP on this gene

EOA83290

hypothetical protein
  
Accession: EOA83291
  
Location: 1101860-1103377
  
  
**BlastP hit with Mycgr3G57362**
  
Percentage identity: 45 %
  
BlastP bit score: 373
  
Sequence coverage: 98 %
  
E-value: 8e-121
  
  
 NCBI BlastP on this gene

EOA83291

hypothetical protein
  
Accession: EOA83292
  
Location: 1103724-1104663
  
 NCBI BlastP on this gene

EOA83292

hypothetical protein
  
Accession: EOA83293
  
Location: 1105138-1106047
  
 NCBI BlastP on this gene

EOA83293

hypothetical protein
  
Accession: EOA83294
  
Location: 1107636-1111510
  
 NCBI BlastP on this gene

EOA83294

208. :  KB733456 Bipolaris maydis ATCC 48331 unplaced genomic scaffold COCC4scaffold\_13     Total score: 1.0     Cumulative Blast bit score: 373

hypothetical protein
  
Accession: ENI04559
  
Location: 86956-89594
  
 NCBI BlastP on this gene

ENI04559

hypothetical protein
  
Accession: ENI04560
  
Location: 89859-91118
  
 NCBI BlastP on this gene

ENI04560

hypothetical protein
  
Accession: ENI04561
  
Location: 92971-93438
  
 NCBI BlastP on this gene

ENI04561

hypothetical protein
  
Accession: ENI04562
  
Location: 95127-96656
  
  
**BlastP hit with Mycgr3G57362**
  
Percentage identity: 45 %
  
BlastP bit score: 373
  
Sequence coverage: 96 %
  
E-value: 8e-121
  
  
 NCBI BlastP on this gene

ENI04562

hypothetical protein
  
Accession: ENI04563
  
Location: 97040-97819
  
 NCBI BlastP on this gene

ENI04563

hypothetical protein
  
Accession: ENI04564
  
Location: 98055-98417
  
 NCBI BlastP on this gene

ENI04564

glycoside hydrolase family 17 protein
  
Accession: ENI04565
  
Location: 98728-100023
  
 NCBI BlastP on this gene

ENI04565

hypothetical protein
  
Accession: ENI04566
  
Location: 100313-102170
  
 NCBI BlastP on this gene

ENI04566

hypothetical protein
  
Accession: ENI04567
  
Location: 104206-104745
  
 NCBI BlastP on this gene

ENI04567

209. :  KB445574 Cochliobolus heterostrophus C5 unplaced genomic scaffold COCHEscaffold\_6     Total score: 1.0     Cumulative Blast bit score: 373

hypothetical protein
  
Accession: EMD93049
  
Location: 883889-885418
  
  
**BlastP hit with Mycgr3G57362**
  
Percentage identity: 45 %
  
BlastP bit score: 373
  
Sequence coverage: 96 %
  
E-value: 8e-121
  
  
 NCBI BlastP on this gene

EMD93049

hypothetical protein
  
Accession: EMD93048
  
Location: 882726-883505
  
 NCBI BlastP on this gene

EMD93048

hypothetical protein
  
Accession: EMD93047
  
Location: 882122-882490
  
 NCBI BlastP on this gene

EMD93047

glycoside hydrolase family 17 protein
  
Accession: EMD93046
  
Location: 880690-881667
  
 NCBI BlastP on this gene

EMD93046

hypothetical protein
  
Accession: EMD93045
  
Location: 878375-880232
  
 NCBI BlastP on this gene

EMD93045

hypothetical protein
  
Accession: EMD93044
  
Location: 875239-876339
  
 NCBI BlastP on this gene

EMD93044

210. :  ABDG02000027 Trichoderma atroviride IMI 206040     Total score: 1.0     Cumulative Blast bit score: 371

high-affinity nickel transport protein
  
Accession: EHK41551
  
Location: 3381317-3382686
  
  
**BlastP hit with Mycgr3G108865**
  
Percentage identity: 52 %
  
BlastP bit score: 371
  
Sequence coverage: 83 %
  
E-value: 1e-120
  
  
 NCBI BlastP on this gene

EHK41551

hypothetical protein
  
Accession: EHK41550
  
Location: 3375780-3378519
  
 NCBI BlastP on this gene

EHK41550

hypothetical protein
  
Accession: EHK41549
  
Location: 3373354-3374853
  
 NCBI BlastP on this gene

EHK41549

hypothetical protein
  
Accession: EHK41548
  
Location: 3371554-3372778
  
 NCBI BlastP on this gene

EHK41548

211. :  CH445339 Phaeosphaeria nodorum SN15 scaffold\_15     Total score: 1.0     Cumulative Blast bit score: 369

hypothetical protein
  
Accession: EAT82811
  
Location: 228887-231809
  
 NCBI BlastP on this gene

EAT82811

hypothetical protein
  
Accession: EAT82810
  
Location: 227501-228363
  
 NCBI BlastP on this gene

EAT82810

hypothetical protein
  
Accession: EAT82809
  
Location: 225258-226256
  
 NCBI BlastP on this gene

EAT82809

hypothetical protein
  
Accession: EAT82808
  
Location: 222829-224357
  
  
**BlastP hit with Mycgr3G57362**
  
Percentage identity: 44 %
  
BlastP bit score: 369
  
Sequence coverage: 96 %
  
E-value: 3e-119
  
  
 NCBI BlastP on this gene

EAT82808

hypothetical protein
  
Accession: EAT82807
  
Location: 221777-222490
  
 NCBI BlastP on this gene

EAT82807

hypothetical protein
  
Accession: EAT82806
  
Location: 220968-221780
  
 NCBI BlastP on this gene

EAT82806

hypothetical protein
  
Accession: EAT82805
  
Location: 219468-220310
  
 NCBI BlastP on this gene

EAT82805

hypothetical protein
  
Accession: EAT82804
  
Location: 217087-218019
  
 NCBI BlastP on this gene

EAT82804

hypothetical protein
  
Accession: EAT82803
  
Location: 213856-216044
  
 NCBI BlastP on this gene

EAT82803

212. :  CP003004 Myceliophthora thermophila ATCC 42464 chromosome 3     Total score: 1.0     Cumulative Blast bit score: 363

hypothetical protein
  
Accession: AEO58193
  
Location: 4129163-4131114
  
  
**BlastP hit with Mycgr3G108865**
  
Percentage identity: 54 %
  
BlastP bit score: 363
  
Sequence coverage: 78 %
  
E-value: 1e-117
  
  
 NCBI BlastP on this gene

MYCTH\_2093133

hypothetical protein
  
Accession: AEO58192
  
Location: 4128260-4128778
  
 NCBI BlastP on this gene

MYCTH\_2110543

hypothetical protein
  
Accession: AEO58191
  
Location: 4126947-4127898
  
 NCBI BlastP on this gene

MYCTH\_2110542

hypothetical protein
  
Accession: AEO58190
  
Location: 4122089-4123429
  
 NCBI BlastP on this gene

MYCTH\_2060238

213. :  GL988041 Chaetomium thermophilum var. thermophilum DSM 1495 unplaced genomic scaffold scf7180000...     Total score: 1.0     Cumulative Blast bit score: 355

hypothetical protein
  
Accession: EGS20367
  
Location: 331967-332986
  
 NCBI BlastP on this gene

EGS20367

hypothetical protein
  
Accession: EGS20368
  
Location: 334320-336175
  
 NCBI BlastP on this gene

EGS20368

hypothetical protein
  
Accession: EGS20369
  
Location: 340000-341305
  
  
**BlastP hit with Mycgr3G108865**
  
Percentage identity: 54 %
  
BlastP bit score: 355
  
Sequence coverage: 72 %
  
E-value: 9e-115
  
  
 NCBI BlastP on this gene

EGS20369

hypothetical protein
  
Accession: EGS20370
  
Location: 341877-345689
  
 NCBI BlastP on this gene

EGS20370

putative UDP-glucose protein
  
Accession: EGS20371
  
Location: 347532-349033
  
 NCBI BlastP on this gene

EGS20371

214. :  DS572815 Paracoccidioides brasiliensis Pb01 supercont1.5 genomic scaffold     Total score: 1.0     Cumulative Blast bit score: 350

predicted protein
  
Accession: EEH40152
  
Location: 61345-62338
  
 NCBI BlastP on this gene

EEH40152

conserved hypothetical protein
  
Accession: EEH40153
  
Location: 62449-64994
  
  
**BlastP hit with Mycgr3G108865**
  
Percentage identity: 48 %
  
BlastP bit score: 350
  
Sequence coverage: 97 %
  
E-value: 2e-111
  
  
 NCBI BlastP on this gene

EEH40153

215. :  DS544809 Paracoccidioides brasiliensis Pb03 supercont1.7 genomic scaffold     Total score: 1.0     Cumulative Blast bit score: 342

DDHD domain-containing protein
  
Accession: EEH22506
  
Location: 387006-390000
  
 NCBI BlastP on this gene

EEH22506

conserved hypothetical protein
  
Accession: EEH22507
  
Location: 390619-391294
  
 NCBI BlastP on this gene

EEH22507

NADH dehydrogenase 29/21K chain
  
Accession: EEH22508
  
Location: 392436-393607
  
 NCBI BlastP on this gene

EEH22508

conserved hypothetical protein
  
Accession: EEH22509
  
Location: 394343-395152
  
 NCBI BlastP on this gene

EEH22509

high-affinity nickel permease
  
Accession: EEH22510
  
Location: 396131-399088
  
  
**BlastP hit with Mycgr3G108865**
  
Percentage identity: 48 %
  
BlastP bit score: 342
  
Sequence coverage: 94 %
  
E-value: 3e-107
  
  
 NCBI BlastP on this gene

EEH22510

DNA polymerase beta
  
Accession: EEH22511
  
Location: 399312-401563
  
 NCBI BlastP on this gene

EEH22511

ATPase
  
Accession: EEH22512
  
Location: 402086-406325
  
 NCBI BlastP on this gene

EEH22512

216. :  JH921428 Marssonina brunnea f. sp. 'multigermtubi' MB\_m1 unplaced genomic scaffold M6\_S00001     Total score: 1.0     Cumulative Blast bit score: 338

integral membrane protein (Pth11)
  
Accession: EKD20892
  
Location: 519567-520980
  
 NCBI BlastP on this gene

EKD20892

hypothetical protein
  
Accession: EKD20893
  
Location: 522615-523930
  
 NCBI BlastP on this gene

EKD20893

allantoate permease
  
Accession: EKD20894
  
Location: 524075-525749
  
 NCBI BlastP on this gene

EKD20894

FAD dependent oxidoreductase
  
Accession: EKD20895
  
Location: 528471-529955
  
  
**BlastP hit with Mycgr3G57362**
  
Percentage identity: 44 %
  
BlastP bit score: 338
  
Sequence coverage: 100 %
  
E-value: 2e-107
  
  
 NCBI BlastP on this gene

EKD20895

amidase family protein
  
Accession: EKD20896
  
Location: 532575-534704
  
 NCBI BlastP on this gene

EKD20896

integral membrane protein DUF92
  
Accession: EKD20897
  
Location: 535679-536937
  
 NCBI BlastP on this gene

EKD20897

hypothetical protein
  
Accession: EKD20898
  
Location: 537208-541135
  
 NCBI BlastP on this gene

EKD20898

217. :  CP003012 Thielavia terrestris NRRL 8126 chromosome 4     Total score: 1.0     Cumulative Blast bit score: 338

hypothetical protein
  
Accession: AEO69085
  
Location: 1954685-1955371
  
 NCBI BlastP on this gene

THITE\_2119095

hypothetical protein
  
Accession: AEO69084
  
Location: 1951707-1953598
  
 NCBI BlastP on this gene

THITE\_43879

hypothetical protein
  
Accession: AEO69083
  
Location: 1947927-1950879
  
 NCBI BlastP on this gene

THITE\_113789

hypothetical protein
  
Accession: AEO69082
  
Location: 1945306-1946824
  
  
**BlastP hit with Mycgr3G108865**
  
Percentage identity: 50 %
  
BlastP bit score: 338
  
Sequence coverage: 78 %
  
E-value: 9e-108
  
  
 NCBI BlastP on this gene

THITE\_2119090

hypothetical protein
  
Accession: AEO69081
  
Location: 1942046-1943150
  
 NCBI BlastP on this gene

THITE\_2119087

hypothetical protein
  
Accession: AEO69080
  
Location: 1941471-1941680
  
 NCBI BlastP on this gene

THITE\_2119083

glycosyltransferase family 2 protein
  
Accession: AEO69079
  
Location: 1934469-1938733
  
 NCBI BlastP on this gene

THITE\_121780

218. :  KB707715 Botryotinia fuckeliana BcDW1 unplaced genomic scaffold Scaffold\_43     Total score: 1.0     Cumulative Blast bit score: 336

putative mediator of rna polymerase ii transcription subunit 22 protein
  
Accession: EMR90153
  
Location: 128927-130664
  
 NCBI BlastP on this gene

EMR90153

putative atp-dependent rna helicase mss116 protein
  
Accession: EMR90152
  
Location: 126633-128477
  
 NCBI BlastP on this gene

EMR90152

putative mannitol dehydrogenase protein
  
Accession: EMR90151
  
Location: 124441-125305
  
 NCBI BlastP on this gene

EMR90151

putative high affinity nickel transport protein nic1 protein
  
Accession: EMR90150
  
Location: 120979-122037
  
  
**BlastP hit with Mycgr3G108865**
  
Percentage identity: 60 %
  
BlastP bit score: 336
  
Sequence coverage: 62 %
  
E-value: 1e-108
  
  
 NCBI BlastP on this gene

EMR90150

putative duf1295 domain protein
  
Accession: EMR90149
  
Location: 119118-120407
  
 NCBI BlastP on this gene

EMR90149

putative btb poz domain containing protein
  
Accession: EMR90148
  
Location: 116272-117693
  
 NCBI BlastP on this gene

EMR90148

putative dihydrofolate reductase protein
  
Accession: EMR90147
  
Location: 113446-114417
  
 NCBI BlastP on this gene

EMR90147

219. :  CP003003 Myceliophthora thermophila ATCC 42464 chromosome 2     Total score: 1.0     Cumulative Blast bit score: 324

hypothetical protein
  
Accession: AEO55901
  
Location: 603300-605691
  
 NCBI BlastP on this gene

MYCTH\_2300199

hypothetical protein
  
Accession: AEO55900
  
Location: 601162-601671
  
 NCBI BlastP on this gene

MYCTH\_2314426

hypothetical protein
  
Accession: AEO55899
  
Location: 600167-600583
  
 NCBI BlastP on this gene

MYCTH\_2058204

hypothetical protein
  
Accession: AEO55898
  
Location: 598108-599518
  
 NCBI BlastP on this gene

MYCTH\_2300192

hypothetical protein
  
Accession: AEO55897
  
Location: 595421-597323
  
  
**BlastP hit with Mycgr3G57362**
  
Percentage identity: 41 %
  
BlastP bit score: 324
  
Sequence coverage: 105 %
  
E-value: 8e-101
  
  
 NCBI BlastP on this gene

MYCTH\_2116566

hypothetical protein
  
Accession: AEO55896
  
Location: 591961-593724
  
 NCBI BlastP on this gene

MYCTH\_93330

hypothetical protein
  
Accession: AEO55895
  
Location: 590091-590683
  
 NCBI BlastP on this gene

MYCTH\_2124857

hypothetical protein
  
Accession: AEO55894
  
Location: 587818-589643
  
 NCBI BlastP on this gene

MYCTH\_2300189

220. :  ACFW01000049 Coccidioides posadasii C735 delta SOWgp     Total score: 1.0     Cumulative Blast bit score: 324

carboxypeptidase A4 precursor, putative
  
Accession: EER24122
  
Location: 3400736-3402551
  
 NCBI BlastP on this gene

EER24122

High-affinity nickel transport protein nic1, putative
  
Accession: EER24123
  
Location: 3409572-3410947
  
  
**BlastP hit with Mycgr3G108865**
  
Percentage identity: 55 %
  
BlastP bit score: 324
  
Sequence coverage: 70 %
  
E-value: 3e-103
  
  
 NCBI BlastP on this gene

EER24123

hypothetical protein
  
Accession: EER24124
  
Location: 3411385-3415345
  
 NCBI BlastP on this gene

EER24124

hypothetical protein
  
Accession: EER24125
  
Location: 3417838-3420033
  
 NCBI BlastP on this gene

EER24125

221. :  KE145369 Glarea lozoyensis ATCC 20868 chromosome Unknown GLAREA5     Total score: 1.0     Cumulative Blast bit score: 319

FAD/NAD(P)-binding protein
  
Accession: EPE27543
  
Location: 411420-412924
  
  
**BlastP hit with Mycgr3G57362**
  
Percentage identity: 43 %
  
BlastP bit score: 319
  
Sequence coverage: 100 %
  
E-value: 8e-100
  
  
 NCBI BlastP on this gene

EPE27543

(Trans)glycosidase
  
Accession: EPE27542
  
Location: 410085-410984
  
 NCBI BlastP on this gene

EPE27542

Di-copper centre-containing
  
Accession: EPE27541
  
Location: 405404-408559
  
 NCBI BlastP on this gene

EPE27541

Cytochrome P450
  
Accession: EPE27540
  
Location: 400310-403844
  
 NCBI BlastP on this gene

EPE27540

222. :  DS989823 Arthroderma gypseum CBS 118893 supercont1.2 genomic scaffold     Total score: 1.0     Cumulative Blast bit score: 318

cytohesin-2
  
Accession: EFQ99133
  
Location: 400681-404941
  
 NCBI BlastP on this gene

EFQ99133

hypothetical protein
  
Accession: EFQ99132
  
Location: 398497-399716
  
 NCBI BlastP on this gene

EFQ99132

hypothetical protein
  
Accession: EFQ99131
  
Location: 396411-397472
  
 NCBI BlastP on this gene

EFQ99131

ECM14
  
Accession: EFQ99130
  
Location: 394251-396100
  
 NCBI BlastP on this gene

EFQ99130

high-affinity nickel transporter nic1
  
Accession: EFQ99129
  
Location: 391564-393317
  
  
**BlastP hit with Mycgr3G108865**
  
Percentage identity: 48 %
  
BlastP bit score: 318
  
Sequence coverage: 95 %
  
E-value: 1e-99
  
  
 NCBI BlastP on this gene

EFQ99129

alkaline proteinase
  
Accession: EFQ99128
  
Location: 389526-391097
  
 NCBI BlastP on this gene

EFQ99128

hypothetical protein
  
Accession: EFQ99127
  
Location: 386917-387285
  
 NCBI BlastP on this gene

EFQ99127

DNA polymerase lambda
  
Accession: EFQ99126
  
Location: 383667-385709
  
 NCBI BlastP on this gene

EFQ99126

223. :  CAGA01000037 Claviceps purpurea 20.1     Total score: 1.0     Cumulative Blast bit score: 317

related to protein kinase SWE1
  
Accession: CCE32052
  
Location: 119662-122403
  
 NCBI BlastP on this gene

CCE32052

uncharacterized protein
  
Accession: CCE32051
  
Location: 114669-117877
  
  
**BlastP hit with Mycgr3G57362**
  
Percentage identity: 41 %
  
BlastP bit score: 317
  
Sequence coverage: 98 %
  
E-value: 1e-95
  
  
 NCBI BlastP on this gene

CCE32051

uncharacterized protein
  
Accession: CCE32050
  
Location: 112256-114061
  
 NCBI BlastP on this gene

CCE32050

probable ribosomal protein L30, cytosolic
  
Accession: CCE32049
  
Location: 110008-110677
  
 NCBI BlastP on this gene

CCE32049

uncharacterized protein
  
Accession: CCE32048
  
Location: 107340-107728
  
 NCBI BlastP on this gene

CCE32048

224. :  ABDF02000003 Trichoderma virens Gv29-8     Total score: 1.0     Cumulative Blast bit score: 317

hypothetical protein
  
Accession: EHK25543
  
Location: 1569554-1569880
  
 NCBI BlastP on this gene

EHK25543

hypothetical protein
  
Accession: EHK25544
  
Location: 1571649-1575228
  
 NCBI BlastP on this gene

EHK25544

hypothetical protein
  
Accession: EHK25545
  
Location: 1576509-1577990
  
  
**BlastP hit with Mycgr3G57362**
  
Percentage identity: 43 %
  
BlastP bit score: 317
  
Sequence coverage: 100 %
  
E-value: 5e-99
  
  
 NCBI BlastP on this gene

EHK25545

glycoside hydrolase family 76 protein
  
Accession: EHK25546
  
Location: 1579003-1580470
  
 NCBI BlastP on this gene

EHK25546

hypothetical protein
  
Accession: EHK25547
  
Location: 1581408-1583454
  
 NCBI BlastP on this gene

EHK25547

hypothetical protein
  
Accession: EHK25548
  
Location: 1584006-1586778
  
 NCBI BlastP on this gene

EHK25548

225. :  KB730323 Fusarium oxysporum f. sp. cubense race 1 unplaced genomic scaffold scaffold253     Total score: 1.0     Cumulative Blast bit score: 316

hypothetical protein
  
Accession: ENH67701
  
Location: 843752-844221
  
 NCBI BlastP on this gene

ENH67701

Mitosis inhibitor protein kinase wee1
  
Accession: ENH67702
  
Location: 846912-850413
  
 NCBI BlastP on this gene

ENH67702

Gamma-glutamylputrescine oxidoreductase
  
Accession: ENH67703
  
Location: 851590-853024
  
  
**BlastP hit with Mycgr3G57362**
  
Percentage identity: 41 %
  
BlastP bit score: 316
  
Sequence coverage: 96 %
  
E-value: 9e-99
  
  
 NCBI BlastP on this gene

ENH67703

226. :  KB726993 Fusarium oxysporum f. sp. cubense race 4 unplaced genomic scaffold scaffold83     Total score: 1.0     Cumulative Blast bit score: 316

hypothetical protein
  
Accession: EMT63343
  
Location: 1172229-1172698
  
 NCBI BlastP on this gene

EMT63343

Mitosis inhibitor protein kinase wee1
  
Accession: EMT63344
  
Location: 1175400-1178901
  
 NCBI BlastP on this gene

EMT63344

Gamma-glutamylputrescine oxidoreductase
  
Accession: EMT63345
  
Location: 1180077-1181511
  
  
**BlastP hit with Mycgr3G57362**
  
Percentage identity: 41 %
  
BlastP bit score: 316
  
Sequence coverage: 96 %
  
E-value: 9e-99
  
  
 NCBI BlastP on this gene

EMT63345

227. :  HF679027 Fusarium fujikuroi IMI 58289 draft genome, chromosome FFUJ\_chr05.     Total score: 1.0     Cumulative Blast bit score: 315

related to protein kinase SWE1
  
Accession: CCT68406
  
Location: 1318145-1321640
  
 NCBI BlastP on this gene

FFUJ\_07186

uncharacterized protein
  
Accession: CCT68407
  
Location: 1322784-1324218
  
  
**BlastP hit with Mycgr3G57362**
  
Percentage identity: 41 %
  
BlastP bit score: 315
  
Sequence coverage: 96 %
  
E-value: 1e-98
  
  
 NCBI BlastP on this gene

FFUJ\_07187

228. :  JH226136 Exophiala dermatitidis NIH/UT8656 unplaced genomic scaffold supercont1.7     Total score: 1.0     Cumulative Blast bit score: 306

high-affinity nickel-transporter
  
Accession: EHY60299
  
Location: 1246501-1248153
  
  
**BlastP hit with Mycgr3G108865**
  
Percentage identity: 59 %
  
BlastP bit score: 306
  
Sequence coverage: 60 %
  
E-value: 6e-94
  
  
 NCBI BlastP on this gene

EHY60299

CTP synthase
  
Accession: EHY60298
  
Location: 1244059-1245977
  
 NCBI BlastP on this gene

EHY60298

alkanesulfonate monooxygenase
  
Accession: EHY60297
  
Location: 1241592-1242920
  
 NCBI BlastP on this gene

EHY60297

hypothetical protein
  
Accession: EHY60296
  
Location: 1237198-1241089
  
 NCBI BlastP on this gene

EHY60296

229. :  JH126399 Cordyceps militaris CM01 unplaced genomic scaffold CCM\_S00001     Total score: 1.0     Cumulative Blast bit score: 301

Protein kinase-like domain
  
Accession: EGX95626
  
Location: 1008510-1011746
  
 NCBI BlastP on this gene

EGX95626

FAD dependent oxidoreductase
  
Accession: EGX95627
  
Location: 1012814-1014300
  
  
**BlastP hit with Mycgr3G57362**
  
Percentage identity: 40 %
  
BlastP bit score: 301
  
Sequence coverage: 99 %
  
E-value: 9e-93
  
  
 NCBI BlastP on this gene

EGX95627

hypothetical protein
  
Accession: EGX95628
  
Location: 1014377-1014746
  
 NCBI BlastP on this gene

EGX95628

hypothetical protein
  
Accession: EGX95629
  
Location: 1015516-1018185
  
 NCBI BlastP on this gene

EGX95629

Protein kinase-like domain
  
Accession: EGX95630
  
Location: 1020658-1021823
  
 NCBI BlastP on this gene

EGX95630

NADH-ubiquinone oxidoreductase 24 kDa subunit
  
Accession: EGX95631
  
Location: 1022030-1023171
  
 NCBI BlastP on this gene

EGX95631

230. :  CP003009 Thielavia terrestris NRRL 8126 chromosome 1     Total score: 1.0     Cumulative Blast bit score: 296

hypothetical protein
  
Accession: AEO62298
  
Location: 646127-648480
  
 NCBI BlastP on this gene

THITE\_2106325

hypothetical protein
  
Accession: AEO62297
  
Location: 643674-644183
  
 NCBI BlastP on this gene

THITE\_2106324

hypothetical protein
  
Accession: AEO62296
  
Location: 642590-643055
  
 NCBI BlastP on this gene

THITE\_2106323

hypothetical protein
  
Accession: AEO62295
  
Location: 640564-641969
  
 NCBI BlastP on this gene

THITE\_153800

hypothetical protein
  
Accession: AEO62294
  
Location: 637784-639803
  
  
**BlastP hit with Mycgr3G57362**
  
Percentage identity: 40 %
  
BlastP bit score: 296
  
Sequence coverage: 104 %
  
E-value: 2e-90
  
  
 NCBI BlastP on this gene

THITE\_2039081

hypothetical protein
  
Accession: AEO62293
  
Location: 633929-634489
  
 NCBI BlastP on this gene

THITE\_2106312

hypothetical protein
  
Accession: AEO62292
  
Location: 629216-630304
  
 NCBI BlastP on this gene

THITE\_2124977

231. :  ABDG02000029 Trichoderma atroviride IMI 206040     Total score: 1.0     Cumulative Blast bit score: 291

hypothetical protein
  
Accession: EHK39641
  
Location: 885633-885987
  
 NCBI BlastP on this gene

EHK39641

hypothetical protein
  
Accession: EHK39642
  
Location: 887671-891218
  
 NCBI BlastP on this gene

EHK39642

hypothetical protein
  
Accession: EHK39643
  
Location: 892507-893914
  
  
**BlastP hit with Mycgr3G57362**
  
Percentage identity: 41 %
  
BlastP bit score: 291
  
Sequence coverage: 95 %
  
E-value: 2e-89
  
  
 NCBI BlastP on this gene

EHK39643

232. :  DS572755 Paracoccidioides brasiliensis Pb18 supercont1.6 genomic scaffold     Total score: 1.0     Cumulative Blast bit score: 271

DDHD domain-containing protein
  
Accession: EEH49263
  
Location: 862141-866666
  
 NCBI BlastP on this gene

EEH49263

NADH-ubiquinone oxidoreductase 21.3 kDa subunit
  
Accession: EEH49264
  
Location: 867809-868516
  
 NCBI BlastP on this gene

EEH49264

conserved hypothetical protein
  
Accession: EEH49265
  
Location: 869716-870525
  
 NCBI BlastP on this gene

EEH49265

high-affinity nickel transport protein
  
Accession: EEH49266
  
Location: 871516-873006
  
  
**BlastP hit with Mycgr3G108865**
  
Percentage identity: 59 %
  
BlastP bit score: 271
  
Sequence coverage: 53 %
  
E-value: 1e-83
  
  
 NCBI BlastP on this gene

EEH49266

DNA polymerase beta
  
Accession: EEH49267
  
Location: 874673-876736
  
 NCBI BlastP on this gene

EEH49267

phospholipid-transporting ATPase
  
Accession: EEH49268
  
Location: 877449-881688
  
 NCBI BlastP on this gene

EEH49268

233. :  GL891302 Neurospora tetrasperma FGSC 2508 unplaced genomic scaffold NEUTE1scaffold\_1     Total score: 1.0     Cumulative Blast bit score: 264

hypothetical protein
  
Accession: EGO61428
  
Location: 4937978-4939924
  
 NCBI BlastP on this gene

EGO61428

hypothetical protein
  
Accession: EGO61429
  
Location: 4942890-4943402
  
 NCBI BlastP on this gene

EGO61429

hypothetical protein
  
Accession: EGO61430
  
Location: 4944023-4944476
  
 NCBI BlastP on this gene

EGO61430

hypothetical protein
  
Accession: EGO61431
  
Location: 4946148-4947236
  
 NCBI BlastP on this gene

EGO61431

hypothetical protein
  
Accession: EGO61432
  
Location: 4948412-4949926
  
  
**BlastP hit with Mycgr3G57362**
  
Percentage identity: 38 %
  
BlastP bit score: 264
  
Sequence coverage: 107 %
  
E-value: 3e-78
  
  
 NCBI BlastP on this gene

EGO61432

234. :  GL891107 Neurospora tetrasperma FGSC 2509 unplaced genomic scaffold NEUTE2scaffold\_2     Total score: 1.0     Cumulative Blast bit score: 264

hypothetical protein
  
Accession: EGZ74544
  
Location: 1217070-1219016
  
 NCBI BlastP on this gene

EGZ74544

hypothetical protein
  
Accession: EGZ74543
  
Location: 1213595-1214107
  
 NCBI BlastP on this gene

EGZ74543

hypothetical protein
  
Accession: EGZ74542
  
Location: 1212521-1212974
  
 NCBI BlastP on this gene

EGZ74542

eukaryotic translation initiation factor 2 beta subunit
  
Accession: EGZ74541
  
Location: 1209761-1210849
  
 NCBI BlastP on this gene

EGZ74541

FAD dependent oxidoreductase
  
Accession: EGZ74540
  
Location: 1207071-1208585
  
  
**BlastP hit with Mycgr3G57362**
  
Percentage identity: 38 %
  
BlastP bit score: 264
  
Sequence coverage: 107 %
  
E-value: 3e-78
  
  
 NCBI BlastP on this gene

EGZ74540

Metallo-dependent hydrolase
  
Accession: EGZ74539
  
Location: 1204129-1206029
  
 NCBI BlastP on this gene

EGZ74539

hypothetical protein
  
Accession: EGZ74538
  
Location: 1200862-1203487
  
 NCBI BlastP on this gene

EGZ74538

hypothetical protein
  
Accession: EGZ74537
  
Location: 1199321-1200245
  
 NCBI BlastP on this gene

EGZ74537

235. :  ADOT01000059 Arthrobotrys oligospora ATCC 24927     Total score: 1.0     Cumulative Blast bit score: 259

hypothetical protein
  
Accession: EGX52096
  
Location: 335880-339502
  
 NCBI BlastP on this gene

EGX52096

hypothetical protein
  
Accession: EGX52097
  
Location: 342478-343694
  
  
**BlastP hit with Mycgr3G108865**
  
Percentage identity: 53 %
  
BlastP bit score: 259
  
Sequence coverage: 58 %
  
E-value: 3e-78
  
  
 NCBI BlastP on this gene

EGX52097

hypothetical protein
  
Accession: EGX52098
  
Location: 344180-345822
  
 NCBI BlastP on this gene

EGX52098

hypothetical protein
  
Accession: EGX52099
  
Location: 347860-349523
  
 NCBI BlastP on this gene

EGX52099

hypothetical protein
  
Accession: EGX52100
  
Location: 350340-351539
  
 NCBI BlastP on this gene

EGX52100

hypothetical protein
  
Accession: EGX52101
  
Location: 351954-354206
  
 NCBI BlastP on this gene

EGX52101

236. :  KB446557 Pseudocercospora fijiensis CIRAD86 unplaced genomic scaffold MYCFIscaffold\_3     Total score: 1.0     Cumulative Blast bit score: 245

hypothetical protein
  
Accession: EME85143
  
Location: 5903096-5903698
  
  
**BlastP hit with Mycgr3G70471**
  
Percentage identity: 90 %
  
BlastP bit score: 245
  
Sequence coverage: 99 %
  
E-value: 8e-81
  
  
 NCBI BlastP on this gene

EME85143

hypothetical protein
  
Accession: EME85142
  
Location: 5901122-5901519
  
 NCBI BlastP on this gene

EME85142

hypothetical protein
  
Accession: EME85141
  
Location: 5898926-5901089
  
 NCBI BlastP on this gene

EME85141

hypothetical protein
  
Accession: EME85140
  
Location: 5896537-5897541
  
 NCBI BlastP on this gene

EME85140

hypothetical protein
  
Accession: EME85139
  
Location: 5894470-5895874
  
 NCBI BlastP on this gene

EME85139

237. :  CP000494 Bradyrhizobium sp. BTAi1     Total score: 1.0     Cumulative Blast bit score: 239

conjugal transfer protein trbE
  
Accession: ABQ35466
  
Location: 3523046-3525490
  
 NCBI BlastP on this gene

trbE

Conjugal transfer protein trbD
  
Accession: ABQ35465
  
Location: 3522754-3523035
  
 NCBI BlastP on this gene

trbD

conjugal transfer protein trbC
  
Accession: ABQ35464
  
Location: 3522425-3522754
  
 NCBI BlastP on this gene

trbC

conjugal transfer protein trbB
  
Accession: ABQ35463
  
Location: 3521448-3522428
  
 NCBI BlastP on this gene

trbB

Putative tonB-dependent receptor family (Outer membrane siderophore receptor) precursor
  
Accession: ABQ35462
  
Location: 3518690-3521131
  
 NCBI BlastP on this gene

BBta\_3362

putative ABC transporter (ATP-binding protein)
  
Accession: ABQ35461
  
Location: 3517807-3518526
  
 NCBI BlastP on this gene

BBta\_3361

putative membrane protein of unknown function
  
Accession: ABQ35460
  
Location: 3516408-3517805
  
 NCBI BlastP on this gene

BBta\_3360

putative membrane protein of unknown function
  
Accession: ABQ35459
  
Location: 3514917-3516392
  
 NCBI BlastP on this gene

BBta\_3359

high-affinity nickel-transport protein
  
Accession: ABQ35458
  
Location: 3513490-3514584
  
  
**BlastP hit with Mycgr3G108865**
  
Percentage identity: 38 %
  
BlastP bit score: 240
  
Sequence coverage: 69 %
  
E-value: 6e-71
  
  
 NCBI BlastP on this gene

nxiA

hypothetical protein
  
Accession: ABQ35457
  
Location: 3513056-3513493
  
 NCBI BlastP on this gene

BBta\_3357

Putative Conjugal transfer protein traG
  
Accession: ABQ35456
  
Location: 3511046-3513046
  
 NCBI BlastP on this gene

BBta\_3356

putative membrane protein of unknown function
  
Accession: ABQ35455
  
Location: 3509626-3510888
  
 NCBI BlastP on this gene

BBta\_3355

hypothetical protein
  
Accession: ABQ35454
  
Location: 3509038-3509433
  
 NCBI BlastP on this gene

BBta\_3353

putative alpha/beta-Hydrolases superfamily
  
Accession: ABQ35453
  
Location: 3508055-3508999
  
 NCBI BlastP on this gene

BBta\_3352

hypothetical protein
  
Accession: ABQ35452
  
Location: 3506148-3507899
  
 NCBI BlastP on this gene

BBta\_3351

putative Lytic transglycosylase
  
Accession: ABQ35451
  
Location: 3505071-3505901
  
 NCBI BlastP on this gene

BBta\_3350

conjugation peptidase TraF, Serine peptidase, MEROPS family S26C
  
Accession: ABQ35450
  
Location: 3504553-3505068
  
 NCBI BlastP on this gene

BBta\_3349

238. :  ACJE01000001 Aspergillus niger ATCC 1015     Total score: 1.0     Cumulative Blast bit score: 237

hypothetical protein
  
Accession: EHA28122
  
Location: 118177-119620
  
  
**BlastP hit with Mycgr3G57362**
  
Percentage identity: 33 %
  
BlastP bit score: 237
  
Sequence coverage: 96 %
  
E-value: 1e-68
  
  
 NCBI BlastP on this gene

EHA28122

hypothetical protein
  
Accession: EHA28121
  
Location: 113639-114925
  
 NCBI BlastP on this gene

EHA28121

hypothetical protein
  
Accession: EHA28120
  
Location: 112757-113329
  
 NCBI BlastP on this gene

EHA28120

hypothetical protein
  
Accession: EHA28119
  
Location: 109829-111562
  
 NCBI BlastP on this gene

EHA28119

239. :  AM920437 Penicillium chrysogenum Wisconsin 54-1255 complete genome, contig Pc00c22.     Total score: 1.0     Cumulative Blast bit score: 228

not annotated
  
Accession: CAP98998
  
Location: 4033866-4036870
  
 NCBI BlastP on this gene

Pc22g17100

not annotated
  
Accession: CAP98999
  
Location: 4037383-4038717
  
 NCBI BlastP on this gene

Pc22g17110

not annotated
  
Accession: CAP99000
  
Location: 4040204-4042665
  
 NCBI BlastP on this gene

Pc22g17120

not annotated
  
Accession: CAP99001
  
Location: 4043053-4044438
  
  
**BlastP hit with Mycgr3G57362**
  
Percentage identity: 33 %
  
BlastP bit score: 228
  
Sequence coverage: 98 %
  
E-value: 3e-65
  
  
 NCBI BlastP on this gene

Pc22g17130

240. :  JH226131 Exophiala dermatitidis NIH/UT8656 unplaced genomic scaffold supercont1.2     Total score: 1.0     Cumulative Blast bit score: 223

hypothetical protein
  
Accession: EHY54734
  
Location: 4002560-4002946
  
 NCBI BlastP on this gene

EHY54734

hypothetical protein
  
Accession: EHY54735
  
Location: 4005396-4006475
  
 NCBI BlastP on this gene

EHY54735

hypothetical protein
  
Accession: EHY54736
  
Location: 4007956-4010238
  
 NCBI BlastP on this gene

EHY54736

hypothetical protein
  
Accession: EHY54737
  
Location: 4011316-4012737
  
  
**BlastP hit with Mycgr3G57362**
  
Percentage identity: 33 %
  
BlastP bit score: 224
  
Sequence coverage: 101 %
  
E-value: 1e-63
  
  
 NCBI BlastP on this gene

EHY54737

241. :  ACJE01000012 Aspergillus niger ATCC 1015     Total score: 1.0     Cumulative Blast bit score: 223

hypothetical protein
  
Accession: EHA22338
  
Location: 476001-477620
  
  
**BlastP hit with Mycgr3G57362**
  
Percentage identity: 32 %
  
BlastP bit score: 224
  
Sequence coverage: 98 %
  
E-value: 1e-63
  
  
 NCBI BlastP on this gene

EHA22338

hypothetical protein
  
Accession: EHA22337
  
Location: 473629-475591
  
 NCBI BlastP on this gene

EHA22337

hypothetical protein
  
Accession: EHA22336
  
Location: 472270-473304
  
 NCBI BlastP on this gene

EHA22336

hypothetical protein
  
Accession: EHA22335
  
Location: 469787-471367
  
 NCBI BlastP on this gene

EHA22335

242. :  KB726307 Fusarium oxysporum f. sp. cubense race 4 unplaced genomic scaffold scaffold26     Total score: 1.0     Cumulative Blast bit score: 223

Purine-cytosine permease FCY21
  
Accession: EMT71627
  
Location: 1729203-1730871
  
 NCBI BlastP on this gene

EMT71627

hypothetical protein
  
Accession: EMT71628
  
Location: 1732899-1733326
  
 NCBI BlastP on this gene

EMT71628

3-oxoadipate enol-lactonase 2
  
Accession: EMT71629
  
Location: 1733856-1734677
  
 NCBI BlastP on this gene

EMT71629

hypothetical protein
  
Accession: EMT71630
  
Location: 1736117-1736455
  
 NCBI BlastP on this gene

EMT71630

hypothetical protein
  
Accession: EMT71631
  
Location: 1737778-1738350
  
 NCBI BlastP on this gene

EMT71631

Gamma-glutamylputrescine oxidoreductase
  
Accession: EMT71632
  
Location: 1739000-1740556
  
  
**BlastP hit with Mycgr3G57362**
  
Percentage identity: 32 %
  
BlastP bit score: 223
  
Sequence coverage: 98 %
  
E-value: 2e-63
  
  
 NCBI BlastP on this gene

EMT71632

243. :  KB644412 Penicillium oxalicum 114-2 unplaced genomic scaffold scaffold\_5     Total score: 1.0     Cumulative Blast bit score: 220

hypothetical protein
  
Accession: EPS30040
  
Location: 1593446-1595954
  
 NCBI BlastP on this gene

EPS30040

hypothetical protein
  
Accession: EPS30041
  
Location: 1596035-1596757
  
 NCBI BlastP on this gene

EPS30041

hypothetical protein
  
Accession: EPS30042
  
Location: 1597849-1599498
  
 NCBI BlastP on this gene

EPS30042

hypothetical protein
  
Accession: EPS30043
  
Location: 1600204-1601751
  
  
**BlastP hit with Mycgr3G57362**
  
Percentage identity: 33 %
  
BlastP bit score: 220
  
Sequence coverage: 96 %
  
E-value: 8e-62
  
  
 NCBI BlastP on this gene

EPS30043

hypothetical protein
  
Accession: EPS30044
  
Location: 1602120-1605245
  
 NCBI BlastP on this gene

EPS30044

hypothetical protein
  
Accession: EPS30045
  
Location: 1606656-1607780
  
 NCBI BlastP on this gene

EPS30045

hypothetical protein
  
Accession: EPS30046
  
Location: 1609063-1612406
  
 NCBI BlastP on this gene

EPS30046

244. :  CP003009 Thielavia terrestris NRRL 8126 chromosome 1     Total score: 1.0     Cumulative Blast bit score: 220

hypothetical protein
  
Accession: AEO64380
  
Location: 8446410-8448225
  
  
**BlastP hit with Mycgr3G57362**
  
Percentage identity: 34 %
  
BlastP bit score: 220
  
Sequence coverage: 100 %
  
E-value: 6e-62
  
  
 NCBI BlastP on this gene

THITE\_2042085

hypothetical protein
  
Accession: AEO64379
  
Location: 8443397-8445086
  
 NCBI BlastP on this gene

THITE\_2041902

hypothetical protein
  
Accession: AEO64378
  
Location: 8441120-8442611
  
 NCBI BlastP on this gene

THITE\_2110483

hypothetical protein
  
Accession: AEO64377
  
Location: 8439218-8439933
  
 NCBI BlastP on this gene

THITE\_2110481

245. :  HF679032 Fusarium fujikuroi IMI 58289 draft genome, chromosome FFUJ\_chr10.     Total score: 1.0     Cumulative Blast bit score: 217

related to oxidoreductase
  
Accession: CCT75015
  
Location: 2273854-2275409
  
  
**BlastP hit with Mycgr3G57362**
  
Percentage identity: 33 %
  
BlastP bit score: 217
  
Sequence coverage: 100 %
  
E-value: 4e-61
  
  
 NCBI BlastP on this gene

FFUJ\_11090

uncharacterized protein
  
Accession: CCT75014
  
Location: 2273137-2273625
  
 NCBI BlastP on this gene

FFUJ\_11089

uncharacterized protein
  
Accession: CCT75013
  
Location: 2271411-2272550
  
 NCBI BlastP on this gene

FFUJ\_11088

246. :  GG704912 Coccidioides immitis RS genomic scaffold supercont3.2     Total score: 1.0     Cumulative Blast bit score: 217

hypothetical protein
  
Accession: EAS31716
  
Location: 3697547-3699137
  
  
**BlastP hit with Mycgr3G57362**
  
Percentage identity: 33 %
  
BlastP bit score: 217
  
Sequence coverage: 98 %
  
E-value: 4e-61
  
  
 NCBI BlastP on this gene

EAS31716

RNA-binding La domain-containing protein
  
Accession: EAS31715
  
Location: 3695690-3697158
  
 NCBI BlastP on this gene

EAS31715

chitin synthase class VI
  
Accession: EAS31713
  
Location: 3690677-3693254
  
 NCBI BlastP on this gene

EAS31713

hsp88-like protein
  
Accession: EAS31711
  
Location: 3686899-3689522
  
 NCBI BlastP on this gene

EAS31711

247. :  ACFW01000025 Coccidioides posadasii C735 delta SOWgp     Total score: 1.0     Cumulative Blast bit score: 217

FAD dependent oxidoreductase family protein
  
Accession: EER27931
  
Location: 3370382-3371971
  
  
**BlastP hit with Mycgr3G57362**
  
Percentage identity: 33 %
  
BlastP bit score: 217
  
Sequence coverage: 98 %
  
E-value: 4e-61
  
  
 NCBI BlastP on this gene

EER27931

La domain containing protein
  
Accession: EER27930
  
Location: 3368520-3369994
  
 NCBI BlastP on this gene

EER27930

Chitin synthase D , putative
  
Accession: EER27929
  
Location: 3363499-3366079
  
 NCBI BlastP on this gene

EER27929

Heat shock protein, putative
  
Accession: EER27928
  
Location: 3359683-3362341
  
 NCBI BlastP on this gene

EER27928

248. :  KE145352 Glarea lozoyensis ATCC 20868 chromosome Unknown GLAREA1     Total score: 1.0     Cumulative Blast bit score: 216

hypothetical protein
  
Accession: EPE36636
  
Location: 433483-435552
  
 NCBI BlastP on this gene

EPE36636

hypothetical protein
  
Accession: EPE36635
  
Location: 430655-432306
  
 NCBI BlastP on this gene

EPE36635

Zn-dependent exopeptidase
  
Accession: EPE36634
  
Location: 428147-429758
  
 NCBI BlastP on this gene

EPE36634

FAD/NAD(P)-binding protein
  
Accession: EPE36633
  
Location: 424122-425658
  
  
**BlastP hit with Mycgr3G57362**
  
Percentage identity: 32 %
  
BlastP bit score: 216
  
Sequence coverage: 100 %
  
E-value: 7e-61
  
  
 NCBI BlastP on this gene

EPE36633

hypothetical protein
  
Accession: EPE36632
  
Location: 423093-423671
  
 NCBI BlastP on this gene

EPE36632

hypothetical protein
  
Accession: EPE36631
  
Location: 420934-422010
  
 NCBI BlastP on this gene

EPE36631

ATP synthase subunit 4, mitochondrial precursor
  
Accession: EPE36630
  
Location: 419331-420229
  
 NCBI BlastP on this gene

EPE36630

hypothetical protein
  
Accession: EPE36629
  
Location: 417386-419122
  
 NCBI BlastP on this gene

EPE36629

HCP-like protein
  
Accession: EPE36628
  
Location: 415396-416949
  
 NCBI BlastP on this gene

EPE36628

249. :  AEOI01000007 Ogataea parapolymorpha DL-1     Total score: 1.0     Cumulative Blast bit score: 216

Na+/Pi cotransporter, active in early growth phase
  
Accession: EFW96479
  
Location: 777-2483
  
 NCBI BlastP on this gene

EFW96479

oxidoreductase, aldo/keto reductase family protein
  
Accession: EFW96480
  
Location: 2882-3814
  
 NCBI BlastP on this gene

EFW96480

pantothenate transporter, putative
  
Accession: EFW96481
  
Location: 3941-5506
  
 NCBI BlastP on this gene

EFW96481

FAD dependent oxidoreductase superfamily
  
Accession: EFW96482
  
Location: 5685-7019
  
  
**BlastP hit with Mycgr3G57362**
  
Percentage identity: 34 %
  
BlastP bit score: 216
  
Sequence coverage: 98 %
  
E-value: 5e-61
  
  
 NCBI BlastP on this gene

EFW96482

2-haloalkanoic acid dehalogenase, putative
  
Accession: EFW96483
  
Location: 7160-7882
  
 NCBI BlastP on this gene

EFW96483

transcriptional activator protein, putative
  
Accession: EFW96484
  
Location: 7898-8977
  
 NCBI BlastP on this gene

EFW96484

aryl-alcohol dehydrogenase (AAD4)
  
Accession: EFW96485
  
Location: 10032-11108
  
 NCBI BlastP on this gene

EFW96485

Histidine acid phosphatase family protein
  
Accession: EFW96486
  
Location: 11490-12704
  
 NCBI BlastP on this gene

EFW96486

Gly-X carboxypeptidase
  
Accession: EFW96487
  
Location: 12750-14408
  
 NCBI BlastP on this gene

EFW96487

siderophore-iron transporter Str3
  
Accession: EFW96488
  
Location: 15219-18052
  
 NCBI BlastP on this gene

EFW96488

250. :  KE148151 Ophiostoma piceae UAMH 11346 chromosome Unknown scf06     Total score: 1.0     Cumulative Blast bit score: 214

amidohydrolase domain containing protein
  
Accession: EPE07448
  
Location: 1405314-1406662
  
 NCBI BlastP on this gene

EPE07448

cytochrome p450
  
Accession: EPE07449
  
Location: 1406927-1408746
  
 NCBI BlastP on this gene

EPE07449

short-chain dehydrogenase
  
Accession: EPE07450
  
Location: 1409217-1410231
  
 NCBI BlastP on this gene

EPE07450

mfs general substrate transporter
  
Accession: EPE07451
  
Location: 1410571-1412603
  
 NCBI BlastP on this gene

EPE07451

fad dependent oxidoreductase
  
Accession: EPE07452
  
Location: 1413198-1414742
  
  
**BlastP hit with Mycgr3G57362**
  
Percentage identity: 35 %
  
BlastP bit score: 214
  
Sequence coverage: 99 %
  
E-value: 8e-60
  
  
 NCBI BlastP on this gene

EPE07452

Detecting sequence homology at the gene cluster level with MultiGeneBlast.
  
Marnix H. Medema, Rainer Breitling & Eriko Takano (2013)
  
*Molecular Biology and Evolution* , 30: 1218-1223.
