# Supplementary material for: In silico prediction and characterization of secondary metabolite biosynthetic gene clusters in the wheat pathogen Zymoseptoria tritici
Source: BMC Genomics. 2017 Aug 17;18:631. doi: 10.1186/s12864-017-3969-y (PMC5561558; doi:10.1186/s12864-017-3969-y)
Supplement: Supplementary file 1 — MultiGeneBLAST analysis of putative secondary metabolite clusters. All encoded amino acid sequences from genes residing in clusters predicted by AntiSMASH are given as FASTA file format. All output data from MultiGeneBLASTs are also provided. (ZIP 42911 kb) [file 12864_2017_3969_MOESM1_ESM.zip › Cluster MultiGene BLAST/out/Clusters_1_34/Cluster_14/displaypage1.xhtml]

xml version="1.0" encoding="UTF-8"?


Search Results
  
  
 Results pages: 1, 2, 3, 4, 5

**MultiGeneBlast hits**

Select gene cluster alignment
1. CM001199\_0 Mycosphaerella graminicola IPO323 chromosome 4, whole genome sh...
2. GG698929\_0 Nectria haematococca mpVI 77-13-4 chromosome 11 genomic scaffol...
3. AFNW01000059\_0 Fusarium pseudograminearum CS3096, whole genome shotgun seq...
4. CH408030\_0 Chaetomium globosum CBS 148.51 scaffold\_2 genomic scaffold, who...
5. KB730104\_0 Fusarium oxysporum f. sp. cubense race 1 unplaced genomic scaff...
6. DF238784\_0 Pseudozyma hubeiensis SY62 DNA, scaffold: PHS21, whole genome s...
7. AACP01000049\_0 Ustilago maydis 521, whole genome shotgun sequencing project.
8. DF196790\_0 Pseudozyma antarctica T-34 DNA, contig: scaffold00024, whole ge...
9. FQ311441\_0 Sporisorium reilianum SRZ2 chromosome 2 complete DNA sequence.
10. JH687382\_0 Stereum hirsutum FP-91666 SS1 unplaced genomic scaffold STEHIs...
11. KB726570\_0 Fusarium oxysporum f. sp. cubense race 4 unplaced genomic scaf...
12. KB456264\_0 Mycosphaerella populorum SO2202 unplaced genomic scaffold SEPM...
13. CH476600\_0 Aspergillus terreus NIH2624 scaffold\_7 genomic scaffold, whole...
14. AACD01000007\_0 Aspergillus nidulans FGSC A4, whole genome shotgun sequenc...
15. AHHD01000417\_0 Macrophomina phaseolina MS6, whole genome shotgun sequenci...
16. KB915996\_0 Neofusicoccum parvum UCRNP2 chromosome Unknown NP2\_03\_scaffold...
17. DS499596\_1 Aspergillus fumigatus A1163 scf\_000003 genomic scaffold, whole...
18. DS027697\_1 Neosartorya fischeri NRRL 181 1099437636265 genomic scaffold, ...
19. AAHF01000010\_1 Aspergillus fumigatus Af293, whole genome shotgun sequenci...
20. HE605208\_0 Candida parapsilosis strain CDC317 annotated contig 006372.
21. GL377303\_0 Schizophyllum commune H4-8 unplaced genomic scaffold SCHCOscaf...
22. EQ963479\_0 Aspergillus flavus NRRL3357 scf\_1106286418500 genomic scaffold...
23. AP007154\_0 Aspergillus oryzae RIB40 DNA, SC001.
24. CAOJ01000067\_0 Rhizoctonia solani AG-1 IB strain isolate 7/3/14, whole ge...
25. EQ963475\_1 Aspergillus flavus NRRL3357 scf\_1106286419142 genomic scaffold...
26. AP007157\_1 Aspergillus oryzae RIB40 DNA, SC023.
27. AKHY01000182\_1 Aspergillus oryzae 3.042, whole genome shotgun sequencing ...
28. JH687874\_0 Auricularia delicata TFB-10046 SS5 unplaced genomic scaffold A...
29. GL985075\_0 Trichoderma reesei QM6a unplaced genomic scaffold TRIREscaffol...
30. AM920431\_1 Penicillium chrysogenum Wisconsin 54-1255 complete genome, con...
31. KB916185\_0 Neofusicoccum parvum UCRNP2 chromosome Unknown NP2\_03\_scaffold...
32. HF679032\_0 Fusarium fujikuroi IMI 58289 draft genome, chromosome FFUJ\_chr10.
33. KB730325\_0 Fusarium oxysporum f. sp. cubense race 1 unplaced genomic scaf...
34. KB726307\_0 Fusarium oxysporum f. sp. cubense race 4 unplaced genomic scaf...
35. CP003002\_0 Myceliophthora thermophila ATCC 42464 chromosome 1, complete s...
36. ABDF02000085\_0 Trichoderma virens Gv29-8, whole genome shotgun sequencing...
37. AFQF01003643\_0 Fusarium oxysporum Fo5176, whole genome shotgun sequencing...
38. GG704911\_1 Coccidioides immitis RS genomic scaffold supercont3.1, whole g...
39. ACFW01000049\_1 Coccidioides posadasii C735 delta SOWgp, whole genome shot...
40. CH476615\_1 Uncinocarpus reesii 1704 scaffold\_1 genomic scaffold, whole ge...
41. KB644411\_1 Penicillium oxalicum 114-2 unplaced genomic scaffold scaffold\_...
42. EQ962653\_1 Talaromyces stipitatus ATCC 10500 scf\_1105507295527 genomic sc...
43. DS995701\_0 Microsporum canis CBS 113480 supercont1.1 genomic scaffold, wh...
44. AM920428\_1 Penicillium chrysogenum Wisconsin 54-1255 complete genome, con...
45. JH126399\_2 Cordyceps militaris CM01 unplaced genomic scaffold CCM\_S00001,...
46. GL377303\_1 Schizophyllum commune H4-8 unplaced genomic scaffold SCHCOscaf...
47. DS995900\_0 Penicillium marneffei ATCC 18224 scf\_1105668340758 genomic sca...
48. DS990636\_1 Ajellomyces capsulatus H88 supercont1.1 genomic scaffold, whol...
49. CP003009\_1 Thielavia terrestris NRRL 8126 chromosome 1, complete sequence.
50. KE375213\_0 Blumeria graminis f. sp. tritici 96224 unplaced genomic scaffo...

Query: Architecture Search FASTA input

CM001199 : Mycosphaerella graminicola IPO323 chromosome 4    Total score: 7.0     Cumulative Blast bit score: 7628

Hit cluster cross-links:

Mycgr3G41235 Mycgr3T
  
Location: 0-4062

Mycgr3G41235\_Mycgr3T

Mycgr3G70577 Mycgr3T
  
Location: 4162-6109

Mycgr3G70577\_Mycgr3T

Mycgr3G40534 Mycgr3T
  
Location: 6209-7166

Mycgr3G40534\_Mycgr3T

Mycgr3G85486 Mycgr3T
  
Location: 7266-8511

Mycgr3G85486\_Mycgr3T

Mycgr3G92221 Mycgr3T
  
Location: 8611-9193

Mycgr3G92221\_Mycgr3T

Mycgr3G39931 Mycgr3T
  
Location: 9293-10157

Mycgr3G39931\_Mycgr3T

Mycgr3G99766 Mycgr3T
  
Location: 10257-11775

Mycgr3G99766\_Mycgr3T

signal-peptide-containing protein
  
Accession: EGP88587
  
Location: 12877-13759
  
  
**BlastP hit with Mycgr3G92221\_Mycgr3T**
  
Percentage identity: 100 %
  
BlastP bit score: 395
  
Sequence coverage: 99 %
  
E-value: 3e-138
  
  
 NCBI BlastP on this gene

EGP88587

Non-ribosomal peptide synthetase
  
Accession: EGP88586
  
Location: 15580-30095
  
  
**BlastP hit with Mycgr3G40534\_Mycgr3T**
  
Percentage identity: 100 %
  
BlastP bit score: 667
  
Sequence coverage: 100 %
  
E-value: 0.0
  
  
  
**BlastP hit with Mycgr3G39931\_Mycgr3T**
  
Percentage identity: 100 %
  
BlastP bit score: 607
  
Sequence coverage: 100 %
  
E-value: 0.0
  
  
 NCBI BlastP on this gene

EGP88586

putative siderophore biosynthesis
  
Accession: EGP87766
  
Location: 30645-32000
  
  
**BlastP hit with Mycgr3G85486\_Mycgr3T**
  
Percentage identity: 100 %
  
BlastP bit score: 860
  
Sequence coverage: 99 %
  
E-value: 0.0
  
  
 NCBI BlastP on this gene

EGP87766

putative siderophore-dependent iron transporter
  
Accession: EGP87767
  
Location: 33143-35151
  
  
**BlastP hit with Mycgr3G70577\_Mycgr3T**
  
Percentage identity: 100 %
  
BlastP bit score: 1330
  
Sequence coverage: 99 %
  
E-value: 0.0
  
  
 NCBI BlastP on this gene

EGP87767

putative ABC transporter
  
Accession: EGP87768
  
Location: 36524-40624
  
  
**BlastP hit with Mycgr3G41235\_Mycgr3T**
  
Percentage identity: 100 %
  
BlastP bit score: 2751
  
Sequence coverage: 99 %
  
E-value: 0.0
  
  
 NCBI BlastP on this gene

EGP87768

MFS1 putative major facilitator superfamily transporter
  
Accession: EGP87769
  
Location: 41788-43948
  
  
**BlastP hit with Mycgr3G99766\_Mycgr3T**
  
Percentage identity: 100 %
  
BlastP bit score: 1018
  
Sequence coverage: 99 %
  
E-value: 0.0
  
  
 NCBI BlastP on this gene

EGP87769

Query: Architecture Search FASTA input

GG698929 : Nectria haematococca mpVI 77-13-4 chromosome 11 genomic scaffold NECHAsca\_39\_chr11\_5\_0    Total score: 5.0     Cumulative Blast bit score: 2803

Hit cluster cross-links:

Mycgr3G41235 Mycgr3T
  
Location: 0-4062

Mycgr3G41235\_Mycgr3T

Mycgr3G70577 Mycgr3T
  
Location: 4162-6109

Mycgr3G70577\_Mycgr3T

Mycgr3G40534 Mycgr3T
  
Location: 6209-7166

Mycgr3G40534\_Mycgr3T

Mycgr3G85486 Mycgr3T
  
Location: 7266-8511

Mycgr3G85486\_Mycgr3T

Mycgr3G92221 Mycgr3T
  
Location: 8611-9193

Mycgr3G92221\_Mycgr3T

Mycgr3G39931 Mycgr3T
  
Location: 9293-10157

Mycgr3G39931\_Mycgr3T

Mycgr3G99766 Mycgr3T
  
Location: 10257-11775

Mycgr3G99766\_Mycgr3T

predicted protein
  
Accession: EEU36395
  
Location: 314386-315252
  
 NCBI BlastP on this gene

EEU36395

hypothetical protein
  
Accession: EEU36328
  
Location: 312227-314170
  
  
**BlastP hit with Mycgr3G70577\_Mycgr3T**
  
Percentage identity: 54 %
  
BlastP bit score: 658
  
Sequence coverage: 91 %
  
E-value: 0.0
  
  
 NCBI BlastP on this gene

EEU36328

hypothetical protein
  
Accession: EEU36327
  
Location: 306959-311185
  
  
**BlastP hit with Mycgr3G41235\_Mycgr3T**
  
Percentage identity: 44 %
  
BlastP bit score: 1117
  
Sequence coverage: 102 %
  
E-value: 0.0
  
  
 NCBI BlastP on this gene

EEU36327

hypothetical protein
  
Accession: EEU36394
  
Location: 304750-306030
  
  
**BlastP hit with Mycgr3G85486\_Mycgr3T**
  
Percentage identity: 57 %
  
BlastP bit score: 482
  
Sequence coverage: 103 %
  
E-value: 3e-165
  
  
 NCBI BlastP on this gene

EEU36394

hypothetical protein
  
Accession: EEU36393
  
Location: 290155-304310
  
  
**BlastP hit with Mycgr3G40534\_Mycgr3T**
  
Percentage identity: 52 %
  
BlastP bit score: 273
  
Sequence coverage: 81 %
  
E-value: 1e-78
  
  
  
**BlastP hit with Mycgr3G39931\_Mycgr3T**
  
Percentage identity: 49 %
  
BlastP bit score: 273
  
Sequence coverage: 96 %
  
E-value: 7e-79
  
  
 NCBI BlastP on this gene

EEU36393

Query: Architecture Search FASTA input

AFNW01000059 : Fusarium pseudograminearum CS3096    Total score: 5.0     Cumulative Blast bit score: 2796

Hit cluster cross-links:

Mycgr3G41235 Mycgr3T
  
Location: 0-4062

Mycgr3G41235\_Mycgr3T

Mycgr3G70577 Mycgr3T
  
Location: 4162-6109

Mycgr3G70577\_Mycgr3T

Mycgr3G40534 Mycgr3T
  
Location: 6209-7166

Mycgr3G40534\_Mycgr3T

Mycgr3G85486 Mycgr3T
  
Location: 7266-8511

Mycgr3G85486\_Mycgr3T

Mycgr3G92221 Mycgr3T
  
Location: 8611-9193

Mycgr3G92221\_Mycgr3T

Mycgr3G39931 Mycgr3T
  
Location: 9293-10157

Mycgr3G39931\_Mycgr3T

Mycgr3G99766 Mycgr3T
  
Location: 10257-11775

Mycgr3G99766\_Mycgr3T

hypothetical protein
  
Accession: EKJ77375
  
Location: 159625-161541
  
  
**BlastP hit with Mycgr3G70577\_Mycgr3T**
  
Percentage identity: 52 %
  
BlastP bit score: 654
  
Sequence coverage: 95 %
  
E-value: 0.0
  
  
 NCBI BlastP on this gene

EKJ77375

hypothetical protein
  
Accession: EKJ77376
  
Location: 162661-166859
  
  
**BlastP hit with Mycgr3G41235\_Mycgr3T**
  
Percentage identity: 44 %
  
BlastP bit score: 1160
  
Sequence coverage: 103 %
  
E-value: 0.0
  
  
 NCBI BlastP on this gene

EKJ77376

hypothetical protein
  
Accession: EKJ77377
  
Location: 167780-169063
  
  
**BlastP hit with Mycgr3G85486\_Mycgr3T**
  
Percentage identity: 53 %
  
BlastP bit score: 439
  
Sequence coverage: 100 %
  
E-value: 3e-148
  
  
 NCBI BlastP on this gene

EKJ77377

NPS1
  
Accession: EKJ77378
  
Location: 169631-183939
  
  
**BlastP hit with Mycgr3G40534\_Mycgr3T**
  
Percentage identity: 53 %
  
BlastP bit score: 268
  
Sequence coverage: 78 %
  
E-value: 1e-76
  
  
  
**BlastP hit with Mycgr3G39931\_Mycgr3T**
  
Percentage identity: 47 %
  
BlastP bit score: 275
  
Sequence coverage: 99 %
  
E-value: 9e-80
  
  
 NCBI BlastP on this gene

EKJ77378

TRI15
  
Accession: EKJ77379
  
Location: 185439-186368
  
 NCBI BlastP on this gene

EKJ77379

Query: Architecture Search FASTA input

CH408030 : Chaetomium globosum CBS 148.51 scaffold\_2 genomic scaffold    Total score: 5.0     Cumulative Blast bit score: 2769

Hit cluster cross-links:

Mycgr3G41235 Mycgr3T
  
Location: 0-4062

Mycgr3G41235\_Mycgr3T

Mycgr3G70577 Mycgr3T
  
Location: 4162-6109

Mycgr3G70577\_Mycgr3T

Mycgr3G40534 Mycgr3T
  
Location: 6209-7166

Mycgr3G40534\_Mycgr3T

Mycgr3G85486 Mycgr3T
  
Location: 7266-8511

Mycgr3G85486\_Mycgr3T

Mycgr3G92221 Mycgr3T
  
Location: 8611-9193

Mycgr3G92221\_Mycgr3T

Mycgr3G39931 Mycgr3T
  
Location: 9293-10157

Mycgr3G39931\_Mycgr3T

Mycgr3G99766 Mycgr3T
  
Location: 10257-11775

Mycgr3G99766\_Mycgr3T

hypothetical protein
  
Accession: EAQ90321
  
Location: 405316-407076
  
 NCBI BlastP on this gene

EAQ90321

predicted protein
  
Accession: EAQ90320
  
Location: 404582-404908
  
 NCBI BlastP on this gene

EAQ90320

hypothetical protein
  
Accession: EAQ90319
  
Location: 396559-402878
  
  
**BlastP hit with Mycgr3G70577\_Mycgr3T**
  
Percentage identity: 55 %
  
BlastP bit score: 684
  
Sequence coverage: 97 %
  
E-value: 0.0
  
  
 NCBI BlastP on this gene

EAQ90319

hypothetical protein
  
Accession: EAQ90318
  
Location: 388577-395459
  
  
**BlastP hit with Mycgr3G41235\_Mycgr3T**
  
Percentage identity: 42 %
  
BlastP bit score: 1117
  
Sequence coverage: 105 %
  
E-value: 0.0
  
  
 NCBI BlastP on this gene

EAQ90318

hypothetical protein
  
Accession: EAQ90317
  
Location: 381683-385419
  
  
**BlastP hit with Mycgr3G85486\_Mycgr3T**
  
Percentage identity: 54 %
  
BlastP bit score: 440
  
Sequence coverage: 102 %
  
E-value: 2e-145
  
  
 NCBI BlastP on this gene

EAQ90317

hypothetical protein
  
Accession: EAQ90316
  
Location: 366073-380944
  
  
**BlastP hit with Mycgr3G40534\_Mycgr3T**
  
Percentage identity: 54 %
  
BlastP bit score: 263
  
Sequence coverage: 80 %
  
E-value: 3e-75
  
  
  
**BlastP hit with Mycgr3G39931\_Mycgr3T**
  
Percentage identity: 48 %
  
BlastP bit score: 265
  
Sequence coverage: 100 %
  
E-value: 5e-76
  
  
 NCBI BlastP on this gene

EAQ90316

predicted protein
  
Accession: EAQ90315
  
Location: 363770-364526
  
 NCBI BlastP on this gene

EAQ90315

Query: Architecture Search FASTA input

KB730104 : Fusarium oxysporum f. sp. cubense race 1 unplaced genomic scaffold scaffold59    Total score: 5.0     Cumulative Blast bit score: 2664

Hit cluster cross-links:

Mycgr3G41235 Mycgr3T
  
Location: 0-4062

Mycgr3G41235\_Mycgr3T

Mycgr3G70577 Mycgr3T
  
Location: 4162-6109

Mycgr3G70577\_Mycgr3T

Mycgr3G40534 Mycgr3T
  
Location: 6209-7166

Mycgr3G40534\_Mycgr3T

Mycgr3G85486 Mycgr3T
  
Location: 7266-8511

Mycgr3G85486\_Mycgr3T

Mycgr3G92221 Mycgr3T
  
Location: 8611-9193

Mycgr3G92221\_Mycgr3T

Mycgr3G39931 Mycgr3T
  
Location: 9293-10157

Mycgr3G39931\_Mycgr3T

Mycgr3G99766 Mycgr3T
  
Location: 10257-11775

Mycgr3G99766\_Mycgr3T

Ferric reductase transmembrane component 5
  
Accession: ENH72071
  
Location: 86430-88576
  
 NCBI BlastP on this gene

ENH72071

Siderophore iron transporter 3
  
Accession: ENH72072
  
Location: 89324-91243
  
  
**BlastP hit with Mycgr3G70577\_Mycgr3T**
  
Percentage identity: 51 %
  
BlastP bit score: 642
  
Sequence coverage: 96 %
  
E-value: 0.0
  
  
 NCBI BlastP on this gene

ENH72072

Multidrug resistance-associated protein 1
  
Accession: ENH72073
  
Location: 92480-96655
  
  
**BlastP hit with Mycgr3G41235\_Mycgr3T**
  
Percentage identity: 42 %
  
BlastP bit score: 1028
  
Sequence coverage: 104 %
  
E-value: 0.0
  
  
 NCBI BlastP on this gene

ENH72073

Putative lysine N-acyltransferase C17G9.06c
  
Accession: ENH72074
  
Location: 97820-99118
  
  
**BlastP hit with Mycgr3G85486\_Mycgr3T**
  
Percentage identity: 56 %
  
BlastP bit score: 462
  
Sequence coverage: 102 %
  
E-value: 4e-157
  
  
 NCBI BlastP on this gene

ENH72074

Ferrichrome siderophore peptide synthetase
  
Accession: ENH72075
  
Location: 99731-114015
  
  
**BlastP hit with Mycgr3G40534\_Mycgr3T**
  
Percentage identity: 52 %
  
BlastP bit score: 266
  
Sequence coverage: 80 %
  
E-value: 5e-76
  
  
  
**BlastP hit with Mycgr3G39931\_Mycgr3T**
  
Percentage identity: 48 %
  
BlastP bit score: 266
  
Sequence coverage: 99 %
  
E-value: 2e-76
  
  
 NCBI BlastP on this gene

ENH72075

Endo-1,4-beta-xylanase
  
Accession: ENH72076
  
Location: 115053-116284
  
 NCBI BlastP on this gene

ENH72076

Query: Architecture Search FASTA input

DF238784 : Pseudozyma hubeiensis SY62 DNA, scaffold: PHS21    Total score: 5.0     Cumulative Blast bit score: 2235

Hit cluster cross-links:

Mycgr3G41235 Mycgr3T
  
Location: 0-4062

Mycgr3G41235\_Mycgr3T

Mycgr3G70577 Mycgr3T
  
Location: 4162-6109

Mycgr3G70577\_Mycgr3T

Mycgr3G40534 Mycgr3T
  
Location: 6209-7166

Mycgr3G40534\_Mycgr3T

Mycgr3G85486 Mycgr3T
  
Location: 7266-8511

Mycgr3G85486\_Mycgr3T

Mycgr3G92221 Mycgr3T
  
Location: 8611-9193

Mycgr3G92221\_Mycgr3T

Mycgr3G39931 Mycgr3T
  
Location: 9293-10157

Mycgr3G39931\_Mycgr3T

Mycgr3G99766 Mycgr3T
  
Location: 10257-11775

Mycgr3G99766\_Mycgr3T

hypothetical protein
  
Accession: GAC94348
  
Location: 5664-7052
  
 NCBI BlastP on this gene

GAC94348

siderophore iron transporter
  
Accession: GAC94349
  
Location: 8011-10057
  
  
**BlastP hit with Mycgr3G70577\_Mycgr3T**
  
Percentage identity: 36 %
  
BlastP bit score: 379
  
Sequence coverage: 98 %
  
E-value: 2e-118
  
  
 NCBI BlastP on this gene

GAC94349

ABC transporter
  
Accession: GAC94350
  
Location: 11714-15805
  
  
**BlastP hit with Mycgr3G41235\_Mycgr3T**
  
Percentage identity: 43 %
  
BlastP bit score: 1094
  
Sequence coverage: 101 %
  
E-value: 0.0
  
  
 NCBI BlastP on this gene

GAC94350

aerobactin siderophore biosynthesis protein
  
Accession: GAC94351
  
Location: 17131-18612
  
  
**BlastP hit with Mycgr3G85486\_Mycgr3T**
  
Percentage identity: 43 %
  
BlastP bit score: 322
  
Sequence coverage: 98 %
  
E-value: 3e-102
  
  
 NCBI BlastP on this gene

GAC94351

enoyl-CoA hydratase/isomerase
  
Accession: GAC94352
  
Location: 18825-19779
  
 NCBI BlastP on this gene

GAC94352

non-ribosomal peptide synthetase
  
Accession: GAC94353
  
Location: 21418-35944
  
  
**BlastP hit with Mycgr3G40534\_Mycgr3T**
  
Percentage identity: 49 %
  
BlastP bit score: 221
  
Sequence coverage: 83 %
  
E-value: 2e-60
  
  
  
**BlastP hit with Mycgr3G39931\_Mycgr3T**
  
Percentage identity: 47 %
  
BlastP bit score: 219
  
Sequence coverage: 89 %
  
E-value: 4e-60
  
  
 NCBI BlastP on this gene

GAC94353

hypothetical protein
  
Accession: GAC94354
  
Location: 36133-37897
  
 NCBI BlastP on this gene

GAC94354

Query: Architecture Search FASTA input

AACP01000049 : Ustilago maydis 521    Total score: 5.0     Cumulative Blast bit score: 2213

Hit cluster cross-links:

Mycgr3G41235 Mycgr3T
  
Location: 0-4062

Mycgr3G41235\_Mycgr3T

Mycgr3G70577 Mycgr3T
  
Location: 4162-6109

Mycgr3G70577\_Mycgr3T

Mycgr3G40534 Mycgr3T
  
Location: 6209-7166

Mycgr3G40534\_Mycgr3T

Mycgr3G85486 Mycgr3T
  
Location: 7266-8511

Mycgr3G85486\_Mycgr3T

Mycgr3G92221 Mycgr3T
  
Location: 8611-9193

Mycgr3G92221\_Mycgr3T

Mycgr3G39931 Mycgr3T
  
Location: 9293-10157

Mycgr3G39931\_Mycgr3T

Mycgr3G99766 Mycgr3T
  
Location: 10257-11775

Mycgr3G99766\_Mycgr3T

hypothetical protein
  
Accession: EAK81763
  
Location: 73480-74970
  
 NCBI BlastP on this gene

EAK81763

hypothetical protein
  
Accession: EAK81764
  
Location: 75648-80066
  
  
**BlastP hit with Mycgr3G70577\_Mycgr3T**
  
Percentage identity: 39 %
  
BlastP bit score: 379
  
Sequence coverage: 93 %
  
E-value: 3e-114
  
  
 NCBI BlastP on this gene

EAK81764

hypothetical protein
  
Accession: EAK81765
  
Location: 82151-86344
  
  
**BlastP hit with Mycgr3G41235\_Mycgr3T**
  
Percentage identity: 42 %
  
BlastP bit score: 1078
  
Sequence coverage: 103 %
  
E-value: 0.0
  
  
 NCBI BlastP on this gene

EAK81765

hypothetical protein
  
Accession: EAK81766
  
Location: 88032-89579
  
  
**BlastP hit with Mycgr3G85486\_Mycgr3T**
  
Percentage identity: 43 %
  
BlastP bit score: 323
  
Sequence coverage: 99 %
  
E-value: 8e-103
  
  
 NCBI BlastP on this gene

EAK81766

hypothetical protein
  
Accession: EAK81767
  
Location: 89862-90867
  
 NCBI BlastP on this gene

EAK81767

hypothetical protein
  
Accession: EAK81768
  
Location: 92548-107141
  
  
**BlastP hit with Mycgr3G40534\_Mycgr3T**
  
Percentage identity: 48 %
  
BlastP bit score: 222
  
Sequence coverage: 82 %
  
E-value: 8e-61
  
  
  
**BlastP hit with Mycgr3G39931\_Mycgr3T**
  
Percentage identity: 45 %
  
BlastP bit score: 211
  
Sequence coverage: 91 %
  
E-value: 2e-57
  
  
 NCBI BlastP on this gene

EAK81768

hypothetical protein
  
Accession: EAK81769
  
Location: 108397-109758
  
 NCBI BlastP on this gene

EAK81769

Query: Architecture Search FASTA input

DF196790 : Pseudozyma antarctica T-34 DNA, contig: scaffold00024    Total score: 5.0     Cumulative Blast bit score: 2201

Hit cluster cross-links:

Mycgr3G41235 Mycgr3T
  
Location: 0-4062

Mycgr3G41235\_Mycgr3T

Mycgr3G70577 Mycgr3T
  
Location: 4162-6109

Mycgr3G70577\_Mycgr3T

Mycgr3G40534 Mycgr3T
  
Location: 6209-7166

Mycgr3G40534\_Mycgr3T

Mycgr3G85486 Mycgr3T
  
Location: 7266-8511

Mycgr3G85486\_Mycgr3T

Mycgr3G92221 Mycgr3T
  
Location: 8611-9193

Mycgr3G92221\_Mycgr3T

Mycgr3G39931 Mycgr3T
  
Location: 9293-10157

Mycgr3G39931\_Mycgr3T

Mycgr3G99766 Mycgr3T
  
Location: 10257-11775

Mycgr3G99766\_Mycgr3T

putative protein methyltransferase
  
Accession: GAC77014
  
Location: 29222-31015
  
 NCBI BlastP on this gene

GAC77014

hypothetical protein
  
Accession: GAC77013
  
Location: 26562-28655
  
  
**BlastP hit with Mycgr3G70577\_Mycgr3T**
  
Percentage identity: 38 %
  
BlastP bit score: 379
  
Sequence coverage: 94 %
  
E-value: 2e-118
  
  
 NCBI BlastP on this gene

GAC77013

multidrug resistance-associated protein
  
Accession: GAC77012
  
Location: 20835-24989
  
  
**BlastP hit with Mycgr3G41235\_Mycgr3T**
  
Percentage identity: 41 %
  
BlastP bit score: 1062
  
Sequence coverage: 102 %
  
E-value: 0.0
  
  
 NCBI BlastP on this gene

GAC77012

hypothetical protein
  
Accession: GAC77011
  
Location: 17657-19026
  
  
**BlastP hit with Mycgr3G85486\_Mycgr3T**
  
Percentage identity: 47 %
  
BlastP bit score: 326
  
Sequence coverage: 86 %
  
E-value: 4e-104
  
  
 NCBI BlastP on this gene

GAC77011

aromatic-l-amino-acid/l-histidine decarboxylase
  
Accession: GAC77010
  
Location: 16255-17494
  
 NCBI BlastP on this gene

GAC77010

non-ribosomal peptide synthetase
  
Accession: GAC77009
  
Location: 469-15048
  
  
**BlastP hit with Mycgr3G40534\_Mycgr3T**
  
Percentage identity: 49 %
  
BlastP bit score: 228
  
Sequence coverage: 83 %
  
E-value: 9e-63
  
  
  
**BlastP hit with Mycgr3G39931\_Mycgr3T**
  
Percentage identity: 44 %
  
BlastP bit score: 206
  
Sequence coverage: 89 %
  
E-value: 8e-56
  
  
 NCBI BlastP on this gene

GAC77009

Query: Architecture Search FASTA input

FQ311441 : Sporisorium reilianum SRZ2 chromosome 2 complete DNA sequence.    Total score: 5.0     Cumulative Blast bit score: 2178

Hit cluster cross-links:

Mycgr3G41235 Mycgr3T
  
Location: 0-4062

Mycgr3G41235\_Mycgr3T

Mycgr3G70577 Mycgr3T
  
Location: 4162-6109

Mycgr3G70577\_Mycgr3T

Mycgr3G40534 Mycgr3T
  
Location: 6209-7166

Mycgr3G40534\_Mycgr3T

Mycgr3G85486 Mycgr3T
  
Location: 7266-8511

Mycgr3G85486\_Mycgr3T

Mycgr3G92221 Mycgr3T
  
Location: 8611-9193

Mycgr3G92221\_Mycgr3T

Mycgr3G39931 Mycgr3T
  
Location: 9293-10157

Mycgr3G39931\_Mycgr3T

Mycgr3G99766 Mycgr3T
  
Location: 10257-11775

Mycgr3G99766\_Mycgr3T

related to MCH4-monocarboxylate transporter
  
Accession: CBQ70843
  
Location: 1781910-1783259
  
 NCBI BlastP on this gene

sr12504

Siderophore peptide synthetase involved in ferrichromeA biosynthesis
  
Accession: CBQ70842
  
Location: 1766153-1780839
  
  
**BlastP hit with Mycgr3G40534\_Mycgr3T**
  
Percentage identity: 49 %
  
BlastP bit score: 231
  
Sequence coverage: 82 %
  
E-value: 5e-64
  
  
  
**BlastP hit with Mycgr3G39931\_Mycgr3T**
  
Percentage identity: 43 %
  
BlastP bit score: 202
  
Sequence coverage: 89 %
  
E-value: 1e-54
  
  
 NCBI BlastP on this gene

Fer3

related to N6-hydroxylysine acetyl transferase
  
Accession: CBQ70841
  
Location: 1763565-1764933
  
  
**BlastP hit with Mycgr3G85486\_Mycgr3T**
  
Percentage identity: 48 %
  
BlastP bit score: 334
  
Sequence coverage: 86 %
  
E-value: 4e-107
  
  
 NCBI BlastP on this gene

Fer5

related to Enoyl-CoA hydratase
  
Accession: CBQ70840
  
Location: 1762340-1763326
  
 NCBI BlastP on this gene

Fer4

related to ATP-binding cassette transporter protein
  
Accession: CBQ70839
  
Location: 1756382-1760341
  
  
**BlastP hit with Mycgr3G41235\_Mycgr3T**
  
Percentage identity: 42 %
  
BlastP bit score: 1056
  
Sequence coverage: 100 %
  
E-value: 0.0
  
  
 NCBI BlastP on this gene

Fer6

related to Siderophore iron transporter 3
  
Accession: CBQ70838
  
Location: 1752864-1754937
  
  
**BlastP hit with Mycgr3G70577\_Mycgr3T**
  
Percentage identity: 36 %
  
BlastP bit score: 355
  
Sequence coverage: 97 %
  
E-value: 3e-109
  
  
 NCBI BlastP on this gene

Fer7

conserved hypothetical protein
  
Accession: CBQ70837
  
Location: 1750657-1751996
  
 NCBI BlastP on this gene

Fer8

Query: Architecture Search FASTA input

JH687382 : Stereum hirsutum FP-91666 SS1 unplaced genomic scaffold STEHIscaffold\_4    Total score: 4.0     Cumulative Blast bit score: 1915

Hit cluster cross-links:

Mycgr3G41235 Mycgr3T
  
Location: 0-4062

Mycgr3G41235\_Mycgr3T

Mycgr3G70577 Mycgr3T
  
Location: 4162-6109

Mycgr3G70577\_Mycgr3T

Mycgr3G40534 Mycgr3T
  
Location: 6209-7166

Mycgr3G40534\_Mycgr3T

Mycgr3G85486 Mycgr3T
  
Location: 7266-8511

Mycgr3G85486\_Mycgr3T

Mycgr3G92221 Mycgr3T
  
Location: 8611-9193

Mycgr3G92221\_Mycgr3T

Mycgr3G39931 Mycgr3T
  
Location: 9293-10157

Mycgr3G39931\_Mycgr3T

Mycgr3G99766 Mycgr3T
  
Location: 10257-11775

Mycgr3G99766\_Mycgr3T

hypothetical protein
  
Accession: EIM88653
  
Location: 55989-58175
  
 NCBI BlastP on this gene

EIM88653

hypothetical protein
  
Accession: EIM88654
  
Location: 58670-67575
  
  
**BlastP hit with Mycgr3G39931\_Mycgr3T**
  
Percentage identity: 30 %
  
BlastP bit score: 117
  
Sequence coverage: 102 %
  
E-value: 1e-25
  
  
 NCBI BlastP on this gene

EIM88654

enoyl-CoA hydratase
  
Accession: EIM88655
  
Location: 68633-69892
  
 NCBI BlastP on this gene

EIM88655

hypothetical protein
  
Accession: EIM88656
  
Location: 70239-70851
  
 NCBI BlastP on this gene

EIM88656

hypothetical protein
  
Accession: EIM88657
  
Location: 71233-71852
  
 NCBI BlastP on this gene

EIM88657

FAD/NAD-P-binding domain-containing protein
  
Accession: EIM88658
  
Location: 72084-74286
  
 NCBI BlastP on this gene

EIM88658

hypothetical protein
  
Accession: EIM88659
  
Location: 75165-77460
  
 NCBI BlastP on this gene

EIM88659

hypothetical protein
  
Accession: EIM88660
  
Location: 78639-80070
  
  
**BlastP hit with Mycgr3G85486\_Mycgr3T**
  
Percentage identity: 49 %
  
BlastP bit score: 333
  
Sequence coverage: 83 %
  
E-value: 4e-107
  
  
 NCBI BlastP on this gene

EIM88660

MFS general substrate transporter
  
Accession: EIM88661
  
Location: 81661-84769
  
  
**BlastP hit with Mycgr3G70577\_Mycgr3T**
  
Percentage identity: 40 %
  
BlastP bit score: 391
  
Sequence coverage: 85 %
  
E-value: 1e-123
  
  
 NCBI BlastP on this gene

EIM88661

hypothetical protein
  
Accession: EIM88662
  
Location: 85425-85766
  
 NCBI BlastP on this gene

EIM88662

MFS general substrate transporter
  
Accession: EIM88663
  
Location: 86731-89687
  
  
**BlastP hit with Mycgr3G70577\_Mycgr3T**
  
Percentage identity: 40 %
  
BlastP bit score: 356
  
Sequence coverage: 78 %
  
E-value: 4e-111
  
  
 NCBI BlastP on this gene

EIM88663

hypothetical protein
  
Accession: EIM88664
  
Location: 91062-92164
  
 NCBI BlastP on this gene

EIM88664

hypothetical protein
  
Accession: EIM88665
  
Location: 93070-94382
  
 NCBI BlastP on this gene

EIM88665

hypothetical protein
  
Accession: EIM88666
  
Location: 96875-97378
  
 NCBI BlastP on this gene

EIM88666

hypothetical protein
  
Accession: EIM88667
  
Location: 99872-101307
  
 NCBI BlastP on this gene

EIM88667

hypothetical protein
  
Accession: EIM88668
  
Location: 105409-105798
  
 NCBI BlastP on this gene

EIM88668

hypothetical protein
  
Accession: EIM88669
  
Location: 107703-110836
  
 NCBI BlastP on this gene

EIM88669

hypothetical protein
  
Accession: EIM88670
  
Location: 112064-118290
  
  
**BlastP hit with Mycgr3G41235\_Mycgr3T**
  
Percentage identity: 41 %
  
BlastP bit score: 718
  
Sequence coverage: 67 %
  
E-value: 0.0
  
  
 NCBI BlastP on this gene

EIM88670

hypothetical protein
  
Accession: EIM88671
  
Location: 118681-120886
  
 NCBI BlastP on this gene

EIM88671

Query: Architecture Search FASTA input

KB726570 : Fusarium oxysporum f. sp. cubense race 4 unplaced genomic scaffold scaffold111    Total score: 4.0     Cumulative Blast bit score: 1782

Hit cluster cross-links:

Mycgr3G41235 Mycgr3T
  
Location: 0-4062

Mycgr3G41235\_Mycgr3T

Mycgr3G70577 Mycgr3T
  
Location: 4162-6109

Mycgr3G70577\_Mycgr3T

Mycgr3G40534 Mycgr3T
  
Location: 6209-7166

Mycgr3G40534\_Mycgr3T

Mycgr3G85486 Mycgr3T
  
Location: 7266-8511

Mycgr3G85486\_Mycgr3T

Mycgr3G92221 Mycgr3T
  
Location: 8611-9193

Mycgr3G92221\_Mycgr3T

Mycgr3G39931 Mycgr3T
  
Location: 9293-10157

Mycgr3G39931\_Mycgr3T

Mycgr3G99766 Mycgr3T
  
Location: 10257-11775

Mycgr3G99766\_Mycgr3T

Ferric reductase transmembrane component 5
  
Accession: EMT67830
  
Location: 1572995-1575394
  
 NCBI BlastP on this gene

EMT67830

Siderophore iron transporter 3
  
Accession: EMT67829
  
Location: 1570580-1572499
  
  
**BlastP hit with Mycgr3G70577\_Mycgr3T**
  
Percentage identity: 51 %
  
BlastP bit score: 642
  
Sequence coverage: 96 %
  
E-value: 0.0
  
  
 NCBI BlastP on this gene

EMT67829

Putative lysine N-acyltransferase C17G9.06c
  
Accession: EMT67828
  
Location: 1560538-1561836
  
  
**BlastP hit with Mycgr3G85486\_Mycgr3T**
  
Percentage identity: 56 %
  
BlastP bit score: 462
  
Sequence coverage: 102 %
  
E-value: 3e-157
  
  
 NCBI BlastP on this gene

EMT67828

hypothetical protein
  
Accession: EMT67827
  
Location: 1558968-1559928
  
  
**BlastP hit with Mycgr3G85486\_Mycgr3T**
  
Percentage identity: 79 %
  
BlastP bit score: 138
  
Sequence coverage: 20 %
  
E-value: 5e-34
  
  
 NCBI BlastP on this gene

EMT67827

Ferrichrome siderophore peptide synthetase
  
Accession: EMT67826
  
Location: 1538511-1558358
  
  
**BlastP hit with Mycgr3G40534\_Mycgr3T**
  
Percentage identity: 52 %
  
BlastP bit score: 270
  
Sequence coverage: 80 %
  
E-value: 2e-77
  
  
  
**BlastP hit with Mycgr3G39931\_Mycgr3T**
  
Percentage identity: 49 %
  
BlastP bit score: 271
  
Sequence coverage: 99 %
  
E-value: 3e-78
  
  
 NCBI BlastP on this gene

EMT67826

Endo-1,4-beta-xylanase
  
Accession: EMT67825
  
Location: 1536240-1537471
  
 NCBI BlastP on this gene

EMT67825

Query: Architecture Search FASTA input

KB456264 : Mycosphaerella populorum SO2202 unplaced genomic scaffold SEPMUscaffold\_5    Total score: 3.0     Cumulative Blast bit score: 3139

Hit cluster cross-links:

Mycgr3G41235 Mycgr3T
  
Location: 0-4062

Mycgr3G41235\_Mycgr3T

Mycgr3G70577 Mycgr3T
  
Location: 4162-6109

Mycgr3G70577\_Mycgr3T

Mycgr3G40534 Mycgr3T
  
Location: 6209-7166

Mycgr3G40534\_Mycgr3T

Mycgr3G85486 Mycgr3T
  
Location: 7266-8511

Mycgr3G85486\_Mycgr3T

Mycgr3G92221 Mycgr3T
  
Location: 8611-9193

Mycgr3G92221\_Mycgr3T

Mycgr3G39931 Mycgr3T
  
Location: 9293-10157

Mycgr3G39931\_Mycgr3T

Mycgr3G99766 Mycgr3T
  
Location: 10257-11775

Mycgr3G99766\_Mycgr3T

Metallo-dependent phosphatase
  
Accession: EMF12689
  
Location: 1069617-1071542
  
 NCBI BlastP on this gene

EMF12689

hypothetical protein
  
Accession: EMF12690
  
Location: 1072175-1082437
  
  
**BlastP hit with Mycgr3G39931\_Mycgr3T**
  
Percentage identity: 71 %
  
BlastP bit score: 407
  
Sequence coverage: 96 %
  
E-value: 2e-125
  
  
 NCBI BlastP on this gene

EMF12690

MFS general substrate transporter
  
Accession: EMF12691
  
Location: 1090315-1092361
  
  
**BlastP hit with Mycgr3G70577\_Mycgr3T**
  
Percentage identity: 75 %
  
BlastP bit score: 892
  
Sequence coverage: 96 %
  
E-value: 0.0
  
  
 NCBI BlastP on this gene

EMF12691

P-loop containing nucleoside triphosphate hydrolase protein
  
Accession: EMF12693
  
Location: 1094798-1099078
  
  
**BlastP hit with Mycgr3G41235\_Mycgr3T**
  
Percentage identity: 66 %
  
BlastP bit score: 1841
  
Sequence coverage: 104 %
  
E-value: 0.0
  
  
 NCBI BlastP on this gene

EMF12693

L-ornithine N5-oxygenase sida
  
Accession: EMF12694
  
Location: 1099791-1101408
  
 NCBI BlastP on this gene

EMF12694

ribonuclease H-like protein
  
Accession: EMF12695
  
Location: 1101430-1102068
  
 NCBI BlastP on this gene

EMF12695

Query: Architecture Search FASTA input

CH476600 : Aspergillus terreus NIH2624 scaffold\_7 genomic scaffold    Total score: 3.0     Cumulative Blast bit score: 613

Hit cluster cross-links:

Mycgr3G41235 Mycgr3T
  
Location: 0-4062

Mycgr3G41235\_Mycgr3T

Mycgr3G70577 Mycgr3T
  
Location: 4162-6109

Mycgr3G70577\_Mycgr3T

Mycgr3G40534 Mycgr3T
  
Location: 6209-7166

Mycgr3G40534\_Mycgr3T

Mycgr3G85486 Mycgr3T
  
Location: 7266-8511

Mycgr3G85486\_Mycgr3T

Mycgr3G92221 Mycgr3T
  
Location: 8611-9193

Mycgr3G92221\_Mycgr3T

Mycgr3G39931 Mycgr3T
  
Location: 9293-10157

Mycgr3G39931\_Mycgr3T

Mycgr3G99766 Mycgr3T
  
Location: 10257-11775

Mycgr3G99766\_Mycgr3T

predicted protein
  
Accession: EAU34145
  
Location: 427905-429124
  
 NCBI BlastP on this gene

EAU34145

conserved hypothetical protein
  
Accession: EAU34144
  
Location: 424658-426063
  
  
**BlastP hit with Mycgr3G85486\_Mycgr3T**
  
Percentage identity: 30 %
  
BlastP bit score: 155
  
Sequence coverage: 84 %
  
E-value: 4e-39
  
  
 NCBI BlastP on this gene

EAU34144

hypothetical protein
  
Accession: EAU34143
  
Location: 422013-423996
  
 NCBI BlastP on this gene

EAU34143

hypothetical protein
  
Accession: EAU34142
  
Location: 405286-419584
  
  
**BlastP hit with Mycgr3G40534\_Mycgr3T**
  
Percentage identity: 52 %
  
BlastP bit score: 247
  
Sequence coverage: 80 %
  
E-value: 2e-69
  
  
  
**BlastP hit with Mycgr3G39931\_Mycgr3T**
  
Percentage identity: 43 %
  
BlastP bit score: 211
  
Sequence coverage: 97 %
  
E-value: 2e-57
  
  
 NCBI BlastP on this gene

EAU34142

conserved hypothetical protein
  
Accession: EAU34141
  
Location: 402007-404439
  
 NCBI BlastP on this gene

EAU34141

Query: Architecture Search FASTA input

AACD01000007 : Aspergillus nidulans FGSC A4    Total score: 3.0     Cumulative Blast bit score: 595

Hit cluster cross-links:

Mycgr3G41235 Mycgr3T
  
Location: 0-4062

Mycgr3G41235\_Mycgr3T

Mycgr3G70577 Mycgr3T
  
Location: 4162-6109

Mycgr3G70577\_Mycgr3T

Mycgr3G40534 Mycgr3T
  
Location: 6209-7166

Mycgr3G40534\_Mycgr3T

Mycgr3G85486 Mycgr3T
  
Location: 7266-8511

Mycgr3G85486\_Mycgr3T

Mycgr3G92221 Mycgr3T
  
Location: 8611-9193

Mycgr3G92221\_Mycgr3T

Mycgr3G39931 Mycgr3T
  
Location: 9293-10157

Mycgr3G39931\_Mycgr3T

Mycgr3G99766 Mycgr3T
  
Location: 10257-11775

Mycgr3G99766\_Mycgr3T

hypothetical protein
  
Accession: EAA66708
  
Location: 739203-741236
  
 NCBI BlastP on this gene

EAA66708

hypothetical protein
  
Accession: EAA66707
  
Location: 737167-738547
  
  
**BlastP hit with Mycgr3G85486\_Mycgr3T**
  
Percentage identity: 30 %
  
BlastP bit score: 141
  
Sequence coverage: 81 %
  
E-value: 5e-34
  
  
 NCBI BlastP on this gene

EAA66707

hypothetical protein
  
Accession: EAA66706
  
Location: 721978-736405
  
  
**BlastP hit with Mycgr3G40534\_Mycgr3T**
  
Percentage identity: 49 %
  
BlastP bit score: 235
  
Sequence coverage: 80 %
  
E-value: 3e-65
  
  
  
**BlastP hit with Mycgr3G39931\_Mycgr3T**
  
Percentage identity: 45 %
  
BlastP bit score: 219
  
Sequence coverage: 90 %
  
E-value: 3e-60
  
  
 NCBI BlastP on this gene

EAA66706

hypothetical protein
  
Accession: EAA66705
  
Location: 718702-720666
  
 NCBI BlastP on this gene

EAA66705

Query: Architecture Search FASTA input

AHHD01000417 : Macrophomina phaseolina MS6    Total score: 3.0     Cumulative Blast bit score: 592

Hit cluster cross-links:

Mycgr3G41235 Mycgr3T
  
Location: 0-4062

Mycgr3G41235\_Mycgr3T

Mycgr3G70577 Mycgr3T
  
Location: 4162-6109

Mycgr3G70577\_Mycgr3T

Mycgr3G40534 Mycgr3T
  
Location: 6209-7166

Mycgr3G40534\_Mycgr3T

Mycgr3G85486 Mycgr3T
  
Location: 7266-8511

Mycgr3G85486\_Mycgr3T

Mycgr3G92221 Mycgr3T
  
Location: 8611-9193

Mycgr3G92221\_Mycgr3T

Mycgr3G39931 Mycgr3T
  
Location: 9293-10157

Mycgr3G39931\_Mycgr3T

Mycgr3G99766 Mycgr3T
  
Location: 10257-11775

Mycgr3G99766\_Mycgr3T

hypothetical protein
  
Accession: EKG13084
  
Location: 26781-27448
  
 NCBI BlastP on this gene

EKG13084

Acyl-CoA N-acyltransferase
  
Accession: EKG13085
  
Location: 29734-31056
  
  
**BlastP hit with Mycgr3G85486\_Mycgr3T**
  
Percentage identity: 33 %
  
BlastP bit score: 156
  
Sequence coverage: 75 %
  
E-value: 2e-39
  
  
 NCBI BlastP on this gene

EKG13085

AMP-dependent synthetase/ligase
  
Accession: EKG13086
  
Location: 32464-33871
  
 NCBI BlastP on this gene

EKG13086

AMP-dependent synthetase/ligase
  
Accession: EKG13087
  
Location: 34954-49462
  
  
**BlastP hit with Mycgr3G40534\_Mycgr3T**
  
Percentage identity: 51 %
  
BlastP bit score: 266
  
Sequence coverage: 82 %
  
E-value: 4e-76
  
  
  
**BlastP hit with Mycgr3G39931\_Mycgr3T**
  
Percentage identity: 40 %
  
BlastP bit score: 170
  
Sequence coverage: 90 %
  
E-value: 2e-43
  
  
 NCBI BlastP on this gene

EKG13087

Bacterial alpha-L-rhamnosidase
  
Accession: EKG13088
  
Location: 49813-51971
  
 NCBI BlastP on this gene

EKG13088

Query: Architecture Search FASTA input

KB915996 : Neofusicoccum parvum UCRNP2 chromosome Unknown NP2\_03\_scaffold\_358    Total score: 3.0     Cumulative Blast bit score: 508

Hit cluster cross-links:

Mycgr3G41235 Mycgr3T
  
Location: 0-4062

Mycgr3G41235\_Mycgr3T

Mycgr3G70577 Mycgr3T
  
Location: 4162-6109

Mycgr3G70577\_Mycgr3T

Mycgr3G40534 Mycgr3T
  
Location: 6209-7166

Mycgr3G40534\_Mycgr3T

Mycgr3G85486 Mycgr3T
  
Location: 7266-8511

Mycgr3G85486\_Mycgr3T

Mycgr3G92221 Mycgr3T
  
Location: 8611-9193

Mycgr3G92221\_Mycgr3T

Mycgr3G39931 Mycgr3T
  
Location: 9293-10157

Mycgr3G39931\_Mycgr3T

Mycgr3G99766 Mycgr3T
  
Location: 10257-11775

Mycgr3G99766\_Mycgr3T

putative aerobactin siderophore biosynthesis protein iucb protein
  
Accession: EOD50324
  
Location: 6418-7743
  
  
**BlastP hit with Mycgr3G85486\_Mycgr3T**
  
Percentage identity: 31 %
  
BlastP bit score: 153
  
Sequence coverage: 76 %
  
E-value: 2e-38
  
  
 NCBI BlastP on this gene

EOD50324

putative long-chain-fatty-acid- ligase protein
  
Accession: EOD50335
  
Location: 8610-10774
  
 NCBI BlastP on this gene

EOD50335

putative amp-dependent synthetase ligase protein
  
Accession: EOD50322
  
Location: 11855-12671
  
 NCBI BlastP on this gene

EOD50322

putative nonribosomal siderophore peptide synthase protein
  
Accession: EOD50346
  
Location: 13291-26075
  
  
**BlastP hit with Mycgr3G40534\_Mycgr3T**
  
Percentage identity: 49 %
  
BlastP bit score: 179
  
Sequence coverage: 60 %
  
E-value: 3e-46
  
  
  
**BlastP hit with Mycgr3G39931\_Mycgr3T**
  
Percentage identity: 41 %
  
BlastP bit score: 176
  
Sequence coverage: 90 %
  
E-value: 2e-45
  
  
 NCBI BlastP on this gene

EOD50346

putative alpha-l-rhamnosidase protein
  
Accession: EOD50311
  
Location: 26648-28824
  
 NCBI BlastP on this gene

EOD50311

Query: Architecture Search FASTA input

DS499596 : Aspergillus fumigatus A1163 scf\_000003 genomic scaffold    Total score: 3.0     Cumulative Blast bit score: 447

Hit cluster cross-links:

Mycgr3G41235 Mycgr3T
  
Location: 0-4062

Mycgr3G41235\_Mycgr3T

Mycgr3G70577 Mycgr3T
  
Location: 4162-6109

Mycgr3G70577\_Mycgr3T

Mycgr3G40534 Mycgr3T
  
Location: 6209-7166

Mycgr3G40534\_Mycgr3T

Mycgr3G85486 Mycgr3T
  
Location: 7266-8511

Mycgr3G85486\_Mycgr3T

Mycgr3G92221 Mycgr3T
  
Location: 8611-9193

Mycgr3G92221\_Mycgr3T

Mycgr3G39931 Mycgr3T
  
Location: 9293-10157

Mycgr3G39931\_Mycgr3T

Mycgr3G99766 Mycgr3T
  
Location: 10257-11775

Mycgr3G99766\_Mycgr3T

mitochondrial enoyl reductase, putative
  
Accession: EDP53317
  
Location: 3222031-3223257
  
 NCBI BlastP on this gene

EDP53317

nonribosomal peptide synthase SidE
  
Accession: EDP53316
  
Location: 3214421-3220750
  
  
**BlastP hit with Mycgr3G40534\_Mycgr3T**
  
Percentage identity: 38 %
  
BlastP bit score: 154
  
Sequence coverage: 87 %
  
E-value: 8e-38
  
  
  
**BlastP hit with Mycgr3G39931\_Mycgr3T**
  
Percentage identity: 33 %
  
BlastP bit score: 121
  
Sequence coverage: 88 %
  
E-value: 6e-27
  
  
 NCBI BlastP on this gene

EDP53316

cell surface protein, putative
  
Accession: EDP53315
  
Location: 3213303-3213863
  
 NCBI BlastP on this gene

EDP53315

sugar O-acetyltransferase, putative
  
Accession: EDP53314
  
Location: 3211178-3211921
  
 NCBI BlastP on this gene

EDP53314

siderophore biosynthesis lipase/esterase, putative
  
Accession: EDP53313
  
Location: 3209863-3210987
  
 NCBI BlastP on this gene

EDP53313

siderophore biosynthesis acetylase AceI, putative
  
Accession: EDP53312
  
Location: 3206927-3208315
  
  
**BlastP hit with Mycgr3G85486\_Mycgr3T**
  
Percentage identity: 34 %
  
BlastP bit score: 173
  
Sequence coverage: 80 %
  
E-value: 2e-45
  
  
 NCBI BlastP on this gene

EDP53312

enoyl-CoA hydratase/isomerase family protein
  
Accession: EDP53311
  
Location: 3205730-3206615
  
 NCBI BlastP on this gene

EDP53311

nonribosomal peptide synthase SidD
  
Accession: EDP53310
  
Location: 3198715-3205021
  
 NCBI BlastP on this gene

EDP53310

Query: Architecture Search FASTA input

DS027697 : Neosartorya fischeri NRRL 181 1099437636265 genomic scaffold    Total score: 3.0     Cumulative Blast bit score: 447

Hit cluster cross-links:

Mycgr3G41235 Mycgr3T
  
Location: 0-4062

Mycgr3G41235\_Mycgr3T

Mycgr3G70577 Mycgr3T
  
Location: 4162-6109

Mycgr3G70577\_Mycgr3T

Mycgr3G40534 Mycgr3T
  
Location: 6209-7166

Mycgr3G40534\_Mycgr3T

Mycgr3G85486 Mycgr3T
  
Location: 7266-8511

Mycgr3G85486\_Mycgr3T

Mycgr3G92221 Mycgr3T
  
Location: 8611-9193

Mycgr3G92221\_Mycgr3T

Mycgr3G39931 Mycgr3T
  
Location: 9293-10157

Mycgr3G39931\_Mycgr3T

Mycgr3G99766 Mycgr3T
  
Location: 10257-11775

Mycgr3G99766\_Mycgr3T

mitochondrial enoyl reductase, putative
  
Accession: EAW17189
  
Location: 1441589-1442812
  
 NCBI BlastP on this gene

EAW17189

nonribosomal peptide synthase SidE
  
Accession: EAW17190
  
Location: 1444078-1450407
  
  
**BlastP hit with Mycgr3G40534\_Mycgr3T**
  
Percentage identity: 40 %
  
BlastP bit score: 155
  
Sequence coverage: 79 %
  
E-value: 4e-38
  
  
  
**BlastP hit with Mycgr3G39931\_Mycgr3T**
  
Percentage identity: 33 %
  
BlastP bit score: 122
  
Sequence coverage: 96 %
  
E-value: 3e-27
  
  
 NCBI BlastP on this gene

EAW17190

cell surface protein, putative
  
Accession: EAW17191
  
Location: 1451158-1451718
  
 NCBI BlastP on this gene

EAW17191

sugar O-acetyltransferase, putative
  
Accession: EAW17192
  
Location: 1453189-1453932
  
 NCBI BlastP on this gene

EAW17192

siderophore biosynthesis lipase/esterase, putative
  
Accession: EAW17193
  
Location: 1454144-1455264
  
 NCBI BlastP on this gene

EAW17193

siderophore biosynthesis acetylase AceI, putative
  
Accession: EAW17194
  
Location: 1456800-1458188
  
  
**BlastP hit with Mycgr3G85486\_Mycgr3T**
  
Percentage identity: 34 %
  
BlastP bit score: 171
  
Sequence coverage: 80 %
  
E-value: 2e-44
  
  
 NCBI BlastP on this gene

EAW17194

enoyl-CoA hydratase/isomerase family protein
  
Accession: EAW17195
  
Location: 1458510-1459376
  
 NCBI BlastP on this gene

EAW17195

nonribosomal peptide synthase SidD
  
Accession: EAW17196
  
Location: 1460111-1466417
  
 NCBI BlastP on this gene

EAW17196

Query: Architecture Search FASTA input

AAHF01000010 : Aspergillus fumigatus Af293    Total score: 3.0     Cumulative Blast bit score: 447

Hit cluster cross-links:

Mycgr3G41235 Mycgr3T
  
Location: 0-4062

Mycgr3G41235\_Mycgr3T

Mycgr3G70577 Mycgr3T
  
Location: 4162-6109

Mycgr3G70577\_Mycgr3T

Mycgr3G40534 Mycgr3T
  
Location: 6209-7166

Mycgr3G40534\_Mycgr3T

Mycgr3G85486 Mycgr3T
  
Location: 7266-8511

Mycgr3G85486\_Mycgr3T

Mycgr3G92221 Mycgr3T
  
Location: 8611-9193

Mycgr3G92221\_Mycgr3T

Mycgr3G39931 Mycgr3T
  
Location: 9293-10157

Mycgr3G39931\_Mycgr3T

Mycgr3G99766 Mycgr3T
  
Location: 10257-11775

Mycgr3G99766\_Mycgr3T

mitochondrial enoyl reductase, putative
  
Accession: EAL86615
  
Location: 889931-891157
  
 NCBI BlastP on this gene

EAL86615

nonribosomal peptide synthase SidE
  
Accession: EAL86616
  
Location: 892438-898767
  
  
**BlastP hit with Mycgr3G40534\_Mycgr3T**
  
Percentage identity: 38 %
  
BlastP bit score: 154
  
Sequence coverage: 87 %
  
E-value: 8e-38
  
  
  
**BlastP hit with Mycgr3G39931\_Mycgr3T**
  
Percentage identity: 33 %
  
BlastP bit score: 121
  
Sequence coverage: 88 %
  
E-value: 6e-27
  
  
 NCBI BlastP on this gene

EAL86616

cell surface protein, putative
  
Accession: EAL86619
  
Location: 899325-899885
  
 NCBI BlastP on this gene

EAL86619

sugar O-acetyltransferase, putative
  
Accession: EAL86620
  
Location: 901268-902011
  
 NCBI BlastP on this gene

EAL86620

siderophore biosynthesis lipase/esterase, putative
  
Accession: EAL86621
  
Location: 902202-903326
  
 NCBI BlastP on this gene

EAL86621

siderophore biosynthesis acetylase AceI, putative
  
Accession: EAL86622
  
Location: 904874-906262
  
  
**BlastP hit with Mycgr3G85486\_Mycgr3T**
  
Percentage identity: 34 %
  
BlastP bit score: 173
  
Sequence coverage: 80 %
  
E-value: 2e-45
  
  
 NCBI BlastP on this gene

EAL86622

enoyl-CoA hydratase/isomerase family protein
  
Accession: EAL86623
  
Location: 906574-907459
  
 NCBI BlastP on this gene

EAL86623

nonribosomal peptide synthase SidD
  
Accession: EAL86624
  
Location: 908168-914474
  
 NCBI BlastP on this gene

EAL86624

Query: Architecture Search FASTA input

HE605208 : Candida parapsilosis strain CDC317 annotated contig 006372.    Total score: 2.0     Cumulative Blast bit score: 854

Hit cluster cross-links:

Mycgr3G41235 Mycgr3T
  
Location: 0-4062

Mycgr3G41235\_Mycgr3T

Mycgr3G70577 Mycgr3T
  
Location: 4162-6109

Mycgr3G70577\_Mycgr3T

Mycgr3G40534 Mycgr3T
  
Location: 6209-7166

Mycgr3G40534\_Mycgr3T

Mycgr3G85486 Mycgr3T
  
Location: 7266-8511

Mycgr3G85486\_Mycgr3T

Mycgr3G92221 Mycgr3T
  
Location: 8611-9193

Mycgr3G92221\_Mycgr3T

Mycgr3G39931 Mycgr3T
  
Location: 9293-10157

Mycgr3G39931\_Mycgr3T

Mycgr3G99766 Mycgr3T
  
Location: 10257-11775

Mycgr3G99766\_Mycgr3T

hypothetical protein
  
Accession: CCE44955
  
Location: 1700332-1702149
  
 NCBI BlastP on this gene

CPAR2\_407570

hypothetical protein
  
Accession: CCE44954
  
Location: 1696040-1697866
  
  
**BlastP hit with Mycgr3G70577\_Mycgr3T**
  
Percentage identity: 26 %
  
BlastP bit score: 196
  
Sequence coverage: 93 %
  
E-value: 7e-51
  
  
 NCBI BlastP on this gene

CPAR2\_407560

hypothetical protein
  
Accession: CCE44953
  
Location: 1694968-1695691
  
 NCBI BlastP on this gene

CPAR2\_407550

hypothetical protein
  
Accession: CCE44952
  
Location: 1693574-1694842
  
 NCBI BlastP on this gene

CPAR2\_407540

hypothetical protein
  
Accession: CCE44951
  
Location: 1686682-1693206
  
 NCBI BlastP on this gene

CPAR2\_407530

hypothetical protein
  
Accession: CCE44950
  
Location: 1684286-1686115
  
 NCBI BlastP on this gene

CPAR2\_407520

not annotated
  
Accession: CPAR2\_407510
  
Location: 1677696-1682048
  
  
**BlastP hit with Mycgr3G41235\_Mycgr3T**
  
Percentage identity: 30 %
  
BlastP bit score: 658
  
Sequence coverage: 98 %
  
E-value: 0.0
  
  
 NCBI BlastP on this gene

CPAR2\_407510

hypothetical protein
  
Accession: CCE44948
  
Location: 1676461-1676796
  
 NCBI BlastP on this gene

CPAR2\_407500

hypothetical protein
  
Accession: CCE44947
  
Location: 1675239-1676006
  
 NCBI BlastP on this gene

CPAR2\_407490

Query: Architecture Search FASTA input

GL377303 : Schizophyllum commune H4-8 unplaced genomic scaffold SCHCOscaffold\_2    Total score: 2.0     Cumulative Blast bit score: 784

Hit cluster cross-links:

Mycgr3G41235 Mycgr3T
  
Location: 0-4062

Mycgr3G41235\_Mycgr3T

Mycgr3G70577 Mycgr3T
  
Location: 4162-6109

Mycgr3G70577\_Mycgr3T

Mycgr3G40534 Mycgr3T
  
Location: 6209-7166

Mycgr3G40534\_Mycgr3T

Mycgr3G85486 Mycgr3T
  
Location: 7266-8511

Mycgr3G85486\_Mycgr3T

Mycgr3G92221 Mycgr3T
  
Location: 8611-9193

Mycgr3G92221\_Mycgr3T

Mycgr3G39931 Mycgr3T
  
Location: 9293-10157

Mycgr3G39931\_Mycgr3T

Mycgr3G99766 Mycgr3T
  
Location: 10257-11775

Mycgr3G99766\_Mycgr3T

hypothetical protein
  
Accession: EFJ00115
  
Location: 1690286-1693992
  
 NCBI BlastP on this gene

EFJ00115

hypothetical protein
  
Accession: EFJ00116
  
Location: 1694494-1695526
  
 NCBI BlastP on this gene

EFJ00116

hypothetical protein
  
Accession: EFJ00117
  
Location: 1695853-1697154
  
  
**BlastP hit with Mycgr3G85486\_Mycgr3T**
  
Percentage identity: 48 %
  
BlastP bit score: 330
  
Sequence coverage: 84 %
  
E-value: 7e-106
  
  
 NCBI BlastP on this gene

EFJ00117

hypothetical protein
  
Accession: EFJ00118
  
Location: 1697461-1699615
  
 NCBI BlastP on this gene

EFJ00118

hypothetical protein
  
Accession: EFJ00119
  
Location: 1700226-1701315
  
 NCBI BlastP on this gene

EFJ00119

hypothetical protein
  
Accession: EFJ00120
  
Location: 1701744-1702852
  
 NCBI BlastP on this gene

EFJ00120

hypothetical protein
  
Accession: EFJ00946
  
Location: 1703027-1706916
  
 NCBI BlastP on this gene

EFJ00946

hypothetical protein
  
Accession: EFJ00121
  
Location: 1707544-1708270
  
 NCBI BlastP on this gene

EFJ00121

hypothetical protein
  
Accession: EFJ00947
  
Location: 1708441-1713759
  
 NCBI BlastP on this gene

EFJ00947

hypothetical protein
  
Accession: EFJ00948
  
Location: 1714164-1715961
  
 NCBI BlastP on this gene

EFJ00948

hypothetical protein
  
Accession: EFJ00122
  
Location: 1716825-1718859
  
  
**BlastP hit with Mycgr3G70577\_Mycgr3T**
  
Percentage identity: 41 %
  
BlastP bit score: 454
  
Sequence coverage: 97 %
  
E-value: 2e-147
  
  
 NCBI BlastP on this gene

EFJ00122

hypothetical protein
  
Accession: EFJ00123
  
Location: 1719469-1721678
  
 NCBI BlastP on this gene

EFJ00123

Query: Architecture Search FASTA input

EQ963479 : Aspergillus flavus NRRL3357 scf\_1106286418500 genomic scaffold    Total score: 2.0     Cumulative Blast bit score: 757

Hit cluster cross-links:

Mycgr3G41235 Mycgr3T
  
Location: 0-4062

Mycgr3G41235\_Mycgr3T

Mycgr3G70577 Mycgr3T
  
Location: 4162-6109

Mycgr3G70577\_Mycgr3T

Mycgr3G40534 Mycgr3T
  
Location: 6209-7166

Mycgr3G40534\_Mycgr3T

Mycgr3G85486 Mycgr3T
  
Location: 7266-8511

Mycgr3G85486\_Mycgr3T

Mycgr3G92221 Mycgr3T
  
Location: 8611-9193

Mycgr3G92221\_Mycgr3T

Mycgr3G39931 Mycgr3T
  
Location: 9293-10157

Mycgr3G39931\_Mycgr3T

Mycgr3G99766 Mycgr3T
  
Location: 10257-11775

Mycgr3G99766\_Mycgr3T

pantothenate transporter, putative
  
Accession: EED49650
  
Location: 143636-145482
  
 NCBI BlastP on this gene

EED49650

sugar transporter, putative
  
Accession: EED49651
  
Location: 146590-148563
  
  
**BlastP hit with Mycgr3G99766\_Mycgr3T**
  
Percentage identity: 34 %
  
BlastP bit score: 286
  
Sequence coverage: 97 %
  
E-value: 6e-86
  
  
 NCBI BlastP on this gene

EED49651

ankyrin repeat domain, putative
  
Accession: EED49652
  
Location: 151230-152975
  
 NCBI BlastP on this gene

EED49652

cell wall glycosyl hydrolase YteR, putative
  
Accession: EED49653
  
Location: 153324-154621
  
 NCBI BlastP on this gene

EED49653

naringenin,2-oxoglutarate 3-dioxygenase, putative
  
Accession: EED49654
  
Location: 155093-156473
  
 NCBI BlastP on this gene

EED49654

siderophore iron transporter, putative
  
Accession: EED49655
  
Location: 156920-158892
  
  
**BlastP hit with Mycgr3G70577\_Mycgr3T**
  
Percentage identity: 42 %
  
BlastP bit score: 471
  
Sequence coverage: 94 %
  
E-value: 4e-154
  
  
 NCBI BlastP on this gene

EED49655

Query: Architecture Search FASTA input

AP007154 : Aspergillus oryzae RIB40 DNA, SC001.    Total score: 2.0     Cumulative Blast bit score: 757

Hit cluster cross-links:

Mycgr3G41235 Mycgr3T
  
Location: 0-4062

Mycgr3G41235\_Mycgr3T

Mycgr3G70577 Mycgr3T
  
Location: 4162-6109

Mycgr3G70577\_Mycgr3T

Mycgr3G40534 Mycgr3T
  
Location: 6209-7166

Mycgr3G40534\_Mycgr3T

Mycgr3G85486 Mycgr3T
  
Location: 7266-8511

Mycgr3G85486\_Mycgr3T

Mycgr3G92221 Mycgr3T
  
Location: 8611-9193

Mycgr3G92221\_Mycgr3T

Mycgr3G39931 Mycgr3T
  
Location: 9293-10157

Mycgr3G39931\_Mycgr3T

Mycgr3G99766 Mycgr3T
  
Location: 10257-11775

Mycgr3G99766\_Mycgr3T

not annotated
  
Accession: BAE56621
  
Location: 130971-134279
  
  
**BlastP hit with Mycgr3G99766\_Mycgr3T**
  
Percentage identity: 34 %
  
BlastP bit score: 286
  
Sequence coverage: 97 %
  
E-value: 3e-86
  
  
 NCBI BlastP on this gene

AO090001000061

not annotated
  
Accession: BAE56622
  
Location: 137092-138699
  
 NCBI BlastP on this gene

AO090001000062

not annotated
  
Accession: BAE56623
  
Location: 139178-140337
  
 NCBI BlastP on this gene

AO090001000063

not annotated
  
Accession: BAE56624
  
Location: 140806-142186
  
 NCBI BlastP on this gene

AO090001000064

not annotated
  
Accession: BAE56625
  
Location: 142632-144604
  
  
**BlastP hit with Mycgr3G70577\_Mycgr3T**
  
Percentage identity: 42 %
  
BlastP bit score: 471
  
Sequence coverage: 94 %
  
E-value: 3e-154
  
  
 NCBI BlastP on this gene

AO090001000065

not annotated
  
Accession: BAE56626
  
Location: 146665-148342
  
 NCBI BlastP on this gene

AO090001000066

Query: Architecture Search FASTA input

CAOJ01000067 : Rhizoctonia solani AG-1 IB strain isolate 7/3/14    Total score: 2.0     Cumulative Blast bit score: 530

Hit cluster cross-links:

Mycgr3G41235 Mycgr3T
  
Location: 0-4062

Mycgr3G41235\_Mycgr3T

Mycgr3G70577 Mycgr3T
  
Location: 4162-6109

Mycgr3G70577\_Mycgr3T

Mycgr3G40534 Mycgr3T
  
Location: 6209-7166

Mycgr3G40534\_Mycgr3T

Mycgr3G85486 Mycgr3T
  
Location: 7266-8511

Mycgr3G85486\_Mycgr3T

Mycgr3G92221 Mycgr3T
  
Location: 8611-9193

Mycgr3G92221\_Mycgr3T

Mycgr3G39931 Mycgr3T
  
Location: 9293-10157

Mycgr3G39931\_Mycgr3T

Mycgr3G99766 Mycgr3T
  
Location: 10257-11775

Mycgr3G99766\_Mycgr3T

putative N-acetyltransferase san
  
Accession: CCO26033
  
Location: 2900-3693
  
 NCBI BlastP on this gene

CCO26033

Putative lysine N-acyltransferase C17G9.06c
  
Accession: CCO26034
  
Location: 3890-5312
  
  
**BlastP hit with Mycgr3G85486\_Mycgr3T**
  
Percentage identity: 51 %
  
BlastP bit score: 323
  
Sequence coverage: 76 %
  
E-value: 2e-103
  
  
 NCBI BlastP on this gene

CCO26034

Siderophore iron transporter 3
  
Accession: CCO26035
  
Location: 5684-6969
  
  
**BlastP hit with Mycgr3G70577\_Mycgr3T**
  
Percentage identity: 36 %
  
BlastP bit score: 207
  
Sequence coverage: 49 %
  
E-value: 1e-57
  
  
 NCBI BlastP on this gene

CCO26035

Query: Architecture Search FASTA input

EQ963475 : Aspergillus flavus NRRL3357 scf\_1106286419142 genomic scaffold    Total score: 2.0     Cumulative Blast bit score: 432

Hit cluster cross-links:

Mycgr3G41235 Mycgr3T
  
Location: 0-4062

Mycgr3G41235\_Mycgr3T

Mycgr3G70577 Mycgr3T
  
Location: 4162-6109

Mycgr3G70577\_Mycgr3T

Mycgr3G40534 Mycgr3T
  
Location: 6209-7166

Mycgr3G40534\_Mycgr3T

Mycgr3G85486 Mycgr3T
  
Location: 7266-8511

Mycgr3G85486\_Mycgr3T

Mycgr3G92221 Mycgr3T
  
Location: 8611-9193

Mycgr3G92221\_Mycgr3T

Mycgr3G39931 Mycgr3T
  
Location: 9293-10157

Mycgr3G39931\_Mycgr3T

Mycgr3G99766 Mycgr3T
  
Location: 10257-11775

Mycgr3G99766\_Mycgr3T

nonribosomal siderophore peptide synthase SidC
  
Accession: EED53566
  
Location: 1401753-1416093
  
  
**BlastP hit with Mycgr3G40534\_Mycgr3T**
  
Percentage identity: 50 %
  
BlastP bit score: 236
  
Sequence coverage: 79 %
  
E-value: 7e-66
  
  
  
**BlastP hit with Mycgr3G39931\_Mycgr3T**
  
Percentage identity: 43 %
  
BlastP bit score: 197
  
Sequence coverage: 91 %
  
E-value: 1e-52
  
  
 NCBI BlastP on this gene

EED53566

actin cytoskeleton organization protein App1, putative
  
Accession: EED53565
  
Location: 1398897-1401293
  
 NCBI BlastP on this gene

EED53565

Query: Architecture Search FASTA input

AP007157 : Aspergillus oryzae RIB40 DNA, SC023.    Total score: 2.0     Cumulative Blast bit score: 430

Hit cluster cross-links:

Mycgr3G41235 Mycgr3T
  
Location: 0-4062

Mycgr3G41235\_Mycgr3T

Mycgr3G70577 Mycgr3T
  
Location: 4162-6109

Mycgr3G70577\_Mycgr3T

Mycgr3G40534 Mycgr3T
  
Location: 6209-7166

Mycgr3G40534\_Mycgr3T

Mycgr3G85486 Mycgr3T
  
Location: 7266-8511

Mycgr3G85486\_Mycgr3T

Mycgr3G92221 Mycgr3T
  
Location: 8611-9193

Mycgr3G92221\_Mycgr3T

Mycgr3G39931 Mycgr3T
  
Location: 9293-10157

Mycgr3G39931\_Mycgr3T

Mycgr3G99766 Mycgr3T
  
Location: 10257-11775

Mycgr3G99766\_Mycgr3T

not annotated
  
Accession: BAE59066
  
Location: 1386059-1400399
  
  
**BlastP hit with Mycgr3G40534\_Mycgr3T**
  
Percentage identity: 49 %
  
BlastP bit score: 236
  
Sequence coverage: 79 %
  
E-value: 1e-65
  
  
  
**BlastP hit with Mycgr3G39931\_Mycgr3T**
  
Percentage identity: 42 %
  
BlastP bit score: 194
  
Sequence coverage: 91 %
  
E-value: 8e-52
  
  
 NCBI BlastP on this gene

AO090023000528

not annotated
  
Accession: BAE59065
  
Location: 1383436-1385598
  
 NCBI BlastP on this gene

AO090023000527

Query: Architecture Search FASTA input

AKHY01000182 : Aspergillus oryzae 3.042    Total score: 2.0     Cumulative Blast bit score: 429

Hit cluster cross-links:

Mycgr3G41235 Mycgr3T
  
Location: 0-4062

Mycgr3G41235\_Mycgr3T

Mycgr3G70577 Mycgr3T
  
Location: 4162-6109

Mycgr3G70577\_Mycgr3T

Mycgr3G40534 Mycgr3T
  
Location: 6209-7166

Mycgr3G40534\_Mycgr3T

Mycgr3G85486 Mycgr3T
  
Location: 7266-8511

Mycgr3G85486\_Mycgr3T

Mycgr3G92221 Mycgr3T
  
Location: 8611-9193

Mycgr3G92221\_Mycgr3T

Mycgr3G39931 Mycgr3T
  
Location: 9293-10157

Mycgr3G39931\_Mycgr3T

Mycgr3G99766 Mycgr3T
  
Location: 10257-11775

Mycgr3G99766\_Mycgr3T

non-ribosomal peptide synthetase/alpha-aminoadipate reductase
  
Accession: EIT75205
  
Location: 1179418-1193758
  
  
**BlastP hit with Mycgr3G40534\_Mycgr3T**
  
Percentage identity: 49 %
  
BlastP bit score: 235
  
Sequence coverage: 80 %
  
E-value: 3e-65
  
  
  
**BlastP hit with Mycgr3G39931\_Mycgr3T**
  
Percentage identity: 43 %
  
BlastP bit score: 194
  
Sequence coverage: 91 %
  
E-value: 9e-52
  
  
 NCBI BlastP on this gene

EIT75205

hypothetical protein
  
Accession: EIT75184
  
Location: 1176795-1178957
  
 NCBI BlastP on this gene

EIT75184

Query: Architecture Search FASTA input

JH687874 : Auricularia delicata TFB-10046 SS5 unplaced genomic scaffold AURDEscaffold\_142    Total score: 2.0     Cumulative Blast bit score: 346

Hit cluster cross-links:

Mycgr3G41235 Mycgr3T
  
Location: 0-4062

Mycgr3G41235\_Mycgr3T

Mycgr3G70577 Mycgr3T
  
Location: 4162-6109

Mycgr3G70577\_Mycgr3T

Mycgr3G40534 Mycgr3T
  
Location: 6209-7166

Mycgr3G40534\_Mycgr3T

Mycgr3G85486 Mycgr3T
  
Location: 7266-8511

Mycgr3G85486\_Mycgr3T

Mycgr3G92221 Mycgr3T
  
Location: 8611-9193

Mycgr3G92221\_Mycgr3T

Mycgr3G39931 Mycgr3T
  
Location: 9293-10157

Mycgr3G39931\_Mycgr3T

Mycgr3G99766 Mycgr3T
  
Location: 10257-11775

Mycgr3G99766\_Mycgr3T

NAD(P)-binding protein
  
Accession: EJD35972
  
Location: 75084-76116
  
 NCBI BlastP on this gene

EJD35972

hypothetical protein
  
Accession: EJD35973
  
Location: 76451-77788
  
 NCBI BlastP on this gene

EJD35973

acetyl-CoA synthetase-like protein
  
Accession: EJD35974
  
Location: 78172-91094
  
  
**BlastP hit with Mycgr3G39931\_Mycgr3T**
  
Percentage identity: 43 %
  
BlastP bit score: 189
  
Sequence coverage: 88 %
  
E-value: 5e-50
  
  
 NCBI BlastP on this gene

EJD35974

hypothetical protein
  
Accession: EJD35975
  
Location: 91566-93659
  
  
**BlastP hit with Mycgr3G85486\_Mycgr3T**
  
Percentage identity: 35 %
  
BlastP bit score: 157
  
Sequence coverage: 71 %
  
E-value: 1e-39
  
  
 NCBI BlastP on this gene

EJD35975

hypothetical protein
  
Accession: EJD35976
  
Location: 93923-95607
  
 NCBI BlastP on this gene

EJD35976

P-loop containing nucleoside triphosphate hydrolase protein
  
Accession: EJD35977
  
Location: 95984-101600
  
 NCBI BlastP on this gene

EJD35977

Query: Architecture Search FASTA input

GL985075 : Trichoderma reesei QM6a unplaced genomic scaffold TRIREscaffold\_20    Total score: 2.0     Cumulative Blast bit score: 346

Hit cluster cross-links:

Mycgr3G41235 Mycgr3T
  
Location: 0-4062

Mycgr3G41235\_Mycgr3T

Mycgr3G70577 Mycgr3T
  
Location: 4162-6109

Mycgr3G70577\_Mycgr3T

Mycgr3G40534 Mycgr3T
  
Location: 6209-7166

Mycgr3G40534\_Mycgr3T

Mycgr3G85486 Mycgr3T
  
Location: 7266-8511

Mycgr3G85486\_Mycgr3T

Mycgr3G92221 Mycgr3T
  
Location: 8611-9193

Mycgr3G92221\_Mycgr3T

Mycgr3G39931 Mycgr3T
  
Location: 9293-10157

Mycgr3G39931\_Mycgr3T

Mycgr3G99766 Mycgr3T
  
Location: 10257-11775

Mycgr3G99766\_Mycgr3T

predicted protein
  
Accession: EGR46096
  
Location: 518013-522275
  
 NCBI BlastP on this gene

EGR46096

predicted protein
  
Accession: EGR46020
  
Location: 523796-525332
  
 NCBI BlastP on this gene

EGR46020

predicted protein
  
Accession: EGR46097
  
Location: 525837-527838
  
  
**BlastP hit with Mycgr3G70577\_Mycgr3T**
  
Percentage identity: 26 %
  
BlastP bit score: 182
  
Sequence coverage: 91 %
  
E-value: 2e-46
  
  
 NCBI BlastP on this gene

EGR46097

predicted protein
  
Accession: EGR46021
  
Location: 530969-532408
  
  
**BlastP hit with Mycgr3G85486\_Mycgr3T**
  
Percentage identity: 31 %
  
BlastP bit score: 164
  
Sequence coverage: 80 %
  
E-value: 5e-42
  
  
 NCBI BlastP on this gene

EGR46021

predicted protein
  
Accession: EGR46098
  
Location: 532659-534585
  
 NCBI BlastP on this gene

EGR46098

non-ribosomal peptide synthetase
  
Accession: EGR46022
  
Location: 536612-542053
  
 NCBI BlastP on this gene

EGR46022

Query: Architecture Search FASTA input

AM920431 : Penicillium chrysogenum Wisconsin 54-1255 complete genome, contig Pc00c16.    Total score: 2.0     Cumulative Blast bit score: 346

Hit cluster cross-links:

Mycgr3G41235 Mycgr3T
  
Location: 0-4062

Mycgr3G41235\_Mycgr3T

Mycgr3G70577 Mycgr3T
  
Location: 4162-6109

Mycgr3G70577\_Mycgr3T

Mycgr3G40534 Mycgr3T
  
Location: 6209-7166

Mycgr3G40534\_Mycgr3T

Mycgr3G85486 Mycgr3T
  
Location: 7266-8511

Mycgr3G85486\_Mycgr3T

Mycgr3G92221 Mycgr3T
  
Location: 8611-9193

Mycgr3G92221\_Mycgr3T

Mycgr3G39931 Mycgr3T
  
Location: 9293-10157

Mycgr3G39931\_Mycgr3T

Mycgr3G99766 Mycgr3T
  
Location: 10257-11775

Mycgr3G99766\_Mycgr3T

not annotated
  
Accession: CAP93055
  
Location: 871894-877643
  
 NCBI BlastP on this gene

Pc16g03850

not annotated
  
Accession: CAP93056
  
Location: 878527-879908
  
  
**BlastP hit with Mycgr3G85486\_Mycgr3T**
  
Percentage identity: 30 %
  
BlastP bit score: 165
  
Sequence coverage: 84 %
  
E-value: 2e-42
  
  
 NCBI BlastP on this gene

Pc16g03860

not annotated
  
Accession: CAP93057
  
Location: 880563-884634
  
 NCBI BlastP on this gene

Pc16g03870

not annotated
  
Accession: CAP93058
  
Location: 884789-886193
  
 NCBI BlastP on this gene

Pc16g03880

not annotated
  
Accession: CAP93059
  
Location: 886545-887288
  
 NCBI BlastP on this gene

Pc16g03890

unnamed
  
Accession: CAP93060
  
Location: 888312-889889
  
 NCBI BlastP on this gene

Pc16g03900

not annotated
  
Accession: CAP93061
  
Location: 890263-892148
  
  
**BlastP hit with Mycgr3G70577\_Mycgr3T**
  
Percentage identity: 26 %
  
BlastP bit score: 181
  
Sequence coverage: 89 %
  
E-value: 5e-46
  
  
 NCBI BlastP on this gene

Pc16g03910

not annotated
  
Accession: CAP93062
  
Location: 893255-893635
  
 NCBI BlastP on this gene

Pc16g03920

hypothetical protein
  
Accession: CAP93063
  
Location: 893980-894758
  
 NCBI BlastP on this gene

Pc16g03930

Query: Architecture Search FASTA input

KB916185 : Neofusicoccum parvum UCRNP2 chromosome Unknown NP2\_03\_scaffold\_547    Total score: 2.0     Cumulative Blast bit score: 345

Hit cluster cross-links:

Mycgr3G41235 Mycgr3T
  
Location: 0-4062

Mycgr3G41235\_Mycgr3T

Mycgr3G70577 Mycgr3T
  
Location: 4162-6109

Mycgr3G70577\_Mycgr3T

Mycgr3G40534 Mycgr3T
  
Location: 6209-7166

Mycgr3G40534\_Mycgr3T

Mycgr3G85486 Mycgr3T
  
Location: 7266-8511

Mycgr3G85486\_Mycgr3T

Mycgr3G92221 Mycgr3T
  
Location: 8611-9193

Mycgr3G92221\_Mycgr3T

Mycgr3G39931 Mycgr3T
  
Location: 9293-10157

Mycgr3G39931\_Mycgr3T

Mycgr3G99766 Mycgr3T
  
Location: 10257-11775

Mycgr3G99766\_Mycgr3T

putative nonribosomal siderophore peptide synthase protein
  
Accession: EOD48642
  
Location: 65978-68784
  
  
**BlastP hit with Mycgr3G40534\_Mycgr3T**
  
Percentage identity: 30 %
  
BlastP bit score: 125
  
Sequence coverage: 100 %
  
E-value: 2e-28
  
  
  
**BlastP hit with Mycgr3G39931\_Mycgr3T**
  
Percentage identity: 32 %
  
BlastP bit score: 111
  
Sequence coverage: 89 %
  
E-value: 5e-24
  
  
 NCBI BlastP on this gene

EOD48642

putative nonribosomal peptide synthetase 2 protein
  
Accession: EOD48651
  
Location: 54514-63750
  
  
**BlastP hit with Mycgr3G39931\_Mycgr3T**
  
Percentage identity: 31 %
  
BlastP bit score: 109
  
Sequence coverage: 91 %
  
E-value: 7e-23
  
  
 NCBI BlastP on this gene

EOD48651

hypothetical protein
  
Accession: EOD48661
  
Location: 53367-54101
  
 NCBI BlastP on this gene

EOD48661

Query: Architecture Search FASTA input

HF679032 : Fusarium fujikuroi IMI 58289 draft genome, chromosome FFUJ\_chr10.    Total score: 2.0     Cumulative Blast bit score: 345

Hit cluster cross-links:

Mycgr3G41235 Mycgr3T
  
Location: 0-4062

Mycgr3G41235\_Mycgr3T

Mycgr3G70577 Mycgr3T
  
Location: 4162-6109

Mycgr3G70577\_Mycgr3T

Mycgr3G40534 Mycgr3T
  
Location: 6209-7166

Mycgr3G40534\_Mycgr3T

Mycgr3G85486 Mycgr3T
  
Location: 7266-8511

Mycgr3G85486\_Mycgr3T

Mycgr3G92221 Mycgr3T
  
Location: 8611-9193

Mycgr3G92221\_Mycgr3T

Mycgr3G39931 Mycgr3T
  
Location: 9293-10157

Mycgr3G39931\_Mycgr3T

Mycgr3G99766 Mycgr3T
  
Location: 10257-11775

Mycgr3G99766\_Mycgr3T

related to AM-toxin synthetase (AMT)
  
Accession: CCT74675
  
Location: 1402333-1408592
  
 NCBI BlastP on this gene

FFUJ\_10736

related to enoyl-CoA hydratase
  
Accession: CCT74674
  
Location: 1400338-1401210
  
 NCBI BlastP on this gene

FFUJ\_10735

related to aerobactin siderophore biosynthesis protein iucB
  
Accession: CCT74673
  
Location: 1396540-1397826
  
  
**BlastP hit with Mycgr3G85486\_Mycgr3T**
  
Percentage identity: 32 %
  
BlastP bit score: 162
  
Sequence coverage: 77 %
  
E-value: 2e-41
  
  
 NCBI BlastP on this gene

FFUJ\_10734

related to major facilitator MirA
  
Accession: CCT74672
  
Location: 1392754-1394638
  
  
**BlastP hit with Mycgr3G70577\_Mycgr3T**
  
Percentage identity: 26 %
  
BlastP bit score: 183
  
Sequence coverage: 93 %
  
E-value: 1e-46
  
  
 NCBI BlastP on this gene

FFUJ\_10733

uncharacterized protein
  
Accession: CCT74671
  
Location: 1391252-1392212
  
 NCBI BlastP on this gene

FFUJ\_10732

related to cellobiose dehydrogenase
  
Accession: CCT74670
  
Location: 1386301-1388682
  
 NCBI BlastP on this gene

FFUJ\_10731

Query: Architecture Search FASTA input

KB730325 : Fusarium oxysporum f. sp. cubense race 1 unplaced genomic scaffold scaffold314    Total score: 2.0     Cumulative Blast bit score: 344

Hit cluster cross-links:

Mycgr3G41235 Mycgr3T
  
Location: 0-4062

Mycgr3G41235\_Mycgr3T

Mycgr3G70577 Mycgr3T
  
Location: 4162-6109

Mycgr3G70577\_Mycgr3T

Mycgr3G40534 Mycgr3T
  
Location: 6209-7166

Mycgr3G40534\_Mycgr3T

Mycgr3G85486 Mycgr3T
  
Location: 7266-8511

Mycgr3G85486\_Mycgr3T

Mycgr3G92221 Mycgr3T
  
Location: 8611-9193

Mycgr3G92221\_Mycgr3T

Mycgr3G39931 Mycgr3T
  
Location: 9293-10157

Mycgr3G39931\_Mycgr3T

Mycgr3G99766 Mycgr3T
  
Location: 10257-11775

Mycgr3G99766\_Mycgr3T

HC-toxin synthetase
  
Accession: ENH67315
  
Location: 345279-351538
  
 NCBI BlastP on this gene

ENH67315

Putative enoyl-CoA hydratase/isomerase yngF
  
Accession: ENH67316
  
Location: 352631-353428
  
 NCBI BlastP on this gene

ENH67316

Putative lysine N-acyltransferase C17G9.06c
  
Accession: ENH67317
  
Location: 356013-357265
  
  
**BlastP hit with Mycgr3G85486\_Mycgr3T**
  
Percentage identity: 32 %
  
BlastP bit score: 162
  
Sequence coverage: 77 %
  
E-value: 1e-41
  
  
 NCBI BlastP on this gene

ENH67317

Siderophore iron transporter mirB
  
Accession: ENH67318
  
Location: 359157-361041
  
  
**BlastP hit with Mycgr3G70577\_Mycgr3T**
  
Percentage identity: 26 %
  
BlastP bit score: 182
  
Sequence coverage: 93 %
  
E-value: 3e-46
  
  
 NCBI BlastP on this gene

ENH67318

Ferri-bacillibactin esterase BesA
  
Accession: ENH67319
  
Location: 361589-362549
  
 NCBI BlastP on this gene

ENH67319

Cellobiose dehydrogenase
  
Accession: ENH67320
  
Location: 365308-367689
  
 NCBI BlastP on this gene

ENH67320

Query: Architecture Search FASTA input

KB726307 : Fusarium oxysporum f. sp. cubense race 4 unplaced genomic scaffold scaffold26    Total score: 2.0     Cumulative Blast bit score: 342

Hit cluster cross-links:

Mycgr3G41235 Mycgr3T
  
Location: 0-4062

Mycgr3G41235\_Mycgr3T

Mycgr3G70577 Mycgr3T
  
Location: 4162-6109

Mycgr3G70577\_Mycgr3T

Mycgr3G40534 Mycgr3T
  
Location: 6209-7166

Mycgr3G40534\_Mycgr3T

Mycgr3G85486 Mycgr3T
  
Location: 7266-8511

Mycgr3G85486\_Mycgr3T

Mycgr3G92221 Mycgr3T
  
Location: 8611-9193

Mycgr3G92221\_Mycgr3T

Mycgr3G39931 Mycgr3T
  
Location: 9293-10157

Mycgr3G39931\_Mycgr3T

Mycgr3G99766 Mycgr3T
  
Location: 10257-11775

Mycgr3G99766\_Mycgr3T

HC-toxin synthetase
  
Accession: EMT71267
  
Location: 775770-782029
  
 NCBI BlastP on this gene

EMT71267

Putative enoyl-CoA hydratase/isomerase yngF
  
Accession: EMT71266
  
Location: 773802-774674
  
 NCBI BlastP on this gene

EMT71266

Putative lysine N-acyltransferase C17G9.06c
  
Accession: EMT71265
  
Location: 770010-770984
  
  
**BlastP hit with Mycgr3G85486\_Mycgr3T**
  
Percentage identity: 35 %
  
BlastP bit score: 159
  
Sequence coverage: 66 %
  
E-value: 4e-41
  
  
 NCBI BlastP on this gene

EMT71265

Siderophore iron transporter mirB
  
Accession: EMT71264
  
Location: 766236-768120
  
  
**BlastP hit with Mycgr3G70577\_Mycgr3T**
  
Percentage identity: 26 %
  
BlastP bit score: 183
  
Sequence coverage: 93 %
  
E-value: 2e-46
  
  
 NCBI BlastP on this gene

EMT71264

Ferri-bacillibactin esterase BesA
  
Accession: EMT71263
  
Location: 762491-765688
  
 NCBI BlastP on this gene

EMT71263

Query: Architecture Search FASTA input

CP003002 : Myceliophthora thermophila ATCC 42464 chromosome 1    Total score: 2.0     Cumulative Blast bit score: 341

Hit cluster cross-links:

Mycgr3G41235 Mycgr3T
  
Location: 0-4062

Mycgr3G41235\_Mycgr3T

Mycgr3G70577 Mycgr3T
  
Location: 4162-6109

Mycgr3G70577\_Mycgr3T

Mycgr3G40534 Mycgr3T
  
Location: 6209-7166

Mycgr3G40534\_Mycgr3T

Mycgr3G85486 Mycgr3T
  
Location: 7266-8511

Mycgr3G85486\_Mycgr3T

Mycgr3G92221 Mycgr3T
  
Location: 8611-9193

Mycgr3G92221\_Mycgr3T

Mycgr3G39931 Mycgr3T
  
Location: 9293-10157

Mycgr3G39931\_Mycgr3T

Mycgr3G99766 Mycgr3T
  
Location: 10257-11775

Mycgr3G99766\_Mycgr3T

ABC transporter-like protein
  
Accession: AEO55702
  
Location: 10627496-10631807
  
 NCBI BlastP on this gene

MYCTH\_104717

siderophore biosynthesis protein
  
Accession: AEO55703
  
Location: 10632494-10633979
  
  
**BlastP hit with Mycgr3G85486\_Mycgr3T**
  
Percentage identity: 33 %
  
BlastP bit score: 158
  
Sequence coverage: 81 %
  
E-value: 5e-40
  
  
 NCBI BlastP on this gene

MYCTH\_59137

Crotonase-like protein
  
Accession: AEO55704
  
Location: 10634403-10635390
  
 NCBI BlastP on this gene

MYCTH\_112802

general substrate transporter
  
Accession: AEO55705
  
Location: 10636416-10638428
  
  
**BlastP hit with Mycgr3G70577\_Mycgr3T**
  
Percentage identity: 26 %
  
BlastP bit score: 183
  
Sequence coverage: 94 %
  
E-value: 1e-46
  
  
 NCBI BlastP on this gene

MYCTH\_109499

hypothetical protein
  
Accession: AEO55706
  
Location: 10640414-10644102
  
 NCBI BlastP on this gene

MYCTH\_2299794

Query: Architecture Search FASTA input

ABDF02000085 : Trichoderma virens Gv29-8    Total score: 2.0     Cumulative Blast bit score: 338

Hit cluster cross-links:

Mycgr3G41235 Mycgr3T
  
Location: 0-4062

Mycgr3G41235\_Mycgr3T

Mycgr3G70577 Mycgr3T
  
Location: 4162-6109

Mycgr3G70577\_Mycgr3T

Mycgr3G40534 Mycgr3T
  
Location: 6209-7166

Mycgr3G40534\_Mycgr3T

Mycgr3G85486 Mycgr3T
  
Location: 7266-8511

Mycgr3G85486\_Mycgr3T

Mycgr3G92221 Mycgr3T
  
Location: 8611-9193

Mycgr3G92221\_Mycgr3T

Mycgr3G39931 Mycgr3T
  
Location: 9293-10157

Mycgr3G39931\_Mycgr3T

Mycgr3G99766 Mycgr3T
  
Location: 10257-11775

Mycgr3G99766\_Mycgr3T

non-ribosomal peptide synthetase
  
Accession: EHK18682
  
Location: 78183-83471
  
 NCBI BlastP on this gene

EHK18682

hypothetical protein
  
Accession: EHK18683
  
Location: 85465-87308
  
 NCBI BlastP on this gene

EHK18683

hypothetical protein
  
Accession: EHK18684
  
Location: 87521-88966
  
  
**BlastP hit with Mycgr3G85486\_Mycgr3T**
  
Percentage identity: 30 %
  
BlastP bit score: 155
  
Sequence coverage: 80 %
  
E-value: 5e-39
  
  
 NCBI BlastP on this gene

EHK18684

hypothetical protein
  
Accession: EHK18685
  
Location: 91207-93161
  
  
**BlastP hit with Mycgr3G70577\_Mycgr3T**
  
Percentage identity: 26 %
  
BlastP bit score: 183
  
Sequence coverage: 87 %
  
E-value: 1e-46
  
  
 NCBI BlastP on this gene

EHK18685

hypothetical protein
  
Accession: EHK18686
  
Location: 93666-95147
  
 NCBI BlastP on this gene

EHK18686

hypothetical protein
  
Accession: EHK18687
  
Location: 96467-100675
  
 NCBI BlastP on this gene

EHK18687

Query: Architecture Search FASTA input

AFQF01003643 : Fusarium oxysporum Fo5176    Total score: 2.0     Cumulative Blast bit score: 326

Hit cluster cross-links:

Mycgr3G41235 Mycgr3T
  
Location: 0-4062

Mycgr3G41235\_Mycgr3T

Mycgr3G70577 Mycgr3T
  
Location: 4162-6109

Mycgr3G70577\_Mycgr3T

Mycgr3G40534 Mycgr3T
  
Location: 6209-7166

Mycgr3G40534\_Mycgr3T

Mycgr3G85486 Mycgr3T
  
Location: 7266-8511

Mycgr3G85486\_Mycgr3T

Mycgr3G92221 Mycgr3T
  
Location: 8611-9193

Mycgr3G92221\_Mycgr3T

Mycgr3G39931 Mycgr3T
  
Location: 9293-10157

Mycgr3G39931\_Mycgr3T

Mycgr3G99766 Mycgr3T
  
Location: 10257-11775

Mycgr3G99766\_Mycgr3T

hypothetical protein
  
Accession: EGU74748
  
Location: 76-822
  
  
**BlastP hit with Mycgr3G85486\_Mycgr3T**
  
Percentage identity: 38 %
  
BlastP bit score: 144
  
Sequence coverage: 49 %
  
E-value: 9e-37
  
  
 NCBI BlastP on this gene

EGU74748

hypothetical protein
  
Accession: EGU74749
  
Location: 2714-4598
  
  
**BlastP hit with Mycgr3G70577\_Mycgr3T**
  
Percentage identity: 26 %
  
BlastP bit score: 182
  
Sequence coverage: 93 %
  
E-value: 4e-46
  
  
 NCBI BlastP on this gene

EGU74749

hypothetical protein
  
Accession: EGU74750
  
Location: 5146-6106
  
 NCBI BlastP on this gene

EGU74750

hypothetical protein
  
Accession: EGU74751
  
Location: 8616-11246
  
 NCBI BlastP on this gene

EGU74751

Query: Architecture Search FASTA input

GG704911 : Coccidioides immitis RS genomic scaffold supercont3.1    Total score: 2.0     Cumulative Blast bit score: 306

Hit cluster cross-links:

Mycgr3G41235 Mycgr3T
  
Location: 0-4062

Mycgr3G41235\_Mycgr3T

Mycgr3G70577 Mycgr3T
  
Location: 4162-6109

Mycgr3G70577\_Mycgr3T

Mycgr3G40534 Mycgr3T
  
Location: 6209-7166

Mycgr3G40534\_Mycgr3T

Mycgr3G85486 Mycgr3T
  
Location: 7266-8511

Mycgr3G85486\_Mycgr3T

Mycgr3G92221 Mycgr3T
  
Location: 8611-9193

Mycgr3G92221\_Mycgr3T

Mycgr3G39931 Mycgr3T
  
Location: 9293-10157

Mycgr3G39931\_Mycgr3T

Mycgr3G99766 Mycgr3T
  
Location: 10257-11775

Mycgr3G99766\_Mycgr3T

L-ornithine 5-monooxygenase
  
Accession: EAS35650
  
Location: 5986030-5987648
  
 NCBI BlastP on this gene

EAS35650

hypothetical protein
  
Accession: EAS35649
  
Location: 5987837-5988561
  
 NCBI BlastP on this gene

EAS35649

amino acid adenylation domain-containing protein
  
Accession: EAS35647
  
Location: 5989172-6004808
  
  
**BlastP hit with Mycgr3G40534\_Mycgr3T**
  
Percentage identity: 42 %
  
BlastP bit score: 172
  
Sequence coverage: 81 %
  
E-value: 8e-44
  
  
  
**BlastP hit with Mycgr3G39931\_Mycgr3T**
  
Percentage identity: 35 %
  
BlastP bit score: 134
  
Sequence coverage: 93 %
  
E-value: 4e-31
  
  
 NCBI BlastP on this gene

EAS35647

protein kinase
  
Accession: EAS35646
  
Location: 6005938-6009291
  
 NCBI BlastP on this gene

EAS35646

Query: Architecture Search FASTA input

ACFW01000049 : Coccidioides posadasii C735 delta SOWgp    Total score: 2.0     Cumulative Blast bit score: 306

Hit cluster cross-links:

Mycgr3G41235 Mycgr3T
  
Location: 0-4062

Mycgr3G41235\_Mycgr3T

Mycgr3G70577 Mycgr3T
  
Location: 4162-6109

Mycgr3G70577\_Mycgr3T

Mycgr3G40534 Mycgr3T
  
Location: 6209-7166

Mycgr3G40534\_Mycgr3T

Mycgr3G85486 Mycgr3T
  
Location: 7266-8511

Mycgr3G85486\_Mycgr3T

Mycgr3G92221 Mycgr3T
  
Location: 8611-9193

Mycgr3G92221\_Mycgr3T

Mycgr3G39931 Mycgr3T
  
Location: 9293-10157

Mycgr3G39931\_Mycgr3T

Mycgr3G99766 Mycgr3T
  
Location: 10257-11775

Mycgr3G99766\_Mycgr3T

L-ornithine 5-monooxygenase, putative
  
Accession: EER24035
  
Location: 3048954-3050561
  
 NCBI BlastP on this gene

EER24035

peptide synthetase, putative
  
Accession: EER24036
  
Location: 3052091-3067586
  
  
**BlastP hit with Mycgr3G40534\_Mycgr3T**
  
Percentage identity: 41 %
  
BlastP bit score: 172
  
Sequence coverage: 80 %
  
E-value: 1e-43
  
  
  
**BlastP hit with Mycgr3G39931\_Mycgr3T**
  
Percentage identity: 35 %
  
BlastP bit score: 134
  
Sequence coverage: 93 %
  
E-value: 3e-31
  
  
 NCBI BlastP on this gene

EER24036

protein kinase, putative
  
Accession: EER24037
  
Location: 3068852-3072205
  
 NCBI BlastP on this gene

EER24037

Query: Architecture Search FASTA input

CH476615 : Uncinocarpus reesii 1704 scaffold\_1 genomic scaffold    Total score: 2.0     Cumulative Blast bit score: 302

Hit cluster cross-links:

Mycgr3G41235 Mycgr3T
  
Location: 0-4062

Mycgr3G41235\_Mycgr3T

Mycgr3G70577 Mycgr3T
  
Location: 4162-6109

Mycgr3G70577\_Mycgr3T

Mycgr3G40534 Mycgr3T
  
Location: 6209-7166

Mycgr3G40534\_Mycgr3T

Mycgr3G85486 Mycgr3T
  
Location: 7266-8511

Mycgr3G85486\_Mycgr3T

Mycgr3G92221 Mycgr3T
  
Location: 8611-9193

Mycgr3G92221\_Mycgr3T

Mycgr3G39931 Mycgr3T
  
Location: 9293-10157

Mycgr3G39931\_Mycgr3T

Mycgr3G99766 Mycgr3T
  
Location: 10257-11775

Mycgr3G99766\_Mycgr3T

predicted protein
  
Accession: EEP76045
  
Location: 2104471-2105278
  
 NCBI BlastP on this gene

EEP76045

conserved hypothetical protein
  
Accession: EEP76044
  
Location: 2103621-2104414
  
 NCBI BlastP on this gene

EEP76044

predicted protein
  
Accession: EEP76043
  
Location: 2098708-2102342
  
  
**BlastP hit with Mycgr3G40534\_Mycgr3T**
  
Percentage identity: 42 %
  
BlastP bit score: 172
  
Sequence coverage: 81 %
  
E-value: 3e-44
  
  
 NCBI BlastP on this gene

EEP76043

predicted protein
  
Accession: EEP76042
  
Location: 2086732-2097150
  
  
**BlastP hit with Mycgr3G39931\_Mycgr3T**
  
Percentage identity: 34 %
  
BlastP bit score: 130
  
Sequence coverage: 93 %
  
E-value: 8e-30
  
  
 NCBI BlastP on this gene

EEP76042

hypothetical protein
  
Accession: EEP76041
  
Location: 2082428-2085782
  
 NCBI BlastP on this gene

EEP76041

Query: Architecture Search FASTA input

KB644411 : Penicillium oxalicum 114-2 unplaced genomic scaffold scaffold\_4    Total score: 2.0     Cumulative Blast bit score: 301

Hit cluster cross-links:

Mycgr3G41235 Mycgr3T
  
Location: 0-4062

Mycgr3G41235\_Mycgr3T

Mycgr3G70577 Mycgr3T
  
Location: 4162-6109

Mycgr3G70577\_Mycgr3T

Mycgr3G40534 Mycgr3T
  
Location: 6209-7166

Mycgr3G40534\_Mycgr3T

Mycgr3G85486 Mycgr3T
  
Location: 7266-8511

Mycgr3G85486\_Mycgr3T

Mycgr3G92221 Mycgr3T
  
Location: 8611-9193

Mycgr3G92221\_Mycgr3T

Mycgr3G39931 Mycgr3T
  
Location: 9293-10157

Mycgr3G39931\_Mycgr3T

Mycgr3G99766 Mycgr3T
  
Location: 10257-11775

Mycgr3G99766\_Mycgr3T

hypothetical protein
  
Accession: EPS29125
  
Location: 2397439-2413126
  
  
**BlastP hit with Mycgr3G40534\_Mycgr3T**
  
Percentage identity: 41 %
  
BlastP bit score: 175
  
Sequence coverage: 82 %
  
E-value: 1e-44
  
  
  
**BlastP hit with Mycgr3G39931\_Mycgr3T**
  
Percentage identity: 34 %
  
BlastP bit score: 126
  
Sequence coverage: 90 %
  
E-value: 1e-28
  
  
 NCBI BlastP on this gene

EPS29125

Query: Architecture Search FASTA input

EQ962653 : Talaromyces stipitatus ATCC 10500 scf\_1105507295527 genomic scaffold    Total score: 2.0     Cumulative Blast bit score: 301

Hit cluster cross-links:

Mycgr3G41235 Mycgr3T
  
Location: 0-4062

Mycgr3G41235\_Mycgr3T

Mycgr3G70577 Mycgr3T
  
Location: 4162-6109

Mycgr3G70577\_Mycgr3T

Mycgr3G40534 Mycgr3T
  
Location: 6209-7166

Mycgr3G40534\_Mycgr3T

Mycgr3G85486 Mycgr3T
  
Location: 7266-8511

Mycgr3G85486\_Mycgr3T

Mycgr3G92221 Mycgr3T
  
Location: 8611-9193

Mycgr3G92221\_Mycgr3T

Mycgr3G39931 Mycgr3T
  
Location: 9293-10157

Mycgr3G39931\_Mycgr3T

Mycgr3G99766 Mycgr3T
  
Location: 10257-11775

Mycgr3G99766\_Mycgr3T

L-ornithine N5-oxygenase SidA
  
Accession: EED21788
  
Location: 2668207-2669704
  
 NCBI BlastP on this gene

EED21788

nonribosomal siderophore peptide synthase SidC
  
Accession: EED21789
  
Location: 2670983-2686751
  
  
**BlastP hit with Mycgr3G40534\_Mycgr3T**
  
Percentage identity: 36 %
  
BlastP bit score: 168
  
Sequence coverage: 102 %
  
E-value: 3e-42
  
  
  
**BlastP hit with Mycgr3G39931\_Mycgr3T**
  
Percentage identity: 34 %
  
BlastP bit score: 133
  
Sequence coverage: 90 %
  
E-value: 1e-30
  
  
 NCBI BlastP on this gene

EED21789

Query: Architecture Search FASTA input

DS995701 : Microsporum canis CBS 113480 supercont1.1 genomic scaffold    Total score: 2.0     Cumulative Blast bit score: 299

Hit cluster cross-links:

Mycgr3G41235 Mycgr3T
  
Location: 0-4062

Mycgr3G41235\_Mycgr3T

Mycgr3G70577 Mycgr3T
  
Location: 4162-6109

Mycgr3G70577\_Mycgr3T

Mycgr3G40534 Mycgr3T
  
Location: 6209-7166

Mycgr3G40534\_Mycgr3T

Mycgr3G85486 Mycgr3T
  
Location: 7266-8511

Mycgr3G85486\_Mycgr3T

Mycgr3G92221 Mycgr3T
  
Location: 8611-9193

Mycgr3G92221\_Mycgr3T

Mycgr3G39931 Mycgr3T
  
Location: 9293-10157

Mycgr3G39931\_Mycgr3T

Mycgr3G99766 Mycgr3T
  
Location: 10257-11775

Mycgr3G99766\_Mycgr3T

L-ornithine 5-monooxygenase
  
Accession: EEQ27378
  
Location: 681825-683396
  
 NCBI BlastP on this gene

EEQ27378

nonribosomal peptide synthetase 2
  
Accession: EEQ27377
  
Location: 664435-680056
  
  
**BlastP hit with Mycgr3G40534\_Mycgr3T**
  
Percentage identity: 40 %
  
BlastP bit score: 169
  
Sequence coverage: 81 %
  
E-value: 1e-42
  
  
  
**BlastP hit with Mycgr3G39931\_Mycgr3T**
  
Percentage identity: 35 %
  
BlastP bit score: 130
  
Sequence coverage: 90 %
  
E-value: 9e-30
  
  
 NCBI BlastP on this gene

EEQ27377

AnkA
  
Accession: EEQ27376
  
Location: 659838-662841
  
 NCBI BlastP on this gene

EEQ27376

Query: Architecture Search FASTA input

AM920428 : Penicillium chrysogenum Wisconsin 54-1255 complete genome, contig Pc00c13.    Total score: 2.0     Cumulative Blast bit score: 297

Hit cluster cross-links:

Mycgr3G41235 Mycgr3T
  
Location: 0-4062

Mycgr3G41235\_Mycgr3T

Mycgr3G70577 Mycgr3T
  
Location: 4162-6109

Mycgr3G70577\_Mycgr3T

Mycgr3G40534 Mycgr3T
  
Location: 6209-7166

Mycgr3G40534\_Mycgr3T

Mycgr3G85486 Mycgr3T
  
Location: 7266-8511

Mycgr3G85486\_Mycgr3T

Mycgr3G92221 Mycgr3T
  
Location: 8611-9193

Mycgr3G92221\_Mycgr3T

Mycgr3G39931 Mycgr3T
  
Location: 9293-10157

Mycgr3G39931\_Mycgr3T

Mycgr3G99766 Mycgr3T
  
Location: 10257-11775

Mycgr3G99766\_Mycgr3T

not annotated
  
Accession: CAP91595
  
Location: 1307856-1309413
  
 NCBI BlastP on this gene

Pc13g05260

not annotated
  
Accession: CAP91594
  
Location: 1290919-1306476
  
  
**BlastP hit with Mycgr3G40534\_Mycgr3T**
  
Percentage identity: 38 %
  
BlastP bit score: 177
  
Sequence coverage: 85 %
  
E-value: 2e-45
  
  
  
**BlastP hit with Mycgr3G39931\_Mycgr3T**
  
Percentage identity: 32 %
  
BlastP bit score: 120
  
Sequence coverage: 90 %
  
E-value: 2e-26
  
  
 NCBI BlastP on this gene

Pc13g05250

not annotated
  
Accession: CAP91593
  
Location: 1285814-1289005
  
 NCBI BlastP on this gene

Pc13g05240

Query: Architecture Search FASTA input

JH126399 : Cordyceps militaris CM01 unplaced genomic scaffold CCM\_S00001    Total score: 2.0     Cumulative Blast bit score: 295

Hit cluster cross-links:

Mycgr3G41235 Mycgr3T
  
Location: 0-4062

Mycgr3G41235\_Mycgr3T

Mycgr3G70577 Mycgr3T
  
Location: 4162-6109

Mycgr3G70577\_Mycgr3T

Mycgr3G40534 Mycgr3T
  
Location: 6209-7166

Mycgr3G40534\_Mycgr3T

Mycgr3G85486 Mycgr3T
  
Location: 7266-8511

Mycgr3G85486\_Mycgr3T

Mycgr3G92221 Mycgr3T
  
Location: 8611-9193

Mycgr3G92221\_Mycgr3T

Mycgr3G39931 Mycgr3T
  
Location: 9293-10157

Mycgr3G39931\_Mycgr3T

Mycgr3G99766 Mycgr3T
  
Location: 10257-11775

Mycgr3G99766\_Mycgr3T

nonribosomal siderophore peptide synthase
  
Accession: EGX97046
  
Location: 5481892-5496446
  
  
**BlastP hit with Mycgr3G40534\_Mycgr3T**
  
Percentage identity: 41 %
  
BlastP bit score: 169
  
Sequence coverage: 81 %
  
E-value: 9e-43
  
  
  
**BlastP hit with Mycgr3G39931\_Mycgr3T**
  
Percentage identity: 33 %
  
BlastP bit score: 126
  
Sequence coverage: 90 %
  
E-value: 2e-28
  
  
 NCBI BlastP on this gene

EGX97046

choline oxidase (CodA), putative
  
Accession: EGX97045
  
Location: 5479825-5481462
  
 NCBI BlastP on this gene

EGX97045

Query: Architecture Search FASTA input

GL377303 : Schizophyllum commune H4-8 unplaced genomic scaffold SCHCOscaffold\_2    Total score: 2.0     Cumulative Blast bit score: 294

Hit cluster cross-links:

Mycgr3G41235 Mycgr3T
  
Location: 0-4062

Mycgr3G41235\_Mycgr3T

Mycgr3G70577 Mycgr3T
  
Location: 4162-6109

Mycgr3G70577\_Mycgr3T

Mycgr3G40534 Mycgr3T
  
Location: 6209-7166

Mycgr3G40534\_Mycgr3T

Mycgr3G85486 Mycgr3T
  
Location: 7266-8511

Mycgr3G85486\_Mycgr3T

Mycgr3G92221 Mycgr3T
  
Location: 8611-9193

Mycgr3G92221\_Mycgr3T

Mycgr3G39931 Mycgr3T
  
Location: 9293-10157

Mycgr3G39931\_Mycgr3T

Mycgr3G99766 Mycgr3T
  
Location: 10257-11775

Mycgr3G99766\_Mycgr3T

hypothetical protein
  
Accession: EFJ00415
  
Location: 3200241-3205280
  
 NCBI BlastP on this gene

EFJ00415

hypothetical protein
  
Accession: EFJ01218
  
Location: 3180710-3199916
  
  
**BlastP hit with Mycgr3G40534\_Mycgr3T**
  
Percentage identity: 37 %
  
BlastP bit score: 187
  
Sequence coverage: 105 %
  
E-value: 7e-49
  
  
  
**BlastP hit with Mycgr3G39931\_Mycgr3T**
  
Percentage identity: 33 %
  
BlastP bit score: 107
  
Sequence coverage: 91 %
  
E-value: 2e-22
  
  
 NCBI BlastP on this gene

EFJ01218

hypothetical protein
  
Accession: EFJ01217
  
Location: 3176250-3179923
  
 NCBI BlastP on this gene

EFJ01217

Query: Architecture Search FASTA input

DS995900 : Penicillium marneffei ATCC 18224 scf\_1105668340758 genomic scaffold    Total score: 2.0     Cumulative Blast bit score: 293

Hit cluster cross-links:

Mycgr3G41235 Mycgr3T
  
Location: 0-4062

Mycgr3G41235\_Mycgr3T

Mycgr3G70577 Mycgr3T
  
Location: 4162-6109

Mycgr3G70577\_Mycgr3T

Mycgr3G40534 Mycgr3T
  
Location: 6209-7166

Mycgr3G40534\_Mycgr3T

Mycgr3G85486 Mycgr3T
  
Location: 7266-8511

Mycgr3G85486\_Mycgr3T

Mycgr3G92221 Mycgr3T
  
Location: 8611-9193

Mycgr3G92221\_Mycgr3T

Mycgr3G39931 Mycgr3T
  
Location: 9293-10157

Mycgr3G39931\_Mycgr3T

Mycgr3G99766 Mycgr3T
  
Location: 10257-11775

Mycgr3G99766\_Mycgr3T

L-ornithine N5-oxygenase SidA
  
Accession: EEA25906
  
Location: 2214865-2216378
  
 NCBI BlastP on this gene

EEA25906

nonribosomal siderophore peptide synthase, putative
  
Accession: EEA25907
  
Location: 2217834-2233637
  
  
**BlastP hit with Mycgr3G40534\_Mycgr3T**
  
Percentage identity: 39 %
  
BlastP bit score: 161
  
Sequence coverage: 81 %
  
E-value: 4e-40
  
  
  
**BlastP hit with Mycgr3G39931\_Mycgr3T**
  
Percentage identity: 33 %
  
BlastP bit score: 132
  
Sequence coverage: 90 %
  
E-value: 1e-30
  
  
 NCBI BlastP on this gene

EEA25907

Query: Architecture Search FASTA input

DS990636 : Ajellomyces capsulatus H88 supercont1.1 genomic scaffold    Total score: 2.0     Cumulative Blast bit score: 289

Hit cluster cross-links:

Mycgr3G41235 Mycgr3T
  
Location: 0-4062

Mycgr3G41235\_Mycgr3T

Mycgr3G70577 Mycgr3T
  
Location: 4162-6109

Mycgr3G70577\_Mycgr3T

Mycgr3G40534 Mycgr3T
  
Location: 6209-7166

Mycgr3G40534\_Mycgr3T

Mycgr3G85486 Mycgr3T
  
Location: 7266-8511

Mycgr3G85486\_Mycgr3T

Mycgr3G92221 Mycgr3T
  
Location: 8611-9193

Mycgr3G92221\_Mycgr3T

Mycgr3G39931 Mycgr3T
  
Location: 9293-10157

Mycgr3G39931\_Mycgr3T

Mycgr3G99766 Mycgr3T
  
Location: 10257-11775

Mycgr3G99766\_Mycgr3T

nonribosomal peptide synthetase
  
Accession: EGC41890
  
Location: 4852900-4866948
  
  
**BlastP hit with Mycgr3G40534\_Mycgr3T**
  
Percentage identity: 39 %
  
BlastP bit score: 167
  
Sequence coverage: 102 %
  
E-value: 5e-42
  
  
  
**BlastP hit with Mycgr3G39931\_Mycgr3T**
  
Percentage identity: 31 %
  
BlastP bit score: 122
  
Sequence coverage: 96 %
  
E-value: 3e-27
  
  
 NCBI BlastP on this gene

EGC41890

nonribosomal peptide synthase
  
Accession: EGC41889
  
Location: 4851004-4852714
  
 NCBI BlastP on this gene

EGC41889

Query: Architecture Search FASTA input

CP003009 : Thielavia terrestris NRRL 8126 chromosome 1    Total score: 2.0     Cumulative Blast bit score: 280

Hit cluster cross-links:

Mycgr3G41235 Mycgr3T
  
Location: 0-4062

Mycgr3G41235\_Mycgr3T

Mycgr3G70577 Mycgr3T
  
Location: 4162-6109

Mycgr3G70577\_Mycgr3T

Mycgr3G40534 Mycgr3T
  
Location: 6209-7166

Mycgr3G40534\_Mycgr3T

Mycgr3G85486 Mycgr3T
  
Location: 7266-8511

Mycgr3G85486\_Mycgr3T

Mycgr3G92221 Mycgr3T
  
Location: 8611-9193

Mycgr3G92221\_Mycgr3T

Mycgr3G39931 Mycgr3T
  
Location: 9293-10157

Mycgr3G39931\_Mycgr3T

Mycgr3G99766 Mycgr3T
  
Location: 10257-11775

Mycgr3G99766\_Mycgr3T

hypothetical protein
  
Accession: AEO63767
  
Location: 6174935-6176671
  
 NCBI BlastP on this gene

THITE\_2109366

non-ribosomal peptide synthetase
  
Accession: AEO63766
  
Location: 6158586-6173589
  
  
**BlastP hit with Mycgr3G40534\_Mycgr3T**
  
Percentage identity: 42 %
  
BlastP bit score: 164
  
Sequence coverage: 81 %
  
E-value: 3e-41
  
  
  
**BlastP hit with Mycgr3G39931\_Mycgr3T**
  
Percentage identity: 30 %
  
BlastP bit score: 116
  
Sequence coverage: 89 %
  
E-value: 3e-25
  
  
 NCBI BlastP on this gene

THITE\_62324

hypothetical protein
  
Accession: AEO63765
  
Location: 6157633-6158444
  
 NCBI BlastP on this gene

THITE\_135405

hypothetical protein
  
Accession: AEO63764
  
Location: 6154333-6156423
  
 NCBI BlastP on this gene

THITE\_2109360

Query: Architecture Search FASTA input

KE375213 : Blumeria graminis f. sp. tritici 96224 unplaced genomic scaffold Scaffold-87    Total score: 2.0     Cumulative Blast bit score: 279

Hit cluster cross-links:

Mycgr3G41235 Mycgr3T
  
Location: 0-4062

Mycgr3G41235\_Mycgr3T

Mycgr3G70577 Mycgr3T
  
Location: 4162-6109

Mycgr3G70577\_Mycgr3T

Mycgr3G40534 Mycgr3T
  
Location: 6209-7166

Mycgr3G40534\_Mycgr3T

Mycgr3G85486 Mycgr3T
  
Location: 7266-8511

Mycgr3G85486\_Mycgr3T

Mycgr3G92221 Mycgr3T
  
Location: 8611-9193

Mycgr3G92221\_Mycgr3T

Mycgr3G39931 Mycgr3T
  
Location: 9293-10157

Mycgr3G39931\_Mycgr3T

Mycgr3G99766 Mycgr3T
  
Location: 10257-11775

Mycgr3G99766\_Mycgr3T

hypothetical protein
  
Accession: EPQ61840
  
Location: 1565561-1566808
  
  
**BlastP hit with Mycgr3G40534\_Mycgr3T**
  
Percentage identity: 37 %
  
BlastP bit score: 140
  
Sequence coverage: 90 %
  
E-value: 1e-34
  
  
 NCBI BlastP on this gene

EPQ61840

Alpha aminoadipate reductase
  
Accession: EPQ61841
  
Location: 1567007-1580442
  
  
**BlastP hit with Mycgr3G39931\_Mycgr3T**
  
Percentage identity: 36 %
  
BlastP bit score: 139
  
Sequence coverage: 90 %
  
E-value: 6e-33
  
  
 NCBI BlastP on this gene

EPQ61841

Query: Architecture Search FASTA input

1. :  CM001199 Mycosphaerella graminicola IPO323 chromosome 4     Total score: 7.0     Cumulative Blast bit score: 7628

Mycgr3G41235 Mycgr3T
  
Location: 0-4062
  
 NCBI BlastP on this gene

Mycgr3G41235\_Mycgr3T

Mycgr3G70577 Mycgr3T
  
Location: 4162-6109
  
 NCBI BlastP on this gene

Mycgr3G70577\_Mycgr3T

Mycgr3G40534 Mycgr3T
  
Location: 6209-7166
  
 NCBI BlastP on this gene

Mycgr3G40534\_Mycgr3T

Mycgr3G85486 Mycgr3T
  
Location: 7266-8511
  
 NCBI BlastP on this gene

Mycgr3G85486\_Mycgr3T

Mycgr3G92221 Mycgr3T
  
Location: 8611-9193
  
 NCBI BlastP on this gene

Mycgr3G92221\_Mycgr3T

Mycgr3G39931 Mycgr3T
  
Location: 9293-10157
  
 NCBI BlastP on this gene

Mycgr3G39931\_Mycgr3T

Mycgr3G99766 Mycgr3T
  
Location: 10257-11775
  
 NCBI BlastP on this gene

Mycgr3G99766\_Mycgr3T

signal-peptide-containing protein
  
Accession: EGP88587
  
Location: 12877-13759
  
  
**BlastP hit with Mycgr3G92221\_Mycgr3T**
  
Percentage identity: 100 %
  
BlastP bit score: 395
  
Sequence coverage: 99 %
  
E-value: 3e-138
  
  
 NCBI BlastP on this gene

EGP88587

Non-ribosomal peptide synthetase
  
Accession: EGP88586
  
Location: 15580-30095
  
  
**BlastP hit with Mycgr3G40534\_Mycgr3T**
  
Percentage identity: 100 %
  
BlastP bit score: 667
  
Sequence coverage: 100 %
  
E-value: 0.0
  
  
  
**BlastP hit with Mycgr3G39931\_Mycgr3T**
  
Percentage identity: 100 %
  
BlastP bit score: 607
  
Sequence coverage: 100 %
  
E-value: 0.0
  
  
 NCBI BlastP on this gene

EGP88586

putative siderophore biosynthesis
  
Accession: EGP87766
  
Location: 30645-32000
  
  
**BlastP hit with Mycgr3G85486\_Mycgr3T**
  
Percentage identity: 100 %
  
BlastP bit score: 860
  
Sequence coverage: 99 %
  
E-value: 0.0
  
  
 NCBI BlastP on this gene

EGP87766

putative siderophore-dependent iron transporter
  
Accession: EGP87767
  
Location: 33143-35151
  
  
**BlastP hit with Mycgr3G70577\_Mycgr3T**
  
Percentage identity: 100 %
  
BlastP bit score: 1330
  
Sequence coverage: 99 %
  
E-value: 0.0
  
  
 NCBI BlastP on this gene

EGP87767

putative ABC transporter
  
Accession: EGP87768
  
Location: 36524-40624
  
  
**BlastP hit with Mycgr3G41235\_Mycgr3T**
  
Percentage identity: 100 %
  
BlastP bit score: 2751
  
Sequence coverage: 99 %
  
E-value: 0.0
  
  
 NCBI BlastP on this gene

EGP87768

MFS1 putative major facilitator superfamily transporter
  
Accession: EGP87769
  
Location: 41788-43948
  
  
**BlastP hit with Mycgr3G99766\_Mycgr3T**
  
Percentage identity: 100 %
  
BlastP bit score: 1018
  
Sequence coverage: 99 %
  
E-value: 0.0
  
  
 NCBI BlastP on this gene

EGP87769

succinate dehydrogenase flavoprotein subunit
  
Accession: EGP88585
  
Location: 48463-49155
  
 NCBI BlastP on this gene

EGP88585

hypothetical protein
  
Accession: EGP87770
  
Location: 49623-51124
  
 NCBI BlastP on this gene

EGP87770

hypothetical protein
  
Accession: EGP88584
  
Location: 51363-52158
  
 NCBI BlastP on this gene

EGP88584

hypothetical protein
  
Accession: EGP87771
  
Location: 52309-53838
  
 NCBI BlastP on this gene

EGP87771

hypothetical protein
  
Accession: EGP88583
  
Location: 54527-55273
  
 NCBI BlastP on this gene

EGP88583

hypothetical protein
  
Accession: EGP88582
  
Location: 56007-58646
  
 NCBI BlastP on this gene

EGP88582

hypothetical protein
  
Accession: EGP87772
  
Location: 59434-60066
  
 NCBI BlastP on this gene

EGP87772

2. :  GG698929 Nectria haematococca mpVI 77-13-4 chromosome 11 genomic scaffold NECHAsca\_39\_chr11\_5\_0     Total score: 5.0     Cumulative Blast bit score: 2803

hypothetical protein
  
Accession: EEU36332
  
Location: 332904-337150
  
 NCBI BlastP on this gene

EEU36332

hypothetical protein
  
Accession: EEU36331
  
Location: 328644-330705
  
 NCBI BlastP on this gene

EEU36331

hypothetical protein
  
Accession: EEU36397
  
Location: 325849-327752
  
 NCBI BlastP on this gene

EEU36397

hypothetical protein
  
Accession: EEU36330
  
Location: 322120-323856
  
 NCBI BlastP on this gene

EEU36330

hypothetical protein
  
Accession: EEU36329
  
Location: 320185-321442
  
 NCBI BlastP on this gene

EEU36329

hypothetical protein
  
Accession: EEU36396
  
Location: 318269-319365
  
 NCBI BlastP on this gene

EEU36396

predicted protein
  
Accession: EEU36395
  
Location: 314386-315252
  
 NCBI BlastP on this gene

EEU36395

hypothetical protein
  
Accession: EEU36328
  
Location: 312227-314170
  
  
**BlastP hit with Mycgr3G70577\_Mycgr3T**
  
Percentage identity: 54 %
  
BlastP bit score: 658
  
Sequence coverage: 91 %
  
E-value: 0.0
  
  
 NCBI BlastP on this gene

EEU36328

hypothetical protein
  
Accession: EEU36327
  
Location: 306959-311185
  
  
**BlastP hit with Mycgr3G41235\_Mycgr3T**
  
Percentage identity: 44 %
  
BlastP bit score: 1117
  
Sequence coverage: 102 %
  
E-value: 0.0
  
  
 NCBI BlastP on this gene

EEU36327

hypothetical protein
  
Accession: EEU36394
  
Location: 304750-306030
  
  
**BlastP hit with Mycgr3G85486\_Mycgr3T**
  
Percentage identity: 57 %
  
BlastP bit score: 482
  
Sequence coverage: 103 %
  
E-value: 3e-165
  
  
 NCBI BlastP on this gene

EEU36394

hypothetical protein
  
Accession: EEU36393
  
Location: 290155-304310
  
  
**BlastP hit with Mycgr3G40534\_Mycgr3T**
  
Percentage identity: 52 %
  
BlastP bit score: 273
  
Sequence coverage: 81 %
  
E-value: 1e-78
  
  
  
**BlastP hit with Mycgr3G39931\_Mycgr3T**
  
Percentage identity: 49 %
  
BlastP bit score: 273
  
Sequence coverage: 96 %
  
E-value: 7e-79
  
  
 NCBI BlastP on this gene

EEU36393

hypothetical protein
  
Accession: EEU36392
  
Location: 287237-287479
  
 NCBI BlastP on this gene

EEU36392

hypothetical protein
  
Accession: EEU36391
  
Location: 283521-285386
  
 NCBI BlastP on this gene

EEU36391

hypothetical protein
  
Accession: EEU36326
  
Location: 281618-283306
  
 NCBI BlastP on this gene

EEU36326

hypothetical protein
  
Accession: EEU36325
  
Location: 279861-281025
  
 NCBI BlastP on this gene

EEU36325

hypothetical protein
  
Accession: EEU36390
  
Location: 277758-278909
  
 NCBI BlastP on this gene

EEU36390

predicted protein
  
Accession: EEU36389
  
Location: 275852-277295
  
 NCBI BlastP on this gene

EEU36389

hypothetical protein
  
Accession: EEU36324
  
Location: 273177-275347
  
 NCBI BlastP on this gene

EEU36324

hypothetical protein
  
Accession: EEU36388
  
Location: 270447-271154
  
 NCBI BlastP on this gene

EEU36388

3. :  AFNW01000059 Fusarium pseudograminearum CS3096     Total score: 5.0     Cumulative Blast bit score: 2796

hypothetical protein
  
Accession: EKJ77368
  
Location: 140688-141935
  
 NCBI BlastP on this gene

EKJ77368

hypothetical protein
  
Accession: EKJ77369
  
Location: 142640-144406
  
 NCBI BlastP on this gene

EKJ77369

hypothetical protein
  
Accession: EKJ77370
  
Location: 145550-145979
  
 NCBI BlastP on this gene

EKJ77370

hypothetical protein
  
Accession: EKJ77371
  
Location: 146211-146399
  
 NCBI BlastP on this gene

EKJ77371

GAOA
  
Accession: EKJ77372
  
Location: 147448-149490
  
 NCBI BlastP on this gene

EKJ77372

hypothetical protein
  
Accession: EKJ77373
  
Location: 151263-152904
  
 NCBI BlastP on this gene

EKJ77373

hypothetical protein
  
Accession: EKJ77374
  
Location: 153641-154303
  
 NCBI BlastP on this gene

EKJ77374

hypothetical protein
  
Accession: EKJ77375
  
Location: 159625-161541
  
  
**BlastP hit with Mycgr3G70577\_Mycgr3T**
  
Percentage identity: 52 %
  
BlastP bit score: 654
  
Sequence coverage: 95 %
  
E-value: 0.0
  
  
 NCBI BlastP on this gene

EKJ77375

hypothetical protein
  
Accession: EKJ77376
  
Location: 162661-166859
  
  
**BlastP hit with Mycgr3G41235\_Mycgr3T**
  
Percentage identity: 44 %
  
BlastP bit score: 1160
  
Sequence coverage: 103 %
  
E-value: 0.0
  
  
 NCBI BlastP on this gene

EKJ77376

hypothetical protein
  
Accession: EKJ77377
  
Location: 167780-169063
  
  
**BlastP hit with Mycgr3G85486\_Mycgr3T**
  
Percentage identity: 53 %
  
BlastP bit score: 439
  
Sequence coverage: 100 %
  
E-value: 3e-148
  
  
 NCBI BlastP on this gene

EKJ77377

NPS1
  
Accession: EKJ77378
  
Location: 169631-183939
  
  
**BlastP hit with Mycgr3G40534\_Mycgr3T**
  
Percentage identity: 53 %
  
BlastP bit score: 268
  
Sequence coverage: 78 %
  
E-value: 1e-76
  
  
  
**BlastP hit with Mycgr3G39931\_Mycgr3T**
  
Percentage identity: 47 %
  
BlastP bit score: 275
  
Sequence coverage: 99 %
  
E-value: 9e-80
  
  
 NCBI BlastP on this gene

EKJ77378

TRI15
  
Accession: EKJ77379
  
Location: 185439-186368
  
 NCBI BlastP on this gene

EKJ77379

hypothetical protein
  
Accession: EKJ77380
  
Location: 186684-187901
  
 NCBI BlastP on this gene

EKJ77380

hypothetical protein
  
Accession: EKJ77381
  
Location: 188308-189962
  
 NCBI BlastP on this gene

EKJ77381

hypothetical protein
  
Accession: EKJ77382
  
Location: 190552-191432
  
 NCBI BlastP on this gene

EKJ77382

hypothetical protein
  
Accession: EKJ77383
  
Location: 191868-192993
  
 NCBI BlastP on this gene

EKJ77383

hypothetical protein
  
Accession: EKJ77384
  
Location: 193359-194210
  
 NCBI BlastP on this gene

EKJ77384

hypothetical protein
  
Accession: EKJ77385
  
Location: 194528-196213
  
 NCBI BlastP on this gene

EKJ77385

hypothetical protein
  
Accession: EKJ77386
  
Location: 198863-200659
  
 NCBI BlastP on this gene

EKJ77386

hypothetical protein
  
Accession: EKJ77387
  
Location: 200769-201041
  
 NCBI BlastP on this gene

EKJ77387

hypothetical protein
  
Accession: EKJ77388
  
Location: 201428-201977
  
 NCBI BlastP on this gene

EKJ77388

hypothetical protein
  
Accession: EKJ77389
  
Location: 202371-203525
  
 NCBI BlastP on this gene

EKJ77389

4. :  CH408030 Chaetomium globosum CBS 148.51 scaffold\_2 genomic scaffold     Total score: 5.0     Cumulative Blast bit score: 2769

hypothetical protein
  
Accession: EAQ90324
  
Location: 415476-417244
  
 NCBI BlastP on this gene

EAQ90324

hypothetical protein
  
Accession: EAQ90323
  
Location: 409153-414227
  
 NCBI BlastP on this gene

EAQ90323

hypothetical protein
  
Accession: EAQ90322
  
Location: 407662-408813
  
 NCBI BlastP on this gene

EAQ90322

hypothetical protein
  
Accession: EAQ90321
  
Location: 405316-407076
  
 NCBI BlastP on this gene

EAQ90321

predicted protein
  
Accession: EAQ90320
  
Location: 404582-404908
  
 NCBI BlastP on this gene

EAQ90320

hypothetical protein
  
Accession: EAQ90319
  
Location: 396559-402878
  
  
**BlastP hit with Mycgr3G70577\_Mycgr3T**
  
Percentage identity: 55 %
  
BlastP bit score: 684
  
Sequence coverage: 97 %
  
E-value: 0.0
  
  
 NCBI BlastP on this gene

EAQ90319

hypothetical protein
  
Accession: EAQ90318
  
Location: 388577-395459
  
  
**BlastP hit with Mycgr3G41235\_Mycgr3T**
  
Percentage identity: 42 %
  
BlastP bit score: 1117
  
Sequence coverage: 105 %
  
E-value: 0.0
  
  
 NCBI BlastP on this gene

EAQ90318

hypothetical protein
  
Accession: EAQ90317
  
Location: 381683-385419
  
  
**BlastP hit with Mycgr3G85486\_Mycgr3T**
  
Percentage identity: 54 %
  
BlastP bit score: 440
  
Sequence coverage: 102 %
  
E-value: 2e-145
  
  
 NCBI BlastP on this gene

EAQ90317

hypothetical protein
  
Accession: EAQ90316
  
Location: 366073-380944
  
  
**BlastP hit with Mycgr3G40534\_Mycgr3T**
  
Percentage identity: 54 %
  
BlastP bit score: 263
  
Sequence coverage: 80 %
  
E-value: 3e-75
  
  
  
**BlastP hit with Mycgr3G39931\_Mycgr3T**
  
Percentage identity: 48 %
  
BlastP bit score: 265
  
Sequence coverage: 100 %
  
E-value: 5e-76
  
  
 NCBI BlastP on this gene

EAQ90316

predicted protein
  
Accession: EAQ90315
  
Location: 363770-364526
  
 NCBI BlastP on this gene

EAQ90315

hypothetical protein
  
Accession: EAQ90314
  
Location: 361487-363198
  
 NCBI BlastP on this gene

EAQ90314

hypothetical protein
  
Accession: EAQ90313
  
Location: 350524-360194
  
 NCBI BlastP on this gene

EAQ90313

5. :  KB730104 Fusarium oxysporum f. sp. cubense race 1 unplaced genomic scaffold scaffold59     Total score: 5.0     Cumulative Blast bit score: 2664

Mitochondrial enolase superfamily member 1
  
Accession: ENH72065
  
Location: 69610-71209
  
 NCBI BlastP on this gene

ENH72065

hypothetical protein
  
Accession: ENH72066
  
Location: 71479-73064
  
 NCBI BlastP on this gene

ENH72066

hypothetical protein
  
Accession: ENH72067
  
Location: 74313-75443
  
 NCBI BlastP on this gene

ENH72067

Sugar transporter STL1
  
Accession: ENH72068
  
Location: 76063-77987
  
 NCBI BlastP on this gene

ENH72068

Sterol 3-beta-glucosyltransferase
  
Accession: ENH72069
  
Location: 78710-81039
  
 NCBI BlastP on this gene

ENH72069

Endo-1,4-beta-xylanase Z
  
Accession: ENH72070
  
Location: 81973-82980
  
 NCBI BlastP on this gene

ENH72070

Ferric reductase transmembrane component 5
  
Accession: ENH72071
  
Location: 86430-88576
  
 NCBI BlastP on this gene

ENH72071

Siderophore iron transporter 3
  
Accession: ENH72072
  
Location: 89324-91243
  
  
**BlastP hit with Mycgr3G70577\_Mycgr3T**
  
Percentage identity: 51 %
  
BlastP bit score: 642
  
Sequence coverage: 96 %
  
E-value: 0.0
  
  
 NCBI BlastP on this gene

ENH72072

Multidrug resistance-associated protein 1
  
Accession: ENH72073
  
Location: 92480-96655
  
  
**BlastP hit with Mycgr3G41235\_Mycgr3T**
  
Percentage identity: 42 %
  
BlastP bit score: 1028
  
Sequence coverage: 104 %
  
E-value: 0.0
  
  
 NCBI BlastP on this gene

ENH72073

Putative lysine N-acyltransferase C17G9.06c
  
Accession: ENH72074
  
Location: 97820-99118
  
  
**BlastP hit with Mycgr3G85486\_Mycgr3T**
  
Percentage identity: 56 %
  
BlastP bit score: 462
  
Sequence coverage: 102 %
  
E-value: 4e-157
  
  
 NCBI BlastP on this gene

ENH72074

Ferrichrome siderophore peptide synthetase
  
Accession: ENH72075
  
Location: 99731-114015
  
  
**BlastP hit with Mycgr3G40534\_Mycgr3T**
  
Percentage identity: 52 %
  
BlastP bit score: 266
  
Sequence coverage: 80 %
  
E-value: 5e-76
  
  
  
**BlastP hit with Mycgr3G39931\_Mycgr3T**
  
Percentage identity: 48 %
  
BlastP bit score: 266
  
Sequence coverage: 99 %
  
E-value: 2e-76
  
  
 NCBI BlastP on this gene

ENH72075

Endo-1,4-beta-xylanase
  
Accession: ENH72076
  
Location: 115053-116284
  
 NCBI BlastP on this gene

ENH72076

Averantin oxidoreductase
  
Accession: ENH72077
  
Location: 116822-118506
  
 NCBI BlastP on this gene

ENH72077

hypothetical protein
  
Accession: ENH72078
  
Location: 118902-119999
  
 NCBI BlastP on this gene

ENH72078

hypothetical protein
  
Accession: ENH72079
  
Location: 121345-122768
  
 NCBI BlastP on this gene

ENH72079

hypothetical protein
  
Accession: ENH72080
  
Location: 123284-124804
  
 NCBI BlastP on this gene

ENH72080

hypothetical protein
  
Accession: ENH72081
  
Location: 125885-127882
  
 NCBI BlastP on this gene

ENH72081

hypothetical protein
  
Accession: ENH72082
  
Location: 128307-130337
  
 NCBI BlastP on this gene

ENH72082

hypothetical protein
  
Accession: ENH72083
  
Location: 130508-132368
  
 NCBI BlastP on this gene

ENH72083

Choline transport protein
  
Accession: ENH72084
  
Location: 133069-134822
  
 NCBI BlastP on this gene

ENH72084

6. :  DF238784 Pseudozyma hubeiensis SY62 DNA, scaffold: PHS21     Total score: 5.0     Cumulative Blast bit score: 2235

hypothetical protein
  
Accession: GAC94346
  
Location: 1770-2045
  
 NCBI BlastP on this gene

GAC94346

likely ferric reductase
  
Accession: GAC94347
  
Location: 2618-5276
  
 NCBI BlastP on this gene

GAC94347

hypothetical protein
  
Accession: GAC94348
  
Location: 5664-7052
  
 NCBI BlastP on this gene

GAC94348

siderophore iron transporter
  
Accession: GAC94349
  
Location: 8011-10057
  
  
**BlastP hit with Mycgr3G70577\_Mycgr3T**
  
Percentage identity: 36 %
  
BlastP bit score: 379
  
Sequence coverage: 98 %
  
E-value: 2e-118
  
  
 NCBI BlastP on this gene

GAC94349

ABC transporter
  
Accession: GAC94350
  
Location: 11714-15805
  
  
**BlastP hit with Mycgr3G41235\_Mycgr3T**
  
Percentage identity: 43 %
  
BlastP bit score: 1094
  
Sequence coverage: 101 %
  
E-value: 0.0
  
  
 NCBI BlastP on this gene

GAC94350

aerobactin siderophore biosynthesis protein
  
Accession: GAC94351
  
Location: 17131-18612
  
  
**BlastP hit with Mycgr3G85486\_Mycgr3T**
  
Percentage identity: 43 %
  
BlastP bit score: 322
  
Sequence coverage: 98 %
  
E-value: 3e-102
  
  
 NCBI BlastP on this gene

GAC94351

enoyl-CoA hydratase/isomerase
  
Accession: GAC94352
  
Location: 18825-19779
  
 NCBI BlastP on this gene

GAC94352

non-ribosomal peptide synthetase
  
Accession: GAC94353
  
Location: 21418-35944
  
  
**BlastP hit with Mycgr3G40534\_Mycgr3T**
  
Percentage identity: 49 %
  
BlastP bit score: 221
  
Sequence coverage: 83 %
  
E-value: 2e-60
  
  
  
**BlastP hit with Mycgr3G39931\_Mycgr3T**
  
Percentage identity: 47 %
  
BlastP bit score: 219
  
Sequence coverage: 89 %
  
E-value: 4e-60
  
  
 NCBI BlastP on this gene

GAC94353

hypothetical protein
  
Accession: GAC94354
  
Location: 36133-37897
  
 NCBI BlastP on this gene

GAC94354

multidrug transporter
  
Accession: GAC94355
  
Location: 39253-40860
  
 NCBI BlastP on this gene

GAC94355

hypothetical protein
  
Accession: GAC94356
  
Location: 41010-42461
  
 NCBI BlastP on this gene

GAC94356

hypothetical protein
  
Accession: GAC94357
  
Location: 43019-45586
  
 NCBI BlastP on this gene

GAC94357

ectomycorrhiza-regulated esterase
  
Accession: GAC94358
  
Location: 46915-47891
  
 NCBI BlastP on this gene

GAC94358

transcription factor
  
Accession: GAC94359
  
Location: 48350-51386
  
 NCBI BlastP on this gene

GAC94359

transporter
  
Accession: GAC94360
  
Location: 51459-54321
  
 NCBI BlastP on this gene

GAC94360

7. :  AACP01000049 Ustilago maydis 521     Total score: 5.0     Cumulative Blast bit score: 2213

hypothetical protein
  
Accession: EAK81756
  
Location: 59307-60317
  
 NCBI BlastP on this gene

EAK81756

hypothetical protein
  
Accession: EAK81757
  
Location: 60565-62383
  
 NCBI BlastP on this gene

EAK81757

hypothetical protein
  
Accession: EAK81758
  
Location: 63038-64612
  
 NCBI BlastP on this gene

EAK81758

hypothetical protein
  
Accession: EAK81759
  
Location: 65428-66858
  
 NCBI BlastP on this gene

EAK81759

hypothetical protein
  
Accession: EAK81760
  
Location: 67058-67973
  
 NCBI BlastP on this gene

EAK81760

hypothetical protein
  
Accession: EAK81761
  
Location: 69723-70580
  
 NCBI BlastP on this gene

EAK81761

hypothetical protein
  
Accession: EAK81762
  
Location: 70879-71988
  
 NCBI BlastP on this gene

EAK81762

hypothetical protein
  
Accession: EAK81763
  
Location: 73480-74970
  
 NCBI BlastP on this gene

EAK81763

hypothetical protein
  
Accession: EAK81764
  
Location: 75648-80066
  
  
**BlastP hit with Mycgr3G70577\_Mycgr3T**
  
Percentage identity: 39 %
  
BlastP bit score: 379
  
Sequence coverage: 93 %
  
E-value: 3e-114
  
  
 NCBI BlastP on this gene

EAK81764

hypothetical protein
  
Accession: EAK81765
  
Location: 82151-86344
  
  
**BlastP hit with Mycgr3G41235\_Mycgr3T**
  
Percentage identity: 42 %
  
BlastP bit score: 1078
  
Sequence coverage: 103 %
  
E-value: 0.0
  
  
 NCBI BlastP on this gene

EAK81765

hypothetical protein
  
Accession: EAK81766
  
Location: 88032-89579
  
  
**BlastP hit with Mycgr3G85486\_Mycgr3T**
  
Percentage identity: 43 %
  
BlastP bit score: 323
  
Sequence coverage: 99 %
  
E-value: 8e-103
  
  
 NCBI BlastP on this gene

EAK81766

hypothetical protein
  
Accession: EAK81767
  
Location: 89862-90867
  
 NCBI BlastP on this gene

EAK81767

hypothetical protein
  
Accession: EAK81768
  
Location: 92548-107141
  
  
**BlastP hit with Mycgr3G40534\_Mycgr3T**
  
Percentage identity: 48 %
  
BlastP bit score: 222
  
Sequence coverage: 82 %
  
E-value: 8e-61
  
  
  
**BlastP hit with Mycgr3G39931\_Mycgr3T**
  
Percentage identity: 45 %
  
BlastP bit score: 211
  
Sequence coverage: 91 %
  
E-value: 2e-57
  
  
 NCBI BlastP on this gene

EAK81768

hypothetical protein
  
Accession: EAK81769
  
Location: 108397-109758
  
 NCBI BlastP on this gene

EAK81769

hypothetical protein
  
Accession: EAK81770
  
Location: 110564-112144
  
 NCBI BlastP on this gene

EAK81770

predicted protein
  
Accession: EAK81771
  
Location: 113359-114193
  
 NCBI BlastP on this gene

EAK81771

8. :  DF196790 Pseudozyma antarctica T-34 DNA, contig: scaffold00024     Total score: 5.0     Cumulative Blast bit score: 2201

hypothetical protein
  
Accession: GAC77019
  
Location: 43768-46545
  
 NCBI BlastP on this gene

GAC77019

hypothetical protein
  
Accession: GAC77018
  
Location: 42351-43600
  
 NCBI BlastP on this gene

GAC77018

RNA polymerase III, large subunit
  
Accession: GAC77017
  
Location: 37918-42120
  
 NCBI BlastP on this gene

GAC77017

hypothetical protein
  
Accession: GAC77016
  
Location: 34336-37083
  
 NCBI BlastP on this gene

GAC77016

hypothetical protein
  
Accession: GAC77015
  
Location: 31778-34231
  
 NCBI BlastP on this gene

GAC77015

putative protein methyltransferase
  
Accession: GAC77014
  
Location: 29222-31015
  
 NCBI BlastP on this gene

GAC77014

hypothetical protein
  
Accession: GAC77013
  
Location: 26562-28655
  
  
**BlastP hit with Mycgr3G70577\_Mycgr3T**
  
Percentage identity: 38 %
  
BlastP bit score: 379
  
Sequence coverage: 94 %
  
E-value: 2e-118
  
  
 NCBI BlastP on this gene

GAC77013

multidrug resistance-associated protein
  
Accession: GAC77012
  
Location: 20835-24989
  
  
**BlastP hit with Mycgr3G41235\_Mycgr3T**
  
Percentage identity: 41 %
  
BlastP bit score: 1062
  
Sequence coverage: 102 %
  
E-value: 0.0
  
  
 NCBI BlastP on this gene

GAC77012

hypothetical protein
  
Accession: GAC77011
  
Location: 17657-19026
  
  
**BlastP hit with Mycgr3G85486\_Mycgr3T**
  
Percentage identity: 47 %
  
BlastP bit score: 326
  
Sequence coverage: 86 %
  
E-value: 4e-104
  
  
 NCBI BlastP on this gene

GAC77011

aromatic-l-amino-acid/l-histidine decarboxylase
  
Accession: GAC77010
  
Location: 16255-17494
  
 NCBI BlastP on this gene

GAC77010

non-ribosomal peptide synthetase
  
Accession: GAC77009
  
Location: 469-15048
  
  
**BlastP hit with Mycgr3G40534\_Mycgr3T**
  
Percentage identity: 49 %
  
BlastP bit score: 228
  
Sequence coverage: 83 %
  
E-value: 9e-63
  
  
  
**BlastP hit with Mycgr3G39931\_Mycgr3T**
  
Percentage identity: 44 %
  
BlastP bit score: 206
  
Sequence coverage: 89 %
  
E-value: 8e-56
  
  
 NCBI BlastP on this gene

GAC77009

9. :  FQ311441 Sporisorium reilianum SRZ2 chromosome 2 complete DNA sequence.     Total score: 5.0     Cumulative Blast bit score: 2178

conserved hypothetical protein
  
Accession: CBQ70845
  
Location: 1788779-1790585
  
 NCBI BlastP on this gene

sr12506

conserved hypothetical protein
  
Accession: CBQ70844
  
Location: 1784537-1786322
  
 NCBI BlastP on this gene

sr12505

related to MCH4-monocarboxylate transporter
  
Accession: CBQ70843
  
Location: 1781910-1783259
  
 NCBI BlastP on this gene

sr12504

Siderophore peptide synthetase involved in ferrichromeA biosynthesis
  
Accession: CBQ70842
  
Location: 1766153-1780839
  
  
**BlastP hit with Mycgr3G40534\_Mycgr3T**
  
Percentage identity: 49 %
  
BlastP bit score: 231
  
Sequence coverage: 82 %
  
E-value: 5e-64
  
  
  
**BlastP hit with Mycgr3G39931\_Mycgr3T**
  
Percentage identity: 43 %
  
BlastP bit score: 202
  
Sequence coverage: 89 %
  
E-value: 1e-54
  
  
 NCBI BlastP on this gene

Fer3

related to N6-hydroxylysine acetyl transferase
  
Accession: CBQ70841
  
Location: 1763565-1764933
  
  
**BlastP hit with Mycgr3G85486\_Mycgr3T**
  
Percentage identity: 48 %
  
BlastP bit score: 334
  
Sequence coverage: 86 %
  
E-value: 4e-107
  
  
 NCBI BlastP on this gene

Fer5

related to Enoyl-CoA hydratase
  
Accession: CBQ70840
  
Location: 1762340-1763326
  
 NCBI BlastP on this gene

Fer4

related to ATP-binding cassette transporter protein
  
Accession: CBQ70839
  
Location: 1756382-1760341
  
  
**BlastP hit with Mycgr3G41235\_Mycgr3T**
  
Percentage identity: 42 %
  
BlastP bit score: 1056
  
Sequence coverage: 100 %
  
E-value: 0.0
  
  
 NCBI BlastP on this gene

Fer6

related to Siderophore iron transporter 3
  
Accession: CBQ70838
  
Location: 1752864-1754937
  
  
**BlastP hit with Mycgr3G70577\_Mycgr3T**
  
Percentage identity: 36 %
  
BlastP bit score: 355
  
Sequence coverage: 97 %
  
E-value: 3e-109
  
  
 NCBI BlastP on this gene

Fer7

conserved hypothetical protein
  
Accession: CBQ70837
  
Location: 1750657-1751996
  
 NCBI BlastP on this gene

Fer8

related to high-affinity nickel transport protein nic1
  
Accession: CBQ70836
  
Location: 1748589-1750115
  
 NCBI BlastP on this gene

sr12497

related to beta-1,3-glucan binding protein
  
Accession: CBQ70835
  
Location: 1746674-1748410
  
 NCBI BlastP on this gene

sr12496

conserved hypothetical protein
  
Accession: CBQ70834
  
Location: 1739173-1741953
  
 NCBI BlastP on this gene

sr12495

conserved hypothetical protein
  
Accession: CBQ70833
  
Location: 1737360-1738439
  
 NCBI BlastP on this gene

sr12494

conserved hypothetical protein
  
Accession: CBQ70832
  
Location: 1735652-1736538
  
 NCBI BlastP on this gene

sr12493

probable homogentisate 1,2-dioxygenase
  
Accession: CBQ70831
  
Location: 1734130-1735550
  
 NCBI BlastP on this gene

sr12492

10. :  JH687382 Stereum hirsutum FP-91666 SS1 unplaced genomic scaffold STEHIscaffold\_4     Total score: 4.0     Cumulative Blast bit score: 1915

hypothetical protein
  
Accession: EIM88653
  
Location: 55989-58175
  
 NCBI BlastP on this gene

EIM88653

hypothetical protein
  
Accession: EIM88654
  
Location: 58670-67575
  
  
**BlastP hit with Mycgr3G39931\_Mycgr3T**
  
Percentage identity: 30 %
  
BlastP bit score: 117
  
Sequence coverage: 102 %
  
E-value: 1e-25
  
  
 NCBI BlastP on this gene

EIM88654

enoyl-CoA hydratase
  
Accession: EIM88655
  
Location: 68633-69892
  
 NCBI BlastP on this gene

EIM88655

hypothetical protein
  
Accession: EIM88656
  
Location: 70239-70851
  
 NCBI BlastP on this gene

EIM88656

hypothetical protein
  
Accession: EIM88657
  
Location: 71233-71852
  
 NCBI BlastP on this gene

EIM88657

FAD/NAD-P-binding domain-containing protein
  
Accession: EIM88658
  
Location: 72084-74286
  
 NCBI BlastP on this gene

EIM88658

hypothetical protein
  
Accession: EIM88659
  
Location: 75165-77460
  
 NCBI BlastP on this gene

EIM88659

hypothetical protein
  
Accession: EIM88660
  
Location: 78639-80070
  
  
**BlastP hit with Mycgr3G85486\_Mycgr3T**
  
Percentage identity: 49 %
  
BlastP bit score: 333
  
Sequence coverage: 83 %
  
E-value: 4e-107
  
  
 NCBI BlastP on this gene

EIM88660

MFS general substrate transporter
  
Accession: EIM88661
  
Location: 81661-84769
  
  
**BlastP hit with Mycgr3G70577\_Mycgr3T**
  
Percentage identity: 40 %
  
BlastP bit score: 391
  
Sequence coverage: 85 %
  
E-value: 1e-123
  
  
 NCBI BlastP on this gene

EIM88661

hypothetical protein
  
Accession: EIM88662
  
Location: 85425-85766
  
 NCBI BlastP on this gene

EIM88662

MFS general substrate transporter
  
Accession: EIM88663
  
Location: 86731-89687
  
  
**BlastP hit with Mycgr3G70577\_Mycgr3T**
  
Percentage identity: 40 %
  
BlastP bit score: 356
  
Sequence coverage: 78 %
  
E-value: 4e-111
  
  
 NCBI BlastP on this gene

EIM88663

hypothetical protein
  
Accession: EIM88664
  
Location: 91062-92164
  
 NCBI BlastP on this gene

EIM88664

hypothetical protein
  
Accession: EIM88665
  
Location: 93070-94382
  
 NCBI BlastP on this gene

EIM88665

hypothetical protein
  
Accession: EIM88666
  
Location: 96875-97378
  
 NCBI BlastP on this gene

EIM88666

hypothetical protein
  
Accession: EIM88667
  
Location: 99872-101307
  
 NCBI BlastP on this gene

EIM88667

hypothetical protein
  
Accession: EIM88668
  
Location: 105409-105798
  
 NCBI BlastP on this gene

EIM88668

hypothetical protein
  
Accession: EIM88669
  
Location: 107703-110836
  
 NCBI BlastP on this gene

EIM88669

hypothetical protein
  
Accession: EIM88670
  
Location: 112064-118290
  
  
**BlastP hit with Mycgr3G41235\_Mycgr3T**
  
Percentage identity: 41 %
  
BlastP bit score: 718
  
Sequence coverage: 67 %
  
E-value: 0.0
  
  
 NCBI BlastP on this gene

EIM88670

hypothetical protein
  
Accession: EIM88671
  
Location: 118681-120886
  
 NCBI BlastP on this gene

EIM88671

11. :  KB726570 Fusarium oxysporum f. sp. cubense race 4 unplaced genomic scaffold scaffold111     Total score: 4.0     Cumulative Blast bit score: 1782

Sugar transporter STL1
  
Accession: EMT67835
  
Location: 1587052-1588976
  
 NCBI BlastP on this gene

EMT67835

Sterol 3-beta-glucosyltransferase
  
Accession: EMT67834
  
Location: 1582969-1585855
  
 NCBI BlastP on this gene

EMT67834

Endo-1,4-beta-xylanase Z
  
Accession: EMT67833
  
Location: 1581018-1582025
  
 NCBI BlastP on this gene

EMT67833

hypothetical protein
  
Accession: EMT67832
  
Location: 1579037-1579702
  
 NCBI BlastP on this gene

EMT67832

Ferric reductase transmembrane component 6
  
Accession: EMT67831
  
Location: 1576893-1577510
  
 NCBI BlastP on this gene

EMT67831

Ferric reductase transmembrane component 5
  
Accession: EMT67830
  
Location: 1572995-1575394
  
 NCBI BlastP on this gene

EMT67830

Siderophore iron transporter 3
  
Accession: EMT67829
  
Location: 1570580-1572499
  
  
**BlastP hit with Mycgr3G70577\_Mycgr3T**
  
Percentage identity: 51 %
  
BlastP bit score: 642
  
Sequence coverage: 96 %
  
E-value: 0.0
  
  
 NCBI BlastP on this gene

EMT67829

Putative lysine N-acyltransferase C17G9.06c
  
Accession: EMT67828
  
Location: 1560538-1561836
  
  
**BlastP hit with Mycgr3G85486\_Mycgr3T**
  
Percentage identity: 56 %
  
BlastP bit score: 462
  
Sequence coverage: 102 %
  
E-value: 3e-157
  
  
 NCBI BlastP on this gene

EMT67828

hypothetical protein
  
Accession: EMT67827
  
Location: 1558968-1559928
  
  
**BlastP hit with Mycgr3G85486\_Mycgr3T**
  
Percentage identity: 79 %
  
BlastP bit score: 138
  
Sequence coverage: 20 %
  
E-value: 5e-34
  
  
 NCBI BlastP on this gene

EMT67827

Ferrichrome siderophore peptide synthetase
  
Accession: EMT67826
  
Location: 1538511-1558358
  
  
**BlastP hit with Mycgr3G40534\_Mycgr3T**
  
Percentage identity: 52 %
  
BlastP bit score: 270
  
Sequence coverage: 80 %
  
E-value: 2e-77
  
  
  
**BlastP hit with Mycgr3G39931\_Mycgr3T**
  
Percentage identity: 49 %
  
BlastP bit score: 271
  
Sequence coverage: 99 %
  
E-value: 3e-78
  
  
 NCBI BlastP on this gene

EMT67826

Endo-1,4-beta-xylanase
  
Accession: EMT67825
  
Location: 1536240-1537471
  
 NCBI BlastP on this gene

EMT67825

Averantin oxidoreductase
  
Accession: EMT67824
  
Location: 1533996-1535681
  
 NCBI BlastP on this gene

EMT67824

hypothetical protein
  
Accession: EMT67823
  
Location: 1532505-1533602
  
 NCBI BlastP on this gene

EMT67823

hypothetical protein
  
Accession: EMT67822
  
Location: 1529736-1531150
  
 NCBI BlastP on this gene

EMT67822

hypothetical protein
  
Accession: EMT67821
  
Location: 1527699-1529219
  
 NCBI BlastP on this gene

EMT67821

hypothetical protein
  
Accession: EMT67820
  
Location: 1524622-1526619
  
 NCBI BlastP on this gene

EMT67820

hypothetical protein
  
Accession: EMT67819
  
Location: 1522167-1524197
  
 NCBI BlastP on this gene

EMT67819

12. :  KB456264 Mycosphaerella populorum SO2202 unplaced genomic scaffold SEPMUscaffold\_5     Total score: 3.0     Cumulative Blast bit score: 3139

cytochrome P450 52A11
  
Accession: EMF12684
  
Location: 1054477-1056714
  
 NCBI BlastP on this gene

EMF12684

hypothetical protein
  
Accession: EMF12685
  
Location: 1059882-1061408
  
 NCBI BlastP on this gene

EMF12685

hypothetical protein
  
Accession: EMF12687
  
Location: 1064485-1065747
  
 NCBI BlastP on this gene

EMF12687

FKBP C-domain-containing protein
  
Accession: EMF12688
  
Location: 1066576-1068172
  
 NCBI BlastP on this gene

EMF12688

Metallo-dependent phosphatase
  
Accession: EMF12689
  
Location: 1069617-1071542
  
 NCBI BlastP on this gene

EMF12689

hypothetical protein
  
Accession: EMF12690
  
Location: 1072175-1082437
  
  
**BlastP hit with Mycgr3G39931\_Mycgr3T**
  
Percentage identity: 71 %
  
BlastP bit score: 407
  
Sequence coverage: 96 %
  
E-value: 2e-125
  
  
 NCBI BlastP on this gene

EMF12690

MFS general substrate transporter
  
Accession: EMF12691
  
Location: 1090315-1092361
  
  
**BlastP hit with Mycgr3G70577\_Mycgr3T**
  
Percentage identity: 75 %
  
BlastP bit score: 892
  
Sequence coverage: 96 %
  
E-value: 0.0
  
  
 NCBI BlastP on this gene

EMF12691

P-loop containing nucleoside triphosphate hydrolase protein
  
Accession: EMF12693
  
Location: 1094798-1099078
  
  
**BlastP hit with Mycgr3G41235\_Mycgr3T**
  
Percentage identity: 66 %
  
BlastP bit score: 1841
  
Sequence coverage: 104 %
  
E-value: 0.0
  
  
 NCBI BlastP on this gene

EMF12693

L-ornithine N5-oxygenase sida
  
Accession: EMF12694
  
Location: 1099791-1101408
  
 NCBI BlastP on this gene

EMF12694

ribonuclease H-like protein
  
Accession: EMF12695
  
Location: 1101430-1102068
  
 NCBI BlastP on this gene

EMF12695

plastidic glucose transporter 4
  
Accession: EMF12696
  
Location: 1103286-1105370
  
 NCBI BlastP on this gene

EMF12696

hypothetical protein
  
Accession: EMF12697
  
Location: 1106011-1106709
  
 NCBI BlastP on this gene

EMF12697

hypothetical protein
  
Accession: EMF12698
  
Location: 1108325-1109085
  
 NCBI BlastP on this gene

EMF12698

oligomeric complex COG6
  
Accession: EMF12699
  
Location: 1110566-1113049
  
 NCBI BlastP on this gene

EMF12699

hypothetical protein
  
Accession: EMF12700
  
Location: 1116184-1116852
  
 NCBI BlastP on this gene

EMF12700

Rpn3 C-domain-containing protein
  
Accession: EMF12702
  
Location: 1117244-1118871
  
 NCBI BlastP on this gene

EMF12702

13. :  CH476600 Aspergillus terreus NIH2624 scaffold\_7 genomic scaffold     Total score: 3.0     Cumulative Blast bit score: 613

conserved hypothetical protein
  
Accession: EAU34152
  
Location: 444157-445747
  
 NCBI BlastP on this gene

EAU34152

conserved hypothetical protein
  
Accession: EAU34151
  
Location: 441866-443585
  
 NCBI BlastP on this gene

EAU34151

conserved hypothetical protein
  
Accession: EAU34150
  
Location: 440306-441346
  
 NCBI BlastP on this gene

EAU34150

voltage-gated potassium channel beta-1 subunit
  
Accession: EAU34149
  
Location: 436699-438003
  
 NCBI BlastP on this gene

EAU34149

conserved hypothetical protein
  
Accession: EAU34148
  
Location: 434428-435850
  
 NCBI BlastP on this gene

EAU34148

conserved hypothetical protein
  
Accession: EAU34147
  
Location: 431785-433673
  
 NCBI BlastP on this gene

EAU34147

hypothetical protein
  
Accession: EAU34146
  
Location: 429559-430835
  
 NCBI BlastP on this gene

EAU34146

predicted protein
  
Accession: EAU34145
  
Location: 427905-429124
  
 NCBI BlastP on this gene

EAU34145

conserved hypothetical protein
  
Accession: EAU34144
  
Location: 424658-426063
  
  
**BlastP hit with Mycgr3G85486\_Mycgr3T**
  
Percentage identity: 30 %
  
BlastP bit score: 155
  
Sequence coverage: 84 %
  
E-value: 4e-39
  
  
 NCBI BlastP on this gene

EAU34144

hypothetical protein
  
Accession: EAU34143
  
Location: 422013-423996
  
 NCBI BlastP on this gene

EAU34143

hypothetical protein
  
Accession: EAU34142
  
Location: 405286-419584
  
  
**BlastP hit with Mycgr3G40534\_Mycgr3T**
  
Percentage identity: 52 %
  
BlastP bit score: 247
  
Sequence coverage: 80 %
  
E-value: 2e-69
  
  
  
**BlastP hit with Mycgr3G39931\_Mycgr3T**
  
Percentage identity: 43 %
  
BlastP bit score: 211
  
Sequence coverage: 97 %
  
E-value: 2e-57
  
  
 NCBI BlastP on this gene

EAU34142

conserved hypothetical protein
  
Accession: EAU34141
  
Location: 402007-404439
  
 NCBI BlastP on this gene

EAU34141

UV-damage endonuclease
  
Accession: EAU34140
  
Location: 398943-400768
  
 NCBI BlastP on this gene

EAU34140

conserved hypothetical protein
  
Accession: EAU34139
  
Location: 396189-397373
  
 NCBI BlastP on this gene

EAU34139

conserved hypothetical protein
  
Accession: EAU34138
  
Location: 394229-395068
  
 NCBI BlastP on this gene

EAU34138

conserved hypothetical protein
  
Accession: EAU34137
  
Location: 391643-393008
  
 NCBI BlastP on this gene

EAU34137

predicted protein
  
Accession: EAU34136
  
Location: 390251-390867
  
 NCBI BlastP on this gene

EAU34136

conserved hypothetical protein
  
Accession: EAU34135
  
Location: 389125-389700
  
 NCBI BlastP on this gene

EAU34135

14. :  AACD01000007 Aspergillus nidulans FGSC A4     Total score: 3.0     Cumulative Blast bit score: 595

hypothetical protein
  
Accession: EAA66714
  
Location: 755383-756502
  
 NCBI BlastP on this gene

EAA66714

hypothetical protein
  
Accession: EAA66713
  
Location: 753471-755122
  
 NCBI BlastP on this gene

EAA66713

hypothetical protein
  
Accession: EAA66712
  
Location: 751685-752959
  
 NCBI BlastP on this gene

EAA66712

hypothetical protein
  
Accession: EAA66711
  
Location: 750351-751206
  
 NCBI BlastP on this gene

EAA66711

predicted protein
  
Accession: EAA66710
  
Location: 748529-749088
  
 NCBI BlastP on this gene

EAA66710

hypothetical protein
  
Accession: EAA66709
  
Location: 745209-746544
  
 NCBI BlastP on this gene

EAA66709

hypothetical protein
  
Accession: EAA66708
  
Location: 739203-741236
  
 NCBI BlastP on this gene

EAA66708

hypothetical protein
  
Accession: EAA66707
  
Location: 737167-738547
  
  
**BlastP hit with Mycgr3G85486\_Mycgr3T**
  
Percentage identity: 30 %
  
BlastP bit score: 141
  
Sequence coverage: 81 %
  
E-value: 5e-34
  
  
 NCBI BlastP on this gene

EAA66707

hypothetical protein
  
Accession: EAA66706
  
Location: 721978-736405
  
  
**BlastP hit with Mycgr3G40534\_Mycgr3T**
  
Percentage identity: 49 %
  
BlastP bit score: 235
  
Sequence coverage: 80 %
  
E-value: 3e-65
  
  
  
**BlastP hit with Mycgr3G39931\_Mycgr3T**
  
Percentage identity: 45 %
  
BlastP bit score: 219
  
Sequence coverage: 90 %
  
E-value: 3e-60
  
  
 NCBI BlastP on this gene

EAA66706

hypothetical protein
  
Accession: EAA66705
  
Location: 718702-720666
  
 NCBI BlastP on this gene

EAA66705

hypothetical protein
  
Accession: EAA66704
  
Location: 715029-718393
  
 NCBI BlastP on this gene

EAA66704

hypothetical protein
  
Accession: EAA66703
  
Location: 712138-713971
  
 NCBI BlastP on this gene

EAA66703

hypothetical protein
  
Accession: EAA66702
  
Location: 710885-710995
  
 NCBI BlastP on this gene

EAA66702

hypothetical protein
  
Accession: EAA66701
  
Location: 709615-710655
  
 NCBI BlastP on this gene

EAA66701

hypothetical protein
  
Accession: EAA66700
  
Location: 705730-707785
  
 NCBI BlastP on this gene

EAA66700

hypothetical protein
  
Accession: EAA66699
  
Location: 703846-704679
  
 NCBI BlastP on this gene

EAA66699

hypothetical protein
  
Accession: EAA66698
  
Location: 701979-703201
  
 NCBI BlastP on this gene

EAA66698

15. :  AHHD01000417 Macrophomina phaseolina MS6     Total score: 3.0     Cumulative Blast bit score: 592

hypothetical protein
  
Accession: EKG13081
  
Location: 12558-13692
  
 NCBI BlastP on this gene

EKG13081

Putative ABC transporter protein
  
Accession: EKG13082
  
Location: 14272-18248
  
 NCBI BlastP on this gene

EKG13082

hypothetical protein
  
Accession: EKG13083
  
Location: 24597-25748
  
 NCBI BlastP on this gene

EKG13083

hypothetical protein
  
Accession: EKG13084
  
Location: 26781-27448
  
 NCBI BlastP on this gene

EKG13084

Acyl-CoA N-acyltransferase
  
Accession: EKG13085
  
Location: 29734-31056
  
  
**BlastP hit with Mycgr3G85486\_Mycgr3T**
  
Percentage identity: 33 %
  
BlastP bit score: 156
  
Sequence coverage: 75 %
  
E-value: 2e-39
  
  
 NCBI BlastP on this gene

EKG13085

AMP-dependent synthetase/ligase
  
Accession: EKG13086
  
Location: 32464-33871
  
 NCBI BlastP on this gene

EKG13086

AMP-dependent synthetase/ligase
  
Accession: EKG13087
  
Location: 34954-49462
  
  
**BlastP hit with Mycgr3G40534\_Mycgr3T**
  
Percentage identity: 51 %
  
BlastP bit score: 266
  
Sequence coverage: 82 %
  
E-value: 4e-76
  
  
  
**BlastP hit with Mycgr3G39931\_Mycgr3T**
  
Percentage identity: 40 %
  
BlastP bit score: 170
  
Sequence coverage: 90 %
  
E-value: 2e-43
  
  
 NCBI BlastP on this gene

EKG13087

Bacterial alpha-L-rhamnosidase
  
Accession: EKG13088
  
Location: 49813-51971
  
 NCBI BlastP on this gene

EKG13088

hypothetical protein
  
Accession: EKG13089
  
Location: 53329-54972
  
 NCBI BlastP on this gene

EKG13089

Tyrosinase
  
Accession: EKG13090
  
Location: 58220-58690
  
 NCBI BlastP on this gene

EKG13090

Inhibitor of apoptosis-promoting Bax1-related protein
  
Accession: EKG13091
  
Location: 60379-61458
  
 NCBI BlastP on this gene

EKG13091

IQ motif EF-hand binding site
  
Accession: EKG13092
  
Location: 62282-67856
  
 NCBI BlastP on this gene

EKG13092

Cystathionine beta-synthase core
  
Accession: EKG13093
  
Location: 68921-70790
  
 NCBI BlastP on this gene

EKG13093

16. :  KB915996 Neofusicoccum parvum UCRNP2 chromosome Unknown NP2\_03\_scaffold\_358     Total score: 3.0     Cumulative Blast bit score: 508

putative siderophore iron transporter mirb protein
  
Accession: EOD50332
  
Location: 133-1740
  
 NCBI BlastP on this gene

EOD50332

putative pyridine nucleotide-disulfide oxidoreductase amid- protein
  
Accession: EOD50310
  
Location: 2370-3857
  
 NCBI BlastP on this gene

EOD50310

putative aerobactin siderophore biosynthesis protein iucb protein
  
Accession: EOD50324
  
Location: 6418-7743
  
  
**BlastP hit with Mycgr3G85486\_Mycgr3T**
  
Percentage identity: 31 %
  
BlastP bit score: 153
  
Sequence coverage: 76 %
  
E-value: 2e-38
  
  
 NCBI BlastP on this gene

EOD50324

putative long-chain-fatty-acid- ligase protein
  
Accession: EOD50335
  
Location: 8610-10774
  
 NCBI BlastP on this gene

EOD50335

putative amp-dependent synthetase ligase protein
  
Accession: EOD50322
  
Location: 11855-12671
  
 NCBI BlastP on this gene

EOD50322

putative nonribosomal siderophore peptide synthase protein
  
Accession: EOD50346
  
Location: 13291-26075
  
  
**BlastP hit with Mycgr3G40534\_Mycgr3T**
  
Percentage identity: 49 %
  
BlastP bit score: 179
  
Sequence coverage: 60 %
  
E-value: 3e-46
  
  
  
**BlastP hit with Mycgr3G39931\_Mycgr3T**
  
Percentage identity: 41 %
  
BlastP bit score: 176
  
Sequence coverage: 90 %
  
E-value: 2e-45
  
  
 NCBI BlastP on this gene

EOD50346

putative alpha-l-rhamnosidase protein
  
Accession: EOD50311
  
Location: 26648-28824
  
 NCBI BlastP on this gene

EOD50311

putative phosphatidylserine decarboxylase protein
  
Accession: EOD50312
  
Location: 30151-31731
  
 NCBI BlastP on this gene

EOD50312

putative tyrosinase central domain protein
  
Accession: EOD50342
  
Location: 35015-36320
  
 NCBI BlastP on this gene

EOD50342

putative bax inhibitor family protein
  
Accession: EOD50340
  
Location: 37294-38481
  
 NCBI BlastP on this gene

EOD50340

putative dihydroxy-acid dehydratase protein
  
Accession: EOD50330
  
Location: 42515-44504
  
 NCBI BlastP on this gene

EOD50330

17. :  DS499596 Aspergillus fumigatus A1163 scf\_000003 genomic scaffold     Total score: 3.0     Cumulative Blast bit score: 447

isochorismatase family hydrolase, putative
  
Accession: EDP53324
  
Location: 3237878-3238739
  
 NCBI BlastP on this gene

EDP53324

FAD binding monooxygenase, putative
  
Accession: EDP53323
  
Location: 3235484-3237031
  
 NCBI BlastP on this gene

EDP53323

conserved hypothetical protein
  
Accession: EDP53322
  
Location: 3233099-3234300
  
 NCBI BlastP on this gene

EDP53322

hypothetical protein
  
Accession: EDP53321
  
Location: 3229027-3231822
  
 NCBI BlastP on this gene

EDP53321

RTA1 domain protein
  
Accession: EDP53320
  
Location: 3227339-3228288
  
 NCBI BlastP on this gene

EDP53320

C6 transcription factor, putative
  
Accession: EDP53319
  
Location: 3225865-3227135
  
 NCBI BlastP on this gene

EDP53319

MFS monocarboxylate transporter, putative
  
Accession: EDP53318
  
Location: 3223962-3225399
  
 NCBI BlastP on this gene

EDP53318

mitochondrial enoyl reductase, putative
  
Accession: EDP53317
  
Location: 3222031-3223257
  
 NCBI BlastP on this gene

EDP53317

nonribosomal peptide synthase SidE
  
Accession: EDP53316
  
Location: 3214421-3220750
  
  
**BlastP hit with Mycgr3G40534\_Mycgr3T**
  
Percentage identity: 38 %
  
BlastP bit score: 154
  
Sequence coverage: 87 %
  
E-value: 8e-38
  
  
  
**BlastP hit with Mycgr3G39931\_Mycgr3T**
  
Percentage identity: 33 %
  
BlastP bit score: 121
  
Sequence coverage: 88 %
  
E-value: 6e-27
  
  
 NCBI BlastP on this gene

EDP53316

cell surface protein, putative
  
Accession: EDP53315
  
Location: 3213303-3213863
  
 NCBI BlastP on this gene

EDP53315

sugar O-acetyltransferase, putative
  
Accession: EDP53314
  
Location: 3211178-3211921
  
 NCBI BlastP on this gene

EDP53314

siderophore biosynthesis lipase/esterase, putative
  
Accession: EDP53313
  
Location: 3209863-3210987
  
 NCBI BlastP on this gene

EDP53313

siderophore biosynthesis acetylase AceI, putative
  
Accession: EDP53312
  
Location: 3206927-3208315
  
  
**BlastP hit with Mycgr3G85486\_Mycgr3T**
  
Percentage identity: 34 %
  
BlastP bit score: 173
  
Sequence coverage: 80 %
  
E-value: 2e-45
  
  
 NCBI BlastP on this gene

EDP53312

enoyl-CoA hydratase/isomerase family protein
  
Accession: EDP53311
  
Location: 3205730-3206615
  
 NCBI BlastP on this gene

EDP53311

nonribosomal peptide synthase SidD
  
Accession: EDP53310
  
Location: 3198715-3205021
  
 NCBI BlastP on this gene

EDP53310

ABC multidrug transporter SitT
  
Accession: EDP53309
  
Location: 3194593-3198587
  
 NCBI BlastP on this gene

EDP53309

MFS siderophore transporter MirB-like, putative
  
Accession: EDP53308
  
Location: 3191863-3193815
  
 NCBI BlastP on this gene

EDP53308

conserved hypothetical protein
  
Accession: EDP53307
  
Location: 3190259-3191042
  
 NCBI BlastP on this gene

EDP53307

cytochrome P450 oxidoreductase, putative
  
Accession: EDP53306
  
Location: 3188078-3189676
  
 NCBI BlastP on this gene

EDP53306

18. :  DS027697 Neosartorya fischeri NRRL 181 1099437636265 genomic scaffold     Total score: 3.0     Cumulative Blast bit score: 447

FAD binding monooxygenase, putative
  
Accession: EAW17179
  
Location: 1425139-1426686
  
 NCBI BlastP on this gene

EAW17179

GNAT family acetyltransferase, putative
  
Accession: EAW17180
  
Location: 1427088-1427821
  
 NCBI BlastP on this gene

EAW17180

conserved hypothetical protein
  
Accession: EAW17181
  
Location: 1428566-1429726
  
 NCBI BlastP on this gene

EAW17181

hypothetical protein
  
Accession: EAW17182
  
Location: 1430369-1430843
  
 NCBI BlastP on this gene

EAW17182

predicted protein
  
Accession: EAW17183
  
Location: 1431944-1432451
  
 NCBI BlastP on this gene

EAW17183

hypothetical protein
  
Accession: EAW17184
  
Location: 1433049-1433950
  
 NCBI BlastP on this gene

EAW17184

FAD binding domain protein
  
Accession: EAW17185
  
Location: 1434228-1435845
  
 NCBI BlastP on this gene

EAW17185

RTA1 like protein
  
Accession: EAW17186
  
Location: 1436588-1437538
  
 NCBI BlastP on this gene

EAW17186

conserved hypothetical protein
  
Accession: EAW17187
  
Location: 1437741-1439035
  
 NCBI BlastP on this gene

EAW17187

MFS monocarboxylate transporter, putative
  
Accession: EAW17188
  
Location: 1439463-1440892
  
 NCBI BlastP on this gene

EAW17188

mitochondrial enoyl reductase, putative
  
Accession: EAW17189
  
Location: 1441589-1442812
  
 NCBI BlastP on this gene

EAW17189

nonribosomal peptide synthase SidE
  
Accession: EAW17190
  
Location: 1444078-1450407
  
  
**BlastP hit with Mycgr3G40534\_Mycgr3T**
  
Percentage identity: 40 %
  
BlastP bit score: 155
  
Sequence coverage: 79 %
  
E-value: 4e-38
  
  
  
**BlastP hit with Mycgr3G39931\_Mycgr3T**
  
Percentage identity: 33 %
  
BlastP bit score: 122
  
Sequence coverage: 96 %
  
E-value: 3e-27
  
  
 NCBI BlastP on this gene

EAW17190

cell surface protein, putative
  
Accession: EAW17191
  
Location: 1451158-1451718
  
 NCBI BlastP on this gene

EAW17191

sugar O-acetyltransferase, putative
  
Accession: EAW17192
  
Location: 1453189-1453932
  
 NCBI BlastP on this gene

EAW17192

siderophore biosynthesis lipase/esterase, putative
  
Accession: EAW17193
  
Location: 1454144-1455264
  
 NCBI BlastP on this gene

EAW17193

siderophore biosynthesis acetylase AceI, putative
  
Accession: EAW17194
  
Location: 1456800-1458188
  
  
**BlastP hit with Mycgr3G85486\_Mycgr3T**
  
Percentage identity: 34 %
  
BlastP bit score: 171
  
Sequence coverage: 80 %
  
E-value: 2e-44
  
  
 NCBI BlastP on this gene

EAW17194

enoyl-CoA hydratase/isomerase family protein
  
Accession: EAW17195
  
Location: 1458510-1459376
  
 NCBI BlastP on this gene

EAW17195

nonribosomal peptide synthase SidD
  
Accession: EAW17196
  
Location: 1460111-1466417
  
 NCBI BlastP on this gene

EAW17196

ABC multidrug transporter SitT
  
Accession: EAW17197
  
Location: 1466545-1470539
  
 NCBI BlastP on this gene

EAW17197

siderophore transporter, putative
  
Accession: EAW17198
  
Location: 1471291-1473240
  
 NCBI BlastP on this gene

EAW17198

NADP(+)-dependent dehydrogenase, putative
  
Accession: EAW17199
  
Location: 1474322-1475103
  
 NCBI BlastP on this gene

EAW17199

cytochrome P450 pisatin demethylase, putative
  
Accession: EAW17200
  
Location: 1475691-1477279
  
 NCBI BlastP on this gene

EAW17200

19. :  AAHF01000010 Aspergillus fumigatus Af293     Total score: 3.0     Cumulative Blast bit score: 447

isochorismatase family hydrolase, putative
  
Accession: EAL86608
  
Location: 874449-875310
  
 NCBI BlastP on this gene

EAL86608

FAD binding monooxygenase, putative
  
Accession: EAL86609
  
Location: 876157-877704
  
 NCBI BlastP on this gene

EAL86609

conserved hypothetical protein
  
Accession: EAL86610
  
Location: 878888-879295
  
 NCBI BlastP on this gene

EAL86610

conserved hypothetical protein
  
Accession: EBA27280
  
Location: 881366-882267
  
 NCBI BlastP on this gene

EBA27280

FAD-binding oxidoreductase, putative
  
Accession: EBA27281
  
Location: 882542-884161
  
 NCBI BlastP on this gene

EBA27281

RTA1 domain protein
  
Accession: EAL86612
  
Location: 884900-885849
  
 NCBI BlastP on this gene

EAL86612

C6 transcription factor, putative
  
Accession: EAL86613
  
Location: 886053-887323
  
 NCBI BlastP on this gene

EAL86613

MFS monocarboxylate transporter, putative
  
Accession: EAL86614
  
Location: 887789-889226
  
 NCBI BlastP on this gene

EAL86614

mitochondrial enoyl reductase, putative
  
Accession: EAL86615
  
Location: 889931-891157
  
 NCBI BlastP on this gene

EAL86615

nonribosomal peptide synthase SidE
  
Accession: EAL86616
  
Location: 892438-898767
  
  
**BlastP hit with Mycgr3G40534\_Mycgr3T**
  
Percentage identity: 38 %
  
BlastP bit score: 154
  
Sequence coverage: 87 %
  
E-value: 8e-38
  
  
  
**BlastP hit with Mycgr3G39931\_Mycgr3T**
  
Percentage identity: 33 %
  
BlastP bit score: 121
  
Sequence coverage: 88 %
  
E-value: 6e-27
  
  
 NCBI BlastP on this gene

EAL86616

cell surface protein, putative
  
Accession: EAL86619
  
Location: 899325-899885
  
 NCBI BlastP on this gene

EAL86619

sugar O-acetyltransferase, putative
  
Accession: EAL86620
  
Location: 901268-902011
  
 NCBI BlastP on this gene

EAL86620

siderophore biosynthesis lipase/esterase, putative
  
Accession: EAL86621
  
Location: 902202-903326
  
 NCBI BlastP on this gene

EAL86621

siderophore biosynthesis acetylase AceI, putative
  
Accession: EAL86622
  
Location: 904874-906262
  
  
**BlastP hit with Mycgr3G85486\_Mycgr3T**
  
Percentage identity: 34 %
  
BlastP bit score: 173
  
Sequence coverage: 80 %
  
E-value: 2e-45
  
  
 NCBI BlastP on this gene

EAL86622

enoyl-CoA hydratase/isomerase family protein
  
Accession: EAL86623
  
Location: 906574-907459
  
 NCBI BlastP on this gene

EAL86623

nonribosomal peptide synthase SidD
  
Accession: EAL86624
  
Location: 908168-914474
  
 NCBI BlastP on this gene

EAL86624

ABC multidrug transporter SitT
  
Accession: EAL86625
  
Location: 914602-918596
  
 NCBI BlastP on this gene

EAL86625

MFS siderophore iron transporter, putative
  
Accession: EAL86626
  
Location: 919374-921326
  
 NCBI BlastP on this gene

EAL86626

short-chain alcohol dehydrogenase, putative
  
Accession: EBA27282
  
Location: 922147-922930
  
 NCBI BlastP on this gene

EBA27282

cytochrome P450 oxidoreductase, putative
  
Accession: EAL86627
  
Location: 923525-925123
  
 NCBI BlastP on this gene

EAL86627

20. :  HE605208 Candida parapsilosis strain CDC317 annotated contig 006372.     Total score: 2.0     Cumulative Blast bit score: 854

hypothetical protein
  
Accession: CCE44960
  
Location: 1709794-1713618
  
 NCBI BlastP on this gene

CPAR2\_407630

hypothetical protein
  
Accession: CCE44959
  
Location: 1708527-1709519
  
 NCBI BlastP on this gene

CPAR2\_407620

hypothetical protein
  
Accession: CCE44958
  
Location: 1706880-1707908
  
 NCBI BlastP on this gene

CPAR2\_407610

hypothetical protein
  
Accession: CCE44957
  
Location: 1705194-1706171
  
 NCBI BlastP on this gene

CPAR2\_407600

hypothetical protein
  
Accession: CCE44956
  
Location: 1704820-1705029
  
 NCBI BlastP on this gene

CPAR2\_407590

hypothetical protein
  
Accession: CCE44955
  
Location: 1700332-1702149
  
 NCBI BlastP on this gene

CPAR2\_407570

hypothetical protein
  
Accession: CCE44954
  
Location: 1696040-1697866
  
  
**BlastP hit with Mycgr3G70577\_Mycgr3T**
  
Percentage identity: 26 %
  
BlastP bit score: 196
  
Sequence coverage: 93 %
  
E-value: 7e-51
  
  
 NCBI BlastP on this gene

CPAR2\_407560

hypothetical protein
  
Accession: CCE44953
  
Location: 1694968-1695691
  
 NCBI BlastP on this gene

CPAR2\_407550

hypothetical protein
  
Accession: CCE44952
  
Location: 1693574-1694842
  
 NCBI BlastP on this gene

CPAR2\_407540

hypothetical protein
  
Accession: CCE44951
  
Location: 1686682-1693206
  
 NCBI BlastP on this gene

CPAR2\_407530

hypothetical protein
  
Accession: CCE44950
  
Location: 1684286-1686115
  
 NCBI BlastP on this gene

CPAR2\_407520

not annotated
  
Accession: CPAR2\_407510
  
Location: 1677696-1682048
  
  
**BlastP hit with Mycgr3G41235\_Mycgr3T**
  
Percentage identity: 30 %
  
BlastP bit score: 658
  
Sequence coverage: 98 %
  
E-value: 0.0
  
  
 NCBI BlastP on this gene

CPAR2\_407510

hypothetical protein
  
Accession: CCE44948
  
Location: 1676461-1676796
  
 NCBI BlastP on this gene

CPAR2\_407500

hypothetical protein
  
Accession: CCE44947
  
Location: 1675239-1676006
  
 NCBI BlastP on this gene

CPAR2\_407490

hypothetical protein
  
Accession: CCE44946
  
Location: 1672342-1675113
  
 NCBI BlastP on this gene

CPAR2\_407480

hypothetical protein
  
Accession: CCE44945
  
Location: 1670143-1671774
  
 NCBI BlastP on this gene

CPAR2\_407470

hypothetical protein
  
Accession: CCE44944
  
Location: 1668112-1669275
  
 NCBI BlastP on this gene

CPAR2\_407460

hypothetical protein
  
Accession: CCE44943
  
Location: 1662938-1667077
  
 NCBI BlastP on this gene

CPAR2\_407450

hypothetical protein
  
Accession: CCE44942
  
Location: 1661945-1662838
  
 NCBI BlastP on this gene

CPAR2\_407440

hypothetical protein
  
Accession: CCE44941
  
Location: 1659206-1661803
  
 NCBI BlastP on this gene

CPAR2\_407430

21. :  GL377303 Schizophyllum commune H4-8 unplaced genomic scaffold SCHCOscaffold\_2     Total score: 2.0     Cumulative Blast bit score: 784

hypothetical protein
  
Accession: EFJ00113
  
Location: 1677713-1680640
  
 NCBI BlastP on this gene

EFJ00113

hypothetical protein
  
Accession: EFJ00945
  
Location: 1680703-1685479
  
 NCBI BlastP on this gene

EFJ00945

hypothetical protein
  
Accession: EFJ00114
  
Location: 1686095-1689484
  
 NCBI BlastP on this gene

EFJ00114

hypothetical protein
  
Accession: EFJ00115
  
Location: 1690286-1693992
  
 NCBI BlastP on this gene

EFJ00115

hypothetical protein
  
Accession: EFJ00116
  
Location: 1694494-1695526
  
 NCBI BlastP on this gene

EFJ00116

hypothetical protein
  
Accession: EFJ00117
  
Location: 1695853-1697154
  
  
**BlastP hit with Mycgr3G85486\_Mycgr3T**
  
Percentage identity: 48 %
  
BlastP bit score: 330
  
Sequence coverage: 84 %
  
E-value: 7e-106
  
  
 NCBI BlastP on this gene

EFJ00117

hypothetical protein
  
Accession: EFJ00118
  
Location: 1697461-1699615
  
 NCBI BlastP on this gene

EFJ00118

hypothetical protein
  
Accession: EFJ00119
  
Location: 1700226-1701315
  
 NCBI BlastP on this gene

EFJ00119

hypothetical protein
  
Accession: EFJ00120
  
Location: 1701744-1702852
  
 NCBI BlastP on this gene

EFJ00120

hypothetical protein
  
Accession: EFJ00946
  
Location: 1703027-1706916
  
 NCBI BlastP on this gene

EFJ00946

hypothetical protein
  
Accession: EFJ00121
  
Location: 1707544-1708270
  
 NCBI BlastP on this gene

EFJ00121

hypothetical protein
  
Accession: EFJ00947
  
Location: 1708441-1713759
  
 NCBI BlastP on this gene

EFJ00947

hypothetical protein
  
Accession: EFJ00948
  
Location: 1714164-1715961
  
 NCBI BlastP on this gene

EFJ00948

hypothetical protein
  
Accession: EFJ00122
  
Location: 1716825-1718859
  
  
**BlastP hit with Mycgr3G70577\_Mycgr3T**
  
Percentage identity: 41 %
  
BlastP bit score: 454
  
Sequence coverage: 97 %
  
E-value: 2e-147
  
  
 NCBI BlastP on this gene

EFJ00122

hypothetical protein
  
Accession: EFJ00123
  
Location: 1719469-1721678
  
 NCBI BlastP on this gene

EFJ00123

hypothetical protein
  
Accession: EFJ00124
  
Location: 1722812-1725100
  
 NCBI BlastP on this gene

EFJ00124

hypothetical protein
  
Accession: EFJ00949
  
Location: 1725878-1727876
  
 NCBI BlastP on this gene

EFJ00949

expressed protein
  
Accession: EFJ00950
  
Location: 1730164-1732267
  
 NCBI BlastP on this gene

EFJ00950

hypothetical protein
  
Accession: EFJ00125
  
Location: 1734942-1737222
  
 NCBI BlastP on this gene

EFJ00125

hypothetical protein
  
Accession: EFJ00951
  
Location: 1737649-1740387
  
 NCBI BlastP on this gene

EFJ00951

22. :  EQ963479 Aspergillus flavus NRRL3357 scf\_1106286418500 genomic scaffold     Total score: 2.0     Cumulative Blast bit score: 757

oxidoreductase, short-chain
  
Accession: EED49643
  
Location: 126602-127323
  
 NCBI BlastP on this gene

EED49643

conserved hypothetical protein
  
Accession: EED49644
  
Location: 129326-129790
  
 NCBI BlastP on this gene

EED49644

conserved hypothetical protein
  
Accession: EED49645
  
Location: 130716-131907
  
 NCBI BlastP on this gene

EED49645

hypothetical protein
  
Accession: EED49646
  
Location: 132556-134032
  
 NCBI BlastP on this gene

EED49646

conserved hypothetical protein
  
Accession: EED49647
  
Location: 135673-137025
  
 NCBI BlastP on this gene

EED49647

conserved hypothetical protein
  
Accession: EED49648
  
Location: 138795-140876
  
 NCBI BlastP on this gene

EED49648

hypothetical protein
  
Accession: EED49649
  
Location: 141865-142570
  
 NCBI BlastP on this gene

EED49649

pantothenate transporter, putative
  
Accession: EED49650
  
Location: 143636-145482
  
 NCBI BlastP on this gene

EED49650

sugar transporter, putative
  
Accession: EED49651
  
Location: 146590-148563
  
  
**BlastP hit with Mycgr3G99766\_Mycgr3T**
  
Percentage identity: 34 %
  
BlastP bit score: 286
  
Sequence coverage: 97 %
  
E-value: 6e-86
  
  
 NCBI BlastP on this gene

EED49651

ankyrin repeat domain, putative
  
Accession: EED49652
  
Location: 151230-152975
  
 NCBI BlastP on this gene

EED49652

cell wall glycosyl hydrolase YteR, putative
  
Accession: EED49653
  
Location: 153324-154621
  
 NCBI BlastP on this gene

EED49653

naringenin,2-oxoglutarate 3-dioxygenase, putative
  
Accession: EED49654
  
Location: 155093-156473
  
 NCBI BlastP on this gene

EED49654

siderophore iron transporter, putative
  
Accession: EED49655
  
Location: 156920-158892
  
  
**BlastP hit with Mycgr3G70577\_Mycgr3T**
  
Percentage identity: 42 %
  
BlastP bit score: 471
  
Sequence coverage: 94 %
  
E-value: 4e-154
  
  
 NCBI BlastP on this gene

EED49655

hypothetical protein
  
Accession: EED49656
  
Location: 162315-162632
  
 NCBI BlastP on this gene

EED49656

conserved hypothetical protein
  
Accession: EED49657
  
Location: 162898-164492
  
 NCBI BlastP on this gene

EED49657

conserved hypothetical protein
  
Accession: EED49658
  
Location: 165165-166289
  
 NCBI BlastP on this gene

EED49658

MFS monosaccharide transporter, putative
  
Accession: EED49659
  
Location: 167845-169990
  
 NCBI BlastP on this gene

EED49659

extracellular proline-serine rich protein
  
Accession: EED49660
  
Location: 173859-174671
  
 NCBI BlastP on this gene

EED49660

conserved hypothetical protein
  
Accession: EED49661
  
Location: 175563-176101
  
 NCBI BlastP on this gene

EED49661

transmembrane protein 14, putative
  
Accession: EED49662
  
Location: 178100-178560
  
 NCBI BlastP on this gene

EED49662

23. :  AP007154 Aspergillus oryzae RIB40 DNA, SC001.     Total score: 2.0     Cumulative Blast bit score: 757

not annotated
  
Accession: BAE56615
  
Location: 112370-113220
  
 NCBI BlastP on this gene

AO090001000052

not annotated
  
Accession: BAE56616
  
Location: 113875-114561
  
 NCBI BlastP on this gene

AO090001000053

not annotated
  
Accession: BAE56617
  
Location: 116394-117101
  
 NCBI BlastP on this gene

AO090001000055

not annotated
  
Accession: BAE56618
  
Location: 118981-119900
  
 NCBI BlastP on this gene

AO090001000056

not annotated
  
Accession: BAE56619
  
Location: 121392-122738
  
 NCBI BlastP on this gene

AO090001000057

not annotated
  
Accession: BAE56620
  
Location: 124509-126590
  
 NCBI BlastP on this gene

AO090001000058

not annotated
  
Accession: BAE56621
  
Location: 130971-134279
  
  
**BlastP hit with Mycgr3G99766\_Mycgr3T**
  
Percentage identity: 34 %
  
BlastP bit score: 286
  
Sequence coverage: 97 %
  
E-value: 3e-86
  
  
 NCBI BlastP on this gene

AO090001000061

not annotated
  
Accession: BAE56622
  
Location: 137092-138699
  
 NCBI BlastP on this gene

AO090001000062

not annotated
  
Accession: BAE56623
  
Location: 139178-140337
  
 NCBI BlastP on this gene

AO090001000063

not annotated
  
Accession: BAE56624
  
Location: 140806-142186
  
 NCBI BlastP on this gene

AO090001000064

not annotated
  
Accession: BAE56625
  
Location: 142632-144604
  
  
**BlastP hit with Mycgr3G70577\_Mycgr3T**
  
Percentage identity: 42 %
  
BlastP bit score: 471
  
Sequence coverage: 94 %
  
E-value: 3e-154
  
  
 NCBI BlastP on this gene

AO090001000065

not annotated
  
Accession: BAE56626
  
Location: 146665-148342
  
 NCBI BlastP on this gene

AO090001000066

not annotated
  
Accession: BAE56627
  
Location: 148608-150201
  
 NCBI BlastP on this gene

AO090001000067

not annotated
  
Accession: BAE56628
  
Location: 150874-151578
  
 NCBI BlastP on this gene

AO090001000068

not annotated
  
Accession: BAE56629
  
Location: 153544-157062
  
 NCBI BlastP on this gene

AO090001000069

not annotated
  
Accession: BAE56630
  
Location: 159560-160372
  
 NCBI BlastP on this gene

AO090001000070

not annotated
  
Accession: BAE56631
  
Location: 161263-161801
  
 NCBI BlastP on this gene

AO090001000071

24. :  CAOJ01000067 Rhizoctonia solani AG-1 IB strain isolate 7/3/14     Total score: 2.0     Cumulative Blast bit score: 530

putative N-acetyltransferase san
  
Accession: CCO26033
  
Location: 2900-3693
  
 NCBI BlastP on this gene

CCO26033

Putative lysine N-acyltransferase C17G9.06c
  
Accession: CCO26034
  
Location: 3890-5312
  
  
**BlastP hit with Mycgr3G85486\_Mycgr3T**
  
Percentage identity: 51 %
  
BlastP bit score: 323
  
Sequence coverage: 76 %
  
E-value: 2e-103
  
  
 NCBI BlastP on this gene

CCO26034

Siderophore iron transporter 3
  
Accession: CCO26035
  
Location: 5684-6969
  
  
**BlastP hit with Mycgr3G70577\_Mycgr3T**
  
Percentage identity: 36 %
  
BlastP bit score: 207
  
Sequence coverage: 49 %
  
E-value: 1e-57
  
  
 NCBI BlastP on this gene

CCO26035

25. :  EQ963475 Aspergillus flavus NRRL3357 scf\_1106286419142 genomic scaffold     Total score: 2.0     Cumulative Blast bit score: 432

nonribosomal siderophore peptide synthase SidC
  
Accession: EED53566
  
Location: 1401753-1416093
  
  
**BlastP hit with Mycgr3G40534\_Mycgr3T**
  
Percentage identity: 50 %
  
BlastP bit score: 236
  
Sequence coverage: 79 %
  
E-value: 7e-66
  
  
  
**BlastP hit with Mycgr3G39931\_Mycgr3T**
  
Percentage identity: 43 %
  
BlastP bit score: 197
  
Sequence coverage: 91 %
  
E-value: 1e-52
  
  
 NCBI BlastP on this gene

EED53566

actin cytoskeleton organization protein App1, putative
  
Accession: EED53565
  
Location: 1398897-1401293
  
 NCBI BlastP on this gene

EED53565

UV-endonuclease UVE-1
  
Accession: EED53564
  
Location: 1395653-1397964
  
 NCBI BlastP on this gene

EED53564

extracellular protein, putative
  
Accession: EED53563
  
Location: 1393611-1394651
  
 NCBI BlastP on this gene

EED53563

conserved hypothetical protein
  
Accession: EED53562
  
Location: 1390969-1391828
  
 NCBI BlastP on this gene

EED53562

alcohol dehydrogenase, putative
  
Accession: EED53561
  
Location: 1388586-1389948
  
 NCBI BlastP on this gene

EED53561

conserved hypothetical protein
  
Accession: EED53560
  
Location: 1386159-1386713
  
 NCBI BlastP on this gene

EED53560

UV-damaged DNA binding protein, putative
  
Accession: EED53559
  
Location: 1382230-1385765
  
 NCBI BlastP on this gene

EED53559

26. :  AP007157 Aspergillus oryzae RIB40 DNA, SC023.     Total score: 2.0     Cumulative Blast bit score: 430

not annotated
  
Accession: BAE59066
  
Location: 1386059-1400399
  
  
**BlastP hit with Mycgr3G40534\_Mycgr3T**
  
Percentage identity: 49 %
  
BlastP bit score: 236
  
Sequence coverage: 79 %
  
E-value: 1e-65
  
  
  
**BlastP hit with Mycgr3G39931\_Mycgr3T**
  
Percentage identity: 42 %
  
BlastP bit score: 194
  
Sequence coverage: 91 %
  
E-value: 8e-52
  
  
 NCBI BlastP on this gene

AO090023000528

not annotated
  
Accession: BAE59065
  
Location: 1383436-1385598
  
 NCBI BlastP on this gene

AO090023000527

not annotated
  
Accession: BAE59064
  
Location: 1380309-1382269
  
 NCBI BlastP on this gene

AO090023000526

not annotated
  
Accession: BAE59063
  
Location: 1377916-1378956
  
 NCBI BlastP on this gene

AO090023000525

not annotated
  
Accession: BAE59062
  
Location: 1375274-1376133
  
 NCBI BlastP on this gene

AO090023000524

not annotated
  
Accession: BAE59061
  
Location: 1372951-1374253
  
 NCBI BlastP on this gene

AO090023000523

not annotated
  
Accession: BAE59060
  
Location: 1370462-1371199
  
 NCBI BlastP on this gene

AO090023000522

not annotated
  
Accession: BAE59059
  
Location: 1366394-1370068
  
 NCBI BlastP on this gene

AO090023000521

27. :  AKHY01000182 Aspergillus oryzae 3.042     Total score: 2.0     Cumulative Blast bit score: 429

non-ribosomal peptide synthetase/alpha-aminoadipate reductase
  
Accession: EIT75205
  
Location: 1179418-1193758
  
  
**BlastP hit with Mycgr3G40534\_Mycgr3T**
  
Percentage identity: 49 %
  
BlastP bit score: 235
  
Sequence coverage: 80 %
  
E-value: 3e-65
  
  
  
**BlastP hit with Mycgr3G39931\_Mycgr3T**
  
Percentage identity: 43 %
  
BlastP bit score: 194
  
Sequence coverage: 91 %
  
E-value: 9e-52
  
  
 NCBI BlastP on this gene

EIT75205

hypothetical protein
  
Accession: EIT75184
  
Location: 1176795-1178957
  
 NCBI BlastP on this gene

EIT75184

UV damage repair endonuclease
  
Accession: EIT75175
  
Location: 1173668-1175540
  
 NCBI BlastP on this gene

EIT75175

extracellular protein
  
Accession: EIT74896
  
Location: 1171275-1172315
  
 NCBI BlastP on this gene

EIT74896

hypothetical protein
  
Accession: EIT74869
  
Location: 1168633-1169492
  
 NCBI BlastP on this gene

EIT74869

threonine dehydrogenase
  
Accession: EIT75018
  
Location: 1166310-1167612
  
 NCBI BlastP on this gene

EIT75018

hypothetical protein
  
Accession: EIT75216
  
Location: 1163820-1164558
  
 NCBI BlastP on this gene

EIT75216

damage-specific DNA binding complex, subunit DDB1
  
Accession: EIT74917
  
Location: 1159754-1163426
  
 NCBI BlastP on this gene

EIT74917

28. :  JH687874 Auricularia delicata TFB-10046 SS5 unplaced genomic scaffold AURDEscaffold\_142     Total score: 2.0     Cumulative Blast bit score: 346

hypothetical protein
  
Accession: EJD35965
  
Location: 59142-59927
  
 NCBI BlastP on this gene

EJD35965

hypothetical protein
  
Accession: EJD35966
  
Location: 60780-62320
  
 NCBI BlastP on this gene

EJD35966

Zn-dependent exopeptidase
  
Accession: EJD35967
  
Location: 63179-64445
  
 NCBI BlastP on this gene

EJD35967

hypothetical protein
  
Accession: EJD35968
  
Location: 64804-65166
  
 NCBI BlastP on this gene

EJD35968

CGI-121-domain-containing protein
  
Accession: EJD35969
  
Location: 65562-66299
  
 NCBI BlastP on this gene

EJD35969

thioredoxin-domain-containing protein
  
Accession: EJD35970
  
Location: 66414-69318
  
 NCBI BlastP on this gene

EJD35970

hypothetical protein
  
Accession: EJD35971
  
Location: 69916-70481
  
 NCBI BlastP on this gene

EJD35971

NAD(P)-binding protein
  
Accession: EJD35972
  
Location: 75084-76116
  
 NCBI BlastP on this gene

EJD35972

hypothetical protein
  
Accession: EJD35973
  
Location: 76451-77788
  
 NCBI BlastP on this gene

EJD35973

acetyl-CoA synthetase-like protein
  
Accession: EJD35974
  
Location: 78172-91094
  
  
**BlastP hit with Mycgr3G39931\_Mycgr3T**
  
Percentage identity: 43 %
  
BlastP bit score: 189
  
Sequence coverage: 88 %
  
E-value: 5e-50
  
  
 NCBI BlastP on this gene

EJD35974

hypothetical protein
  
Accession: EJD35975
  
Location: 91566-93659
  
  
**BlastP hit with Mycgr3G85486\_Mycgr3T**
  
Percentage identity: 35 %
  
BlastP bit score: 157
  
Sequence coverage: 71 %
  
E-value: 1e-39
  
  
 NCBI BlastP on this gene

EJD35975

hypothetical protein
  
Accession: EJD35976
  
Location: 93923-95607
  
 NCBI BlastP on this gene

EJD35976

P-loop containing nucleoside triphosphate hydrolase protein
  
Accession: EJD35977
  
Location: 95984-101600
  
 NCBI BlastP on this gene

EJD35977

cyclin-like protein
  
Accession: EJD35978
  
Location: 101741-103073
  
 NCBI BlastP on this gene

EJD35978

PRTase-like protein
  
Accession: EJD35979
  
Location: 103533-104720
  
 NCBI BlastP on this gene

EJD35979

hypothetical protein
  
Accession: EJD35980
  
Location: 106724-107913
  
 NCBI BlastP on this gene

EJD35980

actin 1
  
Accession: EJD35981
  
Location: 108547-110226
  
 NCBI BlastP on this gene

EJD35981

hypothetical protein
  
Accession: EJD35982
  
Location: 110533-111578
  
 NCBI BlastP on this gene

EJD35982

29. :  GL985075 Trichoderma reesei QM6a unplaced genomic scaffold TRIREscaffold\_20     Total score: 2.0     Cumulative Blast bit score: 346

predicted protein
  
Accession: EGR46019
  
Location: 509385-509681
  
 NCBI BlastP on this gene

EGR46019

predicted protein
  
Accession: EGR46095
  
Location: 509958-516684
  
 NCBI BlastP on this gene

EGR46095

predicted protein
  
Accession: EGR46096
  
Location: 518013-522275
  
 NCBI BlastP on this gene

EGR46096

predicted protein
  
Accession: EGR46020
  
Location: 523796-525332
  
 NCBI BlastP on this gene

EGR46020

predicted protein
  
Accession: EGR46097
  
Location: 525837-527838
  
  
**BlastP hit with Mycgr3G70577\_Mycgr3T**
  
Percentage identity: 26 %
  
BlastP bit score: 182
  
Sequence coverage: 91 %
  
E-value: 2e-46
  
  
 NCBI BlastP on this gene

EGR46097

predicted protein
  
Accession: EGR46021
  
Location: 530969-532408
  
  
**BlastP hit with Mycgr3G85486\_Mycgr3T**
  
Percentage identity: 31 %
  
BlastP bit score: 164
  
Sequence coverage: 80 %
  
E-value: 5e-42
  
  
 NCBI BlastP on this gene

EGR46021

predicted protein
  
Accession: EGR46098
  
Location: 532659-534585
  
 NCBI BlastP on this gene

EGR46098

non-ribosomal peptide synthetase
  
Accession: EGR46022
  
Location: 536612-542053
  
 NCBI BlastP on this gene

EGR46022

predicted protein
  
Accession: EGR46099
  
Location: 542649-545703
  
 NCBI BlastP on this gene

EGR46099

predicted protein
  
Accession: EGR46100
  
Location: 550836-552346
  
 NCBI BlastP on this gene

EGR46100

30. :  AM920431 Penicillium chrysogenum Wisconsin 54-1255 complete genome, contig Pc00c16.     Total score: 2.0     Cumulative Blast bit score: 346

not annotated
  
Accession: CAP93051
  
Location: 864367-866034
  
 NCBI BlastP on this gene

Pc16g03810

not annotated
  
Accession: CAP93052
  
Location: 867608-869295
  
 NCBI BlastP on this gene

Pc16g03820

not annotated
  
Accession: CAP93053
  
Location: 869776-870426
  
 NCBI BlastP on this gene

Pc16g03830

hypothetical protein
  
Accession: CAP93054
  
Location: 870809-871366
  
 NCBI BlastP on this gene

Pc16g03840

not annotated
  
Accession: CAP93055
  
Location: 871894-877643
  
 NCBI BlastP on this gene

Pc16g03850

not annotated
  
Accession: CAP93056
  
Location: 878527-879908
  
  
**BlastP hit with Mycgr3G85486\_Mycgr3T**
  
Percentage identity: 30 %
  
BlastP bit score: 165
  
Sequence coverage: 84 %
  
E-value: 2e-42
  
  
 NCBI BlastP on this gene

Pc16g03860

not annotated
  
Accession: CAP93057
  
Location: 880563-884634
  
 NCBI BlastP on this gene

Pc16g03870

not annotated
  
Accession: CAP93058
  
Location: 884789-886193
  
 NCBI BlastP on this gene

Pc16g03880

not annotated
  
Accession: CAP93059
  
Location: 886545-887288
  
 NCBI BlastP on this gene

Pc16g03890

unnamed
  
Accession: CAP93060
  
Location: 888312-889889
  
 NCBI BlastP on this gene

Pc16g03900

not annotated
  
Accession: CAP93061
  
Location: 890263-892148
  
  
**BlastP hit with Mycgr3G70577\_Mycgr3T**
  
Percentage identity: 26 %
  
BlastP bit score: 181
  
Sequence coverage: 89 %
  
E-value: 5e-46
  
  
 NCBI BlastP on this gene

Pc16g03910

not annotated
  
Accession: CAP93062
  
Location: 893255-893635
  
 NCBI BlastP on this gene

Pc16g03920

hypothetical protein
  
Accession: CAP93063
  
Location: 893980-894758
  
 NCBI BlastP on this gene

Pc16g03930

not annotated
  
Accession: CAP93064
  
Location: 894901-896497
  
 NCBI BlastP on this gene

Pc16g03940

not annotated
  
Accession: CAP93065
  
Location: 897066-897508
  
 NCBI BlastP on this gene

Pc16g03950

not annotated
  
Accession: CAP93066
  
Location: 898326-900913
  
 NCBI BlastP on this gene

Pc16g03960

not annotated
  
Accession: CAP93067
  
Location: 901731-903874
  
 NCBI BlastP on this gene

Pc16g03970

not annotated
  
Accession: CAP93068
  
Location: 904441-905325
  
 NCBI BlastP on this gene

Pc16g03980

not annotated
  
Accession: CAP93069
  
Location: 906101-908616
  
 NCBI BlastP on this gene

Pc16g03990

not annotated
  
Accession: Pc16g04000
  
Location: 909559-911850
  
 NCBI BlastP on this gene

Pc16g04000

not annotated
  
Accession: Pc16g04010
  
Location: 912015-912297
  
 NCBI BlastP on this gene

Pc16g04010

31. :  KB916185 Neofusicoccum parvum UCRNP2 chromosome Unknown NP2\_03\_scaffold\_547     Total score: 2.0     Cumulative Blast bit score: 345

putative oxysterol binding protein
  
Accession: EOD48670
  
Location: 88409-89877
  
 NCBI BlastP on this gene

EOD48670

putative phosphoglucomutase protein
  
Accession: EOD48671
  
Location: 85500-87501
  
 NCBI BlastP on this gene

EOD48671

putative l-ornithine n5-oxygenase protein
  
Accession: EOD48640
  
Location: 78529-80395
  
 NCBI BlastP on this gene

EOD48640

putative abc multidrug transporter mdr1 protein
  
Accession: EOD48645
  
Location: 73006-76896
  
 NCBI BlastP on this gene

EOD48645

putative nonribosomal siderophore peptide synthase protein
  
Accession: EOD48642
  
Location: 65978-68784
  
  
**BlastP hit with Mycgr3G40534\_Mycgr3T**
  
Percentage identity: 30 %
  
BlastP bit score: 125
  
Sequence coverage: 100 %
  
E-value: 2e-28
  
  
  
**BlastP hit with Mycgr3G39931\_Mycgr3T**
  
Percentage identity: 32 %
  
BlastP bit score: 111
  
Sequence coverage: 89 %
  
E-value: 5e-24
  
  
 NCBI BlastP on this gene

EOD48642

putative nonribosomal peptide synthetase 2 protein
  
Accession: EOD48651
  
Location: 54514-63750
  
  
**BlastP hit with Mycgr3G39931\_Mycgr3T**
  
Percentage identity: 31 %
  
BlastP bit score: 109
  
Sequence coverage: 91 %
  
E-value: 7e-23
  
  
 NCBI BlastP on this gene

EOD48651

hypothetical protein
  
Accession: EOD48661
  
Location: 53367-54101
  
 NCBI BlastP on this gene

EOD48661

putative l-psp endoribonuclease family protein
  
Accession: EOD48634
  
Location: 49279-49742
  
 NCBI BlastP on this gene

EOD48634

hypothetical protein
  
Accession: EOD48662
  
Location: 44857-47148
  
 NCBI BlastP on this gene

EOD48662

putative allantoate permease protein
  
Accession: EOD48648
  
Location: 39591-41205
  
 NCBI BlastP on this gene

EOD48648

putative fad-binding protein
  
Accession: EOD48657
  
Location: 35986-39407
  
 NCBI BlastP on this gene

EOD48657

32. :  HF679032 Fusarium fujikuroi IMI 58289 draft genome, chromosome FFUJ\_chr10.     Total score: 2.0     Cumulative Blast bit score: 345

related to isoamyl alcohol oxidase
  
Accession: CCT74678
  
Location: 1415062-1417211
  
 NCBI BlastP on this gene

FFUJ\_10739

uncharacterized protein
  
Accession: CCT74677
  
Location: 1412482-1414528
  
 NCBI BlastP on this gene

FFUJ\_10738

uncharacterized protein
  
Accession: CCT74676
  
Location: 1409484-1410929
  
 NCBI BlastP on this gene

FFUJ\_10737

related to AM-toxin synthetase (AMT)
  
Accession: CCT74675
  
Location: 1402333-1408592
  
 NCBI BlastP on this gene

FFUJ\_10736

related to enoyl-CoA hydratase
  
Accession: CCT74674
  
Location: 1400338-1401210
  
 NCBI BlastP on this gene

FFUJ\_10735

related to aerobactin siderophore biosynthesis protein iucB
  
Accession: CCT74673
  
Location: 1396540-1397826
  
  
**BlastP hit with Mycgr3G85486\_Mycgr3T**
  
Percentage identity: 32 %
  
BlastP bit score: 162
  
Sequence coverage: 77 %
  
E-value: 2e-41
  
  
 NCBI BlastP on this gene

FFUJ\_10734

related to major facilitator MirA
  
Accession: CCT74672
  
Location: 1392754-1394638
  
  
**BlastP hit with Mycgr3G70577\_Mycgr3T**
  
Percentage identity: 26 %
  
BlastP bit score: 183
  
Sequence coverage: 93 %
  
E-value: 1e-46
  
  
 NCBI BlastP on this gene

FFUJ\_10733

uncharacterized protein
  
Accession: CCT74671
  
Location: 1391252-1392212
  
 NCBI BlastP on this gene

FFUJ\_10732

related to cellobiose dehydrogenase
  
Accession: CCT74670
  
Location: 1386301-1388682
  
 NCBI BlastP on this gene

FFUJ\_10731

related to O-methylsterigmatocystin oxidoreductase
  
Accession: CCT74669
  
Location: 1383753-1385449
  
 NCBI BlastP on this gene

FFUJ\_10730

uncharacterized protein
  
Accession: CCT74668
  
Location: 1381694-1382496
  
 NCBI BlastP on this gene

FFUJ\_10729

related to glycine-rich RNA-binding protein
  
Accession: CCT74667
  
Location: 1380282-1380783
  
 NCBI BlastP on this gene

FFUJ\_10728

related to maltose O-acetyltransferase
  
Accession: CCT74666
  
Location: 1379032-1379794
  
 NCBI BlastP on this gene

FFUJ\_10727

uncharacterized protein
  
Accession: CCT74665
  
Location: 1376970-1378352
  
 NCBI BlastP on this gene

FFUJ\_10726

related to alcohol oxidase
  
Accession: CCT74664
  
Location: 1374138-1376325
  
 NCBI BlastP on this gene

FFUJ\_10725

33. :  KB730325 Fusarium oxysporum f. sp. cubense race 1 unplaced genomic scaffold scaffold314     Total score: 2.0     Cumulative Blast bit score: 344

hypothetical protein
  
Accession: ENH67312
  
Location: 338795-340564
  
 NCBI BlastP on this gene

ENH67312

hypothetical protein
  
Accession: ENH67313
  
Location: 341192-341422
  
 NCBI BlastP on this gene

ENH67313

Nitrosoguanidine resistance protein SNG1
  
Accession: ENH67314
  
Location: 342948-344393
  
 NCBI BlastP on this gene

ENH67314

HC-toxin synthetase
  
Accession: ENH67315
  
Location: 345279-351538
  
 NCBI BlastP on this gene

ENH67315

Putative enoyl-CoA hydratase/isomerase yngF
  
Accession: ENH67316
  
Location: 352631-353428
  
 NCBI BlastP on this gene

ENH67316

Putative lysine N-acyltransferase C17G9.06c
  
Accession: ENH67317
  
Location: 356013-357265
  
  
**BlastP hit with Mycgr3G85486\_Mycgr3T**
  
Percentage identity: 32 %
  
BlastP bit score: 162
  
Sequence coverage: 77 %
  
E-value: 1e-41
  
  
 NCBI BlastP on this gene

ENH67317

Siderophore iron transporter mirB
  
Accession: ENH67318
  
Location: 359157-361041
  
  
**BlastP hit with Mycgr3G70577\_Mycgr3T**
  
Percentage identity: 26 %
  
BlastP bit score: 182
  
Sequence coverage: 93 %
  
E-value: 3e-46
  
  
 NCBI BlastP on this gene

ENH67318

Ferri-bacillibactin esterase BesA
  
Accession: ENH67319
  
Location: 361589-362549
  
 NCBI BlastP on this gene

ENH67319

Cellobiose dehydrogenase
  
Accession: ENH67320
  
Location: 365308-367689
  
 NCBI BlastP on this gene

ENH67320

Cytochrome P450 76A2
  
Accession: ENH67321
  
Location: 368527-370242
  
 NCBI BlastP on this gene

ENH67321

hypothetical protein
  
Accession: ENH67322
  
Location: 371453-372256
  
 NCBI BlastP on this gene

ENH67322

Glycine-rich RNA-binding protein 2
  
Accession: ENH67323
  
Location: 373155-373669
  
 NCBI BlastP on this gene

ENH67323

hypothetical protein
  
Accession: ENH67324
  
Location: 374153-374909
  
 NCBI BlastP on this gene

ENH67324

hypothetical protein
  
Accession: ENH67325
  
Location: 375584-376969
  
 NCBI BlastP on this gene

ENH67325

Alcohol dehydrogenase [acceptor]
  
Accession: ENH67326
  
Location: 377464-379799
  
 NCBI BlastP on this gene

ENH67326

hypothetical protein
  
Accession: ENH67327
  
Location: 380777-382233
  
 NCBI BlastP on this gene

ENH67327

34. :  KB726307 Fusarium oxysporum f. sp. cubense race 4 unplaced genomic scaffold scaffold26     Total score: 2.0     Cumulative Blast bit score: 342

6-hydroxy-D-nicotine oxidase
  
Accession: EMT71270
  
Location: 788458-790608
  
 NCBI BlastP on this gene

EMT71270

hypothetical protein
  
Accession: EMT71269
  
Location: 785651-787527
  
 NCBI BlastP on this gene

EMT71269

Nitrosoguanidine resistance protein SNG1
  
Accession: EMT71268
  
Location: 782934-784358
  
 NCBI BlastP on this gene

EMT71268

HC-toxin synthetase
  
Accession: EMT71267
  
Location: 775770-782029
  
 NCBI BlastP on this gene

EMT71267

Putative enoyl-CoA hydratase/isomerase yngF
  
Accession: EMT71266
  
Location: 773802-774674
  
 NCBI BlastP on this gene

EMT71266

Putative lysine N-acyltransferase C17G9.06c
  
Accession: EMT71265
  
Location: 770010-770984
  
  
**BlastP hit with Mycgr3G85486\_Mycgr3T**
  
Percentage identity: 35 %
  
BlastP bit score: 159
  
Sequence coverage: 66 %
  
E-value: 4e-41
  
  
 NCBI BlastP on this gene

EMT71265

Siderophore iron transporter mirB
  
Accession: EMT71264
  
Location: 766236-768120
  
  
**BlastP hit with Mycgr3G70577\_Mycgr3T**
  
Percentage identity: 26 %
  
BlastP bit score: 183
  
Sequence coverage: 93 %
  
E-value: 2e-46
  
  
 NCBI BlastP on this gene

EMT71264

Ferri-bacillibactin esterase BesA
  
Accession: EMT71263
  
Location: 762491-765688
  
 NCBI BlastP on this gene

EMT71263

Cellobiose dehydrogenase
  
Accession: EMT71262
  
Location: 755286-757667
  
 NCBI BlastP on this gene

EMT71262

Cytochrome P450 76A2
  
Accession: EMT71261
  
Location: 752733-754449
  
 NCBI BlastP on this gene

EMT71261

hypothetical protein
  
Accession: EMT71260
  
Location: 750714-751517
  
 NCBI BlastP on this gene

EMT71260

Glycine-rich RNA-binding protein 2, mitochondrial
  
Accession: EMT71259
  
Location: 749297-749814
  
 NCBI BlastP on this gene

EMT71259

hypothetical protein
  
Accession: EMT71258
  
Location: 748059-748815
  
 NCBI BlastP on this gene

EMT71258

35. :  CP003002 Myceliophthora thermophila ATCC 42464 chromosome 1     Total score: 2.0     Cumulative Blast bit score: 341

hypothetical protein
  
Accession: AEO55699
  
Location: 10614079-10615774
  
 NCBI BlastP on this gene

MYCTH\_2116408

hypothetical protein
  
Accession: AEO55700
  
Location: 10616173-10617467
  
 NCBI BlastP on this gene

MYCTH\_40734

non-ribosomal peptide synthetase
  
Accession: AEO55701
  
Location: 10619242-10626902
  
 NCBI BlastP on this gene

MYCTH\_112805

ABC transporter-like protein
  
Accession: AEO55702
  
Location: 10627496-10631807
  
 NCBI BlastP on this gene

MYCTH\_104717

siderophore biosynthesis protein
  
Accession: AEO55703
  
Location: 10632494-10633979
  
  
**BlastP hit with Mycgr3G85486\_Mycgr3T**
  
Percentage identity: 33 %
  
BlastP bit score: 158
  
Sequence coverage: 81 %
  
E-value: 5e-40
  
  
 NCBI BlastP on this gene

MYCTH\_59137

Crotonase-like protein
  
Accession: AEO55704
  
Location: 10634403-10635390
  
 NCBI BlastP on this gene

MYCTH\_112802

general substrate transporter
  
Accession: AEO55705
  
Location: 10636416-10638428
  
  
**BlastP hit with Mycgr3G70577\_Mycgr3T**
  
Percentage identity: 26 %
  
BlastP bit score: 183
  
Sequence coverage: 94 %
  
E-value: 1e-46
  
  
 NCBI BlastP on this gene

MYCTH\_109499

hypothetical protein
  
Accession: AEO55706
  
Location: 10640414-10644102
  
 NCBI BlastP on this gene

MYCTH\_2299794

RTA1 like protein
  
Accession: AEO55707
  
Location: 10645839-10647041
  
 NCBI BlastP on this gene

MYCTH\_97342

hypothetical protein
  
Accession: AEO55708
  
Location: 10647874-10649623
  
 NCBI BlastP on this gene

MYCTH\_2087100

hypothetical protein
  
Accession: AEO55709
  
Location: 10650627-10652444
  
 NCBI BlastP on this gene

MYCTH\_2299804

hypothetical protein
  
Accession: AEO55710
  
Location: 10654388-10655332
  
 NCBI BlastP on this gene

MYCTH\_2299806

non-ribosomal peptide synthetase
  
Accession: AEO55711
  
Location: 10656484-10661973
  
 NCBI BlastP on this gene

MYCTH\_73236

36. :  ABDF02000085 Trichoderma virens Gv29-8     Total score: 2.0     Cumulative Blast bit score: 338

hypothetical protein
  
Accession: EHK18680
  
Location: 69384-70863
  
 NCBI BlastP on this gene

EHK18680

hypothetical protein
  
Accession: EHK18681
  
Location: 76328-77656
  
 NCBI BlastP on this gene

EHK18681

non-ribosomal peptide synthetase
  
Accession: EHK18682
  
Location: 78183-83471
  
 NCBI BlastP on this gene

EHK18682

hypothetical protein
  
Accession: EHK18683
  
Location: 85465-87308
  
 NCBI BlastP on this gene

EHK18683

hypothetical protein
  
Accession: EHK18684
  
Location: 87521-88966
  
  
**BlastP hit with Mycgr3G85486\_Mycgr3T**
  
Percentage identity: 30 %
  
BlastP bit score: 155
  
Sequence coverage: 80 %
  
E-value: 5e-39
  
  
 NCBI BlastP on this gene

EHK18684

hypothetical protein
  
Accession: EHK18685
  
Location: 91207-93161
  
  
**BlastP hit with Mycgr3G70577\_Mycgr3T**
  
Percentage identity: 26 %
  
BlastP bit score: 183
  
Sequence coverage: 87 %
  
E-value: 1e-46
  
  
 NCBI BlastP on this gene

EHK18685

hypothetical protein
  
Accession: EHK18686
  
Location: 93666-95147
  
 NCBI BlastP on this gene

EHK18686

hypothetical protein
  
Accession: EHK18687
  
Location: 96467-100675
  
 NCBI BlastP on this gene

EHK18687

hypothetical protein
  
Accession: EHK18688
  
Location: 101030-108322
  
 NCBI BlastP on this gene

EHK18688

hypothetical protein
  
Accession: EHK18689
  
Location: 108541-108843
  
 NCBI BlastP on this gene

EHK18689

hypothetical protein
  
Accession: EHK18690
  
Location: 110784-113297
  
 NCBI BlastP on this gene

EHK18690

37. :  AFQF01003643 Fusarium oxysporum Fo5176     Total score: 2.0     Cumulative Blast bit score: 326

hypothetical protein
  
Accession: EGU74748
  
Location: 76-822
  
  
**BlastP hit with Mycgr3G85486\_Mycgr3T**
  
Percentage identity: 38 %
  
BlastP bit score: 144
  
Sequence coverage: 49 %
  
E-value: 9e-37
  
  
 NCBI BlastP on this gene

EGU74748

hypothetical protein
  
Accession: EGU74749
  
Location: 2714-4598
  
  
**BlastP hit with Mycgr3G70577\_Mycgr3T**
  
Percentage identity: 26 %
  
BlastP bit score: 182
  
Sequence coverage: 93 %
  
E-value: 4e-46
  
  
 NCBI BlastP on this gene

EGU74749

hypothetical protein
  
Accession: EGU74750
  
Location: 5146-6106
  
 NCBI BlastP on this gene

EGU74750

hypothetical protein
  
Accession: EGU74751
  
Location: 8616-11246
  
 NCBI BlastP on this gene

EGU74751

hypothetical protein
  
Accession: EGU74752
  
Location: 12084-13800
  
 NCBI BlastP on this gene

EGU74752

hypothetical protein
  
Accession: EGU74753
  
Location: 15016-15819
  
 NCBI BlastP on this gene

EGU74753

38. :  GG704911 Coccidioides immitis RS genomic scaffold supercont3.1     Total score: 2.0     Cumulative Blast bit score: 306

hypothetical protein
  
Accession: EAS35657
  
Location: 5970514-5971694
  
 NCBI BlastP on this gene

EAS35657

hypothetical protein
  
Accession: EAS35656
  
Location: 5972483-5973596
  
 NCBI BlastP on this gene

EAS35656

prolidase
  
Accession: EAS35655
  
Location: 5974058-5975636
  
 NCBI BlastP on this gene

EAS35655

hypothetical protein
  
Accession: EAS35654
  
Location: 5976125-5977693
  
 NCBI BlastP on this gene

EAS35654

hypothetical protein
  
Accession: EAS35653
  
Location: 5978192-5979824
  
 NCBI BlastP on this gene

EAS35653

cyclin-dependent protein kinase complex component
  
Accession: EAS35652
  
Location: 5980777-5981427
  
 NCBI BlastP on this gene

EAS35652

palmitoyltransferase akr1
  
Accession: EAS35651
  
Location: 5982178-5984688
  
 NCBI BlastP on this gene

EAS35651

L-ornithine 5-monooxygenase
  
Accession: EAS35650
  
Location: 5986030-5987648
  
 NCBI BlastP on this gene

EAS35650

hypothetical protein
  
Accession: EAS35649
  
Location: 5987837-5988561
  
 NCBI BlastP on this gene

EAS35649

amino acid adenylation domain-containing protein
  
Accession: EAS35647
  
Location: 5989172-6004808
  
  
**BlastP hit with Mycgr3G40534\_Mycgr3T**
  
Percentage identity: 42 %
  
BlastP bit score: 172
  
Sequence coverage: 81 %
  
E-value: 8e-44
  
  
  
**BlastP hit with Mycgr3G39931\_Mycgr3T**
  
Percentage identity: 35 %
  
BlastP bit score: 134
  
Sequence coverage: 93 %
  
E-value: 4e-31
  
  
 NCBI BlastP on this gene

EAS35647

protein kinase
  
Accession: EAS35646
  
Location: 6005938-6009291
  
 NCBI BlastP on this gene

EAS35646

mRNA splicing factor RNA helicase
  
Accession: EAS35645
  
Location: 6010543-6013863
  
 NCBI BlastP on this gene

EAS35645

cytochrome b5
  
Accession: EAS35644
  
Location: 6014454-6014806
  
 NCBI BlastP on this gene

EAS35644

hypothetical protein
  
Accession: EAS35643
  
Location: 6015468-6016073
  
 NCBI BlastP on this gene

EAS35643

nuclear migration protein
  
Accession: EAS35642
  
Location: 6017013-6020978
  
 NCBI BlastP on this gene

EAS35642

hypothetical protein
  
Accession: EAS35641
  
Location: 6021339-6022720
  
 NCBI BlastP on this gene

EAS35641

hypothetical protein
  
Accession: EAS35640
  
Location: 6023282-6024061
  
 NCBI BlastP on this gene

EAS35640

hypothetical protein
  
Accession: EAS35639
  
Location: 6024618-6024959
  
 NCBI BlastP on this gene

EAS35639

39. :  ACFW01000049 Coccidioides posadasii C735 delta SOWgp     Total score: 2.0     Cumulative Blast bit score: 306

Ubiquinol-cytochrome C chaperone family protein
  
Accession: EER24028
  
Location: 3033988-3034596
  
 NCBI BlastP on this gene

EER24028

hypothetical protein
  
Accession: EER24029
  
Location: 3035394-3036507
  
 NCBI BlastP on this gene

EER24029

metallopeptidase M24 family protein
  
Accession: EER24030
  
Location: 3036969-3038547
  
 NCBI BlastP on this gene

EER24030

hypothetical protein
  
Accession: EER24031
  
Location: 3039046-3040614
  
 NCBI BlastP on this gene

EER24031

hypothetical protein
  
Accession: EER24032
  
Location: 3041114-3042742
  
 NCBI BlastP on this gene

EER24032

hypothetical protein
  
Accession: EER24033
  
Location: 3043688-3044338
  
 NCBI BlastP on this gene

EER24033

DHHC zinc finger domain containing protein
  
Accession: EER24034
  
Location: 3045094-3047604
  
 NCBI BlastP on this gene

EER24034

L-ornithine 5-monooxygenase, putative
  
Accession: EER24035
  
Location: 3048954-3050561
  
 NCBI BlastP on this gene

EER24035

peptide synthetase, putative
  
Accession: EER24036
  
Location: 3052091-3067586
  
  
**BlastP hit with Mycgr3G40534\_Mycgr3T**
  
Percentage identity: 41 %
  
BlastP bit score: 172
  
Sequence coverage: 80 %
  
E-value: 1e-43
  
  
  
**BlastP hit with Mycgr3G39931\_Mycgr3T**
  
Percentage identity: 35 %
  
BlastP bit score: 134
  
Sequence coverage: 93 %
  
E-value: 3e-31
  
  
 NCBI BlastP on this gene

EER24036

protein kinase, putative
  
Accession: EER24037
  
Location: 3068852-3072205
  
 NCBI BlastP on this gene

EER24037

ATP-dependent RNA helicase, putative
  
Accession: EER24038
  
Location: 3073461-3076781
  
 NCBI BlastP on this gene

EER24038

ATPase, AAA family protein
  
Accession: EER24039
  
Location: 3078596-3079347
  
 NCBI BlastP on this gene

EER24039

Protein kinase domain containing protein
  
Accession: EER24040
  
Location: 3081163-3082523
  
 NCBI BlastP on this gene

EER24040

Cytochrome b5-like Heme/Steroid binding domain containing protein
  
Accession: EER24041
  
Location: 3083256-3083608
  
 NCBI BlastP on this gene

EER24041

hypothetical protein
  
Accession: EER24042
  
Location: 3084270-3084875
  
 NCBI BlastP on this gene

EER24042

hypothetical protein
  
Accession: EER24043
  
Location: 3085803-3089765
  
 NCBI BlastP on this gene

EER24043

40. :  CH476615 Uncinocarpus reesii 1704 scaffold\_1 genomic scaffold     Total score: 2.0     Cumulative Blast bit score: 302

conserved hypothetical protein
  
Accession: EEP76051
  
Location: 2120337-2121860
  
 NCBI BlastP on this gene

EEP76051

predicted protein
  
Accession: EEP76050
  
Location: 2118303-2119740
  
 NCBI BlastP on this gene

EEP76050

predicted protein
  
Accession: EEP76049
  
Location: 2115713-2117550
  
 NCBI BlastP on this gene

EEP76049

predicted protein
  
Accession: EEP76048
  
Location: 2112296-2115157
  
 NCBI BlastP on this gene

EEP76048

5-oxoprolinase
  
Accession: EEP76047
  
Location: 2106626-2110906
  
 NCBI BlastP on this gene

EEP76047

predicted protein
  
Accession: EEP76046
  
Location: 2105825-2106419
  
 NCBI BlastP on this gene

EEP76046

predicted protein
  
Accession: EEP76045
  
Location: 2104471-2105278
  
 NCBI BlastP on this gene

EEP76045

conserved hypothetical protein
  
Accession: EEP76044
  
Location: 2103621-2104414
  
 NCBI BlastP on this gene

EEP76044

predicted protein
  
Accession: EEP76043
  
Location: 2098708-2102342
  
  
**BlastP hit with Mycgr3G40534\_Mycgr3T**
  
Percentage identity: 42 %
  
BlastP bit score: 172
  
Sequence coverage: 81 %
  
E-value: 3e-44
  
  
 NCBI BlastP on this gene

EEP76043

predicted protein
  
Accession: EEP76042
  
Location: 2086732-2097150
  
  
**BlastP hit with Mycgr3G39931\_Mycgr3T**
  
Percentage identity: 34 %
  
BlastP bit score: 130
  
Sequence coverage: 93 %
  
E-value: 8e-30
  
  
 NCBI BlastP on this gene

EEP76042

hypothetical protein
  
Accession: EEP76041
  
Location: 2082428-2085782
  
 NCBI BlastP on this gene

EEP76041

ATP-dependent RNA helicase DHX8
  
Accession: EEP76040
  
Location: 2075952-2081256
  
 NCBI BlastP on this gene

EEP76040

predicted protein
  
Accession: EEP76039
  
Location: 2071204-2075118
  
 NCBI BlastP on this gene

EEP76039

predicted protein
  
Accession: EEP76038
  
Location: 2069317-2070027
  
 NCBI BlastP on this gene

EEP76038

41. :  KB644411 Penicillium oxalicum 114-2 unplaced genomic scaffold scaffold\_4     Total score: 2.0     Cumulative Blast bit score: 301

hypothetical protein
  
Accession: EPS29118
  
Location: 2377756-2378022
  
 NCBI BlastP on this gene

EPS29118

hypothetical protein
  
Accession: EPS29119
  
Location: 2379605-2380057
  
 NCBI BlastP on this gene

EPS29119

hypothetical protein
  
Accession: EPS29120
  
Location: 2380436-2380825
  
 NCBI BlastP on this gene

EPS29120

hypothetical protein
  
Accession: EPS29121
  
Location: 2383429-2384214
  
 NCBI BlastP on this gene

EPS29121

hypothetical protein
  
Accession: EPS29122
  
Location: 2385035-2387741
  
 NCBI BlastP on this gene

EPS29122

hypothetical protein
  
Accession: EPS29123
  
Location: 2388999-2389100
  
 NCBI BlastP on this gene

EPS29123

hypothetical protein
  
Accession: EPS29124
  
Location: 2389674-2391288
  
 NCBI BlastP on this gene

EPS29124

hypothetical protein
  
Accession: EPS29125
  
Location: 2397439-2413126
  
  
**BlastP hit with Mycgr3G40534\_Mycgr3T**
  
Percentage identity: 41 %
  
BlastP bit score: 175
  
Sequence coverage: 82 %
  
E-value: 1e-44
  
  
  
**BlastP hit with Mycgr3G39931\_Mycgr3T**
  
Percentage identity: 34 %
  
BlastP bit score: 126
  
Sequence coverage: 90 %
  
E-value: 1e-28
  
  
 NCBI BlastP on this gene

EPS29125

42. :  EQ962653 Talaromyces stipitatus ATCC 10500 scf\_1105507295527 genomic scaffold     Total score: 2.0     Cumulative Blast bit score: 301

conserved hypothetical protein
  
Accession: EED21784
  
Location: 2652459-2654179
  
 NCBI BlastP on this gene

EED21784

cyclin-dependent protein kinase complex component, putative
  
Accession: EED21785
  
Location: 2656489-2659379
  
 NCBI BlastP on this gene

EED21785

palmitoyltransferase SidR
  
Accession: EED21786
  
Location: 2660237-2662729
  
 NCBI BlastP on this gene

EED21786

conserved hypothetical protein
  
Accession: EED21787
  
Location: 2664344-2665605
  
 NCBI BlastP on this gene

EED21787

L-ornithine N5-oxygenase SidA
  
Accession: EED21788
  
Location: 2668207-2669704
  
 NCBI BlastP on this gene

EED21788

nonribosomal siderophore peptide synthase SidC
  
Accession: EED21789
  
Location: 2670983-2686751
  
  
**BlastP hit with Mycgr3G40534\_Mycgr3T**
  
Percentage identity: 36 %
  
BlastP bit score: 168
  
Sequence coverage: 102 %
  
E-value: 3e-42
  
  
  
**BlastP hit with Mycgr3G39931\_Mycgr3T**
  
Percentage identity: 34 %
  
BlastP bit score: 133
  
Sequence coverage: 90 %
  
E-value: 1e-30
  
  
 NCBI BlastP on this gene

EED21789

43. :  DS995701 Microsporum canis CBS 113480 supercont1.1 genomic scaffold     Total score: 2.0     Cumulative Blast bit score: 299

conserved hypothetical protein
  
Accession: EEQ27387
  
Location: 699544-703636
  
 NCBI BlastP on this gene

EEQ27387

conserved hypothetical protein
  
Accession: EEQ27386
  
Location: 698296-698910
  
 NCBI BlastP on this gene

EEQ27386

conserved hypothetical protein
  
Accession: EEQ27385
  
Location: 697422-697742
  
 NCBI BlastP on this gene

EEQ27385

conserved hypothetical protein
  
Accession: EEQ27384
  
Location: 696208-696843
  
 NCBI BlastP on this gene

EEQ27384

conserved hypothetical protein
  
Accession: EEQ27383
  
Location: 692804-694442
  
 NCBI BlastP on this gene

EEQ27383

predicted protein
  
Accession: EEQ27382
  
Location: 691768-692163
  
 NCBI BlastP on this gene

EEQ27382

ATP-dependent RNA helicase DHX8
  
Accession: EEQ27381
  
Location: 687778-691074
  
 NCBI BlastP on this gene

EEQ27381

palmitoyltransferase akr1
  
Accession: EEQ27380
  
Location: 685078-686900
  
 NCBI BlastP on this gene

EEQ27380

hypothetical protein
  
Accession: EEQ27379
  
Location: 683811-684452
  
 NCBI BlastP on this gene

EEQ27379

L-ornithine 5-monooxygenase
  
Accession: EEQ27378
  
Location: 681825-683396
  
 NCBI BlastP on this gene

EEQ27378

nonribosomal peptide synthetase 2
  
Accession: EEQ27377
  
Location: 664435-680056
  
  
**BlastP hit with Mycgr3G40534\_Mycgr3T**
  
Percentage identity: 40 %
  
BlastP bit score: 169
  
Sequence coverage: 81 %
  
E-value: 1e-42
  
  
  
**BlastP hit with Mycgr3G39931\_Mycgr3T**
  
Percentage identity: 35 %
  
BlastP bit score: 130
  
Sequence coverage: 90 %
  
E-value: 9e-30
  
  
 NCBI BlastP on this gene

EEQ27377

AnkA
  
Accession: EEQ27376
  
Location: 659838-662841
  
 NCBI BlastP on this gene

EEQ27376

glutamate 5-kinase
  
Accession: EEQ27375
  
Location: 656694-658280
  
 NCBI BlastP on this gene

EEQ27375

wiskott-Aldrich syndrome protein family member 2
  
Accession: EEQ27374
  
Location: 654907-656129
  
 NCBI BlastP on this gene

EEQ27374

signal recognition particle receptor subunit beta
  
Accession: EEQ27373
  
Location: 653414-654375
  
 NCBI BlastP on this gene

EEQ27373

cystathionine beta-synthase
  
Accession: EEQ27372
  
Location: 651099-652857
  
 NCBI BlastP on this gene

EEQ27372

metalloprotease ATP23
  
Accession: EEQ27371
  
Location: 649725-650587
  
 NCBI BlastP on this gene

EEQ27371

serine/threonine-protein kinase 6
  
Accession: EEQ27370
  
Location: 648386-649619
  
 NCBI BlastP on this gene

EEQ27370

type 2A phosphatase activator TIP41
  
Accession: EEQ27369
  
Location: 647065-648010
  
 NCBI BlastP on this gene

EEQ27369

conserved hypothetical protein
  
Accession: EEQ27368
  
Location: 645122-646735
  
 NCBI BlastP on this gene

EEQ27368

44. :  AM920428 Penicillium chrysogenum Wisconsin 54-1255 complete genome, contig Pc00c13.     Total score: 2.0     Cumulative Blast bit score: 297

not annotated
  
Accession: CAP91602
  
Location: 1326191-1327193
  
 NCBI BlastP on this gene

Pc13g05330

not annotated
  
Accession: CAP91601
  
Location: 1323656-1325415
  
 NCBI BlastP on this gene

Pc13g05320

not annotated
  
Accession: CAP91600
  
Location: 1320770-1322204
  
 NCBI BlastP on this gene

Pc13g05310

not annotated
  
Accession: CAP91599
  
Location: 1319842-1320546
  
 NCBI BlastP on this gene

Pc13g05300

not annotated
  
Accession: CAP91598
  
Location: 1315880-1317574
  
 NCBI BlastP on this gene

Pc13g05290

hypothetical protein
  
Accession: CAP91597
  
Location: 1314789-1315310
  
 NCBI BlastP on this gene

Pc13g05280

not annotated
  
Accession: CAP91596
  
Location: 1311780-1314148
  
 NCBI BlastP on this gene

Pc13g05270

not annotated
  
Accession: CAP91595
  
Location: 1307856-1309413
  
 NCBI BlastP on this gene

Pc13g05260

not annotated
  
Accession: CAP91594
  
Location: 1290919-1306476
  
  
**BlastP hit with Mycgr3G40534\_Mycgr3T**
  
Percentage identity: 38 %
  
BlastP bit score: 177
  
Sequence coverage: 85 %
  
E-value: 2e-45
  
  
  
**BlastP hit with Mycgr3G39931\_Mycgr3T**
  
Percentage identity: 32 %
  
BlastP bit score: 120
  
Sequence coverage: 90 %
  
E-value: 2e-26
  
  
 NCBI BlastP on this gene

Pc13g05250

not annotated
  
Accession: CAP91593
  
Location: 1285814-1289005
  
 NCBI BlastP on this gene

Pc13g05240

not annotated
  
Accession: CAP91592
  
Location: 1281174-1284548
  
 NCBI BlastP on this gene

Pc13g05230

not annotated
  
Accession: CAP91591
  
Location: 1280487-1280849
  
 NCBI BlastP on this gene

Pc13g05220

not annotated
  
Accession: CAP91590
  
Location: 1277127-1278325
  
 NCBI BlastP on this gene

Pc13g05210

not annotated
  
Accession: CAP91589
  
Location: 1272398-1276386
  
 NCBI BlastP on this gene

Pc13g05200

45. :  JH126399 Cordyceps militaris CM01 unplaced genomic scaffold CCM\_S00001     Total score: 2.0     Cumulative Blast bit score: 295

nonribosomal siderophore peptide synthase
  
Accession: EGX97046
  
Location: 5481892-5496446
  
  
**BlastP hit with Mycgr3G40534\_Mycgr3T**
  
Percentage identity: 41 %
  
BlastP bit score: 169
  
Sequence coverage: 81 %
  
E-value: 9e-43
  
  
  
**BlastP hit with Mycgr3G39931\_Mycgr3T**
  
Percentage identity: 33 %
  
BlastP bit score: 126
  
Sequence coverage: 90 %
  
E-value: 2e-28
  
  
 NCBI BlastP on this gene

EGX97046

choline oxidase (CodA), putative
  
Accession: EGX97045
  
Location: 5479825-5481462
  
 NCBI BlastP on this gene

EGX97045

betaine aldehyde dehydrogenase
  
Accession: EGX97044
  
Location: 5475843-5477372
  
 NCBI BlastP on this gene

EGX97044

glyoxalase family protein
  
Accession: EGX97043
  
Location: 5475227-5475631
  
 NCBI BlastP on this gene

EGX97043

hypothetical protein
  
Accession: EGX97042
  
Location: 5472306-5474576
  
 NCBI BlastP on this gene

EGX97042

amidase family protein
  
Accession: EGX97041
  
Location: 5467965-5469862
  
 NCBI BlastP on this gene

EGX97041

hypothetical protein
  
Accession: EGX97040
  
Location: 5465650-5466994
  
 NCBI BlastP on this gene

EGX97040

46. :  GL377303 Schizophyllum commune H4-8 unplaced genomic scaffold SCHCOscaffold\_2     Total score: 2.0     Cumulative Blast bit score: 294

hypothetical protein
  
Accession: EFJ00418
  
Location: 3218164-3219373
  
 NCBI BlastP on this gene

EFJ00418

hypothetical protein
  
Accession: EFJ01221
  
Location: 3216924-3218010
  
 NCBI BlastP on this gene

EFJ01221

hypothetical protein
  
Accession: EFJ01220
  
Location: 3215816-3216695
  
 NCBI BlastP on this gene

EFJ01220

hypothetical protein
  
Accession: EFJ00417
  
Location: 3211669-3213237
  
 NCBI BlastP on this gene

EFJ00417

hypothetical protein
  
Accession: EFJ01219
  
Location: 3208544-3210956
  
 NCBI BlastP on this gene

EFJ01219

expressed protein
  
Accession: EFJ00416
  
Location: 3207256-3207732
  
 NCBI BlastP on this gene

EFJ00416

hypothetical protein
  
Accession: EFJ00415
  
Location: 3200241-3205280
  
 NCBI BlastP on this gene

EFJ00415

hypothetical protein
  
Accession: EFJ01218
  
Location: 3180710-3199916
  
  
**BlastP hit with Mycgr3G40534\_Mycgr3T**
  
Percentage identity: 37 %
  
BlastP bit score: 187
  
Sequence coverage: 105 %
  
E-value: 7e-49
  
  
  
**BlastP hit with Mycgr3G39931\_Mycgr3T**
  
Percentage identity: 33 %
  
BlastP bit score: 107
  
Sequence coverage: 91 %
  
E-value: 2e-22
  
  
 NCBI BlastP on this gene

EFJ01218

hypothetical protein
  
Accession: EFJ01217
  
Location: 3176250-3179923
  
 NCBI BlastP on this gene

EFJ01217

hypothetical protein
  
Accession: EFJ01216
  
Location: 3171834-3175877
  
 NCBI BlastP on this gene

EFJ01216

hypothetical protein
  
Accession: EFJ00414
  
Location: 3171204-3171719
  
 NCBI BlastP on this gene

EFJ00414

hypothetical protein
  
Accession: EFJ00413
  
Location: 3168332-3169885
  
 NCBI BlastP on this gene

EFJ00413

hypothetical protein
  
Accession: EFJ00412
  
Location: 3165592-3167173
  
 NCBI BlastP on this gene

EFJ00412

hypothetical protein
  
Accession: EFJ00411
  
Location: 3162700-3165160
  
 NCBI BlastP on this gene

EFJ00411

47. :  DS995900 Penicillium marneffei ATCC 18224 scf\_1105668340758 genomic scaffold     Total score: 2.0     Cumulative Blast bit score: 293

SRP receptor beta subunit (Srp102), putative
  
Accession: EEA25899
  
Location: 2199123-2200173
  
 NCBI BlastP on this gene

EEA25899

cystathionine beta-synthase, putative
  
Accession: EEA25900
  
Location: 2200641-2202411
  
 NCBI BlastP on this gene

EEA25900

conserved hypothetical protein
  
Accession: EEA25901
  
Location: 2202866-2204580
  
 NCBI BlastP on this gene

EEA25901

hypothetical protein
  
Accession: EEA25902
  
Location: 2206857-2208182
  
 NCBI BlastP on this gene

EEA25902

cyclin-dependent protein kinase complex component, putative
  
Accession: EEA25904
  
Location: 2209101-2209913
  
 NCBI BlastP on this gene

EEA25904

palmitoyltransferase SidR
  
Accession: EEA25905
  
Location: 2211232-2213725
  
 NCBI BlastP on this gene

EEA25905

L-ornithine N5-oxygenase SidA
  
Accession: EEA25906
  
Location: 2214865-2216378
  
 NCBI BlastP on this gene

EEA25906

nonribosomal siderophore peptide synthase, putative
  
Accession: EEA25907
  
Location: 2217834-2233637
  
  
**BlastP hit with Mycgr3G40534\_Mycgr3T**
  
Percentage identity: 39 %
  
BlastP bit score: 161
  
Sequence coverage: 81 %
  
E-value: 4e-40
  
  
  
**BlastP hit with Mycgr3G39931\_Mycgr3T**
  
Percentage identity: 33 %
  
BlastP bit score: 132
  
Sequence coverage: 90 %
  
E-value: 1e-30
  
  
 NCBI BlastP on this gene

EEA25907

protein kinase, putative
  
Accession: EEA25908
  
Location: 2237315-2240286
  
 NCBI BlastP on this gene

EEA25908

mRNA splicing factor RNA helicase (Cdc28), putative
  
Accession: EEA25909
  
Location: 2241437-2244832
  
 NCBI BlastP on this gene

EEA25909

cytochrome b5, putative
  
Accession: EEA25911
  
Location: 2245345-2245636
  
 NCBI BlastP on this gene

EEA25911

conserved hypothetical protein
  
Accession: EEA25914
  
Location: 2248094-2248882
  
 NCBI BlastP on this gene

EEA25914

nuclear migration protein, putative
  
Accession: EEA25915
  
Location: 2249753-2253695
  
 NCBI BlastP on this gene

EEA25915

48. :  DS990636 Ajellomyces capsulatus H88 supercont1.1 genomic scaffold     Total score: 2.0     Cumulative Blast bit score: 289

nonribosomal peptide synthetase
  
Accession: EGC41890
  
Location: 4852900-4866948
  
  
**BlastP hit with Mycgr3G40534\_Mycgr3T**
  
Percentage identity: 39 %
  
BlastP bit score: 167
  
Sequence coverage: 102 %
  
E-value: 5e-42
  
  
  
**BlastP hit with Mycgr3G39931\_Mycgr3T**
  
Percentage identity: 31 %
  
BlastP bit score: 122
  
Sequence coverage: 96 %
  
E-value: 3e-27
  
  
 NCBI BlastP on this gene

EGC41890

nonribosomal peptide synthase
  
Accession: EGC41889
  
Location: 4851004-4852714
  
 NCBI BlastP on this gene

EGC41889

conserved hypothetical protein
  
Accession: EGC41888
  
Location: 4844626-4848302
  
 NCBI BlastP on this gene

EGC41888

ATP-dependent RNA helicase DHX8
  
Accession: EGC41887
  
Location: 4839194-4842586
  
 NCBI BlastP on this gene

EGC41887

cytochrome b5
  
Accession: EGC41886
  
Location: 4838220-4838604
  
 NCBI BlastP on this gene

EGC41886

conserved hypothetical protein
  
Accession: EGC41885
  
Location: 4836254-4836910
  
 NCBI BlastP on this gene

EGC41885

predicted protein
  
Accession: EGC41884
  
Location: 4834787-4835706
  
 NCBI BlastP on this gene

EGC41884

49. :  CP003009 Thielavia terrestris NRRL 8126 chromosome 1     Total score: 2.0     Cumulative Blast bit score: 280

hypothetical protein
  
Accession: AEO63772
  
Location: 6190201-6190500
  
 NCBI BlastP on this gene

THITE\_48000

hypothetical protein
  
Accession: AEO63771
  
Location: 6189205-6189366
  
 NCBI BlastP on this gene

THITE\_47801

hypothetical protein
  
Accession: AEO63770
  
Location: 6185289-6188335
  
 NCBI BlastP on this gene

THITE\_2109372

hypothetical protein
  
Accession: AEO63769
  
Location: 6183355-6185013
  
 NCBI BlastP on this gene

THITE\_2034747

hypothetical protein
  
Accession: AEO63768
  
Location: 6180825-6181815
  
 NCBI BlastP on this gene

THITE\_2109370

hypothetical protein
  
Accession: AEO63767
  
Location: 6174935-6176671
  
 NCBI BlastP on this gene

THITE\_2109366

non-ribosomal peptide synthetase
  
Accession: AEO63766
  
Location: 6158586-6173589
  
  
**BlastP hit with Mycgr3G40534\_Mycgr3T**
  
Percentage identity: 42 %
  
BlastP bit score: 164
  
Sequence coverage: 81 %
  
E-value: 3e-41
  
  
  
**BlastP hit with Mycgr3G39931\_Mycgr3T**
  
Percentage identity: 30 %
  
BlastP bit score: 116
  
Sequence coverage: 89 %
  
E-value: 3e-25
  
  
 NCBI BlastP on this gene

THITE\_62324

hypothetical protein
  
Accession: AEO63765
  
Location: 6157633-6158444
  
 NCBI BlastP on this gene

THITE\_135405

hypothetical protein
  
Accession: AEO63764
  
Location: 6154333-6156423
  
 NCBI BlastP on this gene

THITE\_2109360

hypothetical protein
  
Accession: AEO63763
  
Location: 6151398-6153202
  
 NCBI BlastP on this gene

THITE\_2109357

hypothetical protein
  
Accession: AEO63762
  
Location: 6149031-6150051
  
 NCBI BlastP on this gene

THITE\_123303

hypothetical protein
  
Accession: AEO63761
  
Location: 6146794-6148029
  
 NCBI BlastP on this gene

THITE\_2126217

glycoside hydrolase family 16 protein
  
Accession: AEO63760
  
Location: 6143234-6144560
  
 NCBI BlastP on this gene

THITE\_2042795

hypothetical protein
  
Accession: AEO63759
  
Location: 6139732-6141678
  
 NCBI BlastP on this gene

THITE\_2109354

50. :  KE375213 Blumeria graminis f. sp. tritici 96224 unplaced genomic scaffold Scaffold-87     Total score: 2.0     Cumulative Blast bit score: 279

hypothetical protein
  
Accession: EPQ61837
  
Location: 1547557-1548306
  
 NCBI BlastP on this gene

EPQ61837

hypothetical protein
  
Accession: EPQ61838
  
Location: 1548303-1549914
  
 NCBI BlastP on this gene

EPQ61838

L-ornithine 5-monooxygenase
  
Accession: EPQ61839
  
Location: 1560739-1562306
  
 NCBI BlastP on this gene

EPQ61839

hypothetical protein
  
Accession: EPQ61840
  
Location: 1565561-1566808
  
  
**BlastP hit with Mycgr3G40534\_Mycgr3T**
  
Percentage identity: 37 %
  
BlastP bit score: 140
  
Sequence coverage: 90 %
  
E-value: 1e-34
  
  
 NCBI BlastP on this gene

EPQ61840

Alpha aminoadipate reductase
  
Accession: EPQ61841
  
Location: 1567007-1580442
  
  
**BlastP hit with Mycgr3G39931\_Mycgr3T**
  
Percentage identity: 36 %
  
BlastP bit score: 139
  
Sequence coverage: 90 %
  
E-value: 6e-33
  
  
 NCBI BlastP on this gene

EPQ61841

hypothetical protein
  
Accession: EPQ61842
  
Location: 1589498-1590070
  
 NCBI BlastP on this gene

EPQ61842

Detecting sequence homology at the gene cluster level with MultiGeneBlast.
  
Marnix H. Medema, Rainer Breitling & Eriko Takano (2013)
  
*Molecular Biology and Evolution* , 30: 1218-1223.
