# Supplementary material for: In silico prediction and characterization of secondary metabolite biosynthetic gene clusters in the wheat pathogen Zymoseptoria tritici
Source: BMC Genomics. 2017 Aug 17;18:631. doi: 10.1186/s12864-017-3969-y (PMC5561558; doi:10.1186/s12864-017-3969-y)
Supplement: Supplementary file 1 — MultiGeneBLAST analysis of putative secondary metabolite clusters. All encoded amino acid sequences from genes residing in clusters predicted by AntiSMASH are given as FASTA file format. All output data from MultiGeneBLASTs are also provided. (ZIP 42911 kb) [file 12864_2017_3969_MOESM1_ESM.zip › Cluster MultiGene BLAST/out/Clusters_1_34/Cluster_14/displaypage2.xhtml]

xml version="1.0" encoding="UTF-8"?


Search Results
  
  
 Results pages: 1, 2, 3, 4, 5

**MultiGeneBlast hits**

Select gene cluster alignment
51. GL698712\_1 Metarhizium anisopliae ARSEF 23 unplaced genomic scaffold Scf\_...
52. FQ790277\_0 Botryotinia fuckeliana T4 SuperContig\_379\_1 genomic supercontig.
53. CP003002\_1 Myceliophthora thermophila ATCC 42464 chromosome 1, complete s...
54. CM001197\_0 Mycosphaerella graminicola IPO323 chromosome 2, whole genome s...
55. GL988041\_0 Chaetomium thermophilum var. thermophilum DSM 1495 unplaced ge...
56. ABDF02000001\_0 Trichoderma virens Gv29-8, whole genome shotgun sequencing...
57. DS027050\_1 Aspergillus clavatus NRRL 1 1099423829796 genomic scaffold, wh...
58. CU633895\_1 Podospora anserina S mat+ genomic DNA chromosome 4, superconti...
59. CH476662\_0 Ajellomyces capsulatus NAm1 scaffold\_8 genomic scaffold, whole...
60. CH408034\_1 Chaetomium globosum CBS 148.51 scaffold\_6 genomic scaffold, wh...
61. DS989822\_0 Arthroderma gypseum CBS 118893 supercont1.1 genomic scaffold, ...
62. HF679023\_0 Fusarium fujikuroi IMI 58289 draft genome, chromosome FFUJ\_chr01.
63. DS499596\_0 Aspergillus fumigatus A1163 scf\_000003 genomic scaffold, whole...
64. KE145368\_0 Glarea lozoyensis ATCC 20868 chromosome Unknown GLAREA4, whole...
65. KB933064\_0 Togninia minima UCRPA7 unplaced genomic scaffold PA7\_03\_scaffo...
66. CH476616\_1 Uncinocarpus reesii 1704 scaffold\_2 genomic scaffold, whole ge...
67. GL377306\_0 Schizophyllum commune H4-8 unplaced genomic scaffold SCHCOscaf...
68. CAIF01000223\_0 Wickerhamomyces ciferrii strain NRRL Y-1031 F-60-10, whole...
69. CP002685\_0 Arabidopsis thaliana chromosome 2, complete sequence.
70. CP002684\_0 Arabidopsis thaliana chromosome 1, complete sequence.
71. JH930470\_0 Phanerochaete carnosa HHB-10118-sp unplaced genomic scaffold P...
72. JH711789\_0 Trametes versicolor FP-101664 SS1 unplaced genomic scaffold TR...
73. KB445804\_0 Ceriporiopsis subvermispora B unplaced genomic scaffold CERSUs...
74. JH687380\_1 Stereum hirsutum FP-91666 SS1 unplaced genomic scaffold STEHIs...
75. JH687380\_3 Stereum hirsutum FP-91666 SS1 unplaced genomic scaffold STEHIs...
76. JH717971\_0 Fomitiporia mediterranea MF3/22 unplaced genomic scaffold FOMM...
77. JH719400\_1 Dichomitus squalens LYAD-421 SS1 unplaced genomic scaffold DIC...
78. AACS02000004\_0 Coprinopsis cinerea okayama7#130, whole genome shotgun seq...
79. JH931607\_1 Agaricus bisporus var. bisporus H97 unplaced genomic scaffold ...
80. JH687380\_2 Stereum hirsutum FP-91666 SS1 unplaced genomic scaffold STEHIs...
81. JH711575\_1 Coniophora puteana RWD-64-598 SS2 unplaced genomic scaffold CO...
82. JH687379\_0 Stereum hirsutum FP-91666 SS1 unplaced genomic scaffold STEHIs...
83. JH711575\_2 Coniophora puteana RWD-64-598 SS2 unplaced genomic scaffold CO...
84. CAFZ01000285\_0 Piriformospora indica DSM 11827, whole genome shotgun sequ...
85. JH971391\_1 Agaricus bisporus var. burnettii JB137-S8 unplaced genomic sca...
86. JH711789\_1 Trametes versicolor FP-101664 SS1 unplaced genomic scaffold TR...
87. KB445804\_1 Ceriporiopsis subvermispora B unplaced genomic scaffold CERSUs...
88. JH717968\_1 Fomitiporia mediterranea MF3/22 unplaced genomic scaffold FOMM...
89. JH931607\_0 Agaricus bisporus var. bisporus H97 unplaced genomic scaffold ...
90. JH711575\_0 Coniophora puteana RWD-64-598 SS2 unplaced genomic scaffold CO...
91. JH687380\_0 Stereum hirsutum FP-91666 SS1 unplaced genomic scaffold STEHIs...
92. JH971391\_0 Agaricus bisporus var. burnettii JB137-S8 unplaced genomic sca...
93. JH930470\_1 Phanerochaete carnosa HHB-10118-sp unplaced genomic scaffold P...
94. GL377306\_1 Schizophyllum commune H4-8 unplaced genomic scaffold SCHCOscaf...
95. DS027054\_0 Aspergillus clavatus NRRL 1 1099423829800 genomic scaffold, wh...
96. CP003009\_0 Thielavia terrestris NRRL 8126 chromosome 1, complete sequence.
97. CP003003\_0 Myceliophthora thermophila ATCC 42464 chromosome 2, complete s...
98. CM001232\_0 Magnaporthe oryzae 70-15 chromosome 2, whole genome shotgun se...
99. JH793788\_1 Magnaporthe oryzae Y34 unplaced genomic scaffold Y34\_scaffold0...
100. GL891302\_0 Neurospora tetrasperma FGSC 2508 unplaced genomic scaffold NE...

Query: Architecture Search FASTA input

GL698712 : Metarhizium anisopliae ARSEF 23 unplaced genomic scaffold Scf\_002    Total score: 2.0     Cumulative Blast bit score: 277

Hit cluster cross-links:

Mycgr3G41235 Mycgr3T
  
Location: 0-4062

Mycgr3G41235\_Mycgr3T

Mycgr3G70577 Mycgr3T
  
Location: 4162-6109

Mycgr3G70577\_Mycgr3T

Mycgr3G40534 Mycgr3T
  
Location: 6209-7166

Mycgr3G40534\_Mycgr3T

Mycgr3G85486 Mycgr3T
  
Location: 7266-8511

Mycgr3G85486\_Mycgr3T

Mycgr3G92221 Mycgr3T
  
Location: 8611-9193

Mycgr3G92221\_Mycgr3T

Mycgr3G39931 Mycgr3T
  
Location: 9293-10157

Mycgr3G39931\_Mycgr3T

Mycgr3G99766 Mycgr3T
  
Location: 10257-11775

Mycgr3G99766\_Mycgr3T

peptide synthetase
  
Accession: EFZ02308
  
Location: 1073222-1087875
  
  
**BlastP hit with Mycgr3G40534\_Mycgr3T**
  
Percentage identity: 37 %
  
BlastP bit score: 156
  
Sequence coverage: 87 %
  
E-value: 3e-38
  
  
  
**BlastP hit with Mycgr3G39931\_Mycgr3T**
  
Percentage identity: 33 %
  
BlastP bit score: 121
  
Sequence coverage: 89 %
  
E-value: 6e-27
  
  
 NCBI BlastP on this gene

EFZ02308

DUF1479 domain-containing protein
  
Accession: EFZ02307
  
Location: 1071342-1073069
  
 NCBI BlastP on this gene

EFZ02307

Query: Architecture Search FASTA input

FQ790277 : Botryotinia fuckeliana T4 SuperContig\_379\_1 genomic supercontig.    Total score: 2.0     Cumulative Blast bit score: 273

Hit cluster cross-links:

Mycgr3G41235 Mycgr3T
  
Location: 0-4062

Mycgr3G41235\_Mycgr3T

Mycgr3G70577 Mycgr3T
  
Location: 4162-6109

Mycgr3G70577\_Mycgr3T

Mycgr3G40534 Mycgr3T
  
Location: 6209-7166

Mycgr3G40534\_Mycgr3T

Mycgr3G85486 Mycgr3T
  
Location: 7266-8511

Mycgr3G85486\_Mycgr3T

Mycgr3G92221 Mycgr3T
  
Location: 8611-9193

Mycgr3G92221\_Mycgr3T

Mycgr3G39931 Mycgr3T
  
Location: 9293-10157

Mycgr3G39931\_Mycgr3T

Mycgr3G99766 Mycgr3T
  
Location: 10257-11775

Mycgr3G99766\_Mycgr3T

similar to ABC transporter
  
Accession: CCD45294
  
Location: 33266-37575
  
 NCBI BlastP on this gene

BofuT4\_P120000.1

BcNRPS2, nonribosomal peptide synthetase, partial sequence
  
Accession: CCD45295
  
Location: 39844-43181
  
  
**BlastP hit with Mycgr3G40534\_Mycgr3T**
  
Percentage identity: 37 %
  
BlastP bit score: 153
  
Sequence coverage: 81 %
  
E-value: 1e-37
  
  
 NCBI BlastP on this gene

BofuT4\_P120010.1

BcNRPS2, nonribosomal peptide synthetase, partial sequence
  
Accession: CCD45296
  
Location: 44232-52768
  
  
**BlastP hit with Mycgr3G39931\_Mycgr3T**
  
Percentage identity: 31 %
  
BlastP bit score: 120
  
Sequence coverage: 99 %
  
E-value: 1e-26
  
  
 NCBI BlastP on this gene

BofuT4\_P120020.1

hypothetical protein
  
Accession: CCD45297
  
Location: 53142-55183
  
 NCBI BlastP on this gene

BofuT4\_P120030.1

Query: Architecture Search FASTA input

CP003002 : Myceliophthora thermophila ATCC 42464 chromosome 1    Total score: 2.0     Cumulative Blast bit score: 273

Hit cluster cross-links:

Mycgr3G41235 Mycgr3T
  
Location: 0-4062

Mycgr3G41235\_Mycgr3T

Mycgr3G70577 Mycgr3T
  
Location: 4162-6109

Mycgr3G70577\_Mycgr3T

Mycgr3G40534 Mycgr3T
  
Location: 6209-7166

Mycgr3G40534\_Mycgr3T

Mycgr3G85486 Mycgr3T
  
Location: 7266-8511

Mycgr3G85486\_Mycgr3T

Mycgr3G92221 Mycgr3T
  
Location: 8611-9193

Mycgr3G92221\_Mycgr3T

Mycgr3G39931 Mycgr3T
  
Location: 9293-10157

Mycgr3G39931\_Mycgr3T

Mycgr3G99766 Mycgr3T
  
Location: 10257-11775

Mycgr3G99766\_Mycgr3T

non-ribosomal peptide synthetase
  
Accession: AEO55755
  
Location: 10846889-10862191
  
  
**BlastP hit with Mycgr3G40534\_Mycgr3T**
  
Percentage identity: 36 %
  
BlastP bit score: 154
  
Sequence coverage: 99 %
  
E-value: 1e-37
  
  
  
**BlastP hit with Mycgr3G39931\_Mycgr3T**
  
Percentage identity: 30 %
  
BlastP bit score: 119
  
Sequence coverage: 88 %
  
E-value: 3e-26
  
  
 NCBI BlastP on this gene

MYCTH\_97310

hypothetical protein
  
Accession: AEO55754
  
Location: 10845713-10846607
  
 NCBI BlastP on this gene

MYCTH\_2314374

hypothetical protein
  
Accession: AEO55753
  
Location: 10842451-10844658
  
 NCBI BlastP on this gene

MYCTH\_2299891

Query: Architecture Search FASTA input

CM001197 : Mycosphaerella graminicola IPO323 chromosome 2    Total score: 2.0     Cumulative Blast bit score: 273

Hit cluster cross-links:

Mycgr3G41235 Mycgr3T
  
Location: 0-4062

Mycgr3G41235\_Mycgr3T

Mycgr3G70577 Mycgr3T
  
Location: 4162-6109

Mycgr3G70577\_Mycgr3T

Mycgr3G40534 Mycgr3T
  
Location: 6209-7166

Mycgr3G40534\_Mycgr3T

Mycgr3G85486 Mycgr3T
  
Location: 7266-8511

Mycgr3G85486\_Mycgr3T

Mycgr3G92221 Mycgr3T
  
Location: 8611-9193

Mycgr3G92221\_Mycgr3T

Mycgr3G39931 Mycgr3T
  
Location: 9293-10157

Mycgr3G39931\_Mycgr3T

Mycgr3G99766 Mycgr3T
  
Location: 10257-11775

Mycgr3G99766\_Mycgr3T

putative ABC transporter
  
Accession: EGP90754
  
Location: 433742-437840
  
 NCBI BlastP on this gene

EGP90754

putative Non-ribosomal peptide synthetase
  
Accession: EGP89716
  
Location: 439283-453243
  
  
**BlastP hit with Mycgr3G40534\_Mycgr3T**
  
Percentage identity: 37 %
  
BlastP bit score: 139
  
Sequence coverage: 82 %
  
E-value: 2e-32
  
  
  
**BlastP hit with Mycgr3G39931\_Mycgr3T**
  
Percentage identity: 35 %
  
BlastP bit score: 134
  
Sequence coverage: 90 %
  
E-value: 2e-31
  
  
 NCBI BlastP on this gene

EGP89716

hypothetical protein
  
Accession: EGP90753
  
Location: 453825-455477
  
 NCBI BlastP on this gene

EGP90753

Query: Architecture Search FASTA input

GL988041 : Chaetomium thermophilum var. thermophilum DSM 1495 unplaced genomic scaffold scf7180000...    Total score: 2.0     Cumulative Blast bit score: 272

Hit cluster cross-links:

Mycgr3G41235 Mycgr3T
  
Location: 0-4062

Mycgr3G41235\_Mycgr3T

Mycgr3G70577 Mycgr3T
  
Location: 4162-6109

Mycgr3G70577\_Mycgr3T

Mycgr3G40534 Mycgr3T
  
Location: 6209-7166

Mycgr3G40534\_Mycgr3T

Mycgr3G85486 Mycgr3T
  
Location: 7266-8511

Mycgr3G85486\_Mycgr3T

Mycgr3G92221 Mycgr3T
  
Location: 8611-9193

Mycgr3G92221\_Mycgr3T

Mycgr3G39931 Mycgr3T
  
Location: 9293-10157

Mycgr3G39931\_Mycgr3T

Mycgr3G99766 Mycgr3T
  
Location: 10257-11775

Mycgr3G99766\_Mycgr3T

nonribosomal peptide synthase-like protein
  
Accession: EGS20291
  
Location: 6067-19404
  
  
**BlastP hit with Mycgr3G40534\_Mycgr3T**
  
Percentage identity: 36 %
  
BlastP bit score: 157
  
Sequence coverage: 106 %
  
E-value: 8e-39
  
  
  
**BlastP hit with Mycgr3G39931\_Mycgr3T**
  
Percentage identity: 33 %
  
BlastP bit score: 115
  
Sequence coverage: 92 %
  
E-value: 1e-24
  
  
 NCBI BlastP on this gene

EGS20291

hypothetical protein
  
Accession: EGS20290
  
Location: 1255-3822
  
 NCBI BlastP on this gene

EGS20290

Query: Architecture Search FASTA input

ABDF02000001 : Trichoderma virens Gv29-8    Total score: 2.0     Cumulative Blast bit score: 272

Hit cluster cross-links:

Mycgr3G41235 Mycgr3T
  
Location: 0-4062

Mycgr3G41235\_Mycgr3T

Mycgr3G70577 Mycgr3T
  
Location: 4162-6109

Mycgr3G70577\_Mycgr3T

Mycgr3G40534 Mycgr3T
  
Location: 6209-7166

Mycgr3G40534\_Mycgr3T

Mycgr3G85486 Mycgr3T
  
Location: 7266-8511

Mycgr3G85486\_Mycgr3T

Mycgr3G92221 Mycgr3T
  
Location: 8611-9193

Mycgr3G92221\_Mycgr3T

Mycgr3G39931 Mycgr3T
  
Location: 9293-10157

Mycgr3G39931\_Mycgr3T

Mycgr3G99766 Mycgr3T
  
Location: 10257-11775

Mycgr3G99766\_Mycgr3T

putative non-ribosomal peptide synthetase
  
Accession: EHK26839
  
Location: 357960-372711
  
  
**BlastP hit with Mycgr3G40534\_Mycgr3T**
  
Percentage identity: 38 %
  
BlastP bit score: 150
  
Sequence coverage: 81 %
  
E-value: 2e-36
  
  
  
**BlastP hit with Mycgr3G39931\_Mycgr3T**
  
Percentage identity: 33 %
  
BlastP bit score: 122
  
Sequence coverage: 90 %
  
E-value: 3e-27
  
  
 NCBI BlastP on this gene

EHK26839

hypothetical protein
  
Accession: EHK26840
  
Location: 373092-374723
  
 NCBI BlastP on this gene

EHK26840

Query: Architecture Search FASTA input

DS027050 : Aspergillus clavatus NRRL 1 1099423829796 genomic scaffold    Total score: 2.0     Cumulative Blast bit score: 270

Hit cluster cross-links:

Mycgr3G41235 Mycgr3T
  
Location: 0-4062

Mycgr3G41235\_Mycgr3T

Mycgr3G70577 Mycgr3T
  
Location: 4162-6109

Mycgr3G70577\_Mycgr3T

Mycgr3G40534 Mycgr3T
  
Location: 6209-7166

Mycgr3G40534\_Mycgr3T

Mycgr3G85486 Mycgr3T
  
Location: 7266-8511

Mycgr3G85486\_Mycgr3T

Mycgr3G92221 Mycgr3T
  
Location: 8611-9193

Mycgr3G92221\_Mycgr3T

Mycgr3G39931 Mycgr3T
  
Location: 9293-10157

Mycgr3G39931\_Mycgr3T

Mycgr3G99766 Mycgr3T
  
Location: 10257-11775

Mycgr3G99766\_Mycgr3T

MFS monocarboxylate transporter, putative
  
Accession: EAW12156
  
Location: 227609-229056
  
 NCBI BlastP on this gene

EAW12156

mitochondrial enoyl reductase, putative
  
Accession: EAW12157
  
Location: 229852-230931
  
 NCBI BlastP on this gene

EAW12157

nonribosomal peptide synthase SidE
  
Accession: EAW12158
  
Location: 232515-238862
  
  
**BlastP hit with Mycgr3G40534\_Mycgr3T**
  
Percentage identity: 40 %
  
BlastP bit score: 150
  
Sequence coverage: 79 %
  
E-value: 1e-36
  
  
  
**BlastP hit with Mycgr3G39931\_Mycgr3T**
  
Percentage identity: 32 %
  
BlastP bit score: 120
  
Sequence coverage: 88 %
  
E-value: 2e-26
  
  
 NCBI BlastP on this gene

EAW12158

Query: Architecture Search FASTA input

CU633895 : Podospora anserina S mat+ genomic DNA chromosome 4, supercontig 4.    Total score: 2.0     Cumulative Blast bit score: 266

Hit cluster cross-links:

Mycgr3G41235 Mycgr3T
  
Location: 0-4062

Mycgr3G41235\_Mycgr3T

Mycgr3G70577 Mycgr3T
  
Location: 4162-6109

Mycgr3G70577\_Mycgr3T

Mycgr3G40534 Mycgr3T
  
Location: 6209-7166

Mycgr3G40534\_Mycgr3T

Mycgr3G85486 Mycgr3T
  
Location: 7266-8511

Mycgr3G85486\_Mycgr3T

Mycgr3G92221 Mycgr3T
  
Location: 8611-9193

Mycgr3G92221\_Mycgr3T

Mycgr3G39931 Mycgr3T
  
Location: 9293-10157

Mycgr3G39931\_Mycgr3T

Mycgr3G99766 Mycgr3T
  
Location: 10257-11775

Mycgr3G99766\_Mycgr3T

not annotated
  
Accession: CAP67082
  
Location: 2318020-2333277
  
  
**BlastP hit with Mycgr3G40534\_Mycgr3T**
  
Percentage identity: 41 %
  
BlastP bit score: 157
  
Sequence coverage: 81 %
  
E-value: 1e-38
  
  
  
**BlastP hit with Mycgr3G39931\_Mycgr3T**
  
Percentage identity: 26 %
  
BlastP bit score: 109
  
Sequence coverage: 88 %
  
E-value: 7e-23
  
  
 NCBI BlastP on this gene

CAP67082

not annotated
  
Accession: CAP67081
  
Location: 2315657-2317633
  
 NCBI BlastP on this gene

CAP67081

Query: Architecture Search FASTA input

CH476662 : Ajellomyces capsulatus NAm1 scaffold\_8 genomic scaffold    Total score: 2.0     Cumulative Blast bit score: 265

Hit cluster cross-links:

Mycgr3G41235 Mycgr3T
  
Location: 0-4062

Mycgr3G41235\_Mycgr3T

Mycgr3G70577 Mycgr3T
  
Location: 4162-6109

Mycgr3G70577\_Mycgr3T

Mycgr3G40534 Mycgr3T
  
Location: 6209-7166

Mycgr3G40534\_Mycgr3T

Mycgr3G85486 Mycgr3T
  
Location: 7266-8511

Mycgr3G85486\_Mycgr3T

Mycgr3G92221 Mycgr3T
  
Location: 8611-9193

Mycgr3G92221\_Mycgr3T

Mycgr3G39931 Mycgr3T
  
Location: 9293-10157

Mycgr3G39931\_Mycgr3T

Mycgr3G99766 Mycgr3T
  
Location: 10257-11775

Mycgr3G99766\_Mycgr3T

palmitoyltransferase akr1
  
Accession: EDN10969
  
Location: 1686872-1690490
  
 NCBI BlastP on this gene

EDN10969

predicted protein
  
Accession: EDN10968
  
Location: 1682681-1684794
  
  
**BlastP hit with Mycgr3G40534\_Mycgr3T**
  
Percentage identity: 42 %
  
BlastP bit score: 147
  
Sequence coverage: 78 %
  
E-value: 2e-36
  
  
 NCBI BlastP on this gene

EDN10968

predicted protein
  
Accession: EDN10967
  
Location: 1673064-1679253
  
  
**BlastP hit with Mycgr3G39931\_Mycgr3T**
  
Percentage identity: 31 %
  
BlastP bit score: 118
  
Sequence coverage: 96 %
  
E-value: 8e-26
  
  
 NCBI BlastP on this gene

EDN10967

Query: Architecture Search FASTA input

CH408034 : Chaetomium globosum CBS 148.51 scaffold\_6 genomic scaffold    Total score: 2.0     Cumulative Blast bit score: 255

Hit cluster cross-links:

Mycgr3G41235 Mycgr3T
  
Location: 0-4062

Mycgr3G41235\_Mycgr3T

Mycgr3G70577 Mycgr3T
  
Location: 4162-6109

Mycgr3G70577\_Mycgr3T

Mycgr3G40534 Mycgr3T
  
Location: 6209-7166

Mycgr3G40534\_Mycgr3T

Mycgr3G85486 Mycgr3T
  
Location: 7266-8511

Mycgr3G85486\_Mycgr3T

Mycgr3G92221 Mycgr3T
  
Location: 8611-9193

Mycgr3G92221\_Mycgr3T

Mycgr3G39931 Mycgr3T
  
Location: 9293-10157

Mycgr3G39931\_Mycgr3T

Mycgr3G99766 Mycgr3T
  
Location: 10257-11775

Mycgr3G99766\_Mycgr3T

hypothetical protein
  
Accession: EAQ85528
  
Location: 3762503-3764800
  
 NCBI BlastP on this gene

EAQ85528

hypothetical protein
  
Accession: EAQ85529
  
Location: 3766215-3781488
  
  
**BlastP hit with Mycgr3G40534\_Mycgr3T**
  
Percentage identity: 35 %
  
BlastP bit score: 145
  
Sequence coverage: 102 %
  
E-value: 1e-34
  
  
  
**BlastP hit with Mycgr3G39931\_Mycgr3T**
  
Percentage identity: 33 %
  
BlastP bit score: 110
  
Sequence coverage: 89 %
  
E-value: 4e-23
  
  
 NCBI BlastP on this gene

EAQ85529

Query: Architecture Search FASTA input

DS989822 : Arthroderma gypseum CBS 118893 supercont1.1 genomic scaffold    Total score: 2.0     Cumulative Blast bit score: 254

Hit cluster cross-links:

Mycgr3G41235 Mycgr3T
  
Location: 0-4062

Mycgr3G41235\_Mycgr3T

Mycgr3G70577 Mycgr3T
  
Location: 4162-6109

Mycgr3G70577\_Mycgr3T

Mycgr3G40534 Mycgr3T
  
Location: 6209-7166

Mycgr3G40534\_Mycgr3T

Mycgr3G85486 Mycgr3T
  
Location: 7266-8511

Mycgr3G85486\_Mycgr3T

Mycgr3G92221 Mycgr3T
  
Location: 8611-9193

Mycgr3G92221\_Mycgr3T

Mycgr3G39931 Mycgr3T
  
Location: 9293-10157

Mycgr3G39931\_Mycgr3T

Mycgr3G99766 Mycgr3T
  
Location: 10257-11775

Mycgr3G99766\_Mycgr3T

hypothetical protein
  
Accession: EFQ97050
  
Location: 244064-268485
  
  
**BlastP hit with Mycgr3G40534\_Mycgr3T**
  
Percentage identity: 33 %
  
BlastP bit score: 135
  
Sequence coverage: 89 %
  
E-value: 2e-31
  
  
  
**BlastP hit with Mycgr3G39931\_Mycgr3T**
  
Percentage identity: 29 %
  
BlastP bit score: 119
  
Sequence coverage: 92 %
  
E-value: 6e-26
  
  
 NCBI BlastP on this gene

EFQ97050

hypothetical protein
  
Accession: EFQ97049
  
Location: 242865-243245
  
 NCBI BlastP on this gene

EFQ97049

GMP synthase
  
Accession: EFQ97048
  
Location: 240940-242635
  
 NCBI BlastP on this gene

EFQ97048

Query: Architecture Search FASTA input

HF679023 : Fusarium fujikuroi IMI 58289 draft genome, chromosome FFUJ\_chr01.    Total score: 2.0     Cumulative Blast bit score: 245

Hit cluster cross-links:

Mycgr3G41235 Mycgr3T
  
Location: 0-4062

Mycgr3G41235\_Mycgr3T

Mycgr3G70577 Mycgr3T
  
Location: 4162-6109

Mycgr3G70577\_Mycgr3T

Mycgr3G40534 Mycgr3T
  
Location: 6209-7166

Mycgr3G40534\_Mycgr3T

Mycgr3G85486 Mycgr3T
  
Location: 7266-8511

Mycgr3G85486\_Mycgr3T

Mycgr3G92221 Mycgr3T
  
Location: 8611-9193

Mycgr3G92221\_Mycgr3T

Mycgr3G39931 Mycgr3T
  
Location: 9293-10157

Mycgr3G39931\_Mycgr3T

Mycgr3G99766 Mycgr3T
  
Location: 10257-11775

Mycgr3G99766\_Mycgr3T

related to 2,4-dienoyl-CoA reductase precursor
  
Accession: CCT61501
  
Location: 192618-193727
  
 NCBI BlastP on this gene

FFUJ\_02020

uncharacterized protein
  
Accession: CCT61500
  
Location: 190995-191690
  
 NCBI BlastP on this gene

FFUJ\_02021

non-ribosomal peptide synthetase
  
Accession: CCT61499
  
Location: 181990-189795
  
  
**BlastP hit with Mycgr3G40534\_Mycgr3T**
  
Percentage identity: 37 %
  
BlastP bit score: 137
  
Sequence coverage: 80 %
  
E-value: 7e-32
  
  
  
**BlastP hit with Mycgr3G39931\_Mycgr3T**
  
Percentage identity: 30 %
  
BlastP bit score: 108
  
Sequence coverage: 92 %
  
E-value: 2e-22
  
  
 NCBI BlastP on this gene

FFUJ\_02022

uncharacterized protein
  
Accession: CCT61498
  
Location: 180264-181881
  
 NCBI BlastP on this gene

FFUJ\_02023

uncharacterized protein
  
Accession: CCT61497
  
Location: 178451-179516
  
 NCBI BlastP on this gene

FFUJ\_02024

related to xylan 1,4-beta-xylosidase
  
Accession: CCT61496
  
Location: 176018-178366
  
 NCBI BlastP on this gene

FFUJ\_02025

Query: Architecture Search FASTA input

DS499596 : Aspergillus fumigatus A1163 scf\_000003 genomic scaffold    Total score: 2.0     Cumulative Blast bit score: 245

Hit cluster cross-links:

Mycgr3G41235 Mycgr3T
  
Location: 0-4062

Mycgr3G41235\_Mycgr3T

Mycgr3G70577 Mycgr3T
  
Location: 4162-6109

Mycgr3G70577\_Mycgr3T

Mycgr3G40534 Mycgr3T
  
Location: 6209-7166

Mycgr3G40534\_Mycgr3T

Mycgr3G85486 Mycgr3T
  
Location: 7266-8511

Mycgr3G85486\_Mycgr3T

Mycgr3G92221 Mycgr3T
  
Location: 8611-9193

Mycgr3G92221\_Mycgr3T

Mycgr3G39931 Mycgr3T
  
Location: 9293-10157

Mycgr3G39931\_Mycgr3T

Mycgr3G99766 Mycgr3T
  
Location: 10257-11775

Mycgr3G99766\_Mycgr3T

general amidase, putative
  
Accession: EDP52235
  
Location: 57210-59063
  
 NCBI BlastP on this gene

EDP52235

conserved hypothetical protein
  
Accession: EDP52234
  
Location: 56236-56922
  
 NCBI BlastP on this gene

EDP52234

MFS drug efflux transporter, putative
  
Accession: EDP52233
  
Location: 53918-55618
  
 NCBI BlastP on this gene

EDP52233

hypothetical protein
  
Accession: EDP52232
  
Location: 53085-53597
  
 NCBI BlastP on this gene

EDP52232

nonribosomal peptide synthase, putative
  
Accession: EDP52231
  
Location: 45768-52883
  
  
**BlastP hit with Mycgr3G40534\_Mycgr3T**
  
Percentage identity: 37 %
  
BlastP bit score: 133
  
Sequence coverage: 81 %
  
E-value: 1e-30
  
  
  
**BlastP hit with Mycgr3G39931\_Mycgr3T**
  
Percentage identity: 30 %
  
BlastP bit score: 112
  
Sequence coverage: 92 %
  
E-value: 8e-24
  
  
 NCBI BlastP on this gene

EDP52231

methyltransferase, putative
  
Accession: EDP52230
  
Location: 44255-45315
  
 NCBI BlastP on this gene

EDP52230

C6 transcription factor, putative
  
Accession: EDP52229
  
Location: 39937-42165
  
 NCBI BlastP on this gene

EDP52229

Query: Architecture Search FASTA input

KE145368 : Glarea lozoyensis ATCC 20868 chromosome Unknown GLAREA4    Total score: 2.0     Cumulative Blast bit score: 240

Hit cluster cross-links:

Mycgr3G41235 Mycgr3T
  
Location: 0-4062

Mycgr3G41235\_Mycgr3T

Mycgr3G70577 Mycgr3T
  
Location: 4162-6109

Mycgr3G70577\_Mycgr3T

Mycgr3G40534 Mycgr3T
  
Location: 6209-7166

Mycgr3G40534\_Mycgr3T

Mycgr3G85486 Mycgr3T
  
Location: 7266-8511

Mycgr3G85486\_Mycgr3T

Mycgr3G92221 Mycgr3T
  
Location: 8611-9193

Mycgr3G92221\_Mycgr3T

Mycgr3G39931 Mycgr3T
  
Location: 9293-10157

Mycgr3G39931\_Mycgr3T

Mycgr3G99766 Mycgr3T
  
Location: 10257-11775

Mycgr3G99766\_Mycgr3T

FAD/NAD(P)-binding protein
  
Accession: EPE28433
  
Location: 583360-584778
  
 NCBI BlastP on this gene

EPE28433

PLP-dependent transferase
  
Accession: EPE28434
  
Location: 585257-586977
  
 NCBI BlastP on this gene

EPE28434

Acetyl-CoA synthetase-like protein
  
Accession: EPE28435
  
Location: 588414-597802
  
  
**BlastP hit with Mycgr3G40534\_Mycgr3T**
  
Percentage identity: 33 %
  
BlastP bit score: 126
  
Sequence coverage: 81 %
  
E-value: 4e-28
  
  
  
**BlastP hit with Mycgr3G39931\_Mycgr3T**
  
Percentage identity: 30 %
  
BlastP bit score: 115
  
Sequence coverage: 90 %
  
E-value: 1e-24
  
  
 NCBI BlastP on this gene

EPE28435

Clavaminate synthase-like protein
  
Accession: EPE28436
  
Location: 598904-599991
  
 NCBI BlastP on this gene

EPE28436

alpha/beta-Hydrolase
  
Accession: EPE28437
  
Location: 601010-602224
  
 NCBI BlastP on this gene

EPE28437

Query: Architecture Search FASTA input

KB933064 : Togninia minima UCRPA7 unplaced genomic scaffold PA7\_03\_scaffold\_293    Total score: 2.0     Cumulative Blast bit score: 239

Hit cluster cross-links:

Mycgr3G41235 Mycgr3T
  
Location: 0-4062

Mycgr3G41235\_Mycgr3T

Mycgr3G70577 Mycgr3T
  
Location: 4162-6109

Mycgr3G70577\_Mycgr3T

Mycgr3G40534 Mycgr3T
  
Location: 6209-7166

Mycgr3G40534\_Mycgr3T

Mycgr3G85486 Mycgr3T
  
Location: 7266-8511

Mycgr3G85486\_Mycgr3T

Mycgr3G92221 Mycgr3T
  
Location: 8611-9193

Mycgr3G92221\_Mycgr3T

Mycgr3G39931 Mycgr3T
  
Location: 9293-10157

Mycgr3G39931\_Mycgr3T

Mycgr3G99766 Mycgr3T
  
Location: 10257-11775

Mycgr3G99766\_Mycgr3T

hypothetical protein
  
Accession: EOO00601
  
Location: 147741-180254
  
  
**BlastP hit with Mycgr3G40534\_Mycgr3T**
  
Percentage identity: 36 %
  
BlastP bit score: 133
  
Sequence coverage: 83 %
  
E-value: 2e-30
  
  
 NCBI BlastP on this gene

EOO00601

putative alpha beta hydrolase protein
  
Accession: EOO00594
  
Location: 183798-184880
  
 NCBI BlastP on this gene

EOO00594

hypothetical protein
  
Accession: EOO00604
  
Location: 185818-213011
  
  
**BlastP hit with Mycgr3G39931\_Mycgr3T**
  
Percentage identity: 30 %
  
BlastP bit score: 107
  
Sequence coverage: 91 %
  
E-value: 3e-22
  
  
 NCBI BlastP on this gene

EOO00604

Query: Architecture Search FASTA input

CH476616 : Uncinocarpus reesii 1704 scaffold\_2 genomic scaffold    Total score: 2.0     Cumulative Blast bit score: 239

Hit cluster cross-links:

Mycgr3G41235 Mycgr3T
  
Location: 0-4062

Mycgr3G41235\_Mycgr3T

Mycgr3G70577 Mycgr3T
  
Location: 4162-6109

Mycgr3G70577\_Mycgr3T

Mycgr3G40534 Mycgr3T
  
Location: 6209-7166

Mycgr3G40534\_Mycgr3T

Mycgr3G85486 Mycgr3T
  
Location: 7266-8511

Mycgr3G85486\_Mycgr3T

Mycgr3G92221 Mycgr3T
  
Location: 8611-9193

Mycgr3G92221\_Mycgr3T

Mycgr3G39931 Mycgr3T
  
Location: 9293-10157

Mycgr3G39931\_Mycgr3T

Mycgr3G99766 Mycgr3T
  
Location: 10257-11775

Mycgr3G99766\_Mycgr3T

predicted protein
  
Accession: EEP79831
  
Location: 5054874-5072607
  
  
**BlastP hit with Mycgr3G40534\_Mycgr3T**
  
Percentage identity: 33 %
  
BlastP bit score: 131
  
Sequence coverage: 84 %
  
E-value: 5e-30
  
  
  
**BlastP hit with Mycgr3G39931\_Mycgr3T**
  
Percentage identity: 31 %
  
BlastP bit score: 108
  
Sequence coverage: 94 %
  
E-value: 1e-22
  
  
 NCBI BlastP on this gene

EEP79831

predicted protein
  
Accession: EEP79830
  
Location: 5047933-5054073
  
 NCBI BlastP on this gene

EEP79830

Query: Architecture Search FASTA input

GL377306 : Schizophyllum commune H4-8 unplaced genomic scaffold SCHCOscaffold\_5    Total score: 1.0     Cumulative Blast bit score: 2036

Hit cluster cross-links:

Mycgr3G41235 Mycgr3T
  
Location: 0-4062

Mycgr3G41235\_Mycgr3T

Mycgr3G70577 Mycgr3T
  
Location: 4162-6109

Mycgr3G70577\_Mycgr3T

Mycgr3G40534 Mycgr3T
  
Location: 6209-7166

Mycgr3G40534\_Mycgr3T

Mycgr3G85486 Mycgr3T
  
Location: 7266-8511

Mycgr3G85486\_Mycgr3T

Mycgr3G92221 Mycgr3T
  
Location: 8611-9193

Mycgr3G92221\_Mycgr3T

Mycgr3G39931 Mycgr3T
  
Location: 9293-10157

Mycgr3G39931\_Mycgr3T

Mycgr3G99766 Mycgr3T
  
Location: 10257-11775

Mycgr3G99766\_Mycgr3T

hypothetical protein
  
Accession: EFI96709
  
Location: 328978-330318
  
 NCBI BlastP on this gene

EFI96709

hypothetical protein
  
Accession: EFI97147
  
Location: 330844-335699
  
  
**BlastP hit with Mycgr3G41235\_Mycgr3T**
  
Percentage identity: 40 %
  
BlastP bit score: 1035
  
Sequence coverage: 101 %
  
E-value: 0.0
  
  
 NCBI BlastP on this gene

EFI97147

hypothetical protein
  
Accession: EFI97148
  
Location: 336326-339842
  
 NCBI BlastP on this gene

EFI97148

hypothetical protein
  
Accession: EFI96710
  
Location: 340140-340731
  
 NCBI BlastP on this gene

EFI96710

hypothetical protein
  
Accession: EFI96711
  
Location: 341968-347026
  
  
**BlastP hit with Mycgr3G41235\_Mycgr3T**
  
Percentage identity: 39 %
  
BlastP bit score: 1001
  
Sequence coverage: 103 %
  
E-value: 0.0
  
  
 NCBI BlastP on this gene

EFI96711

Query: Architecture Search FASTA input

CAIF01000223 : Wickerhamomyces ciferrii strain NRRL Y-1031 F-60-10    Total score: 1.0     Cumulative Blast bit score: 1642

Hit cluster cross-links:

Mycgr3G41235 Mycgr3T
  
Location: 0-4062

Mycgr3G41235\_Mycgr3T

Mycgr3G70577 Mycgr3T
  
Location: 4162-6109

Mycgr3G70577\_Mycgr3T

Mycgr3G40534 Mycgr3T
  
Location: 6209-7166

Mycgr3G40534\_Mycgr3T

Mycgr3G85486 Mycgr3T
  
Location: 7266-8511

Mycgr3G85486\_Mycgr3T

Mycgr3G92221 Mycgr3T
  
Location: 8611-9193

Mycgr3G92221\_Mycgr3T

Mycgr3G39931 Mycgr3T
  
Location: 9293-10157

Mycgr3G39931\_Mycgr3T

Mycgr3G99766 Mycgr3T
  
Location: 10257-11775

Mycgr3G99766\_Mycgr3T

hypothetical protein
  
Accession: CCH46043
  
Location: 24543-25703
  
 NCBI BlastP on this gene

CCH46043

Y+L amino acid transporter
  
Accession: CCH46044
  
Location: 26084-27574
  
 NCBI BlastP on this gene

CCH46044

Siderophore iron transporter
  
Accession: CCH46045
  
Location: 27746-29680
  
  
**BlastP hit with Mycgr3G70577\_Mycgr3T**
  
Percentage identity: 37 %
  
BlastP bit score: 396
  
Sequence coverage: 98 %
  
E-value: 3e-125
  
  
 NCBI BlastP on this gene

CCH46045

Siderophore iron transporter
  
Accession: CCH46046
  
Location: 31808-33742
  
  
**BlastP hit with Mycgr3G70577\_Mycgr3T**
  
Percentage identity: 37 %
  
BlastP bit score: 426
  
Sequence coverage: 96 %
  
E-value: 7e-137
  
  
 NCBI BlastP on this gene

CCH46046

Siderophore iron transporter
  
Accession: CCH46047
  
Location: 36120-38072
  
  
**BlastP hit with Mycgr3G70577\_Mycgr3T**
  
Percentage identity: 36 %
  
BlastP bit score: 409
  
Sequence coverage: 97 %
  
E-value: 4e-130
  
  
 NCBI BlastP on this gene

CCH46047

Siderophore iron transporter
  
Accession: CCH46048
  
Location: 40043-41986
  
  
**BlastP hit with Mycgr3G70577\_Mycgr3T**
  
Percentage identity: 36 %
  
BlastP bit score: 411
  
Sequence coverage: 97 %
  
E-value: 6e-131
  
  
 NCBI BlastP on this gene

CCH46048

Query: Architecture Search FASTA input

CP002685 : Arabidopsis thaliana chromosome 2    Total score: 1.0     Cumulative Blast bit score: 1316

Hit cluster cross-links:

Mycgr3G41235 Mycgr3T
  
Location: 0-4062

Mycgr3G41235\_Mycgr3T

Mycgr3G70577 Mycgr3T
  
Location: 4162-6109

Mycgr3G70577\_Mycgr3T

Mycgr3G40534 Mycgr3T
  
Location: 6209-7166

Mycgr3G40534\_Mycgr3T

Mycgr3G85486 Mycgr3T
  
Location: 7266-8511

Mycgr3G85486\_Mycgr3T

Mycgr3G92221 Mycgr3T
  
Location: 8611-9193

Mycgr3G92221\_Mycgr3T

Mycgr3G39931 Mycgr3T
  
Location: 9293-10157

Mycgr3G39931\_Mycgr3T

Mycgr3G99766 Mycgr3T
  
Location: 10257-11775

Mycgr3G99766\_Mycgr3T

ABC transporter C family member 2
  
Accession: AEC09005
  
Location: 14603267-14612387
  
  
**BlastP hit with Mycgr3G41235\_Mycgr3T**
  
Percentage identity: 31 %
  
BlastP bit score: 658
  
Sequence coverage: 98 %
  
E-value: 0.0
  
  
 NCBI BlastP on this gene

MRP2

ABC transporter C family member 2
  
Accession: AEC09006
  
Location: 14603267-14612387
  
  
**BlastP hit with Mycgr3G41235\_Mycgr3T**
  
Percentage identity: 31 %
  
BlastP bit score: 658
  
Sequence coverage: 98 %
  
E-value: 0.0
  
  
 NCBI BlastP on this gene

MRP2

uncharacterized protein
  
Accession: AEC09008
  
Location: 14612741-14615231
  
 NCBI BlastP on this gene

AT2G34670

uncharacterized protein
  
Accession: AEC09007
  
Location: 14613239-14615231
  
 NCBI BlastP on this gene

AT2G34670

Query: Architecture Search FASTA input

CP002684 : Arabidopsis thaliana chromosome 1    Total score: 1.0     Cumulative Blast bit score: 1290

Hit cluster cross-links:

Mycgr3G41235 Mycgr3T
  
Location: 0-4062

Mycgr3G41235\_Mycgr3T

Mycgr3G70577 Mycgr3T
  
Location: 4162-6109

Mycgr3G70577\_Mycgr3T

Mycgr3G40534 Mycgr3T
  
Location: 6209-7166

Mycgr3G40534\_Mycgr3T

Mycgr3G85486 Mycgr3T
  
Location: 7266-8511

Mycgr3G85486\_Mycgr3T

Mycgr3G92221 Mycgr3T
  
Location: 8611-9193

Mycgr3G92221\_Mycgr3T

Mycgr3G39931 Mycgr3T
  
Location: 9293-10157

Mycgr3G39931\_Mycgr3T

Mycgr3G99766 Mycgr3T
  
Location: 10257-11775

Mycgr3G99766\_Mycgr3T

glutathione S-conjugate transporting ATPase
  
Accession: AEE31212
  
Location: 10728139-10737697
  
  
**BlastP hit with Mycgr3G41235\_Mycgr3T**
  
Percentage identity: 31 %
  
BlastP bit score: 645
  
Sequence coverage: 98 %
  
E-value: 0.0
  
  
 NCBI BlastP on this gene

MRP1

glutathione S-conjugate transporting ATPase
  
Accession: AEE31213
  
Location: 10728139-10737697
  
  
**BlastP hit with Mycgr3G41235\_Mycgr3T**
  
Percentage identity: 31 %
  
BlastP bit score: 645
  
Sequence coverage: 98 %
  
E-value: 0.0
  
  
 NCBI BlastP on this gene

MRP1

multidrug resistance-associated protein 13
  
Accession: AEE31214
  
Location: 10739357-10747017
  
 NCBI BlastP on this gene

MRP13

Query: Architecture Search FASTA input

JH930470 : Phanerochaete carnosa HHB-10118-sp unplaced genomic scaffold PHACAscaffold\_3    Total score: 1.0     Cumulative Blast bit score: 1058

Hit cluster cross-links:

Mycgr3G41235 Mycgr3T
  
Location: 0-4062

Mycgr3G41235\_Mycgr3T

Mycgr3G70577 Mycgr3T
  
Location: 4162-6109

Mycgr3G70577\_Mycgr3T

Mycgr3G40534 Mycgr3T
  
Location: 6209-7166

Mycgr3G40534\_Mycgr3T

Mycgr3G85486 Mycgr3T
  
Location: 7266-8511

Mycgr3G85486\_Mycgr3T

Mycgr3G92221 Mycgr3T
  
Location: 8611-9193

Mycgr3G92221\_Mycgr3T

Mycgr3G39931 Mycgr3T
  
Location: 9293-10157

Mycgr3G39931\_Mycgr3T

Mycgr3G99766 Mycgr3T
  
Location: 10257-11775

Mycgr3G99766\_Mycgr3T

hypothetical protein
  
Accession: EKM58200
  
Location: 2459263-2461635
  
 NCBI BlastP on this gene

EKM58200

hypothetical protein
  
Accession: EKM58201
  
Location: 2462166-2463179
  
 NCBI BlastP on this gene

EKM58201

hypothetical protein
  
Accession: EKM58202
  
Location: 2464741-2465983
  
 NCBI BlastP on this gene

EKM58202

hypothetical protein
  
Accession: EKM58203
  
Location: 2467399-2472467
  
  
**BlastP hit with Mycgr3G41235\_Mycgr3T**
  
Percentage identity: 41 %
  
BlastP bit score: 1058
  
Sequence coverage: 100 %
  
E-value: 0.0
  
  
 NCBI BlastP on this gene

EKM58203

hypothetical protein
  
Accession: EKM58204
  
Location: 2475912-2476943
  
 NCBI BlastP on this gene

EKM58204

hypothetical protein
  
Accession: EKM58205
  
Location: 2477705-2480581
  
 NCBI BlastP on this gene

EKM58205

Query: Architecture Search FASTA input

JH711789 : Trametes versicolor FP-101664 SS1 unplaced genomic scaffold TRAVEscaffold\_7    Total score: 1.0     Cumulative Blast bit score: 1043

Hit cluster cross-links:

Mycgr3G41235 Mycgr3T
  
Location: 0-4062

Mycgr3G41235\_Mycgr3T

Mycgr3G70577 Mycgr3T
  
Location: 4162-6109

Mycgr3G70577\_Mycgr3T

Mycgr3G40534 Mycgr3T
  
Location: 6209-7166

Mycgr3G40534\_Mycgr3T

Mycgr3G85486 Mycgr3T
  
Location: 7266-8511

Mycgr3G85486\_Mycgr3T

Mycgr3G92221 Mycgr3T
  
Location: 8611-9193

Mycgr3G92221\_Mycgr3T

Mycgr3G39931 Mycgr3T
  
Location: 9293-10157

Mycgr3G39931\_Mycgr3T

Mycgr3G99766 Mycgr3T
  
Location: 10257-11775

Mycgr3G99766\_Mycgr3T

hypothetical protein
  
Accession: EIW57321
  
Location: 627060-627491
  
 NCBI BlastP on this gene

EIW57321

DEAD-domain-containing protein
  
Accession: EIW57320
  
Location: 622592-625229
  
 NCBI BlastP on this gene

EIW57320

P-loop containing nucleoside triphosphate hydrolase protein
  
Accession: EIW57319
  
Location: 616211-621367
  
  
**BlastP hit with Mycgr3G41235\_Mycgr3T**
  
Percentage identity: 40 %
  
BlastP bit score: 1043
  
Sequence coverage: 104 %
  
E-value: 0.0
  
  
 NCBI BlastP on this gene

EIW57319

CoA-transferase family III
  
Accession: EIW57318
  
Location: 614052-615692
  
 NCBI BlastP on this gene

EIW57318

multifunctional beta-oxidation protein
  
Accession: EIW57317
  
Location: 608803-613175
  
 NCBI BlastP on this gene

EIW57317

Query: Architecture Search FASTA input

KB445804 : Ceriporiopsis subvermispora B unplaced genomic scaffold CERSUscaffold\_14    Total score: 1.0     Cumulative Blast bit score: 1041

Hit cluster cross-links:

Mycgr3G41235 Mycgr3T
  
Location: 0-4062

Mycgr3G41235\_Mycgr3T

Mycgr3G70577 Mycgr3T
  
Location: 4162-6109

Mycgr3G70577\_Mycgr3T

Mycgr3G40534 Mycgr3T
  
Location: 6209-7166

Mycgr3G40534\_Mycgr3T

Mycgr3G85486 Mycgr3T
  
Location: 7266-8511

Mycgr3G85486\_Mycgr3T

Mycgr3G92221 Mycgr3T
  
Location: 8611-9193

Mycgr3G92221\_Mycgr3T

Mycgr3G39931 Mycgr3T
  
Location: 9293-10157

Mycgr3G39931\_Mycgr3T

Mycgr3G99766 Mycgr3T
  
Location: 10257-11775

Mycgr3G99766\_Mycgr3T

hypothetical protein
  
Accession: EMD34146
  
Location: 964997-965423
  
 NCBI BlastP on this gene

EMD34146

hypothetical protein
  
Accession: EMD34147
  
Location: 967334-970010
  
 NCBI BlastP on this gene

EMD34147

hypothetical protein
  
Accession: EMD34148
  
Location: 970862-976409
  
  
**BlastP hit with Mycgr3G41235\_Mycgr3T**
  
Percentage identity: 39 %
  
BlastP bit score: 1041
  
Sequence coverage: 103 %
  
E-value: 0.0
  
  
 NCBI BlastP on this gene

EMD34148

hypothetical protein
  
Accession: EMD34149
  
Location: 976890-978647
  
 NCBI BlastP on this gene

EMD34149

hypothetical protein
  
Accession: EMD34150
  
Location: 979045-980773
  
 NCBI BlastP on this gene

EMD34150

Query: Architecture Search FASTA input

JH687380 : Stereum hirsutum FP-91666 SS1 unplaced genomic scaffold STEHIscaffold\_2    Total score: 1.0     Cumulative Blast bit score: 1039

Hit cluster cross-links:

Mycgr3G41235 Mycgr3T
  
Location: 0-4062

Mycgr3G41235\_Mycgr3T

Mycgr3G70577 Mycgr3T
  
Location: 4162-6109

Mycgr3G70577\_Mycgr3T

Mycgr3G40534 Mycgr3T
  
Location: 6209-7166

Mycgr3G40534\_Mycgr3T

Mycgr3G85486 Mycgr3T
  
Location: 7266-8511

Mycgr3G85486\_Mycgr3T

Mycgr3G92221 Mycgr3T
  
Location: 8611-9193

Mycgr3G92221\_Mycgr3T

Mycgr3G39931 Mycgr3T
  
Location: 9293-10157

Mycgr3G39931\_Mycgr3T

Mycgr3G99766 Mycgr3T
  
Location: 10257-11775

Mycgr3G99766\_Mycgr3T

Sodium/hydrogen exchanger
  
Accession: EIM91111
  
Location: 1776869-1778829
  
 NCBI BlastP on this gene

EIM91111

hypothetical protein
  
Accession: EIM91112
  
Location: 1780394-1781412
  
 NCBI BlastP on this gene

EIM91112

ABC transporter
  
Accession: EIM91113
  
Location: 1782241-1787709
  
  
**BlastP hit with Mycgr3G41235\_Mycgr3T**
  
Percentage identity: 40 %
  
BlastP bit score: 1039
  
Sequence coverage: 99 %
  
E-value: 0.0
  
  
 NCBI BlastP on this gene

EIM91113

phosphatidylserine decarboxylase-like protein
  
Accession: EIM91114
  
Location: 1788503-1789902
  
 NCBI BlastP on this gene

EIM91114

hypothetical protein
  
Accession: EIM91115
  
Location: 1790156-1790973
  
 NCBI BlastP on this gene

EIM91115

Query: Architecture Search FASTA input

JH687380 : Stereum hirsutum FP-91666 SS1 unplaced genomic scaffold STEHIscaffold\_2    Total score: 1.0     Cumulative Blast bit score: 1026

Hit cluster cross-links:

Mycgr3G41235 Mycgr3T
  
Location: 0-4062

Mycgr3G41235\_Mycgr3T

Mycgr3G70577 Mycgr3T
  
Location: 4162-6109

Mycgr3G70577\_Mycgr3T

Mycgr3G40534 Mycgr3T
  
Location: 6209-7166

Mycgr3G40534\_Mycgr3T

Mycgr3G85486 Mycgr3T
  
Location: 7266-8511

Mycgr3G85486\_Mycgr3T

Mycgr3G92221 Mycgr3T
  
Location: 8611-9193

Mycgr3G92221\_Mycgr3T

Mycgr3G39931 Mycgr3T
  
Location: 9293-10157

Mycgr3G39931\_Mycgr3T

Mycgr3G99766 Mycgr3T
  
Location: 10257-11775

Mycgr3G99766\_Mycgr3T

ABC transporter
  
Accession: EIM91429
  
Location: 2822120-2828297
  
  
**BlastP hit with Mycgr3G41235\_Mycgr3T**
  
Percentage identity: 40 %
  
BlastP bit score: 1026
  
Sequence coverage: 102 %
  
E-value: 0.0
  
  
 NCBI BlastP on this gene

EIM91429

ankyrin
  
Accession: EIM91428
  
Location: 2815731-2820065
  
 NCBI BlastP on this gene

EIM91428

Query: Architecture Search FASTA input

JH717971 : Fomitiporia mediterranea MF3/22 unplaced genomic scaffold FOMMEscaffold\_5    Total score: 1.0     Cumulative Blast bit score: 1023

Hit cluster cross-links:

Mycgr3G41235 Mycgr3T
  
Location: 0-4062

Mycgr3G41235\_Mycgr3T

Mycgr3G70577 Mycgr3T
  
Location: 4162-6109

Mycgr3G70577\_Mycgr3T

Mycgr3G40534 Mycgr3T
  
Location: 6209-7166

Mycgr3G40534\_Mycgr3T

Mycgr3G85486 Mycgr3T
  
Location: 7266-8511

Mycgr3G85486\_Mycgr3T

Mycgr3G92221 Mycgr3T
  
Location: 8611-9193

Mycgr3G92221\_Mycgr3T

Mycgr3G39931 Mycgr3T
  
Location: 9293-10157

Mycgr3G39931\_Mycgr3T

Mycgr3G99766 Mycgr3T
  
Location: 10257-11775

Mycgr3G99766\_Mycgr3T

hypothetical protein
  
Accession: EJD03687
  
Location: 870318-873863
  
 NCBI BlastP on this gene

EJD03687

ABC transporter
  
Accession: EJD03686
  
Location: 864063-869569
  
  
**BlastP hit with Mycgr3G41235\_Mycgr3T**
  
Percentage identity: 39 %
  
BlastP bit score: 1023
  
Sequence coverage: 103 %
  
E-value: 0.0
  
  
 NCBI BlastP on this gene

EJD03686

hypothetical protein
  
Accession: EJD03685
  
Location: 862943-863872
  
 NCBI BlastP on this gene

EJD03685

hypothetical protein
  
Accession: EJD03684
  
Location: 861869-862695
  
 NCBI BlastP on this gene

EJD03684

hypothetical protein
  
Accession: EJD03683
  
Location: 860817-861633
  
 NCBI BlastP on this gene

EJD03683

phenylacetyl-CoA ligase
  
Accession: EJD03682
  
Location: 856968-859677
  
 NCBI BlastP on this gene

EJD03682

Query: Architecture Search FASTA input

JH719400 : Dichomitus squalens LYAD-421 SS1 unplaced genomic scaffold DICSQscaffold\_4    Total score: 1.0     Cumulative Blast bit score: 1018

Hit cluster cross-links:

Mycgr3G41235 Mycgr3T
  
Location: 0-4062

Mycgr3G41235\_Mycgr3T

Mycgr3G70577 Mycgr3T
  
Location: 4162-6109

Mycgr3G70577\_Mycgr3T

Mycgr3G40534 Mycgr3T
  
Location: 6209-7166

Mycgr3G40534\_Mycgr3T

Mycgr3G85486 Mycgr3T
  
Location: 7266-8511

Mycgr3G85486\_Mycgr3T

Mycgr3G92221 Mycgr3T
  
Location: 8611-9193

Mycgr3G92221\_Mycgr3T

Mycgr3G39931 Mycgr3T
  
Location: 9293-10157

Mycgr3G39931\_Mycgr3T

Mycgr3G99766 Mycgr3T
  
Location: 10257-11775

Mycgr3G99766\_Mycgr3T

ABC transporter
  
Accession: EJF64657
  
Location: 641871-646695
  
  
**BlastP hit with Mycgr3G41235\_Mycgr3T**
  
Percentage identity: 40 %
  
BlastP bit score: 1019
  
Sequence coverage: 101 %
  
E-value: 0.0
  
  
 NCBI BlastP on this gene

EJF64657

Query: Architecture Search FASTA input

AACS02000004 : Coprinopsis cinerea okayama7#130    Total score: 1.0     Cumulative Blast bit score: 1010

Hit cluster cross-links:

Mycgr3G41235 Mycgr3T
  
Location: 0-4062

Mycgr3G41235\_Mycgr3T

Mycgr3G70577 Mycgr3T
  
Location: 4162-6109

Mycgr3G70577\_Mycgr3T

Mycgr3G40534 Mycgr3T
  
Location: 6209-7166

Mycgr3G40534\_Mycgr3T

Mycgr3G85486 Mycgr3T
  
Location: 7266-8511

Mycgr3G85486\_Mycgr3T

Mycgr3G92221 Mycgr3T
  
Location: 8611-9193

Mycgr3G92221\_Mycgr3T

Mycgr3G39931 Mycgr3T
  
Location: 9293-10157

Mycgr3G39931\_Mycgr3T

Mycgr3G99766 Mycgr3T
  
Location: 10257-11775

Mycgr3G99766\_Mycgr3T

hypothetical protein
  
Accession: EFI27926
  
Location: 2082913-2083656
  
 NCBI BlastP on this gene

EFI27926

hypothetical protein
  
Accession: EFI27927
  
Location: 2084004-2086053
  
 NCBI BlastP on this gene

EFI27927

hypothetical protein
  
Accession: EAU85518
  
Location: 2086199-2087349
  
 NCBI BlastP on this gene

EAU85518

ATP-binding cassette transporter YOR1
  
Accession: EAU85519
  
Location: 2087523-2093390
  
  
**BlastP hit with Mycgr3G41235\_Mycgr3T**
  
Percentage identity: 38 %
  
BlastP bit score: 1010
  
Sequence coverage: 105 %
  
E-value: 0.0
  
  
 NCBI BlastP on this gene

EAU85519

histone acetyltransferase mst2
  
Accession: EFI27928
  
Location: 2093703-2101960
  
 NCBI BlastP on this gene

EFI27928

Query: Architecture Search FASTA input

JH931607 : Agaricus bisporus var. bisporus H97 unplaced genomic scaffold AGABI2scaffold\_3    Total score: 1.0     Cumulative Blast bit score: 1008

Hit cluster cross-links:

Mycgr3G41235 Mycgr3T
  
Location: 0-4062

Mycgr3G41235\_Mycgr3T

Mycgr3G70577 Mycgr3T
  
Location: 4162-6109

Mycgr3G70577\_Mycgr3T

Mycgr3G40534 Mycgr3T
  
Location: 6209-7166

Mycgr3G40534\_Mycgr3T

Mycgr3G85486 Mycgr3T
  
Location: 7266-8511

Mycgr3G85486\_Mycgr3T

Mycgr3G92221 Mycgr3T
  
Location: 8611-9193

Mycgr3G92221\_Mycgr3T

Mycgr3G39931 Mycgr3T
  
Location: 9293-10157

Mycgr3G39931\_Mycgr3T

Mycgr3G99766 Mycgr3T
  
Location: 10257-11775

Mycgr3G99766\_Mycgr3T

hypothetical protein
  
Accession: EKV49243
  
Location: 1401950-1407855
  
  
**BlastP hit with Mycgr3G41235\_Mycgr3T**
  
Percentage identity: 39 %
  
BlastP bit score: 1008
  
Sequence coverage: 101 %
  
E-value: 0.0
  
  
 NCBI BlastP on this gene

EKV49243

hypothetical protein
  
Accession: EKV49242
  
Location: 1396389-1401618
  
 NCBI BlastP on this gene

EKV49242

Query: Architecture Search FASTA input

JH687380 : Stereum hirsutum FP-91666 SS1 unplaced genomic scaffold STEHIscaffold\_2    Total score: 1.0     Cumulative Blast bit score: 1008

Hit cluster cross-links:

Mycgr3G41235 Mycgr3T
  
Location: 0-4062

Mycgr3G41235\_Mycgr3T

Mycgr3G70577 Mycgr3T
  
Location: 4162-6109

Mycgr3G70577\_Mycgr3T

Mycgr3G40534 Mycgr3T
  
Location: 6209-7166

Mycgr3G40534\_Mycgr3T

Mycgr3G85486 Mycgr3T
  
Location: 7266-8511

Mycgr3G85486\_Mycgr3T

Mycgr3G92221 Mycgr3T
  
Location: 8611-9193

Mycgr3G92221\_Mycgr3T

Mycgr3G39931 Mycgr3T
  
Location: 9293-10157

Mycgr3G39931\_Mycgr3T

Mycgr3G99766 Mycgr3T
  
Location: 10257-11775

Mycgr3G99766\_Mycgr3T

hypothetical protein
  
Accession: EIM91383
  
Location: 2640665-2644233
  
 NCBI BlastP on this gene

EIM91383

ABC transporter
  
Accession: EIM91382
  
Location: 2632680-2638146
  
  
**BlastP hit with Mycgr3G41235\_Mycgr3T**
  
Percentage identity: 39 %
  
BlastP bit score: 1008
  
Sequence coverage: 103 %
  
E-value: 0.0
  
  
 NCBI BlastP on this gene

EIM91382

hypothetical protein
  
Accession: EIM91381
  
Location: 2630136-2632260
  
 NCBI BlastP on this gene

EIM91381

hypothetical protein
  
Accession: EIM91380
  
Location: 2628152-2629137
  
 NCBI BlastP on this gene

EIM91380

Query: Architecture Search FASTA input

JH711575 : Coniophora puteana RWD-64-598 SS2 unplaced genomic scaffold CONPUscaffold\_3    Total score: 1.0     Cumulative Blast bit score: 997

Hit cluster cross-links:

Mycgr3G41235 Mycgr3T
  
Location: 0-4062

Mycgr3G41235\_Mycgr3T

Mycgr3G70577 Mycgr3T
  
Location: 4162-6109

Mycgr3G70577\_Mycgr3T

Mycgr3G40534 Mycgr3T
  
Location: 6209-7166

Mycgr3G40534\_Mycgr3T

Mycgr3G85486 Mycgr3T
  
Location: 7266-8511

Mycgr3G85486\_Mycgr3T

Mycgr3G92221 Mycgr3T
  
Location: 8611-9193

Mycgr3G92221\_Mycgr3T

Mycgr3G39931 Mycgr3T
  
Location: 9293-10157

Mycgr3G39931\_Mycgr3T

Mycgr3G99766 Mycgr3T
  
Location: 10257-11775

Mycgr3G99766\_Mycgr3T

PAH-inducible cytochrome P450 monooxygenase PC-PAH 1
  
Accession: EIW84209
  
Location: 1962129-1964218
  
 NCBI BlastP on this gene

EIW84209

P-loop containing nucleoside triphosphate hydrolase protein
  
Accession: EIW84208
  
Location: 1959257-1961613
  
 NCBI BlastP on this gene

EIW84208

ABC transporter
  
Accession: EIW84207
  
Location: 1953373-1958654
  
  
**BlastP hit with Mycgr3G41235\_Mycgr3T**
  
Percentage identity: 40 %
  
BlastP bit score: 998
  
Sequence coverage: 101 %
  
E-value: 0.0
  
  
 NCBI BlastP on this gene

EIW84207

hypothetical protein
  
Accession: EIW84206
  
Location: 1951714-1953001
  
 NCBI BlastP on this gene

EIW84206

hypothetical protein
  
Accession: EIW84205
  
Location: 1950155-1951400
  
 NCBI BlastP on this gene

EIW84205

flavo protein
  
Accession: EIW84204
  
Location: 1947526-1949879
  
 NCBI BlastP on this gene

EIW84204

Query: Architecture Search FASTA input

JH687379 : Stereum hirsutum FP-91666 SS1 unplaced genomic scaffold STEHIscaffold\_1    Total score: 1.0     Cumulative Blast bit score: 991

Hit cluster cross-links:

Mycgr3G41235 Mycgr3T
  
Location: 0-4062

Mycgr3G41235\_Mycgr3T

Mycgr3G70577 Mycgr3T
  
Location: 4162-6109

Mycgr3G70577\_Mycgr3T

Mycgr3G40534 Mycgr3T
  
Location: 6209-7166

Mycgr3G40534\_Mycgr3T

Mycgr3G85486 Mycgr3T
  
Location: 7266-8511

Mycgr3G85486\_Mycgr3T

Mycgr3G92221 Mycgr3T
  
Location: 8611-9193

Mycgr3G92221\_Mycgr3T

Mycgr3G39931 Mycgr3T
  
Location: 9293-10157

Mycgr3G39931\_Mycgr3T

Mycgr3G99766 Mycgr3T
  
Location: 10257-11775

Mycgr3G99766\_Mycgr3T

NADH-ubiquinone oxidoreductase
  
Accession: EIM92415
  
Location: 2241497-2243627
  
 NCBI BlastP on this gene

EIM92415

glutaredoxin
  
Accession: EIM92414
  
Location: 2239137-2239735
  
 NCBI BlastP on this gene

EIM92414

ATP-binding cassette transporter YOR1
  
Accession: EIM92413
  
Location: 2231975-2237447
  
  
**BlastP hit with Mycgr3G41235\_Mycgr3T**
  
Percentage identity: 39 %
  
BlastP bit score: 991
  
Sequence coverage: 100 %
  
E-value: 0.0
  
  
 NCBI BlastP on this gene

EIM92413

hypothetical protein
  
Accession: EIM92412
  
Location: 2229672-2231483
  
 NCBI BlastP on this gene

EIM92412

hypothetical protein
  
Accession: EIM92411
  
Location: 2227362-2229150
  
 NCBI BlastP on this gene

EIM92411

Query: Architecture Search FASTA input

JH711575 : Coniophora puteana RWD-64-598 SS2 unplaced genomic scaffold CONPUscaffold\_3    Total score: 1.0     Cumulative Blast bit score: 973

Hit cluster cross-links:

Mycgr3G41235 Mycgr3T
  
Location: 0-4062

Mycgr3G41235\_Mycgr3T

Mycgr3G70577 Mycgr3T
  
Location: 4162-6109

Mycgr3G70577\_Mycgr3T

Mycgr3G40534 Mycgr3T
  
Location: 6209-7166

Mycgr3G40534\_Mycgr3T

Mycgr3G85486 Mycgr3T
  
Location: 7266-8511

Mycgr3G85486\_Mycgr3T

Mycgr3G92221 Mycgr3T
  
Location: 8611-9193

Mycgr3G92221\_Mycgr3T

Mycgr3G39931 Mycgr3T
  
Location: 9293-10157

Mycgr3G39931\_Mycgr3T

Mycgr3G99766 Mycgr3T
  
Location: 10257-11775

Mycgr3G99766\_Mycgr3T

williams-Beuren syndrome critical region protein 20 copy A
  
Accession: EIW84228
  
Location: 2016233-2018035
  
 NCBI BlastP on this gene

EIW84228

DnaJ-domain-containing protein
  
Accession: EIW84229
  
Location: 2018469-2020401
  
 NCBI BlastP on this gene

EIW84229

hypothetical protein
  
Accession: EIW84230
  
Location: 2021076-2021683
  
 NCBI BlastP on this gene

EIW84230

P-loop containing nucleoside triphosphate hydrolase protein
  
Accession: EIW84231
  
Location: 2022418-2027867
  
  
**BlastP hit with Mycgr3G41235\_Mycgr3T**
  
Percentage identity: 38 %
  
BlastP bit score: 973
  
Sequence coverage: 102 %
  
E-value: 0.0
  
  
 NCBI BlastP on this gene

EIW84231

Query: Architecture Search FASTA input

CAFZ01000285 : Piriformospora indica DSM 11827    Total score: 1.0     Cumulative Blast bit score: 948

Hit cluster cross-links:

Mycgr3G41235 Mycgr3T
  
Location: 0-4062

Mycgr3G41235\_Mycgr3T

Mycgr3G70577 Mycgr3T
  
Location: 4162-6109

Mycgr3G70577\_Mycgr3T

Mycgr3G40534 Mycgr3T
  
Location: 6209-7166

Mycgr3G40534\_Mycgr3T

Mycgr3G85486 Mycgr3T
  
Location: 7266-8511

Mycgr3G85486\_Mycgr3T

Mycgr3G92221 Mycgr3T
  
Location: 8611-9193

Mycgr3G92221\_Mycgr3T

Mycgr3G39931 Mycgr3T
  
Location: 9293-10157

Mycgr3G39931\_Mycgr3T

Mycgr3G99766 Mycgr3T
  
Location: 10257-11775

Mycgr3G99766\_Mycgr3T

hypothetical protein
  
Accession: CCA74160
  
Location: 634-2564
  
 NCBI BlastP on this gene

CCA74160

related to major facilitator MirA
  
Accession: CCA74161
  
Location: 2887-5086
  
  
**BlastP hit with Mycgr3G70577\_Mycgr3T**
  
Percentage identity: 33 %
  
BlastP bit score: 338
  
Sequence coverage: 94 %
  
E-value: 3e-103
  
  
 NCBI BlastP on this gene

CCA74161

related to major facilitator MirA
  
Accession: CCA74162
  
Location: 5319-7551
  
  
**BlastP hit with Mycgr3G70577\_Mycgr3T**
  
Percentage identity: 33 %
  
BlastP bit score: 317
  
Sequence coverage: 95 %
  
E-value: 4e-95
  
  
 NCBI BlastP on this gene

CCA74162

related to Siderophore iron transporter 3
  
Accession: CCA74163
  
Location: 7954-10268
  
  
**BlastP hit with Mycgr3G70577\_Mycgr3T**
  
Percentage identity: 30 %
  
BlastP bit score: 293
  
Sequence coverage: 96 %
  
E-value: 4e-86
  
  
 NCBI BlastP on this gene

CCA74163

hypothetical protein
  
Accession: CCA74164
  
Location: 10526-12259
  
 NCBI BlastP on this gene

CCA74164

hypothetical protein
  
Accession: CCA74165
  
Location: 12981-14760
  
 NCBI BlastP on this gene

CCA74165

Query: Architecture Search FASTA input

JH971391 : Agaricus bisporus var. burnettii JB137-S8 unplaced genomic scaffold AGABI1scaffold\_7    Total score: 1.0     Cumulative Blast bit score: 931

Hit cluster cross-links:

Mycgr3G41235 Mycgr3T
  
Location: 0-4062

Mycgr3G41235\_Mycgr3T

Mycgr3G70577 Mycgr3T
  
Location: 4162-6109

Mycgr3G70577\_Mycgr3T

Mycgr3G40534 Mycgr3T
  
Location: 6209-7166

Mycgr3G40534\_Mycgr3T

Mycgr3G85486 Mycgr3T
  
Location: 7266-8511

Mycgr3G85486\_Mycgr3T

Mycgr3G92221 Mycgr3T
  
Location: 8611-9193

Mycgr3G92221\_Mycgr3T

Mycgr3G39931 Mycgr3T
  
Location: 9293-10157

Mycgr3G39931\_Mycgr3T

Mycgr3G99766 Mycgr3T
  
Location: 10257-11775

Mycgr3G99766\_Mycgr3T

hypothetical protein
  
Accession: EKM78690
  
Location: 322845-328958
  
  
**BlastP hit with Mycgr3G41235\_Mycgr3T**
  
Percentage identity: 37 %
  
BlastP bit score: 931
  
Sequence coverage: 104 %
  
E-value: 0.0
  
  
 NCBI BlastP on this gene

EKM78690

hypothetical protein
  
Accession: EKM78689
  
Location: 317301-322517
  
 NCBI BlastP on this gene

EKM78689

Query: Architecture Search FASTA input

JH711789 : Trametes versicolor FP-101664 SS1 unplaced genomic scaffold TRAVEscaffold\_7    Total score: 1.0     Cumulative Blast bit score: 901

Hit cluster cross-links:

Mycgr3G41235 Mycgr3T
  
Location: 0-4062

Mycgr3G41235\_Mycgr3T

Mycgr3G70577 Mycgr3T
  
Location: 4162-6109

Mycgr3G70577\_Mycgr3T

Mycgr3G40534 Mycgr3T
  
Location: 6209-7166

Mycgr3G40534\_Mycgr3T

Mycgr3G85486 Mycgr3T
  
Location: 7266-8511

Mycgr3G85486\_Mycgr3T

Mycgr3G92221 Mycgr3T
  
Location: 8611-9193

Mycgr3G92221\_Mycgr3T

Mycgr3G39931 Mycgr3T
  
Location: 9293-10157

Mycgr3G39931\_Mycgr3T

Mycgr3G99766 Mycgr3T
  
Location: 10257-11775

Mycgr3G99766\_Mycgr3T

hypothetical protein
  
Accession: EIW57393
  
Location: 840769-843731
  
 NCBI BlastP on this gene

EIW57393

hypothetical protein
  
Accession: EIW57394
  
Location: 845698-846215
  
 NCBI BlastP on this gene

EIW57394

ABC protein
  
Accession: EIW57395
  
Location: 846939-853616
  
  
**BlastP hit with Mycgr3G41235\_Mycgr3T**
  
Percentage identity: 36 %
  
BlastP bit score: 901
  
Sequence coverage: 103 %
  
E-value: 0.0
  
  
 NCBI BlastP on this gene

EIW57395

hypothetical protein
  
Accession: EIW57396
  
Location: 853771-856939
  
 NCBI BlastP on this gene

EIW57396

Query: Architecture Search FASTA input

KB445804 : Ceriporiopsis subvermispora B unplaced genomic scaffold CERSUscaffold\_14    Total score: 1.0     Cumulative Blast bit score: 892

Hit cluster cross-links:

Mycgr3G41235 Mycgr3T
  
Location: 0-4062

Mycgr3G41235\_Mycgr3T

Mycgr3G70577 Mycgr3T
  
Location: 4162-6109

Mycgr3G70577\_Mycgr3T

Mycgr3G40534 Mycgr3T
  
Location: 6209-7166

Mycgr3G40534\_Mycgr3T

Mycgr3G85486 Mycgr3T
  
Location: 7266-8511

Mycgr3G85486\_Mycgr3T

Mycgr3G92221 Mycgr3T
  
Location: 8611-9193

Mycgr3G92221\_Mycgr3T

Mycgr3G39931 Mycgr3T
  
Location: 9293-10157

Mycgr3G39931\_Mycgr3T

Mycgr3G99766 Mycgr3T
  
Location: 10257-11775

Mycgr3G99766\_Mycgr3T

CsMn25
  
Accession: EMD34176
  
Location: 1057319-1064202
  
  
**BlastP hit with Mycgr3G41235\_Mycgr3T**
  
Percentage identity: 36 %
  
BlastP bit score: 892
  
Sequence coverage: 104 %
  
E-value: 0.0
  
  
 NCBI BlastP on this gene

EMD34176

hypothetical protein
  
Accession: EMD34175
  
Location: 1053620-1057064
  
 NCBI BlastP on this gene

EMD34175

Query: Architecture Search FASTA input

JH717968 : Fomitiporia mediterranea MF3/22 unplaced genomic scaffold FOMMEscaffold\_2    Total score: 1.0     Cumulative Blast bit score: 885

Hit cluster cross-links:

Mycgr3G41235 Mycgr3T
  
Location: 0-4062

Mycgr3G41235\_Mycgr3T

Mycgr3G70577 Mycgr3T
  
Location: 4162-6109

Mycgr3G70577\_Mycgr3T

Mycgr3G40534 Mycgr3T
  
Location: 6209-7166

Mycgr3G40534\_Mycgr3T

Mycgr3G85486 Mycgr3T
  
Location: 7266-8511

Mycgr3G85486\_Mycgr3T

Mycgr3G92221 Mycgr3T
  
Location: 8611-9193

Mycgr3G92221\_Mycgr3T

Mycgr3G39931 Mycgr3T
  
Location: 9293-10157

Mycgr3G39931\_Mycgr3T

Mycgr3G99766 Mycgr3T
  
Location: 10257-11775

Mycgr3G99766\_Mycgr3T

sugar transporter
  
Accession: EJD07134
  
Location: 4300577-4302693
  
 NCBI BlastP on this gene

EJD07134

zf-DHHC-domain-containing protein
  
Accession: EJD07135
  
Location: 4303521-4305035
  
 NCBI BlastP on this gene

EJD07135

hypothetical protein
  
Accession: EJD07136
  
Location: 4306333-4306912
  
 NCBI BlastP on this gene

EJD07136

ABC protein
  
Accession: EJD07137
  
Location: 4307623-4313942
  
  
**BlastP hit with Mycgr3G41235\_Mycgr3T**
  
Percentage identity: 36 %
  
BlastP bit score: 885
  
Sequence coverage: 104 %
  
E-value: 0.0
  
  
 NCBI BlastP on this gene

EJD07137

hypothetical protein
  
Accession: EJD07138
  
Location: 4314640-4317612
  
 NCBI BlastP on this gene

EJD07138

carboxylesterase
  
Accession: EJD07139
  
Location: 4317981-4320123
  
 NCBI BlastP on this gene

EJD07139

Query: Architecture Search FASTA input

JH931607 : Agaricus bisporus var. bisporus H97 unplaced genomic scaffold AGABI2scaffold\_3    Total score: 1.0     Cumulative Blast bit score: 869

Hit cluster cross-links:

Mycgr3G41235 Mycgr3T
  
Location: 0-4062

Mycgr3G41235\_Mycgr3T

Mycgr3G70577 Mycgr3T
  
Location: 4162-6109

Mycgr3G70577\_Mycgr3T

Mycgr3G40534 Mycgr3T
  
Location: 6209-7166

Mycgr3G40534\_Mycgr3T

Mycgr3G85486 Mycgr3T
  
Location: 7266-8511

Mycgr3G85486\_Mycgr3T

Mycgr3G92221 Mycgr3T
  
Location: 8611-9193

Mycgr3G92221\_Mycgr3T

Mycgr3G39931 Mycgr3T
  
Location: 9293-10157

Mycgr3G39931\_Mycgr3T

Mycgr3G99766 Mycgr3T
  
Location: 10257-11775

Mycgr3G99766\_Mycgr3T

hypothetical protein
  
Accession: EKV49147
  
Location: 1146251-1148447
  
 NCBI BlastP on this gene

EKV49147

hypothetical protein
  
Accession: EKV49148
  
Location: 1149691-1151888
  
 NCBI BlastP on this gene

EKV49148

hypothetical protein
  
Accession: EKV49149
  
Location: 1152320-1158449
  
  
**BlastP hit with Mycgr3G41235\_Mycgr3T**
  
Percentage identity: 36 %
  
BlastP bit score: 869
  
Sequence coverage: 103 %
  
E-value: 0.0
  
  
 NCBI BlastP on this gene

EKV49149

hypothetical protein
  
Accession: EKV49150
  
Location: 1159023-1160216
  
 NCBI BlastP on this gene

EKV49150

hypothetical protein
  
Accession: EKV49151
  
Location: 1160441-1161182
  
 NCBI BlastP on this gene

EKV49151

hypothetical protein
  
Accession: EKV49152
  
Location: 1161454-1162015
  
 NCBI BlastP on this gene

EKV49152

hypothetical protein
  
Accession: EKV49153
  
Location: 1162284-1164552
  
 NCBI BlastP on this gene

EKV49153

Query: Architecture Search FASTA input

JH711575 : Coniophora puteana RWD-64-598 SS2 unplaced genomic scaffold CONPUscaffold\_3    Total score: 1.0     Cumulative Blast bit score: 867

Hit cluster cross-links:

Mycgr3G41235 Mycgr3T
  
Location: 0-4062

Mycgr3G41235\_Mycgr3T

Mycgr3G70577 Mycgr3T
  
Location: 4162-6109

Mycgr3G70577\_Mycgr3T

Mycgr3G40534 Mycgr3T
  
Location: 6209-7166

Mycgr3G40534\_Mycgr3T

Mycgr3G85486 Mycgr3T
  
Location: 7266-8511

Mycgr3G85486\_Mycgr3T

Mycgr3G92221 Mycgr3T
  
Location: 8611-9193

Mycgr3G92221\_Mycgr3T

Mycgr3G39931 Mycgr3T
  
Location: 9293-10157

Mycgr3G39931\_Mycgr3T

Mycgr3G99766 Mycgr3T
  
Location: 10257-11775

Mycgr3G99766\_Mycgr3T

acyl-CoA dehydrogenase NM domain-like protein
  
Accession: EIW84012
  
Location: 1419563-1422094
  
 NCBI BlastP on this gene

EIW84012

hypothetical protein
  
Accession: EIW84011
  
Location: 1417242-1419154
  
 NCBI BlastP on this gene

EIW84011

DHS-like NAD/FAD-binding domain-containing protein
  
Accession: EIW84010
  
Location: 1415223-1416999
  
 NCBI BlastP on this gene

EIW84010

ABC protein
  
Accession: EIW84009
  
Location: 1408082-1414426
  
  
**BlastP hit with Mycgr3G41235\_Mycgr3T**
  
Percentage identity: 35 %
  
BlastP bit score: 867
  
Sequence coverage: 105 %
  
E-value: 0.0
  
  
 NCBI BlastP on this gene

EIW84009

WD40 repeat-like protein
  
Accession: EIW84008
  
Location: 1405464-1406372
  
 NCBI BlastP on this gene

EIW84008

hypothetical protein
  
Accession: EIW84007
  
Location: 1399251-1403044
  
 NCBI BlastP on this gene

EIW84007

Query: Architecture Search FASTA input

JH687380 : Stereum hirsutum FP-91666 SS1 unplaced genomic scaffold STEHIscaffold\_2    Total score: 1.0     Cumulative Blast bit score: 861

Hit cluster cross-links:

Mycgr3G41235 Mycgr3T
  
Location: 0-4062

Mycgr3G41235\_Mycgr3T

Mycgr3G70577 Mycgr3T
  
Location: 4162-6109

Mycgr3G70577\_Mycgr3T

Mycgr3G40534 Mycgr3T
  
Location: 6209-7166

Mycgr3G40534\_Mycgr3T

Mycgr3G85486 Mycgr3T
  
Location: 7266-8511

Mycgr3G85486\_Mycgr3T

Mycgr3G92221 Mycgr3T
  
Location: 8611-9193

Mycgr3G92221\_Mycgr3T

Mycgr3G39931 Mycgr3T
  
Location: 9293-10157

Mycgr3G39931\_Mycgr3T

Mycgr3G99766 Mycgr3T
  
Location: 10257-11775

Mycgr3G99766\_Mycgr3T

DNase I-like protein
  
Accession: EIM90806
  
Location: 866723-871368
  
 NCBI BlastP on this gene

EIM90806

ABC protein
  
Accession: EIM90805
  
Location: 858520-865547
  
  
**BlastP hit with Mycgr3G41235\_Mycgr3T**
  
Percentage identity: 36 %
  
BlastP bit score: 862
  
Sequence coverage: 103 %
  
E-value: 0.0
  
  
 NCBI BlastP on this gene

EIM90805

MFS general substrate transporter
  
Accession: EIM90804
  
Location: 855084-857770
  
 NCBI BlastP on this gene

EIM90804

Query: Architecture Search FASTA input

JH971391 : Agaricus bisporus var. burnettii JB137-S8 unplaced genomic scaffold AGABI1scaffold\_7    Total score: 1.0     Cumulative Blast bit score: 852

Hit cluster cross-links:

Mycgr3G41235 Mycgr3T
  
Location: 0-4062

Mycgr3G41235\_Mycgr3T

Mycgr3G70577 Mycgr3T
  
Location: 4162-6109

Mycgr3G70577\_Mycgr3T

Mycgr3G40534 Mycgr3T
  
Location: 6209-7166

Mycgr3G40534\_Mycgr3T

Mycgr3G85486 Mycgr3T
  
Location: 7266-8511

Mycgr3G85486\_Mycgr3T

Mycgr3G92221 Mycgr3T
  
Location: 8611-9193

Mycgr3G92221\_Mycgr3T

Mycgr3G39931 Mycgr3T
  
Location: 9293-10157

Mycgr3G39931\_Mycgr3T

Mycgr3G99766 Mycgr3T
  
Location: 10257-11775

Mycgr3G99766\_Mycgr3T

hypothetical protein
  
Accession: EKM78593
  
Location: 66370-68566
  
 NCBI BlastP on this gene

EKM78593

hypothetical protein
  
Accession: EKM78594
  
Location: 69806-72003
  
 NCBI BlastP on this gene

EKM78594

hypothetical protein
  
Accession: EKM78595
  
Location: 72435-78567
  
  
**BlastP hit with Mycgr3G41235\_Mycgr3T**
  
Percentage identity: 36 %
  
BlastP bit score: 852
  
Sequence coverage: 103 %
  
E-value: 0.0
  
  
 NCBI BlastP on this gene

EKM78595

hypothetical protein
  
Accession: EKM78596
  
Location: 79142-80338
  
 NCBI BlastP on this gene

EKM78596

hypothetical protein
  
Accession: EKM78597
  
Location: 80563-81304
  
 NCBI BlastP on this gene

EKM78597

hypothetical protein
  
Accession: EKM78598
  
Location: 81580-82141
  
 NCBI BlastP on this gene

EKM78598

hypothetical protein
  
Accession: EKM78599
  
Location: 82410-84673
  
 NCBI BlastP on this gene

EKM78599

Query: Architecture Search FASTA input

JH930470 : Phanerochaete carnosa HHB-10118-sp unplaced genomic scaffold PHACAscaffold\_3    Total score: 1.0     Cumulative Blast bit score: 850

Hit cluster cross-links:

Mycgr3G41235 Mycgr3T
  
Location: 0-4062

Mycgr3G41235\_Mycgr3T

Mycgr3G70577 Mycgr3T
  
Location: 4162-6109

Mycgr3G70577\_Mycgr3T

Mycgr3G40534 Mycgr3T
  
Location: 6209-7166

Mycgr3G40534\_Mycgr3T

Mycgr3G85486 Mycgr3T
  
Location: 7266-8511

Mycgr3G85486\_Mycgr3T

Mycgr3G92221 Mycgr3T
  
Location: 8611-9193

Mycgr3G92221\_Mycgr3T

Mycgr3G39931 Mycgr3T
  
Location: 9293-10157

Mycgr3G39931\_Mycgr3T

Mycgr3G99766 Mycgr3T
  
Location: 10257-11775

Mycgr3G99766\_Mycgr3T

hypothetical protein
  
Accession: EKM58258
  
Location: 2640621-2646585
  
  
**BlastP hit with Mycgr3G41235\_Mycgr3T**
  
Percentage identity: 35 %
  
BlastP bit score: 850
  
Sequence coverage: 104 %
  
E-value: 0.0
  
  
 NCBI BlastP on this gene

EKM58258

hypothetical protein
  
Accession: EKM58257
  
Location: 2636763-2640370
  
 NCBI BlastP on this gene

EKM58257

Query: Architecture Search FASTA input

GL377306 : Schizophyllum commune H4-8 unplaced genomic scaffold SCHCOscaffold\_5    Total score: 1.0     Cumulative Blast bit score: 842

Hit cluster cross-links:

Mycgr3G41235 Mycgr3T
  
Location: 0-4062

Mycgr3G41235\_Mycgr3T

Mycgr3G70577 Mycgr3T
  
Location: 4162-6109

Mycgr3G70577\_Mycgr3T

Mycgr3G40534 Mycgr3T
  
Location: 6209-7166

Mycgr3G40534\_Mycgr3T

Mycgr3G85486 Mycgr3T
  
Location: 7266-8511

Mycgr3G85486\_Mycgr3T

Mycgr3G92221 Mycgr3T
  
Location: 8611-9193

Mycgr3G92221\_Mycgr3T

Mycgr3G39931 Mycgr3T
  
Location: 9293-10157

Mycgr3G39931\_Mycgr3T

Mycgr3G99766 Mycgr3T
  
Location: 10257-11775

Mycgr3G99766\_Mycgr3T

hypothetical protein
  
Accession: EFI97157
  
Location: 410626-412689
  
 NCBI BlastP on this gene

EFI97157

hypothetical protein
  
Accession: EFI97158
  
Location: 413061-414031
  
 NCBI BlastP on this gene

EFI97158

hypothetical protein
  
Accession: EFI96728
  
Location: 415385-421230
  
  
**BlastP hit with Mycgr3G41235\_Mycgr3T**
  
Percentage identity: 35 %
  
BlastP bit score: 842
  
Sequence coverage: 103 %
  
E-value: 0.0
  
  
 NCBI BlastP on this gene

EFI96728

hypothetical protein
  
Accession: EFI96729
  
Location: 424304-426075
  
 NCBI BlastP on this gene

EFI96729

expressed protein
  
Accession: EFI97159
  
Location: 426403-427659
  
 NCBI BlastP on this gene

EFI97159

Query: Architecture Search FASTA input

DS027054 : Aspergillus clavatus NRRL 1 1099423829800 genomic scaffold    Total score: 1.0     Cumulative Blast bit score: 824

Hit cluster cross-links:

Mycgr3G41235 Mycgr3T
  
Location: 0-4062

Mycgr3G41235\_Mycgr3T

Mycgr3G70577 Mycgr3T
  
Location: 4162-6109

Mycgr3G70577\_Mycgr3T

Mycgr3G40534 Mycgr3T
  
Location: 6209-7166

Mycgr3G40534\_Mycgr3T

Mycgr3G85486 Mycgr3T
  
Location: 7266-8511

Mycgr3G85486\_Mycgr3T

Mycgr3G92221 Mycgr3T
  
Location: 8611-9193

Mycgr3G92221\_Mycgr3T

Mycgr3G39931 Mycgr3T
  
Location: 9293-10157

Mycgr3G39931\_Mycgr3T

Mycgr3G99766 Mycgr3T
  
Location: 10257-11775

Mycgr3G99766\_Mycgr3T

L-ornithine aminotransferase Car2, putative
  
Accession: EAW10381
  
Location: 1015294-1016798
  
 NCBI BlastP on this gene

EAW10381

hypothetical protein
  
Accession: EAW10382
  
Location: 1017851-1018198
  
 NCBI BlastP on this gene

EAW10382

hypothetical protein
  
Accession: EAW10383
  
Location: 1020376-1020528
  
 NCBI BlastP on this gene

EAW10383

hypothetical protein
  
Accession: EAW10384
  
Location: 1020563-1021018
  
 NCBI BlastP on this gene

EAW10384

ABC multidrug transporter, putative
  
Accession: EAW10385
  
Location: 1022331-1026680
  
  
**BlastP hit with Mycgr3G41235\_Mycgr3T**
  
Percentage identity: 34 %
  
BlastP bit score: 824
  
Sequence coverage: 102 %
  
E-value: 0.0
  
  
 NCBI BlastP on this gene

EAW10385

S-adenosyl-methionine-sterol-C- methyltransferas
  
Accession: EAW10386
  
Location: 1029795-1030994
  
 NCBI BlastP on this gene

EAW10386

conserved hypothetical protein
  
Accession: EAW10387
  
Location: 1032369-1033689
  
 NCBI BlastP on this gene

EAW10387

Query: Architecture Search FASTA input

CP003009 : Thielavia terrestris NRRL 8126 chromosome 1    Total score: 1.0     Cumulative Blast bit score: 824

Hit cluster cross-links:

Mycgr3G41235 Mycgr3T
  
Location: 0-4062

Mycgr3G41235\_Mycgr3T

Mycgr3G70577 Mycgr3T
  
Location: 4162-6109

Mycgr3G70577\_Mycgr3T

Mycgr3G40534 Mycgr3T
  
Location: 6209-7166

Mycgr3G40534\_Mycgr3T

Mycgr3G85486 Mycgr3T
  
Location: 7266-8511

Mycgr3G85486\_Mycgr3T

Mycgr3G92221 Mycgr3T
  
Location: 8611-9193

Mycgr3G92221\_Mycgr3T

Mycgr3G39931 Mycgr3T
  
Location: 9293-10157

Mycgr3G39931\_Mycgr3T

Mycgr3G99766 Mycgr3T
  
Location: 10257-11775

Mycgr3G99766\_Mycgr3T

hypothetical protein
  
Accession: AEO63439
  
Location: 4983123-4984424
  
 NCBI BlastP on this gene

THITE\_2108702

hypothetical protein
  
Accession: AEO63440
  
Location: 4989344-4993964
  
  
**BlastP hit with Mycgr3G41235\_Mycgr3T**
  
Percentage identity: 34 %
  
BlastP bit score: 824
  
Sequence coverage: 102 %
  
E-value: 0.0
  
  
 NCBI BlastP on this gene

THITE\_2108707

hypothetical protein
  
Accession: AEO63441
  
Location: 4995901-4997103
  
 NCBI BlastP on this gene

THITE\_2108709

hypothetical protein
  
Accession: AEO63442
  
Location: 4998320-4999491
  
 NCBI BlastP on this gene

THITE\_121184

Query: Architecture Search FASTA input

CP003003 : Myceliophthora thermophila ATCC 42464 chromosome 2    Total score: 1.0     Cumulative Blast bit score: 824

Hit cluster cross-links:

Mycgr3G41235 Mycgr3T
  
Location: 0-4062

Mycgr3G41235\_Mycgr3T

Mycgr3G70577 Mycgr3T
  
Location: 4162-6109

Mycgr3G70577\_Mycgr3T

Mycgr3G40534 Mycgr3T
  
Location: 6209-7166

Mycgr3G40534\_Mycgr3T

Mycgr3G85486 Mycgr3T
  
Location: 7266-8511

Mycgr3G85486\_Mycgr3T

Mycgr3G92221 Mycgr3T
  
Location: 8611-9193

Mycgr3G92221\_Mycgr3T

Mycgr3G39931 Mycgr3T
  
Location: 9293-10157

Mycgr3G39931\_Mycgr3T

Mycgr3G99766 Mycgr3T
  
Location: 10257-11775

Mycgr3G99766\_Mycgr3T

hypothetical protein
  
Accession: AEO56927
  
Location: 4499546-4500817
  
 NCBI BlastP on this gene

MYCTH\_2314855

hypothetical protein
  
Accession: AEO56928
  
Location: 4504494-4509120
  
  
**BlastP hit with Mycgr3G41235\_Mycgr3T**
  
Percentage identity: 35 %
  
BlastP bit score: 824
  
Sequence coverage: 104 %
  
E-value: 0.0
  
  
 NCBI BlastP on this gene

MYCTH\_2302447

hypothetical protein
  
Accession: AEO56929
  
Location: 4510292-4511524
  
 NCBI BlastP on this gene

MYCTH\_2302452

hypothetical protein
  
Accession: AEO56930
  
Location: 4512947-4514062
  
 NCBI BlastP on this gene

MYCTH\_2059229

Query: Architecture Search FASTA input

CM001232 : Magnaporthe oryzae 70-15 chromosome 2    Total score: 1.0     Cumulative Blast bit score: 822

Hit cluster cross-links:

Mycgr3G41235 Mycgr3T
  
Location: 0-4062

Mycgr3G41235\_Mycgr3T

Mycgr3G70577 Mycgr3T
  
Location: 4162-6109

Mycgr3G70577\_Mycgr3T

Mycgr3G40534 Mycgr3T
  
Location: 6209-7166

Mycgr3G40534\_Mycgr3T

Mycgr3G85486 Mycgr3T
  
Location: 7266-8511

Mycgr3G85486\_Mycgr3T

Mycgr3G92221 Mycgr3T
  
Location: 8611-9193

Mycgr3G92221\_Mycgr3T

Mycgr3G39931 Mycgr3T
  
Location: 9293-10157

Mycgr3G39931\_Mycgr3T

Mycgr3G99766 Mycgr3T
  
Location: 10257-11775

Mycgr3G99766\_Mycgr3T

hypothetical protein
  
Accession: EHA55958
  
Location: 7909827-7912286
  
 NCBI BlastP on this gene

EHA55958

oxidoreductase
  
Accession: EHA55959
  
Location: 7914670-7915933
  
 NCBI BlastP on this gene

EHA55959

multidrug resistance-associated protein 2
  
Accession: EHA55960
  
Location: 7918051-7922632
  
  
**BlastP hit with Mycgr3G41235\_Mycgr3T**
  
Percentage identity: 34 %
  
BlastP bit score: 822
  
Sequence coverage: 104 %
  
E-value: 0.0
  
  
 NCBI BlastP on this gene

EHA55960

hypothetical protein
  
Accession: EHA55961
  
Location: 7923395-7924353
  
 NCBI BlastP on this gene

EHA55961

hypothetical protein
  
Accession: EHA55962
  
Location: 7925421-7927293
  
 NCBI BlastP on this gene

EHA55962

hypothetical protein
  
Accession: EHA55963
  
Location: 7927431-7928000
  
 NCBI BlastP on this gene

EHA55963

abhydrolase domain-containing protein 12
  
Accession: EHA55964
  
Location: 7928702-7929895
  
 NCBI BlastP on this gene

EHA55964

Query: Architecture Search FASTA input

JH793788 : Magnaporthe oryzae Y34 unplaced genomic scaffold Y34\_scaffold00766    Total score: 1.0     Cumulative Blast bit score: 816

Hit cluster cross-links:

Mycgr3G41235 Mycgr3T
  
Location: 0-4062

Mycgr3G41235\_Mycgr3T

Mycgr3G70577 Mycgr3T
  
Location: 4162-6109

Mycgr3G70577\_Mycgr3T

Mycgr3G40534 Mycgr3T
  
Location: 6209-7166

Mycgr3G40534\_Mycgr3T

Mycgr3G85486 Mycgr3T
  
Location: 7266-8511

Mycgr3G85486\_Mycgr3T

Mycgr3G92221 Mycgr3T
  
Location: 8611-9193

Mycgr3G92221\_Mycgr3T

Mycgr3G39931 Mycgr3T
  
Location: 9293-10157

Mycgr3G39931\_Mycgr3T

Mycgr3G99766 Mycgr3T
  
Location: 10257-11775

Mycgr3G99766\_Mycgr3T

multidrug resistance-associated protein 2
  
Accession: ELQ34450
  
Location: 88833-93414
  
  
**BlastP hit with Mycgr3G41235\_Mycgr3T**
  
Percentage identity: 33 %
  
BlastP bit score: 816
  
Sequence coverage: 104 %
  
E-value: 0.0
  
  
 NCBI BlastP on this gene

ELQ34450

hypothetical protein
  
Accession: ELQ34449
  
Location: 87112-88070
  
 NCBI BlastP on this gene

ELQ34449

hypothetical protein
  
Accession: ELQ34448
  
Location: 83434-86044
  
 NCBI BlastP on this gene

ELQ34448

abhydrolase domain-containing protein 12
  
Accession: ELQ34447
  
Location: 81570-82763
  
 NCBI BlastP on this gene

ELQ34447

Query: Architecture Search FASTA input

GL891302 : Neurospora tetrasperma FGSC 2508 unplaced genomic scaffold NEUTE1scaffold\_1    Total score: 1.0     Cumulative Blast bit score: 816

Hit cluster cross-links:

Mycgr3G41235 Mycgr3T
  
Location: 0-4062

Mycgr3G41235\_Mycgr3T

Mycgr3G70577 Mycgr3T
  
Location: 4162-6109

Mycgr3G70577\_Mycgr3T

Mycgr3G40534 Mycgr3T
  
Location: 6209-7166

Mycgr3G40534\_Mycgr3T

Mycgr3G85486 Mycgr3T
  
Location: 7266-8511

Mycgr3G85486\_Mycgr3T

Mycgr3G92221 Mycgr3T
  
Location: 8611-9193

Mycgr3G92221\_Mycgr3T

Mycgr3G39931 Mycgr3T
  
Location: 9293-10157

Mycgr3G39931\_Mycgr3T

Mycgr3G99766 Mycgr3T
  
Location: 10257-11775

Mycgr3G99766\_Mycgr3T

hypothetical protein
  
Accession: EGO60392
  
Location: 1207498-1208454
  
 NCBI BlastP on this gene

EGO60392

hypothetical protein
  
Accession: EGO60393
  
Location: 1212949-1217476
  
  
**BlastP hit with Mycgr3G41235\_Mycgr3T**
  
Percentage identity: 33 %
  
BlastP bit score: 816
  
Sequence coverage: 104 %
  
E-value: 0.0
  
  
 NCBI BlastP on this gene

EGO60393

hypothetical protein
  
Accession: EGO60394
  
Location: 1219616-1220575
  
 NCBI BlastP on this gene

EGO60394

hypothetical protein
  
Accession: EGO60395
  
Location: 1222472-1223200
  
 NCBI BlastP on this gene

EGO60395

Query: Architecture Search FASTA input

51. :  GL698712 Metarhizium anisopliae ARSEF 23 unplaced genomic scaffold Scf\_002     Total score: 2.0     Cumulative Blast bit score: 277

Mycgr3G41235 Mycgr3T
  
Location: 0-4062
  
 NCBI BlastP on this gene

Mycgr3G41235\_Mycgr3T

Mycgr3G70577 Mycgr3T
  
Location: 4162-6109
  
 NCBI BlastP on this gene

Mycgr3G70577\_Mycgr3T

Mycgr3G40534 Mycgr3T
  
Location: 6209-7166
  
 NCBI BlastP on this gene

Mycgr3G40534\_Mycgr3T

Mycgr3G85486 Mycgr3T
  
Location: 7266-8511
  
 NCBI BlastP on this gene

Mycgr3G85486\_Mycgr3T

Mycgr3G92221 Mycgr3T
  
Location: 8611-9193
  
 NCBI BlastP on this gene

Mycgr3G92221\_Mycgr3T

Mycgr3G39931 Mycgr3T
  
Location: 9293-10157
  
 NCBI BlastP on this gene

Mycgr3G39931\_Mycgr3T

Mycgr3G99766 Mycgr3T
  
Location: 10257-11775
  
 NCBI BlastP on this gene

Mycgr3G99766\_Mycgr3T

peptide synthetase
  
Accession: EFZ02308
  
Location: 1073222-1087875
  
  
**BlastP hit with Mycgr3G40534\_Mycgr3T**
  
Percentage identity: 37 %
  
BlastP bit score: 156
  
Sequence coverage: 87 %
  
E-value: 3e-38
  
  
  
**BlastP hit with Mycgr3G39931\_Mycgr3T**
  
Percentage identity: 33 %
  
BlastP bit score: 121
  
Sequence coverage: 89 %
  
E-value: 6e-27
  
  
 NCBI BlastP on this gene

EFZ02308

DUF1479 domain-containing protein
  
Accession: EFZ02307
  
Location: 1071342-1073069
  
 NCBI BlastP on this gene

EFZ02307

choline oxidase (CodA)
  
Accession: EFZ02306
  
Location: 1068612-1070246
  
 NCBI BlastP on this gene

EFZ02306

betaine aldehyde dehydrogenase
  
Accession: EFZ02305
  
Location: 1063846-1065375
  
 NCBI BlastP on this gene

EFZ02305

glyoxalase/bleomycin resistance
  
Accession: EFZ02304
  
Location: 1062981-1063379
  
 NCBI BlastP on this gene

EFZ02304

hypothetical protein
  
Accession: EFZ02303
  
Location: 1060399-1062335
  
 NCBI BlastP on this gene

EFZ02303

hypothetical protein
  
Accession: EFZ02302
  
Location: 1057191-1058582
  
 NCBI BlastP on this gene

EFZ02302

52. :  FQ790277 Botryotinia fuckeliana T4 SuperContig\_379\_1 genomic supercontig.     Total score: 2.0     Cumulative Blast bit score: 273

hypothetical protein
  
Accession: CCD45288
  
Location: 20035-21345
  
 NCBI BlastP on this gene

BofuT4\_P119940.1

hypothetical protein
  
Accession: CCD45289
  
Location: 21757-22388
  
 NCBI BlastP on this gene

BofuT4\_P119950.1

BcYVC1, Ca2+ channel protein, partial sequence
  
Accession: CCD45290
  
Location: 22451-23806
  
 NCBI BlastP on this gene

BofuT4\_P119960.1

BcYVC1, Ca2+ channel protein, partial sequence
  
Accession: CCD45291
  
Location: 24490-24945
  
 NCBI BlastP on this gene

BofuT4\_P119970.1

similar to l-ornithine 5-monooxygenase
  
Accession: CCD45292
  
Location: 28830-30466
  
 NCBI BlastP on this gene

BofuT4\_P119980.1

predicted protein
  
Accession: CCD45293
  
Location: 31845-32284
  
 NCBI BlastP on this gene

BofuT4\_uP119990.1

similar to ABC transporter
  
Accession: CCD45294
  
Location: 33266-37575
  
 NCBI BlastP on this gene

BofuT4\_P120000.1

BcNRPS2, nonribosomal peptide synthetase, partial sequence
  
Accession: CCD45295
  
Location: 39844-43181
  
  
**BlastP hit with Mycgr3G40534\_Mycgr3T**
  
Percentage identity: 37 %
  
BlastP bit score: 153
  
Sequence coverage: 81 %
  
E-value: 1e-37
  
  
 NCBI BlastP on this gene

BofuT4\_P120010.1

BcNRPS2, nonribosomal peptide synthetase, partial sequence
  
Accession: CCD45296
  
Location: 44232-52768
  
  
**BlastP hit with Mycgr3G39931\_Mycgr3T**
  
Percentage identity: 31 %
  
BlastP bit score: 120
  
Sequence coverage: 99 %
  
E-value: 1e-26
  
  
 NCBI BlastP on this gene

BofuT4\_P120020.1

hypothetical protein
  
Accession: CCD45297
  
Location: 53142-55183
  
 NCBI BlastP on this gene

BofuT4\_P120030.1

similar to C2HC5 finger protein
  
Accession: CCD45298
  
Location: 56018-57694
  
 NCBI BlastP on this gene

BofuT4\_P120040.1

similar to TPR domain-containing protein
  
Accession: CCD45299
  
Location: 57994-60876
  
 NCBI BlastP on this gene

BofuT4\_P120050.1

similar to GYF domain-containing protein
  
Accession: CCD45300
  
Location: 61369-66203
  
 NCBI BlastP on this gene

BofuT4\_P120060.1

hypothetical protein
  
Accession: CCD45301
  
Location: 68218-69471
  
 NCBI BlastP on this gene

BofuT4\_P120070.1

hypothetical protein
  
Accession: CCD45302
  
Location: 71031-71903
  
 NCBI BlastP on this gene

BofuT4\_P120080.1

53. :  CP003002 Myceliophthora thermophila ATCC 42464 chromosome 1     Total score: 2.0     Cumulative Blast bit score: 273

non-ribosomal peptide synthetase
  
Accession: AEO55755
  
Location: 10846889-10862191
  
  
**BlastP hit with Mycgr3G40534\_Mycgr3T**
  
Percentage identity: 36 %
  
BlastP bit score: 154
  
Sequence coverage: 99 %
  
E-value: 1e-37
  
  
  
**BlastP hit with Mycgr3G39931\_Mycgr3T**
  
Percentage identity: 30 %
  
BlastP bit score: 119
  
Sequence coverage: 88 %
  
E-value: 3e-26
  
  
 NCBI BlastP on this gene

MYCTH\_97310

hypothetical protein
  
Accession: AEO55754
  
Location: 10845713-10846607
  
 NCBI BlastP on this gene

MYCTH\_2314374

hypothetical protein
  
Accession: AEO55753
  
Location: 10842451-10844658
  
 NCBI BlastP on this gene

MYCTH\_2299891

hypothetical protein
  
Accession: AEO55752
  
Location: 10840549-10840929
  
 NCBI BlastP on this gene

MYCTH\_2299888

hypothetical protein
  
Accession: AEO55751
  
Location: 10839172-10840329
  
 NCBI BlastP on this gene

MYCTH\_2299887

hypothetical protein
  
Accession: AEO55750
  
Location: 10836047-10837201
  
 NCBI BlastP on this gene

MYCTH\_2299886

glycoside hydrolase family 16 protein
  
Accession: AEO55749
  
Location: 10834002-10835234
  
 NCBI BlastP on this gene

MYCTH\_37570

hypothetical protein
  
Accession: AEO55748
  
Location: 10830620-10832556
  
 NCBI BlastP on this gene

MYCTH\_2299883

hypothetical protein
  
Accession: AEO55747
  
Location: 10827393-10827764
  
 NCBI BlastP on this gene

MYCTH\_2299879

54. :  CM001197 Mycosphaerella graminicola IPO323 chromosome 2     Total score: 2.0     Cumulative Blast bit score: 273

hypothetical protein
  
Accession: EGP90758
  
Location: 420584-422080
  
 NCBI BlastP on this gene

EGP90758

hypothetical protein
  
Accession: EGP89714
  
Location: 422453-423453
  
 NCBI BlastP on this gene

EGP89714

hypothetical protein
  
Accession: EGP90757
  
Location: 423720-424702
  
 NCBI BlastP on this gene

EGP90757

hypothetical protein
  
Accession: EGP90756
  
Location: 427564-428871
  
 NCBI BlastP on this gene

EGP90756

hypothetical protein
  
Accession: EGP89715
  
Location: 429154-431323
  
 NCBI BlastP on this gene

EGP89715

putative L-ornithine 5-monooxygenase
  
Accession: EGP90755
  
Location: 431119-432712
  
 NCBI BlastP on this gene

EGP90755

putative ABC transporter
  
Accession: EGP90754
  
Location: 433742-437840
  
 NCBI BlastP on this gene

EGP90754

putative Non-ribosomal peptide synthetase
  
Accession: EGP89716
  
Location: 439283-453243
  
  
**BlastP hit with Mycgr3G40534\_Mycgr3T**
  
Percentage identity: 37 %
  
BlastP bit score: 139
  
Sequence coverage: 82 %
  
E-value: 2e-32
  
  
  
**BlastP hit with Mycgr3G39931\_Mycgr3T**
  
Percentage identity: 35 %
  
BlastP bit score: 134
  
Sequence coverage: 90 %
  
E-value: 2e-31
  
  
 NCBI BlastP on this gene

EGP89716

hypothetical protein
  
Accession: EGP90753
  
Location: 453825-455477
  
 NCBI BlastP on this gene

EGP90753

hypothetical protein
  
Accession: EGP89717
  
Location: 455916-456450
  
 NCBI BlastP on this gene

EGP89717

hypothetical protein
  
Accession: EGP90752
  
Location: 456769-458519
  
 NCBI BlastP on this gene

EGP90752

hypothetical protein
  
Accession: EGP90751
  
Location: 459143-460416
  
 NCBI BlastP on this gene

EGP90751

TOR1 phosphatidylinositol 3-kinase
  
Accession: EGP90750
  
Location: 461149-468432
  
 NCBI BlastP on this gene

EGP90750

hypothetical protein
  
Accession: EGP89718
  
Location: 468869-471319
  
 NCBI BlastP on this gene

EGP89718

hypothetical protein
  
Accession: EGP89719
  
Location: 471658-471819
  
 NCBI BlastP on this gene

EGP89719

hypothetical protein
  
Accession: EGP90749
  
Location: 472679-473598
  
 NCBI BlastP on this gene

EGP90749

55. :  GL988041 Chaetomium thermophilum var. thermophilum DSM 1495 unplaced genomic scaffold scf7180000...     Total score: 2.0     Cumulative Blast bit score: 272

hypothetical protein
  
Accession: EGS20297
  
Location: 38382-40308
  
 NCBI BlastP on this gene

EGS20297

putative malic acid transport protein
  
Accession: EGS20296
  
Location: 36137-37615
  
 NCBI BlastP on this gene

EGS20296

hypothetical protein
  
Accession: EGS20295
  
Location: 34043-35937
  
 NCBI BlastP on this gene

EGS20295

hypothetical protein
  
Accession: EGS20294
  
Location: 31475-33265
  
 NCBI BlastP on this gene

EGS20294

hypothetical protein
  
Accession: EGS20293
  
Location: 27302-28434
  
 NCBI BlastP on this gene

EGS20293

putative L-ornithine protein
  
Accession: EGS20292
  
Location: 22656-24251
  
 NCBI BlastP on this gene

EGS20292

nonribosomal peptide synthase-like protein
  
Accession: EGS20291
  
Location: 6067-19404
  
  
**BlastP hit with Mycgr3G40534\_Mycgr3T**
  
Percentage identity: 36 %
  
BlastP bit score: 157
  
Sequence coverage: 106 %
  
E-value: 8e-39
  
  
  
**BlastP hit with Mycgr3G39931\_Mycgr3T**
  
Percentage identity: 33 %
  
BlastP bit score: 115
  
Sequence coverage: 92 %
  
E-value: 1e-24
  
  
 NCBI BlastP on this gene

EGS20291

hypothetical protein
  
Accession: EGS20290
  
Location: 1255-3822
  
 NCBI BlastP on this gene

EGS20290

56. :  ABDF02000001 Trichoderma virens Gv29-8     Total score: 2.0     Cumulative Blast bit score: 272

hypothetical protein
  
Accession: EHK26831
  
Location: 338219-339191
  
 NCBI BlastP on this gene

EHK26831

hypothetical protein
  
Accession: EHK26832
  
Location: 339544-340684
  
 NCBI BlastP on this gene

EHK26832

hypothetical protein
  
Accession: EHK26833
  
Location: 341198-342101
  
 NCBI BlastP on this gene

EHK26833

hypothetical protein
  
Accession: EHK26834
  
Location: 342553-343845
  
 NCBI BlastP on this gene

EHK26834

hypothetical protein
  
Accession: EHK26835
  
Location: 344326-346017
  
 NCBI BlastP on this gene

EHK26835

hypothetical protein
  
Accession: EHK26836
  
Location: 346122-347736
  
 NCBI BlastP on this gene

EHK26836

hypothetical protein
  
Accession: EHK26837
  
Location: 348434-350695
  
 NCBI BlastP on this gene

EHK26837

hypothetical protein
  
Accession: EHK26838
  
Location: 353593-355146
  
 NCBI BlastP on this gene

EHK26838

putative non-ribosomal peptide synthetase
  
Accession: EHK26839
  
Location: 357960-372711
  
  
**BlastP hit with Mycgr3G40534\_Mycgr3T**
  
Percentage identity: 38 %
  
BlastP bit score: 150
  
Sequence coverage: 81 %
  
E-value: 2e-36
  
  
  
**BlastP hit with Mycgr3G39931\_Mycgr3T**
  
Percentage identity: 33 %
  
BlastP bit score: 122
  
Sequence coverage: 90 %
  
E-value: 3e-27
  
  
 NCBI BlastP on this gene

EHK26839

hypothetical protein
  
Accession: EHK26840
  
Location: 373092-374723
  
 NCBI BlastP on this gene

EHK26840

hypothetical protein
  
Accession: EHK26841
  
Location: 376878-378404
  
 NCBI BlastP on this gene

EHK26841

hypothetical protein
  
Accession: EHK26842
  
Location: 379817-381097
  
 NCBI BlastP on this gene

EHK26842

hypothetical protein
  
Accession: EHK26843
  
Location: 383409-385697
  
 NCBI BlastP on this gene

EHK26843

hypothetical protein
  
Accession: EHK26844
  
Location: 386262-388125
  
 NCBI BlastP on this gene

EHK26844

hypothetical protein
  
Accession: EHK26845
  
Location: 388877-390034
  
 NCBI BlastP on this gene

EHK26845

hypothetical protein
  
Accession: EHK26846
  
Location: 392184-393857
  
 NCBI BlastP on this gene

EHK26846

57. :  DS027050 Aspergillus clavatus NRRL 1 1099423829796 genomic scaffold     Total score: 2.0     Cumulative Blast bit score: 270

FAD binding monooxygenase, putative
  
Accession: EAW12155
  
Location: 225326-226882
  
 NCBI BlastP on this gene

EAW12155

MFS monocarboxylate transporter, putative
  
Accession: EAW12156
  
Location: 227609-229056
  
 NCBI BlastP on this gene

EAW12156

mitochondrial enoyl reductase, putative
  
Accession: EAW12157
  
Location: 229852-230931
  
 NCBI BlastP on this gene

EAW12157

nonribosomal peptide synthase SidE
  
Accession: EAW12158
  
Location: 232515-238862
  
  
**BlastP hit with Mycgr3G40534\_Mycgr3T**
  
Percentage identity: 40 %
  
BlastP bit score: 150
  
Sequence coverage: 79 %
  
E-value: 1e-36
  
  
  
**BlastP hit with Mycgr3G39931\_Mycgr3T**
  
Percentage identity: 32 %
  
BlastP bit score: 120
  
Sequence coverage: 88 %
  
E-value: 2e-26
  
  
 NCBI BlastP on this gene

EAW12158

58. :  CU633895 Podospora anserina S mat+ genomic DNA chromosome 4, supercontig 4.     Total score: 2.0     Cumulative Blast bit score: 266

not annotated
  
Accession: CAP67082
  
Location: 2318020-2333277
  
  
**BlastP hit with Mycgr3G40534\_Mycgr3T**
  
Percentage identity: 41 %
  
BlastP bit score: 157
  
Sequence coverage: 81 %
  
E-value: 1e-38
  
  
  
**BlastP hit with Mycgr3G39931\_Mycgr3T**
  
Percentage identity: 26 %
  
BlastP bit score: 109
  
Sequence coverage: 88 %
  
E-value: 7e-23
  
  
 NCBI BlastP on this gene

CAP67082

not annotated
  
Accession: CAP67081
  
Location: 2315657-2317633
  
 NCBI BlastP on this gene

CAP67081

not annotated
  
Accession: CAP67080
  
Location: 2311069-2313355
  
 NCBI BlastP on this gene

CAP67080

not annotated
  
Accession: CAP67079
  
Location: 2307353-2309408
  
 NCBI BlastP on this gene

CAP67079

not annotated
  
Accession: CAP67078
  
Location: 2305717-2306427
  
 NCBI BlastP on this gene

CAP67078

not annotated
  
Accession: CAP67077
  
Location: 2304163-2305650
  
 NCBI BlastP on this gene

CAP67077

59. :  CH476662 Ajellomyces capsulatus NAm1 scaffold\_8 genomic scaffold     Total score: 2.0     Cumulative Blast bit score: 265

cystathionine beta-synthase
  
Accession: EDN10973
  
Location: 1698736-1700617
  
 NCBI BlastP on this gene

EDN10973

predicted protein
  
Accession: EDN10972
  
Location: 1697128-1698216
  
 NCBI BlastP on this gene

EDN10972

conserved hypothetical protein
  
Accession: EDN10971
  
Location: 1694127-1695343
  
 NCBI BlastP on this gene

EDN10971

predicted protein
  
Accession: EDN10970
  
Location: 1691918-1693695
  
 NCBI BlastP on this gene

EDN10970

palmitoyltransferase akr1
  
Accession: EDN10969
  
Location: 1686872-1690490
  
 NCBI BlastP on this gene

EDN10969

predicted protein
  
Accession: EDN10968
  
Location: 1682681-1684794
  
  
**BlastP hit with Mycgr3G40534\_Mycgr3T**
  
Percentage identity: 42 %
  
BlastP bit score: 147
  
Sequence coverage: 78 %
  
E-value: 2e-36
  
  
 NCBI BlastP on this gene

EDN10968

predicted protein
  
Accession: EDN10967
  
Location: 1673064-1679253
  
  
**BlastP hit with Mycgr3G39931\_Mycgr3T**
  
Percentage identity: 31 %
  
BlastP bit score: 118
  
Sequence coverage: 96 %
  
E-value: 8e-26
  
  
 NCBI BlastP on this gene

EDN10967

predicted protein
  
Accession: EDN10966
  
Location: 1664219-1665375
  
 NCBI BlastP on this gene

EDN10966

hypothetical protein
  
Accession: EDN10965
  
Location: 1659484-1662064
  
 NCBI BlastP on this gene

EDN10965

conserved hypothetical protein
  
Accession: EDN10964
  
Location: 1657707-1658090
  
 NCBI BlastP on this gene

EDN10964

60. :  CH408034 Chaetomium globosum CBS 148.51 scaffold\_6 genomic scaffold     Total score: 2.0     Cumulative Blast bit score: 255

hypothetical protein
  
Accession: EAQ85524
  
Location: 3746389-3750923
  
 NCBI BlastP on this gene

EAQ85524

predicted protein
  
Accession: EAQ85525
  
Location: 3754106-3754370
  
 NCBI BlastP on this gene

EAQ85525

hypothetical protein
  
Accession: EAQ85526
  
Location: 3756346-3757719
  
 NCBI BlastP on this gene

EAQ85526

predicted protein
  
Accession: EAQ85527
  
Location: 3758907-3759726
  
 NCBI BlastP on this gene

EAQ85527

hypothetical protein
  
Accession: EAQ85528
  
Location: 3762503-3764800
  
 NCBI BlastP on this gene

EAQ85528

hypothetical protein
  
Accession: EAQ85529
  
Location: 3766215-3781488
  
  
**BlastP hit with Mycgr3G40534\_Mycgr3T**
  
Percentage identity: 35 %
  
BlastP bit score: 145
  
Sequence coverage: 102 %
  
E-value: 1e-34
  
  
  
**BlastP hit with Mycgr3G39931\_Mycgr3T**
  
Percentage identity: 33 %
  
BlastP bit score: 110
  
Sequence coverage: 89 %
  
E-value: 4e-23
  
  
 NCBI BlastP on this gene

EAQ85529

61. :  DS989822 Arthroderma gypseum CBS 118893 supercont1.1 genomic scaffold     Total score: 2.0     Cumulative Blast bit score: 254

hypothetical protein
  
Accession: EFQ97055
  
Location: 286345-287595
  
 NCBI BlastP on this gene

EFQ97055

hypothetical protein
  
Accession: EFQ97054
  
Location: 282021-284604
  
 NCBI BlastP on this gene

EFQ97054

hypothetical protein
  
Accession: EFQ97053
  
Location: 280997-281586
  
 NCBI BlastP on this gene

EFQ97053

canalicular multispecific organic anion transporter 1
  
Accession: EFQ97052
  
Location: 275201-280623
  
 NCBI BlastP on this gene

EFQ97052

hypothetical protein
  
Accession: EFQ97051
  
Location: 272653-273331
  
 NCBI BlastP on this gene

EFQ97051

hypothetical protein
  
Accession: EFQ97050
  
Location: 244064-268485
  
  
**BlastP hit with Mycgr3G40534\_Mycgr3T**
  
Percentage identity: 33 %
  
BlastP bit score: 135
  
Sequence coverage: 89 %
  
E-value: 2e-31
  
  
  
**BlastP hit with Mycgr3G39931\_Mycgr3T**
  
Percentage identity: 29 %
  
BlastP bit score: 119
  
Sequence coverage: 92 %
  
E-value: 6e-26
  
  
 NCBI BlastP on this gene

EFQ97050

hypothetical protein
  
Accession: EFQ97049
  
Location: 242865-243245
  
 NCBI BlastP on this gene

EFQ97049

GMP synthase
  
Accession: EFQ97048
  
Location: 240940-242635
  
 NCBI BlastP on this gene

EFQ97048

bcp1
  
Accession: EFQ97047
  
Location: 239570-240560
  
 NCBI BlastP on this gene

EFQ97047

chorismate mutase
  
Accession: EFQ97046
  
Location: 238404-239327
  
 NCBI BlastP on this gene

EFQ97046

mitochondrial division protein 1
  
Accession: EFQ97045
  
Location: 234959-237303
  
 NCBI BlastP on this gene

EFQ97045

hypothetical protein
  
Accession: EFQ97044
  
Location: 231674-233735
  
 NCBI BlastP on this gene

EFQ97044

kinesin family protein
  
Accession: EFQ97043
  
Location: 224435-230101
  
 NCBI BlastP on this gene

EFQ97043

62. :  HF679023 Fusarium fujikuroi IMI 58289 draft genome, chromosome FFUJ\_chr01.     Total score: 2.0     Cumulative Blast bit score: 245

uncharacterized protein
  
Accession: CCT61507
  
Location: 209364-210781
  
 NCBI BlastP on this gene

FFUJ\_02014

uncharacterized protein
  
Accession: CCT61506
  
Location: 206807-208701
  
 NCBI BlastP on this gene

FFUJ\_02015

related to Melibiase subfamily
  
Accession: CCT61505
  
Location: 203742-205883
  
 NCBI BlastP on this gene

FFUJ\_02016

probable alpha-glucoside transport protein
  
Accession: CCT61504
  
Location: 201393-202938
  
 NCBI BlastP on this gene

FFUJ\_02017

related to transcription factor ZMS1
  
Accession: CCT61503
  
Location: 198315-200952
  
 NCBI BlastP on this gene

FFUJ\_02018

aurofusarin/rubrofusarin efflux pump AFLT
  
Accession: CCT61502
  
Location: 194395-196233
  
 NCBI BlastP on this gene

FFUJ\_02019

related to 2,4-dienoyl-CoA reductase precursor
  
Accession: CCT61501
  
Location: 192618-193727
  
 NCBI BlastP on this gene

FFUJ\_02020

uncharacterized protein
  
Accession: CCT61500
  
Location: 190995-191690
  
 NCBI BlastP on this gene

FFUJ\_02021

non-ribosomal peptide synthetase
  
Accession: CCT61499
  
Location: 181990-189795
  
  
**BlastP hit with Mycgr3G40534\_Mycgr3T**
  
Percentage identity: 37 %
  
BlastP bit score: 137
  
Sequence coverage: 80 %
  
E-value: 7e-32
  
  
  
**BlastP hit with Mycgr3G39931\_Mycgr3T**
  
Percentage identity: 30 %
  
BlastP bit score: 108
  
Sequence coverage: 92 %
  
E-value: 2e-22
  
  
 NCBI BlastP on this gene

FFUJ\_02022

uncharacterized protein
  
Accession: CCT61498
  
Location: 180264-181881
  
 NCBI BlastP on this gene

FFUJ\_02023

uncharacterized protein
  
Accession: CCT61497
  
Location: 178451-179516
  
 NCBI BlastP on this gene

FFUJ\_02024

related to xylan 1,4-beta-xylosidase
  
Accession: CCT61496
  
Location: 176018-178366
  
 NCBI BlastP on this gene

FFUJ\_02025

uncharacterized protein
  
Accession: CCT61495
  
Location: 171874-172698
  
 NCBI BlastP on this gene

FFUJ\_02026

related to ARO80-positive transcription regulator of ARO9 and ARO10
  
Accession: CCT61494
  
Location: 168881-171459
  
 NCBI BlastP on this gene

FFUJ\_02027

related to nicotinamide mononucleotide permease
  
Accession: CCT61493
  
Location: 166742-168418
  
 NCBI BlastP on this gene

FFUJ\_02028

related to carboxyphosphonoenolpyruvate phosphonomutase
  
Accession: CCT61492
  
Location: 165067-166230
  
 NCBI BlastP on this gene

FFUJ\_02029

uncharacterized protein
  
Accession: CCT61491
  
Location: 163844-164922
  
 NCBI BlastP on this gene

FFUJ\_02030

related to 2`-hydroxyisoflavone reductase
  
Accession: CCT61490
  
Location: 162224-163457
  
 NCBI BlastP on this gene

FFUJ\_02031

63. :  DS499596 Aspergillus fumigatus A1163 scf\_000003 genomic scaffold     Total score: 2.0     Cumulative Blast bit score: 245

periplasmic nitrate reductase, putative
  
Accession: EDP52239
  
Location: 70530-73321
  
 NCBI BlastP on this gene

EDP52239

conserved hypothetical protein
  
Accession: EDP52238
  
Location: 65177-66810
  
 NCBI BlastP on this gene

EDP52238

endo-1,4-beta-xylanase, putative
  
Accession: EDP52237
  
Location: 62942-64085
  
 NCBI BlastP on this gene

EDP52237

conserved hypothetical protein
  
Accession: EDP52236
  
Location: 59383-60624
  
 NCBI BlastP on this gene

EDP52236

general amidase, putative
  
Accession: EDP52235
  
Location: 57210-59063
  
 NCBI BlastP on this gene

EDP52235

conserved hypothetical protein
  
Accession: EDP52234
  
Location: 56236-56922
  
 NCBI BlastP on this gene

EDP52234

MFS drug efflux transporter, putative
  
Accession: EDP52233
  
Location: 53918-55618
  
 NCBI BlastP on this gene

EDP52233

hypothetical protein
  
Accession: EDP52232
  
Location: 53085-53597
  
 NCBI BlastP on this gene

EDP52232

nonribosomal peptide synthase, putative
  
Accession: EDP52231
  
Location: 45768-52883
  
  
**BlastP hit with Mycgr3G40534\_Mycgr3T**
  
Percentage identity: 37 %
  
BlastP bit score: 133
  
Sequence coverage: 81 %
  
E-value: 1e-30
  
  
  
**BlastP hit with Mycgr3G39931\_Mycgr3T**
  
Percentage identity: 30 %
  
BlastP bit score: 112
  
Sequence coverage: 92 %
  
E-value: 8e-24
  
  
 NCBI BlastP on this gene

EDP52231

methyltransferase, putative
  
Accession: EDP52230
  
Location: 44255-45315
  
 NCBI BlastP on this gene

EDP52230

C6 transcription factor, putative
  
Accession: EDP52229
  
Location: 39937-42165
  
 NCBI BlastP on this gene

EDP52229

amine transporter, putative
  
Accession: EDP52228
  
Location: 35079-35906
  
 NCBI BlastP on this gene

EDP52228

PKS-like enzyme, putative
  
Accession: EDP52227
  
Location: 33190-33949
  
 NCBI BlastP on this gene

EDP52227

hypothetical protein
  
Accession: EDP52226
  
Location: 31734-32093
  
 NCBI BlastP on this gene

EDP52226

hypothetical protein
  
Accession: EDP52225
  
Location: 27735-28916
  
 NCBI BlastP on this gene

EDP52225

64. :  KE145368 Glarea lozoyensis ATCC 20868 chromosome Unknown GLAREA4     Total score: 2.0     Cumulative Blast bit score: 240

Ankyrin repeat-containing protein
  
Accession: EPE28427
  
Location: 571264-572284
  
 NCBI BlastP on this gene

EPE28427

Glutathione synthetase ATP-binding protein
  
Accession: EPE28428
  
Location: 573608-575704
  
 NCBI BlastP on this gene

EPE28428

PLP-dependent transferase
  
Accession: EPE28429
  
Location: 576313-576801
  
 NCBI BlastP on this gene

EPE28429

PLP-dependent transferase
  
Accession: EPE28430
  
Location: 578796-579363
  
 NCBI BlastP on this gene

EPE28430

hypothetical protein
  
Accession: EPE28431
  
Location: 580054-581043
  
 NCBI BlastP on this gene

EPE28431

Clavaminate synthase-like protein
  
Accession: EPE28432
  
Location: 581557-582668
  
 NCBI BlastP on this gene

EPE28432

FAD/NAD(P)-binding protein
  
Accession: EPE28433
  
Location: 583360-584778
  
 NCBI BlastP on this gene

EPE28433

PLP-dependent transferase
  
Accession: EPE28434
  
Location: 585257-586977
  
 NCBI BlastP on this gene

EPE28434

Acetyl-CoA synthetase-like protein
  
Accession: EPE28435
  
Location: 588414-597802
  
  
**BlastP hit with Mycgr3G40534\_Mycgr3T**
  
Percentage identity: 33 %
  
BlastP bit score: 126
  
Sequence coverage: 81 %
  
E-value: 4e-28
  
  
  
**BlastP hit with Mycgr3G39931\_Mycgr3T**
  
Percentage identity: 30 %
  
BlastP bit score: 115
  
Sequence coverage: 90 %
  
E-value: 1e-24
  
  
 NCBI BlastP on this gene

EPE28435

Clavaminate synthase-like protein
  
Accession: EPE28436
  
Location: 598904-599991
  
 NCBI BlastP on this gene

EPE28436

alpha/beta-Hydrolase
  
Accession: EPE28437
  
Location: 601010-602224
  
 NCBI BlastP on this gene

EPE28437

Clavaminate synthase-like protein
  
Accession: EPE28438
  
Location: 603234-604432
  
 NCBI BlastP on this gene

EPE28438

Acetyl-CoA synthetase-like protein
  
Accession: EPE28439
  
Location: 605456-608072
  
 NCBI BlastP on this gene

EPE28439

Acetyl-CoA synthetase-like protein
  
Accession: EPE28440
  
Location: 608150-611569
  
 NCBI BlastP on this gene

EPE28440

alpha/beta-Hydrolase
  
Accession: EPE28441
  
Location: 612330-613430
  
 NCBI BlastP on this gene

EPE28441

MFS general substrate transporter
  
Accession: EPE28442
  
Location: 614165-615961
  
 NCBI BlastP on this gene

EPE28442

65. :  KB933064 Togninia minima UCRPA7 unplaced genomic scaffold PA7\_03\_scaffold\_293     Total score: 2.0     Cumulative Blast bit score: 239

hypothetical protein
  
Accession: EOO00601
  
Location: 147741-180254
  
  
**BlastP hit with Mycgr3G40534\_Mycgr3T**
  
Percentage identity: 36 %
  
BlastP bit score: 133
  
Sequence coverage: 83 %
  
E-value: 2e-30
  
  
 NCBI BlastP on this gene

EOO00601

putative alpha beta hydrolase protein
  
Accession: EOO00594
  
Location: 183798-184880
  
 NCBI BlastP on this gene

EOO00594

hypothetical protein
  
Accession: EOO00604
  
Location: 185818-213011
  
  
**BlastP hit with Mycgr3G39931\_Mycgr3T**
  
Percentage identity: 30 %
  
BlastP bit score: 107
  
Sequence coverage: 91 %
  
E-value: 3e-22
  
  
 NCBI BlastP on this gene

EOO00604

66. :  CH476616 Uncinocarpus reesii 1704 scaffold\_2 genomic scaffold     Total score: 2.0     Cumulative Blast bit score: 239

predicted protein
  
Accession: EEP79831
  
Location: 5054874-5072607
  
  
**BlastP hit with Mycgr3G40534\_Mycgr3T**
  
Percentage identity: 33 %
  
BlastP bit score: 131
  
Sequence coverage: 84 %
  
E-value: 5e-30
  
  
  
**BlastP hit with Mycgr3G39931\_Mycgr3T**
  
Percentage identity: 31 %
  
BlastP bit score: 108
  
Sequence coverage: 94 %
  
E-value: 1e-22
  
  
 NCBI BlastP on this gene

EEP79831

predicted protein
  
Accession: EEP79830
  
Location: 5047933-5054073
  
 NCBI BlastP on this gene

EEP79830

predicted protein
  
Accession: EEP79829
  
Location: 5047129-5047550
  
 NCBI BlastP on this gene

EEP79829

conserved hypothetical protein
  
Accession: EEP79828
  
Location: 5046040-5046509
  
 NCBI BlastP on this gene

EEP79828

chorismate mutase
  
Accession: EEP79827
  
Location: 5044469-5045443
  
 NCBI BlastP on this gene

EEP79827

predicted protein
  
Accession: EEP79826
  
Location: 5041249-5043195
  
 NCBI BlastP on this gene

EEP79826

conserved hypothetical protein
  
Accession: EEP79825
  
Location: 5037062-5039293
  
 NCBI BlastP on this gene

EEP79825

predicted protein
  
Accession: EEP79824
  
Location: 5035588-5036097
  
 NCBI BlastP on this gene

EEP79824

67. :  GL377306 Schizophyllum commune H4-8 unplaced genomic scaffold SCHCOscaffold\_5     Total score: 1.0     Cumulative Blast bit score: 2036

expressed protein
  
Accession: EFI97145
  
Location: 315139-315550
  
 NCBI BlastP on this gene

EFI97145

hypothetical protein
  
Accession: EFI96706
  
Location: 316116-316463
  
 NCBI BlastP on this gene

EFI96706

glycoside hydrolase family 43 protein
  
Accession: EFI96707
  
Location: 318802-319799
  
 NCBI BlastP on this gene

EFI96707

hypothetical protein
  
Accession: EFI97146
  
Location: 320286-324565
  
 NCBI BlastP on this gene

EFI97146

hypothetical protein
  
Accession: EFI96708
  
Location: 325048-325389
  
 NCBI BlastP on this gene

EFI96708

hypothetical protein
  
Accession: EFI96709
  
Location: 328978-330318
  
 NCBI BlastP on this gene

EFI96709

hypothetical protein
  
Accession: EFI97147
  
Location: 330844-335699
  
  
**BlastP hit with Mycgr3G41235\_Mycgr3T**
  
Percentage identity: 40 %
  
BlastP bit score: 1035
  
Sequence coverage: 101 %
  
E-value: 0.0
  
  
 NCBI BlastP on this gene

EFI97147

hypothetical protein
  
Accession: EFI97148
  
Location: 336326-339842
  
 NCBI BlastP on this gene

EFI97148

hypothetical protein
  
Accession: EFI96710
  
Location: 340140-340731
  
 NCBI BlastP on this gene

EFI96710

hypothetical protein
  
Accession: EFI96711
  
Location: 341968-347026
  
  
**BlastP hit with Mycgr3G41235\_Mycgr3T**
  
Percentage identity: 39 %
  
BlastP bit score: 1001
  
Sequence coverage: 103 %
  
E-value: 0.0
  
  
 NCBI BlastP on this gene

EFI96711

hypothetical protein
  
Accession: EFI97149
  
Location: 349631-351695
  
 NCBI BlastP on this gene

EFI97149

hypothetical protein
  
Accession: EFI96713
  
Location: 353041-355161
  
 NCBI BlastP on this gene

EFI96713

hypothetical protein
  
Accession: EFI96714
  
Location: 356166-358283
  
 NCBI BlastP on this gene

EFI96714

hypothetical protein
  
Accession: EFI97150
  
Location: 358679-361365
  
 NCBI BlastP on this gene

EFI97150

alkaline phosphatase
  
Accession: EFI97151
  
Location: 361936-364931
  
 NCBI BlastP on this gene

EFI97151

hypothetical protein
  
Accession: EFI97152
  
Location: 365615-366681
  
 NCBI BlastP on this gene

EFI97152

68. :  CAIF01000223 Wickerhamomyces ciferrii strain NRRL Y-1031 F-60-10     Total score: 1.0     Cumulative Blast bit score: 1642

Inorganic phosphate transport protein PHO88
  
Accession: CCH46036
  
Location: 9520-10092
  
 NCBI BlastP on this gene

CCH46036

putative WD repeat-containing protein
  
Accession: CCH46037
  
Location: 10500-12188
  
 NCBI BlastP on this gene

CCH46037

Metal-activated transcriptional activator protein AMT1
  
Accession: CCH46038
  
Location: 12819-14153
  
 NCBI BlastP on this gene

CCH46038

Ubiquitin-conjugating enzyme E2
  
Accession: CCH46039
  
Location: 14755-15341
  
 NCBI BlastP on this gene

CCH46039

hypothetical protein
  
Accession: CCH46040
  
Location: 15927-19884
  
 NCBI BlastP on this gene

CCH46040

nuclease domain-containing protein
  
Accession: CCH46041
  
Location: 20067-21617
  
 NCBI BlastP on this gene

CCH46041

hypothetical protein
  
Accession: CCH46042
  
Location: 22027-23889
  
 NCBI BlastP on this gene

CCH46042

hypothetical protein
  
Accession: CCH46043
  
Location: 24543-25703
  
 NCBI BlastP on this gene

CCH46043

Y+L amino acid transporter
  
Accession: CCH46044
  
Location: 26084-27574
  
 NCBI BlastP on this gene

CCH46044

Siderophore iron transporter
  
Accession: CCH46045
  
Location: 27746-29680
  
  
**BlastP hit with Mycgr3G70577\_Mycgr3T**
  
Percentage identity: 37 %
  
BlastP bit score: 396
  
Sequence coverage: 98 %
  
E-value: 3e-125
  
  
 NCBI BlastP on this gene

CCH46045

Siderophore iron transporter
  
Accession: CCH46046
  
Location: 31808-33742
  
  
**BlastP hit with Mycgr3G70577\_Mycgr3T**
  
Percentage identity: 37 %
  
BlastP bit score: 426
  
Sequence coverage: 96 %
  
E-value: 7e-137
  
  
 NCBI BlastP on this gene

CCH46046

Siderophore iron transporter
  
Accession: CCH46047
  
Location: 36120-38072
  
  
**BlastP hit with Mycgr3G70577\_Mycgr3T**
  
Percentage identity: 36 %
  
BlastP bit score: 409
  
Sequence coverage: 97 %
  
E-value: 4e-130
  
  
 NCBI BlastP on this gene

CCH46047

Siderophore iron transporter
  
Accession: CCH46048
  
Location: 40043-41986
  
  
**BlastP hit with Mycgr3G70577\_Mycgr3T**
  
Percentage identity: 36 %
  
BlastP bit score: 411
  
Sequence coverage: 97 %
  
E-value: 6e-131
  
  
 NCBI BlastP on this gene

CCH46048

69. :  CP002685 Arabidopsis thaliana chromosome 2     Total score: 1.0     Cumulative Blast bit score: 1316

protein kinase PINOID
  
Accession: AEC09003
  
Location: 14589934-14591557
  
 NCBI BlastP on this gene

PID

uncharacterized protein
  
Accession: AEC09004
  
Location: 14596836-14596904
  
 NCBI BlastP on this gene

AT2G34655

ABC transporter C family member 2
  
Accession: AEC09005
  
Location: 14603267-14612387
  
  
**BlastP hit with Mycgr3G41235\_Mycgr3T**
  
Percentage identity: 31 %
  
BlastP bit score: 658
  
Sequence coverage: 98 %
  
E-value: 0.0
  
  
 NCBI BlastP on this gene

MRP2

ABC transporter C family member 2
  
Accession: AEC09006
  
Location: 14603267-14612387
  
  
**BlastP hit with Mycgr3G41235\_Mycgr3T**
  
Percentage identity: 31 %
  
BlastP bit score: 658
  
Sequence coverage: 98 %
  
E-value: 0.0
  
  
 NCBI BlastP on this gene

MRP2

uncharacterized protein
  
Accession: AEC09008
  
Location: 14612741-14615231
  
 NCBI BlastP on this gene

AT2G34670

uncharacterized protein
  
Accession: AEC09007
  
Location: 14613239-14615231
  
 NCBI BlastP on this gene

AT2G34670

Outer arm dynein light chain 1 protein
  
Accession: AEC09009
  
Location: 14616947-14628300
  
 NCBI BlastP on this gene

AIR9

glycolipid transfer protein
  
Accession: AEC09010
  
Location: 14630425-14631779
  
 NCBI BlastP on this gene

ACD11

70. :  CP002684 Arabidopsis thaliana chromosome 1     Total score: 1.0     Cumulative Blast bit score: 1290

probable pectate lyase 4
  
Accession: AEE31208
  
Location: 10710194-10711664
  
 NCBI BlastP on this gene

AT1G30350

Early-responsive to dehydration stress protein (ERD4)
  
Accession: AEE31209
  
Location: 10715892-10718799
  
 NCBI BlastP on this gene

ERD4

DAD1-like acylhydrolase
  
Accession: AEE31210
  
Location: 10719169-10720758
  
 NCBI BlastP on this gene

AT1G30370

photosystem I reaction center subunit psaK
  
Accession: AEE31211
  
Location: 10722325-10723013
  
 NCBI BlastP on this gene

PSAK

glutathione S-conjugate transporting ATPase
  
Accession: AEE31212
  
Location: 10728139-10737697
  
  
**BlastP hit with Mycgr3G41235\_Mycgr3T**
  
Percentage identity: 31 %
  
BlastP bit score: 645
  
Sequence coverage: 98 %
  
E-value: 0.0
  
  
 NCBI BlastP on this gene

MRP1

glutathione S-conjugate transporting ATPase
  
Accession: AEE31213
  
Location: 10728139-10737697
  
  
**BlastP hit with Mycgr3G41235\_Mycgr3T**
  
Percentage identity: 31 %
  
BlastP bit score: 645
  
Sequence coverage: 98 %
  
E-value: 0.0
  
  
 NCBI BlastP on this gene

MRP1

multidrug resistance-associated protein 13
  
Accession: AEE31214
  
Location: 10739357-10747017
  
 NCBI BlastP on this gene

MRP13

ABC transporter C family member 11
  
Accession: AEE31215
  
Location: 10748816-10756316
  
 NCBI BlastP on this gene

MRP12

71. :  JH930470 Phanerochaete carnosa HHB-10118-sp unplaced genomic scaffold PHACAscaffold\_3     Total score: 1.0     Cumulative Blast bit score: 1058

hypothetical protein
  
Accession: EKM58195
  
Location: 2447875-2449543
  
 NCBI BlastP on this gene

EKM58195

hypothetical protein
  
Accession: EKM58196
  
Location: 2450449-2452254
  
 NCBI BlastP on this gene

EKM58196

hypothetical protein
  
Accession: EKM58197
  
Location: 2452693-2452992
  
 NCBI BlastP on this gene

EKM58197

hypothetical protein
  
Accession: EKM58198
  
Location: 2455232-2455437
  
 NCBI BlastP on this gene

EKM58198

hypothetical protein
  
Accession: EKM58199
  
Location: 2456476-2457592
  
 NCBI BlastP on this gene

EKM58199

hypothetical protein
  
Accession: EKM58200
  
Location: 2459263-2461635
  
 NCBI BlastP on this gene

EKM58200

hypothetical protein
  
Accession: EKM58201
  
Location: 2462166-2463179
  
 NCBI BlastP on this gene

EKM58201

hypothetical protein
  
Accession: EKM58202
  
Location: 2464741-2465983
  
 NCBI BlastP on this gene

EKM58202

hypothetical protein
  
Accession: EKM58203
  
Location: 2467399-2472467
  
  
**BlastP hit with Mycgr3G41235\_Mycgr3T**
  
Percentage identity: 41 %
  
BlastP bit score: 1058
  
Sequence coverage: 100 %
  
E-value: 0.0
  
  
 NCBI BlastP on this gene

EKM58203

hypothetical protein
  
Accession: EKM58204
  
Location: 2475912-2476943
  
 NCBI BlastP on this gene

EKM58204

hypothetical protein
  
Accession: EKM58205
  
Location: 2477705-2480581
  
 NCBI BlastP on this gene

EKM58205

hypothetical protein
  
Accession: EKM58206
  
Location: 2481316-2482024
  
 NCBI BlastP on this gene

EKM58206

hypothetical protein
  
Accession: EKM58207
  
Location: 2486486-2488868
  
 NCBI BlastP on this gene

EKM58207

72. :  JH711789 Trametes versicolor FP-101664 SS1 unplaced genomic scaffold TRAVEscaffold\_7     Total score: 1.0     Cumulative Blast bit score: 1043

acyl-CoA dehydrogenase
  
Accession: EIW57326
  
Location: 636842-639379
  
 NCBI BlastP on this gene

EIW57326

hypothetical protein
  
Accession: EIW57325
  
Location: 633448-634711
  
 NCBI BlastP on this gene

EIW57325

hypothetical protein
  
Accession: EIW57324
  
Location: 632442-632807
  
 NCBI BlastP on this gene

EIW57324

hypothetical protein
  
Accession: EIW57323
  
Location: 630192-631496
  
 NCBI BlastP on this gene

EIW57323

hypothetical protein
  
Accession: EIW57322
  
Location: 628553-629541
  
 NCBI BlastP on this gene

EIW57322

hypothetical protein
  
Accession: EIW57321
  
Location: 627060-627491
  
 NCBI BlastP on this gene

EIW57321

DEAD-domain-containing protein
  
Accession: EIW57320
  
Location: 622592-625229
  
 NCBI BlastP on this gene

EIW57320

P-loop containing nucleoside triphosphate hydrolase protein
  
Accession: EIW57319
  
Location: 616211-621367
  
  
**BlastP hit with Mycgr3G41235\_Mycgr3T**
  
Percentage identity: 40 %
  
BlastP bit score: 1043
  
Sequence coverage: 104 %
  
E-value: 0.0
  
  
 NCBI BlastP on this gene

EIW57319

CoA-transferase family III
  
Accession: EIW57318
  
Location: 614052-615692
  
 NCBI BlastP on this gene

EIW57318

multifunctional beta-oxidation protein
  
Accession: EIW57317
  
Location: 608803-613175
  
 NCBI BlastP on this gene

EIW57317

calcium/proton exchanger
  
Accession: EIW57316
  
Location: 605598-608209
  
 NCBI BlastP on this gene

EIW57316

hypothetical protein
  
Accession: EIW57315
  
Location: 597437-599846
  
 NCBI BlastP on this gene

EIW57315

73. :  KB445804 Ceriporiopsis subvermispora B unplaced genomic scaffold CERSUscaffold\_14     Total score: 1.0     Cumulative Blast bit score: 1041

hypothetical protein
  
Accession: EMD34142
  
Location: 952728-957018
  
 NCBI BlastP on this gene

EMD34142

hypothetical protein
  
Accession: EMD34143
  
Location: 958067-959254
  
 NCBI BlastP on this gene

EMD34143

hypothetical protein
  
Accession: EMD34144
  
Location: 961226-962164
  
 NCBI BlastP on this gene

EMD34144

hypothetical protein
  
Accession: EMD34145
  
Location: 963112-964615
  
 NCBI BlastP on this gene

EMD34145

hypothetical protein
  
Accession: EMD34146
  
Location: 964997-965423
  
 NCBI BlastP on this gene

EMD34146

hypothetical protein
  
Accession: EMD34147
  
Location: 967334-970010
  
 NCBI BlastP on this gene

EMD34147

hypothetical protein
  
Accession: EMD34148
  
Location: 970862-976409
  
  
**BlastP hit with Mycgr3G41235\_Mycgr3T**
  
Percentage identity: 39 %
  
BlastP bit score: 1041
  
Sequence coverage: 103 %
  
E-value: 0.0
  
  
 NCBI BlastP on this gene

EMD34148

hypothetical protein
  
Accession: EMD34149
  
Location: 976890-978647
  
 NCBI BlastP on this gene

EMD34149

hypothetical protein
  
Accession: EMD34150
  
Location: 979045-980773
  
 NCBI BlastP on this gene

EMD34150

hypothetical protein
  
Accession: EMD34151
  
Location: 983559-987490
  
 NCBI BlastP on this gene

EMD34151

hypothetical protein
  
Accession: EMD34152
  
Location: 988168-989650
  
 NCBI BlastP on this gene

EMD34152

hypothetical protein
  
Accession: EMD34153
  
Location: 993358-996825
  
 NCBI BlastP on this gene

EMD34153

74. :  JH687380 Stereum hirsutum FP-91666 SS1 unplaced genomic scaffold STEHIscaffold\_2     Total score: 1.0     Cumulative Blast bit score: 1039

hypothetical protein
  
Accession: EIM91105
  
Location: 1762698-1764537
  
 NCBI BlastP on this gene

EIM91105

actin-domain-containing protein
  
Accession: EIM91106
  
Location: 1764774-1766899
  
 NCBI BlastP on this gene

EIM91106

hypothetical protein
  
Accession: EIM91107
  
Location: 1767450-1769227
  
 NCBI BlastP on this gene

EIM91107

inorganic diphosphatase
  
Accession: EIM91108
  
Location: 1769623-1771039
  
 NCBI BlastP on this gene

EIM91108

hypothetical protein
  
Accession: EIM91109
  
Location: 1772803-1774289
  
 NCBI BlastP on this gene

EIM91109

glycoside hydrolase
  
Accession: EIM91110
  
Location: 1774995-1776533
  
 NCBI BlastP on this gene

EIM91110

Sodium/hydrogen exchanger
  
Accession: EIM91111
  
Location: 1776869-1778829
  
 NCBI BlastP on this gene

EIM91111

hypothetical protein
  
Accession: EIM91112
  
Location: 1780394-1781412
  
 NCBI BlastP on this gene

EIM91112

ABC transporter
  
Accession: EIM91113
  
Location: 1782241-1787709
  
  
**BlastP hit with Mycgr3G41235\_Mycgr3T**
  
Percentage identity: 40 %
  
BlastP bit score: 1039
  
Sequence coverage: 99 %
  
E-value: 0.0
  
  
 NCBI BlastP on this gene

EIM91113

phosphatidylserine decarboxylase-like protein
  
Accession: EIM91114
  
Location: 1788503-1789902
  
 NCBI BlastP on this gene

EIM91114

hypothetical protein
  
Accession: EIM91115
  
Location: 1790156-1790973
  
 NCBI BlastP on this gene

EIM91115

actin-like protein Arp3
  
Accession: EIM91116
  
Location: 1793781-1795515
  
 NCBI BlastP on this gene

EIM91116

TPR-like protein
  
Accession: EIM91117
  
Location: 1795992-1799484
  
 NCBI BlastP on this gene

EIM91117

antifreeze protein
  
Accession: EIM91118
  
Location: 1799947-1800976
  
 NCBI BlastP on this gene

EIM91118

hypothetical protein
  
Accession: EIM91119
  
Location: 1802317-1803631
  
 NCBI BlastP on this gene

EIM91119

hypothetical protein
  
Accession: EIM91120
  
Location: 1806694-1809073
  
 NCBI BlastP on this gene

EIM91120

75. :  JH687380 Stereum hirsutum FP-91666 SS1 unplaced genomic scaffold STEHIscaffold\_2     Total score: 1.0     Cumulative Blast bit score: 1026

ABC transporter
  
Accession: EIM91429
  
Location: 2822120-2828297
  
  
**BlastP hit with Mycgr3G41235\_Mycgr3T**
  
Percentage identity: 40 %
  
BlastP bit score: 1026
  
Sequence coverage: 102 %
  
E-value: 0.0
  
  
 NCBI BlastP on this gene

EIM91429

ankyrin
  
Accession: EIM91428
  
Location: 2815731-2820065
  
 NCBI BlastP on this gene

EIM91428

hypothetical protein
  
Accession: EIM91427
  
Location: 2810570-2814958
  
 NCBI BlastP on this gene

EIM91427

glycoside hydrolase family 81 protein
  
Accession: EIM91426
  
Location: 2806471-2810185
  
 NCBI BlastP on this gene

EIM91426

hypothetical protein
  
Accession: EIM91425
  
Location: 2802740-2804717
  
 NCBI BlastP on this gene

EIM91425

76. :  JH717971 Fomitiporia mediterranea MF3/22 unplaced genomic scaffold FOMMEscaffold\_5     Total score: 1.0     Cumulative Blast bit score: 1023

hypothetical protein
  
Accession: EJD03691
  
Location: 885088-889520
  
 NCBI BlastP on this gene

EJD03691

hypothetical protein
  
Accession: EJD03690
  
Location: 882889-884007
  
 NCBI BlastP on this gene

EJD03690

hypothetical protein
  
Accession: EJD03689
  
Location: 881175-882487
  
 NCBI BlastP on this gene

EJD03689

hypothetical protein
  
Accession: EJD03688
  
Location: 875280-880794
  
 NCBI BlastP on this gene

EJD03688

hypothetical protein
  
Accession: EJD03687
  
Location: 870318-873863
  
 NCBI BlastP on this gene

EJD03687

ABC transporter
  
Accession: EJD03686
  
Location: 864063-869569
  
  
**BlastP hit with Mycgr3G41235\_Mycgr3T**
  
Percentage identity: 39 %
  
BlastP bit score: 1023
  
Sequence coverage: 103 %
  
E-value: 0.0
  
  
 NCBI BlastP on this gene

EJD03686

hypothetical protein
  
Accession: EJD03685
  
Location: 862943-863872
  
 NCBI BlastP on this gene

EJD03685

hypothetical protein
  
Accession: EJD03684
  
Location: 861869-862695
  
 NCBI BlastP on this gene

EJD03684

hypothetical protein
  
Accession: EJD03683
  
Location: 860817-861633
  
 NCBI BlastP on this gene

EJD03683

phenylacetyl-CoA ligase
  
Accession: EJD03682
  
Location: 856968-859677
  
 NCBI BlastP on this gene

EJD03682

hypothetical protein
  
Accession: EJD03681
  
Location: 851777-853557
  
 NCBI BlastP on this gene

EJD03681

hypothetical protein
  
Accession: EJD03680
  
Location: 850163-850633
  
 NCBI BlastP on this gene

EJD03680

acetyl-CoA synthetase-like protein
  
Accession: EJD03679
  
Location: 845884-848607
  
 NCBI BlastP on this gene

EJD03679

77. :  JH719400 Dichomitus squalens LYAD-421 SS1 unplaced genomic scaffold DICSQscaffold\_4     Total score: 1.0     Cumulative Blast bit score: 1018

ABC transporter
  
Accession: EJF64657
  
Location: 641871-646695
  
  
**BlastP hit with Mycgr3G41235\_Mycgr3T**
  
Percentage identity: 40 %
  
BlastP bit score: 1019
  
Sequence coverage: 101 %
  
E-value: 0.0
  
  
 NCBI BlastP on this gene

EJF64657

MFS general substrate transporter
  
Accession: EJF64656
  
Location: 633222-635101
  
 NCBI BlastP on this gene

EJF64656

multifunctional beta-oxidation protein
  
Accession: EJF64655
  
Location: 627573-631734
  
 NCBI BlastP on this gene

EJF64655

calcium/proton exchanger
  
Accession: EJF64654
  
Location: 624231-627039
  
 NCBI BlastP on this gene

EJF64654

78. :  AACS02000004 Coprinopsis cinerea okayama7#130     Total score: 1.0     Cumulative Blast bit score: 1010

hypothetical protein
  
Accession: EAU85510
  
Location: 2067905-2068994
  
 NCBI BlastP on this gene

EAU85510

hypothetical protein
  
Accession: EAU85511
  
Location: 2069973-2071397
  
 NCBI BlastP on this gene

EAU85511

hypothetical protein
  
Accession: EAU85512
  
Location: 2072754-2073783
  
 NCBI BlastP on this gene

EAU85512

metal homeostatis protein bsd2
  
Accession: EAU85513
  
Location: 2074485-2075866
  
 NCBI BlastP on this gene

EAU85513

hypothetical protein
  
Accession: EAU85514
  
Location: 2076069-2077208
  
 NCBI BlastP on this gene

EAU85514

hypothetical protein
  
Accession: EAU85515
  
Location: 2077824-2078574
  
 NCBI BlastP on this gene

EAU85515

CMGC/CLK protein kinase
  
Accession: EAU85516
  
Location: 2079430-2081417
  
 NCBI BlastP on this gene

EAU85516

hypothetical protein
  
Accession: EFI27926
  
Location: 2082913-2083656
  
 NCBI BlastP on this gene

EFI27926

hypothetical protein
  
Accession: EFI27927
  
Location: 2084004-2086053
  
 NCBI BlastP on this gene

EFI27927

hypothetical protein
  
Accession: EAU85518
  
Location: 2086199-2087349
  
 NCBI BlastP on this gene

EAU85518

ATP-binding cassette transporter YOR1
  
Accession: EAU85519
  
Location: 2087523-2093390
  
  
**BlastP hit with Mycgr3G41235\_Mycgr3T**
  
Percentage identity: 38 %
  
BlastP bit score: 1010
  
Sequence coverage: 105 %
  
E-value: 0.0
  
  
 NCBI BlastP on this gene

EAU85519

histone acetyltransferase mst2
  
Accession: EFI27928
  
Location: 2093703-2101960
  
 NCBI BlastP on this gene

EFI27928

actin binding protein
  
Accession: EAU85522
  
Location: 2102418-2104161
  
 NCBI BlastP on this gene

EAU85522

hypothetical protein
  
Accession: EAU85523
  
Location: 2104238-2106788
  
 NCBI BlastP on this gene

EAU85523

TKL/TKL-ccin protein kinase
  
Accession: EAU85524
  
Location: 2107145-2109248
  
 NCBI BlastP on this gene

EAU85524

hypothetical protein
  
Accession: EAU85525
  
Location: 2109688-2110023
  
 NCBI BlastP on this gene

EAU85525

hypothetical protein
  
Accession: EAU85526
  
Location: 2110507-2111731
  
 NCBI BlastP on this gene

EAU85526

hypothetical protein
  
Accession: EFI27929
  
Location: 2112726-2116420
  
 NCBI BlastP on this gene

EFI27929

79. :  JH931607 Agaricus bisporus var. bisporus H97 unplaced genomic scaffold AGABI2scaffold\_3     Total score: 1.0     Cumulative Blast bit score: 1008

hypothetical protein
  
Accession: EKV49243
  
Location: 1401950-1407855
  
  
**BlastP hit with Mycgr3G41235\_Mycgr3T**
  
Percentage identity: 39 %
  
BlastP bit score: 1008
  
Sequence coverage: 101 %
  
E-value: 0.0
  
  
 NCBI BlastP on this gene

EKV49243

hypothetical protein
  
Accession: EKV49242
  
Location: 1396389-1401618
  
 NCBI BlastP on this gene

EKV49242

hypothetical protein
  
Accession: EKV49241
  
Location: 1393879-1395697
  
 NCBI BlastP on this gene

EKV49241

hypothetical protein
  
Accession: EKV49240
  
Location: 1390432-1392378
  
 NCBI BlastP on this gene

EKV49240

hypothetical protein
  
Accession: EKV49239
  
Location: 1387823-1390050
  
 NCBI BlastP on this gene

EKV49239

hypothetical protein
  
Accession: EKV49238
  
Location: 1384135-1385696
  
 NCBI BlastP on this gene

EKV49238

80. :  JH687380 Stereum hirsutum FP-91666 SS1 unplaced genomic scaffold STEHIscaffold\_2     Total score: 1.0     Cumulative Blast bit score: 1008

hypothetical protein
  
Accession: EIM91387
  
Location: 2656018-2657027
  
 NCBI BlastP on this gene

EIM91387

hypothetical protein
  
Accession: EIM91386
  
Location: 2651604-2652729
  
 NCBI BlastP on this gene

EIM91386

UPF0061-domain-containing protein
  
Accession: EIM91385
  
Location: 2647982-2650722
  
 NCBI BlastP on this gene

EIM91385

hypothetical protein
  
Accession: EIM91384
  
Location: 2645548-2647237
  
 NCBI BlastP on this gene

EIM91384

hypothetical protein
  
Accession: EIM91383
  
Location: 2640665-2644233
  
 NCBI BlastP on this gene

EIM91383

ABC transporter
  
Accession: EIM91382
  
Location: 2632680-2638146
  
  
**BlastP hit with Mycgr3G41235\_Mycgr3T**
  
Percentage identity: 39 %
  
BlastP bit score: 1008
  
Sequence coverage: 103 %
  
E-value: 0.0
  
  
 NCBI BlastP on this gene

EIM91382

hypothetical protein
  
Accession: EIM91381
  
Location: 2630136-2632260
  
 NCBI BlastP on this gene

EIM91381

hypothetical protein
  
Accession: EIM91380
  
Location: 2628152-2629137
  
 NCBI BlastP on this gene

EIM91380

hypothetical protein
  
Accession: EIM91379
  
Location: 2625947-2626783
  
 NCBI BlastP on this gene

EIM91379

glutathione S-transferase
  
Accession: EIM91378
  
Location: 2621928-2622976
  
 NCBI BlastP on this gene

EIM91378

hypothetical protein
  
Accession: EIM91377
  
Location: 2619346-2620765
  
 NCBI BlastP on this gene

EIM91377

FAD-binding domain-containing protein
  
Accession: EIM91376
  
Location: 2615965-2618695
  
 NCBI BlastP on this gene

EIM91376

hypothetical protein
  
Accession: EIM91375
  
Location: 2613877-2615604
  
 NCBI BlastP on this gene

EIM91375

81. :  JH711575 Coniophora puteana RWD-64-598 SS2 unplaced genomic scaffold CONPUscaffold\_3     Total score: 1.0     Cumulative Blast bit score: 997

ClpP crotonase
  
Accession: EIW84215
  
Location: 1978421-1979554
  
 NCBI BlastP on this gene

EIW84215

hypothetical protein
  
Accession: EIW84214
  
Location: 1976501-1977642
  
 NCBI BlastP on this gene

EIW84214

CMGC CLK protein kinase
  
Accession: EIW84213
  
Location: 1973568-1975496
  
 NCBI BlastP on this gene

EIW84213

hypothetical protein
  
Accession: EIW84212
  
Location: 1971947-1972153
  
 NCBI BlastP on this gene

EIW84212

hypothetical protein
  
Accession: EIW84211
  
Location: 1970158-1971453
  
 NCBI BlastP on this gene

EIW84211

hypothetical protein
  
Accession: EIW84210
  
Location: 1966560-1968201
  
 NCBI BlastP on this gene

EIW84210

PAH-inducible cytochrome P450 monooxygenase PC-PAH 1
  
Accession: EIW84209
  
Location: 1962129-1964218
  
 NCBI BlastP on this gene

EIW84209

P-loop containing nucleoside triphosphate hydrolase protein
  
Accession: EIW84208
  
Location: 1959257-1961613
  
 NCBI BlastP on this gene

EIW84208

ABC transporter
  
Accession: EIW84207
  
Location: 1953373-1958654
  
  
**BlastP hit with Mycgr3G41235\_Mycgr3T**
  
Percentage identity: 40 %
  
BlastP bit score: 998
  
Sequence coverage: 101 %
  
E-value: 0.0
  
  
 NCBI BlastP on this gene

EIW84207

hypothetical protein
  
Accession: EIW84206
  
Location: 1951714-1953001
  
 NCBI BlastP on this gene

EIW84206

hypothetical protein
  
Accession: EIW84205
  
Location: 1950155-1951400
  
 NCBI BlastP on this gene

EIW84205

flavo protein
  
Accession: EIW84204
  
Location: 1947526-1949879
  
 NCBI BlastP on this gene

EIW84204

DNA polymerase eta
  
Accession: EIW84203
  
Location: 1942097-1944595
  
 NCBI BlastP on this gene

EIW84203

hypothetical protein
  
Accession: EIW84202
  
Location: 1940435-1941287
  
 NCBI BlastP on this gene

EIW84202

complex I intermediate-associated protein CIA30
  
Accession: EIW84201
  
Location: 1937706-1938694
  
 NCBI BlastP on this gene

EIW84201

ras-like protein
  
Accession: EIW84200
  
Location: 1936412-1937413
  
 NCBI BlastP on this gene

EIW84200

DUF1295-domain-containing protein
  
Accession: EIW84199
  
Location: 1934066-1935404
  
 NCBI BlastP on this gene

EIW84199

82. :  JH687379 Stereum hirsutum FP-91666 SS1 unplaced genomic scaffold STEHIscaffold\_1     Total score: 1.0     Cumulative Blast bit score: 991

hypothetical protein
  
Accession: EIM92421
  
Location: 2256231-2258163
  
 NCBI BlastP on this gene

EIM92421

hypothetical protein
  
Accession: EIM92420
  
Location: 2254587-2254855
  
 NCBI BlastP on this gene

EIM92420

enoyl-CoA hydratase/carnithine racemase
  
Accession: EIM92419
  
Location: 2251737-2253749
  
 NCBI BlastP on this gene

EIM92419

metalloprotease ATP23
  
Accession: EIM92418
  
Location: 2247309-2248264
  
 NCBI BlastP on this gene

EIM92418

hypothetical protein
  
Accession: EIM92417
  
Location: 2245916-2246841
  
 NCBI BlastP on this gene

EIM92417

mannose 6-phosphate receptor domain-containing protein
  
Accession: EIM92416
  
Location: 2243772-2245160
  
 NCBI BlastP on this gene

EIM92416

NADH-ubiquinone oxidoreductase
  
Accession: EIM92415
  
Location: 2241497-2243627
  
 NCBI BlastP on this gene

EIM92415

glutaredoxin
  
Accession: EIM92414
  
Location: 2239137-2239735
  
 NCBI BlastP on this gene

EIM92414

ATP-binding cassette transporter YOR1
  
Accession: EIM92413
  
Location: 2231975-2237447
  
  
**BlastP hit with Mycgr3G41235\_Mycgr3T**
  
Percentage identity: 39 %
  
BlastP bit score: 991
  
Sequence coverage: 100 %
  
E-value: 0.0
  
  
 NCBI BlastP on this gene

EIM92413

hypothetical protein
  
Accession: EIM92412
  
Location: 2229672-2231483
  
 NCBI BlastP on this gene

EIM92412

hypothetical protein
  
Accession: EIM92411
  
Location: 2227362-2229150
  
 NCBI BlastP on this gene

EIM92411

CDF-like metal transporter
  
Accession: EIM92410
  
Location: 2223570-2225861
  
 NCBI BlastP on this gene

EIM92410

hypothetical protein
  
Accession: EIM92409
  
Location: 2218239-2222919
  
 NCBI BlastP on this gene

EIM92409

Pkinase-domain-containing protein
  
Accession: EIM92408
  
Location: 2214342-2217631
  
 NCBI BlastP on this gene

EIM92408

83. :  JH711575 Coniophora puteana RWD-64-598 SS2 unplaced genomic scaffold CONPUscaffold\_3     Total score: 1.0     Cumulative Blast bit score: 973

hypothetical protein
  
Accession: EIW84223
  
Location: 2002727-2005651
  
 NCBI BlastP on this gene

EIW84223

uracil-DNA glycosylase
  
Accession: EIW84224
  
Location: 2006360-2007771
  
 NCBI BlastP on this gene

EIW84224

hypothetical protein
  
Accession: EIW84225
  
Location: 2008492-2009638
  
 NCBI BlastP on this gene

EIW84225

DNA helicase
  
Accession: EIW84226
  
Location: 2010541-2013876
  
 NCBI BlastP on this gene

EIW84226

hypothetical protein
  
Accession: EIW84227
  
Location: 2014922-2015953
  
 NCBI BlastP on this gene

EIW84227

williams-Beuren syndrome critical region protein 20 copy A
  
Accession: EIW84228
  
Location: 2016233-2018035
  
 NCBI BlastP on this gene

EIW84228

DnaJ-domain-containing protein
  
Accession: EIW84229
  
Location: 2018469-2020401
  
 NCBI BlastP on this gene

EIW84229

hypothetical protein
  
Accession: EIW84230
  
Location: 2021076-2021683
  
 NCBI BlastP on this gene

EIW84230

P-loop containing nucleoside triphosphate hydrolase protein
  
Accession: EIW84231
  
Location: 2022418-2027867
  
  
**BlastP hit with Mycgr3G41235\_Mycgr3T**
  
Percentage identity: 38 %
  
BlastP bit score: 973
  
Sequence coverage: 102 %
  
E-value: 0.0
  
  
 NCBI BlastP on this gene

EIW84231

84. :  CAFZ01000285 Piriformospora indica DSM 11827     Total score: 1.0     Cumulative Blast bit score: 948

hypothetical protein
  
Accession: CCA74160
  
Location: 634-2564
  
 NCBI BlastP on this gene

CCA74160

related to major facilitator MirA
  
Accession: CCA74161
  
Location: 2887-5086
  
  
**BlastP hit with Mycgr3G70577\_Mycgr3T**
  
Percentage identity: 33 %
  
BlastP bit score: 338
  
Sequence coverage: 94 %
  
E-value: 3e-103
  
  
 NCBI BlastP on this gene

CCA74161

related to major facilitator MirA
  
Accession: CCA74162
  
Location: 5319-7551
  
  
**BlastP hit with Mycgr3G70577\_Mycgr3T**
  
Percentage identity: 33 %
  
BlastP bit score: 317
  
Sequence coverage: 95 %
  
E-value: 4e-95
  
  
 NCBI BlastP on this gene

CCA74162

related to Siderophore iron transporter 3
  
Accession: CCA74163
  
Location: 7954-10268
  
  
**BlastP hit with Mycgr3G70577\_Mycgr3T**
  
Percentage identity: 30 %
  
BlastP bit score: 293
  
Sequence coverage: 96 %
  
E-value: 4e-86
  
  
 NCBI BlastP on this gene

CCA74163

hypothetical protein
  
Accession: CCA74164
  
Location: 10526-12259
  
 NCBI BlastP on this gene

CCA74164

hypothetical protein
  
Accession: CCA74165
  
Location: 12981-14760
  
 NCBI BlastP on this gene

CCA74165

hypothetical protein
  
Accession: CCA74166
  
Location: 15288-17966
  
 NCBI BlastP on this gene

CCA74166

related to kinesin light chain
  
Accession: CCA74167
  
Location: 18678-21594
  
 NCBI BlastP on this gene

CCA74167

related to TY4B-Putative pseudo-TY4B
  
Accession: CCA74168
  
Location: 21771-24040
  
 NCBI BlastP on this gene

CCA74168

85. :  JH971391 Agaricus bisporus var. burnettii JB137-S8 unplaced genomic scaffold AGABI1scaffold\_7     Total score: 1.0     Cumulative Blast bit score: 931

hypothetical protein
  
Accession: EKM78690
  
Location: 322845-328958
  
  
**BlastP hit with Mycgr3G41235\_Mycgr3T**
  
Percentage identity: 37 %
  
BlastP bit score: 931
  
Sequence coverage: 104 %
  
E-value: 0.0
  
  
 NCBI BlastP on this gene

EKM78690

hypothetical protein
  
Accession: EKM78689
  
Location: 317301-322517
  
 NCBI BlastP on this gene

EKM78689

hypothetical protein
  
Accession: EKM78688
  
Location: 314790-316725
  
 NCBI BlastP on this gene

EKM78688

hypothetical protein
  
Accession: EKM78687
  
Location: 312600-313287
  
 NCBI BlastP on this gene

EKM78687

hypothetical protein
  
Accession: EKM78686
  
Location: 308962-310962
  
 NCBI BlastP on this gene

EKM78686

hypothetical protein
  
Accession: EKM78685
  
Location: 306215-307776
  
 NCBI BlastP on this gene

EKM78685

86. :  JH711789 Trametes versicolor FP-101664 SS1 unplaced genomic scaffold TRAVEscaffold\_7     Total score: 1.0     Cumulative Blast bit score: 901

DNA helicase
  
Accession: EIW57389
  
Location: 829878-833214
  
 NCBI BlastP on this gene

EIW57389

hypothetical protein
  
Accession: EIW57390
  
Location: 833492-835398
  
 NCBI BlastP on this gene

EIW57390

S-adenosyl-L-methionine-dependent methyltransferase
  
Accession: EIW57391
  
Location: 835741-837661
  
 NCBI BlastP on this gene

EIW57391

DnaJ-domain-containing protein
  
Accession: EIW57392
  
Location: 838169-840094
  
 NCBI BlastP on this gene

EIW57392

hypothetical protein
  
Accession: EIW57393
  
Location: 840769-843731
  
 NCBI BlastP on this gene

EIW57393

hypothetical protein
  
Accession: EIW57394
  
Location: 845698-846215
  
 NCBI BlastP on this gene

EIW57394

ABC protein
  
Accession: EIW57395
  
Location: 846939-853616
  
  
**BlastP hit with Mycgr3G41235\_Mycgr3T**
  
Percentage identity: 36 %
  
BlastP bit score: 901
  
Sequence coverage: 103 %
  
E-value: 0.0
  
  
 NCBI BlastP on this gene

EIW57395

hypothetical protein
  
Accession: EIW57396
  
Location: 853771-856939
  
 NCBI BlastP on this gene

EIW57396

NAD-P-binding protein
  
Accession: EIW57397
  
Location: 859187-860745
  
 NCBI BlastP on this gene

EIW57397

hypothetical protein
  
Accession: EIW57398
  
Location: 861673-863061
  
 NCBI BlastP on this gene

EIW57398

hypothetical protein
  
Accession: EIW57399
  
Location: 865108-865988
  
 NCBI BlastP on this gene

EIW57399

WD-40 repeat-containing protein
  
Accession: EIW57400
  
Location: 866921-868475
  
 NCBI BlastP on this gene

EIW57400

hypothetical protein
  
Accession: EIW57401
  
Location: 869610-870661
  
 NCBI BlastP on this gene

EIW57401

cyclophilin-like protein
  
Accession: EIW57402
  
Location: 872598-873534
  
 NCBI BlastP on this gene

EIW57402

87. :  KB445804 Ceriporiopsis subvermispora B unplaced genomic scaffold CERSUscaffold\_14     Total score: 1.0     Cumulative Blast bit score: 892

CsMn25
  
Accession: EMD34176
  
Location: 1057319-1064202
  
  
**BlastP hit with Mycgr3G41235\_Mycgr3T**
  
Percentage identity: 36 %
  
BlastP bit score: 892
  
Sequence coverage: 104 %
  
E-value: 0.0
  
  
 NCBI BlastP on this gene

EMD34176

hypothetical protein
  
Accession: EMD34175
  
Location: 1053620-1057064
  
 NCBI BlastP on this gene

EMD34175

hypothetical protein
  
Accession: EMD34174
  
Location: 1051122-1052081
  
 NCBI BlastP on this gene

EMD34174

hypothetical protein
  
Accession: EMD34173
  
Location: 1049519-1050983
  
 NCBI BlastP on this gene

EMD34173

hypothetical protein
  
Accession: EMD34172
  
Location: 1048364-1048858
  
 NCBI BlastP on this gene

EMD34172

hypothetical protein
  
Accession: EMD34171
  
Location: 1043818-1045233
  
 NCBI BlastP on this gene

EMD34171

hypothetical protein
  
Accession: EMD34170
  
Location: 1040009-1042652
  
 NCBI BlastP on this gene

EMD34170

88. :  JH717968 Fomitiporia mediterranea MF3/22 unplaced genomic scaffold FOMMEscaffold\_2     Total score: 1.0     Cumulative Blast bit score: 885

A1pp-domain-containing protein
  
Accession: EJD07133
  
Location: 4298860-4299921
  
 NCBI BlastP on this gene

EJD07133

sugar transporter
  
Accession: EJD07134
  
Location: 4300577-4302693
  
 NCBI BlastP on this gene

EJD07134

zf-DHHC-domain-containing protein
  
Accession: EJD07135
  
Location: 4303521-4305035
  
 NCBI BlastP on this gene

EJD07135

hypothetical protein
  
Accession: EJD07136
  
Location: 4306333-4306912
  
 NCBI BlastP on this gene

EJD07136

ABC protein
  
Accession: EJD07137
  
Location: 4307623-4313942
  
  
**BlastP hit with Mycgr3G41235\_Mycgr3T**
  
Percentage identity: 36 %
  
BlastP bit score: 885
  
Sequence coverage: 104 %
  
E-value: 0.0
  
  
 NCBI BlastP on this gene

EJD07137

hypothetical protein
  
Accession: EJD07138
  
Location: 4314640-4317612
  
 NCBI BlastP on this gene

EJD07138

carboxylesterase
  
Accession: EJD07139
  
Location: 4317981-4320123
  
 NCBI BlastP on this gene

EJD07139

hypothetical protein
  
Accession: EJD07140
  
Location: 4320244-4323058
  
 NCBI BlastP on this gene

EJD07140

hypothetical protein
  
Accession: EJD07141
  
Location: 4324882-4325939
  
 NCBI BlastP on this gene

EJD07141

hypothetical protein
  
Accession: EJD07142
  
Location: 4327509-4328009
  
 NCBI BlastP on this gene

EJD07142

TPR-like protein
  
Accession: EJD07143
  
Location: 4331459-4334613
  
 NCBI BlastP on this gene

EJD07143

89. :  JH931607 Agaricus bisporus var. bisporus H97 unplaced genomic scaffold AGABI2scaffold\_3     Total score: 1.0     Cumulative Blast bit score: 869

hypothetical protein
  
Accession: EKV49142
  
Location: 1133685-1135335
  
 NCBI BlastP on this gene

EKV49142

hypothetical protein
  
Accession: EKV49143
  
Location: 1135334-1137138
  
 NCBI BlastP on this gene

EKV49143

hypothetical protein
  
Accession: EKV49144
  
Location: 1137287-1138260
  
 NCBI BlastP on this gene

EKV49144

hypothetical protein
  
Accession: EKV49145
  
Location: 1143415-1144430
  
 NCBI BlastP on this gene

EKV49145

hypothetical protein
  
Accession: EKV49146
  
Location: 1144508-1145953
  
 NCBI BlastP on this gene

EKV49146

hypothetical protein
  
Accession: EKV49147
  
Location: 1146251-1148447
  
 NCBI BlastP on this gene

EKV49147

hypothetical protein
  
Accession: EKV49148
  
Location: 1149691-1151888
  
 NCBI BlastP on this gene

EKV49148

hypothetical protein
  
Accession: EKV49149
  
Location: 1152320-1158449
  
  
**BlastP hit with Mycgr3G41235\_Mycgr3T**
  
Percentage identity: 36 %
  
BlastP bit score: 869
  
Sequence coverage: 103 %
  
E-value: 0.0
  
  
 NCBI BlastP on this gene

EKV49149

hypothetical protein
  
Accession: EKV49150
  
Location: 1159023-1160216
  
 NCBI BlastP on this gene

EKV49150

hypothetical protein
  
Accession: EKV49151
  
Location: 1160441-1161182
  
 NCBI BlastP on this gene

EKV49151

hypothetical protein
  
Accession: EKV49152
  
Location: 1161454-1162015
  
 NCBI BlastP on this gene

EKV49152

hypothetical protein
  
Accession: EKV49153
  
Location: 1162284-1164552
  
 NCBI BlastP on this gene

EKV49153

hypothetical protein
  
Accession: EKV49154
  
Location: 1164591-1166657
  
 NCBI BlastP on this gene

EKV49154

hypothetical protein
  
Accession: EKV49155
  
Location: 1167131-1171925
  
 NCBI BlastP on this gene

EKV49155

hypothetical protein
  
Accession: EKV49156
  
Location: 1172947-1174037
  
 NCBI BlastP on this gene

EKV49156

alpha,alpha-trehalose-phosphate synthase TPS1 subunit
  
Accession: EKV49157
  
Location: 1174600-1176325
  
 NCBI BlastP on this gene

EKV49157

hypothetical protein
  
Accession: EKV49158
  
Location: 1178176-1182441
  
 NCBI BlastP on this gene

EKV49158

90. :  JH711575 Coniophora puteana RWD-64-598 SS2 unplaced genomic scaffold CONPUscaffold\_3     Total score: 1.0     Cumulative Blast bit score: 867

hypothetical protein
  
Accession: EIW84017
  
Location: 1433418-1434176
  
 NCBI BlastP on this gene

EIW84017

peroxisomal acyl-CoA-dehydrogenase
  
Accession: EIW84016
  
Location: 1430612-1432910
  
 NCBI BlastP on this gene

EIW84016

hypothetical protein
  
Accession: EIW84015
  
Location: 1429167-1430054
  
 NCBI BlastP on this gene

EIW84015

coproporphyrinogen III oxidase
  
Accession: EIW84014
  
Location: 1427597-1428646
  
 NCBI BlastP on this gene

EIW84014

hypothetical protein
  
Accession: EIW84013
  
Location: 1425028-1426371
  
 NCBI BlastP on this gene

EIW84013

acyl-CoA dehydrogenase NM domain-like protein
  
Accession: EIW84012
  
Location: 1419563-1422094
  
 NCBI BlastP on this gene

EIW84012

hypothetical protein
  
Accession: EIW84011
  
Location: 1417242-1419154
  
 NCBI BlastP on this gene

EIW84011

DHS-like NAD/FAD-binding domain-containing protein
  
Accession: EIW84010
  
Location: 1415223-1416999
  
 NCBI BlastP on this gene

EIW84010

ABC protein
  
Accession: EIW84009
  
Location: 1408082-1414426
  
  
**BlastP hit with Mycgr3G41235\_Mycgr3T**
  
Percentage identity: 35 %
  
BlastP bit score: 867
  
Sequence coverage: 105 %
  
E-value: 0.0
  
  
 NCBI BlastP on this gene

EIW84009

WD40 repeat-like protein
  
Accession: EIW84008
  
Location: 1405464-1406372
  
 NCBI BlastP on this gene

EIW84008

hypothetical protein
  
Accession: EIW84007
  
Location: 1399251-1403044
  
 NCBI BlastP on this gene

EIW84007

C5-sterol desaturase
  
Accession: EIW84006
  
Location: 1396080-1397192
  
 NCBI BlastP on this gene

EIW84006

GroES-like protein
  
Accession: EIW84005
  
Location: 1394557-1395708
  
 NCBI BlastP on this gene

EIW84005

WD40 repeat-like protein
  
Accession: EIW84004
  
Location: 1392601-1394029
  
 NCBI BlastP on this gene

EIW84004

91. :  JH687380 Stereum hirsutum FP-91666 SS1 unplaced genomic scaffold STEHIscaffold\_2     Total score: 1.0     Cumulative Blast bit score: 861

kinase-like protein
  
Accession: EIM90811
  
Location: 883194-885680
  
 NCBI BlastP on this gene

EIM90811

hypothetical protein
  
Accession: EIM90810
  
Location: 879842-881401
  
 NCBI BlastP on this gene

EIM90810

WD40 repeat-like protein
  
Accession: EIM90809
  
Location: 879240-879623
  
 NCBI BlastP on this gene

EIM90809

thioredoxin-like protein
  
Accession: EIM90808
  
Location: 875235-876609
  
 NCBI BlastP on this gene

EIM90808

SH3-domain-containing protein
  
Accession: EIM90807
  
Location: 872746-874243
  
 NCBI BlastP on this gene

EIM90807

DNase I-like protein
  
Accession: EIM90806
  
Location: 866723-871368
  
 NCBI BlastP on this gene

EIM90806

ABC protein
  
Accession: EIM90805
  
Location: 858520-865547
  
  
**BlastP hit with Mycgr3G41235\_Mycgr3T**
  
Percentage identity: 36 %
  
BlastP bit score: 862
  
Sequence coverage: 103 %
  
E-value: 0.0
  
  
 NCBI BlastP on this gene

EIM90805

MFS general substrate transporter
  
Accession: EIM90804
  
Location: 855084-857770
  
 NCBI BlastP on this gene

EIM90804

hypothetical protein
  
Accession: EIM90803
  
Location: 848195-848512
  
 NCBI BlastP on this gene

EIM90803

hypothetical protein
  
Accession: EIM90802
  
Location: 846058-847397
  
 NCBI BlastP on this gene

EIM90802

hypothetical protein
  
Accession: EIM90801
  
Location: 843632-844894
  
 NCBI BlastP on this gene

EIM90801

glycoside hydrolase
  
Accession: EIM90800
  
Location: 840941-841808
  
 NCBI BlastP on this gene

EIM90800

92. :  JH971391 Agaricus bisporus var. burnettii JB137-S8 unplaced genomic scaffold AGABI1scaffold\_7     Total score: 1.0     Cumulative Blast bit score: 852

hypothetical protein
  
Accession: EKM78588
  
Location: 53834-55485
  
 NCBI BlastP on this gene

EKM78588

hypothetical protein
  
Accession: EKM78589
  
Location: 55484-57288
  
 NCBI BlastP on this gene

EKM78589

hypothetical protein
  
Accession: EKM78590
  
Location: 57437-58408
  
 NCBI BlastP on this gene

EKM78590

hypothetical protein
  
Accession: EKM78591
  
Location: 64123-64504
  
 NCBI BlastP on this gene

EKM78591

hypothetical protein
  
Accession: EKM78592
  
Location: 65359-66072
  
 NCBI BlastP on this gene

EKM78592

hypothetical protein
  
Accession: EKM78593
  
Location: 66370-68566
  
 NCBI BlastP on this gene

EKM78593

hypothetical protein
  
Accession: EKM78594
  
Location: 69806-72003
  
 NCBI BlastP on this gene

EKM78594

hypothetical protein
  
Accession: EKM78595
  
Location: 72435-78567
  
  
**BlastP hit with Mycgr3G41235\_Mycgr3T**
  
Percentage identity: 36 %
  
BlastP bit score: 852
  
Sequence coverage: 103 %
  
E-value: 0.0
  
  
 NCBI BlastP on this gene

EKM78595

hypothetical protein
  
Accession: EKM78596
  
Location: 79142-80338
  
 NCBI BlastP on this gene

EKM78596

hypothetical protein
  
Accession: EKM78597
  
Location: 80563-81304
  
 NCBI BlastP on this gene

EKM78597

hypothetical protein
  
Accession: EKM78598
  
Location: 81580-82141
  
 NCBI BlastP on this gene

EKM78598

hypothetical protein
  
Accession: EKM78599
  
Location: 82410-84673
  
 NCBI BlastP on this gene

EKM78599

hypothetical protein
  
Accession: EKM78600
  
Location: 84743-86525
  
 NCBI BlastP on this gene

EKM78600

hypothetical protein
  
Accession: EKM78601
  
Location: 87245-92015
  
 NCBI BlastP on this gene

EKM78601

hypothetical protein
  
Accession: EKM78602
  
Location: 93038-94128
  
 NCBI BlastP on this gene

EKM78602

hypothetical protein
  
Accession: EKM78603
  
Location: 94691-96416
  
 NCBI BlastP on this gene

EKM78603

hypothetical protein
  
Accession: EKM78604
  
Location: 98252-102518
  
 NCBI BlastP on this gene

EKM78604

93. :  JH930470 Phanerochaete carnosa HHB-10118-sp unplaced genomic scaffold PHACAscaffold\_3     Total score: 1.0     Cumulative Blast bit score: 850

hypothetical protein
  
Accession: EKM58258
  
Location: 2640621-2646585
  
  
**BlastP hit with Mycgr3G41235\_Mycgr3T**
  
Percentage identity: 35 %
  
BlastP bit score: 850
  
Sequence coverage: 104 %
  
E-value: 0.0
  
  
 NCBI BlastP on this gene

EKM58258

hypothetical protein
  
Accession: EKM58257
  
Location: 2636763-2640370
  
 NCBI BlastP on this gene

EKM58257

hypothetical protein
  
Accession: EKM58256
  
Location: 2631731-2633174
  
 NCBI BlastP on this gene

EKM58256

hypothetical protein
  
Accession: EKM58255
  
Location: 2629622-2630635
  
 NCBI BlastP on this gene

EKM58255

hypothetical protein
  
Accession: EKM58254
  
Location: 2627460-2628232
  
 NCBI BlastP on this gene

EKM58254

hypothetical protein
  
Accession: EKM58253
  
Location: 2625784-2626910
  
 NCBI BlastP on this gene

EKM58253

hypothetical protein
  
Accession: EKM58252
  
Location: 2622187-2623112
  
 NCBI BlastP on this gene

EKM58252

94. :  GL377306 Schizophyllum commune H4-8 unplaced genomic scaffold SCHCOscaffold\_5     Total score: 1.0     Cumulative Blast bit score: 842

hypothetical protein
  
Accession: EFI96725
  
Location: 396172-397930
  
 NCBI BlastP on this gene

EFI96725

hypothetical protein
  
Accession: EFI97156
  
Location: 399459-401705
  
 NCBI BlastP on this gene

EFI97156

hypothetical protein
  
Accession: EFI96726
  
Location: 403426-405531
  
 NCBI BlastP on this gene

EFI96726

hypothetical protein
  
Accession: EFI96727
  
Location: 406923-409777
  
 NCBI BlastP on this gene

EFI96727

hypothetical protein
  
Accession: EFI97157
  
Location: 410626-412689
  
 NCBI BlastP on this gene

EFI97157

hypothetical protein
  
Accession: EFI97158
  
Location: 413061-414031
  
 NCBI BlastP on this gene

EFI97158

hypothetical protein
  
Accession: EFI96728
  
Location: 415385-421230
  
  
**BlastP hit with Mycgr3G41235\_Mycgr3T**
  
Percentage identity: 35 %
  
BlastP bit score: 842
  
Sequence coverage: 103 %
  
E-value: 0.0
  
  
 NCBI BlastP on this gene

EFI96728

hypothetical protein
  
Accession: EFI96729
  
Location: 424304-426075
  
 NCBI BlastP on this gene

EFI96729

expressed protein
  
Accession: EFI97159
  
Location: 426403-427659
  
 NCBI BlastP on this gene

EFI97159

expressed protein
  
Accession: EFI97160
  
Location: 429174-430261
  
 NCBI BlastP on this gene

EFI97160

hypothetical protein
  
Accession: EFI97161
  
Location: 430713-431429
  
 NCBI BlastP on this gene

EFI97161

hypothetical protein
  
Accession: EFI96730
  
Location: 432280-432854
  
 NCBI BlastP on this gene

EFI96730

glycoside hydrolase family 5 protein
  
Accession: EFI96731
  
Location: 434533-435882
  
 NCBI BlastP on this gene

EFI96731

glycoside hydrolase family 5 protein
  
Accession: EFI97162
  
Location: 436308-438266
  
 NCBI BlastP on this gene

EFI97162

hypothetical protein
  
Accession: EFI97163
  
Location: 439122-441269
  
 NCBI BlastP on this gene

EFI97163

95. :  DS027054 Aspergillus clavatus NRRL 1 1099423829800 genomic scaffold     Total score: 1.0     Cumulative Blast bit score: 824

DnaJ domain protein
  
Accession: EAW10377
  
Location: 1003992-1004564
  
 NCBI BlastP on this gene

EAW10377

cytochrome c peroxidase Ccp1, putative
  
Accession: EAW10378
  
Location: 1005201-1006367
  
 NCBI BlastP on this gene

EAW10378

GTPase activating protein for Arf, putative
  
Accession: EAW10379
  
Location: 1009197-1011390
  
 NCBI BlastP on this gene

EAW10379

mannosyltransferase, putative
  
Accession: EAW10380
  
Location: 1011833-1014531
  
 NCBI BlastP on this gene

EAW10380

L-ornithine aminotransferase Car2, putative
  
Accession: EAW10381
  
Location: 1015294-1016798
  
 NCBI BlastP on this gene

EAW10381

hypothetical protein
  
Accession: EAW10382
  
Location: 1017851-1018198
  
 NCBI BlastP on this gene

EAW10382

hypothetical protein
  
Accession: EAW10383
  
Location: 1020376-1020528
  
 NCBI BlastP on this gene

EAW10383

hypothetical protein
  
Accession: EAW10384
  
Location: 1020563-1021018
  
 NCBI BlastP on this gene

EAW10384

ABC multidrug transporter, putative
  
Accession: EAW10385
  
Location: 1022331-1026680
  
  
**BlastP hit with Mycgr3G41235\_Mycgr3T**
  
Percentage identity: 34 %
  
BlastP bit score: 824
  
Sequence coverage: 102 %
  
E-value: 0.0
  
  
 NCBI BlastP on this gene

EAW10385

S-adenosyl-methionine-sterol-C- methyltransferas
  
Accession: EAW10386
  
Location: 1029795-1030994
  
 NCBI BlastP on this gene

EAW10386

conserved hypothetical protein
  
Accession: EAW10387
  
Location: 1032369-1033689
  
 NCBI BlastP on this gene

EAW10387

phosphate transporter
  
Accession: EAW10388
  
Location: 1035119-1037280
  
 NCBI BlastP on this gene

EAW10388

conserved hypothetical protein
  
Accession: EAW10389
  
Location: 1039255-1041185
  
 NCBI BlastP on this gene

EAW10389

conserved hypothetical protein
  
Accession: EAW10390
  
Location: 1041238-1041948
  
 NCBI BlastP on this gene

EAW10390

conserved hypothetical protein
  
Accession: EAW10391
  
Location: 1042986-1044379
  
 NCBI BlastP on this gene

EAW10391

conserved hypothetical protein
  
Accession: EAW10392
  
Location: 1045361-1045956
  
 NCBI BlastP on this gene

EAW10392

96. :  CP003009 Thielavia terrestris NRRL 8126 chromosome 1     Total score: 1.0     Cumulative Blast bit score: 824

hypothetical protein
  
Accession: AEO63436
  
Location: 4972441-4974461
  
 NCBI BlastP on this gene

THITE\_2108696

hypothetical protein
  
Accession: AEO63437
  
Location: 4975112-4978347
  
 NCBI BlastP on this gene

THITE\_2108697

hypothetical protein
  
Accession: AEO63438
  
Location: 4979465-4982203
  
 NCBI BlastP on this gene

THITE\_2108701

hypothetical protein
  
Accession: AEO63439
  
Location: 4983123-4984424
  
 NCBI BlastP on this gene

THITE\_2108702

hypothetical protein
  
Accession: AEO63440
  
Location: 4989344-4993964
  
  
**BlastP hit with Mycgr3G41235\_Mycgr3T**
  
Percentage identity: 34 %
  
BlastP bit score: 824
  
Sequence coverage: 102 %
  
E-value: 0.0
  
  
 NCBI BlastP on this gene

THITE\_2108707

hypothetical protein
  
Accession: AEO63441
  
Location: 4995901-4997103
  
 NCBI BlastP on this gene

THITE\_2108709

hypothetical protein
  
Accession: AEO63442
  
Location: 4998320-4999491
  
 NCBI BlastP on this gene

THITE\_121184

hypothetical protein
  
Accession: AEO63443
  
Location: 5004030-5005436
  
 NCBI BlastP on this gene

THITE\_2108713

C-8 sterol isomerase
  
Accession: AEO63444
  
Location: 5006234-5007033
  
 NCBI BlastP on this gene

THITE\_70538

hypothetical protein
  
Accession: AEO63445
  
Location: 5007492-5008269
  
 NCBI BlastP on this gene

THITE\_2074474

hypothetical protein
  
Accession: AEO63446
  
Location: 5009165-5010142
  
 NCBI BlastP on this gene

THITE\_2108720

hypothetical protein
  
Accession: AEO63447
  
Location: 5010686-5012161
  
 NCBI BlastP on this gene

THITE\_2108722

hypothetical protein
  
Accession: AEO63448
  
Location: 5012747-5014904
  
 NCBI BlastP on this gene

THITE\_2108723

97. :  CP003003 Myceliophthora thermophila ATCC 42464 chromosome 2     Total score: 1.0     Cumulative Blast bit score: 824

hypothetical protein
  
Accession: AEO56924
  
Location: 4488126-4490344
  
 NCBI BlastP on this gene

MYCTH\_2302439

hypothetical protein
  
Accession: AEO56925
  
Location: 4491493-4494494
  
 NCBI BlastP on this gene

MYCTH\_2302441

hypothetical protein
  
Accession: AEO56926
  
Location: 4495578-4498324
  
 NCBI BlastP on this gene

MYCTH\_2302442

hypothetical protein
  
Accession: AEO56927
  
Location: 4499546-4500817
  
 NCBI BlastP on this gene

MYCTH\_2314855

hypothetical protein
  
Accession: AEO56928
  
Location: 4504494-4509120
  
  
**BlastP hit with Mycgr3G41235\_Mycgr3T**
  
Percentage identity: 35 %
  
BlastP bit score: 824
  
Sequence coverage: 104 %
  
E-value: 0.0
  
  
 NCBI BlastP on this gene

MYCTH\_2302447

hypothetical protein
  
Accession: AEO56929
  
Location: 4510292-4511524
  
 NCBI BlastP on this gene

MYCTH\_2302452

hypothetical protein
  
Accession: AEO56930
  
Location: 4512947-4514062
  
 NCBI BlastP on this gene

MYCTH\_2059229

hypothetical protein
  
Accession: AEO56931
  
Location: 4519165-4520595
  
 NCBI BlastP on this gene

MYCTH\_2302460

C-8 sterol isomerase
  
Accession: AEO56932
  
Location: 4521542-4522455
  
 NCBI BlastP on this gene

MYCTH\_64893

hypothetical protein
  
Accession: AEO56933
  
Location: 4523236-4524109
  
 NCBI BlastP on this gene

MYCTH\_2302465

hypothetical protein
  
Accession: AEO56934
  
Location: 4525239-4526144
  
 NCBI BlastP on this gene

MYCTH\_2302466

hypothetical protein
  
Accession: AEO56935
  
Location: 4526679-4528136
  
 NCBI BlastP on this gene

MYCTH\_2302467

hypothetical protein
  
Accession: AEO56936
  
Location: 4528853-4531026
  
 NCBI BlastP on this gene

MYCTH\_2302469

98. :  CM001232 Magnaporthe oryzae 70-15 chromosome 2     Total score: 1.0     Cumulative Blast bit score: 822

hypothetical protein
  
Accession: EHA55954
  
Location: 7899981-7900671
  
 NCBI BlastP on this gene

EHA55954

C6 transcription factor
  
Accession: EHA55955
  
Location: 7901673-7904814
  
 NCBI BlastP on this gene

EHA55955

hypothetical protein
  
Accession: EHA55956
  
Location: 7905160-7906544
  
 NCBI BlastP on this gene

EHA55956

hypothetical protein
  
Accession: EHA55957
  
Location: 7907941-7908771
  
 NCBI BlastP on this gene

EHA55957

hypothetical protein
  
Accession: EHA55958
  
Location: 7909827-7912286
  
 NCBI BlastP on this gene

EHA55958

oxidoreductase
  
Accession: EHA55959
  
Location: 7914670-7915933
  
 NCBI BlastP on this gene

EHA55959

multidrug resistance-associated protein 2
  
Accession: EHA55960
  
Location: 7918051-7922632
  
  
**BlastP hit with Mycgr3G41235\_Mycgr3T**
  
Percentage identity: 34 %
  
BlastP bit score: 822
  
Sequence coverage: 104 %
  
E-value: 0.0
  
  
 NCBI BlastP on this gene

EHA55960

hypothetical protein
  
Accession: EHA55961
  
Location: 7923395-7924353
  
 NCBI BlastP on this gene

EHA55961

hypothetical protein
  
Accession: EHA55962
  
Location: 7925421-7927293
  
 NCBI BlastP on this gene

EHA55962

hypothetical protein
  
Accession: EHA55963
  
Location: 7927431-7928000
  
 NCBI BlastP on this gene

EHA55963

abhydrolase domain-containing protein 12
  
Accession: EHA55964
  
Location: 7928702-7929895
  
 NCBI BlastP on this gene

EHA55964

hypothetical protein
  
Accession: EHA55965
  
Location: 7930419-7931920
  
 NCBI BlastP on this gene

EHA55965

mechanosensitive ion channel family protein
  
Accession: EHA55966
  
Location: 7932193-7934877
  
 NCBI BlastP on this gene

EHA55966

XPG domain-containing protein
  
Accession: EHA55967
  
Location: 7938027-7940857
  
 NCBI BlastP on this gene

EHA55967

rRNA-processing protein FCF1
  
Accession: EHA55968
  
Location: 7941747-7942426
  
 NCBI BlastP on this gene

EHA55968

99. :  JH793788 Magnaporthe oryzae Y34 unplaced genomic scaffold Y34\_scaffold00766     Total score: 1.0     Cumulative Blast bit score: 816

multidrug resistance-associated protein 2
  
Accession: ELQ34450
  
Location: 88833-93414
  
  
**BlastP hit with Mycgr3G41235\_Mycgr3T**
  
Percentage identity: 33 %
  
BlastP bit score: 816
  
Sequence coverage: 104 %
  
E-value: 0.0
  
  
 NCBI BlastP on this gene

ELQ34450

hypothetical protein
  
Accession: ELQ34449
  
Location: 87112-88070
  
 NCBI BlastP on this gene

ELQ34449

hypothetical protein
  
Accession: ELQ34448
  
Location: 83434-86044
  
 NCBI BlastP on this gene

ELQ34448

abhydrolase domain-containing protein 12
  
Accession: ELQ34447
  
Location: 81570-82763
  
 NCBI BlastP on this gene

ELQ34447

hypothetical protein
  
Accession: ELQ34446
  
Location: 79545-81046
  
 NCBI BlastP on this gene

ELQ34446

mechanosensitive ion channel family
  
Accession: ELQ34445
  
Location: 76588-79272
  
 NCBI BlastP on this gene

ELQ34445

XPG N-terminal domain-containing protein
  
Accession: ELQ34444
  
Location: 70607-73437
  
 NCBI BlastP on this gene

ELQ34444

rRNA-processing protein FCF1
  
Accession: ELQ34443
  
Location: 69038-69717
  
 NCBI BlastP on this gene

ELQ34443

100. :  GL891302 Neurospora tetrasperma FGSC 2508 unplaced genomic scaffold NEUTE1scaffold\_1     Total score: 1.0     Cumulative Blast bit score: 816

hypothetical protein
  
Accession: EGO60388
  
Location: 1194380-1197648
  
 NCBI BlastP on this gene

EGO60388

hypothetical protein
  
Accession: EGO60389
  
Location: 1198318-1200344
  
 NCBI BlastP on this gene

EGO60389

hypothetical protein
  
Accession: EGO60390
  
Location: 1201122-1202448
  
 NCBI BlastP on this gene

EGO60390

hypothetical protein
  
Accession: EGO60391
  
Location: 1203991-1205419
  
 NCBI BlastP on this gene

EGO60391

hypothetical protein
  
Accession: EGO60392
  
Location: 1207498-1208454
  
 NCBI BlastP on this gene

EGO60392

hypothetical protein
  
Accession: EGO60393
  
Location: 1212949-1217476
  
  
**BlastP hit with Mycgr3G41235\_Mycgr3T**
  
Percentage identity: 33 %
  
BlastP bit score: 816
  
Sequence coverage: 104 %
  
E-value: 0.0
  
  
 NCBI BlastP on this gene

EGO60393

hypothetical protein
  
Accession: EGO60394
  
Location: 1219616-1220575
  
 NCBI BlastP on this gene

EGO60394

hypothetical protein
  
Accession: EGO60395
  
Location: 1222472-1223200
  
 NCBI BlastP on this gene

EGO60395

hypothetical protein
  
Accession: EGO60396
  
Location: 1225634-1226431
  
 NCBI BlastP on this gene

EGO60396

hypothetical protein
  
Accession: EGO60397
  
Location: 1234451-1235305
  
 NCBI BlastP on this gene

EGO60397

hypothetical protein
  
Accession: EGO60398
  
Location: 1236037-1236754
  
 NCBI BlastP on this gene

EGO60398

Detecting sequence homology at the gene cluster level with MultiGeneBlast.
  
Marnix H. Medema, Rainer Breitling & Eriko Takano (2013)
  
*Molecular Biology and Evolution* , 30: 1218-1223.
