# Supplementary material for: In silico prediction and characterization of secondary metabolite biosynthetic gene clusters in the wheat pathogen Zymoseptoria tritici
Source: BMC Genomics. 2017 Aug 17;18:631. doi: 10.1186/s12864-017-3969-y (PMC5561558; doi:10.1186/s12864-017-3969-y)
Supplement: Supplementary file 1 — MultiGeneBLAST analysis of putative secondary metabolite clusters. All encoded amino acid sequences from genes residing in clusters predicted by AntiSMASH are given as FASTA file format. All output data from MultiGeneBLASTs are also provided. (ZIP 42911 kb) [file 12864_2017_3969_MOESM1_ESM.zip › Cluster MultiGene BLAST/out/Clusters_1_34/Cluster_14/displaypage3.xhtml]

xml version="1.0" encoding="UTF-8"?


Search Results
  
  
 Results pages: 1, 2, 3, 4, 5

**MultiGeneBlast hits**

Select gene cluster alignment
101. GL891107\_1 Neurospora tetrasperma FGSC 2509 unplaced genomic scaffold NE...
102. HF679025\_0 Fusarium fujikuroi IMI 58289 draft genome, chromosome FFUJ\_ch...
103. CM001202\_0 Mycosphaerella graminicola IPO323 chromosome 7, whole genome ...
104. GL385397\_1 Gaeumannomyces graminis var. tritici R3-111a-1 unplaced genom...
105. EQ962656\_1 Talaromyces stipitatus ATCC 10500 scf\_1105507295549 genomic s...
106. DS989823\_1 Arthroderma gypseum CBS 118893 supercont1.2 genomic scaffold,...
107. GG700652\_0 Trichophyton rubrum CBS 118892 genomic scaffold supercont2.5,...
108. JH719400\_0 Dichomitus squalens LYAD-421 SS1 unplaced genomic scaffold DI...
109. DS995903\_2 Penicillium marneffei ATCC 18224 scf\_1105668340984 genomic sc...
110. DS995721\_0 Trichophyton equinum CBS 127.97 supercont1.4 genomic scaffold...
111. DS499598\_0 Aspergillus fumigatus A1163 scf\_000005 genomic scaffold, whol...
112. AAHF01000005\_1 Aspergillus fumigatus Af293, whole genome shotgun sequenc...
113. DS995901\_1 Penicillium marneffei ATCC 18224 scf\_1105668340960 genomic sc...
114. JH719402\_0 Dichomitus squalens LYAD-421 SS1 unplaced genomic scaffold DI...
115. DS027685\_1 Neosartorya fischeri NRRL 181 1099437636245 genomic scaffold,...
116. ABDF02000001\_1 Trichoderma virens Gv29-8, whole genome shotgun sequencin...
117. DS995906\_0 Penicillium marneffei ATCC 18224 scf\_1105668340770 genomic sc...
118. GL985056\_1 Trichoderma reesei QM6a unplaced genomic scaffold TRIREscaffo...
119. KB445647\_1 Cochliobolus sativus ND90Pr unplaced genomic scaffold COCSAsc...
120. AACS02000012\_0 Coprinopsis cinerea okayama7#130, whole genome shotgun se...
121. CM001197\_1 Mycosphaerella graminicola IPO323 chromosome 2, whole genome ...
122. GL377310\_0 Schizophyllum commune H4-8 unplaced genomic scaffold SCHCOsca...
123. FP929130\_1 Leptosphaeria maculans JN3 lm\_SuperContig\_17\_v2 genomic super...
124. JH711791\_1 Trametes versicolor FP-101664 SS1 unplaced genomic scaffold T...
125. EQ962655\_1 Talaromyces stipitatus ATCC 10500 scf\_1105507295555 genomic s...
126. CH476605\_0 Aspergillus terreus NIH2624 scaffold\_12 genomic scaffold, who...
127. CP003009\_2 Thielavia terrestris NRRL 8126 chromosome 1, complete sequence.
128. JH226130\_0 Exophiala dermatitidis NIH/UT8656 unplaced genomic scaffold s...
129. AACS02000004\_1 Coprinopsis cinerea okayama7#130, whole genome shotgun se...
130. JH687393\_0 Stereum hirsutum FP-91666 SS1 unplaced genomic scaffold STEHI...
131. CH476615\_3 Uncinocarpus reesii 1704 scaffold\_1 genomic scaffold, whole g...
132. AP007174\_1 Aspergillus oryzae RIB40 DNA, SC103.
133. AKHY01000199\_0 Aspergillus oryzae 3.042, whole genome shotgun sequencing...
134. AACS02000012\_1 Coprinopsis cinerea okayama7#130, whole genome shotgun se...
135. JH687764\_1 Auricularia delicata TFB-10046 SS5 unplaced genomic scaffold ...
136. DS995903\_0 Penicillium marneffei ATCC 18224 scf\_1105668340984 genomic sc...
137. EQ963486\_0 Aspergillus flavus NRRL3357 scf\_1106286417242 genomic scaffol...
138. JH687764\_0 Auricularia delicata TFB-10046 SS5 unplaced genomic scaffold ...
139. FQ311430\_1 Sporisorium reilianum SRZ2 chromosome 1 complete DNA sequence.
140. DF196775\_1 Pseudozyma antarctica T-34 DNA, contig: scaffold00009, whole ...
141. AE017342\_0 Cryptococcus neoformans var. neoformans JEC21 chromosome 2, c...
142. CR382134\_0 Debaryomyces hansenii CBS767 chromosome B complete sequence.
143. GL996527\_1 Candida tenuis ATCC 10573 unplaced genomic scaffold CANTEscaf...
144. GL996527\_0 Candida tenuis ATCC 10573 unplaced genomic scaffold CANTEscaf...
145. CU928166\_1 Lachancea thermotolerans CBS 6340 chromosome B complete seque...
146. HE681721\_1 Candida orthopsilosis Co 90-125, chromosome 3 draft sequence.
147. CH477324\_0 Aedes aegypti strain Liverpool supercont1.139 genomic scaffol...
148. HE681721\_0 Candida orthopsilosis Co 90-125, chromosome 3 draft sequence.
149. CR382134\_2 Debaryomyces hansenii CBS767 chromosome B complete sequence.
150. CH477324\_1 Aedes aegypti strain Liverpool supercont1.139 genomic scaffol...

Query: Architecture Search FASTA input

GL891107 : Neurospora tetrasperma FGSC 2509 unplaced genomic scaffold NEUTE2scaffold\_2    Total score: 1.0     Cumulative Blast bit score: 816

Hit cluster cross-links:

Mycgr3G41235 Mycgr3T
  
Location: 0-4062

Mycgr3G41235\_Mycgr3T

Mycgr3G70577 Mycgr3T
  
Location: 4162-6109

Mycgr3G70577\_Mycgr3T

Mycgr3G40534 Mycgr3T
  
Location: 6209-7166

Mycgr3G40534\_Mycgr3T

Mycgr3G85486 Mycgr3T
  
Location: 7266-8511

Mycgr3G85486\_Mycgr3T

Mycgr3G92221 Mycgr3T
  
Location: 8611-9193

Mycgr3G92221\_Mycgr3T

Mycgr3G39931 Mycgr3T
  
Location: 9293-10157

Mycgr3G39931\_Mycgr3T

Mycgr3G99766 Mycgr3T
  
Location: 10257-11775

Mycgr3G99766\_Mycgr3T

P-loop containing nucleoside triphosphate hydrolase protein
  
Accession: EGZ75631
  
Location: 4961220-4965747
  
  
**BlastP hit with Mycgr3G41235\_Mycgr3T**
  
Percentage identity: 33 %
  
BlastP bit score: 816
  
Sequence coverage: 104 %
  
E-value: 0.0
  
  
 NCBI BlastP on this gene

EGZ75631

Query: Architecture Search FASTA input

HF679025 : Fusarium fujikuroi IMI 58289 draft genome, chromosome FFUJ\_chr03.    Total score: 1.0     Cumulative Blast bit score: 814

Hit cluster cross-links:

Mycgr3G41235 Mycgr3T
  
Location: 0-4062

Mycgr3G41235\_Mycgr3T

Mycgr3G70577 Mycgr3T
  
Location: 4162-6109

Mycgr3G70577\_Mycgr3T

Mycgr3G40534 Mycgr3T
  
Location: 6209-7166

Mycgr3G40534\_Mycgr3T

Mycgr3G85486 Mycgr3T
  
Location: 7266-8511

Mycgr3G85486\_Mycgr3T

Mycgr3G92221 Mycgr3T
  
Location: 8611-9193

Mycgr3G92221\_Mycgr3T

Mycgr3G39931 Mycgr3T
  
Location: 9293-10157

Mycgr3G39931\_Mycgr3T

Mycgr3G99766 Mycgr3T
  
Location: 10257-11775

Mycgr3G99766\_Mycgr3T

related to CSI2 protein
  
Accession: CCT65660
  
Location: 1578272-1579474
  
 NCBI BlastP on this gene

FFUJ\_02623

related to TFIID and SAGA subunit TAF61
  
Accession: CCT65659
  
Location: 1576237-1577190
  
 NCBI BlastP on this gene

FFUJ\_02622

probable CSL4-core component of the 3`-5` exosome
  
Accession: CCT65658
  
Location: 1574507-1575187
  
 NCBI BlastP on this gene

FFUJ\_02621

probable ATP-binding cassette transporter protein YOR1
  
Accession: CCT65657
  
Location: 1567992-1572518
  
  
**BlastP hit with Mycgr3G41235\_Mycgr3T**
  
Percentage identity: 34 %
  
BlastP bit score: 814
  
Sequence coverage: 101 %
  
E-value: 0.0
  
  
 NCBI BlastP on this gene

FFUJ\_02620

related to pseudouridine synthase
  
Accession: CCT66662
  
Location: 1565859-1567407
  
 NCBI BlastP on this gene

FFUJ\_14894

uncharacterized protein
  
Accession: CCT65656
  
Location: 1562098-1564611
  
 NCBI BlastP on this gene

FFUJ\_02619

Query: Architecture Search FASTA input

CM001202 : Mycosphaerella graminicola IPO323 chromosome 7    Total score: 1.0     Cumulative Blast bit score: 812

Hit cluster cross-links:

Mycgr3G41235 Mycgr3T
  
Location: 0-4062

Mycgr3G41235\_Mycgr3T

Mycgr3G70577 Mycgr3T
  
Location: 4162-6109

Mycgr3G70577\_Mycgr3T

Mycgr3G40534 Mycgr3T
  
Location: 6209-7166

Mycgr3G40534\_Mycgr3T

Mycgr3G85486 Mycgr3T
  
Location: 7266-8511

Mycgr3G85486\_Mycgr3T

Mycgr3G92221 Mycgr3T
  
Location: 8611-9193

Mycgr3G92221\_Mycgr3T

Mycgr3G39931 Mycgr3T
  
Location: 9293-10157

Mycgr3G39931\_Mycgr3T

Mycgr3G99766 Mycgr3T
  
Location: 10257-11775

Mycgr3G99766\_Mycgr3T

hypothetical protein
  
Accession: EGP86236
  
Location: 435960-436483
  
 NCBI BlastP on this gene

EGP86236

putative ABC transporter
  
Accession: EGP86237
  
Location: 428260-432801
  
  
**BlastP hit with Mycgr3G41235\_Mycgr3T**
  
Percentage identity: 33 %
  
BlastP bit score: 812
  
Sequence coverage: 105 %
  
E-value: 0.0
  
  
 NCBI BlastP on this gene

EGP86237

hypothetical protein
  
Accession: EGP86238
  
Location: 420628-424588
  
 NCBI BlastP on this gene

EGP86238

Query: Architecture Search FASTA input

GL385397 : Gaeumannomyces graminis var. tritici R3-111a-1 unplaced genomic scaffold supercont2.3    Total score: 1.0     Cumulative Blast bit score: 805

Hit cluster cross-links:

Mycgr3G41235 Mycgr3T
  
Location: 0-4062

Mycgr3G41235\_Mycgr3T

Mycgr3G70577 Mycgr3T
  
Location: 4162-6109

Mycgr3G70577\_Mycgr3T

Mycgr3G40534 Mycgr3T
  
Location: 6209-7166

Mycgr3G40534\_Mycgr3T

Mycgr3G85486 Mycgr3T
  
Location: 7266-8511

Mycgr3G85486\_Mycgr3T

Mycgr3G92221 Mycgr3T
  
Location: 8611-9193

Mycgr3G92221\_Mycgr3T

Mycgr3G39931 Mycgr3T
  
Location: 9293-10157

Mycgr3G39931\_Mycgr3T

Mycgr3G99766 Mycgr3T
  
Location: 10257-11775

Mycgr3G99766\_Mycgr3T

hypothetical protein
  
Accession: EJT77408
  
Location: 6544949-6547453
  
 NCBI BlastP on this gene

EJT77408

multidrug resistance-associated protein 2
  
Accession: EJT77410
  
Location: 6549330-6553904
  
  
**BlastP hit with Mycgr3G41235\_Mycgr3T**
  
Percentage identity: 34 %
  
BlastP bit score: 805
  
Sequence coverage: 105 %
  
E-value: 0.0
  
  
 NCBI BlastP on this gene

EJT77410

Query: Architecture Search FASTA input

EQ962656 : Talaromyces stipitatus ATCC 10500 scf\_1105507295549 genomic scaffold    Total score: 1.0     Cumulative Blast bit score: 804

Hit cluster cross-links:

Mycgr3G41235 Mycgr3T
  
Location: 0-4062

Mycgr3G41235\_Mycgr3T

Mycgr3G70577 Mycgr3T
  
Location: 4162-6109

Mycgr3G70577\_Mycgr3T

Mycgr3G40534 Mycgr3T
  
Location: 6209-7166

Mycgr3G40534\_Mycgr3T

Mycgr3G85486 Mycgr3T
  
Location: 7266-8511

Mycgr3G85486\_Mycgr3T

Mycgr3G92221 Mycgr3T
  
Location: 8611-9193

Mycgr3G92221\_Mycgr3T

Mycgr3G39931 Mycgr3T
  
Location: 9293-10157

Mycgr3G39931\_Mycgr3T

Mycgr3G99766 Mycgr3T
  
Location: 10257-11775

Mycgr3G99766\_Mycgr3T

tripeptidyl-peptidase (TppA), putative
  
Accession: EED16525
  
Location: 2024302-2026250
  
 NCBI BlastP on this gene

EED16525

hypothetical protein
  
Accession: EED16526
  
Location: 2026696-2028367
  
 NCBI BlastP on this gene

EED16526

ABC multidrug transporter, putative
  
Accession: EED16527
  
Location: 2029727-2034135
  
  
**BlastP hit with Mycgr3G41235\_Mycgr3T**
  
Percentage identity: 33 %
  
BlastP bit score: 804
  
Sequence coverage: 102 %
  
E-value: 0.0
  
  
 NCBI BlastP on this gene

EED16527

Query: Architecture Search FASTA input

DS989823 : Arthroderma gypseum CBS 118893 supercont1.2 genomic scaffold    Total score: 1.0     Cumulative Blast bit score: 799

Hit cluster cross-links:

Mycgr3G41235 Mycgr3T
  
Location: 0-4062

Mycgr3G41235\_Mycgr3T

Mycgr3G70577 Mycgr3T
  
Location: 4162-6109

Mycgr3G70577\_Mycgr3T

Mycgr3G40534 Mycgr3T
  
Location: 6209-7166

Mycgr3G40534\_Mycgr3T

Mycgr3G85486 Mycgr3T
  
Location: 7266-8511

Mycgr3G85486\_Mycgr3T

Mycgr3G92221 Mycgr3T
  
Location: 8611-9193

Mycgr3G92221\_Mycgr3T

Mycgr3G39931 Mycgr3T
  
Location: 9293-10157

Mycgr3G39931\_Mycgr3T

Mycgr3G99766 Mycgr3T
  
Location: 10257-11775

Mycgr3G99766\_Mycgr3T

GPI mannosyltransferase 3
  
Accession: EFR00025
  
Location: 2677066-2679581
  
 NCBI BlastP on this gene

EFR00025

ornithine aminotransferase
  
Accession: EFR00026
  
Location: 2680202-2681678
  
 NCBI BlastP on this gene

EFR00026

oligomycin resistance ATP-dependent permease YOR1
  
Accession: EFR00027
  
Location: 2685158-2689594
  
  
**BlastP hit with Mycgr3G41235\_Mycgr3T**
  
Percentage identity: 33 %
  
BlastP bit score: 799
  
Sequence coverage: 103 %
  
E-value: 0.0
  
  
 NCBI BlastP on this gene

EFR00027

amino-acid permease 2
  
Accession: EFR00028
  
Location: 2689812-2691677
  
 NCBI BlastP on this gene

EFR00028

tyrosyl-DNA phosphodiesterase 1
  
Accession: EFR00029
  
Location: 2692560-2694517
  
 NCBI BlastP on this gene

EFR00029

hypothetical protein
  
Accession: EFR00030
  
Location: 2694928-2695523
  
 NCBI BlastP on this gene

EFR00030

Query: Architecture Search FASTA input

GG700652 : Trichophyton rubrum CBS 118892 genomic scaffold supercont2.5    Total score: 1.0     Cumulative Blast bit score: 797

Hit cluster cross-links:

Mycgr3G41235 Mycgr3T
  
Location: 0-4062

Mycgr3G41235\_Mycgr3T

Mycgr3G70577 Mycgr3T
  
Location: 4162-6109

Mycgr3G70577\_Mycgr3T

Mycgr3G40534 Mycgr3T
  
Location: 6209-7166

Mycgr3G40534\_Mycgr3T

Mycgr3G85486 Mycgr3T
  
Location: 7266-8511

Mycgr3G85486\_Mycgr3T

Mycgr3G92221 Mycgr3T
  
Location: 8611-9193

Mycgr3G92221\_Mycgr3T

Mycgr3G39931 Mycgr3T
  
Location: 9293-10157

Mycgr3G39931\_Mycgr3T

Mycgr3G99766 Mycgr3T
  
Location: 10257-11775

Mycgr3G99766\_Mycgr3T

mannosyltransferase
  
Accession: EGD88599
  
Location: 335297-337825
  
 NCBI BlastP on this gene

EGD88599

ornithine aminotransferase
  
Accession: EGD88600
  
Location: 338589-340043
  
 NCBI BlastP on this gene

EGD88600

hypothetical protein
  
Accession: EGD88601
  
Location: 342299-342798
  
 NCBI BlastP on this gene

EGD88601

multidrug resistance-associated protein 5
  
Accession: EGD88602
  
Location: 343981-348417
  
  
**BlastP hit with Mycgr3G41235\_Mycgr3T**
  
Percentage identity: 33 %
  
BlastP bit score: 798
  
Sequence coverage: 105 %
  
E-value: 0.0
  
  
 NCBI BlastP on this gene

EGD88602

amino acid permease
  
Accession: EGD88603
  
Location: 348652-350514
  
 NCBI BlastP on this gene

EGD88603

tyrosyl-DNA phosphodiesterase
  
Accession: EGD88604
  
Location: 351328-353267
  
 NCBI BlastP on this gene

EGD88604

hypothetical protein
  
Accession: EGD88605
  
Location: 353698-354164
  
 NCBI BlastP on this gene

EGD88605

Query: Architecture Search FASTA input

JH719400 : Dichomitus squalens LYAD-421 SS1 unplaced genomic scaffold DICSQscaffold\_4    Total score: 1.0     Cumulative Blast bit score: 795

Hit cluster cross-links:

Mycgr3G41235 Mycgr3T
  
Location: 0-4062

Mycgr3G41235\_Mycgr3T

Mycgr3G70577 Mycgr3T
  
Location: 4162-6109

Mycgr3G70577\_Mycgr3T

Mycgr3G40534 Mycgr3T
  
Location: 6209-7166

Mycgr3G40534\_Mycgr3T

Mycgr3G85486 Mycgr3T
  
Location: 7266-8511

Mycgr3G85486\_Mycgr3T

Mycgr3G92221 Mycgr3T
  
Location: 8611-9193

Mycgr3G92221\_Mycgr3T

Mycgr3G39931 Mycgr3T
  
Location: 9293-10157

Mycgr3G39931\_Mycgr3T

Mycgr3G99766 Mycgr3T
  
Location: 10257-11775

Mycgr3G99766\_Mycgr3T

hypothetical protein
  
Accession: EJF64556
  
Location: 323385-326357
  
 NCBI BlastP on this gene

EJF64556

hypothetical protein
  
Accession: EJF64557
  
Location: 327072-328923
  
 NCBI BlastP on this gene

EJF64557

P-loop containing nucleoside triphosphate hydrolase protein
  
Accession: EJF64558
  
Location: 329941-336484
  
  
**BlastP hit with Mycgr3G41235\_Mycgr3T**
  
Percentage identity: 34 %
  
BlastP bit score: 795
  
Sequence coverage: 102 %
  
E-value: 0.0
  
  
 NCBI BlastP on this gene

EJF64558

hypothetical protein
  
Accession: EJF64559
  
Location: 336714-340809
  
 NCBI BlastP on this gene

EJF64559

Query: Architecture Search FASTA input

DS995903 : Penicillium marneffei ATCC 18224 scf\_1105668340984 genomic scaffold    Total score: 1.0     Cumulative Blast bit score: 795

Hit cluster cross-links:

Mycgr3G41235 Mycgr3T
  
Location: 0-4062

Mycgr3G41235\_Mycgr3T

Mycgr3G70577 Mycgr3T
  
Location: 4162-6109

Mycgr3G70577\_Mycgr3T

Mycgr3G40534 Mycgr3T
  
Location: 6209-7166

Mycgr3G40534\_Mycgr3T

Mycgr3G85486 Mycgr3T
  
Location: 7266-8511

Mycgr3G85486\_Mycgr3T

Mycgr3G92221 Mycgr3T
  
Location: 8611-9193

Mycgr3G92221\_Mycgr3T

Mycgr3G39931 Mycgr3T
  
Location: 9293-10157

Mycgr3G39931\_Mycgr3T

Mycgr3G99766 Mycgr3T
  
Location: 10257-11775

Mycgr3G99766\_Mycgr3T

ABC multidrug transporter, putative
  
Accession: EEA21837
  
Location: 1688303-1692687
  
  
**BlastP hit with Mycgr3G41235\_Mycgr3T**
  
Percentage identity: 32 %
  
BlastP bit score: 795
  
Sequence coverage: 102 %
  
E-value: 0.0
  
  
 NCBI BlastP on this gene

EEA21837

alpha/beta hydrolase family protein, putative
  
Accession: EEA21836
  
Location: 1686350-1687636
  
 NCBI BlastP on this gene

EEA21836

hypothetical protein
  
Accession: EEA21835
  
Location: 1685882-1686179
  
 NCBI BlastP on this gene

EEA21835

hypothetical protein
  
Accession: EEA21834
  
Location: 1681739-1682737
  
 NCBI BlastP on this gene

EEA21834

Query: Architecture Search FASTA input

DS995721 : Trichophyton equinum CBS 127.97 supercont1.4 genomic scaffold    Total score: 1.0     Cumulative Blast bit score: 790

Hit cluster cross-links:

Mycgr3G41235 Mycgr3T
  
Location: 0-4062

Mycgr3G41235\_Mycgr3T

Mycgr3G70577 Mycgr3T
  
Location: 4162-6109

Mycgr3G70577\_Mycgr3T

Mycgr3G40534 Mycgr3T
  
Location: 6209-7166

Mycgr3G40534\_Mycgr3T

Mycgr3G85486 Mycgr3T
  
Location: 7266-8511

Mycgr3G85486\_Mycgr3T

Mycgr3G92221 Mycgr3T
  
Location: 8611-9193

Mycgr3G92221\_Mycgr3T

Mycgr3G39931 Mycgr3T
  
Location: 9293-10157

Mycgr3G39931\_Mycgr3T

Mycgr3G99766 Mycgr3T
  
Location: 10257-11775

Mycgr3G99766\_Mycgr3T

mannosyltransferase
  
Accession: EGE02095
  
Location: 17180-20127
  
 NCBI BlastP on this gene

EGE02095

ornithine aminotransferase
  
Accession: EGE02096
  
Location: 20348-21807
  
 NCBI BlastP on this gene

EGE02096

ABC multidrug transporter
  
Accession: EGE02097
  
Location: 25841-30275
  
  
**BlastP hit with Mycgr3G41235\_Mycgr3T**
  
Percentage identity: 33 %
  
BlastP bit score: 790
  
Sequence coverage: 103 %
  
E-value: 0.0
  
  
 NCBI BlastP on this gene

EGE02097

amino acid permease
  
Accession: EGE02098
  
Location: 30500-32360
  
 NCBI BlastP on this gene

EGE02098

tyrosyl-DNA phosphodiesterase
  
Accession: EGE02099
  
Location: 33232-35177
  
 NCBI BlastP on this gene

EGE02099

hypothetical protein
  
Accession: EGE02100
  
Location: 35616-36064
  
 NCBI BlastP on this gene

EGE02100

Query: Architecture Search FASTA input

DS499598 : Aspergillus fumigatus A1163 scf\_000005 genomic scaffold    Total score: 1.0     Cumulative Blast bit score: 786

Hit cluster cross-links:

Mycgr3G41235 Mycgr3T
  
Location: 0-4062

Mycgr3G41235\_Mycgr3T

Mycgr3G70577 Mycgr3T
  
Location: 4162-6109

Mycgr3G70577\_Mycgr3T

Mycgr3G40534 Mycgr3T
  
Location: 6209-7166

Mycgr3G40534\_Mycgr3T

Mycgr3G85486 Mycgr3T
  
Location: 7266-8511

Mycgr3G85486\_Mycgr3T

Mycgr3G92221 Mycgr3T
  
Location: 8611-9193

Mycgr3G92221\_Mycgr3T

Mycgr3G39931 Mycgr3T
  
Location: 9293-10157

Mycgr3G39931\_Mycgr3T

Mycgr3G99766 Mycgr3T
  
Location: 10257-11775

Mycgr3G99766\_Mycgr3T

mannosyltransferase, putative
  
Accession: EDP50290
  
Location: 936365-938963
  
 NCBI BlastP on this gene

EDP50290

L-ornithine aminotransferase Car2, putative
  
Accession: EDP50291
  
Location: 939678-941092
  
 NCBI BlastP on this gene

EDP50291

ABC multidrug transporter, putative
  
Accession: EDP50292
  
Location: 942783-947085
  
  
**BlastP hit with Mycgr3G41235\_Mycgr3T**
  
Percentage identity: 33 %
  
BlastP bit score: 786
  
Sequence coverage: 101 %
  
E-value: 0.0
  
  
 NCBI BlastP on this gene

EDP50292

ankyrin repeat protein
  
Accession: EDP50293
  
Location: 947466-948049
  
 NCBI BlastP on this gene

EDP50293

anion exchange family protein
  
Accession: EDP50294
  
Location: 948328-950355
  
 NCBI BlastP on this gene

EDP50294

SET domain protein
  
Accession: EDP50295
  
Location: 951763-952964
  
 NCBI BlastP on this gene

EDP50295

Query: Architecture Search FASTA input

AAHF01000005 : Aspergillus fumigatus Af293    Total score: 1.0     Cumulative Blast bit score: 786

Hit cluster cross-links:

Mycgr3G41235 Mycgr3T
  
Location: 0-4062

Mycgr3G41235\_Mycgr3T

Mycgr3G70577 Mycgr3T
  
Location: 4162-6109

Mycgr3G70577\_Mycgr3T

Mycgr3G40534 Mycgr3T
  
Location: 6209-7166

Mycgr3G40534\_Mycgr3T

Mycgr3G85486 Mycgr3T
  
Location: 7266-8511

Mycgr3G85486\_Mycgr3T

Mycgr3G92221 Mycgr3T
  
Location: 8611-9193

Mycgr3G92221\_Mycgr3T

Mycgr3G39931 Mycgr3T
  
Location: 9293-10157

Mycgr3G39931\_Mycgr3T

Mycgr3G99766 Mycgr3T
  
Location: 10257-11775

Mycgr3G99766\_Mycgr3T

ABC multidrug transporter, putative
  
Accession: EAL89872
  
Location: 1531528-1535830
  
  
**BlastP hit with Mycgr3G41235\_Mycgr3T**
  
Percentage identity: 33 %
  
BlastP bit score: 786
  
Sequence coverage: 101 %
  
E-value: 0.0
  
  
 NCBI BlastP on this gene

EAL89872

ankyrin repeat protein
  
Accession: EAL89871
  
Location: 1530564-1531147
  
 NCBI BlastP on this gene

EAL89871

anion exchange family protein
  
Accession: EAL89870
  
Location: 1528265-1530286
  
 NCBI BlastP on this gene

EAL89870

SET domain protein
  
Accession: EAL89869
  
Location: 1525656-1526857
  
 NCBI BlastP on this gene

EAL89869

Query: Architecture Search FASTA input

DS995901 : Penicillium marneffei ATCC 18224 scf\_1105668340960 genomic scaffold    Total score: 1.0     Cumulative Blast bit score: 781

Hit cluster cross-links:

Mycgr3G41235 Mycgr3T
  
Location: 0-4062

Mycgr3G41235\_Mycgr3T

Mycgr3G70577 Mycgr3T
  
Location: 4162-6109

Mycgr3G70577\_Mycgr3T

Mycgr3G40534 Mycgr3T
  
Location: 6209-7166

Mycgr3G40534\_Mycgr3T

Mycgr3G85486 Mycgr3T
  
Location: 7266-8511

Mycgr3G85486\_Mycgr3T

Mycgr3G92221 Mycgr3T
  
Location: 8611-9193

Mycgr3G92221\_Mycgr3T

Mycgr3G39931 Mycgr3T
  
Location: 9293-10157

Mycgr3G39931\_Mycgr3T

Mycgr3G99766 Mycgr3T
  
Location: 10257-11775

Mycgr3G99766\_Mycgr3T

oligomycin resistance ATP-dependent permease yor1, putative
  
Accession: EEA24390
  
Location: 1966956-1971293
  
  
**BlastP hit with Mycgr3G41235\_Mycgr3T**
  
Percentage identity: 33 %
  
BlastP bit score: 781
  
Sequence coverage: 103 %
  
E-value: 0.0
  
  
 NCBI BlastP on this gene

EEA24390

cutinase, putative
  
Accession: EEA24389
  
Location: 1964067-1964973
  
 NCBI BlastP on this gene

EEA24389

conserved hypothetical protein
  
Accession: EEA24388
  
Location: 1960982-1963399
  
 NCBI BlastP on this gene

EEA24388

Query: Architecture Search FASTA input

JH719402 : Dichomitus squalens LYAD-421 SS1 unplaced genomic scaffold DICSQscaffold\_6    Total score: 1.0     Cumulative Blast bit score: 776

Hit cluster cross-links:

Mycgr3G41235 Mycgr3T
  
Location: 0-4062

Mycgr3G41235\_Mycgr3T

Mycgr3G70577 Mycgr3T
  
Location: 4162-6109

Mycgr3G70577\_Mycgr3T

Mycgr3G40534 Mycgr3T
  
Location: 6209-7166

Mycgr3G40534\_Mycgr3T

Mycgr3G85486 Mycgr3T
  
Location: 7266-8511

Mycgr3G85486\_Mycgr3T

Mycgr3G92221 Mycgr3T
  
Location: 8611-9193

Mycgr3G92221\_Mycgr3T

Mycgr3G39931 Mycgr3T
  
Location: 9293-10157

Mycgr3G39931\_Mycgr3T

Mycgr3G99766 Mycgr3T
  
Location: 10257-11775

Mycgr3G99766\_Mycgr3T

cytochrome P450
  
Accession: EJF63642
  
Location: 310449-313334
  
 NCBI BlastP on this gene

EJF63642

hypothetical protein
  
Accession: EJF63641
  
Location: 309526-310191
  
 NCBI BlastP on this gene

EJF63641

hypothetical protein
  
Accession: EJF63640
  
Location: 306598-308600
  
 NCBI BlastP on this gene

EJF63640

multidrug resistance-associated ABC transporter
  
Accession: EJF63639
  
Location: 299614-305768
  
  
**BlastP hit with Mycgr3G41235\_Mycgr3T**
  
Percentage identity: 32 %
  
BlastP bit score: 776
  
Sequence coverage: 107 %
  
E-value: 0.0
  
  
 NCBI BlastP on this gene

EJF63639

cytochrome P450
  
Accession: EJF63638
  
Location: 296580-298974
  
 NCBI BlastP on this gene

EJF63638

hypothetical protein
  
Accession: EJF63637
  
Location: 294086-294711
  
 NCBI BlastP on this gene

EJF63637

Query: Architecture Search FASTA input

DS027685 : Neosartorya fischeri NRRL 181 1099437636245 genomic scaffold    Total score: 1.0     Cumulative Blast bit score: 776

Hit cluster cross-links:

Mycgr3G41235 Mycgr3T
  
Location: 0-4062

Mycgr3G41235\_Mycgr3T

Mycgr3G70577 Mycgr3T
  
Location: 4162-6109

Mycgr3G70577\_Mycgr3T

Mycgr3G40534 Mycgr3T
  
Location: 6209-7166

Mycgr3G40534\_Mycgr3T

Mycgr3G85486 Mycgr3T
  
Location: 7266-8511

Mycgr3G85486\_Mycgr3T

Mycgr3G92221 Mycgr3T
  
Location: 8611-9193

Mycgr3G92221\_Mycgr3T

Mycgr3G39931 Mycgr3T
  
Location: 9293-10157

Mycgr3G39931\_Mycgr3T

Mycgr3G99766 Mycgr3T
  
Location: 10257-11775

Mycgr3G99766\_Mycgr3T

ABC multidrug transporter, putative
  
Accession: EAW25211
  
Location: 1612989-1617284
  
  
**BlastP hit with Mycgr3G41235\_Mycgr3T**
  
Percentage identity: 33 %
  
BlastP bit score: 776
  
Sequence coverage: 101 %
  
E-value: 0.0
  
  
 NCBI BlastP on this gene

EAW25211

Ankyrin repeat protein
  
Accession: EAW25210
  
Location: 1604300-1608054
  
 NCBI BlastP on this gene

EAW25210

Query: Architecture Search FASTA input

ABDF02000001 : Trichoderma virens Gv29-8    Total score: 1.0     Cumulative Blast bit score: 776

Hit cluster cross-links:

Mycgr3G41235 Mycgr3T
  
Location: 0-4062

Mycgr3G41235\_Mycgr3T

Mycgr3G70577 Mycgr3T
  
Location: 4162-6109

Mycgr3G70577\_Mycgr3T

Mycgr3G40534 Mycgr3T
  
Location: 6209-7166

Mycgr3G40534\_Mycgr3T

Mycgr3G85486 Mycgr3T
  
Location: 7266-8511

Mycgr3G85486\_Mycgr3T

Mycgr3G92221 Mycgr3T
  
Location: 8611-9193

Mycgr3G92221\_Mycgr3T

Mycgr3G39931 Mycgr3T
  
Location: 9293-10157

Mycgr3G39931\_Mycgr3T

Mycgr3G99766 Mycgr3T
  
Location: 10257-11775

Mycgr3G99766\_Mycgr3T

hypothetical protein
  
Accession: EHK27273
  
Location: 1699237-1700700
  
 NCBI BlastP on this gene

EHK27273

hypothetical protein
  
Accession: EHK27274
  
Location: 1701339-1703715
  
 NCBI BlastP on this gene

EHK27274

hypothetical protein
  
Accession: EHK27275
  
Location: 1705907-1710479
  
  
**BlastP hit with Mycgr3G41235\_Mycgr3T**
  
Percentage identity: 33 %
  
BlastP bit score: 776
  
Sequence coverage: 105 %
  
E-value: 0.0
  
  
 NCBI BlastP on this gene

EHK27275

Query: Architecture Search FASTA input

DS995906 : Penicillium marneffei ATCC 18224 scf\_1105668340770 genomic scaffold    Total score: 1.0     Cumulative Blast bit score: 771

Hit cluster cross-links:

Mycgr3G41235 Mycgr3T
  
Location: 0-4062

Mycgr3G41235\_Mycgr3T

Mycgr3G70577 Mycgr3T
  
Location: 4162-6109

Mycgr3G70577\_Mycgr3T

Mycgr3G40534 Mycgr3T
  
Location: 6209-7166

Mycgr3G40534\_Mycgr3T

Mycgr3G85486 Mycgr3T
  
Location: 7266-8511

Mycgr3G85486\_Mycgr3T

Mycgr3G92221 Mycgr3T
  
Location: 8611-9193

Mycgr3G92221\_Mycgr3T

Mycgr3G39931 Mycgr3T
  
Location: 9293-10157

Mycgr3G39931\_Mycgr3T

Mycgr3G99766 Mycgr3T
  
Location: 10257-11775

Mycgr3G99766\_Mycgr3T

nonribosomal peptide synthase, putative
  
Accession: EEA19149
  
Location: 1517822-1523002
  
 NCBI BlastP on this gene

EEA19149

conserved hypothetical protein
  
Accession: EEA19148
  
Location: 1516488-1517582
  
 NCBI BlastP on this gene

EEA19148

conserved hypothetical protein
  
Accession: EEA19147
  
Location: 1514040-1515981
  
 NCBI BlastP on this gene

EEA19147

oligomycin resistance ATP-dependent permease yor1, putative
  
Accession: EEA19146
  
Location: 1509447-1513663
  
  
**BlastP hit with Mycgr3G41235\_Mycgr3T**
  
Percentage identity: 34 %
  
BlastP bit score: 771
  
Sequence coverage: 100 %
  
E-value: 0.0
  
  
 NCBI BlastP on this gene

EEA19146

conserved hypothetical protein
  
Accession: EEA19145
  
Location: 1507194-1509399
  
 NCBI BlastP on this gene

EEA19145

conserved hypothetical protein
  
Accession: EEA19143
  
Location: 1501299-1506454
  
 NCBI BlastP on this gene

EEA19143

Query: Architecture Search FASTA input

GL985056 : Trichoderma reesei QM6a unplaced genomic scaffold TRIREscaffold\_1    Total score: 1.0     Cumulative Blast bit score: 768

Hit cluster cross-links:

Mycgr3G41235 Mycgr3T
  
Location: 0-4062

Mycgr3G41235\_Mycgr3T

Mycgr3G70577 Mycgr3T
  
Location: 4162-6109

Mycgr3G70577\_Mycgr3T

Mycgr3G40534 Mycgr3T
  
Location: 6209-7166

Mycgr3G40534\_Mycgr3T

Mycgr3G85486 Mycgr3T
  
Location: 7266-8511

Mycgr3G85486\_Mycgr3T

Mycgr3G92221 Mycgr3T
  
Location: 8611-9193

Mycgr3G92221\_Mycgr3T

Mycgr3G39931 Mycgr3T
  
Location: 9293-10157

Mycgr3G39931\_Mycgr3T

Mycgr3G99766 Mycgr3T
  
Location: 10257-11775

Mycgr3G99766\_Mycgr3T

predicted protein
  
Accession: EGR53081
  
Location: 3199556-3203833
  
  
**BlastP hit with Mycgr3G41235\_Mycgr3T**
  
Percentage identity: 32 %
  
BlastP bit score: 768
  
Sequence coverage: 103 %
  
E-value: 0.0
  
  
 NCBI BlastP on this gene

EGR53081

hypothetical protein
  
Accession: EGR52545
  
Location: 3197856-3198878
  
 NCBI BlastP on this gene

EGR52545

predicted protein
  
Accession: EGR53080
  
Location: 3195668-3196390
  
 NCBI BlastP on this gene

EGR53080

predicted protein
  
Accession: EGR53079
  
Location: 3194459-3194884
  
 NCBI BlastP on this gene

EGR53079

predicted protein
  
Accession: EGR52544
  
Location: 3192759-3193907
  
 NCBI BlastP on this gene

EGR52544

Query: Architecture Search FASTA input

KB445647 : Cochliobolus sativus ND90Pr unplaced genomic scaffold COCSAscaffold\_11    Total score: 1.0     Cumulative Blast bit score: 767

Hit cluster cross-links:

Mycgr3G41235 Mycgr3T
  
Location: 0-4062

Mycgr3G41235\_Mycgr3T

Mycgr3G70577 Mycgr3T
  
Location: 4162-6109

Mycgr3G70577\_Mycgr3T

Mycgr3G40534 Mycgr3T
  
Location: 6209-7166

Mycgr3G40534\_Mycgr3T

Mycgr3G85486 Mycgr3T
  
Location: 7266-8511

Mycgr3G85486\_Mycgr3T

Mycgr3G92221 Mycgr3T
  
Location: 8611-9193

Mycgr3G92221\_Mycgr3T

Mycgr3G39931 Mycgr3T
  
Location: 9293-10157

Mycgr3G39931\_Mycgr3T

Mycgr3G99766 Mycgr3T
  
Location: 10257-11775

Mycgr3G99766\_Mycgr3T

hypothetical protein
  
Accession: EMD61888
  
Location: 380779-384617
  
 NCBI BlastP on this gene

EMD61888

hypothetical protein
  
Accession: EMD61889
  
Location: 386418-387972
  
 NCBI BlastP on this gene

EMD61889

hypothetical protein
  
Accession: EMD61890
  
Location: 389583-394173
  
  
**BlastP hit with Mycgr3G41235\_Mycgr3T**
  
Percentage identity: 32 %
  
BlastP bit score: 767
  
Sequence coverage: 103 %
  
E-value: 0.0
  
  
 NCBI BlastP on this gene

EMD61890

Query: Architecture Search FASTA input

AACS02000012 : Coprinopsis cinerea okayama7#130    Total score: 1.0     Cumulative Blast bit score: 767

Hit cluster cross-links:

Mycgr3G41235 Mycgr3T
  
Location: 0-4062

Mycgr3G41235\_Mycgr3T

Mycgr3G70577 Mycgr3T
  
Location: 4162-6109

Mycgr3G70577\_Mycgr3T

Mycgr3G40534 Mycgr3T
  
Location: 6209-7166

Mycgr3G40534\_Mycgr3T

Mycgr3G85486 Mycgr3T
  
Location: 7266-8511

Mycgr3G85486\_Mycgr3T

Mycgr3G92221 Mycgr3T
  
Location: 8611-9193

Mycgr3G92221\_Mycgr3T

Mycgr3G39931 Mycgr3T
  
Location: 9293-10157

Mycgr3G39931\_Mycgr3T

Mycgr3G99766 Mycgr3T
  
Location: 10257-11775

Mycgr3G99766\_Mycgr3T

ribosomal processing
  
Accession: EAU86772
  
Location: 1493052-1496397
  
 NCBI BlastP on this gene

EAU86772

ATP-dependent bile acid permease
  
Accession: EAU86771
  
Location: 1486151-1492069
  
  
**BlastP hit with Mycgr3G41235\_Mycgr3T**
  
Percentage identity: 34 %
  
BlastP bit score: 767
  
Sequence coverage: 103 %
  
E-value: 0.0
  
  
 NCBI BlastP on this gene

EAU86771

hypothetical protein
  
Accession: EAU86770
  
Location: 1484949-1485807
  
 NCBI BlastP on this gene

EAU86770

hypothetical protein
  
Accession: EAU86769
  
Location: 1480119-1481843
  
 NCBI BlastP on this gene

EAU86769

Query: Architecture Search FASTA input

CM001197 : Mycosphaerella graminicola IPO323 chromosome 2    Total score: 1.0     Cumulative Blast bit score: 765

Hit cluster cross-links:

Mycgr3G41235 Mycgr3T
  
Location: 0-4062

Mycgr3G41235\_Mycgr3T

Mycgr3G70577 Mycgr3T
  
Location: 4162-6109

Mycgr3G70577\_Mycgr3T

Mycgr3G40534 Mycgr3T
  
Location: 6209-7166

Mycgr3G40534\_Mycgr3T

Mycgr3G85486 Mycgr3T
  
Location: 7266-8511

Mycgr3G85486\_Mycgr3T

Mycgr3G92221 Mycgr3T
  
Location: 8611-9193

Mycgr3G92221\_Mycgr3T

Mycgr3G39931 Mycgr3T
  
Location: 9293-10157

Mycgr3G39931\_Mycgr3T

Mycgr3G99766 Mycgr3T
  
Location: 10257-11775

Mycgr3G99766\_Mycgr3T

putative ABC transporter
  
Accession: EGP90265
  
Location: 3393870-3398338
  
  
**BlastP hit with Mycgr3G41235\_Mycgr3T**
  
Percentage identity: 33 %
  
BlastP bit score: 765
  
Sequence coverage: 103 %
  
E-value: 0.0
  
  
 NCBI BlastP on this gene

EGP90265

hypothetical protein
  
Accession: EGP90156
  
Location: 3391482-3392087
  
 NCBI BlastP on this gene

EGP90156

hypothetical protein
  
Accession: EGP90266
  
Location: 3387815-3390661
  
 NCBI BlastP on this gene

EGP90266

Query: Architecture Search FASTA input

GL377310 : Schizophyllum commune H4-8 unplaced genomic scaffold SCHCOscaffold\_9    Total score: 1.0     Cumulative Blast bit score: 763

Hit cluster cross-links:

Mycgr3G41235 Mycgr3T
  
Location: 0-4062

Mycgr3G41235\_Mycgr3T

Mycgr3G70577 Mycgr3T
  
Location: 4162-6109

Mycgr3G70577\_Mycgr3T

Mycgr3G40534 Mycgr3T
  
Location: 6209-7166

Mycgr3G40534\_Mycgr3T

Mycgr3G85486 Mycgr3T
  
Location: 7266-8511

Mycgr3G85486\_Mycgr3T

Mycgr3G92221 Mycgr3T
  
Location: 8611-9193

Mycgr3G92221\_Mycgr3T

Mycgr3G39931 Mycgr3T
  
Location: 9293-10157

Mycgr3G39931\_Mycgr3T

Mycgr3G99766 Mycgr3T
  
Location: 10257-11775

Mycgr3G99766\_Mycgr3T

hypothetical protein
  
Accession: EFI93734
  
Location: 690700-691095
  
 NCBI BlastP on this gene

EFI93734

hypothetical protein
  
Accession: EFI94034
  
Location: 688574-690480
  
 NCBI BlastP on this gene

EFI94034

hypothetical protein
  
Accession: EFI94033
  
Location: 687417-688205
  
 NCBI BlastP on this gene

EFI94033

hypothetical protein
  
Accession: EFI94032
  
Location: 686032-686995
  
 NCBI BlastP on this gene

EFI94032

hypothetical protein
  
Accession: EFI94031
  
Location: 679703-685413
  
  
**BlastP hit with Mycgr3G41235\_Mycgr3T**
  
Percentage identity: 34 %
  
BlastP bit score: 763
  
Sequence coverage: 104 %
  
E-value: 0.0
  
  
 NCBI BlastP on this gene

EFI94031

expressed protein
  
Accession: EFI94030
  
Location: 676215-678220
  
 NCBI BlastP on this gene

EFI94030

hypothetical protein
  
Accession: EFI94029
  
Location: 674467-675346
  
 NCBI BlastP on this gene

EFI94029

Query: Architecture Search FASTA input

FP929130 : Leptosphaeria maculans JN3 lm\_SuperContig\_17\_v2 genomic supercontig    Total score: 1.0     Cumulative Blast bit score: 763

Hit cluster cross-links:

Mycgr3G41235 Mycgr3T
  
Location: 0-4062

Mycgr3G41235\_Mycgr3T

Mycgr3G70577 Mycgr3T
  
Location: 4162-6109

Mycgr3G70577\_Mycgr3T

Mycgr3G40534 Mycgr3T
  
Location: 6209-7166

Mycgr3G40534\_Mycgr3T

Mycgr3G85486 Mycgr3T
  
Location: 7266-8511

Mycgr3G85486\_Mycgr3T

Mycgr3G92221 Mycgr3T
  
Location: 8611-9193

Mycgr3G92221\_Mycgr3T

Mycgr3G39931 Mycgr3T
  
Location: 9293-10157

Mycgr3G39931\_Mycgr3T

Mycgr3G99766 Mycgr3T
  
Location: 10257-11775

Mycgr3G99766\_Mycgr3T

similar to ornithine aminotransferase
  
Accession: CBX97087
  
Location: 1085650-1087257
  
 NCBI BlastP on this gene

LEMA\_P102180.1

hypothetical protein
  
Accession: CBX97088
  
Location: 1088086-1089330
  
 NCBI BlastP on this gene

LEMA\_P102190.1

similar to ABC multidrug transporter
  
Accession: CBX97089
  
Location: 1091682-1096279
  
  
**BlastP hit with Mycgr3G41235\_Mycgr3T**
  
Percentage identity: 32 %
  
BlastP bit score: 763
  
Sequence coverage: 105 %
  
E-value: 0.0
  
  
 NCBI BlastP on this gene

LEMA\_P102200.1

Query: Architecture Search FASTA input

JH711791 : Trametes versicolor FP-101664 SS1 unplaced genomic scaffold TRAVEscaffold\_9    Total score: 1.0     Cumulative Blast bit score: 759

Hit cluster cross-links:

Mycgr3G41235 Mycgr3T
  
Location: 0-4062

Mycgr3G41235\_Mycgr3T

Mycgr3G70577 Mycgr3T
  
Location: 4162-6109

Mycgr3G70577\_Mycgr3T

Mycgr3G40534 Mycgr3T
  
Location: 6209-7166

Mycgr3G40534\_Mycgr3T

Mycgr3G85486 Mycgr3T
  
Location: 7266-8511

Mycgr3G85486\_Mycgr3T

Mycgr3G92221 Mycgr3T
  
Location: 8611-9193

Mycgr3G92221\_Mycgr3T

Mycgr3G39931 Mycgr3T
  
Location: 9293-10157

Mycgr3G39931\_Mycgr3T

Mycgr3G99766 Mycgr3T
  
Location: 10257-11775

Mycgr3G99766\_Mycgr3T

hypothetical protein
  
Accession: EIW55761
  
Location: 1015122-1016951
  
 NCBI BlastP on this gene

EIW55761

hypothetical protein
  
Accession: EIW55760
  
Location: 1012549-1014505
  
 NCBI BlastP on this gene

EIW55760

multidrug resistance-associated ABC transporter
  
Accession: EIW55759
  
Location: 1004647-1011806
  
  
**BlastP hit with Mycgr3G41235\_Mycgr3T**
  
Percentage identity: 32 %
  
BlastP bit score: 759
  
Sequence coverage: 105 %
  
E-value: 0.0
  
  
 NCBI BlastP on this gene

EIW55759

cytochrome P450
  
Accession: EIW55758
  
Location: 1001508-1003803
  
 NCBI BlastP on this gene

EIW55758

hypothetical protein
  
Accession: EIW55757
  
Location: 999261-1000424
  
 NCBI BlastP on this gene

EIW55757

Query: Architecture Search FASTA input

EQ962655 : Talaromyces stipitatus ATCC 10500 scf\_1105507295555 genomic scaffold    Total score: 1.0     Cumulative Blast bit score: 759

Hit cluster cross-links:

Mycgr3G41235 Mycgr3T
  
Location: 0-4062

Mycgr3G41235\_Mycgr3T

Mycgr3G70577 Mycgr3T
  
Location: 4162-6109

Mycgr3G70577\_Mycgr3T

Mycgr3G40534 Mycgr3T
  
Location: 6209-7166

Mycgr3G40534\_Mycgr3T

Mycgr3G85486 Mycgr3T
  
Location: 7266-8511

Mycgr3G85486\_Mycgr3T

Mycgr3G92221 Mycgr3T
  
Location: 8611-9193

Mycgr3G92221\_Mycgr3T

Mycgr3G39931 Mycgr3T
  
Location: 9293-10157

Mycgr3G39931\_Mycgr3T

Mycgr3G99766 Mycgr3T
  
Location: 10257-11775

Mycgr3G99766\_Mycgr3T

oligomycin resistance ATP-dependent permease yor1, putative
  
Accession: EED18119
  
Location: 2083865-2088172
  
  
**BlastP hit with Mycgr3G41235\_Mycgr3T**
  
Percentage identity: 33 %
  
BlastP bit score: 759
  
Sequence coverage: 104 %
  
E-value: 0.0
  
  
 NCBI BlastP on this gene

EED18119

cutinase, putative
  
Accession: EED18118
  
Location: 2081157-2082037
  
 NCBI BlastP on this gene

EED18118

conserved hypothetical protein
  
Accession: EED18117
  
Location: 2078109-2080369
  
 NCBI BlastP on this gene

EED18117

Query: Architecture Search FASTA input

CH476605 : Aspergillus terreus NIH2624 scaffold\_12 genomic scaffold    Total score: 1.0     Cumulative Blast bit score: 755

Hit cluster cross-links:

Mycgr3G41235 Mycgr3T
  
Location: 0-4062

Mycgr3G41235\_Mycgr3T

Mycgr3G70577 Mycgr3T
  
Location: 4162-6109

Mycgr3G70577\_Mycgr3T

Mycgr3G40534 Mycgr3T
  
Location: 6209-7166

Mycgr3G40534\_Mycgr3T

Mycgr3G85486 Mycgr3T
  
Location: 7266-8511

Mycgr3G85486\_Mycgr3T

Mycgr3G92221 Mycgr3T
  
Location: 8611-9193

Mycgr3G92221\_Mycgr3T

Mycgr3G39931 Mycgr3T
  
Location: 9293-10157

Mycgr3G39931\_Mycgr3T

Mycgr3G99766 Mycgr3T
  
Location: 10257-11775

Mycgr3G99766\_Mycgr3T

alpha-glucosidase precursor
  
Accession: EAU31451
  
Location: 810125-813278
  
 NCBI BlastP on this gene

EAU31451

alpha-amylase A precursor
  
Accession: EAU31452
  
Location: 815081-817051
  
 NCBI BlastP on this gene

EAU31452

hypothetical protein
  
Accession: EAU31453
  
Location: 818774-822927
  
  
**BlastP hit with Mycgr3G41235\_Mycgr3T**
  
Percentage identity: 34 %
  
BlastP bit score: 755
  
Sequence coverage: 98 %
  
E-value: 0.0
  
  
 NCBI BlastP on this gene

EAU31453

predicted protein
  
Accession: EAU31454
  
Location: 823477-826148
  
 NCBI BlastP on this gene

EAU31454

predicted protein
  
Accession: EAU31455
  
Location: 826554-828153
  
 NCBI BlastP on this gene

EAU31455

predicted protein
  
Accession: EAU31456
  
Location: 829100-829639
  
 NCBI BlastP on this gene

EAU31456

Query: Architecture Search FASTA input

CP003009 : Thielavia terrestris NRRL 8126 chromosome 1    Total score: 1.0     Cumulative Blast bit score: 748

Hit cluster cross-links:

Mycgr3G41235 Mycgr3T
  
Location: 0-4062

Mycgr3G41235\_Mycgr3T

Mycgr3G70577 Mycgr3T
  
Location: 4162-6109

Mycgr3G70577\_Mycgr3T

Mycgr3G40534 Mycgr3T
  
Location: 6209-7166

Mycgr3G40534\_Mycgr3T

Mycgr3G85486 Mycgr3T
  
Location: 7266-8511

Mycgr3G85486\_Mycgr3T

Mycgr3G92221 Mycgr3T
  
Location: 8611-9193

Mycgr3G92221\_Mycgr3T

Mycgr3G39931 Mycgr3T
  
Location: 9293-10157

Mycgr3G39931\_Mycgr3T

Mycgr3G99766 Mycgr3T
  
Location: 10257-11775

Mycgr3G99766\_Mycgr3T

hypothetical protein
  
Accession: AEO64821
  
Location: 10080223-10081877
  
 NCBI BlastP on this gene

THITE\_2142624

hypothetical protein
  
Accession: AEO64822
  
Location: 10086001-10090387
  
  
**BlastP hit with Mycgr3G41235\_Mycgr3T**
  
Percentage identity: 32 %
  
BlastP bit score: 748
  
Sequence coverage: 100 %
  
E-value: 0.0
  
  
 NCBI BlastP on this gene

THITE\_2142625

Query: Architecture Search FASTA input

JH226130 : Exophiala dermatitidis NIH/UT8656 unplaced genomic scaffold supercont1.1    Total score: 1.0     Cumulative Blast bit score: 741

Hit cluster cross-links:

Mycgr3G41235 Mycgr3T
  
Location: 0-4062

Mycgr3G41235\_Mycgr3T

Mycgr3G70577 Mycgr3T
  
Location: 4162-6109

Mycgr3G70577\_Mycgr3T

Mycgr3G40534 Mycgr3T
  
Location: 6209-7166

Mycgr3G40534\_Mycgr3T

Mycgr3G85486 Mycgr3T
  
Location: 7266-8511

Mycgr3G85486\_Mycgr3T

Mycgr3G92221 Mycgr3T
  
Location: 8611-9193

Mycgr3G92221\_Mycgr3T

Mycgr3G39931 Mycgr3T
  
Location: 9293-10157

Mycgr3G39931\_Mycgr3T

Mycgr3G99766 Mycgr3T
  
Location: 10257-11775

Mycgr3G99766\_Mycgr3T

hypothetical protein
  
Accession: EHY51902
  
Location: 340526-341611
  
 NCBI BlastP on this gene

EHY51902

succinate dehydrogenase [ubiquinone] iron-sulfur subunit, mitochondrial
  
Accession: EHY51903
  
Location: 342119-343119
  
 NCBI BlastP on this gene

EHY51903

hypothetical protein
  
Accession: EHY51904
  
Location: 345354-345686
  
 NCBI BlastP on this gene

EHY51904

ABC multidrug transporter
  
Accession: EHY51906
  
Location: 346523-351022
  
  
**BlastP hit with Mycgr3G41235\_Mycgr3T**
  
Percentage identity: 35 %
  
BlastP bit score: 741
  
Sequence coverage: 85 %
  
E-value: 0.0
  
  
 NCBI BlastP on this gene

EHY51906

hypothetical protein
  
Accession: EHY51907
  
Location: 352784-353263
  
 NCBI BlastP on this gene

EHY51907

Pin2-interacting protein X1
  
Accession: EHY51908
  
Location: 354116-355069
  
 NCBI BlastP on this gene

EHY51908

serine/threonine-protein kinase Chk2
  
Accession: EHY51909
  
Location: 355861-358077
  
 NCBI BlastP on this gene

EHY51909

Query: Architecture Search FASTA input

AACS02000004 : Coprinopsis cinerea okayama7#130    Total score: 1.0     Cumulative Blast bit score: 740

Hit cluster cross-links:

Mycgr3G41235 Mycgr3T
  
Location: 0-4062

Mycgr3G41235\_Mycgr3T

Mycgr3G70577 Mycgr3T
  
Location: 4162-6109

Mycgr3G70577\_Mycgr3T

Mycgr3G40534 Mycgr3T
  
Location: 6209-7166

Mycgr3G40534\_Mycgr3T

Mycgr3G85486 Mycgr3T
  
Location: 7266-8511

Mycgr3G85486\_Mycgr3T

Mycgr3G92221 Mycgr3T
  
Location: 8611-9193

Mycgr3G92221\_Mycgr3T

Mycgr3G39931 Mycgr3T
  
Location: 9293-10157

Mycgr3G39931\_Mycgr3T

Mycgr3G99766 Mycgr3T
  
Location: 10257-11775

Mycgr3G99766\_Mycgr3T

ABC protein
  
Accession: EFI27939
  
Location: 2317594-2323541
  
  
**BlastP hit with Mycgr3G41235\_Mycgr3T**
  
Percentage identity: 33 %
  
BlastP bit score: 740
  
Sequence coverage: 101 %
  
E-value: 0.0
  
  
 NCBI BlastP on this gene

EFI27939

fatty acid synthetase alpha subunit
  
Accession: EAU85370
  
Location: 2304613-2317403
  
 NCBI BlastP on this gene

EAU85370

Query: Architecture Search FASTA input

JH687393 : Stereum hirsutum FP-91666 SS1 unplaced genomic scaffold STEHIscaffold\_15    Total score: 1.0     Cumulative Blast bit score: 736

Hit cluster cross-links:

Mycgr3G41235 Mycgr3T
  
Location: 0-4062

Mycgr3G41235\_Mycgr3T

Mycgr3G70577 Mycgr3T
  
Location: 4162-6109

Mycgr3G70577\_Mycgr3T

Mycgr3G40534 Mycgr3T
  
Location: 6209-7166

Mycgr3G40534\_Mycgr3T

Mycgr3G85486 Mycgr3T
  
Location: 7266-8511

Mycgr3G85486\_Mycgr3T

Mycgr3G92221 Mycgr3T
  
Location: 8611-9193

Mycgr3G92221\_Mycgr3T

Mycgr3G39931 Mycgr3T
  
Location: 9293-10157

Mycgr3G39931\_Mycgr3T

Mycgr3G99766 Mycgr3T
  
Location: 10257-11775

Mycgr3G99766\_Mycgr3T

hypothetical protein
  
Accession: EIM82567
  
Location: 1321755-1323180
  
 NCBI BlastP on this gene

EIM82567

MFS general substrate transporter
  
Accession: EIM82568
  
Location: 1324892-1327690
  
  
**BlastP hit with Mycgr3G70577\_Mycgr3T**
  
Percentage identity: 35 %
  
BlastP bit score: 367
  
Sequence coverage: 91 %
  
E-value: 2e-114
  
  
 NCBI BlastP on this gene

EIM82568

MFS general substrate transporter
  
Accession: EIM82569
  
Location: 1329736-1332579
  
  
**BlastP hit with Mycgr3G70577\_Mycgr3T**
  
Percentage identity: 36 %
  
BlastP bit score: 369
  
Sequence coverage: 85 %
  
E-value: 4e-115
  
  
 NCBI BlastP on this gene

EIM82569

hypothetical protein
  
Accession: EIM82570
  
Location: 1333404-1333847
  
 NCBI BlastP on this gene

EIM82570

cytochrome P450
  
Accession: EIM82571
  
Location: 1334694-1335357
  
 NCBI BlastP on this gene

EIM82571

cytochrome P450
  
Accession: EIM82572
  
Location: 1335493-1336648
  
 NCBI BlastP on this gene

EIM82572

Query: Architecture Search FASTA input

CH476615 : Uncinocarpus reesii 1704 scaffold\_1 genomic scaffold    Total score: 1.0     Cumulative Blast bit score: 729

Hit cluster cross-links:

Mycgr3G41235 Mycgr3T
  
Location: 0-4062

Mycgr3G41235\_Mycgr3T

Mycgr3G70577 Mycgr3T
  
Location: 4162-6109

Mycgr3G70577\_Mycgr3T

Mycgr3G40534 Mycgr3T
  
Location: 6209-7166

Mycgr3G40534\_Mycgr3T

Mycgr3G85486 Mycgr3T
  
Location: 7266-8511

Mycgr3G85486\_Mycgr3T

Mycgr3G92221 Mycgr3T
  
Location: 8611-9193

Mycgr3G92221\_Mycgr3T

Mycgr3G39931 Mycgr3T
  
Location: 9293-10157

Mycgr3G39931\_Mycgr3T

Mycgr3G99766 Mycgr3T
  
Location: 10257-11775

Mycgr3G99766\_Mycgr3T

hypothetical protein
  
Accession: EEP77403
  
Location: 5926782-5931308
  
  
**BlastP hit with Mycgr3G41235\_Mycgr3T**
  
Percentage identity: 36 %
  
BlastP bit score: 729
  
Sequence coverage: 84 %
  
E-value: 0.0
  
  
 NCBI BlastP on this gene

EEP77403

conserved hypothetical protein
  
Accession: EEP77402
  
Location: 5924440-5926241
  
 NCBI BlastP on this gene

EEP77402

Query: Architecture Search FASTA input

AP007174 : Aspergillus oryzae RIB40 DNA, SC103.    Total score: 1.0     Cumulative Blast bit score: 726

Hit cluster cross-links:

Mycgr3G41235 Mycgr3T
  
Location: 0-4062

Mycgr3G41235\_Mycgr3T

Mycgr3G70577 Mycgr3T
  
Location: 4162-6109

Mycgr3G70577\_Mycgr3T

Mycgr3G40534 Mycgr3T
  
Location: 6209-7166

Mycgr3G40534\_Mycgr3T

Mycgr3G85486 Mycgr3T
  
Location: 7266-8511

Mycgr3G85486\_Mycgr3T

Mycgr3G92221 Mycgr3T
  
Location: 8611-9193

Mycgr3G92221\_Mycgr3T

Mycgr3G39931 Mycgr3T
  
Location: 9293-10157

Mycgr3G39931\_Mycgr3T

Mycgr3G99766 Mycgr3T
  
Location: 10257-11775

Mycgr3G99766\_Mycgr3T

not annotated
  
Accession: BAE65685
  
Location: 596318-600535
  
  
**BlastP hit with Mycgr3G41235\_Mycgr3T**
  
Percentage identity: 32 %
  
BlastP bit score: 726
  
Sequence coverage: 101 %
  
E-value: 0.0
  
  
 NCBI BlastP on this gene

AO090103000226

not annotated
  
Accession: BAE65684
  
Location: 594689-595931
  
 NCBI BlastP on this gene

AO090103000225

not annotated
  
Accession: BAE65683
  
Location: 581629-593329
  
 NCBI BlastP on this gene

AO090103000224

Query: Architecture Search FASTA input

AKHY01000199 : Aspergillus oryzae 3.042    Total score: 1.0     Cumulative Blast bit score: 717

Hit cluster cross-links:

Mycgr3G41235 Mycgr3T
  
Location: 0-4062

Mycgr3G41235\_Mycgr3T

Mycgr3G70577 Mycgr3T
  
Location: 4162-6109

Mycgr3G70577\_Mycgr3T

Mycgr3G40534 Mycgr3T
  
Location: 6209-7166

Mycgr3G40534\_Mycgr3T

Mycgr3G85486 Mycgr3T
  
Location: 7266-8511

Mycgr3G85486\_Mycgr3T

Mycgr3G92221 Mycgr3T
  
Location: 8611-9193

Mycgr3G92221\_Mycgr3T

Mycgr3G39931 Mycgr3T
  
Location: 9293-10157

Mycgr3G39931\_Mycgr3T

Mycgr3G99766 Mycgr3T
  
Location: 10257-11775

Mycgr3G99766\_Mycgr3T

hypothetical protein
  
Accession: EIT73727
  
Location: 401090-401937
  
 NCBI BlastP on this gene

EIT73727

hypothetical protein
  
Accession: EIT73630
  
Location: 404776-405486
  
 NCBI BlastP on this gene

EIT73630

multidrug resistance-associated protein
  
Accession: EIT73554
  
Location: 406470-410699
  
  
**BlastP hit with Mycgr3G41235\_Mycgr3T**
  
Percentage identity: 32 %
  
BlastP bit score: 717
  
Sequence coverage: 101 %
  
E-value: 0.0
  
  
 NCBI BlastP on this gene

EIT73554

isopenicillin N synthase
  
Accession: EIT73763
  
Location: 411086-412328
  
 NCBI BlastP on this gene

EIT73763

polyketide synthase module
  
Accession: EIT73707
  
Location: 413688-425388
  
 NCBI BlastP on this gene

EIT73707

Query: Architecture Search FASTA input

AACS02000012 : Coprinopsis cinerea okayama7#130    Total score: 1.0     Cumulative Blast bit score: 714

Hit cluster cross-links:

Mycgr3G41235 Mycgr3T
  
Location: 0-4062

Mycgr3G41235\_Mycgr3T

Mycgr3G70577 Mycgr3T
  
Location: 4162-6109

Mycgr3G70577\_Mycgr3T

Mycgr3G40534 Mycgr3T
  
Location: 6209-7166

Mycgr3G40534\_Mycgr3T

Mycgr3G85486 Mycgr3T
  
Location: 7266-8511

Mycgr3G85486\_Mycgr3T

Mycgr3G92221 Mycgr3T
  
Location: 8611-9193

Mycgr3G92221\_Mycgr3T

Mycgr3G39931 Mycgr3T
  
Location: 9293-10157

Mycgr3G39931\_Mycgr3T

Mycgr3G99766 Mycgr3T
  
Location: 10257-11775

Mycgr3G99766\_Mycgr3T

cadmium ion transporter
  
Accession: EAU86674
  
Location: 1678680-1684579
  
  
**BlastP hit with Mycgr3G41235\_Mycgr3T**
  
Percentage identity: 31 %
  
BlastP bit score: 714
  
Sequence coverage: 105 %
  
E-value: 0.0
  
  
 NCBI BlastP on this gene

EAU86674

cytoplasmic protein
  
Accession: EAU86673
  
Location: 1676097-1677687
  
 NCBI BlastP on this gene

EAU86673

Query: Architecture Search FASTA input

JH687764 : Auricularia delicata TFB-10046 SS5 unplaced genomic scaffold AURDEscaffold\_32    Total score: 1.0     Cumulative Blast bit score: 712

Hit cluster cross-links:

Mycgr3G41235 Mycgr3T
  
Location: 0-4062

Mycgr3G41235\_Mycgr3T

Mycgr3G70577 Mycgr3T
  
Location: 4162-6109

Mycgr3G70577\_Mycgr3T

Mycgr3G40534 Mycgr3T
  
Location: 6209-7166

Mycgr3G40534\_Mycgr3T

Mycgr3G85486 Mycgr3T
  
Location: 7266-8511

Mycgr3G85486\_Mycgr3T

Mycgr3G92221 Mycgr3T
  
Location: 8611-9193

Mycgr3G92221\_Mycgr3T

Mycgr3G39931 Mycgr3T
  
Location: 9293-10157

Mycgr3G39931\_Mycgr3T

Mycgr3G99766 Mycgr3T
  
Location: 10257-11775

Mycgr3G99766\_Mycgr3T

hypothetical protein
  
Accession: EJD45473
  
Location: 94889-97271
  
 NCBI BlastP on this gene

EJD45473

cadmium ion transporter
  
Accession: EJD45474
  
Location: 100161-105938
  
  
**BlastP hit with Mycgr3G41235\_Mycgr3T**
  
Percentage identity: 31 %
  
BlastP bit score: 712
  
Sequence coverage: 102 %
  
E-value: 0.0
  
  
 NCBI BlastP on this gene

EJD45474

Query: Architecture Search FASTA input

DS995903 : Penicillium marneffei ATCC 18224 scf\_1105668340984 genomic scaffold    Total score: 1.0     Cumulative Blast bit score: 699

Hit cluster cross-links:

Mycgr3G41235 Mycgr3T
  
Location: 0-4062

Mycgr3G41235\_Mycgr3T

Mycgr3G70577 Mycgr3T
  
Location: 4162-6109

Mycgr3G70577\_Mycgr3T

Mycgr3G40534 Mycgr3T
  
Location: 6209-7166

Mycgr3G40534\_Mycgr3T

Mycgr3G85486 Mycgr3T
  
Location: 7266-8511

Mycgr3G85486\_Mycgr3T

Mycgr3G92221 Mycgr3T
  
Location: 8611-9193

Mycgr3G92221\_Mycgr3T

Mycgr3G39931 Mycgr3T
  
Location: 9293-10157

Mycgr3G39931\_Mycgr3T

Mycgr3G99766 Mycgr3T
  
Location: 10257-11775

Mycgr3G99766\_Mycgr3T

alcohol dehydrogenase, putative
  
Accession: EEA21216
  
Location: 33030-34233
  
 NCBI BlastP on this gene

EEA21216

N-alkane-inducible cytochrome P450, putative
  
Accession: EEA21215
  
Location: 30654-32357
  
 NCBI BlastP on this gene

EEA21215

conserved hypothetical protein
  
Accession: EEA21214
  
Location: 29530-30171
  
 NCBI BlastP on this gene

EEA21214

oligomycin resistance ATP-dependent permease yor1, putative
  
Accession: EEA21213
  
Location: 24639-28887
  
  
**BlastP hit with Mycgr3G41235\_Mycgr3T**
  
Percentage identity: 31 %
  
BlastP bit score: 699
  
Sequence coverage: 103 %
  
E-value: 0.0
  
  
 NCBI BlastP on this gene

EEA21213

ankyrin repeat-containing protein, putative
  
Accession: EEA21212
  
Location: 22112-22870
  
 NCBI BlastP on this gene

EEA21212

conserved hypothetical protein
  
Accession: EEA21211
  
Location: 18978-19334
  
 NCBI BlastP on this gene

EEA21211

Query: Architecture Search FASTA input

EQ963486 : Aspergillus flavus NRRL3357 scf\_1106286417242 genomic scaffold    Total score: 1.0     Cumulative Blast bit score: 697

Hit cluster cross-links:

Mycgr3G41235 Mycgr3T
  
Location: 0-4062

Mycgr3G41235\_Mycgr3T

Mycgr3G70577 Mycgr3T
  
Location: 4162-6109

Mycgr3G70577\_Mycgr3T

Mycgr3G40534 Mycgr3T
  
Location: 6209-7166

Mycgr3G40534\_Mycgr3T

Mycgr3G85486 Mycgr3T
  
Location: 7266-8511

Mycgr3G85486\_Mycgr3T

Mycgr3G92221 Mycgr3T
  
Location: 8611-9193

Mycgr3G92221\_Mycgr3T

Mycgr3G39931 Mycgr3T
  
Location: 9293-10157

Mycgr3G39931\_Mycgr3T

Mycgr3G99766 Mycgr3T
  
Location: 10257-11775

Mycgr3G99766\_Mycgr3T

hypothetical protein
  
Accession: EED45111
  
Location: 671847-674725
  
 NCBI BlastP on this gene

EED45111

hypothetical protein
  
Accession: EED45112
  
Location: 674887-675566
  
 NCBI BlastP on this gene

EED45112

hypothetical protein
  
Accession: EED45113
  
Location: 676291-676683
  
 NCBI BlastP on this gene

EED45113

ABC multidrug transporter, putative
  
Accession: EED45114
  
Location: 680721-684644
  
  
**BlastP hit with Mycgr3G41235\_Mycgr3T**
  
Percentage identity: 32 %
  
BlastP bit score: 697
  
Sequence coverage: 97 %
  
E-value: 0.0
  
  
 NCBI BlastP on this gene

EED45114

1-aminocyclopropane-1-carboxylate oxidase, putative
  
Accession: EED45115
  
Location: 685466-686495
  
 NCBI BlastP on this gene

EED45115

polyketide synthase, putative
  
Accession: EED45116
  
Location: 687862-693859
  
 NCBI BlastP on this gene

EED45116

Query: Architecture Search FASTA input

JH687764 : Auricularia delicata TFB-10046 SS5 unplaced genomic scaffold AURDEscaffold\_32    Total score: 1.0     Cumulative Blast bit score: 695

Hit cluster cross-links:

Mycgr3G41235 Mycgr3T
  
Location: 0-4062

Mycgr3G41235\_Mycgr3T

Mycgr3G70577 Mycgr3T
  
Location: 4162-6109

Mycgr3G70577\_Mycgr3T

Mycgr3G40534 Mycgr3T
  
Location: 6209-7166

Mycgr3G40534\_Mycgr3T

Mycgr3G85486 Mycgr3T
  
Location: 7266-8511

Mycgr3G85486\_Mycgr3T

Mycgr3G92221 Mycgr3T
  
Location: 8611-9193

Mycgr3G92221\_Mycgr3T

Mycgr3G39931 Mycgr3T
  
Location: 9293-10157

Mycgr3G39931\_Mycgr3T

Mycgr3G99766 Mycgr3T
  
Location: 10257-11775

Mycgr3G99766\_Mycgr3T

hypothetical protein
  
Accession: EJD45441
  
Location: 11755-15254
  
 NCBI BlastP on this gene

EJD45441

hypothetical protein
  
Accession: EJD45442
  
Location: 15908-16305
  
 NCBI BlastP on this gene

EJD45442

hypothetical protein
  
Accession: EJD45443
  
Location: 16923-18003
  
 NCBI BlastP on this gene

EJD45443

ATP-dependent bile acid permease
  
Accession: EJD45444
  
Location: 19709-25146
  
  
**BlastP hit with Mycgr3G41235\_Mycgr3T**
  
Percentage identity: 31 %
  
BlastP bit score: 695
  
Sequence coverage: 106 %
  
E-value: 0.0
  
  
 NCBI BlastP on this gene

EJD45444

hypothetical protein
  
Accession: EJD45445
  
Location: 25501-26626
  
 NCBI BlastP on this gene

EJD45445

alpha/beta-hydrolase
  
Accession: EJD45446
  
Location: 27094-28207
  
 NCBI BlastP on this gene

EJD45446

zincin
  
Accession: EJD45447
  
Location: 29127-30301
  
 NCBI BlastP on this gene

EJD45447

hypothetical protein
  
Accession: EJD45448
  
Location: 30813-32026
  
 NCBI BlastP on this gene

EJD45448

Query: Architecture Search FASTA input

FQ311430 : Sporisorium reilianum SRZ2 chromosome 1 complete DNA sequence.    Total score: 1.0     Cumulative Blast bit score: 694

Hit cluster cross-links:

Mycgr3G41235 Mycgr3T
  
Location: 0-4062

Mycgr3G41235\_Mycgr3T

Mycgr3G70577 Mycgr3T
  
Location: 4162-6109

Mycgr3G70577\_Mycgr3T

Mycgr3G40534 Mycgr3T
  
Location: 6209-7166

Mycgr3G40534\_Mycgr3T

Mycgr3G85486 Mycgr3T
  
Location: 7266-8511

Mycgr3G85486\_Mycgr3T

Mycgr3G92221 Mycgr3T
  
Location: 8611-9193

Mycgr3G92221\_Mycgr3T

Mycgr3G39931 Mycgr3T
  
Location: 9293-10157

Mycgr3G39931\_Mycgr3T

Mycgr3G99766 Mycgr3T
  
Location: 10257-11775

Mycgr3G99766\_Mycgr3T

probable YOR1-ABC transporter
  
Accession: CBQ68135
  
Location: 2089203-2094056
  
  
**BlastP hit with Mycgr3G41235\_Mycgr3T**
  
Percentage identity: 39 %
  
BlastP bit score: 694
  
Sequence coverage: 72 %
  
E-value: 0.0
  
  
 NCBI BlastP on this gene

sr12000

related to Proteasome activator complex subunit 3
  
Accession: CBQ68134
  
Location: 2088204-2088989
  
 NCBI BlastP on this gene

sr11999

related to Exocyst complex component Sec5
  
Accession: CBQ68133
  
Location: 2085167-2087842
  
 NCBI BlastP on this gene

sr11998

probable TIM17-mitochondrial inner membrane import translocase subunit
  
Accession: CBQ68132
  
Location: 2084449-2084943
  
 NCBI BlastP on this gene

sr11997

related to monooxygenase
  
Accession: CBQ68131
  
Location: 2081886-2083652
  
 NCBI BlastP on this gene

sr11996

Query: Architecture Search FASTA input

DF196775 : Pseudozyma antarctica T-34 DNA, contig: scaffold00009    Total score: 1.0     Cumulative Blast bit score: 682

Hit cluster cross-links:

Mycgr3G41235 Mycgr3T
  
Location: 0-4062

Mycgr3G41235\_Mycgr3T

Mycgr3G70577 Mycgr3T
  
Location: 4162-6109

Mycgr3G70577\_Mycgr3T

Mycgr3G40534 Mycgr3T
  
Location: 6209-7166

Mycgr3G40534\_Mycgr3T

Mycgr3G85486 Mycgr3T
  
Location: 7266-8511

Mycgr3G85486\_Mycgr3T

Mycgr3G92221 Mycgr3T
  
Location: 8611-9193

Mycgr3G92221\_Mycgr3T

Mycgr3G39931 Mycgr3T
  
Location: 9293-10157

Mycgr3G39931\_Mycgr3T

Mycgr3G99766 Mycgr3T
  
Location: 10257-11775

Mycgr3G99766\_Mycgr3T

multidrug resistance-associated protein
  
Accession: GAC73965
  
Location: 2042126-2046958
  
  
**BlastP hit with Mycgr3G41235\_Mycgr3T**
  
Percentage identity: 39 %
  
BlastP bit score: 682
  
Sequence coverage: 68 %
  
E-value: 0.0
  
  
 NCBI BlastP on this gene

GAC73965

proteasome activator subunit
  
Accession: GAC73964
  
Location: 2041125-2041922
  
 NCBI BlastP on this gene

GAC73964

sec5 subunit of exocyst complex
  
Accession: GAC73963
  
Location: 2038097-2040734
  
 NCBI BlastP on this gene

GAC73963

mitochondrial import inner membrane translocase, subunit TIM17
  
Accession: GAC73962
  
Location: 2037355-2037849
  
 NCBI BlastP on this gene

GAC73962

hypothetical protein
  
Accession: GAC73961
  
Location: 2034693-2036539
  
 NCBI BlastP on this gene

GAC73961

Query: Architecture Search FASTA input

AE017342 : Cryptococcus neoformans var. neoformans JEC21 chromosome 2    Total score: 1.0     Cumulative Blast bit score: 678

Hit cluster cross-links:

Mycgr3G41235 Mycgr3T
  
Location: 0-4062

Mycgr3G41235\_Mycgr3T

Mycgr3G70577 Mycgr3T
  
Location: 4162-6109

Mycgr3G70577\_Mycgr3T

Mycgr3G40534 Mycgr3T
  
Location: 6209-7166

Mycgr3G40534\_Mycgr3T

Mycgr3G85486 Mycgr3T
  
Location: 7266-8511

Mycgr3G85486\_Mycgr3T

Mycgr3G92221 Mycgr3T
  
Location: 8611-9193

Mycgr3G92221\_Mycgr3T

Mycgr3G39931 Mycgr3T
  
Location: 9293-10157

Mycgr3G39931\_Mycgr3T

Mycgr3G99766 Mycgr3T
  
Location: 10257-11775

Mycgr3G99766\_Mycgr3T

dehydrogenase, putative
  
Accession: AAW41745
  
Location: 120276-121739
  
 NCBI BlastP on this gene

CNB00420

conserved hypothetical protein
  
Accession: AAW41477
  
Location: 116287-117232
  
 NCBI BlastP on this gene

CNB00410

conserved hypothetical protein
  
Accession: AAW41476
  
Location: 116287-117704
  
 NCBI BlastP on this gene

CNB00410

conserved hypothetical protein
  
Accession: AAW41475
  
Location: 112785-115457
  
  
**BlastP hit with Mycgr3G70577\_Mycgr3T**
  
Percentage identity: 32 %
  
BlastP bit score: 342
  
Sequence coverage: 102 %
  
E-value: 2e-104
  
  
 NCBI BlastP on this gene

CNB00400

conserved hypothetical protein
  
Accession: AAW41474
  
Location: 112785-115457
  
  
**BlastP hit with Mycgr3G70577\_Mycgr3T**
  
Percentage identity: 31 %
  
BlastP bit score: 336
  
Sequence coverage: 104 %
  
E-value: 6e-102
  
  
 NCBI BlastP on this gene

CNB00400

hypothetical protein
  
Accession: AAW41473
  
Location: 110234-111942
  
 NCBI BlastP on this gene

CNB00390

seryl-tRNA synthetase, putative
  
Accession: AAW41472
  
Location: 107240-108937
  
 NCBI BlastP on this gene

CNB00380

fatty acid beta-oxidation-related protein, putative
  
Accession: AAW41471
  
Location: 105323-106617
  
 NCBI BlastP on this gene

CNB00370

Query: Architecture Search FASTA input

CR382134 : Debaryomyces hansenii CBS767 chromosome B complete sequence.    Total score: 1.0     Cumulative Blast bit score: 674

Hit cluster cross-links:

Mycgr3G41235 Mycgr3T
  
Location: 0-4062

Mycgr3G41235\_Mycgr3T

Mycgr3G70577 Mycgr3T
  
Location: 4162-6109

Mycgr3G70577\_Mycgr3T

Mycgr3G40534 Mycgr3T
  
Location: 6209-7166

Mycgr3G40534\_Mycgr3T

Mycgr3G85486 Mycgr3T
  
Location: 7266-8511

Mycgr3G85486\_Mycgr3T

Mycgr3G92221 Mycgr3T
  
Location: 8611-9193

Mycgr3G92221\_Mycgr3T

Mycgr3G39931 Mycgr3T
  
Location: 9293-10157

Mycgr3G39931\_Mycgr3T

Mycgr3G99766 Mycgr3T
  
Location: 10257-11775

Mycgr3G99766\_Mycgr3T

DEHA2B01452p
  
Accession: CAR65428
  
Location: 111106-115899
  
 NCBI BlastP on this gene

DEHA2B01452g

DEHA2B01430p
  
Accession: CAG85014
  
Location: 105614-109774
  
  
**BlastP hit with Mycgr3G41235\_Mycgr3T**
  
Percentage identity: 29 %
  
BlastP bit score: 674
  
Sequence coverage: 103 %
  
E-value: 0.0
  
  
 NCBI BlastP on this gene

DEHA2B01430g

DEHA2B01408p
  
Accession: CAG85013
  
Location: 104374-105504
  
 NCBI BlastP on this gene

DEHA2B01408g

DEHA2B01386p
  
Accession: CAG85012
  
Location: 100817-104209
  
 NCBI BlastP on this gene

DEHA2B01386g

DEHA2B01364p
  
Accession: CAG85011
  
Location: 99881-100267
  
 NCBI BlastP on this gene

DEHA2B01364g

Query: Architecture Search FASTA input

GL996527 : Candida tenuis ATCC 10573 unplaced genomic scaffold CANTEscaffold\_00021    Total score: 1.0     Cumulative Blast bit score: 669

Hit cluster cross-links:

Mycgr3G41235 Mycgr3T
  
Location: 0-4062

Mycgr3G41235\_Mycgr3T

Mycgr3G70577 Mycgr3T
  
Location: 4162-6109

Mycgr3G70577\_Mycgr3T

Mycgr3G40534 Mycgr3T
  
Location: 6209-7166

Mycgr3G40534\_Mycgr3T

Mycgr3G85486 Mycgr3T
  
Location: 7266-8511

Mycgr3G85486\_Mycgr3T

Mycgr3G92221 Mycgr3T
  
Location: 8611-9193

Mycgr3G92221\_Mycgr3T

Mycgr3G39931 Mycgr3T
  
Location: 9293-10157

Mycgr3G39931\_Mycgr3T

Mycgr3G99766 Mycgr3T
  
Location: 10257-11775

Mycgr3G99766\_Mycgr3T

hypothetical protein
  
Accession: EGV61734
  
Location: 826770-830936
  
  
**BlastP hit with Mycgr3G41235\_Mycgr3T**
  
Percentage identity: 28 %
  
BlastP bit score: 669
  
Sequence coverage: 105 %
  
E-value: 0.0
  
  
 NCBI BlastP on this gene

EGV61734

hypothetical protein
  
Accession: EGV62638
  
Location: 825576-826733
  
 NCBI BlastP on this gene

EGV62638

hypothetical protein
  
Accession: EGV62637
  
Location: 824946-826733
  
 NCBI BlastP on this gene

EGV62637

dihydroxyacetone synthase
  
Accession: EGV61733
  
Location: 822340-824433
  
 NCBI BlastP on this gene

EGV61733

hypothetical protein
  
Accession: EGV62954
  
Location: 820096-821670
  
 NCBI BlastP on this gene

EGV62954

Query: Architecture Search FASTA input

GL996527 : Candida tenuis ATCC 10573 unplaced genomic scaffold CANTEscaffold\_00021    Total score: 1.0     Cumulative Blast bit score: 667

Hit cluster cross-links:

Mycgr3G41235 Mycgr3T
  
Location: 0-4062

Mycgr3G41235\_Mycgr3T

Mycgr3G70577 Mycgr3T
  
Location: 4162-6109

Mycgr3G70577\_Mycgr3T

Mycgr3G40534 Mycgr3T
  
Location: 6209-7166

Mycgr3G40534\_Mycgr3T

Mycgr3G85486 Mycgr3T
  
Location: 7266-8511

Mycgr3G85486\_Mycgr3T

Mycgr3G92221 Mycgr3T
  
Location: 8611-9193

Mycgr3G92221\_Mycgr3T

Mycgr3G39931 Mycgr3T
  
Location: 9293-10157

Mycgr3G39931\_Mycgr3T

Mycgr3G99766 Mycgr3T
  
Location: 10257-11775

Mycgr3G99766\_Mycgr3T

hypothetical protein
  
Accession: EGV61462
  
Location: 238684-239280
  
 NCBI BlastP on this gene

EGV61462

hypothetical protein
  
Accession: EGV62531
  
Location: 239270-239827
  
 NCBI BlastP on this gene

EGV62531

hypothetical protein
  
Accession: EGV61463
  
Location: 239997-241409
  
 NCBI BlastP on this gene

EGV61463

hypothetical protein
  
Accession: EGV61464
  
Location: 242666-243514
  
 NCBI BlastP on this gene

EGV61464

hypothetical protein
  
Accession: EGV61465
  
Location: 245286-249293
  
  
**BlastP hit with Mycgr3G41235\_Mycgr3T**
  
Percentage identity: 30 %
  
BlastP bit score: 667
  
Sequence coverage: 102 %
  
E-value: 0.0
  
  
 NCBI BlastP on this gene

EGV61465

hypothetical protein
  
Accession: EGV61466
  
Location: 249320-252370
  
 NCBI BlastP on this gene

EGV61466

mitochondrial import inner membrane translocase subunit TIM16
  
Accession: EGV61467
  
Location: 252717-253097
  
 NCBI BlastP on this gene

EGV61467

Query: Architecture Search FASTA input

CU928166 : Lachancea thermotolerans CBS 6340 chromosome B complete sequence.    Total score: 1.0     Cumulative Blast bit score: 667

Hit cluster cross-links:

Mycgr3G41235 Mycgr3T
  
Location: 0-4062

Mycgr3G41235\_Mycgr3T

Mycgr3G70577 Mycgr3T
  
Location: 4162-6109

Mycgr3G70577\_Mycgr3T

Mycgr3G40534 Mycgr3T
  
Location: 6209-7166

Mycgr3G40534\_Mycgr3T

Mycgr3G85486 Mycgr3T
  
Location: 7266-8511

Mycgr3G85486\_Mycgr3T

Mycgr3G92221 Mycgr3T
  
Location: 8611-9193

Mycgr3G92221\_Mycgr3T

Mycgr3G39931 Mycgr3T
  
Location: 9293-10157

Mycgr3G39931\_Mycgr3T

Mycgr3G99766 Mycgr3T
  
Location: 10257-11775

Mycgr3G99766\_Mycgr3T

KLTH0B09724p
  
Accession: CAR21771
  
Location: 796023-800483
  
  
**BlastP hit with Mycgr3G41235\_Mycgr3T**
  
Percentage identity: 30 %
  
BlastP bit score: 667
  
Sequence coverage: 102 %
  
E-value: 0.0
  
  
 NCBI BlastP on this gene

KLTH0B09724g

KLTH0B09680p
  
Accession: CAR21770
  
Location: 790481-792934
  
 NCBI BlastP on this gene

KLTH0B09680g

KLTH0B09658p
  
Accession: CAR21769
  
Location: 789016-789939
  
 NCBI BlastP on this gene

KLTH0B09658g

Query: Architecture Search FASTA input

HE681721 : Candida orthopsilosis Co 90-125, chromosome 3 draft sequence.    Total score: 1.0     Cumulative Blast bit score: 665

Hit cluster cross-links:

Mycgr3G41235 Mycgr3T
  
Location: 0-4062

Mycgr3G41235\_Mycgr3T

Mycgr3G70577 Mycgr3T
  
Location: 4162-6109

Mycgr3G70577\_Mycgr3T

Mycgr3G40534 Mycgr3T
  
Location: 6209-7166

Mycgr3G40534\_Mycgr3T

Mycgr3G85486 Mycgr3T
  
Location: 7266-8511

Mycgr3G85486\_Mycgr3T

Mycgr3G92221 Mycgr3T
  
Location: 8611-9193

Mycgr3G92221\_Mycgr3T

Mycgr3G39931 Mycgr3T
  
Location: 9293-10157

Mycgr3G39931\_Mycgr3T

Mycgr3G99766 Mycgr3T
  
Location: 10257-11775

Mycgr3G99766\_Mycgr3T

hypothetical protein
  
Accession: CCG26058
  
Location: 1532971-1537332
  
  
**BlastP hit with Mycgr3G41235\_Mycgr3T**
  
Percentage identity: 30 %
  
BlastP bit score: 665
  
Sequence coverage: 100 %
  
E-value: 0.0
  
  
 NCBI BlastP on this gene

CORT\_0C06860

Cyc1 cytochrome c
  
Accession: CCG26057
  
Location: 1531856-1532191
  
 NCBI BlastP on this gene

CORT\_0C06850

Plp2 protein
  
Accession: CCG26056
  
Location: 1530655-1531419
  
 NCBI BlastP on this gene

CORT\_0C06840

hypothetical protein
  
Accession: CCG26055
  
Location: 1527735-1530518
  
 NCBI BlastP on this gene

CORT\_0C06830

Ubp8 protein
  
Accession: CCG26054
  
Location: 1525590-1527221
  
 NCBI BlastP on this gene

CORT\_0C06820

Query: Architecture Search FASTA input

CH477324 : Aedes aegypti strain Liverpool supercont1.139 genomic scaffold    Total score: 1.0     Cumulative Blast bit score: 663

Hit cluster cross-links:

Mycgr3G41235 Mycgr3T
  
Location: 0-4062

Mycgr3G41235\_Mycgr3T

Mycgr3G70577 Mycgr3T
  
Location: 4162-6109

Mycgr3G70577\_Mycgr3T

Mycgr3G40534 Mycgr3T
  
Location: 6209-7166

Mycgr3G40534\_Mycgr3T

Mycgr3G85486 Mycgr3T
  
Location: 7266-8511

Mycgr3G85486\_Mycgr3T

Mycgr3G92221 Mycgr3T
  
Location: 8611-9193

Mycgr3G92221\_Mycgr3T

Mycgr3G39931 Mycgr3T
  
Location: 9293-10157

Mycgr3G39931\_Mycgr3T

Mycgr3G99766 Mycgr3T
  
Location: 10257-11775

Mycgr3G99766\_Mycgr3T

AAEL005043-PA
  
Accession: EAT43548
  
Location: 1140679-1145502
  
  
**BlastP hit with Mycgr3G41235\_Mycgr3T**
  
Percentage identity: 30 %
  
BlastP bit score: 663
  
Sequence coverage: 101 %
  
E-value: 0.0
  
  
 NCBI BlastP on this gene

EAT43548

Query: Architecture Search FASTA input

HE681721 : Candida orthopsilosis Co 90-125, chromosome 3 draft sequence.    Total score: 1.0     Cumulative Blast bit score: 659

Hit cluster cross-links:

Mycgr3G41235 Mycgr3T
  
Location: 0-4062

Mycgr3G41235\_Mycgr3T

Mycgr3G70577 Mycgr3T
  
Location: 4162-6109

Mycgr3G70577\_Mycgr3T

Mycgr3G40534 Mycgr3T
  
Location: 6209-7166

Mycgr3G40534\_Mycgr3T

Mycgr3G85486 Mycgr3T
  
Location: 7266-8511

Mycgr3G85486\_Mycgr3T

Mycgr3G92221 Mycgr3T
  
Location: 8611-9193

Mycgr3G92221\_Mycgr3T

Mycgr3G39931 Mycgr3T
  
Location: 9293-10157

Mycgr3G39931\_Mycgr3T

Mycgr3G99766 Mycgr3T
  
Location: 10257-11775

Mycgr3G99766\_Mycgr3T

hypothetical protein
  
Accession: CCG26033
  
Location: 1477220-1478065
  
 NCBI BlastP on this gene

CORT\_0C06600

hypothetical protein
  
Accession: CCG26032
  
Location: 1475902-1477149
  
 NCBI BlastP on this gene

CORT\_0C06590

monooxygenase
  
Accession: CCG26031
  
Location: 1474126-1475628
  
 NCBI BlastP on this gene

CORT\_0C06580

Yor1 protein
  
Accession: CCG26030
  
Location: 1467217-1471587
  
  
**BlastP hit with Mycgr3G41235\_Mycgr3T**
  
Percentage identity: 30 %
  
BlastP bit score: 659
  
Sequence coverage: 106 %
  
E-value: 0.0
  
  
 NCBI BlastP on this gene

CORT\_0C06570

calmodulin-dependent protein kinase
  
Accession: CCG26029
  
Location: 1465140-1466474
  
 NCBI BlastP on this gene

CORT\_0C06560

Set2 protein
  
Accession: CCG26028
  
Location: 1462475-1464895
  
 NCBI BlastP on this gene

CORT\_0C06550

Gpd1 protein
  
Accession: CCG26027
  
Location: 1461106-1462260
  
 NCBI BlastP on this gene

CORT\_0C06540

Query: Architecture Search FASTA input

CR382134 : Debaryomyces hansenii CBS767 chromosome B complete sequence.    Total score: 1.0     Cumulative Blast bit score: 658

Hit cluster cross-links:

Mycgr3G41235 Mycgr3T
  
Location: 0-4062

Mycgr3G41235\_Mycgr3T

Mycgr3G70577 Mycgr3T
  
Location: 4162-6109

Mycgr3G70577\_Mycgr3T

Mycgr3G40534 Mycgr3T
  
Location: 6209-7166

Mycgr3G40534\_Mycgr3T

Mycgr3G85486 Mycgr3T
  
Location: 7266-8511

Mycgr3G85486\_Mycgr3T

Mycgr3G92221 Mycgr3T
  
Location: 8611-9193

Mycgr3G92221\_Mycgr3T

Mycgr3G39931 Mycgr3T
  
Location: 9293-10157

Mycgr3G39931\_Mycgr3T

Mycgr3G99766 Mycgr3T
  
Location: 10257-11775

Mycgr3G99766\_Mycgr3T

DEHA2B13970p
  
Accession: CAR65493
  
Location: 1092795-1094852
  
 NCBI BlastP on this gene

DEHA2B13970g

DEHA2B13992p
  
Accession: CAG85565
  
Location: 1095412-1097451
  
 NCBI BlastP on this gene

DEHA2B13992g

DEHA2B14014p
  
Accession: CAR65494
  
Location: 1098064-1098171
  
 NCBI BlastP on this gene

DEHA2B14014g

DEHA2B14058p
  
Accession: CAR65495
  
Location: 1099134-1099469
  
 NCBI BlastP on this gene

DEHA2B14058g

DEHA2B14080p
  
Accession: CAR65496
  
Location: 1100131-1104420
  
  
**BlastP hit with Mycgr3G41235\_Mycgr3T**
  
Percentage identity: 30 %
  
BlastP bit score: 658
  
Sequence coverage: 104 %
  
E-value: 0.0
  
  
 NCBI BlastP on this gene

DEHA2B14080g

DEHA2B14102p
  
Accession: CAG85569
  
Location: 1105067-1107091
  
 NCBI BlastP on this gene

DEHA2B14102g

DEHA2B14124p
  
Accession: CAG85570
  
Location: 1107741-1109789
  
 NCBI BlastP on this gene

DEHA2B14124g

DEHA2B14146p
  
Accession: CAR65497
  
Location: 1110498-1112510
  
 NCBI BlastP on this gene

DEHA2B14146g

Query: Architecture Search FASTA input

CH477324 : Aedes aegypti strain Liverpool supercont1.139 genomic scaffold    Total score: 1.0     Cumulative Blast bit score: 644

Hit cluster cross-links:

Mycgr3G41235 Mycgr3T
  
Location: 0-4062

Mycgr3G41235\_Mycgr3T

Mycgr3G70577 Mycgr3T
  
Location: 4162-6109

Mycgr3G70577\_Mycgr3T

Mycgr3G40534 Mycgr3T
  
Location: 6209-7166

Mycgr3G40534\_Mycgr3T

Mycgr3G85486 Mycgr3T
  
Location: 7266-8511

Mycgr3G85486\_Mycgr3T

Mycgr3G92221 Mycgr3T
  
Location: 8611-9193

Mycgr3G92221\_Mycgr3T

Mycgr3G39931 Mycgr3T
  
Location: 9293-10157

Mycgr3G39931\_Mycgr3T

Mycgr3G99766 Mycgr3T
  
Location: 10257-11775

Mycgr3G99766\_Mycgr3T

AAEL005045-PA
  
Accession: EAT43550
  
Location: 1184563-1195380
  
  
**BlastP hit with Mycgr3G41235\_Mycgr3T**
  
Percentage identity: 30 %
  
BlastP bit score: 644
  
Sequence coverage: 101 %
  
E-value: 0.0
  
  
 NCBI BlastP on this gene

EAT43550

AAEL005026-PA
  
Accession: EAT43549
  
Location: 1168407-1184363
  
 NCBI BlastP on this gene

EAT43549

Query: Architecture Search FASTA input

101. :  GL891107 Neurospora tetrasperma FGSC 2509 unplaced genomic scaffold NEUTE2scaffold\_2     Total score: 1.0     Cumulative Blast bit score: 816

Mycgr3G41235 Mycgr3T
  
Location: 0-4062
  
 NCBI BlastP on this gene

Mycgr3G41235\_Mycgr3T

Mycgr3G70577 Mycgr3T
  
Location: 4162-6109
  
 NCBI BlastP on this gene

Mycgr3G70577\_Mycgr3T

Mycgr3G40534 Mycgr3T
  
Location: 6209-7166
  
 NCBI BlastP on this gene

Mycgr3G40534\_Mycgr3T

Mycgr3G85486 Mycgr3T
  
Location: 7266-8511
  
 NCBI BlastP on this gene

Mycgr3G85486\_Mycgr3T

Mycgr3G92221 Mycgr3T
  
Location: 8611-9193
  
 NCBI BlastP on this gene

Mycgr3G92221\_Mycgr3T

Mycgr3G39931 Mycgr3T
  
Location: 9293-10157
  
 NCBI BlastP on this gene

Mycgr3G39931\_Mycgr3T

Mycgr3G99766 Mycgr3T
  
Location: 10257-11775
  
 NCBI BlastP on this gene

Mycgr3G99766\_Mycgr3T

P-loop containing nucleoside triphosphate hydrolase protein
  
Accession: EGZ75631
  
Location: 4961220-4965747
  
  
**BlastP hit with Mycgr3G41235\_Mycgr3T**
  
Percentage identity: 33 %
  
BlastP bit score: 816
  
Sequence coverage: 104 %
  
E-value: 0.0
  
  
 NCBI BlastP on this gene

EGZ75631

102. :  HF679025 Fusarium fujikuroi IMI 58289 draft genome, chromosome FFUJ\_chr03.     Total score: 1.0     Cumulative Blast bit score: 814

related to CSI2 protein
  
Accession: CCT65660
  
Location: 1578272-1579474
  
 NCBI BlastP on this gene

FFUJ\_02623

related to TFIID and SAGA subunit TAF61
  
Accession: CCT65659
  
Location: 1576237-1577190
  
 NCBI BlastP on this gene

FFUJ\_02622

probable CSL4-core component of the 3`-5` exosome
  
Accession: CCT65658
  
Location: 1574507-1575187
  
 NCBI BlastP on this gene

FFUJ\_02621

probable ATP-binding cassette transporter protein YOR1
  
Accession: CCT65657
  
Location: 1567992-1572518
  
  
**BlastP hit with Mycgr3G41235\_Mycgr3T**
  
Percentage identity: 34 %
  
BlastP bit score: 814
  
Sequence coverage: 101 %
  
E-value: 0.0
  
  
 NCBI BlastP on this gene

FFUJ\_02620

related to pseudouridine synthase
  
Accession: CCT66662
  
Location: 1565859-1567407
  
 NCBI BlastP on this gene

FFUJ\_14894

uncharacterized protein
  
Accession: CCT65656
  
Location: 1562098-1564611
  
 NCBI BlastP on this gene

FFUJ\_02619

103. :  CM001202 Mycosphaerella graminicola IPO323 chromosome 7     Total score: 1.0     Cumulative Blast bit score: 812

hypothetical protein
  
Accession: EGP86236
  
Location: 435960-436483
  
 NCBI BlastP on this gene

EGP86236

putative ABC transporter
  
Accession: EGP86237
  
Location: 428260-432801
  
  
**BlastP hit with Mycgr3G41235\_Mycgr3T**
  
Percentage identity: 33 %
  
BlastP bit score: 812
  
Sequence coverage: 105 %
  
E-value: 0.0
  
  
 NCBI BlastP on this gene

EGP86237

hypothetical protein
  
Accession: EGP86238
  
Location: 420628-424588
  
 NCBI BlastP on this gene

EGP86238

104. :  GL385397 Gaeumannomyces graminis var. tritici R3-111a-1 unplaced genomic scaffold supercont2.3     Total score: 1.0     Cumulative Blast bit score: 805

hypothetical protein
  
Accession: EJT77408
  
Location: 6544949-6547453
  
 NCBI BlastP on this gene

EJT77408

multidrug resistance-associated protein 2
  
Accession: EJT77410
  
Location: 6549330-6553904
  
  
**BlastP hit with Mycgr3G41235\_Mycgr3T**
  
Percentage identity: 34 %
  
BlastP bit score: 805
  
Sequence coverage: 105 %
  
E-value: 0.0
  
  
 NCBI BlastP on this gene

EJT77410

105. :  EQ962656 Talaromyces stipitatus ATCC 10500 scf\_1105507295549 genomic scaffold     Total score: 1.0     Cumulative Blast bit score: 804

tripeptidyl-peptidase (TppA), putative
  
Accession: EED16525
  
Location: 2024302-2026250
  
 NCBI BlastP on this gene

EED16525

hypothetical protein
  
Accession: EED16526
  
Location: 2026696-2028367
  
 NCBI BlastP on this gene

EED16526

ABC multidrug transporter, putative
  
Accession: EED16527
  
Location: 2029727-2034135
  
  
**BlastP hit with Mycgr3G41235\_Mycgr3T**
  
Percentage identity: 33 %
  
BlastP bit score: 804
  
Sequence coverage: 102 %
  
E-value: 0.0
  
  
 NCBI BlastP on this gene

EED16527

106. :  DS989823 Arthroderma gypseum CBS 118893 supercont1.2 genomic scaffold     Total score: 1.0     Cumulative Blast bit score: 799

GPI mannosyltransferase 3
  
Accession: EFR00025
  
Location: 2677066-2679581
  
 NCBI BlastP on this gene

EFR00025

ornithine aminotransferase
  
Accession: EFR00026
  
Location: 2680202-2681678
  
 NCBI BlastP on this gene

EFR00026

oligomycin resistance ATP-dependent permease YOR1
  
Accession: EFR00027
  
Location: 2685158-2689594
  
  
**BlastP hit with Mycgr3G41235\_Mycgr3T**
  
Percentage identity: 33 %
  
BlastP bit score: 799
  
Sequence coverage: 103 %
  
E-value: 0.0
  
  
 NCBI BlastP on this gene

EFR00027

amino-acid permease 2
  
Accession: EFR00028
  
Location: 2689812-2691677
  
 NCBI BlastP on this gene

EFR00028

tyrosyl-DNA phosphodiesterase 1
  
Accession: EFR00029
  
Location: 2692560-2694517
  
 NCBI BlastP on this gene

EFR00029

hypothetical protein
  
Accession: EFR00030
  
Location: 2694928-2695523
  
 NCBI BlastP on this gene

EFR00030

107. :  GG700652 Trichophyton rubrum CBS 118892 genomic scaffold supercont2.5     Total score: 1.0     Cumulative Blast bit score: 797

mannosyltransferase
  
Accession: EGD88599
  
Location: 335297-337825
  
 NCBI BlastP on this gene

EGD88599

ornithine aminotransferase
  
Accession: EGD88600
  
Location: 338589-340043
  
 NCBI BlastP on this gene

EGD88600

hypothetical protein
  
Accession: EGD88601
  
Location: 342299-342798
  
 NCBI BlastP on this gene

EGD88601

multidrug resistance-associated protein 5
  
Accession: EGD88602
  
Location: 343981-348417
  
  
**BlastP hit with Mycgr3G41235\_Mycgr3T**
  
Percentage identity: 33 %
  
BlastP bit score: 798
  
Sequence coverage: 105 %
  
E-value: 0.0
  
  
 NCBI BlastP on this gene

EGD88602

amino acid permease
  
Accession: EGD88603
  
Location: 348652-350514
  
 NCBI BlastP on this gene

EGD88603

tyrosyl-DNA phosphodiesterase
  
Accession: EGD88604
  
Location: 351328-353267
  
 NCBI BlastP on this gene

EGD88604

hypothetical protein
  
Accession: EGD88605
  
Location: 353698-354164
  
 NCBI BlastP on this gene

EGD88605

108. :  JH719400 Dichomitus squalens LYAD-421 SS1 unplaced genomic scaffold DICSQscaffold\_4     Total score: 1.0     Cumulative Blast bit score: 795

hypothetical protein
  
Accession: EJF64556
  
Location: 323385-326357
  
 NCBI BlastP on this gene

EJF64556

hypothetical protein
  
Accession: EJF64557
  
Location: 327072-328923
  
 NCBI BlastP on this gene

EJF64557

P-loop containing nucleoside triphosphate hydrolase protein
  
Accession: EJF64558
  
Location: 329941-336484
  
  
**BlastP hit with Mycgr3G41235\_Mycgr3T**
  
Percentage identity: 34 %
  
BlastP bit score: 795
  
Sequence coverage: 102 %
  
E-value: 0.0
  
  
 NCBI BlastP on this gene

EJF64558

hypothetical protein
  
Accession: EJF64559
  
Location: 336714-340809
  
 NCBI BlastP on this gene

EJF64559

109. :  DS995903 Penicillium marneffei ATCC 18224 scf\_1105668340984 genomic scaffold     Total score: 1.0     Cumulative Blast bit score: 795

ABC multidrug transporter, putative
  
Accession: EEA21837
  
Location: 1688303-1692687
  
  
**BlastP hit with Mycgr3G41235\_Mycgr3T**
  
Percentage identity: 32 %
  
BlastP bit score: 795
  
Sequence coverage: 102 %
  
E-value: 0.0
  
  
 NCBI BlastP on this gene

EEA21837

alpha/beta hydrolase family protein, putative
  
Accession: EEA21836
  
Location: 1686350-1687636
  
 NCBI BlastP on this gene

EEA21836

hypothetical protein
  
Accession: EEA21835
  
Location: 1685882-1686179
  
 NCBI BlastP on this gene

EEA21835

hypothetical protein
  
Accession: EEA21834
  
Location: 1681739-1682737
  
 NCBI BlastP on this gene

EEA21834

110. :  DS995721 Trichophyton equinum CBS 127.97 supercont1.4 genomic scaffold     Total score: 1.0     Cumulative Blast bit score: 790

mannosyltransferase
  
Accession: EGE02095
  
Location: 17180-20127
  
 NCBI BlastP on this gene

EGE02095

ornithine aminotransferase
  
Accession: EGE02096
  
Location: 20348-21807
  
 NCBI BlastP on this gene

EGE02096

ABC multidrug transporter
  
Accession: EGE02097
  
Location: 25841-30275
  
  
**BlastP hit with Mycgr3G41235\_Mycgr3T**
  
Percentage identity: 33 %
  
BlastP bit score: 790
  
Sequence coverage: 103 %
  
E-value: 0.0
  
  
 NCBI BlastP on this gene

EGE02097

amino acid permease
  
Accession: EGE02098
  
Location: 30500-32360
  
 NCBI BlastP on this gene

EGE02098

tyrosyl-DNA phosphodiesterase
  
Accession: EGE02099
  
Location: 33232-35177
  
 NCBI BlastP on this gene

EGE02099

hypothetical protein
  
Accession: EGE02100
  
Location: 35616-36064
  
 NCBI BlastP on this gene

EGE02100

111. :  DS499598 Aspergillus fumigatus A1163 scf\_000005 genomic scaffold     Total score: 1.0     Cumulative Blast bit score: 786

mannosyltransferase, putative
  
Accession: EDP50290
  
Location: 936365-938963
  
 NCBI BlastP on this gene

EDP50290

L-ornithine aminotransferase Car2, putative
  
Accession: EDP50291
  
Location: 939678-941092
  
 NCBI BlastP on this gene

EDP50291

ABC multidrug transporter, putative
  
Accession: EDP50292
  
Location: 942783-947085
  
  
**BlastP hit with Mycgr3G41235\_Mycgr3T**
  
Percentage identity: 33 %
  
BlastP bit score: 786
  
Sequence coverage: 101 %
  
E-value: 0.0
  
  
 NCBI BlastP on this gene

EDP50292

ankyrin repeat protein
  
Accession: EDP50293
  
Location: 947466-948049
  
 NCBI BlastP on this gene

EDP50293

anion exchange family protein
  
Accession: EDP50294
  
Location: 948328-950355
  
 NCBI BlastP on this gene

EDP50294

SET domain protein
  
Accession: EDP50295
  
Location: 951763-952964
  
 NCBI BlastP on this gene

EDP50295

112. :  AAHF01000005 Aspergillus fumigatus Af293     Total score: 1.0     Cumulative Blast bit score: 786

ABC multidrug transporter, putative
  
Accession: EAL89872
  
Location: 1531528-1535830
  
  
**BlastP hit with Mycgr3G41235\_Mycgr3T**
  
Percentage identity: 33 %
  
BlastP bit score: 786
  
Sequence coverage: 101 %
  
E-value: 0.0
  
  
 NCBI BlastP on this gene

EAL89872

ankyrin repeat protein
  
Accession: EAL89871
  
Location: 1530564-1531147
  
 NCBI BlastP on this gene

EAL89871

anion exchange family protein
  
Accession: EAL89870
  
Location: 1528265-1530286
  
 NCBI BlastP on this gene

EAL89870

SET domain protein
  
Accession: EAL89869
  
Location: 1525656-1526857
  
 NCBI BlastP on this gene

EAL89869

113. :  DS995901 Penicillium marneffei ATCC 18224 scf\_1105668340960 genomic scaffold     Total score: 1.0     Cumulative Blast bit score: 781

oligomycin resistance ATP-dependent permease yor1, putative
  
Accession: EEA24390
  
Location: 1966956-1971293
  
  
**BlastP hit with Mycgr3G41235\_Mycgr3T**
  
Percentage identity: 33 %
  
BlastP bit score: 781
  
Sequence coverage: 103 %
  
E-value: 0.0
  
  
 NCBI BlastP on this gene

EEA24390

cutinase, putative
  
Accession: EEA24389
  
Location: 1964067-1964973
  
 NCBI BlastP on this gene

EEA24389

conserved hypothetical protein
  
Accession: EEA24388
  
Location: 1960982-1963399
  
 NCBI BlastP on this gene

EEA24388

114. :  JH719402 Dichomitus squalens LYAD-421 SS1 unplaced genomic scaffold DICSQscaffold\_6     Total score: 1.0     Cumulative Blast bit score: 776

cytochrome P450
  
Accession: EJF63642
  
Location: 310449-313334
  
 NCBI BlastP on this gene

EJF63642

hypothetical protein
  
Accession: EJF63641
  
Location: 309526-310191
  
 NCBI BlastP on this gene

EJF63641

hypothetical protein
  
Accession: EJF63640
  
Location: 306598-308600
  
 NCBI BlastP on this gene

EJF63640

multidrug resistance-associated ABC transporter
  
Accession: EJF63639
  
Location: 299614-305768
  
  
**BlastP hit with Mycgr3G41235\_Mycgr3T**
  
Percentage identity: 32 %
  
BlastP bit score: 776
  
Sequence coverage: 107 %
  
E-value: 0.0
  
  
 NCBI BlastP on this gene

EJF63639

cytochrome P450
  
Accession: EJF63638
  
Location: 296580-298974
  
 NCBI BlastP on this gene

EJF63638

hypothetical protein
  
Accession: EJF63637
  
Location: 294086-294711
  
 NCBI BlastP on this gene

EJF63637

115. :  DS027685 Neosartorya fischeri NRRL 181 1099437636245 genomic scaffold     Total score: 1.0     Cumulative Blast bit score: 776

ABC multidrug transporter, putative
  
Accession: EAW25211
  
Location: 1612989-1617284
  
  
**BlastP hit with Mycgr3G41235\_Mycgr3T**
  
Percentage identity: 33 %
  
BlastP bit score: 776
  
Sequence coverage: 101 %
  
E-value: 0.0
  
  
 NCBI BlastP on this gene

EAW25211

Ankyrin repeat protein
  
Accession: EAW25210
  
Location: 1604300-1608054
  
 NCBI BlastP on this gene

EAW25210

116. :  ABDF02000001 Trichoderma virens Gv29-8     Total score: 1.0     Cumulative Blast bit score: 776

hypothetical protein
  
Accession: EHK27273
  
Location: 1699237-1700700
  
 NCBI BlastP on this gene

EHK27273

hypothetical protein
  
Accession: EHK27274
  
Location: 1701339-1703715
  
 NCBI BlastP on this gene

EHK27274

hypothetical protein
  
Accession: EHK27275
  
Location: 1705907-1710479
  
  
**BlastP hit with Mycgr3G41235\_Mycgr3T**
  
Percentage identity: 33 %
  
BlastP bit score: 776
  
Sequence coverage: 105 %
  
E-value: 0.0
  
  
 NCBI BlastP on this gene

EHK27275

117. :  DS995906 Penicillium marneffei ATCC 18224 scf\_1105668340770 genomic scaffold     Total score: 1.0     Cumulative Blast bit score: 771

nonribosomal peptide synthase, putative
  
Accession: EEA19149
  
Location: 1517822-1523002
  
 NCBI BlastP on this gene

EEA19149

conserved hypothetical protein
  
Accession: EEA19148
  
Location: 1516488-1517582
  
 NCBI BlastP on this gene

EEA19148

conserved hypothetical protein
  
Accession: EEA19147
  
Location: 1514040-1515981
  
 NCBI BlastP on this gene

EEA19147

oligomycin resistance ATP-dependent permease yor1, putative
  
Accession: EEA19146
  
Location: 1509447-1513663
  
  
**BlastP hit with Mycgr3G41235\_Mycgr3T**
  
Percentage identity: 34 %
  
BlastP bit score: 771
  
Sequence coverage: 100 %
  
E-value: 0.0
  
  
 NCBI BlastP on this gene

EEA19146

conserved hypothetical protein
  
Accession: EEA19145
  
Location: 1507194-1509399
  
 NCBI BlastP on this gene

EEA19145

conserved hypothetical protein
  
Accession: EEA19143
  
Location: 1501299-1506454
  
 NCBI BlastP on this gene

EEA19143

118. :  GL985056 Trichoderma reesei QM6a unplaced genomic scaffold TRIREscaffold\_1     Total score: 1.0     Cumulative Blast bit score: 768

predicted protein
  
Accession: EGR53081
  
Location: 3199556-3203833
  
  
**BlastP hit with Mycgr3G41235\_Mycgr3T**
  
Percentage identity: 32 %
  
BlastP bit score: 768
  
Sequence coverage: 103 %
  
E-value: 0.0
  
  
 NCBI BlastP on this gene

EGR53081

hypothetical protein
  
Accession: EGR52545
  
Location: 3197856-3198878
  
 NCBI BlastP on this gene

EGR52545

predicted protein
  
Accession: EGR53080
  
Location: 3195668-3196390
  
 NCBI BlastP on this gene

EGR53080

predicted protein
  
Accession: EGR53079
  
Location: 3194459-3194884
  
 NCBI BlastP on this gene

EGR53079

predicted protein
  
Accession: EGR52544
  
Location: 3192759-3193907
  
 NCBI BlastP on this gene

EGR52544

119. :  KB445647 Cochliobolus sativus ND90Pr unplaced genomic scaffold COCSAscaffold\_11     Total score: 1.0     Cumulative Blast bit score: 767

hypothetical protein
  
Accession: EMD61888
  
Location: 380779-384617
  
 NCBI BlastP on this gene

EMD61888

hypothetical protein
  
Accession: EMD61889
  
Location: 386418-387972
  
 NCBI BlastP on this gene

EMD61889

hypothetical protein
  
Accession: EMD61890
  
Location: 389583-394173
  
  
**BlastP hit with Mycgr3G41235\_Mycgr3T**
  
Percentage identity: 32 %
  
BlastP bit score: 767
  
Sequence coverage: 103 %
  
E-value: 0.0
  
  
 NCBI BlastP on this gene

EMD61890

120. :  AACS02000012 Coprinopsis cinerea okayama7#130     Total score: 1.0     Cumulative Blast bit score: 767

ribosomal processing
  
Accession: EAU86772
  
Location: 1493052-1496397
  
 NCBI BlastP on this gene

EAU86772

ATP-dependent bile acid permease
  
Accession: EAU86771
  
Location: 1486151-1492069
  
  
**BlastP hit with Mycgr3G41235\_Mycgr3T**
  
Percentage identity: 34 %
  
BlastP bit score: 767
  
Sequence coverage: 103 %
  
E-value: 0.0
  
  
 NCBI BlastP on this gene

EAU86771

hypothetical protein
  
Accession: EAU86770
  
Location: 1484949-1485807
  
 NCBI BlastP on this gene

EAU86770

hypothetical protein
  
Accession: EAU86769
  
Location: 1480119-1481843
  
 NCBI BlastP on this gene

EAU86769

121. :  CM001197 Mycosphaerella graminicola IPO323 chromosome 2     Total score: 1.0     Cumulative Blast bit score: 765

putative ABC transporter
  
Accession: EGP90265
  
Location: 3393870-3398338
  
  
**BlastP hit with Mycgr3G41235\_Mycgr3T**
  
Percentage identity: 33 %
  
BlastP bit score: 765
  
Sequence coverage: 103 %
  
E-value: 0.0
  
  
 NCBI BlastP on this gene

EGP90265

hypothetical protein
  
Accession: EGP90156
  
Location: 3391482-3392087
  
 NCBI BlastP on this gene

EGP90156

hypothetical protein
  
Accession: EGP90266
  
Location: 3387815-3390661
  
 NCBI BlastP on this gene

EGP90266

122. :  GL377310 Schizophyllum commune H4-8 unplaced genomic scaffold SCHCOscaffold\_9     Total score: 1.0     Cumulative Blast bit score: 763

hypothetical protein
  
Accession: EFI93734
  
Location: 690700-691095
  
 NCBI BlastP on this gene

EFI93734

hypothetical protein
  
Accession: EFI94034
  
Location: 688574-690480
  
 NCBI BlastP on this gene

EFI94034

hypothetical protein
  
Accession: EFI94033
  
Location: 687417-688205
  
 NCBI BlastP on this gene

EFI94033

hypothetical protein
  
Accession: EFI94032
  
Location: 686032-686995
  
 NCBI BlastP on this gene

EFI94032

hypothetical protein
  
Accession: EFI94031
  
Location: 679703-685413
  
  
**BlastP hit with Mycgr3G41235\_Mycgr3T**
  
Percentage identity: 34 %
  
BlastP bit score: 763
  
Sequence coverage: 104 %
  
E-value: 0.0
  
  
 NCBI BlastP on this gene

EFI94031

expressed protein
  
Accession: EFI94030
  
Location: 676215-678220
  
 NCBI BlastP on this gene

EFI94030

hypothetical protein
  
Accession: EFI94029
  
Location: 674467-675346
  
 NCBI BlastP on this gene

EFI94029

123. :  FP929130 Leptosphaeria maculans JN3 lm\_SuperContig\_17\_v2 genomic supercontig     Total score: 1.0     Cumulative Blast bit score: 763

similar to ornithine aminotransferase
  
Accession: CBX97087
  
Location: 1085650-1087257
  
 NCBI BlastP on this gene

LEMA\_P102180.1

hypothetical protein
  
Accession: CBX97088
  
Location: 1088086-1089330
  
 NCBI BlastP on this gene

LEMA\_P102190.1

similar to ABC multidrug transporter
  
Accession: CBX97089
  
Location: 1091682-1096279
  
  
**BlastP hit with Mycgr3G41235\_Mycgr3T**
  
Percentage identity: 32 %
  
BlastP bit score: 763
  
Sequence coverage: 105 %
  
E-value: 0.0
  
  
 NCBI BlastP on this gene

LEMA\_P102200.1

124. :  JH711791 Trametes versicolor FP-101664 SS1 unplaced genomic scaffold TRAVEscaffold\_9     Total score: 1.0     Cumulative Blast bit score: 759

hypothetical protein
  
Accession: EIW55761
  
Location: 1015122-1016951
  
 NCBI BlastP on this gene

EIW55761

hypothetical protein
  
Accession: EIW55760
  
Location: 1012549-1014505
  
 NCBI BlastP on this gene

EIW55760

multidrug resistance-associated ABC transporter
  
Accession: EIW55759
  
Location: 1004647-1011806
  
  
**BlastP hit with Mycgr3G41235\_Mycgr3T**
  
Percentage identity: 32 %
  
BlastP bit score: 759
  
Sequence coverage: 105 %
  
E-value: 0.0
  
  
 NCBI BlastP on this gene

EIW55759

cytochrome P450
  
Accession: EIW55758
  
Location: 1001508-1003803
  
 NCBI BlastP on this gene

EIW55758

hypothetical protein
  
Accession: EIW55757
  
Location: 999261-1000424
  
 NCBI BlastP on this gene

EIW55757

125. :  EQ962655 Talaromyces stipitatus ATCC 10500 scf\_1105507295555 genomic scaffold     Total score: 1.0     Cumulative Blast bit score: 759

oligomycin resistance ATP-dependent permease yor1, putative
  
Accession: EED18119
  
Location: 2083865-2088172
  
  
**BlastP hit with Mycgr3G41235\_Mycgr3T**
  
Percentage identity: 33 %
  
BlastP bit score: 759
  
Sequence coverage: 104 %
  
E-value: 0.0
  
  
 NCBI BlastP on this gene

EED18119

cutinase, putative
  
Accession: EED18118
  
Location: 2081157-2082037
  
 NCBI BlastP on this gene

EED18118

conserved hypothetical protein
  
Accession: EED18117
  
Location: 2078109-2080369
  
 NCBI BlastP on this gene

EED18117

126. :  CH476605 Aspergillus terreus NIH2624 scaffold\_12 genomic scaffold     Total score: 1.0     Cumulative Blast bit score: 755

alpha-glucosidase precursor
  
Accession: EAU31451
  
Location: 810125-813278
  
 NCBI BlastP on this gene

EAU31451

alpha-amylase A precursor
  
Accession: EAU31452
  
Location: 815081-817051
  
 NCBI BlastP on this gene

EAU31452

hypothetical protein
  
Accession: EAU31453
  
Location: 818774-822927
  
  
**BlastP hit with Mycgr3G41235\_Mycgr3T**
  
Percentage identity: 34 %
  
BlastP bit score: 755
  
Sequence coverage: 98 %
  
E-value: 0.0
  
  
 NCBI BlastP on this gene

EAU31453

predicted protein
  
Accession: EAU31454
  
Location: 823477-826148
  
 NCBI BlastP on this gene

EAU31454

predicted protein
  
Accession: EAU31455
  
Location: 826554-828153
  
 NCBI BlastP on this gene

EAU31455

predicted protein
  
Accession: EAU31456
  
Location: 829100-829639
  
 NCBI BlastP on this gene

EAU31456

127. :  CP003009 Thielavia terrestris NRRL 8126 chromosome 1     Total score: 1.0     Cumulative Blast bit score: 748

hypothetical protein
  
Accession: AEO64821
  
Location: 10080223-10081877
  
 NCBI BlastP on this gene

THITE\_2142624

hypothetical protein
  
Accession: AEO64822
  
Location: 10086001-10090387
  
  
**BlastP hit with Mycgr3G41235\_Mycgr3T**
  
Percentage identity: 32 %
  
BlastP bit score: 748
  
Sequence coverage: 100 %
  
E-value: 0.0
  
  
 NCBI BlastP on this gene

THITE\_2142625

128. :  JH226130 Exophiala dermatitidis NIH/UT8656 unplaced genomic scaffold supercont1.1     Total score: 1.0     Cumulative Blast bit score: 741

hypothetical protein
  
Accession: EHY51902
  
Location: 340526-341611
  
 NCBI BlastP on this gene

EHY51902

succinate dehydrogenase [ubiquinone] iron-sulfur subunit, mitochondrial
  
Accession: EHY51903
  
Location: 342119-343119
  
 NCBI BlastP on this gene

EHY51903

hypothetical protein
  
Accession: EHY51904
  
Location: 345354-345686
  
 NCBI BlastP on this gene

EHY51904

ABC multidrug transporter
  
Accession: EHY51906
  
Location: 346523-351022
  
  
**BlastP hit with Mycgr3G41235\_Mycgr3T**
  
Percentage identity: 35 %
  
BlastP bit score: 741
  
Sequence coverage: 85 %
  
E-value: 0.0
  
  
 NCBI BlastP on this gene

EHY51906

hypothetical protein
  
Accession: EHY51907
  
Location: 352784-353263
  
 NCBI BlastP on this gene

EHY51907

Pin2-interacting protein X1
  
Accession: EHY51908
  
Location: 354116-355069
  
 NCBI BlastP on this gene

EHY51908

serine/threonine-protein kinase Chk2
  
Accession: EHY51909
  
Location: 355861-358077
  
 NCBI BlastP on this gene

EHY51909

129. :  AACS02000004 Coprinopsis cinerea okayama7#130     Total score: 1.0     Cumulative Blast bit score: 740

ABC protein
  
Accession: EFI27939
  
Location: 2317594-2323541
  
  
**BlastP hit with Mycgr3G41235\_Mycgr3T**
  
Percentage identity: 33 %
  
BlastP bit score: 740
  
Sequence coverage: 101 %
  
E-value: 0.0
  
  
 NCBI BlastP on this gene

EFI27939

fatty acid synthetase alpha subunit
  
Accession: EAU85370
  
Location: 2304613-2317403
  
 NCBI BlastP on this gene

EAU85370

130. :  JH687393 Stereum hirsutum FP-91666 SS1 unplaced genomic scaffold STEHIscaffold\_15     Total score: 1.0     Cumulative Blast bit score: 736

hypothetical protein
  
Accession: EIM82567
  
Location: 1321755-1323180
  
 NCBI BlastP on this gene

EIM82567

MFS general substrate transporter
  
Accession: EIM82568
  
Location: 1324892-1327690
  
  
**BlastP hit with Mycgr3G70577\_Mycgr3T**
  
Percentage identity: 35 %
  
BlastP bit score: 367
  
Sequence coverage: 91 %
  
E-value: 2e-114
  
  
 NCBI BlastP on this gene

EIM82568

MFS general substrate transporter
  
Accession: EIM82569
  
Location: 1329736-1332579
  
  
**BlastP hit with Mycgr3G70577\_Mycgr3T**
  
Percentage identity: 36 %
  
BlastP bit score: 369
  
Sequence coverage: 85 %
  
E-value: 4e-115
  
  
 NCBI BlastP on this gene

EIM82569

hypothetical protein
  
Accession: EIM82570
  
Location: 1333404-1333847
  
 NCBI BlastP on this gene

EIM82570

cytochrome P450
  
Accession: EIM82571
  
Location: 1334694-1335357
  
 NCBI BlastP on this gene

EIM82571

cytochrome P450
  
Accession: EIM82572
  
Location: 1335493-1336648
  
 NCBI BlastP on this gene

EIM82572

131. :  CH476615 Uncinocarpus reesii 1704 scaffold\_1 genomic scaffold     Total score: 1.0     Cumulative Blast bit score: 729

hypothetical protein
  
Accession: EEP77403
  
Location: 5926782-5931308
  
  
**BlastP hit with Mycgr3G41235\_Mycgr3T**
  
Percentage identity: 36 %
  
BlastP bit score: 729
  
Sequence coverage: 84 %
  
E-value: 0.0
  
  
 NCBI BlastP on this gene

EEP77403

conserved hypothetical protein
  
Accession: EEP77402
  
Location: 5924440-5926241
  
 NCBI BlastP on this gene

EEP77402

132. :  AP007174 Aspergillus oryzae RIB40 DNA, SC103.     Total score: 1.0     Cumulative Blast bit score: 726

not annotated
  
Accession: BAE65685
  
Location: 596318-600535
  
  
**BlastP hit with Mycgr3G41235\_Mycgr3T**
  
Percentage identity: 32 %
  
BlastP bit score: 726
  
Sequence coverage: 101 %
  
E-value: 0.0
  
  
 NCBI BlastP on this gene

AO090103000226

not annotated
  
Accession: BAE65684
  
Location: 594689-595931
  
 NCBI BlastP on this gene

AO090103000225

not annotated
  
Accession: BAE65683
  
Location: 581629-593329
  
 NCBI BlastP on this gene

AO090103000224

133. :  AKHY01000199 Aspergillus oryzae 3.042     Total score: 1.0     Cumulative Blast bit score: 717

hypothetical protein
  
Accession: EIT73727
  
Location: 401090-401937
  
 NCBI BlastP on this gene

EIT73727

hypothetical protein
  
Accession: EIT73630
  
Location: 404776-405486
  
 NCBI BlastP on this gene

EIT73630

multidrug resistance-associated protein
  
Accession: EIT73554
  
Location: 406470-410699
  
  
**BlastP hit with Mycgr3G41235\_Mycgr3T**
  
Percentage identity: 32 %
  
BlastP bit score: 717
  
Sequence coverage: 101 %
  
E-value: 0.0
  
  
 NCBI BlastP on this gene

EIT73554

isopenicillin N synthase
  
Accession: EIT73763
  
Location: 411086-412328
  
 NCBI BlastP on this gene

EIT73763

polyketide synthase module
  
Accession: EIT73707
  
Location: 413688-425388
  
 NCBI BlastP on this gene

EIT73707

134. :  AACS02000012 Coprinopsis cinerea okayama7#130     Total score: 1.0     Cumulative Blast bit score: 714

cadmium ion transporter
  
Accession: EAU86674
  
Location: 1678680-1684579
  
  
**BlastP hit with Mycgr3G41235\_Mycgr3T**
  
Percentage identity: 31 %
  
BlastP bit score: 714
  
Sequence coverage: 105 %
  
E-value: 0.0
  
  
 NCBI BlastP on this gene

EAU86674

cytoplasmic protein
  
Accession: EAU86673
  
Location: 1676097-1677687
  
 NCBI BlastP on this gene

EAU86673

135. :  JH687764 Auricularia delicata TFB-10046 SS5 unplaced genomic scaffold AURDEscaffold\_32     Total score: 1.0     Cumulative Blast bit score: 712

hypothetical protein
  
Accession: EJD45473
  
Location: 94889-97271
  
 NCBI BlastP on this gene

EJD45473

cadmium ion transporter
  
Accession: EJD45474
  
Location: 100161-105938
  
  
**BlastP hit with Mycgr3G41235\_Mycgr3T**
  
Percentage identity: 31 %
  
BlastP bit score: 712
  
Sequence coverage: 102 %
  
E-value: 0.0
  
  
 NCBI BlastP on this gene

EJD45474

136. :  DS995903 Penicillium marneffei ATCC 18224 scf\_1105668340984 genomic scaffold     Total score: 1.0     Cumulative Blast bit score: 699

alcohol dehydrogenase, putative
  
Accession: EEA21216
  
Location: 33030-34233
  
 NCBI BlastP on this gene

EEA21216

N-alkane-inducible cytochrome P450, putative
  
Accession: EEA21215
  
Location: 30654-32357
  
 NCBI BlastP on this gene

EEA21215

conserved hypothetical protein
  
Accession: EEA21214
  
Location: 29530-30171
  
 NCBI BlastP on this gene

EEA21214

oligomycin resistance ATP-dependent permease yor1, putative
  
Accession: EEA21213
  
Location: 24639-28887
  
  
**BlastP hit with Mycgr3G41235\_Mycgr3T**
  
Percentage identity: 31 %
  
BlastP bit score: 699
  
Sequence coverage: 103 %
  
E-value: 0.0
  
  
 NCBI BlastP on this gene

EEA21213

ankyrin repeat-containing protein, putative
  
Accession: EEA21212
  
Location: 22112-22870
  
 NCBI BlastP on this gene

EEA21212

conserved hypothetical protein
  
Accession: EEA21211
  
Location: 18978-19334
  
 NCBI BlastP on this gene

EEA21211

137. :  EQ963486 Aspergillus flavus NRRL3357 scf\_1106286417242 genomic scaffold     Total score: 1.0     Cumulative Blast bit score: 697

hypothetical protein
  
Accession: EED45111
  
Location: 671847-674725
  
 NCBI BlastP on this gene

EED45111

hypothetical protein
  
Accession: EED45112
  
Location: 674887-675566
  
 NCBI BlastP on this gene

EED45112

hypothetical protein
  
Accession: EED45113
  
Location: 676291-676683
  
 NCBI BlastP on this gene

EED45113

ABC multidrug transporter, putative
  
Accession: EED45114
  
Location: 680721-684644
  
  
**BlastP hit with Mycgr3G41235\_Mycgr3T**
  
Percentage identity: 32 %
  
BlastP bit score: 697
  
Sequence coverage: 97 %
  
E-value: 0.0
  
  
 NCBI BlastP on this gene

EED45114

1-aminocyclopropane-1-carboxylate oxidase, putative
  
Accession: EED45115
  
Location: 685466-686495
  
 NCBI BlastP on this gene

EED45115

polyketide synthase, putative
  
Accession: EED45116
  
Location: 687862-693859
  
 NCBI BlastP on this gene

EED45116

138. :  JH687764 Auricularia delicata TFB-10046 SS5 unplaced genomic scaffold AURDEscaffold\_32     Total score: 1.0     Cumulative Blast bit score: 695

hypothetical protein
  
Accession: EJD45441
  
Location: 11755-15254
  
 NCBI BlastP on this gene

EJD45441

hypothetical protein
  
Accession: EJD45442
  
Location: 15908-16305
  
 NCBI BlastP on this gene

EJD45442

hypothetical protein
  
Accession: EJD45443
  
Location: 16923-18003
  
 NCBI BlastP on this gene

EJD45443

ATP-dependent bile acid permease
  
Accession: EJD45444
  
Location: 19709-25146
  
  
**BlastP hit with Mycgr3G41235\_Mycgr3T**
  
Percentage identity: 31 %
  
BlastP bit score: 695
  
Sequence coverage: 106 %
  
E-value: 0.0
  
  
 NCBI BlastP on this gene

EJD45444

hypothetical protein
  
Accession: EJD45445
  
Location: 25501-26626
  
 NCBI BlastP on this gene

EJD45445

alpha/beta-hydrolase
  
Accession: EJD45446
  
Location: 27094-28207
  
 NCBI BlastP on this gene

EJD45446

zincin
  
Accession: EJD45447
  
Location: 29127-30301
  
 NCBI BlastP on this gene

EJD45447

hypothetical protein
  
Accession: EJD45448
  
Location: 30813-32026
  
 NCBI BlastP on this gene

EJD45448

139. :  FQ311430 Sporisorium reilianum SRZ2 chromosome 1 complete DNA sequence.     Total score: 1.0     Cumulative Blast bit score: 694

probable YOR1-ABC transporter
  
Accession: CBQ68135
  
Location: 2089203-2094056
  
  
**BlastP hit with Mycgr3G41235\_Mycgr3T**
  
Percentage identity: 39 %
  
BlastP bit score: 694
  
Sequence coverage: 72 %
  
E-value: 0.0
  
  
 NCBI BlastP on this gene

sr12000

related to Proteasome activator complex subunit 3
  
Accession: CBQ68134
  
Location: 2088204-2088989
  
 NCBI BlastP on this gene

sr11999

related to Exocyst complex component Sec5
  
Accession: CBQ68133
  
Location: 2085167-2087842
  
 NCBI BlastP on this gene

sr11998

probable TIM17-mitochondrial inner membrane import translocase subunit
  
Accession: CBQ68132
  
Location: 2084449-2084943
  
 NCBI BlastP on this gene

sr11997

related to monooxygenase
  
Accession: CBQ68131
  
Location: 2081886-2083652
  
 NCBI BlastP on this gene

sr11996

140. :  DF196775 Pseudozyma antarctica T-34 DNA, contig: scaffold00009     Total score: 1.0     Cumulative Blast bit score: 682

multidrug resistance-associated protein
  
Accession: GAC73965
  
Location: 2042126-2046958
  
  
**BlastP hit with Mycgr3G41235\_Mycgr3T**
  
Percentage identity: 39 %
  
BlastP bit score: 682
  
Sequence coverage: 68 %
  
E-value: 0.0
  
  
 NCBI BlastP on this gene

GAC73965

proteasome activator subunit
  
Accession: GAC73964
  
Location: 2041125-2041922
  
 NCBI BlastP on this gene

GAC73964

sec5 subunit of exocyst complex
  
Accession: GAC73963
  
Location: 2038097-2040734
  
 NCBI BlastP on this gene

GAC73963

mitochondrial import inner membrane translocase, subunit TIM17
  
Accession: GAC73962
  
Location: 2037355-2037849
  
 NCBI BlastP on this gene

GAC73962

hypothetical protein
  
Accession: GAC73961
  
Location: 2034693-2036539
  
 NCBI BlastP on this gene

GAC73961

141. :  AE017342 Cryptococcus neoformans var. neoformans JEC21 chromosome 2     Total score: 1.0     Cumulative Blast bit score: 678

dehydrogenase, putative
  
Accession: AAW41745
  
Location: 120276-121739
  
 NCBI BlastP on this gene

CNB00420

conserved hypothetical protein
  
Accession: AAW41477
  
Location: 116287-117232
  
 NCBI BlastP on this gene

CNB00410

conserved hypothetical protein
  
Accession: AAW41476
  
Location: 116287-117704
  
 NCBI BlastP on this gene

CNB00410

conserved hypothetical protein
  
Accession: AAW41475
  
Location: 112785-115457
  
  
**BlastP hit with Mycgr3G70577\_Mycgr3T**
  
Percentage identity: 32 %
  
BlastP bit score: 342
  
Sequence coverage: 102 %
  
E-value: 2e-104
  
  
 NCBI BlastP on this gene

CNB00400

conserved hypothetical protein
  
Accession: AAW41474
  
Location: 112785-115457
  
  
**BlastP hit with Mycgr3G70577\_Mycgr3T**
  
Percentage identity: 31 %
  
BlastP bit score: 336
  
Sequence coverage: 104 %
  
E-value: 6e-102
  
  
 NCBI BlastP on this gene

CNB00400

hypothetical protein
  
Accession: AAW41473
  
Location: 110234-111942
  
 NCBI BlastP on this gene

CNB00390

seryl-tRNA synthetase, putative
  
Accession: AAW41472
  
Location: 107240-108937
  
 NCBI BlastP on this gene

CNB00380

fatty acid beta-oxidation-related protein, putative
  
Accession: AAW41471
  
Location: 105323-106617
  
 NCBI BlastP on this gene

CNB00370

142. :  CR382134 Debaryomyces hansenii CBS767 chromosome B complete sequence.     Total score: 1.0     Cumulative Blast bit score: 674

DEHA2B01452p
  
Accession: CAR65428
  
Location: 111106-115899
  
 NCBI BlastP on this gene

DEHA2B01452g

DEHA2B01430p
  
Accession: CAG85014
  
Location: 105614-109774
  
  
**BlastP hit with Mycgr3G41235\_Mycgr3T**
  
Percentage identity: 29 %
  
BlastP bit score: 674
  
Sequence coverage: 103 %
  
E-value: 0.0
  
  
 NCBI BlastP on this gene

DEHA2B01430g

DEHA2B01408p
  
Accession: CAG85013
  
Location: 104374-105504
  
 NCBI BlastP on this gene

DEHA2B01408g

DEHA2B01386p
  
Accession: CAG85012
  
Location: 100817-104209
  
 NCBI BlastP on this gene

DEHA2B01386g

DEHA2B01364p
  
Accession: CAG85011
  
Location: 99881-100267
  
 NCBI BlastP on this gene

DEHA2B01364g

143. :  GL996527 Candida tenuis ATCC 10573 unplaced genomic scaffold CANTEscaffold\_00021     Total score: 1.0     Cumulative Blast bit score: 669

hypothetical protein
  
Accession: EGV61734
  
Location: 826770-830936
  
  
**BlastP hit with Mycgr3G41235\_Mycgr3T**
  
Percentage identity: 28 %
  
BlastP bit score: 669
  
Sequence coverage: 105 %
  
E-value: 0.0
  
  
 NCBI BlastP on this gene

EGV61734

hypothetical protein
  
Accession: EGV62638
  
Location: 825576-826733
  
 NCBI BlastP on this gene

EGV62638

hypothetical protein
  
Accession: EGV62637
  
Location: 824946-826733
  
 NCBI BlastP on this gene

EGV62637

dihydroxyacetone synthase
  
Accession: EGV61733
  
Location: 822340-824433
  
 NCBI BlastP on this gene

EGV61733

hypothetical protein
  
Accession: EGV62954
  
Location: 820096-821670
  
 NCBI BlastP on this gene

EGV62954

144. :  GL996527 Candida tenuis ATCC 10573 unplaced genomic scaffold CANTEscaffold\_00021     Total score: 1.0     Cumulative Blast bit score: 667

hypothetical protein
  
Accession: EGV61462
  
Location: 238684-239280
  
 NCBI BlastP on this gene

EGV61462

hypothetical protein
  
Accession: EGV62531
  
Location: 239270-239827
  
 NCBI BlastP on this gene

EGV62531

hypothetical protein
  
Accession: EGV61463
  
Location: 239997-241409
  
 NCBI BlastP on this gene

EGV61463

hypothetical protein
  
Accession: EGV61464
  
Location: 242666-243514
  
 NCBI BlastP on this gene

EGV61464

hypothetical protein
  
Accession: EGV61465
  
Location: 245286-249293
  
  
**BlastP hit with Mycgr3G41235\_Mycgr3T**
  
Percentage identity: 30 %
  
BlastP bit score: 667
  
Sequence coverage: 102 %
  
E-value: 0.0
  
  
 NCBI BlastP on this gene

EGV61465

hypothetical protein
  
Accession: EGV61466
  
Location: 249320-252370
  
 NCBI BlastP on this gene

EGV61466

mitochondrial import inner membrane translocase subunit TIM16
  
Accession: EGV61467
  
Location: 252717-253097
  
 NCBI BlastP on this gene

EGV61467

145. :  CU928166 Lachancea thermotolerans CBS 6340 chromosome B complete sequence.     Total score: 1.0     Cumulative Blast bit score: 667

KLTH0B09724p
  
Accession: CAR21771
  
Location: 796023-800483
  
  
**BlastP hit with Mycgr3G41235\_Mycgr3T**
  
Percentage identity: 30 %
  
BlastP bit score: 667
  
Sequence coverage: 102 %
  
E-value: 0.0
  
  
 NCBI BlastP on this gene

KLTH0B09724g

KLTH0B09680p
  
Accession: CAR21770
  
Location: 790481-792934
  
 NCBI BlastP on this gene

KLTH0B09680g

KLTH0B09658p
  
Accession: CAR21769
  
Location: 789016-789939
  
 NCBI BlastP on this gene

KLTH0B09658g

146. :  HE681721 Candida orthopsilosis Co 90-125, chromosome 3 draft sequence.     Total score: 1.0     Cumulative Blast bit score: 665

hypothetical protein
  
Accession: CCG26058
  
Location: 1532971-1537332
  
  
**BlastP hit with Mycgr3G41235\_Mycgr3T**
  
Percentage identity: 30 %
  
BlastP bit score: 665
  
Sequence coverage: 100 %
  
E-value: 0.0
  
  
 NCBI BlastP on this gene

CORT\_0C06860

Cyc1 cytochrome c
  
Accession: CCG26057
  
Location: 1531856-1532191
  
 NCBI BlastP on this gene

CORT\_0C06850

Plp2 protein
  
Accession: CCG26056
  
Location: 1530655-1531419
  
 NCBI BlastP on this gene

CORT\_0C06840

hypothetical protein
  
Accession: CCG26055
  
Location: 1527735-1530518
  
 NCBI BlastP on this gene

CORT\_0C06830

Ubp8 protein
  
Accession: CCG26054
  
Location: 1525590-1527221
  
 NCBI BlastP on this gene

CORT\_0C06820

147. :  CH477324 Aedes aegypti strain Liverpool supercont1.139 genomic scaffold     Total score: 1.0     Cumulative Blast bit score: 663

AAEL005043-PA
  
Accession: EAT43548
  
Location: 1140679-1145502
  
  
**BlastP hit with Mycgr3G41235\_Mycgr3T**
  
Percentage identity: 30 %
  
BlastP bit score: 663
  
Sequence coverage: 101 %
  
E-value: 0.0
  
  
 NCBI BlastP on this gene

EAT43548

148. :  HE681721 Candida orthopsilosis Co 90-125, chromosome 3 draft sequence.     Total score: 1.0     Cumulative Blast bit score: 659

hypothetical protein
  
Accession: CCG26033
  
Location: 1477220-1478065
  
 NCBI BlastP on this gene

CORT\_0C06600

hypothetical protein
  
Accession: CCG26032
  
Location: 1475902-1477149
  
 NCBI BlastP on this gene

CORT\_0C06590

monooxygenase
  
Accession: CCG26031
  
Location: 1474126-1475628
  
 NCBI BlastP on this gene

CORT\_0C06580

Yor1 protein
  
Accession: CCG26030
  
Location: 1467217-1471587
  
  
**BlastP hit with Mycgr3G41235\_Mycgr3T**
  
Percentage identity: 30 %
  
BlastP bit score: 659
  
Sequence coverage: 106 %
  
E-value: 0.0
  
  
 NCBI BlastP on this gene

CORT\_0C06570

calmodulin-dependent protein kinase
  
Accession: CCG26029
  
Location: 1465140-1466474
  
 NCBI BlastP on this gene

CORT\_0C06560

Set2 protein
  
Accession: CCG26028
  
Location: 1462475-1464895
  
 NCBI BlastP on this gene

CORT\_0C06550

Gpd1 protein
  
Accession: CCG26027
  
Location: 1461106-1462260
  
 NCBI BlastP on this gene

CORT\_0C06540

149. :  CR382134 Debaryomyces hansenii CBS767 chromosome B complete sequence.     Total score: 1.0     Cumulative Blast bit score: 658

DEHA2B13970p
  
Accession: CAR65493
  
Location: 1092795-1094852
  
 NCBI BlastP on this gene

DEHA2B13970g

DEHA2B13992p
  
Accession: CAG85565
  
Location: 1095412-1097451
  
 NCBI BlastP on this gene

DEHA2B13992g

DEHA2B14014p
  
Accession: CAR65494
  
Location: 1098064-1098171
  
 NCBI BlastP on this gene

DEHA2B14014g

DEHA2B14058p
  
Accession: CAR65495
  
Location: 1099134-1099469
  
 NCBI BlastP on this gene

DEHA2B14058g

DEHA2B14080p
  
Accession: CAR65496
  
Location: 1100131-1104420
  
  
**BlastP hit with Mycgr3G41235\_Mycgr3T**
  
Percentage identity: 30 %
  
BlastP bit score: 658
  
Sequence coverage: 104 %
  
E-value: 0.0
  
  
 NCBI BlastP on this gene

DEHA2B14080g

DEHA2B14102p
  
Accession: CAG85569
  
Location: 1105067-1107091
  
 NCBI BlastP on this gene

DEHA2B14102g

DEHA2B14124p
  
Accession: CAG85570
  
Location: 1107741-1109789
  
 NCBI BlastP on this gene

DEHA2B14124g

DEHA2B14146p
  
Accession: CAR65497
  
Location: 1110498-1112510
  
 NCBI BlastP on this gene

DEHA2B14146g

150. :  CH477324 Aedes aegypti strain Liverpool supercont1.139 genomic scaffold     Total score: 1.0     Cumulative Blast bit score: 644

AAEL005045-PA
  
Accession: EAT43550
  
Location: 1184563-1195380
  
  
**BlastP hit with Mycgr3G41235\_Mycgr3T**
  
Percentage identity: 30 %
  
BlastP bit score: 644
  
Sequence coverage: 101 %
  
E-value: 0.0
  
  
 NCBI BlastP on this gene

EAT43550

AAEL005026-PA
  
Accession: EAT43549
  
Location: 1168407-1184363
  
 NCBI BlastP on this gene

EAT43549

Detecting sequence homology at the gene cluster level with MultiGeneBlast.
  
Marnix H. Medema, Rainer Breitling & Eriko Takano (2013)
  
*Molecular Biology and Evolution* , 30: 1218-1223.
