# Supplementary material for: In silico prediction and characterization of secondary metabolite biosynthetic gene clusters in the wheat pathogen Zymoseptoria tritici
Source: BMC Genomics. 2017 Aug 17;18:631. doi: 10.1186/s12864-017-3969-y (PMC5561558; doi:10.1186/s12864-017-3969-y)
Supplement: Supplementary file 1 — MultiGeneBLAST analysis of putative secondary metabolite clusters. All encoded amino acid sequences from genes residing in clusters predicted by AntiSMASH are given as FASTA file format. All output data from MultiGeneBLASTs are also provided. (ZIP 42911 kb) [file 12864_2017_3969_MOESM1_ESM.zip › Cluster MultiGene BLAST/out/Clusters_1_34/Cluster_14/displaypage4.xhtml]

xml version="1.0" encoding="UTF-8"?


Search Results
  
  
 Results pages: 1, 2, 3, 4, 5

**MultiGeneBlast hits**

Select gene cluster alignment
151. GL883167\_0 Melampsora larici-populina 98AG31 unplaced genomic scaffold M...
152. GL985056\_0 Trichoderma reesei QM6a unplaced genomic scaffold TRIREscaffo...
153. EQ963474\_1 Aspergillus flavus NRRL3357 scf\_1106286417496 genomic scaffol...
154. AKHY01000171\_1 Aspergillus oryzae 3.042, whole genome shotgun sequencing...
155. CU329670\_0 Schizosaccharomyces pombe chromosome I, complete sequence.
156. AP007161\_1 Aspergillus oryzae RIB40 DNA, SC012.
157. GL377306\_2 Schizophyllum commune H4-8 unplaced genomic scaffold SCHCOsca...
158. CR382133\_1 Debaryomyces hansenii CBS767 chromosome A complete sequence.
159. KB644409\_1 Penicillium oxalicum 114-2 unplaced genomic scaffold scaffold...
160. DS027696\_1 Neosartorya fischeri NRRL 181 1099437636264 genomic scaffold,...
161. CR382137\_1 Debaryomyces hansenii CBS767 chromosome E complete sequence.
162. DS995906\_1 Penicillium marneffei ATCC 18224 scf\_1105668340770 genomic sc...
163. AM920431\_2 Penicillium chrysogenum Wisconsin 54-1255 complete genome, co...
164. KB644408\_1 Penicillium oxalicum 114-2 unplaced genomic scaffold scaffold...
165. JH687379\_1 Stereum hirsutum FP-91666 SS1 unplaced genomic scaffold STEHI...
166. FO082046\_1 Pichia sorbitophila strain CBS 7064 chromosome N complete seq...
167. GL698717\_1 Metarhizium anisopliae ARSEF 23 unplaced genomic scaffold Scf...
168. AM920435\_2 Penicillium chrysogenum Wisconsin 54-1255 complete genome, co...
169. AM920435\_1 Penicillium chrysogenum Wisconsin 54-1255 complete genome, co...
170. EQ962652\_0 Talaromyces stipitatus ATCC 10500 scf\_1105507295523 genomic s...
171. DS027045\_1 Aspergillus clavatus NRRL 1 1099423829791 genomic scaffold, w...
172. CH408159\_0 Pichia guilliermondii ATCC 6260 scaffold\_5 genomic scaffold, ...
173. AM920437\_1 Penicillium chrysogenum Wisconsin 54-1255 complete genome, co...
174. CH408159\_1 Pichia guilliermondii ATCC 6260 scaffold\_5 genomic scaffold, ...
175. AM920437\_5 Penicillium chrysogenum Wisconsin 54-1255 complete genome, co...
176. CR382134\_3 Debaryomyces hansenii CBS767 chromosome B complete sequence.
177. JH126400\_0 Cordyceps militaris CM01 unplaced genomic scaffold CCM\_S00002...
178. HF679023\_1 Fusarium fujikuroi IMI 58289 draft genome, chromosome FFUJ\_ch...
179. JH711791\_2 Trametes versicolor FP-101664 SS1 unplaced genomic scaffold T...
180. AKCT01000319\_1 Penicillium digitatum PHI26, whole genome shotgun sequenc...
181. JH711584\_0 Coniophora puteana RWD-64-598 SS2 unplaced genomic scaffold C...
182. AM920428\_0 Penicillium chrysogenum Wisconsin 54-1255 complete genome, co...
183. JH711791\_3 Trametes versicolor FP-101664 SS1 unplaced genomic scaffold T...
184. EQ962652\_1 Talaromyces stipitatus ATCC 10500 scf\_1105507295523 genomic s...
185. GL377310\_1 Schizophyllum commune H4-8 unplaced genomic scaffold SCHCOsca...
186. KE145363\_1 Glarea lozoyensis ATCC 20868 chromosome Unknown GLAREA2, whol...
187. FP929130\_0 Leptosphaeria maculans JN3 lm\_SuperContig\_17\_v2 genomic super...
188. DS499598\_1 Aspergillus fumigatus A1163 scf\_000005 genomic scaffold, whol...
189. AAHF01000005\_0 Aspergillus fumigatus Af293, whole genome shotgun sequenc...
190. JH711573\_0 Coniophora puteana RWD-64-598 SS2 unplaced genomic scaffold C...
191. DS027054\_1 Aspergillus clavatus NRRL 1 1099423829800 genomic scaffold, w...
192. GL377303\_2 Schizophyllum commune H4-8 unplaced genomic scaffold SCHCOsca...
193. DS027685\_0 Neosartorya fischeri NRRL 181 1099437636245 genomic scaffold,...
194. GG704911\_2 Coccidioides immitis RS genomic scaffold supercont3.1, whole ...
195. ACFW01000049\_2 Coccidioides posadasii C735 delta SOWgp, whole genome sho...
196. EQ962655\_0 Talaromyces stipitatus ATCC 10500 scf\_1105507295555 genomic s...
197. CM001198\_1 Mycosphaerella graminicola IPO323 chromosome 3, whole genome ...
198. HF679025\_1 Fusarium fujikuroi IMI 58289 draft genome, chromosome FFUJ\_ch...
199. KB446557\_1 Pseudocercospora fijiensis CIRAD86 unplaced genomic scaffold ...
200. KB644410\_1 Penicillium oxalicum 114-2 unplaced genomic scaffold scaffold...

Query: Architecture Search FASTA input

GL883167 : Melampsora larici-populina 98AG31 unplaced genomic scaffold MELLAscaffold\_78    Total score: 1.0     Cumulative Blast bit score: 607

Hit cluster cross-links:

Mycgr3G41235 Mycgr3T
  
Location: 0-4062

Mycgr3G41235\_Mycgr3T

Mycgr3G70577 Mycgr3T
  
Location: 4162-6109

Mycgr3G70577\_Mycgr3T

Mycgr3G40534 Mycgr3T
  
Location: 6209-7166

Mycgr3G40534\_Mycgr3T

Mycgr3G85486 Mycgr3T
  
Location: 7266-8511

Mycgr3G85486\_Mycgr3T

Mycgr3G92221 Mycgr3T
  
Location: 8611-9193

Mycgr3G92221\_Mycgr3T

Mycgr3G39931 Mycgr3T
  
Location: 9293-10157

Mycgr3G39931\_Mycgr3T

Mycgr3G99766 Mycgr3T
  
Location: 10257-11775

Mycgr3G99766\_Mycgr3T

hypothetical protein
  
Accession: EGF98912
  
Location: 231101-233768
  
  
**BlastP hit with Mycgr3G99766\_Mycgr3T**
  
Percentage identity: 36 %
  
BlastP bit score: 304
  
Sequence coverage: 95 %
  
E-value: 4e-93
  
  
 NCBI BlastP on this gene

EGF98912

hypothetical protein
  
Accession: EGF98886
  
Location: 241851-242636
  
 NCBI BlastP on this gene

EGF98886

hypothetical protein
  
Accession: EGF98911
  
Location: 242878-243763
  
 NCBI BlastP on this gene

EGF98911

hypothetical protein
  
Accession: EGF98887
  
Location: 244199-246851
  
  
**BlastP hit with Mycgr3G99766\_Mycgr3T**
  
Percentage identity: 36 %
  
BlastP bit score: 303
  
Sequence coverage: 95 %
  
E-value: 1e-92
  
  
 NCBI BlastP on this gene

EGF98887

Query: Architecture Search FASTA input

GL985056 : Trichoderma reesei QM6a unplaced genomic scaffold TRIREscaffold\_1    Total score: 1.0     Cumulative Blast bit score: 466

Hit cluster cross-links:

Mycgr3G41235 Mycgr3T
  
Location: 0-4062

Mycgr3G41235\_Mycgr3T

Mycgr3G70577 Mycgr3T
  
Location: 4162-6109

Mycgr3G70577\_Mycgr3T

Mycgr3G40534 Mycgr3T
  
Location: 6209-7166

Mycgr3G40534\_Mycgr3T

Mycgr3G85486 Mycgr3T
  
Location: 7266-8511

Mycgr3G85486\_Mycgr3T

Mycgr3G92221 Mycgr3T
  
Location: 8611-9193

Mycgr3G92221\_Mycgr3T

Mycgr3G39931 Mycgr3T
  
Location: 9293-10157

Mycgr3G39931\_Mycgr3T

Mycgr3G99766 Mycgr3T
  
Location: 10257-11775

Mycgr3G99766\_Mycgr3T

predicted protein
  
Accession: EGR52982
  
Location: 2446167-2447126
  
 NCBI BlastP on this gene

EGR52982

predicted protein
  
Accession: EGR52981
  
Location: 2441731-2443506
  
 NCBI BlastP on this gene

EGR52981

predicted protein
  
Accession: EGR52429
  
Location: 2440405-2441265
  
 NCBI BlastP on this gene

EGR52429

predicted protein
  
Accession: EGR52980
  
Location: 2437048-2439031
  
  
**BlastP hit with Mycgr3G70577\_Mycgr3T**
  
Percentage identity: 42 %
  
BlastP bit score: 466
  
Sequence coverage: 90 %
  
E-value: 1e-152
  
  
 NCBI BlastP on this gene

EGR52980

predicted protein
  
Accession: EGR52979
  
Location: 2434650-2435924
  
 NCBI BlastP on this gene

EGR52979

Query: Architecture Search FASTA input

EQ963474 : Aspergillus flavus NRRL3357 scf\_1106286417496 genomic scaffold    Total score: 1.0     Cumulative Blast bit score: 396

Hit cluster cross-links:

Mycgr3G41235 Mycgr3T
  
Location: 0-4062

Mycgr3G41235\_Mycgr3T

Mycgr3G70577 Mycgr3T
  
Location: 4162-6109

Mycgr3G70577\_Mycgr3T

Mycgr3G40534 Mycgr3T
  
Location: 6209-7166

Mycgr3G40534\_Mycgr3T

Mycgr3G85486 Mycgr3T
  
Location: 7266-8511

Mycgr3G85486\_Mycgr3T

Mycgr3G92221 Mycgr3T
  
Location: 8611-9193

Mycgr3G92221\_Mycgr3T

Mycgr3G39931 Mycgr3T
  
Location: 9293-10157

Mycgr3G39931\_Mycgr3T

Mycgr3G99766 Mycgr3T
  
Location: 10257-11775

Mycgr3G99766\_Mycgr3T

conserved hypothetical protein
  
Accession: EED54301
  
Location: 734526-736301
  
 NCBI BlastP on this gene

EED54301

amino acid transporter, putative
  
Accession: EED54302
  
Location: 737286-738920
  
 NCBI BlastP on this gene

EED54302

MFS sugar transporter, putative
  
Accession: EED54303
  
Location: 743091-744696
  
  
**BlastP hit with Mycgr3G99766\_Mycgr3T**
  
Percentage identity: 41 %
  
BlastP bit score: 397
  
Sequence coverage: 97 %
  
E-value: 3e-129
  
  
 NCBI BlastP on this gene

EED54303

Query: Architecture Search FASTA input

AKHY01000171 : Aspergillus oryzae 3.042    Total score: 1.0     Cumulative Blast bit score: 396

Hit cluster cross-links:

Mycgr3G41235 Mycgr3T
  
Location: 0-4062

Mycgr3G41235\_Mycgr3T

Mycgr3G70577 Mycgr3T
  
Location: 4162-6109

Mycgr3G70577\_Mycgr3T

Mycgr3G40534 Mycgr3T
  
Location: 6209-7166

Mycgr3G40534\_Mycgr3T

Mycgr3G85486 Mycgr3T
  
Location: 7266-8511

Mycgr3G85486\_Mycgr3T

Mycgr3G92221 Mycgr3T
  
Location: 8611-9193

Mycgr3G92221\_Mycgr3T

Mycgr3G39931 Mycgr3T
  
Location: 9293-10157

Mycgr3G39931\_Mycgr3T

Mycgr3G99766 Mycgr3T
  
Location: 10257-11775

Mycgr3G99766\_Mycgr3T

hypothetical protein
  
Accession: EIT76106
  
Location: 687859-689241
  
 NCBI BlastP on this gene

EIT76106

hypothetical protein
  
Accession: EIT76062
  
Location: 690723-692278
  
 NCBI BlastP on this gene

EIT76062

putative transporter
  
Accession: EIT75984
  
Location: 696425-698030
  
  
**BlastP hit with Mycgr3G99766\_Mycgr3T**
  
Percentage identity: 41 %
  
BlastP bit score: 397
  
Sequence coverage: 97 %
  
E-value: 3e-129
  
  
 NCBI BlastP on this gene

EIT75984

Query: Architecture Search FASTA input

CU329670 : Schizosaccharomyces pombe chromosome I    Total score: 1.0     Cumulative Blast bit score: 396

Hit cluster cross-links:

Mycgr3G41235 Mycgr3T
  
Location: 0-4062

Mycgr3G41235\_Mycgr3T

Mycgr3G70577 Mycgr3T
  
Location: 4162-6109

Mycgr3G70577\_Mycgr3T

Mycgr3G40534 Mycgr3T
  
Location: 6209-7166

Mycgr3G40534\_Mycgr3T

Mycgr3G85486 Mycgr3T
  
Location: 7266-8511

Mycgr3G85486\_Mycgr3T

Mycgr3G92221 Mycgr3T
  
Location: 8611-9193

Mycgr3G92221\_Mycgr3T

Mycgr3G39931 Mycgr3T
  
Location: 9293-10157

Mycgr3G39931\_Mycgr3T

Mycgr3G99766 Mycgr3T
  
Location: 10257-11775

Mycgr3G99766\_Mycgr3T

sequence orphan
  
Accession: CAB03599
  
Location: 96000-96548
  
 NCBI BlastP on this gene

isp3

hydrolase (predicted)
  
Accession: CAB03598
  
Location: 92480-93871
  
 NCBI BlastP on this gene

SPAC1F8.04c

siderophore-iron transporter Str3
  
Accession: CAB03597
  
Location: 88367-90259
  
  
**BlastP hit with Mycgr3G70577\_Mycgr3T**
  
Percentage identity: 37 %
  
BlastP bit score: 396
  
Sequence coverage: 90 %
  
E-value: 2e-125
  
  
 NCBI BlastP on this gene

str3

sequence orphan
  
Accession: CAB03596
  
Location: 85598-86278
  
 NCBI BlastP on this gene

SPAC1F8.02c

hexose transporter Ght3
  
Accession: CAB03595
  
Location: 82936-84603
  
 NCBI BlastP on this gene

ght3

Query: Architecture Search FASTA input

AP007161 : Aspergillus oryzae RIB40 DNA, SC012.    Total score: 1.0     Cumulative Blast bit score: 395

Hit cluster cross-links:

Mycgr3G41235 Mycgr3T
  
Location: 0-4062

Mycgr3G41235\_Mycgr3T

Mycgr3G70577 Mycgr3T
  
Location: 4162-6109

Mycgr3G70577\_Mycgr3T

Mycgr3G40534 Mycgr3T
  
Location: 6209-7166

Mycgr3G40534\_Mycgr3T

Mycgr3G85486 Mycgr3T
  
Location: 7266-8511

Mycgr3G85486\_Mycgr3T

Mycgr3G92221 Mycgr3T
  
Location: 8611-9193

Mycgr3G92221\_Mycgr3T

Mycgr3G39931 Mycgr3T
  
Location: 9293-10157

Mycgr3G39931\_Mycgr3T

Mycgr3G99766 Mycgr3T
  
Location: 10257-11775

Mycgr3G99766\_Mycgr3T

not annotated
  
Accession: BAE60485
  
Location: 698341-699723
  
 NCBI BlastP on this gene

AO090012000281

not annotated
  
Accession: BAE60486
  
Location: 701205-702760
  
 NCBI BlastP on this gene

AO090012000283

not annotated
  
Accession: BAE60487
  
Location: 706906-708511
  
  
**BlastP hit with Mycgr3G99766\_Mycgr3T**
  
Percentage identity: 41 %
  
BlastP bit score: 395
  
Sequence coverage: 97 %
  
E-value: 2e-128
  
  
 NCBI BlastP on this gene

AO090012000284

Query: Architecture Search FASTA input

GL377306 : Schizophyllum commune H4-8 unplaced genomic scaffold SCHCOscaffold\_5    Total score: 1.0     Cumulative Blast bit score: 393

Hit cluster cross-links:

Mycgr3G41235 Mycgr3T
  
Location: 0-4062

Mycgr3G41235\_Mycgr3T

Mycgr3G70577 Mycgr3T
  
Location: 4162-6109

Mycgr3G70577\_Mycgr3T

Mycgr3G40534 Mycgr3T
  
Location: 6209-7166

Mycgr3G40534\_Mycgr3T

Mycgr3G85486 Mycgr3T
  
Location: 7266-8511

Mycgr3G85486\_Mycgr3T

Mycgr3G92221 Mycgr3T
  
Location: 8611-9193

Mycgr3G92221\_Mycgr3T

Mycgr3G39931 Mycgr3T
  
Location: 9293-10157

Mycgr3G39931\_Mycgr3T

Mycgr3G99766 Mycgr3T
  
Location: 10257-11775

Mycgr3G99766\_Mycgr3T

hypothetical protein
  
Accession: EFI97305
  
Location: 1187581-1190463
  
 NCBI BlastP on this gene

EFI97305

hypothetical protein
  
Accession: EFI96858
  
Location: 1191716-1193063
  
 NCBI BlastP on this gene

EFI96858

hypothetical protein
  
Accession: EFI96859
  
Location: 1195025-1196993
  
  
**BlastP hit with Mycgr3G70577\_Mycgr3T**
  
Percentage identity: 40 %
  
BlastP bit score: 393
  
Sequence coverage: 91 %
  
E-value: 1e-124
  
  
 NCBI BlastP on this gene

EFI96859

Query: Architecture Search FASTA input

CR382133 : Debaryomyces hansenii CBS767 chromosome A complete sequence.    Total score: 1.0     Cumulative Blast bit score: 392

Hit cluster cross-links:

Mycgr3G41235 Mycgr3T
  
Location: 0-4062

Mycgr3G41235\_Mycgr3T

Mycgr3G70577 Mycgr3T
  
Location: 4162-6109

Mycgr3G70577\_Mycgr3T

Mycgr3G40534 Mycgr3T
  
Location: 6209-7166

Mycgr3G40534\_Mycgr3T

Mycgr3G85486 Mycgr3T
  
Location: 7266-8511

Mycgr3G85486\_Mycgr3T

Mycgr3G92221 Mycgr3T
  
Location: 8611-9193

Mycgr3G92221\_Mycgr3T

Mycgr3G39931 Mycgr3T
  
Location: 9293-10157

Mycgr3G39931\_Mycgr3T

Mycgr3G99766 Mycgr3T
  
Location: 10257-11775

Mycgr3G99766\_Mycgr3T

DEHA2A14652p
  
Accession: CAG84941
  
Location: 1224311-1224649
  
 NCBI BlastP on this gene

DEHA2A14652g

DEHA2A14674p
  
Accession: CAR65419
  
Location: 1226047-1226451
  
 NCBI BlastP on this gene

DEHA2A14674g

DEHA2A14696p
  
Accession: CAG84942
  
Location: 1229756-1231654
  
  
**BlastP hit with Mycgr3G70577\_Mycgr3T**
  
Percentage identity: 36 %
  
BlastP bit score: 392
  
Sequence coverage: 99 %
  
E-value: 8e-124
  
  
 NCBI BlastP on this gene

DEHA2A14696g

Query: Architecture Search FASTA input

KB644409 : Penicillium oxalicum 114-2 unplaced genomic scaffold scaffold\_2    Total score: 1.0     Cumulative Blast bit score: 375

Hit cluster cross-links:

Mycgr3G41235 Mycgr3T
  
Location: 0-4062

Mycgr3G41235\_Mycgr3T

Mycgr3G70577 Mycgr3T
  
Location: 4162-6109

Mycgr3G70577\_Mycgr3T

Mycgr3G40534 Mycgr3T
  
Location: 6209-7166

Mycgr3G40534\_Mycgr3T

Mycgr3G85486 Mycgr3T
  
Location: 7266-8511

Mycgr3G85486\_Mycgr3T

Mycgr3G92221 Mycgr3T
  
Location: 8611-9193

Mycgr3G92221\_Mycgr3T

Mycgr3G39931 Mycgr3T
  
Location: 9293-10157

Mycgr3G39931\_Mycgr3T

Mycgr3G99766 Mycgr3T
  
Location: 10257-11775

Mycgr3G99766\_Mycgr3T

hypothetical protein
  
Accession: EPS27052
  
Location: 2148557-2150199
  
  
**BlastP hit with Mycgr3G99766\_Mycgr3T**
  
Percentage identity: 42 %
  
BlastP bit score: 375
  
Sequence coverage: 96 %
  
E-value: 1e-120
  
  
 NCBI BlastP on this gene

EPS27052

hypothetical protein
  
Accession: EPS27051
  
Location: 2145484-2146724
  
 NCBI BlastP on this gene

EPS27051

hypothetical protein
  
Accession: EPS27050
  
Location: 2143178-2144106
  
 NCBI BlastP on this gene

EPS27050

Query: Architecture Search FASTA input

DS027696 : Neosartorya fischeri NRRL 181 1099437636264 genomic scaffold    Total score: 1.0     Cumulative Blast bit score: 374

Hit cluster cross-links:

Mycgr3G41235 Mycgr3T
  
Location: 0-4062

Mycgr3G41235\_Mycgr3T

Mycgr3G70577 Mycgr3T
  
Location: 4162-6109

Mycgr3G70577\_Mycgr3T

Mycgr3G40534 Mycgr3T
  
Location: 6209-7166

Mycgr3G40534\_Mycgr3T

Mycgr3G85486 Mycgr3T
  
Location: 7266-8511

Mycgr3G85486\_Mycgr3T

Mycgr3G92221 Mycgr3T
  
Location: 8611-9193

Mycgr3G92221\_Mycgr3T

Mycgr3G39931 Mycgr3T
  
Location: 9293-10157

Mycgr3G39931\_Mycgr3T

Mycgr3G99766 Mycgr3T
  
Location: 10257-11775

Mycgr3G99766\_Mycgr3T

hypothetical protein
  
Accession: EAW19103
  
Location: 5454056-5454972
  
 NCBI BlastP on this gene

EAW19103

FluG family protein
  
Accession: EAW19104
  
Location: 5457337-5458680
  
 NCBI BlastP on this gene

EAW19104

hypothetical protein
  
Accession: EAW19105
  
Location: 5459374-5459841
  
 NCBI BlastP on this gene

EAW19105

hypothetical protein
  
Accession: EAW19106
  
Location: 5460079-5460400
  
 NCBI BlastP on this gene

EAW19106

MFS sugar transporter, putative
  
Accession: EAW19107
  
Location: 5461922-5463543
  
  
**BlastP hit with Mycgr3G99766\_Mycgr3T**
  
Percentage identity: 41 %
  
BlastP bit score: 374
  
Sequence coverage: 97 %
  
E-value: 6e-120
  
  
 NCBI BlastP on this gene

EAW19107

Query: Architecture Search FASTA input

CR382137 : Debaryomyces hansenii CBS767 chromosome E complete sequence.    Total score: 1.0     Cumulative Blast bit score: 373

Hit cluster cross-links:

Mycgr3G41235 Mycgr3T
  
Location: 0-4062

Mycgr3G41235\_Mycgr3T

Mycgr3G70577 Mycgr3T
  
Location: 4162-6109

Mycgr3G70577\_Mycgr3T

Mycgr3G40534 Mycgr3T
  
Location: 6209-7166

Mycgr3G40534\_Mycgr3T

Mycgr3G85486 Mycgr3T
  
Location: 7266-8511

Mycgr3G85486\_Mycgr3T

Mycgr3G92221 Mycgr3T
  
Location: 8611-9193

Mycgr3G92221\_Mycgr3T

Mycgr3G39931 Mycgr3T
  
Location: 9293-10157

Mycgr3G39931\_Mycgr3T

Mycgr3G99766 Mycgr3T
  
Location: 10257-11775

Mycgr3G99766\_Mycgr3T

DEHA2E02596p
  
Accession: CAR65745
  
Location: 231504-233441
  
  
**BlastP hit with Mycgr3G70577\_Mycgr3T**
  
Percentage identity: 35 %
  
BlastP bit score: 373
  
Sequence coverage: 94 %
  
E-value: 2e-116
  
  
 NCBI BlastP on this gene

DEHA2E02596g

DEHA2E02574p
  
Accession: CAG87657
  
Location: 229554-230834
  
 NCBI BlastP on this gene

DEHA2E02574g

DEHA2E02552p
  
Accession: CAG87656
  
Location: 228993-229112
  
 NCBI BlastP on this gene

DEHA2E02552g

DEHA2E02530p
  
Accession: CAG87655
  
Location: 226878-228953
  
 NCBI BlastP on this gene

DEHA2E02530g

DEHA2E02508p
  
Accession: CAG87654
  
Location: 224663-226042
  
 NCBI BlastP on this gene

DEHA2E02508g

DEHA2E02464p
  
Accession: CAG87653
  
Location: 220532-224110
  
 NCBI BlastP on this gene

DEHA2E02464g

Query: Architecture Search FASTA input

DS995906 : Penicillium marneffei ATCC 18224 scf\_1105668340770 genomic scaffold    Total score: 1.0     Cumulative Blast bit score: 367

Hit cluster cross-links:

Mycgr3G41235 Mycgr3T
  
Location: 0-4062

Mycgr3G41235\_Mycgr3T

Mycgr3G70577 Mycgr3T
  
Location: 4162-6109

Mycgr3G70577\_Mycgr3T

Mycgr3G40534 Mycgr3T
  
Location: 6209-7166

Mycgr3G40534\_Mycgr3T

Mycgr3G85486 Mycgr3T
  
Location: 7266-8511

Mycgr3G85486\_Mycgr3T

Mycgr3G92221 Mycgr3T
  
Location: 8611-9193

Mycgr3G92221\_Mycgr3T

Mycgr3G39931 Mycgr3T
  
Location: 9293-10157

Mycgr3G39931\_Mycgr3T

Mycgr3G99766 Mycgr3T
  
Location: 10257-11775

Mycgr3G99766\_Mycgr3T

MFS sugar transporter, putative
  
Accession: EEA19218
  
Location: 1688151-1689712
  
  
**BlastP hit with Mycgr3G99766\_Mycgr3T**
  
Percentage identity: 41 %
  
BlastP bit score: 367
  
Sequence coverage: 98 %
  
E-value: 2e-117
  
  
 NCBI BlastP on this gene

EEA19218

DUF895 domain membrane protein
  
Accession: EEA19217
  
Location: 1686110-1687727
  
 NCBI BlastP on this gene

EEA19217

mycelial catalase Cat1
  
Accession: EEA19216
  
Location: 1682724-1685131
  
 NCBI BlastP on this gene

EEA19216

Query: Architecture Search FASTA input

AM920431 : Penicillium chrysogenum Wisconsin 54-1255 complete genome, contig Pc00c16.    Total score: 1.0     Cumulative Blast bit score: 360

Hit cluster cross-links:

Mycgr3G41235 Mycgr3T
  
Location: 0-4062

Mycgr3G41235\_Mycgr3T

Mycgr3G70577 Mycgr3T
  
Location: 4162-6109

Mycgr3G70577\_Mycgr3T

Mycgr3G40534 Mycgr3T
  
Location: 6209-7166

Mycgr3G40534\_Mycgr3T

Mycgr3G85486 Mycgr3T
  
Location: 7266-8511

Mycgr3G85486\_Mycgr3T

Mycgr3G92221 Mycgr3T
  
Location: 8611-9193

Mycgr3G92221\_Mycgr3T

Mycgr3G39931 Mycgr3T
  
Location: 9293-10157

Mycgr3G39931\_Mycgr3T

Mycgr3G99766 Mycgr3T
  
Location: 10257-11775

Mycgr3G99766\_Mycgr3T

unnamed
  
Accession: CAP93792
  
Location: 2699905-2701640
  
  
**BlastP hit with Mycgr3G99766\_Mycgr3T**
  
Percentage identity: 39 %
  
BlastP bit score: 360
  
Sequence coverage: 99 %
  
E-value: 1e-114
  
  
 NCBI BlastP on this gene

Pc16g11220

not annotated
  
Accession: Pc16g11210
  
Location: 2696769-2699306
  
 NCBI BlastP on this gene

Pc16g11210

not annotated
  
Accession: CAP93790
  
Location: 2693622-2695722
  
 NCBI BlastP on this gene

Pc16g11200

not annotated
  
Accession: CAP93789
  
Location: 2690642-2693245
  
 NCBI BlastP on this gene

Pc16g11190

Query: Architecture Search FASTA input

KB644408 : Penicillium oxalicum 114-2 unplaced genomic scaffold scaffold\_1    Total score: 1.0     Cumulative Blast bit score: 359

Hit cluster cross-links:

Mycgr3G41235 Mycgr3T
  
Location: 0-4062

Mycgr3G41235\_Mycgr3T

Mycgr3G70577 Mycgr3T
  
Location: 4162-6109

Mycgr3G70577\_Mycgr3T

Mycgr3G40534 Mycgr3T
  
Location: 6209-7166

Mycgr3G40534\_Mycgr3T

Mycgr3G85486 Mycgr3T
  
Location: 7266-8511

Mycgr3G85486\_Mycgr3T

Mycgr3G92221 Mycgr3T
  
Location: 8611-9193

Mycgr3G92221\_Mycgr3T

Mycgr3G39931 Mycgr3T
  
Location: 9293-10157

Mycgr3G39931\_Mycgr3T

Mycgr3G99766 Mycgr3T
  
Location: 10257-11775

Mycgr3G99766\_Mycgr3T

hypothetical protein
  
Accession: EPS25962
  
Location: 2561223-2563949
  
 NCBI BlastP on this gene

EPS25962

hypothetical protein
  
Accession: EPS25963
  
Location: 2564543-2567751
  
 NCBI BlastP on this gene

EPS25963

hypothetical protein
  
Accession: EPS25964
  
Location: 2571172-2573049
  
  
**BlastP hit with Mycgr3G99766\_Mycgr3T**
  
Percentage identity: 40 %
  
BlastP bit score: 359
  
Sequence coverage: 99 %
  
E-value: 4e-114
  
  
 NCBI BlastP on this gene

EPS25964

hypothetical protein
  
Accession: EPS25965
  
Location: 2574164-2578681
  
 NCBI BlastP on this gene

EPS25965

hypothetical protein
  
Accession: EPS25966
  
Location: 2579561-2580857
  
 NCBI BlastP on this gene

EPS25966

Query: Architecture Search FASTA input

JH687379 : Stereum hirsutum FP-91666 SS1 unplaced genomic scaffold STEHIscaffold\_1    Total score: 1.0     Cumulative Blast bit score: 358

Hit cluster cross-links:

Mycgr3G41235 Mycgr3T
  
Location: 0-4062

Mycgr3G41235\_Mycgr3T

Mycgr3G70577 Mycgr3T
  
Location: 4162-6109

Mycgr3G70577\_Mycgr3T

Mycgr3G40534 Mycgr3T
  
Location: 6209-7166

Mycgr3G40534\_Mycgr3T

Mycgr3G85486 Mycgr3T
  
Location: 7266-8511

Mycgr3G85486\_Mycgr3T

Mycgr3G92221 Mycgr3T
  
Location: 8611-9193

Mycgr3G92221\_Mycgr3T

Mycgr3G39931 Mycgr3T
  
Location: 9293-10157

Mycgr3G39931\_Mycgr3T

Mycgr3G99766 Mycgr3T
  
Location: 10257-11775

Mycgr3G99766\_Mycgr3T

MFS general substrate transporter
  
Accession: EIM92619
  
Location: 2783233-2786258
  
  
**BlastP hit with Mycgr3G70577\_Mycgr3T**
  
Percentage identity: 35 %
  
BlastP bit score: 358
  
Sequence coverage: 89 %
  
E-value: 9e-111
  
  
 NCBI BlastP on this gene

EIM92619

hypothetical protein
  
Accession: EIM92618
  
Location: 2781206-2782268
  
 NCBI BlastP on this gene

EIM92618

hypothetical protein
  
Accession: EIM92617
  
Location: 2778774-2780852
  
 NCBI BlastP on this gene

EIM92617

Query: Architecture Search FASTA input

FO082046 : Pichia sorbitophila strain CBS 7064 chromosome N complete sequence.    Total score: 1.0     Cumulative Blast bit score: 357

Hit cluster cross-links:

Mycgr3G41235 Mycgr3T
  
Location: 0-4062

Mycgr3G41235\_Mycgr3T

Mycgr3G70577 Mycgr3T
  
Location: 4162-6109

Mycgr3G70577\_Mycgr3T

Mycgr3G40534 Mycgr3T
  
Location: 6209-7166

Mycgr3G40534\_Mycgr3T

Mycgr3G85486 Mycgr3T
  
Location: 7266-8511

Mycgr3G85486\_Mycgr3T

Mycgr3G92221 Mycgr3T
  
Location: 8611-9193

Mycgr3G92221\_Mycgr3T

Mycgr3G39931 Mycgr3T
  
Location: 9293-10157

Mycgr3G39931\_Mycgr3T

Mycgr3G99766 Mycgr3T
  
Location: 10257-11775

Mycgr3G99766\_Mycgr3T

not annotated
  
Accession: CCE87355
  
Location: 2098250-2100106
  
  
**BlastP hit with Mycgr3G70577\_Mycgr3T**
  
Percentage identity: 34 %
  
BlastP bit score: 357
  
Sequence coverage: 96 %
  
E-value: 1e-110
  
  
 NCBI BlastP on this gene

Piso0\_005905

not annotated
  
Accession: CCE87354
  
Location: 2096079-2097554
  
 NCBI BlastP on this gene

Piso0\_005904

not annotated
  
Accession: CCE87353
  
Location: 2093933-2095165
  
 NCBI BlastP on this gene

Piso0\_005903

not annotated
  
Accession: CCE87352
  
Location: 2092569-2093423
  
 NCBI BlastP on this gene

Piso0\_005902

not annotated
  
Accession: CCE87351
  
Location: 2091331-2091957
  
 NCBI BlastP on this gene

Piso0\_005901

Query: Architecture Search FASTA input

GL698717 : Metarhizium anisopliae ARSEF 23 unplaced genomic scaffold Scf\_007    Total score: 1.0     Cumulative Blast bit score: 355

Hit cluster cross-links:

Mycgr3G41235 Mycgr3T
  
Location: 0-4062

Mycgr3G41235\_Mycgr3T

Mycgr3G70577 Mycgr3T
  
Location: 4162-6109

Mycgr3G70577\_Mycgr3T

Mycgr3G40534 Mycgr3T
  
Location: 6209-7166

Mycgr3G40534\_Mycgr3T

Mycgr3G85486 Mycgr3T
  
Location: 7266-8511

Mycgr3G85486\_Mycgr3T

Mycgr3G92221 Mycgr3T
  
Location: 8611-9193

Mycgr3G92221\_Mycgr3T

Mycgr3G39931 Mycgr3T
  
Location: 9293-10157

Mycgr3G39931\_Mycgr3T

Mycgr3G99766 Mycgr3T
  
Location: 10257-11775

Mycgr3G99766\_Mycgr3T

DNA kinase/phosphatase Pnk1
  
Accession: EFY99409
  
Location: 1555789-1557298
  
 NCBI BlastP on this gene

EFY99409

hypothetical protein
  
Accession: EFY99410
  
Location: 1557753-1558650
  
 NCBI BlastP on this gene

EFY99410

siderophore iron transporter
  
Accession: EFY99411
  
Location: 1559459-1561228
  
  
**BlastP hit with Mycgr3G70577\_Mycgr3T**
  
Percentage identity: 27 %
  
BlastP bit score: 177
  
Sequence coverage: 85 %
  
E-value: 1e-44
  
  
 NCBI BlastP on this gene

EFY99411

transferase family protein
  
Accession: EFY99412
  
Location: 1561941-1567139
  
 NCBI BlastP on this gene

EFY99412

hypothetical protein
  
Accession: EFY99413
  
Location: 1567963-1569462
  
 NCBI BlastP on this gene

EFY99413

lipase
  
Accession: EFY99414
  
Location: 1570166-1571175
  
 NCBI BlastP on this gene

EFY99414

siderophore iron transporter mirB
  
Accession: EFY99415
  
Location: 1571989-1573758
  
  
**BlastP hit with Mycgr3G70577\_Mycgr3T**
  
Percentage identity: 26 %
  
BlastP bit score: 178
  
Sequence coverage: 91 %
  
E-value: 7e-45
  
  
 NCBI BlastP on this gene

EFY99415

hypothetical protein
  
Accession: EFY99416
  
Location: 1574564-1575069
  
 NCBI BlastP on this gene

EFY99416

Query: Architecture Search FASTA input

AM920435 : Penicillium chrysogenum Wisconsin 54-1255 complete genome, contig Pc00c20.    Total score: 1.0     Cumulative Blast bit score: 355

Hit cluster cross-links:

Mycgr3G41235 Mycgr3T
  
Location: 0-4062

Mycgr3G41235\_Mycgr3T

Mycgr3G70577 Mycgr3T
  
Location: 4162-6109

Mycgr3G70577\_Mycgr3T

Mycgr3G40534 Mycgr3T
  
Location: 6209-7166

Mycgr3G40534\_Mycgr3T

Mycgr3G85486 Mycgr3T
  
Location: 7266-8511

Mycgr3G85486\_Mycgr3T

Mycgr3G92221 Mycgr3T
  
Location: 8611-9193

Mycgr3G92221\_Mycgr3T

Mycgr3G39931 Mycgr3T
  
Location: 9293-10157

Mycgr3G39931\_Mycgr3T

Mycgr3G99766 Mycgr3T
  
Location: 10257-11775

Mycgr3G99766\_Mycgr3T

unnamed
  
Accession: CAP86901
  
Location: 3636003-3637630
  
  
**BlastP hit with Mycgr3G99766\_Mycgr3T**
  
Percentage identity: 38 %
  
BlastP bit score: 355
  
Sequence coverage: 101 %
  
E-value: 6e-113
  
  
 NCBI BlastP on this gene

Pc20g15720

not annotated
  
Accession: CAP86900
  
Location: 3634129-3635630
  
 NCBI BlastP on this gene

Pc20g15710

hypothetical protein
  
Accession: CAP86899
  
Location: 3633480-3633931
  
 NCBI BlastP on this gene

Pc20g15700

not annotated
  
Accession: CAP86898
  
Location: 3631692-3633230
  
 NCBI BlastP on this gene

Pc20g15690

hypothetical protein
  
Accession: CAP86897
  
Location: 3631158-3631618
  
 NCBI BlastP on this gene

Pc20g15680

not annotated
  
Accession: CAP86896
  
Location: 3630500-3631075
  
 NCBI BlastP on this gene

Pc20g15670

not annotated
  
Accession: CAP86895
  
Location: 3628441-3629957
  
 NCBI BlastP on this gene

Pc20g15660

Query: Architecture Search FASTA input

AM920435 : Penicillium chrysogenum Wisconsin 54-1255 complete genome, contig Pc00c20.    Total score: 1.0     Cumulative Blast bit score: 354

Hit cluster cross-links:

Mycgr3G41235 Mycgr3T
  
Location: 0-4062

Mycgr3G41235\_Mycgr3T

Mycgr3G70577 Mycgr3T
  
Location: 4162-6109

Mycgr3G70577\_Mycgr3T

Mycgr3G40534 Mycgr3T
  
Location: 6209-7166

Mycgr3G40534\_Mycgr3T

Mycgr3G85486 Mycgr3T
  
Location: 7266-8511

Mycgr3G85486\_Mycgr3T

Mycgr3G92221 Mycgr3T
  
Location: 8611-9193

Mycgr3G92221\_Mycgr3T

Mycgr3G39931 Mycgr3T
  
Location: 9293-10157

Mycgr3G39931\_Mycgr3T

Mycgr3G99766 Mycgr3T
  
Location: 10257-11775

Mycgr3G99766\_Mycgr3T

hypothetical protein
  
Accession: CAP86479
  
Location: 2644984-2646036
  
 NCBI BlastP on this gene

Pc20g11500

hypothetical protein
  
Accession: CAP86478
  
Location: 2643442-2643922
  
 NCBI BlastP on this gene

Pc20g11490

hypothetical protein
  
Accession: CAP86477
  
Location: 2639691-2641573
  
 NCBI BlastP on this gene

Pc20g11480

not annotated
  
Accession: CAP86476
  
Location: 2639044-2639430
  
 NCBI BlastP on this gene

Pc20g11470

unnamed
  
Accession: CAP86475
  
Location: 2635856-2637515
  
  
**BlastP hit with Mycgr3G99766\_Mycgr3T**
  
Percentage identity: 39 %
  
BlastP bit score: 354
  
Sequence coverage: 100 %
  
E-value: 2e-112
  
  
 NCBI BlastP on this gene

Pc20g11460

not annotated
  
Accession: CAP86474
  
Location: 2634401-2635120
  
 NCBI BlastP on this gene

Pc20g11450

not annotated
  
Accession: CAP86473
  
Location: 2631774-2633619
  
 NCBI BlastP on this gene

Pc20g11440

unnamed
  
Accession: CAP86472
  
Location: 2629170-2631293
  
 NCBI BlastP on this gene

Pc20g11430

Query: Architecture Search FASTA input

EQ962652 : Talaromyces stipitatus ATCC 10500 scf\_1105507295523 genomic scaffold    Total score: 1.0     Cumulative Blast bit score: 352

Hit cluster cross-links:

Mycgr3G41235 Mycgr3T
  
Location: 0-4062

Mycgr3G41235\_Mycgr3T

Mycgr3G70577 Mycgr3T
  
Location: 4162-6109

Mycgr3G70577\_Mycgr3T

Mycgr3G40534 Mycgr3T
  
Location: 6209-7166

Mycgr3G40534\_Mycgr3T

Mycgr3G85486 Mycgr3T
  
Location: 7266-8511

Mycgr3G85486\_Mycgr3T

Mycgr3G92221 Mycgr3T
  
Location: 8611-9193

Mycgr3G92221\_Mycgr3T

Mycgr3G39931 Mycgr3T
  
Location: 9293-10157

Mycgr3G39931\_Mycgr3T

Mycgr3G99766 Mycgr3T
  
Location: 10257-11775

Mycgr3G99766\_Mycgr3T

sugar transport protein, putative
  
Accession: EED23931
  
Location: 3774187-3775986
  
 NCBI BlastP on this gene

EED23931

conserved hypothetical protein
  
Accession: EED23930
  
Location: 3769869-3771264
  
 NCBI BlastP on this gene

EED23930

sugar transporter, putative
  
Accession: EED23929
  
Location: 3767544-3769166
  
  
**BlastP hit with Mycgr3G99766\_Mycgr3T**
  
Percentage identity: 40 %
  
BlastP bit score: 352
  
Sequence coverage: 99 %
  
E-value: 9e-112
  
  
 NCBI BlastP on this gene

EED23929

hypothetical protein
  
Accession: EED23928
  
Location: 3762763-3763970
  
 NCBI BlastP on this gene

EED23928

C6 transcription factor, putative
  
Accession: EED23927
  
Location: 3757544-3760440
  
 NCBI BlastP on this gene

EED23927

Query: Architecture Search FASTA input

DS027045 : Aspergillus clavatus NRRL 1 1099423829791 genomic scaffold    Total score: 1.0     Cumulative Blast bit score: 350

Hit cluster cross-links:

Mycgr3G41235 Mycgr3T
  
Location: 0-4062

Mycgr3G41235\_Mycgr3T

Mycgr3G70577 Mycgr3T
  
Location: 4162-6109

Mycgr3G70577\_Mycgr3T

Mycgr3G40534 Mycgr3T
  
Location: 6209-7166

Mycgr3G40534\_Mycgr3T

Mycgr3G85486 Mycgr3T
  
Location: 7266-8511

Mycgr3G85486\_Mycgr3T

Mycgr3G92221 Mycgr3T
  
Location: 8611-9193

Mycgr3G92221\_Mycgr3T

Mycgr3G39931 Mycgr3T
  
Location: 9293-10157

Mycgr3G39931\_Mycgr3T

Mycgr3G99766 Mycgr3T
  
Location: 10257-11775

Mycgr3G99766\_Mycgr3T

monooxygenase
  
Accession: EAW14397
  
Location: 1996936-1999050
  
 NCBI BlastP on this gene

EAW14397

FluG family protein
  
Accession: EAW14398
  
Location: 2000261-2001607
  
 NCBI BlastP on this gene

EAW14398

conserved hypothetical protein
  
Accession: EAW14399
  
Location: 2002166-2004259
  
 NCBI BlastP on this gene

EAW14399

MFS sugar transporter, putative
  
Accession: EAW14400
  
Location: 2006489-2008113
  
  
**BlastP hit with Mycgr3G99766\_Mycgr3T**
  
Percentage identity: 40 %
  
BlastP bit score: 350
  
Sequence coverage: 97 %
  
E-value: 8e-111
  
  
 NCBI BlastP on this gene

EAW14400

Query: Architecture Search FASTA input

CH408159 : Pichia guilliermondii ATCC 6260 scaffold\_5 genomic scaffold    Total score: 1.0     Cumulative Blast bit score: 346

Hit cluster cross-links:

Mycgr3G41235 Mycgr3T
  
Location: 0-4062

Mycgr3G41235\_Mycgr3T

Mycgr3G70577 Mycgr3T
  
Location: 4162-6109

Mycgr3G70577\_Mycgr3T

Mycgr3G40534 Mycgr3T
  
Location: 6209-7166

Mycgr3G40534\_Mycgr3T

Mycgr3G85486 Mycgr3T
  
Location: 7266-8511

Mycgr3G85486\_Mycgr3T

Mycgr3G92221 Mycgr3T
  
Location: 8611-9193

Mycgr3G92221\_Mycgr3T

Mycgr3G39931 Mycgr3T
  
Location: 9293-10157

Mycgr3G39931\_Mycgr3T

Mycgr3G99766 Mycgr3T
  
Location: 10257-11775

Mycgr3G99766\_Mycgr3T

hypothetical protein
  
Accession: EDK39908
  
Location: 102049-103743
  
 NCBI BlastP on this gene

EDK39908

hypothetical protein
  
Accession: EDK39907
  
Location: 99208-100701
  
 NCBI BlastP on this gene

EDK39907

hypothetical protein
  
Accession: EDK39906
  
Location: 97764-98801
  
 NCBI BlastP on this gene

EDK39906

hypothetical protein
  
Accession: EDK39905
  
Location: 95859-97580
  
 NCBI BlastP on this gene

EDK39905

hypothetical protein
  
Accession: EDK39904
  
Location: 92774-94687
  
  
**BlastP hit with Mycgr3G70577\_Mycgr3T**
  
Percentage identity: 34 %
  
BlastP bit score: 346
  
Sequence coverage: 97 %
  
E-value: 4e-106
  
  
 NCBI BlastP on this gene

EDK39904

hypothetical protein
  
Accession: EDK39903
  
Location: 90884-92603
  
 NCBI BlastP on this gene

EDK39903

hypothetical protein
  
Accession: EDK39902
  
Location: 88174-90366
  
 NCBI BlastP on this gene

EDK39902

hypothetical protein
  
Accession: EDK39901
  
Location: 86654-87304
  
 NCBI BlastP on this gene

EDK39901

hypothetical protein
  
Accession: EDK39900
  
Location: 85520-86188
  
 NCBI BlastP on this gene

EDK39900

Query: Architecture Search FASTA input

AM920437 : Penicillium chrysogenum Wisconsin 54-1255 complete genome, contig Pc00c22.    Total score: 1.0     Cumulative Blast bit score: 345

Hit cluster cross-links:

Mycgr3G41235 Mycgr3T
  
Location: 0-4062

Mycgr3G41235\_Mycgr3T

Mycgr3G70577 Mycgr3T
  
Location: 4162-6109

Mycgr3G70577\_Mycgr3T

Mycgr3G40534 Mycgr3T
  
Location: 6209-7166

Mycgr3G40534\_Mycgr3T

Mycgr3G85486 Mycgr3T
  
Location: 7266-8511

Mycgr3G85486\_Mycgr3T

Mycgr3G92221 Mycgr3T
  
Location: 8611-9193

Mycgr3G92221\_Mycgr3T

Mycgr3G39931 Mycgr3T
  
Location: 9293-10157

Mycgr3G39931\_Mycgr3T

Mycgr3G99766 Mycgr3T
  
Location: 10257-11775

Mycgr3G99766\_Mycgr3T

not annotated
  
Accession: CAP98038
  
Location: 1787213-1789051
  
 NCBI BlastP on this gene

Pc22g07500

not annotated
  
Accession: CAP98037
  
Location: 1785343-1786783
  
 NCBI BlastP on this gene

Pc22g07490

unnamed
  
Accession: CAP98036
  
Location: 1783530-1784858
  
 NCBI BlastP on this gene

Pc22g07480

unnamed
  
Accession: CAP98035
  
Location: 1780777-1782400
  
  
**BlastP hit with Mycgr3G99766\_Mycgr3T**
  
Percentage identity: 40 %
  
BlastP bit score: 345
  
Sequence coverage: 95 %
  
E-value: 3e-109
  
  
 NCBI BlastP on this gene

Pc22g07470

not annotated
  
Accession: CAP98034
  
Location: 1780185-1780540
  
 NCBI BlastP on this gene

Pc22g07460

not annotated
  
Accession: CAP98033
  
Location: 1778951-1779885
  
 NCBI BlastP on this gene

Pc22g07450

unnamed
  
Accession: CAP98032
  
Location: 1776206-1777834
  
 NCBI BlastP on this gene

Pc22g07440

not annotated
  
Accession: Pc22g07430
  
Location: 1774876-1775287
  
 NCBI BlastP on this gene

Pc22g07430

not annotated
  
Accession: CAP98030
  
Location: 1773389-1773888
  
 NCBI BlastP on this gene

Pc22g07420

Query: Architecture Search FASTA input

CH408159 : Pichia guilliermondii ATCC 6260 scaffold\_5 genomic scaffold    Total score: 1.0     Cumulative Blast bit score: 344

Hit cluster cross-links:

Mycgr3G41235 Mycgr3T
  
Location: 0-4062

Mycgr3G41235\_Mycgr3T

Mycgr3G70577 Mycgr3T
  
Location: 4162-6109

Mycgr3G70577\_Mycgr3T

Mycgr3G40534 Mycgr3T
  
Location: 6209-7166

Mycgr3G40534\_Mycgr3T

Mycgr3G85486 Mycgr3T
  
Location: 7266-8511

Mycgr3G85486\_Mycgr3T

Mycgr3G92221 Mycgr3T
  
Location: 8611-9193

Mycgr3G92221\_Mycgr3T

Mycgr3G39931 Mycgr3T
  
Location: 9293-10157

Mycgr3G39931\_Mycgr3T

Mycgr3G99766 Mycgr3T
  
Location: 10257-11775

Mycgr3G99766\_Mycgr3T

hypothetical protein
  
Accession: EDK40415
  
Location: 1047585-1049444
  
  
**BlastP hit with Mycgr3G70577\_Mycgr3T**
  
Percentage identity: 35 %
  
BlastP bit score: 344
  
Sequence coverage: 89 %
  
E-value: 1e-105
  
  
 NCBI BlastP on this gene

EDK40415

hypothetical protein
  
Accession: EDK40414
  
Location: 1046222-1047304
  
 NCBI BlastP on this gene

EDK40414

hypothetical protein
  
Accession: EDK40413
  
Location: 1045691-1046170
  
 NCBI BlastP on this gene

EDK40413

predicted protein
  
Accession: EDK40412
  
Location: 1044542-1045432
  
 NCBI BlastP on this gene

EDK40412

hypothetical protein
  
Accession: EDK40411
  
Location: 1042505-1044325
  
 NCBI BlastP on this gene

EDK40411

hypothetical protein
  
Accession: EDK40410
  
Location: 1040553-1042274
  
 NCBI BlastP on this gene

EDK40410

hypothetical protein
  
Accession: EDK40409
  
Location: 1038654-1040336
  
 NCBI BlastP on this gene

EDK40409

Query: Architecture Search FASTA input

AM920437 : Penicillium chrysogenum Wisconsin 54-1255 complete genome, contig Pc00c22.    Total score: 1.0     Cumulative Blast bit score: 344

Hit cluster cross-links:

Mycgr3G41235 Mycgr3T
  
Location: 0-4062

Mycgr3G41235\_Mycgr3T

Mycgr3G70577 Mycgr3T
  
Location: 4162-6109

Mycgr3G70577\_Mycgr3T

Mycgr3G40534 Mycgr3T
  
Location: 6209-7166

Mycgr3G40534\_Mycgr3T

Mycgr3G85486 Mycgr3T
  
Location: 7266-8511

Mycgr3G85486\_Mycgr3T

Mycgr3G92221 Mycgr3T
  
Location: 8611-9193

Mycgr3G92221\_Mycgr3T

Mycgr3G39931 Mycgr3T
  
Location: 9293-10157

Mycgr3G39931\_Mycgr3T

Mycgr3G99766 Mycgr3T
  
Location: 10257-11775

Mycgr3G99766\_Mycgr3T

not annotated
  
Accession: CAP99789
  
Location: 5905767-5906947
  
 NCBI BlastP on this gene

Pc22g25010

not annotated
  
Accession: CAP99790
  
Location: 5907439-5909551
  
 NCBI BlastP on this gene

Pc22g25020

not annotated
  
Accession: CAP99791
  
Location: 5910726-5912610
  
 NCBI BlastP on this gene

Pc22g25030

unnamed
  
Accession: CAP99792
  
Location: 5913418-5915017
  
  
**BlastP hit with Mycgr3G99766\_Mycgr3T**
  
Percentage identity: 40 %
  
BlastP bit score: 344
  
Sequence coverage: 96 %
  
E-value: 9e-109
  
  
 NCBI BlastP on this gene

Pc22g25040

Query: Architecture Search FASTA input

CR382134 : Debaryomyces hansenii CBS767 chromosome B complete sequence.    Total score: 1.0     Cumulative Blast bit score: 342

Hit cluster cross-links:

Mycgr3G41235 Mycgr3T
  
Location: 0-4062

Mycgr3G41235\_Mycgr3T

Mycgr3G70577 Mycgr3T
  
Location: 4162-6109

Mycgr3G70577\_Mycgr3T

Mycgr3G40534 Mycgr3T
  
Location: 6209-7166

Mycgr3G40534\_Mycgr3T

Mycgr3G85486 Mycgr3T
  
Location: 7266-8511

Mycgr3G85486\_Mycgr3T

Mycgr3G92221 Mycgr3T
  
Location: 8611-9193

Mycgr3G92221\_Mycgr3T

Mycgr3G39931 Mycgr3T
  
Location: 9293-10157

Mycgr3G39931\_Mycgr3T

Mycgr3G99766 Mycgr3T
  
Location: 10257-11775

Mycgr3G99766\_Mycgr3T

DEHA2B16412p
  
Accession: CAG85679
  
Location: 1297723-1298430
  
 NCBI BlastP on this gene

DEHA2B16412g

DEHA2B16434p
  
Accession: CAG85680
  
Location: 1299298-1300329
  
 NCBI BlastP on this gene

DEHA2B16434g

DEHA2B16456p
  
Accession: CAG85681
  
Location: 1301169-1303721
  
 NCBI BlastP on this gene

DEHA2B16456g

DEHA2B16478p
  
Accession: CAG85682
  
Location: 1305810-1307675
  
  
**BlastP hit with Mycgr3G70577\_Mycgr3T**
  
Percentage identity: 33 %
  
BlastP bit score: 342
  
Sequence coverage: 95 %
  
E-value: 7e-105
  
  
 NCBI BlastP on this gene

DEHA2B16478g

Query: Architecture Search FASTA input

JH126400 : Cordyceps militaris CM01 unplaced genomic scaffold CCM\_S00002    Total score: 1.0     Cumulative Blast bit score: 340

Hit cluster cross-links:

Mycgr3G41235 Mycgr3T
  
Location: 0-4062

Mycgr3G41235\_Mycgr3T

Mycgr3G70577 Mycgr3T
  
Location: 4162-6109

Mycgr3G70577\_Mycgr3T

Mycgr3G40534 Mycgr3T
  
Location: 6209-7166

Mycgr3G40534\_Mycgr3T

Mycgr3G85486 Mycgr3T
  
Location: 7266-8511

Mycgr3G85486\_Mycgr3T

Mycgr3G92221 Mycgr3T
  
Location: 8611-9193

Mycgr3G92221\_Mycgr3T

Mycgr3G39931 Mycgr3T
  
Location: 9293-10157

Mycgr3G39931\_Mycgr3T

Mycgr3G99766 Mycgr3T
  
Location: 10257-11775

Mycgr3G99766\_Mycgr3T

Aminoglycoside phosphotransferase
  
Accession: EGX93826
  
Location: 478578-479558
  
 NCBI BlastP on this gene

EGX93826

reticulon-4-interacting protein 1
  
Accession: EGX93827
  
Location: 479937-480950
  
 NCBI BlastP on this gene

EGX93827

phosphorylcholine phosphatase
  
Accession: EGX93828
  
Location: 482303-483406
  
 NCBI BlastP on this gene

EGX93828

MFS sugar transporter, putative
  
Accession: EGX93829
  
Location: 485013-486619
  
  
**BlastP hit with Mycgr3G99766\_Mycgr3T**
  
Percentage identity: 40 %
  
BlastP bit score: 340
  
Sequence coverage: 101 %
  
E-value: 6e-107
  
  
 NCBI BlastP on this gene

EGX93829

integral membrane protein, putative
  
Accession: EGX93830
  
Location: 487078-488423
  
 NCBI BlastP on this gene

EGX93830

Query: Architecture Search FASTA input

HF679023 : Fusarium fujikuroi IMI 58289 draft genome, chromosome FFUJ\_chr01.    Total score: 1.0     Cumulative Blast bit score: 340

Hit cluster cross-links:

Mycgr3G41235 Mycgr3T
  
Location: 0-4062

Mycgr3G41235\_Mycgr3T

Mycgr3G70577 Mycgr3T
  
Location: 4162-6109

Mycgr3G70577\_Mycgr3T

Mycgr3G40534 Mycgr3T
  
Location: 6209-7166

Mycgr3G40534\_Mycgr3T

Mycgr3G85486 Mycgr3T
  
Location: 7266-8511

Mycgr3G85486\_Mycgr3T

Mycgr3G92221 Mycgr3T
  
Location: 8611-9193

Mycgr3G92221\_Mycgr3T

Mycgr3G39931 Mycgr3T
  
Location: 9293-10157

Mycgr3G39931\_Mycgr3T

Mycgr3G99766 Mycgr3T
  
Location: 10257-11775

Mycgr3G99766\_Mycgr3T

related to acyl-CoA thiolesterase
  
Accession: CCT61538
  
Location: 292882-294289
  
 NCBI BlastP on this gene

FFUJ\_01983

uncharacterized protein
  
Accession: CCT61537
  
Location: 291898-292677
  
 NCBI BlastP on this gene

FFUJ\_01984

putative trichothecene biosynthesis gene
  
Accession: CCT61536
  
Location: 288238-289373
  
 NCBI BlastP on this gene

FFUJ\_01985

related to immune-responsive protein 1
  
Accession: CCT61535
  
Location: 286343-287830
  
 NCBI BlastP on this gene

FFUJ\_01986

related to sugar transporter
  
Accession: CCT61534
  
Location: 284028-285884
  
  
**BlastP hit with Mycgr3G99766\_Mycgr3T**
  
Percentage identity: 40 %
  
BlastP bit score: 340
  
Sequence coverage: 95 %
  
E-value: 4e-107
  
  
 NCBI BlastP on this gene

FFUJ\_01987

related to transcription activator protein acu-15
  
Accession: CCT61533
  
Location: 281409-283025
  
 NCBI BlastP on this gene

FFUJ\_01988

related to isoamyl alcohol oxidase
  
Accession: CCT61532
  
Location: 279138-281261
  
 NCBI BlastP on this gene

FFUJ\_01989

uncharacterized protein
  
Accession: CCT61531
  
Location: 278362-278742
  
 NCBI BlastP on this gene

FFUJ\_01990

Query: Architecture Search FASTA input

JH711791 : Trametes versicolor FP-101664 SS1 unplaced genomic scaffold TRAVEscaffold\_9    Total score: 1.0     Cumulative Blast bit score: 338

Hit cluster cross-links:

Mycgr3G41235 Mycgr3T
  
Location: 0-4062

Mycgr3G41235\_Mycgr3T

Mycgr3G70577 Mycgr3T
  
Location: 4162-6109

Mycgr3G70577\_Mycgr3T

Mycgr3G40534 Mycgr3T
  
Location: 6209-7166

Mycgr3G40534\_Mycgr3T

Mycgr3G85486 Mycgr3T
  
Location: 7266-8511

Mycgr3G85486\_Mycgr3T

Mycgr3G92221 Mycgr3T
  
Location: 8611-9193

Mycgr3G92221\_Mycgr3T

Mycgr3G39931 Mycgr3T
  
Location: 9293-10157

Mycgr3G39931\_Mycgr3T

Mycgr3G99766 Mycgr3T
  
Location: 10257-11775

Mycgr3G99766\_Mycgr3T

hypothetical protein
  
Accession: EIW56227
  
Location: 2648598-2649859
  
 NCBI BlastP on this gene

EIW56227

drug:h+ antiporter
  
Accession: EIW56226
  
Location: 2645243-2647513
  
  
**BlastP hit with Mycgr3G70577\_Mycgr3T**
  
Percentage identity: 34 %
  
BlastP bit score: 338
  
Sequence coverage: 95 %
  
E-value: 1e-103
  
  
 NCBI BlastP on this gene

EIW56226

hypothetical protein
  
Accession: EIW56225
  
Location: 2643534-2644707
  
 NCBI BlastP on this gene

EIW56225

hypothetical protein
  
Accession: EIW56224
  
Location: 2641286-2642488
  
 NCBI BlastP on this gene

EIW56224

hypothetical protein
  
Accession: EIW56223
  
Location: 2639002-2640008
  
 NCBI BlastP on this gene

EIW56223

Query: Architecture Search FASTA input

AKCT01000319 : Penicillium digitatum PHI26    Total score: 1.0     Cumulative Blast bit score: 335

Hit cluster cross-links:

Mycgr3G41235 Mycgr3T
  
Location: 0-4062

Mycgr3G41235\_Mycgr3T

Mycgr3G70577 Mycgr3T
  
Location: 4162-6109

Mycgr3G70577\_Mycgr3T

Mycgr3G40534 Mycgr3T
  
Location: 6209-7166

Mycgr3G40534\_Mycgr3T

Mycgr3G85486 Mycgr3T
  
Location: 7266-8511

Mycgr3G85486\_Mycgr3T

Mycgr3G92221 Mycgr3T
  
Location: 8611-9193

Mycgr3G92221\_Mycgr3T

Mycgr3G39931 Mycgr3T
  
Location: 9293-10157

Mycgr3G39931\_Mycgr3T

Mycgr3G99766 Mycgr3T
  
Location: 10257-11775

Mycgr3G99766\_Mycgr3T

hypothetical protein
  
Accession: EKV04880
  
Location: 190790-192619
  
 NCBI BlastP on this gene

EKV04880

LPS glycosyltransferase, putative
  
Accession: EKV04881
  
Location: 193510-194845
  
 NCBI BlastP on this gene

EKV04881

Sugar transporter, putative
  
Accession: EKV04882
  
Location: 196015-197635
  
  
**BlastP hit with Mycgr3G99766\_Mycgr3T**
  
Percentage identity: 38 %
  
BlastP bit score: 335
  
Sequence coverage: 95 %
  
E-value: 5e-105
  
  
 NCBI BlastP on this gene

EKV04882

Query: Architecture Search FASTA input

JH711584 : Coniophora puteana RWD-64-598 SS2 unplaced genomic scaffold CONPUscaffold\_12    Total score: 1.0     Cumulative Blast bit score: 330

Hit cluster cross-links:

Mycgr3G41235 Mycgr3T
  
Location: 0-4062

Mycgr3G41235\_Mycgr3T

Mycgr3G70577 Mycgr3T
  
Location: 4162-6109

Mycgr3G70577\_Mycgr3T

Mycgr3G40534 Mycgr3T
  
Location: 6209-7166

Mycgr3G40534\_Mycgr3T

Mycgr3G85486 Mycgr3T
  
Location: 7266-8511

Mycgr3G85486\_Mycgr3T

Mycgr3G92221 Mycgr3T
  
Location: 8611-9193

Mycgr3G92221\_Mycgr3T

Mycgr3G39931 Mycgr3T
  
Location: 9293-10157

Mycgr3G39931\_Mycgr3T

Mycgr3G99766 Mycgr3T
  
Location: 10257-11775

Mycgr3G99766\_Mycgr3T

hypothetical protein
  
Accession: EIW77301
  
Location: 1073238-1073693
  
 NCBI BlastP on this gene

EIW77301

hypothetical protein
  
Accession: EIW77302
  
Location: 1075016-1076090
  
  
**BlastP hit with Mycgr3G85486\_Mycgr3T**
  
Percentage identity: 38 %
  
BlastP bit score: 170
  
Sequence coverage: 64 %
  
E-value: 4e-46
  
  
 NCBI BlastP on this gene

EIW77302

hypothetical protein
  
Accession: EIW77303
  
Location: 1077361-1079823
  
 NCBI BlastP on this gene

EIW77303

hypothetical protein
  
Accession: EIW77304
  
Location: 1081857-1083395
  
 NCBI BlastP on this gene

EIW77304

hypothetical protein
  
Accession: EIW77305
  
Location: 1083502-1084306
  
 NCBI BlastP on this gene

EIW77305

DnaJ-domain-containing protein
  
Accession: EIW77306
  
Location: 1085968-1087430
  
 NCBI BlastP on this gene

EIW77306

amidase signature enzyme
  
Accession: EIW77307
  
Location: 1087807-1089808
  
 NCBI BlastP on this gene

EIW77307

hypothetical protein
  
Accession: EIW77308
  
Location: 1092875-1093636
  
 NCBI BlastP on this gene

EIW77308

hypothetical protein
  
Accession: EIW77309
  
Location: 1094598-1095876
  
 NCBI BlastP on this gene

EIW77309

hypothetical protein
  
Accession: EIW77310
  
Location: 1096826-1097905
  
  
**BlastP hit with Mycgr3G85486\_Mycgr3T**
  
Percentage identity: 37 %
  
BlastP bit score: 160
  
Sequence coverage: 64 %
  
E-value: 2e-42
  
  
 NCBI BlastP on this gene

EIW77310

hypothetical protein
  
Accession: EIW77311
  
Location: 1098782-1099543
  
 NCBI BlastP on this gene

EIW77311

Query: Architecture Search FASTA input

AM920428 : Penicillium chrysogenum Wisconsin 54-1255 complete genome, contig Pc00c13.    Total score: 1.0     Cumulative Blast bit score: 330

Hit cluster cross-links:

Mycgr3G41235 Mycgr3T
  
Location: 0-4062

Mycgr3G41235\_Mycgr3T

Mycgr3G70577 Mycgr3T
  
Location: 4162-6109

Mycgr3G70577\_Mycgr3T

Mycgr3G40534 Mycgr3T
  
Location: 6209-7166

Mycgr3G40534\_Mycgr3T

Mycgr3G85486 Mycgr3T
  
Location: 7266-8511

Mycgr3G85486\_Mycgr3T

Mycgr3G92221 Mycgr3T
  
Location: 8611-9193

Mycgr3G92221\_Mycgr3T

Mycgr3G39931 Mycgr3T
  
Location: 9293-10157

Mycgr3G39931\_Mycgr3T

Mycgr3G99766 Mycgr3T
  
Location: 10257-11775

Mycgr3G99766\_Mycgr3T

hypothetical protein
  
Accession: CAP91201
  
Location: 308505-309759
  
 NCBI BlastP on this gene

Pc13g01320

hypothetical protein
  
Accession: CAP91202
  
Location: 309937-310554
  
 NCBI BlastP on this gene

Pc13g01330

not annotated
  
Accession: CAP91203
  
Location: 311236-312720
  
 NCBI BlastP on this gene

Pc13g01340

unnamed
  
Accession: CAP91204
  
Location: 313975-315646
  
  
**BlastP hit with Mycgr3G99766\_Mycgr3T**
  
Percentage identity: 39 %
  
BlastP bit score: 330
  
Sequence coverage: 97 %
  
E-value: 4e-103
  
  
 NCBI BlastP on this gene

Pc13g01350

not annotated
  
Accession: CAP91205
  
Location: 316096-316996
  
 NCBI BlastP on this gene

Pc13g01360

not annotated
  
Accession: CAP91206
  
Location: 317309-319209
  
 NCBI BlastP on this gene

Pc13g01370

unnamed
  
Accession: CAP91207
  
Location: 319798-321173
  
 NCBI BlastP on this gene

Pc13g01380

not annotated
  
Accession: CAP91208
  
Location: 321349-323840
  
 NCBI BlastP on this gene

Pc13g01390

Query: Architecture Search FASTA input

JH711791 : Trametes versicolor FP-101664 SS1 unplaced genomic scaffold TRAVEscaffold\_9    Total score: 1.0     Cumulative Blast bit score: 328

Hit cluster cross-links:

Mycgr3G41235 Mycgr3T
  
Location: 0-4062

Mycgr3G41235\_Mycgr3T

Mycgr3G70577 Mycgr3T
  
Location: 4162-6109

Mycgr3G70577\_Mycgr3T

Mycgr3G40534 Mycgr3T
  
Location: 6209-7166

Mycgr3G40534\_Mycgr3T

Mycgr3G85486 Mycgr3T
  
Location: 7266-8511

Mycgr3G85486\_Mycgr3T

Mycgr3G92221 Mycgr3T
  
Location: 8611-9193

Mycgr3G92221\_Mycgr3T

Mycgr3G39931 Mycgr3T
  
Location: 9293-10157

Mycgr3G39931\_Mycgr3T

Mycgr3G99766 Mycgr3T
  
Location: 10257-11775

Mycgr3G99766\_Mycgr3T

drug:h+ antiporter
  
Accession: EIW56249
  
Location: 2710202-2712929
  
  
**BlastP hit with Mycgr3G70577\_Mycgr3T**
  
Percentage identity: 36 %
  
BlastP bit score: 328
  
Sequence coverage: 91 %
  
E-value: 3e-99
  
  
 NCBI BlastP on this gene

EIW56249

hypothetical protein
  
Accession: EIW56248
  
Location: 2707060-2708206
  
 NCBI BlastP on this gene

EIW56248

hypothetical protein
  
Accession: EIW56247
  
Location: 2705868-2706354
  
 NCBI BlastP on this gene

EIW56247

hypothetical protein
  
Accession: EIW56246
  
Location: 2703745-2704927
  
 NCBI BlastP on this gene

EIW56246

Query: Architecture Search FASTA input

EQ962652 : Talaromyces stipitatus ATCC 10500 scf\_1105507295523 genomic scaffold    Total score: 1.0     Cumulative Blast bit score: 327

Hit cluster cross-links:

Mycgr3G41235 Mycgr3T
  
Location: 0-4062

Mycgr3G41235\_Mycgr3T

Mycgr3G70577 Mycgr3T
  
Location: 4162-6109

Mycgr3G70577\_Mycgr3T

Mycgr3G40534 Mycgr3T
  
Location: 6209-7166

Mycgr3G40534\_Mycgr3T

Mycgr3G85486 Mycgr3T
  
Location: 7266-8511

Mycgr3G85486\_Mycgr3T

Mycgr3G92221 Mycgr3T
  
Location: 8611-9193

Mycgr3G92221\_Mycgr3T

Mycgr3G39931 Mycgr3T
  
Location: 9293-10157

Mycgr3G39931\_Mycgr3T

Mycgr3G99766 Mycgr3T
  
Location: 10257-11775

Mycgr3G99766\_Mycgr3T

sugar transporter, putative
  
Accession: EED24029
  
Location: 3998358-4000021
  
  
**BlastP hit with Mycgr3G99766\_Mycgr3T**
  
Percentage identity: 37 %
  
BlastP bit score: 327
  
Sequence coverage: 101 %
  
E-value: 6e-102
  
  
 NCBI BlastP on this gene

EED24029

hydrolase, putative
  
Accession: EED24028
  
Location: 3996601-3997819
  
 NCBI BlastP on this gene

EED24028

feruloyl esterase, putative
  
Accession: EED24027
  
Location: 3994146-3995714
  
 NCBI BlastP on this gene

EED24027

conserved hypothetical protein
  
Accession: EED24026
  
Location: 3992783-3993986
  
 NCBI BlastP on this gene

EED24026

histone transcription regulator Hir1, putative
  
Accession: EED24023
  
Location: 3988492-3991925
  
 NCBI BlastP on this gene

EED24023

Query: Architecture Search FASTA input

GL377310 : Schizophyllum commune H4-8 unplaced genomic scaffold SCHCOscaffold\_9    Total score: 1.0     Cumulative Blast bit score: 325

Hit cluster cross-links:

Mycgr3G41235 Mycgr3T
  
Location: 0-4062

Mycgr3G41235\_Mycgr3T

Mycgr3G70577 Mycgr3T
  
Location: 4162-6109

Mycgr3G70577\_Mycgr3T

Mycgr3G40534 Mycgr3T
  
Location: 6209-7166

Mycgr3G40534\_Mycgr3T

Mycgr3G85486 Mycgr3T
  
Location: 7266-8511

Mycgr3G85486\_Mycgr3T

Mycgr3G92221 Mycgr3T
  
Location: 8611-9193

Mycgr3G92221\_Mycgr3T

Mycgr3G39931 Mycgr3T
  
Location: 9293-10157

Mycgr3G39931\_Mycgr3T

Mycgr3G99766 Mycgr3T
  
Location: 10257-11775

Mycgr3G99766\_Mycgr3T

hypothetical protein
  
Accession: EFI94101
  
Location: 1027388-1028963
  
 NCBI BlastP on this gene

EFI94101

expressed protein
  
Accession: EFI94102
  
Location: 1029628-1030623
  
 NCBI BlastP on this gene

EFI94102

hypothetical protein
  
Accession: EFI93793
  
Location: 1033381-1035772
  
  
**BlastP hit with Mycgr3G70577\_Mycgr3T**
  
Percentage identity: 34 %
  
BlastP bit score: 325
  
Sequence coverage: 97 %
  
E-value: 7e-98
  
  
 NCBI BlastP on this gene

EFI93793

Query: Architecture Search FASTA input

KE145363 : Glarea lozoyensis ATCC 20868 chromosome Unknown GLAREA2    Total score: 1.0     Cumulative Blast bit score: 315

Hit cluster cross-links:

Mycgr3G41235 Mycgr3T
  
Location: 0-4062

Mycgr3G41235\_Mycgr3T

Mycgr3G70577 Mycgr3T
  
Location: 4162-6109

Mycgr3G70577\_Mycgr3T

Mycgr3G40534 Mycgr3T
  
Location: 6209-7166

Mycgr3G40534\_Mycgr3T

Mycgr3G85486 Mycgr3T
  
Location: 7266-8511

Mycgr3G85486\_Mycgr3T

Mycgr3G92221 Mycgr3T
  
Location: 8611-9193

Mycgr3G92221\_Mycgr3T

Mycgr3G39931 Mycgr3T
  
Location: 9293-10157

Mycgr3G39931\_Mycgr3T

Mycgr3G99766 Mycgr3T
  
Location: 10257-11775

Mycgr3G99766\_Mycgr3T

MFS general substrate transporter
  
Accession: EPE30675
  
Location: 941112-943170
  
  
**BlastP hit with Mycgr3G99766\_Mycgr3T**
  
Percentage identity: 36 %
  
BlastP bit score: 315
  
Sequence coverage: 97 %
  
E-value: 5e-97
  
  
 NCBI BlastP on this gene

EPE30675

Protein kinase-like (PK-like)
  
Accession: EPE30674
  
Location: 936962-938802
  
 NCBI BlastP on this gene

EPE30674

Query: Architecture Search FASTA input

FP929130 : Leptosphaeria maculans JN3 lm\_SuperContig\_17\_v2 genomic supercontig    Total score: 1.0     Cumulative Blast bit score: 314

Hit cluster cross-links:

Mycgr3G41235 Mycgr3T
  
Location: 0-4062

Mycgr3G41235\_Mycgr3T

Mycgr3G70577 Mycgr3T
  
Location: 4162-6109

Mycgr3G70577\_Mycgr3T

Mycgr3G40534 Mycgr3T
  
Location: 6209-7166

Mycgr3G40534\_Mycgr3T

Mycgr3G85486 Mycgr3T
  
Location: 7266-8511

Mycgr3G85486\_Mycgr3T

Mycgr3G92221 Mycgr3T
  
Location: 8611-9193

Mycgr3G92221\_Mycgr3T

Mycgr3G39931 Mycgr3T
  
Location: 9293-10157

Mycgr3G39931\_Mycgr3T

Mycgr3G99766 Mycgr3T
  
Location: 10257-11775

Mycgr3G99766\_Mycgr3T

similar to zinc knuckle domain-containing protein
  
Accession: CBX96947
  
Location: 739621-740570
  
 NCBI BlastP on this gene

LEMA\_P100780.1

hypothetical protein
  
Accession: CBX96948
  
Location: 742152-743578
  
 NCBI BlastP on this gene

LEMA\_P100790.1

similar to glycoside hydrolase family 61 protein
  
Accession: CBX96949
  
Location: 744605-745722
  
 NCBI BlastP on this gene

LEMA\_P100800.1

predicted protein
  
Accession: CBX96950
  
Location: 745967-746662
  
 NCBI BlastP on this gene

LEMA\_P100810.1

similar to MFS sugar transporter
  
Accession: CBX96951
  
Location: 747978-749906
  
  
**BlastP hit with Mycgr3G99766\_Mycgr3T**
  
Percentage identity: 37 %
  
BlastP bit score: 314
  
Sequence coverage: 96 %
  
E-value: 2e-96
  
  
 NCBI BlastP on this gene

LEMA\_P100820.1

predicted protein
  
Accession: CBX96952
  
Location: 752482-752873
  
 NCBI BlastP on this gene

LEMA\_P100830.1

similar to NADH:flavin oxidoreductase/NADH oxidase
  
Accession: CBX96953
  
Location: 755345-756694
  
 NCBI BlastP on this gene

LEMA\_P100840.1

Query: Architecture Search FASTA input

DS499598 : Aspergillus fumigatus A1163 scf\_000005 genomic scaffold    Total score: 1.0     Cumulative Blast bit score: 313

Hit cluster cross-links:

Mycgr3G41235 Mycgr3T
  
Location: 0-4062

Mycgr3G41235\_Mycgr3T

Mycgr3G70577 Mycgr3T
  
Location: 4162-6109

Mycgr3G70577\_Mycgr3T

Mycgr3G40534 Mycgr3T
  
Location: 6209-7166

Mycgr3G40534\_Mycgr3T

Mycgr3G85486 Mycgr3T
  
Location: 7266-8511

Mycgr3G85486\_Mycgr3T

Mycgr3G92221 Mycgr3T
  
Location: 8611-9193

Mycgr3G92221\_Mycgr3T

Mycgr3G39931 Mycgr3T
  
Location: 9293-10157

Mycgr3G39931\_Mycgr3T

Mycgr3G99766 Mycgr3T
  
Location: 10257-11775

Mycgr3G99766\_Mycgr3T

zinc knuckle transcription factor (CnjB), putative
  
Accession: EDP50659
  
Location: 1981175-1983451
  
 NCBI BlastP on this gene

EDP50659

alpha/beta hydrolase, putative
  
Accession: EDP50660
  
Location: 1984139-1985107
  
 NCBI BlastP on this gene

EDP50660

MFS monosaccharide transporter, putative
  
Accession: EDP50661
  
Location: 1989997-1991903
  
  
**BlastP hit with Mycgr3G99766\_Mycgr3T**
  
Percentage identity: 35 %
  
BlastP bit score: 313
  
Sequence coverage: 97 %
  
E-value: 4e-96
  
  
 NCBI BlastP on this gene

EDP50661

Query: Architecture Search FASTA input

AAHF01000005 : Aspergillus fumigatus Af293    Total score: 1.0     Cumulative Blast bit score: 313

Hit cluster cross-links:

Mycgr3G41235 Mycgr3T
  
Location: 0-4062

Mycgr3G41235\_Mycgr3T

Mycgr3G70577 Mycgr3T
  
Location: 4162-6109

Mycgr3G70577\_Mycgr3T

Mycgr3G40534 Mycgr3T
  
Location: 6209-7166

Mycgr3G40534\_Mycgr3T

Mycgr3G85486 Mycgr3T
  
Location: 7266-8511

Mycgr3G85486\_Mycgr3T

Mycgr3G92221 Mycgr3T
  
Location: 8611-9193

Mycgr3G92221\_Mycgr3T

Mycgr3G39931 Mycgr3T
  
Location: 9293-10157

Mycgr3G39931\_Mycgr3T

Mycgr3G99766 Mycgr3T
  
Location: 10257-11775

Mycgr3G99766\_Mycgr3T

zinc knuckle transcription factor (CnjB), putative
  
Accession: EAL89487
  
Location: 504356-506632
  
 NCBI BlastP on this gene

EAL89487

alpha/beta hydrolase, putative
  
Accession: EAL89486
  
Location: 502622-503461
  
 NCBI BlastP on this gene

EAL89486

MFS monosaccharide transporter, putative
  
Accession: EAL89485
  
Location: 497930-499836
  
  
**BlastP hit with Mycgr3G99766\_Mycgr3T**
  
Percentage identity: 35 %
  
BlastP bit score: 313
  
Sequence coverage: 97 %
  
E-value: 4e-96
  
  
 NCBI BlastP on this gene

EAL89485

MFS transporter, putative
  
Accession: EAL89484
  
Location: 494692-496502
  
 NCBI BlastP on this gene

EAL89484

serine/proline-rich protein
  
Accession: EAL89482
  
Location: 493650-494234
  
 NCBI BlastP on this gene

EAL89482

glutamine synthetase
  
Accession: EAL89481
  
Location: 490843-492547
  
 NCBI BlastP on this gene

EAL89481

Query: Architecture Search FASTA input

JH711573 : Coniophora puteana RWD-64-598 SS2 unplaced genomic scaffold CONPUscaffold\_1    Total score: 1.0     Cumulative Blast bit score: 311

Hit cluster cross-links:

Mycgr3G41235 Mycgr3T
  
Location: 0-4062

Mycgr3G41235\_Mycgr3T

Mycgr3G70577 Mycgr3T
  
Location: 4162-6109

Mycgr3G70577\_Mycgr3T

Mycgr3G40534 Mycgr3T
  
Location: 6209-7166

Mycgr3G40534\_Mycgr3T

Mycgr3G85486 Mycgr3T
  
Location: 7266-8511

Mycgr3G85486\_Mycgr3T

Mycgr3G92221 Mycgr3T
  
Location: 8611-9193

Mycgr3G92221\_Mycgr3T

Mycgr3G39931 Mycgr3T
  
Location: 9293-10157

Mycgr3G39931\_Mycgr3T

Mycgr3G99766 Mycgr3T
  
Location: 10257-11775

Mycgr3G99766\_Mycgr3T

glycoside hydrolase family 55 protein
  
Accession: EIW85957
  
Location: 253680-256851
  
 NCBI BlastP on this gene

EIW85957

hypothetical protein
  
Accession: EIW85958
  
Location: 257588-258329
  
 NCBI BlastP on this gene

EIW85958

hypothetical protein
  
Accession: EIW85959
  
Location: 258905-260984
  
 NCBI BlastP on this gene

EIW85959

MFS general substrate transporter
  
Accession: EIW85960
  
Location: 261546-264645
  
  
**BlastP hit with Mycgr3G70577\_Mycgr3T**
  
Percentage identity: 33 %
  
BlastP bit score: 311
  
Sequence coverage: 96 %
  
E-value: 2e-92
  
  
 NCBI BlastP on this gene

EIW85960

hypothetical protein
  
Accession: EIW85961
  
Location: 265332-265868
  
 NCBI BlastP on this gene

EIW85961

hypothetical protein
  
Accession: EIW85962
  
Location: 267471-269212
  
 NCBI BlastP on this gene

EIW85962

Query: Architecture Search FASTA input

DS027054 : Aspergillus clavatus NRRL 1 1099423829800 genomic scaffold    Total score: 1.0     Cumulative Blast bit score: 311

Hit cluster cross-links:

Mycgr3G41235 Mycgr3T
  
Location: 0-4062

Mycgr3G41235\_Mycgr3T

Mycgr3G70577 Mycgr3T
  
Location: 4162-6109

Mycgr3G70577\_Mycgr3T

Mycgr3G40534 Mycgr3T
  
Location: 6209-7166

Mycgr3G40534\_Mycgr3T

Mycgr3G85486 Mycgr3T
  
Location: 7266-8511

Mycgr3G85486\_Mycgr3T

Mycgr3G92221 Mycgr3T
  
Location: 8611-9193

Mycgr3G92221\_Mycgr3T

Mycgr3G39931 Mycgr3T
  
Location: 9293-10157

Mycgr3G39931\_Mycgr3T

Mycgr3G99766 Mycgr3T
  
Location: 10257-11775

Mycgr3G99766\_Mycgr3T

zinc knuckle transcription factor (CnjB), putative
  
Accession: EAW10734
  
Location: 2071201-2073484
  
 NCBI BlastP on this gene

EAW10734

MFS monosaccharide transporter, putative
  
Accession: EAW10735
  
Location: 2079693-2081584
  
  
**BlastP hit with Mycgr3G99766\_Mycgr3T**
  
Percentage identity: 34 %
  
BlastP bit score: 311
  
Sequence coverage: 97 %
  
E-value: 1e-95
  
  
 NCBI BlastP on this gene

EAW10735

Query: Architecture Search FASTA input

GL377303 : Schizophyllum commune H4-8 unplaced genomic scaffold SCHCOscaffold\_2    Total score: 1.0     Cumulative Blast bit score: 310

Hit cluster cross-links:

Mycgr3G41235 Mycgr3T
  
Location: 0-4062

Mycgr3G41235\_Mycgr3T

Mycgr3G70577 Mycgr3T
  
Location: 4162-6109

Mycgr3G70577\_Mycgr3T

Mycgr3G40534 Mycgr3T
  
Location: 6209-7166

Mycgr3G40534\_Mycgr3T

Mycgr3G85486 Mycgr3T
  
Location: 7266-8511

Mycgr3G85486\_Mycgr3T

Mycgr3G92221 Mycgr3T
  
Location: 8611-9193

Mycgr3G92221\_Mycgr3T

Mycgr3G39931 Mycgr3T
  
Location: 9293-10157

Mycgr3G39931\_Mycgr3T

Mycgr3G99766 Mycgr3T
  
Location: 10257-11775

Mycgr3G99766\_Mycgr3T

hypothetical protein
  
Accession: EFJ00466
  
Location: 3472152-3474157
  
 NCBI BlastP on this gene

EFJ00466

hypothetical protein
  
Accession: EFJ00467
  
Location: 3475688-3476855
  
 NCBI BlastP on this gene

EFJ00467

hypothetical protein
  
Accession: EFJ01269
  
Location: 3477096-3478404
  
 NCBI BlastP on this gene

EFJ01269

hypothetical protein
  
Accession: EFJ00468
  
Location: 3479270-3481549
  
  
**BlastP hit with Mycgr3G70577\_Mycgr3T**
  
Percentage identity: 32 %
  
BlastP bit score: 310
  
Sequence coverage: 92 %
  
E-value: 8e-93
  
  
 NCBI BlastP on this gene

EFJ00468

Query: Architecture Search FASTA input

DS027685 : Neosartorya fischeri NRRL 181 1099437636245 genomic scaffold    Total score: 1.0     Cumulative Blast bit score: 308

Hit cluster cross-links:

Mycgr3G41235 Mycgr3T
  
Location: 0-4062

Mycgr3G41235\_Mycgr3T

Mycgr3G70577 Mycgr3T
  
Location: 4162-6109

Mycgr3G70577\_Mycgr3T

Mycgr3G40534 Mycgr3T
  
Location: 6209-7166

Mycgr3G40534\_Mycgr3T

Mycgr3G85486 Mycgr3T
  
Location: 7266-8511

Mycgr3G85486\_Mycgr3T

Mycgr3G92221 Mycgr3T
  
Location: 8611-9193

Mycgr3G92221\_Mycgr3T

Mycgr3G39931 Mycgr3T
  
Location: 9293-10157

Mycgr3G39931\_Mycgr3T

Mycgr3G99766 Mycgr3T
  
Location: 10257-11775

Mycgr3G99766\_Mycgr3T

zinc knuckle transcription factor (CnjB), putative
  
Accession: EAW24841
  
Location: 539504-541763
  
 NCBI BlastP on this gene

EAW24841

alpha/beta fold family hydrolase, putative
  
Accession: EAW24840
  
Location: 537726-538589
  
 NCBI BlastP on this gene

EAW24840

MFS monosaccharide transporter, putative
  
Accession: EAW24839
  
Location: 533177-535080
  
  
**BlastP hit with Mycgr3G99766\_Mycgr3T**
  
Percentage identity: 34 %
  
BlastP bit score: 308
  
Sequence coverage: 97 %
  
E-value: 2e-94
  
  
 NCBI BlastP on this gene

EAW24839

MFS transporter, putative
  
Accession: EAW24838
  
Location: 529869-531668
  
 NCBI BlastP on this gene

EAW24838

hypothetical protein
  
Accession: EAW24837
  
Location: 528453-529143
  
 NCBI BlastP on this gene

EAW24837

glutamine synthetase
  
Accession: EAW24836
  
Location: 526015-527697
  
 NCBI BlastP on this gene

EAW24836

Query: Architecture Search FASTA input

GG704911 : Coccidioides immitis RS genomic scaffold supercont3.1    Total score: 1.0     Cumulative Blast bit score: 307

Hit cluster cross-links:

Mycgr3G41235 Mycgr3T
  
Location: 0-4062

Mycgr3G41235\_Mycgr3T

Mycgr3G70577 Mycgr3T
  
Location: 4162-6109

Mycgr3G70577\_Mycgr3T

Mycgr3G40534 Mycgr3T
  
Location: 6209-7166

Mycgr3G40534\_Mycgr3T

Mycgr3G85486 Mycgr3T
  
Location: 7266-8511

Mycgr3G85486\_Mycgr3T

Mycgr3G92221 Mycgr3T
  
Location: 8611-9193

Mycgr3G92221\_Mycgr3T

Mycgr3G39931 Mycgr3T
  
Location: 9293-10157

Mycgr3G39931\_Mycgr3T

Mycgr3G99766 Mycgr3T
  
Location: 10257-11775

Mycgr3G99766\_Mycgr3T

sugar porter (SP) family MFS transporter
  
Accession: EAS35333
  
Location: 6801838-6803798
  
  
**BlastP hit with Mycgr3G99766\_Mycgr3T**
  
Percentage identity: 35 %
  
BlastP bit score: 307
  
Sequence coverage: 99 %
  
E-value: 7e-94
  
  
 NCBI BlastP on this gene

EAS35333

hypothetical protein
  
Accession: EAS35334
  
Location: 6800246-6801294
  
 NCBI BlastP on this gene

EAS35334

COPI-coated vesicle protein
  
Accession: EAS35335
  
Location: 6798825-6799553
  
 NCBI BlastP on this gene

EAS35335

SreP protein
  
Accession: EAS35338
  
Location: 6795022-6796911
  
 NCBI BlastP on this gene

EAS35338

Query: Architecture Search FASTA input

ACFW01000049 : Coccidioides posadasii C735 delta SOWgp    Total score: 1.0     Cumulative Blast bit score: 307

Hit cluster cross-links:

Mycgr3G41235 Mycgr3T
  
Location: 0-4062

Mycgr3G41235\_Mycgr3T

Mycgr3G70577 Mycgr3T
  
Location: 4162-6109

Mycgr3G70577\_Mycgr3T

Mycgr3G40534 Mycgr3T
  
Location: 6209-7166

Mycgr3G40534\_Mycgr3T

Mycgr3G85486 Mycgr3T
  
Location: 7266-8511

Mycgr3G85486\_Mycgr3T

Mycgr3G92221 Mycgr3T
  
Location: 8611-9193

Mycgr3G92221\_Mycgr3T

Mycgr3G39931 Mycgr3T
  
Location: 9293-10157

Mycgr3G39931\_Mycgr3T

Mycgr3G99766 Mycgr3T
  
Location: 10257-11775

Mycgr3G99766\_Mycgr3T

Sugar transporter family protein
  
Accession: EER24249
  
Location: 3796420-3798379
  
  
**BlastP hit with Mycgr3G99766\_Mycgr3T**
  
Percentage identity: 35 %
  
BlastP bit score: 307
  
Sequence coverage: 99 %
  
E-value: 6e-94
  
  
 NCBI BlastP on this gene

EER24249

hypothetical protein
  
Accession: EER24248
  
Location: 3794825-3795873
  
 NCBI BlastP on this gene

EER24248

hypothetical protein
  
Accession: EER24247
  
Location: 3793392-3793959
  
 NCBI BlastP on this gene

EER24247

GATA family transcription factor
  
Accession: EER24246
  
Location: 3789589-3791478
  
 NCBI BlastP on this gene

EER24246

Query: Architecture Search FASTA input

EQ962655 : Talaromyces stipitatus ATCC 10500 scf\_1105507295555 genomic scaffold    Total score: 1.0     Cumulative Blast bit score: 306

Hit cluster cross-links:

Mycgr3G41235 Mycgr3T
  
Location: 0-4062

Mycgr3G41235\_Mycgr3T

Mycgr3G70577 Mycgr3T
  
Location: 4162-6109

Mycgr3G70577\_Mycgr3T

Mycgr3G40534 Mycgr3T
  
Location: 6209-7166

Mycgr3G40534\_Mycgr3T

Mycgr3G85486 Mycgr3T
  
Location: 7266-8511

Mycgr3G85486\_Mycgr3T

Mycgr3G92221 Mycgr3T
  
Location: 8611-9193

Mycgr3G92221\_Mycgr3T

Mycgr3G39931 Mycgr3T
  
Location: 9293-10157

Mycgr3G39931\_Mycgr3T

Mycgr3G99766 Mycgr3T
  
Location: 10257-11775

Mycgr3G99766\_Mycgr3T

3-hydroxyacyl-CoA dehyrogenase, putative
  
Accession: EED17717
  
Location: 1122070-1123091
  
 NCBI BlastP on this gene

EED17717

zinc knuckle transcription factor (CnjB), putative
  
Accession: EED17718
  
Location: 1123571-1125181
  
 NCBI BlastP on this gene

EED17718

monoxygenase, putative
  
Accession: EED17719
  
Location: 1125852-1126715
  
 NCBI BlastP on this gene

EED17719

hypothetical protein
  
Accession: EED17720
  
Location: 1126827-1127745
  
 NCBI BlastP on this gene

EED17720

MFS monosaccharide transporter, putative
  
Accession: EED17721
  
Location: 1130400-1132280
  
  
**BlastP hit with Mycgr3G99766\_Mycgr3T**
  
Percentage identity: 36 %
  
BlastP bit score: 306
  
Sequence coverage: 95 %
  
E-value: 9e-94
  
  
 NCBI BlastP on this gene

EED17721

glutamine synthetase
  
Accession: EED17722
  
Location: 1132932-1134365
  
 NCBI BlastP on this gene

EED17722

conserved hypothetical protein
  
Accession: EED17723
  
Location: 1136513-1138702
  
 NCBI BlastP on this gene

EED17723

conserved hypothetical protein
  
Accession: EED17724
  
Location: 1139055-1139960
  
 NCBI BlastP on this gene

EED17724

Query: Architecture Search FASTA input

CM001198 : Mycosphaerella graminicola IPO323 chromosome 3    Total score: 1.0     Cumulative Blast bit score: 306

Hit cluster cross-links:

Mycgr3G41235 Mycgr3T
  
Location: 0-4062

Mycgr3G41235\_Mycgr3T

Mycgr3G70577 Mycgr3T
  
Location: 4162-6109

Mycgr3G70577\_Mycgr3T

Mycgr3G40534 Mycgr3T
  
Location: 6209-7166

Mycgr3G40534\_Mycgr3T

Mycgr3G85486 Mycgr3T
  
Location: 7266-8511

Mycgr3G85486\_Mycgr3T

Mycgr3G92221 Mycgr3T
  
Location: 8611-9193

Mycgr3G92221\_Mycgr3T

Mycgr3G39931 Mycgr3T
  
Location: 9293-10157

Mycgr3G39931\_Mycgr3T

Mycgr3G99766 Mycgr3T
  
Location: 10257-11775

Mycgr3G99766\_Mycgr3T

hypothetical protein
  
Accession: EGP89094
  
Location: 3306039-3307526
  
 NCBI BlastP on this gene

EGP89094

hypothetical protein
  
Accession: EGP89150
  
Location: 3307831-3309434
  
 NCBI BlastP on this gene

EGP89150

hypothetical protein
  
Accession: EGP89095
  
Location: 3313304-3315585
  
  
**BlastP hit with Mycgr3G99766\_Mycgr3T**
  
Percentage identity: 36 %
  
BlastP bit score: 306
  
Sequence coverage: 97 %
  
E-value: 7e-94
  
  
 NCBI BlastP on this gene

EGP89095

Query: Architecture Search FASTA input

HF679025 : Fusarium fujikuroi IMI 58289 draft genome, chromosome FFUJ\_chr03.    Total score: 1.0     Cumulative Blast bit score: 305

Hit cluster cross-links:

Mycgr3G41235 Mycgr3T
  
Location: 0-4062

Mycgr3G41235\_Mycgr3T

Mycgr3G70577 Mycgr3T
  
Location: 4162-6109

Mycgr3G70577\_Mycgr3T

Mycgr3G40534 Mycgr3T
  
Location: 6209-7166

Mycgr3G40534\_Mycgr3T

Mycgr3G85486 Mycgr3T
  
Location: 7266-8511

Mycgr3G85486\_Mycgr3T

Mycgr3G92221 Mycgr3T
  
Location: 8611-9193

Mycgr3G92221\_Mycgr3T

Mycgr3G39931 Mycgr3T
  
Location: 9293-10157

Mycgr3G39931\_Mycgr3T

Mycgr3G99766 Mycgr3T
  
Location: 10257-11775

Mycgr3G99766\_Mycgr3T

related to sugar transporter
  
Accession: CCT66518
  
Location: 4684187-4685789
  
  
**BlastP hit with Mycgr3G99766\_Mycgr3T**
  
Percentage identity: 35 %
  
BlastP bit score: 305
  
Sequence coverage: 96 %
  
E-value: 1e-93
  
  
 NCBI BlastP on this gene

FFUJ\_03554

related to alpha-L-arabinofuranosidase A precursor
  
Accession: CCT66517
  
Location: 4681306-4683489
  
 NCBI BlastP on this gene

FFUJ\_03553

uncharacterized protein
  
Accession: CCT66516
  
Location: 4678813-4679142
  
 NCBI BlastP on this gene

FFUJ\_03552

uncharacterized protein
  
Accession: CCT66515
  
Location: 4675793-4677349
  
 NCBI BlastP on this gene

FFUJ\_03551

Query: Architecture Search FASTA input

KB446557 : Pseudocercospora fijiensis CIRAD86 unplaced genomic scaffold MYCFIscaffold\_3    Total score: 1.0     Cumulative Blast bit score: 303

Hit cluster cross-links:

Mycgr3G41235 Mycgr3T
  
Location: 0-4062

Mycgr3G41235\_Mycgr3T

Mycgr3G70577 Mycgr3T
  
Location: 4162-6109

Mycgr3G70577\_Mycgr3T

Mycgr3G40534 Mycgr3T
  
Location: 6209-7166

Mycgr3G40534\_Mycgr3T

Mycgr3G85486 Mycgr3T
  
Location: 7266-8511

Mycgr3G85486\_Mycgr3T

Mycgr3G92221 Mycgr3T
  
Location: 8611-9193

Mycgr3G92221\_Mycgr3T

Mycgr3G39931 Mycgr3T
  
Location: 9293-10157

Mycgr3G39931\_Mycgr3T

Mycgr3G99766 Mycgr3T
  
Location: 10257-11775

Mycgr3G99766\_Mycgr3T

hypothetical protein
  
Accession: EME84611
  
Location: 3977599-3979482
  
  
**BlastP hit with Mycgr3G99766\_Mycgr3T**
  
Percentage identity: 36 %
  
BlastP bit score: 303
  
Sequence coverage: 97 %
  
E-value: 2e-92
  
  
 NCBI BlastP on this gene

EME84611

hypothetical protein
  
Accession: EME84610
  
Location: 3973902-3976079
  
 NCBI BlastP on this gene

EME84610

hypothetical protein
  
Accession: EME84609
  
Location: 3972114-3973801
  
 NCBI BlastP on this gene

EME84609

hypothetical protein
  
Accession: EME84608
  
Location: 3970022-3971065
  
 NCBI BlastP on this gene

EME84608

Query: Architecture Search FASTA input

KB644410 : Penicillium oxalicum 114-2 unplaced genomic scaffold scaffold\_3    Total score: 1.0     Cumulative Blast bit score: 301

Hit cluster cross-links:

Mycgr3G41235 Mycgr3T
  
Location: 0-4062

Mycgr3G41235\_Mycgr3T

Mycgr3G70577 Mycgr3T
  
Location: 4162-6109

Mycgr3G70577\_Mycgr3T

Mycgr3G40534 Mycgr3T
  
Location: 6209-7166

Mycgr3G40534\_Mycgr3T

Mycgr3G85486 Mycgr3T
  
Location: 7266-8511

Mycgr3G85486\_Mycgr3T

Mycgr3G92221 Mycgr3T
  
Location: 8611-9193

Mycgr3G92221\_Mycgr3T

Mycgr3G39931 Mycgr3T
  
Location: 9293-10157

Mycgr3G39931\_Mycgr3T

Mycgr3G99766 Mycgr3T
  
Location: 10257-11775

Mycgr3G99766\_Mycgr3T

hypothetical protein
  
Accession: EPS27226
  
Location: 202009-203806
  
  
**BlastP hit with Mycgr3G99766\_Mycgr3T**
  
Percentage identity: 34 %
  
BlastP bit score: 301
  
Sequence coverage: 98 %
  
E-value: 1e-91
  
  
 NCBI BlastP on this gene

EPS27226

hypothetical protein
  
Accession: EPS27225
  
Location: 199036-201025
  
 NCBI BlastP on this gene

EPS27225

hypothetical protein
  
Accession: EPS27224
  
Location: 195611-197874
  
 NCBI BlastP on this gene

EPS27224

Query: Architecture Search FASTA input

151. :  GL883167 Melampsora larici-populina 98AG31 unplaced genomic scaffold MELLAscaffold\_78     Total score: 1.0     Cumulative Blast bit score: 607

Mycgr3G41235 Mycgr3T
  
Location: 0-4062
  
 NCBI BlastP on this gene

Mycgr3G41235\_Mycgr3T

Mycgr3G70577 Mycgr3T
  
Location: 4162-6109
  
 NCBI BlastP on this gene

Mycgr3G70577\_Mycgr3T

Mycgr3G40534 Mycgr3T
  
Location: 6209-7166
  
 NCBI BlastP on this gene

Mycgr3G40534\_Mycgr3T

Mycgr3G85486 Mycgr3T
  
Location: 7266-8511
  
 NCBI BlastP on this gene

Mycgr3G85486\_Mycgr3T

Mycgr3G92221 Mycgr3T
  
Location: 8611-9193
  
 NCBI BlastP on this gene

Mycgr3G92221\_Mycgr3T

Mycgr3G39931 Mycgr3T
  
Location: 9293-10157
  
 NCBI BlastP on this gene

Mycgr3G39931\_Mycgr3T

Mycgr3G99766 Mycgr3T
  
Location: 10257-11775
  
 NCBI BlastP on this gene

Mycgr3G99766\_Mycgr3T

hypothetical protein
  
Accession: EGF98885
  
Location: 226474-227644
  
 NCBI BlastP on this gene

EGF98885

hypothetical protein
  
Accession: EGF98912
  
Location: 231101-233768
  
  
**BlastP hit with Mycgr3G99766\_Mycgr3T**
  
Percentage identity: 36 %
  
BlastP bit score: 304
  
Sequence coverage: 95 %
  
E-value: 4e-93
  
  
 NCBI BlastP on this gene

EGF98912

hypothetical protein
  
Accession: EGF98886
  
Location: 241851-242636
  
 NCBI BlastP on this gene

EGF98886

hypothetical protein
  
Accession: EGF98911
  
Location: 242878-243763
  
 NCBI BlastP on this gene

EGF98911

hypothetical protein
  
Accession: EGF98887
  
Location: 244199-246851
  
  
**BlastP hit with Mycgr3G99766\_Mycgr3T**
  
Percentage identity: 36 %
  
BlastP bit score: 303
  
Sequence coverage: 95 %
  
E-value: 1e-92
  
  
 NCBI BlastP on this gene

EGF98887

hypothetical protein
  
Accession: EGF98910
  
Location: 252276-252920
  
 NCBI BlastP on this gene

EGF98910

152. :  GL985056 Trichoderma reesei QM6a unplaced genomic scaffold TRIREscaffold\_1     Total score: 1.0     Cumulative Blast bit score: 466

predicted protein
  
Accession: EGR52431
  
Location: 2451265-2451984
  
 NCBI BlastP on this gene

EGR52431

predicted protein
  
Accession: EGR52430
  
Location: 2449695-2450069
  
 NCBI BlastP on this gene

EGR52430

predicted protein
  
Accession: EGR52982
  
Location: 2446167-2447126
  
 NCBI BlastP on this gene

EGR52982

predicted protein
  
Accession: EGR52981
  
Location: 2441731-2443506
  
 NCBI BlastP on this gene

EGR52981

predicted protein
  
Accession: EGR52429
  
Location: 2440405-2441265
  
 NCBI BlastP on this gene

EGR52429

predicted protein
  
Accession: EGR52980
  
Location: 2437048-2439031
  
  
**BlastP hit with Mycgr3G70577\_Mycgr3T**
  
Percentage identity: 42 %
  
BlastP bit score: 466
  
Sequence coverage: 90 %
  
E-value: 1e-152
  
  
 NCBI BlastP on this gene

EGR52980

predicted protein
  
Accession: EGR52979
  
Location: 2434650-2435924
  
 NCBI BlastP on this gene

EGR52979

predicted protein
  
Accession: EGR52978
  
Location: 2425165-2425989
  
 NCBI BlastP on this gene

EGR52978

predicted protein
  
Accession: EGR52977
  
Location: 2423105-2424132
  
 NCBI BlastP on this gene

EGR52977

153. :  EQ963474 Aspergillus flavus NRRL3357 scf\_1106286417496 genomic scaffold     Total score: 1.0     Cumulative Blast bit score: 396

hypothetical protein
  
Accession: EED54300
  
Location: 731535-731942
  
 NCBI BlastP on this gene

EED54300

conserved hypothetical protein
  
Accession: EED54301
  
Location: 734526-736301
  
 NCBI BlastP on this gene

EED54301

amino acid transporter, putative
  
Accession: EED54302
  
Location: 737286-738920
  
 NCBI BlastP on this gene

EED54302

MFS sugar transporter, putative
  
Accession: EED54303
  
Location: 743091-744696
  
  
**BlastP hit with Mycgr3G99766\_Mycgr3T**
  
Percentage identity: 41 %
  
BlastP bit score: 397
  
Sequence coverage: 97 %
  
E-value: 3e-129
  
  
 NCBI BlastP on this gene

EED54303

154. :  AKHY01000171 Aspergillus oryzae 3.042     Total score: 1.0     Cumulative Blast bit score: 396

hypothetical protein
  
Accession: EIT75916
  
Location: 683593-685275
  
 NCBI BlastP on this gene

EIT75916

hypothetical protein
  
Accession: EIT76106
  
Location: 687859-689241
  
 NCBI BlastP on this gene

EIT76106

hypothetical protein
  
Accession: EIT76062
  
Location: 690723-692278
  
 NCBI BlastP on this gene

EIT76062

putative transporter
  
Accession: EIT75984
  
Location: 696425-698030
  
  
**BlastP hit with Mycgr3G99766\_Mycgr3T**
  
Percentage identity: 41 %
  
BlastP bit score: 397
  
Sequence coverage: 97 %
  
E-value: 3e-129
  
  
 NCBI BlastP on this gene

EIT75984

155. :  CU329670 Schizosaccharomyces pombe chromosome I     Total score: 1.0     Cumulative Blast bit score: 396

pyruvate decarboxylase (predicted)
  
Accession: CAB03601
  
Location: 101836-103544
  
 NCBI BlastP on this gene

SPAC1F8.07c

cell surface glycoprotein
  
Accession: CAB03600
  
Location: 100011-101168
  
 NCBI BlastP on this gene

fta5

sequence orphan
  
Accession: CAB03599
  
Location: 96000-96548
  
 NCBI BlastP on this gene

isp3

hydrolase (predicted)
  
Accession: CAB03598
  
Location: 92480-93871
  
 NCBI BlastP on this gene

SPAC1F8.04c

siderophore-iron transporter Str3
  
Accession: CAB03597
  
Location: 88367-90259
  
  
**BlastP hit with Mycgr3G70577\_Mycgr3T**
  
Percentage identity: 37 %
  
BlastP bit score: 396
  
Sequence coverage: 90 %
  
E-value: 2e-125
  
  
 NCBI BlastP on this gene

str3

sequence orphan
  
Accession: CAB03596
  
Location: 85598-86278
  
 NCBI BlastP on this gene

SPAC1F8.02c

hexose transporter Ght3
  
Accession: CAB03595
  
Location: 82936-84603
  
 NCBI BlastP on this gene

ght3

S. pombe specific UPF0321 family protein 3
  
Accession: CAB61210
  
Location: 77578-77931
  
 NCBI BlastP on this gene

SPAPJ695.01c

sequence orphan
  
Accession: CCD31308
  
Location: 75698-75910
  
 NCBI BlastP on this gene

SPAPJ695.02

156. :  AP007161 Aspergillus oryzae RIB40 DNA, SC012.     Total score: 1.0     Cumulative Blast bit score: 395

not annotated
  
Accession: BAE60484
  
Location: 694148-695830
  
 NCBI BlastP on this gene

AO090012000280

not annotated
  
Accession: BAE60485
  
Location: 698341-699723
  
 NCBI BlastP on this gene

AO090012000281

not annotated
  
Accession: BAE60486
  
Location: 701205-702760
  
 NCBI BlastP on this gene

AO090012000283

not annotated
  
Accession: BAE60487
  
Location: 706906-708511
  
  
**BlastP hit with Mycgr3G99766\_Mycgr3T**
  
Percentage identity: 41 %
  
BlastP bit score: 395
  
Sequence coverage: 97 %
  
E-value: 2e-128
  
  
 NCBI BlastP on this gene

AO090012000284

157. :  GL377306 Schizophyllum commune H4-8 unplaced genomic scaffold SCHCOscaffold\_5     Total score: 1.0     Cumulative Blast bit score: 393

hypothetical protein
  
Accession: EFI97304
  
Location: 1181070-1183856
  
 NCBI BlastP on this gene

EFI97304

hypothetical protein
  
Accession: EFI96857
  
Location: 1185020-1186242
  
 NCBI BlastP on this gene

EFI96857

hypothetical protein
  
Accession: EFI97305
  
Location: 1187581-1190463
  
 NCBI BlastP on this gene

EFI97305

hypothetical protein
  
Accession: EFI96858
  
Location: 1191716-1193063
  
 NCBI BlastP on this gene

EFI96858

hypothetical protein
  
Accession: EFI96859
  
Location: 1195025-1196993
  
  
**BlastP hit with Mycgr3G70577\_Mycgr3T**
  
Percentage identity: 40 %
  
BlastP bit score: 393
  
Sequence coverage: 91 %
  
E-value: 1e-124
  
  
 NCBI BlastP on this gene

EFI96859

158. :  CR382133 Debaryomyces hansenii CBS767 chromosome A complete sequence.     Total score: 1.0     Cumulative Blast bit score: 392

DEHA2A14586p
  
Accession: CAG84939
  
Location: 1217722-1219350
  
 NCBI BlastP on this gene

DEHA2A14586g

DEHA2A14608p
  
Accession: CAG84940
  
Location: 1220815-1221897
  
 NCBI BlastP on this gene

DEHA2A14608g

DEHA2A14630p
  
Accession: CAR65418
  
Location: 1222157-1222312
  
 NCBI BlastP on this gene

DEHA2A14630g

DEHA2A14652p
  
Accession: CAG84941
  
Location: 1224311-1224649
  
 NCBI BlastP on this gene

DEHA2A14652g

DEHA2A14674p
  
Accession: CAR65419
  
Location: 1226047-1226451
  
 NCBI BlastP on this gene

DEHA2A14674g

DEHA2A14696p
  
Accession: CAG84942
  
Location: 1229756-1231654
  
  
**BlastP hit with Mycgr3G70577\_Mycgr3T**
  
Percentage identity: 36 %
  
BlastP bit score: 392
  
Sequence coverage: 99 %
  
E-value: 8e-124
  
  
 NCBI BlastP on this gene

DEHA2A14696g

159. :  KB644409 Penicillium oxalicum 114-2 unplaced genomic scaffold scaffold\_2     Total score: 1.0     Cumulative Blast bit score: 375

hypothetical protein
  
Accession: EPS27052
  
Location: 2148557-2150199
  
  
**BlastP hit with Mycgr3G99766\_Mycgr3T**
  
Percentage identity: 42 %
  
BlastP bit score: 375
  
Sequence coverage: 96 %
  
E-value: 1e-120
  
  
 NCBI BlastP on this gene

EPS27052

hypothetical protein
  
Accession: EPS27051
  
Location: 2145484-2146724
  
 NCBI BlastP on this gene

EPS27051

hypothetical protein
  
Accession: EPS27050
  
Location: 2143178-2144106
  
 NCBI BlastP on this gene

EPS27050

hypothetical protein
  
Accession: EPS27049
  
Location: 2139476-2139752
  
 NCBI BlastP on this gene

EPS27049

hypothetical protein
  
Accession: EPS27048
  
Location: 2134399-2138974
  
 NCBI BlastP on this gene

EPS27048

160. :  DS027696 Neosartorya fischeri NRRL 181 1099437636264 genomic scaffold     Total score: 1.0     Cumulative Blast bit score: 374

hypothetical protein
  
Accession: EAW19100
  
Location: 5448975-5449610
  
 NCBI BlastP on this gene

EAW19100

conserved hypothetical protein
  
Accession: EAW19101
  
Location: 5449915-5450547
  
 NCBI BlastP on this gene

EAW19101

MFS transporter family protein, putative
  
Accession: EAW19102
  
Location: 5451565-5453538
  
 NCBI BlastP on this gene

EAW19102

hypothetical protein
  
Accession: EAW19103
  
Location: 5454056-5454972
  
 NCBI BlastP on this gene

EAW19103

FluG family protein
  
Accession: EAW19104
  
Location: 5457337-5458680
  
 NCBI BlastP on this gene

EAW19104

hypothetical protein
  
Accession: EAW19105
  
Location: 5459374-5459841
  
 NCBI BlastP on this gene

EAW19105

hypothetical protein
  
Accession: EAW19106
  
Location: 5460079-5460400
  
 NCBI BlastP on this gene

EAW19106

MFS sugar transporter, putative
  
Accession: EAW19107
  
Location: 5461922-5463543
  
  
**BlastP hit with Mycgr3G99766\_Mycgr3T**
  
Percentage identity: 41 %
  
BlastP bit score: 374
  
Sequence coverage: 97 %
  
E-value: 6e-120
  
  
 NCBI BlastP on this gene

EAW19107

161. :  CR382137 Debaryomyces hansenii CBS767 chromosome E complete sequence.     Total score: 1.0     Cumulative Blast bit score: 373

DEHA2E02596p
  
Accession: CAR65745
  
Location: 231504-233441
  
  
**BlastP hit with Mycgr3G70577\_Mycgr3T**
  
Percentage identity: 35 %
  
BlastP bit score: 373
  
Sequence coverage: 94 %
  
E-value: 2e-116
  
  
 NCBI BlastP on this gene

DEHA2E02596g

DEHA2E02574p
  
Accession: CAG87657
  
Location: 229554-230834
  
 NCBI BlastP on this gene

DEHA2E02574g

DEHA2E02552p
  
Accession: CAG87656
  
Location: 228993-229112
  
 NCBI BlastP on this gene

DEHA2E02552g

DEHA2E02530p
  
Accession: CAG87655
  
Location: 226878-228953
  
 NCBI BlastP on this gene

DEHA2E02530g

DEHA2E02508p
  
Accession: CAG87654
  
Location: 224663-226042
  
 NCBI BlastP on this gene

DEHA2E02508g

DEHA2E02464p
  
Accession: CAG87653
  
Location: 220532-224110
  
 NCBI BlastP on this gene

DEHA2E02464g

DEHA2E02420p
  
Accession: CAG87652
  
Location: 218710-219726
  
 NCBI BlastP on this gene

DEHA2E02420g

DEHA2E02398p
  
Accession: CAG87651
  
Location: 217077-218564
  
 NCBI BlastP on this gene

DEHA2E02398g

162. :  DS995906 Penicillium marneffei ATCC 18224 scf\_1105668340770 genomic scaffold     Total score: 1.0     Cumulative Blast bit score: 367

MFS sugar transporter, putative
  
Accession: EEA19218
  
Location: 1688151-1689712
  
  
**BlastP hit with Mycgr3G99766\_Mycgr3T**
  
Percentage identity: 41 %
  
BlastP bit score: 367
  
Sequence coverage: 98 %
  
E-value: 2e-117
  
  
 NCBI BlastP on this gene

EEA19218

DUF895 domain membrane protein
  
Accession: EEA19217
  
Location: 1686110-1687727
  
 NCBI BlastP on this gene

EEA19217

mycelial catalase Cat1
  
Accession: EEA19216
  
Location: 1682724-1685131
  
 NCBI BlastP on this gene

EEA19216

hypothetical protein
  
Accession: EEA19215
  
Location: 1679520-1680083
  
 NCBI BlastP on this gene

EEA19215

beta-glucosidase, putative
  
Accession: EEA19214
  
Location: 1676483-1679070
  
 NCBI BlastP on this gene

EEA19214

integral membrane protein (Pth11), putative
  
Accession: EEA19213
  
Location: 1673564-1675054
  
 NCBI BlastP on this gene

EEA19213

163. :  AM920431 Penicillium chrysogenum Wisconsin 54-1255 complete genome, contig Pc00c16.     Total score: 1.0     Cumulative Blast bit score: 360

unnamed
  
Accession: CAP93792
  
Location: 2699905-2701640
  
  
**BlastP hit with Mycgr3G99766\_Mycgr3T**
  
Percentage identity: 39 %
  
BlastP bit score: 360
  
Sequence coverage: 99 %
  
E-value: 1e-114
  
  
 NCBI BlastP on this gene

Pc16g11220

not annotated
  
Accession: Pc16g11210
  
Location: 2696769-2699306
  
 NCBI BlastP on this gene

Pc16g11210

not annotated
  
Accession: CAP93790
  
Location: 2693622-2695722
  
 NCBI BlastP on this gene

Pc16g11200

not annotated
  
Accession: CAP93789
  
Location: 2690642-2693245
  
 NCBI BlastP on this gene

Pc16g11190

not annotated
  
Accession: CAP93788
  
Location: 2689616-2690185
  
 NCBI BlastP on this gene

Pc16g11180

not annotated
  
Accession: CAP93787
  
Location: 2687676-2689310
  
 NCBI BlastP on this gene

Pc16g11170

164. :  KB644408 Penicillium oxalicum 114-2 unplaced genomic scaffold scaffold\_1     Total score: 1.0     Cumulative Blast bit score: 359

hypothetical protein
  
Accession: EPS25961
  
Location: 2558191-2559843
  
 NCBI BlastP on this gene

EPS25961

hypothetical protein
  
Accession: EPS25962
  
Location: 2561223-2563949
  
 NCBI BlastP on this gene

EPS25962

hypothetical protein
  
Accession: EPS25963
  
Location: 2564543-2567751
  
 NCBI BlastP on this gene

EPS25963

hypothetical protein
  
Accession: EPS25964
  
Location: 2571172-2573049
  
  
**BlastP hit with Mycgr3G99766\_Mycgr3T**
  
Percentage identity: 40 %
  
BlastP bit score: 359
  
Sequence coverage: 99 %
  
E-value: 4e-114
  
  
 NCBI BlastP on this gene

EPS25964

hypothetical protein
  
Accession: EPS25965
  
Location: 2574164-2578681
  
 NCBI BlastP on this gene

EPS25965

hypothetical protein
  
Accession: EPS25966
  
Location: 2579561-2580857
  
 NCBI BlastP on this gene

EPS25966

hypothetical protein
  
Accession: EPS25967
  
Location: 2583724-2586799
  
 NCBI BlastP on this gene

EPS25967

165. :  JH687379 Stereum hirsutum FP-91666 SS1 unplaced genomic scaffold STEHIscaffold\_1     Total score: 1.0     Cumulative Blast bit score: 358

MFS general substrate transporter
  
Accession: EIM92619
  
Location: 2783233-2786258
  
  
**BlastP hit with Mycgr3G70577\_Mycgr3T**
  
Percentage identity: 35 %
  
BlastP bit score: 358
  
Sequence coverage: 89 %
  
E-value: 9e-111
  
  
 NCBI BlastP on this gene

EIM92619

hypothetical protein
  
Accession: EIM92618
  
Location: 2781206-2782268
  
 NCBI BlastP on this gene

EIM92618

hypothetical protein
  
Accession: EIM92617
  
Location: 2778774-2780852
  
 NCBI BlastP on this gene

EIM92617

hypothetical protein
  
Accession: EIM92616
  
Location: 2775838-2776332
  
 NCBI BlastP on this gene

EIM92616

hypothetical protein
  
Accession: EIM92615
  
Location: 2774876-2775437
  
 NCBI BlastP on this gene

EIM92615

nucleoporin
  
Accession: EIM92614
  
Location: 2769287-2774477
  
 NCBI BlastP on this gene

EIM92614

166. :  FO082046 Pichia sorbitophila strain CBS 7064 chromosome N complete sequence.     Total score: 1.0     Cumulative Blast bit score: 357

not annotated
  
Accession: CCE87355
  
Location: 2098250-2100106
  
  
**BlastP hit with Mycgr3G70577\_Mycgr3T**
  
Percentage identity: 34 %
  
BlastP bit score: 357
  
Sequence coverage: 96 %
  
E-value: 1e-110
  
  
 NCBI BlastP on this gene

Piso0\_005905

not annotated
  
Accession: CCE87354
  
Location: 2096079-2097554
  
 NCBI BlastP on this gene

Piso0\_005904

not annotated
  
Accession: CCE87353
  
Location: 2093933-2095165
  
 NCBI BlastP on this gene

Piso0\_005903

not annotated
  
Accession: CCE87352
  
Location: 2092569-2093423
  
 NCBI BlastP on this gene

Piso0\_005902

not annotated
  
Accession: CCE87351
  
Location: 2091331-2091957
  
 NCBI BlastP on this gene

Piso0\_005901

not annotated
  
Accession: CCE87350
  
Location: 2089292-2090656
  
 NCBI BlastP on this gene

Piso0\_005900

not annotated
  
Accession: CCE87349
  
Location: 2084337-2088590
  
 NCBI BlastP on this gene

Piso0\_005899

167. :  GL698717 Metarhizium anisopliae ARSEF 23 unplaced genomic scaffold Scf\_007     Total score: 1.0     Cumulative Blast bit score: 355

MFS quinate transporter
  
Accession: EFY99408
  
Location: 1551042-1553021
  
 NCBI BlastP on this gene

EFY99408

DNA kinase/phosphatase Pnk1
  
Accession: EFY99409
  
Location: 1555789-1557298
  
 NCBI BlastP on this gene

EFY99409

hypothetical protein
  
Accession: EFY99410
  
Location: 1557753-1558650
  
 NCBI BlastP on this gene

EFY99410

siderophore iron transporter
  
Accession: EFY99411
  
Location: 1559459-1561228
  
  
**BlastP hit with Mycgr3G70577\_Mycgr3T**
  
Percentage identity: 27 %
  
BlastP bit score: 177
  
Sequence coverage: 85 %
  
E-value: 1e-44
  
  
 NCBI BlastP on this gene

EFY99411

transferase family protein
  
Accession: EFY99412
  
Location: 1561941-1567139
  
 NCBI BlastP on this gene

EFY99412

hypothetical protein
  
Accession: EFY99413
  
Location: 1567963-1569462
  
 NCBI BlastP on this gene

EFY99413

lipase
  
Accession: EFY99414
  
Location: 1570166-1571175
  
 NCBI BlastP on this gene

EFY99414

siderophore iron transporter mirB
  
Accession: EFY99415
  
Location: 1571989-1573758
  
  
**BlastP hit with Mycgr3G70577\_Mycgr3T**
  
Percentage identity: 26 %
  
BlastP bit score: 178
  
Sequence coverage: 91 %
  
E-value: 7e-45
  
  
 NCBI BlastP on this gene

EFY99415

hypothetical protein
  
Accession: EFY99416
  
Location: 1574564-1575069
  
 NCBI BlastP on this gene

EFY99416

polysaccharide synthase
  
Accession: EFY99417
  
Location: 1578044-1579607
  
 NCBI BlastP on this gene

EFY99417

168. :  AM920435 Penicillium chrysogenum Wisconsin 54-1255 complete genome, contig Pc00c20.     Total score: 1.0     Cumulative Blast bit score: 355

unnamed
  
Accession: CAP86901
  
Location: 3636003-3637630
  
  
**BlastP hit with Mycgr3G99766\_Mycgr3T**
  
Percentage identity: 38 %
  
BlastP bit score: 355
  
Sequence coverage: 101 %
  
E-value: 6e-113
  
  
 NCBI BlastP on this gene

Pc20g15720

not annotated
  
Accession: CAP86900
  
Location: 3634129-3635630
  
 NCBI BlastP on this gene

Pc20g15710

hypothetical protein
  
Accession: CAP86899
  
Location: 3633480-3633931
  
 NCBI BlastP on this gene

Pc20g15700

not annotated
  
Accession: CAP86898
  
Location: 3631692-3633230
  
 NCBI BlastP on this gene

Pc20g15690

hypothetical protein
  
Accession: CAP86897
  
Location: 3631158-3631618
  
 NCBI BlastP on this gene

Pc20g15680

not annotated
  
Accession: CAP86896
  
Location: 3630500-3631075
  
 NCBI BlastP on this gene

Pc20g15670

not annotated
  
Accession: CAP86895
  
Location: 3628441-3629957
  
 NCBI BlastP on this gene

Pc20g15660

not annotated
  
Accession: CAP86894
  
Location: 3627406-3627922
  
 NCBI BlastP on this gene

Pc20g15650

not annotated
  
Accession: CAP86893
  
Location: 3625722-3626996
  
 NCBI BlastP on this gene

Pc20g15640

not annotated
  
Accession: CAP86892
  
Location: 3623772-3625421
  
 NCBI BlastP on this gene

Pc20g15630

169. :  AM920435 Penicillium chrysogenum Wisconsin 54-1255 complete genome, contig Pc00c20.     Total score: 1.0     Cumulative Blast bit score: 354

not annotated
  
Accession: CAP86482
  
Location: 2649783-2654315
  
 NCBI BlastP on this gene

Pc20g11530

not annotated
  
Accession: CAP86481
  
Location: 2647440-2649058
  
 NCBI BlastP on this gene

Pc20g11520

hypothetical protein
  
Accession: CAP86480
  
Location: 2646457-2646924
  
 NCBI BlastP on this gene

Pc20g11510

hypothetical protein
  
Accession: CAP86479
  
Location: 2644984-2646036
  
 NCBI BlastP on this gene

Pc20g11500

hypothetical protein
  
Accession: CAP86478
  
Location: 2643442-2643922
  
 NCBI BlastP on this gene

Pc20g11490

hypothetical protein
  
Accession: CAP86477
  
Location: 2639691-2641573
  
 NCBI BlastP on this gene

Pc20g11480

not annotated
  
Accession: CAP86476
  
Location: 2639044-2639430
  
 NCBI BlastP on this gene

Pc20g11470

unnamed
  
Accession: CAP86475
  
Location: 2635856-2637515
  
  
**BlastP hit with Mycgr3G99766\_Mycgr3T**
  
Percentage identity: 39 %
  
BlastP bit score: 354
  
Sequence coverage: 100 %
  
E-value: 2e-112
  
  
 NCBI BlastP on this gene

Pc20g11460

not annotated
  
Accession: CAP86474
  
Location: 2634401-2635120
  
 NCBI BlastP on this gene

Pc20g11450

not annotated
  
Accession: CAP86473
  
Location: 2631774-2633619
  
 NCBI BlastP on this gene

Pc20g11440

unnamed
  
Accession: CAP86472
  
Location: 2629170-2631293
  
 NCBI BlastP on this gene

Pc20g11430

not annotated
  
Accession: CAP86471
  
Location: 2627322-2627807
  
 NCBI BlastP on this gene

Pc20g11420

not annotated
  
Accession: CAP86470
  
Location: 2626138-2626644
  
 NCBI BlastP on this gene

Pc20g11410

not annotated
  
Accession: CAP86469
  
Location: 2624630-2625164
  
 NCBI BlastP on this gene

Pc20g11400

not annotated
  
Accession: CAP86468
  
Location: 2621432-2624030
  
 NCBI BlastP on this gene

Pc20g11390

170. :  EQ962652 Talaromyces stipitatus ATCC 10500 scf\_1105507295523 genomic scaffold     Total score: 1.0     Cumulative Blast bit score: 352

efflux pump antibiotic resistance protein, putative
  
Accession: EED23932
  
Location: 3777978-3779911
  
 NCBI BlastP on this gene

EED23932

sugar transport protein, putative
  
Accession: EED23931
  
Location: 3774187-3775986
  
 NCBI BlastP on this gene

EED23931

conserved hypothetical protein
  
Accession: EED23930
  
Location: 3769869-3771264
  
 NCBI BlastP on this gene

EED23930

sugar transporter, putative
  
Accession: EED23929
  
Location: 3767544-3769166
  
  
**BlastP hit with Mycgr3G99766\_Mycgr3T**
  
Percentage identity: 40 %
  
BlastP bit score: 352
  
Sequence coverage: 99 %
  
E-value: 9e-112
  
  
 NCBI BlastP on this gene

EED23929

hypothetical protein
  
Accession: EED23928
  
Location: 3762763-3763970
  
 NCBI BlastP on this gene

EED23928

C6 transcription factor, putative
  
Accession: EED23927
  
Location: 3757544-3760440
  
 NCBI BlastP on this gene

EED23927

conserved hypothetical protein
  
Accession: EED23926
  
Location: 3755941-3756477
  
 NCBI BlastP on this gene

EED23926

hypothetical protein
  
Accession: EED23925
  
Location: 3755191-3755544
  
 NCBI BlastP on this gene

EED23925

171. :  DS027045 Aspergillus clavatus NRRL 1 1099423829791 genomic scaffold     Total score: 1.0     Cumulative Blast bit score: 350

monooxygenase
  
Accession: EAW14397
  
Location: 1996936-1999050
  
 NCBI BlastP on this gene

EAW14397

FluG family protein
  
Accession: EAW14398
  
Location: 2000261-2001607
  
 NCBI BlastP on this gene

EAW14398

conserved hypothetical protein
  
Accession: EAW14399
  
Location: 2002166-2004259
  
 NCBI BlastP on this gene

EAW14399

MFS sugar transporter, putative
  
Accession: EAW14400
  
Location: 2006489-2008113
  
  
**BlastP hit with Mycgr3G99766\_Mycgr3T**
  
Percentage identity: 40 %
  
BlastP bit score: 350
  
Sequence coverage: 97 %
  
E-value: 8e-111
  
  
 NCBI BlastP on this gene

EAW14400

172. :  CH408159 Pichia guilliermondii ATCC 6260 scaffold\_5 genomic scaffold     Total score: 1.0     Cumulative Blast bit score: 346

hypothetical protein
  
Accession: EDK39910
  
Location: 105841-108252
  
 NCBI BlastP on this gene

EDK39910

hypothetical protein
  
Accession: EDK39909
  
Location: 103974-105686
  
 NCBI BlastP on this gene

EDK39909

hypothetical protein
  
Accession: EDK39908
  
Location: 102049-103743
  
 NCBI BlastP on this gene

EDK39908

hypothetical protein
  
Accession: EDK39907
  
Location: 99208-100701
  
 NCBI BlastP on this gene

EDK39907

hypothetical protein
  
Accession: EDK39906
  
Location: 97764-98801
  
 NCBI BlastP on this gene

EDK39906

hypothetical protein
  
Accession: EDK39905
  
Location: 95859-97580
  
 NCBI BlastP on this gene

EDK39905

hypothetical protein
  
Accession: EDK39904
  
Location: 92774-94687
  
  
**BlastP hit with Mycgr3G70577\_Mycgr3T**
  
Percentage identity: 34 %
  
BlastP bit score: 346
  
Sequence coverage: 97 %
  
E-value: 4e-106
  
  
 NCBI BlastP on this gene

EDK39904

hypothetical protein
  
Accession: EDK39903
  
Location: 90884-92603
  
 NCBI BlastP on this gene

EDK39903

hypothetical protein
  
Accession: EDK39902
  
Location: 88174-90366
  
 NCBI BlastP on this gene

EDK39902

hypothetical protein
  
Accession: EDK39901
  
Location: 86654-87304
  
 NCBI BlastP on this gene

EDK39901

hypothetical protein
  
Accession: EDK39900
  
Location: 85520-86188
  
 NCBI BlastP on this gene

EDK39900

conserved hypothetical protein
  
Accession: EDK39899
  
Location: 84128-85051
  
 NCBI BlastP on this gene

EDK39899

hypothetical protein
  
Accession: EDK39898
  
Location: 82139-83713
  
 NCBI BlastP on this gene

EDK39898

hypothetical protein
  
Accession: EDK39897
  
Location: 81553-82116
  
 NCBI BlastP on this gene

EDK39897

hypothetical protein
  
Accession: EDK39896
  
Location: 80503-81465
  
 NCBI BlastP on this gene

EDK39896

hypothetical protein
  
Accession: EDK39895
  
Location: 79069-80496
  
 NCBI BlastP on this gene

EDK39895

173. :  AM920437 Penicillium chrysogenum Wisconsin 54-1255 complete genome, contig Pc00c22.     Total score: 1.0     Cumulative Blast bit score: 345

not annotated
  
Accession: CAP98041
  
Location: 1795331-1796949
  
 NCBI BlastP on this gene

Pc22g07530

not annotated
  
Accession: CAP98040
  
Location: 1793184-1794923
  
 NCBI BlastP on this gene

Pc22g07520

not annotated
  
Accession: CAP98039
  
Location: 1790995-1792944
  
 NCBI BlastP on this gene

Pc22g07510

not annotated
  
Accession: CAP98038
  
Location: 1787213-1789051
  
 NCBI BlastP on this gene

Pc22g07500

not annotated
  
Accession: CAP98037
  
Location: 1785343-1786783
  
 NCBI BlastP on this gene

Pc22g07490

unnamed
  
Accession: CAP98036
  
Location: 1783530-1784858
  
 NCBI BlastP on this gene

Pc22g07480

unnamed
  
Accession: CAP98035
  
Location: 1780777-1782400
  
  
**BlastP hit with Mycgr3G99766\_Mycgr3T**
  
Percentage identity: 40 %
  
BlastP bit score: 345
  
Sequence coverage: 95 %
  
E-value: 3e-109
  
  
 NCBI BlastP on this gene

Pc22g07470

not annotated
  
Accession: CAP98034
  
Location: 1780185-1780540
  
 NCBI BlastP on this gene

Pc22g07460

not annotated
  
Accession: CAP98033
  
Location: 1778951-1779885
  
 NCBI BlastP on this gene

Pc22g07450

unnamed
  
Accession: CAP98032
  
Location: 1776206-1777834
  
 NCBI BlastP on this gene

Pc22g07440

not annotated
  
Accession: Pc22g07430
  
Location: 1774876-1775287
  
 NCBI BlastP on this gene

Pc22g07430

not annotated
  
Accession: CAP98030
  
Location: 1773389-1773888
  
 NCBI BlastP on this gene

Pc22g07420

not annotated
  
Accession: CAP98029
  
Location: 1771653-1772730
  
 NCBI BlastP on this gene

Pc22g07410

not annotated
  
Accession: CAP98028
  
Location: 1770400-1771341
  
 NCBI BlastP on this gene

Pc22g07400

not annotated
  
Accession: Pc22g07390
  
Location: 1767456-1768971
  
 NCBI BlastP on this gene

Pc22g07390

174. :  CH408159 Pichia guilliermondii ATCC 6260 scaffold\_5 genomic scaffold     Total score: 1.0     Cumulative Blast bit score: 344

hypothetical protein
  
Accession: EDK40415
  
Location: 1047585-1049444
  
  
**BlastP hit with Mycgr3G70577\_Mycgr3T**
  
Percentage identity: 35 %
  
BlastP bit score: 344
  
Sequence coverage: 89 %
  
E-value: 1e-105
  
  
 NCBI BlastP on this gene

EDK40415

hypothetical protein
  
Accession: EDK40414
  
Location: 1046222-1047304
  
 NCBI BlastP on this gene

EDK40414

hypothetical protein
  
Accession: EDK40413
  
Location: 1045691-1046170
  
 NCBI BlastP on this gene

EDK40413

predicted protein
  
Accession: EDK40412
  
Location: 1044542-1045432
  
 NCBI BlastP on this gene

EDK40412

hypothetical protein
  
Accession: EDK40411
  
Location: 1042505-1044325
  
 NCBI BlastP on this gene

EDK40411

hypothetical protein
  
Accession: EDK40410
  
Location: 1040553-1042274
  
 NCBI BlastP on this gene

EDK40410

hypothetical protein
  
Accession: EDK40409
  
Location: 1038654-1040336
  
 NCBI BlastP on this gene

EDK40409

hypothetical protein
  
Accession: EDK40408
  
Location: 1037680-1038207
  
 NCBI BlastP on this gene

EDK40408

hypothetical protein
  
Accession: EDK40407
  
Location: 1036668-1037609
  
 NCBI BlastP on this gene

EDK40407

hypothetical protein
  
Accession: EDK40406
  
Location: 1034895-1036031
  
 NCBI BlastP on this gene

EDK40406

175. :  AM920437 Penicillium chrysogenum Wisconsin 54-1255 complete genome, contig Pc00c22.     Total score: 1.0     Cumulative Blast bit score: 344

not annotated
  
Accession: CAP99785
  
Location: 5899683-5900569
  
 NCBI BlastP on this gene

Pc22g24970

not annotated
  
Accession: CAP99786
  
Location: 5901044-5901616
  
 NCBI BlastP on this gene

Pc22g24980

not annotated
  
Accession: CAP99787
  
Location: 5901681-5902849
  
 NCBI BlastP on this gene

Pc22g24990

not annotated
  
Accession: Pc22g25000
  
Location: 5903111-5903950
  
 NCBI BlastP on this gene

Pc22g25000

not annotated
  
Accession: CAP99789
  
Location: 5905767-5906947
  
 NCBI BlastP on this gene

Pc22g25010

not annotated
  
Accession: CAP99790
  
Location: 5907439-5909551
  
 NCBI BlastP on this gene

Pc22g25020

not annotated
  
Accession: CAP99791
  
Location: 5910726-5912610
  
 NCBI BlastP on this gene

Pc22g25030

unnamed
  
Accession: CAP99792
  
Location: 5913418-5915017
  
  
**BlastP hit with Mycgr3G99766\_Mycgr3T**
  
Percentage identity: 40 %
  
BlastP bit score: 344
  
Sequence coverage: 96 %
  
E-value: 9e-109
  
  
 NCBI BlastP on this gene

Pc22g25040

176. :  CR382134 Debaryomyces hansenii CBS767 chromosome B complete sequence.     Total score: 1.0     Cumulative Blast bit score: 342

DEHA2B16368p
  
Accession: CAG85677
  
Location: 1293843-1295081
  
 NCBI BlastP on this gene

DEHA2B16368g

DEHA2B16390p
  
Accession: CAG85678
  
Location: 1295685-1296524
  
 NCBI BlastP on this gene

DEHA2B16390g

DEHA2B16412p
  
Accession: CAG85679
  
Location: 1297723-1298430
  
 NCBI BlastP on this gene

DEHA2B16412g

DEHA2B16434p
  
Accession: CAG85680
  
Location: 1299298-1300329
  
 NCBI BlastP on this gene

DEHA2B16434g

DEHA2B16456p
  
Accession: CAG85681
  
Location: 1301169-1303721
  
 NCBI BlastP on this gene

DEHA2B16456g

DEHA2B16478p
  
Accession: CAG85682
  
Location: 1305810-1307675
  
  
**BlastP hit with Mycgr3G70577\_Mycgr3T**
  
Percentage identity: 33 %
  
BlastP bit score: 342
  
Sequence coverage: 95 %
  
E-value: 7e-105
  
  
 NCBI BlastP on this gene

DEHA2B16478g

177. :  JH126400 Cordyceps militaris CM01 unplaced genomic scaffold CCM\_S00002     Total score: 1.0     Cumulative Blast bit score: 340

hypothetical protein
  
Accession: EGX93824
  
Location: 472291-472739
  
 NCBI BlastP on this gene

EGX93824

heat shock protein DnaJ domain protein
  
Accession: EGX93825
  
Location: 473664-474442
  
 NCBI BlastP on this gene

EGX93825

Aminoglycoside phosphotransferase
  
Accession: EGX93826
  
Location: 478578-479558
  
 NCBI BlastP on this gene

EGX93826

reticulon-4-interacting protein 1
  
Accession: EGX93827
  
Location: 479937-480950
  
 NCBI BlastP on this gene

EGX93827

phosphorylcholine phosphatase
  
Accession: EGX93828
  
Location: 482303-483406
  
 NCBI BlastP on this gene

EGX93828

MFS sugar transporter, putative
  
Accession: EGX93829
  
Location: 485013-486619
  
  
**BlastP hit with Mycgr3G99766\_Mycgr3T**
  
Percentage identity: 40 %
  
BlastP bit score: 340
  
Sequence coverage: 101 %
  
E-value: 6e-107
  
  
 NCBI BlastP on this gene

EGX93829

integral membrane protein, putative
  
Accession: EGX93830
  
Location: 487078-488423
  
 NCBI BlastP on this gene

EGX93830

Peptidoglycan-binding lysin domain
  
Accession: EGX93831
  
Location: 499236-501353
  
 NCBI BlastP on this gene

EGX93831

178. :  HF679023 Fusarium fujikuroi IMI 58289 draft genome, chromosome FFUJ\_chr01.     Total score: 1.0     Cumulative Blast bit score: 340

uncharacterized protein
  
Accession: CCT61540
  
Location: 298699-301317
  
 NCBI BlastP on this gene

FFUJ\_01981

related to 6-hydroxy-D-nicotine oxidase
  
Accession: CCT61539
  
Location: 296326-298110
  
 NCBI BlastP on this gene

FFUJ\_01982

related to acyl-CoA thiolesterase
  
Accession: CCT61538
  
Location: 292882-294289
  
 NCBI BlastP on this gene

FFUJ\_01983

uncharacterized protein
  
Accession: CCT61537
  
Location: 291898-292677
  
 NCBI BlastP on this gene

FFUJ\_01984

putative trichothecene biosynthesis gene
  
Accession: CCT61536
  
Location: 288238-289373
  
 NCBI BlastP on this gene

FFUJ\_01985

related to immune-responsive protein 1
  
Accession: CCT61535
  
Location: 286343-287830
  
 NCBI BlastP on this gene

FFUJ\_01986

related to sugar transporter
  
Accession: CCT61534
  
Location: 284028-285884
  
  
**BlastP hit with Mycgr3G99766\_Mycgr3T**
  
Percentage identity: 40 %
  
BlastP bit score: 340
  
Sequence coverage: 95 %
  
E-value: 4e-107
  
  
 NCBI BlastP on this gene

FFUJ\_01987

related to transcription activator protein acu-15
  
Accession: CCT61533
  
Location: 281409-283025
  
 NCBI BlastP on this gene

FFUJ\_01988

related to isoamyl alcohol oxidase
  
Accession: CCT61532
  
Location: 279138-281261
  
 NCBI BlastP on this gene

FFUJ\_01989

uncharacterized protein
  
Accession: CCT61531
  
Location: 278362-278742
  
 NCBI BlastP on this gene

FFUJ\_01990

uncharacterized protein
  
Accession: CCT61530
  
Location: 275776-276181
  
 NCBI BlastP on this gene

FFUJ\_01991

related to hydrophobin
  
Accession: CCT61529
  
Location: 273869-274467
  
 NCBI BlastP on this gene

FFUJ\_01992

179. :  JH711791 Trametes versicolor FP-101664 SS1 unplaced genomic scaffold TRAVEscaffold\_9     Total score: 1.0     Cumulative Blast bit score: 338

cyclophilin
  
Accession: EIW56229
  
Location: 2659821-2660681
  
 NCBI BlastP on this gene

EIW56229

hypothetical protein
  
Accession: EIW56228
  
Location: 2658923-2659495
  
 NCBI BlastP on this gene

EIW56228

hypothetical protein
  
Accession: EIW56227
  
Location: 2648598-2649859
  
 NCBI BlastP on this gene

EIW56227

drug:h+ antiporter
  
Accession: EIW56226
  
Location: 2645243-2647513
  
  
**BlastP hit with Mycgr3G70577\_Mycgr3T**
  
Percentage identity: 34 %
  
BlastP bit score: 338
  
Sequence coverage: 95 %
  
E-value: 1e-103
  
  
 NCBI BlastP on this gene

EIW56226

hypothetical protein
  
Accession: EIW56225
  
Location: 2643534-2644707
  
 NCBI BlastP on this gene

EIW56225

hypothetical protein
  
Accession: EIW56224
  
Location: 2641286-2642488
  
 NCBI BlastP on this gene

EIW56224

hypothetical protein
  
Accession: EIW56223
  
Location: 2639002-2640008
  
 NCBI BlastP on this gene

EIW56223

hypothetical protein
  
Accession: EIW56222
  
Location: 2636583-2637007
  
 NCBI BlastP on this gene

EIW56222

hypothetical protein
  
Accession: EIW56221
  
Location: 2634661-2636059
  
 NCBI BlastP on this gene

EIW56221

180. :  AKCT01000319 Penicillium digitatum PHI26     Total score: 1.0     Cumulative Blast bit score: 335

hypothetical protein
  
Accession: EKV04878
  
Location: 183161-183910
  
 NCBI BlastP on this gene

EKV04878

hypothetical protein
  
Accession: EKV04879
  
Location: 186242-188182
  
 NCBI BlastP on this gene

EKV04879

hypothetical protein
  
Accession: EKV04880
  
Location: 190790-192619
  
 NCBI BlastP on this gene

EKV04880

LPS glycosyltransferase, putative
  
Accession: EKV04881
  
Location: 193510-194845
  
 NCBI BlastP on this gene

EKV04881

Sugar transporter, putative
  
Accession: EKV04882
  
Location: 196015-197635
  
  
**BlastP hit with Mycgr3G99766\_Mycgr3T**
  
Percentage identity: 38 %
  
BlastP bit score: 335
  
Sequence coverage: 95 %
  
E-value: 5e-105
  
  
 NCBI BlastP on this gene

EKV04882

181. :  JH711584 Coniophora puteana RWD-64-598 SS2 unplaced genomic scaffold CONPUscaffold\_12     Total score: 1.0     Cumulative Blast bit score: 330

hypothetical protein
  
Accession: EIW77301
  
Location: 1073238-1073693
  
 NCBI BlastP on this gene

EIW77301

hypothetical protein
  
Accession: EIW77302
  
Location: 1075016-1076090
  
  
**BlastP hit with Mycgr3G85486\_Mycgr3T**
  
Percentage identity: 38 %
  
BlastP bit score: 170
  
Sequence coverage: 64 %
  
E-value: 4e-46
  
  
 NCBI BlastP on this gene

EIW77302

hypothetical protein
  
Accession: EIW77303
  
Location: 1077361-1079823
  
 NCBI BlastP on this gene

EIW77303

hypothetical protein
  
Accession: EIW77304
  
Location: 1081857-1083395
  
 NCBI BlastP on this gene

EIW77304

hypothetical protein
  
Accession: EIW77305
  
Location: 1083502-1084306
  
 NCBI BlastP on this gene

EIW77305

DnaJ-domain-containing protein
  
Accession: EIW77306
  
Location: 1085968-1087430
  
 NCBI BlastP on this gene

EIW77306

amidase signature enzyme
  
Accession: EIW77307
  
Location: 1087807-1089808
  
 NCBI BlastP on this gene

EIW77307

hypothetical protein
  
Accession: EIW77308
  
Location: 1092875-1093636
  
 NCBI BlastP on this gene

EIW77308

hypothetical protein
  
Accession: EIW77309
  
Location: 1094598-1095876
  
 NCBI BlastP on this gene

EIW77309

hypothetical protein
  
Accession: EIW77310
  
Location: 1096826-1097905
  
  
**BlastP hit with Mycgr3G85486\_Mycgr3T**
  
Percentage identity: 37 %
  
BlastP bit score: 160
  
Sequence coverage: 64 %
  
E-value: 2e-42
  
  
 NCBI BlastP on this gene

EIW77310

hypothetical protein
  
Accession: EIW77311
  
Location: 1098782-1099543
  
 NCBI BlastP on this gene

EIW77311

182. :  AM920428 Penicillium chrysogenum Wisconsin 54-1255 complete genome, contig Pc00c13.     Total score: 1.0     Cumulative Blast bit score: 330

not annotated
  
Accession: CAP91199
  
Location: 300926-301906
  
 NCBI BlastP on this gene

Pc13g01300

unnamed
  
Accession: CAP91200
  
Location: 304368-305837
  
 NCBI BlastP on this gene

Pc13g01310

hypothetical protein
  
Accession: CAP91201
  
Location: 308505-309759
  
 NCBI BlastP on this gene

Pc13g01320

hypothetical protein
  
Accession: CAP91202
  
Location: 309937-310554
  
 NCBI BlastP on this gene

Pc13g01330

not annotated
  
Accession: CAP91203
  
Location: 311236-312720
  
 NCBI BlastP on this gene

Pc13g01340

unnamed
  
Accession: CAP91204
  
Location: 313975-315646
  
  
**BlastP hit with Mycgr3G99766\_Mycgr3T**
  
Percentage identity: 39 %
  
BlastP bit score: 330
  
Sequence coverage: 97 %
  
E-value: 4e-103
  
  
 NCBI BlastP on this gene

Pc13g01350

not annotated
  
Accession: CAP91205
  
Location: 316096-316996
  
 NCBI BlastP on this gene

Pc13g01360

not annotated
  
Accession: CAP91206
  
Location: 317309-319209
  
 NCBI BlastP on this gene

Pc13g01370

unnamed
  
Accession: CAP91207
  
Location: 319798-321173
  
 NCBI BlastP on this gene

Pc13g01380

not annotated
  
Accession: CAP91208
  
Location: 321349-323840
  
 NCBI BlastP on this gene

Pc13g01390

not annotated
  
Accession: CAP91209
  
Location: 324242-325753
  
 NCBI BlastP on this gene

Pc13g01400

hypothetical protein
  
Accession: CAP91210
  
Location: 326589-328657
  
 NCBI BlastP on this gene

Pc13g01410

183. :  JH711791 Trametes versicolor FP-101664 SS1 unplaced genomic scaffold TRAVEscaffold\_9     Total score: 1.0     Cumulative Blast bit score: 328

drug:h+ antiporter
  
Accession: EIW56249
  
Location: 2710202-2712929
  
  
**BlastP hit with Mycgr3G70577\_Mycgr3T**
  
Percentage identity: 36 %
  
BlastP bit score: 328
  
Sequence coverage: 91 %
  
E-value: 3e-99
  
  
 NCBI BlastP on this gene

EIW56249

hypothetical protein
  
Accession: EIW56248
  
Location: 2707060-2708206
  
 NCBI BlastP on this gene

EIW56248

hypothetical protein
  
Accession: EIW56247
  
Location: 2705868-2706354
  
 NCBI BlastP on this gene

EIW56247

hypothetical protein
  
Accession: EIW56246
  
Location: 2703745-2704927
  
 NCBI BlastP on this gene

EIW56246

hypothetical protein
  
Accession: EIW56245
  
Location: 2701797-2702966
  
 NCBI BlastP on this gene

EIW56245

hypothetical protein
  
Accession: EIW56244
  
Location: 2698916-2701051
  
 NCBI BlastP on this gene

EIW56244

hypothetical protein
  
Accession: EIW56243
  
Location: 2697043-2698344
  
 NCBI BlastP on this gene

EIW56243

184. :  EQ962652 Talaromyces stipitatus ATCC 10500 scf\_1105507295523 genomic scaffold     Total score: 1.0     Cumulative Blast bit score: 327

sugar transporter, putative
  
Accession: EED24029
  
Location: 3998358-4000021
  
  
**BlastP hit with Mycgr3G99766\_Mycgr3T**
  
Percentage identity: 37 %
  
BlastP bit score: 327
  
Sequence coverage: 101 %
  
E-value: 6e-102
  
  
 NCBI BlastP on this gene

EED24029

hydrolase, putative
  
Accession: EED24028
  
Location: 3996601-3997819
  
 NCBI BlastP on this gene

EED24028

feruloyl esterase, putative
  
Accession: EED24027
  
Location: 3994146-3995714
  
 NCBI BlastP on this gene

EED24027

conserved hypothetical protein
  
Accession: EED24026
  
Location: 3992783-3993986
  
 NCBI BlastP on this gene

EED24026

histone transcription regulator Hir1, putative
  
Accession: EED24023
  
Location: 3988492-3991925
  
 NCBI BlastP on this gene

EED24023

stomatin family protein
  
Accession: EED24022
  
Location: 3986326-3987703
  
 NCBI BlastP on this gene

EED24022

185. :  GL377310 Schizophyllum commune H4-8 unplaced genomic scaffold SCHCOscaffold\_9     Total score: 1.0     Cumulative Blast bit score: 325

hypothetical protein
  
Accession: EFI93791
  
Location: 1019678-1021035
  
 NCBI BlastP on this gene

EFI93791

hypothetical protein
  
Accession: EFI93792
  
Location: 1022816-1024867
  
 NCBI BlastP on this gene

EFI93792

expressed protein
  
Accession: EFI94100
  
Location: 1025721-1026065
  
 NCBI BlastP on this gene

EFI94100

hypothetical protein
  
Accession: EFI94101
  
Location: 1027388-1028963
  
 NCBI BlastP on this gene

EFI94101

expressed protein
  
Accession: EFI94102
  
Location: 1029628-1030623
  
 NCBI BlastP on this gene

EFI94102

hypothetical protein
  
Accession: EFI93793
  
Location: 1033381-1035772
  
  
**BlastP hit with Mycgr3G70577\_Mycgr3T**
  
Percentage identity: 34 %
  
BlastP bit score: 325
  
Sequence coverage: 97 %
  
E-value: 7e-98
  
  
 NCBI BlastP on this gene

EFI93793

186. :  KE145363 Glarea lozoyensis ATCC 20868 chromosome Unknown GLAREA2     Total score: 1.0     Cumulative Blast bit score: 315

MFS general substrate transporter
  
Accession: EPE30675
  
Location: 941112-943170
  
  
**BlastP hit with Mycgr3G99766\_Mycgr3T**
  
Percentage identity: 36 %
  
BlastP bit score: 315
  
Sequence coverage: 97 %
  
E-value: 5e-97
  
  
 NCBI BlastP on this gene

EPE30675

Protein kinase-like (PK-like)
  
Accession: EPE30674
  
Location: 936962-938802
  
 NCBI BlastP on this gene

EPE30674

MFS general substrate transporter
  
Accession: EPE30673
  
Location: 930741-932532
  
 NCBI BlastP on this gene

EPE30673

(Trans)glycosidase
  
Accession: EPE30672
  
Location: 927296-929659
  
 NCBI BlastP on this gene

EPE30672

187. :  FP929130 Leptosphaeria maculans JN3 lm\_SuperContig\_17\_v2 genomic supercontig     Total score: 1.0     Cumulative Blast bit score: 314

similar to cupin domain containing protein
  
Accession: CBX96946
  
Location: 735678-738627
  
 NCBI BlastP on this gene

LEMA\_P100770.1

similar to zinc knuckle domain-containing protein
  
Accession: CBX96947
  
Location: 739621-740570
  
 NCBI BlastP on this gene

LEMA\_P100780.1

hypothetical protein
  
Accession: CBX96948
  
Location: 742152-743578
  
 NCBI BlastP on this gene

LEMA\_P100790.1

similar to glycoside hydrolase family 61 protein
  
Accession: CBX96949
  
Location: 744605-745722
  
 NCBI BlastP on this gene

LEMA\_P100800.1

predicted protein
  
Accession: CBX96950
  
Location: 745967-746662
  
 NCBI BlastP on this gene

LEMA\_P100810.1

similar to MFS sugar transporter
  
Accession: CBX96951
  
Location: 747978-749906
  
  
**BlastP hit with Mycgr3G99766\_Mycgr3T**
  
Percentage identity: 37 %
  
BlastP bit score: 314
  
Sequence coverage: 96 %
  
E-value: 2e-96
  
  
 NCBI BlastP on this gene

LEMA\_P100820.1

predicted protein
  
Accession: CBX96952
  
Location: 752482-752873
  
 NCBI BlastP on this gene

LEMA\_P100830.1

similar to NADH:flavin oxidoreductase/NADH oxidase
  
Accession: CBX96953
  
Location: 755345-756694
  
 NCBI BlastP on this gene

LEMA\_P100840.1

hypothetical protein
  
Accession: CBX96954
  
Location: 760153-761272
  
 NCBI BlastP on this gene

LEMA\_P100850.1

188. :  DS499598 Aspergillus fumigatus A1163 scf\_000005 genomic scaffold     Total score: 1.0     Cumulative Blast bit score: 313

clathrin-coated vesicle protein, putative
  
Accession: EDP50657
  
Location: 1976309-1977029
  
 NCBI BlastP on this gene

EDP50657

conserved hypothetical protein
  
Accession: EDP50658
  
Location: 1978597-1980180
  
 NCBI BlastP on this gene

EDP50658

zinc knuckle transcription factor (CnjB), putative
  
Accession: EDP50659
  
Location: 1981175-1983451
  
 NCBI BlastP on this gene

EDP50659

alpha/beta hydrolase, putative
  
Accession: EDP50660
  
Location: 1984139-1985107
  
 NCBI BlastP on this gene

EDP50660

MFS monosaccharide transporter, putative
  
Accession: EDP50661
  
Location: 1989997-1991903
  
  
**BlastP hit with Mycgr3G99766\_Mycgr3T**
  
Percentage identity: 35 %
  
BlastP bit score: 313
  
Sequence coverage: 97 %
  
E-value: 4e-96
  
  
 NCBI BlastP on this gene

EDP50661

189. :  AAHF01000005 Aspergillus fumigatus Af293     Total score: 1.0     Cumulative Blast bit score: 313

citrate lyase beta subunit, putative
  
Accession: EAL89490
  
Location: 512004-513233
  
 NCBI BlastP on this gene

EAL89490

clathrin-coated vesicle protein, putative
  
Accession: EAL89489
  
Location: 510795-511515
  
 NCBI BlastP on this gene

EAL89489

conserved hypothetical protein
  
Accession: EAL89488
  
Location: 507627-509227
  
 NCBI BlastP on this gene

EAL89488

zinc knuckle transcription factor (CnjB), putative
  
Accession: EAL89487
  
Location: 504356-506632
  
 NCBI BlastP on this gene

EAL89487

alpha/beta hydrolase, putative
  
Accession: EAL89486
  
Location: 502622-503461
  
 NCBI BlastP on this gene

EAL89486

MFS monosaccharide transporter, putative
  
Accession: EAL89485
  
Location: 497930-499836
  
  
**BlastP hit with Mycgr3G99766\_Mycgr3T**
  
Percentage identity: 35 %
  
BlastP bit score: 313
  
Sequence coverage: 97 %
  
E-value: 4e-96
  
  
 NCBI BlastP on this gene

EAL89485

MFS transporter, putative
  
Accession: EAL89484
  
Location: 494692-496502
  
 NCBI BlastP on this gene

EAL89484

serine/proline-rich protein
  
Accession: EAL89482
  
Location: 493650-494234
  
 NCBI BlastP on this gene

EAL89482

glutamine synthetase
  
Accession: EAL89481
  
Location: 490843-492547
  
 NCBI BlastP on this gene

EAL89481

conserved hypothetical protein
  
Accession: EAL89480
  
Location: 484066-486432
  
 NCBI BlastP on this gene

EAL89480

190. :  JH711573 Coniophora puteana RWD-64-598 SS2 unplaced genomic scaffold CONPUscaffold\_1     Total score: 1.0     Cumulative Blast bit score: 311

glycoside hydrolase family 55 protein
  
Accession: EIW85955
  
Location: 247216-250064
  
 NCBI BlastP on this gene

EIW85955

hypothetical protein
  
Accession: EIW85956
  
Location: 251723-253341
  
 NCBI BlastP on this gene

EIW85956

glycoside hydrolase family 55 protein
  
Accession: EIW85957
  
Location: 253680-256851
  
 NCBI BlastP on this gene

EIW85957

hypothetical protein
  
Accession: EIW85958
  
Location: 257588-258329
  
 NCBI BlastP on this gene

EIW85958

hypothetical protein
  
Accession: EIW85959
  
Location: 258905-260984
  
 NCBI BlastP on this gene

EIW85959

MFS general substrate transporter
  
Accession: EIW85960
  
Location: 261546-264645
  
  
**BlastP hit with Mycgr3G70577\_Mycgr3T**
  
Percentage identity: 33 %
  
BlastP bit score: 311
  
Sequence coverage: 96 %
  
E-value: 2e-92
  
  
 NCBI BlastP on this gene

EIW85960

hypothetical protein
  
Accession: EIW85961
  
Location: 265332-265868
  
 NCBI BlastP on this gene

EIW85961

hypothetical protein
  
Accession: EIW85962
  
Location: 267471-269212
  
 NCBI BlastP on this gene

EIW85962

hypothetical protein
  
Accession: EIW85963
  
Location: 271678-272328
  
 NCBI BlastP on this gene

EIW85963

hypothetical protein
  
Accession: EIW85964
  
Location: 273440-275061
  
 NCBI BlastP on this gene

EIW85964

hypothetical protein
  
Accession: EIW85965
  
Location: 275491-276319
  
 NCBI BlastP on this gene

EIW85965

191. :  DS027054 Aspergillus clavatus NRRL 1 1099423829800 genomic scaffold     Total score: 1.0     Cumulative Blast bit score: 311

clathrin-coated vesicle protein, putative
  
Accession: EAW10732
  
Location: 2066592-2067314
  
 NCBI BlastP on this gene

EAW10732

conserved hypothetical protein
  
Accession: EAW10733
  
Location: 2068922-2070259
  
 NCBI BlastP on this gene

EAW10733

zinc knuckle transcription factor (CnjB), putative
  
Accession: EAW10734
  
Location: 2071201-2073484
  
 NCBI BlastP on this gene

EAW10734

MFS monosaccharide transporter, putative
  
Accession: EAW10735
  
Location: 2079693-2081584
  
  
**BlastP hit with Mycgr3G99766\_Mycgr3T**
  
Percentage identity: 34 %
  
BlastP bit score: 311
  
Sequence coverage: 97 %
  
E-value: 1e-95
  
  
 NCBI BlastP on this gene

EAW10735

192. :  GL377303 Schizophyllum commune H4-8 unplaced genomic scaffold SCHCOscaffold\_2     Total score: 1.0     Cumulative Blast bit score: 310

hypothetical protein
  
Accession: EFJ00465
  
Location: 3465452-3467555
  
 NCBI BlastP on this gene

EFJ00465

hypothetical protein
  
Accession: EFJ01267
  
Location: 3467775-3469678
  
 NCBI BlastP on this gene

EFJ01267

hypothetical protein
  
Accession: EFJ01268
  
Location: 3470593-3471787
  
 NCBI BlastP on this gene

EFJ01268

hypothetical protein
  
Accession: EFJ00466
  
Location: 3472152-3474157
  
 NCBI BlastP on this gene

EFJ00466

hypothetical protein
  
Accession: EFJ00467
  
Location: 3475688-3476855
  
 NCBI BlastP on this gene

EFJ00467

hypothetical protein
  
Accession: EFJ01269
  
Location: 3477096-3478404
  
 NCBI BlastP on this gene

EFJ01269

hypothetical protein
  
Accession: EFJ00468
  
Location: 3479270-3481549
  
  
**BlastP hit with Mycgr3G70577\_Mycgr3T**
  
Percentage identity: 32 %
  
BlastP bit score: 310
  
Sequence coverage: 92 %
  
E-value: 8e-93
  
  
 NCBI BlastP on this gene

EFJ00468

193. :  DS027685 Neosartorya fischeri NRRL 181 1099437636245 genomic scaffold     Total score: 1.0     Cumulative Blast bit score: 308

citrate lyase beta subunit, putative
  
Accession: EAW24844
  
Location: 547245-548382
  
 NCBI BlastP on this gene

EAW24844

clathrin-coated vesicle protein, putative
  
Accession: EAW24843
  
Location: 545953-546673
  
 NCBI BlastP on this gene

EAW24843

conserved hypothetical protein
  
Accession: EAW24842
  
Location: 542787-544387
  
 NCBI BlastP on this gene

EAW24842

zinc knuckle transcription factor (CnjB), putative
  
Accession: EAW24841
  
Location: 539504-541763
  
 NCBI BlastP on this gene

EAW24841

alpha/beta fold family hydrolase, putative
  
Accession: EAW24840
  
Location: 537726-538589
  
 NCBI BlastP on this gene

EAW24840

MFS monosaccharide transporter, putative
  
Accession: EAW24839
  
Location: 533177-535080
  
  
**BlastP hit with Mycgr3G99766\_Mycgr3T**
  
Percentage identity: 34 %
  
BlastP bit score: 308
  
Sequence coverage: 97 %
  
E-value: 2e-94
  
  
 NCBI BlastP on this gene

EAW24839

MFS transporter, putative
  
Accession: EAW24838
  
Location: 529869-531668
  
 NCBI BlastP on this gene

EAW24838

hypothetical protein
  
Accession: EAW24837
  
Location: 528453-529143
  
 NCBI BlastP on this gene

EAW24837

glutamine synthetase
  
Accession: EAW24836
  
Location: 526015-527697
  
 NCBI BlastP on this gene

EAW24836

conserved hypothetical protein
  
Accession: EAW24835
  
Location: 519271-521637
  
 NCBI BlastP on this gene

EAW24835

194. :  GG704911 Coccidioides immitis RS genomic scaffold supercont3.1     Total score: 1.0     Cumulative Blast bit score: 307

sugar porter (SP) family MFS transporter
  
Accession: EAS35333
  
Location: 6801838-6803798
  
  
**BlastP hit with Mycgr3G99766\_Mycgr3T**
  
Percentage identity: 35 %
  
BlastP bit score: 307
  
Sequence coverage: 99 %
  
E-value: 7e-94
  
  
 NCBI BlastP on this gene

EAS35333

hypothetical protein
  
Accession: EAS35334
  
Location: 6800246-6801294
  
 NCBI BlastP on this gene

EAS35334

COPI-coated vesicle protein
  
Accession: EAS35335
  
Location: 6798825-6799553
  
 NCBI BlastP on this gene

EAS35335

SreP protein
  
Accession: EAS35338
  
Location: 6795022-6796911
  
 NCBI BlastP on this gene

EAS35338

hypothetical protein
  
Accession: EJB10714
  
Location: 6793884-6794120
  
 NCBI BlastP on this gene

EJB10714

hypothetical protein
  
Accession: EAS35339
  
Location: 6793049-6793687
  
 NCBI BlastP on this gene

EAS35339

hypothetical protein
  
Accession: EAS35341
  
Location: 6789478-6790944
  
 NCBI BlastP on this gene

EAS35341

195. :  ACFW01000049 Coccidioides posadasii C735 delta SOWgp     Total score: 1.0     Cumulative Blast bit score: 307

Sugar transporter family protein
  
Accession: EER24249
  
Location: 3796420-3798379
  
  
**BlastP hit with Mycgr3G99766\_Mycgr3T**
  
Percentage identity: 35 %
  
BlastP bit score: 307
  
Sequence coverage: 99 %
  
E-value: 6e-94
  
  
 NCBI BlastP on this gene

EER24249

hypothetical protein
  
Accession: EER24248
  
Location: 3794825-3795873
  
 NCBI BlastP on this gene

EER24248

hypothetical protein
  
Accession: EER24247
  
Location: 3793392-3793959
  
 NCBI BlastP on this gene

EER24247

GATA family transcription factor
  
Accession: EER24246
  
Location: 3789589-3791478
  
 NCBI BlastP on this gene

EER24246

hypothetical protein
  
Accession: EER24245
  
Location: 3783814-3785385
  
 NCBI BlastP on this gene

EER24245

196. :  EQ962655 Talaromyces stipitatus ATCC 10500 scf\_1105507295555 genomic scaffold     Total score: 1.0     Cumulative Blast bit score: 306

37S ribosomal protein Rsm24, putative
  
Accession: EED17713
  
Location: 1116425-1117677
  
 NCBI BlastP on this gene

EED17713

glycosyltransferase family 28, putative
  
Accession: EED17714
  
Location: 1117981-1118680
  
 NCBI BlastP on this gene

EED17714

actin-related protein ArpA
  
Accession: EED17715
  
Location: 1119332-1120585
  
 NCBI BlastP on this gene

EED17715

GNAT family acetyltransferase, putative
  
Accession: EED17716
  
Location: 1121089-1121866
  
 NCBI BlastP on this gene

EED17716

3-hydroxyacyl-CoA dehyrogenase, putative
  
Accession: EED17717
  
Location: 1122070-1123091
  
 NCBI BlastP on this gene

EED17717

zinc knuckle transcription factor (CnjB), putative
  
Accession: EED17718
  
Location: 1123571-1125181
  
 NCBI BlastP on this gene

EED17718

monoxygenase, putative
  
Accession: EED17719
  
Location: 1125852-1126715
  
 NCBI BlastP on this gene

EED17719

hypothetical protein
  
Accession: EED17720
  
Location: 1126827-1127745
  
 NCBI BlastP on this gene

EED17720

MFS monosaccharide transporter, putative
  
Accession: EED17721
  
Location: 1130400-1132280
  
  
**BlastP hit with Mycgr3G99766\_Mycgr3T**
  
Percentage identity: 36 %
  
BlastP bit score: 306
  
Sequence coverage: 95 %
  
E-value: 9e-94
  
  
 NCBI BlastP on this gene

EED17721

glutamine synthetase
  
Accession: EED17722
  
Location: 1132932-1134365
  
 NCBI BlastP on this gene

EED17722

conserved hypothetical protein
  
Accession: EED17723
  
Location: 1136513-1138702
  
 NCBI BlastP on this gene

EED17723

conserved hypothetical protein
  
Accession: EED17724
  
Location: 1139055-1139960
  
 NCBI BlastP on this gene

EED17724

conserved hypothetical protein
  
Accession: EED17725
  
Location: 1140430-1142355
  
 NCBI BlastP on this gene

EED17725

hypothetical protein
  
Accession: EED17726
  
Location: 1142601-1143028
  
 NCBI BlastP on this gene

EED17726

hypothetical protein
  
Accession: EED17727
  
Location: 1143821-1146069
  
 NCBI BlastP on this gene

EED17727

197. :  CM001198 Mycosphaerella graminicola IPO323 chromosome 3     Total score: 1.0     Cumulative Blast bit score: 306

putative major intrinsic protein superfamily protein
  
Accession: EGP89151
  
Location: 3299747-3300734
  
 NCBI BlastP on this gene

EGP89151

hypothetical protein
  
Accession: EGP89093
  
Location: 3302142-3304022
  
 NCBI BlastP on this gene

EGP89093

hypothetical protein
  
Accession: EGP89094
  
Location: 3306039-3307526
  
 NCBI BlastP on this gene

EGP89094

hypothetical protein
  
Accession: EGP89150
  
Location: 3307831-3309434
  
 NCBI BlastP on this gene

EGP89150

hypothetical protein
  
Accession: EGP89095
  
Location: 3313304-3315585
  
  
**BlastP hit with Mycgr3G99766\_Mycgr3T**
  
Percentage identity: 36 %
  
BlastP bit score: 306
  
Sequence coverage: 97 %
  
E-value: 7e-94
  
  
 NCBI BlastP on this gene

EGP89095

198. :  HF679025 Fusarium fujikuroi IMI 58289 draft genome, chromosome FFUJ\_chr03.     Total score: 1.0     Cumulative Blast bit score: 305

related to sugar transporter
  
Accession: CCT66518
  
Location: 4684187-4685789
  
  
**BlastP hit with Mycgr3G99766\_Mycgr3T**
  
Percentage identity: 35 %
  
BlastP bit score: 305
  
Sequence coverage: 96 %
  
E-value: 1e-93
  
  
 NCBI BlastP on this gene

FFUJ\_03554

related to alpha-L-arabinofuranosidase A precursor
  
Accession: CCT66517
  
Location: 4681306-4683489
  
 NCBI BlastP on this gene

FFUJ\_03553

uncharacterized protein
  
Accession: CCT66516
  
Location: 4678813-4679142
  
 NCBI BlastP on this gene

FFUJ\_03552

uncharacterized protein
  
Accession: CCT66515
  
Location: 4675793-4677349
  
 NCBI BlastP on this gene

FFUJ\_03551

uncharacterized protein
  
Accession: CCT66514
  
Location: 4673685-4674902
  
 NCBI BlastP on this gene

FFUJ\_03550

uncharacterized protein
  
Accession: CCT66513
  
Location: 4671527-4673411
  
 NCBI BlastP on this gene

FFUJ\_03549

199. :  KB446557 Pseudocercospora fijiensis CIRAD86 unplaced genomic scaffold MYCFIscaffold\_3     Total score: 1.0     Cumulative Blast bit score: 303

hypothetical protein
  
Accession: EME84611
  
Location: 3977599-3979482
  
  
**BlastP hit with Mycgr3G99766\_Mycgr3T**
  
Percentage identity: 36 %
  
BlastP bit score: 303
  
Sequence coverage: 97 %
  
E-value: 2e-92
  
  
 NCBI BlastP on this gene

EME84611

hypothetical protein
  
Accession: EME84610
  
Location: 3973902-3976079
  
 NCBI BlastP on this gene

EME84610

hypothetical protein
  
Accession: EME84609
  
Location: 3972114-3973801
  
 NCBI BlastP on this gene

EME84609

hypothetical protein
  
Accession: EME84608
  
Location: 3970022-3971065
  
 NCBI BlastP on this gene

EME84608

hypothetical protein
  
Accession: EME84607
  
Location: 3969270-3969980
  
 NCBI BlastP on this gene

EME84607

hypothetical protein
  
Accession: EME84606
  
Location: 3967043-3967456
  
 NCBI BlastP on this gene

EME84606

hypothetical protein
  
Accession: EME84605
  
Location: 3966292-3966642
  
 NCBI BlastP on this gene

EME84605

hypothetical protein
  
Accession: EME84604
  
Location: 3963968-3965200
  
 NCBI BlastP on this gene

EME84604

200. :  KB644410 Penicillium oxalicum 114-2 unplaced genomic scaffold scaffold\_3     Total score: 1.0     Cumulative Blast bit score: 301

hypothetical protein
  
Accession: EPS27226
  
Location: 202009-203806
  
  
**BlastP hit with Mycgr3G99766\_Mycgr3T**
  
Percentage identity: 34 %
  
BlastP bit score: 301
  
Sequence coverage: 98 %
  
E-value: 1e-91
  
  
 NCBI BlastP on this gene

EPS27226

hypothetical protein
  
Accession: EPS27225
  
Location: 199036-201025
  
 NCBI BlastP on this gene

EPS27225

hypothetical protein
  
Accession: EPS27224
  
Location: 195611-197874
  
 NCBI BlastP on this gene

EPS27224

hypothetical protein
  
Accession: EPS27223
  
Location: 191498-193131
  
 NCBI BlastP on this gene

EPS27223

putative beta-xylosidase
  
Accession: EPS27222
  
Location: 188500-190414
  
 NCBI BlastP on this gene

EPS27222

Detecting sequence homology at the gene cluster level with MultiGeneBlast.
  
Marnix H. Medema, Rainer Breitling & Eriko Takano (2013)
  
*Molecular Biology and Evolution* , 30: 1218-1223.
