# Supplementary material for: In silico prediction and characterization of secondary metabolite biosynthetic gene clusters in the wheat pathogen Zymoseptoria tritici
Source: BMC Genomics. 2017 Aug 17;18:631. doi: 10.1186/s12864-017-3969-y (PMC5561558; doi:10.1186/s12864-017-3969-y)
Supplement: Supplementary file 1 — MultiGeneBLAST analysis of putative secondary metabolite clusters. All encoded amino acid sequences from genes residing in clusters predicted by AntiSMASH are given as FASTA file format. All output data from MultiGeneBLASTs are also provided. (ZIP 42911 kb) [file 12864_2017_3969_MOESM1_ESM.zip › Cluster MultiGene BLAST/out/Clusters_1_34/Cluster_14/displaypage5.xhtml]

xml version="1.0" encoding="UTF-8"?


Search Results
  
  
 Results pages: 1, 2, 3, 4, 5

**MultiGeneBlast hits**

Select gene cluster alignment
201. CR382133\_0 Debaryomyces hansenii CBS767 chromosome A complete sequence.
202. DS995901\_0 Penicillium marneffei ATCC 18224 scf\_1105668340960 genomic sc...
203. KB445812\_0 Ceriporiopsis subvermispora B unplaced genomic scaffold CERSU...
204. DS989822\_2 Arthroderma gypseum CBS 118893 supercont1.1 genomic scaffold,...
205. CH476597\_1 Aspergillus terreus NIH2624 scaffold\_4 genomic scaffold, whol...
206. AP007150\_1 Aspergillus oryzae RIB40 DNA, SC009.
207. KB644408\_0 Penicillium oxalicum 114-2 unplaced genomic scaffold scaffold...
208. KB446538\_0 Dothistroma septosporum NZE10 unplaced genomic scaffold DOTSE...
209. CP003003\_1 Myceliophthora thermophila ATCC 42464 chromosome 2, complete ...
210. AM920435\_0 Penicillium chrysogenum Wisconsin 54-1255 complete genome, co...
211. AM920437\_0 Penicillium chrysogenum Wisconsin 54-1255 complete genome, co...
212. KB445647\_0 Cochliobolus sativus ND90Pr unplaced genomic scaffold COCSAsc...
213. DS995701\_1 Microsporum canis CBS 113480 supercont1.1 genomic scaffold, w...
214. AM920428\_2 Penicillium chrysogenum Wisconsin 54-1255 complete genome, co...
215. KE007245\_1 Wallemia ichthyophaga EXF-994 unplaced genomic scaffold scaff...
216. KB456263\_2 Mycosphaerella populorum SO2202 unplaced genomic scaffold SEP...
217. JH717968\_2 Fomitiporia mediterranea MF3/22 unplaced genomic scaffold FOM...
218. KB445573\_1 Cochliobolus heterostrophus C5 unplaced genomic scaffold COCH...
219. KB908481\_1 Setosphaeria turcica Et28A unplaced genomic scaffold SETTUsca...
220. GL385397\_0 Gaeumannomyces graminis var. tritici R3-111a-1 unplaced genom...
221. CM001202\_1 Mycosphaerella graminicola IPO323 chromosome 7, whole genome ...
222. CH476615\_0 Uncinocarpus reesii 1704 scaffold\_1 genomic scaffold, whole g...
223. CR382129\_0 Yarrowia lipolytica CLIB122 chromosome C complete sequence.
224. CM001199\_1 Mycosphaerella graminicola IPO323 chromosome 4, whole genome ...
225. AKCT01000319\_0 Penicillium digitatum PHI26, whole genome shotgun sequenc...
226. AM920437\_2 Penicillium chrysogenum Wisconsin 54-1255 complete genome, co...
227. KB730093\_0 Fusarium oxysporum f. sp. cubense race 1 unplaced genomic sca...
228. JH668249\_0 Wallemia sebi CBS 633.66 unplaced genomic scaffold WALSEscaff...
229. AACD01000043\_1 Aspergillus nidulans FGSC A4, whole genome shotgun sequen...
230. HF679023\_2 Fusarium fujikuroi IMI 58289 draft genome, chromosome FFUJ\_ch...
231. KB726991\_0 Fusarium oxysporum f. sp. cubense race 4 unplaced genomic sca...
232. FO082046\_0 Pichia sorbitophila strain CBS 7064 chromosome N complete seq...
233. CU928166\_0 Lachancea thermotolerans CBS 6340 chromosome B complete seque...
234. EQ963475\_0 Aspergillus flavus NRRL3357 scf\_1106286419142 genomic scaffol...
235. EQ963474\_0 Aspergillus flavus NRRL3357 scf\_1106286417496 genomic scaffol...
236. AP007161\_0 Aspergillus oryzae RIB40 DNA, SC012.
237. AP007157\_0 Aspergillus oryzae RIB40 DNA, SC023.
238. AKHY01000182\_0 Aspergillus oryzae 3.042, whole genome shotgun sequencing...
239. AKHY01000171\_0 Aspergillus oryzae 3.042, whole genome shotgun sequencing...
240. CR382137\_0 Debaryomyces hansenii CBS767 chromosome E complete sequence.
241. CR382129\_1 Yarrowia lipolytica CLIB122 chromosome C complete sequence.
242. CR382134\_1 Debaryomyces hansenii CBS767 chromosome B complete sequence.
243. GL891302\_1 Neurospora tetrasperma FGSC 2508 unplaced genomic scaffold NE...
244. GL891107\_0 Neurospora tetrasperma FGSC 2509 unplaced genomic scaffold NE...
245. KB731258\_1 Fusarium oxysporum f. sp. cubense race 1 unplaced genomic sca...
246. GL385399\_0 Gaeumannomyces graminis var. tritici R3-111a-1 unplaced genom...
247. CM001235\_0 Magnaporthe oryzae 70-15 chromosome 5, whole genome shotgun s...
248. AMWD01000002\_0 Janthinobacterium sp. HH01, whole genome shotgun sequenci...
249. AP007150\_0 Aspergillus oryzae RIB40 DNA, SC009.
250. CM001198\_0 Mycosphaerella graminicola IPO323 chromosome 3, whole genome ...

Query: Architecture Search FASTA input

CR382133 : Debaryomyces hansenii CBS767 chromosome A complete sequence.    Total score: 1.0     Cumulative Blast bit score: 301

Hit cluster cross-links:

Mycgr3G41235 Mycgr3T
  
Location: 0-4062

Mycgr3G41235\_Mycgr3T

Mycgr3G70577 Mycgr3T
  
Location: 4162-6109

Mycgr3G70577\_Mycgr3T

Mycgr3G40534 Mycgr3T
  
Location: 6209-7166

Mycgr3G40534\_Mycgr3T

Mycgr3G85486 Mycgr3T
  
Location: 7266-8511

Mycgr3G85486\_Mycgr3T

Mycgr3G92221 Mycgr3T
  
Location: 8611-9193

Mycgr3G92221\_Mycgr3T

Mycgr3G39931 Mycgr3T
  
Location: 9293-10157

Mycgr3G39931\_Mycgr3T

Mycgr3G99766 Mycgr3T
  
Location: 10257-11775

Mycgr3G99766\_Mycgr3T

DEHA2A12496p
  
Accession: CAG84848
  
Location: 1054493-1055188
  
 NCBI BlastP on this gene

DEHA2A12496g

DEHA2A12474p
  
Accession: CAG84847
  
Location: 1052666-1053196
  
 NCBI BlastP on this gene

DEHA2A12474g

DEHA2A12452p
  
Accession: CAG84846
  
Location: 1051589-1052455
  
 NCBI BlastP on this gene

DEHA2A12452g

DEHA2A12430p
  
Accession: CAG84845
  
Location: 1050316-1051125
  
 NCBI BlastP on this gene

DEHA2A12430g

DEHA2A12408p
  
Accession: CAR65407
  
Location: 1049206-1049400
  
 NCBI BlastP on this gene

DEHA2A12408g

DEHA2A12386p
  
Accession: CAG84844
  
Location: 1048052-1048921
  
 NCBI BlastP on this gene

DEHA2A12386g

DEHA2A12364p
  
Accession: CAG84843
  
Location: 1045762-1047420
  
  
**BlastP hit with Mycgr3G99766\_Mycgr3T**
  
Percentage identity: 33 %
  
BlastP bit score: 301
  
Sequence coverage: 101 %
  
E-value: 8e-92
  
  
 NCBI BlastP on this gene

DEHA2A12364g

DEHA2A12342p
  
Accession: CAG84842
  
Location: 1043702-1045519
  
 NCBI BlastP on this gene

DEHA2A12342g

DEHA2A12320p
  
Accession: CAG84841
  
Location: 1042691-1043161
  
 NCBI BlastP on this gene

DEHA2A12320g

DEHA2A12298p
  
Accession: CAG84840
  
Location: 1039435-1042515
  
 NCBI BlastP on this gene

DEHA2A12298g

DEHA2A12276p
  
Accession: CAG84839
  
Location: 1036688-1039003
  
 NCBI BlastP on this gene

DEHA2A12276g

Query: Architecture Search FASTA input

DS995901 : Penicillium marneffei ATCC 18224 scf\_1105668340960 genomic scaffold    Total score: 1.0     Cumulative Blast bit score: 300

Hit cluster cross-links:

Mycgr3G41235 Mycgr3T
  
Location: 0-4062

Mycgr3G41235\_Mycgr3T

Mycgr3G70577 Mycgr3T
  
Location: 4162-6109

Mycgr3G70577\_Mycgr3T

Mycgr3G40534 Mycgr3T
  
Location: 6209-7166

Mycgr3G40534\_Mycgr3T

Mycgr3G85486 Mycgr3T
  
Location: 7266-8511

Mycgr3G85486\_Mycgr3T

Mycgr3G92221 Mycgr3T
  
Location: 8611-9193

Mycgr3G92221\_Mycgr3T

Mycgr3G39931 Mycgr3T
  
Location: 9293-10157

Mycgr3G39931\_Mycgr3T

Mycgr3G99766 Mycgr3T
  
Location: 10257-11775

Mycgr3G99766\_Mycgr3T

N-acetyltransferase, GNAT family, putative
  
Accession: EEA24048
  
Location: 1079649-1080197
  
 NCBI BlastP on this gene

EEA24048

3-hydroxyacyl-CoA dehyrogenase, putative
  
Accession: EEA24049
  
Location: 1080474-1081518
  
 NCBI BlastP on this gene

EEA24049

zinc knuckle transcription factor (CnjB), putative
  
Accession: EEA24050
  
Location: 1082192-1083859
  
 NCBI BlastP on this gene

EEA24050

MFS monosaccharide transporter, putative
  
Accession: EEA24051
  
Location: 1087088-1088972
  
  
**BlastP hit with Mycgr3G99766\_Mycgr3T**
  
Percentage identity: 34 %
  
BlastP bit score: 300
  
Sequence coverage: 100 %
  
E-value: 4e-91
  
  
 NCBI BlastP on this gene

EEA24051

glutamine synthetase
  
Accession: EEA24052
  
Location: 1090027-1091271
  
 NCBI BlastP on this gene

EEA24052

conserved hypothetical protein
  
Accession: EEA24053
  
Location: 1094220-1096420
  
 NCBI BlastP on this gene

EEA24053

Query: Architecture Search FASTA input

KB445812 : Ceriporiopsis subvermispora B unplaced genomic scaffold CERSUscaffold\_22    Total score: 1.0     Cumulative Blast bit score: 299

Hit cluster cross-links:

Mycgr3G41235 Mycgr3T
  
Location: 0-4062

Mycgr3G41235\_Mycgr3T

Mycgr3G70577 Mycgr3T
  
Location: 4162-6109

Mycgr3G70577\_Mycgr3T

Mycgr3G40534 Mycgr3T
  
Location: 6209-7166

Mycgr3G40534\_Mycgr3T

Mycgr3G85486 Mycgr3T
  
Location: 7266-8511

Mycgr3G85486\_Mycgr3T

Mycgr3G92221 Mycgr3T
  
Location: 8611-9193

Mycgr3G92221\_Mycgr3T

Mycgr3G39931 Mycgr3T
  
Location: 9293-10157

Mycgr3G39931\_Mycgr3T

Mycgr3G99766 Mycgr3T
  
Location: 10257-11775

Mycgr3G99766\_Mycgr3T

hypothetical protein
  
Accession: EMD32264
  
Location: 338547-339559
  
 NCBI BlastP on this gene

EMD32264

PKS/NRPS enzyme
  
Accession: EMD32324
  
Location: 325094-336915
  
  
**BlastP hit with Mycgr3G40534\_Mycgr3T**
  
Percentage identity: 38 %
  
BlastP bit score: 141
  
Sequence coverage: 79 %
  
E-value: 3e-33
  
  
 NCBI BlastP on this gene

EMD32324

polyketide synthetase
  
Accession: EMD32323
  
Location: 325048-336915
  
  
**BlastP hit with Mycgr3G40534\_Mycgr3T**
  
Percentage identity: 40 %
  
BlastP bit score: 158
  
Sequence coverage: 79 %
  
E-value: 6e-39
  
  
 NCBI BlastP on this gene

EMD32323

hypothetical protein
  
Accession: EMD32263
  
Location: 322498-322855
  
 NCBI BlastP on this gene

EMD32263

Query: Architecture Search FASTA input

DS989822 : Arthroderma gypseum CBS 118893 supercont1.1 genomic scaffold    Total score: 1.0     Cumulative Blast bit score: 298

Hit cluster cross-links:

Mycgr3G41235 Mycgr3T
  
Location: 0-4062

Mycgr3G41235\_Mycgr3T

Mycgr3G70577 Mycgr3T
  
Location: 4162-6109

Mycgr3G70577\_Mycgr3T

Mycgr3G40534 Mycgr3T
  
Location: 6209-7166

Mycgr3G40534\_Mycgr3T

Mycgr3G85486 Mycgr3T
  
Location: 7266-8511

Mycgr3G85486\_Mycgr3T

Mycgr3G92221 Mycgr3T
  
Location: 8611-9193

Mycgr3G92221\_Mycgr3T

Mycgr3G39931 Mycgr3T
  
Location: 9293-10157

Mycgr3G39931\_Mycgr3T

Mycgr3G99766 Mycgr3T
  
Location: 10257-11775

Mycgr3G99766\_Mycgr3T

sugar transporter STL1
  
Accession: EFQ98600
  
Location: 4438455-4440510
  
  
**BlastP hit with Mycgr3G99766\_Mycgr3T**
  
Percentage identity: 36 %
  
BlastP bit score: 298
  
Sequence coverage: 97 %
  
E-value: 2e-90
  
  
 NCBI BlastP on this gene

EFQ98600

hypothetical protein
  
Accession: EFQ98599
  
Location: 4437652-4438026
  
 NCBI BlastP on this gene

EFQ98599

hypothetical protein
  
Accession: EFQ98598
  
Location: 4434489-4436385
  
 NCBI BlastP on this gene

EFQ98598

secreted protein
  
Accession: EFQ98597
  
Location: 4432445-4433143
  
 NCBI BlastP on this gene

EFQ98597

Query: Architecture Search FASTA input

CH476597 : Aspergillus terreus NIH2624 scaffold\_4 genomic scaffold    Total score: 1.0     Cumulative Blast bit score: 298

Hit cluster cross-links:

Mycgr3G41235 Mycgr3T
  
Location: 0-4062

Mycgr3G41235\_Mycgr3T

Mycgr3G70577 Mycgr3T
  
Location: 4162-6109

Mycgr3G70577\_Mycgr3T

Mycgr3G40534 Mycgr3T
  
Location: 6209-7166

Mycgr3G40534\_Mycgr3T

Mycgr3G85486 Mycgr3T
  
Location: 7266-8511

Mycgr3G85486\_Mycgr3T

Mycgr3G92221 Mycgr3T
  
Location: 8611-9193

Mycgr3G92221\_Mycgr3T

Mycgr3G39931 Mycgr3T
  
Location: 9293-10157

Mycgr3G39931\_Mycgr3T

Mycgr3G99766 Mycgr3T
  
Location: 10257-11775

Mycgr3G99766\_Mycgr3T

conserved hypothetical protein
  
Accession: EAU36463
  
Location: 1114997-1117146
  
 NCBI BlastP on this gene

EAU36463

conserved hypothetical protein
  
Accession: EAU36464
  
Location: 1122644-1124442
  
  
**BlastP hit with Mycgr3G99766\_Mycgr3T**
  
Percentage identity: 34 %
  
BlastP bit score: 298
  
Sequence coverage: 96 %
  
E-value: 1e-90
  
  
 NCBI BlastP on this gene

EAU36464

Query: Architecture Search FASTA input

AP007150 : Aspergillus oryzae RIB40 DNA, SC009.    Total score: 1.0     Cumulative Blast bit score: 298

Hit cluster cross-links:

Mycgr3G41235 Mycgr3T
  
Location: 0-4062

Mycgr3G41235\_Mycgr3T

Mycgr3G70577 Mycgr3T
  
Location: 4162-6109

Mycgr3G70577\_Mycgr3T

Mycgr3G40534 Mycgr3T
  
Location: 6209-7166

Mycgr3G40534\_Mycgr3T

Mycgr3G85486 Mycgr3T
  
Location: 7266-8511

Mycgr3G85486\_Mycgr3T

Mycgr3G92221 Mycgr3T
  
Location: 8611-9193

Mycgr3G92221\_Mycgr3T

Mycgr3G39931 Mycgr3T
  
Location: 9293-10157

Mycgr3G39931\_Mycgr3T

Mycgr3G99766 Mycgr3T
  
Location: 10257-11775

Mycgr3G99766\_Mycgr3T

not annotated
  
Accession: BAE54753
  
Location: 723216-724050
  
 NCBI BlastP on this gene

AO090009000272

not annotated
  
Accession: BAE54754
  
Location: 724104-725403
  
 NCBI BlastP on this gene

AO090009000273

not annotated
  
Accession: BAE54755
  
Location: 730858-732654
  
  
**BlastP hit with Mycgr3G99766\_Mycgr3T**
  
Percentage identity: 33 %
  
BlastP bit score: 298
  
Sequence coverage: 96 %
  
E-value: 1e-90
  
  
 NCBI BlastP on this gene

AO090009000275

Query: Architecture Search FASTA input

KB644408 : Penicillium oxalicum 114-2 unplaced genomic scaffold scaffold\_1    Total score: 1.0     Cumulative Blast bit score: 296

Hit cluster cross-links:

Mycgr3G41235 Mycgr3T
  
Location: 0-4062

Mycgr3G41235\_Mycgr3T

Mycgr3G70577 Mycgr3T
  
Location: 4162-6109

Mycgr3G70577\_Mycgr3T

Mycgr3G40534 Mycgr3T
  
Location: 6209-7166

Mycgr3G40534\_Mycgr3T

Mycgr3G85486 Mycgr3T
  
Location: 7266-8511

Mycgr3G85486\_Mycgr3T

Mycgr3G92221 Mycgr3T
  
Location: 8611-9193

Mycgr3G92221\_Mycgr3T

Mycgr3G39931 Mycgr3T
  
Location: 9293-10157

Mycgr3G39931\_Mycgr3T

Mycgr3G99766 Mycgr3T
  
Location: 10257-11775

Mycgr3G99766\_Mycgr3T

hypothetical protein
  
Accession: EPS25756
  
Location: 1978083-1982459
  
 NCBI BlastP on this gene

EPS25756

hypothetical protein
  
Accession: EPS25755
  
Location: 1974349-1975999
  
  
**BlastP hit with Mycgr3G99766\_Mycgr3T**
  
Percentage identity: 34 %
  
BlastP bit score: 296
  
Sequence coverage: 97 %
  
E-value: 3e-90
  
  
 NCBI BlastP on this gene

EPS25755

hypothetical protein
  
Accession: EPS25754
  
Location: 1972674-1973495
  
 NCBI BlastP on this gene

EPS25754

putative UDP-Xyl: (mannosyl)
  
Accession: EPS25753
  
Location: 1969704-1971662
  
 NCBI BlastP on this gene

EPS25753

hypothetical protein
  
Accession: EPS25752
  
Location: 1965848-1968691
  
 NCBI BlastP on this gene

EPS25752

Query: Architecture Search FASTA input

KB446538 : Dothistroma septosporum NZE10 unplaced genomic scaffold DOTSEscaffold\_4    Total score: 1.0     Cumulative Blast bit score: 295

Hit cluster cross-links:

Mycgr3G41235 Mycgr3T
  
Location: 0-4062

Mycgr3G41235\_Mycgr3T

Mycgr3G70577 Mycgr3T
  
Location: 4162-6109

Mycgr3G70577\_Mycgr3T

Mycgr3G40534 Mycgr3T
  
Location: 6209-7166

Mycgr3G40534\_Mycgr3T

Mycgr3G85486 Mycgr3T
  
Location: 7266-8511

Mycgr3G85486\_Mycgr3T

Mycgr3G92221 Mycgr3T
  
Location: 8611-9193

Mycgr3G92221\_Mycgr3T

Mycgr3G39931 Mycgr3T
  
Location: 9293-10157

Mycgr3G39931\_Mycgr3T

Mycgr3G99766 Mycgr3T
  
Location: 10257-11775

Mycgr3G99766\_Mycgr3T

hypothetical protein
  
Accession: EME45756
  
Location: 2313689-2315149
  
 NCBI BlastP on this gene

EME45756

hypothetical protein
  
Accession: EME45755
  
Location: 2312517-2313237
  
 NCBI BlastP on this gene

EME45755

hypothetical protein
  
Accession: EME45753
  
Location: 2309153-2310121
  
 NCBI BlastP on this gene

EME45753

hypothetical protein
  
Accession: EME45752
  
Location: 2305594-2307479
  
  
**BlastP hit with Mycgr3G99766\_Mycgr3T**
  
Percentage identity: 35 %
  
BlastP bit score: 295
  
Sequence coverage: 98 %
  
E-value: 2e-89
  
  
 NCBI BlastP on this gene

EME45752

hypothetical protein
  
Accession: EME45751
  
Location: 2302754-2305443
  
 NCBI BlastP on this gene

EME45751

hypothetical protein
  
Accession: EME45750
  
Location: 2300520-2300699
  
 NCBI BlastP on this gene

EME45750

hypothetical protein
  
Accession: EME45749
  
Location: 2299009-2299650
  
 NCBI BlastP on this gene

EME45749

hypothetical protein
  
Accession: EME45748
  
Location: 2298041-2298779
  
 NCBI BlastP on this gene

EME45748

Query: Architecture Search FASTA input

CP003003 : Myceliophthora thermophila ATCC 42464 chromosome 2    Total score: 1.0     Cumulative Blast bit score: 295

Hit cluster cross-links:

Mycgr3G41235 Mycgr3T
  
Location: 0-4062

Mycgr3G41235\_Mycgr3T

Mycgr3G70577 Mycgr3T
  
Location: 4162-6109

Mycgr3G70577\_Mycgr3T

Mycgr3G40534 Mycgr3T
  
Location: 6209-7166

Mycgr3G40534\_Mycgr3T

Mycgr3G85486 Mycgr3T
  
Location: 7266-8511

Mycgr3G85486\_Mycgr3T

Mycgr3G92221 Mycgr3T
  
Location: 8611-9193

Mycgr3G92221\_Mycgr3T

Mycgr3G39931 Mycgr3T
  
Location: 9293-10157

Mycgr3G39931\_Mycgr3T

Mycgr3G99766 Mycgr3T
  
Location: 10257-11775

Mycgr3G99766\_Mycgr3T

hypothetical protein
  
Accession: AEO57131
  
Location: 5180901-5182804
  
  
**BlastP hit with Mycgr3G99766\_Mycgr3T**
  
Percentage identity: 35 %
  
BlastP bit score: 295
  
Sequence coverage: 97 %
  
E-value: 4e-89
  
  
 NCBI BlastP on this gene

MYCTH\_2302949

metalloprotease
  
Accession: AEO57130
  
Location: 5178173-5179261
  
 NCBI BlastP on this gene

MYCTH\_78093

hypothetical protein
  
Accession: AEO57129
  
Location: 5176947-5177419
  
 NCBI BlastP on this gene

MYCTH\_2126111

Intradiol ring-cleavage dioxygenase-like protein
  
Accession: AEO57128
  
Location: 5175196-5176687
  
 NCBI BlastP on this gene

MYCTH\_100089

Query: Architecture Search FASTA input

AM920435 : Penicillium chrysogenum Wisconsin 54-1255 complete genome, contig Pc00c20.    Total score: 1.0     Cumulative Blast bit score: 295

Hit cluster cross-links:

Mycgr3G41235 Mycgr3T
  
Location: 0-4062

Mycgr3G41235\_Mycgr3T

Mycgr3G70577 Mycgr3T
  
Location: 4162-6109

Mycgr3G70577\_Mycgr3T

Mycgr3G40534 Mycgr3T
  
Location: 6209-7166

Mycgr3G40534\_Mycgr3T

Mycgr3G85486 Mycgr3T
  
Location: 7266-8511

Mycgr3G85486\_Mycgr3T

Mycgr3G92221 Mycgr3T
  
Location: 8611-9193

Mycgr3G92221\_Mycgr3T

Mycgr3G39931 Mycgr3T
  
Location: 9293-10157

Mycgr3G39931\_Mycgr3T

Mycgr3G99766 Mycgr3T
  
Location: 10257-11775

Mycgr3G99766\_Mycgr3T

not annotated
  
Accession: CAP85504
  
Location: 398358-400032
  
 NCBI BlastP on this gene

Pc20g01750

not annotated
  
Accession: CAP85505
  
Location: 400892-402821
  
 NCBI BlastP on this gene

Pc20g01760

hypothetical protein
  
Accession: CAP85506
  
Location: 403481-404312
  
 NCBI BlastP on this gene

Pc20g01770

not annotated
  
Accession: CAP85507
  
Location: 405825-407737
  
  
**BlastP hit with Mycgr3G99766\_Mycgr3T**
  
Percentage identity: 33 %
  
BlastP bit score: 295
  
Sequence coverage: 95 %
  
E-value: 2e-89
  
  
 NCBI BlastP on this gene

Pc20g01780

unnamed
  
Accession: CAP85508
  
Location: 408172-409984
  
 NCBI BlastP on this gene

Pc20g01790

not annotated
  
Accession: CAP85509
  
Location: 412030-414283
  
 NCBI BlastP on this gene

Pc20g01800

not annotated
  
Accession: CAP85510
  
Location: 415010-416565
  
 NCBI BlastP on this gene

Pc20g01810

Query: Architecture Search FASTA input

AM920437 : Penicillium chrysogenum Wisconsin 54-1255 complete genome, contig Pc00c22.    Total score: 1.0     Cumulative Blast bit score: 294

Hit cluster cross-links:

Mycgr3G41235 Mycgr3T
  
Location: 0-4062

Mycgr3G41235\_Mycgr3T

Mycgr3G70577 Mycgr3T
  
Location: 4162-6109

Mycgr3G70577\_Mycgr3T

Mycgr3G40534 Mycgr3T
  
Location: 6209-7166

Mycgr3G40534\_Mycgr3T

Mycgr3G85486 Mycgr3T
  
Location: 7266-8511

Mycgr3G85486\_Mycgr3T

Mycgr3G92221 Mycgr3T
  
Location: 8611-9193

Mycgr3G92221\_Mycgr3T

Mycgr3G39931 Mycgr3T
  
Location: 9293-10157

Mycgr3G39931\_Mycgr3T

Mycgr3G99766 Mycgr3T
  
Location: 10257-11775

Mycgr3G99766\_Mycgr3T

not annotated
  
Accession: CAP97586
  
Location: 684546-685693
  
 NCBI BlastP on this gene

Pc22g02980

not annotated
  
Accession: CAP97587
  
Location: 686818-687567
  
 NCBI BlastP on this gene

Pc22g02990

not annotated
  
Accession: CAP97588
  
Location: 688224-690532
  
 NCBI BlastP on this gene

Pc22g03000

hypothetical protein
  
Accession: CAP97589
  
Location: 691193-692635
  
 NCBI BlastP on this gene

Pc22g03010

not annotated
  
Accession: CAP97590
  
Location: 692712-694682
  
  
**BlastP hit with Mycgr3G99766\_Mycgr3T**
  
Percentage identity: 34 %
  
BlastP bit score: 294
  
Sequence coverage: 97 %
  
E-value: 5e-89
  
  
 NCBI BlastP on this gene

Pc22g03020

unnamed
  
Accession: CAP97591
  
Location: 694933-695983
  
 NCBI BlastP on this gene

Pc22g03030

hypothetical protein
  
Accession: CAP97592
  
Location: 697003-697618
  
 NCBI BlastP on this gene

Pc22g03040

not annotated
  
Accession: Pc22g03050
  
Location: 697686-698607
  
 NCBI BlastP on this gene

Pc22g03050

not annotated
  
Accession: CAP97594
  
Location: 699540-701492
  
 NCBI BlastP on this gene

Pc22g03060

Query: Architecture Search FASTA input

KB445647 : Cochliobolus sativus ND90Pr unplaced genomic scaffold COCSAscaffold\_11    Total score: 1.0     Cumulative Blast bit score: 293

Hit cluster cross-links:

Mycgr3G41235 Mycgr3T
  
Location: 0-4062

Mycgr3G41235\_Mycgr3T

Mycgr3G70577 Mycgr3T
  
Location: 4162-6109

Mycgr3G70577\_Mycgr3T

Mycgr3G40534 Mycgr3T
  
Location: 6209-7166

Mycgr3G40534\_Mycgr3T

Mycgr3G85486 Mycgr3T
  
Location: 7266-8511

Mycgr3G85486\_Mycgr3T

Mycgr3G92221 Mycgr3T
  
Location: 8611-9193

Mycgr3G92221\_Mycgr3T

Mycgr3G39931 Mycgr3T
  
Location: 9293-10157

Mycgr3G39931\_Mycgr3T

Mycgr3G99766 Mycgr3T
  
Location: 10257-11775

Mycgr3G99766\_Mycgr3T

hypothetical protein
  
Accession: EMD61791
  
Location: 98830-99728
  
 NCBI BlastP on this gene

EMD61791

hypothetical protein
  
Accession: EMD61792
  
Location: 102057-104224
  
 NCBI BlastP on this gene

EMD61792

hypothetical protein
  
Accession: EMD61793
  
Location: 104495-104926
  
 NCBI BlastP on this gene

EMD61793

hypothetical protein
  
Accession: EMD61794
  
Location: 107054-108699
  
  
**BlastP hit with Mycgr3G99766\_Mycgr3T**
  
Percentage identity: 35 %
  
BlastP bit score: 293
  
Sequence coverage: 97 %
  
E-value: 6e-89
  
  
 NCBI BlastP on this gene

EMD61794

hypothetical protein
  
Accession: EMD61795
  
Location: 109195-109604
  
 NCBI BlastP on this gene

EMD61795

hypothetical protein
  
Accession: EMD61796
  
Location: 111640-114102
  
 NCBI BlastP on this gene

EMD61796

hypothetical protein
  
Accession: EMD61797
  
Location: 114534-115293
  
 NCBI BlastP on this gene

EMD61797

hypothetical protein
  
Accession: EMD61798
  
Location: 115475-116138
  
 NCBI BlastP on this gene

EMD61798

Query: Architecture Search FASTA input

DS995701 : Microsporum canis CBS 113480 supercont1.1 genomic scaffold    Total score: 1.0     Cumulative Blast bit score: 293

Hit cluster cross-links:

Mycgr3G41235 Mycgr3T
  
Location: 0-4062

Mycgr3G41235\_Mycgr3T

Mycgr3G70577 Mycgr3T
  
Location: 4162-6109

Mycgr3G70577\_Mycgr3T

Mycgr3G40534 Mycgr3T
  
Location: 6209-7166

Mycgr3G40534\_Mycgr3T

Mycgr3G85486 Mycgr3T
  
Location: 7266-8511

Mycgr3G85486\_Mycgr3T

Mycgr3G92221 Mycgr3T
  
Location: 8611-9193

Mycgr3G92221\_Mycgr3T

Mycgr3G39931 Mycgr3T
  
Location: 9293-10157

Mycgr3G39931\_Mycgr3T

Mycgr3G99766 Mycgr3T
  
Location: 10257-11775

Mycgr3G99766\_Mycgr3T

sugar transporter
  
Accession: EEQ28189
  
Location: 2941554-2943524
  
  
**BlastP hit with Mycgr3G99766\_Mycgr3T**
  
Percentage identity: 35 %
  
BlastP bit score: 293
  
Sequence coverage: 97 %
  
E-value: 9e-89
  
  
 NCBI BlastP on this gene

EEQ28189

conserved hypothetical protein
  
Accession: EEQ28188
  
Location: 2939207-2941239
  
 NCBI BlastP on this gene

EEQ28188

high affinity nicotinic acid plasma membrane permease
  
Accession: EEQ28187
  
Location: 2936593-2938171
  
 NCBI BlastP on this gene

EEQ28187

glutamine synthetase
  
Accession: EEQ28186
  
Location: 2934062-2935623
  
 NCBI BlastP on this gene

EEQ28186

Query: Architecture Search FASTA input

AM920428 : Penicillium chrysogenum Wisconsin 54-1255 complete genome, contig Pc00c13.    Total score: 1.0     Cumulative Blast bit score: 292

Hit cluster cross-links:

Mycgr3G41235 Mycgr3T
  
Location: 0-4062

Mycgr3G41235\_Mycgr3T

Mycgr3G70577 Mycgr3T
  
Location: 4162-6109

Mycgr3G70577\_Mycgr3T

Mycgr3G40534 Mycgr3T
  
Location: 6209-7166

Mycgr3G40534\_Mycgr3T

Mycgr3G85486 Mycgr3T
  
Location: 7266-8511

Mycgr3G85486\_Mycgr3T

Mycgr3G92221 Mycgr3T
  
Location: 8611-9193

Mycgr3G92221\_Mycgr3T

Mycgr3G39931 Mycgr3T
  
Location: 9293-10157

Mycgr3G39931\_Mycgr3T

Mycgr3G99766 Mycgr3T
  
Location: 10257-11775

Mycgr3G99766\_Mycgr3T

unnamed
  
Accession: CAP92339
  
Location: 3072376-3074285
  
  
**BlastP hit with Mycgr3G99766\_Mycgr3T**
  
Percentage identity: 34 %
  
BlastP bit score: 292
  
Sequence coverage: 102 %
  
E-value: 3e-88
  
  
 NCBI BlastP on this gene

Pc13g12700

unnamed
  
Accession: CAP92338
  
Location: 3071446-3072185
  
 NCBI BlastP on this gene

Pc13g12690

not annotated
  
Accession: Pc13g12680
  
Location: 3070043-3070708
  
 NCBI BlastP on this gene

Pc13g12680

hypothetical protein
  
Accession: CAP92336
  
Location: 3068669-3069775
  
 NCBI BlastP on this gene

Pc13g12670

hypothetical protein
  
Accession: CAP92335
  
Location: 3067665-3068152
  
 NCBI BlastP on this gene

Pc13g12660

not annotated
  
Accession: CAP92334
  
Location: 3064990-3067293
  
 NCBI BlastP on this gene

Pc13g12650

Query: Architecture Search FASTA input

KE007245 : Wallemia ichthyophaga EXF-994 unplaced genomic scaffold scaffold22    Total score: 1.0     Cumulative Blast bit score: 291

Hit cluster cross-links:

Mycgr3G41235 Mycgr3T
  
Location: 0-4062

Mycgr3G41235\_Mycgr3T

Mycgr3G70577 Mycgr3T
  
Location: 4162-6109

Mycgr3G70577\_Mycgr3T

Mycgr3G40534 Mycgr3T
  
Location: 6209-7166

Mycgr3G40534\_Mycgr3T

Mycgr3G85486 Mycgr3T
  
Location: 7266-8511

Mycgr3G85486\_Mycgr3T

Mycgr3G92221 Mycgr3T
  
Location: 8611-9193

Mycgr3G92221\_Mycgr3T

Mycgr3G39931 Mycgr3T
  
Location: 9293-10157

Mycgr3G39931\_Mycgr3T

Mycgr3G99766 Mycgr3T
  
Location: 10257-11775

Mycgr3G99766\_Mycgr3T

Kinesin-like protein
  
Accession: EOQ99037
  
Location: 56580-61572
  
 NCBI BlastP on this gene

EOQ99037

hypothetical protein
  
Accession: EOQ99038
  
Location: 61867-63862
  
 NCBI BlastP on this gene

EOQ99038

Intermediate cleaving peptidase 55
  
Accession: EOQ99039
  
Location: 64866-66416
  
 NCBI BlastP on this gene

EOQ99039

Putative lysine N-acyltransferase C17G9.06c
  
Accession: EOQ99040
  
Location: 66766-67998
  
  
**BlastP hit with Mycgr3G85486\_Mycgr3T**
  
Percentage identity: 45 %
  
BlastP bit score: 291
  
Sequence coverage: 82 %
  
E-value: 4e-91
  
  
 NCBI BlastP on this gene

EOQ99040

Query: Architecture Search FASTA input

KB456263 : Mycosphaerella populorum SO2202 unplaced genomic scaffold SEPMUscaffold\_4    Total score: 1.0     Cumulative Blast bit score: 290

Hit cluster cross-links:

Mycgr3G41235 Mycgr3T
  
Location: 0-4062

Mycgr3G41235\_Mycgr3T

Mycgr3G70577 Mycgr3T
  
Location: 4162-6109

Mycgr3G70577\_Mycgr3T

Mycgr3G40534 Mycgr3T
  
Location: 6209-7166

Mycgr3G40534\_Mycgr3T

Mycgr3G85486 Mycgr3T
  
Location: 7266-8511

Mycgr3G85486\_Mycgr3T

Mycgr3G92221 Mycgr3T
  
Location: 8611-9193

Mycgr3G92221\_Mycgr3T

Mycgr3G39931 Mycgr3T
  
Location: 9293-10157

Mycgr3G39931\_Mycgr3T

Mycgr3G99766 Mycgr3T
  
Location: 10257-11775

Mycgr3G99766\_Mycgr3T

MFS monosaccharide transporter
  
Accession: EMF13593
  
Location: 1500434-1502719
  
  
**BlastP hit with Mycgr3G99766\_Mycgr3T**
  
Percentage identity: 35 %
  
BlastP bit score: 290
  
Sequence coverage: 98 %
  
E-value: 3e-87
  
  
 NCBI BlastP on this gene

EMF13593

L-amino-acid oxidase
  
Accession: EMF13592
  
Location: 1498212-1500329
  
 NCBI BlastP on this gene

EMF13592

Isy1-like splicing factor
  
Accession: EMF13591
  
Location: 1496224-1496952
  
 NCBI BlastP on this gene

EMF13591

calcium ATPase
  
Accession: EMF13590
  
Location: 1491360-1494621
  
 NCBI BlastP on this gene

EMF13590

Query: Architecture Search FASTA input

JH717968 : Fomitiporia mediterranea MF3/22 unplaced genomic scaffold FOMMEscaffold\_2    Total score: 1.0     Cumulative Blast bit score: 290

Hit cluster cross-links:

Mycgr3G41235 Mycgr3T
  
Location: 0-4062

Mycgr3G41235\_Mycgr3T

Mycgr3G70577 Mycgr3T
  
Location: 4162-6109

Mycgr3G70577\_Mycgr3T

Mycgr3G40534 Mycgr3T
  
Location: 6209-7166

Mycgr3G40534\_Mycgr3T

Mycgr3G85486 Mycgr3T
  
Location: 7266-8511

Mycgr3G85486\_Mycgr3T

Mycgr3G92221 Mycgr3T
  
Location: 8611-9193

Mycgr3G92221\_Mycgr3T

Mycgr3G39931 Mycgr3T
  
Location: 9293-10157

Mycgr3G39931\_Mycgr3T

Mycgr3G99766 Mycgr3T
  
Location: 10257-11775

Mycgr3G99766\_Mycgr3T

drug:h+ antiporter
  
Accession: EJD07174
  
Location: 4566984-4570021
  
  
**BlastP hit with Mycgr3G70577\_Mycgr3T**
  
Percentage identity: 32 %
  
BlastP bit score: 290
  
Sequence coverage: 95 %
  
E-value: 5e-85
  
  
 NCBI BlastP on this gene

EJD07174

Query: Architecture Search FASTA input

KB445573 : Cochliobolus heterostrophus C5 unplaced genomic scaffold COCHEscaffold\_5    Total score: 1.0     Cumulative Blast bit score: 289

Hit cluster cross-links:

Mycgr3G41235 Mycgr3T
  
Location: 0-4062

Mycgr3G41235\_Mycgr3T

Mycgr3G70577 Mycgr3T
  
Location: 4162-6109

Mycgr3G70577\_Mycgr3T

Mycgr3G40534 Mycgr3T
  
Location: 6209-7166

Mycgr3G40534\_Mycgr3T

Mycgr3G85486 Mycgr3T
  
Location: 7266-8511

Mycgr3G85486\_Mycgr3T

Mycgr3G92221 Mycgr3T
  
Location: 8611-9193

Mycgr3G92221\_Mycgr3T

Mycgr3G39931 Mycgr3T
  
Location: 9293-10157

Mycgr3G39931\_Mycgr3T

Mycgr3G99766 Mycgr3T
  
Location: 10257-11775

Mycgr3G99766\_Mycgr3T

hypothetical protein
  
Accession: EMD93842
  
Location: 1871111-1872759
  
  
**BlastP hit with Mycgr3G99766\_Mycgr3T**
  
Percentage identity: 36 %
  
BlastP bit score: 289
  
Sequence coverage: 98 %
  
E-value: 2e-87
  
  
 NCBI BlastP on this gene

EMD93842

hypothetical protein
  
Accession: EMD93841
  
Location: 1869444-1869962
  
 NCBI BlastP on this gene

EMD93841

Query: Architecture Search FASTA input

KB908481 : Setosphaeria turcica Et28A unplaced genomic scaffold SETTUscaffold\_1    Total score: 1.0     Cumulative Blast bit score: 287

Hit cluster cross-links:

Mycgr3G41235 Mycgr3T
  
Location: 0-4062

Mycgr3G41235\_Mycgr3T

Mycgr3G70577 Mycgr3T
  
Location: 4162-6109

Mycgr3G70577\_Mycgr3T

Mycgr3G40534 Mycgr3T
  
Location: 6209-7166

Mycgr3G40534\_Mycgr3T

Mycgr3G85486 Mycgr3T
  
Location: 7266-8511

Mycgr3G85486\_Mycgr3T

Mycgr3G92221 Mycgr3T
  
Location: 8611-9193

Mycgr3G92221\_Mycgr3T

Mycgr3G39931 Mycgr3T
  
Location: 9293-10157

Mycgr3G39931\_Mycgr3T

Mycgr3G99766 Mycgr3T
  
Location: 10257-11775

Mycgr3G99766\_Mycgr3T

hypothetical protein
  
Accession: EOA91983
  
Location: 2788679-2790646
  
 NCBI BlastP on this gene

EOA91983

hypothetical protein
  
Accession: EOA91984
  
Location: 2791612-2792619
  
 NCBI BlastP on this gene

EOA91984

hypothetical protein
  
Accession: EOA91985
  
Location: 2797158-2798799
  
  
**BlastP hit with Mycgr3G99766\_Mycgr3T**
  
Percentage identity: 34 %
  
BlastP bit score: 287
  
Sequence coverage: 98 %
  
E-value: 9e-87
  
  
 NCBI BlastP on this gene

EOA91985

Query: Architecture Search FASTA input

GL385397 : Gaeumannomyces graminis var. tritici R3-111a-1 unplaced genomic scaffold supercont2.3    Total score: 1.0     Cumulative Blast bit score: 287

Hit cluster cross-links:

Mycgr3G41235 Mycgr3T
  
Location: 0-4062

Mycgr3G41235\_Mycgr3T

Mycgr3G70577 Mycgr3T
  
Location: 4162-6109

Mycgr3G70577\_Mycgr3T

Mycgr3G40534 Mycgr3T
  
Location: 6209-7166

Mycgr3G40534\_Mycgr3T

Mycgr3G85486 Mycgr3T
  
Location: 7266-8511

Mycgr3G85486\_Mycgr3T

Mycgr3G92221 Mycgr3T
  
Location: 8611-9193

Mycgr3G92221\_Mycgr3T

Mycgr3G39931 Mycgr3T
  
Location: 9293-10157

Mycgr3G39931\_Mycgr3T

Mycgr3G99766 Mycgr3T
  
Location: 10257-11775

Mycgr3G99766\_Mycgr3T

hypothetical protein
  
Accession: EJT77362
  
Location: 6424662-6427139
  
 NCBI BlastP on this gene

EJT77362

hypothetical protein
  
Accession: EJT77361
  
Location: 6423003-6424358
  
 NCBI BlastP on this gene

EJT77361

hypothetical protein
  
Accession: EJT77360
  
Location: 6420489-6422372
  
 NCBI BlastP on this gene

EJT77360

sugar transporter STL1
  
Accession: EJT77359
  
Location: 6418066-6419824
  
  
**BlastP hit with Mycgr3G99766\_Mycgr3T**
  
Percentage identity: 34 %
  
BlastP bit score: 287
  
Sequence coverage: 97 %
  
E-value: 2e-86
  
  
 NCBI BlastP on this gene

EJT77359

hypothetical protein
  
Accession: EJT77358
  
Location: 6416594-6417531
  
 NCBI BlastP on this gene

EJT77358

hypothetical protein
  
Accession: EJT77357
  
Location: 6414564-6416528
  
 NCBI BlastP on this gene

EJT77357

hypothetical protein
  
Accession: EJT77356
  
Location: 6412291-6413950
  
 NCBI BlastP on this gene

EJT77356

hypothetical protein
  
Accession: EJT77355
  
Location: 6409856-6411814
  
 NCBI BlastP on this gene

EJT77355

Query: Architecture Search FASTA input

CM001202 : Mycosphaerella graminicola IPO323 chromosome 7    Total score: 1.0     Cumulative Blast bit score: 287

Hit cluster cross-links:

Mycgr3G41235 Mycgr3T
  
Location: 0-4062

Mycgr3G41235\_Mycgr3T

Mycgr3G70577 Mycgr3T
  
Location: 4162-6109

Mycgr3G70577\_Mycgr3T

Mycgr3G40534 Mycgr3T
  
Location: 6209-7166

Mycgr3G40534\_Mycgr3T

Mycgr3G85486 Mycgr3T
  
Location: 7266-8511

Mycgr3G85486\_Mycgr3T

Mycgr3G92221 Mycgr3T
  
Location: 8611-9193

Mycgr3G92221\_Mycgr3T

Mycgr3G39931 Mycgr3T
  
Location: 9293-10157

Mycgr3G39931\_Mycgr3T

Mycgr3G99766 Mycgr3T
  
Location: 10257-11775

Mycgr3G99766\_Mycgr3T

hypothetical protein
  
Accession: EGP86225
  
Location: 500337-501938
  
 NCBI BlastP on this gene

EGP86225

hypothetical protein
  
Accession: EGP86224
  
Location: 502233-503902
  
 NCBI BlastP on this gene

EGP86224

hypothetical protein
  
Accession: EGP86223
  
Location: 504739-505457
  
 NCBI BlastP on this gene

EGP86223

hypothetical protein
  
Accession: EGP85599
  
Location: 506022-507050
  
 NCBI BlastP on this gene

EGP85599

hypothetical protein
  
Accession: EGP85600
  
Location: 507879-510119
  
  
**BlastP hit with Mycgr3G99766\_Mycgr3T**
  
Percentage identity: 35 %
  
BlastP bit score: 287
  
Sequence coverage: 97 %
  
E-value: 2e-86
  
  
 NCBI BlastP on this gene

EGP85600

Query: Architecture Search FASTA input

CH476615 : Uncinocarpus reesii 1704 scaffold\_1 genomic scaffold    Total score: 1.0     Cumulative Blast bit score: 287

Hit cluster cross-links:

Mycgr3G41235 Mycgr3T
  
Location: 0-4062

Mycgr3G41235\_Mycgr3T

Mycgr3G70577 Mycgr3T
  
Location: 4162-6109

Mycgr3G70577\_Mycgr3T

Mycgr3G40534 Mycgr3T
  
Location: 6209-7166

Mycgr3G40534\_Mycgr3T

Mycgr3G85486 Mycgr3T
  
Location: 7266-8511

Mycgr3G85486\_Mycgr3T

Mycgr3G92221 Mycgr3T
  
Location: 8611-9193

Mycgr3G92221\_Mycgr3T

Mycgr3G39931 Mycgr3T
  
Location: 9293-10157

Mycgr3G39931\_Mycgr3T

Mycgr3G99766 Mycgr3T
  
Location: 10257-11775

Mycgr3G99766\_Mycgr3T

conserved hypothetical protein
  
Accession: EEP75793
  
Location: 1344176-1345127
  
 NCBI BlastP on this gene

EEP75793

predicted protein
  
Accession: EEP75794
  
Location: 1345522-1346787
  
 NCBI BlastP on this gene

EEP75794

predicted protein
  
Accession: EEP75795
  
Location: 1347774-1348259
  
 NCBI BlastP on this gene

EEP75795

conserved hypothetical protein
  
Accession: EEP75796
  
Location: 1349119-1350031
  
 NCBI BlastP on this gene

EEP75796

conserved hypothetical protein
  
Accession: EEP75797
  
Location: 1351940-1353898
  
  
**BlastP hit with Mycgr3G99766\_Mycgr3T**
  
Percentage identity: 34 %
  
BlastP bit score: 287
  
Sequence coverage: 101 %
  
E-value: 5e-86
  
  
 NCBI BlastP on this gene

EEP75797

predicted protein
  
Accession: EEP75798
  
Location: 1354404-1355397
  
 NCBI BlastP on this gene

EEP75798

conserved hypothetical protein
  
Accession: EEP75799
  
Location: 1355908-1356666
  
 NCBI BlastP on this gene

EEP75799

predicted protein
  
Accession: EEP75800
  
Location: 1358325-1360178
  
 NCBI BlastP on this gene

EEP75800

Query: Architecture Search FASTA input

CR382129 : Yarrowia lipolytica CLIB122 chromosome C complete sequence.    Total score: 1.0     Cumulative Blast bit score: 286

Hit cluster cross-links:

Mycgr3G41235 Mycgr3T
  
Location: 0-4062

Mycgr3G41235\_Mycgr3T

Mycgr3G70577 Mycgr3T
  
Location: 4162-6109

Mycgr3G70577\_Mycgr3T

Mycgr3G40534 Mycgr3T
  
Location: 6209-7166

Mycgr3G40534\_Mycgr3T

Mycgr3G85486 Mycgr3T
  
Location: 7266-8511

Mycgr3G85486\_Mycgr3T

Mycgr3G92221 Mycgr3T
  
Location: 8611-9193

Mycgr3G92221\_Mycgr3T

Mycgr3G39931 Mycgr3T
  
Location: 9293-10157

Mycgr3G39931\_Mycgr3T

Mycgr3G99766 Mycgr3T
  
Location: 10257-11775

Mycgr3G99766\_Mycgr3T

YALI0C04620p
  
Accession: CAG81749
  
Location: 614285-617044
  
 NCBI BlastP on this gene

YALI0\_C04620g

YALI0C04730p
  
Accession: CAG81752
  
Location: 622876-624573
  
  
**BlastP hit with Mycgr3G99766\_Mycgr3T**
  
Percentage identity: 33 %
  
BlastP bit score: 286
  
Sequence coverage: 104 %
  
E-value: 8e-86
  
  
 NCBI BlastP on this gene

YALI0\_C04730g

YALI0C04774p
  
Accession: CAG81753
  
Location: 625342-627255
  
 NCBI BlastP on this gene

YALI0\_C04774g

YALI0C04796p
  
Accession: CAG81754
  
Location: 628618-630402
  
 NCBI BlastP on this gene

YALI0\_C04796g

YALI0C04818p
  
Accession: CAG81755
  
Location: 631617-633371
  
 NCBI BlastP on this gene

YALI0\_C04818g

Query: Architecture Search FASTA input

CM001199 : Mycosphaerella graminicola IPO323 chromosome 4    Total score: 1.0     Cumulative Blast bit score: 286

Hit cluster cross-links:

Mycgr3G41235 Mycgr3T
  
Location: 0-4062

Mycgr3G41235\_Mycgr3T

Mycgr3G70577 Mycgr3T
  
Location: 4162-6109

Mycgr3G70577\_Mycgr3T

Mycgr3G40534 Mycgr3T
  
Location: 6209-7166

Mycgr3G40534\_Mycgr3T

Mycgr3G85486 Mycgr3T
  
Location: 7266-8511

Mycgr3G85486\_Mycgr3T

Mycgr3G92221 Mycgr3T
  
Location: 8611-9193

Mycgr3G92221\_Mycgr3T

Mycgr3G39931 Mycgr3T
  
Location: 9293-10157

Mycgr3G39931\_Mycgr3T

Mycgr3G99766 Mycgr3T
  
Location: 10257-11775

Mycgr3G99766\_Mycgr3T

hypothetical protein
  
Accession: EGP88531
  
Location: 535272-537446
  
  
**BlastP hit with Mycgr3G99766\_Mycgr3T**
  
Percentage identity: 33 %
  
BlastP bit score: 286
  
Sequence coverage: 105 %
  
E-value: 1e-85
  
  
 NCBI BlastP on this gene

EGP88531

Bromodomain-containing AAA ATPase protein
  
Accession: EGP87834
  
Location: 527038-531994
  
 NCBI BlastP on this gene

EGP87834

Query: Architecture Search FASTA input

AKCT01000319 : Penicillium digitatum PHI26    Total score: 1.0     Cumulative Blast bit score: 286

Hit cluster cross-links:

Mycgr3G41235 Mycgr3T
  
Location: 0-4062

Mycgr3G41235\_Mycgr3T

Mycgr3G70577 Mycgr3T
  
Location: 4162-6109

Mycgr3G70577\_Mycgr3T

Mycgr3G40534 Mycgr3T
  
Location: 6209-7166

Mycgr3G40534\_Mycgr3T

Mycgr3G85486 Mycgr3T
  
Location: 7266-8511

Mycgr3G85486\_Mycgr3T

Mycgr3G92221 Mycgr3T
  
Location: 8611-9193

Mycgr3G92221\_Mycgr3T

Mycgr3G39931 Mycgr3T
  
Location: 9293-10157

Mycgr3G39931\_Mycgr3T

Mycgr3G99766 Mycgr3T
  
Location: 10257-11775

Mycgr3G99766\_Mycgr3T

hypothetical protein
  
Accession: EKV04858
  
Location: 124390-125267
  
 NCBI BlastP on this gene

EKV04858

Actin-related protein 4
  
Accession: EKV04859
  
Location: 126099-127684
  
 NCBI BlastP on this gene

EKV04859

hypothetical protein
  
Accession: EKV04860
  
Location: 128707-129799
  
 NCBI BlastP on this gene

EKV04860

MFS sugar transporter, putative
  
Accession: EKV04861
  
Location: 132504-134054
  
  
**BlastP hit with Mycgr3G99766\_Mycgr3T**
  
Percentage identity: 34 %
  
BlastP bit score: 286
  
Sequence coverage: 97 %
  
E-value: 2e-86
  
  
 NCBI BlastP on this gene

EKV04861

hypothetical protein
  
Accession: EKV04862
  
Location: 135328-136455
  
 NCBI BlastP on this gene

EKV04862

hypothetical protein
  
Accession: EKV04863
  
Location: 138164-140084
  
 NCBI BlastP on this gene

EKV04863

hypothetical protein
  
Accession: EKV04864
  
Location: 141219-141959
  
 NCBI BlastP on this gene

EKV04864

Query: Architecture Search FASTA input

AM920437 : Penicillium chrysogenum Wisconsin 54-1255 complete genome, contig Pc00c22.    Total score: 1.0     Cumulative Blast bit score: 285

Hit cluster cross-links:

Mycgr3G41235 Mycgr3T
  
Location: 0-4062

Mycgr3G41235\_Mycgr3T

Mycgr3G70577 Mycgr3T
  
Location: 4162-6109

Mycgr3G70577\_Mycgr3T

Mycgr3G40534 Mycgr3T
  
Location: 6209-7166

Mycgr3G40534\_Mycgr3T

Mycgr3G85486 Mycgr3T
  
Location: 7266-8511

Mycgr3G85486\_Mycgr3T

Mycgr3G92221 Mycgr3T
  
Location: 8611-9193

Mycgr3G92221\_Mycgr3T

Mycgr3G39931 Mycgr3T
  
Location: 9293-10157

Mycgr3G39931\_Mycgr3T

Mycgr3G99766 Mycgr3T
  
Location: 10257-11775

Mycgr3G99766\_Mycgr3T

not annotated
  
Accession: CAP98073
  
Location: 1860917-1861800
  
 NCBI BlastP on this gene

Pc22g07850

not annotated
  
Accession: CAP98072
  
Location: 1858575-1860153
  
 NCBI BlastP on this gene

Pc22g07840

not annotated
  
Accession: CAP98071
  
Location: 1856478-1857571
  
 NCBI BlastP on this gene

Pc22g07830

hypothetical protein
  
Accession: CAP98070
  
Location: 1854097-1854827
  
 NCBI BlastP on this gene

Pc22g07820

unnamed
  
Accession: CAP98069
  
Location: 1852309-1853859
  
  
**BlastP hit with Mycgr3G99766\_Mycgr3T**
  
Percentage identity: 33 %
  
BlastP bit score: 285
  
Sequence coverage: 97 %
  
E-value: 8e-86
  
  
 NCBI BlastP on this gene

Pc22g07810

hypothetical protein
  
Accession: CAP98068
  
Location: 1849887-1851473
  
 NCBI BlastP on this gene

Pc22g07800

unnamed
  
Accession: CAP98067
  
Location: 1847410-1849556
  
 NCBI BlastP on this gene

Pc22g07790

unnamed
  
Accession: CAP98066
  
Location: 1844614-1846288
  
 NCBI BlastP on this gene

Pc22g07780

Query: Architecture Search FASTA input

KB730093 : Fusarium oxysporum f. sp. cubense race 1 unplaced genomic scaffold scaffold48    Total score: 1.0     Cumulative Blast bit score: 281

Hit cluster cross-links:

Mycgr3G41235 Mycgr3T
  
Location: 0-4062

Mycgr3G41235\_Mycgr3T

Mycgr3G70577 Mycgr3T
  
Location: 4162-6109

Mycgr3G70577\_Mycgr3T

Mycgr3G40534 Mycgr3T
  
Location: 6209-7166

Mycgr3G40534\_Mycgr3T

Mycgr3G85486 Mycgr3T
  
Location: 7266-8511

Mycgr3G85486\_Mycgr3T

Mycgr3G92221 Mycgr3T
  
Location: 8611-9193

Mycgr3G92221\_Mycgr3T

Mycgr3G39931 Mycgr3T
  
Location: 9293-10157

Mycgr3G39931\_Mycgr3T

Mycgr3G99766 Mycgr3T
  
Location: 10257-11775

Mycgr3G99766\_Mycgr3T

Telomere length regulator protein rif1
  
Accession: ENH72509
  
Location: 459422-464790
  
 NCBI BlastP on this gene

ENH72509

PiggyBac transposable element-derived protein 4
  
Accession: ENH72508
  
Location: 456104-458041
  
 NCBI BlastP on this gene

ENH72508

Sugar transporter STL1
  
Accession: ENH72507
  
Location: 451689-453457
  
  
**BlastP hit with Mycgr3G99766\_Mycgr3T**
  
Percentage identity: 34 %
  
BlastP bit score: 281
  
Sequence coverage: 97 %
  
E-value: 7e-84
  
  
 NCBI BlastP on this gene

ENH72507

Glucose-6-phosphate 1-epimerase
  
Accession: ENH72506
  
Location: 449808-450856
  
 NCBI BlastP on this gene

ENH72506

Phospho-2-dehydro-3-deoxyheptonate aldolase
  
Accession: ENH72505
  
Location: 448065-449480
  
 NCBI BlastP on this gene

ENH72505

hypothetical protein
  
Accession: ENH72504
  
Location: 446982-447305
  
 NCBI BlastP on this gene

ENH72504

Microcephalin
  
Accession: ENH72503
  
Location: 443142-446589
  
 NCBI BlastP on this gene

ENH72503

Query: Architecture Search FASTA input

JH668249 : Wallemia sebi CBS 633.66 unplaced genomic scaffold WALSEscaffold\_27    Total score: 1.0     Cumulative Blast bit score: 281

Hit cluster cross-links:

Mycgr3G41235 Mycgr3T
  
Location: 0-4062

Mycgr3G41235\_Mycgr3T

Mycgr3G70577 Mycgr3T
  
Location: 4162-6109

Mycgr3G70577\_Mycgr3T

Mycgr3G40534 Mycgr3T
  
Location: 6209-7166

Mycgr3G40534\_Mycgr3T

Mycgr3G85486 Mycgr3T
  
Location: 7266-8511

Mycgr3G85486\_Mycgr3T

Mycgr3G92221 Mycgr3T
  
Location: 8611-9193

Mycgr3G92221\_Mycgr3T

Mycgr3G39931 Mycgr3T
  
Location: 9293-10157

Mycgr3G39931\_Mycgr3T

Mycgr3G99766 Mycgr3T
  
Location: 10257-11775

Mycgr3G99766\_Mycgr3T

kinesin-like protein
  
Accession: EIM19516
  
Location: 48417-53354
  
 NCBI BlastP on this gene

EIM19516

hypothetical protein
  
Accession: EIM19517
  
Location: 53608-54953
  
 NCBI BlastP on this gene

EIM19517

hypothetical protein
  
Accession: EIM19518
  
Location: 55874-57413
  
 NCBI BlastP on this gene

EIM19518

hypothetical protein
  
Accession: EIM19519
  
Location: 57687-58852
  
  
**BlastP hit with Mycgr3G85486\_Mycgr3T**
  
Percentage identity: 44 %
  
BlastP bit score: 281
  
Sequence coverage: 84 %
  
E-value: 9e-88
  
  
 NCBI BlastP on this gene

EIM19519

mitochondrial carrier
  
Accession: EIM19520
  
Location: 58943-60625
  
 NCBI BlastP on this gene

EIM19520

hypothetical protein
  
Accession: EIM19521
  
Location: 60665-61582
  
 NCBI BlastP on this gene

EIM19521

hypothetical protein
  
Accession: EIM19522
  
Location: 61815-64302
  
 NCBI BlastP on this gene

EIM19522

hypothetical protein
  
Accession: EIM19523
  
Location: 64342-68802
  
 NCBI BlastP on this gene

EIM19523

Query: Architecture Search FASTA input

AACD01000043 : Aspergillus nidulans FGSC A4    Total score: 1.0     Cumulative Blast bit score: 281

Hit cluster cross-links:

Mycgr3G41235 Mycgr3T
  
Location: 0-4062

Mycgr3G41235\_Mycgr3T

Mycgr3G70577 Mycgr3T
  
Location: 4162-6109

Mycgr3G70577\_Mycgr3T

Mycgr3G40534 Mycgr3T
  
Location: 6209-7166

Mycgr3G40534\_Mycgr3T

Mycgr3G85486 Mycgr3T
  
Location: 7266-8511

Mycgr3G85486\_Mycgr3T

Mycgr3G92221 Mycgr3T
  
Location: 8611-9193

Mycgr3G92221\_Mycgr3T

Mycgr3G39931 Mycgr3T
  
Location: 9293-10157

Mycgr3G39931\_Mycgr3T

Mycgr3G99766 Mycgr3T
  
Location: 10257-11775

Mycgr3G99766\_Mycgr3T

hypothetical protein
  
Accession: EAA64689
  
Location: 226758-228332
  
  
**BlastP hit with Mycgr3G99766\_Mycgr3T**
  
Percentage identity: 34 %
  
BlastP bit score: 281
  
Sequence coverage: 96 %
  
E-value: 2e-84
  
  
 NCBI BlastP on this gene

EAA64689

hypothetical protein
  
Accession: EAA64688
  
Location: 224392-225635
  
 NCBI BlastP on this gene

EAA64688

hypothetical protein
  
Accession: EAA64687
  
Location: 222212-223601
  
 NCBI BlastP on this gene

EAA64687

hypothetical protein
  
Accession: EAA64686
  
Location: 217545-221325
  
 NCBI BlastP on this gene

EAA64686

Query: Architecture Search FASTA input

HF679023 : Fusarium fujikuroi IMI 58289 draft genome, chromosome FFUJ\_chr01.    Total score: 1.0     Cumulative Blast bit score: 280

Hit cluster cross-links:

Mycgr3G41235 Mycgr3T
  
Location: 0-4062

Mycgr3G41235\_Mycgr3T

Mycgr3G70577 Mycgr3T
  
Location: 4162-6109

Mycgr3G70577\_Mycgr3T

Mycgr3G40534 Mycgr3T
  
Location: 6209-7166

Mycgr3G40534\_Mycgr3T

Mycgr3G85486 Mycgr3T
  
Location: 7266-8511

Mycgr3G85486\_Mycgr3T

Mycgr3G92221 Mycgr3T
  
Location: 8611-9193

Mycgr3G92221\_Mycgr3T

Mycgr3G39931 Mycgr3T
  
Location: 9293-10157

Mycgr3G39931\_Mycgr3T

Mycgr3G99766 Mycgr3T
  
Location: 10257-11775

Mycgr3G99766\_Mycgr3T

related to telomere length regulator protein rif1
  
Accession: CCT61789
  
Location: 1117281-1122658
  
 NCBI BlastP on this gene

FFUJ\_01721

probable sugar transporter
  
Accession: CCT61790
  
Location: 1125937-1127711
  
  
**BlastP hit with Mycgr3G99766\_Mycgr3T**
  
Percentage identity: 34 %
  
BlastP bit score: 280
  
Sequence coverage: 97 %
  
E-value: 1e-83
  
  
 NCBI BlastP on this gene

FFUJ\_01720

Query: Architecture Search FASTA input

KB726991 : Fusarium oxysporum f. sp. cubense race 4 unplaced genomic scaffold scaffold135    Total score: 1.0     Cumulative Blast bit score: 279

Hit cluster cross-links:

Mycgr3G41235 Mycgr3T
  
Location: 0-4062

Mycgr3G41235\_Mycgr3T

Mycgr3G70577 Mycgr3T
  
Location: 4162-6109

Mycgr3G70577\_Mycgr3T

Mycgr3G40534 Mycgr3T
  
Location: 6209-7166

Mycgr3G40534\_Mycgr3T

Mycgr3G85486 Mycgr3T
  
Location: 7266-8511

Mycgr3G85486\_Mycgr3T

Mycgr3G92221 Mycgr3T
  
Location: 8611-9193

Mycgr3G92221\_Mycgr3T

Mycgr3G39931 Mycgr3T
  
Location: 9293-10157

Mycgr3G39931\_Mycgr3T

Mycgr3G99766 Mycgr3T
  
Location: 10257-11775

Mycgr3G99766\_Mycgr3T

Telomere length regulator protein rif1
  
Accession: EMT64558
  
Location: 526179-533911
  
 NCBI BlastP on this gene

EMT64558

Sugar transporter STL1
  
Accession: EMT64557
  
Location: 520950-522718
  
  
**BlastP hit with Mycgr3G99766\_Mycgr3T**
  
Percentage identity: 34 %
  
BlastP bit score: 279
  
Sequence coverage: 97 %
  
E-value: 2e-83
  
  
 NCBI BlastP on this gene

EMT64557

Glucose-6-phosphate 1-epimerase
  
Accession: EMT64556
  
Location: 516926-517974
  
 NCBI BlastP on this gene

EMT64556

Phospho-2-dehydro-3-deoxyheptonate aldolase
  
Accession: EMT64555
  
Location: 515183-516598
  
 NCBI BlastP on this gene

EMT64555

hypothetical protein
  
Accession: EMT64554
  
Location: 514080-514403
  
 NCBI BlastP on this gene

EMT64554

Microcephalin
  
Accession: EMT64553
  
Location: 508331-513687
  
 NCBI BlastP on this gene

EMT64553

Query: Architecture Search FASTA input

FO082046 : Pichia sorbitophila strain CBS 7064 chromosome N complete sequence.    Total score: 1.0     Cumulative Blast bit score: 279

Hit cluster cross-links:

Mycgr3G41235 Mycgr3T
  
Location: 0-4062

Mycgr3G41235\_Mycgr3T

Mycgr3G70577 Mycgr3T
  
Location: 4162-6109

Mycgr3G70577\_Mycgr3T

Mycgr3G40534 Mycgr3T
  
Location: 6209-7166

Mycgr3G40534\_Mycgr3T

Mycgr3G85486 Mycgr3T
  
Location: 7266-8511

Mycgr3G85486\_Mycgr3T

Mycgr3G92221 Mycgr3T
  
Location: 8611-9193

Mycgr3G92221\_Mycgr3T

Mycgr3G39931 Mycgr3T
  
Location: 9293-10157

Mycgr3G39931\_Mycgr3T

Mycgr3G99766 Mycgr3T
  
Location: 10257-11775

Mycgr3G99766\_Mycgr3T

not annotated
  
Accession: CCE86745
  
Location: 919429-926343
  
 NCBI BlastP on this gene

Piso0\_005255

not annotated
  
Accession: CCE86744
  
Location: 917681-918376
  
 NCBI BlastP on this gene

Piso0\_005254

not annotated
  
Accession: CCE86743
  
Location: 916635-916880
  
 NCBI BlastP on this gene

Piso0\_005253

not annotated
  
Accession: CCE86742
  
Location: 915883-916242
  
 NCBI BlastP on this gene

Piso0\_005252

not annotated
  
Accession: CCE86741
  
Location: 913548-915314
  
  
**BlastP hit with Mycgr3G99766\_Mycgr3T**
  
Percentage identity: 32 %
  
BlastP bit score: 279
  
Sequence coverage: 104 %
  
E-value: 1e-82
  
  
 NCBI BlastP on this gene

Piso0\_005250

not annotated
  
Accession: CCE86740
  
Location: 912860-913396
  
 NCBI BlastP on this gene

Piso0\_005249

not annotated
  
Accession: CCE86739
  
Location: 911623-912252
  
 NCBI BlastP on this gene

Piso0\_005248

not annotated
  
Accession: CCE86738
  
Location: 909781-911154
  
 NCBI BlastP on this gene

Piso0\_005247

not annotated
  
Accession: CCE86737
  
Location: 907636-908721
  
 NCBI BlastP on this gene

Piso0\_005246

not annotated
  
Accession: CCE86736
  
Location: 905218-906618
  
 NCBI BlastP on this gene

Piso0\_005245

Query: Architecture Search FASTA input

CU928166 : Lachancea thermotolerans CBS 6340 chromosome B complete sequence.    Total score: 1.0     Cumulative Blast bit score: 279

Hit cluster cross-links:

Mycgr3G41235 Mycgr3T
  
Location: 0-4062

Mycgr3G41235\_Mycgr3T

Mycgr3G70577 Mycgr3T
  
Location: 4162-6109

Mycgr3G70577\_Mycgr3T

Mycgr3G40534 Mycgr3T
  
Location: 6209-7166

Mycgr3G40534\_Mycgr3T

Mycgr3G85486 Mycgr3T
  
Location: 7266-8511

Mycgr3G85486\_Mycgr3T

Mycgr3G92221 Mycgr3T
  
Location: 8611-9193

Mycgr3G92221\_Mycgr3T

Mycgr3G39931 Mycgr3T
  
Location: 9293-10157

Mycgr3G39931\_Mycgr3T

Mycgr3G99766 Mycgr3T
  
Location: 10257-11775

Mycgr3G99766\_Mycgr3T

KLTH0B04576p
  
Accession: CAR21548
  
Location: 370504-373431
  
 NCBI BlastP on this gene

KLTH0B04576g

KLTH0B04554p
  
Accession: CAR21547
  
Location: 367962-370022
  
 NCBI BlastP on this gene

KLTH0B04554g

KLTH0B04532p
  
Accession: CAR21546
  
Location: 367438-367770
  
 NCBI BlastP on this gene

KLTH0B04532g

KLTH0B04510p
  
Accession: CAR21545
  
Location: 364062-365768
  
  
**BlastP hit with Mycgr3G99766\_Mycgr3T**
  
Percentage identity: 32 %
  
BlastP bit score: 279
  
Sequence coverage: 101 %
  
E-value: 5e-83
  
  
 NCBI BlastP on this gene

KLTH0B04510g

KLTH0B04488p
  
Accession: CAR21544
  
Location: 362390-363715
  
 NCBI BlastP on this gene

KLTH0B04488g

KLTH0B04466p
  
Accession: CAR21543
  
Location: 360380-362344
  
 NCBI BlastP on this gene

KLTH0B04466g

KLTH0B04444p
  
Accession: CAR21542
  
Location: 359264-360151
  
 NCBI BlastP on this gene

KLTH0B04444g

KLTH0B04422p
  
Accession: CAR21541
  
Location: 358915-359253
  
 NCBI BlastP on this gene

KLTH0B04422g

KLTH0B04400p
  
Accession: CAR21540
  
Location: 357622-358890
  
 NCBI BlastP on this gene

KLTH0B04400g

KLTH0B04378p
  
Accession: CAR21539
  
Location: 356395-357537
  
 NCBI BlastP on this gene

KLTH0B04378g

Query: Architecture Search FASTA input

EQ963475 : Aspergillus flavus NRRL3357 scf\_1106286419142 genomic scaffold    Total score: 1.0     Cumulative Blast bit score: 278

Hit cluster cross-links:

Mycgr3G41235 Mycgr3T
  
Location: 0-4062

Mycgr3G41235\_Mycgr3T

Mycgr3G70577 Mycgr3T
  
Location: 4162-6109

Mycgr3G70577\_Mycgr3T

Mycgr3G40534 Mycgr3T
  
Location: 6209-7166

Mycgr3G40534\_Mycgr3T

Mycgr3G85486 Mycgr3T
  
Location: 7266-8511

Mycgr3G85486\_Mycgr3T

Mycgr3G92221 Mycgr3T
  
Location: 8611-9193

Mycgr3G92221\_Mycgr3T

Mycgr3G39931 Mycgr3T
  
Location: 9293-10157

Mycgr3G39931\_Mycgr3T

Mycgr3G99766 Mycgr3T
  
Location: 10257-11775

Mycgr3G99766\_Mycgr3T

conserved hypothetical protein
  
Accession: EED53490
  
Location: 1180417-1182188
  
 NCBI BlastP on this gene

EED53490

mitochondrial uncoupling protein, putative
  
Accession: EED53489
  
Location: 1179033-1180125
  
 NCBI BlastP on this gene

EED53489

vitamin H transporter, putative
  
Accession: EED53488
  
Location: 1176795-1178587
  
 NCBI BlastP on this gene

EED53488

MFS sugar transporter, putative
  
Accession: EED53487
  
Location: 1173797-1175393
  
  
**BlastP hit with Mycgr3G99766\_Mycgr3T**
  
Percentage identity: 34 %
  
BlastP bit score: 278
  
Sequence coverage: 95 %
  
E-value: 2e-83
  
  
 NCBI BlastP on this gene

EED53487

Dioxygenase family protein
  
Accession: EED53486
  
Location: 1171710-1172925
  
 NCBI BlastP on this gene

EED53486

cytochrome P450, putative
  
Accession: EED53485
  
Location: 1165381-1166754
  
 NCBI BlastP on this gene

EED53485

Query: Architecture Search FASTA input

EQ963474 : Aspergillus flavus NRRL3357 scf\_1106286417496 genomic scaffold    Total score: 1.0     Cumulative Blast bit score: 278

Hit cluster cross-links:

Mycgr3G41235 Mycgr3T
  
Location: 0-4062

Mycgr3G41235\_Mycgr3T

Mycgr3G70577 Mycgr3T
  
Location: 4162-6109

Mycgr3G70577\_Mycgr3T

Mycgr3G40534 Mycgr3T
  
Location: 6209-7166

Mycgr3G40534\_Mycgr3T

Mycgr3G85486 Mycgr3T
  
Location: 7266-8511

Mycgr3G85486\_Mycgr3T

Mycgr3G92221 Mycgr3T
  
Location: 8611-9193

Mycgr3G92221\_Mycgr3T

Mycgr3G39931 Mycgr3T
  
Location: 9293-10157

Mycgr3G39931\_Mycgr3T

Mycgr3G99766 Mycgr3T
  
Location: 10257-11775

Mycgr3G99766\_Mycgr3T

conserved hypothetical protein
  
Accession: EED54060
  
Location: 70669-72041
  
 NCBI BlastP on this gene

EED54060

efflux pump antibiotic resistance protein, putative
  
Accession: EED54059
  
Location: 68807-69616
  
 NCBI BlastP on this gene

EED54059

conserved hypothetical protein
  
Accession: EED54058
  
Location: 67507-68554
  
 NCBI BlastP on this gene

EED54058

fungal specific transcription factor, putative
  
Accession: EED54057
  
Location: 64970-66434
  
 NCBI BlastP on this gene

EED54057

conserved hypothetical protein
  
Accession: EED54056
  
Location: 64438-64857
  
 NCBI BlastP on this gene

EED54056

sugar transporter, putative
  
Accession: EED54055
  
Location: 61598-63282
  
  
**BlastP hit with Mycgr3G99766\_Mycgr3T**
  
Percentage identity: 33 %
  
BlastP bit score: 278
  
Sequence coverage: 95 %
  
E-value: 3e-83
  
  
 NCBI BlastP on this gene

EED54055

conserved hypothetical protein
  
Accession: EED54054
  
Location: 59044-61101
  
 NCBI BlastP on this gene

EED54054

conserved hypothetical protein
  
Accession: EED54053
  
Location: 57560-58165
  
 NCBI BlastP on this gene

EED54053

conserved hypothetical protein
  
Accession: EED54052
  
Location: 56150-57205
  
 NCBI BlastP on this gene

EED54052

conserved hypothetical protein
  
Accession: EED54051
  
Location: 53343-54595
  
 NCBI BlastP on this gene

EED54051

Query: Architecture Search FASTA input

AP007161 : Aspergillus oryzae RIB40 DNA, SC012.    Total score: 1.0     Cumulative Blast bit score: 278

Hit cluster cross-links:

Mycgr3G41235 Mycgr3T
  
Location: 0-4062

Mycgr3G41235\_Mycgr3T

Mycgr3G70577 Mycgr3T
  
Location: 4162-6109

Mycgr3G70577\_Mycgr3T

Mycgr3G40534 Mycgr3T
  
Location: 6209-7166

Mycgr3G40534\_Mycgr3T

Mycgr3G85486 Mycgr3T
  
Location: 7266-8511

Mycgr3G85486\_Mycgr3T

Mycgr3G92221 Mycgr3T
  
Location: 8611-9193

Mycgr3G92221\_Mycgr3T

Mycgr3G39931 Mycgr3T
  
Location: 9293-10157

Mycgr3G39931\_Mycgr3T

Mycgr3G99766 Mycgr3T
  
Location: 10257-11775

Mycgr3G99766\_Mycgr3T

not annotated
  
Accession: BAE60269
  
Location: 48979-50351
  
 NCBI BlastP on this gene

AO090012000022

not annotated
  
Accession: BAE60268
  
Location: 47117-48667
  
 NCBI BlastP on this gene

AO090012000021

not annotated
  
Accession: BAE60267
  
Location: 45816-46863
  
 NCBI BlastP on this gene

AO090012000020

not annotated
  
Accession: BAE60266
  
Location: 42746-43165
  
 NCBI BlastP on this gene

AO090012000019

not annotated
  
Accession: BAE60265
  
Location: 39901-41585
  
  
**BlastP hit with Mycgr3G99766\_Mycgr3T**
  
Percentage identity: 33 %
  
BlastP bit score: 278
  
Sequence coverage: 95 %
  
E-value: 3e-83
  
  
 NCBI BlastP on this gene

AO090012000018

not annotated
  
Accession: BAE60264
  
Location: 38064-39404
  
 NCBI BlastP on this gene

AO090012000017

not annotated
  
Accession: BAE60263
  
Location: 37353-37911
  
 NCBI BlastP on this gene

AO090012000016

not annotated
  
Accession: BAE60262
  
Location: 35867-36731
  
 NCBI BlastP on this gene

AO090012000015

not annotated
  
Accession: BAE60261
  
Location: 34457-35512
  
 NCBI BlastP on this gene

AO090012000014

not annotated
  
Accession: BAE60260
  
Location: 31650-32902
  
 NCBI BlastP on this gene

AO090012000013

Query: Architecture Search FASTA input

AP007157 : Aspergillus oryzae RIB40 DNA, SC023.    Total score: 1.0     Cumulative Blast bit score: 278

Hit cluster cross-links:

Mycgr3G41235 Mycgr3T
  
Location: 0-4062

Mycgr3G41235\_Mycgr3T

Mycgr3G70577 Mycgr3T
  
Location: 4162-6109

Mycgr3G70577\_Mycgr3T

Mycgr3G40534 Mycgr3T
  
Location: 6209-7166

Mycgr3G40534\_Mycgr3T

Mycgr3G85486 Mycgr3T
  
Location: 7266-8511

Mycgr3G85486\_Mycgr3T

Mycgr3G92221 Mycgr3T
  
Location: 8611-9193

Mycgr3G92221\_Mycgr3T

Mycgr3G39931 Mycgr3T
  
Location: 9293-10157

Mycgr3G39931\_Mycgr3T

Mycgr3G99766 Mycgr3T
  
Location: 10257-11775

Mycgr3G99766\_Mycgr3T

not annotated
  
Accession: BAE58999
  
Location: 1162623-1165206
  
 NCBI BlastP on this gene

AO090023000454

not annotated
  
Accession: BAE58998
  
Location: 1160385-1162177
  
 NCBI BlastP on this gene

AO090023000453

not annotated
  
Accession: BAE58997
  
Location: 1157388-1158984
  
  
**BlastP hit with Mycgr3G99766\_Mycgr3T**
  
Percentage identity: 34 %
  
BlastP bit score: 278
  
Sequence coverage: 95 %
  
E-value: 2e-83
  
  
 NCBI BlastP on this gene

AO090023000452

not annotated
  
Accession: BAE58996
  
Location: 1155287-1156516
  
 NCBI BlastP on this gene

AO090023000451

not annotated
  
Accession: BAE58995
  
Location: 1148972-1150552
  
 NCBI BlastP on this gene

AO090023000450

Query: Architecture Search FASTA input

AKHY01000182 : Aspergillus oryzae 3.042    Total score: 1.0     Cumulative Blast bit score: 278

Hit cluster cross-links:

Mycgr3G41235 Mycgr3T
  
Location: 0-4062

Mycgr3G41235\_Mycgr3T

Mycgr3G70577 Mycgr3T
  
Location: 4162-6109

Mycgr3G70577\_Mycgr3T

Mycgr3G40534 Mycgr3T
  
Location: 6209-7166

Mycgr3G40534\_Mycgr3T

Mycgr3G85486 Mycgr3T
  
Location: 7266-8511

Mycgr3G85486\_Mycgr3T

Mycgr3G92221 Mycgr3T
  
Location: 8611-9193

Mycgr3G92221\_Mycgr3T

Mycgr3G39931 Mycgr3T
  
Location: 9293-10157

Mycgr3G39931\_Mycgr3T

Mycgr3G99766 Mycgr3T
  
Location: 10257-11775

Mycgr3G99766\_Mycgr3T

tricarboxylate carrier protein
  
Accession: EIT74860
  
Location: 956078-958661
  
 NCBI BlastP on this gene

EIT74860

permease of the major facilitator superfamily
  
Accession: EIT74924
  
Location: 953840-955632
  
 NCBI BlastP on this gene

EIT74924

putative transporter
  
Accession: EIT74971
  
Location: 950843-952439
  
  
**BlastP hit with Mycgr3G99766\_Mycgr3T**
  
Percentage identity: 34 %
  
BlastP bit score: 278
  
Sequence coverage: 95 %
  
E-value: 2e-83
  
  
 NCBI BlastP on this gene

EIT74971

hypothetical protein
  
Accession: EIT75040
  
Location: 948742-949944
  
 NCBI BlastP on this gene

EIT75040

cytochrome protein
  
Accession: EIT75146
  
Location: 942427-944079
  
 NCBI BlastP on this gene

EIT75146

Query: Architecture Search FASTA input

AKHY01000171 : Aspergillus oryzae 3.042    Total score: 1.0     Cumulative Blast bit score: 278

Hit cluster cross-links:

Mycgr3G41235 Mycgr3T
  
Location: 0-4062

Mycgr3G41235\_Mycgr3T

Mycgr3G70577 Mycgr3T
  
Location: 4162-6109

Mycgr3G70577\_Mycgr3T

Mycgr3G40534 Mycgr3T
  
Location: 6209-7166

Mycgr3G40534\_Mycgr3T

Mycgr3G85486 Mycgr3T
  
Location: 7266-8511

Mycgr3G85486\_Mycgr3T

Mycgr3G92221 Mycgr3T
  
Location: 8611-9193

Mycgr3G92221\_Mycgr3T

Mycgr3G39931 Mycgr3T
  
Location: 9293-10157

Mycgr3G39931\_Mycgr3T

Mycgr3G99766 Mycgr3T
  
Location: 10257-11775

Mycgr3G99766\_Mycgr3T

hypothetical protein
  
Accession: EIT76142
  
Location: 37633-38680
  
 NCBI BlastP on this gene

EIT76142

hypothetical protein
  
Accession: EIT76108
  
Location: 34563-34982
  
 NCBI BlastP on this gene

EIT76108

putative transporter
  
Accession: EIT75907
  
Location: 31718-33402
  
  
**BlastP hit with Mycgr3G99766\_Mycgr3T**
  
Percentage identity: 33 %
  
BlastP bit score: 278
  
Sequence coverage: 95 %
  
E-value: 3e-83
  
  
 NCBI BlastP on this gene

EIT75907

hypothetical protein
  
Accession: EIT76065
  
Location: 29167-31221
  
 NCBI BlastP on this gene

EIT76065

hypothetical protein
  
Accession: EIT76094
  
Location: 27684-28626
  
 NCBI BlastP on this gene

EIT76094

hypothetical protein
  
Accession: EIT75895
  
Location: 26274-27329
  
 NCBI BlastP on this gene

EIT75895

hypothetical protein
  
Accession: EIT75938
  
Location: 23467-24719
  
 NCBI BlastP on this gene

EIT75938

Query: Architecture Search FASTA input

CR382137 : Debaryomyces hansenii CBS767 chromosome E complete sequence.    Total score: 1.0     Cumulative Blast bit score: 277

Hit cluster cross-links:

Mycgr3G41235 Mycgr3T
  
Location: 0-4062

Mycgr3G41235\_Mycgr3T

Mycgr3G70577 Mycgr3T
  
Location: 4162-6109

Mycgr3G70577\_Mycgr3T

Mycgr3G40534 Mycgr3T
  
Location: 6209-7166

Mycgr3G40534\_Mycgr3T

Mycgr3G85486 Mycgr3T
  
Location: 7266-8511

Mycgr3G85486\_Mycgr3T

Mycgr3G92221 Mycgr3T
  
Location: 8611-9193

Mycgr3G92221\_Mycgr3T

Mycgr3G39931 Mycgr3T
  
Location: 9293-10157

Mycgr3G39931\_Mycgr3T

Mycgr3G99766 Mycgr3T
  
Location: 10257-11775

Mycgr3G99766\_Mycgr3T

DEHA2E01320p
  
Accession: CAG87590
  
Location: 110858-111391
  
 NCBI BlastP on this gene

DEHA2E01320g

DEHA2E01342p
  
Accession: CAG87596
  
Location: 115691-115858
  
 NCBI BlastP on this gene

DEHA2E01342g

DEHA2E01364p
  
Accession: CAG87597
  
Location: 116364-118052
  
 NCBI BlastP on this gene

DEHA2E01364g

DEHA2E01386p
  
Accession: CAG87598
  
Location: 118585-120234
  
  
**BlastP hit with Mycgr3G99766\_Mycgr3T**
  
Percentage identity: 33 %
  
BlastP bit score: 277
  
Sequence coverage: 100 %
  
E-value: 2e-82
  
  
 NCBI BlastP on this gene

DEHA2E01386g

DEHA2E01408p
  
Accession: CAG87599
  
Location: 120488-122218
  
 NCBI BlastP on this gene

DEHA2E01408g

DEHA2E01430p
  
Accession: CAG87600
  
Location: 122529-124964
  
 NCBI BlastP on this gene

DEHA2E01430g

DEHA2E01452p
  
Accession: CAR65737
  
Location: 125226-128138
  
 NCBI BlastP on this gene

DEHA2E01452g

Query: Architecture Search FASTA input

CR382129 : Yarrowia lipolytica CLIB122 chromosome C complete sequence.    Total score: 1.0     Cumulative Blast bit score: 277

Hit cluster cross-links:

Mycgr3G41235 Mycgr3T
  
Location: 0-4062

Mycgr3G41235\_Mycgr3T

Mycgr3G70577 Mycgr3T
  
Location: 4162-6109

Mycgr3G70577\_Mycgr3T

Mycgr3G40534 Mycgr3T
  
Location: 6209-7166

Mycgr3G40534\_Mycgr3T

Mycgr3G85486 Mycgr3T
  
Location: 7266-8511

Mycgr3G85486\_Mycgr3T

Mycgr3G92221 Mycgr3T
  
Location: 8611-9193

Mycgr3G92221\_Mycgr3T

Mycgr3G39931 Mycgr3T
  
Location: 9293-10157

Mycgr3G39931\_Mycgr3T

Mycgr3G99766 Mycgr3T
  
Location: 10257-11775

Mycgr3G99766\_Mycgr3T

YALI0C16456p
  
Accession: CAG82224
  
Location: 2322212-2323362
  
 NCBI BlastP on this gene

YALI0\_C16456g

YALI0C16478p
  
Accession: CAG82225
  
Location: 2324018-2324707
  
 NCBI BlastP on this gene

YALI0\_C16478g

YALI0C16500p
  
Accession: CAG82226
  
Location: 2324805-2326478
  
 NCBI BlastP on this gene

YALI0\_C16500g

YALI0C16522p
  
Accession: CAG82227
  
Location: 2329693-2331429
  
  
**BlastP hit with Mycgr3G99766\_Mycgr3T**
  
Percentage identity: 33 %
  
BlastP bit score: 277
  
Sequence coverage: 107 %
  
E-value: 3e-82
  
  
 NCBI BlastP on this gene

YALI0\_C16522g

Query: Architecture Search FASTA input

CR382134 : Debaryomyces hansenii CBS767 chromosome B complete sequence.    Total score: 1.0     Cumulative Blast bit score: 276

Hit cluster cross-links:

Mycgr3G41235 Mycgr3T
  
Location: 0-4062

Mycgr3G41235\_Mycgr3T

Mycgr3G70577 Mycgr3T
  
Location: 4162-6109

Mycgr3G70577\_Mycgr3T

Mycgr3G40534 Mycgr3T
  
Location: 6209-7166

Mycgr3G40534\_Mycgr3T

Mycgr3G85486 Mycgr3T
  
Location: 7266-8511

Mycgr3G85486\_Mycgr3T

Mycgr3G92221 Mycgr3T
  
Location: 8611-9193

Mycgr3G92221\_Mycgr3T

Mycgr3G39931 Mycgr3T
  
Location: 9293-10157

Mycgr3G39931\_Mycgr3T

Mycgr3G99766 Mycgr3T
  
Location: 10257-11775

Mycgr3G99766\_Mycgr3T

DEHA2B13046p
  
Accession: CAG85518
  
Location: 1023160-1024272
  
 NCBI BlastP on this gene

DEHA2B13046g

DEHA2B13024p
  
Accession: CAG85517
  
Location: 1021278-1022987
  
 NCBI BlastP on this gene

DEHA2B13024g

DEHA2B13002p
  
Accession: CAG85516
  
Location: 1019678-1020972
  
 NCBI BlastP on this gene

DEHA2B13002g

DEHA2B12980p
  
Accession: CAG85515
  
Location: 1018306-1019283
  
 NCBI BlastP on this gene

DEHA2B12980g

DEHA2B12958p
  
Accession: CAG85514
  
Location: 1015230-1016915
  
  
**BlastP hit with Mycgr3G99766\_Mycgr3T**
  
Percentage identity: 33 %
  
BlastP bit score: 276
  
Sequence coverage: 101 %
  
E-value: 5e-82
  
  
 NCBI BlastP on this gene

DEHA2B12958g

DEHA2B12936p
  
Accession: CAG85513
  
Location: 1013453-1014568
  
 NCBI BlastP on this gene

DEHA2B12936g

DEHA2B12914p
  
Accession: CAG85512
  
Location: 1012676-1013167
  
 NCBI BlastP on this gene

DEHA2B12914g

DEHA2B12892p
  
Accession: CAG85511
  
Location: 1010521-1012311
  
 NCBI BlastP on this gene

DEHA2B12892g

DEHA2B12870p
  
Accession: CAR65486
  
Location: 1007416-1009218
  
 NCBI BlastP on this gene

DEHA2B12870g

Query: Architecture Search FASTA input

GL891302 : Neurospora tetrasperma FGSC 2508 unplaced genomic scaffold NEUTE1scaffold\_1    Total score: 1.0     Cumulative Blast bit score: 275

Hit cluster cross-links:

Mycgr3G41235 Mycgr3T
  
Location: 0-4062

Mycgr3G41235\_Mycgr3T

Mycgr3G70577 Mycgr3T
  
Location: 4162-6109

Mycgr3G70577\_Mycgr3T

Mycgr3G40534 Mycgr3T
  
Location: 6209-7166

Mycgr3G40534\_Mycgr3T

Mycgr3G85486 Mycgr3T
  
Location: 7266-8511

Mycgr3G85486\_Mycgr3T

Mycgr3G92221 Mycgr3T
  
Location: 8611-9193

Mycgr3G92221\_Mycgr3T

Mycgr3G39931 Mycgr3T
  
Location: 9293-10157

Mycgr3G39931\_Mycgr3T

Mycgr3G99766 Mycgr3T
  
Location: 10257-11775

Mycgr3G99766\_Mycgr3T

hypothetical protein
  
Accession: EGO60949
  
Location: 3249875-3251665
  
  
**BlastP hit with Mycgr3G99766\_Mycgr3T**
  
Percentage identity: 36 %
  
BlastP bit score: 275
  
Sequence coverage: 98 %
  
E-value: 1e-81
  
  
 NCBI BlastP on this gene

EGO60949

hypothetical protein
  
Accession: EGO60948
  
Location: 3247813-3248467
  
 NCBI BlastP on this gene

EGO60948

hypothetical protein
  
Accession: EGO60947
  
Location: 3245518-3247707
  
 NCBI BlastP on this gene

EGO60947

hypothetical protein
  
Accession: EGO60946
  
Location: 3241775-3244561
  
 NCBI BlastP on this gene

EGO60946

Query: Architecture Search FASTA input

GL891107 : Neurospora tetrasperma FGSC 2509 unplaced genomic scaffold NEUTE2scaffold\_2    Total score: 1.0     Cumulative Blast bit score: 275

Hit cluster cross-links:

Mycgr3G41235 Mycgr3T
  
Location: 0-4062

Mycgr3G41235\_Mycgr3T

Mycgr3G70577 Mycgr3T
  
Location: 4162-6109

Mycgr3G70577\_Mycgr3T

Mycgr3G40534 Mycgr3T
  
Location: 6209-7166

Mycgr3G40534\_Mycgr3T

Mycgr3G85486 Mycgr3T
  
Location: 7266-8511

Mycgr3G85486\_Mycgr3T

Mycgr3G92221 Mycgr3T
  
Location: 8611-9193

Mycgr3G92221\_Mycgr3T

Mycgr3G39931 Mycgr3T
  
Location: 9293-10157

Mycgr3G39931\_Mycgr3T

Mycgr3G99766 Mycgr3T
  
Location: 10257-11775

Mycgr3G99766\_Mycgr3T

DUF636-domain-containing protein
  
Accession: EGZ75046
  
Location: 2910898-2911417
  
 NCBI BlastP on this gene

EGZ75046

alcohol dehydrogenase I
  
Accession: EGZ75047
  
Location: 2912443-2912686
  
 NCBI BlastP on this gene

EGZ75047

hypothetical protein
  
Accession: EGZ75048
  
Location: 2913800-2914123
  
 NCBI BlastP on this gene

EGZ75048

hypothetical protein
  
Accession: EGZ75049
  
Location: 2915316-2915665
  
 NCBI BlastP on this gene

EGZ75049

hypothetical protein
  
Accession: EGZ75050
  
Location: 2915883-2916312
  
 NCBI BlastP on this gene

EGZ75050

hypothetical protein
  
Accession: EGZ75051
  
Location: 2916547-2917300
  
 NCBI BlastP on this gene

EGZ75051

putative sugar transporter
  
Accession: EGZ75052
  
Location: 2917505-2919295
  
  
**BlastP hit with Mycgr3G99766\_Mycgr3T**
  
Percentage identity: 36 %
  
BlastP bit score: 275
  
Sequence coverage: 98 %
  
E-value: 1e-81
  
  
 NCBI BlastP on this gene

EGZ75052

hypothetical protein
  
Accession: EGZ75053
  
Location: 2920703-2921357
  
 NCBI BlastP on this gene

EGZ75053

cytochrome P450
  
Accession: EGZ75054
  
Location: 2921463-2923652
  
 NCBI BlastP on this gene

EGZ75054

hypothetical protein
  
Accession: EGZ75055
  
Location: 2924609-2927395
  
 NCBI BlastP on this gene

EGZ75055

Query: Architecture Search FASTA input

KB731258 : Fusarium oxysporum f. sp. cubense race 1 unplaced genomic scaffold scaffold166    Total score: 1.0     Cumulative Blast bit score: 274

Hit cluster cross-links:

Mycgr3G41235 Mycgr3T
  
Location: 0-4062

Mycgr3G41235\_Mycgr3T

Mycgr3G70577 Mycgr3T
  
Location: 4162-6109

Mycgr3G70577\_Mycgr3T

Mycgr3G40534 Mycgr3T
  
Location: 6209-7166

Mycgr3G40534\_Mycgr3T

Mycgr3G85486 Mycgr3T
  
Location: 7266-8511

Mycgr3G85486\_Mycgr3T

Mycgr3G92221 Mycgr3T
  
Location: 8611-9193

Mycgr3G92221\_Mycgr3T

Mycgr3G39931 Mycgr3T
  
Location: 9293-10157

Mycgr3G39931\_Mycgr3T

Mycgr3G99766 Mycgr3T
  
Location: 10257-11775

Mycgr3G99766\_Mycgr3T

Sugar transporter STL1
  
Accession: ENH62911
  
Location: 342303-343795
  
  
**BlastP hit with Mycgr3G99766\_Mycgr3T**
  
Percentage identity: 34 %
  
BlastP bit score: 274
  
Sequence coverage: 89 %
  
E-value: 3e-82
  
  
 NCBI BlastP on this gene

ENH62911

3-isopropylmalate dehydrogenase
  
Accession: ENH62910
  
Location: 337126-340524
  
 NCBI BlastP on this gene

ENH62910

Carboxyvinyl-carboxyphosphonate phosphorylmutase
  
Accession: ENH62909
  
Location: 335368-336517
  
 NCBI BlastP on this gene

ENH62909

hypothetical protein
  
Accession: ENH62908
  
Location: 332478-334862
  
 NCBI BlastP on this gene

ENH62908

Query: Architecture Search FASTA input

GL385399 : Gaeumannomyces graminis var. tritici R3-111a-1 unplaced genomic scaffold supercont2.5    Total score: 1.0     Cumulative Blast bit score: 273

Hit cluster cross-links:

Mycgr3G41235 Mycgr3T
  
Location: 0-4062

Mycgr3G41235\_Mycgr3T

Mycgr3G70577 Mycgr3T
  
Location: 4162-6109

Mycgr3G70577\_Mycgr3T

Mycgr3G40534 Mycgr3T
  
Location: 6209-7166

Mycgr3G40534\_Mycgr3T

Mycgr3G85486 Mycgr3T
  
Location: 7266-8511

Mycgr3G85486\_Mycgr3T

Mycgr3G92221 Mycgr3T
  
Location: 8611-9193

Mycgr3G92221\_Mycgr3T

Mycgr3G39931 Mycgr3T
  
Location: 9293-10157

Mycgr3G39931\_Mycgr3T

Mycgr3G99766 Mycgr3T
  
Location: 10257-11775

Mycgr3G99766\_Mycgr3T

high-affinity glucose transporter ght2
  
Accession: EJT72421
  
Location: 897562-899391
  
 NCBI BlastP on this gene

EJT72421

hypothetical protein
  
Accession: EJT72422
  
Location: 901896-902267
  
 NCBI BlastP on this gene

EJT72422

hypothetical protein
  
Accession: EJT72423
  
Location: 902730-903180
  
 NCBI BlastP on this gene

EJT72423

hypothetical protein
  
Accession: EJT72424
  
Location: 903993-904589
  
 NCBI BlastP on this gene

EJT72424

hypothetical protein
  
Accession: EJT72425
  
Location: 905020-905304
  
 NCBI BlastP on this gene

EJT72425

sugar transporter STL1
  
Accession: EJT72426
  
Location: 905843-907587
  
  
**BlastP hit with Mycgr3G99766\_Mycgr3T**
  
Percentage identity: 33 %
  
BlastP bit score: 273
  
Sequence coverage: 94 %
  
E-value: 3e-81
  
  
 NCBI BlastP on this gene

EJT72426

hypothetical protein
  
Accession: EJT72427
  
Location: 909148-910239
  
 NCBI BlastP on this gene

EJT72427

hypothetical protein
  
Accession: EJT72428
  
Location: 910855-911506
  
 NCBI BlastP on this gene

EJT72428

hypothetical protein
  
Accession: EJT72429
  
Location: 911944-912552
  
 NCBI BlastP on this gene

EJT72429

hypothetical protein
  
Accession: EJT72430
  
Location: 913288-913908
  
 NCBI BlastP on this gene

EJT72430

Query: Architecture Search FASTA input

CM001235 : Magnaporthe oryzae 70-15 chromosome 5    Total score: 1.0     Cumulative Blast bit score: 273

Hit cluster cross-links:

Mycgr3G41235 Mycgr3T
  
Location: 0-4062

Mycgr3G41235\_Mycgr3T

Mycgr3G70577 Mycgr3T
  
Location: 4162-6109

Mycgr3G70577\_Mycgr3T

Mycgr3G40534 Mycgr3T
  
Location: 6209-7166

Mycgr3G40534\_Mycgr3T

Mycgr3G85486 Mycgr3T
  
Location: 7266-8511

Mycgr3G85486\_Mycgr3T

Mycgr3G92221 Mycgr3T
  
Location: 8611-9193

Mycgr3G92221\_Mycgr3T

Mycgr3G39931 Mycgr3T
  
Location: 9293-10157

Mycgr3G39931\_Mycgr3T

Mycgr3G99766 Mycgr3T
  
Location: 10257-11775

Mycgr3G99766\_Mycgr3T

hypothetical protein
  
Accession: EHA48100
  
Location: 32732-33333
  
 NCBI BlastP on this gene

EHA48100

hypothetical protein
  
Accession: EHA48099
  
Location: 29802-31076
  
 NCBI BlastP on this gene

EHA48099

hypothetical protein
  
Accession: EHA48098
  
Location: 28950-29237
  
 NCBI BlastP on this gene

EHA48098

sugar transporter STL1
  
Accession: EHA48097
  
Location: 25207-26964
  
  
**BlastP hit with Mycgr3G99766\_Mycgr3T**
  
Percentage identity: 33 %
  
BlastP bit score: 273
  
Sequence coverage: 98 %
  
E-value: 3e-81
  
  
 NCBI BlastP on this gene

EHA48097

hypothetical protein
  
Accession: EHA48096
  
Location: 22629-23290
  
 NCBI BlastP on this gene

EHA48096

hypothetical protein
  
Accession: EHA48095
  
Location: 20327-20635
  
 NCBI BlastP on this gene

EHA48095

retinol dehydrogenase 12
  
Accession: EHA48094
  
Location: 17145-18080
  
 NCBI BlastP on this gene

EHA48094

Query: Architecture Search FASTA input

AMWD01000002 : Janthinobacterium sp. HH01    Total score: 1.0     Cumulative Blast bit score: 262

Hit cluster cross-links:

Mycgr3G41235 Mycgr3T
  
Location: 0-4062

Mycgr3G41235\_Mycgr3T

Mycgr3G70577 Mycgr3T
  
Location: 4162-6109

Mycgr3G70577\_Mycgr3T

Mycgr3G40534 Mycgr3T
  
Location: 6209-7166

Mycgr3G40534\_Mycgr3T

Mycgr3G85486 Mycgr3T
  
Location: 7266-8511

Mycgr3G85486\_Mycgr3T

Mycgr3G92221 Mycgr3T
  
Location: 8611-9193

Mycgr3G92221\_Mycgr3T

Mycgr3G39931 Mycgr3T
  
Location: 9293-10157

Mycgr3G39931\_Mycgr3T

Mycgr3G99766 Mycgr3T
  
Location: 10257-11775

Mycgr3G99766\_Mycgr3T

acyl-CoA dehydrogenase type 2 domain containing protein
  
Accession: ELX08663
  
Location: 882509-883687
  
 NCBI BlastP on this gene

ELX08663

linear gramicidin synthase subunit C
  
Accession: ELX08664
  
Location: 883733-885520
  
 NCBI BlastP on this gene

ELX08664

non-ribosomal peptide synthetase
  
Accession: ELX08665
  
Location: 885552-892007
  
  
**BlastP hit with Mycgr3G40534\_Mycgr3T**
  
Percentage identity: 38 %
  
BlastP bit score: 136
  
Sequence coverage: 81 %
  
E-value: 1e-31
  
  
 NCBI BlastP on this gene

ELX08665

2,4-dichlorophenol 6-monooxygenase TfdB
  
Accession: ELX08666
  
Location: 892004-893791
  
 NCBI BlastP on this gene

ELX08666

major facilitator superfamily MFS 1
  
Accession: ELX08667
  
Location: 893788-895092
  
 NCBI BlastP on this gene

ELX08667

tyrocidine synthase 3
  
Accession: ELX08668
  
Location: 895089-907430
  
 NCBI BlastP on this gene

ELX08668

linear gramicidin synthase subunit C
  
Accession: ELX08669
  
Location: 907441-915156
  
  
**BlastP hit with Mycgr3G40534\_Mycgr3T**
  
Percentage identity: 36 %
  
BlastP bit score: 126
  
Sequence coverage: 81 %
  
E-value: 2e-28
  
  
 NCBI BlastP on this gene

ELX08669

integrase family protein
  
Accession: ELX08670
  
Location: 915520-916686
  
 NCBI BlastP on this gene

ELX08670

hypothetical protein
  
Accession: ELX08671
  
Location: 917375-917566
  
 NCBI BlastP on this gene

ELX08671

Query: Architecture Search FASTA input

AP007150 : Aspergillus oryzae RIB40 DNA, SC009.    Total score: 1.0     Cumulative Blast bit score: 228

Hit cluster cross-links:

Mycgr3G41235 Mycgr3T
  
Location: 0-4062

Mycgr3G41235\_Mycgr3T

Mycgr3G70577 Mycgr3T
  
Location: 4162-6109

Mycgr3G70577\_Mycgr3T

Mycgr3G40534 Mycgr3T
  
Location: 6209-7166

Mycgr3G40534\_Mycgr3T

Mycgr3G85486 Mycgr3T
  
Location: 7266-8511

Mycgr3G85486\_Mycgr3T

Mycgr3G92221 Mycgr3T
  
Location: 8611-9193

Mycgr3G92221\_Mycgr3T

Mycgr3G39931 Mycgr3T
  
Location: 9293-10157

Mycgr3G39931\_Mycgr3T

Mycgr3G99766 Mycgr3T
  
Location: 10257-11775

Mycgr3G99766\_Mycgr3T

not annotated
  
Accession: BAE54580
  
Location: 179200-180216
  
 NCBI BlastP on this gene

AO090009000063

not annotated
  
Accession: BAE54579
  
Location: 177787-178235
  
 NCBI BlastP on this gene

AO090009000062

not annotated
  
Accession: BAE54578
  
Location: 174495-176469
  
  
**BlastP hit with Mycgr3G70577\_Mycgr3T**
  
Percentage identity: 28 %
  
BlastP bit score: 228
  
Sequence coverage: 97 %
  
E-value: 3e-62
  
  
 NCBI BlastP on this gene

AO090009000061

not annotated
  
Accession: BAE54577
  
Location: 173530-174363
  
 NCBI BlastP on this gene

AO090009000060

not annotated
  
Accession: BAE54576
  
Location: 167219-168013
  
 NCBI BlastP on this gene

AO090009000057

Query: Architecture Search FASTA input

CM001198 : Mycosphaerella graminicola IPO323 chromosome 3    Total score: 1.0     Cumulative Blast bit score: 223

Hit cluster cross-links:

Mycgr3G41235 Mycgr3T
  
Location: 0-4062

Mycgr3G41235\_Mycgr3T

Mycgr3G70577 Mycgr3T
  
Location: 4162-6109

Mycgr3G70577\_Mycgr3T

Mycgr3G40534 Mycgr3T
  
Location: 6209-7166

Mycgr3G40534\_Mycgr3T

Mycgr3G85486 Mycgr3T
  
Location: 7266-8511

Mycgr3G85486\_Mycgr3T

Mycgr3G92221 Mycgr3T
  
Location: 8611-9193

Mycgr3G92221\_Mycgr3T

Mycgr3G39931 Mycgr3T
  
Location: 9293-10157

Mycgr3G39931\_Mycgr3T

Mycgr3G99766 Mycgr3T
  
Location: 10257-11775

Mycgr3G99766\_Mycgr3T

hypothetical protein
  
Accession: EGP89384
  
Location: 1797929-1799116
  
 NCBI BlastP on this gene

EGP89384

hypothetical protein
  
Accession: EGP88817
  
Location: 1794846-1795948
  
 NCBI BlastP on this gene

EGP88817

DNA polymerase beta-like protein
  
Accession: EGP89385
  
Location: 1792312-1794411
  
 NCBI BlastP on this gene

EGP89385

putative siderophore-dependent iron transporter
  
Accession: EGP89386
  
Location: 1789350-1791305
  
  
**BlastP hit with Mycgr3G70577\_Mycgr3T**
  
Percentage identity: 29 %
  
BlastP bit score: 223
  
Sequence coverage: 93 %
  
E-value: 4e-60
  
  
 NCBI BlastP on this gene

EGP89386

hypothetical protein
  
Accession: EGP89387
  
Location: 1786829-1787747
  
 NCBI BlastP on this gene

EGP89387

Query: Architecture Search FASTA input

201. :  CR382133 Debaryomyces hansenii CBS767 chromosome A complete sequence.     Total score: 1.0     Cumulative Blast bit score: 301

Mycgr3G41235 Mycgr3T
  
Location: 0-4062
  
 NCBI BlastP on this gene

Mycgr3G41235\_Mycgr3T

Mycgr3G70577 Mycgr3T
  
Location: 4162-6109
  
 NCBI BlastP on this gene

Mycgr3G70577\_Mycgr3T

Mycgr3G40534 Mycgr3T
  
Location: 6209-7166
  
 NCBI BlastP on this gene

Mycgr3G40534\_Mycgr3T

Mycgr3G85486 Mycgr3T
  
Location: 7266-8511
  
 NCBI BlastP on this gene

Mycgr3G85486\_Mycgr3T

Mycgr3G92221 Mycgr3T
  
Location: 8611-9193
  
 NCBI BlastP on this gene

Mycgr3G92221\_Mycgr3T

Mycgr3G39931 Mycgr3T
  
Location: 9293-10157
  
 NCBI BlastP on this gene

Mycgr3G39931\_Mycgr3T

Mycgr3G99766 Mycgr3T
  
Location: 10257-11775
  
 NCBI BlastP on this gene

Mycgr3G99766\_Mycgr3T

DEHA2A12628p
  
Accession: CAG84855
  
Location: 1063635-1064166
  
 NCBI BlastP on this gene

DEHA2A12628g

DEHA2A12606p
  
Accession: CAR65408
  
Location: 1059991-1063254
  
 NCBI BlastP on this gene

DEHA2A12606g

DEHA2A12584p
  
Accession: CAG84852
  
Location: 1058361-1059947
  
 NCBI BlastP on this gene

DEHA2A12584g

DEHA2A12562p
  
Accession: CAG84851
  
Location: 1057636-1058172
  
 NCBI BlastP on this gene

DEHA2A12562g

DEHA2A12540p
  
Accession: CAG84850
  
Location: 1055904-1057013
  
 NCBI BlastP on this gene

DEHA2A12540g

DEHA2A12518p
  
Accession: CAG84849
  
Location: 1055276-1055698
  
 NCBI BlastP on this gene

DEHA2A12518g

DEHA2A12496p
  
Accession: CAG84848
  
Location: 1054493-1055188
  
 NCBI BlastP on this gene

DEHA2A12496g

DEHA2A12474p
  
Accession: CAG84847
  
Location: 1052666-1053196
  
 NCBI BlastP on this gene

DEHA2A12474g

DEHA2A12452p
  
Accession: CAG84846
  
Location: 1051589-1052455
  
 NCBI BlastP on this gene

DEHA2A12452g

DEHA2A12430p
  
Accession: CAG84845
  
Location: 1050316-1051125
  
 NCBI BlastP on this gene

DEHA2A12430g

DEHA2A12408p
  
Accession: CAR65407
  
Location: 1049206-1049400
  
 NCBI BlastP on this gene

DEHA2A12408g

DEHA2A12386p
  
Accession: CAG84844
  
Location: 1048052-1048921
  
 NCBI BlastP on this gene

DEHA2A12386g

DEHA2A12364p
  
Accession: CAG84843
  
Location: 1045762-1047420
  
  
**BlastP hit with Mycgr3G99766\_Mycgr3T**
  
Percentage identity: 33 %
  
BlastP bit score: 301
  
Sequence coverage: 101 %
  
E-value: 8e-92
  
  
 NCBI BlastP on this gene

DEHA2A12364g

DEHA2A12342p
  
Accession: CAG84842
  
Location: 1043702-1045519
  
 NCBI BlastP on this gene

DEHA2A12342g

DEHA2A12320p
  
Accession: CAG84841
  
Location: 1042691-1043161
  
 NCBI BlastP on this gene

DEHA2A12320g

DEHA2A12298p
  
Accession: CAG84840
  
Location: 1039435-1042515
  
 NCBI BlastP on this gene

DEHA2A12298g

DEHA2A12276p
  
Accession: CAG84839
  
Location: 1036688-1039003
  
 NCBI BlastP on this gene

DEHA2A12276g

DEHA2A12254p
  
Accession: CAR65406
  
Location: 1034993-1036630
  
 NCBI BlastP on this gene

DEHA2A12254g

DEHA2A12232p
  
Accession: CAG84837
  
Location: 1033831-1034217
  
 NCBI BlastP on this gene

DEHA2A12232g

DEHA2A12210p
  
Accession: CAG84836
  
Location: 1032671-1033675
  
 NCBI BlastP on this gene

DEHA2A12210g

DEHA2A12188p
  
Accession: CAG84835
  
Location: 1032049-1032387
  
 NCBI BlastP on this gene

DEHA2A12188g

DEHA2A12166p
  
Accession: CAG84834
  
Location: 1029657-1031780
  
 NCBI BlastP on this gene

DEHA2A12166g

202. :  DS995901 Penicillium marneffei ATCC 18224 scf\_1105668340960 genomic scaffold     Total score: 1.0     Cumulative Blast bit score: 300

conserved hypothetical protein
  
Accession: EEA24044
  
Location: 1072733-1074027
  
 NCBI BlastP on this gene

EEA24044

37S ribosomal protein Rsm24, putative
  
Accession: EEA24045
  
Location: 1074741-1076005
  
 NCBI BlastP on this gene

EEA24045

glycosyltransferase family 28, putative
  
Accession: EEA24046
  
Location: 1076315-1077019
  
 NCBI BlastP on this gene

EEA24046

actin-related protein ArpA
  
Accession: EEA24047
  
Location: 1077661-1078923
  
 NCBI BlastP on this gene

EEA24047

N-acetyltransferase, GNAT family, putative
  
Accession: EEA24048
  
Location: 1079649-1080197
  
 NCBI BlastP on this gene

EEA24048

3-hydroxyacyl-CoA dehyrogenase, putative
  
Accession: EEA24049
  
Location: 1080474-1081518
  
 NCBI BlastP on this gene

EEA24049

zinc knuckle transcription factor (CnjB), putative
  
Accession: EEA24050
  
Location: 1082192-1083859
  
 NCBI BlastP on this gene

EEA24050

MFS monosaccharide transporter, putative
  
Accession: EEA24051
  
Location: 1087088-1088972
  
  
**BlastP hit with Mycgr3G99766\_Mycgr3T**
  
Percentage identity: 34 %
  
BlastP bit score: 300
  
Sequence coverage: 100 %
  
E-value: 4e-91
  
  
 NCBI BlastP on this gene

EEA24051

glutamine synthetase
  
Accession: EEA24052
  
Location: 1090027-1091271
  
 NCBI BlastP on this gene

EEA24052

conserved hypothetical protein
  
Accession: EEA24053
  
Location: 1094220-1096420
  
 NCBI BlastP on this gene

EEA24053

conserved hypothetical protein
  
Accession: EEA24057
  
Location: 1096804-1097487
  
 NCBI BlastP on this gene

EEA24057

conserved hypothetical protein
  
Accession: EEA24058
  
Location: 1098214-1100109
  
 NCBI BlastP on this gene

EEA24058

conserved hypothetical protein
  
Accession: EEA24059
  
Location: 1101886-1103100
  
 NCBI BlastP on this gene

EEA24059

SNARE domain protein
  
Accession: EEA24060
  
Location: 1103574-1104463
  
 NCBI BlastP on this gene

EEA24060

NAP family protein
  
Accession: EEA24061
  
Location: 1104542-1105787
  
 NCBI BlastP on this gene

EEA24061

203. :  KB445812 Ceriporiopsis subvermispora B unplaced genomic scaffold CERSUscaffold\_22     Total score: 1.0     Cumulative Blast bit score: 299

hypothetical protein
  
Accession: EMD32265
  
Location: 341141-347948
  
 NCBI BlastP on this gene

EMD32265

hypothetical protein
  
Accession: EMD32264
  
Location: 338547-339559
  
 NCBI BlastP on this gene

EMD32264

PKS/NRPS enzyme
  
Accession: EMD32324
  
Location: 325094-336915
  
  
**BlastP hit with Mycgr3G40534\_Mycgr3T**
  
Percentage identity: 38 %
  
BlastP bit score: 141
  
Sequence coverage: 79 %
  
E-value: 3e-33
  
  
 NCBI BlastP on this gene

EMD32324

polyketide synthetase
  
Accession: EMD32323
  
Location: 325048-336915
  
  
**BlastP hit with Mycgr3G40534\_Mycgr3T**
  
Percentage identity: 40 %
  
BlastP bit score: 158
  
Sequence coverage: 79 %
  
E-value: 6e-39
  
  
 NCBI BlastP on this gene

EMD32323

hypothetical protein
  
Accession: EMD32263
  
Location: 322498-322855
  
 NCBI BlastP on this gene

EMD32263

hypothetical protein
  
Accession: EMD32262
  
Location: 320751-321331
  
 NCBI BlastP on this gene

EMD32262

expansin-like protein
  
Accession: EMD32261
  
Location: 319604-320085
  
 NCBI BlastP on this gene

EMD32261

expansin-like protein
  
Accession: EMD32260
  
Location: 317386-317844
  
 NCBI BlastP on this gene

EMD32260

hypothetical protein
  
Accession: EMD32259
  
Location: 314768-315259
  
 NCBI BlastP on this gene

EMD32259

204. :  DS989822 Arthroderma gypseum CBS 118893 supercont1.1 genomic scaffold     Total score: 1.0     Cumulative Blast bit score: 298

sugar transporter STL1
  
Accession: EFQ98600
  
Location: 4438455-4440510
  
  
**BlastP hit with Mycgr3G99766\_Mycgr3T**
  
Percentage identity: 36 %
  
BlastP bit score: 298
  
Sequence coverage: 97 %
  
E-value: 2e-90
  
  
 NCBI BlastP on this gene

EFQ98600

hypothetical protein
  
Accession: EFQ98599
  
Location: 4437652-4438026
  
 NCBI BlastP on this gene

EFQ98599

hypothetical protein
  
Accession: EFQ98598
  
Location: 4434489-4436385
  
 NCBI BlastP on this gene

EFQ98598

secreted protein
  
Accession: EFQ98597
  
Location: 4432445-4433143
  
 NCBI BlastP on this gene

EFQ98597

AGC/PDK1 protein kinase
  
Accession: EFQ98596
  
Location: 4426606-4429125
  
 NCBI BlastP on this gene

EFQ98596

hypothetical protein
  
Accession: EFQ98595
  
Location: 4423250-4424322
  
 NCBI BlastP on this gene

EFQ98595

UDP-galactopyranose mutase
  
Accession: EFQ98594
  
Location: 4420899-4422803
  
 NCBI BlastP on this gene

EFQ98594

205. :  CH476597 Aspergillus terreus NIH2624 scaffold\_4 genomic scaffold     Total score: 1.0     Cumulative Blast bit score: 298

conserved hypothetical protein
  
Accession: EAU36459
  
Location: 1104255-1106560
  
 NCBI BlastP on this gene

EAU36459

conserved hypothetical protein
  
Accession: EAU36460
  
Location: 1106831-1107968
  
 NCBI BlastP on this gene

EAU36460

conserved hypothetical protein
  
Accession: EAU36461
  
Location: 1108520-1109446
  
 NCBI BlastP on this gene

EAU36461

predicted protein
  
Accession: EAU36462
  
Location: 1112886-1114273
  
 NCBI BlastP on this gene

EAU36462

conserved hypothetical protein
  
Accession: EAU36463
  
Location: 1114997-1117146
  
 NCBI BlastP on this gene

EAU36463

conserved hypothetical protein
  
Accession: EAU36464
  
Location: 1122644-1124442
  
  
**BlastP hit with Mycgr3G99766\_Mycgr3T**
  
Percentage identity: 34 %
  
BlastP bit score: 298
  
Sequence coverage: 96 %
  
E-value: 1e-90
  
  
 NCBI BlastP on this gene

EAU36464

206. :  AP007150 Aspergillus oryzae RIB40 DNA, SC009.     Total score: 1.0     Cumulative Blast bit score: 298

not annotated
  
Accession: BAE54751
  
Location: 716165-718410
  
 NCBI BlastP on this gene

AO090009000269

not annotated
  
Accession: BAE54752
  
Location: 718766-720631
  
 NCBI BlastP on this gene

AO090009000270

not annotated
  
Accession: BAE54753
  
Location: 723216-724050
  
 NCBI BlastP on this gene

AO090009000272

not annotated
  
Accession: BAE54754
  
Location: 724104-725403
  
 NCBI BlastP on this gene

AO090009000273

not annotated
  
Accession: BAE54755
  
Location: 730858-732654
  
  
**BlastP hit with Mycgr3G99766\_Mycgr3T**
  
Percentage identity: 33 %
  
BlastP bit score: 298
  
Sequence coverage: 96 %
  
E-value: 1e-90
  
  
 NCBI BlastP on this gene

AO090009000275

207. :  KB644408 Penicillium oxalicum 114-2 unplaced genomic scaffold scaffold\_1     Total score: 1.0     Cumulative Blast bit score: 296

hypothetical protein
  
Accession: EPS25760
  
Location: 1991020-1992418
  
 NCBI BlastP on this gene

EPS25760

hypothetical protein
  
Accession: EPS25759
  
Location: 1989701-1990222
  
 NCBI BlastP on this gene

EPS25759

hypothetical protein
  
Accession: EPS25758
  
Location: 1988015-1988791
  
 NCBI BlastP on this gene

EPS25758

hypothetical protein
  
Accession: EPS25757
  
Location: 1984538-1985233
  
 NCBI BlastP on this gene

EPS25757

hypothetical protein
  
Accession: EPS25756
  
Location: 1978083-1982459
  
 NCBI BlastP on this gene

EPS25756

hypothetical protein
  
Accession: EPS25755
  
Location: 1974349-1975999
  
  
**BlastP hit with Mycgr3G99766\_Mycgr3T**
  
Percentage identity: 34 %
  
BlastP bit score: 296
  
Sequence coverage: 97 %
  
E-value: 3e-90
  
  
 NCBI BlastP on this gene

EPS25755

hypothetical protein
  
Accession: EPS25754
  
Location: 1972674-1973495
  
 NCBI BlastP on this gene

EPS25754

putative UDP-Xyl: (mannosyl)
  
Accession: EPS25753
  
Location: 1969704-1971662
  
 NCBI BlastP on this gene

EPS25753

hypothetical protein
  
Accession: EPS25752
  
Location: 1965848-1968691
  
 NCBI BlastP on this gene

EPS25752

hypothetical protein
  
Accession: EPS25751
  
Location: 1963818-1964808
  
 NCBI BlastP on this gene

EPS25751

hypothetical protein
  
Accession: EPS25750
  
Location: 1957526-1962850
  
 NCBI BlastP on this gene

EPS25750

208. :  KB446538 Dothistroma septosporum NZE10 unplaced genomic scaffold DOTSEscaffold\_4     Total score: 1.0     Cumulative Blast bit score: 295

hypothetical protein
  
Accession: EME45759
  
Location: 2323631-2324630
  
 NCBI BlastP on this gene

EME45759

hypothetical protein
  
Accession: EME45758
  
Location: 2317836-2322731
  
 NCBI BlastP on this gene

EME45758

hypothetical protein
  
Accession: EME45757
  
Location: 2315518-2317404
  
 NCBI BlastP on this gene

EME45757

hypothetical protein
  
Accession: EME45756
  
Location: 2313689-2315149
  
 NCBI BlastP on this gene

EME45756

hypothetical protein
  
Accession: EME45755
  
Location: 2312517-2313237
  
 NCBI BlastP on this gene

EME45755

hypothetical protein
  
Accession: EME45753
  
Location: 2309153-2310121
  
 NCBI BlastP on this gene

EME45753

hypothetical protein
  
Accession: EME45752
  
Location: 2305594-2307479
  
  
**BlastP hit with Mycgr3G99766\_Mycgr3T**
  
Percentage identity: 35 %
  
BlastP bit score: 295
  
Sequence coverage: 98 %
  
E-value: 2e-89
  
  
 NCBI BlastP on this gene

EME45752

hypothetical protein
  
Accession: EME45751
  
Location: 2302754-2305443
  
 NCBI BlastP on this gene

EME45751

hypothetical protein
  
Accession: EME45750
  
Location: 2300520-2300699
  
 NCBI BlastP on this gene

EME45750

hypothetical protein
  
Accession: EME45749
  
Location: 2299009-2299650
  
 NCBI BlastP on this gene

EME45749

hypothetical protein
  
Accession: EME45748
  
Location: 2298041-2298779
  
 NCBI BlastP on this gene

EME45748

hypothetical protein
  
Accession: EME45746
  
Location: 2290851-2292734
  
 NCBI BlastP on this gene

EME45746

hypothetical protein
  
Accession: EME45745
  
Location: 2289875-2290333
  
 NCBI BlastP on this gene

EME45745

hypothetical protein
  
Accession: EME45744
  
Location: 2289575-2289766
  
 NCBI BlastP on this gene

EME45744

209. :  CP003003 Myceliophthora thermophila ATCC 42464 chromosome 2     Total score: 1.0     Cumulative Blast bit score: 295

hypothetical protein
  
Accession: AEO57131
  
Location: 5180901-5182804
  
  
**BlastP hit with Mycgr3G99766\_Mycgr3T**
  
Percentage identity: 35 %
  
BlastP bit score: 295
  
Sequence coverage: 97 %
  
E-value: 4e-89
  
  
 NCBI BlastP on this gene

MYCTH\_2302949

metalloprotease
  
Accession: AEO57130
  
Location: 5178173-5179261
  
 NCBI BlastP on this gene

MYCTH\_78093

hypothetical protein
  
Accession: AEO57129
  
Location: 5176947-5177419
  
 NCBI BlastP on this gene

MYCTH\_2126111

Intradiol ring-cleavage dioxygenase-like protein
  
Accession: AEO57128
  
Location: 5175196-5176687
  
 NCBI BlastP on this gene

MYCTH\_100089

hypothetical protein
  
Accession: AEO57127
  
Location: 5171498-5172269
  
 NCBI BlastP on this gene

MYCTH\_2302941

hypothetical protein
  
Accession: AEO57126
  
Location: 5170151-5170831
  
 NCBI BlastP on this gene

MYCTH\_2302939

hypothetical protein
  
Accession: AEO57125
  
Location: 5168921-5170003
  
 NCBI BlastP on this gene

MYCTH\_2143420

hypothetical protein
  
Accession: AEO57124
  
Location: 5167139-5167776
  
 NCBI BlastP on this gene

MYCTH\_92248

hypothetical protein
  
Accession: AEO57123
  
Location: 5165507-5166448
  
 NCBI BlastP on this gene

MYCTH\_2302938

hypothetical protein
  
Accession: AEO57122
  
Location: 5163298-5164608
  
 NCBI BlastP on this gene

MYCTH\_2302937

210. :  AM920435 Penicillium chrysogenum Wisconsin 54-1255 complete genome, contig Pc00c20.     Total score: 1.0     Cumulative Blast bit score: 295

not annotated
  
Accession: CAP85500
  
Location: 388623-391409
  
 NCBI BlastP on this gene

Pc20g01710

unnamed
  
Accession: CAP85501
  
Location: 391965-393164
  
 NCBI BlastP on this gene

Pc20g01720

not annotated
  
Accession: CAP85502
  
Location: 394968-396651
  
 NCBI BlastP on this gene

Pc20g01730

not annotated
  
Accession: CAP85503
  
Location: 397195-398002
  
 NCBI BlastP on this gene

Pc20g01740

not annotated
  
Accession: CAP85504
  
Location: 398358-400032
  
 NCBI BlastP on this gene

Pc20g01750

not annotated
  
Accession: CAP85505
  
Location: 400892-402821
  
 NCBI BlastP on this gene

Pc20g01760

hypothetical protein
  
Accession: CAP85506
  
Location: 403481-404312
  
 NCBI BlastP on this gene

Pc20g01770

not annotated
  
Accession: CAP85507
  
Location: 405825-407737
  
  
**BlastP hit with Mycgr3G99766\_Mycgr3T**
  
Percentage identity: 33 %
  
BlastP bit score: 295
  
Sequence coverage: 95 %
  
E-value: 2e-89
  
  
 NCBI BlastP on this gene

Pc20g01780

unnamed
  
Accession: CAP85508
  
Location: 408172-409984
  
 NCBI BlastP on this gene

Pc20g01790

not annotated
  
Accession: CAP85509
  
Location: 412030-414283
  
 NCBI BlastP on this gene

Pc20g01800

not annotated
  
Accession: CAP85510
  
Location: 415010-416565
  
 NCBI BlastP on this gene

Pc20g01810

not annotated
  
Accession: CAP85511
  
Location: 417257-418983
  
 NCBI BlastP on this gene

Pc20g01820

not annotated
  
Accession: CAP85512
  
Location: 419250-420148
  
 NCBI BlastP on this gene

Pc20g01830

unnamed
  
Accession: CAP85513
  
Location: 420597-421734
  
 NCBI BlastP on this gene

Pc20g01840

unnamed
  
Accession: CAP85514
  
Location: 422801-424328
  
 NCBI BlastP on this gene

Pc20g01850

211. :  AM920437 Penicillium chrysogenum Wisconsin 54-1255 complete genome, contig Pc00c22.     Total score: 1.0     Cumulative Blast bit score: 294

not annotated
  
Accession: CAP97583
  
Location: 675584-678850
  
 NCBI BlastP on this gene

Pc22g02950

not annotated
  
Accession: CAP97584
  
Location: 679487-680870
  
 NCBI BlastP on this gene

Pc22g02960

not annotated
  
Accession: CAP97585
  
Location: 681831-684163
  
 NCBI BlastP on this gene

Pc22g02970

not annotated
  
Accession: CAP97586
  
Location: 684546-685693
  
 NCBI BlastP on this gene

Pc22g02980

not annotated
  
Accession: CAP97587
  
Location: 686818-687567
  
 NCBI BlastP on this gene

Pc22g02990

not annotated
  
Accession: CAP97588
  
Location: 688224-690532
  
 NCBI BlastP on this gene

Pc22g03000

hypothetical protein
  
Accession: CAP97589
  
Location: 691193-692635
  
 NCBI BlastP on this gene

Pc22g03010

not annotated
  
Accession: CAP97590
  
Location: 692712-694682
  
  
**BlastP hit with Mycgr3G99766\_Mycgr3T**
  
Percentage identity: 34 %
  
BlastP bit score: 294
  
Sequence coverage: 97 %
  
E-value: 5e-89
  
  
 NCBI BlastP on this gene

Pc22g03020

unnamed
  
Accession: CAP97591
  
Location: 694933-695983
  
 NCBI BlastP on this gene

Pc22g03030

hypothetical protein
  
Accession: CAP97592
  
Location: 697003-697618
  
 NCBI BlastP on this gene

Pc22g03040

not annotated
  
Accession: Pc22g03050
  
Location: 697686-698607
  
 NCBI BlastP on this gene

Pc22g03050

not annotated
  
Accession: CAP97594
  
Location: 699540-701492
  
 NCBI BlastP on this gene

Pc22g03060

not annotated
  
Accession: CAP97595
  
Location: 702164-703743
  
 NCBI BlastP on this gene

Pc22g03070

hypothetical protein
  
Accession: CAP97596
  
Location: 704456-705557
  
 NCBI BlastP on this gene

Pc22g03080

not annotated
  
Accession: CAP97597
  
Location: 707987-710655
  
 NCBI BlastP on this gene

Pc22g03090

212. :  KB445647 Cochliobolus sativus ND90Pr unplaced genomic scaffold COCSAscaffold\_11     Total score: 1.0     Cumulative Blast bit score: 293

hypothetical protein
  
Accession: EMD61789
  
Location: 87533-91003
  
 NCBI BlastP on this gene

EMD61789

hypothetical protein
  
Accession: EMD61790
  
Location: 91953-92189
  
 NCBI BlastP on this gene

EMD61790

hypothetical protein
  
Accession: EMD61791
  
Location: 98830-99728
  
 NCBI BlastP on this gene

EMD61791

hypothetical protein
  
Accession: EMD61792
  
Location: 102057-104224
  
 NCBI BlastP on this gene

EMD61792

hypothetical protein
  
Accession: EMD61793
  
Location: 104495-104926
  
 NCBI BlastP on this gene

EMD61793

hypothetical protein
  
Accession: EMD61794
  
Location: 107054-108699
  
  
**BlastP hit with Mycgr3G99766\_Mycgr3T**
  
Percentage identity: 35 %
  
BlastP bit score: 293
  
Sequence coverage: 97 %
  
E-value: 6e-89
  
  
 NCBI BlastP on this gene

EMD61794

hypothetical protein
  
Accession: EMD61795
  
Location: 109195-109604
  
 NCBI BlastP on this gene

EMD61795

hypothetical protein
  
Accession: EMD61796
  
Location: 111640-114102
  
 NCBI BlastP on this gene

EMD61796

hypothetical protein
  
Accession: EMD61797
  
Location: 114534-115293
  
 NCBI BlastP on this gene

EMD61797

hypothetical protein
  
Accession: EMD61798
  
Location: 115475-116138
  
 NCBI BlastP on this gene

EMD61798

hypothetical protein
  
Accession: EMD61799
  
Location: 116426-117796
  
 NCBI BlastP on this gene

EMD61799

hypothetical protein
  
Accession: EMD61800
  
Location: 118004-119290
  
 NCBI BlastP on this gene

EMD61800

hypothetical protein
  
Accession: EMD61801
  
Location: 119545-120734
  
 NCBI BlastP on this gene

EMD61801

hypothetical protein
  
Accession: EMD61802
  
Location: 121443-122845
  
 NCBI BlastP on this gene

EMD61802

hypothetical protein
  
Accession: EMD61803
  
Location: 124781-125146
  
 NCBI BlastP on this gene

EMD61803

213. :  DS995701 Microsporum canis CBS 113480 supercont1.1 genomic scaffold     Total score: 1.0     Cumulative Blast bit score: 293

sugar transporter
  
Accession: EEQ28189
  
Location: 2941554-2943524
  
  
**BlastP hit with Mycgr3G99766\_Mycgr3T**
  
Percentage identity: 35 %
  
BlastP bit score: 293
  
Sequence coverage: 97 %
  
E-value: 9e-89
  
  
 NCBI BlastP on this gene

EEQ28189

conserved hypothetical protein
  
Accession: EEQ28188
  
Location: 2939207-2941239
  
 NCBI BlastP on this gene

EEQ28188

high affinity nicotinic acid plasma membrane permease
  
Accession: EEQ28187
  
Location: 2936593-2938171
  
 NCBI BlastP on this gene

EEQ28187

glutamine synthetase
  
Accession: EEQ28186
  
Location: 2934062-2935623
  
 NCBI BlastP on this gene

EEQ28186

conserved hypothetical protein
  
Accession: EEQ28185
  
Location: 2931006-2933186
  
 NCBI BlastP on this gene

EEQ28185

conserved hypothetical protein
  
Accession: EEQ28184
  
Location: 2929707-2930564
  
 NCBI BlastP on this gene

EEQ28184

DUF159 domain-containing protein
  
Accession: EEQ28183
  
Location: 2928272-2929574
  
 NCBI BlastP on this gene

EEQ28183

TPR domain-containing protein
  
Accession: EEQ28182
  
Location: 2926202-2927632
  
 NCBI BlastP on this gene

EEQ28182

G-protein complex beta subunit CpcB
  
Accession: EEQ28181
  
Location: 2924273-2925664
  
 NCBI BlastP on this gene

EEQ28181

214. :  AM920428 Penicillium chrysogenum Wisconsin 54-1255 complete genome, contig Pc00c13.     Total score: 1.0     Cumulative Blast bit score: 292

unnamed
  
Accession: CAP92339
  
Location: 3072376-3074285
  
  
**BlastP hit with Mycgr3G99766\_Mycgr3T**
  
Percentage identity: 34 %
  
BlastP bit score: 292
  
Sequence coverage: 102 %
  
E-value: 3e-88
  
  
 NCBI BlastP on this gene

Pc13g12700

unnamed
  
Accession: CAP92338
  
Location: 3071446-3072185
  
 NCBI BlastP on this gene

Pc13g12690

not annotated
  
Accession: Pc13g12680
  
Location: 3070043-3070708
  
 NCBI BlastP on this gene

Pc13g12680

hypothetical protein
  
Accession: CAP92336
  
Location: 3068669-3069775
  
 NCBI BlastP on this gene

Pc13g12670

hypothetical protein
  
Accession: CAP92335
  
Location: 3067665-3068152
  
 NCBI BlastP on this gene

Pc13g12660

not annotated
  
Accession: CAP92334
  
Location: 3064990-3067293
  
 NCBI BlastP on this gene

Pc13g12650

not annotated
  
Accession: CAP92333
  
Location: 3062354-3063757
  
 NCBI BlastP on this gene

Pc13g12640

not annotated
  
Accession: CAP92332
  
Location: 3058693-3061842
  
 NCBI BlastP on this gene

Pc13g12630

not annotated
  
Accession: CAP92331
  
Location: 3053811-3056163
  
 NCBI BlastP on this gene

Pc13g12620

215. :  KE007245 Wallemia ichthyophaga EXF-994 unplaced genomic scaffold scaffold22     Total score: 1.0     Cumulative Blast bit score: 291

hypothetical protein
  
Accession: EOQ99034
  
Location: 49761-50492
  
 NCBI BlastP on this gene

EOQ99034

Nuclear pore complex protein
  
Accession: EOQ99035
  
Location: 50559-55017
  
 NCBI BlastP on this gene

EOQ99035

Equilibrative nucleoside transporter 1
  
Accession: EOQ99036
  
Location: 55046-56425
  
 NCBI BlastP on this gene

EOQ99036

Kinesin-like protein
  
Accession: EOQ99037
  
Location: 56580-61572
  
 NCBI BlastP on this gene

EOQ99037

hypothetical protein
  
Accession: EOQ99038
  
Location: 61867-63862
  
 NCBI BlastP on this gene

EOQ99038

Intermediate cleaving peptidase 55
  
Accession: EOQ99039
  
Location: 64866-66416
  
 NCBI BlastP on this gene

EOQ99039

Putative lysine N-acyltransferase C17G9.06c
  
Accession: EOQ99040
  
Location: 66766-67998
  
  
**BlastP hit with Mycgr3G85486\_Mycgr3T**
  
Percentage identity: 45 %
  
BlastP bit score: 291
  
Sequence coverage: 82 %
  
E-value: 4e-91
  
  
 NCBI BlastP on this gene

EOQ99040

216. :  KB456263 Mycosphaerella populorum SO2202 unplaced genomic scaffold SEPMUscaffold\_4     Total score: 1.0     Cumulative Blast bit score: 290

MFS monosaccharide transporter
  
Accession: EMF13593
  
Location: 1500434-1502719
  
  
**BlastP hit with Mycgr3G99766\_Mycgr3T**
  
Percentage identity: 35 %
  
BlastP bit score: 290
  
Sequence coverage: 98 %
  
E-value: 3e-87
  
  
 NCBI BlastP on this gene

EMF13593

L-amino-acid oxidase
  
Accession: EMF13592
  
Location: 1498212-1500329
  
 NCBI BlastP on this gene

EMF13592

Isy1-like splicing factor
  
Accession: EMF13591
  
Location: 1496224-1496952
  
 NCBI BlastP on this gene

EMF13591

calcium ATPase
  
Accession: EMF13590
  
Location: 1491360-1494621
  
 NCBI BlastP on this gene

EMF13590

glycoside hydrolase family 55 protein
  
Accession: EMF13589
  
Location: 1488277-1490905
  
 NCBI BlastP on this gene

EMF13589

SGNH hydrolase
  
Accession: EMF13588
  
Location: 1486105-1486926
  
 NCBI BlastP on this gene

EMF13588

glycosyltransferase family 76 protein
  
Accession: EMF13587
  
Location: 1484139-1485650
  
 NCBI BlastP on this gene

EMF13587

217. :  JH717968 Fomitiporia mediterranea MF3/22 unplaced genomic scaffold FOMMEscaffold\_2     Total score: 1.0     Cumulative Blast bit score: 290

drug:h+ antiporter
  
Accession: EJD07174
  
Location: 4566984-4570021
  
  
**BlastP hit with Mycgr3G70577\_Mycgr3T**
  
Percentage identity: 32 %
  
BlastP bit score: 290
  
Sequence coverage: 95 %
  
E-value: 5e-85
  
  
 NCBI BlastP on this gene

EJD07174

218. :  KB445573 Cochliobolus heterostrophus C5 unplaced genomic scaffold COCHEscaffold\_5     Total score: 1.0     Cumulative Blast bit score: 289

hypothetical protein
  
Accession: EMD93842
  
Location: 1871111-1872759
  
  
**BlastP hit with Mycgr3G99766\_Mycgr3T**
  
Percentage identity: 36 %
  
BlastP bit score: 289
  
Sequence coverage: 98 %
  
E-value: 2e-87
  
  
 NCBI BlastP on this gene

EMD93842

hypothetical protein
  
Accession: EMD93841
  
Location: 1869444-1869962
  
 NCBI BlastP on this gene

EMD93841

219. :  KB908481 Setosphaeria turcica Et28A unplaced genomic scaffold SETTUscaffold\_1     Total score: 1.0     Cumulative Blast bit score: 287

hypothetical protein
  
Accession: EOA91982
  
Location: 2785306-2788559
  
 NCBI BlastP on this gene

EOA91982

hypothetical protein
  
Accession: EOA91983
  
Location: 2788679-2790646
  
 NCBI BlastP on this gene

EOA91983

hypothetical protein
  
Accession: EOA91984
  
Location: 2791612-2792619
  
 NCBI BlastP on this gene

EOA91984

hypothetical protein
  
Accession: EOA91985
  
Location: 2797158-2798799
  
  
**BlastP hit with Mycgr3G99766\_Mycgr3T**
  
Percentage identity: 34 %
  
BlastP bit score: 287
  
Sequence coverage: 98 %
  
E-value: 9e-87
  
  
 NCBI BlastP on this gene

EOA91985

220. :  GL385397 Gaeumannomyces graminis var. tritici R3-111a-1 unplaced genomic scaffold supercont2.3     Total score: 1.0     Cumulative Blast bit score: 287

hypothetical protein
  
Accession: EJT77366
  
Location: 6434600-6436496
  
 NCBI BlastP on this gene

EJT77366

hypothetical protein
  
Accession: EJT77365
  
Location: 6430827-6432710
  
 NCBI BlastP on this gene

EJT77365

hypothetical protein
  
Accession: EJT77364
  
Location: 6429023-6430734
  
 NCBI BlastP on this gene

EJT77364

hypothetical protein
  
Accession: EJT77363
  
Location: 6427877-6428380
  
 NCBI BlastP on this gene

EJT77363

hypothetical protein
  
Accession: EJT77362
  
Location: 6424662-6427139
  
 NCBI BlastP on this gene

EJT77362

hypothetical protein
  
Accession: EJT77361
  
Location: 6423003-6424358
  
 NCBI BlastP on this gene

EJT77361

hypothetical protein
  
Accession: EJT77360
  
Location: 6420489-6422372
  
 NCBI BlastP on this gene

EJT77360

sugar transporter STL1
  
Accession: EJT77359
  
Location: 6418066-6419824
  
  
**BlastP hit with Mycgr3G99766\_Mycgr3T**
  
Percentage identity: 34 %
  
BlastP bit score: 287
  
Sequence coverage: 97 %
  
E-value: 2e-86
  
  
 NCBI BlastP on this gene

EJT77359

hypothetical protein
  
Accession: EJT77358
  
Location: 6416594-6417531
  
 NCBI BlastP on this gene

EJT77358

hypothetical protein
  
Accession: EJT77357
  
Location: 6414564-6416528
  
 NCBI BlastP on this gene

EJT77357

hypothetical protein
  
Accession: EJT77356
  
Location: 6412291-6413950
  
 NCBI BlastP on this gene

EJT77356

hypothetical protein
  
Accession: EJT77355
  
Location: 6409856-6411814
  
 NCBI BlastP on this gene

EJT77355

hypothetical protein
  
Accession: EJT77354
  
Location: 6408782-6409186
  
 NCBI BlastP on this gene

EJT77354

hypothetical protein
  
Accession: EJT77353
  
Location: 6407262-6407927
  
 NCBI BlastP on this gene

EJT77353

hypothetical protein
  
Accession: EJT77352
  
Location: 6405023-6407054
  
 NCBI BlastP on this gene

EJT77352

hypothetical protein
  
Accession: EJT77351
  
Location: 6404186-6404803
  
 NCBI BlastP on this gene

EJT77351

221. :  CM001202 Mycosphaerella graminicola IPO323 chromosome 7     Total score: 1.0     Cumulative Blast bit score: 287

histone methyltransferase
  
Accession: EGP85597
  
Location: 492915-496575
  
 NCBI BlastP on this gene

EGP85597

triacylglycerol hydrolase
  
Accession: EGP85598
  
Location: 497233-498826
  
 NCBI BlastP on this gene

EGP85598

hypothetical protein
  
Accession: EGP86225
  
Location: 500337-501938
  
 NCBI BlastP on this gene

EGP86225

hypothetical protein
  
Accession: EGP86224
  
Location: 502233-503902
  
 NCBI BlastP on this gene

EGP86224

hypothetical protein
  
Accession: EGP86223
  
Location: 504739-505457
  
 NCBI BlastP on this gene

EGP86223

hypothetical protein
  
Accession: EGP85599
  
Location: 506022-507050
  
 NCBI BlastP on this gene

EGP85599

hypothetical protein
  
Accession: EGP85600
  
Location: 507879-510119
  
  
**BlastP hit with Mycgr3G99766\_Mycgr3T**
  
Percentage identity: 35 %
  
BlastP bit score: 287
  
Sequence coverage: 97 %
  
E-value: 2e-86
  
  
 NCBI BlastP on this gene

EGP85600

222. :  CH476615 Uncinocarpus reesii 1704 scaffold\_1 genomic scaffold     Total score: 1.0     Cumulative Blast bit score: 287

conserved hypothetical protein
  
Accession: EEP75789
  
Location: 1335837-1338347
  
 NCBI BlastP on this gene

EEP75789

conserved hypothetical protein
  
Accession: EEP75790
  
Location: 1338677-1339805
  
 NCBI BlastP on this gene

EEP75790

conserved hypothetical protein
  
Accession: EEP75791
  
Location: 1340981-1341797
  
 NCBI BlastP on this gene

EEP75791

conserved hypothetical protein
  
Accession: EEP75792
  
Location: 1342165-1343680
  
 NCBI BlastP on this gene

EEP75792

conserved hypothetical protein
  
Accession: EEP75793
  
Location: 1344176-1345127
  
 NCBI BlastP on this gene

EEP75793

predicted protein
  
Accession: EEP75794
  
Location: 1345522-1346787
  
 NCBI BlastP on this gene

EEP75794

predicted protein
  
Accession: EEP75795
  
Location: 1347774-1348259
  
 NCBI BlastP on this gene

EEP75795

conserved hypothetical protein
  
Accession: EEP75796
  
Location: 1349119-1350031
  
 NCBI BlastP on this gene

EEP75796

conserved hypothetical protein
  
Accession: EEP75797
  
Location: 1351940-1353898
  
  
**BlastP hit with Mycgr3G99766\_Mycgr3T**
  
Percentage identity: 34 %
  
BlastP bit score: 287
  
Sequence coverage: 101 %
  
E-value: 5e-86
  
  
 NCBI BlastP on this gene

EEP75797

predicted protein
  
Accession: EEP75798
  
Location: 1354404-1355397
  
 NCBI BlastP on this gene

EEP75798

conserved hypothetical protein
  
Accession: EEP75799
  
Location: 1355908-1356666
  
 NCBI BlastP on this gene

EEP75799

predicted protein
  
Accession: EEP75800
  
Location: 1358325-1360178
  
 NCBI BlastP on this gene

EEP75800

predicted protein
  
Accession: EEP75801
  
Location: 1361750-1363577
  
 NCBI BlastP on this gene

EEP75801

hypothetical protein
  
Accession: EEP75802
  
Location: 1365249-1366329
  
 NCBI BlastP on this gene

EEP75802

predicted protein
  
Accession: EEP75803
  
Location: 1367415-1368329
  
 NCBI BlastP on this gene

EEP75803

223. :  CR382129 Yarrowia lipolytica CLIB122 chromosome C complete sequence.     Total score: 1.0     Cumulative Blast bit score: 286

YALI0C04543p
  
Accession: CAG81745
  
Location: 604760-606604
  
 NCBI BlastP on this gene

YALI0\_C04543g

YALI0C04565p
  
Accession: CAG81746
  
Location: 606776-607693
  
 NCBI BlastP on this gene

YALI0\_C04565g

YALI0C04587p
  
Accession: CAG81747
  
Location: 609248-611224
  
 NCBI BlastP on this gene

YALI0\_C04587g

YALI0C04620p
  
Accession: CAG81749
  
Location: 614285-617044
  
 NCBI BlastP on this gene

YALI0\_C04620g

YALI0C04730p
  
Accession: CAG81752
  
Location: 622876-624573
  
  
**BlastP hit with Mycgr3G99766\_Mycgr3T**
  
Percentage identity: 33 %
  
BlastP bit score: 286
  
Sequence coverage: 104 %
  
E-value: 8e-86
  
  
 NCBI BlastP on this gene

YALI0\_C04730g

YALI0C04774p
  
Accession: CAG81753
  
Location: 625342-627255
  
 NCBI BlastP on this gene

YALI0\_C04774g

YALI0C04796p
  
Accession: CAG81754
  
Location: 628618-630402
  
 NCBI BlastP on this gene

YALI0\_C04796g

YALI0C04818p
  
Accession: CAG81755
  
Location: 631617-633371
  
 NCBI BlastP on this gene

YALI0\_C04818g

YALI0C04884p
  
Accession: CAG81756
  
Location: 637397-638221
  
 NCBI BlastP on this gene

YALI0\_C04884g

YALI0C04906p
  
Accession: CAG81757
  
Location: 639586-640582
  
 NCBI BlastP on this gene

YALI0\_C04906g

224. :  CM001199 Mycosphaerella graminicola IPO323 chromosome 4     Total score: 1.0     Cumulative Blast bit score: 286

hypothetical protein
  
Accession: EGP88531
  
Location: 535272-537446
  
  
**BlastP hit with Mycgr3G99766\_Mycgr3T**
  
Percentage identity: 33 %
  
BlastP bit score: 286
  
Sequence coverage: 105 %
  
E-value: 1e-85
  
  
 NCBI BlastP on this gene

EGP88531

Bromodomain-containing AAA ATPase protein
  
Accession: EGP87834
  
Location: 527038-531994
  
 NCBI BlastP on this gene

EGP87834

hypothetical protein
  
Accession: EGP88532
  
Location: 523564-525728
  
 NCBI BlastP on this gene

EGP88532

hypothetical protein
  
Accession: EGP87833
  
Location: 519146-520243
  
 NCBI BlastP on this gene

EGP87833

225. :  AKCT01000319 Penicillium digitatum PHI26     Total score: 1.0     Cumulative Blast bit score: 286

Maltase
  
Accession: EKV04855
  
Location: 115588-117321
  
 NCBI BlastP on this gene

EKV04855

hypothetical protein
  
Accession: EKV04856
  
Location: 117695-117808
  
 NCBI BlastP on this gene

EKV04856

DNA-directed RNA polymerase
  
Accession: EKV04857
  
Location: 119614-123420
  
 NCBI BlastP on this gene

EKV04857

hypothetical protein
  
Accession: EKV04858
  
Location: 124390-125267
  
 NCBI BlastP on this gene

EKV04858

Actin-related protein 4
  
Accession: EKV04859
  
Location: 126099-127684
  
 NCBI BlastP on this gene

EKV04859

hypothetical protein
  
Accession: EKV04860
  
Location: 128707-129799
  
 NCBI BlastP on this gene

EKV04860

MFS sugar transporter, putative
  
Accession: EKV04861
  
Location: 132504-134054
  
  
**BlastP hit with Mycgr3G99766\_Mycgr3T**
  
Percentage identity: 34 %
  
BlastP bit score: 286
  
Sequence coverage: 97 %
  
E-value: 2e-86
  
  
 NCBI BlastP on this gene

EKV04861

hypothetical protein
  
Accession: EKV04862
  
Location: 135328-136455
  
 NCBI BlastP on this gene

EKV04862

hypothetical protein
  
Accession: EKV04863
  
Location: 138164-140084
  
 NCBI BlastP on this gene

EKV04863

hypothetical protein
  
Accession: EKV04864
  
Location: 141219-141959
  
 NCBI BlastP on this gene

EKV04864

hypothetical protein
  
Accession: EKV04865
  
Location: 142384-142948
  
 NCBI BlastP on this gene

EKV04865

hypothetical protein
  
Accession: EKV04866
  
Location: 144597-146205
  
 NCBI BlastP on this gene

EKV04866

hypothetical protein
  
Accession: EKV04867
  
Location: 146522-147355
  
 NCBI BlastP on this gene

EKV04867

hypothetical protein
  
Accession: EKV04868
  
Location: 148095-149690
  
 NCBI BlastP on this gene

EKV04868

226. :  AM920437 Penicillium chrysogenum Wisconsin 54-1255 complete genome, contig Pc00c22.     Total score: 1.0     Cumulative Blast bit score: 285

not annotated
  
Accession: CAP98076
  
Location: 1869550-1871283
  
 NCBI BlastP on this gene

Pc22g07880

not annotated
  
Accession: CAP98075
  
Location: 1866930-1868558
  
 NCBI BlastP on this gene

Pc22g07870

not annotated
  
Accession: CAP98074
  
Location: 1862802-1866606
  
 NCBI BlastP on this gene

Pc22g07860

not annotated
  
Accession: CAP98073
  
Location: 1860917-1861800
  
 NCBI BlastP on this gene

Pc22g07850

not annotated
  
Accession: CAP98072
  
Location: 1858575-1860153
  
 NCBI BlastP on this gene

Pc22g07840

not annotated
  
Accession: CAP98071
  
Location: 1856478-1857571
  
 NCBI BlastP on this gene

Pc22g07830

hypothetical protein
  
Accession: CAP98070
  
Location: 1854097-1854827
  
 NCBI BlastP on this gene

Pc22g07820

unnamed
  
Accession: CAP98069
  
Location: 1852309-1853859
  
  
**BlastP hit with Mycgr3G99766\_Mycgr3T**
  
Percentage identity: 33 %
  
BlastP bit score: 285
  
Sequence coverage: 97 %
  
E-value: 8e-86
  
  
 NCBI BlastP on this gene

Pc22g07810

hypothetical protein
  
Accession: CAP98068
  
Location: 1849887-1851473
  
 NCBI BlastP on this gene

Pc22g07800

unnamed
  
Accession: CAP98067
  
Location: 1847410-1849556
  
 NCBI BlastP on this gene

Pc22g07790

unnamed
  
Accession: CAP98066
  
Location: 1844614-1846288
  
 NCBI BlastP on this gene

Pc22g07780

unnamed
  
Accession: CAP98065
  
Location: 1841264-1842933
  
 NCBI BlastP on this gene

Pc22g07770

unnamed
  
Accession: CAP98064
  
Location: 1837931-1839854
  
 NCBI BlastP on this gene

Pc22g07760

227. :  KB730093 Fusarium oxysporum f. sp. cubense race 1 unplaced genomic scaffold scaffold48     Total score: 1.0     Cumulative Blast bit score: 281

hypothetical protein
  
Accession: ENH72512
  
Location: 469085-469652
  
 NCBI BlastP on this gene

ENH72512

Testis-specific chromodomain protein Y 2
  
Accession: ENH72511
  
Location: 467900-468656
  
 NCBI BlastP on this gene

ENH72511

Synembryn-A
  
Accession: ENH72510
  
Location: 465583-467169
  
 NCBI BlastP on this gene

ENH72510

Telomere length regulator protein rif1
  
Accession: ENH72509
  
Location: 459422-464790
  
 NCBI BlastP on this gene

ENH72509

PiggyBac transposable element-derived protein 4
  
Accession: ENH72508
  
Location: 456104-458041
  
 NCBI BlastP on this gene

ENH72508

Sugar transporter STL1
  
Accession: ENH72507
  
Location: 451689-453457
  
  
**BlastP hit with Mycgr3G99766\_Mycgr3T**
  
Percentage identity: 34 %
  
BlastP bit score: 281
  
Sequence coverage: 97 %
  
E-value: 7e-84
  
  
 NCBI BlastP on this gene

ENH72507

Glucose-6-phosphate 1-epimerase
  
Accession: ENH72506
  
Location: 449808-450856
  
 NCBI BlastP on this gene

ENH72506

Phospho-2-dehydro-3-deoxyheptonate aldolase
  
Accession: ENH72505
  
Location: 448065-449480
  
 NCBI BlastP on this gene

ENH72505

hypothetical protein
  
Accession: ENH72504
  
Location: 446982-447305
  
 NCBI BlastP on this gene

ENH72504

Microcephalin
  
Accession: ENH72503
  
Location: 443142-446589
  
 NCBI BlastP on this gene

ENH72503

hypothetical protein
  
Accession: ENH72502
  
Location: 441613-441998
  
 NCBI BlastP on this gene

ENH72502

Putative ribonuclease P protein subunit 3
  
Accession: ENH72501
  
Location: 439934-440827
  
 NCBI BlastP on this gene

ENH72501

hypothetical protein
  
Accession: ENH72500
  
Location: 438308-439706
  
 NCBI BlastP on this gene

ENH72500

hypothetical protein
  
Accession: ENH72499
  
Location: 435619-436310
  
 NCBI BlastP on this gene

ENH72499

228. :  JH668249 Wallemia sebi CBS 633.66 unplaced genomic scaffold WALSEscaffold\_27     Total score: 1.0     Cumulative Blast bit score: 281

hypothetical protein
  
Accession: EIM19512
  
Location: 40779-41134
  
 NCBI BlastP on this gene

EIM19512

hypothetical protein
  
Accession: EIM19513
  
Location: 41162-45148
  
 NCBI BlastP on this gene

EIM19513

mitochondrial pyruvate dehydrogenase E1 component beta subunit
  
Accession: EIM19514
  
Location: 45230-46378
  
 NCBI BlastP on this gene

EIM19514

formaldehyde dehydrogenase
  
Accession: EIM19515
  
Location: 46689-47862
  
 NCBI BlastP on this gene

EIM19515

kinesin-like protein
  
Accession: EIM19516
  
Location: 48417-53354
  
 NCBI BlastP on this gene

EIM19516

hypothetical protein
  
Accession: EIM19517
  
Location: 53608-54953
  
 NCBI BlastP on this gene

EIM19517

hypothetical protein
  
Accession: EIM19518
  
Location: 55874-57413
  
 NCBI BlastP on this gene

EIM19518

hypothetical protein
  
Accession: EIM19519
  
Location: 57687-58852
  
  
**BlastP hit with Mycgr3G85486\_Mycgr3T**
  
Percentage identity: 44 %
  
BlastP bit score: 281
  
Sequence coverage: 84 %
  
E-value: 9e-88
  
  
 NCBI BlastP on this gene

EIM19519

mitochondrial carrier
  
Accession: EIM19520
  
Location: 58943-60625
  
 NCBI BlastP on this gene

EIM19520

hypothetical protein
  
Accession: EIM19521
  
Location: 60665-61582
  
 NCBI BlastP on this gene

EIM19521

hypothetical protein
  
Accession: EIM19522
  
Location: 61815-64302
  
 NCBI BlastP on this gene

EIM19522

hypothetical protein
  
Accession: EIM19523
  
Location: 64342-68802
  
 NCBI BlastP on this gene

EIM19523

hypothetical protein
  
Accession: EIM19524
  
Location: 68968-69512
  
 NCBI BlastP on this gene

EIM19524

hypothetical protein
  
Accession: EIM19525
  
Location: 72919-73452
  
 NCBI BlastP on this gene

EIM19525

hypothetical protein
  
Accession: EIM19526
  
Location: 74921-75727
  
 NCBI BlastP on this gene

EIM19526

229. :  AACD01000043 Aspergillus nidulans FGSC A4     Total score: 1.0     Cumulative Blast bit score: 281

hypothetical protein
  
Accession: EAA64689
  
Location: 226758-228332
  
  
**BlastP hit with Mycgr3G99766\_Mycgr3T**
  
Percentage identity: 34 %
  
BlastP bit score: 281
  
Sequence coverage: 96 %
  
E-value: 2e-84
  
  
 NCBI BlastP on this gene

EAA64689

hypothetical protein
  
Accession: EAA64688
  
Location: 224392-225635
  
 NCBI BlastP on this gene

EAA64688

hypothetical protein
  
Accession: EAA64687
  
Location: 222212-223601
  
 NCBI BlastP on this gene

EAA64687

hypothetical protein
  
Accession: EAA64686
  
Location: 217545-221325
  
 NCBI BlastP on this gene

EAA64686

predicted protein
  
Accession: EAA64685
  
Location: 213629-215170
  
 NCBI BlastP on this gene

EAA64685

hypothetical protein
  
Accession: EAA64684
  
Location: 207467-211265
  
 NCBI BlastP on this gene

EAA64684

230. :  HF679023 Fusarium fujikuroi IMI 58289 draft genome, chromosome FFUJ\_chr01.     Total score: 1.0     Cumulative Blast bit score: 280

uncharacterized protein
  
Accession: CCT61784
  
Location: 1108615-1110735
  
 NCBI BlastP on this gene

FFUJ\_01726

uncharacterized protein
  
Accession: CCT61785
  
Location: 1111503-1112186
  
 NCBI BlastP on this gene

FFUJ\_01725

uncharacterized protein
  
Accession: CCT61786
  
Location: 1112462-1112995
  
 NCBI BlastP on this gene

FFUJ\_01724

uncharacterized protein
  
Accession: CCT61787
  
Location: 1113422-1114172
  
 NCBI BlastP on this gene

FFUJ\_01723

uncharacterized protein
  
Accession: CCT61788
  
Location: 1114918-1116510
  
 NCBI BlastP on this gene

FFUJ\_01722

related to telomere length regulator protein rif1
  
Accession: CCT61789
  
Location: 1117281-1122658
  
 NCBI BlastP on this gene

FFUJ\_01721

probable sugar transporter
  
Accession: CCT61790
  
Location: 1125937-1127711
  
  
**BlastP hit with Mycgr3G99766\_Mycgr3T**
  
Percentage identity: 34 %
  
BlastP bit score: 280
  
Sequence coverage: 97 %
  
E-value: 1e-83
  
  
 NCBI BlastP on this gene

FFUJ\_01720

231. :  KB726991 Fusarium oxysporum f. sp. cubense race 4 unplaced genomic scaffold scaffold135     Total score: 1.0     Cumulative Blast bit score: 279

Testis-specific chromodomain protein Y 1
  
Accession: EMT64561
  
Location: 538871-539633
  
 NCBI BlastP on this gene

EMT64561

Synembryn-A
  
Accession: EMT64560
  
Location: 536553-538139
  
 NCBI BlastP on this gene

EMT64560

hypothetical protein
  
Accession: EMT64559
  
Location: 534704-534996
  
 NCBI BlastP on this gene

EMT64559

Telomere length regulator protein rif1
  
Accession: EMT64558
  
Location: 526179-533911
  
 NCBI BlastP on this gene

EMT64558

Sugar transporter STL1
  
Accession: EMT64557
  
Location: 520950-522718
  
  
**BlastP hit with Mycgr3G99766\_Mycgr3T**
  
Percentage identity: 34 %
  
BlastP bit score: 279
  
Sequence coverage: 97 %
  
E-value: 2e-83
  
  
 NCBI BlastP on this gene

EMT64557

Glucose-6-phosphate 1-epimerase
  
Accession: EMT64556
  
Location: 516926-517974
  
 NCBI BlastP on this gene

EMT64556

Phospho-2-dehydro-3-deoxyheptonate aldolase
  
Accession: EMT64555
  
Location: 515183-516598
  
 NCBI BlastP on this gene

EMT64555

hypothetical protein
  
Accession: EMT64554
  
Location: 514080-514403
  
 NCBI BlastP on this gene

EMT64554

Microcephalin
  
Accession: EMT64553
  
Location: 508331-513687
  
 NCBI BlastP on this gene

EMT64553

hypothetical protein
  
Accession: EMT64552
  
Location: 506800-507185
  
 NCBI BlastP on this gene

EMT64552

Putative ribonuclease P protein subunit 3
  
Accession: EMT64551
  
Location: 505116-506009
  
 NCBI BlastP on this gene

EMT64551

hypothetical protein
  
Accession: EMT64550
  
Location: 503492-504888
  
 NCBI BlastP on this gene

EMT64550

232. :  FO082046 Pichia sorbitophila strain CBS 7064 chromosome N complete sequence.     Total score: 1.0     Cumulative Blast bit score: 279

not annotated
  
Accession: CCE86747
  
Location: 927910-933296
  
 NCBI BlastP on this gene

Piso0\_005257

not annotated
  
Accession: CCE86746
  
Location: 926556-927749
  
 NCBI BlastP on this gene

Piso0\_005256

not annotated
  
Accession: CCE86745
  
Location: 919429-926343
  
 NCBI BlastP on this gene

Piso0\_005255

not annotated
  
Accession: CCE86744
  
Location: 917681-918376
  
 NCBI BlastP on this gene

Piso0\_005254

not annotated
  
Accession: CCE86743
  
Location: 916635-916880
  
 NCBI BlastP on this gene

Piso0\_005253

not annotated
  
Accession: CCE86742
  
Location: 915883-916242
  
 NCBI BlastP on this gene

Piso0\_005252

not annotated
  
Accession: CCE86741
  
Location: 913548-915314
  
  
**BlastP hit with Mycgr3G99766\_Mycgr3T**
  
Percentage identity: 32 %
  
BlastP bit score: 279
  
Sequence coverage: 104 %
  
E-value: 1e-82
  
  
 NCBI BlastP on this gene

Piso0\_005250

not annotated
  
Accession: CCE86740
  
Location: 912860-913396
  
 NCBI BlastP on this gene

Piso0\_005249

not annotated
  
Accession: CCE86739
  
Location: 911623-912252
  
 NCBI BlastP on this gene

Piso0\_005248

not annotated
  
Accession: CCE86738
  
Location: 909781-911154
  
 NCBI BlastP on this gene

Piso0\_005247

not annotated
  
Accession: CCE86737
  
Location: 907636-908721
  
 NCBI BlastP on this gene

Piso0\_005246

not annotated
  
Accession: CCE86736
  
Location: 905218-906618
  
 NCBI BlastP on this gene

Piso0\_005245

not annotated
  
Accession: CCE86735
  
Location: 903609-904541
  
 NCBI BlastP on this gene

Piso0\_005244

not annotated
  
Accession: CCE86734
  
Location: 900537-902993
  
 NCBI BlastP on this gene

Piso0\_005243

not annotated
  
Accession: CCE86733
  
Location: 899474-900388
  
 NCBI BlastP on this gene

Piso0\_005242

not annotated
  
Accession: CCE86732
  
Location: 897995-899392
  
 NCBI BlastP on this gene

Piso0\_005241

not annotated
  
Accession: CCE86731
  
Location: 896938-897674
  
 NCBI BlastP on this gene

Piso0\_005240

233. :  CU928166 Lachancea thermotolerans CBS 6340 chromosome B complete sequence.     Total score: 1.0     Cumulative Blast bit score: 279

KLTH0B04686p
  
Accession: CAR21553
  
Location: 381618-381932
  
 NCBI BlastP on this gene

KLTH0B04686g

KLTH0B04664p
  
Accession: CAR21552
  
Location: 378488-380590
  
 NCBI BlastP on this gene

KLTH0B04664g

KLTH0B04642p
  
Accession: CAR21551
  
Location: 377498-378223
  
 NCBI BlastP on this gene

KLTH0B04642g

KLTH0B04620p
  
Accession: CAR21550
  
Location: 375653-377353
  
 NCBI BlastP on this gene

KLTH0B04620g

KLTH0B04598p
  
Accession: CAR21549
  
Location: 373616-375145
  
 NCBI BlastP on this gene

KLTH0B04598g

KLTH0B04576p
  
Accession: CAR21548
  
Location: 370504-373431
  
 NCBI BlastP on this gene

KLTH0B04576g

KLTH0B04554p
  
Accession: CAR21547
  
Location: 367962-370022
  
 NCBI BlastP on this gene

KLTH0B04554g

KLTH0B04532p
  
Accession: CAR21546
  
Location: 367438-367770
  
 NCBI BlastP on this gene

KLTH0B04532g

KLTH0B04510p
  
Accession: CAR21545
  
Location: 364062-365768
  
  
**BlastP hit with Mycgr3G99766\_Mycgr3T**
  
Percentage identity: 32 %
  
BlastP bit score: 279
  
Sequence coverage: 101 %
  
E-value: 5e-83
  
  
 NCBI BlastP on this gene

KLTH0B04510g

KLTH0B04488p
  
Accession: CAR21544
  
Location: 362390-363715
  
 NCBI BlastP on this gene

KLTH0B04488g

KLTH0B04466p
  
Accession: CAR21543
  
Location: 360380-362344
  
 NCBI BlastP on this gene

KLTH0B04466g

KLTH0B04444p
  
Accession: CAR21542
  
Location: 359264-360151
  
 NCBI BlastP on this gene

KLTH0B04444g

KLTH0B04422p
  
Accession: CAR21541
  
Location: 358915-359253
  
 NCBI BlastP on this gene

KLTH0B04422g

KLTH0B04400p
  
Accession: CAR21540
  
Location: 357622-358890
  
 NCBI BlastP on this gene

KLTH0B04400g

KLTH0B04378p
  
Accession: CAR21539
  
Location: 356395-357537
  
 NCBI BlastP on this gene

KLTH0B04378g

KLTH0B04356p
  
Accession: CAR21538
  
Location: 353523-355418
  
 NCBI BlastP on this gene

KLTH0B04356g

KLTH0B04334p
  
Accession: CAR21537
  
Location: 350971-351879
  
 NCBI BlastP on this gene

KLTH0B04334g

KLTH0B04312p
  
Accession: CAR21536
  
Location: 349547-350941
  
 NCBI BlastP on this gene

KLTH0B04312g

KLTH0B04290p
  
Accession: CAR21535
  
Location: 346686-349340
  
 NCBI BlastP on this gene

KLTH0B04290g

234. :  EQ963475 Aspergillus flavus NRRL3357 scf\_1106286419142 genomic scaffold     Total score: 1.0     Cumulative Blast bit score: 278

conserved hypothetical protein
  
Accession: EED53493
  
Location: 1189258-1192172
  
 NCBI BlastP on this gene

EED53493

cytochrome P450, putative
  
Accession: EED53492
  
Location: 1186835-1188895
  
 NCBI BlastP on this gene

EED53492

conserved hypothetical protein
  
Accession: EED53491
  
Location: 1183626-1184972
  
 NCBI BlastP on this gene

EED53491

conserved hypothetical protein
  
Accession: EED53490
  
Location: 1180417-1182188
  
 NCBI BlastP on this gene

EED53490

mitochondrial uncoupling protein, putative
  
Accession: EED53489
  
Location: 1179033-1180125
  
 NCBI BlastP on this gene

EED53489

vitamin H transporter, putative
  
Accession: EED53488
  
Location: 1176795-1178587
  
 NCBI BlastP on this gene

EED53488

MFS sugar transporter, putative
  
Accession: EED53487
  
Location: 1173797-1175393
  
  
**BlastP hit with Mycgr3G99766\_Mycgr3T**
  
Percentage identity: 34 %
  
BlastP bit score: 278
  
Sequence coverage: 95 %
  
E-value: 2e-83
  
  
 NCBI BlastP on this gene

EED53487

Dioxygenase family protein
  
Accession: EED53486
  
Location: 1171710-1172925
  
 NCBI BlastP on this gene

EED53486

cytochrome P450, putative
  
Accession: EED53485
  
Location: 1165381-1166754
  
 NCBI BlastP on this gene

EED53485

conserved hypothetical protein
  
Accession: EED53484
  
Location: 1161239-1162034
  
 NCBI BlastP on this gene

EED53484

acid phosphatase, putative
  
Accession: EED53483
  
Location: 1160173-1161070
  
 NCBI BlastP on this gene

EED53483

235. :  EQ963474 Aspergillus flavus NRRL3357 scf\_1106286417496 genomic scaffold     Total score: 1.0     Cumulative Blast bit score: 278

methyltransferase family protein
  
Accession: EED54064
  
Location: 79094-80168
  
 NCBI BlastP on this gene

EED54064

conserved hypothetical protein
  
Accession: EED54063
  
Location: 77420-78760
  
 NCBI BlastP on this gene

EED54063

conserved hypothetical protein
  
Accession: EED54062
  
Location: 75909-76452
  
 NCBI BlastP on this gene

EED54062

hypothetical protein
  
Accession: EED54061
  
Location: 72201-73215
  
 NCBI BlastP on this gene

EED54061

conserved hypothetical protein
  
Accession: EED54060
  
Location: 70669-72041
  
 NCBI BlastP on this gene

EED54060

efflux pump antibiotic resistance protein, putative
  
Accession: EED54059
  
Location: 68807-69616
  
 NCBI BlastP on this gene

EED54059

conserved hypothetical protein
  
Accession: EED54058
  
Location: 67507-68554
  
 NCBI BlastP on this gene

EED54058

fungal specific transcription factor, putative
  
Accession: EED54057
  
Location: 64970-66434
  
 NCBI BlastP on this gene

EED54057

conserved hypothetical protein
  
Accession: EED54056
  
Location: 64438-64857
  
 NCBI BlastP on this gene

EED54056

sugar transporter, putative
  
Accession: EED54055
  
Location: 61598-63282
  
  
**BlastP hit with Mycgr3G99766\_Mycgr3T**
  
Percentage identity: 33 %
  
BlastP bit score: 278
  
Sequence coverage: 95 %
  
E-value: 3e-83
  
  
 NCBI BlastP on this gene

EED54055

conserved hypothetical protein
  
Accession: EED54054
  
Location: 59044-61101
  
 NCBI BlastP on this gene

EED54054

conserved hypothetical protein
  
Accession: EED54053
  
Location: 57560-58165
  
 NCBI BlastP on this gene

EED54053

conserved hypothetical protein
  
Accession: EED54052
  
Location: 56150-57205
  
 NCBI BlastP on this gene

EED54052

conserved hypothetical protein
  
Accession: EED54051
  
Location: 53343-54595
  
 NCBI BlastP on this gene

EED54051

efflux pump antibiotic resistance protein, putative
  
Accession: EED54050
  
Location: 51066-52791
  
 NCBI BlastP on this gene

EED54050

conserved hypothetical protein
  
Accession: EED54049
  
Location: 49145-50602
  
 NCBI BlastP on this gene

EED54049

NAD dependent epimerase/dehydratase, putative
  
Accession: EED54048
  
Location: 47947-48729
  
 NCBI BlastP on this gene

EED54048

NAD dependent epimerase/dehydratase, putative
  
Accession: EED54047
  
Location: 47284-47863
  
 NCBI BlastP on this gene

EED54047

conserved hypothetical protein
  
Accession: EED54046
  
Location: 45994-46695
  
 NCBI BlastP on this gene

EED54046

conserved hypothetical protein
  
Accession: EED54045
  
Location: 43851-45735
  
 NCBI BlastP on this gene

EED54045

236. :  AP007161 Aspergillus oryzae RIB40 DNA, SC012.     Total score: 1.0     Cumulative Blast bit score: 278

not annotated
  
Accession: BAE60272
  
Location: 57407-58481
  
 NCBI BlastP on this gene

AO090012000027

not annotated
  
Accession: BAE60271
  
Location: 55555-57072
  
 NCBI BlastP on this gene

AO090012000026

not annotated
  
Accession: BAE60270
  
Location: 54219-54762
  
 NCBI BlastP on this gene

AO090012000025

not annotated
  
Accession: BAE60269
  
Location: 48979-50351
  
 NCBI BlastP on this gene

AO090012000022

not annotated
  
Accession: BAE60268
  
Location: 47117-48667
  
 NCBI BlastP on this gene

AO090012000021

not annotated
  
Accession: BAE60267
  
Location: 45816-46863
  
 NCBI BlastP on this gene

AO090012000020

not annotated
  
Accession: BAE60266
  
Location: 42746-43165
  
 NCBI BlastP on this gene

AO090012000019

not annotated
  
Accession: BAE60265
  
Location: 39901-41585
  
  
**BlastP hit with Mycgr3G99766\_Mycgr3T**
  
Percentage identity: 33 %
  
BlastP bit score: 278
  
Sequence coverage: 95 %
  
E-value: 3e-83
  
  
 NCBI BlastP on this gene

AO090012000018

not annotated
  
Accession: BAE60264
  
Location: 38064-39404
  
 NCBI BlastP on this gene

AO090012000017

not annotated
  
Accession: BAE60263
  
Location: 37353-37911
  
 NCBI BlastP on this gene

AO090012000016

not annotated
  
Accession: BAE60262
  
Location: 35867-36731
  
 NCBI BlastP on this gene

AO090012000015

not annotated
  
Accession: BAE60261
  
Location: 34457-35512
  
 NCBI BlastP on this gene

AO090012000014

not annotated
  
Accession: BAE60260
  
Location: 31650-32902
  
 NCBI BlastP on this gene

AO090012000013

not annotated
  
Accession: BAE60259
  
Location: 29373-31098
  
 NCBI BlastP on this gene

AO090012000012

not annotated
  
Accession: BAE60258
  
Location: 27677-28910
  
 NCBI BlastP on this gene

AO090012000011

not annotated
  
Accession: BAE60257
  
Location: 26255-27037
  
 NCBI BlastP on this gene

AO090012000010

not annotated
  
Accession: BAE60256
  
Location: 25592-25942
  
 NCBI BlastP on this gene

AO090012000009

not annotated
  
Accession: BAE60255
  
Location: 24289-24990
  
 NCBI BlastP on this gene

AO090012000008

not annotated
  
Accession: BAE60254
  
Location: 22146-23566
  
 NCBI BlastP on this gene

AO090012000007

237. :  AP007157 Aspergillus oryzae RIB40 DNA, SC023.     Total score: 1.0     Cumulative Blast bit score: 278

not annotated
  
Accession: BAE59003
  
Location: 1174739-1177580
  
 NCBI BlastP on this gene

AO090023000458

not annotated
  
Accession: BAE59002
  
Location: 1172850-1173800
  
 NCBI BlastP on this gene

AO090023000457

not annotated
  
Accession: BAE59001
  
Location: 1170838-1172488
  
 NCBI BlastP on this gene

AO090023000456

not annotated
  
Accession: BAE59000
  
Location: 1167214-1168560
  
 NCBI BlastP on this gene

AO090023000455

not annotated
  
Accession: BAE58999
  
Location: 1162623-1165206
  
 NCBI BlastP on this gene

AO090023000454

not annotated
  
Accession: BAE58998
  
Location: 1160385-1162177
  
 NCBI BlastP on this gene

AO090023000453

not annotated
  
Accession: BAE58997
  
Location: 1157388-1158984
  
  
**BlastP hit with Mycgr3G99766\_Mycgr3T**
  
Percentage identity: 34 %
  
BlastP bit score: 278
  
Sequence coverage: 95 %
  
E-value: 2e-83
  
  
 NCBI BlastP on this gene

AO090023000452

not annotated
  
Accession: BAE58996
  
Location: 1155287-1156516
  
 NCBI BlastP on this gene

AO090023000451

not annotated
  
Accession: BAE58995
  
Location: 1148972-1150552
  
 NCBI BlastP on this gene

AO090023000450

not annotated
  
Accession: BAE58994
  
Location: 1142969-1145207
  
 NCBI BlastP on this gene

AO090023000448

238. :  AKHY01000182 Aspergillus oryzae 3.042     Total score: 1.0     Cumulative Blast bit score: 278

1-aminocyclopropane-1-carboxylate synthase
  
Accession: EIT75178
  
Location: 968194-971035
  
 NCBI BlastP on this gene

EIT75178

hypothetical protein
  
Accession: EIT75025
  
Location: 966305-967255
  
 NCBI BlastP on this gene

EIT75025

cytochrome protein
  
Accession: EIT75257
  
Location: 964293-965943
  
 NCBI BlastP on this gene

EIT75257

hypothetical protein
  
Accession: EIT75080
  
Location: 960669-962015
  
 NCBI BlastP on this gene

EIT75080

tricarboxylate carrier protein
  
Accession: EIT74860
  
Location: 956078-958661
  
 NCBI BlastP on this gene

EIT74860

permease of the major facilitator superfamily
  
Accession: EIT74924
  
Location: 953840-955632
  
 NCBI BlastP on this gene

EIT74924

putative transporter
  
Accession: EIT74971
  
Location: 950843-952439
  
  
**BlastP hit with Mycgr3G99766\_Mycgr3T**
  
Percentage identity: 34 %
  
BlastP bit score: 278
  
Sequence coverage: 95 %
  
E-value: 2e-83
  
  
 NCBI BlastP on this gene

EIT74971

hypothetical protein
  
Accession: EIT75040
  
Location: 948742-949944
  
 NCBI BlastP on this gene

EIT75040

cytochrome protein
  
Accession: EIT75146
  
Location: 942427-944079
  
 NCBI BlastP on this gene

EIT75146

multiple inositol polyphosphate phosphatase
  
Accession: EIT74952
  
Location: 936443-938681
  
 NCBI BlastP on this gene

EIT74952

239. :  AKHY01000171 Aspergillus oryzae 3.042     Total score: 1.0     Cumulative Blast bit score: 278

methyltransferase family protein
  
Accession: EIT76126
  
Location: 46637-47711
  
 NCBI BlastP on this gene

EIT76126

hypothetical protein
  
Accession: EIT76025
  
Location: 44785-46302
  
 NCBI BlastP on this gene

EIT76025

permease of the major facilitator superfamily
  
Accession: EIT75964
  
Location: 42300-44028
  
 NCBI BlastP on this gene

EIT75964

hypothetical protein
  
Accession: EIT76142
  
Location: 37633-38680
  
 NCBI BlastP on this gene

EIT76142

hypothetical protein
  
Accession: EIT76108
  
Location: 34563-34982
  
 NCBI BlastP on this gene

EIT76108

putative transporter
  
Accession: EIT75907
  
Location: 31718-33402
  
  
**BlastP hit with Mycgr3G99766\_Mycgr3T**
  
Percentage identity: 33 %
  
BlastP bit score: 278
  
Sequence coverage: 95 %
  
E-value: 3e-83
  
  
 NCBI BlastP on this gene

EIT75907

hypothetical protein
  
Accession: EIT76065
  
Location: 29167-31221
  
 NCBI BlastP on this gene

EIT76065

hypothetical protein
  
Accession: EIT76094
  
Location: 27684-28626
  
 NCBI BlastP on this gene

EIT76094

hypothetical protein
  
Accession: EIT75895
  
Location: 26274-27329
  
 NCBI BlastP on this gene

EIT75895

hypothetical protein
  
Accession: EIT75938
  
Location: 23467-24719
  
 NCBI BlastP on this gene

EIT75938

permease of the major facilitator superfamily
  
Accession: EIT76013
  
Location: 21190-22915
  
 NCBI BlastP on this gene

EIT76013

hypothetical protein
  
Accession: EIT76151
  
Location: 19494-20727
  
 NCBI BlastP on this gene

EIT76151

cinnamoyl-CoA reductase
  
Accession: EIT76086
  
Location: 18072-18854
  
 NCBI BlastP on this gene

EIT76086

hypothetical protein
  
Accession: EIT75926
  
Location: 16106-16807
  
 NCBI BlastP on this gene

EIT75926

240. :  CR382137 Debaryomyces hansenii CBS767 chromosome E complete sequence.     Total score: 1.0     Cumulative Blast bit score: 277

DEHA2E01210p
  
Accession: CAG87584
  
Location: 101533-103146
  
 NCBI BlastP on this gene

DEHA2E01210g

DEHA2E01232p
  
Accession: CAG87585
  
Location: 103865-104500
  
 NCBI BlastP on this gene

DEHA2E01232g

DEHA2E01254p
  
Accession: CAG87587
  
Location: 105212-105382
  
 NCBI BlastP on this gene

DEHA2E01254g

DEHA2E01276p
  
Accession: CAG87588
  
Location: 106516-108057
  
 NCBI BlastP on this gene

DEHA2E01276g

DEHA2E01298p
  
Accession: CAG87589
  
Location: 108454-110091
  
 NCBI BlastP on this gene

DEHA2E01298g

DEHA2E01320p
  
Accession: CAG87590
  
Location: 110858-111391
  
 NCBI BlastP on this gene

DEHA2E01320g

DEHA2E01342p
  
Accession: CAG87596
  
Location: 115691-115858
  
 NCBI BlastP on this gene

DEHA2E01342g

DEHA2E01364p
  
Accession: CAG87597
  
Location: 116364-118052
  
 NCBI BlastP on this gene

DEHA2E01364g

DEHA2E01386p
  
Accession: CAG87598
  
Location: 118585-120234
  
  
**BlastP hit with Mycgr3G99766\_Mycgr3T**
  
Percentage identity: 33 %
  
BlastP bit score: 277
  
Sequence coverage: 100 %
  
E-value: 2e-82
  
  
 NCBI BlastP on this gene

DEHA2E01386g

DEHA2E01408p
  
Accession: CAG87599
  
Location: 120488-122218
  
 NCBI BlastP on this gene

DEHA2E01408g

DEHA2E01430p
  
Accession: CAG87600
  
Location: 122529-124964
  
 NCBI BlastP on this gene

DEHA2E01430g

DEHA2E01452p
  
Accession: CAR65737
  
Location: 125226-128138
  
 NCBI BlastP on this gene

DEHA2E01452g

DEHA2E01474p
  
Accession: CAG87602
  
Location: 128377-130447
  
 NCBI BlastP on this gene

DEHA2E01474g

DEHA2E01496p
  
Accession: CAG87604
  
Location: 130607-132109
  
 NCBI BlastP on this gene

DEHA2E01496g

DEHA2E01518p
  
Accession: CAG87605
  
Location: 132415-133647
  
 NCBI BlastP on this gene

DEHA2E01518g

DEHA2E01540p
  
Accession: CAG87606
  
Location: 133865-135556
  
 NCBI BlastP on this gene

DEHA2E01540g

DEHA2E01562p
  
Accession: CAG87607
  
Location: 135773-136435
  
 NCBI BlastP on this gene

DEHA2E01562g

241. :  CR382129 Yarrowia lipolytica CLIB122 chromosome C complete sequence.     Total score: 1.0     Cumulative Blast bit score: 277

YALI0C16390p
  
Accession: CAG82221
  
Location: 2313145-2317957
  
 NCBI BlastP on this gene

YALI0\_C16390g

YALI0C16412p
  
Accession: CAG82222
  
Location: 2319026-2320063
  
 NCBI BlastP on this gene

YALI0\_C16412g

YALI0C16434p
  
Accession: CAG82223
  
Location: 2320971-2321537
  
 NCBI BlastP on this gene

YALI0\_C16434g

YALI0C16456p
  
Accession: CAG82224
  
Location: 2322212-2323362
  
 NCBI BlastP on this gene

YALI0\_C16456g

YALI0C16478p
  
Accession: CAG82225
  
Location: 2324018-2324707
  
 NCBI BlastP on this gene

YALI0\_C16478g

YALI0C16500p
  
Accession: CAG82226
  
Location: 2324805-2326478
  
 NCBI BlastP on this gene

YALI0\_C16500g

YALI0C16522p
  
Accession: CAG82227
  
Location: 2329693-2331429
  
  
**BlastP hit with Mycgr3G99766\_Mycgr3T**
  
Percentage identity: 33 %
  
BlastP bit score: 277
  
Sequence coverage: 107 %
  
E-value: 3e-82
  
  
 NCBI BlastP on this gene

YALI0\_C16522g

242. :  CR382134 Debaryomyces hansenii CBS767 chromosome B complete sequence.     Total score: 1.0     Cumulative Blast bit score: 276

DEHA2B13156p
  
Accession: CAR65487
  
Location: 1029630-1031636
  
 NCBI BlastP on this gene

DEHA2B13156g

DEHA2B13134p
  
Accession: CAG85522
  
Location: 1028455-1029069
  
 NCBI BlastP on this gene

DEHA2B13134g

DEHA2B13112p
  
Accession: CAG85521
  
Location: 1027151-1028158
  
 NCBI BlastP on this gene

DEHA2B13112g

DEHA2B13090p
  
Accession: CAG85520
  
Location: 1026047-1026910
  
 NCBI BlastP on this gene

DEHA2B13090g

DEHA2B13068p
  
Accession: CAG85519
  
Location: 1024534-1025679
  
 NCBI BlastP on this gene

DEHA2B13068g

DEHA2B13046p
  
Accession: CAG85518
  
Location: 1023160-1024272
  
 NCBI BlastP on this gene

DEHA2B13046g

DEHA2B13024p
  
Accession: CAG85517
  
Location: 1021278-1022987
  
 NCBI BlastP on this gene

DEHA2B13024g

DEHA2B13002p
  
Accession: CAG85516
  
Location: 1019678-1020972
  
 NCBI BlastP on this gene

DEHA2B13002g

DEHA2B12980p
  
Accession: CAG85515
  
Location: 1018306-1019283
  
 NCBI BlastP on this gene

DEHA2B12980g

DEHA2B12958p
  
Accession: CAG85514
  
Location: 1015230-1016915
  
  
**BlastP hit with Mycgr3G99766\_Mycgr3T**
  
Percentage identity: 33 %
  
BlastP bit score: 276
  
Sequence coverage: 101 %
  
E-value: 5e-82
  
  
 NCBI BlastP on this gene

DEHA2B12958g

DEHA2B12936p
  
Accession: CAG85513
  
Location: 1013453-1014568
  
 NCBI BlastP on this gene

DEHA2B12936g

DEHA2B12914p
  
Accession: CAG85512
  
Location: 1012676-1013167
  
 NCBI BlastP on this gene

DEHA2B12914g

DEHA2B12892p
  
Accession: CAG85511
  
Location: 1010521-1012311
  
 NCBI BlastP on this gene

DEHA2B12892g

DEHA2B12870p
  
Accession: CAR65486
  
Location: 1007416-1009218
  
 NCBI BlastP on this gene

DEHA2B12870g

DEHA2B12787p
  
Accession: CAR65485
  
Location: 996801-1001291
  
 NCBI BlastP on this gene

DEHA2B12787g

243. :  GL891302 Neurospora tetrasperma FGSC 2508 unplaced genomic scaffold NEUTE1scaffold\_1     Total score: 1.0     Cumulative Blast bit score: 275

hypothetical protein
  
Accession: EGO60949
  
Location: 3249875-3251665
  
  
**BlastP hit with Mycgr3G99766\_Mycgr3T**
  
Percentage identity: 36 %
  
BlastP bit score: 275
  
Sequence coverage: 98 %
  
E-value: 1e-81
  
  
 NCBI BlastP on this gene

EGO60949

hypothetical protein
  
Accession: EGO60948
  
Location: 3247813-3248467
  
 NCBI BlastP on this gene

EGO60948

hypothetical protein
  
Accession: EGO60947
  
Location: 3245518-3247707
  
 NCBI BlastP on this gene

EGO60947

hypothetical protein
  
Accession: EGO60946
  
Location: 3241775-3244561
  
 NCBI BlastP on this gene

EGO60946

hypothetical protein
  
Accession: EGO60945
  
Location: 3239371-3240177
  
 NCBI BlastP on this gene

EGO60945

hypothetical protein
  
Accession: EGO60944
  
Location: 3235641-3236737
  
 NCBI BlastP on this gene

EGO60944

244. :  GL891107 Neurospora tetrasperma FGSC 2509 unplaced genomic scaffold NEUTE2scaffold\_2     Total score: 1.0     Cumulative Blast bit score: 275

hypothetical protein
  
Accession: EGZ75042
  
Location: 2900917-2903670
  
 NCBI BlastP on this gene

EGZ75042

FAD-binding domain-containing protein
  
Accession: EGZ75043
  
Location: 2903940-2905532
  
 NCBI BlastP on this gene

EGZ75043

hypothetical protein
  
Accession: EGZ75044
  
Location: 2905945-2907584
  
 NCBI BlastP on this gene

EGZ75044

hypothetical protein
  
Accession: EGZ75045
  
Location: 2908606-2909644
  
 NCBI BlastP on this gene

EGZ75045

DUF636-domain-containing protein
  
Accession: EGZ75046
  
Location: 2910898-2911417
  
 NCBI BlastP on this gene

EGZ75046

alcohol dehydrogenase I
  
Accession: EGZ75047
  
Location: 2912443-2912686
  
 NCBI BlastP on this gene

EGZ75047

hypothetical protein
  
Accession: EGZ75048
  
Location: 2913800-2914123
  
 NCBI BlastP on this gene

EGZ75048

hypothetical protein
  
Accession: EGZ75049
  
Location: 2915316-2915665
  
 NCBI BlastP on this gene

EGZ75049

hypothetical protein
  
Accession: EGZ75050
  
Location: 2915883-2916312
  
 NCBI BlastP on this gene

EGZ75050

hypothetical protein
  
Accession: EGZ75051
  
Location: 2916547-2917300
  
 NCBI BlastP on this gene

EGZ75051

putative sugar transporter
  
Accession: EGZ75052
  
Location: 2917505-2919295
  
  
**BlastP hit with Mycgr3G99766\_Mycgr3T**
  
Percentage identity: 36 %
  
BlastP bit score: 275
  
Sequence coverage: 98 %
  
E-value: 1e-81
  
  
 NCBI BlastP on this gene

EGZ75052

hypothetical protein
  
Accession: EGZ75053
  
Location: 2920703-2921357
  
 NCBI BlastP on this gene

EGZ75053

cytochrome P450
  
Accession: EGZ75054
  
Location: 2921463-2923652
  
 NCBI BlastP on this gene

EGZ75054

hypothetical protein
  
Accession: EGZ75055
  
Location: 2924609-2927395
  
 NCBI BlastP on this gene

EGZ75055

hypothetical protein
  
Accession: EGZ75056
  
Location: 2928993-2929799
  
 NCBI BlastP on this gene

EGZ75056

NAD(P)-binding protein
  
Accession: EGZ75057
  
Location: 2932433-2933529
  
 NCBI BlastP on this gene

EGZ75057

245. :  KB731258 Fusarium oxysporum f. sp. cubense race 1 unplaced genomic scaffold scaffold166     Total score: 1.0     Cumulative Blast bit score: 274

Sugar transporter STL1
  
Accession: ENH62911
  
Location: 342303-343795
  
  
**BlastP hit with Mycgr3G99766\_Mycgr3T**
  
Percentage identity: 34 %
  
BlastP bit score: 274
  
Sequence coverage: 89 %
  
E-value: 3e-82
  
  
 NCBI BlastP on this gene

ENH62911

3-isopropylmalate dehydrogenase
  
Accession: ENH62910
  
Location: 337126-340524
  
 NCBI BlastP on this gene

ENH62910

Carboxyvinyl-carboxyphosphonate phosphorylmutase
  
Accession: ENH62909
  
Location: 335368-336517
  
 NCBI BlastP on this gene

ENH62909

hypothetical protein
  
Accession: ENH62908
  
Location: 332478-334862
  
 NCBI BlastP on this gene

ENH62908

hypothetical protein
  
Accession: ENH62907
  
Location: 330083-331493
  
 NCBI BlastP on this gene

ENH62907

NAD-dependent alcohol dehydrogenase
  
Accession: ENH62906
  
Location: 328828-329802
  
 NCBI BlastP on this gene

ENH62906

D-amino acid dehydrogenase small subunit
  
Accession: ENH62905
  
Location: 327075-328175
  
 NCBI BlastP on this gene

ENH62905

Peroxisomal membrane protein PMP27
  
Accession: ENH62904
  
Location: 325961-326717
  
 NCBI BlastP on this gene

ENH62904

246. :  GL385399 Gaeumannomyces graminis var. tritici R3-111a-1 unplaced genomic scaffold supercont2.5     Total score: 1.0     Cumulative Blast bit score: 273

hypothetical protein
  
Accession: EJT72417
  
Location: 888931-891203
  
 NCBI BlastP on this gene

EJT72417

hypothetical protein
  
Accession: EJT72418
  
Location: 891689-892441
  
 NCBI BlastP on this gene

EJT72418

hypothetical protein
  
Accession: EJT72419
  
Location: 894508-894873
  
 NCBI BlastP on this gene

EJT72419

hypothetical protein
  
Accession: EJT72420
  
Location: 895450-897198
  
 NCBI BlastP on this gene

EJT72420

high-affinity glucose transporter ght2
  
Accession: EJT72421
  
Location: 897562-899391
  
 NCBI BlastP on this gene

EJT72421

hypothetical protein
  
Accession: EJT72422
  
Location: 901896-902267
  
 NCBI BlastP on this gene

EJT72422

hypothetical protein
  
Accession: EJT72423
  
Location: 902730-903180
  
 NCBI BlastP on this gene

EJT72423

hypothetical protein
  
Accession: EJT72424
  
Location: 903993-904589
  
 NCBI BlastP on this gene

EJT72424

hypothetical protein
  
Accession: EJT72425
  
Location: 905020-905304
  
 NCBI BlastP on this gene

EJT72425

sugar transporter STL1
  
Accession: EJT72426
  
Location: 905843-907587
  
  
**BlastP hit with Mycgr3G99766\_Mycgr3T**
  
Percentage identity: 33 %
  
BlastP bit score: 273
  
Sequence coverage: 94 %
  
E-value: 3e-81
  
  
 NCBI BlastP on this gene

EJT72426

hypothetical protein
  
Accession: EJT72427
  
Location: 909148-910239
  
 NCBI BlastP on this gene

EJT72427

hypothetical protein
  
Accession: EJT72428
  
Location: 910855-911506
  
 NCBI BlastP on this gene

EJT72428

hypothetical protein
  
Accession: EJT72429
  
Location: 911944-912552
  
 NCBI BlastP on this gene

EJT72429

hypothetical protein
  
Accession: EJT72430
  
Location: 913288-913908
  
 NCBI BlastP on this gene

EJT72430

3-phytase
  
Accession: EJT72431
  
Location: 915205-917667
  
 NCBI BlastP on this gene

EJT72431

xylosidase/arabinosidase
  
Accession: EJT72432
  
Location: 919669-921078
  
 NCBI BlastP on this gene

EJT72432

hypothetical protein
  
Accession: EJT72433
  
Location: 922120-922912
  
 NCBI BlastP on this gene

EJT72433

hypothetical protein
  
Accession: EJT72434
  
Location: 923409-925226
  
 NCBI BlastP on this gene

EJT72434

247. :  CM001235 Magnaporthe oryzae 70-15 chromosome 5     Total score: 1.0     Cumulative Blast bit score: 273

hypothetical protein
  
Accession: EHA48101
  
Location: 39582-40105
  
 NCBI BlastP on this gene

EHA48101

hypothetical protein
  
Accession: EHA48100
  
Location: 32732-33333
  
 NCBI BlastP on this gene

EHA48100

hypothetical protein
  
Accession: EHA48099
  
Location: 29802-31076
  
 NCBI BlastP on this gene

EHA48099

hypothetical protein
  
Accession: EHA48098
  
Location: 28950-29237
  
 NCBI BlastP on this gene

EHA48098

sugar transporter STL1
  
Accession: EHA48097
  
Location: 25207-26964
  
  
**BlastP hit with Mycgr3G99766\_Mycgr3T**
  
Percentage identity: 33 %
  
BlastP bit score: 273
  
Sequence coverage: 98 %
  
E-value: 3e-81
  
  
 NCBI BlastP on this gene

EHA48097

hypothetical protein
  
Accession: EHA48096
  
Location: 22629-23290
  
 NCBI BlastP on this gene

EHA48096

hypothetical protein
  
Accession: EHA48095
  
Location: 20327-20635
  
 NCBI BlastP on this gene

EHA48095

retinol dehydrogenase 12
  
Accession: EHA48094
  
Location: 17145-18080
  
 NCBI BlastP on this gene

EHA48094

hypothetical protein
  
Accession: EHA48093
  
Location: 10764-12751
  
 NCBI BlastP on this gene

EHA48093

248. :  AMWD01000002 Janthinobacterium sp. HH01     Total score: 1.0     Cumulative Blast bit score: 262

acyl-CoA dehydrogenase type 2 domain containing protein
  
Accession: ELX08663
  
Location: 882509-883687
  
 NCBI BlastP on this gene

ELX08663

linear gramicidin synthase subunit C
  
Accession: ELX08664
  
Location: 883733-885520
  
 NCBI BlastP on this gene

ELX08664

non-ribosomal peptide synthetase
  
Accession: ELX08665
  
Location: 885552-892007
  
  
**BlastP hit with Mycgr3G40534\_Mycgr3T**
  
Percentage identity: 38 %
  
BlastP bit score: 136
  
Sequence coverage: 81 %
  
E-value: 1e-31
  
  
 NCBI BlastP on this gene

ELX08665

2,4-dichlorophenol 6-monooxygenase TfdB
  
Accession: ELX08666
  
Location: 892004-893791
  
 NCBI BlastP on this gene

ELX08666

major facilitator superfamily MFS 1
  
Accession: ELX08667
  
Location: 893788-895092
  
 NCBI BlastP on this gene

ELX08667

tyrocidine synthase 3
  
Accession: ELX08668
  
Location: 895089-907430
  
 NCBI BlastP on this gene

ELX08668

linear gramicidin synthase subunit C
  
Accession: ELX08669
  
Location: 907441-915156
  
  
**BlastP hit with Mycgr3G40534\_Mycgr3T**
  
Percentage identity: 36 %
  
BlastP bit score: 126
  
Sequence coverage: 81 %
  
E-value: 2e-28
  
  
 NCBI BlastP on this gene

ELX08669

integrase family protein
  
Accession: ELX08670
  
Location: 915520-916686
  
 NCBI BlastP on this gene

ELX08670

hypothetical protein
  
Accession: ELX08671
  
Location: 917375-917566
  
 NCBI BlastP on this gene

ELX08671

249. :  AP007150 Aspergillus oryzae RIB40 DNA, SC009.     Total score: 1.0     Cumulative Blast bit score: 228

not annotated
  
Accession: BAE54583
  
Location: 189563-191379
  
 NCBI BlastP on this gene

AO090009000067

not annotated
  
Accession: BAE54582
  
Location: 186298-187301
  
 NCBI BlastP on this gene

AO090009000066

not annotated
  
Accession: BAE54581
  
Location: 184783-185674
  
 NCBI BlastP on this gene

AO090009000065

not annotated
  
Accession: BAE54580
  
Location: 179200-180216
  
 NCBI BlastP on this gene

AO090009000063

not annotated
  
Accession: BAE54579
  
Location: 177787-178235
  
 NCBI BlastP on this gene

AO090009000062

not annotated
  
Accession: BAE54578
  
Location: 174495-176469
  
  
**BlastP hit with Mycgr3G70577\_Mycgr3T**
  
Percentage identity: 28 %
  
BlastP bit score: 228
  
Sequence coverage: 97 %
  
E-value: 3e-62
  
  
 NCBI BlastP on this gene

AO090009000061

not annotated
  
Accession: BAE54577
  
Location: 173530-174363
  
 NCBI BlastP on this gene

AO090009000060

not annotated
  
Accession: BAE54576
  
Location: 167219-168013
  
 NCBI BlastP on this gene

AO090009000057

not annotated
  
Accession: BAE54575
  
Location: 166436-167064
  
 NCBI BlastP on this gene

AO090009000056

not annotated
  
Accession: BAE54574
  
Location: 159676-164008
  
 NCBI BlastP on this gene

AO090009000055

250. :  CM001198 Mycosphaerella graminicola IPO323 chromosome 3     Total score: 1.0     Cumulative Blast bit score: 223

hypothetical protein
  
Accession: EGP89382
  
Location: 1804499-1805353
  
 NCBI BlastP on this gene

EGP89382

hypothetical protein
  
Accession: EGP88819
  
Location: 1803785-1804312
  
 NCBI BlastP on this gene

EGP88819

hypothetical protein
  
Accession: EGP88818
  
Location: 1802066-1803349
  
 NCBI BlastP on this gene

EGP88818

hypothetical protein
  
Accession: EGP89383
  
Location: 1799525-1801072
  
 NCBI BlastP on this gene

EGP89383

hypothetical protein
  
Accession: EGP89384
  
Location: 1797929-1799116
  
 NCBI BlastP on this gene

EGP89384

hypothetical protein
  
Accession: EGP88817
  
Location: 1794846-1795948
  
 NCBI BlastP on this gene

EGP88817

DNA polymerase beta-like protein
  
Accession: EGP89385
  
Location: 1792312-1794411
  
 NCBI BlastP on this gene

EGP89385

putative siderophore-dependent iron transporter
  
Accession: EGP89386
  
Location: 1789350-1791305
  
  
**BlastP hit with Mycgr3G70577\_Mycgr3T**
  
Percentage identity: 29 %
  
BlastP bit score: 223
  
Sequence coverage: 93 %
  
E-value: 4e-60
  
  
 NCBI BlastP on this gene

EGP89386

hypothetical protein
  
Accession: EGP89387
  
Location: 1786829-1787747
  
 NCBI BlastP on this gene

EGP89387

hypothetical protein
  
Accession: EGP89388
  
Location: 1778961-1779978
  
 NCBI BlastP on this gene

EGP89388

hypothetical protein
  
Accession: EGP89389
  
Location: 1776331-1778094
  
 NCBI BlastP on this gene

EGP89389

hypothetical protein
  
Accession: EGP89390
  
Location: 1772956-1775103
  
 NCBI BlastP on this gene

EGP89390

Detecting sequence homology at the gene cluster level with MultiGeneBlast.
  
Marnix H. Medema, Rainer Breitling & Eriko Takano (2013)
  
*Molecular Biology and Evolution* , 30: 1218-1223.
