# Supplementary material for: In silico prediction and characterization of secondary metabolite biosynthetic gene clusters in the wheat pathogen Zymoseptoria tritici
Source: BMC Genomics. 2017 Aug 17;18:631. doi: 10.1186/s12864-017-3969-y (PMC5561558; doi:10.1186/s12864-017-3969-y)
Supplement: Supplementary file 1 — MultiGeneBLAST analysis of putative secondary metabolite clusters. All encoded amino acid sequences from genes residing in clusters predicted by AntiSMASH are given as FASTA file format. All output data from MultiGeneBLASTs are also provided. (ZIP 42911 kb) [file 12864_2017_3969_MOESM1_ESM.zip › Cluster MultiGene BLAST/out/Clusters_1_34/Cluster_15/displaypage1.xhtml]

xml version="1.0" encoding="UTF-8"?


Search Results
  
  
 Results pages: 1, 2, 3, 4, 5

**MultiGeneBlast hits**

Select gene cluster alignment
1. CM001200\_0 Mycosphaerella graminicola IPO323 chromosome 5, whole genome sh...
2. KB456260\_2 Mycosphaerella populorum SO2202 unplaced genomic scaffold SEPMU...
3. ABDF02000089\_2 Trichoderma virens Gv29-8, whole genome shotgun sequencing ...
4. KE145357\_1 Glarea lozoyensis ATCC 20868 chromosome Unknown GLAREA14, whole...
5. ABDF02000006\_1 Trichoderma virens Gv29-8, whole genome shotgun sequencing ...
6. ABDG02000026\_1 Trichoderma atroviride IMI 206040, whole genome shotgun seq...
7. JH725159\_0 Beauveria bassiana ARSEF 2860 unplaced genomic scaffold BBA\_S00...
8. GG698924\_0 Nectria haematococca mpVI 77-13-4 chromosome 11 genomic scaffol...
9. KB446535\_2 Dothistroma septosporum NZE10 unplaced genomic scaffold DOTSEsc...
10. GL629756\_0 Grosmannia clavigera kw1407 unplaced genomic scaffold GCSC\_132...
11. KB446555\_4 Pseudocercospora fijiensis CIRAD86 unplaced genomic scaffold M...
12. CABT02000055\_0 Sordaria macrospora k-hell, whole genome shotgun sequencin...
13. KB456266\_0 Mycosphaerella populorum SO2202 unplaced genomic scaffold SEPM...
14. KB446560\_2 Pseudocercospora fijiensis CIRAD86 unplaced genomic scaffold M...
15. CM001231\_4 Magnaporthe oryzae 70-15 chromosome 1, whole genome shotgun se...
16. AM270278\_0 Aspergillus niger contig An12c0220, genomic contig.
17. DS995904\_0 Penicillium marneffei ATCC 18224 scf\_1105668340738 genomic sca...
18. AM920436\_3 Penicillium chrysogenum Wisconsin 54-1255 complete genome, con...
19. EQ962652\_4 Talaromyces stipitatus ATCC 10500 scf\_1105507295523 genomic sc...
20. AHHD01000525\_0 Macrophomina phaseolina MS6, whole genome shotgun sequenci...
21. ACJE01000006\_0 Aspergillus niger ATCC 1015, whole genome shotgun sequenci...
22. DF126460\_0 Aspergillus kawachii IFO 4308 DNA, contig: scaffold00014, whol...
23. ADOT01000195\_0 Arthrobotrys oligospora ATCC 24927, whole genome shotgun s...
24. GL531877\_0 Pyrenophora teres f. teres 0-1 unplaced genomic scaffold scaff...
25. KB908481\_1 Setosphaeria turcica Et28A unplaced genomic scaffold SETTUscaf...
26. AQGS01000059\_0 Dactylellina haptotyla CBS 200.50, whole genome shotgun se...
27. DS027045\_0 Aspergillus clavatus NRRL 1 1099423829791 genomic scaffold, wh...
28. KB733458\_0 Bipolaris maydis ATCC 48331 unplaced genomic scaffold COCC4sca...
29. KB445583\_1 Cochliobolus heterostrophus C5 unplaced genomic scaffold COCHE...
30. EQ962652\_3 Talaromyces stipitatus ATCC 10500 scf\_1105507295523 genomic sc...
31. AACD01000123\_0 Aspergillus nidulans FGSC A4, whole genome shotgun sequenc...
32. AM920427\_0 Penicillium chrysogenum Wisconsin 54-1255 complete genome, con...
33. KB445652\_1 Cochliobolus sativus ND90Pr unplaced genomic scaffold COCSAsca...
34. KE145364\_0 Glarea lozoyensis ATCC 20868 chromosome Unknown GLAREA20, whol...
35. KB915896\_0 Neofusicoccum parvum UCRNP2 chromosome Unknown NP2\_03\_scaffold...
36. JH921455\_1 Marssonina brunnea f. sp. 'multigermtubi' MB\_m1 unplaced genom...
37. AMYD01000564\_0 Colletotrichum gloeosporioides Cg-14, whole genome shotgun...
38. KB725756\_0 Colletotrichum orbiculare MAFF 240422 unplaced genomic scaffol...
39. AKCU01000308\_0 Penicillium digitatum Pd1, whole genome shotgun sequencing...
40. AKCT01000265\_0 Penicillium digitatum PHI26, whole genome shotgun sequenci...
41. GG697333\_0 Glomerella graminicola M1.001 genomic scaffold supercont1.3, w...
42. KB446535\_4 Dothistroma septosporum NZE10 unplaced genomic scaffold DOTSEs...
43. KB020741\_0 Colletotrichum gloeosporioides Nara gc5 unplaced genomic scaff...
44. EQ963475\_0 Aspergillus flavus NRRL3357 scf\_1106286419142 genomic scaffold...
45. GG698906\_3 Nectria haematococca mpVI 77-13-4 chromosome 10 genomic scaffo...
46. GL385399\_0 Gaeumannomyces graminis var. tritici R3-111a-1 unplaced genomi...
47. JH725244\_0 Beauveria bassiana ARSEF 2860 unplaced genomic scaffold BBA\_S0...
48. GL891305\_0 Neurospora tetrasperma FGSC 2508 unplaced genomic scaffold NEU...
49. KB445558\_1 Baudoinia compniacensis UAMH 10762 unplaced genomic scaffold B...
50. GL698524\_0 Metarhizium acridum CQMa 102 unplaced genomic scaffold Scf\_055...

Query: Architecture Search FASTA input

CM001200 : Mycosphaerella graminicola IPO323 chromosome 5    Total score: 22.0     Cumulative Blast bit score: 26518

Hit cluster cross-links:

Mycgr3G85918 Mycgr3T
  
Location: 0-1602

Mycgr3G85918\_Mycgr3T

Mycgr3G42010 Mycgr3T
  
Location: 1702-8569

Mycgr3G42010\_Mycgr3T

Mycgr3G29582 Mycgr3T
  
Location: 8669-8915

Mycgr3G29582\_Mycgr3T

Mycgr3G31170 Mycgr3T
  
Location: 9015-9255

Mycgr3G31170\_Mycgr3T

Mycgr3G85924 Mycgr3T
  
Location: 9355-11218

Mycgr3G85924\_Mycgr3T

Mycgr3G71676 Mycgr3T
  
Location: 11318-12494

Mycgr3G71676\_Mycgr3T

Mycgr3G11468 Mycgr3T
  
Location: 12594-13653

Mycgr3G11468\_Mycgr3T

Mycgr3G58567 Mycgr3T
  
Location: 13753-14506

Mycgr3G58567\_Mycgr3T

Mycgr3G100089 Mycgr3
  
Location: 14606-21152

Mycgr3G100089\_Mycgr3

Mycgr3G42698 Mycgr3T
  
Location: 21252-22131

Mycgr3G42698\_Mycgr3T

Mycgr3G71681 Mycgr3T
  
Location: 22231-23461

Mycgr3G71681\_Mycgr3T

Mycgr3G109328 Mycgr3
  
Location: 23561-24239

Mycgr3G109328\_Mycgr3

Mycgr3G104334 Mycgr3
  
Location: 24339-24567

Mycgr3G104334\_Mycgr3

Mycgr3G42715 Mycgr3T
  
Location: 24667-25981

Mycgr3G42715\_Mycgr3T

Mycgr3G92934 Mycgr3T
  
Location: 26081-27593

Mycgr3G92934\_Mycgr3T

Mycgr3G41969 Mycgr3T
  
Location: 27693-29328

Mycgr3G41969\_Mycgr3T

Mycgr3G80635 Mycgr3T
  
Location: 29428-29821

Mycgr3G80635\_Mycgr3T

Mycgr3G41426 Mycgr3T
  
Location: 29921-35255

Mycgr3G41426\_Mycgr3T

Mycgr3G104337 Mycgr3
  
Location: 35355-36108

Mycgr3G104337\_Mycgr3

Mycgr3G71679 Mycgr3T
  
Location: 36208-37300

Mycgr3G71679\_Mycgr3T

Mycgr3G92938 Mycgr3T
  
Location: 37400-38699

Mycgr3G92938\_Mycgr3T

Mycgr3G92941 Mycgr3T
  
Location: 38799-40734

Mycgr3G92941\_Mycgr3T

hypothetical protein
  
Accession: EGP86989
  
Location: 82944-84859
  
 NCBI BlastP on this gene

EGP86989

hypothetical protein
  
Accession: EGP86990
  
Location: 85463-87322
  
  
**BlastP hit with Mycgr3G85918\_Mycgr3T**
  
Percentage identity: 100 %
  
BlastP bit score: 1087
  
Sequence coverage: 99 %
  
E-value: 0.0
  
  
 NCBI BlastP on this gene

EGP86990

hypothetical protein
  
Accession: EGP86991
  
Location: 88348-89691
  
  
**BlastP hit with Mycgr3G71676\_Mycgr3T**
  
Percentage identity: 100 %
  
BlastP bit score: 807
  
Sequence coverage: 99 %
  
E-value: 0.0
  
  
 NCBI BlastP on this gene

EGP86991

hypothetical protein
  
Accession: EGP86992
  
Location: 90138-91442
  
  
**BlastP hit with Mycgr3G71679\_Mycgr3T**
  
Percentage identity: 100 %
  
BlastP bit score: 741
  
Sequence coverage: 99 %
  
E-value: 0.0
  
  
 NCBI BlastP on this gene

EGP86992

hypothetical protein
  
Accession: EGP86993
  
Location: 92609-94060
  
  
**BlastP hit with Mycgr3G71681\_Mycgr3T**
  
Percentage identity: 100 %
  
BlastP bit score: 852
  
Sequence coverage: 99 %
  
E-value: 0.0
  
  
 NCBI BlastP on this gene

EGP86993

hypothetical protein
  
Accession: EGP87764
  
Location: 94206-94958
  
  
**BlastP hit with Mycgr3G58567\_Mycgr3T**
  
Percentage identity: 100 %
  
BlastP bit score: 518
  
Sequence coverage: 100 %
  
E-value: 0.0
  
  
 NCBI BlastP on this gene

EGP87764

hypothetical protein
  
Accession: EGP87763
  
Location: 95307-96818
  
  
**BlastP hit with Mycgr3G92934\_Mycgr3T**
  
Percentage identity: 100 %
  
BlastP bit score: 1023
  
Sequence coverage: 99 %
  
E-value: 0.0
  
  
 NCBI BlastP on this gene

EGP87763

hypothetical protein
  
Accession: EGP86994
  
Location: 96999-99044
  
  
**BlastP hit with Mycgr3G85924\_Mycgr3T**
  
Percentage identity: 100 %
  
BlastP bit score: 1218
  
Sequence coverage: 99 %
  
E-value: 0.0
  
  
 NCBI BlastP on this gene

EGP86994

hypothetical protein
  
Accession: EGP87762
  
Location: 99487-99714
  
  
**BlastP hit with Mycgr3G104334\_Mycgr3**
  
Percentage identity: 100 %
  
BlastP bit score: 153
  
Sequence coverage: 98 %
  
E-value: 3e-46
  
  
 NCBI BlastP on this gene

EGP87762

hypothetical protein
  
Accession: EGP86995
  
Location: 100315-101462
  
  
**BlastP hit with Mycgr3G42698\_Mycgr3T**
  
Percentage identity: 100 %
  
BlastP bit score: 615
  
Sequence coverage: 99 %
  
E-value: 0.0
  
  
 NCBI BlastP on this gene

EGP86995

hypothetical protein
  
Accession: EGP87761
  
Location: 101621-108595
  
  
**BlastP hit with Mycgr3G42010\_Mycgr3T**
  
Percentage identity: 100 %
  
BlastP bit score: 4741
  
Sequence coverage: 99 %
  
E-value: 0.0
  
  
 NCBI BlastP on this gene

EGP87761

hypothetical protein
  
Accession: EGP86996
  
Location: 108946-110334
  
  
**BlastP hit with Mycgr3G92938\_Mycgr3T**
  
Percentage identity: 100 %
  
BlastP bit score: 893
  
Sequence coverage: 99 %
  
E-value: 0.0
  
  
 NCBI BlastP on this gene

EGP86996

hypothetical protein
  
Accession: EGP86997
  
Location: 111722-112639
  
  
**BlastP hit with Mycgr3G109328\_Mycgr3**
  
Percentage identity: 100 %
  
BlastP bit score: 449
  
Sequence coverage: 99 %
  
E-value: 2e-158
  
  
 NCBI BlastP on this gene

EGP86997

hypothetical protein
  
Accession: EGP86998
  
Location: 113332-114768
  
  
**BlastP hit with Mycgr3G42715\_Mycgr3T**
  
Percentage identity: 100 %
  
BlastP bit score: 909
  
Sequence coverage: 99 %
  
E-value: 0.0
  
  
 NCBI BlastP on this gene

EGP86998

hypothetical protein
  
Accession: EGP87760
  
Location: 115565-118586
  
  
**BlastP hit with Mycgr3G92941\_Mycgr3T**
  
Percentage identity: 100 %
  
BlastP bit score: 1328
  
Sequence coverage: 99 %
  
E-value: 0.0
  
  
 NCBI BlastP on this gene

EGP87760

hypothetical protein
  
Accession: EGP86999
  
Location: 120571-121824
  
  
**BlastP hit with Mycgr3G11468\_Mycgr3T**
  
Percentage identity: 100 %
  
BlastP bit score: 728
  
Sequence coverage: 100 %
  
E-value: 0.0
  
  
 NCBI BlastP on this gene

EGP86999

polyketide synthase
  
Accession: EGP87759
  
Location: 122351-129292
  
  
**BlastP hit with Mycgr3G100089\_Mycgr3**
  
Percentage identity: 100 %
  
BlastP bit score: 4525
  
Sequence coverage: 99 %
  
E-value: 0.0
  
  
 NCBI BlastP on this gene

EGP87759

hypothetical protein
  
Accession: EGP87000
  
Location: 130690-132388
  
  
**BlastP hit with Mycgr3G41969\_Mycgr3T**
  
Percentage identity: 100 %
  
BlastP bit score: 1121
  
Sequence coverage: 99 %
  
E-value: 0.0
  
  
 NCBI BlastP on this gene

EGP87000

hypothetical protein
  
Accession: EGP87001
  
Location: 133725-134685
  
  
**BlastP hit with Mycgr3G104337\_Mycgr3**
  
Percentage identity: 100 %
  
BlastP bit score: 521
  
Sequence coverage: 99 %
  
E-value: 0.0
  
  
 NCBI BlastP on this gene

EGP87001

hypothetical protein
  
Accession: EGP87758
  
Location: 136993-137385
  
  
**BlastP hit with Mycgr3G80635\_Mycgr3T**
  
Percentage identity: 100 %
  
BlastP bit score: 268
  
Sequence coverage: 99 %
  
E-value: 5e-90
  
  
 NCBI BlastP on this gene

EGP87758

hypothetical protein
  
Accession: EGP87757
  
Location: 139379-139694
  
  
**BlastP hit with Mycgr3G29582\_Mycgr3T**
  
Percentage identity: 100 %
  
BlastP bit score: 160
  
Sequence coverage: 100 %
  
E-value: 3e-49
  
  
 NCBI BlastP on this gene

EGP87757

hypothetical protein
  
Accession: EGP87756
  
Location: 141738-142115
  
  
**BlastP hit with Mycgr3G31170\_Mycgr3T**
  
Percentage identity: 100 %
  
BlastP bit score: 162
  
Sequence coverage: 100 %
  
E-value: 6e-50
  
  
 NCBI BlastP on this gene

EGP87756

hypothetical protein
  
Accession: EGP87755
  
Location: 144090-149465
  
  
**BlastP hit with Mycgr3G41426\_Mycgr3T**
  
Percentage identity: 100 %
  
BlastP bit score: 3699
  
Sequence coverage: 100 %
  
E-value: 0.0
  
  
 NCBI BlastP on this gene

EGP87755

hypothetical protein
  
Accession: EGP87002
  
Location: 150248-152095
  
 NCBI BlastP on this gene

EGP87002

Query: Architecture Search FASTA input

KB456260 : Mycosphaerella populorum SO2202 unplaced genomic scaffold SEPMUscaffold\_1    Total score: 4.0     Cumulative Blast bit score: 899

Hit cluster cross-links:

Mycgr3G85918 Mycgr3T
  
Location: 0-1602

Mycgr3G85918\_Mycgr3T

Mycgr3G42010 Mycgr3T
  
Location: 1702-8569

Mycgr3G42010\_Mycgr3T

Mycgr3G29582 Mycgr3T
  
Location: 8669-8915

Mycgr3G29582\_Mycgr3T

Mycgr3G31170 Mycgr3T
  
Location: 9015-9255

Mycgr3G31170\_Mycgr3T

Mycgr3G85924 Mycgr3T
  
Location: 9355-11218

Mycgr3G85924\_Mycgr3T

Mycgr3G71676 Mycgr3T
  
Location: 11318-12494

Mycgr3G71676\_Mycgr3T

Mycgr3G11468 Mycgr3T
  
Location: 12594-13653

Mycgr3G11468\_Mycgr3T

Mycgr3G58567 Mycgr3T
  
Location: 13753-14506

Mycgr3G58567\_Mycgr3T

Mycgr3G100089 Mycgr3
  
Location: 14606-21152

Mycgr3G100089\_Mycgr3

Mycgr3G42698 Mycgr3T
  
Location: 21252-22131

Mycgr3G42698\_Mycgr3T

Mycgr3G71681 Mycgr3T
  
Location: 22231-23461

Mycgr3G71681\_Mycgr3T

Mycgr3G109328 Mycgr3
  
Location: 23561-24239

Mycgr3G109328\_Mycgr3

Mycgr3G104334 Mycgr3
  
Location: 24339-24567

Mycgr3G104334\_Mycgr3

Mycgr3G42715 Mycgr3T
  
Location: 24667-25981

Mycgr3G42715\_Mycgr3T

Mycgr3G92934 Mycgr3T
  
Location: 26081-27593

Mycgr3G92934\_Mycgr3T

Mycgr3G41969 Mycgr3T
  
Location: 27693-29328

Mycgr3G41969\_Mycgr3T

Mycgr3G80635 Mycgr3T
  
Location: 29428-29821

Mycgr3G80635\_Mycgr3T

Mycgr3G41426 Mycgr3T
  
Location: 29921-35255

Mycgr3G41426\_Mycgr3T

Mycgr3G104337 Mycgr3
  
Location: 35355-36108

Mycgr3G104337\_Mycgr3

Mycgr3G71679 Mycgr3T
  
Location: 36208-37300

Mycgr3G71679\_Mycgr3T

Mycgr3G92938 Mycgr3T
  
Location: 37400-38699

Mycgr3G92938\_Mycgr3T

Mycgr3G92941 Mycgr3T
  
Location: 38799-40734

Mycgr3G92941\_Mycgr3T

hypothetical protein
  
Accession: EMF16472
  
Location: 878884-879231
  
 NCBI BlastP on this gene

EMF16472

FMN-linked oxidoreductase
  
Accession: EMF16473
  
Location: 879722-881187
  
 NCBI BlastP on this gene

EMF16473

hypothetical protein
  
Accession: EMF16474
  
Location: 882319-883272
  
 NCBI BlastP on this gene

EMF16474

kinase-like protein
  
Accession: EMF16475
  
Location: 884611-887352
  
 NCBI BlastP on this gene

EMF16475

eukaryotic type KH-domain (KH-domain type I)
  
Accession: EMF16476
  
Location: 888633-890373
  
 NCBI BlastP on this gene

EMF16476

P-loop containing nucleoside triphosphate hydrolase protein
  
Accession: EMF16477
  
Location: 890780-891541
  
 NCBI BlastP on this gene

EMF16477

DUF1741-domain-containing protein
  
Accession: EMF16478
  
Location: 891619-893731
  
 NCBI BlastP on this gene

EMF16478

hypothetical protein
  
Accession: EMF16479
  
Location: 894230-895054
  
  
**BlastP hit with Mycgr3G92941\_Mycgr3T**
  
Percentage identity: 64 %
  
BlastP bit score: 362
  
Sequence coverage: 42 %
  
E-value: 4e-117
  
  
 NCBI BlastP on this gene

EMF16479

SRF-TF-domain-containing protein
  
Accession: EMF16480
  
Location: 897129-897927
  
  
**BlastP hit with Mycgr3G31170\_Mycgr3T**
  
Percentage identity: 100 %
  
BlastP bit score: 167
  
Sequence coverage: 100 %
  
E-value: 5e-50
  
  
 NCBI BlastP on this gene

EMF16480

hypothetical protein
  
Accession: EMF16481
  
Location: 899176-899554
  
 NCBI BlastP on this gene

EMF16481

DASH Hsk3-domain-containing protein
  
Accession: EMF16482
  
Location: 899812-900097
  
  
**BlastP hit with Mycgr3G29582\_Mycgr3T**
  
Percentage identity: 89 %
  
BlastP bit score: 134
  
Sequence coverage: 93 %
  
E-value: 6e-39
  
  
 NCBI BlastP on this gene

EMF16482

hypothetical protein
  
Accession: EMF16483
  
Location: 900322-900723
  
 NCBI BlastP on this gene

EMF16483

FAD-binding domain-containing protein
  
Accession: EMF16484
  
Location: 900989-902575
  
 NCBI BlastP on this gene

EMF16484

hypothetical protein
  
Accession: EMF16485
  
Location: 903154-905418
  
 NCBI BlastP on this gene

EMF16485

lysophospholipase Plb2
  
Accession: EMF16486
  
Location: 906405-908536
  
  
**BlastP hit with Mycgr3G41969\_Mycgr3T**
  
Percentage identity: 32 %
  
BlastP bit score: 236
  
Sequence coverage: 107 %
  
E-value: 1e-65
  
  
 NCBI BlastP on this gene

EMF16486

MFS general substrate transporter
  
Accession: EMF16487
  
Location: 909865-911571
  
 NCBI BlastP on this gene

EMF16487

acid phosphatase/Vanadium-dependent haloperoxidase
  
Accession: EMF16488
  
Location: 912036-913014
  
 NCBI BlastP on this gene

EMF16488

MED7-domain-containing protein
  
Accession: EMF16489
  
Location: 913304-914038
  
 NCBI BlastP on this gene

EMF16489

Hexokinase 1-domain-containing protein
  
Accession: EMF16490
  
Location: 915425-920157
  
 NCBI BlastP on this gene

EMF16490

hypothetical protein
  
Accession: EMF16491
  
Location: 920719-923830
  
 NCBI BlastP on this gene

EMF16491

hypothetical protein
  
Accession: EMF16492
  
Location: 924054-924766
  
 NCBI BlastP on this gene

EMF16492

Query: Architecture Search FASTA input

ABDF02000089 : Trichoderma virens Gv29-8    Total score: 3.0     Cumulative Blast bit score: 2425

Hit cluster cross-links:

Mycgr3G85918 Mycgr3T
  
Location: 0-1602

Mycgr3G85918\_Mycgr3T

Mycgr3G42010 Mycgr3T
  
Location: 1702-8569

Mycgr3G42010\_Mycgr3T

Mycgr3G29582 Mycgr3T
  
Location: 8669-8915

Mycgr3G29582\_Mycgr3T

Mycgr3G31170 Mycgr3T
  
Location: 9015-9255

Mycgr3G31170\_Mycgr3T

Mycgr3G85924 Mycgr3T
  
Location: 9355-11218

Mycgr3G85924\_Mycgr3T

Mycgr3G71676 Mycgr3T
  
Location: 11318-12494

Mycgr3G71676\_Mycgr3T

Mycgr3G11468 Mycgr3T
  
Location: 12594-13653

Mycgr3G11468\_Mycgr3T

Mycgr3G58567 Mycgr3T
  
Location: 13753-14506

Mycgr3G58567\_Mycgr3T

Mycgr3G100089 Mycgr3
  
Location: 14606-21152

Mycgr3G100089\_Mycgr3

Mycgr3G42698 Mycgr3T
  
Location: 21252-22131

Mycgr3G42698\_Mycgr3T

Mycgr3G71681 Mycgr3T
  
Location: 22231-23461

Mycgr3G71681\_Mycgr3T

Mycgr3G109328 Mycgr3
  
Location: 23561-24239

Mycgr3G109328\_Mycgr3

Mycgr3G104334 Mycgr3
  
Location: 24339-24567

Mycgr3G104334\_Mycgr3

Mycgr3G42715 Mycgr3T
  
Location: 24667-25981

Mycgr3G42715\_Mycgr3T

Mycgr3G92934 Mycgr3T
  
Location: 26081-27593

Mycgr3G92934\_Mycgr3T

Mycgr3G41969 Mycgr3T
  
Location: 27693-29328

Mycgr3G41969\_Mycgr3T

Mycgr3G80635 Mycgr3T
  
Location: 29428-29821

Mycgr3G80635\_Mycgr3T

Mycgr3G41426 Mycgr3T
  
Location: 29921-35255

Mycgr3G41426\_Mycgr3T

Mycgr3G104337 Mycgr3
  
Location: 35355-36108

Mycgr3G104337\_Mycgr3

Mycgr3G71679 Mycgr3T
  
Location: 36208-37300

Mycgr3G71679\_Mycgr3T

Mycgr3G92938 Mycgr3T
  
Location: 37400-38699

Mycgr3G92938\_Mycgr3T

Mycgr3G92941 Mycgr3T
  
Location: 38799-40734

Mycgr3G92941\_Mycgr3T

carbohydrate esterase family 16 protein
  
Accession: EHK17225
  
Location: 1066690-1067968
  
 NCBI BlastP on this gene

EHK17225

hypothetical protein
  
Accession: EHK17224
  
Location: 1064549-1066570
  
 NCBI BlastP on this gene

EHK17224

glycosyltransferase family 90 protein
  
Accession: EHK17394
  
Location: 1060147-1062751
  
 NCBI BlastP on this gene

EHK17394

hypothetical protein
  
Accession: EHK17223
  
Location: 1057727-1059933
  
 NCBI BlastP on this gene

EHK17223

hypothetical protein
  
Accession: EHK17222
  
Location: 1054725-1055992
  
 NCBI BlastP on this gene

EHK17222

hypothetical protein
  
Accession: EHK17221
  
Location: 1053084-1053710
  
 NCBI BlastP on this gene

EHK17221

hypothetical protein
  
Accession: EHK17220
  
Location: 1050904-1052219
  
  
**BlastP hit with Mycgr3G92938\_Mycgr3T**
  
Percentage identity: 40 %
  
BlastP bit score: 261
  
Sequence coverage: 81 %
  
E-value: 6e-79
  
  
 NCBI BlastP on this gene

EHK17220

hypothetical protein
  
Accession: EHK17219
  
Location: 1042982-1049962
  
  
**BlastP hit with Mycgr3G42010\_Mycgr3T**
  
Percentage identity: 44 %
  
BlastP bit score: 1959
  
Sequence coverage: 102 %
  
E-value: 0.0
  
  
 NCBI BlastP on this gene

EHK17219

hypothetical protein
  
Accession: EHK17218
  
Location: 1040771-1042134
  
  
**BlastP hit with Mycgr3G71676\_Mycgr3T**
  
Percentage identity: 33 %
  
BlastP bit score: 205
  
Sequence coverage: 101 %
  
E-value: 6e-58
  
  
 NCBI BlastP on this gene

EHK17218

hypothetical protein
  
Accession: EHK17217
  
Location: 1038989-1040530
  
 NCBI BlastP on this gene

EHK17217

hypothetical protein
  
Accession: EHK17216
  
Location: 1036657-1037436
  
 NCBI BlastP on this gene

EHK17216

hypothetical protein
  
Accession: EHK17215
  
Location: 1034923-1035342
  
 NCBI BlastP on this gene

EHK17215

hypothetical protein
  
Accession: EHK17214
  
Location: 1032047-1033362
  
 NCBI BlastP on this gene

EHK17214

hypothetical protein
  
Accession: EHK17213
  
Location: 1026103-1028272
  
 NCBI BlastP on this gene

EHK17213

hypothetical protein
  
Accession: EHK17212
  
Location: 1024825-1025905
  
 NCBI BlastP on this gene

EHK17212

hypothetical protein
  
Accession: EHK17211
  
Location: 1022768-1024441
  
 NCBI BlastP on this gene

EHK17211

Query: Architecture Search FASTA input

KE145357 : Glarea lozoyensis ATCC 20868 chromosome Unknown GLAREA14    Total score: 3.0     Cumulative Blast bit score: 2354

Hit cluster cross-links:

Mycgr3G85918 Mycgr3T
  
Location: 0-1602

Mycgr3G85918\_Mycgr3T

Mycgr3G42010 Mycgr3T
  
Location: 1702-8569

Mycgr3G42010\_Mycgr3T

Mycgr3G29582 Mycgr3T
  
Location: 8669-8915

Mycgr3G29582\_Mycgr3T

Mycgr3G31170 Mycgr3T
  
Location: 9015-9255

Mycgr3G31170\_Mycgr3T

Mycgr3G85924 Mycgr3T
  
Location: 9355-11218

Mycgr3G85924\_Mycgr3T

Mycgr3G71676 Mycgr3T
  
Location: 11318-12494

Mycgr3G71676\_Mycgr3T

Mycgr3G11468 Mycgr3T
  
Location: 12594-13653

Mycgr3G11468\_Mycgr3T

Mycgr3G58567 Mycgr3T
  
Location: 13753-14506

Mycgr3G58567\_Mycgr3T

Mycgr3G100089 Mycgr3
  
Location: 14606-21152

Mycgr3G100089\_Mycgr3

Mycgr3G42698 Mycgr3T
  
Location: 21252-22131

Mycgr3G42698\_Mycgr3T

Mycgr3G71681 Mycgr3T
  
Location: 22231-23461

Mycgr3G71681\_Mycgr3T

Mycgr3G109328 Mycgr3
  
Location: 23561-24239

Mycgr3G109328\_Mycgr3

Mycgr3G104334 Mycgr3
  
Location: 24339-24567

Mycgr3G104334\_Mycgr3

Mycgr3G42715 Mycgr3T
  
Location: 24667-25981

Mycgr3G42715\_Mycgr3T

Mycgr3G92934 Mycgr3T
  
Location: 26081-27593

Mycgr3G92934\_Mycgr3T

Mycgr3G41969 Mycgr3T
  
Location: 27693-29328

Mycgr3G41969\_Mycgr3T

Mycgr3G80635 Mycgr3T
  
Location: 29428-29821

Mycgr3G80635\_Mycgr3T

Mycgr3G41426 Mycgr3T
  
Location: 29921-35255

Mycgr3G41426\_Mycgr3T

Mycgr3G104337 Mycgr3
  
Location: 35355-36108

Mycgr3G104337\_Mycgr3

Mycgr3G71679 Mycgr3T
  
Location: 36208-37300

Mycgr3G71679\_Mycgr3T

Mycgr3G92938 Mycgr3T
  
Location: 37400-38699

Mycgr3G92938\_Mycgr3T

Mycgr3G92941 Mycgr3T
  
Location: 38799-40734

Mycgr3G92941\_Mycgr3T

2Fe-2S ferredoxin-like protein
  
Accession: EPE34200
  
Location: 1885647-1886344
  
 NCBI BlastP on this gene

EPE34200

hypothetical protein
  
Accession: EPE34201
  
Location: 1886687-1887472
  
 NCBI BlastP on this gene

EPE34201

P-loop containing nucleoside triphosphate hydrolase
  
Accession: EPE34202
  
Location: 1887675-1889311
  
 NCBI BlastP on this gene

EPE34202

P-loop containing nucleoside triphosphate hydrolase
  
Accession: EPE34203
  
Location: 1891804-1899037
  
  
**BlastP hit with Mycgr3G42010\_Mycgr3T**
  
Percentage identity: 45 %
  
BlastP bit score: 1940
  
Sequence coverage: 101 %
  
E-value: 0.0
  
  
 NCBI BlastP on this gene

EPE34203

geranylgeranyl pyrophosphate synthetase
  
Accession: EPE34204
  
Location: 1899709-1901176
  
  
**BlastP hit with Mycgr3G92938\_Mycgr3T**
  
Percentage identity: 37 %
  
BlastP bit score: 247
  
Sequence coverage: 84 %
  
E-value: 6e-73
  
  
 NCBI BlastP on this gene

EPE34204

hypothetical protein
  
Accession: EPE34205
  
Location: 1902280-1902912
  
 NCBI BlastP on this gene

EPE34205

hypothetical protein
  
Accession: EPE34206
  
Location: 1904241-1905923
  
 NCBI BlastP on this gene

EPE34206

NAD(P)-binding Rossmann-fold containing protein
  
Accession: EPE34207
  
Location: 1906959-1908151
  
 NCBI BlastP on this gene

EPE34207

WD40 repeat-like protein
  
Accession: EPE34208
  
Location: 1908948-1910180
  
 NCBI BlastP on this gene

EPE34208

P-loop containing nucleoside triphosphate hydrolase
  
Accession: EPE34209
  
Location: 1911052-1914078
  
 NCBI BlastP on this gene

EPE34209

hypothetical protein
  
Accession: EPE34210
  
Location: 1915652-1917898
  
 NCBI BlastP on this gene

EPE34210

hypothetical protein
  
Accession: EPE34211
  
Location: 1918626-1919719
  
 NCBI BlastP on this gene

EPE34211

Di-copper centre-containing
  
Accession: EPE34212
  
Location: 1920979-1922646
  
  
**BlastP hit with Mycgr3G42698\_Mycgr3T**
  
Percentage identity: 31 %
  
BlastP bit score: 167
  
Sequence coverage: 113 %
  
E-value: 9e-45
  
  
 NCBI BlastP on this gene

EPE34212

hypothetical protein
  
Accession: EPE34213
  
Location: 1923882-1925735
  
 NCBI BlastP on this gene

EPE34213

hypothetical protein
  
Accession: EPE34214
  
Location: 1926593-1928300
  
 NCBI BlastP on this gene

EPE34214

hypothetical protein
  
Accession: EPE34215
  
Location: 1929601-1934471
  
 NCBI BlastP on this gene

EPE34215

Query: Architecture Search FASTA input

ABDF02000006 : Trichoderma virens Gv29-8    Total score: 3.0     Cumulative Blast bit score: 2223

Hit cluster cross-links:

Mycgr3G85918 Mycgr3T
  
Location: 0-1602

Mycgr3G85918\_Mycgr3T

Mycgr3G42010 Mycgr3T
  
Location: 1702-8569

Mycgr3G42010\_Mycgr3T

Mycgr3G29582 Mycgr3T
  
Location: 8669-8915

Mycgr3G29582\_Mycgr3T

Mycgr3G31170 Mycgr3T
  
Location: 9015-9255

Mycgr3G31170\_Mycgr3T

Mycgr3G85924 Mycgr3T
  
Location: 9355-11218

Mycgr3G85924\_Mycgr3T

Mycgr3G71676 Mycgr3T
  
Location: 11318-12494

Mycgr3G71676\_Mycgr3T

Mycgr3G11468 Mycgr3T
  
Location: 12594-13653

Mycgr3G11468\_Mycgr3T

Mycgr3G58567 Mycgr3T
  
Location: 13753-14506

Mycgr3G58567\_Mycgr3T

Mycgr3G100089 Mycgr3
  
Location: 14606-21152

Mycgr3G100089\_Mycgr3

Mycgr3G42698 Mycgr3T
  
Location: 21252-22131

Mycgr3G42698\_Mycgr3T

Mycgr3G71681 Mycgr3T
  
Location: 22231-23461

Mycgr3G71681\_Mycgr3T

Mycgr3G109328 Mycgr3
  
Location: 23561-24239

Mycgr3G109328\_Mycgr3

Mycgr3G104334 Mycgr3
  
Location: 24339-24567

Mycgr3G104334\_Mycgr3

Mycgr3G42715 Mycgr3T
  
Location: 24667-25981

Mycgr3G42715\_Mycgr3T

Mycgr3G92934 Mycgr3T
  
Location: 26081-27593

Mycgr3G92934\_Mycgr3T

Mycgr3G41969 Mycgr3T
  
Location: 27693-29328

Mycgr3G41969\_Mycgr3T

Mycgr3G80635 Mycgr3T
  
Location: 29428-29821

Mycgr3G80635\_Mycgr3T

Mycgr3G41426 Mycgr3T
  
Location: 29921-35255

Mycgr3G41426\_Mycgr3T

Mycgr3G104337 Mycgr3
  
Location: 35355-36108

Mycgr3G104337\_Mycgr3

Mycgr3G71679 Mycgr3T
  
Location: 36208-37300

Mycgr3G71679\_Mycgr3T

Mycgr3G92938 Mycgr3T
  
Location: 37400-38699

Mycgr3G92938\_Mycgr3T

Mycgr3G92941 Mycgr3T
  
Location: 38799-40734

Mycgr3G92941\_Mycgr3T

hypothetical protein
  
Accession: EHK22781
  
Location: 2255841-2257250
  
 NCBI BlastP on this gene

EHK22781

hypothetical protein
  
Accession: EHK22782
  
Location: 2258075-2258791
  
 NCBI BlastP on this gene

EHK22782

hypothetical protein
  
Accession: EHK22783
  
Location: 2261308-2263799
  
  
**BlastP hit with Mycgr3G11468\_Mycgr3T**
  
Percentage identity: 30 %
  
BlastP bit score: 158
  
Sequence coverage: 101 %
  
E-value: 5e-40
  
  
 NCBI BlastP on this gene

EHK22783

hypothetical protein
  
Accession: EHK22784
  
Location: 2263929-2264534
  
 NCBI BlastP on this gene

EHK22784

hypothetical protein
  
Accession: EHK22785
  
Location: 2266141-2267383
  
 NCBI BlastP on this gene

EHK22785

hypothetical protein
  
Accession: EHK22786
  
Location: 2269628-2271538
  
 NCBI BlastP on this gene

EHK22786

hypothetical protein
  
Accession: EHK22787
  
Location: 2272617-2273284
  
 NCBI BlastP on this gene

EHK22787

hypothetical protein
  
Accession: EHK22788
  
Location: 2273912-2274350
  
 NCBI BlastP on this gene

EHK22788

glycoside hydrolase family 20 protein
  
Accession: EHK22789
  
Location: 2274754-2277014
  
 NCBI BlastP on this gene

EHK22789

hypothetical protein
  
Accession: EHK22790
  
Location: 2280140-2282726
  
 NCBI BlastP on this gene

EHK22790

hypothetical protein
  
Accession: EHK22791
  
Location: 2283688-2284152
  
 NCBI BlastP on this gene

EHK22791

hypothetical protein
  
Accession: EHK22792
  
Location: 2285457-2286064
  
 NCBI BlastP on this gene

EHK22792

putative polyketide synthase
  
Accession: EHK22793
  
Location: 2286988-2293963
  
  
**BlastP hit with Mycgr3G100089\_Mycgr3**
  
Percentage identity: 44 %
  
BlastP bit score: 1847
  
Sequence coverage: 101 %
  
E-value: 0.0
  
  
 NCBI BlastP on this gene

EHK22793

hypothetical protein
  
Accession: EHK22794
  
Location: 2295807-2296765
  
  
**BlastP hit with Mycgr3G104337\_Mycgr3**
  
Percentage identity: 45 %
  
BlastP bit score: 218
  
Sequence coverage: 98 %
  
E-value: 1e-66
  
  
 NCBI BlastP on this gene

EHK22794

glycosyltransferase family 1 protein
  
Accession: EHK22795
  
Location: 2297637-2299297
  
 NCBI BlastP on this gene

EHK22795

Query: Architecture Search FASTA input

ABDG02000026 : Trichoderma atroviride IMI 206040    Total score: 3.0     Cumulative Blast bit score: 2201

Hit cluster cross-links:

Mycgr3G85918 Mycgr3T
  
Location: 0-1602

Mycgr3G85918\_Mycgr3T

Mycgr3G42010 Mycgr3T
  
Location: 1702-8569

Mycgr3G42010\_Mycgr3T

Mycgr3G29582 Mycgr3T
  
Location: 8669-8915

Mycgr3G29582\_Mycgr3T

Mycgr3G31170 Mycgr3T
  
Location: 9015-9255

Mycgr3G31170\_Mycgr3T

Mycgr3G85924 Mycgr3T
  
Location: 9355-11218

Mycgr3G85924\_Mycgr3T

Mycgr3G71676 Mycgr3T
  
Location: 11318-12494

Mycgr3G71676\_Mycgr3T

Mycgr3G11468 Mycgr3T
  
Location: 12594-13653

Mycgr3G11468\_Mycgr3T

Mycgr3G58567 Mycgr3T
  
Location: 13753-14506

Mycgr3G58567\_Mycgr3T

Mycgr3G100089 Mycgr3
  
Location: 14606-21152

Mycgr3G100089\_Mycgr3

Mycgr3G42698 Mycgr3T
  
Location: 21252-22131

Mycgr3G42698\_Mycgr3T

Mycgr3G71681 Mycgr3T
  
Location: 22231-23461

Mycgr3G71681\_Mycgr3T

Mycgr3G109328 Mycgr3
  
Location: 23561-24239

Mycgr3G109328\_Mycgr3

Mycgr3G104334 Mycgr3
  
Location: 24339-24567

Mycgr3G104334\_Mycgr3

Mycgr3G42715 Mycgr3T
  
Location: 24667-25981

Mycgr3G42715\_Mycgr3T

Mycgr3G92934 Mycgr3T
  
Location: 26081-27593

Mycgr3G92934\_Mycgr3T

Mycgr3G41969 Mycgr3T
  
Location: 27693-29328

Mycgr3G41969\_Mycgr3T

Mycgr3G80635 Mycgr3T
  
Location: 29428-29821

Mycgr3G80635\_Mycgr3T

Mycgr3G41426 Mycgr3T
  
Location: 29921-35255

Mycgr3G41426\_Mycgr3T

Mycgr3G104337 Mycgr3
  
Location: 35355-36108

Mycgr3G104337\_Mycgr3

Mycgr3G71679 Mycgr3T
  
Location: 36208-37300

Mycgr3G71679\_Mycgr3T

Mycgr3G92938 Mycgr3T
  
Location: 37400-38699

Mycgr3G92938\_Mycgr3T

Mycgr3G92941 Mycgr3T
  
Location: 38799-40734

Mycgr3G92941\_Mycgr3T

hypothetical protein
  
Accession: EHK42704
  
Location: 1106473-1107593
  
 NCBI BlastP on this gene

EHK42704

sterol 24-C-methyltransferase
  
Accession: EHK42703
  
Location: 1104002-1105296
  
 NCBI BlastP on this gene

EHK42703

hypothetical protein
  
Accession: EHK42702
  
Location: 1099620-1102514
  
  
**BlastP hit with Mycgr3G11468\_Mycgr3T**
  
Percentage identity: 31 %
  
BlastP bit score: 165
  
Sequence coverage: 103 %
  
E-value: 4e-42
  
  
 NCBI BlastP on this gene

EHK42702

hypothetical protein
  
Accession: EHK42701
  
Location: 1098879-1099506
  
 NCBI BlastP on this gene

EHK42701

hypothetical protein
  
Accession: EHK42700
  
Location: 1096314-1097513
  
 NCBI BlastP on this gene

EHK42700

hypothetical protein
  
Accession: EHK42699
  
Location: 1091792-1093774
  
 NCBI BlastP on this gene

EHK42699

hypothetical protein
  
Accession: EHK42698
  
Location: 1089936-1090570
  
 NCBI BlastP on this gene

EHK42698

glycoside hydrolase family 20 protein
  
Accession: EHK43609
  
Location: 1087200-1089476
  
 NCBI BlastP on this gene

EHK43609

hypothetical protein
  
Accession: EHK42697
  
Location: 1080913-1083545
  
 NCBI BlastP on this gene

EHK42697

hypothetical protein
  
Accession: EHK42696
  
Location: 1078644-1079108
  
 NCBI BlastP on this gene

EHK42696

hypothetical protein
  
Accession: EHK42695
  
Location: 1076610-1077198
  
 NCBI BlastP on this gene

EHK42695

polyketide synthase
  
Accession: EHK42694
  
Location: 1068691-1075711
  
  
**BlastP hit with Mycgr3G100089\_Mycgr3**
  
Percentage identity: 43 %
  
BlastP bit score: 1815
  
Sequence coverage: 101 %
  
E-value: 0.0
  
  
 NCBI BlastP on this gene

EHK42694

hypothetical protein
  
Accession: EHK42693
  
Location: 1065925-1066903
  
  
**BlastP hit with Mycgr3G104337\_Mycgr3**
  
Percentage identity: 45 %
  
BlastP bit score: 221
  
Sequence coverage: 100 %
  
E-value: 5e-68
  
  
 NCBI BlastP on this gene

EHK42693

glycosyltransferase family 1 protein
  
Accession: EHK43614
  
Location: 1063433-1065084
  
 NCBI BlastP on this gene

EHK43614

Query: Architecture Search FASTA input

JH725159 : Beauveria bassiana ARSEF 2860 unplaced genomic scaffold BBA\_S00010    Total score: 3.0     Cumulative Blast bit score: 1765

Hit cluster cross-links:

Mycgr3G85918 Mycgr3T
  
Location: 0-1602

Mycgr3G85918\_Mycgr3T

Mycgr3G42010 Mycgr3T
  
Location: 1702-8569

Mycgr3G42010\_Mycgr3T

Mycgr3G29582 Mycgr3T
  
Location: 8669-8915

Mycgr3G29582\_Mycgr3T

Mycgr3G31170 Mycgr3T
  
Location: 9015-9255

Mycgr3G31170\_Mycgr3T

Mycgr3G85924 Mycgr3T
  
Location: 9355-11218

Mycgr3G85924\_Mycgr3T

Mycgr3G71676 Mycgr3T
  
Location: 11318-12494

Mycgr3G71676\_Mycgr3T

Mycgr3G11468 Mycgr3T
  
Location: 12594-13653

Mycgr3G11468\_Mycgr3T

Mycgr3G58567 Mycgr3T
  
Location: 13753-14506

Mycgr3G58567\_Mycgr3T

Mycgr3G100089 Mycgr3
  
Location: 14606-21152

Mycgr3G100089\_Mycgr3

Mycgr3G42698 Mycgr3T
  
Location: 21252-22131

Mycgr3G42698\_Mycgr3T

Mycgr3G71681 Mycgr3T
  
Location: 22231-23461

Mycgr3G71681\_Mycgr3T

Mycgr3G109328 Mycgr3
  
Location: 23561-24239

Mycgr3G109328\_Mycgr3

Mycgr3G104334 Mycgr3
  
Location: 24339-24567

Mycgr3G104334\_Mycgr3

Mycgr3G42715 Mycgr3T
  
Location: 24667-25981

Mycgr3G42715\_Mycgr3T

Mycgr3G92934 Mycgr3T
  
Location: 26081-27593

Mycgr3G92934\_Mycgr3T

Mycgr3G41969 Mycgr3T
  
Location: 27693-29328

Mycgr3G41969\_Mycgr3T

Mycgr3G80635 Mycgr3T
  
Location: 29428-29821

Mycgr3G80635\_Mycgr3T

Mycgr3G41426 Mycgr3T
  
Location: 29921-35255

Mycgr3G41426\_Mycgr3T

Mycgr3G104337 Mycgr3
  
Location: 35355-36108

Mycgr3G104337\_Mycgr3

Mycgr3G71679 Mycgr3T
  
Location: 36208-37300

Mycgr3G71679\_Mycgr3T

Mycgr3G92938 Mycgr3T
  
Location: 37400-38699

Mycgr3G92938\_Mycgr3T

Mycgr3G92941 Mycgr3T
  
Location: 38799-40734

Mycgr3G92941\_Mycgr3T

decapping enzyme Dcp2
  
Accession: EJP66579
  
Location: 584838-587584
  
 NCBI BlastP on this gene

EJP66579

RNA recognition motif containing protein
  
Accession: EJP66580
  
Location: 588112-589382
  
 NCBI BlastP on this gene

EJP66580

hypothetical protein
  
Accession: EJP66581
  
Location: 590955-591847
  
 NCBI BlastP on this gene

EJP66581

histone H3 methyltransferase complex and RNA cleavage factor II complex, subunit SWD2
  
Accession: EJP66582
  
Location: 592412-593699
  
 NCBI BlastP on this gene

EJP66582

mitochondrial oxaloacetate transport protein
  
Accession: EJP66583
  
Location: 594639-596148
  
 NCBI BlastP on this gene

EJP66583

cytoplasmic tRNA 2-thiolation protein 2
  
Accession: EJP66584
  
Location: 596480-597765
  
 NCBI BlastP on this gene

EJP66584

PH domain-containing protein
  
Accession: EJP66585
  
Location: 600015-601287
  
 NCBI BlastP on this gene

EJP66585

cellulase-like protein
  
Accession: EJP66586
  
Location: 602983-604260
  
  
**BlastP hit with Mycgr3G71681\_Mycgr3T**
  
Percentage identity: 32 %
  
BlastP bit score: 187
  
Sequence coverage: 98 %
  
E-value: 9e-51
  
  
 NCBI BlastP on this gene

EJP66586

hypothetical protein
  
Accession: EJP66587
  
Location: 604711-605874
  
 NCBI BlastP on this gene

EJP66587

geranylgeranyl pyrophosphate synthetase
  
Accession: EJP66588
  
Location: 606682-608200
  
  
**BlastP hit with Mycgr3G92938\_Mycgr3T**
  
Percentage identity: 33 %
  
BlastP bit score: 241
  
Sequence coverage: 101 %
  
E-value: 7e-71
  
  
 NCBI BlastP on this gene

EJP66588

ATPase protein
  
Accession: EJP66589
  
Location: 608830-611275
  
  
**BlastP hit with Mycgr3G42010\_Mycgr3T**
  
Percentage identity: 34 %
  
BlastP bit score: 439
  
Sequence coverage: 34 %
  
E-value: 1e-129
  
  
 NCBI BlastP on this gene

EJP66589

cbbX-like protein
  
Accession: EJP66590
  
Location: 612576-615880
  
  
**BlastP hit with Mycgr3G42010\_Mycgr3T**
  
Percentage identity: 46 %
  
BlastP bit score: 899
  
Sequence coverage: 47 %
  
E-value: 0.0
  
  
 NCBI BlastP on this gene

EJP66590

spliceosome associated protein
  
Accession: EJP66591
  
Location: 616725-618560
  
 NCBI BlastP on this gene

EJP66591

hypothetical protein
  
Accession: EJP66592
  
Location: 619098-621375
  
 NCBI BlastP on this gene

EJP66592

hypothetical protein
  
Accession: EJP66593
  
Location: 622658-624844
  
 NCBI BlastP on this gene

EJP66593

hypothetical protein
  
Accession: EJP66594
  
Location: 625194-626387
  
 NCBI BlastP on this gene

EJP66594

serine/arginine repetitive matrix protein 1
  
Accession: EJP66595
  
Location: 627225-629096
  
 NCBI BlastP on this gene

EJP66595

histone deacetylase
  
Accession: EJP66596
  
Location: 629437-631709
  
 NCBI BlastP on this gene

EJP66596

Query: Architecture Search FASTA input

GG698924 : Nectria haematococca mpVI 77-13-4 chromosome 11 genomic scaffold NECHAsca\_32\_chr11\_3\_0    Total score: 3.0     Cumulative Blast bit score: 1648

Hit cluster cross-links:

Mycgr3G85918 Mycgr3T
  
Location: 0-1602

Mycgr3G85918\_Mycgr3T

Mycgr3G42010 Mycgr3T
  
Location: 1702-8569

Mycgr3G42010\_Mycgr3T

Mycgr3G29582 Mycgr3T
  
Location: 8669-8915

Mycgr3G29582\_Mycgr3T

Mycgr3G31170 Mycgr3T
  
Location: 9015-9255

Mycgr3G31170\_Mycgr3T

Mycgr3G85924 Mycgr3T
  
Location: 9355-11218

Mycgr3G85924\_Mycgr3T

Mycgr3G71676 Mycgr3T
  
Location: 11318-12494

Mycgr3G71676\_Mycgr3T

Mycgr3G11468 Mycgr3T
  
Location: 12594-13653

Mycgr3G11468\_Mycgr3T

Mycgr3G58567 Mycgr3T
  
Location: 13753-14506

Mycgr3G58567\_Mycgr3T

Mycgr3G100089 Mycgr3
  
Location: 14606-21152

Mycgr3G100089\_Mycgr3

Mycgr3G42698 Mycgr3T
  
Location: 21252-22131

Mycgr3G42698\_Mycgr3T

Mycgr3G71681 Mycgr3T
  
Location: 22231-23461

Mycgr3G71681\_Mycgr3T

Mycgr3G109328 Mycgr3
  
Location: 23561-24239

Mycgr3G109328\_Mycgr3

Mycgr3G104334 Mycgr3
  
Location: 24339-24567

Mycgr3G104334\_Mycgr3

Mycgr3G42715 Mycgr3T
  
Location: 24667-25981

Mycgr3G42715\_Mycgr3T

Mycgr3G92934 Mycgr3T
  
Location: 26081-27593

Mycgr3G92934\_Mycgr3T

Mycgr3G41969 Mycgr3T
  
Location: 27693-29328

Mycgr3G41969\_Mycgr3T

Mycgr3G80635 Mycgr3T
  
Location: 29428-29821

Mycgr3G80635\_Mycgr3T

Mycgr3G41426 Mycgr3T
  
Location: 29921-35255

Mycgr3G41426\_Mycgr3T

Mycgr3G104337 Mycgr3
  
Location: 35355-36108

Mycgr3G104337\_Mycgr3

Mycgr3G71679 Mycgr3T
  
Location: 36208-37300

Mycgr3G71679\_Mycgr3T

Mycgr3G92938 Mycgr3T
  
Location: 37400-38699

Mycgr3G92938\_Mycgr3T

Mycgr3G92941 Mycgr3T
  
Location: 38799-40734

Mycgr3G92941\_Mycgr3T

hypothetical protein
  
Accession: EEU37133
  
Location: 428536-430872
  
 NCBI BlastP on this gene

EEU37133

hypothetical protein
  
Accession: EEU37132
  
Location: 425610-426938
  
 NCBI BlastP on this gene

EEU37132

predicted protein
  
Accession: EEU37051
  
Location: 423776-425457
  
 NCBI BlastP on this gene

EEU37051

hypothetical protein
  
Accession: EEU37050
  
Location: 418985-422000
  
 NCBI BlastP on this gene

EEU37050

hypothetical protein
  
Accession: EEU37131
  
Location: 415445-416603
  
 NCBI BlastP on this gene

EEU37131

predicted protein
  
Accession: EEU37049
  
Location: 414007-415264
  
  
**BlastP hit with Mycgr3G71679\_Mycgr3T**
  
Percentage identity: 38 %
  
BlastP bit score: 246
  
Sequence coverage: 99 %
  
E-value: 1e-74
  
  
 NCBI BlastP on this gene

EEU37049

hypothetical protein
  
Accession: EEU37048
  
Location: 410831-412473
  
 NCBI BlastP on this gene

EEU37048

hypothetical protein
  
Accession: EEU37130
  
Location: 409559-410459
  
  
**BlastP hit with Mycgr3G104337\_Mycgr3**
  
Percentage identity: 45 %
  
BlastP bit score: 218
  
Sequence coverage: 97 %
  
E-value: 1e-66
  
  
 NCBI BlastP on this gene

EEU37130

hypothetical protein
  
Accession: EEU37129
  
Location: 401232-408233
  
  
**BlastP hit with Mycgr3G100089\_Mycgr3**
  
Percentage identity: 46 %
  
BlastP bit score: 1184
  
Sequence coverage: 57 %
  
E-value: 0.0
  
  
 NCBI BlastP on this gene

EEU37129

hypothetical protein
  
Accession: EEU37128
  
Location: 396393-398308
  
 NCBI BlastP on this gene

EEU37128

predicted protein
  
Accession: EEU37047
  
Location: 394517-396281
  
 NCBI BlastP on this gene

EEU37047

predicted protein
  
Accession: EEU37127
  
Location: 392800-394026
  
 NCBI BlastP on this gene

EEU37127

hypothetical protein
  
Accession: EEU37046
  
Location: 390550-391629
  
 NCBI BlastP on this gene

EEU37046

hypothetical protein
  
Accession: EEU37045
  
Location: 388814-389887
  
 NCBI BlastP on this gene

EEU37045

hypothetical protein
  
Accession: EEU37044
  
Location: 387442-388125
  
 NCBI BlastP on this gene

EEU37044

hypothetical protein
  
Accession: EEU37126
  
Location: 384281-385648
  
 NCBI BlastP on this gene

EEU37126

Query: Architecture Search FASTA input

KB446535 : Dothistroma septosporum NZE10 unplaced genomic scaffold DOTSEscaffold\_1    Total score: 3.0     Cumulative Blast bit score: 623

Hit cluster cross-links:

Mycgr3G85918 Mycgr3T
  
Location: 0-1602

Mycgr3G85918\_Mycgr3T

Mycgr3G42010 Mycgr3T
  
Location: 1702-8569

Mycgr3G42010\_Mycgr3T

Mycgr3G29582 Mycgr3T
  
Location: 8669-8915

Mycgr3G29582\_Mycgr3T

Mycgr3G31170 Mycgr3T
  
Location: 9015-9255

Mycgr3G31170\_Mycgr3T

Mycgr3G85924 Mycgr3T
  
Location: 9355-11218

Mycgr3G85924\_Mycgr3T

Mycgr3G71676 Mycgr3T
  
Location: 11318-12494

Mycgr3G71676\_Mycgr3T

Mycgr3G11468 Mycgr3T
  
Location: 12594-13653

Mycgr3G11468\_Mycgr3T

Mycgr3G58567 Mycgr3T
  
Location: 13753-14506

Mycgr3G58567\_Mycgr3T

Mycgr3G100089 Mycgr3
  
Location: 14606-21152

Mycgr3G100089\_Mycgr3

Mycgr3G42698 Mycgr3T
  
Location: 21252-22131

Mycgr3G42698\_Mycgr3T

Mycgr3G71681 Mycgr3T
  
Location: 22231-23461

Mycgr3G71681\_Mycgr3T

Mycgr3G109328 Mycgr3
  
Location: 23561-24239

Mycgr3G109328\_Mycgr3

Mycgr3G104334 Mycgr3
  
Location: 24339-24567

Mycgr3G104334\_Mycgr3

Mycgr3G42715 Mycgr3T
  
Location: 24667-25981

Mycgr3G42715\_Mycgr3T

Mycgr3G92934 Mycgr3T
  
Location: 26081-27593

Mycgr3G92934\_Mycgr3T

Mycgr3G41969 Mycgr3T
  
Location: 27693-29328

Mycgr3G41969\_Mycgr3T

Mycgr3G80635 Mycgr3T
  
Location: 29428-29821

Mycgr3G80635\_Mycgr3T

Mycgr3G41426 Mycgr3T
  
Location: 29921-35255

Mycgr3G41426\_Mycgr3T

Mycgr3G104337 Mycgr3
  
Location: 35355-36108

Mycgr3G104337\_Mycgr3

Mycgr3G71679 Mycgr3T
  
Location: 36208-37300

Mycgr3G71679\_Mycgr3T

Mycgr3G92938 Mycgr3T
  
Location: 37400-38699

Mycgr3G92938\_Mycgr3T

Mycgr3G92941 Mycgr3T
  
Location: 38799-40734

Mycgr3G92941\_Mycgr3T

hypothetical protein
  
Accession: EME48423
  
Location: 244260-244680
  
 NCBI BlastP on this gene

EME48423

hypothetical protein
  
Accession: EME48422
  
Location: 241716-242972
  
 NCBI BlastP on this gene

EME48422

hypothetical protein
  
Accession: EME48421
  
Location: 240636-241423
  
 NCBI BlastP on this gene

EME48421

hypothetical protein
  
Accession: EME48420
  
Location: 238233-240065
  
 NCBI BlastP on this gene

EME48420

hypothetical protein
  
Accession: EME48419
  
Location: 235098-237792
  
 NCBI BlastP on this gene

EME48419

hypothetical protein
  
Accession: EME48418
  
Location: 232303-233040
  
 NCBI BlastP on this gene

EME48418

hypothetical protein
  
Accession: EME48417
  
Location: 230471-231358
  
 NCBI BlastP on this gene

EME48417

hypothetical protein
  
Accession: EME48416
  
Location: 227646-228397
  
  
**BlastP hit with Mycgr3G31170\_Mycgr3T**
  
Percentage identity: 100 %
  
BlastP bit score: 167
  
Sequence coverage: 100 %
  
E-value: 5e-50
  
  
 NCBI BlastP on this gene

EME48416

hypothetical protein
  
Accession: EME48415
  
Location: 226480-226807
  
 NCBI BlastP on this gene

EME48415

hypothetical protein
  
Accession: EME48414
  
Location: 225993-226289
  
  
**BlastP hit with Mycgr3G29582\_Mycgr3T**
  
Percentage identity: 93 %
  
BlastP bit score: 117
  
Sequence coverage: 79 %
  
E-value: 4e-32
  
  
 NCBI BlastP on this gene

EME48414

hypothetical protein
  
Accession: EME48413
  
Location: 225273-225641
  
 NCBI BlastP on this gene

EME48413

hypothetical protein
  
Accession: EME48412
  
Location: 224691-225143
  
 NCBI BlastP on this gene

EME48412

hypothetical protein
  
Accession: EME48411
  
Location: 224014-224651
  
 NCBI BlastP on this gene

EME48411

hypothetical protein
  
Accession: EME48410
  
Location: 222961-223956
  
 NCBI BlastP on this gene

EME48410

hypothetical protein
  
Accession: EME48409
  
Location: 221770-222747
  
  
**BlastP hit with Mycgr3G92941\_Mycgr3T**
  
Percentage identity: 56 %
  
BlastP bit score: 339
  
Sequence coverage: 48 %
  
E-value: 1e-107
  
  
 NCBI BlastP on this gene

EME48409

hypothetical protein
  
Accession: EME48408
  
Location: 218599-219402
  
 NCBI BlastP on this gene

EME48408

hypothetical protein
  
Accession: EME48406
  
Location: 215154-216488
  
 NCBI BlastP on this gene

EME48406

hypothetical protein
  
Accession: EME48405
  
Location: 212957-213475
  
 NCBI BlastP on this gene

EME48405

hypothetical protein
  
Accession: EME48404
  
Location: 211478-211988
  
 NCBI BlastP on this gene

EME48404

hypothetical protein
  
Accession: EME48403
  
Location: 209369-210952
  
 NCBI BlastP on this gene

EME48403

hypothetical protein
  
Accession: EME48402
  
Location: 205909-207646
  
 NCBI BlastP on this gene

EME48402

hypothetical protein
  
Accession: EME48401
  
Location: 204112-205539
  
 NCBI BlastP on this gene

EME48401

Query: Architecture Search FASTA input

GL629756 : Grosmannia clavigera kw1407 unplaced genomic scaffold GCSC\_132    Total score: 3.0     Cumulative Blast bit score: 602

Hit cluster cross-links:

Mycgr3G85918 Mycgr3T
  
Location: 0-1602

Mycgr3G85918\_Mycgr3T

Mycgr3G42010 Mycgr3T
  
Location: 1702-8569

Mycgr3G42010\_Mycgr3T

Mycgr3G29582 Mycgr3T
  
Location: 8669-8915

Mycgr3G29582\_Mycgr3T

Mycgr3G31170 Mycgr3T
  
Location: 9015-9255

Mycgr3G31170\_Mycgr3T

Mycgr3G85924 Mycgr3T
  
Location: 9355-11218

Mycgr3G85924\_Mycgr3T

Mycgr3G71676 Mycgr3T
  
Location: 11318-12494

Mycgr3G71676\_Mycgr3T

Mycgr3G11468 Mycgr3T
  
Location: 12594-13653

Mycgr3G11468\_Mycgr3T

Mycgr3G58567 Mycgr3T
  
Location: 13753-14506

Mycgr3G58567\_Mycgr3T

Mycgr3G100089 Mycgr3
  
Location: 14606-21152

Mycgr3G100089\_Mycgr3

Mycgr3G42698 Mycgr3T
  
Location: 21252-22131

Mycgr3G42698\_Mycgr3T

Mycgr3G71681 Mycgr3T
  
Location: 22231-23461

Mycgr3G71681\_Mycgr3T

Mycgr3G109328 Mycgr3
  
Location: 23561-24239

Mycgr3G109328\_Mycgr3

Mycgr3G104334 Mycgr3
  
Location: 24339-24567

Mycgr3G104334\_Mycgr3

Mycgr3G42715 Mycgr3T
  
Location: 24667-25981

Mycgr3G42715\_Mycgr3T

Mycgr3G92934 Mycgr3T
  
Location: 26081-27593

Mycgr3G92934\_Mycgr3T

Mycgr3G41969 Mycgr3T
  
Location: 27693-29328

Mycgr3G41969\_Mycgr3T

Mycgr3G80635 Mycgr3T
  
Location: 29428-29821

Mycgr3G80635\_Mycgr3T

Mycgr3G41426 Mycgr3T
  
Location: 29921-35255

Mycgr3G41426\_Mycgr3T

Mycgr3G104337 Mycgr3
  
Location: 35355-36108

Mycgr3G104337\_Mycgr3

Mycgr3G71679 Mycgr3T
  
Location: 36208-37300

Mycgr3G71679\_Mycgr3T

Mycgr3G92938 Mycgr3T
  
Location: 37400-38699

Mycgr3G92938\_Mycgr3T

Mycgr3G92941 Mycgr3T
  
Location: 38799-40734

Mycgr3G92941\_Mycgr3T

hypothetical protein
  
Accession: EFX04908
  
Location: 409240-410023
  
 NCBI BlastP on this gene

EFX04908

taud/tfda taurine catabolism dioxygenase
  
Accession: EFX05037
  
Location: 407910-409094
  
 NCBI BlastP on this gene

EFX05037

hypothetical protein
  
Accession: EFX04959
  
Location: 405283-406668
  
 NCBI BlastP on this gene

EFX04959

hypothetical protein
  
Accession: EFX05022
  
Location: 403391-404764
  
 NCBI BlastP on this gene

EFX05022

tyrosinase central domain containing protein
  
Accession: EFX04978
  
Location: 401544-403089
  
  
**BlastP hit with Mycgr3G42698\_Mycgr3T**
  
Percentage identity: 34 %
  
BlastP bit score: 187
  
Sequence coverage: 115 %
  
E-value: 1e-52
  
  
 NCBI BlastP on this gene

EFX04978

integral membrane protein
  
Accession: EFX04839
  
Location: 399031-400492
  
 NCBI BlastP on this gene

EFX04839

duf895 domain protein membrane protein
  
Accession: EFX04873
  
Location: 395227-396804
  
 NCBI BlastP on this gene

EFX04873

methyltransferase type 11
  
Accession: EFX05074
  
Location: 392223-393038
  
 NCBI BlastP on this gene

EFX05074

hypothetical protein
  
Accession: EFX05081
  
Location: 390988-391962
  
 NCBI BlastP on this gene

EFX05081

major facilitator superfamily transporter quinate
  
Accession: EFX04852
  
Location: 388984-390678
  
 NCBI BlastP on this gene

EFX04852

hypothetical protein
  
Accession: EFX04856
  
Location: 385556-386782
  
 NCBI BlastP on this gene

EFX04856

hypothetical protein
  
Accession: EFX05055
  
Location: 384184-384960
  
 NCBI BlastP on this gene

EFX05055

c6 zinc finger domain containing protein
  
Accession: EFX04895
  
Location: 378994-383259
  
  
**BlastP hit with Mycgr3G11468\_Mycgr3T**
  
Percentage identity: 32 %
  
BlastP bit score: 162
  
Sequence coverage: 100 %
  
E-value: 2e-40
  
  
 NCBI BlastP on this gene

EFX04895

heterokaryon incompatibility protein
  
Accession: EFX04862
  
Location: 376384-377751
  
 NCBI BlastP on this gene

EFX04862

allantoate permease
  
Accession: EFX04995
  
Location: 371938-375070
  
  
**BlastP hit with Mycgr3G71679\_Mycgr3T**
  
Percentage identity: 40 %
  
BlastP bit score: 253
  
Sequence coverage: 101 %
  
E-value: 1e-72
  
  
 NCBI BlastP on this gene

EFX04995

cytoskeleton assembly control protein
  
Accession: EFX04935
  
Location: 366904-370181
  
 NCBI BlastP on this gene

EFX04935

hypothetical protein
  
Accession: EFX05047
  
Location: 364281-365876
  
 NCBI BlastP on this gene

EFX05047

Query: Architecture Search FASTA input

KB446555 : Pseudocercospora fijiensis CIRAD86 unplaced genomic scaffold MYCFIscaffold\_1    Total score: 3.0     Cumulative Blast bit score: 593

Hit cluster cross-links:

Mycgr3G85918 Mycgr3T
  
Location: 0-1602

Mycgr3G85918\_Mycgr3T

Mycgr3G42010 Mycgr3T
  
Location: 1702-8569

Mycgr3G42010\_Mycgr3T

Mycgr3G29582 Mycgr3T
  
Location: 8669-8915

Mycgr3G29582\_Mycgr3T

Mycgr3G31170 Mycgr3T
  
Location: 9015-9255

Mycgr3G31170\_Mycgr3T

Mycgr3G85924 Mycgr3T
  
Location: 9355-11218

Mycgr3G85924\_Mycgr3T

Mycgr3G71676 Mycgr3T
  
Location: 11318-12494

Mycgr3G71676\_Mycgr3T

Mycgr3G11468 Mycgr3T
  
Location: 12594-13653

Mycgr3G11468\_Mycgr3T

Mycgr3G58567 Mycgr3T
  
Location: 13753-14506

Mycgr3G58567\_Mycgr3T

Mycgr3G100089 Mycgr3
  
Location: 14606-21152

Mycgr3G100089\_Mycgr3

Mycgr3G42698 Mycgr3T
  
Location: 21252-22131

Mycgr3G42698\_Mycgr3T

Mycgr3G71681 Mycgr3T
  
Location: 22231-23461

Mycgr3G71681\_Mycgr3T

Mycgr3G109328 Mycgr3
  
Location: 23561-24239

Mycgr3G109328\_Mycgr3

Mycgr3G104334 Mycgr3
  
Location: 24339-24567

Mycgr3G104334\_Mycgr3

Mycgr3G42715 Mycgr3T
  
Location: 24667-25981

Mycgr3G42715\_Mycgr3T

Mycgr3G92934 Mycgr3T
  
Location: 26081-27593

Mycgr3G92934\_Mycgr3T

Mycgr3G41969 Mycgr3T
  
Location: 27693-29328

Mycgr3G41969\_Mycgr3T

Mycgr3G80635 Mycgr3T
  
Location: 29428-29821

Mycgr3G80635\_Mycgr3T

Mycgr3G41426 Mycgr3T
  
Location: 29921-35255

Mycgr3G41426\_Mycgr3T

Mycgr3G104337 Mycgr3
  
Location: 35355-36108

Mycgr3G104337\_Mycgr3

Mycgr3G71679 Mycgr3T
  
Location: 36208-37300

Mycgr3G71679\_Mycgr3T

Mycgr3G92938 Mycgr3T
  
Location: 37400-38699

Mycgr3G92938\_Mycgr3T

Mycgr3G92941 Mycgr3T
  
Location: 38799-40734

Mycgr3G92941\_Mycgr3T

hypothetical protein
  
Accession: EME89653
  
Location: 11214595-11217387
  
 NCBI BlastP on this gene

EME89653

hypothetical protein
  
Accession: EME89651
  
Location: 11213452-11214099
  
 NCBI BlastP on this gene

EME89651

hypothetical protein
  
Accession: EME89650
  
Location: 11211221-11212552
  
 NCBI BlastP on this gene

EME89650

hypothetical protein
  
Accession: EME89649
  
Location: 11208039-11210936
  
 NCBI BlastP on this gene

EME89649

hypothetical protein
  
Accession: EME89648
  
Location: 11204965-11207976
  
 NCBI BlastP on this gene

EME89648

hypothetical protein
  
Accession: EME89647
  
Location: 11203387-11204468
  
 NCBI BlastP on this gene

EME89647

hypothetical protein
  
Accession: EME89646
  
Location: 11202273-11202956
  
  
**BlastP hit with Mycgr3G92941\_Mycgr3T**
  
Percentage identity: 61 %
  
BlastP bit score: 295
  
Sequence coverage: 35 %
  
E-value: 6e-92
  
  
 NCBI BlastP on this gene

EME89646

hypothetical protein
  
Accession: EME89645
  
Location: 11195527-11196063
  
 NCBI BlastP on this gene

EME89645

hypothetical protein
  
Accession: EME89644
  
Location: 11192179-11193209
  
 NCBI BlastP on this gene

EME89644

hypothetical protein
  
Accession: EME89643
  
Location: 11189025-11189714
  
  
**BlastP hit with Mycgr3G31170\_Mycgr3T**
  
Percentage identity: 100 %
  
BlastP bit score: 166
  
Sequence coverage: 100 %
  
E-value: 3e-50
  
  
 NCBI BlastP on this gene

EME89643

hypothetical protein
  
Accession: EME89642
  
Location: 11188678-11188929
  
 NCBI BlastP on this gene

EME89642

hypothetical protein
  
Accession: EME89641
  
Location: 11187181-11187502
  
  
**BlastP hit with Mycgr3G29582\_Mycgr3T**
  
Percentage identity: 89 %
  
BlastP bit score: 132
  
Sequence coverage: 92 %
  
E-value: 3e-38
  
  
 NCBI BlastP on this gene

EME89641

hypothetical protein
  
Accession: EME89640
  
Location: 11186936-11187181
  
 NCBI BlastP on this gene

EME89640

hypothetical protein
  
Accession: EME89639
  
Location: 11181897-11184399
  
 NCBI BlastP on this gene

EME89639

hypothetical protein
  
Accession: EME89638
  
Location: 11180493-11181115
  
 NCBI BlastP on this gene

EME89638

hypothetical protein
  
Accession: EME89637
  
Location: 11178461-11179275
  
 NCBI BlastP on this gene

EME89637

hypothetical protein
  
Accession: EME89635
  
Location: 11174172-11177214
  
 NCBI BlastP on this gene

EME89635

hypothetical protein
  
Accession: EME89634
  
Location: 11171820-11172920
  
 NCBI BlastP on this gene

EME89634

Query: Architecture Search FASTA input

CABT02000055 : Sordaria macrospora k-hell    Total score: 2.0     Cumulative Blast bit score: 3843

Hit cluster cross-links:

Mycgr3G85918 Mycgr3T
  
Location: 0-1602

Mycgr3G85918\_Mycgr3T

Mycgr3G42010 Mycgr3T
  
Location: 1702-8569

Mycgr3G42010\_Mycgr3T

Mycgr3G29582 Mycgr3T
  
Location: 8669-8915

Mycgr3G29582\_Mycgr3T

Mycgr3G31170 Mycgr3T
  
Location: 9015-9255

Mycgr3G31170\_Mycgr3T

Mycgr3G85924 Mycgr3T
  
Location: 9355-11218

Mycgr3G85924\_Mycgr3T

Mycgr3G71676 Mycgr3T
  
Location: 11318-12494

Mycgr3G71676\_Mycgr3T

Mycgr3G11468 Mycgr3T
  
Location: 12594-13653

Mycgr3G11468\_Mycgr3T

Mycgr3G58567 Mycgr3T
  
Location: 13753-14506

Mycgr3G58567\_Mycgr3T

Mycgr3G100089 Mycgr3
  
Location: 14606-21152

Mycgr3G100089\_Mycgr3

Mycgr3G42698 Mycgr3T
  
Location: 21252-22131

Mycgr3G42698\_Mycgr3T

Mycgr3G71681 Mycgr3T
  
Location: 22231-23461

Mycgr3G71681\_Mycgr3T

Mycgr3G109328 Mycgr3
  
Location: 23561-24239

Mycgr3G109328\_Mycgr3

Mycgr3G104334 Mycgr3
  
Location: 24339-24567

Mycgr3G104334\_Mycgr3

Mycgr3G42715 Mycgr3T
  
Location: 24667-25981

Mycgr3G42715\_Mycgr3T

Mycgr3G92934 Mycgr3T
  
Location: 26081-27593

Mycgr3G92934\_Mycgr3T

Mycgr3G41969 Mycgr3T
  
Location: 27693-29328

Mycgr3G41969\_Mycgr3T

Mycgr3G80635 Mycgr3T
  
Location: 29428-29821

Mycgr3G80635\_Mycgr3T

Mycgr3G41426 Mycgr3T
  
Location: 29921-35255

Mycgr3G41426\_Mycgr3T

Mycgr3G104337 Mycgr3
  
Location: 35355-36108

Mycgr3G104337\_Mycgr3

Mycgr3G71679 Mycgr3T
  
Location: 36208-37300

Mycgr3G71679\_Mycgr3T

Mycgr3G92938 Mycgr3T
  
Location: 37400-38699

Mycgr3G92938\_Mycgr3T

Mycgr3G92941 Mycgr3T
  
Location: 38799-40734

Mycgr3G92941\_Mycgr3T

not annotated
  
Accession: CCC05205
  
Location: 3991-11276
  
  
**BlastP hit with Mycgr3G42010\_Mycgr3T**
  
Percentage identity: 45 %
  
BlastP bit score: 1899
  
Sequence coverage: 101 %
  
E-value: 0.0
  
  
 NCBI BlastP on this gene

CCC05205

not annotated
  
Accession: CCC05206
  
Location: 15517-22768
  
  
**BlastP hit with Mycgr3G42010\_Mycgr3T**
  
Percentage identity: 43 %
  
BlastP bit score: 1769
  
Sequence coverage: 101 %
  
E-value: 0.0
  
  
 NCBI BlastP on this gene

CCC05206

not annotated
  
Accession: CCC05207
  
Location: 24027-25918
  
  
**BlastP hit with Mycgr3G92938\_Mycgr3T**
  
Percentage identity: 35 %
  
BlastP bit score: 175
  
Sequence coverage: 72 %
  
E-value: 2e-45
  
  
 NCBI BlastP on this gene

CCC05207

not annotated
  
Accession: CCC05208
  
Location: 30368-31286
  
 NCBI BlastP on this gene

CCC05208

not annotated
  
Accession: CCC05209
  
Location: 32085-32980
  
 NCBI BlastP on this gene

CCC05209

not annotated
  
Accession: CCC05210
  
Location: 34108-34383
  
 NCBI BlastP on this gene

CCC05210

not annotated
  
Accession: CCC05211
  
Location: 35263-43044
  
 NCBI BlastP on this gene

CCC05211

Query: Architecture Search FASTA input

KB456266 : Mycosphaerella populorum SO2202 unplaced genomic scaffold SEPMUscaffold\_7    Total score: 2.0     Cumulative Blast bit score: 3607

Hit cluster cross-links:

Mycgr3G85918 Mycgr3T
  
Location: 0-1602

Mycgr3G85918\_Mycgr3T

Mycgr3G42010 Mycgr3T
  
Location: 1702-8569

Mycgr3G42010\_Mycgr3T

Mycgr3G29582 Mycgr3T
  
Location: 8669-8915

Mycgr3G29582\_Mycgr3T

Mycgr3G31170 Mycgr3T
  
Location: 9015-9255

Mycgr3G31170\_Mycgr3T

Mycgr3G85924 Mycgr3T
  
Location: 9355-11218

Mycgr3G85924\_Mycgr3T

Mycgr3G71676 Mycgr3T
  
Location: 11318-12494

Mycgr3G71676\_Mycgr3T

Mycgr3G11468 Mycgr3T
  
Location: 12594-13653

Mycgr3G11468\_Mycgr3T

Mycgr3G58567 Mycgr3T
  
Location: 13753-14506

Mycgr3G58567\_Mycgr3T

Mycgr3G100089 Mycgr3
  
Location: 14606-21152

Mycgr3G100089\_Mycgr3

Mycgr3G42698 Mycgr3T
  
Location: 21252-22131

Mycgr3G42698\_Mycgr3T

Mycgr3G71681 Mycgr3T
  
Location: 22231-23461

Mycgr3G71681\_Mycgr3T

Mycgr3G109328 Mycgr3
  
Location: 23561-24239

Mycgr3G109328\_Mycgr3

Mycgr3G104334 Mycgr3
  
Location: 24339-24567

Mycgr3G104334\_Mycgr3

Mycgr3G42715 Mycgr3T
  
Location: 24667-25981

Mycgr3G42715\_Mycgr3T

Mycgr3G92934 Mycgr3T
  
Location: 26081-27593

Mycgr3G92934\_Mycgr3T

Mycgr3G41969 Mycgr3T
  
Location: 27693-29328

Mycgr3G41969\_Mycgr3T

Mycgr3G80635 Mycgr3T
  
Location: 29428-29821

Mycgr3G80635\_Mycgr3T

Mycgr3G41426 Mycgr3T
  
Location: 29921-35255

Mycgr3G41426\_Mycgr3T

Mycgr3G104337 Mycgr3
  
Location: 35355-36108

Mycgr3G104337\_Mycgr3

Mycgr3G71679 Mycgr3T
  
Location: 36208-37300

Mycgr3G71679\_Mycgr3T

Mycgr3G92938 Mycgr3T
  
Location: 37400-38699

Mycgr3G92938\_Mycgr3T

Mycgr3G92941 Mycgr3T
  
Location: 38799-40734

Mycgr3G92941\_Mycgr3T

HAD-like protein
  
Accession: EMF11317
  
Location: 1071416-1072138
  
 NCBI BlastP on this gene

EMF11317

hypothetical protein
  
Accession: EMF11318
  
Location: 1072997-1073218
  
 NCBI BlastP on this gene

EMF11318

hypothetical protein
  
Accession: EMF11319
  
Location: 1075103-1075806
  
 NCBI BlastP on this gene

EMF11319

ketoreductase
  
Accession: EMF11320
  
Location: 1076386-1077466
  
 NCBI BlastP on this gene

EMF11320

hypothetical protein
  
Accession: EMF11321
  
Location: 1078601-1079034
  
 NCBI BlastP on this gene

EMF11321

hypothetical protein
  
Accession: EMF11322
  
Location: 1079970-1080869
  
 NCBI BlastP on this gene

EMF11322

hypothetical protein
  
Accession: EMF11323
  
Location: 1081671-1083036
  
 NCBI BlastP on this gene

EMF11323

hypothetical protein
  
Accession: EMF11324
  
Location: 1083700-1084014
  
 NCBI BlastP on this gene

EMF11324

26S protease regulatory subunit 6A
  
Accession: EMF11325
  
Location: 1084780-1086282
  
 NCBI BlastP on this gene

EMF11325

amine oxidase
  
Accession: EMF11326
  
Location: 1087497-1088552
  
 NCBI BlastP on this gene

EMF11326

hypothetical protein
  
Accession: EMF11327
  
Location: 1088862-1090133
  
  
**BlastP hit with Mycgr3G92938\_Mycgr3T**
  
Percentage identity: 46 %
  
BlastP bit score: 370
  
Sequence coverage: 98 %
  
E-value: 4e-121
  
  
 NCBI BlastP on this gene

EMF11327

hypothetical protein
  
Accession: EMF11328
  
Location: 1090257-1090442
  
 NCBI BlastP on this gene

EMF11328

AAA family ATPase
  
Accession: EMF11329
  
Location: 1090710-1097754
  
  
**BlastP hit with Mycgr3G42010\_Mycgr3T**
  
Percentage identity: 68 %
  
BlastP bit score: 3237
  
Sequence coverage: 101 %
  
E-value: 0.0
  
  
 NCBI BlastP on this gene

EMF11329

FAD/NAD(P)-binding domain-containing protein
  
Accession: EMF11330
  
Location: 1107187-1109097
  
 NCBI BlastP on this gene

EMF11330

hypothetical protein
  
Accession: EMF11331
  
Location: 1109583-1110644
  
 NCBI BlastP on this gene

EMF11331

hypothetical protein
  
Accession: EMF11332
  
Location: 1111701-1112987
  
 NCBI BlastP on this gene

EMF11332

hypothetical protein
  
Accession: EMF11333
  
Location: 1113150-1114103
  
 NCBI BlastP on this gene

EMF11333

Query: Architecture Search FASTA input

KB446560 : Pseudocercospora fijiensis CIRAD86 unplaced genomic scaffold MYCFIscaffold\_6    Total score: 2.0     Cumulative Blast bit score: 3286

Hit cluster cross-links:

Mycgr3G85918 Mycgr3T
  
Location: 0-1602

Mycgr3G85918\_Mycgr3T

Mycgr3G42010 Mycgr3T
  
Location: 1702-8569

Mycgr3G42010\_Mycgr3T

Mycgr3G29582 Mycgr3T
  
Location: 8669-8915

Mycgr3G29582\_Mycgr3T

Mycgr3G31170 Mycgr3T
  
Location: 9015-9255

Mycgr3G31170\_Mycgr3T

Mycgr3G85924 Mycgr3T
  
Location: 9355-11218

Mycgr3G85924\_Mycgr3T

Mycgr3G71676 Mycgr3T
  
Location: 11318-12494

Mycgr3G71676\_Mycgr3T

Mycgr3G11468 Mycgr3T
  
Location: 12594-13653

Mycgr3G11468\_Mycgr3T

Mycgr3G58567 Mycgr3T
  
Location: 13753-14506

Mycgr3G58567\_Mycgr3T

Mycgr3G100089 Mycgr3
  
Location: 14606-21152

Mycgr3G100089\_Mycgr3

Mycgr3G42698 Mycgr3T
  
Location: 21252-22131

Mycgr3G42698\_Mycgr3T

Mycgr3G71681 Mycgr3T
  
Location: 22231-23461

Mycgr3G71681\_Mycgr3T

Mycgr3G109328 Mycgr3
  
Location: 23561-24239

Mycgr3G109328\_Mycgr3

Mycgr3G104334 Mycgr3
  
Location: 24339-24567

Mycgr3G104334\_Mycgr3

Mycgr3G42715 Mycgr3T
  
Location: 24667-25981

Mycgr3G42715\_Mycgr3T

Mycgr3G92934 Mycgr3T
  
Location: 26081-27593

Mycgr3G92934\_Mycgr3T

Mycgr3G41969 Mycgr3T
  
Location: 27693-29328

Mycgr3G41969\_Mycgr3T

Mycgr3G80635 Mycgr3T
  
Location: 29428-29821

Mycgr3G80635\_Mycgr3T

Mycgr3G41426 Mycgr3T
  
Location: 29921-35255

Mycgr3G41426\_Mycgr3T

Mycgr3G104337 Mycgr3
  
Location: 35355-36108

Mycgr3G104337\_Mycgr3

Mycgr3G71679 Mycgr3T
  
Location: 36208-37300

Mycgr3G71679\_Mycgr3T

Mycgr3G92938 Mycgr3T
  
Location: 37400-38699

Mycgr3G92938\_Mycgr3T

Mycgr3G92941 Mycgr3T
  
Location: 38799-40734

Mycgr3G92941\_Mycgr3T

hypothetical protein
  
Accession: EME81371
  
Location: 3685179-3685695
  
 NCBI BlastP on this gene

EME81371

hypothetical protein
  
Accession: EME81372
  
Location: 3686296-3693288
  
  
**BlastP hit with Mycgr3G42010\_Mycgr3T**
  
Percentage identity: 62 %
  
BlastP bit score: 2952
  
Sequence coverage: 100 %
  
E-value: 0.0
  
  
 NCBI BlastP on this gene

EME81372

hypothetical protein
  
Accession: EME81373
  
Location: 3693465-3695053
  
  
**BlastP hit with Mycgr3G92938\_Mycgr3T**
  
Percentage identity: 48 %
  
BlastP bit score: 334
  
Sequence coverage: 83 %
  
E-value: 4e-106
  
  
 NCBI BlastP on this gene

EME81373

hypothetical protein
  
Accession: EME81374
  
Location: 3697166-3698138
  
 NCBI BlastP on this gene

EME81374

Query: Architecture Search FASTA input

CM001231 : Magnaporthe oryzae 70-15 chromosome 1    Total score: 2.0     Cumulative Blast bit score: 2996

Hit cluster cross-links:

Mycgr3G85918 Mycgr3T
  
Location: 0-1602

Mycgr3G85918\_Mycgr3T

Mycgr3G42010 Mycgr3T
  
Location: 1702-8569

Mycgr3G42010\_Mycgr3T

Mycgr3G29582 Mycgr3T
  
Location: 8669-8915

Mycgr3G29582\_Mycgr3T

Mycgr3G31170 Mycgr3T
  
Location: 9015-9255

Mycgr3G31170\_Mycgr3T

Mycgr3G85924 Mycgr3T
  
Location: 9355-11218

Mycgr3G85924\_Mycgr3T

Mycgr3G71676 Mycgr3T
  
Location: 11318-12494

Mycgr3G71676\_Mycgr3T

Mycgr3G11468 Mycgr3T
  
Location: 12594-13653

Mycgr3G11468\_Mycgr3T

Mycgr3G58567 Mycgr3T
  
Location: 13753-14506

Mycgr3G58567\_Mycgr3T

Mycgr3G100089 Mycgr3
  
Location: 14606-21152

Mycgr3G100089\_Mycgr3

Mycgr3G42698 Mycgr3T
  
Location: 21252-22131

Mycgr3G42698\_Mycgr3T

Mycgr3G71681 Mycgr3T
  
Location: 22231-23461

Mycgr3G71681\_Mycgr3T

Mycgr3G109328 Mycgr3
  
Location: 23561-24239

Mycgr3G109328\_Mycgr3

Mycgr3G104334 Mycgr3
  
Location: 24339-24567

Mycgr3G104334\_Mycgr3

Mycgr3G42715 Mycgr3T
  
Location: 24667-25981

Mycgr3G42715\_Mycgr3T

Mycgr3G92934 Mycgr3T
  
Location: 26081-27593

Mycgr3G92934\_Mycgr3T

Mycgr3G41969 Mycgr3T
  
Location: 27693-29328

Mycgr3G41969\_Mycgr3T

Mycgr3G80635 Mycgr3T
  
Location: 29428-29821

Mycgr3G80635\_Mycgr3T

Mycgr3G41426 Mycgr3T
  
Location: 29921-35255

Mycgr3G41426\_Mycgr3T

Mycgr3G104337 Mycgr3
  
Location: 35355-36108

Mycgr3G104337\_Mycgr3

Mycgr3G71679 Mycgr3T
  
Location: 36208-37300

Mycgr3G71679\_Mycgr3T

Mycgr3G92938 Mycgr3T
  
Location: 37400-38699

Mycgr3G92938\_Mycgr3T

Mycgr3G92941 Mycgr3T
  
Location: 38799-40734

Mycgr3G92941\_Mycgr3T

hypothetical protein
  
Accession: EHA58173
  
Location: 6758210-6759756
  
 NCBI BlastP on this gene

EHA58173

hypothetical protein
  
Accession: EHA58172
  
Location: 6756806-6757101
  
 NCBI BlastP on this gene

EHA58172

bilirubin oxidase
  
Accession: EHA58171
  
Location: 6753225-6755168
  
 NCBI BlastP on this gene

EHA58171

hypothetical protein
  
Accession: EHA58170
  
Location: 6751299-6752937
  
 NCBI BlastP on this gene

EHA58170

hypothetical protein
  
Accession: EHA58169
  
Location: 6750207-6750563
  
 NCBI BlastP on this gene

EHA58169

glycerate kinase
  
Accession: EHA58168
  
Location: 6748639-6750102
  
 NCBI BlastP on this gene

EHA58168

multidrug resistance protein 3
  
Accession: EHA58167
  
Location: 6743712-6748276
  
 NCBI BlastP on this gene

EHA58167

hypothetical protein
  
Accession: EHA58166
  
Location: 6741720-6742557
  
  
**BlastP hit with Mycgr3G104337\_Mycgr3**
  
Percentage identity: 41 %
  
BlastP bit score: 206
  
Sequence coverage: 100 %
  
E-value: 4e-62
  
  
 NCBI BlastP on this gene

EHA58166

fatty acid synthase S-acetyltransferase
  
Accession: EHA58165
  
Location: 6732693-6739671
  
  
**BlastP hit with Mycgr3G100089\_Mycgr3**
  
Percentage identity: 40 %
  
BlastP bit score: 1403
  
Sequence coverage: 91 %
  
E-value: 0.0
  
  
 NCBI BlastP on this gene

EHA58165

fatty acid synthase S-acetyltransferase, variant
  
Accession: EHA58164
  
Location: 6732693-6738655
  
  
**BlastP hit with Mycgr3G100089\_Mycgr3**
  
Percentage identity: 40 %
  
BlastP bit score: 1387
  
Sequence coverage: 90 %
  
E-value: 0.0
  
  
 NCBI BlastP on this gene

EHA58164

hypothetical protein
  
Accession: EHA58163
  
Location: 6730837-6731355
  
 NCBI BlastP on this gene

EHA58163

hypothetical protein
  
Accession: EHA58162
  
Location: 6728184-6729778
  
 NCBI BlastP on this gene

EHA58162

hypothetical protein
  
Accession: EHA58161
  
Location: 6727111-6728036
  
 NCBI BlastP on this gene

EHA58161

sugar transporter STL1, variant
  
Accession: EHA58159
  
Location: 6725101-6726809
  
 NCBI BlastP on this gene

EHA58159

ankyrin repeat and protein kinase domain-containing protein 1
  
Accession: EHA58158
  
Location: 6717652-6721130
  
 NCBI BlastP on this gene

EHA58158

hypothetical protein
  
Accession: EHA58157
  
Location: 6716655-6717274
  
 NCBI BlastP on this gene

EHA58157

hypothetical protein
  
Accession: EHA58156
  
Location: 6714022-6715109
  
 NCBI BlastP on this gene

EHA58156

Query: Architecture Search FASTA input

AM270278 : Aspergillus niger contig An12c0220, genomic contig.    Total score: 2.0     Cumulative Blast bit score: 2747

Hit cluster cross-links:

Mycgr3G85918 Mycgr3T
  
Location: 0-1602

Mycgr3G85918\_Mycgr3T

Mycgr3G42010 Mycgr3T
  
Location: 1702-8569

Mycgr3G42010\_Mycgr3T

Mycgr3G29582 Mycgr3T
  
Location: 8669-8915

Mycgr3G29582\_Mycgr3T

Mycgr3G31170 Mycgr3T
  
Location: 9015-9255

Mycgr3G31170\_Mycgr3T

Mycgr3G85924 Mycgr3T
  
Location: 9355-11218

Mycgr3G85924\_Mycgr3T

Mycgr3G71676 Mycgr3T
  
Location: 11318-12494

Mycgr3G71676\_Mycgr3T

Mycgr3G11468 Mycgr3T
  
Location: 12594-13653

Mycgr3G11468\_Mycgr3T

Mycgr3G58567 Mycgr3T
  
Location: 13753-14506

Mycgr3G58567\_Mycgr3T

Mycgr3G100089 Mycgr3
  
Location: 14606-21152

Mycgr3G100089\_Mycgr3

Mycgr3G42698 Mycgr3T
  
Location: 21252-22131

Mycgr3G42698\_Mycgr3T

Mycgr3G71681 Mycgr3T
  
Location: 22231-23461

Mycgr3G71681\_Mycgr3T

Mycgr3G109328 Mycgr3
  
Location: 23561-24239

Mycgr3G109328\_Mycgr3

Mycgr3G104334 Mycgr3
  
Location: 24339-24567

Mycgr3G104334\_Mycgr3

Mycgr3G42715 Mycgr3T
  
Location: 24667-25981

Mycgr3G42715\_Mycgr3T

Mycgr3G92934 Mycgr3T
  
Location: 26081-27593

Mycgr3G92934\_Mycgr3T

Mycgr3G41969 Mycgr3T
  
Location: 27693-29328

Mycgr3G41969\_Mycgr3T

Mycgr3G80635 Mycgr3T
  
Location: 29428-29821

Mycgr3G80635\_Mycgr3T

Mycgr3G41426 Mycgr3T
  
Location: 29921-35255

Mycgr3G41426\_Mycgr3T

Mycgr3G104337 Mycgr3
  
Location: 35355-36108

Mycgr3G104337\_Mycgr3

Mycgr3G71679 Mycgr3T
  
Location: 36208-37300

Mycgr3G71679\_Mycgr3T

Mycgr3G92938 Mycgr3T
  
Location: 37400-38699

Mycgr3G92938\_Mycgr3T

Mycgr3G92941 Mycgr3T
  
Location: 38799-40734

Mycgr3G92941\_Mycgr3T

not annotated
  
Accession: CAK46342
  
Location: 52537-54246
  
 NCBI BlastP on this gene

An12g07140

not annotated
  
Accession: CAK46341
  
Location: 50920-51987
  
 NCBI BlastP on this gene

An12g07130

unnamed
  
Accession: CAK46340
  
Location: 48472-50548
  
 NCBI BlastP on this gene

An12g07120

not annotated
  
Accession: CAK46339
  
Location: 41567-43672
  
 NCBI BlastP on this gene

An12g07110

not annotated
  
Accession: CAK46338
  
Location: 38626-39761
  
 NCBI BlastP on this gene

An12g07100

not annotated
  
Accession: CAK46337
  
Location: 35651-37079
  
 NCBI BlastP on this gene

An12g07090

not annotated
  
Accession: CAK46336
  
Location: 28213-35378
  
  
**BlastP hit with Mycgr3G100089\_Mycgr3**
  
Percentage identity: 53 %
  
BlastP bit score: 2295
  
Sequence coverage: 100 %
  
E-value: 0.0
  
  
 NCBI BlastP on this gene

An12g07070

not annotated
  
Accession: CAK46335
  
Location: 26598-27527
  
  
**BlastP hit with Mycgr3G104337\_Mycgr3**
  
Percentage identity: 46 %
  
BlastP bit score: 230
  
Sequence coverage: 99 %
  
E-value: 1e-71
  
  
 NCBI BlastP on this gene

An12g07060

unnamed
  
Accession: CAK46334
  
Location: 25361-26347
  
  
**BlastP hit with Mycgr3G104337\_Mycgr3**
  
Percentage identity: 45 %
  
BlastP bit score: 222
  
Sequence coverage: 98 %
  
E-value: 2e-68
  
  
 NCBI BlastP on this gene

An12g07050

not annotated
  
Accession: CAK46333
  
Location: 24086-24832
  
 NCBI BlastP on this gene

An12g07040

unnamed
  
Accession: CAK46332
  
Location: 22216-23211
  
 NCBI BlastP on this gene

An12g07030

not annotated
  
Accession: CAK46331
  
Location: 20407-21672
  
 NCBI BlastP on this gene

An12g07020

not annotated
  
Accession: CAK46330
  
Location: 18046-19026
  
 NCBI BlastP on this gene

An12g07000

not annotated
  
Accession: CAK46329
  
Location: 17603-17871
  
 NCBI BlastP on this gene

An12g06990

not annotated
  
Accession: CAK46328
  
Location: 15996-16549
  
 NCBI BlastP on this gene

An12g06980

not annotated
  
Accession: CAK46327
  
Location: 13980-14777
  
 NCBI BlastP on this gene

An12g06970

not annotated
  
Accession: CAK46326
  
Location: 12286-13589
  
 NCBI BlastP on this gene

An12g06960

unnamed
  
Accession: CAK46325
  
Location: 10370-11992
  
 NCBI BlastP on this gene

An12g06940

extracellular alpha-amylase
  
Accession: CAK46324
  
Location: 7374-9415
  
 NCBI BlastP on this gene

amyA

Query: Architecture Search FASTA input

DS995904 : Penicillium marneffei ATCC 18224 scf\_1105668340738 genomic scaffold    Total score: 2.0     Cumulative Blast bit score: 2721

Hit cluster cross-links:

Mycgr3G85918 Mycgr3T
  
Location: 0-1602

Mycgr3G85918\_Mycgr3T

Mycgr3G42010 Mycgr3T
  
Location: 1702-8569

Mycgr3G42010\_Mycgr3T

Mycgr3G29582 Mycgr3T
  
Location: 8669-8915

Mycgr3G29582\_Mycgr3T

Mycgr3G31170 Mycgr3T
  
Location: 9015-9255

Mycgr3G31170\_Mycgr3T

Mycgr3G85924 Mycgr3T
  
Location: 9355-11218

Mycgr3G85924\_Mycgr3T

Mycgr3G71676 Mycgr3T
  
Location: 11318-12494

Mycgr3G71676\_Mycgr3T

Mycgr3G11468 Mycgr3T
  
Location: 12594-13653

Mycgr3G11468\_Mycgr3T

Mycgr3G58567 Mycgr3T
  
Location: 13753-14506

Mycgr3G58567\_Mycgr3T

Mycgr3G100089 Mycgr3
  
Location: 14606-21152

Mycgr3G100089\_Mycgr3

Mycgr3G42698 Mycgr3T
  
Location: 21252-22131

Mycgr3G42698\_Mycgr3T

Mycgr3G71681 Mycgr3T
  
Location: 22231-23461

Mycgr3G71681\_Mycgr3T

Mycgr3G109328 Mycgr3
  
Location: 23561-24239

Mycgr3G109328\_Mycgr3

Mycgr3G104334 Mycgr3
  
Location: 24339-24567

Mycgr3G104334\_Mycgr3

Mycgr3G42715 Mycgr3T
  
Location: 24667-25981

Mycgr3G42715\_Mycgr3T

Mycgr3G92934 Mycgr3T
  
Location: 26081-27593

Mycgr3G92934\_Mycgr3T

Mycgr3G41969 Mycgr3T
  
Location: 27693-29328

Mycgr3G41969\_Mycgr3T

Mycgr3G80635 Mycgr3T
  
Location: 29428-29821

Mycgr3G80635\_Mycgr3T

Mycgr3G41426 Mycgr3T
  
Location: 29921-35255

Mycgr3G41426\_Mycgr3T

Mycgr3G104337 Mycgr3
  
Location: 35355-36108

Mycgr3G104337\_Mycgr3

Mycgr3G71679 Mycgr3T
  
Location: 36208-37300

Mycgr3G71679\_Mycgr3T

Mycgr3G92938 Mycgr3T
  
Location: 37400-38699

Mycgr3G92938\_Mycgr3T

Mycgr3G92941 Mycgr3T
  
Location: 38799-40734

Mycgr3G92941\_Mycgr3T

dihydrodipicolinate synthase, putative
  
Accession: EEA20038
  
Location: 68859-70318
  
 NCBI BlastP on this gene

EEA20038

hypothetical protein
  
Accession: EEA20039
  
Location: 71829-72531
  
 NCBI BlastP on this gene

EEA20039

conserved hypothetical protein
  
Accession: EEA20040
  
Location: 73531-74058
  
 NCBI BlastP on this gene

EEA20040

alpha-ketoglutarate-dependent 2,4-dichlorophenoxyacetate dioxygenase, putative
  
Accession: EEA20041
  
Location: 74511-75884
  
 NCBI BlastP on this gene

EEA20041

aminohydrolase, putative
  
Accession: EEA20042
  
Location: 76567-81684
  
 NCBI BlastP on this gene

EEA20042

oxidoreductase, putative
  
Accession: EEA20043
  
Location: 82286-85704
  
 NCBI BlastP on this gene

EEA20043

DUF341 family oxidoreductase, putative
  
Accession: EEA20044
  
Location: 86437-87443
  
  
**BlastP hit with Mycgr3G104337\_Mycgr3**
  
Percentage identity: 58 %
  
BlastP bit score: 335
  
Sequence coverage: 98 %
  
E-value: 1e-112
  
  
 NCBI BlastP on this gene

EEA20044

polyketide synthase, putative
  
Accession: EEA20045
  
Location: 88781-95890
  
  
**BlastP hit with Mycgr3G100089\_Mycgr3**
  
Percentage identity: 53 %
  
BlastP bit score: 2386
  
Sequence coverage: 102 %
  
E-value: 0.0
  
  
 NCBI BlastP on this gene

EEA20045

conserved hypothetical protein
  
Accession: EEA20046
  
Location: 96394-96873
  
 NCBI BlastP on this gene

EEA20046

hypothetical protein
  
Accession: EEA20047
  
Location: 98821-99528
  
 NCBI BlastP on this gene

EEA20047

hypothetical protein
  
Accession: EEA20048
  
Location: 101134-102031
  
 NCBI BlastP on this gene

EEA20048

biphenyl-2,3-diol 1,2-dioxygenase, putative
  
Accession: EEA20049
  
Location: 102367-103014
  
 NCBI BlastP on this gene

EEA20049

conserved hypothetical protein
  
Accession: EEA20050
  
Location: 103701-104284
  
 NCBI BlastP on this gene

EEA20050

phenol 2-monooxygenase, putative
  
Accession: EEA20051
  
Location: 105201-107270
  
 NCBI BlastP on this gene

EEA20051

hypothetical protein
  
Accession: EEA20052
  
Location: 107395-108816
  
 NCBI BlastP on this gene

EEA20052

amino acid permease, putative
  
Accession: EEA20053
  
Location: 112068-113783
  
 NCBI BlastP on this gene

EEA20053

Query: Architecture Search FASTA input

AM920436 : Penicillium chrysogenum Wisconsin 54-1255 complete genome, contig Pc00c21.    Total score: 2.0     Cumulative Blast bit score: 2708

Hit cluster cross-links:

Mycgr3G85918 Mycgr3T
  
Location: 0-1602

Mycgr3G85918\_Mycgr3T

Mycgr3G42010 Mycgr3T
  
Location: 1702-8569

Mycgr3G42010\_Mycgr3T

Mycgr3G29582 Mycgr3T
  
Location: 8669-8915

Mycgr3G29582\_Mycgr3T

Mycgr3G31170 Mycgr3T
  
Location: 9015-9255

Mycgr3G31170\_Mycgr3T

Mycgr3G85924 Mycgr3T
  
Location: 9355-11218

Mycgr3G85924\_Mycgr3T

Mycgr3G71676 Mycgr3T
  
Location: 11318-12494

Mycgr3G71676\_Mycgr3T

Mycgr3G11468 Mycgr3T
  
Location: 12594-13653

Mycgr3G11468\_Mycgr3T

Mycgr3G58567 Mycgr3T
  
Location: 13753-14506

Mycgr3G58567\_Mycgr3T

Mycgr3G100089 Mycgr3
  
Location: 14606-21152

Mycgr3G100089\_Mycgr3

Mycgr3G42698 Mycgr3T
  
Location: 21252-22131

Mycgr3G42698\_Mycgr3T

Mycgr3G71681 Mycgr3T
  
Location: 22231-23461

Mycgr3G71681\_Mycgr3T

Mycgr3G109328 Mycgr3
  
Location: 23561-24239

Mycgr3G109328\_Mycgr3

Mycgr3G104334 Mycgr3
  
Location: 24339-24567

Mycgr3G104334\_Mycgr3

Mycgr3G42715 Mycgr3T
  
Location: 24667-25981

Mycgr3G42715\_Mycgr3T

Mycgr3G92934 Mycgr3T
  
Location: 26081-27593

Mycgr3G92934\_Mycgr3T

Mycgr3G41969 Mycgr3T
  
Location: 27693-29328

Mycgr3G41969\_Mycgr3T

Mycgr3G80635 Mycgr3T
  
Location: 29428-29821

Mycgr3G80635\_Mycgr3T

Mycgr3G41426 Mycgr3T
  
Location: 29921-35255

Mycgr3G41426\_Mycgr3T

Mycgr3G104337 Mycgr3
  
Location: 35355-36108

Mycgr3G104337\_Mycgr3

Mycgr3G71679 Mycgr3T
  
Location: 36208-37300

Mycgr3G71679\_Mycgr3T

Mycgr3G92938 Mycgr3T
  
Location: 37400-38699

Mycgr3G92938\_Mycgr3T

Mycgr3G92941 Mycgr3T
  
Location: 38799-40734

Mycgr3G92941\_Mycgr3T

unnamed
  
Accession: CAP95373
  
Location: 1122985-1123763
  
 NCBI BlastP on this gene

Pc21g04760

unnamed
  
Accession: CAP95374
  
Location: 1124393-1125404
  
 NCBI BlastP on this gene

Pc21g04770

hypothetical protein
  
Accession: CAP95375
  
Location: 1125716-1126669
  
 NCBI BlastP on this gene

Pc21g04780

not annotated
  
Accession: CAP95376
  
Location: 1127334-1129150
  
 NCBI BlastP on this gene

Pc21g04790

unnamed
  
Accession: CAP95377
  
Location: 1130647-1132685
  
 NCBI BlastP on this gene

Pc21g04800

not annotated
  
Accession: CAP95378
  
Location: 1133717-1135038
  
 NCBI BlastP on this gene

Pc21g04810

not annotated
  
Accession: CAP95379
  
Location: 1135671-1135961
  
 NCBI BlastP on this gene

Pc21g04820

not annotated
  
Accession: CAP95380
  
Location: 1137439-1138998
  
 NCBI BlastP on this gene

Pc21g04830

not annotated
  
Accession: CAP95381
  
Location: 1139872-1146768
  
  
**BlastP hit with Mycgr3G100089\_Mycgr3**
  
Percentage identity: 54 %
  
BlastP bit score: 2414
  
Sequence coverage: 100 %
  
E-value: 0.0
  
  
 NCBI BlastP on this gene

Pc21g04840

not annotated
  
Accession: CAP95382
  
Location: 1147328-1148304
  
  
**BlastP hit with Mycgr3G104337\_Mycgr3**
  
Percentage identity: 55 %
  
BlastP bit score: 294
  
Sequence coverage: 97 %
  
E-value: 2e-96
  
  
 NCBI BlastP on this gene

Pc21g04850

hypothetical protein
  
Accession: CAP95383
  
Location: 1150094-1150636
  
 NCBI BlastP on this gene

Pc21g04860

unnamed
  
Accession: CAP95384
  
Location: 1150831-1151460
  
 NCBI BlastP on this gene

Pc21g04870

not annotated
  
Accession: CAP95385
  
Location: 1151931-1153377
  
 NCBI BlastP on this gene

Pc21g04880

unnamed
  
Accession: CAP95386
  
Location: 1153926-1154631
  
 NCBI BlastP on this gene

Pc21g04890

not annotated
  
Accession: CAP95387
  
Location: 1155624-1156319
  
 NCBI BlastP on this gene

Pc21g04900

not annotated
  
Accession: CAP95388
  
Location: 1156536-1157614
  
 NCBI BlastP on this gene

Pc21g04910

not annotated
  
Accession: CAP95389
  
Location: 1158147-1159074
  
 NCBI BlastP on this gene

Pc21g04920

unnamed
  
Accession: CAP95390
  
Location: 1159502-1162477
  
 NCBI BlastP on this gene

Pc21g04930

not annotated
  
Accession: CAP95391
  
Location: 1163044-1164221
  
 NCBI BlastP on this gene

Pc21g04940

not annotated
  
Accession: CAP95392
  
Location: 1164533-1165615
  
 NCBI BlastP on this gene

Pc21g04950

unnamed
  
Accession: CAP95393
  
Location: 1165822-1166396
  
 NCBI BlastP on this gene

Pc21g04960

Query: Architecture Search FASTA input

EQ962652 : Talaromyces stipitatus ATCC 10500 scf\_1105507295523 genomic scaffold    Total score: 2.0     Cumulative Blast bit score: 2697

Hit cluster cross-links:

Mycgr3G85918 Mycgr3T
  
Location: 0-1602

Mycgr3G85918\_Mycgr3T

Mycgr3G42010 Mycgr3T
  
Location: 1702-8569

Mycgr3G42010\_Mycgr3T

Mycgr3G29582 Mycgr3T
  
Location: 8669-8915

Mycgr3G29582\_Mycgr3T

Mycgr3G31170 Mycgr3T
  
Location: 9015-9255

Mycgr3G31170\_Mycgr3T

Mycgr3G85924 Mycgr3T
  
Location: 9355-11218

Mycgr3G85924\_Mycgr3T

Mycgr3G71676 Mycgr3T
  
Location: 11318-12494

Mycgr3G71676\_Mycgr3T

Mycgr3G11468 Mycgr3T
  
Location: 12594-13653

Mycgr3G11468\_Mycgr3T

Mycgr3G58567 Mycgr3T
  
Location: 13753-14506

Mycgr3G58567\_Mycgr3T

Mycgr3G100089 Mycgr3
  
Location: 14606-21152

Mycgr3G100089\_Mycgr3

Mycgr3G42698 Mycgr3T
  
Location: 21252-22131

Mycgr3G42698\_Mycgr3T

Mycgr3G71681 Mycgr3T
  
Location: 22231-23461

Mycgr3G71681\_Mycgr3T

Mycgr3G109328 Mycgr3
  
Location: 23561-24239

Mycgr3G109328\_Mycgr3

Mycgr3G104334 Mycgr3
  
Location: 24339-24567

Mycgr3G104334\_Mycgr3

Mycgr3G42715 Mycgr3T
  
Location: 24667-25981

Mycgr3G42715\_Mycgr3T

Mycgr3G92934 Mycgr3T
  
Location: 26081-27593

Mycgr3G92934\_Mycgr3T

Mycgr3G41969 Mycgr3T
  
Location: 27693-29328

Mycgr3G41969\_Mycgr3T

Mycgr3G80635 Mycgr3T
  
Location: 29428-29821

Mycgr3G80635\_Mycgr3T

Mycgr3G41426 Mycgr3T
  
Location: 29921-35255

Mycgr3G41426\_Mycgr3T

Mycgr3G104337 Mycgr3
  
Location: 35355-36108

Mycgr3G104337\_Mycgr3

Mycgr3G71679 Mycgr3T
  
Location: 36208-37300

Mycgr3G71679\_Mycgr3T

Mycgr3G92938 Mycgr3T
  
Location: 37400-38699

Mycgr3G92938\_Mycgr3T

Mycgr3G92941 Mycgr3T
  
Location: 38799-40734

Mycgr3G92941\_Mycgr3T

amino acid permease, putative
  
Accession: EED24605
  
Location: 5458788-5460143
  
 NCBI BlastP on this gene

EED24605

hypothetical protein
  
Accession: EED24606
  
Location: 5463226-5464665
  
 NCBI BlastP on this gene

EED24606

phenol 2-monooxygenase, putative
  
Accession: EED24607
  
Location: 5464957-5467118
  
 NCBI BlastP on this gene

EED24607

hypothetical protein
  
Accession: EED24608
  
Location: 5467504-5468418
  
 NCBI BlastP on this gene

EED24608

biphenyl-2,3-diol 1,2-dioxygenase, putative
  
Accession: EED24609
  
Location: 5468687-5469467
  
 NCBI BlastP on this gene

EED24609

hypothetical protein
  
Accession: EED24610
  
Location: 5470989-5471558
  
 NCBI BlastP on this gene

EED24610

ferulic acid esterase (FaeA), putative
  
Accession: EED24611
  
Location: 5473398-5474352
  
 NCBI BlastP on this gene

EED24611

conserved hypothetical protein
  
Accession: EED24612
  
Location: 5474887-5475377
  
 NCBI BlastP on this gene

EED24612

conserved hypothetical protein
  
Accession: EED24613
  
Location: 5476633-5477571
  
 NCBI BlastP on this gene

EED24613

polyketide synthase, putative
  
Accession: EED24614
  
Location: 5478431-5485502
  
  
**BlastP hit with Mycgr3G100089\_Mycgr3**
  
Percentage identity: 54 %
  
BlastP bit score: 2408
  
Sequence coverage: 102 %
  
E-value: 0.0
  
  
 NCBI BlastP on this gene

EED24614

conserved hypothetical protein
  
Accession: EED24615
  
Location: 5486576-5487446
  
  
**BlastP hit with Mycgr3G104337\_Mycgr3**
  
Percentage identity: 55 %
  
BlastP bit score: 289
  
Sequence coverage: 92 %
  
E-value: 8e-95
  
  
 NCBI BlastP on this gene

EED24615

acetylxylan esterase precursor, putative
  
Accession: EED24616
  
Location: 5489113-5489980
  
 NCBI BlastP on this gene

EED24616

oxidoreductase, putative
  
Accession: EED24617
  
Location: 5494465-5495801
  
 NCBI BlastP on this gene

EED24617

transposable element tc1 transposase, putative
  
Accession: EED24618
  
Location: 5497026-5498095
  
 NCBI BlastP on this gene

EED24618

reverse transcriptase, putative
  
Accession: EED24619
  
Location: 5499643-5503257
  
 NCBI BlastP on this gene

EED24619

conserved hypothetical protein
  
Accession: EED24620
  
Location: 5503512-5505242
  
 NCBI BlastP on this gene

EED24620

Query: Architecture Search FASTA input

AHHD01000525 : Macrophomina phaseolina MS6    Total score: 2.0     Cumulative Blast bit score: 2617

Hit cluster cross-links:

Mycgr3G85918 Mycgr3T
  
Location: 0-1602

Mycgr3G85918\_Mycgr3T

Mycgr3G42010 Mycgr3T
  
Location: 1702-8569

Mycgr3G42010\_Mycgr3T

Mycgr3G29582 Mycgr3T
  
Location: 8669-8915

Mycgr3G29582\_Mycgr3T

Mycgr3G31170 Mycgr3T
  
Location: 9015-9255

Mycgr3G31170\_Mycgr3T

Mycgr3G85924 Mycgr3T
  
Location: 9355-11218

Mycgr3G85924\_Mycgr3T

Mycgr3G71676 Mycgr3T
  
Location: 11318-12494

Mycgr3G71676\_Mycgr3T

Mycgr3G11468 Mycgr3T
  
Location: 12594-13653

Mycgr3G11468\_Mycgr3T

Mycgr3G58567 Mycgr3T
  
Location: 13753-14506

Mycgr3G58567\_Mycgr3T

Mycgr3G100089 Mycgr3
  
Location: 14606-21152

Mycgr3G100089\_Mycgr3

Mycgr3G42698 Mycgr3T
  
Location: 21252-22131

Mycgr3G42698\_Mycgr3T

Mycgr3G71681 Mycgr3T
  
Location: 22231-23461

Mycgr3G71681\_Mycgr3T

Mycgr3G109328 Mycgr3
  
Location: 23561-24239

Mycgr3G109328\_Mycgr3

Mycgr3G104334 Mycgr3
  
Location: 24339-24567

Mycgr3G104334\_Mycgr3

Mycgr3G42715 Mycgr3T
  
Location: 24667-25981

Mycgr3G42715\_Mycgr3T

Mycgr3G92934 Mycgr3T
  
Location: 26081-27593

Mycgr3G92934\_Mycgr3T

Mycgr3G41969 Mycgr3T
  
Location: 27693-29328

Mycgr3G41969\_Mycgr3T

Mycgr3G80635 Mycgr3T
  
Location: 29428-29821

Mycgr3G80635\_Mycgr3T

Mycgr3G41426 Mycgr3T
  
Location: 29921-35255

Mycgr3G41426\_Mycgr3T

Mycgr3G104337 Mycgr3
  
Location: 35355-36108

Mycgr3G104337\_Mycgr3

Mycgr3G71679 Mycgr3T
  
Location: 36208-37300

Mycgr3G71679\_Mycgr3T

Mycgr3G92938 Mycgr3T
  
Location: 37400-38699

Mycgr3G92938\_Mycgr3T

Mycgr3G92941 Mycgr3T
  
Location: 38799-40734

Mycgr3G92941\_Mycgr3T

hypothetical protein
  
Accession: EKG10217
  
Location: 10348-12016
  
 NCBI BlastP on this gene

EKG10217

Heat shock protein Hsp70
  
Accession: EKG10218
  
Location: 12772-14903
  
 NCBI BlastP on this gene

EKG10218

hypothetical protein
  
Accession: EKG10219
  
Location: 16210-17112
  
 NCBI BlastP on this gene

EKG10219

hypothetical protein
  
Accession: EKG10220
  
Location: 18203-19099
  
 NCBI BlastP on this gene

EKG10220

Carboxylesterase type B
  
Accession: EKG10221
  
Location: 19947-20786
  
 NCBI BlastP on this gene

EKG10221

hypothetical protein
  
Accession: EKG10222
  
Location: 25421-25860
  
 NCBI BlastP on this gene

EKG10222

CbxX/CfqX
  
Accession: EKG10223
  
Location: 27205-34929
  
  
**BlastP hit with Mycgr3G42010\_Mycgr3T**
  
Percentage identity: 53 %
  
BlastP bit score: 2334
  
Sequence coverage: 97 %
  
E-value: 0.0
  
  
 NCBI BlastP on this gene

EKG10223

hypothetical protein
  
Accession: EKG10224
  
Location: 35757-36827
  
  
**BlastP hit with Mycgr3G92938\_Mycgr3T**
  
Percentage identity: 43 %
  
BlastP bit score: 283
  
Sequence coverage: 78 %
  
E-value: 4e-88
  
  
 NCBI BlastP on this gene

EKG10224

hypothetical protein
  
Accession: EKG10225
  
Location: 37271-39836
  
 NCBI BlastP on this gene

EKG10225

hypothetical protein
  
Accession: EKG10226
  
Location: 40850-42252
  
 NCBI BlastP on this gene

EKG10226

hypothetical protein
  
Accession: EKG10227
  
Location: 42989-43910
  
 NCBI BlastP on this gene

EKG10227

hypothetical protein
  
Accession: EKG10228
  
Location: 44495-45007
  
 NCBI BlastP on this gene

EKG10228

Short-chain dehydrogenase/reductase SDR
  
Accession: EKG10229
  
Location: 46770-49935
  
 NCBI BlastP on this gene

EKG10229

hypothetical protein
  
Accession: EKG10230
  
Location: 50378-51657
  
 NCBI BlastP on this gene

EKG10230

Query: Architecture Search FASTA input

ACJE01000006 : Aspergillus niger ATCC 1015    Total score: 2.0     Cumulative Blast bit score: 2589

Hit cluster cross-links:

Mycgr3G85918 Mycgr3T
  
Location: 0-1602

Mycgr3G85918\_Mycgr3T

Mycgr3G42010 Mycgr3T
  
Location: 1702-8569

Mycgr3G42010\_Mycgr3T

Mycgr3G29582 Mycgr3T
  
Location: 8669-8915

Mycgr3G29582\_Mycgr3T

Mycgr3G31170 Mycgr3T
  
Location: 9015-9255

Mycgr3G31170\_Mycgr3T

Mycgr3G85924 Mycgr3T
  
Location: 9355-11218

Mycgr3G85924\_Mycgr3T

Mycgr3G71676 Mycgr3T
  
Location: 11318-12494

Mycgr3G71676\_Mycgr3T

Mycgr3G11468 Mycgr3T
  
Location: 12594-13653

Mycgr3G11468\_Mycgr3T

Mycgr3G58567 Mycgr3T
  
Location: 13753-14506

Mycgr3G58567\_Mycgr3T

Mycgr3G100089 Mycgr3
  
Location: 14606-21152

Mycgr3G100089\_Mycgr3

Mycgr3G42698 Mycgr3T
  
Location: 21252-22131

Mycgr3G42698\_Mycgr3T

Mycgr3G71681 Mycgr3T
  
Location: 22231-23461

Mycgr3G71681\_Mycgr3T

Mycgr3G109328 Mycgr3
  
Location: 23561-24239

Mycgr3G109328\_Mycgr3

Mycgr3G104334 Mycgr3
  
Location: 24339-24567

Mycgr3G104334\_Mycgr3

Mycgr3G42715 Mycgr3T
  
Location: 24667-25981

Mycgr3G42715\_Mycgr3T

Mycgr3G92934 Mycgr3T
  
Location: 26081-27593

Mycgr3G92934\_Mycgr3T

Mycgr3G41969 Mycgr3T
  
Location: 27693-29328

Mycgr3G41969\_Mycgr3T

Mycgr3G80635 Mycgr3T
  
Location: 29428-29821

Mycgr3G80635\_Mycgr3T

Mycgr3G41426 Mycgr3T
  
Location: 29921-35255

Mycgr3G41426\_Mycgr3T

Mycgr3G104337 Mycgr3
  
Location: 35355-36108

Mycgr3G104337\_Mycgr3

Mycgr3G71679 Mycgr3T
  
Location: 36208-37300

Mycgr3G71679\_Mycgr3T

Mycgr3G92938 Mycgr3T
  
Location: 37400-38699

Mycgr3G92938\_Mycgr3T

Mycgr3G92941 Mycgr3T
  
Location: 38799-40734

Mycgr3G92941\_Mycgr3T

hypothetical protein
  
Accession: EHA25429
  
Location: 1090147-1091856
  
 NCBI BlastP on this gene

EHA25429

hypothetical protein
  
Accession: EHA25428
  
Location: 1088530-1089597
  
 NCBI BlastP on this gene

EHA25428

hypothetical protein
  
Accession: EHA25427
  
Location: 1086130-1088158
  
 NCBI BlastP on this gene

EHA25427

hypothetical protein
  
Accession: EHA25426
  
Location: 1079929-1081281
  
 NCBI BlastP on this gene

EHA25426

hypothetical protein
  
Accession: EHA25425
  
Location: 1078663-1079289
  
 NCBI BlastP on this gene

EHA25425

hypothetical protein
  
Accession: EHA25424
  
Location: 1076169-1077370
  
 NCBI BlastP on this gene

EHA25424

hypothetical protein
  
Accession: EHA25423
  
Location: 1073194-1074622
  
 NCBI BlastP on this gene

EHA25423

hypothetical protein
  
Accession: EHA25422
  
Location: 1065786-1072569
  
  
**BlastP hit with Mycgr3G100089\_Mycgr3**
  
Percentage identity: 53 %
  
BlastP bit score: 2290
  
Sequence coverage: 100 %
  
E-value: 0.0
  
  
 NCBI BlastP on this gene

EHA25422

hypothetical protein
  
Accession: EHA25421
  
Location: 1064326-1065070
  
  
**BlastP hit with Mycgr3G104337\_Mycgr3**
  
Percentage identity: 43 %
  
BlastP bit score: 167
  
Sequence coverage: 88 %
  
E-value: 4e-47
  
  
 NCBI BlastP on this gene

EHA25421

hypothetical protein
  
Accession: EHA25420
  
Location: 1063115-1063890
  
  
**BlastP hit with Mycgr3G104337\_Mycgr3**
  
Percentage identity: 36 %
  
BlastP bit score: 132
  
Sequence coverage: 78 %
  
E-value: 2e-34
  
  
 NCBI BlastP on this gene

EHA25420

hypothetical protein
  
Accession: EHA25419
  
Location: 1060276-1060824
  
 NCBI BlastP on this gene

EHA25419

hypothetical protein
  
Accession: EHA25418
  
Location: 1058089-1059132
  
 NCBI BlastP on this gene

EHA25418

hypothetical protein
  
Accession: EHA25417
  
Location: 1053684-1056259
  
 NCBI BlastP on this gene

EHA25417

hypothetical protein
  
Accession: EHA25416
  
Location: 1051631-1053086
  
 NCBI BlastP on this gene

EHA25416

hypothetical protein
  
Accession: EHA25415
  
Location: 1049630-1050883
  
 NCBI BlastP on this gene

EHA25415

hypothetical protein
  
Accession: EHA25414
  
Location: 1047420-1048829
  
 NCBI BlastP on this gene

EHA25414

hypothetical protein
  
Accession: EHA25413
  
Location: 1045450-1047167
  
 NCBI BlastP on this gene

EHA25413

Query: Architecture Search FASTA input

DF126460 : Aspergillus kawachii IFO 4308 DNA, contig: scaffold00014    Total score: 2.0     Cumulative Blast bit score: 2549

Hit cluster cross-links:

Mycgr3G85918 Mycgr3T
  
Location: 0-1602

Mycgr3G85918\_Mycgr3T

Mycgr3G42010 Mycgr3T
  
Location: 1702-8569

Mycgr3G42010\_Mycgr3T

Mycgr3G29582 Mycgr3T
  
Location: 8669-8915

Mycgr3G29582\_Mycgr3T

Mycgr3G31170 Mycgr3T
  
Location: 9015-9255

Mycgr3G31170\_Mycgr3T

Mycgr3G85924 Mycgr3T
  
Location: 9355-11218

Mycgr3G85924\_Mycgr3T

Mycgr3G71676 Mycgr3T
  
Location: 11318-12494

Mycgr3G71676\_Mycgr3T

Mycgr3G11468 Mycgr3T
  
Location: 12594-13653

Mycgr3G11468\_Mycgr3T

Mycgr3G58567 Mycgr3T
  
Location: 13753-14506

Mycgr3G58567\_Mycgr3T

Mycgr3G100089 Mycgr3
  
Location: 14606-21152

Mycgr3G100089\_Mycgr3

Mycgr3G42698 Mycgr3T
  
Location: 21252-22131

Mycgr3G42698\_Mycgr3T

Mycgr3G71681 Mycgr3T
  
Location: 22231-23461

Mycgr3G71681\_Mycgr3T

Mycgr3G109328 Mycgr3
  
Location: 23561-24239

Mycgr3G109328\_Mycgr3

Mycgr3G104334 Mycgr3
  
Location: 24339-24567

Mycgr3G104334\_Mycgr3

Mycgr3G42715 Mycgr3T
  
Location: 24667-25981

Mycgr3G42715\_Mycgr3T

Mycgr3G92934 Mycgr3T
  
Location: 26081-27593

Mycgr3G92934\_Mycgr3T

Mycgr3G41969 Mycgr3T
  
Location: 27693-29328

Mycgr3G41969\_Mycgr3T

Mycgr3G80635 Mycgr3T
  
Location: 29428-29821

Mycgr3G80635\_Mycgr3T

Mycgr3G41426 Mycgr3T
  
Location: 29921-35255

Mycgr3G41426\_Mycgr3T

Mycgr3G104337 Mycgr3
  
Location: 35355-36108

Mycgr3G104337\_Mycgr3

Mycgr3G71679 Mycgr3T
  
Location: 36208-37300

Mycgr3G71679\_Mycgr3T

Mycgr3G92938 Mycgr3T
  
Location: 37400-38699

Mycgr3G92938\_Mycgr3T

Mycgr3G92941 Mycgr3T
  
Location: 38799-40734

Mycgr3G92941\_Mycgr3T

alcohol dehydrogenase
  
Accession: GAA87592
  
Location: 2873-4121
  
 NCBI BlastP on this gene

GAA87592

similar to DUF895 domain membrane protein
  
Accession: GAA87593
  
Location: 5638-7127
  
 NCBI BlastP on this gene

GAA87593

hypothetical protein
  
Accession: GAA87594
  
Location: 7720-10296
  
 NCBI BlastP on this gene

GAA87594

alpha 1,6 mannosyltransferase
  
Accession: GAA87595
  
Location: 11913-13034
  
 NCBI BlastP on this gene

GAA87595

similar to An12g07030
  
Accession: GAA87596
  
Location: 14171-14724
  
 NCBI BlastP on this gene

GAA87596

DUF341 family oxidoreductase
  
Accession: GAA87597
  
Location: 16465-18668
  
  
**BlastP hit with Mycgr3G104337\_Mycgr3**
  
Percentage identity: 52 %
  
BlastP bit score: 251
  
Sequence coverage: 96 %
  
E-value: 9e-77
  
  
 NCBI BlastP on this gene

GAA87597

polyketide synthase
  
Accession: GAA87598
  
Location: 19394-26179
  
  
**BlastP hit with Mycgr3G100089\_Mycgr3**
  
Percentage identity: 53 %
  
BlastP bit score: 2298
  
Sequence coverage: 101 %
  
E-value: 0.0
  
  
 NCBI BlastP on this gene

GAA87598

FAD binding domain protein
  
Accession: GAA87599
  
Location: 26985-28611
  
 NCBI BlastP on this gene

GAA87599

similar to An12g07100
  
Accession: GAA87600
  
Location: 30088-31202
  
 NCBI BlastP on this gene

GAA87600

hypothetical protein
  
Accession: GAA87601
  
Location: 32212-32838
  
 NCBI BlastP on this gene

GAA87601

salicylate synthetase
  
Accession: GAA87602
  
Location: 33317-34669
  
 NCBI BlastP on this gene

GAA87602

cytochrome P450
  
Accession: GAA87603
  
Location: 35356-37374
  
 NCBI BlastP on this gene

GAA87603

isoflavone reductase family protein
  
Accession: GAA87604
  
Location: 37800-38868
  
 NCBI BlastP on this gene

GAA87604

C6 zinc finger domain protein
  
Accession: GAA87605
  
Location: 39411-41132
  
 NCBI BlastP on this gene

GAA87605

similar to An12g07160
  
Accession: GAA87606
  
Location: 42785-44008
  
 NCBI BlastP on this gene

GAA87606

sodium/solute symporter
  
Accession: GAA87607
  
Location: 44109-46296
  
 NCBI BlastP on this gene

GAA87607

Query: Architecture Search FASTA input

ADOT01000195 : Arthrobotrys oligospora ATCC 24927    Total score: 2.0     Cumulative Blast bit score: 2481

Hit cluster cross-links:

Mycgr3G85918 Mycgr3T
  
Location: 0-1602

Mycgr3G85918\_Mycgr3T

Mycgr3G42010 Mycgr3T
  
Location: 1702-8569

Mycgr3G42010\_Mycgr3T

Mycgr3G29582 Mycgr3T
  
Location: 8669-8915

Mycgr3G29582\_Mycgr3T

Mycgr3G31170 Mycgr3T
  
Location: 9015-9255

Mycgr3G31170\_Mycgr3T

Mycgr3G85924 Mycgr3T
  
Location: 9355-11218

Mycgr3G85924\_Mycgr3T

Mycgr3G71676 Mycgr3T
  
Location: 11318-12494

Mycgr3G71676\_Mycgr3T

Mycgr3G11468 Mycgr3T
  
Location: 12594-13653

Mycgr3G11468\_Mycgr3T

Mycgr3G58567 Mycgr3T
  
Location: 13753-14506

Mycgr3G58567\_Mycgr3T

Mycgr3G100089 Mycgr3
  
Location: 14606-21152

Mycgr3G100089\_Mycgr3

Mycgr3G42698 Mycgr3T
  
Location: 21252-22131

Mycgr3G42698\_Mycgr3T

Mycgr3G71681 Mycgr3T
  
Location: 22231-23461

Mycgr3G71681\_Mycgr3T

Mycgr3G109328 Mycgr3
  
Location: 23561-24239

Mycgr3G109328\_Mycgr3

Mycgr3G104334 Mycgr3
  
Location: 24339-24567

Mycgr3G104334\_Mycgr3

Mycgr3G42715 Mycgr3T
  
Location: 24667-25981

Mycgr3G42715\_Mycgr3T

Mycgr3G92934 Mycgr3T
  
Location: 26081-27593

Mycgr3G92934\_Mycgr3T

Mycgr3G41969 Mycgr3T
  
Location: 27693-29328

Mycgr3G41969\_Mycgr3T

Mycgr3G80635 Mycgr3T
  
Location: 29428-29821

Mycgr3G80635\_Mycgr3T

Mycgr3G41426 Mycgr3T
  
Location: 29921-35255

Mycgr3G41426\_Mycgr3T

Mycgr3G104337 Mycgr3
  
Location: 35355-36108

Mycgr3G104337\_Mycgr3

Mycgr3G71679 Mycgr3T
  
Location: 36208-37300

Mycgr3G71679\_Mycgr3T

Mycgr3G92938 Mycgr3T
  
Location: 37400-38699

Mycgr3G92938\_Mycgr3T

Mycgr3G92941 Mycgr3T
  
Location: 38799-40734

Mycgr3G92941\_Mycgr3T

hypothetical protein
  
Accession: EGX46125
  
Location: 371171-372202
  
 NCBI BlastP on this gene

EGX46125

hypothetical protein
  
Accession: EGX46126
  
Location: 373177-374520
  
 NCBI BlastP on this gene

EGX46126

hypothetical protein
  
Accession: EGX46127
  
Location: 377708-378662
  
 NCBI BlastP on this gene

EGX46127

hypothetical protein
  
Accession: EGX46128
  
Location: 379247-379699
  
 NCBI BlastP on this gene

EGX46128

hypothetical protein
  
Accession: EGX46129
  
Location: 380489-381823
  
 NCBI BlastP on this gene

EGX46129

hypothetical protein
  
Accession: EGX46130
  
Location: 383717-385189
  
 NCBI BlastP on this gene

EGX46130

hypothetical protein
  
Accession: EGX46131
  
Location: 386089-389108
  
 NCBI BlastP on this gene

EGX46131

hypothetical protein
  
Accession: EGX46132
  
Location: 389971-397204
  
  
**BlastP hit with Mycgr3G42010\_Mycgr3T**
  
Percentage identity: 49 %
  
BlastP bit score: 2237
  
Sequence coverage: 103 %
  
E-value: 0.0
  
  
 NCBI BlastP on this gene

EGX46132

hypothetical protein
  
Accession: EGX46133
  
Location: 397680-399181
  
  
**BlastP hit with Mycgr3G92938\_Mycgr3T**
  
Percentage identity: 34 %
  
BlastP bit score: 244
  
Sequence coverage: 101 %
  
E-value: 1e-71
  
  
 NCBI BlastP on this gene

EGX46133

hypothetical protein
  
Accession: EGX46134
  
Location: 399443-401644
  
 NCBI BlastP on this gene

EGX46134

hypothetical protein
  
Accession: EGX46135
  
Location: 405848-406789
  
 NCBI BlastP on this gene

EGX46135

hypothetical protein
  
Accession: EGX46136
  
Location: 407568-408123
  
 NCBI BlastP on this gene

EGX46136

hypothetical protein
  
Accession: EGX46137
  
Location: 410119-411201
  
 NCBI BlastP on this gene

EGX46137

hypothetical protein
  
Accession: EGX46138
  
Location: 411301-414525
  
 NCBI BlastP on this gene

EGX46138

hypothetical protein
  
Accession: EGX46139
  
Location: 416956-419025
  
 NCBI BlastP on this gene

EGX46139

Query: Architecture Search FASTA input

GL531877 : Pyrenophora teres f. teres 0-1 unplaced genomic scaffold scaffold\_21635    Total score: 2.0     Cumulative Blast bit score: 2453

Hit cluster cross-links:

Mycgr3G85918 Mycgr3T
  
Location: 0-1602

Mycgr3G85918\_Mycgr3T

Mycgr3G42010 Mycgr3T
  
Location: 1702-8569

Mycgr3G42010\_Mycgr3T

Mycgr3G29582 Mycgr3T
  
Location: 8669-8915

Mycgr3G29582\_Mycgr3T

Mycgr3G31170 Mycgr3T
  
Location: 9015-9255

Mycgr3G31170\_Mycgr3T

Mycgr3G85924 Mycgr3T
  
Location: 9355-11218

Mycgr3G85924\_Mycgr3T

Mycgr3G71676 Mycgr3T
  
Location: 11318-12494

Mycgr3G71676\_Mycgr3T

Mycgr3G11468 Mycgr3T
  
Location: 12594-13653

Mycgr3G11468\_Mycgr3T

Mycgr3G58567 Mycgr3T
  
Location: 13753-14506

Mycgr3G58567\_Mycgr3T

Mycgr3G100089 Mycgr3
  
Location: 14606-21152

Mycgr3G100089\_Mycgr3

Mycgr3G42698 Mycgr3T
  
Location: 21252-22131

Mycgr3G42698\_Mycgr3T

Mycgr3G71681 Mycgr3T
  
Location: 22231-23461

Mycgr3G71681\_Mycgr3T

Mycgr3G109328 Mycgr3
  
Location: 23561-24239

Mycgr3G109328\_Mycgr3

Mycgr3G104334 Mycgr3
  
Location: 24339-24567

Mycgr3G104334\_Mycgr3

Mycgr3G42715 Mycgr3T
  
Location: 24667-25981

Mycgr3G42715\_Mycgr3T

Mycgr3G92934 Mycgr3T
  
Location: 26081-27593

Mycgr3G92934\_Mycgr3T

Mycgr3G41969 Mycgr3T
  
Location: 27693-29328

Mycgr3G41969\_Mycgr3T

Mycgr3G80635 Mycgr3T
  
Location: 29428-29821

Mycgr3G80635\_Mycgr3T

Mycgr3G41426 Mycgr3T
  
Location: 29921-35255

Mycgr3G41426\_Mycgr3T

Mycgr3G104337 Mycgr3
  
Location: 35355-36108

Mycgr3G104337\_Mycgr3

Mycgr3G71679 Mycgr3T
  
Location: 36208-37300

Mycgr3G71679\_Mycgr3T

Mycgr3G92938 Mycgr3T
  
Location: 37400-38699

Mycgr3G92938\_Mycgr3T

Mycgr3G92941 Mycgr3T
  
Location: 38799-40734

Mycgr3G92941\_Mycgr3T

hypothetical protein
  
Accession: EFQ96509
  
Location: 510-884
  
 NCBI BlastP on this gene

EFQ96509

hypothetical protein
  
Accession: EFQ96510
  
Location: 3245-4234
  
 NCBI BlastP on this gene

EFQ96510

hypothetical protein
  
Accession: EFQ96511
  
Location: 4514-11744
  
  
**BlastP hit with Mycgr3G42010\_Mycgr3T**
  
Percentage identity: 48 %
  
BlastP bit score: 2175
  
Sequence coverage: 101 %
  
E-value: 0.0
  
  
 NCBI BlastP on this gene

EFQ96511

hypothetical protein
  
Accession: EFQ96512
  
Location: 12181-13668
  
  
**BlastP hit with Mycgr3G92938\_Mycgr3T**
  
Percentage identity: 38 %
  
BlastP bit score: 278
  
Sequence coverage: 102 %
  
E-value: 2e-84
  
  
 NCBI BlastP on this gene

EFQ96512

hypothetical protein
  
Accession: EFQ96513
  
Location: 15738-16835
  
 NCBI BlastP on this gene

EFQ96513

hypothetical protein
  
Accession: EFQ96514
  
Location: 17580-18407
  
 NCBI BlastP on this gene

EFQ96514

hypothetical protein
  
Accession: EFQ96515
  
Location: 18697-20232
  
 NCBI BlastP on this gene

EFQ96515

Query: Architecture Search FASTA input

KB908481 : Setosphaeria turcica Et28A unplaced genomic scaffold SETTUscaffold\_1    Total score: 2.0     Cumulative Blast bit score: 2448

Hit cluster cross-links:

Mycgr3G85918 Mycgr3T
  
Location: 0-1602

Mycgr3G85918\_Mycgr3T

Mycgr3G42010 Mycgr3T
  
Location: 1702-8569

Mycgr3G42010\_Mycgr3T

Mycgr3G29582 Mycgr3T
  
Location: 8669-8915

Mycgr3G29582\_Mycgr3T

Mycgr3G31170 Mycgr3T
  
Location: 9015-9255

Mycgr3G31170\_Mycgr3T

Mycgr3G85924 Mycgr3T
  
Location: 9355-11218

Mycgr3G85924\_Mycgr3T

Mycgr3G71676 Mycgr3T
  
Location: 11318-12494

Mycgr3G71676\_Mycgr3T

Mycgr3G11468 Mycgr3T
  
Location: 12594-13653

Mycgr3G11468\_Mycgr3T

Mycgr3G58567 Mycgr3T
  
Location: 13753-14506

Mycgr3G58567\_Mycgr3T

Mycgr3G100089 Mycgr3
  
Location: 14606-21152

Mycgr3G100089\_Mycgr3

Mycgr3G42698 Mycgr3T
  
Location: 21252-22131

Mycgr3G42698\_Mycgr3T

Mycgr3G71681 Mycgr3T
  
Location: 22231-23461

Mycgr3G71681\_Mycgr3T

Mycgr3G109328 Mycgr3
  
Location: 23561-24239

Mycgr3G109328\_Mycgr3

Mycgr3G104334 Mycgr3
  
Location: 24339-24567

Mycgr3G104334\_Mycgr3

Mycgr3G42715 Mycgr3T
  
Location: 24667-25981

Mycgr3G42715\_Mycgr3T

Mycgr3G92934 Mycgr3T
  
Location: 26081-27593

Mycgr3G92934\_Mycgr3T

Mycgr3G41969 Mycgr3T
  
Location: 27693-29328

Mycgr3G41969\_Mycgr3T

Mycgr3G80635 Mycgr3T
  
Location: 29428-29821

Mycgr3G80635\_Mycgr3T

Mycgr3G41426 Mycgr3T
  
Location: 29921-35255

Mycgr3G41426\_Mycgr3T

Mycgr3G104337 Mycgr3
  
Location: 35355-36108

Mycgr3G104337\_Mycgr3

Mycgr3G71679 Mycgr3T
  
Location: 36208-37300

Mycgr3G71679\_Mycgr3T

Mycgr3G92938 Mycgr3T
  
Location: 37400-38699

Mycgr3G92938\_Mycgr3T

Mycgr3G92941 Mycgr3T
  
Location: 38799-40734

Mycgr3G92941\_Mycgr3T

hypothetical protein
  
Accession: EOA91500
  
Location: 1181357-1181812
  
 NCBI BlastP on this gene

EOA91500

hypothetical protein
  
Accession: EOA91501
  
Location: 1184267-1184650
  
 NCBI BlastP on this gene

EOA91501

hypothetical protein
  
Accession: EOA91502
  
Location: 1185015-1185746
  
 NCBI BlastP on this gene

EOA91502

hypothetical protein
  
Accession: EOA91503
  
Location: 1187549-1189547
  
 NCBI BlastP on this gene

EOA91503

hypothetical protein
  
Accession: EOA91504
  
Location: 1191934-1192328
  
 NCBI BlastP on this gene

EOA91504

hypothetical protein
  
Accession: EOA91505
  
Location: 1194742-1196353
  
 NCBI BlastP on this gene

EOA91505

hypothetical protein
  
Accession: EOA91506
  
Location: 1197037-1204523
  
  
**BlastP hit with Mycgr3G42010\_Mycgr3T**
  
Percentage identity: 48 %
  
BlastP bit score: 2187
  
Sequence coverage: 102 %
  
E-value: 0.0
  
  
 NCBI BlastP on this gene

EOA91506

hypothetical protein
  
Accession: EOA91507
  
Location: 1204958-1206475
  
  
**BlastP hit with Mycgr3G92938\_Mycgr3T**
  
Percentage identity: 39 %
  
BlastP bit score: 261
  
Sequence coverage: 90 %
  
E-value: 4e-78
  
  
 NCBI BlastP on this gene

EOA91507

hypothetical protein
  
Accession: EOA91508
  
Location: 1208097-1209671
  
 NCBI BlastP on this gene

EOA91508

hypothetical protein
  
Accession: EOA91509
  
Location: 1209873-1210748
  
 NCBI BlastP on this gene

EOA91509

hypothetical protein
  
Accession: EOA91510
  
Location: 1211901-1213454
  
 NCBI BlastP on this gene

EOA91510

hypothetical protein
  
Accession: EOA91511
  
Location: 1213986-1214430
  
 NCBI BlastP on this gene

EOA91511

glycosyltransferase family 4 protein
  
Accession: EOA91512
  
Location: 1216094-1225525
  
 NCBI BlastP on this gene

EOA91512

Query: Architecture Search FASTA input

AQGS01000059 : Dactylellina haptotyla CBS 200.50    Total score: 2.0     Cumulative Blast bit score: 2448

Hit cluster cross-links:

Mycgr3G85918 Mycgr3T
  
Location: 0-1602

Mycgr3G85918\_Mycgr3T

Mycgr3G42010 Mycgr3T
  
Location: 1702-8569

Mycgr3G42010\_Mycgr3T

Mycgr3G29582 Mycgr3T
  
Location: 8669-8915

Mycgr3G29582\_Mycgr3T

Mycgr3G31170 Mycgr3T
  
Location: 9015-9255

Mycgr3G31170\_Mycgr3T

Mycgr3G85924 Mycgr3T
  
Location: 9355-11218

Mycgr3G85924\_Mycgr3T

Mycgr3G71676 Mycgr3T
  
Location: 11318-12494

Mycgr3G71676\_Mycgr3T

Mycgr3G11468 Mycgr3T
  
Location: 12594-13653

Mycgr3G11468\_Mycgr3T

Mycgr3G58567 Mycgr3T
  
Location: 13753-14506

Mycgr3G58567\_Mycgr3T

Mycgr3G100089 Mycgr3
  
Location: 14606-21152

Mycgr3G100089\_Mycgr3

Mycgr3G42698 Mycgr3T
  
Location: 21252-22131

Mycgr3G42698\_Mycgr3T

Mycgr3G71681 Mycgr3T
  
Location: 22231-23461

Mycgr3G71681\_Mycgr3T

Mycgr3G109328 Mycgr3
  
Location: 23561-24239

Mycgr3G109328\_Mycgr3

Mycgr3G104334 Mycgr3
  
Location: 24339-24567

Mycgr3G104334\_Mycgr3

Mycgr3G42715 Mycgr3T
  
Location: 24667-25981

Mycgr3G42715\_Mycgr3T

Mycgr3G92934 Mycgr3T
  
Location: 26081-27593

Mycgr3G92934\_Mycgr3T

Mycgr3G41969 Mycgr3T
  
Location: 27693-29328

Mycgr3G41969\_Mycgr3T

Mycgr3G80635 Mycgr3T
  
Location: 29428-29821

Mycgr3G80635\_Mycgr3T

Mycgr3G41426 Mycgr3T
  
Location: 29921-35255

Mycgr3G41426\_Mycgr3T

Mycgr3G104337 Mycgr3
  
Location: 35355-36108

Mycgr3G104337\_Mycgr3

Mycgr3G71679 Mycgr3T
  
Location: 36208-37300

Mycgr3G71679\_Mycgr3T

Mycgr3G92938 Mycgr3T
  
Location: 37400-38699

Mycgr3G92938\_Mycgr3T

Mycgr3G92941 Mycgr3T
  
Location: 38799-40734

Mycgr3G92941\_Mycgr3T

hypothetical protein
  
Accession: EPS44092
  
Location: 220449-221252
  
 NCBI BlastP on this gene

EPS44092

hypothetical protein
  
Accession: EPS44060
  
Location: 224427-233524
  
 NCBI BlastP on this gene

EPS44060

hypothetical protein
  
Accession: EPS44085
  
Location: 234882-235535
  
 NCBI BlastP on this gene

EPS44085

hypothetical protein
  
Accession: EPS44064
  
Location: 236197-237696
  
  
**BlastP hit with Mycgr3G92938\_Mycgr3T**
  
Percentage identity: 36 %
  
BlastP bit score: 239
  
Sequence coverage: 101 %
  
E-value: 8e-70
  
  
 NCBI BlastP on this gene

EPS44064

hypothetical protein
  
Accession: EPS44029
  
Location: 238105-245295
  
  
**BlastP hit with Mycgr3G42010\_Mycgr3T**
  
Percentage identity: 48 %
  
BlastP bit score: 2209
  
Sequence coverage: 102 %
  
E-value: 0.0
  
  
 NCBI BlastP on this gene

EPS44029

hypothetical protein
  
Accession: EPS44069
  
Location: 247759-252400
  
 NCBI BlastP on this gene

EPS44069

hypothetical protein
  
Accession: EPS44058
  
Location: 253045-254980
  
 NCBI BlastP on this gene

EPS44058

hypothetical protein
  
Accession: EPS44073
  
Location: 258188-259741
  
 NCBI BlastP on this gene

EPS44073

hypothetical protein
  
Accession: EPS44053
  
Location: 259891-261260
  
 NCBI BlastP on this gene

EPS44053

hypothetical protein
  
Accession: EPS44034
  
Location: 263095-264441
  
 NCBI BlastP on this gene

EPS44034

Query: Architecture Search FASTA input

DS027045 : Aspergillus clavatus NRRL 1 1099423829791 genomic scaffold    Total score: 2.0     Cumulative Blast bit score: 2442

Hit cluster cross-links:

Mycgr3G85918 Mycgr3T
  
Location: 0-1602

Mycgr3G85918\_Mycgr3T

Mycgr3G42010 Mycgr3T
  
Location: 1702-8569

Mycgr3G42010\_Mycgr3T

Mycgr3G29582 Mycgr3T
  
Location: 8669-8915

Mycgr3G29582\_Mycgr3T

Mycgr3G31170 Mycgr3T
  
Location: 9015-9255

Mycgr3G31170\_Mycgr3T

Mycgr3G85924 Mycgr3T
  
Location: 9355-11218

Mycgr3G85924\_Mycgr3T

Mycgr3G71676 Mycgr3T
  
Location: 11318-12494

Mycgr3G71676\_Mycgr3T

Mycgr3G11468 Mycgr3T
  
Location: 12594-13653

Mycgr3G11468\_Mycgr3T

Mycgr3G58567 Mycgr3T
  
Location: 13753-14506

Mycgr3G58567\_Mycgr3T

Mycgr3G100089 Mycgr3
  
Location: 14606-21152

Mycgr3G100089\_Mycgr3

Mycgr3G42698 Mycgr3T
  
Location: 21252-22131

Mycgr3G42698\_Mycgr3T

Mycgr3G71681 Mycgr3T
  
Location: 22231-23461

Mycgr3G71681\_Mycgr3T

Mycgr3G109328 Mycgr3
  
Location: 23561-24239

Mycgr3G109328\_Mycgr3

Mycgr3G104334 Mycgr3
  
Location: 24339-24567

Mycgr3G104334\_Mycgr3

Mycgr3G42715 Mycgr3T
  
Location: 24667-25981

Mycgr3G42715\_Mycgr3T

Mycgr3G92934 Mycgr3T
  
Location: 26081-27593

Mycgr3G92934\_Mycgr3T

Mycgr3G41969 Mycgr3T
  
Location: 27693-29328

Mycgr3G41969\_Mycgr3T

Mycgr3G80635 Mycgr3T
  
Location: 29428-29821

Mycgr3G80635\_Mycgr3T

Mycgr3G41426 Mycgr3T
  
Location: 29921-35255

Mycgr3G41426\_Mycgr3T

Mycgr3G104337 Mycgr3
  
Location: 35355-36108

Mycgr3G104337\_Mycgr3

Mycgr3G71679 Mycgr3T
  
Location: 36208-37300

Mycgr3G71679\_Mycgr3T

Mycgr3G92938 Mycgr3T
  
Location: 37400-38699

Mycgr3G92938\_Mycgr3T

Mycgr3G92941 Mycgr3T
  
Location: 38799-40734

Mycgr3G92941\_Mycgr3T

conserved hypothetical protein
  
Accession: EAW14573
  
Location: 2505449-2509427
  
 NCBI BlastP on this gene

EAW14573

conserved hypothetical protein
  
Accession: EAW14574
  
Location: 2509607-2512446
  
 NCBI BlastP on this gene

EAW14574

C2H2 transcription factor (AmdX), putative
  
Accession: EAW14575
  
Location: 2519936-2523629
  
 NCBI BlastP on this gene

EAW14575

AAA family ATPase, putative
  
Accession: EAW14576
  
Location: 2524676-2531986
  
  
**BlastP hit with Mycgr3G42010\_Mycgr3T**
  
Percentage identity: 48 %
  
BlastP bit score: 2156
  
Sequence coverage: 102 %
  
E-value: 0.0
  
  
 NCBI BlastP on this gene

EAW14576

hypothetical protein
  
Accession: EAW14577
  
Location: 2532390-2533906
  
  
**BlastP hit with Mycgr3G92938\_Mycgr3T**
  
Percentage identity: 43 %
  
BlastP bit score: 286
  
Sequence coverage: 84 %
  
E-value: 9e-88
  
  
 NCBI BlastP on this gene

EAW14577

phosphoesterase, putative
  
Accession: EAW14578
  
Location: 2535712-2536668
  
 NCBI BlastP on this gene

EAW14578

conserved hypothetical protein
  
Accession: EAW14579
  
Location: 2537281-2538777
  
 NCBI BlastP on this gene

EAW14579

extracelular serine carboxypeptidase, putative
  
Accession: EAW14580
  
Location: 2539753-2541501
  
 NCBI BlastP on this gene

EAW14580

hypothetical protein
  
Accession: EAW14581
  
Location: 2542075-2542935
  
 NCBI BlastP on this gene

EAW14581

MFS monocarboxylate transporter (Mct), putative
  
Accession: EAW14582
  
Location: 2545874-2547329
  
 NCBI BlastP on this gene

EAW14582

haloacid dehalogenase-like hydrolase, putative
  
Accession: EAW14583
  
Location: 2547482-2548168
  
 NCBI BlastP on this gene

EAW14583

conserved hypothetical protein
  
Accession: EAW14584
  
Location: 2548517-2549078
  
 NCBI BlastP on this gene

EAW14584

hydantoinase/oxoprolinase, putative
  
Accession: EAW14585
  
Location: 2549308-2552511
  
 NCBI BlastP on this gene

EAW14585

Query: Architecture Search FASTA input

KB733458 : Bipolaris maydis ATCC 48331 unplaced genomic scaffold COCC4scaffold\_15    Total score: 2.0     Cumulative Blast bit score: 2422

Hit cluster cross-links:

Mycgr3G85918 Mycgr3T
  
Location: 0-1602

Mycgr3G85918\_Mycgr3T

Mycgr3G42010 Mycgr3T
  
Location: 1702-8569

Mycgr3G42010\_Mycgr3T

Mycgr3G29582 Mycgr3T
  
Location: 8669-8915

Mycgr3G29582\_Mycgr3T

Mycgr3G31170 Mycgr3T
  
Location: 9015-9255

Mycgr3G31170\_Mycgr3T

Mycgr3G85924 Mycgr3T
  
Location: 9355-11218

Mycgr3G85924\_Mycgr3T

Mycgr3G71676 Mycgr3T
  
Location: 11318-12494

Mycgr3G71676\_Mycgr3T

Mycgr3G11468 Mycgr3T
  
Location: 12594-13653

Mycgr3G11468\_Mycgr3T

Mycgr3G58567 Mycgr3T
  
Location: 13753-14506

Mycgr3G58567\_Mycgr3T

Mycgr3G100089 Mycgr3
  
Location: 14606-21152

Mycgr3G100089\_Mycgr3

Mycgr3G42698 Mycgr3T
  
Location: 21252-22131

Mycgr3G42698\_Mycgr3T

Mycgr3G71681 Mycgr3T
  
Location: 22231-23461

Mycgr3G71681\_Mycgr3T

Mycgr3G109328 Mycgr3
  
Location: 23561-24239

Mycgr3G109328\_Mycgr3

Mycgr3G104334 Mycgr3
  
Location: 24339-24567

Mycgr3G104334\_Mycgr3

Mycgr3G42715 Mycgr3T
  
Location: 24667-25981

Mycgr3G42715\_Mycgr3T

Mycgr3G92934 Mycgr3T
  
Location: 26081-27593

Mycgr3G92934\_Mycgr3T

Mycgr3G41969 Mycgr3T
  
Location: 27693-29328

Mycgr3G41969\_Mycgr3T

Mycgr3G80635 Mycgr3T
  
Location: 29428-29821

Mycgr3G80635\_Mycgr3T

Mycgr3G41426 Mycgr3T
  
Location: 29921-35255

Mycgr3G41426\_Mycgr3T

Mycgr3G104337 Mycgr3
  
Location: 35355-36108

Mycgr3G104337\_Mycgr3

Mycgr3G71679 Mycgr3T
  
Location: 36208-37300

Mycgr3G71679\_Mycgr3T

Mycgr3G92938 Mycgr3T
  
Location: 37400-38699

Mycgr3G92938\_Mycgr3T

Mycgr3G92941 Mycgr3T
  
Location: 38799-40734

Mycgr3G92941\_Mycgr3T

hypothetical protein
  
Accession: ENI03896
  
Location: 121610-121771
  
 NCBI BlastP on this gene

ENI03896

hypothetical protein
  
Accession: ENI03897
  
Location: 125074-126593
  
 NCBI BlastP on this gene

ENI03897

hypothetical protein
  
Accession: ENI03898
  
Location: 127411-130230
  
 NCBI BlastP on this gene

ENI03898

hypothetical protein
  
Accession: ENI03899
  
Location: 131366-132262
  
 NCBI BlastP on this gene

ENI03899

hypothetical protein
  
Accession: ENI03900
  
Location: 133013-133857
  
 NCBI BlastP on this gene

ENI03900

hypothetical protein
  
Accession: ENI03901
  
Location: 134690-138015
  
 NCBI BlastP on this gene

ENI03901

hypothetical protein
  
Accession: ENI03902
  
Location: 138022-138228
  
 NCBI BlastP on this gene

ENI03902

hypothetical protein
  
Accession: ENI03903
  
Location: 138846-139412
  
 NCBI BlastP on this gene

ENI03903

hypothetical protein
  
Accession: ENI03904
  
Location: 139851-141408
  
  
**BlastP hit with Mycgr3G92938\_Mycgr3T**
  
Percentage identity: 35 %
  
BlastP bit score: 239
  
Sequence coverage: 113 %
  
E-value: 2e-69
  
  
 NCBI BlastP on this gene

ENI03904

hypothetical protein
  
Accession: ENI03905
  
Location: 141899-149198
  
  
**BlastP hit with Mycgr3G42010\_Mycgr3T**
  
Percentage identity: 48 %
  
BlastP bit score: 2183
  
Sequence coverage: 102 %
  
E-value: 0.0
  
  
 NCBI BlastP on this gene

ENI03905

hypothetical protein
  
Accession: ENI03906
  
Location: 150167-151777
  
 NCBI BlastP on this gene

ENI03906

hypothetical protein
  
Accession: ENI03907
  
Location: 153362-153691
  
 NCBI BlastP on this gene

ENI03907

hypothetical protein
  
Accession: ENI03908
  
Location: 158165-160011
  
 NCBI BlastP on this gene

ENI03908

hypothetical protein
  
Accession: ENI03909
  
Location: 161544-163007
  
 NCBI BlastP on this gene

ENI03909

hypothetical protein
  
Accession: ENI03910
  
Location: 163672-164151
  
 NCBI BlastP on this gene

ENI03910

hypothetical protein
  
Accession: ENI03911
  
Location: 165314-165670
  
 NCBI BlastP on this gene

ENI03911

Query: Architecture Search FASTA input

KB445583 : Cochliobolus heterostrophus C5 unplaced genomic scaffold COCHEscaffold\_15    Total score: 2.0     Cumulative Blast bit score: 2422

Hit cluster cross-links:

Mycgr3G85918 Mycgr3T
  
Location: 0-1602

Mycgr3G85918\_Mycgr3T

Mycgr3G42010 Mycgr3T
  
Location: 1702-8569

Mycgr3G42010\_Mycgr3T

Mycgr3G29582 Mycgr3T
  
Location: 8669-8915

Mycgr3G29582\_Mycgr3T

Mycgr3G31170 Mycgr3T
  
Location: 9015-9255

Mycgr3G31170\_Mycgr3T

Mycgr3G85924 Mycgr3T
  
Location: 9355-11218

Mycgr3G85924\_Mycgr3T

Mycgr3G71676 Mycgr3T
  
Location: 11318-12494

Mycgr3G71676\_Mycgr3T

Mycgr3G11468 Mycgr3T
  
Location: 12594-13653

Mycgr3G11468\_Mycgr3T

Mycgr3G58567 Mycgr3T
  
Location: 13753-14506

Mycgr3G58567\_Mycgr3T

Mycgr3G100089 Mycgr3
  
Location: 14606-21152

Mycgr3G100089\_Mycgr3

Mycgr3G42698 Mycgr3T
  
Location: 21252-22131

Mycgr3G42698\_Mycgr3T

Mycgr3G71681 Mycgr3T
  
Location: 22231-23461

Mycgr3G71681\_Mycgr3T

Mycgr3G109328 Mycgr3
  
Location: 23561-24239

Mycgr3G109328\_Mycgr3

Mycgr3G104334 Mycgr3
  
Location: 24339-24567

Mycgr3G104334\_Mycgr3

Mycgr3G42715 Mycgr3T
  
Location: 24667-25981

Mycgr3G42715\_Mycgr3T

Mycgr3G92934 Mycgr3T
  
Location: 26081-27593

Mycgr3G92934\_Mycgr3T

Mycgr3G41969 Mycgr3T
  
Location: 27693-29328

Mycgr3G41969\_Mycgr3T

Mycgr3G80635 Mycgr3T
  
Location: 29428-29821

Mycgr3G80635\_Mycgr3T

Mycgr3G41426 Mycgr3T
  
Location: 29921-35255

Mycgr3G41426\_Mycgr3T

Mycgr3G104337 Mycgr3
  
Location: 35355-36108

Mycgr3G104337\_Mycgr3

Mycgr3G71679 Mycgr3T
  
Location: 36208-37300

Mycgr3G71679\_Mycgr3T

Mycgr3G92938 Mycgr3T
  
Location: 37400-38699

Mycgr3G92938\_Mycgr3T

Mycgr3G92941 Mycgr3T
  
Location: 38799-40734

Mycgr3G92941\_Mycgr3T

hypothetical protein
  
Accession: EMD87095
  
Location: 701318-701674
  
 NCBI BlastP on this gene

EMD87095

hypothetical protein
  
Accession: EMD87096
  
Location: 702837-703316
  
 NCBI BlastP on this gene

EMD87096

hypothetical protein
  
Accession: EMD87097
  
Location: 703981-705444
  
 NCBI BlastP on this gene

EMD87097

hypothetical protein
  
Accession: EMD87098
  
Location: 706977-708823
  
 NCBI BlastP on this gene

EMD87098

hypothetical protein
  
Accession: EMD87099
  
Location: 713297-713626
  
 NCBI BlastP on this gene

EMD87099

hypothetical protein
  
Accession: EMD87100
  
Location: 715211-716821
  
 NCBI BlastP on this gene

EMD87100

hypothetical protein
  
Accession: EMD87101
  
Location: 717790-725089
  
  
**BlastP hit with Mycgr3G42010\_Mycgr3T**
  
Percentage identity: 48 %
  
BlastP bit score: 2183
  
Sequence coverage: 102 %
  
E-value: 0.0
  
  
 NCBI BlastP on this gene

EMD87101

hypothetical protein
  
Accession: EMD87102
  
Location: 725580-727137
  
  
**BlastP hit with Mycgr3G92938\_Mycgr3T**
  
Percentage identity: 35 %
  
BlastP bit score: 239
  
Sequence coverage: 113 %
  
E-value: 2e-69
  
  
 NCBI BlastP on this gene

EMD87102

hypothetical protein
  
Accession: EMD87103
  
Location: 727576-728142
  
 NCBI BlastP on this gene

EMD87103

hypothetical protein
  
Accession: EMD87104
  
Location: 728973-732298
  
 NCBI BlastP on this gene

EMD87104

hypothetical protein
  
Accession: EMD87106
  
Location: 733131-733975
  
 NCBI BlastP on this gene

EMD87106

hypothetical protein
  
Accession: EMD87107
  
Location: 734726-735622
  
 NCBI BlastP on this gene

EMD87107

hypothetical protein
  
Accession: EMD87108
  
Location: 736758-739577
  
 NCBI BlastP on this gene

EMD87108

hypothetical protein
  
Accession: EMD87109
  
Location: 740395-741914
  
 NCBI BlastP on this gene

EMD87109

hypothetical protein
  
Accession: EMD87110
  
Location: 745217-745378
  
 NCBI BlastP on this gene

EMD87110

Query: Architecture Search FASTA input

EQ962652 : Talaromyces stipitatus ATCC 10500 scf\_1105507295523 genomic scaffold    Total score: 2.0     Cumulative Blast bit score: 2383

Hit cluster cross-links:

Mycgr3G85918 Mycgr3T
  
Location: 0-1602

Mycgr3G85918\_Mycgr3T

Mycgr3G42010 Mycgr3T
  
Location: 1702-8569

Mycgr3G42010\_Mycgr3T

Mycgr3G29582 Mycgr3T
  
Location: 8669-8915

Mycgr3G29582\_Mycgr3T

Mycgr3G31170 Mycgr3T
  
Location: 9015-9255

Mycgr3G31170\_Mycgr3T

Mycgr3G85924 Mycgr3T
  
Location: 9355-11218

Mycgr3G85924\_Mycgr3T

Mycgr3G71676 Mycgr3T
  
Location: 11318-12494

Mycgr3G71676\_Mycgr3T

Mycgr3G11468 Mycgr3T
  
Location: 12594-13653

Mycgr3G11468\_Mycgr3T

Mycgr3G58567 Mycgr3T
  
Location: 13753-14506

Mycgr3G58567\_Mycgr3T

Mycgr3G100089 Mycgr3
  
Location: 14606-21152

Mycgr3G100089\_Mycgr3

Mycgr3G42698 Mycgr3T
  
Location: 21252-22131

Mycgr3G42698\_Mycgr3T

Mycgr3G71681 Mycgr3T
  
Location: 22231-23461

Mycgr3G71681\_Mycgr3T

Mycgr3G109328 Mycgr3
  
Location: 23561-24239

Mycgr3G109328\_Mycgr3

Mycgr3G104334 Mycgr3
  
Location: 24339-24567

Mycgr3G104334\_Mycgr3

Mycgr3G42715 Mycgr3T
  
Location: 24667-25981

Mycgr3G42715\_Mycgr3T

Mycgr3G92934 Mycgr3T
  
Location: 26081-27593

Mycgr3G92934\_Mycgr3T

Mycgr3G41969 Mycgr3T
  
Location: 27693-29328

Mycgr3G41969\_Mycgr3T

Mycgr3G80635 Mycgr3T
  
Location: 29428-29821

Mycgr3G80635\_Mycgr3T

Mycgr3G41426 Mycgr3T
  
Location: 29921-35255

Mycgr3G41426\_Mycgr3T

Mycgr3G104337 Mycgr3
  
Location: 35355-36108

Mycgr3G104337\_Mycgr3

Mycgr3G71679 Mycgr3T
  
Location: 36208-37300

Mycgr3G71679\_Mycgr3T

Mycgr3G92938 Mycgr3T
  
Location: 37400-38699

Mycgr3G92938\_Mycgr3T

Mycgr3G92941 Mycgr3T
  
Location: 38799-40734

Mycgr3G92941\_Mycgr3T

hypothetical protein
  
Accession: EED23730
  
Location: 3265553-3266274
  
 NCBI BlastP on this gene

EED23730

hypothetical protein
  
Accession: EED23731
  
Location: 3266573-3267785
  
 NCBI BlastP on this gene

EED23731

conserved hypothetical protein
  
Accession: EED23732
  
Location: 3268827-3269975
  
 NCBI BlastP on this gene

EED23732

branched-chain amino acid aminotransferase, cytosolic
  
Accession: EED23735
  
Location: 3274693-3276139
  
 NCBI BlastP on this gene

EED23735

G-patch DNA repair protein (Drt111), putative
  
Accession: EED23736
  
Location: 3276725-3278618
  
 NCBI BlastP on this gene

EED23736

SUMO conjugating enzyme (UbcI), putative
  
Accession: EED23737
  
Location: 3279328-3280126
  
 NCBI BlastP on this gene

EED23737

conserved hypothetical protein
  
Accession: EED23738
  
Location: 3282171-3289512
  
  
**BlastP hit with Mycgr3G42010\_Mycgr3T**
  
Percentage identity: 47 %
  
BlastP bit score: 2116
  
Sequence coverage: 102 %
  
E-value: 0.0
  
  
 NCBI BlastP on this gene

EED23738

conserved hypothetical protein
  
Accession: EED23739
  
Location: 3290495-3291822
  
  
**BlastP hit with Mycgr3G92938\_Mycgr3T**
  
Percentage identity: 37 %
  
BlastP bit score: 267
  
Sequence coverage: 97 %
  
E-value: 5e-81
  
  
 NCBI BlastP on this gene

EED23739

conserved hypothetical protein
  
Accession: EED23740
  
Location: 3292137-3293726
  
 NCBI BlastP on this gene

EED23740

hypothetical protein
  
Accession: EED23741
  
Location: 3295033-3295709
  
 NCBI BlastP on this gene

EED23741

asparagine synthetase Asn2, putative
  
Accession: EED23742
  
Location: 3296731-3299006
  
 NCBI BlastP on this gene

EED23742

outer mitochondrial membrane protein porin
  
Accession: EED23743
  
Location: 3299584-3300816
  
 NCBI BlastP on this gene

EED23743

DnaJ domain protein
  
Accession: EED23744
  
Location: 3301330-3302338
  
 NCBI BlastP on this gene

EED23744

methionine aminopeptidase, type II, putative
  
Accession: EED23745
  
Location: 3302638-3304335
  
 NCBI BlastP on this gene

EED23745

sphingolipid desaturase, putative
  
Accession: EED23746
  
Location: 3304663-3306086
  
 NCBI BlastP on this gene

EED23746

hypothetical protein
  
Accession: EED23747
  
Location: 3307638-3308132
  
 NCBI BlastP on this gene

EED23747

hypothetical protein
  
Accession: EED23748
  
Location: 3308324-3310476
  
 NCBI BlastP on this gene

EED23748

Query: Architecture Search FASTA input

AACD01000123 : Aspergillus nidulans FGSC A4    Total score: 2.0     Cumulative Blast bit score: 2343

Hit cluster cross-links:

Mycgr3G85918 Mycgr3T
  
Location: 0-1602

Mycgr3G85918\_Mycgr3T

Mycgr3G42010 Mycgr3T
  
Location: 1702-8569

Mycgr3G42010\_Mycgr3T

Mycgr3G29582 Mycgr3T
  
Location: 8669-8915

Mycgr3G29582\_Mycgr3T

Mycgr3G31170 Mycgr3T
  
Location: 9015-9255

Mycgr3G31170\_Mycgr3T

Mycgr3G85924 Mycgr3T
  
Location: 9355-11218

Mycgr3G85924\_Mycgr3T

Mycgr3G71676 Mycgr3T
  
Location: 11318-12494

Mycgr3G71676\_Mycgr3T

Mycgr3G11468 Mycgr3T
  
Location: 12594-13653

Mycgr3G11468\_Mycgr3T

Mycgr3G58567 Mycgr3T
  
Location: 13753-14506

Mycgr3G58567\_Mycgr3T

Mycgr3G100089 Mycgr3
  
Location: 14606-21152

Mycgr3G100089\_Mycgr3

Mycgr3G42698 Mycgr3T
  
Location: 21252-22131

Mycgr3G42698\_Mycgr3T

Mycgr3G71681 Mycgr3T
  
Location: 22231-23461

Mycgr3G71681\_Mycgr3T

Mycgr3G109328 Mycgr3
  
Location: 23561-24239

Mycgr3G109328\_Mycgr3

Mycgr3G104334 Mycgr3
  
Location: 24339-24567

Mycgr3G104334\_Mycgr3

Mycgr3G42715 Mycgr3T
  
Location: 24667-25981

Mycgr3G42715\_Mycgr3T

Mycgr3G92934 Mycgr3T
  
Location: 26081-27593

Mycgr3G92934\_Mycgr3T

Mycgr3G41969 Mycgr3T
  
Location: 27693-29328

Mycgr3G41969\_Mycgr3T

Mycgr3G80635 Mycgr3T
  
Location: 29428-29821

Mycgr3G80635\_Mycgr3T

Mycgr3G41426 Mycgr3T
  
Location: 29921-35255

Mycgr3G41426\_Mycgr3T

Mycgr3G104337 Mycgr3
  
Location: 35355-36108

Mycgr3G104337\_Mycgr3

Mycgr3G71679 Mycgr3T
  
Location: 36208-37300

Mycgr3G71679\_Mycgr3T

Mycgr3G92938 Mycgr3T
  
Location: 37400-38699

Mycgr3G92938\_Mycgr3T

Mycgr3G92941 Mycgr3T
  
Location: 38799-40734

Mycgr3G92941\_Mycgr3T

hypothetical protein
  
Accession: EAA61284
  
Location: 97408-99248
  
 NCBI BlastP on this gene

EAA61284

hypothetical protein
  
Accession: EAA61285
  
Location: 99966-101404
  
 NCBI BlastP on this gene

EAA61285

hypothetical protein
  
Accession: EAA61286
  
Location: 102737-103509
  
 NCBI BlastP on this gene

EAA61286

hypothetical protein
  
Accession: EAA61287
  
Location: 103751-105256
  
 NCBI BlastP on this gene

EAA61287

hypothetical protein
  
Accession: EAA61288
  
Location: 105872-108046
  
 NCBI BlastP on this gene

EAA61288

hypothetical protein
  
Accession: EAA61289
  
Location: 108982-110676
  
 NCBI BlastP on this gene

EAA61289

hypothetical protein
  
Accession: EAA61290
  
Location: 111356-113098
  
 NCBI BlastP on this gene

EAA61290

predicted protein
  
Accession: EAA61291
  
Location: 114417-114827
  
 NCBI BlastP on this gene

EAA61291

hypothetical protein
  
Accession: EAA61292
  
Location: 115178-116653
  
  
**BlastP hit with Mycgr3G92938\_Mycgr3T**
  
Percentage identity: 32 %
  
BlastP bit score: 228
  
Sequence coverage: 102 %
  
E-value: 9e-66
  
  
 NCBI BlastP on this gene

EAA61292

hypothetical protein
  
Accession: EAA61293
  
Location: 117127-124370
  
  
**BlastP hit with Mycgr3G42010\_Mycgr3T**
  
Percentage identity: 47 %
  
BlastP bit score: 2115
  
Sequence coverage: 102 %
  
E-value: 0.0
  
  
 NCBI BlastP on this gene

EAA61293

predicted protein
  
Accession: EAA61294
  
Location: 126492-127407
  
 NCBI BlastP on this gene

EAA61294

Query: Architecture Search FASTA input

AM920427 : Penicillium chrysogenum Wisconsin 54-1255 complete genome, contig Pc00c12.    Total score: 2.0     Cumulative Blast bit score: 2339

Hit cluster cross-links:

Mycgr3G85918 Mycgr3T
  
Location: 0-1602

Mycgr3G85918\_Mycgr3T

Mycgr3G42010 Mycgr3T
  
Location: 1702-8569

Mycgr3G42010\_Mycgr3T

Mycgr3G29582 Mycgr3T
  
Location: 8669-8915

Mycgr3G29582\_Mycgr3T

Mycgr3G31170 Mycgr3T
  
Location: 9015-9255

Mycgr3G31170\_Mycgr3T

Mycgr3G85924 Mycgr3T
  
Location: 9355-11218

Mycgr3G85924\_Mycgr3T

Mycgr3G71676 Mycgr3T
  
Location: 11318-12494

Mycgr3G71676\_Mycgr3T

Mycgr3G11468 Mycgr3T
  
Location: 12594-13653

Mycgr3G11468\_Mycgr3T

Mycgr3G58567 Mycgr3T
  
Location: 13753-14506

Mycgr3G58567\_Mycgr3T

Mycgr3G100089 Mycgr3
  
Location: 14606-21152

Mycgr3G100089\_Mycgr3

Mycgr3G42698 Mycgr3T
  
Location: 21252-22131

Mycgr3G42698\_Mycgr3T

Mycgr3G71681 Mycgr3T
  
Location: 22231-23461

Mycgr3G71681\_Mycgr3T

Mycgr3G109328 Mycgr3
  
Location: 23561-24239

Mycgr3G109328\_Mycgr3

Mycgr3G104334 Mycgr3
  
Location: 24339-24567

Mycgr3G104334\_Mycgr3

Mycgr3G42715 Mycgr3T
  
Location: 24667-25981

Mycgr3G42715\_Mycgr3T

Mycgr3G92934 Mycgr3T
  
Location: 26081-27593

Mycgr3G92934\_Mycgr3T

Mycgr3G41969 Mycgr3T
  
Location: 27693-29328

Mycgr3G41969\_Mycgr3T

Mycgr3G80635 Mycgr3T
  
Location: 29428-29821

Mycgr3G80635\_Mycgr3T

Mycgr3G41426 Mycgr3T
  
Location: 29921-35255

Mycgr3G41426\_Mycgr3T

Mycgr3G104337 Mycgr3
  
Location: 35355-36108

Mycgr3G104337\_Mycgr3

Mycgr3G71679 Mycgr3T
  
Location: 36208-37300

Mycgr3G71679\_Mycgr3T

Mycgr3G92938 Mycgr3T
  
Location: 37400-38699

Mycgr3G92938\_Mycgr3T

Mycgr3G92941 Mycgr3T
  
Location: 38799-40734

Mycgr3G92941\_Mycgr3T

not annotated
  
Accession: Pc12g01980
  
Location: 452284-452601
  
 NCBI BlastP on this gene

Pc12g01980

hypothetical protein
  
Accession: CAP79824
  
Location: 450166-450507
  
 NCBI BlastP on this gene

Pc12g01970

not annotated
  
Accession: CAP79823
  
Location: 448222-449905
  
 NCBI BlastP on this gene

Pc12g01960

not annotated
  
Accession: CAP79822
  
Location: 445947-447842
  
 NCBI BlastP on this gene

Pc12g01950

not annotated
  
Accession: CAP79821
  
Location: 443540-444431
  
 NCBI BlastP on this gene

Pc12g01940

not annotated
  
Accession: CAP79820
  
Location: 440971-441494
  
 NCBI BlastP on this gene

Pc12g01930

hypothetical protein
  
Accession: CAP79819
  
Location: 439828-440557
  
 NCBI BlastP on this gene

Pc12g01920

not annotated
  
Accession: CAP79818
  
Location: 438697-439277
  
 NCBI BlastP on this gene

Pc12g01910

not annotated
  
Accession: CAP79817
  
Location: 436170-437709
  
 NCBI BlastP on this gene

Pc12g01900

not annotated
  
Accession: CAP79816
  
Location: 433884-435303
  
  
**BlastP hit with Mycgr3G92938\_Mycgr3T**
  
Percentage identity: 37 %
  
BlastP bit score: 268
  
Sequence coverage: 91 %
  
E-value: 2e-81
  
  
 NCBI BlastP on this gene

Pc12g01890

not annotated
  
Accession: CAP79815
  
Location: 429792-433162
  
  
**BlastP hit with Mycgr3G42010\_Mycgr3T**
  
Percentage identity: 45 %
  
BlastP bit score: 939
  
Sequence coverage: 47 %
  
E-value: 0.0
  
  
 NCBI BlastP on this gene

Pc12g01880

not annotated
  
Accession: CAP79814
  
Location: 425863-429740
  
  
**BlastP hit with Mycgr3G42010\_Mycgr3T**
  
Percentage identity: 46 %
  
BlastP bit score: 1132
  
Sequence coverage: 55 %
  
E-value: 0.0
  
  
 NCBI BlastP on this gene

Pc12g01870

not annotated
  
Accession: CAP79813
  
Location: 423432-425188
  
 NCBI BlastP on this gene

Pc12g01860

hypothetical protein
  
Accession: CAP79812
  
Location: 423048-423353
  
 NCBI BlastP on this gene

Pc12g01850

not annotated
  
Accession: CAP79811
  
Location: 421448-422868
  
 NCBI BlastP on this gene

Pc12g01840

not annotated
  
Accession: CAP79810
  
Location: 419029-420975
  
 NCBI BlastP on this gene

Pc12g01830

unnamed
  
Accession: CAP79809
  
Location: 414796-417248
  
 NCBI BlastP on this gene

Pc12g01820

not annotated
  
Accession: CAP79808
  
Location: 412865-414583
  
 NCBI BlastP on this gene

Pc12g01810

not annotated
  
Accession: CAP79807
  
Location: 410537-412087
  
 NCBI BlastP on this gene

Pc12g01800

not annotated
  
Accession: CAP79806
  
Location: 408048-409841
  
 NCBI BlastP on this gene

Pc12g01790

Query: Architecture Search FASTA input

KB445652 : Cochliobolus sativus ND90Pr unplaced genomic scaffold COCSAscaffold\_16    Total score: 2.0     Cumulative Blast bit score: 2330

Hit cluster cross-links:

Mycgr3G85918 Mycgr3T
  
Location: 0-1602

Mycgr3G85918\_Mycgr3T

Mycgr3G42010 Mycgr3T
  
Location: 1702-8569

Mycgr3G42010\_Mycgr3T

Mycgr3G29582 Mycgr3T
  
Location: 8669-8915

Mycgr3G29582\_Mycgr3T

Mycgr3G31170 Mycgr3T
  
Location: 9015-9255

Mycgr3G31170\_Mycgr3T

Mycgr3G85924 Mycgr3T
  
Location: 9355-11218

Mycgr3G85924\_Mycgr3T

Mycgr3G71676 Mycgr3T
  
Location: 11318-12494

Mycgr3G71676\_Mycgr3T

Mycgr3G11468 Mycgr3T
  
Location: 12594-13653

Mycgr3G11468\_Mycgr3T

Mycgr3G58567 Mycgr3T
  
Location: 13753-14506

Mycgr3G58567\_Mycgr3T

Mycgr3G100089 Mycgr3
  
Location: 14606-21152

Mycgr3G100089\_Mycgr3

Mycgr3G42698 Mycgr3T
  
Location: 21252-22131

Mycgr3G42698\_Mycgr3T

Mycgr3G71681 Mycgr3T
  
Location: 22231-23461

Mycgr3G71681\_Mycgr3T

Mycgr3G109328 Mycgr3
  
Location: 23561-24239

Mycgr3G109328\_Mycgr3

Mycgr3G104334 Mycgr3
  
Location: 24339-24567

Mycgr3G104334\_Mycgr3

Mycgr3G42715 Mycgr3T
  
Location: 24667-25981

Mycgr3G42715\_Mycgr3T

Mycgr3G92934 Mycgr3T
  
Location: 26081-27593

Mycgr3G92934\_Mycgr3T

Mycgr3G41969 Mycgr3T
  
Location: 27693-29328

Mycgr3G41969\_Mycgr3T

Mycgr3G80635 Mycgr3T
  
Location: 29428-29821

Mycgr3G80635\_Mycgr3T

Mycgr3G41426 Mycgr3T
  
Location: 29921-35255

Mycgr3G41426\_Mycgr3T

Mycgr3G104337 Mycgr3
  
Location: 35355-36108

Mycgr3G104337\_Mycgr3

Mycgr3G71679 Mycgr3T
  
Location: 36208-37300

Mycgr3G71679\_Mycgr3T

Mycgr3G92938 Mycgr3T
  
Location: 37400-38699

Mycgr3G92938\_Mycgr3T

Mycgr3G92941 Mycgr3T
  
Location: 38799-40734

Mycgr3G92941\_Mycgr3T

hypothetical protein
  
Accession: EMD59813
  
Location: 963050-963502
  
 NCBI BlastP on this gene

EMD59813

hypothetical protein
  
Accession: EMD59814
  
Location: 964664-965152
  
 NCBI BlastP on this gene

EMD59814

hypothetical protein
  
Accession: EMD59815
  
Location: 965824-967289
  
 NCBI BlastP on this gene

EMD59815

hypothetical protein
  
Accession: EMD59816
  
Location: 967949-968557
  
 NCBI BlastP on this gene

EMD59816

hypothetical protein
  
Accession: EMD59817
  
Location: 968821-970609
  
 NCBI BlastP on this gene

EMD59817

hypothetical protein
  
Accession: EMD59818
  
Location: 973339-973740
  
 NCBI BlastP on this gene

EMD59818

hypothetical protein
  
Accession: EMD59819
  
Location: 974979-975312
  
 NCBI BlastP on this gene

EMD59819

hypothetical protein
  
Accession: EMD59820
  
Location: 976884-978485
  
 NCBI BlastP on this gene

EMD59820

hypothetical protein
  
Accession: EMD59821
  
Location: 979448-986746
  
  
**BlastP hit with Mycgr3G42010\_Mycgr3T**
  
Percentage identity: 49 %
  
BlastP bit score: 2169
  
Sequence coverage: 102 %
  
E-value: 0.0
  
  
 NCBI BlastP on this gene

EMD59821

hypothetical protein
  
Accession: EMD59822
  
Location: 987236-988843
  
  
**BlastP hit with Mycgr3G92938\_Mycgr3T**
  
Percentage identity: 28 %
  
BlastP bit score: 161
  
Sequence coverage: 109 %
  
E-value: 2e-40
  
  
 NCBI BlastP on this gene

EMD59822

hypothetical protein
  
Accession: EMD59823
  
Location: 989279-989843
  
 NCBI BlastP on this gene

EMD59823

hypothetical protein
  
Accession: EMD59824
  
Location: 990666-993989
  
 NCBI BlastP on this gene

EMD59824

hypothetical protein
  
Accession: EMD59825
  
Location: 994815-995659
  
 NCBI BlastP on this gene

EMD59825

hypothetical protein
  
Accession: EMD59826
  
Location: 996396-997285
  
 NCBI BlastP on this gene

EMD59826

hypothetical protein
  
Accession: EMD59827
  
Location: 998374-1000687
  
 NCBI BlastP on this gene

EMD59827

hypothetical protein
  
Accession: EMD59828
  
Location: 1002037-1003557
  
 NCBI BlastP on this gene

EMD59828

Query: Architecture Search FASTA input

KE145364 : Glarea lozoyensis ATCC 20868 chromosome Unknown GLAREA20    Total score: 2.0     Cumulative Blast bit score: 2320

Hit cluster cross-links:

Mycgr3G85918 Mycgr3T
  
Location: 0-1602

Mycgr3G85918\_Mycgr3T

Mycgr3G42010 Mycgr3T
  
Location: 1702-8569

Mycgr3G42010\_Mycgr3T

Mycgr3G29582 Mycgr3T
  
Location: 8669-8915

Mycgr3G29582\_Mycgr3T

Mycgr3G31170 Mycgr3T
  
Location: 9015-9255

Mycgr3G31170\_Mycgr3T

Mycgr3G85924 Mycgr3T
  
Location: 9355-11218

Mycgr3G85924\_Mycgr3T

Mycgr3G71676 Mycgr3T
  
Location: 11318-12494

Mycgr3G71676\_Mycgr3T

Mycgr3G11468 Mycgr3T
  
Location: 12594-13653

Mycgr3G11468\_Mycgr3T

Mycgr3G58567 Mycgr3T
  
Location: 13753-14506

Mycgr3G58567\_Mycgr3T

Mycgr3G100089 Mycgr3
  
Location: 14606-21152

Mycgr3G100089\_Mycgr3

Mycgr3G42698 Mycgr3T
  
Location: 21252-22131

Mycgr3G42698\_Mycgr3T

Mycgr3G71681 Mycgr3T
  
Location: 22231-23461

Mycgr3G71681\_Mycgr3T

Mycgr3G109328 Mycgr3
  
Location: 23561-24239

Mycgr3G109328\_Mycgr3

Mycgr3G104334 Mycgr3
  
Location: 24339-24567

Mycgr3G104334\_Mycgr3

Mycgr3G42715 Mycgr3T
  
Location: 24667-25981

Mycgr3G42715\_Mycgr3T

Mycgr3G92934 Mycgr3T
  
Location: 26081-27593

Mycgr3G92934\_Mycgr3T

Mycgr3G41969 Mycgr3T
  
Location: 27693-29328

Mycgr3G41969\_Mycgr3T

Mycgr3G80635 Mycgr3T
  
Location: 29428-29821

Mycgr3G80635\_Mycgr3T

Mycgr3G41426 Mycgr3T
  
Location: 29921-35255

Mycgr3G41426\_Mycgr3T

Mycgr3G104337 Mycgr3
  
Location: 35355-36108

Mycgr3G104337\_Mycgr3

Mycgr3G71679 Mycgr3T
  
Location: 36208-37300

Mycgr3G71679\_Mycgr3T

Mycgr3G92938 Mycgr3T
  
Location: 37400-38699

Mycgr3G92938\_Mycgr3T

Mycgr3G92941 Mycgr3T
  
Location: 38799-40734

Mycgr3G92941\_Mycgr3T

hypothetical protein
  
Accession: EPE30300
  
Location: 168016-169596
  
 NCBI BlastP on this gene

EPE30300

NAD(P)-binding Rossmann-fold containing protein
  
Accession: EPE30301
  
Location: 172841-174090
  
 NCBI BlastP on this gene

EPE30301

hypothetical protein
  
Accession: EPE30302
  
Location: 175219-175809
  
 NCBI BlastP on this gene

EPE30302

Soluble quinoprotein glucose dehydrogenase
  
Accession: EPE30303
  
Location: 178363-179484
  
 NCBI BlastP on this gene

EPE30303

hypothetical protein
  
Accession: EPE30304
  
Location: 180574-181449
  
 NCBI BlastP on this gene

EPE30304

hypothetical protein
  
Accession: EPE30305
  
Location: 182608-184359
  
 NCBI BlastP on this gene

EPE30305

Thiolase-like protein
  
Accession: EPE30306
  
Location: 185108-192662
  
  
**BlastP hit with Mycgr3G100089\_Mycgr3**
  
Percentage identity: 49 %
  
BlastP bit score: 2125
  
Sequence coverage: 101 %
  
E-value: 0.0
  
  
 NCBI BlastP on this gene

EPE30306

hypothetical protein
  
Accession: EPE30307
  
Location: 196530-197377
  
  
**BlastP hit with Mycgr3G104337\_Mycgr3**
  
Percentage identity: 43 %
  
BlastP bit score: 195
  
Sequence coverage: 95 %
  
E-value: 8e-58
  
  
 NCBI BlastP on this gene

EPE30307

hypothetical protein
  
Accession: EPE30308
  
Location: 198822-199404
  
 NCBI BlastP on this gene

EPE30308

hypothetical protein
  
Accession: EPE30309
  
Location: 200384-203918
  
 NCBI BlastP on this gene

EPE30309

hypothetical protein
  
Accession: EPE30310
  
Location: 204404-204778
  
 NCBI BlastP on this gene

EPE30310

hypothetical protein
  
Accession: EPE30311
  
Location: 207297-207851
  
 NCBI BlastP on this gene

EPE30311

hypothetical protein
  
Accession: EPE30312
  
Location: 210979-212567
  
 NCBI BlastP on this gene

EPE30312

Query: Architecture Search FASTA input

KB915896 : Neofusicoccum parvum UCRNP2 chromosome Unknown NP2\_03\_scaffold\_258    Total score: 2.0     Cumulative Blast bit score: 2317

Hit cluster cross-links:

Mycgr3G85918 Mycgr3T
  
Location: 0-1602

Mycgr3G85918\_Mycgr3T

Mycgr3G42010 Mycgr3T
  
Location: 1702-8569

Mycgr3G42010\_Mycgr3T

Mycgr3G29582 Mycgr3T
  
Location: 8669-8915

Mycgr3G29582\_Mycgr3T

Mycgr3G31170 Mycgr3T
  
Location: 9015-9255

Mycgr3G31170\_Mycgr3T

Mycgr3G85924 Mycgr3T
  
Location: 9355-11218

Mycgr3G85924\_Mycgr3T

Mycgr3G71676 Mycgr3T
  
Location: 11318-12494

Mycgr3G71676\_Mycgr3T

Mycgr3G11468 Mycgr3T
  
Location: 12594-13653

Mycgr3G11468\_Mycgr3T

Mycgr3G58567 Mycgr3T
  
Location: 13753-14506

Mycgr3G58567\_Mycgr3T

Mycgr3G100089 Mycgr3
  
Location: 14606-21152

Mycgr3G100089\_Mycgr3

Mycgr3G42698 Mycgr3T
  
Location: 21252-22131

Mycgr3G42698\_Mycgr3T

Mycgr3G71681 Mycgr3T
  
Location: 22231-23461

Mycgr3G71681\_Mycgr3T

Mycgr3G109328 Mycgr3
  
Location: 23561-24239

Mycgr3G109328\_Mycgr3

Mycgr3G104334 Mycgr3
  
Location: 24339-24567

Mycgr3G104334\_Mycgr3

Mycgr3G42715 Mycgr3T
  
Location: 24667-25981

Mycgr3G42715\_Mycgr3T

Mycgr3G92934 Mycgr3T
  
Location: 26081-27593

Mycgr3G92934\_Mycgr3T

Mycgr3G41969 Mycgr3T
  
Location: 27693-29328

Mycgr3G41969\_Mycgr3T

Mycgr3G80635 Mycgr3T
  
Location: 29428-29821

Mycgr3G80635\_Mycgr3T

Mycgr3G41426 Mycgr3T
  
Location: 29921-35255

Mycgr3G41426\_Mycgr3T

Mycgr3G104337 Mycgr3
  
Location: 35355-36108

Mycgr3G104337\_Mycgr3

Mycgr3G71679 Mycgr3T
  
Location: 36208-37300

Mycgr3G71679\_Mycgr3T

Mycgr3G92938 Mycgr3T
  
Location: 37400-38699

Mycgr3G92938\_Mycgr3T

Mycgr3G92941 Mycgr3T
  
Location: 38799-40734

Mycgr3G92941\_Mycgr3T

putative sugar transporter protein
  
Accession: EOD51127
  
Location: 37-1746
  
 NCBI BlastP on this gene

EOD51127

hypothetical protein
  
Accession: EOD51121
  
Location: 6552-7469
  
 NCBI BlastP on this gene

EOD51121

hypothetical protein
  
Accession: EOD51123
  
Location: 9563-10099
  
 NCBI BlastP on this gene

EOD51123

hypothetical protein
  
Accession: EOD51116
  
Location: 12102-13384
  
 NCBI BlastP on this gene

EOD51116

putative polyketide synthase protein
  
Accession: EOD51113
  
Location: 16067-23074
  
  
**BlastP hit with Mycgr3G100089\_Mycgr3**
  
Percentage identity: 48 %
  
BlastP bit score: 2100
  
Sequence coverage: 102 %
  
E-value: 0.0
  
  
 NCBI BlastP on this gene

EOD51113

putative duf341 domain containing protein
  
Accession: EOD51112
  
Location: 24508-25440
  
  
**BlastP hit with Mycgr3G104337\_Mycgr3**
  
Percentage identity: 45 %
  
BlastP bit score: 217
  
Sequence coverage: 96 %
  
E-value: 3e-66
  
  
 NCBI BlastP on this gene

EOD51112

putative fumarylacetoacetate hydrolase protein
  
Accession: EOD51126
  
Location: 28734-29798
  
 NCBI BlastP on this gene

EOD51126

putative glycosyl hydrolases family protein
  
Accession: EOD51125
  
Location: 30209-32018
  
 NCBI BlastP on this gene

EOD51125

putative hexose transporter protein
  
Accession: EOD51118
  
Location: 33172-35139
  
 NCBI BlastP on this gene

EOD51118

putative transcription factor fungi protein
  
Accession: EOD51114
  
Location: 35276-37057
  
 NCBI BlastP on this gene

EOD51114

putative rrna-processing protein efg1 protein
  
Accession: EOD51110
  
Location: 40466-41272
  
 NCBI BlastP on this gene

EOD51110

putative ubiquitin-protein ligase e3 protein
  
Accession: EOD51122
  
Location: 41629-44647
  
 NCBI BlastP on this gene

EOD51122

Query: Architecture Search FASTA input

JH921455 : Marssonina brunnea f. sp. 'multigermtubi' MB\_m1 unplaced genomic scaffold M6\_S00028    Total score: 2.0     Cumulative Blast bit score: 2309

Hit cluster cross-links:

Mycgr3G85918 Mycgr3T
  
Location: 0-1602

Mycgr3G85918\_Mycgr3T

Mycgr3G42010 Mycgr3T
  
Location: 1702-8569

Mycgr3G42010\_Mycgr3T

Mycgr3G29582 Mycgr3T
  
Location: 8669-8915

Mycgr3G29582\_Mycgr3T

Mycgr3G31170 Mycgr3T
  
Location: 9015-9255

Mycgr3G31170\_Mycgr3T

Mycgr3G85924 Mycgr3T
  
Location: 9355-11218

Mycgr3G85924\_Mycgr3T

Mycgr3G71676 Mycgr3T
  
Location: 11318-12494

Mycgr3G71676\_Mycgr3T

Mycgr3G11468 Mycgr3T
  
Location: 12594-13653

Mycgr3G11468\_Mycgr3T

Mycgr3G58567 Mycgr3T
  
Location: 13753-14506

Mycgr3G58567\_Mycgr3T

Mycgr3G100089 Mycgr3
  
Location: 14606-21152

Mycgr3G100089\_Mycgr3

Mycgr3G42698 Mycgr3T
  
Location: 21252-22131

Mycgr3G42698\_Mycgr3T

Mycgr3G71681 Mycgr3T
  
Location: 22231-23461

Mycgr3G71681\_Mycgr3T

Mycgr3G109328 Mycgr3
  
Location: 23561-24239

Mycgr3G109328\_Mycgr3

Mycgr3G104334 Mycgr3
  
Location: 24339-24567

Mycgr3G104334\_Mycgr3

Mycgr3G42715 Mycgr3T
  
Location: 24667-25981

Mycgr3G42715\_Mycgr3T

Mycgr3G92934 Mycgr3T
  
Location: 26081-27593

Mycgr3G92934\_Mycgr3T

Mycgr3G41969 Mycgr3T
  
Location: 27693-29328

Mycgr3G41969\_Mycgr3T

Mycgr3G80635 Mycgr3T
  
Location: 29428-29821

Mycgr3G80635\_Mycgr3T

Mycgr3G41426 Mycgr3T
  
Location: 29921-35255

Mycgr3G41426\_Mycgr3T

Mycgr3G104337 Mycgr3
  
Location: 35355-36108

Mycgr3G104337\_Mycgr3

Mycgr3G71679 Mycgr3T
  
Location: 36208-37300

Mycgr3G71679\_Mycgr3T

Mycgr3G92938 Mycgr3T
  
Location: 37400-38699

Mycgr3G92938\_Mycgr3T

Mycgr3G92941 Mycgr3T
  
Location: 38799-40734

Mycgr3G92941\_Mycgr3T

glyoxylate pathway regulator
  
Accession: EKD12627
  
Location: 282801-284285
  
 NCBI BlastP on this gene

EKD12627

RNA polymerase I specific transcription initiation factor
  
Accession: EKD12628
  
Location: 285638-287515
  
 NCBI BlastP on this gene

EKD12628

hypothetical protein
  
Accession: EKD12629
  
Location: 289628-290170
  
 NCBI BlastP on this gene

EKD12629

hypothetical protein
  
Accession: EKD12630
  
Location: 290405-291765
  
 NCBI BlastP on this gene

EKD12630

pescadillo
  
Accession: EKD12631
  
Location: 292016-294058
  
 NCBI BlastP on this gene

EKD12631

hypothetical protein
  
Accession: EKD12632
  
Location: 294511-298254
  
 NCBI BlastP on this gene

EKD12632

beta-ketoacyl synthase domain-containing protein
  
Accession: EKD12633
  
Location: 299348-306442
  
  
**BlastP hit with Mycgr3G100089\_Mycgr3**
  
Percentage identity: 48 %
  
BlastP bit score: 2108
  
Sequence coverage: 102 %
  
E-value: 0.0
  
  
 NCBI BlastP on this gene

EKD12633

hypothetical protein
  
Accession: EKD12634
  
Location: 309287-310301
  
  
**BlastP hit with Mycgr3G104337\_Mycgr3**
  
Percentage identity: 42 %
  
BlastP bit score: 201
  
Sequence coverage: 95 %
  
E-value: 5e-60
  
  
 NCBI BlastP on this gene

EKD12634

short chain dehydrogenase/reductase SDR
  
Accession: EKD12635
  
Location: 310854-312068
  
 NCBI BlastP on this gene

EKD12635

hypothetical protein
  
Accession: EKD12636
  
Location: 313280-316000
  
 NCBI BlastP on this gene

EKD12636

hypothetical protein
  
Accession: EKD12637
  
Location: 316985-317668
  
 NCBI BlastP on this gene

EKD12637

hypothetical protein
  
Accession: EKD12638
  
Location: 318386-318859
  
 NCBI BlastP on this gene

EKD12638

hypothetical protein
  
Accession: EKD12639
  
Location: 320651-321064
  
 NCBI BlastP on this gene

EKD12639

hypothetical protein
  
Accession: EKD12640
  
Location: 322480-322980
  
 NCBI BlastP on this gene

EKD12640

ethyl tert-butyl ether degradation EthD
  
Accession: EKD12641
  
Location: 326346-326651
  
 NCBI BlastP on this gene

EKD12641

hypothetical protein
  
Accession: EKD12642
  
Location: 327120-327712
  
 NCBI BlastP on this gene

EKD12642

Query: Architecture Search FASTA input

AMYD01000564 : Colletotrichum gloeosporioides Cg-14    Total score: 2.0     Cumulative Blast bit score: 2306

Hit cluster cross-links:

Mycgr3G85918 Mycgr3T
  
Location: 0-1602

Mycgr3G85918\_Mycgr3T

Mycgr3G42010 Mycgr3T
  
Location: 1702-8569

Mycgr3G42010\_Mycgr3T

Mycgr3G29582 Mycgr3T
  
Location: 8669-8915

Mycgr3G29582\_Mycgr3T

Mycgr3G31170 Mycgr3T
  
Location: 9015-9255

Mycgr3G31170\_Mycgr3T

Mycgr3G85924 Mycgr3T
  
Location: 9355-11218

Mycgr3G85924\_Mycgr3T

Mycgr3G71676 Mycgr3T
  
Location: 11318-12494

Mycgr3G71676\_Mycgr3T

Mycgr3G11468 Mycgr3T
  
Location: 12594-13653

Mycgr3G11468\_Mycgr3T

Mycgr3G58567 Mycgr3T
  
Location: 13753-14506

Mycgr3G58567\_Mycgr3T

Mycgr3G100089 Mycgr3
  
Location: 14606-21152

Mycgr3G100089\_Mycgr3

Mycgr3G42698 Mycgr3T
  
Location: 21252-22131

Mycgr3G42698\_Mycgr3T

Mycgr3G71681 Mycgr3T
  
Location: 22231-23461

Mycgr3G71681\_Mycgr3T

Mycgr3G109328 Mycgr3
  
Location: 23561-24239

Mycgr3G109328\_Mycgr3

Mycgr3G104334 Mycgr3
  
Location: 24339-24567

Mycgr3G104334\_Mycgr3

Mycgr3G42715 Mycgr3T
  
Location: 24667-25981

Mycgr3G42715\_Mycgr3T

Mycgr3G92934 Mycgr3T
  
Location: 26081-27593

Mycgr3G92934\_Mycgr3T

Mycgr3G41969 Mycgr3T
  
Location: 27693-29328

Mycgr3G41969\_Mycgr3T

Mycgr3G80635 Mycgr3T
  
Location: 29428-29821

Mycgr3G80635\_Mycgr3T

Mycgr3G41426 Mycgr3T
  
Location: 29921-35255

Mycgr3G41426\_Mycgr3T

Mycgr3G104337 Mycgr3
  
Location: 35355-36108

Mycgr3G104337\_Mycgr3

Mycgr3G71679 Mycgr3T
  
Location: 36208-37300

Mycgr3G71679\_Mycgr3T

Mycgr3G92938 Mycgr3T
  
Location: 37400-38699

Mycgr3G92938\_Mycgr3T

Mycgr3G92941 Mycgr3T
  
Location: 38799-40734

Mycgr3G92941\_Mycgr3T

hypothetical protein
  
Accession: EQB57153
  
Location: 151-563
  
 NCBI BlastP on this gene

EQB57153

FAD binding domain-containing protein
  
Accession: EQB57154
  
Location: 2563-4083
  
 NCBI BlastP on this gene

EQB57154

hypothetical protein
  
Accession: EQB57155
  
Location: 5195-6410
  
 NCBI BlastP on this gene

EQB57155

hypothetical protein
  
Accession: EQB57156
  
Location: 6824-7712
  
 NCBI BlastP on this gene

EQB57156

hypothetical protein
  
Accession: EQB57157
  
Location: 7852-8368
  
 NCBI BlastP on this gene

EQB57157

hypothetical protein
  
Accession: EQB57158
  
Location: 8992-16107
  
  
**BlastP hit with Mycgr3G42010\_Mycgr3T**
  
Percentage identity: 46 %
  
BlastP bit score: 2010
  
Sequence coverage: 100 %
  
E-value: 0.0
  
  
 NCBI BlastP on this gene

EQB57158

geranylgeranyl pyrophosphate synthetase
  
Accession: EQB57159
  
Location: 16660-18061
  
  
**BlastP hit with Mycgr3G92938\_Mycgr3T**
  
Percentage identity: 39 %
  
BlastP bit score: 296
  
Sequence coverage: 104 %
  
E-value: 7e-92
  
  
 NCBI BlastP on this gene

EQB57159

Query: Architecture Search FASTA input

KB725756 : Colletotrichum orbiculare MAFF 240422 unplaced genomic scaffold Scaffold\_209    Total score: 2.0     Cumulative Blast bit score: 2294

Hit cluster cross-links:

Mycgr3G85918 Mycgr3T
  
Location: 0-1602

Mycgr3G85918\_Mycgr3T

Mycgr3G42010 Mycgr3T
  
Location: 1702-8569

Mycgr3G42010\_Mycgr3T

Mycgr3G29582 Mycgr3T
  
Location: 8669-8915

Mycgr3G29582\_Mycgr3T

Mycgr3G31170 Mycgr3T
  
Location: 9015-9255

Mycgr3G31170\_Mycgr3T

Mycgr3G85924 Mycgr3T
  
Location: 9355-11218

Mycgr3G85924\_Mycgr3T

Mycgr3G71676 Mycgr3T
  
Location: 11318-12494

Mycgr3G71676\_Mycgr3T

Mycgr3G11468 Mycgr3T
  
Location: 12594-13653

Mycgr3G11468\_Mycgr3T

Mycgr3G58567 Mycgr3T
  
Location: 13753-14506

Mycgr3G58567\_Mycgr3T

Mycgr3G100089 Mycgr3
  
Location: 14606-21152

Mycgr3G100089\_Mycgr3

Mycgr3G42698 Mycgr3T
  
Location: 21252-22131

Mycgr3G42698\_Mycgr3T

Mycgr3G71681 Mycgr3T
  
Location: 22231-23461

Mycgr3G71681\_Mycgr3T

Mycgr3G109328 Mycgr3
  
Location: 23561-24239

Mycgr3G109328\_Mycgr3

Mycgr3G104334 Mycgr3
  
Location: 24339-24567

Mycgr3G104334\_Mycgr3

Mycgr3G42715 Mycgr3T
  
Location: 24667-25981

Mycgr3G42715\_Mycgr3T

Mycgr3G92934 Mycgr3T
  
Location: 26081-27593

Mycgr3G92934\_Mycgr3T

Mycgr3G41969 Mycgr3T
  
Location: 27693-29328

Mycgr3G41969\_Mycgr3T

Mycgr3G80635 Mycgr3T
  
Location: 29428-29821

Mycgr3G80635\_Mycgr3T

Mycgr3G41426 Mycgr3T
  
Location: 29921-35255

Mycgr3G41426\_Mycgr3T

Mycgr3G104337 Mycgr3
  
Location: 35355-36108

Mycgr3G104337\_Mycgr3

Mycgr3G71679 Mycgr3T
  
Location: 36208-37300

Mycgr3G71679\_Mycgr3T

Mycgr3G92938 Mycgr3T
  
Location: 37400-38699

Mycgr3G92938\_Mycgr3T

Mycgr3G92941 Mycgr3T
  
Location: 38799-40734

Mycgr3G92941\_Mycgr3T

60s ribosomal protein l16
  
Accession: ENH85897
  
Location: 65918-67070
  
 NCBI BlastP on this gene

ENH85897

bag domain protein
  
Accession: ENH85898
  
Location: 68120-70450
  
 NCBI BlastP on this gene

ENH85898

glutaredoxin
  
Accession: ENH85899
  
Location: 71352-71756
  
 NCBI BlastP on this gene

ENH85899

chalcone-flavanone isomerase
  
Accession: ENH85900
  
Location: 72073-73260
  
 NCBI BlastP on this gene

ENH85900

ubiquitin ligase complex f-box protein
  
Accession: ENH85901
  
Location: 78440-80997
  
 NCBI BlastP on this gene

ENH85901

RNA exonuclease
  
Accession: ENH85902
  
Location: 81622-83056
  
 NCBI BlastP on this gene

ENH85902

geranylgeranyl pyrophosphate synthetase
  
Accession: ENH85903
  
Location: 84166-85466
  
  
**BlastP hit with Mycgr3G92938\_Mycgr3T**
  
Percentage identity: 42 %
  
BlastP bit score: 283
  
Sequence coverage: 84 %
  
E-value: 3e-87
  
  
 NCBI BlastP on this gene

ENH85903

nfx1-type zinc finger-containing protein 1
  
Accession: ENH85904
  
Location: 86209-93338
  
  
**BlastP hit with Mycgr3G42010\_Mycgr3T**
  
Percentage identity: 46 %
  
BlastP bit score: 2011
  
Sequence coverage: 101 %
  
E-value: 0.0
  
  
 NCBI BlastP on this gene

ENH85904

hypothetical protein
  
Accession: ENH85905
  
Location: 94083-94534
  
 NCBI BlastP on this gene

ENH85905

aspartate aminotransferase
  
Accession: ENH85906
  
Location: 95892-97600
  
 NCBI BlastP on this gene

ENH85906

oxalate formate antiporter
  
Accession: ENH85907
  
Location: 98078-99611
  
 NCBI BlastP on this gene

ENH85907

C6 zinc finger domain-containing protein
  
Accession: ENH85908
  
Location: 100004-102951
  
 NCBI BlastP on this gene

ENH85908

hypothetical protein
  
Accession: ENH85909
  
Location: 103551-105005
  
 NCBI BlastP on this gene

ENH85909

24-dehydrocholesterol reductase precursor
  
Accession: ENH85910
  
Location: 106673-108265
  
 NCBI BlastP on this gene

ENH85910

cyanide hydratase
  
Accession: ENH85911
  
Location: 109331-110549
  
 NCBI BlastP on this gene

ENH85911

phospholipid methyltransferase
  
Accession: ENH85912
  
Location: 110948-111837
  
 NCBI BlastP on this gene

ENH85912

Query: Architecture Search FASTA input

AKCU01000308 : Penicillium digitatum Pd1    Total score: 2.0     Cumulative Blast bit score: 2290

Hit cluster cross-links:

Mycgr3G85918 Mycgr3T
  
Location: 0-1602

Mycgr3G85918\_Mycgr3T

Mycgr3G42010 Mycgr3T
  
Location: 1702-8569

Mycgr3G42010\_Mycgr3T

Mycgr3G29582 Mycgr3T
  
Location: 8669-8915

Mycgr3G29582\_Mycgr3T

Mycgr3G31170 Mycgr3T
  
Location: 9015-9255

Mycgr3G31170\_Mycgr3T

Mycgr3G85924 Mycgr3T
  
Location: 9355-11218

Mycgr3G85924\_Mycgr3T

Mycgr3G71676 Mycgr3T
  
Location: 11318-12494

Mycgr3G71676\_Mycgr3T

Mycgr3G11468 Mycgr3T
  
Location: 12594-13653

Mycgr3G11468\_Mycgr3T

Mycgr3G58567 Mycgr3T
  
Location: 13753-14506

Mycgr3G58567\_Mycgr3T

Mycgr3G100089 Mycgr3
  
Location: 14606-21152

Mycgr3G100089\_Mycgr3

Mycgr3G42698 Mycgr3T
  
Location: 21252-22131

Mycgr3G42698\_Mycgr3T

Mycgr3G71681 Mycgr3T
  
Location: 22231-23461

Mycgr3G71681\_Mycgr3T

Mycgr3G109328 Mycgr3
  
Location: 23561-24239

Mycgr3G109328\_Mycgr3

Mycgr3G104334 Mycgr3
  
Location: 24339-24567

Mycgr3G104334\_Mycgr3

Mycgr3G42715 Mycgr3T
  
Location: 24667-25981

Mycgr3G42715\_Mycgr3T

Mycgr3G92934 Mycgr3T
  
Location: 26081-27593

Mycgr3G92934\_Mycgr3T

Mycgr3G41969 Mycgr3T
  
Location: 27693-29328

Mycgr3G41969\_Mycgr3T

Mycgr3G80635 Mycgr3T
  
Location: 29428-29821

Mycgr3G80635\_Mycgr3T

Mycgr3G41426 Mycgr3T
  
Location: 29921-35255

Mycgr3G41426\_Mycgr3T

Mycgr3G104337 Mycgr3
  
Location: 35355-36108

Mycgr3G104337\_Mycgr3

Mycgr3G71679 Mycgr3T
  
Location: 36208-37300

Mycgr3G71679\_Mycgr3T

Mycgr3G92938 Mycgr3T
  
Location: 37400-38699

Mycgr3G92938\_Mycgr3T

Mycgr3G92941 Mycgr3T
  
Location: 38799-40734

Mycgr3G92941\_Mycgr3T

hypothetical protein
  
Accession: EKV14359
  
Location: 44347-44815
  
 NCBI BlastP on this gene

EKV14359

hypothetical protein
  
Accession: EKV14360
  
Location: 47327-49074
  
 NCBI BlastP on this gene

EKV14360

37S ribosomal protein Rsm24, putative
  
Accession: EKV14361
  
Location: 49972-51185
  
 NCBI BlastP on this gene

EKV14361

UDP-N-acetylglucosamine transferase subunit alg13
  
Accession: EKV14362
  
Location: 51466-51967
  
 NCBI BlastP on this gene

EKV14362

Actin-related protein ArpA
  
Accession: EKV14363
  
Location: 52723-53991
  
 NCBI BlastP on this gene

EKV14363

hypothetical protein
  
Accession: EKV14364
  
Location: 55619-56107
  
 NCBI BlastP on this gene

EKV14364

hypothetical protein
  
Accession: EKV14365
  
Location: 59043-59828
  
 NCBI BlastP on this gene

EKV14365

hypothetical protein
  
Accession: EKV14366
  
Location: 60183-67514
  
  
**BlastP hit with Mycgr3G42010\_Mycgr3T**
  
Percentage identity: 47 %
  
BlastP bit score: 2078
  
Sequence coverage: 102 %
  
E-value: 0.0
  
  
 NCBI BlastP on this gene

EKV14366

hypothetical protein
  
Accession: EKV14367
  
Location: 68372-69274
  
  
**BlastP hit with Mycgr3G92938\_Mycgr3T**
  
Percentage identity: 37 %
  
BlastP bit score: 212
  
Sequence coverage: 68 %
  
E-value: 1e-61
  
  
 NCBI BlastP on this gene

EKV14367

hypothetical protein
  
Accession: EKV14368
  
Location: 70253-71080
  
 NCBI BlastP on this gene

EKV14368

hypothetical protein
  
Accession: EKV14369
  
Location: 72050-74299
  
 NCBI BlastP on this gene

EKV14369

ATP-dependent RNA helicase DDX18
  
Accession: EKV14370
  
Location: 79517-81524
  
 NCBI BlastP on this gene

EKV14370

hypothetical protein
  
Accession: EKV14371
  
Location: 83475-88279
  
 NCBI BlastP on this gene

EKV14371

Query: Architecture Search FASTA input

AKCT01000265 : Penicillium digitatum PHI26    Total score: 2.0     Cumulative Blast bit score: 2290

Hit cluster cross-links:

Mycgr3G85918 Mycgr3T
  
Location: 0-1602

Mycgr3G85918\_Mycgr3T

Mycgr3G42010 Mycgr3T
  
Location: 1702-8569

Mycgr3G42010\_Mycgr3T

Mycgr3G29582 Mycgr3T
  
Location: 8669-8915

Mycgr3G29582\_Mycgr3T

Mycgr3G31170 Mycgr3T
  
Location: 9015-9255

Mycgr3G31170\_Mycgr3T

Mycgr3G85924 Mycgr3T
  
Location: 9355-11218

Mycgr3G85924\_Mycgr3T

Mycgr3G71676 Mycgr3T
  
Location: 11318-12494

Mycgr3G71676\_Mycgr3T

Mycgr3G11468 Mycgr3T
  
Location: 12594-13653

Mycgr3G11468\_Mycgr3T

Mycgr3G58567 Mycgr3T
  
Location: 13753-14506

Mycgr3G58567\_Mycgr3T

Mycgr3G100089 Mycgr3
  
Location: 14606-21152

Mycgr3G100089\_Mycgr3

Mycgr3G42698 Mycgr3T
  
Location: 21252-22131

Mycgr3G42698\_Mycgr3T

Mycgr3G71681 Mycgr3T
  
Location: 22231-23461

Mycgr3G71681\_Mycgr3T

Mycgr3G109328 Mycgr3
  
Location: 23561-24239

Mycgr3G109328\_Mycgr3

Mycgr3G104334 Mycgr3
  
Location: 24339-24567

Mycgr3G104334\_Mycgr3

Mycgr3G42715 Mycgr3T
  
Location: 24667-25981

Mycgr3G42715\_Mycgr3T

Mycgr3G92934 Mycgr3T
  
Location: 26081-27593

Mycgr3G92934\_Mycgr3T

Mycgr3G41969 Mycgr3T
  
Location: 27693-29328

Mycgr3G41969\_Mycgr3T

Mycgr3G80635 Mycgr3T
  
Location: 29428-29821

Mycgr3G80635\_Mycgr3T

Mycgr3G41426 Mycgr3T
  
Location: 29921-35255

Mycgr3G41426\_Mycgr3T

Mycgr3G104337 Mycgr3
  
Location: 35355-36108

Mycgr3G104337\_Mycgr3

Mycgr3G71679 Mycgr3T
  
Location: 36208-37300

Mycgr3G71679\_Mycgr3T

Mycgr3G92938 Mycgr3T
  
Location: 37400-38699

Mycgr3G92938\_Mycgr3T

Mycgr3G92941 Mycgr3T
  
Location: 38799-40734

Mycgr3G92941\_Mycgr3T

hypothetical protein
  
Accession: EKV07281
  
Location: 42254-42722
  
 NCBI BlastP on this gene

EKV07281

hypothetical protein
  
Accession: EKV07282
  
Location: 45234-46981
  
 NCBI BlastP on this gene

EKV07282

37S ribosomal protein Rsm24, putative
  
Accession: EKV07283
  
Location: 47879-49092
  
 NCBI BlastP on this gene

EKV07283

UDP-N-acetylglucosamine transferase subunit alg13
  
Accession: EKV07284
  
Location: 49373-49874
  
 NCBI BlastP on this gene

EKV07284

Actin-related protein ArpA
  
Accession: EKV07285
  
Location: 50630-51898
  
 NCBI BlastP on this gene

EKV07285

hypothetical protein
  
Accession: EKV07286
  
Location: 53526-54014
  
 NCBI BlastP on this gene

EKV07286

hypothetical protein
  
Accession: EKV07287
  
Location: 56931-57716
  
 NCBI BlastP on this gene

EKV07287

hypothetical protein
  
Accession: EKV07288
  
Location: 57969-65300
  
  
**BlastP hit with Mycgr3G42010\_Mycgr3T**
  
Percentage identity: 47 %
  
BlastP bit score: 2078
  
Sequence coverage: 102 %
  
E-value: 0.0
  
  
 NCBI BlastP on this gene

EKV07288

hypothetical protein
  
Accession: EKV07289
  
Location: 66158-67060
  
  
**BlastP hit with Mycgr3G92938\_Mycgr3T**
  
Percentage identity: 37 %
  
BlastP bit score: 212
  
Sequence coverage: 68 %
  
E-value: 1e-61
  
  
 NCBI BlastP on this gene

EKV07289

hypothetical protein
  
Accession: EKV07290
  
Location: 68039-68866
  
 NCBI BlastP on this gene

EKV07290

hypothetical protein
  
Accession: EKV07291
  
Location: 69836-72085
  
 NCBI BlastP on this gene

EKV07291

Query: Architecture Search FASTA input

GG697333 : Glomerella graminicola M1.001 genomic scaffold supercont1.3    Total score: 2.0     Cumulative Blast bit score: 2283

Hit cluster cross-links:

Mycgr3G85918 Mycgr3T
  
Location: 0-1602

Mycgr3G85918\_Mycgr3T

Mycgr3G42010 Mycgr3T
  
Location: 1702-8569

Mycgr3G42010\_Mycgr3T

Mycgr3G29582 Mycgr3T
  
Location: 8669-8915

Mycgr3G29582\_Mycgr3T

Mycgr3G31170 Mycgr3T
  
Location: 9015-9255

Mycgr3G31170\_Mycgr3T

Mycgr3G85924 Mycgr3T
  
Location: 9355-11218

Mycgr3G85924\_Mycgr3T

Mycgr3G71676 Mycgr3T
  
Location: 11318-12494

Mycgr3G71676\_Mycgr3T

Mycgr3G11468 Mycgr3T
  
Location: 12594-13653

Mycgr3G11468\_Mycgr3T

Mycgr3G58567 Mycgr3T
  
Location: 13753-14506

Mycgr3G58567\_Mycgr3T

Mycgr3G100089 Mycgr3
  
Location: 14606-21152

Mycgr3G100089\_Mycgr3

Mycgr3G42698 Mycgr3T
  
Location: 21252-22131

Mycgr3G42698\_Mycgr3T

Mycgr3G71681 Mycgr3T
  
Location: 22231-23461

Mycgr3G71681\_Mycgr3T

Mycgr3G109328 Mycgr3
  
Location: 23561-24239

Mycgr3G109328\_Mycgr3

Mycgr3G104334 Mycgr3
  
Location: 24339-24567

Mycgr3G104334\_Mycgr3

Mycgr3G42715 Mycgr3T
  
Location: 24667-25981

Mycgr3G42715\_Mycgr3T

Mycgr3G92934 Mycgr3T
  
Location: 26081-27593

Mycgr3G92934\_Mycgr3T

Mycgr3G41969 Mycgr3T
  
Location: 27693-29328

Mycgr3G41969\_Mycgr3T

Mycgr3G80635 Mycgr3T
  
Location: 29428-29821

Mycgr3G80635\_Mycgr3T

Mycgr3G41426 Mycgr3T
  
Location: 29921-35255

Mycgr3G41426\_Mycgr3T

Mycgr3G104337 Mycgr3
  
Location: 35355-36108

Mycgr3G104337\_Mycgr3

Mycgr3G71679 Mycgr3T
  
Location: 36208-37300

Mycgr3G71679\_Mycgr3T

Mycgr3G92938 Mycgr3T
  
Location: 37400-38699

Mycgr3G92938\_Mycgr3T

Mycgr3G92941 Mycgr3T
  
Location: 38799-40734

Mycgr3G92941\_Mycgr3T

BAG domain-containing protein
  
Accession: EFQ25840
  
Location: 246374-247345
  
 NCBI BlastP on this gene

EFQ25840

glutaredoxin
  
Accession: EFQ25841
  
Location: 249834-250307
  
 NCBI BlastP on this gene

EFQ25841

chalcone-flavanone isomerase
  
Accession: EFQ25842
  
Location: 250761-252080
  
 NCBI BlastP on this gene

EFQ25842

F-box domain-containing protein
  
Accession: EFQ25843
  
Location: 258429-261062
  
 NCBI BlastP on this gene

EFQ25843

exonuclease
  
Accession: EFQ25844
  
Location: 261962-263462
  
 NCBI BlastP on this gene

EFQ25844

geranylgeranyl pyrophosphate synthetase
  
Accession: EFQ25845
  
Location: 265127-266559
  
  
**BlastP hit with Mycgr3G92938\_Mycgr3T**
  
Percentage identity: 37 %
  
BlastP bit score: 293
  
Sequence coverage: 104 %
  
E-value: 1e-90
  
  
 NCBI BlastP on this gene

EFQ25845

ATPase
  
Accession: EFQ25846
  
Location: 267176-274326
  
  
**BlastP hit with Mycgr3G42010\_Mycgr3T**
  
Percentage identity: 46 %
  
BlastP bit score: 1990
  
Sequence coverage: 101 %
  
E-value: 0.0
  
  
 NCBI BlastP on this gene

EFQ25846

hypothetical protein
  
Accession: EFQ25847
  
Location: 275926-277174
  
 NCBI BlastP on this gene

EFQ25847

O-methyltransferase
  
Accession: EFQ25848
  
Location: 279676-281044
  
 NCBI BlastP on this gene

EFQ25848

hypothetical protein
  
Accession: EFQ25849
  
Location: 281501-283801
  
 NCBI BlastP on this gene

EFQ25849

hypothetical protein
  
Accession: EFQ25850
  
Location: 285677-286252
  
 NCBI BlastP on this gene

EFQ25850

hypothetical protein
  
Accession: EFQ25851
  
Location: 288547-288993
  
 NCBI BlastP on this gene

EFQ25851

C6 zinc finger domain-containing protein
  
Accession: EFQ25852
  
Location: 289562-291386
  
 NCBI BlastP on this gene

EFQ25852

Query: Architecture Search FASTA input

KB446535 : Dothistroma septosporum NZE10 unplaced genomic scaffold DOTSEscaffold\_1    Total score: 2.0     Cumulative Blast bit score: 2268

Hit cluster cross-links:

Mycgr3G85918 Mycgr3T
  
Location: 0-1602

Mycgr3G85918\_Mycgr3T

Mycgr3G42010 Mycgr3T
  
Location: 1702-8569

Mycgr3G42010\_Mycgr3T

Mycgr3G29582 Mycgr3T
  
Location: 8669-8915

Mycgr3G29582\_Mycgr3T

Mycgr3G31170 Mycgr3T
  
Location: 9015-9255

Mycgr3G31170\_Mycgr3T

Mycgr3G85924 Mycgr3T
  
Location: 9355-11218

Mycgr3G85924\_Mycgr3T

Mycgr3G71676 Mycgr3T
  
Location: 11318-12494

Mycgr3G71676\_Mycgr3T

Mycgr3G11468 Mycgr3T
  
Location: 12594-13653

Mycgr3G11468\_Mycgr3T

Mycgr3G58567 Mycgr3T
  
Location: 13753-14506

Mycgr3G58567\_Mycgr3T

Mycgr3G100089 Mycgr3
  
Location: 14606-21152

Mycgr3G100089\_Mycgr3

Mycgr3G42698 Mycgr3T
  
Location: 21252-22131

Mycgr3G42698\_Mycgr3T

Mycgr3G71681 Mycgr3T
  
Location: 22231-23461

Mycgr3G71681\_Mycgr3T

Mycgr3G109328 Mycgr3
  
Location: 23561-24239

Mycgr3G109328\_Mycgr3

Mycgr3G104334 Mycgr3
  
Location: 24339-24567

Mycgr3G104334\_Mycgr3

Mycgr3G42715 Mycgr3T
  
Location: 24667-25981

Mycgr3G42715\_Mycgr3T

Mycgr3G92934 Mycgr3T
  
Location: 26081-27593

Mycgr3G92934\_Mycgr3T

Mycgr3G41969 Mycgr3T
  
Location: 27693-29328

Mycgr3G41969\_Mycgr3T

Mycgr3G80635 Mycgr3T
  
Location: 29428-29821

Mycgr3G80635\_Mycgr3T

Mycgr3G41426 Mycgr3T
  
Location: 29921-35255

Mycgr3G41426\_Mycgr3T

Mycgr3G104337 Mycgr3
  
Location: 35355-36108

Mycgr3G104337\_Mycgr3

Mycgr3G71679 Mycgr3T
  
Location: 36208-37300

Mycgr3G71679\_Mycgr3T

Mycgr3G92938 Mycgr3T
  
Location: 37400-38699

Mycgr3G92938\_Mycgr3T

Mycgr3G92941 Mycgr3T
  
Location: 38799-40734

Mycgr3G92941\_Mycgr3T

hypothetical protein
  
Accession: EME48882
  
Location: 1340176-1341006
  
 NCBI BlastP on this gene

EME48882

hypothetical protein
  
Accession: EME48881
  
Location: 1339231-1339431
  
 NCBI BlastP on this gene

EME48881

hypothetical protein
  
Accession: EME48880
  
Location: 1335865-1336710
  
 NCBI BlastP on this gene

EME48880

hypothetical protein
  
Accession: EME48879
  
Location: 1332880-1334838
  
 NCBI BlastP on this gene

EME48879

hypothetical protein
  
Accession: EME48878
  
Location: 1326694-1332105
  
  
**BlastP hit with Mycgr3G41426\_Mycgr3T**
  
Percentage identity: 55 %
  
BlastP bit score: 2014
  
Sequence coverage: 100 %
  
E-value: 0.0
  
  
 NCBI BlastP on this gene

EME48878

hypothetical protein
  
Accession: EME48877
  
Location: 1321515-1324153
  
 NCBI BlastP on this gene

EME48877

hypothetical protein
  
Accession: EME48876
  
Location: 1320423-1321439
  
 NCBI BlastP on this gene

EME48876

hypothetical protein
  
Accession: EME48875
  
Location: 1317427-1318572
  
 NCBI BlastP on this gene

EME48875

hypothetical protein
  
Accession: EME48874
  
Location: 1315875-1316821
  
 NCBI BlastP on this gene

EME48874

hypothetical protein
  
Accession: EME48873
  
Location: 1312337-1314642
  
 NCBI BlastP on this gene

EME48873

hypothetical protein
  
Accession: EME48872
  
Location: 1309909-1311752
  
 NCBI BlastP on this gene

EME48872

hypothetical protein
  
Accession: EME48871
  
Location: 1307092-1309291
  
 NCBI BlastP on this gene

EME48871

hypothetical protein
  
Accession: EME48870
  
Location: 1304785-1306669
  
 NCBI BlastP on this gene

EME48870

hypothetical protein
  
Accession: EME48869
  
Location: 1303487-1304290
  
  
**BlastP hit with Mycgr3G58567\_Mycgr3T**
  
Percentage identity: 53 %
  
BlastP bit score: 254
  
Sequence coverage: 98 %
  
E-value: 1e-80
  
  
 NCBI BlastP on this gene

EME48869

hypothetical protein
  
Accession: EME48867
  
Location: 1298681-1301703
  
 NCBI BlastP on this gene

EME48867

hypothetical protein
  
Accession: EME48866
  
Location: 1292927-1296823
  
 NCBI BlastP on this gene

EME48866

Query: Architecture Search FASTA input

KB020741 : Colletotrichum gloeosporioides Nara gc5 unplaced genomic scaffold scaffold364    Total score: 2.0     Cumulative Blast bit score: 2255

Hit cluster cross-links:

Mycgr3G85918 Mycgr3T
  
Location: 0-1602

Mycgr3G85918\_Mycgr3T

Mycgr3G42010 Mycgr3T
  
Location: 1702-8569

Mycgr3G42010\_Mycgr3T

Mycgr3G29582 Mycgr3T
  
Location: 8669-8915

Mycgr3G29582\_Mycgr3T

Mycgr3G31170 Mycgr3T
  
Location: 9015-9255

Mycgr3G31170\_Mycgr3T

Mycgr3G85924 Mycgr3T
  
Location: 9355-11218

Mycgr3G85924\_Mycgr3T

Mycgr3G71676 Mycgr3T
  
Location: 11318-12494

Mycgr3G71676\_Mycgr3T

Mycgr3G11468 Mycgr3T
  
Location: 12594-13653

Mycgr3G11468\_Mycgr3T

Mycgr3G58567 Mycgr3T
  
Location: 13753-14506

Mycgr3G58567\_Mycgr3T

Mycgr3G100089 Mycgr3
  
Location: 14606-21152

Mycgr3G100089\_Mycgr3

Mycgr3G42698 Mycgr3T
  
Location: 21252-22131

Mycgr3G42698\_Mycgr3T

Mycgr3G71681 Mycgr3T
  
Location: 22231-23461

Mycgr3G71681\_Mycgr3T

Mycgr3G109328 Mycgr3
  
Location: 23561-24239

Mycgr3G109328\_Mycgr3

Mycgr3G104334 Mycgr3
  
Location: 24339-24567

Mycgr3G104334\_Mycgr3

Mycgr3G42715 Mycgr3T
  
Location: 24667-25981

Mycgr3G42715\_Mycgr3T

Mycgr3G92934 Mycgr3T
  
Location: 26081-27593

Mycgr3G92934\_Mycgr3T

Mycgr3G41969 Mycgr3T
  
Location: 27693-29328

Mycgr3G41969\_Mycgr3T

Mycgr3G80635 Mycgr3T
  
Location: 29428-29821

Mycgr3G80635\_Mycgr3T

Mycgr3G41426 Mycgr3T
  
Location: 29921-35255

Mycgr3G41426\_Mycgr3T

Mycgr3G104337 Mycgr3
  
Location: 35355-36108

Mycgr3G104337\_Mycgr3

Mycgr3G71679 Mycgr3T
  
Location: 36208-37300

Mycgr3G71679\_Mycgr3T

Mycgr3G92938 Mycgr3T
  
Location: 37400-38699

Mycgr3G92938\_Mycgr3T

Mycgr3G92941 Mycgr3T
  
Location: 38799-40734

Mycgr3G92941\_Mycgr3T

bag domain protein
  
Accession: ELA31574
  
Location: 41324-42289
  
 NCBI BlastP on this gene

ELA31574

glutaredoxin
  
Accession: ELA31575
  
Location: 44913-45306
  
 NCBI BlastP on this gene

ELA31575

chalcone-flavanone isomerase
  
Accession: ELA31576
  
Location: 45665-46885
  
 NCBI BlastP on this gene

ELA31576

ubiquitin ligase complex f-box protein
  
Accession: ELA31577
  
Location: 52091-54618
  
 NCBI BlastP on this gene

ELA31577

3'-5' exonuclease
  
Accession: ELA31578
  
Location: 55444-56966
  
 NCBI BlastP on this gene

ELA31578

geranylgeranyl pyrophosphate synthetase
  
Accession: ELA31579
  
Location: 58901-60302
  
  
**BlastP hit with Mycgr3G92938\_Mycgr3T**
  
Percentage identity: 38 %
  
BlastP bit score: 294
  
Sequence coverage: 103 %
  
E-value: 4e-91
  
  
 NCBI BlastP on this gene

ELA31579

AAA family
  
Accession: ELA31580
  
Location: 60848-68129
  
  
**BlastP hit with Mycgr3G42010\_Mycgr3T**
  
Percentage identity: 46 %
  
BlastP bit score: 1961
  
Sequence coverage: 102 %
  
E-value: 0.0
  
  
 NCBI BlastP on this gene

ELA31580

phospholipid methyltransferase
  
Accession: ELA31581
  
Location: 69436-70326
  
 NCBI BlastP on this gene

ELA31581

cyanide hydratase
  
Accession: ELA31582
  
Location: 70743-71947
  
 NCBI BlastP on this gene

ELA31582

24-dehydrocholesterol reductase precursor
  
Accession: ELA31583
  
Location: 73060-74580
  
 NCBI BlastP on this gene

ELA31583

hypothetical protein
  
Accession: ELA31584
  
Location: 76660-77641
  
 NCBI BlastP on this gene

ELA31584

MFS sugar transporter
  
Accession: ELA31585
  
Location: 78147-79970
  
 NCBI BlastP on this gene

ELA31585

mucin-desulfating sulfatase (n-acetylglucosamine-6-sulfatase)
  
Accession: ELA31586
  
Location: 80745-84828
  
 NCBI BlastP on this gene

ELA31586

hypothetical protein
  
Accession: ELA31587
  
Location: 85639-85939
  
 NCBI BlastP on this gene

ELA31587

periplasmic beta-glucosidase
  
Accession: ELA31588
  
Location: 86288-88753
  
 NCBI BlastP on this gene

ELA31588

Query: Architecture Search FASTA input

EQ963475 : Aspergillus flavus NRRL3357 scf\_1106286419142 genomic scaffold    Total score: 2.0     Cumulative Blast bit score: 2227

Hit cluster cross-links:

Mycgr3G85918 Mycgr3T
  
Location: 0-1602

Mycgr3G85918\_Mycgr3T

Mycgr3G42010 Mycgr3T
  
Location: 1702-8569

Mycgr3G42010\_Mycgr3T

Mycgr3G29582 Mycgr3T
  
Location: 8669-8915

Mycgr3G29582\_Mycgr3T

Mycgr3G31170 Mycgr3T
  
Location: 9015-9255

Mycgr3G31170\_Mycgr3T

Mycgr3G85924 Mycgr3T
  
Location: 9355-11218

Mycgr3G85924\_Mycgr3T

Mycgr3G71676 Mycgr3T
  
Location: 11318-12494

Mycgr3G71676\_Mycgr3T

Mycgr3G11468 Mycgr3T
  
Location: 12594-13653

Mycgr3G11468\_Mycgr3T

Mycgr3G58567 Mycgr3T
  
Location: 13753-14506

Mycgr3G58567\_Mycgr3T

Mycgr3G100089 Mycgr3
  
Location: 14606-21152

Mycgr3G100089\_Mycgr3

Mycgr3G42698 Mycgr3T
  
Location: 21252-22131

Mycgr3G42698\_Mycgr3T

Mycgr3G71681 Mycgr3T
  
Location: 22231-23461

Mycgr3G71681\_Mycgr3T

Mycgr3G109328 Mycgr3
  
Location: 23561-24239

Mycgr3G109328\_Mycgr3

Mycgr3G104334 Mycgr3
  
Location: 24339-24567

Mycgr3G104334\_Mycgr3

Mycgr3G42715 Mycgr3T
  
Location: 24667-25981

Mycgr3G42715\_Mycgr3T

Mycgr3G92934 Mycgr3T
  
Location: 26081-27593

Mycgr3G92934\_Mycgr3T

Mycgr3G41969 Mycgr3T
  
Location: 27693-29328

Mycgr3G41969\_Mycgr3T

Mycgr3G80635 Mycgr3T
  
Location: 29428-29821

Mycgr3G80635\_Mycgr3T

Mycgr3G41426 Mycgr3T
  
Location: 29921-35255

Mycgr3G41426\_Mycgr3T

Mycgr3G104337 Mycgr3
  
Location: 35355-36108

Mycgr3G104337\_Mycgr3

Mycgr3G71679 Mycgr3T
  
Location: 36208-37300

Mycgr3G71679\_Mycgr3T

Mycgr3G92938 Mycgr3T
  
Location: 37400-38699

Mycgr3G92938\_Mycgr3T

Mycgr3G92941 Mycgr3T
  
Location: 38799-40734

Mycgr3G92941\_Mycgr3T

subtilisin, putative
  
Accession: EED53069
  
Location: 72026-74080
  
 NCBI BlastP on this gene

EED53069

conserved hypothetical protein
  
Accession: EED53068
  
Location: 69054-70401
  
 NCBI BlastP on this gene

EED53068

monocarboxylate transporter, putative
  
Accession: EED53067
  
Location: 65668-67179
  
 NCBI BlastP on this gene

EED53067

hypothetical protein
  
Accession: EED53066
  
Location: 64752-64957
  
 NCBI BlastP on this gene

EED53066

amine oxidase, putative
  
Accession: EED53065
  
Location: 62819-64120
  
 NCBI BlastP on this gene

EED53065

conserved hypothetical protein
  
Accession: EED53064
  
Location: 59588-60298
  
  
**BlastP hit with Mycgr3G92938\_Mycgr3T**
  
Percentage identity: 29 %
  
BlastP bit score: 94
  
Sequence coverage: 56 %
  
E-value: 7e-19
  
  
 NCBI BlastP on this gene

EED53064

hypothetical protein
  
Accession: EED53063
  
Location: 58927-59355
  
 NCBI BlastP on this gene

EED53063

nonsense-mediated mRNA decay protein, putative
  
Accession: EED53062
  
Location: 48502-55653
  
  
**BlastP hit with Mycgr3G42010\_Mycgr3T**
  
Percentage identity: 47 %
  
BlastP bit score: 2133
  
Sequence coverage: 102 %
  
E-value: 0.0
  
  
 NCBI BlastP on this gene

EED53062

dynamin, putative
  
Accession: EED53061
  
Location: 44559-46931
  
 NCBI BlastP on this gene

EED53061

conserved hypothetical protein
  
Accession: EED53060
  
Location: 41934-42940
  
 NCBI BlastP on this gene

EED53060

hypothetical protein
  
Accession: EED53059
  
Location: 39446-41257
  
 NCBI BlastP on this gene

EED53059

conserved hypothetical protein
  
Accession: EED53058
  
Location: 38424-39355
  
 NCBI BlastP on this gene

EED53058

DUF567 domain protein
  
Accession: EED53057
  
Location: 37406-38082
  
 NCBI BlastP on this gene

EED53057

fungal alpha-L-arabinofuranosidase, putative
  
Accession: EED53056
  
Location: 35160-36680
  
 NCBI BlastP on this gene

EED53056

hypothetical protein
  
Accession: EED53055
  
Location: 32033-33586
  
 NCBI BlastP on this gene

EED53055

hypothetical protein
  
Accession: EED53054
  
Location: 31473-31778
  
 NCBI BlastP on this gene

EED53054

Query: Architecture Search FASTA input

GG698906 : Nectria haematococca mpVI 77-13-4 chromosome 10 genomic scaffold NECHAsca\_14\_chr10\_3\_0    Total score: 2.0     Cumulative Blast bit score: 2175

Hit cluster cross-links:

Mycgr3G85918 Mycgr3T
  
Location: 0-1602

Mycgr3G85918\_Mycgr3T

Mycgr3G42010 Mycgr3T
  
Location: 1702-8569

Mycgr3G42010\_Mycgr3T

Mycgr3G29582 Mycgr3T
  
Location: 8669-8915

Mycgr3G29582\_Mycgr3T

Mycgr3G31170 Mycgr3T
  
Location: 9015-9255

Mycgr3G31170\_Mycgr3T

Mycgr3G85924 Mycgr3T
  
Location: 9355-11218

Mycgr3G85924\_Mycgr3T

Mycgr3G71676 Mycgr3T
  
Location: 11318-12494

Mycgr3G71676\_Mycgr3T

Mycgr3G11468 Mycgr3T
  
Location: 12594-13653

Mycgr3G11468\_Mycgr3T

Mycgr3G58567 Mycgr3T
  
Location: 13753-14506

Mycgr3G58567\_Mycgr3T

Mycgr3G100089 Mycgr3
  
Location: 14606-21152

Mycgr3G100089\_Mycgr3

Mycgr3G42698 Mycgr3T
  
Location: 21252-22131

Mycgr3G42698\_Mycgr3T

Mycgr3G71681 Mycgr3T
  
Location: 22231-23461

Mycgr3G71681\_Mycgr3T

Mycgr3G109328 Mycgr3
  
Location: 23561-24239

Mycgr3G109328\_Mycgr3

Mycgr3G104334 Mycgr3
  
Location: 24339-24567

Mycgr3G104334\_Mycgr3

Mycgr3G42715 Mycgr3T
  
Location: 24667-25981

Mycgr3G42715\_Mycgr3T

Mycgr3G92934 Mycgr3T
  
Location: 26081-27593

Mycgr3G92934\_Mycgr3T

Mycgr3G41969 Mycgr3T
  
Location: 27693-29328

Mycgr3G41969\_Mycgr3T

Mycgr3G80635 Mycgr3T
  
Location: 29428-29821

Mycgr3G80635\_Mycgr3T

Mycgr3G41426 Mycgr3T
  
Location: 29921-35255

Mycgr3G41426\_Mycgr3T

Mycgr3G104337 Mycgr3
  
Location: 35355-36108

Mycgr3G104337\_Mycgr3

Mycgr3G71679 Mycgr3T
  
Location: 36208-37300

Mycgr3G71679\_Mycgr3T

Mycgr3G92938 Mycgr3T
  
Location: 37400-38699

Mycgr3G92938\_Mycgr3T

Mycgr3G92941 Mycgr3T
  
Location: 38799-40734

Mycgr3G92941\_Mycgr3T

hypothetical protein
  
Accession: EEU41970
  
Location: 873435-874006
  
 NCBI BlastP on this gene

EEU41970

predicted protein
  
Accession: EEU41971
  
Location: 876657-877093
  
 NCBI BlastP on this gene

EEU41971

hypothetical protein
  
Accession: EEU41972
  
Location: 878689-879517
  
 NCBI BlastP on this gene

EEU41972

hypothetical protein
  
Accession: EEU42142
  
Location: 882044-882484
  
 NCBI BlastP on this gene

EEU42142

hypothetical protein
  
Accession: EEU42143
  
Location: 883414-884493
  
 NCBI BlastP on this gene

EEU42143

hypothetical protein
  
Accession: EEU42144
  
Location: 885005-888085
  
 NCBI BlastP on this gene

EEU42144

hypothetical protein
  
Accession: EEU42145
  
Location: 889526-896420
  
  
**BlastP hit with Mycgr3G42010\_Mycgr3T**
  
Percentage identity: 45 %
  
BlastP bit score: 1941
  
Sequence coverage: 101 %
  
E-value: 0.0
  
  
 NCBI BlastP on this gene

EEU42145

hypothetical protein
  
Accession: EEU41973
  
Location: 897098-898443
  
  
**BlastP hit with Mycgr3G92938\_Mycgr3T**
  
Percentage identity: 35 %
  
BlastP bit score: 234
  
Sequence coverage: 101 %
  
E-value: 3e-68
  
  
 NCBI BlastP on this gene

EEU41973

hypothetical protein
  
Accession: EEU41974
  
Location: 901657-902975
  
 NCBI BlastP on this gene

EEU41974

predicted protein
  
Accession: EEU41975
  
Location: 903323-904410
  
 NCBI BlastP on this gene

EEU41975

hypothetical protein
  
Accession: EEU41976
  
Location: 905595-906869
  
 NCBI BlastP on this gene

EEU41976

hypothetical protein
  
Accession: EEU42146
  
Location: 907251-907784
  
 NCBI BlastP on this gene

EEU42146

predicted protein
  
Accession: EEU41977
  
Location: 909044-910256
  
 NCBI BlastP on this gene

EEU41977

hypothetical protein
  
Accession: EEU41978
  
Location: 910745-913000
  
 NCBI BlastP on this gene

EEU41978

hypothetical protein
  
Accession: EEU42147
  
Location: 913041-916262
  
 NCBI BlastP on this gene

EEU42147

hypothetical protein
  
Accession: EEU41979
  
Location: 916601-918746
  
 NCBI BlastP on this gene

EEU41979

Query: Architecture Search FASTA input

GL385399 : Gaeumannomyces graminis var. tritici R3-111a-1 unplaced genomic scaffold supercont2.5    Total score: 2.0     Cumulative Blast bit score: 2171

Hit cluster cross-links:

Mycgr3G85918 Mycgr3T
  
Location: 0-1602

Mycgr3G85918\_Mycgr3T

Mycgr3G42010 Mycgr3T
  
Location: 1702-8569

Mycgr3G42010\_Mycgr3T

Mycgr3G29582 Mycgr3T
  
Location: 8669-8915

Mycgr3G29582\_Mycgr3T

Mycgr3G31170 Mycgr3T
  
Location: 9015-9255

Mycgr3G31170\_Mycgr3T

Mycgr3G85924 Mycgr3T
  
Location: 9355-11218

Mycgr3G85924\_Mycgr3T

Mycgr3G71676 Mycgr3T
  
Location: 11318-12494

Mycgr3G71676\_Mycgr3T

Mycgr3G11468 Mycgr3T
  
Location: 12594-13653

Mycgr3G11468\_Mycgr3T

Mycgr3G58567 Mycgr3T
  
Location: 13753-14506

Mycgr3G58567\_Mycgr3T

Mycgr3G100089 Mycgr3
  
Location: 14606-21152

Mycgr3G100089\_Mycgr3

Mycgr3G42698 Mycgr3T
  
Location: 21252-22131

Mycgr3G42698\_Mycgr3T

Mycgr3G71681 Mycgr3T
  
Location: 22231-23461

Mycgr3G71681\_Mycgr3T

Mycgr3G109328 Mycgr3
  
Location: 23561-24239

Mycgr3G109328\_Mycgr3

Mycgr3G104334 Mycgr3
  
Location: 24339-24567

Mycgr3G104334\_Mycgr3

Mycgr3G42715 Mycgr3T
  
Location: 24667-25981

Mycgr3G42715\_Mycgr3T

Mycgr3G92934 Mycgr3T
  
Location: 26081-27593

Mycgr3G92934\_Mycgr3T

Mycgr3G41969 Mycgr3T
  
Location: 27693-29328

Mycgr3G41969\_Mycgr3T

Mycgr3G80635 Mycgr3T
  
Location: 29428-29821

Mycgr3G80635\_Mycgr3T

Mycgr3G41426 Mycgr3T
  
Location: 29921-35255

Mycgr3G41426\_Mycgr3T

Mycgr3G104337 Mycgr3
  
Location: 35355-36108

Mycgr3G104337\_Mycgr3

Mycgr3G71679 Mycgr3T
  
Location: 36208-37300

Mycgr3G71679\_Mycgr3T

Mycgr3G92938 Mycgr3T
  
Location: 37400-38699

Mycgr3G92938\_Mycgr3T

Mycgr3G92941 Mycgr3T
  
Location: 38799-40734

Mycgr3G92941\_Mycgr3T

hypothetical protein
  
Accession: EJT72262
  
Location: 421866-422746
  
 NCBI BlastP on this gene

EJT72262

hypothetical protein
  
Accession: EJT72263
  
Location: 423408-426641
  
 NCBI BlastP on this gene

EJT72263

methyltransferase-UbiE family protein
  
Accession: EJT72264
  
Location: 427811-428638
  
 NCBI BlastP on this gene

EJT72264

hypothetical protein
  
Accession: EJT72265
  
Location: 429132-433707
  
 NCBI BlastP on this gene

EJT72265

hypothetical protein
  
Accession: EJT72266
  
Location: 434137-436157
  
 NCBI BlastP on this gene

EJT72266

hypothetical protein
  
Accession: EJT72267
  
Location: 439343-446527
  
  
**BlastP hit with Mycgr3G42010\_Mycgr3T**
  
Percentage identity: 47 %
  
BlastP bit score: 1903
  
Sequence coverage: 92 %
  
E-value: 0.0
  
  
 NCBI BlastP on this gene

EJT72267

hypothetical protein
  
Accession: EJT72268
  
Location: 447491-449195
  
  
**BlastP hit with Mycgr3G92938\_Mycgr3T**
  
Percentage identity: 39 %
  
BlastP bit score: 268
  
Sequence coverage: 86 %
  
E-value: 1e-80
  
  
 NCBI BlastP on this gene

EJT72268

hypothetical protein
  
Accession: EJT72269
  
Location: 449913-452271
  
 NCBI BlastP on this gene

EJT72269

hypothetical protein
  
Accession: EJT72270
  
Location: 454675-455610
  
 NCBI BlastP on this gene

EJT72270

glycogen debranching enzyme
  
Accession: EJT72271
  
Location: 456839-461738
  
 NCBI BlastP on this gene

EJT72271

hypothetical protein
  
Accession: EJT72272
  
Location: 462107-463082
  
 NCBI BlastP on this gene

EJT72272

hypothetical protein
  
Accession: EJT72273
  
Location: 464472-465782
  
 NCBI BlastP on this gene

EJT72273

Query: Architecture Search FASTA input

JH725244 : Beauveria bassiana ARSEF 2860 unplaced genomic scaffold BBA\_S00095    Total score: 2.0     Cumulative Blast bit score: 2170

Hit cluster cross-links:

Mycgr3G85918 Mycgr3T
  
Location: 0-1602

Mycgr3G85918\_Mycgr3T

Mycgr3G42010 Mycgr3T
  
Location: 1702-8569

Mycgr3G42010\_Mycgr3T

Mycgr3G29582 Mycgr3T
  
Location: 8669-8915

Mycgr3G29582\_Mycgr3T

Mycgr3G31170 Mycgr3T
  
Location: 9015-9255

Mycgr3G31170\_Mycgr3T

Mycgr3G85924 Mycgr3T
  
Location: 9355-11218

Mycgr3G85924\_Mycgr3T

Mycgr3G71676 Mycgr3T
  
Location: 11318-12494

Mycgr3G71676\_Mycgr3T

Mycgr3G11468 Mycgr3T
  
Location: 12594-13653

Mycgr3G11468\_Mycgr3T

Mycgr3G58567 Mycgr3T
  
Location: 13753-14506

Mycgr3G58567\_Mycgr3T

Mycgr3G100089 Mycgr3
  
Location: 14606-21152

Mycgr3G100089\_Mycgr3

Mycgr3G42698 Mycgr3T
  
Location: 21252-22131

Mycgr3G42698\_Mycgr3T

Mycgr3G71681 Mycgr3T
  
Location: 22231-23461

Mycgr3G71681\_Mycgr3T

Mycgr3G109328 Mycgr3
  
Location: 23561-24239

Mycgr3G109328\_Mycgr3

Mycgr3G104334 Mycgr3
  
Location: 24339-24567

Mycgr3G104334\_Mycgr3

Mycgr3G42715 Mycgr3T
  
Location: 24667-25981

Mycgr3G42715\_Mycgr3T

Mycgr3G92934 Mycgr3T
  
Location: 26081-27593

Mycgr3G92934\_Mycgr3T

Mycgr3G41969 Mycgr3T
  
Location: 27693-29328

Mycgr3G41969\_Mycgr3T

Mycgr3G80635 Mycgr3T
  
Location: 29428-29821

Mycgr3G80635\_Mycgr3T

Mycgr3G41426 Mycgr3T
  
Location: 29921-35255

Mycgr3G41426\_Mycgr3T

Mycgr3G104337 Mycgr3
  
Location: 35355-36108

Mycgr3G104337\_Mycgr3

Mycgr3G71679 Mycgr3T
  
Location: 36208-37300

Mycgr3G71679\_Mycgr3T

Mycgr3G92938 Mycgr3T
  
Location: 37400-38699

Mycgr3G92938\_Mycgr3T

Mycgr3G92941 Mycgr3T
  
Location: 38799-40734

Mycgr3G92941\_Mycgr3T

hypothetical protein
  
Accession: EJP60882
  
Location: 7235-8371
  
 NCBI BlastP on this gene

EJP60882

hypothetical protein
  
Accession: EJP60883
  
Location: 9707-10975
  
 NCBI BlastP on this gene

EJP60883

geranylgeranyl pyrophosphate synthetase
  
Accession: EJP60884
  
Location: 14321-15799
  
  
**BlastP hit with Mycgr3G92938\_Mycgr3T**
  
Percentage identity: 34 %
  
BlastP bit score: 248
  
Sequence coverage: 109 %
  
E-value: 3e-73
  
  
 NCBI BlastP on this gene

EJP60884

ATPase protein
  
Accession: EJP60885
  
Location: 16047-24564
  
  
**BlastP hit with Mycgr3G42010\_Mycgr3T**
  
Percentage identity: 44 %
  
BlastP bit score: 1922
  
Sequence coverage: 102 %
  
E-value: 0.0
  
  
 NCBI BlastP on this gene

EJP60885

Query: Architecture Search FASTA input

GL891305 : Neurospora tetrasperma FGSC 2508 unplaced genomic scaffold NEUTE1scaffold\_4    Total score: 2.0     Cumulative Blast bit score: 2162

Hit cluster cross-links:

Mycgr3G85918 Mycgr3T
  
Location: 0-1602

Mycgr3G85918\_Mycgr3T

Mycgr3G42010 Mycgr3T
  
Location: 1702-8569

Mycgr3G42010\_Mycgr3T

Mycgr3G29582 Mycgr3T
  
Location: 8669-8915

Mycgr3G29582\_Mycgr3T

Mycgr3G31170 Mycgr3T
  
Location: 9015-9255

Mycgr3G31170\_Mycgr3T

Mycgr3G85924 Mycgr3T
  
Location: 9355-11218

Mycgr3G85924\_Mycgr3T

Mycgr3G71676 Mycgr3T
  
Location: 11318-12494

Mycgr3G71676\_Mycgr3T

Mycgr3G11468 Mycgr3T
  
Location: 12594-13653

Mycgr3G11468\_Mycgr3T

Mycgr3G58567 Mycgr3T
  
Location: 13753-14506

Mycgr3G58567\_Mycgr3T

Mycgr3G100089 Mycgr3
  
Location: 14606-21152

Mycgr3G100089\_Mycgr3

Mycgr3G42698 Mycgr3T
  
Location: 21252-22131

Mycgr3G42698\_Mycgr3T

Mycgr3G71681 Mycgr3T
  
Location: 22231-23461

Mycgr3G71681\_Mycgr3T

Mycgr3G109328 Mycgr3
  
Location: 23561-24239

Mycgr3G109328\_Mycgr3

Mycgr3G104334 Mycgr3
  
Location: 24339-24567

Mycgr3G104334\_Mycgr3

Mycgr3G42715 Mycgr3T
  
Location: 24667-25981

Mycgr3G42715\_Mycgr3T

Mycgr3G92934 Mycgr3T
  
Location: 26081-27593

Mycgr3G92934\_Mycgr3T

Mycgr3G41969 Mycgr3T
  
Location: 27693-29328

Mycgr3G41969\_Mycgr3T

Mycgr3G80635 Mycgr3T
  
Location: 29428-29821

Mycgr3G80635\_Mycgr3T

Mycgr3G41426 Mycgr3T
  
Location: 29921-35255

Mycgr3G41426\_Mycgr3T

Mycgr3G104337 Mycgr3
  
Location: 35355-36108

Mycgr3G104337\_Mycgr3

Mycgr3G71679 Mycgr3T
  
Location: 36208-37300

Mycgr3G71679\_Mycgr3T

Mycgr3G92938 Mycgr3T
  
Location: 37400-38699

Mycgr3G92938\_Mycgr3T

Mycgr3G92941 Mycgr3T
  
Location: 38799-40734

Mycgr3G92941\_Mycgr3T

hypothetical protein
  
Accession: EGO56085
  
Location: 196463-196927
  
 NCBI BlastP on this gene

EGO56085

hypothetical protein
  
Accession: EGO56086
  
Location: 197272-199766
  
 NCBI BlastP on this gene

EGO56086

hypothetical protein
  
Accession: EGO56087
  
Location: 200673-201581
  
 NCBI BlastP on this gene

EGO56087

hypothetical protein
  
Accession: EGO56088
  
Location: 202466-203494
  
 NCBI BlastP on this gene

EGO56088

hypothetical protein
  
Accession: EGO56089
  
Location: 204325-205482
  
 NCBI BlastP on this gene

EGO56089

hypothetical protein
  
Accession: EGO56090
  
Location: 205881-207592
  
 NCBI BlastP on this gene

EGO56090

hypothetical protein
  
Accession: EGO56091
  
Location: 208085-209538
  
 NCBI BlastP on this gene

EGO56091

hypothetical protein
  
Accession: EGO56092
  
Location: 210546-212459
  
 NCBI BlastP on this gene

EGO56092

hypothetical protein
  
Accession: EGO56093
  
Location: 214565-221760
  
  
**BlastP hit with Mycgr3G42010\_Mycgr3T**
  
Percentage identity: 45 %
  
BlastP bit score: 1956
  
Sequence coverage: 101 %
  
E-value: 0.0
  
  
 NCBI BlastP on this gene

EGO56093

hypothetical protein
  
Accession: EGO56094
  
Location: 223154-224970
  
  
**BlastP hit with Mycgr3G92938\_Mycgr3T**
  
Percentage identity: 37 %
  
BlastP bit score: 206
  
Sequence coverage: 71 %
  
E-value: 2e-56
  
  
 NCBI BlastP on this gene

EGO56094

hypothetical protein
  
Accession: EGO56095
  
Location: 225718-228139
  
 NCBI BlastP on this gene

EGO56095

hypothetical protein
  
Accession: EGO56096
  
Location: 229266-230202
  
 NCBI BlastP on this gene

EGO56096

hypothetical protein
  
Accession: EGO56097
  
Location: 231056-233440
  
 NCBI BlastP on this gene

EGO56097

hypothetical protein
  
Accession: EGO56098
  
Location: 234675-239337
  
 NCBI BlastP on this gene

EGO56098

hypothetical protein
  
Accession: EGO56099
  
Location: 240796-241074
  
 NCBI BlastP on this gene

EGO56099

hypothetical protein
  
Accession: EGO56100
  
Location: 241698-243625
  
 NCBI BlastP on this gene

EGO56100

Query: Architecture Search FASTA input

KB445558 : Baudoinia compniacensis UAMH 10762 unplaced genomic scaffold BAUCOscaffold\_9    Total score: 2.0     Cumulative Blast bit score: 2158

Hit cluster cross-links:

Mycgr3G85918 Mycgr3T
  
Location: 0-1602

Mycgr3G85918\_Mycgr3T

Mycgr3G42010 Mycgr3T
  
Location: 1702-8569

Mycgr3G42010\_Mycgr3T

Mycgr3G29582 Mycgr3T
  
Location: 8669-8915

Mycgr3G29582\_Mycgr3T

Mycgr3G31170 Mycgr3T
  
Location: 9015-9255

Mycgr3G31170\_Mycgr3T

Mycgr3G85924 Mycgr3T
  
Location: 9355-11218

Mycgr3G85924\_Mycgr3T

Mycgr3G71676 Mycgr3T
  
Location: 11318-12494

Mycgr3G71676\_Mycgr3T

Mycgr3G11468 Mycgr3T
  
Location: 12594-13653

Mycgr3G11468\_Mycgr3T

Mycgr3G58567 Mycgr3T
  
Location: 13753-14506

Mycgr3G58567\_Mycgr3T

Mycgr3G100089 Mycgr3
  
Location: 14606-21152

Mycgr3G100089\_Mycgr3

Mycgr3G42698 Mycgr3T
  
Location: 21252-22131

Mycgr3G42698\_Mycgr3T

Mycgr3G71681 Mycgr3T
  
Location: 22231-23461

Mycgr3G71681\_Mycgr3T

Mycgr3G109328 Mycgr3
  
Location: 23561-24239

Mycgr3G109328\_Mycgr3

Mycgr3G104334 Mycgr3
  
Location: 24339-24567

Mycgr3G104334\_Mycgr3

Mycgr3G42715 Mycgr3T
  
Location: 24667-25981

Mycgr3G42715\_Mycgr3T

Mycgr3G92934 Mycgr3T
  
Location: 26081-27593

Mycgr3G92934\_Mycgr3T

Mycgr3G41969 Mycgr3T
  
Location: 27693-29328

Mycgr3G41969\_Mycgr3T

Mycgr3G80635 Mycgr3T
  
Location: 29428-29821

Mycgr3G80635\_Mycgr3T

Mycgr3G41426 Mycgr3T
  
Location: 29921-35255

Mycgr3G41426\_Mycgr3T

Mycgr3G104337 Mycgr3
  
Location: 35355-36108

Mycgr3G104337\_Mycgr3

Mycgr3G71679 Mycgr3T
  
Location: 36208-37300

Mycgr3G71679\_Mycgr3T

Mycgr3G92938 Mycgr3T
  
Location: 37400-38699

Mycgr3G92938\_Mycgr3T

Mycgr3G92941 Mycgr3T
  
Location: 38799-40734

Mycgr3G92941\_Mycgr3T

hypothetical protein
  
Accession: EMC94831
  
Location: 1079647-1081104
  
 NCBI BlastP on this gene

EMC94831

hypothetical protein
  
Accession: EMC94830
  
Location: 1072686-1074932
  
 NCBI BlastP on this gene

EMC94830

hypothetical protein
  
Accession: EMC94829
  
Location: 1070815-1071960
  
 NCBI BlastP on this gene

EMC94829

hypothetical protein
  
Accession: EMC94828
  
Location: 1067000-1068628
  
 NCBI BlastP on this gene

EMC94828

hypothetical protein
  
Accession: EMC94827
  
Location: 1065477-1066564
  
 NCBI BlastP on this gene

EMC94827

hypothetical protein
  
Accession: EMC94826
  
Location: 1058514-1064026
  
  
**BlastP hit with Mycgr3G41426\_Mycgr3T**
  
Percentage identity: 56 %
  
BlastP bit score: 1992
  
Sequence coverage: 101 %
  
E-value: 0.0
  
  
 NCBI BlastP on this gene

EMC94826

hypothetical protein
  
Accession: EMC94825
  
Location: 1054045-1056734
  
 NCBI BlastP on this gene

EMC94825

hypothetical protein
  
Accession: EMC94824
  
Location: 1052973-1053926
  
 NCBI BlastP on this gene

EMC94824

hypothetical protein
  
Accession: EMC94823
  
Location: 1050802-1051595
  
  
**BlastP hit with Mycgr3G31170\_Mycgr3T**
  
Percentage identity: 100 %
  
BlastP bit score: 166
  
Sequence coverage: 100 %
  
E-value: 7e-50
  
  
 NCBI BlastP on this gene

EMC94823

hypothetical protein
  
Accession: EMC94822
  
Location: 1048283-1050219
  
 NCBI BlastP on this gene

EMC94822

hypothetical protein
  
Accession: EMC94821
  
Location: 1047382-1047864
  
 NCBI BlastP on this gene

EMC94821

hypothetical protein
  
Accession: EMC94820
  
Location: 1043193-1046693
  
 NCBI BlastP on this gene

EMC94820

hypothetical protein
  
Accession: EMC94819
  
Location: 1042159-1042746
  
 NCBI BlastP on this gene

EMC94819

hypothetical protein
  
Accession: EMC94818
  
Location: 1037773-1041234
  
 NCBI BlastP on this gene

EMC94818

hypothetical protein
  
Accession: EMC94817
  
Location: 1037390-1037551
  
 NCBI BlastP on this gene

EMC94817

hypothetical protein
  
Accession: EMC94816
  
Location: 1035164-1036978
  
 NCBI BlastP on this gene

EMC94816

Query: Architecture Search FASTA input

GL698524 : Metarhizium acridum CQMa 102 unplaced genomic scaffold Scf\_055    Total score: 2.0     Cumulative Blast bit score: 2156

Hit cluster cross-links:

Mycgr3G85918 Mycgr3T
  
Location: 0-1602

Mycgr3G85918\_Mycgr3T

Mycgr3G42010 Mycgr3T
  
Location: 1702-8569

Mycgr3G42010\_Mycgr3T

Mycgr3G29582 Mycgr3T
  
Location: 8669-8915

Mycgr3G29582\_Mycgr3T

Mycgr3G31170 Mycgr3T
  
Location: 9015-9255

Mycgr3G31170\_Mycgr3T

Mycgr3G85924 Mycgr3T
  
Location: 9355-11218

Mycgr3G85924\_Mycgr3T

Mycgr3G71676 Mycgr3T
  
Location: 11318-12494

Mycgr3G71676\_Mycgr3T

Mycgr3G11468 Mycgr3T
  
Location: 12594-13653

Mycgr3G11468\_Mycgr3T

Mycgr3G58567 Mycgr3T
  
Location: 13753-14506

Mycgr3G58567\_Mycgr3T

Mycgr3G100089 Mycgr3
  
Location: 14606-21152

Mycgr3G100089\_Mycgr3

Mycgr3G42698 Mycgr3T
  
Location: 21252-22131

Mycgr3G42698\_Mycgr3T

Mycgr3G71681 Mycgr3T
  
Location: 22231-23461

Mycgr3G71681\_Mycgr3T

Mycgr3G109328 Mycgr3
  
Location: 23561-24239

Mycgr3G109328\_Mycgr3

Mycgr3G104334 Mycgr3
  
Location: 24339-24567

Mycgr3G104334\_Mycgr3

Mycgr3G42715 Mycgr3T
  
Location: 24667-25981

Mycgr3G42715\_Mycgr3T

Mycgr3G92934 Mycgr3T
  
Location: 26081-27593

Mycgr3G92934\_Mycgr3T

Mycgr3G41969 Mycgr3T
  
Location: 27693-29328

Mycgr3G41969\_Mycgr3T

Mycgr3G80635 Mycgr3T
  
Location: 29428-29821

Mycgr3G80635\_Mycgr3T

Mycgr3G41426 Mycgr3T
  
Location: 29921-35255

Mycgr3G41426\_Mycgr3T

Mycgr3G104337 Mycgr3
  
Location: 35355-36108

Mycgr3G104337\_Mycgr3

Mycgr3G71679 Mycgr3T
  
Location: 36208-37300

Mycgr3G71679\_Mycgr3T

Mycgr3G92938 Mycgr3T
  
Location: 37400-38699

Mycgr3G92938\_Mycgr3T

Mycgr3G92941 Mycgr3T
  
Location: 38799-40734

Mycgr3G92941\_Mycgr3T

high affinity nickel transport protein nic1
  
Accession: EFY87589
  
Location: 3979-5251
  
 NCBI BlastP on this gene

EFY87589

hypothetical protein
  
Accession: EFY87590
  
Location: 5386-6270
  
 NCBI BlastP on this gene

EFY87590

Autophagy-related protein 17
  
Accession: EFY87591
  
Location: 7711-9299
  
 NCBI BlastP on this gene

EFY87591

proteinase, putative
  
Accession: EFY87592
  
Location: 9705-11594
  
 NCBI BlastP on this gene

EFY87592

AAA family ATPase, putative
  
Accession: EFY87593
  
Location: 15581-22118
  
  
**BlastP hit with Mycgr3G42010\_Mycgr3T**
  
Percentage identity: 46 %
  
BlastP bit score: 1900
  
Sequence coverage: 93 %
  
E-value: 0.0
  
  
 NCBI BlastP on this gene

EFY87593

hypothetical protein
  
Accession: EFY87594
  
Location: 23101-24538
  
  
**BlastP hit with Mycgr3G92938\_Mycgr3T**
  
Percentage identity: 34 %
  
BlastP bit score: 256
  
Sequence coverage: 106 %
  
E-value: 3e-76
  
  
 NCBI BlastP on this gene

EFY87594

hypothetical protein
  
Accession: EFY87595
  
Location: 25882-26775
  
 NCBI BlastP on this gene

EFY87595

hypothetical protein
  
Accession: EFY87596
  
Location: 27754-28392
  
 NCBI BlastP on this gene

EFY87596

hypothetical protein
  
Accession: EFY87597
  
Location: 32752-34104
  
 NCBI BlastP on this gene

EFY87597

hypothetical protein
  
Accession: EFY87598
  
Location: 37207-40757
  
 NCBI BlastP on this gene

EFY87598

hypothetical protein
  
Accession: EFY87599
  
Location: 42063-43252
  
 NCBI BlastP on this gene

EFY87599

Query: Architecture Search FASTA input

1. :  CM001200 Mycosphaerella graminicola IPO323 chromosome 5     Total score: 22.0     Cumulative Blast bit score: 26518

Mycgr3G85918 Mycgr3T
  
Location: 0-1602
  
 NCBI BlastP on this gene

Mycgr3G85918\_Mycgr3T

Mycgr3G42010 Mycgr3T
  
Location: 1702-8569
  
 NCBI BlastP on this gene

Mycgr3G42010\_Mycgr3T

Mycgr3G29582 Mycgr3T
  
Location: 8669-8915
  
 NCBI BlastP on this gene

Mycgr3G29582\_Mycgr3T

Mycgr3G31170 Mycgr3T
  
Location: 9015-9255
  
 NCBI BlastP on this gene

Mycgr3G31170\_Mycgr3T

Mycgr3G85924 Mycgr3T
  
Location: 9355-11218
  
 NCBI BlastP on this gene

Mycgr3G85924\_Mycgr3T

Mycgr3G71676 Mycgr3T
  
Location: 11318-12494
  
 NCBI BlastP on this gene

Mycgr3G71676\_Mycgr3T

Mycgr3G11468 Mycgr3T
  
Location: 12594-13653
  
 NCBI BlastP on this gene

Mycgr3G11468\_Mycgr3T

Mycgr3G58567 Mycgr3T
  
Location: 13753-14506
  
 NCBI BlastP on this gene

Mycgr3G58567\_Mycgr3T

Mycgr3G100089 Mycgr3
  
Location: 14606-21152
  
 NCBI BlastP on this gene

Mycgr3G100089\_Mycgr3

Mycgr3G42698 Mycgr3T
  
Location: 21252-22131
  
 NCBI BlastP on this gene

Mycgr3G42698\_Mycgr3T

Mycgr3G71681 Mycgr3T
  
Location: 22231-23461
  
 NCBI BlastP on this gene

Mycgr3G71681\_Mycgr3T

Mycgr3G109328 Mycgr3
  
Location: 23561-24239
  
 NCBI BlastP on this gene

Mycgr3G109328\_Mycgr3

Mycgr3G104334 Mycgr3
  
Location: 24339-24567
  
 NCBI BlastP on this gene

Mycgr3G104334\_Mycgr3

Mycgr3G42715 Mycgr3T
  
Location: 24667-25981
  
 NCBI BlastP on this gene

Mycgr3G42715\_Mycgr3T

Mycgr3G92934 Mycgr3T
  
Location: 26081-27593
  
 NCBI BlastP on this gene

Mycgr3G92934\_Mycgr3T

Mycgr3G41969 Mycgr3T
  
Location: 27693-29328
  
 NCBI BlastP on this gene

Mycgr3G41969\_Mycgr3T

Mycgr3G80635 Mycgr3T
  
Location: 29428-29821
  
 NCBI BlastP on this gene

Mycgr3G80635\_Mycgr3T

Mycgr3G41426 Mycgr3T
  
Location: 29921-35255
  
 NCBI BlastP on this gene

Mycgr3G41426\_Mycgr3T

Mycgr3G104337 Mycgr3
  
Location: 35355-36108
  
 NCBI BlastP on this gene

Mycgr3G104337\_Mycgr3

Mycgr3G71679 Mycgr3T
  
Location: 36208-37300
  
 NCBI BlastP on this gene

Mycgr3G71679\_Mycgr3T

Mycgr3G92938 Mycgr3T
  
Location: 37400-38699
  
 NCBI BlastP on this gene

Mycgr3G92938\_Mycgr3T

Mycgr3G92941 Mycgr3T
  
Location: 38799-40734
  
 NCBI BlastP on this gene

Mycgr3G92941\_Mycgr3T

hypothetical protein
  
Accession: EGP86989
  
Location: 82944-84859
  
 NCBI BlastP on this gene

EGP86989

hypothetical protein
  
Accession: EGP86990
  
Location: 85463-87322
  
  
**BlastP hit with Mycgr3G85918\_Mycgr3T**
  
Percentage identity: 100 %
  
BlastP bit score: 1087
  
Sequence coverage: 99 %
  
E-value: 0.0
  
  
 NCBI BlastP on this gene

EGP86990

hypothetical protein
  
Accession: EGP86991
  
Location: 88348-89691
  
  
**BlastP hit with Mycgr3G71676\_Mycgr3T**
  
Percentage identity: 100 %
  
BlastP bit score: 807
  
Sequence coverage: 99 %
  
E-value: 0.0
  
  
 NCBI BlastP on this gene

EGP86991

hypothetical protein
  
Accession: EGP86992
  
Location: 90138-91442
  
  
**BlastP hit with Mycgr3G71679\_Mycgr3T**
  
Percentage identity: 100 %
  
BlastP bit score: 741
  
Sequence coverage: 99 %
  
E-value: 0.0
  
  
 NCBI BlastP on this gene

EGP86992

hypothetical protein
  
Accession: EGP86993
  
Location: 92609-94060
  
  
**BlastP hit with Mycgr3G71681\_Mycgr3T**
  
Percentage identity: 100 %
  
BlastP bit score: 852
  
Sequence coverage: 99 %
  
E-value: 0.0
  
  
 NCBI BlastP on this gene

EGP86993

hypothetical protein
  
Accession: EGP87764
  
Location: 94206-94958
  
  
**BlastP hit with Mycgr3G58567\_Mycgr3T**
  
Percentage identity: 100 %
  
BlastP bit score: 518
  
Sequence coverage: 100 %
  
E-value: 0.0
  
  
 NCBI BlastP on this gene

EGP87764

hypothetical protein
  
Accession: EGP87763
  
Location: 95307-96818
  
  
**BlastP hit with Mycgr3G92934\_Mycgr3T**
  
Percentage identity: 100 %
  
BlastP bit score: 1023
  
Sequence coverage: 99 %
  
E-value: 0.0
  
  
 NCBI BlastP on this gene

EGP87763

hypothetical protein
  
Accession: EGP86994
  
Location: 96999-99044
  
  
**BlastP hit with Mycgr3G85924\_Mycgr3T**
  
Percentage identity: 100 %
  
BlastP bit score: 1218
  
Sequence coverage: 99 %
  
E-value: 0.0
  
  
 NCBI BlastP on this gene

EGP86994

hypothetical protein
  
Accession: EGP87762
  
Location: 99487-99714
  
  
**BlastP hit with Mycgr3G104334\_Mycgr3**
  
Percentage identity: 100 %
  
BlastP bit score: 153
  
Sequence coverage: 98 %
  
E-value: 3e-46
  
  
 NCBI BlastP on this gene

EGP87762

hypothetical protein
  
Accession: EGP86995
  
Location: 100315-101462
  
  
**BlastP hit with Mycgr3G42698\_Mycgr3T**
  
Percentage identity: 100 %
  
BlastP bit score: 615
  
Sequence coverage: 99 %
  
E-value: 0.0
  
  
 NCBI BlastP on this gene

EGP86995

hypothetical protein
  
Accession: EGP87761
  
Location: 101621-108595
  
  
**BlastP hit with Mycgr3G42010\_Mycgr3T**
  
Percentage identity: 100 %
  
BlastP bit score: 4741
  
Sequence coverage: 99 %
  
E-value: 0.0
  
  
 NCBI BlastP on this gene

EGP87761

hypothetical protein
  
Accession: EGP86996
  
Location: 108946-110334
  
  
**BlastP hit with Mycgr3G92938\_Mycgr3T**
  
Percentage identity: 100 %
  
BlastP bit score: 893
  
Sequence coverage: 99 %
  
E-value: 0.0
  
  
 NCBI BlastP on this gene

EGP86996

hypothetical protein
  
Accession: EGP86997
  
Location: 111722-112639
  
  
**BlastP hit with Mycgr3G109328\_Mycgr3**
  
Percentage identity: 100 %
  
BlastP bit score: 449
  
Sequence coverage: 99 %
  
E-value: 2e-158
  
  
 NCBI BlastP on this gene

EGP86997

hypothetical protein
  
Accession: EGP86998
  
Location: 113332-114768
  
  
**BlastP hit with Mycgr3G42715\_Mycgr3T**
  
Percentage identity: 100 %
  
BlastP bit score: 909
  
Sequence coverage: 99 %
  
E-value: 0.0
  
  
 NCBI BlastP on this gene

EGP86998

hypothetical protein
  
Accession: EGP87760
  
Location: 115565-118586
  
  
**BlastP hit with Mycgr3G92941\_Mycgr3T**
  
Percentage identity: 100 %
  
BlastP bit score: 1328
  
Sequence coverage: 99 %
  
E-value: 0.0
  
  
 NCBI BlastP on this gene

EGP87760

hypothetical protein
  
Accession: EGP86999
  
Location: 120571-121824
  
  
**BlastP hit with Mycgr3G11468\_Mycgr3T**
  
Percentage identity: 100 %
  
BlastP bit score: 728
  
Sequence coverage: 100 %
  
E-value: 0.0
  
  
 NCBI BlastP on this gene

EGP86999

polyketide synthase
  
Accession: EGP87759
  
Location: 122351-129292
  
  
**BlastP hit with Mycgr3G100089\_Mycgr3**
  
Percentage identity: 100 %
  
BlastP bit score: 4525
  
Sequence coverage: 99 %
  
E-value: 0.0
  
  
 NCBI BlastP on this gene

EGP87759

hypothetical protein
  
Accession: EGP87000
  
Location: 130690-132388
  
  
**BlastP hit with Mycgr3G41969\_Mycgr3T**
  
Percentage identity: 100 %
  
BlastP bit score: 1121
  
Sequence coverage: 99 %
  
E-value: 0.0
  
  
 NCBI BlastP on this gene

EGP87000

hypothetical protein
  
Accession: EGP87001
  
Location: 133725-134685
  
  
**BlastP hit with Mycgr3G104337\_Mycgr3**
  
Percentage identity: 100 %
  
BlastP bit score: 521
  
Sequence coverage: 99 %
  
E-value: 0.0
  
  
 NCBI BlastP on this gene

EGP87001

hypothetical protein
  
Accession: EGP87758
  
Location: 136993-137385
  
  
**BlastP hit with Mycgr3G80635\_Mycgr3T**
  
Percentage identity: 100 %
  
BlastP bit score: 268
  
Sequence coverage: 99 %
  
E-value: 5e-90
  
  
 NCBI BlastP on this gene

EGP87758

hypothetical protein
  
Accession: EGP87757
  
Location: 139379-139694
  
  
**BlastP hit with Mycgr3G29582\_Mycgr3T**
  
Percentage identity: 100 %
  
BlastP bit score: 160
  
Sequence coverage: 100 %
  
E-value: 3e-49
  
  
 NCBI BlastP on this gene

EGP87757

hypothetical protein
  
Accession: EGP87756
  
Location: 141738-142115
  
  
**BlastP hit with Mycgr3G31170\_Mycgr3T**
  
Percentage identity: 100 %
  
BlastP bit score: 162
  
Sequence coverage: 100 %
  
E-value: 6e-50
  
  
 NCBI BlastP on this gene

EGP87756

hypothetical protein
  
Accession: EGP87755
  
Location: 144090-149465
  
  
**BlastP hit with Mycgr3G41426\_Mycgr3T**
  
Percentage identity: 100 %
  
BlastP bit score: 3699
  
Sequence coverage: 100 %
  
E-value: 0.0
  
  
 NCBI BlastP on this gene

EGP87755

hypothetical protein
  
Accession: EGP87002
  
Location: 150248-152095
  
 NCBI BlastP on this gene

EGP87002

2. :  KB456260 Mycosphaerella populorum SO2202 unplaced genomic scaffold SEPMUscaffold\_1     Total score: 4.0     Cumulative Blast bit score: 899

hypothetical protein
  
Accession: EMF16471
  
Location: 876547-877410
  
 NCBI BlastP on this gene

EMF16471

hypothetical protein
  
Accession: EMF16472
  
Location: 878884-879231
  
 NCBI BlastP on this gene

EMF16472

FMN-linked oxidoreductase
  
Accession: EMF16473
  
Location: 879722-881187
  
 NCBI BlastP on this gene

EMF16473

hypothetical protein
  
Accession: EMF16474
  
Location: 882319-883272
  
 NCBI BlastP on this gene

EMF16474

kinase-like protein
  
Accession: EMF16475
  
Location: 884611-887352
  
 NCBI BlastP on this gene

EMF16475

eukaryotic type KH-domain (KH-domain type I)
  
Accession: EMF16476
  
Location: 888633-890373
  
 NCBI BlastP on this gene

EMF16476

P-loop containing nucleoside triphosphate hydrolase protein
  
Accession: EMF16477
  
Location: 890780-891541
  
 NCBI BlastP on this gene

EMF16477

DUF1741-domain-containing protein
  
Accession: EMF16478
  
Location: 891619-893731
  
 NCBI BlastP on this gene

EMF16478

hypothetical protein
  
Accession: EMF16479
  
Location: 894230-895054
  
  
**BlastP hit with Mycgr3G92941\_Mycgr3T**
  
Percentage identity: 64 %
  
BlastP bit score: 362
  
Sequence coverage: 42 %
  
E-value: 4e-117
  
  
 NCBI BlastP on this gene

EMF16479

SRF-TF-domain-containing protein
  
Accession: EMF16480
  
Location: 897129-897927
  
  
**BlastP hit with Mycgr3G31170\_Mycgr3T**
  
Percentage identity: 100 %
  
BlastP bit score: 167
  
Sequence coverage: 100 %
  
E-value: 5e-50
  
  
 NCBI BlastP on this gene

EMF16480

hypothetical protein
  
Accession: EMF16481
  
Location: 899176-899554
  
 NCBI BlastP on this gene

EMF16481

DASH Hsk3-domain-containing protein
  
Accession: EMF16482
  
Location: 899812-900097
  
  
**BlastP hit with Mycgr3G29582\_Mycgr3T**
  
Percentage identity: 89 %
  
BlastP bit score: 134
  
Sequence coverage: 93 %
  
E-value: 6e-39
  
  
 NCBI BlastP on this gene

EMF16482

hypothetical protein
  
Accession: EMF16483
  
Location: 900322-900723
  
 NCBI BlastP on this gene

EMF16483

FAD-binding domain-containing protein
  
Accession: EMF16484
  
Location: 900989-902575
  
 NCBI BlastP on this gene

EMF16484

hypothetical protein
  
Accession: EMF16485
  
Location: 903154-905418
  
 NCBI BlastP on this gene

EMF16485

lysophospholipase Plb2
  
Accession: EMF16486
  
Location: 906405-908536
  
  
**BlastP hit with Mycgr3G41969\_Mycgr3T**
  
Percentage identity: 32 %
  
BlastP bit score: 236
  
Sequence coverage: 107 %
  
E-value: 1e-65
  
  
 NCBI BlastP on this gene

EMF16486

MFS general substrate transporter
  
Accession: EMF16487
  
Location: 909865-911571
  
 NCBI BlastP on this gene

EMF16487

acid phosphatase/Vanadium-dependent haloperoxidase
  
Accession: EMF16488
  
Location: 912036-913014
  
 NCBI BlastP on this gene

EMF16488

MED7-domain-containing protein
  
Accession: EMF16489
  
Location: 913304-914038
  
 NCBI BlastP on this gene

EMF16489

Hexokinase 1-domain-containing protein
  
Accession: EMF16490
  
Location: 915425-920157
  
 NCBI BlastP on this gene

EMF16490

hypothetical protein
  
Accession: EMF16491
  
Location: 920719-923830
  
 NCBI BlastP on this gene

EMF16491

hypothetical protein
  
Accession: EMF16492
  
Location: 924054-924766
  
 NCBI BlastP on this gene

EMF16492

glutathione S-transferase domain-containing protein
  
Accession: EMF16493
  
Location: 925393-926455
  
 NCBI BlastP on this gene

EMF16493

GABA permease
  
Accession: EMF16494
  
Location: 926527-928498
  
 NCBI BlastP on this gene

EMF16494

3. :  ABDF02000089 Trichoderma virens Gv29-8     Total score: 3.0     Cumulative Blast bit score: 2425

hypothetical protein
  
Accession: EHK17227
  
Location: 1071778-1073113
  
 NCBI BlastP on this gene

EHK17227

hypothetical protein
  
Accession: EHK17226
  
Location: 1069379-1070452
  
 NCBI BlastP on this gene

EHK17226

carbohydrate esterase family 16 protein
  
Accession: EHK17225
  
Location: 1066690-1067968
  
 NCBI BlastP on this gene

EHK17225

hypothetical protein
  
Accession: EHK17224
  
Location: 1064549-1066570
  
 NCBI BlastP on this gene

EHK17224

glycosyltransferase family 90 protein
  
Accession: EHK17394
  
Location: 1060147-1062751
  
 NCBI BlastP on this gene

EHK17394

hypothetical protein
  
Accession: EHK17223
  
Location: 1057727-1059933
  
 NCBI BlastP on this gene

EHK17223

hypothetical protein
  
Accession: EHK17222
  
Location: 1054725-1055992
  
 NCBI BlastP on this gene

EHK17222

hypothetical protein
  
Accession: EHK17221
  
Location: 1053084-1053710
  
 NCBI BlastP on this gene

EHK17221

hypothetical protein
  
Accession: EHK17220
  
Location: 1050904-1052219
  
  
**BlastP hit with Mycgr3G92938\_Mycgr3T**
  
Percentage identity: 40 %
  
BlastP bit score: 261
  
Sequence coverage: 81 %
  
E-value: 6e-79
  
  
 NCBI BlastP on this gene

EHK17220

hypothetical protein
  
Accession: EHK17219
  
Location: 1042982-1049962
  
  
**BlastP hit with Mycgr3G42010\_Mycgr3T**
  
Percentage identity: 44 %
  
BlastP bit score: 1959
  
Sequence coverage: 102 %
  
E-value: 0.0
  
  
 NCBI BlastP on this gene

EHK17219

hypothetical protein
  
Accession: EHK17218
  
Location: 1040771-1042134
  
  
**BlastP hit with Mycgr3G71676\_Mycgr3T**
  
Percentage identity: 33 %
  
BlastP bit score: 205
  
Sequence coverage: 101 %
  
E-value: 6e-58
  
  
 NCBI BlastP on this gene

EHK17218

hypothetical protein
  
Accession: EHK17217
  
Location: 1038989-1040530
  
 NCBI BlastP on this gene

EHK17217

hypothetical protein
  
Accession: EHK17216
  
Location: 1036657-1037436
  
 NCBI BlastP on this gene

EHK17216

hypothetical protein
  
Accession: EHK17215
  
Location: 1034923-1035342
  
 NCBI BlastP on this gene

EHK17215

hypothetical protein
  
Accession: EHK17214
  
Location: 1032047-1033362
  
 NCBI BlastP on this gene

EHK17214

hypothetical protein
  
Accession: EHK17213
  
Location: 1026103-1028272
  
 NCBI BlastP on this gene

EHK17213

hypothetical protein
  
Accession: EHK17212
  
Location: 1024825-1025905
  
 NCBI BlastP on this gene

EHK17212

hypothetical protein
  
Accession: EHK17211
  
Location: 1022768-1024441
  
 NCBI BlastP on this gene

EHK17211

4. :  KE145357 Glarea lozoyensis ATCC 20868 chromosome Unknown GLAREA14     Total score: 3.0     Cumulative Blast bit score: 2354

Ferredoxin reductase-like, C-terminal NADP-linked
  
Accession: EPE34197
  
Location: 1873318-1875462
  
 NCBI BlastP on this gene

EPE34197

Calcium ATPase, transmembrane M
  
Accession: EPE34198
  
Location: 1877312-1881273
  
 NCBI BlastP on this gene

EPE34198

hypothetical protein
  
Accession: EPE34199
  
Location: 1883265-1883684
  
 NCBI BlastP on this gene

EPE34199

2Fe-2S ferredoxin-like protein
  
Accession: EPE34200
  
Location: 1885647-1886344
  
 NCBI BlastP on this gene

EPE34200

hypothetical protein
  
Accession: EPE34201
  
Location: 1886687-1887472
  
 NCBI BlastP on this gene

EPE34201

P-loop containing nucleoside triphosphate hydrolase
  
Accession: EPE34202
  
Location: 1887675-1889311
  
 NCBI BlastP on this gene

EPE34202

P-loop containing nucleoside triphosphate hydrolase
  
Accession: EPE34203
  
Location: 1891804-1899037
  
  
**BlastP hit with Mycgr3G42010\_Mycgr3T**
  
Percentage identity: 45 %
  
BlastP bit score: 1940
  
Sequence coverage: 101 %
  
E-value: 0.0
  
  
 NCBI BlastP on this gene

EPE34203

geranylgeranyl pyrophosphate synthetase
  
Accession: EPE34204
  
Location: 1899709-1901176
  
  
**BlastP hit with Mycgr3G92938\_Mycgr3T**
  
Percentage identity: 37 %
  
BlastP bit score: 247
  
Sequence coverage: 84 %
  
E-value: 6e-73
  
  
 NCBI BlastP on this gene

EPE34204

hypothetical protein
  
Accession: EPE34205
  
Location: 1902280-1902912
  
 NCBI BlastP on this gene

EPE34205

hypothetical protein
  
Accession: EPE34206
  
Location: 1904241-1905923
  
 NCBI BlastP on this gene

EPE34206

NAD(P)-binding Rossmann-fold containing protein
  
Accession: EPE34207
  
Location: 1906959-1908151
  
 NCBI BlastP on this gene

EPE34207

WD40 repeat-like protein
  
Accession: EPE34208
  
Location: 1908948-1910180
  
 NCBI BlastP on this gene

EPE34208

P-loop containing nucleoside triphosphate hydrolase
  
Accession: EPE34209
  
Location: 1911052-1914078
  
 NCBI BlastP on this gene

EPE34209

hypothetical protein
  
Accession: EPE34210
  
Location: 1915652-1917898
  
 NCBI BlastP on this gene

EPE34210

hypothetical protein
  
Accession: EPE34211
  
Location: 1918626-1919719
  
 NCBI BlastP on this gene

EPE34211

Di-copper centre-containing
  
Accession: EPE34212
  
Location: 1920979-1922646
  
  
**BlastP hit with Mycgr3G42698\_Mycgr3T**
  
Percentage identity: 31 %
  
BlastP bit score: 167
  
Sequence coverage: 113 %
  
E-value: 9e-45
  
  
 NCBI BlastP on this gene

EPE34212

hypothetical protein
  
Accession: EPE34213
  
Location: 1923882-1925735
  
 NCBI BlastP on this gene

EPE34213

hypothetical protein
  
Accession: EPE34214
  
Location: 1926593-1928300
  
 NCBI BlastP on this gene

EPE34214

hypothetical protein
  
Accession: EPE34215
  
Location: 1929601-1934471
  
 NCBI BlastP on this gene

EPE34215

hypothetical protein
  
Accession: EPE34216
  
Location: 1935205-1936040
  
 NCBI BlastP on this gene

EPE34216

hypothetical protein
  
Accession: EPE34217
  
Location: 1937166-1937744
  
 NCBI BlastP on this gene

EPE34217

hypothetical protein
  
Accession: EPE34218
  
Location: 1937938-1938321
  
 NCBI BlastP on this gene

EPE34218

hypothetical protein
  
Accession: EPE34219
  
Location: 1939037-1939969
  
 NCBI BlastP on this gene

EPE34219

hypothetical protein
  
Accession: EPE34220
  
Location: 1941294-1944601
  
 NCBI BlastP on this gene

EPE34220

5. :  ABDF02000006 Trichoderma virens Gv29-8     Total score: 3.0     Cumulative Blast bit score: 2223

hypothetical protein
  
Accession: EHK22776
  
Location: 2244216-2248104
  
 NCBI BlastP on this gene

EHK22776

hypothetical protein
  
Accession: EHK22777
  
Location: 2248854-2250180
  
 NCBI BlastP on this gene

EHK22777

hypothetical protein
  
Accession: EHK22778
  
Location: 2251103-2252414
  
 NCBI BlastP on this gene

EHK22778

hypothetical protein
  
Accession: EHK22779
  
Location: 2253633-2254807
  
 NCBI BlastP on this gene

EHK22779

hypothetical protein
  
Accession: EHK22780
  
Location: 2255037-2255516
  
 NCBI BlastP on this gene

EHK22780

hypothetical protein
  
Accession: EHK22781
  
Location: 2255841-2257250
  
 NCBI BlastP on this gene

EHK22781

hypothetical protein
  
Accession: EHK22782
  
Location: 2258075-2258791
  
 NCBI BlastP on this gene

EHK22782

hypothetical protein
  
Accession: EHK22783
  
Location: 2261308-2263799
  
  
**BlastP hit with Mycgr3G11468\_Mycgr3T**
  
Percentage identity: 30 %
  
BlastP bit score: 158
  
Sequence coverage: 101 %
  
E-value: 5e-40
  
  
 NCBI BlastP on this gene

EHK22783

hypothetical protein
  
Accession: EHK22784
  
Location: 2263929-2264534
  
 NCBI BlastP on this gene

EHK22784

hypothetical protein
  
Accession: EHK22785
  
Location: 2266141-2267383
  
 NCBI BlastP on this gene

EHK22785

hypothetical protein
  
Accession: EHK22786
  
Location: 2269628-2271538
  
 NCBI BlastP on this gene

EHK22786

hypothetical protein
  
Accession: EHK22787
  
Location: 2272617-2273284
  
 NCBI BlastP on this gene

EHK22787

hypothetical protein
  
Accession: EHK22788
  
Location: 2273912-2274350
  
 NCBI BlastP on this gene

EHK22788

glycoside hydrolase family 20 protein
  
Accession: EHK22789
  
Location: 2274754-2277014
  
 NCBI BlastP on this gene

EHK22789

hypothetical protein
  
Accession: EHK22790
  
Location: 2280140-2282726
  
 NCBI BlastP on this gene

EHK22790

hypothetical protein
  
Accession: EHK22791
  
Location: 2283688-2284152
  
 NCBI BlastP on this gene

EHK22791

hypothetical protein
  
Accession: EHK22792
  
Location: 2285457-2286064
  
 NCBI BlastP on this gene

EHK22792

putative polyketide synthase
  
Accession: EHK22793
  
Location: 2286988-2293963
  
  
**BlastP hit with Mycgr3G100089\_Mycgr3**
  
Percentage identity: 44 %
  
BlastP bit score: 1847
  
Sequence coverage: 101 %
  
E-value: 0.0
  
  
 NCBI BlastP on this gene

EHK22793

hypothetical protein
  
Accession: EHK22794
  
Location: 2295807-2296765
  
  
**BlastP hit with Mycgr3G104337\_Mycgr3**
  
Percentage identity: 45 %
  
BlastP bit score: 218
  
Sequence coverage: 98 %
  
E-value: 1e-66
  
  
 NCBI BlastP on this gene

EHK22794

glycosyltransferase family 1 protein
  
Accession: EHK22795
  
Location: 2297637-2299297
  
 NCBI BlastP on this gene

EHK22795

hypothetical protein
  
Accession: EHK22796
  
Location: 2307969-2309129
  
 NCBI BlastP on this gene

EHK22796

hypothetical protein
  
Accession: EHK22797
  
Location: 2311666-2315106
  
 NCBI BlastP on this gene

EHK22797

6. :  ABDG02000026 Trichoderma atroviride IMI 206040     Total score: 3.0     Cumulative Blast bit score: 2201

hypothetical protein
  
Accession: EHK42710
  
Location: 1118691-1119338
  
 NCBI BlastP on this gene

EHK42710

hypothetical protein
  
Accession: EHK42709
  
Location: 1117630-1118306
  
 NCBI BlastP on this gene

EHK42709

hypothetical protein
  
Accession: EHK42708
  
Location: 1112978-1116943
  
 NCBI BlastP on this gene

EHK42708

hypothetical protein
  
Accession: EHK42707
  
Location: 1111063-1112273
  
 NCBI BlastP on this gene

EHK42707

hypothetical protein
  
Accession: EHK42706
  
Location: 1108739-1110161
  
 NCBI BlastP on this gene

EHK42706

hypothetical protein
  
Accession: EHK42705
  
Location: 1107870-1108379
  
 NCBI BlastP on this gene

EHK42705

hypothetical protein
  
Accession: EHK42704
  
Location: 1106473-1107593
  
 NCBI BlastP on this gene

EHK42704

sterol 24-C-methyltransferase
  
Accession: EHK42703
  
Location: 1104002-1105296
  
 NCBI BlastP on this gene

EHK42703

hypothetical protein
  
Accession: EHK42702
  
Location: 1099620-1102514
  
  
**BlastP hit with Mycgr3G11468\_Mycgr3T**
  
Percentage identity: 31 %
  
BlastP bit score: 165
  
Sequence coverage: 103 %
  
E-value: 4e-42
  
  
 NCBI BlastP on this gene

EHK42702

hypothetical protein
  
Accession: EHK42701
  
Location: 1098879-1099506
  
 NCBI BlastP on this gene

EHK42701

hypothetical protein
  
Accession: EHK42700
  
Location: 1096314-1097513
  
 NCBI BlastP on this gene

EHK42700

hypothetical protein
  
Accession: EHK42699
  
Location: 1091792-1093774
  
 NCBI BlastP on this gene

EHK42699

hypothetical protein
  
Accession: EHK42698
  
Location: 1089936-1090570
  
 NCBI BlastP on this gene

EHK42698

glycoside hydrolase family 20 protein
  
Accession: EHK43609
  
Location: 1087200-1089476
  
 NCBI BlastP on this gene

EHK43609

hypothetical protein
  
Accession: EHK42697
  
Location: 1080913-1083545
  
 NCBI BlastP on this gene

EHK42697

hypothetical protein
  
Accession: EHK42696
  
Location: 1078644-1079108
  
 NCBI BlastP on this gene

EHK42696

hypothetical protein
  
Accession: EHK42695
  
Location: 1076610-1077198
  
 NCBI BlastP on this gene

EHK42695

polyketide synthase
  
Accession: EHK42694
  
Location: 1068691-1075711
  
  
**BlastP hit with Mycgr3G100089\_Mycgr3**
  
Percentage identity: 43 %
  
BlastP bit score: 1815
  
Sequence coverage: 101 %
  
E-value: 0.0
  
  
 NCBI BlastP on this gene

EHK42694

hypothetical protein
  
Accession: EHK42693
  
Location: 1065925-1066903
  
  
**BlastP hit with Mycgr3G104337\_Mycgr3**
  
Percentage identity: 45 %
  
BlastP bit score: 221
  
Sequence coverage: 100 %
  
E-value: 5e-68
  
  
 NCBI BlastP on this gene

EHK42693

glycosyltransferase family 1 protein
  
Accession: EHK43614
  
Location: 1063433-1065084
  
 NCBI BlastP on this gene

EHK43614

hypothetical protein
  
Accession: EHK42692
  
Location: 1052204-1053478
  
 NCBI BlastP on this gene

EHK42692

hypothetical protein
  
Accession: EHK42691
  
Location: 1051014-1051232
  
 NCBI BlastP on this gene

EHK42691

7. :  JH725159 Beauveria bassiana ARSEF 2860 unplaced genomic scaffold BBA\_S00010     Total score: 3.0     Cumulative Blast bit score: 1765

hypothetical protein
  
Accession: EJP66578
  
Location: 583407-584039
  
 NCBI BlastP on this gene

EJP66578

decapping enzyme Dcp2
  
Accession: EJP66579
  
Location: 584838-587584
  
 NCBI BlastP on this gene

EJP66579

RNA recognition motif containing protein
  
Accession: EJP66580
  
Location: 588112-589382
  
 NCBI BlastP on this gene

EJP66580

hypothetical protein
  
Accession: EJP66581
  
Location: 590955-591847
  
 NCBI BlastP on this gene

EJP66581

histone H3 methyltransferase complex and RNA cleavage factor II complex, subunit SWD2
  
Accession: EJP66582
  
Location: 592412-593699
  
 NCBI BlastP on this gene

EJP66582

mitochondrial oxaloacetate transport protein
  
Accession: EJP66583
  
Location: 594639-596148
  
 NCBI BlastP on this gene

EJP66583

cytoplasmic tRNA 2-thiolation protein 2
  
Accession: EJP66584
  
Location: 596480-597765
  
 NCBI BlastP on this gene

EJP66584

PH domain-containing protein
  
Accession: EJP66585
  
Location: 600015-601287
  
 NCBI BlastP on this gene

EJP66585

cellulase-like protein
  
Accession: EJP66586
  
Location: 602983-604260
  
  
**BlastP hit with Mycgr3G71681\_Mycgr3T**
  
Percentage identity: 32 %
  
BlastP bit score: 187
  
Sequence coverage: 98 %
  
E-value: 9e-51
  
  
 NCBI BlastP on this gene

EJP66586

hypothetical protein
  
Accession: EJP66587
  
Location: 604711-605874
  
 NCBI BlastP on this gene

EJP66587

geranylgeranyl pyrophosphate synthetase
  
Accession: EJP66588
  
Location: 606682-608200
  
  
**BlastP hit with Mycgr3G92938\_Mycgr3T**
  
Percentage identity: 33 %
  
BlastP bit score: 241
  
Sequence coverage: 101 %
  
E-value: 7e-71
  
  
 NCBI BlastP on this gene

EJP66588

ATPase protein
  
Accession: EJP66589
  
Location: 608830-611275
  
  
**BlastP hit with Mycgr3G42010\_Mycgr3T**
  
Percentage identity: 34 %
  
BlastP bit score: 439
  
Sequence coverage: 34 %
  
E-value: 1e-129
  
  
 NCBI BlastP on this gene

EJP66589

cbbX-like protein
  
Accession: EJP66590
  
Location: 612576-615880
  
  
**BlastP hit with Mycgr3G42010\_Mycgr3T**
  
Percentage identity: 46 %
  
BlastP bit score: 899
  
Sequence coverage: 47 %
  
E-value: 0.0
  
  
 NCBI BlastP on this gene

EJP66590

spliceosome associated protein
  
Accession: EJP66591
  
Location: 616725-618560
  
 NCBI BlastP on this gene

EJP66591

hypothetical protein
  
Accession: EJP66592
  
Location: 619098-621375
  
 NCBI BlastP on this gene

EJP66592

hypothetical protein
  
Accession: EJP66593
  
Location: 622658-624844
  
 NCBI BlastP on this gene

EJP66593

hypothetical protein
  
Accession: EJP66594
  
Location: 625194-626387
  
 NCBI BlastP on this gene

EJP66594

serine/arginine repetitive matrix protein 1
  
Accession: EJP66595
  
Location: 627225-629096
  
 NCBI BlastP on this gene

EJP66595

histone deacetylase
  
Accession: EJP66596
  
Location: 629437-631709
  
 NCBI BlastP on this gene

EJP66596

hypothetical protein
  
Accession: EJP66597
  
Location: 632920-634033
  
 NCBI BlastP on this gene

EJP66597

F-box domain-containing protein
  
Accession: EJP66598
  
Location: 634185-635532
  
 NCBI BlastP on this gene

EJP66598

8. :  GG698924 Nectria haematococca mpVI 77-13-4 chromosome 11 genomic scaffold NECHAsca\_32\_chr11\_3\_0     Total score: 3.0     Cumulative Blast bit score: 1648

hypothetical protein
  
Accession: EEU37134
  
Location: 435067-437256
  
 NCBI BlastP on this gene

EEU37134

predicted protein
  
Accession: EEU37053
  
Location: 433502-434980
  
 NCBI BlastP on this gene

EEU37053

hypothetical protein
  
Accession: EEU37052
  
Location: 431592-431963
  
 NCBI BlastP on this gene

EEU37052

hypothetical protein
  
Accession: EEU37133
  
Location: 428536-430872
  
 NCBI BlastP on this gene

EEU37133

hypothetical protein
  
Accession: EEU37132
  
Location: 425610-426938
  
 NCBI BlastP on this gene

EEU37132

predicted protein
  
Accession: EEU37051
  
Location: 423776-425457
  
 NCBI BlastP on this gene

EEU37051

hypothetical protein
  
Accession: EEU37050
  
Location: 418985-422000
  
 NCBI BlastP on this gene

EEU37050

hypothetical protein
  
Accession: EEU37131
  
Location: 415445-416603
  
 NCBI BlastP on this gene

EEU37131

predicted protein
  
Accession: EEU37049
  
Location: 414007-415264
  
  
**BlastP hit with Mycgr3G71679\_Mycgr3T**
  
Percentage identity: 38 %
  
BlastP bit score: 246
  
Sequence coverage: 99 %
  
E-value: 1e-74
  
  
 NCBI BlastP on this gene

EEU37049

hypothetical protein
  
Accession: EEU37048
  
Location: 410831-412473
  
 NCBI BlastP on this gene

EEU37048

hypothetical protein
  
Accession: EEU37130
  
Location: 409559-410459
  
  
**BlastP hit with Mycgr3G104337\_Mycgr3**
  
Percentage identity: 45 %
  
BlastP bit score: 218
  
Sequence coverage: 97 %
  
E-value: 1e-66
  
  
 NCBI BlastP on this gene

EEU37130

hypothetical protein
  
Accession: EEU37129
  
Location: 401232-408233
  
  
**BlastP hit with Mycgr3G100089\_Mycgr3**
  
Percentage identity: 46 %
  
BlastP bit score: 1184
  
Sequence coverage: 57 %
  
E-value: 0.0
  
  
 NCBI BlastP on this gene

EEU37129

hypothetical protein
  
Accession: EEU37128
  
Location: 396393-398308
  
 NCBI BlastP on this gene

EEU37128

predicted protein
  
Accession: EEU37047
  
Location: 394517-396281
  
 NCBI BlastP on this gene

EEU37047

predicted protein
  
Accession: EEU37127
  
Location: 392800-394026
  
 NCBI BlastP on this gene

EEU37127

hypothetical protein
  
Accession: EEU37046
  
Location: 390550-391629
  
 NCBI BlastP on this gene

EEU37046

hypothetical protein
  
Accession: EEU37045
  
Location: 388814-389887
  
 NCBI BlastP on this gene

EEU37045

hypothetical protein
  
Accession: EEU37044
  
Location: 387442-388125
  
 NCBI BlastP on this gene

EEU37044

hypothetical protein
  
Accession: EEU37126
  
Location: 384281-385648
  
 NCBI BlastP on this gene

EEU37126

hypothetical protein
  
Accession: EEU37043
  
Location: 382672-384197
  
 NCBI BlastP on this gene

EEU37043

9. :  KB446535 Dothistroma septosporum NZE10 unplaced genomic scaffold DOTSEscaffold\_1     Total score: 3.0     Cumulative Blast bit score: 623

hypothetical protein
  
Accession: EME48423
  
Location: 244260-244680
  
 NCBI BlastP on this gene

EME48423

hypothetical protein
  
Accession: EME48422
  
Location: 241716-242972
  
 NCBI BlastP on this gene

EME48422

hypothetical protein
  
Accession: EME48421
  
Location: 240636-241423
  
 NCBI BlastP on this gene

EME48421

hypothetical protein
  
Accession: EME48420
  
Location: 238233-240065
  
 NCBI BlastP on this gene

EME48420

hypothetical protein
  
Accession: EME48419
  
Location: 235098-237792
  
 NCBI BlastP on this gene

EME48419

hypothetical protein
  
Accession: EME48418
  
Location: 232303-233040
  
 NCBI BlastP on this gene

EME48418

hypothetical protein
  
Accession: EME48417
  
Location: 230471-231358
  
 NCBI BlastP on this gene

EME48417

hypothetical protein
  
Accession: EME48416
  
Location: 227646-228397
  
  
**BlastP hit with Mycgr3G31170\_Mycgr3T**
  
Percentage identity: 100 %
  
BlastP bit score: 167
  
Sequence coverage: 100 %
  
E-value: 5e-50
  
  
 NCBI BlastP on this gene

EME48416

hypothetical protein
  
Accession: EME48415
  
Location: 226480-226807
  
 NCBI BlastP on this gene

EME48415

hypothetical protein
  
Accession: EME48414
  
Location: 225993-226289
  
  
**BlastP hit with Mycgr3G29582\_Mycgr3T**
  
Percentage identity: 93 %
  
BlastP bit score: 117
  
Sequence coverage: 79 %
  
E-value: 4e-32
  
  
 NCBI BlastP on this gene

EME48414

hypothetical protein
  
Accession: EME48413
  
Location: 225273-225641
  
 NCBI BlastP on this gene

EME48413

hypothetical protein
  
Accession: EME48412
  
Location: 224691-225143
  
 NCBI BlastP on this gene

EME48412

hypothetical protein
  
Accession: EME48411
  
Location: 224014-224651
  
 NCBI BlastP on this gene

EME48411

hypothetical protein
  
Accession: EME48410
  
Location: 222961-223956
  
 NCBI BlastP on this gene

EME48410

hypothetical protein
  
Accession: EME48409
  
Location: 221770-222747
  
  
**BlastP hit with Mycgr3G92941\_Mycgr3T**
  
Percentage identity: 56 %
  
BlastP bit score: 339
  
Sequence coverage: 48 %
  
E-value: 1e-107
  
  
 NCBI BlastP on this gene

EME48409

hypothetical protein
  
Accession: EME48408
  
Location: 218599-219402
  
 NCBI BlastP on this gene

EME48408

hypothetical protein
  
Accession: EME48406
  
Location: 215154-216488
  
 NCBI BlastP on this gene

EME48406

hypothetical protein
  
Accession: EME48405
  
Location: 212957-213475
  
 NCBI BlastP on this gene

EME48405

hypothetical protein
  
Accession: EME48404
  
Location: 211478-211988
  
 NCBI BlastP on this gene

EME48404

hypothetical protein
  
Accession: EME48403
  
Location: 209369-210952
  
 NCBI BlastP on this gene

EME48403

hypothetical protein
  
Accession: EME48402
  
Location: 205909-207646
  
 NCBI BlastP on this gene

EME48402

hypothetical protein
  
Accession: EME48401
  
Location: 204112-205539
  
 NCBI BlastP on this gene

EME48401

10. :  GL629756 Grosmannia clavigera kw1407 unplaced genomic scaffold GCSC\_132     Total score: 3.0     Cumulative Blast bit score: 602

glycoside hydrolase family 3 domain containing protein
  
Accession: EFX05115
  
Location: 421203-423828
  
 NCBI BlastP on this gene

EFX05115

maltose permease
  
Accession: EFX04971
  
Location: 419469-421129
  
 NCBI BlastP on this gene

EFX04971

hypothetical protein
  
Accession: EFX04841
  
Location: 418173-418871
  
 NCBI BlastP on this gene

EFX04841

alpha-l-rhamnosidase
  
Accession: EFX04866
  
Location: 415554-417711
  
 NCBI BlastP on this gene

EFX04866

fungal specific transcription factor domain containing protein
  
Accession: EFX05109
  
Location: 413819-414977
  
 NCBI BlastP on this gene

EFX05109

hypothetical protein
  
Accession: EFX04908
  
Location: 409240-410023
  
 NCBI BlastP on this gene

EFX04908

taud/tfda taurine catabolism dioxygenase
  
Accession: EFX05037
  
Location: 407910-409094
  
 NCBI BlastP on this gene

EFX05037

hypothetical protein
  
Accession: EFX04959
  
Location: 405283-406668
  
 NCBI BlastP on this gene

EFX04959

hypothetical protein
  
Accession: EFX05022
  
Location: 403391-404764
  
 NCBI BlastP on this gene

EFX05022

tyrosinase central domain containing protein
  
Accession: EFX04978
  
Location: 401544-403089
  
  
**BlastP hit with Mycgr3G42698\_Mycgr3T**
  
Percentage identity: 34 %
  
BlastP bit score: 187
  
Sequence coverage: 115 %
  
E-value: 1e-52
  
  
 NCBI BlastP on this gene

EFX04978

integral membrane protein
  
Accession: EFX04839
  
Location: 399031-400492
  
 NCBI BlastP on this gene

EFX04839

duf895 domain protein membrane protein
  
Accession: EFX04873
  
Location: 395227-396804
  
 NCBI BlastP on this gene

EFX04873

methyltransferase type 11
  
Accession: EFX05074
  
Location: 392223-393038
  
 NCBI BlastP on this gene

EFX05074

hypothetical protein
  
Accession: EFX05081
  
Location: 390988-391962
  
 NCBI BlastP on this gene

EFX05081

major facilitator superfamily transporter quinate
  
Accession: EFX04852
  
Location: 388984-390678
  
 NCBI BlastP on this gene

EFX04852

hypothetical protein
  
Accession: EFX04856
  
Location: 385556-386782
  
 NCBI BlastP on this gene

EFX04856

hypothetical protein
  
Accession: EFX05055
  
Location: 384184-384960
  
 NCBI BlastP on this gene

EFX05055

c6 zinc finger domain containing protein
  
Accession: EFX04895
  
Location: 378994-383259
  
  
**BlastP hit with Mycgr3G11468\_Mycgr3T**
  
Percentage identity: 32 %
  
BlastP bit score: 162
  
Sequence coverage: 100 %
  
E-value: 2e-40
  
  
 NCBI BlastP on this gene

EFX04895

heterokaryon incompatibility protein
  
Accession: EFX04862
  
Location: 376384-377751
  
 NCBI BlastP on this gene

EFX04862

allantoate permease
  
Accession: EFX04995
  
Location: 371938-375070
  
  
**BlastP hit with Mycgr3G71679\_Mycgr3T**
  
Percentage identity: 40 %
  
BlastP bit score: 253
  
Sequence coverage: 101 %
  
E-value: 1e-72
  
  
 NCBI BlastP on this gene

EFX04995

cytoskeleton assembly control protein
  
Accession: EFX04935
  
Location: 366904-370181
  
 NCBI BlastP on this gene

EFX04935

hypothetical protein
  
Accession: EFX05047
  
Location: 364281-365876
  
 NCBI BlastP on this gene

EFX05047

mating type protein 1-2-1
  
Accession: EFX05114
  
Location: 362628-363607
  
 NCBI BlastP on this gene

EFX05114

hypothetical protein
  
Accession: EFX04946
  
Location: 359634-360749
  
 NCBI BlastP on this gene

EFX04946

hypothetical protein
  
Accession: EFX05097
  
Location: 357361-357917
  
 NCBI BlastP on this gene

EFX05097

11. :  KB446555 Pseudocercospora fijiensis CIRAD86 unplaced genomic scaffold MYCFIscaffold\_1     Total score: 3.0     Cumulative Blast bit score: 593

hypothetical protein
  
Accession: EME89655
  
Location: 11222151-11223422
  
 NCBI BlastP on this gene

EME89655

hypothetical protein
  
Accession: EME89654
  
Location: 11221023-11221998
  
 NCBI BlastP on this gene

EME89654

hypothetical protein
  
Accession: EME89653
  
Location: 11214595-11217387
  
 NCBI BlastP on this gene

EME89653

hypothetical protein
  
Accession: EME89651
  
Location: 11213452-11214099
  
 NCBI BlastP on this gene

EME89651

hypothetical protein
  
Accession: EME89650
  
Location: 11211221-11212552
  
 NCBI BlastP on this gene

EME89650

hypothetical protein
  
Accession: EME89649
  
Location: 11208039-11210936
  
 NCBI BlastP on this gene

EME89649

hypothetical protein
  
Accession: EME89648
  
Location: 11204965-11207976
  
 NCBI BlastP on this gene

EME89648

hypothetical protein
  
Accession: EME89647
  
Location: 11203387-11204468
  
 NCBI BlastP on this gene

EME89647

hypothetical protein
  
Accession: EME89646
  
Location: 11202273-11202956
  
  
**BlastP hit with Mycgr3G92941\_Mycgr3T**
  
Percentage identity: 61 %
  
BlastP bit score: 295
  
Sequence coverage: 35 %
  
E-value: 6e-92
  
  
 NCBI BlastP on this gene

EME89646

hypothetical protein
  
Accession: EME89645
  
Location: 11195527-11196063
  
 NCBI BlastP on this gene

EME89645

hypothetical protein
  
Accession: EME89644
  
Location: 11192179-11193209
  
 NCBI BlastP on this gene

EME89644

hypothetical protein
  
Accession: EME89643
  
Location: 11189025-11189714
  
  
**BlastP hit with Mycgr3G31170\_Mycgr3T**
  
Percentage identity: 100 %
  
BlastP bit score: 166
  
Sequence coverage: 100 %
  
E-value: 3e-50
  
  
 NCBI BlastP on this gene

EME89643

hypothetical protein
  
Accession: EME89642
  
Location: 11188678-11188929
  
 NCBI BlastP on this gene

EME89642

hypothetical protein
  
Accession: EME89641
  
Location: 11187181-11187502
  
  
**BlastP hit with Mycgr3G29582\_Mycgr3T**
  
Percentage identity: 89 %
  
BlastP bit score: 132
  
Sequence coverage: 92 %
  
E-value: 3e-38
  
  
 NCBI BlastP on this gene

EME89641

hypothetical protein
  
Accession: EME89640
  
Location: 11186936-11187181
  
 NCBI BlastP on this gene

EME89640

hypothetical protein
  
Accession: EME89639
  
Location: 11181897-11184399
  
 NCBI BlastP on this gene

EME89639

hypothetical protein
  
Accession: EME89638
  
Location: 11180493-11181115
  
 NCBI BlastP on this gene

EME89638

hypothetical protein
  
Accession: EME89637
  
Location: 11178461-11179275
  
 NCBI BlastP on this gene

EME89637

hypothetical protein
  
Accession: EME89635
  
Location: 11174172-11177214
  
 NCBI BlastP on this gene

EME89635

hypothetical protein
  
Accession: EME89634
  
Location: 11171820-11172920
  
 NCBI BlastP on this gene

EME89634

hypothetical protein
  
Accession: EME89633
  
Location: 11168918-11171641
  
 NCBI BlastP on this gene

EME89633

hypothetical protein
  
Accession: EME89632
  
Location: 11167690-11168880
  
 NCBI BlastP on this gene

EME89632

12. :  CABT02000055 Sordaria macrospora k-hell     Total score: 2.0     Cumulative Blast bit score: 3843

not annotated
  
Accession: CCC05205
  
Location: 3991-11276
  
  
**BlastP hit with Mycgr3G42010\_Mycgr3T**
  
Percentage identity: 45 %
  
BlastP bit score: 1899
  
Sequence coverage: 101 %
  
E-value: 0.0
  
  
 NCBI BlastP on this gene

CCC05205

not annotated
  
Accession: CCC05206
  
Location: 15517-22768
  
  
**BlastP hit with Mycgr3G42010\_Mycgr3T**
  
Percentage identity: 43 %
  
BlastP bit score: 1769
  
Sequence coverage: 101 %
  
E-value: 0.0
  
  
 NCBI BlastP on this gene

CCC05206

not annotated
  
Accession: CCC05207
  
Location: 24027-25918
  
  
**BlastP hit with Mycgr3G92938\_Mycgr3T**
  
Percentage identity: 35 %
  
BlastP bit score: 175
  
Sequence coverage: 72 %
  
E-value: 2e-45
  
  
 NCBI BlastP on this gene

CCC05207

not annotated
  
Accession: CCC05208
  
Location: 30368-31286
  
 NCBI BlastP on this gene

CCC05208

not annotated
  
Accession: CCC05209
  
Location: 32085-32980
  
 NCBI BlastP on this gene

CCC05209

not annotated
  
Accession: CCC05210
  
Location: 34108-34383
  
 NCBI BlastP on this gene

CCC05210

not annotated
  
Accession: CCC05211
  
Location: 35263-43044
  
 NCBI BlastP on this gene

CCC05211

not annotated
  
Accession: CCC05212
  
Location: 44339-45644
  
 NCBI BlastP on this gene

CCC05212

not annotated
  
Accession: CCC05213
  
Location: 45868-50109
  
 NCBI BlastP on this gene

CCC05213

13. :  KB456266 Mycosphaerella populorum SO2202 unplaced genomic scaffold SEPMUscaffold\_7     Total score: 2.0     Cumulative Blast bit score: 3607

HAD-like protein
  
Accession: EMF11317
  
Location: 1071416-1072138
  
 NCBI BlastP on this gene

EMF11317

hypothetical protein
  
Accession: EMF11318
  
Location: 1072997-1073218
  
 NCBI BlastP on this gene

EMF11318

hypothetical protein
  
Accession: EMF11319
  
Location: 1075103-1075806
  
 NCBI BlastP on this gene

EMF11319

ketoreductase
  
Accession: EMF11320
  
Location: 1076386-1077466
  
 NCBI BlastP on this gene

EMF11320

hypothetical protein
  
Accession: EMF11321
  
Location: 1078601-1079034
  
 NCBI BlastP on this gene

EMF11321

hypothetical protein
  
Accession: EMF11322
  
Location: 1079970-1080869
  
 NCBI BlastP on this gene

EMF11322

hypothetical protein
  
Accession: EMF11323
  
Location: 1081671-1083036
  
 NCBI BlastP on this gene

EMF11323

hypothetical protein
  
Accession: EMF11324
  
Location: 1083700-1084014
  
 NCBI BlastP on this gene

EMF11324

26S protease regulatory subunit 6A
  
Accession: EMF11325
  
Location: 1084780-1086282
  
 NCBI BlastP on this gene

EMF11325

amine oxidase
  
Accession: EMF11326
  
Location: 1087497-1088552
  
 NCBI BlastP on this gene

EMF11326

hypothetical protein
  
Accession: EMF11327
  
Location: 1088862-1090133
  
  
**BlastP hit with Mycgr3G92938\_Mycgr3T**
  
Percentage identity: 46 %
  
BlastP bit score: 370
  
Sequence coverage: 98 %
  
E-value: 4e-121
  
  
 NCBI BlastP on this gene

EMF11327

hypothetical protein
  
Accession: EMF11328
  
Location: 1090257-1090442
  
 NCBI BlastP on this gene

EMF11328

AAA family ATPase
  
Accession: EMF11329
  
Location: 1090710-1097754
  
  
**BlastP hit with Mycgr3G42010\_Mycgr3T**
  
Percentage identity: 68 %
  
BlastP bit score: 3237
  
Sequence coverage: 101 %
  
E-value: 0.0
  
  
 NCBI BlastP on this gene

EMF11329

FAD/NAD(P)-binding domain-containing protein
  
Accession: EMF11330
  
Location: 1107187-1109097
  
 NCBI BlastP on this gene

EMF11330

hypothetical protein
  
Accession: EMF11331
  
Location: 1109583-1110644
  
 NCBI BlastP on this gene

EMF11331

hypothetical protein
  
Accession: EMF11332
  
Location: 1111701-1112987
  
 NCBI BlastP on this gene

EMF11332

hypothetical protein
  
Accession: EMF11333
  
Location: 1113150-1114103
  
 NCBI BlastP on this gene

EMF11333

hypothetical protein
  
Accession: EMF11334
  
Location: 1116859-1117617
  
 NCBI BlastP on this gene

EMF11334

14. :  KB446560 Pseudocercospora fijiensis CIRAD86 unplaced genomic scaffold MYCFIscaffold\_6     Total score: 2.0     Cumulative Blast bit score: 3286

hypothetical protein
  
Accession: EME81371
  
Location: 3685179-3685695
  
 NCBI BlastP on this gene

EME81371

hypothetical protein
  
Accession: EME81372
  
Location: 3686296-3693288
  
  
**BlastP hit with Mycgr3G42010\_Mycgr3T**
  
Percentage identity: 62 %
  
BlastP bit score: 2952
  
Sequence coverage: 100 %
  
E-value: 0.0
  
  
 NCBI BlastP on this gene

EME81372

hypothetical protein
  
Accession: EME81373
  
Location: 3693465-3695053
  
  
**BlastP hit with Mycgr3G92938\_Mycgr3T**
  
Percentage identity: 48 %
  
BlastP bit score: 334
  
Sequence coverage: 83 %
  
E-value: 4e-106
  
  
 NCBI BlastP on this gene

EME81373

hypothetical protein
  
Accession: EME81374
  
Location: 3697166-3698138
  
 NCBI BlastP on this gene

EME81374

15. :  CM001231 Magnaporthe oryzae 70-15 chromosome 1     Total score: 2.0     Cumulative Blast bit score: 2996

glutamyl-tRNA(Gln) amidotransferase subunit A
  
Accession: EHA58174
  
Location: 6761163-6762894
  
 NCBI BlastP on this gene

EHA58174

hypothetical protein
  
Accession: EHA58173
  
Location: 6758210-6759756
  
 NCBI BlastP on this gene

EHA58173

hypothetical protein
  
Accession: EHA58172
  
Location: 6756806-6757101
  
 NCBI BlastP on this gene

EHA58172

bilirubin oxidase
  
Accession: EHA58171
  
Location: 6753225-6755168
  
 NCBI BlastP on this gene

EHA58171

hypothetical protein
  
Accession: EHA58170
  
Location: 6751299-6752937
  
 NCBI BlastP on this gene

EHA58170

hypothetical protein
  
Accession: EHA58169
  
Location: 6750207-6750563
  
 NCBI BlastP on this gene

EHA58169

glycerate kinase
  
Accession: EHA58168
  
Location: 6748639-6750102
  
 NCBI BlastP on this gene

EHA58168

multidrug resistance protein 3
  
Accession: EHA58167
  
Location: 6743712-6748276
  
 NCBI BlastP on this gene

EHA58167

hypothetical protein
  
Accession: EHA58166
  
Location: 6741720-6742557
  
  
**BlastP hit with Mycgr3G104337\_Mycgr3**
  
Percentage identity: 41 %
  
BlastP bit score: 206
  
Sequence coverage: 100 %
  
E-value: 4e-62
  
  
 NCBI BlastP on this gene

EHA58166

fatty acid synthase S-acetyltransferase
  
Accession: EHA58165
  
Location: 6732693-6739671
  
  
**BlastP hit with Mycgr3G100089\_Mycgr3**
  
Percentage identity: 40 %
  
BlastP bit score: 1403
  
Sequence coverage: 91 %
  
E-value: 0.0
  
  
 NCBI BlastP on this gene

EHA58165

fatty acid synthase S-acetyltransferase, variant
  
Accession: EHA58164
  
Location: 6732693-6738655
  
  
**BlastP hit with Mycgr3G100089\_Mycgr3**
  
Percentage identity: 40 %
  
BlastP bit score: 1387
  
Sequence coverage: 90 %
  
E-value: 0.0
  
  
 NCBI BlastP on this gene

EHA58164

hypothetical protein
  
Accession: EHA58163
  
Location: 6730837-6731355
  
 NCBI BlastP on this gene

EHA58163

hypothetical protein
  
Accession: EHA58162
  
Location: 6728184-6729778
  
 NCBI BlastP on this gene

EHA58162

hypothetical protein
  
Accession: EHA58161
  
Location: 6727111-6728036
  
 NCBI BlastP on this gene

EHA58161

sugar transporter STL1, variant
  
Accession: EHA58159
  
Location: 6725101-6726809
  
 NCBI BlastP on this gene

EHA58159

ankyrin repeat and protein kinase domain-containing protein 1
  
Accession: EHA58158
  
Location: 6717652-6721130
  
 NCBI BlastP on this gene

EHA58158

hypothetical protein
  
Accession: EHA58157
  
Location: 6716655-6717274
  
 NCBI BlastP on this gene

EHA58157

hypothetical protein
  
Accession: EHA58156
  
Location: 6714022-6715109
  
 NCBI BlastP on this gene

EHA58156

16. :  AM270278 Aspergillus niger contig An12c0220, genomic contig.     Total score: 2.0     Cumulative Blast bit score: 2747

not annotated
  
Accession: CAK46342
  
Location: 52537-54246
  
 NCBI BlastP on this gene

An12g07140

not annotated
  
Accession: CAK46341
  
Location: 50920-51987
  
 NCBI BlastP on this gene

An12g07130

unnamed
  
Accession: CAK46340
  
Location: 48472-50548
  
 NCBI BlastP on this gene

An12g07120

not annotated
  
Accession: CAK46339
  
Location: 41567-43672
  
 NCBI BlastP on this gene

An12g07110

not annotated
  
Accession: CAK46338
  
Location: 38626-39761
  
 NCBI BlastP on this gene

An12g07100

not annotated
  
Accession: CAK46337
  
Location: 35651-37079
  
 NCBI BlastP on this gene

An12g07090

not annotated
  
Accession: CAK46336
  
Location: 28213-35378
  
  
**BlastP hit with Mycgr3G100089\_Mycgr3**
  
Percentage identity: 53 %
  
BlastP bit score: 2295
  
Sequence coverage: 100 %
  
E-value: 0.0
  
  
 NCBI BlastP on this gene

An12g07070

not annotated
  
Accession: CAK46335
  
Location: 26598-27527
  
  
**BlastP hit with Mycgr3G104337\_Mycgr3**
  
Percentage identity: 46 %
  
BlastP bit score: 230
  
Sequence coverage: 99 %
  
E-value: 1e-71
  
  
 NCBI BlastP on this gene

An12g07060

unnamed
  
Accession: CAK46334
  
Location: 25361-26347
  
  
**BlastP hit with Mycgr3G104337\_Mycgr3**
  
Percentage identity: 45 %
  
BlastP bit score: 222
  
Sequence coverage: 98 %
  
E-value: 2e-68
  
  
 NCBI BlastP on this gene

An12g07050

not annotated
  
Accession: CAK46333
  
Location: 24086-24832
  
 NCBI BlastP on this gene

An12g07040

unnamed
  
Accession: CAK46332
  
Location: 22216-23211
  
 NCBI BlastP on this gene

An12g07030

not annotated
  
Accession: CAK46331
  
Location: 20407-21672
  
 NCBI BlastP on this gene

An12g07020

not annotated
  
Accession: CAK46330
  
Location: 18046-19026
  
 NCBI BlastP on this gene

An12g07000

not annotated
  
Accession: CAK46329
  
Location: 17603-17871
  
 NCBI BlastP on this gene

An12g06990

not annotated
  
Accession: CAK46328
  
Location: 15996-16549
  
 NCBI BlastP on this gene

An12g06980

not annotated
  
Accession: CAK46327
  
Location: 13980-14777
  
 NCBI BlastP on this gene

An12g06970

not annotated
  
Accession: CAK46326
  
Location: 12286-13589
  
 NCBI BlastP on this gene

An12g06960

unnamed
  
Accession: CAK46325
  
Location: 10370-11992
  
 NCBI BlastP on this gene

An12g06940

extracellular alpha-amylase
  
Accession: CAK46324
  
Location: 7374-9415
  
 NCBI BlastP on this gene

amyA

17. :  DS995904 Penicillium marneffei ATCC 18224 scf\_1105668340738 genomic scaffold     Total score: 2.0     Cumulative Blast bit score: 2721

hypothetical protein
  
Accession: EEA20037
  
Location: 67191-67532
  
 NCBI BlastP on this gene

EEA20037

dihydrodipicolinate synthase, putative
  
Accession: EEA20038
  
Location: 68859-70318
  
 NCBI BlastP on this gene

EEA20038

hypothetical protein
  
Accession: EEA20039
  
Location: 71829-72531
  
 NCBI BlastP on this gene

EEA20039

conserved hypothetical protein
  
Accession: EEA20040
  
Location: 73531-74058
  
 NCBI BlastP on this gene

EEA20040

alpha-ketoglutarate-dependent 2,4-dichlorophenoxyacetate dioxygenase, putative
  
Accession: EEA20041
  
Location: 74511-75884
  
 NCBI BlastP on this gene

EEA20041

aminohydrolase, putative
  
Accession: EEA20042
  
Location: 76567-81684
  
 NCBI BlastP on this gene

EEA20042

oxidoreductase, putative
  
Accession: EEA20043
  
Location: 82286-85704
  
 NCBI BlastP on this gene

EEA20043

DUF341 family oxidoreductase, putative
  
Accession: EEA20044
  
Location: 86437-87443
  
  
**BlastP hit with Mycgr3G104337\_Mycgr3**
  
Percentage identity: 58 %
  
BlastP bit score: 335
  
Sequence coverage: 98 %
  
E-value: 1e-112
  
  
 NCBI BlastP on this gene

EEA20044

polyketide synthase, putative
  
Accession: EEA20045
  
Location: 88781-95890
  
  
**BlastP hit with Mycgr3G100089\_Mycgr3**
  
Percentage identity: 53 %
  
BlastP bit score: 2386
  
Sequence coverage: 102 %
  
E-value: 0.0
  
  
 NCBI BlastP on this gene

EEA20045

conserved hypothetical protein
  
Accession: EEA20046
  
Location: 96394-96873
  
 NCBI BlastP on this gene

EEA20046

hypothetical protein
  
Accession: EEA20047
  
Location: 98821-99528
  
 NCBI BlastP on this gene

EEA20047

hypothetical protein
  
Accession: EEA20048
  
Location: 101134-102031
  
 NCBI BlastP on this gene

EEA20048

biphenyl-2,3-diol 1,2-dioxygenase, putative
  
Accession: EEA20049
  
Location: 102367-103014
  
 NCBI BlastP on this gene

EEA20049

conserved hypothetical protein
  
Accession: EEA20050
  
Location: 103701-104284
  
 NCBI BlastP on this gene

EEA20050

phenol 2-monooxygenase, putative
  
Accession: EEA20051
  
Location: 105201-107270
  
 NCBI BlastP on this gene

EEA20051

hypothetical protein
  
Accession: EEA20052
  
Location: 107395-108816
  
 NCBI BlastP on this gene

EEA20052

amino acid permease, putative
  
Accession: EEA20053
  
Location: 112068-113783
  
 NCBI BlastP on this gene

EEA20053

developmental regulator flbA, putative
  
Accession: EEA20054
  
Location: 114324-116091
  
 NCBI BlastP on this gene

EEA20054

18. :  AM920436 Penicillium chrysogenum Wisconsin 54-1255 complete genome, contig Pc00c21.     Total score: 2.0     Cumulative Blast bit score: 2708

unnamed
  
Accession: CAP95373
  
Location: 1122985-1123763
  
 NCBI BlastP on this gene

Pc21g04760

unnamed
  
Accession: CAP95374
  
Location: 1124393-1125404
  
 NCBI BlastP on this gene

Pc21g04770

hypothetical protein
  
Accession: CAP95375
  
Location: 1125716-1126669
  
 NCBI BlastP on this gene

Pc21g04780

not annotated
  
Accession: CAP95376
  
Location: 1127334-1129150
  
 NCBI BlastP on this gene

Pc21g04790

unnamed
  
Accession: CAP95377
  
Location: 1130647-1132685
  
 NCBI BlastP on this gene

Pc21g04800

not annotated
  
Accession: CAP95378
  
Location: 1133717-1135038
  
 NCBI BlastP on this gene

Pc21g04810

not annotated
  
Accession: CAP95379
  
Location: 1135671-1135961
  
 NCBI BlastP on this gene

Pc21g04820

not annotated
  
Accession: CAP95380
  
Location: 1137439-1138998
  
 NCBI BlastP on this gene

Pc21g04830

not annotated
  
Accession: CAP95381
  
Location: 1139872-1146768
  
  
**BlastP hit with Mycgr3G100089\_Mycgr3**
  
Percentage identity: 54 %
  
BlastP bit score: 2414
  
Sequence coverage: 100 %
  
E-value: 0.0
  
  
 NCBI BlastP on this gene

Pc21g04840

not annotated
  
Accession: CAP95382
  
Location: 1147328-1148304
  
  
**BlastP hit with Mycgr3G104337\_Mycgr3**
  
Percentage identity: 55 %
  
BlastP bit score: 294
  
Sequence coverage: 97 %
  
E-value: 2e-96
  
  
 NCBI BlastP on this gene

Pc21g04850

hypothetical protein
  
Accession: CAP95383
  
Location: 1150094-1150636
  
 NCBI BlastP on this gene

Pc21g04860

unnamed
  
Accession: CAP95384
  
Location: 1150831-1151460
  
 NCBI BlastP on this gene

Pc21g04870

not annotated
  
Accession: CAP95385
  
Location: 1151931-1153377
  
 NCBI BlastP on this gene

Pc21g04880

unnamed
  
Accession: CAP95386
  
Location: 1153926-1154631
  
 NCBI BlastP on this gene

Pc21g04890

not annotated
  
Accession: CAP95387
  
Location: 1155624-1156319
  
 NCBI BlastP on this gene

Pc21g04900

not annotated
  
Accession: CAP95388
  
Location: 1156536-1157614
  
 NCBI BlastP on this gene

Pc21g04910

not annotated
  
Accession: CAP95389
  
Location: 1158147-1159074
  
 NCBI BlastP on this gene

Pc21g04920

unnamed
  
Accession: CAP95390
  
Location: 1159502-1162477
  
 NCBI BlastP on this gene

Pc21g04930

not annotated
  
Accession: CAP95391
  
Location: 1163044-1164221
  
 NCBI BlastP on this gene

Pc21g04940

not annotated
  
Accession: CAP95392
  
Location: 1164533-1165615
  
 NCBI BlastP on this gene

Pc21g04950

unnamed
  
Accession: CAP95393
  
Location: 1165822-1166396
  
 NCBI BlastP on this gene

Pc21g04960

hypothetical protein
  
Accession: CAP95394
  
Location: 1166979-1167924
  
 NCBI BlastP on this gene

Pc21g04970

19. :  EQ962652 Talaromyces stipitatus ATCC 10500 scf\_1105507295523 genomic scaffold     Total score: 2.0     Cumulative Blast bit score: 2697

amino acid permease, putative
  
Accession: EED24605
  
Location: 5458788-5460143
  
 NCBI BlastP on this gene

EED24605

hypothetical protein
  
Accession: EED24606
  
Location: 5463226-5464665
  
 NCBI BlastP on this gene

EED24606

phenol 2-monooxygenase, putative
  
Accession: EED24607
  
Location: 5464957-5467118
  
 NCBI BlastP on this gene

EED24607

hypothetical protein
  
Accession: EED24608
  
Location: 5467504-5468418
  
 NCBI BlastP on this gene

EED24608

biphenyl-2,3-diol 1,2-dioxygenase, putative
  
Accession: EED24609
  
Location: 5468687-5469467
  
 NCBI BlastP on this gene

EED24609

hypothetical protein
  
Accession: EED24610
  
Location: 5470989-5471558
  
 NCBI BlastP on this gene

EED24610

ferulic acid esterase (FaeA), putative
  
Accession: EED24611
  
Location: 5473398-5474352
  
 NCBI BlastP on this gene

EED24611

conserved hypothetical protein
  
Accession: EED24612
  
Location: 5474887-5475377
  
 NCBI BlastP on this gene

EED24612

conserved hypothetical protein
  
Accession: EED24613
  
Location: 5476633-5477571
  
 NCBI BlastP on this gene

EED24613

polyketide synthase, putative
  
Accession: EED24614
  
Location: 5478431-5485502
  
  
**BlastP hit with Mycgr3G100089\_Mycgr3**
  
Percentage identity: 54 %
  
BlastP bit score: 2408
  
Sequence coverage: 102 %
  
E-value: 0.0
  
  
 NCBI BlastP on this gene

EED24614

conserved hypothetical protein
  
Accession: EED24615
  
Location: 5486576-5487446
  
  
**BlastP hit with Mycgr3G104337\_Mycgr3**
  
Percentage identity: 55 %
  
BlastP bit score: 289
  
Sequence coverage: 92 %
  
E-value: 8e-95
  
  
 NCBI BlastP on this gene

EED24615

acetylxylan esterase precursor, putative
  
Accession: EED24616
  
Location: 5489113-5489980
  
 NCBI BlastP on this gene

EED24616

oxidoreductase, putative
  
Accession: EED24617
  
Location: 5494465-5495801
  
 NCBI BlastP on this gene

EED24617

transposable element tc1 transposase, putative
  
Accession: EED24618
  
Location: 5497026-5498095
  
 NCBI BlastP on this gene

EED24618

reverse transcriptase, putative
  
Accession: EED24619
  
Location: 5499643-5503257
  
 NCBI BlastP on this gene

EED24619

conserved hypothetical protein
  
Accession: EED24620
  
Location: 5503512-5505242
  
 NCBI BlastP on this gene

EED24620

conserved hypothetical protein
  
Accession: EED24621
  
Location: 5506478-5507475
  
 NCBI BlastP on this gene

EED24621

20. :  AHHD01000525 Macrophomina phaseolina MS6     Total score: 2.0     Cumulative Blast bit score: 2617

hypothetical protein
  
Accession: EKG10216
  
Location: 7370-8927
  
 NCBI BlastP on this gene

EKG10216

hypothetical protein
  
Accession: EKG10217
  
Location: 10348-12016
  
 NCBI BlastP on this gene

EKG10217

Heat shock protein Hsp70
  
Accession: EKG10218
  
Location: 12772-14903
  
 NCBI BlastP on this gene

EKG10218

hypothetical protein
  
Accession: EKG10219
  
Location: 16210-17112
  
 NCBI BlastP on this gene

EKG10219

hypothetical protein
  
Accession: EKG10220
  
Location: 18203-19099
  
 NCBI BlastP on this gene

EKG10220

Carboxylesterase type B
  
Accession: EKG10221
  
Location: 19947-20786
  
 NCBI BlastP on this gene

EKG10221

hypothetical protein
  
Accession: EKG10222
  
Location: 25421-25860
  
 NCBI BlastP on this gene

EKG10222

CbxX/CfqX
  
Accession: EKG10223
  
Location: 27205-34929
  
  
**BlastP hit with Mycgr3G42010\_Mycgr3T**
  
Percentage identity: 53 %
  
BlastP bit score: 2334
  
Sequence coverage: 97 %
  
E-value: 0.0
  
  
 NCBI BlastP on this gene

EKG10223

hypothetical protein
  
Accession: EKG10224
  
Location: 35757-36827
  
  
**BlastP hit with Mycgr3G92938\_Mycgr3T**
  
Percentage identity: 43 %
  
BlastP bit score: 283
  
Sequence coverage: 78 %
  
E-value: 4e-88
  
  
 NCBI BlastP on this gene

EKG10224

hypothetical protein
  
Accession: EKG10225
  
Location: 37271-39836
  
 NCBI BlastP on this gene

EKG10225

hypothetical protein
  
Accession: EKG10226
  
Location: 40850-42252
  
 NCBI BlastP on this gene

EKG10226

hypothetical protein
  
Accession: EKG10227
  
Location: 42989-43910
  
 NCBI BlastP on this gene

EKG10227

hypothetical protein
  
Accession: EKG10228
  
Location: 44495-45007
  
 NCBI BlastP on this gene

EKG10228

Short-chain dehydrogenase/reductase SDR
  
Accession: EKG10229
  
Location: 46770-49935
  
 NCBI BlastP on this gene

EKG10229

hypothetical protein
  
Accession: EKG10230
  
Location: 50378-51657
  
 NCBI BlastP on this gene

EKG10230

Methyltransferase type 11
  
Accession: EKG10231
  
Location: 56417-57746
  
 NCBI BlastP on this gene

EKG10231

21. :  ACJE01000006 Aspergillus niger ATCC 1015     Total score: 2.0     Cumulative Blast bit score: 2589

hypothetical protein
  
Accession: EHA25429
  
Location: 1090147-1091856
  
 NCBI BlastP on this gene

EHA25429

hypothetical protein
  
Accession: EHA25428
  
Location: 1088530-1089597
  
 NCBI BlastP on this gene

EHA25428

hypothetical protein
  
Accession: EHA25427
  
Location: 1086130-1088158
  
 NCBI BlastP on this gene

EHA25427

hypothetical protein
  
Accession: EHA25426
  
Location: 1079929-1081281
  
 NCBI BlastP on this gene

EHA25426

hypothetical protein
  
Accession: EHA25425
  
Location: 1078663-1079289
  
 NCBI BlastP on this gene

EHA25425

hypothetical protein
  
Accession: EHA25424
  
Location: 1076169-1077370
  
 NCBI BlastP on this gene

EHA25424

hypothetical protein
  
Accession: EHA25423
  
Location: 1073194-1074622
  
 NCBI BlastP on this gene

EHA25423

hypothetical protein
  
Accession: EHA25422
  
Location: 1065786-1072569
  
  
**BlastP hit with Mycgr3G100089\_Mycgr3**
  
Percentage identity: 53 %
  
BlastP bit score: 2290
  
Sequence coverage: 100 %
  
E-value: 0.0
  
  
 NCBI BlastP on this gene

EHA25422

hypothetical protein
  
Accession: EHA25421
  
Location: 1064326-1065070
  
  
**BlastP hit with Mycgr3G104337\_Mycgr3**
  
Percentage identity: 43 %
  
BlastP bit score: 167
  
Sequence coverage: 88 %
  
E-value: 4e-47
  
  
 NCBI BlastP on this gene

EHA25421

hypothetical protein
  
Accession: EHA25420
  
Location: 1063115-1063890
  
  
**BlastP hit with Mycgr3G104337\_Mycgr3**
  
Percentage identity: 36 %
  
BlastP bit score: 132
  
Sequence coverage: 78 %
  
E-value: 2e-34
  
  
 NCBI BlastP on this gene

EHA25420

hypothetical protein
  
Accession: EHA25419
  
Location: 1060276-1060824
  
 NCBI BlastP on this gene

EHA25419

hypothetical protein
  
Accession: EHA25418
  
Location: 1058089-1059132
  
 NCBI BlastP on this gene

EHA25418

hypothetical protein
  
Accession: EHA25417
  
Location: 1053684-1056259
  
 NCBI BlastP on this gene

EHA25417

hypothetical protein
  
Accession: EHA25416
  
Location: 1051631-1053086
  
 NCBI BlastP on this gene

EHA25416

hypothetical protein
  
Accession: EHA25415
  
Location: 1049630-1050883
  
 NCBI BlastP on this gene

EHA25415

hypothetical protein
  
Accession: EHA25414
  
Location: 1047420-1048829
  
 NCBI BlastP on this gene

EHA25414

hypothetical protein
  
Accession: EHA25413
  
Location: 1045450-1047167
  
 NCBI BlastP on this gene

EHA25413

hypothetical protein
  
Accession: EHA25412
  
Location: 1043795-1044559
  
 NCBI BlastP on this gene

EHA25412

22. :  DF126460 Aspergillus kawachii IFO 4308 DNA, contig: scaffold00014     Total score: 2.0     Cumulative Blast bit score: 2549

alcohol dehydrogenase
  
Accession: GAA87592
  
Location: 2873-4121
  
 NCBI BlastP on this gene

GAA87592

similar to DUF895 domain membrane protein
  
Accession: GAA87593
  
Location: 5638-7127
  
 NCBI BlastP on this gene

GAA87593

hypothetical protein
  
Accession: GAA87594
  
Location: 7720-10296
  
 NCBI BlastP on this gene

GAA87594

alpha 1,6 mannosyltransferase
  
Accession: GAA87595
  
Location: 11913-13034
  
 NCBI BlastP on this gene

GAA87595

similar to An12g07030
  
Accession: GAA87596
  
Location: 14171-14724
  
 NCBI BlastP on this gene

GAA87596

DUF341 family oxidoreductase
  
Accession: GAA87597
  
Location: 16465-18668
  
  
**BlastP hit with Mycgr3G104337\_Mycgr3**
  
Percentage identity: 52 %
  
BlastP bit score: 251
  
Sequence coverage: 96 %
  
E-value: 9e-77
  
  
 NCBI BlastP on this gene

GAA87597

polyketide synthase
  
Accession: GAA87598
  
Location: 19394-26179
  
  
**BlastP hit with Mycgr3G100089\_Mycgr3**
  
Percentage identity: 53 %
  
BlastP bit score: 2298
  
Sequence coverage: 101 %
  
E-value: 0.0
  
  
 NCBI BlastP on this gene

GAA87598

FAD binding domain protein
  
Accession: GAA87599
  
Location: 26985-28611
  
 NCBI BlastP on this gene

GAA87599

similar to An12g07100
  
Accession: GAA87600
  
Location: 30088-31202
  
 NCBI BlastP on this gene

GAA87600

hypothetical protein
  
Accession: GAA87601
  
Location: 32212-32838
  
 NCBI BlastP on this gene

GAA87601

salicylate synthetase
  
Accession: GAA87602
  
Location: 33317-34669
  
 NCBI BlastP on this gene

GAA87602

cytochrome P450
  
Accession: GAA87603
  
Location: 35356-37374
  
 NCBI BlastP on this gene

GAA87603

isoflavone reductase family protein
  
Accession: GAA87604
  
Location: 37800-38868
  
 NCBI BlastP on this gene

GAA87604

C6 zinc finger domain protein
  
Accession: GAA87605
  
Location: 39411-41132
  
 NCBI BlastP on this gene

GAA87605

similar to An12g07160
  
Accession: GAA87606
  
Location: 42785-44008
  
 NCBI BlastP on this gene

GAA87606

sodium/solute symporter
  
Accession: GAA87607
  
Location: 44109-46296
  
 NCBI BlastP on this gene

GAA87607

23. :  ADOT01000195 Arthrobotrys oligospora ATCC 24927     Total score: 2.0     Cumulative Blast bit score: 2481

hypothetical protein
  
Accession: EGX46125
  
Location: 371171-372202
  
 NCBI BlastP on this gene

EGX46125

hypothetical protein
  
Accession: EGX46126
  
Location: 373177-374520
  
 NCBI BlastP on this gene

EGX46126

hypothetical protein
  
Accession: EGX46127
  
Location: 377708-378662
  
 NCBI BlastP on this gene

EGX46127

hypothetical protein
  
Accession: EGX46128
  
Location: 379247-379699
  
 NCBI BlastP on this gene

EGX46128

hypothetical protein
  
Accession: EGX46129
  
Location: 380489-381823
  
 NCBI BlastP on this gene

EGX46129

hypothetical protein
  
Accession: EGX46130
  
Location: 383717-385189
  
 NCBI BlastP on this gene

EGX46130

hypothetical protein
  
Accession: EGX46131
  
Location: 386089-389108
  
 NCBI BlastP on this gene

EGX46131

hypothetical protein
  
Accession: EGX46132
  
Location: 389971-397204
  
  
**BlastP hit with Mycgr3G42010\_Mycgr3T**
  
Percentage identity: 49 %
  
BlastP bit score: 2237
  
Sequence coverage: 103 %
  
E-value: 0.0
  
  
 NCBI BlastP on this gene

EGX46132

hypothetical protein
  
Accession: EGX46133
  
Location: 397680-399181
  
  
**BlastP hit with Mycgr3G92938\_Mycgr3T**
  
Percentage identity: 34 %
  
BlastP bit score: 244
  
Sequence coverage: 101 %
  
E-value: 1e-71
  
  
 NCBI BlastP on this gene

EGX46133

hypothetical protein
  
Accession: EGX46134
  
Location: 399443-401644
  
 NCBI BlastP on this gene

EGX46134

hypothetical protein
  
Accession: EGX46135
  
Location: 405848-406789
  
 NCBI BlastP on this gene

EGX46135

hypothetical protein
  
Accession: EGX46136
  
Location: 407568-408123
  
 NCBI BlastP on this gene

EGX46136

hypothetical protein
  
Accession: EGX46137
  
Location: 410119-411201
  
 NCBI BlastP on this gene

EGX46137

hypothetical protein
  
Accession: EGX46138
  
Location: 411301-414525
  
 NCBI BlastP on this gene

EGX46138

hypothetical protein
  
Accession: EGX46139
  
Location: 416956-419025
  
 NCBI BlastP on this gene

EGX46139

24. :  GL531877 Pyrenophora teres f. teres 0-1 unplaced genomic scaffold scaffold\_21635     Total score: 2.0     Cumulative Blast bit score: 2453

hypothetical protein
  
Accession: EFQ96509
  
Location: 510-884
  
 NCBI BlastP on this gene

EFQ96509

hypothetical protein
  
Accession: EFQ96510
  
Location: 3245-4234
  
 NCBI BlastP on this gene

EFQ96510

hypothetical protein
  
Accession: EFQ96511
  
Location: 4514-11744
  
  
**BlastP hit with Mycgr3G42010\_Mycgr3T**
  
Percentage identity: 48 %
  
BlastP bit score: 2175
  
Sequence coverage: 101 %
  
E-value: 0.0
  
  
 NCBI BlastP on this gene

EFQ96511

hypothetical protein
  
Accession: EFQ96512
  
Location: 12181-13668
  
  
**BlastP hit with Mycgr3G92938\_Mycgr3T**
  
Percentage identity: 38 %
  
BlastP bit score: 278
  
Sequence coverage: 102 %
  
E-value: 2e-84
  
  
 NCBI BlastP on this gene

EFQ96512

hypothetical protein
  
Accession: EFQ96513
  
Location: 15738-16835
  
 NCBI BlastP on this gene

EFQ96513

hypothetical protein
  
Accession: EFQ96514
  
Location: 17580-18407
  
 NCBI BlastP on this gene

EFQ96514

hypothetical protein
  
Accession: EFQ96515
  
Location: 18697-20232
  
 NCBI BlastP on this gene

EFQ96515

25. :  KB908481 Setosphaeria turcica Et28A unplaced genomic scaffold SETTUscaffold\_1     Total score: 2.0     Cumulative Blast bit score: 2448

hypothetical protein
  
Accession: EOA91500
  
Location: 1181357-1181812
  
 NCBI BlastP on this gene

EOA91500

hypothetical protein
  
Accession: EOA91501
  
Location: 1184267-1184650
  
 NCBI BlastP on this gene

EOA91501

hypothetical protein
  
Accession: EOA91502
  
Location: 1185015-1185746
  
 NCBI BlastP on this gene

EOA91502

hypothetical protein
  
Accession: EOA91503
  
Location: 1187549-1189547
  
 NCBI BlastP on this gene

EOA91503

hypothetical protein
  
Accession: EOA91504
  
Location: 1191934-1192328
  
 NCBI BlastP on this gene

EOA91504

hypothetical protein
  
Accession: EOA91505
  
Location: 1194742-1196353
  
 NCBI BlastP on this gene

EOA91505

hypothetical protein
  
Accession: EOA91506
  
Location: 1197037-1204523
  
  
**BlastP hit with Mycgr3G42010\_Mycgr3T**
  
Percentage identity: 48 %
  
BlastP bit score: 2187
  
Sequence coverage: 102 %
  
E-value: 0.0
  
  
 NCBI BlastP on this gene

EOA91506

hypothetical protein
  
Accession: EOA91507
  
Location: 1204958-1206475
  
  
**BlastP hit with Mycgr3G92938\_Mycgr3T**
  
Percentage identity: 39 %
  
BlastP bit score: 261
  
Sequence coverage: 90 %
  
E-value: 4e-78
  
  
 NCBI BlastP on this gene

EOA91507

hypothetical protein
  
Accession: EOA91508
  
Location: 1208097-1209671
  
 NCBI BlastP on this gene

EOA91508

hypothetical protein
  
Accession: EOA91509
  
Location: 1209873-1210748
  
 NCBI BlastP on this gene

EOA91509

hypothetical protein
  
Accession: EOA91510
  
Location: 1211901-1213454
  
 NCBI BlastP on this gene

EOA91510

hypothetical protein
  
Accession: EOA91511
  
Location: 1213986-1214430
  
 NCBI BlastP on this gene

EOA91511

glycosyltransferase family 4 protein
  
Accession: EOA91512
  
Location: 1216094-1225525
  
 NCBI BlastP on this gene

EOA91512

26. :  AQGS01000059 Dactylellina haptotyla CBS 200.50     Total score: 2.0     Cumulative Blast bit score: 2448

hypothetical protein
  
Accession: EPS44106
  
Location: 216699-217380
  
 NCBI BlastP on this gene

EPS44106

hypothetical protein
  
Accession: EPS44092
  
Location: 220449-221252
  
 NCBI BlastP on this gene

EPS44092

hypothetical protein
  
Accession: EPS44060
  
Location: 224427-233524
  
 NCBI BlastP on this gene

EPS44060

hypothetical protein
  
Accession: EPS44085
  
Location: 234882-235535
  
 NCBI BlastP on this gene

EPS44085

hypothetical protein
  
Accession: EPS44064
  
Location: 236197-237696
  
  
**BlastP hit with Mycgr3G92938\_Mycgr3T**
  
Percentage identity: 36 %
  
BlastP bit score: 239
  
Sequence coverage: 101 %
  
E-value: 8e-70
  
  
 NCBI BlastP on this gene

EPS44064

hypothetical protein
  
Accession: EPS44029
  
Location: 238105-245295
  
  
**BlastP hit with Mycgr3G42010\_Mycgr3T**
  
Percentage identity: 48 %
  
BlastP bit score: 2209
  
Sequence coverage: 102 %
  
E-value: 0.0
  
  
 NCBI BlastP on this gene

EPS44029

hypothetical protein
  
Accession: EPS44069
  
Location: 247759-252400
  
 NCBI BlastP on this gene

EPS44069

hypothetical protein
  
Accession: EPS44058
  
Location: 253045-254980
  
 NCBI BlastP on this gene

EPS44058

hypothetical protein
  
Accession: EPS44073
  
Location: 258188-259741
  
 NCBI BlastP on this gene

EPS44073

hypothetical protein
  
Accession: EPS44053
  
Location: 259891-261260
  
 NCBI BlastP on this gene

EPS44053

hypothetical protein
  
Accession: EPS44034
  
Location: 263095-264441
  
 NCBI BlastP on this gene

EPS44034

27. :  DS027045 Aspergillus clavatus NRRL 1 1099423829791 genomic scaffold     Total score: 2.0     Cumulative Blast bit score: 2442

conserved hypothetical protein
  
Accession: EAW14573
  
Location: 2505449-2509427
  
 NCBI BlastP on this gene

EAW14573

conserved hypothetical protein
  
Accession: EAW14574
  
Location: 2509607-2512446
  
 NCBI BlastP on this gene

EAW14574

C2H2 transcription factor (AmdX), putative
  
Accession: EAW14575
  
Location: 2519936-2523629
  
 NCBI BlastP on this gene

EAW14575

AAA family ATPase, putative
  
Accession: EAW14576
  
Location: 2524676-2531986
  
  
**BlastP hit with Mycgr3G42010\_Mycgr3T**
  
Percentage identity: 48 %
  
BlastP bit score: 2156
  
Sequence coverage: 102 %
  
E-value: 0.0
  
  
 NCBI BlastP on this gene

EAW14576

hypothetical protein
  
Accession: EAW14577
  
Location: 2532390-2533906
  
  
**BlastP hit with Mycgr3G92938\_Mycgr3T**
  
Percentage identity: 43 %
  
BlastP bit score: 286
  
Sequence coverage: 84 %
  
E-value: 9e-88
  
  
 NCBI BlastP on this gene

EAW14577

phosphoesterase, putative
  
Accession: EAW14578
  
Location: 2535712-2536668
  
 NCBI BlastP on this gene

EAW14578

conserved hypothetical protein
  
Accession: EAW14579
  
Location: 2537281-2538777
  
 NCBI BlastP on this gene

EAW14579

extracelular serine carboxypeptidase, putative
  
Accession: EAW14580
  
Location: 2539753-2541501
  
 NCBI BlastP on this gene

EAW14580

hypothetical protein
  
Accession: EAW14581
  
Location: 2542075-2542935
  
 NCBI BlastP on this gene

EAW14581

MFS monocarboxylate transporter (Mct), putative
  
Accession: EAW14582
  
Location: 2545874-2547329
  
 NCBI BlastP on this gene

EAW14582

haloacid dehalogenase-like hydrolase, putative
  
Accession: EAW14583
  
Location: 2547482-2548168
  
 NCBI BlastP on this gene

EAW14583

conserved hypothetical protein
  
Accession: EAW14584
  
Location: 2548517-2549078
  
 NCBI BlastP on this gene

EAW14584

hydantoinase/oxoprolinase, putative
  
Accession: EAW14585
  
Location: 2549308-2552511
  
 NCBI BlastP on this gene

EAW14585

NCS1 nucleoside transporter family protein
  
Accession: EAW14586
  
Location: 2552887-2554945
  
 NCBI BlastP on this gene

EAW14586

28. :  KB733458 Bipolaris maydis ATCC 48331 unplaced genomic scaffold COCC4scaffold\_15     Total score: 2.0     Cumulative Blast bit score: 2422

hypothetical protein
  
Accession: ENI03896
  
Location: 121610-121771
  
 NCBI BlastP on this gene

ENI03896

hypothetical protein
  
Accession: ENI03897
  
Location: 125074-126593
  
 NCBI BlastP on this gene

ENI03897

hypothetical protein
  
Accession: ENI03898
  
Location: 127411-130230
  
 NCBI BlastP on this gene

ENI03898

hypothetical protein
  
Accession: ENI03899
  
Location: 131366-132262
  
 NCBI BlastP on this gene

ENI03899

hypothetical protein
  
Accession: ENI03900
  
Location: 133013-133857
  
 NCBI BlastP on this gene

ENI03900

hypothetical protein
  
Accession: ENI03901
  
Location: 134690-138015
  
 NCBI BlastP on this gene

ENI03901

hypothetical protein
  
Accession: ENI03902
  
Location: 138022-138228
  
 NCBI BlastP on this gene

ENI03902

hypothetical protein
  
Accession: ENI03903
  
Location: 138846-139412
  
 NCBI BlastP on this gene

ENI03903

hypothetical protein
  
Accession: ENI03904
  
Location: 139851-141408
  
  
**BlastP hit with Mycgr3G92938\_Mycgr3T**
  
Percentage identity: 35 %
  
BlastP bit score: 239
  
Sequence coverage: 113 %
  
E-value: 2e-69
  
  
 NCBI BlastP on this gene

ENI03904

hypothetical protein
  
Accession: ENI03905
  
Location: 141899-149198
  
  
**BlastP hit with Mycgr3G42010\_Mycgr3T**
  
Percentage identity: 48 %
  
BlastP bit score: 2183
  
Sequence coverage: 102 %
  
E-value: 0.0
  
  
 NCBI BlastP on this gene

ENI03905

hypothetical protein
  
Accession: ENI03906
  
Location: 150167-151777
  
 NCBI BlastP on this gene

ENI03906

hypothetical protein
  
Accession: ENI03907
  
Location: 153362-153691
  
 NCBI BlastP on this gene

ENI03907

hypothetical protein
  
Accession: ENI03908
  
Location: 158165-160011
  
 NCBI BlastP on this gene

ENI03908

hypothetical protein
  
Accession: ENI03909
  
Location: 161544-163007
  
 NCBI BlastP on this gene

ENI03909

hypothetical protein
  
Accession: ENI03910
  
Location: 163672-164151
  
 NCBI BlastP on this gene

ENI03910

hypothetical protein
  
Accession: ENI03911
  
Location: 165314-165670
  
 NCBI BlastP on this gene

ENI03911

glycoside hydrolase family 1 protein
  
Accession: ENI03912
  
Location: 168326-170269
  
 NCBI BlastP on this gene

ENI03912

29. :  KB445583 Cochliobolus heterostrophus C5 unplaced genomic scaffold COCHEscaffold\_15     Total score: 2.0     Cumulative Blast bit score: 2422

hypothetical protein
  
Accession: EMD87095
  
Location: 701318-701674
  
 NCBI BlastP on this gene

EMD87095

hypothetical protein
  
Accession: EMD87096
  
Location: 702837-703316
  
 NCBI BlastP on this gene

EMD87096

hypothetical protein
  
Accession: EMD87097
  
Location: 703981-705444
  
 NCBI BlastP on this gene

EMD87097

hypothetical protein
  
Accession: EMD87098
  
Location: 706977-708823
  
 NCBI BlastP on this gene

EMD87098

hypothetical protein
  
Accession: EMD87099
  
Location: 713297-713626
  
 NCBI BlastP on this gene

EMD87099

hypothetical protein
  
Accession: EMD87100
  
Location: 715211-716821
  
 NCBI BlastP on this gene

EMD87100

hypothetical protein
  
Accession: EMD87101
  
Location: 717790-725089
  
  
**BlastP hit with Mycgr3G42010\_Mycgr3T**
  
Percentage identity: 48 %
  
BlastP bit score: 2183
  
Sequence coverage: 102 %
  
E-value: 0.0
  
  
 NCBI BlastP on this gene

EMD87101

hypothetical protein
  
Accession: EMD87102
  
Location: 725580-727137
  
  
**BlastP hit with Mycgr3G92938\_Mycgr3T**
  
Percentage identity: 35 %
  
BlastP bit score: 239
  
Sequence coverage: 113 %
  
E-value: 2e-69
  
  
 NCBI BlastP on this gene

EMD87102

hypothetical protein
  
Accession: EMD87103
  
Location: 727576-728142
  
 NCBI BlastP on this gene

EMD87103

hypothetical protein
  
Accession: EMD87104
  
Location: 728973-732298
  
 NCBI BlastP on this gene

EMD87104

hypothetical protein
  
Accession: EMD87106
  
Location: 733131-733975
  
 NCBI BlastP on this gene

EMD87106

hypothetical protein
  
Accession: EMD87107
  
Location: 734726-735622
  
 NCBI BlastP on this gene

EMD87107

hypothetical protein
  
Accession: EMD87108
  
Location: 736758-739577
  
 NCBI BlastP on this gene

EMD87108

hypothetical protein
  
Accession: EMD87109
  
Location: 740395-741914
  
 NCBI BlastP on this gene

EMD87109

hypothetical protein
  
Accession: EMD87110
  
Location: 745217-745378
  
 NCBI BlastP on this gene

EMD87110

glycoside hydrolase family 114 protein
  
Accession: EMD87111
  
Location: 746959-748147
  
 NCBI BlastP on this gene

EMD87111

30. :  EQ962652 Talaromyces stipitatus ATCC 10500 scf\_1105507295523 genomic scaffold     Total score: 2.0     Cumulative Blast bit score: 2383

hypothetical protein
  
Accession: EED23730
  
Location: 3265553-3266274
  
 NCBI BlastP on this gene

EED23730

hypothetical protein
  
Accession: EED23731
  
Location: 3266573-3267785
  
 NCBI BlastP on this gene

EED23731

conserved hypothetical protein
  
Accession: EED23732
  
Location: 3268827-3269975
  
 NCBI BlastP on this gene

EED23732

branched-chain amino acid aminotransferase, cytosolic
  
Accession: EED23735
  
Location: 3274693-3276139
  
 NCBI BlastP on this gene

EED23735

G-patch DNA repair protein (Drt111), putative
  
Accession: EED23736
  
Location: 3276725-3278618
  
 NCBI BlastP on this gene

EED23736

SUMO conjugating enzyme (UbcI), putative
  
Accession: EED23737
  
Location: 3279328-3280126
  
 NCBI BlastP on this gene

EED23737

conserved hypothetical protein
  
Accession: EED23738
  
Location: 3282171-3289512
  
  
**BlastP hit with Mycgr3G42010\_Mycgr3T**
  
Percentage identity: 47 %
  
BlastP bit score: 2116
  
Sequence coverage: 102 %
  
E-value: 0.0
  
  
 NCBI BlastP on this gene

EED23738

conserved hypothetical protein
  
Accession: EED23739
  
Location: 3290495-3291822
  
  
**BlastP hit with Mycgr3G92938\_Mycgr3T**
  
Percentage identity: 37 %
  
BlastP bit score: 267
  
Sequence coverage: 97 %
  
E-value: 5e-81
  
  
 NCBI BlastP on this gene

EED23739

conserved hypothetical protein
  
Accession: EED23740
  
Location: 3292137-3293726
  
 NCBI BlastP on this gene

EED23740

hypothetical protein
  
Accession: EED23741
  
Location: 3295033-3295709
  
 NCBI BlastP on this gene

EED23741

asparagine synthetase Asn2, putative
  
Accession: EED23742
  
Location: 3296731-3299006
  
 NCBI BlastP on this gene

EED23742

outer mitochondrial membrane protein porin
  
Accession: EED23743
  
Location: 3299584-3300816
  
 NCBI BlastP on this gene

EED23743

DnaJ domain protein
  
Accession: EED23744
  
Location: 3301330-3302338
  
 NCBI BlastP on this gene

EED23744

methionine aminopeptidase, type II, putative
  
Accession: EED23745
  
Location: 3302638-3304335
  
 NCBI BlastP on this gene

EED23745

sphingolipid desaturase, putative
  
Accession: EED23746
  
Location: 3304663-3306086
  
 NCBI BlastP on this gene

EED23746

hypothetical protein
  
Accession: EED23747
  
Location: 3307638-3308132
  
 NCBI BlastP on this gene

EED23747

hypothetical protein
  
Accession: EED23748
  
Location: 3308324-3310476
  
 NCBI BlastP on this gene

EED23748

hypothetical protein
  
Accession: EED23749
  
Location: 3310493-3310759
  
 NCBI BlastP on this gene

EED23749

31. :  AACD01000123 Aspergillus nidulans FGSC A4     Total score: 2.0     Cumulative Blast bit score: 2343

predicted protein
  
Accession: EAA61283
  
Location: 95349-95987
  
 NCBI BlastP on this gene

EAA61283

hypothetical protein
  
Accession: EAA61284
  
Location: 97408-99248
  
 NCBI BlastP on this gene

EAA61284

hypothetical protein
  
Accession: EAA61285
  
Location: 99966-101404
  
 NCBI BlastP on this gene

EAA61285

hypothetical protein
  
Accession: EAA61286
  
Location: 102737-103509
  
 NCBI BlastP on this gene

EAA61286

hypothetical protein
  
Accession: EAA61287
  
Location: 103751-105256
  
 NCBI BlastP on this gene

EAA61287

hypothetical protein
  
Accession: EAA61288
  
Location: 105872-108046
  
 NCBI BlastP on this gene

EAA61288

hypothetical protein
  
Accession: EAA61289
  
Location: 108982-110676
  
 NCBI BlastP on this gene

EAA61289

hypothetical protein
  
Accession: EAA61290
  
Location: 111356-113098
  
 NCBI BlastP on this gene

EAA61290

predicted protein
  
Accession: EAA61291
  
Location: 114417-114827
  
 NCBI BlastP on this gene

EAA61291

hypothetical protein
  
Accession: EAA61292
  
Location: 115178-116653
  
  
**BlastP hit with Mycgr3G92938\_Mycgr3T**
  
Percentage identity: 32 %
  
BlastP bit score: 228
  
Sequence coverage: 102 %
  
E-value: 9e-66
  
  
 NCBI BlastP on this gene

EAA61292

hypothetical protein
  
Accession: EAA61293
  
Location: 117127-124370
  
  
**BlastP hit with Mycgr3G42010\_Mycgr3T**
  
Percentage identity: 47 %
  
BlastP bit score: 2115
  
Sequence coverage: 102 %
  
E-value: 0.0
  
  
 NCBI BlastP on this gene

EAA61293

predicted protein
  
Accession: EAA61294
  
Location: 126492-127407
  
 NCBI BlastP on this gene

EAA61294

32. :  AM920427 Penicillium chrysogenum Wisconsin 54-1255 complete genome, contig Pc00c12.     Total score: 2.0     Cumulative Blast bit score: 2339

unnamed
  
Accession: CAP79827
  
Location: 455100-456671
  
 NCBI BlastP on this gene

Pc12g02000

not annotated
  
Accession: CAP79826
  
Location: 454198-454485
  
 NCBI BlastP on this gene

Pc12g01990

not annotated
  
Accession: Pc12g01980
  
Location: 452284-452601
  
 NCBI BlastP on this gene

Pc12g01980

hypothetical protein
  
Accession: CAP79824
  
Location: 450166-450507
  
 NCBI BlastP on this gene

Pc12g01970

not annotated
  
Accession: CAP79823
  
Location: 448222-449905
  
 NCBI BlastP on this gene

Pc12g01960

not annotated
  
Accession: CAP79822
  
Location: 445947-447842
  
 NCBI BlastP on this gene

Pc12g01950

not annotated
  
Accession: CAP79821
  
Location: 443540-444431
  
 NCBI BlastP on this gene

Pc12g01940

not annotated
  
Accession: CAP79820
  
Location: 440971-441494
  
 NCBI BlastP on this gene

Pc12g01930

hypothetical protein
  
Accession: CAP79819
  
Location: 439828-440557
  
 NCBI BlastP on this gene

Pc12g01920

not annotated
  
Accession: CAP79818
  
Location: 438697-439277
  
 NCBI BlastP on this gene

Pc12g01910

not annotated
  
Accession: CAP79817
  
Location: 436170-437709
  
 NCBI BlastP on this gene

Pc12g01900

not annotated
  
Accession: CAP79816
  
Location: 433884-435303
  
  
**BlastP hit with Mycgr3G92938\_Mycgr3T**
  
Percentage identity: 37 %
  
BlastP bit score: 268
  
Sequence coverage: 91 %
  
E-value: 2e-81
  
  
 NCBI BlastP on this gene

Pc12g01890

not annotated
  
Accession: CAP79815
  
Location: 429792-433162
  
  
**BlastP hit with Mycgr3G42010\_Mycgr3T**
  
Percentage identity: 45 %
  
BlastP bit score: 939
  
Sequence coverage: 47 %
  
E-value: 0.0
  
  
 NCBI BlastP on this gene

Pc12g01880

not annotated
  
Accession: CAP79814
  
Location: 425863-429740
  
  
**BlastP hit with Mycgr3G42010\_Mycgr3T**
  
Percentage identity: 46 %
  
BlastP bit score: 1132
  
Sequence coverage: 55 %
  
E-value: 0.0
  
  
 NCBI BlastP on this gene

Pc12g01870

not annotated
  
Accession: CAP79813
  
Location: 423432-425188
  
 NCBI BlastP on this gene

Pc12g01860

hypothetical protein
  
Accession: CAP79812
  
Location: 423048-423353
  
 NCBI BlastP on this gene

Pc12g01850

not annotated
  
Accession: CAP79811
  
Location: 421448-422868
  
 NCBI BlastP on this gene

Pc12g01840

not annotated
  
Accession: CAP79810
  
Location: 419029-420975
  
 NCBI BlastP on this gene

Pc12g01830

unnamed
  
Accession: CAP79809
  
Location: 414796-417248
  
 NCBI BlastP on this gene

Pc12g01820

not annotated
  
Accession: CAP79808
  
Location: 412865-414583
  
 NCBI BlastP on this gene

Pc12g01810

not annotated
  
Accession: CAP79807
  
Location: 410537-412087
  
 NCBI BlastP on this gene

Pc12g01800

not annotated
  
Accession: CAP79806
  
Location: 408048-409841
  
 NCBI BlastP on this gene

Pc12g01790

not annotated
  
Accession: CAP79805
  
Location: 406523-407362
  
 NCBI BlastP on this gene

Pc12g01780

33. :  KB445652 Cochliobolus sativus ND90Pr unplaced genomic scaffold COCSAscaffold\_16     Total score: 2.0     Cumulative Blast bit score: 2330

hypothetical protein
  
Accession: EMD59813
  
Location: 963050-963502
  
 NCBI BlastP on this gene

EMD59813

hypothetical protein
  
Accession: EMD59814
  
Location: 964664-965152
  
 NCBI BlastP on this gene

EMD59814

hypothetical protein
  
Accession: EMD59815
  
Location: 965824-967289
  
 NCBI BlastP on this gene

EMD59815

hypothetical protein
  
Accession: EMD59816
  
Location: 967949-968557
  
 NCBI BlastP on this gene

EMD59816

hypothetical protein
  
Accession: EMD59817
  
Location: 968821-970609
  
 NCBI BlastP on this gene

EMD59817

hypothetical protein
  
Accession: EMD59818
  
Location: 973339-973740
  
 NCBI BlastP on this gene

EMD59818

hypothetical protein
  
Accession: EMD59819
  
Location: 974979-975312
  
 NCBI BlastP on this gene

EMD59819

hypothetical protein
  
Accession: EMD59820
  
Location: 976884-978485
  
 NCBI BlastP on this gene

EMD59820

hypothetical protein
  
Accession: EMD59821
  
Location: 979448-986746
  
  
**BlastP hit with Mycgr3G42010\_Mycgr3T**
  
Percentage identity: 49 %
  
BlastP bit score: 2169
  
Sequence coverage: 102 %
  
E-value: 0.0
  
  
 NCBI BlastP on this gene

EMD59821

hypothetical protein
  
Accession: EMD59822
  
Location: 987236-988843
  
  
**BlastP hit with Mycgr3G92938\_Mycgr3T**
  
Percentage identity: 28 %
  
BlastP bit score: 161
  
Sequence coverage: 109 %
  
E-value: 2e-40
  
  
 NCBI BlastP on this gene

EMD59822

hypothetical protein
  
Accession: EMD59823
  
Location: 989279-989843
  
 NCBI BlastP on this gene

EMD59823

hypothetical protein
  
Accession: EMD59824
  
Location: 990666-993989
  
 NCBI BlastP on this gene

EMD59824

hypothetical protein
  
Accession: EMD59825
  
Location: 994815-995659
  
 NCBI BlastP on this gene

EMD59825

hypothetical protein
  
Accession: EMD59826
  
Location: 996396-997285
  
 NCBI BlastP on this gene

EMD59826

hypothetical protein
  
Accession: EMD59827
  
Location: 998374-1000687
  
 NCBI BlastP on this gene

EMD59827

hypothetical protein
  
Accession: EMD59828
  
Location: 1002037-1003557
  
 NCBI BlastP on this gene

EMD59828

glycoside hydrolase family 114 protein
  
Accession: EMD59829
  
Location: 1008573-1009772
  
 NCBI BlastP on this gene

EMD59829

34. :  KE145364 Glarea lozoyensis ATCC 20868 chromosome Unknown GLAREA20     Total score: 2.0     Cumulative Blast bit score: 2320

hypothetical protein
  
Accession: EPE30300
  
Location: 168016-169596
  
 NCBI BlastP on this gene

EPE30300

NAD(P)-binding Rossmann-fold containing protein
  
Accession: EPE30301
  
Location: 172841-174090
  
 NCBI BlastP on this gene

EPE30301

hypothetical protein
  
Accession: EPE30302
  
Location: 175219-175809
  
 NCBI BlastP on this gene

EPE30302

Soluble quinoprotein glucose dehydrogenase
  
Accession: EPE30303
  
Location: 178363-179484
  
 NCBI BlastP on this gene

EPE30303

hypothetical protein
  
Accession: EPE30304
  
Location: 180574-181449
  
 NCBI BlastP on this gene

EPE30304

hypothetical protein
  
Accession: EPE30305
  
Location: 182608-184359
  
 NCBI BlastP on this gene

EPE30305

Thiolase-like protein
  
Accession: EPE30306
  
Location: 185108-192662
  
  
**BlastP hit with Mycgr3G100089\_Mycgr3**
  
Percentage identity: 49 %
  
BlastP bit score: 2125
  
Sequence coverage: 101 %
  
E-value: 0.0
  
  
 NCBI BlastP on this gene

EPE30306

hypothetical protein
  
Accession: EPE30307
  
Location: 196530-197377
  
  
**BlastP hit with Mycgr3G104337\_Mycgr3**
  
Percentage identity: 43 %
  
BlastP bit score: 195
  
Sequence coverage: 95 %
  
E-value: 8e-58
  
  
 NCBI BlastP on this gene

EPE30307

hypothetical protein
  
Accession: EPE30308
  
Location: 198822-199404
  
 NCBI BlastP on this gene

EPE30308

hypothetical protein
  
Accession: EPE30309
  
Location: 200384-203918
  
 NCBI BlastP on this gene

EPE30309

hypothetical protein
  
Accession: EPE30310
  
Location: 204404-204778
  
 NCBI BlastP on this gene

EPE30310

hypothetical protein
  
Accession: EPE30311
  
Location: 207297-207851
  
 NCBI BlastP on this gene

EPE30311

hypothetical protein
  
Accession: EPE30312
  
Location: 210979-212567
  
 NCBI BlastP on this gene

EPE30312

hypothetical protein
  
Accession: EPE30313
  
Location: 215010-215675
  
 NCBI BlastP on this gene

EPE30313

vWA-like protein
  
Accession: EPE30314
  
Location: 217250-220414
  
 NCBI BlastP on this gene

EPE30314

35. :  KB915896 Neofusicoccum parvum UCRNP2 chromosome Unknown NP2\_03\_scaffold\_258     Total score: 2.0     Cumulative Blast bit score: 2317

putative sugar transporter protein
  
Accession: EOD51127
  
Location: 37-1746
  
 NCBI BlastP on this gene

EOD51127

hypothetical protein
  
Accession: EOD51121
  
Location: 6552-7469
  
 NCBI BlastP on this gene

EOD51121

hypothetical protein
  
Accession: EOD51123
  
Location: 9563-10099
  
 NCBI BlastP on this gene

EOD51123

hypothetical protein
  
Accession: EOD51116
  
Location: 12102-13384
  
 NCBI BlastP on this gene

EOD51116

putative polyketide synthase protein
  
Accession: EOD51113
  
Location: 16067-23074
  
  
**BlastP hit with Mycgr3G100089\_Mycgr3**
  
Percentage identity: 48 %
  
BlastP bit score: 2100
  
Sequence coverage: 102 %
  
E-value: 0.0
  
  
 NCBI BlastP on this gene

EOD51113

putative duf341 domain containing protein
  
Accession: EOD51112
  
Location: 24508-25440
  
  
**BlastP hit with Mycgr3G104337\_Mycgr3**
  
Percentage identity: 45 %
  
BlastP bit score: 217
  
Sequence coverage: 96 %
  
E-value: 3e-66
  
  
 NCBI BlastP on this gene

EOD51112

putative fumarylacetoacetate hydrolase protein
  
Accession: EOD51126
  
Location: 28734-29798
  
 NCBI BlastP on this gene

EOD51126

putative glycosyl hydrolases family protein
  
Accession: EOD51125
  
Location: 30209-32018
  
 NCBI BlastP on this gene

EOD51125

putative hexose transporter protein
  
Accession: EOD51118
  
Location: 33172-35139
  
 NCBI BlastP on this gene

EOD51118

putative transcription factor fungi protein
  
Accession: EOD51114
  
Location: 35276-37057
  
 NCBI BlastP on this gene

EOD51114

putative rrna-processing protein efg1 protein
  
Accession: EOD51110
  
Location: 40466-41272
  
 NCBI BlastP on this gene

EOD51110

putative ubiquitin-protein ligase e3 protein
  
Accession: EOD51122
  
Location: 41629-44647
  
 NCBI BlastP on this gene

EOD51122

36. :  JH921455 Marssonina brunnea f. sp. 'multigermtubi' MB\_m1 unplaced genomic scaffold M6\_S00028     Total score: 2.0     Cumulative Blast bit score: 2309

hypothetical protein
  
Accession: EKD12626
  
Location: 279514-280118
  
 NCBI BlastP on this gene

EKD12626

glyoxylate pathway regulator
  
Accession: EKD12627
  
Location: 282801-284285
  
 NCBI BlastP on this gene

EKD12627

RNA polymerase I specific transcription initiation factor
  
Accession: EKD12628
  
Location: 285638-287515
  
 NCBI BlastP on this gene

EKD12628

hypothetical protein
  
Accession: EKD12629
  
Location: 289628-290170
  
 NCBI BlastP on this gene

EKD12629

hypothetical protein
  
Accession: EKD12630
  
Location: 290405-291765
  
 NCBI BlastP on this gene

EKD12630

pescadillo
  
Accession: EKD12631
  
Location: 292016-294058
  
 NCBI BlastP on this gene

EKD12631

hypothetical protein
  
Accession: EKD12632
  
Location: 294511-298254
  
 NCBI BlastP on this gene

EKD12632

beta-ketoacyl synthase domain-containing protein
  
Accession: EKD12633
  
Location: 299348-306442
  
  
**BlastP hit with Mycgr3G100089\_Mycgr3**
  
Percentage identity: 48 %
  
BlastP bit score: 2108
  
Sequence coverage: 102 %
  
E-value: 0.0
  
  
 NCBI BlastP on this gene

EKD12633

hypothetical protein
  
Accession: EKD12634
  
Location: 309287-310301
  
  
**BlastP hit with Mycgr3G104337\_Mycgr3**
  
Percentage identity: 42 %
  
BlastP bit score: 201
  
Sequence coverage: 95 %
  
E-value: 5e-60
  
  
 NCBI BlastP on this gene

EKD12634

short chain dehydrogenase/reductase SDR
  
Accession: EKD12635
  
Location: 310854-312068
  
 NCBI BlastP on this gene

EKD12635

hypothetical protein
  
Accession: EKD12636
  
Location: 313280-316000
  
 NCBI BlastP on this gene

EKD12636

hypothetical protein
  
Accession: EKD12637
  
Location: 316985-317668
  
 NCBI BlastP on this gene

EKD12637

hypothetical protein
  
Accession: EKD12638
  
Location: 318386-318859
  
 NCBI BlastP on this gene

EKD12638

hypothetical protein
  
Accession: EKD12639
  
Location: 320651-321064
  
 NCBI BlastP on this gene

EKD12639

hypothetical protein
  
Accession: EKD12640
  
Location: 322480-322980
  
 NCBI BlastP on this gene

EKD12640

ethyl tert-butyl ether degradation EthD
  
Accession: EKD12641
  
Location: 326346-326651
  
 NCBI BlastP on this gene

EKD12641

hypothetical protein
  
Accession: EKD12642
  
Location: 327120-327712
  
 NCBI BlastP on this gene

EKD12642

hypothetical protein
  
Accession: EKD12643
  
Location: 330086-330971
  
 NCBI BlastP on this gene

EKD12643

37. :  AMYD01000564 Colletotrichum gloeosporioides Cg-14     Total score: 2.0     Cumulative Blast bit score: 2306

hypothetical protein
  
Accession: EQB57153
  
Location: 151-563
  
 NCBI BlastP on this gene

EQB57153

FAD binding domain-containing protein
  
Accession: EQB57154
  
Location: 2563-4083
  
 NCBI BlastP on this gene

EQB57154

hypothetical protein
  
Accession: EQB57155
  
Location: 5195-6410
  
 NCBI BlastP on this gene

EQB57155

hypothetical protein
  
Accession: EQB57156
  
Location: 6824-7712
  
 NCBI BlastP on this gene

EQB57156

hypothetical protein
  
Accession: EQB57157
  
Location: 7852-8368
  
 NCBI BlastP on this gene

EQB57157

hypothetical protein
  
Accession: EQB57158
  
Location: 8992-16107
  
  
**BlastP hit with Mycgr3G42010\_Mycgr3T**
  
Percentage identity: 46 %
  
BlastP bit score: 2010
  
Sequence coverage: 100 %
  
E-value: 0.0
  
  
 NCBI BlastP on this gene

EQB57158

geranylgeranyl pyrophosphate synthetase
  
Accession: EQB57159
  
Location: 16660-18061
  
  
**BlastP hit with Mycgr3G92938\_Mycgr3T**
  
Percentage identity: 39 %
  
BlastP bit score: 296
  
Sequence coverage: 104 %
  
E-value: 7e-92
  
  
 NCBI BlastP on this gene

EQB57159

38. :  KB725756 Colletotrichum orbiculare MAFF 240422 unplaced genomic scaffold Scaffold\_209     Total score: 2.0     Cumulative Blast bit score: 2294

60s ribosomal protein l16
  
Accession: ENH85897
  
Location: 65918-67070
  
 NCBI BlastP on this gene

ENH85897

bag domain protein
  
Accession: ENH85898
  
Location: 68120-70450
  
 NCBI BlastP on this gene

ENH85898

glutaredoxin
  
Accession: ENH85899
  
Location: 71352-71756
  
 NCBI BlastP on this gene

ENH85899

chalcone-flavanone isomerase
  
Accession: ENH85900
  
Location: 72073-73260
  
 NCBI BlastP on this gene

ENH85900

ubiquitin ligase complex f-box protein
  
Accession: ENH85901
  
Location: 78440-80997
  
 NCBI BlastP on this gene

ENH85901

RNA exonuclease
  
Accession: ENH85902
  
Location: 81622-83056
  
 NCBI BlastP on this gene

ENH85902

geranylgeranyl pyrophosphate synthetase
  
Accession: ENH85903
  
Location: 84166-85466
  
  
**BlastP hit with Mycgr3G92938\_Mycgr3T**
  
Percentage identity: 42 %
  
BlastP bit score: 283
  
Sequence coverage: 84 %
  
E-value: 3e-87
  
  
 NCBI BlastP on this gene

ENH85903

nfx1-type zinc finger-containing protein 1
  
Accession: ENH85904
  
Location: 86209-93338
  
  
**BlastP hit with Mycgr3G42010\_Mycgr3T**
  
Percentage identity: 46 %
  
BlastP bit score: 2011
  
Sequence coverage: 101 %
  
E-value: 0.0
  
  
 NCBI BlastP on this gene

ENH85904

hypothetical protein
  
Accession: ENH85905
  
Location: 94083-94534
  
 NCBI BlastP on this gene

ENH85905

aspartate aminotransferase
  
Accession: ENH85906
  
Location: 95892-97600
  
 NCBI BlastP on this gene

ENH85906

oxalate formate antiporter
  
Accession: ENH85907
  
Location: 98078-99611
  
 NCBI BlastP on this gene

ENH85907

C6 zinc finger domain-containing protein
  
Accession: ENH85908
  
Location: 100004-102951
  
 NCBI BlastP on this gene

ENH85908

hypothetical protein
  
Accession: ENH85909
  
Location: 103551-105005
  
 NCBI BlastP on this gene

ENH85909

24-dehydrocholesterol reductase precursor
  
Accession: ENH85910
  
Location: 106673-108265
  
 NCBI BlastP on this gene

ENH85910

cyanide hydratase
  
Accession: ENH85911
  
Location: 109331-110549
  
 NCBI BlastP on this gene

ENH85911

phospholipid methyltransferase
  
Accession: ENH85912
  
Location: 110948-111837
  
 NCBI BlastP on this gene

ENH85912

MFS sugar transporter
  
Accession: ENH85913
  
Location: 112100-113949
  
 NCBI BlastP on this gene

ENH85913

39. :  AKCU01000308 Penicillium digitatum Pd1     Total score: 2.0     Cumulative Blast bit score: 2290

hypothetical protein
  
Accession: EKV14359
  
Location: 44347-44815
  
 NCBI BlastP on this gene

EKV14359

hypothetical protein
  
Accession: EKV14360
  
Location: 47327-49074
  
 NCBI BlastP on this gene

EKV14360

37S ribosomal protein Rsm24, putative
  
Accession: EKV14361
  
Location: 49972-51185
  
 NCBI BlastP on this gene

EKV14361

UDP-N-acetylglucosamine transferase subunit alg13
  
Accession: EKV14362
  
Location: 51466-51967
  
 NCBI BlastP on this gene

EKV14362

Actin-related protein ArpA
  
Accession: EKV14363
  
Location: 52723-53991
  
 NCBI BlastP on this gene

EKV14363

hypothetical protein
  
Accession: EKV14364
  
Location: 55619-56107
  
 NCBI BlastP on this gene

EKV14364

hypothetical protein
  
Accession: EKV14365
  
Location: 59043-59828
  
 NCBI BlastP on this gene

EKV14365

hypothetical protein
  
Accession: EKV14366
  
Location: 60183-67514
  
  
**BlastP hit with Mycgr3G42010\_Mycgr3T**
  
Percentage identity: 47 %
  
BlastP bit score: 2078
  
Sequence coverage: 102 %
  
E-value: 0.0
  
  
 NCBI BlastP on this gene

EKV14366

hypothetical protein
  
Accession: EKV14367
  
Location: 68372-69274
  
  
**BlastP hit with Mycgr3G92938\_Mycgr3T**
  
Percentage identity: 37 %
  
BlastP bit score: 212
  
Sequence coverage: 68 %
  
E-value: 1e-61
  
  
 NCBI BlastP on this gene

EKV14367

hypothetical protein
  
Accession: EKV14368
  
Location: 70253-71080
  
 NCBI BlastP on this gene

EKV14368

hypothetical protein
  
Accession: EKV14369
  
Location: 72050-74299
  
 NCBI BlastP on this gene

EKV14369

ATP-dependent RNA helicase DDX18
  
Accession: EKV14370
  
Location: 79517-81524
  
 NCBI BlastP on this gene

EKV14370

hypothetical protein
  
Accession: EKV14371
  
Location: 83475-88279
  
 NCBI BlastP on this gene

EKV14371

40. :  AKCT01000265 Penicillium digitatum PHI26     Total score: 2.0     Cumulative Blast bit score: 2290

hypothetical protein
  
Accession: EKV07281
  
Location: 42254-42722
  
 NCBI BlastP on this gene

EKV07281

hypothetical protein
  
Accession: EKV07282
  
Location: 45234-46981
  
 NCBI BlastP on this gene

EKV07282

37S ribosomal protein Rsm24, putative
  
Accession: EKV07283
  
Location: 47879-49092
  
 NCBI BlastP on this gene

EKV07283

UDP-N-acetylglucosamine transferase subunit alg13
  
Accession: EKV07284
  
Location: 49373-49874
  
 NCBI BlastP on this gene

EKV07284

Actin-related protein ArpA
  
Accession: EKV07285
  
Location: 50630-51898
  
 NCBI BlastP on this gene

EKV07285

hypothetical protein
  
Accession: EKV07286
  
Location: 53526-54014
  
 NCBI BlastP on this gene

EKV07286

hypothetical protein
  
Accession: EKV07287
  
Location: 56931-57716
  
 NCBI BlastP on this gene

EKV07287

hypothetical protein
  
Accession: EKV07288
  
Location: 57969-65300
  
  
**BlastP hit with Mycgr3G42010\_Mycgr3T**
  
Percentage identity: 47 %
  
BlastP bit score: 2078
  
Sequence coverage: 102 %
  
E-value: 0.0
  
  
 NCBI BlastP on this gene

EKV07288

hypothetical protein
  
Accession: EKV07289
  
Location: 66158-67060
  
  
**BlastP hit with Mycgr3G92938\_Mycgr3T**
  
Percentage identity: 37 %
  
BlastP bit score: 212
  
Sequence coverage: 68 %
  
E-value: 1e-61
  
  
 NCBI BlastP on this gene

EKV07289

hypothetical protein
  
Accession: EKV07290
  
Location: 68039-68866
  
 NCBI BlastP on this gene

EKV07290

hypothetical protein
  
Accession: EKV07291
  
Location: 69836-72085
  
 NCBI BlastP on this gene

EKV07291

41. :  GG697333 Glomerella graminicola M1.001 genomic scaffold supercont1.3     Total score: 2.0     Cumulative Blast bit score: 2283

BAG domain-containing protein
  
Accession: EFQ25840
  
Location: 246374-247345
  
 NCBI BlastP on this gene

EFQ25840

glutaredoxin
  
Accession: EFQ25841
  
Location: 249834-250307
  
 NCBI BlastP on this gene

EFQ25841

chalcone-flavanone isomerase
  
Accession: EFQ25842
  
Location: 250761-252080
  
 NCBI BlastP on this gene

EFQ25842

F-box domain-containing protein
  
Accession: EFQ25843
  
Location: 258429-261062
  
 NCBI BlastP on this gene

EFQ25843

exonuclease
  
Accession: EFQ25844
  
Location: 261962-263462
  
 NCBI BlastP on this gene

EFQ25844

geranylgeranyl pyrophosphate synthetase
  
Accession: EFQ25845
  
Location: 265127-266559
  
  
**BlastP hit with Mycgr3G92938\_Mycgr3T**
  
Percentage identity: 37 %
  
BlastP bit score: 293
  
Sequence coverage: 104 %
  
E-value: 1e-90
  
  
 NCBI BlastP on this gene

EFQ25845

ATPase
  
Accession: EFQ25846
  
Location: 267176-274326
  
  
**BlastP hit with Mycgr3G42010\_Mycgr3T**
  
Percentage identity: 46 %
  
BlastP bit score: 1990
  
Sequence coverage: 101 %
  
E-value: 0.0
  
  
 NCBI BlastP on this gene

EFQ25846

hypothetical protein
  
Accession: EFQ25847
  
Location: 275926-277174
  
 NCBI BlastP on this gene

EFQ25847

O-methyltransferase
  
Accession: EFQ25848
  
Location: 279676-281044
  
 NCBI BlastP on this gene

EFQ25848

hypothetical protein
  
Accession: EFQ25849
  
Location: 281501-283801
  
 NCBI BlastP on this gene

EFQ25849

hypothetical protein
  
Accession: EFQ25850
  
Location: 285677-286252
  
 NCBI BlastP on this gene

EFQ25850

hypothetical protein
  
Accession: EFQ25851
  
Location: 288547-288993
  
 NCBI BlastP on this gene

EFQ25851

C6 zinc finger domain-containing protein
  
Accession: EFQ25852
  
Location: 289562-291386
  
 NCBI BlastP on this gene

EFQ25852

GMC oxidoreductase
  
Accession: EFQ25853
  
Location: 292947-295195
  
 NCBI BlastP on this gene

EFQ25853

42. :  KB446535 Dothistroma septosporum NZE10 unplaced genomic scaffold DOTSEscaffold\_1     Total score: 2.0     Cumulative Blast bit score: 2268

hypothetical protein
  
Accession: EME48887
  
Location: 1351655-1353570
  
 NCBI BlastP on this gene

EME48887

hypothetical protein
  
Accession: EME48886
  
Location: 1350086-1350581
  
 NCBI BlastP on this gene

EME48886

hypothetical protein
  
Accession: EME48885
  
Location: 1349638-1349832
  
 NCBI BlastP on this gene

EME48885

hypothetical protein
  
Accession: EME48884
  
Location: 1346959-1349166
  
 NCBI BlastP on this gene

EME48884

glycoside hydrolase family 16 protein
  
Accession: EME48883
  
Location: 1344891-1346183
  
 NCBI BlastP on this gene

EME48883

hypothetical protein
  
Accession: EME48882
  
Location: 1340176-1341006
  
 NCBI BlastP on this gene

EME48882

hypothetical protein
  
Accession: EME48881
  
Location: 1339231-1339431
  
 NCBI BlastP on this gene

EME48881

hypothetical protein
  
Accession: EME48880
  
Location: 1335865-1336710
  
 NCBI BlastP on this gene

EME48880

hypothetical protein
  
Accession: EME48879
  
Location: 1332880-1334838
  
 NCBI BlastP on this gene

EME48879

hypothetical protein
  
Accession: EME48878
  
Location: 1326694-1332105
  
  
**BlastP hit with Mycgr3G41426\_Mycgr3T**
  
Percentage identity: 55 %
  
BlastP bit score: 2014
  
Sequence coverage: 100 %
  
E-value: 0.0
  
  
 NCBI BlastP on this gene

EME48878

hypothetical protein
  
Accession: EME48877
  
Location: 1321515-1324153
  
 NCBI BlastP on this gene

EME48877

hypothetical protein
  
Accession: EME48876
  
Location: 1320423-1321439
  
 NCBI BlastP on this gene

EME48876

hypothetical protein
  
Accession: EME48875
  
Location: 1317427-1318572
  
 NCBI BlastP on this gene

EME48875

hypothetical protein
  
Accession: EME48874
  
Location: 1315875-1316821
  
 NCBI BlastP on this gene

EME48874

hypothetical protein
  
Accession: EME48873
  
Location: 1312337-1314642
  
 NCBI BlastP on this gene

EME48873

hypothetical protein
  
Accession: EME48872
  
Location: 1309909-1311752
  
 NCBI BlastP on this gene

EME48872

hypothetical protein
  
Accession: EME48871
  
Location: 1307092-1309291
  
 NCBI BlastP on this gene

EME48871

hypothetical protein
  
Accession: EME48870
  
Location: 1304785-1306669
  
 NCBI BlastP on this gene

EME48870

hypothetical protein
  
Accession: EME48869
  
Location: 1303487-1304290
  
  
**BlastP hit with Mycgr3G58567\_Mycgr3T**
  
Percentage identity: 53 %
  
BlastP bit score: 254
  
Sequence coverage: 98 %
  
E-value: 1e-80
  
  
 NCBI BlastP on this gene

EME48869

hypothetical protein
  
Accession: EME48867
  
Location: 1298681-1301703
  
 NCBI BlastP on this gene

EME48867

hypothetical protein
  
Accession: EME48866
  
Location: 1292927-1296823
  
 NCBI BlastP on this gene

EME48866

hypothetical protein
  
Accession: EME48865
  
Location: 1287791-1290595
  
 NCBI BlastP on this gene

EME48865

hypothetical protein
  
Accession: EME48864
  
Location: 1286203-1287175
  
 NCBI BlastP on this gene

EME48864

43. :  KB020741 Colletotrichum gloeosporioides Nara gc5 unplaced genomic scaffold scaffold364     Total score: 2.0     Cumulative Blast bit score: 2255

60s ribosomal protein l16
  
Accession: ELA31573
  
Location: 39138-40308
  
 NCBI BlastP on this gene

ELA31573

bag domain protein
  
Accession: ELA31574
  
Location: 41324-42289
  
 NCBI BlastP on this gene

ELA31574

glutaredoxin
  
Accession: ELA31575
  
Location: 44913-45306
  
 NCBI BlastP on this gene

ELA31575

chalcone-flavanone isomerase
  
Accession: ELA31576
  
Location: 45665-46885
  
 NCBI BlastP on this gene

ELA31576

ubiquitin ligase complex f-box protein
  
Accession: ELA31577
  
Location: 52091-54618
  
 NCBI BlastP on this gene

ELA31577

3'-5' exonuclease
  
Accession: ELA31578
  
Location: 55444-56966
  
 NCBI BlastP on this gene

ELA31578

geranylgeranyl pyrophosphate synthetase
  
Accession: ELA31579
  
Location: 58901-60302
  
  
**BlastP hit with Mycgr3G92938\_Mycgr3T**
  
Percentage identity: 38 %
  
BlastP bit score: 294
  
Sequence coverage: 103 %
  
E-value: 4e-91
  
  
 NCBI BlastP on this gene

ELA31579

AAA family
  
Accession: ELA31580
  
Location: 60848-68129
  
  
**BlastP hit with Mycgr3G42010\_Mycgr3T**
  
Percentage identity: 46 %
  
BlastP bit score: 1961
  
Sequence coverage: 102 %
  
E-value: 0.0
  
  
 NCBI BlastP on this gene

ELA31580

phospholipid methyltransferase
  
Accession: ELA31581
  
Location: 69436-70326
  
 NCBI BlastP on this gene

ELA31581

cyanide hydratase
  
Accession: ELA31582
  
Location: 70743-71947
  
 NCBI BlastP on this gene

ELA31582

24-dehydrocholesterol reductase precursor
  
Accession: ELA31583
  
Location: 73060-74580
  
 NCBI BlastP on this gene

ELA31583

hypothetical protein
  
Accession: ELA31584
  
Location: 76660-77641
  
 NCBI BlastP on this gene

ELA31584

MFS sugar transporter
  
Accession: ELA31585
  
Location: 78147-79970
  
 NCBI BlastP on this gene

ELA31585

mucin-desulfating sulfatase (n-acetylglucosamine-6-sulfatase)
  
Accession: ELA31586
  
Location: 80745-84828
  
 NCBI BlastP on this gene

ELA31586

hypothetical protein
  
Accession: ELA31587
  
Location: 85639-85939
  
 NCBI BlastP on this gene

ELA31587

periplasmic beta-glucosidase
  
Accession: ELA31588
  
Location: 86288-88753
  
 NCBI BlastP on this gene

ELA31588

44. :  EQ963475 Aspergillus flavus NRRL3357 scf\_1106286419142 genomic scaffold     Total score: 2.0     Cumulative Blast bit score: 2227

conserved hypothetical protein
  
Accession: EED53070
  
Location: 80222-81403
  
 NCBI BlastP on this gene

EED53070

subtilisin, putative
  
Accession: EED53069
  
Location: 72026-74080
  
 NCBI BlastP on this gene

EED53069

conserved hypothetical protein
  
Accession: EED53068
  
Location: 69054-70401
  
 NCBI BlastP on this gene

EED53068

monocarboxylate transporter, putative
  
Accession: EED53067
  
Location: 65668-67179
  
 NCBI BlastP on this gene

EED53067

hypothetical protein
  
Accession: EED53066
  
Location: 64752-64957
  
 NCBI BlastP on this gene

EED53066

amine oxidase, putative
  
Accession: EED53065
  
Location: 62819-64120
  
 NCBI BlastP on this gene

EED53065

conserved hypothetical protein
  
Accession: EED53064
  
Location: 59588-60298
  
  
**BlastP hit with Mycgr3G92938\_Mycgr3T**
  
Percentage identity: 29 %
  
BlastP bit score: 94
  
Sequence coverage: 56 %
  
E-value: 7e-19
  
  
 NCBI BlastP on this gene

EED53064

hypothetical protein
  
Accession: EED53063
  
Location: 58927-59355
  
 NCBI BlastP on this gene

EED53063

nonsense-mediated mRNA decay protein, putative
  
Accession: EED53062
  
Location: 48502-55653
  
  
**BlastP hit with Mycgr3G42010\_Mycgr3T**
  
Percentage identity: 47 %
  
BlastP bit score: 2133
  
Sequence coverage: 102 %
  
E-value: 0.0
  
  
 NCBI BlastP on this gene

EED53062

dynamin, putative
  
Accession: EED53061
  
Location: 44559-46931
  
 NCBI BlastP on this gene

EED53061

conserved hypothetical protein
  
Accession: EED53060
  
Location: 41934-42940
  
 NCBI BlastP on this gene

EED53060

hypothetical protein
  
Accession: EED53059
  
Location: 39446-41257
  
 NCBI BlastP on this gene

EED53059

conserved hypothetical protein
  
Accession: EED53058
  
Location: 38424-39355
  
 NCBI BlastP on this gene

EED53058

DUF567 domain protein
  
Accession: EED53057
  
Location: 37406-38082
  
 NCBI BlastP on this gene

EED53057

fungal alpha-L-arabinofuranosidase, putative
  
Accession: EED53056
  
Location: 35160-36680
  
 NCBI BlastP on this gene

EED53056

hypothetical protein
  
Accession: EED53055
  
Location: 32033-33586
  
 NCBI BlastP on this gene

EED53055

hypothetical protein
  
Accession: EED53054
  
Location: 31473-31778
  
 NCBI BlastP on this gene

EED53054

acyl-CoA dehydrogenase family protein
  
Accession: EED53053
  
Location: 28874-30333
  
 NCBI BlastP on this gene

EED53053

45. :  GG698906 Nectria haematococca mpVI 77-13-4 chromosome 10 genomic scaffold NECHAsca\_14\_chr10\_3\_0     Total score: 2.0     Cumulative Blast bit score: 2175

hypothetical protein
  
Accession: EEU41970
  
Location: 873435-874006
  
 NCBI BlastP on this gene

EEU41970

predicted protein
  
Accession: EEU41971
  
Location: 876657-877093
  
 NCBI BlastP on this gene

EEU41971

hypothetical protein
  
Accession: EEU41972
  
Location: 878689-879517
  
 NCBI BlastP on this gene

EEU41972

hypothetical protein
  
Accession: EEU42142
  
Location: 882044-882484
  
 NCBI BlastP on this gene

EEU42142

hypothetical protein
  
Accession: EEU42143
  
Location: 883414-884493
  
 NCBI BlastP on this gene

EEU42143

hypothetical protein
  
Accession: EEU42144
  
Location: 885005-888085
  
 NCBI BlastP on this gene

EEU42144

hypothetical protein
  
Accession: EEU42145
  
Location: 889526-896420
  
  
**BlastP hit with Mycgr3G42010\_Mycgr3T**
  
Percentage identity: 45 %
  
BlastP bit score: 1941
  
Sequence coverage: 101 %
  
E-value: 0.0
  
  
 NCBI BlastP on this gene

EEU42145

hypothetical protein
  
Accession: EEU41973
  
Location: 897098-898443
  
  
**BlastP hit with Mycgr3G92938\_Mycgr3T**
  
Percentage identity: 35 %
  
BlastP bit score: 234
  
Sequence coverage: 101 %
  
E-value: 3e-68
  
  
 NCBI BlastP on this gene

EEU41973

hypothetical protein
  
Accession: EEU41974
  
Location: 901657-902975
  
 NCBI BlastP on this gene

EEU41974

predicted protein
  
Accession: EEU41975
  
Location: 903323-904410
  
 NCBI BlastP on this gene

EEU41975

hypothetical protein
  
Accession: EEU41976
  
Location: 905595-906869
  
 NCBI BlastP on this gene

EEU41976

hypothetical protein
  
Accession: EEU42146
  
Location: 907251-907784
  
 NCBI BlastP on this gene

EEU42146

predicted protein
  
Accession: EEU41977
  
Location: 909044-910256
  
 NCBI BlastP on this gene

EEU41977

hypothetical protein
  
Accession: EEU41978
  
Location: 910745-913000
  
 NCBI BlastP on this gene

EEU41978

hypothetical protein
  
Accession: EEU42147
  
Location: 913041-916262
  
 NCBI BlastP on this gene

EEU42147

hypothetical protein
  
Accession: EEU41979
  
Location: 916601-918746
  
 NCBI BlastP on this gene

EEU41979

46. :  GL385399 Gaeumannomyces graminis var. tritici R3-111a-1 unplaced genomic scaffold supercont2.5     Total score: 2.0     Cumulative Blast bit score: 2171

hypothetical protein
  
Accession: EJT72262
  
Location: 421866-422746
  
 NCBI BlastP on this gene

EJT72262

hypothetical protein
  
Accession: EJT72263
  
Location: 423408-426641
  
 NCBI BlastP on this gene

EJT72263

methyltransferase-UbiE family protein
  
Accession: EJT72264
  
Location: 427811-428638
  
 NCBI BlastP on this gene

EJT72264

hypothetical protein
  
Accession: EJT72265
  
Location: 429132-433707
  
 NCBI BlastP on this gene

EJT72265

hypothetical protein
  
Accession: EJT72266
  
Location: 434137-436157
  
 NCBI BlastP on this gene

EJT72266

hypothetical protein
  
Accession: EJT72267
  
Location: 439343-446527
  
  
**BlastP hit with Mycgr3G42010\_Mycgr3T**
  
Percentage identity: 47 %
  
BlastP bit score: 1903
  
Sequence coverage: 92 %
  
E-value: 0.0
  
  
 NCBI BlastP on this gene

EJT72267

hypothetical protein
  
Accession: EJT72268
  
Location: 447491-449195
  
  
**BlastP hit with Mycgr3G92938\_Mycgr3T**
  
Percentage identity: 39 %
  
BlastP bit score: 268
  
Sequence coverage: 86 %
  
E-value: 1e-80
  
  
 NCBI BlastP on this gene

EJT72268

hypothetical protein
  
Accession: EJT72269
  
Location: 449913-452271
  
 NCBI BlastP on this gene

EJT72269

hypothetical protein
  
Accession: EJT72270
  
Location: 454675-455610
  
 NCBI BlastP on this gene

EJT72270

glycogen debranching enzyme
  
Accession: EJT72271
  
Location: 456839-461738
  
 NCBI BlastP on this gene

EJT72271

hypothetical protein
  
Accession: EJT72272
  
Location: 462107-463082
  
 NCBI BlastP on this gene

EJT72272

hypothetical protein
  
Accession: EJT72273
  
Location: 464472-465782
  
 NCBI BlastP on this gene

EJT72273

glycerol-3-phosphate dehydrogenase
  
Accession: EJT72274
  
Location: 467622-469224
  
 NCBI BlastP on this gene

EJT72274

47. :  JH725244 Beauveria bassiana ARSEF 2860 unplaced genomic scaffold BBA\_S00095     Total score: 2.0     Cumulative Blast bit score: 2170

hypothetical protein
  
Accession: EJP60882
  
Location: 7235-8371
  
 NCBI BlastP on this gene

EJP60882

hypothetical protein
  
Accession: EJP60883
  
Location: 9707-10975
  
 NCBI BlastP on this gene

EJP60883

geranylgeranyl pyrophosphate synthetase
  
Accession: EJP60884
  
Location: 14321-15799
  
  
**BlastP hit with Mycgr3G92938\_Mycgr3T**
  
Percentage identity: 34 %
  
BlastP bit score: 248
  
Sequence coverage: 109 %
  
E-value: 3e-73
  
  
 NCBI BlastP on this gene

EJP60884

ATPase protein
  
Accession: EJP60885
  
Location: 16047-24564
  
  
**BlastP hit with Mycgr3G42010\_Mycgr3T**
  
Percentage identity: 44 %
  
BlastP bit score: 1922
  
Sequence coverage: 102 %
  
E-value: 0.0
  
  
 NCBI BlastP on this gene

EJP60885

48. :  GL891305 Neurospora tetrasperma FGSC 2508 unplaced genomic scaffold NEUTE1scaffold\_4     Total score: 2.0     Cumulative Blast bit score: 2162

hypothetical protein
  
Accession: EGO56085
  
Location: 196463-196927
  
 NCBI BlastP on this gene

EGO56085

hypothetical protein
  
Accession: EGO56086
  
Location: 197272-199766
  
 NCBI BlastP on this gene

EGO56086

hypothetical protein
  
Accession: EGO56087
  
Location: 200673-201581
  
 NCBI BlastP on this gene

EGO56087

hypothetical protein
  
Accession: EGO56088
  
Location: 202466-203494
  
 NCBI BlastP on this gene

EGO56088

hypothetical protein
  
Accession: EGO56089
  
Location: 204325-205482
  
 NCBI BlastP on this gene

EGO56089

hypothetical protein
  
Accession: EGO56090
  
Location: 205881-207592
  
 NCBI BlastP on this gene

EGO56090

hypothetical protein
  
Accession: EGO56091
  
Location: 208085-209538
  
 NCBI BlastP on this gene

EGO56091

hypothetical protein
  
Accession: EGO56092
  
Location: 210546-212459
  
 NCBI BlastP on this gene

EGO56092

hypothetical protein
  
Accession: EGO56093
  
Location: 214565-221760
  
  
**BlastP hit with Mycgr3G42010\_Mycgr3T**
  
Percentage identity: 45 %
  
BlastP bit score: 1956
  
Sequence coverage: 101 %
  
E-value: 0.0
  
  
 NCBI BlastP on this gene

EGO56093

hypothetical protein
  
Accession: EGO56094
  
Location: 223154-224970
  
  
**BlastP hit with Mycgr3G92938\_Mycgr3T**
  
Percentage identity: 37 %
  
BlastP bit score: 206
  
Sequence coverage: 71 %
  
E-value: 2e-56
  
  
 NCBI BlastP on this gene

EGO56094

hypothetical protein
  
Accession: EGO56095
  
Location: 225718-228139
  
 NCBI BlastP on this gene

EGO56095

hypothetical protein
  
Accession: EGO56096
  
Location: 229266-230202
  
 NCBI BlastP on this gene

EGO56096

hypothetical protein
  
Accession: EGO56097
  
Location: 231056-233440
  
 NCBI BlastP on this gene

EGO56097

hypothetical protein
  
Accession: EGO56098
  
Location: 234675-239337
  
 NCBI BlastP on this gene

EGO56098

hypothetical protein
  
Accession: EGO56099
  
Location: 240796-241074
  
 NCBI BlastP on this gene

EGO56099

hypothetical protein
  
Accession: EGO56100
  
Location: 241698-243625
  
 NCBI BlastP on this gene

EGO56100

49. :  KB445558 Baudoinia compniacensis UAMH 10762 unplaced genomic scaffold BAUCOscaffold\_9     Total score: 2.0     Cumulative Blast bit score: 2158

hypothetical protein
  
Accession: EMC94834
  
Location: 1083250-1084069
  
 NCBI BlastP on this gene

EMC94834

hypothetical protein
  
Accession: EMC94833
  
Location: 1082043-1082846
  
 NCBI BlastP on this gene

EMC94833

hypothetical protein
  
Accession: EMC94832
  
Location: 1081658-1081831
  
 NCBI BlastP on this gene

EMC94832

hypothetical protein
  
Accession: EMC94831
  
Location: 1079647-1081104
  
 NCBI BlastP on this gene

EMC94831

hypothetical protein
  
Accession: EMC94830
  
Location: 1072686-1074932
  
 NCBI BlastP on this gene

EMC94830

hypothetical protein
  
Accession: EMC94829
  
Location: 1070815-1071960
  
 NCBI BlastP on this gene

EMC94829

hypothetical protein
  
Accession: EMC94828
  
Location: 1067000-1068628
  
 NCBI BlastP on this gene

EMC94828

hypothetical protein
  
Accession: EMC94827
  
Location: 1065477-1066564
  
 NCBI BlastP on this gene

EMC94827

hypothetical protein
  
Accession: EMC94826
  
Location: 1058514-1064026
  
  
**BlastP hit with Mycgr3G41426\_Mycgr3T**
  
Percentage identity: 56 %
  
BlastP bit score: 1992
  
Sequence coverage: 101 %
  
E-value: 0.0
  
  
 NCBI BlastP on this gene

EMC94826

hypothetical protein
  
Accession: EMC94825
  
Location: 1054045-1056734
  
 NCBI BlastP on this gene

EMC94825

hypothetical protein
  
Accession: EMC94824
  
Location: 1052973-1053926
  
 NCBI BlastP on this gene

EMC94824

hypothetical protein
  
Accession: EMC94823
  
Location: 1050802-1051595
  
  
**BlastP hit with Mycgr3G31170\_Mycgr3T**
  
Percentage identity: 100 %
  
BlastP bit score: 166
  
Sequence coverage: 100 %
  
E-value: 7e-50
  
  
 NCBI BlastP on this gene

EMC94823

hypothetical protein
  
Accession: EMC94822
  
Location: 1048283-1050219
  
 NCBI BlastP on this gene

EMC94822

hypothetical protein
  
Accession: EMC94821
  
Location: 1047382-1047864
  
 NCBI BlastP on this gene

EMC94821

hypothetical protein
  
Accession: EMC94820
  
Location: 1043193-1046693
  
 NCBI BlastP on this gene

EMC94820

hypothetical protein
  
Accession: EMC94819
  
Location: 1042159-1042746
  
 NCBI BlastP on this gene

EMC94819

hypothetical protein
  
Accession: EMC94818
  
Location: 1037773-1041234
  
 NCBI BlastP on this gene

EMC94818

hypothetical protein
  
Accession: EMC94817
  
Location: 1037390-1037551
  
 NCBI BlastP on this gene

EMC94817

hypothetical protein
  
Accession: EMC94816
  
Location: 1035164-1036978
  
 NCBI BlastP on this gene

EMC94816

hypothetical protein
  
Accession: EMC94815
  
Location: 1032343-1034208
  
 NCBI BlastP on this gene

EMC94815

50. :  GL698524 Metarhizium acridum CQMa 102 unplaced genomic scaffold Scf\_055     Total score: 2.0     Cumulative Blast bit score: 2156

high affinity nickel transport protein nic1
  
Accession: EFY87589
  
Location: 3979-5251
  
 NCBI BlastP on this gene

EFY87589

hypothetical protein
  
Accession: EFY87590
  
Location: 5386-6270
  
 NCBI BlastP on this gene

EFY87590

Autophagy-related protein 17
  
Accession: EFY87591
  
Location: 7711-9299
  
 NCBI BlastP on this gene

EFY87591

proteinase, putative
  
Accession: EFY87592
  
Location: 9705-11594
  
 NCBI BlastP on this gene

EFY87592

AAA family ATPase, putative
  
Accession: EFY87593
  
Location: 15581-22118
  
  
**BlastP hit with Mycgr3G42010\_Mycgr3T**
  
Percentage identity: 46 %
  
BlastP bit score: 1900
  
Sequence coverage: 93 %
  
E-value: 0.0
  
  
 NCBI BlastP on this gene

EFY87593

hypothetical protein
  
Accession: EFY87594
  
Location: 23101-24538
  
  
**BlastP hit with Mycgr3G92938\_Mycgr3T**
  
Percentage identity: 34 %
  
BlastP bit score: 256
  
Sequence coverage: 106 %
  
E-value: 3e-76
  
  
 NCBI BlastP on this gene

EFY87594

hypothetical protein
  
Accession: EFY87595
  
Location: 25882-26775
  
 NCBI BlastP on this gene

EFY87595

hypothetical protein
  
Accession: EFY87596
  
Location: 27754-28392
  
 NCBI BlastP on this gene

EFY87596

hypothetical protein
  
Accession: EFY87597
  
Location: 32752-34104
  
 NCBI BlastP on this gene

EFY87597

hypothetical protein
  
Accession: EFY87598
  
Location: 37207-40757
  
 NCBI BlastP on this gene

EFY87598

hypothetical protein
  
Accession: EFY87599
  
Location: 42063-43252
  
 NCBI BlastP on this gene

EFY87599

Detecting sequence homology at the gene cluster level with MultiGeneBlast.
  
Marnix H. Medema, Rainer Breitling & Eriko Takano (2013)
  
*Molecular Biology and Evolution* , 30: 1218-1223.
