# Supplementary material for: In silico prediction and characterization of secondary metabolite biosynthetic gene clusters in the wheat pathogen Zymoseptoria tritici
Source: BMC Genomics. 2017 Aug 17;18:631. doi: 10.1186/s12864-017-3969-y (PMC5561558; doi:10.1186/s12864-017-3969-y)
Supplement: Supplementary file 1 — MultiGeneBLAST analysis of putative secondary metabolite clusters. All encoded amino acid sequences from genes residing in clusters predicted by AntiSMASH are given as FASTA file format. All output data from MultiGeneBLASTs are also provided. (ZIP 42911 kb) [file 12864_2017_3969_MOESM1_ESM.zip › Cluster MultiGene BLAST/out/Clusters_1_34/Cluster_15/displaypage2.xhtml]

xml version="1.0" encoding="UTF-8"?


Search Results
  
  
 Results pages: 1, 2, 3, 4, 5

**MultiGeneBlast hits**

Select gene cluster alignment
51. GL891247\_0 Neurospora tetrasperma FGSC 2509 unplaced genomic scaffold NEU...
52. GL985074\_0 Trichoderma reesei QM6a unplaced genomic scaffold TRIREscaffol...
53. CAGA01000005\_0 Claviceps purpurea 20.1, whole genome shotgun sequencing p...
54. AABX02000071\_0 Neurospora crassa OR74A, whole genome shotgun sequencing p...
55. AFQF01000625\_0 Fusarium oxysporum Fo5176, whole genome shotgun sequencing...
56. KB726184\_0 Fusarium oxysporum f. sp. cubense race 4 unplaced genomic scaf...
57. ABDG02000020\_2 Trichoderma atroviride IMI 206040, whole genome shotgun se...
58. AFNW01000011\_0 Fusarium pseudograminearum CS3096, whole genome shotgun se...
59. DS995706\_0 Microsporum canis CBS 113480 supercont1.6 genomic scaffold, wh...
60. GL629801\_0 Grosmannia clavigera kw1407 unplaced genomic scaffold GCSC\_173...
61. KE145354\_1 Glarea lozoyensis ATCC 20868 chromosome Unknown GLAREA11, whol...
62. KB725947\_0 Colletotrichum orbiculare MAFF 240422 unplaced genomic scaffol...
63. KB707707\_0 Botryotinia fuckeliana BcDW1 unplaced genomic scaffold Scaffol...
64. CAGA01000033\_0 Claviceps purpurea 20.1, whole genome shotgun sequencing p...
65. KB730345\_1 Fusarium oxysporum f. sp. cubense race 1 unplaced genomic scaf...
66. CABT02000029\_0 Sordaria macrospora k-hell, whole genome shotgun sequencin...
67. GL985061\_0 Trichoderma reesei QM6a unplaced genomic scaffold TRIREscaffol...
68. GG697340\_0 Glomerella graminicola M1.001 genomic scaffold supercont1.10, ...
69. GL698722\_0 Metarhizium anisopliae ARSEF 23 unplaced genomic scaffold Scf\_...
70. KB708021\_2 Botryotinia fuckeliana BcDW1 unplaced genomic scaffold Scaffol...
71. FQ790281\_1 Botryotinia fuckeliana T4 SuperContig\_330\_1 genomic supercontig.
72. GL385396\_0 Gaeumannomyces graminis var. tritici R3-111a-1 unplaced genomi...
73. GL891307\_0 Neurospora tetrasperma FGSC 2508 unplaced genomic scaffold NEU...
74. GL891269\_1 Neurospora tetrasperma FGSC 2509 unplaced genomic scaffold NEU...
75. KB021009\_0 Colletotrichum gloeosporioides Nara gc5 unplaced genomic scaff...
76. CH476603\_0 Aspergillus terreus NIH2624 scaffold\_10 genomic scaffold, whol...
77. KE148164\_0 Ophiostoma piceae UAMH 11346 chromosome Unknown scf19, whole g...
78. ACJE01000003\_1 Aspergillus niger ATCC 1015, whole genome shotgun sequenci...
79. HF679030\_3 Fusarium fujikuroi IMI 58289 draft genome, chromosome FFUJ\_chr08.
80. KE148164\_1 Ophiostoma piceae UAMH 11346 chromosome Unknown scf19, whole g...
81. JH921436\_0 Marssonina brunnea f. sp. 'multigermtubi' MB\_m1 unplaced genom...
82. GL385397\_2 Gaeumannomyces graminis var. tritici R3-111a-1 unplaced genomi...
83. CP003009\_0 Thielavia terrestris NRRL 8126 chromosome 1, complete sequence.
84. DS572699\_0 Verticillium dahliae VdLs.17 supercont1.5 genomic scaffold, wh...
85. CM001231\_3 Magnaporthe oryzae 70-15 chromosome 1, whole genome shotgun se...
86. AM270302\_0 Aspergillus niger contig An13c0080, genomic contig.
87. DF126480\_0 Aspergillus kawachii IFO 4308 DNA, contig: scaffold00034, whol...
88. JH725151\_1 Beauveria bassiana ARSEF 2860 unplaced genomic scaffold BBA\_S0...
89. CH408034\_1 Chaetomium globosum CBS 148.51 scaffold\_6 genomic scaffold, wh...
90. AABX02000004\_0 Neurospora crassa OR74A, whole genome shotgun sequencing p...
91. JH921449\_0 Marssonina brunnea f. sp. 'multigermtubi' MB\_m1 unplaced genom...
92. DF126457\_1 Aspergillus kawachii IFO 4308 DNA, contig: scaffold00011, whol...
93. CP003008\_0 Myceliophthora thermophila ATCC 42464 chromosome 7, complete s...
94. CAGA01000048\_0 Claviceps purpurea 20.1, whole genome shotgun sequencing p...
95. JH795568\_0 Magnaporthe oryzae P131 unplaced genomic scaffold P131\_scaffol...
96. JH793928\_0 Magnaporthe oryzae Y34 unplaced genomic scaffold Y34\_scaffold0...
97. FP929139\_2 Leptosphaeria maculans JN3 lm\_SuperContig\_0\_v2 genomic superco...
98. DS572714\_0 Verticillium dahliae VdLs.17 supercont1.20 genomic scaffold, w...
99. FQ790354\_0 Botryotinia fuckeliana T4 SupSuperContig\_210\_20\_1 genomic supe...
100. CH476622\_0 Sclerotinia sclerotiorum 1980 scaffold\_2 genomic scaffold, wh...

Query: Architecture Search FASTA input

GL891247 : Neurospora tetrasperma FGSC 2509 unplaced genomic scaffold NEUTE2scaffold\_5    Total score: 2.0     Cumulative Blast bit score: 2149

Hit cluster cross-links:

Mycgr3G85918 Mycgr3T
  
Location: 0-1602

Mycgr3G85918\_Mycgr3T

Mycgr3G42010 Mycgr3T
  
Location: 1702-8569

Mycgr3G42010\_Mycgr3T

Mycgr3G29582 Mycgr3T
  
Location: 8669-8915

Mycgr3G29582\_Mycgr3T

Mycgr3G31170 Mycgr3T
  
Location: 9015-9255

Mycgr3G31170\_Mycgr3T

Mycgr3G85924 Mycgr3T
  
Location: 9355-11218

Mycgr3G85924\_Mycgr3T

Mycgr3G71676 Mycgr3T
  
Location: 11318-12494

Mycgr3G71676\_Mycgr3T

Mycgr3G11468 Mycgr3T
  
Location: 12594-13653

Mycgr3G11468\_Mycgr3T

Mycgr3G58567 Mycgr3T
  
Location: 13753-14506

Mycgr3G58567\_Mycgr3T

Mycgr3G100089 Mycgr3
  
Location: 14606-21152

Mycgr3G100089\_Mycgr3

Mycgr3G42698 Mycgr3T
  
Location: 21252-22131

Mycgr3G42698\_Mycgr3T

Mycgr3G71681 Mycgr3T
  
Location: 22231-23461

Mycgr3G71681\_Mycgr3T

Mycgr3G109328 Mycgr3
  
Location: 23561-24239

Mycgr3G109328\_Mycgr3

Mycgr3G104334 Mycgr3
  
Location: 24339-24567

Mycgr3G104334\_Mycgr3

Mycgr3G42715 Mycgr3T
  
Location: 24667-25981

Mycgr3G42715\_Mycgr3T

Mycgr3G92934 Mycgr3T
  
Location: 26081-27593

Mycgr3G92934\_Mycgr3T

Mycgr3G41969 Mycgr3T
  
Location: 27693-29328

Mycgr3G41969\_Mycgr3T

Mycgr3G80635 Mycgr3T
  
Location: 29428-29821

Mycgr3G80635\_Mycgr3T

Mycgr3G41426 Mycgr3T
  
Location: 29921-35255

Mycgr3G41426\_Mycgr3T

Mycgr3G104337 Mycgr3
  
Location: 35355-36108

Mycgr3G104337\_Mycgr3

Mycgr3G71679 Mycgr3T
  
Location: 36208-37300

Mycgr3G71679\_Mycgr3T

Mycgr3G92938 Mycgr3T
  
Location: 37400-38699

Mycgr3G92938\_Mycgr3T

Mycgr3G92941 Mycgr3T
  
Location: 38799-40734

Mycgr3G92941\_Mycgr3T

hypothetical protein
  
Accession: EGZ71050
  
Location: 3960096-3962023
  
 NCBI BlastP on this gene

EGZ71050

hypothetical protein
  
Accession: EGZ71051
  
Location: 3962647-3962925
  
 NCBI BlastP on this gene

EGZ71051

hypothetical protein
  
Accession: EGZ71052
  
Location: 3964379-3968993
  
 NCBI BlastP on this gene

EGZ71052

hypothetical protein
  
Accession: EGZ71053
  
Location: 3970276-3972660
  
 NCBI BlastP on this gene

EGZ71053

hypothetical protein
  
Accession: EGZ71054
  
Location: 3973514-3974450
  
 NCBI BlastP on this gene

EGZ71054

HET-domain-containing protein
  
Accession: EGZ71055
  
Location: 3975577-3977998
  
 NCBI BlastP on this gene

EGZ71055

hypothetical protein
  
Accession: EGZ71056
  
Location: 3979283-3980453
  
  
**BlastP hit with Mycgr3G92938\_Mycgr3T**
  
Percentage identity: 39 %
  
BlastP bit score: 201
  
Sequence coverage: 64 %
  
E-value: 1e-56
  
  
 NCBI BlastP on this gene

EGZ71056

P-loop containing nucleoside triphosphate hydrolase protein
  
Accession: EGZ71057
  
Location: 3981955-3989150
  
  
**BlastP hit with Mycgr3G42010\_Mycgr3T**
  
Percentage identity: 45 %
  
BlastP bit score: 1948
  
Sequence coverage: 101 %
  
E-value: 0.0
  
  
 NCBI BlastP on this gene

EGZ71057

general substrate transporter
  
Accession: EGZ71058
  
Location: 3990242-3992155
  
 NCBI BlastP on this gene

EGZ71058

UPF0075-domain-containing protein
  
Accession: EGZ71059
  
Location: 3993163-3994615
  
 NCBI BlastP on this gene

EGZ71059

C6 zinc finger domain protein
  
Accession: EGZ71060
  
Location: 3995108-3996819
  
 NCBI BlastP on this gene

EGZ71060

hypothetical protein
  
Accession: EGZ71061
  
Location: 3997218-3998375
  
 NCBI BlastP on this gene

EGZ71061

alpha-L-arabinofuranosidase B
  
Accession: EGZ71062
  
Location: 3999206-4000234
  
 NCBI BlastP on this gene

EGZ71062

alpha/beta-hydrolase
  
Accession: EGZ71063
  
Location: 4001119-4002027
  
 NCBI BlastP on this gene

EGZ71063

OPT superfamily oligopeptide transporter
  
Accession: EGZ71064
  
Location: 4002934-4005428
  
 NCBI BlastP on this gene

EGZ71064

hypothetical protein
  
Accession: EGZ71065
  
Location: 4005773-4006237
  
 NCBI BlastP on this gene

EGZ71065

Query: Architecture Search FASTA input

GL985074 : Trichoderma reesei QM6a unplaced genomic scaffold TRIREscaffold\_19    Total score: 2.0     Cumulative Blast bit score: 2139

Hit cluster cross-links:

Mycgr3G85918 Mycgr3T
  
Location: 0-1602

Mycgr3G85918\_Mycgr3T

Mycgr3G42010 Mycgr3T
  
Location: 1702-8569

Mycgr3G42010\_Mycgr3T

Mycgr3G29582 Mycgr3T
  
Location: 8669-8915

Mycgr3G29582\_Mycgr3T

Mycgr3G31170 Mycgr3T
  
Location: 9015-9255

Mycgr3G31170\_Mycgr3T

Mycgr3G85924 Mycgr3T
  
Location: 9355-11218

Mycgr3G85924\_Mycgr3T

Mycgr3G71676 Mycgr3T
  
Location: 11318-12494

Mycgr3G71676\_Mycgr3T

Mycgr3G11468 Mycgr3T
  
Location: 12594-13653

Mycgr3G11468\_Mycgr3T

Mycgr3G58567 Mycgr3T
  
Location: 13753-14506

Mycgr3G58567\_Mycgr3T

Mycgr3G100089 Mycgr3
  
Location: 14606-21152

Mycgr3G100089\_Mycgr3

Mycgr3G42698 Mycgr3T
  
Location: 21252-22131

Mycgr3G42698\_Mycgr3T

Mycgr3G71681 Mycgr3T
  
Location: 22231-23461

Mycgr3G71681\_Mycgr3T

Mycgr3G109328 Mycgr3
  
Location: 23561-24239

Mycgr3G109328\_Mycgr3

Mycgr3G104334 Mycgr3
  
Location: 24339-24567

Mycgr3G104334\_Mycgr3

Mycgr3G42715 Mycgr3T
  
Location: 24667-25981

Mycgr3G42715\_Mycgr3T

Mycgr3G92934 Mycgr3T
  
Location: 26081-27593

Mycgr3G92934\_Mycgr3T

Mycgr3G41969 Mycgr3T
  
Location: 27693-29328

Mycgr3G41969\_Mycgr3T

Mycgr3G80635 Mycgr3T
  
Location: 29428-29821

Mycgr3G80635\_Mycgr3T

Mycgr3G41426 Mycgr3T
  
Location: 29921-35255

Mycgr3G41426\_Mycgr3T

Mycgr3G104337 Mycgr3
  
Location: 35355-36108

Mycgr3G104337\_Mycgr3

Mycgr3G71679 Mycgr3T
  
Location: 36208-37300

Mycgr3G71679\_Mycgr3T

Mycgr3G92938 Mycgr3T
  
Location: 37400-38699

Mycgr3G92938\_Mycgr3T

Mycgr3G92941 Mycgr3T
  
Location: 38799-40734

Mycgr3G92941\_Mycgr3T

proteinase T-like protein
  
Accession: EGR46243
  
Location: 264600-265955
  
 NCBI BlastP on this gene

EGR46243

predicted protein
  
Accession: EGR46150
  
Location: 270707-272048
  
 NCBI BlastP on this gene

EGR46150

predicted protein
  
Accession: EGR46151
  
Location: 272362-273457
  
 NCBI BlastP on this gene

EGR46151

predicted protein
  
Accession: EGR46152
  
Location: 274371-274899
  
 NCBI BlastP on this gene

EGR46152

predicted protein
  
Accession: EGR46244
  
Location: 275437-275882
  
 NCBI BlastP on this gene

EGR46244

predicted protein
  
Accession: EGR46153
  
Location: 277379-278168
  
 NCBI BlastP on this gene

EGR46153

predicted protein
  
Accession: EGR46154
  
Location: 280159-281463
  
 NCBI BlastP on this gene

EGR46154

predicted protein
  
Accession: EGR46245
  
Location: 281700-283047
  
 NCBI BlastP on this gene

EGR46245

predicted protein
  
Accession: EGR46246
  
Location: 283689-290663
  
  
**BlastP hit with Mycgr3G42010\_Mycgr3T**
  
Percentage identity: 43 %
  
BlastP bit score: 1910
  
Sequence coverage: 101 %
  
E-value: 0.0
  
  
 NCBI BlastP on this gene

EGR46246

predicted protein
  
Accession: EGR46155
  
Location: 291551-292927
  
  
**BlastP hit with Mycgr3G92938\_Mycgr3T**
  
Percentage identity: 35 %
  
BlastP bit score: 229
  
Sequence coverage: 81 %
  
E-value: 2e-66
  
  
 NCBI BlastP on this gene

EGR46155

predicted protein
  
Accession: EGR46156
  
Location: 293602-294471
  
 NCBI BlastP on this gene

EGR46156

predicted protein
  
Accession: EGR46247
  
Location: 294588-295520
  
 NCBI BlastP on this gene

EGR46247

predicted protein
  
Accession: EGR46157
  
Location: 296086-296424
  
 NCBI BlastP on this gene

EGR46157

predicted protein
  
Accession: EGR46248
  
Location: 299445-300625
  
 NCBI BlastP on this gene

EGR46248

amino acid transporter
  
Accession: EGR46158
  
Location: 301270-303137
  
 NCBI BlastP on this gene

EGR46158

predicted protein
  
Accession: EGR46249
  
Location: 303380-305011
  
 NCBI BlastP on this gene

EGR46249

predicted protein
  
Accession: EGR46159
  
Location: 307694-309682
  
 NCBI BlastP on this gene

EGR46159

Query: Architecture Search FASTA input

CAGA01000005 : Claviceps purpurea 20.1    Total score: 2.0     Cumulative Blast bit score: 2137

Hit cluster cross-links:

Mycgr3G85918 Mycgr3T
  
Location: 0-1602

Mycgr3G85918\_Mycgr3T

Mycgr3G42010 Mycgr3T
  
Location: 1702-8569

Mycgr3G42010\_Mycgr3T

Mycgr3G29582 Mycgr3T
  
Location: 8669-8915

Mycgr3G29582\_Mycgr3T

Mycgr3G31170 Mycgr3T
  
Location: 9015-9255

Mycgr3G31170\_Mycgr3T

Mycgr3G85924 Mycgr3T
  
Location: 9355-11218

Mycgr3G85924\_Mycgr3T

Mycgr3G71676 Mycgr3T
  
Location: 11318-12494

Mycgr3G71676\_Mycgr3T

Mycgr3G11468 Mycgr3T
  
Location: 12594-13653

Mycgr3G11468\_Mycgr3T

Mycgr3G58567 Mycgr3T
  
Location: 13753-14506

Mycgr3G58567\_Mycgr3T

Mycgr3G100089 Mycgr3
  
Location: 14606-21152

Mycgr3G100089\_Mycgr3

Mycgr3G42698 Mycgr3T
  
Location: 21252-22131

Mycgr3G42698\_Mycgr3T

Mycgr3G71681 Mycgr3T
  
Location: 22231-23461

Mycgr3G71681\_Mycgr3T

Mycgr3G109328 Mycgr3
  
Location: 23561-24239

Mycgr3G109328\_Mycgr3

Mycgr3G104334 Mycgr3
  
Location: 24339-24567

Mycgr3G104334\_Mycgr3

Mycgr3G42715 Mycgr3T
  
Location: 24667-25981

Mycgr3G42715\_Mycgr3T

Mycgr3G92934 Mycgr3T
  
Location: 26081-27593

Mycgr3G92934\_Mycgr3T

Mycgr3G41969 Mycgr3T
  
Location: 27693-29328

Mycgr3G41969\_Mycgr3T

Mycgr3G80635 Mycgr3T
  
Location: 29428-29821

Mycgr3G80635\_Mycgr3T

Mycgr3G41426 Mycgr3T
  
Location: 29921-35255

Mycgr3G41426\_Mycgr3T

Mycgr3G104337 Mycgr3
  
Location: 35355-36108

Mycgr3G104337\_Mycgr3

Mycgr3G71679 Mycgr3T
  
Location: 36208-37300

Mycgr3G71679\_Mycgr3T

Mycgr3G92938 Mycgr3T
  
Location: 37400-38699

Mycgr3G92938\_Mycgr3T

Mycgr3G92941 Mycgr3T
  
Location: 38799-40734

Mycgr3G92941\_Mycgr3T

related to L-serine dehydratase
  
Accession: CCE27610
  
Location: 133256-134221
  
 NCBI BlastP on this gene

CCE27610

related to Carboxymuconolactone decarboxylase
  
Accession: CCE27611
  
Location: 135410-136137
  
 NCBI BlastP on this gene

CCE27611

uncharacterized protein
  
Accession: CCE27612
  
Location: 138458-140245
  
 NCBI BlastP on this gene

CCE27612

uncharacterized protein
  
Accession: CCE27613
  
Location: 141214-143013
  
 NCBI BlastP on this gene

CCE27613

uncharacterized protein
  
Accession: CCE27614
  
Location: 144169-145685
  
 NCBI BlastP on this gene

CCE27614

related to ECM32-DNA dependent ATPase/DNA helicase B
  
Accession: CCE27615
  
Location: 146163-153324
  
  
**BlastP hit with Mycgr3G42010\_Mycgr3T**
  
Percentage identity: 44 %
  
BlastP bit score: 1892
  
Sequence coverage: 101 %
  
E-value: 0.0
  
  
 NCBI BlastP on this gene

CCE27615

uncharacterized protein
  
Accession: CCE27616
  
Location: 153715-155292
  
  
**BlastP hit with Mycgr3G92938\_Mycgr3T**
  
Percentage identity: 33 %
  
BlastP bit score: 245
  
Sequence coverage: 104 %
  
E-value: 1e-71
  
  
 NCBI BlastP on this gene

CCE27616

uncharacterized protein
  
Accession: CCE27617
  
Location: 157286-159176
  
 NCBI BlastP on this gene

CCE27617

uncharacterized protein
  
Accession: CCE27618
  
Location: 160151-162365
  
 NCBI BlastP on this gene

CCE27618

uncharacterized protein
  
Accession: CCE27619
  
Location: 162899-164896
  
 NCBI BlastP on this gene

CCE27619

uncharacterized protein
  
Accession: CCE27620
  
Location: 166613-168333
  
 NCBI BlastP on this gene

CCE27620

Query: Architecture Search FASTA input

AABX02000071 : Neurospora crassa OR74A    Total score: 2.0     Cumulative Blast bit score: 2136

Hit cluster cross-links:

Mycgr3G85918 Mycgr3T
  
Location: 0-1602

Mycgr3G85918\_Mycgr3T

Mycgr3G42010 Mycgr3T
  
Location: 1702-8569

Mycgr3G42010\_Mycgr3T

Mycgr3G29582 Mycgr3T
  
Location: 8669-8915

Mycgr3G29582\_Mycgr3T

Mycgr3G31170 Mycgr3T
  
Location: 9015-9255

Mycgr3G31170\_Mycgr3T

Mycgr3G85924 Mycgr3T
  
Location: 9355-11218

Mycgr3G85924\_Mycgr3T

Mycgr3G71676 Mycgr3T
  
Location: 11318-12494

Mycgr3G71676\_Mycgr3T

Mycgr3G11468 Mycgr3T
  
Location: 12594-13653

Mycgr3G11468\_Mycgr3T

Mycgr3G58567 Mycgr3T
  
Location: 13753-14506

Mycgr3G58567\_Mycgr3T

Mycgr3G100089 Mycgr3
  
Location: 14606-21152

Mycgr3G100089\_Mycgr3

Mycgr3G42698 Mycgr3T
  
Location: 21252-22131

Mycgr3G42698\_Mycgr3T

Mycgr3G71681 Mycgr3T
  
Location: 22231-23461

Mycgr3G71681\_Mycgr3T

Mycgr3G109328 Mycgr3
  
Location: 23561-24239

Mycgr3G109328\_Mycgr3

Mycgr3G104334 Mycgr3
  
Location: 24339-24567

Mycgr3G104334\_Mycgr3

Mycgr3G42715 Mycgr3T
  
Location: 24667-25981

Mycgr3G42715\_Mycgr3T

Mycgr3G92934 Mycgr3T
  
Location: 26081-27593

Mycgr3G92934\_Mycgr3T

Mycgr3G41969 Mycgr3T
  
Location: 27693-29328

Mycgr3G41969\_Mycgr3T

Mycgr3G80635 Mycgr3T
  
Location: 29428-29821

Mycgr3G80635\_Mycgr3T

Mycgr3G41426 Mycgr3T
  
Location: 29921-35255

Mycgr3G41426\_Mycgr3T

Mycgr3G104337 Mycgr3
  
Location: 35355-36108

Mycgr3G104337\_Mycgr3

Mycgr3G71679 Mycgr3T
  
Location: 36208-37300

Mycgr3G71679\_Mycgr3T

Mycgr3G92938 Mycgr3T
  
Location: 37400-38699

Mycgr3G92938\_Mycgr3T

Mycgr3G92941 Mycgr3T
  
Location: 38799-40734

Mycgr3G92941\_Mycgr3T

conserved hypothetical protein
  
Accession: EAA29695
  
Location: 97025-98963
  
 NCBI BlastP on this gene

EAA29695

predicted protein
  
Accession: EAA29696
  
Location: 100898-105622
  
 NCBI BlastP on this gene

EAA29696

predicted protein
  
Accession: EAA29697
  
Location: 106576-108984
  
 NCBI BlastP on this gene

EAA29697

predicted protein
  
Accession: EAA29698
  
Location: 109816-110726
  
 NCBI BlastP on this gene

EAA29698

predicted protein
  
Accession: EAA29699
  
Location: 115132-116926
  
  
**BlastP hit with Mycgr3G92938\_Mycgr3T**
  
Percentage identity: 49 %
  
BlastP bit score: 156
  
Sequence coverage: 35 %
  
E-value: 1e-38
  
  
 NCBI BlastP on this gene

EAA29699

predicted protein
  
Accession: EAA29700
  
Location: 118319-125575
  
  
**BlastP hit with Mycgr3G42010\_Mycgr3T**
  
Percentage identity: 45 %
  
BlastP bit score: 1980
  
Sequence coverage: 102 %
  
E-value: 0.0
  
  
 NCBI BlastP on this gene

EAA29700

hypothetical protein
  
Accession: EAA29701
  
Location: 126433-128355
  
 NCBI BlastP on this gene

EAA29701

Query: Architecture Search FASTA input

AFQF01000625 : Fusarium oxysporum Fo5176    Total score: 2.0     Cumulative Blast bit score: 2116

Hit cluster cross-links:

Mycgr3G85918 Mycgr3T
  
Location: 0-1602

Mycgr3G85918\_Mycgr3T

Mycgr3G42010 Mycgr3T
  
Location: 1702-8569

Mycgr3G42010\_Mycgr3T

Mycgr3G29582 Mycgr3T
  
Location: 8669-8915

Mycgr3G29582\_Mycgr3T

Mycgr3G31170 Mycgr3T
  
Location: 9015-9255

Mycgr3G31170\_Mycgr3T

Mycgr3G85924 Mycgr3T
  
Location: 9355-11218

Mycgr3G85924\_Mycgr3T

Mycgr3G71676 Mycgr3T
  
Location: 11318-12494

Mycgr3G71676\_Mycgr3T

Mycgr3G11468 Mycgr3T
  
Location: 12594-13653

Mycgr3G11468\_Mycgr3T

Mycgr3G58567 Mycgr3T
  
Location: 13753-14506

Mycgr3G58567\_Mycgr3T

Mycgr3G100089 Mycgr3
  
Location: 14606-21152

Mycgr3G100089\_Mycgr3

Mycgr3G42698 Mycgr3T
  
Location: 21252-22131

Mycgr3G42698\_Mycgr3T

Mycgr3G71681 Mycgr3T
  
Location: 22231-23461

Mycgr3G71681\_Mycgr3T

Mycgr3G109328 Mycgr3
  
Location: 23561-24239

Mycgr3G109328\_Mycgr3

Mycgr3G104334 Mycgr3
  
Location: 24339-24567

Mycgr3G104334\_Mycgr3

Mycgr3G42715 Mycgr3T
  
Location: 24667-25981

Mycgr3G42715\_Mycgr3T

Mycgr3G92934 Mycgr3T
  
Location: 26081-27593

Mycgr3G92934\_Mycgr3T

Mycgr3G41969 Mycgr3T
  
Location: 27693-29328

Mycgr3G41969\_Mycgr3T

Mycgr3G80635 Mycgr3T
  
Location: 29428-29821

Mycgr3G80635\_Mycgr3T

Mycgr3G41426 Mycgr3T
  
Location: 29921-35255

Mycgr3G41426\_Mycgr3T

Mycgr3G104337 Mycgr3
  
Location: 35355-36108

Mycgr3G104337\_Mycgr3

Mycgr3G71679 Mycgr3T
  
Location: 36208-37300

Mycgr3G71679\_Mycgr3T

Mycgr3G92938 Mycgr3T
  
Location: 37400-38699

Mycgr3G92938\_Mycgr3T

Mycgr3G92941 Mycgr3T
  
Location: 38799-40734

Mycgr3G92941\_Mycgr3T

hypothetical protein
  
Accession: EGU87652
  
Location: 26242-29504
  
 NCBI BlastP on this gene

EGU87652

hypothetical protein
  
Accession: EGU87653
  
Location: 29808-31082
  
 NCBI BlastP on this gene

EGU87653

hypothetical protein
  
Accession: EGU87654
  
Location: 32115-33619
  
 NCBI BlastP on this gene

EGU87654

hypothetical protein
  
Accession: EGU87655
  
Location: 33896-34738
  
 NCBI BlastP on this gene

EGU87655

hypothetical protein
  
Accession: EGU87656
  
Location: 36340-36691
  
 NCBI BlastP on this gene

EGU87656

hypothetical protein
  
Accession: EGU87657
  
Location: 37598-39279
  
 NCBI BlastP on this gene

EGU87657

hypothetical protein
  
Accession: EGU87658
  
Location: 41134-42756
  
 NCBI BlastP on this gene

EGU87658

hypothetical protein
  
Accession: EGU87659
  
Location: 43290-44441
  
 NCBI BlastP on this gene

EGU87659

hypothetical protein
  
Accession: EGU87660
  
Location: 45662-52690
  
  
**BlastP hit with Mycgr3G42010\_Mycgr3T**
  
Percentage identity: 44 %
  
BlastP bit score: 1880
  
Sequence coverage: 102 %
  
E-value: 0.0
  
  
 NCBI BlastP on this gene

EGU87660

hypothetical protein
  
Accession: EGU87661
  
Location: 53495-54846
  
  
**BlastP hit with Mycgr3G92938\_Mycgr3T**
  
Percentage identity: 36 %
  
BlastP bit score: 236
  
Sequence coverage: 87 %
  
E-value: 5e-69
  
  
 NCBI BlastP on this gene

EGU87661

hypothetical protein
  
Accession: EGU87662
  
Location: 57217-58524
  
 NCBI BlastP on this gene

EGU87662

hypothetical protein
  
Accession: EGU87663
  
Location: 58771-62120
  
 NCBI BlastP on this gene

EGU87663

hypothetical protein
  
Accession: EGU87664
  
Location: 62284-62817
  
 NCBI BlastP on this gene

EGU87664

hypothetical protein
  
Accession: EGU87665
  
Location: 65756-68679
  
 NCBI BlastP on this gene

EGU87665

hypothetical protein
  
Accession: EGU87666
  
Location: 68953-70495
  
 NCBI BlastP on this gene

EGU87666

hypothetical protein
  
Accession: EGU87667
  
Location: 70916-73450
  
 NCBI BlastP on this gene

EGU87667

Query: Architecture Search FASTA input

KB726184 : Fusarium oxysporum f. sp. cubense race 4 unplaced genomic scaffold scaffold30    Total score: 2.0     Cumulative Blast bit score: 2105

Hit cluster cross-links:

Mycgr3G85918 Mycgr3T
  
Location: 0-1602

Mycgr3G85918\_Mycgr3T

Mycgr3G42010 Mycgr3T
  
Location: 1702-8569

Mycgr3G42010\_Mycgr3T

Mycgr3G29582 Mycgr3T
  
Location: 8669-8915

Mycgr3G29582\_Mycgr3T

Mycgr3G31170 Mycgr3T
  
Location: 9015-9255

Mycgr3G31170\_Mycgr3T

Mycgr3G85924 Mycgr3T
  
Location: 9355-11218

Mycgr3G85924\_Mycgr3T

Mycgr3G71676 Mycgr3T
  
Location: 11318-12494

Mycgr3G71676\_Mycgr3T

Mycgr3G11468 Mycgr3T
  
Location: 12594-13653

Mycgr3G11468\_Mycgr3T

Mycgr3G58567 Mycgr3T
  
Location: 13753-14506

Mycgr3G58567\_Mycgr3T

Mycgr3G100089 Mycgr3
  
Location: 14606-21152

Mycgr3G100089\_Mycgr3

Mycgr3G42698 Mycgr3T
  
Location: 21252-22131

Mycgr3G42698\_Mycgr3T

Mycgr3G71681 Mycgr3T
  
Location: 22231-23461

Mycgr3G71681\_Mycgr3T

Mycgr3G109328 Mycgr3
  
Location: 23561-24239

Mycgr3G109328\_Mycgr3

Mycgr3G104334 Mycgr3
  
Location: 24339-24567

Mycgr3G104334\_Mycgr3

Mycgr3G42715 Mycgr3T
  
Location: 24667-25981

Mycgr3G42715\_Mycgr3T

Mycgr3G92934 Mycgr3T
  
Location: 26081-27593

Mycgr3G92934\_Mycgr3T

Mycgr3G41969 Mycgr3T
  
Location: 27693-29328

Mycgr3G41969\_Mycgr3T

Mycgr3G80635 Mycgr3T
  
Location: 29428-29821

Mycgr3G80635\_Mycgr3T

Mycgr3G41426 Mycgr3T
  
Location: 29921-35255

Mycgr3G41426\_Mycgr3T

Mycgr3G104337 Mycgr3
  
Location: 35355-36108

Mycgr3G104337\_Mycgr3

Mycgr3G71679 Mycgr3T
  
Location: 36208-37300

Mycgr3G71679\_Mycgr3T

Mycgr3G92938 Mycgr3T
  
Location: 37400-38699

Mycgr3G92938\_Mycgr3T

Mycgr3G92941 Mycgr3T
  
Location: 38799-40734

Mycgr3G92941\_Mycgr3T

hypothetical protein
  
Accession: EMT74590
  
Location: 40275-43046
  
 NCBI BlastP on this gene

EMT74590

hypothetical protein
  
Accession: EMT74591
  
Location: 43441-46241
  
 NCBI BlastP on this gene

EMT74591

Putative protein yjlB
  
Accession: EMT74592
  
Location: 49130-49663
  
 NCBI BlastP on this gene

EMT74592

hypothetical protein
  
Accession: EMT74593
  
Location: 49826-50122
  
 NCBI BlastP on this gene

EMT74593

Putative protein yjlB
  
Accession: EMT74594
  
Location: 50746-51279
  
 NCBI BlastP on this gene

EMT74594

Stabilin-2
  
Accession: EMT74595
  
Location: 51442-52710
  
 NCBI BlastP on this gene

EMT74595

Cyclin CCL1
  
Accession: EMT74596
  
Location: 53703-54792
  
 NCBI BlastP on this gene

EMT74596

hypothetical protein
  
Accession: EMT74597
  
Location: 55039-56346
  
 NCBI BlastP on this gene

EMT74597

hypothetical protein
  
Accession: EMT74598
  
Location: 58719-60076
  
  
**BlastP hit with Mycgr3G92938\_Mycgr3T**
  
Percentage identity: 34 %
  
BlastP bit score: 230
  
Sequence coverage: 100 %
  
E-value: 1e-66
  
  
 NCBI BlastP on this gene

EMT74598

NFX1-type zinc finger-containing protein 1
  
Accession: EMT74599
  
Location: 60865-67901
  
  
**BlastP hit with Mycgr3G42010\_Mycgr3T**
  
Percentage identity: 44 %
  
BlastP bit score: 1875
  
Sequence coverage: 102 %
  
E-value: 0.0
  
  
 NCBI BlastP on this gene

EMT74599

hypothetical protein
  
Accession: EMT74600
  
Location: 68874-69953
  
 NCBI BlastP on this gene

EMT74600

hypothetical protein
  
Accession: EMT74601
  
Location: 70699-71598
  
 NCBI BlastP on this gene

EMT74601

hypothetical protein
  
Accession: EMT74602
  
Location: 74208-75509
  
 NCBI BlastP on this gene

EMT74602

Canalicular multispecific organic anion transporter 2
  
Accession: EMT74603
  
Location: 76851-81524
  
 NCBI BlastP on this gene

EMT74603

Cytochrome P450 4F4
  
Accession: EMT74604
  
Location: 81624-83315
  
 NCBI BlastP on this gene

EMT74604

NADH-cytochrome b5 reductase 1
  
Accession: EMT74605
  
Location: 83967-85988
  
 NCBI BlastP on this gene

EMT74605

Query: Architecture Search FASTA input

ABDG02000020 : Trichoderma atroviride IMI 206040    Total score: 2.0     Cumulative Blast bit score: 2101

Hit cluster cross-links:

Mycgr3G85918 Mycgr3T
  
Location: 0-1602

Mycgr3G85918\_Mycgr3T

Mycgr3G42010 Mycgr3T
  
Location: 1702-8569

Mycgr3G42010\_Mycgr3T

Mycgr3G29582 Mycgr3T
  
Location: 8669-8915

Mycgr3G29582\_Mycgr3T

Mycgr3G31170 Mycgr3T
  
Location: 9015-9255

Mycgr3G31170\_Mycgr3T

Mycgr3G85924 Mycgr3T
  
Location: 9355-11218

Mycgr3G85924\_Mycgr3T

Mycgr3G71676 Mycgr3T
  
Location: 11318-12494

Mycgr3G71676\_Mycgr3T

Mycgr3G11468 Mycgr3T
  
Location: 12594-13653

Mycgr3G11468\_Mycgr3T

Mycgr3G58567 Mycgr3T
  
Location: 13753-14506

Mycgr3G58567\_Mycgr3T

Mycgr3G100089 Mycgr3
  
Location: 14606-21152

Mycgr3G100089\_Mycgr3

Mycgr3G42698 Mycgr3T
  
Location: 21252-22131

Mycgr3G42698\_Mycgr3T

Mycgr3G71681 Mycgr3T
  
Location: 22231-23461

Mycgr3G71681\_Mycgr3T

Mycgr3G109328 Mycgr3
  
Location: 23561-24239

Mycgr3G109328\_Mycgr3

Mycgr3G104334 Mycgr3
  
Location: 24339-24567

Mycgr3G104334\_Mycgr3

Mycgr3G42715 Mycgr3T
  
Location: 24667-25981

Mycgr3G42715\_Mycgr3T

Mycgr3G92934 Mycgr3T
  
Location: 26081-27593

Mycgr3G92934\_Mycgr3T

Mycgr3G41969 Mycgr3T
  
Location: 27693-29328

Mycgr3G41969\_Mycgr3T

Mycgr3G80635 Mycgr3T
  
Location: 29428-29821

Mycgr3G80635\_Mycgr3T

Mycgr3G41426 Mycgr3T
  
Location: 29921-35255

Mycgr3G41426\_Mycgr3T

Mycgr3G104337 Mycgr3
  
Location: 35355-36108

Mycgr3G104337\_Mycgr3

Mycgr3G71679 Mycgr3T
  
Location: 36208-37300

Mycgr3G71679\_Mycgr3T

Mycgr3G92938 Mycgr3T
  
Location: 37400-38699

Mycgr3G92938\_Mycgr3T

Mycgr3G92941 Mycgr3T
  
Location: 38799-40734

Mycgr3G92941\_Mycgr3T

hypothetical protein
  
Accession: EHK47659
  
Location: 1007295-1008968
  
 NCBI BlastP on this gene

EHK47659

hypothetical protein
  
Accession: EHK47660
  
Location: 1009249-1010319
  
 NCBI BlastP on this gene

EHK47660

hypothetical protein
  
Accession: EHK47661
  
Location: 1010546-1012616
  
 NCBI BlastP on this gene

EHK47661

hypothetical protein
  
Accession: EHK47662
  
Location: 1015138-1017628
  
 NCBI BlastP on this gene

EHK47662

hypothetical protein
  
Accession: EHK47663
  
Location: 1018706-1019118
  
 NCBI BlastP on this gene

EHK47663

hypothetical protein
  
Accession: EHK47664
  
Location: 1020834-1021609
  
 NCBI BlastP on this gene

EHK47664

hypothetical protein
  
Accession: EHK47665
  
Location: 1023555-1024730
  
 NCBI BlastP on this gene

EHK47665

hypothetical protein
  
Accession: EHK47666
  
Location: 1025258-1032304
  
  
**BlastP hit with Mycgr3G42010\_Mycgr3T**
  
Percentage identity: 43 %
  
BlastP bit score: 1875
  
Sequence coverage: 102 %
  
E-value: 0.0
  
  
 NCBI BlastP on this gene

EHK47666

hypothetical protein
  
Accession: EHK47667
  
Location: 1032982-1034381
  
  
**BlastP hit with Mycgr3G92938\_Mycgr3T**
  
Percentage identity: 36 %
  
BlastP bit score: 226
  
Sequence coverage: 81 %
  
E-value: 3e-65
  
  
 NCBI BlastP on this gene

EHK47667

hypothetical protein
  
Accession: EHK47668
  
Location: 1035154-1035777
  
 NCBI BlastP on this gene

EHK47668

hypothetical protein
  
Accession: EHK47669
  
Location: 1036323-1037574
  
 NCBI BlastP on this gene

EHK47669

hypothetical protein
  
Accession: EHK47670
  
Location: 1039287-1041499
  
 NCBI BlastP on this gene

EHK47670

glycosyltransferase family 90 protein
  
Accession: EHK47881
  
Location: 1041717-1044844
  
 NCBI BlastP on this gene

EHK47881

hypothetical protein
  
Accession: EHK47671
  
Location: 1045765-1047846
  
 NCBI BlastP on this gene

EHK47671

hypothetical protein
  
Accession: EHK47672
  
Location: 1050293-1051507
  
 NCBI BlastP on this gene

EHK47672

hypothetical protein
  
Accession: EHK47673
  
Location: 1052252-1054182
  
 NCBI BlastP on this gene

EHK47673

Query: Architecture Search FASTA input

AFNW01000011 : Fusarium pseudograminearum CS3096    Total score: 2.0     Cumulative Blast bit score: 2099

Hit cluster cross-links:

Mycgr3G85918 Mycgr3T
  
Location: 0-1602

Mycgr3G85918\_Mycgr3T

Mycgr3G42010 Mycgr3T
  
Location: 1702-8569

Mycgr3G42010\_Mycgr3T

Mycgr3G29582 Mycgr3T
  
Location: 8669-8915

Mycgr3G29582\_Mycgr3T

Mycgr3G31170 Mycgr3T
  
Location: 9015-9255

Mycgr3G31170\_Mycgr3T

Mycgr3G85924 Mycgr3T
  
Location: 9355-11218

Mycgr3G85924\_Mycgr3T

Mycgr3G71676 Mycgr3T
  
Location: 11318-12494

Mycgr3G71676\_Mycgr3T

Mycgr3G11468 Mycgr3T
  
Location: 12594-13653

Mycgr3G11468\_Mycgr3T

Mycgr3G58567 Mycgr3T
  
Location: 13753-14506

Mycgr3G58567\_Mycgr3T

Mycgr3G100089 Mycgr3
  
Location: 14606-21152

Mycgr3G100089\_Mycgr3

Mycgr3G42698 Mycgr3T
  
Location: 21252-22131

Mycgr3G42698\_Mycgr3T

Mycgr3G71681 Mycgr3T
  
Location: 22231-23461

Mycgr3G71681\_Mycgr3T

Mycgr3G109328 Mycgr3
  
Location: 23561-24239

Mycgr3G109328\_Mycgr3

Mycgr3G104334 Mycgr3
  
Location: 24339-24567

Mycgr3G104334\_Mycgr3

Mycgr3G42715 Mycgr3T
  
Location: 24667-25981

Mycgr3G42715\_Mycgr3T

Mycgr3G92934 Mycgr3T
  
Location: 26081-27593

Mycgr3G92934\_Mycgr3T

Mycgr3G41969 Mycgr3T
  
Location: 27693-29328

Mycgr3G41969\_Mycgr3T

Mycgr3G80635 Mycgr3T
  
Location: 29428-29821

Mycgr3G80635\_Mycgr3T

Mycgr3G41426 Mycgr3T
  
Location: 29921-35255

Mycgr3G41426\_Mycgr3T

Mycgr3G104337 Mycgr3
  
Location: 35355-36108

Mycgr3G104337\_Mycgr3

Mycgr3G71679 Mycgr3T
  
Location: 36208-37300

Mycgr3G71679\_Mycgr3T

Mycgr3G92938 Mycgr3T
  
Location: 37400-38699

Mycgr3G92938\_Mycgr3T

Mycgr3G92941 Mycgr3T
  
Location: 38799-40734

Mycgr3G92941\_Mycgr3T

hypothetical protein
  
Accession: EKJ79238
  
Location: 88162-88695
  
 NCBI BlastP on this gene

EKJ79238

hypothetical protein
  
Accession: EKJ79239
  
Location: 88887-90160
  
 NCBI BlastP on this gene

EKJ79239

hypothetical protein
  
Accession: EKJ79240
  
Location: 91272-92371
  
 NCBI BlastP on this gene

EKJ79240

hypothetical protein
  
Accession: EKJ79241
  
Location: 92621-93936
  
 NCBI BlastP on this gene

EKJ79241

hypothetical protein
  
Accession: EKJ79242
  
Location: 95879-96411
  
 NCBI BlastP on this gene

EKJ79242

hypothetical protein
  
Accession: EKJ79243
  
Location: 97173-98685
  
 NCBI BlastP on this gene

EKJ79243

hypothetical protein
  
Accession: EKJ79244
  
Location: 99066-104567
  
 NCBI BlastP on this gene

EKJ79244

hypothetical protein
  
Accession: EKJ79245
  
Location: 104797-111801
  
  
**BlastP hit with Mycgr3G42010\_Mycgr3T**
  
Percentage identity: 44 %
  
BlastP bit score: 1882
  
Sequence coverage: 101 %
  
E-value: 0.0
  
  
 NCBI BlastP on this gene

EKJ79245

hypothetical protein
  
Accession: EKJ79246
  
Location: 112506-113895
  
  
**BlastP hit with Mycgr3G92938\_Mycgr3T**
  
Percentage identity: 33 %
  
BlastP bit score: 217
  
Sequence coverage: 82 %
  
E-value: 1e-61
  
  
 NCBI BlastP on this gene

EKJ79246

hypothetical protein
  
Accession: EKJ79247
  
Location: 115095-116558
  
 NCBI BlastP on this gene

EKJ79247

hypothetical protein
  
Accession: EKJ79248
  
Location: 117484-118605
  
 NCBI BlastP on this gene

EKJ79248

hypothetical protein
  
Accession: EKJ79249
  
Location: 119778-121478
  
 NCBI BlastP on this gene

EKJ79249

hypothetical protein
  
Accession: EKJ79250
  
Location: 124725-125572
  
 NCBI BlastP on this gene

EKJ79250

hypothetical protein
  
Accession: EKJ79251
  
Location: 125811-126161
  
 NCBI BlastP on this gene

EKJ79251

hypothetical protein
  
Accession: EKJ79252
  
Location: 126455-128203
  
 NCBI BlastP on this gene

EKJ79252

hypothetical protein
  
Accession: EKJ79253
  
Location: 128580-129990
  
 NCBI BlastP on this gene

EKJ79253

hypothetical protein
  
Accession: EKJ79254
  
Location: 130856-132556
  
 NCBI BlastP on this gene

EKJ79254

Query: Architecture Search FASTA input

DS995706 : Microsporum canis CBS 113480 supercont1.6 genomic scaffold    Total score: 2.0     Cumulative Blast bit score: 2097

Hit cluster cross-links:

Mycgr3G85918 Mycgr3T
  
Location: 0-1602

Mycgr3G85918\_Mycgr3T

Mycgr3G42010 Mycgr3T
  
Location: 1702-8569

Mycgr3G42010\_Mycgr3T

Mycgr3G29582 Mycgr3T
  
Location: 8669-8915

Mycgr3G29582\_Mycgr3T

Mycgr3G31170 Mycgr3T
  
Location: 9015-9255

Mycgr3G31170\_Mycgr3T

Mycgr3G85924 Mycgr3T
  
Location: 9355-11218

Mycgr3G85924\_Mycgr3T

Mycgr3G71676 Mycgr3T
  
Location: 11318-12494

Mycgr3G71676\_Mycgr3T

Mycgr3G11468 Mycgr3T
  
Location: 12594-13653

Mycgr3G11468\_Mycgr3T

Mycgr3G58567 Mycgr3T
  
Location: 13753-14506

Mycgr3G58567\_Mycgr3T

Mycgr3G100089 Mycgr3
  
Location: 14606-21152

Mycgr3G100089\_Mycgr3

Mycgr3G42698 Mycgr3T
  
Location: 21252-22131

Mycgr3G42698\_Mycgr3T

Mycgr3G71681 Mycgr3T
  
Location: 22231-23461

Mycgr3G71681\_Mycgr3T

Mycgr3G109328 Mycgr3
  
Location: 23561-24239

Mycgr3G109328\_Mycgr3

Mycgr3G104334 Mycgr3
  
Location: 24339-24567

Mycgr3G104334\_Mycgr3

Mycgr3G42715 Mycgr3T
  
Location: 24667-25981

Mycgr3G42715\_Mycgr3T

Mycgr3G92934 Mycgr3T
  
Location: 26081-27593

Mycgr3G92934\_Mycgr3T

Mycgr3G41969 Mycgr3T
  
Location: 27693-29328

Mycgr3G41969\_Mycgr3T

Mycgr3G80635 Mycgr3T
  
Location: 29428-29821

Mycgr3G80635\_Mycgr3T

Mycgr3G41426 Mycgr3T
  
Location: 29921-35255

Mycgr3G41426\_Mycgr3T

Mycgr3G104337 Mycgr3
  
Location: 35355-36108

Mycgr3G104337\_Mycgr3

Mycgr3G71679 Mycgr3T
  
Location: 36208-37300

Mycgr3G71679\_Mycgr3T

Mycgr3G92938 Mycgr3T
  
Location: 37400-38699

Mycgr3G92938\_Mycgr3T

Mycgr3G92941 Mycgr3T
  
Location: 38799-40734

Mycgr3G92941\_Mycgr3T

conserved hypothetical protein
  
Accession: EEQ33491
  
Location: 455197-457545
  
 NCBI BlastP on this gene

EEQ33491

glyoxylate reductase
  
Accession: EEQ33492
  
Location: 458012-459636
  
 NCBI BlastP on this gene

EEQ33492

cell cycle control protein cwf19
  
Accession: EEQ33493
  
Location: 460417-462601
  
 NCBI BlastP on this gene

EEQ33493

ATPase NPA3
  
Accession: EEQ33494
  
Location: 462854-464095
  
 NCBI BlastP on this gene

EEQ33494

conserved hypothetical protein
  
Accession: EEQ33495
  
Location: 464373-465012
  
 NCBI BlastP on this gene

EEQ33495

conserved hypothetical protein
  
Accession: EEQ33496
  
Location: 465401-467758
  
 NCBI BlastP on this gene

EEQ33496

conserved hypothetical protein
  
Accession: EEQ33497
  
Location: 468388-469423
  
 NCBI BlastP on this gene

EEQ33497

conserved hypothetical protein
  
Accession: EEQ33498
  
Location: 469891-470471
  
 NCBI BlastP on this gene

EEQ33498

conserved hypothetical protein
  
Accession: EEQ33499
  
Location: 470929-472916
  
 NCBI BlastP on this gene

EEQ33499

NFX1-type zinc finger-containing protein 1
  
Accession: EEQ33500
  
Location: 473557-480978
  
  
**BlastP hit with Mycgr3G42010\_Mycgr3T**
  
Percentage identity: 44 %
  
BlastP bit score: 1891
  
Sequence coverage: 102 %
  
E-value: 0.0
  
  
 NCBI BlastP on this gene

EEQ33500

conserved hypothetical protein
  
Accession: EEQ33501
  
Location: 481360-482668
  
  
**BlastP hit with Mycgr3G92938\_Mycgr3T**
  
Percentage identity: 41 %
  
BlastP bit score: 206
  
Sequence coverage: 58 %
  
E-value: 7e-58
  
  
 NCBI BlastP on this gene

EEQ33501

predicted protein
  
Accession: EEQ33502
  
Location: 483382-484364
  
 NCBI BlastP on this gene

EEQ33502

leucine Rich Repeat domain-containing protein
  
Accession: EEQ33503
  
Location: 486886-488966
  
 NCBI BlastP on this gene

EEQ33503

conserved hypothetical protein
  
Accession: EEQ33504
  
Location: 489478-491280
  
 NCBI BlastP on this gene

EEQ33504

choline transport protein
  
Accession: EEQ33505
  
Location: 494102-496154
  
 NCBI BlastP on this gene

EEQ33505

cytochrome P450 3A8
  
Accession: EEQ33506
  
Location: 497087-498729
  
 NCBI BlastP on this gene

EEQ33506

conserved hypothetical protein
  
Accession: EEQ33507
  
Location: 498993-500314
  
 NCBI BlastP on this gene

EEQ33507

SpRPA12
  
Accession: EEQ33508
  
Location: 500529-501004
  
 NCBI BlastP on this gene

EEQ33508

Query: Architecture Search FASTA input

GL629801 : Grosmannia clavigera kw1407 unplaced genomic scaffold GCSC\_173    Total score: 2.0     Cumulative Blast bit score: 2094

Hit cluster cross-links:

Mycgr3G85918 Mycgr3T
  
Location: 0-1602

Mycgr3G85918\_Mycgr3T

Mycgr3G42010 Mycgr3T
  
Location: 1702-8569

Mycgr3G42010\_Mycgr3T

Mycgr3G29582 Mycgr3T
  
Location: 8669-8915

Mycgr3G29582\_Mycgr3T

Mycgr3G31170 Mycgr3T
  
Location: 9015-9255

Mycgr3G31170\_Mycgr3T

Mycgr3G85924 Mycgr3T
  
Location: 9355-11218

Mycgr3G85924\_Mycgr3T

Mycgr3G71676 Mycgr3T
  
Location: 11318-12494

Mycgr3G71676\_Mycgr3T

Mycgr3G11468 Mycgr3T
  
Location: 12594-13653

Mycgr3G11468\_Mycgr3T

Mycgr3G58567 Mycgr3T
  
Location: 13753-14506

Mycgr3G58567\_Mycgr3T

Mycgr3G100089 Mycgr3
  
Location: 14606-21152

Mycgr3G100089\_Mycgr3

Mycgr3G42698 Mycgr3T
  
Location: 21252-22131

Mycgr3G42698\_Mycgr3T

Mycgr3G71681 Mycgr3T
  
Location: 22231-23461

Mycgr3G71681\_Mycgr3T

Mycgr3G109328 Mycgr3
  
Location: 23561-24239

Mycgr3G109328\_Mycgr3

Mycgr3G104334 Mycgr3
  
Location: 24339-24567

Mycgr3G104334\_Mycgr3

Mycgr3G42715 Mycgr3T
  
Location: 24667-25981

Mycgr3G42715\_Mycgr3T

Mycgr3G92934 Mycgr3T
  
Location: 26081-27593

Mycgr3G92934\_Mycgr3T

Mycgr3G41969 Mycgr3T
  
Location: 27693-29328

Mycgr3G41969\_Mycgr3T

Mycgr3G80635 Mycgr3T
  
Location: 29428-29821

Mycgr3G80635\_Mycgr3T

Mycgr3G41426 Mycgr3T
  
Location: 29921-35255

Mycgr3G41426\_Mycgr3T

Mycgr3G104337 Mycgr3
  
Location: 35355-36108

Mycgr3G104337\_Mycgr3

Mycgr3G71679 Mycgr3T
  
Location: 36208-37300

Mycgr3G71679\_Mycgr3T

Mycgr3G92938 Mycgr3T
  
Location: 37400-38699

Mycgr3G92938\_Mycgr3T

Mycgr3G92941 Mycgr3T
  
Location: 38799-40734

Mycgr3G92941\_Mycgr3T

acetolactate synthase
  
Accession: EFX00095
  
Location: 1544756-1551620
  
 NCBI BlastP on this gene

EFX00095

hypothetical protein
  
Accession: EFX00586
  
Location: 1554180-1555976
  
 NCBI BlastP on this gene

EFX00586

hypothetical protein
  
Accession: EFX00683
  
Location: 1556620-1556994
  
 NCBI BlastP on this gene

EFX00683

hypothetical protein
  
Accession: EFX00457
  
Location: 1557139-1557750
  
 NCBI BlastP on this gene

EFX00457

umta methyltransferase family protein
  
Accession: EFX00489
  
Location: 1559231-1560795
  
 NCBI BlastP on this gene

EFX00489

hypothetical protein
  
Accession: EFX00224
  
Location: 1561184-1562421
  
 NCBI BlastP on this gene

EFX00224

duf341 domain containing protein
  
Accession: EFX00520
  
Location: 1564325-1565346
  
  
**BlastP hit with Mycgr3G104337\_Mycgr3**
  
Percentage identity: 47 %
  
BlastP bit score: 214
  
Sequence coverage: 92 %
  
E-value: 1e-64
  
  
 NCBI BlastP on this gene

EFX00520

polyketide synthase
  
Accession: EFX00060
  
Location: 1566165-1573137
  
  
**BlastP hit with Mycgr3G100089\_Mycgr3**
  
Percentage identity: 45 %
  
BlastP bit score: 1880
  
Sequence coverage: 101 %
  
E-value: 0.0
  
  
 NCBI BlastP on this gene

EFX00060

hypothetical protein
  
Accession: EFX00687
  
Location: 1576602-1577205
  
 NCBI BlastP on this gene

EFX00687

hypothetical protein
  
Accession: EFX00732
  
Location: 1580879-1581964
  
 NCBI BlastP on this gene

EFX00732

extradiol ring-cleavage class 3 subunit b
  
Accession: EFX00625
  
Location: 1583364-1584362
  
 NCBI BlastP on this gene

EFX00625

hypothetical protein
  
Accession: EFX00254
  
Location: 1586167-1587262
  
 NCBI BlastP on this gene

EFX00254

integral membrane protein
  
Accession: EFX00537
  
Location: 1589167-1590542
  
 NCBI BlastP on this gene

EFX00537

Query: Architecture Search FASTA input

KE145354 : Glarea lozoyensis ATCC 20868 chromosome Unknown GLAREA11    Total score: 2.0     Cumulative Blast bit score: 2083

Hit cluster cross-links:

Mycgr3G85918 Mycgr3T
  
Location: 0-1602

Mycgr3G85918\_Mycgr3T

Mycgr3G42010 Mycgr3T
  
Location: 1702-8569

Mycgr3G42010\_Mycgr3T

Mycgr3G29582 Mycgr3T
  
Location: 8669-8915

Mycgr3G29582\_Mycgr3T

Mycgr3G31170 Mycgr3T
  
Location: 9015-9255

Mycgr3G31170\_Mycgr3T

Mycgr3G85924 Mycgr3T
  
Location: 9355-11218

Mycgr3G85924\_Mycgr3T

Mycgr3G71676 Mycgr3T
  
Location: 11318-12494

Mycgr3G71676\_Mycgr3T

Mycgr3G11468 Mycgr3T
  
Location: 12594-13653

Mycgr3G11468\_Mycgr3T

Mycgr3G58567 Mycgr3T
  
Location: 13753-14506

Mycgr3G58567\_Mycgr3T

Mycgr3G100089 Mycgr3
  
Location: 14606-21152

Mycgr3G100089\_Mycgr3

Mycgr3G42698 Mycgr3T
  
Location: 21252-22131

Mycgr3G42698\_Mycgr3T

Mycgr3G71681 Mycgr3T
  
Location: 22231-23461

Mycgr3G71681\_Mycgr3T

Mycgr3G109328 Mycgr3
  
Location: 23561-24239

Mycgr3G109328\_Mycgr3

Mycgr3G104334 Mycgr3
  
Location: 24339-24567

Mycgr3G104334\_Mycgr3

Mycgr3G42715 Mycgr3T
  
Location: 24667-25981

Mycgr3G42715\_Mycgr3T

Mycgr3G92934 Mycgr3T
  
Location: 26081-27593

Mycgr3G92934\_Mycgr3T

Mycgr3G41969 Mycgr3T
  
Location: 27693-29328

Mycgr3G41969\_Mycgr3T

Mycgr3G80635 Mycgr3T
  
Location: 29428-29821

Mycgr3G80635\_Mycgr3T

Mycgr3G41426 Mycgr3T
  
Location: 29921-35255

Mycgr3G41426\_Mycgr3T

Mycgr3G104337 Mycgr3
  
Location: 35355-36108

Mycgr3G104337\_Mycgr3

Mycgr3G71679 Mycgr3T
  
Location: 36208-37300

Mycgr3G71679\_Mycgr3T

Mycgr3G92938 Mycgr3T
  
Location: 37400-38699

Mycgr3G92938\_Mycgr3T

Mycgr3G92941 Mycgr3T
  
Location: 38799-40734

Mycgr3G92941\_Mycgr3T

Protein kinase-like (PK-like)
  
Accession: EPE35645
  
Location: 1401316-1403213
  
 NCBI BlastP on this gene

EPE35645

hypothetical protein
  
Accession: EPE35646
  
Location: 1406600-1407383
  
 NCBI BlastP on this gene

EPE35646

hypothetical protein
  
Accession: EPE35647
  
Location: 1410330-1411220
  
 NCBI BlastP on this gene

EPE35647

P-loop containing nucleoside triphosphate hydrolase
  
Accession: EPE35648
  
Location: 1411713-1416535
  
 NCBI BlastP on this gene

EPE35648

hypothetical protein
  
Accession: EPE35649
  
Location: 1417685-1418671
  
  
**BlastP hit with Mycgr3G104337\_Mycgr3**
  
Percentage identity: 39 %
  
BlastP bit score: 210
  
Sequence coverage: 109 %
  
E-value: 2e-63
  
  
 NCBI BlastP on this gene

EPE35649

Thiolase-like protein
  
Accession: EPE35650
  
Location: 1420170-1427192
  
  
**BlastP hit with Mycgr3G100089\_Mycgr3**
  
Percentage identity: 44 %
  
BlastP bit score: 1873
  
Sequence coverage: 100 %
  
E-value: 0.0
  
  
 NCBI BlastP on this gene

EPE35650

NAD kinase
  
Accession: EPE35651
  
Location: 1427895-1429196
  
 NCBI BlastP on this gene

EPE35651

Glycerol-3-phosphate (1)-acyltransferase
  
Accession: EPE35652
  
Location: 1429820-1431217
  
 NCBI BlastP on this gene

EPE35652

hypothetical protein
  
Accession: EPE35653
  
Location: 1432866-1434359
  
 NCBI BlastP on this gene

EPE35653

Protein kinase-like (PK-like)
  
Accession: EPE35654
  
Location: 1434599-1439263
  
 NCBI BlastP on this gene

EPE35654

Cytochrome P450
  
Accession: EPE35655
  
Location: 1440949-1442646
  
 NCBI BlastP on this gene

EPE35655

ClpP/crotonase
  
Accession: EPE35656
  
Location: 1444351-1445742
  
 NCBI BlastP on this gene

EPE35656

Query: Architecture Search FASTA input

KB725947 : Colletotrichum orbiculare MAFF 240422 unplaced genomic scaffold Scaffold\_381    Total score: 2.0     Cumulative Blast bit score: 2078

Hit cluster cross-links:

Mycgr3G85918 Mycgr3T
  
Location: 0-1602

Mycgr3G85918\_Mycgr3T

Mycgr3G42010 Mycgr3T
  
Location: 1702-8569

Mycgr3G42010\_Mycgr3T

Mycgr3G29582 Mycgr3T
  
Location: 8669-8915

Mycgr3G29582\_Mycgr3T

Mycgr3G31170 Mycgr3T
  
Location: 9015-9255

Mycgr3G31170\_Mycgr3T

Mycgr3G85924 Mycgr3T
  
Location: 9355-11218

Mycgr3G85924\_Mycgr3T

Mycgr3G71676 Mycgr3T
  
Location: 11318-12494

Mycgr3G71676\_Mycgr3T

Mycgr3G11468 Mycgr3T
  
Location: 12594-13653

Mycgr3G11468\_Mycgr3T

Mycgr3G58567 Mycgr3T
  
Location: 13753-14506

Mycgr3G58567\_Mycgr3T

Mycgr3G100089 Mycgr3
  
Location: 14606-21152

Mycgr3G100089\_Mycgr3

Mycgr3G42698 Mycgr3T
  
Location: 21252-22131

Mycgr3G42698\_Mycgr3T

Mycgr3G71681 Mycgr3T
  
Location: 22231-23461

Mycgr3G71681\_Mycgr3T

Mycgr3G109328 Mycgr3
  
Location: 23561-24239

Mycgr3G109328\_Mycgr3

Mycgr3G104334 Mycgr3
  
Location: 24339-24567

Mycgr3G104334\_Mycgr3

Mycgr3G42715 Mycgr3T
  
Location: 24667-25981

Mycgr3G42715\_Mycgr3T

Mycgr3G92934 Mycgr3T
  
Location: 26081-27593

Mycgr3G92934\_Mycgr3T

Mycgr3G41969 Mycgr3T
  
Location: 27693-29328

Mycgr3G41969\_Mycgr3T

Mycgr3G80635 Mycgr3T
  
Location: 29428-29821

Mycgr3G80635\_Mycgr3T

Mycgr3G41426 Mycgr3T
  
Location: 29921-35255

Mycgr3G41426\_Mycgr3T

Mycgr3G104337 Mycgr3
  
Location: 35355-36108

Mycgr3G104337\_Mycgr3

Mycgr3G71679 Mycgr3T
  
Location: 36208-37300

Mycgr3G71679\_Mycgr3T

Mycgr3G92938 Mycgr3T
  
Location: 37400-38699

Mycgr3G92938\_Mycgr3T

Mycgr3G92941 Mycgr3T
  
Location: 38799-40734

Mycgr3G92941\_Mycgr3T

choline oxidase
  
Accession: ENH82084
  
Location: 1857655-1859434
  
 NCBI BlastP on this gene

ENH82084

fungal specific transcription factor
  
Accession: ENH82085
  
Location: 1861775-1864309
  
 NCBI BlastP on this gene

ENH82085

short-chain dehydrogenase
  
Accession: ENH82086
  
Location: 1866487-1868562
  
 NCBI BlastP on this gene

ENH82086

hypothetical protein
  
Accession: ENH82087
  
Location: 1869225-1870116
  
 NCBI BlastP on this gene

ENH82087

hypothetical protein
  
Accession: ENH82088
  
Location: 1870973-1874533
  
 NCBI BlastP on this gene

ENH82088

polyketide synthase
  
Accession: ENH82089
  
Location: 1875690-1882772
  
  
**BlastP hit with Mycgr3G100089\_Mycgr3**
  
Percentage identity: 44 %
  
BlastP bit score: 1868
  
Sequence coverage: 101 %
  
E-value: 0.0
  
  
 NCBI BlastP on this gene

ENH82089

duf341 family
  
Accession: ENH82090
  
Location: 1887558-1888556
  
  
**BlastP hit with Mycgr3G104337\_Mycgr3**
  
Percentage identity: 42 %
  
BlastP bit score: 210
  
Sequence coverage: 99 %
  
E-value: 1e-63
  
  
 NCBI BlastP on this gene

ENH82090

ABC multidrug transporter mdr1
  
Accession: ENH82091
  
Location: 1891297-1896167
  
 NCBI BlastP on this gene

ENH82091

cytochrome b5 type b
  
Accession: ENH82092
  
Location: 1896728-1898824
  
 NCBI BlastP on this gene

ENH82092

sulfite reductase subunit alpha
  
Accession: ENH82093
  
Location: 1898864-1901793
  
 NCBI BlastP on this gene

ENH82093

lipase b precursor
  
Accession: ENH82094
  
Location: 1904568-1905833
  
 NCBI BlastP on this gene

ENH82094

Query: Architecture Search FASTA input

KB707707 : Botryotinia fuckeliana BcDW1 unplaced genomic scaffold Scaffold\_35    Total score: 2.0     Cumulative Blast bit score: 2077

Hit cluster cross-links:

Mycgr3G85918 Mycgr3T
  
Location: 0-1602

Mycgr3G85918\_Mycgr3T

Mycgr3G42010 Mycgr3T
  
Location: 1702-8569

Mycgr3G42010\_Mycgr3T

Mycgr3G29582 Mycgr3T
  
Location: 8669-8915

Mycgr3G29582\_Mycgr3T

Mycgr3G31170 Mycgr3T
  
Location: 9015-9255

Mycgr3G31170\_Mycgr3T

Mycgr3G85924 Mycgr3T
  
Location: 9355-11218

Mycgr3G85924\_Mycgr3T

Mycgr3G71676 Mycgr3T
  
Location: 11318-12494

Mycgr3G71676\_Mycgr3T

Mycgr3G11468 Mycgr3T
  
Location: 12594-13653

Mycgr3G11468\_Mycgr3T

Mycgr3G58567 Mycgr3T
  
Location: 13753-14506

Mycgr3G58567\_Mycgr3T

Mycgr3G100089 Mycgr3
  
Location: 14606-21152

Mycgr3G100089\_Mycgr3

Mycgr3G42698 Mycgr3T
  
Location: 21252-22131

Mycgr3G42698\_Mycgr3T

Mycgr3G71681 Mycgr3T
  
Location: 22231-23461

Mycgr3G71681\_Mycgr3T

Mycgr3G109328 Mycgr3
  
Location: 23561-24239

Mycgr3G109328\_Mycgr3

Mycgr3G104334 Mycgr3
  
Location: 24339-24567

Mycgr3G104334\_Mycgr3

Mycgr3G42715 Mycgr3T
  
Location: 24667-25981

Mycgr3G42715\_Mycgr3T

Mycgr3G92934 Mycgr3T
  
Location: 26081-27593

Mycgr3G92934\_Mycgr3T

Mycgr3G41969 Mycgr3T
  
Location: 27693-29328

Mycgr3G41969\_Mycgr3T

Mycgr3G80635 Mycgr3T
  
Location: 29428-29821

Mycgr3G80635\_Mycgr3T

Mycgr3G41426 Mycgr3T
  
Location: 29921-35255

Mycgr3G41426\_Mycgr3T

Mycgr3G104337 Mycgr3
  
Location: 35355-36108

Mycgr3G104337\_Mycgr3

Mycgr3G71679 Mycgr3T
  
Location: 36208-37300

Mycgr3G71679\_Mycgr3T

Mycgr3G92938 Mycgr3T
  
Location: 37400-38699

Mycgr3G92938\_Mycgr3T

Mycgr3G92941 Mycgr3T
  
Location: 38799-40734

Mycgr3G92941\_Mycgr3T

putative calmodulin protein
  
Accession: EMR90205
  
Location: 75660-76690
  
 NCBI BlastP on this gene

EMR90205

putative snare domain-containing protein
  
Accession: EMR90206
  
Location: 77100-78287
  
 NCBI BlastP on this gene

EMR90206

putative ser thr protein phosphatase family protein
  
Accession: EMR90207
  
Location: 80319-82062
  
 NCBI BlastP on this gene

EMR90207

putative nucleoside transporter family protein
  
Accession: EMR90208
  
Location: 84425-85893
  
 NCBI BlastP on this gene

EMR90208

putative transcription factor c2h2 protein
  
Accession: EMR90209
  
Location: 87689-91382
  
 NCBI BlastP on this gene

EMR90209

putative nfx1-type zinc finger-containing protein 1 protein
  
Accession: EMR90210
  
Location: 92388-100652
  
  
**BlastP hit with Mycgr3G42010\_Mycgr3T**
  
Percentage identity: 44 %
  
BlastP bit score: 1867
  
Sequence coverage: 103 %
  
E-value: 0.0
  
  
 NCBI BlastP on this gene

EMR90210

putative geranylgeranyl pyrophosphate synthetase protein
  
Accession: EMR90211
  
Location: 101324-102691
  
  
**BlastP hit with Mycgr3G92938\_Mycgr3T**
  
Percentage identity: 39 %
  
BlastP bit score: 210
  
Sequence coverage: 69 %
  
E-value: 2e-59
  
  
 NCBI BlastP on this gene

EMR90211

putative hexose transporter protein
  
Accession: EMR90212
  
Location: 103116-104914
  
 NCBI BlastP on this gene

EMR90212

putative glycoside hydrolase subgroup catalytic core protein
  
Accession: EMR90213
  
Location: 105592-107370
  
 NCBI BlastP on this gene

EMR90213

putative cytochrome p450 monooxygenase protein
  
Accession: EMR90214
  
Location: 108784-110154
  
 NCBI BlastP on this gene

EMR90214

hypothetical protein
  
Accession: EMR90215
  
Location: 110510-110767
  
 NCBI BlastP on this gene

EMR90215

hypothetical protein
  
Accession: EMR90216
  
Location: 111032-111469
  
 NCBI BlastP on this gene

EMR90216

putative maltose permease mal61 protein
  
Accession: EMR90217
  
Location: 112262-114044
  
 NCBI BlastP on this gene

EMR90217

putative glycoside hydrolase family 78 sequence protein
  
Accession: EMR90218
  
Location: 115006-117480
  
 NCBI BlastP on this gene

EMR90218

hypothetical protein
  
Accession: EMR90219
  
Location: 118089-119038
  
 NCBI BlastP on this gene

EMR90219

hypothetical protein
  
Accession: EMR90220
  
Location: 120173-121050
  
 NCBI BlastP on this gene

EMR90220

Query: Architecture Search FASTA input

CAGA01000033 : Claviceps purpurea 20.1    Total score: 2.0     Cumulative Blast bit score: 2075

Hit cluster cross-links:

Mycgr3G85918 Mycgr3T
  
Location: 0-1602

Mycgr3G85918\_Mycgr3T

Mycgr3G42010 Mycgr3T
  
Location: 1702-8569

Mycgr3G42010\_Mycgr3T

Mycgr3G29582 Mycgr3T
  
Location: 8669-8915

Mycgr3G29582\_Mycgr3T

Mycgr3G31170 Mycgr3T
  
Location: 9015-9255

Mycgr3G31170\_Mycgr3T

Mycgr3G85924 Mycgr3T
  
Location: 9355-11218

Mycgr3G85924\_Mycgr3T

Mycgr3G71676 Mycgr3T
  
Location: 11318-12494

Mycgr3G71676\_Mycgr3T

Mycgr3G11468 Mycgr3T
  
Location: 12594-13653

Mycgr3G11468\_Mycgr3T

Mycgr3G58567 Mycgr3T
  
Location: 13753-14506

Mycgr3G58567\_Mycgr3T

Mycgr3G100089 Mycgr3
  
Location: 14606-21152

Mycgr3G100089\_Mycgr3

Mycgr3G42698 Mycgr3T
  
Location: 21252-22131

Mycgr3G42698\_Mycgr3T

Mycgr3G71681 Mycgr3T
  
Location: 22231-23461

Mycgr3G71681\_Mycgr3T

Mycgr3G109328 Mycgr3
  
Location: 23561-24239

Mycgr3G109328\_Mycgr3

Mycgr3G104334 Mycgr3
  
Location: 24339-24567

Mycgr3G104334\_Mycgr3

Mycgr3G42715 Mycgr3T
  
Location: 24667-25981

Mycgr3G42715\_Mycgr3T

Mycgr3G92934 Mycgr3T
  
Location: 26081-27593

Mycgr3G92934\_Mycgr3T

Mycgr3G41969 Mycgr3T
  
Location: 27693-29328

Mycgr3G41969\_Mycgr3T

Mycgr3G80635 Mycgr3T
  
Location: 29428-29821

Mycgr3G80635\_Mycgr3T

Mycgr3G41426 Mycgr3T
  
Location: 29921-35255

Mycgr3G41426\_Mycgr3T

Mycgr3G104337 Mycgr3
  
Location: 35355-36108

Mycgr3G104337\_Mycgr3

Mycgr3G71679 Mycgr3T
  
Location: 36208-37300

Mycgr3G71679\_Mycgr3T

Mycgr3G92938 Mycgr3T
  
Location: 37400-38699

Mycgr3G92938\_Mycgr3T

Mycgr3G92941 Mycgr3T
  
Location: 38799-40734

Mycgr3G92941\_Mycgr3T

uncharacterized protein
  
Accession: CCE31711
  
Location: 259105-260046
  
 NCBI BlastP on this gene

CCE31711

related to YER185w, Rta1p
  
Accession: CCE31712
  
Location: 265670-266742
  
 NCBI BlastP on this gene

CCE31712

probable aspartyl aminopeptidase
  
Accession: CCE31713
  
Location: 267374-268910
  
 NCBI BlastP on this gene

CCE31713

uncharacterized protein
  
Accession: CCE31714
  
Location: 270057-271052
  
 NCBI BlastP on this gene

CCE31714

uncharacterized protein
  
Accession: CCE31715
  
Location: 273940-275033
  
  
**BlastP hit with Mycgr3G104337\_Mycgr3**
  
Percentage identity: 41 %
  
BlastP bit score: 204
  
Sequence coverage: 101 %
  
E-value: 1e-60
  
  
 NCBI BlastP on this gene

CCE31715

related to polyketide synthase
  
Accession: CCE31716
  
Location: 277091-283978
  
  
**BlastP hit with Mycgr3G100089\_Mycgr3**
  
Percentage identity: 44 %
  
BlastP bit score: 1871
  
Sequence coverage: 100 %
  
E-value: 0.0
  
  
 NCBI BlastP on this gene

CCE31716

uncharacterized protein
  
Accession: CCE31717
  
Location: 287276-292937
  
 NCBI BlastP on this gene

CCE31717

Query: Architecture Search FASTA input

KB730345 : Fusarium oxysporum f. sp. cubense race 1 unplaced genomic scaffold scaffold299    Total score: 2.0     Cumulative Blast bit score: 2068

Hit cluster cross-links:

Mycgr3G85918 Mycgr3T
  
Location: 0-1602

Mycgr3G85918\_Mycgr3T

Mycgr3G42010 Mycgr3T
  
Location: 1702-8569

Mycgr3G42010\_Mycgr3T

Mycgr3G29582 Mycgr3T
  
Location: 8669-8915

Mycgr3G29582\_Mycgr3T

Mycgr3G31170 Mycgr3T
  
Location: 9015-9255

Mycgr3G31170\_Mycgr3T

Mycgr3G85924 Mycgr3T
  
Location: 9355-11218

Mycgr3G85924\_Mycgr3T

Mycgr3G71676 Mycgr3T
  
Location: 11318-12494

Mycgr3G71676\_Mycgr3T

Mycgr3G11468 Mycgr3T
  
Location: 12594-13653

Mycgr3G11468\_Mycgr3T

Mycgr3G58567 Mycgr3T
  
Location: 13753-14506

Mycgr3G58567\_Mycgr3T

Mycgr3G100089 Mycgr3
  
Location: 14606-21152

Mycgr3G100089\_Mycgr3

Mycgr3G42698 Mycgr3T
  
Location: 21252-22131

Mycgr3G42698\_Mycgr3T

Mycgr3G71681 Mycgr3T
  
Location: 22231-23461

Mycgr3G71681\_Mycgr3T

Mycgr3G109328 Mycgr3
  
Location: 23561-24239

Mycgr3G109328\_Mycgr3

Mycgr3G104334 Mycgr3
  
Location: 24339-24567

Mycgr3G104334\_Mycgr3

Mycgr3G42715 Mycgr3T
  
Location: 24667-25981

Mycgr3G42715\_Mycgr3T

Mycgr3G92934 Mycgr3T
  
Location: 26081-27593

Mycgr3G92934\_Mycgr3T

Mycgr3G41969 Mycgr3T
  
Location: 27693-29328

Mycgr3G41969\_Mycgr3T

Mycgr3G80635 Mycgr3T
  
Location: 29428-29821

Mycgr3G80635\_Mycgr3T

Mycgr3G41426 Mycgr3T
  
Location: 29921-35255

Mycgr3G41426\_Mycgr3T

Mycgr3G104337 Mycgr3
  
Location: 35355-36108

Mycgr3G104337\_Mycgr3

Mycgr3G71679 Mycgr3T
  
Location: 36208-37300

Mycgr3G71679\_Mycgr3T

Mycgr3G92938 Mycgr3T
  
Location: 37400-38699

Mycgr3G92938\_Mycgr3T

Mycgr3G92941 Mycgr3T
  
Location: 38799-40734

Mycgr3G92941\_Mycgr3T

hypothetical protein
  
Accession: ENH66882
  
Location: 420859-423235
  
 NCBI BlastP on this gene

ENH66882

hypothetical protein
  
Accession: ENH66883
  
Location: 423814-425323
  
 NCBI BlastP on this gene

ENH66883

hypothetical protein
  
Accession: ENH66884
  
Location: 425765-428520
  
 NCBI BlastP on this gene

ENH66884

Putative protein yjlB
  
Accession: ENH66885
  
Location: 431459-431992
  
 NCBI BlastP on this gene

ENH66885

Stabilin-2
  
Accession: ENH66886
  
Location: 432156-433424
  
 NCBI BlastP on this gene

ENH66886

Cyclin CCL1
  
Accession: ENH66887
  
Location: 434416-435505
  
 NCBI BlastP on this gene

ENH66887

hypothetical protein
  
Accession: ENH66888
  
Location: 435752-437059
  
 NCBI BlastP on this gene

ENH66888

hypothetical protein
  
Accession: ENH66889
  
Location: 439433-440790
  
  
**BlastP hit with Mycgr3G92938\_Mycgr3T**
  
Percentage identity: 37 %
  
BlastP bit score: 230
  
Sequence coverage: 80 %
  
E-value: 1e-66
  
  
 NCBI BlastP on this gene

ENH66889

NFX1-type zinc finger-containing protein 1
  
Accession: ENH66890
  
Location: 441586-448935
  
  
**BlastP hit with Mycgr3G42010\_Mycgr3T**
  
Percentage identity: 44 %
  
BlastP bit score: 1838
  
Sequence coverage: 100 %
  
E-value: 0.0
  
  
 NCBI BlastP on this gene

ENH66890

hypothetical protein
  
Accession: ENH66891
  
Location: 449827-450873
  
 NCBI BlastP on this gene

ENH66891

hypothetical protein
  
Accession: ENH66892
  
Location: 452230-453519
  
 NCBI BlastP on this gene

ENH66892

hypothetical protein
  
Accession: ENH66893
  
Location: 454485-454673
  
 NCBI BlastP on this gene

ENH66893

hypothetical protein
  
Accession: ENH66894
  
Location: 455795-457366
  
 NCBI BlastP on this gene

ENH66894

hypothetical protein
  
Accession: ENH66895
  
Location: 458284-458634
  
 NCBI BlastP on this gene

ENH66895

hypothetical protein
  
Accession: ENH66896
  
Location: 458861-460417
  
 NCBI BlastP on this gene

ENH66896

hypothetical protein
  
Accession: ENH66897
  
Location: 463637-465202
  
 NCBI BlastP on this gene

ENH66897

Killer toxin subunits alpha/beta
  
Accession: ENH66898
  
Location: 465455-468361
  
 NCBI BlastP on this gene

ENH66898

Query: Architecture Search FASTA input

CABT02000029 : Sordaria macrospora k-hell    Total score: 2.0     Cumulative Blast bit score: 2068

Hit cluster cross-links:

Mycgr3G85918 Mycgr3T
  
Location: 0-1602

Mycgr3G85918\_Mycgr3T

Mycgr3G42010 Mycgr3T
  
Location: 1702-8569

Mycgr3G42010\_Mycgr3T

Mycgr3G29582 Mycgr3T
  
Location: 8669-8915

Mycgr3G29582\_Mycgr3T

Mycgr3G31170 Mycgr3T
  
Location: 9015-9255

Mycgr3G31170\_Mycgr3T

Mycgr3G85924 Mycgr3T
  
Location: 9355-11218

Mycgr3G85924\_Mycgr3T

Mycgr3G71676 Mycgr3T
  
Location: 11318-12494

Mycgr3G71676\_Mycgr3T

Mycgr3G11468 Mycgr3T
  
Location: 12594-13653

Mycgr3G11468\_Mycgr3T

Mycgr3G58567 Mycgr3T
  
Location: 13753-14506

Mycgr3G58567\_Mycgr3T

Mycgr3G100089 Mycgr3
  
Location: 14606-21152

Mycgr3G100089\_Mycgr3

Mycgr3G42698 Mycgr3T
  
Location: 21252-22131

Mycgr3G42698\_Mycgr3T

Mycgr3G71681 Mycgr3T
  
Location: 22231-23461

Mycgr3G71681\_Mycgr3T

Mycgr3G109328 Mycgr3
  
Location: 23561-24239

Mycgr3G109328\_Mycgr3

Mycgr3G104334 Mycgr3
  
Location: 24339-24567

Mycgr3G104334\_Mycgr3

Mycgr3G42715 Mycgr3T
  
Location: 24667-25981

Mycgr3G42715\_Mycgr3T

Mycgr3G92934 Mycgr3T
  
Location: 26081-27593

Mycgr3G92934\_Mycgr3T

Mycgr3G41969 Mycgr3T
  
Location: 27693-29328

Mycgr3G41969\_Mycgr3T

Mycgr3G80635 Mycgr3T
  
Location: 29428-29821

Mycgr3G80635\_Mycgr3T

Mycgr3G41426 Mycgr3T
  
Location: 29921-35255

Mycgr3G41426\_Mycgr3T

Mycgr3G104337 Mycgr3
  
Location: 35355-36108

Mycgr3G104337\_Mycgr3

Mycgr3G71679 Mycgr3T
  
Location: 36208-37300

Mycgr3G71679\_Mycgr3T

Mycgr3G92938 Mycgr3T
  
Location: 37400-38699

Mycgr3G92938\_Mycgr3T

Mycgr3G92941 Mycgr3T
  
Location: 38799-40734

Mycgr3G92941\_Mycgr3T

not annotated
  
Accession: CCC12635
  
Location: 392935-394218
  
 NCBI BlastP on this gene

CCC12635

not annotated
  
Accession: CCC12636
  
Location: 395325-396472
  
 NCBI BlastP on this gene

CCC12636

not annotated
  
Accession: CCC12637
  
Location: 396641-399317
  
 NCBI BlastP on this gene

CCC12637

not annotated
  
Accession: CCC12638
  
Location: 400001-401041
  
 NCBI BlastP on this gene

CCC12638

not annotated
  
Accession: CCC12639
  
Location: 401251-401771
  
 NCBI BlastP on this gene

CCC12639

not annotated
  
Accession: CCC12640
  
Location: 404183-408198
  
 NCBI BlastP on this gene

CCC12640

not annotated
  
Accession: CCC12641
  
Location: 408670-409329
  
 NCBI BlastP on this gene

CCC12641

not annotated
  
Accession: CCC12642
  
Location: 411007-411966
  
  
**BlastP hit with Mycgr3G104337\_Mycgr3**
  
Percentage identity: 41 %
  
BlastP bit score: 216
  
Sequence coverage: 100 %
  
E-value: 4e-66
  
  
 NCBI BlastP on this gene

CCC12642

not annotated
  
Accession: CCC12643
  
Location: 414023-421384
  
  
**BlastP hit with Mycgr3G100089\_Mycgr3**
  
Percentage identity: 44 %
  
BlastP bit score: 1852
  
Sequence coverage: 100 %
  
E-value: 0.0
  
  
 NCBI BlastP on this gene

CCC12643

Query: Architecture Search FASTA input

GL985061 : Trichoderma reesei QM6a unplaced genomic scaffold TRIREscaffold\_6    Total score: 2.0     Cumulative Blast bit score: 2067

Hit cluster cross-links:

Mycgr3G85918 Mycgr3T
  
Location: 0-1602

Mycgr3G85918\_Mycgr3T

Mycgr3G42010 Mycgr3T
  
Location: 1702-8569

Mycgr3G42010\_Mycgr3T

Mycgr3G29582 Mycgr3T
  
Location: 8669-8915

Mycgr3G29582\_Mycgr3T

Mycgr3G31170 Mycgr3T
  
Location: 9015-9255

Mycgr3G31170\_Mycgr3T

Mycgr3G85924 Mycgr3T
  
Location: 9355-11218

Mycgr3G85924\_Mycgr3T

Mycgr3G71676 Mycgr3T
  
Location: 11318-12494

Mycgr3G71676\_Mycgr3T

Mycgr3G11468 Mycgr3T
  
Location: 12594-13653

Mycgr3G11468\_Mycgr3T

Mycgr3G58567 Mycgr3T
  
Location: 13753-14506

Mycgr3G58567\_Mycgr3T

Mycgr3G100089 Mycgr3
  
Location: 14606-21152

Mycgr3G100089\_Mycgr3

Mycgr3G42698 Mycgr3T
  
Location: 21252-22131

Mycgr3G42698\_Mycgr3T

Mycgr3G71681 Mycgr3T
  
Location: 22231-23461

Mycgr3G71681\_Mycgr3T

Mycgr3G109328 Mycgr3
  
Location: 23561-24239

Mycgr3G109328\_Mycgr3

Mycgr3G104334 Mycgr3
  
Location: 24339-24567

Mycgr3G104334\_Mycgr3

Mycgr3G42715 Mycgr3T
  
Location: 24667-25981

Mycgr3G42715\_Mycgr3T

Mycgr3G92934 Mycgr3T
  
Location: 26081-27593

Mycgr3G92934\_Mycgr3T

Mycgr3G41969 Mycgr3T
  
Location: 27693-29328

Mycgr3G41969\_Mycgr3T

Mycgr3G80635 Mycgr3T
  
Location: 29428-29821

Mycgr3G80635\_Mycgr3T

Mycgr3G41426 Mycgr3T
  
Location: 29921-35255

Mycgr3G41426\_Mycgr3T

Mycgr3G104337 Mycgr3
  
Location: 35355-36108

Mycgr3G104337\_Mycgr3

Mycgr3G71679 Mycgr3T
  
Location: 36208-37300

Mycgr3G71679\_Mycgr3T

Mycgr3G92938 Mycgr3T
  
Location: 37400-38699

Mycgr3G92938\_Mycgr3T

Mycgr3G92941 Mycgr3T
  
Location: 38799-40734

Mycgr3G92941\_Mycgr3T

vesicular transport protein
  
Accession: EGR49750
  
Location: 467138-470596
  
 NCBI BlastP on this gene

EGR49750

predicted protein
  
Accession: EGR49941
  
Location: 472904-474067
  
 NCBI BlastP on this gene

EGR49941

glycosyltransferase family 1
  
Accession: EGR49942
  
Location: 484124-485739
  
 NCBI BlastP on this gene

EGR49942

hypothetical protein
  
Accession: EGR49943
  
Location: 486801-487766
  
  
**BlastP hit with Mycgr3G104337\_Mycgr3**
  
Percentage identity: 43 %
  
BlastP bit score: 206
  
Sequence coverage: 98 %
  
E-value: 8e-62
  
  
 NCBI BlastP on this gene

EGR49943

polyketide synthase
  
Accession: EGR49751
  
Location: 489709-496750
  
  
**BlastP hit with Mycgr3G100089\_Mycgr3**
  
Percentage identity: 45 %
  
BlastP bit score: 1861
  
Sequence coverage: 101 %
  
E-value: 0.0
  
  
 NCBI BlastP on this gene

EGR49751

predicted protein
  
Accession: EGR49752
  
Location: 500672-501274
  
 NCBI BlastP on this gene

EGR49752

predicted protein
  
Accession: EGR49753
  
Location: 502741-503205
  
 NCBI BlastP on this gene

EGR49753

predicted protein
  
Accession: EGR49944
  
Location: 504033-506795
  
 NCBI BlastP on this gene

EGR49944

glycoside hydrolase family 20
  
Accession: EGR49754
  
Location: 509949-512211
  
 NCBI BlastP on this gene

EGR49754

predicted protein
  
Accession: EGR49755
  
Location: 512920-513568
  
 NCBI BlastP on this gene

EGR49755

predicted protein
  
Accession: EGR49945
  
Location: 514286-516064
  
 NCBI BlastP on this gene

EGR49945

Query: Architecture Search FASTA input

GG697340 : Glomerella graminicola M1.001 genomic scaffold supercont1.10    Total score: 2.0     Cumulative Blast bit score: 2055

Hit cluster cross-links:

Mycgr3G85918 Mycgr3T
  
Location: 0-1602

Mycgr3G85918\_Mycgr3T

Mycgr3G42010 Mycgr3T
  
Location: 1702-8569

Mycgr3G42010\_Mycgr3T

Mycgr3G29582 Mycgr3T
  
Location: 8669-8915

Mycgr3G29582\_Mycgr3T

Mycgr3G31170 Mycgr3T
  
Location: 9015-9255

Mycgr3G31170\_Mycgr3T

Mycgr3G85924 Mycgr3T
  
Location: 9355-11218

Mycgr3G85924\_Mycgr3T

Mycgr3G71676 Mycgr3T
  
Location: 11318-12494

Mycgr3G71676\_Mycgr3T

Mycgr3G11468 Mycgr3T
  
Location: 12594-13653

Mycgr3G11468\_Mycgr3T

Mycgr3G58567 Mycgr3T
  
Location: 13753-14506

Mycgr3G58567\_Mycgr3T

Mycgr3G100089 Mycgr3
  
Location: 14606-21152

Mycgr3G100089\_Mycgr3

Mycgr3G42698 Mycgr3T
  
Location: 21252-22131

Mycgr3G42698\_Mycgr3T

Mycgr3G71681 Mycgr3T
  
Location: 22231-23461

Mycgr3G71681\_Mycgr3T

Mycgr3G109328 Mycgr3
  
Location: 23561-24239

Mycgr3G109328\_Mycgr3

Mycgr3G104334 Mycgr3
  
Location: 24339-24567

Mycgr3G104334\_Mycgr3

Mycgr3G42715 Mycgr3T
  
Location: 24667-25981

Mycgr3G42715\_Mycgr3T

Mycgr3G92934 Mycgr3T
  
Location: 26081-27593

Mycgr3G92934\_Mycgr3T

Mycgr3G41969 Mycgr3T
  
Location: 27693-29328

Mycgr3G41969\_Mycgr3T

Mycgr3G80635 Mycgr3T
  
Location: 29428-29821

Mycgr3G80635\_Mycgr3T

Mycgr3G41426 Mycgr3T
  
Location: 29921-35255

Mycgr3G41426\_Mycgr3T

Mycgr3G104337 Mycgr3
  
Location: 35355-36108

Mycgr3G104337\_Mycgr3

Mycgr3G71679 Mycgr3T
  
Location: 36208-37300

Mycgr3G71679\_Mycgr3T

Mycgr3G92938 Mycgr3T
  
Location: 37400-38699

Mycgr3G92938\_Mycgr3T

Mycgr3G92941 Mycgr3T
  
Location: 38799-40734

Mycgr3G92941\_Mycgr3T

hypothetical protein
  
Accession: EFQ28213
  
Location: 203918-207661
  
 NCBI BlastP on this gene

EFQ28213

GMC oxidoreductase
  
Accession: EFQ28214
  
Location: 210383-212149
  
 NCBI BlastP on this gene

EFQ28214

aldehyde dehydrogenase
  
Accession: EFQ28215
  
Location: 215183-216697
  
 NCBI BlastP on this gene

EFQ28215

beta-ketoacyl synthase domain-containing protein
  
Accession: EFQ28216
  
Location: 218621-225746
  
  
**BlastP hit with Mycgr3G100089\_Mycgr3**
  
Percentage identity: 44 %
  
BlastP bit score: 1851
  
Sequence coverage: 101 %
  
E-value: 0.0
  
  
 NCBI BlastP on this gene

EFQ28216

hypothetical protein
  
Accession: EFQ28217
  
Location: 230427-231402
  
  
**BlastP hit with Mycgr3G104337\_Mycgr3**
  
Percentage identity: 41 %
  
BlastP bit score: 204
  
Sequence coverage: 99 %
  
E-value: 3e-61
  
  
 NCBI BlastP on this gene

EFQ28217

ABC transporter
  
Accession: EFQ28218
  
Location: 233613-238526
  
 NCBI BlastP on this gene

EFQ28218

cytochrome b5-like Heme/Steroid binding domain-containing protein
  
Accession: EFQ28219
  
Location: 241391-246688
  
 NCBI BlastP on this gene

EFQ28219

Query: Architecture Search FASTA input

GL698722 : Metarhizium anisopliae ARSEF 23 unplaced genomic scaffold Scf\_012    Total score: 2.0     Cumulative Blast bit score: 2046

Hit cluster cross-links:

Mycgr3G85918 Mycgr3T
  
Location: 0-1602

Mycgr3G85918\_Mycgr3T

Mycgr3G42010 Mycgr3T
  
Location: 1702-8569

Mycgr3G42010\_Mycgr3T

Mycgr3G29582 Mycgr3T
  
Location: 8669-8915

Mycgr3G29582\_Mycgr3T

Mycgr3G31170 Mycgr3T
  
Location: 9015-9255

Mycgr3G31170\_Mycgr3T

Mycgr3G85924 Mycgr3T
  
Location: 9355-11218

Mycgr3G85924\_Mycgr3T

Mycgr3G71676 Mycgr3T
  
Location: 11318-12494

Mycgr3G71676\_Mycgr3T

Mycgr3G11468 Mycgr3T
  
Location: 12594-13653

Mycgr3G11468\_Mycgr3T

Mycgr3G58567 Mycgr3T
  
Location: 13753-14506

Mycgr3G58567\_Mycgr3T

Mycgr3G100089 Mycgr3
  
Location: 14606-21152

Mycgr3G100089\_Mycgr3

Mycgr3G42698 Mycgr3T
  
Location: 21252-22131

Mycgr3G42698\_Mycgr3T

Mycgr3G71681 Mycgr3T
  
Location: 22231-23461

Mycgr3G71681\_Mycgr3T

Mycgr3G109328 Mycgr3
  
Location: 23561-24239

Mycgr3G109328\_Mycgr3

Mycgr3G104334 Mycgr3
  
Location: 24339-24567

Mycgr3G104334\_Mycgr3

Mycgr3G42715 Mycgr3T
  
Location: 24667-25981

Mycgr3G42715\_Mycgr3T

Mycgr3G92934 Mycgr3T
  
Location: 26081-27593

Mycgr3G92934\_Mycgr3T

Mycgr3G41969 Mycgr3T
  
Location: 27693-29328

Mycgr3G41969\_Mycgr3T

Mycgr3G80635 Mycgr3T
  
Location: 29428-29821

Mycgr3G80635\_Mycgr3T

Mycgr3G41426 Mycgr3T
  
Location: 29921-35255

Mycgr3G41426\_Mycgr3T

Mycgr3G104337 Mycgr3
  
Location: 35355-36108

Mycgr3G104337\_Mycgr3

Mycgr3G71679 Mycgr3T
  
Location: 36208-37300

Mycgr3G71679\_Mycgr3T

Mycgr3G92938 Mycgr3T
  
Location: 37400-38699

Mycgr3G92938\_Mycgr3T

Mycgr3G92941 Mycgr3T
  
Location: 38799-40734

Mycgr3G92941\_Mycgr3T

thioesterase family protein
  
Accession: EFY97476
  
Location: 486292-486798
  
 NCBI BlastP on this gene

EFY97476

C6 transcription factor, putative
  
Accession: EFY97477
  
Location: 486925-488983
  
 NCBI BlastP on this gene

EFY97477

malate/L-lactate dehydrogenase
  
Accession: EFY97478
  
Location: 489437-490507
  
 NCBI BlastP on this gene

EFY97478

stress responsive A/B barrel domain protein
  
Accession: EFY97479
  
Location: 490997-491570
  
 NCBI BlastP on this gene

EFY97479

high affinity nickel transport protein nic1
  
Accession: EFY97480
  
Location: 492114-493385
  
 NCBI BlastP on this gene

EFY97480

Autophagy- protein 17
  
Accession: EFY97481
  
Location: 495875-497463
  
 NCBI BlastP on this gene

EFY97481

proteinase, putative
  
Accession: EFY97482
  
Location: 497891-499784
  
 NCBI BlastP on this gene

EFY97482

hypothetical protein
  
Accession: EFY97483
  
Location: 500744-502180
  
 NCBI BlastP on this gene

EFY97483

nonsense-mediated mRNA decay protein, putative
  
Accession: EFY97484
  
Location: 503322-510301
  
  
**BlastP hit with Mycgr3G42010\_Mycgr3T**
  
Percentage identity: 42 %
  
BlastP bit score: 1813
  
Sequence coverage: 101 %
  
E-value: 0.0
  
  
 NCBI BlastP on this gene

EFY97484

hypothetical protein
  
Accession: EFY97485
  
Location: 511091-512508
  
  
**BlastP hit with Mycgr3G92938\_Mycgr3T**
  
Percentage identity: 36 %
  
BlastP bit score: 233
  
Sequence coverage: 81 %
  
E-value: 1e-67
  
  
 NCBI BlastP on this gene

EFY97485

hypothetical protein
  
Accession: EFY97486
  
Location: 513879-514778
  
 NCBI BlastP on this gene

EFY97486

F-box domain containing protein
  
Accession: EFY97487
  
Location: 516980-519522
  
 NCBI BlastP on this gene

EFY97487

5-hydroxyisourate hydrolase
  
Accession: EFY97488
  
Location: 523614-524072
  
 NCBI BlastP on this gene

EFY97488

short-chain dehydrogenase, putative
  
Accession: EFY97489
  
Location: 524970-525989
  
 NCBI BlastP on this gene

EFY97489

major facilitator superfamily MFS 1
  
Accession: EFY97490
  
Location: 526786-528561
  
 NCBI BlastP on this gene

EFY97490

hypothetical protein
  
Accession: EFY97491
  
Location: 529344-529754
  
 NCBI BlastP on this gene

EFY97491

cytochrome P450, putative
  
Accession: EFY97492
  
Location: 530247-532446
  
 NCBI BlastP on this gene

EFY97492

Query: Architecture Search FASTA input

KB708021 : Botryotinia fuckeliana BcDW1 unplaced genomic scaffold Scaffold\_349    Total score: 2.0     Cumulative Blast bit score: 2042

Hit cluster cross-links:

Mycgr3G85918 Mycgr3T
  
Location: 0-1602

Mycgr3G85918\_Mycgr3T

Mycgr3G42010 Mycgr3T
  
Location: 1702-8569

Mycgr3G42010\_Mycgr3T

Mycgr3G29582 Mycgr3T
  
Location: 8669-8915

Mycgr3G29582\_Mycgr3T

Mycgr3G31170 Mycgr3T
  
Location: 9015-9255

Mycgr3G31170\_Mycgr3T

Mycgr3G85924 Mycgr3T
  
Location: 9355-11218

Mycgr3G85924\_Mycgr3T

Mycgr3G71676 Mycgr3T
  
Location: 11318-12494

Mycgr3G71676\_Mycgr3T

Mycgr3G11468 Mycgr3T
  
Location: 12594-13653

Mycgr3G11468\_Mycgr3T

Mycgr3G58567 Mycgr3T
  
Location: 13753-14506

Mycgr3G58567\_Mycgr3T

Mycgr3G100089 Mycgr3
  
Location: 14606-21152

Mycgr3G100089\_Mycgr3

Mycgr3G42698 Mycgr3T
  
Location: 21252-22131

Mycgr3G42698\_Mycgr3T

Mycgr3G71681 Mycgr3T
  
Location: 22231-23461

Mycgr3G71681\_Mycgr3T

Mycgr3G109328 Mycgr3
  
Location: 23561-24239

Mycgr3G109328\_Mycgr3

Mycgr3G104334 Mycgr3
  
Location: 24339-24567

Mycgr3G104334\_Mycgr3

Mycgr3G42715 Mycgr3T
  
Location: 24667-25981

Mycgr3G42715\_Mycgr3T

Mycgr3G92934 Mycgr3T
  
Location: 26081-27593

Mycgr3G92934\_Mycgr3T

Mycgr3G41969 Mycgr3T
  
Location: 27693-29328

Mycgr3G41969\_Mycgr3T

Mycgr3G80635 Mycgr3T
  
Location: 29428-29821

Mycgr3G80635\_Mycgr3T

Mycgr3G41426 Mycgr3T
  
Location: 29921-35255

Mycgr3G41426\_Mycgr3T

Mycgr3G104337 Mycgr3
  
Location: 35355-36108

Mycgr3G104337\_Mycgr3

Mycgr3G71679 Mycgr3T
  
Location: 36208-37300

Mycgr3G71679\_Mycgr3T

Mycgr3G92938 Mycgr3T
  
Location: 37400-38699

Mycgr3G92938\_Mycgr3T

Mycgr3G92941 Mycgr3T
  
Location: 38799-40734

Mycgr3G92941\_Mycgr3T

putative gmp synthase protein
  
Accession: EMR83048
  
Location: 192481-193329
  
 NCBI BlastP on this gene

EMR83048

putative glycoside hydrolase family 25 protein
  
Accession: EMR83049
  
Location: 194453-195580
  
 NCBI BlastP on this gene

EMR83049

putative gtp-binding protein gtr1 protein
  
Accession: EMR83050
  
Location: 196231-197592
  
 NCBI BlastP on this gene

EMR83050

putative het domain-containing protein
  
Accession: EMR83051
  
Location: 199126-200892
  
 NCBI BlastP on this gene

EMR83051

hypothetical protein
  
Accession: EMR83052
  
Location: 202536-203468
  
 NCBI BlastP on this gene

EMR83052

putative abc transporter protein
  
Accession: EMR83053
  
Location: 205230-209964
  
 NCBI BlastP on this gene

EMR83053

putative ef-hand calcium-binding domain protein
  
Accession: EMR83054
  
Location: 210979-211937
  
  
**BlastP hit with Mycgr3G104337\_Mycgr3**
  
Percentage identity: 45 %
  
BlastP bit score: 215
  
Sequence coverage: 95 %
  
E-value: 2e-65
  
  
 NCBI BlastP on this gene

EMR83054

putative polyketide synthase protein
  
Accession: EMR83055
  
Location: 213423-220647
  
  
**BlastP hit with Mycgr3G100089\_Mycgr3**
  
Percentage identity: 43 %
  
BlastP bit score: 1827
  
Sequence coverage: 100 %
  
E-value: 0.0
  
  
 NCBI BlastP on this gene

EMR83055

putative low temperature requirement a protein
  
Accession: EMR83056
  
Location: 221791-223614
  
 NCBI BlastP on this gene

EMR83056

putative kelch repeat-containing protein
  
Accession: EMR83057
  
Location: 224766-226046
  
 NCBI BlastP on this gene

EMR83057

Query: Architecture Search FASTA input

FQ790281 : Botryotinia fuckeliana T4 SuperContig\_330\_1 genomic supercontig.    Total score: 2.0     Cumulative Blast bit score: 2037

Hit cluster cross-links:

Mycgr3G85918 Mycgr3T
  
Location: 0-1602

Mycgr3G85918\_Mycgr3T

Mycgr3G42010 Mycgr3T
  
Location: 1702-8569

Mycgr3G42010\_Mycgr3T

Mycgr3G29582 Mycgr3T
  
Location: 8669-8915

Mycgr3G29582\_Mycgr3T

Mycgr3G31170 Mycgr3T
  
Location: 9015-9255

Mycgr3G31170\_Mycgr3T

Mycgr3G85924 Mycgr3T
  
Location: 9355-11218

Mycgr3G85924\_Mycgr3T

Mycgr3G71676 Mycgr3T
  
Location: 11318-12494

Mycgr3G71676\_Mycgr3T

Mycgr3G11468 Mycgr3T
  
Location: 12594-13653

Mycgr3G11468\_Mycgr3T

Mycgr3G58567 Mycgr3T
  
Location: 13753-14506

Mycgr3G58567\_Mycgr3T

Mycgr3G100089 Mycgr3
  
Location: 14606-21152

Mycgr3G100089\_Mycgr3

Mycgr3G42698 Mycgr3T
  
Location: 21252-22131

Mycgr3G42698\_Mycgr3T

Mycgr3G71681 Mycgr3T
  
Location: 22231-23461

Mycgr3G71681\_Mycgr3T

Mycgr3G109328 Mycgr3
  
Location: 23561-24239

Mycgr3G109328\_Mycgr3

Mycgr3G104334 Mycgr3
  
Location: 24339-24567

Mycgr3G104334\_Mycgr3

Mycgr3G42715 Mycgr3T
  
Location: 24667-25981

Mycgr3G42715\_Mycgr3T

Mycgr3G92934 Mycgr3T
  
Location: 26081-27593

Mycgr3G92934\_Mycgr3T

Mycgr3G41969 Mycgr3T
  
Location: 27693-29328

Mycgr3G41969\_Mycgr3T

Mycgr3G80635 Mycgr3T
  
Location: 29428-29821

Mycgr3G80635\_Mycgr3T

Mycgr3G41426 Mycgr3T
  
Location: 29921-35255

Mycgr3G41426\_Mycgr3T

Mycgr3G104337 Mycgr3
  
Location: 35355-36108

Mycgr3G104337\_Mycgr3

Mycgr3G71679 Mycgr3T
  
Location: 36208-37300

Mycgr3G71679\_Mycgr3T

Mycgr3G92938 Mycgr3T
  
Location: 37400-38699

Mycgr3G92938\_Mycgr3T

Mycgr3G92941 Mycgr3T
  
Location: 38799-40734

Mycgr3G92941\_Mycgr3T

similar to glutamine amidotransferase class-I
  
Accession: CCD46344
  
Location: 723734-724582
  
 NCBI BlastP on this gene

BofuT4\_P118940.1

glycoside hydrolase family 25 protein
  
Accession: CCD46345
  
Location: 725706-726833
  
 NCBI BlastP on this gene

BofuT4\_P118950.1

hypothetical protein
  
Accession: CCD46346
  
Location: 727484-728845
  
 NCBI BlastP on this gene

BofuT4\_P118960.1

hypothetical protein
  
Accession: CCD46347
  
Location: 729711-732145
  
 NCBI BlastP on this gene

BofuT4\_P118970.1

hypothetical protein
  
Accession: CDF43968
  
Location: 733789-734721
  
 NCBI BlastP on this gene

BofuT4P330000018001

similar to ABC transporter
  
Accession: CCD46349
  
Location: 736483-740451
  
 NCBI BlastP on this gene

BofuT4P330000028001

hypothetical protein
  
Accession: CCD46350
  
Location: 740511-741217
  
 NCBI BlastP on this gene

BofuT4P330000029001

similar to EF-hand calcium-binding domain protein
  
Accession: CCD46351
  
Location: 742232-743190
  
  
**BlastP hit with Mycgr3G104337\_Mycgr3**
  
Percentage identity: 45 %
  
BlastP bit score: 215
  
Sequence coverage: 95 %
  
E-value: 2e-65
  
  
 NCBI BlastP on this gene

BofuT4P330000030001

predicted protein
  
Accession: CCD46352
  
Location: 743951-744513
  
 NCBI BlastP on this gene

BofuT4P330000031001

BcPKS8, polyketide synthase
  
Accession: CCD46353
  
Location: 744676-751900
  
  
**BlastP hit with Mycgr3G100089\_Mycgr3**
  
Percentage identity: 43 %
  
BlastP bit score: 1823
  
Sequence coverage: 101 %
  
E-value: 0.0
  
  
 NCBI BlastP on this gene

BofuT4P330000032001

hypothetical protein
  
Accession: CCD46354
  
Location: 752966-755358
  
 NCBI BlastP on this gene

BofuT4P330000033001

similar to kelch repeat-containing protein
  
Accession: CCD46355
  
Location: 756019-757299
  
 NCBI BlastP on this gene

BofuT4\_P119050.1

hypothetical protein
  
Accession: CCD46356
  
Location: 757710-758181
  
 NCBI BlastP on this gene

BofuT4\_P119060.1

similar to transcription factor bHLH
  
Accession: CCD46357
  
Location: 761137-762777
  
 NCBI BlastP on this gene

BofuT4\_P119070.1

predicted protein
  
Accession: CCD46358
  
Location: 768459-768950
  
 NCBI BlastP on this gene

BofuT4\_uP119080.1

Query: Architecture Search FASTA input

GL385396 : Gaeumannomyces graminis var. tritici R3-111a-1 unplaced genomic scaffold supercont2.2    Total score: 2.0     Cumulative Blast bit score: 2032

Hit cluster cross-links:

Mycgr3G85918 Mycgr3T
  
Location: 0-1602

Mycgr3G85918\_Mycgr3T

Mycgr3G42010 Mycgr3T
  
Location: 1702-8569

Mycgr3G42010\_Mycgr3T

Mycgr3G29582 Mycgr3T
  
Location: 8669-8915

Mycgr3G29582\_Mycgr3T

Mycgr3G31170 Mycgr3T
  
Location: 9015-9255

Mycgr3G31170\_Mycgr3T

Mycgr3G85924 Mycgr3T
  
Location: 9355-11218

Mycgr3G85924\_Mycgr3T

Mycgr3G71676 Mycgr3T
  
Location: 11318-12494

Mycgr3G71676\_Mycgr3T

Mycgr3G11468 Mycgr3T
  
Location: 12594-13653

Mycgr3G11468\_Mycgr3T

Mycgr3G58567 Mycgr3T
  
Location: 13753-14506

Mycgr3G58567\_Mycgr3T

Mycgr3G100089 Mycgr3
  
Location: 14606-21152

Mycgr3G100089\_Mycgr3

Mycgr3G42698 Mycgr3T
  
Location: 21252-22131

Mycgr3G42698\_Mycgr3T

Mycgr3G71681 Mycgr3T
  
Location: 22231-23461

Mycgr3G71681\_Mycgr3T

Mycgr3G109328 Mycgr3
  
Location: 23561-24239

Mycgr3G109328\_Mycgr3

Mycgr3G104334 Mycgr3
  
Location: 24339-24567

Mycgr3G104334\_Mycgr3

Mycgr3G42715 Mycgr3T
  
Location: 24667-25981

Mycgr3G42715\_Mycgr3T

Mycgr3G92934 Mycgr3T
  
Location: 26081-27593

Mycgr3G92934\_Mycgr3T

Mycgr3G41969 Mycgr3T
  
Location: 27693-29328

Mycgr3G41969\_Mycgr3T

Mycgr3G80635 Mycgr3T
  
Location: 29428-29821

Mycgr3G80635\_Mycgr3T

Mycgr3G41426 Mycgr3T
  
Location: 29921-35255

Mycgr3G41426\_Mycgr3T

Mycgr3G104337 Mycgr3
  
Location: 35355-36108

Mycgr3G104337\_Mycgr3

Mycgr3G71679 Mycgr3T
  
Location: 36208-37300

Mycgr3G71679\_Mycgr3T

Mycgr3G92938 Mycgr3T
  
Location: 37400-38699

Mycgr3G92938\_Mycgr3T

Mycgr3G92941 Mycgr3T
  
Location: 38799-40734

Mycgr3G92941\_Mycgr3T

hypothetical protein
  
Accession: EJT78057
  
Location: 1902638-1906339
  
 NCBI BlastP on this gene

EJT78057

hypothetical protein
  
Accession: EJT78058
  
Location: 1907875-1908772
  
 NCBI BlastP on this gene

EJT78058

hypothetical protein
  
Accession: EJT78059
  
Location: 1909011-1910769
  
 NCBI BlastP on this gene

EJT78059

hypothetical protein
  
Accession: EJT78060
  
Location: 1911383-1917225
  
 NCBI BlastP on this gene

EJT78060

hypothetical protein
  
Accession: EJT78061
  
Location: 1919096-1920062
  
  
**BlastP hit with Mycgr3G104337\_Mycgr3**
  
Percentage identity: 44 %
  
BlastP bit score: 207
  
Sequence coverage: 95 %
  
E-value: 2e-62
  
  
 NCBI BlastP on this gene

EJT78061

hypothetical protein
  
Accession: EJT78062
  
Location: 1922426-1929709
  
  
**BlastP hit with Mycgr3G100089\_Mycgr3**
  
Percentage identity: 44 %
  
BlastP bit score: 1825
  
Sequence coverage: 101 %
  
E-value: 0.0
  
  
 NCBI BlastP on this gene

EJT78062

DNA polymerase sigma
  
Accession: EJT78063
  
Location: 1931219-1933617
  
 NCBI BlastP on this gene

EJT78063

hypothetical protein
  
Accession: EJT78064
  
Location: 1935060-1937230
  
 NCBI BlastP on this gene

EJT78064

hypothetical protein
  
Accession: EJT78065
  
Location: 1937863-1938974
  
 NCBI BlastP on this gene

EJT78065

hypothetical protein
  
Accession: EJT78066
  
Location: 1939619-1943218
  
 NCBI BlastP on this gene

EJT78066

hypothetical protein
  
Accession: EJT78067
  
Location: 1945916-1946430
  
 NCBI BlastP on this gene

EJT78067

hypothetical protein
  
Accession: EJT78068
  
Location: 1947126-1948065
  
 NCBI BlastP on this gene

EJT78068

Query: Architecture Search FASTA input

GL891307 : Neurospora tetrasperma FGSC 2508 unplaced genomic scaffold NEUTE1scaffold\_6    Total score: 2.0     Cumulative Blast bit score: 2028

Hit cluster cross-links:

Mycgr3G85918 Mycgr3T
  
Location: 0-1602

Mycgr3G85918\_Mycgr3T

Mycgr3G42010 Mycgr3T
  
Location: 1702-8569

Mycgr3G42010\_Mycgr3T

Mycgr3G29582 Mycgr3T
  
Location: 8669-8915

Mycgr3G29582\_Mycgr3T

Mycgr3G31170 Mycgr3T
  
Location: 9015-9255

Mycgr3G31170\_Mycgr3T

Mycgr3G85924 Mycgr3T
  
Location: 9355-11218

Mycgr3G85924\_Mycgr3T

Mycgr3G71676 Mycgr3T
  
Location: 11318-12494

Mycgr3G71676\_Mycgr3T

Mycgr3G11468 Mycgr3T
  
Location: 12594-13653

Mycgr3G11468\_Mycgr3T

Mycgr3G58567 Mycgr3T
  
Location: 13753-14506

Mycgr3G58567\_Mycgr3T

Mycgr3G100089 Mycgr3
  
Location: 14606-21152

Mycgr3G100089\_Mycgr3

Mycgr3G42698 Mycgr3T
  
Location: 21252-22131

Mycgr3G42698\_Mycgr3T

Mycgr3G71681 Mycgr3T
  
Location: 22231-23461

Mycgr3G71681\_Mycgr3T

Mycgr3G109328 Mycgr3
  
Location: 23561-24239

Mycgr3G109328\_Mycgr3

Mycgr3G104334 Mycgr3
  
Location: 24339-24567

Mycgr3G104334\_Mycgr3

Mycgr3G42715 Mycgr3T
  
Location: 24667-25981

Mycgr3G42715\_Mycgr3T

Mycgr3G92934 Mycgr3T
  
Location: 26081-27593

Mycgr3G92934\_Mycgr3T

Mycgr3G41969 Mycgr3T
  
Location: 27693-29328

Mycgr3G41969\_Mycgr3T

Mycgr3G80635 Mycgr3T
  
Location: 29428-29821

Mycgr3G80635\_Mycgr3T

Mycgr3G41426 Mycgr3T
  
Location: 29921-35255

Mycgr3G41426\_Mycgr3T

Mycgr3G104337 Mycgr3
  
Location: 35355-36108

Mycgr3G104337\_Mycgr3

Mycgr3G71679 Mycgr3T
  
Location: 36208-37300

Mycgr3G71679\_Mycgr3T

Mycgr3G92938 Mycgr3T
  
Location: 37400-38699

Mycgr3G92938\_Mycgr3T

Mycgr3G92941 Mycgr3T
  
Location: 38799-40734

Mycgr3G92941\_Mycgr3T

hypothetical protein
  
Accession: EGO54652
  
Location: 2856255-2871377
  
  
**BlastP hit with Mycgr3G100089\_Mycgr3**
  
Percentage identity: 44 %
  
BlastP bit score: 1815
  
Sequence coverage: 100 %
  
E-value: 0.0
  
  
 NCBI BlastP on this gene

EGO54652

hypothetical protein
  
Accession: EGO54653
  
Location: 2871926-2872129
  
 NCBI BlastP on this gene

EGO54653

hypothetical protein
  
Accession: EGO54654
  
Location: 2873404-2874360
  
  
**BlastP hit with Mycgr3G104337\_Mycgr3**
  
Percentage identity: 41 %
  
BlastP bit score: 213
  
Sequence coverage: 99 %
  
E-value: 1e-64
  
  
 NCBI BlastP on this gene

EGO54654

hypothetical protein
  
Accession: EGO54655
  
Location: 2876087-2881260
  
 NCBI BlastP on this gene

EGO54655

hypothetical protein
  
Accession: EGO54656
  
Location: 2882806-2884738
  
 NCBI BlastP on this gene

EGO54656

hypothetical protein
  
Accession: EGO54657
  
Location: 2886231-2887387
  
 NCBI BlastP on this gene

EGO54657

Query: Architecture Search FASTA input

GL891269 : Neurospora tetrasperma FGSC 2509 unplaced genomic scaffold NEUTE2scaffold\_7    Total score: 2.0     Cumulative Blast bit score: 2028

Hit cluster cross-links:

Mycgr3G85918 Mycgr3T
  
Location: 0-1602

Mycgr3G85918\_Mycgr3T

Mycgr3G42010 Mycgr3T
  
Location: 1702-8569

Mycgr3G42010\_Mycgr3T

Mycgr3G29582 Mycgr3T
  
Location: 8669-8915

Mycgr3G29582\_Mycgr3T

Mycgr3G31170 Mycgr3T
  
Location: 9015-9255

Mycgr3G31170\_Mycgr3T

Mycgr3G85924 Mycgr3T
  
Location: 9355-11218

Mycgr3G85924\_Mycgr3T

Mycgr3G71676 Mycgr3T
  
Location: 11318-12494

Mycgr3G71676\_Mycgr3T

Mycgr3G11468 Mycgr3T
  
Location: 12594-13653

Mycgr3G11468\_Mycgr3T

Mycgr3G58567 Mycgr3T
  
Location: 13753-14506

Mycgr3G58567\_Mycgr3T

Mycgr3G100089 Mycgr3
  
Location: 14606-21152

Mycgr3G100089\_Mycgr3

Mycgr3G42698 Mycgr3T
  
Location: 21252-22131

Mycgr3G42698\_Mycgr3T

Mycgr3G71681 Mycgr3T
  
Location: 22231-23461

Mycgr3G71681\_Mycgr3T

Mycgr3G109328 Mycgr3
  
Location: 23561-24239

Mycgr3G109328\_Mycgr3

Mycgr3G104334 Mycgr3
  
Location: 24339-24567

Mycgr3G104334\_Mycgr3

Mycgr3G42715 Mycgr3T
  
Location: 24667-25981

Mycgr3G42715\_Mycgr3T

Mycgr3G92934 Mycgr3T
  
Location: 26081-27593

Mycgr3G92934\_Mycgr3T

Mycgr3G41969 Mycgr3T
  
Location: 27693-29328

Mycgr3G41969\_Mycgr3T

Mycgr3G80635 Mycgr3T
  
Location: 29428-29821

Mycgr3G80635\_Mycgr3T

Mycgr3G41426 Mycgr3T
  
Location: 29921-35255

Mycgr3G41426\_Mycgr3T

Mycgr3G104337 Mycgr3
  
Location: 35355-36108

Mycgr3G104337\_Mycgr3

Mycgr3G71679 Mycgr3T
  
Location: 36208-37300

Mycgr3G71679\_Mycgr3T

Mycgr3G92938 Mycgr3T
  
Location: 37400-38699

Mycgr3G92938\_Mycgr3T

Mycgr3G92941 Mycgr3T
  
Location: 38799-40734

Mycgr3G92941\_Mycgr3T

Aldo/keto reductase
  
Accession: EGZ67872
  
Location: 996538-998279
  
 NCBI BlastP on this gene

EGZ67872

hypothetical protein
  
Accession: EGZ67873
  
Location: 1000848-1002780
  
 NCBI BlastP on this gene

EGZ67873

P-loop containing nucleoside triphosphate hydrolase protein
  
Accession: EGZ67874
  
Location: 1004326-1009499
  
 NCBI BlastP on this gene

EGZ67874

hypothetical protein
  
Accession: EGZ67875
  
Location: 1011224-1012180
  
  
**BlastP hit with Mycgr3G104337\_Mycgr3**
  
Percentage identity: 41 %
  
BlastP bit score: 213
  
Sequence coverage: 99 %
  
E-value: 1e-64
  
  
 NCBI BlastP on this gene

EGZ67875

hypothetical protein
  
Accession: EGZ67876
  
Location: 1013453-1013656
  
 NCBI BlastP on this gene

EGZ67876

ketoacyl-synt-domain-containing protein
  
Accession: EGZ67877
  
Location: 1014205-1029328
  
  
**BlastP hit with Mycgr3G100089\_Mycgr3**
  
Percentage identity: 44 %
  
BlastP bit score: 1815
  
Sequence coverage: 100 %
  
E-value: 0.0
  
  
 NCBI BlastP on this gene

EGZ67877

hypothetical protein
  
Accession: EGZ67878
  
Location: 1030624-1030782
  
 NCBI BlastP on this gene

EGZ67878

hypothetical protein
  
Accession: EGZ67879
  
Location: 1032370-1032525
  
 NCBI BlastP on this gene

EGZ67879

hypothetical protein
  
Accession: EGZ67880
  
Location: 1035220-1036071
  
 NCBI BlastP on this gene

EGZ67880

Query: Architecture Search FASTA input

KB021009 : Colletotrichum gloeosporioides Nara gc5 unplaced genomic scaffold scaffold605    Total score: 2.0     Cumulative Blast bit score: 2026

Hit cluster cross-links:

Mycgr3G85918 Mycgr3T
  
Location: 0-1602

Mycgr3G85918\_Mycgr3T

Mycgr3G42010 Mycgr3T
  
Location: 1702-8569

Mycgr3G42010\_Mycgr3T

Mycgr3G29582 Mycgr3T
  
Location: 8669-8915

Mycgr3G29582\_Mycgr3T

Mycgr3G31170 Mycgr3T
  
Location: 9015-9255

Mycgr3G31170\_Mycgr3T

Mycgr3G85924 Mycgr3T
  
Location: 9355-11218

Mycgr3G85924\_Mycgr3T

Mycgr3G71676 Mycgr3T
  
Location: 11318-12494

Mycgr3G71676\_Mycgr3T

Mycgr3G11468 Mycgr3T
  
Location: 12594-13653

Mycgr3G11468\_Mycgr3T

Mycgr3G58567 Mycgr3T
  
Location: 13753-14506

Mycgr3G58567\_Mycgr3T

Mycgr3G100089 Mycgr3
  
Location: 14606-21152

Mycgr3G100089\_Mycgr3

Mycgr3G42698 Mycgr3T
  
Location: 21252-22131

Mycgr3G42698\_Mycgr3T

Mycgr3G71681 Mycgr3T
  
Location: 22231-23461

Mycgr3G71681\_Mycgr3T

Mycgr3G109328 Mycgr3
  
Location: 23561-24239

Mycgr3G109328\_Mycgr3

Mycgr3G104334 Mycgr3
  
Location: 24339-24567

Mycgr3G104334\_Mycgr3

Mycgr3G42715 Mycgr3T
  
Location: 24667-25981

Mycgr3G42715\_Mycgr3T

Mycgr3G92934 Mycgr3T
  
Location: 26081-27593

Mycgr3G92934\_Mycgr3T

Mycgr3G41969 Mycgr3T
  
Location: 27693-29328

Mycgr3G41969\_Mycgr3T

Mycgr3G80635 Mycgr3T
  
Location: 29428-29821

Mycgr3G80635\_Mycgr3T

Mycgr3G41426 Mycgr3T
  
Location: 29921-35255

Mycgr3G41426\_Mycgr3T

Mycgr3G104337 Mycgr3
  
Location: 35355-36108

Mycgr3G104337\_Mycgr3

Mycgr3G71679 Mycgr3T
  
Location: 36208-37300

Mycgr3G71679\_Mycgr3T

Mycgr3G92938 Mycgr3T
  
Location: 37400-38699

Mycgr3G92938\_Mycgr3T

Mycgr3G92941 Mycgr3T
  
Location: 38799-40734

Mycgr3G92941\_Mycgr3T

lipid a export atp-binding permease protein msba
  
Accession: ELA26921
  
Location: 343-3758
  
 NCBI BlastP on this gene

ELA26921

duf341 family
  
Accession: ELA26922
  
Location: 7465-8478
  
  
**BlastP hit with Mycgr3G104337\_Mycgr3**
  
Percentage identity: 42 %
  
BlastP bit score: 209
  
Sequence coverage: 99 %
  
E-value: 5e-63
  
  
 NCBI BlastP on this gene

ELA26922

polyketide synthase
  
Accession: ELA26923
  
Location: 13784-20850
  
  
**BlastP hit with Mycgr3G100089\_Mycgr3**
  
Percentage identity: 43 %
  
BlastP bit score: 1817
  
Sequence coverage: 101 %
  
E-value: 0.0
  
  
 NCBI BlastP on this gene

ELA26923

ankyrin repeat protein
  
Accession: ELA26924
  
Location: 21563-24444
  
 NCBI BlastP on this gene

ELA26924

MFS multidrug transporter
  
Accession: ELA26925
  
Location: 26251-27839
  
 NCBI BlastP on this gene

ELA26925

C6 finger domain-containing protein
  
Accession: ELA26926
  
Location: 28970-30073
  
 NCBI BlastP on this gene

ELA26926

short-chain dehydrogenase, putative
  
Accession: ELA26927
  
Location: 30178-31158
  
 NCBI BlastP on this gene

ELA26927

NADPH--cytochrome p450 reductase
  
Accession: ELA26928
  
Location: 31501-32842
  
 NCBI BlastP on this gene

ELA26928

fungal specific transcription factor
  
Accession: ELA26929
  
Location: 33435-35918
  
 NCBI BlastP on this gene

ELA26929

Query: Architecture Search FASTA input

CH476603 : Aspergillus terreus NIH2624 scaffold\_10 genomic scaffold    Total score: 2.0     Cumulative Blast bit score: 2020

Hit cluster cross-links:

Mycgr3G85918 Mycgr3T
  
Location: 0-1602

Mycgr3G85918\_Mycgr3T

Mycgr3G42010 Mycgr3T
  
Location: 1702-8569

Mycgr3G42010\_Mycgr3T

Mycgr3G29582 Mycgr3T
  
Location: 8669-8915

Mycgr3G29582\_Mycgr3T

Mycgr3G31170 Mycgr3T
  
Location: 9015-9255

Mycgr3G31170\_Mycgr3T

Mycgr3G85924 Mycgr3T
  
Location: 9355-11218

Mycgr3G85924\_Mycgr3T

Mycgr3G71676 Mycgr3T
  
Location: 11318-12494

Mycgr3G71676\_Mycgr3T

Mycgr3G11468 Mycgr3T
  
Location: 12594-13653

Mycgr3G11468\_Mycgr3T

Mycgr3G58567 Mycgr3T
  
Location: 13753-14506

Mycgr3G58567\_Mycgr3T

Mycgr3G100089 Mycgr3
  
Location: 14606-21152

Mycgr3G100089\_Mycgr3

Mycgr3G42698 Mycgr3T
  
Location: 21252-22131

Mycgr3G42698\_Mycgr3T

Mycgr3G71681 Mycgr3T
  
Location: 22231-23461

Mycgr3G71681\_Mycgr3T

Mycgr3G109328 Mycgr3
  
Location: 23561-24239

Mycgr3G109328\_Mycgr3

Mycgr3G104334 Mycgr3
  
Location: 24339-24567

Mycgr3G104334\_Mycgr3

Mycgr3G42715 Mycgr3T
  
Location: 24667-25981

Mycgr3G42715\_Mycgr3T

Mycgr3G92934 Mycgr3T
  
Location: 26081-27593

Mycgr3G92934\_Mycgr3T

Mycgr3G41969 Mycgr3T
  
Location: 27693-29328

Mycgr3G41969\_Mycgr3T

Mycgr3G80635 Mycgr3T
  
Location: 29428-29821

Mycgr3G80635\_Mycgr3T

Mycgr3G41426 Mycgr3T
  
Location: 29921-35255

Mycgr3G41426\_Mycgr3T

Mycgr3G104337 Mycgr3
  
Location: 35355-36108

Mycgr3G104337\_Mycgr3

Mycgr3G71679 Mycgr3T
  
Location: 36208-37300

Mycgr3G71679\_Mycgr3T

Mycgr3G92938 Mycgr3T
  
Location: 37400-38699

Mycgr3G92938\_Mycgr3T

Mycgr3G92941 Mycgr3T
  
Location: 38799-40734

Mycgr3G92941\_Mycgr3T

GTPase-activating protein GYP7
  
Accession: EAU32442
  
Location: 604913-607649
  
 NCBI BlastP on this gene

EAU32442

predicted protein
  
Accession: EAU32443
  
Location: 608041-608618
  
 NCBI BlastP on this gene

EAU32443

conserved hypothetical protein
  
Accession: EAU32444
  
Location: 610355-611524
  
 NCBI BlastP on this gene

EAU32444

conserved hypothetical protein
  
Accession: EAU32445
  
Location: 612716-613472
  
 NCBI BlastP on this gene

EAU32445

hypothetical protein
  
Accession: EAU32446
  
Location: 614039-615196
  
 NCBI BlastP on this gene

EAU32446

hypothetical protein
  
Accession: EAU32447
  
Location: 615788-617205
  
 NCBI BlastP on this gene

EAU32447

conserved hypothetical protein
  
Accession: EAU32448
  
Location: 617708-618290
  
 NCBI BlastP on this gene

EAU32448

predicted protein
  
Accession: EAU32449
  
Location: 618833-620758
  
 NCBI BlastP on this gene

EAU32449

predicted protein
  
Accession: EAU32450
  
Location: 621328-622182
  
  
**BlastP hit with Mycgr3G104337\_Mycgr3**
  
Percentage identity: 32 %
  
BlastP bit score: 131
  
Sequence coverage: 96 %
  
E-value: 1e-33
  
  
 NCBI BlastP on this gene

EAU32450

hypothetical protein
  
Accession: EAU32451
  
Location: 623328-630255
  
  
**BlastP hit with Mycgr3G100089\_Mycgr3**
  
Percentage identity: 46 %
  
BlastP bit score: 1889
  
Sequence coverage: 101 %
  
E-value: 0.0
  
  
 NCBI BlastP on this gene

EAU32451

predicted protein
  
Accession: EAU32452
  
Location: 631817-634928
  
 NCBI BlastP on this gene

EAU32452

conserved hypothetical protein
  
Accession: EAU32453
  
Location: 635506-637438
  
 NCBI BlastP on this gene

EAU32453

conserved hypothetical protein
  
Accession: EAU32454
  
Location: 638596-639852
  
 NCBI BlastP on this gene

EAU32454

conserved hypothetical protein
  
Accession: EAU32455
  
Location: 641804-645435
  
 NCBI BlastP on this gene

EAU32455

predicted protein
  
Accession: EAU32456
  
Location: 647069-651135
  
 NCBI BlastP on this gene

EAU32456

Query: Architecture Search FASTA input

KE148164 : Ophiostoma piceae UAMH 11346 chromosome Unknown scf19    Total score: 2.0     Cumulative Blast bit score: 2014

Hit cluster cross-links:

Mycgr3G85918 Mycgr3T
  
Location: 0-1602

Mycgr3G85918\_Mycgr3T

Mycgr3G42010 Mycgr3T
  
Location: 1702-8569

Mycgr3G42010\_Mycgr3T

Mycgr3G29582 Mycgr3T
  
Location: 8669-8915

Mycgr3G29582\_Mycgr3T

Mycgr3G31170 Mycgr3T
  
Location: 9015-9255

Mycgr3G31170\_Mycgr3T

Mycgr3G85924 Mycgr3T
  
Location: 9355-11218

Mycgr3G85924\_Mycgr3T

Mycgr3G71676 Mycgr3T
  
Location: 11318-12494

Mycgr3G71676\_Mycgr3T

Mycgr3G11468 Mycgr3T
  
Location: 12594-13653

Mycgr3G11468\_Mycgr3T

Mycgr3G58567 Mycgr3T
  
Location: 13753-14506

Mycgr3G58567\_Mycgr3T

Mycgr3G100089 Mycgr3
  
Location: 14606-21152

Mycgr3G100089\_Mycgr3

Mycgr3G42698 Mycgr3T
  
Location: 21252-22131

Mycgr3G42698\_Mycgr3T

Mycgr3G71681 Mycgr3T
  
Location: 22231-23461

Mycgr3G71681\_Mycgr3T

Mycgr3G109328 Mycgr3
  
Location: 23561-24239

Mycgr3G109328\_Mycgr3

Mycgr3G104334 Mycgr3
  
Location: 24339-24567

Mycgr3G104334\_Mycgr3

Mycgr3G42715 Mycgr3T
  
Location: 24667-25981

Mycgr3G42715\_Mycgr3T

Mycgr3G92934 Mycgr3T
  
Location: 26081-27593

Mycgr3G92934\_Mycgr3T

Mycgr3G41969 Mycgr3T
  
Location: 27693-29328

Mycgr3G41969\_Mycgr3T

Mycgr3G80635 Mycgr3T
  
Location: 29428-29821

Mycgr3G80635\_Mycgr3T

Mycgr3G41426 Mycgr3T
  
Location: 29921-35255

Mycgr3G41426\_Mycgr3T

Mycgr3G104337 Mycgr3
  
Location: 35355-36108

Mycgr3G104337\_Mycgr3

Mycgr3G71679 Mycgr3T
  
Location: 36208-37300

Mycgr3G71679\_Mycgr3T

Mycgr3G92938 Mycgr3T
  
Location: 37400-38699

Mycgr3G92938\_Mycgr3T

Mycgr3G92941 Mycgr3T
  
Location: 38799-40734

Mycgr3G92941\_Mycgr3T

potassium channel
  
Accession: EPE03864
  
Location: 208792-211719
  
 NCBI BlastP on this gene

EPE03864

ubiquitin fusion degradation protein
  
Accession: EPE03865
  
Location: 212620-215121
  
 NCBI BlastP on this gene

EPE03865

hypothetical protein
  
Accession: EPE03866
  
Location: 215186-216292
  
 NCBI BlastP on this gene

EPE03866

hypothetical protein
  
Accession: EPE03867
  
Location: 217305-217771
  
 NCBI BlastP on this gene

EPE03867

hypothetical protein
  
Accession: EPE03868
  
Location: 217864-218200
  
 NCBI BlastP on this gene

EPE03868

abc transporter
  
Accession: EPE03869
  
Location: 218525-223040
  
 NCBI BlastP on this gene

EPE03869

ef-hand calcium-binding domain protein
  
Accession: EPE03870
  
Location: 225996-226821
  
  
**BlastP hit with Mycgr3G104337\_Mycgr3**
  
Percentage identity: 42 %
  
BlastP bit score: 207
  
Sequence coverage: 98 %
  
E-value: 1e-62
  
  
 NCBI BlastP on this gene

EPE03870

polyketide synthase
  
Accession: EPE03871
  
Location: 233951-240763
  
  
**BlastP hit with Mycgr3G100089\_Mycgr3**
  
Percentage identity: 43 %
  
BlastP bit score: 1807
  
Sequence coverage: 102 %
  
E-value: 0.0
  
  
 NCBI BlastP on this gene

EPE03871

hypothetical protein
  
Accession: EPE03872
  
Location: 241505-246112
  
 NCBI BlastP on this gene

EPE03872

hypothetical protein
  
Accession: EPE03873
  
Location: 253736-254971
  
 NCBI BlastP on this gene

EPE03873

glucosidase 2 subunit beta
  
Accession: EPE03874
  
Location: 255461-257371
  
 NCBI BlastP on this gene

EPE03874

Query: Architecture Search FASTA input

ACJE01000003 : Aspergillus niger ATCC 1015    Total score: 2.0     Cumulative Blast bit score: 2003

Hit cluster cross-links:

Mycgr3G85918 Mycgr3T
  
Location: 0-1602

Mycgr3G85918\_Mycgr3T

Mycgr3G42010 Mycgr3T
  
Location: 1702-8569

Mycgr3G42010\_Mycgr3T

Mycgr3G29582 Mycgr3T
  
Location: 8669-8915

Mycgr3G29582\_Mycgr3T

Mycgr3G31170 Mycgr3T
  
Location: 9015-9255

Mycgr3G31170\_Mycgr3T

Mycgr3G85924 Mycgr3T
  
Location: 9355-11218

Mycgr3G85924\_Mycgr3T

Mycgr3G71676 Mycgr3T
  
Location: 11318-12494

Mycgr3G71676\_Mycgr3T

Mycgr3G11468 Mycgr3T
  
Location: 12594-13653

Mycgr3G11468\_Mycgr3T

Mycgr3G58567 Mycgr3T
  
Location: 13753-14506

Mycgr3G58567\_Mycgr3T

Mycgr3G100089 Mycgr3
  
Location: 14606-21152

Mycgr3G100089\_Mycgr3

Mycgr3G42698 Mycgr3T
  
Location: 21252-22131

Mycgr3G42698\_Mycgr3T

Mycgr3G71681 Mycgr3T
  
Location: 22231-23461

Mycgr3G71681\_Mycgr3T

Mycgr3G109328 Mycgr3
  
Location: 23561-24239

Mycgr3G109328\_Mycgr3

Mycgr3G104334 Mycgr3
  
Location: 24339-24567

Mycgr3G104334\_Mycgr3

Mycgr3G42715 Mycgr3T
  
Location: 24667-25981

Mycgr3G42715\_Mycgr3T

Mycgr3G92934 Mycgr3T
  
Location: 26081-27593

Mycgr3G92934\_Mycgr3T

Mycgr3G41969 Mycgr3T
  
Location: 27693-29328

Mycgr3G41969\_Mycgr3T

Mycgr3G80635 Mycgr3T
  
Location: 29428-29821

Mycgr3G80635\_Mycgr3T

Mycgr3G41426 Mycgr3T
  
Location: 29921-35255

Mycgr3G41426\_Mycgr3T

Mycgr3G104337 Mycgr3
  
Location: 35355-36108

Mycgr3G104337\_Mycgr3

Mycgr3G71679 Mycgr3T
  
Location: 36208-37300

Mycgr3G71679\_Mycgr3T

Mycgr3G92938 Mycgr3T
  
Location: 37400-38699

Mycgr3G92938\_Mycgr3T

Mycgr3G92941 Mycgr3T
  
Location: 38799-40734

Mycgr3G92941\_Mycgr3T

hypothetical protein
  
Accession: EHA27400
  
Location: 583414-585327
  
 NCBI BlastP on this gene

EHA27400

hypothetical protein
  
Accession: EHA27401
  
Location: 586604-587545
  
 NCBI BlastP on this gene

EHA27401

hypothetical protein
  
Accession: EHA27402
  
Location: 588726-589930
  
 NCBI BlastP on this gene

EHA27402

hypothetical protein
  
Accession: EHA27403
  
Location: 590777-592618
  
 NCBI BlastP on this gene

EHA27403

hypothetical protein
  
Accession: EHA27404
  
Location: 593136-595006
  
 NCBI BlastP on this gene

EHA27404

hypothetical protein
  
Accession: EHA27405
  
Location: 596222-598235
  
 NCBI BlastP on this gene

EHA27405

hypothetical protein
  
Accession: EHA27406
  
Location: 599395-600497
  
 NCBI BlastP on this gene

EHA27406

hypothetical protein
  
Accession: EHA27407
  
Location: 602133-603048
  
  
**BlastP hit with Mycgr3G104337\_Mycgr3**
  
Percentage identity: 44 %
  
BlastP bit score: 209
  
Sequence coverage: 98 %
  
E-value: 3e-63
  
  
 NCBI BlastP on this gene

EHA27407

hypothetical protein
  
Accession: EHA27408
  
Location: 604912-611896
  
  
**BlastP hit with Mycgr3G100089\_Mycgr3**
  
Percentage identity: 44 %
  
BlastP bit score: 1794
  
Sequence coverage: 102 %
  
E-value: 0.0
  
  
 NCBI BlastP on this gene

EHA27408

hypothetical protein
  
Accession: EHA27409
  
Location: 613108-615065
  
 NCBI BlastP on this gene

EHA27409

hypothetical protein
  
Accession: EHA27410
  
Location: 616750-620047
  
 NCBI BlastP on this gene

EHA27410

amine oxidase
  
Accession: EHA27411
  
Location: 621045-622887
  
 NCBI BlastP on this gene

EHA27411

hypothetical protein
  
Accession: EHA27412
  
Location: 627011-628099
  
 NCBI BlastP on this gene

EHA27412

hypothetical protein
  
Accession: EHA27413
  
Location: 629480-630363
  
 NCBI BlastP on this gene

EHA27413

Query: Architecture Search FASTA input

HF679030 : Fusarium fujikuroi IMI 58289 draft genome, chromosome FFUJ\_chr08.    Total score: 2.0     Cumulative Blast bit score: 1998

Hit cluster cross-links:

Mycgr3G85918 Mycgr3T
  
Location: 0-1602

Mycgr3G85918\_Mycgr3T

Mycgr3G42010 Mycgr3T
  
Location: 1702-8569

Mycgr3G42010\_Mycgr3T

Mycgr3G29582 Mycgr3T
  
Location: 8669-8915

Mycgr3G29582\_Mycgr3T

Mycgr3G31170 Mycgr3T
  
Location: 9015-9255

Mycgr3G31170\_Mycgr3T

Mycgr3G85924 Mycgr3T
  
Location: 9355-11218

Mycgr3G85924\_Mycgr3T

Mycgr3G71676 Mycgr3T
  
Location: 11318-12494

Mycgr3G71676\_Mycgr3T

Mycgr3G11468 Mycgr3T
  
Location: 12594-13653

Mycgr3G11468\_Mycgr3T

Mycgr3G58567 Mycgr3T
  
Location: 13753-14506

Mycgr3G58567\_Mycgr3T

Mycgr3G100089 Mycgr3
  
Location: 14606-21152

Mycgr3G100089\_Mycgr3

Mycgr3G42698 Mycgr3T
  
Location: 21252-22131

Mycgr3G42698\_Mycgr3T

Mycgr3G71681 Mycgr3T
  
Location: 22231-23461

Mycgr3G71681\_Mycgr3T

Mycgr3G109328 Mycgr3
  
Location: 23561-24239

Mycgr3G109328\_Mycgr3

Mycgr3G104334 Mycgr3
  
Location: 24339-24567

Mycgr3G104334\_Mycgr3

Mycgr3G42715 Mycgr3T
  
Location: 24667-25981

Mycgr3G42715\_Mycgr3T

Mycgr3G92934 Mycgr3T
  
Location: 26081-27593

Mycgr3G92934\_Mycgr3T

Mycgr3G41969 Mycgr3T
  
Location: 27693-29328

Mycgr3G41969\_Mycgr3T

Mycgr3G80635 Mycgr3T
  
Location: 29428-29821

Mycgr3G80635\_Mycgr3T

Mycgr3G41426 Mycgr3T
  
Location: 29921-35255

Mycgr3G41426\_Mycgr3T

Mycgr3G104337 Mycgr3
  
Location: 35355-36108

Mycgr3G104337\_Mycgr3

Mycgr3G71679 Mycgr3T
  
Location: 36208-37300

Mycgr3G71679\_Mycgr3T

Mycgr3G92938 Mycgr3T
  
Location: 37400-38699

Mycgr3G92938\_Mycgr3T

Mycgr3G92941 Mycgr3T
  
Location: 38799-40734

Mycgr3G92941\_Mycgr3T

probable NADH cytb-reductase
  
Accession: CCT72092
  
Location: 449878-451277
  
 NCBI BlastP on this gene

FFUJ\_13910

probable high affinity methionine permease
  
Accession: CCT72093
  
Location: 451542-453266
  
 NCBI BlastP on this gene

FFUJ\_13909

probable saccharopine dehydrogenase (NAD, L-lysine-forming)
  
Accession: CCT72094
  
Location: 453666-454868
  
 NCBI BlastP on this gene

FFUJ\_13908

related to allantoate permease
  
Accession: CCT72095
  
Location: 455030-456542
  
 NCBI BlastP on this gene

FFUJ\_13907

related to D-arabinitol 2-dehydrogenase
  
Accession: CCT72096
  
Location: 457101-458085
  
 NCBI BlastP on this gene

FFUJ\_13906

related to ribose-5-phosphate isomerase
  
Accession: CCT72097
  
Location: 458540-459087
  
 NCBI BlastP on this gene

FFUJ\_13905

related to fructose-bisphosphate aldolase
  
Accession: CCT72098
  
Location: 459508-460424
  
 NCBI BlastP on this gene

FFUJ\_13904

uncharacterized protein
  
Accession: CCT72099
  
Location: 460490-462352
  
 NCBI BlastP on this gene

FFUJ\_13903

related to dihydroxyacetone kinase
  
Accession: CCT72100
  
Location: 462818-464651
  
 NCBI BlastP on this gene

FFUJ\_13902

related to beta transducin-like protein
  
Accession: CCT72101
  
Location: 465995-467203
  
 NCBI BlastP on this gene

FFUJ\_13901

related to ECM32-DNA dependent ATPase/DNA helicase B
  
Accession: CCT73103
  
Location: 467577-474600
  
  
**BlastP hit with Mycgr3G42010\_Mycgr3T**
  
Percentage identity: 43 %
  
BlastP bit score: 1771
  
Sequence coverage: 100 %
  
E-value: 0.0
  
  
 NCBI BlastP on this gene

FFUJ\_13900

uncharacterized protein
  
Accession: CCT72102
  
Location: 475384-476735
  
  
**BlastP hit with Mycgr3G92938\_Mycgr3T**
  
Percentage identity: 32 %
  
BlastP bit score: 227
  
Sequence coverage: 100 %
  
E-value: 2e-65
  
  
 NCBI BlastP on this gene

FFUJ\_13899

related to lysophosphatidic acid acyltransferase
  
Accession: CCT72103
  
Location: 479133-480439
  
 NCBI BlastP on this gene

FFUJ\_13898

related to cyclin CCL1
  
Accession: CCT72104
  
Location: 480683-481776
  
 NCBI BlastP on this gene

FFUJ\_13897

related to TGF beta induced protein ig-h3 precursor
  
Accession: CCT72105
  
Location: 482778-484045
  
 NCBI BlastP on this gene

FFUJ\_13896

uncharacterized protein
  
Accession: CCT72106
  
Location: 484206-484739
  
 NCBI BlastP on this gene

FFUJ\_13895

related to integral membrane protein PTH11
  
Accession: CCT72107
  
Location: 487670-489217
  
 NCBI BlastP on this gene

FFUJ\_13894

uncharacterized protein
  
Accession: CCT72108
  
Location: 489830-490460
  
 NCBI BlastP on this gene

FFUJ\_13893

related to DUF895 domain membrane protein
  
Accession: CCT72109
  
Location: 490828-492369
  
 NCBI BlastP on this gene

FFUJ\_13892

uncharacterized protein
  
Accession: CCT72110
  
Location: 492956-495313
  
 NCBI BlastP on this gene

FFUJ\_13891

Query: Architecture Search FASTA input

KE148164 : Ophiostoma piceae UAMH 11346 chromosome Unknown scf19    Total score: 2.0     Cumulative Blast bit score: 1985

Hit cluster cross-links:

Mycgr3G85918 Mycgr3T
  
Location: 0-1602

Mycgr3G85918\_Mycgr3T

Mycgr3G42010 Mycgr3T
  
Location: 1702-8569

Mycgr3G42010\_Mycgr3T

Mycgr3G29582 Mycgr3T
  
Location: 8669-8915

Mycgr3G29582\_Mycgr3T

Mycgr3G31170 Mycgr3T
  
Location: 9015-9255

Mycgr3G31170\_Mycgr3T

Mycgr3G85924 Mycgr3T
  
Location: 9355-11218

Mycgr3G85924\_Mycgr3T

Mycgr3G71676 Mycgr3T
  
Location: 11318-12494

Mycgr3G71676\_Mycgr3T

Mycgr3G11468 Mycgr3T
  
Location: 12594-13653

Mycgr3G11468\_Mycgr3T

Mycgr3G58567 Mycgr3T
  
Location: 13753-14506

Mycgr3G58567\_Mycgr3T

Mycgr3G100089 Mycgr3
  
Location: 14606-21152

Mycgr3G100089\_Mycgr3

Mycgr3G42698 Mycgr3T
  
Location: 21252-22131

Mycgr3G42698\_Mycgr3T

Mycgr3G71681 Mycgr3T
  
Location: 22231-23461

Mycgr3G71681\_Mycgr3T

Mycgr3G109328 Mycgr3
  
Location: 23561-24239

Mycgr3G109328\_Mycgr3

Mycgr3G104334 Mycgr3
  
Location: 24339-24567

Mycgr3G104334\_Mycgr3

Mycgr3G42715 Mycgr3T
  
Location: 24667-25981

Mycgr3G42715\_Mycgr3T

Mycgr3G92934 Mycgr3T
  
Location: 26081-27593

Mycgr3G92934\_Mycgr3T

Mycgr3G41969 Mycgr3T
  
Location: 27693-29328

Mycgr3G41969\_Mycgr3T

Mycgr3G80635 Mycgr3T
  
Location: 29428-29821

Mycgr3G80635\_Mycgr3T

Mycgr3G41426 Mycgr3T
  
Location: 29921-35255

Mycgr3G41426\_Mycgr3T

Mycgr3G104337 Mycgr3
  
Location: 35355-36108

Mycgr3G104337\_Mycgr3

Mycgr3G71679 Mycgr3T
  
Location: 36208-37300

Mycgr3G71679\_Mycgr3T

Mycgr3G92938 Mycgr3T
  
Location: 37400-38699

Mycgr3G92938\_Mycgr3T

Mycgr3G92941 Mycgr3T
  
Location: 38799-40734

Mycgr3G92941\_Mycgr3T

transcription factor
  
Accession: EPE03903
  
Location: 344428-346924
  
 NCBI BlastP on this gene

EPE03903

fluconazole resistance protein 1
  
Accession: EPE03904
  
Location: 358498-360216
  
 NCBI BlastP on this gene

EPE03904

hypothetical protein
  
Accession: EPE03905
  
Location: 361732-363267
  
 NCBI BlastP on this gene

EPE03905

aaa family ATPase
  
Accession: EPE03906
  
Location: 363979-371364
  
  
**BlastP hit with Mycgr3G42010\_Mycgr3T**
  
Percentage identity: 41 %
  
BlastP bit score: 1766
  
Sequence coverage: 108 %
  
E-value: 0.0
  
  
 NCBI BlastP on this gene

EPE03906

geranylgeranyl pyrophosphate synthetase
  
Accession: EPE03907
  
Location: 372017-373771
  
  
**BlastP hit with Mycgr3G92938\_Mycgr3T**
  
Percentage identity: 35 %
  
BlastP bit score: 219
  
Sequence coverage: 93 %
  
E-value: 2e-61
  
  
 NCBI BlastP on this gene

EPE03907

hexose transporter
  
Accession: EPE03908
  
Location: 383334-385001
  
 NCBI BlastP on this gene

EPE03908

hypothetical protein
  
Accession: EPE03909
  
Location: 386154-388971
  
 NCBI BlastP on this gene

EPE03909

aldehyde dehydrogenase
  
Accession: EPE03910
  
Location: 389766-391319
  
 NCBI BlastP on this gene

EPE03910

isotrichodermin c-15 hydroxylase
  
Accession: EPE03911
  
Location: 391688-393286
  
 NCBI BlastP on this gene

EPE03911

Query: Architecture Search FASTA input

JH921436 : Marssonina brunnea f. sp. 'multigermtubi' MB\_m1 unplaced genomic scaffold M6\_S00009    Total score: 2.0     Cumulative Blast bit score: 1982

Hit cluster cross-links:

Mycgr3G85918 Mycgr3T
  
Location: 0-1602

Mycgr3G85918\_Mycgr3T

Mycgr3G42010 Mycgr3T
  
Location: 1702-8569

Mycgr3G42010\_Mycgr3T

Mycgr3G29582 Mycgr3T
  
Location: 8669-8915

Mycgr3G29582\_Mycgr3T

Mycgr3G31170 Mycgr3T
  
Location: 9015-9255

Mycgr3G31170\_Mycgr3T

Mycgr3G85924 Mycgr3T
  
Location: 9355-11218

Mycgr3G85924\_Mycgr3T

Mycgr3G71676 Mycgr3T
  
Location: 11318-12494

Mycgr3G71676\_Mycgr3T

Mycgr3G11468 Mycgr3T
  
Location: 12594-13653

Mycgr3G11468\_Mycgr3T

Mycgr3G58567 Mycgr3T
  
Location: 13753-14506

Mycgr3G58567\_Mycgr3T

Mycgr3G100089 Mycgr3
  
Location: 14606-21152

Mycgr3G100089\_Mycgr3

Mycgr3G42698 Mycgr3T
  
Location: 21252-22131

Mycgr3G42698\_Mycgr3T

Mycgr3G71681 Mycgr3T
  
Location: 22231-23461

Mycgr3G71681\_Mycgr3T

Mycgr3G109328 Mycgr3
  
Location: 23561-24239

Mycgr3G109328\_Mycgr3

Mycgr3G104334 Mycgr3
  
Location: 24339-24567

Mycgr3G104334\_Mycgr3

Mycgr3G42715 Mycgr3T
  
Location: 24667-25981

Mycgr3G42715\_Mycgr3T

Mycgr3G92934 Mycgr3T
  
Location: 26081-27593

Mycgr3G92934\_Mycgr3T

Mycgr3G41969 Mycgr3T
  
Location: 27693-29328

Mycgr3G41969\_Mycgr3T

Mycgr3G80635 Mycgr3T
  
Location: 29428-29821

Mycgr3G80635\_Mycgr3T

Mycgr3G41426 Mycgr3T
  
Location: 29921-35255

Mycgr3G41426\_Mycgr3T

Mycgr3G104337 Mycgr3
  
Location: 35355-36108

Mycgr3G104337\_Mycgr3

Mycgr3G71679 Mycgr3T
  
Location: 36208-37300

Mycgr3G71679\_Mycgr3T

Mycgr3G92938 Mycgr3T
  
Location: 37400-38699

Mycgr3G92938\_Mycgr3T

Mycgr3G92941 Mycgr3T
  
Location: 38799-40734

Mycgr3G92941\_Mycgr3T

autophagy protein
  
Accession: EKD17570
  
Location: 1810086-1812240
  
 NCBI BlastP on this gene

EKD17570

peroxisomal adenine nucleotide transporter 1
  
Accession: EKD17571
  
Location: 1812797-1814366
  
 NCBI BlastP on this gene

EKD17571

hypothetical protein
  
Accession: EKD17572
  
Location: 1814921-1816122
  
 NCBI BlastP on this gene

EKD17572

zinc finger containing protein
  
Accession: EKD17573
  
Location: 1818089-1819299
  
 NCBI BlastP on this gene

EKD17573

putative tafazzin
  
Accession: EKD17574
  
Location: 1820213-1821530
  
 NCBI BlastP on this gene

EKD17574

Phosphomannomutase
  
Accession: EKD17575
  
Location: 1822559-1824358
  
 NCBI BlastP on this gene

EKD17575

pyridoxine
  
Accession: EKD17576
  
Location: 1825800-1826720
  
 NCBI BlastP on this gene

EKD17576

AtaAp protein
  
Accession: EKD17577
  
Location: 1827024-1827981
  
 NCBI BlastP on this gene

EKD17577

hypothetical protein
  
Accession: EKD17578
  
Location: 1829718-1831221
  
  
**BlastP hit with Mycgr3G92938\_Mycgr3T**
  
Percentage identity: 36 %
  
BlastP bit score: 205
  
Sequence coverage: 68 %
  
E-value: 4e-57
  
  
 NCBI BlastP on this gene

EKD17578

NFX1-type zinc finger-containing protein 1
  
Accession: EKD17579
  
Location: 1831726-1839437
  
  
**BlastP hit with Mycgr3G42010\_Mycgr3T**
  
Percentage identity: 42 %
  
BlastP bit score: 1777
  
Sequence coverage: 105 %
  
E-value: 0.0
  
  
 NCBI BlastP on this gene

EKD17579

hypothetical protein
  
Accession: EKD17580
  
Location: 1840823-1841364
  
 NCBI BlastP on this gene

EKD17580

Ni2+-Co2+ transporter transition metal uptake transporter
  
Accession: EKD17581
  
Location: 1842789-1844103
  
 NCBI BlastP on this gene

EKD17581

hypothetical protein
  
Accession: EKD17582
  
Location: 1849122-1850397
  
 NCBI BlastP on this gene

EKD17582

Query: Architecture Search FASTA input

GL385397 : Gaeumannomyces graminis var. tritici R3-111a-1 unplaced genomic scaffold supercont2.3    Total score: 2.0     Cumulative Blast bit score: 1980

Hit cluster cross-links:

Mycgr3G85918 Mycgr3T
  
Location: 0-1602

Mycgr3G85918\_Mycgr3T

Mycgr3G42010 Mycgr3T
  
Location: 1702-8569

Mycgr3G42010\_Mycgr3T

Mycgr3G29582 Mycgr3T
  
Location: 8669-8915

Mycgr3G29582\_Mycgr3T

Mycgr3G31170 Mycgr3T
  
Location: 9015-9255

Mycgr3G31170\_Mycgr3T

Mycgr3G85924 Mycgr3T
  
Location: 9355-11218

Mycgr3G85924\_Mycgr3T

Mycgr3G71676 Mycgr3T
  
Location: 11318-12494

Mycgr3G71676\_Mycgr3T

Mycgr3G11468 Mycgr3T
  
Location: 12594-13653

Mycgr3G11468\_Mycgr3T

Mycgr3G58567 Mycgr3T
  
Location: 13753-14506

Mycgr3G58567\_Mycgr3T

Mycgr3G100089 Mycgr3
  
Location: 14606-21152

Mycgr3G100089\_Mycgr3

Mycgr3G42698 Mycgr3T
  
Location: 21252-22131

Mycgr3G42698\_Mycgr3T

Mycgr3G71681 Mycgr3T
  
Location: 22231-23461

Mycgr3G71681\_Mycgr3T

Mycgr3G109328 Mycgr3
  
Location: 23561-24239

Mycgr3G109328\_Mycgr3

Mycgr3G104334 Mycgr3
  
Location: 24339-24567

Mycgr3G104334\_Mycgr3

Mycgr3G42715 Mycgr3T
  
Location: 24667-25981

Mycgr3G42715\_Mycgr3T

Mycgr3G92934 Mycgr3T
  
Location: 26081-27593

Mycgr3G92934\_Mycgr3T

Mycgr3G41969 Mycgr3T
  
Location: 27693-29328

Mycgr3G41969\_Mycgr3T

Mycgr3G80635 Mycgr3T
  
Location: 29428-29821

Mycgr3G80635\_Mycgr3T

Mycgr3G41426 Mycgr3T
  
Location: 29921-35255

Mycgr3G41426\_Mycgr3T

Mycgr3G104337 Mycgr3
  
Location: 35355-36108

Mycgr3G104337\_Mycgr3

Mycgr3G71679 Mycgr3T
  
Location: 36208-37300

Mycgr3G71679\_Mycgr3T

Mycgr3G92938 Mycgr3T
  
Location: 37400-38699

Mycgr3G92938\_Mycgr3T

Mycgr3G92941 Mycgr3T
  
Location: 38799-40734

Mycgr3G92941\_Mycgr3T

hypothetical protein
  
Accession: EJT77322
  
Location: 6324702-6325847
  
 NCBI BlastP on this gene

EJT77322

hypothetical protein
  
Accession: EJT77323
  
Location: 6326427-6328872
  
 NCBI BlastP on this gene

EJT77323

hypothetical protein
  
Accession: EJT77324
  
Location: 6329256-6329957
  
 NCBI BlastP on this gene

EJT77324

hypothetical protein
  
Accession: EJT77325
  
Location: 6330409-6331728
  
 NCBI BlastP on this gene

EJT77325

hypothetical protein
  
Accession: EJT77326
  
Location: 6342367-6349492
  
  
**BlastP hit with Mycgr3G100089\_Mycgr3**
  
Percentage identity: 43 %
  
BlastP bit score: 1763
  
Sequence coverage: 102 %
  
E-value: 0.0
  
  
 NCBI BlastP on this gene

EJT77326

hypothetical protein
  
Accession: EJT77327
  
Location: 6351500-6352493
  
  
**BlastP hit with Mycgr3G104337\_Mycgr3**
  
Percentage identity: 42 %
  
BlastP bit score: 217
  
Sequence coverage: 99 %
  
E-value: 2e-66
  
  
 NCBI BlastP on this gene

EJT77327

hypothetical protein
  
Accession: EJT77328
  
Location: 6353944-6358977
  
 NCBI BlastP on this gene

EJT77328

hypothetical protein
  
Accession: EJT77329
  
Location: 6359346-6360665
  
 NCBI BlastP on this gene

EJT77329

hypothetical protein
  
Accession: EJT77330
  
Location: 6360761-6361480
  
 NCBI BlastP on this gene

EJT77330

hypothetical protein
  
Accession: EJT77331
  
Location: 6361779-6362366
  
 NCBI BlastP on this gene

EJT77331

hypothetical protein
  
Accession: EJT77332
  
Location: 6362728-6364400
  
 NCBI BlastP on this gene

EJT77332

hypothetical protein
  
Accession: EJT77333
  
Location: 6364684-6365331
  
 NCBI BlastP on this gene

EJT77333

hypothetical protein
  
Accession: EJT77334
  
Location: 6366741-6367950
  
 NCBI BlastP on this gene

EJT77334

hypothetical protein
  
Accession: EJT77335
  
Location: 6368928-6370189
  
 NCBI BlastP on this gene

EJT77335

Query: Architecture Search FASTA input

CP003009 : Thielavia terrestris NRRL 8126 chromosome 1    Total score: 2.0     Cumulative Blast bit score: 1976

Hit cluster cross-links:

Mycgr3G85918 Mycgr3T
  
Location: 0-1602

Mycgr3G85918\_Mycgr3T

Mycgr3G42010 Mycgr3T
  
Location: 1702-8569

Mycgr3G42010\_Mycgr3T

Mycgr3G29582 Mycgr3T
  
Location: 8669-8915

Mycgr3G29582\_Mycgr3T

Mycgr3G31170 Mycgr3T
  
Location: 9015-9255

Mycgr3G31170\_Mycgr3T

Mycgr3G85924 Mycgr3T
  
Location: 9355-11218

Mycgr3G85924\_Mycgr3T

Mycgr3G71676 Mycgr3T
  
Location: 11318-12494

Mycgr3G71676\_Mycgr3T

Mycgr3G11468 Mycgr3T
  
Location: 12594-13653

Mycgr3G11468\_Mycgr3T

Mycgr3G58567 Mycgr3T
  
Location: 13753-14506

Mycgr3G58567\_Mycgr3T

Mycgr3G100089 Mycgr3
  
Location: 14606-21152

Mycgr3G100089\_Mycgr3

Mycgr3G42698 Mycgr3T
  
Location: 21252-22131

Mycgr3G42698\_Mycgr3T

Mycgr3G71681 Mycgr3T
  
Location: 22231-23461

Mycgr3G71681\_Mycgr3T

Mycgr3G109328 Mycgr3
  
Location: 23561-24239

Mycgr3G109328\_Mycgr3

Mycgr3G104334 Mycgr3
  
Location: 24339-24567

Mycgr3G104334\_Mycgr3

Mycgr3G42715 Mycgr3T
  
Location: 24667-25981

Mycgr3G42715\_Mycgr3T

Mycgr3G92934 Mycgr3T
  
Location: 26081-27593

Mycgr3G92934\_Mycgr3T

Mycgr3G41969 Mycgr3T
  
Location: 27693-29328

Mycgr3G41969\_Mycgr3T

Mycgr3G80635 Mycgr3T
  
Location: 29428-29821

Mycgr3G80635\_Mycgr3T

Mycgr3G41426 Mycgr3T
  
Location: 29921-35255

Mycgr3G41426\_Mycgr3T

Mycgr3G104337 Mycgr3
  
Location: 35355-36108

Mycgr3G104337\_Mycgr3

Mycgr3G71679 Mycgr3T
  
Location: 36208-37300

Mycgr3G71679\_Mycgr3T

Mycgr3G92938 Mycgr3T
  
Location: 37400-38699

Mycgr3G92938\_Mycgr3T

Mycgr3G92941 Mycgr3T
  
Location: 38799-40734

Mycgr3G92941\_Mycgr3T

hypothetical protein
  
Accession: AEO64470
  
Location: 8720085-8722442
  
 NCBI BlastP on this gene

THITE\_2110633

hypothetical protein
  
Accession: AEO64471
  
Location: 8723685-8725866
  
 NCBI BlastP on this gene

THITE\_2110636

glycoside hydrolase family 2 protein
  
Accession: AEO64472
  
Location: 8728063-8730708
  
 NCBI BlastP on this gene

THITE\_2110638

hypothetical protein
  
Accession: AEO64473
  
Location: 8730831-8732004
  
 NCBI BlastP on this gene

THITE\_2110640

hypothetical protein
  
Accession: AEO64474
  
Location: 8732550-8737419
  
 NCBI BlastP on this gene

THITE\_2040380

hypothetical protein
  
Accession: AEO64475
  
Location: 8739834-8740796
  
  
**BlastP hit with Mycgr3G104337\_Mycgr3**
  
Percentage identity: 43 %
  
BlastP bit score: 221
  
Sequence coverage: 99 %
  
E-value: 1e-67
  
  
 NCBI BlastP on this gene

THITE\_2037371

polyketide synthase
  
Accession: AEO64476
  
Location: 8743360-8750632
  
  
**BlastP hit with Mycgr3G100089\_Mycgr3**
  
Percentage identity: 42 %
  
BlastP bit score: 1755
  
Sequence coverage: 101 %
  
E-value: 0.0
  
  
 NCBI BlastP on this gene

THITE\_123823

hypothetical protein
  
Accession: AEO64477
  
Location: 8751428-8753560
  
 NCBI BlastP on this gene

THITE\_2142385

hypothetical protein
  
Accession: AEO64478
  
Location: 8755334-8756280
  
 NCBI BlastP on this gene

THITE\_74555

hypothetical protein
  
Accession: AEO64479
  
Location: 8756804-8758561
  
 NCBI BlastP on this gene

THITE\_2110648

hypothetical protein
  
Accession: AEO64480
  
Location: 8761149-8766032
  
 NCBI BlastP on this gene

THITE\_2110651

hypothetical protein
  
Accession: AEO64481
  
Location: 8766998-8767633
  
 NCBI BlastP on this gene

THITE\_2110654

Query: Architecture Search FASTA input

DS572699 : Verticillium dahliae VdLs.17 supercont1.5 genomic scaffold    Total score: 2.0     Cumulative Blast bit score: 1975

Hit cluster cross-links:

Mycgr3G85918 Mycgr3T
  
Location: 0-1602

Mycgr3G85918\_Mycgr3T

Mycgr3G42010 Mycgr3T
  
Location: 1702-8569

Mycgr3G42010\_Mycgr3T

Mycgr3G29582 Mycgr3T
  
Location: 8669-8915

Mycgr3G29582\_Mycgr3T

Mycgr3G31170 Mycgr3T
  
Location: 9015-9255

Mycgr3G31170\_Mycgr3T

Mycgr3G85924 Mycgr3T
  
Location: 9355-11218

Mycgr3G85924\_Mycgr3T

Mycgr3G71676 Mycgr3T
  
Location: 11318-12494

Mycgr3G71676\_Mycgr3T

Mycgr3G11468 Mycgr3T
  
Location: 12594-13653

Mycgr3G11468\_Mycgr3T

Mycgr3G58567 Mycgr3T
  
Location: 13753-14506

Mycgr3G58567\_Mycgr3T

Mycgr3G100089 Mycgr3
  
Location: 14606-21152

Mycgr3G100089\_Mycgr3

Mycgr3G42698 Mycgr3T
  
Location: 21252-22131

Mycgr3G42698\_Mycgr3T

Mycgr3G71681 Mycgr3T
  
Location: 22231-23461

Mycgr3G71681\_Mycgr3T

Mycgr3G109328 Mycgr3
  
Location: 23561-24239

Mycgr3G109328\_Mycgr3

Mycgr3G104334 Mycgr3
  
Location: 24339-24567

Mycgr3G104334\_Mycgr3

Mycgr3G42715 Mycgr3T
  
Location: 24667-25981

Mycgr3G42715\_Mycgr3T

Mycgr3G92934 Mycgr3T
  
Location: 26081-27593

Mycgr3G92934\_Mycgr3T

Mycgr3G41969 Mycgr3T
  
Location: 27693-29328

Mycgr3G41969\_Mycgr3T

Mycgr3G80635 Mycgr3T
  
Location: 29428-29821

Mycgr3G80635\_Mycgr3T

Mycgr3G41426 Mycgr3T
  
Location: 29921-35255

Mycgr3G41426\_Mycgr3T

Mycgr3G104337 Mycgr3
  
Location: 35355-36108

Mycgr3G104337\_Mycgr3

Mycgr3G71679 Mycgr3T
  
Location: 36208-37300

Mycgr3G71679\_Mycgr3T

Mycgr3G92938 Mycgr3T
  
Location: 37400-38699

Mycgr3G92938\_Mycgr3T

Mycgr3G92941 Mycgr3T
  
Location: 38799-40734

Mycgr3G92941\_Mycgr3T

hypothetical protein
  
Accession: EGY22017
  
Location: 1500448-1501474
  
 NCBI BlastP on this gene

EGY22017

hypothetical protein
  
Accession: EGY22018
  
Location: 1501949-1502650
  
 NCBI BlastP on this gene

EGY22018

mitochondrial inner membrane translocase subunit TIM44
  
Accession: EGY22019
  
Location: 1503228-1504912
  
 NCBI BlastP on this gene

EGY22019

mitochondrial ribosomal protein S18
  
Accession: EGY22020
  
Location: 1505384-1506130
  
 NCBI BlastP on this gene

EGY22020

hag1
  
Accession: EGY22021
  
Location: 1506436-1507278
  
 NCBI BlastP on this gene

EGY22021

GTP-dependent nucleic acid-binding protein engD
  
Accession: EGY22022
  
Location: 1508332-1509968
  
 NCBI BlastP on this gene

EGY22022

exopolygalacturonase
  
Accession: EGY22023
  
Location: 1510190-1512025
  
 NCBI BlastP on this gene

EGY22023

thioredoxin
  
Accession: EGY22024
  
Location: 1512752-1513198
  
 NCBI BlastP on this gene

EGY22024

drug resistance protein
  
Accession: EGY22025
  
Location: 1514042-1515821
  
 NCBI BlastP on this gene

EGY22025

fatty acid synthase S-acetyltransferase
  
Accession: EGY22026
  
Location: 1516713-1523486
  
  
**BlastP hit with Mycgr3G100089\_Mycgr3**
  
Percentage identity: 44 %
  
BlastP bit score: 1794
  
Sequence coverage: 100 %
  
E-value: 0.0
  
  
 NCBI BlastP on this gene

EGY22026

hypothetical protein
  
Accession: EGY22027
  
Location: 1524238-1525299
  
  
**BlastP hit with Mycgr3G104337\_Mycgr3**
  
Percentage identity: 38 %
  
BlastP bit score: 181
  
Sequence coverage: 101 %
  
E-value: 6e-52
  
  
 NCBI BlastP on this gene

EGY22027

3-hydroxyisobutyrate dehydrogenase
  
Accession: EGY22028
  
Location: 1525907-1529426
  
 NCBI BlastP on this gene

EGY22028

hypothetical protein
  
Accession: EGY22029
  
Location: 1530013-1532403
  
 NCBI BlastP on this gene

EGY22029

phthalate transporter
  
Accession: EGY22030
  
Location: 1533491-1535100
  
 NCBI BlastP on this gene

EGY22030

hypothetical protein
  
Accession: EGY22031
  
Location: 1535841-1536458
  
 NCBI BlastP on this gene

EGY22031

hypothetical protein
  
Accession: EGY22032
  
Location: 1537220-1538028
  
 NCBI BlastP on this gene

EGY22032

cutinase-2
  
Accession: EGY22033
  
Location: 1539140-1539869
  
 NCBI BlastP on this gene

EGY22033

vacuolar protein sorting-associated protein
  
Accession: EGY22034
  
Location: 1542234-1544693
  
 NCBI BlastP on this gene

EGY22034

Query: Architecture Search FASTA input

CM001231 : Magnaporthe oryzae 70-15 chromosome 1    Total score: 2.0     Cumulative Blast bit score: 1970

Hit cluster cross-links:

Mycgr3G85918 Mycgr3T
  
Location: 0-1602

Mycgr3G85918\_Mycgr3T

Mycgr3G42010 Mycgr3T
  
Location: 1702-8569

Mycgr3G42010\_Mycgr3T

Mycgr3G29582 Mycgr3T
  
Location: 8669-8915

Mycgr3G29582\_Mycgr3T

Mycgr3G31170 Mycgr3T
  
Location: 9015-9255

Mycgr3G31170\_Mycgr3T

Mycgr3G85924 Mycgr3T
  
Location: 9355-11218

Mycgr3G85924\_Mycgr3T

Mycgr3G71676 Mycgr3T
  
Location: 11318-12494

Mycgr3G71676\_Mycgr3T

Mycgr3G11468 Mycgr3T
  
Location: 12594-13653

Mycgr3G11468\_Mycgr3T

Mycgr3G58567 Mycgr3T
  
Location: 13753-14506

Mycgr3G58567\_Mycgr3T

Mycgr3G100089 Mycgr3
  
Location: 14606-21152

Mycgr3G100089\_Mycgr3

Mycgr3G42698 Mycgr3T
  
Location: 21252-22131

Mycgr3G42698\_Mycgr3T

Mycgr3G71681 Mycgr3T
  
Location: 22231-23461

Mycgr3G71681\_Mycgr3T

Mycgr3G109328 Mycgr3
  
Location: 23561-24239

Mycgr3G109328\_Mycgr3

Mycgr3G104334 Mycgr3
  
Location: 24339-24567

Mycgr3G104334\_Mycgr3

Mycgr3G42715 Mycgr3T
  
Location: 24667-25981

Mycgr3G42715\_Mycgr3T

Mycgr3G92934 Mycgr3T
  
Location: 26081-27593

Mycgr3G92934\_Mycgr3T

Mycgr3G41969 Mycgr3T
  
Location: 27693-29328

Mycgr3G41969\_Mycgr3T

Mycgr3G80635 Mycgr3T
  
Location: 29428-29821

Mycgr3G80635\_Mycgr3T

Mycgr3G41426 Mycgr3T
  
Location: 29921-35255

Mycgr3G41426\_Mycgr3T

Mycgr3G104337 Mycgr3
  
Location: 35355-36108

Mycgr3G104337\_Mycgr3

Mycgr3G71679 Mycgr3T
  
Location: 36208-37300

Mycgr3G71679\_Mycgr3T

Mycgr3G92938 Mycgr3T
  
Location: 37400-38699

Mycgr3G92938\_Mycgr3T

Mycgr3G92941 Mycgr3T
  
Location: 38799-40734

Mycgr3G92941\_Mycgr3T

lovastatin nonaketide synthase
  
Accession: EHA57854
  
Location: 5748736-5755754
  
  
**BlastP hit with Mycgr3G100089\_Mycgr3**
  
Percentage identity: 42 %
  
BlastP bit score: 1755
  
Sequence coverage: 101 %
  
E-value: 0.0
  
  
 NCBI BlastP on this gene

EHA57854

zeaxanthin epoxidase
  
Accession: EHA57855
  
Location: 5757154-5758404
  
 NCBI BlastP on this gene

EHA57855

hypothetical protein
  
Accession: EHA57856
  
Location: 5759786-5760533
  
 NCBI BlastP on this gene

EHA57856

hypothetical protein
  
Accession: EHA57857
  
Location: 5761734-5763376
  
 NCBI BlastP on this gene

EHA57857

hypothetical protein
  
Accession: EHA57858
  
Location: 5763555-5764268
  
 NCBI BlastP on this gene

EHA57858

hypothetical protein
  
Accession: EHA57859
  
Location: 5764790-5765659
  
 NCBI BlastP on this gene

EHA57859

hypothetical protein
  
Accession: EHA57860
  
Location: 5766500-5767507
  
  
**BlastP hit with Mycgr3G104337\_Mycgr3**
  
Percentage identity: 43 %
  
BlastP bit score: 216
  
Sequence coverage: 95 %
  
E-value: 1e-65
  
  
 NCBI BlastP on this gene

EHA57860

hypothetical protein
  
Accession: EHA57861
  
Location: 5768694-5769176
  
 NCBI BlastP on this gene

EHA57861

hypothetical protein
  
Accession: EHA57862
  
Location: 5775475-5776069
  
 NCBI BlastP on this gene

EHA57862

hypothetical protein
  
Accession: EHA57863
  
Location: 5776651-5778599
  
 NCBI BlastP on this gene

EHA57863

Query: Architecture Search FASTA input

AM270302 : Aspergillus niger contig An13c0080, genomic contig.    Total score: 2.0     Cumulative Blast bit score: 1965

Hit cluster cross-links:

Mycgr3G85918 Mycgr3T
  
Location: 0-1602

Mycgr3G85918\_Mycgr3T

Mycgr3G42010 Mycgr3T
  
Location: 1702-8569

Mycgr3G42010\_Mycgr3T

Mycgr3G29582 Mycgr3T
  
Location: 8669-8915

Mycgr3G29582\_Mycgr3T

Mycgr3G31170 Mycgr3T
  
Location: 9015-9255

Mycgr3G31170\_Mycgr3T

Mycgr3G85924 Mycgr3T
  
Location: 9355-11218

Mycgr3G85924\_Mycgr3T

Mycgr3G71676 Mycgr3T
  
Location: 11318-12494

Mycgr3G71676\_Mycgr3T

Mycgr3G11468 Mycgr3T
  
Location: 12594-13653

Mycgr3G11468\_Mycgr3T

Mycgr3G58567 Mycgr3T
  
Location: 13753-14506

Mycgr3G58567\_Mycgr3T

Mycgr3G100089 Mycgr3
  
Location: 14606-21152

Mycgr3G100089\_Mycgr3

Mycgr3G42698 Mycgr3T
  
Location: 21252-22131

Mycgr3G42698\_Mycgr3T

Mycgr3G71681 Mycgr3T
  
Location: 22231-23461

Mycgr3G71681\_Mycgr3T

Mycgr3G109328 Mycgr3
  
Location: 23561-24239

Mycgr3G109328\_Mycgr3

Mycgr3G104334 Mycgr3
  
Location: 24339-24567

Mycgr3G104334\_Mycgr3

Mycgr3G42715 Mycgr3T
  
Location: 24667-25981

Mycgr3G42715\_Mycgr3T

Mycgr3G92934 Mycgr3T
  
Location: 26081-27593

Mycgr3G92934\_Mycgr3T

Mycgr3G41969 Mycgr3T
  
Location: 27693-29328

Mycgr3G41969\_Mycgr3T

Mycgr3G80635 Mycgr3T
  
Location: 29428-29821

Mycgr3G80635\_Mycgr3T

Mycgr3G41426 Mycgr3T
  
Location: 29921-35255

Mycgr3G41426\_Mycgr3T

Mycgr3G104337 Mycgr3
  
Location: 35355-36108

Mycgr3G104337\_Mycgr3

Mycgr3G71679 Mycgr3T
  
Location: 36208-37300

Mycgr3G71679\_Mycgr3T

Mycgr3G92938 Mycgr3T
  
Location: 37400-38699

Mycgr3G92938\_Mycgr3T

Mycgr3G92941 Mycgr3T
  
Location: 38799-40734

Mycgr3G92941\_Mycgr3T

unnamed
  
Accession: CAK41634
  
Location: 3850-6559
  
 NCBI BlastP on this gene

An13g02350

not annotated
  
Accession: CAK41635
  
Location: 7192-9105
  
 NCBI BlastP on this gene

An13g02360

not annotated
  
Accession: CAK41636
  
Location: 9739-11410
  
 NCBI BlastP on this gene

An13g02370

unnamed
  
Accession: CAK41637
  
Location: 12504-13708
  
 NCBI BlastP on this gene

An13g02380

not annotated
  
Accession: CAK41638
  
Location: 14555-16597
  
 NCBI BlastP on this gene

An13g02390

not annotated
  
Accession: CAK41639
  
Location: 17814-19902
  
 NCBI BlastP on this gene

An13g02400

not annotated
  
Accession: CAK41640
  
Location: 20694-22152
  
 NCBI BlastP on this gene

An13g02410

not annotated
  
Accession: CAK41641
  
Location: 23726-24641
  
  
**BlastP hit with Mycgr3G104337\_Mycgr3**
  
Percentage identity: 44 %
  
BlastP bit score: 209
  
Sequence coverage: 98 %
  
E-value: 3e-63
  
  
 NCBI BlastP on this gene

An13g02420

not annotated
  
Accession: CAK41642
  
Location: 26479-33983
  
  
**BlastP hit with Mycgr3G100089\_Mycgr3**
  
Percentage identity: 44 %
  
BlastP bit score: 1756
  
Sequence coverage: 102 %
  
E-value: 0.0
  
  
 NCBI BlastP on this gene

An13g02430

unnamed
  
Accession: CAK41643
  
Location: 34703-37042
  
 NCBI BlastP on this gene

An13g02450

not annotated
  
Accession: CAK41644
  
Location: 38345-41642
  
 NCBI BlastP on this gene

An13g02460

hypothetical protein
  
Accession: CAK41645
  
Location: 42070-42457
  
 NCBI BlastP on this gene

An13g02470

unnamed
  
Accession: CAK41646
  
Location: 42640-44476
  
 NCBI BlastP on this gene

An13g02480

hypothetical protein
  
Accession: CAK41647
  
Location: 45634-48439
  
 NCBI BlastP on this gene

An13g02500

unnamed
  
Accession: CAK41648
  
Location: 48606-49694
  
 NCBI BlastP on this gene

An13g02510

hypothetical protein
  
Accession: CAK41649
  
Location: 49837-50534
  
 NCBI BlastP on this gene

An13g02520

unnamed
  
Accession: CAK41650
  
Location: 51075-51799
  
 NCBI BlastP on this gene

An13g02530

Query: Architecture Search FASTA input

DF126480 : Aspergillus kawachii IFO 4308 DNA, contig: scaffold00034    Total score: 2.0     Cumulative Blast bit score: 1958

Hit cluster cross-links:

Mycgr3G85918 Mycgr3T
  
Location: 0-1602

Mycgr3G85918\_Mycgr3T

Mycgr3G42010 Mycgr3T
  
Location: 1702-8569

Mycgr3G42010\_Mycgr3T

Mycgr3G29582 Mycgr3T
  
Location: 8669-8915

Mycgr3G29582\_Mycgr3T

Mycgr3G31170 Mycgr3T
  
Location: 9015-9255

Mycgr3G31170\_Mycgr3T

Mycgr3G85924 Mycgr3T
  
Location: 9355-11218

Mycgr3G85924\_Mycgr3T

Mycgr3G71676 Mycgr3T
  
Location: 11318-12494

Mycgr3G71676\_Mycgr3T

Mycgr3G11468 Mycgr3T
  
Location: 12594-13653

Mycgr3G11468\_Mycgr3T

Mycgr3G58567 Mycgr3T
  
Location: 13753-14506

Mycgr3G58567\_Mycgr3T

Mycgr3G100089 Mycgr3
  
Location: 14606-21152

Mycgr3G100089\_Mycgr3

Mycgr3G42698 Mycgr3T
  
Location: 21252-22131

Mycgr3G42698\_Mycgr3T

Mycgr3G71681 Mycgr3T
  
Location: 22231-23461

Mycgr3G71681\_Mycgr3T

Mycgr3G109328 Mycgr3
  
Location: 23561-24239

Mycgr3G109328\_Mycgr3

Mycgr3G104334 Mycgr3
  
Location: 24339-24567

Mycgr3G104334\_Mycgr3

Mycgr3G42715 Mycgr3T
  
Location: 24667-25981

Mycgr3G42715\_Mycgr3T

Mycgr3G92934 Mycgr3T
  
Location: 26081-27593

Mycgr3G92934\_Mycgr3T

Mycgr3G41969 Mycgr3T
  
Location: 27693-29328

Mycgr3G41969\_Mycgr3T

Mycgr3G80635 Mycgr3T
  
Location: 29428-29821

Mycgr3G80635\_Mycgr3T

Mycgr3G41426 Mycgr3T
  
Location: 29921-35255

Mycgr3G41426\_Mycgr3T

Mycgr3G104337 Mycgr3
  
Location: 35355-36108

Mycgr3G104337\_Mycgr3

Mycgr3G71679 Mycgr3T
  
Location: 36208-37300

Mycgr3G71679\_Mycgr3T

Mycgr3G92938 Mycgr3T
  
Location: 37400-38699

Mycgr3G92938\_Mycgr3T

Mycgr3G92941 Mycgr3T
  
Location: 38799-40734

Mycgr3G92941\_Mycgr3T

hypothetical protein
  
Accession: GAA91454
  
Location: 327273-329399
  
 NCBI BlastP on this gene

GAA91454

MFS transporter
  
Accession: GAA91455
  
Location: 330224-331858
  
 NCBI BlastP on this gene

GAA91455

MATE efflux family protein subfamily
  
Accession: GAA91456
  
Location: 333116-334687
  
 NCBI BlastP on this gene

GAA91456

agmatinase
  
Accession: GAA91457
  
Location: 335618-337221
  
 NCBI BlastP on this gene

GAA91457

similar to An14g05950
  
Accession: GAA91458
  
Location: 338299-339522
  
 NCBI BlastP on this gene

GAA91458

similar to An10g00090
  
Accession: GAA91459
  
Location: 341582-342124
  
 NCBI BlastP on this gene

GAA91459

AAA family ATPase
  
Accession: GAA91460
  
Location: 343999-351140
  
  
**BlastP hit with Mycgr3G42010\_Mycgr3T**
  
Percentage identity: 47 %
  
BlastP bit score: 1768
  
Sequence coverage: 85 %
  
E-value: 0.0
  
  
 NCBI BlastP on this gene

GAA91460

geranylgeranyl pyrophosphate synthetase
  
Accession: GAA91461
  
Location: 351465-352917
  
  
**BlastP hit with Mycgr3G92938\_Mycgr3T**
  
Percentage identity: 31 %
  
BlastP bit score: 190
  
Sequence coverage: 95 %
  
E-value: 2e-51
  
  
 NCBI BlastP on this gene

GAA91461

hypothetical protein
  
Accession: GAA91462
  
Location: 354134-354984
  
 NCBI BlastP on this gene

GAA91462

similar to An14g07400
  
Accession: GAA91463
  
Location: 355973-357072
  
 NCBI BlastP on this gene

GAA91463

class V chitinase
  
Accession: GAA91464
  
Location: 357261-360935
  
 NCBI BlastP on this gene

GAA91464

hypothetical protein
  
Accession: GAA91465
  
Location: 363401-370204
  
 NCBI BlastP on this gene

GAA91465

Query: Architecture Search FASTA input

JH725151 : Beauveria bassiana ARSEF 2860 unplaced genomic scaffold BBA\_S00002    Total score: 2.0     Cumulative Blast bit score: 1952

Hit cluster cross-links:

Mycgr3G85918 Mycgr3T
  
Location: 0-1602

Mycgr3G85918\_Mycgr3T

Mycgr3G42010 Mycgr3T
  
Location: 1702-8569

Mycgr3G42010\_Mycgr3T

Mycgr3G29582 Mycgr3T
  
Location: 8669-8915

Mycgr3G29582\_Mycgr3T

Mycgr3G31170 Mycgr3T
  
Location: 9015-9255

Mycgr3G31170\_Mycgr3T

Mycgr3G85924 Mycgr3T
  
Location: 9355-11218

Mycgr3G85924\_Mycgr3T

Mycgr3G71676 Mycgr3T
  
Location: 11318-12494

Mycgr3G71676\_Mycgr3T

Mycgr3G11468 Mycgr3T
  
Location: 12594-13653

Mycgr3G11468\_Mycgr3T

Mycgr3G58567 Mycgr3T
  
Location: 13753-14506

Mycgr3G58567\_Mycgr3T

Mycgr3G100089 Mycgr3
  
Location: 14606-21152

Mycgr3G100089\_Mycgr3

Mycgr3G42698 Mycgr3T
  
Location: 21252-22131

Mycgr3G42698\_Mycgr3T

Mycgr3G71681 Mycgr3T
  
Location: 22231-23461

Mycgr3G71681\_Mycgr3T

Mycgr3G109328 Mycgr3
  
Location: 23561-24239

Mycgr3G109328\_Mycgr3

Mycgr3G104334 Mycgr3
  
Location: 24339-24567

Mycgr3G104334\_Mycgr3

Mycgr3G42715 Mycgr3T
  
Location: 24667-25981

Mycgr3G42715\_Mycgr3T

Mycgr3G92934 Mycgr3T
  
Location: 26081-27593

Mycgr3G92934\_Mycgr3T

Mycgr3G41969 Mycgr3T
  
Location: 27693-29328

Mycgr3G41969\_Mycgr3T

Mycgr3G80635 Mycgr3T
  
Location: 29428-29821

Mycgr3G80635\_Mycgr3T

Mycgr3G41426 Mycgr3T
  
Location: 29921-35255

Mycgr3G41426\_Mycgr3T

Mycgr3G104337 Mycgr3
  
Location: 35355-36108

Mycgr3G104337\_Mycgr3

Mycgr3G71679 Mycgr3T
  
Location: 36208-37300

Mycgr3G71679\_Mycgr3T

Mycgr3G92938 Mycgr3T
  
Location: 37400-38699

Mycgr3G92938\_Mycgr3T

Mycgr3G92941 Mycgr3T
  
Location: 38799-40734

Mycgr3G92941\_Mycgr3T

small nucleolar ribonucleoprotein complex component (Utp5)
  
Accession: EJP70137
  
Location: 1194086-1195267
  
 NCBI BlastP on this gene

EJP70137

hypothetical protein
  
Accession: EJP70138
  
Location: 1196004-1197902
  
 NCBI BlastP on this gene

EJP70138

hypothetical protein
  
Accession: EJP70139
  
Location: 1202330-1206037
  
 NCBI BlastP on this gene

EJP70139

amino acid permease
  
Accession: EJP70140
  
Location: 1206587-1208305
  
 NCBI BlastP on this gene

EJP70140

lovastatin nonaketide synthase
  
Accession: EJP70141
  
Location: 1209213-1216452
  
  
**BlastP hit with Mycgr3G100089\_Mycgr3**
  
Percentage identity: 43 %
  
BlastP bit score: 1758
  
Sequence coverage: 104 %
  
E-value: 0.0
  
  
 NCBI BlastP on this gene

EJP70141

EF-hand calcium-binding domain protein
  
Accession: EJP70142
  
Location: 1221799-1222797
  
  
**BlastP hit with Mycgr3G104337\_Mycgr3**
  
Percentage identity: 41 %
  
BlastP bit score: 195
  
Sequence coverage: 100 %
  
E-value: 7e-58
  
  
 NCBI BlastP on this gene

EJP70142

UDP-glucoronosyl and UDP-glucosyl transferase family protein
  
Accession: EJP70143
  
Location: 1224748-1226436
  
 NCBI BlastP on this gene

EJP70143

protein (fungal and bacterial)
  
Accession: EJP70144
  
Location: 1226724-1228871
  
 NCBI BlastP on this gene

EJP70144

C6 transcription factor, putative
  
Accession: EJP70145
  
Location: 1232367-1233773
  
 NCBI BlastP on this gene

EJP70145

major facilitator superfamily transporter
  
Accession: EJP70146
  
Location: 1235553-1237305
  
 NCBI BlastP on this gene

EJP70146

Ribonuclease/ribotoxin
  
Accession: EJP70147
  
Location: 1237900-1238351
  
 NCBI BlastP on this gene

EJP70147

Query: Architecture Search FASTA input

CH408034 : Chaetomium globosum CBS 148.51 scaffold\_6 genomic scaffold    Total score: 2.0     Cumulative Blast bit score: 1948

Hit cluster cross-links:

Mycgr3G85918 Mycgr3T
  
Location: 0-1602

Mycgr3G85918\_Mycgr3T

Mycgr3G42010 Mycgr3T
  
Location: 1702-8569

Mycgr3G42010\_Mycgr3T

Mycgr3G29582 Mycgr3T
  
Location: 8669-8915

Mycgr3G29582\_Mycgr3T

Mycgr3G31170 Mycgr3T
  
Location: 9015-9255

Mycgr3G31170\_Mycgr3T

Mycgr3G85924 Mycgr3T
  
Location: 9355-11218

Mycgr3G85924\_Mycgr3T

Mycgr3G71676 Mycgr3T
  
Location: 11318-12494

Mycgr3G71676\_Mycgr3T

Mycgr3G11468 Mycgr3T
  
Location: 12594-13653

Mycgr3G11468\_Mycgr3T

Mycgr3G58567 Mycgr3T
  
Location: 13753-14506

Mycgr3G58567\_Mycgr3T

Mycgr3G100089 Mycgr3
  
Location: 14606-21152

Mycgr3G100089\_Mycgr3

Mycgr3G42698 Mycgr3T
  
Location: 21252-22131

Mycgr3G42698\_Mycgr3T

Mycgr3G71681 Mycgr3T
  
Location: 22231-23461

Mycgr3G71681\_Mycgr3T

Mycgr3G109328 Mycgr3
  
Location: 23561-24239

Mycgr3G109328\_Mycgr3

Mycgr3G104334 Mycgr3
  
Location: 24339-24567

Mycgr3G104334\_Mycgr3

Mycgr3G42715 Mycgr3T
  
Location: 24667-25981

Mycgr3G42715\_Mycgr3T

Mycgr3G92934 Mycgr3T
  
Location: 26081-27593

Mycgr3G92934\_Mycgr3T

Mycgr3G41969 Mycgr3T
  
Location: 27693-29328

Mycgr3G41969\_Mycgr3T

Mycgr3G80635 Mycgr3T
  
Location: 29428-29821

Mycgr3G80635\_Mycgr3T

Mycgr3G41426 Mycgr3T
  
Location: 29921-35255

Mycgr3G41426\_Mycgr3T

Mycgr3G104337 Mycgr3
  
Location: 35355-36108

Mycgr3G104337\_Mycgr3

Mycgr3G71679 Mycgr3T
  
Location: 36208-37300

Mycgr3G71679\_Mycgr3T

Mycgr3G92938 Mycgr3T
  
Location: 37400-38699

Mycgr3G92938\_Mycgr3T

Mycgr3G92941 Mycgr3T
  
Location: 38799-40734

Mycgr3G92941\_Mycgr3T

hypothetical protein
  
Accession: EAQ84725
  
Location: 1222984-1224827
  
 NCBI BlastP on this gene

EAQ84725

hypothetical protein
  
Accession: EAQ84726
  
Location: 1225272-1226805
  
 NCBI BlastP on this gene

EAQ84726

hypothetical protein
  
Accession: EAQ84727
  
Location: 1228303-1230898
  
 NCBI BlastP on this gene

EAQ84727

conserved hypothetical protein
  
Accession: EAQ84728
  
Location: 1231000-1232153
  
 NCBI BlastP on this gene

EAQ84728

hypothetical protein
  
Accession: EAQ84729
  
Location: 1233922-1234341
  
 NCBI BlastP on this gene

EAQ84729

hypothetical protein
  
Accession: EAQ84730
  
Location: 1235132-1239454
  
 NCBI BlastP on this gene

EAQ84730

hypothetical protein
  
Accession: EAQ84731
  
Location: 1241032-1242593
  
  
**BlastP hit with Mycgr3G104337\_Mycgr3**
  
Percentage identity: 41 %
  
BlastP bit score: 161
  
Sequence coverage: 78 %
  
E-value: 2e-44
  
  
 NCBI BlastP on this gene

EAQ84731

hypothetical protein
  
Accession: EAQ84732
  
Location: 1245180-1252426
  
  
**BlastP hit with Mycgr3G100089\_Mycgr3**
  
Percentage identity: 43 %
  
BlastP bit score: 1787
  
Sequence coverage: 100 %
  
E-value: 0.0
  
  
 NCBI BlastP on this gene

EAQ84732

hypothetical protein
  
Accession: EAQ84733
  
Location: 1252965-1255119
  
 NCBI BlastP on this gene

EAQ84733

hypothetical protein
  
Accession: EAQ84734
  
Location: 1258048-1260516
  
 NCBI BlastP on this gene

EAQ84734

conserved hypothetical protein
  
Accession: EAQ84735
  
Location: 1261328-1262727
  
 NCBI BlastP on this gene

EAQ84735

hypothetical protein
  
Accession: EAQ84736
  
Location: 1263118-1264250
  
 NCBI BlastP on this gene

EAQ84736

hypothetical protein
  
Accession: EAQ84737
  
Location: 1265463-1267300
  
 NCBI BlastP on this gene

EAQ84737

hypothetical protein
  
Accession: EAQ84738
  
Location: 1269509-1270613
  
 NCBI BlastP on this gene

EAQ84738

Query: Architecture Search FASTA input

AABX02000004 : Neurospora crassa OR74A    Total score: 2.0     Cumulative Blast bit score: 1940

Hit cluster cross-links:

Mycgr3G85918 Mycgr3T
  
Location: 0-1602

Mycgr3G85918\_Mycgr3T

Mycgr3G42010 Mycgr3T
  
Location: 1702-8569

Mycgr3G42010\_Mycgr3T

Mycgr3G29582 Mycgr3T
  
Location: 8669-8915

Mycgr3G29582\_Mycgr3T

Mycgr3G31170 Mycgr3T
  
Location: 9015-9255

Mycgr3G31170\_Mycgr3T

Mycgr3G85924 Mycgr3T
  
Location: 9355-11218

Mycgr3G85924\_Mycgr3T

Mycgr3G71676 Mycgr3T
  
Location: 11318-12494

Mycgr3G71676\_Mycgr3T

Mycgr3G11468 Mycgr3T
  
Location: 12594-13653

Mycgr3G11468\_Mycgr3T

Mycgr3G58567 Mycgr3T
  
Location: 13753-14506

Mycgr3G58567\_Mycgr3T

Mycgr3G100089 Mycgr3
  
Location: 14606-21152

Mycgr3G100089\_Mycgr3

Mycgr3G42698 Mycgr3T
  
Location: 21252-22131

Mycgr3G42698\_Mycgr3T

Mycgr3G71681 Mycgr3T
  
Location: 22231-23461

Mycgr3G71681\_Mycgr3T

Mycgr3G109328 Mycgr3
  
Location: 23561-24239

Mycgr3G109328\_Mycgr3

Mycgr3G104334 Mycgr3
  
Location: 24339-24567

Mycgr3G104334\_Mycgr3

Mycgr3G42715 Mycgr3T
  
Location: 24667-25981

Mycgr3G42715\_Mycgr3T

Mycgr3G92934 Mycgr3T
  
Location: 26081-27593

Mycgr3G92934\_Mycgr3T

Mycgr3G41969 Mycgr3T
  
Location: 27693-29328

Mycgr3G41969\_Mycgr3T

Mycgr3G80635 Mycgr3T
  
Location: 29428-29821

Mycgr3G80635\_Mycgr3T

Mycgr3G41426 Mycgr3T
  
Location: 29921-35255

Mycgr3G41426\_Mycgr3T

Mycgr3G104337 Mycgr3
  
Location: 35355-36108

Mycgr3G104337\_Mycgr3

Mycgr3G71679 Mycgr3T
  
Location: 36208-37300

Mycgr3G71679\_Mycgr3T

Mycgr3G92938 Mycgr3T
  
Location: 37400-38699

Mycgr3G92938\_Mycgr3T

Mycgr3G92941 Mycgr3T
  
Location: 38799-40734

Mycgr3G92941\_Mycgr3T

hypothetical protein
  
Accession: EAA28899
  
Location: 124736-132387
  
  
**BlastP hit with Mycgr3G100089\_Mycgr3**
  
Percentage identity: 43 %
  
BlastP bit score: 1726
  
Sequence coverage: 100 %
  
E-value: 0.0
  
  
 NCBI BlastP on this gene

EAA28899

conserved hypothetical protein
  
Accession: EAA29584
  
Location: 134461-135421
  
  
**BlastP hit with Mycgr3G104337\_Mycgr3**
  
Percentage identity: 41 %
  
BlastP bit score: 214
  
Sequence coverage: 100 %
  
E-value: 5e-65
  
  
 NCBI BlastP on this gene

EAA29584

hypothetical protein
  
Accession: EAA29583
  
Location: 137203-142380
  
 NCBI BlastP on this gene

EAA29583

hypothetical protein
  
Accession: EAA29582
  
Location: 143944-145882
  
 NCBI BlastP on this gene

EAA29582

hypothetical protein
  
Accession: EAA29581
  
Location: 147338-148498
  
 NCBI BlastP on this gene

EAA29581

hypothetical protein
  
Accession: EAA29580
  
Location: 149690-150970
  
 NCBI BlastP on this gene

EAA29580

predicted protein
  
Accession: EAA29579
  
Location: 151285-152181
  
 NCBI BlastP on this gene

EAA29579

predicted protein
  
Accession: EAA29578
  
Location: 152739-154373
  
 NCBI BlastP on this gene

EAA29578

Query: Architecture Search FASTA input

JH921449 : Marssonina brunnea f. sp. 'multigermtubi' MB\_m1 unplaced genomic scaffold M6\_S00022    Total score: 2.0     Cumulative Blast bit score: 1935

Hit cluster cross-links:

Mycgr3G85918 Mycgr3T
  
Location: 0-1602

Mycgr3G85918\_Mycgr3T

Mycgr3G42010 Mycgr3T
  
Location: 1702-8569

Mycgr3G42010\_Mycgr3T

Mycgr3G29582 Mycgr3T
  
Location: 8669-8915

Mycgr3G29582\_Mycgr3T

Mycgr3G31170 Mycgr3T
  
Location: 9015-9255

Mycgr3G31170\_Mycgr3T

Mycgr3G85924 Mycgr3T
  
Location: 9355-11218

Mycgr3G85924\_Mycgr3T

Mycgr3G71676 Mycgr3T
  
Location: 11318-12494

Mycgr3G71676\_Mycgr3T

Mycgr3G11468 Mycgr3T
  
Location: 12594-13653

Mycgr3G11468\_Mycgr3T

Mycgr3G58567 Mycgr3T
  
Location: 13753-14506

Mycgr3G58567\_Mycgr3T

Mycgr3G100089 Mycgr3
  
Location: 14606-21152

Mycgr3G100089\_Mycgr3

Mycgr3G42698 Mycgr3T
  
Location: 21252-22131

Mycgr3G42698\_Mycgr3T

Mycgr3G71681 Mycgr3T
  
Location: 22231-23461

Mycgr3G71681\_Mycgr3T

Mycgr3G109328 Mycgr3
  
Location: 23561-24239

Mycgr3G109328\_Mycgr3

Mycgr3G104334 Mycgr3
  
Location: 24339-24567

Mycgr3G104334\_Mycgr3

Mycgr3G42715 Mycgr3T
  
Location: 24667-25981

Mycgr3G42715\_Mycgr3T

Mycgr3G92934 Mycgr3T
  
Location: 26081-27593

Mycgr3G92934\_Mycgr3T

Mycgr3G41969 Mycgr3T
  
Location: 27693-29328

Mycgr3G41969\_Mycgr3T

Mycgr3G80635 Mycgr3T
  
Location: 29428-29821

Mycgr3G80635\_Mycgr3T

Mycgr3G41426 Mycgr3T
  
Location: 29921-35255

Mycgr3G41426\_Mycgr3T

Mycgr3G104337 Mycgr3
  
Location: 35355-36108

Mycgr3G104337\_Mycgr3

Mycgr3G71679 Mycgr3T
  
Location: 36208-37300

Mycgr3G71679\_Mycgr3T

Mycgr3G92938 Mycgr3T
  
Location: 37400-38699

Mycgr3G92938\_Mycgr3T

Mycgr3G92941 Mycgr3T
  
Location: 38799-40734

Mycgr3G92941\_Mycgr3T

arrestin domain-containing protein
  
Accession: EKD13594
  
Location: 504727-507230
  
 NCBI BlastP on this gene

EKD13594

hypothetical protein
  
Accession: EKD13595
  
Location: 514017-514472
  
 NCBI BlastP on this gene

EKD13595

ABC transporter
  
Accession: EKD13596
  
Location: 515959-521009
  
 NCBI BlastP on this gene

EKD13596

putative EF-hand calcium-binding domain protein
  
Accession: EKD13597
  
Location: 522874-523854
  
  
**BlastP hit with Mycgr3G104337\_Mycgr3**
  
Percentage identity: 43 %
  
BlastP bit score: 223
  
Sequence coverage: 106 %
  
E-value: 2e-68
  
  
 NCBI BlastP on this gene

EKD13597

polyketide synthase
  
Accession: EKD13598
  
Location: 525884-533571
  
  
**BlastP hit with Mycgr3G100089\_Mycgr3**
  
Percentage identity: 41 %
  
BlastP bit score: 1712
  
Sequence coverage: 101 %
  
E-value: 0.0
  
  
 NCBI BlastP on this gene

EKD13598

hypothetical protein
  
Accession: EKD13599
  
Location: 535310-536182
  
 NCBI BlastP on this gene

EKD13599

hypothetical protein
  
Accession: EKD13600
  
Location: 537175-537411
  
 NCBI BlastP on this gene

EKD13600

hypothetical protein
  
Accession: EKD13601
  
Location: 538647-539769
  
 NCBI BlastP on this gene

EKD13601

hypothetical protein
  
Accession: EKD13602
  
Location: 541793-543132
  
 NCBI BlastP on this gene

EKD13602

DNA replication licensing factor mcm7
  
Accession: EKD13603
  
Location: 544605-547268
  
 NCBI BlastP on this gene

EKD13603

ATPase
  
Accession: EKD13604
  
Location: 547679-551456
  
 NCBI BlastP on this gene

EKD13604

Query: Architecture Search FASTA input

DF126457 : Aspergillus kawachii IFO 4308 DNA, contig: scaffold00011    Total score: 2.0     Cumulative Blast bit score: 1925

Hit cluster cross-links:

Mycgr3G85918 Mycgr3T
  
Location: 0-1602

Mycgr3G85918\_Mycgr3T

Mycgr3G42010 Mycgr3T
  
Location: 1702-8569

Mycgr3G42010\_Mycgr3T

Mycgr3G29582 Mycgr3T
  
Location: 8669-8915

Mycgr3G29582\_Mycgr3T

Mycgr3G31170 Mycgr3T
  
Location: 9015-9255

Mycgr3G31170\_Mycgr3T

Mycgr3G85924 Mycgr3T
  
Location: 9355-11218

Mycgr3G85924\_Mycgr3T

Mycgr3G71676 Mycgr3T
  
Location: 11318-12494

Mycgr3G71676\_Mycgr3T

Mycgr3G11468 Mycgr3T
  
Location: 12594-13653

Mycgr3G11468\_Mycgr3T

Mycgr3G58567 Mycgr3T
  
Location: 13753-14506

Mycgr3G58567\_Mycgr3T

Mycgr3G100089 Mycgr3
  
Location: 14606-21152

Mycgr3G100089\_Mycgr3

Mycgr3G42698 Mycgr3T
  
Location: 21252-22131

Mycgr3G42698\_Mycgr3T

Mycgr3G71681 Mycgr3T
  
Location: 22231-23461

Mycgr3G71681\_Mycgr3T

Mycgr3G109328 Mycgr3
  
Location: 23561-24239

Mycgr3G109328\_Mycgr3

Mycgr3G104334 Mycgr3
  
Location: 24339-24567

Mycgr3G104334\_Mycgr3

Mycgr3G42715 Mycgr3T
  
Location: 24667-25981

Mycgr3G42715\_Mycgr3T

Mycgr3G92934 Mycgr3T
  
Location: 26081-27593

Mycgr3G92934\_Mycgr3T

Mycgr3G41969 Mycgr3T
  
Location: 27693-29328

Mycgr3G41969\_Mycgr3T

Mycgr3G80635 Mycgr3T
  
Location: 29428-29821

Mycgr3G80635\_Mycgr3T

Mycgr3G41426 Mycgr3T
  
Location: 29921-35255

Mycgr3G41426\_Mycgr3T

Mycgr3G104337 Mycgr3
  
Location: 35355-36108

Mycgr3G104337\_Mycgr3

Mycgr3G71679 Mycgr3T
  
Location: 36208-37300

Mycgr3G71679\_Mycgr3T

Mycgr3G92938 Mycgr3T
  
Location: 37400-38699

Mycgr3G92938\_Mycgr3T

Mycgr3G92941 Mycgr3T
  
Location: 38799-40734

Mycgr3G92941\_Mycgr3T

hypothetical protein
  
Accession: GAA86901
  
Location: 817651-819567
  
 NCBI BlastP on this gene

GAA86901

C-x8-C-x5-C-x3-H type zinc finger protein
  
Accession: GAA86902
  
Location: 820216-821887
  
 NCBI BlastP on this gene

GAA86902

hypothetical protein
  
Accession: GAA86903
  
Location: 822804-824005
  
 NCBI BlastP on this gene

GAA86903

MFS multidrug transporter
  
Accession: GAA86904
  
Location: 824802-826661
  
 NCBI BlastP on this gene

GAA86904

nucleoside transporter
  
Accession: GAA86905
  
Location: 827182-829048
  
 NCBI BlastP on this gene

GAA86905

C6 transcription factor
  
Accession: GAA86906
  
Location: 830332-832428
  
 NCBI BlastP on this gene

GAA86906

similar to An13g02410
  
Accession: GAA86907
  
Location: 833183-834654
  
 NCBI BlastP on this gene

GAA86907

DUF341 family oxidoreductase
  
Accession: GAA86908
  
Location: 835404-836360
  
  
**BlastP hit with Mycgr3G104337\_Mycgr3**
  
Percentage identity: 37 %
  
BlastP bit score: 183
  
Sequence coverage: 98 %
  
E-value: 3e-53
  
  
 NCBI BlastP on this gene

GAA86908

polyketide synthase
  
Accession: GAA86909
  
Location: 838044-845051
  
  
**BlastP hit with Mycgr3G100089\_Mycgr3**
  
Percentage identity: 43 %
  
BlastP bit score: 1742
  
Sequence coverage: 103 %
  
E-value: 0.0
  
  
 NCBI BlastP on this gene

GAA86909

six-hairpin glycosidase
  
Accession: GAA86910
  
Location: 846344-848383
  
 NCBI BlastP on this gene

GAA86910

NRPS-like enzyme
  
Accession: GAA86911
  
Location: 849961-853241
  
 NCBI BlastP on this gene

GAA86911

similar to An13g02470
  
Accession: GAA86912
  
Location: 853705-854061
  
 NCBI BlastP on this gene

GAA86912

flavin containing polyamine oxidase
  
Accession: GAA86913
  
Location: 854252-856088
  
 NCBI BlastP on this gene

GAA86913

cell wall glucanase/allergen F16-like protein
  
Accession: GAA86914
  
Location: 860166-861261
  
 NCBI BlastP on this gene

GAA86914

carbonic anhydrase
  
Accession: GAA86915
  
Location: 862601-863584
  
 NCBI BlastP on this gene

GAA86915

Query: Architecture Search FASTA input

CP003008 : Myceliophthora thermophila ATCC 42464 chromosome 7    Total score: 2.0     Cumulative Blast bit score: 1922

Hit cluster cross-links:

Mycgr3G85918 Mycgr3T
  
Location: 0-1602

Mycgr3G85918\_Mycgr3T

Mycgr3G42010 Mycgr3T
  
Location: 1702-8569

Mycgr3G42010\_Mycgr3T

Mycgr3G29582 Mycgr3T
  
Location: 8669-8915

Mycgr3G29582\_Mycgr3T

Mycgr3G31170 Mycgr3T
  
Location: 9015-9255

Mycgr3G31170\_Mycgr3T

Mycgr3G85924 Mycgr3T
  
Location: 9355-11218

Mycgr3G85924\_Mycgr3T

Mycgr3G71676 Mycgr3T
  
Location: 11318-12494

Mycgr3G71676\_Mycgr3T

Mycgr3G11468 Mycgr3T
  
Location: 12594-13653

Mycgr3G11468\_Mycgr3T

Mycgr3G58567 Mycgr3T
  
Location: 13753-14506

Mycgr3G58567\_Mycgr3T

Mycgr3G100089 Mycgr3
  
Location: 14606-21152

Mycgr3G100089\_Mycgr3

Mycgr3G42698 Mycgr3T
  
Location: 21252-22131

Mycgr3G42698\_Mycgr3T

Mycgr3G71681 Mycgr3T
  
Location: 22231-23461

Mycgr3G71681\_Mycgr3T

Mycgr3G109328 Mycgr3
  
Location: 23561-24239

Mycgr3G109328\_Mycgr3

Mycgr3G104334 Mycgr3
  
Location: 24339-24567

Mycgr3G104334\_Mycgr3

Mycgr3G42715 Mycgr3T
  
Location: 24667-25981

Mycgr3G42715\_Mycgr3T

Mycgr3G92934 Mycgr3T
  
Location: 26081-27593

Mycgr3G92934\_Mycgr3T

Mycgr3G41969 Mycgr3T
  
Location: 27693-29328

Mycgr3G41969\_Mycgr3T

Mycgr3G80635 Mycgr3T
  
Location: 29428-29821

Mycgr3G80635\_Mycgr3T

Mycgr3G41426 Mycgr3T
  
Location: 29921-35255

Mycgr3G41426\_Mycgr3T

Mycgr3G104337 Mycgr3
  
Location: 35355-36108

Mycgr3G104337\_Mycgr3

Mycgr3G71679 Mycgr3T
  
Location: 36208-37300

Mycgr3G71679\_Mycgr3T

Mycgr3G92938 Mycgr3T
  
Location: 37400-38699

Mycgr3G92938\_Mycgr3T

Mycgr3G92941 Mycgr3T
  
Location: 38799-40734

Mycgr3G92941\_Mycgr3T

hypothetical protein
  
Accession: AEO62133
  
Location: 3896272-3897504
  
 NCBI BlastP on this gene

MYCTH\_2113991

hypothetical protein
  
Accession: AEO62134
  
Location: 3897968-3898660
  
 NCBI BlastP on this gene

MYCTH\_2313149

hypothetical protein
  
Accession: AEO62135
  
Location: 3899823-3902683
  
 NCBI BlastP on this gene

MYCTH\_2313151

hypothetical protein
  
Accession: AEO62136
  
Location: 3910728-3912516
  
  
**BlastP hit with Mycgr3G92938\_Mycgr3T**
  
Percentage identity: 50 %
  
BlastP bit score: 150
  
Sequence coverage: 34 %
  
E-value: 1e-36
  
  
 NCBI BlastP on this gene

MYCTH\_2313154

hypothetical protein
  
Accession: AEO62137
  
Location: 3913944-3921784
  
  
**BlastP hit with Mycgr3G42010\_Mycgr3T**
  
Percentage identity: 45 %
  
BlastP bit score: 1772
  
Sequence coverage: 96 %
  
E-value: 0.0
  
  
 NCBI BlastP on this gene

MYCTH\_2313156

glycoside hydrolase family 47 protein
  
Accession: AEO62138
  
Location: 3922910-3924892
  
 NCBI BlastP on this gene

MYCTH\_2313159

hypothetical protein
  
Accession: AEO62139
  
Location: 3926181-3927482
  
 NCBI BlastP on this gene

MYCTH\_2313161

hypothetical protein
  
Accession: AEO62140
  
Location: 3928368-3929523
  
 NCBI BlastP on this gene

MYCTH\_103818

hypothetical protein
  
Accession: AEO62141
  
Location: 3930955-3933897
  
 NCBI BlastP on this gene

MYCTH\_2313163

hypothetical protein
  
Accession: AEO62142
  
Location: 3934837-3937159
  
 NCBI BlastP on this gene

MYCTH\_2313164

Query: Architecture Search FASTA input

CAGA01000048 : Claviceps purpurea 20.1    Total score: 2.0     Cumulative Blast bit score: 1901

Hit cluster cross-links:

Mycgr3G85918 Mycgr3T
  
Location: 0-1602

Mycgr3G85918\_Mycgr3T

Mycgr3G42010 Mycgr3T
  
Location: 1702-8569

Mycgr3G42010\_Mycgr3T

Mycgr3G29582 Mycgr3T
  
Location: 8669-8915

Mycgr3G29582\_Mycgr3T

Mycgr3G31170 Mycgr3T
  
Location: 9015-9255

Mycgr3G31170\_Mycgr3T

Mycgr3G85924 Mycgr3T
  
Location: 9355-11218

Mycgr3G85924\_Mycgr3T

Mycgr3G71676 Mycgr3T
  
Location: 11318-12494

Mycgr3G71676\_Mycgr3T

Mycgr3G11468 Mycgr3T
  
Location: 12594-13653

Mycgr3G11468\_Mycgr3T

Mycgr3G58567 Mycgr3T
  
Location: 13753-14506

Mycgr3G58567\_Mycgr3T

Mycgr3G100089 Mycgr3
  
Location: 14606-21152

Mycgr3G100089\_Mycgr3

Mycgr3G42698 Mycgr3T
  
Location: 21252-22131

Mycgr3G42698\_Mycgr3T

Mycgr3G71681 Mycgr3T
  
Location: 22231-23461

Mycgr3G71681\_Mycgr3T

Mycgr3G109328 Mycgr3
  
Location: 23561-24239

Mycgr3G109328\_Mycgr3

Mycgr3G104334 Mycgr3
  
Location: 24339-24567

Mycgr3G104334\_Mycgr3

Mycgr3G42715 Mycgr3T
  
Location: 24667-25981

Mycgr3G42715\_Mycgr3T

Mycgr3G92934 Mycgr3T
  
Location: 26081-27593

Mycgr3G92934\_Mycgr3T

Mycgr3G41969 Mycgr3T
  
Location: 27693-29328

Mycgr3G41969\_Mycgr3T

Mycgr3G80635 Mycgr3T
  
Location: 29428-29821

Mycgr3G80635\_Mycgr3T

Mycgr3G41426 Mycgr3T
  
Location: 29921-35255

Mycgr3G41426\_Mycgr3T

Mycgr3G104337 Mycgr3
  
Location: 35355-36108

Mycgr3G104337\_Mycgr3

Mycgr3G71679 Mycgr3T
  
Location: 36208-37300

Mycgr3G71679\_Mycgr3T

Mycgr3G92938 Mycgr3T
  
Location: 37400-38699

Mycgr3G92938\_Mycgr3T

Mycgr3G92941 Mycgr3T
  
Location: 38799-40734

Mycgr3G92941\_Mycgr3T

probable cytochrome P450 monooxygenase (lovA)
  
Accession: CCE32887
  
Location: 9826-10941
  
 NCBI BlastP on this gene

CCE32887

uncharacterized protein
  
Accession: CCE32888
  
Location: 16360-17719
  
 NCBI BlastP on this gene

CCE32888

related to gibberellin 20-oxidase
  
Accession: CCE32889
  
Location: 22454-23706
  
 NCBI BlastP on this gene

CCE32889

related to 7alpha-cephem-methoxylase P8 chain
  
Accession: CCE32890
  
Location: 23915-24736
  
 NCBI BlastP on this gene

CCE32890

related to amidohydrolase
  
Accession: CCE32891
  
Location: 26940-28389
  
 NCBI BlastP on this gene

CCE32891

related to polyketide synthase
  
Accession: CCE32892
  
Location: 28674-35696
  
  
**BlastP hit with Mycgr3G100089\_Mycgr3**
  
Percentage identity: 43 %
  
BlastP bit score: 1773
  
Sequence coverage: 103 %
  
E-value: 0.0
  
  
 NCBI BlastP on this gene

CCE32892

uncharacterized protein
  
Accession: CCE32893
  
Location: 36690-37424
  
  
**BlastP hit with Mycgr3G104337\_Mycgr3**
  
Percentage identity: 41 %
  
BlastP bit score: 129
  
Sequence coverage: 63 %
  
E-value: 3e-33
  
  
 NCBI BlastP on this gene

CCE32893

uncharacterized protein
  
Accession: CCE32894
  
Location: 40768-41580
  
 NCBI BlastP on this gene

CCE32894

uncharacterized protein
  
Accession: CCE32895
  
Location: 42999-43538
  
 NCBI BlastP on this gene

CCE32895

related to carboxyphosphonoenolpyruvate phosphonomutase
  
Accession: CCE32896
  
Location: 47467-48648
  
 NCBI BlastP on this gene

CCE32896

uncharacterized protein
  
Accession: CCE32897
  
Location: 50442-51394
  
 NCBI BlastP on this gene

CCE32897

related to protein histidine kinase
  
Accession: CCE32898
  
Location: 53836-57234
  
 NCBI BlastP on this gene

CCE32898

Query: Architecture Search FASTA input

JH795568 : Magnaporthe oryzae P131 unplaced genomic scaffold P131\_scaffold00435    Total score: 2.0     Cumulative Blast bit score: 1878

Hit cluster cross-links:

Mycgr3G85918 Mycgr3T
  
Location: 0-1602

Mycgr3G85918\_Mycgr3T

Mycgr3G42010 Mycgr3T
  
Location: 1702-8569

Mycgr3G42010\_Mycgr3T

Mycgr3G29582 Mycgr3T
  
Location: 8669-8915

Mycgr3G29582\_Mycgr3T

Mycgr3G31170 Mycgr3T
  
Location: 9015-9255

Mycgr3G31170\_Mycgr3T

Mycgr3G85924 Mycgr3T
  
Location: 9355-11218

Mycgr3G85924\_Mycgr3T

Mycgr3G71676 Mycgr3T
  
Location: 11318-12494

Mycgr3G71676\_Mycgr3T

Mycgr3G11468 Mycgr3T
  
Location: 12594-13653

Mycgr3G11468\_Mycgr3T

Mycgr3G58567 Mycgr3T
  
Location: 13753-14506

Mycgr3G58567\_Mycgr3T

Mycgr3G100089 Mycgr3
  
Location: 14606-21152

Mycgr3G100089\_Mycgr3

Mycgr3G42698 Mycgr3T
  
Location: 21252-22131

Mycgr3G42698\_Mycgr3T

Mycgr3G71681 Mycgr3T
  
Location: 22231-23461

Mycgr3G71681\_Mycgr3T

Mycgr3G109328 Mycgr3
  
Location: 23561-24239

Mycgr3G109328\_Mycgr3

Mycgr3G104334 Mycgr3
  
Location: 24339-24567

Mycgr3G104334\_Mycgr3

Mycgr3G42715 Mycgr3T
  
Location: 24667-25981

Mycgr3G42715\_Mycgr3T

Mycgr3G92934 Mycgr3T
  
Location: 26081-27593

Mycgr3G92934\_Mycgr3T

Mycgr3G41969 Mycgr3T
  
Location: 27693-29328

Mycgr3G41969\_Mycgr3T

Mycgr3G80635 Mycgr3T
  
Location: 29428-29821

Mycgr3G80635\_Mycgr3T

Mycgr3G41426 Mycgr3T
  
Location: 29921-35255

Mycgr3G41426\_Mycgr3T

Mycgr3G104337 Mycgr3
  
Location: 35355-36108

Mycgr3G104337\_Mycgr3

Mycgr3G71679 Mycgr3T
  
Location: 36208-37300

Mycgr3G71679\_Mycgr3T

Mycgr3G92938 Mycgr3T
  
Location: 37400-38699

Mycgr3G92938\_Mycgr3T

Mycgr3G92941 Mycgr3T
  
Location: 38799-40734

Mycgr3G92941\_Mycgr3T

hypothetical protein
  
Accession: ELQ66019
  
Location: 22248-22340
  
 NCBI BlastP on this gene

ELQ66019

hypothetical protein
  
Accession: ELQ66020
  
Location: 22922-24468
  
 NCBI BlastP on this gene

ELQ66020

bilirubin oxidase
  
Accession: ELQ66021
  
Location: 27510-29453
  
 NCBI BlastP on this gene

ELQ66021

mannan endo-1,6-alpha-mannosidase DCW1
  
Accession: ELQ66022
  
Location: 30350-31379
  
 NCBI BlastP on this gene

ELQ66022

glycerate kinase
  
Accession: ELQ66023
  
Location: 32576-34039
  
 NCBI BlastP on this gene

ELQ66023

multidrug resistance protein 3
  
Accession: ELQ66024
  
Location: 34325-38964
  
 NCBI BlastP on this gene

ELQ66024

hypothetical protein
  
Accession: ELQ66025
  
Location: 40119-40956
  
  
**BlastP hit with Mycgr3G104337\_Mycgr3**
  
Percentage identity: 41 %
  
BlastP bit score: 206
  
Sequence coverage: 100 %
  
E-value: 4e-62
  
  
 NCBI BlastP on this gene

ELQ66025

fatty acid synthase S-acetyltransferase
  
Accession: ELQ66026
  
Location: 43006-49984
  
  
**BlastP hit with Mycgr3G100089\_Mycgr3**
  
Percentage identity: 42 %
  
BlastP bit score: 1672
  
Sequence coverage: 102 %
  
E-value: 0.0
  
  
 NCBI BlastP on this gene

ELQ66026

hypothetical protein
  
Accession: ELQ66027
  
Location: 51305-51823
  
 NCBI BlastP on this gene

ELQ66027

antibiotic biosynthesis monooxygenase
  
Accession: ELQ66028
  
Location: 52158-52805
  
 NCBI BlastP on this gene

ELQ66028

hypothetical protein
  
Accession: ELQ66029
  
Location: 53047-54477
  
 NCBI BlastP on this gene

ELQ66029

sugar transporter STL1
  
Accession: ELQ66030
  
Location: 54625-57560
  
 NCBI BlastP on this gene

ELQ66030

hypothetical protein
  
Accession: ELQ66031
  
Location: 58646-58939
  
 NCBI BlastP on this gene

ELQ66031

ankyrin repeat and protein kinase domain-containing protein 1
  
Accession: ELQ66032
  
Location: 59101-66533
  
 NCBI BlastP on this gene

ELQ66032

Query: Architecture Search FASTA input

JH793928 : Magnaporthe oryzae Y34 unplaced genomic scaffold Y34\_scaffold00214    Total score: 2.0     Cumulative Blast bit score: 1878

Hit cluster cross-links:

Mycgr3G85918 Mycgr3T
  
Location: 0-1602

Mycgr3G85918\_Mycgr3T

Mycgr3G42010 Mycgr3T
  
Location: 1702-8569

Mycgr3G42010\_Mycgr3T

Mycgr3G29582 Mycgr3T
  
Location: 8669-8915

Mycgr3G29582\_Mycgr3T

Mycgr3G31170 Mycgr3T
  
Location: 9015-9255

Mycgr3G31170\_Mycgr3T

Mycgr3G85924 Mycgr3T
  
Location: 9355-11218

Mycgr3G85924\_Mycgr3T

Mycgr3G71676 Mycgr3T
  
Location: 11318-12494

Mycgr3G71676\_Mycgr3T

Mycgr3G11468 Mycgr3T
  
Location: 12594-13653

Mycgr3G11468\_Mycgr3T

Mycgr3G58567 Mycgr3T
  
Location: 13753-14506

Mycgr3G58567\_Mycgr3T

Mycgr3G100089 Mycgr3
  
Location: 14606-21152

Mycgr3G100089\_Mycgr3

Mycgr3G42698 Mycgr3T
  
Location: 21252-22131

Mycgr3G42698\_Mycgr3T

Mycgr3G71681 Mycgr3T
  
Location: 22231-23461

Mycgr3G71681\_Mycgr3T

Mycgr3G109328 Mycgr3
  
Location: 23561-24239

Mycgr3G109328\_Mycgr3

Mycgr3G104334 Mycgr3
  
Location: 24339-24567

Mycgr3G104334\_Mycgr3

Mycgr3G42715 Mycgr3T
  
Location: 24667-25981

Mycgr3G42715\_Mycgr3T

Mycgr3G92934 Mycgr3T
  
Location: 26081-27593

Mycgr3G92934\_Mycgr3T

Mycgr3G41969 Mycgr3T
  
Location: 27693-29328

Mycgr3G41969\_Mycgr3T

Mycgr3G80635 Mycgr3T
  
Location: 29428-29821

Mycgr3G80635\_Mycgr3T

Mycgr3G41426 Mycgr3T
  
Location: 29921-35255

Mycgr3G41426\_Mycgr3T

Mycgr3G104337 Mycgr3
  
Location: 35355-36108

Mycgr3G104337\_Mycgr3

Mycgr3G71679 Mycgr3T
  
Location: 36208-37300

Mycgr3G71679\_Mycgr3T

Mycgr3G92938 Mycgr3T
  
Location: 37400-38699

Mycgr3G92938\_Mycgr3T

Mycgr3G92941 Mycgr3T
  
Location: 38799-40734

Mycgr3G92941\_Mycgr3T

hypothetical protein
  
Accession: ELQ42347
  
Location: 22050-22142
  
 NCBI BlastP on this gene

ELQ42347

hypothetical protein
  
Accession: ELQ42348
  
Location: 22724-24270
  
 NCBI BlastP on this gene

ELQ42348

bilirubin oxidase
  
Accession: ELQ42349
  
Location: 27312-29255
  
 NCBI BlastP on this gene

ELQ42349

mannan endo-1,6-alpha-mannosidase DCW1
  
Accession: ELQ42350
  
Location: 30152-31181
  
 NCBI BlastP on this gene

ELQ42350

glycerate kinase
  
Accession: ELQ42351
  
Location: 32378-33841
  
 NCBI BlastP on this gene

ELQ42351

multidrug resistance protein 3
  
Accession: ELQ42352
  
Location: 34127-38766
  
 NCBI BlastP on this gene

ELQ42352

hypothetical protein
  
Accession: ELQ42353
  
Location: 39921-40758
  
  
**BlastP hit with Mycgr3G104337\_Mycgr3**
  
Percentage identity: 41 %
  
BlastP bit score: 206
  
Sequence coverage: 100 %
  
E-value: 4e-62
  
  
 NCBI BlastP on this gene

ELQ42353

fatty acid synthase S-acetyltransferase
  
Accession: ELQ42354
  
Location: 42807-49785
  
  
**BlastP hit with Mycgr3G100089\_Mycgr3**
  
Percentage identity: 42 %
  
BlastP bit score: 1672
  
Sequence coverage: 102 %
  
E-value: 0.0
  
  
 NCBI BlastP on this gene

ELQ42354

hypothetical protein
  
Accession: ELQ42355
  
Location: 51122-51640
  
 NCBI BlastP on this gene

ELQ42355

antibiotic biosynthesis monooxygenase
  
Accession: ELQ42356
  
Location: 51975-52622
  
 NCBI BlastP on this gene

ELQ42356

hypothetical protein
  
Accession: ELQ42357
  
Location: 52864-54294
  
 NCBI BlastP on this gene

ELQ42357

sugar transporter STL1
  
Accession: ELQ42358
  
Location: 54442-57377
  
 NCBI BlastP on this gene

ELQ42358

hypothetical protein
  
Accession: ELQ42359
  
Location: 58463-58756
  
 NCBI BlastP on this gene

ELQ42359

ankyrin repeat and protein kinase domain-containing protein 1
  
Accession: ELQ42360
  
Location: 58918-67067
  
 NCBI BlastP on this gene

ELQ42360

hypothetical protein
  
Accession: ELQ42361
  
Location: 67367-68843
  
 NCBI BlastP on this gene

ELQ42361

Query: Architecture Search FASTA input

FP929139 : Leptosphaeria maculans JN3 lm\_SuperContig\_0\_v2 genomic supercontig    Total score: 2.0     Cumulative Blast bit score: 1827

Hit cluster cross-links:

Mycgr3G85918 Mycgr3T
  
Location: 0-1602

Mycgr3G85918\_Mycgr3T

Mycgr3G42010 Mycgr3T
  
Location: 1702-8569

Mycgr3G42010\_Mycgr3T

Mycgr3G29582 Mycgr3T
  
Location: 8669-8915

Mycgr3G29582\_Mycgr3T

Mycgr3G31170 Mycgr3T
  
Location: 9015-9255

Mycgr3G31170\_Mycgr3T

Mycgr3G85924 Mycgr3T
  
Location: 9355-11218

Mycgr3G85924\_Mycgr3T

Mycgr3G71676 Mycgr3T
  
Location: 11318-12494

Mycgr3G71676\_Mycgr3T

Mycgr3G11468 Mycgr3T
  
Location: 12594-13653

Mycgr3G11468\_Mycgr3T

Mycgr3G58567 Mycgr3T
  
Location: 13753-14506

Mycgr3G58567\_Mycgr3T

Mycgr3G100089 Mycgr3
  
Location: 14606-21152

Mycgr3G100089\_Mycgr3

Mycgr3G42698 Mycgr3T
  
Location: 21252-22131

Mycgr3G42698\_Mycgr3T

Mycgr3G71681 Mycgr3T
  
Location: 22231-23461

Mycgr3G71681\_Mycgr3T

Mycgr3G109328 Mycgr3
  
Location: 23561-24239

Mycgr3G109328\_Mycgr3

Mycgr3G104334 Mycgr3
  
Location: 24339-24567

Mycgr3G104334\_Mycgr3

Mycgr3G42715 Mycgr3T
  
Location: 24667-25981

Mycgr3G42715\_Mycgr3T

Mycgr3G92934 Mycgr3T
  
Location: 26081-27593

Mycgr3G92934\_Mycgr3T

Mycgr3G41969 Mycgr3T
  
Location: 27693-29328

Mycgr3G41969\_Mycgr3T

Mycgr3G80635 Mycgr3T
  
Location: 29428-29821

Mycgr3G80635\_Mycgr3T

Mycgr3G41426 Mycgr3T
  
Location: 29921-35255

Mycgr3G41426\_Mycgr3T

Mycgr3G104337 Mycgr3
  
Location: 35355-36108

Mycgr3G104337\_Mycgr3

Mycgr3G71679 Mycgr3T
  
Location: 36208-37300

Mycgr3G71679\_Mycgr3T

Mycgr3G92938 Mycgr3T
  
Location: 37400-38699

Mycgr3G92938\_Mycgr3T

Mycgr3G92941 Mycgr3T
  
Location: 38799-40734

Mycgr3G92941\_Mycgr3T

hypothetical protein
  
Accession: CBY01874
  
Location: 2503452-2513155
  
  
**BlastP hit with Mycgr3G100089\_Mycgr3**
  
Percentage identity: 40 %
  
BlastP bit score: 1618
  
Sequence coverage: 101 %
  
E-value: 0.0
  
  
 NCBI BlastP on this gene

LEMA\_P006610.1

similar to EF-hand calcium-binding domain protein
  
Accession: CBY01875
  
Location: 2514273-2515241
  
  
**BlastP hit with Mycgr3G104337\_Mycgr3**
  
Percentage identity: 43 %
  
BlastP bit score: 209
  
Sequence coverage: 100 %
  
E-value: 2e-63
  
  
 NCBI BlastP on this gene

LEMA\_P006620.1

similar to ABC multidrug transporter
  
Accession: CBY01876
  
Location: 2515904-2520524
  
 NCBI BlastP on this gene

LEMA\_P006630.1

similar to beta-lactamase family protein
  
Accession: CBY01877
  
Location: 2521380-2523855
  
 NCBI BlastP on this gene

LEMA\_P006640.1

similar to ATP dependent RNA helicase
  
Accession: CBY01878
  
Location: 2525250-2530309
  
 NCBI BlastP on this gene

LEMA\_P006650.1

similar to translational activator
  
Accession: CBY01879
  
Location: 2530701-2539025
  
 NCBI BlastP on this gene

LEMA\_P006660.1

Query: Architecture Search FASTA input

DS572714 : Verticillium dahliae VdLs.17 supercont1.20 genomic scaffold    Total score: 2.0     Cumulative Blast bit score: 1769

Hit cluster cross-links:

Mycgr3G85918 Mycgr3T
  
Location: 0-1602

Mycgr3G85918\_Mycgr3T

Mycgr3G42010 Mycgr3T
  
Location: 1702-8569

Mycgr3G42010\_Mycgr3T

Mycgr3G29582 Mycgr3T
  
Location: 8669-8915

Mycgr3G29582\_Mycgr3T

Mycgr3G31170 Mycgr3T
  
Location: 9015-9255

Mycgr3G31170\_Mycgr3T

Mycgr3G85924 Mycgr3T
  
Location: 9355-11218

Mycgr3G85924\_Mycgr3T

Mycgr3G71676 Mycgr3T
  
Location: 11318-12494

Mycgr3G71676\_Mycgr3T

Mycgr3G11468 Mycgr3T
  
Location: 12594-13653

Mycgr3G11468\_Mycgr3T

Mycgr3G58567 Mycgr3T
  
Location: 13753-14506

Mycgr3G58567\_Mycgr3T

Mycgr3G100089 Mycgr3
  
Location: 14606-21152

Mycgr3G100089\_Mycgr3

Mycgr3G42698 Mycgr3T
  
Location: 21252-22131

Mycgr3G42698\_Mycgr3T

Mycgr3G71681 Mycgr3T
  
Location: 22231-23461

Mycgr3G71681\_Mycgr3T

Mycgr3G109328 Mycgr3
  
Location: 23561-24239

Mycgr3G109328\_Mycgr3

Mycgr3G104334 Mycgr3
  
Location: 24339-24567

Mycgr3G104334\_Mycgr3

Mycgr3G42715 Mycgr3T
  
Location: 24667-25981

Mycgr3G42715\_Mycgr3T

Mycgr3G92934 Mycgr3T
  
Location: 26081-27593

Mycgr3G92934\_Mycgr3T

Mycgr3G41969 Mycgr3T
  
Location: 27693-29328

Mycgr3G41969\_Mycgr3T

Mycgr3G80635 Mycgr3T
  
Location: 29428-29821

Mycgr3G80635\_Mycgr3T

Mycgr3G41426 Mycgr3T
  
Location: 29921-35255

Mycgr3G41426\_Mycgr3T

Mycgr3G104337 Mycgr3
  
Location: 35355-36108

Mycgr3G104337\_Mycgr3

Mycgr3G71679 Mycgr3T
  
Location: 36208-37300

Mycgr3G71679\_Mycgr3T

Mycgr3G92938 Mycgr3T
  
Location: 37400-38699

Mycgr3G92938\_Mycgr3T

Mycgr3G92941 Mycgr3T
  
Location: 38799-40734

Mycgr3G92941\_Mycgr3T

ubiquitin C-terminal hydrolase
  
Accession: EGY18108
  
Location: 457337-459982
  
 NCBI BlastP on this gene

EGY18108

54S ribosomal protein L7
  
Accession: EGY18109
  
Location: 461868-463047
  
 NCBI BlastP on this gene

EGY18109

Hsp70 nucleotide exchange factor FES1
  
Accession: EGY18110
  
Location: 463348-463977
  
 NCBI BlastP on this gene

EGY18110

actin
  
Accession: EGY18111
  
Location: 466404-467516
  
 NCBI BlastP on this gene

EGY18111

multidrug resistance protein
  
Accession: EGY18112
  
Location: 469569-474381
  
 NCBI BlastP on this gene

EGY18112

hypothetical protein
  
Accession: EGY18113
  
Location: 475077-476013
  
  
**BlastP hit with Mycgr3G104337\_Mycgr3**
  
Percentage identity: 40 %
  
BlastP bit score: 205
  
Sequence coverage: 101 %
  
E-value: 2e-61
  
  
 NCBI BlastP on this gene

EGY18113

lovastatin nonaketide synthase
  
Accession: EGY18114
  
Location: 477875-484921
  
  
**BlastP hit with Mycgr3G100089\_Mycgr3**
  
Percentage identity: 39 %
  
BlastP bit score: 1564
  
Sequence coverage: 101 %
  
E-value: 0.0
  
  
 NCBI BlastP on this gene

EGY18114

hypothetical protein
  
Accession: EGY18115
  
Location: 485368-485787
  
 NCBI BlastP on this gene

EGY18115

elongator complex protein
  
Accession: EGY18116
  
Location: 486187-488797
  
 NCBI BlastP on this gene

EGY18116

hypothetical protein
  
Accession: EGY18117
  
Location: 489422-489940
  
 NCBI BlastP on this gene

EGY18117

zinc finger protein
  
Accession: EGY18118
  
Location: 495011-496489
  
 NCBI BlastP on this gene

EGY18118

Query: Architecture Search FASTA input

FQ790354 : Botryotinia fuckeliana T4 SupSuperContig\_210\_20\_1 genomic supercontig.    Total score: 2.0     Cumulative Blast bit score: 1764

Hit cluster cross-links:

Mycgr3G85918 Mycgr3T
  
Location: 0-1602

Mycgr3G85918\_Mycgr3T

Mycgr3G42010 Mycgr3T
  
Location: 1702-8569

Mycgr3G42010\_Mycgr3T

Mycgr3G29582 Mycgr3T
  
Location: 8669-8915

Mycgr3G29582\_Mycgr3T

Mycgr3G31170 Mycgr3T
  
Location: 9015-9255

Mycgr3G31170\_Mycgr3T

Mycgr3G85924 Mycgr3T
  
Location: 9355-11218

Mycgr3G85924\_Mycgr3T

Mycgr3G71676 Mycgr3T
  
Location: 11318-12494

Mycgr3G71676\_Mycgr3T

Mycgr3G11468 Mycgr3T
  
Location: 12594-13653

Mycgr3G11468\_Mycgr3T

Mycgr3G58567 Mycgr3T
  
Location: 13753-14506

Mycgr3G58567\_Mycgr3T

Mycgr3G100089 Mycgr3
  
Location: 14606-21152

Mycgr3G100089\_Mycgr3

Mycgr3G42698 Mycgr3T
  
Location: 21252-22131

Mycgr3G42698\_Mycgr3T

Mycgr3G71681 Mycgr3T
  
Location: 22231-23461

Mycgr3G71681\_Mycgr3T

Mycgr3G109328 Mycgr3
  
Location: 23561-24239

Mycgr3G109328\_Mycgr3

Mycgr3G104334 Mycgr3
  
Location: 24339-24567

Mycgr3G104334\_Mycgr3

Mycgr3G42715 Mycgr3T
  
Location: 24667-25981

Mycgr3G42715\_Mycgr3T

Mycgr3G92934 Mycgr3T
  
Location: 26081-27593

Mycgr3G92934\_Mycgr3T

Mycgr3G41969 Mycgr3T
  
Location: 27693-29328

Mycgr3G41969\_Mycgr3T

Mycgr3G80635 Mycgr3T
  
Location: 29428-29821

Mycgr3G80635\_Mycgr3T

Mycgr3G41426 Mycgr3T
  
Location: 29921-35255

Mycgr3G41426\_Mycgr3T

Mycgr3G104337 Mycgr3
  
Location: 35355-36108

Mycgr3G104337\_Mycgr3

Mycgr3G71679 Mycgr3T
  
Location: 36208-37300

Mycgr3G71679\_Mycgr3T

Mycgr3G92938 Mycgr3T
  
Location: 37400-38699

Mycgr3G92938\_Mycgr3T

Mycgr3G92941 Mycgr3T
  
Location: 38799-40734

Mycgr3G92941\_Mycgr3T

hypothetical protein
  
Accession: CCD55452
  
Location: 668813-669763
  
 NCBI BlastP on this gene

BofuT4\_P158480.1

glycoside hydrolase family 78 protein, partial sequence
  
Accession: CCD55451
  
Location: 666518-668221
  
 NCBI BlastP on this gene

BofuT4\_P158470.1

glycoside hydrolase family 78 protein, partial sequence
  
Accession: CCD55450
  
Location: 665174-666117
  
 NCBI BlastP on this gene

BofuT4\_P158460.1

similar to MFS sugar transporter
  
Accession: CCD55449
  
Location: 662421-664203
  
 NCBI BlastP on this gene

BofuT4P210000010001

hypothetical protein
  
Accession: CCD55448
  
Location: 660662-661621
  
 NCBI BlastP on this gene

BofuT4\_P158440.1

similar to cytochrome P450 monooxygenase
  
Accession: CCD55447
  
Location: 658528-660306
  
 NCBI BlastP on this gene

BofuT4P210000009001

hypothetical protein
  
Accession: CCD55446
  
Location: 655744-657642
  
 NCBI BlastP on this gene

BofuT4\_P158420.1

similar to MFS sugar transporter
  
Accession: CCD55445
  
Location: 653276-655074
  
 NCBI BlastP on this gene

BofuT4P210000008001

hypothetical protein
  
Accession: CCD55444
  
Location: 651483-652850
  
  
**BlastP hit with Mycgr3G92938\_Mycgr3T**
  
Percentage identity: 39 %
  
BlastP bit score: 209
  
Sequence coverage: 69 %
  
E-value: 3e-59
  
  
 NCBI BlastP on this gene

BofuT4\_P158400.1

similar to helicase required for RNAi-mediated heterochromatin assembly 1
  
Accession: CCD55443
  
Location: 647844-650811
  
  
**BlastP hit with Mycgr3G42010\_Mycgr3T**
  
Percentage identity: 41 %
  
BlastP bit score: 654
  
Sequence coverage: 39 %
  
E-value: 0.0
  
  
 NCBI BlastP on this gene

BofuT4\_P158390.1

hypothetical protein
  
Accession: CCD55442
  
Location: 642630-646724
  
  
**BlastP hit with Mycgr3G42010\_Mycgr3T**
  
Percentage identity: 46 %
  
BlastP bit score: 901
  
Sequence coverage: 47 %
  
E-value: 0.0
  
  
 NCBI BlastP on this gene

BofuT4\_P158380.1

similar to transcription factor Zn, C2H2
  
Accession: CCD55441
  
Location: 637940-641624
  
 NCBI BlastP on this gene

BofuT4\_P158370.1

similar to nucleoside transporter family
  
Accession: CCD55440
  
Location: 634672-636140
  
 NCBI BlastP on this gene

BofuT4\_P158360.1

hypothetical protein
  
Accession: CCD55439
  
Location: 633333-633777
  
 NCBI BlastP on this gene

BofuT4\_P158350.1

similar to phosphatase, partial sequence
  
Accession: CCD55438
  
Location: 630859-632305
  
 NCBI BlastP on this gene

BofuT4\_P158340.1

similar to phosphatase, partial sequence
  
Accession: CCD55437
  
Location: 630023-630439
  
 NCBI BlastP on this gene

BofuT4\_P158330.1

hypothetical protein
  
Accession: CCD55436
  
Location: 627237-627710
  
 NCBI BlastP on this gene

BofuT4\_P158320.1

BC4, calmodulin
  
Accession: CCD55435
  
Location: 625064-626093
  
 NCBI BlastP on this gene

BofuT4P210000007001

Query: Architecture Search FASTA input

CH476622 : Sclerotinia sclerotiorum 1980 scaffold\_2 genomic scaffold    Total score: 2.0     Cumulative Blast bit score: 1755

Hit cluster cross-links:

Mycgr3G85918 Mycgr3T
  
Location: 0-1602

Mycgr3G85918\_Mycgr3T

Mycgr3G42010 Mycgr3T
  
Location: 1702-8569

Mycgr3G42010\_Mycgr3T

Mycgr3G29582 Mycgr3T
  
Location: 8669-8915

Mycgr3G29582\_Mycgr3T

Mycgr3G31170 Mycgr3T
  
Location: 9015-9255

Mycgr3G31170\_Mycgr3T

Mycgr3G85924 Mycgr3T
  
Location: 9355-11218

Mycgr3G85924\_Mycgr3T

Mycgr3G71676 Mycgr3T
  
Location: 11318-12494

Mycgr3G71676\_Mycgr3T

Mycgr3G11468 Mycgr3T
  
Location: 12594-13653

Mycgr3G11468\_Mycgr3T

Mycgr3G58567 Mycgr3T
  
Location: 13753-14506

Mycgr3G58567\_Mycgr3T

Mycgr3G100089 Mycgr3
  
Location: 14606-21152

Mycgr3G100089\_Mycgr3

Mycgr3G42698 Mycgr3T
  
Location: 21252-22131

Mycgr3G42698\_Mycgr3T

Mycgr3G71681 Mycgr3T
  
Location: 22231-23461

Mycgr3G71681\_Mycgr3T

Mycgr3G109328 Mycgr3
  
Location: 23561-24239

Mycgr3G109328\_Mycgr3

Mycgr3G104334 Mycgr3
  
Location: 24339-24567

Mycgr3G104334\_Mycgr3

Mycgr3G42715 Mycgr3T
  
Location: 24667-25981

Mycgr3G42715\_Mycgr3T

Mycgr3G92934 Mycgr3T
  
Location: 26081-27593

Mycgr3G92934\_Mycgr3T

Mycgr3G41969 Mycgr3T
  
Location: 27693-29328

Mycgr3G41969\_Mycgr3T

Mycgr3G80635 Mycgr3T
  
Location: 29428-29821

Mycgr3G80635\_Mycgr3T

Mycgr3G41426 Mycgr3T
  
Location: 29921-35255

Mycgr3G41426\_Mycgr3T

Mycgr3G104337 Mycgr3
  
Location: 35355-36108

Mycgr3G104337\_Mycgr3

Mycgr3G71679 Mycgr3T
  
Location: 36208-37300

Mycgr3G71679\_Mycgr3T

Mycgr3G92938 Mycgr3T
  
Location: 37400-38699

Mycgr3G92938\_Mycgr3T

Mycgr3G92941 Mycgr3T
  
Location: 38799-40734

Mycgr3G92941\_Mycgr3T

hypothetical protein
  
Accession: EDN97064
  
Location: 2396682-2398373
  
 NCBI BlastP on this gene

EDN97064

predicted protein
  
Accession: EDN97065
  
Location: 2405854-2406385
  
 NCBI BlastP on this gene

EDN97065

predicted protein
  
Accession: EDN97066
  
Location: 2406542-2407604
  
 NCBI BlastP on this gene

EDN97066

hypothetical protein
  
Accession: EDN97067
  
Location: 2408856-2410498
  
 NCBI BlastP on this gene

EDN97067

hypothetical protein
  
Accession: EDN97068
  
Location: 2412280-2414701
  
 NCBI BlastP on this gene

EDN97068

hypothetical protein
  
Accession: EDN97069
  
Location: 2415739-2423304
  
  
**BlastP hit with Mycgr3G100089\_Mycgr3**
  
Percentage identity: 39 %
  
BlastP bit score: 1537
  
Sequence coverage: 101 %
  
E-value: 0.0
  
  
 NCBI BlastP on this gene

EDN97069

predicted protein
  
Accession: EDN97070
  
Location: 2423748-2424243
  
 NCBI BlastP on this gene

EDN97070

hypothetical protein
  
Accession: EDN97071
  
Location: 2424790-2425751
  
  
**BlastP hit with Mycgr3G104337\_Mycgr3**
  
Percentage identity: 46 %
  
BlastP bit score: 218
  
Sequence coverage: 95 %
  
E-value: 2e-66
  
  
 NCBI BlastP on this gene

EDN97071

hypothetical protein
  
Accession: EDN97072
  
Location: 2426747-2431478
  
 NCBI BlastP on this gene

EDN97072

predicted protein
  
Accession: EDN97073
  
Location: 2433188-2434118
  
 NCBI BlastP on this gene

EDN97073

hypothetical protein
  
Accession: EDN97074
  
Location: 2435499-2436873
  
 NCBI BlastP on this gene

EDN97074

hypothetical protein
  
Accession: EDN97075
  
Location: 2437311-2438460
  
 NCBI BlastP on this gene

EDN97075

hypothetical protein
  
Accession: EDN97076
  
Location: 2439863-2440732
  
 NCBI BlastP on this gene

EDN97076

hypothetical protein
  
Accession: EDN97077
  
Location: 2441243-2443659
  
 NCBI BlastP on this gene

EDN97077

Query: Architecture Search FASTA input

51. :  GL891247 Neurospora tetrasperma FGSC 2509 unplaced genomic scaffold NEUTE2scaffold\_5     Total score: 2.0     Cumulative Blast bit score: 2149

Mycgr3G85918 Mycgr3T
  
Location: 0-1602
  
 NCBI BlastP on this gene

Mycgr3G85918\_Mycgr3T

Mycgr3G42010 Mycgr3T
  
Location: 1702-8569
  
 NCBI BlastP on this gene

Mycgr3G42010\_Mycgr3T

Mycgr3G29582 Mycgr3T
  
Location: 8669-8915
  
 NCBI BlastP on this gene

Mycgr3G29582\_Mycgr3T

Mycgr3G31170 Mycgr3T
  
Location: 9015-9255
  
 NCBI BlastP on this gene

Mycgr3G31170\_Mycgr3T

Mycgr3G85924 Mycgr3T
  
Location: 9355-11218
  
 NCBI BlastP on this gene

Mycgr3G85924\_Mycgr3T

Mycgr3G71676 Mycgr3T
  
Location: 11318-12494
  
 NCBI BlastP on this gene

Mycgr3G71676\_Mycgr3T

Mycgr3G11468 Mycgr3T
  
Location: 12594-13653
  
 NCBI BlastP on this gene

Mycgr3G11468\_Mycgr3T

Mycgr3G58567 Mycgr3T
  
Location: 13753-14506
  
 NCBI BlastP on this gene

Mycgr3G58567\_Mycgr3T

Mycgr3G100089 Mycgr3
  
Location: 14606-21152
  
 NCBI BlastP on this gene

Mycgr3G100089\_Mycgr3

Mycgr3G42698 Mycgr3T
  
Location: 21252-22131
  
 NCBI BlastP on this gene

Mycgr3G42698\_Mycgr3T

Mycgr3G71681 Mycgr3T
  
Location: 22231-23461
  
 NCBI BlastP on this gene

Mycgr3G71681\_Mycgr3T

Mycgr3G109328 Mycgr3
  
Location: 23561-24239
  
 NCBI BlastP on this gene

Mycgr3G109328\_Mycgr3

Mycgr3G104334 Mycgr3
  
Location: 24339-24567
  
 NCBI BlastP on this gene

Mycgr3G104334\_Mycgr3

Mycgr3G42715 Mycgr3T
  
Location: 24667-25981
  
 NCBI BlastP on this gene

Mycgr3G42715\_Mycgr3T

Mycgr3G92934 Mycgr3T
  
Location: 26081-27593
  
 NCBI BlastP on this gene

Mycgr3G92934\_Mycgr3T

Mycgr3G41969 Mycgr3T
  
Location: 27693-29328
  
 NCBI BlastP on this gene

Mycgr3G41969\_Mycgr3T

Mycgr3G80635 Mycgr3T
  
Location: 29428-29821
  
 NCBI BlastP on this gene

Mycgr3G80635\_Mycgr3T

Mycgr3G41426 Mycgr3T
  
Location: 29921-35255
  
 NCBI BlastP on this gene

Mycgr3G41426\_Mycgr3T

Mycgr3G104337 Mycgr3
  
Location: 35355-36108
  
 NCBI BlastP on this gene

Mycgr3G104337\_Mycgr3

Mycgr3G71679 Mycgr3T
  
Location: 36208-37300
  
 NCBI BlastP on this gene

Mycgr3G71679\_Mycgr3T

Mycgr3G92938 Mycgr3T
  
Location: 37400-38699
  
 NCBI BlastP on this gene

Mycgr3G92938\_Mycgr3T

Mycgr3G92941 Mycgr3T
  
Location: 38799-40734
  
 NCBI BlastP on this gene

Mycgr3G92941\_Mycgr3T

hypothetical protein
  
Accession: EGZ71050
  
Location: 3960096-3962023
  
 NCBI BlastP on this gene

EGZ71050

hypothetical protein
  
Accession: EGZ71051
  
Location: 3962647-3962925
  
 NCBI BlastP on this gene

EGZ71051

hypothetical protein
  
Accession: EGZ71052
  
Location: 3964379-3968993
  
 NCBI BlastP on this gene

EGZ71052

hypothetical protein
  
Accession: EGZ71053
  
Location: 3970276-3972660
  
 NCBI BlastP on this gene

EGZ71053

hypothetical protein
  
Accession: EGZ71054
  
Location: 3973514-3974450
  
 NCBI BlastP on this gene

EGZ71054

HET-domain-containing protein
  
Accession: EGZ71055
  
Location: 3975577-3977998
  
 NCBI BlastP on this gene

EGZ71055

hypothetical protein
  
Accession: EGZ71056
  
Location: 3979283-3980453
  
  
**BlastP hit with Mycgr3G92938\_Mycgr3T**
  
Percentage identity: 39 %
  
BlastP bit score: 201
  
Sequence coverage: 64 %
  
E-value: 1e-56
  
  
 NCBI BlastP on this gene

EGZ71056

P-loop containing nucleoside triphosphate hydrolase protein
  
Accession: EGZ71057
  
Location: 3981955-3989150
  
  
**BlastP hit with Mycgr3G42010\_Mycgr3T**
  
Percentage identity: 45 %
  
BlastP bit score: 1948
  
Sequence coverage: 101 %
  
E-value: 0.0
  
  
 NCBI BlastP on this gene

EGZ71057

general substrate transporter
  
Accession: EGZ71058
  
Location: 3990242-3992155
  
 NCBI BlastP on this gene

EGZ71058

UPF0075-domain-containing protein
  
Accession: EGZ71059
  
Location: 3993163-3994615
  
 NCBI BlastP on this gene

EGZ71059

C6 zinc finger domain protein
  
Accession: EGZ71060
  
Location: 3995108-3996819
  
 NCBI BlastP on this gene

EGZ71060

hypothetical protein
  
Accession: EGZ71061
  
Location: 3997218-3998375
  
 NCBI BlastP on this gene

EGZ71061

alpha-L-arabinofuranosidase B
  
Accession: EGZ71062
  
Location: 3999206-4000234
  
 NCBI BlastP on this gene

EGZ71062

alpha/beta-hydrolase
  
Accession: EGZ71063
  
Location: 4001119-4002027
  
 NCBI BlastP on this gene

EGZ71063

OPT superfamily oligopeptide transporter
  
Accession: EGZ71064
  
Location: 4002934-4005428
  
 NCBI BlastP on this gene

EGZ71064

hypothetical protein
  
Accession: EGZ71065
  
Location: 4005773-4006237
  
 NCBI BlastP on this gene

EGZ71065

52. :  GL985074 Trichoderma reesei QM6a unplaced genomic scaffold TRIREscaffold\_19     Total score: 2.0     Cumulative Blast bit score: 2139

proteinase T-like protein
  
Accession: EGR46243
  
Location: 264600-265955
  
 NCBI BlastP on this gene

EGR46243

predicted protein
  
Accession: EGR46150
  
Location: 270707-272048
  
 NCBI BlastP on this gene

EGR46150

predicted protein
  
Accession: EGR46151
  
Location: 272362-273457
  
 NCBI BlastP on this gene

EGR46151

predicted protein
  
Accession: EGR46152
  
Location: 274371-274899
  
 NCBI BlastP on this gene

EGR46152

predicted protein
  
Accession: EGR46244
  
Location: 275437-275882
  
 NCBI BlastP on this gene

EGR46244

predicted protein
  
Accession: EGR46153
  
Location: 277379-278168
  
 NCBI BlastP on this gene

EGR46153

predicted protein
  
Accession: EGR46154
  
Location: 280159-281463
  
 NCBI BlastP on this gene

EGR46154

predicted protein
  
Accession: EGR46245
  
Location: 281700-283047
  
 NCBI BlastP on this gene

EGR46245

predicted protein
  
Accession: EGR46246
  
Location: 283689-290663
  
  
**BlastP hit with Mycgr3G42010\_Mycgr3T**
  
Percentage identity: 43 %
  
BlastP bit score: 1910
  
Sequence coverage: 101 %
  
E-value: 0.0
  
  
 NCBI BlastP on this gene

EGR46246

predicted protein
  
Accession: EGR46155
  
Location: 291551-292927
  
  
**BlastP hit with Mycgr3G92938\_Mycgr3T**
  
Percentage identity: 35 %
  
BlastP bit score: 229
  
Sequence coverage: 81 %
  
E-value: 2e-66
  
  
 NCBI BlastP on this gene

EGR46155

predicted protein
  
Accession: EGR46156
  
Location: 293602-294471
  
 NCBI BlastP on this gene

EGR46156

predicted protein
  
Accession: EGR46247
  
Location: 294588-295520
  
 NCBI BlastP on this gene

EGR46247

predicted protein
  
Accession: EGR46157
  
Location: 296086-296424
  
 NCBI BlastP on this gene

EGR46157

predicted protein
  
Accession: EGR46248
  
Location: 299445-300625
  
 NCBI BlastP on this gene

EGR46248

amino acid transporter
  
Accession: EGR46158
  
Location: 301270-303137
  
 NCBI BlastP on this gene

EGR46158

predicted protein
  
Accession: EGR46249
  
Location: 303380-305011
  
 NCBI BlastP on this gene

EGR46249

predicted protein
  
Accession: EGR46159
  
Location: 307694-309682
  
 NCBI BlastP on this gene

EGR46159

53. :  CAGA01000005 Claviceps purpurea 20.1     Total score: 2.0     Cumulative Blast bit score: 2137

related to L-serine dehydratase
  
Accession: CCE27610
  
Location: 133256-134221
  
 NCBI BlastP on this gene

CCE27610

related to Carboxymuconolactone decarboxylase
  
Accession: CCE27611
  
Location: 135410-136137
  
 NCBI BlastP on this gene

CCE27611

uncharacterized protein
  
Accession: CCE27612
  
Location: 138458-140245
  
 NCBI BlastP on this gene

CCE27612

uncharacterized protein
  
Accession: CCE27613
  
Location: 141214-143013
  
 NCBI BlastP on this gene

CCE27613

uncharacterized protein
  
Accession: CCE27614
  
Location: 144169-145685
  
 NCBI BlastP on this gene

CCE27614

related to ECM32-DNA dependent ATPase/DNA helicase B
  
Accession: CCE27615
  
Location: 146163-153324
  
  
**BlastP hit with Mycgr3G42010\_Mycgr3T**
  
Percentage identity: 44 %
  
BlastP bit score: 1892
  
Sequence coverage: 101 %
  
E-value: 0.0
  
  
 NCBI BlastP on this gene

CCE27615

uncharacterized protein
  
Accession: CCE27616
  
Location: 153715-155292
  
  
**BlastP hit with Mycgr3G92938\_Mycgr3T**
  
Percentage identity: 33 %
  
BlastP bit score: 245
  
Sequence coverage: 104 %
  
E-value: 1e-71
  
  
 NCBI BlastP on this gene

CCE27616

uncharacterized protein
  
Accession: CCE27617
  
Location: 157286-159176
  
 NCBI BlastP on this gene

CCE27617

uncharacterized protein
  
Accession: CCE27618
  
Location: 160151-162365
  
 NCBI BlastP on this gene

CCE27618

uncharacterized protein
  
Accession: CCE27619
  
Location: 162899-164896
  
 NCBI BlastP on this gene

CCE27619

uncharacterized protein
  
Accession: CCE27620
  
Location: 166613-168333
  
 NCBI BlastP on this gene

CCE27620

54. :  AABX02000071 Neurospora crassa OR74A     Total score: 2.0     Cumulative Blast bit score: 2136

conserved hypothetical protein
  
Accession: EAA29695
  
Location: 97025-98963
  
 NCBI BlastP on this gene

EAA29695

predicted protein
  
Accession: EAA29696
  
Location: 100898-105622
  
 NCBI BlastP on this gene

EAA29696

predicted protein
  
Accession: EAA29697
  
Location: 106576-108984
  
 NCBI BlastP on this gene

EAA29697

predicted protein
  
Accession: EAA29698
  
Location: 109816-110726
  
 NCBI BlastP on this gene

EAA29698

predicted protein
  
Accession: EAA29699
  
Location: 115132-116926
  
  
**BlastP hit with Mycgr3G92938\_Mycgr3T**
  
Percentage identity: 49 %
  
BlastP bit score: 156
  
Sequence coverage: 35 %
  
E-value: 1e-38
  
  
 NCBI BlastP on this gene

EAA29699

predicted protein
  
Accession: EAA29700
  
Location: 118319-125575
  
  
**BlastP hit with Mycgr3G42010\_Mycgr3T**
  
Percentage identity: 45 %
  
BlastP bit score: 1980
  
Sequence coverage: 102 %
  
E-value: 0.0
  
  
 NCBI BlastP on this gene

EAA29700

hypothetical protein
  
Accession: EAA29701
  
Location: 126433-128355
  
 NCBI BlastP on this gene

EAA29701

55. :  AFQF01000625 Fusarium oxysporum Fo5176     Total score: 2.0     Cumulative Blast bit score: 2116

hypothetical protein
  
Accession: EGU87652
  
Location: 26242-29504
  
 NCBI BlastP on this gene

EGU87652

hypothetical protein
  
Accession: EGU87653
  
Location: 29808-31082
  
 NCBI BlastP on this gene

EGU87653

hypothetical protein
  
Accession: EGU87654
  
Location: 32115-33619
  
 NCBI BlastP on this gene

EGU87654

hypothetical protein
  
Accession: EGU87655
  
Location: 33896-34738
  
 NCBI BlastP on this gene

EGU87655

hypothetical protein
  
Accession: EGU87656
  
Location: 36340-36691
  
 NCBI BlastP on this gene

EGU87656

hypothetical protein
  
Accession: EGU87657
  
Location: 37598-39279
  
 NCBI BlastP on this gene

EGU87657

hypothetical protein
  
Accession: EGU87658
  
Location: 41134-42756
  
 NCBI BlastP on this gene

EGU87658

hypothetical protein
  
Accession: EGU87659
  
Location: 43290-44441
  
 NCBI BlastP on this gene

EGU87659

hypothetical protein
  
Accession: EGU87660
  
Location: 45662-52690
  
  
**BlastP hit with Mycgr3G42010\_Mycgr3T**
  
Percentage identity: 44 %
  
BlastP bit score: 1880
  
Sequence coverage: 102 %
  
E-value: 0.0
  
  
 NCBI BlastP on this gene

EGU87660

hypothetical protein
  
Accession: EGU87661
  
Location: 53495-54846
  
  
**BlastP hit with Mycgr3G92938\_Mycgr3T**
  
Percentage identity: 36 %
  
BlastP bit score: 236
  
Sequence coverage: 87 %
  
E-value: 5e-69
  
  
 NCBI BlastP on this gene

EGU87661

hypothetical protein
  
Accession: EGU87662
  
Location: 57217-58524
  
 NCBI BlastP on this gene

EGU87662

hypothetical protein
  
Accession: EGU87663
  
Location: 58771-62120
  
 NCBI BlastP on this gene

EGU87663

hypothetical protein
  
Accession: EGU87664
  
Location: 62284-62817
  
 NCBI BlastP on this gene

EGU87664

hypothetical protein
  
Accession: EGU87665
  
Location: 65756-68679
  
 NCBI BlastP on this gene

EGU87665

hypothetical protein
  
Accession: EGU87666
  
Location: 68953-70495
  
 NCBI BlastP on this gene

EGU87666

hypothetical protein
  
Accession: EGU87667
  
Location: 70916-73450
  
 NCBI BlastP on this gene

EGU87667

56. :  KB726184 Fusarium oxysporum f. sp. cubense race 4 unplaced genomic scaffold scaffold30     Total score: 2.0     Cumulative Blast bit score: 2105

hypothetical protein
  
Accession: EMT74590
  
Location: 40275-43046
  
 NCBI BlastP on this gene

EMT74590

hypothetical protein
  
Accession: EMT74591
  
Location: 43441-46241
  
 NCBI BlastP on this gene

EMT74591

Putative protein yjlB
  
Accession: EMT74592
  
Location: 49130-49663
  
 NCBI BlastP on this gene

EMT74592

hypothetical protein
  
Accession: EMT74593
  
Location: 49826-50122
  
 NCBI BlastP on this gene

EMT74593

Putative protein yjlB
  
Accession: EMT74594
  
Location: 50746-51279
  
 NCBI BlastP on this gene

EMT74594

Stabilin-2
  
Accession: EMT74595
  
Location: 51442-52710
  
 NCBI BlastP on this gene

EMT74595

Cyclin CCL1
  
Accession: EMT74596
  
Location: 53703-54792
  
 NCBI BlastP on this gene

EMT74596

hypothetical protein
  
Accession: EMT74597
  
Location: 55039-56346
  
 NCBI BlastP on this gene

EMT74597

hypothetical protein
  
Accession: EMT74598
  
Location: 58719-60076
  
  
**BlastP hit with Mycgr3G92938\_Mycgr3T**
  
Percentage identity: 34 %
  
BlastP bit score: 230
  
Sequence coverage: 100 %
  
E-value: 1e-66
  
  
 NCBI BlastP on this gene

EMT74598

NFX1-type zinc finger-containing protein 1
  
Accession: EMT74599
  
Location: 60865-67901
  
  
**BlastP hit with Mycgr3G42010\_Mycgr3T**
  
Percentage identity: 44 %
  
BlastP bit score: 1875
  
Sequence coverage: 102 %
  
E-value: 0.0
  
  
 NCBI BlastP on this gene

EMT74599

hypothetical protein
  
Accession: EMT74600
  
Location: 68874-69953
  
 NCBI BlastP on this gene

EMT74600

hypothetical protein
  
Accession: EMT74601
  
Location: 70699-71598
  
 NCBI BlastP on this gene

EMT74601

hypothetical protein
  
Accession: EMT74602
  
Location: 74208-75509
  
 NCBI BlastP on this gene

EMT74602

Canalicular multispecific organic anion transporter 2
  
Accession: EMT74603
  
Location: 76851-81524
  
 NCBI BlastP on this gene

EMT74603

Cytochrome P450 4F4
  
Accession: EMT74604
  
Location: 81624-83315
  
 NCBI BlastP on this gene

EMT74604

NADH-cytochrome b5 reductase 1
  
Accession: EMT74605
  
Location: 83967-85988
  
 NCBI BlastP on this gene

EMT74605

57. :  ABDG02000020 Trichoderma atroviride IMI 206040     Total score: 2.0     Cumulative Blast bit score: 2101

hypothetical protein
  
Accession: EHK47659
  
Location: 1007295-1008968
  
 NCBI BlastP on this gene

EHK47659

hypothetical protein
  
Accession: EHK47660
  
Location: 1009249-1010319
  
 NCBI BlastP on this gene

EHK47660

hypothetical protein
  
Accession: EHK47661
  
Location: 1010546-1012616
  
 NCBI BlastP on this gene

EHK47661

hypothetical protein
  
Accession: EHK47662
  
Location: 1015138-1017628
  
 NCBI BlastP on this gene

EHK47662

hypothetical protein
  
Accession: EHK47663
  
Location: 1018706-1019118
  
 NCBI BlastP on this gene

EHK47663

hypothetical protein
  
Accession: EHK47664
  
Location: 1020834-1021609
  
 NCBI BlastP on this gene

EHK47664

hypothetical protein
  
Accession: EHK47665
  
Location: 1023555-1024730
  
 NCBI BlastP on this gene

EHK47665

hypothetical protein
  
Accession: EHK47666
  
Location: 1025258-1032304
  
  
**BlastP hit with Mycgr3G42010\_Mycgr3T**
  
Percentage identity: 43 %
  
BlastP bit score: 1875
  
Sequence coverage: 102 %
  
E-value: 0.0
  
  
 NCBI BlastP on this gene

EHK47666

hypothetical protein
  
Accession: EHK47667
  
Location: 1032982-1034381
  
  
**BlastP hit with Mycgr3G92938\_Mycgr3T**
  
Percentage identity: 36 %
  
BlastP bit score: 226
  
Sequence coverage: 81 %
  
E-value: 3e-65
  
  
 NCBI BlastP on this gene

EHK47667

hypothetical protein
  
Accession: EHK47668
  
Location: 1035154-1035777
  
 NCBI BlastP on this gene

EHK47668

hypothetical protein
  
Accession: EHK47669
  
Location: 1036323-1037574
  
 NCBI BlastP on this gene

EHK47669

hypothetical protein
  
Accession: EHK47670
  
Location: 1039287-1041499
  
 NCBI BlastP on this gene

EHK47670

glycosyltransferase family 90 protein
  
Accession: EHK47881
  
Location: 1041717-1044844
  
 NCBI BlastP on this gene

EHK47881

hypothetical protein
  
Accession: EHK47671
  
Location: 1045765-1047846
  
 NCBI BlastP on this gene

EHK47671

hypothetical protein
  
Accession: EHK47672
  
Location: 1050293-1051507
  
 NCBI BlastP on this gene

EHK47672

hypothetical protein
  
Accession: EHK47673
  
Location: 1052252-1054182
  
 NCBI BlastP on this gene

EHK47673

58. :  AFNW01000011 Fusarium pseudograminearum CS3096     Total score: 2.0     Cumulative Blast bit score: 2099

hypothetical protein
  
Accession: EKJ79238
  
Location: 88162-88695
  
 NCBI BlastP on this gene

EKJ79238

hypothetical protein
  
Accession: EKJ79239
  
Location: 88887-90160
  
 NCBI BlastP on this gene

EKJ79239

hypothetical protein
  
Accession: EKJ79240
  
Location: 91272-92371
  
 NCBI BlastP on this gene

EKJ79240

hypothetical protein
  
Accession: EKJ79241
  
Location: 92621-93936
  
 NCBI BlastP on this gene

EKJ79241

hypothetical protein
  
Accession: EKJ79242
  
Location: 95879-96411
  
 NCBI BlastP on this gene

EKJ79242

hypothetical protein
  
Accession: EKJ79243
  
Location: 97173-98685
  
 NCBI BlastP on this gene

EKJ79243

hypothetical protein
  
Accession: EKJ79244
  
Location: 99066-104567
  
 NCBI BlastP on this gene

EKJ79244

hypothetical protein
  
Accession: EKJ79245
  
Location: 104797-111801
  
  
**BlastP hit with Mycgr3G42010\_Mycgr3T**
  
Percentage identity: 44 %
  
BlastP bit score: 1882
  
Sequence coverage: 101 %
  
E-value: 0.0
  
  
 NCBI BlastP on this gene

EKJ79245

hypothetical protein
  
Accession: EKJ79246
  
Location: 112506-113895
  
  
**BlastP hit with Mycgr3G92938\_Mycgr3T**
  
Percentage identity: 33 %
  
BlastP bit score: 217
  
Sequence coverage: 82 %
  
E-value: 1e-61
  
  
 NCBI BlastP on this gene

EKJ79246

hypothetical protein
  
Accession: EKJ79247
  
Location: 115095-116558
  
 NCBI BlastP on this gene

EKJ79247

hypothetical protein
  
Accession: EKJ79248
  
Location: 117484-118605
  
 NCBI BlastP on this gene

EKJ79248

hypothetical protein
  
Accession: EKJ79249
  
Location: 119778-121478
  
 NCBI BlastP on this gene

EKJ79249

hypothetical protein
  
Accession: EKJ79250
  
Location: 124725-125572
  
 NCBI BlastP on this gene

EKJ79250

hypothetical protein
  
Accession: EKJ79251
  
Location: 125811-126161
  
 NCBI BlastP on this gene

EKJ79251

hypothetical protein
  
Accession: EKJ79252
  
Location: 126455-128203
  
 NCBI BlastP on this gene

EKJ79252

hypothetical protein
  
Accession: EKJ79253
  
Location: 128580-129990
  
 NCBI BlastP on this gene

EKJ79253

hypothetical protein
  
Accession: EKJ79254
  
Location: 130856-132556
  
 NCBI BlastP on this gene

EKJ79254

59. :  DS995706 Microsporum canis CBS 113480 supercont1.6 genomic scaffold     Total score: 2.0     Cumulative Blast bit score: 2097

conserved hypothetical protein
  
Accession: EEQ33491
  
Location: 455197-457545
  
 NCBI BlastP on this gene

EEQ33491

glyoxylate reductase
  
Accession: EEQ33492
  
Location: 458012-459636
  
 NCBI BlastP on this gene

EEQ33492

cell cycle control protein cwf19
  
Accession: EEQ33493
  
Location: 460417-462601
  
 NCBI BlastP on this gene

EEQ33493

ATPase NPA3
  
Accession: EEQ33494
  
Location: 462854-464095
  
 NCBI BlastP on this gene

EEQ33494

conserved hypothetical protein
  
Accession: EEQ33495
  
Location: 464373-465012
  
 NCBI BlastP on this gene

EEQ33495

conserved hypothetical protein
  
Accession: EEQ33496
  
Location: 465401-467758
  
 NCBI BlastP on this gene

EEQ33496

conserved hypothetical protein
  
Accession: EEQ33497
  
Location: 468388-469423
  
 NCBI BlastP on this gene

EEQ33497

conserved hypothetical protein
  
Accession: EEQ33498
  
Location: 469891-470471
  
 NCBI BlastP on this gene

EEQ33498

conserved hypothetical protein
  
Accession: EEQ33499
  
Location: 470929-472916
  
 NCBI BlastP on this gene

EEQ33499

NFX1-type zinc finger-containing protein 1
  
Accession: EEQ33500
  
Location: 473557-480978
  
  
**BlastP hit with Mycgr3G42010\_Mycgr3T**
  
Percentage identity: 44 %
  
BlastP bit score: 1891
  
Sequence coverage: 102 %
  
E-value: 0.0
  
  
 NCBI BlastP on this gene

EEQ33500

conserved hypothetical protein
  
Accession: EEQ33501
  
Location: 481360-482668
  
  
**BlastP hit with Mycgr3G92938\_Mycgr3T**
  
Percentage identity: 41 %
  
BlastP bit score: 206
  
Sequence coverage: 58 %
  
E-value: 7e-58
  
  
 NCBI BlastP on this gene

EEQ33501

predicted protein
  
Accession: EEQ33502
  
Location: 483382-484364
  
 NCBI BlastP on this gene

EEQ33502

leucine Rich Repeat domain-containing protein
  
Accession: EEQ33503
  
Location: 486886-488966
  
 NCBI BlastP on this gene

EEQ33503

conserved hypothetical protein
  
Accession: EEQ33504
  
Location: 489478-491280
  
 NCBI BlastP on this gene

EEQ33504

choline transport protein
  
Accession: EEQ33505
  
Location: 494102-496154
  
 NCBI BlastP on this gene

EEQ33505

cytochrome P450 3A8
  
Accession: EEQ33506
  
Location: 497087-498729
  
 NCBI BlastP on this gene

EEQ33506

conserved hypothetical protein
  
Accession: EEQ33507
  
Location: 498993-500314
  
 NCBI BlastP on this gene

EEQ33507

SpRPA12
  
Accession: EEQ33508
  
Location: 500529-501004
  
 NCBI BlastP on this gene

EEQ33508

60. :  GL629801 Grosmannia clavigera kw1407 unplaced genomic scaffold GCSC\_173     Total score: 2.0     Cumulative Blast bit score: 2094

acetolactate synthase
  
Accession: EFX00095
  
Location: 1544756-1551620
  
 NCBI BlastP on this gene

EFX00095

hypothetical protein
  
Accession: EFX00586
  
Location: 1554180-1555976
  
 NCBI BlastP on this gene

EFX00586

hypothetical protein
  
Accession: EFX00683
  
Location: 1556620-1556994
  
 NCBI BlastP on this gene

EFX00683

hypothetical protein
  
Accession: EFX00457
  
Location: 1557139-1557750
  
 NCBI BlastP on this gene

EFX00457

umta methyltransferase family protein
  
Accession: EFX00489
  
Location: 1559231-1560795
  
 NCBI BlastP on this gene

EFX00489

hypothetical protein
  
Accession: EFX00224
  
Location: 1561184-1562421
  
 NCBI BlastP on this gene

EFX00224

duf341 domain containing protein
  
Accession: EFX00520
  
Location: 1564325-1565346
  
  
**BlastP hit with Mycgr3G104337\_Mycgr3**
  
Percentage identity: 47 %
  
BlastP bit score: 214
  
Sequence coverage: 92 %
  
E-value: 1e-64
  
  
 NCBI BlastP on this gene

EFX00520

polyketide synthase
  
Accession: EFX00060
  
Location: 1566165-1573137
  
  
**BlastP hit with Mycgr3G100089\_Mycgr3**
  
Percentage identity: 45 %
  
BlastP bit score: 1880
  
Sequence coverage: 101 %
  
E-value: 0.0
  
  
 NCBI BlastP on this gene

EFX00060

hypothetical protein
  
Accession: EFX00687
  
Location: 1576602-1577205
  
 NCBI BlastP on this gene

EFX00687

hypothetical protein
  
Accession: EFX00732
  
Location: 1580879-1581964
  
 NCBI BlastP on this gene

EFX00732

extradiol ring-cleavage class 3 subunit b
  
Accession: EFX00625
  
Location: 1583364-1584362
  
 NCBI BlastP on this gene

EFX00625

hypothetical protein
  
Accession: EFX00254
  
Location: 1586167-1587262
  
 NCBI BlastP on this gene

EFX00254

integral membrane protein
  
Accession: EFX00537
  
Location: 1589167-1590542
  
 NCBI BlastP on this gene

EFX00537

61. :  KE145354 Glarea lozoyensis ATCC 20868 chromosome Unknown GLAREA11     Total score: 2.0     Cumulative Blast bit score: 2083

Protein kinase-like (PK-like)
  
Accession: EPE35645
  
Location: 1401316-1403213
  
 NCBI BlastP on this gene

EPE35645

hypothetical protein
  
Accession: EPE35646
  
Location: 1406600-1407383
  
 NCBI BlastP on this gene

EPE35646

hypothetical protein
  
Accession: EPE35647
  
Location: 1410330-1411220
  
 NCBI BlastP on this gene

EPE35647

P-loop containing nucleoside triphosphate hydrolase
  
Accession: EPE35648
  
Location: 1411713-1416535
  
 NCBI BlastP on this gene

EPE35648

hypothetical protein
  
Accession: EPE35649
  
Location: 1417685-1418671
  
  
**BlastP hit with Mycgr3G104337\_Mycgr3**
  
Percentage identity: 39 %
  
BlastP bit score: 210
  
Sequence coverage: 109 %
  
E-value: 2e-63
  
  
 NCBI BlastP on this gene

EPE35649

Thiolase-like protein
  
Accession: EPE35650
  
Location: 1420170-1427192
  
  
**BlastP hit with Mycgr3G100089\_Mycgr3**
  
Percentage identity: 44 %
  
BlastP bit score: 1873
  
Sequence coverage: 100 %
  
E-value: 0.0
  
  
 NCBI BlastP on this gene

EPE35650

NAD kinase
  
Accession: EPE35651
  
Location: 1427895-1429196
  
 NCBI BlastP on this gene

EPE35651

Glycerol-3-phosphate (1)-acyltransferase
  
Accession: EPE35652
  
Location: 1429820-1431217
  
 NCBI BlastP on this gene

EPE35652

hypothetical protein
  
Accession: EPE35653
  
Location: 1432866-1434359
  
 NCBI BlastP on this gene

EPE35653

Protein kinase-like (PK-like)
  
Accession: EPE35654
  
Location: 1434599-1439263
  
 NCBI BlastP on this gene

EPE35654

Cytochrome P450
  
Accession: EPE35655
  
Location: 1440949-1442646
  
 NCBI BlastP on this gene

EPE35655

ClpP/crotonase
  
Accession: EPE35656
  
Location: 1444351-1445742
  
 NCBI BlastP on this gene

EPE35656

62. :  KB725947 Colletotrichum orbiculare MAFF 240422 unplaced genomic scaffold Scaffold\_381     Total score: 2.0     Cumulative Blast bit score: 2078

choline oxidase
  
Accession: ENH82084
  
Location: 1857655-1859434
  
 NCBI BlastP on this gene

ENH82084

fungal specific transcription factor
  
Accession: ENH82085
  
Location: 1861775-1864309
  
 NCBI BlastP on this gene

ENH82085

short-chain dehydrogenase
  
Accession: ENH82086
  
Location: 1866487-1868562
  
 NCBI BlastP on this gene

ENH82086

hypothetical protein
  
Accession: ENH82087
  
Location: 1869225-1870116
  
 NCBI BlastP on this gene

ENH82087

hypothetical protein
  
Accession: ENH82088
  
Location: 1870973-1874533
  
 NCBI BlastP on this gene

ENH82088

polyketide synthase
  
Accession: ENH82089
  
Location: 1875690-1882772
  
  
**BlastP hit with Mycgr3G100089\_Mycgr3**
  
Percentage identity: 44 %
  
BlastP bit score: 1868
  
Sequence coverage: 101 %
  
E-value: 0.0
  
  
 NCBI BlastP on this gene

ENH82089

duf341 family
  
Accession: ENH82090
  
Location: 1887558-1888556
  
  
**BlastP hit with Mycgr3G104337\_Mycgr3**
  
Percentage identity: 42 %
  
BlastP bit score: 210
  
Sequence coverage: 99 %
  
E-value: 1e-63
  
  
 NCBI BlastP on this gene

ENH82090

ABC multidrug transporter mdr1
  
Accession: ENH82091
  
Location: 1891297-1896167
  
 NCBI BlastP on this gene

ENH82091

cytochrome b5 type b
  
Accession: ENH82092
  
Location: 1896728-1898824
  
 NCBI BlastP on this gene

ENH82092

sulfite reductase subunit alpha
  
Accession: ENH82093
  
Location: 1898864-1901793
  
 NCBI BlastP on this gene

ENH82093

lipase b precursor
  
Accession: ENH82094
  
Location: 1904568-1905833
  
 NCBI BlastP on this gene

ENH82094

63. :  KB707707 Botryotinia fuckeliana BcDW1 unplaced genomic scaffold Scaffold\_35     Total score: 2.0     Cumulative Blast bit score: 2077

putative calmodulin protein
  
Accession: EMR90205
  
Location: 75660-76690
  
 NCBI BlastP on this gene

EMR90205

putative snare domain-containing protein
  
Accession: EMR90206
  
Location: 77100-78287
  
 NCBI BlastP on this gene

EMR90206

putative ser thr protein phosphatase family protein
  
Accession: EMR90207
  
Location: 80319-82062
  
 NCBI BlastP on this gene

EMR90207

putative nucleoside transporter family protein
  
Accession: EMR90208
  
Location: 84425-85893
  
 NCBI BlastP on this gene

EMR90208

putative transcription factor c2h2 protein
  
Accession: EMR90209
  
Location: 87689-91382
  
 NCBI BlastP on this gene

EMR90209

putative nfx1-type zinc finger-containing protein 1 protein
  
Accession: EMR90210
  
Location: 92388-100652
  
  
**BlastP hit with Mycgr3G42010\_Mycgr3T**
  
Percentage identity: 44 %
  
BlastP bit score: 1867
  
Sequence coverage: 103 %
  
E-value: 0.0
  
  
 NCBI BlastP on this gene

EMR90210

putative geranylgeranyl pyrophosphate synthetase protein
  
Accession: EMR90211
  
Location: 101324-102691
  
  
**BlastP hit with Mycgr3G92938\_Mycgr3T**
  
Percentage identity: 39 %
  
BlastP bit score: 210
  
Sequence coverage: 69 %
  
E-value: 2e-59
  
  
 NCBI BlastP on this gene

EMR90211

putative hexose transporter protein
  
Accession: EMR90212
  
Location: 103116-104914
  
 NCBI BlastP on this gene

EMR90212

putative glycoside hydrolase subgroup catalytic core protein
  
Accession: EMR90213
  
Location: 105592-107370
  
 NCBI BlastP on this gene

EMR90213

putative cytochrome p450 monooxygenase protein
  
Accession: EMR90214
  
Location: 108784-110154
  
 NCBI BlastP on this gene

EMR90214

hypothetical protein
  
Accession: EMR90215
  
Location: 110510-110767
  
 NCBI BlastP on this gene

EMR90215

hypothetical protein
  
Accession: EMR90216
  
Location: 111032-111469
  
 NCBI BlastP on this gene

EMR90216

putative maltose permease mal61 protein
  
Accession: EMR90217
  
Location: 112262-114044
  
 NCBI BlastP on this gene

EMR90217

putative glycoside hydrolase family 78 sequence protein
  
Accession: EMR90218
  
Location: 115006-117480
  
 NCBI BlastP on this gene

EMR90218

hypothetical protein
  
Accession: EMR90219
  
Location: 118089-119038
  
 NCBI BlastP on this gene

EMR90219

hypothetical protein
  
Accession: EMR90220
  
Location: 120173-121050
  
 NCBI BlastP on this gene

EMR90220

64. :  CAGA01000033 Claviceps purpurea 20.1     Total score: 2.0     Cumulative Blast bit score: 2075

uncharacterized protein
  
Accession: CCE31711
  
Location: 259105-260046
  
 NCBI BlastP on this gene

CCE31711

related to YER185w, Rta1p
  
Accession: CCE31712
  
Location: 265670-266742
  
 NCBI BlastP on this gene

CCE31712

probable aspartyl aminopeptidase
  
Accession: CCE31713
  
Location: 267374-268910
  
 NCBI BlastP on this gene

CCE31713

uncharacterized protein
  
Accession: CCE31714
  
Location: 270057-271052
  
 NCBI BlastP on this gene

CCE31714

uncharacterized protein
  
Accession: CCE31715
  
Location: 273940-275033
  
  
**BlastP hit with Mycgr3G104337\_Mycgr3**
  
Percentage identity: 41 %
  
BlastP bit score: 204
  
Sequence coverage: 101 %
  
E-value: 1e-60
  
  
 NCBI BlastP on this gene

CCE31715

related to polyketide synthase
  
Accession: CCE31716
  
Location: 277091-283978
  
  
**BlastP hit with Mycgr3G100089\_Mycgr3**
  
Percentage identity: 44 %
  
BlastP bit score: 1871
  
Sequence coverage: 100 %
  
E-value: 0.0
  
  
 NCBI BlastP on this gene

CCE31716

uncharacterized protein
  
Accession: CCE31717
  
Location: 287276-292937
  
 NCBI BlastP on this gene

CCE31717

65. :  KB730345 Fusarium oxysporum f. sp. cubense race 1 unplaced genomic scaffold scaffold299     Total score: 2.0     Cumulative Blast bit score: 2068

hypothetical protein
  
Accession: ENH66882
  
Location: 420859-423235
  
 NCBI BlastP on this gene

ENH66882

hypothetical protein
  
Accession: ENH66883
  
Location: 423814-425323
  
 NCBI BlastP on this gene

ENH66883

hypothetical protein
  
Accession: ENH66884
  
Location: 425765-428520
  
 NCBI BlastP on this gene

ENH66884

Putative protein yjlB
  
Accession: ENH66885
  
Location: 431459-431992
  
 NCBI BlastP on this gene

ENH66885

Stabilin-2
  
Accession: ENH66886
  
Location: 432156-433424
  
 NCBI BlastP on this gene

ENH66886

Cyclin CCL1
  
Accession: ENH66887
  
Location: 434416-435505
  
 NCBI BlastP on this gene

ENH66887

hypothetical protein
  
Accession: ENH66888
  
Location: 435752-437059
  
 NCBI BlastP on this gene

ENH66888

hypothetical protein
  
Accession: ENH66889
  
Location: 439433-440790
  
  
**BlastP hit with Mycgr3G92938\_Mycgr3T**
  
Percentage identity: 37 %
  
BlastP bit score: 230
  
Sequence coverage: 80 %
  
E-value: 1e-66
  
  
 NCBI BlastP on this gene

ENH66889

NFX1-type zinc finger-containing protein 1
  
Accession: ENH66890
  
Location: 441586-448935
  
  
**BlastP hit with Mycgr3G42010\_Mycgr3T**
  
Percentage identity: 44 %
  
BlastP bit score: 1838
  
Sequence coverage: 100 %
  
E-value: 0.0
  
  
 NCBI BlastP on this gene

ENH66890

hypothetical protein
  
Accession: ENH66891
  
Location: 449827-450873
  
 NCBI BlastP on this gene

ENH66891

hypothetical protein
  
Accession: ENH66892
  
Location: 452230-453519
  
 NCBI BlastP on this gene

ENH66892

hypothetical protein
  
Accession: ENH66893
  
Location: 454485-454673
  
 NCBI BlastP on this gene

ENH66893

hypothetical protein
  
Accession: ENH66894
  
Location: 455795-457366
  
 NCBI BlastP on this gene

ENH66894

hypothetical protein
  
Accession: ENH66895
  
Location: 458284-458634
  
 NCBI BlastP on this gene

ENH66895

hypothetical protein
  
Accession: ENH66896
  
Location: 458861-460417
  
 NCBI BlastP on this gene

ENH66896

hypothetical protein
  
Accession: ENH66897
  
Location: 463637-465202
  
 NCBI BlastP on this gene

ENH66897

Killer toxin subunits alpha/beta
  
Accession: ENH66898
  
Location: 465455-468361
  
 NCBI BlastP on this gene

ENH66898

66. :  CABT02000029 Sordaria macrospora k-hell     Total score: 2.0     Cumulative Blast bit score: 2068

not annotated
  
Accession: CCC12635
  
Location: 392935-394218
  
 NCBI BlastP on this gene

CCC12635

not annotated
  
Accession: CCC12636
  
Location: 395325-396472
  
 NCBI BlastP on this gene

CCC12636

not annotated
  
Accession: CCC12637
  
Location: 396641-399317
  
 NCBI BlastP on this gene

CCC12637

not annotated
  
Accession: CCC12638
  
Location: 400001-401041
  
 NCBI BlastP on this gene

CCC12638

not annotated
  
Accession: CCC12639
  
Location: 401251-401771
  
 NCBI BlastP on this gene

CCC12639

not annotated
  
Accession: CCC12640
  
Location: 404183-408198
  
 NCBI BlastP on this gene

CCC12640

not annotated
  
Accession: CCC12641
  
Location: 408670-409329
  
 NCBI BlastP on this gene

CCC12641

not annotated
  
Accession: CCC12642
  
Location: 411007-411966
  
  
**BlastP hit with Mycgr3G104337\_Mycgr3**
  
Percentage identity: 41 %
  
BlastP bit score: 216
  
Sequence coverage: 100 %
  
E-value: 4e-66
  
  
 NCBI BlastP on this gene

CCC12642

not annotated
  
Accession: CCC12643
  
Location: 414023-421384
  
  
**BlastP hit with Mycgr3G100089\_Mycgr3**
  
Percentage identity: 44 %
  
BlastP bit score: 1852
  
Sequence coverage: 100 %
  
E-value: 0.0
  
  
 NCBI BlastP on this gene

CCC12643

67. :  GL985061 Trichoderma reesei QM6a unplaced genomic scaffold TRIREscaffold\_6     Total score: 2.0     Cumulative Blast bit score: 2067

vesicular transport protein
  
Accession: EGR49750
  
Location: 467138-470596
  
 NCBI BlastP on this gene

EGR49750

predicted protein
  
Accession: EGR49941
  
Location: 472904-474067
  
 NCBI BlastP on this gene

EGR49941

glycosyltransferase family 1
  
Accession: EGR49942
  
Location: 484124-485739
  
 NCBI BlastP on this gene

EGR49942

hypothetical protein
  
Accession: EGR49943
  
Location: 486801-487766
  
  
**BlastP hit with Mycgr3G104337\_Mycgr3**
  
Percentage identity: 43 %
  
BlastP bit score: 206
  
Sequence coverage: 98 %
  
E-value: 8e-62
  
  
 NCBI BlastP on this gene

EGR49943

polyketide synthase
  
Accession: EGR49751
  
Location: 489709-496750
  
  
**BlastP hit with Mycgr3G100089\_Mycgr3**
  
Percentage identity: 45 %
  
BlastP bit score: 1861
  
Sequence coverage: 101 %
  
E-value: 0.0
  
  
 NCBI BlastP on this gene

EGR49751

predicted protein
  
Accession: EGR49752
  
Location: 500672-501274
  
 NCBI BlastP on this gene

EGR49752

predicted protein
  
Accession: EGR49753
  
Location: 502741-503205
  
 NCBI BlastP on this gene

EGR49753

predicted protein
  
Accession: EGR49944
  
Location: 504033-506795
  
 NCBI BlastP on this gene

EGR49944

glycoside hydrolase family 20
  
Accession: EGR49754
  
Location: 509949-512211
  
 NCBI BlastP on this gene

EGR49754

predicted protein
  
Accession: EGR49755
  
Location: 512920-513568
  
 NCBI BlastP on this gene

EGR49755

predicted protein
  
Accession: EGR49945
  
Location: 514286-516064
  
 NCBI BlastP on this gene

EGR49945

68. :  GG697340 Glomerella graminicola M1.001 genomic scaffold supercont1.10     Total score: 2.0     Cumulative Blast bit score: 2055

hypothetical protein
  
Accession: EFQ28213
  
Location: 203918-207661
  
 NCBI BlastP on this gene

EFQ28213

GMC oxidoreductase
  
Accession: EFQ28214
  
Location: 210383-212149
  
 NCBI BlastP on this gene

EFQ28214

aldehyde dehydrogenase
  
Accession: EFQ28215
  
Location: 215183-216697
  
 NCBI BlastP on this gene

EFQ28215

beta-ketoacyl synthase domain-containing protein
  
Accession: EFQ28216
  
Location: 218621-225746
  
  
**BlastP hit with Mycgr3G100089\_Mycgr3**
  
Percentage identity: 44 %
  
BlastP bit score: 1851
  
Sequence coverage: 101 %
  
E-value: 0.0
  
  
 NCBI BlastP on this gene

EFQ28216

hypothetical protein
  
Accession: EFQ28217
  
Location: 230427-231402
  
  
**BlastP hit with Mycgr3G104337\_Mycgr3**
  
Percentage identity: 41 %
  
BlastP bit score: 204
  
Sequence coverage: 99 %
  
E-value: 3e-61
  
  
 NCBI BlastP on this gene

EFQ28217

ABC transporter
  
Accession: EFQ28218
  
Location: 233613-238526
  
 NCBI BlastP on this gene

EFQ28218

cytochrome b5-like Heme/Steroid binding domain-containing protein
  
Accession: EFQ28219
  
Location: 241391-246688
  
 NCBI BlastP on this gene

EFQ28219

69. :  GL698722 Metarhizium anisopliae ARSEF 23 unplaced genomic scaffold Scf\_012     Total score: 2.0     Cumulative Blast bit score: 2046

thioesterase family protein
  
Accession: EFY97476
  
Location: 486292-486798
  
 NCBI BlastP on this gene

EFY97476

C6 transcription factor, putative
  
Accession: EFY97477
  
Location: 486925-488983
  
 NCBI BlastP on this gene

EFY97477

malate/L-lactate dehydrogenase
  
Accession: EFY97478
  
Location: 489437-490507
  
 NCBI BlastP on this gene

EFY97478

stress responsive A/B barrel domain protein
  
Accession: EFY97479
  
Location: 490997-491570
  
 NCBI BlastP on this gene

EFY97479

high affinity nickel transport protein nic1
  
Accession: EFY97480
  
Location: 492114-493385
  
 NCBI BlastP on this gene

EFY97480

Autophagy- protein 17
  
Accession: EFY97481
  
Location: 495875-497463
  
 NCBI BlastP on this gene

EFY97481

proteinase, putative
  
Accession: EFY97482
  
Location: 497891-499784
  
 NCBI BlastP on this gene

EFY97482

hypothetical protein
  
Accession: EFY97483
  
Location: 500744-502180
  
 NCBI BlastP on this gene

EFY97483

nonsense-mediated mRNA decay protein, putative
  
Accession: EFY97484
  
Location: 503322-510301
  
  
**BlastP hit with Mycgr3G42010\_Mycgr3T**
  
Percentage identity: 42 %
  
BlastP bit score: 1813
  
Sequence coverage: 101 %
  
E-value: 0.0
  
  
 NCBI BlastP on this gene

EFY97484

hypothetical protein
  
Accession: EFY97485
  
Location: 511091-512508
  
  
**BlastP hit with Mycgr3G92938\_Mycgr3T**
  
Percentage identity: 36 %
  
BlastP bit score: 233
  
Sequence coverage: 81 %
  
E-value: 1e-67
  
  
 NCBI BlastP on this gene

EFY97485

hypothetical protein
  
Accession: EFY97486
  
Location: 513879-514778
  
 NCBI BlastP on this gene

EFY97486

F-box domain containing protein
  
Accession: EFY97487
  
Location: 516980-519522
  
 NCBI BlastP on this gene

EFY97487

5-hydroxyisourate hydrolase
  
Accession: EFY97488
  
Location: 523614-524072
  
 NCBI BlastP on this gene

EFY97488

short-chain dehydrogenase, putative
  
Accession: EFY97489
  
Location: 524970-525989
  
 NCBI BlastP on this gene

EFY97489

major facilitator superfamily MFS 1
  
Accession: EFY97490
  
Location: 526786-528561
  
 NCBI BlastP on this gene

EFY97490

hypothetical protein
  
Accession: EFY97491
  
Location: 529344-529754
  
 NCBI BlastP on this gene

EFY97491

cytochrome P450, putative
  
Accession: EFY97492
  
Location: 530247-532446
  
 NCBI BlastP on this gene

EFY97492

70. :  KB708021 Botryotinia fuckeliana BcDW1 unplaced genomic scaffold Scaffold\_349     Total score: 2.0     Cumulative Blast bit score: 2042

putative gmp synthase protein
  
Accession: EMR83048
  
Location: 192481-193329
  
 NCBI BlastP on this gene

EMR83048

putative glycoside hydrolase family 25 protein
  
Accession: EMR83049
  
Location: 194453-195580
  
 NCBI BlastP on this gene

EMR83049

putative gtp-binding protein gtr1 protein
  
Accession: EMR83050
  
Location: 196231-197592
  
 NCBI BlastP on this gene

EMR83050

putative het domain-containing protein
  
Accession: EMR83051
  
Location: 199126-200892
  
 NCBI BlastP on this gene

EMR83051

hypothetical protein
  
Accession: EMR83052
  
Location: 202536-203468
  
 NCBI BlastP on this gene

EMR83052

putative abc transporter protein
  
Accession: EMR83053
  
Location: 205230-209964
  
 NCBI BlastP on this gene

EMR83053

putative ef-hand calcium-binding domain protein
  
Accession: EMR83054
  
Location: 210979-211937
  
  
**BlastP hit with Mycgr3G104337\_Mycgr3**
  
Percentage identity: 45 %
  
BlastP bit score: 215
  
Sequence coverage: 95 %
  
E-value: 2e-65
  
  
 NCBI BlastP on this gene

EMR83054

putative polyketide synthase protein
  
Accession: EMR83055
  
Location: 213423-220647
  
  
**BlastP hit with Mycgr3G100089\_Mycgr3**
  
Percentage identity: 43 %
  
BlastP bit score: 1827
  
Sequence coverage: 100 %
  
E-value: 0.0
  
  
 NCBI BlastP on this gene

EMR83055

putative low temperature requirement a protein
  
Accession: EMR83056
  
Location: 221791-223614
  
 NCBI BlastP on this gene

EMR83056

putative kelch repeat-containing protein
  
Accession: EMR83057
  
Location: 224766-226046
  
 NCBI BlastP on this gene

EMR83057

71. :  FQ790281 Botryotinia fuckeliana T4 SuperContig\_330\_1 genomic supercontig.     Total score: 2.0     Cumulative Blast bit score: 2037

similar to glutamine amidotransferase class-I
  
Accession: CCD46344
  
Location: 723734-724582
  
 NCBI BlastP on this gene

BofuT4\_P118940.1

glycoside hydrolase family 25 protein
  
Accession: CCD46345
  
Location: 725706-726833
  
 NCBI BlastP on this gene

BofuT4\_P118950.1

hypothetical protein
  
Accession: CCD46346
  
Location: 727484-728845
  
 NCBI BlastP on this gene

BofuT4\_P118960.1

hypothetical protein
  
Accession: CCD46347
  
Location: 729711-732145
  
 NCBI BlastP on this gene

BofuT4\_P118970.1

hypothetical protein
  
Accession: CDF43968
  
Location: 733789-734721
  
 NCBI BlastP on this gene

BofuT4P330000018001

similar to ABC transporter
  
Accession: CCD46349
  
Location: 736483-740451
  
 NCBI BlastP on this gene

BofuT4P330000028001

hypothetical protein
  
Accession: CCD46350
  
Location: 740511-741217
  
 NCBI BlastP on this gene

BofuT4P330000029001

similar to EF-hand calcium-binding domain protein
  
Accession: CCD46351
  
Location: 742232-743190
  
  
**BlastP hit with Mycgr3G104337\_Mycgr3**
  
Percentage identity: 45 %
  
BlastP bit score: 215
  
Sequence coverage: 95 %
  
E-value: 2e-65
  
  
 NCBI BlastP on this gene

BofuT4P330000030001

predicted protein
  
Accession: CCD46352
  
Location: 743951-744513
  
 NCBI BlastP on this gene

BofuT4P330000031001

BcPKS8, polyketide synthase
  
Accession: CCD46353
  
Location: 744676-751900
  
  
**BlastP hit with Mycgr3G100089\_Mycgr3**
  
Percentage identity: 43 %
  
BlastP bit score: 1823
  
Sequence coverage: 101 %
  
E-value: 0.0
  
  
 NCBI BlastP on this gene

BofuT4P330000032001

hypothetical protein
  
Accession: CCD46354
  
Location: 752966-755358
  
 NCBI BlastP on this gene

BofuT4P330000033001

similar to kelch repeat-containing protein
  
Accession: CCD46355
  
Location: 756019-757299
  
 NCBI BlastP on this gene

BofuT4\_P119050.1

hypothetical protein
  
Accession: CCD46356
  
Location: 757710-758181
  
 NCBI BlastP on this gene

BofuT4\_P119060.1

similar to transcription factor bHLH
  
Accession: CCD46357
  
Location: 761137-762777
  
 NCBI BlastP on this gene

BofuT4\_P119070.1

predicted protein
  
Accession: CCD46358
  
Location: 768459-768950
  
 NCBI BlastP on this gene

BofuT4\_uP119080.1

72. :  GL385396 Gaeumannomyces graminis var. tritici R3-111a-1 unplaced genomic scaffold supercont2.2     Total score: 2.0     Cumulative Blast bit score: 2032

hypothetical protein
  
Accession: EJT78057
  
Location: 1902638-1906339
  
 NCBI BlastP on this gene

EJT78057

hypothetical protein
  
Accession: EJT78058
  
Location: 1907875-1908772
  
 NCBI BlastP on this gene

EJT78058

hypothetical protein
  
Accession: EJT78059
  
Location: 1909011-1910769
  
 NCBI BlastP on this gene

EJT78059

hypothetical protein
  
Accession: EJT78060
  
Location: 1911383-1917225
  
 NCBI BlastP on this gene

EJT78060

hypothetical protein
  
Accession: EJT78061
  
Location: 1919096-1920062
  
  
**BlastP hit with Mycgr3G104337\_Mycgr3**
  
Percentage identity: 44 %
  
BlastP bit score: 207
  
Sequence coverage: 95 %
  
E-value: 2e-62
  
  
 NCBI BlastP on this gene

EJT78061

hypothetical protein
  
Accession: EJT78062
  
Location: 1922426-1929709
  
  
**BlastP hit with Mycgr3G100089\_Mycgr3**
  
Percentage identity: 44 %
  
BlastP bit score: 1825
  
Sequence coverage: 101 %
  
E-value: 0.0
  
  
 NCBI BlastP on this gene

EJT78062

DNA polymerase sigma
  
Accession: EJT78063
  
Location: 1931219-1933617
  
 NCBI BlastP on this gene

EJT78063

hypothetical protein
  
Accession: EJT78064
  
Location: 1935060-1937230
  
 NCBI BlastP on this gene

EJT78064

hypothetical protein
  
Accession: EJT78065
  
Location: 1937863-1938974
  
 NCBI BlastP on this gene

EJT78065

hypothetical protein
  
Accession: EJT78066
  
Location: 1939619-1943218
  
 NCBI BlastP on this gene

EJT78066

hypothetical protein
  
Accession: EJT78067
  
Location: 1945916-1946430
  
 NCBI BlastP on this gene

EJT78067

hypothetical protein
  
Accession: EJT78068
  
Location: 1947126-1948065
  
 NCBI BlastP on this gene

EJT78068

73. :  GL891307 Neurospora tetrasperma FGSC 2508 unplaced genomic scaffold NEUTE1scaffold\_6     Total score: 2.0     Cumulative Blast bit score: 2028

hypothetical protein
  
Accession: EGO54652
  
Location: 2856255-2871377
  
  
**BlastP hit with Mycgr3G100089\_Mycgr3**
  
Percentage identity: 44 %
  
BlastP bit score: 1815
  
Sequence coverage: 100 %
  
E-value: 0.0
  
  
 NCBI BlastP on this gene

EGO54652

hypothetical protein
  
Accession: EGO54653
  
Location: 2871926-2872129
  
 NCBI BlastP on this gene

EGO54653

hypothetical protein
  
Accession: EGO54654
  
Location: 2873404-2874360
  
  
**BlastP hit with Mycgr3G104337\_Mycgr3**
  
Percentage identity: 41 %
  
BlastP bit score: 213
  
Sequence coverage: 99 %
  
E-value: 1e-64
  
  
 NCBI BlastP on this gene

EGO54654

hypothetical protein
  
Accession: EGO54655
  
Location: 2876087-2881260
  
 NCBI BlastP on this gene

EGO54655

hypothetical protein
  
Accession: EGO54656
  
Location: 2882806-2884738
  
 NCBI BlastP on this gene

EGO54656

hypothetical protein
  
Accession: EGO54657
  
Location: 2886231-2887387
  
 NCBI BlastP on this gene

EGO54657

74. :  GL891269 Neurospora tetrasperma FGSC 2509 unplaced genomic scaffold NEUTE2scaffold\_7     Total score: 2.0     Cumulative Blast bit score: 2028

Aldo/keto reductase
  
Accession: EGZ67872
  
Location: 996538-998279
  
 NCBI BlastP on this gene

EGZ67872

hypothetical protein
  
Accession: EGZ67873
  
Location: 1000848-1002780
  
 NCBI BlastP on this gene

EGZ67873

P-loop containing nucleoside triphosphate hydrolase protein
  
Accession: EGZ67874
  
Location: 1004326-1009499
  
 NCBI BlastP on this gene

EGZ67874

hypothetical protein
  
Accession: EGZ67875
  
Location: 1011224-1012180
  
  
**BlastP hit with Mycgr3G104337\_Mycgr3**
  
Percentage identity: 41 %
  
BlastP bit score: 213
  
Sequence coverage: 99 %
  
E-value: 1e-64
  
  
 NCBI BlastP on this gene

EGZ67875

hypothetical protein
  
Accession: EGZ67876
  
Location: 1013453-1013656
  
 NCBI BlastP on this gene

EGZ67876

ketoacyl-synt-domain-containing protein
  
Accession: EGZ67877
  
Location: 1014205-1029328
  
  
**BlastP hit with Mycgr3G100089\_Mycgr3**
  
Percentage identity: 44 %
  
BlastP bit score: 1815
  
Sequence coverage: 100 %
  
E-value: 0.0
  
  
 NCBI BlastP on this gene

EGZ67877

hypothetical protein
  
Accession: EGZ67878
  
Location: 1030624-1030782
  
 NCBI BlastP on this gene

EGZ67878

hypothetical protein
  
Accession: EGZ67879
  
Location: 1032370-1032525
  
 NCBI BlastP on this gene

EGZ67879

hypothetical protein
  
Accession: EGZ67880
  
Location: 1035220-1036071
  
 NCBI BlastP on this gene

EGZ67880

75. :  KB021009 Colletotrichum gloeosporioides Nara gc5 unplaced genomic scaffold scaffold605     Total score: 2.0     Cumulative Blast bit score: 2026

lipid a export atp-binding permease protein msba
  
Accession: ELA26921
  
Location: 343-3758
  
 NCBI BlastP on this gene

ELA26921

duf341 family
  
Accession: ELA26922
  
Location: 7465-8478
  
  
**BlastP hit with Mycgr3G104337\_Mycgr3**
  
Percentage identity: 42 %
  
BlastP bit score: 209
  
Sequence coverage: 99 %
  
E-value: 5e-63
  
  
 NCBI BlastP on this gene

ELA26922

polyketide synthase
  
Accession: ELA26923
  
Location: 13784-20850
  
  
**BlastP hit with Mycgr3G100089\_Mycgr3**
  
Percentage identity: 43 %
  
BlastP bit score: 1817
  
Sequence coverage: 101 %
  
E-value: 0.0
  
  
 NCBI BlastP on this gene

ELA26923

ankyrin repeat protein
  
Accession: ELA26924
  
Location: 21563-24444
  
 NCBI BlastP on this gene

ELA26924

MFS multidrug transporter
  
Accession: ELA26925
  
Location: 26251-27839
  
 NCBI BlastP on this gene

ELA26925

C6 finger domain-containing protein
  
Accession: ELA26926
  
Location: 28970-30073
  
 NCBI BlastP on this gene

ELA26926

short-chain dehydrogenase, putative
  
Accession: ELA26927
  
Location: 30178-31158
  
 NCBI BlastP on this gene

ELA26927

NADPH--cytochrome p450 reductase
  
Accession: ELA26928
  
Location: 31501-32842
  
 NCBI BlastP on this gene

ELA26928

fungal specific transcription factor
  
Accession: ELA26929
  
Location: 33435-35918
  
 NCBI BlastP on this gene

ELA26929

76. :  CH476603 Aspergillus terreus NIH2624 scaffold\_10 genomic scaffold     Total score: 2.0     Cumulative Blast bit score: 2020

GTPase-activating protein GYP7
  
Accession: EAU32442
  
Location: 604913-607649
  
 NCBI BlastP on this gene

EAU32442

predicted protein
  
Accession: EAU32443
  
Location: 608041-608618
  
 NCBI BlastP on this gene

EAU32443

conserved hypothetical protein
  
Accession: EAU32444
  
Location: 610355-611524
  
 NCBI BlastP on this gene

EAU32444

conserved hypothetical protein
  
Accession: EAU32445
  
Location: 612716-613472
  
 NCBI BlastP on this gene

EAU32445

hypothetical protein
  
Accession: EAU32446
  
Location: 614039-615196
  
 NCBI BlastP on this gene

EAU32446

hypothetical protein
  
Accession: EAU32447
  
Location: 615788-617205
  
 NCBI BlastP on this gene

EAU32447

conserved hypothetical protein
  
Accession: EAU32448
  
Location: 617708-618290
  
 NCBI BlastP on this gene

EAU32448

predicted protein
  
Accession: EAU32449
  
Location: 618833-620758
  
 NCBI BlastP on this gene

EAU32449

predicted protein
  
Accession: EAU32450
  
Location: 621328-622182
  
  
**BlastP hit with Mycgr3G104337\_Mycgr3**
  
Percentage identity: 32 %
  
BlastP bit score: 131
  
Sequence coverage: 96 %
  
E-value: 1e-33
  
  
 NCBI BlastP on this gene

EAU32450

hypothetical protein
  
Accession: EAU32451
  
Location: 623328-630255
  
  
**BlastP hit with Mycgr3G100089\_Mycgr3**
  
Percentage identity: 46 %
  
BlastP bit score: 1889
  
Sequence coverage: 101 %
  
E-value: 0.0
  
  
 NCBI BlastP on this gene

EAU32451

predicted protein
  
Accession: EAU32452
  
Location: 631817-634928
  
 NCBI BlastP on this gene

EAU32452

conserved hypothetical protein
  
Accession: EAU32453
  
Location: 635506-637438
  
 NCBI BlastP on this gene

EAU32453

conserved hypothetical protein
  
Accession: EAU32454
  
Location: 638596-639852
  
 NCBI BlastP on this gene

EAU32454

conserved hypothetical protein
  
Accession: EAU32455
  
Location: 641804-645435
  
 NCBI BlastP on this gene

EAU32455

predicted protein
  
Accession: EAU32456
  
Location: 647069-651135
  
 NCBI BlastP on this gene

EAU32456

77. :  KE148164 Ophiostoma piceae UAMH 11346 chromosome Unknown scf19     Total score: 2.0     Cumulative Blast bit score: 2014

potassium channel
  
Accession: EPE03864
  
Location: 208792-211719
  
 NCBI BlastP on this gene

EPE03864

ubiquitin fusion degradation protein
  
Accession: EPE03865
  
Location: 212620-215121
  
 NCBI BlastP on this gene

EPE03865

hypothetical protein
  
Accession: EPE03866
  
Location: 215186-216292
  
 NCBI BlastP on this gene

EPE03866

hypothetical protein
  
Accession: EPE03867
  
Location: 217305-217771
  
 NCBI BlastP on this gene

EPE03867

hypothetical protein
  
Accession: EPE03868
  
Location: 217864-218200
  
 NCBI BlastP on this gene

EPE03868

abc transporter
  
Accession: EPE03869
  
Location: 218525-223040
  
 NCBI BlastP on this gene

EPE03869

ef-hand calcium-binding domain protein
  
Accession: EPE03870
  
Location: 225996-226821
  
  
**BlastP hit with Mycgr3G104337\_Mycgr3**
  
Percentage identity: 42 %
  
BlastP bit score: 207
  
Sequence coverage: 98 %
  
E-value: 1e-62
  
  
 NCBI BlastP on this gene

EPE03870

polyketide synthase
  
Accession: EPE03871
  
Location: 233951-240763
  
  
**BlastP hit with Mycgr3G100089\_Mycgr3**
  
Percentage identity: 43 %
  
BlastP bit score: 1807
  
Sequence coverage: 102 %
  
E-value: 0.0
  
  
 NCBI BlastP on this gene

EPE03871

hypothetical protein
  
Accession: EPE03872
  
Location: 241505-246112
  
 NCBI BlastP on this gene

EPE03872

hypothetical protein
  
Accession: EPE03873
  
Location: 253736-254971
  
 NCBI BlastP on this gene

EPE03873

glucosidase 2 subunit beta
  
Accession: EPE03874
  
Location: 255461-257371
  
 NCBI BlastP on this gene

EPE03874

78. :  ACJE01000003 Aspergillus niger ATCC 1015     Total score: 2.0     Cumulative Blast bit score: 2003

hypothetical protein
  
Accession: EHA27400
  
Location: 583414-585327
  
 NCBI BlastP on this gene

EHA27400

hypothetical protein
  
Accession: EHA27401
  
Location: 586604-587545
  
 NCBI BlastP on this gene

EHA27401

hypothetical protein
  
Accession: EHA27402
  
Location: 588726-589930
  
 NCBI BlastP on this gene

EHA27402

hypothetical protein
  
Accession: EHA27403
  
Location: 590777-592618
  
 NCBI BlastP on this gene

EHA27403

hypothetical protein
  
Accession: EHA27404
  
Location: 593136-595006
  
 NCBI BlastP on this gene

EHA27404

hypothetical protein
  
Accession: EHA27405
  
Location: 596222-598235
  
 NCBI BlastP on this gene

EHA27405

hypothetical protein
  
Accession: EHA27406
  
Location: 599395-600497
  
 NCBI BlastP on this gene

EHA27406

hypothetical protein
  
Accession: EHA27407
  
Location: 602133-603048
  
  
**BlastP hit with Mycgr3G104337\_Mycgr3**
  
Percentage identity: 44 %
  
BlastP bit score: 209
  
Sequence coverage: 98 %
  
E-value: 3e-63
  
  
 NCBI BlastP on this gene

EHA27407

hypothetical protein
  
Accession: EHA27408
  
Location: 604912-611896
  
  
**BlastP hit with Mycgr3G100089\_Mycgr3**
  
Percentage identity: 44 %
  
BlastP bit score: 1794
  
Sequence coverage: 102 %
  
E-value: 0.0
  
  
 NCBI BlastP on this gene

EHA27408

hypothetical protein
  
Accession: EHA27409
  
Location: 613108-615065
  
 NCBI BlastP on this gene

EHA27409

hypothetical protein
  
Accession: EHA27410
  
Location: 616750-620047
  
 NCBI BlastP on this gene

EHA27410

amine oxidase
  
Accession: EHA27411
  
Location: 621045-622887
  
 NCBI BlastP on this gene

EHA27411

hypothetical protein
  
Accession: EHA27412
  
Location: 627011-628099
  
 NCBI BlastP on this gene

EHA27412

hypothetical protein
  
Accession: EHA27413
  
Location: 629480-630363
  
 NCBI BlastP on this gene

EHA27413

79. :  HF679030 Fusarium fujikuroi IMI 58289 draft genome, chromosome FFUJ\_chr08.     Total score: 2.0     Cumulative Blast bit score: 1998

probable NADH cytb-reductase
  
Accession: CCT72092
  
Location: 449878-451277
  
 NCBI BlastP on this gene

FFUJ\_13910

probable high affinity methionine permease
  
Accession: CCT72093
  
Location: 451542-453266
  
 NCBI BlastP on this gene

FFUJ\_13909

probable saccharopine dehydrogenase (NAD, L-lysine-forming)
  
Accession: CCT72094
  
Location: 453666-454868
  
 NCBI BlastP on this gene

FFUJ\_13908

related to allantoate permease
  
Accession: CCT72095
  
Location: 455030-456542
  
 NCBI BlastP on this gene

FFUJ\_13907

related to D-arabinitol 2-dehydrogenase
  
Accession: CCT72096
  
Location: 457101-458085
  
 NCBI BlastP on this gene

FFUJ\_13906

related to ribose-5-phosphate isomerase
  
Accession: CCT72097
  
Location: 458540-459087
  
 NCBI BlastP on this gene

FFUJ\_13905

related to fructose-bisphosphate aldolase
  
Accession: CCT72098
  
Location: 459508-460424
  
 NCBI BlastP on this gene

FFUJ\_13904

uncharacterized protein
  
Accession: CCT72099
  
Location: 460490-462352
  
 NCBI BlastP on this gene

FFUJ\_13903

related to dihydroxyacetone kinase
  
Accession: CCT72100
  
Location: 462818-464651
  
 NCBI BlastP on this gene

FFUJ\_13902

related to beta transducin-like protein
  
Accession: CCT72101
  
Location: 465995-467203
  
 NCBI BlastP on this gene

FFUJ\_13901

related to ECM32-DNA dependent ATPase/DNA helicase B
  
Accession: CCT73103
  
Location: 467577-474600
  
  
**BlastP hit with Mycgr3G42010\_Mycgr3T**
  
Percentage identity: 43 %
  
BlastP bit score: 1771
  
Sequence coverage: 100 %
  
E-value: 0.0
  
  
 NCBI BlastP on this gene

FFUJ\_13900

uncharacterized protein
  
Accession: CCT72102
  
Location: 475384-476735
  
  
**BlastP hit with Mycgr3G92938\_Mycgr3T**
  
Percentage identity: 32 %
  
BlastP bit score: 227
  
Sequence coverage: 100 %
  
E-value: 2e-65
  
  
 NCBI BlastP on this gene

FFUJ\_13899

related to lysophosphatidic acid acyltransferase
  
Accession: CCT72103
  
Location: 479133-480439
  
 NCBI BlastP on this gene

FFUJ\_13898

related to cyclin CCL1
  
Accession: CCT72104
  
Location: 480683-481776
  
 NCBI BlastP on this gene

FFUJ\_13897

related to TGF beta induced protein ig-h3 precursor
  
Accession: CCT72105
  
Location: 482778-484045
  
 NCBI BlastP on this gene

FFUJ\_13896

uncharacterized protein
  
Accession: CCT72106
  
Location: 484206-484739
  
 NCBI BlastP on this gene

FFUJ\_13895

related to integral membrane protein PTH11
  
Accession: CCT72107
  
Location: 487670-489217
  
 NCBI BlastP on this gene

FFUJ\_13894

uncharacterized protein
  
Accession: CCT72108
  
Location: 489830-490460
  
 NCBI BlastP on this gene

FFUJ\_13893

related to DUF895 domain membrane protein
  
Accession: CCT72109
  
Location: 490828-492369
  
 NCBI BlastP on this gene

FFUJ\_13892

uncharacterized protein
  
Accession: CCT72110
  
Location: 492956-495313
  
 NCBI BlastP on this gene

FFUJ\_13891

80. :  KE148164 Ophiostoma piceae UAMH 11346 chromosome Unknown scf19     Total score: 2.0     Cumulative Blast bit score: 1985

transcription factor
  
Accession: EPE03903
  
Location: 344428-346924
  
 NCBI BlastP on this gene

EPE03903

fluconazole resistance protein 1
  
Accession: EPE03904
  
Location: 358498-360216
  
 NCBI BlastP on this gene

EPE03904

hypothetical protein
  
Accession: EPE03905
  
Location: 361732-363267
  
 NCBI BlastP on this gene

EPE03905

aaa family ATPase
  
Accession: EPE03906
  
Location: 363979-371364
  
  
**BlastP hit with Mycgr3G42010\_Mycgr3T**
  
Percentage identity: 41 %
  
BlastP bit score: 1766
  
Sequence coverage: 108 %
  
E-value: 0.0
  
  
 NCBI BlastP on this gene

EPE03906

geranylgeranyl pyrophosphate synthetase
  
Accession: EPE03907
  
Location: 372017-373771
  
  
**BlastP hit with Mycgr3G92938\_Mycgr3T**
  
Percentage identity: 35 %
  
BlastP bit score: 219
  
Sequence coverage: 93 %
  
E-value: 2e-61
  
  
 NCBI BlastP on this gene

EPE03907

hexose transporter
  
Accession: EPE03908
  
Location: 383334-385001
  
 NCBI BlastP on this gene

EPE03908

hypothetical protein
  
Accession: EPE03909
  
Location: 386154-388971
  
 NCBI BlastP on this gene

EPE03909

aldehyde dehydrogenase
  
Accession: EPE03910
  
Location: 389766-391319
  
 NCBI BlastP on this gene

EPE03910

isotrichodermin c-15 hydroxylase
  
Accession: EPE03911
  
Location: 391688-393286
  
 NCBI BlastP on this gene

EPE03911

81. :  JH921436 Marssonina brunnea f. sp. 'multigermtubi' MB\_m1 unplaced genomic scaffold M6\_S00009     Total score: 2.0     Cumulative Blast bit score: 1982

autophagy protein
  
Accession: EKD17570
  
Location: 1810086-1812240
  
 NCBI BlastP on this gene

EKD17570

peroxisomal adenine nucleotide transporter 1
  
Accession: EKD17571
  
Location: 1812797-1814366
  
 NCBI BlastP on this gene

EKD17571

hypothetical protein
  
Accession: EKD17572
  
Location: 1814921-1816122
  
 NCBI BlastP on this gene

EKD17572

zinc finger containing protein
  
Accession: EKD17573
  
Location: 1818089-1819299
  
 NCBI BlastP on this gene

EKD17573

putative tafazzin
  
Accession: EKD17574
  
Location: 1820213-1821530
  
 NCBI BlastP on this gene

EKD17574

Phosphomannomutase
  
Accession: EKD17575
  
Location: 1822559-1824358
  
 NCBI BlastP on this gene

EKD17575

pyridoxine
  
Accession: EKD17576
  
Location: 1825800-1826720
  
 NCBI BlastP on this gene

EKD17576

AtaAp protein
  
Accession: EKD17577
  
Location: 1827024-1827981
  
 NCBI BlastP on this gene

EKD17577

hypothetical protein
  
Accession: EKD17578
  
Location: 1829718-1831221
  
  
**BlastP hit with Mycgr3G92938\_Mycgr3T**
  
Percentage identity: 36 %
  
BlastP bit score: 205
  
Sequence coverage: 68 %
  
E-value: 4e-57
  
  
 NCBI BlastP on this gene

EKD17578

NFX1-type zinc finger-containing protein 1
  
Accession: EKD17579
  
Location: 1831726-1839437
  
  
**BlastP hit with Mycgr3G42010\_Mycgr3T**
  
Percentage identity: 42 %
  
BlastP bit score: 1777
  
Sequence coverage: 105 %
  
E-value: 0.0
  
  
 NCBI BlastP on this gene

EKD17579

hypothetical protein
  
Accession: EKD17580
  
Location: 1840823-1841364
  
 NCBI BlastP on this gene

EKD17580

Ni2+-Co2+ transporter transition metal uptake transporter
  
Accession: EKD17581
  
Location: 1842789-1844103
  
 NCBI BlastP on this gene

EKD17581

hypothetical protein
  
Accession: EKD17582
  
Location: 1849122-1850397
  
 NCBI BlastP on this gene

EKD17582

82. :  GL385397 Gaeumannomyces graminis var. tritici R3-111a-1 unplaced genomic scaffold supercont2.3     Total score: 2.0     Cumulative Blast bit score: 1980

hypothetical protein
  
Accession: EJT77322
  
Location: 6324702-6325847
  
 NCBI BlastP on this gene

EJT77322

hypothetical protein
  
Accession: EJT77323
  
Location: 6326427-6328872
  
 NCBI BlastP on this gene

EJT77323

hypothetical protein
  
Accession: EJT77324
  
Location: 6329256-6329957
  
 NCBI BlastP on this gene

EJT77324

hypothetical protein
  
Accession: EJT77325
  
Location: 6330409-6331728
  
 NCBI BlastP on this gene

EJT77325

hypothetical protein
  
Accession: EJT77326
  
Location: 6342367-6349492
  
  
**BlastP hit with Mycgr3G100089\_Mycgr3**
  
Percentage identity: 43 %
  
BlastP bit score: 1763
  
Sequence coverage: 102 %
  
E-value: 0.0
  
  
 NCBI BlastP on this gene

EJT77326

hypothetical protein
  
Accession: EJT77327
  
Location: 6351500-6352493
  
  
**BlastP hit with Mycgr3G104337\_Mycgr3**
  
Percentage identity: 42 %
  
BlastP bit score: 217
  
Sequence coverage: 99 %
  
E-value: 2e-66
  
  
 NCBI BlastP on this gene

EJT77327

hypothetical protein
  
Accession: EJT77328
  
Location: 6353944-6358977
  
 NCBI BlastP on this gene

EJT77328

hypothetical protein
  
Accession: EJT77329
  
Location: 6359346-6360665
  
 NCBI BlastP on this gene

EJT77329

hypothetical protein
  
Accession: EJT77330
  
Location: 6360761-6361480
  
 NCBI BlastP on this gene

EJT77330

hypothetical protein
  
Accession: EJT77331
  
Location: 6361779-6362366
  
 NCBI BlastP on this gene

EJT77331

hypothetical protein
  
Accession: EJT77332
  
Location: 6362728-6364400
  
 NCBI BlastP on this gene

EJT77332

hypothetical protein
  
Accession: EJT77333
  
Location: 6364684-6365331
  
 NCBI BlastP on this gene

EJT77333

hypothetical protein
  
Accession: EJT77334
  
Location: 6366741-6367950
  
 NCBI BlastP on this gene

EJT77334

hypothetical protein
  
Accession: EJT77335
  
Location: 6368928-6370189
  
 NCBI BlastP on this gene

EJT77335

83. :  CP003009 Thielavia terrestris NRRL 8126 chromosome 1     Total score: 2.0     Cumulative Blast bit score: 1976

hypothetical protein
  
Accession: AEO64470
  
Location: 8720085-8722442
  
 NCBI BlastP on this gene

THITE\_2110633

hypothetical protein
  
Accession: AEO64471
  
Location: 8723685-8725866
  
 NCBI BlastP on this gene

THITE\_2110636

glycoside hydrolase family 2 protein
  
Accession: AEO64472
  
Location: 8728063-8730708
  
 NCBI BlastP on this gene

THITE\_2110638

hypothetical protein
  
Accession: AEO64473
  
Location: 8730831-8732004
  
 NCBI BlastP on this gene

THITE\_2110640

hypothetical protein
  
Accession: AEO64474
  
Location: 8732550-8737419
  
 NCBI BlastP on this gene

THITE\_2040380

hypothetical protein
  
Accession: AEO64475
  
Location: 8739834-8740796
  
  
**BlastP hit with Mycgr3G104337\_Mycgr3**
  
Percentage identity: 43 %
  
BlastP bit score: 221
  
Sequence coverage: 99 %
  
E-value: 1e-67
  
  
 NCBI BlastP on this gene

THITE\_2037371

polyketide synthase
  
Accession: AEO64476
  
Location: 8743360-8750632
  
  
**BlastP hit with Mycgr3G100089\_Mycgr3**
  
Percentage identity: 42 %
  
BlastP bit score: 1755
  
Sequence coverage: 101 %
  
E-value: 0.0
  
  
 NCBI BlastP on this gene

THITE\_123823

hypothetical protein
  
Accession: AEO64477
  
Location: 8751428-8753560
  
 NCBI BlastP on this gene

THITE\_2142385

hypothetical protein
  
Accession: AEO64478
  
Location: 8755334-8756280
  
 NCBI BlastP on this gene

THITE\_74555

hypothetical protein
  
Accession: AEO64479
  
Location: 8756804-8758561
  
 NCBI BlastP on this gene

THITE\_2110648

hypothetical protein
  
Accession: AEO64480
  
Location: 8761149-8766032
  
 NCBI BlastP on this gene

THITE\_2110651

hypothetical protein
  
Accession: AEO64481
  
Location: 8766998-8767633
  
 NCBI BlastP on this gene

THITE\_2110654

84. :  DS572699 Verticillium dahliae VdLs.17 supercont1.5 genomic scaffold     Total score: 2.0     Cumulative Blast bit score: 1975

hypothetical protein
  
Accession: EGY22017
  
Location: 1500448-1501474
  
 NCBI BlastP on this gene

EGY22017

hypothetical protein
  
Accession: EGY22018
  
Location: 1501949-1502650
  
 NCBI BlastP on this gene

EGY22018

mitochondrial inner membrane translocase subunit TIM44
  
Accession: EGY22019
  
Location: 1503228-1504912
  
 NCBI BlastP on this gene

EGY22019

mitochondrial ribosomal protein S18
  
Accession: EGY22020
  
Location: 1505384-1506130
  
 NCBI BlastP on this gene

EGY22020

hag1
  
Accession: EGY22021
  
Location: 1506436-1507278
  
 NCBI BlastP on this gene

EGY22021

GTP-dependent nucleic acid-binding protein engD
  
Accession: EGY22022
  
Location: 1508332-1509968
  
 NCBI BlastP on this gene

EGY22022

exopolygalacturonase
  
Accession: EGY22023
  
Location: 1510190-1512025
  
 NCBI BlastP on this gene

EGY22023

thioredoxin
  
Accession: EGY22024
  
Location: 1512752-1513198
  
 NCBI BlastP on this gene

EGY22024

drug resistance protein
  
Accession: EGY22025
  
Location: 1514042-1515821
  
 NCBI BlastP on this gene

EGY22025

fatty acid synthase S-acetyltransferase
  
Accession: EGY22026
  
Location: 1516713-1523486
  
  
**BlastP hit with Mycgr3G100089\_Mycgr3**
  
Percentage identity: 44 %
  
BlastP bit score: 1794
  
Sequence coverage: 100 %
  
E-value: 0.0
  
  
 NCBI BlastP on this gene

EGY22026

hypothetical protein
  
Accession: EGY22027
  
Location: 1524238-1525299
  
  
**BlastP hit with Mycgr3G104337\_Mycgr3**
  
Percentage identity: 38 %
  
BlastP bit score: 181
  
Sequence coverage: 101 %
  
E-value: 6e-52
  
  
 NCBI BlastP on this gene

EGY22027

3-hydroxyisobutyrate dehydrogenase
  
Accession: EGY22028
  
Location: 1525907-1529426
  
 NCBI BlastP on this gene

EGY22028

hypothetical protein
  
Accession: EGY22029
  
Location: 1530013-1532403
  
 NCBI BlastP on this gene

EGY22029

phthalate transporter
  
Accession: EGY22030
  
Location: 1533491-1535100
  
 NCBI BlastP on this gene

EGY22030

hypothetical protein
  
Accession: EGY22031
  
Location: 1535841-1536458
  
 NCBI BlastP on this gene

EGY22031

hypothetical protein
  
Accession: EGY22032
  
Location: 1537220-1538028
  
 NCBI BlastP on this gene

EGY22032

cutinase-2
  
Accession: EGY22033
  
Location: 1539140-1539869
  
 NCBI BlastP on this gene

EGY22033

vacuolar protein sorting-associated protein
  
Accession: EGY22034
  
Location: 1542234-1544693
  
 NCBI BlastP on this gene

EGY22034

85. :  CM001231 Magnaporthe oryzae 70-15 chromosome 1     Total score: 2.0     Cumulative Blast bit score: 1970

lovastatin nonaketide synthase
  
Accession: EHA57854
  
Location: 5748736-5755754
  
  
**BlastP hit with Mycgr3G100089\_Mycgr3**
  
Percentage identity: 42 %
  
BlastP bit score: 1755
  
Sequence coverage: 101 %
  
E-value: 0.0
  
  
 NCBI BlastP on this gene

EHA57854

zeaxanthin epoxidase
  
Accession: EHA57855
  
Location: 5757154-5758404
  
 NCBI BlastP on this gene

EHA57855

hypothetical protein
  
Accession: EHA57856
  
Location: 5759786-5760533
  
 NCBI BlastP on this gene

EHA57856

hypothetical protein
  
Accession: EHA57857
  
Location: 5761734-5763376
  
 NCBI BlastP on this gene

EHA57857

hypothetical protein
  
Accession: EHA57858
  
Location: 5763555-5764268
  
 NCBI BlastP on this gene

EHA57858

hypothetical protein
  
Accession: EHA57859
  
Location: 5764790-5765659
  
 NCBI BlastP on this gene

EHA57859

hypothetical protein
  
Accession: EHA57860
  
Location: 5766500-5767507
  
  
**BlastP hit with Mycgr3G104337\_Mycgr3**
  
Percentage identity: 43 %
  
BlastP bit score: 216
  
Sequence coverage: 95 %
  
E-value: 1e-65
  
  
 NCBI BlastP on this gene

EHA57860

hypothetical protein
  
Accession: EHA57861
  
Location: 5768694-5769176
  
 NCBI BlastP on this gene

EHA57861

hypothetical protein
  
Accession: EHA57862
  
Location: 5775475-5776069
  
 NCBI BlastP on this gene

EHA57862

hypothetical protein
  
Accession: EHA57863
  
Location: 5776651-5778599
  
 NCBI BlastP on this gene

EHA57863

86. :  AM270302 Aspergillus niger contig An13c0080, genomic contig.     Total score: 2.0     Cumulative Blast bit score: 1965

unnamed
  
Accession: CAK41634
  
Location: 3850-6559
  
 NCBI BlastP on this gene

An13g02350

not annotated
  
Accession: CAK41635
  
Location: 7192-9105
  
 NCBI BlastP on this gene

An13g02360

not annotated
  
Accession: CAK41636
  
Location: 9739-11410
  
 NCBI BlastP on this gene

An13g02370

unnamed
  
Accession: CAK41637
  
Location: 12504-13708
  
 NCBI BlastP on this gene

An13g02380

not annotated
  
Accession: CAK41638
  
Location: 14555-16597
  
 NCBI BlastP on this gene

An13g02390

not annotated
  
Accession: CAK41639
  
Location: 17814-19902
  
 NCBI BlastP on this gene

An13g02400

not annotated
  
Accession: CAK41640
  
Location: 20694-22152
  
 NCBI BlastP on this gene

An13g02410

not annotated
  
Accession: CAK41641
  
Location: 23726-24641
  
  
**BlastP hit with Mycgr3G104337\_Mycgr3**
  
Percentage identity: 44 %
  
BlastP bit score: 209
  
Sequence coverage: 98 %
  
E-value: 3e-63
  
  
 NCBI BlastP on this gene

An13g02420

not annotated
  
Accession: CAK41642
  
Location: 26479-33983
  
  
**BlastP hit with Mycgr3G100089\_Mycgr3**
  
Percentage identity: 44 %
  
BlastP bit score: 1756
  
Sequence coverage: 102 %
  
E-value: 0.0
  
  
 NCBI BlastP on this gene

An13g02430

unnamed
  
Accession: CAK41643
  
Location: 34703-37042
  
 NCBI BlastP on this gene

An13g02450

not annotated
  
Accession: CAK41644
  
Location: 38345-41642
  
 NCBI BlastP on this gene

An13g02460

hypothetical protein
  
Accession: CAK41645
  
Location: 42070-42457
  
 NCBI BlastP on this gene

An13g02470

unnamed
  
Accession: CAK41646
  
Location: 42640-44476
  
 NCBI BlastP on this gene

An13g02480

hypothetical protein
  
Accession: CAK41647
  
Location: 45634-48439
  
 NCBI BlastP on this gene

An13g02500

unnamed
  
Accession: CAK41648
  
Location: 48606-49694
  
 NCBI BlastP on this gene

An13g02510

hypothetical protein
  
Accession: CAK41649
  
Location: 49837-50534
  
 NCBI BlastP on this gene

An13g02520

unnamed
  
Accession: CAK41650
  
Location: 51075-51799
  
 NCBI BlastP on this gene

An13g02530

87. :  DF126480 Aspergillus kawachii IFO 4308 DNA, contig: scaffold00034     Total score: 2.0     Cumulative Blast bit score: 1958

hypothetical protein
  
Accession: GAA91454
  
Location: 327273-329399
  
 NCBI BlastP on this gene

GAA91454

MFS transporter
  
Accession: GAA91455
  
Location: 330224-331858
  
 NCBI BlastP on this gene

GAA91455

MATE efflux family protein subfamily
  
Accession: GAA91456
  
Location: 333116-334687
  
 NCBI BlastP on this gene

GAA91456

agmatinase
  
Accession: GAA91457
  
Location: 335618-337221
  
 NCBI BlastP on this gene

GAA91457

similar to An14g05950
  
Accession: GAA91458
  
Location: 338299-339522
  
 NCBI BlastP on this gene

GAA91458

similar to An10g00090
  
Accession: GAA91459
  
Location: 341582-342124
  
 NCBI BlastP on this gene

GAA91459

AAA family ATPase
  
Accession: GAA91460
  
Location: 343999-351140
  
  
**BlastP hit with Mycgr3G42010\_Mycgr3T**
  
Percentage identity: 47 %
  
BlastP bit score: 1768
  
Sequence coverage: 85 %
  
E-value: 0.0
  
  
 NCBI BlastP on this gene

GAA91460

geranylgeranyl pyrophosphate synthetase
  
Accession: GAA91461
  
Location: 351465-352917
  
  
**BlastP hit with Mycgr3G92938\_Mycgr3T**
  
Percentage identity: 31 %
  
BlastP bit score: 190
  
Sequence coverage: 95 %
  
E-value: 2e-51
  
  
 NCBI BlastP on this gene

GAA91461

hypothetical protein
  
Accession: GAA91462
  
Location: 354134-354984
  
 NCBI BlastP on this gene

GAA91462

similar to An14g07400
  
Accession: GAA91463
  
Location: 355973-357072
  
 NCBI BlastP on this gene

GAA91463

class V chitinase
  
Accession: GAA91464
  
Location: 357261-360935
  
 NCBI BlastP on this gene

GAA91464

hypothetical protein
  
Accession: GAA91465
  
Location: 363401-370204
  
 NCBI BlastP on this gene

GAA91465

88. :  JH725151 Beauveria bassiana ARSEF 2860 unplaced genomic scaffold BBA\_S00002     Total score: 2.0     Cumulative Blast bit score: 1952

small nucleolar ribonucleoprotein complex component (Utp5)
  
Accession: EJP70137
  
Location: 1194086-1195267
  
 NCBI BlastP on this gene

EJP70137

hypothetical protein
  
Accession: EJP70138
  
Location: 1196004-1197902
  
 NCBI BlastP on this gene

EJP70138

hypothetical protein
  
Accession: EJP70139
  
Location: 1202330-1206037
  
 NCBI BlastP on this gene

EJP70139

amino acid permease
  
Accession: EJP70140
  
Location: 1206587-1208305
  
 NCBI BlastP on this gene

EJP70140

lovastatin nonaketide synthase
  
Accession: EJP70141
  
Location: 1209213-1216452
  
  
**BlastP hit with Mycgr3G100089\_Mycgr3**
  
Percentage identity: 43 %
  
BlastP bit score: 1758
  
Sequence coverage: 104 %
  
E-value: 0.0
  
  
 NCBI BlastP on this gene

EJP70141

EF-hand calcium-binding domain protein
  
Accession: EJP70142
  
Location: 1221799-1222797
  
  
**BlastP hit with Mycgr3G104337\_Mycgr3**
  
Percentage identity: 41 %
  
BlastP bit score: 195
  
Sequence coverage: 100 %
  
E-value: 7e-58
  
  
 NCBI BlastP on this gene

EJP70142

UDP-glucoronosyl and UDP-glucosyl transferase family protein
  
Accession: EJP70143
  
Location: 1224748-1226436
  
 NCBI BlastP on this gene

EJP70143

protein (fungal and bacterial)
  
Accession: EJP70144
  
Location: 1226724-1228871
  
 NCBI BlastP on this gene

EJP70144

C6 transcription factor, putative
  
Accession: EJP70145
  
Location: 1232367-1233773
  
 NCBI BlastP on this gene

EJP70145

major facilitator superfamily transporter
  
Accession: EJP70146
  
Location: 1235553-1237305
  
 NCBI BlastP on this gene

EJP70146

Ribonuclease/ribotoxin
  
Accession: EJP70147
  
Location: 1237900-1238351
  
 NCBI BlastP on this gene

EJP70147

89. :  CH408034 Chaetomium globosum CBS 148.51 scaffold\_6 genomic scaffold     Total score: 2.0     Cumulative Blast bit score: 1948

hypothetical protein
  
Accession: EAQ84725
  
Location: 1222984-1224827
  
 NCBI BlastP on this gene

EAQ84725

hypothetical protein
  
Accession: EAQ84726
  
Location: 1225272-1226805
  
 NCBI BlastP on this gene

EAQ84726

hypothetical protein
  
Accession: EAQ84727
  
Location: 1228303-1230898
  
 NCBI BlastP on this gene

EAQ84727

conserved hypothetical protein
  
Accession: EAQ84728
  
Location: 1231000-1232153
  
 NCBI BlastP on this gene

EAQ84728

hypothetical protein
  
Accession: EAQ84729
  
Location: 1233922-1234341
  
 NCBI BlastP on this gene

EAQ84729

hypothetical protein
  
Accession: EAQ84730
  
Location: 1235132-1239454
  
 NCBI BlastP on this gene

EAQ84730

hypothetical protein
  
Accession: EAQ84731
  
Location: 1241032-1242593
  
  
**BlastP hit with Mycgr3G104337\_Mycgr3**
  
Percentage identity: 41 %
  
BlastP bit score: 161
  
Sequence coverage: 78 %
  
E-value: 2e-44
  
  
 NCBI BlastP on this gene

EAQ84731

hypothetical protein
  
Accession: EAQ84732
  
Location: 1245180-1252426
  
  
**BlastP hit with Mycgr3G100089\_Mycgr3**
  
Percentage identity: 43 %
  
BlastP bit score: 1787
  
Sequence coverage: 100 %
  
E-value: 0.0
  
  
 NCBI BlastP on this gene

EAQ84732

hypothetical protein
  
Accession: EAQ84733
  
Location: 1252965-1255119
  
 NCBI BlastP on this gene

EAQ84733

hypothetical protein
  
Accession: EAQ84734
  
Location: 1258048-1260516
  
 NCBI BlastP on this gene

EAQ84734

conserved hypothetical protein
  
Accession: EAQ84735
  
Location: 1261328-1262727
  
 NCBI BlastP on this gene

EAQ84735

hypothetical protein
  
Accession: EAQ84736
  
Location: 1263118-1264250
  
 NCBI BlastP on this gene

EAQ84736

hypothetical protein
  
Accession: EAQ84737
  
Location: 1265463-1267300
  
 NCBI BlastP on this gene

EAQ84737

hypothetical protein
  
Accession: EAQ84738
  
Location: 1269509-1270613
  
 NCBI BlastP on this gene

EAQ84738

90. :  AABX02000004 Neurospora crassa OR74A     Total score: 2.0     Cumulative Blast bit score: 1940

hypothetical protein
  
Accession: EAA28899
  
Location: 124736-132387
  
  
**BlastP hit with Mycgr3G100089\_Mycgr3**
  
Percentage identity: 43 %
  
BlastP bit score: 1726
  
Sequence coverage: 100 %
  
E-value: 0.0
  
  
 NCBI BlastP on this gene

EAA28899

conserved hypothetical protein
  
Accession: EAA29584
  
Location: 134461-135421
  
  
**BlastP hit with Mycgr3G104337\_Mycgr3**
  
Percentage identity: 41 %
  
BlastP bit score: 214
  
Sequence coverage: 100 %
  
E-value: 5e-65
  
  
 NCBI BlastP on this gene

EAA29584

hypothetical protein
  
Accession: EAA29583
  
Location: 137203-142380
  
 NCBI BlastP on this gene

EAA29583

hypothetical protein
  
Accession: EAA29582
  
Location: 143944-145882
  
 NCBI BlastP on this gene

EAA29582

hypothetical protein
  
Accession: EAA29581
  
Location: 147338-148498
  
 NCBI BlastP on this gene

EAA29581

hypothetical protein
  
Accession: EAA29580
  
Location: 149690-150970
  
 NCBI BlastP on this gene

EAA29580

predicted protein
  
Accession: EAA29579
  
Location: 151285-152181
  
 NCBI BlastP on this gene

EAA29579

predicted protein
  
Accession: EAA29578
  
Location: 152739-154373
  
 NCBI BlastP on this gene

EAA29578

91. :  JH921449 Marssonina brunnea f. sp. 'multigermtubi' MB\_m1 unplaced genomic scaffold M6\_S00022     Total score: 2.0     Cumulative Blast bit score: 1935

arrestin domain-containing protein
  
Accession: EKD13594
  
Location: 504727-507230
  
 NCBI BlastP on this gene

EKD13594

hypothetical protein
  
Accession: EKD13595
  
Location: 514017-514472
  
 NCBI BlastP on this gene

EKD13595

ABC transporter
  
Accession: EKD13596
  
Location: 515959-521009
  
 NCBI BlastP on this gene

EKD13596

putative EF-hand calcium-binding domain protein
  
Accession: EKD13597
  
Location: 522874-523854
  
  
**BlastP hit with Mycgr3G104337\_Mycgr3**
  
Percentage identity: 43 %
  
BlastP bit score: 223
  
Sequence coverage: 106 %
  
E-value: 2e-68
  
  
 NCBI BlastP on this gene

EKD13597

polyketide synthase
  
Accession: EKD13598
  
Location: 525884-533571
  
  
**BlastP hit with Mycgr3G100089\_Mycgr3**
  
Percentage identity: 41 %
  
BlastP bit score: 1712
  
Sequence coverage: 101 %
  
E-value: 0.0
  
  
 NCBI BlastP on this gene

EKD13598

hypothetical protein
  
Accession: EKD13599
  
Location: 535310-536182
  
 NCBI BlastP on this gene

EKD13599

hypothetical protein
  
Accession: EKD13600
  
Location: 537175-537411
  
 NCBI BlastP on this gene

EKD13600

hypothetical protein
  
Accession: EKD13601
  
Location: 538647-539769
  
 NCBI BlastP on this gene

EKD13601

hypothetical protein
  
Accession: EKD13602
  
Location: 541793-543132
  
 NCBI BlastP on this gene

EKD13602

DNA replication licensing factor mcm7
  
Accession: EKD13603
  
Location: 544605-547268
  
 NCBI BlastP on this gene

EKD13603

ATPase
  
Accession: EKD13604
  
Location: 547679-551456
  
 NCBI BlastP on this gene

EKD13604

92. :  DF126457 Aspergillus kawachii IFO 4308 DNA, contig: scaffold00011     Total score: 2.0     Cumulative Blast bit score: 1925

hypothetical protein
  
Accession: GAA86901
  
Location: 817651-819567
  
 NCBI BlastP on this gene

GAA86901

C-x8-C-x5-C-x3-H type zinc finger protein
  
Accession: GAA86902
  
Location: 820216-821887
  
 NCBI BlastP on this gene

GAA86902

hypothetical protein
  
Accession: GAA86903
  
Location: 822804-824005
  
 NCBI BlastP on this gene

GAA86903

MFS multidrug transporter
  
Accession: GAA86904
  
Location: 824802-826661
  
 NCBI BlastP on this gene

GAA86904

nucleoside transporter
  
Accession: GAA86905
  
Location: 827182-829048
  
 NCBI BlastP on this gene

GAA86905

C6 transcription factor
  
Accession: GAA86906
  
Location: 830332-832428
  
 NCBI BlastP on this gene

GAA86906

similar to An13g02410
  
Accession: GAA86907
  
Location: 833183-834654
  
 NCBI BlastP on this gene

GAA86907

DUF341 family oxidoreductase
  
Accession: GAA86908
  
Location: 835404-836360
  
  
**BlastP hit with Mycgr3G104337\_Mycgr3**
  
Percentage identity: 37 %
  
BlastP bit score: 183
  
Sequence coverage: 98 %
  
E-value: 3e-53
  
  
 NCBI BlastP on this gene

GAA86908

polyketide synthase
  
Accession: GAA86909
  
Location: 838044-845051
  
  
**BlastP hit with Mycgr3G100089\_Mycgr3**
  
Percentage identity: 43 %
  
BlastP bit score: 1742
  
Sequence coverage: 103 %
  
E-value: 0.0
  
  
 NCBI BlastP on this gene

GAA86909

six-hairpin glycosidase
  
Accession: GAA86910
  
Location: 846344-848383
  
 NCBI BlastP on this gene

GAA86910

NRPS-like enzyme
  
Accession: GAA86911
  
Location: 849961-853241
  
 NCBI BlastP on this gene

GAA86911

similar to An13g02470
  
Accession: GAA86912
  
Location: 853705-854061
  
 NCBI BlastP on this gene

GAA86912

flavin containing polyamine oxidase
  
Accession: GAA86913
  
Location: 854252-856088
  
 NCBI BlastP on this gene

GAA86913

cell wall glucanase/allergen F16-like protein
  
Accession: GAA86914
  
Location: 860166-861261
  
 NCBI BlastP on this gene

GAA86914

carbonic anhydrase
  
Accession: GAA86915
  
Location: 862601-863584
  
 NCBI BlastP on this gene

GAA86915

93. :  CP003008 Myceliophthora thermophila ATCC 42464 chromosome 7     Total score: 2.0     Cumulative Blast bit score: 1922

hypothetical protein
  
Accession: AEO62133
  
Location: 3896272-3897504
  
 NCBI BlastP on this gene

MYCTH\_2113991

hypothetical protein
  
Accession: AEO62134
  
Location: 3897968-3898660
  
 NCBI BlastP on this gene

MYCTH\_2313149

hypothetical protein
  
Accession: AEO62135
  
Location: 3899823-3902683
  
 NCBI BlastP on this gene

MYCTH\_2313151

hypothetical protein
  
Accession: AEO62136
  
Location: 3910728-3912516
  
  
**BlastP hit with Mycgr3G92938\_Mycgr3T**
  
Percentage identity: 50 %
  
BlastP bit score: 150
  
Sequence coverage: 34 %
  
E-value: 1e-36
  
  
 NCBI BlastP on this gene

MYCTH\_2313154

hypothetical protein
  
Accession: AEO62137
  
Location: 3913944-3921784
  
  
**BlastP hit with Mycgr3G42010\_Mycgr3T**
  
Percentage identity: 45 %
  
BlastP bit score: 1772
  
Sequence coverage: 96 %
  
E-value: 0.0
  
  
 NCBI BlastP on this gene

MYCTH\_2313156

glycoside hydrolase family 47 protein
  
Accession: AEO62138
  
Location: 3922910-3924892
  
 NCBI BlastP on this gene

MYCTH\_2313159

hypothetical protein
  
Accession: AEO62139
  
Location: 3926181-3927482
  
 NCBI BlastP on this gene

MYCTH\_2313161

hypothetical protein
  
Accession: AEO62140
  
Location: 3928368-3929523
  
 NCBI BlastP on this gene

MYCTH\_103818

hypothetical protein
  
Accession: AEO62141
  
Location: 3930955-3933897
  
 NCBI BlastP on this gene

MYCTH\_2313163

hypothetical protein
  
Accession: AEO62142
  
Location: 3934837-3937159
  
 NCBI BlastP on this gene

MYCTH\_2313164

94. :  CAGA01000048 Claviceps purpurea 20.1     Total score: 2.0     Cumulative Blast bit score: 1901

probable cytochrome P450 monooxygenase (lovA)
  
Accession: CCE32887
  
Location: 9826-10941
  
 NCBI BlastP on this gene

CCE32887

uncharacterized protein
  
Accession: CCE32888
  
Location: 16360-17719
  
 NCBI BlastP on this gene

CCE32888

related to gibberellin 20-oxidase
  
Accession: CCE32889
  
Location: 22454-23706
  
 NCBI BlastP on this gene

CCE32889

related to 7alpha-cephem-methoxylase P8 chain
  
Accession: CCE32890
  
Location: 23915-24736
  
 NCBI BlastP on this gene

CCE32890

related to amidohydrolase
  
Accession: CCE32891
  
Location: 26940-28389
  
 NCBI BlastP on this gene

CCE32891

related to polyketide synthase
  
Accession: CCE32892
  
Location: 28674-35696
  
  
**BlastP hit with Mycgr3G100089\_Mycgr3**
  
Percentage identity: 43 %
  
BlastP bit score: 1773
  
Sequence coverage: 103 %
  
E-value: 0.0
  
  
 NCBI BlastP on this gene

CCE32892

uncharacterized protein
  
Accession: CCE32893
  
Location: 36690-37424
  
  
**BlastP hit with Mycgr3G104337\_Mycgr3**
  
Percentage identity: 41 %
  
BlastP bit score: 129
  
Sequence coverage: 63 %
  
E-value: 3e-33
  
  
 NCBI BlastP on this gene

CCE32893

uncharacterized protein
  
Accession: CCE32894
  
Location: 40768-41580
  
 NCBI BlastP on this gene

CCE32894

uncharacterized protein
  
Accession: CCE32895
  
Location: 42999-43538
  
 NCBI BlastP on this gene

CCE32895

related to carboxyphosphonoenolpyruvate phosphonomutase
  
Accession: CCE32896
  
Location: 47467-48648
  
 NCBI BlastP on this gene

CCE32896

uncharacterized protein
  
Accession: CCE32897
  
Location: 50442-51394
  
 NCBI BlastP on this gene

CCE32897

related to protein histidine kinase
  
Accession: CCE32898
  
Location: 53836-57234
  
 NCBI BlastP on this gene

CCE32898

95. :  JH795568 Magnaporthe oryzae P131 unplaced genomic scaffold P131\_scaffold00435     Total score: 2.0     Cumulative Blast bit score: 1878

hypothetical protein
  
Accession: ELQ66019
  
Location: 22248-22340
  
 NCBI BlastP on this gene

ELQ66019

hypothetical protein
  
Accession: ELQ66020
  
Location: 22922-24468
  
 NCBI BlastP on this gene

ELQ66020

bilirubin oxidase
  
Accession: ELQ66021
  
Location: 27510-29453
  
 NCBI BlastP on this gene

ELQ66021

mannan endo-1,6-alpha-mannosidase DCW1
  
Accession: ELQ66022
  
Location: 30350-31379
  
 NCBI BlastP on this gene

ELQ66022

glycerate kinase
  
Accession: ELQ66023
  
Location: 32576-34039
  
 NCBI BlastP on this gene

ELQ66023

multidrug resistance protein 3
  
Accession: ELQ66024
  
Location: 34325-38964
  
 NCBI BlastP on this gene

ELQ66024

hypothetical protein
  
Accession: ELQ66025
  
Location: 40119-40956
  
  
**BlastP hit with Mycgr3G104337\_Mycgr3**
  
Percentage identity: 41 %
  
BlastP bit score: 206
  
Sequence coverage: 100 %
  
E-value: 4e-62
  
  
 NCBI BlastP on this gene

ELQ66025

fatty acid synthase S-acetyltransferase
  
Accession: ELQ66026
  
Location: 43006-49984
  
  
**BlastP hit with Mycgr3G100089\_Mycgr3**
  
Percentage identity: 42 %
  
BlastP bit score: 1672
  
Sequence coverage: 102 %
  
E-value: 0.0
  
  
 NCBI BlastP on this gene

ELQ66026

hypothetical protein
  
Accession: ELQ66027
  
Location: 51305-51823
  
 NCBI BlastP on this gene

ELQ66027

antibiotic biosynthesis monooxygenase
  
Accession: ELQ66028
  
Location: 52158-52805
  
 NCBI BlastP on this gene

ELQ66028

hypothetical protein
  
Accession: ELQ66029
  
Location: 53047-54477
  
 NCBI BlastP on this gene

ELQ66029

sugar transporter STL1
  
Accession: ELQ66030
  
Location: 54625-57560
  
 NCBI BlastP on this gene

ELQ66030

hypothetical protein
  
Accession: ELQ66031
  
Location: 58646-58939
  
 NCBI BlastP on this gene

ELQ66031

ankyrin repeat and protein kinase domain-containing protein 1
  
Accession: ELQ66032
  
Location: 59101-66533
  
 NCBI BlastP on this gene

ELQ66032

96. :  JH793928 Magnaporthe oryzae Y34 unplaced genomic scaffold Y34\_scaffold00214     Total score: 2.0     Cumulative Blast bit score: 1878

hypothetical protein
  
Accession: ELQ42347
  
Location: 22050-22142
  
 NCBI BlastP on this gene

ELQ42347

hypothetical protein
  
Accession: ELQ42348
  
Location: 22724-24270
  
 NCBI BlastP on this gene

ELQ42348

bilirubin oxidase
  
Accession: ELQ42349
  
Location: 27312-29255
  
 NCBI BlastP on this gene

ELQ42349

mannan endo-1,6-alpha-mannosidase DCW1
  
Accession: ELQ42350
  
Location: 30152-31181
  
 NCBI BlastP on this gene

ELQ42350

glycerate kinase
  
Accession: ELQ42351
  
Location: 32378-33841
  
 NCBI BlastP on this gene

ELQ42351

multidrug resistance protein 3
  
Accession: ELQ42352
  
Location: 34127-38766
  
 NCBI BlastP on this gene

ELQ42352

hypothetical protein
  
Accession: ELQ42353
  
Location: 39921-40758
  
  
**BlastP hit with Mycgr3G104337\_Mycgr3**
  
Percentage identity: 41 %
  
BlastP bit score: 206
  
Sequence coverage: 100 %
  
E-value: 4e-62
  
  
 NCBI BlastP on this gene

ELQ42353

fatty acid synthase S-acetyltransferase
  
Accession: ELQ42354
  
Location: 42807-49785
  
  
**BlastP hit with Mycgr3G100089\_Mycgr3**
  
Percentage identity: 42 %
  
BlastP bit score: 1672
  
Sequence coverage: 102 %
  
E-value: 0.0
  
  
 NCBI BlastP on this gene

ELQ42354

hypothetical protein
  
Accession: ELQ42355
  
Location: 51122-51640
  
 NCBI BlastP on this gene

ELQ42355

antibiotic biosynthesis monooxygenase
  
Accession: ELQ42356
  
Location: 51975-52622
  
 NCBI BlastP on this gene

ELQ42356

hypothetical protein
  
Accession: ELQ42357
  
Location: 52864-54294
  
 NCBI BlastP on this gene

ELQ42357

sugar transporter STL1
  
Accession: ELQ42358
  
Location: 54442-57377
  
 NCBI BlastP on this gene

ELQ42358

hypothetical protein
  
Accession: ELQ42359
  
Location: 58463-58756
  
 NCBI BlastP on this gene

ELQ42359

ankyrin repeat and protein kinase domain-containing protein 1
  
Accession: ELQ42360
  
Location: 58918-67067
  
 NCBI BlastP on this gene

ELQ42360

hypothetical protein
  
Accession: ELQ42361
  
Location: 67367-68843
  
 NCBI BlastP on this gene

ELQ42361

97. :  FP929139 Leptosphaeria maculans JN3 lm\_SuperContig\_0\_v2 genomic supercontig     Total score: 2.0     Cumulative Blast bit score: 1827

hypothetical protein
  
Accession: CBY01874
  
Location: 2503452-2513155
  
  
**BlastP hit with Mycgr3G100089\_Mycgr3**
  
Percentage identity: 40 %
  
BlastP bit score: 1618
  
Sequence coverage: 101 %
  
E-value: 0.0
  
  
 NCBI BlastP on this gene

LEMA\_P006610.1

similar to EF-hand calcium-binding domain protein
  
Accession: CBY01875
  
Location: 2514273-2515241
  
  
**BlastP hit with Mycgr3G104337\_Mycgr3**
  
Percentage identity: 43 %
  
BlastP bit score: 209
  
Sequence coverage: 100 %
  
E-value: 2e-63
  
  
 NCBI BlastP on this gene

LEMA\_P006620.1

similar to ABC multidrug transporter
  
Accession: CBY01876
  
Location: 2515904-2520524
  
 NCBI BlastP on this gene

LEMA\_P006630.1

similar to beta-lactamase family protein
  
Accession: CBY01877
  
Location: 2521380-2523855
  
 NCBI BlastP on this gene

LEMA\_P006640.1

similar to ATP dependent RNA helicase
  
Accession: CBY01878
  
Location: 2525250-2530309
  
 NCBI BlastP on this gene

LEMA\_P006650.1

similar to translational activator
  
Accession: CBY01879
  
Location: 2530701-2539025
  
 NCBI BlastP on this gene

LEMA\_P006660.1

98. :  DS572714 Verticillium dahliae VdLs.17 supercont1.20 genomic scaffold     Total score: 2.0     Cumulative Blast bit score: 1769

ubiquitin C-terminal hydrolase
  
Accession: EGY18108
  
Location: 457337-459982
  
 NCBI BlastP on this gene

EGY18108

54S ribosomal protein L7
  
Accession: EGY18109
  
Location: 461868-463047
  
 NCBI BlastP on this gene

EGY18109

Hsp70 nucleotide exchange factor FES1
  
Accession: EGY18110
  
Location: 463348-463977
  
 NCBI BlastP on this gene

EGY18110

actin
  
Accession: EGY18111
  
Location: 466404-467516
  
 NCBI BlastP on this gene

EGY18111

multidrug resistance protein
  
Accession: EGY18112
  
Location: 469569-474381
  
 NCBI BlastP on this gene

EGY18112

hypothetical protein
  
Accession: EGY18113
  
Location: 475077-476013
  
  
**BlastP hit with Mycgr3G104337\_Mycgr3**
  
Percentage identity: 40 %
  
BlastP bit score: 205
  
Sequence coverage: 101 %
  
E-value: 2e-61
  
  
 NCBI BlastP on this gene

EGY18113

lovastatin nonaketide synthase
  
Accession: EGY18114
  
Location: 477875-484921
  
  
**BlastP hit with Mycgr3G100089\_Mycgr3**
  
Percentage identity: 39 %
  
BlastP bit score: 1564
  
Sequence coverage: 101 %
  
E-value: 0.0
  
  
 NCBI BlastP on this gene

EGY18114

hypothetical protein
  
Accession: EGY18115
  
Location: 485368-485787
  
 NCBI BlastP on this gene

EGY18115

elongator complex protein
  
Accession: EGY18116
  
Location: 486187-488797
  
 NCBI BlastP on this gene

EGY18116

hypothetical protein
  
Accession: EGY18117
  
Location: 489422-489940
  
 NCBI BlastP on this gene

EGY18117

zinc finger protein
  
Accession: EGY18118
  
Location: 495011-496489
  
 NCBI BlastP on this gene

EGY18118

99. :  FQ790354 Botryotinia fuckeliana T4 SupSuperContig\_210\_20\_1 genomic supercontig.     Total score: 2.0     Cumulative Blast bit score: 1764

hypothetical protein
  
Accession: CCD55452
  
Location: 668813-669763
  
 NCBI BlastP on this gene

BofuT4\_P158480.1

glycoside hydrolase family 78 protein, partial sequence
  
Accession: CCD55451
  
Location: 666518-668221
  
 NCBI BlastP on this gene

BofuT4\_P158470.1

glycoside hydrolase family 78 protein, partial sequence
  
Accession: CCD55450
  
Location: 665174-666117
  
 NCBI BlastP on this gene

BofuT4\_P158460.1

similar to MFS sugar transporter
  
Accession: CCD55449
  
Location: 662421-664203
  
 NCBI BlastP on this gene

BofuT4P210000010001

hypothetical protein
  
Accession: CCD55448
  
Location: 660662-661621
  
 NCBI BlastP on this gene

BofuT4\_P158440.1

similar to cytochrome P450 monooxygenase
  
Accession: CCD55447
  
Location: 658528-660306
  
 NCBI BlastP on this gene

BofuT4P210000009001

hypothetical protein
  
Accession: CCD55446
  
Location: 655744-657642
  
 NCBI BlastP on this gene

BofuT4\_P158420.1

similar to MFS sugar transporter
  
Accession: CCD55445
  
Location: 653276-655074
  
 NCBI BlastP on this gene

BofuT4P210000008001

hypothetical protein
  
Accession: CCD55444
  
Location: 651483-652850
  
  
**BlastP hit with Mycgr3G92938\_Mycgr3T**
  
Percentage identity: 39 %
  
BlastP bit score: 209
  
Sequence coverage: 69 %
  
E-value: 3e-59
  
  
 NCBI BlastP on this gene

BofuT4\_P158400.1

similar to helicase required for RNAi-mediated heterochromatin assembly 1
  
Accession: CCD55443
  
Location: 647844-650811
  
  
**BlastP hit with Mycgr3G42010\_Mycgr3T**
  
Percentage identity: 41 %
  
BlastP bit score: 654
  
Sequence coverage: 39 %
  
E-value: 0.0
  
  
 NCBI BlastP on this gene

BofuT4\_P158390.1

hypothetical protein
  
Accession: CCD55442
  
Location: 642630-646724
  
  
**BlastP hit with Mycgr3G42010\_Mycgr3T**
  
Percentage identity: 46 %
  
BlastP bit score: 901
  
Sequence coverage: 47 %
  
E-value: 0.0
  
  
 NCBI BlastP on this gene

BofuT4\_P158380.1

similar to transcription factor Zn, C2H2
  
Accession: CCD55441
  
Location: 637940-641624
  
 NCBI BlastP on this gene

BofuT4\_P158370.1

similar to nucleoside transporter family
  
Accession: CCD55440
  
Location: 634672-636140
  
 NCBI BlastP on this gene

BofuT4\_P158360.1

hypothetical protein
  
Accession: CCD55439
  
Location: 633333-633777
  
 NCBI BlastP on this gene

BofuT4\_P158350.1

similar to phosphatase, partial sequence
  
Accession: CCD55438
  
Location: 630859-632305
  
 NCBI BlastP on this gene

BofuT4\_P158340.1

similar to phosphatase, partial sequence
  
Accession: CCD55437
  
Location: 630023-630439
  
 NCBI BlastP on this gene

BofuT4\_P158330.1

hypothetical protein
  
Accession: CCD55436
  
Location: 627237-627710
  
 NCBI BlastP on this gene

BofuT4\_P158320.1

BC4, calmodulin
  
Accession: CCD55435
  
Location: 625064-626093
  
 NCBI BlastP on this gene

BofuT4P210000007001

100. :  CH476622 Sclerotinia sclerotiorum 1980 scaffold\_2 genomic scaffold     Total score: 2.0     Cumulative Blast bit score: 1755

hypothetical protein
  
Accession: EDN97064
  
Location: 2396682-2398373
  
 NCBI BlastP on this gene

EDN97064

predicted protein
  
Accession: EDN97065
  
Location: 2405854-2406385
  
 NCBI BlastP on this gene

EDN97065

predicted protein
  
Accession: EDN97066
  
Location: 2406542-2407604
  
 NCBI BlastP on this gene

EDN97066

hypothetical protein
  
Accession: EDN97067
  
Location: 2408856-2410498
  
 NCBI BlastP on this gene

EDN97067

hypothetical protein
  
Accession: EDN97068
  
Location: 2412280-2414701
  
 NCBI BlastP on this gene

EDN97068

hypothetical protein
  
Accession: EDN97069
  
Location: 2415739-2423304
  
  
**BlastP hit with Mycgr3G100089\_Mycgr3**
  
Percentage identity: 39 %
  
BlastP bit score: 1537
  
Sequence coverage: 101 %
  
E-value: 0.0
  
  
 NCBI BlastP on this gene

EDN97069

predicted protein
  
Accession: EDN97070
  
Location: 2423748-2424243
  
 NCBI BlastP on this gene

EDN97070

hypothetical protein
  
Accession: EDN97071
  
Location: 2424790-2425751
  
  
**BlastP hit with Mycgr3G104337\_Mycgr3**
  
Percentage identity: 46 %
  
BlastP bit score: 218
  
Sequence coverage: 95 %
  
E-value: 2e-66
  
  
 NCBI BlastP on this gene

EDN97071

hypothetical protein
  
Accession: EDN97072
  
Location: 2426747-2431478
  
 NCBI BlastP on this gene

EDN97072

predicted protein
  
Accession: EDN97073
  
Location: 2433188-2434118
  
 NCBI BlastP on this gene

EDN97073

hypothetical protein
  
Accession: EDN97074
  
Location: 2435499-2436873
  
 NCBI BlastP on this gene

EDN97074

hypothetical protein
  
Accession: EDN97075
  
Location: 2437311-2438460
  
 NCBI BlastP on this gene

EDN97075

hypothetical protein
  
Accession: EDN97076
  
Location: 2439863-2440732
  
 NCBI BlastP on this gene

EDN97076

hypothetical protein
  
Accession: EDN97077
  
Location: 2441243-2443659
  
 NCBI BlastP on this gene

EDN97077

Detecting sequence homology at the gene cluster level with MultiGeneBlast.
  
Marnix H. Medema, Rainer Breitling & Eriko Takano (2013)
  
*Molecular Biology and Evolution* , 30: 1218-1223.
