# Supplementary material for: In silico prediction and characterization of secondary metabolite biosynthetic gene clusters in the wheat pathogen Zymoseptoria tritici
Source: BMC Genomics. 2017 Aug 17;18:631. doi: 10.1186/s12864-017-3969-y (PMC5561558; doi:10.1186/s12864-017-3969-y)
Supplement: Supplementary file 1 — MultiGeneBLAST analysis of putative secondary metabolite clusters. All encoded amino acid sequences from genes residing in clusters predicted by AntiSMASH are given as FASTA file format. All output data from MultiGeneBLASTs are also provided. (ZIP 42911 kb) [file 12864_2017_3969_MOESM1_ESM.zip › Cluster MultiGene BLAST/out/Clusters_1_34/Cluster_15/displaypage3.xhtml]

xml version="1.0" encoding="UTF-8"?


Search Results
  
  
 Results pages: 1, 2, 3, 4, 5

**MultiGeneBlast hits**

Select gene cluster alignment
101. GL385398\_1 Gaeumannomyces graminis var. tritici R3-111a-1 unplaced genom...
102. DS231618\_1 Pyrenophora tritici-repentis Pt-1C-BFP supercont1.4 genomic s...
103. DS985214\_0 Verticillium albo-atrum VaMs.102 supercont1.1 genomic scaffol...
104. JH767580\_0 Coniosporium apollinis CBS 100218 chromosome Unknown supercon...
105. GL385401\_1 Gaeumannomyces graminis var. tritici R3-111a-1 unplaced genom...
106. CU633870\_1 Podospora anserina S mat+ genomic DNA chromosome 5, supercont...
107. AGUE01000230\_0 Glarea lozoyensis 74030, whole genome shotgun sequencing ...
108. KB445637\_1 Cochliobolus sativus ND90Pr unplaced genomic scaffold COCSAsc...
109. KB733444\_3 Bipolaris maydis ATCC 48331 unplaced genomic scaffold COCC4sc...
110. KB445570\_3 Cochliobolus heterostrophus C5 unplaced genomic scaffold COCH...
111. KB908482\_1 Setosphaeria turcica Et28A unplaced genomic scaffold SETTUsca...
112. DS985227\_0 Verticillium albo-atrum VaMs.102 supercont1.14 genomic scaffo...
113. CP003006\_3 Myceliophthora thermophila ATCC 42464 chromosome 5, complete ...
114. KB707649\_0 Eutypa lata UCREL1 unplaced genomic scaffold EL1\_03\_scaffold\_...
115. GL537139\_0 Pyrenophora teres f. teres 0-1 unplaced genomic scaffold scaf...
116. GL533442\_0 Pyrenophora teres f. teres 0-1 unplaced genomic scaffold scaf...
117. CP003011\_1 Thielavia terrestris NRRL 8126 chromosome 3, complete sequence.
118. CH408029\_0 Chaetomium globosum CBS 148.51 scaffold\_1 genomic scaffold, w...
119. DS231619\_0 Pyrenophora tritici-repentis Pt-1C-BFP supercont1.5 genomic s...
120. DS027698\_0 Neosartorya fischeri NRRL 181 1099437636266 genomic scaffold,...
121. DS499602\_1 Aspergillus fumigatus A1163 scf\_000009 genomic scaffold, whol...
122. AAHF01000012\_1 Aspergillus fumigatus Af293, whole genome shotgun sequenc...
123. AGUE01000275\_0 Glarea lozoyensis 74030, whole genome shotgun sequencing ...
124. GG698510\_0 Trichophyton tonsurans CBS 112818 genomic scaffold supercont1...
125. DS995899\_1 Penicillium marneffei ATCC 18224 scf\_1105668340764 genomic sc...
126. EQ999974\_0 Ajellomyces dermatitidis ER-3 genomic scaffold supercont1.2, ...
127. DS989822\_3 Arthroderma gypseum CBS 118893 supercont1.1 genomic scaffold,...
128. AACD01000159\_0 Aspergillus nidulans FGSC A4, whole genome shotgun sequen...
129. AM270285\_0 Aspergillus niger contig An12c0290, genomic contig.
130. ACJE01000006\_2 Aspergillus niger ATCC 1015, whole genome shotgun sequenc...
131. DF126460\_3 Aspergillus kawachii IFO 4308 DNA, contig: scaffold00014, who...
132. CH476603\_3 Aspergillus terreus NIH2624 scaffold\_10 genomic scaffold, who...
133. GG700648\_1 Trichophyton rubrum CBS 118892 genomic scaffold supercont2.1,...
134. CH476657\_0 Ajellomyces capsulatus NAm1 scaffold\_3 genomic scaffold, whol...
135. ABSU01000024\_0 Arthroderma benhamiae CBS 112371, whole genome shotgun se...
136. ACYE01000336\_0 Trichophyton verrucosum HKI 0517, whole genome shotgun se...
137. CH445343\_1 Phaeosphaeria nodorum SN15 scaffold\_19, whole genome shotgun ...
138. AKHY01000183\_0 Aspergillus oryzae 3.042, whole genome shotgun sequencing...
139. KB644411\_0 Penicillium oxalicum 114-2 unplaced genomic scaffold scaffold...
140. JH226133\_1 Exophiala dermatitidis NIH/UT8656 unplaced genomic scaffold s...
141. GL636491\_0 Coccidioides posadasii str. Silveira unplaced genomic scaffol...
142. JH921445\_1 Marssonina brunnea f. sp. 'multigermtubi' MB\_m1 unplaced geno...
143. DS544803\_0 Paracoccidioides brasiliensis Pb03 supercont1.1 genomic scaff...
144. DS572752\_0 Paracoccidioides brasiliensis Pb18 supercont1.3 genomic scaff...
145. ABDG02000027\_2 Trichoderma atroviride IMI 206040, whole genome shotgun s...
146. JH126400\_1 Cordyceps militaris CM01 unplaced genomic scaffold CCM\_S00002...
147. KB446555\_2 Pseudocercospora fijiensis CIRAD86 unplaced genomic scaffold ...
148. AM920428\_1 Penicillium chrysogenum Wisconsin 54-1255 complete genome, co...
149. KB446535\_0 Dothistroma septosporum NZE10 unplaced genomic scaffold DOTSE...
150. AKCU01000203\_0 Penicillium digitatum Pd1, whole genome shotgun sequencin...

Query: Architecture Search FASTA input

GL385398 : Gaeumannomyces graminis var. tritici R3-111a-1 unplaced genomic scaffold supercont2.4    Total score: 2.0     Cumulative Blast bit score: 1750

Hit cluster cross-links:

Mycgr3G85918 Mycgr3T
  
Location: 0-1602

Mycgr3G85918\_Mycgr3T

Mycgr3G42010 Mycgr3T
  
Location: 1702-8569

Mycgr3G42010\_Mycgr3T

Mycgr3G29582 Mycgr3T
  
Location: 8669-8915

Mycgr3G29582\_Mycgr3T

Mycgr3G31170 Mycgr3T
  
Location: 9015-9255

Mycgr3G31170\_Mycgr3T

Mycgr3G85924 Mycgr3T
  
Location: 9355-11218

Mycgr3G85924\_Mycgr3T

Mycgr3G71676 Mycgr3T
  
Location: 11318-12494

Mycgr3G71676\_Mycgr3T

Mycgr3G11468 Mycgr3T
  
Location: 12594-13653

Mycgr3G11468\_Mycgr3T

Mycgr3G58567 Mycgr3T
  
Location: 13753-14506

Mycgr3G58567\_Mycgr3T

Mycgr3G100089 Mycgr3
  
Location: 14606-21152

Mycgr3G100089\_Mycgr3

Mycgr3G42698 Mycgr3T
  
Location: 21252-22131

Mycgr3G42698\_Mycgr3T

Mycgr3G71681 Mycgr3T
  
Location: 22231-23461

Mycgr3G71681\_Mycgr3T

Mycgr3G109328 Mycgr3
  
Location: 23561-24239

Mycgr3G109328\_Mycgr3

Mycgr3G104334 Mycgr3
  
Location: 24339-24567

Mycgr3G104334\_Mycgr3

Mycgr3G42715 Mycgr3T
  
Location: 24667-25981

Mycgr3G42715\_Mycgr3T

Mycgr3G92934 Mycgr3T
  
Location: 26081-27593

Mycgr3G92934\_Mycgr3T

Mycgr3G41969 Mycgr3T
  
Location: 27693-29328

Mycgr3G41969\_Mycgr3T

Mycgr3G80635 Mycgr3T
  
Location: 29428-29821

Mycgr3G80635\_Mycgr3T

Mycgr3G41426 Mycgr3T
  
Location: 29921-35255

Mycgr3G41426\_Mycgr3T

Mycgr3G104337 Mycgr3
  
Location: 35355-36108

Mycgr3G104337\_Mycgr3

Mycgr3G71679 Mycgr3T
  
Location: 36208-37300

Mycgr3G71679\_Mycgr3T

Mycgr3G92938 Mycgr3T
  
Location: 37400-38699

Mycgr3G92938\_Mycgr3T

Mycgr3G92941 Mycgr3T
  
Location: 38799-40734

Mycgr3G92941\_Mycgr3T

hypothetical protein
  
Accession: EJT74836
  
Location: 3789867-3790455
  
 NCBI BlastP on this gene

EJT74836

hypothetical protein
  
Accession: EJT74837
  
Location: 3792122-3792301
  
 NCBI BlastP on this gene

EJT74837

hypothetical protein
  
Accession: EJT74838
  
Location: 3792683-3794116
  
 NCBI BlastP on this gene

EJT74838

hypothetical protein
  
Accession: EJT74839
  
Location: 3794658-3795331
  
 NCBI BlastP on this gene

EJT74839

hypothetical protein
  
Accession: EJT74840
  
Location: 3796051-3801488
  
 NCBI BlastP on this gene

EJT74840

hypothetical protein
  
Accession: EJT74841
  
Location: 3802551-3804030
  
 NCBI BlastP on this gene

EJT74841

hypothetical protein
  
Accession: EJT74842
  
Location: 3805447-3807160
  
  
**BlastP hit with Mycgr3G42698\_Mycgr3T**
  
Percentage identity: 30 %
  
BlastP bit score: 152
  
Sequence coverage: 116 %
  
E-value: 4e-39
  
  
 NCBI BlastP on this gene

EJT74842

hypothetical protein
  
Accession: EJT74843
  
Location: 3808724-3811516
  
 NCBI BlastP on this gene

EJT74843

hypothetical protein
  
Accession: EJT74844
  
Location: 3811990-3812214
  
 NCBI BlastP on this gene

EJT74844

hypothetical protein
  
Accession: EJT74845
  
Location: 3813386-3820649
  
  
**BlastP hit with Mycgr3G100089\_Mycgr3**
  
Percentage identity: 43 %
  
BlastP bit score: 1598
  
Sequence coverage: 92 %
  
E-value: 0.0
  
  
 NCBI BlastP on this gene

EJT74845

hypothetical protein
  
Accession: EJT74846
  
Location: 3821818-3823059
  
 NCBI BlastP on this gene

EJT74846

hypothetical protein
  
Accession: EJT74847
  
Location: 3824537-3826272
  
 NCBI BlastP on this gene

EJT74847

hypothetical protein
  
Accession: EJT74848
  
Location: 3827929-3828573
  
 NCBI BlastP on this gene

EJT74848

hypothetical protein
  
Accession: EJT74849
  
Location: 3829635-3830404
  
 NCBI BlastP on this gene

EJT74849

hypothetical protein
  
Accession: EJT74850
  
Location: 3830755-3831813
  
 NCBI BlastP on this gene

EJT74850

hypothetical protein
  
Accession: EJT74851
  
Location: 3832406-3833396
  
 NCBI BlastP on this gene

EJT74851

hypothetical protein
  
Accession: EJT74852
  
Location: 3833471-3838560
  
 NCBI BlastP on this gene

EJT74852

Query: Architecture Search FASTA input

DS231618 : Pyrenophora tritici-repentis Pt-1C-BFP supercont1.4 genomic scaffold    Total score: 2.0     Cumulative Blast bit score: 1726

Hit cluster cross-links:

Mycgr3G85918 Mycgr3T
  
Location: 0-1602

Mycgr3G85918\_Mycgr3T

Mycgr3G42010 Mycgr3T
  
Location: 1702-8569

Mycgr3G42010\_Mycgr3T

Mycgr3G29582 Mycgr3T
  
Location: 8669-8915

Mycgr3G29582\_Mycgr3T

Mycgr3G31170 Mycgr3T
  
Location: 9015-9255

Mycgr3G31170\_Mycgr3T

Mycgr3G85924 Mycgr3T
  
Location: 9355-11218

Mycgr3G85924\_Mycgr3T

Mycgr3G71676 Mycgr3T
  
Location: 11318-12494

Mycgr3G71676\_Mycgr3T

Mycgr3G11468 Mycgr3T
  
Location: 12594-13653

Mycgr3G11468\_Mycgr3T

Mycgr3G58567 Mycgr3T
  
Location: 13753-14506

Mycgr3G58567\_Mycgr3T

Mycgr3G100089 Mycgr3
  
Location: 14606-21152

Mycgr3G100089\_Mycgr3

Mycgr3G42698 Mycgr3T
  
Location: 21252-22131

Mycgr3G42698\_Mycgr3T

Mycgr3G71681 Mycgr3T
  
Location: 22231-23461

Mycgr3G71681\_Mycgr3T

Mycgr3G109328 Mycgr3
  
Location: 23561-24239

Mycgr3G109328\_Mycgr3

Mycgr3G104334 Mycgr3
  
Location: 24339-24567

Mycgr3G104334\_Mycgr3

Mycgr3G42715 Mycgr3T
  
Location: 24667-25981

Mycgr3G42715\_Mycgr3T

Mycgr3G92934 Mycgr3T
  
Location: 26081-27593

Mycgr3G92934\_Mycgr3T

Mycgr3G41969 Mycgr3T
  
Location: 27693-29328

Mycgr3G41969\_Mycgr3T

Mycgr3G80635 Mycgr3T
  
Location: 29428-29821

Mycgr3G80635\_Mycgr3T

Mycgr3G41426 Mycgr3T
  
Location: 29921-35255

Mycgr3G41426\_Mycgr3T

Mycgr3G104337 Mycgr3
  
Location: 35355-36108

Mycgr3G104337\_Mycgr3

Mycgr3G71679 Mycgr3T
  
Location: 36208-37300

Mycgr3G71679\_Mycgr3T

Mycgr3G92938 Mycgr3T
  
Location: 37400-38699

Mycgr3G92938\_Mycgr3T

Mycgr3G92941 Mycgr3T
  
Location: 38799-40734

Mycgr3G92941\_Mycgr3T

predicted protein
  
Accession: EDU47629
  
Location: 858974-859438
  
 NCBI BlastP on this gene

EDU47629

predicted protein
  
Accession: EDU47630
  
Location: 873868-874580
  
 NCBI BlastP on this gene

EDU47630

stage V sporulation protein K
  
Accession: EDU47631
  
Location: 874875-880745
  
  
**BlastP hit with Mycgr3G42010\_Mycgr3T**
  
Percentage identity: 48 %
  
BlastP bit score: 1436
  
Sequence coverage: 68 %
  
E-value: 0.0
  
  
 NCBI BlastP on this gene

EDU47631

conserved hypothetical protein
  
Accession: EDU47632
  
Location: 882420-883913
  
  
**BlastP hit with Mycgr3G92938\_Mycgr3T**
  
Percentage identity: 40 %
  
BlastP bit score: 290
  
Sequence coverage: 101 %
  
E-value: 4e-89
  
  
 NCBI BlastP on this gene

EDU47632

conserved hypothetical protein
  
Accession: EDU47633
  
Location: 885945-887042
  
 NCBI BlastP on this gene

EDU47633

HPP family protein
  
Accession: EDU47634
  
Location: 887801-888661
  
 NCBI BlastP on this gene

EDU47634

galactose oxidase precursor
  
Accession: EDU47635
  
Location: 888939-890474
  
 NCBI BlastP on this gene

EDU47635

F-box domain containing protein
  
Accession: EDU47636
  
Location: 895971-897931
  
 NCBI BlastP on this gene

EDU47636

conserved hypothetical protein
  
Accession: EDU47637
  
Location: 898388-899847
  
 NCBI BlastP on this gene

EDU47637

proteasome-activating nucleotidase
  
Accession: EDU47638
  
Location: 901742-903448
  
 NCBI BlastP on this gene

EDU47638

Query: Architecture Search FASTA input

DS985214 : Verticillium albo-atrum VaMs.102 supercont1.1 genomic scaffold    Total score: 2.0     Cumulative Blast bit score: 1710

Hit cluster cross-links:

Mycgr3G85918 Mycgr3T
  
Location: 0-1602

Mycgr3G85918\_Mycgr3T

Mycgr3G42010 Mycgr3T
  
Location: 1702-8569

Mycgr3G42010\_Mycgr3T

Mycgr3G29582 Mycgr3T
  
Location: 8669-8915

Mycgr3G29582\_Mycgr3T

Mycgr3G31170 Mycgr3T
  
Location: 9015-9255

Mycgr3G31170\_Mycgr3T

Mycgr3G85924 Mycgr3T
  
Location: 9355-11218

Mycgr3G85924\_Mycgr3T

Mycgr3G71676 Mycgr3T
  
Location: 11318-12494

Mycgr3G71676\_Mycgr3T

Mycgr3G11468 Mycgr3T
  
Location: 12594-13653

Mycgr3G11468\_Mycgr3T

Mycgr3G58567 Mycgr3T
  
Location: 13753-14506

Mycgr3G58567\_Mycgr3T

Mycgr3G100089 Mycgr3
  
Location: 14606-21152

Mycgr3G100089\_Mycgr3

Mycgr3G42698 Mycgr3T
  
Location: 21252-22131

Mycgr3G42698\_Mycgr3T

Mycgr3G71681 Mycgr3T
  
Location: 22231-23461

Mycgr3G71681\_Mycgr3T

Mycgr3G109328 Mycgr3
  
Location: 23561-24239

Mycgr3G109328\_Mycgr3

Mycgr3G104334 Mycgr3
  
Location: 24339-24567

Mycgr3G104334\_Mycgr3

Mycgr3G42715 Mycgr3T
  
Location: 24667-25981

Mycgr3G42715\_Mycgr3T

Mycgr3G92934 Mycgr3T
  
Location: 26081-27593

Mycgr3G92934\_Mycgr3T

Mycgr3G41969 Mycgr3T
  
Location: 27693-29328

Mycgr3G41969\_Mycgr3T

Mycgr3G80635 Mycgr3T
  
Location: 29428-29821

Mycgr3G80635\_Mycgr3T

Mycgr3G41426 Mycgr3T
  
Location: 29921-35255

Mycgr3G41426\_Mycgr3T

Mycgr3G104337 Mycgr3
  
Location: 35355-36108

Mycgr3G104337\_Mycgr3

Mycgr3G71679 Mycgr3T
  
Location: 36208-37300

Mycgr3G71679\_Mycgr3T

Mycgr3G92938 Mycgr3T
  
Location: 37400-38699

Mycgr3G92938\_Mycgr3T

Mycgr3G92941 Mycgr3T
  
Location: 38799-40734

Mycgr3G92941\_Mycgr3T

ubiquitin C-terminal hydrolase family protein
  
Accession: EEY14048
  
Location: 475230-477937
  
 NCBI BlastP on this gene

EEY14048

54S ribosomal protein L7
  
Accession: EEY14049
  
Location: 479798-480977
  
 NCBI BlastP on this gene

EEY14049

Hsp70 nucleotide exchange factor FES1
  
Accession: EEY14050
  
Location: 481241-481759
  
 NCBI BlastP on this gene

EEY14050

actin
  
Accession: EEY14051
  
Location: 484296-485863
  
 NCBI BlastP on this gene

EEY14051

peptidyl-tRNA hydrolase domain-containing protein
  
Accession: EEY14052
  
Location: 486325-486933
  
 NCBI BlastP on this gene

EEY14052

ATP-binding cassette sub-family B member 5
  
Accession: EEY14053
  
Location: 487356-492271
  
 NCBI BlastP on this gene

EEY14053

conserved hypothetical protein
  
Accession: EEY14054
  
Location: 492973-493909
  
  
**BlastP hit with Mycgr3G104337\_Mycgr3**
  
Percentage identity: 40 %
  
BlastP bit score: 206
  
Sequence coverage: 101 %
  
E-value: 5e-62
  
  
 NCBI BlastP on this gene

EEY14054

fatty acid synthase S-acetyltransferase
  
Accession: EEY14055
  
Location: 495873-502926
  
  
**BlastP hit with Mycgr3G100089\_Mycgr3**
  
Percentage identity: 40 %
  
BlastP bit score: 1504
  
Sequence coverage: 91 %
  
E-value: 0.0
  
  
 NCBI BlastP on this gene

EEY14055

conserved hypothetical protein
  
Accession: EEY14056
  
Location: 503348-503776
  
 NCBI BlastP on this gene

EEY14056

elongator complex protein
  
Accession: EEY14057
  
Location: 504176-506869
  
 NCBI BlastP on this gene

EEY14057

conserved hypothetical protein
  
Accession: EEY14058
  
Location: 507493-507988
  
 NCBI BlastP on this gene

EEY14058

zinc finger protein
  
Accession: EEY14059
  
Location: 512644-514119
  
 NCBI BlastP on this gene

EEY14059

Query: Architecture Search FASTA input

JH767580 : Coniosporium apollinis CBS 100218 chromosome Unknown supercont1.27    Total score: 2.0     Cumulative Blast bit score: 1687

Hit cluster cross-links:

Mycgr3G85918 Mycgr3T
  
Location: 0-1602

Mycgr3G85918\_Mycgr3T

Mycgr3G42010 Mycgr3T
  
Location: 1702-8569

Mycgr3G42010\_Mycgr3T

Mycgr3G29582 Mycgr3T
  
Location: 8669-8915

Mycgr3G29582\_Mycgr3T

Mycgr3G31170 Mycgr3T
  
Location: 9015-9255

Mycgr3G31170\_Mycgr3T

Mycgr3G85924 Mycgr3T
  
Location: 9355-11218

Mycgr3G85924\_Mycgr3T

Mycgr3G71676 Mycgr3T
  
Location: 11318-12494

Mycgr3G71676\_Mycgr3T

Mycgr3G11468 Mycgr3T
  
Location: 12594-13653

Mycgr3G11468\_Mycgr3T

Mycgr3G58567 Mycgr3T
  
Location: 13753-14506

Mycgr3G58567\_Mycgr3T

Mycgr3G100089 Mycgr3
  
Location: 14606-21152

Mycgr3G100089\_Mycgr3

Mycgr3G42698 Mycgr3T
  
Location: 21252-22131

Mycgr3G42698\_Mycgr3T

Mycgr3G71681 Mycgr3T
  
Location: 22231-23461

Mycgr3G71681\_Mycgr3T

Mycgr3G109328 Mycgr3
  
Location: 23561-24239

Mycgr3G109328\_Mycgr3

Mycgr3G104334 Mycgr3
  
Location: 24339-24567

Mycgr3G104334\_Mycgr3

Mycgr3G42715 Mycgr3T
  
Location: 24667-25981

Mycgr3G42715\_Mycgr3T

Mycgr3G92934 Mycgr3T
  
Location: 26081-27593

Mycgr3G92934\_Mycgr3T

Mycgr3G41969 Mycgr3T
  
Location: 27693-29328

Mycgr3G41969\_Mycgr3T

Mycgr3G80635 Mycgr3T
  
Location: 29428-29821

Mycgr3G80635\_Mycgr3T

Mycgr3G41426 Mycgr3T
  
Location: 29921-35255

Mycgr3G41426\_Mycgr3T

Mycgr3G104337 Mycgr3
  
Location: 35355-36108

Mycgr3G104337\_Mycgr3

Mycgr3G71679 Mycgr3T
  
Location: 36208-37300

Mycgr3G71679\_Mycgr3T

Mycgr3G92938 Mycgr3T
  
Location: 37400-38699

Mycgr3G92938\_Mycgr3T

Mycgr3G92941 Mycgr3T
  
Location: 38799-40734

Mycgr3G92941\_Mycgr3T

hypothetical protein
  
Accession: EON66363
  
Location: 91730-93778
  
 NCBI BlastP on this gene

EON66363

phosphoglycerate mutase
  
Accession: EON66362
  
Location: 90194-91108
  
 NCBI BlastP on this gene

EON66362

hypothetical protein
  
Accession: EON66361
  
Location: 87609-88719
  
 NCBI BlastP on this gene

EON66361

hypothetical protein
  
Accession: EON66360
  
Location: 84619-86356
  
 NCBI BlastP on this gene

EON66360

hypothetical protein
  
Accession: EON66359
  
Location: 82693-83513
  
 NCBI BlastP on this gene

EON66359

hypothetical protein
  
Accession: EON66358
  
Location: 80299-81139
  
  
**BlastP hit with Mycgr3G31170\_Mycgr3T**
  
Percentage identity: 98 %
  
BlastP bit score: 162
  
Sequence coverage: 100 %
  
E-value: 5e-48
  
  
 NCBI BlastP on this gene

EON66358

hypothetical protein
  
Accession: EON66357
  
Location: 76583-78322
  
 NCBI BlastP on this gene

EON66357

hypothetical protein
  
Accession: EON66356
  
Location: 74654-76148
  
 NCBI BlastP on this gene

EON66356

phospholipid-translocating ATPase
  
Accession: EON66355
  
Location: 66517-71262
  
 NCBI BlastP on this gene

EON66355

hypothetical protein
  
Accession: EON66354
  
Location: 59223-65407
  
  
**BlastP hit with Mycgr3G41426\_Mycgr3T**
  
Percentage identity: 44 %
  
BlastP bit score: 1525
  
Sequence coverage: 103 %
  
E-value: 0.0
  
  
 NCBI BlastP on this gene

EON66354

hypothetical protein
  
Accession: EON66353
  
Location: 54947-57498
  
 NCBI BlastP on this gene

EON66353

hypothetical protein
  
Accession: EON66352
  
Location: 52582-54544
  
 NCBI BlastP on this gene

EON66352

hypothetical protein
  
Accession: EON66351
  
Location: 51157-51979
  
 NCBI BlastP on this gene

EON66351

hypothetical protein
  
Accession: EON66350
  
Location: 50328-50836
  
 NCBI BlastP on this gene

EON66350

hypothetical protein
  
Accession: EON66349
  
Location: 49686-50200
  
 NCBI BlastP on this gene

EON66349

hypothetical protein
  
Accession: EON66348
  
Location: 48270-49325
  
 NCBI BlastP on this gene

EON66348

Query: Architecture Search FASTA input

GL385401 : Gaeumannomyces graminis var. tritici R3-111a-1 unplaced genomic scaffold supercont2.7    Total score: 2.0     Cumulative Blast bit score: 1683

Hit cluster cross-links:

Mycgr3G85918 Mycgr3T
  
Location: 0-1602

Mycgr3G85918\_Mycgr3T

Mycgr3G42010 Mycgr3T
  
Location: 1702-8569

Mycgr3G42010\_Mycgr3T

Mycgr3G29582 Mycgr3T
  
Location: 8669-8915

Mycgr3G29582\_Mycgr3T

Mycgr3G31170 Mycgr3T
  
Location: 9015-9255

Mycgr3G31170\_Mycgr3T

Mycgr3G85924 Mycgr3T
  
Location: 9355-11218

Mycgr3G85924\_Mycgr3T

Mycgr3G71676 Mycgr3T
  
Location: 11318-12494

Mycgr3G71676\_Mycgr3T

Mycgr3G11468 Mycgr3T
  
Location: 12594-13653

Mycgr3G11468\_Mycgr3T

Mycgr3G58567 Mycgr3T
  
Location: 13753-14506

Mycgr3G58567\_Mycgr3T

Mycgr3G100089 Mycgr3
  
Location: 14606-21152

Mycgr3G100089\_Mycgr3

Mycgr3G42698 Mycgr3T
  
Location: 21252-22131

Mycgr3G42698\_Mycgr3T

Mycgr3G71681 Mycgr3T
  
Location: 22231-23461

Mycgr3G71681\_Mycgr3T

Mycgr3G109328 Mycgr3
  
Location: 23561-24239

Mycgr3G109328\_Mycgr3

Mycgr3G104334 Mycgr3
  
Location: 24339-24567

Mycgr3G104334\_Mycgr3

Mycgr3G42715 Mycgr3T
  
Location: 24667-25981

Mycgr3G42715\_Mycgr3T

Mycgr3G92934 Mycgr3T
  
Location: 26081-27593

Mycgr3G92934\_Mycgr3T

Mycgr3G41969 Mycgr3T
  
Location: 27693-29328

Mycgr3G41969\_Mycgr3T

Mycgr3G80635 Mycgr3T
  
Location: 29428-29821

Mycgr3G80635\_Mycgr3T

Mycgr3G41426 Mycgr3T
  
Location: 29921-35255

Mycgr3G41426\_Mycgr3T

Mycgr3G104337 Mycgr3
  
Location: 35355-36108

Mycgr3G104337\_Mycgr3

Mycgr3G71679 Mycgr3T
  
Location: 36208-37300

Mycgr3G71679\_Mycgr3T

Mycgr3G92938 Mycgr3T
  
Location: 37400-38699

Mycgr3G92938\_Mycgr3T

Mycgr3G92941 Mycgr3T
  
Location: 38799-40734

Mycgr3G92941\_Mycgr3T

hypothetical protein
  
Accession: EJT70646
  
Location: 1057927-1060380
  
 NCBI BlastP on this gene

EJT70646

1-aminocyclopropane-1-carboxylate deaminase
  
Accession: EJT70645
  
Location: 1056079-1057220
  
 NCBI BlastP on this gene

EJT70645

hypothetical protein
  
Accession: EJT70644
  
Location: 1055504-1055768
  
 NCBI BlastP on this gene

EJT70644

amidophosphoribosyltransferase
  
Accession: EJT70643
  
Location: 1052747-1054769
  
 NCBI BlastP on this gene

EJT70643

hypothetical protein
  
Accession: EJT70642
  
Location: 1049416-1051380
  
 NCBI BlastP on this gene

EJT70642

DNA-binding protein SMUBP-2
  
Accession: EJT70641
  
Location: 1041985-1048462
  
  
**BlastP hit with Mycgr3G41426\_Mycgr3T**
  
Percentage identity: 33 %
  
BlastP bit score: 922
  
Sequence coverage: 106 %
  
E-value: 0.0
  
  
 NCBI BlastP on this gene

EJT70641

hypothetical protein
  
Accession: EJT70640
  
Location: 1040156-1040809
  
 NCBI BlastP on this gene

EJT70640

hypothetical protein
  
Accession: EJT70639
  
Location: 1038058-1039388
  
 NCBI BlastP on this gene

EJT70639

hypothetical protein
  
Accession: EJT70638
  
Location: 1036415-1037864
  
 NCBI BlastP on this gene

EJT70638

hypothetical protein
  
Accession: EJT70637
  
Location: 1035627-1036258
  
 NCBI BlastP on this gene

EJT70637

hypothetical protein
  
Accession: EJT70636
  
Location: 1033911-1035087
  
 NCBI BlastP on this gene

EJT70636

hypothetical protein
  
Accession: EJT70635
  
Location: 1030771-1032717
  
 NCBI BlastP on this gene

EJT70635

hypothetical protein
  
Accession: EJT70634
  
Location: 1025775-1029803
  
  
**BlastP hit with Mycgr3G42010\_Mycgr3T**
  
Percentage identity: 40 %
  
BlastP bit score: 761
  
Sequence coverage: 48 %
  
E-value: 0.0
  
  
 NCBI BlastP on this gene

EJT70634

hypothetical protein
  
Accession: EJT70633
  
Location: 1024594-1025754
  
 NCBI BlastP on this gene

EJT70633

hypothetical protein
  
Accession: EJT70632
  
Location: 1024224-1024536
  
 NCBI BlastP on this gene

EJT70632

hypothetical protein
  
Accession: EJT70631
  
Location: 1017782-1022084
  
 NCBI BlastP on this gene

EJT70631

hypothetical protein
  
Accession: EJT70630
  
Location: 1013801-1017514
  
 NCBI BlastP on this gene

EJT70630

Query: Architecture Search FASTA input

CU633870 : Podospora anserina S mat+ genomic DNA chromosome 5, supercontig 8.    Total score: 2.0     Cumulative Blast bit score: 1665

Hit cluster cross-links:

Mycgr3G85918 Mycgr3T
  
Location: 0-1602

Mycgr3G85918\_Mycgr3T

Mycgr3G42010 Mycgr3T
  
Location: 1702-8569

Mycgr3G42010\_Mycgr3T

Mycgr3G29582 Mycgr3T
  
Location: 8669-8915

Mycgr3G29582\_Mycgr3T

Mycgr3G31170 Mycgr3T
  
Location: 9015-9255

Mycgr3G31170\_Mycgr3T

Mycgr3G85924 Mycgr3T
  
Location: 9355-11218

Mycgr3G85924\_Mycgr3T

Mycgr3G71676 Mycgr3T
  
Location: 11318-12494

Mycgr3G71676\_Mycgr3T

Mycgr3G11468 Mycgr3T
  
Location: 12594-13653

Mycgr3G11468\_Mycgr3T

Mycgr3G58567 Mycgr3T
  
Location: 13753-14506

Mycgr3G58567\_Mycgr3T

Mycgr3G100089 Mycgr3
  
Location: 14606-21152

Mycgr3G100089\_Mycgr3

Mycgr3G42698 Mycgr3T
  
Location: 21252-22131

Mycgr3G42698\_Mycgr3T

Mycgr3G71681 Mycgr3T
  
Location: 22231-23461

Mycgr3G71681\_Mycgr3T

Mycgr3G109328 Mycgr3
  
Location: 23561-24239

Mycgr3G109328\_Mycgr3

Mycgr3G104334 Mycgr3
  
Location: 24339-24567

Mycgr3G104334\_Mycgr3

Mycgr3G42715 Mycgr3T
  
Location: 24667-25981

Mycgr3G42715\_Mycgr3T

Mycgr3G92934 Mycgr3T
  
Location: 26081-27593

Mycgr3G92934\_Mycgr3T

Mycgr3G41969 Mycgr3T
  
Location: 27693-29328

Mycgr3G41969\_Mycgr3T

Mycgr3G80635 Mycgr3T
  
Location: 29428-29821

Mycgr3G80635\_Mycgr3T

Mycgr3G41426 Mycgr3T
  
Location: 29921-35255

Mycgr3G41426\_Mycgr3T

Mycgr3G104337 Mycgr3
  
Location: 35355-36108

Mycgr3G104337\_Mycgr3

Mycgr3G71679 Mycgr3T
  
Location: 36208-37300

Mycgr3G71679\_Mycgr3T

Mycgr3G92938 Mycgr3T
  
Location: 37400-38699

Mycgr3G92938\_Mycgr3T

Mycgr3G92941 Mycgr3T
  
Location: 38799-40734

Mycgr3G92941\_Mycgr3T

not annotated
  
Accession: CAP65179
  
Location: 371658-372986
  
 NCBI BlastP on this gene

CAP65179

not annotated
  
Accession: CAP65180
  
Location: 375153-375682
  
 NCBI BlastP on this gene

CAP65180

not annotated
  
Accession: CAP65181
  
Location: 377132-378190
  
 NCBI BlastP on this gene

CAP65181

not annotated
  
Accession: CAP65182
  
Location: 381477-383442
  
 NCBI BlastP on this gene

CAP65182

not annotated
  
Accession: CAP65183
  
Location: 384465-385198
  
 NCBI BlastP on this gene

CAP65183

not annotated
  
Accession: CAP65184
  
Location: 385644-386566
  
 NCBI BlastP on this gene

CAP65184

not annotated
  
Accession: CAP65185
  
Location: 387049-389244
  
 NCBI BlastP on this gene

CAP65185

not annotated
  
Accession: CAP65186
  
Location: 389833-396580
  
  
**BlastP hit with Mycgr3G100089\_Mycgr3**
  
Percentage identity: 38 %
  
BlastP bit score: 1466
  
Sequence coverage: 103 %
  
E-value: 0.0
  
  
 NCBI BlastP on this gene

CAP65186

not annotated
  
Accession: CAP65187
  
Location: 398271-399340
  
  
**BlastP hit with Mycgr3G104337\_Mycgr3**
  
Percentage identity: 42 %
  
BlastP bit score: 199
  
Sequence coverage: 100 %
  
E-value: 9e-59
  
  
 NCBI BlastP on this gene

CAP65187

not annotated
  
Accession: CAP65188
  
Location: 403437-404936
  
 NCBI BlastP on this gene

CAP65188

not annotated
  
Accession: CAP65189
  
Location: 407649-410144
  
 NCBI BlastP on this gene

CAP65189

not annotated
  
Accession: CAP65190
  
Location: 412031-412612
  
 NCBI BlastP on this gene

CAP65190

not annotated
  
Accession: CAP65191
  
Location: 412798-413184
  
 NCBI BlastP on this gene

CAP65191

not annotated
  
Accession: CAP65192
  
Location: 413517-414914
  
 NCBI BlastP on this gene

CAP65192

Query: Architecture Search FASTA input

AGUE01000230 : Glarea lozoyensis 74030    Total score: 2.0     Cumulative Blast bit score: 1657

Hit cluster cross-links:

Mycgr3G85918 Mycgr3T
  
Location: 0-1602

Mycgr3G85918\_Mycgr3T

Mycgr3G42010 Mycgr3T
  
Location: 1702-8569

Mycgr3G42010\_Mycgr3T

Mycgr3G29582 Mycgr3T
  
Location: 8669-8915

Mycgr3G29582\_Mycgr3T

Mycgr3G31170 Mycgr3T
  
Location: 9015-9255

Mycgr3G31170\_Mycgr3T

Mycgr3G85924 Mycgr3T
  
Location: 9355-11218

Mycgr3G85924\_Mycgr3T

Mycgr3G71676 Mycgr3T
  
Location: 11318-12494

Mycgr3G71676\_Mycgr3T

Mycgr3G11468 Mycgr3T
  
Location: 12594-13653

Mycgr3G11468\_Mycgr3T

Mycgr3G58567 Mycgr3T
  
Location: 13753-14506

Mycgr3G58567\_Mycgr3T

Mycgr3G100089 Mycgr3
  
Location: 14606-21152

Mycgr3G100089\_Mycgr3

Mycgr3G42698 Mycgr3T
  
Location: 21252-22131

Mycgr3G42698\_Mycgr3T

Mycgr3G71681 Mycgr3T
  
Location: 22231-23461

Mycgr3G71681\_Mycgr3T

Mycgr3G109328 Mycgr3
  
Location: 23561-24239

Mycgr3G109328\_Mycgr3

Mycgr3G104334 Mycgr3
  
Location: 24339-24567

Mycgr3G104334\_Mycgr3

Mycgr3G42715 Mycgr3T
  
Location: 24667-25981

Mycgr3G42715\_Mycgr3T

Mycgr3G92934 Mycgr3T
  
Location: 26081-27593

Mycgr3G92934\_Mycgr3T

Mycgr3G41969 Mycgr3T
  
Location: 27693-29328

Mycgr3G41969\_Mycgr3T

Mycgr3G80635 Mycgr3T
  
Location: 29428-29821

Mycgr3G80635\_Mycgr3T

Mycgr3G41426 Mycgr3T
  
Location: 29921-35255

Mycgr3G41426\_Mycgr3T

Mycgr3G104337 Mycgr3
  
Location: 35355-36108

Mycgr3G104337\_Mycgr3

Mycgr3G71679 Mycgr3T
  
Location: 36208-37300

Mycgr3G71679\_Mycgr3T

Mycgr3G92938 Mycgr3T
  
Location: 37400-38699

Mycgr3G92938\_Mycgr3T

Mycgr3G92941 Mycgr3T
  
Location: 38799-40734

Mycgr3G92941\_Mycgr3T

putative Retinol-binding protein 3
  
Accession: EHK96639
  
Location: 39028-40185
  
 NCBI BlastP on this gene

EHK96639

hypothetical protein
  
Accession: EHK96640
  
Location: 49320-50538
  
 NCBI BlastP on this gene

EHK96640

hypothetical protein
  
Accession: EHK96641
  
Location: 51256-51600
  
 NCBI BlastP on this gene

EHK96641

hypothetical protein
  
Accession: EHK96642
  
Location: 53855-54950
  
 NCBI BlastP on this gene

EHK96642

hypothetical protein
  
Accession: EHK96643
  
Location: 55574-55758
  
 NCBI BlastP on this gene

EHK96643

putative Phthioceranic/hydroxyphthioceranic acid synthase
  
Accession: EHK96644
  
Location: 57302-63976
  
  
**BlastP hit with Mycgr3G100089\_Mycgr3**
  
Percentage identity: 42 %
  
BlastP bit score: 1468
  
Sequence coverage: 83 %
  
E-value: 0.0
  
  
 NCBI BlastP on this gene

EHK96644

putative Uncharacterized hydrolase C22A12.06c
  
Accession: EHK96645
  
Location: 65599-67085
  
  
**BlastP hit with Mycgr3G104337\_Mycgr3**
  
Percentage identity: 40 %
  
BlastP bit score: 189
  
Sequence coverage: 93 %
  
E-value: 2e-55
  
  
 NCBI BlastP on this gene

EHK96645

putative ABC transporter B family member 11
  
Accession: EHK96646
  
Location: 70014-70404
  
 NCBI BlastP on this gene

EHK96646

hypothetical protein
  
Accession: EHK96647
  
Location: 73784-74155
  
 NCBI BlastP on this gene

EHK96647

putative UPF0364 protein
  
Accession: EHK96648
  
Location: 84393-86120
  
 NCBI BlastP on this gene

EHK96648

Query: Architecture Search FASTA input

KB445637 : Cochliobolus sativus ND90Pr unplaced genomic scaffold COCSAscaffold\_1    Total score: 2.0     Cumulative Blast bit score: 1654

Hit cluster cross-links:

Mycgr3G85918 Mycgr3T
  
Location: 0-1602

Mycgr3G85918\_Mycgr3T

Mycgr3G42010 Mycgr3T
  
Location: 1702-8569

Mycgr3G42010\_Mycgr3T

Mycgr3G29582 Mycgr3T
  
Location: 8669-8915

Mycgr3G29582\_Mycgr3T

Mycgr3G31170 Mycgr3T
  
Location: 9015-9255

Mycgr3G31170\_Mycgr3T

Mycgr3G85924 Mycgr3T
  
Location: 9355-11218

Mycgr3G85924\_Mycgr3T

Mycgr3G71676 Mycgr3T
  
Location: 11318-12494

Mycgr3G71676\_Mycgr3T

Mycgr3G11468 Mycgr3T
  
Location: 12594-13653

Mycgr3G11468\_Mycgr3T

Mycgr3G58567 Mycgr3T
  
Location: 13753-14506

Mycgr3G58567\_Mycgr3T

Mycgr3G100089 Mycgr3
  
Location: 14606-21152

Mycgr3G100089\_Mycgr3

Mycgr3G42698 Mycgr3T
  
Location: 21252-22131

Mycgr3G42698\_Mycgr3T

Mycgr3G71681 Mycgr3T
  
Location: 22231-23461

Mycgr3G71681\_Mycgr3T

Mycgr3G109328 Mycgr3
  
Location: 23561-24239

Mycgr3G109328\_Mycgr3

Mycgr3G104334 Mycgr3
  
Location: 24339-24567

Mycgr3G104334\_Mycgr3

Mycgr3G42715 Mycgr3T
  
Location: 24667-25981

Mycgr3G42715\_Mycgr3T

Mycgr3G92934 Mycgr3T
  
Location: 26081-27593

Mycgr3G92934\_Mycgr3T

Mycgr3G41969 Mycgr3T
  
Location: 27693-29328

Mycgr3G41969\_Mycgr3T

Mycgr3G80635 Mycgr3T
  
Location: 29428-29821

Mycgr3G80635\_Mycgr3T

Mycgr3G41426 Mycgr3T
  
Location: 29921-35255

Mycgr3G41426\_Mycgr3T

Mycgr3G104337 Mycgr3
  
Location: 35355-36108

Mycgr3G104337\_Mycgr3

Mycgr3G71679 Mycgr3T
  
Location: 36208-37300

Mycgr3G71679\_Mycgr3T

Mycgr3G92938 Mycgr3T
  
Location: 37400-38699

Mycgr3G92938\_Mycgr3T

Mycgr3G92941 Mycgr3T
  
Location: 38799-40734

Mycgr3G92941\_Mycgr3T

polysaccharide lyase family 4 protein
  
Accession: EMD69104
  
Location: 448468-450608
  
 NCBI BlastP on this gene

EMD69104

hypothetical protein
  
Accession: EMD69103
  
Location: 447658-448035
  
 NCBI BlastP on this gene

EMD69103

hypothetical protein
  
Accession: EMD69102
  
Location: 442526-447493
  
 NCBI BlastP on this gene

EMD69102

hypothetical protein
  
Accession: EMD69101
  
Location: 438829-440928
  
 NCBI BlastP on this gene

EMD69101

hypothetical protein
  
Accession: EMD69100
  
Location: 435828-436693
  
 NCBI BlastP on this gene

EMD69100

hypothetical protein
  
Accession: EMD69099
  
Location: 432399-433267
  
  
**BlastP hit with Mycgr3G31170\_Mycgr3T**
  
Percentage identity: 100 %
  
BlastP bit score: 166
  
Sequence coverage: 100 %
  
E-value: 1e-49
  
  
 NCBI BlastP on this gene

EMD69099

hypothetical protein
  
Accession: EMD69098
  
Location: 427831-429792
  
 NCBI BlastP on this gene

EMD69098

hypothetical protein
  
Accession: EMD69097
  
Location: 420899-427015
  
  
**BlastP hit with Mycgr3G41426\_Mycgr3T**
  
Percentage identity: 43 %
  
BlastP bit score: 1488
  
Sequence coverage: 102 %
  
E-value: 0.0
  
  
 NCBI BlastP on this gene

EMD69097

hypothetical protein
  
Accession: EMD69096
  
Location: 416917-419046
  
 NCBI BlastP on this gene

EMD69096

hypothetical protein
  
Accession: EMD69095
  
Location: 413384-416463
  
 NCBI BlastP on this gene

EMD69095

hypothetical protein
  
Accession: EMD69094
  
Location: 412288-413120
  
 NCBI BlastP on this gene

EMD69094

hypothetical protein
  
Accession: EMD69093
  
Location: 411127-411945
  
 NCBI BlastP on this gene

EMD69093

hypothetical protein
  
Accession: EMD69092
  
Location: 409018-410586
  
 NCBI BlastP on this gene

EMD69092

hypothetical protein
  
Accession: EMD69091
  
Location: 408416-408688
  
 NCBI BlastP on this gene

EMD69091

hypothetical protein
  
Accession: EMD69090
  
Location: 406869-408007
  
 NCBI BlastP on this gene

EMD69090

hypothetical protein
  
Accession: EMD69089
  
Location: 403239-405117
  
 NCBI BlastP on this gene

EMD69089

Query: Architecture Search FASTA input

KB733444 : Bipolaris maydis ATCC 48331 unplaced genomic scaffold COCC4scaffold\_1    Total score: 2.0     Cumulative Blast bit score: 1650

Hit cluster cross-links:

Mycgr3G85918 Mycgr3T
  
Location: 0-1602

Mycgr3G85918\_Mycgr3T

Mycgr3G42010 Mycgr3T
  
Location: 1702-8569

Mycgr3G42010\_Mycgr3T

Mycgr3G29582 Mycgr3T
  
Location: 8669-8915

Mycgr3G29582\_Mycgr3T

Mycgr3G31170 Mycgr3T
  
Location: 9015-9255

Mycgr3G31170\_Mycgr3T

Mycgr3G85924 Mycgr3T
  
Location: 9355-11218

Mycgr3G85924\_Mycgr3T

Mycgr3G71676 Mycgr3T
  
Location: 11318-12494

Mycgr3G71676\_Mycgr3T

Mycgr3G11468 Mycgr3T
  
Location: 12594-13653

Mycgr3G11468\_Mycgr3T

Mycgr3G58567 Mycgr3T
  
Location: 13753-14506

Mycgr3G58567\_Mycgr3T

Mycgr3G100089 Mycgr3
  
Location: 14606-21152

Mycgr3G100089\_Mycgr3

Mycgr3G42698 Mycgr3T
  
Location: 21252-22131

Mycgr3G42698\_Mycgr3T

Mycgr3G71681 Mycgr3T
  
Location: 22231-23461

Mycgr3G71681\_Mycgr3T

Mycgr3G109328 Mycgr3
  
Location: 23561-24239

Mycgr3G109328\_Mycgr3

Mycgr3G104334 Mycgr3
  
Location: 24339-24567

Mycgr3G104334\_Mycgr3

Mycgr3G42715 Mycgr3T
  
Location: 24667-25981

Mycgr3G42715\_Mycgr3T

Mycgr3G92934 Mycgr3T
  
Location: 26081-27593

Mycgr3G92934\_Mycgr3T

Mycgr3G41969 Mycgr3T
  
Location: 27693-29328

Mycgr3G41969\_Mycgr3T

Mycgr3G80635 Mycgr3T
  
Location: 29428-29821

Mycgr3G80635\_Mycgr3T

Mycgr3G41426 Mycgr3T
  
Location: 29921-35255

Mycgr3G41426\_Mycgr3T

Mycgr3G104337 Mycgr3
  
Location: 35355-36108

Mycgr3G104337\_Mycgr3

Mycgr3G71679 Mycgr3T
  
Location: 36208-37300

Mycgr3G71679\_Mycgr3T

Mycgr3G92938 Mycgr3T
  
Location: 37400-38699

Mycgr3G92938\_Mycgr3T

Mycgr3G92941 Mycgr3T
  
Location: 38799-40734

Mycgr3G92941\_Mycgr3T

polysaccharide lyase family 4 protein
  
Accession: ENI11027
  
Location: 1889630-1891830
  
 NCBI BlastP on this gene

ENI11027

hypothetical protein
  
Accession: ENI11028
  
Location: 1892162-1892380
  
 NCBI BlastP on this gene

ENI11028

hypothetical protein
  
Accession: ENI11029
  
Location: 1892897-1897868
  
 NCBI BlastP on this gene

ENI11029

hypothetical protein
  
Accession: ENI11030
  
Location: 1899488-1901539
  
 NCBI BlastP on this gene

ENI11030

hypothetical protein
  
Accession: ENI11031
  
Location: 1901644-1901843
  
 NCBI BlastP on this gene

ENI11031

hypothetical protein
  
Accession: ENI11032
  
Location: 1903905-1904796
  
 NCBI BlastP on this gene

ENI11032

hypothetical protein
  
Accession: ENI11033
  
Location: 1906114-1906530
  
 NCBI BlastP on this gene

ENI11033

hypothetical protein
  
Accession: ENI11034
  
Location: 1907443-1908327
  
  
**BlastP hit with Mycgr3G31170\_Mycgr3T**
  
Percentage identity: 100 %
  
BlastP bit score: 166
  
Sequence coverage: 100 %
  
E-value: 1e-49
  
  
 NCBI BlastP on this gene

ENI11034

hypothetical protein
  
Accession: ENI11035
  
Location: 1911048-1912930
  
 NCBI BlastP on this gene

ENI11035

hypothetical protein
  
Accession: ENI11036
  
Location: 1913885-1919857
  
  
**BlastP hit with Mycgr3G41426\_Mycgr3T**
  
Percentage identity: 43 %
  
BlastP bit score: 1484
  
Sequence coverage: 102 %
  
E-value: 0.0
  
  
 NCBI BlastP on this gene

ENI11036

hypothetical protein
  
Accession: ENI11037
  
Location: 1921887-1924007
  
 NCBI BlastP on this gene

ENI11037

hypothetical protein
  
Accession: ENI11038
  
Location: 1924476-1925087
  
 NCBI BlastP on this gene

ENI11038

hypothetical protein
  
Accession: ENI11039
  
Location: 1925689-1927578
  
 NCBI BlastP on this gene

ENI11039

hypothetical protein
  
Accession: ENI11040
  
Location: 1927844-1928678
  
 NCBI BlastP on this gene

ENI11040

hypothetical protein
  
Accession: ENI11041
  
Location: 1929008-1929823
  
 NCBI BlastP on this gene

ENI11041

hypothetical protein
  
Accession: ENI11042
  
Location: 1930410-1931978
  
 NCBI BlastP on this gene

ENI11042

hypothetical protein
  
Accession: ENI11043
  
Location: 1932999-1934135
  
 NCBI BlastP on this gene

ENI11043

hypothetical protein
  
Accession: ENI11044
  
Location: 1935516-1936842
  
 NCBI BlastP on this gene

ENI11044

Query: Architecture Search FASTA input

KB445570 : Cochliobolus heterostrophus C5 unplaced genomic scaffold COCHEscaffold\_2    Total score: 2.0     Cumulative Blast bit score: 1650

Hit cluster cross-links:

Mycgr3G85918 Mycgr3T
  
Location: 0-1602

Mycgr3G85918\_Mycgr3T

Mycgr3G42010 Mycgr3T
  
Location: 1702-8569

Mycgr3G42010\_Mycgr3T

Mycgr3G29582 Mycgr3T
  
Location: 8669-8915

Mycgr3G29582\_Mycgr3T

Mycgr3G31170 Mycgr3T
  
Location: 9015-9255

Mycgr3G31170\_Mycgr3T

Mycgr3G85924 Mycgr3T
  
Location: 9355-11218

Mycgr3G85924\_Mycgr3T

Mycgr3G71676 Mycgr3T
  
Location: 11318-12494

Mycgr3G71676\_Mycgr3T

Mycgr3G11468 Mycgr3T
  
Location: 12594-13653

Mycgr3G11468\_Mycgr3T

Mycgr3G58567 Mycgr3T
  
Location: 13753-14506

Mycgr3G58567\_Mycgr3T

Mycgr3G100089 Mycgr3
  
Location: 14606-21152

Mycgr3G100089\_Mycgr3

Mycgr3G42698 Mycgr3T
  
Location: 21252-22131

Mycgr3G42698\_Mycgr3T

Mycgr3G71681 Mycgr3T
  
Location: 22231-23461

Mycgr3G71681\_Mycgr3T

Mycgr3G109328 Mycgr3
  
Location: 23561-24239

Mycgr3G109328\_Mycgr3

Mycgr3G104334 Mycgr3
  
Location: 24339-24567

Mycgr3G104334\_Mycgr3

Mycgr3G42715 Mycgr3T
  
Location: 24667-25981

Mycgr3G42715\_Mycgr3T

Mycgr3G92934 Mycgr3T
  
Location: 26081-27593

Mycgr3G92934\_Mycgr3T

Mycgr3G41969 Mycgr3T
  
Location: 27693-29328

Mycgr3G41969\_Mycgr3T

Mycgr3G80635 Mycgr3T
  
Location: 29428-29821

Mycgr3G80635\_Mycgr3T

Mycgr3G41426 Mycgr3T
  
Location: 29921-35255

Mycgr3G41426\_Mycgr3T

Mycgr3G104337 Mycgr3
  
Location: 35355-36108

Mycgr3G104337\_Mycgr3

Mycgr3G71679 Mycgr3T
  
Location: 36208-37300

Mycgr3G71679\_Mycgr3T

Mycgr3G92938 Mycgr3T
  
Location: 37400-38699

Mycgr3G92938\_Mycgr3T

Mycgr3G92941 Mycgr3T
  
Location: 38799-40734

Mycgr3G92941\_Mycgr3T

polysaccharide lyase family 4 protein
  
Accession: EMD96168
  
Location: 1911294-1913494
  
 NCBI BlastP on this gene

EMD96168

hypothetical protein
  
Accession: EMD96169
  
Location: 1913826-1914044
  
 NCBI BlastP on this gene

EMD96169

hypothetical protein
  
Accession: EMD96170
  
Location: 1914561-1919532
  
 NCBI BlastP on this gene

EMD96170

hypothetical protein
  
Accession: EMD96171
  
Location: 1921152-1923203
  
 NCBI BlastP on this gene

EMD96171

hypothetical protein
  
Accession: EMD96172
  
Location: 1923308-1923507
  
 NCBI BlastP on this gene

EMD96172

hypothetical protein
  
Accession: EMD96173
  
Location: 1925569-1926460
  
 NCBI BlastP on this gene

EMD96173

hypothetical protein
  
Accession: EMD96174
  
Location: 1927778-1928194
  
 NCBI BlastP on this gene

EMD96174

hypothetical protein
  
Accession: EMD96175
  
Location: 1929107-1929991
  
  
**BlastP hit with Mycgr3G31170\_Mycgr3T**
  
Percentage identity: 100 %
  
BlastP bit score: 166
  
Sequence coverage: 100 %
  
E-value: 1e-49
  
  
 NCBI BlastP on this gene

EMD96175

hypothetical protein
  
Accession: EMD96176
  
Location: 1932712-1934594
  
 NCBI BlastP on this gene

EMD96176

hypothetical protein
  
Accession: EMD96177
  
Location: 1935549-1941521
  
  
**BlastP hit with Mycgr3G41426\_Mycgr3T**
  
Percentage identity: 43 %
  
BlastP bit score: 1484
  
Sequence coverage: 102 %
  
E-value: 0.0
  
  
 NCBI BlastP on this gene

EMD96177

hypothetical protein
  
Accession: EMD96178
  
Location: 1943590-1945686
  
 NCBI BlastP on this gene

EMD96178

hypothetical protein
  
Accession: EMD96179
  
Location: 1946140-1946751
  
 NCBI BlastP on this gene

EMD96179

hypothetical protein
  
Accession: EMD96180
  
Location: 1947353-1949242
  
 NCBI BlastP on this gene

EMD96180

hypothetical protein
  
Accession: EMD96181
  
Location: 1949508-1950342
  
 NCBI BlastP on this gene

EMD96181

hypothetical protein
  
Accession: EMD96182
  
Location: 1950672-1951487
  
 NCBI BlastP on this gene

EMD96182

hypothetical protein
  
Accession: EMD96183
  
Location: 1952074-1953642
  
 NCBI BlastP on this gene

EMD96183

hypothetical protein
  
Accession: EMD96184
  
Location: 1954738-1955751
  
 NCBI BlastP on this gene

EMD96184

hypothetical protein
  
Accession: EMD96185
  
Location: 1957180-1958506
  
 NCBI BlastP on this gene

EMD96185

Query: Architecture Search FASTA input

KB908482 : Setosphaeria turcica Et28A unplaced genomic scaffold SETTUscaffold\_10    Total score: 2.0     Cumulative Blast bit score: 1641

Hit cluster cross-links:

Mycgr3G85918 Mycgr3T
  
Location: 0-1602

Mycgr3G85918\_Mycgr3T

Mycgr3G42010 Mycgr3T
  
Location: 1702-8569

Mycgr3G42010\_Mycgr3T

Mycgr3G29582 Mycgr3T
  
Location: 8669-8915

Mycgr3G29582\_Mycgr3T

Mycgr3G31170 Mycgr3T
  
Location: 9015-9255

Mycgr3G31170\_Mycgr3T

Mycgr3G85924 Mycgr3T
  
Location: 9355-11218

Mycgr3G85924\_Mycgr3T

Mycgr3G71676 Mycgr3T
  
Location: 11318-12494

Mycgr3G71676\_Mycgr3T

Mycgr3G11468 Mycgr3T
  
Location: 12594-13653

Mycgr3G11468\_Mycgr3T

Mycgr3G58567 Mycgr3T
  
Location: 13753-14506

Mycgr3G58567\_Mycgr3T

Mycgr3G100089 Mycgr3
  
Location: 14606-21152

Mycgr3G100089\_Mycgr3

Mycgr3G42698 Mycgr3T
  
Location: 21252-22131

Mycgr3G42698\_Mycgr3T

Mycgr3G71681 Mycgr3T
  
Location: 22231-23461

Mycgr3G71681\_Mycgr3T

Mycgr3G109328 Mycgr3
  
Location: 23561-24239

Mycgr3G109328\_Mycgr3

Mycgr3G104334 Mycgr3
  
Location: 24339-24567

Mycgr3G104334\_Mycgr3

Mycgr3G42715 Mycgr3T
  
Location: 24667-25981

Mycgr3G42715\_Mycgr3T

Mycgr3G92934 Mycgr3T
  
Location: 26081-27593

Mycgr3G92934\_Mycgr3T

Mycgr3G41969 Mycgr3T
  
Location: 27693-29328

Mycgr3G41969\_Mycgr3T

Mycgr3G80635 Mycgr3T
  
Location: 29428-29821

Mycgr3G80635\_Mycgr3T

Mycgr3G41426 Mycgr3T
  
Location: 29921-35255

Mycgr3G41426\_Mycgr3T

Mycgr3G104337 Mycgr3
  
Location: 35355-36108

Mycgr3G104337\_Mycgr3

Mycgr3G71679 Mycgr3T
  
Location: 36208-37300

Mycgr3G71679\_Mycgr3T

Mycgr3G92938 Mycgr3T
  
Location: 37400-38699

Mycgr3G92938\_Mycgr3T

Mycgr3G92941 Mycgr3T
  
Location: 38799-40734

Mycgr3G92941\_Mycgr3T

hypothetical protein
  
Accession: EOA90926
  
Location: 1155867-1160804
  
 NCBI BlastP on this gene

EOA90926

hypothetical protein
  
Accession: EOA90927
  
Location: 1162792-1164961
  
 NCBI BlastP on this gene

EOA90927

hypothetical protein
  
Accession: EOA90928
  
Location: 1168587-1169517
  
 NCBI BlastP on this gene

EOA90928

hypothetical protein
  
Accession: EOA90929
  
Location: 1172598-1173543
  
  
**BlastP hit with Mycgr3G31170\_Mycgr3T**
  
Percentage identity: 100 %
  
BlastP bit score: 166
  
Sequence coverage: 100 %
  
E-value: 1e-49
  
  
 NCBI BlastP on this gene

EOA90929

hypothetical protein
  
Accession: EOA90930
  
Location: 1176615-1182675
  
  
**BlastP hit with Mycgr3G41426\_Mycgr3T**
  
Percentage identity: 44 %
  
BlastP bit score: 1475
  
Sequence coverage: 102 %
  
E-value: 0.0
  
  
 NCBI BlastP on this gene

EOA90930

hypothetical protein
  
Accession: EOA90931
  
Location: 1184496-1186721
  
 NCBI BlastP on this gene

EOA90931

hypothetical protein
  
Accession: EOA90932
  
Location: 1187207-1187837
  
 NCBI BlastP on this gene

EOA90932

hypothetical protein
  
Accession: EOA90933
  
Location: 1188380-1190284
  
 NCBI BlastP on this gene

EOA90933

hypothetical protein
  
Accession: EOA90934
  
Location: 1190578-1191404
  
 NCBI BlastP on this gene

EOA90934

hypothetical protein
  
Accession: EOA90935
  
Location: 1191740-1192558
  
 NCBI BlastP on this gene

EOA90935

hypothetical protein
  
Accession: EOA90936
  
Location: 1193008-1194576
  
 NCBI BlastP on this gene

EOA90936

hypothetical protein
  
Accession: EOA90937
  
Location: 1198165-1198854
  
 NCBI BlastP on this gene

EOA90937

hypothetical protein
  
Accession: EOA90938
  
Location: 1199008-1200855
  
 NCBI BlastP on this gene

EOA90938

Query: Architecture Search FASTA input

DS985227 : Verticillium albo-atrum VaMs.102 supercont1.14 genomic scaffold    Total score: 2.0     Cumulative Blast bit score: 1621

Hit cluster cross-links:

Mycgr3G85918 Mycgr3T
  
Location: 0-1602

Mycgr3G85918\_Mycgr3T

Mycgr3G42010 Mycgr3T
  
Location: 1702-8569

Mycgr3G42010\_Mycgr3T

Mycgr3G29582 Mycgr3T
  
Location: 8669-8915

Mycgr3G29582\_Mycgr3T

Mycgr3G31170 Mycgr3T
  
Location: 9015-9255

Mycgr3G31170\_Mycgr3T

Mycgr3G85924 Mycgr3T
  
Location: 9355-11218

Mycgr3G85924\_Mycgr3T

Mycgr3G71676 Mycgr3T
  
Location: 11318-12494

Mycgr3G71676\_Mycgr3T

Mycgr3G11468 Mycgr3T
  
Location: 12594-13653

Mycgr3G11468\_Mycgr3T

Mycgr3G58567 Mycgr3T
  
Location: 13753-14506

Mycgr3G58567\_Mycgr3T

Mycgr3G100089 Mycgr3
  
Location: 14606-21152

Mycgr3G100089\_Mycgr3

Mycgr3G42698 Mycgr3T
  
Location: 21252-22131

Mycgr3G42698\_Mycgr3T

Mycgr3G71681 Mycgr3T
  
Location: 22231-23461

Mycgr3G71681\_Mycgr3T

Mycgr3G109328 Mycgr3
  
Location: 23561-24239

Mycgr3G109328\_Mycgr3

Mycgr3G104334 Mycgr3
  
Location: 24339-24567

Mycgr3G104334\_Mycgr3

Mycgr3G42715 Mycgr3T
  
Location: 24667-25981

Mycgr3G42715\_Mycgr3T

Mycgr3G92934 Mycgr3T
  
Location: 26081-27593

Mycgr3G92934\_Mycgr3T

Mycgr3G41969 Mycgr3T
  
Location: 27693-29328

Mycgr3G41969\_Mycgr3T

Mycgr3G80635 Mycgr3T
  
Location: 29428-29821

Mycgr3G80635\_Mycgr3T

Mycgr3G41426 Mycgr3T
  
Location: 29921-35255

Mycgr3G41426\_Mycgr3T

Mycgr3G104337 Mycgr3
  
Location: 35355-36108

Mycgr3G104337\_Mycgr3

Mycgr3G71679 Mycgr3T
  
Location: 36208-37300

Mycgr3G71679\_Mycgr3T

Mycgr3G92938 Mycgr3T
  
Location: 37400-38699

Mycgr3G92938\_Mycgr3T

Mycgr3G92941 Mycgr3T
  
Location: 38799-40734

Mycgr3G92941\_Mycgr3T

hypothetical protein
  
Accession: EEY22933
  
Location: 260362-261003
  
 NCBI BlastP on this gene

EEY22933

alcohol dehydrogenase
  
Accession: EEY22934
  
Location: 262099-264788
  
 NCBI BlastP on this gene

EEY22934

sugar transporter
  
Accession: EEY22935
  
Location: 265629-267504
  
 NCBI BlastP on this gene

EEY22935

thermostable beta-glucosidase B
  
Accession: EEY22936
  
Location: 268353-270950
  
 NCBI BlastP on this gene

EEY22936

predicted protein
  
Accession: EEY22937
  
Location: 275617-276410
  
 NCBI BlastP on this gene

EEY22937

hexose transporter protein
  
Accession: EEY22938
  
Location: 276481-277171
  
 NCBI BlastP on this gene

EEY22938

cbbX
  
Accession: EEY22939
  
Location: 277707-284840
  
  
**BlastP hit with Mycgr3G42010\_Mycgr3T**
  
Percentage identity: 43 %
  
BlastP bit score: 1514
  
Sequence coverage: 87 %
  
E-value: 0.0
  
  
 NCBI BlastP on this gene

EEY22939

conserved hypothetical protein
  
Accession: EEY22940
  
Location: 285610-287045
  
  
**BlastP hit with Mycgr3G92938\_Mycgr3T**
  
Percentage identity: 26 %
  
BlastP bit score: 108
  
Sequence coverage: 80 %
  
E-value: 3e-23
  
  
 NCBI BlastP on this gene

EEY22940

SCF E3 ubiquitin ligase complex F-box protein grrA
  
Accession: EEY22941
  
Location: 288762-291254
  
 NCBI BlastP on this gene

EEY22941

glutaredoxin
  
Accession: EEY22942
  
Location: 295712-296183
  
 NCBI BlastP on this gene

EEY22942

BAG domain-containing protein
  
Accession: EEY22943
  
Location: 296594-299081
  
 NCBI BlastP on this gene

EEY22943

60S ribosomal protein L16
  
Accession: EEY22944
  
Location: 300155-301294
  
 NCBI BlastP on this gene

EEY22944

hydrolase
  
Accession: EEY22945
  
Location: 302099-303127
  
 NCBI BlastP on this gene

EEY22945

Query: Architecture Search FASTA input

CP003006 : Myceliophthora thermophila ATCC 42464 chromosome 5    Total score: 2.0     Cumulative Blast bit score: 1620

Hit cluster cross-links:

Mycgr3G85918 Mycgr3T
  
Location: 0-1602

Mycgr3G85918\_Mycgr3T

Mycgr3G42010 Mycgr3T
  
Location: 1702-8569

Mycgr3G42010\_Mycgr3T

Mycgr3G29582 Mycgr3T
  
Location: 8669-8915

Mycgr3G29582\_Mycgr3T

Mycgr3G31170 Mycgr3T
  
Location: 9015-9255

Mycgr3G31170\_Mycgr3T

Mycgr3G85924 Mycgr3T
  
Location: 9355-11218

Mycgr3G85924\_Mycgr3T

Mycgr3G71676 Mycgr3T
  
Location: 11318-12494

Mycgr3G71676\_Mycgr3T

Mycgr3G11468 Mycgr3T
  
Location: 12594-13653

Mycgr3G11468\_Mycgr3T

Mycgr3G58567 Mycgr3T
  
Location: 13753-14506

Mycgr3G58567\_Mycgr3T

Mycgr3G100089 Mycgr3
  
Location: 14606-21152

Mycgr3G100089\_Mycgr3

Mycgr3G42698 Mycgr3T
  
Location: 21252-22131

Mycgr3G42698\_Mycgr3T

Mycgr3G71681 Mycgr3T
  
Location: 22231-23461

Mycgr3G71681\_Mycgr3T

Mycgr3G109328 Mycgr3
  
Location: 23561-24239

Mycgr3G109328\_Mycgr3

Mycgr3G104334 Mycgr3
  
Location: 24339-24567

Mycgr3G104334\_Mycgr3

Mycgr3G42715 Mycgr3T
  
Location: 24667-25981

Mycgr3G42715\_Mycgr3T

Mycgr3G92934 Mycgr3T
  
Location: 26081-27593

Mycgr3G92934\_Mycgr3T

Mycgr3G41969 Mycgr3T
  
Location: 27693-29328

Mycgr3G41969\_Mycgr3T

Mycgr3G80635 Mycgr3T
  
Location: 29428-29821

Mycgr3G80635\_Mycgr3T

Mycgr3G41426 Mycgr3T
  
Location: 29921-35255

Mycgr3G41426\_Mycgr3T

Mycgr3G104337 Mycgr3
  
Location: 35355-36108

Mycgr3G104337\_Mycgr3

Mycgr3G71679 Mycgr3T
  
Location: 36208-37300

Mycgr3G71679\_Mycgr3T

Mycgr3G92938 Mycgr3T
  
Location: 37400-38699

Mycgr3G92938\_Mycgr3T

Mycgr3G92941 Mycgr3T
  
Location: 38799-40734

Mycgr3G92941\_Mycgr3T

hypothetical protein
  
Accession: AEO60432
  
Location: 4224330-4224798
  
 NCBI BlastP on this gene

MYCTH\_2129532

hypothetical protein
  
Accession: AEO60433
  
Location: 4225839-4227517
  
 NCBI BlastP on this gene

MYCTH\_2309630

hypothetical protein
  
Accession: AEO60434
  
Location: 4228877-4232972
  
 NCBI BlastP on this gene

MYCTH\_2309634

hypothetical protein
  
Accession: AEO60435
  
Location: 4234592-4236469
  
 NCBI BlastP on this gene

MYCTH\_2309636

hypothetical protein
  
Accession: AEO60436
  
Location: 4240613-4241545
  
 NCBI BlastP on this gene

MYCTH\_2309637

hypothetical protein
  
Accession: AEO60437
  
Location: 4241930-4242937
  
  
**BlastP hit with Mycgr3G104337\_Mycgr3**
  
Percentage identity: 40 %
  
BlastP bit score: 184
  
Sequence coverage: 93 %
  
E-value: 4e-53
  
  
 NCBI BlastP on this gene

MYCTH\_54987

polyketide synthase
  
Accession: AEO60438
  
Location: 4244361-4251153
  
  
**BlastP hit with Mycgr3G100089\_Mycgr3**
  
Percentage identity: 38 %
  
BlastP bit score: 1436
  
Sequence coverage: 102 %
  
E-value: 0.0
  
  
 NCBI BlastP on this gene

MYCTH\_103061

hypothetical protein
  
Accession: AEO60439
  
Location: 4252450-4254451
  
 NCBI BlastP on this gene

MYCTH\_2309641

hypothetical protein
  
Accession: AEO60440
  
Location: 4255049-4255991
  
 NCBI BlastP on this gene

MYCTH\_2309644

hypothetical protein
  
Accession: AEO60441
  
Location: 4256449-4257299
  
 NCBI BlastP on this gene

MYCTH\_2316112

hypothetical protein
  
Accession: AEO60442
  
Location: 4258397-4260525
  
 NCBI BlastP on this gene

MYCTH\_2309647

glycosyltransferase family 2 protein
  
Accession: AEO60443
  
Location: 4263729-4265552
  
 NCBI BlastP on this gene

MYCTH\_10713

glycosyltransferase family 31 protein
  
Accession: AEO60444
  
Location: 4266718-4268287
  
 NCBI BlastP on this gene

MYCTH\_2309651

Query: Architecture Search FASTA input

KB707649 : Eutypa lata UCREL1 unplaced genomic scaffold EL1\_03\_scaffold\_2311    Total score: 2.0     Cumulative Blast bit score: 1618

Hit cluster cross-links:

Mycgr3G85918 Mycgr3T
  
Location: 0-1602

Mycgr3G85918\_Mycgr3T

Mycgr3G42010 Mycgr3T
  
Location: 1702-8569

Mycgr3G42010\_Mycgr3T

Mycgr3G29582 Mycgr3T
  
Location: 8669-8915

Mycgr3G29582\_Mycgr3T

Mycgr3G31170 Mycgr3T
  
Location: 9015-9255

Mycgr3G31170\_Mycgr3T

Mycgr3G85924 Mycgr3T
  
Location: 9355-11218

Mycgr3G85924\_Mycgr3T

Mycgr3G71676 Mycgr3T
  
Location: 11318-12494

Mycgr3G71676\_Mycgr3T

Mycgr3G11468 Mycgr3T
  
Location: 12594-13653

Mycgr3G11468\_Mycgr3T

Mycgr3G58567 Mycgr3T
  
Location: 13753-14506

Mycgr3G58567\_Mycgr3T

Mycgr3G100089 Mycgr3
  
Location: 14606-21152

Mycgr3G100089\_Mycgr3

Mycgr3G42698 Mycgr3T
  
Location: 21252-22131

Mycgr3G42698\_Mycgr3T

Mycgr3G71681 Mycgr3T
  
Location: 22231-23461

Mycgr3G71681\_Mycgr3T

Mycgr3G109328 Mycgr3
  
Location: 23561-24239

Mycgr3G109328\_Mycgr3

Mycgr3G104334 Mycgr3
  
Location: 24339-24567

Mycgr3G104334\_Mycgr3

Mycgr3G42715 Mycgr3T
  
Location: 24667-25981

Mycgr3G42715\_Mycgr3T

Mycgr3G92934 Mycgr3T
  
Location: 26081-27593

Mycgr3G92934\_Mycgr3T

Mycgr3G41969 Mycgr3T
  
Location: 27693-29328

Mycgr3G41969\_Mycgr3T

Mycgr3G80635 Mycgr3T
  
Location: 29428-29821

Mycgr3G80635\_Mycgr3T

Mycgr3G41426 Mycgr3T
  
Location: 29921-35255

Mycgr3G41426\_Mycgr3T

Mycgr3G104337 Mycgr3
  
Location: 35355-36108

Mycgr3G104337\_Mycgr3

Mycgr3G71679 Mycgr3T
  
Location: 36208-37300

Mycgr3G71679\_Mycgr3T

Mycgr3G92938 Mycgr3T
  
Location: 37400-38699

Mycgr3G92938\_Mycgr3T

Mycgr3G92941 Mycgr3T
  
Location: 38799-40734

Mycgr3G92941\_Mycgr3T

putative cytochrome p450 protein
  
Accession: EMR61340
  
Location: 92-1763
  
 NCBI BlastP on this gene

EMR61340

putative pectinesterase precursor protein
  
Accession: EMR61338
  
Location: 3061-4129
  
 NCBI BlastP on this gene

EMR61338

putative methyltransferase type 11 protein
  
Accession: EMR61348
  
Location: 6678-7544
  
 NCBI BlastP on this gene

EMR61348

putative cytochrome p450 protein
  
Accession: EMR61346
  
Location: 7947-9158
  
 NCBI BlastP on this gene

EMR61346

putative polyketide synthase protein
  
Accession: EMR61344
  
Location: 10824-17766
  
  
**BlastP hit with Mycgr3G100089\_Mycgr3**
  
Percentage identity: 41 %
  
BlastP bit score: 1443
  
Sequence coverage: 89 %
  
E-value: 0.0
  
  
 NCBI BlastP on this gene

EMR61344

putative enoyl- hydratase isomerase family protein
  
Accession: EMR61341
  
Location: 18276-19583
  
 NCBI BlastP on this gene

EMR61341

putative ef-hand calcium-binding domain protein
  
Accession: EMR61339
  
Location: 20129-21010
  
  
**BlastP hit with Mycgr3G104337\_Mycgr3**
  
Percentage identity: 36 %
  
BlastP bit score: 175
  
Sequence coverage: 96 %
  
E-value: 4e-50
  
  
 NCBI BlastP on this gene

EMR61339

putative capsule polysaccharide biosynthesis protein
  
Accession: EMR61347
  
Location: 21600-22832
  
 NCBI BlastP on this gene

EMR61347

putative -like methyltransferase protein
  
Accession: EMR61345
  
Location: 23914-24869
  
 NCBI BlastP on this gene

EMR61345

putative poly(aspartic acid) hydrolase protein
  
Accession: EMR61342
  
Location: 30184-31236
  
 NCBI BlastP on this gene

EMR61342

Query: Architecture Search FASTA input

GL537139 : Pyrenophora teres f. teres 0-1 unplaced genomic scaffold scaffold\_193442    Total score: 2.0     Cumulative Blast bit score: 1525

Hit cluster cross-links:

Mycgr3G85918 Mycgr3T
  
Location: 0-1602

Mycgr3G85918\_Mycgr3T

Mycgr3G42010 Mycgr3T
  
Location: 1702-8569

Mycgr3G42010\_Mycgr3T

Mycgr3G29582 Mycgr3T
  
Location: 8669-8915

Mycgr3G29582\_Mycgr3T

Mycgr3G31170 Mycgr3T
  
Location: 9015-9255

Mycgr3G31170\_Mycgr3T

Mycgr3G85924 Mycgr3T
  
Location: 9355-11218

Mycgr3G85924\_Mycgr3T

Mycgr3G71676 Mycgr3T
  
Location: 11318-12494

Mycgr3G71676\_Mycgr3T

Mycgr3G11468 Mycgr3T
  
Location: 12594-13653

Mycgr3G11468\_Mycgr3T

Mycgr3G58567 Mycgr3T
  
Location: 13753-14506

Mycgr3G58567\_Mycgr3T

Mycgr3G100089 Mycgr3
  
Location: 14606-21152

Mycgr3G100089\_Mycgr3

Mycgr3G42698 Mycgr3T
  
Location: 21252-22131

Mycgr3G42698\_Mycgr3T

Mycgr3G71681 Mycgr3T
  
Location: 22231-23461

Mycgr3G71681\_Mycgr3T

Mycgr3G109328 Mycgr3
  
Location: 23561-24239

Mycgr3G109328\_Mycgr3

Mycgr3G104334 Mycgr3
  
Location: 24339-24567

Mycgr3G104334\_Mycgr3

Mycgr3G42715 Mycgr3T
  
Location: 24667-25981

Mycgr3G42715\_Mycgr3T

Mycgr3G92934 Mycgr3T
  
Location: 26081-27593

Mycgr3G92934\_Mycgr3T

Mycgr3G41969 Mycgr3T
  
Location: 27693-29328

Mycgr3G41969\_Mycgr3T

Mycgr3G80635 Mycgr3T
  
Location: 29428-29821

Mycgr3G80635\_Mycgr3T

Mycgr3G41426 Mycgr3T
  
Location: 29921-35255

Mycgr3G41426\_Mycgr3T

Mycgr3G104337 Mycgr3
  
Location: 35355-36108

Mycgr3G104337\_Mycgr3

Mycgr3G71679 Mycgr3T
  
Location: 36208-37300

Mycgr3G71679\_Mycgr3T

Mycgr3G92938 Mycgr3T
  
Location: 37400-38699

Mycgr3G92938\_Mycgr3T

Mycgr3G92941 Mycgr3T
  
Location: 38799-40734

Mycgr3G92941\_Mycgr3T

hypothetical protein
  
Accession: EFQ87089
  
Location: 14592-15452
  
  
**BlastP hit with Mycgr3G31170\_Mycgr3T**
  
Percentage identity: 100 %
  
BlastP bit score: 166
  
Sequence coverage: 100 %
  
E-value: 1e-49
  
  
 NCBI BlastP on this gene

EFQ87089

hypothetical protein
  
Accession: EFQ87088
  
Location: 10210-12071
  
 NCBI BlastP on this gene

EFQ87088

hypothetical protein
  
Accession: EFQ87087
  
Location: 3363-9317
  
  
**BlastP hit with Mycgr3G41426\_Mycgr3T**
  
Percentage identity: 40 %
  
BlastP bit score: 1359
  
Sequence coverage: 102 %
  
E-value: 0.0
  
  
 NCBI BlastP on this gene

EFQ87087

hypothetical protein
  
Accession: EFQ87086
  
Location: 31-1932
  
 NCBI BlastP on this gene

EFQ87086

Query: Architecture Search FASTA input

GL533442 : Pyrenophora teres f. teres 0-1 unplaced genomic scaffold scaffold\_189679    Total score: 2.0     Cumulative Blast bit score: 1525

Hit cluster cross-links:

Mycgr3G85918 Mycgr3T
  
Location: 0-1602

Mycgr3G85918\_Mycgr3T

Mycgr3G42010 Mycgr3T
  
Location: 1702-8569

Mycgr3G42010\_Mycgr3T

Mycgr3G29582 Mycgr3T
  
Location: 8669-8915

Mycgr3G29582\_Mycgr3T

Mycgr3G31170 Mycgr3T
  
Location: 9015-9255

Mycgr3G31170\_Mycgr3T

Mycgr3G85924 Mycgr3T
  
Location: 9355-11218

Mycgr3G85924\_Mycgr3T

Mycgr3G71676 Mycgr3T
  
Location: 11318-12494

Mycgr3G71676\_Mycgr3T

Mycgr3G11468 Mycgr3T
  
Location: 12594-13653

Mycgr3G11468\_Mycgr3T

Mycgr3G58567 Mycgr3T
  
Location: 13753-14506

Mycgr3G58567\_Mycgr3T

Mycgr3G100089 Mycgr3
  
Location: 14606-21152

Mycgr3G100089\_Mycgr3

Mycgr3G42698 Mycgr3T
  
Location: 21252-22131

Mycgr3G42698\_Mycgr3T

Mycgr3G71681 Mycgr3T
  
Location: 22231-23461

Mycgr3G71681\_Mycgr3T

Mycgr3G109328 Mycgr3
  
Location: 23561-24239

Mycgr3G109328\_Mycgr3

Mycgr3G104334 Mycgr3
  
Location: 24339-24567

Mycgr3G104334\_Mycgr3

Mycgr3G42715 Mycgr3T
  
Location: 24667-25981

Mycgr3G42715\_Mycgr3T

Mycgr3G92934 Mycgr3T
  
Location: 26081-27593

Mycgr3G92934\_Mycgr3T

Mycgr3G41969 Mycgr3T
  
Location: 27693-29328

Mycgr3G41969\_Mycgr3T

Mycgr3G80635 Mycgr3T
  
Location: 29428-29821

Mycgr3G80635\_Mycgr3T

Mycgr3G41426 Mycgr3T
  
Location: 29921-35255

Mycgr3G41426\_Mycgr3T

Mycgr3G104337 Mycgr3
  
Location: 35355-36108

Mycgr3G104337\_Mycgr3

Mycgr3G71679 Mycgr3T
  
Location: 36208-37300

Mycgr3G71679\_Mycgr3T

Mycgr3G92938 Mycgr3T
  
Location: 37400-38699

Mycgr3G92938\_Mycgr3T

Mycgr3G92941 Mycgr3T
  
Location: 38799-40734

Mycgr3G92941\_Mycgr3T

hypothetical protein
  
Accession: EFQ94251
  
Location: 24060-24920
  
  
**BlastP hit with Mycgr3G31170\_Mycgr3T**
  
Percentage identity: 100 %
  
BlastP bit score: 166
  
Sequence coverage: 100 %
  
E-value: 1e-49
  
  
 NCBI BlastP on this gene

EFQ94251

hypothetical protein
  
Accession: EFQ94250
  
Location: 19678-21539
  
 NCBI BlastP on this gene

EFQ94250

hypothetical protein
  
Accession: EFQ94249
  
Location: 12831-18785
  
  
**BlastP hit with Mycgr3G41426\_Mycgr3T**
  
Percentage identity: 40 %
  
BlastP bit score: 1359
  
Sequence coverage: 102 %
  
E-value: 0.0
  
  
 NCBI BlastP on this gene

EFQ94249

hypothetical protein
  
Accession: EFQ94248
  
Location: 9220-11400
  
 NCBI BlastP on this gene

EFQ94248

hypothetical protein
  
Accession: EFQ94247
  
Location: 8089-8655
  
 NCBI BlastP on this gene

EFQ94247

hypothetical protein
  
Accession: EFQ94246
  
Location: 5793-7694
  
 NCBI BlastP on this gene

EFQ94246

hypothetical protein
  
Accession: EFQ94245
  
Location: 4696-5520
  
 NCBI BlastP on this gene

EFQ94245

hypothetical protein
  
Accession: EFQ94244
  
Location: 3612-4427
  
 NCBI BlastP on this gene

EFQ94244

hypothetical protein
  
Accession: EFQ94243
  
Location: 1518-3133
  
 NCBI BlastP on this gene

EFQ94243

hypothetical protein
  
Accession: EFQ94242
  
Location: 208-1368
  
 NCBI BlastP on this gene

EFQ94242

Query: Architecture Search FASTA input

CP003011 : Thielavia terrestris NRRL 8126 chromosome 3    Total score: 2.0     Cumulative Blast bit score: 1512

Hit cluster cross-links:

Mycgr3G85918 Mycgr3T
  
Location: 0-1602

Mycgr3G85918\_Mycgr3T

Mycgr3G42010 Mycgr3T
  
Location: 1702-8569

Mycgr3G42010\_Mycgr3T

Mycgr3G29582 Mycgr3T
  
Location: 8669-8915

Mycgr3G29582\_Mycgr3T

Mycgr3G31170 Mycgr3T
  
Location: 9015-9255

Mycgr3G31170\_Mycgr3T

Mycgr3G85924 Mycgr3T
  
Location: 9355-11218

Mycgr3G85924\_Mycgr3T

Mycgr3G71676 Mycgr3T
  
Location: 11318-12494

Mycgr3G71676\_Mycgr3T

Mycgr3G11468 Mycgr3T
  
Location: 12594-13653

Mycgr3G11468\_Mycgr3T

Mycgr3G58567 Mycgr3T
  
Location: 13753-14506

Mycgr3G58567\_Mycgr3T

Mycgr3G100089 Mycgr3
  
Location: 14606-21152

Mycgr3G100089\_Mycgr3

Mycgr3G42698 Mycgr3T
  
Location: 21252-22131

Mycgr3G42698\_Mycgr3T

Mycgr3G71681 Mycgr3T
  
Location: 22231-23461

Mycgr3G71681\_Mycgr3T

Mycgr3G109328 Mycgr3
  
Location: 23561-24239

Mycgr3G109328\_Mycgr3

Mycgr3G104334 Mycgr3
  
Location: 24339-24567

Mycgr3G104334\_Mycgr3

Mycgr3G42715 Mycgr3T
  
Location: 24667-25981

Mycgr3G42715\_Mycgr3T

Mycgr3G92934 Mycgr3T
  
Location: 26081-27593

Mycgr3G92934\_Mycgr3T

Mycgr3G41969 Mycgr3T
  
Location: 27693-29328

Mycgr3G41969\_Mycgr3T

Mycgr3G80635 Mycgr3T
  
Location: 29428-29821

Mycgr3G80635\_Mycgr3T

Mycgr3G41426 Mycgr3T
  
Location: 29921-35255

Mycgr3G41426\_Mycgr3T

Mycgr3G104337 Mycgr3
  
Location: 35355-36108

Mycgr3G104337\_Mycgr3

Mycgr3G71679 Mycgr3T
  
Location: 36208-37300

Mycgr3G71679\_Mycgr3T

Mycgr3G92938 Mycgr3T
  
Location: 37400-38699

Mycgr3G92938\_Mycgr3T

Mycgr3G92941 Mycgr3T
  
Location: 38799-40734

Mycgr3G92941\_Mycgr3T

hypothetical protein
  
Accession: AEO67426
  
Location: 110647-112697
  
 NCBI BlastP on this gene

THITE\_2116314

polyketide synthase
  
Accession: AEO67427
  
Location: 113172-120431
  
  
**BlastP hit with Mycgr3G100089\_Mycgr3**
  
Percentage identity: 36 %
  
BlastP bit score: 1318
  
Sequence coverage: 102 %
  
E-value: 0.0
  
  
 NCBI BlastP on this gene

THITE\_132390

hypothetical protein
  
Accession: AEO67428
  
Location: 121100-122115
  
  
**BlastP hit with Mycgr3G104337\_Mycgr3**
  
Percentage identity: 40 %
  
BlastP bit score: 194
  
Sequence coverage: 100 %
  
E-value: 3e-57
  
  
 NCBI BlastP on this gene

THITE\_125661

hypothetical protein
  
Accession: AEO67429
  
Location: 123526-123883
  
 NCBI BlastP on this gene

THITE\_2088916

hypothetical protein
  
Accession: AEO67430
  
Location: 123944-127096
  
 NCBI BlastP on this gene

THITE\_2144801

hypothetical protein
  
Accession: AEO67431
  
Location: 129543-130997
  
 NCBI BlastP on this gene

THITE\_2050923

hypothetical protein
  
Accession: AEO67432
  
Location: 132518-133794
  
 NCBI BlastP on this gene

THITE\_2088919

Query: Architecture Search FASTA input

CH408029 : Chaetomium globosum CBS 148.51 scaffold\_1 genomic scaffold    Total score: 2.0     Cumulative Blast bit score: 1506

Hit cluster cross-links:

Mycgr3G85918 Mycgr3T
  
Location: 0-1602

Mycgr3G85918\_Mycgr3T

Mycgr3G42010 Mycgr3T
  
Location: 1702-8569

Mycgr3G42010\_Mycgr3T

Mycgr3G29582 Mycgr3T
  
Location: 8669-8915

Mycgr3G29582\_Mycgr3T

Mycgr3G31170 Mycgr3T
  
Location: 9015-9255

Mycgr3G31170\_Mycgr3T

Mycgr3G85924 Mycgr3T
  
Location: 9355-11218

Mycgr3G85924\_Mycgr3T

Mycgr3G71676 Mycgr3T
  
Location: 11318-12494

Mycgr3G71676\_Mycgr3T

Mycgr3G11468 Mycgr3T
  
Location: 12594-13653

Mycgr3G11468\_Mycgr3T

Mycgr3G58567 Mycgr3T
  
Location: 13753-14506

Mycgr3G58567\_Mycgr3T

Mycgr3G100089 Mycgr3
  
Location: 14606-21152

Mycgr3G100089\_Mycgr3

Mycgr3G42698 Mycgr3T
  
Location: 21252-22131

Mycgr3G42698\_Mycgr3T

Mycgr3G71681 Mycgr3T
  
Location: 22231-23461

Mycgr3G71681\_Mycgr3T

Mycgr3G109328 Mycgr3
  
Location: 23561-24239

Mycgr3G109328\_Mycgr3

Mycgr3G104334 Mycgr3
  
Location: 24339-24567

Mycgr3G104334\_Mycgr3

Mycgr3G42715 Mycgr3T
  
Location: 24667-25981

Mycgr3G42715\_Mycgr3T

Mycgr3G92934 Mycgr3T
  
Location: 26081-27593

Mycgr3G92934\_Mycgr3T

Mycgr3G41969 Mycgr3T
  
Location: 27693-29328

Mycgr3G41969\_Mycgr3T

Mycgr3G80635 Mycgr3T
  
Location: 29428-29821

Mycgr3G80635\_Mycgr3T

Mycgr3G41426 Mycgr3T
  
Location: 29921-35255

Mycgr3G41426\_Mycgr3T

Mycgr3G104337 Mycgr3
  
Location: 35355-36108

Mycgr3G104337\_Mycgr3

Mycgr3G71679 Mycgr3T
  
Location: 36208-37300

Mycgr3G71679\_Mycgr3T

Mycgr3G92938 Mycgr3T
  
Location: 37400-38699

Mycgr3G92938\_Mycgr3T

Mycgr3G92941 Mycgr3T
  
Location: 38799-40734

Mycgr3G92941\_Mycgr3T

hypothetical protein
  
Accession: EAQ91803
  
Location: 107127-108854
  
 NCBI BlastP on this gene

EAQ91803

hypothetical protein
  
Accession: EAQ91804
  
Location: 110092-112068
  
 NCBI BlastP on this gene

EAQ91804

hypothetical protein
  
Accession: EAQ91805
  
Location: 112620-114191
  
 NCBI BlastP on this gene

EAQ91805

hypothetical protein
  
Accession: EAQ91806
  
Location: 114718-118048
  
 NCBI BlastP on this gene

EAQ91806

hypothetical protein
  
Accession: EAQ91807
  
Location: 118404-120302
  
 NCBI BlastP on this gene

EAQ91807

predicted protein
  
Accession: EAQ91808
  
Location: 122121-122777
  
 NCBI BlastP on this gene

EAQ91808

hypothetical protein
  
Accession: EAQ91809
  
Location: 123217-124699
  
 NCBI BlastP on this gene

EAQ91809

hypothetical protein
  
Accession: EAQ91810
  
Location: 125085-127158
  
  
**BlastP hit with Mycgr3G104337\_Mycgr3**
  
Percentage identity: 37 %
  
BlastP bit score: 179
  
Sequence coverage: 100 %
  
E-value: 1e-49
  
  
 NCBI BlastP on this gene

EAQ91810

hypothetical protein
  
Accession: EAQ91811
  
Location: 127830-134360
  
  
**BlastP hit with Mycgr3G100089\_Mycgr3**
  
Percentage identity: 37 %
  
BlastP bit score: 1327
  
Sequence coverage: 101 %
  
E-value: 0.0
  
  
 NCBI BlastP on this gene

EAQ91811

NADH-ubiquinone oxidoreductase 23 kDa subunit
  
Accession: EAQ91812
  
Location: 137783-138679
  
 NCBI BlastP on this gene

EAQ91812

hypothetical protein
  
Accession: EAQ91813
  
Location: 139279-140139
  
 NCBI BlastP on this gene

EAQ91813

hypothetical protein
  
Accession: EAQ91814
  
Location: 140868-142977
  
 NCBI BlastP on this gene

EAQ91814

hypothetical protein
  
Accession: EAQ91815
  
Location: 144521-146376
  
 NCBI BlastP on this gene

EAQ91815

hypothetical protein
  
Accession: EAQ91816
  
Location: 147108-148693
  
 NCBI BlastP on this gene

EAQ91816

hypothetical protein
  
Accession: EAQ91817
  
Location: 151188-152356
  
 NCBI BlastP on this gene

EAQ91817

Query: Architecture Search FASTA input

DS231619 : Pyrenophora tritici-repentis Pt-1C-BFP supercont1.5 genomic scaffold    Total score: 2.0     Cumulative Blast bit score: 1500

Hit cluster cross-links:

Mycgr3G85918 Mycgr3T
  
Location: 0-1602

Mycgr3G85918\_Mycgr3T

Mycgr3G42010 Mycgr3T
  
Location: 1702-8569

Mycgr3G42010\_Mycgr3T

Mycgr3G29582 Mycgr3T
  
Location: 8669-8915

Mycgr3G29582\_Mycgr3T

Mycgr3G31170 Mycgr3T
  
Location: 9015-9255

Mycgr3G31170\_Mycgr3T

Mycgr3G85924 Mycgr3T
  
Location: 9355-11218

Mycgr3G85924\_Mycgr3T

Mycgr3G71676 Mycgr3T
  
Location: 11318-12494

Mycgr3G71676\_Mycgr3T

Mycgr3G11468 Mycgr3T
  
Location: 12594-13653

Mycgr3G11468\_Mycgr3T

Mycgr3G58567 Mycgr3T
  
Location: 13753-14506

Mycgr3G58567\_Mycgr3T

Mycgr3G100089 Mycgr3
  
Location: 14606-21152

Mycgr3G100089\_Mycgr3

Mycgr3G42698 Mycgr3T
  
Location: 21252-22131

Mycgr3G42698\_Mycgr3T

Mycgr3G71681 Mycgr3T
  
Location: 22231-23461

Mycgr3G71681\_Mycgr3T

Mycgr3G109328 Mycgr3
  
Location: 23561-24239

Mycgr3G109328\_Mycgr3

Mycgr3G104334 Mycgr3
  
Location: 24339-24567

Mycgr3G104334\_Mycgr3

Mycgr3G42715 Mycgr3T
  
Location: 24667-25981

Mycgr3G42715\_Mycgr3T

Mycgr3G92934 Mycgr3T
  
Location: 26081-27593

Mycgr3G92934\_Mycgr3T

Mycgr3G41969 Mycgr3T
  
Location: 27693-29328

Mycgr3G41969\_Mycgr3T

Mycgr3G80635 Mycgr3T
  
Location: 29428-29821

Mycgr3G80635\_Mycgr3T

Mycgr3G41426 Mycgr3T
  
Location: 29921-35255

Mycgr3G41426\_Mycgr3T

Mycgr3G104337 Mycgr3
  
Location: 35355-36108

Mycgr3G104337\_Mycgr3

Mycgr3G71679 Mycgr3T
  
Location: 36208-37300

Mycgr3G71679\_Mycgr3T

Mycgr3G92938 Mycgr3T
  
Location: 37400-38699

Mycgr3G92938\_Mycgr3T

Mycgr3G92941 Mycgr3T
  
Location: 38799-40734

Mycgr3G92941\_Mycgr3T

predicted protein
  
Accession: EDU48270
  
Location: 170417-171253
  
 NCBI BlastP on this gene

EDU48270

phospholipid-translocating P-type ATPase domain containing protein
  
Accession: EDU48271
  
Location: 172508-177295
  
 NCBI BlastP on this gene

EDU48271

predicted protein
  
Accession: EDU48272
  
Location: 178706-181019
  
 NCBI BlastP on this gene

EDU48272

tetraspanin
  
Accession: EDU48273
  
Location: 183713-184634
  
 NCBI BlastP on this gene

EDU48273

MADS box transcription factor Mcm1
  
Accession: EDU48274
  
Location: 186836-187667
  
  
**BlastP hit with Mycgr3G31170\_Mycgr3T**
  
Percentage identity: 100 %
  
BlastP bit score: 166
  
Sequence coverage: 100 %
  
E-value: 1e-49
  
  
 NCBI BlastP on this gene

EDU48274

choline transport protein
  
Accession: EDU48275
  
Location: 190148-192002
  
 NCBI BlastP on this gene

EDU48275

DNA-binding protein SMUBP-2
  
Accession: EDU48276
  
Location: 192904-198819
  
  
**BlastP hit with Mycgr3G41426\_Mycgr3T**
  
Percentage identity: 40 %
  
BlastP bit score: 1334
  
Sequence coverage: 102 %
  
E-value: 0.0
  
  
 NCBI BlastP on this gene

EDU48276

conserved hypothetical protein
  
Accession: EDU48277
  
Location: 200196-202409
  
 NCBI BlastP on this gene

EDU48277

glucosamine 6-phosphate N-acetyltransferase
  
Accession: EDU48278
  
Location: 202981-203545
  
 NCBI BlastP on this gene

EDU48278

conserved hypothetical protein
  
Accession: EDU48279
  
Location: 203967-205877
  
 NCBI BlastP on this gene

EDU48279

40S ribosomal protein S10
  
Accession: EDU48280
  
Location: 206151-206975
  
 NCBI BlastP on this gene

EDU48280

conserved hypothetical protein
  
Accession: EDU48281
  
Location: 207248-208063
  
 NCBI BlastP on this gene

EDU48281

N-carbamoyl-L-amino acid hydrolase
  
Accession: EDU48282
  
Location: 208543-210156
  
 NCBI BlastP on this gene

EDU48282

phospholipase
  
Accession: EDU48283
  
Location: 211989-213773
  
 NCBI BlastP on this gene

EDU48283

conserved hypothetical protein
  
Accession: EDU48284
  
Location: 214398-214943
  
 NCBI BlastP on this gene

EDU48284

conserved hypothetical protein
  
Accession: EDU48285
  
Location: 215148-216611
  
 NCBI BlastP on this gene

EDU48285

Query: Architecture Search FASTA input

DS027698 : Neosartorya fischeri NRRL 181 1099437636266 genomic scaffold    Total score: 2.0     Cumulative Blast bit score: 1477

Hit cluster cross-links:

Mycgr3G85918 Mycgr3T
  
Location: 0-1602

Mycgr3G85918\_Mycgr3T

Mycgr3G42010 Mycgr3T
  
Location: 1702-8569

Mycgr3G42010\_Mycgr3T

Mycgr3G29582 Mycgr3T
  
Location: 8669-8915

Mycgr3G29582\_Mycgr3T

Mycgr3G31170 Mycgr3T
  
Location: 9015-9255

Mycgr3G31170\_Mycgr3T

Mycgr3G85924 Mycgr3T
  
Location: 9355-11218

Mycgr3G85924\_Mycgr3T

Mycgr3G71676 Mycgr3T
  
Location: 11318-12494

Mycgr3G71676\_Mycgr3T

Mycgr3G11468 Mycgr3T
  
Location: 12594-13653

Mycgr3G11468\_Mycgr3T

Mycgr3G58567 Mycgr3T
  
Location: 13753-14506

Mycgr3G58567\_Mycgr3T

Mycgr3G100089 Mycgr3
  
Location: 14606-21152

Mycgr3G100089\_Mycgr3

Mycgr3G42698 Mycgr3T
  
Location: 21252-22131

Mycgr3G42698\_Mycgr3T

Mycgr3G71681 Mycgr3T
  
Location: 22231-23461

Mycgr3G71681\_Mycgr3T

Mycgr3G109328 Mycgr3
  
Location: 23561-24239

Mycgr3G109328\_Mycgr3

Mycgr3G104334 Mycgr3
  
Location: 24339-24567

Mycgr3G104334\_Mycgr3

Mycgr3G42715 Mycgr3T
  
Location: 24667-25981

Mycgr3G42715\_Mycgr3T

Mycgr3G92934 Mycgr3T
  
Location: 26081-27593

Mycgr3G92934\_Mycgr3T

Mycgr3G41969 Mycgr3T
  
Location: 27693-29328

Mycgr3G41969\_Mycgr3T

Mycgr3G80635 Mycgr3T
  
Location: 29428-29821

Mycgr3G80635\_Mycgr3T

Mycgr3G41426 Mycgr3T
  
Location: 29921-35255

Mycgr3G41426\_Mycgr3T

Mycgr3G104337 Mycgr3
  
Location: 35355-36108

Mycgr3G104337\_Mycgr3

Mycgr3G71679 Mycgr3T
  
Location: 36208-37300

Mycgr3G71679\_Mycgr3T

Mycgr3G92938 Mycgr3T
  
Location: 37400-38699

Mycgr3G92938\_Mycgr3T

Mycgr3G92941 Mycgr3T
  
Location: 38799-40734

Mycgr3G92941\_Mycgr3T

Rab geranylgeranyl transferase escort protein, putative
  
Accession: EAW15497
  
Location: 366869-368762
  
 NCBI BlastP on this gene

EAW15497

conserved hypothetical protein
  
Accession: EAW15496
  
Location: 360462-362036
  
 NCBI BlastP on this gene

EAW15496

SRF-type transcription factor (Umc1), putative
  
Accession: EAW15495
  
Location: 357403-358305
  
  
**BlastP hit with Mycgr3G31170\_Mycgr3T**
  
Percentage identity: 98 %
  
BlastP bit score: 162
  
Sequence coverage: 100 %
  
E-value: 3e-48
  
  
 NCBI BlastP on this gene

EAW15495

conserved hypothetical protein
  
Accession: EAW15494
  
Location: 354247-356126
  
 NCBI BlastP on this gene

EAW15494

ATP synthase subunit E, putative
  
Accession: EAW15493
  
Location: 352873-353688
  
 NCBI BlastP on this gene

EAW15493

conserved hypothetical protein
  
Accession: EAW15492
  
Location: 349924-351829
  
 NCBI BlastP on this gene

EAW15492

phospholipid-translocating P-type ATPase domain-containing protein
  
Accession: EAW15491
  
Location: 341351-346052
  
 NCBI BlastP on this gene

EAW15491

tRNA-splicing endonuclease, putative
  
Accession: EAW15490
  
Location: 333714-340039
  
  
**BlastP hit with Mycgr3G41426\_Mycgr3T**
  
Percentage identity: 40 %
  
BlastP bit score: 1315
  
Sequence coverage: 103 %
  
E-value: 0.0
  
  
 NCBI BlastP on this gene

EAW15490

conserved hypothetical protein
  
Accession: EAW15489
  
Location: 327234-330045
  
 NCBI BlastP on this gene

EAW15489

conserved hypothetical protein
  
Accession: EAW15488
  
Location: 325852-326543
  
 NCBI BlastP on this gene

EAW15488

aminotransferase, putative
  
Accession: EAW15487
  
Location: 323906-325448
  
 NCBI BlastP on this gene

EAW15487

Query: Architecture Search FASTA input

DS499602 : Aspergillus fumigatus A1163 scf\_000009 genomic scaffold    Total score: 2.0     Cumulative Blast bit score: 1474

Hit cluster cross-links:

Mycgr3G85918 Mycgr3T
  
Location: 0-1602

Mycgr3G85918\_Mycgr3T

Mycgr3G42010 Mycgr3T
  
Location: 1702-8569

Mycgr3G42010\_Mycgr3T

Mycgr3G29582 Mycgr3T
  
Location: 8669-8915

Mycgr3G29582\_Mycgr3T

Mycgr3G31170 Mycgr3T
  
Location: 9015-9255

Mycgr3G31170\_Mycgr3T

Mycgr3G85924 Mycgr3T
  
Location: 9355-11218

Mycgr3G85924\_Mycgr3T

Mycgr3G71676 Mycgr3T
  
Location: 11318-12494

Mycgr3G71676\_Mycgr3T

Mycgr3G11468 Mycgr3T
  
Location: 12594-13653

Mycgr3G11468\_Mycgr3T

Mycgr3G58567 Mycgr3T
  
Location: 13753-14506

Mycgr3G58567\_Mycgr3T

Mycgr3G100089 Mycgr3
  
Location: 14606-21152

Mycgr3G100089\_Mycgr3

Mycgr3G42698 Mycgr3T
  
Location: 21252-22131

Mycgr3G42698\_Mycgr3T

Mycgr3G71681 Mycgr3T
  
Location: 22231-23461

Mycgr3G71681\_Mycgr3T

Mycgr3G109328 Mycgr3
  
Location: 23561-24239

Mycgr3G109328\_Mycgr3

Mycgr3G104334 Mycgr3
  
Location: 24339-24567

Mycgr3G104334\_Mycgr3

Mycgr3G42715 Mycgr3T
  
Location: 24667-25981

Mycgr3G42715\_Mycgr3T

Mycgr3G92934 Mycgr3T
  
Location: 26081-27593

Mycgr3G92934\_Mycgr3T

Mycgr3G41969 Mycgr3T
  
Location: 27693-29328

Mycgr3G41969\_Mycgr3T

Mycgr3G80635 Mycgr3T
  
Location: 29428-29821

Mycgr3G80635\_Mycgr3T

Mycgr3G41426 Mycgr3T
  
Location: 29921-35255

Mycgr3G41426\_Mycgr3T

Mycgr3G104337 Mycgr3
  
Location: 35355-36108

Mycgr3G104337\_Mycgr3

Mycgr3G71679 Mycgr3T
  
Location: 36208-37300

Mycgr3G71679\_Mycgr3T

Mycgr3G92938 Mycgr3T
  
Location: 37400-38699

Mycgr3G92938\_Mycgr3T

Mycgr3G92941 Mycgr3T
  
Location: 38799-40734

Mycgr3G92941\_Mycgr3T

Rab geranylgeranyl transferase escort protein, putative
  
Accession: EDP47769
  
Location: 936856-938751
  
 NCBI BlastP on this gene

EDP47769

conserved hypothetical protein
  
Accession: EDP47770
  
Location: 943484-945097
  
 NCBI BlastP on this gene

EDP47770

SRF-type transcription factor (Umc1), putative
  
Accession: EDP47771
  
Location: 947169-948069
  
  
**BlastP hit with Mycgr3G31170\_Mycgr3T**
  
Percentage identity: 98 %
  
BlastP bit score: 162
  
Sequence coverage: 100 %
  
E-value: 3e-48
  
  
 NCBI BlastP on this gene

EDP47771

DUF803 domain protein
  
Accession: EDP47772
  
Location: 949233-951228
  
 NCBI BlastP on this gene

EDP47772

ATP synthase subunit E, putative
  
Accession: EDP47773
  
Location: 951844-952603
  
 NCBI BlastP on this gene

EDP47773

hypothetical protein
  
Accession: EDP47774
  
Location: 953164-953646
  
 NCBI BlastP on this gene

EDP47774

phospholipid-translocating P-type ATPase domain-containing protein
  
Accession: EDP47775
  
Location: 959487-964188
  
 NCBI BlastP on this gene

EDP47775

tRNA-splicing endonuclease, putative
  
Accession: EDP47776
  
Location: 965476-971799
  
  
**BlastP hit with Mycgr3G41426\_Mycgr3T**
  
Percentage identity: 39 %
  
BlastP bit score: 1312
  
Sequence coverage: 103 %
  
E-value: 0.0
  
  
 NCBI BlastP on this gene

EDP47776

conserved hypothetical protein
  
Accession: EDP47777
  
Location: 975310-978100
  
 NCBI BlastP on this gene

EDP47777

conserved hypothetical protein
  
Accession: EDP47778
  
Location: 978842-979533
  
 NCBI BlastP on this gene

EDP47778

aminotransferase, putative
  
Accession: EDP47779
  
Location: 979951-981466
  
 NCBI BlastP on this gene

EDP47779

Query: Architecture Search FASTA input

AAHF01000012 : Aspergillus fumigatus Af293    Total score: 2.0     Cumulative Blast bit score: 1472

Hit cluster cross-links:

Mycgr3G85918 Mycgr3T
  
Location: 0-1602

Mycgr3G85918\_Mycgr3T

Mycgr3G42010 Mycgr3T
  
Location: 1702-8569

Mycgr3G42010\_Mycgr3T

Mycgr3G29582 Mycgr3T
  
Location: 8669-8915

Mycgr3G29582\_Mycgr3T

Mycgr3G31170 Mycgr3T
  
Location: 9015-9255

Mycgr3G31170\_Mycgr3T

Mycgr3G85924 Mycgr3T
  
Location: 9355-11218

Mycgr3G85924\_Mycgr3T

Mycgr3G71676 Mycgr3T
  
Location: 11318-12494

Mycgr3G71676\_Mycgr3T

Mycgr3G11468 Mycgr3T
  
Location: 12594-13653

Mycgr3G11468\_Mycgr3T

Mycgr3G58567 Mycgr3T
  
Location: 13753-14506

Mycgr3G58567\_Mycgr3T

Mycgr3G100089 Mycgr3
  
Location: 14606-21152

Mycgr3G100089\_Mycgr3

Mycgr3G42698 Mycgr3T
  
Location: 21252-22131

Mycgr3G42698\_Mycgr3T

Mycgr3G71681 Mycgr3T
  
Location: 22231-23461

Mycgr3G71681\_Mycgr3T

Mycgr3G109328 Mycgr3
  
Location: 23561-24239

Mycgr3G109328\_Mycgr3

Mycgr3G104334 Mycgr3
  
Location: 24339-24567

Mycgr3G104334\_Mycgr3

Mycgr3G42715 Mycgr3T
  
Location: 24667-25981

Mycgr3G42715\_Mycgr3T

Mycgr3G92934 Mycgr3T
  
Location: 26081-27593

Mycgr3G92934\_Mycgr3T

Mycgr3G41969 Mycgr3T
  
Location: 27693-29328

Mycgr3G41969\_Mycgr3T

Mycgr3G80635 Mycgr3T
  
Location: 29428-29821

Mycgr3G80635\_Mycgr3T

Mycgr3G41426 Mycgr3T
  
Location: 29921-35255

Mycgr3G41426\_Mycgr3T

Mycgr3G104337 Mycgr3
  
Location: 35355-36108

Mycgr3G104337\_Mycgr3

Mycgr3G71679 Mycgr3T
  
Location: 36208-37300

Mycgr3G71679\_Mycgr3T

Mycgr3G92938 Mycgr3T
  
Location: 37400-38699

Mycgr3G92938\_Mycgr3T

Mycgr3G92941 Mycgr3T
  
Location: 38799-40734

Mycgr3G92941\_Mycgr3T

Rab geranylgeranyl transferase escort protein, putative
  
Accession: EAL85826
  
Location: 925051-926946
  
 NCBI BlastP on this gene

EAL85826

conserved hypothetical protein
  
Accession: EAL85827
  
Location: 931656-933269
  
 NCBI BlastP on this gene

EAL85827

MADS box transcription factor Mcm1
  
Accession: EAL85828
  
Location: 935341-936241
  
  
**BlastP hit with Mycgr3G31170\_Mycgr3T**
  
Percentage identity: 98 %
  
BlastP bit score: 162
  
Sequence coverage: 100 %
  
E-value: 3e-48
  
  
 NCBI BlastP on this gene

EAL85828

DUF803 domain protein
  
Accession: EAL85829
  
Location: 937405-939400
  
 NCBI BlastP on this gene

EAL85829

ATP synthase subunit E, putative
  
Accession: EAL85830
  
Location: 940015-940774
  
 NCBI BlastP on this gene

EAL85830

hypothetical protein
  
Accession: EAL85831
  
Location: 941335-941817
  
 NCBI BlastP on this gene

EAL85831

phospholipid-translocating P-type ATPase domain-containing protein
  
Accession: EAL85832
  
Location: 947663-952364
  
 NCBI BlastP on this gene

EAL85832

tRNA-splicing endonuclease, putative
  
Accession: EAL85833
  
Location: 953652-959975
  
  
**BlastP hit with Mycgr3G41426\_Mycgr3T**
  
Percentage identity: 39 %
  
BlastP bit score: 1310
  
Sequence coverage: 103 %
  
E-value: 0.0
  
  
 NCBI BlastP on this gene

EAL85833

conserved hypothetical protein
  
Accession: EAL85834
  
Location: 963483-966273
  
 NCBI BlastP on this gene

EAL85834

conserved hypothetical protein
  
Accession: EAL85835
  
Location: 967015-967706
  
 NCBI BlastP on this gene

EAL85835

aminotransferase, putative
  
Accession: EAL85836
  
Location: 968124-969639
  
 NCBI BlastP on this gene

EAL85836

Query: Architecture Search FASTA input

AGUE01000275 : Glarea lozoyensis 74030    Total score: 2.0     Cumulative Blast bit score: 1464

Hit cluster cross-links:

Mycgr3G85918 Mycgr3T
  
Location: 0-1602

Mycgr3G85918\_Mycgr3T

Mycgr3G42010 Mycgr3T
  
Location: 1702-8569

Mycgr3G42010\_Mycgr3T

Mycgr3G29582 Mycgr3T
  
Location: 8669-8915

Mycgr3G29582\_Mycgr3T

Mycgr3G31170 Mycgr3T
  
Location: 9015-9255

Mycgr3G31170\_Mycgr3T

Mycgr3G85924 Mycgr3T
  
Location: 9355-11218

Mycgr3G85924\_Mycgr3T

Mycgr3G71676 Mycgr3T
  
Location: 11318-12494

Mycgr3G71676\_Mycgr3T

Mycgr3G11468 Mycgr3T
  
Location: 12594-13653

Mycgr3G11468\_Mycgr3T

Mycgr3G58567 Mycgr3T
  
Location: 13753-14506

Mycgr3G58567\_Mycgr3T

Mycgr3G100089 Mycgr3
  
Location: 14606-21152

Mycgr3G100089\_Mycgr3

Mycgr3G42698 Mycgr3T
  
Location: 21252-22131

Mycgr3G42698\_Mycgr3T

Mycgr3G71681 Mycgr3T
  
Location: 22231-23461

Mycgr3G71681\_Mycgr3T

Mycgr3G109328 Mycgr3
  
Location: 23561-24239

Mycgr3G109328\_Mycgr3

Mycgr3G104334 Mycgr3
  
Location: 24339-24567

Mycgr3G104334\_Mycgr3

Mycgr3G42715 Mycgr3T
  
Location: 24667-25981

Mycgr3G42715\_Mycgr3T

Mycgr3G92934 Mycgr3T
  
Location: 26081-27593

Mycgr3G92934\_Mycgr3T

Mycgr3G41969 Mycgr3T
  
Location: 27693-29328

Mycgr3G41969\_Mycgr3T

Mycgr3G80635 Mycgr3T
  
Location: 29428-29821

Mycgr3G80635\_Mycgr3T

Mycgr3G41426 Mycgr3T
  
Location: 29921-35255

Mycgr3G41426\_Mycgr3T

Mycgr3G104337 Mycgr3
  
Location: 35355-36108

Mycgr3G104337\_Mycgr3

Mycgr3G71679 Mycgr3T
  
Location: 36208-37300

Mycgr3G71679\_Mycgr3T

Mycgr3G92938 Mycgr3T
  
Location: 37400-38699

Mycgr3G92938\_Mycgr3T

Mycgr3G92941 Mycgr3T
  
Location: 38799-40734

Mycgr3G92941\_Mycgr3T

putative Ferric/cupric reductase transmembrane component 1
  
Accession: EHK96143
  
Location: 28237-30381
  
 NCBI BlastP on this gene

EHK96143

putative Sodium/potassium-transporting ATPase subunit alpha-1
  
Accession: EHK96144
  
Location: 32850-35504
  
 NCBI BlastP on this gene

EHK96144

putative Ribosomal RNA-processing protein 15
  
Accession: EHK96145
  
Location: 40634-42390
  
 NCBI BlastP on this gene

EHK96145

putative Signal recognition particle 54 kDa protein like protein
  
Accession: EHK96146
  
Location: 42464-44229
  
 NCBI BlastP on this gene

EHK96146

putative protein CbxX, chromosomal
  
Accession: EHK96147
  
Location: 46722-53955
  
  
**BlastP hit with Mycgr3G42010\_Mycgr3T**
  
Percentage identity: 50 %
  
BlastP bit score: 1226
  
Sequence coverage: 58 %
  
E-value: 0.0
  
  
 NCBI BlastP on this gene

EHK96147

hypothetical protein
  
Accession: EHK96148
  
Location: 55057-56094
  
  
**BlastP hit with Mycgr3G92938\_Mycgr3T**
  
Percentage identity: 37 %
  
BlastP bit score: 238
  
Sequence coverage: 83 %
  
E-value: 9e-71
  
  
 NCBI BlastP on this gene

EHK96148

hypothetical protein
  
Accession: EHK96149
  
Location: 56358-56928
  
 NCBI BlastP on this gene

EHK96149

putative Quinone oxidoreductase PIG3
  
Accession: EHK96150
  
Location: 62618-63069
  
 NCBI BlastP on this gene

EHK96150

putative Eukaryotic translation initiation factor 3 subunit I
  
Accession: EHK96151
  
Location: 63866-65098
  
 NCBI BlastP on this gene

EHK96151

putative C-1-tetrahydrofolate synthase, cytoplasmic
  
Accession: EHK96152
  
Location: 65970-68996
  
 NCBI BlastP on this gene

EHK96152

Query: Architecture Search FASTA input

GG698510 : Trichophyton tonsurans CBS 112818 genomic scaffold supercont1.34    Total score: 2.0     Cumulative Blast bit score: 1460

Hit cluster cross-links:

Mycgr3G85918 Mycgr3T
  
Location: 0-1602

Mycgr3G85918\_Mycgr3T

Mycgr3G42010 Mycgr3T
  
Location: 1702-8569

Mycgr3G42010\_Mycgr3T

Mycgr3G29582 Mycgr3T
  
Location: 8669-8915

Mycgr3G29582\_Mycgr3T

Mycgr3G31170 Mycgr3T
  
Location: 9015-9255

Mycgr3G31170\_Mycgr3T

Mycgr3G85924 Mycgr3T
  
Location: 9355-11218

Mycgr3G85924\_Mycgr3T

Mycgr3G71676 Mycgr3T
  
Location: 11318-12494

Mycgr3G71676\_Mycgr3T

Mycgr3G11468 Mycgr3T
  
Location: 12594-13653

Mycgr3G11468\_Mycgr3T

Mycgr3G58567 Mycgr3T
  
Location: 13753-14506

Mycgr3G58567\_Mycgr3T

Mycgr3G100089 Mycgr3
  
Location: 14606-21152

Mycgr3G100089\_Mycgr3

Mycgr3G42698 Mycgr3T
  
Location: 21252-22131

Mycgr3G42698\_Mycgr3T

Mycgr3G71681 Mycgr3T
  
Location: 22231-23461

Mycgr3G71681\_Mycgr3T

Mycgr3G109328 Mycgr3
  
Location: 23561-24239

Mycgr3G109328\_Mycgr3

Mycgr3G104334 Mycgr3
  
Location: 24339-24567

Mycgr3G104334\_Mycgr3

Mycgr3G42715 Mycgr3T
  
Location: 24667-25981

Mycgr3G42715\_Mycgr3T

Mycgr3G92934 Mycgr3T
  
Location: 26081-27593

Mycgr3G92934\_Mycgr3T

Mycgr3G41969 Mycgr3T
  
Location: 27693-29328

Mycgr3G41969\_Mycgr3T

Mycgr3G80635 Mycgr3T
  
Location: 29428-29821

Mycgr3G80635\_Mycgr3T

Mycgr3G41426 Mycgr3T
  
Location: 29921-35255

Mycgr3G41426\_Mycgr3T

Mycgr3G104337 Mycgr3
  
Location: 35355-36108

Mycgr3G104337\_Mycgr3

Mycgr3G71679 Mycgr3T
  
Location: 36208-37300

Mycgr3G71679\_Mycgr3T

Mycgr3G92938 Mycgr3T
  
Location: 37400-38699

Mycgr3G92938\_Mycgr3T

Mycgr3G92941 Mycgr3T
  
Location: 38799-40734

Mycgr3G92941\_Mycgr3T

hypothetical protein
  
Accession: EGD98391
  
Location: 181447-184411
  
 NCBI BlastP on this gene

EGD98391

hypothetical protein
  
Accession: EGD98392
  
Location: 187100-188846
  
 NCBI BlastP on this gene

EGD98392

tRNA-splicing endonuclease
  
Accession: EGD98393
  
Location: 192175-199008
  
  
**BlastP hit with Mycgr3G41426\_Mycgr3T**
  
Percentage identity: 40 %
  
BlastP bit score: 1306
  
Sequence coverage: 103 %
  
E-value: 0.0
  
  
 NCBI BlastP on this gene

EGD98393

phospholipid-translocating P-type ATPase
  
Accession: EGD98394
  
Location: 200566-205138
  
 NCBI BlastP on this gene

EGD98394

hypothetical protein
  
Accession: EGD98395
  
Location: 205924-206795
  
 NCBI BlastP on this gene

EGD98395

hypothetical protein
  
Accession: EGD98396
  
Location: 210632-213002
  
 NCBI BlastP on this gene

EGD98396

vacuolar ATP synthase subunit E
  
Accession: EGD98397
  
Location: 213382-214223
  
 NCBI BlastP on this gene

EGD98397

hypothetical protein
  
Accession: EGD98398
  
Location: 214850-216634
  
 NCBI BlastP on this gene

EGD98398

hypothetical protein
  
Accession: EGD98399
  
Location: 217648-218899
  
 NCBI BlastP on this gene

EGD98399

MADS box transcription factor
  
Accession: EGD98400
  
Location: 220310-221191
  
  
**BlastP hit with Mycgr3G31170\_Mycgr3T**
  
Percentage identity: 96 %
  
BlastP bit score: 154
  
Sequence coverage: 98 %
  
E-value: 5e-45
  
  
 NCBI BlastP on this gene

EGD98400

hypothetical protein
  
Accession: EGD98401
  
Location: 222607-223533
  
 NCBI BlastP on this gene

EGD98401

transcriptional activator
  
Accession: EGD98402
  
Location: 224368-225439
  
 NCBI BlastP on this gene

EGD98402

GNAT family acetyltransferase
  
Accession: EGD98403
  
Location: 226566-227684
  
 NCBI BlastP on this gene

EGD98403

metalloreductase transmembrane component
  
Accession: EGD98404
  
Location: 228206-230494
  
 NCBI BlastP on this gene

EGD98404

Query: Architecture Search FASTA input

DS995899 : Penicillium marneffei ATCC 18224 scf\_1105668340764 genomic scaffold    Total score: 2.0     Cumulative Blast bit score: 1459

Hit cluster cross-links:

Mycgr3G85918 Mycgr3T
  
Location: 0-1602

Mycgr3G85918\_Mycgr3T

Mycgr3G42010 Mycgr3T
  
Location: 1702-8569

Mycgr3G42010\_Mycgr3T

Mycgr3G29582 Mycgr3T
  
Location: 8669-8915

Mycgr3G29582\_Mycgr3T

Mycgr3G31170 Mycgr3T
  
Location: 9015-9255

Mycgr3G31170\_Mycgr3T

Mycgr3G85924 Mycgr3T
  
Location: 9355-11218

Mycgr3G85924\_Mycgr3T

Mycgr3G71676 Mycgr3T
  
Location: 11318-12494

Mycgr3G71676\_Mycgr3T

Mycgr3G11468 Mycgr3T
  
Location: 12594-13653

Mycgr3G11468\_Mycgr3T

Mycgr3G58567 Mycgr3T
  
Location: 13753-14506

Mycgr3G58567\_Mycgr3T

Mycgr3G100089 Mycgr3
  
Location: 14606-21152

Mycgr3G100089\_Mycgr3

Mycgr3G42698 Mycgr3T
  
Location: 21252-22131

Mycgr3G42698\_Mycgr3T

Mycgr3G71681 Mycgr3T
  
Location: 22231-23461

Mycgr3G71681\_Mycgr3T

Mycgr3G109328 Mycgr3
  
Location: 23561-24239

Mycgr3G109328\_Mycgr3

Mycgr3G104334 Mycgr3
  
Location: 24339-24567

Mycgr3G104334\_Mycgr3

Mycgr3G42715 Mycgr3T
  
Location: 24667-25981

Mycgr3G42715\_Mycgr3T

Mycgr3G92934 Mycgr3T
  
Location: 26081-27593

Mycgr3G92934\_Mycgr3T

Mycgr3G41969 Mycgr3T
  
Location: 27693-29328

Mycgr3G41969\_Mycgr3T

Mycgr3G80635 Mycgr3T
  
Location: 29428-29821

Mycgr3G80635\_Mycgr3T

Mycgr3G41426 Mycgr3T
  
Location: 29921-35255

Mycgr3G41426\_Mycgr3T

Mycgr3G104337 Mycgr3
  
Location: 35355-36108

Mycgr3G104337\_Mycgr3

Mycgr3G71679 Mycgr3T
  
Location: 36208-37300

Mycgr3G71679\_Mycgr3T

Mycgr3G92938 Mycgr3T
  
Location: 37400-38699

Mycgr3G92938\_Mycgr3T

Mycgr3G92941 Mycgr3T
  
Location: 38799-40734

Mycgr3G92941\_Mycgr3T

conserved hypothetical protein
  
Accession: EEA26966
  
Location: 905998-907635
  
 NCBI BlastP on this gene

EEA26966

C6 transcription factor, putative
  
Accession: EEA26967
  
Location: 909048-911836
  
 NCBI BlastP on this gene

EEA26967

conserved hypothetical protein
  
Accession: EEA26968
  
Location: 912049-912894
  
 NCBI BlastP on this gene

EEA26968

MADS box transcription factor Mcm1
  
Accession: EEA26969
  
Location: 915864-916695
  
  
**BlastP hit with Mycgr3G31170\_Mycgr3T**
  
Percentage identity: 100 %
  
BlastP bit score: 167
  
Sequence coverage: 100 %
  
E-value: 8e-50
  
  
 NCBI BlastP on this gene

EEA26969

DUF803 domain protein
  
Accession: EEA26970
  
Location: 918047-919982
  
 NCBI BlastP on this gene

EEA26970

ATP synthase subunit E, putative
  
Accession: EEA26971
  
Location: 920591-921405
  
 NCBI BlastP on this gene

EEA26971

hypothetical protein
  
Accession: EEA26972
  
Location: 921787-924110
  
 NCBI BlastP on this gene

EEA26972

paraoxonase, putative
  
Accession: EEA26973
  
Location: 924613-925844
  
 NCBI BlastP on this gene

EEA26973

hypothetical protein
  
Accession: EEA26974
  
Location: 926055-926473
  
 NCBI BlastP on this gene

EEA26974

phospholipid-translocating P-type ATPase domain-containing protein
  
Accession: EEA26976
  
Location: 928749-933167
  
 NCBI BlastP on this gene

EEA26976

tRNA-splicing endonuclease, putative
  
Accession: EEA26977
  
Location: 934494-940990
  
  
**BlastP hit with Mycgr3G41426\_Mycgr3T**
  
Percentage identity: 39 %
  
BlastP bit score: 1292
  
Sequence coverage: 103 %
  
E-value: 0.0
  
  
 NCBI BlastP on this gene

EEA26977

conserved hypothetical protein
  
Accession: EEA26978
  
Location: 944055-947180
  
 NCBI BlastP on this gene

EEA26978

conserved hypothetical protein
  
Accession: EEA26979
  
Location: 947500-948840
  
 NCBI BlastP on this gene

EEA26979

chitin biosynthesis protein (Chs5), putative
  
Accession: EEA26980
  
Location: 950133-951465
  
 NCBI BlastP on this gene

EEA26980

Query: Architecture Search FASTA input

EQ999974 : Ajellomyces dermatitidis ER-3 genomic scaffold supercont1.2    Total score: 2.0     Cumulative Blast bit score: 1458

Hit cluster cross-links:

Mycgr3G85918 Mycgr3T
  
Location: 0-1602

Mycgr3G85918\_Mycgr3T

Mycgr3G42010 Mycgr3T
  
Location: 1702-8569

Mycgr3G42010\_Mycgr3T

Mycgr3G29582 Mycgr3T
  
Location: 8669-8915

Mycgr3G29582\_Mycgr3T

Mycgr3G31170 Mycgr3T
  
Location: 9015-9255

Mycgr3G31170\_Mycgr3T

Mycgr3G85924 Mycgr3T
  
Location: 9355-11218

Mycgr3G85924\_Mycgr3T

Mycgr3G71676 Mycgr3T
  
Location: 11318-12494

Mycgr3G71676\_Mycgr3T

Mycgr3G11468 Mycgr3T
  
Location: 12594-13653

Mycgr3G11468\_Mycgr3T

Mycgr3G58567 Mycgr3T
  
Location: 13753-14506

Mycgr3G58567\_Mycgr3T

Mycgr3G100089 Mycgr3
  
Location: 14606-21152

Mycgr3G100089\_Mycgr3

Mycgr3G42698 Mycgr3T
  
Location: 21252-22131

Mycgr3G42698\_Mycgr3T

Mycgr3G71681 Mycgr3T
  
Location: 22231-23461

Mycgr3G71681\_Mycgr3T

Mycgr3G109328 Mycgr3
  
Location: 23561-24239

Mycgr3G109328\_Mycgr3

Mycgr3G104334 Mycgr3
  
Location: 24339-24567

Mycgr3G104334\_Mycgr3

Mycgr3G42715 Mycgr3T
  
Location: 24667-25981

Mycgr3G42715\_Mycgr3T

Mycgr3G92934 Mycgr3T
  
Location: 26081-27593

Mycgr3G92934\_Mycgr3T

Mycgr3G41969 Mycgr3T
  
Location: 27693-29328

Mycgr3G41969\_Mycgr3T

Mycgr3G80635 Mycgr3T
  
Location: 29428-29821

Mycgr3G80635\_Mycgr3T

Mycgr3G41426 Mycgr3T
  
Location: 29921-35255

Mycgr3G41426\_Mycgr3T

Mycgr3G104337 Mycgr3
  
Location: 35355-36108

Mycgr3G104337\_Mycgr3

Mycgr3G71679 Mycgr3T
  
Location: 36208-37300

Mycgr3G71679\_Mycgr3T

Mycgr3G92938 Mycgr3T
  
Location: 37400-38699

Mycgr3G92938\_Mycgr3T

Mycgr3G92941 Mycgr3T
  
Location: 38799-40734

Mycgr3G92941\_Mycgr3T

serine protein kinase Sky1
  
Accession: EEQ87178
  
Location: 7018349-7019826
  
 NCBI BlastP on this gene

EEQ87178

conserved hypothetical protein
  
Accession: EEQ87179
  
Location: 7021138-7022393
  
 NCBI BlastP on this gene

EEQ87179

predicted protein
  
Accession: EEQ87180
  
Location: 7023631-7024179
  
 NCBI BlastP on this gene

EEQ87180

MADS box transcription factor Mcm1
  
Accession: EEQ87181
  
Location: 7025081-7026058
  
  
**BlastP hit with Mycgr3G31170\_Mycgr3T**
  
Percentage identity: 100 %
  
BlastP bit score: 166
  
Sequence coverage: 100 %
  
E-value: 1e-49
  
  
 NCBI BlastP on this gene

EEQ87181

DUF803 domain-containing protein
  
Accession: EEQ87182
  
Location: 7029356-7031506
  
 NCBI BlastP on this gene

EEQ87182

vacuolar ATP synthase subunit E
  
Accession: EEQ87183
  
Location: 7032039-7032963
  
 NCBI BlastP on this gene

EEQ87183

conserved hypothetical protein
  
Accession: EEQ87184
  
Location: 7033660-7036143
  
 NCBI BlastP on this gene

EEQ87184

conserved hypothetical protein
  
Accession: EEQ87185
  
Location: 7037329-7038743
  
 NCBI BlastP on this gene

EEQ87185

phospholipid-translocating P-type ATPase domain-containing protein
  
Accession: EEQ87186
  
Location: 7043295-7047950
  
 NCBI BlastP on this gene

EEQ87186

tRNA-splicing endonuclease
  
Accession: EEQ87187
  
Location: 7049839-7056709
  
  
**BlastP hit with Mycgr3G41426\_Mycgr3T**
  
Percentage identity: 38 %
  
BlastP bit score: 1292
  
Sequence coverage: 103 %
  
E-value: 0.0
  
  
 NCBI BlastP on this gene

EEQ87187

predicted protein
  
Accession: EEQ87188
  
Location: 7061682-7063862
  
 NCBI BlastP on this gene

EEQ87188

Query: Architecture Search FASTA input

DS989822 : Arthroderma gypseum CBS 118893 supercont1.1 genomic scaffold    Total score: 2.0     Cumulative Blast bit score: 1457

Hit cluster cross-links:

Mycgr3G85918 Mycgr3T
  
Location: 0-1602

Mycgr3G85918\_Mycgr3T

Mycgr3G42010 Mycgr3T
  
Location: 1702-8569

Mycgr3G42010\_Mycgr3T

Mycgr3G29582 Mycgr3T
  
Location: 8669-8915

Mycgr3G29582\_Mycgr3T

Mycgr3G31170 Mycgr3T
  
Location: 9015-9255

Mycgr3G31170\_Mycgr3T

Mycgr3G85924 Mycgr3T
  
Location: 9355-11218

Mycgr3G85924\_Mycgr3T

Mycgr3G71676 Mycgr3T
  
Location: 11318-12494

Mycgr3G71676\_Mycgr3T

Mycgr3G11468 Mycgr3T
  
Location: 12594-13653

Mycgr3G11468\_Mycgr3T

Mycgr3G58567 Mycgr3T
  
Location: 13753-14506

Mycgr3G58567\_Mycgr3T

Mycgr3G100089 Mycgr3
  
Location: 14606-21152

Mycgr3G100089\_Mycgr3

Mycgr3G42698 Mycgr3T
  
Location: 21252-22131

Mycgr3G42698\_Mycgr3T

Mycgr3G71681 Mycgr3T
  
Location: 22231-23461

Mycgr3G71681\_Mycgr3T

Mycgr3G109328 Mycgr3
  
Location: 23561-24239

Mycgr3G109328\_Mycgr3

Mycgr3G104334 Mycgr3
  
Location: 24339-24567

Mycgr3G104334\_Mycgr3

Mycgr3G42715 Mycgr3T
  
Location: 24667-25981

Mycgr3G42715\_Mycgr3T

Mycgr3G92934 Mycgr3T
  
Location: 26081-27593

Mycgr3G92934\_Mycgr3T

Mycgr3G41969 Mycgr3T
  
Location: 27693-29328

Mycgr3G41969\_Mycgr3T

Mycgr3G80635 Mycgr3T
  
Location: 29428-29821

Mycgr3G80635\_Mycgr3T

Mycgr3G41426 Mycgr3T
  
Location: 29921-35255

Mycgr3G41426\_Mycgr3T

Mycgr3G104337 Mycgr3
  
Location: 35355-36108

Mycgr3G104337\_Mycgr3

Mycgr3G71679 Mycgr3T
  
Location: 36208-37300

Mycgr3G71679\_Mycgr3T

Mycgr3G92938 Mycgr3T
  
Location: 37400-38699

Mycgr3G92938\_Mycgr3T

Mycgr3G92941 Mycgr3T
  
Location: 38799-40734

Mycgr3G92941\_Mycgr3T

hypothetical protein
  
Accession: EFQ98323
  
Location: 3701066-3703987
  
 NCBI BlastP on this gene

EFQ98323

hypothetical protein
  
Accession: EFQ98324
  
Location: 3705926-3706540
  
 NCBI BlastP on this gene

EFQ98324

hypothetical protein
  
Accession: EFQ98325
  
Location: 3706820-3708578
  
 NCBI BlastP on this gene

EFQ98325

hypothetical protein
  
Accession: EFQ98326
  
Location: 3709258-3710130
  
 NCBI BlastP on this gene

EFQ98326

helicase SEN1
  
Accession: EFQ98327
  
Location: 3711978-3718887
  
  
**BlastP hit with Mycgr3G41426\_Mycgr3T**
  
Percentage identity: 39 %
  
BlastP bit score: 1303
  
Sequence coverage: 103 %
  
E-value: 0.0
  
  
 NCBI BlastP on this gene

EFQ98327

phospholipid-transporting ATPase 1
  
Accession: EFQ98328
  
Location: 3720239-3724971
  
 NCBI BlastP on this gene

EFQ98328

hypothetical protein
  
Accession: EFQ98329
  
Location: 3725489-3726353
  
 NCBI BlastP on this gene

EFQ98329

hypothetical protein
  
Accession: EFQ98330
  
Location: 3727761-3730115
  
 NCBI BlastP on this gene

EFQ98330

vacuolar ATP synthase subunit E
  
Accession: EFQ98331
  
Location: 3730534-3731380
  
 NCBI BlastP on this gene

EFQ98331

DUF803 domain-containing protein
  
Accession: EFQ98332
  
Location: 3732069-3733851
  
 NCBI BlastP on this gene

EFQ98332

carboxy-cis,cis-muconate cyclase
  
Accession: EFQ98333
  
Location: 3734986-3736228
  
 NCBI BlastP on this gene

EFQ98333

MADS box transcription factor Mcm1
  
Accession: EFQ98334
  
Location: 3737424-3738317
  
  
**BlastP hit with Mycgr3G31170\_Mycgr3T**
  
Percentage identity: 96 %
  
BlastP bit score: 154
  
Sequence coverage: 98 %
  
E-value: 6e-45
  
  
 NCBI BlastP on this gene

EFQ98334

tetraspanin
  
Accession: EFQ98335
  
Location: 3739722-3740647
  
 NCBI BlastP on this gene

EFQ98335

PTAC beta
  
Accession: EFQ98336
  
Location: 3741509-3742611
  
 NCBI BlastP on this gene

EFQ98336

hypothetical protein
  
Accession: EFQ98337
  
Location: 3743575-3744864
  
 NCBI BlastP on this gene

EFQ98337

hypothetical protein
  
Accession: EFQ98338
  
Location: 3745387-3747675
  
 NCBI BlastP on this gene

EFQ98338

Query: Architecture Search FASTA input

AACD01000159 : Aspergillus nidulans FGSC A4    Total score: 2.0     Cumulative Blast bit score: 1453

Hit cluster cross-links:

Mycgr3G85918 Mycgr3T
  
Location: 0-1602

Mycgr3G85918\_Mycgr3T

Mycgr3G42010 Mycgr3T
  
Location: 1702-8569

Mycgr3G42010\_Mycgr3T

Mycgr3G29582 Mycgr3T
  
Location: 8669-8915

Mycgr3G29582\_Mycgr3T

Mycgr3G31170 Mycgr3T
  
Location: 9015-9255

Mycgr3G31170\_Mycgr3T

Mycgr3G85924 Mycgr3T
  
Location: 9355-11218

Mycgr3G85924\_Mycgr3T

Mycgr3G71676 Mycgr3T
  
Location: 11318-12494

Mycgr3G71676\_Mycgr3T

Mycgr3G11468 Mycgr3T
  
Location: 12594-13653

Mycgr3G11468\_Mycgr3T

Mycgr3G58567 Mycgr3T
  
Location: 13753-14506

Mycgr3G58567\_Mycgr3T

Mycgr3G100089 Mycgr3
  
Location: 14606-21152

Mycgr3G100089\_Mycgr3

Mycgr3G42698 Mycgr3T
  
Location: 21252-22131

Mycgr3G42698\_Mycgr3T

Mycgr3G71681 Mycgr3T
  
Location: 22231-23461

Mycgr3G71681\_Mycgr3T

Mycgr3G109328 Mycgr3
  
Location: 23561-24239

Mycgr3G109328\_Mycgr3

Mycgr3G104334 Mycgr3
  
Location: 24339-24567

Mycgr3G104334\_Mycgr3

Mycgr3G42715 Mycgr3T
  
Location: 24667-25981

Mycgr3G42715\_Mycgr3T

Mycgr3G92934 Mycgr3T
  
Location: 26081-27593

Mycgr3G92934\_Mycgr3T

Mycgr3G41969 Mycgr3T
  
Location: 27693-29328

Mycgr3G41969\_Mycgr3T

Mycgr3G80635 Mycgr3T
  
Location: 29428-29821

Mycgr3G80635\_Mycgr3T

Mycgr3G41426 Mycgr3T
  
Location: 29921-35255

Mycgr3G41426\_Mycgr3T

Mycgr3G104337 Mycgr3
  
Location: 35355-36108

Mycgr3G104337\_Mycgr3

Mycgr3G71679 Mycgr3T
  
Location: 36208-37300

Mycgr3G71679\_Mycgr3T

Mycgr3G92938 Mycgr3T
  
Location: 37400-38699

Mycgr3G92938\_Mycgr3T

Mycgr3G92941 Mycgr3T
  
Location: 38799-40734

Mycgr3G92941\_Mycgr3T

hypothetical protein
  
Accession: EAA60101
  
Location: 42437-44270
  
 NCBI BlastP on this gene

EAA60101

predicted protein
  
Accession: EAA60100
  
Location: 41295-41745
  
 NCBI BlastP on this gene

EAA60100

hypothetical protein
  
Accession: EAA60099
  
Location: 36913-38478
  
 NCBI BlastP on this gene

EAA60099

hypothetical protein
  
Accession: EAA60098
  
Location: 33815-34722
  
  
**BlastP hit with Mycgr3G31170\_Mycgr3T**
  
Percentage identity: 100 %
  
BlastP bit score: 167
  
Sequence coverage: 100 %
  
E-value: 9e-50
  
  
 NCBI BlastP on this gene

EAA60098

hypothetical protein
  
Accession: EAA60097
  
Location: 30695-32722
  
 NCBI BlastP on this gene

EAA60097

hypothetical protein
  
Accession: EAA60096
  
Location: 29344-30165
  
 NCBI BlastP on this gene

EAA60096

hypothetical protein
  
Accession: EAA60095
  
Location: 25150-28502
  
 NCBI BlastP on this gene

EAA60095

hypothetical protein
  
Accession: EAA60094
  
Location: 18483-23159
  
 NCBI BlastP on this gene

EAA60094

hypothetical protein
  
Accession: EAA60093
  
Location: 9974-17399
  
  
**BlastP hit with Mycgr3G41426\_Mycgr3T**
  
Percentage identity: 39 %
  
BlastP bit score: 1286
  
Sequence coverage: 103 %
  
E-value: 0.0
  
  
 NCBI BlastP on this gene

EAA60093

hypothetical protein
  
Accession: EAA60092
  
Location: 4793-7527
  
 NCBI BlastP on this gene

EAA60092

hypothetical protein
  
Accession: EAA60091
  
Location: 3362-4153
  
 NCBI BlastP on this gene

EAA60091

Query: Architecture Search FASTA input

AM270285 : Aspergillus niger contig An12c0290, genomic contig.    Total score: 2.0     Cumulative Blast bit score: 1452

Hit cluster cross-links:

Mycgr3G85918 Mycgr3T
  
Location: 0-1602

Mycgr3G85918\_Mycgr3T

Mycgr3G42010 Mycgr3T
  
Location: 1702-8569

Mycgr3G42010\_Mycgr3T

Mycgr3G29582 Mycgr3T
  
Location: 8669-8915

Mycgr3G29582\_Mycgr3T

Mycgr3G31170 Mycgr3T
  
Location: 9015-9255

Mycgr3G31170\_Mycgr3T

Mycgr3G85924 Mycgr3T
  
Location: 9355-11218

Mycgr3G85924\_Mycgr3T

Mycgr3G71676 Mycgr3T
  
Location: 11318-12494

Mycgr3G71676\_Mycgr3T

Mycgr3G11468 Mycgr3T
  
Location: 12594-13653

Mycgr3G11468\_Mycgr3T

Mycgr3G58567 Mycgr3T
  
Location: 13753-14506

Mycgr3G58567\_Mycgr3T

Mycgr3G100089 Mycgr3
  
Location: 14606-21152

Mycgr3G100089\_Mycgr3

Mycgr3G42698 Mycgr3T
  
Location: 21252-22131

Mycgr3G42698\_Mycgr3T

Mycgr3G71681 Mycgr3T
  
Location: 22231-23461

Mycgr3G71681\_Mycgr3T

Mycgr3G109328 Mycgr3
  
Location: 23561-24239

Mycgr3G109328\_Mycgr3

Mycgr3G104334 Mycgr3
  
Location: 24339-24567

Mycgr3G104334\_Mycgr3

Mycgr3G42715 Mycgr3T
  
Location: 24667-25981

Mycgr3G42715\_Mycgr3T

Mycgr3G92934 Mycgr3T
  
Location: 26081-27593

Mycgr3G92934\_Mycgr3T

Mycgr3G41969 Mycgr3T
  
Location: 27693-29328

Mycgr3G41969\_Mycgr3T

Mycgr3G80635 Mycgr3T
  
Location: 29428-29821

Mycgr3G80635\_Mycgr3T

Mycgr3G41426 Mycgr3T
  
Location: 29921-35255

Mycgr3G41426\_Mycgr3T

Mycgr3G104337 Mycgr3
  
Location: 35355-36108

Mycgr3G104337\_Mycgr3

Mycgr3G71679 Mycgr3T
  
Location: 36208-37300

Mycgr3G71679\_Mycgr3T

Mycgr3G92938 Mycgr3T
  
Location: 37400-38699

Mycgr3G92938\_Mycgr3T

Mycgr3G92941 Mycgr3T
  
Location: 38799-40734

Mycgr3G92941\_Mycgr3T

hypothetical protein
  
Accession: CAK41321
  
Location: 41584-44480
  
 NCBI BlastP on this gene

An12g08710

unnamed
  
Accession: CAK41322
  
Location: 45435-47000
  
 NCBI BlastP on this gene

An12g08720

not annotated
  
Accession: CAK41323
  
Location: 51270-52404
  
  
**BlastP hit with Mycgr3G31170\_Mycgr3T**
  
Percentage identity: 98 %
  
BlastP bit score: 162
  
Sequence coverage: 100 %
  
E-value: 5e-48
  
  
 NCBI BlastP on this gene

An12g08730

hypothetical protein
  
Accession: CAK41324
  
Location: 52877-53398
  
 NCBI BlastP on this gene

An12g08740

not annotated
  
Accession: CAK41325
  
Location: 53992-55662
  
 NCBI BlastP on this gene

An12g08750

not annotated
  
Accession: CAK41326
  
Location: 56585-57429
  
 NCBI BlastP on this gene

An12g08760

hypothetical protein
  
Accession: CAK41327
  
Location: 58187-60690
  
 NCBI BlastP on this gene

An12g08770

hypothetical protein
  
Accession: CAK41328
  
Location: 61408-61635
  
 NCBI BlastP on this gene

An12g08780

not annotated
  
Accession: CAK41329
  
Location: 63988-68709
  
 NCBI BlastP on this gene

An12g08790

not annotated
  
Accession: CAK41330
  
Location: 69882-76413
  
  
**BlastP hit with Mycgr3G41426\_Mycgr3T**
  
Percentage identity: 39 %
  
BlastP bit score: 1290
  
Sequence coverage: 103 %
  
E-value: 0.0
  
  
 NCBI BlastP on this gene

An12g08800

hypothetical protein
  
Accession: CAK41331
  
Location: 79291-80112
  
 NCBI BlastP on this gene

An12g08810

not annotated
  
Accession: CAK41332
  
Location: 81270-82880
  
 NCBI BlastP on this gene

An12g08820

Query: Architecture Search FASTA input

ACJE01000006 : Aspergillus niger ATCC 1015    Total score: 2.0     Cumulative Blast bit score: 1452

Hit cluster cross-links:

Mycgr3G85918 Mycgr3T
  
Location: 0-1602

Mycgr3G85918\_Mycgr3T

Mycgr3G42010 Mycgr3T
  
Location: 1702-8569

Mycgr3G42010\_Mycgr3T

Mycgr3G29582 Mycgr3T
  
Location: 8669-8915

Mycgr3G29582\_Mycgr3T

Mycgr3G31170 Mycgr3T
  
Location: 9015-9255

Mycgr3G31170\_Mycgr3T

Mycgr3G85924 Mycgr3T
  
Location: 9355-11218

Mycgr3G85924\_Mycgr3T

Mycgr3G71676 Mycgr3T
  
Location: 11318-12494

Mycgr3G71676\_Mycgr3T

Mycgr3G11468 Mycgr3T
  
Location: 12594-13653

Mycgr3G11468\_Mycgr3T

Mycgr3G58567 Mycgr3T
  
Location: 13753-14506

Mycgr3G58567\_Mycgr3T

Mycgr3G100089 Mycgr3
  
Location: 14606-21152

Mycgr3G100089\_Mycgr3

Mycgr3G42698 Mycgr3T
  
Location: 21252-22131

Mycgr3G42698\_Mycgr3T

Mycgr3G71681 Mycgr3T
  
Location: 22231-23461

Mycgr3G71681\_Mycgr3T

Mycgr3G109328 Mycgr3
  
Location: 23561-24239

Mycgr3G109328\_Mycgr3

Mycgr3G104334 Mycgr3
  
Location: 24339-24567

Mycgr3G104334\_Mycgr3

Mycgr3G42715 Mycgr3T
  
Location: 24667-25981

Mycgr3G42715\_Mycgr3T

Mycgr3G92934 Mycgr3T
  
Location: 26081-27593

Mycgr3G92934\_Mycgr3T

Mycgr3G41969 Mycgr3T
  
Location: 27693-29328

Mycgr3G41969\_Mycgr3T

Mycgr3G80635 Mycgr3T
  
Location: 29428-29821

Mycgr3G80635\_Mycgr3T

Mycgr3G41426 Mycgr3T
  
Location: 29921-35255

Mycgr3G41426\_Mycgr3T

Mycgr3G104337 Mycgr3
  
Location: 35355-36108

Mycgr3G104337\_Mycgr3

Mycgr3G71679 Mycgr3T
  
Location: 36208-37300

Mycgr3G71679\_Mycgr3T

Mycgr3G92938 Mycgr3T
  
Location: 37400-38699

Mycgr3G92938\_Mycgr3T

Mycgr3G92941 Mycgr3T
  
Location: 38799-40734

Mycgr3G92941\_Mycgr3T

hypothetical protein
  
Accession: EHA25541
  
Location: 1451908-1453473
  
 NCBI BlastP on this gene

EHA25541

hypothetical protein
  
Accession: EHA25542
  
Location: 1457747-1458670
  
  
**BlastP hit with Mycgr3G31170\_Mycgr3T**
  
Percentage identity: 98 %
  
BlastP bit score: 162
  
Sequence coverage: 100 %
  
E-value: 3e-48
  
  
 NCBI BlastP on this gene

EHA25542

hypothetical protein
  
Accession: EHA25543
  
Location: 1460491-1462463
  
 NCBI BlastP on this gene

EHA25543

hypothetical protein
  
Accession: EHA25544
  
Location: 1463063-1463907
  
 NCBI BlastP on this gene

EHA25544

hypothetical protein
  
Accession: EHA25545
  
Location: 1464753-1467024
  
 NCBI BlastP on this gene

EHA25545

hypothetical protein
  
Accession: EHA25546
  
Location: 1470466-1475184
  
 NCBI BlastP on this gene

EHA25546

hypothetical protein
  
Accession: EHA25547
  
Location: 1476357-1482581
  
  
**BlastP hit with Mycgr3G41426\_Mycgr3T**
  
Percentage identity: 39 %
  
BlastP bit score: 1290
  
Sequence coverage: 103 %
  
E-value: 0.0
  
  
 NCBI BlastP on this gene

EHA25547

hypothetical protein
  
Accession: EHA25548
  
Location: 1487649-1490420
  
 NCBI BlastP on this gene

EHA25548

hypothetical protein
  
Accession: EHA25549
  
Location: 1491031-1491870
  
 NCBI BlastP on this gene

EHA25549

Query: Architecture Search FASTA input

DF126460 : Aspergillus kawachii IFO 4308 DNA, contig: scaffold00014    Total score: 2.0     Cumulative Blast bit score: 1449

Hit cluster cross-links:

Mycgr3G85918 Mycgr3T
  
Location: 0-1602

Mycgr3G85918\_Mycgr3T

Mycgr3G42010 Mycgr3T
  
Location: 1702-8569

Mycgr3G42010\_Mycgr3T

Mycgr3G29582 Mycgr3T
  
Location: 8669-8915

Mycgr3G29582\_Mycgr3T

Mycgr3G31170 Mycgr3T
  
Location: 9015-9255

Mycgr3G31170\_Mycgr3T

Mycgr3G85924 Mycgr3T
  
Location: 9355-11218

Mycgr3G85924\_Mycgr3T

Mycgr3G71676 Mycgr3T
  
Location: 11318-12494

Mycgr3G71676\_Mycgr3T

Mycgr3G11468 Mycgr3T
  
Location: 12594-13653

Mycgr3G11468\_Mycgr3T

Mycgr3G58567 Mycgr3T
  
Location: 13753-14506

Mycgr3G58567\_Mycgr3T

Mycgr3G100089 Mycgr3
  
Location: 14606-21152

Mycgr3G100089\_Mycgr3

Mycgr3G42698 Mycgr3T
  
Location: 21252-22131

Mycgr3G42698\_Mycgr3T

Mycgr3G71681 Mycgr3T
  
Location: 22231-23461

Mycgr3G71681\_Mycgr3T

Mycgr3G109328 Mycgr3
  
Location: 23561-24239

Mycgr3G109328\_Mycgr3

Mycgr3G104334 Mycgr3
  
Location: 24339-24567

Mycgr3G104334\_Mycgr3

Mycgr3G42715 Mycgr3T
  
Location: 24667-25981

Mycgr3G42715\_Mycgr3T

Mycgr3G92934 Mycgr3T
  
Location: 26081-27593

Mycgr3G92934\_Mycgr3T

Mycgr3G41969 Mycgr3T
  
Location: 27693-29328

Mycgr3G41969\_Mycgr3T

Mycgr3G80635 Mycgr3T
  
Location: 29428-29821

Mycgr3G80635\_Mycgr3T

Mycgr3G41426 Mycgr3T
  
Location: 29921-35255

Mycgr3G41426\_Mycgr3T

Mycgr3G104337 Mycgr3
  
Location: 35355-36108

Mycgr3G104337\_Mycgr3

Mycgr3G71679 Mycgr3T
  
Location: 36208-37300

Mycgr3G71679\_Mycgr3T

Mycgr3G92938 Mycgr3T
  
Location: 37400-38699

Mycgr3G92938\_Mycgr3T

Mycgr3G92941 Mycgr3T
  
Location: 38799-40734

Mycgr3G92941\_Mycgr3T

similar to An12g08720
  
Accession: GAA87727
  
Location: 418126-419691
  
 NCBI BlastP on this gene

GAA87727

MADS box transcription factor Mcm1
  
Accession: GAA87728
  
Location: 424006-424926
  
  
**BlastP hit with Mycgr3G31170\_Mycgr3T**
  
Percentage identity: 98 %
  
BlastP bit score: 162
  
Sequence coverage: 100 %
  
E-value: 3e-48
  
  
 NCBI BlastP on this gene

GAA87728

DUF803 domain protein
  
Accession: GAA87729
  
Location: 426799-428794
  
 NCBI BlastP on this gene

GAA87729

vacuolar ATP synthase subunit E
  
Accession: GAA87730
  
Location: 429440-430290
  
 NCBI BlastP on this gene

GAA87730

similar to An12g08770
  
Accession: GAA87731
  
Location: 431041-433401
  
 NCBI BlastP on this gene

GAA87731

phospholipid-translocating P-type ATPase domain-containing protein
  
Accession: GAA87732
  
Location: 436921-441627
  
 NCBI BlastP on this gene

GAA87732

tRNA-splicing endonuclease
  
Accession: GAA87733
  
Location: 442798-449325
  
  
**BlastP hit with Mycgr3G41426\_Mycgr3T**
  
Percentage identity: 39 %
  
BlastP bit score: 1287
  
Sequence coverage: 103 %
  
E-value: 0.0
  
  
 NCBI BlastP on this gene

GAA87733

similar to An12g08820
  
Accession: GAA87734
  
Location: 454664-457335
  
 NCBI BlastP on this gene

GAA87734

aminotransferase
  
Accession: GAA87735
  
Location: 457944-458790
  
 NCBI BlastP on this gene

GAA87735

Query: Architecture Search FASTA input

CH476603 : Aspergillus terreus NIH2624 scaffold\_10 genomic scaffold    Total score: 2.0     Cumulative Blast bit score: 1447

Hit cluster cross-links:

Mycgr3G85918 Mycgr3T
  
Location: 0-1602

Mycgr3G85918\_Mycgr3T

Mycgr3G42010 Mycgr3T
  
Location: 1702-8569

Mycgr3G42010\_Mycgr3T

Mycgr3G29582 Mycgr3T
  
Location: 8669-8915

Mycgr3G29582\_Mycgr3T

Mycgr3G31170 Mycgr3T
  
Location: 9015-9255

Mycgr3G31170\_Mycgr3T

Mycgr3G85924 Mycgr3T
  
Location: 9355-11218

Mycgr3G85924\_Mycgr3T

Mycgr3G71676 Mycgr3T
  
Location: 11318-12494

Mycgr3G71676\_Mycgr3T

Mycgr3G11468 Mycgr3T
  
Location: 12594-13653

Mycgr3G11468\_Mycgr3T

Mycgr3G58567 Mycgr3T
  
Location: 13753-14506

Mycgr3G58567\_Mycgr3T

Mycgr3G100089 Mycgr3
  
Location: 14606-21152

Mycgr3G100089\_Mycgr3

Mycgr3G42698 Mycgr3T
  
Location: 21252-22131

Mycgr3G42698\_Mycgr3T

Mycgr3G71681 Mycgr3T
  
Location: 22231-23461

Mycgr3G71681\_Mycgr3T

Mycgr3G109328 Mycgr3
  
Location: 23561-24239

Mycgr3G109328\_Mycgr3

Mycgr3G104334 Mycgr3
  
Location: 24339-24567

Mycgr3G104334\_Mycgr3

Mycgr3G42715 Mycgr3T
  
Location: 24667-25981

Mycgr3G42715\_Mycgr3T

Mycgr3G92934 Mycgr3T
  
Location: 26081-27593

Mycgr3G92934\_Mycgr3T

Mycgr3G41969 Mycgr3T
  
Location: 27693-29328

Mycgr3G41969\_Mycgr3T

Mycgr3G80635 Mycgr3T
  
Location: 29428-29821

Mycgr3G80635\_Mycgr3T

Mycgr3G41426 Mycgr3T
  
Location: 29921-35255

Mycgr3G41426\_Mycgr3T

Mycgr3G104337 Mycgr3
  
Location: 35355-36108

Mycgr3G104337\_Mycgr3

Mycgr3G71679 Mycgr3T
  
Location: 36208-37300

Mycgr3G71679\_Mycgr3T

Mycgr3G92938 Mycgr3T
  
Location: 37400-38699

Mycgr3G92938\_Mycgr3T

Mycgr3G92941 Mycgr3T
  
Location: 38799-40734

Mycgr3G92941\_Mycgr3T

conserved hypothetical protein
  
Accession: EAU32622
  
Location: 1100543-1102414
  
 NCBI BlastP on this gene

EAU32622

conserved hypothetical protein
  
Accession: EAU32623
  
Location: 1107857-1109311
  
 NCBI BlastP on this gene

EAU32623

hypothetical protein
  
Accession: EAU32624
  
Location: 1111982-1112918
  
  
**BlastP hit with Mycgr3G31170\_Mycgr3T**
  
Percentage identity: 98 %
  
BlastP bit score: 162
  
Sequence coverage: 100 %
  
E-value: 3e-48
  
  
 NCBI BlastP on this gene

EAU32624

conserved hypothetical protein
  
Accession: EAU32625
  
Location: 1114195-1116254
  
 NCBI BlastP on this gene

EAU32625

vacuolar ATP synthase subunit E
  
Accession: EAU32626
  
Location: 1116765-1117511
  
 NCBI BlastP on this gene

EAU32626

predicted protein
  
Accession: EAU32627
  
Location: 1118057-1120064
  
 NCBI BlastP on this gene

EAU32627

hypothetical protein
  
Accession: EAU32628
  
Location: 1123523-1128220
  
 NCBI BlastP on this gene

EAU32628

conserved hypothetical protein
  
Accession: EAU32629
  
Location: 1129328-1135881
  
  
**BlastP hit with Mycgr3G41426\_Mycgr3T**
  
Percentage identity: 39 %
  
BlastP bit score: 1285
  
Sequence coverage: 104 %
  
E-value: 0.0
  
  
 NCBI BlastP on this gene

EAU32629

predicted protein
  
Accession: EAU32630
  
Location: 1139359-1139712
  
 NCBI BlastP on this gene

EAU32630

predicted protein
  
Accession: EAU32631
  
Location: 1139947-1142040
  
 NCBI BlastP on this gene

EAU32631

predicted protein
  
Accession: EAU32632
  
Location: 1142578-1143444
  
 NCBI BlastP on this gene

EAU32632

conserved hypothetical protein
  
Accession: EAU32633
  
Location: 1144039-1145285
  
 NCBI BlastP on this gene

EAU32633

predicted protein
  
Accession: EAU32634
  
Location: 1145800-1148303
  
 NCBI BlastP on this gene

EAU32634

Query: Architecture Search FASTA input

GG700648 : Trichophyton rubrum CBS 118892 genomic scaffold supercont2.1    Total score: 2.0     Cumulative Blast bit score: 1441

Hit cluster cross-links:

Mycgr3G85918 Mycgr3T
  
Location: 0-1602

Mycgr3G85918\_Mycgr3T

Mycgr3G42010 Mycgr3T
  
Location: 1702-8569

Mycgr3G42010\_Mycgr3T

Mycgr3G29582 Mycgr3T
  
Location: 8669-8915

Mycgr3G29582\_Mycgr3T

Mycgr3G31170 Mycgr3T
  
Location: 9015-9255

Mycgr3G31170\_Mycgr3T

Mycgr3G85924 Mycgr3T
  
Location: 9355-11218

Mycgr3G85924\_Mycgr3T

Mycgr3G71676 Mycgr3T
  
Location: 11318-12494

Mycgr3G71676\_Mycgr3T

Mycgr3G11468 Mycgr3T
  
Location: 12594-13653

Mycgr3G11468\_Mycgr3T

Mycgr3G58567 Mycgr3T
  
Location: 13753-14506

Mycgr3G58567\_Mycgr3T

Mycgr3G100089 Mycgr3
  
Location: 14606-21152

Mycgr3G100089\_Mycgr3

Mycgr3G42698 Mycgr3T
  
Location: 21252-22131

Mycgr3G42698\_Mycgr3T

Mycgr3G71681 Mycgr3T
  
Location: 22231-23461

Mycgr3G71681\_Mycgr3T

Mycgr3G109328 Mycgr3
  
Location: 23561-24239

Mycgr3G109328\_Mycgr3

Mycgr3G104334 Mycgr3
  
Location: 24339-24567

Mycgr3G104334\_Mycgr3

Mycgr3G42715 Mycgr3T
  
Location: 24667-25981

Mycgr3G42715\_Mycgr3T

Mycgr3G92934 Mycgr3T
  
Location: 26081-27593

Mycgr3G92934\_Mycgr3T

Mycgr3G41969 Mycgr3T
  
Location: 27693-29328

Mycgr3G41969\_Mycgr3T

Mycgr3G80635 Mycgr3T
  
Location: 29428-29821

Mycgr3G80635\_Mycgr3T

Mycgr3G41426 Mycgr3T
  
Location: 29921-35255

Mycgr3G41426\_Mycgr3T

Mycgr3G104337 Mycgr3
  
Location: 35355-36108

Mycgr3G104337\_Mycgr3

Mycgr3G71679 Mycgr3T
  
Location: 36208-37300

Mycgr3G71679\_Mycgr3T

Mycgr3G92938 Mycgr3T
  
Location: 37400-38699

Mycgr3G92938\_Mycgr3T

Mycgr3G92941 Mycgr3T
  
Location: 38799-40734

Mycgr3G92941\_Mycgr3T

metalloreductase transmembrane component
  
Accession: EGD83852
  
Location: 344325-346613
  
 NCBI BlastP on this gene

EGD83852

GNAT family acetyltransferase
  
Accession: EGD83853
  
Location: 347065-348186
  
 NCBI BlastP on this gene

EGD83853

hypothetical protein
  
Accession: EGD83854
  
Location: 349272-350370
  
 NCBI BlastP on this gene

EGD83854

tetraspanin Pls1 family protein
  
Accession: EGD83855
  
Location: 351229-352146
  
 NCBI BlastP on this gene

EGD83855

MADS box transcription factor
  
Accession: EGD83856
  
Location: 353392-354349
  
  
**BlastP hit with Mycgr3G31170\_Mycgr3T**
  
Percentage identity: 96 %
  
BlastP bit score: 154
  
Sequence coverage: 98 %
  
E-value: 5e-45
  
  
 NCBI BlastP on this gene

EGD83856

hypothetical protein
  
Accession: EGD83857
  
Location: 355720-356966
  
 NCBI BlastP on this gene

EGD83857

hypothetical protein
  
Accession: EGD83858
  
Location: 358083-359862
  
 NCBI BlastP on this gene

EGD83858

vacuolar ATP synthase subunit E
  
Accession: EGD83859
  
Location: 360486-361326
  
 NCBI BlastP on this gene

EGD83859

hypothetical protein
  
Accession: EGD83860
  
Location: 361721-364092
  
 NCBI BlastP on this gene

EGD83860

hypothetical protein
  
Accession: EGD83861
  
Location: 364624-364894
  
 NCBI BlastP on this gene

EGD83861

hypothetical protein
  
Accession: EGD83862
  
Location: 366055-366426
  
 NCBI BlastP on this gene

EGD83862

phospholipid-translocating P-type ATPase
  
Accession: EGD83863
  
Location: 367457-372157
  
 NCBI BlastP on this gene

EGD83863

tRNA-splicing endonuclease
  
Accession: EGD83864
  
Location: 373316-380209
  
  
**BlastP hit with Mycgr3G41426\_Mycgr3T**
  
Percentage identity: 39 %
  
BlastP bit score: 1287
  
Sequence coverage: 103 %
  
E-value: 0.0
  
  
 NCBI BlastP on this gene

EGD83864

hypothetical protein
  
Accession: EGD83865
  
Location: 380643-381283
  
 NCBI BlastP on this gene

EGD83865

hypothetical protein
  
Accession: EGD83866
  
Location: 383517-385273
  
 NCBI BlastP on this gene

EGD83866

hypothetical protein
  
Accession: EGD83867
  
Location: 385897-386172
  
 NCBI BlastP on this gene

EGD83867

hypothetical protein
  
Accession: EGD83868
  
Location: 387764-390712
  
 NCBI BlastP on this gene

EGD83868

Query: Architecture Search FASTA input

CH476657 : Ajellomyces capsulatus NAm1 scaffold\_3 genomic scaffold    Total score: 2.0     Cumulative Blast bit score: 1435

Hit cluster cross-links:

Mycgr3G85918 Mycgr3T
  
Location: 0-1602

Mycgr3G85918\_Mycgr3T

Mycgr3G42010 Mycgr3T
  
Location: 1702-8569

Mycgr3G42010\_Mycgr3T

Mycgr3G29582 Mycgr3T
  
Location: 8669-8915

Mycgr3G29582\_Mycgr3T

Mycgr3G31170 Mycgr3T
  
Location: 9015-9255

Mycgr3G31170\_Mycgr3T

Mycgr3G85924 Mycgr3T
  
Location: 9355-11218

Mycgr3G85924\_Mycgr3T

Mycgr3G71676 Mycgr3T
  
Location: 11318-12494

Mycgr3G71676\_Mycgr3T

Mycgr3G11468 Mycgr3T
  
Location: 12594-13653

Mycgr3G11468\_Mycgr3T

Mycgr3G58567 Mycgr3T
  
Location: 13753-14506

Mycgr3G58567\_Mycgr3T

Mycgr3G100089 Mycgr3
  
Location: 14606-21152

Mycgr3G100089\_Mycgr3

Mycgr3G42698 Mycgr3T
  
Location: 21252-22131

Mycgr3G42698\_Mycgr3T

Mycgr3G71681 Mycgr3T
  
Location: 22231-23461

Mycgr3G71681\_Mycgr3T

Mycgr3G109328 Mycgr3
  
Location: 23561-24239

Mycgr3G109328\_Mycgr3

Mycgr3G104334 Mycgr3
  
Location: 24339-24567

Mycgr3G104334\_Mycgr3

Mycgr3G42715 Mycgr3T
  
Location: 24667-25981

Mycgr3G42715\_Mycgr3T

Mycgr3G92934 Mycgr3T
  
Location: 26081-27593

Mycgr3G92934\_Mycgr3T

Mycgr3G41969 Mycgr3T
  
Location: 27693-29328

Mycgr3G41969\_Mycgr3T

Mycgr3G80635 Mycgr3T
  
Location: 29428-29821

Mycgr3G80635\_Mycgr3T

Mycgr3G41426 Mycgr3T
  
Location: 29921-35255

Mycgr3G41426\_Mycgr3T

Mycgr3G104337 Mycgr3
  
Location: 35355-36108

Mycgr3G104337\_Mycgr3

Mycgr3G71679 Mycgr3T
  
Location: 36208-37300

Mycgr3G71679\_Mycgr3T

Mycgr3G92938 Mycgr3T
  
Location: 37400-38699

Mycgr3G92938\_Mycgr3T

Mycgr3G92941 Mycgr3T
  
Location: 38799-40734

Mycgr3G92941\_Mycgr3T

predicted protein
  
Accession: EDN06675
  
Location: 244789-246834
  
 NCBI BlastP on this gene

EDN06675

predicted protein
  
Accession: EDN06676
  
Location: 249218-250398
  
 NCBI BlastP on this gene

EDN06676

conserved hypothetical protein
  
Accession: EDN06677
  
Location: 251153-252083
  
  
**BlastP hit with Mycgr3G31170\_Mycgr3T**
  
Percentage identity: 98 %
  
BlastP bit score: 164
  
Sequence coverage: 100 %
  
E-value: 2e-48
  
  
 NCBI BlastP on this gene

EDN06677

predicted protein
  
Accession: EDN06678
  
Location: 254847-257060
  
 NCBI BlastP on this gene

EDN06678

vacuolar ATP synthase subunit E
  
Accession: EDN06679
  
Location: 257663-258447
  
 NCBI BlastP on this gene

EDN06679

predicted protein
  
Accession: EDN06680
  
Location: 259023-263841
  
 NCBI BlastP on this gene

EDN06680

predicted protein
  
Accession: EDN06681
  
Location: 264431-265257
  
 NCBI BlastP on this gene

EDN06681

hypothetical protein
  
Accession: EDN06682
  
Location: 265869-270537
  
 NCBI BlastP on this gene

EDN06682

predicted protein
  
Accession: EDN06683
  
Location: 271607-273047
  
 NCBI BlastP on this gene

EDN06683

conserved hypothetical protein
  
Accession: EDN06684
  
Location: 274264-281010
  
  
**BlastP hit with Mycgr3G41426\_Mycgr3T**
  
Percentage identity: 38 %
  
BlastP bit score: 1271
  
Sequence coverage: 103 %
  
E-value: 0.0
  
  
 NCBI BlastP on this gene

EDN06684

predicted protein
  
Accession: EDN06685
  
Location: 288746-289042
  
 NCBI BlastP on this gene

EDN06685

Query: Architecture Search FASTA input

ABSU01000024 : Arthroderma benhamiae CBS 112371    Total score: 2.0     Cumulative Blast bit score: 1432

Hit cluster cross-links:

Mycgr3G85918 Mycgr3T
  
Location: 0-1602

Mycgr3G85918\_Mycgr3T

Mycgr3G42010 Mycgr3T
  
Location: 1702-8569

Mycgr3G42010\_Mycgr3T

Mycgr3G29582 Mycgr3T
  
Location: 8669-8915

Mycgr3G29582\_Mycgr3T

Mycgr3G31170 Mycgr3T
  
Location: 9015-9255

Mycgr3G31170\_Mycgr3T

Mycgr3G85924 Mycgr3T
  
Location: 9355-11218

Mycgr3G85924\_Mycgr3T

Mycgr3G71676 Mycgr3T
  
Location: 11318-12494

Mycgr3G71676\_Mycgr3T

Mycgr3G11468 Mycgr3T
  
Location: 12594-13653

Mycgr3G11468\_Mycgr3T

Mycgr3G58567 Mycgr3T
  
Location: 13753-14506

Mycgr3G58567\_Mycgr3T

Mycgr3G100089 Mycgr3
  
Location: 14606-21152

Mycgr3G100089\_Mycgr3

Mycgr3G42698 Mycgr3T
  
Location: 21252-22131

Mycgr3G42698\_Mycgr3T

Mycgr3G71681 Mycgr3T
  
Location: 22231-23461

Mycgr3G71681\_Mycgr3T

Mycgr3G109328 Mycgr3
  
Location: 23561-24239

Mycgr3G109328\_Mycgr3

Mycgr3G104334 Mycgr3
  
Location: 24339-24567

Mycgr3G104334\_Mycgr3

Mycgr3G42715 Mycgr3T
  
Location: 24667-25981

Mycgr3G42715\_Mycgr3T

Mycgr3G92934 Mycgr3T
  
Location: 26081-27593

Mycgr3G92934\_Mycgr3T

Mycgr3G41969 Mycgr3T
  
Location: 27693-29328

Mycgr3G41969\_Mycgr3T

Mycgr3G80635 Mycgr3T
  
Location: 29428-29821

Mycgr3G80635\_Mycgr3T

Mycgr3G41426 Mycgr3T
  
Location: 29921-35255

Mycgr3G41426\_Mycgr3T

Mycgr3G104337 Mycgr3
  
Location: 35355-36108

Mycgr3G104337\_Mycgr3

Mycgr3G71679 Mycgr3T
  
Location: 36208-37300

Mycgr3G71679\_Mycgr3T

Mycgr3G92938 Mycgr3T
  
Location: 37400-38699

Mycgr3G92938\_Mycgr3T

Mycgr3G92941 Mycgr3T
  
Location: 38799-40734

Mycgr3G92941\_Mycgr3T

hypothetical protein
  
Accession: EFE31140
  
Location: 216105-218393
  
 NCBI BlastP on this gene

EFE31140

hypothetical protein
  
Accession: EFE31141
  
Location: 218878-219999
  
 NCBI BlastP on this gene

EFE31141

transcriptional activator (PtaC), putative
  
Accession: EFE31142
  
Location: 221062-222159
  
 NCBI BlastP on this gene

EFE31142

hypothetical protein
  
Accession: EFE31143
  
Location: 222989-223905
  
 NCBI BlastP on this gene

EFE31143

hypothetical protein
  
Accession: EFE31144
  
Location: 225291-226172
  
  
**BlastP hit with Mycgr3G31170\_Mycgr3T**
  
Percentage identity: 82 %
  
BlastP bit score: 125
  
Sequence coverage: 98 %
  
E-value: 2e-33
  
  
 NCBI BlastP on this gene

EFE31144

hypothetical protein
  
Accession: EFE31145
  
Location: 227012-227380
  
 NCBI BlastP on this gene

EFE31145

conserved hypothetical protein
  
Accession: EFE31146
  
Location: 227626-228888
  
 NCBI BlastP on this gene

EFE31146

DUF803 domain protein
  
Accession: EFE31147
  
Location: 229962-231032
  
 NCBI BlastP on this gene

EFE31147

hypothetical protein
  
Accession: EFE31148
  
Location: 231898-232321
  
 NCBI BlastP on this gene

EFE31148

hypothetical protein
  
Accession: EFE31149
  
Location: 232372-233218
  
 NCBI BlastP on this gene

EFE31149

hypothetical protein
  
Accession: EFE31150
  
Location: 233605-234730
  
 NCBI BlastP on this gene

EFE31150

hypothetical protein
  
Accession: EFE31151
  
Location: 235037-236387
  
 NCBI BlastP on this gene

EFE31151

hypothetical protein
  
Accession: EFE31152
  
Location: 238935-243634
  
 NCBI BlastP on this gene

EFE31152

hypothetical protein
  
Accession: EFE31153
  
Location: 245117-251532
  
  
**BlastP hit with Mycgr3G41426\_Mycgr3T**
  
Percentage identity: 40 %
  
BlastP bit score: 1307
  
Sequence coverage: 103 %
  
E-value: 0.0
  
  
 NCBI BlastP on this gene

EFE31153

hypothetical protein
  
Accession: EFE31154
  
Location: 255131-255650
  
 NCBI BlastP on this gene

EFE31154

hypothetical protein
  
Accession: EFE31155
  
Location: 256228-257049
  
 NCBI BlastP on this gene

EFE31155

hypothetical protein
  
Accession: EFE31156
  
Location: 259745-262678
  
 NCBI BlastP on this gene

EFE31156

Query: Architecture Search FASTA input

ACYE01000336 : Trichophyton verrucosum HKI 0517    Total score: 2.0     Cumulative Blast bit score: 1428

Hit cluster cross-links:

Mycgr3G85918 Mycgr3T
  
Location: 0-1602

Mycgr3G85918\_Mycgr3T

Mycgr3G42010 Mycgr3T
  
Location: 1702-8569

Mycgr3G42010\_Mycgr3T

Mycgr3G29582 Mycgr3T
  
Location: 8669-8915

Mycgr3G29582\_Mycgr3T

Mycgr3G31170 Mycgr3T
  
Location: 9015-9255

Mycgr3G31170\_Mycgr3T

Mycgr3G85924 Mycgr3T
  
Location: 9355-11218

Mycgr3G85924\_Mycgr3T

Mycgr3G71676 Mycgr3T
  
Location: 11318-12494

Mycgr3G71676\_Mycgr3T

Mycgr3G11468 Mycgr3T
  
Location: 12594-13653

Mycgr3G11468\_Mycgr3T

Mycgr3G58567 Mycgr3T
  
Location: 13753-14506

Mycgr3G58567\_Mycgr3T

Mycgr3G100089 Mycgr3
  
Location: 14606-21152

Mycgr3G100089\_Mycgr3

Mycgr3G42698 Mycgr3T
  
Location: 21252-22131

Mycgr3G42698\_Mycgr3T

Mycgr3G71681 Mycgr3T
  
Location: 22231-23461

Mycgr3G71681\_Mycgr3T

Mycgr3G109328 Mycgr3
  
Location: 23561-24239

Mycgr3G109328\_Mycgr3

Mycgr3G104334 Mycgr3
  
Location: 24339-24567

Mycgr3G104334\_Mycgr3

Mycgr3G42715 Mycgr3T
  
Location: 24667-25981

Mycgr3G42715\_Mycgr3T

Mycgr3G92934 Mycgr3T
  
Location: 26081-27593

Mycgr3G92934\_Mycgr3T

Mycgr3G41969 Mycgr3T
  
Location: 27693-29328

Mycgr3G41969\_Mycgr3T

Mycgr3G80635 Mycgr3T
  
Location: 29428-29821

Mycgr3G80635\_Mycgr3T

Mycgr3G41426 Mycgr3T
  
Location: 29921-35255

Mycgr3G41426\_Mycgr3T

Mycgr3G104337 Mycgr3
  
Location: 35355-36108

Mycgr3G104337\_Mycgr3

Mycgr3G71679 Mycgr3T
  
Location: 36208-37300

Mycgr3G71679\_Mycgr3T

Mycgr3G92938 Mycgr3T
  
Location: 37400-38699

Mycgr3G92938\_Mycgr3T

Mycgr3G92941 Mycgr3T
  
Location: 38799-40734

Mycgr3G92941\_Mycgr3T

hypothetical protein
  
Accession: EFE39442
  
Location: 40638-41507
  
 NCBI BlastP on this gene

EFE39442

hypothetical protein
  
Accession: EFE39443
  
Location: 41577-42926
  
 NCBI BlastP on this gene

EFE39443

hypothetical protein
  
Accession: EFE39444
  
Location: 43306-44548
  
 NCBI BlastP on this gene

EFE39444

transcriptional activator (PtaC), putative
  
Accession: EFE39445
  
Location: 45344-46728
  
 NCBI BlastP on this gene

EFE39445

hypothetical protein
  
Accession: EFE39446
  
Location: 47580-48497
  
 NCBI BlastP on this gene

EFE39446

hypothetical protein
  
Accession: EFE39447
  
Location: 49864-50781
  
  
**BlastP hit with Mycgr3G31170\_Mycgr3T**
  
Percentage identity: 82 %
  
BlastP bit score: 125
  
Sequence coverage: 98 %
  
E-value: 2e-33
  
  
 NCBI BlastP on this gene

EFE39447

conserved hypothetical protein
  
Accession: EFE39448
  
Location: 52191-53446
  
 NCBI BlastP on this gene

EFE39448

DUF803 domain protein
  
Accession: EFE39449
  
Location: 54580-55650
  
 NCBI BlastP on this gene

EFE39449

hypothetical protein
  
Accession: EFE39450
  
Location: 56516-56946
  
 NCBI BlastP on this gene

EFE39450

hypothetical protein
  
Accession: EFE39451
  
Location: 56997-57840
  
 NCBI BlastP on this gene

EFE39451

hypothetical protein
  
Accession: EFE39452
  
Location: 58229-60600
  
 NCBI BlastP on this gene

EFE39452

hypothetical protein
  
Accession: EFE39453
  
Location: 63467-68162
  
 NCBI BlastP on this gene

EFE39453

hypothetical protein
  
Accession: EFE39454
  
Location: 69603-76408
  
  
**BlastP hit with Mycgr3G41426\_Mycgr3T**
  
Percentage identity: 40 %
  
BlastP bit score: 1303
  
Sequence coverage: 103 %
  
E-value: 0.0
  
  
 NCBI BlastP on this gene

EFE39454

hypothetical protein
  
Accession: EFE39455
  
Location: 79732-80418
  
 NCBI BlastP on this gene

EFE39455

hypothetical protein
  
Accession: EFE39456
  
Location: 80674-81495
  
 NCBI BlastP on this gene

EFE39456

hypothetical protein
  
Accession: EFE39457
  
Location: 84194-87127
  
 NCBI BlastP on this gene

EFE39457

Query: Architecture Search FASTA input

CH445343 : Phaeosphaeria nodorum SN15 scaffold\_19    Total score: 2.0     Cumulative Blast bit score: 1424

Hit cluster cross-links:

Mycgr3G85918 Mycgr3T
  
Location: 0-1602

Mycgr3G85918\_Mycgr3T

Mycgr3G42010 Mycgr3T
  
Location: 1702-8569

Mycgr3G42010\_Mycgr3T

Mycgr3G29582 Mycgr3T
  
Location: 8669-8915

Mycgr3G29582\_Mycgr3T

Mycgr3G31170 Mycgr3T
  
Location: 9015-9255

Mycgr3G31170\_Mycgr3T

Mycgr3G85924 Mycgr3T
  
Location: 9355-11218

Mycgr3G85924\_Mycgr3T

Mycgr3G71676 Mycgr3T
  
Location: 11318-12494

Mycgr3G71676\_Mycgr3T

Mycgr3G11468 Mycgr3T
  
Location: 12594-13653

Mycgr3G11468\_Mycgr3T

Mycgr3G58567 Mycgr3T
  
Location: 13753-14506

Mycgr3G58567\_Mycgr3T

Mycgr3G100089 Mycgr3
  
Location: 14606-21152

Mycgr3G100089\_Mycgr3

Mycgr3G42698 Mycgr3T
  
Location: 21252-22131

Mycgr3G42698\_Mycgr3T

Mycgr3G71681 Mycgr3T
  
Location: 22231-23461

Mycgr3G71681\_Mycgr3T

Mycgr3G109328 Mycgr3
  
Location: 23561-24239

Mycgr3G109328\_Mycgr3

Mycgr3G104334 Mycgr3
  
Location: 24339-24567

Mycgr3G104334\_Mycgr3

Mycgr3G42715 Mycgr3T
  
Location: 24667-25981

Mycgr3G42715\_Mycgr3T

Mycgr3G92934 Mycgr3T
  
Location: 26081-27593

Mycgr3G92934\_Mycgr3T

Mycgr3G41969 Mycgr3T
  
Location: 27693-29328

Mycgr3G41969\_Mycgr3T

Mycgr3G80635 Mycgr3T
  
Location: 29428-29821

Mycgr3G80635\_Mycgr3T

Mycgr3G41426 Mycgr3T
  
Location: 29921-35255

Mycgr3G41426\_Mycgr3T

Mycgr3G104337 Mycgr3
  
Location: 35355-36108

Mycgr3G104337\_Mycgr3

Mycgr3G71679 Mycgr3T
  
Location: 36208-37300

Mycgr3G71679\_Mycgr3T

Mycgr3G92938 Mycgr3T
  
Location: 37400-38699

Mycgr3G92938\_Mycgr3T

Mycgr3G92941 Mycgr3T
  
Location: 38799-40734

Mycgr3G92941\_Mycgr3T

hypothetical protein
  
Accession: EAT81272
  
Location: 635850-638039
  
 NCBI BlastP on this gene

EAT81272

hypothetical protein
  
Accession: EAT81273
  
Location: 638463-639159
  
 NCBI BlastP on this gene

EAT81273

hypothetical protein
  
Accession: EAT81274
  
Location: 639787-640568
  
 NCBI BlastP on this gene

EAT81274

hypothetical protein
  
Accession: EAT81275
  
Location: 641106-641785
  
 NCBI BlastP on this gene

EAT81275

hypothetical protein
  
Accession: EAT81276
  
Location: 641906-642441
  
 NCBI BlastP on this gene

EAT81276

hypothetical protein
  
Accession: EAT81277
  
Location: 642991-648116
  
 NCBI BlastP on this gene

EAT81277

hypothetical protein
  
Accession: EAT81278
  
Location: 649601-650905
  
 NCBI BlastP on this gene

EAT81278

hypothetical protein
  
Accession: EAT81279
  
Location: 651136-651792
  
 NCBI BlastP on this gene

EAT81279

hypothetical protein
  
Accession: EAT81280
  
Location: 652429-653193
  
 NCBI BlastP on this gene

EAT81280

hypothetical protein
  
Accession: EAT81281
  
Location: 653706-655589
  
 NCBI BlastP on this gene

EAT81281

hypothetical protein
  
Accession: EAT81282
  
Location: 655816-663166
  
  
**BlastP hit with Mycgr3G42010\_Mycgr3T**
  
Percentage identity: 50 %
  
BlastP bit score: 1150
  
Sequence coverage: 52 %
  
E-value: 0.0
  
  
 NCBI BlastP on this gene

EAT81282

hypothetical protein
  
Accession: EAT81283
  
Location: 663606-665069
  
  
**BlastP hit with Mycgr3G92938\_Mycgr3T**
  
Percentage identity: 35 %
  
BlastP bit score: 274
  
Sequence coverage: 113 %
  
E-value: 5e-83
  
  
 NCBI BlastP on this gene

EAT81283

hypothetical protein
  
Accession: EAT81284
  
Location: 667601-669127
  
 NCBI BlastP on this gene

EAT81284

hypothetical protein
  
Accession: EAT81285
  
Location: 669336-669585
  
 NCBI BlastP on this gene

EAT81285

hypothetical protein
  
Accession: EAT81286
  
Location: 670977-672116
  
 NCBI BlastP on this gene

EAT81286

hypothetical protein
  
Accession: EAT81287
  
Location: 673047-673852
  
 NCBI BlastP on this gene

EAT81287

hypothetical protein
  
Accession: EAT81288
  
Location: 674310-676543
  
 NCBI BlastP on this gene

EAT81288

hypothetical protein
  
Accession: EAT81289
  
Location: 676603-677972
  
 NCBI BlastP on this gene

EAT81289

hypothetical protein
  
Accession: EAT81290
  
Location: 678920-680056
  
 NCBI BlastP on this gene

EAT81290

hypothetical protein
  
Accession: EAT81291
  
Location: 680853-683081
  
 NCBI BlastP on this gene

EAT81291

Query: Architecture Search FASTA input

AKHY01000183 : Aspergillus oryzae 3.042    Total score: 2.0     Cumulative Blast bit score: 1421

Hit cluster cross-links:

Mycgr3G85918 Mycgr3T
  
Location: 0-1602

Mycgr3G85918\_Mycgr3T

Mycgr3G42010 Mycgr3T
  
Location: 1702-8569

Mycgr3G42010\_Mycgr3T

Mycgr3G29582 Mycgr3T
  
Location: 8669-8915

Mycgr3G29582\_Mycgr3T

Mycgr3G31170 Mycgr3T
  
Location: 9015-9255

Mycgr3G31170\_Mycgr3T

Mycgr3G85924 Mycgr3T
  
Location: 9355-11218

Mycgr3G85924\_Mycgr3T

Mycgr3G71676 Mycgr3T
  
Location: 11318-12494

Mycgr3G71676\_Mycgr3T

Mycgr3G11468 Mycgr3T
  
Location: 12594-13653

Mycgr3G11468\_Mycgr3T

Mycgr3G58567 Mycgr3T
  
Location: 13753-14506

Mycgr3G58567\_Mycgr3T

Mycgr3G100089 Mycgr3
  
Location: 14606-21152

Mycgr3G100089\_Mycgr3

Mycgr3G42698 Mycgr3T
  
Location: 21252-22131

Mycgr3G42698\_Mycgr3T

Mycgr3G71681 Mycgr3T
  
Location: 22231-23461

Mycgr3G71681\_Mycgr3T

Mycgr3G109328 Mycgr3
  
Location: 23561-24239

Mycgr3G109328\_Mycgr3

Mycgr3G104334 Mycgr3
  
Location: 24339-24567

Mycgr3G104334\_Mycgr3

Mycgr3G42715 Mycgr3T
  
Location: 24667-25981

Mycgr3G42715\_Mycgr3T

Mycgr3G92934 Mycgr3T
  
Location: 26081-27593

Mycgr3G92934\_Mycgr3T

Mycgr3G41969 Mycgr3T
  
Location: 27693-29328

Mycgr3G41969\_Mycgr3T

Mycgr3G80635 Mycgr3T
  
Location: 29428-29821

Mycgr3G80635\_Mycgr3T

Mycgr3G41426 Mycgr3T
  
Location: 29921-35255

Mycgr3G41426\_Mycgr3T

Mycgr3G104337 Mycgr3
  
Location: 35355-36108

Mycgr3G104337\_Mycgr3

Mycgr3G71679 Mycgr3T
  
Location: 36208-37300

Mycgr3G71679\_Mycgr3T

Mycgr3G92938 Mycgr3T
  
Location: 37400-38699

Mycgr3G92938\_Mycgr3T

Mycgr3G92941 Mycgr3T
  
Location: 38799-40734

Mycgr3G92941\_Mycgr3T

carboxylesterase type B
  
Accession: EIT74797
  
Location: 69883-72741
  
 NCBI BlastP on this gene

EIT74797

hypothetical protein
  
Accession: EIT74815
  
Location: 67258-68652
  
 NCBI BlastP on this gene

EIT74815

hypothetical protein
  
Accession: EIT74770
  
Location: 64956-65599
  
 NCBI BlastP on this gene

EIT74770

hypothetical protein
  
Accession: EIT74811
  
Location: 63197-64543
  
 NCBI BlastP on this gene

EIT74811

hypothetical protein
  
Accession: EIT74777
  
Location: 61828-62466
  
 NCBI BlastP on this gene

EIT74777

monocarboxylate transporter
  
Accession: EIT74807
  
Location: 59096-61355
  
 NCBI BlastP on this gene

EIT74807

amine oxidase
  
Accession: EIT74800
  
Location: 56981-58282
  
 NCBI BlastP on this gene

EIT74800

hypothetical protein
  
Accession: EIT74812
  
Location: 55364-56092
  
 NCBI BlastP on this gene

EIT74812

hypothetical protein
  
Accession: EIT74759
  
Location: 53758-54918
  
  
**BlastP hit with Mycgr3G92938\_Mycgr3T**
  
Percentage identity: 29 %
  
BlastP bit score: 114
  
Sequence coverage: 75 %
  
E-value: 5e-25
  
  
 NCBI BlastP on this gene

EIT74759

nonsense-mediated mRNA decay protein, putative
  
Accession: EIT74785
  
Location: 47501-49830
  
  
**BlastP hit with Mycgr3G42010\_Mycgr3T**
  
Percentage identity: 36 %
  
BlastP bit score: 323
  
Sequence coverage: 20 %
  
E-value: 6e-93
  
  
 NCBI BlastP on this gene

EIT74785

ATPases of the AAA+ class
  
Accession: EIT74772
  
Location: 43169-45933
  
  
**BlastP hit with Mycgr3G42010\_Mycgr3T**
  
Percentage identity: 56 %
  
BlastP bit score: 984
  
Sequence coverage: 37 %
  
E-value: 0.0
  
  
 NCBI BlastP on this gene

EIT74772

vacuolar sorting protein VPS1, dynamin
  
Accession: EIT74773
  
Location: 38401-41107
  
 NCBI BlastP on this gene

EIT74773

hypothetical protein
  
Accession: EIT74799
  
Location: 35167-37614
  
 NCBI BlastP on this gene

EIT74799

hypothetical protein
  
Accession: EIT74801
  
Location: 33616-34308
  
 NCBI BlastP on this gene

EIT74801

hypothetical protein
  
Accession: EIT74762
  
Location: 32594-33525
  
 NCBI BlastP on this gene

EIT74762

hypothetical protein
  
Accession: EIT74775
  
Location: 31576-32353
  
 NCBI BlastP on this gene

EIT74775

fungal alpha-L-arabinofuranosidase, putative
  
Accession: EIT74798
  
Location: 29328-30848
  
 NCBI BlastP on this gene

EIT74798

Query: Architecture Search FASTA input

KB644411 : Penicillium oxalicum 114-2 unplaced genomic scaffold scaffold\_4    Total score: 2.0     Cumulative Blast bit score: 1398

Hit cluster cross-links:

Mycgr3G85918 Mycgr3T
  
Location: 0-1602

Mycgr3G85918\_Mycgr3T

Mycgr3G42010 Mycgr3T
  
Location: 1702-8569

Mycgr3G42010\_Mycgr3T

Mycgr3G29582 Mycgr3T
  
Location: 8669-8915

Mycgr3G29582\_Mycgr3T

Mycgr3G31170 Mycgr3T
  
Location: 9015-9255

Mycgr3G31170\_Mycgr3T

Mycgr3G85924 Mycgr3T
  
Location: 9355-11218

Mycgr3G85924\_Mycgr3T

Mycgr3G71676 Mycgr3T
  
Location: 11318-12494

Mycgr3G71676\_Mycgr3T

Mycgr3G11468 Mycgr3T
  
Location: 12594-13653

Mycgr3G11468\_Mycgr3T

Mycgr3G58567 Mycgr3T
  
Location: 13753-14506

Mycgr3G58567\_Mycgr3T

Mycgr3G100089 Mycgr3
  
Location: 14606-21152

Mycgr3G100089\_Mycgr3

Mycgr3G42698 Mycgr3T
  
Location: 21252-22131

Mycgr3G42698\_Mycgr3T

Mycgr3G71681 Mycgr3T
  
Location: 22231-23461

Mycgr3G71681\_Mycgr3T

Mycgr3G109328 Mycgr3
  
Location: 23561-24239

Mycgr3G109328\_Mycgr3

Mycgr3G104334 Mycgr3
  
Location: 24339-24567

Mycgr3G104334\_Mycgr3

Mycgr3G42715 Mycgr3T
  
Location: 24667-25981

Mycgr3G42715\_Mycgr3T

Mycgr3G92934 Mycgr3T
  
Location: 26081-27593

Mycgr3G92934\_Mycgr3T

Mycgr3G41969 Mycgr3T
  
Location: 27693-29328

Mycgr3G41969\_Mycgr3T

Mycgr3G80635 Mycgr3T
  
Location: 29428-29821

Mycgr3G80635\_Mycgr3T

Mycgr3G41426 Mycgr3T
  
Location: 29921-35255

Mycgr3G41426\_Mycgr3T

Mycgr3G104337 Mycgr3
  
Location: 35355-36108

Mycgr3G104337\_Mycgr3

Mycgr3G71679 Mycgr3T
  
Location: 36208-37300

Mycgr3G71679\_Mycgr3T

Mycgr3G92938 Mycgr3T
  
Location: 37400-38699

Mycgr3G92938\_Mycgr3T

Mycgr3G92941 Mycgr3T
  
Location: 38799-40734

Mycgr3G92941\_Mycgr3T

putative chondroitin sulfate synthase
  
Accession: EPS28408
  
Location: 165190-166785
  
 NCBI BlastP on this gene

EPS28408

hypothetical protein
  
Accession: EPS28407
  
Location: 160626-161578
  
  
**BlastP hit with Mycgr3G31170\_Mycgr3T**
  
Percentage identity: 100 %
  
BlastP bit score: 166
  
Sequence coverage: 100 %
  
E-value: 1e-49
  
  
 NCBI BlastP on this gene

EPS28407

hypothetical protein
  
Accession: EPS28406
  
Location: 156430-158309
  
 NCBI BlastP on this gene

EPS28406

hypothetical protein
  
Accession: EPS28405
  
Location: 155128-156000
  
 NCBI BlastP on this gene

EPS28405

hypothetical protein
  
Accession: EPS28404
  
Location: 152365-154758
  
 NCBI BlastP on this gene

EPS28404

hypothetical protein
  
Accession: EPS28403
  
Location: 142386-147085
  
 NCBI BlastP on this gene

EPS28403

hypothetical protein
  
Accession: EPS28402
  
Location: 139464-139938
  
 NCBI BlastP on this gene

EPS28402

hypothetical protein
  
Accession: EPS28401
  
Location: 131741-138269
  
  
**BlastP hit with Mycgr3G41426\_Mycgr3T**
  
Percentage identity: 38 %
  
BlastP bit score: 1232
  
Sequence coverage: 103 %
  
E-value: 0.0
  
  
 NCBI BlastP on this gene

EPS28401

hypothetical protein
  
Accession: EPS28400
  
Location: 125901-128708
  
 NCBI BlastP on this gene

EPS28400

hypothetical protein
  
Accession: EPS28399
  
Location: 124020-124718
  
 NCBI BlastP on this gene

EPS28399

Query: Architecture Search FASTA input

JH226133 : Exophiala dermatitidis NIH/UT8656 unplaced genomic scaffold supercont1.4    Total score: 2.0     Cumulative Blast bit score: 1394

Hit cluster cross-links:

Mycgr3G85918 Mycgr3T
  
Location: 0-1602

Mycgr3G85918\_Mycgr3T

Mycgr3G42010 Mycgr3T
  
Location: 1702-8569

Mycgr3G42010\_Mycgr3T

Mycgr3G29582 Mycgr3T
  
Location: 8669-8915

Mycgr3G29582\_Mycgr3T

Mycgr3G31170 Mycgr3T
  
Location: 9015-9255

Mycgr3G31170\_Mycgr3T

Mycgr3G85924 Mycgr3T
  
Location: 9355-11218

Mycgr3G85924\_Mycgr3T

Mycgr3G71676 Mycgr3T
  
Location: 11318-12494

Mycgr3G71676\_Mycgr3T

Mycgr3G11468 Mycgr3T
  
Location: 12594-13653

Mycgr3G11468\_Mycgr3T

Mycgr3G58567 Mycgr3T
  
Location: 13753-14506

Mycgr3G58567\_Mycgr3T

Mycgr3G100089 Mycgr3
  
Location: 14606-21152

Mycgr3G100089\_Mycgr3

Mycgr3G42698 Mycgr3T
  
Location: 21252-22131

Mycgr3G42698\_Mycgr3T

Mycgr3G71681 Mycgr3T
  
Location: 22231-23461

Mycgr3G71681\_Mycgr3T

Mycgr3G109328 Mycgr3
  
Location: 23561-24239

Mycgr3G109328\_Mycgr3

Mycgr3G104334 Mycgr3
  
Location: 24339-24567

Mycgr3G104334\_Mycgr3

Mycgr3G42715 Mycgr3T
  
Location: 24667-25981

Mycgr3G42715\_Mycgr3T

Mycgr3G92934 Mycgr3T
  
Location: 26081-27593

Mycgr3G92934\_Mycgr3T

Mycgr3G41969 Mycgr3T
  
Location: 27693-29328

Mycgr3G41969\_Mycgr3T

Mycgr3G80635 Mycgr3T
  
Location: 29428-29821

Mycgr3G80635\_Mycgr3T

Mycgr3G41426 Mycgr3T
  
Location: 29921-35255

Mycgr3G41426\_Mycgr3T

Mycgr3G104337 Mycgr3
  
Location: 35355-36108

Mycgr3G104337\_Mycgr3

Mycgr3G71679 Mycgr3T
  
Location: 36208-37300

Mycgr3G71679\_Mycgr3T

Mycgr3G92938 Mycgr3T
  
Location: 37400-38699

Mycgr3G92938\_Mycgr3T

Mycgr3G92941 Mycgr3T
  
Location: 38799-40734

Mycgr3G92941\_Mycgr3T

carbamoyl-phosphate synthase arginine-specific small chain
  
Accession: EHY56996
  
Location: 2038206-2039752
  
 NCBI BlastP on this gene

EHY56996

hypothetical protein
  
Accession: EHY56997
  
Location: 2040527-2040922
  
 NCBI BlastP on this gene

EHY56997

hypothetical protein
  
Accession: EHY56998
  
Location: 2044357-2047737
  
 NCBI BlastP on this gene

EHY56998

senataxin
  
Accession: EHY56999
  
Location: 2048646-2055497
  
  
**BlastP hit with Mycgr3G41426\_Mycgr3T**
  
Percentage identity: 40 %
  
BlastP bit score: 1310
  
Sequence coverage: 104 %
  
E-value: 0.0
  
  
 NCBI BlastP on this gene

EHY56999

phospholipid-translocating ATPase
  
Accession: EHY57000
  
Location: 2056377-2061222
  
 NCBI BlastP on this gene

EHY57000

hypothetical protein
  
Accession: EHY57001
  
Location: 2064903-2065265
  
 NCBI BlastP on this gene

EHY57001

2-dehydropantoate 2-reductase, variant
  
Accession: EHY57002
  
Location: 2067094-2069592
  
 NCBI BlastP on this gene

EHY57002

hypothetical protein
  
Accession: EHY57004
  
Location: 2071108-2071434
  
  
**BlastP hit with Mycgr3G29582\_Mycgr3T**
  
Percentage identity: 64 %
  
BlastP bit score: 84
  
Sequence coverage: 75 %
  
E-value: 6e-19
  
  
 NCBI BlastP on this gene

EHY57004

hypothetical protein
  
Accession: EHY57005
  
Location: 2071859-2072305
  
 NCBI BlastP on this gene

EHY57005

hypothetical protein
  
Accession: EHY57006
  
Location: 2073817-2075467
  
 NCBI BlastP on this gene

EHY57006

NADP-dependent mannitol dehydrogenase
  
Accession: EHY57007
  
Location: 2076427-2077290
  
 NCBI BlastP on this gene

EHY57007

phosphatidylinositol glycan, class O
  
Accession: EHY57008
  
Location: 2078092-2081205
  
 NCBI BlastP on this gene

EHY57008

hypothetical protein
  
Accession: EHY57009
  
Location: 2081666-2082637
  
 NCBI BlastP on this gene

EHY57009

Query: Architecture Search FASTA input

GL636491 : Coccidioides posadasii str. Silveira unplaced genomic scaffold supercont2.6    Total score: 2.0     Cumulative Blast bit score: 1385

Hit cluster cross-links:

Mycgr3G85918 Mycgr3T
  
Location: 0-1602

Mycgr3G85918\_Mycgr3T

Mycgr3G42010 Mycgr3T
  
Location: 1702-8569

Mycgr3G42010\_Mycgr3T

Mycgr3G29582 Mycgr3T
  
Location: 8669-8915

Mycgr3G29582\_Mycgr3T

Mycgr3G31170 Mycgr3T
  
Location: 9015-9255

Mycgr3G31170\_Mycgr3T

Mycgr3G85924 Mycgr3T
  
Location: 9355-11218

Mycgr3G85924\_Mycgr3T

Mycgr3G71676 Mycgr3T
  
Location: 11318-12494

Mycgr3G71676\_Mycgr3T

Mycgr3G11468 Mycgr3T
  
Location: 12594-13653

Mycgr3G11468\_Mycgr3T

Mycgr3G58567 Mycgr3T
  
Location: 13753-14506

Mycgr3G58567\_Mycgr3T

Mycgr3G100089 Mycgr3
  
Location: 14606-21152

Mycgr3G100089\_Mycgr3

Mycgr3G42698 Mycgr3T
  
Location: 21252-22131

Mycgr3G42698\_Mycgr3T

Mycgr3G71681 Mycgr3T
  
Location: 22231-23461

Mycgr3G71681\_Mycgr3T

Mycgr3G109328 Mycgr3
  
Location: 23561-24239

Mycgr3G109328\_Mycgr3

Mycgr3G104334 Mycgr3
  
Location: 24339-24567

Mycgr3G104334\_Mycgr3

Mycgr3G42715 Mycgr3T
  
Location: 24667-25981

Mycgr3G42715\_Mycgr3T

Mycgr3G92934 Mycgr3T
  
Location: 26081-27593

Mycgr3G92934\_Mycgr3T

Mycgr3G41969 Mycgr3T
  
Location: 27693-29328

Mycgr3G41969\_Mycgr3T

Mycgr3G80635 Mycgr3T
  
Location: 29428-29821

Mycgr3G80635\_Mycgr3T

Mycgr3G41426 Mycgr3T
  
Location: 29921-35255

Mycgr3G41426\_Mycgr3T

Mycgr3G104337 Mycgr3
  
Location: 35355-36108

Mycgr3G104337\_Mycgr3

Mycgr3G71679 Mycgr3T
  
Location: 36208-37300

Mycgr3G71679\_Mycgr3T

Mycgr3G92938 Mycgr3T
  
Location: 37400-38699

Mycgr3G92938\_Mycgr3T

Mycgr3G92941 Mycgr3T
  
Location: 38799-40734

Mycgr3G92941\_Mycgr3T

conserved hypothetical protein
  
Accession: EFW18852
  
Location: 453928-454682
  
 NCBI BlastP on this gene

EFW18852

MADS box transcription factor Mcm1
  
Accession: EFW18851
  
Location: 451437-452655
  
  
**BlastP hit with Mycgr3G31170\_Mycgr3T**
  
Percentage identity: 97 %
  
BlastP bit score: 160
  
Sequence coverage: 100 %
  
E-value: 2e-47
  
  
 NCBI BlastP on this gene

EFW18851

hypothetical protein
  
Accession: EFW18850
  
Location: 448889-450607
  
 NCBI BlastP on this gene

EFW18850

vacuolar ATP synthase subunit E
  
Accession: EFW18849
  
Location: 446966-447846
  
 NCBI BlastP on this gene

EFW18849

conserved hypothetical protein
  
Accession: EFW18848
  
Location: 444295-445952
  
 NCBI BlastP on this gene

EFW18848

P-type ATPase
  
Accession: EFW18847
  
Location: 437877-442625
  
 NCBI BlastP on this gene

EFW18847

tRNA-splicing endonuclease
  
Accession: EFW18846
  
Location: 433172-435748
  
  
**BlastP hit with Mycgr3G41426\_Mycgr3T**
  
Percentage identity: 29 %
  
BlastP bit score: 363
  
Sequence coverage: 44 %
  
E-value: 1e-103
  
  
 NCBI BlastP on this gene

EFW18846

tRNA-splicing endonuclease
  
Accession: EFW18845
  
Location: 429533-433022
  
  
**BlastP hit with Mycgr3G41426\_Mycgr3T**
  
Percentage identity: 54 %
  
BlastP bit score: 862
  
Sequence coverage: 42 %
  
E-value: 0.0
  
  
 NCBI BlastP on this gene

EFW18845

conserved hypothetical protein
  
Accession: EFW18844
  
Location: 426846-427341
  
 NCBI BlastP on this gene

EFW18844

predicted protein
  
Accession: EFW18843
  
Location: 425824-426244
  
 NCBI BlastP on this gene

EFW18843

hypothetical protein
  
Accession: EFW18841
  
Location: 425318-425749
  
 NCBI BlastP on this gene

EFW18841

conserved hypothetical protein
  
Accession: EFW18842
  
Location: 422421-425049
  
 NCBI BlastP on this gene

EFW18842

inner membrane magnesium transporter MRS2
  
Accession: EFW18840
  
Location: 419562-421570
  
 NCBI BlastP on this gene

EFW18840

conserved hypothetical protein
  
Accession: EFW18839
  
Location: 416810-418773
  
 NCBI BlastP on this gene

EFW18839

Query: Architecture Search FASTA input

JH921445 : Marssonina brunnea f. sp. 'multigermtubi' MB\_m1 unplaced genomic scaffold M6\_S00018    Total score: 2.0     Cumulative Blast bit score: 1315

Hit cluster cross-links:

Mycgr3G85918 Mycgr3T
  
Location: 0-1602

Mycgr3G85918\_Mycgr3T

Mycgr3G42010 Mycgr3T
  
Location: 1702-8569

Mycgr3G42010\_Mycgr3T

Mycgr3G29582 Mycgr3T
  
Location: 8669-8915

Mycgr3G29582\_Mycgr3T

Mycgr3G31170 Mycgr3T
  
Location: 9015-9255

Mycgr3G31170\_Mycgr3T

Mycgr3G85924 Mycgr3T
  
Location: 9355-11218

Mycgr3G85924\_Mycgr3T

Mycgr3G71676 Mycgr3T
  
Location: 11318-12494

Mycgr3G71676\_Mycgr3T

Mycgr3G11468 Mycgr3T
  
Location: 12594-13653

Mycgr3G11468\_Mycgr3T

Mycgr3G58567 Mycgr3T
  
Location: 13753-14506

Mycgr3G58567\_Mycgr3T

Mycgr3G100089 Mycgr3
  
Location: 14606-21152

Mycgr3G100089\_Mycgr3

Mycgr3G42698 Mycgr3T
  
Location: 21252-22131

Mycgr3G42698\_Mycgr3T

Mycgr3G71681 Mycgr3T
  
Location: 22231-23461

Mycgr3G71681\_Mycgr3T

Mycgr3G109328 Mycgr3
  
Location: 23561-24239

Mycgr3G109328\_Mycgr3

Mycgr3G104334 Mycgr3
  
Location: 24339-24567

Mycgr3G104334\_Mycgr3

Mycgr3G42715 Mycgr3T
  
Location: 24667-25981

Mycgr3G42715\_Mycgr3T

Mycgr3G92934 Mycgr3T
  
Location: 26081-27593

Mycgr3G92934\_Mycgr3T

Mycgr3G41969 Mycgr3T
  
Location: 27693-29328

Mycgr3G41969\_Mycgr3T

Mycgr3G80635 Mycgr3T
  
Location: 29428-29821

Mycgr3G80635\_Mycgr3T

Mycgr3G41426 Mycgr3T
  
Location: 29921-35255

Mycgr3G41426\_Mycgr3T

Mycgr3G104337 Mycgr3
  
Location: 35355-36108

Mycgr3G104337\_Mycgr3

Mycgr3G71679 Mycgr3T
  
Location: 36208-37300

Mycgr3G71679\_Mycgr3T

Mycgr3G92938 Mycgr3T
  
Location: 37400-38699

Mycgr3G92938\_Mycgr3T

Mycgr3G92941 Mycgr3T
  
Location: 38799-40734

Mycgr3G92941\_Mycgr3T

C-5 cytosine-specific DNA methylase
  
Accession: EKD14577
  
Location: 755212-758372
  
 NCBI BlastP on this gene

EKD14577

amidophosphoribosyltransferase
  
Accession: EKD14578
  
Location: 760108-761947
  
 NCBI BlastP on this gene

EKD14578

base excision DNA repair protein
  
Accession: EKD14579
  
Location: 762805-764628
  
 NCBI BlastP on this gene

EKD14579

hypothetical protein
  
Accession: EKD14580
  
Location: 765452-766144
  
 NCBI BlastP on this gene

EKD14580

SRP54-type protein
  
Accession: EKD14581
  
Location: 766297-768557
  
 NCBI BlastP on this gene

EKD14581

hypothetical protein
  
Accession: EKD14582
  
Location: 768836-769920
  
 NCBI BlastP on this gene

EKD14582

hypothetical protein
  
Accession: EKD14583
  
Location: 771363-777673
  
  
**BlastP hit with Mycgr3G41426\_Mycgr3T**
  
Percentage identity: 34 %
  
BlastP bit score: 1030
  
Sequence coverage: 104 %
  
E-value: 0.0
  
  
 NCBI BlastP on this gene

EKD14583

hypothetical protein
  
Accession: EKD14584
  
Location: 779724-782198
  
 NCBI BlastP on this gene

EKD14584

sorbitol dehydrogenase
  
Accession: EKD14585
  
Location: 782692-784392
  
  
**BlastP hit with Mycgr3G71679\_Mycgr3T**
  
Percentage identity: 45 %
  
BlastP bit score: 285
  
Sequence coverage: 101 %
  
E-value: 2e-89
  
  
 NCBI BlastP on this gene

EKD14585

BRCA1 C Terminus domain-containing protein
  
Accession: EKD14586
  
Location: 785050-789513
  
 NCBI BlastP on this gene

EKD14586

eukaryotic translation initiation factor eIF-1A
  
Accession: EKD14587
  
Location: 790000-790539
  
 NCBI BlastP on this gene

EKD14587

WH1 domain-containing protein
  
Accession: EKD14588
  
Location: 791036-793208
  
 NCBI BlastP on this gene

EKD14588

hypothetical protein
  
Accession: EKD14589
  
Location: 794094-794705
  
 NCBI BlastP on this gene

EKD14589

MUS38-like protein
  
Accession: EKD14590
  
Location: 794773-797622
  
 NCBI BlastP on this gene

EKD14590

actin cortical patch protein
  
Accession: EKD14591
  
Location: 798567-799934
  
 NCBI BlastP on this gene

EKD14591

hypothetical protein
  
Accession: EKD14592
  
Location: 800260-801162
  
 NCBI BlastP on this gene

EKD14592

Query: Architecture Search FASTA input

DS544803 : Paracoccidioides brasiliensis Pb03 supercont1.1 genomic scaffold    Total score: 2.0     Cumulative Blast bit score: 1285

Hit cluster cross-links:

Mycgr3G85918 Mycgr3T
  
Location: 0-1602

Mycgr3G85918\_Mycgr3T

Mycgr3G42010 Mycgr3T
  
Location: 1702-8569

Mycgr3G42010\_Mycgr3T

Mycgr3G29582 Mycgr3T
  
Location: 8669-8915

Mycgr3G29582\_Mycgr3T

Mycgr3G31170 Mycgr3T
  
Location: 9015-9255

Mycgr3G31170\_Mycgr3T

Mycgr3G85924 Mycgr3T
  
Location: 9355-11218

Mycgr3G85924\_Mycgr3T

Mycgr3G71676 Mycgr3T
  
Location: 11318-12494

Mycgr3G71676\_Mycgr3T

Mycgr3G11468 Mycgr3T
  
Location: 12594-13653

Mycgr3G11468\_Mycgr3T

Mycgr3G58567 Mycgr3T
  
Location: 13753-14506

Mycgr3G58567\_Mycgr3T

Mycgr3G100089 Mycgr3
  
Location: 14606-21152

Mycgr3G100089\_Mycgr3

Mycgr3G42698 Mycgr3T
  
Location: 21252-22131

Mycgr3G42698\_Mycgr3T

Mycgr3G71681 Mycgr3T
  
Location: 22231-23461

Mycgr3G71681\_Mycgr3T

Mycgr3G109328 Mycgr3
  
Location: 23561-24239

Mycgr3G109328\_Mycgr3

Mycgr3G104334 Mycgr3
  
Location: 24339-24567

Mycgr3G104334\_Mycgr3

Mycgr3G42715 Mycgr3T
  
Location: 24667-25981

Mycgr3G42715\_Mycgr3T

Mycgr3G92934 Mycgr3T
  
Location: 26081-27593

Mycgr3G92934\_Mycgr3T

Mycgr3G41969 Mycgr3T
  
Location: 27693-29328

Mycgr3G41969\_Mycgr3T

Mycgr3G80635 Mycgr3T
  
Location: 29428-29821

Mycgr3G80635\_Mycgr3T

Mycgr3G41426 Mycgr3T
  
Location: 29921-35255

Mycgr3G41426\_Mycgr3T

Mycgr3G104337 Mycgr3
  
Location: 35355-36108

Mycgr3G104337\_Mycgr3

Mycgr3G71679 Mycgr3T
  
Location: 36208-37300

Mycgr3G71679\_Mycgr3T

Mycgr3G92938 Mycgr3T
  
Location: 37400-38699

Mycgr3G92938\_Mycgr3T

Mycgr3G92941 Mycgr3T
  
Location: 38799-40734

Mycgr3G92941\_Mycgr3T

cytosolic Cu/Zn superoxide dismutase
  
Accession: EEH17868
  
Location: 1549894-1550642
  
 NCBI BlastP on this gene

EEH17868

predicted protein
  
Accession: EEH17869
  
Location: 1552184-1553567
  
 NCBI BlastP on this gene

EEH17869

conserved hypothetical protein
  
Accession: EEH17870
  
Location: 1554094-1555957
  
 NCBI BlastP on this gene

EEH17870

CORD and CS domain-containing protein
  
Accession: EEH17871
  
Location: 1557992-1559188
  
 NCBI BlastP on this gene

EEH17871

conserved hypothetical protein
  
Accession: EEH17872
  
Location: 1559633-1559959
  
 NCBI BlastP on this gene

EEH17872

2,4-dihydroxyhept-2-ene-1,7-dioic acid aldolase
  
Accession: EEH17873
  
Location: 1560053-1561195
  
 NCBI BlastP on this gene

EEH17873

conserved hypothetical protein
  
Accession: EEH17874
  
Location: 1561965-1564211
  
  
**BlastP hit with Mycgr3G104337\_Mycgr3**
  
Percentage identity: 35 %
  
BlastP bit score: 169
  
Sequence coverage: 104 %
  
E-value: 3e-46
  
  
 NCBI BlastP on this gene

EEH17874

fatty acid synthase S-acetyltransferase
  
Accession: EEH17875
  
Location: 1564688-1572713
  
  
**BlastP hit with Mycgr3G100089\_Mycgr3**
  
Percentage identity: 44 %
  
BlastP bit score: 1116
  
Sequence coverage: 58 %
  
E-value: 0.0
  
  
 NCBI BlastP on this gene

EEH17875

conserved hypothetical protein
  
Accession: EEH17876
  
Location: 1573656-1576532
  
 NCBI BlastP on this gene

EEH17876

predicted protein
  
Accession: EEH17877
  
Location: 1578138-1578942
  
 NCBI BlastP on this gene

EEH17877

medium-chain specific acyl-CoA dehydrogenase
  
Accession: EEH17878
  
Location: 1580302-1581883
  
 NCBI BlastP on this gene

EEH17878

conserved hypothetical protein
  
Accession: EEH17879
  
Location: 1583446-1584468
  
 NCBI BlastP on this gene

EEH17879

predicted protein
  
Accession: EEH17880
  
Location: 1589386-1590417
  
 NCBI BlastP on this gene

EEH17880

Query: Architecture Search FASTA input

DS572752 : Paracoccidioides brasiliensis Pb18 supercont1.3 genomic scaffold    Total score: 2.0     Cumulative Blast bit score: 1283

Hit cluster cross-links:

Mycgr3G85918 Mycgr3T
  
Location: 0-1602

Mycgr3G85918\_Mycgr3T

Mycgr3G42010 Mycgr3T
  
Location: 1702-8569

Mycgr3G42010\_Mycgr3T

Mycgr3G29582 Mycgr3T
  
Location: 8669-8915

Mycgr3G29582\_Mycgr3T

Mycgr3G31170 Mycgr3T
  
Location: 9015-9255

Mycgr3G31170\_Mycgr3T

Mycgr3G85924 Mycgr3T
  
Location: 9355-11218

Mycgr3G85924\_Mycgr3T

Mycgr3G71676 Mycgr3T
  
Location: 11318-12494

Mycgr3G71676\_Mycgr3T

Mycgr3G11468 Mycgr3T
  
Location: 12594-13653

Mycgr3G11468\_Mycgr3T

Mycgr3G58567 Mycgr3T
  
Location: 13753-14506

Mycgr3G58567\_Mycgr3T

Mycgr3G100089 Mycgr3
  
Location: 14606-21152

Mycgr3G100089\_Mycgr3

Mycgr3G42698 Mycgr3T
  
Location: 21252-22131

Mycgr3G42698\_Mycgr3T

Mycgr3G71681 Mycgr3T
  
Location: 22231-23461

Mycgr3G71681\_Mycgr3T

Mycgr3G109328 Mycgr3
  
Location: 23561-24239

Mycgr3G109328\_Mycgr3

Mycgr3G104334 Mycgr3
  
Location: 24339-24567

Mycgr3G104334\_Mycgr3

Mycgr3G42715 Mycgr3T
  
Location: 24667-25981

Mycgr3G42715\_Mycgr3T

Mycgr3G92934 Mycgr3T
  
Location: 26081-27593

Mycgr3G92934\_Mycgr3T

Mycgr3G41969 Mycgr3T
  
Location: 27693-29328

Mycgr3G41969\_Mycgr3T

Mycgr3G80635 Mycgr3T
  
Location: 29428-29821

Mycgr3G80635\_Mycgr3T

Mycgr3G41426 Mycgr3T
  
Location: 29921-35255

Mycgr3G41426\_Mycgr3T

Mycgr3G104337 Mycgr3
  
Location: 35355-36108

Mycgr3G104337\_Mycgr3

Mycgr3G71679 Mycgr3T
  
Location: 36208-37300

Mycgr3G71679\_Mycgr3T

Mycgr3G92938 Mycgr3T
  
Location: 37400-38699

Mycgr3G92938\_Mycgr3T

Mycgr3G92941 Mycgr3T
  
Location: 38799-40734

Mycgr3G92941\_Mycgr3T

conserved hypothetical protein
  
Accession: EEH46744
  
Location: 1543163-1543911
  
 NCBI BlastP on this gene

EEH46744

predicted protein
  
Accession: EEH46745
  
Location: 1546206-1546837
  
 NCBI BlastP on this gene

EEH46745

conserved hypothetical protein
  
Accession: EEH46746
  
Location: 1547335-1548914
  
 NCBI BlastP on this gene

EEH46746

integrin beta-1-binding protein
  
Accession: EEH46747
  
Location: 1551230-1552426
  
 NCBI BlastP on this gene

EEH46747

glutaredoxin domain-containing protein
  
Accession: EEH46748
  
Location: 1552835-1553161
  
 NCBI BlastP on this gene

EEH46748

HpcH/HpaI aldolase/citrate lyase family protein
  
Accession: EEH46749
  
Location: 1553323-1554398
  
 NCBI BlastP on this gene

EEH46749

conserved hypothetical protein
  
Accession: EEH46750
  
Location: 1555652-1556446
  
  
**BlastP hit with Mycgr3G104337\_Mycgr3**
  
Percentage identity: 38 %
  
BlastP bit score: 169
  
Sequence coverage: 93 %
  
E-value: 8e-48
  
  
 NCBI BlastP on this gene

EEH46750

6-methylsalicylic acid synthase
  
Accession: EEH46751
  
Location: 1557888-1565913
  
  
**BlastP hit with Mycgr3G100089\_Mycgr3**
  
Percentage identity: 44 %
  
BlastP bit score: 1114
  
Sequence coverage: 57 %
  
E-value: 0.0
  
  
 NCBI BlastP on this gene

EEH46751

conserved hypothetical protein
  
Accession: EEH46752
  
Location: 1566757-1570854
  
 NCBI BlastP on this gene

EEH46752

predicted protein
  
Accession: EEH46753
  
Location: 1571376-1572180
  
 NCBI BlastP on this gene

EEH46753

acyl-CoA dehydrogenase
  
Accession: EEH46754
  
Location: 1573525-1575106
  
 NCBI BlastP on this gene

EEH46754

conserved hypothetical protein
  
Accession: EEH46755
  
Location: 1576504-1577866
  
 NCBI BlastP on this gene

EEH46755

predicted protein
  
Accession: EEH46756
  
Location: 1578838-1579023
  
 NCBI BlastP on this gene

EEH46756

predicted protein
  
Accession: EEH46757
  
Location: 1581754-1582767
  
 NCBI BlastP on this gene

EEH46757

predicted protein
  
Accession: EEH46758
  
Location: 1582897-1583238
  
 NCBI BlastP on this gene

EEH46758

endopolyphosphatase
  
Accession: EEH46759
  
Location: 1583560-1585697
  
 NCBI BlastP on this gene

EEH46759

Query: Architecture Search FASTA input

ABDG02000027 : Trichoderma atroviride IMI 206040    Total score: 2.0     Cumulative Blast bit score: 1269

Hit cluster cross-links:

Mycgr3G85918 Mycgr3T
  
Location: 0-1602

Mycgr3G85918\_Mycgr3T

Mycgr3G42010 Mycgr3T
  
Location: 1702-8569

Mycgr3G42010\_Mycgr3T

Mycgr3G29582 Mycgr3T
  
Location: 8669-8915

Mycgr3G29582\_Mycgr3T

Mycgr3G31170 Mycgr3T
  
Location: 9015-9255

Mycgr3G31170\_Mycgr3T

Mycgr3G85924 Mycgr3T
  
Location: 9355-11218

Mycgr3G85924\_Mycgr3T

Mycgr3G71676 Mycgr3T
  
Location: 11318-12494

Mycgr3G71676\_Mycgr3T

Mycgr3G11468 Mycgr3T
  
Location: 12594-13653

Mycgr3G11468\_Mycgr3T

Mycgr3G58567 Mycgr3T
  
Location: 13753-14506

Mycgr3G58567\_Mycgr3T

Mycgr3G100089 Mycgr3
  
Location: 14606-21152

Mycgr3G100089\_Mycgr3

Mycgr3G42698 Mycgr3T
  
Location: 21252-22131

Mycgr3G42698\_Mycgr3T

Mycgr3G71681 Mycgr3T
  
Location: 22231-23461

Mycgr3G71681\_Mycgr3T

Mycgr3G109328 Mycgr3
  
Location: 23561-24239

Mycgr3G109328\_Mycgr3

Mycgr3G104334 Mycgr3
  
Location: 24339-24567

Mycgr3G104334\_Mycgr3

Mycgr3G42715 Mycgr3T
  
Location: 24667-25981

Mycgr3G42715\_Mycgr3T

Mycgr3G92934 Mycgr3T
  
Location: 26081-27593

Mycgr3G92934\_Mycgr3T

Mycgr3G41969 Mycgr3T
  
Location: 27693-29328

Mycgr3G41969\_Mycgr3T

Mycgr3G80635 Mycgr3T
  
Location: 29428-29821

Mycgr3G80635\_Mycgr3T

Mycgr3G41426 Mycgr3T
  
Location: 29921-35255

Mycgr3G41426\_Mycgr3T

Mycgr3G104337 Mycgr3
  
Location: 35355-36108

Mycgr3G104337\_Mycgr3

Mycgr3G71679 Mycgr3T
  
Location: 36208-37300

Mycgr3G71679\_Mycgr3T

Mycgr3G92938 Mycgr3T
  
Location: 37400-38699

Mycgr3G92938\_Mycgr3T

Mycgr3G92941 Mycgr3T
  
Location: 38799-40734

Mycgr3G92941\_Mycgr3T

hypothetical protein
  
Accession: EHK42026
  
Location: 4856752-4857859
  
 NCBI BlastP on this gene

EHK42026

hypothetical protein
  
Accession: EHK42027
  
Location: 4860765-4861868
  
 NCBI BlastP on this gene

EHK42027

hypothetical protein
  
Accession: EHK42028
  
Location: 4864237-4866321
  
  
**BlastP hit with Mycgr3G42010\_Mycgr3T**
  
Percentage identity: 42 %
  
BlastP bit score: 521
  
Sequence coverage: 28 %
  
E-value: 2e-160
  
  
 NCBI BlastP on this gene

EHK42028

hypothetical protein
  
Accession: EHK42029
  
Location: 4866743-4866907
  
 NCBI BlastP on this gene

EHK42029

hypothetical protein
  
Accession: EHK42030
  
Location: 4868186-4871012
  
 NCBI BlastP on this gene

EHK42030

hypothetical protein
  
Accession: EHK42031
  
Location: 4871127-4872032
  
 NCBI BlastP on this gene

EHK42031

hypothetical protein
  
Accession: EHK42032
  
Location: 4872372-4874756
  
 NCBI BlastP on this gene

EHK42032

hypothetical protein
  
Accession: EHK42033
  
Location: 4876158-4876319
  
 NCBI BlastP on this gene

EHK42033

hypothetical protein
  
Accession: EHK42034
  
Location: 4876691-4878104
  
 NCBI BlastP on this gene

EHK42034

putative gamma-butyrobetaine hydroxylase
  
Accession: EHK42035
  
Location: 4878395-4879642
  
 NCBI BlastP on this gene

EHK42035

hypothetical protein
  
Accession: EHK42036
  
Location: 4880772-4882490
  
 NCBI BlastP on this gene

EHK42036

glycosyltransferase family 21 protein
  
Accession: EHK42037
  
Location: 4884170-4885819
  
 NCBI BlastP on this gene

EHK42037

polyketide synthase
  
Accession: EHK42038
  
Location: 4886179-4894430
  
  
**BlastP hit with Mycgr3G100089\_Mycgr3**
  
Percentage identity: 36 %
  
BlastP bit score: 748
  
Sequence coverage: 56 %
  
E-value: 0.0
  
  
 NCBI BlastP on this gene

EHK42038

hypothetical protein
  
Accession: EHK42039
  
Location: 4894943-4896379
  
 NCBI BlastP on this gene

EHK42039

Query: Architecture Search FASTA input

JH126400 : Cordyceps militaris CM01 unplaced genomic scaffold CCM\_S00002    Total score: 2.0     Cumulative Blast bit score: 1249

Hit cluster cross-links:

Mycgr3G85918 Mycgr3T
  
Location: 0-1602

Mycgr3G85918\_Mycgr3T

Mycgr3G42010 Mycgr3T
  
Location: 1702-8569

Mycgr3G42010\_Mycgr3T

Mycgr3G29582 Mycgr3T
  
Location: 8669-8915

Mycgr3G29582\_Mycgr3T

Mycgr3G31170 Mycgr3T
  
Location: 9015-9255

Mycgr3G31170\_Mycgr3T

Mycgr3G85924 Mycgr3T
  
Location: 9355-11218

Mycgr3G85924\_Mycgr3T

Mycgr3G71676 Mycgr3T
  
Location: 11318-12494

Mycgr3G71676\_Mycgr3T

Mycgr3G11468 Mycgr3T
  
Location: 12594-13653

Mycgr3G11468\_Mycgr3T

Mycgr3G58567 Mycgr3T
  
Location: 13753-14506

Mycgr3G58567\_Mycgr3T

Mycgr3G100089 Mycgr3
  
Location: 14606-21152

Mycgr3G100089\_Mycgr3

Mycgr3G42698 Mycgr3T
  
Location: 21252-22131

Mycgr3G42698\_Mycgr3T

Mycgr3G71681 Mycgr3T
  
Location: 22231-23461

Mycgr3G71681\_Mycgr3T

Mycgr3G109328 Mycgr3
  
Location: 23561-24239

Mycgr3G109328\_Mycgr3

Mycgr3G104334 Mycgr3
  
Location: 24339-24567

Mycgr3G104334\_Mycgr3

Mycgr3G42715 Mycgr3T
  
Location: 24667-25981

Mycgr3G42715\_Mycgr3T

Mycgr3G92934 Mycgr3T
  
Location: 26081-27593

Mycgr3G92934\_Mycgr3T

Mycgr3G41969 Mycgr3T
  
Location: 27693-29328

Mycgr3G41969\_Mycgr3T

Mycgr3G80635 Mycgr3T
  
Location: 29428-29821

Mycgr3G80635\_Mycgr3T

Mycgr3G41426 Mycgr3T
  
Location: 29921-35255

Mycgr3G41426\_Mycgr3T

Mycgr3G104337 Mycgr3
  
Location: 35355-36108

Mycgr3G104337\_Mycgr3

Mycgr3G71679 Mycgr3T
  
Location: 36208-37300

Mycgr3G71679\_Mycgr3T

Mycgr3G92938 Mycgr3T
  
Location: 37400-38699

Mycgr3G92938\_Mycgr3T

Mycgr3G92941 Mycgr3T
  
Location: 38799-40734

Mycgr3G92941\_Mycgr3T

hypothetical protein
  
Accession: EGX94099
  
Location: 1384963-1386147
  
 NCBI BlastP on this gene

EGX94099

hypothetical protein
  
Accession: EGX94100
  
Location: 1387259-1388416
  
 NCBI BlastP on this gene

EGX94100

hypothetical protein
  
Accession: EGX94101
  
Location: 1392197-1396931
  
 NCBI BlastP on this gene

EGX94101

amino acid permease, putative
  
Accession: EGX94102
  
Location: 1397487-1399217
  
 NCBI BlastP on this gene

EGX94102

polyketide synthase, putative
  
Accession: EGX94103
  
Location: 1399952-1408884
  
  
**BlastP hit with Mycgr3G100089\_Mycgr3**
  
Percentage identity: 42 %
  
BlastP bit score: 1052
  
Sequence coverage: 59 %
  
E-value: 0.0
  
  
 NCBI BlastP on this gene

EGX94103

DUF341 family oxidoreductase, putative
  
Accession: EGX94104
  
Location: 1412699-1413649
  
  
**BlastP hit with Mycgr3G104337\_Mycgr3**
  
Percentage identity: 40 %
  
BlastP bit score: 197
  
Sequence coverage: 100 %
  
E-value: 1e-58
  
  
 NCBI BlastP on this gene

EGX94104

diacylglycerol o-acyltransferase
  
Accession: EGX94105
  
Location: 1415843-1417519
  
 NCBI BlastP on this gene

EGX94105

Major facilitator superfamily transporter
  
Accession: EGX94106
  
Location: 1420270-1422026
  
 NCBI BlastP on this gene

EGX94106

guanyl-specific ribonuclease Pb1
  
Accession: EGX94107
  
Location: 1422597-1423050
  
 NCBI BlastP on this gene

EGX94107

hypothetical protein
  
Accession: EGX94108
  
Location: 1424609-1425922
  
 NCBI BlastP on this gene

EGX94108

hypothetical protein
  
Accession: EGX94109
  
Location: 1427004-1429745
  
 NCBI BlastP on this gene

EGX94109

Query: Architecture Search FASTA input

KB446555 : Pseudocercospora fijiensis CIRAD86 unplaced genomic scaffold MYCFIscaffold\_1    Total score: 2.0     Cumulative Blast bit score: 1231

Hit cluster cross-links:

Mycgr3G85918 Mycgr3T
  
Location: 0-1602

Mycgr3G85918\_Mycgr3T

Mycgr3G42010 Mycgr3T
  
Location: 1702-8569

Mycgr3G42010\_Mycgr3T

Mycgr3G29582 Mycgr3T
  
Location: 8669-8915

Mycgr3G29582\_Mycgr3T

Mycgr3G31170 Mycgr3T
  
Location: 9015-9255

Mycgr3G31170\_Mycgr3T

Mycgr3G85924 Mycgr3T
  
Location: 9355-11218

Mycgr3G85924\_Mycgr3T

Mycgr3G71676 Mycgr3T
  
Location: 11318-12494

Mycgr3G71676\_Mycgr3T

Mycgr3G11468 Mycgr3T
  
Location: 12594-13653

Mycgr3G11468\_Mycgr3T

Mycgr3G58567 Mycgr3T
  
Location: 13753-14506

Mycgr3G58567\_Mycgr3T

Mycgr3G100089 Mycgr3
  
Location: 14606-21152

Mycgr3G100089\_Mycgr3

Mycgr3G42698 Mycgr3T
  
Location: 21252-22131

Mycgr3G42698\_Mycgr3T

Mycgr3G71681 Mycgr3T
  
Location: 22231-23461

Mycgr3G71681\_Mycgr3T

Mycgr3G109328 Mycgr3
  
Location: 23561-24239

Mycgr3G109328\_Mycgr3

Mycgr3G104334 Mycgr3
  
Location: 24339-24567

Mycgr3G104334\_Mycgr3

Mycgr3G42715 Mycgr3T
  
Location: 24667-25981

Mycgr3G42715\_Mycgr3T

Mycgr3G92934 Mycgr3T
  
Location: 26081-27593

Mycgr3G92934\_Mycgr3T

Mycgr3G41969 Mycgr3T
  
Location: 27693-29328

Mycgr3G41969\_Mycgr3T

Mycgr3G80635 Mycgr3T
  
Location: 29428-29821

Mycgr3G80635\_Mycgr3T

Mycgr3G41426 Mycgr3T
  
Location: 29921-35255

Mycgr3G41426\_Mycgr3T

Mycgr3G104337 Mycgr3
  
Location: 35355-36108

Mycgr3G104337\_Mycgr3

Mycgr3G71679 Mycgr3T
  
Location: 36208-37300

Mycgr3G71679\_Mycgr3T

Mycgr3G92938 Mycgr3T
  
Location: 37400-38699

Mycgr3G92938\_Mycgr3T

Mycgr3G92941 Mycgr3T
  
Location: 38799-40734

Mycgr3G92941\_Mycgr3T

hypothetical protein
  
Accession: EME88234
  
Location: 4514629-4517144
  
 NCBI BlastP on this gene

EME88234

hypothetical protein
  
Accession: EME88235
  
Location: 4517174-4521585
  
 NCBI BlastP on this gene

EME88235

hypothetical protein
  
Accession: EME88236
  
Location: 4522263-4523208
  
 NCBI BlastP on this gene

EME88236

hypothetical protein
  
Accession: EME88237
  
Location: 4526412-4527799
  
 NCBI BlastP on this gene

EME88237

hypothetical protein
  
Accession: EME88239
  
Location: 4527979-4529325
  
 NCBI BlastP on this gene

EME88239

hypothetical protein
  
Accession: EME88240
  
Location: 4530267-4532277
  
 NCBI BlastP on this gene

EME88240

hypothetical protein
  
Accession: EME88241
  
Location: 4532302-4534215
  
  
**BlastP hit with Mycgr3G85924\_Mycgr3T**
  
Percentage identity: 57 %
  
BlastP bit score: 545
  
Sequence coverage: 100 %
  
E-value: 0.0
  
  
 NCBI BlastP on this gene

EME88241

hypothetical protein
  
Accession: EME88242
  
Location: 4534506-4535909
  
  
**BlastP hit with Mycgr3G92934\_Mycgr3T**
  
Percentage identity: 73 %
  
BlastP bit score: 686
  
Sequence coverage: 93 %
  
E-value: 0.0
  
  
 NCBI BlastP on this gene

EME88242

hypothetical protein
  
Accession: EME88243
  
Location: 4535889-4537250
  
 NCBI BlastP on this gene

EME88243

hypothetical protein
  
Accession: EME88244
  
Location: 4537275-4538559
  
 NCBI BlastP on this gene

EME88244

hypothetical protein
  
Accession: EME88245
  
Location: 4540242-4543405
  
 NCBI BlastP on this gene

EME88245

pyruvate kinase
  
Accession: EME88246
  
Location: 4544207-4545908
  
 NCBI BlastP on this gene

EME88246

hypothetical protein
  
Accession: EME88247
  
Location: 4549659-4551776
  
 NCBI BlastP on this gene

EME88247

hypothetical protein
  
Accession: EME88248
  
Location: 4552447-4553023
  
 NCBI BlastP on this gene

EME88248

hypothetical protein
  
Accession: EME88249
  
Location: 4553311-4554218
  
 NCBI BlastP on this gene

EME88249

casein kinase II, beta subunit
  
Accession: EME88250
  
Location: 4554519-4555693
  
 NCBI BlastP on this gene

EME88250

Query: Architecture Search FASTA input

AM920428 : Penicillium chrysogenum Wisconsin 54-1255 complete genome, contig Pc00c13.    Total score: 2.0     Cumulative Blast bit score: 1226

Hit cluster cross-links:

Mycgr3G85918 Mycgr3T
  
Location: 0-1602

Mycgr3G85918\_Mycgr3T

Mycgr3G42010 Mycgr3T
  
Location: 1702-8569

Mycgr3G42010\_Mycgr3T

Mycgr3G29582 Mycgr3T
  
Location: 8669-8915

Mycgr3G29582\_Mycgr3T

Mycgr3G31170 Mycgr3T
  
Location: 9015-9255

Mycgr3G31170\_Mycgr3T

Mycgr3G85924 Mycgr3T
  
Location: 9355-11218

Mycgr3G85924\_Mycgr3T

Mycgr3G71676 Mycgr3T
  
Location: 11318-12494

Mycgr3G71676\_Mycgr3T

Mycgr3G11468 Mycgr3T
  
Location: 12594-13653

Mycgr3G11468\_Mycgr3T

Mycgr3G58567 Mycgr3T
  
Location: 13753-14506

Mycgr3G58567\_Mycgr3T

Mycgr3G100089 Mycgr3
  
Location: 14606-21152

Mycgr3G100089\_Mycgr3

Mycgr3G42698 Mycgr3T
  
Location: 21252-22131

Mycgr3G42698\_Mycgr3T

Mycgr3G71681 Mycgr3T
  
Location: 22231-23461

Mycgr3G71681\_Mycgr3T

Mycgr3G109328 Mycgr3
  
Location: 23561-24239

Mycgr3G109328\_Mycgr3

Mycgr3G104334 Mycgr3
  
Location: 24339-24567

Mycgr3G104334\_Mycgr3

Mycgr3G42715 Mycgr3T
  
Location: 24667-25981

Mycgr3G42715\_Mycgr3T

Mycgr3G92934 Mycgr3T
  
Location: 26081-27593

Mycgr3G92934\_Mycgr3T

Mycgr3G41969 Mycgr3T
  
Location: 27693-29328

Mycgr3G41969\_Mycgr3T

Mycgr3G80635 Mycgr3T
  
Location: 29428-29821

Mycgr3G80635\_Mycgr3T

Mycgr3G41426 Mycgr3T
  
Location: 29921-35255

Mycgr3G41426\_Mycgr3T

Mycgr3G104337 Mycgr3
  
Location: 35355-36108

Mycgr3G104337\_Mycgr3

Mycgr3G71679 Mycgr3T
  
Location: 36208-37300

Mycgr3G71679\_Mycgr3T

Mycgr3G92938 Mycgr3T
  
Location: 37400-38699

Mycgr3G92938\_Mycgr3T

Mycgr3G92941 Mycgr3T
  
Location: 38799-40734

Mycgr3G92941\_Mycgr3T

unnamed
  
Accession: CAP91930
  
Location: 2073749-2075700
  
 NCBI BlastP on this gene

Pc13g08610

hypothetical protein
  
Accession: CAP91931
  
Location: 2076390-2077813
  
 NCBI BlastP on this gene

Pc13g08620

not annotated
  
Accession: CAP91932
  
Location: 2081162-2083335
  
 NCBI BlastP on this gene

Pc13g08630

not annotated
  
Accession: CAP91933
  
Location: 2084282-2086141
  
 NCBI BlastP on this gene

Pc13g08640

not annotated
  
Accession: CAP91934
  
Location: 2086330-2087551
  
 NCBI BlastP on this gene

Pc13g08650

hypothetical protein
  
Accession: CAP91935
  
Location: 2088816-2089313
  
 NCBI BlastP on this gene

Pc13g08660

not annotated
  
Accession: CAP91936
  
Location: 2089563-2091214
  
 NCBI BlastP on this gene

Pc13g08670

not annotated
  
Accession: CAP91937
  
Location: 2091632-2093092
  
 NCBI BlastP on this gene

Pc13g08680

not annotated
  
Accession: CAP91938
  
Location: 2093606-2101605
  
  
**BlastP hit with Mycgr3G100089\_Mycgr3**
  
Percentage identity: 43 %
  
BlastP bit score: 1062
  
Sequence coverage: 58 %
  
E-value: 0.0
  
  
 NCBI BlastP on this gene

Pc13g08690

not annotated
  
Accession: CAP91939
  
Location: 2102331-2103184
  
  
**BlastP hit with Mycgr3G104337\_Mycgr3**
  
Percentage identity: 39 %
  
BlastP bit score: 164
  
Sequence coverage: 98 %
  
E-value: 4e-46
  
  
 NCBI BlastP on this gene

Pc13g08700

unnamed
  
Accession: CAP91940
  
Location: 2104634-2105583
  
 NCBI BlastP on this gene

Pc13g08710

not annotated
  
Accession: CAP91941
  
Location: 2106573-2113325
  
 NCBI BlastP on this gene

Pc13g08720

unnamed
  
Accession: CAP91942
  
Location: 2114599-2115663
  
 NCBI BlastP on this gene

Pc13g08730

hypothetical protein
  
Accession: CAP91943
  
Location: 2115756-2117286
  
 NCBI BlastP on this gene

Pc13g08740

not annotated
  
Accession: CAP91944
  
Location: 2117592-2119232
  
 NCBI BlastP on this gene

Pc13g08750

Query: Architecture Search FASTA input

KB446535 : Dothistroma septosporum NZE10 unplaced genomic scaffold DOTSEscaffold\_1    Total score: 2.0     Cumulative Blast bit score: 1220

Hit cluster cross-links:

Mycgr3G85918 Mycgr3T
  
Location: 0-1602

Mycgr3G85918\_Mycgr3T

Mycgr3G42010 Mycgr3T
  
Location: 1702-8569

Mycgr3G42010\_Mycgr3T

Mycgr3G29582 Mycgr3T
  
Location: 8669-8915

Mycgr3G29582\_Mycgr3T

Mycgr3G31170 Mycgr3T
  
Location: 9015-9255

Mycgr3G31170\_Mycgr3T

Mycgr3G85924 Mycgr3T
  
Location: 9355-11218

Mycgr3G85924\_Mycgr3T

Mycgr3G71676 Mycgr3T
  
Location: 11318-12494

Mycgr3G71676\_Mycgr3T

Mycgr3G11468 Mycgr3T
  
Location: 12594-13653

Mycgr3G11468\_Mycgr3T

Mycgr3G58567 Mycgr3T
  
Location: 13753-14506

Mycgr3G58567\_Mycgr3T

Mycgr3G100089 Mycgr3
  
Location: 14606-21152

Mycgr3G100089\_Mycgr3

Mycgr3G42698 Mycgr3T
  
Location: 21252-22131

Mycgr3G42698\_Mycgr3T

Mycgr3G71681 Mycgr3T
  
Location: 22231-23461

Mycgr3G71681\_Mycgr3T

Mycgr3G109328 Mycgr3
  
Location: 23561-24239

Mycgr3G109328\_Mycgr3

Mycgr3G104334 Mycgr3
  
Location: 24339-24567

Mycgr3G104334\_Mycgr3

Mycgr3G42715 Mycgr3T
  
Location: 24667-25981

Mycgr3G42715\_Mycgr3T

Mycgr3G92934 Mycgr3T
  
Location: 26081-27593

Mycgr3G92934\_Mycgr3T

Mycgr3G41969 Mycgr3T
  
Location: 27693-29328

Mycgr3G41969\_Mycgr3T

Mycgr3G80635 Mycgr3T
  
Location: 29428-29821

Mycgr3G80635\_Mycgr3T

Mycgr3G41426 Mycgr3T
  
Location: 29921-35255

Mycgr3G41426\_Mycgr3T

Mycgr3G104337 Mycgr3
  
Location: 35355-36108

Mycgr3G104337\_Mycgr3

Mycgr3G71679 Mycgr3T
  
Location: 36208-37300

Mycgr3G71679\_Mycgr3T

Mycgr3G92938 Mycgr3T
  
Location: 37400-38699

Mycgr3G92938\_Mycgr3T

Mycgr3G92941 Mycgr3T
  
Location: 38799-40734

Mycgr3G92941\_Mycgr3T

glycoside hydrolase family 3 protein
  
Accession: EME48327
  
Location: 39310-42090
  
 NCBI BlastP on this gene

EME48327

hypothetical protein
  
Accession: EME48328
  
Location: 42979-43481
  
 NCBI BlastP on this gene

EME48328

glycoside hydrolase family 54 protein
  
Accession: EME48329
  
Location: 46959-48525
  
 NCBI BlastP on this gene

EME48329

hypothetical protein
  
Accession: EME48330
  
Location: 50587-52626
  
 NCBI BlastP on this gene

EME48330

hypothetical protein
  
Accession: EME48331
  
Location: 53246-53704
  
 NCBI BlastP on this gene

EME48331

hypothetical protein
  
Accession: EME48332
  
Location: 54209-55834
  
 NCBI BlastP on this gene

EME48332

hypothetical protein
  
Accession: EME48333
  
Location: 57200-59049
  
  
**BlastP hit with Mycgr3G92934\_Mycgr3T**
  
Percentage identity: 77 %
  
BlastP bit score: 702
  
Sequence coverage: 88 %
  
E-value: 0.0
  
  
 NCBI BlastP on this gene

EME48333

hypothetical protein
  
Accession: EME48334
  
Location: 59373-61262
  
  
**BlastP hit with Mycgr3G85924\_Mycgr3T**
  
Percentage identity: 56 %
  
BlastP bit score: 518
  
Sequence coverage: 100 %
  
E-value: 4e-173
  
  
 NCBI BlastP on this gene

EME48334

hypothetical protein
  
Accession: EME48335
  
Location: 62847-63128
  
 NCBI BlastP on this gene

EME48335

hypothetical protein
  
Accession: EME48336
  
Location: 64347-64823
  
 NCBI BlastP on this gene

EME48336

hypothetical protein
  
Accession: EME48337
  
Location: 65187-65564
  
 NCBI BlastP on this gene

EME48337

hypothetical protein
  
Accession: EME48338
  
Location: 66101-67452
  
 NCBI BlastP on this gene

EME48338

hypothetical protein
  
Accession: EME48339
  
Location: 68579-69091
  
 NCBI BlastP on this gene

EME48339

hypothetical protein
  
Accession: EME48340
  
Location: 70223-71845
  
 NCBI BlastP on this gene

EME48340

hypothetical protein
  
Accession: EME48341
  
Location: 72444-73388
  
 NCBI BlastP on this gene

EME48341

hypothetical protein
  
Accession: EME48342
  
Location: 73454-73768
  
 NCBI BlastP on this gene

EME48342

hypothetical protein
  
Accession: EME48343
  
Location: 74058-75148
  
 NCBI BlastP on this gene

EME48343

hypothetical protein
  
Accession: EME48344
  
Location: 75364-77694
  
 NCBI BlastP on this gene

EME48344

hypothetical protein
  
Accession: EME48345
  
Location: 78546-80393
  
 NCBI BlastP on this gene

EME48345

Query: Architecture Search FASTA input

AKCU01000203 : Penicillium digitatum Pd1    Total score: 2.0     Cumulative Blast bit score: 1210

Hit cluster cross-links:

Mycgr3G85918 Mycgr3T
  
Location: 0-1602

Mycgr3G85918\_Mycgr3T

Mycgr3G42010 Mycgr3T
  
Location: 1702-8569

Mycgr3G42010\_Mycgr3T

Mycgr3G29582 Mycgr3T
  
Location: 8669-8915

Mycgr3G29582\_Mycgr3T

Mycgr3G31170 Mycgr3T
  
Location: 9015-9255

Mycgr3G31170\_Mycgr3T

Mycgr3G85924 Mycgr3T
  
Location: 9355-11218

Mycgr3G85924\_Mycgr3T

Mycgr3G71676 Mycgr3T
  
Location: 11318-12494

Mycgr3G71676\_Mycgr3T

Mycgr3G11468 Mycgr3T
  
Location: 12594-13653

Mycgr3G11468\_Mycgr3T

Mycgr3G58567 Mycgr3T
  
Location: 13753-14506

Mycgr3G58567\_Mycgr3T

Mycgr3G100089 Mycgr3
  
Location: 14606-21152

Mycgr3G100089\_Mycgr3

Mycgr3G42698 Mycgr3T
  
Location: 21252-22131

Mycgr3G42698\_Mycgr3T

Mycgr3G71681 Mycgr3T
  
Location: 22231-23461

Mycgr3G71681\_Mycgr3T

Mycgr3G109328 Mycgr3
  
Location: 23561-24239

Mycgr3G109328\_Mycgr3

Mycgr3G104334 Mycgr3
  
Location: 24339-24567

Mycgr3G104334\_Mycgr3

Mycgr3G42715 Mycgr3T
  
Location: 24667-25981

Mycgr3G42715\_Mycgr3T

Mycgr3G92934 Mycgr3T
  
Location: 26081-27593

Mycgr3G92934\_Mycgr3T

Mycgr3G41969 Mycgr3T
  
Location: 27693-29328

Mycgr3G41969\_Mycgr3T

Mycgr3G80635 Mycgr3T
  
Location: 29428-29821

Mycgr3G80635\_Mycgr3T

Mycgr3G41426 Mycgr3T
  
Location: 29921-35255

Mycgr3G41426\_Mycgr3T

Mycgr3G104337 Mycgr3
  
Location: 35355-36108

Mycgr3G104337\_Mycgr3

Mycgr3G71679 Mycgr3T
  
Location: 36208-37300

Mycgr3G71679\_Mycgr3T

Mycgr3G92938 Mycgr3T
  
Location: 37400-38699

Mycgr3G92938\_Mycgr3T

Mycgr3G92941 Mycgr3T
  
Location: 38799-40734

Mycgr3G92941\_Mycgr3T

hypothetical protein
  
Accession: EKV17643
  
Location: 54916-55116
  
 NCBI BlastP on this gene

EKV17643

hypothetical protein
  
Accession: EKV17644
  
Location: 55547-55684
  
 NCBI BlastP on this gene

EKV17644

Myosin heavy chain-like protein, putative
  
Accession: EKV17645
  
Location: 60660-67406
  
 NCBI BlastP on this gene

EKV17645

Endosomal cargo receptor (P24), putative
  
Accession: EKV17646
  
Location: 68571-69511
  
 NCBI BlastP on this gene

EKV17646

hypothetical protein
  
Accession: EKV17647
  
Location: 70102-70939
  
 NCBI BlastP on this gene

EKV17647

hypothetical protein
  
Accession: EKV17648
  
Location: 71030-71891
  
  
**BlastP hit with Mycgr3G104337\_Mycgr3**
  
Percentage identity: 39 %
  
BlastP bit score: 160
  
Sequence coverage: 98 %
  
E-value: 2e-44
  
  
 NCBI BlastP on this gene

EKV17648

hypothetical protein
  
Accession: EKV17649
  
Location: 72623-80609
  
  
**BlastP hit with Mycgr3G100089\_Mycgr3**
  
Percentage identity: 43 %
  
BlastP bit score: 1050
  
Sequence coverage: 58 %
  
E-value: 0.0
  
  
 NCBI BlastP on this gene

EKV17649

hypothetical protein
  
Accession: EKV17650
  
Location: 81973-83624
  
 NCBI BlastP on this gene

EKV17650

hypothetical protein
  
Accession: EKV17651
  
Location: 84814-86027
  
 NCBI BlastP on this gene

EKV17651

MFS lactose permease, putative
  
Accession: EKV17652
  
Location: 87470-89636
  
 NCBI BlastP on this gene

EKV17652

Endopolyphosphatase
  
Accession: EKV17653
  
Location: 93667-95616
  
 NCBI BlastP on this gene

EKV17653

hypothetical protein
  
Accession: EKV17654
  
Location: 97541-98614
  
 NCBI BlastP on this gene

EKV17654

Query: Architecture Search FASTA input

101. :  GL385398 Gaeumannomyces graminis var. tritici R3-111a-1 unplaced genomic scaffold supercont2.4     Total score: 2.0     Cumulative Blast bit score: 1750

Mycgr3G85918 Mycgr3T
  
Location: 0-1602
  
 NCBI BlastP on this gene

Mycgr3G85918\_Mycgr3T

Mycgr3G42010 Mycgr3T
  
Location: 1702-8569
  
 NCBI BlastP on this gene

Mycgr3G42010\_Mycgr3T

Mycgr3G29582 Mycgr3T
  
Location: 8669-8915
  
 NCBI BlastP on this gene

Mycgr3G29582\_Mycgr3T

Mycgr3G31170 Mycgr3T
  
Location: 9015-9255
  
 NCBI BlastP on this gene

Mycgr3G31170\_Mycgr3T

Mycgr3G85924 Mycgr3T
  
Location: 9355-11218
  
 NCBI BlastP on this gene

Mycgr3G85924\_Mycgr3T

Mycgr3G71676 Mycgr3T
  
Location: 11318-12494
  
 NCBI BlastP on this gene

Mycgr3G71676\_Mycgr3T

Mycgr3G11468 Mycgr3T
  
Location: 12594-13653
  
 NCBI BlastP on this gene

Mycgr3G11468\_Mycgr3T

Mycgr3G58567 Mycgr3T
  
Location: 13753-14506
  
 NCBI BlastP on this gene

Mycgr3G58567\_Mycgr3T

Mycgr3G100089 Mycgr3
  
Location: 14606-21152
  
 NCBI BlastP on this gene

Mycgr3G100089\_Mycgr3

Mycgr3G42698 Mycgr3T
  
Location: 21252-22131
  
 NCBI BlastP on this gene

Mycgr3G42698\_Mycgr3T

Mycgr3G71681 Mycgr3T
  
Location: 22231-23461
  
 NCBI BlastP on this gene

Mycgr3G71681\_Mycgr3T

Mycgr3G109328 Mycgr3
  
Location: 23561-24239
  
 NCBI BlastP on this gene

Mycgr3G109328\_Mycgr3

Mycgr3G104334 Mycgr3
  
Location: 24339-24567
  
 NCBI BlastP on this gene

Mycgr3G104334\_Mycgr3

Mycgr3G42715 Mycgr3T
  
Location: 24667-25981
  
 NCBI BlastP on this gene

Mycgr3G42715\_Mycgr3T

Mycgr3G92934 Mycgr3T
  
Location: 26081-27593
  
 NCBI BlastP on this gene

Mycgr3G92934\_Mycgr3T

Mycgr3G41969 Mycgr3T
  
Location: 27693-29328
  
 NCBI BlastP on this gene

Mycgr3G41969\_Mycgr3T

Mycgr3G80635 Mycgr3T
  
Location: 29428-29821
  
 NCBI BlastP on this gene

Mycgr3G80635\_Mycgr3T

Mycgr3G41426 Mycgr3T
  
Location: 29921-35255
  
 NCBI BlastP on this gene

Mycgr3G41426\_Mycgr3T

Mycgr3G104337 Mycgr3
  
Location: 35355-36108
  
 NCBI BlastP on this gene

Mycgr3G104337\_Mycgr3

Mycgr3G71679 Mycgr3T
  
Location: 36208-37300
  
 NCBI BlastP on this gene

Mycgr3G71679\_Mycgr3T

Mycgr3G92938 Mycgr3T
  
Location: 37400-38699
  
 NCBI BlastP on this gene

Mycgr3G92938\_Mycgr3T

Mycgr3G92941 Mycgr3T
  
Location: 38799-40734
  
 NCBI BlastP on this gene

Mycgr3G92941\_Mycgr3T

hypothetical protein
  
Accession: EJT74836
  
Location: 3789867-3790455
  
 NCBI BlastP on this gene

EJT74836

hypothetical protein
  
Accession: EJT74837
  
Location: 3792122-3792301
  
 NCBI BlastP on this gene

EJT74837

hypothetical protein
  
Accession: EJT74838
  
Location: 3792683-3794116
  
 NCBI BlastP on this gene

EJT74838

hypothetical protein
  
Accession: EJT74839
  
Location: 3794658-3795331
  
 NCBI BlastP on this gene

EJT74839

hypothetical protein
  
Accession: EJT74840
  
Location: 3796051-3801488
  
 NCBI BlastP on this gene

EJT74840

hypothetical protein
  
Accession: EJT74841
  
Location: 3802551-3804030
  
 NCBI BlastP on this gene

EJT74841

hypothetical protein
  
Accession: EJT74842
  
Location: 3805447-3807160
  
  
**BlastP hit with Mycgr3G42698\_Mycgr3T**
  
Percentage identity: 30 %
  
BlastP bit score: 152
  
Sequence coverage: 116 %
  
E-value: 4e-39
  
  
 NCBI BlastP on this gene

EJT74842

hypothetical protein
  
Accession: EJT74843
  
Location: 3808724-3811516
  
 NCBI BlastP on this gene

EJT74843

hypothetical protein
  
Accession: EJT74844
  
Location: 3811990-3812214
  
 NCBI BlastP on this gene

EJT74844

hypothetical protein
  
Accession: EJT74845
  
Location: 3813386-3820649
  
  
**BlastP hit with Mycgr3G100089\_Mycgr3**
  
Percentage identity: 43 %
  
BlastP bit score: 1598
  
Sequence coverage: 92 %
  
E-value: 0.0
  
  
 NCBI BlastP on this gene

EJT74845

hypothetical protein
  
Accession: EJT74846
  
Location: 3821818-3823059
  
 NCBI BlastP on this gene

EJT74846

hypothetical protein
  
Accession: EJT74847
  
Location: 3824537-3826272
  
 NCBI BlastP on this gene

EJT74847

hypothetical protein
  
Accession: EJT74848
  
Location: 3827929-3828573
  
 NCBI BlastP on this gene

EJT74848

hypothetical protein
  
Accession: EJT74849
  
Location: 3829635-3830404
  
 NCBI BlastP on this gene

EJT74849

hypothetical protein
  
Accession: EJT74850
  
Location: 3830755-3831813
  
 NCBI BlastP on this gene

EJT74850

hypothetical protein
  
Accession: EJT74851
  
Location: 3832406-3833396
  
 NCBI BlastP on this gene

EJT74851

hypothetical protein
  
Accession: EJT74852
  
Location: 3833471-3838560
  
 NCBI BlastP on this gene

EJT74852

102. :  DS231618 Pyrenophora tritici-repentis Pt-1C-BFP supercont1.4 genomic scaffold     Total score: 2.0     Cumulative Blast bit score: 1726

predicted protein
  
Accession: EDU47629
  
Location: 858974-859438
  
 NCBI BlastP on this gene

EDU47629

predicted protein
  
Accession: EDU47630
  
Location: 873868-874580
  
 NCBI BlastP on this gene

EDU47630

stage V sporulation protein K
  
Accession: EDU47631
  
Location: 874875-880745
  
  
**BlastP hit with Mycgr3G42010\_Mycgr3T**
  
Percentage identity: 48 %
  
BlastP bit score: 1436
  
Sequence coverage: 68 %
  
E-value: 0.0
  
  
 NCBI BlastP on this gene

EDU47631

conserved hypothetical protein
  
Accession: EDU47632
  
Location: 882420-883913
  
  
**BlastP hit with Mycgr3G92938\_Mycgr3T**
  
Percentage identity: 40 %
  
BlastP bit score: 290
  
Sequence coverage: 101 %
  
E-value: 4e-89
  
  
 NCBI BlastP on this gene

EDU47632

conserved hypothetical protein
  
Accession: EDU47633
  
Location: 885945-887042
  
 NCBI BlastP on this gene

EDU47633

HPP family protein
  
Accession: EDU47634
  
Location: 887801-888661
  
 NCBI BlastP on this gene

EDU47634

galactose oxidase precursor
  
Accession: EDU47635
  
Location: 888939-890474
  
 NCBI BlastP on this gene

EDU47635

F-box domain containing protein
  
Accession: EDU47636
  
Location: 895971-897931
  
 NCBI BlastP on this gene

EDU47636

conserved hypothetical protein
  
Accession: EDU47637
  
Location: 898388-899847
  
 NCBI BlastP on this gene

EDU47637

proteasome-activating nucleotidase
  
Accession: EDU47638
  
Location: 901742-903448
  
 NCBI BlastP on this gene

EDU47638

103. :  DS985214 Verticillium albo-atrum VaMs.102 supercont1.1 genomic scaffold     Total score: 2.0     Cumulative Blast bit score: 1710

ubiquitin C-terminal hydrolase family protein
  
Accession: EEY14048
  
Location: 475230-477937
  
 NCBI BlastP on this gene

EEY14048

54S ribosomal protein L7
  
Accession: EEY14049
  
Location: 479798-480977
  
 NCBI BlastP on this gene

EEY14049

Hsp70 nucleotide exchange factor FES1
  
Accession: EEY14050
  
Location: 481241-481759
  
 NCBI BlastP on this gene

EEY14050

actin
  
Accession: EEY14051
  
Location: 484296-485863
  
 NCBI BlastP on this gene

EEY14051

peptidyl-tRNA hydrolase domain-containing protein
  
Accession: EEY14052
  
Location: 486325-486933
  
 NCBI BlastP on this gene

EEY14052

ATP-binding cassette sub-family B member 5
  
Accession: EEY14053
  
Location: 487356-492271
  
 NCBI BlastP on this gene

EEY14053

conserved hypothetical protein
  
Accession: EEY14054
  
Location: 492973-493909
  
  
**BlastP hit with Mycgr3G104337\_Mycgr3**
  
Percentage identity: 40 %
  
BlastP bit score: 206
  
Sequence coverage: 101 %
  
E-value: 5e-62
  
  
 NCBI BlastP on this gene

EEY14054

fatty acid synthase S-acetyltransferase
  
Accession: EEY14055
  
Location: 495873-502926
  
  
**BlastP hit with Mycgr3G100089\_Mycgr3**
  
Percentage identity: 40 %
  
BlastP bit score: 1504
  
Sequence coverage: 91 %
  
E-value: 0.0
  
  
 NCBI BlastP on this gene

EEY14055

conserved hypothetical protein
  
Accession: EEY14056
  
Location: 503348-503776
  
 NCBI BlastP on this gene

EEY14056

elongator complex protein
  
Accession: EEY14057
  
Location: 504176-506869
  
 NCBI BlastP on this gene

EEY14057

conserved hypothetical protein
  
Accession: EEY14058
  
Location: 507493-507988
  
 NCBI BlastP on this gene

EEY14058

zinc finger protein
  
Accession: EEY14059
  
Location: 512644-514119
  
 NCBI BlastP on this gene

EEY14059

104. :  JH767580 Coniosporium apollinis CBS 100218 chromosome Unknown supercont1.27     Total score: 2.0     Cumulative Blast bit score: 1687

hypothetical protein
  
Accession: EON66363
  
Location: 91730-93778
  
 NCBI BlastP on this gene

EON66363

phosphoglycerate mutase
  
Accession: EON66362
  
Location: 90194-91108
  
 NCBI BlastP on this gene

EON66362

hypothetical protein
  
Accession: EON66361
  
Location: 87609-88719
  
 NCBI BlastP on this gene

EON66361

hypothetical protein
  
Accession: EON66360
  
Location: 84619-86356
  
 NCBI BlastP on this gene

EON66360

hypothetical protein
  
Accession: EON66359
  
Location: 82693-83513
  
 NCBI BlastP on this gene

EON66359

hypothetical protein
  
Accession: EON66358
  
Location: 80299-81139
  
  
**BlastP hit with Mycgr3G31170\_Mycgr3T**
  
Percentage identity: 98 %
  
BlastP bit score: 162
  
Sequence coverage: 100 %
  
E-value: 5e-48
  
  
 NCBI BlastP on this gene

EON66358

hypothetical protein
  
Accession: EON66357
  
Location: 76583-78322
  
 NCBI BlastP on this gene

EON66357

hypothetical protein
  
Accession: EON66356
  
Location: 74654-76148
  
 NCBI BlastP on this gene

EON66356

phospholipid-translocating ATPase
  
Accession: EON66355
  
Location: 66517-71262
  
 NCBI BlastP on this gene

EON66355

hypothetical protein
  
Accession: EON66354
  
Location: 59223-65407
  
  
**BlastP hit with Mycgr3G41426\_Mycgr3T**
  
Percentage identity: 44 %
  
BlastP bit score: 1525
  
Sequence coverage: 103 %
  
E-value: 0.0
  
  
 NCBI BlastP on this gene

EON66354

hypothetical protein
  
Accession: EON66353
  
Location: 54947-57498
  
 NCBI BlastP on this gene

EON66353

hypothetical protein
  
Accession: EON66352
  
Location: 52582-54544
  
 NCBI BlastP on this gene

EON66352

hypothetical protein
  
Accession: EON66351
  
Location: 51157-51979
  
 NCBI BlastP on this gene

EON66351

hypothetical protein
  
Accession: EON66350
  
Location: 50328-50836
  
 NCBI BlastP on this gene

EON66350

hypothetical protein
  
Accession: EON66349
  
Location: 49686-50200
  
 NCBI BlastP on this gene

EON66349

hypothetical protein
  
Accession: EON66348
  
Location: 48270-49325
  
 NCBI BlastP on this gene

EON66348

105. :  GL385401 Gaeumannomyces graminis var. tritici R3-111a-1 unplaced genomic scaffold supercont2.7     Total score: 2.0     Cumulative Blast bit score: 1683

hypothetical protein
  
Accession: EJT70646
  
Location: 1057927-1060380
  
 NCBI BlastP on this gene

EJT70646

1-aminocyclopropane-1-carboxylate deaminase
  
Accession: EJT70645
  
Location: 1056079-1057220
  
 NCBI BlastP on this gene

EJT70645

hypothetical protein
  
Accession: EJT70644
  
Location: 1055504-1055768
  
 NCBI BlastP on this gene

EJT70644

amidophosphoribosyltransferase
  
Accession: EJT70643
  
Location: 1052747-1054769
  
 NCBI BlastP on this gene

EJT70643

hypothetical protein
  
Accession: EJT70642
  
Location: 1049416-1051380
  
 NCBI BlastP on this gene

EJT70642

DNA-binding protein SMUBP-2
  
Accession: EJT70641
  
Location: 1041985-1048462
  
  
**BlastP hit with Mycgr3G41426\_Mycgr3T**
  
Percentage identity: 33 %
  
BlastP bit score: 922
  
Sequence coverage: 106 %
  
E-value: 0.0
  
  
 NCBI BlastP on this gene

EJT70641

hypothetical protein
  
Accession: EJT70640
  
Location: 1040156-1040809
  
 NCBI BlastP on this gene

EJT70640

hypothetical protein
  
Accession: EJT70639
  
Location: 1038058-1039388
  
 NCBI BlastP on this gene

EJT70639

hypothetical protein
  
Accession: EJT70638
  
Location: 1036415-1037864
  
 NCBI BlastP on this gene

EJT70638

hypothetical protein
  
Accession: EJT70637
  
Location: 1035627-1036258
  
 NCBI BlastP on this gene

EJT70637

hypothetical protein
  
Accession: EJT70636
  
Location: 1033911-1035087
  
 NCBI BlastP on this gene

EJT70636

hypothetical protein
  
Accession: EJT70635
  
Location: 1030771-1032717
  
 NCBI BlastP on this gene

EJT70635

hypothetical protein
  
Accession: EJT70634
  
Location: 1025775-1029803
  
  
**BlastP hit with Mycgr3G42010\_Mycgr3T**
  
Percentage identity: 40 %
  
BlastP bit score: 761
  
Sequence coverage: 48 %
  
E-value: 0.0
  
  
 NCBI BlastP on this gene

EJT70634

hypothetical protein
  
Accession: EJT70633
  
Location: 1024594-1025754
  
 NCBI BlastP on this gene

EJT70633

hypothetical protein
  
Accession: EJT70632
  
Location: 1024224-1024536
  
 NCBI BlastP on this gene

EJT70632

hypothetical protein
  
Accession: EJT70631
  
Location: 1017782-1022084
  
 NCBI BlastP on this gene

EJT70631

hypothetical protein
  
Accession: EJT70630
  
Location: 1013801-1017514
  
 NCBI BlastP on this gene

EJT70630

106. :  CU633870 Podospora anserina S mat+ genomic DNA chromosome 5, supercontig 8.     Total score: 2.0     Cumulative Blast bit score: 1665

not annotated
  
Accession: CAP65179
  
Location: 371658-372986
  
 NCBI BlastP on this gene

CAP65179

not annotated
  
Accession: CAP65180
  
Location: 375153-375682
  
 NCBI BlastP on this gene

CAP65180

not annotated
  
Accession: CAP65181
  
Location: 377132-378190
  
 NCBI BlastP on this gene

CAP65181

not annotated
  
Accession: CAP65182
  
Location: 381477-383442
  
 NCBI BlastP on this gene

CAP65182

not annotated
  
Accession: CAP65183
  
Location: 384465-385198
  
 NCBI BlastP on this gene

CAP65183

not annotated
  
Accession: CAP65184
  
Location: 385644-386566
  
 NCBI BlastP on this gene

CAP65184

not annotated
  
Accession: CAP65185
  
Location: 387049-389244
  
 NCBI BlastP on this gene

CAP65185

not annotated
  
Accession: CAP65186
  
Location: 389833-396580
  
  
**BlastP hit with Mycgr3G100089\_Mycgr3**
  
Percentage identity: 38 %
  
BlastP bit score: 1466
  
Sequence coverage: 103 %
  
E-value: 0.0
  
  
 NCBI BlastP on this gene

CAP65186

not annotated
  
Accession: CAP65187
  
Location: 398271-399340
  
  
**BlastP hit with Mycgr3G104337\_Mycgr3**
  
Percentage identity: 42 %
  
BlastP bit score: 199
  
Sequence coverage: 100 %
  
E-value: 9e-59
  
  
 NCBI BlastP on this gene

CAP65187

not annotated
  
Accession: CAP65188
  
Location: 403437-404936
  
 NCBI BlastP on this gene

CAP65188

not annotated
  
Accession: CAP65189
  
Location: 407649-410144
  
 NCBI BlastP on this gene

CAP65189

not annotated
  
Accession: CAP65190
  
Location: 412031-412612
  
 NCBI BlastP on this gene

CAP65190

not annotated
  
Accession: CAP65191
  
Location: 412798-413184
  
 NCBI BlastP on this gene

CAP65191

not annotated
  
Accession: CAP65192
  
Location: 413517-414914
  
 NCBI BlastP on this gene

CAP65192

107. :  AGUE01000230 Glarea lozoyensis 74030     Total score: 2.0     Cumulative Blast bit score: 1657

putative Retinol-binding protein 3
  
Accession: EHK96639
  
Location: 39028-40185
  
 NCBI BlastP on this gene

EHK96639

hypothetical protein
  
Accession: EHK96640
  
Location: 49320-50538
  
 NCBI BlastP on this gene

EHK96640

hypothetical protein
  
Accession: EHK96641
  
Location: 51256-51600
  
 NCBI BlastP on this gene

EHK96641

hypothetical protein
  
Accession: EHK96642
  
Location: 53855-54950
  
 NCBI BlastP on this gene

EHK96642

hypothetical protein
  
Accession: EHK96643
  
Location: 55574-55758
  
 NCBI BlastP on this gene

EHK96643

putative Phthioceranic/hydroxyphthioceranic acid synthase
  
Accession: EHK96644
  
Location: 57302-63976
  
  
**BlastP hit with Mycgr3G100089\_Mycgr3**
  
Percentage identity: 42 %
  
BlastP bit score: 1468
  
Sequence coverage: 83 %
  
E-value: 0.0
  
  
 NCBI BlastP on this gene

EHK96644

putative Uncharacterized hydrolase C22A12.06c
  
Accession: EHK96645
  
Location: 65599-67085
  
  
**BlastP hit with Mycgr3G104337\_Mycgr3**
  
Percentage identity: 40 %
  
BlastP bit score: 189
  
Sequence coverage: 93 %
  
E-value: 2e-55
  
  
 NCBI BlastP on this gene

EHK96645

putative ABC transporter B family member 11
  
Accession: EHK96646
  
Location: 70014-70404
  
 NCBI BlastP on this gene

EHK96646

hypothetical protein
  
Accession: EHK96647
  
Location: 73784-74155
  
 NCBI BlastP on this gene

EHK96647

putative UPF0364 protein
  
Accession: EHK96648
  
Location: 84393-86120
  
 NCBI BlastP on this gene

EHK96648

108. :  KB445637 Cochliobolus sativus ND90Pr unplaced genomic scaffold COCSAscaffold\_1     Total score: 2.0     Cumulative Blast bit score: 1654

polysaccharide lyase family 4 protein
  
Accession: EMD69104
  
Location: 448468-450608
  
 NCBI BlastP on this gene

EMD69104

hypothetical protein
  
Accession: EMD69103
  
Location: 447658-448035
  
 NCBI BlastP on this gene

EMD69103

hypothetical protein
  
Accession: EMD69102
  
Location: 442526-447493
  
 NCBI BlastP on this gene

EMD69102

hypothetical protein
  
Accession: EMD69101
  
Location: 438829-440928
  
 NCBI BlastP on this gene

EMD69101

hypothetical protein
  
Accession: EMD69100
  
Location: 435828-436693
  
 NCBI BlastP on this gene

EMD69100

hypothetical protein
  
Accession: EMD69099
  
Location: 432399-433267
  
  
**BlastP hit with Mycgr3G31170\_Mycgr3T**
  
Percentage identity: 100 %
  
BlastP bit score: 166
  
Sequence coverage: 100 %
  
E-value: 1e-49
  
  
 NCBI BlastP on this gene

EMD69099

hypothetical protein
  
Accession: EMD69098
  
Location: 427831-429792
  
 NCBI BlastP on this gene

EMD69098

hypothetical protein
  
Accession: EMD69097
  
Location: 420899-427015
  
  
**BlastP hit with Mycgr3G41426\_Mycgr3T**
  
Percentage identity: 43 %
  
BlastP bit score: 1488
  
Sequence coverage: 102 %
  
E-value: 0.0
  
  
 NCBI BlastP on this gene

EMD69097

hypothetical protein
  
Accession: EMD69096
  
Location: 416917-419046
  
 NCBI BlastP on this gene

EMD69096

hypothetical protein
  
Accession: EMD69095
  
Location: 413384-416463
  
 NCBI BlastP on this gene

EMD69095

hypothetical protein
  
Accession: EMD69094
  
Location: 412288-413120
  
 NCBI BlastP on this gene

EMD69094

hypothetical protein
  
Accession: EMD69093
  
Location: 411127-411945
  
 NCBI BlastP on this gene

EMD69093

hypothetical protein
  
Accession: EMD69092
  
Location: 409018-410586
  
 NCBI BlastP on this gene

EMD69092

hypothetical protein
  
Accession: EMD69091
  
Location: 408416-408688
  
 NCBI BlastP on this gene

EMD69091

hypothetical protein
  
Accession: EMD69090
  
Location: 406869-408007
  
 NCBI BlastP on this gene

EMD69090

hypothetical protein
  
Accession: EMD69089
  
Location: 403239-405117
  
 NCBI BlastP on this gene

EMD69089

109. :  KB733444 Bipolaris maydis ATCC 48331 unplaced genomic scaffold COCC4scaffold\_1     Total score: 2.0     Cumulative Blast bit score: 1650

polysaccharide lyase family 4 protein
  
Accession: ENI11027
  
Location: 1889630-1891830
  
 NCBI BlastP on this gene

ENI11027

hypothetical protein
  
Accession: ENI11028
  
Location: 1892162-1892380
  
 NCBI BlastP on this gene

ENI11028

hypothetical protein
  
Accession: ENI11029
  
Location: 1892897-1897868
  
 NCBI BlastP on this gene

ENI11029

hypothetical protein
  
Accession: ENI11030
  
Location: 1899488-1901539
  
 NCBI BlastP on this gene

ENI11030

hypothetical protein
  
Accession: ENI11031
  
Location: 1901644-1901843
  
 NCBI BlastP on this gene

ENI11031

hypothetical protein
  
Accession: ENI11032
  
Location: 1903905-1904796
  
 NCBI BlastP on this gene

ENI11032

hypothetical protein
  
Accession: ENI11033
  
Location: 1906114-1906530
  
 NCBI BlastP on this gene

ENI11033

hypothetical protein
  
Accession: ENI11034
  
Location: 1907443-1908327
  
  
**BlastP hit with Mycgr3G31170\_Mycgr3T**
  
Percentage identity: 100 %
  
BlastP bit score: 166
  
Sequence coverage: 100 %
  
E-value: 1e-49
  
  
 NCBI BlastP on this gene

ENI11034

hypothetical protein
  
Accession: ENI11035
  
Location: 1911048-1912930
  
 NCBI BlastP on this gene

ENI11035

hypothetical protein
  
Accession: ENI11036
  
Location: 1913885-1919857
  
  
**BlastP hit with Mycgr3G41426\_Mycgr3T**
  
Percentage identity: 43 %
  
BlastP bit score: 1484
  
Sequence coverage: 102 %
  
E-value: 0.0
  
  
 NCBI BlastP on this gene

ENI11036

hypothetical protein
  
Accession: ENI11037
  
Location: 1921887-1924007
  
 NCBI BlastP on this gene

ENI11037

hypothetical protein
  
Accession: ENI11038
  
Location: 1924476-1925087
  
 NCBI BlastP on this gene

ENI11038

hypothetical protein
  
Accession: ENI11039
  
Location: 1925689-1927578
  
 NCBI BlastP on this gene

ENI11039

hypothetical protein
  
Accession: ENI11040
  
Location: 1927844-1928678
  
 NCBI BlastP on this gene

ENI11040

hypothetical protein
  
Accession: ENI11041
  
Location: 1929008-1929823
  
 NCBI BlastP on this gene

ENI11041

hypothetical protein
  
Accession: ENI11042
  
Location: 1930410-1931978
  
 NCBI BlastP on this gene

ENI11042

hypothetical protein
  
Accession: ENI11043
  
Location: 1932999-1934135
  
 NCBI BlastP on this gene

ENI11043

hypothetical protein
  
Accession: ENI11044
  
Location: 1935516-1936842
  
 NCBI BlastP on this gene

ENI11044

110. :  KB445570 Cochliobolus heterostrophus C5 unplaced genomic scaffold COCHEscaffold\_2     Total score: 2.0     Cumulative Blast bit score: 1650

polysaccharide lyase family 4 protein
  
Accession: EMD96168
  
Location: 1911294-1913494
  
 NCBI BlastP on this gene

EMD96168

hypothetical protein
  
Accession: EMD96169
  
Location: 1913826-1914044
  
 NCBI BlastP on this gene

EMD96169

hypothetical protein
  
Accession: EMD96170
  
Location: 1914561-1919532
  
 NCBI BlastP on this gene

EMD96170

hypothetical protein
  
Accession: EMD96171
  
Location: 1921152-1923203
  
 NCBI BlastP on this gene

EMD96171

hypothetical protein
  
Accession: EMD96172
  
Location: 1923308-1923507
  
 NCBI BlastP on this gene

EMD96172

hypothetical protein
  
Accession: EMD96173
  
Location: 1925569-1926460
  
 NCBI BlastP on this gene

EMD96173

hypothetical protein
  
Accession: EMD96174
  
Location: 1927778-1928194
  
 NCBI BlastP on this gene

EMD96174

hypothetical protein
  
Accession: EMD96175
  
Location: 1929107-1929991
  
  
**BlastP hit with Mycgr3G31170\_Mycgr3T**
  
Percentage identity: 100 %
  
BlastP bit score: 166
  
Sequence coverage: 100 %
  
E-value: 1e-49
  
  
 NCBI BlastP on this gene

EMD96175

hypothetical protein
  
Accession: EMD96176
  
Location: 1932712-1934594
  
 NCBI BlastP on this gene

EMD96176

hypothetical protein
  
Accession: EMD96177
  
Location: 1935549-1941521
  
  
**BlastP hit with Mycgr3G41426\_Mycgr3T**
  
Percentage identity: 43 %
  
BlastP bit score: 1484
  
Sequence coverage: 102 %
  
E-value: 0.0
  
  
 NCBI BlastP on this gene

EMD96177

hypothetical protein
  
Accession: EMD96178
  
Location: 1943590-1945686
  
 NCBI BlastP on this gene

EMD96178

hypothetical protein
  
Accession: EMD96179
  
Location: 1946140-1946751
  
 NCBI BlastP on this gene

EMD96179

hypothetical protein
  
Accession: EMD96180
  
Location: 1947353-1949242
  
 NCBI BlastP on this gene

EMD96180

hypothetical protein
  
Accession: EMD96181
  
Location: 1949508-1950342
  
 NCBI BlastP on this gene

EMD96181

hypothetical protein
  
Accession: EMD96182
  
Location: 1950672-1951487
  
 NCBI BlastP on this gene

EMD96182

hypothetical protein
  
Accession: EMD96183
  
Location: 1952074-1953642
  
 NCBI BlastP on this gene

EMD96183

hypothetical protein
  
Accession: EMD96184
  
Location: 1954738-1955751
  
 NCBI BlastP on this gene

EMD96184

hypothetical protein
  
Accession: EMD96185
  
Location: 1957180-1958506
  
 NCBI BlastP on this gene

EMD96185

111. :  KB908482 Setosphaeria turcica Et28A unplaced genomic scaffold SETTUscaffold\_10     Total score: 2.0     Cumulative Blast bit score: 1641

hypothetical protein
  
Accession: EOA90926
  
Location: 1155867-1160804
  
 NCBI BlastP on this gene

EOA90926

hypothetical protein
  
Accession: EOA90927
  
Location: 1162792-1164961
  
 NCBI BlastP on this gene

EOA90927

hypothetical protein
  
Accession: EOA90928
  
Location: 1168587-1169517
  
 NCBI BlastP on this gene

EOA90928

hypothetical protein
  
Accession: EOA90929
  
Location: 1172598-1173543
  
  
**BlastP hit with Mycgr3G31170\_Mycgr3T**
  
Percentage identity: 100 %
  
BlastP bit score: 166
  
Sequence coverage: 100 %
  
E-value: 1e-49
  
  
 NCBI BlastP on this gene

EOA90929

hypothetical protein
  
Accession: EOA90930
  
Location: 1176615-1182675
  
  
**BlastP hit with Mycgr3G41426\_Mycgr3T**
  
Percentage identity: 44 %
  
BlastP bit score: 1475
  
Sequence coverage: 102 %
  
E-value: 0.0
  
  
 NCBI BlastP on this gene

EOA90930

hypothetical protein
  
Accession: EOA90931
  
Location: 1184496-1186721
  
 NCBI BlastP on this gene

EOA90931

hypothetical protein
  
Accession: EOA90932
  
Location: 1187207-1187837
  
 NCBI BlastP on this gene

EOA90932

hypothetical protein
  
Accession: EOA90933
  
Location: 1188380-1190284
  
 NCBI BlastP on this gene

EOA90933

hypothetical protein
  
Accession: EOA90934
  
Location: 1190578-1191404
  
 NCBI BlastP on this gene

EOA90934

hypothetical protein
  
Accession: EOA90935
  
Location: 1191740-1192558
  
 NCBI BlastP on this gene

EOA90935

hypothetical protein
  
Accession: EOA90936
  
Location: 1193008-1194576
  
 NCBI BlastP on this gene

EOA90936

hypothetical protein
  
Accession: EOA90937
  
Location: 1198165-1198854
  
 NCBI BlastP on this gene

EOA90937

hypothetical protein
  
Accession: EOA90938
  
Location: 1199008-1200855
  
 NCBI BlastP on this gene

EOA90938

112. :  DS985227 Verticillium albo-atrum VaMs.102 supercont1.14 genomic scaffold     Total score: 2.0     Cumulative Blast bit score: 1621

hypothetical protein
  
Accession: EEY22933
  
Location: 260362-261003
  
 NCBI BlastP on this gene

EEY22933

alcohol dehydrogenase
  
Accession: EEY22934
  
Location: 262099-264788
  
 NCBI BlastP on this gene

EEY22934

sugar transporter
  
Accession: EEY22935
  
Location: 265629-267504
  
 NCBI BlastP on this gene

EEY22935

thermostable beta-glucosidase B
  
Accession: EEY22936
  
Location: 268353-270950
  
 NCBI BlastP on this gene

EEY22936

predicted protein
  
Accession: EEY22937
  
Location: 275617-276410
  
 NCBI BlastP on this gene

EEY22937

hexose transporter protein
  
Accession: EEY22938
  
Location: 276481-277171
  
 NCBI BlastP on this gene

EEY22938

cbbX
  
Accession: EEY22939
  
Location: 277707-284840
  
  
**BlastP hit with Mycgr3G42010\_Mycgr3T**
  
Percentage identity: 43 %
  
BlastP bit score: 1514
  
Sequence coverage: 87 %
  
E-value: 0.0
  
  
 NCBI BlastP on this gene

EEY22939

conserved hypothetical protein
  
Accession: EEY22940
  
Location: 285610-287045
  
  
**BlastP hit with Mycgr3G92938\_Mycgr3T**
  
Percentage identity: 26 %
  
BlastP bit score: 108
  
Sequence coverage: 80 %
  
E-value: 3e-23
  
  
 NCBI BlastP on this gene

EEY22940

SCF E3 ubiquitin ligase complex F-box protein grrA
  
Accession: EEY22941
  
Location: 288762-291254
  
 NCBI BlastP on this gene

EEY22941

glutaredoxin
  
Accession: EEY22942
  
Location: 295712-296183
  
 NCBI BlastP on this gene

EEY22942

BAG domain-containing protein
  
Accession: EEY22943
  
Location: 296594-299081
  
 NCBI BlastP on this gene

EEY22943

60S ribosomal protein L16
  
Accession: EEY22944
  
Location: 300155-301294
  
 NCBI BlastP on this gene

EEY22944

hydrolase
  
Accession: EEY22945
  
Location: 302099-303127
  
 NCBI BlastP on this gene

EEY22945

113. :  CP003006 Myceliophthora thermophila ATCC 42464 chromosome 5     Total score: 2.0     Cumulative Blast bit score: 1620

hypothetical protein
  
Accession: AEO60432
  
Location: 4224330-4224798
  
 NCBI BlastP on this gene

MYCTH\_2129532

hypothetical protein
  
Accession: AEO60433
  
Location: 4225839-4227517
  
 NCBI BlastP on this gene

MYCTH\_2309630

hypothetical protein
  
Accession: AEO60434
  
Location: 4228877-4232972
  
 NCBI BlastP on this gene

MYCTH\_2309634

hypothetical protein
  
Accession: AEO60435
  
Location: 4234592-4236469
  
 NCBI BlastP on this gene

MYCTH\_2309636

hypothetical protein
  
Accession: AEO60436
  
Location: 4240613-4241545
  
 NCBI BlastP on this gene

MYCTH\_2309637

hypothetical protein
  
Accession: AEO60437
  
Location: 4241930-4242937
  
  
**BlastP hit with Mycgr3G104337\_Mycgr3**
  
Percentage identity: 40 %
  
BlastP bit score: 184
  
Sequence coverage: 93 %
  
E-value: 4e-53
  
  
 NCBI BlastP on this gene

MYCTH\_54987

polyketide synthase
  
Accession: AEO60438
  
Location: 4244361-4251153
  
  
**BlastP hit with Mycgr3G100089\_Mycgr3**
  
Percentage identity: 38 %
  
BlastP bit score: 1436
  
Sequence coverage: 102 %
  
E-value: 0.0
  
  
 NCBI BlastP on this gene

MYCTH\_103061

hypothetical protein
  
Accession: AEO60439
  
Location: 4252450-4254451
  
 NCBI BlastP on this gene

MYCTH\_2309641

hypothetical protein
  
Accession: AEO60440
  
Location: 4255049-4255991
  
 NCBI BlastP on this gene

MYCTH\_2309644

hypothetical protein
  
Accession: AEO60441
  
Location: 4256449-4257299
  
 NCBI BlastP on this gene

MYCTH\_2316112

hypothetical protein
  
Accession: AEO60442
  
Location: 4258397-4260525
  
 NCBI BlastP on this gene

MYCTH\_2309647

glycosyltransferase family 2 protein
  
Accession: AEO60443
  
Location: 4263729-4265552
  
 NCBI BlastP on this gene

MYCTH\_10713

glycosyltransferase family 31 protein
  
Accession: AEO60444
  
Location: 4266718-4268287
  
 NCBI BlastP on this gene

MYCTH\_2309651

114. :  KB707649 Eutypa lata UCREL1 unplaced genomic scaffold EL1\_03\_scaffold\_2311     Total score: 2.0     Cumulative Blast bit score: 1618

putative cytochrome p450 protein
  
Accession: EMR61340
  
Location: 92-1763
  
 NCBI BlastP on this gene

EMR61340

putative pectinesterase precursor protein
  
Accession: EMR61338
  
Location: 3061-4129
  
 NCBI BlastP on this gene

EMR61338

putative methyltransferase type 11 protein
  
Accession: EMR61348
  
Location: 6678-7544
  
 NCBI BlastP on this gene

EMR61348

putative cytochrome p450 protein
  
Accession: EMR61346
  
Location: 7947-9158
  
 NCBI BlastP on this gene

EMR61346

putative polyketide synthase protein
  
Accession: EMR61344
  
Location: 10824-17766
  
  
**BlastP hit with Mycgr3G100089\_Mycgr3**
  
Percentage identity: 41 %
  
BlastP bit score: 1443
  
Sequence coverage: 89 %
  
E-value: 0.0
  
  
 NCBI BlastP on this gene

EMR61344

putative enoyl- hydratase isomerase family protein
  
Accession: EMR61341
  
Location: 18276-19583
  
 NCBI BlastP on this gene

EMR61341

putative ef-hand calcium-binding domain protein
  
Accession: EMR61339
  
Location: 20129-21010
  
  
**BlastP hit with Mycgr3G104337\_Mycgr3**
  
Percentage identity: 36 %
  
BlastP bit score: 175
  
Sequence coverage: 96 %
  
E-value: 4e-50
  
  
 NCBI BlastP on this gene

EMR61339

putative capsule polysaccharide biosynthesis protein
  
Accession: EMR61347
  
Location: 21600-22832
  
 NCBI BlastP on this gene

EMR61347

putative -like methyltransferase protein
  
Accession: EMR61345
  
Location: 23914-24869
  
 NCBI BlastP on this gene

EMR61345

putative poly(aspartic acid) hydrolase protein
  
Accession: EMR61342
  
Location: 30184-31236
  
 NCBI BlastP on this gene

EMR61342

115. :  GL537139 Pyrenophora teres f. teres 0-1 unplaced genomic scaffold scaffold\_193442     Total score: 2.0     Cumulative Blast bit score: 1525

hypothetical protein
  
Accession: EFQ87089
  
Location: 14592-15452
  
  
**BlastP hit with Mycgr3G31170\_Mycgr3T**
  
Percentage identity: 100 %
  
BlastP bit score: 166
  
Sequence coverage: 100 %
  
E-value: 1e-49
  
  
 NCBI BlastP on this gene

EFQ87089

hypothetical protein
  
Accession: EFQ87088
  
Location: 10210-12071
  
 NCBI BlastP on this gene

EFQ87088

hypothetical protein
  
Accession: EFQ87087
  
Location: 3363-9317
  
  
**BlastP hit with Mycgr3G41426\_Mycgr3T**
  
Percentage identity: 40 %
  
BlastP bit score: 1359
  
Sequence coverage: 102 %
  
E-value: 0.0
  
  
 NCBI BlastP on this gene

EFQ87087

hypothetical protein
  
Accession: EFQ87086
  
Location: 31-1932
  
 NCBI BlastP on this gene

EFQ87086

116. :  GL533442 Pyrenophora teres f. teres 0-1 unplaced genomic scaffold scaffold\_189679     Total score: 2.0     Cumulative Blast bit score: 1525

hypothetical protein
  
Accession: EFQ94251
  
Location: 24060-24920
  
  
**BlastP hit with Mycgr3G31170\_Mycgr3T**
  
Percentage identity: 100 %
  
BlastP bit score: 166
  
Sequence coverage: 100 %
  
E-value: 1e-49
  
  
 NCBI BlastP on this gene

EFQ94251

hypothetical protein
  
Accession: EFQ94250
  
Location: 19678-21539
  
 NCBI BlastP on this gene

EFQ94250

hypothetical protein
  
Accession: EFQ94249
  
Location: 12831-18785
  
  
**BlastP hit with Mycgr3G41426\_Mycgr3T**
  
Percentage identity: 40 %
  
BlastP bit score: 1359
  
Sequence coverage: 102 %
  
E-value: 0.0
  
  
 NCBI BlastP on this gene

EFQ94249

hypothetical protein
  
Accession: EFQ94248
  
Location: 9220-11400
  
 NCBI BlastP on this gene

EFQ94248

hypothetical protein
  
Accession: EFQ94247
  
Location: 8089-8655
  
 NCBI BlastP on this gene

EFQ94247

hypothetical protein
  
Accession: EFQ94246
  
Location: 5793-7694
  
 NCBI BlastP on this gene

EFQ94246

hypothetical protein
  
Accession: EFQ94245
  
Location: 4696-5520
  
 NCBI BlastP on this gene

EFQ94245

hypothetical protein
  
Accession: EFQ94244
  
Location: 3612-4427
  
 NCBI BlastP on this gene

EFQ94244

hypothetical protein
  
Accession: EFQ94243
  
Location: 1518-3133
  
 NCBI BlastP on this gene

EFQ94243

hypothetical protein
  
Accession: EFQ94242
  
Location: 208-1368
  
 NCBI BlastP on this gene

EFQ94242

117. :  CP003011 Thielavia terrestris NRRL 8126 chromosome 3     Total score: 2.0     Cumulative Blast bit score: 1512

hypothetical protein
  
Accession: AEO67426
  
Location: 110647-112697
  
 NCBI BlastP on this gene

THITE\_2116314

polyketide synthase
  
Accession: AEO67427
  
Location: 113172-120431
  
  
**BlastP hit with Mycgr3G100089\_Mycgr3**
  
Percentage identity: 36 %
  
BlastP bit score: 1318
  
Sequence coverage: 102 %
  
E-value: 0.0
  
  
 NCBI BlastP on this gene

THITE\_132390

hypothetical protein
  
Accession: AEO67428
  
Location: 121100-122115
  
  
**BlastP hit with Mycgr3G104337\_Mycgr3**
  
Percentage identity: 40 %
  
BlastP bit score: 194
  
Sequence coverage: 100 %
  
E-value: 3e-57
  
  
 NCBI BlastP on this gene

THITE\_125661

hypothetical protein
  
Accession: AEO67429
  
Location: 123526-123883
  
 NCBI BlastP on this gene

THITE\_2088916

hypothetical protein
  
Accession: AEO67430
  
Location: 123944-127096
  
 NCBI BlastP on this gene

THITE\_2144801

hypothetical protein
  
Accession: AEO67431
  
Location: 129543-130997
  
 NCBI BlastP on this gene

THITE\_2050923

hypothetical protein
  
Accession: AEO67432
  
Location: 132518-133794
  
 NCBI BlastP on this gene

THITE\_2088919

118. :  CH408029 Chaetomium globosum CBS 148.51 scaffold\_1 genomic scaffold     Total score: 2.0     Cumulative Blast bit score: 1506

hypothetical protein
  
Accession: EAQ91803
  
Location: 107127-108854
  
 NCBI BlastP on this gene

EAQ91803

hypothetical protein
  
Accession: EAQ91804
  
Location: 110092-112068
  
 NCBI BlastP on this gene

EAQ91804

hypothetical protein
  
Accession: EAQ91805
  
Location: 112620-114191
  
 NCBI BlastP on this gene

EAQ91805

hypothetical protein
  
Accession: EAQ91806
  
Location: 114718-118048
  
 NCBI BlastP on this gene

EAQ91806

hypothetical protein
  
Accession: EAQ91807
  
Location: 118404-120302
  
 NCBI BlastP on this gene

EAQ91807

predicted protein
  
Accession: EAQ91808
  
Location: 122121-122777
  
 NCBI BlastP on this gene

EAQ91808

hypothetical protein
  
Accession: EAQ91809
  
Location: 123217-124699
  
 NCBI BlastP on this gene

EAQ91809

hypothetical protein
  
Accession: EAQ91810
  
Location: 125085-127158
  
  
**BlastP hit with Mycgr3G104337\_Mycgr3**
  
Percentage identity: 37 %
  
BlastP bit score: 179
  
Sequence coverage: 100 %
  
E-value: 1e-49
  
  
 NCBI BlastP on this gene

EAQ91810

hypothetical protein
  
Accession: EAQ91811
  
Location: 127830-134360
  
  
**BlastP hit with Mycgr3G100089\_Mycgr3**
  
Percentage identity: 37 %
  
BlastP bit score: 1327
  
Sequence coverage: 101 %
  
E-value: 0.0
  
  
 NCBI BlastP on this gene

EAQ91811

NADH-ubiquinone oxidoreductase 23 kDa subunit
  
Accession: EAQ91812
  
Location: 137783-138679
  
 NCBI BlastP on this gene

EAQ91812

hypothetical protein
  
Accession: EAQ91813
  
Location: 139279-140139
  
 NCBI BlastP on this gene

EAQ91813

hypothetical protein
  
Accession: EAQ91814
  
Location: 140868-142977
  
 NCBI BlastP on this gene

EAQ91814

hypothetical protein
  
Accession: EAQ91815
  
Location: 144521-146376
  
 NCBI BlastP on this gene

EAQ91815

hypothetical protein
  
Accession: EAQ91816
  
Location: 147108-148693
  
 NCBI BlastP on this gene

EAQ91816

hypothetical protein
  
Accession: EAQ91817
  
Location: 151188-152356
  
 NCBI BlastP on this gene

EAQ91817

119. :  DS231619 Pyrenophora tritici-repentis Pt-1C-BFP supercont1.5 genomic scaffold     Total score: 2.0     Cumulative Blast bit score: 1500

predicted protein
  
Accession: EDU48270
  
Location: 170417-171253
  
 NCBI BlastP on this gene

EDU48270

phospholipid-translocating P-type ATPase domain containing protein
  
Accession: EDU48271
  
Location: 172508-177295
  
 NCBI BlastP on this gene

EDU48271

predicted protein
  
Accession: EDU48272
  
Location: 178706-181019
  
 NCBI BlastP on this gene

EDU48272

tetraspanin
  
Accession: EDU48273
  
Location: 183713-184634
  
 NCBI BlastP on this gene

EDU48273

MADS box transcription factor Mcm1
  
Accession: EDU48274
  
Location: 186836-187667
  
  
**BlastP hit with Mycgr3G31170\_Mycgr3T**
  
Percentage identity: 100 %
  
BlastP bit score: 166
  
Sequence coverage: 100 %
  
E-value: 1e-49
  
  
 NCBI BlastP on this gene

EDU48274

choline transport protein
  
Accession: EDU48275
  
Location: 190148-192002
  
 NCBI BlastP on this gene

EDU48275

DNA-binding protein SMUBP-2
  
Accession: EDU48276
  
Location: 192904-198819
  
  
**BlastP hit with Mycgr3G41426\_Mycgr3T**
  
Percentage identity: 40 %
  
BlastP bit score: 1334
  
Sequence coverage: 102 %
  
E-value: 0.0
  
  
 NCBI BlastP on this gene

EDU48276

conserved hypothetical protein
  
Accession: EDU48277
  
Location: 200196-202409
  
 NCBI BlastP on this gene

EDU48277

glucosamine 6-phosphate N-acetyltransferase
  
Accession: EDU48278
  
Location: 202981-203545
  
 NCBI BlastP on this gene

EDU48278

conserved hypothetical protein
  
Accession: EDU48279
  
Location: 203967-205877
  
 NCBI BlastP on this gene

EDU48279

40S ribosomal protein S10
  
Accession: EDU48280
  
Location: 206151-206975
  
 NCBI BlastP on this gene

EDU48280

conserved hypothetical protein
  
Accession: EDU48281
  
Location: 207248-208063
  
 NCBI BlastP on this gene

EDU48281

N-carbamoyl-L-amino acid hydrolase
  
Accession: EDU48282
  
Location: 208543-210156
  
 NCBI BlastP on this gene

EDU48282

phospholipase
  
Accession: EDU48283
  
Location: 211989-213773
  
 NCBI BlastP on this gene

EDU48283

conserved hypothetical protein
  
Accession: EDU48284
  
Location: 214398-214943
  
 NCBI BlastP on this gene

EDU48284

conserved hypothetical protein
  
Accession: EDU48285
  
Location: 215148-216611
  
 NCBI BlastP on this gene

EDU48285

120. :  DS027698 Neosartorya fischeri NRRL 181 1099437636266 genomic scaffold     Total score: 2.0     Cumulative Blast bit score: 1477

Rab geranylgeranyl transferase escort protein, putative
  
Accession: EAW15497
  
Location: 366869-368762
  
 NCBI BlastP on this gene

EAW15497

conserved hypothetical protein
  
Accession: EAW15496
  
Location: 360462-362036
  
 NCBI BlastP on this gene

EAW15496

SRF-type transcription factor (Umc1), putative
  
Accession: EAW15495
  
Location: 357403-358305
  
  
**BlastP hit with Mycgr3G31170\_Mycgr3T**
  
Percentage identity: 98 %
  
BlastP bit score: 162
  
Sequence coverage: 100 %
  
E-value: 3e-48
  
  
 NCBI BlastP on this gene

EAW15495

conserved hypothetical protein
  
Accession: EAW15494
  
Location: 354247-356126
  
 NCBI BlastP on this gene

EAW15494

ATP synthase subunit E, putative
  
Accession: EAW15493
  
Location: 352873-353688
  
 NCBI BlastP on this gene

EAW15493

conserved hypothetical protein
  
Accession: EAW15492
  
Location: 349924-351829
  
 NCBI BlastP on this gene

EAW15492

phospholipid-translocating P-type ATPase domain-containing protein
  
Accession: EAW15491
  
Location: 341351-346052
  
 NCBI BlastP on this gene

EAW15491

tRNA-splicing endonuclease, putative
  
Accession: EAW15490
  
Location: 333714-340039
  
  
**BlastP hit with Mycgr3G41426\_Mycgr3T**
  
Percentage identity: 40 %
  
BlastP bit score: 1315
  
Sequence coverage: 103 %
  
E-value: 0.0
  
  
 NCBI BlastP on this gene

EAW15490

conserved hypothetical protein
  
Accession: EAW15489
  
Location: 327234-330045
  
 NCBI BlastP on this gene

EAW15489

conserved hypothetical protein
  
Accession: EAW15488
  
Location: 325852-326543
  
 NCBI BlastP on this gene

EAW15488

aminotransferase, putative
  
Accession: EAW15487
  
Location: 323906-325448
  
 NCBI BlastP on this gene

EAW15487

121. :  DS499602 Aspergillus fumigatus A1163 scf\_000009 genomic scaffold     Total score: 2.0     Cumulative Blast bit score: 1474

Rab geranylgeranyl transferase escort protein, putative
  
Accession: EDP47769
  
Location: 936856-938751
  
 NCBI BlastP on this gene

EDP47769

conserved hypothetical protein
  
Accession: EDP47770
  
Location: 943484-945097
  
 NCBI BlastP on this gene

EDP47770

SRF-type transcription factor (Umc1), putative
  
Accession: EDP47771
  
Location: 947169-948069
  
  
**BlastP hit with Mycgr3G31170\_Mycgr3T**
  
Percentage identity: 98 %
  
BlastP bit score: 162
  
Sequence coverage: 100 %
  
E-value: 3e-48
  
  
 NCBI BlastP on this gene

EDP47771

DUF803 domain protein
  
Accession: EDP47772
  
Location: 949233-951228
  
 NCBI BlastP on this gene

EDP47772

ATP synthase subunit E, putative
  
Accession: EDP47773
  
Location: 951844-952603
  
 NCBI BlastP on this gene

EDP47773

hypothetical protein
  
Accession: EDP47774
  
Location: 953164-953646
  
 NCBI BlastP on this gene

EDP47774

phospholipid-translocating P-type ATPase domain-containing protein
  
Accession: EDP47775
  
Location: 959487-964188
  
 NCBI BlastP on this gene

EDP47775

tRNA-splicing endonuclease, putative
  
Accession: EDP47776
  
Location: 965476-971799
  
  
**BlastP hit with Mycgr3G41426\_Mycgr3T**
  
Percentage identity: 39 %
  
BlastP bit score: 1312
  
Sequence coverage: 103 %
  
E-value: 0.0
  
  
 NCBI BlastP on this gene

EDP47776

conserved hypothetical protein
  
Accession: EDP47777
  
Location: 975310-978100
  
 NCBI BlastP on this gene

EDP47777

conserved hypothetical protein
  
Accession: EDP47778
  
Location: 978842-979533
  
 NCBI BlastP on this gene

EDP47778

aminotransferase, putative
  
Accession: EDP47779
  
Location: 979951-981466
  
 NCBI BlastP on this gene

EDP47779

122. :  AAHF01000012 Aspergillus fumigatus Af293     Total score: 2.0     Cumulative Blast bit score: 1472

Rab geranylgeranyl transferase escort protein, putative
  
Accession: EAL85826
  
Location: 925051-926946
  
 NCBI BlastP on this gene

EAL85826

conserved hypothetical protein
  
Accession: EAL85827
  
Location: 931656-933269
  
 NCBI BlastP on this gene

EAL85827

MADS box transcription factor Mcm1
  
Accession: EAL85828
  
Location: 935341-936241
  
  
**BlastP hit with Mycgr3G31170\_Mycgr3T**
  
Percentage identity: 98 %
  
BlastP bit score: 162
  
Sequence coverage: 100 %
  
E-value: 3e-48
  
  
 NCBI BlastP on this gene

EAL85828

DUF803 domain protein
  
Accession: EAL85829
  
Location: 937405-939400
  
 NCBI BlastP on this gene

EAL85829

ATP synthase subunit E, putative
  
Accession: EAL85830
  
Location: 940015-940774
  
 NCBI BlastP on this gene

EAL85830

hypothetical protein
  
Accession: EAL85831
  
Location: 941335-941817
  
 NCBI BlastP on this gene

EAL85831

phospholipid-translocating P-type ATPase domain-containing protein
  
Accession: EAL85832
  
Location: 947663-952364
  
 NCBI BlastP on this gene

EAL85832

tRNA-splicing endonuclease, putative
  
Accession: EAL85833
  
Location: 953652-959975
  
  
**BlastP hit with Mycgr3G41426\_Mycgr3T**
  
Percentage identity: 39 %
  
BlastP bit score: 1310
  
Sequence coverage: 103 %
  
E-value: 0.0
  
  
 NCBI BlastP on this gene

EAL85833

conserved hypothetical protein
  
Accession: EAL85834
  
Location: 963483-966273
  
 NCBI BlastP on this gene

EAL85834

conserved hypothetical protein
  
Accession: EAL85835
  
Location: 967015-967706
  
 NCBI BlastP on this gene

EAL85835

aminotransferase, putative
  
Accession: EAL85836
  
Location: 968124-969639
  
 NCBI BlastP on this gene

EAL85836

123. :  AGUE01000275 Glarea lozoyensis 74030     Total score: 2.0     Cumulative Blast bit score: 1464

putative Ferric/cupric reductase transmembrane component 1
  
Accession: EHK96143
  
Location: 28237-30381
  
 NCBI BlastP on this gene

EHK96143

putative Sodium/potassium-transporting ATPase subunit alpha-1
  
Accession: EHK96144
  
Location: 32850-35504
  
 NCBI BlastP on this gene

EHK96144

putative Ribosomal RNA-processing protein 15
  
Accession: EHK96145
  
Location: 40634-42390
  
 NCBI BlastP on this gene

EHK96145

putative Signal recognition particle 54 kDa protein like protein
  
Accession: EHK96146
  
Location: 42464-44229
  
 NCBI BlastP on this gene

EHK96146

putative protein CbxX, chromosomal
  
Accession: EHK96147
  
Location: 46722-53955
  
  
**BlastP hit with Mycgr3G42010\_Mycgr3T**
  
Percentage identity: 50 %
  
BlastP bit score: 1226
  
Sequence coverage: 58 %
  
E-value: 0.0
  
  
 NCBI BlastP on this gene

EHK96147

hypothetical protein
  
Accession: EHK96148
  
Location: 55057-56094
  
  
**BlastP hit with Mycgr3G92938\_Mycgr3T**
  
Percentage identity: 37 %
  
BlastP bit score: 238
  
Sequence coverage: 83 %
  
E-value: 9e-71
  
  
 NCBI BlastP on this gene

EHK96148

hypothetical protein
  
Accession: EHK96149
  
Location: 56358-56928
  
 NCBI BlastP on this gene

EHK96149

putative Quinone oxidoreductase PIG3
  
Accession: EHK96150
  
Location: 62618-63069
  
 NCBI BlastP on this gene

EHK96150

putative Eukaryotic translation initiation factor 3 subunit I
  
Accession: EHK96151
  
Location: 63866-65098
  
 NCBI BlastP on this gene

EHK96151

putative C-1-tetrahydrofolate synthase, cytoplasmic
  
Accession: EHK96152
  
Location: 65970-68996
  
 NCBI BlastP on this gene

EHK96152

124. :  GG698510 Trichophyton tonsurans CBS 112818 genomic scaffold supercont1.34     Total score: 2.0     Cumulative Blast bit score: 1460

hypothetical protein
  
Accession: EGD98391
  
Location: 181447-184411
  
 NCBI BlastP on this gene

EGD98391

hypothetical protein
  
Accession: EGD98392
  
Location: 187100-188846
  
 NCBI BlastP on this gene

EGD98392

tRNA-splicing endonuclease
  
Accession: EGD98393
  
Location: 192175-199008
  
  
**BlastP hit with Mycgr3G41426\_Mycgr3T**
  
Percentage identity: 40 %
  
BlastP bit score: 1306
  
Sequence coverage: 103 %
  
E-value: 0.0
  
  
 NCBI BlastP on this gene

EGD98393

phospholipid-translocating P-type ATPase
  
Accession: EGD98394
  
Location: 200566-205138
  
 NCBI BlastP on this gene

EGD98394

hypothetical protein
  
Accession: EGD98395
  
Location: 205924-206795
  
 NCBI BlastP on this gene

EGD98395

hypothetical protein
  
Accession: EGD98396
  
Location: 210632-213002
  
 NCBI BlastP on this gene

EGD98396

vacuolar ATP synthase subunit E
  
Accession: EGD98397
  
Location: 213382-214223
  
 NCBI BlastP on this gene

EGD98397

hypothetical protein
  
Accession: EGD98398
  
Location: 214850-216634
  
 NCBI BlastP on this gene

EGD98398

hypothetical protein
  
Accession: EGD98399
  
Location: 217648-218899
  
 NCBI BlastP on this gene

EGD98399

MADS box transcription factor
  
Accession: EGD98400
  
Location: 220310-221191
  
  
**BlastP hit with Mycgr3G31170\_Mycgr3T**
  
Percentage identity: 96 %
  
BlastP bit score: 154
  
Sequence coverage: 98 %
  
E-value: 5e-45
  
  
 NCBI BlastP on this gene

EGD98400

hypothetical protein
  
Accession: EGD98401
  
Location: 222607-223533
  
 NCBI BlastP on this gene

EGD98401

transcriptional activator
  
Accession: EGD98402
  
Location: 224368-225439
  
 NCBI BlastP on this gene

EGD98402

GNAT family acetyltransferase
  
Accession: EGD98403
  
Location: 226566-227684
  
 NCBI BlastP on this gene

EGD98403

metalloreductase transmembrane component
  
Accession: EGD98404
  
Location: 228206-230494
  
 NCBI BlastP on this gene

EGD98404

125. :  DS995899 Penicillium marneffei ATCC 18224 scf\_1105668340764 genomic scaffold     Total score: 2.0     Cumulative Blast bit score: 1459

conserved hypothetical protein
  
Accession: EEA26966
  
Location: 905998-907635
  
 NCBI BlastP on this gene

EEA26966

C6 transcription factor, putative
  
Accession: EEA26967
  
Location: 909048-911836
  
 NCBI BlastP on this gene

EEA26967

conserved hypothetical protein
  
Accession: EEA26968
  
Location: 912049-912894
  
 NCBI BlastP on this gene

EEA26968

MADS box transcription factor Mcm1
  
Accession: EEA26969
  
Location: 915864-916695
  
  
**BlastP hit with Mycgr3G31170\_Mycgr3T**
  
Percentage identity: 100 %
  
BlastP bit score: 167
  
Sequence coverage: 100 %
  
E-value: 8e-50
  
  
 NCBI BlastP on this gene

EEA26969

DUF803 domain protein
  
Accession: EEA26970
  
Location: 918047-919982
  
 NCBI BlastP on this gene

EEA26970

ATP synthase subunit E, putative
  
Accession: EEA26971
  
Location: 920591-921405
  
 NCBI BlastP on this gene

EEA26971

hypothetical protein
  
Accession: EEA26972
  
Location: 921787-924110
  
 NCBI BlastP on this gene

EEA26972

paraoxonase, putative
  
Accession: EEA26973
  
Location: 924613-925844
  
 NCBI BlastP on this gene

EEA26973

hypothetical protein
  
Accession: EEA26974
  
Location: 926055-926473
  
 NCBI BlastP on this gene

EEA26974

phospholipid-translocating P-type ATPase domain-containing protein
  
Accession: EEA26976
  
Location: 928749-933167
  
 NCBI BlastP on this gene

EEA26976

tRNA-splicing endonuclease, putative
  
Accession: EEA26977
  
Location: 934494-940990
  
  
**BlastP hit with Mycgr3G41426\_Mycgr3T**
  
Percentage identity: 39 %
  
BlastP bit score: 1292
  
Sequence coverage: 103 %
  
E-value: 0.0
  
  
 NCBI BlastP on this gene

EEA26977

conserved hypothetical protein
  
Accession: EEA26978
  
Location: 944055-947180
  
 NCBI BlastP on this gene

EEA26978

conserved hypothetical protein
  
Accession: EEA26979
  
Location: 947500-948840
  
 NCBI BlastP on this gene

EEA26979

chitin biosynthesis protein (Chs5), putative
  
Accession: EEA26980
  
Location: 950133-951465
  
 NCBI BlastP on this gene

EEA26980

126. :  EQ999974 Ajellomyces dermatitidis ER-3 genomic scaffold supercont1.2     Total score: 2.0     Cumulative Blast bit score: 1458

serine protein kinase Sky1
  
Accession: EEQ87178
  
Location: 7018349-7019826
  
 NCBI BlastP on this gene

EEQ87178

conserved hypothetical protein
  
Accession: EEQ87179
  
Location: 7021138-7022393
  
 NCBI BlastP on this gene

EEQ87179

predicted protein
  
Accession: EEQ87180
  
Location: 7023631-7024179
  
 NCBI BlastP on this gene

EEQ87180

MADS box transcription factor Mcm1
  
Accession: EEQ87181
  
Location: 7025081-7026058
  
  
**BlastP hit with Mycgr3G31170\_Mycgr3T**
  
Percentage identity: 100 %
  
BlastP bit score: 166
  
Sequence coverage: 100 %
  
E-value: 1e-49
  
  
 NCBI BlastP on this gene

EEQ87181

DUF803 domain-containing protein
  
Accession: EEQ87182
  
Location: 7029356-7031506
  
 NCBI BlastP on this gene

EEQ87182

vacuolar ATP synthase subunit E
  
Accession: EEQ87183
  
Location: 7032039-7032963
  
 NCBI BlastP on this gene

EEQ87183

conserved hypothetical protein
  
Accession: EEQ87184
  
Location: 7033660-7036143
  
 NCBI BlastP on this gene

EEQ87184

conserved hypothetical protein
  
Accession: EEQ87185
  
Location: 7037329-7038743
  
 NCBI BlastP on this gene

EEQ87185

phospholipid-translocating P-type ATPase domain-containing protein
  
Accession: EEQ87186
  
Location: 7043295-7047950
  
 NCBI BlastP on this gene

EEQ87186

tRNA-splicing endonuclease
  
Accession: EEQ87187
  
Location: 7049839-7056709
  
  
**BlastP hit with Mycgr3G41426\_Mycgr3T**
  
Percentage identity: 38 %
  
BlastP bit score: 1292
  
Sequence coverage: 103 %
  
E-value: 0.0
  
  
 NCBI BlastP on this gene

EEQ87187

predicted protein
  
Accession: EEQ87188
  
Location: 7061682-7063862
  
 NCBI BlastP on this gene

EEQ87188

127. :  DS989822 Arthroderma gypseum CBS 118893 supercont1.1 genomic scaffold     Total score: 2.0     Cumulative Blast bit score: 1457

hypothetical protein
  
Accession: EFQ98323
  
Location: 3701066-3703987
  
 NCBI BlastP on this gene

EFQ98323

hypothetical protein
  
Accession: EFQ98324
  
Location: 3705926-3706540
  
 NCBI BlastP on this gene

EFQ98324

hypothetical protein
  
Accession: EFQ98325
  
Location: 3706820-3708578
  
 NCBI BlastP on this gene

EFQ98325

hypothetical protein
  
Accession: EFQ98326
  
Location: 3709258-3710130
  
 NCBI BlastP on this gene

EFQ98326

helicase SEN1
  
Accession: EFQ98327
  
Location: 3711978-3718887
  
  
**BlastP hit with Mycgr3G41426\_Mycgr3T**
  
Percentage identity: 39 %
  
BlastP bit score: 1303
  
Sequence coverage: 103 %
  
E-value: 0.0
  
  
 NCBI BlastP on this gene

EFQ98327

phospholipid-transporting ATPase 1
  
Accession: EFQ98328
  
Location: 3720239-3724971
  
 NCBI BlastP on this gene

EFQ98328

hypothetical protein
  
Accession: EFQ98329
  
Location: 3725489-3726353
  
 NCBI BlastP on this gene

EFQ98329

hypothetical protein
  
Accession: EFQ98330
  
Location: 3727761-3730115
  
 NCBI BlastP on this gene

EFQ98330

vacuolar ATP synthase subunit E
  
Accession: EFQ98331
  
Location: 3730534-3731380
  
 NCBI BlastP on this gene

EFQ98331

DUF803 domain-containing protein
  
Accession: EFQ98332
  
Location: 3732069-3733851
  
 NCBI BlastP on this gene

EFQ98332

carboxy-cis,cis-muconate cyclase
  
Accession: EFQ98333
  
Location: 3734986-3736228
  
 NCBI BlastP on this gene

EFQ98333

MADS box transcription factor Mcm1
  
Accession: EFQ98334
  
Location: 3737424-3738317
  
  
**BlastP hit with Mycgr3G31170\_Mycgr3T**
  
Percentage identity: 96 %
  
BlastP bit score: 154
  
Sequence coverage: 98 %
  
E-value: 6e-45
  
  
 NCBI BlastP on this gene

EFQ98334

tetraspanin
  
Accession: EFQ98335
  
Location: 3739722-3740647
  
 NCBI BlastP on this gene

EFQ98335

PTAC beta
  
Accession: EFQ98336
  
Location: 3741509-3742611
  
 NCBI BlastP on this gene

EFQ98336

hypothetical protein
  
Accession: EFQ98337
  
Location: 3743575-3744864
  
 NCBI BlastP on this gene

EFQ98337

hypothetical protein
  
Accession: EFQ98338
  
Location: 3745387-3747675
  
 NCBI BlastP on this gene

EFQ98338

128. :  AACD01000159 Aspergillus nidulans FGSC A4     Total score: 2.0     Cumulative Blast bit score: 1453

hypothetical protein
  
Accession: EAA60101
  
Location: 42437-44270
  
 NCBI BlastP on this gene

EAA60101

predicted protein
  
Accession: EAA60100
  
Location: 41295-41745
  
 NCBI BlastP on this gene

EAA60100

hypothetical protein
  
Accession: EAA60099
  
Location: 36913-38478
  
 NCBI BlastP on this gene

EAA60099

hypothetical protein
  
Accession: EAA60098
  
Location: 33815-34722
  
  
**BlastP hit with Mycgr3G31170\_Mycgr3T**
  
Percentage identity: 100 %
  
BlastP bit score: 167
  
Sequence coverage: 100 %
  
E-value: 9e-50
  
  
 NCBI BlastP on this gene

EAA60098

hypothetical protein
  
Accession: EAA60097
  
Location: 30695-32722
  
 NCBI BlastP on this gene

EAA60097

hypothetical protein
  
Accession: EAA60096
  
Location: 29344-30165
  
 NCBI BlastP on this gene

EAA60096

hypothetical protein
  
Accession: EAA60095
  
Location: 25150-28502
  
 NCBI BlastP on this gene

EAA60095

hypothetical protein
  
Accession: EAA60094
  
Location: 18483-23159
  
 NCBI BlastP on this gene

EAA60094

hypothetical protein
  
Accession: EAA60093
  
Location: 9974-17399
  
  
**BlastP hit with Mycgr3G41426\_Mycgr3T**
  
Percentage identity: 39 %
  
BlastP bit score: 1286
  
Sequence coverage: 103 %
  
E-value: 0.0
  
  
 NCBI BlastP on this gene

EAA60093

hypothetical protein
  
Accession: EAA60092
  
Location: 4793-7527
  
 NCBI BlastP on this gene

EAA60092

hypothetical protein
  
Accession: EAA60091
  
Location: 3362-4153
  
 NCBI BlastP on this gene

EAA60091

129. :  AM270285 Aspergillus niger contig An12c0290, genomic contig.     Total score: 2.0     Cumulative Blast bit score: 1452

hypothetical protein
  
Accession: CAK41321
  
Location: 41584-44480
  
 NCBI BlastP on this gene

An12g08710

unnamed
  
Accession: CAK41322
  
Location: 45435-47000
  
 NCBI BlastP on this gene

An12g08720

not annotated
  
Accession: CAK41323
  
Location: 51270-52404
  
  
**BlastP hit with Mycgr3G31170\_Mycgr3T**
  
Percentage identity: 98 %
  
BlastP bit score: 162
  
Sequence coverage: 100 %
  
E-value: 5e-48
  
  
 NCBI BlastP on this gene

An12g08730

hypothetical protein
  
Accession: CAK41324
  
Location: 52877-53398
  
 NCBI BlastP on this gene

An12g08740

not annotated
  
Accession: CAK41325
  
Location: 53992-55662
  
 NCBI BlastP on this gene

An12g08750

not annotated
  
Accession: CAK41326
  
Location: 56585-57429
  
 NCBI BlastP on this gene

An12g08760

hypothetical protein
  
Accession: CAK41327
  
Location: 58187-60690
  
 NCBI BlastP on this gene

An12g08770

hypothetical protein
  
Accession: CAK41328
  
Location: 61408-61635
  
 NCBI BlastP on this gene

An12g08780

not annotated
  
Accession: CAK41329
  
Location: 63988-68709
  
 NCBI BlastP on this gene

An12g08790

not annotated
  
Accession: CAK41330
  
Location: 69882-76413
  
  
**BlastP hit with Mycgr3G41426\_Mycgr3T**
  
Percentage identity: 39 %
  
BlastP bit score: 1290
  
Sequence coverage: 103 %
  
E-value: 0.0
  
  
 NCBI BlastP on this gene

An12g08800

hypothetical protein
  
Accession: CAK41331
  
Location: 79291-80112
  
 NCBI BlastP on this gene

An12g08810

not annotated
  
Accession: CAK41332
  
Location: 81270-82880
  
 NCBI BlastP on this gene

An12g08820

130. :  ACJE01000006 Aspergillus niger ATCC 1015     Total score: 2.0     Cumulative Blast bit score: 1452

hypothetical protein
  
Accession: EHA25541
  
Location: 1451908-1453473
  
 NCBI BlastP on this gene

EHA25541

hypothetical protein
  
Accession: EHA25542
  
Location: 1457747-1458670
  
  
**BlastP hit with Mycgr3G31170\_Mycgr3T**
  
Percentage identity: 98 %
  
BlastP bit score: 162
  
Sequence coverage: 100 %
  
E-value: 3e-48
  
  
 NCBI BlastP on this gene

EHA25542

hypothetical protein
  
Accession: EHA25543
  
Location: 1460491-1462463
  
 NCBI BlastP on this gene

EHA25543

hypothetical protein
  
Accession: EHA25544
  
Location: 1463063-1463907
  
 NCBI BlastP on this gene

EHA25544

hypothetical protein
  
Accession: EHA25545
  
Location: 1464753-1467024
  
 NCBI BlastP on this gene

EHA25545

hypothetical protein
  
Accession: EHA25546
  
Location: 1470466-1475184
  
 NCBI BlastP on this gene

EHA25546

hypothetical protein
  
Accession: EHA25547
  
Location: 1476357-1482581
  
  
**BlastP hit with Mycgr3G41426\_Mycgr3T**
  
Percentage identity: 39 %
  
BlastP bit score: 1290
  
Sequence coverage: 103 %
  
E-value: 0.0
  
  
 NCBI BlastP on this gene

EHA25547

hypothetical protein
  
Accession: EHA25548
  
Location: 1487649-1490420
  
 NCBI BlastP on this gene

EHA25548

hypothetical protein
  
Accession: EHA25549
  
Location: 1491031-1491870
  
 NCBI BlastP on this gene

EHA25549

131. :  DF126460 Aspergillus kawachii IFO 4308 DNA, contig: scaffold00014     Total score: 2.0     Cumulative Blast bit score: 1449

similar to An12g08720
  
Accession: GAA87727
  
Location: 418126-419691
  
 NCBI BlastP on this gene

GAA87727

MADS box transcription factor Mcm1
  
Accession: GAA87728
  
Location: 424006-424926
  
  
**BlastP hit with Mycgr3G31170\_Mycgr3T**
  
Percentage identity: 98 %
  
BlastP bit score: 162
  
Sequence coverage: 100 %
  
E-value: 3e-48
  
  
 NCBI BlastP on this gene

GAA87728

DUF803 domain protein
  
Accession: GAA87729
  
Location: 426799-428794
  
 NCBI BlastP on this gene

GAA87729

vacuolar ATP synthase subunit E
  
Accession: GAA87730
  
Location: 429440-430290
  
 NCBI BlastP on this gene

GAA87730

similar to An12g08770
  
Accession: GAA87731
  
Location: 431041-433401
  
 NCBI BlastP on this gene

GAA87731

phospholipid-translocating P-type ATPase domain-containing protein
  
Accession: GAA87732
  
Location: 436921-441627
  
 NCBI BlastP on this gene

GAA87732

tRNA-splicing endonuclease
  
Accession: GAA87733
  
Location: 442798-449325
  
  
**BlastP hit with Mycgr3G41426\_Mycgr3T**
  
Percentage identity: 39 %
  
BlastP bit score: 1287
  
Sequence coverage: 103 %
  
E-value: 0.0
  
  
 NCBI BlastP on this gene

GAA87733

similar to An12g08820
  
Accession: GAA87734
  
Location: 454664-457335
  
 NCBI BlastP on this gene

GAA87734

aminotransferase
  
Accession: GAA87735
  
Location: 457944-458790
  
 NCBI BlastP on this gene

GAA87735

132. :  CH476603 Aspergillus terreus NIH2624 scaffold\_10 genomic scaffold     Total score: 2.0     Cumulative Blast bit score: 1447

conserved hypothetical protein
  
Accession: EAU32622
  
Location: 1100543-1102414
  
 NCBI BlastP on this gene

EAU32622

conserved hypothetical protein
  
Accession: EAU32623
  
Location: 1107857-1109311
  
 NCBI BlastP on this gene

EAU32623

hypothetical protein
  
Accession: EAU32624
  
Location: 1111982-1112918
  
  
**BlastP hit with Mycgr3G31170\_Mycgr3T**
  
Percentage identity: 98 %
  
BlastP bit score: 162
  
Sequence coverage: 100 %
  
E-value: 3e-48
  
  
 NCBI BlastP on this gene

EAU32624

conserved hypothetical protein
  
Accession: EAU32625
  
Location: 1114195-1116254
  
 NCBI BlastP on this gene

EAU32625

vacuolar ATP synthase subunit E
  
Accession: EAU32626
  
Location: 1116765-1117511
  
 NCBI BlastP on this gene

EAU32626

predicted protein
  
Accession: EAU32627
  
Location: 1118057-1120064
  
 NCBI BlastP on this gene

EAU32627

hypothetical protein
  
Accession: EAU32628
  
Location: 1123523-1128220
  
 NCBI BlastP on this gene

EAU32628

conserved hypothetical protein
  
Accession: EAU32629
  
Location: 1129328-1135881
  
  
**BlastP hit with Mycgr3G41426\_Mycgr3T**
  
Percentage identity: 39 %
  
BlastP bit score: 1285
  
Sequence coverage: 104 %
  
E-value: 0.0
  
  
 NCBI BlastP on this gene

EAU32629

predicted protein
  
Accession: EAU32630
  
Location: 1139359-1139712
  
 NCBI BlastP on this gene

EAU32630

predicted protein
  
Accession: EAU32631
  
Location: 1139947-1142040
  
 NCBI BlastP on this gene

EAU32631

predicted protein
  
Accession: EAU32632
  
Location: 1142578-1143444
  
 NCBI BlastP on this gene

EAU32632

conserved hypothetical protein
  
Accession: EAU32633
  
Location: 1144039-1145285
  
 NCBI BlastP on this gene

EAU32633

predicted protein
  
Accession: EAU32634
  
Location: 1145800-1148303
  
 NCBI BlastP on this gene

EAU32634

133. :  GG700648 Trichophyton rubrum CBS 118892 genomic scaffold supercont2.1     Total score: 2.0     Cumulative Blast bit score: 1441

metalloreductase transmembrane component
  
Accession: EGD83852
  
Location: 344325-346613
  
 NCBI BlastP on this gene

EGD83852

GNAT family acetyltransferase
  
Accession: EGD83853
  
Location: 347065-348186
  
 NCBI BlastP on this gene

EGD83853

hypothetical protein
  
Accession: EGD83854
  
Location: 349272-350370
  
 NCBI BlastP on this gene

EGD83854

tetraspanin Pls1 family protein
  
Accession: EGD83855
  
Location: 351229-352146
  
 NCBI BlastP on this gene

EGD83855

MADS box transcription factor
  
Accession: EGD83856
  
Location: 353392-354349
  
  
**BlastP hit with Mycgr3G31170\_Mycgr3T**
  
Percentage identity: 96 %
  
BlastP bit score: 154
  
Sequence coverage: 98 %
  
E-value: 5e-45
  
  
 NCBI BlastP on this gene

EGD83856

hypothetical protein
  
Accession: EGD83857
  
Location: 355720-356966
  
 NCBI BlastP on this gene

EGD83857

hypothetical protein
  
Accession: EGD83858
  
Location: 358083-359862
  
 NCBI BlastP on this gene

EGD83858

vacuolar ATP synthase subunit E
  
Accession: EGD83859
  
Location: 360486-361326
  
 NCBI BlastP on this gene

EGD83859

hypothetical protein
  
Accession: EGD83860
  
Location: 361721-364092
  
 NCBI BlastP on this gene

EGD83860

hypothetical protein
  
Accession: EGD83861
  
Location: 364624-364894
  
 NCBI BlastP on this gene

EGD83861

hypothetical protein
  
Accession: EGD83862
  
Location: 366055-366426
  
 NCBI BlastP on this gene

EGD83862

phospholipid-translocating P-type ATPase
  
Accession: EGD83863
  
Location: 367457-372157
  
 NCBI BlastP on this gene

EGD83863

tRNA-splicing endonuclease
  
Accession: EGD83864
  
Location: 373316-380209
  
  
**BlastP hit with Mycgr3G41426\_Mycgr3T**
  
Percentage identity: 39 %
  
BlastP bit score: 1287
  
Sequence coverage: 103 %
  
E-value: 0.0
  
  
 NCBI BlastP on this gene

EGD83864

hypothetical protein
  
Accession: EGD83865
  
Location: 380643-381283
  
 NCBI BlastP on this gene

EGD83865

hypothetical protein
  
Accession: EGD83866
  
Location: 383517-385273
  
 NCBI BlastP on this gene

EGD83866

hypothetical protein
  
Accession: EGD83867
  
Location: 385897-386172
  
 NCBI BlastP on this gene

EGD83867

hypothetical protein
  
Accession: EGD83868
  
Location: 387764-390712
  
 NCBI BlastP on this gene

EGD83868

134. :  CH476657 Ajellomyces capsulatus NAm1 scaffold\_3 genomic scaffold     Total score: 2.0     Cumulative Blast bit score: 1435

predicted protein
  
Accession: EDN06675
  
Location: 244789-246834
  
 NCBI BlastP on this gene

EDN06675

predicted protein
  
Accession: EDN06676
  
Location: 249218-250398
  
 NCBI BlastP on this gene

EDN06676

conserved hypothetical protein
  
Accession: EDN06677
  
Location: 251153-252083
  
  
**BlastP hit with Mycgr3G31170\_Mycgr3T**
  
Percentage identity: 98 %
  
BlastP bit score: 164
  
Sequence coverage: 100 %
  
E-value: 2e-48
  
  
 NCBI BlastP on this gene

EDN06677

predicted protein
  
Accession: EDN06678
  
Location: 254847-257060
  
 NCBI BlastP on this gene

EDN06678

vacuolar ATP synthase subunit E
  
Accession: EDN06679
  
Location: 257663-258447
  
 NCBI BlastP on this gene

EDN06679

predicted protein
  
Accession: EDN06680
  
Location: 259023-263841
  
 NCBI BlastP on this gene

EDN06680

predicted protein
  
Accession: EDN06681
  
Location: 264431-265257
  
 NCBI BlastP on this gene

EDN06681

hypothetical protein
  
Accession: EDN06682
  
Location: 265869-270537
  
 NCBI BlastP on this gene

EDN06682

predicted protein
  
Accession: EDN06683
  
Location: 271607-273047
  
 NCBI BlastP on this gene

EDN06683

conserved hypothetical protein
  
Accession: EDN06684
  
Location: 274264-281010
  
  
**BlastP hit with Mycgr3G41426\_Mycgr3T**
  
Percentage identity: 38 %
  
BlastP bit score: 1271
  
Sequence coverage: 103 %
  
E-value: 0.0
  
  
 NCBI BlastP on this gene

EDN06684

predicted protein
  
Accession: EDN06685
  
Location: 288746-289042
  
 NCBI BlastP on this gene

EDN06685

135. :  ABSU01000024 Arthroderma benhamiae CBS 112371     Total score: 2.0     Cumulative Blast bit score: 1432

hypothetical protein
  
Accession: EFE31140
  
Location: 216105-218393
  
 NCBI BlastP on this gene

EFE31140

hypothetical protein
  
Accession: EFE31141
  
Location: 218878-219999
  
 NCBI BlastP on this gene

EFE31141

transcriptional activator (PtaC), putative
  
Accession: EFE31142
  
Location: 221062-222159
  
 NCBI BlastP on this gene

EFE31142

hypothetical protein
  
Accession: EFE31143
  
Location: 222989-223905
  
 NCBI BlastP on this gene

EFE31143

hypothetical protein
  
Accession: EFE31144
  
Location: 225291-226172
  
  
**BlastP hit with Mycgr3G31170\_Mycgr3T**
  
Percentage identity: 82 %
  
BlastP bit score: 125
  
Sequence coverage: 98 %
  
E-value: 2e-33
  
  
 NCBI BlastP on this gene

EFE31144

hypothetical protein
  
Accession: EFE31145
  
Location: 227012-227380
  
 NCBI BlastP on this gene

EFE31145

conserved hypothetical protein
  
Accession: EFE31146
  
Location: 227626-228888
  
 NCBI BlastP on this gene

EFE31146

DUF803 domain protein
  
Accession: EFE31147
  
Location: 229962-231032
  
 NCBI BlastP on this gene

EFE31147

hypothetical protein
  
Accession: EFE31148
  
Location: 231898-232321
  
 NCBI BlastP on this gene

EFE31148

hypothetical protein
  
Accession: EFE31149
  
Location: 232372-233218
  
 NCBI BlastP on this gene

EFE31149

hypothetical protein
  
Accession: EFE31150
  
Location: 233605-234730
  
 NCBI BlastP on this gene

EFE31150

hypothetical protein
  
Accession: EFE31151
  
Location: 235037-236387
  
 NCBI BlastP on this gene

EFE31151

hypothetical protein
  
Accession: EFE31152
  
Location: 238935-243634
  
 NCBI BlastP on this gene

EFE31152

hypothetical protein
  
Accession: EFE31153
  
Location: 245117-251532
  
  
**BlastP hit with Mycgr3G41426\_Mycgr3T**
  
Percentage identity: 40 %
  
BlastP bit score: 1307
  
Sequence coverage: 103 %
  
E-value: 0.0
  
  
 NCBI BlastP on this gene

EFE31153

hypothetical protein
  
Accession: EFE31154
  
Location: 255131-255650
  
 NCBI BlastP on this gene

EFE31154

hypothetical protein
  
Accession: EFE31155
  
Location: 256228-257049
  
 NCBI BlastP on this gene

EFE31155

hypothetical protein
  
Accession: EFE31156
  
Location: 259745-262678
  
 NCBI BlastP on this gene

EFE31156

136. :  ACYE01000336 Trichophyton verrucosum HKI 0517     Total score: 2.0     Cumulative Blast bit score: 1428

hypothetical protein
  
Accession: EFE39442
  
Location: 40638-41507
  
 NCBI BlastP on this gene

EFE39442

hypothetical protein
  
Accession: EFE39443
  
Location: 41577-42926
  
 NCBI BlastP on this gene

EFE39443

hypothetical protein
  
Accession: EFE39444
  
Location: 43306-44548
  
 NCBI BlastP on this gene

EFE39444

transcriptional activator (PtaC), putative
  
Accession: EFE39445
  
Location: 45344-46728
  
 NCBI BlastP on this gene

EFE39445

hypothetical protein
  
Accession: EFE39446
  
Location: 47580-48497
  
 NCBI BlastP on this gene

EFE39446

hypothetical protein
  
Accession: EFE39447
  
Location: 49864-50781
  
  
**BlastP hit with Mycgr3G31170\_Mycgr3T**
  
Percentage identity: 82 %
  
BlastP bit score: 125
  
Sequence coverage: 98 %
  
E-value: 2e-33
  
  
 NCBI BlastP on this gene

EFE39447

conserved hypothetical protein
  
Accession: EFE39448
  
Location: 52191-53446
  
 NCBI BlastP on this gene

EFE39448

DUF803 domain protein
  
Accession: EFE39449
  
Location: 54580-55650
  
 NCBI BlastP on this gene

EFE39449

hypothetical protein
  
Accession: EFE39450
  
Location: 56516-56946
  
 NCBI BlastP on this gene

EFE39450

hypothetical protein
  
Accession: EFE39451
  
Location: 56997-57840
  
 NCBI BlastP on this gene

EFE39451

hypothetical protein
  
Accession: EFE39452
  
Location: 58229-60600
  
 NCBI BlastP on this gene

EFE39452

hypothetical protein
  
Accession: EFE39453
  
Location: 63467-68162
  
 NCBI BlastP on this gene

EFE39453

hypothetical protein
  
Accession: EFE39454
  
Location: 69603-76408
  
  
**BlastP hit with Mycgr3G41426\_Mycgr3T**
  
Percentage identity: 40 %
  
BlastP bit score: 1303
  
Sequence coverage: 103 %
  
E-value: 0.0
  
  
 NCBI BlastP on this gene

EFE39454

hypothetical protein
  
Accession: EFE39455
  
Location: 79732-80418
  
 NCBI BlastP on this gene

EFE39455

hypothetical protein
  
Accession: EFE39456
  
Location: 80674-81495
  
 NCBI BlastP on this gene

EFE39456

hypothetical protein
  
Accession: EFE39457
  
Location: 84194-87127
  
 NCBI BlastP on this gene

EFE39457

137. :  CH445343 Phaeosphaeria nodorum SN15 scaffold\_19     Total score: 2.0     Cumulative Blast bit score: 1424

hypothetical protein
  
Accession: EAT81272
  
Location: 635850-638039
  
 NCBI BlastP on this gene

EAT81272

hypothetical protein
  
Accession: EAT81273
  
Location: 638463-639159
  
 NCBI BlastP on this gene

EAT81273

hypothetical protein
  
Accession: EAT81274
  
Location: 639787-640568
  
 NCBI BlastP on this gene

EAT81274

hypothetical protein
  
Accession: EAT81275
  
Location: 641106-641785
  
 NCBI BlastP on this gene

EAT81275

hypothetical protein
  
Accession: EAT81276
  
Location: 641906-642441
  
 NCBI BlastP on this gene

EAT81276

hypothetical protein
  
Accession: EAT81277
  
Location: 642991-648116
  
 NCBI BlastP on this gene

EAT81277

hypothetical protein
  
Accession: EAT81278
  
Location: 649601-650905
  
 NCBI BlastP on this gene

EAT81278

hypothetical protein
  
Accession: EAT81279
  
Location: 651136-651792
  
 NCBI BlastP on this gene

EAT81279

hypothetical protein
  
Accession: EAT81280
  
Location: 652429-653193
  
 NCBI BlastP on this gene

EAT81280

hypothetical protein
  
Accession: EAT81281
  
Location: 653706-655589
  
 NCBI BlastP on this gene

EAT81281

hypothetical protein
  
Accession: EAT81282
  
Location: 655816-663166
  
  
**BlastP hit with Mycgr3G42010\_Mycgr3T**
  
Percentage identity: 50 %
  
BlastP bit score: 1150
  
Sequence coverage: 52 %
  
E-value: 0.0
  
  
 NCBI BlastP on this gene

EAT81282

hypothetical protein
  
Accession: EAT81283
  
Location: 663606-665069
  
  
**BlastP hit with Mycgr3G92938\_Mycgr3T**
  
Percentage identity: 35 %
  
BlastP bit score: 274
  
Sequence coverage: 113 %
  
E-value: 5e-83
  
  
 NCBI BlastP on this gene

EAT81283

hypothetical protein
  
Accession: EAT81284
  
Location: 667601-669127
  
 NCBI BlastP on this gene

EAT81284

hypothetical protein
  
Accession: EAT81285
  
Location: 669336-669585
  
 NCBI BlastP on this gene

EAT81285

hypothetical protein
  
Accession: EAT81286
  
Location: 670977-672116
  
 NCBI BlastP on this gene

EAT81286

hypothetical protein
  
Accession: EAT81287
  
Location: 673047-673852
  
 NCBI BlastP on this gene

EAT81287

hypothetical protein
  
Accession: EAT81288
  
Location: 674310-676543
  
 NCBI BlastP on this gene

EAT81288

hypothetical protein
  
Accession: EAT81289
  
Location: 676603-677972
  
 NCBI BlastP on this gene

EAT81289

hypothetical protein
  
Accession: EAT81290
  
Location: 678920-680056
  
 NCBI BlastP on this gene

EAT81290

hypothetical protein
  
Accession: EAT81291
  
Location: 680853-683081
  
 NCBI BlastP on this gene

EAT81291

138. :  AKHY01000183 Aspergillus oryzae 3.042     Total score: 2.0     Cumulative Blast bit score: 1421

carboxylesterase type B
  
Accession: EIT74797
  
Location: 69883-72741
  
 NCBI BlastP on this gene

EIT74797

hypothetical protein
  
Accession: EIT74815
  
Location: 67258-68652
  
 NCBI BlastP on this gene

EIT74815

hypothetical protein
  
Accession: EIT74770
  
Location: 64956-65599
  
 NCBI BlastP on this gene

EIT74770

hypothetical protein
  
Accession: EIT74811
  
Location: 63197-64543
  
 NCBI BlastP on this gene

EIT74811

hypothetical protein
  
Accession: EIT74777
  
Location: 61828-62466
  
 NCBI BlastP on this gene

EIT74777

monocarboxylate transporter
  
Accession: EIT74807
  
Location: 59096-61355
  
 NCBI BlastP on this gene

EIT74807

amine oxidase
  
Accession: EIT74800
  
Location: 56981-58282
  
 NCBI BlastP on this gene

EIT74800

hypothetical protein
  
Accession: EIT74812
  
Location: 55364-56092
  
 NCBI BlastP on this gene

EIT74812

hypothetical protein
  
Accession: EIT74759
  
Location: 53758-54918
  
  
**BlastP hit with Mycgr3G92938\_Mycgr3T**
  
Percentage identity: 29 %
  
BlastP bit score: 114
  
Sequence coverage: 75 %
  
E-value: 5e-25
  
  
 NCBI BlastP on this gene

EIT74759

nonsense-mediated mRNA decay protein, putative
  
Accession: EIT74785
  
Location: 47501-49830
  
  
**BlastP hit with Mycgr3G42010\_Mycgr3T**
  
Percentage identity: 36 %
  
BlastP bit score: 323
  
Sequence coverage: 20 %
  
E-value: 6e-93
  
  
 NCBI BlastP on this gene

EIT74785

ATPases of the AAA+ class
  
Accession: EIT74772
  
Location: 43169-45933
  
  
**BlastP hit with Mycgr3G42010\_Mycgr3T**
  
Percentage identity: 56 %
  
BlastP bit score: 984
  
Sequence coverage: 37 %
  
E-value: 0.0
  
  
 NCBI BlastP on this gene

EIT74772

vacuolar sorting protein VPS1, dynamin
  
Accession: EIT74773
  
Location: 38401-41107
  
 NCBI BlastP on this gene

EIT74773

hypothetical protein
  
Accession: EIT74799
  
Location: 35167-37614
  
 NCBI BlastP on this gene

EIT74799

hypothetical protein
  
Accession: EIT74801
  
Location: 33616-34308
  
 NCBI BlastP on this gene

EIT74801

hypothetical protein
  
Accession: EIT74762
  
Location: 32594-33525
  
 NCBI BlastP on this gene

EIT74762

hypothetical protein
  
Accession: EIT74775
  
Location: 31576-32353
  
 NCBI BlastP on this gene

EIT74775

fungal alpha-L-arabinofuranosidase, putative
  
Accession: EIT74798
  
Location: 29328-30848
  
 NCBI BlastP on this gene

EIT74798

139. :  KB644411 Penicillium oxalicum 114-2 unplaced genomic scaffold scaffold\_4     Total score: 2.0     Cumulative Blast bit score: 1398

putative chondroitin sulfate synthase
  
Accession: EPS28408
  
Location: 165190-166785
  
 NCBI BlastP on this gene

EPS28408

hypothetical protein
  
Accession: EPS28407
  
Location: 160626-161578
  
  
**BlastP hit with Mycgr3G31170\_Mycgr3T**
  
Percentage identity: 100 %
  
BlastP bit score: 166
  
Sequence coverage: 100 %
  
E-value: 1e-49
  
  
 NCBI BlastP on this gene

EPS28407

hypothetical protein
  
Accession: EPS28406
  
Location: 156430-158309
  
 NCBI BlastP on this gene

EPS28406

hypothetical protein
  
Accession: EPS28405
  
Location: 155128-156000
  
 NCBI BlastP on this gene

EPS28405

hypothetical protein
  
Accession: EPS28404
  
Location: 152365-154758
  
 NCBI BlastP on this gene

EPS28404

hypothetical protein
  
Accession: EPS28403
  
Location: 142386-147085
  
 NCBI BlastP on this gene

EPS28403

hypothetical protein
  
Accession: EPS28402
  
Location: 139464-139938
  
 NCBI BlastP on this gene

EPS28402

hypothetical protein
  
Accession: EPS28401
  
Location: 131741-138269
  
  
**BlastP hit with Mycgr3G41426\_Mycgr3T**
  
Percentage identity: 38 %
  
BlastP bit score: 1232
  
Sequence coverage: 103 %
  
E-value: 0.0
  
  
 NCBI BlastP on this gene

EPS28401

hypothetical protein
  
Accession: EPS28400
  
Location: 125901-128708
  
 NCBI BlastP on this gene

EPS28400

hypothetical protein
  
Accession: EPS28399
  
Location: 124020-124718
  
 NCBI BlastP on this gene

EPS28399

140. :  JH226133 Exophiala dermatitidis NIH/UT8656 unplaced genomic scaffold supercont1.4     Total score: 2.0     Cumulative Blast bit score: 1394

carbamoyl-phosphate synthase arginine-specific small chain
  
Accession: EHY56996
  
Location: 2038206-2039752
  
 NCBI BlastP on this gene

EHY56996

hypothetical protein
  
Accession: EHY56997
  
Location: 2040527-2040922
  
 NCBI BlastP on this gene

EHY56997

hypothetical protein
  
Accession: EHY56998
  
Location: 2044357-2047737
  
 NCBI BlastP on this gene

EHY56998

senataxin
  
Accession: EHY56999
  
Location: 2048646-2055497
  
  
**BlastP hit with Mycgr3G41426\_Mycgr3T**
  
Percentage identity: 40 %
  
BlastP bit score: 1310
  
Sequence coverage: 104 %
  
E-value: 0.0
  
  
 NCBI BlastP on this gene

EHY56999

phospholipid-translocating ATPase
  
Accession: EHY57000
  
Location: 2056377-2061222
  
 NCBI BlastP on this gene

EHY57000

hypothetical protein
  
Accession: EHY57001
  
Location: 2064903-2065265
  
 NCBI BlastP on this gene

EHY57001

2-dehydropantoate 2-reductase, variant
  
Accession: EHY57002
  
Location: 2067094-2069592
  
 NCBI BlastP on this gene

EHY57002

hypothetical protein
  
Accession: EHY57004
  
Location: 2071108-2071434
  
  
**BlastP hit with Mycgr3G29582\_Mycgr3T**
  
Percentage identity: 64 %
  
BlastP bit score: 84
  
Sequence coverage: 75 %
  
E-value: 6e-19
  
  
 NCBI BlastP on this gene

EHY57004

hypothetical protein
  
Accession: EHY57005
  
Location: 2071859-2072305
  
 NCBI BlastP on this gene

EHY57005

hypothetical protein
  
Accession: EHY57006
  
Location: 2073817-2075467
  
 NCBI BlastP on this gene

EHY57006

NADP-dependent mannitol dehydrogenase
  
Accession: EHY57007
  
Location: 2076427-2077290
  
 NCBI BlastP on this gene

EHY57007

phosphatidylinositol glycan, class O
  
Accession: EHY57008
  
Location: 2078092-2081205
  
 NCBI BlastP on this gene

EHY57008

hypothetical protein
  
Accession: EHY57009
  
Location: 2081666-2082637
  
 NCBI BlastP on this gene

EHY57009

141. :  GL636491 Coccidioides posadasii str. Silveira unplaced genomic scaffold supercont2.6     Total score: 2.0     Cumulative Blast bit score: 1385

conserved hypothetical protein
  
Accession: EFW18852
  
Location: 453928-454682
  
 NCBI BlastP on this gene

EFW18852

MADS box transcription factor Mcm1
  
Accession: EFW18851
  
Location: 451437-452655
  
  
**BlastP hit with Mycgr3G31170\_Mycgr3T**
  
Percentage identity: 97 %
  
BlastP bit score: 160
  
Sequence coverage: 100 %
  
E-value: 2e-47
  
  
 NCBI BlastP on this gene

EFW18851

hypothetical protein
  
Accession: EFW18850
  
Location: 448889-450607
  
 NCBI BlastP on this gene

EFW18850

vacuolar ATP synthase subunit E
  
Accession: EFW18849
  
Location: 446966-447846
  
 NCBI BlastP on this gene

EFW18849

conserved hypothetical protein
  
Accession: EFW18848
  
Location: 444295-445952
  
 NCBI BlastP on this gene

EFW18848

P-type ATPase
  
Accession: EFW18847
  
Location: 437877-442625
  
 NCBI BlastP on this gene

EFW18847

tRNA-splicing endonuclease
  
Accession: EFW18846
  
Location: 433172-435748
  
  
**BlastP hit with Mycgr3G41426\_Mycgr3T**
  
Percentage identity: 29 %
  
BlastP bit score: 363
  
Sequence coverage: 44 %
  
E-value: 1e-103
  
  
 NCBI BlastP on this gene

EFW18846

tRNA-splicing endonuclease
  
Accession: EFW18845
  
Location: 429533-433022
  
  
**BlastP hit with Mycgr3G41426\_Mycgr3T**
  
Percentage identity: 54 %
  
BlastP bit score: 862
  
Sequence coverage: 42 %
  
E-value: 0.0
  
  
 NCBI BlastP on this gene

EFW18845

conserved hypothetical protein
  
Accession: EFW18844
  
Location: 426846-427341
  
 NCBI BlastP on this gene

EFW18844

predicted protein
  
Accession: EFW18843
  
Location: 425824-426244
  
 NCBI BlastP on this gene

EFW18843

hypothetical protein
  
Accession: EFW18841
  
Location: 425318-425749
  
 NCBI BlastP on this gene

EFW18841

conserved hypothetical protein
  
Accession: EFW18842
  
Location: 422421-425049
  
 NCBI BlastP on this gene

EFW18842

inner membrane magnesium transporter MRS2
  
Accession: EFW18840
  
Location: 419562-421570
  
 NCBI BlastP on this gene

EFW18840

conserved hypothetical protein
  
Accession: EFW18839
  
Location: 416810-418773
  
 NCBI BlastP on this gene

EFW18839

142. :  JH921445 Marssonina brunnea f. sp. 'multigermtubi' MB\_m1 unplaced genomic scaffold M6\_S00018     Total score: 2.0     Cumulative Blast bit score: 1315

C-5 cytosine-specific DNA methylase
  
Accession: EKD14577
  
Location: 755212-758372
  
 NCBI BlastP on this gene

EKD14577

amidophosphoribosyltransferase
  
Accession: EKD14578
  
Location: 760108-761947
  
 NCBI BlastP on this gene

EKD14578

base excision DNA repair protein
  
Accession: EKD14579
  
Location: 762805-764628
  
 NCBI BlastP on this gene

EKD14579

hypothetical protein
  
Accession: EKD14580
  
Location: 765452-766144
  
 NCBI BlastP on this gene

EKD14580

SRP54-type protein
  
Accession: EKD14581
  
Location: 766297-768557
  
 NCBI BlastP on this gene

EKD14581

hypothetical protein
  
Accession: EKD14582
  
Location: 768836-769920
  
 NCBI BlastP on this gene

EKD14582

hypothetical protein
  
Accession: EKD14583
  
Location: 771363-777673
  
  
**BlastP hit with Mycgr3G41426\_Mycgr3T**
  
Percentage identity: 34 %
  
BlastP bit score: 1030
  
Sequence coverage: 104 %
  
E-value: 0.0
  
  
 NCBI BlastP on this gene

EKD14583

hypothetical protein
  
Accession: EKD14584
  
Location: 779724-782198
  
 NCBI BlastP on this gene

EKD14584

sorbitol dehydrogenase
  
Accession: EKD14585
  
Location: 782692-784392
  
  
**BlastP hit with Mycgr3G71679\_Mycgr3T**
  
Percentage identity: 45 %
  
BlastP bit score: 285
  
Sequence coverage: 101 %
  
E-value: 2e-89
  
  
 NCBI BlastP on this gene

EKD14585

BRCA1 C Terminus domain-containing protein
  
Accession: EKD14586
  
Location: 785050-789513
  
 NCBI BlastP on this gene

EKD14586

eukaryotic translation initiation factor eIF-1A
  
Accession: EKD14587
  
Location: 790000-790539
  
 NCBI BlastP on this gene

EKD14587

WH1 domain-containing protein
  
Accession: EKD14588
  
Location: 791036-793208
  
 NCBI BlastP on this gene

EKD14588

hypothetical protein
  
Accession: EKD14589
  
Location: 794094-794705
  
 NCBI BlastP on this gene

EKD14589

MUS38-like protein
  
Accession: EKD14590
  
Location: 794773-797622
  
 NCBI BlastP on this gene

EKD14590

actin cortical patch protein
  
Accession: EKD14591
  
Location: 798567-799934
  
 NCBI BlastP on this gene

EKD14591

hypothetical protein
  
Accession: EKD14592
  
Location: 800260-801162
  
 NCBI BlastP on this gene

EKD14592

143. :  DS544803 Paracoccidioides brasiliensis Pb03 supercont1.1 genomic scaffold     Total score: 2.0     Cumulative Blast bit score: 1285

cytosolic Cu/Zn superoxide dismutase
  
Accession: EEH17868
  
Location: 1549894-1550642
  
 NCBI BlastP on this gene

EEH17868

predicted protein
  
Accession: EEH17869
  
Location: 1552184-1553567
  
 NCBI BlastP on this gene

EEH17869

conserved hypothetical protein
  
Accession: EEH17870
  
Location: 1554094-1555957
  
 NCBI BlastP on this gene

EEH17870

CORD and CS domain-containing protein
  
Accession: EEH17871
  
Location: 1557992-1559188
  
 NCBI BlastP on this gene

EEH17871

conserved hypothetical protein
  
Accession: EEH17872
  
Location: 1559633-1559959
  
 NCBI BlastP on this gene

EEH17872

2,4-dihydroxyhept-2-ene-1,7-dioic acid aldolase
  
Accession: EEH17873
  
Location: 1560053-1561195
  
 NCBI BlastP on this gene

EEH17873

conserved hypothetical protein
  
Accession: EEH17874
  
Location: 1561965-1564211
  
  
**BlastP hit with Mycgr3G104337\_Mycgr3**
  
Percentage identity: 35 %
  
BlastP bit score: 169
  
Sequence coverage: 104 %
  
E-value: 3e-46
  
  
 NCBI BlastP on this gene

EEH17874

fatty acid synthase S-acetyltransferase
  
Accession: EEH17875
  
Location: 1564688-1572713
  
  
**BlastP hit with Mycgr3G100089\_Mycgr3**
  
Percentage identity: 44 %
  
BlastP bit score: 1116
  
Sequence coverage: 58 %
  
E-value: 0.0
  
  
 NCBI BlastP on this gene

EEH17875

conserved hypothetical protein
  
Accession: EEH17876
  
Location: 1573656-1576532
  
 NCBI BlastP on this gene

EEH17876

predicted protein
  
Accession: EEH17877
  
Location: 1578138-1578942
  
 NCBI BlastP on this gene

EEH17877

medium-chain specific acyl-CoA dehydrogenase
  
Accession: EEH17878
  
Location: 1580302-1581883
  
 NCBI BlastP on this gene

EEH17878

conserved hypothetical protein
  
Accession: EEH17879
  
Location: 1583446-1584468
  
 NCBI BlastP on this gene

EEH17879

predicted protein
  
Accession: EEH17880
  
Location: 1589386-1590417
  
 NCBI BlastP on this gene

EEH17880

144. :  DS572752 Paracoccidioides brasiliensis Pb18 supercont1.3 genomic scaffold     Total score: 2.0     Cumulative Blast bit score: 1283

conserved hypothetical protein
  
Accession: EEH46744
  
Location: 1543163-1543911
  
 NCBI BlastP on this gene

EEH46744

predicted protein
  
Accession: EEH46745
  
Location: 1546206-1546837
  
 NCBI BlastP on this gene

EEH46745

conserved hypothetical protein
  
Accession: EEH46746
  
Location: 1547335-1548914
  
 NCBI BlastP on this gene

EEH46746

integrin beta-1-binding protein
  
Accession: EEH46747
  
Location: 1551230-1552426
  
 NCBI BlastP on this gene

EEH46747

glutaredoxin domain-containing protein
  
Accession: EEH46748
  
Location: 1552835-1553161
  
 NCBI BlastP on this gene

EEH46748

HpcH/HpaI aldolase/citrate lyase family protein
  
Accession: EEH46749
  
Location: 1553323-1554398
  
 NCBI BlastP on this gene

EEH46749

conserved hypothetical protein
  
Accession: EEH46750
  
Location: 1555652-1556446
  
  
**BlastP hit with Mycgr3G104337\_Mycgr3**
  
Percentage identity: 38 %
  
BlastP bit score: 169
  
Sequence coverage: 93 %
  
E-value: 8e-48
  
  
 NCBI BlastP on this gene

EEH46750

6-methylsalicylic acid synthase
  
Accession: EEH46751
  
Location: 1557888-1565913
  
  
**BlastP hit with Mycgr3G100089\_Mycgr3**
  
Percentage identity: 44 %
  
BlastP bit score: 1114
  
Sequence coverage: 57 %
  
E-value: 0.0
  
  
 NCBI BlastP on this gene

EEH46751

conserved hypothetical protein
  
Accession: EEH46752
  
Location: 1566757-1570854
  
 NCBI BlastP on this gene

EEH46752

predicted protein
  
Accession: EEH46753
  
Location: 1571376-1572180
  
 NCBI BlastP on this gene

EEH46753

acyl-CoA dehydrogenase
  
Accession: EEH46754
  
Location: 1573525-1575106
  
 NCBI BlastP on this gene

EEH46754

conserved hypothetical protein
  
Accession: EEH46755
  
Location: 1576504-1577866
  
 NCBI BlastP on this gene

EEH46755

predicted protein
  
Accession: EEH46756
  
Location: 1578838-1579023
  
 NCBI BlastP on this gene

EEH46756

predicted protein
  
Accession: EEH46757
  
Location: 1581754-1582767
  
 NCBI BlastP on this gene

EEH46757

predicted protein
  
Accession: EEH46758
  
Location: 1582897-1583238
  
 NCBI BlastP on this gene

EEH46758

endopolyphosphatase
  
Accession: EEH46759
  
Location: 1583560-1585697
  
 NCBI BlastP on this gene

EEH46759

145. :  ABDG02000027 Trichoderma atroviride IMI 206040     Total score: 2.0     Cumulative Blast bit score: 1269

hypothetical protein
  
Accession: EHK42026
  
Location: 4856752-4857859
  
 NCBI BlastP on this gene

EHK42026

hypothetical protein
  
Accession: EHK42027
  
Location: 4860765-4861868
  
 NCBI BlastP on this gene

EHK42027

hypothetical protein
  
Accession: EHK42028
  
Location: 4864237-4866321
  
  
**BlastP hit with Mycgr3G42010\_Mycgr3T**
  
Percentage identity: 42 %
  
BlastP bit score: 521
  
Sequence coverage: 28 %
  
E-value: 2e-160
  
  
 NCBI BlastP on this gene

EHK42028

hypothetical protein
  
Accession: EHK42029
  
Location: 4866743-4866907
  
 NCBI BlastP on this gene

EHK42029

hypothetical protein
  
Accession: EHK42030
  
Location: 4868186-4871012
  
 NCBI BlastP on this gene

EHK42030

hypothetical protein
  
Accession: EHK42031
  
Location: 4871127-4872032
  
 NCBI BlastP on this gene

EHK42031

hypothetical protein
  
Accession: EHK42032
  
Location: 4872372-4874756
  
 NCBI BlastP on this gene

EHK42032

hypothetical protein
  
Accession: EHK42033
  
Location: 4876158-4876319
  
 NCBI BlastP on this gene

EHK42033

hypothetical protein
  
Accession: EHK42034
  
Location: 4876691-4878104
  
 NCBI BlastP on this gene

EHK42034

putative gamma-butyrobetaine hydroxylase
  
Accession: EHK42035
  
Location: 4878395-4879642
  
 NCBI BlastP on this gene

EHK42035

hypothetical protein
  
Accession: EHK42036
  
Location: 4880772-4882490
  
 NCBI BlastP on this gene

EHK42036

glycosyltransferase family 21 protein
  
Accession: EHK42037
  
Location: 4884170-4885819
  
 NCBI BlastP on this gene

EHK42037

polyketide synthase
  
Accession: EHK42038
  
Location: 4886179-4894430
  
  
**BlastP hit with Mycgr3G100089\_Mycgr3**
  
Percentage identity: 36 %
  
BlastP bit score: 748
  
Sequence coverage: 56 %
  
E-value: 0.0
  
  
 NCBI BlastP on this gene

EHK42038

hypothetical protein
  
Accession: EHK42039
  
Location: 4894943-4896379
  
 NCBI BlastP on this gene

EHK42039

146. :  JH126400 Cordyceps militaris CM01 unplaced genomic scaffold CCM\_S00002     Total score: 2.0     Cumulative Blast bit score: 1249

hypothetical protein
  
Accession: EGX94099
  
Location: 1384963-1386147
  
 NCBI BlastP on this gene

EGX94099

hypothetical protein
  
Accession: EGX94100
  
Location: 1387259-1388416
  
 NCBI BlastP on this gene

EGX94100

hypothetical protein
  
Accession: EGX94101
  
Location: 1392197-1396931
  
 NCBI BlastP on this gene

EGX94101

amino acid permease, putative
  
Accession: EGX94102
  
Location: 1397487-1399217
  
 NCBI BlastP on this gene

EGX94102

polyketide synthase, putative
  
Accession: EGX94103
  
Location: 1399952-1408884
  
  
**BlastP hit with Mycgr3G100089\_Mycgr3**
  
Percentage identity: 42 %
  
BlastP bit score: 1052
  
Sequence coverage: 59 %
  
E-value: 0.0
  
  
 NCBI BlastP on this gene

EGX94103

DUF341 family oxidoreductase, putative
  
Accession: EGX94104
  
Location: 1412699-1413649
  
  
**BlastP hit with Mycgr3G104337\_Mycgr3**
  
Percentage identity: 40 %
  
BlastP bit score: 197
  
Sequence coverage: 100 %
  
E-value: 1e-58
  
  
 NCBI BlastP on this gene

EGX94104

diacylglycerol o-acyltransferase
  
Accession: EGX94105
  
Location: 1415843-1417519
  
 NCBI BlastP on this gene

EGX94105

Major facilitator superfamily transporter
  
Accession: EGX94106
  
Location: 1420270-1422026
  
 NCBI BlastP on this gene

EGX94106

guanyl-specific ribonuclease Pb1
  
Accession: EGX94107
  
Location: 1422597-1423050
  
 NCBI BlastP on this gene

EGX94107

hypothetical protein
  
Accession: EGX94108
  
Location: 1424609-1425922
  
 NCBI BlastP on this gene

EGX94108

hypothetical protein
  
Accession: EGX94109
  
Location: 1427004-1429745
  
 NCBI BlastP on this gene

EGX94109

147. :  KB446555 Pseudocercospora fijiensis CIRAD86 unplaced genomic scaffold MYCFIscaffold\_1     Total score: 2.0     Cumulative Blast bit score: 1231

hypothetical protein
  
Accession: EME88234
  
Location: 4514629-4517144
  
 NCBI BlastP on this gene

EME88234

hypothetical protein
  
Accession: EME88235
  
Location: 4517174-4521585
  
 NCBI BlastP on this gene

EME88235

hypothetical protein
  
Accession: EME88236
  
Location: 4522263-4523208
  
 NCBI BlastP on this gene

EME88236

hypothetical protein
  
Accession: EME88237
  
Location: 4526412-4527799
  
 NCBI BlastP on this gene

EME88237

hypothetical protein
  
Accession: EME88239
  
Location: 4527979-4529325
  
 NCBI BlastP on this gene

EME88239

hypothetical protein
  
Accession: EME88240
  
Location: 4530267-4532277
  
 NCBI BlastP on this gene

EME88240

hypothetical protein
  
Accession: EME88241
  
Location: 4532302-4534215
  
  
**BlastP hit with Mycgr3G85924\_Mycgr3T**
  
Percentage identity: 57 %
  
BlastP bit score: 545
  
Sequence coverage: 100 %
  
E-value: 0.0
  
  
 NCBI BlastP on this gene

EME88241

hypothetical protein
  
Accession: EME88242
  
Location: 4534506-4535909
  
  
**BlastP hit with Mycgr3G92934\_Mycgr3T**
  
Percentage identity: 73 %
  
BlastP bit score: 686
  
Sequence coverage: 93 %
  
E-value: 0.0
  
  
 NCBI BlastP on this gene

EME88242

hypothetical protein
  
Accession: EME88243
  
Location: 4535889-4537250
  
 NCBI BlastP on this gene

EME88243

hypothetical protein
  
Accession: EME88244
  
Location: 4537275-4538559
  
 NCBI BlastP on this gene

EME88244

hypothetical protein
  
Accession: EME88245
  
Location: 4540242-4543405
  
 NCBI BlastP on this gene

EME88245

pyruvate kinase
  
Accession: EME88246
  
Location: 4544207-4545908
  
 NCBI BlastP on this gene

EME88246

hypothetical protein
  
Accession: EME88247
  
Location: 4549659-4551776
  
 NCBI BlastP on this gene

EME88247

hypothetical protein
  
Accession: EME88248
  
Location: 4552447-4553023
  
 NCBI BlastP on this gene

EME88248

hypothetical protein
  
Accession: EME88249
  
Location: 4553311-4554218
  
 NCBI BlastP on this gene

EME88249

casein kinase II, beta subunit
  
Accession: EME88250
  
Location: 4554519-4555693
  
 NCBI BlastP on this gene

EME88250

148. :  AM920428 Penicillium chrysogenum Wisconsin 54-1255 complete genome, contig Pc00c13.     Total score: 2.0     Cumulative Blast bit score: 1226

unnamed
  
Accession: CAP91930
  
Location: 2073749-2075700
  
 NCBI BlastP on this gene

Pc13g08610

hypothetical protein
  
Accession: CAP91931
  
Location: 2076390-2077813
  
 NCBI BlastP on this gene

Pc13g08620

not annotated
  
Accession: CAP91932
  
Location: 2081162-2083335
  
 NCBI BlastP on this gene

Pc13g08630

not annotated
  
Accession: CAP91933
  
Location: 2084282-2086141
  
 NCBI BlastP on this gene

Pc13g08640

not annotated
  
Accession: CAP91934
  
Location: 2086330-2087551
  
 NCBI BlastP on this gene

Pc13g08650

hypothetical protein
  
Accession: CAP91935
  
Location: 2088816-2089313
  
 NCBI BlastP on this gene

Pc13g08660

not annotated
  
Accession: CAP91936
  
Location: 2089563-2091214
  
 NCBI BlastP on this gene

Pc13g08670

not annotated
  
Accession: CAP91937
  
Location: 2091632-2093092
  
 NCBI BlastP on this gene

Pc13g08680

not annotated
  
Accession: CAP91938
  
Location: 2093606-2101605
  
  
**BlastP hit with Mycgr3G100089\_Mycgr3**
  
Percentage identity: 43 %
  
BlastP bit score: 1062
  
Sequence coverage: 58 %
  
E-value: 0.0
  
  
 NCBI BlastP on this gene

Pc13g08690

not annotated
  
Accession: CAP91939
  
Location: 2102331-2103184
  
  
**BlastP hit with Mycgr3G104337\_Mycgr3**
  
Percentage identity: 39 %
  
BlastP bit score: 164
  
Sequence coverage: 98 %
  
E-value: 4e-46
  
  
 NCBI BlastP on this gene

Pc13g08700

unnamed
  
Accession: CAP91940
  
Location: 2104634-2105583
  
 NCBI BlastP on this gene

Pc13g08710

not annotated
  
Accession: CAP91941
  
Location: 2106573-2113325
  
 NCBI BlastP on this gene

Pc13g08720

unnamed
  
Accession: CAP91942
  
Location: 2114599-2115663
  
 NCBI BlastP on this gene

Pc13g08730

hypothetical protein
  
Accession: CAP91943
  
Location: 2115756-2117286
  
 NCBI BlastP on this gene

Pc13g08740

not annotated
  
Accession: CAP91944
  
Location: 2117592-2119232
  
 NCBI BlastP on this gene

Pc13g08750

149. :  KB446535 Dothistroma septosporum NZE10 unplaced genomic scaffold DOTSEscaffold\_1     Total score: 2.0     Cumulative Blast bit score: 1220

glycoside hydrolase family 3 protein
  
Accession: EME48327
  
Location: 39310-42090
  
 NCBI BlastP on this gene

EME48327

hypothetical protein
  
Accession: EME48328
  
Location: 42979-43481
  
 NCBI BlastP on this gene

EME48328

glycoside hydrolase family 54 protein
  
Accession: EME48329
  
Location: 46959-48525
  
 NCBI BlastP on this gene

EME48329

hypothetical protein
  
Accession: EME48330
  
Location: 50587-52626
  
 NCBI BlastP on this gene

EME48330

hypothetical protein
  
Accession: EME48331
  
Location: 53246-53704
  
 NCBI BlastP on this gene

EME48331

hypothetical protein
  
Accession: EME48332
  
Location: 54209-55834
  
 NCBI BlastP on this gene

EME48332

hypothetical protein
  
Accession: EME48333
  
Location: 57200-59049
  
  
**BlastP hit with Mycgr3G92934\_Mycgr3T**
  
Percentage identity: 77 %
  
BlastP bit score: 702
  
Sequence coverage: 88 %
  
E-value: 0.0
  
  
 NCBI BlastP on this gene

EME48333

hypothetical protein
  
Accession: EME48334
  
Location: 59373-61262
  
  
**BlastP hit with Mycgr3G85924\_Mycgr3T**
  
Percentage identity: 56 %
  
BlastP bit score: 518
  
Sequence coverage: 100 %
  
E-value: 4e-173
  
  
 NCBI BlastP on this gene

EME48334

hypothetical protein
  
Accession: EME48335
  
Location: 62847-63128
  
 NCBI BlastP on this gene

EME48335

hypothetical protein
  
Accession: EME48336
  
Location: 64347-64823
  
 NCBI BlastP on this gene

EME48336

hypothetical protein
  
Accession: EME48337
  
Location: 65187-65564
  
 NCBI BlastP on this gene

EME48337

hypothetical protein
  
Accession: EME48338
  
Location: 66101-67452
  
 NCBI BlastP on this gene

EME48338

hypothetical protein
  
Accession: EME48339
  
Location: 68579-69091
  
 NCBI BlastP on this gene

EME48339

hypothetical protein
  
Accession: EME48340
  
Location: 70223-71845
  
 NCBI BlastP on this gene

EME48340

hypothetical protein
  
Accession: EME48341
  
Location: 72444-73388
  
 NCBI BlastP on this gene

EME48341

hypothetical protein
  
Accession: EME48342
  
Location: 73454-73768
  
 NCBI BlastP on this gene

EME48342

hypothetical protein
  
Accession: EME48343
  
Location: 74058-75148
  
 NCBI BlastP on this gene

EME48343

hypothetical protein
  
Accession: EME48344
  
Location: 75364-77694
  
 NCBI BlastP on this gene

EME48344

hypothetical protein
  
Accession: EME48345
  
Location: 78546-80393
  
 NCBI BlastP on this gene

EME48345

150. :  AKCU01000203 Penicillium digitatum Pd1     Total score: 2.0     Cumulative Blast bit score: 1210

hypothetical protein
  
Accession: EKV17643
  
Location: 54916-55116
  
 NCBI BlastP on this gene

EKV17643

hypothetical protein
  
Accession: EKV17644
  
Location: 55547-55684
  
 NCBI BlastP on this gene

EKV17644

Myosin heavy chain-like protein, putative
  
Accession: EKV17645
  
Location: 60660-67406
  
 NCBI BlastP on this gene

EKV17645

Endosomal cargo receptor (P24), putative
  
Accession: EKV17646
  
Location: 68571-69511
  
 NCBI BlastP on this gene

EKV17646

hypothetical protein
  
Accession: EKV17647
  
Location: 70102-70939
  
 NCBI BlastP on this gene

EKV17647

hypothetical protein
  
Accession: EKV17648
  
Location: 71030-71891
  
  
**BlastP hit with Mycgr3G104337\_Mycgr3**
  
Percentage identity: 39 %
  
BlastP bit score: 160
  
Sequence coverage: 98 %
  
E-value: 2e-44
  
  
 NCBI BlastP on this gene

EKV17648

hypothetical protein
  
Accession: EKV17649
  
Location: 72623-80609
  
  
**BlastP hit with Mycgr3G100089\_Mycgr3**
  
Percentage identity: 43 %
  
BlastP bit score: 1050
  
Sequence coverage: 58 %
  
E-value: 0.0
  
  
 NCBI BlastP on this gene

EKV17649

hypothetical protein
  
Accession: EKV17650
  
Location: 81973-83624
  
 NCBI BlastP on this gene

EKV17650

hypothetical protein
  
Accession: EKV17651
  
Location: 84814-86027
  
 NCBI BlastP on this gene

EKV17651

MFS lactose permease, putative
  
Accession: EKV17652
  
Location: 87470-89636
  
 NCBI BlastP on this gene

EKV17652

Endopolyphosphatase
  
Accession: EKV17653
  
Location: 93667-95616
  
 NCBI BlastP on this gene

EKV17653

hypothetical protein
  
Accession: EKV17654
  
Location: 97541-98614
  
 NCBI BlastP on this gene

EKV17654

Detecting sequence homology at the gene cluster level with MultiGeneBlast.
  
Marnix H. Medema, Rainer Breitling & Eriko Takano (2013)
  
*Molecular Biology and Evolution* , 30: 1218-1223.
