# Supplementary material for: In silico prediction and characterization of secondary metabolite biosynthetic gene clusters in the wheat pathogen Zymoseptoria tritici
Source: BMC Genomics. 2017 Aug 17;18:631. doi: 10.1186/s12864-017-3969-y (PMC5561558; doi:10.1186/s12864-017-3969-y)
Supplement: Supplementary file 1 — MultiGeneBLAST analysis of putative secondary metabolite clusters. All encoded amino acid sequences from genes residing in clusters predicted by AntiSMASH are given as FASTA file format. All output data from MultiGeneBLASTs are also provided. (ZIP 42911 kb) [file 12864_2017_3969_MOESM1_ESM.zip › Cluster MultiGene BLAST/out/Clusters_1_34/Cluster_15/displaypage4.xhtml]

xml version="1.0" encoding="UTF-8"?


Search Results
  
  
 Results pages: 1, 2, 3, 4, 5

**MultiGeneBlast hits**

Select gene cluster alignment
151. AKCT01000066\_0 Penicillium digitatum PHI26, whole genome shotgun sequenc...
152. KB456260\_0 Mycosphaerella populorum SO2202 unplaced genomic scaffold SEP...
153. CH476634\_0 Sclerotinia sclerotiorum 1980 scaffold\_14 genomic scaffold, w...
154. CH476626\_1 Sclerotinia sclerotiorum 1980 scaffold\_6 genomic scaffold, wh...
155. AKHY01000142\_0 Aspergillus oryzae 3.042, whole genome shotgun sequencing...
156. DS989828\_0 Arthroderma gypseum CBS 118893 supercont1.7 genomic scaffold,...
157. KB644411\_4 Penicillium oxalicum 114-2 unplaced genomic scaffold scaffold...
158. AP007167\_0 Aspergillus oryzae RIB40 DNA, SC020.
159. DS995701\_0 Microsporum canis CBS 113480 supercont1.1 genomic scaffold, w...
160. AKCU01000499\_0 Penicillium digitatum Pd1, whole genome shotgun sequencin...
161. AKCT01000041\_0 Penicillium digitatum PHI26, whole genome shotgun sequenc...
162. KB707685\_0 Botryotinia fuckeliana BcDW1 unplaced genomic scaffold Scaffo...
163. FQ790288\_0 Botryotinia fuckeliana T4 SuperContig\_299\_1 genomic supercontig.
164. AABX02000004\_1 Neurospora crassa OR74A, whole genome shotgun sequencing ...
165. GL891307\_1 Neurospora tetrasperma FGSC 2508 unplaced genomic scaffold NE...
166. GL891269\_0 Neurospora tetrasperma FGSC 2509 unplaced genomic scaffold NE...
167. AFWA01000005\_0 Pneumocystis murina B123, whole genome shotgun sequencing...
168. CABT02000027\_0 Sordaria macrospora k-hell, whole genome shotgun sequenci...
169. EQ962654\_1 Talaromyces stipitatus ATCC 10500 scf\_1105507295541 genomic s...
170. GL988044\_0 Chaetomium thermophilum var. thermophilum DSM 1495 unplaced g...
171. DS027058\_2 Aspergillus clavatus NRRL 1 1099423829804 genomic scaffold, w...
172. GG692437\_0 Ajellomyces capsulatus H143 genomic scaffold supercont2.19, w...
173. AM920437\_7 Penicillium chrysogenum Wisconsin 54-1255 complete genome, co...
174. AKCT01000122\_0 Penicillium digitatum PHI26, whole genome shotgun sequenc...
175. AKCU01000418\_0 Penicillium digitatum Pd1, whole genome shotgun sequencin...
176. DS544814\_1 Paracoccidioides brasiliensis Pb03 supercont1.12 genomic scaf...
177. CH476616\_1 Uncinocarpus reesii 1704 scaffold\_2 genomic scaffold, whole g...
178. DS572762\_0 Paracoccidioides brasiliensis Pb18 supercont1.13 genomic scaf...
179. AFQF01002886\_0 Fusarium oxysporum Fo5176, whole genome shotgun sequencin...
180. KB733479\_0 Bipolaris maydis ATCC 48331 unplaced genomic scaffold COCC4sc...
181. KB445588\_0 Cochliobolus heterostrophus C5 unplaced genomic scaffold COCH...
182. DS499596\_4 Aspergillus fumigatus A1163 scf\_000003 genomic scaffold, whol...
183. AAHF01000010\_2 Aspergillus fumigatus Af293, whole genome shotgun sequenc...
184. DS027697\_2 Neosartorya fischeri NRRL 181 1099437636265 genomic scaffold,...
185. DS572827\_0 Paracoccidioides brasiliensis Pb01 supercont1.17 genomic scaf...
186. DS572697\_2 Verticillium dahliae VdLs.17 supercont1.3 genomic scaffold, w...
187. GL988047\_0 Chaetomium thermophilum var. thermophilum DSM 1495 unplaced g...
188. GL891304\_0 Neurospora tetrasperma FGSC 2508 unplaced genomic scaffold NE...
189. GL891236\_1 Neurospora tetrasperma FGSC 2509 unplaced genomic scaffold NE...
190. AABX02000027\_0 Neurospora crassa OR74A, whole genome shotgun sequencing ...
191. KB932812\_0 Togninia minima UCRPA7 unplaced genomic scaffold PA7\_03\_scaff...
192. CU633900\_0 Podospora anserina S mat+ genomic DNA chromosome 7, supercont...
193. DS985215\_0 Verticillium albo-atrum VaMs.102 supercont1.2 genomic scaffol...
194. CH476607\_0 Aspergillus terreus NIH2624 scaffold\_14 genomic scaffold, who...
195. DF126459\_0 Aspergillus kawachii IFO 4308 DNA, contig: scaffold00013, who...
196. AP007157\_0 Aspergillus oryzae RIB40 DNA, SC023.
197. DS547093\_0 Laccaria bicolor S238N-H82 LACBIscaffold\_3 genomic scaffold, ...
198. CACQ02006690\_0 Colletotrichum higginsianum strain IMI 349063, whole geno...
199. GG663369\_0 Ajellomyces capsulatus G186AR genomic scaffold supercont2.7, ...
200. DS990642\_0 Ajellomyces capsulatus H88 supercont1.7 genomic scaffold, who...

Query: Architecture Search FASTA input

AKCT01000066 : Penicillium digitatum PHI26    Total score: 2.0     Cumulative Blast bit score: 1210

Hit cluster cross-links:

Mycgr3G85918 Mycgr3T
  
Location: 0-1602

Mycgr3G85918\_Mycgr3T

Mycgr3G42010 Mycgr3T
  
Location: 1702-8569

Mycgr3G42010\_Mycgr3T

Mycgr3G29582 Mycgr3T
  
Location: 8669-8915

Mycgr3G29582\_Mycgr3T

Mycgr3G31170 Mycgr3T
  
Location: 9015-9255

Mycgr3G31170\_Mycgr3T

Mycgr3G85924 Mycgr3T
  
Location: 9355-11218

Mycgr3G85924\_Mycgr3T

Mycgr3G71676 Mycgr3T
  
Location: 11318-12494

Mycgr3G71676\_Mycgr3T

Mycgr3G11468 Mycgr3T
  
Location: 12594-13653

Mycgr3G11468\_Mycgr3T

Mycgr3G58567 Mycgr3T
  
Location: 13753-14506

Mycgr3G58567\_Mycgr3T

Mycgr3G100089 Mycgr3
  
Location: 14606-21152

Mycgr3G100089\_Mycgr3

Mycgr3G42698 Mycgr3T
  
Location: 21252-22131

Mycgr3G42698\_Mycgr3T

Mycgr3G71681 Mycgr3T
  
Location: 22231-23461

Mycgr3G71681\_Mycgr3T

Mycgr3G109328 Mycgr3
  
Location: 23561-24239

Mycgr3G109328\_Mycgr3

Mycgr3G104334 Mycgr3
  
Location: 24339-24567

Mycgr3G104334\_Mycgr3

Mycgr3G42715 Mycgr3T
  
Location: 24667-25981

Mycgr3G42715\_Mycgr3T

Mycgr3G92934 Mycgr3T
  
Location: 26081-27593

Mycgr3G92934\_Mycgr3T

Mycgr3G41969 Mycgr3T
  
Location: 27693-29328

Mycgr3G41969\_Mycgr3T

Mycgr3G80635 Mycgr3T
  
Location: 29428-29821

Mycgr3G80635\_Mycgr3T

Mycgr3G41426 Mycgr3T
  
Location: 29921-35255

Mycgr3G41426\_Mycgr3T

Mycgr3G104337 Mycgr3
  
Location: 35355-36108

Mycgr3G104337\_Mycgr3

Mycgr3G71679 Mycgr3T
  
Location: 36208-37300

Mycgr3G71679\_Mycgr3T

Mycgr3G92938 Mycgr3T
  
Location: 37400-38699

Mycgr3G92938\_Mycgr3T

Mycgr3G92941 Mycgr3T
  
Location: 38799-40734

Mycgr3G92941\_Mycgr3T

hypothetical protein
  
Accession: EKV17380
  
Location: 56318-56455
  
 NCBI BlastP on this gene

EKV17380

Myosin heavy chain-like protein, putative
  
Accession: EKV17381
  
Location: 61582-68328
  
 NCBI BlastP on this gene

EKV17381

Endosomal cargo receptor (P24), putative
  
Accession: EKV17382
  
Location: 69493-70433
  
 NCBI BlastP on this gene

EKV17382

hypothetical protein
  
Accession: EKV17383
  
Location: 71023-71860
  
 NCBI BlastP on this gene

EKV17383

hypothetical protein
  
Accession: EKV17384
  
Location: 71950-72811
  
  
**BlastP hit with Mycgr3G104337\_Mycgr3**
  
Percentage identity: 39 %
  
BlastP bit score: 160
  
Sequence coverage: 98 %
  
E-value: 2e-44
  
  
 NCBI BlastP on this gene

EKV17384

hypothetical protein
  
Accession: EKV17385
  
Location: 73543-81529
  
  
**BlastP hit with Mycgr3G100089\_Mycgr3**
  
Percentage identity: 43 %
  
BlastP bit score: 1050
  
Sequence coverage: 58 %
  
E-value: 0.0
  
  
 NCBI BlastP on this gene

EKV17385

hypothetical protein
  
Accession: EKV17386
  
Location: 82893-83908
  
 NCBI BlastP on this gene

EKV17386

hypothetical protein
  
Accession: EKV17387
  
Location: 84281-84544
  
 NCBI BlastP on this gene

EKV17387

hypothetical protein
  
Accession: EKV17388
  
Location: 85806-87019
  
 NCBI BlastP on this gene

EKV17388

MFS lactose permease, putative
  
Accession: EKV17389
  
Location: 88505-90671
  
 NCBI BlastP on this gene

EKV17389

Endopolyphosphatase
  
Accession: EKV17390
  
Location: 94685-96634
  
 NCBI BlastP on this gene

EKV17390

hypothetical protein
  
Accession: EKV17391
  
Location: 98560-99633
  
 NCBI BlastP on this gene

EKV17391

Query: Architecture Search FASTA input

KB456260 : Mycosphaerella populorum SO2202 unplaced genomic scaffold SEPMUscaffold\_1    Total score: 2.0     Cumulative Blast bit score: 1208

Hit cluster cross-links:

Mycgr3G85918 Mycgr3T
  
Location: 0-1602

Mycgr3G85918\_Mycgr3T

Mycgr3G42010 Mycgr3T
  
Location: 1702-8569

Mycgr3G42010\_Mycgr3T

Mycgr3G29582 Mycgr3T
  
Location: 8669-8915

Mycgr3G29582\_Mycgr3T

Mycgr3G31170 Mycgr3T
  
Location: 9015-9255

Mycgr3G31170\_Mycgr3T

Mycgr3G85924 Mycgr3T
  
Location: 9355-11218

Mycgr3G85924\_Mycgr3T

Mycgr3G71676 Mycgr3T
  
Location: 11318-12494

Mycgr3G71676\_Mycgr3T

Mycgr3G11468 Mycgr3T
  
Location: 12594-13653

Mycgr3G11468\_Mycgr3T

Mycgr3G58567 Mycgr3T
  
Location: 13753-14506

Mycgr3G58567\_Mycgr3T

Mycgr3G100089 Mycgr3
  
Location: 14606-21152

Mycgr3G100089\_Mycgr3

Mycgr3G42698 Mycgr3T
  
Location: 21252-22131

Mycgr3G42698\_Mycgr3T

Mycgr3G71681 Mycgr3T
  
Location: 22231-23461

Mycgr3G71681\_Mycgr3T

Mycgr3G109328 Mycgr3
  
Location: 23561-24239

Mycgr3G109328\_Mycgr3

Mycgr3G104334 Mycgr3
  
Location: 24339-24567

Mycgr3G104334\_Mycgr3

Mycgr3G42715 Mycgr3T
  
Location: 24667-25981

Mycgr3G42715\_Mycgr3T

Mycgr3G92934 Mycgr3T
  
Location: 26081-27593

Mycgr3G92934\_Mycgr3T

Mycgr3G41969 Mycgr3T
  
Location: 27693-29328

Mycgr3G41969\_Mycgr3T

Mycgr3G80635 Mycgr3T
  
Location: 29428-29821

Mycgr3G80635\_Mycgr3T

Mycgr3G41426 Mycgr3T
  
Location: 29921-35255

Mycgr3G41426\_Mycgr3T

Mycgr3G104337 Mycgr3
  
Location: 35355-36108

Mycgr3G104337\_Mycgr3

Mycgr3G71679 Mycgr3T
  
Location: 36208-37300

Mycgr3G71679\_Mycgr3T

Mycgr3G92938 Mycgr3T
  
Location: 37400-38699

Mycgr3G92938\_Mycgr3T

Mycgr3G92941 Mycgr3T
  
Location: 38799-40734

Mycgr3G92941\_Mycgr3T

acyl-CoA N-acyltransferase
  
Accession: EMF16352
  
Location: 554524-555006
  
 NCBI BlastP on this gene

EMF16352

hypothetical protein
  
Accession: EMF16353
  
Location: 556230-556733
  
 NCBI BlastP on this gene

EMF16353

hypothetical protein
  
Accession: EMF16355
  
Location: 560467-561345
  
 NCBI BlastP on this gene

EMF16355

hypothetical protein
  
Accession: EMF16356
  
Location: 562147-563825
  
 NCBI BlastP on this gene

EMF16356

hypothetical protein
  
Accession: EMF16357
  
Location: 564310-565674
  
 NCBI BlastP on this gene

EMF16357

metalloprotease
  
Accession: EMF16358
  
Location: 566495-569078
  
 NCBI BlastP on this gene

EMF16358

elongation factor G, mitochondrial
  
Accession: EMF16359
  
Location: 569915-572347
  
 NCBI BlastP on this gene

EMF16359

DEAD-domain-containing protein
  
Accession: EMF16360
  
Location: 572715-574295
  
  
**BlastP hit with Mycgr3G92934\_Mycgr3T**
  
Percentage identity: 70 %
  
BlastP bit score: 690
  
Sequence coverage: 98 %
  
E-value: 0.0
  
  
 NCBI BlastP on this gene

EMF16360

Krr1-domain-containing protein
  
Accession: EMF16361
  
Location: 574487-576457
  
  
**BlastP hit with Mycgr3G85924\_Mycgr3T**
  
Percentage identity: 56 %
  
BlastP bit score: 518
  
Sequence coverage: 99 %
  
E-value: 9e-173
  
  
 NCBI BlastP on this gene

EMF16361

hypothetical protein
  
Accession: EMF16362
  
Location: 577564-577893
  
 NCBI BlastP on this gene

EMF16362

hypothetical protein
  
Accession: EMF16363
  
Location: 578602-579195
  
 NCBI BlastP on this gene

EMF16363

hypothetical protein
  
Accession: EMF16364
  
Location: 580687-584181
  
 NCBI BlastP on this gene

EMF16364

hypothetical protein
  
Accession: EMF16365
  
Location: 586317-587819
  
 NCBI BlastP on this gene

EMF16365

hypothetical protein
  
Accession: EMF16366
  
Location: 588585-589721
  
 NCBI BlastP on this gene

EMF16366

CDC91 cell division cycle 91-like protein
  
Accession: EMF16367
  
Location: 590213-591815
  
 NCBI BlastP on this gene

EMF16367

Pescadillo N-domain-containing protein
  
Accession: EMF16368
  
Location: 592098-594328
  
 NCBI BlastP on this gene

EMF16368

hypothetical protein
  
Accession: EMF16369
  
Location: 594842-595237
  
 NCBI BlastP on this gene

EMF16369

Query: Architecture Search FASTA input

CH476634 : Sclerotinia sclerotiorum 1980 scaffold\_14 genomic scaffold    Total score: 2.0     Cumulative Blast bit score: 1184

Hit cluster cross-links:

Mycgr3G85918 Mycgr3T
  
Location: 0-1602

Mycgr3G85918\_Mycgr3T

Mycgr3G42010 Mycgr3T
  
Location: 1702-8569

Mycgr3G42010\_Mycgr3T

Mycgr3G29582 Mycgr3T
  
Location: 8669-8915

Mycgr3G29582\_Mycgr3T

Mycgr3G31170 Mycgr3T
  
Location: 9015-9255

Mycgr3G31170\_Mycgr3T

Mycgr3G85924 Mycgr3T
  
Location: 9355-11218

Mycgr3G85924\_Mycgr3T

Mycgr3G71676 Mycgr3T
  
Location: 11318-12494

Mycgr3G71676\_Mycgr3T

Mycgr3G11468 Mycgr3T
  
Location: 12594-13653

Mycgr3G11468\_Mycgr3T

Mycgr3G58567 Mycgr3T
  
Location: 13753-14506

Mycgr3G58567\_Mycgr3T

Mycgr3G100089 Mycgr3
  
Location: 14606-21152

Mycgr3G100089\_Mycgr3

Mycgr3G42698 Mycgr3T
  
Location: 21252-22131

Mycgr3G42698\_Mycgr3T

Mycgr3G71681 Mycgr3T
  
Location: 22231-23461

Mycgr3G71681\_Mycgr3T

Mycgr3G109328 Mycgr3
  
Location: 23561-24239

Mycgr3G109328\_Mycgr3

Mycgr3G104334 Mycgr3
  
Location: 24339-24567

Mycgr3G104334\_Mycgr3

Mycgr3G42715 Mycgr3T
  
Location: 24667-25981

Mycgr3G42715\_Mycgr3T

Mycgr3G92934 Mycgr3T
  
Location: 26081-27593

Mycgr3G92934\_Mycgr3T

Mycgr3G41969 Mycgr3T
  
Location: 27693-29328

Mycgr3G41969\_Mycgr3T

Mycgr3G80635 Mycgr3T
  
Location: 29428-29821

Mycgr3G80635\_Mycgr3T

Mycgr3G41426 Mycgr3T
  
Location: 29921-35255

Mycgr3G41426\_Mycgr3T

Mycgr3G104337 Mycgr3
  
Location: 35355-36108

Mycgr3G104337\_Mycgr3

Mycgr3G71679 Mycgr3T
  
Location: 36208-37300

Mycgr3G71679\_Mycgr3T

Mycgr3G92938 Mycgr3T
  
Location: 37400-38699

Mycgr3G92938\_Mycgr3T

Mycgr3G92941 Mycgr3T
  
Location: 38799-40734

Mycgr3G92941\_Mycgr3T

hypothetical protein
  
Accession: EDN93761
  
Location: 46587-47911
  
 NCBI BlastP on this gene

EDN93761

hypothetical protein
  
Accession: EDN93762
  
Location: 48482-50198
  
 NCBI BlastP on this gene

EDN93762

hypothetical protein
  
Accession: EDN93763
  
Location: 50591-51544
  
 NCBI BlastP on this gene

EDN93763

predicted protein
  
Accession: EDN93764
  
Location: 53243-53341
  
 NCBI BlastP on this gene

EDN93764

hypothetical protein
  
Accession: EDN93765
  
Location: 57844-60564
  
 NCBI BlastP on this gene

EDN93765

predicted protein
  
Accession: EDN93766
  
Location: 61960-62143
  
 NCBI BlastP on this gene

EDN93766

hypothetical protein
  
Accession: EDN93767
  
Location: 63381-70210
  
  
**BlastP hit with Mycgr3G42010\_Mycgr3T**
  
Percentage identity: 45 %
  
BlastP bit score: 978
  
Sequence coverage: 54 %
  
E-value: 0.0
  
  
 NCBI BlastP on this gene

EDN93767

hypothetical protein
  
Accession: EDN93768
  
Location: 70731-73500
  
  
**BlastP hit with Mycgr3G92938\_Mycgr3T**
  
Percentage identity: 38 %
  
BlastP bit score: 206
  
Sequence coverage: 68 %
  
E-value: 4e-57
  
  
 NCBI BlastP on this gene

EDN93768

hypothetical protein
  
Accession: EDN93769
  
Location: 73773-75349
  
 NCBI BlastP on this gene

EDN93769

hypothetical protein
  
Accession: EDN93770
  
Location: 76378-78036
  
 NCBI BlastP on this gene

EDN93770

hypothetical protein
  
Accession: EDN93771
  
Location: 78345-80157
  
 NCBI BlastP on this gene

EDN93771

predicted protein
  
Accession: EDN93772
  
Location: 81048-81233
  
 NCBI BlastP on this gene

EDN93772

predicted protein
  
Accession: EDN93773
  
Location: 81703-82122
  
 NCBI BlastP on this gene

EDN93773

predicted protein
  
Accession: EDN93774
  
Location: 82682-83549
  
 NCBI BlastP on this gene

EDN93774

predicted protein
  
Accession: EDN93775
  
Location: 85537-86922
  
 NCBI BlastP on this gene

EDN93775

hypothetical protein
  
Accession: EDN93776
  
Location: 87336-87479
  
 NCBI BlastP on this gene

EDN93776

hypothetical protein
  
Accession: EDN93777
  
Location: 88177-88524
  
 NCBI BlastP on this gene

EDN93777

predicted protein
  
Accession: EDN93778
  
Location: 90986-91195
  
 NCBI BlastP on this gene

EDN93778

Query: Architecture Search FASTA input

CH476626 : Sclerotinia sclerotiorum 1980 scaffold\_6 genomic scaffold    Total score: 2.0     Cumulative Blast bit score: 1174

Hit cluster cross-links:

Mycgr3G85918 Mycgr3T
  
Location: 0-1602

Mycgr3G85918\_Mycgr3T

Mycgr3G42010 Mycgr3T
  
Location: 1702-8569

Mycgr3G42010\_Mycgr3T

Mycgr3G29582 Mycgr3T
  
Location: 8669-8915

Mycgr3G29582\_Mycgr3T

Mycgr3G31170 Mycgr3T
  
Location: 9015-9255

Mycgr3G31170\_Mycgr3T

Mycgr3G85924 Mycgr3T
  
Location: 9355-11218

Mycgr3G85924\_Mycgr3T

Mycgr3G71676 Mycgr3T
  
Location: 11318-12494

Mycgr3G71676\_Mycgr3T

Mycgr3G11468 Mycgr3T
  
Location: 12594-13653

Mycgr3G11468\_Mycgr3T

Mycgr3G58567 Mycgr3T
  
Location: 13753-14506

Mycgr3G58567\_Mycgr3T

Mycgr3G100089 Mycgr3
  
Location: 14606-21152

Mycgr3G100089\_Mycgr3

Mycgr3G42698 Mycgr3T
  
Location: 21252-22131

Mycgr3G42698\_Mycgr3T

Mycgr3G71681 Mycgr3T
  
Location: 22231-23461

Mycgr3G71681\_Mycgr3T

Mycgr3G109328 Mycgr3
  
Location: 23561-24239

Mycgr3G109328\_Mycgr3

Mycgr3G104334 Mycgr3
  
Location: 24339-24567

Mycgr3G104334\_Mycgr3

Mycgr3G42715 Mycgr3T
  
Location: 24667-25981

Mycgr3G42715\_Mycgr3T

Mycgr3G92934 Mycgr3T
  
Location: 26081-27593

Mycgr3G92934\_Mycgr3T

Mycgr3G41969 Mycgr3T
  
Location: 27693-29328

Mycgr3G41969\_Mycgr3T

Mycgr3G80635 Mycgr3T
  
Location: 29428-29821

Mycgr3G80635\_Mycgr3T

Mycgr3G41426 Mycgr3T
  
Location: 29921-35255

Mycgr3G41426\_Mycgr3T

Mycgr3G104337 Mycgr3
  
Location: 35355-36108

Mycgr3G104337\_Mycgr3

Mycgr3G71679 Mycgr3T
  
Location: 36208-37300

Mycgr3G71679\_Mycgr3T

Mycgr3G92938 Mycgr3T
  
Location: 37400-38699

Mycgr3G92938\_Mycgr3T

Mycgr3G92941 Mycgr3T
  
Location: 38799-40734

Mycgr3G92941\_Mycgr3T

malate synthase
  
Accession: EDO03104
  
Location: 1968878-1970633
  
 NCBI BlastP on this gene

EDO03104

predicted protein
  
Accession: EDO03105
  
Location: 1972100-1972285
  
 NCBI BlastP on this gene

EDO03105

predicted protein
  
Accession: EDO03106
  
Location: 1974067-1974882
  
 NCBI BlastP on this gene

EDO03106

hypothetical protein
  
Accession: EDO03107
  
Location: 1975463-1976386
  
 NCBI BlastP on this gene

EDO03107

predicted protein
  
Accession: EDO03108
  
Location: 1978012-1978536
  
 NCBI BlastP on this gene

EDO03108

hypothetical protein
  
Accession: EDO03109
  
Location: 1981916-1982762
  
  
**BlastP hit with Mycgr3G31170\_Mycgr3T**
  
Percentage identity: 97 %
  
BlastP bit score: 159
  
Sequence coverage: 100 %
  
E-value: 9e-47
  
  
 NCBI BlastP on this gene

EDO03109

vacuolar ATP synthase subunit E
  
Accession: EDO03110
  
Location: 1986634-1987557
  
 NCBI BlastP on this gene

EDO03110

hypothetical protein
  
Accession: EDO03111
  
Location: 1987797-1989784
  
 NCBI BlastP on this gene

EDO03111

predicted protein
  
Accession: EDO03112
  
Location: 1990043-1991105
  
 NCBI BlastP on this gene

EDO03112

hypothetical protein
  
Accession: EDO03113
  
Location: 1996827-1997474
  
 NCBI BlastP on this gene

EDO03113

hypothetical protein
  
Accession: EDO03114
  
Location: 1998616-2004906
  
  
**BlastP hit with Mycgr3G41426\_Mycgr3T**
  
Percentage identity: 34 %
  
BlastP bit score: 1015
  
Sequence coverage: 105 %
  
E-value: 0.0
  
  
 NCBI BlastP on this gene

EDO03114

predicted protein
  
Accession: EDO03115
  
Location: 2005351-2005882
  
 NCBI BlastP on this gene

EDO03115

predicted protein
  
Accession: EDO03116
  
Location: 2006400-2007178
  
 NCBI BlastP on this gene

EDO03116

predicted protein
  
Accession: EDO03117
  
Location: 2008103-2008552
  
 NCBI BlastP on this gene

EDO03117

hypothetical protein
  
Accession: EDO03118
  
Location: 2009507-2009923
  
 NCBI BlastP on this gene

EDO03118

hypothetical protein
  
Accession: EDO03119
  
Location: 2012361-2015200
  
 NCBI BlastP on this gene

EDO03119

hypothetical protein
  
Accession: EDO03120
  
Location: 2015676-2015936
  
 NCBI BlastP on this gene

EDO03120

Query: Architecture Search FASTA input

AKHY01000142 : Aspergillus oryzae 3.042    Total score: 2.0     Cumulative Blast bit score: 1173

Hit cluster cross-links:

Mycgr3G85918 Mycgr3T
  
Location: 0-1602

Mycgr3G85918\_Mycgr3T

Mycgr3G42010 Mycgr3T
  
Location: 1702-8569

Mycgr3G42010\_Mycgr3T

Mycgr3G29582 Mycgr3T
  
Location: 8669-8915

Mycgr3G29582\_Mycgr3T

Mycgr3G31170 Mycgr3T
  
Location: 9015-9255

Mycgr3G31170\_Mycgr3T

Mycgr3G85924 Mycgr3T
  
Location: 9355-11218

Mycgr3G85924\_Mycgr3T

Mycgr3G71676 Mycgr3T
  
Location: 11318-12494

Mycgr3G71676\_Mycgr3T

Mycgr3G11468 Mycgr3T
  
Location: 12594-13653

Mycgr3G11468\_Mycgr3T

Mycgr3G58567 Mycgr3T
  
Location: 13753-14506

Mycgr3G58567\_Mycgr3T

Mycgr3G100089 Mycgr3
  
Location: 14606-21152

Mycgr3G100089\_Mycgr3

Mycgr3G42698 Mycgr3T
  
Location: 21252-22131

Mycgr3G42698\_Mycgr3T

Mycgr3G71681 Mycgr3T
  
Location: 22231-23461

Mycgr3G71681\_Mycgr3T

Mycgr3G109328 Mycgr3
  
Location: 23561-24239

Mycgr3G109328\_Mycgr3

Mycgr3G104334 Mycgr3
  
Location: 24339-24567

Mycgr3G104334\_Mycgr3

Mycgr3G42715 Mycgr3T
  
Location: 24667-25981

Mycgr3G42715\_Mycgr3T

Mycgr3G92934 Mycgr3T
  
Location: 26081-27593

Mycgr3G92934\_Mycgr3T

Mycgr3G41969 Mycgr3T
  
Location: 27693-29328

Mycgr3G41969\_Mycgr3T

Mycgr3G80635 Mycgr3T
  
Location: 29428-29821

Mycgr3G80635\_Mycgr3T

Mycgr3G41426 Mycgr3T
  
Location: 29921-35255

Mycgr3G41426\_Mycgr3T

Mycgr3G104337 Mycgr3
  
Location: 35355-36108

Mycgr3G104337\_Mycgr3

Mycgr3G71679 Mycgr3T
  
Location: 36208-37300

Mycgr3G71679\_Mycgr3T

Mycgr3G92938 Mycgr3T
  
Location: 37400-38699

Mycgr3G92938\_Mycgr3T

Mycgr3G92941 Mycgr3T
  
Location: 38799-40734

Mycgr3G92941\_Mycgr3T

hypothetical protein
  
Accession: EIT77858
  
Location: 31565-32365
  
 NCBI BlastP on this gene

EIT77858

permease of the major facilitator superfamily
  
Accession: EIT77947
  
Location: 35832-37569
  
 NCBI BlastP on this gene

EIT77947

hypothetical protein
  
Accession: EIT77966
  
Location: 40674-41624
  
 NCBI BlastP on this gene

EIT77966

hypothetical protein
  
Accession: EIT77877
  
Location: 47206-48984
  
 NCBI BlastP on this gene

EIT77877

polyketide synthase module
  
Accession: EIT77964
  
Location: 50338-58371
  
  
**BlastP hit with Mycgr3G100089\_Mycgr3**
  
Percentage identity: 42 %
  
BlastP bit score: 1052
  
Sequence coverage: 58 %
  
E-value: 0.0
  
  
 NCBI BlastP on this gene

EIT77964

hypothetical protein
  
Accession: EIT77840
  
Location: 59360-59854
  
  
**BlastP hit with Mycgr3G104337\_Mycgr3**
  
Percentage identity: 43 %
  
BlastP bit score: 121
  
Sequence coverage: 56 %
  
E-value: 3e-30
  
  
 NCBI BlastP on this gene

EIT77840

FAD binding oxidoreductase, putative
  
Accession: EIT77828
  
Location: 62822-64224
  
 NCBI BlastP on this gene

EIT77828

RING finger protein
  
Accession: EIT77899
  
Location: 65164-65836
  
 NCBI BlastP on this gene

EIT77899

putative cytochrome C oxidase assembly protein
  
Accession: EIT77873
  
Location: 66677-67903
  
 NCBI BlastP on this gene

EIT77873

molybdopterin synthase large subunit CnxH
  
Accession: EIT77933
  
Location: 67908-68447
  
 NCBI BlastP on this gene

EIT77933

hypothetical protein
  
Accession: EIT77917
  
Location: 68795-69584
  
 NCBI BlastP on this gene

EIT77917

short-chain dehydrogenase/reductase family protein, putative
  
Accession: EIT77923
  
Location: 71059-72051
  
 NCBI BlastP on this gene

EIT77923

alpha-amylase
  
Accession: EIT77864
  
Location: 72796-74690
  
 NCBI BlastP on this gene

EIT77864

hypothetical protein
  
Accession: EIT77911
  
Location: 75014-75688
  
 NCBI BlastP on this gene

EIT77911

LPS glycosyltransferase
  
Accession: EIT77929
  
Location: 75858-77203
  
 NCBI BlastP on this gene

EIT77929

Query: Architecture Search FASTA input

DS989828 : Arthroderma gypseum CBS 118893 supercont1.7 genomic scaffold    Total score: 2.0     Cumulative Blast bit score: 1171

Hit cluster cross-links:

Mycgr3G85918 Mycgr3T
  
Location: 0-1602

Mycgr3G85918\_Mycgr3T

Mycgr3G42010 Mycgr3T
  
Location: 1702-8569

Mycgr3G42010\_Mycgr3T

Mycgr3G29582 Mycgr3T
  
Location: 8669-8915

Mycgr3G29582\_Mycgr3T

Mycgr3G31170 Mycgr3T
  
Location: 9015-9255

Mycgr3G31170\_Mycgr3T

Mycgr3G85924 Mycgr3T
  
Location: 9355-11218

Mycgr3G85924\_Mycgr3T

Mycgr3G71676 Mycgr3T
  
Location: 11318-12494

Mycgr3G71676\_Mycgr3T

Mycgr3G11468 Mycgr3T
  
Location: 12594-13653

Mycgr3G11468\_Mycgr3T

Mycgr3G58567 Mycgr3T
  
Location: 13753-14506

Mycgr3G58567\_Mycgr3T

Mycgr3G100089 Mycgr3
  
Location: 14606-21152

Mycgr3G100089\_Mycgr3

Mycgr3G42698 Mycgr3T
  
Location: 21252-22131

Mycgr3G42698\_Mycgr3T

Mycgr3G71681 Mycgr3T
  
Location: 22231-23461

Mycgr3G71681\_Mycgr3T

Mycgr3G109328 Mycgr3
  
Location: 23561-24239

Mycgr3G109328\_Mycgr3

Mycgr3G104334 Mycgr3
  
Location: 24339-24567

Mycgr3G104334\_Mycgr3

Mycgr3G42715 Mycgr3T
  
Location: 24667-25981

Mycgr3G42715\_Mycgr3T

Mycgr3G92934 Mycgr3T
  
Location: 26081-27593

Mycgr3G92934\_Mycgr3T

Mycgr3G41969 Mycgr3T
  
Location: 27693-29328

Mycgr3G41969\_Mycgr3T

Mycgr3G80635 Mycgr3T
  
Location: 29428-29821

Mycgr3G80635\_Mycgr3T

Mycgr3G41426 Mycgr3T
  
Location: 29921-35255

Mycgr3G41426\_Mycgr3T

Mycgr3G104337 Mycgr3
  
Location: 35355-36108

Mycgr3G104337\_Mycgr3

Mycgr3G71679 Mycgr3T
  
Location: 36208-37300

Mycgr3G71679\_Mycgr3T

Mycgr3G92938 Mycgr3T
  
Location: 37400-38699

Mycgr3G92938\_Mycgr3T

Mycgr3G92941 Mycgr3T
  
Location: 38799-40734

Mycgr3G92941\_Mycgr3T

hypothetical protein
  
Accession: EFR04823
  
Location: 1378330-1378827
  
 NCBI BlastP on this gene

EFR04823

hypothetical protein
  
Accession: EFR04824
  
Location: 1381280-1381949
  
 NCBI BlastP on this gene

EFR04824

cell division control protein 25
  
Accession: EFR04825
  
Location: 1382184-1385852
  
 NCBI BlastP on this gene

EFR04825

hypothetical protein
  
Accession: EFR04826
  
Location: 1389449-1391056
  
 NCBI BlastP on this gene

EFR04826

fatty-acid-CoA ligase
  
Accession: EFR04827
  
Location: 1392766-1394695
  
 NCBI BlastP on this gene

EFR04827

lovastatin nonaketide synthase
  
Accession: EFR04828
  
Location: 1395051-1402076
  
  
**BlastP hit with Mycgr3G100089\_Mycgr3**
  
Percentage identity: 40 %
  
BlastP bit score: 987
  
Sequence coverage: 60 %
  
E-value: 0.0
  
  
 NCBI BlastP on this gene

EFR04828

hypothetical protein
  
Accession: EFR04829
  
Location: 1402897-1403929
  
  
**BlastP hit with Mycgr3G104337\_Mycgr3**
  
Percentage identity: 40 %
  
BlastP bit score: 184
  
Sequence coverage: 100 %
  
E-value: 9e-54
  
  
 NCBI BlastP on this gene

EFR04829

hypothetical protein
  
Accession: EFR04830
  
Location: 1404240-1405769
  
 NCBI BlastP on this gene

EFR04830

hypothetical protein
  
Accession: EFR04831
  
Location: 1406299-1407499
  
 NCBI BlastP on this gene

EFR04831

Query: Architecture Search FASTA input

KB644411 : Penicillium oxalicum 114-2 unplaced genomic scaffold scaffold\_4    Total score: 2.0     Cumulative Blast bit score: 1162

Hit cluster cross-links:

Mycgr3G85918 Mycgr3T
  
Location: 0-1602

Mycgr3G85918\_Mycgr3T

Mycgr3G42010 Mycgr3T
  
Location: 1702-8569

Mycgr3G42010\_Mycgr3T

Mycgr3G29582 Mycgr3T
  
Location: 8669-8915

Mycgr3G29582\_Mycgr3T

Mycgr3G31170 Mycgr3T
  
Location: 9015-9255

Mycgr3G31170\_Mycgr3T

Mycgr3G85924 Mycgr3T
  
Location: 9355-11218

Mycgr3G85924\_Mycgr3T

Mycgr3G71676 Mycgr3T
  
Location: 11318-12494

Mycgr3G71676\_Mycgr3T

Mycgr3G11468 Mycgr3T
  
Location: 12594-13653

Mycgr3G11468\_Mycgr3T

Mycgr3G58567 Mycgr3T
  
Location: 13753-14506

Mycgr3G58567\_Mycgr3T

Mycgr3G100089 Mycgr3
  
Location: 14606-21152

Mycgr3G100089\_Mycgr3

Mycgr3G42698 Mycgr3T
  
Location: 21252-22131

Mycgr3G42698\_Mycgr3T

Mycgr3G71681 Mycgr3T
  
Location: 22231-23461

Mycgr3G71681\_Mycgr3T

Mycgr3G109328 Mycgr3
  
Location: 23561-24239

Mycgr3G109328\_Mycgr3

Mycgr3G104334 Mycgr3
  
Location: 24339-24567

Mycgr3G104334\_Mycgr3

Mycgr3G42715 Mycgr3T
  
Location: 24667-25981

Mycgr3G42715\_Mycgr3T

Mycgr3G92934 Mycgr3T
  
Location: 26081-27593

Mycgr3G92934\_Mycgr3T

Mycgr3G41969 Mycgr3T
  
Location: 27693-29328

Mycgr3G41969\_Mycgr3T

Mycgr3G80635 Mycgr3T
  
Location: 29428-29821

Mycgr3G80635\_Mycgr3T

Mycgr3G41426 Mycgr3T
  
Location: 29921-35255

Mycgr3G41426\_Mycgr3T

Mycgr3G104337 Mycgr3
  
Location: 35355-36108

Mycgr3G104337\_Mycgr3

Mycgr3G71679 Mycgr3T
  
Location: 36208-37300

Mycgr3G71679\_Mycgr3T

Mycgr3G92938 Mycgr3T
  
Location: 37400-38699

Mycgr3G92938\_Mycgr3T

Mycgr3G92941 Mycgr3T
  
Location: 38799-40734

Mycgr3G92941\_Mycgr3T

hypothetical protein
  
Accession: EPS28502
  
Location: 439387-440261
  
 NCBI BlastP on this gene

EPS28502

hypothetical protein
  
Accession: EPS28503
  
Location: 441174-442702
  
 NCBI BlastP on this gene

EPS28503

hypothetical protein
  
Accession: EPS28504
  
Location: 443123-445676
  
 NCBI BlastP on this gene

EPS28504

hypothetical protein
  
Accession: EPS28505
  
Location: 447788-449904
  
 NCBI BlastP on this gene

EPS28505

putative beta-1,3-glucanosyltransglycosylase
  
Accession: EPS28506
  
Location: 450874-452715
  
 NCBI BlastP on this gene

EPS28506

hypothetical protein
  
Accession: EPS28507
  
Location: 454616-456146
  
 NCBI BlastP on this gene

EPS28507

hypothetical protein
  
Accession: EPS28508
  
Location: 457201-458070
  
  
**BlastP hit with Mycgr3G104337\_Mycgr3**
  
Percentage identity: 39 %
  
BlastP bit score: 169
  
Sequence coverage: 100 %
  
E-value: 6e-48
  
  
 NCBI BlastP on this gene

EPS28508

hypothetical protein
  
Accession: EPS28509
  
Location: 458799-466755
  
  
**BlastP hit with Mycgr3G100089\_Mycgr3**
  
Percentage identity: 41 %
  
BlastP bit score: 993
  
Sequence coverage: 58 %
  
E-value: 0.0
  
  
 NCBI BlastP on this gene

EPS28509

hypothetical protein
  
Accession: EPS28510
  
Location: 469817-471357
  
 NCBI BlastP on this gene

EPS28510

hypothetical protein
  
Accession: EPS28511
  
Location: 473337-475644
  
 NCBI BlastP on this gene

EPS28511

hypothetical protein
  
Accession: EPS28512
  
Location: 476925-479736
  
 NCBI BlastP on this gene

EPS28512

hypothetical protein
  
Accession: EPS28513
  
Location: 481522-482192
  
 NCBI BlastP on this gene

EPS28513

hypothetical protein
  
Accession: EPS28514
  
Location: 483534-483890
  
 NCBI BlastP on this gene

EPS28514

Query: Architecture Search FASTA input

AP007167 : Aspergillus oryzae RIB40 DNA, SC020.    Total score: 2.0     Cumulative Blast bit score: 1145

Hit cluster cross-links:

Mycgr3G85918 Mycgr3T
  
Location: 0-1602

Mycgr3G85918\_Mycgr3T

Mycgr3G42010 Mycgr3T
  
Location: 1702-8569

Mycgr3G42010\_Mycgr3T

Mycgr3G29582 Mycgr3T
  
Location: 8669-8915

Mycgr3G29582\_Mycgr3T

Mycgr3G31170 Mycgr3T
  
Location: 9015-9255

Mycgr3G31170\_Mycgr3T

Mycgr3G85924 Mycgr3T
  
Location: 9355-11218

Mycgr3G85924\_Mycgr3T

Mycgr3G71676 Mycgr3T
  
Location: 11318-12494

Mycgr3G71676\_Mycgr3T

Mycgr3G11468 Mycgr3T
  
Location: 12594-13653

Mycgr3G11468\_Mycgr3T

Mycgr3G58567 Mycgr3T
  
Location: 13753-14506

Mycgr3G58567\_Mycgr3T

Mycgr3G100089 Mycgr3
  
Location: 14606-21152

Mycgr3G100089\_Mycgr3

Mycgr3G42698 Mycgr3T
  
Location: 21252-22131

Mycgr3G42698\_Mycgr3T

Mycgr3G71681 Mycgr3T
  
Location: 22231-23461

Mycgr3G71681\_Mycgr3T

Mycgr3G109328 Mycgr3
  
Location: 23561-24239

Mycgr3G109328\_Mycgr3

Mycgr3G104334 Mycgr3
  
Location: 24339-24567

Mycgr3G104334\_Mycgr3

Mycgr3G42715 Mycgr3T
  
Location: 24667-25981

Mycgr3G42715\_Mycgr3T

Mycgr3G92934 Mycgr3T
  
Location: 26081-27593

Mycgr3G92934\_Mycgr3T

Mycgr3G41969 Mycgr3T
  
Location: 27693-29328

Mycgr3G41969\_Mycgr3T

Mycgr3G80635 Mycgr3T
  
Location: 29428-29821

Mycgr3G80635\_Mycgr3T

Mycgr3G41426 Mycgr3T
  
Location: 29921-35255

Mycgr3G41426\_Mycgr3T

Mycgr3G104337 Mycgr3
  
Location: 35355-36108

Mycgr3G104337\_Mycgr3

Mycgr3G71679 Mycgr3T
  
Location: 36208-37300

Mycgr3G71679\_Mycgr3T

Mycgr3G92938 Mycgr3T
  
Location: 37400-38699

Mycgr3G92938\_Mycgr3T

Mycgr3G92941 Mycgr3T
  
Location: 38799-40734

Mycgr3G92941\_Mycgr3T

not annotated
  
Accession: BAE63398
  
Location: 438971-440316
  
 NCBI BlastP on this gene

AO090020000174

not annotated
  
Accession: BAE63399
  
Location: 440486-441160
  
 NCBI BlastP on this gene

AO090020000175

not annotated
  
Accession: BAE63400
  
Location: 441484-443378
  
 NCBI BlastP on this gene

AO090020000176

not annotated
  
Accession: BAE63401
  
Location: 444123-445040
  
 NCBI BlastP on this gene

AO090020000177

not annotated
  
Accession: BAE63402
  
Location: 446590-447364
  
 NCBI BlastP on this gene

AO090020000178

not annotated
  
Accession: BAE63403
  
Location: 447712-448251
  
 NCBI BlastP on this gene

AO090020000179

not annotated
  
Accession: BAE63404
  
Location: 448256-449483
  
 NCBI BlastP on this gene

AO090020000180

not annotated
  
Accession: BAE63405
  
Location: 450324-450996
  
 NCBI BlastP on this gene

AO090020000182

not annotated
  
Accession: BAE63406
  
Location: 451936-453338
  
 NCBI BlastP on this gene

AO090020000183

not annotated
  
Accession: BAE63407
  
Location: 456308-456802
  
  
**BlastP hit with Mycgr3G104337\_Mycgr3**
  
Percentage identity: 41 %
  
BlastP bit score: 90
  
Sequence coverage: 41 %
  
E-value: 4e-19
  
  
 NCBI BlastP on this gene

AO090020000185

not annotated
  
Accession: BAE63408
  
Location: 457790-465823
  
  
**BlastP hit with Mycgr3G100089\_Mycgr3**
  
Percentage identity: 42 %
  
BlastP bit score: 1055
  
Sequence coverage: 58 %
  
E-value: 0.0
  
  
 NCBI BlastP on this gene

AO090020000186

not annotated
  
Accession: BAE63409
  
Location: 467193-470773
  
 NCBI BlastP on this gene

AO090020000188

not annotated
  
Accession: BAE63410
  
Location: 474553-475503
  
 NCBI BlastP on this gene

AO090020000189

not annotated
  
Accession: BAE63411
  
Location: 478597-480899
  
 NCBI BlastP on this gene

AO090020000190

not annotated
  
Accession: BAE63412
  
Location: 483803-484603
  
 NCBI BlastP on this gene

AO090020000191

Query: Architecture Search FASTA input

DS995701 : Microsporum canis CBS 113480 supercont1.1 genomic scaffold    Total score: 2.0     Cumulative Blast bit score: 1141

Hit cluster cross-links:

Mycgr3G85918 Mycgr3T
  
Location: 0-1602

Mycgr3G85918\_Mycgr3T

Mycgr3G42010 Mycgr3T
  
Location: 1702-8569

Mycgr3G42010\_Mycgr3T

Mycgr3G29582 Mycgr3T
  
Location: 8669-8915

Mycgr3G29582\_Mycgr3T

Mycgr3G31170 Mycgr3T
  
Location: 9015-9255

Mycgr3G31170\_Mycgr3T

Mycgr3G85924 Mycgr3T
  
Location: 9355-11218

Mycgr3G85924\_Mycgr3T

Mycgr3G71676 Mycgr3T
  
Location: 11318-12494

Mycgr3G71676\_Mycgr3T

Mycgr3G11468 Mycgr3T
  
Location: 12594-13653

Mycgr3G11468\_Mycgr3T

Mycgr3G58567 Mycgr3T
  
Location: 13753-14506

Mycgr3G58567\_Mycgr3T

Mycgr3G100089 Mycgr3
  
Location: 14606-21152

Mycgr3G100089\_Mycgr3

Mycgr3G42698 Mycgr3T
  
Location: 21252-22131

Mycgr3G42698\_Mycgr3T

Mycgr3G71681 Mycgr3T
  
Location: 22231-23461

Mycgr3G71681\_Mycgr3T

Mycgr3G109328 Mycgr3
  
Location: 23561-24239

Mycgr3G109328\_Mycgr3

Mycgr3G104334 Mycgr3
  
Location: 24339-24567

Mycgr3G104334\_Mycgr3

Mycgr3G42715 Mycgr3T
  
Location: 24667-25981

Mycgr3G42715\_Mycgr3T

Mycgr3G92934 Mycgr3T
  
Location: 26081-27593

Mycgr3G92934\_Mycgr3T

Mycgr3G41969 Mycgr3T
  
Location: 27693-29328

Mycgr3G41969\_Mycgr3T

Mycgr3G80635 Mycgr3T
  
Location: 29428-29821

Mycgr3G80635\_Mycgr3T

Mycgr3G41426 Mycgr3T
  
Location: 29921-35255

Mycgr3G41426\_Mycgr3T

Mycgr3G104337 Mycgr3
  
Location: 35355-36108

Mycgr3G104337\_Mycgr3

Mycgr3G71679 Mycgr3T
  
Location: 36208-37300

Mycgr3G71679\_Mycgr3T

Mycgr3G92938 Mycgr3T
  
Location: 37400-38699

Mycgr3G92938\_Mycgr3T

Mycgr3G92941 Mycgr3T
  
Location: 38799-40734

Mycgr3G92941\_Mycgr3T

ferric reductase
  
Accession: EEQ27701
  
Location: 1606261-1608552
  
 NCBI BlastP on this gene

EEQ27701

conserved hypothetical protein
  
Accession: EEQ27702
  
Location: 1609085-1610203
  
 NCBI BlastP on this gene

EEQ27702

PTAC beta
  
Accession: EEQ27703
  
Location: 1611307-1612414
  
 NCBI BlastP on this gene

EEQ27703

tetraspanin
  
Accession: EEQ27704
  
Location: 1613225-1614125
  
 NCBI BlastP on this gene

EEQ27704

MADS box transcription factor Mcm1
  
Accession: EEQ27705
  
Location: 1615187-1616041
  
  
**BlastP hit with Mycgr3G31170\_Mycgr3T**
  
Percentage identity: 96 %
  
BlastP bit score: 154
  
Sequence coverage: 98 %
  
E-value: 5e-45
  
  
 NCBI BlastP on this gene

EEQ27705

C6 zinc finger domain-containing protein
  
Accession: EEQ27706
  
Location: 1617422-1618819
  
 NCBI BlastP on this gene

EEQ27706

carboxy-cis,cis-muconate cyclase
  
Accession: EEQ27707
  
Location: 1619069-1620313
  
 NCBI BlastP on this gene

EEQ27707

DUF803 domain-containing protein
  
Accession: EEQ27708
  
Location: 1622203-1624287
  
 NCBI BlastP on this gene

EEQ27708

vacuolar ATP synthase subunit E
  
Accession: EEQ27709
  
Location: 1624890-1625739
  
 NCBI BlastP on this gene

EEQ27709

conserved hypothetical protein
  
Accession: EEQ27710
  
Location: 1626283-1628429
  
 NCBI BlastP on this gene

EEQ27710

predicted protein
  
Accession: EEQ27711
  
Location: 1628972-1629797
  
 NCBI BlastP on this gene

EEQ27711

phospholipid-translocating P-type ATPase domain-containing protein
  
Accession: EEQ27712
  
Location: 1630885-1635526
  
 NCBI BlastP on this gene

EEQ27712

conserved hypothetical protein
  
Accession: EEQ27713
  
Location: 1636672-1638128
  
 NCBI BlastP on this gene

EEQ27713

helicase SEN1
  
Accession: EEQ27714
  
Location: 1638703-1643541
  
  
**BlastP hit with Mycgr3G41426\_Mycgr3T**
  
Percentage identity: 45 %
  
BlastP bit score: 987
  
Sequence coverage: 67 %
  
E-value: 0.0
  
  
 NCBI BlastP on this gene

EEQ27714

predicted protein
  
Accession: EEQ27715
  
Location: 1644194-1644763
  
 NCBI BlastP on this gene

EEQ27715

conserved hypothetical protein
  
Accession: EEQ27716
  
Location: 1646514-1648187
  
 NCBI BlastP on this gene

EEQ27716

hypothetical protein
  
Accession: EEQ27717
  
Location: 1650441-1653404
  
 NCBI BlastP on this gene

EEQ27717

Query: Architecture Search FASTA input

AKCU01000499 : Penicillium digitatum Pd1    Total score: 2.0     Cumulative Blast bit score: 1141

Hit cluster cross-links:

Mycgr3G85918 Mycgr3T
  
Location: 0-1602

Mycgr3G85918\_Mycgr3T

Mycgr3G42010 Mycgr3T
  
Location: 1702-8569

Mycgr3G42010\_Mycgr3T

Mycgr3G29582 Mycgr3T
  
Location: 8669-8915

Mycgr3G29582\_Mycgr3T

Mycgr3G31170 Mycgr3T
  
Location: 9015-9255

Mycgr3G31170\_Mycgr3T

Mycgr3G85924 Mycgr3T
  
Location: 9355-11218

Mycgr3G85924\_Mycgr3T

Mycgr3G71676 Mycgr3T
  
Location: 11318-12494

Mycgr3G71676\_Mycgr3T

Mycgr3G11468 Mycgr3T
  
Location: 12594-13653

Mycgr3G11468\_Mycgr3T

Mycgr3G58567 Mycgr3T
  
Location: 13753-14506

Mycgr3G58567\_Mycgr3T

Mycgr3G100089 Mycgr3
  
Location: 14606-21152

Mycgr3G100089\_Mycgr3

Mycgr3G42698 Mycgr3T
  
Location: 21252-22131

Mycgr3G42698\_Mycgr3T

Mycgr3G71681 Mycgr3T
  
Location: 22231-23461

Mycgr3G71681\_Mycgr3T

Mycgr3G109328 Mycgr3
  
Location: 23561-24239

Mycgr3G109328\_Mycgr3

Mycgr3G104334 Mycgr3
  
Location: 24339-24567

Mycgr3G104334\_Mycgr3

Mycgr3G42715 Mycgr3T
  
Location: 24667-25981

Mycgr3G42715\_Mycgr3T

Mycgr3G92934 Mycgr3T
  
Location: 26081-27593

Mycgr3G92934\_Mycgr3T

Mycgr3G41969 Mycgr3T
  
Location: 27693-29328

Mycgr3G41969\_Mycgr3T

Mycgr3G80635 Mycgr3T
  
Location: 29428-29821

Mycgr3G80635\_Mycgr3T

Mycgr3G41426 Mycgr3T
  
Location: 29921-35255

Mycgr3G41426\_Mycgr3T

Mycgr3G104337 Mycgr3
  
Location: 35355-36108

Mycgr3G104337\_Mycgr3

Mycgr3G71679 Mycgr3T
  
Location: 36208-37300

Mycgr3G71679\_Mycgr3T

Mycgr3G92938 Mycgr3T
  
Location: 37400-38699

Mycgr3G92938\_Mycgr3T

Mycgr3G92941 Mycgr3T
  
Location: 38799-40734

Mycgr3G92941\_Mycgr3T

Hexokinase-1
  
Accession: EKV05518
  
Location: 2550-4166
  
 NCBI BlastP on this gene

EKV05518

hypothetical protein
  
Accession: EKV05519
  
Location: 5409-5756
  
 NCBI BlastP on this gene

EKV05519

Polyketide synthase, putative
  
Accession: EKV05520
  
Location: 5797-8814
  
 NCBI BlastP on this gene

EKV05520

Mycocerosic acid synthase
  
Accession: EKV05521
  
Location: 8977-11136
  
  
**BlastP hit with Mycgr3G100089\_Mycgr3**
  
Percentage identity: 59 %
  
BlastP bit score: 845
  
Sequence coverage: 30 %
  
E-value: 0.0
  
  
 NCBI BlastP on this gene

EKV05521

hypothetical protein
  
Accession: EKV05522
  
Location: 11663-12626
  
  
**BlastP hit with Mycgr3G104337\_Mycgr3**
  
Percentage identity: 54 %
  
BlastP bit score: 296
  
Sequence coverage: 99 %
  
E-value: 3e-97
  
  
 NCBI BlastP on this gene

EKV05522

Acetylxylan esterase, putative
  
Accession: EKV05523
  
Location: 13464-13847
  
 NCBI BlastP on this gene

EKV05523

Uridylate kinase Ura6
  
Accession: EKV05524
  
Location: 15765-16394
  
 NCBI BlastP on this gene

EKV05524

Eukaryotic translation initiation factor 3 subunit M
  
Accession: EKV05525
  
Location: 16869-18331
  
 NCBI BlastP on this gene

EKV05525

hypothetical protein
  
Accession: EKV05526
  
Location: 18874-19586
  
 NCBI BlastP on this gene

EKV05526

Thioesterase family protein
  
Accession: EKV05527
  
Location: 20554-21249
  
 NCBI BlastP on this gene

EKV05527

hypothetical protein
  
Accession: EKV05528
  
Location: 21799-21960
  
 NCBI BlastP on this gene

EKV05528

Query: Architecture Search FASTA input

AKCT01000041 : Penicillium digitatum PHI26    Total score: 2.0     Cumulative Blast bit score: 1141

Hit cluster cross-links:

Mycgr3G85918 Mycgr3T
  
Location: 0-1602

Mycgr3G85918\_Mycgr3T

Mycgr3G42010 Mycgr3T
  
Location: 1702-8569

Mycgr3G42010\_Mycgr3T

Mycgr3G29582 Mycgr3T
  
Location: 8669-8915

Mycgr3G29582\_Mycgr3T

Mycgr3G31170 Mycgr3T
  
Location: 9015-9255

Mycgr3G31170\_Mycgr3T

Mycgr3G85924 Mycgr3T
  
Location: 9355-11218

Mycgr3G85924\_Mycgr3T

Mycgr3G71676 Mycgr3T
  
Location: 11318-12494

Mycgr3G71676\_Mycgr3T

Mycgr3G11468 Mycgr3T
  
Location: 12594-13653

Mycgr3G11468\_Mycgr3T

Mycgr3G58567 Mycgr3T
  
Location: 13753-14506

Mycgr3G58567\_Mycgr3T

Mycgr3G100089 Mycgr3
  
Location: 14606-21152

Mycgr3G100089\_Mycgr3

Mycgr3G42698 Mycgr3T
  
Location: 21252-22131

Mycgr3G42698\_Mycgr3T

Mycgr3G71681 Mycgr3T
  
Location: 22231-23461

Mycgr3G71681\_Mycgr3T

Mycgr3G109328 Mycgr3
  
Location: 23561-24239

Mycgr3G109328\_Mycgr3

Mycgr3G104334 Mycgr3
  
Location: 24339-24567

Mycgr3G104334\_Mycgr3

Mycgr3G42715 Mycgr3T
  
Location: 24667-25981

Mycgr3G42715\_Mycgr3T

Mycgr3G92934 Mycgr3T
  
Location: 26081-27593

Mycgr3G92934\_Mycgr3T

Mycgr3G41969 Mycgr3T
  
Location: 27693-29328

Mycgr3G41969\_Mycgr3T

Mycgr3G80635 Mycgr3T
  
Location: 29428-29821

Mycgr3G80635\_Mycgr3T

Mycgr3G41426 Mycgr3T
  
Location: 29921-35255

Mycgr3G41426\_Mycgr3T

Mycgr3G104337 Mycgr3
  
Location: 35355-36108

Mycgr3G104337\_Mycgr3

Mycgr3G71679 Mycgr3T
  
Location: 36208-37300

Mycgr3G71679\_Mycgr3T

Mycgr3G92938 Mycgr3T
  
Location: 37400-38699

Mycgr3G92938\_Mycgr3T

Mycgr3G92941 Mycgr3T
  
Location: 38799-40734

Mycgr3G92941\_Mycgr3T

hypothetical protein
  
Accession: EKV18169
  
Location: 81185-82141
  
 NCBI BlastP on this gene

EKV18169

hypothetical protein
  
Accession: EKV18170
  
Location: 82928-83525
  
 NCBI BlastP on this gene

EKV18170

C6 transcription factor, putative
  
Accession: EKV18171
  
Location: 84874-86352
  
 NCBI BlastP on this gene

EKV18171

hypothetical protein
  
Accession: EKV18172
  
Location: 87683-88461
  
 NCBI BlastP on this gene

EKV18172

hypothetical protein
  
Accession: EKV18173
  
Location: 89671-92196
  
 NCBI BlastP on this gene

EKV18173

Hexokinase-1
  
Accession: EKV18174
  
Location: 94693-96309
  
 NCBI BlastP on this gene

EKV18174

hypothetical protein
  
Accession: EKV18175
  
Location: 97553-97900
  
 NCBI BlastP on this gene

EKV18175

Polyketide synthase, putative
  
Accession: EKV18176
  
Location: 97941-100958
  
 NCBI BlastP on this gene

EKV18176

Mycocerosic acid synthase
  
Accession: EKV18177
  
Location: 101121-103280
  
  
**BlastP hit with Mycgr3G100089\_Mycgr3**
  
Percentage identity: 59 %
  
BlastP bit score: 845
  
Sequence coverage: 30 %
  
E-value: 0.0
  
  
 NCBI BlastP on this gene

EKV18177

hypothetical protein
  
Accession: EKV18178
  
Location: 103807-104770
  
  
**BlastP hit with Mycgr3G104337\_Mycgr3**
  
Percentage identity: 54 %
  
BlastP bit score: 296
  
Sequence coverage: 99 %
  
E-value: 3e-97
  
  
 NCBI BlastP on this gene

EKV18178

Acetylxylan esterase, putative
  
Accession: EKV18179
  
Location: 105608-105991
  
 NCBI BlastP on this gene

EKV18179

Uridylate kinase Ura6
  
Accession: EKV18180
  
Location: 107927-108556
  
 NCBI BlastP on this gene

EKV18180

Eukaryotic translation initiation factor 3 subunit M
  
Accession: EKV18181
  
Location: 109032-110494
  
 NCBI BlastP on this gene

EKV18181

hypothetical protein
  
Accession: EKV18182
  
Location: 111037-111749
  
 NCBI BlastP on this gene

EKV18182

Thioesterase family protein
  
Accession: EKV18183
  
Location: 112717-113412
  
 NCBI BlastP on this gene

EKV18183

hypothetical protein
  
Accession: EKV18184
  
Location: 113962-114123
  
 NCBI BlastP on this gene

EKV18184

hypothetical protein
  
Accession: EKV18185
  
Location: 115059-116861
  
 NCBI BlastP on this gene

EKV18185

Alpha,alpha-trehalose phosphate synthase subunit TPS3, putative
  
Accession: EKV18186
  
Location: 118918-121832
  
 NCBI BlastP on this gene

EKV18186

hypothetical protein
  
Accession: EKV18187
  
Location: 122386-123577
  
 NCBI BlastP on this gene

EKV18187

hypothetical protein
  
Accession: EKV18188
  
Location: 123883-124984
  
 NCBI BlastP on this gene

EKV18188

Query: Architecture Search FASTA input

KB707685 : Botryotinia fuckeliana BcDW1 unplaced genomic scaffold Scaffold\_13    Total score: 2.0     Cumulative Blast bit score: 1140

Hit cluster cross-links:

Mycgr3G85918 Mycgr3T
  
Location: 0-1602

Mycgr3G85918\_Mycgr3T

Mycgr3G42010 Mycgr3T
  
Location: 1702-8569

Mycgr3G42010\_Mycgr3T

Mycgr3G29582 Mycgr3T
  
Location: 8669-8915

Mycgr3G29582\_Mycgr3T

Mycgr3G31170 Mycgr3T
  
Location: 9015-9255

Mycgr3G31170\_Mycgr3T

Mycgr3G85924 Mycgr3T
  
Location: 9355-11218

Mycgr3G85924\_Mycgr3T

Mycgr3G71676 Mycgr3T
  
Location: 11318-12494

Mycgr3G71676\_Mycgr3T

Mycgr3G11468 Mycgr3T
  
Location: 12594-13653

Mycgr3G11468\_Mycgr3T

Mycgr3G58567 Mycgr3T
  
Location: 13753-14506

Mycgr3G58567\_Mycgr3T

Mycgr3G100089 Mycgr3
  
Location: 14606-21152

Mycgr3G100089\_Mycgr3

Mycgr3G42698 Mycgr3T
  
Location: 21252-22131

Mycgr3G42698\_Mycgr3T

Mycgr3G71681 Mycgr3T
  
Location: 22231-23461

Mycgr3G71681\_Mycgr3T

Mycgr3G109328 Mycgr3
  
Location: 23561-24239

Mycgr3G109328\_Mycgr3

Mycgr3G104334 Mycgr3
  
Location: 24339-24567

Mycgr3G104334\_Mycgr3

Mycgr3G42715 Mycgr3T
  
Location: 24667-25981

Mycgr3G42715\_Mycgr3T

Mycgr3G92934 Mycgr3T
  
Location: 26081-27593

Mycgr3G92934\_Mycgr3T

Mycgr3G41969 Mycgr3T
  
Location: 27693-29328

Mycgr3G41969\_Mycgr3T

Mycgr3G80635 Mycgr3T
  
Location: 29428-29821

Mycgr3G80635\_Mycgr3T

Mycgr3G41426 Mycgr3T
  
Location: 29921-35255

Mycgr3G41426\_Mycgr3T

Mycgr3G104337 Mycgr3
  
Location: 35355-36108

Mycgr3G104337\_Mycgr3

Mycgr3G71679 Mycgr3T
  
Location: 36208-37300

Mycgr3G71679\_Mycgr3T

Mycgr3G92938 Mycgr3T
  
Location: 37400-38699

Mycgr3G92938\_Mycgr3T

Mycgr3G92941 Mycgr3T
  
Location: 38799-40734

Mycgr3G92941\_Mycgr3T

putative tetraspanin protein
  
Accession: EMR90826
  
Location: 110789-111547
  
 NCBI BlastP on this gene

EMR90826

hypothetical protein
  
Accession: EMR90827
  
Location: 112997-113206
  
 NCBI BlastP on this gene

EMR90827

putative mads box protein
  
Accession: EMR90828
  
Location: 117395-118244
  
  
**BlastP hit with Mycgr3G31170\_Mycgr3T**
  
Percentage identity: 97 %
  
BlastP bit score: 159
  
Sequence coverage: 100 %
  
E-value: 1e-46
  
  
 NCBI BlastP on this gene

EMR90828

putative duf803 domain-containing protein
  
Accession: EMR90829
  
Location: 120310-122007
  
 NCBI BlastP on this gene

EMR90829

putative vacuolar atp synthase subunit e protein
  
Accession: EMR90830
  
Location: 122592-123519
  
 NCBI BlastP on this gene

EMR90830

hypothetical protein
  
Accession: EMR90831
  
Location: 124344-125768
  
 NCBI BlastP on this gene

EMR90831

hypothetical protein
  
Accession: EMR90832
  
Location: 128938-133488
  
 NCBI BlastP on this gene

EMR90832

putative helicase sen1 protein
  
Accession: EMR90833
  
Location: 134590-140880
  
  
**BlastP hit with Mycgr3G41426\_Mycgr3T**
  
Percentage identity: 33 %
  
BlastP bit score: 981
  
Sequence coverage: 105 %
  
E-value: 0.0
  
  
 NCBI BlastP on this gene

EMR90833

hypothetical protein
  
Accession: EMR90834
  
Location: 142352-144763
  
 NCBI BlastP on this gene

EMR90834

putative magnesium ion transporter protein
  
Accession: EMR90835
  
Location: 146858-147180
  
 NCBI BlastP on this gene

EMR90835

putative mus38-like protein
  
Accession: EMR90836
  
Location: 149525-152363
  
 NCBI BlastP on this gene

EMR90836

Query: Architecture Search FASTA input

FQ790288 : Botryotinia fuckeliana T4 SuperContig\_299\_1 genomic supercontig.    Total score: 2.0     Cumulative Blast bit score: 1140

Hit cluster cross-links:

Mycgr3G85918 Mycgr3T
  
Location: 0-1602

Mycgr3G85918\_Mycgr3T

Mycgr3G42010 Mycgr3T
  
Location: 1702-8569

Mycgr3G42010\_Mycgr3T

Mycgr3G29582 Mycgr3T
  
Location: 8669-8915

Mycgr3G29582\_Mycgr3T

Mycgr3G31170 Mycgr3T
  
Location: 9015-9255

Mycgr3G31170\_Mycgr3T

Mycgr3G85924 Mycgr3T
  
Location: 9355-11218

Mycgr3G85924\_Mycgr3T

Mycgr3G71676 Mycgr3T
  
Location: 11318-12494

Mycgr3G71676\_Mycgr3T

Mycgr3G11468 Mycgr3T
  
Location: 12594-13653

Mycgr3G11468\_Mycgr3T

Mycgr3G58567 Mycgr3T
  
Location: 13753-14506

Mycgr3G58567\_Mycgr3T

Mycgr3G100089 Mycgr3
  
Location: 14606-21152

Mycgr3G100089\_Mycgr3

Mycgr3G42698 Mycgr3T
  
Location: 21252-22131

Mycgr3G42698\_Mycgr3T

Mycgr3G71681 Mycgr3T
  
Location: 22231-23461

Mycgr3G71681\_Mycgr3T

Mycgr3G109328 Mycgr3
  
Location: 23561-24239

Mycgr3G109328\_Mycgr3

Mycgr3G104334 Mycgr3
  
Location: 24339-24567

Mycgr3G104334\_Mycgr3

Mycgr3G42715 Mycgr3T
  
Location: 24667-25981

Mycgr3G42715\_Mycgr3T

Mycgr3G92934 Mycgr3T
  
Location: 26081-27593

Mycgr3G92934\_Mycgr3T

Mycgr3G41969 Mycgr3T
  
Location: 27693-29328

Mycgr3G41969\_Mycgr3T

Mycgr3G80635 Mycgr3T
  
Location: 29428-29821

Mycgr3G80635\_Mycgr3T

Mycgr3G41426 Mycgr3T
  
Location: 29921-35255

Mycgr3G41426\_Mycgr3T

Mycgr3G104337 Mycgr3
  
Location: 35355-36108

Mycgr3G104337\_Mycgr3

Mycgr3G71679 Mycgr3T
  
Location: 36208-37300

Mycgr3G71679\_Mycgr3T

Mycgr3G92938 Mycgr3T
  
Location: 37400-38699

Mycgr3G92938\_Mycgr3T

Mycgr3G92941 Mycgr3T
  
Location: 38799-40734

Mycgr3G92941\_Mycgr3T

BcPLS1, tetraspanin
  
Accession: CCD47897
  
Location: 134703-135374
  
 NCBI BlastP on this gene

BofuT4\_P114010.1

hypothetical protein
  
Accession: CCD47898
  
Location: 135828-136383
  
 NCBI BlastP on this gene

BofuT4\_uP114020.1

hypothetical protein
  
Accession: CCD47899
  
Location: 136820-137029
  
 NCBI BlastP on this gene

BofuT4\_uP114030.1

hypothetical protein
  
Accession: CCD47900
  
Location: 137429-137819
  
 NCBI BlastP on this gene

BofuT4\_P114040.1

hypothetical protein
  
Accession: CCD47901
  
Location: 138616-138765
  
 NCBI BlastP on this gene

BofuT4\_uP114050.1

hypothetical protein
  
Accession: CCD47902
  
Location: 138884-139057
  
 NCBI BlastP on this gene

BofuT4\_uP114060.1

similar to transcription factor MADS
  
Accession: CCD47903
  
Location: 141468-142317
  
  
**BlastP hit with Mycgr3G31170\_Mycgr3T**
  
Percentage identity: 97 %
  
BlastP bit score: 159
  
Sequence coverage: 100 %
  
E-value: 1e-46
  
  
 NCBI BlastP on this gene

BofuT4\_P114070.1

similar to DUF803 domain-containing protein
  
Accession: CCD47904
  
Location: 144419-146116
  
 NCBI BlastP on this gene

BofuT4\_P114080.1

similar to vacuolar ATP synthase subunit E (V-ATPase E subunit) (Vacuolar proton pump E subunit)
  
Accession: CCD47905
  
Location: 146701-147628
  
 NCBI BlastP on this gene

BofuT4\_P114090.1

hypothetical protein
  
Accession: CCD47906
  
Location: 147856-149877
  
 NCBI BlastP on this gene

BofuT4\_P114100.1

hypothetical protein
  
Accession: CCD47907
  
Location: 151155-151845
  
 NCBI BlastP on this gene

BofuT4\_uP114110.1

similar to P-type ATPase
  
Accession: CCD47908
  
Location: 153047-157597
  
 NCBI BlastP on this gene

BofuT4\_P114120.1

similar to similar to tRNA-splicing endonuclease
  
Accession: CCD47909
  
Location: 158699-164989
  
  
**BlastP hit with Mycgr3G41426\_Mycgr3T**
  
Percentage identity: 33 %
  
BlastP bit score: 981
  
Sequence coverage: 105 %
  
E-value: 0.0
  
  
 NCBI BlastP on this gene

BofuT4\_P114130.1

hypothetical protein
  
Accession: CCD47910
  
Location: 166461-168325
  
 NCBI BlastP on this gene

BofuT4\_P114140.1

hypothetical protein
  
Accession: CCD47911
  
Location: 171058-171380
  
 NCBI BlastP on this gene

BofuT4\_P114150.1

hypothetical protein
  
Accession: CCD47912
  
Location: 172042-172538
  
 NCBI BlastP on this gene

BofuT4\_uP114160.1

predicted protein
  
Accession: CCD47913
  
Location: 172582-172755
  
 NCBI BlastP on this gene

BofuT4\_uP114170.1

hypothetical protein
  
Accession: CCD47914
  
Location: 173682-174860
  
 NCBI BlastP on this gene

BofuT4\_P114180.1

hypothetical protein
  
Accession: CCD47915
  
Location: 175056-176672
  
 NCBI BlastP on this gene

BofuT4\_P114190.1

Query: Architecture Search FASTA input

AABX02000004 : Neurospora crassa OR74A    Total score: 2.0     Cumulative Blast bit score: 1127

Hit cluster cross-links:

Mycgr3G85918 Mycgr3T
  
Location: 0-1602

Mycgr3G85918\_Mycgr3T

Mycgr3G42010 Mycgr3T
  
Location: 1702-8569

Mycgr3G42010\_Mycgr3T

Mycgr3G29582 Mycgr3T
  
Location: 8669-8915

Mycgr3G29582\_Mycgr3T

Mycgr3G31170 Mycgr3T
  
Location: 9015-9255

Mycgr3G31170\_Mycgr3T

Mycgr3G85924 Mycgr3T
  
Location: 9355-11218

Mycgr3G85924\_Mycgr3T

Mycgr3G71676 Mycgr3T
  
Location: 11318-12494

Mycgr3G71676\_Mycgr3T

Mycgr3G11468 Mycgr3T
  
Location: 12594-13653

Mycgr3G11468\_Mycgr3T

Mycgr3G58567 Mycgr3T
  
Location: 13753-14506

Mycgr3G58567\_Mycgr3T

Mycgr3G100089 Mycgr3
  
Location: 14606-21152

Mycgr3G100089\_Mycgr3

Mycgr3G42698 Mycgr3T
  
Location: 21252-22131

Mycgr3G42698\_Mycgr3T

Mycgr3G71681 Mycgr3T
  
Location: 22231-23461

Mycgr3G71681\_Mycgr3T

Mycgr3G109328 Mycgr3
  
Location: 23561-24239

Mycgr3G109328\_Mycgr3

Mycgr3G104334 Mycgr3
  
Location: 24339-24567

Mycgr3G104334\_Mycgr3

Mycgr3G42715 Mycgr3T
  
Location: 24667-25981

Mycgr3G42715\_Mycgr3T

Mycgr3G92934 Mycgr3T
  
Location: 26081-27593

Mycgr3G92934\_Mycgr3T

Mycgr3G41969 Mycgr3T
  
Location: 27693-29328

Mycgr3G41969\_Mycgr3T

Mycgr3G80635 Mycgr3T
  
Location: 29428-29821

Mycgr3G80635\_Mycgr3T

Mycgr3G41426 Mycgr3T
  
Location: 29921-35255

Mycgr3G41426\_Mycgr3T

Mycgr3G104337 Mycgr3
  
Location: 35355-36108

Mycgr3G104337\_Mycgr3

Mycgr3G71679 Mycgr3T
  
Location: 36208-37300

Mycgr3G71679\_Mycgr3T

Mycgr3G92938 Mycgr3T
  
Location: 37400-38699

Mycgr3G92938\_Mycgr3T

Mycgr3G92941 Mycgr3T
  
Location: 38799-40734

Mycgr3G92941\_Mycgr3T

predicted protein
  
Accession: EAA27048
  
Location: 1094567-1095774
  
 NCBI BlastP on this gene

EAA27048

hypothetical protein
  
Accession: EAA27047
  
Location: 1100531-1101790
  
 NCBI BlastP on this gene

EAA27047

predicted protein
  
Accession: EAA27046
  
Location: 1102317-1106049
  
 NCBI BlastP on this gene

EAA27046

predicted protein
  
Accession: EAA27045
  
Location: 1109962-1111637
  
  
**BlastP hit with Mycgr3G104337\_Mycgr3**
  
Percentage identity: 41 %
  
BlastP bit score: 185
  
Sequence coverage: 94 %
  
E-value: 3e-52
  
  
 NCBI BlastP on this gene

EAA27045

conserved hypothetical protein
  
Accession: EAA26702
  
Location: 1114980-1122290
  
  
**BlastP hit with Mycgr3G100089\_Mycgr3**
  
Percentage identity: 44 %
  
BlastP bit score: 942
  
Sequence coverage: 49 %
  
E-value: 0.0
  
  
 NCBI BlastP on this gene

EAA26702

predicted protein
  
Accession: EAA26701
  
Location: 1123929-1126123
  
 NCBI BlastP on this gene

EAA26701

NADH:ubiquinone oxidoreductase 21.3c kD subunit
  
Accession: EAA26700
  
Location: 1127978-1129037
  
 NCBI BlastP on this gene

EAA26700

NADH:ubiquinone oxidoreductase 9.6kD subunit
  
Accession: EAA26699
  
Location: 1129961-1131330
  
 NCBI BlastP on this gene

EAA26699

conserved hypothetical protein
  
Accession: EAA26698
  
Location: 1133088-1135338
  
 NCBI BlastP on this gene

EAA26698

Query: Architecture Search FASTA input

GL891307 : Neurospora tetrasperma FGSC 2508 unplaced genomic scaffold NEUTE1scaffold\_6    Total score: 2.0     Cumulative Blast bit score: 1126

Hit cluster cross-links:

Mycgr3G85918 Mycgr3T
  
Location: 0-1602

Mycgr3G85918\_Mycgr3T

Mycgr3G42010 Mycgr3T
  
Location: 1702-8569

Mycgr3G42010\_Mycgr3T

Mycgr3G29582 Mycgr3T
  
Location: 8669-8915

Mycgr3G29582\_Mycgr3T

Mycgr3G31170 Mycgr3T
  
Location: 9015-9255

Mycgr3G31170\_Mycgr3T

Mycgr3G85924 Mycgr3T
  
Location: 9355-11218

Mycgr3G85924\_Mycgr3T

Mycgr3G71676 Mycgr3T
  
Location: 11318-12494

Mycgr3G71676\_Mycgr3T

Mycgr3G11468 Mycgr3T
  
Location: 12594-13653

Mycgr3G11468\_Mycgr3T

Mycgr3G58567 Mycgr3T
  
Location: 13753-14506

Mycgr3G58567\_Mycgr3T

Mycgr3G100089 Mycgr3
  
Location: 14606-21152

Mycgr3G100089\_Mycgr3

Mycgr3G42698 Mycgr3T
  
Location: 21252-22131

Mycgr3G42698\_Mycgr3T

Mycgr3G71681 Mycgr3T
  
Location: 22231-23461

Mycgr3G71681\_Mycgr3T

Mycgr3G109328 Mycgr3
  
Location: 23561-24239

Mycgr3G109328\_Mycgr3

Mycgr3G104334 Mycgr3
  
Location: 24339-24567

Mycgr3G104334\_Mycgr3

Mycgr3G42715 Mycgr3T
  
Location: 24667-25981

Mycgr3G42715\_Mycgr3T

Mycgr3G92934 Mycgr3T
  
Location: 26081-27593

Mycgr3G92934\_Mycgr3T

Mycgr3G41969 Mycgr3T
  
Location: 27693-29328

Mycgr3G41969\_Mycgr3T

Mycgr3G80635 Mycgr3T
  
Location: 29428-29821

Mycgr3G80635\_Mycgr3T

Mycgr3G41426 Mycgr3T
  
Location: 29921-35255

Mycgr3G41426\_Mycgr3T

Mycgr3G104337 Mycgr3
  
Location: 35355-36108

Mycgr3G104337\_Mycgr3

Mycgr3G71679 Mycgr3T
  
Location: 36208-37300

Mycgr3G71679\_Mycgr3T

Mycgr3G92938 Mycgr3T
  
Location: 37400-38699

Mycgr3G92938\_Mycgr3T

Mycgr3G92941 Mycgr3T
  
Location: 38799-40734

Mycgr3G92941\_Mycgr3T

hypothetical protein
  
Accession: EGO54915
  
Location: 3780495-3782872
  
 NCBI BlastP on this gene

EGO54915

hypothetical protein
  
Accession: EGO54916
  
Location: 3785099-3786305
  
 NCBI BlastP on this gene

EGO54916

hypothetical protein
  
Accession: EGO54917
  
Location: 3787858-3789130
  
 NCBI BlastP on this gene

EGO54917

hypothetical protein
  
Accession: EGO54918
  
Location: 3789650-3793378
  
 NCBI BlastP on this gene

EGO54918

hypothetical protein
  
Accession: EGO54919
  
Location: 3797254-3798952
  
  
**BlastP hit with Mycgr3G104337\_Mycgr3**
  
Percentage identity: 41 %
  
BlastP bit score: 187
  
Sequence coverage: 94 %
  
E-value: 8e-53
  
  
 NCBI BlastP on this gene

EGO54919

hypothetical protein
  
Accession: EGO54920
  
Location: 3800103-3800267
  
 NCBI BlastP on this gene

EGO54920

hypothetical protein
  
Accession: EGO54921
  
Location: 3802200-3809510
  
  
**BlastP hit with Mycgr3G100089\_Mycgr3**
  
Percentage identity: 43 %
  
BlastP bit score: 939
  
Sequence coverage: 49 %
  
E-value: 0.0
  
  
 NCBI BlastP on this gene

EGO54921

hypothetical protein
  
Accession: EGO54922
  
Location: 3811904-3814087
  
 NCBI BlastP on this gene

EGO54922

hypothetical protein
  
Accession: EGO54923
  
Location: 3815945-3816978
  
 NCBI BlastP on this gene

EGO54923

hypothetical protein
  
Accession: EGO54924
  
Location: 3817894-3819369
  
 NCBI BlastP on this gene

EGO54924

hypothetical protein
  
Accession: EGO54925
  
Location: 3821204-3823451
  
 NCBI BlastP on this gene

EGO54925

Query: Architecture Search FASTA input

GL891269 : Neurospora tetrasperma FGSC 2509 unplaced genomic scaffold NEUTE2scaffold\_7    Total score: 2.0     Cumulative Blast bit score: 1126

Hit cluster cross-links:

Mycgr3G85918 Mycgr3T
  
Location: 0-1602

Mycgr3G85918\_Mycgr3T

Mycgr3G42010 Mycgr3T
  
Location: 1702-8569

Mycgr3G42010\_Mycgr3T

Mycgr3G29582 Mycgr3T
  
Location: 8669-8915

Mycgr3G29582\_Mycgr3T

Mycgr3G31170 Mycgr3T
  
Location: 9015-9255

Mycgr3G31170\_Mycgr3T

Mycgr3G85924 Mycgr3T
  
Location: 9355-11218

Mycgr3G85924\_Mycgr3T

Mycgr3G71676 Mycgr3T
  
Location: 11318-12494

Mycgr3G71676\_Mycgr3T

Mycgr3G11468 Mycgr3T
  
Location: 12594-13653

Mycgr3G11468\_Mycgr3T

Mycgr3G58567 Mycgr3T
  
Location: 13753-14506

Mycgr3G58567\_Mycgr3T

Mycgr3G100089 Mycgr3
  
Location: 14606-21152

Mycgr3G100089\_Mycgr3

Mycgr3G42698 Mycgr3T
  
Location: 21252-22131

Mycgr3G42698\_Mycgr3T

Mycgr3G71681 Mycgr3T
  
Location: 22231-23461

Mycgr3G71681\_Mycgr3T

Mycgr3G109328 Mycgr3
  
Location: 23561-24239

Mycgr3G109328\_Mycgr3

Mycgr3G104334 Mycgr3
  
Location: 24339-24567

Mycgr3G104334\_Mycgr3

Mycgr3G42715 Mycgr3T
  
Location: 24667-25981

Mycgr3G42715\_Mycgr3T

Mycgr3G92934 Mycgr3T
  
Location: 26081-27593

Mycgr3G92934\_Mycgr3T

Mycgr3G41969 Mycgr3T
  
Location: 27693-29328

Mycgr3G41969\_Mycgr3T

Mycgr3G80635 Mycgr3T
  
Location: 29428-29821

Mycgr3G80635\_Mycgr3T

Mycgr3G41426 Mycgr3T
  
Location: 29921-35255

Mycgr3G41426\_Mycgr3T

Mycgr3G104337 Mycgr3
  
Location: 35355-36108

Mycgr3G104337\_Mycgr3

Mycgr3G71679 Mycgr3T
  
Location: 36208-37300

Mycgr3G71679\_Mycgr3T

Mycgr3G92938 Mycgr3T
  
Location: 37400-38699

Mycgr3G92938\_Mycgr3T

Mycgr3G92941 Mycgr3T
  
Location: 38799-40734

Mycgr3G92941\_Mycgr3T

hypothetical protein
  
Accession: EGZ67583
  
Location: 60147-62394
  
 NCBI BlastP on this gene

EGZ67583

acyl carrier protein
  
Accession: EGZ67584
  
Location: 64849-66324
  
 NCBI BlastP on this gene

EGZ67584

ferredoxin-like iron-sulfur subunit of mitochondrial complex I
  
Accession: EGZ67585
  
Location: 67240-68273
  
 NCBI BlastP on this gene

EGZ67585

hypothetical protein
  
Accession: EGZ67586
  
Location: 70130-72313
  
 NCBI BlastP on this gene

EGZ67586

ketoacyl-synt-domain-containing protein
  
Accession: EGZ67587
  
Location: 74706-82016
  
  
**BlastP hit with Mycgr3G100089\_Mycgr3**
  
Percentage identity: 43 %
  
BlastP bit score: 939
  
Sequence coverage: 49 %
  
E-value: 0.0
  
  
 NCBI BlastP on this gene

EGZ67587

hypothetical protein
  
Accession: EGZ67588
  
Location: 83949-84113
  
 NCBI BlastP on this gene

EGZ67588

hypothetical protein
  
Accession: EGZ67589
  
Location: 85259-86956
  
  
**BlastP hit with Mycgr3G104337\_Mycgr3**
  
Percentage identity: 41 %
  
BlastP bit score: 187
  
Sequence coverage: 94 %
  
E-value: 8e-53
  
  
 NCBI BlastP on this gene

EGZ67589

hypothetical protein
  
Accession: EGZ67590
  
Location: 90832-94560
  
 NCBI BlastP on this gene

EGZ67590

hypothetical protein
  
Accession: EGZ67591
  
Location: 95080-96352
  
 NCBI BlastP on this gene

EGZ67591

hypothetical protein
  
Accession: EGZ67592
  
Location: 97904-99110
  
 NCBI BlastP on this gene

EGZ67592

hypothetical protein
  
Accession: EGZ67593
  
Location: 101336-103713
  
 NCBI BlastP on this gene

EGZ67593

Query: Architecture Search FASTA input

AFWA01000005 : Pneumocystis murina B123    Total score: 2.0     Cumulative Blast bit score: 1125

Hit cluster cross-links:

Mycgr3G85918 Mycgr3T
  
Location: 0-1602

Mycgr3G85918\_Mycgr3T

Mycgr3G42010 Mycgr3T
  
Location: 1702-8569

Mycgr3G42010\_Mycgr3T

Mycgr3G29582 Mycgr3T
  
Location: 8669-8915

Mycgr3G29582\_Mycgr3T

Mycgr3G31170 Mycgr3T
  
Location: 9015-9255

Mycgr3G31170\_Mycgr3T

Mycgr3G85924 Mycgr3T
  
Location: 9355-11218

Mycgr3G85924\_Mycgr3T

Mycgr3G71676 Mycgr3T
  
Location: 11318-12494

Mycgr3G71676\_Mycgr3T

Mycgr3G11468 Mycgr3T
  
Location: 12594-13653

Mycgr3G11468\_Mycgr3T

Mycgr3G58567 Mycgr3T
  
Location: 13753-14506

Mycgr3G58567\_Mycgr3T

Mycgr3G100089 Mycgr3
  
Location: 14606-21152

Mycgr3G100089\_Mycgr3

Mycgr3G42698 Mycgr3T
  
Location: 21252-22131

Mycgr3G42698\_Mycgr3T

Mycgr3G71681 Mycgr3T
  
Location: 22231-23461

Mycgr3G71681\_Mycgr3T

Mycgr3G109328 Mycgr3
  
Location: 23561-24239

Mycgr3G109328\_Mycgr3

Mycgr3G104334 Mycgr3
  
Location: 24339-24567

Mycgr3G104334\_Mycgr3

Mycgr3G42715 Mycgr3T
  
Location: 24667-25981

Mycgr3G42715\_Mycgr3T

Mycgr3G92934 Mycgr3T
  
Location: 26081-27593

Mycgr3G92934\_Mycgr3T

Mycgr3G41969 Mycgr3T
  
Location: 27693-29328

Mycgr3G41969\_Mycgr3T

Mycgr3G80635 Mycgr3T
  
Location: 29428-29821

Mycgr3G80635\_Mycgr3T

Mycgr3G41426 Mycgr3T
  
Location: 29921-35255

Mycgr3G41426\_Mycgr3T

Mycgr3G104337 Mycgr3
  
Location: 35355-36108

Mycgr3G104337\_Mycgr3

Mycgr3G71679 Mycgr3T
  
Location: 36208-37300

Mycgr3G71679\_Mycgr3T

Mycgr3G92938 Mycgr3T
  
Location: 37400-38699

Mycgr3G92938\_Mycgr3T

Mycgr3G92941 Mycgr3T
  
Location: 38799-40734

Mycgr3G92941\_Mycgr3T

hypothetical protein
  
Accession: EMR10503
  
Location: 213414-214453
  
 NCBI BlastP on this gene

EMR10503

hypothetical protein
  
Accession: EMR10504
  
Location: 216311-217133
  
 NCBI BlastP on this gene

EMR10504

hypothetical protein
  
Accession: EMR10505
  
Location: 218169-219067
  
 NCBI BlastP on this gene

EMR10505

hypothetical protein
  
Accession: EMR10506
  
Location: 219236-220372
  
 NCBI BlastP on this gene

EMR10506

hypothetical protein
  
Accession: EMR10507
  
Location: 220559-221712
  
 NCBI BlastP on this gene

EMR10507

hypothetical protein
  
Accession: EMR10508
  
Location: 222146-223686
  
  
**BlastP hit with Mycgr3G92934\_Mycgr3T**
  
Percentage identity: 48 %
  
BlastP bit score: 419
  
Sequence coverage: 84 %
  
E-value: 3e-138
  
  
 NCBI BlastP on this gene

EMR10508

hypothetical protein, variant
  
Accession: EMR10509
  
Location: 222479-223686
  
  
**BlastP hit with Mycgr3G92934\_Mycgr3T**
  
Percentage identity: 48 %
  
BlastP bit score: 358
  
Sequence coverage: 74 %
  
E-value: 6e-116
  
  
 NCBI BlastP on this gene

EMR10509

hypothetical protein
  
Accession: EMR10510
  
Location: 224658-225864
  
 NCBI BlastP on this gene

EMR10510

hypothetical protein
  
Accession: EMR10511
  
Location: 226185-227882
  
 NCBI BlastP on this gene

EMR10511

hypothetical protein
  
Accession: EMR10512
  
Location: 228304-230503
  
 NCBI BlastP on this gene

EMR10512

hypothetical protein
  
Accession: EMR10513
  
Location: 231033-232931
  
 NCBI BlastP on this gene

EMR10513

hypothetical protein
  
Accession: EMR10514
  
Location: 233097-234529
  
 NCBI BlastP on this gene

EMR10514

hypothetical protein, variant
  
Accession: EMR10515
  
Location: 233097-234529
  
 NCBI BlastP on this gene

EMR10515

hypothetical protein
  
Accession: EMR10516
  
Location: 234906-237397
  
 NCBI BlastP on this gene

EMR10516

30S ribosomal protein S7e
  
Accession: EMR10517
  
Location: 237800-238517
  
 NCBI BlastP on this gene

EMR10517

hypothetical protein
  
Accession: EMR10518
  
Location: 239384-240025
  
 NCBI BlastP on this gene

EMR10518

hypothetical protein
  
Accession: EMR10519
  
Location: 240079-240792
  
 NCBI BlastP on this gene

EMR10519

hypothetical protein
  
Accession: EMR10520
  
Location: 241246-242452
  
 NCBI BlastP on this gene

EMR10520

hypothetical protein
  
Accession: EMR10521
  
Location: 242621-243652
  
 NCBI BlastP on this gene

EMR10521

hypothetical protein
  
Accession: EMR10522
  
Location: 244011-245516
  
 NCBI BlastP on this gene

EMR10522

hypothetical protein
  
Accession: EMR10523
  
Location: 246259-246833
  
 NCBI BlastP on this gene

EMR10523

hypothetical protein
  
Accession: EMR10524
  
Location: 247159-247876
  
 NCBI BlastP on this gene

EMR10524

hypothetical protein
  
Accession: EMR10525
  
Location: 247924-249824
  
  
**BlastP hit with Mycgr3G85924\_Mycgr3T**
  
Percentage identity: 30 %
  
BlastP bit score: 188
  
Sequence coverage: 95 %
  
E-value: 1e-48
  
  
 NCBI BlastP on this gene

EMR10525

hypothetical protein, variant
  
Accession: EMR10526
  
Location: 247924-249672
  
  
**BlastP hit with Mycgr3G85924\_Mycgr3T**
  
Percentage identity: 29 %
  
BlastP bit score: 160
  
Sequence coverage: 91 %
  
E-value: 2e-39
  
  
 NCBI BlastP on this gene

EMR10526

hypothetical protein
  
Accession: EMR10527
  
Location: 250212-250801
  
 NCBI BlastP on this gene

EMR10527

STE/STE7/MKK protein kinase
  
Accession: EMR10528
  
Location: 251372-252760
  
 NCBI BlastP on this gene

EMR10528

hypothetical protein
  
Accession: EMR10529
  
Location: 253582-254534
  
 NCBI BlastP on this gene

EMR10529

hypothetical protein
  
Accession: EMR10530
  
Location: 255312-256082
  
 NCBI BlastP on this gene

EMR10530

hypothetical protein, variant
  
Accession: EMR10531
  
Location: 255367-256082
  
 NCBI BlastP on this gene

EMR10531

hypothetical protein
  
Accession: EMR10532
  
Location: 256210-257804
  
 NCBI BlastP on this gene

EMR10532

hypothetical protein
  
Accession: EMR10533
  
Location: 257925-259025
  
 NCBI BlastP on this gene

EMR10533

Query: Architecture Search FASTA input

CABT02000027 : Sordaria macrospora k-hell    Total score: 2.0     Cumulative Blast bit score: 1122

Hit cluster cross-links:

Mycgr3G85918 Mycgr3T
  
Location: 0-1602

Mycgr3G85918\_Mycgr3T

Mycgr3G42010 Mycgr3T
  
Location: 1702-8569

Mycgr3G42010\_Mycgr3T

Mycgr3G29582 Mycgr3T
  
Location: 8669-8915

Mycgr3G29582\_Mycgr3T

Mycgr3G31170 Mycgr3T
  
Location: 9015-9255

Mycgr3G31170\_Mycgr3T

Mycgr3G85924 Mycgr3T
  
Location: 9355-11218

Mycgr3G85924\_Mycgr3T

Mycgr3G71676 Mycgr3T
  
Location: 11318-12494

Mycgr3G71676\_Mycgr3T

Mycgr3G11468 Mycgr3T
  
Location: 12594-13653

Mycgr3G11468\_Mycgr3T

Mycgr3G58567 Mycgr3T
  
Location: 13753-14506

Mycgr3G58567\_Mycgr3T

Mycgr3G100089 Mycgr3
  
Location: 14606-21152

Mycgr3G100089\_Mycgr3

Mycgr3G42698 Mycgr3T
  
Location: 21252-22131

Mycgr3G42698\_Mycgr3T

Mycgr3G71681 Mycgr3T
  
Location: 22231-23461

Mycgr3G71681\_Mycgr3T

Mycgr3G109328 Mycgr3
  
Location: 23561-24239

Mycgr3G109328\_Mycgr3

Mycgr3G104334 Mycgr3
  
Location: 24339-24567

Mycgr3G104334\_Mycgr3

Mycgr3G42715 Mycgr3T
  
Location: 24667-25981

Mycgr3G42715\_Mycgr3T

Mycgr3G92934 Mycgr3T
  
Location: 26081-27593

Mycgr3G92934\_Mycgr3T

Mycgr3G41969 Mycgr3T
  
Location: 27693-29328

Mycgr3G41969\_Mycgr3T

Mycgr3G80635 Mycgr3T
  
Location: 29428-29821

Mycgr3G80635\_Mycgr3T

Mycgr3G41426 Mycgr3T
  
Location: 29921-35255

Mycgr3G41426\_Mycgr3T

Mycgr3G104337 Mycgr3
  
Location: 35355-36108

Mycgr3G104337\_Mycgr3

Mycgr3G71679 Mycgr3T
  
Location: 36208-37300

Mycgr3G71679\_Mycgr3T

Mycgr3G92938 Mycgr3T
  
Location: 37400-38699

Mycgr3G92938\_Mycgr3T

Mycgr3G92941 Mycgr3T
  
Location: 38799-40734

Mycgr3G92941\_Mycgr3T

not annotated
  
Accession: CCC14912
  
Location: 322198-326407
  
 NCBI BlastP on this gene

CCC14912

not annotated
  
Accession: CCC14913
  
Location: 328769-329809
  
 NCBI BlastP on this gene

CCC14913

not annotated
  
Accession: CCC14914
  
Location: 330759-331325
  
 NCBI BlastP on this gene

CCC14914

not annotated
  
Accession: CCC14915
  
Location: 331763-333536
  
 NCBI BlastP on this gene

CCC14915

not annotated
  
Accession: CCC14916
  
Location: 334663-336147
  
  
**BlastP hit with Mycgr3G104337\_Mycgr3**
  
Percentage identity: 42 %
  
BlastP bit score: 191
  
Sequence coverage: 94 %
  
E-value: 6e-55
  
  
 NCBI BlastP on this gene

CCC14916

not annotated
  
Accession: CCC14917
  
Location: 338884-346158
  
  
**BlastP hit with Mycgr3G100089\_Mycgr3**
  
Percentage identity: 43 %
  
BlastP bit score: 931
  
Sequence coverage: 49 %
  
E-value: 0.0
  
  
 NCBI BlastP on this gene

CCC14917

not annotated
  
Accession: CCC14918
  
Location: 347848-350062
  
 NCBI BlastP on this gene

CCC14918

not annotated
  
Accession: CCC14919
  
Location: 356433-358570
  
 NCBI BlastP on this gene

CCC14919

not annotated
  
Accession: CCC14920
  
Location: 361043-362443
  
 NCBI BlastP on this gene

CCC14920

Query: Architecture Search FASTA input

EQ962654 : Talaromyces stipitatus ATCC 10500 scf\_1105507295541 genomic scaffold    Total score: 2.0     Cumulative Blast bit score: 1120

Hit cluster cross-links:

Mycgr3G85918 Mycgr3T
  
Location: 0-1602

Mycgr3G85918\_Mycgr3T

Mycgr3G42010 Mycgr3T
  
Location: 1702-8569

Mycgr3G42010\_Mycgr3T

Mycgr3G29582 Mycgr3T
  
Location: 8669-8915

Mycgr3G29582\_Mycgr3T

Mycgr3G31170 Mycgr3T
  
Location: 9015-9255

Mycgr3G31170\_Mycgr3T

Mycgr3G85924 Mycgr3T
  
Location: 9355-11218

Mycgr3G85924\_Mycgr3T

Mycgr3G71676 Mycgr3T
  
Location: 11318-12494

Mycgr3G71676\_Mycgr3T

Mycgr3G11468 Mycgr3T
  
Location: 12594-13653

Mycgr3G11468\_Mycgr3T

Mycgr3G58567 Mycgr3T
  
Location: 13753-14506

Mycgr3G58567\_Mycgr3T

Mycgr3G100089 Mycgr3
  
Location: 14606-21152

Mycgr3G100089\_Mycgr3

Mycgr3G42698 Mycgr3T
  
Location: 21252-22131

Mycgr3G42698\_Mycgr3T

Mycgr3G71681 Mycgr3T
  
Location: 22231-23461

Mycgr3G71681\_Mycgr3T

Mycgr3G109328 Mycgr3
  
Location: 23561-24239

Mycgr3G109328\_Mycgr3

Mycgr3G104334 Mycgr3
  
Location: 24339-24567

Mycgr3G104334\_Mycgr3

Mycgr3G42715 Mycgr3T
  
Location: 24667-25981

Mycgr3G42715\_Mycgr3T

Mycgr3G92934 Mycgr3T
  
Location: 26081-27593

Mycgr3G92934\_Mycgr3T

Mycgr3G41969 Mycgr3T
  
Location: 27693-29328

Mycgr3G41969\_Mycgr3T

Mycgr3G80635 Mycgr3T
  
Location: 29428-29821

Mycgr3G80635\_Mycgr3T

Mycgr3G41426 Mycgr3T
  
Location: 29921-35255

Mycgr3G41426\_Mycgr3T

Mycgr3G104337 Mycgr3
  
Location: 35355-36108

Mycgr3G104337\_Mycgr3

Mycgr3G71679 Mycgr3T
  
Location: 36208-37300

Mycgr3G71679\_Mycgr3T

Mycgr3G92938 Mycgr3T
  
Location: 37400-38699

Mycgr3G92938\_Mycgr3T

Mycgr3G92941 Mycgr3T
  
Location: 38799-40734

Mycgr3G92941\_Mycgr3T

Rab geranylgeranyl transferase escort protein, putative
  
Accession: EED19368
  
Location: 930471-932333
  
 NCBI BlastP on this gene

EED19368

conserved hypothetical protein
  
Accession: EED19369
  
Location: 934457-936076
  
 NCBI BlastP on this gene

EED19369

C6 transcription factor, putative
  
Accession: EED19370
  
Location: 937415-940188
  
 NCBI BlastP on this gene

EED19370

conserved hypothetical protein
  
Accession: EED19371
  
Location: 940426-941262
  
 NCBI BlastP on this gene

EED19371

MADS box transcription factor Mcm1
  
Accession: EED19372
  
Location: 943533-944362
  
  
**BlastP hit with Mycgr3G31170\_Mycgr3T**
  
Percentage identity: 100 %
  
BlastP bit score: 166
  
Sequence coverage: 100 %
  
E-value: 1e-49
  
  
 NCBI BlastP on this gene

EED19372

DUF803 domain protein
  
Accession: EED19373
  
Location: 945565-947530
  
 NCBI BlastP on this gene

EED19373

ATP synthase subunit E, putative
  
Accession: EED19374
  
Location: 948061-948875
  
 NCBI BlastP on this gene

EED19374

hypothetical protein
  
Accession: EED19375
  
Location: 949263-951562
  
 NCBI BlastP on this gene

EED19375

conserved hypothetical protein
  
Accession: EED19376
  
Location: 952002-952886
  
 NCBI BlastP on this gene

EED19376

phospholipid-translocating P-type ATPase domain-containing protein
  
Accession: EED19378
  
Location: 954455-958936
  
 NCBI BlastP on this gene

EED19378

hypothetical protein
  
Accession: EED19379
  
Location: 960230-962166
  
 NCBI BlastP on this gene

EED19379

tRNA-splicing endonuclease, putative
  
Accession: EED19380
  
Location: 962389-966693
  
  
**BlastP hit with Mycgr3G41426\_Mycgr3T**
  
Percentage identity: 46 %
  
BlastP bit score: 954
  
Sequence coverage: 62 %
  
E-value: 0.0
  
  
 NCBI BlastP on this gene

EED19380

conserved hypothetical protein
  
Accession: EED19381
  
Location: 969307-972345
  
 NCBI BlastP on this gene

EED19381

conserved hypothetical protein
  
Accession: EED19382
  
Location: 972576-973916
  
 NCBI BlastP on this gene

EED19382

chitin biosynthesis protein (Chs5), putative
  
Accession: EED19383
  
Location: 975974-977321
  
 NCBI BlastP on this gene

EED19383

Query: Architecture Search FASTA input

GL988044 : Chaetomium thermophilum var. thermophilum DSM 1495 unplaced genomic scaffold scf7180000...    Total score: 2.0     Cumulative Blast bit score: 1101

Hit cluster cross-links:

Mycgr3G85918 Mycgr3T
  
Location: 0-1602

Mycgr3G85918\_Mycgr3T

Mycgr3G42010 Mycgr3T
  
Location: 1702-8569

Mycgr3G42010\_Mycgr3T

Mycgr3G29582 Mycgr3T
  
Location: 8669-8915

Mycgr3G29582\_Mycgr3T

Mycgr3G31170 Mycgr3T
  
Location: 9015-9255

Mycgr3G31170\_Mycgr3T

Mycgr3G85924 Mycgr3T
  
Location: 9355-11218

Mycgr3G85924\_Mycgr3T

Mycgr3G71676 Mycgr3T
  
Location: 11318-12494

Mycgr3G71676\_Mycgr3T

Mycgr3G11468 Mycgr3T
  
Location: 12594-13653

Mycgr3G11468\_Mycgr3T

Mycgr3G58567 Mycgr3T
  
Location: 13753-14506

Mycgr3G58567\_Mycgr3T

Mycgr3G100089 Mycgr3
  
Location: 14606-21152

Mycgr3G100089\_Mycgr3

Mycgr3G42698 Mycgr3T
  
Location: 21252-22131

Mycgr3G42698\_Mycgr3T

Mycgr3G71681 Mycgr3T
  
Location: 22231-23461

Mycgr3G71681\_Mycgr3T

Mycgr3G109328 Mycgr3
  
Location: 23561-24239

Mycgr3G109328\_Mycgr3

Mycgr3G104334 Mycgr3
  
Location: 24339-24567

Mycgr3G104334\_Mycgr3

Mycgr3G42715 Mycgr3T
  
Location: 24667-25981

Mycgr3G42715\_Mycgr3T

Mycgr3G92934 Mycgr3T
  
Location: 26081-27593

Mycgr3G92934\_Mycgr3T

Mycgr3G41969 Mycgr3T
  
Location: 27693-29328

Mycgr3G41969\_Mycgr3T

Mycgr3G80635 Mycgr3T
  
Location: 29428-29821

Mycgr3G80635\_Mycgr3T

Mycgr3G41426 Mycgr3T
  
Location: 29921-35255

Mycgr3G41426\_Mycgr3T

Mycgr3G104337 Mycgr3
  
Location: 35355-36108

Mycgr3G104337\_Mycgr3

Mycgr3G71679 Mycgr3T
  
Location: 36208-37300

Mycgr3G71679\_Mycgr3T

Mycgr3G92938 Mycgr3T
  
Location: 37400-38699

Mycgr3G92938\_Mycgr3T

Mycgr3G92941 Mycgr3T
  
Location: 38799-40734

Mycgr3G92941\_Mycgr3T

putative cellulose binding protein
  
Accession: EGS19539
  
Location: 995262-996433
  
 NCBI BlastP on this gene

EGS19539

hypothetical protein
  
Accession: EGS19540
  
Location: 998209-999672
  
 NCBI BlastP on this gene

EGS19540

hypothetical protein
  
Accession: EGS19541
  
Location: 1000399-1001476
  
 NCBI BlastP on this gene

EGS19541

hypothetical protein
  
Accession: EGS19542
  
Location: 1002584-1003019
  
 NCBI BlastP on this gene

EGS19542

putative fatty acid protein
  
Accession: EGS19543
  
Location: 1005384-1006851
  
 NCBI BlastP on this gene

EGS19543

hypothetical protein
  
Accession: EGS19544
  
Location: 1007633-1008031
  
 NCBI BlastP on this gene

EGS19544

hypothetical protein
  
Accession: EGS19545
  
Location: 1008659-1011685
  
 NCBI BlastP on this gene

EGS19545

hypothetical protein
  
Accession: EGS19546
  
Location: 1014304-1015314
  
  
**BlastP hit with Mycgr3G104337\_Mycgr3**
  
Percentage identity: 37 %
  
BlastP bit score: 177
  
Sequence coverage: 103 %
  
E-value: 2e-50
  
  
 NCBI BlastP on this gene

EGS19546

hypothetical protein
  
Accession: EGS19547
  
Location: 1016981-1022602
  
  
**BlastP hit with Mycgr3G100089\_Mycgr3**
  
Percentage identity: 44 %
  
BlastP bit score: 924
  
Sequence coverage: 48 %
  
E-value: 0.0
  
  
 NCBI BlastP on this gene

EGS19547

oxidoreductase-like protein
  
Accession: EGS19548
  
Location: 1023653-1027185
  
 NCBI BlastP on this gene

EGS19548

hypothetical protein
  
Accession: EGS19549
  
Location: 1027609-1028479
  
 NCBI BlastP on this gene

EGS19549

zinc finger domain-containing protein
  
Accession: EGS19550
  
Location: 1029415-1031625
  
 NCBI BlastP on this gene

EGS19550

hypothetical protein
  
Accession: EGS19551
  
Location: 1034582-1036196
  
 NCBI BlastP on this gene

EGS19551

hypothetical protein
  
Accession: EGS19552
  
Location: 1040522-1042198
  
 NCBI BlastP on this gene

EGS19552

Query: Architecture Search FASTA input

DS027058 : Aspergillus clavatus NRRL 1 1099423829804 genomic scaffold    Total score: 2.0     Cumulative Blast bit score: 1062

Hit cluster cross-links:

Mycgr3G85918 Mycgr3T
  
Location: 0-1602

Mycgr3G85918\_Mycgr3T

Mycgr3G42010 Mycgr3T
  
Location: 1702-8569

Mycgr3G42010\_Mycgr3T

Mycgr3G29582 Mycgr3T
  
Location: 8669-8915

Mycgr3G29582\_Mycgr3T

Mycgr3G31170 Mycgr3T
  
Location: 9015-9255

Mycgr3G31170\_Mycgr3T

Mycgr3G85924 Mycgr3T
  
Location: 9355-11218

Mycgr3G85924\_Mycgr3T

Mycgr3G71676 Mycgr3T
  
Location: 11318-12494

Mycgr3G71676\_Mycgr3T

Mycgr3G11468 Mycgr3T
  
Location: 12594-13653

Mycgr3G11468\_Mycgr3T

Mycgr3G58567 Mycgr3T
  
Location: 13753-14506

Mycgr3G58567\_Mycgr3T

Mycgr3G100089 Mycgr3
  
Location: 14606-21152

Mycgr3G100089\_Mycgr3

Mycgr3G42698 Mycgr3T
  
Location: 21252-22131

Mycgr3G42698\_Mycgr3T

Mycgr3G71681 Mycgr3T
  
Location: 22231-23461

Mycgr3G71681\_Mycgr3T

Mycgr3G109328 Mycgr3
  
Location: 23561-24239

Mycgr3G109328\_Mycgr3

Mycgr3G104334 Mycgr3
  
Location: 24339-24567

Mycgr3G104334\_Mycgr3

Mycgr3G42715 Mycgr3T
  
Location: 24667-25981

Mycgr3G42715\_Mycgr3T

Mycgr3G92934 Mycgr3T
  
Location: 26081-27593

Mycgr3G92934\_Mycgr3T

Mycgr3G41969 Mycgr3T
  
Location: 27693-29328

Mycgr3G41969\_Mycgr3T

Mycgr3G80635 Mycgr3T
  
Location: 29428-29821

Mycgr3G80635\_Mycgr3T

Mycgr3G41426 Mycgr3T
  
Location: 29921-35255

Mycgr3G41426\_Mycgr3T

Mycgr3G104337 Mycgr3
  
Location: 35355-36108

Mycgr3G104337\_Mycgr3

Mycgr3G71679 Mycgr3T
  
Location: 36208-37300

Mycgr3G71679\_Mycgr3T

Mycgr3G92938 Mycgr3T
  
Location: 37400-38699

Mycgr3G92938\_Mycgr3T

Mycgr3G92941 Mycgr3T
  
Location: 38799-40734

Mycgr3G92941\_Mycgr3T

nonribosomal peptide synthase, putative
  
Accession: EAW08900
  
Location: 919134-944153
  
 NCBI BlastP on this gene

EAW08900

SRF-type transcription factor (Umc1), putative
  
Accession: EAW08901
  
Location: 946418-947301
  
  
**BlastP hit with Mycgr3G31170\_Mycgr3T**
  
Percentage identity: 98 %
  
BlastP bit score: 162
  
Sequence coverage: 100 %
  
E-value: 3e-48
  
  
 NCBI BlastP on this gene

EAW08901

DUF803 domain protein
  
Accession: EAW08902
  
Location: 948473-950445
  
 NCBI BlastP on this gene

EAW08902

ATP synthase subunit E, putative
  
Accession: EAW08903
  
Location: 951055-951877
  
 NCBI BlastP on this gene

EAW08903

hypothetical protein
  
Accession: EAW08904
  
Location: 952346-954740
  
 NCBI BlastP on this gene

EAW08904

phospholipid-translocating P-type ATPase domain-containing protein
  
Accession: EAW08905
  
Location: 958692-963397
  
 NCBI BlastP on this gene

EAW08905

tRNA-splicing endonuclease, putative
  
Accession: EAW08906
  
Location: 964758-971399
  
  
**BlastP hit with Mycgr3G41426\_Mycgr3T**
  
Percentage identity: 52 %
  
BlastP bit score: 900
  
Sequence coverage: 48 %
  
E-value: 0.0
  
  
 NCBI BlastP on this gene

EAW08906

conserved hypothetical protein
  
Accession: EAW08907
  
Location: 975010-977736
  
 NCBI BlastP on this gene

EAW08907

conserved hypothetical protein
  
Accession: EAW08908
  
Location: 978488-979219
  
 NCBI BlastP on this gene

EAW08908

aminotransferase, putative
  
Accession: EAW08909
  
Location: 979638-981241
  
 NCBI BlastP on this gene

EAW08909

Query: Architecture Search FASTA input

GG692437 : Ajellomyces capsulatus H143 genomic scaffold supercont2.19    Total score: 2.0     Cumulative Blast bit score: 1018

Hit cluster cross-links:

Mycgr3G85918 Mycgr3T
  
Location: 0-1602

Mycgr3G85918\_Mycgr3T

Mycgr3G42010 Mycgr3T
  
Location: 1702-8569

Mycgr3G42010\_Mycgr3T

Mycgr3G29582 Mycgr3T
  
Location: 8669-8915

Mycgr3G29582\_Mycgr3T

Mycgr3G31170 Mycgr3T
  
Location: 9015-9255

Mycgr3G31170\_Mycgr3T

Mycgr3G85924 Mycgr3T
  
Location: 9355-11218

Mycgr3G85924\_Mycgr3T

Mycgr3G71676 Mycgr3T
  
Location: 11318-12494

Mycgr3G71676\_Mycgr3T

Mycgr3G11468 Mycgr3T
  
Location: 12594-13653

Mycgr3G11468\_Mycgr3T

Mycgr3G58567 Mycgr3T
  
Location: 13753-14506

Mycgr3G58567\_Mycgr3T

Mycgr3G100089 Mycgr3
  
Location: 14606-21152

Mycgr3G100089\_Mycgr3

Mycgr3G42698 Mycgr3T
  
Location: 21252-22131

Mycgr3G42698\_Mycgr3T

Mycgr3G71681 Mycgr3T
  
Location: 22231-23461

Mycgr3G71681\_Mycgr3T

Mycgr3G109328 Mycgr3
  
Location: 23561-24239

Mycgr3G109328\_Mycgr3

Mycgr3G104334 Mycgr3
  
Location: 24339-24567

Mycgr3G104334\_Mycgr3

Mycgr3G42715 Mycgr3T
  
Location: 24667-25981

Mycgr3G42715\_Mycgr3T

Mycgr3G92934 Mycgr3T
  
Location: 26081-27593

Mycgr3G92934\_Mycgr3T

Mycgr3G41969 Mycgr3T
  
Location: 27693-29328

Mycgr3G41969\_Mycgr3T

Mycgr3G80635 Mycgr3T
  
Location: 29428-29821

Mycgr3G80635\_Mycgr3T

Mycgr3G41426 Mycgr3T
  
Location: 29921-35255

Mycgr3G41426\_Mycgr3T

Mycgr3G104337 Mycgr3
  
Location: 35355-36108

Mycgr3G104337\_Mycgr3

Mycgr3G71679 Mycgr3T
  
Location: 36208-37300

Mycgr3G71679\_Mycgr3T

Mycgr3G92938 Mycgr3T
  
Location: 37400-38699

Mycgr3G92938\_Mycgr3T

Mycgr3G92941 Mycgr3T
  
Location: 38799-40734

Mycgr3G92941\_Mycgr3T

tetraspanin
  
Accession: EER36814
  
Location: 168129-169156
  
 NCBI BlastP on this gene

EER36814

C6 zinc finger protein
  
Accession: EER36815
  
Location: 170997-172335
  
 NCBI BlastP on this gene

EER36815

conserved hypothetical protein
  
Accession: EER36816
  
Location: 174087-174523
  
 NCBI BlastP on this gene

EER36816

conserved hypothetical protein
  
Accession: EER36817
  
Location: 175383-176567
  
 NCBI BlastP on this gene

EER36817

MADS box transcription factor Mcm1
  
Accession: EER36818
  
Location: 177381-178311
  
  
**BlastP hit with Mycgr3G31170\_Mycgr3T**
  
Percentage identity: 98 %
  
BlastP bit score: 164
  
Sequence coverage: 100 %
  
E-value: 2e-48
  
  
 NCBI BlastP on this gene

EER36818

hypothetical protein
  
Accession: EER36819
  
Location: 179522-179766
  
 NCBI BlastP on this gene

EER36819

DUF803 domain-containing protein
  
Accession: EER36820
  
Location: 181051-183234
  
 NCBI BlastP on this gene

EER36820

vacuolar ATP synthase subunit E
  
Accession: EER36821
  
Location: 183686-184613
  
 NCBI BlastP on this gene

EER36821

conserved hypothetical protein
  
Accession: EER36822
  
Location: 185182-187664
  
 NCBI BlastP on this gene

EER36822

serine/threonine protein phosphatase
  
Accession: EER36823
  
Location: 188857-189943
  
 NCBI BlastP on this gene

EER36823

phospholipid-translocating P-type ATPase domain-containing protein
  
Accession: EER36824
  
Location: 191986-196653
  
 NCBI BlastP on this gene

EER36824

helicase SEN1
  
Accession: EER36825
  
Location: 198510-205256
  
  
**BlastP hit with Mycgr3G41426\_Mycgr3T**
  
Percentage identity: 53 %
  
BlastP bit score: 855
  
Sequence coverage: 43 %
  
E-value: 0.0
  
  
 NCBI BlastP on this gene

EER36825

predicted protein
  
Accession: EER36826
  
Location: 206141-206990
  
 NCBI BlastP on this gene

EER36826

predicted protein
  
Accession: EER36827
  
Location: 210111-213257
  
 NCBI BlastP on this gene

EER36827

dihydroorotase
  
Accession: EER36828
  
Location: 213889-215140
  
 NCBI BlastP on this gene

EER36828

Query: Architecture Search FASTA input

AM920437 : Penicillium chrysogenum Wisconsin 54-1255 complete genome, contig Pc00c22.    Total score: 2.0     Cumulative Blast bit score: 1012

Hit cluster cross-links:

Mycgr3G85918 Mycgr3T
  
Location: 0-1602

Mycgr3G85918\_Mycgr3T

Mycgr3G42010 Mycgr3T
  
Location: 1702-8569

Mycgr3G42010\_Mycgr3T

Mycgr3G29582 Mycgr3T
  
Location: 8669-8915

Mycgr3G29582\_Mycgr3T

Mycgr3G31170 Mycgr3T
  
Location: 9015-9255

Mycgr3G31170\_Mycgr3T

Mycgr3G85924 Mycgr3T
  
Location: 9355-11218

Mycgr3G85924\_Mycgr3T

Mycgr3G71676 Mycgr3T
  
Location: 11318-12494

Mycgr3G71676\_Mycgr3T

Mycgr3G11468 Mycgr3T
  
Location: 12594-13653

Mycgr3G11468\_Mycgr3T

Mycgr3G58567 Mycgr3T
  
Location: 13753-14506

Mycgr3G58567\_Mycgr3T

Mycgr3G100089 Mycgr3
  
Location: 14606-21152

Mycgr3G100089\_Mycgr3

Mycgr3G42698 Mycgr3T
  
Location: 21252-22131

Mycgr3G42698\_Mycgr3T

Mycgr3G71681 Mycgr3T
  
Location: 22231-23461

Mycgr3G71681\_Mycgr3T

Mycgr3G109328 Mycgr3
  
Location: 23561-24239

Mycgr3G109328\_Mycgr3

Mycgr3G104334 Mycgr3
  
Location: 24339-24567

Mycgr3G104334\_Mycgr3

Mycgr3G42715 Mycgr3T
  
Location: 24667-25981

Mycgr3G42715\_Mycgr3T

Mycgr3G92934 Mycgr3T
  
Location: 26081-27593

Mycgr3G92934\_Mycgr3T

Mycgr3G41969 Mycgr3T
  
Location: 27693-29328

Mycgr3G41969\_Mycgr3T

Mycgr3G80635 Mycgr3T
  
Location: 29428-29821

Mycgr3G80635\_Mycgr3T

Mycgr3G41426 Mycgr3T
  
Location: 29921-35255

Mycgr3G41426\_Mycgr3T

Mycgr3G104337 Mycgr3
  
Location: 35355-36108

Mycgr3G104337\_Mycgr3

Mycgr3G71679 Mycgr3T
  
Location: 36208-37300

Mycgr3G71679\_Mycgr3T

Mycgr3G92938 Mycgr3T
  
Location: 37400-38699

Mycgr3G92938\_Mycgr3T

Mycgr3G92941 Mycgr3T
  
Location: 38799-40734

Mycgr3G92941\_Mycgr3T

not annotated
  
Accession: CAP99718
  
Location: 5724438-5725292
  
 NCBI BlastP on this gene

Pc22g24300

not annotated
  
Accession: CAP99719
  
Location: 5725511-5726842
  
 NCBI BlastP on this gene

Pc22g24310

not annotated
  
Accession: CAP99720
  
Location: 5731268-5732863
  
 NCBI BlastP on this gene

Pc22g24320

hypothetical protein
  
Accession: CAP99721
  
Location: 5733676-5734809
  
 NCBI BlastP on this gene

Pc22g24330

not annotated
  
Accession: CAP99722
  
Location: 5735120-5735914
  
  
**BlastP hit with Mycgr3G31170\_Mycgr3T**
  
Percentage identity: 98 %
  
BlastP bit score: 162
  
Sequence coverage: 100 %
  
E-value: 3e-48
  
  
 NCBI BlastP on this gene

Pc22g24340

not annotated
  
Accession: CAP99723
  
Location: 5737550-5739301
  
 NCBI BlastP on this gene

Pc22g24350

not annotated
  
Accession: CAP99724
  
Location: 5739818-5740634
  
 NCBI BlastP on this gene

Pc22g24360

not annotated
  
Accession: CAP99725
  
Location: 5741006-5743376
  
 NCBI BlastP on this gene

Pc22g24370

not annotated
  
Accession: CAP99726
  
Location: 5745881-5750590
  
 NCBI BlastP on this gene

Pc22g24380

not annotated
  
Accession: CAP99727
  
Location: 5751745-5758443
  
  
**BlastP hit with Mycgr3G41426\_Mycgr3T**
  
Percentage identity: 50 %
  
BlastP bit score: 850
  
Sequence coverage: 48 %
  
E-value: 0.0
  
  
 NCBI BlastP on this gene

Pc22g24390

unnamed
  
Accession: CAP99728
  
Location: 5759388-5760239
  
 NCBI BlastP on this gene

Pc22g24400

not annotated
  
Accession: CAP99729
  
Location: 5760684-5762431
  
 NCBI BlastP on this gene

Pc22g24410

not annotated
  
Accession: CAP99730
  
Location: 5763884-5766004
  
 NCBI BlastP on this gene

Pc22g24420

not annotated
  
Accession: CAP99731
  
Location: 5767681-5769430
  
 NCBI BlastP on this gene

Pc22g24430

Query: Architecture Search FASTA input

AKCT01000122 : Penicillium digitatum PHI26    Total score: 2.0     Cumulative Blast bit score: 1012

Hit cluster cross-links:

Mycgr3G85918 Mycgr3T
  
Location: 0-1602

Mycgr3G85918\_Mycgr3T

Mycgr3G42010 Mycgr3T
  
Location: 1702-8569

Mycgr3G42010\_Mycgr3T

Mycgr3G29582 Mycgr3T
  
Location: 8669-8915

Mycgr3G29582\_Mycgr3T

Mycgr3G31170 Mycgr3T
  
Location: 9015-9255

Mycgr3G31170\_Mycgr3T

Mycgr3G85924 Mycgr3T
  
Location: 9355-11218

Mycgr3G85924\_Mycgr3T

Mycgr3G71676 Mycgr3T
  
Location: 11318-12494

Mycgr3G71676\_Mycgr3T

Mycgr3G11468 Mycgr3T
  
Location: 12594-13653

Mycgr3G11468\_Mycgr3T

Mycgr3G58567 Mycgr3T
  
Location: 13753-14506

Mycgr3G58567\_Mycgr3T

Mycgr3G100089 Mycgr3
  
Location: 14606-21152

Mycgr3G100089\_Mycgr3

Mycgr3G42698 Mycgr3T
  
Location: 21252-22131

Mycgr3G42698\_Mycgr3T

Mycgr3G71681 Mycgr3T
  
Location: 22231-23461

Mycgr3G71681\_Mycgr3T

Mycgr3G109328 Mycgr3
  
Location: 23561-24239

Mycgr3G109328\_Mycgr3

Mycgr3G104334 Mycgr3
  
Location: 24339-24567

Mycgr3G104334\_Mycgr3

Mycgr3G42715 Mycgr3T
  
Location: 24667-25981

Mycgr3G42715\_Mycgr3T

Mycgr3G92934 Mycgr3T
  
Location: 26081-27593

Mycgr3G92934\_Mycgr3T

Mycgr3G41969 Mycgr3T
  
Location: 27693-29328

Mycgr3G41969\_Mycgr3T

Mycgr3G80635 Mycgr3T
  
Location: 29428-29821

Mycgr3G80635\_Mycgr3T

Mycgr3G41426 Mycgr3T
  
Location: 29921-35255

Mycgr3G41426\_Mycgr3T

Mycgr3G104337 Mycgr3
  
Location: 35355-36108

Mycgr3G104337\_Mycgr3

Mycgr3G71679 Mycgr3T
  
Location: 36208-37300

Mycgr3G71679\_Mycgr3T

Mycgr3G92938 Mycgr3T
  
Location: 37400-38699

Mycgr3G92938\_Mycgr3T

Mycgr3G92941 Mycgr3T
  
Location: 38799-40734

Mycgr3G92941\_Mycgr3T

hypothetical protein
  
Accession: EKV15273
  
Location: 54373-55968
  
 NCBI BlastP on this gene

EKV15273

MADS box transcription factor Mcm1
  
Accession: EKV15272
  
Location: 51313-52098
  
  
**BlastP hit with Mycgr3G31170\_Mycgr3T**
  
Percentage identity: 98 %
  
BlastP bit score: 162
  
Sequence coverage: 100 %
  
E-value: 4e-48
  
  
 NCBI BlastP on this gene

EKV15272

hypothetical protein
  
Accession: EKV15271
  
Location: 47634-49624
  
 NCBI BlastP on this gene

EKV15271

ATP synthase subunit E, putative
  
Accession: EKV15270
  
Location: 46549-47363
  
 NCBI BlastP on this gene

EKV15270

hypothetical protein
  
Accession: EKV15269
  
Location: 43794-46167
  
 NCBI BlastP on this gene

EKV15269

hypothetical protein
  
Accession: EKV15268
  
Location: 36417-41149
  
 NCBI BlastP on this gene

EKV15268

tRNA-splicing endonuclease, putative
  
Accession: EKV15267
  
Location: 28635-35287
  
  
**BlastP hit with Mycgr3G41426\_Mycgr3T**
  
Percentage identity: 49 %
  
BlastP bit score: 850
  
Sequence coverage: 48 %
  
E-value: 0.0
  
  
 NCBI BlastP on this gene

EKV15267

hypothetical protein
  
Accession: EKV15266
  
Location: 26885-27730
  
 NCBI BlastP on this gene

EKV15266

NCS1 allantoate transporter
  
Accession: EKV15265
  
Location: 24629-26422
  
 NCBI BlastP on this gene

EKV15265

G1/S-specific cyclin Pcl5, putative
  
Accession: EKV15264
  
Location: 21222-23301
  
 NCBI BlastP on this gene

EKV15264

hypothetical protein
  
Accession: EKV15263
  
Location: 18799-19161
  
 NCBI BlastP on this gene

EKV15263

hypothetical protein
  
Accession: EKV15262
  
Location: 17596-17849
  
 NCBI BlastP on this gene

EKV15262

Query: Architecture Search FASTA input

AKCU01000418 : Penicillium digitatum Pd1    Total score: 2.0     Cumulative Blast bit score: 1010

Hit cluster cross-links:

Mycgr3G85918 Mycgr3T
  
Location: 0-1602

Mycgr3G85918\_Mycgr3T

Mycgr3G42010 Mycgr3T
  
Location: 1702-8569

Mycgr3G42010\_Mycgr3T

Mycgr3G29582 Mycgr3T
  
Location: 8669-8915

Mycgr3G29582\_Mycgr3T

Mycgr3G31170 Mycgr3T
  
Location: 9015-9255

Mycgr3G31170\_Mycgr3T

Mycgr3G85924 Mycgr3T
  
Location: 9355-11218

Mycgr3G85924\_Mycgr3T

Mycgr3G71676 Mycgr3T
  
Location: 11318-12494

Mycgr3G71676\_Mycgr3T

Mycgr3G11468 Mycgr3T
  
Location: 12594-13653

Mycgr3G11468\_Mycgr3T

Mycgr3G58567 Mycgr3T
  
Location: 13753-14506

Mycgr3G58567\_Mycgr3T

Mycgr3G100089 Mycgr3
  
Location: 14606-21152

Mycgr3G100089\_Mycgr3

Mycgr3G42698 Mycgr3T
  
Location: 21252-22131

Mycgr3G42698\_Mycgr3T

Mycgr3G71681 Mycgr3T
  
Location: 22231-23461

Mycgr3G71681\_Mycgr3T

Mycgr3G109328 Mycgr3
  
Location: 23561-24239

Mycgr3G109328\_Mycgr3

Mycgr3G104334 Mycgr3
  
Location: 24339-24567

Mycgr3G104334\_Mycgr3

Mycgr3G42715 Mycgr3T
  
Location: 24667-25981

Mycgr3G42715\_Mycgr3T

Mycgr3G92934 Mycgr3T
  
Location: 26081-27593

Mycgr3G92934\_Mycgr3T

Mycgr3G41969 Mycgr3T
  
Location: 27693-29328

Mycgr3G41969\_Mycgr3T

Mycgr3G80635 Mycgr3T
  
Location: 29428-29821

Mycgr3G80635\_Mycgr3T

Mycgr3G41426 Mycgr3T
  
Location: 29921-35255

Mycgr3G41426\_Mycgr3T

Mycgr3G104337 Mycgr3
  
Location: 35355-36108

Mycgr3G104337\_Mycgr3

Mycgr3G71679 Mycgr3T
  
Location: 36208-37300

Mycgr3G71679\_Mycgr3T

Mycgr3G92938 Mycgr3T
  
Location: 37400-38699

Mycgr3G92938\_Mycgr3T

Mycgr3G92941 Mycgr3T
  
Location: 38799-40734

Mycgr3G92941\_Mycgr3T

hypothetical protein
  
Accession: EKV10017
  
Location: 38002-39597
  
 NCBI BlastP on this gene

EKV10017

MADS box transcription factor Mcm1
  
Accession: EKV10016
  
Location: 34945-35730
  
  
**BlastP hit with Mycgr3G31170\_Mycgr3T**
  
Percentage identity: 98 %
  
BlastP bit score: 162
  
Sequence coverage: 100 %
  
E-value: 4e-48
  
  
 NCBI BlastP on this gene

EKV10016

hypothetical protein
  
Accession: EKV10015
  
Location: 31265-33255
  
 NCBI BlastP on this gene

EKV10015

ATP synthase subunit E, putative
  
Accession: EKV10014
  
Location: 30180-30994
  
 NCBI BlastP on this gene

EKV10014

hypothetical protein
  
Accession: EKV10013
  
Location: 27425-29798
  
 NCBI BlastP on this gene

EKV10013

hypothetical protein
  
Accession: EKV10012
  
Location: 20049-24781
  
 NCBI BlastP on this gene

EKV10012

tRNA-splicing endonuclease, putative
  
Accession: EKV10011
  
Location: 12267-18919
  
  
**BlastP hit with Mycgr3G41426\_Mycgr3T**
  
Percentage identity: 49 %
  
BlastP bit score: 849
  
Sequence coverage: 48 %
  
E-value: 0.0
  
  
 NCBI BlastP on this gene

EKV10011

hypothetical protein
  
Accession: EKV10010
  
Location: 10517-11362
  
 NCBI BlastP on this gene

EKV10010

NCS1 allantoate transporter
  
Accession: EKV10009
  
Location: 8259-10054
  
 NCBI BlastP on this gene

EKV10009

G1/S-specific cyclin Pcl5, putative
  
Accession: EKV10008
  
Location: 4852-6931
  
 NCBI BlastP on this gene

EKV10008

hypothetical protein
  
Accession: EKV10007
  
Location: 2429-2791
  
 NCBI BlastP on this gene

EKV10007

hypothetical protein
  
Accession: EKV10006
  
Location: 1226-1479
  
 NCBI BlastP on this gene

EKV10006

Query: Architecture Search FASTA input

DS544814 : Paracoccidioides brasiliensis Pb03 supercont1.12 genomic scaffold    Total score: 2.0     Cumulative Blast bit score: 1001

Hit cluster cross-links:

Mycgr3G85918 Mycgr3T
  
Location: 0-1602

Mycgr3G85918\_Mycgr3T

Mycgr3G42010 Mycgr3T
  
Location: 1702-8569

Mycgr3G42010\_Mycgr3T

Mycgr3G29582 Mycgr3T
  
Location: 8669-8915

Mycgr3G29582\_Mycgr3T

Mycgr3G31170 Mycgr3T
  
Location: 9015-9255

Mycgr3G31170\_Mycgr3T

Mycgr3G85924 Mycgr3T
  
Location: 9355-11218

Mycgr3G85924\_Mycgr3T

Mycgr3G71676 Mycgr3T
  
Location: 11318-12494

Mycgr3G71676\_Mycgr3T

Mycgr3G11468 Mycgr3T
  
Location: 12594-13653

Mycgr3G11468\_Mycgr3T

Mycgr3G58567 Mycgr3T
  
Location: 13753-14506

Mycgr3G58567\_Mycgr3T

Mycgr3G100089 Mycgr3
  
Location: 14606-21152

Mycgr3G100089\_Mycgr3

Mycgr3G42698 Mycgr3T
  
Location: 21252-22131

Mycgr3G42698\_Mycgr3T

Mycgr3G71681 Mycgr3T
  
Location: 22231-23461

Mycgr3G71681\_Mycgr3T

Mycgr3G109328 Mycgr3
  
Location: 23561-24239

Mycgr3G109328\_Mycgr3

Mycgr3G104334 Mycgr3
  
Location: 24339-24567

Mycgr3G104334\_Mycgr3

Mycgr3G42715 Mycgr3T
  
Location: 24667-25981

Mycgr3G42715\_Mycgr3T

Mycgr3G92934 Mycgr3T
  
Location: 26081-27593

Mycgr3G92934\_Mycgr3T

Mycgr3G41969 Mycgr3T
  
Location: 27693-29328

Mycgr3G41969\_Mycgr3T

Mycgr3G80635 Mycgr3T
  
Location: 29428-29821

Mycgr3G80635\_Mycgr3T

Mycgr3G41426 Mycgr3T
  
Location: 29921-35255

Mycgr3G41426\_Mycgr3T

Mycgr3G104337 Mycgr3
  
Location: 35355-36108

Mycgr3G104337\_Mycgr3

Mycgr3G71679 Mycgr3T
  
Location: 36208-37300

Mycgr3G71679\_Mycgr3T

Mycgr3G92938 Mycgr3T
  
Location: 37400-38699

Mycgr3G92938\_Mycgr3T

Mycgr3G92941 Mycgr3T
  
Location: 38799-40734

Mycgr3G92941\_Mycgr3T

tetraspanin
  
Accession: EEH16414
  
Location: 443623-447578
  
 NCBI BlastP on this gene

EEH16414

MADS box transcription factor Mcm1
  
Accession: EEH16415
  
Location: 450142-451180
  
  
**BlastP hit with Mycgr3G31170\_Mycgr3T**
  
Percentage identity: 100 %
  
BlastP bit score: 167
  
Sequence coverage: 100 %
  
E-value: 5e-50
  
  
 NCBI BlastP on this gene

EEH16415

conserved hypothetical protein
  
Accession: EEH16416
  
Location: 454132-456380
  
 NCBI BlastP on this gene

EEH16416

vacuolar ATP synthase subunit E
  
Accession: EEH16417
  
Location: 457142-457950
  
 NCBI BlastP on this gene

EEH16417

conserved hypothetical protein
  
Accession: EEH16418
  
Location: 458505-460962
  
 NCBI BlastP on this gene

EEH16418

metallophosphoesterase domain-containing protein
  
Accession: EEH16419
  
Location: 462822-463826
  
 NCBI BlastP on this gene

EEH16419

P-type ATPase
  
Accession: EEH16420
  
Location: 466173-470843
  
 NCBI BlastP on this gene

EEH16420

conserved hypothetical protein
  
Accession: EEH16421
  
Location: 473405-481190
  
  
**BlastP hit with Mycgr3G41426\_Mycgr3T**
  
Percentage identity: 50 %
  
BlastP bit score: 835
  
Sequence coverage: 48 %
  
E-value: 0.0
  
  
 NCBI BlastP on this gene

EEH16421

predicted protein
  
Accession: EEH16422
  
Location: 485315-488494
  
 NCBI BlastP on this gene

EEH16422

Query: Architecture Search FASTA input

CH476616 : Uncinocarpus reesii 1704 scaffold\_2 genomic scaffold    Total score: 2.0     Cumulative Blast bit score: 1001

Hit cluster cross-links:

Mycgr3G85918 Mycgr3T
  
Location: 0-1602

Mycgr3G85918\_Mycgr3T

Mycgr3G42010 Mycgr3T
  
Location: 1702-8569

Mycgr3G42010\_Mycgr3T

Mycgr3G29582 Mycgr3T
  
Location: 8669-8915

Mycgr3G29582\_Mycgr3T

Mycgr3G31170 Mycgr3T
  
Location: 9015-9255

Mycgr3G31170\_Mycgr3T

Mycgr3G85924 Mycgr3T
  
Location: 9355-11218

Mycgr3G85924\_Mycgr3T

Mycgr3G71676 Mycgr3T
  
Location: 11318-12494

Mycgr3G71676\_Mycgr3T

Mycgr3G11468 Mycgr3T
  
Location: 12594-13653

Mycgr3G11468\_Mycgr3T

Mycgr3G58567 Mycgr3T
  
Location: 13753-14506

Mycgr3G58567\_Mycgr3T

Mycgr3G100089 Mycgr3
  
Location: 14606-21152

Mycgr3G100089\_Mycgr3

Mycgr3G42698 Mycgr3T
  
Location: 21252-22131

Mycgr3G42698\_Mycgr3T

Mycgr3G71681 Mycgr3T
  
Location: 22231-23461

Mycgr3G71681\_Mycgr3T

Mycgr3G109328 Mycgr3
  
Location: 23561-24239

Mycgr3G109328\_Mycgr3

Mycgr3G104334 Mycgr3
  
Location: 24339-24567

Mycgr3G104334\_Mycgr3

Mycgr3G42715 Mycgr3T
  
Location: 24667-25981

Mycgr3G42715\_Mycgr3T

Mycgr3G92934 Mycgr3T
  
Location: 26081-27593

Mycgr3G92934\_Mycgr3T

Mycgr3G41969 Mycgr3T
  
Location: 27693-29328

Mycgr3G41969\_Mycgr3T

Mycgr3G80635 Mycgr3T
  
Location: 29428-29821

Mycgr3G80635\_Mycgr3T

Mycgr3G41426 Mycgr3T
  
Location: 29921-35255

Mycgr3G41426\_Mycgr3T

Mycgr3G104337 Mycgr3
  
Location: 35355-36108

Mycgr3G104337\_Mycgr3

Mycgr3G71679 Mycgr3T
  
Location: 36208-37300

Mycgr3G71679\_Mycgr3T

Mycgr3G92938 Mycgr3T
  
Location: 37400-38699

Mycgr3G92938\_Mycgr3T

Mycgr3G92941 Mycgr3T
  
Location: 38799-40734

Mycgr3G92941\_Mycgr3T

predicted protein
  
Accession: EEP79660
  
Location: 4565645-4566716
  
 NCBI BlastP on this gene

EEP79660

conserved hypothetical protein
  
Accession: EEP79661
  
Location: 4567101-4568592
  
 NCBI BlastP on this gene

EEP79661

predicted protein
  
Accession: EEP79662
  
Location: 4569101-4569979
  
 NCBI BlastP on this gene

EEP79662

conserved hypothetical protein
  
Accession: EEP79663
  
Location: 4570757-4572663
  
 NCBI BlastP on this gene

EEP79663

predicted protein
  
Accession: EEP79664
  
Location: 4575048-4575899
  
 NCBI BlastP on this gene

EEP79664

MCMA protein
  
Accession: EEP79665
  
Location: 4576943-4577833
  
  
**BlastP hit with Mycgr3G31170\_Mycgr3T**
  
Percentage identity: 82 %
  
BlastP bit score: 129
  
Sequence coverage: 100 %
  
E-value: 2e-35
  
  
 NCBI BlastP on this gene

EEP79665

conserved hypothetical protein
  
Accession: EEP79666
  
Location: 4579067-4580995
  
 NCBI BlastP on this gene

EEP79666

vacuolar ATP synthase subunit E
  
Accession: EEP79667
  
Location: 4581737-4582391
  
 NCBI BlastP on this gene

EEP79667

predicted protein
  
Accession: EEP79668
  
Location: 4582923-4585219
  
 NCBI BlastP on this gene

EEP79668

hypothetical protein
  
Accession: EEP79669
  
Location: 4586552-4591298
  
 NCBI BlastP on this gene

EEP79669

conserved hypothetical protein
  
Accession: EEP79670
  
Location: 4592600-4599396
  
  
**BlastP hit with Mycgr3G41426\_Mycgr3T**
  
Percentage identity: 54 %
  
BlastP bit score: 872
  
Sequence coverage: 43 %
  
E-value: 0.0
  
  
 NCBI BlastP on this gene

EEP79670

predicted protein
  
Accession: EEP79671
  
Location: 4600305-4604496
  
 NCBI BlastP on this gene

EEP79671

conserved hypothetical protein
  
Accession: EEP79672
  
Location: 4604962-4607058
  
 NCBI BlastP on this gene

EEP79672

conserved hypothetical protein
  
Accession: EEP79673
  
Location: 4607328-4609332
  
 NCBI BlastP on this gene

EEP79673

eukaryotic translation initiation factor 1A
  
Accession: EEP79674
  
Location: 4610328-4611010
  
 NCBI BlastP on this gene

EEP79674

Query: Architecture Search FASTA input

DS572762 : Paracoccidioides brasiliensis Pb18 supercont1.13 genomic scaffold    Total score: 2.0     Cumulative Blast bit score: 999

Hit cluster cross-links:

Mycgr3G85918 Mycgr3T
  
Location: 0-1602

Mycgr3G85918\_Mycgr3T

Mycgr3G42010 Mycgr3T
  
Location: 1702-8569

Mycgr3G42010\_Mycgr3T

Mycgr3G29582 Mycgr3T
  
Location: 8669-8915

Mycgr3G29582\_Mycgr3T

Mycgr3G31170 Mycgr3T
  
Location: 9015-9255

Mycgr3G31170\_Mycgr3T

Mycgr3G85924 Mycgr3T
  
Location: 9355-11218

Mycgr3G85924\_Mycgr3T

Mycgr3G71676 Mycgr3T
  
Location: 11318-12494

Mycgr3G71676\_Mycgr3T

Mycgr3G11468 Mycgr3T
  
Location: 12594-13653

Mycgr3G11468\_Mycgr3T

Mycgr3G58567 Mycgr3T
  
Location: 13753-14506

Mycgr3G58567\_Mycgr3T

Mycgr3G100089 Mycgr3
  
Location: 14606-21152

Mycgr3G100089\_Mycgr3

Mycgr3G42698 Mycgr3T
  
Location: 21252-22131

Mycgr3G42698\_Mycgr3T

Mycgr3G71681 Mycgr3T
  
Location: 22231-23461

Mycgr3G71681\_Mycgr3T

Mycgr3G109328 Mycgr3
  
Location: 23561-24239

Mycgr3G109328\_Mycgr3

Mycgr3G104334 Mycgr3
  
Location: 24339-24567

Mycgr3G104334\_Mycgr3

Mycgr3G42715 Mycgr3T
  
Location: 24667-25981

Mycgr3G42715\_Mycgr3T

Mycgr3G92934 Mycgr3T
  
Location: 26081-27593

Mycgr3G92934\_Mycgr3T

Mycgr3G41969 Mycgr3T
  
Location: 27693-29328

Mycgr3G41969\_Mycgr3T

Mycgr3G80635 Mycgr3T
  
Location: 29428-29821

Mycgr3G80635\_Mycgr3T

Mycgr3G41426 Mycgr3T
  
Location: 29921-35255

Mycgr3G41426\_Mycgr3T

Mycgr3G104337 Mycgr3
  
Location: 35355-36108

Mycgr3G104337\_Mycgr3

Mycgr3G71679 Mycgr3T
  
Location: 36208-37300

Mycgr3G71679\_Mycgr3T

Mycgr3G92938 Mycgr3T
  
Location: 37400-38699

Mycgr3G92938\_Mycgr3T

Mycgr3G92941 Mycgr3T
  
Location: 38799-40734

Mycgr3G92941\_Mycgr3T

conserved hypothetical protein
  
Accession: EEH43139
  
Location: 453166-454373
  
 NCBI BlastP on this gene

EEH43139

tetraspanin
  
Accession: EEH43140
  
Location: 455544-456600
  
 NCBI BlastP on this gene

EEH43140

conserved hypothetical protein
  
Accession: EEH43141
  
Location: 457321-458794
  
 NCBI BlastP on this gene

EEH43141

MADS box transcription factor Mcm1
  
Accession: EEH43142
  
Location: 462082-463123
  
  
**BlastP hit with Mycgr3G31170\_Mycgr3T**
  
Percentage identity: 100 %
  
BlastP bit score: 167
  
Sequence coverage: 100 %
  
E-value: 5e-50
  
  
 NCBI BlastP on this gene

EEH43142

DUF803 domain-containing protein
  
Accession: EEH43143
  
Location: 466041-468319
  
 NCBI BlastP on this gene

EEH43143

vacuolar ATP synthase subunit E
  
Accession: EEH43144
  
Location: 469073-469881
  
 NCBI BlastP on this gene

EEH43144

conserved hypothetical protein
  
Accession: EEH43145
  
Location: 470439-472896
  
 NCBI BlastP on this gene

EEH43145

ser/Thr protein phosphatase family protein
  
Accession: EEH43146
  
Location: 473638-475772
  
 NCBI BlastP on this gene

EEH43146

phospholipid-transporting ATPase
  
Accession: EEH43147
  
Location: 478156-482826
  
 NCBI BlastP on this gene

EEH43147

DEAD-box type RNA helicase
  
Accession: EEH43148
  
Location: 485389-492226
  
  
**BlastP hit with Mycgr3G41426\_Mycgr3T**
  
Percentage identity: 49 %
  
BlastP bit score: 832
  
Sequence coverage: 48 %
  
E-value: 0.0
  
  
 NCBI BlastP on this gene

EEH43148

predicted protein
  
Accession: EEH43149
  
Location: 495310-500451
  
 NCBI BlastP on this gene

EEH43149

Query: Architecture Search FASTA input

AFQF01002886 : Fusarium oxysporum Fo5176    Total score: 2.0     Cumulative Blast bit score: 992

Hit cluster cross-links:

Mycgr3G85918 Mycgr3T
  
Location: 0-1602

Mycgr3G85918\_Mycgr3T

Mycgr3G42010 Mycgr3T
  
Location: 1702-8569

Mycgr3G42010\_Mycgr3T

Mycgr3G29582 Mycgr3T
  
Location: 8669-8915

Mycgr3G29582\_Mycgr3T

Mycgr3G31170 Mycgr3T
  
Location: 9015-9255

Mycgr3G31170\_Mycgr3T

Mycgr3G85924 Mycgr3T
  
Location: 9355-11218

Mycgr3G85924\_Mycgr3T

Mycgr3G71676 Mycgr3T
  
Location: 11318-12494

Mycgr3G71676\_Mycgr3T

Mycgr3G11468 Mycgr3T
  
Location: 12594-13653

Mycgr3G11468\_Mycgr3T

Mycgr3G58567 Mycgr3T
  
Location: 13753-14506

Mycgr3G58567\_Mycgr3T

Mycgr3G100089 Mycgr3
  
Location: 14606-21152

Mycgr3G100089\_Mycgr3

Mycgr3G42698 Mycgr3T
  
Location: 21252-22131

Mycgr3G42698\_Mycgr3T

Mycgr3G71681 Mycgr3T
  
Location: 22231-23461

Mycgr3G71681\_Mycgr3T

Mycgr3G109328 Mycgr3
  
Location: 23561-24239

Mycgr3G109328\_Mycgr3

Mycgr3G104334 Mycgr3
  
Location: 24339-24567

Mycgr3G104334\_Mycgr3

Mycgr3G42715 Mycgr3T
  
Location: 24667-25981

Mycgr3G42715\_Mycgr3T

Mycgr3G92934 Mycgr3T
  
Location: 26081-27593

Mycgr3G92934\_Mycgr3T

Mycgr3G41969 Mycgr3T
  
Location: 27693-29328

Mycgr3G41969\_Mycgr3T

Mycgr3G80635 Mycgr3T
  
Location: 29428-29821

Mycgr3G80635\_Mycgr3T

Mycgr3G41426 Mycgr3T
  
Location: 29921-35255

Mycgr3G41426\_Mycgr3T

Mycgr3G104337 Mycgr3
  
Location: 35355-36108

Mycgr3G104337\_Mycgr3

Mycgr3G71679 Mycgr3T
  
Location: 36208-37300

Mycgr3G71679\_Mycgr3T

Mycgr3G92938 Mycgr3T
  
Location: 37400-38699

Mycgr3G92938\_Mycgr3T

Mycgr3G92941 Mycgr3T
  
Location: 38799-40734

Mycgr3G92941\_Mycgr3T

hypothetical protein
  
Accession: EGU78026
  
Location: 4184-5259
  
 NCBI BlastP on this gene

EGU78026

hypothetical protein
  
Accession: EGU78027
  
Location: 5426-7136
  
 NCBI BlastP on this gene

EGU78027

hypothetical protein
  
Accession: EGU78028
  
Location: 8780-10244
  
 NCBI BlastP on this gene

EGU78028

hypothetical protein
  
Accession: EGU78029
  
Location: 11467-14417
  
 NCBI BlastP on this gene

EGU78029

hypothetical protein
  
Accession: EGU78030
  
Location: 14802-17716
  
 NCBI BlastP on this gene

EGU78030

hypothetical protein
  
Accession: EGU78031
  
Location: 18079-19684
  
 NCBI BlastP on this gene

EGU78031

hypothetical protein
  
Accession: EGU78032
  
Location: 19903-21724
  
 NCBI BlastP on this gene

EGU78032

hypothetical protein
  
Accession: EGU78033
  
Location: 22102-22731
  
  
**BlastP hit with Mycgr3G104337\_Mycgr3**
  
Percentage identity: 27 %
  
BlastP bit score: 69
  
Sequence coverage: 54 %
  
E-value: 2e-11
  
  
 NCBI BlastP on this gene

EGU78033

hypothetical protein
  
Accession: EGU78034
  
Location: 23087-24148
  
 NCBI BlastP on this gene

EGU78034

hypothetical protein
  
Accession: EGU78035
  
Location: 24297-25976
  
 NCBI BlastP on this gene

EGU78035

hypothetical protein
  
Accession: EGU78036
  
Location: 26431-32436
  
  
**BlastP hit with Mycgr3G100089\_Mycgr3**
  
Percentage identity: 40 %
  
BlastP bit score: 923
  
Sequence coverage: 58 %
  
E-value: 0.0
  
  
 NCBI BlastP on this gene

EGU78036

Query: Architecture Search FASTA input

KB733479 : Bipolaris maydis ATCC 48331 unplaced genomic scaffold COCC4scaffold\_36    Total score: 2.0     Cumulative Blast bit score: 990

Hit cluster cross-links:

Mycgr3G85918 Mycgr3T
  
Location: 0-1602

Mycgr3G85918\_Mycgr3T

Mycgr3G42010 Mycgr3T
  
Location: 1702-8569

Mycgr3G42010\_Mycgr3T

Mycgr3G29582 Mycgr3T
  
Location: 8669-8915

Mycgr3G29582\_Mycgr3T

Mycgr3G31170 Mycgr3T
  
Location: 9015-9255

Mycgr3G31170\_Mycgr3T

Mycgr3G85924 Mycgr3T
  
Location: 9355-11218

Mycgr3G85924\_Mycgr3T

Mycgr3G71676 Mycgr3T
  
Location: 11318-12494

Mycgr3G71676\_Mycgr3T

Mycgr3G11468 Mycgr3T
  
Location: 12594-13653

Mycgr3G11468\_Mycgr3T

Mycgr3G58567 Mycgr3T
  
Location: 13753-14506

Mycgr3G58567\_Mycgr3T

Mycgr3G100089 Mycgr3
  
Location: 14606-21152

Mycgr3G100089\_Mycgr3

Mycgr3G42698 Mycgr3T
  
Location: 21252-22131

Mycgr3G42698\_Mycgr3T

Mycgr3G71681 Mycgr3T
  
Location: 22231-23461

Mycgr3G71681\_Mycgr3T

Mycgr3G109328 Mycgr3
  
Location: 23561-24239

Mycgr3G109328\_Mycgr3

Mycgr3G104334 Mycgr3
  
Location: 24339-24567

Mycgr3G104334\_Mycgr3

Mycgr3G42715 Mycgr3T
  
Location: 24667-25981

Mycgr3G42715\_Mycgr3T

Mycgr3G92934 Mycgr3T
  
Location: 26081-27593

Mycgr3G92934\_Mycgr3T

Mycgr3G41969 Mycgr3T
  
Location: 27693-29328

Mycgr3G41969\_Mycgr3T

Mycgr3G80635 Mycgr3T
  
Location: 29428-29821

Mycgr3G80635\_Mycgr3T

Mycgr3G41426 Mycgr3T
  
Location: 29921-35255

Mycgr3G41426\_Mycgr3T

Mycgr3G104337 Mycgr3
  
Location: 35355-36108

Mycgr3G104337\_Mycgr3

Mycgr3G71679 Mycgr3T
  
Location: 36208-37300

Mycgr3G71679\_Mycgr3T

Mycgr3G92938 Mycgr3T
  
Location: 37400-38699

Mycgr3G92938\_Mycgr3T

Mycgr3G92941 Mycgr3T
  
Location: 38799-40734

Mycgr3G92941\_Mycgr3T

glycoside hydrolase family 10 protein
  
Accession: ENH99962
  
Location: 26057-27649
  
 NCBI BlastP on this gene

ENH99962

hypothetical protein
  
Accession: ENH99963
  
Location: 28749-30662
  
 NCBI BlastP on this gene

ENH99963

hypothetical protein
  
Accession: ENH99964
  
Location: 30844-32148
  
 NCBI BlastP on this gene

ENH99964

hypothetical protein
  
Accession: ENH99965
  
Location: 32559-34580
  
 NCBI BlastP on this gene

ENH99965

hypothetical protein
  
Accession: ENH99966
  
Location: 34828-35504
  
 NCBI BlastP on this gene

ENH99966

hypothetical protein
  
Accession: ENH99967
  
Location: 35926-36687
  
  
**BlastP hit with Mycgr3G104337\_Mycgr3**
  
Percentage identity: 30 %
  
BlastP bit score: 93
  
Sequence coverage: 91 %
  
E-value: 2e-19
  
  
 NCBI BlastP on this gene

ENH99967

hypothetical protein
  
Accession: ENH99968
  
Location: 37625-39040
  
 NCBI BlastP on this gene

ENH99968

hypothetical protein
  
Accession: ENH99969
  
Location: 39445-40960
  
 NCBI BlastP on this gene

ENH99969

hypothetical protein
  
Accession: ENH99970
  
Location: 41421-43013
  
 NCBI BlastP on this gene

ENH99970

hypothetical protein
  
Accession: ENH99971
  
Location: 43634-44236
  
 NCBI BlastP on this gene

ENH99971

hypothetical protein
  
Accession: ENH99972
  
Location: 46047-47312
  
 NCBI BlastP on this gene

ENH99972

hypothetical protein
  
Accession: ENH99973
  
Location: 47313-47492
  
 NCBI BlastP on this gene

ENH99973

hypothetical protein
  
Accession: ENH99974
  
Location: 51033-51626
  
 NCBI BlastP on this gene

ENH99974

hypothetical protein
  
Accession: ENH99975
  
Location: 52102-52992
  
 NCBI BlastP on this gene

ENH99975

hypothetical protein
  
Accession: ENH99976
  
Location: 53733-54502
  
 NCBI BlastP on this gene

ENH99976

hypothetical protein
  
Accession: ENH99977
  
Location: 54941-62407
  
  
**BlastP hit with Mycgr3G100089\_Mycgr3**
  
Percentage identity: 39 %
  
BlastP bit score: 897
  
Sequence coverage: 56 %
  
E-value: 0.0
  
  
 NCBI BlastP on this gene

ENH99977

hypothetical protein
  
Accession: ENH99978
  
Location: 62947-64764
  
 NCBI BlastP on this gene

ENH99978

hypothetical protein
  
Accession: ENH99979
  
Location: 65236-67800
  
 NCBI BlastP on this gene

ENH99979

hypothetical protein
  
Accession: ENH99980
  
Location: 68507-69154
  
 NCBI BlastP on this gene

ENH99980

hypothetical protein
  
Accession: ENH99981
  
Location: 69807-71949
  
 NCBI BlastP on this gene

ENH99981

Query: Architecture Search FASTA input

KB445588 : Cochliobolus heterostrophus C5 unplaced genomic scaffold COCHEscaffold\_20    Total score: 2.0     Cumulative Blast bit score: 990

Hit cluster cross-links:

Mycgr3G85918 Mycgr3T
  
Location: 0-1602

Mycgr3G85918\_Mycgr3T

Mycgr3G42010 Mycgr3T
  
Location: 1702-8569

Mycgr3G42010\_Mycgr3T

Mycgr3G29582 Mycgr3T
  
Location: 8669-8915

Mycgr3G29582\_Mycgr3T

Mycgr3G31170 Mycgr3T
  
Location: 9015-9255

Mycgr3G31170\_Mycgr3T

Mycgr3G85924 Mycgr3T
  
Location: 9355-11218

Mycgr3G85924\_Mycgr3T

Mycgr3G71676 Mycgr3T
  
Location: 11318-12494

Mycgr3G71676\_Mycgr3T

Mycgr3G11468 Mycgr3T
  
Location: 12594-13653

Mycgr3G11468\_Mycgr3T

Mycgr3G58567 Mycgr3T
  
Location: 13753-14506

Mycgr3G58567\_Mycgr3T

Mycgr3G100089 Mycgr3
  
Location: 14606-21152

Mycgr3G100089\_Mycgr3

Mycgr3G42698 Mycgr3T
  
Location: 21252-22131

Mycgr3G42698\_Mycgr3T

Mycgr3G71681 Mycgr3T
  
Location: 22231-23461

Mycgr3G71681\_Mycgr3T

Mycgr3G109328 Mycgr3
  
Location: 23561-24239

Mycgr3G109328\_Mycgr3

Mycgr3G104334 Mycgr3
  
Location: 24339-24567

Mycgr3G104334\_Mycgr3

Mycgr3G42715 Mycgr3T
  
Location: 24667-25981

Mycgr3G42715\_Mycgr3T

Mycgr3G92934 Mycgr3T
  
Location: 26081-27593

Mycgr3G92934\_Mycgr3T

Mycgr3G41969 Mycgr3T
  
Location: 27693-29328

Mycgr3G41969\_Mycgr3T

Mycgr3G80635 Mycgr3T
  
Location: 29428-29821

Mycgr3G80635\_Mycgr3T

Mycgr3G41426 Mycgr3T
  
Location: 29921-35255

Mycgr3G41426\_Mycgr3T

Mycgr3G104337 Mycgr3
  
Location: 35355-36108

Mycgr3G104337\_Mycgr3

Mycgr3G71679 Mycgr3T
  
Location: 36208-37300

Mycgr3G71679\_Mycgr3T

Mycgr3G92938 Mycgr3T
  
Location: 37400-38699

Mycgr3G92938\_Mycgr3T

Mycgr3G92941 Mycgr3T
  
Location: 38799-40734

Mycgr3G92941\_Mycgr3T

glycoside hydrolase family 10 protein
  
Accession: EMD85586
  
Location: 350486-352078
  
 NCBI BlastP on this gene

EMD85586

hypothetical protein
  
Accession: EMD85585
  
Location: 347473-349386
  
 NCBI BlastP on this gene

EMD85585

hypothetical protein
  
Accession: EMD85584
  
Location: 345987-347291
  
 NCBI BlastP on this gene

EMD85584

hypothetical protein
  
Accession: EMD85583
  
Location: 343555-345576
  
 NCBI BlastP on this gene

EMD85583

hypothetical protein
  
Accession: EMD85582
  
Location: 342631-343307
  
 NCBI BlastP on this gene

EMD85582

hypothetical protein
  
Accession: EMD85581
  
Location: 341448-342209
  
  
**BlastP hit with Mycgr3G104337\_Mycgr3**
  
Percentage identity: 30 %
  
BlastP bit score: 93
  
Sequence coverage: 91 %
  
E-value: 2e-19
  
  
 NCBI BlastP on this gene

EMD85581

hypothetical protein
  
Accession: EMD85580
  
Location: 339095-340510
  
 NCBI BlastP on this gene

EMD85580

hypothetical protein
  
Accession: EMD85579
  
Location: 337175-338690
  
 NCBI BlastP on this gene

EMD85579

hypothetical protein
  
Accession: EMD85578
  
Location: 335122-336714
  
 NCBI BlastP on this gene

EMD85578

hypothetical protein
  
Accession: EMD85577
  
Location: 333896-334588
  
 NCBI BlastP on this gene

EMD85577

hypothetical protein
  
Accession: EMD85576
  
Location: 333283-333522
  
 NCBI BlastP on this gene

EMD85576

hypothetical protein
  
Accession: EMD85575
  
Location: 330438-331421
  
 NCBI BlastP on this gene

EMD85575

hypothetical protein
  
Accession: EMD85574
  
Location: 330258-330437
  
 NCBI BlastP on this gene

EMD85574

hypothetical protein
  
Accession: EMD85573
  
Location: 326124-326717
  
 NCBI BlastP on this gene

EMD85573

hypothetical protein
  
Accession: EMD85572
  
Location: 324758-325648
  
 NCBI BlastP on this gene

EMD85572

hypothetical protein
  
Accession: EMD85571
  
Location: 323248-324017
  
 NCBI BlastP on this gene

EMD85571

hypothetical protein
  
Accession: EMD85570
  
Location: 315343-322809
  
  
**BlastP hit with Mycgr3G100089\_Mycgr3**
  
Percentage identity: 39 %
  
BlastP bit score: 897
  
Sequence coverage: 56 %
  
E-value: 0.0
  
  
 NCBI BlastP on this gene

EMD85570

hypothetical protein
  
Accession: EMD85569
  
Location: 312986-314803
  
 NCBI BlastP on this gene

EMD85569

hypothetical protein
  
Accession: EMD85568
  
Location: 309950-312514
  
 NCBI BlastP on this gene

EMD85568

hypothetical protein
  
Accession: EMD85567
  
Location: 308596-309243
  
 NCBI BlastP on this gene

EMD85567

hypothetical protein
  
Accession: EMD85566
  
Location: 307703-308011
  
 NCBI BlastP on this gene

EMD85566

hypothetical protein
  
Accession: EMD85565
  
Location: 305785-307399
  
 NCBI BlastP on this gene

EMD85565

Query: Architecture Search FASTA input

DS499596 : Aspergillus fumigatus A1163 scf\_000003 genomic scaffold    Total score: 2.0     Cumulative Blast bit score: 989

Hit cluster cross-links:

Mycgr3G85918 Mycgr3T
  
Location: 0-1602

Mycgr3G85918\_Mycgr3T

Mycgr3G42010 Mycgr3T
  
Location: 1702-8569

Mycgr3G42010\_Mycgr3T

Mycgr3G29582 Mycgr3T
  
Location: 8669-8915

Mycgr3G29582\_Mycgr3T

Mycgr3G31170 Mycgr3T
  
Location: 9015-9255

Mycgr3G31170\_Mycgr3T

Mycgr3G85924 Mycgr3T
  
Location: 9355-11218

Mycgr3G85924\_Mycgr3T

Mycgr3G71676 Mycgr3T
  
Location: 11318-12494

Mycgr3G71676\_Mycgr3T

Mycgr3G11468 Mycgr3T
  
Location: 12594-13653

Mycgr3G11468\_Mycgr3T

Mycgr3G58567 Mycgr3T
  
Location: 13753-14506

Mycgr3G58567\_Mycgr3T

Mycgr3G100089 Mycgr3
  
Location: 14606-21152

Mycgr3G100089\_Mycgr3

Mycgr3G42698 Mycgr3T
  
Location: 21252-22131

Mycgr3G42698\_Mycgr3T

Mycgr3G71681 Mycgr3T
  
Location: 22231-23461

Mycgr3G71681\_Mycgr3T

Mycgr3G109328 Mycgr3
  
Location: 23561-24239

Mycgr3G109328\_Mycgr3

Mycgr3G104334 Mycgr3
  
Location: 24339-24567

Mycgr3G104334\_Mycgr3

Mycgr3G42715 Mycgr3T
  
Location: 24667-25981

Mycgr3G42715\_Mycgr3T

Mycgr3G92934 Mycgr3T
  
Location: 26081-27593

Mycgr3G92934\_Mycgr3T

Mycgr3G41969 Mycgr3T
  
Location: 27693-29328

Mycgr3G41969\_Mycgr3T

Mycgr3G80635 Mycgr3T
  
Location: 29428-29821

Mycgr3G80635\_Mycgr3T

Mycgr3G41426 Mycgr3T
  
Location: 29921-35255

Mycgr3G41426\_Mycgr3T

Mycgr3G104337 Mycgr3
  
Location: 35355-36108

Mycgr3G104337\_Mycgr3

Mycgr3G71679 Mycgr3T
  
Location: 36208-37300

Mycgr3G71679\_Mycgr3T

Mycgr3G92938 Mycgr3T
  
Location: 37400-38699

Mycgr3G92938\_Mycgr3T

Mycgr3G92941 Mycgr3T
  
Location: 38799-40734

Mycgr3G92941\_Mycgr3T

alcohol dehydrogenase, putative
  
Accession: EDP53510
  
Location: 3736754-3737924
  
 NCBI BlastP on this gene

EDP53510

conserved hypothetical protein
  
Accession: EDP53511
  
Location: 3741340-3743238
  
 NCBI BlastP on this gene

EDP53511

conserved hypothetical protein
  
Accession: EDP53512
  
Location: 3743461-3744473
  
 NCBI BlastP on this gene

EDP53512

salicylate hydroxylase, putative
  
Accession: EDP53513
  
Location: 3745387-3746733
  
 NCBI BlastP on this gene

EDP53513

3-methyl-2-oxobutanoate dehydrogenase, putative
  
Accession: EDP53514
  
Location: 3747121-3748408
  
 NCBI BlastP on this gene

EDP53514

DUF1212 domain membrane protein
  
Accession: EDP53515
  
Location: 3749984-3752521
  
 NCBI BlastP on this gene

EDP53515

benzodiazepine receptor family protein
  
Accession: EDP53516
  
Location: 3753761-3754392
  
 NCBI BlastP on this gene

EDP53516

DUF341 domain oxidoreductase, putative
  
Accession: EDP53517
  
Location: 3755108-3755883
  
  
**BlastP hit with Mycgr3G104337\_Mycgr3**
  
Percentage identity: 26 %
  
BlastP bit score: 92
  
Sequence coverage: 91 %
  
E-value: 3e-19
  
  
 NCBI BlastP on this gene

EDP53517

polyketide synthase, putative
  
Accession: EDP53518
  
Location: 3756174-3764303
  
  
**BlastP hit with Mycgr3G100089\_Mycgr3**
  
Percentage identity: 40 %
  
BlastP bit score: 897
  
Sequence coverage: 56 %
  
E-value: 0.0
  
  
 NCBI BlastP on this gene

EDP53518

ABC multidrug transporter, putative
  
Accession: EDP53519
  
Location: 3764538-3768960
  
 NCBI BlastP on this gene

EDP53519

MFS transporter, putative
  
Accession: EDP53520
  
Location: 3777918-3779569
  
 NCBI BlastP on this gene

EDP53520

siderochrome-iron transporter, putative
  
Accession: EDP53521
  
Location: 3781715-3783946
  
 NCBI BlastP on this gene

EDP53521

Query: Architecture Search FASTA input

AAHF01000010 : Aspergillus fumigatus Af293    Total score: 2.0     Cumulative Blast bit score: 988

Hit cluster cross-links:

Mycgr3G85918 Mycgr3T
  
Location: 0-1602

Mycgr3G85918\_Mycgr3T

Mycgr3G42010 Mycgr3T
  
Location: 1702-8569

Mycgr3G42010\_Mycgr3T

Mycgr3G29582 Mycgr3T
  
Location: 8669-8915

Mycgr3G29582\_Mycgr3T

Mycgr3G31170 Mycgr3T
  
Location: 9015-9255

Mycgr3G31170\_Mycgr3T

Mycgr3G85924 Mycgr3T
  
Location: 9355-11218

Mycgr3G85924\_Mycgr3T

Mycgr3G71676 Mycgr3T
  
Location: 11318-12494

Mycgr3G71676\_Mycgr3T

Mycgr3G11468 Mycgr3T
  
Location: 12594-13653

Mycgr3G11468\_Mycgr3T

Mycgr3G58567 Mycgr3T
  
Location: 13753-14506

Mycgr3G58567\_Mycgr3T

Mycgr3G100089 Mycgr3
  
Location: 14606-21152

Mycgr3G100089\_Mycgr3

Mycgr3G42698 Mycgr3T
  
Location: 21252-22131

Mycgr3G42698\_Mycgr3T

Mycgr3G71681 Mycgr3T
  
Location: 22231-23461

Mycgr3G71681\_Mycgr3T

Mycgr3G109328 Mycgr3
  
Location: 23561-24239

Mycgr3G109328\_Mycgr3

Mycgr3G104334 Mycgr3
  
Location: 24339-24567

Mycgr3G104334\_Mycgr3

Mycgr3G42715 Mycgr3T
  
Location: 24667-25981

Mycgr3G42715\_Mycgr3T

Mycgr3G92934 Mycgr3T
  
Location: 26081-27593

Mycgr3G92934\_Mycgr3T

Mycgr3G41969 Mycgr3T
  
Location: 27693-29328

Mycgr3G41969\_Mycgr3T

Mycgr3G80635 Mycgr3T
  
Location: 29428-29821

Mycgr3G80635\_Mycgr3T

Mycgr3G41426 Mycgr3T
  
Location: 29921-35255

Mycgr3G41426\_Mycgr3T

Mycgr3G104337 Mycgr3
  
Location: 35355-36108

Mycgr3G104337\_Mycgr3

Mycgr3G71679 Mycgr3T
  
Location: 36208-37300

Mycgr3G71679\_Mycgr3T

Mycgr3G92938 Mycgr3T
  
Location: 37400-38699

Mycgr3G92938\_Mycgr3T

Mycgr3G92941 Mycgr3T
  
Location: 38799-40734

Mycgr3G92941\_Mycgr3T

alcohol dehydrogenase, putative
  
Accession: EAL86432
  
Location: 379234-380404
  
 NCBI BlastP on this gene

EAL86432

conserved hypothetical protein
  
Accession: EAL86431
  
Location: 373921-375819
  
 NCBI BlastP on this gene

EAL86431

conserved hypothetical protein
  
Accession: EAL86430
  
Location: 372686-373698
  
 NCBI BlastP on this gene

EAL86430

salicylate hydroxylase, putative
  
Accession: EAL86429
  
Location: 370426-371772
  
 NCBI BlastP on this gene

EAL86429

3-methyl-2-oxobutanoate dehydrogenase, putative
  
Accession: EAL86428
  
Location: 368751-370038
  
 NCBI BlastP on this gene

EAL86428

DUF1212 domain membrane protein
  
Accession: EAL86427
  
Location: 364638-367175
  
 NCBI BlastP on this gene

EAL86427

benzodiazepine receptor family protein
  
Accession: EAL86426
  
Location: 362767-363398
  
 NCBI BlastP on this gene

EAL86426

DUF341 family oxidoreductase, putative
  
Accession: EAL86425
  
Location: 361276-362051
  
  
**BlastP hit with Mycgr3G104337\_Mycgr3**
  
Percentage identity: 26 %
  
BlastP bit score: 92
  
Sequence coverage: 91 %
  
E-value: 3e-19
  
  
 NCBI BlastP on this gene

EAL86425

polyketide synthase, putative
  
Accession: EAL86424
  
Location: 352856-360985
  
  
**BlastP hit with Mycgr3G100089\_Mycgr3**
  
Percentage identity: 40 %
  
BlastP bit score: 896
  
Sequence coverage: 56 %
  
E-value: 0.0
  
  
 NCBI BlastP on this gene

EAL86424

ABC multidrug transporter, putative
  
Accession: EAL86423
  
Location: 348200-352621
  
 NCBI BlastP on this gene

EAL86423

MFS transporter, putative
  
Accession: EAL86420
  
Location: 337589-339241
  
 NCBI BlastP on this gene

EAL86420

siderochrome-iron transporter, putative
  
Accession: EAL86419
  
Location: 333213-335443
  
 NCBI BlastP on this gene

EAL86419

Query: Architecture Search FASTA input

DS027697 : Neosartorya fischeri NRRL 181 1099437636265 genomic scaffold    Total score: 2.0     Cumulative Blast bit score: 969

Hit cluster cross-links:

Mycgr3G85918 Mycgr3T
  
Location: 0-1602

Mycgr3G85918\_Mycgr3T

Mycgr3G42010 Mycgr3T
  
Location: 1702-8569

Mycgr3G42010\_Mycgr3T

Mycgr3G29582 Mycgr3T
  
Location: 8669-8915

Mycgr3G29582\_Mycgr3T

Mycgr3G31170 Mycgr3T
  
Location: 9015-9255

Mycgr3G31170\_Mycgr3T

Mycgr3G85924 Mycgr3T
  
Location: 9355-11218

Mycgr3G85924\_Mycgr3T

Mycgr3G71676 Mycgr3T
  
Location: 11318-12494

Mycgr3G71676\_Mycgr3T

Mycgr3G11468 Mycgr3T
  
Location: 12594-13653

Mycgr3G11468\_Mycgr3T

Mycgr3G58567 Mycgr3T
  
Location: 13753-14506

Mycgr3G58567\_Mycgr3T

Mycgr3G100089 Mycgr3
  
Location: 14606-21152

Mycgr3G100089\_Mycgr3

Mycgr3G42698 Mycgr3T
  
Location: 21252-22131

Mycgr3G42698\_Mycgr3T

Mycgr3G71681 Mycgr3T
  
Location: 22231-23461

Mycgr3G71681\_Mycgr3T

Mycgr3G109328 Mycgr3
  
Location: 23561-24239

Mycgr3G109328\_Mycgr3

Mycgr3G104334 Mycgr3
  
Location: 24339-24567

Mycgr3G104334\_Mycgr3

Mycgr3G42715 Mycgr3T
  
Location: 24667-25981

Mycgr3G42715\_Mycgr3T

Mycgr3G92934 Mycgr3T
  
Location: 26081-27593

Mycgr3G92934\_Mycgr3T

Mycgr3G41969 Mycgr3T
  
Location: 27693-29328

Mycgr3G41969\_Mycgr3T

Mycgr3G80635 Mycgr3T
  
Location: 29428-29821

Mycgr3G80635\_Mycgr3T

Mycgr3G41426 Mycgr3T
  
Location: 29921-35255

Mycgr3G41426\_Mycgr3T

Mycgr3G104337 Mycgr3
  
Location: 35355-36108

Mycgr3G104337\_Mycgr3

Mycgr3G71679 Mycgr3T
  
Location: 36208-37300

Mycgr3G71679\_Mycgr3T

Mycgr3G92938 Mycgr3T
  
Location: 37400-38699

Mycgr3G92938\_Mycgr3T

Mycgr3G92941 Mycgr3T
  
Location: 38799-40734

Mycgr3G92941\_Mycgr3T

protein kinase, putative
  
Accession: EAW16894
  
Location: 548385-549274
  
 NCBI BlastP on this gene

EAW16894

conserved hypothetical protein
  
Accession: EAW16893
  
Location: 545197-547093
  
 NCBI BlastP on this gene

EAW16893

conserved hypothetical protein
  
Accession: EAW16892
  
Location: 543963-544970
  
 NCBI BlastP on this gene

EAW16892

salicylate hydroxylase, putative
  
Accession: EAW16891
  
Location: 541711-543057
  
 NCBI BlastP on this gene

EAW16891

2-oxoisovalerate dehydrogenase
  
Accession: EAW16890
  
Location: 540045-541033
  
 NCBI BlastP on this gene

EAW16890

conserved hypothetical protein
  
Accession: EAW16889
  
Location: 533861-536401
  
 NCBI BlastP on this gene

EAW16889

benzodiazepine receptor family protein
  
Accession: EAW16888
  
Location: 532012-532579
  
 NCBI BlastP on this gene

EAW16888

conserved hypothetical protein
  
Accession: EAW16887
  
Location: 530518-531292
  
  
**BlastP hit with Mycgr3G104337\_Mycgr3**
  
Percentage identity: 26 %
  
BlastP bit score: 90
  
Sequence coverage: 91 %
  
E-value: 1e-18
  
  
 NCBI BlastP on this gene

EAW16887

polyketide synthase, putative
  
Accession: EAW16886
  
Location: 522093-530226
  
  
**BlastP hit with Mycgr3G100089\_Mycgr3**
  
Percentage identity: 38 %
  
BlastP bit score: 879
  
Sequence coverage: 59 %
  
E-value: 0.0
  
  
 NCBI BlastP on this gene

EAW16886

ABC multidrug transporter, putative
  
Accession: EAW16885
  
Location: 517448-521866
  
 NCBI BlastP on this gene

EAW16885

MFS transporter, putative
  
Accession: EAW16884
  
Location: 508541-510186
  
 NCBI BlastP on this gene

EAW16884

Query: Architecture Search FASTA input

DS572827 : Paracoccidioides brasiliensis Pb01 supercont1.17 genomic scaffold    Total score: 2.0     Cumulative Blast bit score: 963

Hit cluster cross-links:

Mycgr3G85918 Mycgr3T
  
Location: 0-1602

Mycgr3G85918\_Mycgr3T

Mycgr3G42010 Mycgr3T
  
Location: 1702-8569

Mycgr3G42010\_Mycgr3T

Mycgr3G29582 Mycgr3T
  
Location: 8669-8915

Mycgr3G29582\_Mycgr3T

Mycgr3G31170 Mycgr3T
  
Location: 9015-9255

Mycgr3G31170\_Mycgr3T

Mycgr3G85924 Mycgr3T
  
Location: 9355-11218

Mycgr3G85924\_Mycgr3T

Mycgr3G71676 Mycgr3T
  
Location: 11318-12494

Mycgr3G71676\_Mycgr3T

Mycgr3G11468 Mycgr3T
  
Location: 12594-13653

Mycgr3G11468\_Mycgr3T

Mycgr3G58567 Mycgr3T
  
Location: 13753-14506

Mycgr3G58567\_Mycgr3T

Mycgr3G100089 Mycgr3
  
Location: 14606-21152

Mycgr3G100089\_Mycgr3

Mycgr3G42698 Mycgr3T
  
Location: 21252-22131

Mycgr3G42698\_Mycgr3T

Mycgr3G71681 Mycgr3T
  
Location: 22231-23461

Mycgr3G71681\_Mycgr3T

Mycgr3G109328 Mycgr3
  
Location: 23561-24239

Mycgr3G109328\_Mycgr3

Mycgr3G104334 Mycgr3
  
Location: 24339-24567

Mycgr3G104334\_Mycgr3

Mycgr3G42715 Mycgr3T
  
Location: 24667-25981

Mycgr3G42715\_Mycgr3T

Mycgr3G92934 Mycgr3T
  
Location: 26081-27593

Mycgr3G92934\_Mycgr3T

Mycgr3G41969 Mycgr3T
  
Location: 27693-29328

Mycgr3G41969\_Mycgr3T

Mycgr3G80635 Mycgr3T
  
Location: 29428-29821

Mycgr3G80635\_Mycgr3T

Mycgr3G41426 Mycgr3T
  
Location: 29921-35255

Mycgr3G41426\_Mycgr3T

Mycgr3G104337 Mycgr3
  
Location: 35355-36108

Mycgr3G104337\_Mycgr3

Mycgr3G71679 Mycgr3T
  
Location: 36208-37300

Mycgr3G71679\_Mycgr3T

Mycgr3G92938 Mycgr3T
  
Location: 37400-38699

Mycgr3G92938\_Mycgr3T

Mycgr3G92941 Mycgr3T
  
Location: 38799-40734

Mycgr3G92941\_Mycgr3T

conserved hypothetical protein
  
Accession: EEH35115
  
Location: 313391-314598
  
 NCBI BlastP on this gene

EEH35115

tetraspanin
  
Accession: EEH35114
  
Location: 311054-312095
  
 NCBI BlastP on this gene

EEH35114

predicted protein
  
Accession: EEH35113
  
Location: 310226-310632
  
 NCBI BlastP on this gene

EEH35113

C6 zinc finger domain-containing protein
  
Accession: EEH35112
  
Location: 307786-309192
  
 NCBI BlastP on this gene

EEH35112

MADS box transcription factor Mcm1
  
Accession: EEH35111
  
Location: 304225-306738
  
  
**BlastP hit with Mycgr3G31170\_Mycgr3T**
  
Percentage identity: 100 %
  
BlastP bit score: 164
  
Sequence coverage: 100 %
  
E-value: 3e-46
  
  
 NCBI BlastP on this gene

EEH35111

predicted protein
  
Accession: EEH35110
  
Location: 303149-303555
  
 NCBI BlastP on this gene

EEH35110

DUF803 domain-containing protein
  
Accession: EEH35109
  
Location: 298925-301194
  
 NCBI BlastP on this gene

EEH35109

vacuolar ATP synthase subunit E
  
Accession: EEH35108
  
Location: 297364-298325
  
 NCBI BlastP on this gene

EEH35108

conserved hypothetical protein
  
Accession: EEH35107
  
Location: 294355-297198
  
 NCBI BlastP on this gene

EEH35107

ser/Thr protein phosphatase family protein
  
Accession: EEH35106
  
Location: 291449-292471
  
 NCBI BlastP on this gene

EEH35106

predicted protein
  
Accession: EEH35104
  
Location: 289544-290489
  
 NCBI BlastP on this gene

EEH35104

phospholipid-translocating P-type ATPase domain-containing protein
  
Accession: EEH35105
  
Location: 284504-289177
  
 NCBI BlastP on this gene

EEH35105

conserved hypothetical protein
  
Accession: EEH35103
  
Location: 275041-281805
  
  
**BlastP hit with Mycgr3G41426\_Mycgr3T**
  
Percentage identity: 48 %
  
BlastP bit score: 799
  
Sequence coverage: 48 %
  
E-value: 0.0
  
  
 NCBI BlastP on this gene

EEH35103

predicted protein
  
Accession: EEH35102
  
Location: 273554-274067
  
 NCBI BlastP on this gene

EEH35102

predicted protein
  
Accession: EEH35101
  
Location: 266601-269921
  
 NCBI BlastP on this gene

EEH35101

Query: Architecture Search FASTA input

DS572697 : Verticillium dahliae VdLs.17 supercont1.3 genomic scaffold    Total score: 2.0     Cumulative Blast bit score: 942

Hit cluster cross-links:

Mycgr3G85918 Mycgr3T
  
Location: 0-1602

Mycgr3G85918\_Mycgr3T

Mycgr3G42010 Mycgr3T
  
Location: 1702-8569

Mycgr3G42010\_Mycgr3T

Mycgr3G29582 Mycgr3T
  
Location: 8669-8915

Mycgr3G29582\_Mycgr3T

Mycgr3G31170 Mycgr3T
  
Location: 9015-9255

Mycgr3G31170\_Mycgr3T

Mycgr3G85924 Mycgr3T
  
Location: 9355-11218

Mycgr3G85924\_Mycgr3T

Mycgr3G71676 Mycgr3T
  
Location: 11318-12494

Mycgr3G71676\_Mycgr3T

Mycgr3G11468 Mycgr3T
  
Location: 12594-13653

Mycgr3G11468\_Mycgr3T

Mycgr3G58567 Mycgr3T
  
Location: 13753-14506

Mycgr3G58567\_Mycgr3T

Mycgr3G100089 Mycgr3
  
Location: 14606-21152

Mycgr3G100089\_Mycgr3

Mycgr3G42698 Mycgr3T
  
Location: 21252-22131

Mycgr3G42698\_Mycgr3T

Mycgr3G71681 Mycgr3T
  
Location: 22231-23461

Mycgr3G71681\_Mycgr3T

Mycgr3G109328 Mycgr3
  
Location: 23561-24239

Mycgr3G109328\_Mycgr3

Mycgr3G104334 Mycgr3
  
Location: 24339-24567

Mycgr3G104334\_Mycgr3

Mycgr3G42715 Mycgr3T
  
Location: 24667-25981

Mycgr3G42715\_Mycgr3T

Mycgr3G92934 Mycgr3T
  
Location: 26081-27593

Mycgr3G92934\_Mycgr3T

Mycgr3G41969 Mycgr3T
  
Location: 27693-29328

Mycgr3G41969\_Mycgr3T

Mycgr3G80635 Mycgr3T
  
Location: 29428-29821

Mycgr3G80635\_Mycgr3T

Mycgr3G41426 Mycgr3T
  
Location: 29921-35255

Mycgr3G41426\_Mycgr3T

Mycgr3G104337 Mycgr3
  
Location: 35355-36108

Mycgr3G104337\_Mycgr3

Mycgr3G71679 Mycgr3T
  
Location: 36208-37300

Mycgr3G71679\_Mycgr3T

Mycgr3G92938 Mycgr3T
  
Location: 37400-38699

Mycgr3G92938\_Mycgr3T

Mycgr3G92941 Mycgr3T
  
Location: 38799-40734

Mycgr3G92941\_Mycgr3T

hypothetical protein
  
Accession: EGY19761
  
Location: 488538-491077
  
 NCBI BlastP on this gene

EGY19761

hypothetical protein
  
Accession: EGY19762
  
Location: 492319-492745
  
  
**BlastP hit with Mycgr3G29582\_Mycgr3T**
  
Percentage identity: 68 %
  
BlastP bit score: 97
  
Sequence coverage: 85 %
  
E-value: 4e-24
  
  
 NCBI BlastP on this gene

EGY19762

hypothetical protein
  
Accession: EGY19763
  
Location: 493283-493774
  
 NCBI BlastP on this gene

EGY19763

ATP-dependent RNA helicase SUB2
  
Accession: EGY19764
  
Location: 494395-496461
  
 NCBI BlastP on this gene

EGY19764

polygalacturonase
  
Accession: EGY19765
  
Location: 497618-498925
  
 NCBI BlastP on this gene

EGY19765

pectinesterase family protein
  
Accession: EGY19766
  
Location: 500454-501801
  
 NCBI BlastP on this gene

EGY19766

modification methylase Sau96I
  
Accession: EGY19767
  
Location: 502176-505049
  
 NCBI BlastP on this gene

EGY19767

HhH-GPD family base excision DNA repair protein
  
Accession: EGY19768
  
Location: 506530-508161
  
 NCBI BlastP on this gene

EGY19768

helicase SEN1
  
Accession: EGY19769
  
Location: 508949-515383
  
  
**BlastP hit with Mycgr3G41426\_Mycgr3T**
  
Percentage identity: 33 %
  
BlastP bit score: 845
  
Sequence coverage: 99 %
  
E-value: 0.0
  
  
 NCBI BlastP on this gene

EGY19769

hypothetical protein
  
Accession: EGY19770
  
Location: 516467-516769
  
 NCBI BlastP on this gene

EGY19770

hypothetical protein
  
Accession: EGY19771
  
Location: 518487-520382
  
 NCBI BlastP on this gene

EGY19771

eukaryotic translation initiation factor 1A
  
Accession: EGY19772
  
Location: 521291-521972
  
 NCBI BlastP on this gene

EGY19772

hypothetical protein
  
Accession: EGY19773
  
Location: 522701-525219
  
 NCBI BlastP on this gene

EGY19773

chitin biosynthesis protein CHS5
  
Accession: EGY19774
  
Location: 526379-527702
  
 NCBI BlastP on this gene

EGY19774

Query: Architecture Search FASTA input

GL988047 : Chaetomium thermophilum var. thermophilum DSM 1495 unplaced genomic scaffold scf7180000...    Total score: 2.0     Cumulative Blast bit score: 940

Hit cluster cross-links:

Mycgr3G85918 Mycgr3T
  
Location: 0-1602

Mycgr3G85918\_Mycgr3T

Mycgr3G42010 Mycgr3T
  
Location: 1702-8569

Mycgr3G42010\_Mycgr3T

Mycgr3G29582 Mycgr3T
  
Location: 8669-8915

Mycgr3G29582\_Mycgr3T

Mycgr3G31170 Mycgr3T
  
Location: 9015-9255

Mycgr3G31170\_Mycgr3T

Mycgr3G85924 Mycgr3T
  
Location: 9355-11218

Mycgr3G85924\_Mycgr3T

Mycgr3G71676 Mycgr3T
  
Location: 11318-12494

Mycgr3G71676\_Mycgr3T

Mycgr3G11468 Mycgr3T
  
Location: 12594-13653

Mycgr3G11468\_Mycgr3T

Mycgr3G58567 Mycgr3T
  
Location: 13753-14506

Mycgr3G58567\_Mycgr3T

Mycgr3G100089 Mycgr3
  
Location: 14606-21152

Mycgr3G100089\_Mycgr3

Mycgr3G42698 Mycgr3T
  
Location: 21252-22131

Mycgr3G42698\_Mycgr3T

Mycgr3G71681 Mycgr3T
  
Location: 22231-23461

Mycgr3G71681\_Mycgr3T

Mycgr3G109328 Mycgr3
  
Location: 23561-24239

Mycgr3G109328\_Mycgr3

Mycgr3G104334 Mycgr3
  
Location: 24339-24567

Mycgr3G104334\_Mycgr3

Mycgr3G42715 Mycgr3T
  
Location: 24667-25981

Mycgr3G42715\_Mycgr3T

Mycgr3G92934 Mycgr3T
  
Location: 26081-27593

Mycgr3G92934\_Mycgr3T

Mycgr3G41969 Mycgr3T
  
Location: 27693-29328

Mycgr3G41969\_Mycgr3T

Mycgr3G80635 Mycgr3T
  
Location: 29428-29821

Mycgr3G80635\_Mycgr3T

Mycgr3G41426 Mycgr3T
  
Location: 29921-35255

Mycgr3G41426\_Mycgr3T

Mycgr3G104337 Mycgr3
  
Location: 35355-36108

Mycgr3G104337\_Mycgr3

Mycgr3G71679 Mycgr3T
  
Location: 36208-37300

Mycgr3G71679\_Mycgr3T

Mycgr3G92938 Mycgr3T
  
Location: 37400-38699

Mycgr3G92938\_Mycgr3T

Mycgr3G92941 Mycgr3T
  
Location: 38799-40734

Mycgr3G92941\_Mycgr3T

putative GTP binding protein
  
Accession: EGS17717
  
Location: 2257598-2260374
  
 NCBI BlastP on this gene

EGS17717

hypothetical protein
  
Accession: EGS17718
  
Location: 2262039-2265725
  
 NCBI BlastP on this gene

EGS17718

vacuolar protein sorting-associated protein 21-like protein
  
Accession: EGS17719
  
Location: 2266576-2267436
  
 NCBI BlastP on this gene

EGS17719

hypothetical protein
  
Accession: EGS17720
  
Location: 2268014-2270125
  
  
**BlastP hit with Mycgr3G85924\_Mycgr3T**
  
Percentage identity: 47 %
  
BlastP bit score: 351
  
Sequence coverage: 93 %
  
E-value: 1e-107
  
  
 NCBI BlastP on this gene

EGS17720

hypothetical protein
  
Accession: EGS17721
  
Location: 2271165-2272250
  
 NCBI BlastP on this gene

EGS17721

transferase-like protein
  
Accession: EGS17722
  
Location: 2272393-2273823
  
 NCBI BlastP on this gene

EGS17722

hypothetical protein
  
Accession: EGS17723
  
Location: 2278589-2279560
  
 NCBI BlastP on this gene

EGS17723

hypothetical protein
  
Accession: EGS17724
  
Location: 2284698-2287367
  
 NCBI BlastP on this gene

EGS17724

hypothetical protein
  
Accession: EGS17725
  
Location: 2289388-2291289
  
  
**BlastP hit with Mycgr3G92934\_Mycgr3T**
  
Percentage identity: 60 %
  
BlastP bit score: 589
  
Sequence coverage: 101 %
  
E-value: 0.0
  
  
 NCBI BlastP on this gene

EGS17725

hypothetical protein
  
Accession: EGS17726
  
Location: 2291361-2292560
  
 NCBI BlastP on this gene

EGS17726

oxidoreductase-like protein
  
Accession: EGS17727
  
Location: 2296511-2297657
  
 NCBI BlastP on this gene

EGS17727

pseudouridine synthase-like protein
  
Accession: EGS17728
  
Location: 2299152-2302397
  
 NCBI BlastP on this gene

EGS17728

Query: Architecture Search FASTA input

GL891304 : Neurospora tetrasperma FGSC 2508 unplaced genomic scaffold NEUTE1scaffold\_3    Total score: 2.0     Cumulative Blast bit score: 938

Hit cluster cross-links:

Mycgr3G85918 Mycgr3T
  
Location: 0-1602

Mycgr3G85918\_Mycgr3T

Mycgr3G42010 Mycgr3T
  
Location: 1702-8569

Mycgr3G42010\_Mycgr3T

Mycgr3G29582 Mycgr3T
  
Location: 8669-8915

Mycgr3G29582\_Mycgr3T

Mycgr3G31170 Mycgr3T
  
Location: 9015-9255

Mycgr3G31170\_Mycgr3T

Mycgr3G85924 Mycgr3T
  
Location: 9355-11218

Mycgr3G85924\_Mycgr3T

Mycgr3G71676 Mycgr3T
  
Location: 11318-12494

Mycgr3G71676\_Mycgr3T

Mycgr3G11468 Mycgr3T
  
Location: 12594-13653

Mycgr3G11468\_Mycgr3T

Mycgr3G58567 Mycgr3T
  
Location: 13753-14506

Mycgr3G58567\_Mycgr3T

Mycgr3G100089 Mycgr3
  
Location: 14606-21152

Mycgr3G100089\_Mycgr3

Mycgr3G42698 Mycgr3T
  
Location: 21252-22131

Mycgr3G42698\_Mycgr3T

Mycgr3G71681 Mycgr3T
  
Location: 22231-23461

Mycgr3G71681\_Mycgr3T

Mycgr3G109328 Mycgr3
  
Location: 23561-24239

Mycgr3G109328\_Mycgr3

Mycgr3G104334 Mycgr3
  
Location: 24339-24567

Mycgr3G104334\_Mycgr3

Mycgr3G42715 Mycgr3T
  
Location: 24667-25981

Mycgr3G42715\_Mycgr3T

Mycgr3G92934 Mycgr3T
  
Location: 26081-27593

Mycgr3G92934\_Mycgr3T

Mycgr3G41969 Mycgr3T
  
Location: 27693-29328

Mycgr3G41969\_Mycgr3T

Mycgr3G80635 Mycgr3T
  
Location: 29428-29821

Mycgr3G80635\_Mycgr3T

Mycgr3G41426 Mycgr3T
  
Location: 29921-35255

Mycgr3G41426\_Mycgr3T

Mycgr3G104337 Mycgr3
  
Location: 35355-36108

Mycgr3G104337\_Mycgr3

Mycgr3G71679 Mycgr3T
  
Location: 36208-37300

Mycgr3G71679\_Mycgr3T

Mycgr3G92938 Mycgr3T
  
Location: 37400-38699

Mycgr3G92938\_Mycgr3T

Mycgr3G92941 Mycgr3T
  
Location: 38799-40734

Mycgr3G92941\_Mycgr3T

hypothetical protein
  
Accession: EGO57466
  
Location: 1099898-1102062
  
 NCBI BlastP on this gene

EGO57466

MAP kinase
  
Accession: EGO57465
  
Location: 1096030-1097618
  
 NCBI BlastP on this gene

EGO57465

hypothetical protein
  
Accession: EGO57464
  
Location: 1090288-1092219
  
  
**BlastP hit with Mycgr3G92934\_Mycgr3T**
  
Percentage identity: 60 %
  
BlastP bit score: 590
  
Sequence coverage: 101 %
  
E-value: 0.0
  
  
 NCBI BlastP on this gene

EGO57464

uracil-5-carboxylate decarboxylase
  
Accession: EGO57463
  
Location: 1088643-1089824
  
 NCBI BlastP on this gene

EGO57463

thymine dioxygenase
  
Accession: EGO57462
  
Location: 1086625-1087772
  
 NCBI BlastP on this gene

EGO57462

hypothetical protein
  
Accession: EGO57461
  
Location: 1085094-1086128
  
 NCBI BlastP on this gene

EGO57461

hypothetical protein
  
Accession: EGO57460
  
Location: 1081159-1083690
  
 NCBI BlastP on this gene

EGO57460

hypothetical protein
  
Accession: EGO57459
  
Location: 1069324-1072844
  
 NCBI BlastP on this gene

EGO57459

hypothetical protein
  
Accession: EGO57458
  
Location: 1067077-1067915
  
 NCBI BlastP on this gene

EGO57458

hypothetical protein
  
Accession: EGO57457
  
Location: 1064284-1066440
  
  
**BlastP hit with Mycgr3G85924\_Mycgr3T**
  
Percentage identity: 48 %
  
BlastP bit score: 348
  
Sequence coverage: 97 %
  
E-value: 2e-106
  
  
 NCBI BlastP on this gene

EGO57457

hypothetical protein
  
Accession: EGO57456
  
Location: 1054140-1057607
  
 NCBI BlastP on this gene

EGO57456

Query: Architecture Search FASTA input

GL891236 : Neurospora tetrasperma FGSC 2509 unplaced genomic scaffold NEUTE2scaffold\_4    Total score: 2.0     Cumulative Blast bit score: 938

Hit cluster cross-links:

Mycgr3G85918 Mycgr3T
  
Location: 0-1602

Mycgr3G85918\_Mycgr3T

Mycgr3G42010 Mycgr3T
  
Location: 1702-8569

Mycgr3G42010\_Mycgr3T

Mycgr3G29582 Mycgr3T
  
Location: 8669-8915

Mycgr3G29582\_Mycgr3T

Mycgr3G31170 Mycgr3T
  
Location: 9015-9255

Mycgr3G31170\_Mycgr3T

Mycgr3G85924 Mycgr3T
  
Location: 9355-11218

Mycgr3G85924\_Mycgr3T

Mycgr3G71676 Mycgr3T
  
Location: 11318-12494

Mycgr3G71676\_Mycgr3T

Mycgr3G11468 Mycgr3T
  
Location: 12594-13653

Mycgr3G11468\_Mycgr3T

Mycgr3G58567 Mycgr3T
  
Location: 13753-14506

Mycgr3G58567\_Mycgr3T

Mycgr3G100089 Mycgr3
  
Location: 14606-21152

Mycgr3G100089\_Mycgr3

Mycgr3G42698 Mycgr3T
  
Location: 21252-22131

Mycgr3G42698\_Mycgr3T

Mycgr3G71681 Mycgr3T
  
Location: 22231-23461

Mycgr3G71681\_Mycgr3T

Mycgr3G109328 Mycgr3
  
Location: 23561-24239

Mycgr3G109328\_Mycgr3

Mycgr3G104334 Mycgr3
  
Location: 24339-24567

Mycgr3G104334\_Mycgr3

Mycgr3G42715 Mycgr3T
  
Location: 24667-25981

Mycgr3G42715\_Mycgr3T

Mycgr3G92934 Mycgr3T
  
Location: 26081-27593

Mycgr3G92934\_Mycgr3T

Mycgr3G41969 Mycgr3T
  
Location: 27693-29328

Mycgr3G41969\_Mycgr3T

Mycgr3G80635 Mycgr3T
  
Location: 29428-29821

Mycgr3G80635\_Mycgr3T

Mycgr3G41426 Mycgr3T
  
Location: 29921-35255

Mycgr3G41426\_Mycgr3T

Mycgr3G104337 Mycgr3
  
Location: 35355-36108

Mycgr3G104337\_Mycgr3

Mycgr3G71679 Mycgr3T
  
Location: 36208-37300

Mycgr3G71679\_Mycgr3T

Mycgr3G92938 Mycgr3T
  
Location: 37400-38699

Mycgr3G92938\_Mycgr3T

Mycgr3G92941 Mycgr3T
  
Location: 38799-40734

Mycgr3G92941\_Mycgr3T

hypothetical protein
  
Accession: EGZ72275
  
Location: 3904469-3904810
  
 NCBI BlastP on this gene

EGZ72275

hypothetical protein
  
Accession: EGZ72276
  
Location: 3906252-3906633
  
 NCBI BlastP on this gene

EGZ72276

MAP kinase
  
Accession: EGZ72277
  
Location: 3908913-3910501
  
 NCBI BlastP on this gene

EGZ72277

ATP-dependent RNA helicase dbp-8
  
Accession: EGZ72278
  
Location: 3914306-3916237
  
  
**BlastP hit with Mycgr3G92934\_Mycgr3T**
  
Percentage identity: 60 %
  
BlastP bit score: 590
  
Sequence coverage: 101 %
  
E-value: 0.0
  
  
 NCBI BlastP on this gene

EGZ72278

uracil-5-carboxylate decarboxylase
  
Accession: EGZ72279
  
Location: 3916701-3917882
  
 NCBI BlastP on this gene

EGZ72279

thymine dioxygenase
  
Accession: EGZ72280
  
Location: 3918753-3919900
  
 NCBI BlastP on this gene

EGZ72280

hypothetical protein
  
Accession: EGZ72281
  
Location: 3920397-3921431
  
 NCBI BlastP on this gene

EGZ72281

hypothetical protein
  
Accession: EGZ72282
  
Location: 3922835-3925366
  
 NCBI BlastP on this gene

EGZ72282

hypothetical protein
  
Accession: EGZ72283
  
Location: 3932272-3935792
  
 NCBI BlastP on this gene

EGZ72283

Rab5-like protein ypt51
  
Accession: EGZ72284
  
Location: 3937201-3938039
  
 NCBI BlastP on this gene

EGZ72284

hypothetical protein
  
Accession: EGZ72285
  
Location: 3938676-3940832
  
  
**BlastP hit with Mycgr3G85924\_Mycgr3T**
  
Percentage identity: 48 %
  
BlastP bit score: 348
  
Sequence coverage: 97 %
  
E-value: 2e-106
  
  
 NCBI BlastP on this gene

EGZ72285

hypothetical protein
  
Accession: EGZ72286
  
Location: 3947508-3950975
  
 NCBI BlastP on this gene

EGZ72286

Query: Architecture Search FASTA input

AABX02000027 : Neurospora crassa OR74A    Total score: 2.0     Cumulative Blast bit score: 918

Hit cluster cross-links:

Mycgr3G85918 Mycgr3T
  
Location: 0-1602

Mycgr3G85918\_Mycgr3T

Mycgr3G42010 Mycgr3T
  
Location: 1702-8569

Mycgr3G42010\_Mycgr3T

Mycgr3G29582 Mycgr3T
  
Location: 8669-8915

Mycgr3G29582\_Mycgr3T

Mycgr3G31170 Mycgr3T
  
Location: 9015-9255

Mycgr3G31170\_Mycgr3T

Mycgr3G85924 Mycgr3T
  
Location: 9355-11218

Mycgr3G85924\_Mycgr3T

Mycgr3G71676 Mycgr3T
  
Location: 11318-12494

Mycgr3G71676\_Mycgr3T

Mycgr3G11468 Mycgr3T
  
Location: 12594-13653

Mycgr3G11468\_Mycgr3T

Mycgr3G58567 Mycgr3T
  
Location: 13753-14506

Mycgr3G58567\_Mycgr3T

Mycgr3G100089 Mycgr3
  
Location: 14606-21152

Mycgr3G100089\_Mycgr3

Mycgr3G42698 Mycgr3T
  
Location: 21252-22131

Mycgr3G42698\_Mycgr3T

Mycgr3G71681 Mycgr3T
  
Location: 22231-23461

Mycgr3G71681\_Mycgr3T

Mycgr3G109328 Mycgr3
  
Location: 23561-24239

Mycgr3G109328\_Mycgr3

Mycgr3G104334 Mycgr3
  
Location: 24339-24567

Mycgr3G104334\_Mycgr3

Mycgr3G42715 Mycgr3T
  
Location: 24667-25981

Mycgr3G42715\_Mycgr3T

Mycgr3G92934 Mycgr3T
  
Location: 26081-27593

Mycgr3G92934\_Mycgr3T

Mycgr3G41969 Mycgr3T
  
Location: 27693-29328

Mycgr3G41969\_Mycgr3T

Mycgr3G80635 Mycgr3T
  
Location: 29428-29821

Mycgr3G80635\_Mycgr3T

Mycgr3G41426 Mycgr3T
  
Location: 29921-35255

Mycgr3G41426\_Mycgr3T

Mycgr3G104337 Mycgr3
  
Location: 35355-36108

Mycgr3G104337\_Mycgr3

Mycgr3G71679 Mycgr3T
  
Location: 36208-37300

Mycgr3G71679\_Mycgr3T

Mycgr3G92938 Mycgr3T
  
Location: 37400-38699

Mycgr3G92938\_Mycgr3T

Mycgr3G92941 Mycgr3T
  
Location: 38799-40734

Mycgr3G92941\_Mycgr3T

MAP kinase kinase
  
Accession: EAA28074
  
Location: 62587-64175
  
 NCBI BlastP on this gene

EAA28074

hypothetical protein
  
Accession: EAA28073
  
Location: 56842-58795
  
  
**BlastP hit with Mycgr3G92934\_Mycgr3T**
  
Percentage identity: 60 %
  
BlastP bit score: 590
  
Sequence coverage: 101 %
  
E-value: 0.0
  
  
 NCBI BlastP on this gene

EAA28073

uracil-5-carboxylate decarboxylase
  
Accession: EAA28072
  
Location: 55193-56377
  
 NCBI BlastP on this gene

EAA28072

thymine dioxygenase
  
Accession: EAA28071
  
Location: 53174-54321
  
 NCBI BlastP on this gene

EAA28071

predicted protein
  
Accession: EAA28070
  
Location: 51593-52648
  
 NCBI BlastP on this gene

EAA28070

predicted protein
  
Accession: EAA26720
  
Location: 46914-49456
  
 NCBI BlastP on this gene

EAA26720

predicted protein
  
Accession: EAA26719
  
Location: 42247-45178
  
 NCBI BlastP on this gene

EAA26719

predicted protein
  
Accession: EAA26718
  
Location: 40050-41407
  
 NCBI BlastP on this gene

EAA26718

predicted protein
  
Accession: EAA26717
  
Location: 34961-38476
  
 NCBI BlastP on this gene

EAA26717

Rab5-like protein ypt51
  
Accession: EAA26716
  
Location: 32671-33509
  
 NCBI BlastP on this gene

EAA26716

predicted protein
  
Accession: EAA26715
  
Location: 29824-31995
  
  
**BlastP hit with Mycgr3G85924\_Mycgr3T**
  
Percentage identity: 45 %
  
BlastP bit score: 328
  
Sequence coverage: 104 %
  
E-value: 2e-98
  
  
 NCBI BlastP on this gene

EAA26715

predicted protein
  
Accession: EAA26714
  
Location: 25998-26350
  
 NCBI BlastP on this gene

EAA26714

hypothetical protein
  
Accession: EAA26713
  
Location: 19590-23045
  
 NCBI BlastP on this gene

EAA26713

Query: Architecture Search FASTA input

KB932812 : Togninia minima UCRPA7 unplaced genomic scaffold PA7\_03\_scaffold\_39    Total score: 2.0     Cumulative Blast bit score: 914

Hit cluster cross-links:

Mycgr3G85918 Mycgr3T
  
Location: 0-1602

Mycgr3G85918\_Mycgr3T

Mycgr3G42010 Mycgr3T
  
Location: 1702-8569

Mycgr3G42010\_Mycgr3T

Mycgr3G29582 Mycgr3T
  
Location: 8669-8915

Mycgr3G29582\_Mycgr3T

Mycgr3G31170 Mycgr3T
  
Location: 9015-9255

Mycgr3G31170\_Mycgr3T

Mycgr3G85924 Mycgr3T
  
Location: 9355-11218

Mycgr3G85924\_Mycgr3T

Mycgr3G71676 Mycgr3T
  
Location: 11318-12494

Mycgr3G71676\_Mycgr3T

Mycgr3G11468 Mycgr3T
  
Location: 12594-13653

Mycgr3G11468\_Mycgr3T

Mycgr3G58567 Mycgr3T
  
Location: 13753-14506

Mycgr3G58567\_Mycgr3T

Mycgr3G100089 Mycgr3
  
Location: 14606-21152

Mycgr3G100089\_Mycgr3

Mycgr3G42698 Mycgr3T
  
Location: 21252-22131

Mycgr3G42698\_Mycgr3T

Mycgr3G71681 Mycgr3T
  
Location: 22231-23461

Mycgr3G71681\_Mycgr3T

Mycgr3G109328 Mycgr3
  
Location: 23561-24239

Mycgr3G109328\_Mycgr3

Mycgr3G104334 Mycgr3
  
Location: 24339-24567

Mycgr3G104334\_Mycgr3

Mycgr3G42715 Mycgr3T
  
Location: 24667-25981

Mycgr3G42715\_Mycgr3T

Mycgr3G92934 Mycgr3T
  
Location: 26081-27593

Mycgr3G92934\_Mycgr3T

Mycgr3G41969 Mycgr3T
  
Location: 27693-29328

Mycgr3G41969\_Mycgr3T

Mycgr3G80635 Mycgr3T
  
Location: 29428-29821

Mycgr3G80635\_Mycgr3T

Mycgr3G41426 Mycgr3T
  
Location: 29921-35255

Mycgr3G41426\_Mycgr3T

Mycgr3G104337 Mycgr3
  
Location: 35355-36108

Mycgr3G104337\_Mycgr3

Mycgr3G71679 Mycgr3T
  
Location: 36208-37300

Mycgr3G71679\_Mycgr3T

Mycgr3G92938 Mycgr3T
  
Location: 37400-38699

Mycgr3G92938\_Mycgr3T

Mycgr3G92941 Mycgr3T
  
Location: 38799-40734

Mycgr3G92941\_Mycgr3T

putative mfs hexose protein
  
Accession: EOO03938
  
Location: 607153-608627
  
 NCBI BlastP on this gene

EOO03938

putative diacylglycerol acyltransferase family protein
  
Accession: EOO03753
  
Location: 611523-613076
  
 NCBI BlastP on this gene

EOO03753

putative ef-hand calcium-binding domain protein
  
Accession: EOO03858
  
Location: 614249-615296
  
  
**BlastP hit with Mycgr3G104337\_Mycgr3**
  
Percentage identity: 45 %
  
BlastP bit score: 232
  
Sequence coverage: 96 %
  
E-value: 1e-71
  
  
 NCBI BlastP on this gene

EOO03858

putative polyketide synthase protein
  
Accession: EOO03896
  
Location: 617892-618864
  
 NCBI BlastP on this gene

EOO03896

putative polyketide synthase protein
  
Accession: EOO03974
  
Location: 621680-624201
  
  
**BlastP hit with Mycgr3G100089\_Mycgr3**
  
Percentage identity: 48 %
  
BlastP bit score: 682
  
Sequence coverage: 37 %
  
E-value: 0.0
  
  
 NCBI BlastP on this gene

EOO03974

Query: Architecture Search FASTA input

CU633900 : Podospora anserina S mat+ genomic DNA chromosome 7, supercontig 1.    Total score: 2.0     Cumulative Blast bit score: 873

Hit cluster cross-links:

Mycgr3G85918 Mycgr3T
  
Location: 0-1602

Mycgr3G85918\_Mycgr3T

Mycgr3G42010 Mycgr3T
  
Location: 1702-8569

Mycgr3G42010\_Mycgr3T

Mycgr3G29582 Mycgr3T
  
Location: 8669-8915

Mycgr3G29582\_Mycgr3T

Mycgr3G31170 Mycgr3T
  
Location: 9015-9255

Mycgr3G31170\_Mycgr3T

Mycgr3G85924 Mycgr3T
  
Location: 9355-11218

Mycgr3G85924\_Mycgr3T

Mycgr3G71676 Mycgr3T
  
Location: 11318-12494

Mycgr3G71676\_Mycgr3T

Mycgr3G11468 Mycgr3T
  
Location: 12594-13653

Mycgr3G11468\_Mycgr3T

Mycgr3G58567 Mycgr3T
  
Location: 13753-14506

Mycgr3G58567\_Mycgr3T

Mycgr3G100089 Mycgr3
  
Location: 14606-21152

Mycgr3G100089\_Mycgr3

Mycgr3G42698 Mycgr3T
  
Location: 21252-22131

Mycgr3G42698\_Mycgr3T

Mycgr3G71681 Mycgr3T
  
Location: 22231-23461

Mycgr3G71681\_Mycgr3T

Mycgr3G109328 Mycgr3
  
Location: 23561-24239

Mycgr3G109328\_Mycgr3

Mycgr3G104334 Mycgr3
  
Location: 24339-24567

Mycgr3G104334\_Mycgr3

Mycgr3G42715 Mycgr3T
  
Location: 24667-25981

Mycgr3G42715\_Mycgr3T

Mycgr3G92934 Mycgr3T
  
Location: 26081-27593

Mycgr3G92934\_Mycgr3T

Mycgr3G41969 Mycgr3T
  
Location: 27693-29328

Mycgr3G41969\_Mycgr3T

Mycgr3G80635 Mycgr3T
  
Location: 29428-29821

Mycgr3G80635\_Mycgr3T

Mycgr3G41426 Mycgr3T
  
Location: 29921-35255

Mycgr3G41426\_Mycgr3T

Mycgr3G104337 Mycgr3
  
Location: 35355-36108

Mycgr3G104337\_Mycgr3

Mycgr3G71679 Mycgr3T
  
Location: 36208-37300

Mycgr3G71679\_Mycgr3T

Mycgr3G92938 Mycgr3T
  
Location: 37400-38699

Mycgr3G92938\_Mycgr3T

Mycgr3G92941 Mycgr3T
  
Location: 38799-40734

Mycgr3G92941\_Mycgr3T

not annotated
  
Accession: CAP69064
  
Location: 2925096-2927537
  
 NCBI BlastP on this gene

CAP69064

not annotated
  
Accession: CAP69065
  
Location: 2928147-2931094
  
 NCBI BlastP on this gene

CAP69065

not annotated
  
Accession: CAP69066
  
Location: 2933590-2936202
  
 NCBI BlastP on this gene

CAP69066

not annotated
  
Accession: CAP69067
  
Location: 2937938-2939843
  
 NCBI BlastP on this gene

CAP69067

not annotated
  
Accession: CAP69068
  
Location: 2941183-2942972
  
  
**BlastP hit with Mycgr3G92934\_Mycgr3T**
  
Percentage identity: 62 %
  
BlastP bit score: 577
  
Sequence coverage: 93 %
  
E-value: 0.0
  
  
 NCBI BlastP on this gene

CAP69068

not annotated
  
Accession: CAP69069
  
Location: 2943152-2944299
  
 NCBI BlastP on this gene

CAP69069

not annotated
  
Accession: CAP69070
  
Location: 2945097-2945489
  
 NCBI BlastP on this gene

CAP69070

not annotated
  
Accession: CAP69071
  
Location: 2947650-2949287
  
 NCBI BlastP on this gene

CAP69071

not annotated
  
Accession: CAP69072
  
Location: 2952267-2952942
  
 NCBI BlastP on this gene

CAP69072

not annotated
  
Accession: CAP69073
  
Location: 2955867-2957966
  
  
**BlastP hit with Mycgr3G85924\_Mycgr3T**
  
Percentage identity: 44 %
  
BlastP bit score: 296
  
Sequence coverage: 94 %
  
E-value: 9e-87
  
  
 NCBI BlastP on this gene

CAP69073

not annotated
  
Accession: CAP69074
  
Location: 2958987-2959826
  
 NCBI BlastP on this gene

CAP69074

not annotated
  
Accession: CAP69075
  
Location: 2961453-2963032
  
 NCBI BlastP on this gene

CAP69075

not annotated
  
Accession: CAP69076
  
Location: 2964064-2966538
  
 NCBI BlastP on this gene

CAP69076

not annotated
  
Accession: CAP69077
  
Location: 2968077-2972405
  
 NCBI BlastP on this gene

CAP69077

Query: Architecture Search FASTA input

DS985215 : Verticillium albo-atrum VaMs.102 supercont1.2 genomic scaffold    Total score: 2.0     Cumulative Blast bit score: 847

Hit cluster cross-links:

Mycgr3G85918 Mycgr3T
  
Location: 0-1602

Mycgr3G85918\_Mycgr3T

Mycgr3G42010 Mycgr3T
  
Location: 1702-8569

Mycgr3G42010\_Mycgr3T

Mycgr3G29582 Mycgr3T
  
Location: 8669-8915

Mycgr3G29582\_Mycgr3T

Mycgr3G31170 Mycgr3T
  
Location: 9015-9255

Mycgr3G31170\_Mycgr3T

Mycgr3G85924 Mycgr3T
  
Location: 9355-11218

Mycgr3G85924\_Mycgr3T

Mycgr3G71676 Mycgr3T
  
Location: 11318-12494

Mycgr3G71676\_Mycgr3T

Mycgr3G11468 Mycgr3T
  
Location: 12594-13653

Mycgr3G11468\_Mycgr3T

Mycgr3G58567 Mycgr3T
  
Location: 13753-14506

Mycgr3G58567\_Mycgr3T

Mycgr3G100089 Mycgr3
  
Location: 14606-21152

Mycgr3G100089\_Mycgr3

Mycgr3G42698 Mycgr3T
  
Location: 21252-22131

Mycgr3G42698\_Mycgr3T

Mycgr3G71681 Mycgr3T
  
Location: 22231-23461

Mycgr3G71681\_Mycgr3T

Mycgr3G109328 Mycgr3
  
Location: 23561-24239

Mycgr3G109328\_Mycgr3

Mycgr3G104334 Mycgr3
  
Location: 24339-24567

Mycgr3G104334\_Mycgr3

Mycgr3G42715 Mycgr3T
  
Location: 24667-25981

Mycgr3G42715\_Mycgr3T

Mycgr3G92934 Mycgr3T
  
Location: 26081-27593

Mycgr3G92934\_Mycgr3T

Mycgr3G41969 Mycgr3T
  
Location: 27693-29328

Mycgr3G41969\_Mycgr3T

Mycgr3G80635 Mycgr3T
  
Location: 29428-29821

Mycgr3G80635\_Mycgr3T

Mycgr3G41426 Mycgr3T
  
Location: 29921-35255

Mycgr3G41426\_Mycgr3T

Mycgr3G104337 Mycgr3
  
Location: 35355-36108

Mycgr3G104337\_Mycgr3

Mycgr3G71679 Mycgr3T
  
Location: 36208-37300

Mycgr3G71679\_Mycgr3T

Mycgr3G92938 Mycgr3T
  
Location: 37400-38699

Mycgr3G92938\_Mycgr3T

Mycgr3G92941 Mycgr3T
  
Location: 38799-40734

Mycgr3G92941\_Mycgr3T

conserved hypothetical protein
  
Accession: EEY15665
  
Location: 983392-985252
  
 NCBI BlastP on this gene

EEY15665

conserved hypothetical protein
  
Accession: EEY15664
  
Location: 982703-982972
  
 NCBI BlastP on this gene

EEY15664

conserved hypothetical protein
  
Accession: EEY15663
  
Location: 981022-981452
  
  
**BlastP hit with Mycgr3G29582\_Mycgr3T**
  
Percentage identity: 67 %
  
BlastP bit score: 96
  
Sequence coverage: 85 %
  
E-value: 1e-23
  
  
 NCBI BlastP on this gene

EEY15663

conserved hypothetical protein
  
Accession: EEY15662
  
Location: 979987-980478
  
 NCBI BlastP on this gene

EEY15662

ATP-dependent RNA helicase SUB2
  
Accession: EEY15661
  
Location: 977327-979403
  
 NCBI BlastP on this gene

EEY15661

polygalacturonase
  
Accession: EEY15660
  
Location: 975514-976826
  
 NCBI BlastP on this gene

EEY15660

predicted protein
  
Accession: EEY15659
  
Location: 973830-974456
  
 NCBI BlastP on this gene

EEY15659

pectinesterase family protein
  
Accession: EEY15658
  
Location: 972338-973345
  
 NCBI BlastP on this gene

EEY15658

RIP defective
  
Accession: EEY15657
  
Location: 969110-971839
  
 NCBI BlastP on this gene

EEY15657

HhH-GPD family base excision DNA repair protein
  
Accession: EEY15656
  
Location: 966001-967628
  
 NCBI BlastP on this gene

EEY15656

helicase sen1
  
Accession: EEY15655
  
Location: 958721-965197
  
  
**BlastP hit with Mycgr3G41426\_Mycgr3T**
  
Percentage identity: 32 %
  
BlastP bit score: 751
  
Sequence coverage: 95 %
  
E-value: 0.0
  
  
 NCBI BlastP on this gene

EEY15655

conserved hypothetical protein
  
Accession: EEY15654
  
Location: 955459-955695
  
 NCBI BlastP on this gene

EEY15654

eukaryotic translation initiation factor eIF-1A
  
Accession: EEY15653
  
Location: 951856-952659
  
 NCBI BlastP on this gene

EEY15653

predicted protein
  
Accession: EEY15652
  
Location: 950150-951118
  
 NCBI BlastP on this gene

EEY15652

chitin biosynthesis protein CHS5
  
Accession: EEY15651
  
Location: 949006-949570
  
 NCBI BlastP on this gene

EEY15651

oxidoreductase
  
Accession: EEY15650
  
Location: 947116-947964
  
 NCBI BlastP on this gene

EEY15650

Query: Architecture Search FASTA input

CH476607 : Aspergillus terreus NIH2624 scaffold\_14 genomic scaffold    Total score: 2.0     Cumulative Blast bit score: 834

Hit cluster cross-links:

Mycgr3G85918 Mycgr3T
  
Location: 0-1602

Mycgr3G85918\_Mycgr3T

Mycgr3G42010 Mycgr3T
  
Location: 1702-8569

Mycgr3G42010\_Mycgr3T

Mycgr3G29582 Mycgr3T
  
Location: 8669-8915

Mycgr3G29582\_Mycgr3T

Mycgr3G31170 Mycgr3T
  
Location: 9015-9255

Mycgr3G31170\_Mycgr3T

Mycgr3G85924 Mycgr3T
  
Location: 9355-11218

Mycgr3G85924\_Mycgr3T

Mycgr3G71676 Mycgr3T
  
Location: 11318-12494

Mycgr3G71676\_Mycgr3T

Mycgr3G11468 Mycgr3T
  
Location: 12594-13653

Mycgr3G11468\_Mycgr3T

Mycgr3G58567 Mycgr3T
  
Location: 13753-14506

Mycgr3G58567\_Mycgr3T

Mycgr3G100089 Mycgr3
  
Location: 14606-21152

Mycgr3G100089\_Mycgr3

Mycgr3G42698 Mycgr3T
  
Location: 21252-22131

Mycgr3G42698\_Mycgr3T

Mycgr3G71681 Mycgr3T
  
Location: 22231-23461

Mycgr3G71681\_Mycgr3T

Mycgr3G109328 Mycgr3
  
Location: 23561-24239

Mycgr3G109328\_Mycgr3

Mycgr3G104334 Mycgr3
  
Location: 24339-24567

Mycgr3G104334\_Mycgr3

Mycgr3G42715 Mycgr3T
  
Location: 24667-25981

Mycgr3G42715\_Mycgr3T

Mycgr3G92934 Mycgr3T
  
Location: 26081-27593

Mycgr3G92934\_Mycgr3T

Mycgr3G41969 Mycgr3T
  
Location: 27693-29328

Mycgr3G41969\_Mycgr3T

Mycgr3G80635 Mycgr3T
  
Location: 29428-29821

Mycgr3G80635\_Mycgr3T

Mycgr3G41426 Mycgr3T
  
Location: 29921-35255

Mycgr3G41426\_Mycgr3T

Mycgr3G104337 Mycgr3
  
Location: 35355-36108

Mycgr3G104337\_Mycgr3

Mycgr3G71679 Mycgr3T
  
Location: 36208-37300

Mycgr3G71679\_Mycgr3T

Mycgr3G92938 Mycgr3T
  
Location: 37400-38699

Mycgr3G92938\_Mycgr3T

Mycgr3G92941 Mycgr3T
  
Location: 38799-40734

Mycgr3G92941\_Mycgr3T

conserved hypothetical protein
  
Accession: EAU30232
  
Location: 255246-258145
  
 NCBI BlastP on this gene

EAU30232

conserved hypothetical protein
  
Accession: EAU30231
  
Location: 252506-254705
  
 NCBI BlastP on this gene

EAU30231

conserved hypothetical protein
  
Accession: EAU30230
  
Location: 248661-250236
  
 NCBI BlastP on this gene

EAU30230

predicted protein
  
Accession: EAU30229
  
Location: 246888-247413
  
 NCBI BlastP on this gene

EAU30229

hypothetical protein
  
Accession: EAU30228
  
Location: 241147-245391
  
 NCBI BlastP on this gene

EAU30228

conserved hypothetical protein
  
Accession: EAU30227
  
Location: 238223-240341
  
 NCBI BlastP on this gene

EAU30227

predicted protein
  
Accession: EAU30226
  
Location: 236167-236960
  
  
**BlastP hit with Mycgr3G104337\_Mycgr3**
  
Percentage identity: 26 %
  
BlastP bit score: 84
  
Sequence coverage: 91 %
  
E-value: 2e-16
  
  
 NCBI BlastP on this gene

EAU30226

hypothetical protein
  
Accession: EAU30225
  
Location: 228330-235615
  
  
**BlastP hit with Mycgr3G100089\_Mycgr3**
  
Percentage identity: 37 %
  
BlastP bit score: 750
  
Sequence coverage: 52 %
  
E-value: 0.0
  
  
 NCBI BlastP on this gene

EAU30225

2-oxoisovalerate dehydrogenase beta subunit, mitochondrial precursor
  
Accession: EAU30224
  
Location: 226777-227793
  
 NCBI BlastP on this gene

EAU30224

conserved hypothetical protein
  
Accession: EAU30223
  
Location: 225294-226274
  
 NCBI BlastP on this gene

EAU30223

conserved hypothetical protein
  
Accession: EAU30222
  
Location: 222712-224704
  
 NCBI BlastP on this gene

EAU30222

hypothetical protein
  
Accession: EAU30221
  
Location: 220897-222399
  
 NCBI BlastP on this gene

EAU30221

predicted protein
  
Accession: EAU30220
  
Location: 219051-220037
  
 NCBI BlastP on this gene

EAU30220

hypothetical protein
  
Accession: EAU30219
  
Location: 217702-218516
  
 NCBI BlastP on this gene

EAU30219

dihydroxyacetone synthase
  
Accession: EAU30218
  
Location: 214627-216858
  
 NCBI BlastP on this gene

EAU30218

predicted protein
  
Accession: EAU30217
  
Location: 213198-214045
  
 NCBI BlastP on this gene

EAU30217

hypothetical protein
  
Accession: EAU30216
  
Location: 211340-212632
  
 NCBI BlastP on this gene

EAU30216

conserved hypothetical protein
  
Accession: EAU30215
  
Location: 209359-211186
  
 NCBI BlastP on this gene

EAU30215

Query: Architecture Search FASTA input

DF126459 : Aspergillus kawachii IFO 4308 DNA, contig: scaffold00013    Total score: 2.0     Cumulative Blast bit score: 830

Hit cluster cross-links:

Mycgr3G85918 Mycgr3T
  
Location: 0-1602

Mycgr3G85918\_Mycgr3T

Mycgr3G42010 Mycgr3T
  
Location: 1702-8569

Mycgr3G42010\_Mycgr3T

Mycgr3G29582 Mycgr3T
  
Location: 8669-8915

Mycgr3G29582\_Mycgr3T

Mycgr3G31170 Mycgr3T
  
Location: 9015-9255

Mycgr3G31170\_Mycgr3T

Mycgr3G85924 Mycgr3T
  
Location: 9355-11218

Mycgr3G85924\_Mycgr3T

Mycgr3G71676 Mycgr3T
  
Location: 11318-12494

Mycgr3G71676\_Mycgr3T

Mycgr3G11468 Mycgr3T
  
Location: 12594-13653

Mycgr3G11468\_Mycgr3T

Mycgr3G58567 Mycgr3T
  
Location: 13753-14506

Mycgr3G58567\_Mycgr3T

Mycgr3G100089 Mycgr3
  
Location: 14606-21152

Mycgr3G100089\_Mycgr3

Mycgr3G42698 Mycgr3T
  
Location: 21252-22131

Mycgr3G42698\_Mycgr3T

Mycgr3G71681 Mycgr3T
  
Location: 22231-23461

Mycgr3G71681\_Mycgr3T

Mycgr3G109328 Mycgr3
  
Location: 23561-24239

Mycgr3G109328\_Mycgr3

Mycgr3G104334 Mycgr3
  
Location: 24339-24567

Mycgr3G104334\_Mycgr3

Mycgr3G42715 Mycgr3T
  
Location: 24667-25981

Mycgr3G42715\_Mycgr3T

Mycgr3G92934 Mycgr3T
  
Location: 26081-27593

Mycgr3G92934\_Mycgr3T

Mycgr3G41969 Mycgr3T
  
Location: 27693-29328

Mycgr3G41969\_Mycgr3T

Mycgr3G80635 Mycgr3T
  
Location: 29428-29821

Mycgr3G80635\_Mycgr3T

Mycgr3G41426 Mycgr3T
  
Location: 29921-35255

Mycgr3G41426\_Mycgr3T

Mycgr3G104337 Mycgr3
  
Location: 35355-36108

Mycgr3G104337\_Mycgr3

Mycgr3G71679 Mycgr3T
  
Location: 36208-37300

Mycgr3G71679\_Mycgr3T

Mycgr3G92938 Mycgr3T
  
Location: 37400-38699

Mycgr3G92938\_Mycgr3T

Mycgr3G92941 Mycgr3T
  
Location: 38799-40734

Mycgr3G92941\_Mycgr3T

FAD binding domain protein
  
Accession: GAA87324
  
Location: 150234-152309
  
 NCBI BlastP on this gene

GAA87324

arylsulfatase A
  
Accession: GAA87323
  
Location: 147303-148841
  
 NCBI BlastP on this gene

GAA87323

polygalacturonase (PgaI)
  
Accession: GAA87322
  
Location: 143299-144521
  
 NCBI BlastP on this gene

GAA87322

NRPS-like enzyme
  
Accession: GAA87321
  
Location: 136163-139535
  
 NCBI BlastP on this gene

GAA87321

integral membrane protein
  
Accession: GAA87320
  
Location: 134189-135368
  
 NCBI BlastP on this gene

GAA87320

hypothetical protein
  
Accession: GAA87319
  
Location: 132780-133536
  
  
**BlastP hit with Mycgr3G104337\_Mycgr3**
  
Percentage identity: 30 %
  
BlastP bit score: 55
  
Sequence coverage: 59 %
  
E-value: 4e-06
  
  
 NCBI BlastP on this gene

GAA87319

polyketide synthase
  
Accession: GAA87318
  
Location: 123689-131921
  
  
**BlastP hit with Mycgr3G100089\_Mycgr3**
  
Percentage identity: 38 %
  
BlastP bit score: 775
  
Sequence coverage: 58 %
  
E-value: 0.0
  
  
 NCBI BlastP on this gene

GAA87318

flavin-containing amine oxidasedehydrogenase
  
Accession: GAA87317
  
Location: 121019-122527
  
 NCBI BlastP on this gene

GAA87317

feruloyl esterase B precursor
  
Accession: GAA87316
  
Location: 119092-120654
  
 NCBI BlastP on this gene

GAA87316

similar to An01g11590
  
Accession: GAA87315
  
Location: 114134-115490
  
 NCBI BlastP on this gene

GAA87315

pantothenate transporter
  
Accession: GAA87314
  
Location: 111992-113842
  
 NCBI BlastP on this gene

GAA87314

amidohydrolase
  
Accession: GAA87313
  
Location: 109432-110907
  
 NCBI BlastP on this gene

GAA87313

Zn(II)2Cys6 transcription factor
  
Accession: GAA87312
  
Location: 106786-108743
  
 NCBI BlastP on this gene

GAA87312

hypothetical protein
  
Accession: GAA87311
  
Location: 106065-106706
  
 NCBI BlastP on this gene

GAA87311

Query: Architecture Search FASTA input

AP007157 : Aspergillus oryzae RIB40 DNA, SC023.    Total score: 2.0     Cumulative Blast bit score: 718

Hit cluster cross-links:

Mycgr3G85918 Mycgr3T
  
Location: 0-1602

Mycgr3G85918\_Mycgr3T

Mycgr3G42010 Mycgr3T
  
Location: 1702-8569

Mycgr3G42010\_Mycgr3T

Mycgr3G29582 Mycgr3T
  
Location: 8669-8915

Mycgr3G29582\_Mycgr3T

Mycgr3G31170 Mycgr3T
  
Location: 9015-9255

Mycgr3G31170\_Mycgr3T

Mycgr3G85924 Mycgr3T
  
Location: 9355-11218

Mycgr3G85924\_Mycgr3T

Mycgr3G71676 Mycgr3T
  
Location: 11318-12494

Mycgr3G71676\_Mycgr3T

Mycgr3G11468 Mycgr3T
  
Location: 12594-13653

Mycgr3G11468\_Mycgr3T

Mycgr3G58567 Mycgr3T
  
Location: 13753-14506

Mycgr3G58567\_Mycgr3T

Mycgr3G100089 Mycgr3
  
Location: 14606-21152

Mycgr3G100089\_Mycgr3

Mycgr3G42698 Mycgr3T
  
Location: 21252-22131

Mycgr3G42698\_Mycgr3T

Mycgr3G71681 Mycgr3T
  
Location: 22231-23461

Mycgr3G71681\_Mycgr3T

Mycgr3G109328 Mycgr3
  
Location: 23561-24239

Mycgr3G109328\_Mycgr3

Mycgr3G104334 Mycgr3
  
Location: 24339-24567

Mycgr3G104334\_Mycgr3

Mycgr3G42715 Mycgr3T
  
Location: 24667-25981

Mycgr3G42715\_Mycgr3T

Mycgr3G92934 Mycgr3T
  
Location: 26081-27593

Mycgr3G92934\_Mycgr3T

Mycgr3G41969 Mycgr3T
  
Location: 27693-29328

Mycgr3G41969\_Mycgr3T

Mycgr3G80635 Mycgr3T
  
Location: 29428-29821

Mycgr3G80635\_Mycgr3T

Mycgr3G41426 Mycgr3T
  
Location: 29921-35255

Mycgr3G41426\_Mycgr3T

Mycgr3G104337 Mycgr3
  
Location: 35355-36108

Mycgr3G104337\_Mycgr3

Mycgr3G71679 Mycgr3T
  
Location: 36208-37300

Mycgr3G71679\_Mycgr3T

Mycgr3G92938 Mycgr3T
  
Location: 37400-38699

Mycgr3G92938\_Mycgr3T

Mycgr3G92941 Mycgr3T
  
Location: 38799-40734

Mycgr3G92941\_Mycgr3T

not annotated
  
Accession: BAE58606
  
Location: 43863-46717
  
 NCBI BlastP on this gene

AO090023000016

not annotated
  
Accession: BAE58605
  
Location: 41238-42632
  
 NCBI BlastP on this gene

AO090023000015

not annotated
  
Accession: BAE58604
  
Location: 38934-39578
  
 NCBI BlastP on this gene

AO090023000014

not annotated
  
Accession: BAE58603
  
Location: 37175-38521
  
 NCBI BlastP on this gene

AO090023000013

not annotated
  
Accession: BAE58602
  
Location: 32362-35300
  
 NCBI BlastP on this gene

AO090023000012

not annotated
  
Accession: BAE58601
  
Location: 29813-31114
  
 NCBI BlastP on this gene

AO090023000011

not annotated
  
Accession: BAE58600
  
Location: 26595-27755
  
  
**BlastP hit with Mycgr3G92938\_Mycgr3T**
  
Percentage identity: 29 %
  
BlastP bit score: 114
  
Sequence coverage: 75 %
  
E-value: 4e-25
  
  
 NCBI BlastP on this gene

AO090023000010

not annotated
  
Accession: BAE58599
  
Location: 21131-22662
  
  
**BlastP hit with Mycgr3G42010\_Mycgr3T**
  
Percentage identity: 36 %
  
BlastP bit score: 322
  
Sequence coverage: 20 %
  
E-value: 8e-93
  
  
 NCBI BlastP on this gene

AO090023000008

not annotated
  
Accession: BAE58598
  
Location: 16001-19716
  
  
**BlastP hit with Mycgr3G42010\_Mycgr3T**
  
Percentage identity: 50 %
  
BlastP bit score: 282
  
Sequence coverage: 11 %
  
E-value: 7e-81
  
  
 NCBI BlastP on this gene

AO090023000007

not annotated
  
Accession: BAE58597
  
Location: 11234-13940
  
 NCBI BlastP on this gene

AO090023000006

not annotated
  
Accession: BAE58596
  
Location: 7651-8039
  
 NCBI BlastP on this gene

AO090023000004

not annotated
  
Accession: BAE58595
  
Location: 6228-6920
  
 NCBI BlastP on this gene

AO090023000003

not annotated
  
Accession: BAE58594
  
Location: 4189-4966
  
 NCBI BlastP on this gene

AO090023000002

not annotated
  
Accession: BAE58593
  
Location: 1943-3463
  
 NCBI BlastP on this gene

AO090023000001

Query: Architecture Search FASTA input

DS547093 : Laccaria bicolor S238N-H82 LACBIscaffold\_3 genomic scaffold    Total score: 2.0     Cumulative Blast bit score: 695

Hit cluster cross-links:

Mycgr3G85918 Mycgr3T
  
Location: 0-1602

Mycgr3G85918\_Mycgr3T

Mycgr3G42010 Mycgr3T
  
Location: 1702-8569

Mycgr3G42010\_Mycgr3T

Mycgr3G29582 Mycgr3T
  
Location: 8669-8915

Mycgr3G29582\_Mycgr3T

Mycgr3G31170 Mycgr3T
  
Location: 9015-9255

Mycgr3G31170\_Mycgr3T

Mycgr3G85924 Mycgr3T
  
Location: 9355-11218

Mycgr3G85924\_Mycgr3T

Mycgr3G71676 Mycgr3T
  
Location: 11318-12494

Mycgr3G71676\_Mycgr3T

Mycgr3G11468 Mycgr3T
  
Location: 12594-13653

Mycgr3G11468\_Mycgr3T

Mycgr3G58567 Mycgr3T
  
Location: 13753-14506

Mycgr3G58567\_Mycgr3T

Mycgr3G100089 Mycgr3
  
Location: 14606-21152

Mycgr3G100089\_Mycgr3

Mycgr3G42698 Mycgr3T
  
Location: 21252-22131

Mycgr3G42698\_Mycgr3T

Mycgr3G71681 Mycgr3T
  
Location: 22231-23461

Mycgr3G71681\_Mycgr3T

Mycgr3G109328 Mycgr3
  
Location: 23561-24239

Mycgr3G109328\_Mycgr3

Mycgr3G104334 Mycgr3
  
Location: 24339-24567

Mycgr3G104334\_Mycgr3

Mycgr3G42715 Mycgr3T
  
Location: 24667-25981

Mycgr3G42715\_Mycgr3T

Mycgr3G92934 Mycgr3T
  
Location: 26081-27593

Mycgr3G92934\_Mycgr3T

Mycgr3G41969 Mycgr3T
  
Location: 27693-29328

Mycgr3G41969\_Mycgr3T

Mycgr3G80635 Mycgr3T
  
Location: 29428-29821

Mycgr3G80635\_Mycgr3T

Mycgr3G41426 Mycgr3T
  
Location: 29921-35255

Mycgr3G41426\_Mycgr3T

Mycgr3G104337 Mycgr3
  
Location: 35355-36108

Mycgr3G104337\_Mycgr3

Mycgr3G71679 Mycgr3T
  
Location: 36208-37300

Mycgr3G71679\_Mycgr3T

Mycgr3G92938 Mycgr3T
  
Location: 37400-38699

Mycgr3G92938\_Mycgr3T

Mycgr3G92941 Mycgr3T
  
Location: 38799-40734

Mycgr3G92941\_Mycgr3T

predicted protein
  
Accession: EDR13704
  
Location: 1950107-1951478
  
 NCBI BlastP on this gene

EDR13704

predicted protein
  
Accession: EDR13288
  
Location: 1951864-1954054
  
 NCBI BlastP on this gene

EDR13288

predicted protein
  
Accession: EDR13289
  
Location: 1955047-1955571
  
 NCBI BlastP on this gene

EDR13289

predicted protein
  
Accession: EDR13290
  
Location: 1956040-1956216
  
 NCBI BlastP on this gene

EDR13290

predicted protein
  
Accession: EDR13291
  
Location: 1957634-1957986
  
  
**BlastP hit with Mycgr3G31170\_Mycgr3T**
  
Percentage identity: 93 %
  
BlastP bit score: 154
  
Sequence coverage: 100 %
  
E-value: 1e-46
  
  
 NCBI BlastP on this gene

EDR13291

predicted protein
  
Accession: EDR13705
  
Location: 1958883-1959394
  
 NCBI BlastP on this gene

EDR13705

predicted protein
  
Accession: EDR13706
  
Location: 1959734-1960216
  
 NCBI BlastP on this gene

EDR13706

predicted protein
  
Accession: EDR13707
  
Location: 1960321-1963184
  
 NCBI BlastP on this gene

EDR13707

predicted protein
  
Accession: EDR13708
  
Location: 1966716-1968482
  
 NCBI BlastP on this gene

EDR13708

predicted protein
  
Accession: EDR13292
  
Location: 1968759-1970981
  
 NCBI BlastP on this gene

EDR13292

predicted protein
  
Accession: EDR13709
  
Location: 1971148-1972811
  
 NCBI BlastP on this gene

EDR13709

predicted protein
  
Accession: EDR13710
  
Location: 1973331-1974025
  
 NCBI BlastP on this gene

EDR13710

predicted protein
  
Accession: EDR13711
  
Location: 1974241-1975830
  
 NCBI BlastP on this gene

EDR13711

predicted protein
  
Accession: EDR13712
  
Location: 1975998-1977523
  
 NCBI BlastP on this gene

EDR13712

predicted protein
  
Accession: EDR13713
  
Location: 1977645-1978883
  
 NCBI BlastP on this gene

EDR13713

predicted protein
  
Accession: EDR13714
  
Location: 1979115-1979724
  
 NCBI BlastP on this gene

EDR13714

predicted protein
  
Accession: EDR13715
  
Location: 1979897-1981182
  
 NCBI BlastP on this gene

EDR13715

predicted protein
  
Accession: EDR13716
  
Location: 1981475-1982495
  
 NCBI BlastP on this gene

EDR13716

predicted protein
  
Accession: EDR13293
  
Location: 1982675-1988992
  
  
**BlastP hit with Mycgr3G41426\_Mycgr3T**
  
Percentage identity: 31 %
  
BlastP bit score: 541
  
Sequence coverage: 67 %
  
E-value: 3e-157
  
  
 NCBI BlastP on this gene

EDR13293

predicted protein
  
Accession: EDR13717
  
Location: 1989092-1992898
  
 NCBI BlastP on this gene

EDR13717

predicted protein
  
Accession: EDR13294
  
Location: 1993493-1993901
  
 NCBI BlastP on this gene

EDR13294

predicted protein
  
Accession: EDR13718
  
Location: 1994244-1999983
  
 NCBI BlastP on this gene

EDR13718

Query: Architecture Search FASTA input

CACQ02006690 : Colletotrichum higginsianum strain IMI 349063    Total score: 2.0     Cumulative Blast bit score: 631

Hit cluster cross-links:

Mycgr3G85918 Mycgr3T
  
Location: 0-1602

Mycgr3G85918\_Mycgr3T

Mycgr3G42010 Mycgr3T
  
Location: 1702-8569

Mycgr3G42010\_Mycgr3T

Mycgr3G29582 Mycgr3T
  
Location: 8669-8915

Mycgr3G29582\_Mycgr3T

Mycgr3G31170 Mycgr3T
  
Location: 9015-9255

Mycgr3G31170\_Mycgr3T

Mycgr3G85924 Mycgr3T
  
Location: 9355-11218

Mycgr3G85924\_Mycgr3T

Mycgr3G71676 Mycgr3T
  
Location: 11318-12494

Mycgr3G71676\_Mycgr3T

Mycgr3G11468 Mycgr3T
  
Location: 12594-13653

Mycgr3G11468\_Mycgr3T

Mycgr3G58567 Mycgr3T
  
Location: 13753-14506

Mycgr3G58567\_Mycgr3T

Mycgr3G100089 Mycgr3
  
Location: 14606-21152

Mycgr3G100089\_Mycgr3

Mycgr3G42698 Mycgr3T
  
Location: 21252-22131

Mycgr3G42698\_Mycgr3T

Mycgr3G71681 Mycgr3T
  
Location: 22231-23461

Mycgr3G71681\_Mycgr3T

Mycgr3G109328 Mycgr3
  
Location: 23561-24239

Mycgr3G109328\_Mycgr3

Mycgr3G104334 Mycgr3
  
Location: 24339-24567

Mycgr3G104334\_Mycgr3

Mycgr3G42715 Mycgr3T
  
Location: 24667-25981

Mycgr3G42715\_Mycgr3T

Mycgr3G92934 Mycgr3T
  
Location: 26081-27593

Mycgr3G92934\_Mycgr3T

Mycgr3G41969 Mycgr3T
  
Location: 27693-29328

Mycgr3G41969\_Mycgr3T

Mycgr3G80635 Mycgr3T
  
Location: 29428-29821

Mycgr3G80635\_Mycgr3T

Mycgr3G41426 Mycgr3T
  
Location: 29921-35255

Mycgr3G41426\_Mycgr3T

Mycgr3G104337 Mycgr3
  
Location: 35355-36108

Mycgr3G104337\_Mycgr3

Mycgr3G71679 Mycgr3T
  
Location: 36208-37300

Mycgr3G71679\_Mycgr3T

Mycgr3G92938 Mycgr3T
  
Location: 37400-38699

Mycgr3G92938\_Mycgr3T

Mycgr3G92941 Mycgr3T
  
Location: 38799-40734

Mycgr3G92941\_Mycgr3T

F-box domain-containing protein
  
Accession: CCF44193
  
Location: 203-2840
  
 NCBI BlastP on this gene

CCF44193

RNA exonuclease
  
Accession: CCF44194
  
Location: 3738-5277
  
 NCBI BlastP on this gene

CCF44194

geranylgeranyl pyrophosphate synthetase
  
Accession: CCF44195
  
Location: 7242-8681
  
  
**BlastP hit with Mycgr3G92938\_Mycgr3T**
  
Percentage identity: 40 %
  
BlastP bit score: 271
  
Sequence coverage: 82 %
  
E-value: 5e-82
  
  
 NCBI BlastP on this gene

CCF44195

helicase required for RNAi-mediated heterochromatin assembly 1
  
Accession: CCF44196
  
Location: 9380-11270
  
  
**BlastP hit with Mycgr3G42010\_Mycgr3T**
  
Percentage identity: 37 %
  
BlastP bit score: 360
  
Sequence coverage: 26 %
  
E-value: 3e-104
  
  
 NCBI BlastP on this gene

CCF44196

Query: Architecture Search FASTA input

GG663369 : Ajellomyces capsulatus G186AR genomic scaffold supercont2.7    Total score: 2.0     Cumulative Blast bit score: 624

Hit cluster cross-links:

Mycgr3G85918 Mycgr3T
  
Location: 0-1602

Mycgr3G85918\_Mycgr3T

Mycgr3G42010 Mycgr3T
  
Location: 1702-8569

Mycgr3G42010\_Mycgr3T

Mycgr3G29582 Mycgr3T
  
Location: 8669-8915

Mycgr3G29582\_Mycgr3T

Mycgr3G31170 Mycgr3T
  
Location: 9015-9255

Mycgr3G31170\_Mycgr3T

Mycgr3G85924 Mycgr3T
  
Location: 9355-11218

Mycgr3G85924\_Mycgr3T

Mycgr3G71676 Mycgr3T
  
Location: 11318-12494

Mycgr3G71676\_Mycgr3T

Mycgr3G11468 Mycgr3T
  
Location: 12594-13653

Mycgr3G11468\_Mycgr3T

Mycgr3G58567 Mycgr3T
  
Location: 13753-14506

Mycgr3G58567\_Mycgr3T

Mycgr3G100089 Mycgr3
  
Location: 14606-21152

Mycgr3G100089\_Mycgr3

Mycgr3G42698 Mycgr3T
  
Location: 21252-22131

Mycgr3G42698\_Mycgr3T

Mycgr3G71681 Mycgr3T
  
Location: 22231-23461

Mycgr3G71681\_Mycgr3T

Mycgr3G109328 Mycgr3
  
Location: 23561-24239

Mycgr3G109328\_Mycgr3

Mycgr3G104334 Mycgr3
  
Location: 24339-24567

Mycgr3G104334\_Mycgr3

Mycgr3G42715 Mycgr3T
  
Location: 24667-25981

Mycgr3G42715\_Mycgr3T

Mycgr3G92934 Mycgr3T
  
Location: 26081-27593

Mycgr3G92934\_Mycgr3T

Mycgr3G41969 Mycgr3T
  
Location: 27693-29328

Mycgr3G41969\_Mycgr3T

Mycgr3G80635 Mycgr3T
  
Location: 29428-29821

Mycgr3G80635\_Mycgr3T

Mycgr3G41426 Mycgr3T
  
Location: 29921-35255

Mycgr3G41426\_Mycgr3T

Mycgr3G104337 Mycgr3
  
Location: 35355-36108

Mycgr3G104337\_Mycgr3

Mycgr3G71679 Mycgr3T
  
Location: 36208-37300

Mycgr3G71679\_Mycgr3T

Mycgr3G92938 Mycgr3T
  
Location: 37400-38699

Mycgr3G92938\_Mycgr3T

Mycgr3G92941 Mycgr3T
  
Location: 38799-40734

Mycgr3G92941\_Mycgr3T

C6 zinc finger domain-containing protein
  
Accession: EEH06335
  
Location: 1072470-1073774
  
 NCBI BlastP on this gene

EEH06335

predicted protein
  
Accession: EEH06336
  
Location: 1074536-1076052
  
 NCBI BlastP on this gene

EEH06336

conserved hypothetical protein
  
Accession: EEH06337
  
Location: 1076931-1078115
  
 NCBI BlastP on this gene

EEH06337

MADS box transcription factor Mcm1
  
Accession: EEH06338
  
Location: 1078923-1079856
  
  
**BlastP hit with Mycgr3G31170\_Mycgr3T**
  
Percentage identity: 98 %
  
BlastP bit score: 164
  
Sequence coverage: 100 %
  
E-value: 2e-48
  
  
 NCBI BlastP on this gene

EEH06338

DUF803 domain-containing protein
  
Accession: EEH06339
  
Location: 1082686-1084760
  
 NCBI BlastP on this gene

EEH06339

vacuolar ATP synthase subunit E
  
Accession: EEH06340
  
Location: 1085279-1086206
  
 NCBI BlastP on this gene

EEH06340

conserved hypothetical protein
  
Accession: EEH06341
  
Location: 1086816-1089296
  
 NCBI BlastP on this gene

EEH06341

serine/threonine phosphatase
  
Accession: EEH06342
  
Location: 1090472-1091506
  
 NCBI BlastP on this gene

EEH06342

phospholipid-translocating P-type ATPase domain-containing protein
  
Accession: EEH06343
  
Location: 1093672-1098340
  
 NCBI BlastP on this gene

EEH06343

protein kinase
  
Accession: EEH06344
  
Location: 1099801-1101292
  
 NCBI BlastP on this gene

EEH06344

DEAD-box type RNA helicase
  
Accession: EEH06345
  
Location: 1102134-1108881
  
  
**BlastP hit with Mycgr3G41426\_Mycgr3T**
  
Percentage identity: 30 %
  
BlastP bit score: 460
  
Sequence coverage: 49 %
  
E-value: 1e-129
  
  
 NCBI BlastP on this gene

EEH06345

predicted protein
  
Accession: EEH06346
  
Location: 1110086-1110684
  
 NCBI BlastP on this gene

EEH06346

predicted protein
  
Accession: EEH06347
  
Location: 1113777-1117046
  
 NCBI BlastP on this gene

EEH06347

Query: Architecture Search FASTA input

DS990642 : Ajellomyces capsulatus H88 supercont1.7 genomic scaffold    Total score: 2.0     Cumulative Blast bit score: 624

Hit cluster cross-links:

Mycgr3G85918 Mycgr3T
  
Location: 0-1602

Mycgr3G85918\_Mycgr3T

Mycgr3G42010 Mycgr3T
  
Location: 1702-8569

Mycgr3G42010\_Mycgr3T

Mycgr3G29582 Mycgr3T
  
Location: 8669-8915

Mycgr3G29582\_Mycgr3T

Mycgr3G31170 Mycgr3T
  
Location: 9015-9255

Mycgr3G31170\_Mycgr3T

Mycgr3G85924 Mycgr3T
  
Location: 9355-11218

Mycgr3G85924\_Mycgr3T

Mycgr3G71676 Mycgr3T
  
Location: 11318-12494

Mycgr3G71676\_Mycgr3T

Mycgr3G11468 Mycgr3T
  
Location: 12594-13653

Mycgr3G11468\_Mycgr3T

Mycgr3G58567 Mycgr3T
  
Location: 13753-14506

Mycgr3G58567\_Mycgr3T

Mycgr3G100089 Mycgr3
  
Location: 14606-21152

Mycgr3G100089\_Mycgr3

Mycgr3G42698 Mycgr3T
  
Location: 21252-22131

Mycgr3G42698\_Mycgr3T

Mycgr3G71681 Mycgr3T
  
Location: 22231-23461

Mycgr3G71681\_Mycgr3T

Mycgr3G109328 Mycgr3
  
Location: 23561-24239

Mycgr3G109328\_Mycgr3

Mycgr3G104334 Mycgr3
  
Location: 24339-24567

Mycgr3G104334\_Mycgr3

Mycgr3G42715 Mycgr3T
  
Location: 24667-25981

Mycgr3G42715\_Mycgr3T

Mycgr3G92934 Mycgr3T
  
Location: 26081-27593

Mycgr3G92934\_Mycgr3T

Mycgr3G41969 Mycgr3T
  
Location: 27693-29328

Mycgr3G41969\_Mycgr3T

Mycgr3G80635 Mycgr3T
  
Location: 29428-29821

Mycgr3G80635\_Mycgr3T

Mycgr3G41426 Mycgr3T
  
Location: 29921-35255

Mycgr3G41426\_Mycgr3T

Mycgr3G104337 Mycgr3
  
Location: 35355-36108

Mycgr3G104337\_Mycgr3

Mycgr3G71679 Mycgr3T
  
Location: 36208-37300

Mycgr3G71679\_Mycgr3T

Mycgr3G92938 Mycgr3T
  
Location: 37400-38699

Mycgr3G92938\_Mycgr3T

Mycgr3G92941 Mycgr3T
  
Location: 38799-40734

Mycgr3G92941\_Mycgr3T

tetraspanin
  
Accession: EGC49086
  
Location: 1725745-1726772
  
 NCBI BlastP on this gene

EGC49086

C6 zinc finger domain-containing protein
  
Accession: EGC49087
  
Location: 1728665-1730003
  
 NCBI BlastP on this gene

EGC49087

conserved hypothetical protein
  
Accession: EGC49088
  
Location: 1731831-1732267
  
 NCBI BlastP on this gene

EGC49088

conserved hypothetical protein
  
Accession: EGC49089
  
Location: 1733118-1734302
  
 NCBI BlastP on this gene

EGC49089

MADS box transcription factor Mcm1
  
Accession: EGC49090
  
Location: 1735126-1736056
  
  
**BlastP hit with Mycgr3G31170\_Mycgr3T**
  
Percentage identity: 98 %
  
BlastP bit score: 164
  
Sequence coverage: 100 %
  
E-value: 2e-48
  
  
 NCBI BlastP on this gene

EGC49090

DUF803 domain-containing protein
  
Accession: EGC49091
  
Location: 1738791-1740974
  
 NCBI BlastP on this gene

EGC49091

vacuolar ATP synthase subunit E
  
Accession: EGC49092
  
Location: 1741426-1742353
  
 NCBI BlastP on this gene

EGC49092

conserved hypothetical protein
  
Accession: EGC49093
  
Location: 1742922-1745404
  
 NCBI BlastP on this gene

EGC49093

serine/threonine protein phosphatase
  
Accession: EGC49094
  
Location: 1746597-1747683
  
 NCBI BlastP on this gene

EGC49094

phospholipid-translocating P-type ATPase
  
Accession: EGC49095
  
Location: 1749735-1754402
  
 NCBI BlastP on this gene

EGC49095

helicase SEN1
  
Accession: EGC49096
  
Location: 1756257-1763003
  
  
**BlastP hit with Mycgr3G41426\_Mycgr3T**
  
Percentage identity: 30 %
  
BlastP bit score: 460
  
Sequence coverage: 49 %
  
E-value: 1e-129
  
  
 NCBI BlastP on this gene

EGC49096

predicted protein
  
Accession: EGC49097
  
Location: 1763884-1764736
  
 NCBI BlastP on this gene

EGC49097

predicted protein
  
Accession: EGC49098
  
Location: 1767857-1771015
  
 NCBI BlastP on this gene

EGC49098

dihydroorotase
  
Accession: EGC49099
  
Location: 1771623-1772874
  
 NCBI BlastP on this gene

EGC49099

Query: Architecture Search FASTA input

151. :  AKCT01000066 Penicillium digitatum PHI26     Total score: 2.0     Cumulative Blast bit score: 1210

Mycgr3G85918 Mycgr3T
  
Location: 0-1602
  
 NCBI BlastP on this gene

Mycgr3G85918\_Mycgr3T

Mycgr3G42010 Mycgr3T
  
Location: 1702-8569
  
 NCBI BlastP on this gene

Mycgr3G42010\_Mycgr3T

Mycgr3G29582 Mycgr3T
  
Location: 8669-8915
  
 NCBI BlastP on this gene

Mycgr3G29582\_Mycgr3T

Mycgr3G31170 Mycgr3T
  
Location: 9015-9255
  
 NCBI BlastP on this gene

Mycgr3G31170\_Mycgr3T

Mycgr3G85924 Mycgr3T
  
Location: 9355-11218
  
 NCBI BlastP on this gene

Mycgr3G85924\_Mycgr3T

Mycgr3G71676 Mycgr3T
  
Location: 11318-12494
  
 NCBI BlastP on this gene

Mycgr3G71676\_Mycgr3T

Mycgr3G11468 Mycgr3T
  
Location: 12594-13653
  
 NCBI BlastP on this gene

Mycgr3G11468\_Mycgr3T

Mycgr3G58567 Mycgr3T
  
Location: 13753-14506
  
 NCBI BlastP on this gene

Mycgr3G58567\_Mycgr3T

Mycgr3G100089 Mycgr3
  
Location: 14606-21152
  
 NCBI BlastP on this gene

Mycgr3G100089\_Mycgr3

Mycgr3G42698 Mycgr3T
  
Location: 21252-22131
  
 NCBI BlastP on this gene

Mycgr3G42698\_Mycgr3T

Mycgr3G71681 Mycgr3T
  
Location: 22231-23461
  
 NCBI BlastP on this gene

Mycgr3G71681\_Mycgr3T

Mycgr3G109328 Mycgr3
  
Location: 23561-24239
  
 NCBI BlastP on this gene

Mycgr3G109328\_Mycgr3

Mycgr3G104334 Mycgr3
  
Location: 24339-24567
  
 NCBI BlastP on this gene

Mycgr3G104334\_Mycgr3

Mycgr3G42715 Mycgr3T
  
Location: 24667-25981
  
 NCBI BlastP on this gene

Mycgr3G42715\_Mycgr3T

Mycgr3G92934 Mycgr3T
  
Location: 26081-27593
  
 NCBI BlastP on this gene

Mycgr3G92934\_Mycgr3T

Mycgr3G41969 Mycgr3T
  
Location: 27693-29328
  
 NCBI BlastP on this gene

Mycgr3G41969\_Mycgr3T

Mycgr3G80635 Mycgr3T
  
Location: 29428-29821
  
 NCBI BlastP on this gene

Mycgr3G80635\_Mycgr3T

Mycgr3G41426 Mycgr3T
  
Location: 29921-35255
  
 NCBI BlastP on this gene

Mycgr3G41426\_Mycgr3T

Mycgr3G104337 Mycgr3
  
Location: 35355-36108
  
 NCBI BlastP on this gene

Mycgr3G104337\_Mycgr3

Mycgr3G71679 Mycgr3T
  
Location: 36208-37300
  
 NCBI BlastP on this gene

Mycgr3G71679\_Mycgr3T

Mycgr3G92938 Mycgr3T
  
Location: 37400-38699
  
 NCBI BlastP on this gene

Mycgr3G92938\_Mycgr3T

Mycgr3G92941 Mycgr3T
  
Location: 38799-40734
  
 NCBI BlastP on this gene

Mycgr3G92941\_Mycgr3T

hypothetical protein
  
Accession: EKV17380
  
Location: 56318-56455
  
 NCBI BlastP on this gene

EKV17380

Myosin heavy chain-like protein, putative
  
Accession: EKV17381
  
Location: 61582-68328
  
 NCBI BlastP on this gene

EKV17381

Endosomal cargo receptor (P24), putative
  
Accession: EKV17382
  
Location: 69493-70433
  
 NCBI BlastP on this gene

EKV17382

hypothetical protein
  
Accession: EKV17383
  
Location: 71023-71860
  
 NCBI BlastP on this gene

EKV17383

hypothetical protein
  
Accession: EKV17384
  
Location: 71950-72811
  
  
**BlastP hit with Mycgr3G104337\_Mycgr3**
  
Percentage identity: 39 %
  
BlastP bit score: 160
  
Sequence coverage: 98 %
  
E-value: 2e-44
  
  
 NCBI BlastP on this gene

EKV17384

hypothetical protein
  
Accession: EKV17385
  
Location: 73543-81529
  
  
**BlastP hit with Mycgr3G100089\_Mycgr3**
  
Percentage identity: 43 %
  
BlastP bit score: 1050
  
Sequence coverage: 58 %
  
E-value: 0.0
  
  
 NCBI BlastP on this gene

EKV17385

hypothetical protein
  
Accession: EKV17386
  
Location: 82893-83908
  
 NCBI BlastP on this gene

EKV17386

hypothetical protein
  
Accession: EKV17387
  
Location: 84281-84544
  
 NCBI BlastP on this gene

EKV17387

hypothetical protein
  
Accession: EKV17388
  
Location: 85806-87019
  
 NCBI BlastP on this gene

EKV17388

MFS lactose permease, putative
  
Accession: EKV17389
  
Location: 88505-90671
  
 NCBI BlastP on this gene

EKV17389

Endopolyphosphatase
  
Accession: EKV17390
  
Location: 94685-96634
  
 NCBI BlastP on this gene

EKV17390

hypothetical protein
  
Accession: EKV17391
  
Location: 98560-99633
  
 NCBI BlastP on this gene

EKV17391

152. :  KB456260 Mycosphaerella populorum SO2202 unplaced genomic scaffold SEPMUscaffold\_1     Total score: 2.0     Cumulative Blast bit score: 1208

acyl-CoA N-acyltransferase
  
Accession: EMF16352
  
Location: 554524-555006
  
 NCBI BlastP on this gene

EMF16352

hypothetical protein
  
Accession: EMF16353
  
Location: 556230-556733
  
 NCBI BlastP on this gene

EMF16353

hypothetical protein
  
Accession: EMF16355
  
Location: 560467-561345
  
 NCBI BlastP on this gene

EMF16355

hypothetical protein
  
Accession: EMF16356
  
Location: 562147-563825
  
 NCBI BlastP on this gene

EMF16356

hypothetical protein
  
Accession: EMF16357
  
Location: 564310-565674
  
 NCBI BlastP on this gene

EMF16357

metalloprotease
  
Accession: EMF16358
  
Location: 566495-569078
  
 NCBI BlastP on this gene

EMF16358

elongation factor G, mitochondrial
  
Accession: EMF16359
  
Location: 569915-572347
  
 NCBI BlastP on this gene

EMF16359

DEAD-domain-containing protein
  
Accession: EMF16360
  
Location: 572715-574295
  
  
**BlastP hit with Mycgr3G92934\_Mycgr3T**
  
Percentage identity: 70 %
  
BlastP bit score: 690
  
Sequence coverage: 98 %
  
E-value: 0.0
  
  
 NCBI BlastP on this gene

EMF16360

Krr1-domain-containing protein
  
Accession: EMF16361
  
Location: 574487-576457
  
  
**BlastP hit with Mycgr3G85924\_Mycgr3T**
  
Percentage identity: 56 %
  
BlastP bit score: 518
  
Sequence coverage: 99 %
  
E-value: 9e-173
  
  
 NCBI BlastP on this gene

EMF16361

hypothetical protein
  
Accession: EMF16362
  
Location: 577564-577893
  
 NCBI BlastP on this gene

EMF16362

hypothetical protein
  
Accession: EMF16363
  
Location: 578602-579195
  
 NCBI BlastP on this gene

EMF16363

hypothetical protein
  
Accession: EMF16364
  
Location: 580687-584181
  
 NCBI BlastP on this gene

EMF16364

hypothetical protein
  
Accession: EMF16365
  
Location: 586317-587819
  
 NCBI BlastP on this gene

EMF16365

hypothetical protein
  
Accession: EMF16366
  
Location: 588585-589721
  
 NCBI BlastP on this gene

EMF16366

CDC91 cell division cycle 91-like protein
  
Accession: EMF16367
  
Location: 590213-591815
  
 NCBI BlastP on this gene

EMF16367

Pescadillo N-domain-containing protein
  
Accession: EMF16368
  
Location: 592098-594328
  
 NCBI BlastP on this gene

EMF16368

hypothetical protein
  
Accession: EMF16369
  
Location: 594842-595237
  
 NCBI BlastP on this gene

EMF16369

153. :  CH476634 Sclerotinia sclerotiorum 1980 scaffold\_14 genomic scaffold     Total score: 2.0     Cumulative Blast bit score: 1184

hypothetical protein
  
Accession: EDN93761
  
Location: 46587-47911
  
 NCBI BlastP on this gene

EDN93761

hypothetical protein
  
Accession: EDN93762
  
Location: 48482-50198
  
 NCBI BlastP on this gene

EDN93762

hypothetical protein
  
Accession: EDN93763
  
Location: 50591-51544
  
 NCBI BlastP on this gene

EDN93763

predicted protein
  
Accession: EDN93764
  
Location: 53243-53341
  
 NCBI BlastP on this gene

EDN93764

hypothetical protein
  
Accession: EDN93765
  
Location: 57844-60564
  
 NCBI BlastP on this gene

EDN93765

predicted protein
  
Accession: EDN93766
  
Location: 61960-62143
  
 NCBI BlastP on this gene

EDN93766

hypothetical protein
  
Accession: EDN93767
  
Location: 63381-70210
  
  
**BlastP hit with Mycgr3G42010\_Mycgr3T**
  
Percentage identity: 45 %
  
BlastP bit score: 978
  
Sequence coverage: 54 %
  
E-value: 0.0
  
  
 NCBI BlastP on this gene

EDN93767

hypothetical protein
  
Accession: EDN93768
  
Location: 70731-73500
  
  
**BlastP hit with Mycgr3G92938\_Mycgr3T**
  
Percentage identity: 38 %
  
BlastP bit score: 206
  
Sequence coverage: 68 %
  
E-value: 4e-57
  
  
 NCBI BlastP on this gene

EDN93768

hypothetical protein
  
Accession: EDN93769
  
Location: 73773-75349
  
 NCBI BlastP on this gene

EDN93769

hypothetical protein
  
Accession: EDN93770
  
Location: 76378-78036
  
 NCBI BlastP on this gene

EDN93770

hypothetical protein
  
Accession: EDN93771
  
Location: 78345-80157
  
 NCBI BlastP on this gene

EDN93771

predicted protein
  
Accession: EDN93772
  
Location: 81048-81233
  
 NCBI BlastP on this gene

EDN93772

predicted protein
  
Accession: EDN93773
  
Location: 81703-82122
  
 NCBI BlastP on this gene

EDN93773

predicted protein
  
Accession: EDN93774
  
Location: 82682-83549
  
 NCBI BlastP on this gene

EDN93774

predicted protein
  
Accession: EDN93775
  
Location: 85537-86922
  
 NCBI BlastP on this gene

EDN93775

hypothetical protein
  
Accession: EDN93776
  
Location: 87336-87479
  
 NCBI BlastP on this gene

EDN93776

hypothetical protein
  
Accession: EDN93777
  
Location: 88177-88524
  
 NCBI BlastP on this gene

EDN93777

predicted protein
  
Accession: EDN93778
  
Location: 90986-91195
  
 NCBI BlastP on this gene

EDN93778

154. :  CH476626 Sclerotinia sclerotiorum 1980 scaffold\_6 genomic scaffold     Total score: 2.0     Cumulative Blast bit score: 1174

malate synthase
  
Accession: EDO03104
  
Location: 1968878-1970633
  
 NCBI BlastP on this gene

EDO03104

predicted protein
  
Accession: EDO03105
  
Location: 1972100-1972285
  
 NCBI BlastP on this gene

EDO03105

predicted protein
  
Accession: EDO03106
  
Location: 1974067-1974882
  
 NCBI BlastP on this gene

EDO03106

hypothetical protein
  
Accession: EDO03107
  
Location: 1975463-1976386
  
 NCBI BlastP on this gene

EDO03107

predicted protein
  
Accession: EDO03108
  
Location: 1978012-1978536
  
 NCBI BlastP on this gene

EDO03108

hypothetical protein
  
Accession: EDO03109
  
Location: 1981916-1982762
  
  
**BlastP hit with Mycgr3G31170\_Mycgr3T**
  
Percentage identity: 97 %
  
BlastP bit score: 159
  
Sequence coverage: 100 %
  
E-value: 9e-47
  
  
 NCBI BlastP on this gene

EDO03109

vacuolar ATP synthase subunit E
  
Accession: EDO03110
  
Location: 1986634-1987557
  
 NCBI BlastP on this gene

EDO03110

hypothetical protein
  
Accession: EDO03111
  
Location: 1987797-1989784
  
 NCBI BlastP on this gene

EDO03111

predicted protein
  
Accession: EDO03112
  
Location: 1990043-1991105
  
 NCBI BlastP on this gene

EDO03112

hypothetical protein
  
Accession: EDO03113
  
Location: 1996827-1997474
  
 NCBI BlastP on this gene

EDO03113

hypothetical protein
  
Accession: EDO03114
  
Location: 1998616-2004906
  
  
**BlastP hit with Mycgr3G41426\_Mycgr3T**
  
Percentage identity: 34 %
  
BlastP bit score: 1015
  
Sequence coverage: 105 %
  
E-value: 0.0
  
  
 NCBI BlastP on this gene

EDO03114

predicted protein
  
Accession: EDO03115
  
Location: 2005351-2005882
  
 NCBI BlastP on this gene

EDO03115

predicted protein
  
Accession: EDO03116
  
Location: 2006400-2007178
  
 NCBI BlastP on this gene

EDO03116

predicted protein
  
Accession: EDO03117
  
Location: 2008103-2008552
  
 NCBI BlastP on this gene

EDO03117

hypothetical protein
  
Accession: EDO03118
  
Location: 2009507-2009923
  
 NCBI BlastP on this gene

EDO03118

hypothetical protein
  
Accession: EDO03119
  
Location: 2012361-2015200
  
 NCBI BlastP on this gene

EDO03119

hypothetical protein
  
Accession: EDO03120
  
Location: 2015676-2015936
  
 NCBI BlastP on this gene

EDO03120

155. :  AKHY01000142 Aspergillus oryzae 3.042     Total score: 2.0     Cumulative Blast bit score: 1173

hypothetical protein
  
Accession: EIT77858
  
Location: 31565-32365
  
 NCBI BlastP on this gene

EIT77858

permease of the major facilitator superfamily
  
Accession: EIT77947
  
Location: 35832-37569
  
 NCBI BlastP on this gene

EIT77947

hypothetical protein
  
Accession: EIT77966
  
Location: 40674-41624
  
 NCBI BlastP on this gene

EIT77966

hypothetical protein
  
Accession: EIT77877
  
Location: 47206-48984
  
 NCBI BlastP on this gene

EIT77877

polyketide synthase module
  
Accession: EIT77964
  
Location: 50338-58371
  
  
**BlastP hit with Mycgr3G100089\_Mycgr3**
  
Percentage identity: 42 %
  
BlastP bit score: 1052
  
Sequence coverage: 58 %
  
E-value: 0.0
  
  
 NCBI BlastP on this gene

EIT77964

hypothetical protein
  
Accession: EIT77840
  
Location: 59360-59854
  
  
**BlastP hit with Mycgr3G104337\_Mycgr3**
  
Percentage identity: 43 %
  
BlastP bit score: 121
  
Sequence coverage: 56 %
  
E-value: 3e-30
  
  
 NCBI BlastP on this gene

EIT77840

FAD binding oxidoreductase, putative
  
Accession: EIT77828
  
Location: 62822-64224
  
 NCBI BlastP on this gene

EIT77828

RING finger protein
  
Accession: EIT77899
  
Location: 65164-65836
  
 NCBI BlastP on this gene

EIT77899

putative cytochrome C oxidase assembly protein
  
Accession: EIT77873
  
Location: 66677-67903
  
 NCBI BlastP on this gene

EIT77873

molybdopterin synthase large subunit CnxH
  
Accession: EIT77933
  
Location: 67908-68447
  
 NCBI BlastP on this gene

EIT77933

hypothetical protein
  
Accession: EIT77917
  
Location: 68795-69584
  
 NCBI BlastP on this gene

EIT77917

short-chain dehydrogenase/reductase family protein, putative
  
Accession: EIT77923
  
Location: 71059-72051
  
 NCBI BlastP on this gene

EIT77923

alpha-amylase
  
Accession: EIT77864
  
Location: 72796-74690
  
 NCBI BlastP on this gene

EIT77864

hypothetical protein
  
Accession: EIT77911
  
Location: 75014-75688
  
 NCBI BlastP on this gene

EIT77911

LPS glycosyltransferase
  
Accession: EIT77929
  
Location: 75858-77203
  
 NCBI BlastP on this gene

EIT77929

156. :  DS989828 Arthroderma gypseum CBS 118893 supercont1.7 genomic scaffold     Total score: 2.0     Cumulative Blast bit score: 1171

hypothetical protein
  
Accession: EFR04823
  
Location: 1378330-1378827
  
 NCBI BlastP on this gene

EFR04823

hypothetical protein
  
Accession: EFR04824
  
Location: 1381280-1381949
  
 NCBI BlastP on this gene

EFR04824

cell division control protein 25
  
Accession: EFR04825
  
Location: 1382184-1385852
  
 NCBI BlastP on this gene

EFR04825

hypothetical protein
  
Accession: EFR04826
  
Location: 1389449-1391056
  
 NCBI BlastP on this gene

EFR04826

fatty-acid-CoA ligase
  
Accession: EFR04827
  
Location: 1392766-1394695
  
 NCBI BlastP on this gene

EFR04827

lovastatin nonaketide synthase
  
Accession: EFR04828
  
Location: 1395051-1402076
  
  
**BlastP hit with Mycgr3G100089\_Mycgr3**
  
Percentage identity: 40 %
  
BlastP bit score: 987
  
Sequence coverage: 60 %
  
E-value: 0.0
  
  
 NCBI BlastP on this gene

EFR04828

hypothetical protein
  
Accession: EFR04829
  
Location: 1402897-1403929
  
  
**BlastP hit with Mycgr3G104337\_Mycgr3**
  
Percentage identity: 40 %
  
BlastP bit score: 184
  
Sequence coverage: 100 %
  
E-value: 9e-54
  
  
 NCBI BlastP on this gene

EFR04829

hypothetical protein
  
Accession: EFR04830
  
Location: 1404240-1405769
  
 NCBI BlastP on this gene

EFR04830

hypothetical protein
  
Accession: EFR04831
  
Location: 1406299-1407499
  
 NCBI BlastP on this gene

EFR04831

157. :  KB644411 Penicillium oxalicum 114-2 unplaced genomic scaffold scaffold\_4     Total score: 2.0     Cumulative Blast bit score: 1162

hypothetical protein
  
Accession: EPS28502
  
Location: 439387-440261
  
 NCBI BlastP on this gene

EPS28502

hypothetical protein
  
Accession: EPS28503
  
Location: 441174-442702
  
 NCBI BlastP on this gene

EPS28503

hypothetical protein
  
Accession: EPS28504
  
Location: 443123-445676
  
 NCBI BlastP on this gene

EPS28504

hypothetical protein
  
Accession: EPS28505
  
Location: 447788-449904
  
 NCBI BlastP on this gene

EPS28505

putative beta-1,3-glucanosyltransglycosylase
  
Accession: EPS28506
  
Location: 450874-452715
  
 NCBI BlastP on this gene

EPS28506

hypothetical protein
  
Accession: EPS28507
  
Location: 454616-456146
  
 NCBI BlastP on this gene

EPS28507

hypothetical protein
  
Accession: EPS28508
  
Location: 457201-458070
  
  
**BlastP hit with Mycgr3G104337\_Mycgr3**
  
Percentage identity: 39 %
  
BlastP bit score: 169
  
Sequence coverage: 100 %
  
E-value: 6e-48
  
  
 NCBI BlastP on this gene

EPS28508

hypothetical protein
  
Accession: EPS28509
  
Location: 458799-466755
  
  
**BlastP hit with Mycgr3G100089\_Mycgr3**
  
Percentage identity: 41 %
  
BlastP bit score: 993
  
Sequence coverage: 58 %
  
E-value: 0.0
  
  
 NCBI BlastP on this gene

EPS28509

hypothetical protein
  
Accession: EPS28510
  
Location: 469817-471357
  
 NCBI BlastP on this gene

EPS28510

hypothetical protein
  
Accession: EPS28511
  
Location: 473337-475644
  
 NCBI BlastP on this gene

EPS28511

hypothetical protein
  
Accession: EPS28512
  
Location: 476925-479736
  
 NCBI BlastP on this gene

EPS28512

hypothetical protein
  
Accession: EPS28513
  
Location: 481522-482192
  
 NCBI BlastP on this gene

EPS28513

hypothetical protein
  
Accession: EPS28514
  
Location: 483534-483890
  
 NCBI BlastP on this gene

EPS28514

158. :  AP007167 Aspergillus oryzae RIB40 DNA, SC020.     Total score: 2.0     Cumulative Blast bit score: 1145

not annotated
  
Accession: BAE63398
  
Location: 438971-440316
  
 NCBI BlastP on this gene

AO090020000174

not annotated
  
Accession: BAE63399
  
Location: 440486-441160
  
 NCBI BlastP on this gene

AO090020000175

not annotated
  
Accession: BAE63400
  
Location: 441484-443378
  
 NCBI BlastP on this gene

AO090020000176

not annotated
  
Accession: BAE63401
  
Location: 444123-445040
  
 NCBI BlastP on this gene

AO090020000177

not annotated
  
Accession: BAE63402
  
Location: 446590-447364
  
 NCBI BlastP on this gene

AO090020000178

not annotated
  
Accession: BAE63403
  
Location: 447712-448251
  
 NCBI BlastP on this gene

AO090020000179

not annotated
  
Accession: BAE63404
  
Location: 448256-449483
  
 NCBI BlastP on this gene

AO090020000180

not annotated
  
Accession: BAE63405
  
Location: 450324-450996
  
 NCBI BlastP on this gene

AO090020000182

not annotated
  
Accession: BAE63406
  
Location: 451936-453338
  
 NCBI BlastP on this gene

AO090020000183

not annotated
  
Accession: BAE63407
  
Location: 456308-456802
  
  
**BlastP hit with Mycgr3G104337\_Mycgr3**
  
Percentage identity: 41 %
  
BlastP bit score: 90
  
Sequence coverage: 41 %
  
E-value: 4e-19
  
  
 NCBI BlastP on this gene

AO090020000185

not annotated
  
Accession: BAE63408
  
Location: 457790-465823
  
  
**BlastP hit with Mycgr3G100089\_Mycgr3**
  
Percentage identity: 42 %
  
BlastP bit score: 1055
  
Sequence coverage: 58 %
  
E-value: 0.0
  
  
 NCBI BlastP on this gene

AO090020000186

not annotated
  
Accession: BAE63409
  
Location: 467193-470773
  
 NCBI BlastP on this gene

AO090020000188

not annotated
  
Accession: BAE63410
  
Location: 474553-475503
  
 NCBI BlastP on this gene

AO090020000189

not annotated
  
Accession: BAE63411
  
Location: 478597-480899
  
 NCBI BlastP on this gene

AO090020000190

not annotated
  
Accession: BAE63412
  
Location: 483803-484603
  
 NCBI BlastP on this gene

AO090020000191

159. :  DS995701 Microsporum canis CBS 113480 supercont1.1 genomic scaffold     Total score: 2.0     Cumulative Blast bit score: 1141

ferric reductase
  
Accession: EEQ27701
  
Location: 1606261-1608552
  
 NCBI BlastP on this gene

EEQ27701

conserved hypothetical protein
  
Accession: EEQ27702
  
Location: 1609085-1610203
  
 NCBI BlastP on this gene

EEQ27702

PTAC beta
  
Accession: EEQ27703
  
Location: 1611307-1612414
  
 NCBI BlastP on this gene

EEQ27703

tetraspanin
  
Accession: EEQ27704
  
Location: 1613225-1614125
  
 NCBI BlastP on this gene

EEQ27704

MADS box transcription factor Mcm1
  
Accession: EEQ27705
  
Location: 1615187-1616041
  
  
**BlastP hit with Mycgr3G31170\_Mycgr3T**
  
Percentage identity: 96 %
  
BlastP bit score: 154
  
Sequence coverage: 98 %
  
E-value: 5e-45
  
  
 NCBI BlastP on this gene

EEQ27705

C6 zinc finger domain-containing protein
  
Accession: EEQ27706
  
Location: 1617422-1618819
  
 NCBI BlastP on this gene

EEQ27706

carboxy-cis,cis-muconate cyclase
  
Accession: EEQ27707
  
Location: 1619069-1620313
  
 NCBI BlastP on this gene

EEQ27707

DUF803 domain-containing protein
  
Accession: EEQ27708
  
Location: 1622203-1624287
  
 NCBI BlastP on this gene

EEQ27708

vacuolar ATP synthase subunit E
  
Accession: EEQ27709
  
Location: 1624890-1625739
  
 NCBI BlastP on this gene

EEQ27709

conserved hypothetical protein
  
Accession: EEQ27710
  
Location: 1626283-1628429
  
 NCBI BlastP on this gene

EEQ27710

predicted protein
  
Accession: EEQ27711
  
Location: 1628972-1629797
  
 NCBI BlastP on this gene

EEQ27711

phospholipid-translocating P-type ATPase domain-containing protein
  
Accession: EEQ27712
  
Location: 1630885-1635526
  
 NCBI BlastP on this gene

EEQ27712

conserved hypothetical protein
  
Accession: EEQ27713
  
Location: 1636672-1638128
  
 NCBI BlastP on this gene

EEQ27713

helicase SEN1
  
Accession: EEQ27714
  
Location: 1638703-1643541
  
  
**BlastP hit with Mycgr3G41426\_Mycgr3T**
  
Percentage identity: 45 %
  
BlastP bit score: 987
  
Sequence coverage: 67 %
  
E-value: 0.0
  
  
 NCBI BlastP on this gene

EEQ27714

predicted protein
  
Accession: EEQ27715
  
Location: 1644194-1644763
  
 NCBI BlastP on this gene

EEQ27715

conserved hypothetical protein
  
Accession: EEQ27716
  
Location: 1646514-1648187
  
 NCBI BlastP on this gene

EEQ27716

hypothetical protein
  
Accession: EEQ27717
  
Location: 1650441-1653404
  
 NCBI BlastP on this gene

EEQ27717

160. :  AKCU01000499 Penicillium digitatum Pd1     Total score: 2.0     Cumulative Blast bit score: 1141

Hexokinase-1
  
Accession: EKV05518
  
Location: 2550-4166
  
 NCBI BlastP on this gene

EKV05518

hypothetical protein
  
Accession: EKV05519
  
Location: 5409-5756
  
 NCBI BlastP on this gene

EKV05519

Polyketide synthase, putative
  
Accession: EKV05520
  
Location: 5797-8814
  
 NCBI BlastP on this gene

EKV05520

Mycocerosic acid synthase
  
Accession: EKV05521
  
Location: 8977-11136
  
  
**BlastP hit with Mycgr3G100089\_Mycgr3**
  
Percentage identity: 59 %
  
BlastP bit score: 845
  
Sequence coverage: 30 %
  
E-value: 0.0
  
  
 NCBI BlastP on this gene

EKV05521

hypothetical protein
  
Accession: EKV05522
  
Location: 11663-12626
  
  
**BlastP hit with Mycgr3G104337\_Mycgr3**
  
Percentage identity: 54 %
  
BlastP bit score: 296
  
Sequence coverage: 99 %
  
E-value: 3e-97
  
  
 NCBI BlastP on this gene

EKV05522

Acetylxylan esterase, putative
  
Accession: EKV05523
  
Location: 13464-13847
  
 NCBI BlastP on this gene

EKV05523

Uridylate kinase Ura6
  
Accession: EKV05524
  
Location: 15765-16394
  
 NCBI BlastP on this gene

EKV05524

Eukaryotic translation initiation factor 3 subunit M
  
Accession: EKV05525
  
Location: 16869-18331
  
 NCBI BlastP on this gene

EKV05525

hypothetical protein
  
Accession: EKV05526
  
Location: 18874-19586
  
 NCBI BlastP on this gene

EKV05526

Thioesterase family protein
  
Accession: EKV05527
  
Location: 20554-21249
  
 NCBI BlastP on this gene

EKV05527

hypothetical protein
  
Accession: EKV05528
  
Location: 21799-21960
  
 NCBI BlastP on this gene

EKV05528

161. :  AKCT01000041 Penicillium digitatum PHI26     Total score: 2.0     Cumulative Blast bit score: 1141

hypothetical protein
  
Accession: EKV18169
  
Location: 81185-82141
  
 NCBI BlastP on this gene

EKV18169

hypothetical protein
  
Accession: EKV18170
  
Location: 82928-83525
  
 NCBI BlastP on this gene

EKV18170

C6 transcription factor, putative
  
Accession: EKV18171
  
Location: 84874-86352
  
 NCBI BlastP on this gene

EKV18171

hypothetical protein
  
Accession: EKV18172
  
Location: 87683-88461
  
 NCBI BlastP on this gene

EKV18172

hypothetical protein
  
Accession: EKV18173
  
Location: 89671-92196
  
 NCBI BlastP on this gene

EKV18173

Hexokinase-1
  
Accession: EKV18174
  
Location: 94693-96309
  
 NCBI BlastP on this gene

EKV18174

hypothetical protein
  
Accession: EKV18175
  
Location: 97553-97900
  
 NCBI BlastP on this gene

EKV18175

Polyketide synthase, putative
  
Accession: EKV18176
  
Location: 97941-100958
  
 NCBI BlastP on this gene

EKV18176

Mycocerosic acid synthase
  
Accession: EKV18177
  
Location: 101121-103280
  
  
**BlastP hit with Mycgr3G100089\_Mycgr3**
  
Percentage identity: 59 %
  
BlastP bit score: 845
  
Sequence coverage: 30 %
  
E-value: 0.0
  
  
 NCBI BlastP on this gene

EKV18177

hypothetical protein
  
Accession: EKV18178
  
Location: 103807-104770
  
  
**BlastP hit with Mycgr3G104337\_Mycgr3**
  
Percentage identity: 54 %
  
BlastP bit score: 296
  
Sequence coverage: 99 %
  
E-value: 3e-97
  
  
 NCBI BlastP on this gene

EKV18178

Acetylxylan esterase, putative
  
Accession: EKV18179
  
Location: 105608-105991
  
 NCBI BlastP on this gene

EKV18179

Uridylate kinase Ura6
  
Accession: EKV18180
  
Location: 107927-108556
  
 NCBI BlastP on this gene

EKV18180

Eukaryotic translation initiation factor 3 subunit M
  
Accession: EKV18181
  
Location: 109032-110494
  
 NCBI BlastP on this gene

EKV18181

hypothetical protein
  
Accession: EKV18182
  
Location: 111037-111749
  
 NCBI BlastP on this gene

EKV18182

Thioesterase family protein
  
Accession: EKV18183
  
Location: 112717-113412
  
 NCBI BlastP on this gene

EKV18183

hypothetical protein
  
Accession: EKV18184
  
Location: 113962-114123
  
 NCBI BlastP on this gene

EKV18184

hypothetical protein
  
Accession: EKV18185
  
Location: 115059-116861
  
 NCBI BlastP on this gene

EKV18185

Alpha,alpha-trehalose phosphate synthase subunit TPS3, putative
  
Accession: EKV18186
  
Location: 118918-121832
  
 NCBI BlastP on this gene

EKV18186

hypothetical protein
  
Accession: EKV18187
  
Location: 122386-123577
  
 NCBI BlastP on this gene

EKV18187

hypothetical protein
  
Accession: EKV18188
  
Location: 123883-124984
  
 NCBI BlastP on this gene

EKV18188

162. :  KB707685 Botryotinia fuckeliana BcDW1 unplaced genomic scaffold Scaffold\_13     Total score: 2.0     Cumulative Blast bit score: 1140

putative tetraspanin protein
  
Accession: EMR90826
  
Location: 110789-111547
  
 NCBI BlastP on this gene

EMR90826

hypothetical protein
  
Accession: EMR90827
  
Location: 112997-113206
  
 NCBI BlastP on this gene

EMR90827

putative mads box protein
  
Accession: EMR90828
  
Location: 117395-118244
  
  
**BlastP hit with Mycgr3G31170\_Mycgr3T**
  
Percentage identity: 97 %
  
BlastP bit score: 159
  
Sequence coverage: 100 %
  
E-value: 1e-46
  
  
 NCBI BlastP on this gene

EMR90828

putative duf803 domain-containing protein
  
Accession: EMR90829
  
Location: 120310-122007
  
 NCBI BlastP on this gene

EMR90829

putative vacuolar atp synthase subunit e protein
  
Accession: EMR90830
  
Location: 122592-123519
  
 NCBI BlastP on this gene

EMR90830

hypothetical protein
  
Accession: EMR90831
  
Location: 124344-125768
  
 NCBI BlastP on this gene

EMR90831

hypothetical protein
  
Accession: EMR90832
  
Location: 128938-133488
  
 NCBI BlastP on this gene

EMR90832

putative helicase sen1 protein
  
Accession: EMR90833
  
Location: 134590-140880
  
  
**BlastP hit with Mycgr3G41426\_Mycgr3T**
  
Percentage identity: 33 %
  
BlastP bit score: 981
  
Sequence coverage: 105 %
  
E-value: 0.0
  
  
 NCBI BlastP on this gene

EMR90833

hypothetical protein
  
Accession: EMR90834
  
Location: 142352-144763
  
 NCBI BlastP on this gene

EMR90834

putative magnesium ion transporter protein
  
Accession: EMR90835
  
Location: 146858-147180
  
 NCBI BlastP on this gene

EMR90835

putative mus38-like protein
  
Accession: EMR90836
  
Location: 149525-152363
  
 NCBI BlastP on this gene

EMR90836

163. :  FQ790288 Botryotinia fuckeliana T4 SuperContig\_299\_1 genomic supercontig.     Total score: 2.0     Cumulative Blast bit score: 1140

BcPLS1, tetraspanin
  
Accession: CCD47897
  
Location: 134703-135374
  
 NCBI BlastP on this gene

BofuT4\_P114010.1

hypothetical protein
  
Accession: CCD47898
  
Location: 135828-136383
  
 NCBI BlastP on this gene

BofuT4\_uP114020.1

hypothetical protein
  
Accession: CCD47899
  
Location: 136820-137029
  
 NCBI BlastP on this gene

BofuT4\_uP114030.1

hypothetical protein
  
Accession: CCD47900
  
Location: 137429-137819
  
 NCBI BlastP on this gene

BofuT4\_P114040.1

hypothetical protein
  
Accession: CCD47901
  
Location: 138616-138765
  
 NCBI BlastP on this gene

BofuT4\_uP114050.1

hypothetical protein
  
Accession: CCD47902
  
Location: 138884-139057
  
 NCBI BlastP on this gene

BofuT4\_uP114060.1

similar to transcription factor MADS
  
Accession: CCD47903
  
Location: 141468-142317
  
  
**BlastP hit with Mycgr3G31170\_Mycgr3T**
  
Percentage identity: 97 %
  
BlastP bit score: 159
  
Sequence coverage: 100 %
  
E-value: 1e-46
  
  
 NCBI BlastP on this gene

BofuT4\_P114070.1

similar to DUF803 domain-containing protein
  
Accession: CCD47904
  
Location: 144419-146116
  
 NCBI BlastP on this gene

BofuT4\_P114080.1

similar to vacuolar ATP synthase subunit E (V-ATPase E subunit) (Vacuolar proton pump E subunit)
  
Accession: CCD47905
  
Location: 146701-147628
  
 NCBI BlastP on this gene

BofuT4\_P114090.1

hypothetical protein
  
Accession: CCD47906
  
Location: 147856-149877
  
 NCBI BlastP on this gene

BofuT4\_P114100.1

hypothetical protein
  
Accession: CCD47907
  
Location: 151155-151845
  
 NCBI BlastP on this gene

BofuT4\_uP114110.1

similar to P-type ATPase
  
Accession: CCD47908
  
Location: 153047-157597
  
 NCBI BlastP on this gene

BofuT4\_P114120.1

similar to similar to tRNA-splicing endonuclease
  
Accession: CCD47909
  
Location: 158699-164989
  
  
**BlastP hit with Mycgr3G41426\_Mycgr3T**
  
Percentage identity: 33 %
  
BlastP bit score: 981
  
Sequence coverage: 105 %
  
E-value: 0.0
  
  
 NCBI BlastP on this gene

BofuT4\_P114130.1

hypothetical protein
  
Accession: CCD47910
  
Location: 166461-168325
  
 NCBI BlastP on this gene

BofuT4\_P114140.1

hypothetical protein
  
Accession: CCD47911
  
Location: 171058-171380
  
 NCBI BlastP on this gene

BofuT4\_P114150.1

hypothetical protein
  
Accession: CCD47912
  
Location: 172042-172538
  
 NCBI BlastP on this gene

BofuT4\_uP114160.1

predicted protein
  
Accession: CCD47913
  
Location: 172582-172755
  
 NCBI BlastP on this gene

BofuT4\_uP114170.1

hypothetical protein
  
Accession: CCD47914
  
Location: 173682-174860
  
 NCBI BlastP on this gene

BofuT4\_P114180.1

hypothetical protein
  
Accession: CCD47915
  
Location: 175056-176672
  
 NCBI BlastP on this gene

BofuT4\_P114190.1

164. :  AABX02000004 Neurospora crassa OR74A     Total score: 2.0     Cumulative Blast bit score: 1127

predicted protein
  
Accession: EAA27048
  
Location: 1094567-1095774
  
 NCBI BlastP on this gene

EAA27048

hypothetical protein
  
Accession: EAA27047
  
Location: 1100531-1101790
  
 NCBI BlastP on this gene

EAA27047

predicted protein
  
Accession: EAA27046
  
Location: 1102317-1106049
  
 NCBI BlastP on this gene

EAA27046

predicted protein
  
Accession: EAA27045
  
Location: 1109962-1111637
  
  
**BlastP hit with Mycgr3G104337\_Mycgr3**
  
Percentage identity: 41 %
  
BlastP bit score: 185
  
Sequence coverage: 94 %
  
E-value: 3e-52
  
  
 NCBI BlastP on this gene

EAA27045

conserved hypothetical protein
  
Accession: EAA26702
  
Location: 1114980-1122290
  
  
**BlastP hit with Mycgr3G100089\_Mycgr3**
  
Percentage identity: 44 %
  
BlastP bit score: 942
  
Sequence coverage: 49 %
  
E-value: 0.0
  
  
 NCBI BlastP on this gene

EAA26702

predicted protein
  
Accession: EAA26701
  
Location: 1123929-1126123
  
 NCBI BlastP on this gene

EAA26701

NADH:ubiquinone oxidoreductase 21.3c kD subunit
  
Accession: EAA26700
  
Location: 1127978-1129037
  
 NCBI BlastP on this gene

EAA26700

NADH:ubiquinone oxidoreductase 9.6kD subunit
  
Accession: EAA26699
  
Location: 1129961-1131330
  
 NCBI BlastP on this gene

EAA26699

conserved hypothetical protein
  
Accession: EAA26698
  
Location: 1133088-1135338
  
 NCBI BlastP on this gene

EAA26698

165. :  GL891307 Neurospora tetrasperma FGSC 2508 unplaced genomic scaffold NEUTE1scaffold\_6     Total score: 2.0     Cumulative Blast bit score: 1126

hypothetical protein
  
Accession: EGO54915
  
Location: 3780495-3782872
  
 NCBI BlastP on this gene

EGO54915

hypothetical protein
  
Accession: EGO54916
  
Location: 3785099-3786305
  
 NCBI BlastP on this gene

EGO54916

hypothetical protein
  
Accession: EGO54917
  
Location: 3787858-3789130
  
 NCBI BlastP on this gene

EGO54917

hypothetical protein
  
Accession: EGO54918
  
Location: 3789650-3793378
  
 NCBI BlastP on this gene

EGO54918

hypothetical protein
  
Accession: EGO54919
  
Location: 3797254-3798952
  
  
**BlastP hit with Mycgr3G104337\_Mycgr3**
  
Percentage identity: 41 %
  
BlastP bit score: 187
  
Sequence coverage: 94 %
  
E-value: 8e-53
  
  
 NCBI BlastP on this gene

EGO54919

hypothetical protein
  
Accession: EGO54920
  
Location: 3800103-3800267
  
 NCBI BlastP on this gene

EGO54920

hypothetical protein
  
Accession: EGO54921
  
Location: 3802200-3809510
  
  
**BlastP hit with Mycgr3G100089\_Mycgr3**
  
Percentage identity: 43 %
  
BlastP bit score: 939
  
Sequence coverage: 49 %
  
E-value: 0.0
  
  
 NCBI BlastP on this gene

EGO54921

hypothetical protein
  
Accession: EGO54922
  
Location: 3811904-3814087
  
 NCBI BlastP on this gene

EGO54922

hypothetical protein
  
Accession: EGO54923
  
Location: 3815945-3816978
  
 NCBI BlastP on this gene

EGO54923

hypothetical protein
  
Accession: EGO54924
  
Location: 3817894-3819369
  
 NCBI BlastP on this gene

EGO54924

hypothetical protein
  
Accession: EGO54925
  
Location: 3821204-3823451
  
 NCBI BlastP on this gene

EGO54925

166. :  GL891269 Neurospora tetrasperma FGSC 2509 unplaced genomic scaffold NEUTE2scaffold\_7     Total score: 2.0     Cumulative Blast bit score: 1126

hypothetical protein
  
Accession: EGZ67583
  
Location: 60147-62394
  
 NCBI BlastP on this gene

EGZ67583

acyl carrier protein
  
Accession: EGZ67584
  
Location: 64849-66324
  
 NCBI BlastP on this gene

EGZ67584

ferredoxin-like iron-sulfur subunit of mitochondrial complex I
  
Accession: EGZ67585
  
Location: 67240-68273
  
 NCBI BlastP on this gene

EGZ67585

hypothetical protein
  
Accession: EGZ67586
  
Location: 70130-72313
  
 NCBI BlastP on this gene

EGZ67586

ketoacyl-synt-domain-containing protein
  
Accession: EGZ67587
  
Location: 74706-82016
  
  
**BlastP hit with Mycgr3G100089\_Mycgr3**
  
Percentage identity: 43 %
  
BlastP bit score: 939
  
Sequence coverage: 49 %
  
E-value: 0.0
  
  
 NCBI BlastP on this gene

EGZ67587

hypothetical protein
  
Accession: EGZ67588
  
Location: 83949-84113
  
 NCBI BlastP on this gene

EGZ67588

hypothetical protein
  
Accession: EGZ67589
  
Location: 85259-86956
  
  
**BlastP hit with Mycgr3G104337\_Mycgr3**
  
Percentage identity: 41 %
  
BlastP bit score: 187
  
Sequence coverage: 94 %
  
E-value: 8e-53
  
  
 NCBI BlastP on this gene

EGZ67589

hypothetical protein
  
Accession: EGZ67590
  
Location: 90832-94560
  
 NCBI BlastP on this gene

EGZ67590

hypothetical protein
  
Accession: EGZ67591
  
Location: 95080-96352
  
 NCBI BlastP on this gene

EGZ67591

hypothetical protein
  
Accession: EGZ67592
  
Location: 97904-99110
  
 NCBI BlastP on this gene

EGZ67592

hypothetical protein
  
Accession: EGZ67593
  
Location: 101336-103713
  
 NCBI BlastP on this gene

EGZ67593

167. :  AFWA01000005 Pneumocystis murina B123     Total score: 2.0     Cumulative Blast bit score: 1125

hypothetical protein
  
Accession: EMR10503
  
Location: 213414-214453
  
 NCBI BlastP on this gene

EMR10503

hypothetical protein
  
Accession: EMR10504
  
Location: 216311-217133
  
 NCBI BlastP on this gene

EMR10504

hypothetical protein
  
Accession: EMR10505
  
Location: 218169-219067
  
 NCBI BlastP on this gene

EMR10505

hypothetical protein
  
Accession: EMR10506
  
Location: 219236-220372
  
 NCBI BlastP on this gene

EMR10506

hypothetical protein
  
Accession: EMR10507
  
Location: 220559-221712
  
 NCBI BlastP on this gene

EMR10507

hypothetical protein
  
Accession: EMR10508
  
Location: 222146-223686
  
  
**BlastP hit with Mycgr3G92934\_Mycgr3T**
  
Percentage identity: 48 %
  
BlastP bit score: 419
  
Sequence coverage: 84 %
  
E-value: 3e-138
  
  
 NCBI BlastP on this gene

EMR10508

hypothetical protein, variant
  
Accession: EMR10509
  
Location: 222479-223686
  
  
**BlastP hit with Mycgr3G92934\_Mycgr3T**
  
Percentage identity: 48 %
  
BlastP bit score: 358
  
Sequence coverage: 74 %
  
E-value: 6e-116
  
  
 NCBI BlastP on this gene

EMR10509

hypothetical protein
  
Accession: EMR10510
  
Location: 224658-225864
  
 NCBI BlastP on this gene

EMR10510

hypothetical protein
  
Accession: EMR10511
  
Location: 226185-227882
  
 NCBI BlastP on this gene

EMR10511

hypothetical protein
  
Accession: EMR10512
  
Location: 228304-230503
  
 NCBI BlastP on this gene

EMR10512

hypothetical protein
  
Accession: EMR10513
  
Location: 231033-232931
  
 NCBI BlastP on this gene

EMR10513

hypothetical protein
  
Accession: EMR10514
  
Location: 233097-234529
  
 NCBI BlastP on this gene

EMR10514

hypothetical protein, variant
  
Accession: EMR10515
  
Location: 233097-234529
  
 NCBI BlastP on this gene

EMR10515

hypothetical protein
  
Accession: EMR10516
  
Location: 234906-237397
  
 NCBI BlastP on this gene

EMR10516

30S ribosomal protein S7e
  
Accession: EMR10517
  
Location: 237800-238517
  
 NCBI BlastP on this gene

EMR10517

hypothetical protein
  
Accession: EMR10518
  
Location: 239384-240025
  
 NCBI BlastP on this gene

EMR10518

hypothetical protein
  
Accession: EMR10519
  
Location: 240079-240792
  
 NCBI BlastP on this gene

EMR10519

hypothetical protein
  
Accession: EMR10520
  
Location: 241246-242452
  
 NCBI BlastP on this gene

EMR10520

hypothetical protein
  
Accession: EMR10521
  
Location: 242621-243652
  
 NCBI BlastP on this gene

EMR10521

hypothetical protein
  
Accession: EMR10522
  
Location: 244011-245516
  
 NCBI BlastP on this gene

EMR10522

hypothetical protein
  
Accession: EMR10523
  
Location: 246259-246833
  
 NCBI BlastP on this gene

EMR10523

hypothetical protein
  
Accession: EMR10524
  
Location: 247159-247876
  
 NCBI BlastP on this gene

EMR10524

hypothetical protein
  
Accession: EMR10525
  
Location: 247924-249824
  
  
**BlastP hit with Mycgr3G85924\_Mycgr3T**
  
Percentage identity: 30 %
  
BlastP bit score: 188
  
Sequence coverage: 95 %
  
E-value: 1e-48
  
  
 NCBI BlastP on this gene

EMR10525

hypothetical protein, variant
  
Accession: EMR10526
  
Location: 247924-249672
  
  
**BlastP hit with Mycgr3G85924\_Mycgr3T**
  
Percentage identity: 29 %
  
BlastP bit score: 160
  
Sequence coverage: 91 %
  
E-value: 2e-39
  
  
 NCBI BlastP on this gene

EMR10526

hypothetical protein
  
Accession: EMR10527
  
Location: 250212-250801
  
 NCBI BlastP on this gene

EMR10527

STE/STE7/MKK protein kinase
  
Accession: EMR10528
  
Location: 251372-252760
  
 NCBI BlastP on this gene

EMR10528

hypothetical protein
  
Accession: EMR10529
  
Location: 253582-254534
  
 NCBI BlastP on this gene

EMR10529

hypothetical protein
  
Accession: EMR10530
  
Location: 255312-256082
  
 NCBI BlastP on this gene

EMR10530

hypothetical protein, variant
  
Accession: EMR10531
  
Location: 255367-256082
  
 NCBI BlastP on this gene

EMR10531

hypothetical protein
  
Accession: EMR10532
  
Location: 256210-257804
  
 NCBI BlastP on this gene

EMR10532

hypothetical protein
  
Accession: EMR10533
  
Location: 257925-259025
  
 NCBI BlastP on this gene

EMR10533

168. :  CABT02000027 Sordaria macrospora k-hell     Total score: 2.0     Cumulative Blast bit score: 1122

not annotated
  
Accession: CCC14912
  
Location: 322198-326407
  
 NCBI BlastP on this gene

CCC14912

not annotated
  
Accession: CCC14913
  
Location: 328769-329809
  
 NCBI BlastP on this gene

CCC14913

not annotated
  
Accession: CCC14914
  
Location: 330759-331325
  
 NCBI BlastP on this gene

CCC14914

not annotated
  
Accession: CCC14915
  
Location: 331763-333536
  
 NCBI BlastP on this gene

CCC14915

not annotated
  
Accession: CCC14916
  
Location: 334663-336147
  
  
**BlastP hit with Mycgr3G104337\_Mycgr3**
  
Percentage identity: 42 %
  
BlastP bit score: 191
  
Sequence coverage: 94 %
  
E-value: 6e-55
  
  
 NCBI BlastP on this gene

CCC14916

not annotated
  
Accession: CCC14917
  
Location: 338884-346158
  
  
**BlastP hit with Mycgr3G100089\_Mycgr3**
  
Percentage identity: 43 %
  
BlastP bit score: 931
  
Sequence coverage: 49 %
  
E-value: 0.0
  
  
 NCBI BlastP on this gene

CCC14917

not annotated
  
Accession: CCC14918
  
Location: 347848-350062
  
 NCBI BlastP on this gene

CCC14918

not annotated
  
Accession: CCC14919
  
Location: 356433-358570
  
 NCBI BlastP on this gene

CCC14919

not annotated
  
Accession: CCC14920
  
Location: 361043-362443
  
 NCBI BlastP on this gene

CCC14920

169. :  EQ962654 Talaromyces stipitatus ATCC 10500 scf\_1105507295541 genomic scaffold     Total score: 2.0     Cumulative Blast bit score: 1120

Rab geranylgeranyl transferase escort protein, putative
  
Accession: EED19368
  
Location: 930471-932333
  
 NCBI BlastP on this gene

EED19368

conserved hypothetical protein
  
Accession: EED19369
  
Location: 934457-936076
  
 NCBI BlastP on this gene

EED19369

C6 transcription factor, putative
  
Accession: EED19370
  
Location: 937415-940188
  
 NCBI BlastP on this gene

EED19370

conserved hypothetical protein
  
Accession: EED19371
  
Location: 940426-941262
  
 NCBI BlastP on this gene

EED19371

MADS box transcription factor Mcm1
  
Accession: EED19372
  
Location: 943533-944362
  
  
**BlastP hit with Mycgr3G31170\_Mycgr3T**
  
Percentage identity: 100 %
  
BlastP bit score: 166
  
Sequence coverage: 100 %
  
E-value: 1e-49
  
  
 NCBI BlastP on this gene

EED19372

DUF803 domain protein
  
Accession: EED19373
  
Location: 945565-947530
  
 NCBI BlastP on this gene

EED19373

ATP synthase subunit E, putative
  
Accession: EED19374
  
Location: 948061-948875
  
 NCBI BlastP on this gene

EED19374

hypothetical protein
  
Accession: EED19375
  
Location: 949263-951562
  
 NCBI BlastP on this gene

EED19375

conserved hypothetical protein
  
Accession: EED19376
  
Location: 952002-952886
  
 NCBI BlastP on this gene

EED19376

phospholipid-translocating P-type ATPase domain-containing protein
  
Accession: EED19378
  
Location: 954455-958936
  
 NCBI BlastP on this gene

EED19378

hypothetical protein
  
Accession: EED19379
  
Location: 960230-962166
  
 NCBI BlastP on this gene

EED19379

tRNA-splicing endonuclease, putative
  
Accession: EED19380
  
Location: 962389-966693
  
  
**BlastP hit with Mycgr3G41426\_Mycgr3T**
  
Percentage identity: 46 %
  
BlastP bit score: 954
  
Sequence coverage: 62 %
  
E-value: 0.0
  
  
 NCBI BlastP on this gene

EED19380

conserved hypothetical protein
  
Accession: EED19381
  
Location: 969307-972345
  
 NCBI BlastP on this gene

EED19381

conserved hypothetical protein
  
Accession: EED19382
  
Location: 972576-973916
  
 NCBI BlastP on this gene

EED19382

chitin biosynthesis protein (Chs5), putative
  
Accession: EED19383
  
Location: 975974-977321
  
 NCBI BlastP on this gene

EED19383

170. :  GL988044 Chaetomium thermophilum var. thermophilum DSM 1495 unplaced genomic scaffold scf7180000...     Total score: 2.0     Cumulative Blast bit score: 1101

putative cellulose binding protein
  
Accession: EGS19539
  
Location: 995262-996433
  
 NCBI BlastP on this gene

EGS19539

hypothetical protein
  
Accession: EGS19540
  
Location: 998209-999672
  
 NCBI BlastP on this gene

EGS19540

hypothetical protein
  
Accession: EGS19541
  
Location: 1000399-1001476
  
 NCBI BlastP on this gene

EGS19541

hypothetical protein
  
Accession: EGS19542
  
Location: 1002584-1003019
  
 NCBI BlastP on this gene

EGS19542

putative fatty acid protein
  
Accession: EGS19543
  
Location: 1005384-1006851
  
 NCBI BlastP on this gene

EGS19543

hypothetical protein
  
Accession: EGS19544
  
Location: 1007633-1008031
  
 NCBI BlastP on this gene

EGS19544

hypothetical protein
  
Accession: EGS19545
  
Location: 1008659-1011685
  
 NCBI BlastP on this gene

EGS19545

hypothetical protein
  
Accession: EGS19546
  
Location: 1014304-1015314
  
  
**BlastP hit with Mycgr3G104337\_Mycgr3**
  
Percentage identity: 37 %
  
BlastP bit score: 177
  
Sequence coverage: 103 %
  
E-value: 2e-50
  
  
 NCBI BlastP on this gene

EGS19546

hypothetical protein
  
Accession: EGS19547
  
Location: 1016981-1022602
  
  
**BlastP hit with Mycgr3G100089\_Mycgr3**
  
Percentage identity: 44 %
  
BlastP bit score: 924
  
Sequence coverage: 48 %
  
E-value: 0.0
  
  
 NCBI BlastP on this gene

EGS19547

oxidoreductase-like protein
  
Accession: EGS19548
  
Location: 1023653-1027185
  
 NCBI BlastP on this gene

EGS19548

hypothetical protein
  
Accession: EGS19549
  
Location: 1027609-1028479
  
 NCBI BlastP on this gene

EGS19549

zinc finger domain-containing protein
  
Accession: EGS19550
  
Location: 1029415-1031625
  
 NCBI BlastP on this gene

EGS19550

hypothetical protein
  
Accession: EGS19551
  
Location: 1034582-1036196
  
 NCBI BlastP on this gene

EGS19551

hypothetical protein
  
Accession: EGS19552
  
Location: 1040522-1042198
  
 NCBI BlastP on this gene

EGS19552

171. :  DS027058 Aspergillus clavatus NRRL 1 1099423829804 genomic scaffold     Total score: 2.0     Cumulative Blast bit score: 1062

nonribosomal peptide synthase, putative
  
Accession: EAW08900
  
Location: 919134-944153
  
 NCBI BlastP on this gene

EAW08900

SRF-type transcription factor (Umc1), putative
  
Accession: EAW08901
  
Location: 946418-947301
  
  
**BlastP hit with Mycgr3G31170\_Mycgr3T**
  
Percentage identity: 98 %
  
BlastP bit score: 162
  
Sequence coverage: 100 %
  
E-value: 3e-48
  
  
 NCBI BlastP on this gene

EAW08901

DUF803 domain protein
  
Accession: EAW08902
  
Location: 948473-950445
  
 NCBI BlastP on this gene

EAW08902

ATP synthase subunit E, putative
  
Accession: EAW08903
  
Location: 951055-951877
  
 NCBI BlastP on this gene

EAW08903

hypothetical protein
  
Accession: EAW08904
  
Location: 952346-954740
  
 NCBI BlastP on this gene

EAW08904

phospholipid-translocating P-type ATPase domain-containing protein
  
Accession: EAW08905
  
Location: 958692-963397
  
 NCBI BlastP on this gene

EAW08905

tRNA-splicing endonuclease, putative
  
Accession: EAW08906
  
Location: 964758-971399
  
  
**BlastP hit with Mycgr3G41426\_Mycgr3T**
  
Percentage identity: 52 %
  
BlastP bit score: 900
  
Sequence coverage: 48 %
  
E-value: 0.0
  
  
 NCBI BlastP on this gene

EAW08906

conserved hypothetical protein
  
Accession: EAW08907
  
Location: 975010-977736
  
 NCBI BlastP on this gene

EAW08907

conserved hypothetical protein
  
Accession: EAW08908
  
Location: 978488-979219
  
 NCBI BlastP on this gene

EAW08908

aminotransferase, putative
  
Accession: EAW08909
  
Location: 979638-981241
  
 NCBI BlastP on this gene

EAW08909

172. :  GG692437 Ajellomyces capsulatus H143 genomic scaffold supercont2.19     Total score: 2.0     Cumulative Blast bit score: 1018

tetraspanin
  
Accession: EER36814
  
Location: 168129-169156
  
 NCBI BlastP on this gene

EER36814

C6 zinc finger protein
  
Accession: EER36815
  
Location: 170997-172335
  
 NCBI BlastP on this gene

EER36815

conserved hypothetical protein
  
Accession: EER36816
  
Location: 174087-174523
  
 NCBI BlastP on this gene

EER36816

conserved hypothetical protein
  
Accession: EER36817
  
Location: 175383-176567
  
 NCBI BlastP on this gene

EER36817

MADS box transcription factor Mcm1
  
Accession: EER36818
  
Location: 177381-178311
  
  
**BlastP hit with Mycgr3G31170\_Mycgr3T**
  
Percentage identity: 98 %
  
BlastP bit score: 164
  
Sequence coverage: 100 %
  
E-value: 2e-48
  
  
 NCBI BlastP on this gene

EER36818

hypothetical protein
  
Accession: EER36819
  
Location: 179522-179766
  
 NCBI BlastP on this gene

EER36819

DUF803 domain-containing protein
  
Accession: EER36820
  
Location: 181051-183234
  
 NCBI BlastP on this gene

EER36820

vacuolar ATP synthase subunit E
  
Accession: EER36821
  
Location: 183686-184613
  
 NCBI BlastP on this gene

EER36821

conserved hypothetical protein
  
Accession: EER36822
  
Location: 185182-187664
  
 NCBI BlastP on this gene

EER36822

serine/threonine protein phosphatase
  
Accession: EER36823
  
Location: 188857-189943
  
 NCBI BlastP on this gene

EER36823

phospholipid-translocating P-type ATPase domain-containing protein
  
Accession: EER36824
  
Location: 191986-196653
  
 NCBI BlastP on this gene

EER36824

helicase SEN1
  
Accession: EER36825
  
Location: 198510-205256
  
  
**BlastP hit with Mycgr3G41426\_Mycgr3T**
  
Percentage identity: 53 %
  
BlastP bit score: 855
  
Sequence coverage: 43 %
  
E-value: 0.0
  
  
 NCBI BlastP on this gene

EER36825

predicted protein
  
Accession: EER36826
  
Location: 206141-206990
  
 NCBI BlastP on this gene

EER36826

predicted protein
  
Accession: EER36827
  
Location: 210111-213257
  
 NCBI BlastP on this gene

EER36827

dihydroorotase
  
Accession: EER36828
  
Location: 213889-215140
  
 NCBI BlastP on this gene

EER36828

173. :  AM920437 Penicillium chrysogenum Wisconsin 54-1255 complete genome, contig Pc00c22.     Total score: 2.0     Cumulative Blast bit score: 1012

not annotated
  
Accession: CAP99718
  
Location: 5724438-5725292
  
 NCBI BlastP on this gene

Pc22g24300

not annotated
  
Accession: CAP99719
  
Location: 5725511-5726842
  
 NCBI BlastP on this gene

Pc22g24310

not annotated
  
Accession: CAP99720
  
Location: 5731268-5732863
  
 NCBI BlastP on this gene

Pc22g24320

hypothetical protein
  
Accession: CAP99721
  
Location: 5733676-5734809
  
 NCBI BlastP on this gene

Pc22g24330

not annotated
  
Accession: CAP99722
  
Location: 5735120-5735914
  
  
**BlastP hit with Mycgr3G31170\_Mycgr3T**
  
Percentage identity: 98 %
  
BlastP bit score: 162
  
Sequence coverage: 100 %
  
E-value: 3e-48
  
  
 NCBI BlastP on this gene

Pc22g24340

not annotated
  
Accession: CAP99723
  
Location: 5737550-5739301
  
 NCBI BlastP on this gene

Pc22g24350

not annotated
  
Accession: CAP99724
  
Location: 5739818-5740634
  
 NCBI BlastP on this gene

Pc22g24360

not annotated
  
Accession: CAP99725
  
Location: 5741006-5743376
  
 NCBI BlastP on this gene

Pc22g24370

not annotated
  
Accession: CAP99726
  
Location: 5745881-5750590
  
 NCBI BlastP on this gene

Pc22g24380

not annotated
  
Accession: CAP99727
  
Location: 5751745-5758443
  
  
**BlastP hit with Mycgr3G41426\_Mycgr3T**
  
Percentage identity: 50 %
  
BlastP bit score: 850
  
Sequence coverage: 48 %
  
E-value: 0.0
  
  
 NCBI BlastP on this gene

Pc22g24390

unnamed
  
Accession: CAP99728
  
Location: 5759388-5760239
  
 NCBI BlastP on this gene

Pc22g24400

not annotated
  
Accession: CAP99729
  
Location: 5760684-5762431
  
 NCBI BlastP on this gene

Pc22g24410

not annotated
  
Accession: CAP99730
  
Location: 5763884-5766004
  
 NCBI BlastP on this gene

Pc22g24420

not annotated
  
Accession: CAP99731
  
Location: 5767681-5769430
  
 NCBI BlastP on this gene

Pc22g24430

174. :  AKCT01000122 Penicillium digitatum PHI26     Total score: 2.0     Cumulative Blast bit score: 1012

hypothetical protein
  
Accession: EKV15273
  
Location: 54373-55968
  
 NCBI BlastP on this gene

EKV15273

MADS box transcription factor Mcm1
  
Accession: EKV15272
  
Location: 51313-52098
  
  
**BlastP hit with Mycgr3G31170\_Mycgr3T**
  
Percentage identity: 98 %
  
BlastP bit score: 162
  
Sequence coverage: 100 %
  
E-value: 4e-48
  
  
 NCBI BlastP on this gene

EKV15272

hypothetical protein
  
Accession: EKV15271
  
Location: 47634-49624
  
 NCBI BlastP on this gene

EKV15271

ATP synthase subunit E, putative
  
Accession: EKV15270
  
Location: 46549-47363
  
 NCBI BlastP on this gene

EKV15270

hypothetical protein
  
Accession: EKV15269
  
Location: 43794-46167
  
 NCBI BlastP on this gene

EKV15269

hypothetical protein
  
Accession: EKV15268
  
Location: 36417-41149
  
 NCBI BlastP on this gene

EKV15268

tRNA-splicing endonuclease, putative
  
Accession: EKV15267
  
Location: 28635-35287
  
  
**BlastP hit with Mycgr3G41426\_Mycgr3T**
  
Percentage identity: 49 %
  
BlastP bit score: 850
  
Sequence coverage: 48 %
  
E-value: 0.0
  
  
 NCBI BlastP on this gene

EKV15267

hypothetical protein
  
Accession: EKV15266
  
Location: 26885-27730
  
 NCBI BlastP on this gene

EKV15266

NCS1 allantoate transporter
  
Accession: EKV15265
  
Location: 24629-26422
  
 NCBI BlastP on this gene

EKV15265

G1/S-specific cyclin Pcl5, putative
  
Accession: EKV15264
  
Location: 21222-23301
  
 NCBI BlastP on this gene

EKV15264

hypothetical protein
  
Accession: EKV15263
  
Location: 18799-19161
  
 NCBI BlastP on this gene

EKV15263

hypothetical protein
  
Accession: EKV15262
  
Location: 17596-17849
  
 NCBI BlastP on this gene

EKV15262

175. :  AKCU01000418 Penicillium digitatum Pd1     Total score: 2.0     Cumulative Blast bit score: 1010

hypothetical protein
  
Accession: EKV10017
  
Location: 38002-39597
  
 NCBI BlastP on this gene

EKV10017

MADS box transcription factor Mcm1
  
Accession: EKV10016
  
Location: 34945-35730
  
  
**BlastP hit with Mycgr3G31170\_Mycgr3T**
  
Percentage identity: 98 %
  
BlastP bit score: 162
  
Sequence coverage: 100 %
  
E-value: 4e-48
  
  
 NCBI BlastP on this gene

EKV10016

hypothetical protein
  
Accession: EKV10015
  
Location: 31265-33255
  
 NCBI BlastP on this gene

EKV10015

ATP synthase subunit E, putative
  
Accession: EKV10014
  
Location: 30180-30994
  
 NCBI BlastP on this gene

EKV10014

hypothetical protein
  
Accession: EKV10013
  
Location: 27425-29798
  
 NCBI BlastP on this gene

EKV10013

hypothetical protein
  
Accession: EKV10012
  
Location: 20049-24781
  
 NCBI BlastP on this gene

EKV10012

tRNA-splicing endonuclease, putative
  
Accession: EKV10011
  
Location: 12267-18919
  
  
**BlastP hit with Mycgr3G41426\_Mycgr3T**
  
Percentage identity: 49 %
  
BlastP bit score: 849
  
Sequence coverage: 48 %
  
E-value: 0.0
  
  
 NCBI BlastP on this gene

EKV10011

hypothetical protein
  
Accession: EKV10010
  
Location: 10517-11362
  
 NCBI BlastP on this gene

EKV10010

NCS1 allantoate transporter
  
Accession: EKV10009
  
Location: 8259-10054
  
 NCBI BlastP on this gene

EKV10009

G1/S-specific cyclin Pcl5, putative
  
Accession: EKV10008
  
Location: 4852-6931
  
 NCBI BlastP on this gene

EKV10008

hypothetical protein
  
Accession: EKV10007
  
Location: 2429-2791
  
 NCBI BlastP on this gene

EKV10007

hypothetical protein
  
Accession: EKV10006
  
Location: 1226-1479
  
 NCBI BlastP on this gene

EKV10006

176. :  DS544814 Paracoccidioides brasiliensis Pb03 supercont1.12 genomic scaffold     Total score: 2.0     Cumulative Blast bit score: 1001

tetraspanin
  
Accession: EEH16414
  
Location: 443623-447578
  
 NCBI BlastP on this gene

EEH16414

MADS box transcription factor Mcm1
  
Accession: EEH16415
  
Location: 450142-451180
  
  
**BlastP hit with Mycgr3G31170\_Mycgr3T**
  
Percentage identity: 100 %
  
BlastP bit score: 167
  
Sequence coverage: 100 %
  
E-value: 5e-50
  
  
 NCBI BlastP on this gene

EEH16415

conserved hypothetical protein
  
Accession: EEH16416
  
Location: 454132-456380
  
 NCBI BlastP on this gene

EEH16416

vacuolar ATP synthase subunit E
  
Accession: EEH16417
  
Location: 457142-457950
  
 NCBI BlastP on this gene

EEH16417

conserved hypothetical protein
  
Accession: EEH16418
  
Location: 458505-460962
  
 NCBI BlastP on this gene

EEH16418

metallophosphoesterase domain-containing protein
  
Accession: EEH16419
  
Location: 462822-463826
  
 NCBI BlastP on this gene

EEH16419

P-type ATPase
  
Accession: EEH16420
  
Location: 466173-470843
  
 NCBI BlastP on this gene

EEH16420

conserved hypothetical protein
  
Accession: EEH16421
  
Location: 473405-481190
  
  
**BlastP hit with Mycgr3G41426\_Mycgr3T**
  
Percentage identity: 50 %
  
BlastP bit score: 835
  
Sequence coverage: 48 %
  
E-value: 0.0
  
  
 NCBI BlastP on this gene

EEH16421

predicted protein
  
Accession: EEH16422
  
Location: 485315-488494
  
 NCBI BlastP on this gene

EEH16422

177. :  CH476616 Uncinocarpus reesii 1704 scaffold\_2 genomic scaffold     Total score: 2.0     Cumulative Blast bit score: 1001

predicted protein
  
Accession: EEP79660
  
Location: 4565645-4566716
  
 NCBI BlastP on this gene

EEP79660

conserved hypothetical protein
  
Accession: EEP79661
  
Location: 4567101-4568592
  
 NCBI BlastP on this gene

EEP79661

predicted protein
  
Accession: EEP79662
  
Location: 4569101-4569979
  
 NCBI BlastP on this gene

EEP79662

conserved hypothetical protein
  
Accession: EEP79663
  
Location: 4570757-4572663
  
 NCBI BlastP on this gene

EEP79663

predicted protein
  
Accession: EEP79664
  
Location: 4575048-4575899
  
 NCBI BlastP on this gene

EEP79664

MCMA protein
  
Accession: EEP79665
  
Location: 4576943-4577833
  
  
**BlastP hit with Mycgr3G31170\_Mycgr3T**
  
Percentage identity: 82 %
  
BlastP bit score: 129
  
Sequence coverage: 100 %
  
E-value: 2e-35
  
  
 NCBI BlastP on this gene

EEP79665

conserved hypothetical protein
  
Accession: EEP79666
  
Location: 4579067-4580995
  
 NCBI BlastP on this gene

EEP79666

vacuolar ATP synthase subunit E
  
Accession: EEP79667
  
Location: 4581737-4582391
  
 NCBI BlastP on this gene

EEP79667

predicted protein
  
Accession: EEP79668
  
Location: 4582923-4585219
  
 NCBI BlastP on this gene

EEP79668

hypothetical protein
  
Accession: EEP79669
  
Location: 4586552-4591298
  
 NCBI BlastP on this gene

EEP79669

conserved hypothetical protein
  
Accession: EEP79670
  
Location: 4592600-4599396
  
  
**BlastP hit with Mycgr3G41426\_Mycgr3T**
  
Percentage identity: 54 %
  
BlastP bit score: 872
  
Sequence coverage: 43 %
  
E-value: 0.0
  
  
 NCBI BlastP on this gene

EEP79670

predicted protein
  
Accession: EEP79671
  
Location: 4600305-4604496
  
 NCBI BlastP on this gene

EEP79671

conserved hypothetical protein
  
Accession: EEP79672
  
Location: 4604962-4607058
  
 NCBI BlastP on this gene

EEP79672

conserved hypothetical protein
  
Accession: EEP79673
  
Location: 4607328-4609332
  
 NCBI BlastP on this gene

EEP79673

eukaryotic translation initiation factor 1A
  
Accession: EEP79674
  
Location: 4610328-4611010
  
 NCBI BlastP on this gene

EEP79674

178. :  DS572762 Paracoccidioides brasiliensis Pb18 supercont1.13 genomic scaffold     Total score: 2.0     Cumulative Blast bit score: 999

conserved hypothetical protein
  
Accession: EEH43139
  
Location: 453166-454373
  
 NCBI BlastP on this gene

EEH43139

tetraspanin
  
Accession: EEH43140
  
Location: 455544-456600
  
 NCBI BlastP on this gene

EEH43140

conserved hypothetical protein
  
Accession: EEH43141
  
Location: 457321-458794
  
 NCBI BlastP on this gene

EEH43141

MADS box transcription factor Mcm1
  
Accession: EEH43142
  
Location: 462082-463123
  
  
**BlastP hit with Mycgr3G31170\_Mycgr3T**
  
Percentage identity: 100 %
  
BlastP bit score: 167
  
Sequence coverage: 100 %
  
E-value: 5e-50
  
  
 NCBI BlastP on this gene

EEH43142

DUF803 domain-containing protein
  
Accession: EEH43143
  
Location: 466041-468319
  
 NCBI BlastP on this gene

EEH43143

vacuolar ATP synthase subunit E
  
Accession: EEH43144
  
Location: 469073-469881
  
 NCBI BlastP on this gene

EEH43144

conserved hypothetical protein
  
Accession: EEH43145
  
Location: 470439-472896
  
 NCBI BlastP on this gene

EEH43145

ser/Thr protein phosphatase family protein
  
Accession: EEH43146
  
Location: 473638-475772
  
 NCBI BlastP on this gene

EEH43146

phospholipid-transporting ATPase
  
Accession: EEH43147
  
Location: 478156-482826
  
 NCBI BlastP on this gene

EEH43147

DEAD-box type RNA helicase
  
Accession: EEH43148
  
Location: 485389-492226
  
  
**BlastP hit with Mycgr3G41426\_Mycgr3T**
  
Percentage identity: 49 %
  
BlastP bit score: 832
  
Sequence coverage: 48 %
  
E-value: 0.0
  
  
 NCBI BlastP on this gene

EEH43148

predicted protein
  
Accession: EEH43149
  
Location: 495310-500451
  
 NCBI BlastP on this gene

EEH43149

179. :  AFQF01002886 Fusarium oxysporum Fo5176     Total score: 2.0     Cumulative Blast bit score: 992

hypothetical protein
  
Accession: EGU78026
  
Location: 4184-5259
  
 NCBI BlastP on this gene

EGU78026

hypothetical protein
  
Accession: EGU78027
  
Location: 5426-7136
  
 NCBI BlastP on this gene

EGU78027

hypothetical protein
  
Accession: EGU78028
  
Location: 8780-10244
  
 NCBI BlastP on this gene

EGU78028

hypothetical protein
  
Accession: EGU78029
  
Location: 11467-14417
  
 NCBI BlastP on this gene

EGU78029

hypothetical protein
  
Accession: EGU78030
  
Location: 14802-17716
  
 NCBI BlastP on this gene

EGU78030

hypothetical protein
  
Accession: EGU78031
  
Location: 18079-19684
  
 NCBI BlastP on this gene

EGU78031

hypothetical protein
  
Accession: EGU78032
  
Location: 19903-21724
  
 NCBI BlastP on this gene

EGU78032

hypothetical protein
  
Accession: EGU78033
  
Location: 22102-22731
  
  
**BlastP hit with Mycgr3G104337\_Mycgr3**
  
Percentage identity: 27 %
  
BlastP bit score: 69
  
Sequence coverage: 54 %
  
E-value: 2e-11
  
  
 NCBI BlastP on this gene

EGU78033

hypothetical protein
  
Accession: EGU78034
  
Location: 23087-24148
  
 NCBI BlastP on this gene

EGU78034

hypothetical protein
  
Accession: EGU78035
  
Location: 24297-25976
  
 NCBI BlastP on this gene

EGU78035

hypothetical protein
  
Accession: EGU78036
  
Location: 26431-32436
  
  
**BlastP hit with Mycgr3G100089\_Mycgr3**
  
Percentage identity: 40 %
  
BlastP bit score: 923
  
Sequence coverage: 58 %
  
E-value: 0.0
  
  
 NCBI BlastP on this gene

EGU78036

180. :  KB733479 Bipolaris maydis ATCC 48331 unplaced genomic scaffold COCC4scaffold\_36     Total score: 2.0     Cumulative Blast bit score: 990

glycoside hydrolase family 10 protein
  
Accession: ENH99962
  
Location: 26057-27649
  
 NCBI BlastP on this gene

ENH99962

hypothetical protein
  
Accession: ENH99963
  
Location: 28749-30662
  
 NCBI BlastP on this gene

ENH99963

hypothetical protein
  
Accession: ENH99964
  
Location: 30844-32148
  
 NCBI BlastP on this gene

ENH99964

hypothetical protein
  
Accession: ENH99965
  
Location: 32559-34580
  
 NCBI BlastP on this gene

ENH99965

hypothetical protein
  
Accession: ENH99966
  
Location: 34828-35504
  
 NCBI BlastP on this gene

ENH99966

hypothetical protein
  
Accession: ENH99967
  
Location: 35926-36687
  
  
**BlastP hit with Mycgr3G104337\_Mycgr3**
  
Percentage identity: 30 %
  
BlastP bit score: 93
  
Sequence coverage: 91 %
  
E-value: 2e-19
  
  
 NCBI BlastP on this gene

ENH99967

hypothetical protein
  
Accession: ENH99968
  
Location: 37625-39040
  
 NCBI BlastP on this gene

ENH99968

hypothetical protein
  
Accession: ENH99969
  
Location: 39445-40960
  
 NCBI BlastP on this gene

ENH99969

hypothetical protein
  
Accession: ENH99970
  
Location: 41421-43013
  
 NCBI BlastP on this gene

ENH99970

hypothetical protein
  
Accession: ENH99971
  
Location: 43634-44236
  
 NCBI BlastP on this gene

ENH99971

hypothetical protein
  
Accession: ENH99972
  
Location: 46047-47312
  
 NCBI BlastP on this gene

ENH99972

hypothetical protein
  
Accession: ENH99973
  
Location: 47313-47492
  
 NCBI BlastP on this gene

ENH99973

hypothetical protein
  
Accession: ENH99974
  
Location: 51033-51626
  
 NCBI BlastP on this gene

ENH99974

hypothetical protein
  
Accession: ENH99975
  
Location: 52102-52992
  
 NCBI BlastP on this gene

ENH99975

hypothetical protein
  
Accession: ENH99976
  
Location: 53733-54502
  
 NCBI BlastP on this gene

ENH99976

hypothetical protein
  
Accession: ENH99977
  
Location: 54941-62407
  
  
**BlastP hit with Mycgr3G100089\_Mycgr3**
  
Percentage identity: 39 %
  
BlastP bit score: 897
  
Sequence coverage: 56 %
  
E-value: 0.0
  
  
 NCBI BlastP on this gene

ENH99977

hypothetical protein
  
Accession: ENH99978
  
Location: 62947-64764
  
 NCBI BlastP on this gene

ENH99978

hypothetical protein
  
Accession: ENH99979
  
Location: 65236-67800
  
 NCBI BlastP on this gene

ENH99979

hypothetical protein
  
Accession: ENH99980
  
Location: 68507-69154
  
 NCBI BlastP on this gene

ENH99980

hypothetical protein
  
Accession: ENH99981
  
Location: 69807-71949
  
 NCBI BlastP on this gene

ENH99981

181. :  KB445588 Cochliobolus heterostrophus C5 unplaced genomic scaffold COCHEscaffold\_20     Total score: 2.0     Cumulative Blast bit score: 990

glycoside hydrolase family 10 protein
  
Accession: EMD85586
  
Location: 350486-352078
  
 NCBI BlastP on this gene

EMD85586

hypothetical protein
  
Accession: EMD85585
  
Location: 347473-349386
  
 NCBI BlastP on this gene

EMD85585

hypothetical protein
  
Accession: EMD85584
  
Location: 345987-347291
  
 NCBI BlastP on this gene

EMD85584

hypothetical protein
  
Accession: EMD85583
  
Location: 343555-345576
  
 NCBI BlastP on this gene

EMD85583

hypothetical protein
  
Accession: EMD85582
  
Location: 342631-343307
  
 NCBI BlastP on this gene

EMD85582

hypothetical protein
  
Accession: EMD85581
  
Location: 341448-342209
  
  
**BlastP hit with Mycgr3G104337\_Mycgr3**
  
Percentage identity: 30 %
  
BlastP bit score: 93
  
Sequence coverage: 91 %
  
E-value: 2e-19
  
  
 NCBI BlastP on this gene

EMD85581

hypothetical protein
  
Accession: EMD85580
  
Location: 339095-340510
  
 NCBI BlastP on this gene

EMD85580

hypothetical protein
  
Accession: EMD85579
  
Location: 337175-338690
  
 NCBI BlastP on this gene

EMD85579

hypothetical protein
  
Accession: EMD85578
  
Location: 335122-336714
  
 NCBI BlastP on this gene

EMD85578

hypothetical protein
  
Accession: EMD85577
  
Location: 333896-334588
  
 NCBI BlastP on this gene

EMD85577

hypothetical protein
  
Accession: EMD85576
  
Location: 333283-333522
  
 NCBI BlastP on this gene

EMD85576

hypothetical protein
  
Accession: EMD85575
  
Location: 330438-331421
  
 NCBI BlastP on this gene

EMD85575

hypothetical protein
  
Accession: EMD85574
  
Location: 330258-330437
  
 NCBI BlastP on this gene

EMD85574

hypothetical protein
  
Accession: EMD85573
  
Location: 326124-326717
  
 NCBI BlastP on this gene

EMD85573

hypothetical protein
  
Accession: EMD85572
  
Location: 324758-325648
  
 NCBI BlastP on this gene

EMD85572

hypothetical protein
  
Accession: EMD85571
  
Location: 323248-324017
  
 NCBI BlastP on this gene

EMD85571

hypothetical protein
  
Accession: EMD85570
  
Location: 315343-322809
  
  
**BlastP hit with Mycgr3G100089\_Mycgr3**
  
Percentage identity: 39 %
  
BlastP bit score: 897
  
Sequence coverage: 56 %
  
E-value: 0.0
  
  
 NCBI BlastP on this gene

EMD85570

hypothetical protein
  
Accession: EMD85569
  
Location: 312986-314803
  
 NCBI BlastP on this gene

EMD85569

hypothetical protein
  
Accession: EMD85568
  
Location: 309950-312514
  
 NCBI BlastP on this gene

EMD85568

hypothetical protein
  
Accession: EMD85567
  
Location: 308596-309243
  
 NCBI BlastP on this gene

EMD85567

hypothetical protein
  
Accession: EMD85566
  
Location: 307703-308011
  
 NCBI BlastP on this gene

EMD85566

hypothetical protein
  
Accession: EMD85565
  
Location: 305785-307399
  
 NCBI BlastP on this gene

EMD85565

182. :  DS499596 Aspergillus fumigatus A1163 scf\_000003 genomic scaffold     Total score: 2.0     Cumulative Blast bit score: 989

alcohol dehydrogenase, putative
  
Accession: EDP53510
  
Location: 3736754-3737924
  
 NCBI BlastP on this gene

EDP53510

conserved hypothetical protein
  
Accession: EDP53511
  
Location: 3741340-3743238
  
 NCBI BlastP on this gene

EDP53511

conserved hypothetical protein
  
Accession: EDP53512
  
Location: 3743461-3744473
  
 NCBI BlastP on this gene

EDP53512

salicylate hydroxylase, putative
  
Accession: EDP53513
  
Location: 3745387-3746733
  
 NCBI BlastP on this gene

EDP53513

3-methyl-2-oxobutanoate dehydrogenase, putative
  
Accession: EDP53514
  
Location: 3747121-3748408
  
 NCBI BlastP on this gene

EDP53514

DUF1212 domain membrane protein
  
Accession: EDP53515
  
Location: 3749984-3752521
  
 NCBI BlastP on this gene

EDP53515

benzodiazepine receptor family protein
  
Accession: EDP53516
  
Location: 3753761-3754392
  
 NCBI BlastP on this gene

EDP53516

DUF341 domain oxidoreductase, putative
  
Accession: EDP53517
  
Location: 3755108-3755883
  
  
**BlastP hit with Mycgr3G104337\_Mycgr3**
  
Percentage identity: 26 %
  
BlastP bit score: 92
  
Sequence coverage: 91 %
  
E-value: 3e-19
  
  
 NCBI BlastP on this gene

EDP53517

polyketide synthase, putative
  
Accession: EDP53518
  
Location: 3756174-3764303
  
  
**BlastP hit with Mycgr3G100089\_Mycgr3**
  
Percentage identity: 40 %
  
BlastP bit score: 897
  
Sequence coverage: 56 %
  
E-value: 0.0
  
  
 NCBI BlastP on this gene

EDP53518

ABC multidrug transporter, putative
  
Accession: EDP53519
  
Location: 3764538-3768960
  
 NCBI BlastP on this gene

EDP53519

MFS transporter, putative
  
Accession: EDP53520
  
Location: 3777918-3779569
  
 NCBI BlastP on this gene

EDP53520

siderochrome-iron transporter, putative
  
Accession: EDP53521
  
Location: 3781715-3783946
  
 NCBI BlastP on this gene

EDP53521

183. :  AAHF01000010 Aspergillus fumigatus Af293     Total score: 2.0     Cumulative Blast bit score: 988

alcohol dehydrogenase, putative
  
Accession: EAL86432
  
Location: 379234-380404
  
 NCBI BlastP on this gene

EAL86432

conserved hypothetical protein
  
Accession: EAL86431
  
Location: 373921-375819
  
 NCBI BlastP on this gene

EAL86431

conserved hypothetical protein
  
Accession: EAL86430
  
Location: 372686-373698
  
 NCBI BlastP on this gene

EAL86430

salicylate hydroxylase, putative
  
Accession: EAL86429
  
Location: 370426-371772
  
 NCBI BlastP on this gene

EAL86429

3-methyl-2-oxobutanoate dehydrogenase, putative
  
Accession: EAL86428
  
Location: 368751-370038
  
 NCBI BlastP on this gene

EAL86428

DUF1212 domain membrane protein
  
Accession: EAL86427
  
Location: 364638-367175
  
 NCBI BlastP on this gene

EAL86427

benzodiazepine receptor family protein
  
Accession: EAL86426
  
Location: 362767-363398
  
 NCBI BlastP on this gene

EAL86426

DUF341 family oxidoreductase, putative
  
Accession: EAL86425
  
Location: 361276-362051
  
  
**BlastP hit with Mycgr3G104337\_Mycgr3**
  
Percentage identity: 26 %
  
BlastP bit score: 92
  
Sequence coverage: 91 %
  
E-value: 3e-19
  
  
 NCBI BlastP on this gene

EAL86425

polyketide synthase, putative
  
Accession: EAL86424
  
Location: 352856-360985
  
  
**BlastP hit with Mycgr3G100089\_Mycgr3**
  
Percentage identity: 40 %
  
BlastP bit score: 896
  
Sequence coverage: 56 %
  
E-value: 0.0
  
  
 NCBI BlastP on this gene

EAL86424

ABC multidrug transporter, putative
  
Accession: EAL86423
  
Location: 348200-352621
  
 NCBI BlastP on this gene

EAL86423

MFS transporter, putative
  
Accession: EAL86420
  
Location: 337589-339241
  
 NCBI BlastP on this gene

EAL86420

siderochrome-iron transporter, putative
  
Accession: EAL86419
  
Location: 333213-335443
  
 NCBI BlastP on this gene

EAL86419

184. :  DS027697 Neosartorya fischeri NRRL 181 1099437636265 genomic scaffold     Total score: 2.0     Cumulative Blast bit score: 969

protein kinase, putative
  
Accession: EAW16894
  
Location: 548385-549274
  
 NCBI BlastP on this gene

EAW16894

conserved hypothetical protein
  
Accession: EAW16893
  
Location: 545197-547093
  
 NCBI BlastP on this gene

EAW16893

conserved hypothetical protein
  
Accession: EAW16892
  
Location: 543963-544970
  
 NCBI BlastP on this gene

EAW16892

salicylate hydroxylase, putative
  
Accession: EAW16891
  
Location: 541711-543057
  
 NCBI BlastP on this gene

EAW16891

2-oxoisovalerate dehydrogenase
  
Accession: EAW16890
  
Location: 540045-541033
  
 NCBI BlastP on this gene

EAW16890

conserved hypothetical protein
  
Accession: EAW16889
  
Location: 533861-536401
  
 NCBI BlastP on this gene

EAW16889

benzodiazepine receptor family protein
  
Accession: EAW16888
  
Location: 532012-532579
  
 NCBI BlastP on this gene

EAW16888

conserved hypothetical protein
  
Accession: EAW16887
  
Location: 530518-531292
  
  
**BlastP hit with Mycgr3G104337\_Mycgr3**
  
Percentage identity: 26 %
  
BlastP bit score: 90
  
Sequence coverage: 91 %
  
E-value: 1e-18
  
  
 NCBI BlastP on this gene

EAW16887

polyketide synthase, putative
  
Accession: EAW16886
  
Location: 522093-530226
  
  
**BlastP hit with Mycgr3G100089\_Mycgr3**
  
Percentage identity: 38 %
  
BlastP bit score: 879
  
Sequence coverage: 59 %
  
E-value: 0.0
  
  
 NCBI BlastP on this gene

EAW16886

ABC multidrug transporter, putative
  
Accession: EAW16885
  
Location: 517448-521866
  
 NCBI BlastP on this gene

EAW16885

MFS transporter, putative
  
Accession: EAW16884
  
Location: 508541-510186
  
 NCBI BlastP on this gene

EAW16884

185. :  DS572827 Paracoccidioides brasiliensis Pb01 supercont1.17 genomic scaffold     Total score: 2.0     Cumulative Blast bit score: 963

conserved hypothetical protein
  
Accession: EEH35115
  
Location: 313391-314598
  
 NCBI BlastP on this gene

EEH35115

tetraspanin
  
Accession: EEH35114
  
Location: 311054-312095
  
 NCBI BlastP on this gene

EEH35114

predicted protein
  
Accession: EEH35113
  
Location: 310226-310632
  
 NCBI BlastP on this gene

EEH35113

C6 zinc finger domain-containing protein
  
Accession: EEH35112
  
Location: 307786-309192
  
 NCBI BlastP on this gene

EEH35112

MADS box transcription factor Mcm1
  
Accession: EEH35111
  
Location: 304225-306738
  
  
**BlastP hit with Mycgr3G31170\_Mycgr3T**
  
Percentage identity: 100 %
  
BlastP bit score: 164
  
Sequence coverage: 100 %
  
E-value: 3e-46
  
  
 NCBI BlastP on this gene

EEH35111

predicted protein
  
Accession: EEH35110
  
Location: 303149-303555
  
 NCBI BlastP on this gene

EEH35110

DUF803 domain-containing protein
  
Accession: EEH35109
  
Location: 298925-301194
  
 NCBI BlastP on this gene

EEH35109

vacuolar ATP synthase subunit E
  
Accession: EEH35108
  
Location: 297364-298325
  
 NCBI BlastP on this gene

EEH35108

conserved hypothetical protein
  
Accession: EEH35107
  
Location: 294355-297198
  
 NCBI BlastP on this gene

EEH35107

ser/Thr protein phosphatase family protein
  
Accession: EEH35106
  
Location: 291449-292471
  
 NCBI BlastP on this gene

EEH35106

predicted protein
  
Accession: EEH35104
  
Location: 289544-290489
  
 NCBI BlastP on this gene

EEH35104

phospholipid-translocating P-type ATPase domain-containing protein
  
Accession: EEH35105
  
Location: 284504-289177
  
 NCBI BlastP on this gene

EEH35105

conserved hypothetical protein
  
Accession: EEH35103
  
Location: 275041-281805
  
  
**BlastP hit with Mycgr3G41426\_Mycgr3T**
  
Percentage identity: 48 %
  
BlastP bit score: 799
  
Sequence coverage: 48 %
  
E-value: 0.0
  
  
 NCBI BlastP on this gene

EEH35103

predicted protein
  
Accession: EEH35102
  
Location: 273554-274067
  
 NCBI BlastP on this gene

EEH35102

predicted protein
  
Accession: EEH35101
  
Location: 266601-269921
  
 NCBI BlastP on this gene

EEH35101

186. :  DS572697 Verticillium dahliae VdLs.17 supercont1.3 genomic scaffold     Total score: 2.0     Cumulative Blast bit score: 942

hypothetical protein
  
Accession: EGY19761
  
Location: 488538-491077
  
 NCBI BlastP on this gene

EGY19761

hypothetical protein
  
Accession: EGY19762
  
Location: 492319-492745
  
  
**BlastP hit with Mycgr3G29582\_Mycgr3T**
  
Percentage identity: 68 %
  
BlastP bit score: 97
  
Sequence coverage: 85 %
  
E-value: 4e-24
  
  
 NCBI BlastP on this gene

EGY19762

hypothetical protein
  
Accession: EGY19763
  
Location: 493283-493774
  
 NCBI BlastP on this gene

EGY19763

ATP-dependent RNA helicase SUB2
  
Accession: EGY19764
  
Location: 494395-496461
  
 NCBI BlastP on this gene

EGY19764

polygalacturonase
  
Accession: EGY19765
  
Location: 497618-498925
  
 NCBI BlastP on this gene

EGY19765

pectinesterase family protein
  
Accession: EGY19766
  
Location: 500454-501801
  
 NCBI BlastP on this gene

EGY19766

modification methylase Sau96I
  
Accession: EGY19767
  
Location: 502176-505049
  
 NCBI BlastP on this gene

EGY19767

HhH-GPD family base excision DNA repair protein
  
Accession: EGY19768
  
Location: 506530-508161
  
 NCBI BlastP on this gene

EGY19768

helicase SEN1
  
Accession: EGY19769
  
Location: 508949-515383
  
  
**BlastP hit with Mycgr3G41426\_Mycgr3T**
  
Percentage identity: 33 %
  
BlastP bit score: 845
  
Sequence coverage: 99 %
  
E-value: 0.0
  
  
 NCBI BlastP on this gene

EGY19769

hypothetical protein
  
Accession: EGY19770
  
Location: 516467-516769
  
 NCBI BlastP on this gene

EGY19770

hypothetical protein
  
Accession: EGY19771
  
Location: 518487-520382
  
 NCBI BlastP on this gene

EGY19771

eukaryotic translation initiation factor 1A
  
Accession: EGY19772
  
Location: 521291-521972
  
 NCBI BlastP on this gene

EGY19772

hypothetical protein
  
Accession: EGY19773
  
Location: 522701-525219
  
 NCBI BlastP on this gene

EGY19773

chitin biosynthesis protein CHS5
  
Accession: EGY19774
  
Location: 526379-527702
  
 NCBI BlastP on this gene

EGY19774

187. :  GL988047 Chaetomium thermophilum var. thermophilum DSM 1495 unplaced genomic scaffold scf7180000...     Total score: 2.0     Cumulative Blast bit score: 940

putative GTP binding protein
  
Accession: EGS17717
  
Location: 2257598-2260374
  
 NCBI BlastP on this gene

EGS17717

hypothetical protein
  
Accession: EGS17718
  
Location: 2262039-2265725
  
 NCBI BlastP on this gene

EGS17718

vacuolar protein sorting-associated protein 21-like protein
  
Accession: EGS17719
  
Location: 2266576-2267436
  
 NCBI BlastP on this gene

EGS17719

hypothetical protein
  
Accession: EGS17720
  
Location: 2268014-2270125
  
  
**BlastP hit with Mycgr3G85924\_Mycgr3T**
  
Percentage identity: 47 %
  
BlastP bit score: 351
  
Sequence coverage: 93 %
  
E-value: 1e-107
  
  
 NCBI BlastP on this gene

EGS17720

hypothetical protein
  
Accession: EGS17721
  
Location: 2271165-2272250
  
 NCBI BlastP on this gene

EGS17721

transferase-like protein
  
Accession: EGS17722
  
Location: 2272393-2273823
  
 NCBI BlastP on this gene

EGS17722

hypothetical protein
  
Accession: EGS17723
  
Location: 2278589-2279560
  
 NCBI BlastP on this gene

EGS17723

hypothetical protein
  
Accession: EGS17724
  
Location: 2284698-2287367
  
 NCBI BlastP on this gene

EGS17724

hypothetical protein
  
Accession: EGS17725
  
Location: 2289388-2291289
  
  
**BlastP hit with Mycgr3G92934\_Mycgr3T**
  
Percentage identity: 60 %
  
BlastP bit score: 589
  
Sequence coverage: 101 %
  
E-value: 0.0
  
  
 NCBI BlastP on this gene

EGS17725

hypothetical protein
  
Accession: EGS17726
  
Location: 2291361-2292560
  
 NCBI BlastP on this gene

EGS17726

oxidoreductase-like protein
  
Accession: EGS17727
  
Location: 2296511-2297657
  
 NCBI BlastP on this gene

EGS17727

pseudouridine synthase-like protein
  
Accession: EGS17728
  
Location: 2299152-2302397
  
 NCBI BlastP on this gene

EGS17728

188. :  GL891304 Neurospora tetrasperma FGSC 2508 unplaced genomic scaffold NEUTE1scaffold\_3     Total score: 2.0     Cumulative Blast bit score: 938

hypothetical protein
  
Accession: EGO57466
  
Location: 1099898-1102062
  
 NCBI BlastP on this gene

EGO57466

MAP kinase
  
Accession: EGO57465
  
Location: 1096030-1097618
  
 NCBI BlastP on this gene

EGO57465

hypothetical protein
  
Accession: EGO57464
  
Location: 1090288-1092219
  
  
**BlastP hit with Mycgr3G92934\_Mycgr3T**
  
Percentage identity: 60 %
  
BlastP bit score: 590
  
Sequence coverage: 101 %
  
E-value: 0.0
  
  
 NCBI BlastP on this gene

EGO57464

uracil-5-carboxylate decarboxylase
  
Accession: EGO57463
  
Location: 1088643-1089824
  
 NCBI BlastP on this gene

EGO57463

thymine dioxygenase
  
Accession: EGO57462
  
Location: 1086625-1087772
  
 NCBI BlastP on this gene

EGO57462

hypothetical protein
  
Accession: EGO57461
  
Location: 1085094-1086128
  
 NCBI BlastP on this gene

EGO57461

hypothetical protein
  
Accession: EGO57460
  
Location: 1081159-1083690
  
 NCBI BlastP on this gene

EGO57460

hypothetical protein
  
Accession: EGO57459
  
Location: 1069324-1072844
  
 NCBI BlastP on this gene

EGO57459

hypothetical protein
  
Accession: EGO57458
  
Location: 1067077-1067915
  
 NCBI BlastP on this gene

EGO57458

hypothetical protein
  
Accession: EGO57457
  
Location: 1064284-1066440
  
  
**BlastP hit with Mycgr3G85924\_Mycgr3T**
  
Percentage identity: 48 %
  
BlastP bit score: 348
  
Sequence coverage: 97 %
  
E-value: 2e-106
  
  
 NCBI BlastP on this gene

EGO57457

hypothetical protein
  
Accession: EGO57456
  
Location: 1054140-1057607
  
 NCBI BlastP on this gene

EGO57456

189. :  GL891236 Neurospora tetrasperma FGSC 2509 unplaced genomic scaffold NEUTE2scaffold\_4     Total score: 2.0     Cumulative Blast bit score: 938

hypothetical protein
  
Accession: EGZ72275
  
Location: 3904469-3904810
  
 NCBI BlastP on this gene

EGZ72275

hypothetical protein
  
Accession: EGZ72276
  
Location: 3906252-3906633
  
 NCBI BlastP on this gene

EGZ72276

MAP kinase
  
Accession: EGZ72277
  
Location: 3908913-3910501
  
 NCBI BlastP on this gene

EGZ72277

ATP-dependent RNA helicase dbp-8
  
Accession: EGZ72278
  
Location: 3914306-3916237
  
  
**BlastP hit with Mycgr3G92934\_Mycgr3T**
  
Percentage identity: 60 %
  
BlastP bit score: 590
  
Sequence coverage: 101 %
  
E-value: 0.0
  
  
 NCBI BlastP on this gene

EGZ72278

uracil-5-carboxylate decarboxylase
  
Accession: EGZ72279
  
Location: 3916701-3917882
  
 NCBI BlastP on this gene

EGZ72279

thymine dioxygenase
  
Accession: EGZ72280
  
Location: 3918753-3919900
  
 NCBI BlastP on this gene

EGZ72280

hypothetical protein
  
Accession: EGZ72281
  
Location: 3920397-3921431
  
 NCBI BlastP on this gene

EGZ72281

hypothetical protein
  
Accession: EGZ72282
  
Location: 3922835-3925366
  
 NCBI BlastP on this gene

EGZ72282

hypothetical protein
  
Accession: EGZ72283
  
Location: 3932272-3935792
  
 NCBI BlastP on this gene

EGZ72283

Rab5-like protein ypt51
  
Accession: EGZ72284
  
Location: 3937201-3938039
  
 NCBI BlastP on this gene

EGZ72284

hypothetical protein
  
Accession: EGZ72285
  
Location: 3938676-3940832
  
  
**BlastP hit with Mycgr3G85924\_Mycgr3T**
  
Percentage identity: 48 %
  
BlastP bit score: 348
  
Sequence coverage: 97 %
  
E-value: 2e-106
  
  
 NCBI BlastP on this gene

EGZ72285

hypothetical protein
  
Accession: EGZ72286
  
Location: 3947508-3950975
  
 NCBI BlastP on this gene

EGZ72286

190. :  AABX02000027 Neurospora crassa OR74A     Total score: 2.0     Cumulative Blast bit score: 918

MAP kinase kinase
  
Accession: EAA28074
  
Location: 62587-64175
  
 NCBI BlastP on this gene

EAA28074

hypothetical protein
  
Accession: EAA28073
  
Location: 56842-58795
  
  
**BlastP hit with Mycgr3G92934\_Mycgr3T**
  
Percentage identity: 60 %
  
BlastP bit score: 590
  
Sequence coverage: 101 %
  
E-value: 0.0
  
  
 NCBI BlastP on this gene

EAA28073

uracil-5-carboxylate decarboxylase
  
Accession: EAA28072
  
Location: 55193-56377
  
 NCBI BlastP on this gene

EAA28072

thymine dioxygenase
  
Accession: EAA28071
  
Location: 53174-54321
  
 NCBI BlastP on this gene

EAA28071

predicted protein
  
Accession: EAA28070
  
Location: 51593-52648
  
 NCBI BlastP on this gene

EAA28070

predicted protein
  
Accession: EAA26720
  
Location: 46914-49456
  
 NCBI BlastP on this gene

EAA26720

predicted protein
  
Accession: EAA26719
  
Location: 42247-45178
  
 NCBI BlastP on this gene

EAA26719

predicted protein
  
Accession: EAA26718
  
Location: 40050-41407
  
 NCBI BlastP on this gene

EAA26718

predicted protein
  
Accession: EAA26717
  
Location: 34961-38476
  
 NCBI BlastP on this gene

EAA26717

Rab5-like protein ypt51
  
Accession: EAA26716
  
Location: 32671-33509
  
 NCBI BlastP on this gene

EAA26716

predicted protein
  
Accession: EAA26715
  
Location: 29824-31995
  
  
**BlastP hit with Mycgr3G85924\_Mycgr3T**
  
Percentage identity: 45 %
  
BlastP bit score: 328
  
Sequence coverage: 104 %
  
E-value: 2e-98
  
  
 NCBI BlastP on this gene

EAA26715

predicted protein
  
Accession: EAA26714
  
Location: 25998-26350
  
 NCBI BlastP on this gene

EAA26714

hypothetical protein
  
Accession: EAA26713
  
Location: 19590-23045
  
 NCBI BlastP on this gene

EAA26713

191. :  KB932812 Togninia minima UCRPA7 unplaced genomic scaffold PA7\_03\_scaffold\_39     Total score: 2.0     Cumulative Blast bit score: 914

putative mfs hexose protein
  
Accession: EOO03938
  
Location: 607153-608627
  
 NCBI BlastP on this gene

EOO03938

putative diacylglycerol acyltransferase family protein
  
Accession: EOO03753
  
Location: 611523-613076
  
 NCBI BlastP on this gene

EOO03753

putative ef-hand calcium-binding domain protein
  
Accession: EOO03858
  
Location: 614249-615296
  
  
**BlastP hit with Mycgr3G104337\_Mycgr3**
  
Percentage identity: 45 %
  
BlastP bit score: 232
  
Sequence coverage: 96 %
  
E-value: 1e-71
  
  
 NCBI BlastP on this gene

EOO03858

putative polyketide synthase protein
  
Accession: EOO03896
  
Location: 617892-618864
  
 NCBI BlastP on this gene

EOO03896

putative polyketide synthase protein
  
Accession: EOO03974
  
Location: 621680-624201
  
  
**BlastP hit with Mycgr3G100089\_Mycgr3**
  
Percentage identity: 48 %
  
BlastP bit score: 682
  
Sequence coverage: 37 %
  
E-value: 0.0
  
  
 NCBI BlastP on this gene

EOO03974

192. :  CU633900 Podospora anserina S mat+ genomic DNA chromosome 7, supercontig 1.     Total score: 2.0     Cumulative Blast bit score: 873

not annotated
  
Accession: CAP69064
  
Location: 2925096-2927537
  
 NCBI BlastP on this gene

CAP69064

not annotated
  
Accession: CAP69065
  
Location: 2928147-2931094
  
 NCBI BlastP on this gene

CAP69065

not annotated
  
Accession: CAP69066
  
Location: 2933590-2936202
  
 NCBI BlastP on this gene

CAP69066

not annotated
  
Accession: CAP69067
  
Location: 2937938-2939843
  
 NCBI BlastP on this gene

CAP69067

not annotated
  
Accession: CAP69068
  
Location: 2941183-2942972
  
  
**BlastP hit with Mycgr3G92934\_Mycgr3T**
  
Percentage identity: 62 %
  
BlastP bit score: 577
  
Sequence coverage: 93 %
  
E-value: 0.0
  
  
 NCBI BlastP on this gene

CAP69068

not annotated
  
Accession: CAP69069
  
Location: 2943152-2944299
  
 NCBI BlastP on this gene

CAP69069

not annotated
  
Accession: CAP69070
  
Location: 2945097-2945489
  
 NCBI BlastP on this gene

CAP69070

not annotated
  
Accession: CAP69071
  
Location: 2947650-2949287
  
 NCBI BlastP on this gene

CAP69071

not annotated
  
Accession: CAP69072
  
Location: 2952267-2952942
  
 NCBI BlastP on this gene

CAP69072

not annotated
  
Accession: CAP69073
  
Location: 2955867-2957966
  
  
**BlastP hit with Mycgr3G85924\_Mycgr3T**
  
Percentage identity: 44 %
  
BlastP bit score: 296
  
Sequence coverage: 94 %
  
E-value: 9e-87
  
  
 NCBI BlastP on this gene

CAP69073

not annotated
  
Accession: CAP69074
  
Location: 2958987-2959826
  
 NCBI BlastP on this gene

CAP69074

not annotated
  
Accession: CAP69075
  
Location: 2961453-2963032
  
 NCBI BlastP on this gene

CAP69075

not annotated
  
Accession: CAP69076
  
Location: 2964064-2966538
  
 NCBI BlastP on this gene

CAP69076

not annotated
  
Accession: CAP69077
  
Location: 2968077-2972405
  
 NCBI BlastP on this gene

CAP69077

193. :  DS985215 Verticillium albo-atrum VaMs.102 supercont1.2 genomic scaffold     Total score: 2.0     Cumulative Blast bit score: 847

conserved hypothetical protein
  
Accession: EEY15665
  
Location: 983392-985252
  
 NCBI BlastP on this gene

EEY15665

conserved hypothetical protein
  
Accession: EEY15664
  
Location: 982703-982972
  
 NCBI BlastP on this gene

EEY15664

conserved hypothetical protein
  
Accession: EEY15663
  
Location: 981022-981452
  
  
**BlastP hit with Mycgr3G29582\_Mycgr3T**
  
Percentage identity: 67 %
  
BlastP bit score: 96
  
Sequence coverage: 85 %
  
E-value: 1e-23
  
  
 NCBI BlastP on this gene

EEY15663

conserved hypothetical protein
  
Accession: EEY15662
  
Location: 979987-980478
  
 NCBI BlastP on this gene

EEY15662

ATP-dependent RNA helicase SUB2
  
Accession: EEY15661
  
Location: 977327-979403
  
 NCBI BlastP on this gene

EEY15661

polygalacturonase
  
Accession: EEY15660
  
Location: 975514-976826
  
 NCBI BlastP on this gene

EEY15660

predicted protein
  
Accession: EEY15659
  
Location: 973830-974456
  
 NCBI BlastP on this gene

EEY15659

pectinesterase family protein
  
Accession: EEY15658
  
Location: 972338-973345
  
 NCBI BlastP on this gene

EEY15658

RIP defective
  
Accession: EEY15657
  
Location: 969110-971839
  
 NCBI BlastP on this gene

EEY15657

HhH-GPD family base excision DNA repair protein
  
Accession: EEY15656
  
Location: 966001-967628
  
 NCBI BlastP on this gene

EEY15656

helicase sen1
  
Accession: EEY15655
  
Location: 958721-965197
  
  
**BlastP hit with Mycgr3G41426\_Mycgr3T**
  
Percentage identity: 32 %
  
BlastP bit score: 751
  
Sequence coverage: 95 %
  
E-value: 0.0
  
  
 NCBI BlastP on this gene

EEY15655

conserved hypothetical protein
  
Accession: EEY15654
  
Location: 955459-955695
  
 NCBI BlastP on this gene

EEY15654

eukaryotic translation initiation factor eIF-1A
  
Accession: EEY15653
  
Location: 951856-952659
  
 NCBI BlastP on this gene

EEY15653

predicted protein
  
Accession: EEY15652
  
Location: 950150-951118
  
 NCBI BlastP on this gene

EEY15652

chitin biosynthesis protein CHS5
  
Accession: EEY15651
  
Location: 949006-949570
  
 NCBI BlastP on this gene

EEY15651

oxidoreductase
  
Accession: EEY15650
  
Location: 947116-947964
  
 NCBI BlastP on this gene

EEY15650

194. :  CH476607 Aspergillus terreus NIH2624 scaffold\_14 genomic scaffold     Total score: 2.0     Cumulative Blast bit score: 834

conserved hypothetical protein
  
Accession: EAU30232
  
Location: 255246-258145
  
 NCBI BlastP on this gene

EAU30232

conserved hypothetical protein
  
Accession: EAU30231
  
Location: 252506-254705
  
 NCBI BlastP on this gene

EAU30231

conserved hypothetical protein
  
Accession: EAU30230
  
Location: 248661-250236
  
 NCBI BlastP on this gene

EAU30230

predicted protein
  
Accession: EAU30229
  
Location: 246888-247413
  
 NCBI BlastP on this gene

EAU30229

hypothetical protein
  
Accession: EAU30228
  
Location: 241147-245391
  
 NCBI BlastP on this gene

EAU30228

conserved hypothetical protein
  
Accession: EAU30227
  
Location: 238223-240341
  
 NCBI BlastP on this gene

EAU30227

predicted protein
  
Accession: EAU30226
  
Location: 236167-236960
  
  
**BlastP hit with Mycgr3G104337\_Mycgr3**
  
Percentage identity: 26 %
  
BlastP bit score: 84
  
Sequence coverage: 91 %
  
E-value: 2e-16
  
  
 NCBI BlastP on this gene

EAU30226

hypothetical protein
  
Accession: EAU30225
  
Location: 228330-235615
  
  
**BlastP hit with Mycgr3G100089\_Mycgr3**
  
Percentage identity: 37 %
  
BlastP bit score: 750
  
Sequence coverage: 52 %
  
E-value: 0.0
  
  
 NCBI BlastP on this gene

EAU30225

2-oxoisovalerate dehydrogenase beta subunit, mitochondrial precursor
  
Accession: EAU30224
  
Location: 226777-227793
  
 NCBI BlastP on this gene

EAU30224

conserved hypothetical protein
  
Accession: EAU30223
  
Location: 225294-226274
  
 NCBI BlastP on this gene

EAU30223

conserved hypothetical protein
  
Accession: EAU30222
  
Location: 222712-224704
  
 NCBI BlastP on this gene

EAU30222

hypothetical protein
  
Accession: EAU30221
  
Location: 220897-222399
  
 NCBI BlastP on this gene

EAU30221

predicted protein
  
Accession: EAU30220
  
Location: 219051-220037
  
 NCBI BlastP on this gene

EAU30220

hypothetical protein
  
Accession: EAU30219
  
Location: 217702-218516
  
 NCBI BlastP on this gene

EAU30219

dihydroxyacetone synthase
  
Accession: EAU30218
  
Location: 214627-216858
  
 NCBI BlastP on this gene

EAU30218

predicted protein
  
Accession: EAU30217
  
Location: 213198-214045
  
 NCBI BlastP on this gene

EAU30217

hypothetical protein
  
Accession: EAU30216
  
Location: 211340-212632
  
 NCBI BlastP on this gene

EAU30216

conserved hypothetical protein
  
Accession: EAU30215
  
Location: 209359-211186
  
 NCBI BlastP on this gene

EAU30215

195. :  DF126459 Aspergillus kawachii IFO 4308 DNA, contig: scaffold00013     Total score: 2.0     Cumulative Blast bit score: 830

FAD binding domain protein
  
Accession: GAA87324
  
Location: 150234-152309
  
 NCBI BlastP on this gene

GAA87324

arylsulfatase A
  
Accession: GAA87323
  
Location: 147303-148841
  
 NCBI BlastP on this gene

GAA87323

polygalacturonase (PgaI)
  
Accession: GAA87322
  
Location: 143299-144521
  
 NCBI BlastP on this gene

GAA87322

NRPS-like enzyme
  
Accession: GAA87321
  
Location: 136163-139535
  
 NCBI BlastP on this gene

GAA87321

integral membrane protein
  
Accession: GAA87320
  
Location: 134189-135368
  
 NCBI BlastP on this gene

GAA87320

hypothetical protein
  
Accession: GAA87319
  
Location: 132780-133536
  
  
**BlastP hit with Mycgr3G104337\_Mycgr3**
  
Percentage identity: 30 %
  
BlastP bit score: 55
  
Sequence coverage: 59 %
  
E-value: 4e-06
  
  
 NCBI BlastP on this gene

GAA87319

polyketide synthase
  
Accession: GAA87318
  
Location: 123689-131921
  
  
**BlastP hit with Mycgr3G100089\_Mycgr3**
  
Percentage identity: 38 %
  
BlastP bit score: 775
  
Sequence coverage: 58 %
  
E-value: 0.0
  
  
 NCBI BlastP on this gene

GAA87318

flavin-containing amine oxidasedehydrogenase
  
Accession: GAA87317
  
Location: 121019-122527
  
 NCBI BlastP on this gene

GAA87317

feruloyl esterase B precursor
  
Accession: GAA87316
  
Location: 119092-120654
  
 NCBI BlastP on this gene

GAA87316

similar to An01g11590
  
Accession: GAA87315
  
Location: 114134-115490
  
 NCBI BlastP on this gene

GAA87315

pantothenate transporter
  
Accession: GAA87314
  
Location: 111992-113842
  
 NCBI BlastP on this gene

GAA87314

amidohydrolase
  
Accession: GAA87313
  
Location: 109432-110907
  
 NCBI BlastP on this gene

GAA87313

Zn(II)2Cys6 transcription factor
  
Accession: GAA87312
  
Location: 106786-108743
  
 NCBI BlastP on this gene

GAA87312

hypothetical protein
  
Accession: GAA87311
  
Location: 106065-106706
  
 NCBI BlastP on this gene

GAA87311

196. :  AP007157 Aspergillus oryzae RIB40 DNA, SC023.     Total score: 2.0     Cumulative Blast bit score: 718

not annotated
  
Accession: BAE58606
  
Location: 43863-46717
  
 NCBI BlastP on this gene

AO090023000016

not annotated
  
Accession: BAE58605
  
Location: 41238-42632
  
 NCBI BlastP on this gene

AO090023000015

not annotated
  
Accession: BAE58604
  
Location: 38934-39578
  
 NCBI BlastP on this gene

AO090023000014

not annotated
  
Accession: BAE58603
  
Location: 37175-38521
  
 NCBI BlastP on this gene

AO090023000013

not annotated
  
Accession: BAE58602
  
Location: 32362-35300
  
 NCBI BlastP on this gene

AO090023000012

not annotated
  
Accession: BAE58601
  
Location: 29813-31114
  
 NCBI BlastP on this gene

AO090023000011

not annotated
  
Accession: BAE58600
  
Location: 26595-27755
  
  
**BlastP hit with Mycgr3G92938\_Mycgr3T**
  
Percentage identity: 29 %
  
BlastP bit score: 114
  
Sequence coverage: 75 %
  
E-value: 4e-25
  
  
 NCBI BlastP on this gene

AO090023000010

not annotated
  
Accession: BAE58599
  
Location: 21131-22662
  
  
**BlastP hit with Mycgr3G42010\_Mycgr3T**
  
Percentage identity: 36 %
  
BlastP bit score: 322
  
Sequence coverage: 20 %
  
E-value: 8e-93
  
  
 NCBI BlastP on this gene

AO090023000008

not annotated
  
Accession: BAE58598
  
Location: 16001-19716
  
  
**BlastP hit with Mycgr3G42010\_Mycgr3T**
  
Percentage identity: 50 %
  
BlastP bit score: 282
  
Sequence coverage: 11 %
  
E-value: 7e-81
  
  
 NCBI BlastP on this gene

AO090023000007

not annotated
  
Accession: BAE58597
  
Location: 11234-13940
  
 NCBI BlastP on this gene

AO090023000006

not annotated
  
Accession: BAE58596
  
Location: 7651-8039
  
 NCBI BlastP on this gene

AO090023000004

not annotated
  
Accession: BAE58595
  
Location: 6228-6920
  
 NCBI BlastP on this gene

AO090023000003

not annotated
  
Accession: BAE58594
  
Location: 4189-4966
  
 NCBI BlastP on this gene

AO090023000002

not annotated
  
Accession: BAE58593
  
Location: 1943-3463
  
 NCBI BlastP on this gene

AO090023000001

197. :  DS547093 Laccaria bicolor S238N-H82 LACBIscaffold\_3 genomic scaffold     Total score: 2.0     Cumulative Blast bit score: 695

predicted protein
  
Accession: EDR13704
  
Location: 1950107-1951478
  
 NCBI BlastP on this gene

EDR13704

predicted protein
  
Accession: EDR13288
  
Location: 1951864-1954054
  
 NCBI BlastP on this gene

EDR13288

predicted protein
  
Accession: EDR13289
  
Location: 1955047-1955571
  
 NCBI BlastP on this gene

EDR13289

predicted protein
  
Accession: EDR13290
  
Location: 1956040-1956216
  
 NCBI BlastP on this gene

EDR13290

predicted protein
  
Accession: EDR13291
  
Location: 1957634-1957986
  
  
**BlastP hit with Mycgr3G31170\_Mycgr3T**
  
Percentage identity: 93 %
  
BlastP bit score: 154
  
Sequence coverage: 100 %
  
E-value: 1e-46
  
  
 NCBI BlastP on this gene

EDR13291

predicted protein
  
Accession: EDR13705
  
Location: 1958883-1959394
  
 NCBI BlastP on this gene

EDR13705

predicted protein
  
Accession: EDR13706
  
Location: 1959734-1960216
  
 NCBI BlastP on this gene

EDR13706

predicted protein
  
Accession: EDR13707
  
Location: 1960321-1963184
  
 NCBI BlastP on this gene

EDR13707

predicted protein
  
Accession: EDR13708
  
Location: 1966716-1968482
  
 NCBI BlastP on this gene

EDR13708

predicted protein
  
Accession: EDR13292
  
Location: 1968759-1970981
  
 NCBI BlastP on this gene

EDR13292

predicted protein
  
Accession: EDR13709
  
Location: 1971148-1972811
  
 NCBI BlastP on this gene

EDR13709

predicted protein
  
Accession: EDR13710
  
Location: 1973331-1974025
  
 NCBI BlastP on this gene

EDR13710

predicted protein
  
Accession: EDR13711
  
Location: 1974241-1975830
  
 NCBI BlastP on this gene

EDR13711

predicted protein
  
Accession: EDR13712
  
Location: 1975998-1977523
  
 NCBI BlastP on this gene

EDR13712

predicted protein
  
Accession: EDR13713
  
Location: 1977645-1978883
  
 NCBI BlastP on this gene

EDR13713

predicted protein
  
Accession: EDR13714
  
Location: 1979115-1979724
  
 NCBI BlastP on this gene

EDR13714

predicted protein
  
Accession: EDR13715
  
Location: 1979897-1981182
  
 NCBI BlastP on this gene

EDR13715

predicted protein
  
Accession: EDR13716
  
Location: 1981475-1982495
  
 NCBI BlastP on this gene

EDR13716

predicted protein
  
Accession: EDR13293
  
Location: 1982675-1988992
  
  
**BlastP hit with Mycgr3G41426\_Mycgr3T**
  
Percentage identity: 31 %
  
BlastP bit score: 541
  
Sequence coverage: 67 %
  
E-value: 3e-157
  
  
 NCBI BlastP on this gene

EDR13293

predicted protein
  
Accession: EDR13717
  
Location: 1989092-1992898
  
 NCBI BlastP on this gene

EDR13717

predicted protein
  
Accession: EDR13294
  
Location: 1993493-1993901
  
 NCBI BlastP on this gene

EDR13294

predicted protein
  
Accession: EDR13718
  
Location: 1994244-1999983
  
 NCBI BlastP on this gene

EDR13718

198. :  CACQ02006690 Colletotrichum higginsianum strain IMI 349063     Total score: 2.0     Cumulative Blast bit score: 631

F-box domain-containing protein
  
Accession: CCF44193
  
Location: 203-2840
  
 NCBI BlastP on this gene

CCF44193

RNA exonuclease
  
Accession: CCF44194
  
Location: 3738-5277
  
 NCBI BlastP on this gene

CCF44194

geranylgeranyl pyrophosphate synthetase
  
Accession: CCF44195
  
Location: 7242-8681
  
  
**BlastP hit with Mycgr3G92938\_Mycgr3T**
  
Percentage identity: 40 %
  
BlastP bit score: 271
  
Sequence coverage: 82 %
  
E-value: 5e-82
  
  
 NCBI BlastP on this gene

CCF44195

helicase required for RNAi-mediated heterochromatin assembly 1
  
Accession: CCF44196
  
Location: 9380-11270
  
  
**BlastP hit with Mycgr3G42010\_Mycgr3T**
  
Percentage identity: 37 %
  
BlastP bit score: 360
  
Sequence coverage: 26 %
  
E-value: 3e-104
  
  
 NCBI BlastP on this gene

CCF44196

199. :  GG663369 Ajellomyces capsulatus G186AR genomic scaffold supercont2.7     Total score: 2.0     Cumulative Blast bit score: 624

C6 zinc finger domain-containing protein
  
Accession: EEH06335
  
Location: 1072470-1073774
  
 NCBI BlastP on this gene

EEH06335

predicted protein
  
Accession: EEH06336
  
Location: 1074536-1076052
  
 NCBI BlastP on this gene

EEH06336

conserved hypothetical protein
  
Accession: EEH06337
  
Location: 1076931-1078115
  
 NCBI BlastP on this gene

EEH06337

MADS box transcription factor Mcm1
  
Accession: EEH06338
  
Location: 1078923-1079856
  
  
**BlastP hit with Mycgr3G31170\_Mycgr3T**
  
Percentage identity: 98 %
  
BlastP bit score: 164
  
Sequence coverage: 100 %
  
E-value: 2e-48
  
  
 NCBI BlastP on this gene

EEH06338

DUF803 domain-containing protein
  
Accession: EEH06339
  
Location: 1082686-1084760
  
 NCBI BlastP on this gene

EEH06339

vacuolar ATP synthase subunit E
  
Accession: EEH06340
  
Location: 1085279-1086206
  
 NCBI BlastP on this gene

EEH06340

conserved hypothetical protein
  
Accession: EEH06341
  
Location: 1086816-1089296
  
 NCBI BlastP on this gene

EEH06341

serine/threonine phosphatase
  
Accession: EEH06342
  
Location: 1090472-1091506
  
 NCBI BlastP on this gene

EEH06342

phospholipid-translocating P-type ATPase domain-containing protein
  
Accession: EEH06343
  
Location: 1093672-1098340
  
 NCBI BlastP on this gene

EEH06343

protein kinase
  
Accession: EEH06344
  
Location: 1099801-1101292
  
 NCBI BlastP on this gene

EEH06344

DEAD-box type RNA helicase
  
Accession: EEH06345
  
Location: 1102134-1108881
  
  
**BlastP hit with Mycgr3G41426\_Mycgr3T**
  
Percentage identity: 30 %
  
BlastP bit score: 460
  
Sequence coverage: 49 %
  
E-value: 1e-129
  
  
 NCBI BlastP on this gene

EEH06345

predicted protein
  
Accession: EEH06346
  
Location: 1110086-1110684
  
 NCBI BlastP on this gene

EEH06346

predicted protein
  
Accession: EEH06347
  
Location: 1113777-1117046
  
 NCBI BlastP on this gene

EEH06347

200. :  DS990642 Ajellomyces capsulatus H88 supercont1.7 genomic scaffold     Total score: 2.0     Cumulative Blast bit score: 624

tetraspanin
  
Accession: EGC49086
  
Location: 1725745-1726772
  
 NCBI BlastP on this gene

EGC49086

C6 zinc finger domain-containing protein
  
Accession: EGC49087
  
Location: 1728665-1730003
  
 NCBI BlastP on this gene

EGC49087

conserved hypothetical protein
  
Accession: EGC49088
  
Location: 1731831-1732267
  
 NCBI BlastP on this gene

EGC49088

conserved hypothetical protein
  
Accession: EGC49089
  
Location: 1733118-1734302
  
 NCBI BlastP on this gene

EGC49089

MADS box transcription factor Mcm1
  
Accession: EGC49090
  
Location: 1735126-1736056
  
  
**BlastP hit with Mycgr3G31170\_Mycgr3T**
  
Percentage identity: 98 %
  
BlastP bit score: 164
  
Sequence coverage: 100 %
  
E-value: 2e-48
  
  
 NCBI BlastP on this gene

EGC49090

DUF803 domain-containing protein
  
Accession: EGC49091
  
Location: 1738791-1740974
  
 NCBI BlastP on this gene

EGC49091

vacuolar ATP synthase subunit E
  
Accession: EGC49092
  
Location: 1741426-1742353
  
 NCBI BlastP on this gene

EGC49092

conserved hypothetical protein
  
Accession: EGC49093
  
Location: 1742922-1745404
  
 NCBI BlastP on this gene

EGC49093

serine/threonine protein phosphatase
  
Accession: EGC49094
  
Location: 1746597-1747683
  
 NCBI BlastP on this gene

EGC49094

phospholipid-translocating P-type ATPase
  
Accession: EGC49095
  
Location: 1749735-1754402
  
 NCBI BlastP on this gene

EGC49095

helicase SEN1
  
Accession: EGC49096
  
Location: 1756257-1763003
  
  
**BlastP hit with Mycgr3G41426\_Mycgr3T**
  
Percentage identity: 30 %
  
BlastP bit score: 460
  
Sequence coverage: 49 %
  
E-value: 1e-129
  
  
 NCBI BlastP on this gene

EGC49096

predicted protein
  
Accession: EGC49097
  
Location: 1763884-1764736
  
 NCBI BlastP on this gene

EGC49097

predicted protein
  
Accession: EGC49098
  
Location: 1767857-1771015
  
 NCBI BlastP on this gene

EGC49098

dihydroorotase
  
Accession: EGC49099
  
Location: 1771623-1772874
  
 NCBI BlastP on this gene

EGC49099

Detecting sequence homology at the gene cluster level with MultiGeneBlast.
  
Marnix H. Medema, Rainer Breitling & Eriko Takano (2013)
  
*Molecular Biology and Evolution* , 30: 1218-1223.
