# Supplementary material for: In silico prediction and characterization of secondary metabolite biosynthetic gene clusters in the wheat pathogen Zymoseptoria tritici
Source: BMC Genomics. 2017 Aug 17;18:631. doi: 10.1186/s12864-017-3969-y (PMC5561558; doi:10.1186/s12864-017-3969-y)
Supplement: Supplementary file 1 — MultiGeneBLAST analysis of putative secondary metabolite clusters. All encoded amino acid sequences from genes residing in clusters predicted by AntiSMASH are given as FASTA file format. All output data from MultiGeneBLASTs are also provided. (ZIP 42911 kb) [file 12864_2017_3969_MOESM1_ESM.zip › Cluster MultiGene BLAST/out/Clusters_1_34/Cluster_15/displaypage5.xhtml]

xml version="1.0" encoding="UTF-8"?


Search Results
  
  
 Results pages: 1, 2, 3, 4, 5

**MultiGeneBlast hits**

Select gene cluster alignment
201. GG749462\_0 Ajellomyces dermatitidis ATCC 18188 genomic scaffold supercon...
202. GG657463\_0 Ajellomyces dermatitidis SLH14081 genomic scaffold supercont1...
203. HE978324\_0 Kazachstania naganishii CBS 8797 chromosome 11, complete genome.
204. GG704912\_1 Coccidioides immitis RS genomic scaffold supercont3.2, whole ...
205. ACFW01000025\_1 Coccidioides posadasii C735 delta SOWgp, whole genome sho...
206. AKHY01000076\_1 Aspergillus oryzae 3.042, whole genome shotgun sequencing...
207. HF679028\_0 Fusarium fujikuroi IMI 58289 draft genome, chromosome FFUJ\_ch...
208. KB708021\_0 Botryotinia fuckeliana BcDW1 unplaced genomic scaffold Scaffo...
209. EQ963480\_0 Aspergillus flavus NRRL3357 scf\_1106286418846 genomic scaffol...
210. CP000518\_1 Mycobacterium sp. KMS, complete genome.
211. CP000384\_1 Mycobacterium sp. MCS, complete genome.
212. CP000580\_2 Mycobacterium sp. JLS, complete genome.
213. CP003078\_0 Mycobacterium smegmatis JS623, complete genome.
214. JH126399\_0 Cordyceps militaris CM01 unplaced genomic scaffold CCM\_S00001...
215. KE145369\_0 Glarea lozoyensis ATCC 20868 chromosome Unknown GLAREA5, whol...
216. HF679032\_0 Fusarium fujikuroi IMI 58289 draft genome, chromosome FFUJ\_ch...
217. AP007166\_0 Aspergillus oryzae RIB40 DNA, SC113.
218. KB908855\_0 Setosphaeria turcica Et28A unplaced genomic scaffold SETTUsca...
219. FQ790251\_0 Botryotinia fuckeliana T4 SuperContig\_87\_1 genomic supercontig.
220. KE145357\_0 Glarea lozoyensis ATCC 20868 chromosome Unknown GLAREA14, who...
221. KB726994\_0 Fusarium oxysporum f. sp. cubense race 4 unplaced genomic sca...
222. AFQF01000390\_0 Fusarium oxysporum Fo5176, whole genome shotgun sequencin...
223. KB730083\_0 Fusarium oxysporum f. sp. cubense race 1 unplaced genomic sca...
224. AQGS01001233\_1 Dactylellina haptotyla CBS 200.50, whole genome shotgun s...
225. ADOT01000322\_0 Arthrobotrys oligospora ATCC 24927, whole genome shotgun ...
226. JH795283\_0 Magnaporthe oryzae P131 unplaced genomic scaffold P131\_scaffo...
227. JH793600\_0 Magnaporthe oryzae Y34 unplaced genomic scaffold Y34\_scaffold...
228. CM001235\_1 Magnaporthe oryzae 70-15 chromosome 5, whole genome shotgun s...
229. JH767594\_0 Coniosporium apollinis CBS 100218 chromosome Unknown supercon...
230. GG698901\_0 Nectria haematococca mpVI 77-13-4 chromosome 1 genomic scaffo...
231. DS231619\_1 Pyrenophora tritici-repentis Pt-1C-BFP supercont1.5 genomic s...
232. DF126500\_0 Aspergillus kawachii IFO 4308 DNA, contig: scaffold00054, who...
233. ACJE01000015\_0 Aspergillus niger ATCC 1015, whole genome shotgun sequenc...
234. CABT02000006\_0 Sordaria macrospora k-hell, whole genome shotgun sequenci...
235. DS231616\_2 Pyrenophora tritici-repentis Pt-1C-BFP supercont1.2 genomic s...
236. KB445638\_1 Cochliobolus sativus ND90Pr unplaced genomic scaffold COCSAsc...
237. KB733450\_2 Bipolaris maydis ATCC 48331 unplaced genomic scaffold COCC4sc...
238. KB445573\_1 Cochliobolus heterostrophus C5 unplaced genomic scaffold COCH...
239. AHMM02000023\_0 Leptospira inadai serovar Lyme str. 10, whole genome shot...
240. AHMO02000004\_0 Leptospira broomii serovar Hurstbridge str. 5399, whole g...
241. JH921445\_0 Marssonina brunnea f. sp. 'multigermtubi' MB\_m1 unplaced geno...
242. CH408031\_7 Chaetomium globosum CBS 148.51 scaffold\_3 genomic scaffold, w...
243. CU633897\_0 Podospora anserina S mat+ genomic DNA chromosome 1, supercont...
244. AEYX01000033\_1 Streptomyces griseoaurantiacus M045, whole genome shotgun...
245. CAUH01001128\_0 Blumeria graminis f. sp. hordei DH14, whole genome shotgu...
246. JH711588\_0 Coniophora puteana RWD-64-598 SS2 unplaced genomic scaffold C...
247. KB446555\_3 Pseudocercospora fijiensis CIRAD86 unplaced genomic scaffold ...
248. KB456260\_3 Mycosphaerella populorum SO2202 unplaced genomic scaffold SEP...
249. KB469300\_0 Gloeophyllum trabeum ATCC 11539 unplaced genomic scaffold GLO...
250. JH687546\_0 Punctularia strigosozonata HHB-11173 SS5 unplaced genomic sca...

Query: Architecture Search FASTA input

GG749462 : Ajellomyces dermatitidis ATCC 18188 genomic scaffold supercont1.56    Total score: 2.0     Cumulative Blast bit score: 622

Hit cluster cross-links:

Mycgr3G85918 Mycgr3T
  
Location: 0-1602

Mycgr3G85918\_Mycgr3T

Mycgr3G42010 Mycgr3T
  
Location: 1702-8569

Mycgr3G42010\_Mycgr3T

Mycgr3G29582 Mycgr3T
  
Location: 8669-8915

Mycgr3G29582\_Mycgr3T

Mycgr3G31170 Mycgr3T
  
Location: 9015-9255

Mycgr3G31170\_Mycgr3T

Mycgr3G85924 Mycgr3T
  
Location: 9355-11218

Mycgr3G85924\_Mycgr3T

Mycgr3G71676 Mycgr3T
  
Location: 11318-12494

Mycgr3G71676\_Mycgr3T

Mycgr3G11468 Mycgr3T
  
Location: 12594-13653

Mycgr3G11468\_Mycgr3T

Mycgr3G58567 Mycgr3T
  
Location: 13753-14506

Mycgr3G58567\_Mycgr3T

Mycgr3G100089 Mycgr3
  
Location: 14606-21152

Mycgr3G100089\_Mycgr3

Mycgr3G42698 Mycgr3T
  
Location: 21252-22131

Mycgr3G42698\_Mycgr3T

Mycgr3G71681 Mycgr3T
  
Location: 22231-23461

Mycgr3G71681\_Mycgr3T

Mycgr3G109328 Mycgr3
  
Location: 23561-24239

Mycgr3G109328\_Mycgr3

Mycgr3G104334 Mycgr3
  
Location: 24339-24567

Mycgr3G104334\_Mycgr3

Mycgr3G42715 Mycgr3T
  
Location: 24667-25981

Mycgr3G42715\_Mycgr3T

Mycgr3G92934 Mycgr3T
  
Location: 26081-27593

Mycgr3G92934\_Mycgr3T

Mycgr3G41969 Mycgr3T
  
Location: 27693-29328

Mycgr3G41969\_Mycgr3T

Mycgr3G80635 Mycgr3T
  
Location: 29428-29821

Mycgr3G80635\_Mycgr3T

Mycgr3G41426 Mycgr3T
  
Location: 29921-35255

Mycgr3G41426\_Mycgr3T

Mycgr3G104337 Mycgr3
  
Location: 35355-36108

Mycgr3G104337\_Mycgr3

Mycgr3G71679 Mycgr3T
  
Location: 36208-37300

Mycgr3G71679\_Mycgr3T

Mycgr3G92938 Mycgr3T
  
Location: 37400-38699

Mycgr3G92938\_Mycgr3T

Mycgr3G92941 Mycgr3T
  
Location: 38799-40734

Mycgr3G92941\_Mycgr3T

serine protein kinase Sky1
  
Accession: EGE84164
  
Location: 207730-209207
  
 NCBI BlastP on this gene

EGE84164

hypothetical protein
  
Accession: EGE84163
  
Location: 206805-207141
  
 NCBI BlastP on this gene

EGE84163

hypothetical protein
  
Accession: EGE84162
  
Location: 205163-206418
  
 NCBI BlastP on this gene

EGE84162

MADS box transcription factor Mcm1
  
Accession: EGE84161
  
Location: 201512-202489
  
  
**BlastP hit with Mycgr3G31170\_Mycgr3T**
  
Percentage identity: 100 %
  
BlastP bit score: 166
  
Sequence coverage: 100 %
  
E-value: 1e-49
  
  
 NCBI BlastP on this gene

EGE84161

DUF803 domain-containing protein
  
Accession: EGE84160
  
Location: 196049-198199
  
 NCBI BlastP on this gene

EGE84160

vacuolar ATP synthase subunit E
  
Accession: EGE84159
  
Location: 194599-195523
  
 NCBI BlastP on this gene

EGE84159

hypothetical protein
  
Accession: EGE84158
  
Location: 194213-194563
  
 NCBI BlastP on this gene

EGE84158

hypothetical protein
  
Accession: EGE84157
  
Location: 191419-193902
  
 NCBI BlastP on this gene

EGE84157

serine/threonine protein phosphatase
  
Accession: EGE84156
  
Location: 189477-190256
  
 NCBI BlastP on this gene

EGE84156

hypothetical protein
  
Accession: EGE84155
  
Location: 188220-188710
  
 NCBI BlastP on this gene

EGE84155

phospholipid-translocating P-type ATPase domain-containing protein
  
Accession: EGE84154
  
Location: 182229-186884
  
 NCBI BlastP on this gene

EGE84154

tRNA-splicing endonuclease
  
Accession: EGE84153
  
Location: 172887-180343
  
  
**BlastP hit with Mycgr3G41426\_Mycgr3T**
  
Percentage identity: 30 %
  
BlastP bit score: 456
  
Sequence coverage: 49 %
  
E-value: 3e-128
  
  
 NCBI BlastP on this gene

EGE84153

hypothetical protein
  
Accession: EGE84152
  
Location: 164434-167775
  
 NCBI BlastP on this gene

EGE84152

Query: Architecture Search FASTA input

GG657463 : Ajellomyces dermatitidis SLH14081 genomic scaffold supercont1.16    Total score: 2.0     Cumulative Blast bit score: 620

Hit cluster cross-links:

Mycgr3G85918 Mycgr3T
  
Location: 0-1602

Mycgr3G85918\_Mycgr3T

Mycgr3G42010 Mycgr3T
  
Location: 1702-8569

Mycgr3G42010\_Mycgr3T

Mycgr3G29582 Mycgr3T
  
Location: 8669-8915

Mycgr3G29582\_Mycgr3T

Mycgr3G31170 Mycgr3T
  
Location: 9015-9255

Mycgr3G31170\_Mycgr3T

Mycgr3G85924 Mycgr3T
  
Location: 9355-11218

Mycgr3G85924\_Mycgr3T

Mycgr3G71676 Mycgr3T
  
Location: 11318-12494

Mycgr3G71676\_Mycgr3T

Mycgr3G11468 Mycgr3T
  
Location: 12594-13653

Mycgr3G11468\_Mycgr3T

Mycgr3G58567 Mycgr3T
  
Location: 13753-14506

Mycgr3G58567\_Mycgr3T

Mycgr3G100089 Mycgr3
  
Location: 14606-21152

Mycgr3G100089\_Mycgr3

Mycgr3G42698 Mycgr3T
  
Location: 21252-22131

Mycgr3G42698\_Mycgr3T

Mycgr3G71681 Mycgr3T
  
Location: 22231-23461

Mycgr3G71681\_Mycgr3T

Mycgr3G109328 Mycgr3
  
Location: 23561-24239

Mycgr3G109328\_Mycgr3

Mycgr3G104334 Mycgr3
  
Location: 24339-24567

Mycgr3G104334\_Mycgr3

Mycgr3G42715 Mycgr3T
  
Location: 24667-25981

Mycgr3G42715\_Mycgr3T

Mycgr3G92934 Mycgr3T
  
Location: 26081-27593

Mycgr3G92934\_Mycgr3T

Mycgr3G41969 Mycgr3T
  
Location: 27693-29328

Mycgr3G41969\_Mycgr3T

Mycgr3G80635 Mycgr3T
  
Location: 29428-29821

Mycgr3G80635\_Mycgr3T

Mycgr3G41426 Mycgr3T
  
Location: 29921-35255

Mycgr3G41426\_Mycgr3T

Mycgr3G104337 Mycgr3
  
Location: 35355-36108

Mycgr3G104337\_Mycgr3

Mycgr3G71679 Mycgr3T
  
Location: 36208-37300

Mycgr3G71679\_Mycgr3T

Mycgr3G92938 Mycgr3T
  
Location: 37400-38699

Mycgr3G92938\_Mycgr3T

Mycgr3G92941 Mycgr3T
  
Location: 38799-40734

Mycgr3G92941\_Mycgr3T

conserved hypothetical protein
  
Accession: EEQ71914
  
Location: 1357784-1359039
  
 NCBI BlastP on this gene

EEQ71914

predicted protein
  
Accession: EEQ71913
  
Location: 1350192-1350740
  
 NCBI BlastP on this gene

EEQ71913

MADS box transcription factor Mcm1
  
Accession: EEQ71912
  
Location: 1348343-1349320
  
  
**BlastP hit with Mycgr3G31170\_Mycgr3T**
  
Percentage identity: 100 %
  
BlastP bit score: 166
  
Sequence coverage: 100 %
  
E-value: 1e-49
  
  
 NCBI BlastP on this gene

EEQ71912

DUF803 domain-containing protein
  
Accession: EEQ71911
  
Location: 1343307-1345457
  
 NCBI BlastP on this gene

EEQ71911

vacuolar ATP synthase subunit E
  
Accession: EEQ71910
  
Location: 1341850-1342774
  
 NCBI BlastP on this gene

EEQ71910

conserved hypothetical protein
  
Accession: EEQ71909
  
Location: 1338670-1341153
  
 NCBI BlastP on this gene

EEQ71909

conserved hypothetical protein
  
Accession: EEQ71908
  
Location: 1336707-1337486
  
 NCBI BlastP on this gene

EEQ71908

phospholipid-translocating P-type ATPase domain-containing protein
  
Accession: EEQ71907
  
Location: 1329515-1334169
  
 NCBI BlastP on this gene

EEQ71907

tRNA-splicing endonuclease
  
Accession: EEQ71906
  
Location: 1320794-1327634
  
  
**BlastP hit with Mycgr3G41426\_Mycgr3T**
  
Percentage identity: 30 %
  
BlastP bit score: 454
  
Sequence coverage: 49 %
  
E-value: 9e-128
  
  
 NCBI BlastP on this gene

EEQ71906

hypothetical protein
  
Accession: EEQ71905
  
Location: 1312278-1315517
  
 NCBI BlastP on this gene

EEQ71905

Query: Architecture Search FASTA input

HE978324 : Kazachstania naganishii CBS 8797 chromosome 11    Total score: 2.0     Cumulative Blast bit score: 598

Hit cluster cross-links:

Mycgr3G85918 Mycgr3T
  
Location: 0-1602

Mycgr3G85918\_Mycgr3T

Mycgr3G42010 Mycgr3T
  
Location: 1702-8569

Mycgr3G42010\_Mycgr3T

Mycgr3G29582 Mycgr3T
  
Location: 8669-8915

Mycgr3G29582\_Mycgr3T

Mycgr3G31170 Mycgr3T
  
Location: 9015-9255

Mycgr3G31170\_Mycgr3T

Mycgr3G85924 Mycgr3T
  
Location: 9355-11218

Mycgr3G85924\_Mycgr3T

Mycgr3G71676 Mycgr3T
  
Location: 11318-12494

Mycgr3G71676\_Mycgr3T

Mycgr3G11468 Mycgr3T
  
Location: 12594-13653

Mycgr3G11468\_Mycgr3T

Mycgr3G58567 Mycgr3T
  
Location: 13753-14506

Mycgr3G58567\_Mycgr3T

Mycgr3G100089 Mycgr3
  
Location: 14606-21152

Mycgr3G100089\_Mycgr3

Mycgr3G42698 Mycgr3T
  
Location: 21252-22131

Mycgr3G42698\_Mycgr3T

Mycgr3G71681 Mycgr3T
  
Location: 22231-23461

Mycgr3G71681\_Mycgr3T

Mycgr3G109328 Mycgr3
  
Location: 23561-24239

Mycgr3G109328\_Mycgr3

Mycgr3G104334 Mycgr3
  
Location: 24339-24567

Mycgr3G104334\_Mycgr3

Mycgr3G42715 Mycgr3T
  
Location: 24667-25981

Mycgr3G42715\_Mycgr3T

Mycgr3G92934 Mycgr3T
  
Location: 26081-27593

Mycgr3G92934\_Mycgr3T

Mycgr3G41969 Mycgr3T
  
Location: 27693-29328

Mycgr3G41969\_Mycgr3T

Mycgr3G80635 Mycgr3T
  
Location: 29428-29821

Mycgr3G80635\_Mycgr3T

Mycgr3G41426 Mycgr3T
  
Location: 29921-35255

Mycgr3G41426\_Mycgr3T

Mycgr3G104337 Mycgr3
  
Location: 35355-36108

Mycgr3G104337\_Mycgr3

Mycgr3G71679 Mycgr3T
  
Location: 36208-37300

Mycgr3G71679\_Mycgr3T

Mycgr3G92938 Mycgr3T
  
Location: 37400-38699

Mycgr3G92938\_Mycgr3T

Mycgr3G92941 Mycgr3T
  
Location: 38799-40734

Mycgr3G92941\_Mycgr3T

hypothetical protein
  
Accession: CCK72404
  
Location: 58785-60377
  
 NCBI BlastP on this gene

KNAG0K00360

hypothetical protein
  
Accession: CCK72405
  
Location: 62596-65535
  
 NCBI BlastP on this gene

KNAG0K00370

hypothetical protein
  
Accession: CCK72406
  
Location: 65728-66825
  
 NCBI BlastP on this gene

KNAG0K00380

hypothetical protein
  
Accession: CCK72407
  
Location: 66964-68445
  
 NCBI BlastP on this gene

KNAG0K00390

hypothetical protein
  
Accession: CCK72408
  
Location: 69795-71093
  
  
**BlastP hit with Mycgr3G92934\_Mycgr3T**
  
Percentage identity: 45 %
  
BlastP bit score: 363
  
Sequence coverage: 89 %
  
E-value: 6e-117
  
  
 NCBI BlastP on this gene

KNAG0K00400

hypothetical protein
  
Accession: CCK72409
  
Location: 71166-71738
  
 NCBI BlastP on this gene

KNAG0K00410

hypothetical protein
  
Accession: CCK72410
  
Location: 71859-73397
  
 NCBI BlastP on this gene

KNAG0K00420

hypothetical protein
  
Accession: CCK72411
  
Location: 73566-74159
  
 NCBI BlastP on this gene

KNAG0K00430

hypothetical protein
  
Accession: CCK72412
  
Location: 74289-75608
  
 NCBI BlastP on this gene

KNAG0K00440

hypothetical protein
  
Accession: CCK72413
  
Location: 75675-76478
  
 NCBI BlastP on this gene

KNAG0K00450

hypothetical protein
  
Accession: CCK72414
  
Location: 77388-79508
  
 NCBI BlastP on this gene

KNAG0K00460

hypothetical protein
  
Accession: CCK72415
  
Location: 79664-80908
  
 NCBI BlastP on this gene

KNAG0K00470

hypothetical protein
  
Accession: CCK72416
  
Location: 82114-82653
  
 NCBI BlastP on this gene

KNAG0K00480

hypothetical protein
  
Accession: CCK72417
  
Location: 84367-85047
  
 NCBI BlastP on this gene

KNAG0K00490

hypothetical protein
  
Accession: CCK72418
  
Location: 85328-85981
  
 NCBI BlastP on this gene

KNAG0K00500

hypothetical protein
  
Accession: CCK72419
  
Location: 86667-88943
  
 NCBI BlastP on this gene

KNAG0K00510

hypothetical protein
  
Accession: CCK72420
  
Location: 89428-90687
  
 NCBI BlastP on this gene

KNAG0K00520

hypothetical protein
  
Accession: CCK72421
  
Location: 91791-93842
  
  
**BlastP hit with Mycgr3G41969\_Mycgr3T**
  
Percentage identity: 33 %
  
BlastP bit score: 235
  
Sequence coverage: 104 %
  
E-value: 3e-65
  
  
 NCBI BlastP on this gene

KNAG0K00530

hypothetical protein
  
Accession: CCK72422
  
Location: 94316-95308
  
 NCBI BlastP on this gene

KNAG0K00540

hypothetical protein
  
Accession: CCK72423
  
Location: 96000-96491
  
 NCBI BlastP on this gene

KNAG0K00550

hypothetical protein
  
Accession: CCK72424
  
Location: 96678-99992
  
 NCBI BlastP on this gene

KNAG0K00560

hypothetical protein
  
Accession: CCK72425
  
Location: 100355-101266
  
 NCBI BlastP on this gene

KNAG0K00570

hypothetical protein
  
Accession: CCK72426
  
Location: 101850-104819
  
 NCBI BlastP on this gene

KNAG0K00580

Query: Architecture Search FASTA input

GG704912 : Coccidioides immitis RS genomic scaffold supercont3.2    Total score: 2.0     Cumulative Blast bit score: 594

Hit cluster cross-links:

Mycgr3G85918 Mycgr3T
  
Location: 0-1602

Mycgr3G85918\_Mycgr3T

Mycgr3G42010 Mycgr3T
  
Location: 1702-8569

Mycgr3G42010\_Mycgr3T

Mycgr3G29582 Mycgr3T
  
Location: 8669-8915

Mycgr3G29582\_Mycgr3T

Mycgr3G31170 Mycgr3T
  
Location: 9015-9255

Mycgr3G31170\_Mycgr3T

Mycgr3G85924 Mycgr3T
  
Location: 9355-11218

Mycgr3G85924\_Mycgr3T

Mycgr3G71676 Mycgr3T
  
Location: 11318-12494

Mycgr3G71676\_Mycgr3T

Mycgr3G11468 Mycgr3T
  
Location: 12594-13653

Mycgr3G11468\_Mycgr3T

Mycgr3G58567 Mycgr3T
  
Location: 13753-14506

Mycgr3G58567\_Mycgr3T

Mycgr3G100089 Mycgr3
  
Location: 14606-21152

Mycgr3G100089\_Mycgr3

Mycgr3G42698 Mycgr3T
  
Location: 21252-22131

Mycgr3G42698\_Mycgr3T

Mycgr3G71681 Mycgr3T
  
Location: 22231-23461

Mycgr3G71681\_Mycgr3T

Mycgr3G109328 Mycgr3
  
Location: 23561-24239

Mycgr3G109328\_Mycgr3

Mycgr3G104334 Mycgr3
  
Location: 24339-24567

Mycgr3G104334\_Mycgr3

Mycgr3G42715 Mycgr3T
  
Location: 24667-25981

Mycgr3G42715\_Mycgr3T

Mycgr3G92934 Mycgr3T
  
Location: 26081-27593

Mycgr3G92934\_Mycgr3T

Mycgr3G41969 Mycgr3T
  
Location: 27693-29328

Mycgr3G41969\_Mycgr3T

Mycgr3G80635 Mycgr3T
  
Location: 29428-29821

Mycgr3G80635\_Mycgr3T

Mycgr3G41426 Mycgr3T
  
Location: 29921-35255

Mycgr3G41426\_Mycgr3T

Mycgr3G104337 Mycgr3
  
Location: 35355-36108

Mycgr3G104337\_Mycgr3

Mycgr3G71679 Mycgr3T
  
Location: 36208-37300

Mycgr3G71679\_Mycgr3T

Mycgr3G92938 Mycgr3T
  
Location: 37400-38699

Mycgr3G92938\_Mycgr3T

Mycgr3G92941 Mycgr3T
  
Location: 38799-40734

Mycgr3G92941\_Mycgr3T

hypothetical protein
  
Accession: EAS30546
  
Location: 939085-940075
  
 NCBI BlastP on this gene

EAS30546

hypothetical protein
  
Accession: EJB10891
  
Location: 937802-938205
  
 NCBI BlastP on this gene

EJB10891

hypothetical protein
  
Accession: EAS30545
  
Location: 936011-936408
  
 NCBI BlastP on this gene

EAS30545

hypothetical protein
  
Accession: EAS30544
  
Location: 933419-934840
  
 NCBI BlastP on this gene

EAS30544

hypothetical protein
  
Accession: EAS30542
  
Location: 931619-932556
  
 NCBI BlastP on this gene

EAS30542

MADS box transcription factor Mcm1, variant
  
Accession: EJB10890
  
Location: 929475-930349
  
  
**BlastP hit with Mycgr3G31170\_Mycgr3T**
  
Percentage identity: 97 %
  
BlastP bit score: 160
  
Sequence coverage: 100 %
  
E-value: 4e-47
  
  
 NCBI BlastP on this gene

EJB10890

hypothetical protein, variant
  
Accession: EJB10888
  
Location: 926753-928297
  
 NCBI BlastP on this gene

EJB10888

vacuolar ATP synthase subunit E
  
Accession: EAS30539
  
Location: 924663-925538
  
 NCBI BlastP on this gene

EAS30539

hypothetical protein
  
Accession: EAS30538
  
Location: 921975-924332
  
 NCBI BlastP on this gene

EAS30538

phospholipid-translocating P-type ATPase, flippase
  
Accession: EAS30537
  
Location: 915546-920294
  
 NCBI BlastP on this gene

EAS30537

hypothetical protein
  
Accession: EJB10886
  
Location: 914541-915269
  
 NCBI BlastP on this gene

EJB10886

tRNA-splicing endonuclease
  
Accession: EAS30536
  
Location: 907178-913959
  
  
**BlastP hit with Mycgr3G41426\_Mycgr3T**
  
Percentage identity: 30 %
  
BlastP bit score: 434
  
Sequence coverage: 50 %
  
E-value: 3e-121
  
  
 NCBI BlastP on this gene

EAS30536

hypothetical protein
  
Accession: EAS30533
  
Location: 900054-902693
  
 NCBI BlastP on this gene

EAS30533

mitochondrial inner membrane magnesium transporter mrs2
  
Accession: EAS30532
  
Location: 897178-899185
  
 NCBI BlastP on this gene

EAS30532

Query: Architecture Search FASTA input

ACFW01000025 : Coccidioides posadasii C735 delta SOWgp    Total score: 2.0     Cumulative Blast bit score: 594

Hit cluster cross-links:

Mycgr3G85918 Mycgr3T
  
Location: 0-1602

Mycgr3G85918\_Mycgr3T

Mycgr3G42010 Mycgr3T
  
Location: 1702-8569

Mycgr3G42010\_Mycgr3T

Mycgr3G29582 Mycgr3T
  
Location: 8669-8915

Mycgr3G29582\_Mycgr3T

Mycgr3G31170 Mycgr3T
  
Location: 9015-9255

Mycgr3G31170\_Mycgr3T

Mycgr3G85924 Mycgr3T
  
Location: 9355-11218

Mycgr3G85924\_Mycgr3T

Mycgr3G71676 Mycgr3T
  
Location: 11318-12494

Mycgr3G71676\_Mycgr3T

Mycgr3G11468 Mycgr3T
  
Location: 12594-13653

Mycgr3G11468\_Mycgr3T

Mycgr3G58567 Mycgr3T
  
Location: 13753-14506

Mycgr3G58567\_Mycgr3T

Mycgr3G100089 Mycgr3
  
Location: 14606-21152

Mycgr3G100089\_Mycgr3

Mycgr3G42698 Mycgr3T
  
Location: 21252-22131

Mycgr3G42698\_Mycgr3T

Mycgr3G71681 Mycgr3T
  
Location: 22231-23461

Mycgr3G71681\_Mycgr3T

Mycgr3G109328 Mycgr3
  
Location: 23561-24239

Mycgr3G109328\_Mycgr3

Mycgr3G104334 Mycgr3
  
Location: 24339-24567

Mycgr3G104334\_Mycgr3

Mycgr3G42715 Mycgr3T
  
Location: 24667-25981

Mycgr3G42715\_Mycgr3T

Mycgr3G92934 Mycgr3T
  
Location: 26081-27593

Mycgr3G92934\_Mycgr3T

Mycgr3G41969 Mycgr3T
  
Location: 27693-29328

Mycgr3G41969\_Mycgr3T

Mycgr3G80635 Mycgr3T
  
Location: 29428-29821

Mycgr3G80635\_Mycgr3T

Mycgr3G41426 Mycgr3T
  
Location: 29921-35255

Mycgr3G41426\_Mycgr3T

Mycgr3G104337 Mycgr3
  
Location: 35355-36108

Mycgr3G104337\_Mycgr3

Mycgr3G71679 Mycgr3T
  
Location: 36208-37300

Mycgr3G71679\_Mycgr3T

Mycgr3G92938 Mycgr3T
  
Location: 37400-38699

Mycgr3G92938\_Mycgr3T

Mycgr3G92941 Mycgr3T
  
Location: 38799-40734

Mycgr3G92941\_Mycgr3T

hypothetical protein
  
Accession: EER27116
  
Location: 691566-692199
  
 NCBI BlastP on this gene

EER27116

Phospholipase/Carboxylesterase family protein
  
Accession: EER27115
  
Location: 685586-686437
  
 NCBI BlastP on this gene

EER27115

SRF-type transcription factor family protein
  
Accession: EER27114
  
Location: 683443-684313
  
  
**BlastP hit with Mycgr3G31170\_Mycgr3T**
  
Percentage identity: 97 %
  
BlastP bit score: 160
  
Sequence coverage: 100 %
  
E-value: 3e-47
  
  
 NCBI BlastP on this gene

EER27114

hypothetical protein
  
Accession: EER27113
  
Location: 680547-682265
  
 NCBI BlastP on this gene

EER27113

vacuolar ATP synthase subunit E, putative
  
Accession: EER27112
  
Location: 678624-679491
  
 NCBI BlastP on this gene

EER27112

hypothetical protein
  
Accession: EER27111
  
Location: 675945-678293
  
 NCBI BlastP on this gene

EER27111

phospholipid-translocating P-type ATPase domain-containing protein, putative
  
Accession: EER27110
  
Location: 669526-674274
  
 NCBI BlastP on this gene

EER27110

Zinc knuckle domain containing protein
  
Accession: EER27109
  
Location: 661179-667960
  
  
**BlastP hit with Mycgr3G41426\_Mycgr3T**
  
Percentage identity: 30 %
  
BlastP bit score: 434
  
Sequence coverage: 51 %
  
E-value: 4e-121
  
  
 NCBI BlastP on this gene

EER27109

hypothetical protein
  
Accession: EER27108
  
Location: 654064-656235
  
 NCBI BlastP on this gene

EER27108

CorA-like Mg2+ transporter family protein
  
Accession: EER27107
  
Location: 651205-653213
  
 NCBI BlastP on this gene

EER27107

WH1 domain containing protein
  
Accession: EER27106
  
Location: 648452-650416
  
 NCBI BlastP on this gene

EER27106

Query: Architecture Search FASTA input

AKHY01000076 : Aspergillus oryzae 3.042    Total score: 2.0     Cumulative Blast bit score: 589

Hit cluster cross-links:

Mycgr3G85918 Mycgr3T
  
Location: 0-1602

Mycgr3G85918\_Mycgr3T

Mycgr3G42010 Mycgr3T
  
Location: 1702-8569

Mycgr3G42010\_Mycgr3T

Mycgr3G29582 Mycgr3T
  
Location: 8669-8915

Mycgr3G29582\_Mycgr3T

Mycgr3G31170 Mycgr3T
  
Location: 9015-9255

Mycgr3G31170\_Mycgr3T

Mycgr3G85924 Mycgr3T
  
Location: 9355-11218

Mycgr3G85924\_Mycgr3T

Mycgr3G71676 Mycgr3T
  
Location: 11318-12494

Mycgr3G71676\_Mycgr3T

Mycgr3G11468 Mycgr3T
  
Location: 12594-13653

Mycgr3G11468\_Mycgr3T

Mycgr3G58567 Mycgr3T
  
Location: 13753-14506

Mycgr3G58567\_Mycgr3T

Mycgr3G100089 Mycgr3
  
Location: 14606-21152

Mycgr3G100089\_Mycgr3

Mycgr3G42698 Mycgr3T
  
Location: 21252-22131

Mycgr3G42698\_Mycgr3T

Mycgr3G71681 Mycgr3T
  
Location: 22231-23461

Mycgr3G71681\_Mycgr3T

Mycgr3G109328 Mycgr3
  
Location: 23561-24239

Mycgr3G109328\_Mycgr3

Mycgr3G104334 Mycgr3
  
Location: 24339-24567

Mycgr3G104334\_Mycgr3

Mycgr3G42715 Mycgr3T
  
Location: 24667-25981

Mycgr3G42715\_Mycgr3T

Mycgr3G92934 Mycgr3T
  
Location: 26081-27593

Mycgr3G92934\_Mycgr3T

Mycgr3G41969 Mycgr3T
  
Location: 27693-29328

Mycgr3G41969\_Mycgr3T

Mycgr3G80635 Mycgr3T
  
Location: 29428-29821

Mycgr3G80635\_Mycgr3T

Mycgr3G41426 Mycgr3T
  
Location: 29921-35255

Mycgr3G41426\_Mycgr3T

Mycgr3G104337 Mycgr3
  
Location: 35355-36108

Mycgr3G104337\_Mycgr3

Mycgr3G71679 Mycgr3T
  
Location: 36208-37300

Mycgr3G71679\_Mycgr3T

Mycgr3G92938 Mycgr3T
  
Location: 37400-38699

Mycgr3G92938\_Mycgr3T

Mycgr3G92941 Mycgr3T
  
Location: 38799-40734

Mycgr3G92941\_Mycgr3T

hypothetical protein
  
Accession: EIT82520
  
Location: 257968-259581
  
 NCBI BlastP on this gene

EIT82520

regulator of arginine metabolism
  
Accession: EIT82519
  
Location: 263169-264079
  
  
**BlastP hit with Mycgr3G31170\_Mycgr3T**
  
Percentage identity: 98 %
  
BlastP bit score: 163
  
Sequence coverage: 100 %
  
E-value: 2e-48
  
  
 NCBI BlastP on this gene

EIT82519

hypothetical protein
  
Accession: EIT82523
  
Location: 265424-267096
  
 NCBI BlastP on this gene

EIT82523

vacuolar H+-ATPase V1 sector, subunit E
  
Accession: EIT82514
  
Location: 267985-268801
  
 NCBI BlastP on this gene

EIT82514

hypothetical protein
  
Accession: EIT82513
  
Location: 269238-271616
  
 NCBI BlastP on this gene

EIT82513

P-type ATPase
  
Accession: EIT82534
  
Location: 276107-280822
  
 NCBI BlastP on this gene

EIT82534

tRNA-splicing endonuclease positive effector
  
Accession: EIT82535
  
Location: 281996-288554
  
  
**BlastP hit with Mycgr3G41426\_Mycgr3T**
  
Percentage identity: 30 %
  
BlastP bit score: 426
  
Sequence coverage: 48 %
  
E-value: 1e-118
  
  
 NCBI BlastP on this gene

EIT82535

Query: Architecture Search FASTA input

HF679028 : Fusarium fujikuroi IMI 58289 draft genome, chromosome FFUJ\_chr06.    Total score: 2.0     Cumulative Blast bit score: 583

Hit cluster cross-links:

Mycgr3G85918 Mycgr3T
  
Location: 0-1602

Mycgr3G85918\_Mycgr3T

Mycgr3G42010 Mycgr3T
  
Location: 1702-8569

Mycgr3G42010\_Mycgr3T

Mycgr3G29582 Mycgr3T
  
Location: 8669-8915

Mycgr3G29582\_Mycgr3T

Mycgr3G31170 Mycgr3T
  
Location: 9015-9255

Mycgr3G31170\_Mycgr3T

Mycgr3G85924 Mycgr3T
  
Location: 9355-11218

Mycgr3G85924\_Mycgr3T

Mycgr3G71676 Mycgr3T
  
Location: 11318-12494

Mycgr3G71676\_Mycgr3T

Mycgr3G11468 Mycgr3T
  
Location: 12594-13653

Mycgr3G11468\_Mycgr3T

Mycgr3G58567 Mycgr3T
  
Location: 13753-14506

Mycgr3G58567\_Mycgr3T

Mycgr3G100089 Mycgr3
  
Location: 14606-21152

Mycgr3G100089\_Mycgr3

Mycgr3G42698 Mycgr3T
  
Location: 21252-22131

Mycgr3G42698\_Mycgr3T

Mycgr3G71681 Mycgr3T
  
Location: 22231-23461

Mycgr3G71681\_Mycgr3T

Mycgr3G109328 Mycgr3
  
Location: 23561-24239

Mycgr3G109328\_Mycgr3

Mycgr3G104334 Mycgr3
  
Location: 24339-24567

Mycgr3G104334\_Mycgr3

Mycgr3G42715 Mycgr3T
  
Location: 24667-25981

Mycgr3G42715\_Mycgr3T

Mycgr3G92934 Mycgr3T
  
Location: 26081-27593

Mycgr3G92934\_Mycgr3T

Mycgr3G41969 Mycgr3T
  
Location: 27693-29328

Mycgr3G41969\_Mycgr3T

Mycgr3G80635 Mycgr3T
  
Location: 29428-29821

Mycgr3G80635\_Mycgr3T

Mycgr3G41426 Mycgr3T
  
Location: 29921-35255

Mycgr3G41426\_Mycgr3T

Mycgr3G104337 Mycgr3
  
Location: 35355-36108

Mycgr3G104337\_Mycgr3

Mycgr3G71679 Mycgr3T
  
Location: 36208-37300

Mycgr3G71679\_Mycgr3T

Mycgr3G92938 Mycgr3T
  
Location: 37400-38699

Mycgr3G92938\_Mycgr3T

Mycgr3G92941 Mycgr3T
  
Location: 38799-40734

Mycgr3G92941\_Mycgr3T

related to monooxigenase
  
Accession: CCT69506
  
Location: 179324-181348
  
 NCBI BlastP on this gene

FFUJ\_05397

related to heterokaryon incompatibility protein het-6
  
Accession: CCT69505
  
Location: 176625-179255
  
 NCBI BlastP on this gene

FFUJ\_05396

related to triacylglycerol lipase V precursor
  
Accession: CCT69504
  
Location: 174436-176217
  
 NCBI BlastP on this gene

FFUJ\_05395

uncharacterized protein
  
Accession: CCT69503
  
Location: 171429-173879
  
 NCBI BlastP on this gene

FFUJ\_05394

probable NmrA-like family protein
  
Accession: CCT69502
  
Location: 170438-171385
  
 NCBI BlastP on this gene

FFUJ\_05393

related to NADPH-dependent beta-ketoacyl reductase (rhlG)
  
Accession: CCT69501
  
Location: 168958-170028
  
 NCBI BlastP on this gene

FFUJ\_05392

related to short chain dehydrogenase/reductase
  
Accession: CCT69500
  
Location: 167564-168554
  
 NCBI BlastP on this gene

FFUJ\_05391

related to transporter protein HOL1
  
Accession: CCT69499
  
Location: 164989-166747
  
  
**BlastP hit with Mycgr3G85918\_Mycgr3T**
  
Percentage identity: 31 %
  
BlastP bit score: 207
  
Sequence coverage: 98 %
  
E-value: 3e-56
  
  
 NCBI BlastP on this gene

FFUJ\_05390

uncharacterized protein
  
Accession: CCT69498
  
Location: 164251-164694
  
 NCBI BlastP on this gene

FFUJ\_05389

uncharacterized protein
  
Accession: CCT69497
  
Location: 158509-158979
  
 NCBI BlastP on this gene

FFUJ\_05388

probable L-amino-acid oxidase
  
Accession: CCT69496
  
Location: 155528-157063
  
 NCBI BlastP on this gene

FFUJ\_05387

uncharacterized protein
  
Accession: CCT69495
  
Location: 152504-153615
  
 NCBI BlastP on this gene

FFUJ\_05386

related to aldehyde reductase II
  
Accession: CCT69494
  
Location: 150641-151812
  
 NCBI BlastP on this gene

FFUJ\_05385

uncharacterized protein
  
Accession: CCT69493
  
Location: 149354-150613
  
 NCBI BlastP on this gene

FFUJ\_05384

related to epoxide hydrolase
  
Accession: CCT69492
  
Location: 147396-148653
  
  
**BlastP hit with Mycgr3G71676\_Mycgr3T**
  
Percentage identity: 48 %
  
BlastP bit score: 376
  
Sequence coverage: 99 %
  
E-value: 3e-124
  
  
 NCBI BlastP on this gene

FFUJ\_05383

related to vegetatible incompatibility protein HET-E-1
  
Accession: CCT69491
  
Location: 146265-146942
  
 NCBI BlastP on this gene

FFUJ\_05382

uncharacterized protein
  
Accession: CCT70801
  
Location: 142758-145361
  
 NCBI BlastP on this gene

FFUJ\_05381

uncharacterized protein
  
Accession: CCT69490
  
Location: 141526-142706
  
 NCBI BlastP on this gene

FFUJ\_05380

related to protein TOL
  
Accession: CCT70722
  
Location: 138914-141059
  
 NCBI BlastP on this gene

FFUJ\_05379

related to sugar transporter
  
Accession: CCT69489
  
Location: 137042-138842
  
 NCBI BlastP on this gene

FFUJ\_05378

uncharacterized protein
  
Accession: CCT70792
  
Location: 135275-135942
  
 NCBI BlastP on this gene

FFUJ\_05377

related to methyltransferase
  
Accession: CCT69488
  
Location: 133737-135131
  
 NCBI BlastP on this gene

FFUJ\_05376

Query: Architecture Search FASTA input

KB708021 : Botryotinia fuckeliana BcDW1 unplaced genomic scaffold Scaffold\_349    Total score: 2.0     Cumulative Blast bit score: 571

Hit cluster cross-links:

Mycgr3G85918 Mycgr3T
  
Location: 0-1602

Mycgr3G85918\_Mycgr3T

Mycgr3G42010 Mycgr3T
  
Location: 1702-8569

Mycgr3G42010\_Mycgr3T

Mycgr3G29582 Mycgr3T
  
Location: 8669-8915

Mycgr3G29582\_Mycgr3T

Mycgr3G31170 Mycgr3T
  
Location: 9015-9255

Mycgr3G31170\_Mycgr3T

Mycgr3G85924 Mycgr3T
  
Location: 9355-11218

Mycgr3G85924\_Mycgr3T

Mycgr3G71676 Mycgr3T
  
Location: 11318-12494

Mycgr3G71676\_Mycgr3T

Mycgr3G11468 Mycgr3T
  
Location: 12594-13653

Mycgr3G11468\_Mycgr3T

Mycgr3G58567 Mycgr3T
  
Location: 13753-14506

Mycgr3G58567\_Mycgr3T

Mycgr3G100089 Mycgr3
  
Location: 14606-21152

Mycgr3G100089\_Mycgr3

Mycgr3G42698 Mycgr3T
  
Location: 21252-22131

Mycgr3G42698\_Mycgr3T

Mycgr3G71681 Mycgr3T
  
Location: 22231-23461

Mycgr3G71681\_Mycgr3T

Mycgr3G109328 Mycgr3
  
Location: 23561-24239

Mycgr3G109328\_Mycgr3

Mycgr3G104334 Mycgr3
  
Location: 24339-24567

Mycgr3G104334\_Mycgr3

Mycgr3G42715 Mycgr3T
  
Location: 24667-25981

Mycgr3G42715\_Mycgr3T

Mycgr3G92934 Mycgr3T
  
Location: 26081-27593

Mycgr3G92934\_Mycgr3T

Mycgr3G41969 Mycgr3T
  
Location: 27693-29328

Mycgr3G41969\_Mycgr3T

Mycgr3G80635 Mycgr3T
  
Location: 29428-29821

Mycgr3G80635\_Mycgr3T

Mycgr3G41426 Mycgr3T
  
Location: 29921-35255

Mycgr3G41426\_Mycgr3T

Mycgr3G104337 Mycgr3
  
Location: 35355-36108

Mycgr3G104337\_Mycgr3

Mycgr3G71679 Mycgr3T
  
Location: 36208-37300

Mycgr3G71679\_Mycgr3T

Mycgr3G92938 Mycgr3T
  
Location: 37400-38699

Mycgr3G92938\_Mycgr3T

Mycgr3G92941 Mycgr3T
  
Location: 38799-40734

Mycgr3G92941\_Mycgr3T

putative glycoside hydrolase family 62 protein
  
Accession: EMR82994
  
Location: 32457-33419
  
 NCBI BlastP on this gene

EMR82994

putative major allergen asp f 2-like protein
  
Accession: EMR82993
  
Location: 25877-27386
  
 NCBI BlastP on this gene

EMR82993

putative zip zinc protein
  
Accession: EMR82992
  
Location: 23181-25020
  
 NCBI BlastP on this gene

EMR82992

putative epoxide hydrolase protein
  
Accession: EMR82991
  
Location: 13212-14489
  
  
**BlastP hit with Mycgr3G71676\_Mycgr3T**
  
Percentage identity: 48 %
  
BlastP bit score: 384
  
Sequence coverage: 97 %
  
E-value: 1e-127
  
  
 NCBI BlastP on this gene

EMR82991

putative tyrosinase central domain protein
  
Accession: EMR82990
  
Location: 5981-7352
  
  
**BlastP hit with Mycgr3G42698\_Mycgr3T**
  
Percentage identity: 36 %
  
BlastP bit score: 187
  
Sequence coverage: 108 %
  
E-value: 1e-52
  
  
 NCBI BlastP on this gene

EMR82990

putative fad-dependent monooxygenase protein
  
Accession: EMR82989
  
Location: 2246-3745
  
 NCBI BlastP on this gene

EMR82989

Query: Architecture Search FASTA input

EQ963480 : Aspergillus flavus NRRL3357 scf\_1106286418846 genomic scaffold    Total score: 2.0     Cumulative Blast bit score: 560

Hit cluster cross-links:

Mycgr3G85918 Mycgr3T
  
Location: 0-1602

Mycgr3G85918\_Mycgr3T

Mycgr3G42010 Mycgr3T
  
Location: 1702-8569

Mycgr3G42010\_Mycgr3T

Mycgr3G29582 Mycgr3T
  
Location: 8669-8915

Mycgr3G29582\_Mycgr3T

Mycgr3G31170 Mycgr3T
  
Location: 9015-9255

Mycgr3G31170\_Mycgr3T

Mycgr3G85924 Mycgr3T
  
Location: 9355-11218

Mycgr3G85924\_Mycgr3T

Mycgr3G71676 Mycgr3T
  
Location: 11318-12494

Mycgr3G71676\_Mycgr3T

Mycgr3G11468 Mycgr3T
  
Location: 12594-13653

Mycgr3G11468\_Mycgr3T

Mycgr3G58567 Mycgr3T
  
Location: 13753-14506

Mycgr3G58567\_Mycgr3T

Mycgr3G100089 Mycgr3
  
Location: 14606-21152

Mycgr3G100089\_Mycgr3

Mycgr3G42698 Mycgr3T
  
Location: 21252-22131

Mycgr3G42698\_Mycgr3T

Mycgr3G71681 Mycgr3T
  
Location: 22231-23461

Mycgr3G71681\_Mycgr3T

Mycgr3G109328 Mycgr3
  
Location: 23561-24239

Mycgr3G109328\_Mycgr3

Mycgr3G104334 Mycgr3
  
Location: 24339-24567

Mycgr3G104334\_Mycgr3

Mycgr3G42715 Mycgr3T
  
Location: 24667-25981

Mycgr3G42715\_Mycgr3T

Mycgr3G92934 Mycgr3T
  
Location: 26081-27593

Mycgr3G92934\_Mycgr3T

Mycgr3G41969 Mycgr3T
  
Location: 27693-29328

Mycgr3G41969\_Mycgr3T

Mycgr3G80635 Mycgr3T
  
Location: 29428-29821

Mycgr3G80635\_Mycgr3T

Mycgr3G41426 Mycgr3T
  
Location: 29921-35255

Mycgr3G41426\_Mycgr3T

Mycgr3G104337 Mycgr3
  
Location: 35355-36108

Mycgr3G104337\_Mycgr3

Mycgr3G71679 Mycgr3T
  
Location: 36208-37300

Mycgr3G71679\_Mycgr3T

Mycgr3G92938 Mycgr3T
  
Location: 37400-38699

Mycgr3G92938\_Mycgr3T

Mycgr3G92941 Mycgr3T
  
Location: 38799-40734

Mycgr3G92941\_Mycgr3T

conserved hypothetical protein
  
Accession: EED49011
  
Location: 475842-477455
  
 NCBI BlastP on this gene

EED49011

MADS box transcription factor Mcm1
  
Accession: EED49010
  
Location: 471345-472255
  
  
**BlastP hit with Mycgr3G31170\_Mycgr3T**
  
Percentage identity: 98 %
  
BlastP bit score: 163
  
Sequence coverage: 100 %
  
E-value: 2e-48
  
  
 NCBI BlastP on this gene

EED49010

DUF803 domain protein
  
Accession: EED49009
  
Location: 468265-469998
  
 NCBI BlastP on this gene

EED49009

ATP synthase subunit E, putative
  
Accession: EED49008
  
Location: 466621-467437
  
 NCBI BlastP on this gene

EED49008

conserved hypothetical protein
  
Accession: EED49007
  
Location: 463738-464729
  
 NCBI BlastP on this gene

EED49007

hypothetical protein
  
Accession: EED49006
  
Location: 461026-461606
  
 NCBI BlastP on this gene

EED49006

phospholipid-translocating P-type ATPase domain-containing protein
  
Accession: EED49005
  
Location: 454569-459284
  
 NCBI BlastP on this gene

EED49005

tRNA-splicing endonuclease, putative
  
Accession: EED49004
  
Location: 446718-453137
  
  
**BlastP hit with Mycgr3G41426\_Mycgr3T**
  
Percentage identity: 30 %
  
BlastP bit score: 397
  
Sequence coverage: 45 %
  
E-value: 3e-109
  
  
 NCBI BlastP on this gene

EED49004

conserved hypothetical protein
  
Accession: EED49003
  
Location: 440848-443558
  
 NCBI BlastP on this gene

EED49003

conserved hypothetical protein
  
Accession: EED49002
  
Location: 439548-440247
  
 NCBI BlastP on this gene

EED49002

aminotransferase, putative
  
Accession: EED49001
  
Location: 437546-439115
  
 NCBI BlastP on this gene

EED49001

Query: Architecture Search FASTA input

CP000518 : Mycobacterium sp. KMS    Total score: 2.0     Cumulative Blast bit score: 559

Hit cluster cross-links:

Mycgr3G85918 Mycgr3T
  
Location: 0-1602

Mycgr3G85918\_Mycgr3T

Mycgr3G42010 Mycgr3T
  
Location: 1702-8569

Mycgr3G42010\_Mycgr3T

Mycgr3G29582 Mycgr3T
  
Location: 8669-8915

Mycgr3G29582\_Mycgr3T

Mycgr3G31170 Mycgr3T
  
Location: 9015-9255

Mycgr3G31170\_Mycgr3T

Mycgr3G85924 Mycgr3T
  
Location: 9355-11218

Mycgr3G85924\_Mycgr3T

Mycgr3G71676 Mycgr3T
  
Location: 11318-12494

Mycgr3G71676\_Mycgr3T

Mycgr3G11468 Mycgr3T
  
Location: 12594-13653

Mycgr3G11468\_Mycgr3T

Mycgr3G58567 Mycgr3T
  
Location: 13753-14506

Mycgr3G58567\_Mycgr3T

Mycgr3G100089 Mycgr3
  
Location: 14606-21152

Mycgr3G100089\_Mycgr3

Mycgr3G42698 Mycgr3T
  
Location: 21252-22131

Mycgr3G42698\_Mycgr3T

Mycgr3G71681 Mycgr3T
  
Location: 22231-23461

Mycgr3G71681\_Mycgr3T

Mycgr3G109328 Mycgr3
  
Location: 23561-24239

Mycgr3G109328\_Mycgr3

Mycgr3G104334 Mycgr3
  
Location: 24339-24567

Mycgr3G104334\_Mycgr3

Mycgr3G42715 Mycgr3T
  
Location: 24667-25981

Mycgr3G42715\_Mycgr3T

Mycgr3G92934 Mycgr3T
  
Location: 26081-27593

Mycgr3G92934\_Mycgr3T

Mycgr3G41969 Mycgr3T
  
Location: 27693-29328

Mycgr3G41969\_Mycgr3T

Mycgr3G80635 Mycgr3T
  
Location: 29428-29821

Mycgr3G80635\_Mycgr3T

Mycgr3G41426 Mycgr3T
  
Location: 29921-35255

Mycgr3G41426\_Mycgr3T

Mycgr3G104337 Mycgr3
  
Location: 35355-36108

Mycgr3G104337\_Mycgr3

Mycgr3G71679 Mycgr3T
  
Location: 36208-37300

Mycgr3G71679\_Mycgr3T

Mycgr3G92938 Mycgr3T
  
Location: 37400-38699

Mycgr3G92938\_Mycgr3T

Mycgr3G92941 Mycgr3T
  
Location: 38799-40734

Mycgr3G92941\_Mycgr3T

conserved hypothetical protein
  
Accession: ABL93697
  
Location: 4739620-4740366
  
 NCBI BlastP on this gene

Mkms\_4506

conserved hypothetical protein
  
Accession: ABL93698
  
Location: 4740377-4740787
  
 NCBI BlastP on this gene

Mkms\_4507

dihydrodipicolinate reductase
  
Accession: ABL93699
  
Location: 4740791-4741882
  
 NCBI BlastP on this gene

Mkms\_4508

cytochrome P450
  
Accession: ABL93700
  
Location: 4741879-4743063
  
 NCBI BlastP on this gene

Mkms\_4509

transcriptional regulator, TetR family
  
Accession: ABL93701
  
Location: 4743300-4743923
  
 NCBI BlastP on this gene

Mkms\_4510

NAD-dependent epimerase/dehydratase
  
Accession: ABL93702
  
Location: 4744102-4744761
  
 NCBI BlastP on this gene

Mkms\_4511

alpha/beta hydrolase fold protein
  
Accession: ABL93703
  
Location: 4744785-4745750
  
 NCBI BlastP on this gene

Mkms\_4512

conserved hypothetical protein
  
Accession: ABL93704
  
Location: 4745777-4746208
  
 NCBI BlastP on this gene

Mkms\_4513

conserved hypothetical protein
  
Accession: ABL93705
  
Location: 4746212-4746442
  
 NCBI BlastP on this gene

Mkms\_4514

ATPase, P-type (transporting), HAD superfamily, subfamily IC
  
Accession: ABL93706
  
Location: 4746466-4748856
  
 NCBI BlastP on this gene

Mkms\_4515

beta-lactamase
  
Accession: ABL93707
  
Location: 4748917-4750518
  
 NCBI BlastP on this gene

Mkms\_4516

conserved hypothetical protein
  
Accession: ABL93708
  
Location: 4750528-4751646
  
 NCBI BlastP on this gene

Mkms\_4517

Enoyl-CoA hydratase/isomerase
  
Accession: ABL93709
  
Location: 4751649-4752383
  
 NCBI BlastP on this gene

Mkms\_4518

carboxyl transferase
  
Accession: ABL93710
  
Location: 4752410-4753918
  
 NCBI BlastP on this gene

Mkms\_4519

conserved hypothetical protein
  
Accession: ABL93711
  
Location: 4753919-4754167
  
 NCBI BlastP on this gene

Mkms\_4520

conserved hypothetical protein
  
Accession: ABL93712
  
Location: 4754164-4754379
  
 NCBI BlastP on this gene

Mkms\_4521

conserved hypothetical protein
  
Accession: ABL93713
  
Location: 4754418-4754849
  
 NCBI BlastP on this gene

Mkms\_4522

conserved hypothetical protein
  
Accession: ABL93714
  
Location: 4754859-4755266
  
 NCBI BlastP on this gene

Mkms\_4523

AMP-dependent synthetase and ligase
  
Accession: ABL93715
  
Location: 4755317-4756939
  
 NCBI BlastP on this gene

Mkms\_4524

AMP-dependent synthetase and ligase
  
Accession: ABL93716
  
Location: 4756936-4758633
  
 NCBI BlastP on this gene

Mkms\_4525

putative GAF sensor protein
  
Accession: ABL93717
  
Location: 4758682-4759539
  
 NCBI BlastP on this gene

Mkms\_4526

Epoxide hydrolase domain protein
  
Accession: ABL93718
  
Location: 4759562-4760665
  
  
**BlastP hit with Mycgr3G71676\_Mycgr3T**
  
Percentage identity: 34 %
  
BlastP bit score: 209
  
Sequence coverage: 98 %
  
E-value: 7e-60
  
  
 NCBI BlastP on this gene

Mkms\_4527

conserved hypothetical protein
  
Accession: ABL93719
  
Location: 4760667-4762037
  
  
**BlastP hit with Mycgr3G42715\_Mycgr3T**
  
Percentage identity: 41 %
  
BlastP bit score: 350
  
Sequence coverage: 105 %
  
E-value: 1e-112
  
  
 NCBI BlastP on this gene

Mkms\_4528

diguanylate cyclase
  
Accession: ABL93720
  
Location: 4762122-4763207
  
 NCBI BlastP on this gene

Mkms\_4529

conserved hypothetical protein
  
Accession: ABL93721
  
Location: 4763239-4764114
  
 NCBI BlastP on this gene

Mkms\_4530

ABC transporter related protein
  
Accession: ABL93722
  
Location: 4764238-4766070
  
 NCBI BlastP on this gene

Mkms\_4531

ABC transporter related protein
  
Accession: ABL93723
  
Location: 4766067-4768019
  
 NCBI BlastP on this gene

Mkms\_4532

ABC transporter related protein
  
Accession: ABL93724
  
Location: 4768071-4769954
  
 NCBI BlastP on this gene

Mkms\_4533

Ion transport 2 domain protein
  
Accession: ABL93725
  
Location: 4770030-4770812
  
 NCBI BlastP on this gene

Mkms\_4534

two component transcriptional regulator, winged helix family
  
Accession: ABL93726
  
Location: 4770839-4771540
  
 NCBI BlastP on this gene

Mkms\_4535

integral membrane sensor signal transduction histidine kinase
  
Accession: ABL93727
  
Location: 4771543-4772889
  
 NCBI BlastP on this gene

Mkms\_4536

peptidylprolyl isomerase, FKBP-type
  
Accession: ABL93728
  
Location: 4772961-4773329
  
 NCBI BlastP on this gene

Mkms\_4537

conserved hypothetical protein
  
Accession: ABL93729
  
Location: 4773366-4773614
  
 NCBI BlastP on this gene

Mkms\_4538

conserved hypothetical protein
  
Accession: ABL93730
  
Location: 4773659-4775158
  
 NCBI BlastP on this gene

Mkms\_4539

conserved hypothetical protein
  
Accession: ABL93731
  
Location: 4775190-4775537
  
 NCBI BlastP on this gene

Mkms\_4540

transcriptional regulator, MarR family
  
Accession: ABL93732
  
Location: 4775542-4775961
  
 NCBI BlastP on this gene

Mkms\_4541

drug resistance transporter, EmrB/QacA subfamily
  
Accession: ABL93733
  
Location: 4775951-4777405
  
 NCBI BlastP on this gene

Mkms\_4542

citrate synthase
  
Accession: ABL93734
  
Location: 4777473-4778777
  
 NCBI BlastP on this gene

Mkms\_4543

transcriptional regulator, TetR family
  
Accession: ABL93735
  
Location: 4778873-4779520
  
 NCBI BlastP on this gene

Mkms\_4544

Pyridoxamine 5'-phosphate oxidase
  
Accession: ABL93736
  
Location: 4779648-4780310
  
 NCBI BlastP on this gene

Mkms\_4545

citrate synthase
  
Accession: ABL93737
  
Location: 4780371-4781501
  
 NCBI BlastP on this gene

Mkms\_4546

conserved hypothetical protein
  
Accession: ABL93738
  
Location: 4781498-4782115
  
 NCBI BlastP on this gene

Mkms\_4547

Query: Architecture Search FASTA input

CP000384 : Mycobacterium sp. MCS    Total score: 2.0     Cumulative Blast bit score: 559

Hit cluster cross-links:

Mycgr3G85918 Mycgr3T
  
Location: 0-1602

Mycgr3G85918\_Mycgr3T

Mycgr3G42010 Mycgr3T
  
Location: 1702-8569

Mycgr3G42010\_Mycgr3T

Mycgr3G29582 Mycgr3T
  
Location: 8669-8915

Mycgr3G29582\_Mycgr3T

Mycgr3G31170 Mycgr3T
  
Location: 9015-9255

Mycgr3G31170\_Mycgr3T

Mycgr3G85924 Mycgr3T
  
Location: 9355-11218

Mycgr3G85924\_Mycgr3T

Mycgr3G71676 Mycgr3T
  
Location: 11318-12494

Mycgr3G71676\_Mycgr3T

Mycgr3G11468 Mycgr3T
  
Location: 12594-13653

Mycgr3G11468\_Mycgr3T

Mycgr3G58567 Mycgr3T
  
Location: 13753-14506

Mycgr3G58567\_Mycgr3T

Mycgr3G100089 Mycgr3
  
Location: 14606-21152

Mycgr3G100089\_Mycgr3

Mycgr3G42698 Mycgr3T
  
Location: 21252-22131

Mycgr3G42698\_Mycgr3T

Mycgr3G71681 Mycgr3T
  
Location: 22231-23461

Mycgr3G71681\_Mycgr3T

Mycgr3G109328 Mycgr3
  
Location: 23561-24239

Mycgr3G109328\_Mycgr3

Mycgr3G104334 Mycgr3
  
Location: 24339-24567

Mycgr3G104334\_Mycgr3

Mycgr3G42715 Mycgr3T
  
Location: 24667-25981

Mycgr3G42715\_Mycgr3T

Mycgr3G92934 Mycgr3T
  
Location: 26081-27593

Mycgr3G92934\_Mycgr3T

Mycgr3G41969 Mycgr3T
  
Location: 27693-29328

Mycgr3G41969\_Mycgr3T

Mycgr3G80635 Mycgr3T
  
Location: 29428-29821

Mycgr3G80635\_Mycgr3T

Mycgr3G41426 Mycgr3T
  
Location: 29921-35255

Mycgr3G41426\_Mycgr3T

Mycgr3G104337 Mycgr3
  
Location: 35355-36108

Mycgr3G104337\_Mycgr3

Mycgr3G71679 Mycgr3T
  
Location: 36208-37300

Mycgr3G71679\_Mycgr3T

Mycgr3G92938 Mycgr3T
  
Location: 37400-38699

Mycgr3G92938\_Mycgr3T

Mycgr3G92941 Mycgr3T
  
Location: 38799-40734

Mycgr3G92941\_Mycgr3T

conserved hypothetical protein
  
Accession: ABG10524
  
Location: 4701173-4701583
  
 NCBI BlastP on this gene

Mmcs\_4420

dihydrodipicolinate reductase
  
Accession: ABG10525
  
Location: 4701587-4702678
  
 NCBI BlastP on this gene

Mmcs\_4421

cytochrome P450
  
Accession: ABG10526
  
Location: 4702675-4703859
  
 NCBI BlastP on this gene

Mmcs\_4422

transcriptional regulator, TetR family
  
Accession: ABG10527
  
Location: 4704096-4704719
  
 NCBI BlastP on this gene

Mmcs\_4423

NAD-dependent epimerase/dehydratase
  
Accession: ABG10528
  
Location: 4704898-4705557
  
 NCBI BlastP on this gene

Mmcs\_4424

alpha/beta hydrolase fold protein
  
Accession: ABG10529
  
Location: 4705581-4706546
  
 NCBI BlastP on this gene

Mmcs\_4425

conserved hypothetical protein
  
Accession: ABG10530
  
Location: 4706573-4707004
  
 NCBI BlastP on this gene

Mmcs\_4426

hypothetical protein
  
Accession: ABG10531
  
Location: 4707008-4707238
  
 NCBI BlastP on this gene

Mmcs\_4427

ATPase, P-type,
  
Accession: ABG10532
  
Location: 4707262-4709652
  
 NCBI BlastP on this gene

Mmcs\_4428

beta-lactamase
  
Accession: ABG10533
  
Location: 4709777-4711378
  
 NCBI BlastP on this gene

Mmcs\_4429

conserved hypothetical protein
  
Accession: ABG10534
  
Location: 4711388-4712506
  
 NCBI BlastP on this gene

Mmcs\_4430

Enoyl-CoA hydratase/isomerase
  
Accession: ABG10535
  
Location: 4712509-4713243
  
 NCBI BlastP on this gene

Mmcs\_4431

carboxyl transferase
  
Accession: ABG10536
  
Location: 4713270-4714778
  
 NCBI BlastP on this gene

Mmcs\_4432

hypothetical protein
  
Accession: ABG10537
  
Location: 4714779-4715027
  
 NCBI BlastP on this gene

Mmcs\_4433

conserved hypothetical protein
  
Accession: ABG10538
  
Location: 4715024-4715239
  
 NCBI BlastP on this gene

Mmcs\_4434

hypothetical protein
  
Accession: ABG10539
  
Location: 4715278-4715709
  
 NCBI BlastP on this gene

Mmcs\_4435

hypothetical protein
  
Accession: ABG10540
  
Location: 4715719-4716126
  
 NCBI BlastP on this gene

Mmcs\_4436

AMP-dependent synthetase and ligase
  
Accession: ABG10541
  
Location: 4716177-4717799
  
 NCBI BlastP on this gene

Mmcs\_4437

AMP-dependent synthetase and ligase
  
Accession: ABG10542
  
Location: 4717796-4719493
  
 NCBI BlastP on this gene

Mmcs\_4438

ATP-dependent transcriptional regulator, MalT-like, LuxR family
  
Accession: ABG10543
  
Location: 4719542-4720399
  
 NCBI BlastP on this gene

Mmcs\_4439

Epoxide hydrolase-like protein
  
Accession: ABG10544
  
Location: 4720422-4721525
  
  
**BlastP hit with Mycgr3G71676\_Mycgr3T**
  
Percentage identity: 34 %
  
BlastP bit score: 209
  
Sequence coverage: 98 %
  
E-value: 7e-60
  
  
 NCBI BlastP on this gene

Mmcs\_4440

conserved hypothetical protein
  
Accession: ABG10545
  
Location: 4721527-4722897
  
  
**BlastP hit with Mycgr3G42715\_Mycgr3T**
  
Percentage identity: 41 %
  
BlastP bit score: 350
  
Sequence coverage: 105 %
  
E-value: 1e-112
  
  
 NCBI BlastP on this gene

Mmcs\_4441

diguanylate cyclase
  
Accession: ABG10546
  
Location: 4722982-4724067
  
 NCBI BlastP on this gene

Mmcs\_4442

conserved hypothetical protein
  
Accession: ABG10547
  
Location: 4724099-4724974
  
 NCBI BlastP on this gene

Mmcs\_4443

ABC transporter related protein
  
Accession: ABG10548
  
Location: 4725098-4726930
  
 NCBI BlastP on this gene

Mmcs\_4444

ABC transporter related protein
  
Accession: ABG10549
  
Location: 4726927-4728879
  
 NCBI BlastP on this gene

Mmcs\_4445

ABC transporter related protein
  
Accession: ABG10550
  
Location: 4728931-4730814
  
 NCBI BlastP on this gene

Mmcs\_4446

Ion transport 2
  
Accession: ABG10551
  
Location: 4730890-4731672
  
 NCBI BlastP on this gene

Mmcs\_4447

two component transcriptional regulator, winged helix family
  
Accession: ABG10552
  
Location: 4731699-4732400
  
 NCBI BlastP on this gene

Mmcs\_4448

periplasmic sensor signal transduction histidine kinase
  
Accession: ABG10553
  
Location: 4732403-4733749
  
 NCBI BlastP on this gene

Mmcs\_4449

peptidylprolyl isomerase, FKBP-type
  
Accession: ABG10554
  
Location: 4733821-4734189
  
 NCBI BlastP on this gene

Mmcs\_4450

conserved hypothetical protein
  
Accession: ABG10555
  
Location: 4734226-4734474
  
 NCBI BlastP on this gene

Mmcs\_4451

conserved hypothetical protein
  
Accession: ABG10556
  
Location: 4734519-4736018
  
 NCBI BlastP on this gene

Mmcs\_4452

hypothetical protein
  
Accession: ABG10557
  
Location: 4736050-4736397
  
 NCBI BlastP on this gene

Mmcs\_4453

transcriptional regulator, MarR family
  
Accession: ABG10558
  
Location: 4736402-4736821
  
 NCBI BlastP on this gene

Mmcs\_4454

Drug resistance transporter EmrB/QacA subfamily
  
Accession: ABG10559
  
Location: 4736811-4738247
  
 NCBI BlastP on this gene

Mmcs\_4455

citrate synthase
  
Accession: ABG10560
  
Location: 4738333-4739637
  
 NCBI BlastP on this gene

Mmcs\_4456

transcriptional regulator, TetR family
  
Accession: ABG10561
  
Location: 4739733-4740380
  
 NCBI BlastP on this gene

Mmcs\_4457

Pyridoxamine 5'-phosphate oxidase
  
Accession: ABG10562
  
Location: 4740508-4741170
  
 NCBI BlastP on this gene

Mmcs\_4458

citrate synthase
  
Accession: ABG10563
  
Location: 4741231-4742361
  
 NCBI BlastP on this gene

Mmcs\_4459

hypothetical protein
  
Accession: ABG10564
  
Location: 4742358-4742975
  
 NCBI BlastP on this gene

Mmcs\_4460

Query: Architecture Search FASTA input

CP000580 : Mycobacterium sp. JLS    Total score: 2.0     Cumulative Blast bit score: 557

Hit cluster cross-links:

Mycgr3G85918 Mycgr3T
  
Location: 0-1602

Mycgr3G85918\_Mycgr3T

Mycgr3G42010 Mycgr3T
  
Location: 1702-8569

Mycgr3G42010\_Mycgr3T

Mycgr3G29582 Mycgr3T
  
Location: 8669-8915

Mycgr3G29582\_Mycgr3T

Mycgr3G31170 Mycgr3T
  
Location: 9015-9255

Mycgr3G31170\_Mycgr3T

Mycgr3G85924 Mycgr3T
  
Location: 9355-11218

Mycgr3G85924\_Mycgr3T

Mycgr3G71676 Mycgr3T
  
Location: 11318-12494

Mycgr3G71676\_Mycgr3T

Mycgr3G11468 Mycgr3T
  
Location: 12594-13653

Mycgr3G11468\_Mycgr3T

Mycgr3G58567 Mycgr3T
  
Location: 13753-14506

Mycgr3G58567\_Mycgr3T

Mycgr3G100089 Mycgr3
  
Location: 14606-21152

Mycgr3G100089\_Mycgr3

Mycgr3G42698 Mycgr3T
  
Location: 21252-22131

Mycgr3G42698\_Mycgr3T

Mycgr3G71681 Mycgr3T
  
Location: 22231-23461

Mycgr3G71681\_Mycgr3T

Mycgr3G109328 Mycgr3
  
Location: 23561-24239

Mycgr3G109328\_Mycgr3

Mycgr3G104334 Mycgr3
  
Location: 24339-24567

Mycgr3G104334\_Mycgr3

Mycgr3G42715 Mycgr3T
  
Location: 24667-25981

Mycgr3G42715\_Mycgr3T

Mycgr3G92934 Mycgr3T
  
Location: 26081-27593

Mycgr3G92934\_Mycgr3T

Mycgr3G41969 Mycgr3T
  
Location: 27693-29328

Mycgr3G41969\_Mycgr3T

Mycgr3G80635 Mycgr3T
  
Location: 29428-29821

Mycgr3G80635\_Mycgr3T

Mycgr3G41426 Mycgr3T
  
Location: 29921-35255

Mycgr3G41426\_Mycgr3T

Mycgr3G104337 Mycgr3
  
Location: 35355-36108

Mycgr3G104337\_Mycgr3

Mycgr3G71679 Mycgr3T
  
Location: 36208-37300

Mycgr3G71679\_Mycgr3T

Mycgr3G92938 Mycgr3T
  
Location: 37400-38699

Mycgr3G92938\_Mycgr3T

Mycgr3G92941 Mycgr3T
  
Location: 38799-40734

Mycgr3G92941\_Mycgr3T

conserved hypothetical protein
  
Accession: ABO00566
  
Location: 5048915-5049649
  
 NCBI BlastP on this gene

Mjls\_4800

conserved hypothetical protein
  
Accession: ABO00567
  
Location: 5049660-5050070
  
 NCBI BlastP on this gene

Mjls\_4801

dihydrodipicolinate reductase
  
Accession: ABO00568
  
Location: 5050074-5051165
  
 NCBI BlastP on this gene

Mjls\_4802

cytochrome P450
  
Accession: ABO00569
  
Location: 5051162-5052346
  
 NCBI BlastP on this gene

Mjls\_4803

transcriptional regulator, TetR family
  
Accession: ABO00570
  
Location: 5052583-5053206
  
 NCBI BlastP on this gene

Mjls\_4804

NAD-dependent epimerase/dehydratase
  
Accession: ABO00571
  
Location: 5053385-5054044
  
 NCBI BlastP on this gene

Mjls\_4805

alpha/beta hydrolase fold protein
  
Accession: ABO00572
  
Location: 5054068-5055033
  
 NCBI BlastP on this gene

Mjls\_4806

conserved hypothetical protein
  
Accession: ABO00573
  
Location: 5055060-5055491
  
 NCBI BlastP on this gene

Mjls\_4807

hypothetical protein
  
Accession: ABO00574
  
Location: 5055495-5055725
  
 NCBI BlastP on this gene

Mjls\_4808

ATPase, P-type (transporting), HAD superfamily, subfamily IC
  
Accession: ABO00575
  
Location: 5055749-5058139
  
 NCBI BlastP on this gene

Mjls\_4809

beta-lactamase
  
Accession: ABO00576
  
Location: 5058264-5059865
  
 NCBI BlastP on this gene

Mjls\_4810

conserved hypothetical protein
  
Accession: ABO00577
  
Location: 5059875-5060993
  
 NCBI BlastP on this gene

Mjls\_4811

Enoyl-CoA hydratase/isomerase
  
Accession: ABO00578
  
Location: 5060996-5061730
  
 NCBI BlastP on this gene

Mjls\_4812

carboxyl transferase
  
Accession: ABO00579
  
Location: 5061757-5063265
  
 NCBI BlastP on this gene

Mjls\_4813

conserved hypothetical protein
  
Accession: ABO00580
  
Location: 5063266-5063514
  
 NCBI BlastP on this gene

Mjls\_4814

conserved hypothetical protein
  
Accession: ABO00581
  
Location: 5063511-5063726
  
 NCBI BlastP on this gene

Mjls\_4815

conserved hypothetical protein
  
Accession: ABO00582
  
Location: 5063765-5064196
  
 NCBI BlastP on this gene

Mjls\_4816

conserved hypothetical protein
  
Accession: ABO00583
  
Location: 5064206-5064613
  
 NCBI BlastP on this gene

Mjls\_4817

AMP-dependent synthetase and ligase
  
Accession: ABO00584
  
Location: 5064664-5066286
  
 NCBI BlastP on this gene

Mjls\_4818

AMP-dependent synthetase and ligase
  
Accession: ABO00585
  
Location: 5066283-5067980
  
 NCBI BlastP on this gene

Mjls\_4819

putative GAF sensor protein
  
Accession: ABO00586
  
Location: 5068030-5068887
  
 NCBI BlastP on this gene

Mjls\_4820

Epoxide hydrolase domain protein
  
Accession: ABO00587
  
Location: 5068910-5070013
  
  
**BlastP hit with Mycgr3G71676\_Mycgr3T**
  
Percentage identity: 34 %
  
BlastP bit score: 209
  
Sequence coverage: 98 %
  
E-value: 7e-60
  
  
 NCBI BlastP on this gene

Mjls\_4821

conserved hypothetical protein
  
Accession: ABO00588
  
Location: 5070015-5071385
  
  
**BlastP hit with Mycgr3G42715\_Mycgr3T**
  
Percentage identity: 41 %
  
BlastP bit score: 348
  
Sequence coverage: 105 %
  
E-value: 6e-112
  
  
 NCBI BlastP on this gene

Mjls\_4822

diguanylate cyclase
  
Accession: ABO00589
  
Location: 5071696-5072556
  
 NCBI BlastP on this gene

Mjls\_4823

conserved hypothetical protein
  
Accession: ABO00590
  
Location: 5072588-5072917
  
 NCBI BlastP on this gene

Mjls\_4824

conserved hypothetical protein
  
Accession: ABO00591
  
Location: 5072875-5073462
  
 NCBI BlastP on this gene

Mjls\_4825

ABC transporter related protein
  
Accession: ABO00592
  
Location: 5073585-5075417
  
 NCBI BlastP on this gene

Mjls\_4826

ABC transporter related protein
  
Accession: ABO00593
  
Location: 5075414-5077339
  
 NCBI BlastP on this gene

Mjls\_4827

ABC transporter related protein
  
Accession: ABO00594
  
Location: 5077391-5079271
  
 NCBI BlastP on this gene

Mjls\_4828

Ion transport 2 domain protein
  
Accession: ABO00595
  
Location: 5079346-5080128
  
 NCBI BlastP on this gene

Mjls\_4829

two component transcriptional regulator, winged helix family
  
Accession: ABO00596
  
Location: 5080155-5080856
  
 NCBI BlastP on this gene

Mjls\_4830

integral membrane sensor signal transduction histidine kinase
  
Accession: ABO00597
  
Location: 5080859-5082205
  
 NCBI BlastP on this gene

Mjls\_4831

peptidylprolyl isomerase, FKBP-type
  
Accession: ABO00598
  
Location: 5082277-5082645
  
 NCBI BlastP on this gene

Mjls\_4832

conserved hypothetical protein
  
Accession: ABO00599
  
Location: 5082682-5082930
  
 NCBI BlastP on this gene

Mjls\_4833

conserved hypothetical protein
  
Accession: ABO00600
  
Location: 5082975-5084474
  
 NCBI BlastP on this gene

Mjls\_4834

conserved hypothetical protein
  
Accession: ABO00601
  
Location: 5084506-5084853
  
 NCBI BlastP on this gene

Mjls\_4835

transcriptional regulator, MarR family
  
Accession: ABO00602
  
Location: 5084858-5085277
  
 NCBI BlastP on this gene

Mjls\_4836

drug resistance transporter, EmrB/QacA subfamily
  
Accession: ABO00603
  
Location: 5085267-5086703
  
 NCBI BlastP on this gene

Mjls\_4837

citrate synthase
  
Accession: ABO00604
  
Location: 5086789-5088093
  
 NCBI BlastP on this gene

Mjls\_4838

transcriptional regulator, TetR family
  
Accession: ABO00605
  
Location: 5088189-5088836
  
 NCBI BlastP on this gene

Mjls\_4839

Pyridoxamine 5'-phosphate oxidase
  
Accession: ABO00606
  
Location: 5088964-5089593
  
 NCBI BlastP on this gene

Mjls\_4840

citrate synthase
  
Accession: ABO00607
  
Location: 5089687-5090817
  
 NCBI BlastP on this gene

Mjls\_4841

conserved hypothetical protein
  
Accession: ABO00608
  
Location: 5090814-5091431
  
 NCBI BlastP on this gene

Mjls\_4842

Query: Architecture Search FASTA input

CP003078 : Mycobacterium smegmatis JS623    Total score: 2.0     Cumulative Blast bit score: 548

Hit cluster cross-links:

Mycgr3G85918 Mycgr3T
  
Location: 0-1602

Mycgr3G85918\_Mycgr3T

Mycgr3G42010 Mycgr3T
  
Location: 1702-8569

Mycgr3G42010\_Mycgr3T

Mycgr3G29582 Mycgr3T
  
Location: 8669-8915

Mycgr3G29582\_Mycgr3T

Mycgr3G31170 Mycgr3T
  
Location: 9015-9255

Mycgr3G31170\_Mycgr3T

Mycgr3G85924 Mycgr3T
  
Location: 9355-11218

Mycgr3G85924\_Mycgr3T

Mycgr3G71676 Mycgr3T
  
Location: 11318-12494

Mycgr3G71676\_Mycgr3T

Mycgr3G11468 Mycgr3T
  
Location: 12594-13653

Mycgr3G11468\_Mycgr3T

Mycgr3G58567 Mycgr3T
  
Location: 13753-14506

Mycgr3G58567\_Mycgr3T

Mycgr3G100089 Mycgr3
  
Location: 14606-21152

Mycgr3G100089\_Mycgr3

Mycgr3G42698 Mycgr3T
  
Location: 21252-22131

Mycgr3G42698\_Mycgr3T

Mycgr3G71681 Mycgr3T
  
Location: 22231-23461

Mycgr3G71681\_Mycgr3T

Mycgr3G109328 Mycgr3
  
Location: 23561-24239

Mycgr3G109328\_Mycgr3

Mycgr3G104334 Mycgr3
  
Location: 24339-24567

Mycgr3G104334\_Mycgr3

Mycgr3G42715 Mycgr3T
  
Location: 24667-25981

Mycgr3G42715\_Mycgr3T

Mycgr3G92934 Mycgr3T
  
Location: 26081-27593

Mycgr3G92934\_Mycgr3T

Mycgr3G41969 Mycgr3T
  
Location: 27693-29328

Mycgr3G41969\_Mycgr3T

Mycgr3G80635 Mycgr3T
  
Location: 29428-29821

Mycgr3G80635\_Mycgr3T

Mycgr3G41426 Mycgr3T
  
Location: 29921-35255

Mycgr3G41426\_Mycgr3T

Mycgr3G104337 Mycgr3
  
Location: 35355-36108

Mycgr3G104337\_Mycgr3

Mycgr3G71679 Mycgr3T
  
Location: 36208-37300

Mycgr3G71679\_Mycgr3T

Mycgr3G92938 Mycgr3T
  
Location: 37400-38699

Mycgr3G92938\_Mycgr3T

Mycgr3G92941 Mycgr3T
  
Location: 38799-40734

Mycgr3G92941\_Mycgr3T

putative hydrolase or acyltransferase of
  
Accession: AGB25596
  
Location: 5360224-5360922
  
 NCBI BlastP on this gene

Mycsm\_05405

hypothetical protein
  
Accession: AGB25597
  
Location: 5360938-5361630
  
 NCBI BlastP on this gene

Mycsm\_05406

gluconolactonase
  
Accession: AGB25598
  
Location: 5361687-5362562
  
 NCBI BlastP on this gene

Mycsm\_05407

hypothetical protein
  
Accession: AGB25599
  
Location: 5362569-5362991
  
 NCBI BlastP on this gene

Mycsm\_05408

transcriptional regulator
  
Accession: AGB25600
  
Location: 5363022-5363567
  
 NCBI BlastP on this gene

Mycsm\_05409

2-polyprenyl-6-methoxyphenol hydroxylase-like oxidoreductase
  
Accession: AGB25601
  
Location: 5363662-5364801
  
 NCBI BlastP on this gene

Mycsm\_05410

hypothetical protein
  
Accession: AGB25602
  
Location: 5364779-5365186
  
 NCBI BlastP on this gene

Mycsm\_05411

Cu2+-containing amine oxidase
  
Accession: AGB25603
  
Location: 5365314-5367248
  
 NCBI BlastP on this gene

Mycsm\_05412

amino acid transporter
  
Accession: AGB25604
  
Location: 5367245-5368696
  
 NCBI BlastP on this gene

Mycsm\_05413

NAD-dependent aldehyde dehydrogenase
  
Accession: AGB25605
  
Location: 5368808-5370310
  
 NCBI BlastP on this gene

Mycsm\_05414

amino acid transporter
  
Accession: AGB25606
  
Location: 5370542-5371990
  
 NCBI BlastP on this gene

Mycsm\_05415

transcriptional regulator
  
Accession: AGB25607
  
Location: 5371987-5372703
  
 NCBI BlastP on this gene

Mycsm\_05416

hypothetical protein
  
Accession: AGB25608
  
Location: 5372774-5373694
  
 NCBI BlastP on this gene

Mycsm\_05417

acyl-CoA synthetase (AMP-forming)/AMP-acid ligase II
  
Accession: AGB25609
  
Location: 5373748-5375361
  
 NCBI BlastP on this gene

Mycsm\_05418

acyl-CoA synthetase/AMP-acid ligase
  
Accession: AGB25610
  
Location: 5375358-5377049
  
 NCBI BlastP on this gene

Mycsm\_05419

response regulator containing a CheY-like receiver domain and an HTH DNA-binding domain
  
Accession: AGB25611
  
Location: 5377108-5377944
  
 NCBI BlastP on this gene

Mycsm\_05420

molecular chaperone (small heat shock protein)
  
Accession: AGB25612
  
Location: 5377999-5378481
  
 NCBI BlastP on this gene

Mycsm\_05421

hypothetical protein
  
Accession: AGB25613
  
Location: 5378758-5379390
  
 NCBI BlastP on this gene

Mycsm\_05422

putative hydrolase or acyltransferase of
  
Accession: AGB25614
  
Location: 5379394-5380503
  
  
**BlastP hit with Mycgr3G71676\_Mycgr3T**
  
Percentage identity: 33 %
  
BlastP bit score: 206
  
Sequence coverage: 98 %
  
E-value: 6e-59
  
  
 NCBI BlastP on this gene

Mycsm\_05423

hypothetical protein
  
Accession: AGB25615
  
Location: 5380504-5381877
  
  
**BlastP hit with Mycgr3G42715\_Mycgr3T**
  
Percentage identity: 39 %
  
BlastP bit score: 342
  
Sequence coverage: 105 %
  
E-value: 1e-109
  
  
 NCBI BlastP on this gene

Mycsm\_05424

ABC-type multidrug transport system, ATPase and permease component
  
Accession: AGB25616
  
Location: 5382007-5383830
  
 NCBI BlastP on this gene

Mycsm\_05425

ABC-type multidrug transport system, ATPase and permease component
  
Accession: AGB25617
  
Location: 5383827-5385746
  
 NCBI BlastP on this gene

Mycsm\_05426

chromate transport protein ChrA
  
Accession: AGB25618
  
Location: 5385973-5386521
  
 NCBI BlastP on this gene

Mycsm\_05427

chromate transport protein ChrA
  
Accession: AGB25619
  
Location: 5386518-5387081
  
 NCBI BlastP on this gene

Mycsm\_05428

phosphatidylserine/phosphatidylglycerophosphate/ cardiolipin synthase
  
Accession: AGB25620
  
Location: 5387074-5388063
  
 NCBI BlastP on this gene

Mycsm\_05429

response regulator with CheY-like receiver domain and winged-helix DNA-binding domain
  
Accession: AGB25621
  
Location: 5388224-5388925
  
 NCBI BlastP on this gene

Mycsm\_05430

signal transduction histidine kinase
  
Accession: AGB25622
  
Location: 5388929-5390269
  
 NCBI BlastP on this gene

Mycsm\_05431

FKBP-type peptidyl-prolyl cis-trans isomerase
  
Accession: AGB25623
  
Location: 5390350-5390712
  
 NCBI BlastP on this gene

Mycsm\_05432

Protein of unknown function (DUF2630)
  
Accession: AGB25624
  
Location: 5390736-5390984
  
 NCBI BlastP on this gene

Mycsm\_05433

phytoene dehydrogenase-like oxidoreductase
  
Accession: AGB25625
  
Location: 5390981-5392549
  
 NCBI BlastP on this gene

Mycsm\_05434

adenylate/guanylate cyclase family protein
  
Accession: AGB25626
  
Location: 5392546-5395686
  
 NCBI BlastP on this gene

Mycsm\_05435

transcriptional regulator
  
Accession: AGB25627
  
Location: 5395735-5396154
  
 NCBI BlastP on this gene

Mycsm\_05436

drug resistance transporter, EmrB/QacA subfamily
  
Accession: AGB25628
  
Location: 5396144-5397598
  
 NCBI BlastP on this gene

Mycsm\_05437

citrate synthase I, hexameric type
  
Accession: AGB25629
  
Location: 5397739-5399046
  
 NCBI BlastP on this gene

Mycsm\_05438

transcriptional regulator
  
Accession: AGB25630
  
Location: 5399142-5399804
  
 NCBI BlastP on this gene

Mycsm\_05439

protein of unknown function (DUF894)
  
Accession: AGB25631
  
Location: 5399865-5401157
  
 NCBI BlastP on this gene

Mycsm\_05440

Pyridoxamine 5'-phosphate oxidase
  
Accession: AGB25632
  
Location: 5401157-5401813
  
 NCBI BlastP on this gene

Mycsm\_05441

Query: Architecture Search FASTA input

JH126399 : Cordyceps militaris CM01 unplaced genomic scaffold CCM\_S00001    Total score: 2.0     Cumulative Blast bit score: 524

Hit cluster cross-links:

Mycgr3G85918 Mycgr3T
  
Location: 0-1602

Mycgr3G85918\_Mycgr3T

Mycgr3G42010 Mycgr3T
  
Location: 1702-8569

Mycgr3G42010\_Mycgr3T

Mycgr3G29582 Mycgr3T
  
Location: 8669-8915

Mycgr3G29582\_Mycgr3T

Mycgr3G31170 Mycgr3T
  
Location: 9015-9255

Mycgr3G31170\_Mycgr3T

Mycgr3G85924 Mycgr3T
  
Location: 9355-11218

Mycgr3G85924\_Mycgr3T

Mycgr3G71676 Mycgr3T
  
Location: 11318-12494

Mycgr3G71676\_Mycgr3T

Mycgr3G11468 Mycgr3T
  
Location: 12594-13653

Mycgr3G11468\_Mycgr3T

Mycgr3G58567 Mycgr3T
  
Location: 13753-14506

Mycgr3G58567\_Mycgr3T

Mycgr3G100089 Mycgr3
  
Location: 14606-21152

Mycgr3G100089\_Mycgr3

Mycgr3G42698 Mycgr3T
  
Location: 21252-22131

Mycgr3G42698\_Mycgr3T

Mycgr3G71681 Mycgr3T
  
Location: 22231-23461

Mycgr3G71681\_Mycgr3T

Mycgr3G109328 Mycgr3
  
Location: 23561-24239

Mycgr3G109328\_Mycgr3

Mycgr3G104334 Mycgr3
  
Location: 24339-24567

Mycgr3G104334\_Mycgr3

Mycgr3G42715 Mycgr3T
  
Location: 24667-25981

Mycgr3G42715\_Mycgr3T

Mycgr3G92934 Mycgr3T
  
Location: 26081-27593

Mycgr3G92934\_Mycgr3T

Mycgr3G41969 Mycgr3T
  
Location: 27693-29328

Mycgr3G41969\_Mycgr3T

Mycgr3G80635 Mycgr3T
  
Location: 29428-29821

Mycgr3G80635\_Mycgr3T

Mycgr3G41426 Mycgr3T
  
Location: 29921-35255

Mycgr3G41426\_Mycgr3T

Mycgr3G104337 Mycgr3
  
Location: 35355-36108

Mycgr3G104337\_Mycgr3

Mycgr3G71679 Mycgr3T
  
Location: 36208-37300

Mycgr3G71679\_Mycgr3T

Mycgr3G92938 Mycgr3T
  
Location: 37400-38699

Mycgr3G92938\_Mycgr3T

Mycgr3G92941 Mycgr3T
  
Location: 38799-40734

Mycgr3G92941\_Mycgr3T

Protein kinase-like domain
  
Accession: EGX95385
  
Location: 125630-126577
  
 NCBI BlastP on this gene

EGX95385

uracil permease
  
Accession: EGX95386
  
Location: 128275-130069
  
 NCBI BlastP on this gene

EGX95386

importin beta-5 subunit, putative
  
Accession: EGX95387
  
Location: 132457-135716
  
 NCBI BlastP on this gene

EGX95387

PI31 proteasome regulator
  
Accession: EGX95388
  
Location: 136207-137515
  
 NCBI BlastP on this gene

EGX95388

Major facilitator superfamily transporter
  
Accession: EGX95389
  
Location: 138751-140433
  
 NCBI BlastP on this gene

EGX95389

ankyrin repeat and SAM domain containing protein 6
  
Accession: EGX95390
  
Location: 140873-143164
  
 NCBI BlastP on this gene

EGX95390

mitotic spindle checkpoint protein (Mad2B), putative
  
Accession: EGX95391
  
Location: 144474-145236
  
 NCBI BlastP on this gene

EGX95391

AAA family ATPase, putative
  
Accession: EGX95392
  
Location: 145455-147779
  
  
**BlastP hit with Mycgr3G42010\_Mycgr3T**
  
Percentage identity: 55 %
  
BlastP bit score: 291
  
Sequence coverage: 11 %
  
E-value: 1e-79
  
  
 NCBI BlastP on this gene

EGX95392

hypothetical protein
  
Accession: EGX95393
  
Location: 147915-149562
  
  
**BlastP hit with Mycgr3G92938\_Mycgr3T**
  
Percentage identity: 34 %
  
BlastP bit score: 233
  
Sequence coverage: 97 %
  
E-value: 7e-68
  
  
 NCBI BlastP on this gene

EGX95393

hypothetical protein
  
Accession: EGX95394
  
Location: 149912-150841
  
 NCBI BlastP on this gene

EGX95394

hypothetical protein
  
Accession: EGX95395
  
Location: 166189-167156
  
 NCBI BlastP on this gene

EGX95395

Query: Architecture Search FASTA input

KE145369 : Glarea lozoyensis ATCC 20868 chromosome Unknown GLAREA5    Total score: 2.0     Cumulative Blast bit score: 518

Hit cluster cross-links:

Mycgr3G85918 Mycgr3T
  
Location: 0-1602

Mycgr3G85918\_Mycgr3T

Mycgr3G42010 Mycgr3T
  
Location: 1702-8569

Mycgr3G42010\_Mycgr3T

Mycgr3G29582 Mycgr3T
  
Location: 8669-8915

Mycgr3G29582\_Mycgr3T

Mycgr3G31170 Mycgr3T
  
Location: 9015-9255

Mycgr3G31170\_Mycgr3T

Mycgr3G85924 Mycgr3T
  
Location: 9355-11218

Mycgr3G85924\_Mycgr3T

Mycgr3G71676 Mycgr3T
  
Location: 11318-12494

Mycgr3G71676\_Mycgr3T

Mycgr3G11468 Mycgr3T
  
Location: 12594-13653

Mycgr3G11468\_Mycgr3T

Mycgr3G58567 Mycgr3T
  
Location: 13753-14506

Mycgr3G58567\_Mycgr3T

Mycgr3G100089 Mycgr3
  
Location: 14606-21152

Mycgr3G100089\_Mycgr3

Mycgr3G42698 Mycgr3T
  
Location: 21252-22131

Mycgr3G42698\_Mycgr3T

Mycgr3G71681 Mycgr3T
  
Location: 22231-23461

Mycgr3G71681\_Mycgr3T

Mycgr3G109328 Mycgr3
  
Location: 23561-24239

Mycgr3G109328\_Mycgr3

Mycgr3G104334 Mycgr3
  
Location: 24339-24567

Mycgr3G104334\_Mycgr3

Mycgr3G42715 Mycgr3T
  
Location: 24667-25981

Mycgr3G42715\_Mycgr3T

Mycgr3G92934 Mycgr3T
  
Location: 26081-27593

Mycgr3G92934\_Mycgr3T

Mycgr3G41969 Mycgr3T
  
Location: 27693-29328

Mycgr3G41969\_Mycgr3T

Mycgr3G80635 Mycgr3T
  
Location: 29428-29821

Mycgr3G80635\_Mycgr3T

Mycgr3G41426 Mycgr3T
  
Location: 29921-35255

Mycgr3G41426\_Mycgr3T

Mycgr3G104337 Mycgr3
  
Location: 35355-36108

Mycgr3G104337\_Mycgr3

Mycgr3G71679 Mycgr3T
  
Location: 36208-37300

Mycgr3G71679\_Mycgr3T

Mycgr3G92938 Mycgr3T
  
Location: 37400-38699

Mycgr3G92938\_Mycgr3T

Mycgr3G92941 Mycgr3T
  
Location: 38799-40734

Mycgr3G92941\_Mycgr3T

CheY-like protein
  
Accession: EPE27507
  
Location: 317465-321368
  
 NCBI BlastP on this gene

EPE27507

hypothetical protein
  
Accession: EPE27506
  
Location: 315803-316321
  
 NCBI BlastP on this gene

EPE27506

Ribonuclease H-like protein
  
Accession: EPE27505
  
Location: 311868-315363
  
 NCBI BlastP on this gene

EPE27505

S-adenosyl-L-methionine-dependent methyltransferase
  
Accession: EPE27504
  
Location: 308466-309868
  
 NCBI BlastP on this gene

EPE27504

MFS general substrate transporter
  
Accession: EPE27503
  
Location: 304097-306007
  
  
**BlastP hit with Mycgr3G85918\_Mycgr3T**
  
Percentage identity: 36 %
  
BlastP bit score: 347
  
Sequence coverage: 97 %
  
E-value: 4e-109
  
  
 NCBI BlastP on this gene

EPE27503

hypothetical protein
  
Accession: EPE27502
  
Location: 301946-303676
  
 NCBI BlastP on this gene

EPE27502

hypothetical protein
  
Accession: EPE27501
  
Location: 300215-301531
  
 NCBI BlastP on this gene

EPE27501

hypothetical protein
  
Accession: EPE27500
  
Location: 298159-298911
  
 NCBI BlastP on this gene

EPE27500

hypothetical protein
  
Accession: EPE27499
  
Location: 293387-297823
  
 NCBI BlastP on this gene

EPE27499

Di-copper centre-containing
  
Accession: EPE27498
  
Location: 289770-291650
  
  
**BlastP hit with Mycgr3G42698\_Mycgr3T**
  
Percentage identity: 33 %
  
BlastP bit score: 171
  
Sequence coverage: 108 %
  
E-value: 2e-46
  
  
 NCBI BlastP on this gene

EPE27498

Zn2/Cys6 DNA-binding protein
  
Accession: EPE27497
  
Location: 285663-288405
  
 NCBI BlastP on this gene

EPE27497

NAD(P)-binding Rossmann-fold containing protein
  
Accession: EPE27496
  
Location: 284250-285376
  
 NCBI BlastP on this gene

EPE27496

peptidase M48 Ste24p
  
Accession: EPE27495
  
Location: 282744-283427
  
 NCBI BlastP on this gene

EPE27495

S-adenosyl-L-methionine-dependent methyltransferase
  
Accession: EPE27494
  
Location: 280283-281744
  
 NCBI BlastP on this gene

EPE27494

hypothetical protein
  
Accession: EPE27493
  
Location: 278442-279233
  
 NCBI BlastP on this gene

EPE27493

P-loop containing nucleoside triphosphate hydrolase
  
Accession: EPE27492
  
Location: 275167-278214
  
 NCBI BlastP on this gene

EPE27492

Query: Architecture Search FASTA input

HF679032 : Fusarium fujikuroi IMI 58289 draft genome, chromosome FFUJ\_chr10.    Total score: 2.0     Cumulative Blast bit score: 517

Hit cluster cross-links:

Mycgr3G85918 Mycgr3T
  
Location: 0-1602

Mycgr3G85918\_Mycgr3T

Mycgr3G42010 Mycgr3T
  
Location: 1702-8569

Mycgr3G42010\_Mycgr3T

Mycgr3G29582 Mycgr3T
  
Location: 8669-8915

Mycgr3G29582\_Mycgr3T

Mycgr3G31170 Mycgr3T
  
Location: 9015-9255

Mycgr3G31170\_Mycgr3T

Mycgr3G85924 Mycgr3T
  
Location: 9355-11218

Mycgr3G85924\_Mycgr3T

Mycgr3G71676 Mycgr3T
  
Location: 11318-12494

Mycgr3G71676\_Mycgr3T

Mycgr3G11468 Mycgr3T
  
Location: 12594-13653

Mycgr3G11468\_Mycgr3T

Mycgr3G58567 Mycgr3T
  
Location: 13753-14506

Mycgr3G58567\_Mycgr3T

Mycgr3G100089 Mycgr3
  
Location: 14606-21152

Mycgr3G100089\_Mycgr3

Mycgr3G42698 Mycgr3T
  
Location: 21252-22131

Mycgr3G42698\_Mycgr3T

Mycgr3G71681 Mycgr3T
  
Location: 22231-23461

Mycgr3G71681\_Mycgr3T

Mycgr3G109328 Mycgr3
  
Location: 23561-24239

Mycgr3G109328\_Mycgr3

Mycgr3G104334 Mycgr3
  
Location: 24339-24567

Mycgr3G104334\_Mycgr3

Mycgr3G42715 Mycgr3T
  
Location: 24667-25981

Mycgr3G42715\_Mycgr3T

Mycgr3G92934 Mycgr3T
  
Location: 26081-27593

Mycgr3G92934\_Mycgr3T

Mycgr3G41969 Mycgr3T
  
Location: 27693-29328

Mycgr3G41969\_Mycgr3T

Mycgr3G80635 Mycgr3T
  
Location: 29428-29821

Mycgr3G80635\_Mycgr3T

Mycgr3G41426 Mycgr3T
  
Location: 29921-35255

Mycgr3G41426\_Mycgr3T

Mycgr3G104337 Mycgr3
  
Location: 35355-36108

Mycgr3G104337\_Mycgr3

Mycgr3G71679 Mycgr3T
  
Location: 36208-37300

Mycgr3G71679\_Mycgr3T

Mycgr3G92938 Mycgr3T
  
Location: 37400-38699

Mycgr3G92938\_Mycgr3T

Mycgr3G92941 Mycgr3T
  
Location: 38799-40734

Mycgr3G92941\_Mycgr3T

related to peroxisomal short-chain alcohol dehydrogenase
  
Accession: CCT74200
  
Location: 97598-98494
  
 NCBI BlastP on this gene

FFUJ\_10242

related to chitinase
  
Accession: CCT74201
  
Location: 99450-102382
  
 NCBI BlastP on this gene

FFUJ\_10243

uncharacterized protein
  
Accession: CCT74202
  
Location: 102936-104123
  
 NCBI BlastP on this gene

FFUJ\_10244

uncharacterized protein
  
Accession: CCT74203
  
Location: 104778-106023
  
 NCBI BlastP on this gene

FFUJ\_10245

related to proteoglycan
  
Accession: CCT74204
  
Location: 106593-108572
  
 NCBI BlastP on this gene

FFUJ\_10246

uncharacterized protein
  
Accession: CCT74205
  
Location: 110008-111020
  
 NCBI BlastP on this gene

FFUJ\_10247

related to salicylate 1-monooxygenase
  
Accession: CCT74206
  
Location: 112007-113400
  
 NCBI BlastP on this gene

FFUJ\_10248

related to monooxigenase
  
Accession: CCT74207
  
Location: 113534-115814
  
 NCBI BlastP on this gene

FFUJ\_10249

related to alcohol/sorbitol dehydrogenase
  
Accession: CCT74208
  
Location: 116367-117696
  
  
**BlastP hit with Mycgr3G71679\_Mycgr3T**
  
Percentage identity: 44 %
  
BlastP bit score: 324
  
Sequence coverage: 101 %
  
E-value: 9e-105
  
  
 NCBI BlastP on this gene

FFUJ\_10250

related to reductases
  
Accession: CCT74209
  
Location: 118087-119259
  
 NCBI BlastP on this gene

FFUJ\_10251

related to Copper amine oxidase 1
  
Accession: CCT74210
  
Location: 120051-122263
  
 NCBI BlastP on this gene

FFUJ\_10252

uncharacterized protein
  
Accession: CCT74211
  
Location: 122674-124932
  
  
**BlastP hit with Mycgr3G11468\_Mycgr3T**
  
Percentage identity: 34 %
  
BlastP bit score: 193
  
Sequence coverage: 100 %
  
E-value: 5e-52
  
  
 NCBI BlastP on this gene

FFUJ\_10253

related to permease of the major facilitator superfamily
  
Accession: CCT74212
  
Location: 125643-127234
  
 NCBI BlastP on this gene

FFUJ\_10254

uncharacterized protein
  
Accession: CCT74213
  
Location: 127627-128842
  
 NCBI BlastP on this gene

FFUJ\_10255

related to 3-hydroxybutyryl-CoA dehydrogenase
  
Accession: CCT74214
  
Location: 129359-131170
  
 NCBI BlastP on this gene

FFUJ\_10256

putative NADH cytb-reductase
  
Accession: CCT74215
  
Location: 132717-134124
  
 NCBI BlastP on this gene

FFUJ\_10257

monooxygenase
  
Accession: CCT74216
  
Location: 134987-136748
  
 NCBI BlastP on this gene

FFUJ\_10258

related to tetracycline efflux protein (otrb)
  
Accession: CCT74217
  
Location: 137026-138713
  
 NCBI BlastP on this gene

FFUJ\_10259

related to 6-hydroxy-D-nicotine oxidase
  
Accession: CCT74218
  
Location: 138921-140458
  
 NCBI BlastP on this gene

FFUJ\_10260

probable glutathione S-transferase
  
Accession: CCT75122
  
Location: 141200-142157
  
 NCBI BlastP on this gene

FFUJ\_10261

uncharacterized protein
  
Accession: CCT74219
  
Location: 142412-143443
  
 NCBI BlastP on this gene

FFUJ\_10262

Query: Architecture Search FASTA input

AP007166 : Aspergillus oryzae RIB40 DNA, SC113.    Total score: 2.0     Cumulative Blast bit score: 517

Hit cluster cross-links:

Mycgr3G85918 Mycgr3T
  
Location: 0-1602

Mycgr3G85918\_Mycgr3T

Mycgr3G42010 Mycgr3T
  
Location: 1702-8569

Mycgr3G42010\_Mycgr3T

Mycgr3G29582 Mycgr3T
  
Location: 8669-8915

Mycgr3G29582\_Mycgr3T

Mycgr3G31170 Mycgr3T
  
Location: 9015-9255

Mycgr3G31170\_Mycgr3T

Mycgr3G85924 Mycgr3T
  
Location: 9355-11218

Mycgr3G85924\_Mycgr3T

Mycgr3G71676 Mycgr3T
  
Location: 11318-12494

Mycgr3G71676\_Mycgr3T

Mycgr3G11468 Mycgr3T
  
Location: 12594-13653

Mycgr3G11468\_Mycgr3T

Mycgr3G58567 Mycgr3T
  
Location: 13753-14506

Mycgr3G58567\_Mycgr3T

Mycgr3G100089 Mycgr3
  
Location: 14606-21152

Mycgr3G100089\_Mycgr3

Mycgr3G42698 Mycgr3T
  
Location: 21252-22131

Mycgr3G42698\_Mycgr3T

Mycgr3G71681 Mycgr3T
  
Location: 22231-23461

Mycgr3G71681\_Mycgr3T

Mycgr3G109328 Mycgr3
  
Location: 23561-24239

Mycgr3G109328\_Mycgr3

Mycgr3G104334 Mycgr3
  
Location: 24339-24567

Mycgr3G104334\_Mycgr3

Mycgr3G42715 Mycgr3T
  
Location: 24667-25981

Mycgr3G42715\_Mycgr3T

Mycgr3G92934 Mycgr3T
  
Location: 26081-27593

Mycgr3G92934\_Mycgr3T

Mycgr3G41969 Mycgr3T
  
Location: 27693-29328

Mycgr3G41969\_Mycgr3T

Mycgr3G80635 Mycgr3T
  
Location: 29428-29821

Mycgr3G80635\_Mycgr3T

Mycgr3G41426 Mycgr3T
  
Location: 29921-35255

Mycgr3G41426\_Mycgr3T

Mycgr3G104337 Mycgr3
  
Location: 35355-36108

Mycgr3G104337\_Mycgr3

Mycgr3G71679 Mycgr3T
  
Location: 36208-37300

Mycgr3G71679\_Mycgr3T

Mycgr3G92938 Mycgr3T
  
Location: 37400-38699

Mycgr3G92938\_Mycgr3T

Mycgr3G92941 Mycgr3T
  
Location: 38799-40734

Mycgr3G92941\_Mycgr3T

not annotated
  
Accession: BAE62723
  
Location: 225659-227272
  
 NCBI BlastP on this gene

AO090120000096

not annotated
  
Accession: BAE62722
  
Location: 221157-222072
  
  
**BlastP hit with Mycgr3G31170\_Mycgr3T**
  
Percentage identity: 98 %
  
BlastP bit score: 163
  
Sequence coverage: 100 %
  
E-value: 2e-48
  
  
 NCBI BlastP on this gene

AO090120000095

not annotated
  
Accession: BAE62721
  
Location: 217829-219808
  
 NCBI BlastP on this gene

AO090120000093

not annotated
  
Accession: BAE62720
  
Location: 216431-217247
  
 NCBI BlastP on this gene

AO090120000091

not annotated
  
Accession: BAE62719
  
Location: 213616-215994
  
 NCBI BlastP on this gene

AO090120000090

not annotated
  
Accession: BAE62718
  
Location: 204340-209055
  
 NCBI BlastP on this gene

AO090120000088

not annotated
  
Accession: BAE62717
  
Location: 195790-202723
  
  
**BlastP hit with Mycgr3G41426\_Mycgr3T**
  
Percentage identity: 30 %
  
BlastP bit score: 354
  
Sequence coverage: 41 %
  
E-value: 8e-96
  
  
 NCBI BlastP on this gene

AO090120000087

not annotated
  
Accession: BAE62716
  
Location: 190620-193330
  
 NCBI BlastP on this gene

AO090120000086

not annotated
  
Accession: BAE62715
  
Location: 189320-189730
  
 NCBI BlastP on this gene

AO090120000085

not annotated
  
Accession: BAE62714
  
Location: 187356-188887
  
 NCBI BlastP on this gene

AO090120000084

Query: Architecture Search FASTA input

KB908855 : Setosphaeria turcica Et28A unplaced genomic scaffold SETTUscaffold\_7    Total score: 2.0     Cumulative Blast bit score: 507

Hit cluster cross-links:

Mycgr3G85918 Mycgr3T
  
Location: 0-1602

Mycgr3G85918\_Mycgr3T

Mycgr3G42010 Mycgr3T
  
Location: 1702-8569

Mycgr3G42010\_Mycgr3T

Mycgr3G29582 Mycgr3T
  
Location: 8669-8915

Mycgr3G29582\_Mycgr3T

Mycgr3G31170 Mycgr3T
  
Location: 9015-9255

Mycgr3G31170\_Mycgr3T

Mycgr3G85924 Mycgr3T
  
Location: 9355-11218

Mycgr3G85924\_Mycgr3T

Mycgr3G71676 Mycgr3T
  
Location: 11318-12494

Mycgr3G71676\_Mycgr3T

Mycgr3G11468 Mycgr3T
  
Location: 12594-13653

Mycgr3G11468\_Mycgr3T

Mycgr3G58567 Mycgr3T
  
Location: 13753-14506

Mycgr3G58567\_Mycgr3T

Mycgr3G100089 Mycgr3
  
Location: 14606-21152

Mycgr3G100089\_Mycgr3

Mycgr3G42698 Mycgr3T
  
Location: 21252-22131

Mycgr3G42698\_Mycgr3T

Mycgr3G71681 Mycgr3T
  
Location: 22231-23461

Mycgr3G71681\_Mycgr3T

Mycgr3G109328 Mycgr3
  
Location: 23561-24239

Mycgr3G109328\_Mycgr3

Mycgr3G104334 Mycgr3
  
Location: 24339-24567

Mycgr3G104334\_Mycgr3

Mycgr3G42715 Mycgr3T
  
Location: 24667-25981

Mycgr3G42715\_Mycgr3T

Mycgr3G92934 Mycgr3T
  
Location: 26081-27593

Mycgr3G92934\_Mycgr3T

Mycgr3G41969 Mycgr3T
  
Location: 27693-29328

Mycgr3G41969\_Mycgr3T

Mycgr3G80635 Mycgr3T
  
Location: 29428-29821

Mycgr3G80635\_Mycgr3T

Mycgr3G41426 Mycgr3T
  
Location: 29921-35255

Mycgr3G41426\_Mycgr3T

Mycgr3G104337 Mycgr3
  
Location: 35355-36108

Mycgr3G104337\_Mycgr3

Mycgr3G71679 Mycgr3T
  
Location: 36208-37300

Mycgr3G71679\_Mycgr3T

Mycgr3G92938 Mycgr3T
  
Location: 37400-38699

Mycgr3G92938\_Mycgr3T

Mycgr3G92941 Mycgr3T
  
Location: 38799-40734

Mycgr3G92941\_Mycgr3T

hypothetical protein
  
Accession: EOA82011
  
Location: 974049-974912
  
 NCBI BlastP on this gene

EOA82011

hypothetical protein
  
Accession: EOA82012
  
Location: 975720-977771
  
 NCBI BlastP on this gene

EOA82012

hypothetical protein
  
Accession: EOA82013
  
Location: 979519-981718
  
 NCBI BlastP on this gene

EOA82013

hypothetical protein
  
Accession: EOA82014
  
Location: 984682-985500
  
 NCBI BlastP on this gene

EOA82014

hypothetical protein
  
Accession: EOA82015
  
Location: 986440-992415
  
  
**BlastP hit with Mycgr3G42010\_Mycgr3T**
  
Percentage identity: 28 %
  
BlastP bit score: 342
  
Sequence coverage: 46 %
  
E-value: 1e-91
  
  
 NCBI BlastP on this gene

EOA82015

hypothetical protein
  
Accession: EOA82016
  
Location: 993139-996942
  
 NCBI BlastP on this gene

EOA82016

hypothetical protein
  
Accession: EOA82017
  
Location: 997318-998644
  
 NCBI BlastP on this gene

EOA82017

hypothetical protein
  
Accession: EOA82018
  
Location: 1000710-1001312
  
 NCBI BlastP on this gene

EOA82018

hypothetical protein
  
Accession: EOA82019
  
Location: 1002401-1003757
  
  
**BlastP hit with Mycgr3G42698\_Mycgr3T**
  
Percentage identity: 31 %
  
BlastP bit score: 165
  
Sequence coverage: 112 %
  
E-value: 4e-44
  
  
 NCBI BlastP on this gene

EOA82019

hypothetical protein
  
Accession: EOA82020
  
Location: 1004603-1005278
  
 NCBI BlastP on this gene

EOA82020

hypothetical protein
  
Accession: EOA82021
  
Location: 1009149-1010700
  
 NCBI BlastP on this gene

EOA82021

hypothetical protein
  
Accession: EOA82022
  
Location: 1011262-1014391
  
 NCBI BlastP on this gene

EOA82022

hypothetical protein
  
Accession: EOA82023
  
Location: 1014930-1016157
  
 NCBI BlastP on this gene

EOA82023

Query: Architecture Search FASTA input

FQ790251 : Botryotinia fuckeliana T4 SuperContig\_87\_1 genomic supercontig.    Total score: 2.0     Cumulative Blast bit score: 500

Hit cluster cross-links:

Mycgr3G85918 Mycgr3T
  
Location: 0-1602

Mycgr3G85918\_Mycgr3T

Mycgr3G42010 Mycgr3T
  
Location: 1702-8569

Mycgr3G42010\_Mycgr3T

Mycgr3G29582 Mycgr3T
  
Location: 8669-8915

Mycgr3G29582\_Mycgr3T

Mycgr3G31170 Mycgr3T
  
Location: 9015-9255

Mycgr3G31170\_Mycgr3T

Mycgr3G85924 Mycgr3T
  
Location: 9355-11218

Mycgr3G85924\_Mycgr3T

Mycgr3G71676 Mycgr3T
  
Location: 11318-12494

Mycgr3G71676\_Mycgr3T

Mycgr3G11468 Mycgr3T
  
Location: 12594-13653

Mycgr3G11468\_Mycgr3T

Mycgr3G58567 Mycgr3T
  
Location: 13753-14506

Mycgr3G58567\_Mycgr3T

Mycgr3G100089 Mycgr3
  
Location: 14606-21152

Mycgr3G100089\_Mycgr3

Mycgr3G42698 Mycgr3T
  
Location: 21252-22131

Mycgr3G42698\_Mycgr3T

Mycgr3G71681 Mycgr3T
  
Location: 22231-23461

Mycgr3G71681\_Mycgr3T

Mycgr3G109328 Mycgr3
  
Location: 23561-24239

Mycgr3G109328\_Mycgr3

Mycgr3G104334 Mycgr3
  
Location: 24339-24567

Mycgr3G104334\_Mycgr3

Mycgr3G42715 Mycgr3T
  
Location: 24667-25981

Mycgr3G42715\_Mycgr3T

Mycgr3G92934 Mycgr3T
  
Location: 26081-27593

Mycgr3G92934\_Mycgr3T

Mycgr3G41969 Mycgr3T
  
Location: 27693-29328

Mycgr3G41969\_Mycgr3T

Mycgr3G80635 Mycgr3T
  
Location: 29428-29821

Mycgr3G80635\_Mycgr3T

Mycgr3G41426 Mycgr3T
  
Location: 29921-35255

Mycgr3G41426\_Mycgr3T

Mycgr3G104337 Mycgr3
  
Location: 35355-36108

Mycgr3G104337\_Mycgr3

Mycgr3G71679 Mycgr3T
  
Location: 36208-37300

Mycgr3G71679\_Mycgr3T

Mycgr3G92938 Mycgr3T
  
Location: 37400-38699

Mycgr3G92938\_Mycgr3T

Mycgr3G92941 Mycgr3T
  
Location: 38799-40734

Mycgr3G92941\_Mycgr3T

hypothetical protein
  
Accession: CCD43012
  
Location: 211510-212708
  
 NCBI BlastP on this gene

BofuT4\_P070880.1

hypothetical protein
  
Accession: CCD43013
  
Location: 213255-213557
  
 NCBI BlastP on this gene

BofuT4\_P070890.1

carbohydrate esterase family 5 protein
  
Accession: CCD43014
  
Location: 214213-215056
  
 NCBI BlastP on this gene

BofuT4P87000007001

hypothetical protein
  
Accession: CCD43015
  
Location: 217665-218261
  
 NCBI BlastP on this gene

BofuT4\_P070910.1

hypothetical protein
  
Accession: CCD43016
  
Location: 227148-227440
  
 NCBI BlastP on this gene

BofuT4\_uP070920.1

similar to prolyl aminopeptidase
  
Accession: CCD43017
  
Location: 227688-228775
  
  
**BlastP hit with Mycgr3G71676\_Mycgr3T**
  
Percentage identity: 50 %
  
BlastP bit score: 313
  
Sequence coverage: 77 %
  
E-value: 7e-101
  
  
 NCBI BlastP on this gene

BofuT4\_P070930.1

similar to tyrosinase central domain protein
  
Accession: CCD43018
  
Location: 234635-236006
  
  
**BlastP hit with Mycgr3G42698\_Mycgr3T**
  
Percentage identity: 36 %
  
BlastP bit score: 187
  
Sequence coverage: 108 %
  
E-value: 1e-52
  
  
 NCBI BlastP on this gene

BofuT4\_P070940.1

similar to monooxygenase FAD-binding
  
Accession: CCD43019
  
Location: 238216-239715
  
 NCBI BlastP on this gene

BofuT4\_P070950.1

hypothetical protein
  
Accession: CCD43020
  
Location: 241893-242458
  
 NCBI BlastP on this gene

BofuT4\_P070960.1

hypothetical protein
  
Accession: CCD43021
  
Location: 243654-244762
  
 NCBI BlastP on this gene

BofuT4\_P070970.1

hypothetical protein
  
Accession: CCD43022
  
Location: 252888-253767
  
 NCBI BlastP on this gene

BofuT4\_P070980.1

Query: Architecture Search FASTA input

KE145357 : Glarea lozoyensis ATCC 20868 chromosome Unknown GLAREA14    Total score: 2.0     Cumulative Blast bit score: 487

Hit cluster cross-links:

Mycgr3G85918 Mycgr3T
  
Location: 0-1602

Mycgr3G85918\_Mycgr3T

Mycgr3G42010 Mycgr3T
  
Location: 1702-8569

Mycgr3G42010\_Mycgr3T

Mycgr3G29582 Mycgr3T
  
Location: 8669-8915

Mycgr3G29582\_Mycgr3T

Mycgr3G31170 Mycgr3T
  
Location: 9015-9255

Mycgr3G31170\_Mycgr3T

Mycgr3G85924 Mycgr3T
  
Location: 9355-11218

Mycgr3G85924\_Mycgr3T

Mycgr3G71676 Mycgr3T
  
Location: 11318-12494

Mycgr3G71676\_Mycgr3T

Mycgr3G11468 Mycgr3T
  
Location: 12594-13653

Mycgr3G11468\_Mycgr3T

Mycgr3G58567 Mycgr3T
  
Location: 13753-14506

Mycgr3G58567\_Mycgr3T

Mycgr3G100089 Mycgr3
  
Location: 14606-21152

Mycgr3G100089\_Mycgr3

Mycgr3G42698 Mycgr3T
  
Location: 21252-22131

Mycgr3G42698\_Mycgr3T

Mycgr3G71681 Mycgr3T
  
Location: 22231-23461

Mycgr3G71681\_Mycgr3T

Mycgr3G109328 Mycgr3
  
Location: 23561-24239

Mycgr3G109328\_Mycgr3

Mycgr3G104334 Mycgr3
  
Location: 24339-24567

Mycgr3G104334\_Mycgr3

Mycgr3G42715 Mycgr3T
  
Location: 24667-25981

Mycgr3G42715\_Mycgr3T

Mycgr3G92934 Mycgr3T
  
Location: 26081-27593

Mycgr3G92934\_Mycgr3T

Mycgr3G41969 Mycgr3T
  
Location: 27693-29328

Mycgr3G41969\_Mycgr3T

Mycgr3G80635 Mycgr3T
  
Location: 29428-29821

Mycgr3G80635\_Mycgr3T

Mycgr3G41426 Mycgr3T
  
Location: 29921-35255

Mycgr3G41426\_Mycgr3T

Mycgr3G104337 Mycgr3
  
Location: 35355-36108

Mycgr3G104337\_Mycgr3

Mycgr3G71679 Mycgr3T
  
Location: 36208-37300

Mycgr3G71679\_Mycgr3T

Mycgr3G92938 Mycgr3T
  
Location: 37400-38699

Mycgr3G92938\_Mycgr3T

Mycgr3G92941 Mycgr3T
  
Location: 38799-40734

Mycgr3G92941\_Mycgr3T

protein of unknown function UPF0157
  
Accession: EPE33993
  
Location: 1264799-1265368
  
 NCBI BlastP on this gene

EPE33993

hypothetical protein
  
Accession: EPE33994
  
Location: 1265848-1267668
  
 NCBI BlastP on this gene

EPE33994

ARM repeat-containing protein
  
Accession: EPE33995
  
Location: 1268795-1272362
  
 NCBI BlastP on this gene

EPE33995

CYTH-like phosphatase
  
Accession: EPE33996
  
Location: 1273482-1275927
  
 NCBI BlastP on this gene

EPE33996

MFS general substrate transporter
  
Accession: EPE33997
  
Location: 1276462-1278357
  
 NCBI BlastP on this gene

EPE33997

(Trans)glycosidase
  
Accession: EPE33998
  
Location: 1279770-1283070
  
 NCBI BlastP on this gene

EPE33998

(Trans)glycosidase
  
Accession: EPE33999
  
Location: 1283830-1285192
  
  
**BlastP hit with Mycgr3G71681\_Mycgr3T**
  
Percentage identity: 48 %
  
BlastP bit score: 405
  
Sequence coverage: 95 %
  
E-value: 5e-135
  
  
 NCBI BlastP on this gene

EPE33999

Acyl-CoA dehydrogenase NM
  
Accession: EPE34000
  
Location: 1285283-1286909
  
 NCBI BlastP on this gene

EPE34000

hypothetical protein
  
Accession: EPE34001
  
Location: 1288145-1289125
  
  
**BlastP hit with Mycgr3G92938\_Mycgr3T**
  
Percentage identity: 29 %
  
BlastP bit score: 82
  
Sequence coverage: 52 %
  
E-value: 2e-14
  
  
 NCBI BlastP on this gene

EPE34001

Six-hairpin glycosidase
  
Accession: EPE34002
  
Location: 1289891-1291353
  
 NCBI BlastP on this gene

EPE34002

FAD/NAD(P)-binding protein
  
Accession: EPE34003
  
Location: 1292836-1294182
  
 NCBI BlastP on this gene

EPE34003

GroES-like protein
  
Accession: EPE34004
  
Location: 1294863-1296047
  
 NCBI BlastP on this gene

EPE34004

hypothetical protein
  
Accession: EPE34005
  
Location: 1297261-1298338
  
 NCBI BlastP on this gene

EPE34005

hypothetical protein
  
Accession: EPE34006
  
Location: 1298808-1299674
  
 NCBI BlastP on this gene

EPE34006

hypothetical protein
  
Accession: EPE34007
  
Location: 1300785-1301447
  
 NCBI BlastP on this gene

EPE34007

hypothetical protein
  
Accession: EPE34008
  
Location: 1301989-1302849
  
 NCBI BlastP on this gene

EPE34008

hypothetical protein
  
Accession: EPE34009
  
Location: 1303987-1306887
  
 NCBI BlastP on this gene

EPE34009

hypothetical protein
  
Accession: EPE34010
  
Location: 1308415-1310471
  
 NCBI BlastP on this gene

EPE34010

Query: Architecture Search FASTA input

KB726994 : Fusarium oxysporum f. sp. cubense race 4 unplaced genomic scaffold scaffold63    Total score: 2.0     Cumulative Blast bit score: 481

Hit cluster cross-links:

Mycgr3G85918 Mycgr3T
  
Location: 0-1602

Mycgr3G85918\_Mycgr3T

Mycgr3G42010 Mycgr3T
  
Location: 1702-8569

Mycgr3G42010\_Mycgr3T

Mycgr3G29582 Mycgr3T
  
Location: 8669-8915

Mycgr3G29582\_Mycgr3T

Mycgr3G31170 Mycgr3T
  
Location: 9015-9255

Mycgr3G31170\_Mycgr3T

Mycgr3G85924 Mycgr3T
  
Location: 9355-11218

Mycgr3G85924\_Mycgr3T

Mycgr3G71676 Mycgr3T
  
Location: 11318-12494

Mycgr3G71676\_Mycgr3T

Mycgr3G11468 Mycgr3T
  
Location: 12594-13653

Mycgr3G11468\_Mycgr3T

Mycgr3G58567 Mycgr3T
  
Location: 13753-14506

Mycgr3G58567\_Mycgr3T

Mycgr3G100089 Mycgr3
  
Location: 14606-21152

Mycgr3G100089\_Mycgr3

Mycgr3G42698 Mycgr3T
  
Location: 21252-22131

Mycgr3G42698\_Mycgr3T

Mycgr3G71681 Mycgr3T
  
Location: 22231-23461

Mycgr3G71681\_Mycgr3T

Mycgr3G109328 Mycgr3
  
Location: 23561-24239

Mycgr3G109328\_Mycgr3

Mycgr3G104334 Mycgr3
  
Location: 24339-24567

Mycgr3G104334\_Mycgr3

Mycgr3G42715 Mycgr3T
  
Location: 24667-25981

Mycgr3G42715\_Mycgr3T

Mycgr3G92934 Mycgr3T
  
Location: 26081-27593

Mycgr3G92934\_Mycgr3T

Mycgr3G41969 Mycgr3T
  
Location: 27693-29328

Mycgr3G41969\_Mycgr3T

Mycgr3G80635 Mycgr3T
  
Location: 29428-29821

Mycgr3G80635\_Mycgr3T

Mycgr3G41426 Mycgr3T
  
Location: 29921-35255

Mycgr3G41426\_Mycgr3T

Mycgr3G104337 Mycgr3
  
Location: 35355-36108

Mycgr3G104337\_Mycgr3

Mycgr3G71679 Mycgr3T
  
Location: 36208-37300

Mycgr3G71679\_Mycgr3T

Mycgr3G92938 Mycgr3T
  
Location: 37400-38699

Mycgr3G92938\_Mycgr3T

Mycgr3G92941 Mycgr3T
  
Location: 38799-40734

Mycgr3G92941\_Mycgr3T

Cytochrome P450 4F6
  
Accession: EMT62888
  
Location: 44200-45957
  
 NCBI BlastP on this gene

EMT62888

NADH-cytochrome b5 reductase 1
  
Accession: EMT62889
  
Location: 46812-48219
  
 NCBI BlastP on this gene

EMT62889

Plasma membrane proteolipid 3
  
Accession: EMT62890
  
Location: 48957-49161
  
 NCBI BlastP on this gene

EMT62890

hypothetical protein
  
Accession: EMT62891
  
Location: 49883-51010
  
 NCBI BlastP on this gene

EMT62891

Plasma membrane proteolipid 3
  
Accession: EMT62892
  
Location: 51124-51328
  
 NCBI BlastP on this gene

EMT62892

hypothetical protein
  
Accession: EMT62893
  
Location: 52050-53648
  
 NCBI BlastP on this gene

EMT62893

3-hydroxybutyryl-CoA dehydrogenase
  
Accession: EMT62894
  
Location: 54696-56507
  
 NCBI BlastP on this gene

EMT62894

3-hydroxybutyryl-CoA dehydrogenase
  
Accession: EMT62895
  
Location: 56761-58409
  
 NCBI BlastP on this gene

EMT62895

hypothetical protein
  
Accession: EMT62896
  
Location: 58702-59915
  
 NCBI BlastP on this gene

EMT62896

Major facilitator superfamily domain-containing protein 7-a
  
Accession: EMT62897
  
Location: 60285-61911
  
 NCBI BlastP on this gene

EMT62897

hypothetical protein
  
Accession: EMT62898
  
Location: 62402-64871
  
  
**BlastP hit with Mycgr3G11468\_Mycgr3T**
  
Percentage identity: 33 %
  
BlastP bit score: 192
  
Sequence coverage: 100 %
  
E-value: 5e-52
  
  
 NCBI BlastP on this gene

EMT62898

Copper amine oxidase 1
  
Accession: EMT62899
  
Location: 65581-67797
  
 NCBI BlastP on this gene

EMT62899

hypothetical protein
  
Accession: EMT62900
  
Location: 68458-69657
  
 NCBI BlastP on this gene

EMT62900

Diacetyl reductase [(R)-acetoin forming]
  
Accession: EMT62901
  
Location: 70059-71378
  
  
**BlastP hit with Mycgr3G71679\_Mycgr3T**
  
Percentage identity: 44 %
  
BlastP bit score: 290
  
Sequence coverage: 100 %
  
E-value: 1e-91
  
  
 NCBI BlastP on this gene

EMT62901

Putative sterigmatocystin biosynthesis monooxygenase stcW
  
Accession: EMT62902
  
Location: 71948-74253
  
 NCBI BlastP on this gene

EMT62902

Salicylate hydroxylase
  
Accession: EMT62903
  
Location: 74374-75589
  
 NCBI BlastP on this gene

EMT62903

Polygalacturonase
  
Accession: EMT62904
  
Location: 77113-78546
  
 NCBI BlastP on this gene

EMT62904

hypothetical protein
  
Accession: EMT62905
  
Location: 82611-83842
  
 NCBI BlastP on this gene

EMT62905

hypothetical protein
  
Accession: EMT62906
  
Location: 84440-85336
  
 NCBI BlastP on this gene

EMT62906

Chitotriosidase-1
  
Accession: EMT62907
  
Location: 86025-89629
  
 NCBI BlastP on this gene

EMT62907

Query: Architecture Search FASTA input

AFQF01000390 : Fusarium oxysporum Fo5176    Total score: 2.0     Cumulative Blast bit score: 481

Hit cluster cross-links:

Mycgr3G85918 Mycgr3T
  
Location: 0-1602

Mycgr3G85918\_Mycgr3T

Mycgr3G42010 Mycgr3T
  
Location: 1702-8569

Mycgr3G42010\_Mycgr3T

Mycgr3G29582 Mycgr3T
  
Location: 8669-8915

Mycgr3G29582\_Mycgr3T

Mycgr3G31170 Mycgr3T
  
Location: 9015-9255

Mycgr3G31170\_Mycgr3T

Mycgr3G85924 Mycgr3T
  
Location: 9355-11218

Mycgr3G85924\_Mycgr3T

Mycgr3G71676 Mycgr3T
  
Location: 11318-12494

Mycgr3G71676\_Mycgr3T

Mycgr3G11468 Mycgr3T
  
Location: 12594-13653

Mycgr3G11468\_Mycgr3T

Mycgr3G58567 Mycgr3T
  
Location: 13753-14506

Mycgr3G58567\_Mycgr3T

Mycgr3G100089 Mycgr3
  
Location: 14606-21152

Mycgr3G100089\_Mycgr3

Mycgr3G42698 Mycgr3T
  
Location: 21252-22131

Mycgr3G42698\_Mycgr3T

Mycgr3G71681 Mycgr3T
  
Location: 22231-23461

Mycgr3G71681\_Mycgr3T

Mycgr3G109328 Mycgr3
  
Location: 23561-24239

Mycgr3G109328\_Mycgr3

Mycgr3G104334 Mycgr3
  
Location: 24339-24567

Mycgr3G104334\_Mycgr3

Mycgr3G42715 Mycgr3T
  
Location: 24667-25981

Mycgr3G42715\_Mycgr3T

Mycgr3G92934 Mycgr3T
  
Location: 26081-27593

Mycgr3G92934\_Mycgr3T

Mycgr3G41969 Mycgr3T
  
Location: 27693-29328

Mycgr3G41969\_Mycgr3T

Mycgr3G80635 Mycgr3T
  
Location: 29428-29821

Mycgr3G80635\_Mycgr3T

Mycgr3G41426 Mycgr3T
  
Location: 29921-35255

Mycgr3G41426\_Mycgr3T

Mycgr3G104337 Mycgr3
  
Location: 35355-36108

Mycgr3G104337\_Mycgr3

Mycgr3G71679 Mycgr3T
  
Location: 36208-37300

Mycgr3G71679\_Mycgr3T

Mycgr3G92938 Mycgr3T
  
Location: 37400-38699

Mycgr3G92938\_Mycgr3T

Mycgr3G92941 Mycgr3T
  
Location: 38799-40734

Mycgr3G92941\_Mycgr3T

hypothetical protein
  
Accession: EGU88480
  
Location: 1039-2644
  
 NCBI BlastP on this gene

EGU88480

hypothetical protein
  
Accession: EGU88481
  
Location: 3135-5602
  
  
**BlastP hit with Mycgr3G11468\_Mycgr3T**
  
Percentage identity: 34 %
  
BlastP bit score: 187
  
Sequence coverage: 92 %
  
E-value: 3e-50
  
  
 NCBI BlastP on this gene

EGU88481

hypothetical protein
  
Accession: EGU88482
  
Location: 6226-8525
  
 NCBI BlastP on this gene

EGU88482

hypothetical protein
  
Accession: EGU88483
  
Location: 9353-10553
  
 NCBI BlastP on this gene

EGU88483

hypothetical protein
  
Accession: EGU88484
  
Location: 10956-12288
  
  
**BlastP hit with Mycgr3G71679\_Mycgr3T**
  
Percentage identity: 43 %
  
BlastP bit score: 295
  
Sequence coverage: 103 %
  
E-value: 2e-93
  
  
 NCBI BlastP on this gene

EGU88484

hypothetical protein
  
Accession: EGU88485
  
Location: 12847-15152
  
 NCBI BlastP on this gene

EGU88485

hypothetical protein
  
Accession: EGU88486
  
Location: 15273-16662
  
 NCBI BlastP on this gene

EGU88486

hypothetical protein
  
Accession: EGU88487
  
Location: 18010-19443
  
 NCBI BlastP on this gene

EGU88487

hypothetical protein
  
Accession: EGU88488
  
Location: 20280-21292
  
 NCBI BlastP on this gene

EGU88488

hypothetical protein
  
Accession: EGU88489
  
Location: 21786-23017
  
 NCBI BlastP on this gene

EGU88489

hypothetical protein
  
Accession: EGU88490
  
Location: 23615-24511
  
 NCBI BlastP on this gene

EGU88490

hypothetical protein
  
Accession: EGU88491
  
Location: 25203-28837
  
 NCBI BlastP on this gene

EGU88491

hypothetical protein
  
Accession: EGU88492
  
Location: 30123-31926
  
 NCBI BlastP on this gene

EGU88492

Query: Architecture Search FASTA input

KB730083 : Fusarium oxysporum f. sp. cubense race 1 unplaced genomic scaffold scaffold97    Total score: 2.0     Cumulative Blast bit score: 477

Hit cluster cross-links:

Mycgr3G85918 Mycgr3T
  
Location: 0-1602

Mycgr3G85918\_Mycgr3T

Mycgr3G42010 Mycgr3T
  
Location: 1702-8569

Mycgr3G42010\_Mycgr3T

Mycgr3G29582 Mycgr3T
  
Location: 8669-8915

Mycgr3G29582\_Mycgr3T

Mycgr3G31170 Mycgr3T
  
Location: 9015-9255

Mycgr3G31170\_Mycgr3T

Mycgr3G85924 Mycgr3T
  
Location: 9355-11218

Mycgr3G85924\_Mycgr3T

Mycgr3G71676 Mycgr3T
  
Location: 11318-12494

Mycgr3G71676\_Mycgr3T

Mycgr3G11468 Mycgr3T
  
Location: 12594-13653

Mycgr3G11468\_Mycgr3T

Mycgr3G58567 Mycgr3T
  
Location: 13753-14506

Mycgr3G58567\_Mycgr3T

Mycgr3G100089 Mycgr3
  
Location: 14606-21152

Mycgr3G100089\_Mycgr3

Mycgr3G42698 Mycgr3T
  
Location: 21252-22131

Mycgr3G42698\_Mycgr3T

Mycgr3G71681 Mycgr3T
  
Location: 22231-23461

Mycgr3G71681\_Mycgr3T

Mycgr3G109328 Mycgr3
  
Location: 23561-24239

Mycgr3G109328\_Mycgr3

Mycgr3G104334 Mycgr3
  
Location: 24339-24567

Mycgr3G104334\_Mycgr3

Mycgr3G42715 Mycgr3T
  
Location: 24667-25981

Mycgr3G42715\_Mycgr3T

Mycgr3G92934 Mycgr3T
  
Location: 26081-27593

Mycgr3G92934\_Mycgr3T

Mycgr3G41969 Mycgr3T
  
Location: 27693-29328

Mycgr3G41969\_Mycgr3T

Mycgr3G80635 Mycgr3T
  
Location: 29428-29821

Mycgr3G80635\_Mycgr3T

Mycgr3G41426 Mycgr3T
  
Location: 29921-35255

Mycgr3G41426\_Mycgr3T

Mycgr3G104337 Mycgr3
  
Location: 35355-36108

Mycgr3G104337\_Mycgr3

Mycgr3G71679 Mycgr3T
  
Location: 36208-37300

Mycgr3G71679\_Mycgr3T

Mycgr3G92938 Mycgr3T
  
Location: 37400-38699

Mycgr3G92938\_Mycgr3T

Mycgr3G92941 Mycgr3T
  
Location: 38799-40734

Mycgr3G92941\_Mycgr3T

Putative transporter C3H1.06c
  
Accession: ENH73486
  
Location: 537867-539559
  
 NCBI BlastP on this gene

ENH73486

Cytochrome P450 4F6
  
Accession: ENH73487
  
Location: 539834-541591
  
 NCBI BlastP on this gene

ENH73487

NADH-cytochrome b5 reductase 1
  
Accession: ENH73488
  
Location: 542448-543855
  
 NCBI BlastP on this gene

ENH73488

Plasma membrane proteolipid 3
  
Accession: ENH73489
  
Location: 544615-544819
  
 NCBI BlastP on this gene

ENH73489

hypothetical protein
  
Accession: ENH73490
  
Location: 545539-547135
  
 NCBI BlastP on this gene

ENH73490

3-hydroxybutyryl-CoA dehydrogenase
  
Accession: ENH73491
  
Location: 548192-550003
  
 NCBI BlastP on this gene

ENH73491

hypothetical protein
  
Accession: ENH73492
  
Location: 550296-551513
  
 NCBI BlastP on this gene

ENH73492

Major facilitator superfamily domain-containing protein 7-a
  
Accession: ENH73493
  
Location: 551904-553509
  
 NCBI BlastP on this gene

ENH73493

hypothetical protein
  
Accession: ENH73494
  
Location: 554000-556430
  
  
**BlastP hit with Mycgr3G11468\_Mycgr3T**
  
Percentage identity: 34 %
  
BlastP bit score: 187
  
Sequence coverage: 92 %
  
E-value: 4e-50
  
  
 NCBI BlastP on this gene

ENH73494

Copper amine oxidase 1
  
Accession: ENH73495
  
Location: 557145-559359
  
 NCBI BlastP on this gene

ENH73495

hypothetical protein
  
Accession: ENH73496
  
Location: 560192-561392
  
 NCBI BlastP on this gene

ENH73496

Diacetyl reductase [(R)-acetoin forming]
  
Accession: ENH73497
  
Location: 561796-563116
  
  
**BlastP hit with Mycgr3G71679\_Mycgr3T**
  
Percentage identity: 44 %
  
BlastP bit score: 290
  
Sequence coverage: 99 %
  
E-value: 1e-91
  
  
 NCBI BlastP on this gene

ENH73497

Putative sterigmatocystin biosynthesis monooxygenase stcW
  
Accession: ENH73498
  
Location: 563687-565992
  
 NCBI BlastP on this gene

ENH73498

Salicylate hydroxylase
  
Accession: ENH73499
  
Location: 566113-567328
  
 NCBI BlastP on this gene

ENH73499

hypothetical protein
  
Accession: ENH73500
  
Location: 568863-570296
  
 NCBI BlastP on this gene

ENH73500

hypothetical protein
  
Accession: ENH73501
  
Location: 571126-572106
  
 NCBI BlastP on this gene

ENH73501

hypothetical protein
  
Accession: ENH73502
  
Location: 572617-573848
  
 NCBI BlastP on this gene

ENH73502

hypothetical protein
  
Accession: ENH73503
  
Location: 574423-575316
  
 NCBI BlastP on this gene

ENH73503

Putative chitinase 3
  
Accession: ENH73504
  
Location: 576020-579590
  
 NCBI BlastP on this gene

ENH73504

Lectin-B
  
Accession: ENH73505
  
Location: 580906-582666
  
 NCBI BlastP on this gene

ENH73505

Query: Architecture Search FASTA input

AQGS01001233 : Dactylellina haptotyla CBS 200.50    Total score: 2.0     Cumulative Blast bit score: 424

Hit cluster cross-links:

Mycgr3G85918 Mycgr3T
  
Location: 0-1602

Mycgr3G85918\_Mycgr3T

Mycgr3G42010 Mycgr3T
  
Location: 1702-8569

Mycgr3G42010\_Mycgr3T

Mycgr3G29582 Mycgr3T
  
Location: 8669-8915

Mycgr3G29582\_Mycgr3T

Mycgr3G31170 Mycgr3T
  
Location: 9015-9255

Mycgr3G31170\_Mycgr3T

Mycgr3G85924 Mycgr3T
  
Location: 9355-11218

Mycgr3G85924\_Mycgr3T

Mycgr3G71676 Mycgr3T
  
Location: 11318-12494

Mycgr3G71676\_Mycgr3T

Mycgr3G11468 Mycgr3T
  
Location: 12594-13653

Mycgr3G11468\_Mycgr3T

Mycgr3G58567 Mycgr3T
  
Location: 13753-14506

Mycgr3G58567\_Mycgr3T

Mycgr3G100089 Mycgr3
  
Location: 14606-21152

Mycgr3G100089\_Mycgr3

Mycgr3G42698 Mycgr3T
  
Location: 21252-22131

Mycgr3G42698\_Mycgr3T

Mycgr3G71681 Mycgr3T
  
Location: 22231-23461

Mycgr3G71681\_Mycgr3T

Mycgr3G109328 Mycgr3
  
Location: 23561-24239

Mycgr3G109328\_Mycgr3

Mycgr3G104334 Mycgr3
  
Location: 24339-24567

Mycgr3G104334\_Mycgr3

Mycgr3G42715 Mycgr3T
  
Location: 24667-25981

Mycgr3G42715\_Mycgr3T

Mycgr3G92934 Mycgr3T
  
Location: 26081-27593

Mycgr3G92934\_Mycgr3T

Mycgr3G41969 Mycgr3T
  
Location: 27693-29328

Mycgr3G41969\_Mycgr3T

Mycgr3G80635 Mycgr3T
  
Location: 29428-29821

Mycgr3G80635\_Mycgr3T

Mycgr3G41426 Mycgr3T
  
Location: 29921-35255

Mycgr3G41426\_Mycgr3T

Mycgr3G104337 Mycgr3
  
Location: 35355-36108

Mycgr3G104337\_Mycgr3

Mycgr3G71679 Mycgr3T
  
Location: 36208-37300

Mycgr3G71679\_Mycgr3T

Mycgr3G92938 Mycgr3T
  
Location: 37400-38699

Mycgr3G92938\_Mycgr3T

Mycgr3G92941 Mycgr3T
  
Location: 38799-40734

Mycgr3G92941\_Mycgr3T

hypothetical protein
  
Accession: EPS35104
  
Location: 526683-528662
  
 NCBI BlastP on this gene

EPS35104

hypothetical protein
  
Accession: EPS35120
  
Location: 523879-524976
  
 NCBI BlastP on this gene

EPS35120

hypothetical protein
  
Accession: EPS35101
  
Location: 522209-522894
  
 NCBI BlastP on this gene

EPS35101

hypothetical protein
  
Accession: EPS35211
  
Location: 518856-520495
  
 NCBI BlastP on this gene

EPS35211

hypothetical protein
  
Accession: EPS35063
  
Location: 514581-515090
  
 NCBI BlastP on this gene

EPS35063

hypothetical protein
  
Accession: EPS35189
  
Location: 509923-513603
  
 NCBI BlastP on this gene

EPS35189

hypothetical protein
  
Accession: EPS35114
  
Location: 506883-508427
  
  
**BlastP hit with Mycgr3G71679\_Mycgr3T**
  
Percentage identity: 41 %
  
BlastP bit score: 273
  
Sequence coverage: 102 %
  
E-value: 4e-85
  
  
 NCBI BlastP on this gene

EPS35114

hypothetical protein
  
Accession: EPS35277
  
Location: 500798-502462
  
  
**BlastP hit with Mycgr3G109328\_Mycgr3**
  
Percentage identity: 40 %
  
BlastP bit score: 151
  
Sequence coverage: 98 %
  
E-value: 1e-40
  
  
 NCBI BlastP on this gene

EPS35277

hypothetical protein
  
Accession: EPS35065
  
Location: 493471-493895
  
 NCBI BlastP on this gene

EPS35065

hypothetical protein
  
Accession: EPS35222
  
Location: 486985-488935
  
 NCBI BlastP on this gene

EPS35222

hypothetical protein
  
Accession: EPS35158
  
Location: 484168-485715
  
 NCBI BlastP on this gene

EPS35158

Query: Architecture Search FASTA input

ADOT01000322 : Arthrobotrys oligospora ATCC 24927    Total score: 2.0     Cumulative Blast bit score: 424

Hit cluster cross-links:

Mycgr3G85918 Mycgr3T
  
Location: 0-1602

Mycgr3G85918\_Mycgr3T

Mycgr3G42010 Mycgr3T
  
Location: 1702-8569

Mycgr3G42010\_Mycgr3T

Mycgr3G29582 Mycgr3T
  
Location: 8669-8915

Mycgr3G29582\_Mycgr3T

Mycgr3G31170 Mycgr3T
  
Location: 9015-9255

Mycgr3G31170\_Mycgr3T

Mycgr3G85924 Mycgr3T
  
Location: 9355-11218

Mycgr3G85924\_Mycgr3T

Mycgr3G71676 Mycgr3T
  
Location: 11318-12494

Mycgr3G71676\_Mycgr3T

Mycgr3G11468 Mycgr3T
  
Location: 12594-13653

Mycgr3G11468\_Mycgr3T

Mycgr3G58567 Mycgr3T
  
Location: 13753-14506

Mycgr3G58567\_Mycgr3T

Mycgr3G100089 Mycgr3
  
Location: 14606-21152

Mycgr3G100089\_Mycgr3

Mycgr3G42698 Mycgr3T
  
Location: 21252-22131

Mycgr3G42698\_Mycgr3T

Mycgr3G71681 Mycgr3T
  
Location: 22231-23461

Mycgr3G71681\_Mycgr3T

Mycgr3G109328 Mycgr3
  
Location: 23561-24239

Mycgr3G109328\_Mycgr3

Mycgr3G104334 Mycgr3
  
Location: 24339-24567

Mycgr3G104334\_Mycgr3

Mycgr3G42715 Mycgr3T
  
Location: 24667-25981

Mycgr3G42715\_Mycgr3T

Mycgr3G92934 Mycgr3T
  
Location: 26081-27593

Mycgr3G92934\_Mycgr3T

Mycgr3G41969 Mycgr3T
  
Location: 27693-29328

Mycgr3G41969\_Mycgr3T

Mycgr3G80635 Mycgr3T
  
Location: 29428-29821

Mycgr3G80635\_Mycgr3T

Mycgr3G41426 Mycgr3T
  
Location: 29921-35255

Mycgr3G41426\_Mycgr3T

Mycgr3G104337 Mycgr3
  
Location: 35355-36108

Mycgr3G104337\_Mycgr3

Mycgr3G71679 Mycgr3T
  
Location: 36208-37300

Mycgr3G71679\_Mycgr3T

Mycgr3G92938 Mycgr3T
  
Location: 37400-38699

Mycgr3G92938\_Mycgr3T

Mycgr3G92941 Mycgr3T
  
Location: 38799-40734

Mycgr3G92941\_Mycgr3T

hypothetical protein
  
Accession: EGX43038
  
Location: 244651-248227
  
 NCBI BlastP on this gene

EGX43038

hypothetical protein
  
Accession: EGX43039
  
Location: 248684-249401
  
 NCBI BlastP on this gene

EGX43039

hypothetical protein
  
Accession: EGX43040
  
Location: 251006-252876
  
 NCBI BlastP on this gene

EGX43040

hypothetical protein
  
Accession: EGX43041
  
Location: 257035-257587
  
 NCBI BlastP on this gene

EGX43041

hypothetical protein
  
Accession: EGX43042
  
Location: 258723-262362
  
 NCBI BlastP on this gene

EGX43042

hypothetical protein
  
Accession: EGX43043
  
Location: 263610-265233
  
  
**BlastP hit with Mycgr3G71679\_Mycgr3T**
  
Percentage identity: 40 %
  
BlastP bit score: 265
  
Sequence coverage: 102 %
  
E-value: 8e-82
  
  
 NCBI BlastP on this gene

EGX43043

hypothetical protein
  
Accession: EGX43044
  
Location: 268381-270039
  
  
**BlastP hit with Mycgr3G109328\_Mycgr3**
  
Percentage identity: 38 %
  
BlastP bit score: 159
  
Sequence coverage: 110 %
  
E-value: 2e-43
  
  
 NCBI BlastP on this gene

EGX43044

hypothetical protein
  
Accession: EGX43045
  
Location: 282221-284255
  
 NCBI BlastP on this gene

EGX43045

hypothetical protein
  
Accession: EGX43046
  
Location: 284927-285871
  
 NCBI BlastP on this gene

EGX43046

hypothetical protein
  
Accession: EGX43047
  
Location: 288454-289708
  
 NCBI BlastP on this gene

EGX43047

Query: Architecture Search FASTA input

JH795283 : Magnaporthe oryzae P131 unplaced genomic scaffold P131\_scaffold00916    Total score: 2.0     Cumulative Blast bit score: 415

Hit cluster cross-links:

Mycgr3G85918 Mycgr3T
  
Location: 0-1602

Mycgr3G85918\_Mycgr3T

Mycgr3G42010 Mycgr3T
  
Location: 1702-8569

Mycgr3G42010\_Mycgr3T

Mycgr3G29582 Mycgr3T
  
Location: 8669-8915

Mycgr3G29582\_Mycgr3T

Mycgr3G31170 Mycgr3T
  
Location: 9015-9255

Mycgr3G31170\_Mycgr3T

Mycgr3G85924 Mycgr3T
  
Location: 9355-11218

Mycgr3G85924\_Mycgr3T

Mycgr3G71676 Mycgr3T
  
Location: 11318-12494

Mycgr3G71676\_Mycgr3T

Mycgr3G11468 Mycgr3T
  
Location: 12594-13653

Mycgr3G11468\_Mycgr3T

Mycgr3G58567 Mycgr3T
  
Location: 13753-14506

Mycgr3G58567\_Mycgr3T

Mycgr3G100089 Mycgr3
  
Location: 14606-21152

Mycgr3G100089\_Mycgr3

Mycgr3G42698 Mycgr3T
  
Location: 21252-22131

Mycgr3G42698\_Mycgr3T

Mycgr3G71681 Mycgr3T
  
Location: 22231-23461

Mycgr3G71681\_Mycgr3T

Mycgr3G109328 Mycgr3
  
Location: 23561-24239

Mycgr3G109328\_Mycgr3

Mycgr3G104334 Mycgr3
  
Location: 24339-24567

Mycgr3G104334\_Mycgr3

Mycgr3G42715 Mycgr3T
  
Location: 24667-25981

Mycgr3G42715\_Mycgr3T

Mycgr3G92934 Mycgr3T
  
Location: 26081-27593

Mycgr3G92934\_Mycgr3T

Mycgr3G41969 Mycgr3T
  
Location: 27693-29328

Mycgr3G41969\_Mycgr3T

Mycgr3G80635 Mycgr3T
  
Location: 29428-29821

Mycgr3G80635\_Mycgr3T

Mycgr3G41426 Mycgr3T
  
Location: 29921-35255

Mycgr3G41426\_Mycgr3T

Mycgr3G104337 Mycgr3
  
Location: 35355-36108

Mycgr3G104337\_Mycgr3

Mycgr3G71679 Mycgr3T
  
Location: 36208-37300

Mycgr3G71679\_Mycgr3T

Mycgr3G92938 Mycgr3T
  
Location: 37400-38699

Mycgr3G92938\_Mycgr3T

Mycgr3G92941 Mycgr3T
  
Location: 38799-40734

Mycgr3G92941\_Mycgr3T

hypothetical protein
  
Accession: ELQ63939
  
Location: 34774-35448
  
 NCBI BlastP on this gene

ELQ63939

pre-rRNA-processing protein ESF1
  
Accession: ELQ63940
  
Location: 36386-38871
  
 NCBI BlastP on this gene

ELQ63940

hypothetical protein
  
Accession: ELQ63941
  
Location: 39458-41518
  
 NCBI BlastP on this gene

ELQ63941

phenylacetone monooxygenase
  
Accession: ELQ63942
  
Location: 42381-46001
  
 NCBI BlastP on this gene

ELQ63942

xanthoxin dehydrogenase
  
Accession: ELQ63943
  
Location: 46686-47618
  
 NCBI BlastP on this gene

ELQ63943

(R)-specific carbonyl reductase
  
Accession: ELQ63944
  
Location: 47670-49079
  
 NCBI BlastP on this gene

ELQ63944

hypothetical protein
  
Accession: ELQ63945
  
Location: 49990-51778
  
 NCBI BlastP on this gene

ELQ63945

isotrichodermin C-15 hydroxylase
  
Accession: ELQ63946
  
Location: 51952-53776
  
 NCBI BlastP on this gene

ELQ63946

AAA family ATPase
  
Accession: ELQ63947
  
Location: 54743-59360
  
  
**BlastP hit with Mycgr3G42010\_Mycgr3T**
  
Percentage identity: 42 %
  
BlastP bit score: 343
  
Sequence coverage: 21 %
  
E-value: 2e-94
  
  
 NCBI BlastP on this gene

ELQ63947

hypothetical protein
  
Accession: ELQ63948
  
Location: 60798-61506
  
  
**BlastP hit with Mycgr3G92938\_Mycgr3T**
  
Percentage identity: 28 %
  
BlastP bit score: 72
  
Sequence coverage: 48 %
  
E-value: 2e-11
  
  
 NCBI BlastP on this gene

ELQ63948

Query: Architecture Search FASTA input

JH793600 : Magnaporthe oryzae Y34 unplaced genomic scaffold Y34\_scaffold00217    Total score: 2.0     Cumulative Blast bit score: 415

Hit cluster cross-links:

Mycgr3G85918 Mycgr3T
  
Location: 0-1602

Mycgr3G85918\_Mycgr3T

Mycgr3G42010 Mycgr3T
  
Location: 1702-8569

Mycgr3G42010\_Mycgr3T

Mycgr3G29582 Mycgr3T
  
Location: 8669-8915

Mycgr3G29582\_Mycgr3T

Mycgr3G31170 Mycgr3T
  
Location: 9015-9255

Mycgr3G31170\_Mycgr3T

Mycgr3G85924 Mycgr3T
  
Location: 9355-11218

Mycgr3G85924\_Mycgr3T

Mycgr3G71676 Mycgr3T
  
Location: 11318-12494

Mycgr3G71676\_Mycgr3T

Mycgr3G11468 Mycgr3T
  
Location: 12594-13653

Mycgr3G11468\_Mycgr3T

Mycgr3G58567 Mycgr3T
  
Location: 13753-14506

Mycgr3G58567\_Mycgr3T

Mycgr3G100089 Mycgr3
  
Location: 14606-21152

Mycgr3G100089\_Mycgr3

Mycgr3G42698 Mycgr3T
  
Location: 21252-22131

Mycgr3G42698\_Mycgr3T

Mycgr3G71681 Mycgr3T
  
Location: 22231-23461

Mycgr3G71681\_Mycgr3T

Mycgr3G109328 Mycgr3
  
Location: 23561-24239

Mycgr3G109328\_Mycgr3

Mycgr3G104334 Mycgr3
  
Location: 24339-24567

Mycgr3G104334\_Mycgr3

Mycgr3G42715 Mycgr3T
  
Location: 24667-25981

Mycgr3G42715\_Mycgr3T

Mycgr3G92934 Mycgr3T
  
Location: 26081-27593

Mycgr3G92934\_Mycgr3T

Mycgr3G41969 Mycgr3T
  
Location: 27693-29328

Mycgr3G41969\_Mycgr3T

Mycgr3G80635 Mycgr3T
  
Location: 29428-29821

Mycgr3G80635\_Mycgr3T

Mycgr3G41426 Mycgr3T
  
Location: 29921-35255

Mycgr3G41426\_Mycgr3T

Mycgr3G104337 Mycgr3
  
Location: 35355-36108

Mycgr3G104337\_Mycgr3

Mycgr3G71679 Mycgr3T
  
Location: 36208-37300

Mycgr3G71679\_Mycgr3T

Mycgr3G92938 Mycgr3T
  
Location: 37400-38699

Mycgr3G92938\_Mycgr3T

Mycgr3G92941 Mycgr3T
  
Location: 38799-40734

Mycgr3G92941\_Mycgr3T

glycerol-3-phosphate dehydrogenase
  
Accession: ELQ42264
  
Location: 15484-16855
  
 NCBI BlastP on this gene

ELQ42264

hypothetical protein
  
Accession: ELQ42265
  
Location: 18467-18697
  
 NCBI BlastP on this gene

ELQ42265

hypothetical protein
  
Accession: ELQ42266
  
Location: 18980-20384
  
 NCBI BlastP on this gene

ELQ42266

hypothetical protein
  
Accession: ELQ42267
  
Location: 21055-22410
  
 NCBI BlastP on this gene

ELQ42267

glycogen debranching enzyme
  
Accession: ELQ42268
  
Location: 24524-29770
  
 NCBI BlastP on this gene

ELQ42268

hypothetical protein
  
Accession: ELQ42269
  
Location: 30908-31776
  
 NCBI BlastP on this gene

ELQ42269

hypothetical protein
  
Accession: ELQ42270
  
Location: 33562-33708
  
 NCBI BlastP on this gene

ELQ42270

hypothetical protein
  
Accession: ELQ42271
  
Location: 35058-35766
  
  
**BlastP hit with Mycgr3G92938\_Mycgr3T**
  
Percentage identity: 28 %
  
BlastP bit score: 72
  
Sequence coverage: 48 %
  
E-value: 2e-11
  
  
 NCBI BlastP on this gene

ELQ42271

AAA family ATPase
  
Accession: ELQ42272
  
Location: 37204-41821
  
  
**BlastP hit with Mycgr3G42010\_Mycgr3T**
  
Percentage identity: 42 %
  
BlastP bit score: 343
  
Sequence coverage: 21 %
  
E-value: 2e-94
  
  
 NCBI BlastP on this gene

ELQ42272

isotrichodermin C-15 hydroxylase
  
Accession: ELQ42273
  
Location: 42788-44612
  
 NCBI BlastP on this gene

ELQ42273

hypothetical protein
  
Accession: ELQ42274
  
Location: 44786-46244
  
 NCBI BlastP on this gene

ELQ42274

(R)-specific carbonyl reductase
  
Accession: ELQ42275
  
Location: 47155-48563
  
 NCBI BlastP on this gene

ELQ42275

xanthoxin dehydrogenase
  
Accession: ELQ42276
  
Location: 48615-49547
  
 NCBI BlastP on this gene

ELQ42276

phenylacetone monooxygenase
  
Accession: ELQ42277
  
Location: 50232-53853
  
 NCBI BlastP on this gene

ELQ42277

hypothetical protein
  
Accession: ELQ42278
  
Location: 54716-56776
  
 NCBI BlastP on this gene

ELQ42278

pre-rRNA-processing protein ESF1
  
Accession: ELQ42279
  
Location: 57363-59848
  
 NCBI BlastP on this gene

ELQ42279

hypothetical protein
  
Accession: ELQ42280
  
Location: 60786-61460
  
 NCBI BlastP on this gene

ELQ42280

Query: Architecture Search FASTA input

CM001235 : Magnaporthe oryzae 70-15 chromosome 5    Total score: 2.0     Cumulative Blast bit score: 403

Hit cluster cross-links:

Mycgr3G85918 Mycgr3T
  
Location: 0-1602

Mycgr3G85918\_Mycgr3T

Mycgr3G42010 Mycgr3T
  
Location: 1702-8569

Mycgr3G42010\_Mycgr3T

Mycgr3G29582 Mycgr3T
  
Location: 8669-8915

Mycgr3G29582\_Mycgr3T

Mycgr3G31170 Mycgr3T
  
Location: 9015-9255

Mycgr3G31170\_Mycgr3T

Mycgr3G85924 Mycgr3T
  
Location: 9355-11218

Mycgr3G85924\_Mycgr3T

Mycgr3G71676 Mycgr3T
  
Location: 11318-12494

Mycgr3G71676\_Mycgr3T

Mycgr3G11468 Mycgr3T
  
Location: 12594-13653

Mycgr3G11468\_Mycgr3T

Mycgr3G58567 Mycgr3T
  
Location: 13753-14506

Mycgr3G58567\_Mycgr3T

Mycgr3G100089 Mycgr3
  
Location: 14606-21152

Mycgr3G100089\_Mycgr3

Mycgr3G42698 Mycgr3T
  
Location: 21252-22131

Mycgr3G42698\_Mycgr3T

Mycgr3G71681 Mycgr3T
  
Location: 22231-23461

Mycgr3G71681\_Mycgr3T

Mycgr3G109328 Mycgr3
  
Location: 23561-24239

Mycgr3G109328\_Mycgr3

Mycgr3G104334 Mycgr3
  
Location: 24339-24567

Mycgr3G104334\_Mycgr3

Mycgr3G42715 Mycgr3T
  
Location: 24667-25981

Mycgr3G42715\_Mycgr3T

Mycgr3G92934 Mycgr3T
  
Location: 26081-27593

Mycgr3G92934\_Mycgr3T

Mycgr3G41969 Mycgr3T
  
Location: 27693-29328

Mycgr3G41969\_Mycgr3T

Mycgr3G80635 Mycgr3T
  
Location: 29428-29821

Mycgr3G80635\_Mycgr3T

Mycgr3G41426 Mycgr3T
  
Location: 29921-35255

Mycgr3G41426\_Mycgr3T

Mycgr3G104337 Mycgr3
  
Location: 35355-36108

Mycgr3G104337\_Mycgr3

Mycgr3G71679 Mycgr3T
  
Location: 36208-37300

Mycgr3G71679\_Mycgr3T

Mycgr3G92938 Mycgr3T
  
Location: 37400-38699

Mycgr3G92938\_Mycgr3T

Mycgr3G92941 Mycgr3T
  
Location: 38799-40734

Mycgr3G92941\_Mycgr3T

hypothetical protein
  
Accession: EHA49492
  
Location: 4241623-4242903
  
 NCBI BlastP on this gene

EHA49492

hypothetical protein
  
Accession: EHA49493
  
Location: 4243574-4244929
  
 NCBI BlastP on this gene

EHA49493

glycogen debranching enzymye
  
Accession: EHA49494
  
Location: 4249730-4254721
  
 NCBI BlastP on this gene

EHA49494

hypothetical protein
  
Accession: EHA49495
  
Location: 4255861-4256729
  
 NCBI BlastP on this gene

EHA49495

hypothetical protein
  
Accession: EHA49496
  
Location: 4259675-4260722
  
  
**BlastP hit with Mycgr3G92938\_Mycgr3T**
  
Percentage identity: 28 %
  
BlastP bit score: 62
  
Sequence coverage: 49 %
  
E-value: 7e-08
  
  
 NCBI BlastP on this gene

EHA49496

AAA family ATPase
  
Accession: EHA49497
  
Location: 4262160-4264340
  
  
**BlastP hit with Mycgr3G42010\_Mycgr3T**
  
Percentage identity: 46 %
  
BlastP bit score: 341
  
Sequence coverage: 16 %
  
E-value: 8e-97
  
  
 NCBI BlastP on this gene

EHA49497

hypothetical protein
  
Accession: EHA49498
  
Location: 4265299-4266062
  
 NCBI BlastP on this gene

EHA49498

isotrichodermin C-15 hydroxylase
  
Accession: EHA49499
  
Location: 4267744-4269568
  
 NCBI BlastP on this gene

EHA49499

hypothetical protein
  
Accession: EHA49500
  
Location: 4269742-4271203
  
 NCBI BlastP on this gene

EHA49500

R-specific carbonyl reductase
  
Accession: EHA49501
  
Location: 4272554-4273522
  
 NCBI BlastP on this gene

EHA49501

short-chain dehydrogenase/reductase SDR
  
Accession: EHA49502
  
Location: 4273574-4274506
  
 NCBI BlastP on this gene

EHA49502

phenylacetone monooxygenase
  
Accession: EHA49503
  
Location: 4275191-4277014
  
 NCBI BlastP on this gene

EHA49503

metallo-beta-lactamase superfamily protein
  
Accession: EHA49504
  
Location: 4277634-4278812
  
 NCBI BlastP on this gene

EHA49504

hypothetical protein
  
Accession: EHA49505
  
Location: 4279675-4281735
  
 NCBI BlastP on this gene

EHA49505

hypothetical protein
  
Accession: EHA49506
  
Location: 4282323-4284509
  
 NCBI BlastP on this gene

EHA49506

Query: Architecture Search FASTA input

JH767594 : Coniosporium apollinis CBS 100218 chromosome Unknown supercont1.41    Total score: 2.0     Cumulative Blast bit score: 393

Hit cluster cross-links:

Mycgr3G85918 Mycgr3T
  
Location: 0-1602

Mycgr3G85918\_Mycgr3T

Mycgr3G42010 Mycgr3T
  
Location: 1702-8569

Mycgr3G42010\_Mycgr3T

Mycgr3G29582 Mycgr3T
  
Location: 8669-8915

Mycgr3G29582\_Mycgr3T

Mycgr3G31170 Mycgr3T
  
Location: 9015-9255

Mycgr3G31170\_Mycgr3T

Mycgr3G85924 Mycgr3T
  
Location: 9355-11218

Mycgr3G85924\_Mycgr3T

Mycgr3G71676 Mycgr3T
  
Location: 11318-12494

Mycgr3G71676\_Mycgr3T

Mycgr3G11468 Mycgr3T
  
Location: 12594-13653

Mycgr3G11468\_Mycgr3T

Mycgr3G58567 Mycgr3T
  
Location: 13753-14506

Mycgr3G58567\_Mycgr3T

Mycgr3G100089 Mycgr3
  
Location: 14606-21152

Mycgr3G100089\_Mycgr3

Mycgr3G42698 Mycgr3T
  
Location: 21252-22131

Mycgr3G42698\_Mycgr3T

Mycgr3G71681 Mycgr3T
  
Location: 22231-23461

Mycgr3G71681\_Mycgr3T

Mycgr3G109328 Mycgr3
  
Location: 23561-24239

Mycgr3G109328\_Mycgr3

Mycgr3G104334 Mycgr3
  
Location: 24339-24567

Mycgr3G104334\_Mycgr3

Mycgr3G42715 Mycgr3T
  
Location: 24667-25981

Mycgr3G42715\_Mycgr3T

Mycgr3G92934 Mycgr3T
  
Location: 26081-27593

Mycgr3G92934\_Mycgr3T

Mycgr3G41969 Mycgr3T
  
Location: 27693-29328

Mycgr3G41969\_Mycgr3T

Mycgr3G80635 Mycgr3T
  
Location: 29428-29821

Mycgr3G80635\_Mycgr3T

Mycgr3G41426 Mycgr3T
  
Location: 29921-35255

Mycgr3G41426\_Mycgr3T

Mycgr3G104337 Mycgr3
  
Location: 35355-36108

Mycgr3G104337\_Mycgr3

Mycgr3G71679 Mycgr3T
  
Location: 36208-37300

Mycgr3G71679\_Mycgr3T

Mycgr3G92938 Mycgr3T
  
Location: 37400-38699

Mycgr3G92938\_Mycgr3T

Mycgr3G92941 Mycgr3T
  
Location: 38799-40734

Mycgr3G92941\_Mycgr3T

3-isopropylmalate dehydratase
  
Accession: EON68214
  
Location: 56175-58819
  
 NCBI BlastP on this gene

EON68214

hypothetical protein
  
Accession: EON68213
  
Location: 54227-55033
  
 NCBI BlastP on this gene

EON68213

hypothetical protein
  
Accession: EON68212
  
Location: 50668-52239
  
 NCBI BlastP on this gene

EON68212

hypothetical protein
  
Accession: EON68211
  
Location: 48192-49868
  
 NCBI BlastP on this gene

EON68211

AGC/AKT protein kinase
  
Accession: EON68210
  
Location: 45316-47504
  
 NCBI BlastP on this gene

EON68210

glucose-6-phosphate isomerase
  
Accession: EON68209
  
Location: 42719-44553
  
 NCBI BlastP on this gene

EON68209

hypothetical protein
  
Accession: EON68208
  
Location: 41224-42403
  
 NCBI BlastP on this gene

EON68208

hypothetical protein
  
Accession: EON68207
  
Location: 39935-40417
  
 NCBI BlastP on this gene

EON68207

hypothetical protein
  
Accession: EON68206
  
Location: 38826-39748
  
 NCBI BlastP on this gene

EON68206

hypothetical protein
  
Accession: EON68205
  
Location: 37320-38605
  
  
**BlastP hit with Mycgr3G71676\_Mycgr3T**
  
Percentage identity: 35 %
  
BlastP bit score: 204
  
Sequence coverage: 101 %
  
E-value: 8e-58
  
  
 NCBI BlastP on this gene

EON68205

3-oxoacyl-[acyl-carrier protein] reductase
  
Accession: EON68204
  
Location: 36128-36892
  
 NCBI BlastP on this gene

EON68204

hypothetical protein
  
Accession: EON68203
  
Location: 34038-35597
  
 NCBI BlastP on this gene

EON68203

hypothetical protein
  
Accession: EON68202
  
Location: 31497-33429
  
  
**BlastP hit with Mycgr3G85918\_Mycgr3T**
  
Percentage identity: 27 %
  
BlastP bit score: 189
  
Sequence coverage: 106 %
  
E-value: 1e-49
  
  
 NCBI BlastP on this gene

EON68202

Query: Architecture Search FASTA input

GG698901 : Nectria haematococca mpVI 77-13-4 chromosome 1 genomic scaffold NECHAsca\_8\_chr1\_1\_0    Total score: 2.0     Cumulative Blast bit score: 391

Hit cluster cross-links:

Mycgr3G85918 Mycgr3T
  
Location: 0-1602

Mycgr3G85918\_Mycgr3T

Mycgr3G42010 Mycgr3T
  
Location: 1702-8569

Mycgr3G42010\_Mycgr3T

Mycgr3G29582 Mycgr3T
  
Location: 8669-8915

Mycgr3G29582\_Mycgr3T

Mycgr3G31170 Mycgr3T
  
Location: 9015-9255

Mycgr3G31170\_Mycgr3T

Mycgr3G85924 Mycgr3T
  
Location: 9355-11218

Mycgr3G85924\_Mycgr3T

Mycgr3G71676 Mycgr3T
  
Location: 11318-12494

Mycgr3G71676\_Mycgr3T

Mycgr3G11468 Mycgr3T
  
Location: 12594-13653

Mycgr3G11468\_Mycgr3T

Mycgr3G58567 Mycgr3T
  
Location: 13753-14506

Mycgr3G58567\_Mycgr3T

Mycgr3G100089 Mycgr3
  
Location: 14606-21152

Mycgr3G100089\_Mycgr3

Mycgr3G42698 Mycgr3T
  
Location: 21252-22131

Mycgr3G42698\_Mycgr3T

Mycgr3G71681 Mycgr3T
  
Location: 22231-23461

Mycgr3G71681\_Mycgr3T

Mycgr3G109328 Mycgr3
  
Location: 23561-24239

Mycgr3G109328\_Mycgr3

Mycgr3G104334 Mycgr3
  
Location: 24339-24567

Mycgr3G104334\_Mycgr3

Mycgr3G42715 Mycgr3T
  
Location: 24667-25981

Mycgr3G42715\_Mycgr3T

Mycgr3G92934 Mycgr3T
  
Location: 26081-27593

Mycgr3G92934\_Mycgr3T

Mycgr3G41969 Mycgr3T
  
Location: 27693-29328

Mycgr3G41969\_Mycgr3T

Mycgr3G80635 Mycgr3T
  
Location: 29428-29821

Mycgr3G80635\_Mycgr3T

Mycgr3G41426 Mycgr3T
  
Location: 29921-35255

Mycgr3G41426\_Mycgr3T

Mycgr3G104337 Mycgr3
  
Location: 35355-36108

Mycgr3G104337\_Mycgr3

Mycgr3G71679 Mycgr3T
  
Location: 36208-37300

Mycgr3G71679\_Mycgr3T

Mycgr3G92938 Mycgr3T
  
Location: 37400-38699

Mycgr3G92938\_Mycgr3T

Mycgr3G92941 Mycgr3T
  
Location: 38799-40734

Mycgr3G92941\_Mycgr3T

hypothetical protein
  
Accession: EEU44020
  
Location: 97945-99980
  
 NCBI BlastP on this gene

EEU44020

hypothetical protein
  
Accession: EEU44021
  
Location: 100473-101307
  
 NCBI BlastP on this gene

EEU44021

hypothetical protein
  
Accession: EEU43816
  
Location: 101972-103695
  
 NCBI BlastP on this gene

EEU43816

hypothetical protein
  
Accession: EEU44022
  
Location: 104491-106489
  
 NCBI BlastP on this gene

EEU44022

hypothetical protein
  
Accession: EEU44023
  
Location: 108259-108675
  
 NCBI BlastP on this gene

EEU44023

hypothetical protein
  
Accession: EEU43817
  
Location: 109087-110563
  
 NCBI BlastP on this gene

EEU43817

hypothetical protein
  
Accession: EEU43818
  
Location: 111036-112182
  
 NCBI BlastP on this gene

EEU43818

predicted protein
  
Accession: EEU44024
  
Location: 112643-114919
  
 NCBI BlastP on this gene

EEU44024

hypothetical protein
  
Accession: EEU44025
  
Location: 115648-117750
  
  
**BlastP hit with Mycgr3G85918\_Mycgr3T**
  
Percentage identity: 26 %
  
BlastP bit score: 165
  
Sequence coverage: 93 %
  
E-value: 3e-41
  
  
 NCBI BlastP on this gene

EEU44025

hypothetical protein
  
Accession: EEU43819
  
Location: 118742-121136
  
 NCBI BlastP on this gene

EEU43819

hypothetical protein
  
Accession: EEU44026
  
Location: 121696-122965
  
 NCBI BlastP on this gene

EEU44026

hypothetical protein
  
Accession: EEU44027
  
Location: 123445-124696
  
 NCBI BlastP on this gene

EEU44027

hypothetical protein
  
Accession: EEU43820
  
Location: 125309-127397
  
 NCBI BlastP on this gene

EEU43820

hypothetical protein
  
Accession: EEU43821
  
Location: 128020-129344
  
  
**BlastP hit with Mycgr3G71676\_Mycgr3T**
  
Percentage identity: 37 %
  
BlastP bit score: 226
  
Sequence coverage: 100 %
  
E-value: 6e-66
  
  
 NCBI BlastP on this gene

EEU43821

hypothetical protein
  
Accession: EEU44028
  
Location: 129504-132864
  
 NCBI BlastP on this gene

EEU44028

hypothetical protein
  
Accession: EEU43822
  
Location: 134399-135816
  
 NCBI BlastP on this gene

EEU43822

predicted protein
  
Accession: EEU44029
  
Location: 135906-137682
  
 NCBI BlastP on this gene

EEU44029

predicted protein
  
Accession: EEU44030
  
Location: 138773-141960
  
 NCBI BlastP on this gene

EEU44030

hypothetical protein
  
Accession: EEU43823
  
Location: 142511-147286
  
 NCBI BlastP on this gene

EEU43823

Query: Architecture Search FASTA input

DS231619 : Pyrenophora tritici-repentis Pt-1C-BFP supercont1.5 genomic scaffold    Total score: 2.0     Cumulative Blast bit score: 386

Hit cluster cross-links:

Mycgr3G85918 Mycgr3T
  
Location: 0-1602

Mycgr3G85918\_Mycgr3T

Mycgr3G42010 Mycgr3T
  
Location: 1702-8569

Mycgr3G42010\_Mycgr3T

Mycgr3G29582 Mycgr3T
  
Location: 8669-8915

Mycgr3G29582\_Mycgr3T

Mycgr3G31170 Mycgr3T
  
Location: 9015-9255

Mycgr3G31170\_Mycgr3T

Mycgr3G85924 Mycgr3T
  
Location: 9355-11218

Mycgr3G85924\_Mycgr3T

Mycgr3G71676 Mycgr3T
  
Location: 11318-12494

Mycgr3G71676\_Mycgr3T

Mycgr3G11468 Mycgr3T
  
Location: 12594-13653

Mycgr3G11468\_Mycgr3T

Mycgr3G58567 Mycgr3T
  
Location: 13753-14506

Mycgr3G58567\_Mycgr3T

Mycgr3G100089 Mycgr3
  
Location: 14606-21152

Mycgr3G100089\_Mycgr3

Mycgr3G42698 Mycgr3T
  
Location: 21252-22131

Mycgr3G42698\_Mycgr3T

Mycgr3G71681 Mycgr3T
  
Location: 22231-23461

Mycgr3G71681\_Mycgr3T

Mycgr3G109328 Mycgr3
  
Location: 23561-24239

Mycgr3G109328\_Mycgr3

Mycgr3G104334 Mycgr3
  
Location: 24339-24567

Mycgr3G104334\_Mycgr3

Mycgr3G42715 Mycgr3T
  
Location: 24667-25981

Mycgr3G42715\_Mycgr3T

Mycgr3G92934 Mycgr3T
  
Location: 26081-27593

Mycgr3G92934\_Mycgr3T

Mycgr3G41969 Mycgr3T
  
Location: 27693-29328

Mycgr3G41969\_Mycgr3T

Mycgr3G80635 Mycgr3T
  
Location: 29428-29821

Mycgr3G80635\_Mycgr3T

Mycgr3G41426 Mycgr3T
  
Location: 29921-35255

Mycgr3G41426\_Mycgr3T

Mycgr3G104337 Mycgr3
  
Location: 35355-36108

Mycgr3G104337\_Mycgr3

Mycgr3G71679 Mycgr3T
  
Location: 36208-37300

Mycgr3G71679\_Mycgr3T

Mycgr3G92938 Mycgr3T
  
Location: 37400-38699

Mycgr3G92938\_Mycgr3T

Mycgr3G92941 Mycgr3T
  
Location: 38799-40734

Mycgr3G92941\_Mycgr3T

pisatin demethylase
  
Accession: EDU48575
  
Location: 1018451-1020107
  
 NCBI BlastP on this gene

EDU48575

ADP-ribose pyrophosphatase
  
Accession: EDU48576
  
Location: 1020494-1021099
  
 NCBI BlastP on this gene

EDU48576

conserved hypothetical protein
  
Accession: EDU48577
  
Location: 1021683-1022873
  
 NCBI BlastP on this gene

EDU48577

predicted protein
  
Accession: EDU48578
  
Location: 1023273-1023679
  
 NCBI BlastP on this gene

EDU48578

conserved hypothetical protein
  
Accession: EDU48579
  
Location: 1025053-1025905
  
 NCBI BlastP on this gene

EDU48579

conserved hypothetical protein
  
Accession: EDU48580
  
Location: 1026490-1026975
  
 NCBI BlastP on this gene

EDU48580

NADP-dependent alcohol dehydrogenase C
  
Accession: EDU48581
  
Location: 1027225-1028432
  
 NCBI BlastP on this gene

EDU48581

sorbitol dehydrogenase
  
Accession: EDU48582
  
Location: 1029654-1030821
  
  
**BlastP hit with Mycgr3G71679\_Mycgr3T**
  
Percentage identity: 42 %
  
BlastP bit score: 283
  
Sequence coverage: 100 %
  
E-value: 4e-89
  
  
 NCBI BlastP on this gene

EDU48582

serine/threonine-protein kinase GIN4
  
Accession: EDU48583
  
Location: 1031698-1035781
  
 NCBI BlastP on this gene

EDU48583

specific RNA polymerase II transcription factor
  
Accession: EDU48584
  
Location: 1037851-1039074
  
 NCBI BlastP on this gene

EDU48584

predicted protein
  
Accession: EDU48585
  
Location: 1040865-1041158
  
 NCBI BlastP on this gene

EDU48585

predicted protein
  
Accession: EDU48586
  
Location: 1041928-1042847
  
 NCBI BlastP on this gene

EDU48586

predicted protein
  
Accession: EDU48587
  
Location: 1045903-1046459
  
 NCBI BlastP on this gene

EDU48587

conserved hypothetical protein
  
Accession: EDU48588
  
Location: 1050816-1051841
  
  
**BlastP hit with Mycgr3G92941\_Mycgr3T**
  
Percentage identity: 28 %
  
BlastP bit score: 103
  
Sequence coverage: 49 %
  
E-value: 6e-21
  
  
 NCBI BlastP on this gene

EDU48588

conserved hypothetical protein
  
Accession: EDU48589
  
Location: 1052859-1053108
  
 NCBI BlastP on this gene

EDU48589

conserved hypothetical protein
  
Accession: EDU48590
  
Location: 1054794-1057607
  
 NCBI BlastP on this gene

EDU48590

predicted protein
  
Accession: EDU48591
  
Location: 1058730-1059447
  
 NCBI BlastP on this gene

EDU48591

conserved hypothetical protein
  
Accession: EDU48592
  
Location: 1060173-1060850
  
 NCBI BlastP on this gene

EDU48592

structural maintenance of chromosomes protein 5
  
Accession: EDU48593
  
Location: 1061780-1065217
  
 NCBI BlastP on this gene

EDU48593

Query: Architecture Search FASTA input

DF126500 : Aspergillus kawachii IFO 4308 DNA, contig: scaffold00054    Total score: 2.0     Cumulative Blast bit score: 379

Hit cluster cross-links:

Mycgr3G85918 Mycgr3T
  
Location: 0-1602

Mycgr3G85918\_Mycgr3T

Mycgr3G42010 Mycgr3T
  
Location: 1702-8569

Mycgr3G42010\_Mycgr3T

Mycgr3G29582 Mycgr3T
  
Location: 8669-8915

Mycgr3G29582\_Mycgr3T

Mycgr3G31170 Mycgr3T
  
Location: 9015-9255

Mycgr3G31170\_Mycgr3T

Mycgr3G85924 Mycgr3T
  
Location: 9355-11218

Mycgr3G85924\_Mycgr3T

Mycgr3G71676 Mycgr3T
  
Location: 11318-12494

Mycgr3G71676\_Mycgr3T

Mycgr3G11468 Mycgr3T
  
Location: 12594-13653

Mycgr3G11468\_Mycgr3T

Mycgr3G58567 Mycgr3T
  
Location: 13753-14506

Mycgr3G58567\_Mycgr3T

Mycgr3G100089 Mycgr3
  
Location: 14606-21152

Mycgr3G100089\_Mycgr3

Mycgr3G42698 Mycgr3T
  
Location: 21252-22131

Mycgr3G42698\_Mycgr3T

Mycgr3G71681 Mycgr3T
  
Location: 22231-23461

Mycgr3G71681\_Mycgr3T

Mycgr3G109328 Mycgr3
  
Location: 23561-24239

Mycgr3G109328\_Mycgr3

Mycgr3G104334 Mycgr3
  
Location: 24339-24567

Mycgr3G104334\_Mycgr3

Mycgr3G42715 Mycgr3T
  
Location: 24667-25981

Mycgr3G42715\_Mycgr3T

Mycgr3G92934 Mycgr3T
  
Location: 26081-27593

Mycgr3G92934\_Mycgr3T

Mycgr3G41969 Mycgr3T
  
Location: 27693-29328

Mycgr3G41969\_Mycgr3T

Mycgr3G80635 Mycgr3T
  
Location: 29428-29821

Mycgr3G80635\_Mycgr3T

Mycgr3G41426 Mycgr3T
  
Location: 29921-35255

Mycgr3G41426\_Mycgr3T

Mycgr3G104337 Mycgr3
  
Location: 35355-36108

Mycgr3G104337\_Mycgr3

Mycgr3G71679 Mycgr3T
  
Location: 36208-37300

Mycgr3G71679\_Mycgr3T

Mycgr3G92938 Mycgr3T
  
Location: 37400-38699

Mycgr3G92938\_Mycgr3T

Mycgr3G92941 Mycgr3T
  
Location: 38799-40734

Mycgr3G92941\_Mycgr3T

FAD-dependent oxygenase
  
Accession: GAA92674
  
Location: 40182-41803
  
 NCBI BlastP on this gene

GAA92674

decarboxylase Dec1
  
Accession: GAA92675
  
Location: 42160-42912
  
 NCBI BlastP on this gene

GAA92675

GABA permease
  
Accession: GAA92676
  
Location: 44241-46097
  
 NCBI BlastP on this gene

GAA92676

integral membrane protein
  
Accession: GAA92677
  
Location: 47091-48455
  
 NCBI BlastP on this gene

GAA92677

amidase
  
Accession: GAA92678
  
Location: 48662-50316
  
 NCBI BlastP on this gene

GAA92678

hypothetical protein
  
Accession: GAA92679
  
Location: 51836-52840
  
 NCBI BlastP on this gene

GAA92679

hypothetical protein
  
Accession: GAA92680
  
Location: 54071-54490
  
 NCBI BlastP on this gene

GAA92680

MFS transporter
  
Accession: GAA92681
  
Location: 54773-56545
  
  
**BlastP hit with Mycgr3G85918\_Mycgr3T**
  
Percentage identity: 27 %
  
BlastP bit score: 196
  
Sequence coverage: 98 %
  
E-value: 4e-52
  
  
 NCBI BlastP on this gene

GAA92681

similar to An03g00310
  
Accession: GAA92682
  
Location: 57521-58897
  
 NCBI BlastP on this gene

GAA92682

hypothetical protein
  
Accession: GAA92683
  
Location: 60912-63545
  
 NCBI BlastP on this gene

GAA92683

oxidoreductase
  
Accession: GAA92684
  
Location: 69255-70741
  
 NCBI BlastP on this gene

GAA92684

tyrosinase
  
Accession: GAA92685
  
Location: 72315-73693
  
  
**BlastP hit with Mycgr3G42698\_Mycgr3T**
  
Percentage identity: 33 %
  
BlastP bit score: 183
  
Sequence coverage: 111 %
  
E-value: 4e-51
  
  
 NCBI BlastP on this gene

GAA92685

hypothetical protein
  
Accession: GAA92686
  
Location: 73815-74636
  
 NCBI BlastP on this gene

GAA92686

similar to An03g00260
  
Accession: GAA92687
  
Location: 75975-77150
  
 NCBI BlastP on this gene

GAA92687

hypothetical protein
  
Accession: GAA92688
  
Location: 78148-78917
  
 NCBI BlastP on this gene

GAA92688

similar to An03g00240
  
Accession: GAA92689
  
Location: 79814-81848
  
 NCBI BlastP on this gene

GAA92689

amino acid permease
  
Accession: GAA92690
  
Location: 82117-84093
  
 NCBI BlastP on this gene

GAA92690

similar to An03g00210
  
Accession: GAA92691
  
Location: 85207-86077
  
 NCBI BlastP on this gene

GAA92691

hypothetical protein
  
Accession: GAA92692
  
Location: 86148-86846
  
 NCBI BlastP on this gene

GAA92692

Query: Architecture Search FASTA input

ACJE01000015 : Aspergillus niger ATCC 1015    Total score: 2.0     Cumulative Blast bit score: 359

Hit cluster cross-links:

Mycgr3G85918 Mycgr3T
  
Location: 0-1602

Mycgr3G85918\_Mycgr3T

Mycgr3G42010 Mycgr3T
  
Location: 1702-8569

Mycgr3G42010\_Mycgr3T

Mycgr3G29582 Mycgr3T
  
Location: 8669-8915

Mycgr3G29582\_Mycgr3T

Mycgr3G31170 Mycgr3T
  
Location: 9015-9255

Mycgr3G31170\_Mycgr3T

Mycgr3G85924 Mycgr3T
  
Location: 9355-11218

Mycgr3G85924\_Mycgr3T

Mycgr3G71676 Mycgr3T
  
Location: 11318-12494

Mycgr3G71676\_Mycgr3T

Mycgr3G11468 Mycgr3T
  
Location: 12594-13653

Mycgr3G11468\_Mycgr3T

Mycgr3G58567 Mycgr3T
  
Location: 13753-14506

Mycgr3G58567\_Mycgr3T

Mycgr3G100089 Mycgr3
  
Location: 14606-21152

Mycgr3G100089\_Mycgr3

Mycgr3G42698 Mycgr3T
  
Location: 21252-22131

Mycgr3G42698\_Mycgr3T

Mycgr3G71681 Mycgr3T
  
Location: 22231-23461

Mycgr3G71681\_Mycgr3T

Mycgr3G109328 Mycgr3
  
Location: 23561-24239

Mycgr3G109328\_Mycgr3

Mycgr3G104334 Mycgr3
  
Location: 24339-24567

Mycgr3G104334\_Mycgr3

Mycgr3G42715 Mycgr3T
  
Location: 24667-25981

Mycgr3G42715\_Mycgr3T

Mycgr3G92934 Mycgr3T
  
Location: 26081-27593

Mycgr3G92934\_Mycgr3T

Mycgr3G41969 Mycgr3T
  
Location: 27693-29328

Mycgr3G41969\_Mycgr3T

Mycgr3G80635 Mycgr3T
  
Location: 29428-29821

Mycgr3G80635\_Mycgr3T

Mycgr3G41426 Mycgr3T
  
Location: 29921-35255

Mycgr3G41426\_Mycgr3T

Mycgr3G104337 Mycgr3
  
Location: 35355-36108

Mycgr3G104337\_Mycgr3

Mycgr3G71679 Mycgr3T
  
Location: 36208-37300

Mycgr3G71679\_Mycgr3T

Mycgr3G92938 Mycgr3T
  
Location: 37400-38699

Mycgr3G92938\_Mycgr3T

Mycgr3G92941 Mycgr3T
  
Location: 38799-40734

Mycgr3G92941\_Mycgr3T

hypothetical protein
  
Accession: EHA20919
  
Location: 94384-95755
  
 NCBI BlastP on this gene

EHA20919

hypothetical protein
  
Accession: EHA20920
  
Location: 97297-97980
  
 NCBI BlastP on this gene

EHA20920

hypothetical protein
  
Accession: EHA20921
  
Location: 98036-98650
  
 NCBI BlastP on this gene

EHA20921

hypothetical protein
  
Accession: EHA20922
  
Location: 100096-102007
  
 NCBI BlastP on this gene

EHA20922

hypothetical protein
  
Accession: EHA20923
  
Location: 102279-104304
  
 NCBI BlastP on this gene

EHA20923

hypothetical protein
  
Accession: EHA20924
  
Location: 105192-106458
  
 NCBI BlastP on this gene

EHA20924

hypothetical protein
  
Accession: EHA20925
  
Location: 107932-108315
  
 NCBI BlastP on this gene

EHA20925

hypothetical protein
  
Accession: EHA20926
  
Location: 110930-112867
  
  
**BlastP hit with Mycgr3G42698\_Mycgr3T**
  
Percentage identity: 32 %
  
BlastP bit score: 174
  
Sequence coverage: 108 %
  
E-value: 2e-46
  
  
 NCBI BlastP on this gene

EHA20926

hypothetical protein
  
Accession: EHA20927
  
Location: 114451-115946
  
 NCBI BlastP on this gene

EHA20927

hypothetical protein
  
Accession: EHA20928
  
Location: 116436-117707
  
 NCBI BlastP on this gene

EHA20928

hypothetical protein
  
Accession: EHA20929
  
Location: 119093-120469
  
 NCBI BlastP on this gene

EHA20929

hypothetical protein
  
Accession: EHA20930
  
Location: 121375-123204
  
  
**BlastP hit with Mycgr3G85918\_Mycgr3T**
  
Percentage identity: 27 %
  
BlastP bit score: 185
  
Sequence coverage: 100 %
  
E-value: 4e-48
  
  
 NCBI BlastP on this gene

EHA20930

hypothetical protein
  
Accession: EHA20931
  
Location: 123559-123978
  
 NCBI BlastP on this gene

EHA20931

hypothetical protein
  
Accession: EHA20932
  
Location: 124955-126058
  
 NCBI BlastP on this gene

EHA20932

hypothetical protein
  
Accession: EHA20933
  
Location: 126425-127507
  
 NCBI BlastP on this gene

EHA20933

hypothetical protein
  
Accession: EHA20934
  
Location: 127589-128755
  
 NCBI BlastP on this gene

EHA20934

flavo protein monooxygenase
  
Accession: EHA20935
  
Location: 129174-130639
  
 NCBI BlastP on this gene

EHA20935

dehydrogenase
  
Accession: EHA20936
  
Location: 131285-134305
  
 NCBI BlastP on this gene

EHA20936

hypothetical protein
  
Accession: EHA20937
  
Location: 135559-136566
  
 NCBI BlastP on this gene

EHA20937

amidase
  
Accession: EHA20938
  
Location: 137960-139613
  
 NCBI BlastP on this gene

EHA20938

Query: Architecture Search FASTA input

CABT02000006 : Sordaria macrospora k-hell    Total score: 2.0     Cumulative Blast bit score: 350

Hit cluster cross-links:

Mycgr3G85918 Mycgr3T
  
Location: 0-1602

Mycgr3G85918\_Mycgr3T

Mycgr3G42010 Mycgr3T
  
Location: 1702-8569

Mycgr3G42010\_Mycgr3T

Mycgr3G29582 Mycgr3T
  
Location: 8669-8915

Mycgr3G29582\_Mycgr3T

Mycgr3G31170 Mycgr3T
  
Location: 9015-9255

Mycgr3G31170\_Mycgr3T

Mycgr3G85924 Mycgr3T
  
Location: 9355-11218

Mycgr3G85924\_Mycgr3T

Mycgr3G71676 Mycgr3T
  
Location: 11318-12494

Mycgr3G71676\_Mycgr3T

Mycgr3G11468 Mycgr3T
  
Location: 12594-13653

Mycgr3G11468\_Mycgr3T

Mycgr3G58567 Mycgr3T
  
Location: 13753-14506

Mycgr3G58567\_Mycgr3T

Mycgr3G100089 Mycgr3
  
Location: 14606-21152

Mycgr3G100089\_Mycgr3

Mycgr3G42698 Mycgr3T
  
Location: 21252-22131

Mycgr3G42698\_Mycgr3T

Mycgr3G71681 Mycgr3T
  
Location: 22231-23461

Mycgr3G71681\_Mycgr3T

Mycgr3G109328 Mycgr3
  
Location: 23561-24239

Mycgr3G109328\_Mycgr3

Mycgr3G104334 Mycgr3
  
Location: 24339-24567

Mycgr3G104334\_Mycgr3

Mycgr3G42715 Mycgr3T
  
Location: 24667-25981

Mycgr3G42715\_Mycgr3T

Mycgr3G92934 Mycgr3T
  
Location: 26081-27593

Mycgr3G92934\_Mycgr3T

Mycgr3G41969 Mycgr3T
  
Location: 27693-29328

Mycgr3G41969\_Mycgr3T

Mycgr3G80635 Mycgr3T
  
Location: 29428-29821

Mycgr3G80635\_Mycgr3T

Mycgr3G41426 Mycgr3T
  
Location: 29921-35255

Mycgr3G41426\_Mycgr3T

Mycgr3G104337 Mycgr3
  
Location: 35355-36108

Mycgr3G104337\_Mycgr3

Mycgr3G71679 Mycgr3T
  
Location: 36208-37300

Mycgr3G71679\_Mycgr3T

Mycgr3G92938 Mycgr3T
  
Location: 37400-38699

Mycgr3G92938\_Mycgr3T

Mycgr3G92941 Mycgr3T
  
Location: 38799-40734

Mycgr3G92941\_Mycgr3T

not annotated
  
Accession: CCC08656
  
Location: 681209-682253
  
 NCBI BlastP on this gene

CCC08656

putative polyketide synthase
  
Accession: CCC08657
  
Location: 686705-695145
  
 NCBI BlastP on this gene

CCC08657

not annotated
  
Accession: CCC08658
  
Location: 695464-696860
  
  
**BlastP hit with Mycgr3G42698\_Mycgr3T**
  
Percentage identity: 33 %
  
BlastP bit score: 181
  
Sequence coverage: 109 %
  
E-value: 2e-50
  
  
 NCBI BlastP on this gene

CCC08658

not annotated
  
Accession: CCC08659
  
Location: 699699-700597
  
 NCBI BlastP on this gene

CCC08659

not annotated
  
Accession: CCC08660
  
Location: 701218-705508
  
 NCBI BlastP on this gene

CCC08660

not annotated
  
Accession: CCC08661
  
Location: 706548-709476
  
  
**BlastP hit with Mycgr3G11468\_Mycgr3T**
  
Percentage identity: 31 %
  
BlastP bit score: 169
  
Sequence coverage: 103 %
  
E-value: 1e-43
  
  
 NCBI BlastP on this gene

CCC08661

not annotated
  
Accession: CCC08662
  
Location: 709815-710402
  
 NCBI BlastP on this gene

CCC08662

not annotated
  
Accession: CCC08663
  
Location: 711057-712245
  
 NCBI BlastP on this gene

CCC08663

not annotated
  
Accession: CCC08664
  
Location: 713818-714609
  
 NCBI BlastP on this gene

CCC08664

not annotated
  
Accession: CCC08665
  
Location: 721492-722112
  
 NCBI BlastP on this gene

CCC08665

not annotated
  
Accession: CCC08666
  
Location: 725226-726466
  
 NCBI BlastP on this gene

CCC08666

Query: Architecture Search FASTA input

DS231616 : Pyrenophora tritici-repentis Pt-1C-BFP supercont1.2 genomic scaffold    Total score: 2.0     Cumulative Blast bit score: 316

Hit cluster cross-links:

Mycgr3G85918 Mycgr3T
  
Location: 0-1602

Mycgr3G85918\_Mycgr3T

Mycgr3G42010 Mycgr3T
  
Location: 1702-8569

Mycgr3G42010\_Mycgr3T

Mycgr3G29582 Mycgr3T
  
Location: 8669-8915

Mycgr3G29582\_Mycgr3T

Mycgr3G31170 Mycgr3T
  
Location: 9015-9255

Mycgr3G31170\_Mycgr3T

Mycgr3G85924 Mycgr3T
  
Location: 9355-11218

Mycgr3G85924\_Mycgr3T

Mycgr3G71676 Mycgr3T
  
Location: 11318-12494

Mycgr3G71676\_Mycgr3T

Mycgr3G11468 Mycgr3T
  
Location: 12594-13653

Mycgr3G11468\_Mycgr3T

Mycgr3G58567 Mycgr3T
  
Location: 13753-14506

Mycgr3G58567\_Mycgr3T

Mycgr3G100089 Mycgr3
  
Location: 14606-21152

Mycgr3G100089\_Mycgr3

Mycgr3G42698 Mycgr3T
  
Location: 21252-22131

Mycgr3G42698\_Mycgr3T

Mycgr3G71681 Mycgr3T
  
Location: 22231-23461

Mycgr3G71681\_Mycgr3T

Mycgr3G109328 Mycgr3
  
Location: 23561-24239

Mycgr3G109328\_Mycgr3

Mycgr3G104334 Mycgr3
  
Location: 24339-24567

Mycgr3G104334\_Mycgr3

Mycgr3G42715 Mycgr3T
  
Location: 24667-25981

Mycgr3G42715\_Mycgr3T

Mycgr3G92934 Mycgr3T
  
Location: 26081-27593

Mycgr3G92934\_Mycgr3T

Mycgr3G41969 Mycgr3T
  
Location: 27693-29328

Mycgr3G41969\_Mycgr3T

Mycgr3G80635 Mycgr3T
  
Location: 29428-29821

Mycgr3G80635\_Mycgr3T

Mycgr3G41426 Mycgr3T
  
Location: 29921-35255

Mycgr3G41426\_Mycgr3T

Mycgr3G104337 Mycgr3
  
Location: 35355-36108

Mycgr3G104337\_Mycgr3

Mycgr3G71679 Mycgr3T
  
Location: 36208-37300

Mycgr3G71679\_Mycgr3T

Mycgr3G92938 Mycgr3T
  
Location: 37400-38699

Mycgr3G92938\_Mycgr3T

Mycgr3G92941 Mycgr3T
  
Location: 38799-40734

Mycgr3G92941\_Mycgr3T

acetylcholinesterase precursor
  
Accession: EDU44888
  
Location: 224498-226197
  
 NCBI BlastP on this gene

EDU44888

allantoate permease
  
Accession: EDU44889
  
Location: 226761-228473
  
 NCBI BlastP on this gene

EDU44889

alpha-glucosidase precursor
  
Accession: EDU44890
  
Location: 230348-233132
  
 NCBI BlastP on this gene

EDU44890

hypothetical protein
  
Accession: EDU44891
  
Location: 234054-235428
  
 NCBI BlastP on this gene

EDU44891

conserved hypothetical protein
  
Accession: EDU44892
  
Location: 237168-239092
  
 NCBI BlastP on this gene

EDU44892

hypothetical protein
  
Accession: EDU44893
  
Location: 239553-240329
  
  
**BlastP hit with Mycgr3G58567\_Mycgr3T**
  
Percentage identity: 33 %
  
BlastP bit score: 137
  
Sequence coverage: 95 %
  
E-value: 1e-35
  
  
 NCBI BlastP on this gene

EDU44893

SH3 domain containing protein
  
Accession: EDU44894
  
Location: 240809-241602
  
 NCBI BlastP on this gene

EDU44894

isochorismatase hydrolase
  
Accession: EDU44895
  
Location: 244292-245296
  
 NCBI BlastP on this gene

EDU44895

predicted protein
  
Accession: EDU44896
  
Location: 245917-246790
  
 NCBI BlastP on this gene

EDU44896

conserved hypothetical protein
  
Accession: EDU44897
  
Location: 247689-248980
  
  
**BlastP hit with Mycgr3G42698\_Mycgr3T**
  
Percentage identity: 33 %
  
BlastP bit score: 179
  
Sequence coverage: 112 %
  
E-value: 2e-49
  
  
 NCBI BlastP on this gene

EDU44897

conserved hypothetical protein
  
Accession: EDU44898
  
Location: 251224-252665
  
 NCBI BlastP on this gene

EDU44898

predicted protein
  
Accession: EDU44899
  
Location: 254151-255137
  
 NCBI BlastP on this gene

EDU44899

glucan 1,3-beta-glucosidase precursor
  
Accession: EDU44900
  
Location: 259129-260772
  
 NCBI BlastP on this gene

EDU44900

conserved hypothetical protein
  
Accession: EDU44901
  
Location: 261838-262546
  
 NCBI BlastP on this gene

EDU44901

conserved hypothetical protein
  
Accession: EDU44902
  
Location: 262768-264034
  
 NCBI BlastP on this gene

EDU44902

conserved hypothetical protein
  
Accession: EDU44903
  
Location: 266335-267495
  
 NCBI BlastP on this gene

EDU44903

Query: Architecture Search FASTA input

KB445638 : Cochliobolus sativus ND90Pr unplaced genomic scaffold COCSAscaffold\_2    Total score: 2.0     Cumulative Blast bit score: 305

Hit cluster cross-links:

Mycgr3G85918 Mycgr3T
  
Location: 0-1602

Mycgr3G85918\_Mycgr3T

Mycgr3G42010 Mycgr3T
  
Location: 1702-8569

Mycgr3G42010\_Mycgr3T

Mycgr3G29582 Mycgr3T
  
Location: 8669-8915

Mycgr3G29582\_Mycgr3T

Mycgr3G31170 Mycgr3T
  
Location: 9015-9255

Mycgr3G31170\_Mycgr3T

Mycgr3G85924 Mycgr3T
  
Location: 9355-11218

Mycgr3G85924\_Mycgr3T

Mycgr3G71676 Mycgr3T
  
Location: 11318-12494

Mycgr3G71676\_Mycgr3T

Mycgr3G11468 Mycgr3T
  
Location: 12594-13653

Mycgr3G11468\_Mycgr3T

Mycgr3G58567 Mycgr3T
  
Location: 13753-14506

Mycgr3G58567\_Mycgr3T

Mycgr3G100089 Mycgr3
  
Location: 14606-21152

Mycgr3G100089\_Mycgr3

Mycgr3G42698 Mycgr3T
  
Location: 21252-22131

Mycgr3G42698\_Mycgr3T

Mycgr3G71681 Mycgr3T
  
Location: 22231-23461

Mycgr3G71681\_Mycgr3T

Mycgr3G109328 Mycgr3
  
Location: 23561-24239

Mycgr3G109328\_Mycgr3

Mycgr3G104334 Mycgr3
  
Location: 24339-24567

Mycgr3G104334\_Mycgr3

Mycgr3G42715 Mycgr3T
  
Location: 24667-25981

Mycgr3G42715\_Mycgr3T

Mycgr3G92934 Mycgr3T
  
Location: 26081-27593

Mycgr3G92934\_Mycgr3T

Mycgr3G41969 Mycgr3T
  
Location: 27693-29328

Mycgr3G41969\_Mycgr3T

Mycgr3G80635 Mycgr3T
  
Location: 29428-29821

Mycgr3G80635\_Mycgr3T

Mycgr3G41426 Mycgr3T
  
Location: 29921-35255

Mycgr3G41426\_Mycgr3T

Mycgr3G104337 Mycgr3
  
Location: 35355-36108

Mycgr3G104337\_Mycgr3

Mycgr3G71679 Mycgr3T
  
Location: 36208-37300

Mycgr3G71679\_Mycgr3T

Mycgr3G92938 Mycgr3T
  
Location: 37400-38699

Mycgr3G92938\_Mycgr3T

Mycgr3G92941 Mycgr3T
  
Location: 38799-40734

Mycgr3G92941\_Mycgr3T

hypothetical protein
  
Accession: EMD68067
  
Location: 576839-578844
  
 NCBI BlastP on this gene

EMD68067

hypothetical protein
  
Accession: EMD68068
  
Location: 586432-587287
  
 NCBI BlastP on this gene

EMD68068

hypothetical protein
  
Accession: EMD68069
  
Location: 588622-590195
  
 NCBI BlastP on this gene

EMD68069

hypothetical protein
  
Accession: EMD68070
  
Location: 596697-597473
  
  
**BlastP hit with Mycgr3G58567\_Mycgr3T**
  
Percentage identity: 33 %
  
BlastP bit score: 140
  
Sequence coverage: 95 %
  
E-value: 1e-36
  
  
 NCBI BlastP on this gene

EMD68070

hypothetical protein
  
Accession: EMD68071
  
Location: 598001-598770
  
 NCBI BlastP on this gene

EMD68071

hypothetical protein
  
Accession: EMD68072
  
Location: 599328-600635
  
  
**BlastP hit with Mycgr3G42698\_Mycgr3T**
  
Percentage identity: 31 %
  
BlastP bit score: 165
  
Sequence coverage: 112 %
  
E-value: 4e-44
  
  
 NCBI BlastP on this gene

EMD68072

hypothetical protein
  
Accession: EMD68073
  
Location: 603220-604757
  
 NCBI BlastP on this gene

EMD68073

hypothetical protein
  
Accession: EMD68074
  
Location: 605771-606586
  
 NCBI BlastP on this gene

EMD68074

hypothetical protein
  
Accession: EMD68075
  
Location: 608705-608983
  
 NCBI BlastP on this gene

EMD68075

hypothetical protein
  
Accession: EMD68076
  
Location: 610676-611098
  
 NCBI BlastP on this gene

EMD68076

hypothetical protein
  
Accession: EMD68077
  
Location: 611623-612072
  
 NCBI BlastP on this gene

EMD68077

glycosyltransferase family 1 protein
  
Accession: EMD68078
  
Location: 612894-613597
  
 NCBI BlastP on this gene

EMD68078

hypothetical protein
  
Accession: EMD68079
  
Location: 613846-615062
  
 NCBI BlastP on this gene

EMD68079

hypothetical protein
  
Accession: EMD68080
  
Location: 615753-616953
  
 NCBI BlastP on this gene

EMD68080

hypothetical protein
  
Accession: EMD68081
  
Location: 617732-618387
  
 NCBI BlastP on this gene

EMD68081

hypothetical protein
  
Accession: EMD68082
  
Location: 619081-620612
  
 NCBI BlastP on this gene

EMD68082

Query: Architecture Search FASTA input

KB733450 : Bipolaris maydis ATCC 48331 unplaced genomic scaffold COCC4scaffold\_7    Total score: 2.0     Cumulative Blast bit score: 292

Hit cluster cross-links:

Mycgr3G85918 Mycgr3T
  
Location: 0-1602

Mycgr3G85918\_Mycgr3T

Mycgr3G42010 Mycgr3T
  
Location: 1702-8569

Mycgr3G42010\_Mycgr3T

Mycgr3G29582 Mycgr3T
  
Location: 8669-8915

Mycgr3G29582\_Mycgr3T

Mycgr3G31170 Mycgr3T
  
Location: 9015-9255

Mycgr3G31170\_Mycgr3T

Mycgr3G85924 Mycgr3T
  
Location: 9355-11218

Mycgr3G85924\_Mycgr3T

Mycgr3G71676 Mycgr3T
  
Location: 11318-12494

Mycgr3G71676\_Mycgr3T

Mycgr3G11468 Mycgr3T
  
Location: 12594-13653

Mycgr3G11468\_Mycgr3T

Mycgr3G58567 Mycgr3T
  
Location: 13753-14506

Mycgr3G58567\_Mycgr3T

Mycgr3G100089 Mycgr3
  
Location: 14606-21152

Mycgr3G100089\_Mycgr3

Mycgr3G42698 Mycgr3T
  
Location: 21252-22131

Mycgr3G42698\_Mycgr3T

Mycgr3G71681 Mycgr3T
  
Location: 22231-23461

Mycgr3G71681\_Mycgr3T

Mycgr3G109328 Mycgr3
  
Location: 23561-24239

Mycgr3G109328\_Mycgr3

Mycgr3G104334 Mycgr3
  
Location: 24339-24567

Mycgr3G104334\_Mycgr3

Mycgr3G42715 Mycgr3T
  
Location: 24667-25981

Mycgr3G42715\_Mycgr3T

Mycgr3G92934 Mycgr3T
  
Location: 26081-27593

Mycgr3G92934\_Mycgr3T

Mycgr3G41969 Mycgr3T
  
Location: 27693-29328

Mycgr3G41969\_Mycgr3T

Mycgr3G80635 Mycgr3T
  
Location: 29428-29821

Mycgr3G80635\_Mycgr3T

Mycgr3G41426 Mycgr3T
  
Location: 29921-35255

Mycgr3G41426\_Mycgr3T

Mycgr3G104337 Mycgr3
  
Location: 35355-36108

Mycgr3G104337\_Mycgr3

Mycgr3G71679 Mycgr3T
  
Location: 36208-37300

Mycgr3G71679\_Mycgr3T

Mycgr3G92938 Mycgr3T
  
Location: 37400-38699

Mycgr3G92938\_Mycgr3T

Mycgr3G92941 Mycgr3T
  
Location: 38799-40734

Mycgr3G92941\_Mycgr3T

hypothetical protein
  
Accession: ENI07141
  
Location: 664408-665611
  
 NCBI BlastP on this gene

ENI07141

hypothetical protein
  
Accession: ENI07142
  
Location: 666369-667590
  
 NCBI BlastP on this gene

ENI07142

glycosyltransferase family 1 protein
  
Accession: ENI07143
  
Location: 667840-668543
  
 NCBI BlastP on this gene

ENI07143

hypothetical protein
  
Accession: ENI07144
  
Location: 671847-672065
  
 NCBI BlastP on this gene

ENI07144

hypothetical protein
  
Accession: ENI07145
  
Location: 673290-674140
  
 NCBI BlastP on this gene

ENI07145

hypothetical protein
  
Accession: ENI07146
  
Location: 675134-675949
  
 NCBI BlastP on this gene

ENI07146

hypothetical protein
  
Accession: ENI07147
  
Location: 676990-678538
  
 NCBI BlastP on this gene

ENI07147

hypothetical protein
  
Accession: ENI07148
  
Location: 681157-682438
  
  
**BlastP hit with Mycgr3G42698\_Mycgr3T**
  
Percentage identity: 30 %
  
BlastP bit score: 160
  
Sequence coverage: 112 %
  
E-value: 2e-42
  
  
 NCBI BlastP on this gene

ENI07148

hypothetical protein
  
Accession: ENI07149
  
Location: 682986-683819
  
 NCBI BlastP on this gene

ENI07149

hypothetical protein
  
Accession: ENI07150
  
Location: 684350-685126
  
  
**BlastP hit with Mycgr3G58567\_Mycgr3T**
  
Percentage identity: 32 %
  
BlastP bit score: 132
  
Sequence coverage: 95 %
  
E-value: 1e-33
  
  
 NCBI BlastP on this gene

ENI07150

hypothetical protein
  
Accession: ENI07151
  
Location: 690911-692468
  
 NCBI BlastP on this gene

ENI07151

hypothetical protein
  
Accession: ENI07152
  
Location: 692762-693327
  
 NCBI BlastP on this gene

ENI07152

hypothetical protein
  
Accession: ENI07153
  
Location: 693802-694658
  
 NCBI BlastP on this gene

ENI07153

hypothetical protein
  
Accession: ENI07154
  
Location: 701806-702009
  
 NCBI BlastP on this gene

ENI07154

hypothetical protein
  
Accession: ENI07155
  
Location: 702264-704269
  
 NCBI BlastP on this gene

ENI07155

Query: Architecture Search FASTA input

KB445573 : Cochliobolus heterostrophus C5 unplaced genomic scaffold COCHEscaffold\_5    Total score: 2.0     Cumulative Blast bit score: 292

Hit cluster cross-links:

Mycgr3G85918 Mycgr3T
  
Location: 0-1602

Mycgr3G85918\_Mycgr3T

Mycgr3G42010 Mycgr3T
  
Location: 1702-8569

Mycgr3G42010\_Mycgr3T

Mycgr3G29582 Mycgr3T
  
Location: 8669-8915

Mycgr3G29582\_Mycgr3T

Mycgr3G31170 Mycgr3T
  
Location: 9015-9255

Mycgr3G31170\_Mycgr3T

Mycgr3G85924 Mycgr3T
  
Location: 9355-11218

Mycgr3G85924\_Mycgr3T

Mycgr3G71676 Mycgr3T
  
Location: 11318-12494

Mycgr3G71676\_Mycgr3T

Mycgr3G11468 Mycgr3T
  
Location: 12594-13653

Mycgr3G11468\_Mycgr3T

Mycgr3G58567 Mycgr3T
  
Location: 13753-14506

Mycgr3G58567\_Mycgr3T

Mycgr3G100089 Mycgr3
  
Location: 14606-21152

Mycgr3G100089\_Mycgr3

Mycgr3G42698 Mycgr3T
  
Location: 21252-22131

Mycgr3G42698\_Mycgr3T

Mycgr3G71681 Mycgr3T
  
Location: 22231-23461

Mycgr3G71681\_Mycgr3T

Mycgr3G109328 Mycgr3
  
Location: 23561-24239

Mycgr3G109328\_Mycgr3

Mycgr3G104334 Mycgr3
  
Location: 24339-24567

Mycgr3G104334\_Mycgr3

Mycgr3G42715 Mycgr3T
  
Location: 24667-25981

Mycgr3G42715\_Mycgr3T

Mycgr3G92934 Mycgr3T
  
Location: 26081-27593

Mycgr3G92934\_Mycgr3T

Mycgr3G41969 Mycgr3T
  
Location: 27693-29328

Mycgr3G41969\_Mycgr3T

Mycgr3G80635 Mycgr3T
  
Location: 29428-29821

Mycgr3G80635\_Mycgr3T

Mycgr3G41426 Mycgr3T
  
Location: 29921-35255

Mycgr3G41426\_Mycgr3T

Mycgr3G104337 Mycgr3
  
Location: 35355-36108

Mycgr3G104337\_Mycgr3

Mycgr3G71679 Mycgr3T
  
Location: 36208-37300

Mycgr3G71679\_Mycgr3T

Mycgr3G92938 Mycgr3T
  
Location: 37400-38699

Mycgr3G92938\_Mycgr3T

Mycgr3G92941 Mycgr3T
  
Location: 38799-40734

Mycgr3G92941\_Mycgr3T

hypothetical protein
  
Accession: EMD93397
  
Location: 597616-599621
  
 NCBI BlastP on this gene

EMD93397

hypothetical protein
  
Accession: EMD93398
  
Location: 599876-600079
  
 NCBI BlastP on this gene

EMD93398

hypothetical protein
  
Accession: EMD93399
  
Location: 607227-608083
  
 NCBI BlastP on this gene

EMD93399

hypothetical protein
  
Accession: EMD93400
  
Location: 608558-609123
  
 NCBI BlastP on this gene

EMD93400

hypothetical protein
  
Accession: EMD93401
  
Location: 609417-610897
  
 NCBI BlastP on this gene

EMD93401

hypothetical protein
  
Accession: EMD93402
  
Location: 616759-617535
  
  
**BlastP hit with Mycgr3G58567\_Mycgr3T**
  
Percentage identity: 32 %
  
BlastP bit score: 132
  
Sequence coverage: 95 %
  
E-value: 1e-33
  
  
 NCBI BlastP on this gene

EMD93402

hypothetical protein
  
Accession: EMD93403
  
Location: 618066-618899
  
 NCBI BlastP on this gene

EMD93403

hypothetical protein
  
Accession: EMD93404
  
Location: 619447-620728
  
  
**BlastP hit with Mycgr3G42698\_Mycgr3T**
  
Percentage identity: 30 %
  
BlastP bit score: 160
  
Sequence coverage: 112 %
  
E-value: 2e-42
  
  
 NCBI BlastP on this gene

EMD93404

hypothetical protein
  
Accession: EMD93405
  
Location: 623362-624430
  
 NCBI BlastP on this gene

EMD93405

hypothetical protein
  
Accession: EMD93406
  
Location: 625936-626751
  
 NCBI BlastP on this gene

EMD93406

hypothetical protein
  
Accession: EMD93407
  
Location: 627745-628595
  
 NCBI BlastP on this gene

EMD93407

hypothetical protein
  
Accession: EMD93408
  
Location: 629829-630038
  
 NCBI BlastP on this gene

EMD93408

glycosyltransferase family 1 protein
  
Accession: EMD93409
  
Location: 633342-634045
  
 NCBI BlastP on this gene

EMD93409

hypothetical protein
  
Accession: EMD93410
  
Location: 634295-635516
  
 NCBI BlastP on this gene

EMD93410

hypothetical protein
  
Accession: EMD93411
  
Location: 636274-637477
  
 NCBI BlastP on this gene

EMD93411

hypothetical protein
  
Accession: EMD93412
  
Location: 638250-638913
  
 NCBI BlastP on this gene

EMD93412

hypothetical protein
  
Accession: EMD93413
  
Location: 639622-641153
  
 NCBI BlastP on this gene

EMD93413

Query: Architecture Search FASTA input

AHMM02000023 : Leptospira inadai serovar Lyme str. 10    Total score: 2.0     Cumulative Blast bit score: 223

Hit cluster cross-links:

Mycgr3G85918 Mycgr3T
  
Location: 0-1602

Mycgr3G85918\_Mycgr3T

Mycgr3G42010 Mycgr3T
  
Location: 1702-8569

Mycgr3G42010\_Mycgr3T

Mycgr3G29582 Mycgr3T
  
Location: 8669-8915

Mycgr3G29582\_Mycgr3T

Mycgr3G31170 Mycgr3T
  
Location: 9015-9255

Mycgr3G31170\_Mycgr3T

Mycgr3G85924 Mycgr3T
  
Location: 9355-11218

Mycgr3G85924\_Mycgr3T

Mycgr3G71676 Mycgr3T
  
Location: 11318-12494

Mycgr3G71676\_Mycgr3T

Mycgr3G11468 Mycgr3T
  
Location: 12594-13653

Mycgr3G11468\_Mycgr3T

Mycgr3G58567 Mycgr3T
  
Location: 13753-14506

Mycgr3G58567\_Mycgr3T

Mycgr3G100089 Mycgr3
  
Location: 14606-21152

Mycgr3G100089\_Mycgr3

Mycgr3G42698 Mycgr3T
  
Location: 21252-22131

Mycgr3G42698\_Mycgr3T

Mycgr3G71681 Mycgr3T
  
Location: 22231-23461

Mycgr3G71681\_Mycgr3T

Mycgr3G109328 Mycgr3
  
Location: 23561-24239

Mycgr3G109328\_Mycgr3

Mycgr3G104334 Mycgr3
  
Location: 24339-24567

Mycgr3G104334\_Mycgr3

Mycgr3G42715 Mycgr3T
  
Location: 24667-25981

Mycgr3G42715\_Mycgr3T

Mycgr3G92934 Mycgr3T
  
Location: 26081-27593

Mycgr3G92934\_Mycgr3T

Mycgr3G41969 Mycgr3T
  
Location: 27693-29328

Mycgr3G41969\_Mycgr3T

Mycgr3G80635 Mycgr3T
  
Location: 29428-29821

Mycgr3G80635\_Mycgr3T

Mycgr3G41426 Mycgr3T
  
Location: 29921-35255

Mycgr3G41426\_Mycgr3T

Mycgr3G104337 Mycgr3
  
Location: 35355-36108

Mycgr3G104337\_Mycgr3

Mycgr3G71679 Mycgr3T
  
Location: 36208-37300

Mycgr3G71679\_Mycgr3T

Mycgr3G92938 Mycgr3T
  
Location: 37400-38699

Mycgr3G92938\_Mycgr3T

Mycgr3G92941 Mycgr3T
  
Location: 38799-40734

Mycgr3G92941\_Mycgr3T

tetratricopeptide repeat protein
  
Accession: EQA36150
  
Location: 118111-120126
  
 NCBI BlastP on this gene

EQA36150

hypothetical protein
  
Accession: EQA36189
  
Location: 120158-120967
  
 NCBI BlastP on this gene

EQA36189

enoyl-CoA hydratase/isomerase family protein
  
Accession: EQA36172
  
Location: 120988-121752
  
 NCBI BlastP on this gene

EQA36172

FAD binding domain protein
  
Accession: EQA36137
  
Location: 121821-123413
  
 NCBI BlastP on this gene

EQA36137

SpoIIE-like protein phosphatase domain protein
  
Accession: EQA36181
  
Location: 123672-125699
  
 NCBI BlastP on this gene

EQA36181

FAD dependent oxidoreductase
  
Accession: EQA36196
  
Location: 125863-127506
  
 NCBI BlastP on this gene

EQA36196

transcriptional regulator, TetR family
  
Accession: EQA36195
  
Location: 127610-128188
  
 NCBI BlastP on this gene

EQA36195

PF06127 family protein
  
Accession: EQA36203
  
Location: 128311-128781
  
 NCBI BlastP on this gene

EQA36203

phospholipase, patatin family
  
Accession: EQA36153
  
Location: 128805-129806
  
 NCBI BlastP on this gene

EQA36153

hypothetical protein
  
Accession: EQA36207
  
Location: 129964-130431
  
 NCBI BlastP on this gene

EQA36207

hypothetical protein
  
Accession: EQA36218
  
Location: 130454-131296
  
 NCBI BlastP on this gene

EQA36218

YacP-like NYN domain protein
  
Accession: EQA36167
  
Location: 131398-131862
  
 NCBI BlastP on this gene

EQA36167

putative alginate O-acetyltransferase AlgI
  
Accession: EQA36154
  
Location: 131866-133365
  
 NCBI BlastP on this gene

EQA36154

PF07611 family protein
  
Accession: EQA36210
  
Location: 133384-134469
  
 NCBI BlastP on this gene

EQA36210

DJ-1 family protein
  
Accession: EQA36101
  
Location: 134493-135038
  
 NCBI BlastP on this gene

EQA36101

GtrA-like protein
  
Accession: EQA36215
  
Location: 135095-136249
  
 NCBI BlastP on this gene

EQA36215

glycoside hydrolase, family 5
  
Accession: EQA36129
  
Location: 136589-138493
  
  
**BlastP hit with Mycgr3G71681\_Mycgr3T**
  
Percentage identity: 29 %
  
BlastP bit score: 107
  
Sequence coverage: 84 %
  
E-value: 5e-22
  
  
 NCBI BlastP on this gene

EQA36129

DoxX family protein
  
Accession: EQA36121
  
Location: 138929-139408
  
 NCBI BlastP on this gene

EQA36121

hypothetical protein
  
Accession: EQA36176
  
Location: 139645-139791
  
 NCBI BlastP on this gene

EQA36176

KR domain protein
  
Accession: EQA36109
  
Location: 139849-140628
  
  
**BlastP hit with Mycgr3G58567\_Mycgr3T**
  
Percentage identity: 32 %
  
BlastP bit score: 117
  
Sequence coverage: 94 %
  
E-value: 5e-28
  
  
 NCBI BlastP on this gene

EQA36109

hypothetical protein
  
Accession: EQA36217
  
Location: 141042-141155
  
 NCBI BlastP on this gene

EQA36217

peptidase, S8/S53 family
  
Accession: EQA36087
  
Location: 141230-143059
  
 NCBI BlastP on this gene

EQA36087

VCBS repeat protein
  
Accession: EQA36174
  
Location: 143037-143771
  
 NCBI BlastP on this gene

EQA36174

hypothetical protein
  
Accession: EQA36086
  
Location: 143813-144109
  
 NCBI BlastP on this gene

EQA36086

hypothetical protein
  
Accession: EQA36141
  
Location: 144130-144870
  
 NCBI BlastP on this gene

EQA36141

hypothetical protein
  
Accession: EQA36228
  
Location: 145811-146872
  
 NCBI BlastP on this gene

EQA36228

hypothetical protein
  
Accession: EQA36223
  
Location: 146839-147066
  
 NCBI BlastP on this gene

EQA36223

sigma factor regulatory protein, FecR/PupR family
  
Accession: EQA36211
  
Location: 148477-149421
  
 NCBI BlastP on this gene

EQA36211

hypothetical protein
  
Accession: EQA36116
  
Location: 149479-150771
  
 NCBI BlastP on this gene

EQA36116

hypothetical protein
  
Accession: EQA36168
  
Location: 151189-151404
  
 NCBI BlastP on this gene

EQA36168

Query: Architecture Search FASTA input

AHMO02000004 : Leptospira broomii serovar Hurstbridge str. 5399    Total score: 2.0     Cumulative Blast bit score: 220

Hit cluster cross-links:

Mycgr3G85918 Mycgr3T
  
Location: 0-1602

Mycgr3G85918\_Mycgr3T

Mycgr3G42010 Mycgr3T
  
Location: 1702-8569

Mycgr3G42010\_Mycgr3T

Mycgr3G29582 Mycgr3T
  
Location: 8669-8915

Mycgr3G29582\_Mycgr3T

Mycgr3G31170 Mycgr3T
  
Location: 9015-9255

Mycgr3G31170\_Mycgr3T

Mycgr3G85924 Mycgr3T
  
Location: 9355-11218

Mycgr3G85924\_Mycgr3T

Mycgr3G71676 Mycgr3T
  
Location: 11318-12494

Mycgr3G71676\_Mycgr3T

Mycgr3G11468 Mycgr3T
  
Location: 12594-13653

Mycgr3G11468\_Mycgr3T

Mycgr3G58567 Mycgr3T
  
Location: 13753-14506

Mycgr3G58567\_Mycgr3T

Mycgr3G100089 Mycgr3
  
Location: 14606-21152

Mycgr3G100089\_Mycgr3

Mycgr3G42698 Mycgr3T
  
Location: 21252-22131

Mycgr3G42698\_Mycgr3T

Mycgr3G71681 Mycgr3T
  
Location: 22231-23461

Mycgr3G71681\_Mycgr3T

Mycgr3G109328 Mycgr3
  
Location: 23561-24239

Mycgr3G109328\_Mycgr3

Mycgr3G104334 Mycgr3
  
Location: 24339-24567

Mycgr3G104334\_Mycgr3

Mycgr3G42715 Mycgr3T
  
Location: 24667-25981

Mycgr3G42715\_Mycgr3T

Mycgr3G92934 Mycgr3T
  
Location: 26081-27593

Mycgr3G92934\_Mycgr3T

Mycgr3G41969 Mycgr3T
  
Location: 27693-29328

Mycgr3G41969\_Mycgr3T

Mycgr3G80635 Mycgr3T
  
Location: 29428-29821

Mycgr3G80635\_Mycgr3T

Mycgr3G41426 Mycgr3T
  
Location: 29921-35255

Mycgr3G41426\_Mycgr3T

Mycgr3G104337 Mycgr3
  
Location: 35355-36108

Mycgr3G104337\_Mycgr3

Mycgr3G71679 Mycgr3T
  
Location: 36208-37300

Mycgr3G71679\_Mycgr3T

Mycgr3G92938 Mycgr3T
  
Location: 37400-38699

Mycgr3G92938\_Mycgr3T

Mycgr3G92941 Mycgr3T
  
Location: 38799-40734

Mycgr3G92941\_Mycgr3T

tetratricopeptide repeat protein
  
Accession: EQA46946
  
Location: 661705-663762
  
 NCBI BlastP on this gene

EQA46946

putative lipoprotein
  
Accession: EQA46846
  
Location: 663792-664598
  
 NCBI BlastP on this gene

EQA46846

enoyl-CoA hydratase/isomerase family protein
  
Accession: EQA47072
  
Location: 664619-665383
  
 NCBI BlastP on this gene

EQA47072

FAD binding domain protein
  
Accession: EQA46708
  
Location: 665452-667044
  
 NCBI BlastP on this gene

EQA46708

hypothetical protein
  
Accession: EQA46793
  
Location: 667066-667251
  
 NCBI BlastP on this gene

EQA46793

SpoIIE-like protein phosphatase domain protein
  
Accession: EQA47204
  
Location: 667303-669330
  
 NCBI BlastP on this gene

EQA47204

FAD dependent oxidoreductase
  
Accession: EQA47090
  
Location: 669495-671138
  
 NCBI BlastP on this gene

EQA47090

transcriptional regulator, TetR family
  
Accession: EQA47222
  
Location: 671226-671804
  
 NCBI BlastP on this gene

EQA47222

PF06127 family protein
  
Accession: EQA46957
  
Location: 671926-672396
  
 NCBI BlastP on this gene

EQA46957

phospholipase, patatin family
  
Accession: EQA47097
  
Location: 672420-673460
  
 NCBI BlastP on this gene

EQA47097

hypothetical protein
  
Accession: EQA46844
  
Location: 673580-674047
  
 NCBI BlastP on this gene

EQA46844

hypothetical protein
  
Accession: EQA46956
  
Location: 674069-674911
  
 NCBI BlastP on this gene

EQA46956

YacP-like NYN domain protein
  
Accession: EQA46686
  
Location: 675013-675477
  
 NCBI BlastP on this gene

EQA46686

putative alginate O-acetyltransferase AlgI
  
Accession: EQA46755
  
Location: 675481-676980
  
 NCBI BlastP on this gene

EQA46755

PF07611 family protein
  
Accession: EQA46974
  
Location: 676999-678084
  
 NCBI BlastP on this gene

EQA46974

DJ-1 family protein
  
Accession: EQA47073
  
Location: 678108-678653
  
 NCBI BlastP on this gene

EQA47073

GtrA-like protein
  
Accession: EQA46967
  
Location: 678710-679864
  
 NCBI BlastP on this gene

EQA46967

glycoside hydrolase, family 5
  
Accession: EQA46723
  
Location: 680192-682093
  
  
**BlastP hit with Mycgr3G71681\_Mycgr3T**
  
Percentage identity: 29 %
  
BlastP bit score: 104
  
Sequence coverage: 85 %
  
E-value: 5e-21
  
  
 NCBI BlastP on this gene

EQA46723

DoxX family protein
  
Accession: EQA47213
  
Location: 682510-682989
  
 NCBI BlastP on this gene

EQA47213

KR domain protein
  
Accession: EQA47235
  
Location: 683828-684607
  
  
**BlastP hit with Mycgr3G58567\_Mycgr3T**
  
Percentage identity: 31 %
  
BlastP bit score: 116
  
Sequence coverage: 94 %
  
E-value: 2e-27
  
  
 NCBI BlastP on this gene

EQA47235

hypothetical protein
  
Accession: EQA47290
  
Location: 684614-684826
  
 NCBI BlastP on this gene

EQA47290

hypothetical protein
  
Accession: EQA46897
  
Location: 685177-686169
  
 NCBI BlastP on this gene

EQA46897

hypothetical protein
  
Accession: EQA47192
  
Location: 686206-686382
  
 NCBI BlastP on this gene

EQA47192

hypothetical protein
  
Accession: EQA47121
  
Location: 686786-687022
  
 NCBI BlastP on this gene

EQA47121

sigma factor regulatory protein, FecR/PupR family
  
Accession: EQA46986
  
Location: 688025-688969
  
 NCBI BlastP on this gene

EQA46986

hypothetical protein
  
Accession: EQA46678
  
Location: 689028-690314
  
 NCBI BlastP on this gene

EQA46678

hypothetical protein
  
Accession: EQA47030
  
Location: 690497-690667
  
 NCBI BlastP on this gene

EQA47030

Query: Architecture Search FASTA input

JH921445 : Marssonina brunnea f. sp. 'multigermtubi' MB\_m1 unplaced genomic scaffold M6\_S00018    Total score: 2.0     Cumulative Blast bit score: 203

Hit cluster cross-links:

Mycgr3G85918 Mycgr3T
  
Location: 0-1602

Mycgr3G85918\_Mycgr3T

Mycgr3G42010 Mycgr3T
  
Location: 1702-8569

Mycgr3G42010\_Mycgr3T

Mycgr3G29582 Mycgr3T
  
Location: 8669-8915

Mycgr3G29582\_Mycgr3T

Mycgr3G31170 Mycgr3T
  
Location: 9015-9255

Mycgr3G31170\_Mycgr3T

Mycgr3G85924 Mycgr3T
  
Location: 9355-11218

Mycgr3G85924\_Mycgr3T

Mycgr3G71676 Mycgr3T
  
Location: 11318-12494

Mycgr3G71676\_Mycgr3T

Mycgr3G11468 Mycgr3T
  
Location: 12594-13653

Mycgr3G11468\_Mycgr3T

Mycgr3G58567 Mycgr3T
  
Location: 13753-14506

Mycgr3G58567\_Mycgr3T

Mycgr3G100089 Mycgr3
  
Location: 14606-21152

Mycgr3G100089\_Mycgr3

Mycgr3G42698 Mycgr3T
  
Location: 21252-22131

Mycgr3G42698\_Mycgr3T

Mycgr3G71681 Mycgr3T
  
Location: 22231-23461

Mycgr3G71681\_Mycgr3T

Mycgr3G109328 Mycgr3
  
Location: 23561-24239

Mycgr3G109328\_Mycgr3

Mycgr3G104334 Mycgr3
  
Location: 24339-24567

Mycgr3G104334\_Mycgr3

Mycgr3G42715 Mycgr3T
  
Location: 24667-25981

Mycgr3G42715\_Mycgr3T

Mycgr3G92934 Mycgr3T
  
Location: 26081-27593

Mycgr3G92934\_Mycgr3T

Mycgr3G41969 Mycgr3T
  
Location: 27693-29328

Mycgr3G41969\_Mycgr3T

Mycgr3G80635 Mycgr3T
  
Location: 29428-29821

Mycgr3G80635\_Mycgr3T

Mycgr3G41426 Mycgr3T
  
Location: 29921-35255

Mycgr3G41426\_Mycgr3T

Mycgr3G104337 Mycgr3
  
Location: 35355-36108

Mycgr3G104337\_Mycgr3

Mycgr3G71679 Mycgr3T
  
Location: 36208-37300

Mycgr3G71679\_Mycgr3T

Mycgr3G92938 Mycgr3T
  
Location: 37400-38699

Mycgr3G92938\_Mycgr3T

Mycgr3G92941 Mycgr3T
  
Location: 38799-40734

Mycgr3G92941\_Mycgr3T

NADH-ubiquinone oxidoreductase 10.5 kDa subunit
  
Accession: EKD14475
  
Location: 324908-325585
  
 NCBI BlastP on this gene

EKD14475

oxysterol-binding protein
  
Accession: EKD14476
  
Location: 327453-331368
  
 NCBI BlastP on this gene

EKD14476

hypothetical protein
  
Accession: EKD14477
  
Location: 331808-333072
  
 NCBI BlastP on this gene

EKD14477

DASH family cryptochrome
  
Accession: EKD14478
  
Location: 336087-338116
  
 NCBI BlastP on this gene

EKD14478

Fasciclin domain family protein
  
Accession: EKD14479
  
Location: 341444-342920
  
 NCBI BlastP on this gene

EKD14479

thioesterase family protein
  
Accession: EKD14480
  
Location: 344495-345359
  
  
**BlastP hit with Mycgr3G92941\_Mycgr3T**
  
Percentage identity: 27 %
  
BlastP bit score: 94
  
Sequence coverage: 39 %
  
E-value: 5e-18
  
  
 NCBI BlastP on this gene

EKD14480

hypothetical protein
  
Accession: EKD14481
  
Location: 348231-348880
  
  
**BlastP hit with Mycgr3G29582\_Mycgr3T**
  
Percentage identity: 72 %
  
BlastP bit score: 109
  
Sequence coverage: 92 %
  
E-value: 1e-28
  
  
 NCBI BlastP on this gene

EKD14481

GATA zinc finger protein
  
Accession: EKD14482
  
Location: 351500-361792
  
 NCBI BlastP on this gene

EKD14482

hypothetical protein
  
Accession: EKD14483
  
Location: 368397-368735
  
 NCBI BlastP on this gene

EKD14483

Query: Architecture Search FASTA input

CH408031 : Chaetomium globosum CBS 148.51 scaffold\_3 genomic scaffold    Total score: 2.0     Cumulative Blast bit score: 201

Hit cluster cross-links:

Mycgr3G85918 Mycgr3T
  
Location: 0-1602

Mycgr3G85918\_Mycgr3T

Mycgr3G42010 Mycgr3T
  
Location: 1702-8569

Mycgr3G42010\_Mycgr3T

Mycgr3G29582 Mycgr3T
  
Location: 8669-8915

Mycgr3G29582\_Mycgr3T

Mycgr3G31170 Mycgr3T
  
Location: 9015-9255

Mycgr3G31170\_Mycgr3T

Mycgr3G85924 Mycgr3T
  
Location: 9355-11218

Mycgr3G85924\_Mycgr3T

Mycgr3G71676 Mycgr3T
  
Location: 11318-12494

Mycgr3G71676\_Mycgr3T

Mycgr3G11468 Mycgr3T
  
Location: 12594-13653

Mycgr3G11468\_Mycgr3T

Mycgr3G58567 Mycgr3T
  
Location: 13753-14506

Mycgr3G58567\_Mycgr3T

Mycgr3G100089 Mycgr3
  
Location: 14606-21152

Mycgr3G100089\_Mycgr3

Mycgr3G42698 Mycgr3T
  
Location: 21252-22131

Mycgr3G42698\_Mycgr3T

Mycgr3G71681 Mycgr3T
  
Location: 22231-23461

Mycgr3G71681\_Mycgr3T

Mycgr3G109328 Mycgr3
  
Location: 23561-24239

Mycgr3G109328\_Mycgr3

Mycgr3G104334 Mycgr3
  
Location: 24339-24567

Mycgr3G104334\_Mycgr3

Mycgr3G42715 Mycgr3T
  
Location: 24667-25981

Mycgr3G42715\_Mycgr3T

Mycgr3G92934 Mycgr3T
  
Location: 26081-27593

Mycgr3G92934\_Mycgr3T

Mycgr3G41969 Mycgr3T
  
Location: 27693-29328

Mycgr3G41969\_Mycgr3T

Mycgr3G80635 Mycgr3T
  
Location: 29428-29821

Mycgr3G80635\_Mycgr3T

Mycgr3G41426 Mycgr3T
  
Location: 29921-35255

Mycgr3G41426\_Mycgr3T

Mycgr3G104337 Mycgr3
  
Location: 35355-36108

Mycgr3G104337\_Mycgr3

Mycgr3G71679 Mycgr3T
  
Location: 36208-37300

Mycgr3G71679\_Mycgr3T

Mycgr3G92938 Mycgr3T
  
Location: 37400-38699

Mycgr3G92938\_Mycgr3T

Mycgr3G92941 Mycgr3T
  
Location: 38799-40734

Mycgr3G92941\_Mycgr3T

hypothetical protein
  
Accession: EAQ90101
  
Location: 4360609-4362408
  
 NCBI BlastP on this gene

EAQ90101

hypothetical protein
  
Accession: EAQ90100
  
Location: 4358899-4359871
  
 NCBI BlastP on this gene

EAQ90100

hypothetical protein
  
Accession: EAQ90099
  
Location: 4356341-4357796
  
 NCBI BlastP on this gene

EAQ90099

hypothetical protein
  
Accession: EAQ90098
  
Location: 4353569-4355066
  
 NCBI BlastP on this gene

EAQ90098

hypothetical protein
  
Accession: EAQ90097
  
Location: 4352202-4353273
  
 NCBI BlastP on this gene

EAQ90097

hypothetical protein
  
Accession: EAQ90096
  
Location: 4349110-4351424
  
 NCBI BlastP on this gene

EAQ90096

hypothetical protein
  
Accession: EAQ90095
  
Location: 4346971-4347812
  
 NCBI BlastP on this gene

EAQ90095

hypothetical protein
  
Accession: EAQ90094
  
Location: 4345214-4346422
  
 NCBI BlastP on this gene

EAQ90094

hypothetical protein
  
Accession: EAQ90093
  
Location: 4342482-4344715
  
 NCBI BlastP on this gene

EAQ90093

hypothetical protein
  
Accession: EAQ90092
  
Location: 4339190-4341942
  
  
**BlastP hit with Mycgr3G11468\_Mycgr3T**
  
Percentage identity: 28 %
  
BlastP bit score: 127
  
Sequence coverage: 101 %
  
E-value: 5e-29
  
  
 NCBI BlastP on this gene

EAQ90092

hypothetical protein
  
Accession: EAQ90091
  
Location: 4338336-4338928
  
 NCBI BlastP on this gene

EAQ90091

hypothetical protein
  
Accession: EAQ90090
  
Location: 4337324-4337987
  
 NCBI BlastP on this gene

EAQ90090

hypothetical protein
  
Accession: EAQ90089
  
Location: 4334209-4335206
  
  
**BlastP hit with Mycgr3G109328\_Mycgr3**
  
Percentage identity: 31 %
  
BlastP bit score: 74
  
Sequence coverage: 78 %
  
E-value: 5e-13
  
  
 NCBI BlastP on this gene

EAQ90089

hypothetical protein
  
Accession: EAQ90088
  
Location: 4332527-4333423
  
 NCBI BlastP on this gene

EAQ90088

hypothetical protein
  
Accession: EAQ90087
  
Location: 4329943-4331214
  
 NCBI BlastP on this gene

EAQ90087

predicted protein
  
Accession: EAQ90086
  
Location: 4328891-4329361
  
 NCBI BlastP on this gene

EAQ90086

hypothetical protein
  
Accession: EAQ90085
  
Location: 4322621-4323868
  
 NCBI BlastP on this gene

EAQ90085

hypothetical protein
  
Accession: EAQ90084
  
Location: 4318264-4320992
  
 NCBI BlastP on this gene

EAQ90084

hypothetical protein
  
Accession: EAQ90083
  
Location: 4316383-4317498
  
 NCBI BlastP on this gene

EAQ90083

hypothetical protein
  
Accession: EAQ90082
  
Location: 4314900-4315948
  
 NCBI BlastP on this gene

EAQ90082

Query: Architecture Search FASTA input

CU633897 : Podospora anserina S mat+ genomic DNA chromosome 1, supercontig 6.    Total score: 2.0     Cumulative Blast bit score: 199

Hit cluster cross-links:

Mycgr3G85918 Mycgr3T
  
Location: 0-1602

Mycgr3G85918\_Mycgr3T

Mycgr3G42010 Mycgr3T
  
Location: 1702-8569

Mycgr3G42010\_Mycgr3T

Mycgr3G29582 Mycgr3T
  
Location: 8669-8915

Mycgr3G29582\_Mycgr3T

Mycgr3G31170 Mycgr3T
  
Location: 9015-9255

Mycgr3G31170\_Mycgr3T

Mycgr3G85924 Mycgr3T
  
Location: 9355-11218

Mycgr3G85924\_Mycgr3T

Mycgr3G71676 Mycgr3T
  
Location: 11318-12494

Mycgr3G71676\_Mycgr3T

Mycgr3G11468 Mycgr3T
  
Location: 12594-13653

Mycgr3G11468\_Mycgr3T

Mycgr3G58567 Mycgr3T
  
Location: 13753-14506

Mycgr3G58567\_Mycgr3T

Mycgr3G100089 Mycgr3
  
Location: 14606-21152

Mycgr3G100089\_Mycgr3

Mycgr3G42698 Mycgr3T
  
Location: 21252-22131

Mycgr3G42698\_Mycgr3T

Mycgr3G71681 Mycgr3T
  
Location: 22231-23461

Mycgr3G71681\_Mycgr3T

Mycgr3G109328 Mycgr3
  
Location: 23561-24239

Mycgr3G109328\_Mycgr3

Mycgr3G104334 Mycgr3
  
Location: 24339-24567

Mycgr3G104334\_Mycgr3

Mycgr3G42715 Mycgr3T
  
Location: 24667-25981

Mycgr3G42715\_Mycgr3T

Mycgr3G92934 Mycgr3T
  
Location: 26081-27593

Mycgr3G92934\_Mycgr3T

Mycgr3G41969 Mycgr3T
  
Location: 27693-29328

Mycgr3G41969\_Mycgr3T

Mycgr3G80635 Mycgr3T
  
Location: 29428-29821

Mycgr3G80635\_Mycgr3T

Mycgr3G41426 Mycgr3T
  
Location: 29921-35255

Mycgr3G41426\_Mycgr3T

Mycgr3G104337 Mycgr3
  
Location: 35355-36108

Mycgr3G104337\_Mycgr3

Mycgr3G71679 Mycgr3T
  
Location: 36208-37300

Mycgr3G71679\_Mycgr3T

Mycgr3G92938 Mycgr3T
  
Location: 37400-38699

Mycgr3G92938\_Mycgr3T

Mycgr3G92941 Mycgr3T
  
Location: 38799-40734

Mycgr3G92941\_Mycgr3T

not annotated
  
Accession: CAP67185
  
Location: 231675-232446
  
 NCBI BlastP on this gene

CAP67185

not annotated
  
Accession: CAP67186
  
Location: 233171-233958
  
 NCBI BlastP on this gene

CAP67186

not annotated
  
Accession: CAP67187
  
Location: 234473-238489
  
 NCBI BlastP on this gene

CAP67187

not annotated
  
Accession: CAP67188
  
Location: 238702-240036
  
 NCBI BlastP on this gene

CAP67188

not annotated
  
Accession: CAP67189
  
Location: 240140-241245
  
 NCBI BlastP on this gene

CAP67189

not annotated
  
Accession: CAP67190
  
Location: 241730-242880
  
 NCBI BlastP on this gene

CAP67190

tRNA-Met
  
Accession: CAP67191
  
Location: 244230-244610
  
 NCBI BlastP on this gene

CAP67191

not annotated
  
Accession: CAP67192
  
Location: 246419-248635
  
 NCBI BlastP on this gene

CAP67192

not annotated
  
Accession: CAP67193
  
Location: 249796-251999
  
  
**BlastP hit with Mycgr3G11468\_Mycgr3T**
  
Percentage identity: 30 %
  
BlastP bit score: 132
  
Sequence coverage: 100 %
  
E-value: 7e-31
  
  
 NCBI BlastP on this gene

CAP67193

not annotated
  
Accession: CAP67194
  
Location: 252121-252621
  
 NCBI BlastP on this gene

CAP67194

not annotated
  
Accession: CAP67195
  
Location: 253258-254119
  
 NCBI BlastP on this gene

CAP67195

not annotated
  
Accession: CAP67196
  
Location: 255770-257539
  
 NCBI BlastP on this gene

CAP67196

not annotated
  
Accession: CAP67197
  
Location: 258272-259228
  
  
**BlastP hit with Mycgr3G109328\_Mycgr3**
  
Percentage identity: 26 %
  
BlastP bit score: 67
  
Sequence coverage: 106 %
  
E-value: 2e-10
  
  
 NCBI BlastP on this gene

CAP67197

not annotated
  
Accession: CAP67198
  
Location: 259935-261069
  
 NCBI BlastP on this gene

CAP67198

not annotated
  
Accession: CAP67199
  
Location: 262617-263891
  
 NCBI BlastP on this gene

CAP67199

not annotated
  
Accession: CAP67200
  
Location: 270791-272902
  
 NCBI BlastP on this gene

CAP67200

not annotated
  
Accession: CAP67201
  
Location: 273854-274543
  
 NCBI BlastP on this gene

CAP67201

not annotated
  
Accession: CAP67202
  
Location: 275222-276792
  
 NCBI BlastP on this gene

CAP67202

Query: Architecture Search FASTA input

AEYX01000033 : Streptomyces griseoaurantiacus M045    Total score: 2.0     Cumulative Blast bit score: 196

Hit cluster cross-links:

Mycgr3G85918 Mycgr3T
  
Location: 0-1602

Mycgr3G85918\_Mycgr3T

Mycgr3G42010 Mycgr3T
  
Location: 1702-8569

Mycgr3G42010\_Mycgr3T

Mycgr3G29582 Mycgr3T
  
Location: 8669-8915

Mycgr3G29582\_Mycgr3T

Mycgr3G31170 Mycgr3T
  
Location: 9015-9255

Mycgr3G31170\_Mycgr3T

Mycgr3G85924 Mycgr3T
  
Location: 9355-11218

Mycgr3G85924\_Mycgr3T

Mycgr3G71676 Mycgr3T
  
Location: 11318-12494

Mycgr3G71676\_Mycgr3T

Mycgr3G11468 Mycgr3T
  
Location: 12594-13653

Mycgr3G11468\_Mycgr3T

Mycgr3G58567 Mycgr3T
  
Location: 13753-14506

Mycgr3G58567\_Mycgr3T

Mycgr3G100089 Mycgr3
  
Location: 14606-21152

Mycgr3G100089\_Mycgr3

Mycgr3G42698 Mycgr3T
  
Location: 21252-22131

Mycgr3G42698\_Mycgr3T

Mycgr3G71681 Mycgr3T
  
Location: 22231-23461

Mycgr3G71681\_Mycgr3T

Mycgr3G109328 Mycgr3
  
Location: 23561-24239

Mycgr3G109328\_Mycgr3

Mycgr3G104334 Mycgr3
  
Location: 24339-24567

Mycgr3G104334\_Mycgr3

Mycgr3G42715 Mycgr3T
  
Location: 24667-25981

Mycgr3G42715\_Mycgr3T

Mycgr3G92934 Mycgr3T
  
Location: 26081-27593

Mycgr3G92934\_Mycgr3T

Mycgr3G41969 Mycgr3T
  
Location: 27693-29328

Mycgr3G41969\_Mycgr3T

Mycgr3G80635 Mycgr3T
  
Location: 29428-29821

Mycgr3G80635\_Mycgr3T

Mycgr3G41426 Mycgr3T
  
Location: 29921-35255

Mycgr3G41426\_Mycgr3T

Mycgr3G104337 Mycgr3
  
Location: 35355-36108

Mycgr3G104337\_Mycgr3

Mycgr3G71679 Mycgr3T
  
Location: 36208-37300

Mycgr3G71679\_Mycgr3T

Mycgr3G92938 Mycgr3T
  
Location: 37400-38699

Mycgr3G92938\_Mycgr3T

Mycgr3G92941 Mycgr3T
  
Location: 38799-40734

Mycgr3G92941\_Mycgr3T

anti-anti-sigma-factor
  
Accession: EGG47266
  
Location: 176781-177110
  
 NCBI BlastP on this gene

EGG47266

regulatory protein/ATPase
  
Accession: EGG47267
  
Location: 177287-177754
  
 NCBI BlastP on this gene

EGG47267

Stage II sporulation E family protein
  
Accession: EGG47268
  
Location: 177968-180730
  
 NCBI BlastP on this gene

EGG47268

two component sensor kinase
  
Accession: EGG47269
  
Location: 180796-182010
  
 NCBI BlastP on this gene

EGG47269

two-component response regulator
  
Accession: EGG47270
  
Location: 182007-182666
  
 NCBI BlastP on this gene

EGG47270

integral membrane protein
  
Accession: EGG47271
  
Location: 182805-183440
  
 NCBI BlastP on this gene

EGG47271

hypothetical protein
  
Accession: EGG47272
  
Location: 183540-184232
  
 NCBI BlastP on this gene

EGG47272

Transcriptional regulator, TetR family
  
Accession: EGG47273
  
Location: 184374-184976
  
 NCBI BlastP on this gene

EGG47273

hypothetical protein
  
Accession: EGG47274
  
Location: 185046-185891
  
 NCBI BlastP on this gene

EGG47274

glycosyl hydrolase
  
Accession: EGG47275
  
Location: 186023-187930
  
  
**BlastP hit with Mycgr3G71681\_Mycgr3T**
  
Percentage identity: 28 %
  
BlastP bit score: 82
  
Sequence coverage: 62 %
  
E-value: 9e-14
  
  
 NCBI BlastP on this gene

EGG47275

NADP-dependent alcohol dehydrogenase
  
Accession: EGG47276
  
Location: 187948-188988
  
 NCBI BlastP on this gene

EGG47276

transcriptional regulator
  
Accession: EGG47277
  
Location: 189093-189680
  
 NCBI BlastP on this gene

EGG47277

lipid hydrolase
  
Accession: EGG47278
  
Location: 189879-192080
  
 NCBI BlastP on this gene

EGG47278

TetR family transcriptional regulator
  
Accession: EGG47279
  
Location: 192172-192795
  
 NCBI BlastP on this gene

EGG47279

oxidoreductase
  
Accession: EGG47280
  
Location: 192904-193866
  
 NCBI BlastP on this gene

EGG47280

mini-circle protein
  
Accession: EGG47281
  
Location: 193927-194511
  
 NCBI BlastP on this gene

EGG47281

alcohol dehydrogenase
  
Accession: EGG47282
  
Location: 194633-195685
  
 NCBI BlastP on this gene

EGG47282

catechol 1,2-dioxygenase
  
Accession: EGG47283
  
Location: 195682-196620
  
 NCBI BlastP on this gene

EGG47283

non-ribosomal peptide synthetase
  
Accession: EGG47284
  
Location: 197187-201269
  
 NCBI BlastP on this gene

EGG47284

putative NRPS
  
Accession: EGG47285
  
Location: 201266-210745
  
 NCBI BlastP on this gene

EGG47285

Alpha-ketoglutarate-dependent taurine dioxygenase
  
Accession: EGG47286
  
Location: 210855-211727
  
 NCBI BlastP on this gene

EGG47286

DNA-binding protein
  
Accession: EGG47287
  
Location: 211753-212601
  
 NCBI BlastP on this gene

EGG47287

oxidoreductase
  
Accession: EGG47288
  
Location: 212703-213467
  
  
**BlastP hit with Mycgr3G58567\_Mycgr3T**
  
Percentage identity: 37 %
  
BlastP bit score: 114
  
Sequence coverage: 74 %
  
E-value: 1e-26
  
  
 NCBI BlastP on this gene

EGG47288

NAD(P)H dehydrogenase
  
Accession: EGG47289
  
Location: 213522-214286
  
 NCBI BlastP on this gene

EGG47289

AdpA family transcriptional regulator
  
Accession: EGG47290
  
Location: 214331-215359
  
 NCBI BlastP on this gene

EGG47290

putative chloramphenicol 3-O phosphotransferase
  
Accession: EGG47291
  
Location: 215412-215945
  
 NCBI BlastP on this gene

EGG47291

putative RNA polymerase sigma factor
  
Accession: EGG47292
  
Location: 216065-216643
  
 NCBI BlastP on this gene

EGG47292

hypothetical protein
  
Accession: EGG47293
  
Location: 216636-217478
  
 NCBI BlastP on this gene

EGG47293

lipoprotein
  
Accession: EGG47294
  
Location: 217549-218334
  
 NCBI BlastP on this gene

EGG47294

hypothetical protein
  
Accession: EGG47295
  
Location: 218430-219128
  
 NCBI BlastP on this gene

EGG47295

LysR family transcriptional regulator
  
Accession: EGG47296
  
Location: 219219-220106
  
 NCBI BlastP on this gene

EGG47296

GCN5-related N-acetyltransferase
  
Accession: EGG47297
  
Location: 220228-220683
  
 NCBI BlastP on this gene

EGG47297

hypothetical protein
  
Accession: EGG47298
  
Location: 220963-221280
  
 NCBI BlastP on this gene

EGG47298

hypothetical protein
  
Accession: EGG47299
  
Location: 221253-221681
  
 NCBI BlastP on this gene

EGG47299

transposase
  
Accession: EGG47300
  
Location: 221716-222369
  
 NCBI BlastP on this gene

EGG47300

Query: Architecture Search FASTA input

CAUH01001128 : Blumeria graminis f. sp. hordei DH14    Total score: 2.0     Cumulative Blast bit score: 175

Hit cluster cross-links:

Mycgr3G85918 Mycgr3T
  
Location: 0-1602

Mycgr3G85918\_Mycgr3T

Mycgr3G42010 Mycgr3T
  
Location: 1702-8569

Mycgr3G42010\_Mycgr3T

Mycgr3G29582 Mycgr3T
  
Location: 8669-8915

Mycgr3G29582\_Mycgr3T

Mycgr3G31170 Mycgr3T
  
Location: 9015-9255

Mycgr3G31170\_Mycgr3T

Mycgr3G85924 Mycgr3T
  
Location: 9355-11218

Mycgr3G85924\_Mycgr3T

Mycgr3G71676 Mycgr3T
  
Location: 11318-12494

Mycgr3G71676\_Mycgr3T

Mycgr3G11468 Mycgr3T
  
Location: 12594-13653

Mycgr3G11468\_Mycgr3T

Mycgr3G58567 Mycgr3T
  
Location: 13753-14506

Mycgr3G58567\_Mycgr3T

Mycgr3G100089 Mycgr3
  
Location: 14606-21152

Mycgr3G100089\_Mycgr3

Mycgr3G42698 Mycgr3T
  
Location: 21252-22131

Mycgr3G42698\_Mycgr3T

Mycgr3G71681 Mycgr3T
  
Location: 22231-23461

Mycgr3G71681\_Mycgr3T

Mycgr3G109328 Mycgr3
  
Location: 23561-24239

Mycgr3G109328\_Mycgr3

Mycgr3G104334 Mycgr3
  
Location: 24339-24567

Mycgr3G104334\_Mycgr3

Mycgr3G42715 Mycgr3T
  
Location: 24667-25981

Mycgr3G42715\_Mycgr3T

Mycgr3G92934 Mycgr3T
  
Location: 26081-27593

Mycgr3G92934\_Mycgr3T

Mycgr3G41969 Mycgr3T
  
Location: 27693-29328

Mycgr3G41969\_Mycgr3T

Mycgr3G80635 Mycgr3T
  
Location: 29428-29821

Mycgr3G80635\_Mycgr3T

Mycgr3G41426 Mycgr3T
  
Location: 29921-35255

Mycgr3G41426\_Mycgr3T

Mycgr3G104337 Mycgr3
  
Location: 35355-36108

Mycgr3G104337\_Mycgr3

Mycgr3G71679 Mycgr3T
  
Location: 36208-37300

Mycgr3G71679\_Mycgr3T

Mycgr3G92938 Mycgr3T
  
Location: 37400-38699

Mycgr3G92938\_Mycgr3T

Mycgr3G92941 Mycgr3T
  
Location: 38799-40734

Mycgr3G92941\_Mycgr3T

thioesterase family protein
  
Accession: CCU75257
  
Location: 1042-1938
  
  
**BlastP hit with Mycgr3G92941\_Mycgr3T**
  
Percentage identity: 33 %
  
BlastP bit score: 80
  
Sequence coverage: 25 %
  
E-value: 2e-13
  
  
 NCBI BlastP on this gene

CCU75257

oxysterol-binding protein
  
Accession: CCU75258
  
Location: 12594-16474
  
 NCBI BlastP on this gene

CCU75258

hypothetical protein
  
Accession: CCU75259
  
Location: 18986-19427
  
  
**BlastP hit with Mycgr3G29582\_Mycgr3T**
  
Percentage identity: 60 %
  
BlastP bit score: 95
  
Sequence coverage: 92 %
  
E-value: 2e-23
  
  
 NCBI BlastP on this gene

CCU75259

Query: Architecture Search FASTA input

JH711588 : Coniophora puteana RWD-64-598 SS2 unplaced genomic scaffold CONPUscaffold\_16    Total score: 1.0     Cumulative Blast bit score: 3125

Hit cluster cross-links:

Mycgr3G85918 Mycgr3T
  
Location: 0-1602

Mycgr3G85918\_Mycgr3T

Mycgr3G42010 Mycgr3T
  
Location: 1702-8569

Mycgr3G42010\_Mycgr3T

Mycgr3G29582 Mycgr3T
  
Location: 8669-8915

Mycgr3G29582\_Mycgr3T

Mycgr3G31170 Mycgr3T
  
Location: 9015-9255

Mycgr3G31170\_Mycgr3T

Mycgr3G85924 Mycgr3T
  
Location: 9355-11218

Mycgr3G85924\_Mycgr3T

Mycgr3G71676 Mycgr3T
  
Location: 11318-12494

Mycgr3G71676\_Mycgr3T

Mycgr3G11468 Mycgr3T
  
Location: 12594-13653

Mycgr3G11468\_Mycgr3T

Mycgr3G58567 Mycgr3T
  
Location: 13753-14506

Mycgr3G58567\_Mycgr3T

Mycgr3G100089 Mycgr3
  
Location: 14606-21152

Mycgr3G100089\_Mycgr3

Mycgr3G42698 Mycgr3T
  
Location: 21252-22131

Mycgr3G42698\_Mycgr3T

Mycgr3G71681 Mycgr3T
  
Location: 22231-23461

Mycgr3G71681\_Mycgr3T

Mycgr3G109328 Mycgr3
  
Location: 23561-24239

Mycgr3G109328\_Mycgr3

Mycgr3G104334 Mycgr3
  
Location: 24339-24567

Mycgr3G104334\_Mycgr3

Mycgr3G42715 Mycgr3T
  
Location: 24667-25981

Mycgr3G42715\_Mycgr3T

Mycgr3G92934 Mycgr3T
  
Location: 26081-27593

Mycgr3G92934\_Mycgr3T

Mycgr3G41969 Mycgr3T
  
Location: 27693-29328

Mycgr3G41969\_Mycgr3T

Mycgr3G80635 Mycgr3T
  
Location: 29428-29821

Mycgr3G80635\_Mycgr3T

Mycgr3G41426 Mycgr3T
  
Location: 29921-35255

Mycgr3G41426\_Mycgr3T

Mycgr3G104337 Mycgr3
  
Location: 35355-36108

Mycgr3G104337\_Mycgr3

Mycgr3G71679 Mycgr3T
  
Location: 36208-37300

Mycgr3G71679\_Mycgr3T

Mycgr3G92938 Mycgr3T
  
Location: 37400-38699

Mycgr3G92938\_Mycgr3T

Mycgr3G92941 Mycgr3T
  
Location: 38799-40734

Mycgr3G92941\_Mycgr3T

phospholipid-translocating P-type ATPase
  
Accession: EIW75489
  
Location: 316542-322410
  
 NCBI BlastP on this gene

EIW75489

hypothetical protein
  
Accession: EIW75488
  
Location: 313717-315514
  
 NCBI BlastP on this gene

EIW75488

hypothetical protein
  
Accession: EIW75487
  
Location: 310455-311410
  
 NCBI BlastP on this gene

EIW75487

mitochondrial carrier
  
Accession: EIW75486
  
Location: 307207-309279
  
 NCBI BlastP on this gene

EIW75486

P-loop containing nucleoside triphosphate hydrolase protein
  
Accession: EIW75485
  
Location: 295346-302828
  
  
**BlastP hit with Mycgr3G42010\_Mycgr3T**
  
Percentage identity: 45 %
  
BlastP bit score: 1934
  
Sequence coverage: 102 %
  
E-value: 0.0
  
  
 NCBI BlastP on this gene

EIW75485

hypothetical protein
  
Accession: EIW75484
  
Location: 286107-293188
  
  
**BlastP hit with Mycgr3G42010\_Mycgr3T**
  
Percentage identity: 43 %
  
BlastP bit score: 1191
  
Sequence coverage: 69 %
  
E-value: 0.0
  
  
 NCBI BlastP on this gene

EIW75484

cytochrome P450
  
Accession: EIW75483
  
Location: 283601-285762
  
 NCBI BlastP on this gene

EIW75483

cytochrome P450
  
Accession: EIW75482
  
Location: 280640-282920
  
 NCBI BlastP on this gene

EIW75482

hypothetical protein
  
Accession: EIW75481
  
Location: 278873-280192
  
 NCBI BlastP on this gene

EIW75481

NAD(P)-binding protein
  
Accession: EIW75480
  
Location: 277005-278684
  
 NCBI BlastP on this gene

EIW75480

iron reductase
  
Accession: EIW75479
  
Location: 272617-274877
  
 NCBI BlastP on this gene

EIW75479

Query: Architecture Search FASTA input

KB446555 : Pseudocercospora fijiensis CIRAD86 unplaced genomic scaffold MYCFIscaffold\_1    Total score: 1.0     Cumulative Blast bit score: 2089

Hit cluster cross-links:

Mycgr3G85918 Mycgr3T
  
Location: 0-1602

Mycgr3G85918\_Mycgr3T

Mycgr3G42010 Mycgr3T
  
Location: 1702-8569

Mycgr3G42010\_Mycgr3T

Mycgr3G29582 Mycgr3T
  
Location: 8669-8915

Mycgr3G29582\_Mycgr3T

Mycgr3G31170 Mycgr3T
  
Location: 9015-9255

Mycgr3G31170\_Mycgr3T

Mycgr3G85924 Mycgr3T
  
Location: 9355-11218

Mycgr3G85924\_Mycgr3T

Mycgr3G71676 Mycgr3T
  
Location: 11318-12494

Mycgr3G71676\_Mycgr3T

Mycgr3G11468 Mycgr3T
  
Location: 12594-13653

Mycgr3G11468\_Mycgr3T

Mycgr3G58567 Mycgr3T
  
Location: 13753-14506

Mycgr3G58567\_Mycgr3T

Mycgr3G100089 Mycgr3
  
Location: 14606-21152

Mycgr3G100089\_Mycgr3

Mycgr3G42698 Mycgr3T
  
Location: 21252-22131

Mycgr3G42698\_Mycgr3T

Mycgr3G71681 Mycgr3T
  
Location: 22231-23461

Mycgr3G71681\_Mycgr3T

Mycgr3G109328 Mycgr3
  
Location: 23561-24239

Mycgr3G109328\_Mycgr3

Mycgr3G104334 Mycgr3
  
Location: 24339-24567

Mycgr3G104334\_Mycgr3

Mycgr3G42715 Mycgr3T
  
Location: 24667-25981

Mycgr3G42715\_Mycgr3T

Mycgr3G92934 Mycgr3T
  
Location: 26081-27593

Mycgr3G92934\_Mycgr3T

Mycgr3G41969 Mycgr3T
  
Location: 27693-29328

Mycgr3G41969\_Mycgr3T

Mycgr3G80635 Mycgr3T
  
Location: 29428-29821

Mycgr3G80635\_Mycgr3T

Mycgr3G41426 Mycgr3T
  
Location: 29921-35255

Mycgr3G41426\_Mycgr3T

Mycgr3G104337 Mycgr3
  
Location: 35355-36108

Mycgr3G104337\_Mycgr3

Mycgr3G71679 Mycgr3T
  
Location: 36208-37300

Mycgr3G71679\_Mycgr3T

Mycgr3G92938 Mycgr3T
  
Location: 37400-38699

Mycgr3G92938\_Mycgr3T

Mycgr3G92941 Mycgr3T
  
Location: 38799-40734

Mycgr3G92941\_Mycgr3T

hypothetical protein
  
Accession: EME89031
  
Location: 8485738-8487778
  
 NCBI BlastP on this gene

EME89031

hypothetical protein
  
Accession: EME89030
  
Location: 8484824-8485360
  
 NCBI BlastP on this gene

EME89030

hypothetical protein
  
Accession: EME89029
  
Location: 8480796-8480999
  
 NCBI BlastP on this gene

EME89029

hypothetical protein
  
Accession: EME89028
  
Location: 8478486-8479655
  
 NCBI BlastP on this gene

EME89028

hypothetical protein
  
Accession: EME89027
  
Location: 8469649-8471007
  
 NCBI BlastP on this gene

EME89027

hypothetical protein
  
Accession: EME89026
  
Location: 8466881-8469115
  
 NCBI BlastP on this gene

EME89026

hypothetical protein
  
Accession: EME89025
  
Location: 8460428-8466427
  
  
**BlastP hit with Mycgr3G41426\_Mycgr3T**
  
Percentage identity: 56 %
  
BlastP bit score: 2089
  
Sequence coverage: 101 %
  
E-value: 0.0
  
  
 NCBI BlastP on this gene

EME89025

hypothetical protein
  
Accession: EME89024
  
Location: 8455639-8456064
  
 NCBI BlastP on this gene

EME89024

hypothetical protein
  
Accession: EME89023
  
Location: 8454544-8455509
  
 NCBI BlastP on this gene

EME89023

hypothetical protein
  
Accession: EME89022
  
Location: 8453453-8453991
  
 NCBI BlastP on this gene

EME89022

Query: Architecture Search FASTA input

KB456260 : Mycosphaerella populorum SO2202 unplaced genomic scaffold SEPMUscaffold\_1    Total score: 1.0     Cumulative Blast bit score: 1968

Hit cluster cross-links:

Mycgr3G85918 Mycgr3T
  
Location: 0-1602

Mycgr3G85918\_Mycgr3T

Mycgr3G42010 Mycgr3T
  
Location: 1702-8569

Mycgr3G42010\_Mycgr3T

Mycgr3G29582 Mycgr3T
  
Location: 8669-8915

Mycgr3G29582\_Mycgr3T

Mycgr3G31170 Mycgr3T
  
Location: 9015-9255

Mycgr3G31170\_Mycgr3T

Mycgr3G85924 Mycgr3T
  
Location: 9355-11218

Mycgr3G85924\_Mycgr3T

Mycgr3G71676 Mycgr3T
  
Location: 11318-12494

Mycgr3G71676\_Mycgr3T

Mycgr3G11468 Mycgr3T
  
Location: 12594-13653

Mycgr3G11468\_Mycgr3T

Mycgr3G58567 Mycgr3T
  
Location: 13753-14506

Mycgr3G58567\_Mycgr3T

Mycgr3G100089 Mycgr3
  
Location: 14606-21152

Mycgr3G100089\_Mycgr3

Mycgr3G42698 Mycgr3T
  
Location: 21252-22131

Mycgr3G42698\_Mycgr3T

Mycgr3G71681 Mycgr3T
  
Location: 22231-23461

Mycgr3G71681\_Mycgr3T

Mycgr3G109328 Mycgr3
  
Location: 23561-24239

Mycgr3G109328\_Mycgr3

Mycgr3G104334 Mycgr3
  
Location: 24339-24567

Mycgr3G104334\_Mycgr3

Mycgr3G42715 Mycgr3T
  
Location: 24667-25981

Mycgr3G42715\_Mycgr3T

Mycgr3G92934 Mycgr3T
  
Location: 26081-27593

Mycgr3G92934\_Mycgr3T

Mycgr3G41969 Mycgr3T
  
Location: 27693-29328

Mycgr3G41969\_Mycgr3T

Mycgr3G80635 Mycgr3T
  
Location: 29428-29821

Mycgr3G80635\_Mycgr3T

Mycgr3G41426 Mycgr3T
  
Location: 29921-35255

Mycgr3G41426\_Mycgr3T

Mycgr3G104337 Mycgr3
  
Location: 35355-36108

Mycgr3G104337\_Mycgr3

Mycgr3G71679 Mycgr3T
  
Location: 36208-37300

Mycgr3G71679\_Mycgr3T

Mycgr3G92938 Mycgr3T
  
Location: 37400-38699

Mycgr3G92938\_Mycgr3T

Mycgr3G92941 Mycgr3T
  
Location: 38799-40734

Mycgr3G92941\_Mycgr3T

elongation factor 1 beta subunit
  
Accession: EMF16680
  
Location: 1505292-1506298
  
 NCBI BlastP on this gene

EMF16680

hypothetical protein
  
Accession: EMF16681
  
Location: 1506822-1507097
  
 NCBI BlastP on this gene

EMF16681

mismatch repair protein 5
  
Accession: EMF16682
  
Location: 1507808-1510476
  
 NCBI BlastP on this gene

EMF16682

ribosome biogenesis ATPase RIX7
  
Accession: EMF16683
  
Location: 1510879-1513188
  
 NCBI BlastP on this gene

EMF16683

hypothetical protein
  
Accession: EMF16684
  
Location: 1513610-1514331
  
 NCBI BlastP on this gene

EMF16684

hypothetical protein
  
Accession: EMF16685
  
Location: 1515331-1515753
  
 NCBI BlastP on this gene

EMF16685

hypothetical protein
  
Accession: EMF16686
  
Location: 1519154-1520032
  
 NCBI BlastP on this gene

EMF16686

hypothetical protein
  
Accession: EMF16687
  
Location: 1521201-1522742
  
 NCBI BlastP on this gene

EMF16687

hypothetical protein
  
Accession: EMF16688
  
Location: 1523903-1529914
  
  
**BlastP hit with Mycgr3G41426\_Mycgr3T**
  
Percentage identity: 55 %
  
BlastP bit score: 1968
  
Sequence coverage: 102 %
  
E-value: 0.0
  
  
 NCBI BlastP on this gene

EMF16688

Query: Architecture Search FASTA input

KB469300 : Gloeophyllum trabeum ATCC 11539 unplaced genomic scaffold GLOTRscaffold\_00005    Total score: 1.0     Cumulative Blast bit score: 1955

Hit cluster cross-links:

Mycgr3G85918 Mycgr3T
  
Location: 0-1602

Mycgr3G85918\_Mycgr3T

Mycgr3G42010 Mycgr3T
  
Location: 1702-8569

Mycgr3G42010\_Mycgr3T

Mycgr3G29582 Mycgr3T
  
Location: 8669-8915

Mycgr3G29582\_Mycgr3T

Mycgr3G31170 Mycgr3T
  
Location: 9015-9255

Mycgr3G31170\_Mycgr3T

Mycgr3G85924 Mycgr3T
  
Location: 9355-11218

Mycgr3G85924\_Mycgr3T

Mycgr3G71676 Mycgr3T
  
Location: 11318-12494

Mycgr3G71676\_Mycgr3T

Mycgr3G11468 Mycgr3T
  
Location: 12594-13653

Mycgr3G11468\_Mycgr3T

Mycgr3G58567 Mycgr3T
  
Location: 13753-14506

Mycgr3G58567\_Mycgr3T

Mycgr3G100089 Mycgr3
  
Location: 14606-21152

Mycgr3G100089\_Mycgr3

Mycgr3G42698 Mycgr3T
  
Location: 21252-22131

Mycgr3G42698\_Mycgr3T

Mycgr3G71681 Mycgr3T
  
Location: 22231-23461

Mycgr3G71681\_Mycgr3T

Mycgr3G109328 Mycgr3
  
Location: 23561-24239

Mycgr3G109328\_Mycgr3

Mycgr3G104334 Mycgr3
  
Location: 24339-24567

Mycgr3G104334\_Mycgr3

Mycgr3G42715 Mycgr3T
  
Location: 24667-25981

Mycgr3G42715\_Mycgr3T

Mycgr3G92934 Mycgr3T
  
Location: 26081-27593

Mycgr3G92934\_Mycgr3T

Mycgr3G41969 Mycgr3T
  
Location: 27693-29328

Mycgr3G41969\_Mycgr3T

Mycgr3G80635 Mycgr3T
  
Location: 29428-29821

Mycgr3G80635\_Mycgr3T

Mycgr3G41426 Mycgr3T
  
Location: 29921-35255

Mycgr3G41426\_Mycgr3T

Mycgr3G104337 Mycgr3
  
Location: 35355-36108

Mycgr3G104337\_Mycgr3

Mycgr3G71679 Mycgr3T
  
Location: 36208-37300

Mycgr3G71679\_Mycgr3T

Mycgr3G92938 Mycgr3T
  
Location: 37400-38699

Mycgr3G92938\_Mycgr3T

Mycgr3G92941 Mycgr3T
  
Location: 38799-40734

Mycgr3G92941\_Mycgr3T

hypothetical protein
  
Accession: EPQ56106
  
Location: 151613-152377
  
 NCBI BlastP on this gene

EPQ56106

P-loop containing nucleoside triphosphate hydrolase protein
  
Accession: EPQ56105
  
Location: 147339-151096
  
 NCBI BlastP on this gene

EPQ56105

hypothetical protein
  
Accession: EPQ56104
  
Location: 142745-147235
  
 NCBI BlastP on this gene

EPQ56104

hypothetical protein
  
Accession: EPQ56103
  
Location: 141616-141822
  
 NCBI BlastP on this gene

EPQ56103

hypothetical protein
  
Accession: EPQ56102
  
Location: 138543-139465
  
 NCBI BlastP on this gene

EPQ56102

hypothetical protein
  
Accession: EPQ56101
  
Location: 136456-138237
  
 NCBI BlastP on this gene

EPQ56101

P-loop containing nucleoside triphosphate hydrolase protein
  
Accession: EPQ56100
  
Location: 128005-135685
  
  
**BlastP hit with Mycgr3G42010\_Mycgr3T**
  
Percentage identity: 44 %
  
BlastP bit score: 1955
  
Sequence coverage: 105 %
  
E-value: 0.0
  
  
 NCBI BlastP on this gene

EPQ56100

cobW-domain-containing protein
  
Accession: EPQ56099
  
Location: 123554-125462
  
 NCBI BlastP on this gene

EPQ56099

hypothetical protein
  
Accession: EPQ56098
  
Location: 119021-122633
  
 NCBI BlastP on this gene

EPQ56098

FAD/NAD P-binding domain-containing protein
  
Accession: EPQ56097
  
Location: 116851-118597
  
 NCBI BlastP on this gene

EPQ56097

hypothetical protein
  
Accession: EPQ56096
  
Location: 113642-116697
  
 NCBI BlastP on this gene

EPQ56096

Query: Architecture Search FASTA input

JH687546 : Punctularia strigosozonata HHB-11173 SS5 unplaced genomic scaffold PUNSTscaffold\_9    Total score: 1.0     Cumulative Blast bit score: 1875

Hit cluster cross-links:

Mycgr3G85918 Mycgr3T
  
Location: 0-1602

Mycgr3G85918\_Mycgr3T

Mycgr3G42010 Mycgr3T
  
Location: 1702-8569

Mycgr3G42010\_Mycgr3T

Mycgr3G29582 Mycgr3T
  
Location: 8669-8915

Mycgr3G29582\_Mycgr3T

Mycgr3G31170 Mycgr3T
  
Location: 9015-9255

Mycgr3G31170\_Mycgr3T

Mycgr3G85924 Mycgr3T
  
Location: 9355-11218

Mycgr3G85924\_Mycgr3T

Mycgr3G71676 Mycgr3T
  
Location: 11318-12494

Mycgr3G71676\_Mycgr3T

Mycgr3G11468 Mycgr3T
  
Location: 12594-13653

Mycgr3G11468\_Mycgr3T

Mycgr3G58567 Mycgr3T
  
Location: 13753-14506

Mycgr3G58567\_Mycgr3T

Mycgr3G100089 Mycgr3
  
Location: 14606-21152

Mycgr3G100089\_Mycgr3

Mycgr3G42698 Mycgr3T
  
Location: 21252-22131

Mycgr3G42698\_Mycgr3T

Mycgr3G71681 Mycgr3T
  
Location: 22231-23461

Mycgr3G71681\_Mycgr3T

Mycgr3G109328 Mycgr3
  
Location: 23561-24239

Mycgr3G109328\_Mycgr3

Mycgr3G104334 Mycgr3
  
Location: 24339-24567

Mycgr3G104334\_Mycgr3

Mycgr3G42715 Mycgr3T
  
Location: 24667-25981

Mycgr3G42715\_Mycgr3T

Mycgr3G92934 Mycgr3T
  
Location: 26081-27593

Mycgr3G92934\_Mycgr3T

Mycgr3G41969 Mycgr3T
  
Location: 27693-29328

Mycgr3G41969\_Mycgr3T

Mycgr3G80635 Mycgr3T
  
Location: 29428-29821

Mycgr3G80635\_Mycgr3T

Mycgr3G41426 Mycgr3T
  
Location: 29921-35255

Mycgr3G41426\_Mycgr3T

Mycgr3G104337 Mycgr3
  
Location: 35355-36108

Mycgr3G104337\_Mycgr3

Mycgr3G71679 Mycgr3T
  
Location: 36208-37300

Mycgr3G71679\_Mycgr3T

Mycgr3G92938 Mycgr3T
  
Location: 37400-38699

Mycgr3G92938\_Mycgr3T

Mycgr3G92941 Mycgr3T
  
Location: 38799-40734

Mycgr3G92941\_Mycgr3T

S-adenosyl-L-methionine-dependent methyltransferase
  
Accession: EIN07303
  
Location: 1039044-1040528
  
 NCBI BlastP on this gene

EIN07303

scamp-domain-containing protein
  
Accession: EIN07304
  
Location: 1042752-1044074
  
 NCBI BlastP on this gene

EIN07304

polyadenylate binding protein
  
Accession: EIN07305
  
Location: 1045138-1047309
  
 NCBI BlastP on this gene

EIN07305

alpha/beta-hydrolase
  
Accession: EIN07306
  
Location: 1047721-1049010
  
 NCBI BlastP on this gene

EIN07306

mitochondrial protein
  
Accession: EIN07307
  
Location: 1049453-1050771
  
 NCBI BlastP on this gene

EIN07307

adenylate kinase
  
Accession: EIN07308
  
Location: 1051329-1052231
  
 NCBI BlastP on this gene

EIN07308

alpha/beta-hydrolase
  
Accession: EIN07309
  
Location: 1052666-1054781
  
 NCBI BlastP on this gene

EIN07309

Swi3-domain-containing protein
  
Accession: EIN07310
  
Location: 1055187-1056729
  
 NCBI BlastP on this gene

EIN07310

methyltransferase domain-containing protein
  
Accession: EIN07311
  
Location: 1057081-1058292
  
 NCBI BlastP on this gene

EIN07311

P-loop containing nucleoside triphosphate hydrolase protein
  
Accession: EIN07312
  
Location: 1058678-1066395
  
  
**BlastP hit with Mycgr3G42010\_Mycgr3T**
  
Percentage identity: 43 %
  
BlastP bit score: 1875
  
Sequence coverage: 103 %
  
E-value: 0.0
  
  
 NCBI BlastP on this gene

EIN07312

hypothetical protein
  
Accession: EIN07313
  
Location: 1067427-1071641
  
 NCBI BlastP on this gene

EIN07313

MFS general substrate transporter
  
Accession: EIN07314
  
Location: 1071895-1073944
  
 NCBI BlastP on this gene

EIN07314

hypothetical protein
  
Accession: EIN07315
  
Location: 1074391-1076885
  
 NCBI BlastP on this gene

EIN07315

WD40 repeat-like protein
  
Accession: EIN07316
  
Location: 1077135-1079438
  
 NCBI BlastP on this gene

EIN07316

N2,N2-dimethylguanosine tRNA methyltransferase
  
Accession: EIN07317
  
Location: 1080132-1082487
  
 NCBI BlastP on this gene

EIN07317

Pkinase-domain-containing protein
  
Accession: EIN07318
  
Location: 1082623-1086557
  
 NCBI BlastP on this gene

EIN07318

Query: Architecture Search FASTA input

201. :  GG749462 Ajellomyces dermatitidis ATCC 18188 genomic scaffold supercont1.56     Total score: 2.0     Cumulative Blast bit score: 622

Mycgr3G85918 Mycgr3T
  
Location: 0-1602
  
 NCBI BlastP on this gene

Mycgr3G85918\_Mycgr3T

Mycgr3G42010 Mycgr3T
  
Location: 1702-8569
  
 NCBI BlastP on this gene

Mycgr3G42010\_Mycgr3T

Mycgr3G29582 Mycgr3T
  
Location: 8669-8915
  
 NCBI BlastP on this gene

Mycgr3G29582\_Mycgr3T

Mycgr3G31170 Mycgr3T
  
Location: 9015-9255
  
 NCBI BlastP on this gene

Mycgr3G31170\_Mycgr3T

Mycgr3G85924 Mycgr3T
  
Location: 9355-11218
  
 NCBI BlastP on this gene

Mycgr3G85924\_Mycgr3T

Mycgr3G71676 Mycgr3T
  
Location: 11318-12494
  
 NCBI BlastP on this gene

Mycgr3G71676\_Mycgr3T

Mycgr3G11468 Mycgr3T
  
Location: 12594-13653
  
 NCBI BlastP on this gene

Mycgr3G11468\_Mycgr3T

Mycgr3G58567 Mycgr3T
  
Location: 13753-14506
  
 NCBI BlastP on this gene

Mycgr3G58567\_Mycgr3T

Mycgr3G100089 Mycgr3
  
Location: 14606-21152
  
 NCBI BlastP on this gene

Mycgr3G100089\_Mycgr3

Mycgr3G42698 Mycgr3T
  
Location: 21252-22131
  
 NCBI BlastP on this gene

Mycgr3G42698\_Mycgr3T

Mycgr3G71681 Mycgr3T
  
Location: 22231-23461
  
 NCBI BlastP on this gene

Mycgr3G71681\_Mycgr3T

Mycgr3G109328 Mycgr3
  
Location: 23561-24239
  
 NCBI BlastP on this gene

Mycgr3G109328\_Mycgr3

Mycgr3G104334 Mycgr3
  
Location: 24339-24567
  
 NCBI BlastP on this gene

Mycgr3G104334\_Mycgr3

Mycgr3G42715 Mycgr3T
  
Location: 24667-25981
  
 NCBI BlastP on this gene

Mycgr3G42715\_Mycgr3T

Mycgr3G92934 Mycgr3T
  
Location: 26081-27593
  
 NCBI BlastP on this gene

Mycgr3G92934\_Mycgr3T

Mycgr3G41969 Mycgr3T
  
Location: 27693-29328
  
 NCBI BlastP on this gene

Mycgr3G41969\_Mycgr3T

Mycgr3G80635 Mycgr3T
  
Location: 29428-29821
  
 NCBI BlastP on this gene

Mycgr3G80635\_Mycgr3T

Mycgr3G41426 Mycgr3T
  
Location: 29921-35255
  
 NCBI BlastP on this gene

Mycgr3G41426\_Mycgr3T

Mycgr3G104337 Mycgr3
  
Location: 35355-36108
  
 NCBI BlastP on this gene

Mycgr3G104337\_Mycgr3

Mycgr3G71679 Mycgr3T
  
Location: 36208-37300
  
 NCBI BlastP on this gene

Mycgr3G71679\_Mycgr3T

Mycgr3G92938 Mycgr3T
  
Location: 37400-38699
  
 NCBI BlastP on this gene

Mycgr3G92938\_Mycgr3T

Mycgr3G92941 Mycgr3T
  
Location: 38799-40734
  
 NCBI BlastP on this gene

Mycgr3G92941\_Mycgr3T

serine protein kinase Sky1
  
Accession: EGE84164
  
Location: 207730-209207
  
 NCBI BlastP on this gene

EGE84164

hypothetical protein
  
Accession: EGE84163
  
Location: 206805-207141
  
 NCBI BlastP on this gene

EGE84163

hypothetical protein
  
Accession: EGE84162
  
Location: 205163-206418
  
 NCBI BlastP on this gene

EGE84162

MADS box transcription factor Mcm1
  
Accession: EGE84161
  
Location: 201512-202489
  
  
**BlastP hit with Mycgr3G31170\_Mycgr3T**
  
Percentage identity: 100 %
  
BlastP bit score: 166
  
Sequence coverage: 100 %
  
E-value: 1e-49
  
  
 NCBI BlastP on this gene

EGE84161

DUF803 domain-containing protein
  
Accession: EGE84160
  
Location: 196049-198199
  
 NCBI BlastP on this gene

EGE84160

vacuolar ATP synthase subunit E
  
Accession: EGE84159
  
Location: 194599-195523
  
 NCBI BlastP on this gene

EGE84159

hypothetical protein
  
Accession: EGE84158
  
Location: 194213-194563
  
 NCBI BlastP on this gene

EGE84158

hypothetical protein
  
Accession: EGE84157
  
Location: 191419-193902
  
 NCBI BlastP on this gene

EGE84157

serine/threonine protein phosphatase
  
Accession: EGE84156
  
Location: 189477-190256
  
 NCBI BlastP on this gene

EGE84156

hypothetical protein
  
Accession: EGE84155
  
Location: 188220-188710
  
 NCBI BlastP on this gene

EGE84155

phospholipid-translocating P-type ATPase domain-containing protein
  
Accession: EGE84154
  
Location: 182229-186884
  
 NCBI BlastP on this gene

EGE84154

tRNA-splicing endonuclease
  
Accession: EGE84153
  
Location: 172887-180343
  
  
**BlastP hit with Mycgr3G41426\_Mycgr3T**
  
Percentage identity: 30 %
  
BlastP bit score: 456
  
Sequence coverage: 49 %
  
E-value: 3e-128
  
  
 NCBI BlastP on this gene

EGE84153

hypothetical protein
  
Accession: EGE84152
  
Location: 164434-167775
  
 NCBI BlastP on this gene

EGE84152

202. :  GG657463 Ajellomyces dermatitidis SLH14081 genomic scaffold supercont1.16     Total score: 2.0     Cumulative Blast bit score: 620

conserved hypothetical protein
  
Accession: EEQ71914
  
Location: 1357784-1359039
  
 NCBI BlastP on this gene

EEQ71914

predicted protein
  
Accession: EEQ71913
  
Location: 1350192-1350740
  
 NCBI BlastP on this gene

EEQ71913

MADS box transcription factor Mcm1
  
Accession: EEQ71912
  
Location: 1348343-1349320
  
  
**BlastP hit with Mycgr3G31170\_Mycgr3T**
  
Percentage identity: 100 %
  
BlastP bit score: 166
  
Sequence coverage: 100 %
  
E-value: 1e-49
  
  
 NCBI BlastP on this gene

EEQ71912

DUF803 domain-containing protein
  
Accession: EEQ71911
  
Location: 1343307-1345457
  
 NCBI BlastP on this gene

EEQ71911

vacuolar ATP synthase subunit E
  
Accession: EEQ71910
  
Location: 1341850-1342774
  
 NCBI BlastP on this gene

EEQ71910

conserved hypothetical protein
  
Accession: EEQ71909
  
Location: 1338670-1341153
  
 NCBI BlastP on this gene

EEQ71909

conserved hypothetical protein
  
Accession: EEQ71908
  
Location: 1336707-1337486
  
 NCBI BlastP on this gene

EEQ71908

phospholipid-translocating P-type ATPase domain-containing protein
  
Accession: EEQ71907
  
Location: 1329515-1334169
  
 NCBI BlastP on this gene

EEQ71907

tRNA-splicing endonuclease
  
Accession: EEQ71906
  
Location: 1320794-1327634
  
  
**BlastP hit with Mycgr3G41426\_Mycgr3T**
  
Percentage identity: 30 %
  
BlastP bit score: 454
  
Sequence coverage: 49 %
  
E-value: 9e-128
  
  
 NCBI BlastP on this gene

EEQ71906

hypothetical protein
  
Accession: EEQ71905
  
Location: 1312278-1315517
  
 NCBI BlastP on this gene

EEQ71905

203. :  HE978324 Kazachstania naganishii CBS 8797 chromosome 11     Total score: 2.0     Cumulative Blast bit score: 598

hypothetical protein
  
Accession: CCK72404
  
Location: 58785-60377
  
 NCBI BlastP on this gene

KNAG0K00360

hypothetical protein
  
Accession: CCK72405
  
Location: 62596-65535
  
 NCBI BlastP on this gene

KNAG0K00370

hypothetical protein
  
Accession: CCK72406
  
Location: 65728-66825
  
 NCBI BlastP on this gene

KNAG0K00380

hypothetical protein
  
Accession: CCK72407
  
Location: 66964-68445
  
 NCBI BlastP on this gene

KNAG0K00390

hypothetical protein
  
Accession: CCK72408
  
Location: 69795-71093
  
  
**BlastP hit with Mycgr3G92934\_Mycgr3T**
  
Percentage identity: 45 %
  
BlastP bit score: 363
  
Sequence coverage: 89 %
  
E-value: 6e-117
  
  
 NCBI BlastP on this gene

KNAG0K00400

hypothetical protein
  
Accession: CCK72409
  
Location: 71166-71738
  
 NCBI BlastP on this gene

KNAG0K00410

hypothetical protein
  
Accession: CCK72410
  
Location: 71859-73397
  
 NCBI BlastP on this gene

KNAG0K00420

hypothetical protein
  
Accession: CCK72411
  
Location: 73566-74159
  
 NCBI BlastP on this gene

KNAG0K00430

hypothetical protein
  
Accession: CCK72412
  
Location: 74289-75608
  
 NCBI BlastP on this gene

KNAG0K00440

hypothetical protein
  
Accession: CCK72413
  
Location: 75675-76478
  
 NCBI BlastP on this gene

KNAG0K00450

hypothetical protein
  
Accession: CCK72414
  
Location: 77388-79508
  
 NCBI BlastP on this gene

KNAG0K00460

hypothetical protein
  
Accession: CCK72415
  
Location: 79664-80908
  
 NCBI BlastP on this gene

KNAG0K00470

hypothetical protein
  
Accession: CCK72416
  
Location: 82114-82653
  
 NCBI BlastP on this gene

KNAG0K00480

hypothetical protein
  
Accession: CCK72417
  
Location: 84367-85047
  
 NCBI BlastP on this gene

KNAG0K00490

hypothetical protein
  
Accession: CCK72418
  
Location: 85328-85981
  
 NCBI BlastP on this gene

KNAG0K00500

hypothetical protein
  
Accession: CCK72419
  
Location: 86667-88943
  
 NCBI BlastP on this gene

KNAG0K00510

hypothetical protein
  
Accession: CCK72420
  
Location: 89428-90687
  
 NCBI BlastP on this gene

KNAG0K00520

hypothetical protein
  
Accession: CCK72421
  
Location: 91791-93842
  
  
**BlastP hit with Mycgr3G41969\_Mycgr3T**
  
Percentage identity: 33 %
  
BlastP bit score: 235
  
Sequence coverage: 104 %
  
E-value: 3e-65
  
  
 NCBI BlastP on this gene

KNAG0K00530

hypothetical protein
  
Accession: CCK72422
  
Location: 94316-95308
  
 NCBI BlastP on this gene

KNAG0K00540

hypothetical protein
  
Accession: CCK72423
  
Location: 96000-96491
  
 NCBI BlastP on this gene

KNAG0K00550

hypothetical protein
  
Accession: CCK72424
  
Location: 96678-99992
  
 NCBI BlastP on this gene

KNAG0K00560

hypothetical protein
  
Accession: CCK72425
  
Location: 100355-101266
  
 NCBI BlastP on this gene

KNAG0K00570

hypothetical protein
  
Accession: CCK72426
  
Location: 101850-104819
  
 NCBI BlastP on this gene

KNAG0K00580

204. :  GG704912 Coccidioides immitis RS genomic scaffold supercont3.2     Total score: 2.0     Cumulative Blast bit score: 594

hypothetical protein
  
Accession: EAS30546
  
Location: 939085-940075
  
 NCBI BlastP on this gene

EAS30546

hypothetical protein
  
Accession: EJB10891
  
Location: 937802-938205
  
 NCBI BlastP on this gene

EJB10891

hypothetical protein
  
Accession: EAS30545
  
Location: 936011-936408
  
 NCBI BlastP on this gene

EAS30545

hypothetical protein
  
Accession: EAS30544
  
Location: 933419-934840
  
 NCBI BlastP on this gene

EAS30544

hypothetical protein
  
Accession: EAS30542
  
Location: 931619-932556
  
 NCBI BlastP on this gene

EAS30542

MADS box transcription factor Mcm1, variant
  
Accession: EJB10890
  
Location: 929475-930349
  
  
**BlastP hit with Mycgr3G31170\_Mycgr3T**
  
Percentage identity: 97 %
  
BlastP bit score: 160
  
Sequence coverage: 100 %
  
E-value: 4e-47
  
  
 NCBI BlastP on this gene

EJB10890

hypothetical protein, variant
  
Accession: EJB10888
  
Location: 926753-928297
  
 NCBI BlastP on this gene

EJB10888

vacuolar ATP synthase subunit E
  
Accession: EAS30539
  
Location: 924663-925538
  
 NCBI BlastP on this gene

EAS30539

hypothetical protein
  
Accession: EAS30538
  
Location: 921975-924332
  
 NCBI BlastP on this gene

EAS30538

phospholipid-translocating P-type ATPase, flippase
  
Accession: EAS30537
  
Location: 915546-920294
  
 NCBI BlastP on this gene

EAS30537

hypothetical protein
  
Accession: EJB10886
  
Location: 914541-915269
  
 NCBI BlastP on this gene

EJB10886

tRNA-splicing endonuclease
  
Accession: EAS30536
  
Location: 907178-913959
  
  
**BlastP hit with Mycgr3G41426\_Mycgr3T**
  
Percentage identity: 30 %
  
BlastP bit score: 434
  
Sequence coverage: 50 %
  
E-value: 3e-121
  
  
 NCBI BlastP on this gene

EAS30536

hypothetical protein
  
Accession: EAS30533
  
Location: 900054-902693
  
 NCBI BlastP on this gene

EAS30533

mitochondrial inner membrane magnesium transporter mrs2
  
Accession: EAS30532
  
Location: 897178-899185
  
 NCBI BlastP on this gene

EAS30532

205. :  ACFW01000025 Coccidioides posadasii C735 delta SOWgp     Total score: 2.0     Cumulative Blast bit score: 594

hypothetical protein
  
Accession: EER27116
  
Location: 691566-692199
  
 NCBI BlastP on this gene

EER27116

Phospholipase/Carboxylesterase family protein
  
Accession: EER27115
  
Location: 685586-686437
  
 NCBI BlastP on this gene

EER27115

SRF-type transcription factor family protein
  
Accession: EER27114
  
Location: 683443-684313
  
  
**BlastP hit with Mycgr3G31170\_Mycgr3T**
  
Percentage identity: 97 %
  
BlastP bit score: 160
  
Sequence coverage: 100 %
  
E-value: 3e-47
  
  
 NCBI BlastP on this gene

EER27114

hypothetical protein
  
Accession: EER27113
  
Location: 680547-682265
  
 NCBI BlastP on this gene

EER27113

vacuolar ATP synthase subunit E, putative
  
Accession: EER27112
  
Location: 678624-679491
  
 NCBI BlastP on this gene

EER27112

hypothetical protein
  
Accession: EER27111
  
Location: 675945-678293
  
 NCBI BlastP on this gene

EER27111

phospholipid-translocating P-type ATPase domain-containing protein, putative
  
Accession: EER27110
  
Location: 669526-674274
  
 NCBI BlastP on this gene

EER27110

Zinc knuckle domain containing protein
  
Accession: EER27109
  
Location: 661179-667960
  
  
**BlastP hit with Mycgr3G41426\_Mycgr3T**
  
Percentage identity: 30 %
  
BlastP bit score: 434
  
Sequence coverage: 51 %
  
E-value: 4e-121
  
  
 NCBI BlastP on this gene

EER27109

hypothetical protein
  
Accession: EER27108
  
Location: 654064-656235
  
 NCBI BlastP on this gene

EER27108

CorA-like Mg2+ transporter family protein
  
Accession: EER27107
  
Location: 651205-653213
  
 NCBI BlastP on this gene

EER27107

WH1 domain containing protein
  
Accession: EER27106
  
Location: 648452-650416
  
 NCBI BlastP on this gene

EER27106

206. :  AKHY01000076 Aspergillus oryzae 3.042     Total score: 2.0     Cumulative Blast bit score: 589

hypothetical protein
  
Accession: EIT82520
  
Location: 257968-259581
  
 NCBI BlastP on this gene

EIT82520

regulator of arginine metabolism
  
Accession: EIT82519
  
Location: 263169-264079
  
  
**BlastP hit with Mycgr3G31170\_Mycgr3T**
  
Percentage identity: 98 %
  
BlastP bit score: 163
  
Sequence coverage: 100 %
  
E-value: 2e-48
  
  
 NCBI BlastP on this gene

EIT82519

hypothetical protein
  
Accession: EIT82523
  
Location: 265424-267096
  
 NCBI BlastP on this gene

EIT82523

vacuolar H+-ATPase V1 sector, subunit E
  
Accession: EIT82514
  
Location: 267985-268801
  
 NCBI BlastP on this gene

EIT82514

hypothetical protein
  
Accession: EIT82513
  
Location: 269238-271616
  
 NCBI BlastP on this gene

EIT82513

P-type ATPase
  
Accession: EIT82534
  
Location: 276107-280822
  
 NCBI BlastP on this gene

EIT82534

tRNA-splicing endonuclease positive effector
  
Accession: EIT82535
  
Location: 281996-288554
  
  
**BlastP hit with Mycgr3G41426\_Mycgr3T**
  
Percentage identity: 30 %
  
BlastP bit score: 426
  
Sequence coverage: 48 %
  
E-value: 1e-118
  
  
 NCBI BlastP on this gene

EIT82535

207. :  HF679028 Fusarium fujikuroi IMI 58289 draft genome, chromosome FFUJ\_chr06.     Total score: 2.0     Cumulative Blast bit score: 583

related to monooxigenase
  
Accession: CCT69506
  
Location: 179324-181348
  
 NCBI BlastP on this gene

FFUJ\_05397

related to heterokaryon incompatibility protein het-6
  
Accession: CCT69505
  
Location: 176625-179255
  
 NCBI BlastP on this gene

FFUJ\_05396

related to triacylglycerol lipase V precursor
  
Accession: CCT69504
  
Location: 174436-176217
  
 NCBI BlastP on this gene

FFUJ\_05395

uncharacterized protein
  
Accession: CCT69503
  
Location: 171429-173879
  
 NCBI BlastP on this gene

FFUJ\_05394

probable NmrA-like family protein
  
Accession: CCT69502
  
Location: 170438-171385
  
 NCBI BlastP on this gene

FFUJ\_05393

related to NADPH-dependent beta-ketoacyl reductase (rhlG)
  
Accession: CCT69501
  
Location: 168958-170028
  
 NCBI BlastP on this gene

FFUJ\_05392

related to short chain dehydrogenase/reductase
  
Accession: CCT69500
  
Location: 167564-168554
  
 NCBI BlastP on this gene

FFUJ\_05391

related to transporter protein HOL1
  
Accession: CCT69499
  
Location: 164989-166747
  
  
**BlastP hit with Mycgr3G85918\_Mycgr3T**
  
Percentage identity: 31 %
  
BlastP bit score: 207
  
Sequence coverage: 98 %
  
E-value: 3e-56
  
  
 NCBI BlastP on this gene

FFUJ\_05390

uncharacterized protein
  
Accession: CCT69498
  
Location: 164251-164694
  
 NCBI BlastP on this gene

FFUJ\_05389

uncharacterized protein
  
Accession: CCT69497
  
Location: 158509-158979
  
 NCBI BlastP on this gene

FFUJ\_05388

probable L-amino-acid oxidase
  
Accession: CCT69496
  
Location: 155528-157063
  
 NCBI BlastP on this gene

FFUJ\_05387

uncharacterized protein
  
Accession: CCT69495
  
Location: 152504-153615
  
 NCBI BlastP on this gene

FFUJ\_05386

related to aldehyde reductase II
  
Accession: CCT69494
  
Location: 150641-151812
  
 NCBI BlastP on this gene

FFUJ\_05385

uncharacterized protein
  
Accession: CCT69493
  
Location: 149354-150613
  
 NCBI BlastP on this gene

FFUJ\_05384

related to epoxide hydrolase
  
Accession: CCT69492
  
Location: 147396-148653
  
  
**BlastP hit with Mycgr3G71676\_Mycgr3T**
  
Percentage identity: 48 %
  
BlastP bit score: 376
  
Sequence coverage: 99 %
  
E-value: 3e-124
  
  
 NCBI BlastP on this gene

FFUJ\_05383

related to vegetatible incompatibility protein HET-E-1
  
Accession: CCT69491
  
Location: 146265-146942
  
 NCBI BlastP on this gene

FFUJ\_05382

uncharacterized protein
  
Accession: CCT70801
  
Location: 142758-145361
  
 NCBI BlastP on this gene

FFUJ\_05381

uncharacterized protein
  
Accession: CCT69490
  
Location: 141526-142706
  
 NCBI BlastP on this gene

FFUJ\_05380

related to protein TOL
  
Accession: CCT70722
  
Location: 138914-141059
  
 NCBI BlastP on this gene

FFUJ\_05379

related to sugar transporter
  
Accession: CCT69489
  
Location: 137042-138842
  
 NCBI BlastP on this gene

FFUJ\_05378

uncharacterized protein
  
Accession: CCT70792
  
Location: 135275-135942
  
 NCBI BlastP on this gene

FFUJ\_05377

related to methyltransferase
  
Accession: CCT69488
  
Location: 133737-135131
  
 NCBI BlastP on this gene

FFUJ\_05376

208. :  KB708021 Botryotinia fuckeliana BcDW1 unplaced genomic scaffold Scaffold\_349     Total score: 2.0     Cumulative Blast bit score: 571

putative glycoside hydrolase family 62 protein
  
Accession: EMR82994
  
Location: 32457-33419
  
 NCBI BlastP on this gene

EMR82994

putative major allergen asp f 2-like protein
  
Accession: EMR82993
  
Location: 25877-27386
  
 NCBI BlastP on this gene

EMR82993

putative zip zinc protein
  
Accession: EMR82992
  
Location: 23181-25020
  
 NCBI BlastP on this gene

EMR82992

putative epoxide hydrolase protein
  
Accession: EMR82991
  
Location: 13212-14489
  
  
**BlastP hit with Mycgr3G71676\_Mycgr3T**
  
Percentage identity: 48 %
  
BlastP bit score: 384
  
Sequence coverage: 97 %
  
E-value: 1e-127
  
  
 NCBI BlastP on this gene

EMR82991

putative tyrosinase central domain protein
  
Accession: EMR82990
  
Location: 5981-7352
  
  
**BlastP hit with Mycgr3G42698\_Mycgr3T**
  
Percentage identity: 36 %
  
BlastP bit score: 187
  
Sequence coverage: 108 %
  
E-value: 1e-52
  
  
 NCBI BlastP on this gene

EMR82990

putative fad-dependent monooxygenase protein
  
Accession: EMR82989
  
Location: 2246-3745
  
 NCBI BlastP on this gene

EMR82989

209. :  EQ963480 Aspergillus flavus NRRL3357 scf\_1106286418846 genomic scaffold     Total score: 2.0     Cumulative Blast bit score: 560

conserved hypothetical protein
  
Accession: EED49011
  
Location: 475842-477455
  
 NCBI BlastP on this gene

EED49011

MADS box transcription factor Mcm1
  
Accession: EED49010
  
Location: 471345-472255
  
  
**BlastP hit with Mycgr3G31170\_Mycgr3T**
  
Percentage identity: 98 %
  
BlastP bit score: 163
  
Sequence coverage: 100 %
  
E-value: 2e-48
  
  
 NCBI BlastP on this gene

EED49010

DUF803 domain protein
  
Accession: EED49009
  
Location: 468265-469998
  
 NCBI BlastP on this gene

EED49009

ATP synthase subunit E, putative
  
Accession: EED49008
  
Location: 466621-467437
  
 NCBI BlastP on this gene

EED49008

conserved hypothetical protein
  
Accession: EED49007
  
Location: 463738-464729
  
 NCBI BlastP on this gene

EED49007

hypothetical protein
  
Accession: EED49006
  
Location: 461026-461606
  
 NCBI BlastP on this gene

EED49006

phospholipid-translocating P-type ATPase domain-containing protein
  
Accession: EED49005
  
Location: 454569-459284
  
 NCBI BlastP on this gene

EED49005

tRNA-splicing endonuclease, putative
  
Accession: EED49004
  
Location: 446718-453137
  
  
**BlastP hit with Mycgr3G41426\_Mycgr3T**
  
Percentage identity: 30 %
  
BlastP bit score: 397
  
Sequence coverage: 45 %
  
E-value: 3e-109
  
  
 NCBI BlastP on this gene

EED49004

conserved hypothetical protein
  
Accession: EED49003
  
Location: 440848-443558
  
 NCBI BlastP on this gene

EED49003

conserved hypothetical protein
  
Accession: EED49002
  
Location: 439548-440247
  
 NCBI BlastP on this gene

EED49002

aminotransferase, putative
  
Accession: EED49001
  
Location: 437546-439115
  
 NCBI BlastP on this gene

EED49001

210. :  CP000518 Mycobacterium sp. KMS     Total score: 2.0     Cumulative Blast bit score: 559

conserved hypothetical protein
  
Accession: ABL93697
  
Location: 4739620-4740366
  
 NCBI BlastP on this gene

Mkms\_4506

conserved hypothetical protein
  
Accession: ABL93698
  
Location: 4740377-4740787
  
 NCBI BlastP on this gene

Mkms\_4507

dihydrodipicolinate reductase
  
Accession: ABL93699
  
Location: 4740791-4741882
  
 NCBI BlastP on this gene

Mkms\_4508

cytochrome P450
  
Accession: ABL93700
  
Location: 4741879-4743063
  
 NCBI BlastP on this gene

Mkms\_4509

transcriptional regulator, TetR family
  
Accession: ABL93701
  
Location: 4743300-4743923
  
 NCBI BlastP on this gene

Mkms\_4510

NAD-dependent epimerase/dehydratase
  
Accession: ABL93702
  
Location: 4744102-4744761
  
 NCBI BlastP on this gene

Mkms\_4511

alpha/beta hydrolase fold protein
  
Accession: ABL93703
  
Location: 4744785-4745750
  
 NCBI BlastP on this gene

Mkms\_4512

conserved hypothetical protein
  
Accession: ABL93704
  
Location: 4745777-4746208
  
 NCBI BlastP on this gene

Mkms\_4513

conserved hypothetical protein
  
Accession: ABL93705
  
Location: 4746212-4746442
  
 NCBI BlastP on this gene

Mkms\_4514

ATPase, P-type (transporting), HAD superfamily, subfamily IC
  
Accession: ABL93706
  
Location: 4746466-4748856
  
 NCBI BlastP on this gene

Mkms\_4515

beta-lactamase
  
Accession: ABL93707
  
Location: 4748917-4750518
  
 NCBI BlastP on this gene

Mkms\_4516

conserved hypothetical protein
  
Accession: ABL93708
  
Location: 4750528-4751646
  
 NCBI BlastP on this gene

Mkms\_4517

Enoyl-CoA hydratase/isomerase
  
Accession: ABL93709
  
Location: 4751649-4752383
  
 NCBI BlastP on this gene

Mkms\_4518

carboxyl transferase
  
Accession: ABL93710
  
Location: 4752410-4753918
  
 NCBI BlastP on this gene

Mkms\_4519

conserved hypothetical protein
  
Accession: ABL93711
  
Location: 4753919-4754167
  
 NCBI BlastP on this gene

Mkms\_4520

conserved hypothetical protein
  
Accession: ABL93712
  
Location: 4754164-4754379
  
 NCBI BlastP on this gene

Mkms\_4521

conserved hypothetical protein
  
Accession: ABL93713
  
Location: 4754418-4754849
  
 NCBI BlastP on this gene

Mkms\_4522

conserved hypothetical protein
  
Accession: ABL93714
  
Location: 4754859-4755266
  
 NCBI BlastP on this gene

Mkms\_4523

AMP-dependent synthetase and ligase
  
Accession: ABL93715
  
Location: 4755317-4756939
  
 NCBI BlastP on this gene

Mkms\_4524

AMP-dependent synthetase and ligase
  
Accession: ABL93716
  
Location: 4756936-4758633
  
 NCBI BlastP on this gene

Mkms\_4525

putative GAF sensor protein
  
Accession: ABL93717
  
Location: 4758682-4759539
  
 NCBI BlastP on this gene

Mkms\_4526

Epoxide hydrolase domain protein
  
Accession: ABL93718
  
Location: 4759562-4760665
  
  
**BlastP hit with Mycgr3G71676\_Mycgr3T**
  
Percentage identity: 34 %
  
BlastP bit score: 209
  
Sequence coverage: 98 %
  
E-value: 7e-60
  
  
 NCBI BlastP on this gene

Mkms\_4527

conserved hypothetical protein
  
Accession: ABL93719
  
Location: 4760667-4762037
  
  
**BlastP hit with Mycgr3G42715\_Mycgr3T**
  
Percentage identity: 41 %
  
BlastP bit score: 350
  
Sequence coverage: 105 %
  
E-value: 1e-112
  
  
 NCBI BlastP on this gene

Mkms\_4528

diguanylate cyclase
  
Accession: ABL93720
  
Location: 4762122-4763207
  
 NCBI BlastP on this gene

Mkms\_4529

conserved hypothetical protein
  
Accession: ABL93721
  
Location: 4763239-4764114
  
 NCBI BlastP on this gene

Mkms\_4530

ABC transporter related protein
  
Accession: ABL93722
  
Location: 4764238-4766070
  
 NCBI BlastP on this gene

Mkms\_4531

ABC transporter related protein
  
Accession: ABL93723
  
Location: 4766067-4768019
  
 NCBI BlastP on this gene

Mkms\_4532

ABC transporter related protein
  
Accession: ABL93724
  
Location: 4768071-4769954
  
 NCBI BlastP on this gene

Mkms\_4533

Ion transport 2 domain protein
  
Accession: ABL93725
  
Location: 4770030-4770812
  
 NCBI BlastP on this gene

Mkms\_4534

two component transcriptional regulator, winged helix family
  
Accession: ABL93726
  
Location: 4770839-4771540
  
 NCBI BlastP on this gene

Mkms\_4535

integral membrane sensor signal transduction histidine kinase
  
Accession: ABL93727
  
Location: 4771543-4772889
  
 NCBI BlastP on this gene

Mkms\_4536

peptidylprolyl isomerase, FKBP-type
  
Accession: ABL93728
  
Location: 4772961-4773329
  
 NCBI BlastP on this gene

Mkms\_4537

conserved hypothetical protein
  
Accession: ABL93729
  
Location: 4773366-4773614
  
 NCBI BlastP on this gene

Mkms\_4538

conserved hypothetical protein
  
Accession: ABL93730
  
Location: 4773659-4775158
  
 NCBI BlastP on this gene

Mkms\_4539

conserved hypothetical protein
  
Accession: ABL93731
  
Location: 4775190-4775537
  
 NCBI BlastP on this gene

Mkms\_4540

transcriptional regulator, MarR family
  
Accession: ABL93732
  
Location: 4775542-4775961
  
 NCBI BlastP on this gene

Mkms\_4541

drug resistance transporter, EmrB/QacA subfamily
  
Accession: ABL93733
  
Location: 4775951-4777405
  
 NCBI BlastP on this gene

Mkms\_4542

citrate synthase
  
Accession: ABL93734
  
Location: 4777473-4778777
  
 NCBI BlastP on this gene

Mkms\_4543

transcriptional regulator, TetR family
  
Accession: ABL93735
  
Location: 4778873-4779520
  
 NCBI BlastP on this gene

Mkms\_4544

Pyridoxamine 5'-phosphate oxidase
  
Accession: ABL93736
  
Location: 4779648-4780310
  
 NCBI BlastP on this gene

Mkms\_4545

citrate synthase
  
Accession: ABL93737
  
Location: 4780371-4781501
  
 NCBI BlastP on this gene

Mkms\_4546

conserved hypothetical protein
  
Accession: ABL93738
  
Location: 4781498-4782115
  
 NCBI BlastP on this gene

Mkms\_4547

211. :  CP000384 Mycobacterium sp. MCS     Total score: 2.0     Cumulative Blast bit score: 559

conserved hypothetical protein
  
Accession: ABG10524
  
Location: 4701173-4701583
  
 NCBI BlastP on this gene

Mmcs\_4420

dihydrodipicolinate reductase
  
Accession: ABG10525
  
Location: 4701587-4702678
  
 NCBI BlastP on this gene

Mmcs\_4421

cytochrome P450
  
Accession: ABG10526
  
Location: 4702675-4703859
  
 NCBI BlastP on this gene

Mmcs\_4422

transcriptional regulator, TetR family
  
Accession: ABG10527
  
Location: 4704096-4704719
  
 NCBI BlastP on this gene

Mmcs\_4423

NAD-dependent epimerase/dehydratase
  
Accession: ABG10528
  
Location: 4704898-4705557
  
 NCBI BlastP on this gene

Mmcs\_4424

alpha/beta hydrolase fold protein
  
Accession: ABG10529
  
Location: 4705581-4706546
  
 NCBI BlastP on this gene

Mmcs\_4425

conserved hypothetical protein
  
Accession: ABG10530
  
Location: 4706573-4707004
  
 NCBI BlastP on this gene

Mmcs\_4426

hypothetical protein
  
Accession: ABG10531
  
Location: 4707008-4707238
  
 NCBI BlastP on this gene

Mmcs\_4427

ATPase, P-type,
  
Accession: ABG10532
  
Location: 4707262-4709652
  
 NCBI BlastP on this gene

Mmcs\_4428

beta-lactamase
  
Accession: ABG10533
  
Location: 4709777-4711378
  
 NCBI BlastP on this gene

Mmcs\_4429

conserved hypothetical protein
  
Accession: ABG10534
  
Location: 4711388-4712506
  
 NCBI BlastP on this gene

Mmcs\_4430

Enoyl-CoA hydratase/isomerase
  
Accession: ABG10535
  
Location: 4712509-4713243
  
 NCBI BlastP on this gene

Mmcs\_4431

carboxyl transferase
  
Accession: ABG10536
  
Location: 4713270-4714778
  
 NCBI BlastP on this gene

Mmcs\_4432

hypothetical protein
  
Accession: ABG10537
  
Location: 4714779-4715027
  
 NCBI BlastP on this gene

Mmcs\_4433

conserved hypothetical protein
  
Accession: ABG10538
  
Location: 4715024-4715239
  
 NCBI BlastP on this gene

Mmcs\_4434

hypothetical protein
  
Accession: ABG10539
  
Location: 4715278-4715709
  
 NCBI BlastP on this gene

Mmcs\_4435

hypothetical protein
  
Accession: ABG10540
  
Location: 4715719-4716126
  
 NCBI BlastP on this gene

Mmcs\_4436

AMP-dependent synthetase and ligase
  
Accession: ABG10541
  
Location: 4716177-4717799
  
 NCBI BlastP on this gene

Mmcs\_4437

AMP-dependent synthetase and ligase
  
Accession: ABG10542
  
Location: 4717796-4719493
  
 NCBI BlastP on this gene

Mmcs\_4438

ATP-dependent transcriptional regulator, MalT-like, LuxR family
  
Accession: ABG10543
  
Location: 4719542-4720399
  
 NCBI BlastP on this gene

Mmcs\_4439

Epoxide hydrolase-like protein
  
Accession: ABG10544
  
Location: 4720422-4721525
  
  
**BlastP hit with Mycgr3G71676\_Mycgr3T**
  
Percentage identity: 34 %
  
BlastP bit score: 209
  
Sequence coverage: 98 %
  
E-value: 7e-60
  
  
 NCBI BlastP on this gene

Mmcs\_4440

conserved hypothetical protein
  
Accession: ABG10545
  
Location: 4721527-4722897
  
  
**BlastP hit with Mycgr3G42715\_Mycgr3T**
  
Percentage identity: 41 %
  
BlastP bit score: 350
  
Sequence coverage: 105 %
  
E-value: 1e-112
  
  
 NCBI BlastP on this gene

Mmcs\_4441

diguanylate cyclase
  
Accession: ABG10546
  
Location: 4722982-4724067
  
 NCBI BlastP on this gene

Mmcs\_4442

conserved hypothetical protein
  
Accession: ABG10547
  
Location: 4724099-4724974
  
 NCBI BlastP on this gene

Mmcs\_4443

ABC transporter related protein
  
Accession: ABG10548
  
Location: 4725098-4726930
  
 NCBI BlastP on this gene

Mmcs\_4444

ABC transporter related protein
  
Accession: ABG10549
  
Location: 4726927-4728879
  
 NCBI BlastP on this gene

Mmcs\_4445

ABC transporter related protein
  
Accession: ABG10550
  
Location: 4728931-4730814
  
 NCBI BlastP on this gene

Mmcs\_4446

Ion transport 2
  
Accession: ABG10551
  
Location: 4730890-4731672
  
 NCBI BlastP on this gene

Mmcs\_4447

two component transcriptional regulator, winged helix family
  
Accession: ABG10552
  
Location: 4731699-4732400
  
 NCBI BlastP on this gene

Mmcs\_4448

periplasmic sensor signal transduction histidine kinase
  
Accession: ABG10553
  
Location: 4732403-4733749
  
 NCBI BlastP on this gene

Mmcs\_4449

peptidylprolyl isomerase, FKBP-type
  
Accession: ABG10554
  
Location: 4733821-4734189
  
 NCBI BlastP on this gene

Mmcs\_4450

conserved hypothetical protein
  
Accession: ABG10555
  
Location: 4734226-4734474
  
 NCBI BlastP on this gene

Mmcs\_4451

conserved hypothetical protein
  
Accession: ABG10556
  
Location: 4734519-4736018
  
 NCBI BlastP on this gene

Mmcs\_4452

hypothetical protein
  
Accession: ABG10557
  
Location: 4736050-4736397
  
 NCBI BlastP on this gene

Mmcs\_4453

transcriptional regulator, MarR family
  
Accession: ABG10558
  
Location: 4736402-4736821
  
 NCBI BlastP on this gene

Mmcs\_4454

Drug resistance transporter EmrB/QacA subfamily
  
Accession: ABG10559
  
Location: 4736811-4738247
  
 NCBI BlastP on this gene

Mmcs\_4455

citrate synthase
  
Accession: ABG10560
  
Location: 4738333-4739637
  
 NCBI BlastP on this gene

Mmcs\_4456

transcriptional regulator, TetR family
  
Accession: ABG10561
  
Location: 4739733-4740380
  
 NCBI BlastP on this gene

Mmcs\_4457

Pyridoxamine 5'-phosphate oxidase
  
Accession: ABG10562
  
Location: 4740508-4741170
  
 NCBI BlastP on this gene

Mmcs\_4458

citrate synthase
  
Accession: ABG10563
  
Location: 4741231-4742361
  
 NCBI BlastP on this gene

Mmcs\_4459

hypothetical protein
  
Accession: ABG10564
  
Location: 4742358-4742975
  
 NCBI BlastP on this gene

Mmcs\_4460

212. :  CP000580 Mycobacterium sp. JLS     Total score: 2.0     Cumulative Blast bit score: 557

conserved hypothetical protein
  
Accession: ABO00566
  
Location: 5048915-5049649
  
 NCBI BlastP on this gene

Mjls\_4800

conserved hypothetical protein
  
Accession: ABO00567
  
Location: 5049660-5050070
  
 NCBI BlastP on this gene

Mjls\_4801

dihydrodipicolinate reductase
  
Accession: ABO00568
  
Location: 5050074-5051165
  
 NCBI BlastP on this gene

Mjls\_4802

cytochrome P450
  
Accession: ABO00569
  
Location: 5051162-5052346
  
 NCBI BlastP on this gene

Mjls\_4803

transcriptional regulator, TetR family
  
Accession: ABO00570
  
Location: 5052583-5053206
  
 NCBI BlastP on this gene

Mjls\_4804

NAD-dependent epimerase/dehydratase
  
Accession: ABO00571
  
Location: 5053385-5054044
  
 NCBI BlastP on this gene

Mjls\_4805

alpha/beta hydrolase fold protein
  
Accession: ABO00572
  
Location: 5054068-5055033
  
 NCBI BlastP on this gene

Mjls\_4806

conserved hypothetical protein
  
Accession: ABO00573
  
Location: 5055060-5055491
  
 NCBI BlastP on this gene

Mjls\_4807

hypothetical protein
  
Accession: ABO00574
  
Location: 5055495-5055725
  
 NCBI BlastP on this gene

Mjls\_4808

ATPase, P-type (transporting), HAD superfamily, subfamily IC
  
Accession: ABO00575
  
Location: 5055749-5058139
  
 NCBI BlastP on this gene

Mjls\_4809

beta-lactamase
  
Accession: ABO00576
  
Location: 5058264-5059865
  
 NCBI BlastP on this gene

Mjls\_4810

conserved hypothetical protein
  
Accession: ABO00577
  
Location: 5059875-5060993
  
 NCBI BlastP on this gene

Mjls\_4811

Enoyl-CoA hydratase/isomerase
  
Accession: ABO00578
  
Location: 5060996-5061730
  
 NCBI BlastP on this gene

Mjls\_4812

carboxyl transferase
  
Accession: ABO00579
  
Location: 5061757-5063265
  
 NCBI BlastP on this gene

Mjls\_4813

conserved hypothetical protein
  
Accession: ABO00580
  
Location: 5063266-5063514
  
 NCBI BlastP on this gene

Mjls\_4814

conserved hypothetical protein
  
Accession: ABO00581
  
Location: 5063511-5063726
  
 NCBI BlastP on this gene

Mjls\_4815

conserved hypothetical protein
  
Accession: ABO00582
  
Location: 5063765-5064196
  
 NCBI BlastP on this gene

Mjls\_4816

conserved hypothetical protein
  
Accession: ABO00583
  
Location: 5064206-5064613
  
 NCBI BlastP on this gene

Mjls\_4817

AMP-dependent synthetase and ligase
  
Accession: ABO00584
  
Location: 5064664-5066286
  
 NCBI BlastP on this gene

Mjls\_4818

AMP-dependent synthetase and ligase
  
Accession: ABO00585
  
Location: 5066283-5067980
  
 NCBI BlastP on this gene

Mjls\_4819

putative GAF sensor protein
  
Accession: ABO00586
  
Location: 5068030-5068887
  
 NCBI BlastP on this gene

Mjls\_4820

Epoxide hydrolase domain protein
  
Accession: ABO00587
  
Location: 5068910-5070013
  
  
**BlastP hit with Mycgr3G71676\_Mycgr3T**
  
Percentage identity: 34 %
  
BlastP bit score: 209
  
Sequence coverage: 98 %
  
E-value: 7e-60
  
  
 NCBI BlastP on this gene

Mjls\_4821

conserved hypothetical protein
  
Accession: ABO00588
  
Location: 5070015-5071385
  
  
**BlastP hit with Mycgr3G42715\_Mycgr3T**
  
Percentage identity: 41 %
  
BlastP bit score: 348
  
Sequence coverage: 105 %
  
E-value: 6e-112
  
  
 NCBI BlastP on this gene

Mjls\_4822

diguanylate cyclase
  
Accession: ABO00589
  
Location: 5071696-5072556
  
 NCBI BlastP on this gene

Mjls\_4823

conserved hypothetical protein
  
Accession: ABO00590
  
Location: 5072588-5072917
  
 NCBI BlastP on this gene

Mjls\_4824

conserved hypothetical protein
  
Accession: ABO00591
  
Location: 5072875-5073462
  
 NCBI BlastP on this gene

Mjls\_4825

ABC transporter related protein
  
Accession: ABO00592
  
Location: 5073585-5075417
  
 NCBI BlastP on this gene

Mjls\_4826

ABC transporter related protein
  
Accession: ABO00593
  
Location: 5075414-5077339
  
 NCBI BlastP on this gene

Mjls\_4827

ABC transporter related protein
  
Accession: ABO00594
  
Location: 5077391-5079271
  
 NCBI BlastP on this gene

Mjls\_4828

Ion transport 2 domain protein
  
Accession: ABO00595
  
Location: 5079346-5080128
  
 NCBI BlastP on this gene

Mjls\_4829

two component transcriptional regulator, winged helix family
  
Accession: ABO00596
  
Location: 5080155-5080856
  
 NCBI BlastP on this gene

Mjls\_4830

integral membrane sensor signal transduction histidine kinase
  
Accession: ABO00597
  
Location: 5080859-5082205
  
 NCBI BlastP on this gene

Mjls\_4831

peptidylprolyl isomerase, FKBP-type
  
Accession: ABO00598
  
Location: 5082277-5082645
  
 NCBI BlastP on this gene

Mjls\_4832

conserved hypothetical protein
  
Accession: ABO00599
  
Location: 5082682-5082930
  
 NCBI BlastP on this gene

Mjls\_4833

conserved hypothetical protein
  
Accession: ABO00600
  
Location: 5082975-5084474
  
 NCBI BlastP on this gene

Mjls\_4834

conserved hypothetical protein
  
Accession: ABO00601
  
Location: 5084506-5084853
  
 NCBI BlastP on this gene

Mjls\_4835

transcriptional regulator, MarR family
  
Accession: ABO00602
  
Location: 5084858-5085277
  
 NCBI BlastP on this gene

Mjls\_4836

drug resistance transporter, EmrB/QacA subfamily
  
Accession: ABO00603
  
Location: 5085267-5086703
  
 NCBI BlastP on this gene

Mjls\_4837

citrate synthase
  
Accession: ABO00604
  
Location: 5086789-5088093
  
 NCBI BlastP on this gene

Mjls\_4838

transcriptional regulator, TetR family
  
Accession: ABO00605
  
Location: 5088189-5088836
  
 NCBI BlastP on this gene

Mjls\_4839

Pyridoxamine 5'-phosphate oxidase
  
Accession: ABO00606
  
Location: 5088964-5089593
  
 NCBI BlastP on this gene

Mjls\_4840

citrate synthase
  
Accession: ABO00607
  
Location: 5089687-5090817
  
 NCBI BlastP on this gene

Mjls\_4841

conserved hypothetical protein
  
Accession: ABO00608
  
Location: 5090814-5091431
  
 NCBI BlastP on this gene

Mjls\_4842

213. :  CP003078 Mycobacterium smegmatis JS623     Total score: 2.0     Cumulative Blast bit score: 548

putative hydrolase or acyltransferase of
  
Accession: AGB25596
  
Location: 5360224-5360922
  
 NCBI BlastP on this gene

Mycsm\_05405

hypothetical protein
  
Accession: AGB25597
  
Location: 5360938-5361630
  
 NCBI BlastP on this gene

Mycsm\_05406

gluconolactonase
  
Accession: AGB25598
  
Location: 5361687-5362562
  
 NCBI BlastP on this gene

Mycsm\_05407

hypothetical protein
  
Accession: AGB25599
  
Location: 5362569-5362991
  
 NCBI BlastP on this gene

Mycsm\_05408

transcriptional regulator
  
Accession: AGB25600
  
Location: 5363022-5363567
  
 NCBI BlastP on this gene

Mycsm\_05409

2-polyprenyl-6-methoxyphenol hydroxylase-like oxidoreductase
  
Accession: AGB25601
  
Location: 5363662-5364801
  
 NCBI BlastP on this gene

Mycsm\_05410

hypothetical protein
  
Accession: AGB25602
  
Location: 5364779-5365186
  
 NCBI BlastP on this gene

Mycsm\_05411

Cu2+-containing amine oxidase
  
Accession: AGB25603
  
Location: 5365314-5367248
  
 NCBI BlastP on this gene

Mycsm\_05412

amino acid transporter
  
Accession: AGB25604
  
Location: 5367245-5368696
  
 NCBI BlastP on this gene

Mycsm\_05413

NAD-dependent aldehyde dehydrogenase
  
Accession: AGB25605
  
Location: 5368808-5370310
  
 NCBI BlastP on this gene

Mycsm\_05414

amino acid transporter
  
Accession: AGB25606
  
Location: 5370542-5371990
  
 NCBI BlastP on this gene

Mycsm\_05415

transcriptional regulator
  
Accession: AGB25607
  
Location: 5371987-5372703
  
 NCBI BlastP on this gene

Mycsm\_05416

hypothetical protein
  
Accession: AGB25608
  
Location: 5372774-5373694
  
 NCBI BlastP on this gene

Mycsm\_05417

acyl-CoA synthetase (AMP-forming)/AMP-acid ligase II
  
Accession: AGB25609
  
Location: 5373748-5375361
  
 NCBI BlastP on this gene

Mycsm\_05418

acyl-CoA synthetase/AMP-acid ligase
  
Accession: AGB25610
  
Location: 5375358-5377049
  
 NCBI BlastP on this gene

Mycsm\_05419

response regulator containing a CheY-like receiver domain and an HTH DNA-binding domain
  
Accession: AGB25611
  
Location: 5377108-5377944
  
 NCBI BlastP on this gene

Mycsm\_05420

molecular chaperone (small heat shock protein)
  
Accession: AGB25612
  
Location: 5377999-5378481
  
 NCBI BlastP on this gene

Mycsm\_05421

hypothetical protein
  
Accession: AGB25613
  
Location: 5378758-5379390
  
 NCBI BlastP on this gene

Mycsm\_05422

putative hydrolase or acyltransferase of
  
Accession: AGB25614
  
Location: 5379394-5380503
  
  
**BlastP hit with Mycgr3G71676\_Mycgr3T**
  
Percentage identity: 33 %
  
BlastP bit score: 206
  
Sequence coverage: 98 %
  
E-value: 6e-59
  
  
 NCBI BlastP on this gene

Mycsm\_05423

hypothetical protein
  
Accession: AGB25615
  
Location: 5380504-5381877
  
  
**BlastP hit with Mycgr3G42715\_Mycgr3T**
  
Percentage identity: 39 %
  
BlastP bit score: 342
  
Sequence coverage: 105 %
  
E-value: 1e-109
  
  
 NCBI BlastP on this gene

Mycsm\_05424

ABC-type multidrug transport system, ATPase and permease component
  
Accession: AGB25616
  
Location: 5382007-5383830
  
 NCBI BlastP on this gene

Mycsm\_05425

ABC-type multidrug transport system, ATPase and permease component
  
Accession: AGB25617
  
Location: 5383827-5385746
  
 NCBI BlastP on this gene

Mycsm\_05426

chromate transport protein ChrA
  
Accession: AGB25618
  
Location: 5385973-5386521
  
 NCBI BlastP on this gene

Mycsm\_05427

chromate transport protein ChrA
  
Accession: AGB25619
  
Location: 5386518-5387081
  
 NCBI BlastP on this gene

Mycsm\_05428

phosphatidylserine/phosphatidylglycerophosphate/ cardiolipin synthase
  
Accession: AGB25620
  
Location: 5387074-5388063
  
 NCBI BlastP on this gene

Mycsm\_05429

response regulator with CheY-like receiver domain and winged-helix DNA-binding domain
  
Accession: AGB25621
  
Location: 5388224-5388925
  
 NCBI BlastP on this gene

Mycsm\_05430

signal transduction histidine kinase
  
Accession: AGB25622
  
Location: 5388929-5390269
  
 NCBI BlastP on this gene

Mycsm\_05431

FKBP-type peptidyl-prolyl cis-trans isomerase
  
Accession: AGB25623
  
Location: 5390350-5390712
  
 NCBI BlastP on this gene

Mycsm\_05432

Protein of unknown function (DUF2630)
  
Accession: AGB25624
  
Location: 5390736-5390984
  
 NCBI BlastP on this gene

Mycsm\_05433

phytoene dehydrogenase-like oxidoreductase
  
Accession: AGB25625
  
Location: 5390981-5392549
  
 NCBI BlastP on this gene

Mycsm\_05434

adenylate/guanylate cyclase family protein
  
Accession: AGB25626
  
Location: 5392546-5395686
  
 NCBI BlastP on this gene

Mycsm\_05435

transcriptional regulator
  
Accession: AGB25627
  
Location: 5395735-5396154
  
 NCBI BlastP on this gene

Mycsm\_05436

drug resistance transporter, EmrB/QacA subfamily
  
Accession: AGB25628
  
Location: 5396144-5397598
  
 NCBI BlastP on this gene

Mycsm\_05437

citrate synthase I, hexameric type
  
Accession: AGB25629
  
Location: 5397739-5399046
  
 NCBI BlastP on this gene

Mycsm\_05438

transcriptional regulator
  
Accession: AGB25630
  
Location: 5399142-5399804
  
 NCBI BlastP on this gene

Mycsm\_05439

protein of unknown function (DUF894)
  
Accession: AGB25631
  
Location: 5399865-5401157
  
 NCBI BlastP on this gene

Mycsm\_05440

Pyridoxamine 5'-phosphate oxidase
  
Accession: AGB25632
  
Location: 5401157-5401813
  
 NCBI BlastP on this gene

Mycsm\_05441

214. :  JH126399 Cordyceps militaris CM01 unplaced genomic scaffold CCM\_S00001     Total score: 2.0     Cumulative Blast bit score: 524

Protein kinase-like domain
  
Accession: EGX95385
  
Location: 125630-126577
  
 NCBI BlastP on this gene

EGX95385

uracil permease
  
Accession: EGX95386
  
Location: 128275-130069
  
 NCBI BlastP on this gene

EGX95386

importin beta-5 subunit, putative
  
Accession: EGX95387
  
Location: 132457-135716
  
 NCBI BlastP on this gene

EGX95387

PI31 proteasome regulator
  
Accession: EGX95388
  
Location: 136207-137515
  
 NCBI BlastP on this gene

EGX95388

Major facilitator superfamily transporter
  
Accession: EGX95389
  
Location: 138751-140433
  
 NCBI BlastP on this gene

EGX95389

ankyrin repeat and SAM domain containing protein 6
  
Accession: EGX95390
  
Location: 140873-143164
  
 NCBI BlastP on this gene

EGX95390

mitotic spindle checkpoint protein (Mad2B), putative
  
Accession: EGX95391
  
Location: 144474-145236
  
 NCBI BlastP on this gene

EGX95391

AAA family ATPase, putative
  
Accession: EGX95392
  
Location: 145455-147779
  
  
**BlastP hit with Mycgr3G42010\_Mycgr3T**
  
Percentage identity: 55 %
  
BlastP bit score: 291
  
Sequence coverage: 11 %
  
E-value: 1e-79
  
  
 NCBI BlastP on this gene

EGX95392

hypothetical protein
  
Accession: EGX95393
  
Location: 147915-149562
  
  
**BlastP hit with Mycgr3G92938\_Mycgr3T**
  
Percentage identity: 34 %
  
BlastP bit score: 233
  
Sequence coverage: 97 %
  
E-value: 7e-68
  
  
 NCBI BlastP on this gene

EGX95393

hypothetical protein
  
Accession: EGX95394
  
Location: 149912-150841
  
 NCBI BlastP on this gene

EGX95394

hypothetical protein
  
Accession: EGX95395
  
Location: 166189-167156
  
 NCBI BlastP on this gene

EGX95395

215. :  KE145369 Glarea lozoyensis ATCC 20868 chromosome Unknown GLAREA5     Total score: 2.0     Cumulative Blast bit score: 518

CheY-like protein
  
Accession: EPE27507
  
Location: 317465-321368
  
 NCBI BlastP on this gene

EPE27507

hypothetical protein
  
Accession: EPE27506
  
Location: 315803-316321
  
 NCBI BlastP on this gene

EPE27506

Ribonuclease H-like protein
  
Accession: EPE27505
  
Location: 311868-315363
  
 NCBI BlastP on this gene

EPE27505

S-adenosyl-L-methionine-dependent methyltransferase
  
Accession: EPE27504
  
Location: 308466-309868
  
 NCBI BlastP on this gene

EPE27504

MFS general substrate transporter
  
Accession: EPE27503
  
Location: 304097-306007
  
  
**BlastP hit with Mycgr3G85918\_Mycgr3T**
  
Percentage identity: 36 %
  
BlastP bit score: 347
  
Sequence coverage: 97 %
  
E-value: 4e-109
  
  
 NCBI BlastP on this gene

EPE27503

hypothetical protein
  
Accession: EPE27502
  
Location: 301946-303676
  
 NCBI BlastP on this gene

EPE27502

hypothetical protein
  
Accession: EPE27501
  
Location: 300215-301531
  
 NCBI BlastP on this gene

EPE27501

hypothetical protein
  
Accession: EPE27500
  
Location: 298159-298911
  
 NCBI BlastP on this gene

EPE27500

hypothetical protein
  
Accession: EPE27499
  
Location: 293387-297823
  
 NCBI BlastP on this gene

EPE27499

Di-copper centre-containing
  
Accession: EPE27498
  
Location: 289770-291650
  
  
**BlastP hit with Mycgr3G42698\_Mycgr3T**
  
Percentage identity: 33 %
  
BlastP bit score: 171
  
Sequence coverage: 108 %
  
E-value: 2e-46
  
  
 NCBI BlastP on this gene

EPE27498

Zn2/Cys6 DNA-binding protein
  
Accession: EPE27497
  
Location: 285663-288405
  
 NCBI BlastP on this gene

EPE27497

NAD(P)-binding Rossmann-fold containing protein
  
Accession: EPE27496
  
Location: 284250-285376
  
 NCBI BlastP on this gene

EPE27496

peptidase M48 Ste24p
  
Accession: EPE27495
  
Location: 282744-283427
  
 NCBI BlastP on this gene

EPE27495

S-adenosyl-L-methionine-dependent methyltransferase
  
Accession: EPE27494
  
Location: 280283-281744
  
 NCBI BlastP on this gene

EPE27494

hypothetical protein
  
Accession: EPE27493
  
Location: 278442-279233
  
 NCBI BlastP on this gene

EPE27493

P-loop containing nucleoside triphosphate hydrolase
  
Accession: EPE27492
  
Location: 275167-278214
  
 NCBI BlastP on this gene

EPE27492

216. :  HF679032 Fusarium fujikuroi IMI 58289 draft genome, chromosome FFUJ\_chr10.     Total score: 2.0     Cumulative Blast bit score: 517

related to peroxisomal short-chain alcohol dehydrogenase
  
Accession: CCT74200
  
Location: 97598-98494
  
 NCBI BlastP on this gene

FFUJ\_10242

related to chitinase
  
Accession: CCT74201
  
Location: 99450-102382
  
 NCBI BlastP on this gene

FFUJ\_10243

uncharacterized protein
  
Accession: CCT74202
  
Location: 102936-104123
  
 NCBI BlastP on this gene

FFUJ\_10244

uncharacterized protein
  
Accession: CCT74203
  
Location: 104778-106023
  
 NCBI BlastP on this gene

FFUJ\_10245

related to proteoglycan
  
Accession: CCT74204
  
Location: 106593-108572
  
 NCBI BlastP on this gene

FFUJ\_10246

uncharacterized protein
  
Accession: CCT74205
  
Location: 110008-111020
  
 NCBI BlastP on this gene

FFUJ\_10247

related to salicylate 1-monooxygenase
  
Accession: CCT74206
  
Location: 112007-113400
  
 NCBI BlastP on this gene

FFUJ\_10248

related to monooxigenase
  
Accession: CCT74207
  
Location: 113534-115814
  
 NCBI BlastP on this gene

FFUJ\_10249

related to alcohol/sorbitol dehydrogenase
  
Accession: CCT74208
  
Location: 116367-117696
  
  
**BlastP hit with Mycgr3G71679\_Mycgr3T**
  
Percentage identity: 44 %
  
BlastP bit score: 324
  
Sequence coverage: 101 %
  
E-value: 9e-105
  
  
 NCBI BlastP on this gene

FFUJ\_10250

related to reductases
  
Accession: CCT74209
  
Location: 118087-119259
  
 NCBI BlastP on this gene

FFUJ\_10251

related to Copper amine oxidase 1
  
Accession: CCT74210
  
Location: 120051-122263
  
 NCBI BlastP on this gene

FFUJ\_10252

uncharacterized protein
  
Accession: CCT74211
  
Location: 122674-124932
  
  
**BlastP hit with Mycgr3G11468\_Mycgr3T**
  
Percentage identity: 34 %
  
BlastP bit score: 193
  
Sequence coverage: 100 %
  
E-value: 5e-52
  
  
 NCBI BlastP on this gene

FFUJ\_10253

related to permease of the major facilitator superfamily
  
Accession: CCT74212
  
Location: 125643-127234
  
 NCBI BlastP on this gene

FFUJ\_10254

uncharacterized protein
  
Accession: CCT74213
  
Location: 127627-128842
  
 NCBI BlastP on this gene

FFUJ\_10255

related to 3-hydroxybutyryl-CoA dehydrogenase
  
Accession: CCT74214
  
Location: 129359-131170
  
 NCBI BlastP on this gene

FFUJ\_10256

putative NADH cytb-reductase
  
Accession: CCT74215
  
Location: 132717-134124
  
 NCBI BlastP on this gene

FFUJ\_10257

monooxygenase
  
Accession: CCT74216
  
Location: 134987-136748
  
 NCBI BlastP on this gene

FFUJ\_10258

related to tetracycline efflux protein (otrb)
  
Accession: CCT74217
  
Location: 137026-138713
  
 NCBI BlastP on this gene

FFUJ\_10259

related to 6-hydroxy-D-nicotine oxidase
  
Accession: CCT74218
  
Location: 138921-140458
  
 NCBI BlastP on this gene

FFUJ\_10260

probable glutathione S-transferase
  
Accession: CCT75122
  
Location: 141200-142157
  
 NCBI BlastP on this gene

FFUJ\_10261

uncharacterized protein
  
Accession: CCT74219
  
Location: 142412-143443
  
 NCBI BlastP on this gene

FFUJ\_10262

217. :  AP007166 Aspergillus oryzae RIB40 DNA, SC113.     Total score: 2.0     Cumulative Blast bit score: 517

not annotated
  
Accession: BAE62723
  
Location: 225659-227272
  
 NCBI BlastP on this gene

AO090120000096

not annotated
  
Accession: BAE62722
  
Location: 221157-222072
  
  
**BlastP hit with Mycgr3G31170\_Mycgr3T**
  
Percentage identity: 98 %
  
BlastP bit score: 163
  
Sequence coverage: 100 %
  
E-value: 2e-48
  
  
 NCBI BlastP on this gene

AO090120000095

not annotated
  
Accession: BAE62721
  
Location: 217829-219808
  
 NCBI BlastP on this gene

AO090120000093

not annotated
  
Accession: BAE62720
  
Location: 216431-217247
  
 NCBI BlastP on this gene

AO090120000091

not annotated
  
Accession: BAE62719
  
Location: 213616-215994
  
 NCBI BlastP on this gene

AO090120000090

not annotated
  
Accession: BAE62718
  
Location: 204340-209055
  
 NCBI BlastP on this gene

AO090120000088

not annotated
  
Accession: BAE62717
  
Location: 195790-202723
  
  
**BlastP hit with Mycgr3G41426\_Mycgr3T**
  
Percentage identity: 30 %
  
BlastP bit score: 354
  
Sequence coverage: 41 %
  
E-value: 8e-96
  
  
 NCBI BlastP on this gene

AO090120000087

not annotated
  
Accession: BAE62716
  
Location: 190620-193330
  
 NCBI BlastP on this gene

AO090120000086

not annotated
  
Accession: BAE62715
  
Location: 189320-189730
  
 NCBI BlastP on this gene

AO090120000085

not annotated
  
Accession: BAE62714
  
Location: 187356-188887
  
 NCBI BlastP on this gene

AO090120000084

218. :  KB908855 Setosphaeria turcica Et28A unplaced genomic scaffold SETTUscaffold\_7     Total score: 2.0     Cumulative Blast bit score: 507

hypothetical protein
  
Accession: EOA82011
  
Location: 974049-974912
  
 NCBI BlastP on this gene

EOA82011

hypothetical protein
  
Accession: EOA82012
  
Location: 975720-977771
  
 NCBI BlastP on this gene

EOA82012

hypothetical protein
  
Accession: EOA82013
  
Location: 979519-981718
  
 NCBI BlastP on this gene

EOA82013

hypothetical protein
  
Accession: EOA82014
  
Location: 984682-985500
  
 NCBI BlastP on this gene

EOA82014

hypothetical protein
  
Accession: EOA82015
  
Location: 986440-992415
  
  
**BlastP hit with Mycgr3G42010\_Mycgr3T**
  
Percentage identity: 28 %
  
BlastP bit score: 342
  
Sequence coverage: 46 %
  
E-value: 1e-91
  
  
 NCBI BlastP on this gene

EOA82015

hypothetical protein
  
Accession: EOA82016
  
Location: 993139-996942
  
 NCBI BlastP on this gene

EOA82016

hypothetical protein
  
Accession: EOA82017
  
Location: 997318-998644
  
 NCBI BlastP on this gene

EOA82017

hypothetical protein
  
Accession: EOA82018
  
Location: 1000710-1001312
  
 NCBI BlastP on this gene

EOA82018

hypothetical protein
  
Accession: EOA82019
  
Location: 1002401-1003757
  
  
**BlastP hit with Mycgr3G42698\_Mycgr3T**
  
Percentage identity: 31 %
  
BlastP bit score: 165
  
Sequence coverage: 112 %
  
E-value: 4e-44
  
  
 NCBI BlastP on this gene

EOA82019

hypothetical protein
  
Accession: EOA82020
  
Location: 1004603-1005278
  
 NCBI BlastP on this gene

EOA82020

hypothetical protein
  
Accession: EOA82021
  
Location: 1009149-1010700
  
 NCBI BlastP on this gene

EOA82021

hypothetical protein
  
Accession: EOA82022
  
Location: 1011262-1014391
  
 NCBI BlastP on this gene

EOA82022

hypothetical protein
  
Accession: EOA82023
  
Location: 1014930-1016157
  
 NCBI BlastP on this gene

EOA82023

219. :  FQ790251 Botryotinia fuckeliana T4 SuperContig\_87\_1 genomic supercontig.     Total score: 2.0     Cumulative Blast bit score: 500

hypothetical protein
  
Accession: CCD43012
  
Location: 211510-212708
  
 NCBI BlastP on this gene

BofuT4\_P070880.1

hypothetical protein
  
Accession: CCD43013
  
Location: 213255-213557
  
 NCBI BlastP on this gene

BofuT4\_P070890.1

carbohydrate esterase family 5 protein
  
Accession: CCD43014
  
Location: 214213-215056
  
 NCBI BlastP on this gene

BofuT4P87000007001

hypothetical protein
  
Accession: CCD43015
  
Location: 217665-218261
  
 NCBI BlastP on this gene

BofuT4\_P070910.1

hypothetical protein
  
Accession: CCD43016
  
Location: 227148-227440
  
 NCBI BlastP on this gene

BofuT4\_uP070920.1

similar to prolyl aminopeptidase
  
Accession: CCD43017
  
Location: 227688-228775
  
  
**BlastP hit with Mycgr3G71676\_Mycgr3T**
  
Percentage identity: 50 %
  
BlastP bit score: 313
  
Sequence coverage: 77 %
  
E-value: 7e-101
  
  
 NCBI BlastP on this gene

BofuT4\_P070930.1

similar to tyrosinase central domain protein
  
Accession: CCD43018
  
Location: 234635-236006
  
  
**BlastP hit with Mycgr3G42698\_Mycgr3T**
  
Percentage identity: 36 %
  
BlastP bit score: 187
  
Sequence coverage: 108 %
  
E-value: 1e-52
  
  
 NCBI BlastP on this gene

BofuT4\_P070940.1

similar to monooxygenase FAD-binding
  
Accession: CCD43019
  
Location: 238216-239715
  
 NCBI BlastP on this gene

BofuT4\_P070950.1

hypothetical protein
  
Accession: CCD43020
  
Location: 241893-242458
  
 NCBI BlastP on this gene

BofuT4\_P070960.1

hypothetical protein
  
Accession: CCD43021
  
Location: 243654-244762
  
 NCBI BlastP on this gene

BofuT4\_P070970.1

hypothetical protein
  
Accession: CCD43022
  
Location: 252888-253767
  
 NCBI BlastP on this gene

BofuT4\_P070980.1

220. :  KE145357 Glarea lozoyensis ATCC 20868 chromosome Unknown GLAREA14     Total score: 2.0     Cumulative Blast bit score: 487

protein of unknown function UPF0157
  
Accession: EPE33993
  
Location: 1264799-1265368
  
 NCBI BlastP on this gene

EPE33993

hypothetical protein
  
Accession: EPE33994
  
Location: 1265848-1267668
  
 NCBI BlastP on this gene

EPE33994

ARM repeat-containing protein
  
Accession: EPE33995
  
Location: 1268795-1272362
  
 NCBI BlastP on this gene

EPE33995

CYTH-like phosphatase
  
Accession: EPE33996
  
Location: 1273482-1275927
  
 NCBI BlastP on this gene

EPE33996

MFS general substrate transporter
  
Accession: EPE33997
  
Location: 1276462-1278357
  
 NCBI BlastP on this gene

EPE33997

(Trans)glycosidase
  
Accession: EPE33998
  
Location: 1279770-1283070
  
 NCBI BlastP on this gene

EPE33998

(Trans)glycosidase
  
Accession: EPE33999
  
Location: 1283830-1285192
  
  
**BlastP hit with Mycgr3G71681\_Mycgr3T**
  
Percentage identity: 48 %
  
BlastP bit score: 405
  
Sequence coverage: 95 %
  
E-value: 5e-135
  
  
 NCBI BlastP on this gene

EPE33999

Acyl-CoA dehydrogenase NM
  
Accession: EPE34000
  
Location: 1285283-1286909
  
 NCBI BlastP on this gene

EPE34000

hypothetical protein
  
Accession: EPE34001
  
Location: 1288145-1289125
  
  
**BlastP hit with Mycgr3G92938\_Mycgr3T**
  
Percentage identity: 29 %
  
BlastP bit score: 82
  
Sequence coverage: 52 %
  
E-value: 2e-14
  
  
 NCBI BlastP on this gene

EPE34001

Six-hairpin glycosidase
  
Accession: EPE34002
  
Location: 1289891-1291353
  
 NCBI BlastP on this gene

EPE34002

FAD/NAD(P)-binding protein
  
Accession: EPE34003
  
Location: 1292836-1294182
  
 NCBI BlastP on this gene

EPE34003

GroES-like protein
  
Accession: EPE34004
  
Location: 1294863-1296047
  
 NCBI BlastP on this gene

EPE34004

hypothetical protein
  
Accession: EPE34005
  
Location: 1297261-1298338
  
 NCBI BlastP on this gene

EPE34005

hypothetical protein
  
Accession: EPE34006
  
Location: 1298808-1299674
  
 NCBI BlastP on this gene

EPE34006

hypothetical protein
  
Accession: EPE34007
  
Location: 1300785-1301447
  
 NCBI BlastP on this gene

EPE34007

hypothetical protein
  
Accession: EPE34008
  
Location: 1301989-1302849
  
 NCBI BlastP on this gene

EPE34008

hypothetical protein
  
Accession: EPE34009
  
Location: 1303987-1306887
  
 NCBI BlastP on this gene

EPE34009

hypothetical protein
  
Accession: EPE34010
  
Location: 1308415-1310471
  
 NCBI BlastP on this gene

EPE34010

221. :  KB726994 Fusarium oxysporum f. sp. cubense race 4 unplaced genomic scaffold scaffold63     Total score: 2.0     Cumulative Blast bit score: 481

Cytochrome P450 4F6
  
Accession: EMT62888
  
Location: 44200-45957
  
 NCBI BlastP on this gene

EMT62888

NADH-cytochrome b5 reductase 1
  
Accession: EMT62889
  
Location: 46812-48219
  
 NCBI BlastP on this gene

EMT62889

Plasma membrane proteolipid 3
  
Accession: EMT62890
  
Location: 48957-49161
  
 NCBI BlastP on this gene

EMT62890

hypothetical protein
  
Accession: EMT62891
  
Location: 49883-51010
  
 NCBI BlastP on this gene

EMT62891

Plasma membrane proteolipid 3
  
Accession: EMT62892
  
Location: 51124-51328
  
 NCBI BlastP on this gene

EMT62892

hypothetical protein
  
Accession: EMT62893
  
Location: 52050-53648
  
 NCBI BlastP on this gene

EMT62893

3-hydroxybutyryl-CoA dehydrogenase
  
Accession: EMT62894
  
Location: 54696-56507
  
 NCBI BlastP on this gene

EMT62894

3-hydroxybutyryl-CoA dehydrogenase
  
Accession: EMT62895
  
Location: 56761-58409
  
 NCBI BlastP on this gene

EMT62895

hypothetical protein
  
Accession: EMT62896
  
Location: 58702-59915
  
 NCBI BlastP on this gene

EMT62896

Major facilitator superfamily domain-containing protein 7-a
  
Accession: EMT62897
  
Location: 60285-61911
  
 NCBI BlastP on this gene

EMT62897

hypothetical protein
  
Accession: EMT62898
  
Location: 62402-64871
  
  
**BlastP hit with Mycgr3G11468\_Mycgr3T**
  
Percentage identity: 33 %
  
BlastP bit score: 192
  
Sequence coverage: 100 %
  
E-value: 5e-52
  
  
 NCBI BlastP on this gene

EMT62898

Copper amine oxidase 1
  
Accession: EMT62899
  
Location: 65581-67797
  
 NCBI BlastP on this gene

EMT62899

hypothetical protein
  
Accession: EMT62900
  
Location: 68458-69657
  
 NCBI BlastP on this gene

EMT62900

Diacetyl reductase [(R)-acetoin forming]
  
Accession: EMT62901
  
Location: 70059-71378
  
  
**BlastP hit with Mycgr3G71679\_Mycgr3T**
  
Percentage identity: 44 %
  
BlastP bit score: 290
  
Sequence coverage: 100 %
  
E-value: 1e-91
  
  
 NCBI BlastP on this gene

EMT62901

Putative sterigmatocystin biosynthesis monooxygenase stcW
  
Accession: EMT62902
  
Location: 71948-74253
  
 NCBI BlastP on this gene

EMT62902

Salicylate hydroxylase
  
Accession: EMT62903
  
Location: 74374-75589
  
 NCBI BlastP on this gene

EMT62903

Polygalacturonase
  
Accession: EMT62904
  
Location: 77113-78546
  
 NCBI BlastP on this gene

EMT62904

hypothetical protein
  
Accession: EMT62905
  
Location: 82611-83842
  
 NCBI BlastP on this gene

EMT62905

hypothetical protein
  
Accession: EMT62906
  
Location: 84440-85336
  
 NCBI BlastP on this gene

EMT62906

Chitotriosidase-1
  
Accession: EMT62907
  
Location: 86025-89629
  
 NCBI BlastP on this gene

EMT62907

222. :  AFQF01000390 Fusarium oxysporum Fo5176     Total score: 2.0     Cumulative Blast bit score: 481

hypothetical protein
  
Accession: EGU88480
  
Location: 1039-2644
  
 NCBI BlastP on this gene

EGU88480

hypothetical protein
  
Accession: EGU88481
  
Location: 3135-5602
  
  
**BlastP hit with Mycgr3G11468\_Mycgr3T**
  
Percentage identity: 34 %
  
BlastP bit score: 187
  
Sequence coverage: 92 %
  
E-value: 3e-50
  
  
 NCBI BlastP on this gene

EGU88481

hypothetical protein
  
Accession: EGU88482
  
Location: 6226-8525
  
 NCBI BlastP on this gene

EGU88482

hypothetical protein
  
Accession: EGU88483
  
Location: 9353-10553
  
 NCBI BlastP on this gene

EGU88483

hypothetical protein
  
Accession: EGU88484
  
Location: 10956-12288
  
  
**BlastP hit with Mycgr3G71679\_Mycgr3T**
  
Percentage identity: 43 %
  
BlastP bit score: 295
  
Sequence coverage: 103 %
  
E-value: 2e-93
  
  
 NCBI BlastP on this gene

EGU88484

hypothetical protein
  
Accession: EGU88485
  
Location: 12847-15152
  
 NCBI BlastP on this gene

EGU88485

hypothetical protein
  
Accession: EGU88486
  
Location: 15273-16662
  
 NCBI BlastP on this gene

EGU88486

hypothetical protein
  
Accession: EGU88487
  
Location: 18010-19443
  
 NCBI BlastP on this gene

EGU88487

hypothetical protein
  
Accession: EGU88488
  
Location: 20280-21292
  
 NCBI BlastP on this gene

EGU88488

hypothetical protein
  
Accession: EGU88489
  
Location: 21786-23017
  
 NCBI BlastP on this gene

EGU88489

hypothetical protein
  
Accession: EGU88490
  
Location: 23615-24511
  
 NCBI BlastP on this gene

EGU88490

hypothetical protein
  
Accession: EGU88491
  
Location: 25203-28837
  
 NCBI BlastP on this gene

EGU88491

hypothetical protein
  
Accession: EGU88492
  
Location: 30123-31926
  
 NCBI BlastP on this gene

EGU88492

223. :  KB730083 Fusarium oxysporum f. sp. cubense race 1 unplaced genomic scaffold scaffold97     Total score: 2.0     Cumulative Blast bit score: 477

Putative transporter C3H1.06c
  
Accession: ENH73486
  
Location: 537867-539559
  
 NCBI BlastP on this gene

ENH73486

Cytochrome P450 4F6
  
Accession: ENH73487
  
Location: 539834-541591
  
 NCBI BlastP on this gene

ENH73487

NADH-cytochrome b5 reductase 1
  
Accession: ENH73488
  
Location: 542448-543855
  
 NCBI BlastP on this gene

ENH73488

Plasma membrane proteolipid 3
  
Accession: ENH73489
  
Location: 544615-544819
  
 NCBI BlastP on this gene

ENH73489

hypothetical protein
  
Accession: ENH73490
  
Location: 545539-547135
  
 NCBI BlastP on this gene

ENH73490

3-hydroxybutyryl-CoA dehydrogenase
  
Accession: ENH73491
  
Location: 548192-550003
  
 NCBI BlastP on this gene

ENH73491

hypothetical protein
  
Accession: ENH73492
  
Location: 550296-551513
  
 NCBI BlastP on this gene

ENH73492

Major facilitator superfamily domain-containing protein 7-a
  
Accession: ENH73493
  
Location: 551904-553509
  
 NCBI BlastP on this gene

ENH73493

hypothetical protein
  
Accession: ENH73494
  
Location: 554000-556430
  
  
**BlastP hit with Mycgr3G11468\_Mycgr3T**
  
Percentage identity: 34 %
  
BlastP bit score: 187
  
Sequence coverage: 92 %
  
E-value: 4e-50
  
  
 NCBI BlastP on this gene

ENH73494

Copper amine oxidase 1
  
Accession: ENH73495
  
Location: 557145-559359
  
 NCBI BlastP on this gene

ENH73495

hypothetical protein
  
Accession: ENH73496
  
Location: 560192-561392
  
 NCBI BlastP on this gene

ENH73496

Diacetyl reductase [(R)-acetoin forming]
  
Accession: ENH73497
  
Location: 561796-563116
  
  
**BlastP hit with Mycgr3G71679\_Mycgr3T**
  
Percentage identity: 44 %
  
BlastP bit score: 290
  
Sequence coverage: 99 %
  
E-value: 1e-91
  
  
 NCBI BlastP on this gene

ENH73497

Putative sterigmatocystin biosynthesis monooxygenase stcW
  
Accession: ENH73498
  
Location: 563687-565992
  
 NCBI BlastP on this gene

ENH73498

Salicylate hydroxylase
  
Accession: ENH73499
  
Location: 566113-567328
  
 NCBI BlastP on this gene

ENH73499

hypothetical protein
  
Accession: ENH73500
  
Location: 568863-570296
  
 NCBI BlastP on this gene

ENH73500

hypothetical protein
  
Accession: ENH73501
  
Location: 571126-572106
  
 NCBI BlastP on this gene

ENH73501

hypothetical protein
  
Accession: ENH73502
  
Location: 572617-573848
  
 NCBI BlastP on this gene

ENH73502

hypothetical protein
  
Accession: ENH73503
  
Location: 574423-575316
  
 NCBI BlastP on this gene

ENH73503

Putative chitinase 3
  
Accession: ENH73504
  
Location: 576020-579590
  
 NCBI BlastP on this gene

ENH73504

Lectin-B
  
Accession: ENH73505
  
Location: 580906-582666
  
 NCBI BlastP on this gene

ENH73505

224. :  AQGS01001233 Dactylellina haptotyla CBS 200.50     Total score: 2.0     Cumulative Blast bit score: 424

hypothetical protein
  
Accession: EPS35104
  
Location: 526683-528662
  
 NCBI BlastP on this gene

EPS35104

hypothetical protein
  
Accession: EPS35120
  
Location: 523879-524976
  
 NCBI BlastP on this gene

EPS35120

hypothetical protein
  
Accession: EPS35101
  
Location: 522209-522894
  
 NCBI BlastP on this gene

EPS35101

hypothetical protein
  
Accession: EPS35211
  
Location: 518856-520495
  
 NCBI BlastP on this gene

EPS35211

hypothetical protein
  
Accession: EPS35063
  
Location: 514581-515090
  
 NCBI BlastP on this gene

EPS35063

hypothetical protein
  
Accession: EPS35189
  
Location: 509923-513603
  
 NCBI BlastP on this gene

EPS35189

hypothetical protein
  
Accession: EPS35114
  
Location: 506883-508427
  
  
**BlastP hit with Mycgr3G71679\_Mycgr3T**
  
Percentage identity: 41 %
  
BlastP bit score: 273
  
Sequence coverage: 102 %
  
E-value: 4e-85
  
  
 NCBI BlastP on this gene

EPS35114

hypothetical protein
  
Accession: EPS35277
  
Location: 500798-502462
  
  
**BlastP hit with Mycgr3G109328\_Mycgr3**
  
Percentage identity: 40 %
  
BlastP bit score: 151
  
Sequence coverage: 98 %
  
E-value: 1e-40
  
  
 NCBI BlastP on this gene

EPS35277

hypothetical protein
  
Accession: EPS35065
  
Location: 493471-493895
  
 NCBI BlastP on this gene

EPS35065

hypothetical protein
  
Accession: EPS35222
  
Location: 486985-488935
  
 NCBI BlastP on this gene

EPS35222

hypothetical protein
  
Accession: EPS35158
  
Location: 484168-485715
  
 NCBI BlastP on this gene

EPS35158

225. :  ADOT01000322 Arthrobotrys oligospora ATCC 24927     Total score: 2.0     Cumulative Blast bit score: 424

hypothetical protein
  
Accession: EGX43038
  
Location: 244651-248227
  
 NCBI BlastP on this gene

EGX43038

hypothetical protein
  
Accession: EGX43039
  
Location: 248684-249401
  
 NCBI BlastP on this gene

EGX43039

hypothetical protein
  
Accession: EGX43040
  
Location: 251006-252876
  
 NCBI BlastP on this gene

EGX43040

hypothetical protein
  
Accession: EGX43041
  
Location: 257035-257587
  
 NCBI BlastP on this gene

EGX43041

hypothetical protein
  
Accession: EGX43042
  
Location: 258723-262362
  
 NCBI BlastP on this gene

EGX43042

hypothetical protein
  
Accession: EGX43043
  
Location: 263610-265233
  
  
**BlastP hit with Mycgr3G71679\_Mycgr3T**
  
Percentage identity: 40 %
  
BlastP bit score: 265
  
Sequence coverage: 102 %
  
E-value: 8e-82
  
  
 NCBI BlastP on this gene

EGX43043

hypothetical protein
  
Accession: EGX43044
  
Location: 268381-270039
  
  
**BlastP hit with Mycgr3G109328\_Mycgr3**
  
Percentage identity: 38 %
  
BlastP bit score: 159
  
Sequence coverage: 110 %
  
E-value: 2e-43
  
  
 NCBI BlastP on this gene

EGX43044

hypothetical protein
  
Accession: EGX43045
  
Location: 282221-284255
  
 NCBI BlastP on this gene

EGX43045

hypothetical protein
  
Accession: EGX43046
  
Location: 284927-285871
  
 NCBI BlastP on this gene

EGX43046

hypothetical protein
  
Accession: EGX43047
  
Location: 288454-289708
  
 NCBI BlastP on this gene

EGX43047

226. :  JH795283 Magnaporthe oryzae P131 unplaced genomic scaffold P131\_scaffold00916     Total score: 2.0     Cumulative Blast bit score: 415

hypothetical protein
  
Accession: ELQ63939
  
Location: 34774-35448
  
 NCBI BlastP on this gene

ELQ63939

pre-rRNA-processing protein ESF1
  
Accession: ELQ63940
  
Location: 36386-38871
  
 NCBI BlastP on this gene

ELQ63940

hypothetical protein
  
Accession: ELQ63941
  
Location: 39458-41518
  
 NCBI BlastP on this gene

ELQ63941

phenylacetone monooxygenase
  
Accession: ELQ63942
  
Location: 42381-46001
  
 NCBI BlastP on this gene

ELQ63942

xanthoxin dehydrogenase
  
Accession: ELQ63943
  
Location: 46686-47618
  
 NCBI BlastP on this gene

ELQ63943

(R)-specific carbonyl reductase
  
Accession: ELQ63944
  
Location: 47670-49079
  
 NCBI BlastP on this gene

ELQ63944

hypothetical protein
  
Accession: ELQ63945
  
Location: 49990-51778
  
 NCBI BlastP on this gene

ELQ63945

isotrichodermin C-15 hydroxylase
  
Accession: ELQ63946
  
Location: 51952-53776
  
 NCBI BlastP on this gene

ELQ63946

AAA family ATPase
  
Accession: ELQ63947
  
Location: 54743-59360
  
  
**BlastP hit with Mycgr3G42010\_Mycgr3T**
  
Percentage identity: 42 %
  
BlastP bit score: 343
  
Sequence coverage: 21 %
  
E-value: 2e-94
  
  
 NCBI BlastP on this gene

ELQ63947

hypothetical protein
  
Accession: ELQ63948
  
Location: 60798-61506
  
  
**BlastP hit with Mycgr3G92938\_Mycgr3T**
  
Percentage identity: 28 %
  
BlastP bit score: 72
  
Sequence coverage: 48 %
  
E-value: 2e-11
  
  
 NCBI BlastP on this gene

ELQ63948

227. :  JH793600 Magnaporthe oryzae Y34 unplaced genomic scaffold Y34\_scaffold00217     Total score: 2.0     Cumulative Blast bit score: 415

glycerol-3-phosphate dehydrogenase
  
Accession: ELQ42264
  
Location: 15484-16855
  
 NCBI BlastP on this gene

ELQ42264

hypothetical protein
  
Accession: ELQ42265
  
Location: 18467-18697
  
 NCBI BlastP on this gene

ELQ42265

hypothetical protein
  
Accession: ELQ42266
  
Location: 18980-20384
  
 NCBI BlastP on this gene

ELQ42266

hypothetical protein
  
Accession: ELQ42267
  
Location: 21055-22410
  
 NCBI BlastP on this gene

ELQ42267

glycogen debranching enzyme
  
Accession: ELQ42268
  
Location: 24524-29770
  
 NCBI BlastP on this gene

ELQ42268

hypothetical protein
  
Accession: ELQ42269
  
Location: 30908-31776
  
 NCBI BlastP on this gene

ELQ42269

hypothetical protein
  
Accession: ELQ42270
  
Location: 33562-33708
  
 NCBI BlastP on this gene

ELQ42270

hypothetical protein
  
Accession: ELQ42271
  
Location: 35058-35766
  
  
**BlastP hit with Mycgr3G92938\_Mycgr3T**
  
Percentage identity: 28 %
  
BlastP bit score: 72
  
Sequence coverage: 48 %
  
E-value: 2e-11
  
  
 NCBI BlastP on this gene

ELQ42271

AAA family ATPase
  
Accession: ELQ42272
  
Location: 37204-41821
  
  
**BlastP hit with Mycgr3G42010\_Mycgr3T**
  
Percentage identity: 42 %
  
BlastP bit score: 343
  
Sequence coverage: 21 %
  
E-value: 2e-94
  
  
 NCBI BlastP on this gene

ELQ42272

isotrichodermin C-15 hydroxylase
  
Accession: ELQ42273
  
Location: 42788-44612
  
 NCBI BlastP on this gene

ELQ42273

hypothetical protein
  
Accession: ELQ42274
  
Location: 44786-46244
  
 NCBI BlastP on this gene

ELQ42274

(R)-specific carbonyl reductase
  
Accession: ELQ42275
  
Location: 47155-48563
  
 NCBI BlastP on this gene

ELQ42275

xanthoxin dehydrogenase
  
Accession: ELQ42276
  
Location: 48615-49547
  
 NCBI BlastP on this gene

ELQ42276

phenylacetone monooxygenase
  
Accession: ELQ42277
  
Location: 50232-53853
  
 NCBI BlastP on this gene

ELQ42277

hypothetical protein
  
Accession: ELQ42278
  
Location: 54716-56776
  
 NCBI BlastP on this gene

ELQ42278

pre-rRNA-processing protein ESF1
  
Accession: ELQ42279
  
Location: 57363-59848
  
 NCBI BlastP on this gene

ELQ42279

hypothetical protein
  
Accession: ELQ42280
  
Location: 60786-61460
  
 NCBI BlastP on this gene

ELQ42280

228. :  CM001235 Magnaporthe oryzae 70-15 chromosome 5     Total score: 2.0     Cumulative Blast bit score: 403

hypothetical protein
  
Accession: EHA49492
  
Location: 4241623-4242903
  
 NCBI BlastP on this gene

EHA49492

hypothetical protein
  
Accession: EHA49493
  
Location: 4243574-4244929
  
 NCBI BlastP on this gene

EHA49493

glycogen debranching enzymye
  
Accession: EHA49494
  
Location: 4249730-4254721
  
 NCBI BlastP on this gene

EHA49494

hypothetical protein
  
Accession: EHA49495
  
Location: 4255861-4256729
  
 NCBI BlastP on this gene

EHA49495

hypothetical protein
  
Accession: EHA49496
  
Location: 4259675-4260722
  
  
**BlastP hit with Mycgr3G92938\_Mycgr3T**
  
Percentage identity: 28 %
  
BlastP bit score: 62
  
Sequence coverage: 49 %
  
E-value: 7e-08
  
  
 NCBI BlastP on this gene

EHA49496

AAA family ATPase
  
Accession: EHA49497
  
Location: 4262160-4264340
  
  
**BlastP hit with Mycgr3G42010\_Mycgr3T**
  
Percentage identity: 46 %
  
BlastP bit score: 341
  
Sequence coverage: 16 %
  
E-value: 8e-97
  
  
 NCBI BlastP on this gene

EHA49497

hypothetical protein
  
Accession: EHA49498
  
Location: 4265299-4266062
  
 NCBI BlastP on this gene

EHA49498

isotrichodermin C-15 hydroxylase
  
Accession: EHA49499
  
Location: 4267744-4269568
  
 NCBI BlastP on this gene

EHA49499

hypothetical protein
  
Accession: EHA49500
  
Location: 4269742-4271203
  
 NCBI BlastP on this gene

EHA49500

R-specific carbonyl reductase
  
Accession: EHA49501
  
Location: 4272554-4273522
  
 NCBI BlastP on this gene

EHA49501

short-chain dehydrogenase/reductase SDR
  
Accession: EHA49502
  
Location: 4273574-4274506
  
 NCBI BlastP on this gene

EHA49502

phenylacetone monooxygenase
  
Accession: EHA49503
  
Location: 4275191-4277014
  
 NCBI BlastP on this gene

EHA49503

metallo-beta-lactamase superfamily protein
  
Accession: EHA49504
  
Location: 4277634-4278812
  
 NCBI BlastP on this gene

EHA49504

hypothetical protein
  
Accession: EHA49505
  
Location: 4279675-4281735
  
 NCBI BlastP on this gene

EHA49505

hypothetical protein
  
Accession: EHA49506
  
Location: 4282323-4284509
  
 NCBI BlastP on this gene

EHA49506

229. :  JH767594 Coniosporium apollinis CBS 100218 chromosome Unknown supercont1.41     Total score: 2.0     Cumulative Blast bit score: 393

3-isopropylmalate dehydratase
  
Accession: EON68214
  
Location: 56175-58819
  
 NCBI BlastP on this gene

EON68214

hypothetical protein
  
Accession: EON68213
  
Location: 54227-55033
  
 NCBI BlastP on this gene

EON68213

hypothetical protein
  
Accession: EON68212
  
Location: 50668-52239
  
 NCBI BlastP on this gene

EON68212

hypothetical protein
  
Accession: EON68211
  
Location: 48192-49868
  
 NCBI BlastP on this gene

EON68211

AGC/AKT protein kinase
  
Accession: EON68210
  
Location: 45316-47504
  
 NCBI BlastP on this gene

EON68210

glucose-6-phosphate isomerase
  
Accession: EON68209
  
Location: 42719-44553
  
 NCBI BlastP on this gene

EON68209

hypothetical protein
  
Accession: EON68208
  
Location: 41224-42403
  
 NCBI BlastP on this gene

EON68208

hypothetical protein
  
Accession: EON68207
  
Location: 39935-40417
  
 NCBI BlastP on this gene

EON68207

hypothetical protein
  
Accession: EON68206
  
Location: 38826-39748
  
 NCBI BlastP on this gene

EON68206

hypothetical protein
  
Accession: EON68205
  
Location: 37320-38605
  
  
**BlastP hit with Mycgr3G71676\_Mycgr3T**
  
Percentage identity: 35 %
  
BlastP bit score: 204
  
Sequence coverage: 101 %
  
E-value: 8e-58
  
  
 NCBI BlastP on this gene

EON68205

3-oxoacyl-[acyl-carrier protein] reductase
  
Accession: EON68204
  
Location: 36128-36892
  
 NCBI BlastP on this gene

EON68204

hypothetical protein
  
Accession: EON68203
  
Location: 34038-35597
  
 NCBI BlastP on this gene

EON68203

hypothetical protein
  
Accession: EON68202
  
Location: 31497-33429
  
  
**BlastP hit with Mycgr3G85918\_Mycgr3T**
  
Percentage identity: 27 %
  
BlastP bit score: 189
  
Sequence coverage: 106 %
  
E-value: 1e-49
  
  
 NCBI BlastP on this gene

EON68202

230. :  GG698901 Nectria haematococca mpVI 77-13-4 chromosome 1 genomic scaffold NECHAsca\_8\_chr1\_1\_0     Total score: 2.0     Cumulative Blast bit score: 391

hypothetical protein
  
Accession: EEU44020
  
Location: 97945-99980
  
 NCBI BlastP on this gene

EEU44020

hypothetical protein
  
Accession: EEU44021
  
Location: 100473-101307
  
 NCBI BlastP on this gene

EEU44021

hypothetical protein
  
Accession: EEU43816
  
Location: 101972-103695
  
 NCBI BlastP on this gene

EEU43816

hypothetical protein
  
Accession: EEU44022
  
Location: 104491-106489
  
 NCBI BlastP on this gene

EEU44022

hypothetical protein
  
Accession: EEU44023
  
Location: 108259-108675
  
 NCBI BlastP on this gene

EEU44023

hypothetical protein
  
Accession: EEU43817
  
Location: 109087-110563
  
 NCBI BlastP on this gene

EEU43817

hypothetical protein
  
Accession: EEU43818
  
Location: 111036-112182
  
 NCBI BlastP on this gene

EEU43818

predicted protein
  
Accession: EEU44024
  
Location: 112643-114919
  
 NCBI BlastP on this gene

EEU44024

hypothetical protein
  
Accession: EEU44025
  
Location: 115648-117750
  
  
**BlastP hit with Mycgr3G85918\_Mycgr3T**
  
Percentage identity: 26 %
  
BlastP bit score: 165
  
Sequence coverage: 93 %
  
E-value: 3e-41
  
  
 NCBI BlastP on this gene

EEU44025

hypothetical protein
  
Accession: EEU43819
  
Location: 118742-121136
  
 NCBI BlastP on this gene

EEU43819

hypothetical protein
  
Accession: EEU44026
  
Location: 121696-122965
  
 NCBI BlastP on this gene

EEU44026

hypothetical protein
  
Accession: EEU44027
  
Location: 123445-124696
  
 NCBI BlastP on this gene

EEU44027

hypothetical protein
  
Accession: EEU43820
  
Location: 125309-127397
  
 NCBI BlastP on this gene

EEU43820

hypothetical protein
  
Accession: EEU43821
  
Location: 128020-129344
  
  
**BlastP hit with Mycgr3G71676\_Mycgr3T**
  
Percentage identity: 37 %
  
BlastP bit score: 226
  
Sequence coverage: 100 %
  
E-value: 6e-66
  
  
 NCBI BlastP on this gene

EEU43821

hypothetical protein
  
Accession: EEU44028
  
Location: 129504-132864
  
 NCBI BlastP on this gene

EEU44028

hypothetical protein
  
Accession: EEU43822
  
Location: 134399-135816
  
 NCBI BlastP on this gene

EEU43822

predicted protein
  
Accession: EEU44029
  
Location: 135906-137682
  
 NCBI BlastP on this gene

EEU44029

predicted protein
  
Accession: EEU44030
  
Location: 138773-141960
  
 NCBI BlastP on this gene

EEU44030

hypothetical protein
  
Accession: EEU43823
  
Location: 142511-147286
  
 NCBI BlastP on this gene

EEU43823

231. :  DS231619 Pyrenophora tritici-repentis Pt-1C-BFP supercont1.5 genomic scaffold     Total score: 2.0     Cumulative Blast bit score: 386

pisatin demethylase
  
Accession: EDU48575
  
Location: 1018451-1020107
  
 NCBI BlastP on this gene

EDU48575

ADP-ribose pyrophosphatase
  
Accession: EDU48576
  
Location: 1020494-1021099
  
 NCBI BlastP on this gene

EDU48576

conserved hypothetical protein
  
Accession: EDU48577
  
Location: 1021683-1022873
  
 NCBI BlastP on this gene

EDU48577

predicted protein
  
Accession: EDU48578
  
Location: 1023273-1023679
  
 NCBI BlastP on this gene

EDU48578

conserved hypothetical protein
  
Accession: EDU48579
  
Location: 1025053-1025905
  
 NCBI BlastP on this gene

EDU48579

conserved hypothetical protein
  
Accession: EDU48580
  
Location: 1026490-1026975
  
 NCBI BlastP on this gene

EDU48580

NADP-dependent alcohol dehydrogenase C
  
Accession: EDU48581
  
Location: 1027225-1028432
  
 NCBI BlastP on this gene

EDU48581

sorbitol dehydrogenase
  
Accession: EDU48582
  
Location: 1029654-1030821
  
  
**BlastP hit with Mycgr3G71679\_Mycgr3T**
  
Percentage identity: 42 %
  
BlastP bit score: 283
  
Sequence coverage: 100 %
  
E-value: 4e-89
  
  
 NCBI BlastP on this gene

EDU48582

serine/threonine-protein kinase GIN4
  
Accession: EDU48583
  
Location: 1031698-1035781
  
 NCBI BlastP on this gene

EDU48583

specific RNA polymerase II transcription factor
  
Accession: EDU48584
  
Location: 1037851-1039074
  
 NCBI BlastP on this gene

EDU48584

predicted protein
  
Accession: EDU48585
  
Location: 1040865-1041158
  
 NCBI BlastP on this gene

EDU48585

predicted protein
  
Accession: EDU48586
  
Location: 1041928-1042847
  
 NCBI BlastP on this gene

EDU48586

predicted protein
  
Accession: EDU48587
  
Location: 1045903-1046459
  
 NCBI BlastP on this gene

EDU48587

conserved hypothetical protein
  
Accession: EDU48588
  
Location: 1050816-1051841
  
  
**BlastP hit with Mycgr3G92941\_Mycgr3T**
  
Percentage identity: 28 %
  
BlastP bit score: 103
  
Sequence coverage: 49 %
  
E-value: 6e-21
  
  
 NCBI BlastP on this gene

EDU48588

conserved hypothetical protein
  
Accession: EDU48589
  
Location: 1052859-1053108
  
 NCBI BlastP on this gene

EDU48589

conserved hypothetical protein
  
Accession: EDU48590
  
Location: 1054794-1057607
  
 NCBI BlastP on this gene

EDU48590

predicted protein
  
Accession: EDU48591
  
Location: 1058730-1059447
  
 NCBI BlastP on this gene

EDU48591

conserved hypothetical protein
  
Accession: EDU48592
  
Location: 1060173-1060850
  
 NCBI BlastP on this gene

EDU48592

structural maintenance of chromosomes protein 5
  
Accession: EDU48593
  
Location: 1061780-1065217
  
 NCBI BlastP on this gene

EDU48593

232. :  DF126500 Aspergillus kawachii IFO 4308 DNA, contig: scaffold00054     Total score: 2.0     Cumulative Blast bit score: 379

FAD-dependent oxygenase
  
Accession: GAA92674
  
Location: 40182-41803
  
 NCBI BlastP on this gene

GAA92674

decarboxylase Dec1
  
Accession: GAA92675
  
Location: 42160-42912
  
 NCBI BlastP on this gene

GAA92675

GABA permease
  
Accession: GAA92676
  
Location: 44241-46097
  
 NCBI BlastP on this gene

GAA92676

integral membrane protein
  
Accession: GAA92677
  
Location: 47091-48455
  
 NCBI BlastP on this gene

GAA92677

amidase
  
Accession: GAA92678
  
Location: 48662-50316
  
 NCBI BlastP on this gene

GAA92678

hypothetical protein
  
Accession: GAA92679
  
Location: 51836-52840
  
 NCBI BlastP on this gene

GAA92679

hypothetical protein
  
Accession: GAA92680
  
Location: 54071-54490
  
 NCBI BlastP on this gene

GAA92680

MFS transporter
  
Accession: GAA92681
  
Location: 54773-56545
  
  
**BlastP hit with Mycgr3G85918\_Mycgr3T**
  
Percentage identity: 27 %
  
BlastP bit score: 196
  
Sequence coverage: 98 %
  
E-value: 4e-52
  
  
 NCBI BlastP on this gene

GAA92681

similar to An03g00310
  
Accession: GAA92682
  
Location: 57521-58897
  
 NCBI BlastP on this gene

GAA92682

hypothetical protein
  
Accession: GAA92683
  
Location: 60912-63545
  
 NCBI BlastP on this gene

GAA92683

oxidoreductase
  
Accession: GAA92684
  
Location: 69255-70741
  
 NCBI BlastP on this gene

GAA92684

tyrosinase
  
Accession: GAA92685
  
Location: 72315-73693
  
  
**BlastP hit with Mycgr3G42698\_Mycgr3T**
  
Percentage identity: 33 %
  
BlastP bit score: 183
  
Sequence coverage: 111 %
  
E-value: 4e-51
  
  
 NCBI BlastP on this gene

GAA92685

hypothetical protein
  
Accession: GAA92686
  
Location: 73815-74636
  
 NCBI BlastP on this gene

GAA92686

similar to An03g00260
  
Accession: GAA92687
  
Location: 75975-77150
  
 NCBI BlastP on this gene

GAA92687

hypothetical protein
  
Accession: GAA92688
  
Location: 78148-78917
  
 NCBI BlastP on this gene

GAA92688

similar to An03g00240
  
Accession: GAA92689
  
Location: 79814-81848
  
 NCBI BlastP on this gene

GAA92689

amino acid permease
  
Accession: GAA92690
  
Location: 82117-84093
  
 NCBI BlastP on this gene

GAA92690

similar to An03g00210
  
Accession: GAA92691
  
Location: 85207-86077
  
 NCBI BlastP on this gene

GAA92691

hypothetical protein
  
Accession: GAA92692
  
Location: 86148-86846
  
 NCBI BlastP on this gene

GAA92692

233. :  ACJE01000015 Aspergillus niger ATCC 1015     Total score: 2.0     Cumulative Blast bit score: 359

hypothetical protein
  
Accession: EHA20919
  
Location: 94384-95755
  
 NCBI BlastP on this gene

EHA20919

hypothetical protein
  
Accession: EHA20920
  
Location: 97297-97980
  
 NCBI BlastP on this gene

EHA20920

hypothetical protein
  
Accession: EHA20921
  
Location: 98036-98650
  
 NCBI BlastP on this gene

EHA20921

hypothetical protein
  
Accession: EHA20922
  
Location: 100096-102007
  
 NCBI BlastP on this gene

EHA20922

hypothetical protein
  
Accession: EHA20923
  
Location: 102279-104304
  
 NCBI BlastP on this gene

EHA20923

hypothetical protein
  
Accession: EHA20924
  
Location: 105192-106458
  
 NCBI BlastP on this gene

EHA20924

hypothetical protein
  
Accession: EHA20925
  
Location: 107932-108315
  
 NCBI BlastP on this gene

EHA20925

hypothetical protein
  
Accession: EHA20926
  
Location: 110930-112867
  
  
**BlastP hit with Mycgr3G42698\_Mycgr3T**
  
Percentage identity: 32 %
  
BlastP bit score: 174
  
Sequence coverage: 108 %
  
E-value: 2e-46
  
  
 NCBI BlastP on this gene

EHA20926

hypothetical protein
  
Accession: EHA20927
  
Location: 114451-115946
  
 NCBI BlastP on this gene

EHA20927

hypothetical protein
  
Accession: EHA20928
  
Location: 116436-117707
  
 NCBI BlastP on this gene

EHA20928

hypothetical protein
  
Accession: EHA20929
  
Location: 119093-120469
  
 NCBI BlastP on this gene

EHA20929

hypothetical protein
  
Accession: EHA20930
  
Location: 121375-123204
  
  
**BlastP hit with Mycgr3G85918\_Mycgr3T**
  
Percentage identity: 27 %
  
BlastP bit score: 185
  
Sequence coverage: 100 %
  
E-value: 4e-48
  
  
 NCBI BlastP on this gene

EHA20930

hypothetical protein
  
Accession: EHA20931
  
Location: 123559-123978
  
 NCBI BlastP on this gene

EHA20931

hypothetical protein
  
Accession: EHA20932
  
Location: 124955-126058
  
 NCBI BlastP on this gene

EHA20932

hypothetical protein
  
Accession: EHA20933
  
Location: 126425-127507
  
 NCBI BlastP on this gene

EHA20933

hypothetical protein
  
Accession: EHA20934
  
Location: 127589-128755
  
 NCBI BlastP on this gene

EHA20934

flavo protein monooxygenase
  
Accession: EHA20935
  
Location: 129174-130639
  
 NCBI BlastP on this gene

EHA20935

dehydrogenase
  
Accession: EHA20936
  
Location: 131285-134305
  
 NCBI BlastP on this gene

EHA20936

hypothetical protein
  
Accession: EHA20937
  
Location: 135559-136566
  
 NCBI BlastP on this gene

EHA20937

amidase
  
Accession: EHA20938
  
Location: 137960-139613
  
 NCBI BlastP on this gene

EHA20938

234. :  CABT02000006 Sordaria macrospora k-hell     Total score: 2.0     Cumulative Blast bit score: 350

not annotated
  
Accession: CCC08656
  
Location: 681209-682253
  
 NCBI BlastP on this gene

CCC08656

putative polyketide synthase
  
Accession: CCC08657
  
Location: 686705-695145
  
 NCBI BlastP on this gene

CCC08657

not annotated
  
Accession: CCC08658
  
Location: 695464-696860
  
  
**BlastP hit with Mycgr3G42698\_Mycgr3T**
  
Percentage identity: 33 %
  
BlastP bit score: 181
  
Sequence coverage: 109 %
  
E-value: 2e-50
  
  
 NCBI BlastP on this gene

CCC08658

not annotated
  
Accession: CCC08659
  
Location: 699699-700597
  
 NCBI BlastP on this gene

CCC08659

not annotated
  
Accession: CCC08660
  
Location: 701218-705508
  
 NCBI BlastP on this gene

CCC08660

not annotated
  
Accession: CCC08661
  
Location: 706548-709476
  
  
**BlastP hit with Mycgr3G11468\_Mycgr3T**
  
Percentage identity: 31 %
  
BlastP bit score: 169
  
Sequence coverage: 103 %
  
E-value: 1e-43
  
  
 NCBI BlastP on this gene

CCC08661

not annotated
  
Accession: CCC08662
  
Location: 709815-710402
  
 NCBI BlastP on this gene

CCC08662

not annotated
  
Accession: CCC08663
  
Location: 711057-712245
  
 NCBI BlastP on this gene

CCC08663

not annotated
  
Accession: CCC08664
  
Location: 713818-714609
  
 NCBI BlastP on this gene

CCC08664

not annotated
  
Accession: CCC08665
  
Location: 721492-722112
  
 NCBI BlastP on this gene

CCC08665

not annotated
  
Accession: CCC08666
  
Location: 725226-726466
  
 NCBI BlastP on this gene

CCC08666

235. :  DS231616 Pyrenophora tritici-repentis Pt-1C-BFP supercont1.2 genomic scaffold     Total score: 2.0     Cumulative Blast bit score: 316

acetylcholinesterase precursor
  
Accession: EDU44888
  
Location: 224498-226197
  
 NCBI BlastP on this gene

EDU44888

allantoate permease
  
Accession: EDU44889
  
Location: 226761-228473
  
 NCBI BlastP on this gene

EDU44889

alpha-glucosidase precursor
  
Accession: EDU44890
  
Location: 230348-233132
  
 NCBI BlastP on this gene

EDU44890

hypothetical protein
  
Accession: EDU44891
  
Location: 234054-235428
  
 NCBI BlastP on this gene

EDU44891

conserved hypothetical protein
  
Accession: EDU44892
  
Location: 237168-239092
  
 NCBI BlastP on this gene

EDU44892

hypothetical protein
  
Accession: EDU44893
  
Location: 239553-240329
  
  
**BlastP hit with Mycgr3G58567\_Mycgr3T**
  
Percentage identity: 33 %
  
BlastP bit score: 137
  
Sequence coverage: 95 %
  
E-value: 1e-35
  
  
 NCBI BlastP on this gene

EDU44893

SH3 domain containing protein
  
Accession: EDU44894
  
Location: 240809-241602
  
 NCBI BlastP on this gene

EDU44894

isochorismatase hydrolase
  
Accession: EDU44895
  
Location: 244292-245296
  
 NCBI BlastP on this gene

EDU44895

predicted protein
  
Accession: EDU44896
  
Location: 245917-246790
  
 NCBI BlastP on this gene

EDU44896

conserved hypothetical protein
  
Accession: EDU44897
  
Location: 247689-248980
  
  
**BlastP hit with Mycgr3G42698\_Mycgr3T**
  
Percentage identity: 33 %
  
BlastP bit score: 179
  
Sequence coverage: 112 %
  
E-value: 2e-49
  
  
 NCBI BlastP on this gene

EDU44897

conserved hypothetical protein
  
Accession: EDU44898
  
Location: 251224-252665
  
 NCBI BlastP on this gene

EDU44898

predicted protein
  
Accession: EDU44899
  
Location: 254151-255137
  
 NCBI BlastP on this gene

EDU44899

glucan 1,3-beta-glucosidase precursor
  
Accession: EDU44900
  
Location: 259129-260772
  
 NCBI BlastP on this gene

EDU44900

conserved hypothetical protein
  
Accession: EDU44901
  
Location: 261838-262546
  
 NCBI BlastP on this gene

EDU44901

conserved hypothetical protein
  
Accession: EDU44902
  
Location: 262768-264034
  
 NCBI BlastP on this gene

EDU44902

conserved hypothetical protein
  
Accession: EDU44903
  
Location: 266335-267495
  
 NCBI BlastP on this gene

EDU44903

236. :  KB445638 Cochliobolus sativus ND90Pr unplaced genomic scaffold COCSAscaffold\_2     Total score: 2.0     Cumulative Blast bit score: 305

hypothetical protein
  
Accession: EMD68067
  
Location: 576839-578844
  
 NCBI BlastP on this gene

EMD68067

hypothetical protein
  
Accession: EMD68068
  
Location: 586432-587287
  
 NCBI BlastP on this gene

EMD68068

hypothetical protein
  
Accession: EMD68069
  
Location: 588622-590195
  
 NCBI BlastP on this gene

EMD68069

hypothetical protein
  
Accession: EMD68070
  
Location: 596697-597473
  
  
**BlastP hit with Mycgr3G58567\_Mycgr3T**
  
Percentage identity: 33 %
  
BlastP bit score: 140
  
Sequence coverage: 95 %
  
E-value: 1e-36
  
  
 NCBI BlastP on this gene

EMD68070

hypothetical protein
  
Accession: EMD68071
  
Location: 598001-598770
  
 NCBI BlastP on this gene

EMD68071

hypothetical protein
  
Accession: EMD68072
  
Location: 599328-600635
  
  
**BlastP hit with Mycgr3G42698\_Mycgr3T**
  
Percentage identity: 31 %
  
BlastP bit score: 165
  
Sequence coverage: 112 %
  
E-value: 4e-44
  
  
 NCBI BlastP on this gene

EMD68072

hypothetical protein
  
Accession: EMD68073
  
Location: 603220-604757
  
 NCBI BlastP on this gene

EMD68073

hypothetical protein
  
Accession: EMD68074
  
Location: 605771-606586
  
 NCBI BlastP on this gene

EMD68074

hypothetical protein
  
Accession: EMD68075
  
Location: 608705-608983
  
 NCBI BlastP on this gene

EMD68075

hypothetical protein
  
Accession: EMD68076
  
Location: 610676-611098
  
 NCBI BlastP on this gene

EMD68076

hypothetical protein
  
Accession: EMD68077
  
Location: 611623-612072
  
 NCBI BlastP on this gene

EMD68077

glycosyltransferase family 1 protein
  
Accession: EMD68078
  
Location: 612894-613597
  
 NCBI BlastP on this gene

EMD68078

hypothetical protein
  
Accession: EMD68079
  
Location: 613846-615062
  
 NCBI BlastP on this gene

EMD68079

hypothetical protein
  
Accession: EMD68080
  
Location: 615753-616953
  
 NCBI BlastP on this gene

EMD68080

hypothetical protein
  
Accession: EMD68081
  
Location: 617732-618387
  
 NCBI BlastP on this gene

EMD68081

hypothetical protein
  
Accession: EMD68082
  
Location: 619081-620612
  
 NCBI BlastP on this gene

EMD68082

237. :  KB733450 Bipolaris maydis ATCC 48331 unplaced genomic scaffold COCC4scaffold\_7     Total score: 2.0     Cumulative Blast bit score: 292

hypothetical protein
  
Accession: ENI07141
  
Location: 664408-665611
  
 NCBI BlastP on this gene

ENI07141

hypothetical protein
  
Accession: ENI07142
  
Location: 666369-667590
  
 NCBI BlastP on this gene

ENI07142

glycosyltransferase family 1 protein
  
Accession: ENI07143
  
Location: 667840-668543
  
 NCBI BlastP on this gene

ENI07143

hypothetical protein
  
Accession: ENI07144
  
Location: 671847-672065
  
 NCBI BlastP on this gene

ENI07144

hypothetical protein
  
Accession: ENI07145
  
Location: 673290-674140
  
 NCBI BlastP on this gene

ENI07145

hypothetical protein
  
Accession: ENI07146
  
Location: 675134-675949
  
 NCBI BlastP on this gene

ENI07146

hypothetical protein
  
Accession: ENI07147
  
Location: 676990-678538
  
 NCBI BlastP on this gene

ENI07147

hypothetical protein
  
Accession: ENI07148
  
Location: 681157-682438
  
  
**BlastP hit with Mycgr3G42698\_Mycgr3T**
  
Percentage identity: 30 %
  
BlastP bit score: 160
  
Sequence coverage: 112 %
  
E-value: 2e-42
  
  
 NCBI BlastP on this gene

ENI07148

hypothetical protein
  
Accession: ENI07149
  
Location: 682986-683819
  
 NCBI BlastP on this gene

ENI07149

hypothetical protein
  
Accession: ENI07150
  
Location: 684350-685126
  
  
**BlastP hit with Mycgr3G58567\_Mycgr3T**
  
Percentage identity: 32 %
  
BlastP bit score: 132
  
Sequence coverage: 95 %
  
E-value: 1e-33
  
  
 NCBI BlastP on this gene

ENI07150

hypothetical protein
  
Accession: ENI07151
  
Location: 690911-692468
  
 NCBI BlastP on this gene

ENI07151

hypothetical protein
  
Accession: ENI07152
  
Location: 692762-693327
  
 NCBI BlastP on this gene

ENI07152

hypothetical protein
  
Accession: ENI07153
  
Location: 693802-694658
  
 NCBI BlastP on this gene

ENI07153

hypothetical protein
  
Accession: ENI07154
  
Location: 701806-702009
  
 NCBI BlastP on this gene

ENI07154

hypothetical protein
  
Accession: ENI07155
  
Location: 702264-704269
  
 NCBI BlastP on this gene

ENI07155

238. :  KB445573 Cochliobolus heterostrophus C5 unplaced genomic scaffold COCHEscaffold\_5     Total score: 2.0     Cumulative Blast bit score: 292

hypothetical protein
  
Accession: EMD93397
  
Location: 597616-599621
  
 NCBI BlastP on this gene

EMD93397

hypothetical protein
  
Accession: EMD93398
  
Location: 599876-600079
  
 NCBI BlastP on this gene

EMD93398

hypothetical protein
  
Accession: EMD93399
  
Location: 607227-608083
  
 NCBI BlastP on this gene

EMD93399

hypothetical protein
  
Accession: EMD93400
  
Location: 608558-609123
  
 NCBI BlastP on this gene

EMD93400

hypothetical protein
  
Accession: EMD93401
  
Location: 609417-610897
  
 NCBI BlastP on this gene

EMD93401

hypothetical protein
  
Accession: EMD93402
  
Location: 616759-617535
  
  
**BlastP hit with Mycgr3G58567\_Mycgr3T**
  
Percentage identity: 32 %
  
BlastP bit score: 132
  
Sequence coverage: 95 %
  
E-value: 1e-33
  
  
 NCBI BlastP on this gene

EMD93402

hypothetical protein
  
Accession: EMD93403
  
Location: 618066-618899
  
 NCBI BlastP on this gene

EMD93403

hypothetical protein
  
Accession: EMD93404
  
Location: 619447-620728
  
  
**BlastP hit with Mycgr3G42698\_Mycgr3T**
  
Percentage identity: 30 %
  
BlastP bit score: 160
  
Sequence coverage: 112 %
  
E-value: 2e-42
  
  
 NCBI BlastP on this gene

EMD93404

hypothetical protein
  
Accession: EMD93405
  
Location: 623362-624430
  
 NCBI BlastP on this gene

EMD93405

hypothetical protein
  
Accession: EMD93406
  
Location: 625936-626751
  
 NCBI BlastP on this gene

EMD93406

hypothetical protein
  
Accession: EMD93407
  
Location: 627745-628595
  
 NCBI BlastP on this gene

EMD93407

hypothetical protein
  
Accession: EMD93408
  
Location: 629829-630038
  
 NCBI BlastP on this gene

EMD93408

glycosyltransferase family 1 protein
  
Accession: EMD93409
  
Location: 633342-634045
  
 NCBI BlastP on this gene

EMD93409

hypothetical protein
  
Accession: EMD93410
  
Location: 634295-635516
  
 NCBI BlastP on this gene

EMD93410

hypothetical protein
  
Accession: EMD93411
  
Location: 636274-637477
  
 NCBI BlastP on this gene

EMD93411

hypothetical protein
  
Accession: EMD93412
  
Location: 638250-638913
  
 NCBI BlastP on this gene

EMD93412

hypothetical protein
  
Accession: EMD93413
  
Location: 639622-641153
  
 NCBI BlastP on this gene

EMD93413

239. :  AHMM02000023 Leptospira inadai serovar Lyme str. 10     Total score: 2.0     Cumulative Blast bit score: 223

tetratricopeptide repeat protein
  
Accession: EQA36150
  
Location: 118111-120126
  
 NCBI BlastP on this gene

EQA36150

hypothetical protein
  
Accession: EQA36189
  
Location: 120158-120967
  
 NCBI BlastP on this gene

EQA36189

enoyl-CoA hydratase/isomerase family protein
  
Accession: EQA36172
  
Location: 120988-121752
  
 NCBI BlastP on this gene

EQA36172

FAD binding domain protein
  
Accession: EQA36137
  
Location: 121821-123413
  
 NCBI BlastP on this gene

EQA36137

SpoIIE-like protein phosphatase domain protein
  
Accession: EQA36181
  
Location: 123672-125699
  
 NCBI BlastP on this gene

EQA36181

FAD dependent oxidoreductase
  
Accession: EQA36196
  
Location: 125863-127506
  
 NCBI BlastP on this gene

EQA36196

transcriptional regulator, TetR family
  
Accession: EQA36195
  
Location: 127610-128188
  
 NCBI BlastP on this gene

EQA36195

PF06127 family protein
  
Accession: EQA36203
  
Location: 128311-128781
  
 NCBI BlastP on this gene

EQA36203

phospholipase, patatin family
  
Accession: EQA36153
  
Location: 128805-129806
  
 NCBI BlastP on this gene

EQA36153

hypothetical protein
  
Accession: EQA36207
  
Location: 129964-130431
  
 NCBI BlastP on this gene

EQA36207

hypothetical protein
  
Accession: EQA36218
  
Location: 130454-131296
  
 NCBI BlastP on this gene

EQA36218

YacP-like NYN domain protein
  
Accession: EQA36167
  
Location: 131398-131862
  
 NCBI BlastP on this gene

EQA36167

putative alginate O-acetyltransferase AlgI
  
Accession: EQA36154
  
Location: 131866-133365
  
 NCBI BlastP on this gene

EQA36154

PF07611 family protein
  
Accession: EQA36210
  
Location: 133384-134469
  
 NCBI BlastP on this gene

EQA36210

DJ-1 family protein
  
Accession: EQA36101
  
Location: 134493-135038
  
 NCBI BlastP on this gene

EQA36101

GtrA-like protein
  
Accession: EQA36215
  
Location: 135095-136249
  
 NCBI BlastP on this gene

EQA36215

glycoside hydrolase, family 5
  
Accession: EQA36129
  
Location: 136589-138493
  
  
**BlastP hit with Mycgr3G71681\_Mycgr3T**
  
Percentage identity: 29 %
  
BlastP bit score: 107
  
Sequence coverage: 84 %
  
E-value: 5e-22
  
  
 NCBI BlastP on this gene

EQA36129

DoxX family protein
  
Accession: EQA36121
  
Location: 138929-139408
  
 NCBI BlastP on this gene

EQA36121

hypothetical protein
  
Accession: EQA36176
  
Location: 139645-139791
  
 NCBI BlastP on this gene

EQA36176

KR domain protein
  
Accession: EQA36109
  
Location: 139849-140628
  
  
**BlastP hit with Mycgr3G58567\_Mycgr3T**
  
Percentage identity: 32 %
  
BlastP bit score: 117
  
Sequence coverage: 94 %
  
E-value: 5e-28
  
  
 NCBI BlastP on this gene

EQA36109

hypothetical protein
  
Accession: EQA36217
  
Location: 141042-141155
  
 NCBI BlastP on this gene

EQA36217

peptidase, S8/S53 family
  
Accession: EQA36087
  
Location: 141230-143059
  
 NCBI BlastP on this gene

EQA36087

VCBS repeat protein
  
Accession: EQA36174
  
Location: 143037-143771
  
 NCBI BlastP on this gene

EQA36174

hypothetical protein
  
Accession: EQA36086
  
Location: 143813-144109
  
 NCBI BlastP on this gene

EQA36086

hypothetical protein
  
Accession: EQA36141
  
Location: 144130-144870
  
 NCBI BlastP on this gene

EQA36141

hypothetical protein
  
Accession: EQA36228
  
Location: 145811-146872
  
 NCBI BlastP on this gene

EQA36228

hypothetical protein
  
Accession: EQA36223
  
Location: 146839-147066
  
 NCBI BlastP on this gene

EQA36223

sigma factor regulatory protein, FecR/PupR family
  
Accession: EQA36211
  
Location: 148477-149421
  
 NCBI BlastP on this gene

EQA36211

hypothetical protein
  
Accession: EQA36116
  
Location: 149479-150771
  
 NCBI BlastP on this gene

EQA36116

hypothetical protein
  
Accession: EQA36168
  
Location: 151189-151404
  
 NCBI BlastP on this gene

EQA36168

240. :  AHMO02000004 Leptospira broomii serovar Hurstbridge str. 5399     Total score: 2.0     Cumulative Blast bit score: 220

tetratricopeptide repeat protein
  
Accession: EQA46946
  
Location: 661705-663762
  
 NCBI BlastP on this gene

EQA46946

putative lipoprotein
  
Accession: EQA46846
  
Location: 663792-664598
  
 NCBI BlastP on this gene

EQA46846

enoyl-CoA hydratase/isomerase family protein
  
Accession: EQA47072
  
Location: 664619-665383
  
 NCBI BlastP on this gene

EQA47072

FAD binding domain protein
  
Accession: EQA46708
  
Location: 665452-667044
  
 NCBI BlastP on this gene

EQA46708

hypothetical protein
  
Accession: EQA46793
  
Location: 667066-667251
  
 NCBI BlastP on this gene

EQA46793

SpoIIE-like protein phosphatase domain protein
  
Accession: EQA47204
  
Location: 667303-669330
  
 NCBI BlastP on this gene

EQA47204

FAD dependent oxidoreductase
  
Accession: EQA47090
  
Location: 669495-671138
  
 NCBI BlastP on this gene

EQA47090

transcriptional regulator, TetR family
  
Accession: EQA47222
  
Location: 671226-671804
  
 NCBI BlastP on this gene

EQA47222

PF06127 family protein
  
Accession: EQA46957
  
Location: 671926-672396
  
 NCBI BlastP on this gene

EQA46957

phospholipase, patatin family
  
Accession: EQA47097
  
Location: 672420-673460
  
 NCBI BlastP on this gene

EQA47097

hypothetical protein
  
Accession: EQA46844
  
Location: 673580-674047
  
 NCBI BlastP on this gene

EQA46844

hypothetical protein
  
Accession: EQA46956
  
Location: 674069-674911
  
 NCBI BlastP on this gene

EQA46956

YacP-like NYN domain protein
  
Accession: EQA46686
  
Location: 675013-675477
  
 NCBI BlastP on this gene

EQA46686

putative alginate O-acetyltransferase AlgI
  
Accession: EQA46755
  
Location: 675481-676980
  
 NCBI BlastP on this gene

EQA46755

PF07611 family protein
  
Accession: EQA46974
  
Location: 676999-678084
  
 NCBI BlastP on this gene

EQA46974

DJ-1 family protein
  
Accession: EQA47073
  
Location: 678108-678653
  
 NCBI BlastP on this gene

EQA47073

GtrA-like protein
  
Accession: EQA46967
  
Location: 678710-679864
  
 NCBI BlastP on this gene

EQA46967

glycoside hydrolase, family 5
  
Accession: EQA46723
  
Location: 680192-682093
  
  
**BlastP hit with Mycgr3G71681\_Mycgr3T**
  
Percentage identity: 29 %
  
BlastP bit score: 104
  
Sequence coverage: 85 %
  
E-value: 5e-21
  
  
 NCBI BlastP on this gene

EQA46723

DoxX family protein
  
Accession: EQA47213
  
Location: 682510-682989
  
 NCBI BlastP on this gene

EQA47213

KR domain protein
  
Accession: EQA47235
  
Location: 683828-684607
  
  
**BlastP hit with Mycgr3G58567\_Mycgr3T**
  
Percentage identity: 31 %
  
BlastP bit score: 116
  
Sequence coverage: 94 %
  
E-value: 2e-27
  
  
 NCBI BlastP on this gene

EQA47235

hypothetical protein
  
Accession: EQA47290
  
Location: 684614-684826
  
 NCBI BlastP on this gene

EQA47290

hypothetical protein
  
Accession: EQA46897
  
Location: 685177-686169
  
 NCBI BlastP on this gene

EQA46897

hypothetical protein
  
Accession: EQA47192
  
Location: 686206-686382
  
 NCBI BlastP on this gene

EQA47192

hypothetical protein
  
Accession: EQA47121
  
Location: 686786-687022
  
 NCBI BlastP on this gene

EQA47121

sigma factor regulatory protein, FecR/PupR family
  
Accession: EQA46986
  
Location: 688025-688969
  
 NCBI BlastP on this gene

EQA46986

hypothetical protein
  
Accession: EQA46678
  
Location: 689028-690314
  
 NCBI BlastP on this gene

EQA46678

hypothetical protein
  
Accession: EQA47030
  
Location: 690497-690667
  
 NCBI BlastP on this gene

EQA47030

241. :  JH921445 Marssonina brunnea f. sp. 'multigermtubi' MB\_m1 unplaced genomic scaffold M6\_S00018     Total score: 2.0     Cumulative Blast bit score: 203

NADH-ubiquinone oxidoreductase 10.5 kDa subunit
  
Accession: EKD14475
  
Location: 324908-325585
  
 NCBI BlastP on this gene

EKD14475

oxysterol-binding protein
  
Accession: EKD14476
  
Location: 327453-331368
  
 NCBI BlastP on this gene

EKD14476

hypothetical protein
  
Accession: EKD14477
  
Location: 331808-333072
  
 NCBI BlastP on this gene

EKD14477

DASH family cryptochrome
  
Accession: EKD14478
  
Location: 336087-338116
  
 NCBI BlastP on this gene

EKD14478

Fasciclin domain family protein
  
Accession: EKD14479
  
Location: 341444-342920
  
 NCBI BlastP on this gene

EKD14479

thioesterase family protein
  
Accession: EKD14480
  
Location: 344495-345359
  
  
**BlastP hit with Mycgr3G92941\_Mycgr3T**
  
Percentage identity: 27 %
  
BlastP bit score: 94
  
Sequence coverage: 39 %
  
E-value: 5e-18
  
  
 NCBI BlastP on this gene

EKD14480

hypothetical protein
  
Accession: EKD14481
  
Location: 348231-348880
  
  
**BlastP hit with Mycgr3G29582\_Mycgr3T**
  
Percentage identity: 72 %
  
BlastP bit score: 109
  
Sequence coverage: 92 %
  
E-value: 1e-28
  
  
 NCBI BlastP on this gene

EKD14481

GATA zinc finger protein
  
Accession: EKD14482
  
Location: 351500-361792
  
 NCBI BlastP on this gene

EKD14482

hypothetical protein
  
Accession: EKD14483
  
Location: 368397-368735
  
 NCBI BlastP on this gene

EKD14483

242. :  CH408031 Chaetomium globosum CBS 148.51 scaffold\_3 genomic scaffold     Total score: 2.0     Cumulative Blast bit score: 201

hypothetical protein
  
Accession: EAQ90101
  
Location: 4360609-4362408
  
 NCBI BlastP on this gene

EAQ90101

hypothetical protein
  
Accession: EAQ90100
  
Location: 4358899-4359871
  
 NCBI BlastP on this gene

EAQ90100

hypothetical protein
  
Accession: EAQ90099
  
Location: 4356341-4357796
  
 NCBI BlastP on this gene

EAQ90099

hypothetical protein
  
Accession: EAQ90098
  
Location: 4353569-4355066
  
 NCBI BlastP on this gene

EAQ90098

hypothetical protein
  
Accession: EAQ90097
  
Location: 4352202-4353273
  
 NCBI BlastP on this gene

EAQ90097

hypothetical protein
  
Accession: EAQ90096
  
Location: 4349110-4351424
  
 NCBI BlastP on this gene

EAQ90096

hypothetical protein
  
Accession: EAQ90095
  
Location: 4346971-4347812
  
 NCBI BlastP on this gene

EAQ90095

hypothetical protein
  
Accession: EAQ90094
  
Location: 4345214-4346422
  
 NCBI BlastP on this gene

EAQ90094

hypothetical protein
  
Accession: EAQ90093
  
Location: 4342482-4344715
  
 NCBI BlastP on this gene

EAQ90093

hypothetical protein
  
Accession: EAQ90092
  
Location: 4339190-4341942
  
  
**BlastP hit with Mycgr3G11468\_Mycgr3T**
  
Percentage identity: 28 %
  
BlastP bit score: 127
  
Sequence coverage: 101 %
  
E-value: 5e-29
  
  
 NCBI BlastP on this gene

EAQ90092

hypothetical protein
  
Accession: EAQ90091
  
Location: 4338336-4338928
  
 NCBI BlastP on this gene

EAQ90091

hypothetical protein
  
Accession: EAQ90090
  
Location: 4337324-4337987
  
 NCBI BlastP on this gene

EAQ90090

hypothetical protein
  
Accession: EAQ90089
  
Location: 4334209-4335206
  
  
**BlastP hit with Mycgr3G109328\_Mycgr3**
  
Percentage identity: 31 %
  
BlastP bit score: 74
  
Sequence coverage: 78 %
  
E-value: 5e-13
  
  
 NCBI BlastP on this gene

EAQ90089

hypothetical protein
  
Accession: EAQ90088
  
Location: 4332527-4333423
  
 NCBI BlastP on this gene

EAQ90088

hypothetical protein
  
Accession: EAQ90087
  
Location: 4329943-4331214
  
 NCBI BlastP on this gene

EAQ90087

predicted protein
  
Accession: EAQ90086
  
Location: 4328891-4329361
  
 NCBI BlastP on this gene

EAQ90086

hypothetical protein
  
Accession: EAQ90085
  
Location: 4322621-4323868
  
 NCBI BlastP on this gene

EAQ90085

hypothetical protein
  
Accession: EAQ90084
  
Location: 4318264-4320992
  
 NCBI BlastP on this gene

EAQ90084

hypothetical protein
  
Accession: EAQ90083
  
Location: 4316383-4317498
  
 NCBI BlastP on this gene

EAQ90083

hypothetical protein
  
Accession: EAQ90082
  
Location: 4314900-4315948
  
 NCBI BlastP on this gene

EAQ90082

243. :  CU633897 Podospora anserina S mat+ genomic DNA chromosome 1, supercontig 6.     Total score: 2.0     Cumulative Blast bit score: 199

not annotated
  
Accession: CAP67185
  
Location: 231675-232446
  
 NCBI BlastP on this gene

CAP67185

not annotated
  
Accession: CAP67186
  
Location: 233171-233958
  
 NCBI BlastP on this gene

CAP67186

not annotated
  
Accession: CAP67187
  
Location: 234473-238489
  
 NCBI BlastP on this gene

CAP67187

not annotated
  
Accession: CAP67188
  
Location: 238702-240036
  
 NCBI BlastP on this gene

CAP67188

not annotated
  
Accession: CAP67189
  
Location: 240140-241245
  
 NCBI BlastP on this gene

CAP67189

not annotated
  
Accession: CAP67190
  
Location: 241730-242880
  
 NCBI BlastP on this gene

CAP67190

tRNA-Met
  
Accession: CAP67191
  
Location: 244230-244610
  
 NCBI BlastP on this gene

CAP67191

not annotated
  
Accession: CAP67192
  
Location: 246419-248635
  
 NCBI BlastP on this gene

CAP67192

not annotated
  
Accession: CAP67193
  
Location: 249796-251999
  
  
**BlastP hit with Mycgr3G11468\_Mycgr3T**
  
Percentage identity: 30 %
  
BlastP bit score: 132
  
Sequence coverage: 100 %
  
E-value: 7e-31
  
  
 NCBI BlastP on this gene

CAP67193

not annotated
  
Accession: CAP67194
  
Location: 252121-252621
  
 NCBI BlastP on this gene

CAP67194

not annotated
  
Accession: CAP67195
  
Location: 253258-254119
  
 NCBI BlastP on this gene

CAP67195

not annotated
  
Accession: CAP67196
  
Location: 255770-257539
  
 NCBI BlastP on this gene

CAP67196

not annotated
  
Accession: CAP67197
  
Location: 258272-259228
  
  
**BlastP hit with Mycgr3G109328\_Mycgr3**
  
Percentage identity: 26 %
  
BlastP bit score: 67
  
Sequence coverage: 106 %
  
E-value: 2e-10
  
  
 NCBI BlastP on this gene

CAP67197

not annotated
  
Accession: CAP67198
  
Location: 259935-261069
  
 NCBI BlastP on this gene

CAP67198

not annotated
  
Accession: CAP67199
  
Location: 262617-263891
  
 NCBI BlastP on this gene

CAP67199

not annotated
  
Accession: CAP67200
  
Location: 270791-272902
  
 NCBI BlastP on this gene

CAP67200

not annotated
  
Accession: CAP67201
  
Location: 273854-274543
  
 NCBI BlastP on this gene

CAP67201

not annotated
  
Accession: CAP67202
  
Location: 275222-276792
  
 NCBI BlastP on this gene

CAP67202

244. :  AEYX01000033 Streptomyces griseoaurantiacus M045     Total score: 2.0     Cumulative Blast bit score: 196

anti-anti-sigma-factor
  
Accession: EGG47266
  
Location: 176781-177110
  
 NCBI BlastP on this gene

EGG47266

regulatory protein/ATPase
  
Accession: EGG47267
  
Location: 177287-177754
  
 NCBI BlastP on this gene

EGG47267

Stage II sporulation E family protein
  
Accession: EGG47268
  
Location: 177968-180730
  
 NCBI BlastP on this gene

EGG47268

two component sensor kinase
  
Accession: EGG47269
  
Location: 180796-182010
  
 NCBI BlastP on this gene

EGG47269

two-component response regulator
  
Accession: EGG47270
  
Location: 182007-182666
  
 NCBI BlastP on this gene

EGG47270

integral membrane protein
  
Accession: EGG47271
  
Location: 182805-183440
  
 NCBI BlastP on this gene

EGG47271

hypothetical protein
  
Accession: EGG47272
  
Location: 183540-184232
  
 NCBI BlastP on this gene

EGG47272

Transcriptional regulator, TetR family
  
Accession: EGG47273
  
Location: 184374-184976
  
 NCBI BlastP on this gene

EGG47273

hypothetical protein
  
Accession: EGG47274
  
Location: 185046-185891
  
 NCBI BlastP on this gene

EGG47274

glycosyl hydrolase
  
Accession: EGG47275
  
Location: 186023-187930
  
  
**BlastP hit with Mycgr3G71681\_Mycgr3T**
  
Percentage identity: 28 %
  
BlastP bit score: 82
  
Sequence coverage: 62 %
  
E-value: 9e-14
  
  
 NCBI BlastP on this gene

EGG47275

NADP-dependent alcohol dehydrogenase
  
Accession: EGG47276
  
Location: 187948-188988
  
 NCBI BlastP on this gene

EGG47276

transcriptional regulator
  
Accession: EGG47277
  
Location: 189093-189680
  
 NCBI BlastP on this gene

EGG47277

lipid hydrolase
  
Accession: EGG47278
  
Location: 189879-192080
  
 NCBI BlastP on this gene

EGG47278

TetR family transcriptional regulator
  
Accession: EGG47279
  
Location: 192172-192795
  
 NCBI BlastP on this gene

EGG47279

oxidoreductase
  
Accession: EGG47280
  
Location: 192904-193866
  
 NCBI BlastP on this gene

EGG47280

mini-circle protein
  
Accession: EGG47281
  
Location: 193927-194511
  
 NCBI BlastP on this gene

EGG47281

alcohol dehydrogenase
  
Accession: EGG47282
  
Location: 194633-195685
  
 NCBI BlastP on this gene

EGG47282

catechol 1,2-dioxygenase
  
Accession: EGG47283
  
Location: 195682-196620
  
 NCBI BlastP on this gene

EGG47283

non-ribosomal peptide synthetase
  
Accession: EGG47284
  
Location: 197187-201269
  
 NCBI BlastP on this gene

EGG47284

putative NRPS
  
Accession: EGG47285
  
Location: 201266-210745
  
 NCBI BlastP on this gene

EGG47285

Alpha-ketoglutarate-dependent taurine dioxygenase
  
Accession: EGG47286
  
Location: 210855-211727
  
 NCBI BlastP on this gene

EGG47286

DNA-binding protein
  
Accession: EGG47287
  
Location: 211753-212601
  
 NCBI BlastP on this gene

EGG47287

oxidoreductase
  
Accession: EGG47288
  
Location: 212703-213467
  
  
**BlastP hit with Mycgr3G58567\_Mycgr3T**
  
Percentage identity: 37 %
  
BlastP bit score: 114
  
Sequence coverage: 74 %
  
E-value: 1e-26
  
  
 NCBI BlastP on this gene

EGG47288

NAD(P)H dehydrogenase
  
Accession: EGG47289
  
Location: 213522-214286
  
 NCBI BlastP on this gene

EGG47289

AdpA family transcriptional regulator
  
Accession: EGG47290
  
Location: 214331-215359
  
 NCBI BlastP on this gene

EGG47290

putative chloramphenicol 3-O phosphotransferase
  
Accession: EGG47291
  
Location: 215412-215945
  
 NCBI BlastP on this gene

EGG47291

putative RNA polymerase sigma factor
  
Accession: EGG47292
  
Location: 216065-216643
  
 NCBI BlastP on this gene

EGG47292

hypothetical protein
  
Accession: EGG47293
  
Location: 216636-217478
  
 NCBI BlastP on this gene

EGG47293

lipoprotein
  
Accession: EGG47294
  
Location: 217549-218334
  
 NCBI BlastP on this gene

EGG47294

hypothetical protein
  
Accession: EGG47295
  
Location: 218430-219128
  
 NCBI BlastP on this gene

EGG47295

LysR family transcriptional regulator
  
Accession: EGG47296
  
Location: 219219-220106
  
 NCBI BlastP on this gene

EGG47296

GCN5-related N-acetyltransferase
  
Accession: EGG47297
  
Location: 220228-220683
  
 NCBI BlastP on this gene

EGG47297

hypothetical protein
  
Accession: EGG47298
  
Location: 220963-221280
  
 NCBI BlastP on this gene

EGG47298

hypothetical protein
  
Accession: EGG47299
  
Location: 221253-221681
  
 NCBI BlastP on this gene

EGG47299

transposase
  
Accession: EGG47300
  
Location: 221716-222369
  
 NCBI BlastP on this gene

EGG47300

245. :  CAUH01001128 Blumeria graminis f. sp. hordei DH14     Total score: 2.0     Cumulative Blast bit score: 175

thioesterase family protein
  
Accession: CCU75257
  
Location: 1042-1938
  
  
**BlastP hit with Mycgr3G92941\_Mycgr3T**
  
Percentage identity: 33 %
  
BlastP bit score: 80
  
Sequence coverage: 25 %
  
E-value: 2e-13
  
  
 NCBI BlastP on this gene

CCU75257

oxysterol-binding protein
  
Accession: CCU75258
  
Location: 12594-16474
  
 NCBI BlastP on this gene

CCU75258

hypothetical protein
  
Accession: CCU75259
  
Location: 18986-19427
  
  
**BlastP hit with Mycgr3G29582\_Mycgr3T**
  
Percentage identity: 60 %
  
BlastP bit score: 95
  
Sequence coverage: 92 %
  
E-value: 2e-23
  
  
 NCBI BlastP on this gene

CCU75259

246. :  JH711588 Coniophora puteana RWD-64-598 SS2 unplaced genomic scaffold CONPUscaffold\_16     Total score: 1.0     Cumulative Blast bit score: 3125

phospholipid-translocating P-type ATPase
  
Accession: EIW75489
  
Location: 316542-322410
  
 NCBI BlastP on this gene

EIW75489

hypothetical protein
  
Accession: EIW75488
  
Location: 313717-315514
  
 NCBI BlastP on this gene

EIW75488

hypothetical protein
  
Accession: EIW75487
  
Location: 310455-311410
  
 NCBI BlastP on this gene

EIW75487

mitochondrial carrier
  
Accession: EIW75486
  
Location: 307207-309279
  
 NCBI BlastP on this gene

EIW75486

P-loop containing nucleoside triphosphate hydrolase protein
  
Accession: EIW75485
  
Location: 295346-302828
  
  
**BlastP hit with Mycgr3G42010\_Mycgr3T**
  
Percentage identity: 45 %
  
BlastP bit score: 1934
  
Sequence coverage: 102 %
  
E-value: 0.0
  
  
 NCBI BlastP on this gene

EIW75485

hypothetical protein
  
Accession: EIW75484
  
Location: 286107-293188
  
  
**BlastP hit with Mycgr3G42010\_Mycgr3T**
  
Percentage identity: 43 %
  
BlastP bit score: 1191
  
Sequence coverage: 69 %
  
E-value: 0.0
  
  
 NCBI BlastP on this gene

EIW75484

cytochrome P450
  
Accession: EIW75483
  
Location: 283601-285762
  
 NCBI BlastP on this gene

EIW75483

cytochrome P450
  
Accession: EIW75482
  
Location: 280640-282920
  
 NCBI BlastP on this gene

EIW75482

hypothetical protein
  
Accession: EIW75481
  
Location: 278873-280192
  
 NCBI BlastP on this gene

EIW75481

NAD(P)-binding protein
  
Accession: EIW75480
  
Location: 277005-278684
  
 NCBI BlastP on this gene

EIW75480

iron reductase
  
Accession: EIW75479
  
Location: 272617-274877
  
 NCBI BlastP on this gene

EIW75479

247. :  KB446555 Pseudocercospora fijiensis CIRAD86 unplaced genomic scaffold MYCFIscaffold\_1     Total score: 1.0     Cumulative Blast bit score: 2089

hypothetical protein
  
Accession: EME89031
  
Location: 8485738-8487778
  
 NCBI BlastP on this gene

EME89031

hypothetical protein
  
Accession: EME89030
  
Location: 8484824-8485360
  
 NCBI BlastP on this gene

EME89030

hypothetical protein
  
Accession: EME89029
  
Location: 8480796-8480999
  
 NCBI BlastP on this gene

EME89029

hypothetical protein
  
Accession: EME89028
  
Location: 8478486-8479655
  
 NCBI BlastP on this gene

EME89028

hypothetical protein
  
Accession: EME89027
  
Location: 8469649-8471007
  
 NCBI BlastP on this gene

EME89027

hypothetical protein
  
Accession: EME89026
  
Location: 8466881-8469115
  
 NCBI BlastP on this gene

EME89026

hypothetical protein
  
Accession: EME89025
  
Location: 8460428-8466427
  
  
**BlastP hit with Mycgr3G41426\_Mycgr3T**
  
Percentage identity: 56 %
  
BlastP bit score: 2089
  
Sequence coverage: 101 %
  
E-value: 0.0
  
  
 NCBI BlastP on this gene

EME89025

hypothetical protein
  
Accession: EME89024
  
Location: 8455639-8456064
  
 NCBI BlastP on this gene

EME89024

hypothetical protein
  
Accession: EME89023
  
Location: 8454544-8455509
  
 NCBI BlastP on this gene

EME89023

hypothetical protein
  
Accession: EME89022
  
Location: 8453453-8453991
  
 NCBI BlastP on this gene

EME89022

248. :  KB456260 Mycosphaerella populorum SO2202 unplaced genomic scaffold SEPMUscaffold\_1     Total score: 1.0     Cumulative Blast bit score: 1968

elongation factor 1 beta subunit
  
Accession: EMF16680
  
Location: 1505292-1506298
  
 NCBI BlastP on this gene

EMF16680

hypothetical protein
  
Accession: EMF16681
  
Location: 1506822-1507097
  
 NCBI BlastP on this gene

EMF16681

mismatch repair protein 5
  
Accession: EMF16682
  
Location: 1507808-1510476
  
 NCBI BlastP on this gene

EMF16682

ribosome biogenesis ATPase RIX7
  
Accession: EMF16683
  
Location: 1510879-1513188
  
 NCBI BlastP on this gene

EMF16683

hypothetical protein
  
Accession: EMF16684
  
Location: 1513610-1514331
  
 NCBI BlastP on this gene

EMF16684

hypothetical protein
  
Accession: EMF16685
  
Location: 1515331-1515753
  
 NCBI BlastP on this gene

EMF16685

hypothetical protein
  
Accession: EMF16686
  
Location: 1519154-1520032
  
 NCBI BlastP on this gene

EMF16686

hypothetical protein
  
Accession: EMF16687
  
Location: 1521201-1522742
  
 NCBI BlastP on this gene

EMF16687

hypothetical protein
  
Accession: EMF16688
  
Location: 1523903-1529914
  
  
**BlastP hit with Mycgr3G41426\_Mycgr3T**
  
Percentage identity: 55 %
  
BlastP bit score: 1968
  
Sequence coverage: 102 %
  
E-value: 0.0
  
  
 NCBI BlastP on this gene

EMF16688

249. :  KB469300 Gloeophyllum trabeum ATCC 11539 unplaced genomic scaffold GLOTRscaffold\_00005     Total score: 1.0     Cumulative Blast bit score: 1955

hypothetical protein
  
Accession: EPQ56106
  
Location: 151613-152377
  
 NCBI BlastP on this gene

EPQ56106

P-loop containing nucleoside triphosphate hydrolase protein
  
Accession: EPQ56105
  
Location: 147339-151096
  
 NCBI BlastP on this gene

EPQ56105

hypothetical protein
  
Accession: EPQ56104
  
Location: 142745-147235
  
 NCBI BlastP on this gene

EPQ56104

hypothetical protein
  
Accession: EPQ56103
  
Location: 141616-141822
  
 NCBI BlastP on this gene

EPQ56103

hypothetical protein
  
Accession: EPQ56102
  
Location: 138543-139465
  
 NCBI BlastP on this gene

EPQ56102

hypothetical protein
  
Accession: EPQ56101
  
Location: 136456-138237
  
 NCBI BlastP on this gene

EPQ56101

P-loop containing nucleoside triphosphate hydrolase protein
  
Accession: EPQ56100
  
Location: 128005-135685
  
  
**BlastP hit with Mycgr3G42010\_Mycgr3T**
  
Percentage identity: 44 %
  
BlastP bit score: 1955
  
Sequence coverage: 105 %
  
E-value: 0.0
  
  
 NCBI BlastP on this gene

EPQ56100

cobW-domain-containing protein
  
Accession: EPQ56099
  
Location: 123554-125462
  
 NCBI BlastP on this gene

EPQ56099

hypothetical protein
  
Accession: EPQ56098
  
Location: 119021-122633
  
 NCBI BlastP on this gene

EPQ56098

FAD/NAD P-binding domain-containing protein
  
Accession: EPQ56097
  
Location: 116851-118597
  
 NCBI BlastP on this gene

EPQ56097

hypothetical protein
  
Accession: EPQ56096
  
Location: 113642-116697
  
 NCBI BlastP on this gene

EPQ56096

250. :  JH687546 Punctularia strigosozonata HHB-11173 SS5 unplaced genomic scaffold PUNSTscaffold\_9     Total score: 1.0     Cumulative Blast bit score: 1875

S-adenosyl-L-methionine-dependent methyltransferase
  
Accession: EIN07303
  
Location: 1039044-1040528
  
 NCBI BlastP on this gene

EIN07303

scamp-domain-containing protein
  
Accession: EIN07304
  
Location: 1042752-1044074
  
 NCBI BlastP on this gene

EIN07304

polyadenylate binding protein
  
Accession: EIN07305
  
Location: 1045138-1047309
  
 NCBI BlastP on this gene

EIN07305

alpha/beta-hydrolase
  
Accession: EIN07306
  
Location: 1047721-1049010
  
 NCBI BlastP on this gene

EIN07306

mitochondrial protein
  
Accession: EIN07307
  
Location: 1049453-1050771
  
 NCBI BlastP on this gene

EIN07307

adenylate kinase
  
Accession: EIN07308
  
Location: 1051329-1052231
  
 NCBI BlastP on this gene

EIN07308

alpha/beta-hydrolase
  
Accession: EIN07309
  
Location: 1052666-1054781
  
 NCBI BlastP on this gene

EIN07309

Swi3-domain-containing protein
  
Accession: EIN07310
  
Location: 1055187-1056729
  
 NCBI BlastP on this gene

EIN07310

methyltransferase domain-containing protein
  
Accession: EIN07311
  
Location: 1057081-1058292
  
 NCBI BlastP on this gene

EIN07311

P-loop containing nucleoside triphosphate hydrolase protein
  
Accession: EIN07312
  
Location: 1058678-1066395
  
  
**BlastP hit with Mycgr3G42010\_Mycgr3T**
  
Percentage identity: 43 %
  
BlastP bit score: 1875
  
Sequence coverage: 103 %
  
E-value: 0.0
  
  
 NCBI BlastP on this gene

EIN07312

hypothetical protein
  
Accession: EIN07313
  
Location: 1067427-1071641
  
 NCBI BlastP on this gene

EIN07313

MFS general substrate transporter
  
Accession: EIN07314
  
Location: 1071895-1073944
  
 NCBI BlastP on this gene

EIN07314

hypothetical protein
  
Accession: EIN07315
  
Location: 1074391-1076885
  
 NCBI BlastP on this gene

EIN07315

WD40 repeat-like protein
  
Accession: EIN07316
  
Location: 1077135-1079438
  
 NCBI BlastP on this gene

EIN07316

N2,N2-dimethylguanosine tRNA methyltransferase
  
Accession: EIN07317
  
Location: 1080132-1082487
  
 NCBI BlastP on this gene

EIN07317

Pkinase-domain-containing protein
  
Accession: EIN07318
  
Location: 1082623-1086557
  
 NCBI BlastP on this gene

EIN07318

Detecting sequence homology at the gene cluster level with MultiGeneBlast.
  
Marnix H. Medema, Rainer Breitling & Eriko Takano (2013)
  
*Molecular Biology and Evolution* , 30: 1218-1223.
